# Supplementary material for: On the Use of Bone Remodelling Models to Estimate the Density Distribution of Bones. Uniqueness of the Solution
Source: PLoS One. 2016 Feb 9;11(2):e0148603. doi: 10.1371/journal.pone.0148603 (PMC4747586; doi:10.1371/journal.pone.0148603)
Supplement: S8 File — (PDF) [file pone.0148603.s008.pdf]

N° Elem	Volume (mm3)	Density (g/cm3)
1	1.32415	2.1
2	1.75823	2.1
3	0.984099	2.1
4	1.46442	2.1
5	1.53082	2.1
6	1.32068	2.1
7	1.91404	0.38104
8	1.17036	0.333152
9	0.929209	0.221725
10	1.27254	0.175416
11	2.0926	0.18949
12	2.20407	0.18865
13	1.21488	0.184983
14	1.26024	0.154533
15	0.794454	0.147818
16	1.95087	0.118283
17	0.953925	2.1
18	0.98949	2.1
19	1.27875	2.1
20	1.04598	2.1
21	0.594005	2.1
22	0.578861	2.1
23	1.09506	2.1
24	1.45327	2.1
25	2.58396	1.79343
26	1.58581	2.1
27	1.5858	2.1
28	0.636946	2.03447
29	0.94202	2.1
30	0.946119	1.76517
31	0.636953	2.1
32	0.946107	2.1
33	1.09505	2.1
34	1.27389	1.56383
35	1.00232	2.1
36	1.00231	2.1
37	1.83583	2.1
38	1.00231	2.1
39	1.28828	0.184074
40	1.81696	0.165514
41	1.28828	0.150932
42	1.28828	0.130994
43	1.0044	0.30862
44	0.85148	0.296667
45	0.851539	0.31504
46	1.57767	0.172527
47	1.52227	0.157393
48	0.730657	0.153064
49	0.94613	0.290794
50	1.28228	2.1
51	1.64268	2.1
52	1.10101	2.1
53	1.3939	2.1
54	0.959471	2.1
55	1.10101	2.1

56	1.2873	2.1
57	1.59659	2.1
58	1.80841	0.752238
59	1.80841	0.889735
60	0.946129	0.756742
61	1.36991	0.727723
62	1.32262	0.921068
63	1.32261	0.797429
64	1.38464	0.814892
65	0.962877	0.739374
66	0.962878	0.712248
67	1.82129	0.720758
68	1.82129	0.688628
69	0.94613	0.726355
70	1.39751	0.710787
71	0.946131	0.736555
72	1.34493	0.695871
73	1.39751	0.707297
74	0.837577	0.740252
75	0.823478	0.701443
76	1.10889	1.09752
77	1.01686	0.721955
78	1.1942	1.1885
79	0.836129	0.73725
80	0.937165	0.697124
81	0.969438	0.794546
82	0.895386	0.71779
83	1.30554	1.17696
84	1.15798	0.746838
85	1.22578	1.45559
86	1.15255	0.785836
87	0.9765	0.705348
88	2.02178	1.35127
89	1.63755	1.25426
90	0.727713	0.824685
91	0.810945	0.794131
92	1.00559	1.33795
93	0.879098	0.896304
94	1.07852	1.35902
95	0.657764	0.801184
96	0.829406	0.737029
97	0.720201	0.417986
98	0.946131	0.641356
99	1.11827	0.562891
100	1.50754	0.431786
101	1.64835	0.247119
102	1.71903	0.616396
103	0.851554	0.54006
104	1.75626	0.229581
105	2.1646	0.307979
106	1.5563	0.35277
107	1.5563	0.214794
108	2.10876	0.261608
109	1.05438	0.469289
110	0.946133	0.815752
111	1.05438	0.837855
112	1.20256	0.476969
113	1.47879	0.265033

114	1.47879	1.04265
115	0.77815	0.696372
116	0.947114	2.1
117	1.31257	2.1
118	1.09235	2.1
119	0.946132	2.1
120	1.54246	2.1
121	1.54246	2.1
122	1.09235	2.1
123	0.946131	2.1
124	1.29507	1.58958
125	1.0353	2.1
126	1.03529	2.1
127	1.28667	2.1
128	1.44777	2.1
129	1.61121	2.1
130	1.22176	0.7
131	0.946129	0.692773
132	1.08848	0.622064
133	0.946131	0.700263
134	1.61201	0.694653
135	1.61201	0.640886
136	1.19993	0.675516
137	1.35822	0.683716
138	1.1539	0.212789
139	1.00765	0.142319
140	1.28228	2.1
141	2.65339	2.1
142	1.28228	2.1
143	1.55998	2.1
144	1.00509	2.1
145	0.93845	2.1
146	1.27674	2.1
147	0.810958	2.1
148	1.91381	2.1
149	1.1861	2.1
150	1.24886	2.1
151	2.02842	2.1
152	0.991436	0.134869
153	1.34588	0.12247
154	2.02729	0.126309
155	0.72904	0.184279
156	1.06139	0.1997
157	0.835368	0.209408
158	1.63819	0.120835
159	1.10745	0.132111
160	1.79925	0.131747
161	1.00085	0.19536
162	0.946133	2.1
163	1.41123	1.79226
164	1.8327	2.0357
165	1.8327	2.1
166	0.946132	1.0732
167	1.41123	2.1
168	1.3676	2.1
169	1.11048	2.1
170	1.11048	2.1
171	1.65815	2.1

172	1.9004	2.1
173	1.88691	2.1
174	1.82232	2.1
175	1.46591	2.1
176	1.09115	2.1
177	1.02051	1.06741
178	1.03373	1.12224
179	1.10307	2.1
180	1.05556	2.1
181	1.82533	0.997595
182	1.19496	0.92611
183	1.28597	2.1
184	1.20421	2.1
185	2.23644	2.1
186	1.08378	2.1
187	0.983724	2.1
188	0.602075	2.1
189	1.45876	2.1
190	1.45875	2.1
191	0.602103	1.95721
192	1.11822	2.1
193	0.946133	2.1
194	0.602103	2.1
195	1.20421	2.1
196	1.26371	0.991934
197	1.55179	0.759132
198	1.14042	0.707839
199	1.56876	0.730805
200	2.27496	0.863729
201	1.13748	0.779592
202	1.02884	0.888728
203	1.25124	0.852864
204	0.82703	0.788197
205	0.607775	0.806411
206	1.49242	0.848956
207	1.35674	0.842548
208	1.6697	2.1
209	1.81655	2.1
210	0.900108	1.13407
211	0.851537	1.00042
212	0.703076	1.17937
213	0.639143	1.05199
214	1.42358	1.14691
215	1.4976	1.27562
216	0.86284	1.07417
217	1.2965	1.12599
218	1.27835	1.98409
219	1.00012	1.01651
220	1.74332	1.26274
221	1.08576	2.1
222	1.19	2.1
223	1.08576	2.1
224	1.20854	2.1
225	0.946131	2.1
226	0.946131	2.1
227	1.75985	2.1
228	1.75985	2.1
229	1.41232	2.1

230	1.29365	2.1
231	0.973559	2.1
232	1.23698	0.103612
233	1.45055	0.121597
234	1.26947	0.16125
235	0.856218	0.157751
236	1.75303	0.116819
237	1.14938	0.147969
238	0.984711	0.105155
239	2.05771	0.140379
240	0.625171	0.104245
241	0.965675	0.0962409
242	1.17631	0.121214
243	1.4677	0.169029
244	1.35403	0.332143
245	0.975149	0.345426
246	0.946131	0.371386
247	1.80893	0.187559
248	1.80893	0.16796
249	0.975149	0.182126
250	0.946132	0.345782
251	1.9503	0.328494
252	2.80207	0.26584
253	1.76411	0.327983
254	2.77469	0.268791
255	1.9503	0.242966
256	1.26784	0.270172
257	2.31093	0.437821
258	0.851541	0.564247
259	1.39521	0.319912
260	1.45613	0.492822
261	0.851541	0.618417
262	0.996013	0.38485
263	1.07989	0.575102
264	0.946131	0.605916
265	1.08213	0.390859
266	1.74289	0.386384
267	1.75838	0.395052
268	2.11163	0.541933
269	1.23773	2.1
270	1.25398	2.1
271	1.0304	2.1
272	1.32368	2.1
273	1.11712	0.90238
274	1.07999	0.86124
275	1.1766	2.1
276	1.35735	2.1
277	1.089 0.172631	
278	1.81193	0.23248
279	1.11081	0.0860166
280	1.78069	0.0898551
281	0.717512	0.0935256
282	0.810946	0.088289
283	0.703901	0.0940722
284	1.09028	0.0846205
285	0.958031	0.0884988
286	0.892585	0.0888523
287	0.873431	0.0914648

288	0.997755	0.0786649
289	1.85479	0.076876
290	0.983029	0.741272
291	1.27922	0.746946
292	1.64346	0.924557
293	1.27922	0.831959
294	1.09795	1.56511
295	1.07508	1.06279
296	1.02975	1.26297
297	1.01121	1.59999
298	1.72369	0.103653
299	1.12035	0.0833619
300	2.13511	0.101124
301	0.9705	0.103683
302	0.946131	0.12177
303	0.86182	0.0996653
304	1.51379	0.0975451
305	1.51379	0.0942852
306	0.946132	0.102688
307	0.861849	0.104389
308	1.34591	0.823938
309	1.14721	0.575905
310	1.15539	2.1
311	1.90938	2.1
312	2.19436	1.24553
313	1.41563	2.1
314	0.785641	2.1
315	0.736384	2.1
316	1.46489	2.1
317	1.46488	2.1
318	0.736372	2.1
319	0.946132	2.1
320	1.90941	1.13133
321	1.18907	2.1
322	1.20409	2.1
323	0.973559	2.1
324	1.29461	2.1
325	0.946129	2.1
326	0.94613	2.1
327	1.78751	2.1
328	1.78751	2.1
329	1.38885	2.1
330	1.34479	2.1
331	1.07283	1.16915
332	1.6869	2.1
333	1.52544	2.1
334	1.07797	2.1
335	1.13819	1.008
336	0.776179	2.1
337	1.61092	1.10827
338	1.36155	2.1
339	0.77618	2.1
340	1.2933	1.06082
341	1.15946	2.1
342	0.69857	2.1
343	1.87607	2.1
344	1.07612	2.1
345	1.31976	1.08259

346	1.01243	1.81612
347	1.41279	1.14239
348	1.35146	0.636908
349	1.90664	0.617574
350	1.35145	0.697755
351	1.22811	0.660833
352	1.93147	0.649534
353	1.42486	2.1
354	1.56558	2.1
355	1.0476	0.0857415
356	2.31605	0.0735174
357	1.89367	0.0841156
358	0.804706	0.0752931
359	0.783919	0.0644842
360	1.3116	0.056255
361	1.03891	0.0561932
362	0.960473	0.0616765
363	1.08462	0.0682613
364	0.785994	0.0618207
365	1.1318	0.0567095
366	1.29576	0.0535321
367	1.3946	0.0842168
368	1.11924	0.075654
369	0.834322	2.1
370	1.0256	2.1
371	1.74321	1.0474
372	1.41798	1.02724
373	0.871608	1.0385
374	1.47253	1.04422
375	1.47252	1.04248
376	0.817064	0.994849
377	0.817064	1.01052
378	0.946129	1.00622
379	1.63413	0.994002
380	1.9194	0.363211
381	1.93562	0.322525
382	2.06307	0.38207
383	1.46021	0.291761
384	0.955002	1.5063
385	0.833409	1.66113
386	0.838039	1.40719
387	1.48702	1.55427
388	1.1697	1.45417
389	1.23549	1.59933
390	1.18373	1.4739
391	2.02254	1.51841
392	1.68867	1.61377
393	0.875481	1.2221
394	0.821635	1.53671
395	0.858878	1.09569
396	1.30245	1.27205
397	1.07476	1.20326
398	1.11907	1.29287
399	1.10453	1.21222
400	1.70233	1.30504
401	1.70233	1.18622
402	2.6558	1.45131
403	1.7818	2.1

404	1.87059	2.1
405	1.34412	0.209865
406	1.98068	0.293753
407	2.56369	0.24501
408	1.49512	0.162099
409	1.35977	0.686428
410	1.34254	0.760727
411	1.75618	0.624184
412	1.75618	0.579928
413	0.946129	0.818512
414	0.946129	0.701181
415	1.17665	0.732351
416	1.01976	0.590868
417	1.27376	0.537449
418	1.35978	0.581027
419	1.68741	0.456547
420	1.68741	0.425944
421	0.946132	0.663232
422	0.946133	0.545901
423	1.14241	0.572989
424	0.996831	0.422609
425	0.996828	0.591731
426	1.12774	0.638005
427	1.13664	2.1
428	1.09627	2.1
429	2.8814	2.1
430	3.49751	2.1
431	1.79109	0.768133
432	1.54197	0.64308
433	2.75153	0.613232
434	1.93019	0.467768
435	1.51569	1.10028
436	1.55145	1.28261
437	1.78595	2.1
438	3.74452	2.1
439	1.66537	2.1
440	3.55161	2.1
441	1.35002	0.812634
442	1.19901	0.850977
443	1.61028	1.88907
444	1.35002	0.873979
445	1.65712	0.825472
446	1.10585	0.760836
447	0.961347	0.745281
448	0.810944	0.744235
449	1.16148	1.47834
450	1.08066	1.03737
451	0.934577	0.745214
452	1.05164	1.12213
453	0.79216	0.732716
454	2.99194	2.1
455	1.2832	2.1
456	2.97842	2.1
457	1.39517	2.1
458	1.03391	2.1
459	1.74416	2.1
460	1.05071	2.1
461	0.923782	2.1

462	1.55369	2.1
463	1.86444	2.1
464	1.01617	2.1
465	1.27174	2.1
466	1.20573	2.1
467	1.01616	2.1
468	1.13621	2.07102
469	1.75366	2.04958
470	2.27009	1.33608
471	1.6813	1.64796
472	1.47904	0.160046
473	2.62714	0.25515
474	1.80085	0.21935
475	1.80085	0.134137
476	2.74104	0.298902
477	1.37052	0.304544
478	0.946131	0.546777
479	1.37052	0.390673
480	1.31357	0.392799
481	1.7837	0.235893
482	1.7837	0.48897
483	0.900397	0.354256
484	1.74487	0.293003
485	2.88672	2.1
486	2.88249	2.1
487	1.0961	0.545138
488	1.20143	0.536628
489	1.66739	0.508527
490	1.85795	0.274369
491	1.45789	0.166298
492	2.11407	0.225203
493	0.713524	0.201197
494	1.58391	0.236983
495	1.58391	0.138659
496	1.04765	0.190113
497	1.24979	0.262896
498	0.713551	0.146002
499	0.946127	0.271867
500	1.36768	0.221486
501	1.14433	0.0941965
502	1.66923	0.0993431
503	0.699479	0.108325
504	0.70351	0.108014
505	0.758416	0.104734
506	1.07678	0.098431
507	0.909874	0.100944
508	0.940218	0.105947
509	0.876604	0.118572
510	1.27148	0.111491
511	2.42348	0.111382
512	1.19837	0.0942062
513	0.764764	0.103607
514	0.829259	0.111702
515	0.740584	0.101383
516	1.2772	0.0953586
517	0.964465	0.0984182
518	1.06231	0.0983871
519	1.06924	0.110269

520	1.17566	0.28245
521	1.3063	0.259651
522	2.51253	0.402524
523	2.42267	0.437112
524	1.89226	0.322619
525	1.46492	0.506997
526	0.946132	0.376802
527	0.653151	0.437203
528	0.653124	0.571189
529	1.53995	0.284155
530	1.53995	0.243989
531	0.936806	0.393242
532	1.25626	0.334611
533	1.1893	0.513319
534	1.62206	0.603328
535	1.04921	2.1
536	1.22927	2.1
537	0.801765	2.1
538	0.774917	2.1
539	0.844623	2.1
540	0.737481	2.1
541	3.24379	2.1
542	2.48573	2.1
543	1.40967	1.14933
544	1.47203	1.30582
545	2.07659	1.0033
546	1.40967	1.38046
547	2.06638	1.0677
548	1.17418	2.07844
549	1.04921	2.1
550	1.74593	2.1
551	1.74593	1.42619
552	0.946132	1.064
553	1.38651	2.1
554	1.20761	0.832309
555	1.20761	1.00601
556	1.30555	0.954921
557	1.46812	1.05639
558	2.41602	1.26086
559	1.46812	1.404
560	1.46812	0.995726
561	0.734059	0.973591
562	0.946132	1.52434
563	1.21261	1.22949
564	1.34225	1.27996
565	0.734084	1.12874
566	1.82077	1.18517
567	1.82077	1.18616
568	1.13751	0.837617
569	0.946131	1.05461
570	1.13751	0.960402
571	1.13748	0.944529
572	0.784392	0.910814
573	1.4906	0.932141
574	1.4906	0.823842
575	1.17879	1.05642
576	1.28291	1.03136
577	0.626495	2.1

578	1.45334	2.1
579	1.45334	2.1
580	0.626494	2.1
581	1.3852	2.1
582	0.946116	2.1
583	0.626499	2.1
584	1.47203	1.01301
585	1.42639	2.1
586	1.42639	2.1
587	0.795469	2.1
588	0.795469	2.1
589	0.946132	2.1
590	0.850877	2.1
591	1.37098	2.1
592	1.87194	2.1
593	1.76988	2.1
594	3.16295	0.0985499
595	3.26725	0.0772393
596	1.13322	2.1
597	1.20761	2.1
598	0.686933	0.771078
599	0.493248	0.705482
600	1.2053	1.45974
601	0.947149	0.734912
602	1.27591	0.784762
603	1.75732	1.58586
604	0.493261	0.800371
605	1.34337	1.76934
606	2.73207	0.710891
607	1.19857	0.769353
608	0.671688	0.698795
609	0.946134	1.51986
610	1.36603	0.713211
611	2.07817	0.778614
612	1.82573	1.31004
613	0.671699	0.841935
614	1.47772	0.702986
615	0.975349	1.54825
616	1.22615	2.1
617	1.47868	2.1
618	2.29351	1.24096
619	1.47642	2.1
620	2.94443	1.15454
621	1.80454	2.1
622	0.949135	1.80119
623	0.949134	2.0011
624	1.10476	0.18341
625	0.982857	0.594778
626	1.10938	0.314307
627	1.19052	0.685452
628	1.01012	0.0687191
629	1.35562	0.0671121
630	0.691742	0.0636554
631	2.39596	0.0668421
632	2.35604	0.0688979
633	0.974081	0.0554552
634	3.22444	0.053454
635	1.3294	0.777673

636	1.37265	0.890479
637	1.3294	0.932253
638	1.51113	2.1
639	1.43643	2.1
640	1.72726	2.1
641	1.634	2.1
642	0.755566	2.1
643	1.29319	2.1
644	0.755567	2.1
645	0.946131	2.1
646	1.29319	2.1
647	0.973563	2.1
648	0.904398	2.1
649	0.904406	2.1
650	1.1538	2.1
651	1.10437	2.1
652	0.935418	2.1
653	1.16649	2.1
654	0.975667	2.1
655	1.17952	2.1
656	1.95053	2.1
657	0.948676	2.1
658	1.44287	2.1
659	0.746095	2.08598
660	1.51224	2.1
661	1.27354	2.1
662	0.746066	1.93596
663	1.20573	2.1
664	1.12442	2.1
665	0.671487	2.1
666	1.04355	2.1
667	0.948675	2.1
668	0.967673	0.984559
669	1.12931	0.109872
670	1.38212	0.129153
671	2.40113	0.116567
672	1.08054	0.143386
673	1.04072	0.169704
674	0.691062	0.119897
675	1.58052	0.122736
676	1.33502	0.124245
677	0.946131	0.143178
678	0.691062	0.164461
679	1.02663	0.120156
680	1.80569	0.106293
681	1.26062	0.0912625
682	1.18203	0.114863
683	0.946131	0.146831
684	0.795065	0.110989
685	1.58753	0.10327
686	1.58753	0.0963413
687	0.946131	0.117367
688	0.795093	0.105187
689	0.805057	2.1
690	0.812415	2.1
691	1.23868	0.665811
692	1.34448	0.655872
693	1.67201	2.1

694	1.61202	2.1
695	1.41103	2.1
696	1.41103	2.1
697	0.841088	2.1
698	1.38302	2.1
699	0.841089	2.1
700	0.869097	2.1
701	1.38302	2.1
702	0.949941	2.1
703	0.94994	2.1
704	1.88608	2.1
705	0.854942	2.1
706	1.68893	2.1
707	1.70002	2.1
708	2.02107	0.531825
709	2.04982	0.508678
710	3.11063	0.710389
711	2.03805	0.54927
712	1.67638	2.1
713	0.967492	2.1
714	2.00209	2.1
715	0.967491	2.1
716	0.870742	2.1
717	1.30266	0.290426
718	0.651334	0.325616
719	1.69413	0.209258
720	1.69413	0.356956
721	0.946132	0.332003
722	1.03079	0.334207
723	1.31468	0.275009
724	0.946132	0.400048
725	0.919055	0.228706
726	1.16815	0.246836
727	2.79004	0.26489
728	1.2136	0.171303
729	2.62935	0.22143
730	0.947692	0.73955
731	1.51835	0.710323
732	0.947693	0.434589
733	0.947692	0.512105
734	0.980841	2.1
735	1.43577	2.1
736	0.980841	2.1
737	1.67858	2.1
738	1.34124	0.777052
739	1.39818	0.73364
740	1.2065	2.1
741	0.946133	2.1
742	1.2079	2.1
743	1.65229	2.1
744	1.65229	2.1
745	0.946132	2.1
746	1.2079	2.1
747	1.39053	2.1
748	1.20651	2.1
749	1.36919	2.1
750	1.12717	2.1
751	1.09483	2.1

752	0.994294	2.1
753	0.97356	2.1
754	1.06827	2.1
755	1.40571	2.1
756	1.29259	2.1
757	0.946131	2.1
758	1.7787	2.1
759	1.7787	2.1
760	1.29259	2.1
761	0.946132	2.1
762	1.09408	0.985002
763	1.03824	0.804597
764	0.851541	0.654083
765	1.23608	0.698644
766	0.851539	0.683187
767	1.5021	0.803369
768	1.54839	0.813574
769	1.12373	0.646752
770	1.22617	0.69922
771	1.42762	0.850691
772	2.24746	0.848772
773	1.05475	0.705325
774	1.46845	0.701264
775	1.05475	0.260479
776	0.929726	0.627593
777	1.52072	0.576549
778	1.82488	0.486388
779	1.02271	0.629094
780	1.19225	0.560389
781	1.43071	0.527987
782	0.94613	0.646698
783	1.05475	0.241745
784	1.39223	2.1
785	1.71255	2.1
786	0.946132	0.885828
787	1.10531	0.93567
788	1.46355	0.922725
789	1.46355	0.879727
790	0.78086	1.01331
791	1.10531	0.926673
792	1.1391	0.900178
793	1.56166	0.994687
794	2.21062	0.931803
795	1.0361	2.08913
796	1.73577	2.1
797	1.73577	2.1
798	0.946116	2.1
799	1.28174	2.1
800	0.946118	2.1
801	1.22883	2.1
802	1.28172	2.1
803	1.03609	2.1
804	0.553567	0.0826243
805	1.60581	0.0845705
806	1.60581	0.0732616
807	0.553594	0.0803537
808	0.966748	0.0894564
809	0.966748	0.0899548

810	0.946133	0.0978228
811	1.05602	0.0825331
812	2.38525	0.0928116
813	1.14503	0.0650777
814	1.24678	0.0654946
815	0.56262	0.0685162
816	1.24678	0.0701976
817	0.978467	0.071454
818	1.66262	0.0654646
819	1.66262	0.0633184
820	0.562633	0.067623
821	1.09522	0.0721197
822	2.49356	0.0703152
823	0.592547	0.0737242
824	1.04321	1.07948
825	1.25133	1.21717
826	1.70508	1.10527
827	1.81043	0.905264
828	0.494934	0.983259
829	0.601047	0.956582
830	0.993428	0.935442
831	0.946573	1.05168
832	1.35039	0.911078
833	0.816895	1.31359
834	1.5063	0.873057
835	0.816895	0.831687
836	0.946132	0.821659
837	1.9067	1.13018
838	2.14504	1.24619
839	0.816895	0.81794
840	2.29624	0.829564
841	2.76933	0.836328
842	1.254 1.41883	
843	0.946128	2.1
844	1.38813	2.1
845	1.79998	2.1
846	1.79998	2.1
847	0.94613	2.1
848	1.38813	2.1
849	1.35798	2.1
850	1.27809	2.1
851	1.35798	2.1
852	1.68994	2.1
853	1.68994	2.1
854	0.946132	2.1
855	0.946132	2.1
856	1.18067	2.1
857	0.958256	2.1
858	0.958255	2.1
859	0.958257	2.1
860	0.958255	2.1
861	1.06457	2.1
862	1.14195	2.1
863	1.70423	0.820615
864	1.14195	2.1
865	1.64285	2.1
866	2.02057	2.1
867	0.946132	2.1

868	1.44186	2.1
869	0.946133	2.1
870	1.16366	2.1
871	1.17233	2.1
872	1.31259	0.822555
873	1.2016	2.1
874	1.17129	2.1
875	1.05724	0.747991
876	1.32697	0.929969
877	1.05724	0.759899
878	1.04292	0.985564
879	3.39037	2.1
880	2.57275	2.1
881	0.629212	2.1
882	2.32525	2.1
883	1.76949	0.747983
884	1.65809	0.83308
885	1.27665	2.1
886	1.19861	2.1
887	1.20045	2.1
888	1.1635	2.1
889	0.953633	0.547589
890	0.953634	0.721672
891	1.21408	2.1
892	1.25716	2.1
893	2.01791	2.1
894	1.89318	2.1
895	0.947921	1.24203
896	0.939049	2.1
897	0.947921	1.68133
898	0.740753	2.1
899	0.916104	2.08583
900	1.16941	2.1
901	1.72038	2.1
902	2.04441	2.1
903	0.973182	2.1
904	1.11751	2.09259
905	0.611089	2.1
906	1.53584	0.7
907	0.767923	0.707403
908	1.38925	0.702644
909	1.38926	0.701712
910	0.946131	0.712152
911	0.961347	0.709747
912	1.19583	0.70118
913	0.946131	0.70539
914	1.3663	0.720981
915	1.21067	0.7
916	1.00166	0.156981
917	1.40707	0.18042
918	1.50435	0.170382
919	1.47954	0.833395
920	1.1233	2.1
921	2.36036	1.2262
922	1.04024	0.814789
923	0.739729	0.843376
924	1.45533	0.795634
925	1.45532	0.78347

926	0.946118	0.830325
927	1.15163	0.743167
928	1.15165	0.852314
929	0.739748	0.813222
930	1.04025	0.955668
931	1.47608	0.71258
932	0.946131	0.737629
933	1.02028	0.669548
934	0.946129	0.668587
935	1.78223	0.660978
936	1.78223	0.632981
937	1.34153	0.638072
938	1.38683	0.523845
939	1.3407	0.705791
940	1.3407	0.705005
941	2.04729	0.689666
942	0.851529	0.722917
943	1.26671	0.712825
944	0.946116	0.719952
945	1.4869	0.63175
946	1.29553	0.653714
947	1.03645	0.652246
948	1.20731	0.569834
949	0.73665	2.1
950	0.693596	2.1
951	2.11152	1.93826
952	1.73627	1.38985
953	1.06191	2.1
954	1.52226	2.1
955	1.76869	2.1
956	1.1881	2.1
957	1.32349	2.1
958	1.51041	2.1
959	1.61591	2.1
960	0.946132	2.1
961	0.946132	2.1
962	1.13537	2.1
963	3.0048	2.1
964	0.985109	2.1
965	1.68899	2.1
966	3.23261	2.1
967	1.08831	2.1
968	2.20623	2.1
969	0.91749	2.1
970	1.15515	2.1
971	0.550438	2.1
972	1.66294	2.1
973	1.08257	2.1
974	0.87851	2.1
975	2.09046	2.1
976	0.738711	2.1
977	1.31242	0.142629
978	1.26311	0.15517
979	1.67652	0.0829324
980	0.968594	2.1
981	0.968594	2.1
982	0.981099	0.0917507
983	1.32418	0.117279

984	2.67175	0.101291
985	2.09678	0.0858341
986	1.2511	0.0960492
987	0.912698	0.121234
988	0.810946	0.129251
989	0.810959	0.129621
990	1.67479	0.120918
991	1.65677	0.116287
992	1.28469	0.115855
993	1.48817	0.123197
994	1.08131	0.136299
995	1.04349	0.746858
996	1.42492	0.737958
997	1.05724	0.727244
998	1.65723	0.500346
999	1.95313	0.578019
1000	1.9293	0.502415
1001	2.02729	0.630418
1002	0.963393	1.01909
1003	0.963392	0.949649
1004	2.90171	2.1
1005	2.65447	2.1
1006	1.64158	2.1
1007	1.6488	2.1
1008	1.15991	0.728527
1009	0.946132	0.757443
1010	1.58199	0.7357
1011	1.58199	0.750456
1012	1.03333	0.759481
1013	1.25436	0.737936
1014	1.25436	0.755353
1015	0.946132	0.758065
1016	1.3771	0.725409
1017	0.687745	0.720911
1018	1.37709	0.694071
1019	0.812386	0.732711
1020	1.50172	0.726247
1021	1.50172	0.613241
1022	0.687758	0.721771
1023	1.17506	0.613154
1024	2.00539	0.713175
1025	2.2441	0.743345
1026	1.09667	0.641935
1027	1.43577	2.1
1028	2.24741	2.1
1029	1.26103	2.1
1030	0.851544	2.1
1031	1.5543	2.1
1032	1.59756	2.1
1033	0.851543	2.1
1034	1.44673	2.1
1035	1.31947	2.1
1036	1.1237	2.1
1037	1.26103	2.1
1038	1.089	0.202289
1039	0.946128	0.304563
1040	0.965824	0.322915
1041	0.94613	0.422005

1042	1.51344	0.251703
1043	1.51344	0.228799
1044	1.13508	0.29491
1045	1.32449	0.286649
1046	1.089	0.32446
1047	3.33706	0.532842
1048	2.7542	0.705937
1049	1.27256	0.749884
1050	0.954172	0.7308
1051	1.55527	0.726727
1052	1.05765	0.770374
1053	1.57326	0.752736
1054	1.38251	0.622676
1055	0.94613	0.737539
1056	2.38829	0.598014
1057	2.00539	0.705372
1058	1.53798	0.695443
1059	1.20969	2.1
1060	1.15864	2.1
1061	1.7986	2.1
1062	1.74533	2.1
1063	0.944827	1.54412
1064	0.946131	0.945096
1065	0.851543	0.893706
1066	1.52816	1.00705
1067	1.5314	1.30711
1068	1.39266	0.837029
1069	0.851528	0.816391
1070	1.39589	0.812924
1071	1.7348	0.989757
1072	1.85439	1.56049
1073	1.86367	1.20113
1074	1.90905	1.05834
1075	1.22176	0.7
1076	1.59492	0.700246
1077	2.97929	2.1
1078	2.1905	2.1
1079	1.24128	2.1
1080	0.994293	2.1
1081	2.49767	0.0552344
1082	2.37105	0.0581051
1083	1.10834	0.95351
1084	1.53214	0.860015
1085	1.417	1.80647
1086	2.13599	0.775172
1087	1.61727	2.1
1088	1.75004	2.1
1089	1.96301	2.1
1090	0.966631	2.1
1091	1.37791	2.1
1092	1.47302	2.1
1093	1.47302	2.1
1094	0.871519	2.1
1095	0.94613	2.1
1096	0.87155	2.1
1097	2.38731	2.1
1098	2.12399	1.48445
1099	1.74865	1.46292

1100	1.46724	1.06312
1101	2.38056	1.00349
1102	1.46724	1.07474
1103	0.818887	1.07704
1104	0.810944	1.54451
1105	0.805227	1.02344
1106	1.06558	1.21914
1107	0.841487	1.06333
1108	1.08456	1.27753
1109	0.903083	1.21651
1110	1.08929	0.100089
1111	1.68454	0.120542
1112	1.08929	0.107292
1113	1.07658	0.161683
1114	0.904439	0.164866
1115	1.58501	0.114289
1116	1.19729	0.12895
1117	1.05427	0.114046
1118	1.47764	0.160437
1119	0.810959	0.129789
1120	1.60946	1.02237
1121	2.28887	0.997505
1122	0.946133	1.01683
1123	1.14444	0.957917
1124	1.67964	0.906325
1125	1.67964	0.925195
1126	0.755334	1.06481
1127	1.14444	1.01638
1128	1.29054	0.966896
1129	1.37762	0.962252
1130	0.946132	1.01009
1131	0.533645	0.903654
1132	1.48361	0.86701
1133	1.48361	0.92281
1134	0.533645	0.983234
1135	0.872823	1.01488
1136	1.14444	0.910522
1137	1.37762	0.947657
1138	2.10101	0.938215
1139	1.02311	0.956187
1140	1.04083	2.1
1141	1.04084	2.1
1142	1.04084	2.1
1143	1.30116	2.1
1144	1.23477	0.143612
1145	1.81893	0.206859
1146	1.23477	0.147639
1147	1.23477	0.164829
1148	1.11967	0.155373
1149	0.946131	0.191084
1150	1.11966	0.206425
1151	1.12628	0.158174
1152	0.629641	0.200532
1153	1.6163	0.195695
1154	1.6163	0.116832
1155	1.11117	2.1
1156	1.13006	2.1
1157	1.15149	2.1

1158	1.13179	2.1
1159	0.585368	0.790533
1160	0.986036	0.903622
1161	1.94788	0.712427
1162	1.34857	0.77996
1163	2.31806	2.1
1164	2.06363	2.1
1165	1.00423	2.1
1166	0.829065	2.1
1167	0.551816	2.1
1168	0.99406	2.1
1169	1.98339	2.1
1170	1.68073	2.1
1171	2.558	2.1
1172	0.711814	1.10743
1173	2.97316	1.85971
1174	2.41579	2.1
1175	1.15248	0.525317
1176	1.14305	0.577265
1177	1.15483	0.0951317
1178	1.90981	0.0991915
1179	0.998957	0.089293
1180	1.83742	0.100668
1181	1.27581	2.1
1182	0.94613	2.1
1183	1.6315	2.1
1184	1.6315	2.1
1185	0.946132	2.1
1186	1.28441	2.1
1187	1.28441	2.1
1188	1.00365	2.1
1189	0.968536	2.1
1190	1.57575	2.1
1191	1.27581	2.1
1192	1.99297	2.1
1193	1.32784	2.1
1194	1.4259	2.1
1195	2.27952	2.1
1196	1.5685	2.1
1197	1.54231	2.1
1198	3.54638	2.1
1199	1.3294	2.1
1200	2.98237	2.1
1201	2.01633	1.11199
1202	2.0985	1.1446
1203	2.26047	0.855861
1204	1.7913	0.808308
1205	1.09504	2.1
1206	1.74126	2.1
1207	1.05983	2.1
1208	2.37664	2.1
1209	2.28084	2.1
1210	2.19948	2.1
1211	1.33593	0.0851011
1212	1.31508	0.0974928
1213	1.30647	2.1
1214	1.28846	2.1
1215	1.2955	2.1

1216	1.09044	1.83728
1217	1.4099	1.62328
1218	1.14984	2.03504
1219	0.946132	1.06206
1220	1.70727	1.69987
1221	1.70727	2.1
1222	1.14984	1.22711
1223	0.946132	1.60584
1224	0.911818	2.1
1225	2.12209	2.1
1226	2.4158	2.1
1227	1.20867	2.1
1228	2.84085	2.1
1229	1.42284	2.1
1230	2.45689	2.1
1231	1.41562	2.1
1232	0.918358	2.1
1233	1.11222	2.1
1234	0.999083	2.1
1235	0.696781	2.1
1236	1.30869	2.1
1237	1.66132	2.1
1238	0.696807	2.1
1239	0.745315	2.1
1240	1.23296	2.1
1241	0.780548	2.1
1242	1.93242	1.05677
1243	1.27362	1.09929
1244	2.77828	1.34625
1245	1.02653	0.857037
1246	0.808576	0.900052
1247	0.744012	0.969716
1248	0.605174	0.814963
1249	1.16501	0.788799
1250	0.803432	0.803562
1251	0.961016	0.794889
1252	1.10425	0.844256
1253	1.3756	0.903439
1254	2.17765	1.01109
1255	1.84578	0.91857
1256	1.32801	0.797791
1257	1.76377	0.808282
1258	0.946131	0.77269
1259	1.3022	0.844204
1260	0.694814	0.874456
1261	1.53028	0.779403
1262	1.53028	0.780707
1263	1.3022	0.823339
1264	0.922889	0.838585
1265	1.57766	2.1
1266	2.06911	1.37024
1267	1.57766	1.39195
1268	1.37721	0.189423
1269	1.72174	0.245775
1270	1.4468	0.301349
1271	1.37721	0.118892
1272	1.73972	0.704752
1273	1.62324	0.709409

1274	0.946125	0.7
1275	1.34259	0.699237
1276	1.04187	0.644529
1277	1.05019	0.7
1278	1.2361	0.700691
1279	1.69055	0.593485
1280	2.25056	0.67294
1281	1.10574	0.892089
1282	1.21632	0.972463
1283	1.10574	1.0054
1284	1.46181	1.23119
1285	0.946131	0.894622
1286	1.74111	1.18006
1287	1.74111	0.979693
1288	0.94613	0.975475
1289	1.26891	0.923333
1290	1.26891	0.891246
1291	1.23452	1.16103
1292	1.00255	0.949794
1293	1.72286	0.633091
1294	1.12233	0.4126
1295	1.30682	0.770078
1296	1.97397	0.815567
1297	1.67425	2.1
1298	1.77775	2.1
1299	1.73571	1.67652
1300	0.426265	1.9866
1301	1.31817	1.3392
1302	1.81042	2.1
1303	1.85456	2.1
1304	0.466532	1.37599
1305	1.28025	1.28262
1306	0.964467	1.26682
1307	1.27921	2.1
1308	2.11851	1.331
1309	2.37121	1.78641
1310	1.49357	0.716829
1311	1.42486	2.1
1312	1.00278	2.1
1313	1.00278	2.1
1314	1.12503	2.1
1315	0.568697	2.1
1316	0.555211	2.1
1317	0.982272	1.27732
1318	1.57558	1.15178
1319	0.851539	2.1
1320	1.07307	2.1
1321	1.58292	2.1
1322	1.50731	2.1
1323	0.851541	1.07033
1324	1.00304	2.1
1325	1.36003	2.1
1326	1.13127	2.1
1327	0.672234	2.1
1328	0.763044	2.1
1329	0.966979	2.1
1330	1.31502	2.1
1331	1.67969	2.1

1332	1.62408	2.1
1333	0.661955	2.1
1334	1.35048	2.1
1335	1.40447	2.1
1336	1.40447	2.1
1337	0.607961	2.1
1338	0.946131	2.1
1339	0.607963	2.1
1340	1.01531	2.1
1341	1.01531	2.1
1342	1.91802	2.1
1343	0.777749	2.1
1344	1.22585	0.267701
1345	1.20672	0.236537
1346	1.03166	0.581463
1347	0.553805	2.1
1348	2.00551	2.1
1349	2.07203	1.47851
1350	0.589479	2.1
1351	1.22352	2.1
1352	0.984227	2.1
1353	0.802455	2.1
1354	1.40942	2.1
1355	1.6662	2.1
1356	0.802485	2.1
1357	0.936669	2.1
1358	1.31127	0.841238
1359	0.946132	0.803297
1360	1.09729	0.736925
1361	0.946133	0.810892
1362	1.68703	0.765685
1363	1.68703	0.733087
1364	1.3689	0.786593
1365	1.26426	0.800559
1366	1.26923	2.1
1367	1.23614	2.1
1368	0.828371	2.1
1369	2.04556	2.1
1370	0.772648	2.1
1371	0.791059	2.1
1372	0.809958	2.1
1373	1.70012	2.1
1374	0.267032	2.1
1375	1.67604	2.1
1376	0.803823	2.1
1377	0.267305	2.1
1378	0.80246	2.1
1379	0.874582	2.1
1380	1.00188	2.1
1381	0.709588	2.1
1382	1.50679	2.1
1383	1.22456	2.09019
1384	0.870622	2.1
1385	1.05754	2.07918
1386	1.1689	2.1
1387	1.54556	2.1
1388	1.89226	2.1
1389	0.995311	2.1

1390	1.26195	2.1
1391	1.9	2.1
1392	0.845055	2.1
1393	0.737836	2.1
1394	0.823707	2.1
1395	0.626587	2.1
1396	1.16109	2.1
1397	0.85517	2.1
1398	0.932036	2.1
1399	1.08321	2.1
1400	1.43863	2.1
1401	2.72297	2.1
1402	2.90798	2.1
1403	1.47818	1.07263
1404	1.47819	1.0827
1405	0.694357	1.01948
1406	0.694382	1.03995
1407	0.946132	0.853461
1408	0.694382	0.964012
1409	1.22641	0.918384
1410	1.31155	1.37625
1411	2.45282	1.08478
1412	0.787843	0.913217
1413	0.808984	0.816048
1414	1.14232	1.06872
1415	0.795037	1.04182
1416	0.887382	0.944023
1417	1.14493	0.926339
1418	0.78393	0.870864
1419	1.09129	1.01936
1420	0.791482	0.959626
1421	1.1137	1.25905
1422	1.11819	1.07647
1423	0.960173	0.965249
1424	1.09447	1.24509
1425	0.874365	0.96413
1426	1.2474	0.449256
1427	1.75038	0.759037
1428	1.75038	0.810094
1429	0.94613	0.928854
1430	1.30076	0.771713
1431	1.12817	1.06913
1432	0.94613	0.862765
1433	1.30076	0.853977
1434	1.51841	0.767554
1435	1.51841	0.856538
1436	0.946132	1.05838
1437	1.39575	0.80688
1438	0.970358	1.23626
1439	0.946133	0.968985
1440	1.39575	0.936932
1441	1.06486	0.536572
1442	1.06486	0.546145
1443	1.38622	0.630453
1444	1.04521	0.113513
1445	1.00809	0.181725
1446	1.28952	0.189056
1447	0.849316	0.17287

1448	0.946132	0.173467
1449	0.849344	0.239093
1450	1.53631	0.18863
1451	1.53631	0.201588
1452	1.77843	0.106277
1453	1.69863	0.115649
1454	1.91646	2.1
1455	1.8582	2.1
1456	2.8349	0.0692344
1457	1.36307	0.0677529
1458	1.23886	0.112706
1459	1.12971	0.130528
1460	1.59662	0.131783
1461	1.59662	0.125073
1462	0.640304	0.243595
1463	0.640333	0.114135
1464	0.886276	0.256589
1465	0.886276	0.254088
1466	1.35065	0.165127
1467	2.09002	0.141202
1468	1.35543	0.176926
1469	1.94688	2.1
1470	1.97221	2.1
1471	0.719563	2.1
1472	1.20358	2.1
1473	0.874264	2.1
1474	1.27676	2.1
1475	1.46557	2.1
1476	1.46557	2.1
1477	0.68546	2.1
1478	0.94613	2.1
1479	0.685489	2.1
1480	1.39615	0.381156
1481	1.05404	0.18545
1482	1.06474	0.260626
1483	1.00713	0.269097
1484	0.714535	0.385188
1485	0.714563	0.427505
1486	1.41646	0.208123
1487	1.52582	0.263972
1488	0.721694	0.43723
1489	0.93665	0.392957
1490	1.21067	0.7
1491	1.21067	0.7
1492	1.73577	0.7
1493	1.17799	0.7
1494	3.21432	0.705588
1495	2.41452	0.694837
1496	1.36208	0.585701
1497	2.53256	0.616091
1498	0.834593	0.426189
1499	2.5853	0.616586
1500	1.77259	0.509433
1501	0.615724	0.856452
1502	1.11374	0.999888
1503	0.615726	0.825088
1504	0.946129	1.10798
1505	0.615714	1.06163

1506	1.75489	1.00655
1507	1.75489	0.946648
1508	1.23146	0.812891
1509	2.2772	0.856776
1510	0.74463	0.819755
1511	1.3546	0.81673
1512	1.80252	0.688445
1513	2.38304	0.733275
1514	1.19151	0.836336
1515	1.1231	0.878035
1516	1.82345	0.825776
1517	1.79805	0.793179
1518	0.761337	0.956885
1519	0.946127	0.853182
1520	0.557726	0.854629
1521	1.05353	0.584917
1522	1.05353	0.616627
1523	1.68599	0.596447
1524	1.05353	0.499149
1525	1.40588	2.1
1526	1.09373	2.1
1527	1.18382	0.998738
1528	1.46903	0.94806
1529	1.25103	2.1
1530	1.10378	2.1
1531	1.58941	2.1
1532	1.29639	2.1
1533	1.14874	0.122246
1534	0.899817	0.117694
1535	0.666898	0.123206
1536	1.76519	0.116369
1537	1.17289	0.121897
1538	1.03526	0.117142
1539	1.97708	0.121406
1540	1.31711	0.101711
1541	0.992517	0.100699
1542	1.70193	0.104041
1543	1.13852	0.111263
1544	1.23943	0.571819
1545	1.40014	0.989004
1546	1.04598	2.1
1547	1.1483	1.14418
1548	1.11433	0.927156
1549	1.32037	0.754822
1550	1.00381	0.873486
1551	1.11435	0.731033
1552	0.973502	0.976674
1553	0.946128	1.47098
1554	1.83697	1.13833
1555	1.83697	2.1
1556	1.31721	1.49854
1557	1.38614	1.18655
1558	1.39696	1.10589
1559	1.31721	1.50783
1560	1.0756	1.6676
1561	1.08542	1.00054
1562	0.946127	1.94293
1563	1.81052	0.952859

1564	1.81052	2.1
1565	1.24315	1.80749
1566	1.39696	0.909158
1567	1.35969	1.179
1568	1.24315	1.18814
1569	1.0756	0.829675
1570	1.20669	0.570876
1571	1.18508	0.634559
1572	1.71059	2.1
1573	1.66762	2.1
1574	2.50853	2.1
1575	1.8236	2.1
1576	1.78872	2.1
1577	1.41721	2.1
1578	1.01335	1.30025
1579	1.42439	1.00382
1580	0.946129	1.10908
1581	1.33266	1.15205
1582	1.79776	1.28345
1583	1.79776	1.6112
1584	0.946131	0.913375
1585	1.33266	1.26811
1586	1.41123	1.19247
1587	1.25346	2.1
1588	0.946132	0.908252
1589	0.827287	1.67179
1590	1.57878	1.11173
1591	1.57878	1.09417
1592	0.827287	1.01446
1593	1.07341	1.03175
1594	1.33266	1.0963
1595	1.25346	1.11862
1596	1.61149	2.1
1597	1.15644	1.49522
1598	1.27558	0.953358
1599	1.3764	0.80534
1600	1.89526	1.03951
1601	1.37641	0.798798
1602	1.2064	0.899053
1603	1.38426	2.1
1604	1.01595	2.1
1605	1.15067	2.1
1606	1.82276	2.1
1607	1.82276	0.770625
1608	0.946131	0.859625
1609	1.40609	0.878558
1610	0.946132	2.03443
1611	1.29143	1.01796
1612	1.40609	0.987415
1613	1.30272	1.84408
1614	1.19548	1.33258
1615	2.95451	2.1
1616	1.46124	2.1
1617	2.93215	2.1
1618	1.42105	2.1
1619	1.75477	1.12481
1620	2.31301	1.17758
1621	2.10275	0.830445

1622	1.80099	0.887311
1623	0.97541	2.1
1624	1.49733	2.1
1625	0.97541	2.1
1626	1.30751	2.1
1627	1.28614	0.188036
1628	1.74759	0.161378
1629	1.19756	0.589573
1630	1.71053	0.504663
1631	1.19757	0.7
1632	1.28954	0.548567
1633	2.02703	0.552071
1634	2.29453	0.259385
1635	1.28265	0.175177
1636	0.793267	0.289543
1637	1.19974	0.506284
1638	0.509551	0.248246
1639	1.04072	0.489276
1640	1.35302	0.178124
1641	1.53133	0.502135
1642	0.566153	0.308342
1643	1.09634	0.288599
1644	1.12792	0.337368
1645	1.68908	2.1
1646	1.68908	2.1
1647	0.768212	2.02029
1648	0.768213	2.1
1649	0.946132	2.1
1650	0.768212	2.01759
1651	1.32461	2.02228
1652	1.87291	1.97658
1653	2.64921	2.06545
1654	1.53642	2.1
1655	2.26537	2.1
1656	1.53642	2.1
1657	1.79874	2.1
1658	1.79874	2.1
1659	0.726635	1.38435
1660	0.726636	2.1
1661	0.946128	1.45313
1662	0.726634	1.43004
1663	1.23339	1.26197
1664	0.992674	2.1
1665	1.8023	2.1
1666	1.12324	0.0644258
1667	1.29163	0.0572222
1668	0.996831	0.383435
1669	1.0019	0.359882
1670	0.993831	0.66321
1671	1.07283	1.24471
1672	1.04788	2.1
1673	1.44489	1.07985
1674	1.04788	2.1
1675	1.07652	2.1
1676	0.991406	2.1
1677	1.37	2.1
1678	1.14195	2.1
1679	1.47904	0.164225

1680	1.26593	1.4198
1681	1.75366	1.30922
1682	1.57621	0.707425
1683	1.24538	0.668164
1684	0.969118	2.1
1685	0.946133	1.07978
1686	1.69387	2.1
1687	1.69387	2.1
1688	0.94613	1.51877
1689	1.28713	2.1
1690	1.28713	2.1
1691	1.23877	2.1
1692	0.969118	2.1
1693	1.22615	2.1
1694	0.98084	2.1
1695	1.01843	2.1
1696	1.50876	2.1
1697	1.85391	2.1
1698	1.84322	2.1
1699	1.70377	2.1
1700	1.78919	2.1
1701	2.66787	2.1
1702	1.41549	2.1
1703	1.41549	2.1
1704	0.86707	2.1
1705	0.896172	2.1
1706	0.946126	2.1
1707	0.867074	2.1
1708	0.946118	2.1
1709	1.54468	0.418851
1710	1.54468	0.410153
1711	2.27259	0.444501
1712	0.946131	0.647397
1713	1.25837	0.618358
1714	0.663171	0.704912
1715	1.68931	0.560344
1716	1.68931	0.52479
1717	1.25837	0.62855
1718	1.09411	0.643763
1719	1.73571	0.34608
1720	2.34894	0.420318
1721	0.946128	0.469002
1722	1.30497	0.4688
1723	0.772312	0.62634
1724	1.66895	0.399629
1725	1.66895	0.301427
1726	1.30497	0.444489
1727	1.13629	0.392779
1728	1.22852	0.835499
1729	1.578	0.833909
1730	2.07781	2.1
1731	1.5478	2.1
1732	1.70048	1.9903
1733	1.42329	1.78553
1734	1.05481	1.53533
1735	1.1032	1.07367
1736	1.19267	2.1
1737	1.77324	2.1

1738	1.01121	0.643025
1739	0.991867	0.587003
1740	2.0744	1.14563
1741	1.75142	2.1
1742	1.35418	1.16841
1743	1.40041	2.1
1744	1.50045	2.1
1745	1.37704	2.1
1746	1.50045	2.1
1747	1.87875	2.1
1748	1.35308	0.770584
1749	1.29983	0.762313
1750	1.62784	2.1
1751	1.44807	2.1
1752	1.00965	2.1
1753	2.69264	2.1
1754	1.04075	2.1
1755	0.946131	2.1
1756	0.946132	2.1
1757	1.52704	2.1
1758	1.83246	2.1
1759	1.56757	2.1
1760	1.16687	2.1
1761	2.04857	2.1
1762	1.32707	1.19531
1763	1.25519	0.285415
1764	1.32101	0.548759
1765	1.25055	0.966057
1766	0.827853	0.833018
1767	1.11835	0.466085
1768	1.07281	1.18929
1769	1.54302	0.852743
1770	0.724064	0.968303
1771	1.32709	2.1
1772	3.44123	1.06161
1773	2.46889	0.513166
1774	0.730076	0.327678
1775	1.25809	0.679693
1776	0.730077	0.450595
1777	0.946129	0.652079
1778	1.72777	0.230998
1779	1.72777	0.646446
1780	0.73005	0.487509
1781	1.7522	0.219961
1782	2.51618	0.358197
1783	0.628177	0.328463
1784	1.1068	0.702503
1785	0.747435	0.387888
1786	0.946133	0.675115
1787	1.84668	0.323253
1788	1.8563	0.642896
1789	0.628177	0.567562
1790	1.45501	0.375835
1791	2.19809	0.66197
1792	1.45501	0.42855
1793	1.48974	0.345823
1794	1.7709	2.0602
1795	2.63288	1.9415

1796	1.5362	2.1
1797	1.56327	2.1
1798	3.03945	0.237448
1799	0.899979	0.312367
1800	0.869386	0.258458
1801	1.85864	0.283419
1802	1.78028	0.29502
1803	1.28719	0.330028
1804	1.09532	0.240159
1805	0.881898	0.286791
1806	1.28911	0.309805
1807	1.39644	0.360051
1808	0.688829	0.269854
1809	2.14611	0.259789
1810	1.80313	0.25207
1811	0.68883	0.350661
1812	0.946131	0.395138
1813	1.25334	0.17694
1814	1.88987	0.272453
1815	1.94432	0.231798
1816	1.37592	0.218162
1817	1.11691	1.29724
1818	1.02923	1.46692
1819	0.45824	0.0983121
1820	0.646512	0.271893
1821	2.46292	0.15371
1822	0.493451	0.154079
1823	2.00826	2.1
1824	2.49784	2.1
1825	1.17054	2.1
1826	1.01485	2.1
1827	1.26045	2.1
1828	1.17054	2.1
1829	1.44247	2.1
1830	2.02687	0.104148
1831	1.66365	0.123138
1832	1.64656	0.0970603
1833	1.56597	0.125256
1834	1.21419	2.1
1835	1.5008	2.1
1836	1.10711	2.1
1837	1.44287	2.1
1838	1.44287	2.1
1839	0.750368	2.1
1840	1.25876	2.1
1841	0.82542	2.1
1842	1.04074	2.1
1843	1.25874	2.1
1844	1.04159	0.0922077
1845	1.2489	0.100158
1846	0.980547	0.708954
1847	1.62235	0.656144
1848	1.15991	0.698085
1849	0.980547	0.567162
1850	0.94613	0.716675
1851	1.54662	0.691172
1852	1.54661	0.699057
1853	1.07912	0.720252

1854	1.1032	0.694985
1855	1.1032	0.708226
1856	0.94613	0.710261
1857	1.12468	2.1
1858	1.2724	2.1
1859	1.06521	1.6482
1860	1.0752	1.83552
1861	1.94936	0.486301
1862	1.62772	0.598074
1863	1.94733	0.395509
1864	1.42901	0.552513
1865	1.33022	2.1
1866	1.70839	2.1
1867	1.75048	2.1
1868	1.41201	2.1
1869	1.52314	2.1
1870	1.44039	2.1
1871	1.51113	2.1
1872	2.58638	2.1
1873	0.987247	0.36517
1874	1.38622	0.692726
1875	0.858359	0.5964
1876	1.19967	0.693134
1877	1.06414	0.472979
1878	0.946132	0.75235
1879	0.94613	0.553865
1880	0.687079	0.794557
1881	0.946132	0.745059
1882	0.822611	0.722594
1883	1.70242	0.497885
1884	1.70242	0.73449
1885	0.946132	0.800085
1886	1.11155	0.720614
1887	0.946132	0.851283
1888	1.11155	0.766148
1889	1.37981	0.786087
1890	1.18348	0.466421
1891	2.44179	0.739271
1892	2.00403	0.781725
1893	1.10961	2.1
1894	1.85415	2.1
1895	1.44825	2.1
1896	0.932272	2.1
1897	0.810942	2.1
1898	1.00189	2.1
1899	1.11779	2.1
1900	1.3359	2.1
1901	0.965279	2.1
1902	0.833779	2.1
1903	1.05337	2.1
1904	0.526656	2.1
1905	1.44599	2.1
1906	1.44599	2.1
1907	1.24853	2.1
1908	0.946132	2.1
1909	1.24853	2.1
1910	0.724122	2.1
1911	1.91547	2.1

1912	2.49705	2.1
1913	1.05931	2.1
1914	1.1729	2.1
1915	1.34526	2.1
1916	1.5379	2.1
1917	1.90855	2.1
1918	2.08597	2.1
1919	1.68165	2.1
1920	1.45887	2.1
1921	1.50022	2.1
1922	1.4748	2.1
1923	2.63645	1.99989
1924	1.74431	2.1
1925	1.25288	2.02848
1926	1.25289	1.89106
1927	0.970025	1.78574
1928	0.747542	2.1
1929	0.810959	1.66494
1930	1.15369	1.90337
1931	0.977472	1.78052
1932	1.12695	1.91794
1933	0.996762	1.77658
1934	1.28249	0.87465
1935	0.946132	0.886041
1936	0.528621	0.936977
1937	1.59186	0.835319
1938	1.44072	0.841566
1939	0.633488	0.854714
1940	0.598506	0.900669
1941	1.51618	0.920989
1942	1.19999	0.817369
1943	1.46511	0.794637
1944	1.44056	0.786005
1945	0.912754	0.757298
1946	1.4725	0.720521
1947	1.4725	0.676473
1948	0.912753	0.758674
1949	1.23351	0.752661
1950	1.23351	0.75975
1951	0.946131	0.775657
1952	1.17129	0.680749
1953	1.82551	0.597302
1954	1.27575	1.94478
1955	0.946129	2.09061
1956	0.946129	2.1
1957	0.946128	2.05264
1958	0.792754	2.1
1959	0.94613	1.7245
1960	0.792752	1.66909
1961	2.08054	2.1
1962	1.20091	2.1
1963	2.1669	2.09066
1964	1.24383	1.79225
1965	0.904669	1.95575
1966	0.83804	1.55549
1967	1.90486	1.89248
1968	1.29162	1.70637
1969	1.37383	1.97013

1970	1.86533	1.7087
1971	1.76472	2.00565
1972	1.60428	1.69448
1973	2.43993	1.98071
1974	1.37833	2.1
1975	1.18632	2.1
1976	0.946132	2.1
1977	1.27544	2.1
1978	1.05085	2.1
1979	0.961178	2.1
1980	0.859844	2.1
1981	1.0499	2.1
1982	1.75592	2.1
1983	2.08668	2.1
1984	1.75592	2.1
1985	1.4402	2.1
1986	1.72285	2.1
1987	0.83312	0.252433
1988	1.07295	0.212938
1989	1.37607	0.2528
1990	1.32097	0.210814
1991	1.11332	0.276596
1992	1.18568	0.178607
1993	0.877482	0.181354
1994	1.4458	0.1338
1995	2.34112	0.10416
1996	2.68021	0.22114
1997	0.681677	0.0934438
1998	0.946131	0.135824
1999	0.681649	0.105421
2000	1.83115	0.110429
2001	1.68029	0.119153
2002	1.05482	0.095966
2003	1.31485	0.115594
2004	2.08247	1.30166
2005	1.33153	1.27431
2006	0.935062	1.36242
2007	0.806958	1.72446
2008	0.827683	1.22607
2009	1.01041	1.49779
2010	0.741832	1.22462
2011	1.15668	1.5663
2012	0.908649	1.35921
2013	0.59561	0.813166
2014	1.19028	1.04047
2015	0.59561	1.02991
2016	0.946132	1.07516
2017	0.595623	1.2726
2018	1.58164	1.07163
2019	1.58164	1.12697
2020	0.946131	1.88737
2021	1.55201	2.1
2022	1.55201	2.1
2023	0.946127	2.1
2024	1.13312	2.1
2025	1.13312	2.1
2026	1.08108	2.1
2027	1.04973	2.1

2028	1.20149	2.1
2029	1.86902	1.10654
2030	0.613447	2.1
2031	1.22618	1.17748
2032	1.38663	1.31749
2033	1.38662	2.1
2034	0.613447	1.24464
2035	1.22617	1.95784
2036	0.773899	1.80923
2037	1.23515	1.4751
2038	1.87823	1.17774
2039	1.70303	0.836559
2040	1.4714	2.1
2041	1.74067	2.1
2042	0.735699	2.1
2043	1.73461	2.1
2044	1.47224	2.1
2045	1.22827	2.1
2046	1.1634	2.1
2047	1.36471	2.1
2048	0.735701	2.1
2049	2.8386	0.877024
2050	2.39056	2.1
2051	2.44128	1.00419
2052	1.54102	2.1
2053	1.54102	2.1
2054	0.936141	1.22337
2055	1.40594	2.1
2056	1.07121	0.977675
2057	0.936142	1.65443
2058	1.07121	1.20016
2059	1.22691	0.308007
2060	1.42907	0.338406
2061	2.18048	0.214066
2062	0.947662	0.250777
2063	2.18394	2.1
2064	1.21292	2.1
2065	2.18394	2.1
2066	2.19064	2.1
2067	1.27229	2.1
2068	1.20859	0.866658
2069	1.21073	0.819434
2070	1.73899	2.1
2071	1.3164	2.1
2072	1.61245	2.1
2073	1.41444	2.1
2074	2.55116	2.1
2075	1.26193	2.1
2076	1.89553	2.1
2077	0.916438	2.1
2078	1.26113	2.1
2079	0.810934	2.1
2080	2.00419	2.1
2081	1.27872	2.1
2082	1.35306	2.1
2083	1.96749	2.1
2084	1.07955	2.1
2085	1.31854	2.1

2086	1.29505	0.846843
2087	1.29504	0.840945
2088	0.612469	0.877709
2089	0.990053	0.889704
2090	1.04072	0.840194
2091	0.985266	0.837148
2092	0.990039	0.826187
2093	1.06892	0.967826
2094	1.57158	0.777032
2095	1.895	0.851313
2096	1.05698	0.797341
2097	0.925053	2.1
2098	0.624712	2.1
2099	1.42465	2.1
2100	1.49014	2.1
2101	0.687198	2.1
2102	1.22058	2.1
2103	0.851488	2.1
2104	0.588128	2.1
2105	1.12452	2.1
2106	1.02783	2.1
2107	1.7083	2.1
2108	1.06977	0.867993
2109	0.818569	0.781711
2110	1.14386	1.14862
2111	0.983791	0.849412
2112	1.12214	1.26115
2113	1.0915	0.837666
2114	0.810934	0.803989
2115	0.764114	0.90192
2116	1.87956	0.873078
2117	1.87956	1.12621
2118	0.764141	0.915875
2119	1.37675	0.822193
2120	0.764141	0.888806
2121	0.946132	0.854163
2122	1.9637	1.42049
2123	2.10107	0.823696
2124	1.67814	0.958413
2125	3.02886	0.945002
2126	0.908982	0.457537
2127	0.621727	0.621262
2128	0.525963	0.586469
2129	0.946128	0.663798
2130	1.60979	0.499746
2131	1.45596	0.587113
2132	0.556172	0.565195
2133	2.06779	0.350407
2134	2.17911	0.502856
2135	1.00205	0.236961
2136	1.30685	0.292352
2137	1.16534	0.35132
2138	1.65532	2.1
2139	2.20346	2.1
2140	0.993889	2.1
2141	1.09328	2.1
2142	2.09372	2.1
2143	2.10036	2.1

2144	1.33727	1.11236
2145	2.53383	1.23901
2146	0.893379	1.49069
2147	1.52828	1.20908
2148	1.09599	0.0892071
2149	1.09599	0.0951132
2150	1.50524	2.1
2151	1.54711	2.1
2152	1.41469	2.1
2153	1.99459	1.80229
2154	1.79805	1.97722
2155	1.79805	2.1
2156	0.707344	2.1
2157	1.30318	1.80873
2158	0.707344	2.1
2159	0.946129	2.1
2160	1.30318	2.1
2161	2.40442	2.1
2162	1.89226	2.1
2163	1.40794	2.07317
2164	0.898721	2.06379
2165	0.791921	1.23707
2166	0.810933	1.81308
2167	1.10904	1.45257
2168	0.991102	2.02171
2169	0.808502	1.17649
2170	1.1398	1.61972
2171	1.31346	2.1
2172	1.11833	2.1
2173	1.669 2.1	
2174	1.25715	2.1
2175	1.3199	2.1
2176	1.63092	2.1
2177	1.63092	2.1
2178	0.946131	2.1
2179	0.946131	2.1
2180	1.13534	2.1
2181	0.979853	2.1
2182	2.19994	2.1
2183	0.725844	2.1
2184	2.19994	2.1
2185	2.35055	2.1
2186	1.52204	2.1
2187	1.40118	2.1
2188	3.42285	2.05364
2189	2.05217	2.1
2190	1.50319	2.1
2191	0.947516	1.25877
2192	0.903572	1.23383
2193	0.76996	1.29278
2194	1.90959	2.1
2195	1.18897	1.96231
2196	1.24166	1.73836
2197	2.26785	1.52684
2198	2.05217	2.1
2199	2.73306	2.1
2200	1.42729	0.54852
2201	1.387 0.546453	

2202	1.59458	0.762573
2203	1.91051	0.775267
2204	1.68108	0.732344
2205	2.03357	0.754818
2206	0.928982	0.5957
2207	1.55687	0.658958
2208	1.55687	0.278943
2209	0.92898	0.546255
2210	1.3495	0.693628
2211	1.3495	0.585171
2212	0.946133	0.697198
2213	1.85795	0.451861
2214	2.2727	0.631947
2215	1.20324	0.897331
2216	1.07784	0.657706
2217	0.957792	0.409729
2218	1.2745	0.418678
2219	1.4659	0.450516
2220	0.946129	0.689423
2221	0.95778	0.648885
2222	0.851553	0.752218
2223	1.74103	0.508706
2224	2.28994	0.501721
2225	2.08213	0.466862
2226	2.1556	0.4791
2227	2.20372	0.371582
2228	0.838027	0.169605
2229	1.11373	0.148143
2230	1.2909	0.0992394
2231	0.998424	0.160989
2232	1.40621	0.102623
2233	1.19478	0.119883
2234	1.11783	0.134098
2235	1.6695	0.101823
2236	1.7498	0.121608
2237	0.477652	0.129002
2238	1.77291	0.145445
2239	0.590448	0.124041
2240	1.07668	0.135141
2241	0.810946	0.23816
2242	1.1946	0.196988
2243	2.03914	0.149666
2244	1.25585	0.241558
2245	2.02622	0.141564
2246	1.39091	0.167663
2247	1.31871	0.180573
2248	1.15789	2.1
2249	1.15755	2.1
2250	1.82268	2.1
2251	1.87119	2.1
2252	3.10636	2.1
2253	2.96285	2.1
2254	1.73443	0.851297
2255	2.33812	0.856905
2256	1.59457	0.779795
2257	1.73443	0.993927
2258	2.00665	2.1
2259	1.99841	2.1

2260	1.19901	2.1
2261	1.16661	2.1
2262	2.15883	0.483389
2263	1.20951	0.451819
2264	2.49192	0.67769
2265	0.65112	0.401684
2266	2.45317	0.0983775
2267	1.73091	0.0901226
2268	2.0795	0.0963652
2269	1.45891	0.0795803
2270	1.6531	0.085779
2271	1.78304	0.080849
2272	0.775132	0.117791
2273	1.35051	0.100886
2274	1.0461	0.096766
2275	1.37837	0.0953257
2276	1.00259	0.0983481
2277	1.25796	0.7
2278	1.10719	0.724437
2279	1.51067	2.1
2280	2.65632	1.40461
2281	1.30521	2.1
2282	2.456 2.1	
2283	3.19218	2.1
2284	2.67387	1.5575
2285	0.988862	2.1
2286	1.917 2.1	
2287	0.946132	2.1
2288	1.16202	2.1
2289	1.53871	2.1
2290	1.53871	2.1
2291	0.946132	2.1
2292	1.16202	2.1
2293	1.32282	2.1
2294	2.46356	2.1
2295	2.32404	2.1
2296	1.37562	0.351566
2297	0.985546	0.39768
2298	1.28983	0.292867
2299	1.05566	0.366492
2300	0.895312	0.361182
2301	1.53325	0.316897
2302	1.04342	0.394036
2303	1.80881	0.354928
2304	0.876865	0.333069
2305	0.5689	0.301836
2306	1.09506	0.378892
2307	1.51789	2.1
2308	1.49872	2.1
2309	0.949364	0.704078
2310	2.01233	0.701539
2311	1.55063	0.702788
2312	1.57783	0.756183
2313	1.57783	0.755698
2314	0.674605	0.707988
2315	0.98537	0.703586
2316	0.674633	0.701002
2317	0.98537	0.700252

2318	1.26706	0.741419
2319	0.980664	0.789555
2320	1.03298	0.701073
2321	1.84936	0.766803
2322	1.51696	0.7
2323	1.97074	0.7
2324	1.03298	0.7
2325	1.51324	0.621509
2326	1.51324	0.678868
2327	0.758454	0.676669
2328	1.28632	0.487594
2329	0.758482	0.409402
2330	0.758482	0.524818
2331	0.985372	0.656271
2332	1.14105	0.279862
2333	1.59787	0.774303
2334	2.46244	0.837267
2335	1.70367	0.476923
2336	2.13757	1.21175
2337	1.98142	1.22096
2338	1.35878	0.19541
2339	2.39947	0.333354
2340	1.24554	0.215082
2341	1.7522	0.246176
2342	1.49693	1.89319
2343	1.49652	2.01753
2344	1.6211	1.87226
2345	1.45938	1.98936
2346	0.737431	2.02108
2347	1.18643	1.99646
2348	1.04072	2.1
2349	0.9459	1.89036
2350	0.66364	2.03885
2351	0.985109	1.68817
2352	0.638368	1.94277
2353	0.83804	1.5796
2354	0.998024	1.85198
2355	0.914471	1.54335
2356	1.14281	1.90551
2357	0.833972	1.68477
2358	0.991865	0.7
2359	1.42208	0.7
2360	1.70718	2.1
2361	1.79888	2.1
2362	1.16851	0.692902
2363	1.16851	0.633222
2364	1.51021	0.669001
2365	0.946132	0.385553
2366	1.14897	0.267632
2367	0.946132	0.45497
2368	1.77177	0.453838
2369	1.77177	0.338254
2370	1.33038	0.353157
2371	1.38752	0.313446
2372	1.1726	0.522609
2373	1.51688	0.50716
2374	1.06455	2.1
2375	0.98511	2.1

2376	1.31144	2.1
2377	1.79825	2.1
2378	1.79825	2.1
2379	1.73044	2.1
2380	1.59589	0.127677
2381	1.59589	0.133409
2382	2.78009	2.1
2383	1.68761	2.1
2384	3.05511	2.1
2385	2.25318	2.1
2386	0.578998	2.1
2387	1.03512	2.1
2388	2.26156	2.1
2389	1.44859	0.911213
2390	1.35897	1.13347
2391	1.18301	1.99894
2392	1.25173	1.93337
2393	1.98851	2.1
2394	2.70685	2.1
2395	0.682342	2.1
2396	1.52491	2.1
2397	1.37119	2.1
2398	0.682313	2.1
2399	1.35342	2.1
2400	0.947555	2.1
2401	0.614107	2.1
2402	1.23007	2.1
2403	1.5735	2.1
2404	1.58713	2.1
2405	1.57979	0.0811937
2406	1.30723	0.0821593
2407	0.935658	0.857017
2408	0.8952	0.822954
2409	1.27352	0.991296
2410	1.0985	0.934782
2411	1.07163	0.841256
2412	1.15823	0.899382
2413	0.836451	0.829283
2414	2.08051	0.757234
2415	1.92601	1.00223
2416	2.0644	0.789686
2417	1.37259	2.1
2418	1.37259	2.1
2419	0.752217	2.1
2420	1.28569	2.1
2421	0.752186	2.1
2422	0.839124	2.1
2423	1.28568	2.1
2424	1.04199	2.1
2425	1.04199	2.1
2426	2.59268	2.1
2427	1.04199	2.1
2428	1.5098	2.1
2429	1.5098	2.1
2430	0.520997	2.1
2431	1.29634	2.1
2432	0.520996	2.1
2433	0.73446	2.1

2434	1.29634	2.1
2435	0.983724	2.1
2436	1.23914	2.1
2437	2.65095	2.1
2438	1.52851	2.1
2439	2.399	2.1
2440	1.68137	2.1
2441	1.11285	2.1
2442	1.04921	2.1
2443	1.30711	2.1
2444	1.04921	2.1
2445	1.07496	1.03631
2446	1.55831	0.821669
2447	1.07497	0.788221
2448	1.07497	0.927542
2449	1.27391	2.1
2450	2.5615	2.1
2451	1.2739	2.1
2452	1.75253	2.03201
2453	1.12907	0.50982
2454	1.9194	0.7
2455	1.96617	0.690523
2456	1.01225	2.1
2457	0.947979	2.1
2458	1.01225	1.0872
2459	1.06561	2.1
2460	1.01225	1.72137
2461	3.56876	2.1
2462	3.68767	2.1
2463	1.52301	2.1
2464	1.61013	2.1
2465	1.43726	2.1
2466	2.04275	2.1
2467	0.995734	2.1
2468	1.16199	2.1
2469	1.73704	0.26551
2470	1.04019	0.351999
2471	0.956294	2.1
2472	1.81488	2.1
2473	1.63719	2.1
2474	1.50103	0.0829418
2475	1.75289	0.0927665
2476	1.31491	0.0912861
2477	1.03567	0.0857521
2478	0.672257	0.107938
2479	1.04072	0.150588
2480	0.746903	0.133029
2481	1.43317	0.114576
2482	1.39986	0.0982593
2483	1.06385	0.135151
2484	1.1624	0.134969
2485	1.61667	0.0986172
2486	1.27258	0.092247
2487	2.559	0.0990253
2488	1.66093	0.0868047
2489	1.10719	0.728932
2490	2.03235	0.742699
2491	1.17105	0.991314

2492	1.10719	0.762475
2493	1.3028	2.1
2494	0.946131	2.03243
2495	0.946127	1.97194
2496	1.52912	2.1
2497	1.52912	2.1
2498	0.946128	1.44218
2499	0.949997	1.78521
2500	1.31565	2.1
2501	1.46643	2.1
2502	1.5482	2.1
2503	1.47102	2.1
2504	1.47102	2.1
2505	0.774101	2.1
2506	1.30583	2.1
2507	0.774103	2.1
2508	0.939287	2.1
2509	1.30584	2.1
2510	0.958661	2.1
2511	2.28835	2.1
2512	1.43213	0.217262
2513	2.13255	0.305246
2514	1.43213	0.221637
2515	1.10476	0.195782
2516	1.93165	0.307798
2517	1.03235	0.967707
2518	1.31	0.980402
2519	1.22082	2.1
2520	1.35827	2.1
2521	0.790936	2.1
2522	0.716644	2.1
2523	1.43257	2.1
2524	1.43257	2.1
2525	0.716615	2.1
2526	0.946131	2.1
2527	0.730157	2.1
2528	1.47212	2.1
2529	1.47212	2.1
2530	0.946131	2.1
2531	0.790792	2.1
2532	1.41149	2.1
2533	0.790791	2.1
2534	1.01006	2.1
2535	1.53045	0.697191
2536	1.53045	0.865185
2537	0.946132	1.04921
2538	1.40779	0.813014
2539	1.06879	1.18854
2540	0.990713	0.935715
2541	1.06879	0.986898
2542	1.06486	0.562097
2543	1.18347	0.57258
2544	1.38622	0.621769
2545	1.18347	0.454094
2546	1.7304	0.605713
2547	1.7304	0.745248
2548	0.946131	0.876853
2549	1.26874	0.766361

2550	1.13337	1.05678
2551	1.13337	0.844278
2552	1.40779	0.849446
2553	1.695 2.1	
2554	1.77071	2.1
2555	0.649313	2.1
2556	0.692829	2.1
2557	1.40395	2.1
2558	1.34243	2.1
2559	0.813292	0.684315
2560	0.632691	0.620682
2561	1.75766	0.73448
2562	2.25727	0.844355
2563	1.74206	0.702411
2564	0.826073	0.704539
2565	1.30022	0.701837
2566	1.45641	0.701097
2567	0.946131	0.704966
2568	0.752245	0.700486
2569	0.960952	0.710019
2570	0.743477	0.703269
2571	1.04355	0.7
2572	1.6253	0.703973
2573	0.960577	0.702217
2574	1.34852	0.917491
2575	1.03235	0.977419
2576	1.78168	0.810142
2577	1.2966	0.145324
2578	2.21116	0.12204
2579	1.6395	0.156973
2580	1.6395	0.152728
2581	0.94613	0.205509
2582	1.22187	0.212806
2583	0.962737	0.252339
2584	0.962735	0.192405
2585	1.36376	0.18733
2586	1.2966	0.0943711
2587	1.2966	0.0975509
2588	1.19669	0.93219
2589	2.37636	0.992009
2590	1.08894	1.0706
2591	1.62551	1.14127
2592	2.51472	1.11028
2593	1.89226	0.979271
2594	0.980038	1.14985
2595	1.10187	0.904417
2596	1.01479	0.90127
2597	1.81663	2.1
2598	1.83247	2.1
2599	0.921851	0.815891
2600	0.983148	2.1
2601	1.82816	2.1
2602	1.26103	2.1
2603	2.34818	2.1
2604	1.23152	2.1
2605	2.15738	2.1
2606	1.23152	2.1
2607	1.51042	2.1

2608	0.842995	2.1
2609	1.22257	2.1
2610	0.810959	2.1
2611	1.68242	2.1
2612	0.95701	2.1
2613	1.12389	2.1
2614	1.95255	2.1
2615	1.57974	2.1
2616	1.94632	2.1
2617	0.81534	2.1
2618	1.13996	2.1
2619	1.18958	0.141948
2620	1.25929	0.118654
2621	3.02933	0.836447
2622	2.37434	0.383064
2623	1.10609	0.81734
2624	1.64132	0.781813
2625	1.64132	0.800532
2626	0.946132	0.721814
2627	1.40029	0.705443
2628	1.08946	0.707804
2629	1.08946	0.727822
2630	1.18717	0.403171
2631	2.71421	0.338328
2632	1.08189	0.7
2633	0.94613	0.7
2634	1.37109	0.7
2635	1.17925	0.56664
2636	0.851541	0.7
2637	0.942512	0.7
2638	1.18541	0.7
2639	0.851526	0.7
2640	1.0819	0.7
2641	1.86009	0.7
2642	0.946132	0.7
2643	1.67409	0.266228
2644	1.67409	0.7
2645	0.946131	0.7
2646	1.31716	0.665969
2647	1.30307	0.591677
2648	0.946131	0.7
2649	1.18601	0.552618
2650	3.15646	2.1
2651	0.985109	2.1
2652	3.37048	2.1
2653	1.10667	2.1
2654	1.77065	1.26754
2655	1.45837	1.0757
2656	1.77063	1.09249
2657	2.58104	1.03818
2658	1.37652	1.76275
2659	1.54168	1.77795
2660	1.00224	0.0950839
2661	1.05851	0.105309
2662	1.39093	0.0925227
2663	1.43358	0.10625
2664	0.575741	0.100521
2665	0.518161	0.0953865

2666	1.04071	0.111618
2667	1.13092	1.13538
2668	1.17042	1.71771
2669	1.53347	1.38113
2670	1.52938	1.17055
2671	1.00613	0.709923
2672	1.81618	0.848781
2673	1.81618	0.719539
2674	0.946127	0.823974
2675	1.36479	0.823097
2676	0.94613	0.719842
2677	1.31724	0.760023
2678	1.3648	0.762028
2679	1.00613	0.719046
2680	0.781375	0.371061
2681	1.13431	0.59085
2682	1.03547	0.663523
2683	1.40668	0.657662
2684	0.94613	0.656542
2685	0.713285	0.704462
2686	1.72885	0.695667
2687	1.72885	0.679328
2688	0.713298	0.668062
2689	3.30075	0.436123
2690	2.81337	0.615109
2691	1.39296	0.7205
2692	0.830164	1.2091
2693	1.50493	2.1
2694	1.50494	2.1
2695	0.830136	1.16824
2696	1.28298	2.1
2697	1.0521	2.1
2698	0.830147	2.1
2699	2.0799	1.31417
2700	1.52115	1.14034
2701	2.5142	1.22495
2702	1.31253	0.827522
2703	1.94446	0.957972
2704	1.18817	0.94443
2705	0.598348	1.0675
2706	1.69315	0.935909
2707	1.69315	1.0977
2708	0.598377	0.860327
2709	0.598347	0.866771
2710	0.94613	0.844968
2711	1.28418	0.972743
2712	2.05205	1.05659
2713	1.28418	1.08469
2714	1.0737	2.1
2715	1.81024	2.1
2716	2.73834	2.1
2717	1.71369	2.1
2718	1.71368	2.1
2719	0.946118	2.1
2720	1.29063	2.1
2721	1.36917	2.1
2722	0.94613	2.1
2723	1.29062	2.1

2724	0.947921	2.03897
2725	1.58661	0.941077
2726	1.37658	1.25826
2727	1.2364	1.22386
2728	1.66685	0.821506
2729	1.66685	1.57117
2730	0.946133	0.792412
2731	0.946133	0.943254
2732	1.11978	1.12217
2733	1.09194	0.846163
2734	1.21858	1.44812
2735	1.37658	0.997851
2736	1.64903	0.824109
2737	1.64903	1.9127
2738	0.946132	0.823305
2739	0.946132	0.927059
2740	1.05496	0.917496
2741	1.22851	0.821289
2742	1.22852	1.76569
2743	1.58482	0.755333
2744	1.60578	0.221533
2745	1.06336	0.223931
2746	1.68062	0.233759
2747	1.14721	0.181304
2748	1.11924	0.0834923
2749	1.18421	0.0895342
2750	2.01276	0.0848148
2751	1.23502	1.86112
2752	1.34926	1.62462
2753	0.546751	1.2866
2754	0.790446	1.82862
2755	1.04072	1.24992
2756	0.555868	1.24312
2757	0.930305	1.22077
2758	1.09344	1.34973
2759	1.18351	1.7937
2760	1.97215	1.30444
2761	1.07416	1.88812
2762	1.33204	1.26005
2763	1.22038	1.23831
2764	0.597449	1.01818
2765	0.821422	1.50536
2766	1.04071	1.14354
2767	0.608026	0.961968
2768	0.929427	1.09462
2769	1.64929	0.0874844
2770	1.68541	0.0816467
2771	0.496093	0.138635
2772	1.3385	0.1081
2773	0.790798	0.100277
2774	1.22971	0.100828
2775	0.898853	0.0990129
2776	2.14361	0.0995831
2777	2.10813	0.0925412
2778	1.05417	0.133085
2779	1.80768	0.238119
2780	1.09725	2.1
2781	1.41131	2.1

2782	1.1656	2.1
2783	0.946129	2.1
2784	1.68365	2.1
2785	1.68365	2.1
2786	1.1656	2.1
2787	0.94613	2.1
2788	1.21269	0.0732853
2789	1.48867	0.0781446
2790	1.27806	0.0787019
2791	1.68264	0.0760661
2792	1.37813	1.44409
2793	1.50743	1.04978
2794	1.04121	1.01273
2795	1.12434	0.791636
2796	1.39553	0.78952
2797	1.44721	1.12499
2798	0.946132	0.834884
2799	1.23943	0.851186
2800	1.05292	0.879081
2801	1.28607	0.782197
2802	1.16915	0.747327
2803	1.76306	1.02965
2804	1.42266	1.01776
2805	1.36844	2.1
2806	1.36844	2.1
2807	1.67352	2.1
2808	1.11256	1.15384
2809	1.03217	1.11239
2810	1.18422	0.0907205
2811	1.04159	0.108046
2812	1.60139	0.0896442
2813	1.04159	0.0891025
2814	0.988805	2.1
2815	1.05481	2.1
2816	1.52938	1.53558
2817	1.94724	1.24532
2818	0.896336	0.296967
2819	0.911484	0.302591
2820	1.18315	0.162237
2821	1.10515	0.211119
2822	1.04067	0.15526
2823	1.12758	0.270856
2824	0.904106	0.230323
2825	1.6901	0.148638
2826	1.68671	0.142162
2827	1.99908	0.202256
2828	0.772426	0.84009
2829	0.810945	1.54773
2830	0.667562	1.09243
2831	0.496617	0.746339
2832	0.847689	0.997678
2833	0.914378	1.17906
2834	0.934003	0.807666
2835	1.07459	0.927166
2836	1.40225	2.1
2837	1.07043	2.1
2838	0.94613	2.1
2839	1.63072	2.1

2840	1.63072	2.1
2841	1.07043	2.1
2842	0.946131	2.1
2843	1.53082	2.1
2844	1.69916	2.1
2845	1.75211	0.814048
2846	1.96831	1.93596
2847	1.75211	0.931435
2848	0.946127	0.938445
2849	0.817065	0.969034
2850	1.41798	0.971462
2851	1.41798	0.910705
2852	0.791656	0.947099
2853	0.791656	0.96444
2854	1.41798	0.948736
2855	1.58331	0.936949
2856	1.41776	0.869632
2857	0.791647	0.868921
2858	1.4512	0.860366
2859	1.45119	0.864125
2860	0.75821	0.884053
2861	0.758248	0.876459
2862	0.946104	0.877223
2863	1.36482	0.874886
2864	1.29389	1.98956
2865	1.04164	1.38059
2866	1.40574	1.39194
2867	1.64833	1.27598
2868	1.71183	0.58868
2869	1.71183	0.658588
2870	0.946131	0.833076
2871	1.35721	0.739817
2872	0.946131	0.951776
2873	0.946131	0.839799
2874	1.30076	0.842811
2875	1.2474	0.575542
2876	2.85225	0.770412
2877	2.71441	0.717258
2878	1.2474	0.44962
2879	1.38622	0.697937
2880	0.858359	0.473254
2881	1.3682	0.55209
2882	1.57364	0.542844
2883	0.729695	0.752845
2884	0.729723	0.498197
2885	0.822611	0.822425
2886	0.946133	0.754016
2887	0.729723	0.670473
2888	0.974704	0.854167
2889	0.974702	0.834907
2890	1.81603	2.1
2891	1.96348	2.1
2892	1.43612	0.994271
2893	1.43612	1.07689
2894	0.634295	0.952768
2895	0.93796	1.36484
2896	0.946133	1.01967
2897	0.634326	0.877214

2898	0.946133	0.956552
2899	1.2654	0.825341
2900	1.26865	1.1342
2901	2.2649	0.953021
2902	1.00864	1.00606
2903	1.09689	0.802925
2904	0.832021	0.813615
2905	1.53229	0.789911
2906	1.1145	0.799004
2907	0.929583	0.819386
2908	1.613	0.784729
2909	0.644729	0.807218
2910	1.03471	1.1211
2911	2.7739	2.1
2912	1.36133	1.12824
2913	2.50215	2.1
2914	2.52195	2.1
2915	1.0465	2.1
2916	1.42673	2.1
2917	1.42673	2.1
2918	0.946132	2.1
2919	1.31904	2.1
2920	0.986499	2.1
2921	0.946132	2.1
2922	1.31904	2.1
2923	0.966978	2.1
2924	1.42237	0.341111
2925	2.60925	0.470651
2926	0.753659	0.566768
2927	1.77241	0.407797
2928	1.77241	0.490372
2929	0.946129	0.620051
2930	0.75366	0.415894
2931	1.22144	0.563327
2932	0.94613	0.615488
2933	1.50732	0.399255
2934	1.18514	0.503267
2935	0.902187	0.545463
2936	1.89858	0.347151
2937	1.24294	0.417956
2938	1.67211	0.491898
2939	1.41786	0.37257
2940	0.810959	0.551532
2941	1.50732	0.417342
2942	2.44288	0.514728
2943	1.40969	0.132371
2944	2.06654	0.161032
2945	1.40969	0.139078
2946	1.40882	0.211249
2947	1.94063	0.227948
2948	0.867921	0.158141
2949	1.29289	0.166862
2950	0.867918	0.166401
2951	0.946128	0.160564
2952	0.867944	0.177167
2953	1.66589	0.168768
2954	1.66589	0.146368
2955	0.968595	2.1

2956	1.5534	1.94502
2957	1.27793	2.1
2958	1.13433	2.1
2959	1.3439	0.0856577
2960	2.53709	0.0914521
2961	0.571998	0.0744298
2962	0.64837	0.0764342
2963	1.99755	0.079933
2964	1.99854	0.0788618
2965	0.764229	2.1
2966	1.61842	2.1
2967	1.61842	2.1
2968	1.18315	2.1
2969	1.1995	2.1
2970	1.18315	2.1
2971	0.764256	2.1
2972	1.52364	2.1
2973	2.36631	2.1
2974	0.641912	1.67173
2975	1.7181	2.1
2976	1.459 2.1	
2977	0.634845	1.53295
2978	1.18315	2.1
2979	1.17615	2.1
2980	0.571367	2.1
2981	1.3324	0.712505
2982	0.798214	0.715073
2983	0.946128	0.718989
2984	0.627252	0.710519
2985	1.50337	0.708521
2986	1.50337	0.635588
2987	0.62725	0.722806
2988	1.07763	0.613121
2989	1.07763	0.707191
2990	2.16109	0.718351
2991	1.07763	0.627804
2992	1.12486	2.1
2993	1.11827	2.1
2994	1.0491	2.1
2995	1.09034	2.1
2996	1.09431	2.1
2997	0.946131	2.1
2998	0.748607	2.1
2999	1.44002	2.1
3000	1.44002	2.1
3001	0.748635	2.1
3002	0.782505	2.1
3003	1.40615	2.1
3004	1.19539	2.1
3005	1.29389	1.88412
3006	1.4867	1.10248
3007	1.50103	0.0803053
3008	1.63025	0.0877693
3009	2.72109	0.11587
3010	0.915755	0.105009
3011	0.946132	0.129613
3012	0.915755	0.137554
3013	1.70236	0.103357

3014	1.70236	0.086979
3015	1.26155	0.121349
3016	1.35657	0.124847
3017	1.83151	0.0867606
3018	1.83151	0.089563
3019	2.52309	0.110344
3020	0.983607	2.1
3021	1.06838	0.924032
3022	0.983609	1.08015
3023	0.983596	1.03638
3024	2.74384	0.341932
3025	1.6261	0.261729
3026	1.15477	2.1
3027	1.02136	2.1
3028	1.83561	2.1
3029	1.83561	2.1
3030	0.946131	2.1
3031	1.39523	2.1
3032	1.29313	1.00509
3033	1.29313	2.1
3034	1.38651	2.1
3035	1.06313	2.1
3036	0.974888	2.1
3037	1.0894	2.1
3038	0.974888	2.1
3039	1.82282	2.1
3040	1.82282	2.1
3041	0.946131	2.1
3042	1.37372	2.1
3043	1.32374	2.1
3044	1.32374	2.1
3045	1.39523	2.1
3046	1.40882	0.22036
3047	0.861998	0.248022
3048	1.24448	0.266631
3049	0.861999	0.245393
3050	0.946129	0.274049
3051	0.862011	0.297828
3052	1.67537	0.271163
3053	1.67537	0.220583
3054	1.72399	0.134876
3055	2.58579	0.148577
3056	1.72399	0.135171
3057	1.50951	0.179452
3058	2.48897	0.218042
3059	1.26755	2.1
3060	1.03824	2.1
3061	1.27875	2.1
3062	1.48901	2.1
3063	1.18902	2.1
3064	1.11764	2.1
3065	1.87436	2.1
3066	0.95345	2.02402
3067	0.724191	1.53533
3068	0.86668	1.47032
3069	0.811523	1.69232
3070	1.04074	1.16542
3071	0.843097	1.35731

3072	0.833434	1.56486
3073	0.965727	1.36819
3074	0.85346	1.64432
3075	1.00185	1.00072
3076	1.00184	1.34752
3077	0.980651	1.53977
3078	0.980664	1.33629
3079	0.475737	1.1153
3080	0.719696	1.16678
3081	0.946109	1.09913
3082	0.946116	1.20817
3083	0.719693	1.11874
3084	0.946117	1.21104
3085	1.14879	1.53003
3086	0.719683	1.44671
3087	0.900977	1.14964
3088	0.972364	1.26154
3089	1.22961	1.24606
3090	0.405384	1.15371
3091	1.34406	2.1
3092	1.2187	2.1
3093	0.963571	0.786055
3094	0.946131	0.811296
3095	1.57246	0.892732
3096	1.57246	2.1
3097	0.946131	0.902557
3098	1.1235	1.09596
3099	1.1235	1.03985
3100	1.07439	1.16801
3101	1.19612	1.11124
3102	1.24844	1.08858
3103	1.00007	1.30963
3104	1.02429	2.1
3105	1.10459	2.1
3106	1.06724	0.733219
3107	0.67519	0.825959
3108	0.49762	0.749376
3109	1.43435	0.715345
3110	0.833315	0.783373
3111	0.58135	0.764761
3112	1.14749	0.931945
3113	1.85091	1.15082
3114	1.2802	0.887875
3115	1.27737	1.01767
3116	2.3655	0.993222
3117	1.30584	2.1
3118	2.06424	2.1
3119	0.946131	0.354837
3120	1.04257	0.316976
3121	0.94613	0.397916
3122	1.67201	0.503598
3123	1.67201	0.368043
3124	1.27696	0.335114
3125	1.34118	0.21517
3126	1.3413	0.279911
3127	2.36238	0.202129
3128	0.6036	0.35892
3129	1.11745	0.364215

3130	1.46126	0.260589
3131	1.39612	0.230746
3132	0.670678	0.298298
3133	1.03402	0.385811
3134	1.11271	0.299052
3135	0.838028	0.252673
3136	0.807019	0.369725
3137	1.27787	0.191565
3138	0.994705	0.239591
3139	1.20014	0.199227
3140	0.964793	0.348972
3141	0.863868	0.266332
3142	1.3413	0.194182
3143	1.79549	0.197452
3144	1.35877	2.1
3145	1.63648	2.1
3146	1.08171	2.1
3147	1.27839	2.1
3148	1.52601	2.1
3149	1.27841	2.1
3150	1.71255	2.1
3151	2.16307	2.1
3152	1.91592	2.1
3153	1.78508	2.1
3154	0.946128	2.1
3155	1.41199	2.1
3156	0.946128	2.1
3157	0.901088	2.1
3158	1.55244	2.1
3159	1.59475	2.1
3160	1.40921	2.1
3161	0.960106	2.1
3162	1.40921	2.1
3163	2.0073	2.1
3164	2.2973	0.709686
3165	1.90705	0.713273
3166	1.53671	0.596159
3167	1.86035	0.57271
3168	1.33747	2.1
3169	1.14007	2.1
3170	1.3212	2.1
3171	1.66867	0.7
3172	1.30203	0.657944
3173	1.27696	2.1
3174	1.05025	2.1
3175	1.05025	2.1
3176	1.3704	2.1
3177	1.15677	2.1
3178	0.946129	2.1
3179	1.65879	2.1
3180	1.65879	2.1
3181	1.15676	2.1
3182	0.94613	2.1
3183	0.867217	1.03917
3184	1.28684	1.00416
3185	1.58866	1.01786
3186	1.58866	0.894224
3187	0.867246	1.54407

3188	1.28684	0.962706
3189	1.16906	1.0248
3190	1.13438	1.01199
3191	1.37224	1.04813
3192	1.61519	1.11259
3193	0.883939	1.46958
3194	1.40092	1.09606
3195	1.80383	1.69837
3196	1.80383	1.31237
3197	0.883935	1.50414
3198	1.40092	1.05873
3199	1.28684	1.49135
3200	1.76788	0.965662
3201	2.57368	1.05039
3202	1.37224	1.75413
3203	1.65411	0.990636
3204	1.77042	2.1
3205	1.77042	2.1
3206	1.90446	2.1
3207	1.28667	2.1
3208	1.55635	2.1
3209	1.30646	0.72609
3210	2.31836	0.993706
3211	1.30645	0.75969
3212	1.86429	1.33947
3213	1.48676	0.704365
3214	0.743379	0.704824
3215	1.41848	0.705014
3216	1.41848	0.703261
3217	0.946131	0.704742
3218	0.812649	0.704052
3219	1.34921	0.704423
3220	0.812649	0.704978
3221	0.949366	0.704234
3222	1.32974	0.703249
3223	0.794204	0.05
3224	0.634352	2.1
3225	0.838025	2.1
3226	1.08802	1.98634
3227	0.838038	1.91485
3228	1.18314	2.1
3229	1.12495	2.1
3230	1.22774	2.1
3231	1.04343	2.09197
3232	0.921111	0.0877777
3233	2.1609	0.0780119
3234	1.09987	0.0739237
3235	2.5043	0.0666259
3236	1.05492	0.0622191
3237	0.590344	0.0709815
3238	1.05492	0.0691748
3239	1.25215	0.0677766
3240	1.71674	0.060645
3241	1.71674	0.061895
3242	0.590331	0.0651631
3243	1.17078	0.0628209
3244	0.625542	0.0678879
3245	0.724673	2.1

3246	1.08011	2.1
3247	1.87598	2.1
3248	1.50738	2.1
3249	1.44951	0.429372
3250	1.50102	0.391951
3251	1.8042	0.774205
3252	0.687769	0.856927
3253	1.66483	0.778443
3254	1.66483	0.799085
3255	0.946132	0.847243
3256	0.687798	0.781129
3257	1.20885	0.848372
3258	0.687799	0.829068
3259	0.886063	0.736867
3260	0.798751	0.734954
3261	0.584813	0.716754
3262	1.49483	0.704963
3263	0.888005	0.712218
3264	1.03589	0.711479
3265	1.70435	0.719644
3266	0.710414	2.1
3267	0.917609	1.22744
3268	0.810947	1.22646
3269	0.81096	1.22222
3270	1.48658	1.27184
3271	1.53653	1.28093
3272	1.19329	1.2768
3273	0.946153	1.2614
3274	1.50798	1.27755
3275	1.85017	2.1
3276	1.19768	2.1
3277	0.864304	1.63537
3278	0.792906	1.23014
3279	0.989025	1.24835
3280	1.19768	1.44912
3281	1.54548	1.30804
3282	1.18798	1.46667
3283	1.31875	1.19758
3284	1.05725	1.28079
3285	2.17365	0.101365
3286	2.48106	0.0875258
3287	1.87451	0.108705
3288	1.27189	1.17479
3289	2.79399	1.71273
3290	0.859565	1.23725
3291	0.926174	1.14339
3292	0.604744	1.10587
3293	1.41832	0.897097
3294	1.05494	1.02449
3295	0.874749	1.05191
3296	1.50067	0.970302
3297	0.917817	1.31103
3298	0.63591	1.31638
3299	1.72849	1.2643
3300	1.72849	1.04712
3301	0.635943	1.13278
3302	1.39699	1.29877
3303	0.967411	1.72649

3304	0.635943	1.21268
3305	1.39908	1.1421
3306	1.26971	1.28389
3307	1.02969	1.1014
3308	1.15326	1.9703
3309	2.30741	1.3135
3310	0.93545	1.08969
3311	0.802747	0.958995
3312	1.15466	1.11661
3313	0.859164	1.36075
3314	1.02616	1.16644
3315	1.23149	1.06351
3316	0.83804	1.00485
3317	1.13266	1.6321
3318	1.64142	1.69734
3319	1.21223	2.1
3320	1.43051	2.1
3321	1.32418	0.904845
3322	0.621319	0.985163
3323	1.47134	1.42053
3324	1.79103	0.817261
3325	3.25951	0.208708
3326	3.16764	0.173879
3327	1.65583	0.198177
3328	1.4732	0.302339
3329	1.76476	2.1
3330	1.80114	1.11231
3331	0.754121	1.73026
3332	0.94613	0.903848
3333	0.754119	1.74361
3334	1.45636	1.62152
3335	1.45636	0.837487
3336	1.30029	1.04925
3337	0.910183	1.01966
3338	1.29099	1.44694
3339	1.08039	2.1
3340	1.05498	2.08267
3341	1.24445	1.82337
3342	1.2873	2.1
3343	0.987703	1.82622
3344	0.964782	1.4538
3345	1.73645	0.338426
3346	0.871279	0.50724
3347	0.884744	0.531596
3348	1.85193	0.337991
3349	1.68681	0.417362
3350	1.33689	0.497957
3351	1.23493	0.359097
3352	0.892482	0.530978
3353	2.95185	0.391109
3354	2.10496	0.427728
3355	1.87334	2.1
3356	0.94411	2.1
3357	0.754196	2.1
3358	1.43122	2.1
3359	1.58684	2.1
3360	0.838027	2.1
3361	1.1536	2.1

3362	1.24878	2.1
3363	1.23125	2.1
3364	2.86862	2.1
3365	1.57636	2.1
3366	0.998451	0.873306
3367	1.5176	0.843462
3368	1.13271	0.162467
3369	1.60248	0.145737
3370	1.22083	2.1
3371	1.79996	2.1
3372	1.19087	2.1
3373	2.00342	2.1
3374	1.3715	2.1
3375	2.79227	2.1
3376	2.7873	2.1
3377	0.987265	0.071792
3378	1.62715	2.1
3379	1.70117	1.79233
3380	1.27806	0.0715232
3381	1.45246	2.1
3382	1.45244	2.1
3383	1.87214	2.1
3384	0.995796	2.1
3385	1.68189	0.748189
3386	1.49155	1.28743
3387	1.58054	2.1
3388	1.59169	2.1
3389	0.736364	0.0796257
3390	0.70236	0.0783594
3391	2.24367	0.0848348
3392	2.57085	0.0903555
3393	1.70952	0.0811133
3394	2.60516	0.0862004
3395	1.45158	2.1
3396	1.00371	2.1
3397	1.65748	2.1
3398	1.23706	0.705995
3399	1.55834	0.805522
3400	1.72671	0.785725
3401	0.597656	0.726531
3402	1.39137	0.793132
3403	0.855578	0.703866
3404	1.48398	0.755385
3405	0.745117	0.705154
3406	1.23706	0.744295
3407	1.57916	0.766452
3408	0.893025	0.715194
3409	3.22433	0.738691
3410	2.47324	0.888687
3411	1.63852	2.1
3412	1.63852	2.1
3413	0.946132	2.1
3414	1.19095	2.1
3415	0.946133	2.1
3416	1.16684	2.1
3417	1.19095	2.1
3418	1.06698	0.707614
3419	1.48692	0.70412

3420	1.08334	1.02904
3421	1.07283	2.1
3422	1.28517	0.985608
3423	1.2746	0.0776829
3424	1.30723	0.0794535
3425	2.02069	0.0820816
3426	1.05579	0.0844842
3427	1.59475	2.1
3428	2.40113	2.1
3429	1.58875	2.1
3430	1.6881	2.1
3431	1.6881	2.1
3432	0.819953	2.1
3433	0.819954	2.1
3434	0.946131	2.1
3435	0.946131	2.1
3436	1.34782	2.1
3437	1.65619	2.1
3438	1.65619	2.1
3439	0.79437	2.1
3440	1.25	2.1
3441	0.94613	2.1
3442	0.946131	2.1
3443	0.794371	2.1
3444	1.58875	2.1
3445	2.5	2.1
3446	1.04147	2.1
3447	0.946131	0.915682
3448	1.79686	2.08089
3449	1.79686	2.1
3450	1.25144	0.920842
3451	1.35714	1.28386
3452	1.38585	1.30102
3453	1.25144	1.96913
3454	1.15564	1.9041
3455	1.34389	1.44326
3456	0.946133	1.0752
3457	1.74353	1.70936
3458	1.74353	2.1
3459	1.14553	1.02846
3460	1.38585	1.02365
3461	1.30382	1.09814
3462	1.14553	1.78822
3463	1.15564	1.54515
3464	1.90618	2.1
3465	2.11056	2.1
3466	1.032	2.1
3467	0.953636	2.1
3468	1.23131	1.37781
3469	1.39763	1.40957
3470	2.01024	1.11008
3471	1.9186	1.13567
3472	1.83658	0.532051
3473	1.81656	0.523245
3474	2.2049	0.611523
3475	2.16081	0.592577
3476	1.12219	0.652991
3477	0.599771	0.693245

3478	1.61401	0.540538
3479	1.05912	0.622308
3480	0.951648	0.649613
3481	1.89328	0.548357
3482	0.913682	0.698359
3483	1.00763	0.516399
3484	1.29129	2.1
3485	1.37138	2.1
3486	1.41986	0.256339
3487	1.41986	0.257566
3488	0.944166	0.362284
3489	1.41789	0.329408
3490	0.944167	0.45923
3491	0.944166	0.317934
3492	0.946132	0.284973
3493	1.98713	0.183993
3494	1.23626	0.1275
3495	0.932791	0.240276
3496	0.810251	0.155606
3497	0.986748	0.179555
3498	0.898155	0.262819
3499	0.92624	0.17782
3500	1.1349	0.150987
3501	0.934572	0.183773
3502	1.23104	1.38392
3503	1.23104	1.37998
3504	0.799994	0.89255
3505	0.94613	1.13554
3506	0.802822	0.786716
3507	1.40944	1.27362
3508	0.800035	1.01147
3509	1.41223	1.08733
3510	1.41223	1.08744
3511	1.6	0.953916
3512	1.60565	0.96495
3513	1.23042	2.1
3514	1.0645	2.1
3515	1.00319	2.1
3516	1.29435	2.1
3517	1.2243	2.1
3518	1.2243	2.1
3519	1.05655	1.515
3520	1.29764	1.66057
3521	2.55252	2.1
3522	1.15676	2.1
3523	0.855295	2.1
3524	0.810959	2.1
3525	1.65857	2.1
3526	1.00849	2.1
3527	1.16021	2.1
3528	1.75869	2.1
3529	1.13775	2.1
3530	1.20091	2.1
3531	1.61582	2.1
3532	1.88031	2.1
3533	0.968536	2.1
3534	0.946131	2.1
3535	1.61481	2.1

3536	1.61481	2.1
3537	1.09547	2.1
3538	1.18486	2.1
3539	1.37609	2.1
3540	1.09546	2.1
3541	1.02461	2.1
3542	1.83223	2.1
3543	1.74002	2.1
3544	1.70939	1.66421
3545	1.7094	2.1
3546	0.726287	2.1
3547	1.01712	1.59993
3548	1.30318	2.1
3549	0.946131	2.1
3550	0.87155	2.1
3551	1.77093	1.77727
3552	2.02356	1.78412
3553	1.32449	2.1
3554	1.10003	2.1
3555	1.5161	2.1
3556	1.5161	2.1
3557	0.946132	2.1
3558	1.09206	2.1
3559	0.946132	2.1
3560	1.04404	2.1
3561	1.09206	2.1
3562	0.635628	0.923341
3563	1.14623	0.767735
3564	1.73197	0.821523
3565	1.73197	0.968283
3566	0.529655	0.817551
3567	1.14623	0.845762
3568	0.992085	0.894526
3569	1.16915	1.1827
3570	2.29245	0.765704
3571	0.584573	0.874777
3572	0.956756	0.738909
3573	1.52471	0.762533
3574	1.51409	1.29254
3575	0.584575	0.74083
3576	0.94613	0.768509
3577	1.14622	0.757578
3578	1.28096	1.04896
3579	1.34695	1.15189
3580	1.04193	2.1
3581	1.05378	2.1
3582	0.798209	2.1
3583	1.32007	1.99303
3584	1.64327	0.0966866
3585	2.64674	1.64231
3586	2.38936	0.403269
3587	2.88475	0.374443
3588	1.07745	1.967
3589	1.08911	2.05814
3590	1.49127	2.1
3591	1.49127	2.1
3592	0.709714	2.1
3593	1.37199	2.1

3594	0.82899	2.1
3595	0.709714	2.1
3596	0.946132	2.1
3597	1.00133	2.1
3598	1.40182	2.1
3599	1.53278	2.1
3600	1.40182	2.1
3601	1.31764	0.098407
3602	1.00691	0.106074
3603	1.24094	0.115872
3604	0.64868	0.112621
3605	0.946133	0.112862
3606	0.64868	0.119408
3607	1.71762	0.113425
3608	1.71762	0.102929
3609	1.41662	0.0909966
3610	0.975477	0.0853837
3611	1.73584	0.100242
3612	2.48188	0.105175
3613	2.2045	0.813309
3614	2.08134	0.796958
3615	0.993366	0.592468
3616	0.993368	0.849609
3617	1.14187	0.737846
3618	0.946132	0.758389
3619	0.946133	0.840046
3620	1.68951	0.815509
3621	1.68951	0.871794
3622	1.34612	0.793168
3623	1.28953	0.747233
3624	1.34611	0.909679
3625	1.31892	0.872139
3626	0.946131	0.82901
3627	1.80988	0.862955
3628	1.80988	1.67747
3629	1.31892	0.854678
3630	0.946131	0.858503
3631	2.13214	2.1
3632	1.94691	2.1
3633	1.52661	0.0811577
3634	1.53175	0.0905808
3635	0.956005	0.0880902
3636	0.955991	0.0856007
3637	1.40572	0.0996827
3638	0.955992	0.0852375
3639	1.51084	1.01847
3640	1.67115	1.08802
3641	1.73143	2.1
3642	1.55234	2.1
3643	1.69377	2.1
3644	2.51483	2.1
3645	1.25987	2.1
3646	1.4472	2.1
3647	1.83151	0.829201
3648	2.01001	1.27015
3649	0.993998	0.869002
3650	1.83151	0.783768
3651	1.40915	0.43559

3652	1.24653	0.763496
3653	1.74472	2.1
3654	1.74472	2.1
3655	1.74244	2.1
3656	1.04788	2.09853
3657	1.28522	1.58603
3658	1.03785	2.1
3659	0.896519	2.1
3660	1.60428	2.1
3661	1.73305	2.1
3662	1.39072	0.737894
3663	2.27501	0.891597
3664	1.39073	0.829618
3665	1.39073	0.827774
3666	1.06838	1.99142
3667	0.968462	0.985067
3668	1.06837	1.34561
3669	1.13455	1.27325
3670	1.23527	0.807873
3671	2.71686	0.734061
3672	1.86881	0.760622
3673	1.10586	1.12233
3674	1.03852	1.21833
3675	1.03899	2.1
3676	0.963973	2.1
3677	1.00319	2.1
3678	1.00319	2.1
3679	0.987132	2.1
3680	0.974889	2.1
3681	0.987131	2.1
3682	1.7677	2.1
3683	1.7677	2.1
3684	0.946131	2.1
3685	1.31185	2.1
3686	0.946132	2.1
3687	1.30203	2.1
3688	1.31185	2.1
3689	1.82958	2.1
3690	1.82958	2.1
3691	0.946132	2.1
3692	1.40199	2.1
3693	0.946133	2.1
3694	1.36994	2.1
3695	1.40199	2.1
3696	0.97489	2.1
3697	1.05939	1.85118
3698	1.01922	1.80412
3699	0.810941	1.3798
3700	1.05372	2.1
3701	0.754065	2.1
3702	0.926131	1.88922
3703	1.35905	1.82337
3704	0.810931	1.67602
3705	1.75647	1.09715
3706	1.68265	2.1
3707	1.41995	2.1
3708	1.36457	1.2146
3709	0.933392	1.15446

3710	1.42255	2.1
3711	1.3868	2.1
3712	1.08074	2.1
3713	1.07196	2.1
3714	1.13537	0.858371
3715	1.01179	0.775234
3716	1.60196	0.736975
3717	1.72798	0.741476
3718	1.3715	0.74355
3719	1.56409	0.984427
3720	1.56409	0.816701
3721	1.97819	1.02213
3722	1.47954	0.825105
3723	0.798655	0.719375
3724	1.47952	0.775342
3725	2.30327	0.73248
3726	1.49111	2.1
3727	1.96013	2.0085
3728	1.54599	2.1
3729	2.06632	2.1
3730	1.54601	2.1
3731	1.10557	0.748943
3732	1.17624	0.709458
3733	1.60497	2.1
3734	1.61587	2.1
3735	1.65977	0.0779292
3736	1.69044	0.0799013
3737	1.00659	2.1
3738	1.06226	2.1
3739	1.15171	2.1
3740	1.50853	2.1
3741	1.80252	2.1
3742	1.41432	2.1
3743	1.04687	2.1
3744	0.940878	2.1
3745	1.52031	2.1
3746	1.52031	2.1
3747	0.940877	2.1
3748	0.946132	2.1
3749	1.50853	2.1
3750	0.963973	2.1
3751	1.70562	0.325486
3752	2.43203	0.259639
3753	1.36004	0.165747
3754	1.06648	0.197612
3755	1.66009	0.127903
3756	1.66009	0.159057
3757	0.766427	0.193238
3758	0.766452	0.166311
3759	0.946132	0.218054
3760	1.98012	0.121029
3761	1.20357	2.1
3762	1.20356	2.1
3763	1.67324	2.1
3764	1.52008	2.1
3765	1.19964	2.1
3766	1.36861	2.1
3767	1.19964	2.1

3768	2.85415	2.1
3769	1.43687	2.1
3770	0.935852	2.1
3771	1.09972	2.1
3772	0.810933	2.01578
3773	2.25974	2.1
3774	1.74149	2.1
3775	1.36984	2.1
3776	1.87602	2.1
3777	0.890748	2.1
3778	1.20587	1.87899
3779	0.810956	1.70183
3780	2.03174	1.99197
3781	1.34403	2.05177
3782	1.58825	1.94943
3783	1.63096	1.90259
3784	2.53735	2.1
3785	1.98241	2.02262
3786	2.53014	2.1
3787	1.1775	1.12598
3788	1.06538	1.0149
3789	0.946132	1.01688
3790	1.4101	1.79787
3791	1.75162	2.1
3792	1.75162	0.974333
3793	0.946132	1.05223
3794	1.4101	0.97431
3795	1.28765	0.999747
3796	1.1427	0.926563
3797	1.1427	0.828326
3798	1.3109	2.1
3799	1.02721	1.3978
3800	0.846447	1.5389
3801	0.862076	2.1
3802	0.644098	1.59169
3803	0.581308	1.57842
3804	1.02306	1.45631
3805	0.322641	1.03651
3806	0.589346	1.04939
3807	2.25572	1.37216
3808	1.31372	2.1
3809	1.18873	2.1
3810	1.78225	2.1
3811	1.764 2.1	
3812	1.17503	2.1
3813	1.06821	2.1
3814	1.06821	1.75007
3815	1.7777	1.96257
3816	1.9096	0.905728
3817	1.70239	1.05704
3818	1.16182	0.117118
3819	1.20132	0.132765
3820	1.23475	2.1
3821	1.56859	1.22049
3822	2.46263	2.1
3823	0.528744	2.1
3824	2.93337	2.1
3825	2.42504	2.1

3826	1.38646	0.580153
3827	1.61963	0.729518
3828	1.88016	0.726898
3829	0.622283	0.840109
3830	1.03243	0.76796
3831	0.710307	0.979373
3832	0.798124	0.825026
3833	0.931073	0.85064
3834	0.892862	1.45048
3835	1.06791	0.774464
3836	0.552787	0.938692
3837	0.946132	0.763228
3838	1.36665	0.841399
3839	1.36665	1.36118
3840	0.506966	0.771163
3841	0.506964	1.23561
3842	1.36665	0.785989
3843	0.653469	0.775309
3844	1.36665	0.700383
3845	1.21339	0.715998
3846	1.21339	0.983125
3847	0.552757	0.73864
3848	0.552786	1.16322
3849	1.21336	0.714294
3850	1.70744	1.5926
3851	1.0775	0.752433
3852	1.59527	0.930316
3853	1.48312	0.891875
3854	2.01868	0.771024
3855	1.18947	0.341067
3856	1.48312	0.891479
3857	0.785626	0.43849
3858	1.19601	0.50054
3859	0.785626	0.525201
3860	0.946132	0.471827
3861	0.7856	0.620173
3862	1.59087	0.523461
3863	1.51158	0.51322
3864	1.74098	0.527313
3865	2.39202	0.466093
3866	0.870489	0.732894
3867	1.34534	0.825758
3868	0.870488	0.621195
3869	0.946133	0.822554
3870	0.870489	0.902476
3871	1.67086	0.856411
3872	1.67086	0.866318
3873	2.11311	2.04977
3874	1.98765	1.18362
3875	0.946133	0.933281
3876	1.30881	1.80057
3877	1.72211	2.1
3878	1.72211	1.11176
3879	0.94613	1.4665
3880	1.30881	1.12722
3881	1.35943	1.00582
3882	1.01294	0.699645
3883	1.01294	0.127927

3884	1.38963	0.862709
3885	1.01294	0.732189
3886	1.60056	0.718198
3887	1.60056	0.643983
3888	0.946132	0.79344
3889	1.204	0.738427
3890	0.946132	0.689678
3891	1.11288	0.435984
3892	1.204	0.700691
3893	1.14599	0.92033
3894	1.00065	0.890001
3895	1.74685	0.924462
3896	1.74685	2.1
3897	0.946128	0.917603
3898	1.38463	0.873619
3899	1.2275	2.1
3900	1.2275	1.03195
3901	1.30835	1.07374
3902	2.04407	2.1
3903	2.50425	2.1
3904	1.19647	0.788646
3905	1.19647	0.795103
3906	1.19645	0.834061
3907	1.75446	0.78439
3908	1.1315	0.768343
3909	1.1315	0.760536
3910	1.45569	0.741491
3911	1.1315	0.740817
3912	1.14357	2.1
3913	1.25793	2.1
3914	1.14357	2.1
3915	2.08181	2.1
3916	1.4714	2.1
3917	0.71295	2.1
3918	1.68285	2.1
3919	1.68285	2.1
3920	1.25603	2.1
3921	1.13976	2.1
3922	1.25603	2.1
3923	0.71295	2.1
3924	1.61095	2.1
3925	2.51207	2.1
3926	1.63762	2.1
3927	2.74451	2.1
3928	0.671255	1.66367
3929	1.87601	2.1
3930	1.87601	2.1
3931	0.671226	1.69864
3932	1.25603	2.09298
3933	1.29123	2.1
3934	0.671226	2.09334
3935	1.63723	2.1
3936	0.872928	0.116001
3937	1.47904	0.241796
3938	1.18793	0.192243
3939	2.57464	0.249525
3940	1.14986	1.78404
3941	1.19611	1.73843

3942	0.96029	0.790685
3943	1.7961	2.1
3944	0.928721	2.1
3945	1.34352	2.1
3946	1.37935	2.1
3947	1.37935	2.1
3948	0.892888	2.1
3949	0.946131	2.1
3950	0.892888	2.1
3951	1.75422	2.1
3952	1.05556	2.1
3953	1.27362	0.876447
3954	1.27421	0.465451
3955	1.3003	0.99585
3956	1.31415	0.966223
3957	1.01431	2.1
3958	0.673951	2.1
3959	1.63384	2.1
3960	1.63661	2.1
3961	1.52245	2.1
3962	1.50714	2.1
3963	0.996778	0.0882022
3964	1.56091	0.132925
3965	1.06543	2.1
3966	2.66689	2.1
3967	1.01474	2.1
3968	2.76422	2.1
3969	1.45321	0.71302
3970	0.974716	0.601563
3971	0.810943	0.650899
3972	0.909982	0.715401
3973	0.937793	0.67259
3974	1.05138	0.655111
3975	1.25044	0.643212
3976	0.92095	0.65539
3977	0.830414	0.706382
3978	2.1402	0.672526
3979	1.75938	2.1
3980	1.05556	2.1
3981	1.78558	2.1
3982	1.379 2.1	
3983	0.970214	0.779646
3984	0.9702	0.771636
3985	1.08894	1.11189
3986	2.17251	1.07914
3987	1.51569	1.10623
3988	1.06012	1.05177
3989	0.98966	1.07058
3990	1.55926	0.868465
3991	1.92645	0.967569
3992	1.37427	0.241695
3993	2.16338	0.359873
3994	1.5456	0.216014
3995	1.5456	0.148313
3996	1.94002	0.194277
3997	1.33135	0.286074
3998	1.31948	0.263281
3999	2.23255	0.258052

4000	2.34061	0.268448
4001	1.52903	2.1
4002	3.38327	2.1
4003	1.35977	2.1
4004	2.22414	2.1
4005	1.6812	0.32849
4006	2.33397	0.312965
4007	1.16699	0.467012
4008	0.946131	0.381437
4009	1.58115	0.42062
4010	1.58115	0.415759
4011	0.519726	0.59296
4012	0.946131	0.492047
4013	0.519697	0.49677
4014	1.26868	0.336273
4015	1.16699	0.339756
4016	1.5951	0.272022
4017	1.5951	0.281223
4018	0.840568	0.356673
4019	0.946131	0.320438
4020	0.840598	0.323799
4021	1.30047	2.1
4022	1.5586	2.1
4023	1.31825	0.991932
4024	1.31924	0.96966
4025	1.15818	1.7348
4026	1.52782	0.912569
4027	1.15818	1.0517
4028	1.15818	1.06533
4029	0.99518	0.7
4030	0.94613	0.68594
4031	0.922045	0.7
4032	1.29322	0.699618
4033	1.29321	0.616031
4034	1.29321	0.699938
4035	0.922056	0.7
4036	1.06848	0.7
4037	1.84409	0.7
4038	1.9186	1.32472
4039	2.72244	1.05786
4040	0.946132	0.955973
4041	1.36122	1.07878
4042	1.79658	1.09123
4043	1.79658	0.967022
4044	0.946133	1.21136
4045	1.36122	1.02303
4046	1.38149	0.971534
4047	1.59106	0.965315
4048	0.946116	0.977393
4049	1.29052	1.00873
4050	1.76641	0.981507
4051	1.76641	0.954256
4052	0.885309	1.17766
4053	1.29054	1.02749
4054	1.36118	0.980886
4055	1.59103	1.02477
4056	2.27816	0.921883
4057	1.56164	0.970894

4058	1.41114	0.0706063
4059	2.04128	0.0564811
4060	0.986723	2.1
4061	1.03645	0.652656
4062	1.79224	0.496822
4063	1.2627	2.1
4064	1.23036	2.1
4065	1.45257	2.1
4066	1.41469	2.1
4067	1.41469	2.1
4068	2.60636	2.1
4069	1.45789	0.153301
4070	0.851541	0.137413
4071	1.57877	0.149306
4072	1.53134	0.100558
4073	0.851479	0.136656
4074	1.08982	0.165676
4075	0.716284	0.107862
4076	0.946131	0.173561
4077	1.73193	0.12517
4078	2.57309	0.154339
4079	1.36307	0.104722
4080	2.42175	0.110004
4081	1.72948	0.118533
4082	0.978698	2.1
4083	0.978699	2.1
4084	2.08398	2.1
4085	0.978698	2.1
4086	1.44096	2.1
4087	1.44096	2.1
4088	0.638164	2.1
4089	0.681588	2.1
4090	0.946133	2.1
4091	0.638165	2.1
4092	0.94613	2.1
4093	1.55432	2.1
4094	0.901147	2.1
4095	0.85777	2.1
4096	1.36073	2.1
4097	1.44004	2.1
4098	0.946133	2.1
4099	1.09982	2.1
4100	1.27562	2.1
4101	1.55432	2.1
4102	1.62229	2.1
4103	1.0101	2.1
4104	2.28459	2.07826
4105	2.50554	1.70843
4106	0.946131	1.04823
4107	1.25276	2.09032
4108	1.73721	2.1
4109	1.73721	1.1987
4110	0.93406	1.59541
4111	1.25277	1.30618
4112	1.4185	1.15487
4113	1.53064	1.17839
4114	0.946133	1.21285
4115	0.609462	2.09884

4116	1.65123	2.09352
4117	1.65123	1.46971
4118	0.609466	1.90178
4119	1.00792	1.54334
4120	1.25277	1.34374
4121	1.53064	1.81797
4122	2.15223	1.16126
4123	1.30664	1.58034
4124	1.10674	2.1
4125	1.22862	2.1
4126	1.49358	0.703824
4127	2.0207	0.671587
4128	2.09374	2.1
4129	1.88175	2.1
4130	1.10938	0.334943
4131	1.02478	0.748934
4132	1.13272	0.128137
4133	1.13272	0.140629
4134	1.03935	1.46622
4135	0.810945	1.63344
4136	0.527573	1.11042
4137	0.925011	1.37494
4138	1.03193	1.23735
4139	1.38587	1.34012
4140	0.703469	1.26925
4141	0.912282	1.45708
4142	1.75505	2.1
4143	1.78529	2.1
4144	1.60017	0.972297
4145	1.35608	0.775424
4146	1.01479	0.931868
4147	1.47561	0.760303
4148	1.01479	0.900096
4149	1.28418	0.759814
4150	2.62605	2.1
4151	1.71233	2.1
4152	3.22222	2.1
4153	1.97526	2.1
4154	0.949712	0.704905
4155	1.78986	0.70322
4156	2.72879	0.668667
4157	2.66428	0.700532
4158	1.68108	0.723609
4159	1.67265	0.708619
4160	2.24877	0.7226
4161	1.15558	0.749178
4162	1.39708	2.1
4163	1.39327	2.1
4164	1.69788	0.284196
4165	1.1273	0.241382
4166	1.17302	0.748699
4167	1.04349	0.714823
4168	0.979853	2.1
4169	1.19094	2.1
4170	1.39408	2.1
4171	1.61857	2.1
4172	1.66523	2.1
4173	0.946132	2.1

4174	0.838554	2.1
4175	1.05151	2.1
4176	1.18901	2.1
4177	1.18901	2.1
4178	1.61149	2.1
4179	1.22857	0.826099
4180	1.25848	0.820969
4181	1.09154	2.1
4182	2.20358	2.1
4183	1.23013	2.1
4184	1.55698	2.1
4185	1.55828	1.64348
4186	1.52391	2.03132
4187	0.60346	1.3271
4188	1.06336	2.08871
4189	0.870714	1.16082
4190	0.543109	1.3283
4191	1.10367	1.15871
4192	1.20686	1.32646
4193	1.20684	2.04
4194	2.5118	1.24146
4195	1.20684	2.1
4196	1.52468	1.53578
4197	1.52469	1.89543
4198	0.532308	1.35784
4199	0.903301	2.02106
4200	0.946119	1.21422
4201	0.532315	1.28542
4202	0.946107	1.29022
4203	1.46129	2.1
4204	0.804766	2.1
4205	1.4809	2.1
4206	1.4809	2.1
4207	1.11822	2.1
4208	1.16744	2.1
4209	1.11822	2.1
4210	0.804737	2.1
4211	1.04193	2.1
4212	0.983723	2.1
4213	1.8014	2.1
4214	1.1729	2.1
4215	1.03951	2.1
4216	1.03951	2.1
4217	1.17583	2.1
4218	1.42866	2.1
4219	1.78503	2.1
4220	1.17822	0.986923
4221	1.89121	2.1
4222	1.44472	2.1
4223	1.62143	2.1
4224	1.43837	2.1
4225	1.01121	0.642771
4226	0.962703	0.890202
4227	0.946129	0.813978
4228	1.10941	0.753854
4229	0.946131	0.825375
4230	1.60326	0.851051
4231	1.60326	0.768894

4232	1.14215	0.798511
4233	1.40724	0.789599
4234	0.962703	0.842037
4235	1.53336	0.288363
4236	1.40272	0.255832
4237	1.64984	0.201565
4238	1.67345	0.22831
4239	1.08212	2.1
4240	1.40905	2.1
4241	0.946132	0.100396
4242	0.946132	0.10148
4243	0.81268	0.116497
4244	0.946131	0.0888673
4245	0.812681	0.106187
4246	0.946131	0.101485
4247	0.946132	0.10714
4248	0.997755	0.0874924
4249	1.94585	0.0817246
4250	1.2925	0.0843034
4251	2.52454	0.084049
4252	1.69182	0.0832907
4253	1.69182	0.0806519
4254	0.92361	0.0958558
4255	1.35316	0.0854287
4256	1.26227	0.0922198
4257	0.946131	0.0896301
4258	1.35316	0.0836122
4259	1.48676	0.413422
4260	1.33917	0.410265
4261	0.757292	2.1
4262	1.02415	2.1
4263	1.48495	2.1
4264	0.97587	2.1
4265	0.946132	2.1
4266	0.747365	2.1
4267	1.55432	2.1
4268	1.55432	2.1
4269	0.946132	2.1
4270	0.747394	2.1
4271	1.17464	0.403454
4272	1.0372	0.400194
4273	2.16553	0.149047
4274	2.00616	0.293045
4275	1.12184	0.150165
4276	1.33177	0.146298
4277	0.936674	0.137475
4278	0.946129	0.146551
4279	0.851552	0.184818
4280	1.60205	0.144098
4281	1.77391	0.144697
4282	1.03327	0.270225
4283	1.41512	0.283569
4284	1.03327	0.229645
4285	0.946133	0.286612
4286	0.946131	0.33995
4287	1.80077	0.310059
4288	1.80077	0.286052
4289	1.54848	2.1

4290	1.29931	2.1
4291	1.04482	0.71757
4292	2.08069	0.726129
4293	0.946127	0.713268
4294	0.515539	0.732238
4295	0.515566	0.757279
4296	1.38423	0.713465
4297	1.38423	0.711025
4298	0.972058	0.732058
4299	0.927737	0.739364
4300	0.94971	0.69732
4301	2.19682	0.709194
4302	0.946132	0.727277
4303	1.09841	0.711026
4304	0.522382	0.738501
4305	1.5962	0.70276
4306	1.72791	0.692859
4307	1.07428	0.718245
4308	1.05215	0.714563
4309	1.54632	1.25333
4310	1.15693	1.33564
4311	1.41238	2.1
4312	1.41238	2.1
4313	1.77966	2.1
4314	1.77966	2.1
4315	0.946129	2.1
4316	1.40525	2.1
4317	0.946131	2.1
4318	0.94613	2.1
4319	1.32054	2.1
4320	1.41238	2.1
4321	1.94026	2.1
4322	1.0171	0.778857
4323	1.1211	0.629548
4324	0.993022	2.1
4325	0.773813	2.1
4326	1.4073	2.1
4327	1.4073	2.1
4328	0.94613	2.1
4329	0.787615	2.1
4330	0.94613	2.1
4331	0.787615	2.1
4332	1.764	2.1
4333	1.90964	2.1
4334	1.05787	2.1
4335	1.403	0.769478
4336	0.744189	0.820939
4337	2.32834	2.1
4338	2.34986	2.1
4339	1.17106	1.18865
4340	1.94826	0.394366
4341	2.40606	0.315812
4342	1.26387	0.431409
4343	0.954013	0.452469
4344	0.946131	0.489684
4345	1.71	0.263147
4346	1.51926	0.242546
4347	0.953997	0.258298

4348	0.851554	0.426676
4349	1.96502	0.317998
4350	1.72954	0.434833
4351	2.89117	0.353086
4352	0.971958	0.462097
4353	0.985575	0.497658
4354	1.41943	0.308893
4355	1.20021	0.383918
4356	1.29599	0.442153
4357	1.15687	0.327079
4358	0.810958	0.494142
4359	1.12919	0.0809247
4360	1.98859	2.1
4361	1.82044	2.1
4362	1.90488	2.1
4363	0.851542	2.1
4364	1.02973	2.1
4365	1.39286	2.1
4366	1.44873	2.1
4367	0.946132	2.1
4368	1.11826	2.1
4369	1.26842	2.1
4370	2.09461	2.1
4371	1.76952	0.0907902
4372	1.6473	0.0834537
4373	0.946131	0.152744
4374	1.36486	0.122183
4375	1.04243	0.133548
4376	1.05017	0.124016
4377	1.24059	0.115491
4378	2.11646	0.0858505
4379	1.50368	0.119292
4380	2.38796	0.0981157
4381	1.50368	0.0977153
4382	1.71844	0.102757
4383	1.71844	0.0971812
4384	0.914836	0.182419
4385	1.24416	0.161491
4386	0.914805	0.176891
4387	1.24416	0.152456
4388	1.38911	0.129302
4389	1.46859	0.178022
4390	1.35348	0.176809
4391	0.946131	0.211021
4392	1.1226	0.191965
4393	0.857078	0.246332
4394	0.9746	0.217105
4395	1.37624	0.193531
4396	1.15131	0.084469
4397	1.139	0.0986234
4398	1.90456	0.113795
4399	2.09692	0.172462
4400	1.16655	2.1
4401	1.23017	2.1
4402	2.14592	2.1
4403	1.23017	2.1
4404	1.0752	1.06767
4405	1.52482	0.74133

4406	1.09252	2.1
4407	1.10725	2.1
4408	1.14305	0.451679
4409	0.851541	0.447067
4410	1.48073	0.446227
4411	1.45607	0.268474
4412	0.946131	0.557255
4413	0.932823	0.269752
4414	1.45828	0.508248
4415	0.851528	0.502155
4416	1.14305	0.370235
4417	1.92737	0.591268
4418	1.08211	0.556278
4419	1.69831	0.05
4420	1.69831	0.05
4421	0.946131	0.05
4422	1.26868	0.05
4423	1.23377	0.05
4424	1.23377	0.05
4425	0.94723	0.05
4426	1.64322	0.0711792
4427	1.64322	0.0578968
4428	0.946131	0.59529
4429	1.32068	0.0788649
4430	0.946129	0.0788644
4431	1.18789	0.763048
4432	1.32068	0.079731
4433	1.32813	2.1
4434	1.32461	2.1
4435	1.42729	0.419643
4436	2.47846	0.59984
4437	1.00024	0.693818
4438	1.23923	0.579875
4439	0.946131	0.69967
4440	0.712304	0.527439
4441	1.77501	0.695227
4442	1.77501	0.451009
4443	0.593593	0.71111
4444	1.23923	0.706992
4445	1.23175	0.653213
4446	0.94613	0.712779
4447	0.713646	0.655189
4448	1.75735	0.710396
4449	1.75735	0.497825
4450	0.713633	0.728299
4451	1.37157	0.579853
4452	1.47169	0.591876
4453	2.21808	0.693124
4454	1.47169	0.674774
4455	1.04759	2.1
4456	0.987132	2.1
4457	1.11181	2.1
4458	2.61682	1.53815
4459	1.11181	2.1
4460	1.06461	1.19986
4461	1.69733	2.1
4462	2.79705	2.1
4463	1.08245	2.1

4464	1.40583	2.03546
4465	1.39852	1.94676
4466	2.0239	1.76251
4467	2.0239	1.87814
4468	0.780455	2.1
4469	0.946132	2.1
4470	0.780455	2.1
4471	1.01885	2.1
4472	0.564217	2.1
4473	0.733461	2.1
4474	0.537126	2.1
4475	0.683838	2.1
4476	0.779662	2.1
4477	0.634141	2.1
4478	0.685337	2.1
4479	1.58848	1.64702
4480	1.17129	0.396052
4481	0.663168	0.695291
4482	1.55587	0.570233
4483	1.31342	0.524641
4484	0.596868	0.707375
4485	1.1666	0.734484
4486	0.867261	0.73685
4487	0.946132	0.746252
4488	1.28843	0.471087
4489	1.31248	0.717508
4490	2.59921	0.674573
4491	1.32634	0.385509
4492	1.35564	0.082032
4493	1.52661	0.0909109
4494	1.12323	0.0676252
4495	1.17701	1.13912
4496	1.36405	1.11765
4497	1.19837	1.10392
4498	2.25636	1.05768
4499	1.0823	0.416323
4500	1.31774	0.655881
4501	1.0823	0.665829
4502	1.01121	0.477421
4503	1.27408	0.659277
4504	0.946131	0.731544
4505	0.566074	0.685433
4506	0.79576	0.711017
4507	1.24596	0.725906
4508	1.47567	0.729578
4509	1.47567	0.508877
4510	0.56605	0.715055
4511	1.6567	0.532368
4512	0.967792	0.482889
4513	1.00366	0.680443
4514	1.00366	0.668827
4515	1.56409	0.124146
4516	1.56409	0.11793
4517	0.774911	0.199405
4518	1.09483	0.201814
4519	0.774912	0.177707
4520	1.09483	0.187541
4521	1.24417	0.146195

4522	1.13387	0.104877
4523	1.13387	0.102991
4524	1.30572	0.206133
4525	2.18966	0.168249
4526	0.98575	0.115455
4527	1.54982	0.166423
4528	2.48833	0.135378
4529	1.54982	0.127643
4530	1.28954	0.7
4531	1.64002	0.11287
4532	1.64002	0.0957352
4533	1.85594	0.0898274
4534	1.12968	2.1
4535	1.23536	1.67362
4536	1.4882	2.01026
4537	1.4882	2.1
4538	0.876836	1.91186
4539	0.946131	2.1
4540	0.876835	2.1
4541	1.43166	2.1
4542	2.25936	2.1
4543	1.0305	1.87643
4544	0.992095	2.1
4545	1.12968	2.1
4546	1.53813	2.1
4547	1.53812	2.1
4548	0.583653	2.1
4549	0.94613	2.1
4550	0.583651	2.1
4551	0.964033	2.1
4552	0.964034	2.1
4553	1.54457	1.6711
4554	3.54109	0.450568
4555	0.99974	0.586431
4556	0.78814	0.631691
4557	1.65729	0.438632
4558	1.1283	0.511041
4559	1.09407	0.583167
4560	1.55091	0.449861
4561	0.870773	0.623409
4562	1.19967	0.456006
4563	1.56521	0.507281
4564	1.30896	1.18017
4565	1.17989	2.1
4566	1.72526	1.98669
4567	0.851537	1.53169
4568	1.58944	1.93857
4569	1.56915	2.07128
4570	0.891039	1.39031
4571	1.4172	1.36697
4572	0.963818	1.25986
4573	0.851474	1.78511
4574	1.17989	2.1
4575	1.56261	2.1
4576	1.56261	2.1
4577	0.761574	2.1
4578	1.31323	2.1
4579	0.761573	2.1

4580	0.946133	2.1
4581	1.31323	2.1
4582	1.16759	2.1
4583	1.16759	2.1
4584	2.11829	2.1
4585	1.16759	2.1
4586	1.10748	0.669295
4587	1.47688	0.50037
4588	0.946133	0.445627
4589	0.953319	0.481562
4590	0.946132	0.544166
4591	1.51168	0.584166
4592	1.51168	0.386013
4593	1.25797	0.394432
4594	1.19984	0.222124
4595	1.95729	2.1
4596	2.34853	2.1
4597	1.94343	1.86213
4598	2.00551	1.94233
4599	1.20067	0.856193
4600	1.87244	0.901383
4601	1.4178	2.1
4602	1.33538	2.1
4603	0.946131	2.1
4604	1.22895	1.76861
4605	1.12433	2.1
4606	0.956757	2.1
4607	0.91826	2.1
4608	1.44221	2.1
4609	1.14206	2.1
4610	1.80514	0.964123
4611	0.890304	1.25229
4612	1.1362	0.90695
4613	1.32819	0.945212
4614	1.46311	1.71423
4615	0.8654	0.965596
4616	1.1699	1.13428
4617	1.00267	1.13178
4618	1.02882	0.984069
4619	1.00832	2.1
4620	0.974137	2.1
4621	1.02368	2.1
4622	0.974138	2.1
4623	0.995734	0.429114
4624	1.44605	0.706544
4625	1.18549	0.700108
4626	1.33621	0.842706
4627	1.5176	0.845062
4628	1.91762	0.833485
4629	1.33621	0.828806
4630	1.6638	0.822389
4631	1.66774	2.1
4632	1.11833	2.1
4633	0.926499	2.1
4634	1.51229	2.1
4635	1.51229	2.1
4636	0.926528	2.1
4637	1.40335	2.1

4638	1.03544	2.1
4639	0.926527	2.1
4640	1.85305	2.1
4641	1.5812	2.1
4642	1.15529	2.1
4643	1.31086	2.1
4644	0.999206	2.1
4645	1.46714	2.1
4646	1.73087	2.1
4647	0.772299	2.1
4648	1.24341	2.1
4649	0.997867	2.1
4650	0.772311	2.1
4651	0.936657	2.1
4652	1.35986	2.1
4653	0.959414	2.1
4654	1.36541	2.1
4655	1.49268	2.1
4656	1.49268	2.1
4657	0.832137	2.1
4658	0.946132	2.1
4659	0.832138	2.1
4660	1.64523	2.1
4661	1.11764	2.1
4662	1.05776	2.1
4663	0.972465	2.1
4664	0.972464	2.1
4665	1.98425	2.1
4666	0.969822	2.1
4667	1.30566	2.1
4668	1.52724	2.1
4669	2.32237	0.70413
4670	2.30274	0.711234
4671	2.27761	0.512215
4672	3.33012	0.495658
4673	1.24001	0.826699
4674	1.41821	0.948316
4675	0.978584	2.1
4676	1.18076	2.1
4677	1.07645	2.1
4678	0.877351	0.589502
4679	0.946132	1.03781
4680	0.877353	0.888406
4681	1.58444	2.1
4682	1.49614	1.51305
4683	1.28355	1.10611
4684	1.13491	1.0651
4685	1.56875	0.792058
4686	0.499592	0.434765
4687	0.946131	0.284563
4688	1.20538	0.367736
4689	1.09913	0.313415
4690	1.8049	0.383613
4691	1.8049	0.342841
4692	0.499606	0.475317
4693	2.41076	0.380403
4694	0.860202	0.36191
4695	0.906798	0.393941

4696	1.8626	0.459285
4697	1.33986	0.334901
4698	1.2992	0.537651
4699	1.58702	0.283959
4700	1.05939	0.338507
4701	1.02074	1.90687
4702	1.07866	1.85751
4703	1.5082	0.384845
4704	1.5522	0.286711
4705	1.65903	0.504739
4706	1.86766	2.1
4707	2.11684	2.1
4708	0.946132	2.1
4709	1.6585	2.1
4710	1.6585	2.1
4711	0.94613	2.1
4712	1.1872	2.1
4713	1.1872	2.1
4714	1.05842	2.1
4715	1.20455	2.1
4716	1.34141	2.1
4717	1.34141	2.1
4718	1.42416	2.1
4719	1.75818	1.70441
4720	2.63715	1.02208
4721	1.80454	2.1
4722	2.26198	2.1
4723	0.696255	2.1
4724	0.624075	1.83733
4725	1.50905	0.926119
4726	1.50905	1.95311
4727	0.624075	1.99234
4728	1.31857	0.884052
4729	0.946131	1.43798
4730	0.624075	1.07707
4731	0.914628	1.84886
4732	1.14721	2.1
4733	1.16153	2.1
4734	1.47664	2.1
4735	1.54791	2.1
4736	1.19098	2.1
4737	1.13341	2.1
4738	1.00503	0.804159
4739	1.00503	1.01618
4740	1.22655	2.1
4741	0.999489	2.1
4742	1.3268	1.99963
4743	0.946132	2.1
4744	1.60061	2.1
4745	1.60061	2.1
4746	1.08034	2.1
4747	1.40372	2.1
4748	1.14302	2.1
4749	1.08034	2.1
4750	0.999491	2.1
4751	1.17152	0.564774
4752	1.17152	0.351487
4753	1.17152	0.459706

4754	2.05423	0.475402
4755	1.40984	1.88343
4756	1.2489	2.1
4757	0.885089	2.1
4758	0.810931	2.1
4759	1.84128	2.1
4760	1.11206	2.1
4761	1.21926	2.1
4762	1.99084	2.1
4763	0.976334	0.998132
4764	0.599363	0.903691
4765	1.02621	0.781292
4766	0.462959	0.875592
4767	1.42612	0.759003
4768	0.299682	0.801125
4769	1.51379	2.1
4770	0.719531	2.1
4771	1.3702	2.1
4772	1.33293	2.1
4773	0.851539	2.1
4774	0.62998	2.1
4775	0.851543	2.1
4776	0.629966	2.1
4777	1.43132	2.1
4778	1.13328	1.11441
4779	1.93212	0.941015
4780	1.62345	1.53017
4781	1.38511	1.6563
4782	1.40013	1.19875
4783	1.04132	1.18922
4784	0.83473	1.44147
4785	0.868732	1.43897
4786	0.810595	1.68542
4787	0.729919	1.37304
4788	0.992428	1.55186
4789	0.577107	1.23441
4790	1.18464	1.56627
4791	0.791199	1.47641
4792	0.810943	0.355968
4793	0.860723	0.380696
4794	1.33348	0.209521
4795	1.57467	0.241192
4796	0.981032	0.367058
4797	0.731569	0.235797
4798	0.770312	0.400938
4799	1.46055	2.1
4800	1.42249	2.1
4801	1.75999	0.410911
4802	1.6868	0.307334
4803	1.17042	0.911348
4804	1.01485	2.1
4805	1.19952	0.377447
4806	1.40592	0.547725
4807	1.19952	0.445714
4808	2.20145	0.453619
4809	1.33649	0.654777
4810	1.11612	0.880291
4811	0.957588	0.812576

4812	0.946126	0.854124
4813	1.50648	0.630524
4814	1.50648	0.844293
4815	0.946128	0.80662
4816	2.10671	0.311096
4817	1.91518	0.559965
4818	2.23223	0.624751
4819	1.53556	2.1
4820	1.56253	2.1
4821	1.27806	0.0814568
4822	1.44593	0.0851388
4823	1.03818	0.786108
4824	1.03818	0.788205
4825	1.38969	0.890009
4826	1.85548	0.778785
4827	2.6044	0.835273
4828	1.85548	0.764493
4829	1.49346	2.06751
4830	1.83192	1.89107
4831	0.834043	1.90414
4832	0.810943	1.42754
4833	0.887113	1.4362
4834	0.893827	1.57105
4835	0.991061	1.56936
4836	0.91939	1.63291
4837	0.793307	1.58199
4838	1.75291	1.83245
4839	1.35435	1.63337
4840	1.16989	0.788054
4841	1.36717	0.753687
4842	2.12276	0.701967
4843	2.30493	0.707533
4844	2.47857	0.707888
4845	1.92269	0.701021
4846	1.66426	0.705329
4847	0.956698	0.996439
4848	0.956698	1.70279
4849	0.857957	2.1
4850	1.4706	1.76717
4851	1.65706	2.1
4852	1.47059	2.1
4853	1.04713	0.704449
4854	0.946131	0.707788
4855	1.47374	0.718195
4856	1.47374	0.706743
4857	0.951675	0.707349
4858	1.2582	0.718165
4859	1.2582	0.706473
4860	0.94613	0.716992
4861	1.4468	0.472787
4862	1.90335	0.645382
4863	1.14886	2.1
4864	2.59689	2.1
4865	1.02747	2.1
4866	0.946131	2.1
4867	0.934061	2.1
4868	1.63979	2.1
4869	1.65206	2.1

4870	1.48	2.1
4871	1.27467	2.1
4872	2.20449	2.1
4873	1.1333	0.0997948
4874	1.95334	2.1
4875	2.20984	2.1
4876	1.46389	0.496034
4877	1.4639	0.526449
4878	1.4639	0.658338
4879	0.946132	0.682162
4880	1.5144	0.545988
4881	1.5144	0.647654
4882	0.946132	0.703525
4883	1.35732	0.547918
4884	1.10321	0.674696
4885	0.946132	0.689797
4886	0.980549	0.536867
4887	1.32819	2.1
4888	1.30171	1.14328
4889	1.68377	1.01718
4890	1.68377	2.1
4891	0.946132	0.941125
4892	0.946132	1.73051
4893	1.1952	1.17531
4894	0.990019	1.20345
4895	1.08195	1.53075
4896	1.2269	2.1
4897	1.19219	1.98221
4898	1.01485	2.1
4899	1.57125	0.239056
4900	2.10696	0.211429
4901	1.29666	0.0991654
4902	1.57125	0.243539
4903	1.47712	2.1
4904	1.45182	2.1
4905	1.17816	0.112583
4906	1.4516	0.124507
4907	1.17816	0.0828046
4908	1.17816	0.0840986
4909	0.596954	1.01191
4910	1.65133	0.898625
4911	0.583859	0.84972
4912	1.04072	1.07444
4913	1.28795	0.892558
4914	1.59423	0.89189
4915	0.483559	0.9119
4916	1.74088	0.857507
4917	1.09545	0.995131
4918	1.02519	1.14592
4919	2.47579	0.942341
4920	1.54732	0.997851
4921	2.17663	0.926094
4922	1.75006	0.960267
4923	1.32354	0.992904
4924	1.09962	2.1
4925	1.85057	2.1
4926	1.91046	2.1
4927	2.17037	2.1

4928	1.99528	0.499283
4929	1.97969	0.441438
4930	3.82765	2.1
4931	1.85767	2.1
4932	2.77042	2.1
4933	1.37236	0.254732
4934	2.23254	0.260132
4935	1.10089	0.210899
4936	0.994294	2.1
4937	1.81921	2.1
4938	1.00073	2.1
4939	0.681043	2.1
4940	0.946131	2.1
4941	0.681073	2.1
4942	1.51804	2.1
4943	1.60653	2.1
4944	1.30188	2.1
4945	0.919622	2.1
4946	1.00965	2.1
4947	1.93821	2.1
4948	2.60377	2.1
4949	1.36416	0.133966
4950	1.36416	0.166581
4951	1.36416	0.184758
4952	2.44132	0.15366
4953	2.15871	0.829494
4954	2.22171	0.769687
4955	1.70747	0.717595
4956	1.49515	0.709497
4957	0.773007	0.714792
4958	0.946125	0.727915
4959	0.946131	0.726644
4960	0.773034	0.704777
4961	1.15844	0.718626
4962	1.98104	0.704402
4963	2.44283	0.713854
4964	1.05417	0.109379
4965	1.1596	0.138541
4966	0.829921	0.140159
4967	0.946131	0.1704
4968	0.951911	0.168736
4969	1.39027	0.122631
4970	0.829946	0.172291
4971	1.51223	0.153781
4972	1.51223	0.0938269
4973	1.65984	0.0997763
4974	1.90382	0.0935347
4975	1.93228	1.10525
4976	2.47924	1.02267
4977	1.73904	1.35723
4978	1.40547	0.851271
4979	2.37589	0.930923
4980	0.662605	1.14663
4981	2.33293	1.06306
4982	1.72249	2.1
4983	1.68478	2.1
4984	1.00514	0.733995
4985	1.4537	2.1

4986	1.92698	2.1
4987	1.46041	0.0514803
4988	2.04817	0.05
4989	1.65954	2.1
4990	1.42342	2.1
4991	1.04405	0.125343
4992	1.17056	0.0692419
4993	1.11339	0.062666
4994	0.946133	0.0799881
4995	0.929724	0.0741607
4996	1.35424	0.0673891
4997	1.35424	0.0710057
4998	0.929713	0.0880864
4999	1.85945	0.0854018
5000	1.38905	2.1
5001	1.39056	2.1
5002	1.52852	2.1
5003	1.06472	2.1
5004	0.749096	2.1
5005	1.46076	2.1
5006	1.29263	2.1
5007	0.94613	2.1
5008	0.69097	2.1
5009	0.982284	2.1
5010	0.67419	2.1
5011	1.52852	2.1
5012	1.91121	2.1
5013	1.00775	1.84298
5014	1.61201	2.07734
5015	0.529987	2.1
5016	0.506125	2.1
5017	1.44696	2.1
5018	2.32981	2.1
5019	1.32784	2.1
5020	1.94158	2.1
5021	1.31595	2.1
5022	0.663922	2.1
5023	1.44663	2.1
5024	1.44663	2.1
5025	1.13976	2.1
5026	0.970788	2.1
5027	1.13976	2.1
5028	0.663921	2.1
5029	0.966114	2.1
5030	1.05498	1.02863
5031	1.38086	2.1
5032	0.946127	2.1
5033	0.922513	2.1
5034	1.49903	2.1
5035	1.49903	2.1
5036	0.92251	2.1
5037	1.06105	2.1
5038	1.36049	2.1
5039	1.38086	2.1
5040	1.52534	2.1
5041	1.04101	2.1
5042	1.30861	0.227726
5043	0.949112	0.302557

5044	0.894275	0.37651
5045	1.10232	0.223374
5046	0.830889	0.280646
5047	1.26554	0.285642
5048	0.948347	0.245662
5049	0.81096	0.280344
5050	1.18338	0.332348
5051	0.883764	0.414829
5052	1.54793	0.232313
5053	1.02867	0.326275
5054	1.8573	0.321959
5055	1.06065	0.249469
5056	0.64842	0.29935
5057	1.99319	0.381922
5058	1.9476	0.424439
5059	2.51436	0.05
5060	1.68337	0.31514
5061	1.92321	0.346265
5062	0.810942	0.124905
5063	0.921001	0.202354
5064	0.99758	0.270334
5065	0.960785	0.223959
5066	1.01565	0.26446
5067	0.908624	0.188786
5068	0.912835	0.184572
5069	1.18351	0.237864
5070	1.40239	2.1
5071	1.26091	2.1
5072	0.947979	2.1
5073	0.946131	2.05464
5074	1.81959	1.96653
5075	1.81959	1.02314
5076	0.94613	2.1
5077	1.38415	0.999016
5078	1.38414	1.53877
5079	1.33266	1.80293
5080	0.946132	2.1
5081	1.76225	2.1
5082	1.76225	2.1
5083	1.27599	2.1
5084	1.38158	2.02667
5085	1.3268	2.1
5086	1.27599	2.1
5087	0.947979	2.1
5088	1.0142	0.748138
5089	1.01422	0.74123
5090	1.11435	0.797834
5091	1.42186	0.915037
5092	1.24001	1.00047
5093	1.77342	0.990444
5094	1.42186	1.15887
5095	1.99817	0.933458
5096	1.27361	2.1
5097	1.68914	2.1
5098	1.30116	2.1
5099	1.32819	1.20161
5100	1.68322	1.18402
5101	1.68322	2.1

5102	0.94613	0.988796
5103	0.94613	1.84893
5104	1.14666	1.2288
5105	1.07612	1.1628
5106	1.31151	2.1
5107	1.22095	1.31835
5108	1.57256	1.48916
5109	1.6679	2.1
5110	0.946132	1.15069
5111	0.946133	2.00805
5112	1.13537	1.93671
5113	1.07855	1.3697
5114	1.07855	2.1
5115	1.43895	1.11972
5116	1.38481	0.162477
5117	1.72385	0.156292
5118	0.550441	0.151296
5119	0.79807	0.164519
5120	1.11627	0.194232
5121	1.30349	0.191893
5122	0.94613	0.227373
5123	0.686176	0.235104
5124	1.11627	0.196199
5125	1.73363	0.172071
5126	1.73363	0.14699
5127	0.686139	0.285529
5128	1.24932	0.147867
5129	0.899582	0.154541
5130	2.00472	0.128276
5131	1.17121	0.172907
5132	1.2874	0.133095
5133	2.0194	0.150909
5134	0.88601	0.21902
5135	1.77409	0.220303
5136	1.73293	0.148951
5137	2.74045	0.210681
5138	1.83787	0.155864
5139	1.45448	0.18229
5140	1.27778	0.174924
5141	1.1192	2.1
5142	1.25953	2.1
5143	1.93611	2.1
5144	1.92104	2.1
5145	1.15506	0.107349
5146	1.15506	0.122528
5147	1.23943	0.783857
5148	0.851543	2.1
5149	1.6061	2.1
5150	1.80058	2.1
5151	0.869471	2.1
5152	1.3931	2.1
5153	1.06454	2.1
5154	0.93667	2.1
5155	2.16916	2.1
5156	3.10246	2.1
5157	1.91539	1.99312
5158	1.91539	1.64464
5159	0.620263	1.31829

5160	0.620265	1.9519
5161	0.946132	1.33557
5162	0.620265	1.29176
5163	1.30226	1.20687
5164	1.87292	1.9928
5165	1.18769	2.1
5166	2.60452	1.81121
5167	1.26574	2.1
5168	2.46679	1.47363
5169	1.36459	2.1
5170	1.26865	1.00362
5171	1.1744	0.894299
5172	1.15841	0.756204
5173	1.02247	0.728093
5174	1.93649	2.1
5175	1.64718	2.1
5176	1.59654	2.1
5177	1.57823	2.1
5178	1.00665	0.519484
5179	1.28239	0.447133
5180	0.887895	2.1
5181	1.22083	2.1
5182	1.42127	2.1
5183	1.07374	2.1
5184	1.00503	1.2571
5185	1.23082	0.959788
5186	2.78663	2.1
5187	2.20883	2.1
5188	2.54588	2.1
5189	0.946128	2.1
5190	1.13716	2.1
5191	1.58724	2.1
5192	1.58724	2.1
5193	0.946131	2.1
5194	1.13716	2.1
5195	1.39621	2.1
5196	1.02438	2.1
5197	1.49543	0.178024
5198	2.78336	0.156587
5199	1.27148	0.123689
5200	1.0906	0.135734
5201	1.04072	0.154315
5202	0.63574	0.115038
5203	1.45506	0.117169
5204	1.5863	0.11949
5205	0.928352	0.144737
5206	0.647687	0.152001
5207	2.2375	1.97182
5208	1.50998	1.61635
5209	2.75021	1.61646
5210	1.91912	1.54693
5211	1.99872	1.57324
5212	0.918127	1.79982
5213	1.03031	1.39507
5214	0.951169	1.24729
5215	1.27975	1.42904
5216	1.12735	1.52774
5217	1.15741	1.4192

5218	1.24998	1.39671
5219	1.65953	0.820331
5220	0.967555	0.944391
5221	0.967542	0.712647
5222	1.03824	0.704247
5223	1.48693	0.725304
5224	1.48693	0.681851
5225	2.02901	0.622927
5226	0.946127	0.716935
5227	1.07139	0.588847
5228	0.946126	0.599282
5229	1.84644	0.683481
5230	1.84644	0.617083
5231	1.38683	0.564154
5232	1.40574	0.44539
5233	1.36866	0.776923
5234	0.649325	0.772734
5235	1.93631	2.1
5236	1.50145	2.1
5237	1.6287	2.1
5238	1.31302	2.1
5239	1.18041	2.1
5240	1.18041	2.1
5241	0.974196	2.1
5242	0.946131	2.1
5243	1.74169	2.1
5244	1.74169	2.1
5245	1.23163	2.1
5246	1.27451	2.1
5247	1.41331	2.1
5248	1.23163	2.1
5249	1.28037	2.1
5250	1.28158	2.1
5251	1.28037	2.1
5252	2.28015	0.10328
5253	2.06561	0.0887559
5254	2.61343	0.124567
5255	1.11712	0.115423
5256	1.63689	0.876374
5257	1.01393	0.780279
5258	0.996775	2.1
5259	1.54277	2.1
5260	1.26397	2.1
5261	1.46129	2.1
5262	1.62391	2.1
5263	1.08848	0.137807
5264	1.11833	2.1
5265	1.24041	2.1
5266	2.31884	2.1
5267	1.335 2.1	
5268	1.48589	0.21624
5269	1.77131	0.185939
5270	1.8422	1.42399
5271	2.47453	1.76349
5272	1.23726	1.51067
5273	1.21996	1.5239
5274	1.65512	1.6737
5275	1.65512	1.64574

5276	0.802107	1.67421
5277	0.946129	1.3367
5278	0.802137	1.36959
5279	1.3279	1.30844
5280	1.23726	1.34777
5281	1.79759	1.40117
5282	1.79759	1.32192
5283	0.767577	1.53348
5284	0.946131	1.21971
5285	0.767577	1.22206
5286	1.03534	0.274974
5287	1.09962	0.804255
5288	0.929217	1.12831
5289	1.70126	1.14828
5290	0.991407	2.1
5291	1.41077	2.1
5292	0.991406	2.1
5293	1.62966	2.1
5294	1.62966	2.1
5295	0.946128	2.1
5296	1.18364	2.1
5297	0.946131	2.1
5298	1.14186	2.1
5299	1.18364	2.1
5300	1.16858	2.1
5301	1.66137	0.619433
5302	1.90165	0.202133
5303	1.66139	0.159166
5304	1.26893	0.673684
5305	1.15918	0.7
5306	0.653228	0.700544
5307	0.946133	0.7
5308	1.77488	1.59056
5309	1.77489	0.843303
5310	0.653228	0.7
5311	1.56043	0.702428
5312	1.60894	0.771637
5313	1.56043	0.689472
5314	1.06848	1.4523
5315	1.04307	2.1
5316	1.14188	2.1
5317	1.09195	2.1
5318	0.615121	2.1
5319	1.50867	2.1
5320	1.27152	2.1
5321	0.615094	1.66466
5322	1.07297	2.1
5323	1.1372	2.1
5324	0.553608	2.1
5325	1.23019	2.1
5326	0.992675	2.1
5327	1.40984	1.2992
5328	2.55682	2.1
5329	3.66069	2.1
5330	3.58726	2.1
5331	1.4375	0.699873
5332	1.88424	0.704789
5333	1.56714	2.1

5334	1.6721	2.1
5335	1.18474	2.1
5336	1.2295	2.1
5337	1.28754	2.1
5338	1.36838	2.1
5339	2.78661	2.1
5340	2.64055	2.1
5341	1.35065	1.14979
5342	2.26675	0.844563
5343	1.35065	0.985415
5344	1.21558	0.868983
5345	1.49709	0.0971167
5346	1.4971	0.0931417
5347	0.85472	0.15044
5348	0.854718	0.0904612
5349	0.946129	0.157964
5350	0.854717	0.143928
5351	1.1604	0.125711
5352	1.25265	0.130609
5353	2.32081	0.107106
5354	1.15371	0.0609476
5355	1.14945	0.827187
5356	0.995725	0.806617
5357	1.00307	0.732953
5358	1.10907	0.71257
5359	2.30315	0.71445
5360	1.10908	0.712562
5361	1.27558	0.952125
5362	1.71613	1.20814
5363	1.32977	2.1
5364	0.815276	2.1
5365	1.41697	2.1
5366	1.41697	2.1
5367	0.815276	2.1
5368	1.38897	2.1
5369	0.946131	2.1
5370	0.815275	2.1
5371	1.44684	2.1
5372	1.21489	1.561
5373	1.22476	1.47611
5374	0.870366	0.993993
5375	0.862572	0.930904
5376	0.835435	0.891434
5377	1.6993	0.877528
5378	1.23618	0.878067
5379	1.18624	0.882822
5380	1.53058	0.906587
5381	2.16108	1.0082
5382	2.21589	0.968178
5383	1.04037	1.47192
5384	1.03933	1.50628
5385	1.57753	0.362698
5386	1.87644	0.503695
5387	1.24728	0.664629
5388	1.30394	0.415944
5389	1.3538	2.1
5390	1.69858	2.1
5391	1.1726	0.633191

5392	0.94612	0.354902
5393	1.09805	0.26528
5394	0.946118	0.447579
5395	1.72576	0.412456
5396	1.72575	0.304302
5397	1.34152	0.321637
5398	1.33034	0.243286
5399	0.784151	1.27074
5400	0.946131	2.1
5401	0.784151	1.76013
5402	1.47963	2.1
5403	1.47963	2.1
5404	1.3335	2.1
5405	0.930281	2.1
5406	1.29435	1.18217
5407	0.883308	1.10122
5408	1.31589	2.1
5409	1.67846	2.1
5410	0.993598	0.268844
5411	2.23193	0.229442
5412	0.9936	0.166136
5413	0.615629	0.187728
5414	1.65925	2.1
5415	2.05992	2.1
5416	1.65925	2.1
5417	4.08735	1.30826
5418	2.72856	1.51177
5419	0.869602	2.1
5420	3.42833	1.00576
5421	1.14397	0.844475
5422	1.34563	0.700994
5423	1.08189	0.700075
5424	0.782128	1.83746
5425	1.0022	2.07218
5426	0.900427	1.82864
5427	1.51499	1.93413
5428	1.05588	1.86983
5429	1.13739	2.1
5430	1.37004	1.88854
5431	1.14603	2.08009
5432	0.851541	1.30948
5433	0.946799	1.86337
5434	1.14854	1.76846
5435	1.33793	1.40454
5436	0.94613	1.39802
5437	1.24336	1.51913
5438	0.851473	1.49422
5439	2.08792	1.68516
5440	1.01869	1.79734
5441	0.986953	0.442205
5442	0.896238	0.578682
5443	0.906574	0.653526
5444	0.852145	0.548791
5445	2.14191	0.488666
5446	1.15979	0.465383
5447	1.18327	0.516752
5448	2.32742	0.526666
5449	2.35961	0.648091

5450	2.38126	0.642737
5451	1.13632	0.549294
5452	1.17897	0.126497
5453	1.76199	0.0984716
5454	1.00573	1.47551
5455	0.946127	0.882243
5456	1.7077	1.30689
5457	1.7077	2.1
5458	0.946127	1.18043
5459	1.24887	2.1
5460	1.24887	1.6896
5461	1.22441	1.6799
5462	1.08542	1.62197
5463	1.21743	1.5256
5464	1.12572	2.1
5465	1.05499	0.97169
5466	1.00521	0.949787
5467	2.86191	0.0664116
5468	2.27972	0.0658446
5469	1.01169	2.1
5470	1.03373	2.1
5471	1.39319	2.1
5472	0.636837	1.99467
5473	1.63908	1.78933
5474	1.45713	1.81129
5475	1.04072	1.82623
5476	1.25025	1.82863
5477	1.38914	1.74209
5478	0.636807	1.53776
5479	1.50211	2.1
5480	1.36555	2.01902
5481	2.77545	1.27829
5482	0.682805	2.1
5483	1.50267	1.98187
5484	1.50267	2.1
5485	0.797749	2.1
5486	1.38773	1.86304
5487	1.38773	2.1
5488	0.682776	2.1
5489	2.01245	0.0876196
5490	0.925918	0.15072
5491	0.75689	0.0939729
5492	1.25823	0.0965342
5493	1.16505	0.144704
5494	0.838779	0.105556
5495	1.14722	0.107301
5496	0.756849	0.114877
5497	1.52298	0.140702
5498	2.11034	0.121018
5499	1.92174	0.11702
5500	1.92174	0.117183
5501	0.576083	0.212878
5502	1.23146	0.19736
5503	1.26637	0.153692
5504	1.23146	0.187074
5505	1.26637	0.154211
5506	0.946133	0.946803
5507	1.37034	0.908709

5508	1.84145	0.928671
5509	1.84145	0.850067
5510	0.946132	0.998886
5511	1.37034	0.922593
5512	1.41724	0.929414
5513	1.0685	0.83981
5514	1.14547	0.85524
5515	1.24976	2.1
5516	1.30682	2.1
5517	2.12095	2.1
5518	1.46392	2.1
5519	1.46392	2.1
5520	0.935738	2.1
5521	1.32949	2.1
5522	1.07017	2.1
5523	0.935766	2.1
5524	1.07017	2.1
5525	1.78095	2.1
5526	1.05619	0.828385
5527	1.05793	0.818672
5528	1.77677	2.1
5529	2.04766	2.1
5530	1.02524	2.1
5531	1.18803	2.1
5532	1.70047	2.1
5533	1.556 2.1	
5534	1.24201	0.918756
5535	1.3592	2.1
5536	2.11824	0.411235
5537	2.20248	0.649327
5538	3.43878	0.387752
5539	3.12466	0.308536
5540	2.09351	2.1
5541	0.946128	2.1
5542	1.51269	2.1
5543	1.51269	2.1
5544	0.946131	2.1
5545	1.01306	2.1
5546	1.04676	2.1
5547	1.01306	2.1
5548	1.07444	2.1
5549	2.17465	2.1
5550	1.14808	2.1
5551	1.39067	1.08276
5552	1.20958	1.05338
5553	0.662832	0.666667
5554	1.93512	0.7333
5555	1.15471	2.1
5556	1.75119	2.1
5557	1.75118	2.1
5558	0.946131	2.1
5559	1.28606	2.1
5560	1.27087	2.1
5561	0.946131	2.1
5562	1.28606	2.1
5563	1.58916	0.19087
5564	0.927822	0.505818
5565	2.24367	0.0994054

5566	1.8734	0.101324
5567	3.51668	0.0869638
5568	2.68986	0.0886268
5569	2.32395	2.1
5570	1.39564	2.1
5571	2.10862	2.1
5572	1.36763	2.1
5573	0.970963	0.09488
5574	0.97096	0.110629
5575	1.11897	0.404217
5576	1.26885	0.678999
5577	1.11605	0.484338
5578	2.06612	0.346814
5579	1.717	0.373259
5580	0.59502	0.652164
5581	0.825755	0.269953
5582	0.709624	0.736636
5583	1.65741	0.305953
5584	0.307486	0.370477
5585	1.4785	0.453114
5586	0.983143	2.1
5587	1.08247	2.1
5588	1.42353	2.1
5589	1.42353	2.1
5590	0.781667	2.1
5591	0.797634	2.1
5592	0.946131	2.1
5593	0.781638	2.1
5594	0.946131	2.1
5595	0.98315	2.1
5596	1.083	2.1
5597	1.61975	0.05
5598	1.11946	0.05
5599	1.18977	0.241787
5600	0.996484	0.25557
5601	2.24343	0.130009
5602	1.4787	0.15576
5603	1.66127	0.148518
5604	1.86028	0.222309
5605	1.09128	0.184993
5606	1.03115	0.304347
5607	0.838025	0.278512
5608	1.3736	0.134283
5609	1.44009	0.157014
5610	0.92917	0.149457
5611	1.03889	0.301967
5612	0.956974	0.185967
5613	3.23468	0.17934
5614	0.953922	0.341065
5615	0.957104	0.349948
5616	1.66231	0.166387
5617	1.26778	0.19997
5618	1.47615	0.195095
5619	1.28621	0.318151
5620	1.08043	0.249253
5621	2.35026	2.1
5622	2.35025	2.1
5623	2.60659	2.1

5624	1.57627	2.1
5625	1.5493	2.1
5626	1.24682	0.818689
5627	2.14277	0.823547
5628	1.24682	0.884477
5629	1.00307	0.779023
5630	2.04056	0.781431
5631	1.45408	2.1
5632	1.54249	2.1
5633	1.24474	1.20604
5634	1.37813	1.47608
5635	0.924158	0.315355
5636	0.916623	0.319153
5637	1.35344	0.335898
5638	1.00859	0.346632
5639	1.34786	0.350533
5640	0.952457	0.298068
5641	0.85404	0.297234
5642	1.06341	2.1
5643	1.09194	2.1
5644	1.93707	2.1
5645	2.14087	2.1
5646	1.93707	2.1
5647	1.07519	1.00755
5648	1.07519	0.937085
5649	1.62518	2.1
5650	1.3592	2.1
5651	1.13183	2.1
5652	1.20357	2.1
5653	1.4858	2.1
5654	1.23694	2.1
5655	1.44011	2.1
5656	1.23989	2.1
5657	2.21604	1.38105
5658	1.85574	0.438707
5659	1.55649	1.22244
5660	1.48861	0.293574
5661	1.15968	0.11671
5662	0.946132	0.135527
5663	0.663056	0.0995808
5664	1.70837	0.106325
5665	1.70837	0.106391
5666	0.946131	0.112826
5667	0.663026	0.106783
5668	2.09604	0.110204
5669	0.81094	0.112092
5670	1.06559	0.107187
5671	0.810956	0.102282
5672	1.15474	0.0925091
5673	0.990051	0.10154
5674	0.894971	0.0983133
5675	1.32536	0.0994803
5676	0.946133	2.1
5677	1.33125	2.1
5678	1.75823	2.1
5679	1.75823	2.1
5680	0.946133	2.1
5681	1.33125	2.1

5682	1.37312	2.1
5683	1.27985	2.1
5684	1.37312	2.1
5685	1.70684	2.1
5686	1.70684	2.1
5687	0.946132	2.1
5688	0.946132	2.1
5689	1.25081	2.1
5690	0.956293	2.1
5691	0.956294	2.1
5692	1.36035	2.1
5693	1.41944	2.1
5694	0.830638	2.1
5695	0.747568	2.1
5696	0.888498	2.1
5697	1.04071	2.1
5698	1.17128	2.1
5699	1.92339	2.1
5700	2.91208	2.1
5701	1.82208	0.871785
5702	1.89653	1.55589
5703	1.82588	1.2693
5704	1.26703	0.754526
5705	1.26703	1.12637
5706	0.6157	0.725175
5707	1.26706	1.21561
5708	0.61567	0.705266
5709	0.615671	0.71306
5710	1.26706	0.754884
5711	1.89158	1.03297
5712	1.23134	0.720579
5713	2.49497	0.171579
5714	1.68103	0.112944
5715	2.0554	2.1
5716	1.72647	2.1
5717	1.67375	2.1
5718	1.65037	2.1
5719	1.04349	0.739743
5720	1.69055	0.358528
5721	1.53463	0.277296
5722	1.19022	2.04864
5723	1.89226	1.54142
5724	1.15009	0.915951
5725	1.7054	1.11062
5726	1.31612	0.310101
5727	1.31611	0.647687
5728	1.67345	0.897342
5729	1.06577	0.714904
5730	1.40193	0.521735
5731	1.72411	0.441336
5732	1.46123	0.472449
5733	0.808808	0.699855
5734	0.897714	0.527709
5735	0.851554	0.738513
5736	1.3499	0.517422
5737	1.29001	0.494409
5738	1.22083	2.1
5739	1.06191	2.1

5740	1.05758	1.23467
5741	1.348	1.72951
5742	1.43485	1.18826
5743	1.34799	1.65435
5744	1.36307	0.084561
5745	2.28188	0.36927
5746	1.7926	0.325616
5747	1.38957	2.1
5748	1.47446	2.1
5749	0.953638	2.1
5750	1.11868	2.02826
5751	0.953636	2.1
5752	1.03408	2.1
5753	1.52443	0.112401
5754	1.38539	0.112486
5755	0.946132	0.146454
5756	1.12747	0.126491
5757	1.04393	0.176782
5758	1.05017	0.14299
5759	1.20529	0.136337
5760	1.3663	0.728616
5761	1.89226	0.716533
5762	1.17885	0.739717
5763	0.867881	0.702238
5764	1.32917	0.700061
5765	1.32917	0.7
5766	0.946127	0.707234
5767	1.19583	0.702793
5768	1.00122	0.7
5769	0.946128	0.703667
5770	1.71405	0.76624
5771	0.871815	1.51574
5772	1.16435	2.1
5773	0.686619	2.1
5774	1.63102	2.1
5775	1.06589	1.99531
5776	1.05375	2.1
5777	1.9023	2.1
5778	1.27394	0.917254
5779	1.88345	0.884069
5780	2.05636	0.904635
5781	0.672977	0.887483
5782	1.41274	0.885121
5783	1.65606	0.886938
5784	0.869329	0.889941
5785	1.2207	0.890281
5786	1.35631	0.893197
5787	0.908776	0.886963
5788	0.955081	0.856573
5789	1.2243	2.1
5790	1.23169	2.1
5791	1.52228	2.1
5792	1.23169	2.1
5793	0.990892	0.865391
5794	1.08376	0.750198
5795	1.79348	1.28323
5796	1.50043	0.805169
5797	1.32599	1.62623

5798	1.37924	0.815021
5799	0.838014	0.741561
5800	0.87737	1.0028
5801	0.947086	0.823469
5802	1.27199	1.05401
5803	1.04918	0.806377
5804	1.04851	1.35901
5805	1.1699	0.929818
5806	0.810934	0.952596
5807	1.83602	1.74051
5808	2.21909	1.60441
5809	0.619441	0.932984
5810	2.03755	0.855757
5811	1.83472	1.44302
5812	1.20416	1.15274
5813	1.26691	0.809588
5814	0.655795	0.954151
5815	0.94613	0.892687
5816	2.35169	1.13249
5817	3.54705	2.1
5818	2.60741	2.1
5819	1.59019	0.0989942
5820	1.24526	0.0947223
5821	1.59019	0.108079
5822	2.36406	0.0948404
5823	1.16776	0.907992
5824	1.67311	0.996757
5825	1.67311	0.917718
5826	0.94613	0.81073
5827	1.38645	0.938999
5828	1.13563	0.73455
5829	1.13563	0.817073
5830	1.23279	0.772684
5831	1.05042	1.50364
5832	1.17418	1.01612
5833	1.28909	2.1
5834	0.973448	2.1
5835	1.02958	2.1
5836	3.3005	2.1
5837	2.86325	2.1
5838	1.35879	2.1
5839	1.69332	2.1
5840	1.69332	2.1
5841	0.946128	2.1
5842	1.39659	2.05753
5843	1.12035	2.1
5844	1.12035	2.1
5845	1.24286	2.1
5846	1.10407	2.1
5847	1.14599	1.35753
5848	1.32259	0.907819
5849	0.788592	0.104369
5850	2.4588	2.1
5851	0.909581	1.93581
5852	1.35656	1.38002
5853	0.986895	1.68623
5854	2.30602	1.39844
5855	1.46873	1.71189

5856	1.55584	1.35882
5857	2.1547	1.42107
5858	1.87794	1.99677
5859	0.808286	1.96436
5860	0.928464	1.63881
5861	0.810959	1.62304
5862	1.50962	1.25428
5863	1.1053	1.48452
5864	1.0712	1.43243
5865	1.40504	1.41819
5866	1.87794	1.30047
5867	2.80496	1.5203
5868	1.41479	1.83192
5869	1.38634	2.1
5870	1.38634	2.1
5871	1.67762	2.1
5872	0.946131	2.1
5873	1.188 2.1	
5874	1.62585	2.1
5875	1.62585	2.1
5876	0.946129	2.1
5877	1.18801	2.1
5878	1.38397	2.1
5879	1.04546	2.1
5880	1.16817	2.1
5881	1.06007	2.1
5882	1.19785	2.1
5883	1.43652	2.1
5884	1.06994	2.1
5885	1.89111	1.6219
5886	1.07144	2.1
5887	1.51754	2.1
5888	2.0873	2.1
5889	0.758799	2.1
5890	1.59518	2.1
5891	1.59518	2.1
5892	0.946132	2.1
5893	0.758771	2.1
5894	1.06957	2.1
5895	0.758771	2.1
5896	1.33916	2.1
5897	2.03045	1.13032
5898	2.80184	1.31463
5899	1.76788	1.76567
5900	1.27442	1.75004
5901	1.06756	2.1
5902	0.966979	2.1
5903	1.7054	2.1
5904	1.07156	2.1
5905	0.986096	2.1
5906	1.32813	2.1
5907	1.3331	2.1
5908	2.08369	0.731989
5909	2.23818	1.33646
5910	1.10335	0.810633
5911	0.622314	1.07438
5912	1.72451	0.765043
5913	1.72451	1.20014

5914	0.622283	0.758206
5915	0.622314	0.757032
5916	0.946131	0.745945
5917	1.31254	1.71942
5918	1.15704	1.85765
5919	1.24463	0.789473
5920	2.2067	0.983453
5921	1.30001	0.923398
5922	1.29175	0.963625
5923	1.1233	0.0897846
5924	1.8689	0.149323
5925	1.29494	0.122732
5926	1.11895	0.109332
5927	0.851539	0.146645
5928	0.851474	0.134141
5929	1.58352	0.109788
5930	1.58228	0.104878
5931	0.851527	0.168191
5932	2.6389	0.115782
5933	1.77548	0.216909
5934	1.24555	2.1
5935	2.0512	2.1
5936	1.46101	2.1
5937	2.60635	2.1
5938	1.10476	0.388563
5939	1.44103	0.340341
5940	0.652391	2.1
5941	0.618322	2.1
5942	2.22915	2.1
5943	1.39067	2.1
5944	1.99737	2.1
5945	1.42174	2.1
5946	0.946132	0.862983
5947	1.29735	1.24512
5948	1.61765	2.1
5949	1.61765	0.977341
5950	0.946132	1.56218
5951	1.29735	0.966113
5952	1.26643	0.959808
5953	1.01335	1.11308
5954	1.01335	0.76297
5955	0.947669	2.1
5956	2.17167	2.1
5957	2.59733	1.84728
5958	1.50954	1.96982
5959	1.02638	1.35041
5960	0.555785	2.05709
5961	1.91101	2.03071
5962	2.09809	1.76631
5963	0.641974	1.86435
5964	1.2022	1.69547
5965	1.36359	1.53198
5966	1.8128	2.03079
5967	2.34348	0.711672
5968	1.66343	0.702917
5969	0.974717	0.894975
5970	1.53908	0.838772
5971	1.27737	1.11869

5972	1.09408	0.979005
5973	2.07382	0.966668
5974	1.10466	1.25426
5975	1.05654	1.72961
5976	1.23498	1.30596
5977	1.48104	1.48906
5978	1.23498	1.42235
5979	1.17261	2.1
5980	1.18059	2.1
5981	2.43005	2.1
5982	1.04546	2.1
5983	2.81222	2.1
5984	1.16817	2.1
5985	1.75766	0.749139
5986	1.47021	0.791294
5987	1.07236	0.732238
5988	1.81759	0.86005
5989	1.42913	0.834301
5990	1.00636	0.882943
5991	1.00636	0.777013
5992	1.79397	0.793304
5993	0.946131	0.81432
5994	1.03691	0.858569
5995	0.851539	0.806535
5996	1.46001	0.875996
5997	1.37878	0.893038
5998	0.933258	0.819193
5999	1.32423	0.836698
6000	1.32614	0.0524867
6001	1.11356	0.0653372
6002	1.32614	0.0527913
6003	1.28895	0.053295
6004	1.66895	0.05
6005	1.66895	0.05
6006	0.946132	0.056335
6007	1.29576	0.05
6008	1.1318	0.0516093
6009	1.37621	1.34654
6010	1.94071	1.25043
6011	1.10517	2.1
6012	1.97098	2.1
6013	0.811 2.1	
6014	0.956127	2.1
6015	1.18647	2.00623
6016	0.988455	1.26821
6017	1.12457	0.325057
6018	1.7453	0.305204
6019	0.92492	2.1
6020	1.24353	2.1
6021	1.84536	0.934005
6022	1.67662	0.875831
6023	1.38247	0.147829
6024	1.02663	0.12429
6025	0.946132	0.906228
6026	1.41723	0.874876
6027	1.72976	0.902159
6028	1.72976	0.820042
6029	0.946131	1.00009

6030	1.41723	0.905347
6031	1.25866	0.898322
6032	1.34254	0.784991
6033	1.25866	0.851742
6034	1.65507	0.79635
6035	1.65507	0.746943
6036	0.946132	0.920856
6037	0.946132	0.836911
6038	1.11929	0.838459
6039	0.961779	0.782933
6040	0.961779	0.796636
6041	0.946132	2.1
6042	1.69361	2.1
6043	1.69361	2.1
6044	0.94613	2.1
6045	1.36523	2.1
6046	1.36523	2.1
6047	1.19802	2.1
6048	0.974196	2.1
6049	1.09518	2.1
6050	0.946131	2.1
6051	1.7161	2.1
6052	1.7161	2.1
6053	0.94613	2.1
6054	1.297 2.1	
6055	1.297 2.1	
6056	1.14599	2.1
6057	0.974197	2.1
6058	0.974197	2.1
6059	1.80778	2.1
6060	2.20473	2.1
6061	1.80777	2.1
6062	1.3515	2.1
6063	0.583237	2.1
6064	1.39359	2.1
6065	1.39359	2.1
6066	1.07296	2.1
6067	0.946114	2.1
6068	1.07297	2.1
6069	0.903862	2.1
6070	0.956572	2.1
6071	1.03437	2.1
6072	0.975409	2.1
6073	1.31167	0.456377
6074	2.70246	0.376617
6075	1.12018	0.433575
6076	0.655836	0.474213
6077	0.655837	0.428126
6078	1.81557	0.241484
6079	1.81557	0.234086
6080	0.655836	0.408806
6081	0.946131	0.482202
6082	1.01847	0.33677
6083	1.61334	0.283058
6084	0.810946	0.508856
6085	0.8311	0.54288
6086	1.2466	0.33653
6087	1.20286	0.383418

6088	0.97508	0.540403
6089	0.870805	0.365453
6090	0.760087	0.558254
6091	0.853389	2.1
6092	0.820894	2.1
6093	0.928291	2.1
6094	1.06922	2.1
6095	1.32829	2.1
6096	1.12173	2.1
6097	1.16615	2.1
6098	0.957748	2.1
6099	0.612491	1.85723
6100	0.548141	2.1
6101	0.979614	2.1
6102	0.658184	2.1
6103	0.678456	1.57446
6104	0.529544	1.71758
6105	0.739013	2.1
6106	0.694037	2.08948
6107	0.475716	1.7468
6108	1.58794	1.54127
6109	1.58794	1.30471
6110	0.475743	1.39791
6111	1.08301	1.99807
6112	0.980664	2.1
6113	0.475737	1.86963
6114	1.32709	2.1
6115	1.59396	2.1
6116	1.19719	0.471112
6117	1.34921	0.461414
6118	1.69061	0.473698
6119	1.69061	0.332121
6120	0.946132	0.510715
6121	1.34921	0.504623
6122	1.28753	0.492443
6123	0.95895	0.329774
6124	1.01051	0.389321
6125	1.12468	0.412897
6126	1.22588	0.595152
6127	1.34254	0.528679
6128	1.74561	0.522494
6129	1.74561	0.421344
6130	0.946128	0.61137
6131	1.34253	0.571001
6132	1.3492	0.56427
6133	1.01051	0.3218
6134	1.922	2.1
6135	1.922	2.1
6136	2.12556	2.1
6137	0.946131	2.1
6138	1.06278	2.1
6139	1.47631	2.1
6140	1.47631	2.1
6141	0.946129	2.1
6142	1.06278	2.1
6143	1.35966	2.1
6144	0.991985	2.1
6145	0.738507	1.57105

6146	0.536935	1.85595
6147	0.799004	1.67542
6148	1.04281	1.49043
6149	0.879197	1.3676
6150	1.06538	1.47221
6151	0.715942	1.58004
6152	1.00636	0.797617
6153	1.03824	0.704276
6154	1.70147	0.747731
6155	1.21223	2.1
6156	1.01243	1.39591
6157	1.01242	2.1
6158	1.06935	2.1
6159	1.54241	2.1
6160	0.998971	0.0762359
6161	1.60312	0.0960384
6162	1.44836	0.0755241
6163	2.05632	0.0999718
6164	1.44836	0.0742217
6165	0.973765	0.0919491
6166	0.946131	0.111753
6167	0.851541	0.106772
6168	1.73854	0.094431
6169	1.91752	0.08549
6170	1.49622	0.105466
6171	1.23351	0.108458
6172	3.33289	2.1
6173	1.86606	2.1
6174	2.71233	2.1
6175	0.788816	2.1
6176	0.963972	2.1
6177	1.25866	2.1
6178	1.10817	2.1
6179	1.41908	2.1
6180	1.48734	2.1
6181	1.40822	0.734666
6182	1.40822	0.729746
6183	2.09871	0.78437
6184	0.946133	0.728065
6185	1.14926	0.656247
6186	0.946133	0.732263
6187	1.78098	0.708093
6188	1.78098	0.692708
6189	1.35822	0.719822
6190	1.3689	0.722326
6191	1.22176	0.7
6192	2.03426	0.716927
6193	0.946131	0.751293
6194	1.01713	0.715191
6195	0.704112	0.754405
6196	1.36237	0.722429
6197	1.36237	0.709371
6198	1.01713	0.747302
6199	1.04936	0.747752
6200	1.87377	2.1
6201	1.60501	2.1
6202	0.981584	0.106486
6203	1.69435	0.0977728

6204	0.85154	2.1
6205	1.47841	2.1
6206	1.4952	2.1
6207	1.31941	2.1
6208	1.04023	2.1
6209	1.1995	2.1
6210	0.914791	2.1
6211	1.1427	1.22804
6212	1.64787	1.02669
6213	1.07526	0.771279
6214	1.07527	0.797817
6215	1.07527	0.754206
6216	1.58551	0.845303
6217	2.77362	1.23792
6218	2.17974	0.971728
6219	2.71723	1.08566
6220	1.29868	0.610093
6221	1.3594	0.439252
6222	1.04786	0.480272
6223	0.527876	0.39486
6224	1.4085	0.608696
6225	1.48538	0.272661
6226	0.76829	0.344571
6227	1.18514	0.601973
6228	0.617954	0.267509
6229	0.864161	0.375657
6230	1.18835	0.254599
6231	1.16817	2.1
6232	1.04263	2.1
6233	1.50524	2.1
6234	1.85355	2.1
6235	1.23963	2.1
6236	1.06279	2.1
6237	0.94613	2.1
6238	1.35229	2.1
6239	1.15947	2.1
6240	0.965593	2.1
6241	0.851553	2.1
6242	2.21296	2.1
6243	1.11764	2.1
6244	2.46399	2.1
6245	1.39292	2.1
6246	1.34701	2.00437
6247	1.34701	2.1
6248	1.47001	2.1
6249	0.963166	0.0949952
6250	0.893445	0.0956296
6251	1.21056	0.0878971
6252	1.67375	2.1
6253	2.0977	2.1
6254	1.09154	2.1
6255	1.09154	2.1
6256	1.57122	0.936353
6257	1.60976	0.739356
6258	1.69533	0.716194
6259	1.6951	0.705109
6260	1.5831	0.701008
6261	1.20969	2.1

6262	1.12076	2.1
6263	1.49808	2.1
6264	1.50201	2.1
6265	1.00278	2.1
6266	1.22811	0.674646
6267	1.11545	2.1
6268	1.09646	2.1
6269	1.03107	0.05
6270	1.44293	0.266517
6271	1.66752	1.12082
6272	1.11023	1.13834
6273	2.57831	1.6701
6274	2.20191	1.1588
6275	2.18051	1.90119
6276	1.48035	0.740961
6277	1.0961	0.668331
6278	1.63241	0.596729
6279	1.0961	0.731298
6280	1.20959	2.1
6281	2.86136	2.1
6282	1.64933	2.1
6283	1.47217	2.1
6284	0.915178	2.1
6285	1.19074	2.1
6286	0.897276	2.1
6287	0.915177	2.1
6288	0.987037	2.1
6289	1.04288	2.1
6290	2.39323	2.1
6291	0.976218	0.095846
6292	2.23933	0.115552
6293	0.976215	0.0965076
6294	1.07384	0.0964767
6295	1.07652	2.1
6296	1.55678	2.1
6297	1.54168	2.1
6298	1.69136	1.95056
6299	1.64391	1.45248
6300	1.76477	1.18503
6301	0.611486	1.43724
6302	0.683421	1.07317
6303	0.946131	1.11377
6304	1.1019	1.17935
6305	1.12946	1.05995
6306	1.54226	1.76675
6307	1.19496	1.33708
6308	1.96199	1.14826
6309	2.0775	1.68166
6310	1.22297	1.26486
6311	1.3469	0.967851
6312	1.25366	1.06783
6313	1.65443	1.07544
6314	1.65443	1.06423
6315	0.946132	1.24299
6316	0.946132	1.35677
6317	1.12873	1.15932
6318	1.07468	1.02916
6319	1.2854	0.840671

6320	1.3469	0.983192
6321	1.68616	0.961089
6322	1.68616	0.885339
6323	0.946132	1.16474
6324	0.946132	1.05936
6325	1.16109	1.01819
6326	1.17879	0.943299
6327	1.17879	0.945132
6328	1.45304	1.09852
6329	2.21058	2.1
6330	2.81552	2.1
6331	1.45535	0.609866
6332	1.49906	0.456104
6333	1.04413	2.1
6334	1.74586	2.1
6335	1.34526	2.1
6336	1.17402	1.06222
6337	1.74564	0.981
6338	1.52724	2.1
6339	1.68142	2.1
6340	1.09651	2.1
6341	1.49149	2.1
6342	1.36814	2.1
6343	1.46783	1.18272
6344	2.18116	1.39757
6345	1.23602	1.11739
6346	1.74538	0.968393
6347	0.851539	2.1
6348	1.45775	1.21677
6349	1.28919	2.1
6350	0.851483	2.1
6351	1.26102	1.20725
6352	0.869889	2.1
6353	0.85151	2.1
6354	1.23451	1.00312
6355	1.23602	1.14868
6356	1.11955	2.1
6357	1.08022	2.1
6358	0.977776	2.1
6359	1.27896	2.1
6360	1.64268	2.1
6361	1.97548	2.1
6362	1.72527	1.65456
6363	0.946132	2.1
6364	1.69702	1.4956
6365	1.69702	2.1
6366	1.13635	2.1
6367	1.40274	1.18669
6368	1.24041	2.1
6369	1.13635	2.1
6370	1.78427	0.17506
6371	1.68338	0.249926
6372	1.00681	0.106614
6373	1.12988	0.103969
6374	0.946132	0.122858
6375	0.957543	0.114325
6376	1.40515	0.107959
6377	1.40515	0.111838

6378	0.797932	0.139405
6379	1.30187	0.168475
6380	1.14846	0.145848
6381	0.665137	0.638014
6382	1.09575	0.767338
6383	0.596894	0.707547
6384	1.04072	0.796094
6385	0.663171	0.817657
6386	1.47218	0.794595
6387	1.32889	0.734741
6388	1.32639	0.387808
6389	1.94887	0.426065
6390	1.32639	0.39893
6391	1.19323	0.591641
6392	2.19794	0.696006
6393	0.754756	0.375146
6394	1.11015	0.432018
6395	0.754755	0.376752
6396	0.946133	0.431127
6397	0.754768	0.480805
6398	1.59986	0.440625
6399	1.59986	0.343491
6400	1.84082	2.1
6401	1.79044	2.1
6402	1.75922	2.1
6403	1.86766	2.1
6404	1.58995	2.1
6405	1.58995	2.1
6406	1.74351	2.1
6407	1.04442	2.1
6408	1.42278	2.1
6409	1.08576	2.1
6410	0.994294	2.1
6411	1.44884	1.34571
6412	1.71036	1.08873
6413	0.714101	0.852346
6414	1.17541	1.18124
6415	1.00872	0.764387
6416	0.714072	0.901914
6417	0.936677	0.866333
6418	0.966979	2.1
6419	1.4908	2.1
6420	1.03108	0.733904
6421	0.949712	0.699959
6422	0.810946	0.72181
6423	0.88209	0.72034
6424	0.646702	0.70427
6425	0.972663	0.7
6426	0.862301	0.699374
6427	0.868143	0.691203
6428	1.11094	0.703323
6429	1.35809	0.701446
6430	1.32455	2.1
6431	1.32328	2.1
6432	1.69586	2.1
6433	1.78301	2.1
6434	1.67906	2.1
6435	1.60635	2.1

6436	1.57234	2.1
6437	1.47012	2.1
6438	1.22811	0.448245
6439	1.08732	0.65525
6440	2.08849	0.555966
6441	1.08732	0.613104
6442	1.30635	0.0938386
6443	0.77315	0.0859793
6444	1.25732	0.0742008
6445	2.43845	2.1
6446	2.38577	1.16796
6447	0.946132	0.936243
6448	1.19288	2.1
6449	1.54508	2.1
6450	1.54508	1.27813
6451	0.94515	1.98507
6452	1.19288	1.52602
6453	1.29735	1.11244
6454	1.12087	0.899139
6455	1.54699	1.07059
6456	1.43635	2.1
6457	1.33385	2.1
6458	1.43635	2.1
6459	1.02368	2.1
6460	1.035 2.1	
6461	1.00128	2.1
6462	1.04009	2.1
6463	1.14052	2.1
6464	0.443552	2.1
6465	0.984351	2.1
6466	0.591443	2.1
6467	0.929561	2.1
6468	1.52079	2.1
6469	1.035 2.1	
6470	3.5282	1.95466
6471	1.88288	1.95448
6472	2.73877	1.19045
6473	0.906059	2.1
6474	1.23107	0.301987
6475	0.906712	0.390637
6476	0.841322	0.443883
6477	1.53005	0.304985
6478	1.10007	0.37608
6479	1.38033	0.390886
6480	1.03442	0.302183
6481	0.787223	0.338558
6482	1.42589	0.327366
6483	2.35665	0.352934
6484	1.17793	0.538624
6485	1.81356	0.595223
6486	1.42964	0.479841
6487	1.95641	0.568277
6488	1.39139	0.701409
6489	1.39139	0.708146
6490	0.564533	0.7
6491	1.20016	0.706739
6492	0.755765	0.7
6493	0.755766	0.7

6494	1.20016	0.700711
6495	1.91011	0.7
6496	1.4695	0.7
6497	1.70048	2.1
6498	1.93765	1.82984
6499	0.741307	2.1
6500	0.780371	2.1
6501	0.946133	0.797672
6502	1.28918	1.07848
6503	1.5111	1.72292
6504	1.5111	0.837252
6505	0.946131	1.18567
6506	1.28918	0.847733
6507	1.16805	0.806712
6508	1.01179	0.787356
6509	0.946132	0.834786
6510	1.35328	1.15038
6511	1.69633	1.60063
6512	1.69633	0.880092
6513	0.946133	1.25979
6514	1.35328	0.889752
6515	1.28918	0.848584
6516	1.01179	0.913054
6517	1.58147	0.939509
6518	1.27737	0.844739
6519	2.17223	0.270346
6520	0.697168	0.276294
6521	1.62495	0.429438
6522	1.62495	0.530869
6523	1.93948	0.712301
6524	0.601592	2.1
6525	1.93528	2.1
6526	2.13757	2.1
6527	1.67561	2.1
6528	2.70875	2.1
6529	1.93528	2.1
6530	3.98475	2.1
6531	1.12376	2.1
6532	1.19016	2.1
6533	1.67323	0.440362
6534	1.74019	0.433077
6535	1.84719	0.432684
6536	1.00764	0.382517
6537	1.23475	2.1
6538	1.06757	2.1
6539	1.57916	1.90362
6540	1.06758	2.1
6541	1.28014	2.1
6542	1.33339	2.1
6543	1.2679	2.1
6544	1.23504	2.1
6545	1.50108	2.1
6546	1.3715	2.1
6547	0.58831	0.972645
6548	0.865971	0.964457
6549	0.813704	1.1062
6550	0.658856	1.00793
6551	0.495869	1.10469

6552	1.13845	1.02288
6553	0.780804	1.0458
6554	0.642023	1.06741
6555	0.707748	0.954295
6556	1.64476	0.919681
6557	1.64476	1.00334
6558	0.99611	0.964774
6559	1.35639	0.90724
6560	1.3564	0.977544
6561	0.946129	0.965339
6562	1.99222	0.949891
6563	1.4777	2.1
6564	1.03968	2.1
6565	1.82516	2.1
6566	0.851539	2.1
6567	1.63693	2.1
6568	1.59416	2.1
6569	0.878566	2.1
6570	1.40796	2.1
6571	1.13172	2.0509
6572	0.851527	1.24059
6573	1.14366	2.1
6574	0.823822	0.737394
6575	0.882001	0.73939
6576	1.09756	0.345338
6577	1.11857	0.546856
6578	1.0934	0.519141
6579	0.960104	0.732503
6580	0.982001	0.762268
6581	1.74319	0.690246
6582	1.03043	0.689186
6583	0.967194	0.589401
6584	1.16938	2.1
6585	2.48377	2.1
6586	1.1181	2.1
6587	2.57349	2.1
6588	1.0465	2.1
6589	1.44709	2.1
6590	0.946132	2.1
6591	1.29302	2.1
6592	1.73502	2.1
6593	1.73502	2.1
6594	0.946132	2.1
6595	1.29302	2.1
6596	1.38813	2.1
6597	1.03933	2.1
6598	1.01976	0.753869
6599	1.11764	0.790799
6600	0.961779	0.861956
6601	1.0685	0.803196
6602	1.08426	1.15999
6603	0.99695	1.28337
6604	2.25593	1.41453
6605	1.47992	1.69732
6606	1.88989	1.12018
6607	1.65813	1.11926
6608	1.20278	1.20071
6609	0.981842	1.23395

6610	0.802655	1.9318
6611	0.953489	1.88885
6612	0.961719	1.74777
6613	0.975614	1.6238
6614	0.991617	1.25047
6615	0.940748	1.32643
6616	2.25019	1.91503
6617	2.30712	1.24496
6618	1.98823	1.72537
6619	0.522844	1.24717
6620	0.946122	1.7025
6621	0.946132	1.34641
6622	0.593846	1.22148
6623	1.26501	1.21582
6624	1.21171	0.206604
6625	0.851542	0.317947
6626	0.940327	0.419593
6627	0.940356	0.437235
6628	1.52117	0.287891
6629	1.58072	0.216397
6630	1.35169	0.311431
6631	1.13889	0.274351
6632	1.35007	0.180657
6633	1.35007	0.172977
6634	1.78381	0.21792
6635	0.946132	0.325832
6636	1.06627	0.332451
6637	0.946131	0.452882
6638	1.73005	0.264075
6639	1.73005	0.247544
6640	1.32449	0.307675
6641	1.35169	0.324361
6642	1.42446	0.805833
6643	1.86697	0.78668
6644	1.13413	0.792465
6645	1.42446	0.75398
6646	1.33575	0.54149
6647	1.20473	0.559158
6648	1.87517	0.137511
6649	1.87517	0.145358
6650	0.488712	0.293178
6651	1.13242	0.271492
6652	0.488713	0.344232
6653	0.488713	0.25348
6654	1.23146	0.196655
6655	1.57932	0.13842
6656	1.01698	0.137084
6657	1.42085	0.135378
6658	0.246725	0.113639
6659	0.996283	0.125244
6660	1.36965	0.156676
6661	0.86361	0.279244
6662	0.810945	0.135931
6663	1.72938	0.155069
6664	1.21649	0.27772
6665	1.1865	0.141085
6666	1.62387	0.157313
6667	1.05302	0.174591

6668	1.10049	0.842814
6669	0.946129	2.1
6670	1.70184	0.998127
6671	1.70184	2.1
6672	1.12538	2.1
6673	1.35969	0.946936
6674	1.28828	1.64098
6675	1.12538	1.2069
6676	0.99747	1.33198
6677	1.34851	0.918939
6678	0.923724	2.1
6679	1.47154	1.03001
6680	1.47154	2.1
6681	1.10698	2.1
6682	1.28828	0.977138
6683	1.10698	2.07073
6684	0.923695	1.63661
6685	0.99747	0.832934
6686	1.65677	2.1
6687	1.77677	2.1
6688	1.05689	0.710597
6689	2.11031	0.653824
6690	1.07367	0.425378
6691	0.656239	0.688458
6692	1.4563	0.616075
6693	1.4563	0.570902
6694	0.656238	0.67057
6695	0.946133	0.698059
6696	0.812937	0.727962
6697	0.946131	0.731045
6698	1.05689	0.45514
6699	1.34009	2.1
6700	1.398 2.1	
6701	3.29047	2.1
6702	2.53897	2.1
6703	1.24509	2.1
6704	3.06162	2.1
6705	1.01595	2.1
6706	1.52661	2.1
6707	1.5266	2.1
6708	0.946131	2.1
6709	1.31049	2.1
6710	1.16225	2.1
6711	1.05978	2.1
6712	1.16225	2.1
6713	1.06561	1.66982
6714	0.953927	2.1
6715	1.15114	1.44528
6716	1.1807	0.777357
6717	0.507259	0.736686
6718	1.47709	0.70455
6719	1.36192	0.64974
6720	0.487293	0.666485
6721	1.65569	0.793045
6722	0.679725	0.560295
6723	0.721928	0.686606
6724	1.03931	0.646599
6725	1.0775	0.810681

6726	1.0775	0.863563
6727	2.30207	0.954763
6728	1.01814	0.904513
6729	2.33963	0.882073
6730	2.42589	0.931885
6731	1.33962	2.1
6732	1.27956	2.1
6733	2.14624	0.139211
6734	1.6812	0.159803
6735	1.25698	0.15479
6736	1.35725	0.170306
6737	1.86387	0.140551
6738	1.86387	0.133652
6739	0.750365	0.162419
6740	0.946128	0.154061
6741	0.750365	0.132557
6742	1.72271	0.123651
6743	1.72272	0.222162
6744	2.53736	0.223354
6745	1.07327	2.1
6746	1.05376	2.1
6747	1.00387	1.08926
6748	2.0554	1.07928
6749	1.00387	1.02636
6750	1.00387	0.989419
6751	1.27119	2.1
6752	1.51748	2.1
6753	3.15271	2.1
6754	3.45801	2.1
6755	1.99871	2.1
6756	1.41186	2.1
6757	1.07624	2.1
6758	1.5954	2.1
6759	1.19323	0.964472
6760	1.21286	0.900077
6761	0.946114	2.1
6762	1.38395	2.1
6763	1.73389	2.1
6764	1.7339	2.1
6765	0.946115	2.1
6766	1.38397	2.1
6767	1.29604	2.1
6768	1.04544	2.1
6769	1.60578	0.231901
6770	1.93407	0.215122
6771	1.0525	0.931177
6772	1.0525	1.16099
6773	1.0525	1.45295
6774	1.38506	1.56347
6775	2.34737	0.0975018
6776	1.3531	0.0874539
6777	1.2925	0.0820895
6778	0.871466	0.0990928
6779	1.26623	0.0932423
6780	0.700631	0.0974716
6781	1.88774	0.0921754
6782	1.14167	0.0950577
6783	1.18606	0.0927038

6784	2.1238	0.0928322
6785	1.42762	0.974048
6786	1.67791	0.440382
6787	1.47118	0.406209
6788	0.946128	2.1
6789	1.31641	2.1
6790	1.75933	2.1
6791	1.75933	2.1
6792	0.946131	2.1
6793	1.31641	2.1
6794	1.38905	2.1
6795	1.38437	2.1
6796	1.51829	2.1
6797	0.947114	2.1
6798	1.13335	2.1
6799	1.47521	2.1
6800	1.14911	2.1
6801	1.53822	2.1
6802	1.39702	2.1
6803	0.739166	2.1
6804	0.706056	2.1
6805	0.964033	2.1
6806	1.58993	1.98284
6807	2.51892	1.64199
6808	0.595905	0.27021
6809	1.18155	0.152479
6810	1.73364	0.145293
6811	0.952735	0.179931
6812	2.10381	0.13704
6813	1.12115	0.160466
6814	1.27826	0.154489
6815	2.74021	0.135794
6816	1.78416	2.1
6817	1.78416	2.1
6818	0.946132	2.1
6819	1.41126	2.1
6820	1.21275	2.1
6821	0.946133	2.1
6822	1.41126	2.1
6823	1.33125	2.1
6824	1.33125	2.1
6825	1.78918	2.1
6826	1.0465	2.1
6827	1.99715	2.1
6828	1.17683	2.1
6829	1.25889	2.1
6830	0.860834	0.532479
6831	0.946131	0.401535
6832	1.05008	0.31026
6833	0.970444	0.490838
6834	1.19401	0.464275
6835	1.25989	0.294469
6836	0.702942	0.688575
6837	1.40591	0.640203
6838	1.72168	0.648994
6839	0.446352	0.691232
6840	1.04072	0.49728
6841	0.711038	0.7

6842	0.980737	0.692661
6843	1.05099	0.616761
6844	1.26003	0.450502
6845	0.495934	0.7
6846	1.03114	0.387554
6847	1.09911	0.550565
6848	1.24825	0.390862
6849	2.19827	0.355199
6850	2.0953	0.204434
6851	2.30437	0.290909
6852	0.775923	0.248695
6853	2.2283	0.158229
6854	1.19583	2.1
6855	1.14496	2.1
6856	1.62183	2.1
6857	1.62316	2.1
6858	1.02305	0.73729
6859	1.21136	0.55429
6860	1.44426	0.713616
6861	1.21137	0.519212
6862	1.20143	0.685686
6863	1.15009	0.523321
6864	1.24965	2.1
6865	1.41077	2.1
6866	1.00052	0.302001
6867	1.79553	0.289822
6868	1.06336	0.171978
6869	1.00053	0.211067
6870	1.18041	2.1
6871	1.80818	1.22685
6872	1.73571	1.13556
6873	1.94429	1.26726
6874	1.4375	0.418709
6875	1.44421	0.340137
6876	1.66953	0.468516
6877	1.09529	0.413015
6878	0.952395	2.1
6879	0.717337	2.1
6880	1.03609	2.1
6881	1.33949	0.802167
6882	1.2774	0.719712
6883	2.24983	0.693457
6884	1.28727	0.710076
6885	0.583592	0.702053
6886	0.583591	0.711361
6887	1.82859	0.718154
6888	1.82859	0.725308
6889	0.583593	0.728109
6890	0.946128	0.692829
6891	0.692233	0.749546
6892	1.12226	0.732089
6893	2.57454	0.727882
6894	0.810944	0.719334
6895	0.810945	0.703843
6896	1.12226	0.726568
6897	1.45867	0.727424
6898	1.22403	0.711083
6899	1.15262	0.72245

6900	1.08132	0.720509
6901	0.846671	0.703069
6902	0.480954	0.736353
6903	0.917975	0.725303
6904	0.917975	0.745876
6905	0.720957	0.743152
6906	0.795998	0.710054
6907	0.69316	0.7475
6908	0.641306	0.745479
6909	0.690817	0.706788
6910	0.961899	2.1
6911	2.49579	2.1
6912	2.31106	2.1
6913	1.70482	0.7
6914	1.93156	0.689962
6915	1.23868	0.668667
6916	1.73878	0.700281
6917	1.26267	2.1
6918	1.26441	2.1
6919	1.58095	2.1
6920	1.58095	2.1
6921	0.946132	2.1
6922	0.946132	2.1
6923	1.11077	2.1
6924	1.62905	2.1
6925	1.49692	2.1
6926	3.49966	2.1
6927	2.39213	2.1
6928	2.12603	2.1
6929	0.946129	2.1
6930	0.461661	2.1
6931	1.52841	2.1
6932	1.39091	2.1
6933	0.550679	2.1
6934	0.509894	2.1
6935	1.49508	2.1
6936	2.05862	2.1
6937	2.47851	2.1
6938	1.07213	2.1
6939	1.09148	0.896143
6940	1.99776	0.935383
6941	1.38333	0.932974
6942	1.04412	2.1
6943	1.04412	2.1
6944	1.1893	0.65081
6945	0.946132	0.540492
6946	0.965737	0.568897
6947	0.946132	0.633133
6948	1.54061	0.619844
6949	1.54061	0.421323
6950	1.22878	0.474088
6951	1.25797	0.291346
6952	1.00665	0.593389
6953	1.00665	0.560171
6954	1.80369	0.61132
6955	0.946128	0.528255
6956	1.04424	0.561122
6957	0.85154	0.641149

6958	1.39322	0.550956
6959	1.35464	0.466133
6960	0.939857	0.431036
6961	1.28272	0.363047
6962	1.19323	0.626343
6963	1.03498	0.30476
6964	2.22029	0.340043
6965	0.540005	0.289723
6966	0.970382	0.3871
6967	2.14514	0.635813
6968	1.31542	0.622328
6969	1.05575	0.710026
6970	0.810943	0.716445
6971	1.31542	0.557178
6972	1.70493	0.661721
6973	1.22582	0.696956
6974	1.31681	0.710083
6975	1.40775	0.666072
6976	0.928633	0.722716
6977	1.2545	0.623365
6978	2.66481	0.686953
6979	1.32051	0.668002
6980	1.32051	0.713423
6981	2.66305	2.1
6982	1.3551	2.1
6983	2.45325	2.1
6984	1.3685	2.1
6985	1.51748	2.1
6986	1.78941	2.1
6987	1.02963	0.186576
6988	1.28643	0.307949
6989	0.86131	0.324822
6990	0.851539	0.362693
6991	1.46585	0.19422
6992	1.38822	0.169488
6993	0.996402	0.208034
6994	0.851553	0.305228
6995	1.73156	0.292026
6996	1.91695	0.235484
6997	1.92535	0.31096
6998	2.57287	0.252555
6999	1.77743	0.241844
7000	1.02836	0.684847
7001	1.36362	0.766204
7002	1.36936	0.700389
7003	1.78684	0.5412
7004	1.78685	0.576765
7005	0.946132	0.792262
7006	1.27581	0.776855
7007	0.946132	0.803184
7008	1.02836	0.47575
7009	1.55398	2.1
7010	1.07167	2.1
7011	1.87557	2.1
7012	0.751639	2.1
7013	1.50884	2.1
7014	1.33793	2.1
7015	0.751664	2.1

7016	1.37277	2.1
7017	0.979515	2.1
7018	0.676508	2.1
7019	1.09578	2.1
7020	0.953925	2.1
7021	0.94613	2.1
7022	1.64707	2.1
7023	1.64707	2.1
7024	1.16658	2.1
7025	1.40638	2.1
7026	1.18682	2.1
7027	1.16658	2.1
7028	0.543765	0.507836
7029	1.57462	0.494116
7030	1.57462	0.432396
7031	1.00083	0.512635
7032	1.16834	0.670093
7033	1.16834	0.62936
7034	0.889626	0.573434
7035	1.25944	0.681823
7036	0.824061	0.702753
7037	1.9397	0.414191
7038	1.21893	0.494701
7039	1.91364	0.478983
7040	1.33672	0.67438
7041	0.928069	0.739955
7042	1.41484	0.662041
7043	0.538379	0.587683
7044	1.41484	0.605872
7045	1.35594	0.69168
7046	2.11414	0.668517
7047	2.11414	0.394524
7048	0.538378	0.655425
7049	1.38532	0.376502
7050	0.962877	0.84653
7051	1.07497	0.746283
7052	1.00613	0.68946
7053	0.962878	0.721146
7054	0.711447	2.1
7055	1.40008	0.529275
7056	2.51674	0.606642
7057	1.40008	0.552268
7058	1.7263	0.355129
7059	1.40008	0.686248
7060	1.9216	0.148524
7061	2.7013	0.136434
7062	2.58584	0.121861
7063	0.979296	0.1463
7064	1.46459	0.507934
7065	0.899254	0.425861
7066	1.15038	1.93476
7067	1.24687	1.43591
7068	1.16738	1.85035
7069	0.942323	2.1
7070	1.65573	1.49192
7071	0.844791	2.1
7072	0.54674	1.58837
7073	0.795811	1.82047

7074	1.08131	1.98804
7075	1.03553	1.89418
7076	0.758334	2.05989
7077	0.708665	1.59669
7078	1.22626	1.61661
7079	0.835439	1.60734
7080	0.861131	1.44995
7081	0.68296	1.72701
7082	1.65224	1.61809
7083	2.82624	1.81941
7084	1.0151	1.25726
7085	1.15905	1.41995
7086	1.71076	1.32816
7087	1.4529	1.16395
7088	0.494895	2.04124
7089	0.946132	1.41133
7090	0.613132	1.31289
7091	0.863631	1.33155
7092	1.78391	2.1
7093	1.1706	0.107956
7094	2.02734	0.126721
7095	1.1706	0.0702484
7096	0.681532	0.0924272
7097	1.50316	0.0874026
7098	1.50316	0.0759861
7099	0.681534	0.0891803
7100	0.973819	0.0978123
7101	0.97382	0.103232
7102	0.94613	0.10715
7103	1.1706	0.0802788
7104	1.67727	0.185052
7105	1.60329	0.158172
7106	1.3227	2.1
7107	1.40846	2.1
7108	1.07889	2.1
7109	1.07721	2.1
7110	0.953523	0.978605
7111	1.79675	0.969469
7112	2.09859	0.944588
7113	1.21309	2.1
7114	1.54567	0.872775
7115	1.19722	0.814733
7116	1.82793	0.789797
7117	1.1599	0.764267
7118	1.73621	0.733295
7119	0.933474	0.798075
7120	1.51477	0.772006
7121	1.51477	0.802274
7122	0.93347	0.798079
7123	1.27374	0.771856
7124	1.27376	0.800251
7125	0.946105	0.804433
7126	1.1972	0.763573
7127	0.851541	0.0941937
7128	1.36971	0.0858222
7129	1.27832	0.0849304
7130	0.853489	0.0949574
7131	0.973847	0.0934464

7132	0.976308	0.0999763
7133	0.851528	0.0977456
7134	2.72654	0.0946722
7135	1.77867	0.0793273
7136	3.23032	2.1
7137	2.78208	2.1
7138	1.0227	0.112479
7139	1.02269	0.0892166
7140	1.02269	0.0869376
7141	1.24293	0.108489
7142	0.831695	2.1
7143	0.67513	2.1
7144	0.956294	2.1
7145	1.08623	2.1
7146	1.05556	2.1
7147	1.38859	2.1
7148	1.13179	2.1
7149	1.02438	2.1
7150	1.06659	2.1
7151	1.36595	2.1
7152	1.06659	2.1
7153	1.06659	0.987839
7154	0.946131	1.12812
7155	0.875912	2.1
7156	1.65971	2.1
7157	1.65971	1.32791
7158	0.875912	2.1
7159	1.1229	1.99445
7160	1.41273	1.89785
7161	1.54699	1.30521
7162	1.93655	1.31802
7163	1.42307	1.08728
7164	1.97907	1.22633
7165	0.996774	2.1
7166	1.15505	0.136579
7167	1.28463	0.123992
7168	1.86353	0.152153
7169	2.71398	0.18674
7170	1.56787	0.151828
7171	1.66295	0.205884
7172	1.73432	1.78444
7173	1.05019	0.113065
7174	1.42117	0.108472
7175	0.888299	0.108555
7176	1.38127	0.105014
7177	1.72993	0.107233
7178	0.664129	0.112691
7179	0.99302	0.927179
7180	0.993023	0.919386
7181	1.13491	2.1
7182	0.876316	1.53431
7183	1.2588	2.1
7184	0.895613	2.1
7185	2.07526	2.1
7186	1.23369	1.96514
7187	1.25146	2.1
7188	2.15282	2.1
7189	1.22239	1.51155

7190	1.10962	2.1
7191	1.77402	1.55346
7192	1.0305	2.1
7193	1.50108	1.26317
7194	1.20761	2.1
7195	1.3626	2.1
7196	1.14825	2.1
7197	1.78711	2.1
7198	1.17933	2.1
7199	1.14824	2.1
7200	1.09651	0.402983
7201	1.23851	0.416324
7202	1.83284	0.423278
7203	1.23851	0.25041
7204	1.53521	2.1
7205	1.46279	2.1
7206	1.9233	0.611579
7207	1.2966	0.613358
7208	2.11443	0.597003
7209	1.30265	0.642058
7210	1.13528	0.688108
7211	0.946131	0.759477
7212	1.41169	0.737008
7213	1.33879	0.639153
7214	0.946128	0.695174
7215	1.80435	0.730785
7216	1.80435	0.489512
7217	1.234 2.1	
7218	1.45633	2.1
7219	1.234 2.1	
7220	1.98448	2.1
7221	2.02612	2.1
7222	1.23527	0.858871
7223	1.44472	0.890915
7224	0.993366	1.03212
7225	1.22025	0.981886
7226	0.993368	0.864214
7227	2.43669	2.1
7228	2.56707	2.1
7229	1.80348	1.43293
7230	1.74406	1.03714
7231	1.90937	1.28933
7232	1.49357	0.70824
7233	2.5832	0.779715
7234	1.82274	0.812727
7235	1.6957	0.783339
7236	2.22495	0.78911
7237	2.49959	0.792683
7238	2.01183	0.7
7239	1.37427	0.413912
7240	1.15069	1.27984
7241	1.39379	1.12454
7242	1.69661	0.882067
7243	1.39379	0.9043
7244	1.39654	2.1
7245	1.93505	2.1
7246	1.5525	2.1
7247	1.5525	2.1

7248	0.615065	2.1
7249	1.38908	2.1
7250	0.615065	2.1
7251	0.77849	2.1
7252	1.38908	2.1
7253	1.56839	1.10739
7254	1.09142	1.24059
7255	2.07313	1.0312
7256	0.998288	0.231095
7257	1.31481	0.229956
7258	1.88175	0.320166
7259	2.23267	0.417977
7260	1.06935	2.1
7261	1.06936	2.1
7262	1.17989	1.30143
7263	1.00757	0.988756
7264	1.65318	0.995238
7265	1.4185	0.693212
7266	2.22495	0.742383
7267	1.02011	0.453645
7268	0.59676	0.677542
7269	1.43606	0.596291
7270	1.43606	0.532881
7271	0.89771	0.591808
7272	1.13511	0.734781
7273	0.59676	0.438098
7274	0.897709	0.634957
7275	1.79542	0.56621
7276	2.38566	2.1
7277	1.63838	1.05465
7278	1.63837	1.33831
7279	0.916275	2.03526
7280	1.36183	1.10653
7281	1.19282	2.1
7282	0.916275	1.53339
7283	1.19283	1.78411
7284	2.01582	2.1
7285	1.00215	0.228101
7286	2.60643	2.1
7287	2.15971	2.1
7288	1.28267	2.1
7289	1.29954	2.1
7290	3.40327	2.1
7291	3.49128	2.1
7292	1.3864	2.1
7293	1.3201	2.1
7294	1.45666	0.0845313
7295	1.55798	0.0825405
7296	0.857202	0.0886888
7297	1.24374	0.0874002
7298	1.04157	0.0969445
7299	0.85723	0.0887326
7300	1.02722	0.0874132
7301	1.01361	0.104357
7302	0.974077	0.0721985
7303	1.01814	0.0840753
7304	0.974077	0.070471
7305	1.43987	0.0647547

7306	1.43986	0.0597528
7307	0.927881	0.0600282
7308	0.946132	0.0671739
7309	0.927849	0.0588754
7310	0.927852	0.0582623
7311	1.2493	0.0614996
7312	1.22482	0.827627
7313	1.41319	0.925828
7314	1.22482	0.758256
7315	0.999492	2.1
7316	1.14657	2.1
7317	1.3617	0.110484
7318	0.983269	0.118789
7319	1.33174	0.130206
7320	1.62816	0.13065
7321	1.62817	0.116828
7322	0.81938	0.109295
7323	1.33174	0.118748
7324	1.01332	0.128897
7325	0.874788	0.0867173
7326	1.16725	2.1
7327	1.63991	2.1
7328	1.08848	0.175753
7329	1.15354	0.132425
7330	1.12665	2.1
7331	1.58742	2.1
7332	1.57523	2.1
7333	1.32201	1.00707
7334	1.32097	0.976989
7335	0.851542	0.714149
7336	0.877188	0.699844
7337	0.797489	0.711644
7338	1.44721	0.69999
7339	1.61761	0.7
7340	0.943799	0.712729
7341	1.36079	0.704557
7342	1.16361	1.99205
7343	1.15852	1.24971
7344	1.07624	2.1
7345	1.07624	2.1
7346	0.946132	0.873727
7347	1.37938	1.18202
7348	1.78653	1.51125
7349	1.78653	0.92908
7350	0.946131	1.34707
7351	1.37938	0.945966
7352	1.35328	0.894134
7353	1.44847	0.893861
7354	0.946133	0.918911
7355	1.38149	1.153
7356	1.81474	1.30492
7357	1.81474	0.964684
7358	0.946133	1.39173
7359	1.38149	0.995034
7360	1.37938	0.938729
7361	1.44847	0.98713
7362	1.93665	0.887919
7363	1.5515	0.931479

7364	1.05025	2.1
7365	1.05025	2.1
7366	1.71468	2.1
7367	1.04759	2.1
7368	1.04646	0.133986
7369	1.00739	0.164658
7370	1.37086	0.119147
7371	1.12547	0.161884
7372	1.03853	0.130261
7373	1.34824	0.114671
7374	0.799786	0.121715
7375	2.10105	0.155726
7376	2.72007	0.125924
7377	0.589918	0.0934676
7378	0.589918	0.12238
7379	1.1509	0.104778
7380	0.619832	0.0919136
7381	1.1509	0.115828
7382	0.705903	0.105869
7383	1.53703	0.0933418
7384	1.53703	0.112457
7385	0.705904	0.106782
7386	0.946133	0.117081
7387	0.946132	0.111839
7388	0.946132	0.124148
7389	0.588467	0.0822842
7390	1.1078	0.0912139
7391	0.408061	0.0837662
7392	1.38703	0.107196
7393	1.68328	0.250606
7394	1.87031	0.284568
7395	1.86268	0.373705
7396	1.27472	0.543153
7397	0.947862	1.04202
7398	1.19854	1.19353
7399	0.946129	0.924865
7400	0.963111	0.830525
7401	0.946131	0.941713
7402	1.39055	0.875319
7403	1.39055	0.829121
7404	1.26426	0.894163
7405	1.07242	0.912877
7406	1.31126	0.827972
7407	1.76597	0.944369
7408	0.94613	1.25995
7409	1.27214	1.09303
7410	1.90751	1.0929
7411	1.90751	1.04862
7412	0.767114	1.19104
7413	1.27214	1.18921
7414	1.40248	1.13281
7415	1.41479	1.24106
7416	2.22625	1.26928
7417	0.913373	1.29255
7418	1.0158	1.03046
7419	1.61259	0.999729
7420	1.69051	0.940381
7421	0.560949	1.19082

7422	0.898559	1.22076
7423	1.28011	1.12726
7424	1.51026	0.452027
7425	1.98252	0.549649
7426	1.05129	0.477847
7427	1.2951	0.481656
7428	1.46041	0.0519421
7429	1.7155	2.1
7430	1.72196	2.1
7431	0.933885	2.1
7432	0.986723	2.1
7433	1.28072	0.808484
7434	1.40765	1.05667
7435	1.28072	0.927771
7436	1.61687	2.1
7437	1.60965	2.1
7438	1.21424	2.1
7439	2.07297	2.1
7440	1.19943	2.1
7441	2.38784	2.1
7442	1.10278	0.535543
7443	1.43678	0.547153
7444	1.1142	0.706186
7445	1.18034	0.692357
7446	0.94613	0.706876
7447	0.752356	0.703607
7448	0.752329	0.706512
7449	1.46634	0.634695
7450	1.31789	0.52782
7451	0.958159	0.689094
7452	1.22752	0.475816
7453	1.50472	0.622097
7454	1.98974	2.1
7455	2.54186	2.1
7456	0.76512	2.1
7457	2.08777	2.1
7458	1.7753	2.1
7459	3.07372	2.1
7460	1.9756	2.1
7461	1.48162	2.1
7462	1.23204	2.1
7463	0.946131	0.990102
7464	1.13505	2.1
7465	1.47432	2.1
7466	1.47432	1.10673
7467	0.876831	1.53694
7468	1.13505	1.35647
7469	1.2161	1.10322
7470	1.05267	1.25554
7471	1.05267	1.0628
7472	1.5795	1.05955
7473	0.98186	2.1
7474	1.56735	2.1
7475	0.859099	2.1
7476	1.69858	2.1
7477	1.93586	2.1
7478	1.09233	2.1
7479	1.08732	0.501851

7480	1.3357	0.498323
7481	1.84826	2.1
7482	0.924132	1.96148
7483	1.39188	2.1
7484	1.43685	2.1
7485	0.851541	2.1
7486	1.16251	2.1
7487	0.946131	2.1
7488	1.05685	2.1
7489	2.13781	2.1
7490	1.98859	2.1
7491	1.99731	2.1
7492	1.51379	2.1
7493	1.48796	2.1
7494	0.851542	2.1
7495	0.802558	2.1
7496	1.09244	2.1
7497	1.33891	2.1
7498	0.844958	2.1
7499	0.710864	2.1
7500	1.26087	2.1
7501	1.03679	2.1
7502	1.51026	0.444434
7503	1.05248	0.842754
7504	0.827258	0.807557
7505	1.05248	0.813782
7506	1.28435	0.841132
7507	1.50953	0.860472
7508	1.50953	0.732984
7509	0.827298	0.819906
7510	1.00366	0.704059
7511	2.10495	0.76241
7512	1.17885	2.1
7513	1.25185	2.1
7514	1.02784	2.1
7515	1.76764	2.1
7516	1.23746	0.488486
7517	1.24082	0.527615
7518	1.46506	0.336923
7519	1.33061	0.228643
7520	0.768407	0.866107
7521	0.972058	0.736596
7522	1.16536	1.06782
7523	1.13325	0.797934
7524	0.978906	1.68844
7525	1.02291	0.789374
7526	0.838015	0.732586
7527	1.01052	0.447323
7528	1.08166	0.520366
7529	1.42607	0.225456
7530	1.47354	0.272372
7531	1.21703	2.1
7532	1.29897	2.1
7533	1.83293	2.1
7534	1.29897	2.1
7535	1.24688	0.358708
7536	0.782563	2.1
7537	0.946129	2.1

7538	0.782591	2.1
7539	1.49487	2.1
7540	1.49487	2.1
7541	1.37713	2.1
7542	0.900337	2.1
7543	1.31284	2.1
7544	0.924468	2.1
7545	1.40602	2.0768
7546	1.25669	2.1
7547	0.986901	2.1
7548	0.958565	2.1
7549	1.37266	2.1
7550	1.64146	2.1
7551	0.49345	2.1
7552	1.08276	2.1
7553	1.08276	2.1
7554	1.26929	2.1
7555	1.09847	2.1
7556	0.827081	0.729081
7557	1.07236	0.761463
7558	1.25063	0.796828
7559	1.43548	0.548423
7560	1.25063	0.311758
7561	1.3659	0.217381
7562	1.72441	0.207989
7563	1.3659	0.180156
7564	1.4677	0.217242
7565	1.86114	0.206445
7566	1.40104	0.323866
7567	0.962675	0.348011
7568	0.946131	0.382044
7569	1.74134	0.196501
7570	1.74134	0.175745
7571	0.962676	0.200568
7572	0.946131	0.31742
7573	1.05548	0.561509
7574	0.902187	0.612172
7575	2.14178	0.408392
7576	1.74998	0.499145
7577	1.38541	0.568287
7578	1.64468	0.398626
7579	0.949317	0.598322
7580	2.06654	0.498374
7581	0.839626	0.608164
7582	0.810945	0.634097
7583	1.29203	0.392254
7584	1.18091	0.499115
7585	1.06872	0.604839
7586	1.11956	0.440998
7587	0.926911	0.65674
7588	0.901716	0.636578
7589	0.909146	0.659885
7590	1.33776	0.470813
7591	1.02048	0.560754
7592	1.08365	0.634894
7593	1.21042	0.492066
7594	0.865634	0.672334
7595	0.590354	2.1

7596	0.61173	2.1
7597	3.46825	2.1
7598	3.28404	2.1
7599	1.56004	0.193387
7600	1.69344	0.315097
7601	1.7151	0.816242
7602	0.946131	0.259337
7603	1.40265	0.258045
7604	1.13537	0.499996
7605	0.965708	0.385472
7606	1.13537	0.29402
7607	1.29681	0.179838
7608	1.40915	0.632097
7609	1.61652	0.660991
7610	1.33171	0.809492
7611	1.37681	2.1
7612	0.81094	0.105165
7613	0.598606	0.0988699
7614	0.723111	0.111559
7615	1.04562	0.0947898
7616	0.979052	0.102551
7617	0.798185	0.0974711
7618	0.880117	0.112989
7619	0.979341	0.745722
7620	0.800229	0.725274
7621	1.39126	0.742102
7622	1.13005	0.746886
7623	0.913877	0.762881
7624	1.26959	0.738414
7625	0.699741	0.75909
7626	1.19496	1.52336
7627	1.13392	0.22215
7628	1.11913	0.185145
7629	1.06866	0.184995
7630	0.615546	0.232612
7631	0.831006	0.293525
7632	1.53286	0.198675
7633	0.690977	0.235995
7634	1.01956	0.214013
7635	2.33818	0.162939
7636	1.6585	2.1
7637	1.67061	2.1
7638	1.41414	2.1
7639	1.16756	2.1
7640	1.00898	0.142349
7641	0.966964	2.1
7642	2.85443	2.1
7643	0.966977	2.1
7644	2.08427	2.1
7645	2.94901	2.1
7646	1.10735	0.351497
7647	1.65815	2.1
7648	1.4502	2.1
7649	1.45026	2.1
7650	1.53446	2.1
7651	0.946129	0.984951
7652	1.81075	2.1
7653	1.81075	1.01169

7654	0.946131	1.35649
7655	1.37075	2.1
7656	1.37075	1.00861
7657	1.34245	1.49032
7658	0.973502	1.03434
7659	1.08542	2.00715
7660	1.5814	0.0985464
7661	1.43836	0.0902409
7662	0.946132	0.139316
7663	1.13747	0.123042
7664	1.04429	0.152476
7665	1.05017	0.126284
7666	1.24732	0.121257
7667	1.13387	0.116649
7668	2.19489	0.113341
7669	1.10748	2.1
7670	1.25006	2.1
7671	1.58834	2.1
7672	1.58834	0.795868
7673	0.94613	2.1
7674	1.16548	2.05123
7675	0.946132	0.799506
7676	1.08998	0.894349
7677	1.16548	0.870644
7678	0.995732	0.783997
7679	0.657014	2.1
7680	0.957173	2.1
7681	1.01485	2.1
7682	1.12468	2.1
7683	1.43871	2.1
7684	1.23716	2.1
7685	1.89277	0.959659
7686	2.06653	0.878334
7687	0.967544	0.739816
7688	1.89016	0.834018
7689	2.22165	1.86313
7690	0.83514	1.56299
7691	1.76565	2.1
7692	1.76565	2.1
7693	0.94613	1.48834
7694	0.835112	2.1
7695	1.23426	1.59404
7696	1.36653	1.95255
7697	0.895892	1.18524
7698	0.859278	1.1324
7699	0.791461	1.17641
7700	1.86249	1.29518
7701	1.14607	1.1938
7702	1.19904	1.25545
7703	1.84898	1.21639
7704	1.03452	0.941508
7705	0.946131	0.838114
7706	0.940472	0.765914
7707	1.53478	1.00111
7708	1.84175	0.895642
7709	1.55687	0.877397
7710	1.17787	0.819752
7711	1.60589	0.725078

7712	2.35574	0.669776
7713	1.3305	0.416402
7714	1.15008	1.16583
7715	1.12457	0.186277
7716	1.12457	0.178614
7717	1.01778	2.1
7718	1.35733	2.1
7719	1.50683	2.1
7720	1.12064	2.1
7721	1.42716	0.759036
7722	1.42717	0.892087
7723	1.45927	1.03632
7724	1.04321	1.17081
7725	1.32634	0.67405
7726	2.27022	0.582044
7727	1.19352	0.391132
7728	1.32634	0.40718
7729	1.40924	1.57322
7730	1.22927	1.08192
7731	1.69237	1.31502
7732	1.69237	2.1
7733	0.946132	1.00459
7734	0.946133	1.3757
7735	1.16912	1.21339
7736	1.0417	1.11341
7737	1.14605	1.63071
7738	1.1233	2.1
7739	1.96882	1.94374
7740	1.03804	0.701862
7741	1.15338	0.687897
7742	1.15338	0.699686
7743	2.33051	0.7
7744	1.81507	1.26862
7745	2.42949	2.1
7746	0.725879	1.01391
7747	1.88394	1.07357
7748	2.2699	0.425899
7749	2.78952	2.1
7750	1.88394	0.534758
7751	1.79606	2.1
7752	1.20091	2.1
7753	1.9614	2.1
7754	1.19901	1.32757
7755	3.07915	2.1
7756	1.32592	2.1
7757	2.86461	2.1
7758	1.34817	2.1
7759	1.44859	2.1
7760	2.03969	2.1
7761	0.946132	2.1
7762	1.50709	2.1
7763	1.50709	2.1
7764	0.946132	2.1
7765	1.29322	2.1
7766	1.29322	2.1
7767	1.01985	2.1
7768	0.968537	2.1
7769	1.401	2.1

7770	1.28936	2.1
7771	1.56002	0.0546806
7772	1.92548	0.0525663
7773	1.46041	0.0565582
7774	2.22712	0.0529675
7775	1.56001	0.0542595
7776	1.50524	2.1
7777	1.21304	2.1
7778	1.60665	1.48668
7779	1.85178	1.15222
7780	2.17534	2.1
7781	2.31012	2.1
7782	2.17533	1.41002
7783	0.986724	2.1
7784	2.01895	2.1
7785	2.00433	2.1
7786	1.45752	0.511445
7787	1.12695	0.447814
7788	0.652605	2.0998
7789	1.5133	2.1
7790	1.5133	2.1
7791	0.652603	1.82431
7792	1.228	2.1
7793	0.946132	2.1
7794	0.652604	2.1
7795	0.946132	1.22155
7796	0.716815	2.1
7797	1.62203	1.46512
7798	1.62203	2.1
7799	0.716814	2.1
7800	1.00191	1.86553
7801	1.33694	1.42655
7802	1.38216	2.1
7803	2.53338	2.03577
7804	1.46932	0.122212
7805	1.50848	0.114738
7806	1.50108	1.0659
7807	1.18382	0.889214
7808	2.02115	0.813277
7809	0.921823	0.768516
7810	2.08356	0.124769
7811	1.543	0.0847248
7812	1.00763	0.768243
7813	1.4185	0.795276
7814	1.08028	0.757737
7815	0.946131	0.834888
7816	1.56599	0.770217
7817	1.56599	0.851695
7818	1.08028	0.801785
7819	0.946131	0.73924
7820	1.37518	2.1
7821	1.63453	2.1
7822	1.04627	1.19412
7823	1.04626	1.18429
7824	1.35111	1.26511
7825	1.22297	1.22231
7826	1.64499	1.2738
7827	1.87598	1.06826

7828	1.06994	2.1
7829	1.37063	2.1
7830	1.99943	0.15433
7831	2.38807	0.138438
7832	0.99972	0.215837
7833	1.41388	0.2222
7834	1.66179	0.208124
7835	1.66179	0.161859
7836	0.946129	0.221072
7837	1.41388	0.229408
7838	1.19404	0.21384
7839	1.31023	1.08136
7840	1.20871	1.10484
7841	1.69731	2.1
7842	1.67971	2.1
7843	0.998971	0.0792102
7844	1.32988	0.108304
7845	0.701079	0.112187
7846	0.946131	0.102702
7847	0.582785	0.100497
7848	1.48592	0.0910059
7849	1.48592	0.0831181
7850	1.23351	0.0964629
7851	0.835199	0.0976491
7852	0.92427	0.0774566
7853	1.68262	0.0818793
7854	2.46702	0.0922008
7855	1.08715	1.98571
7856	1.01606	1.71772
7857	1.29579	1.65881
7858	1.01606	2.03516
7859	1.63823	2.1
7860	1.67687	2.1
7861	1.11048	2.09014
7862	1.39477	2.1
7863	0.991114	2.1
7864	2.90978	0.866706
7865	0.906811	0.947523
7866	0.872849	0.728868
7867	1.17091	0.849825
7868	0.685763	0.815892
7869	1.6386	1.08663
7870	1.06907	0.824079
7871	1.05516	0.915249
7872	1.9189	0.907857
7873	1.04615	0.76236
7874	1.2966	2.1
7875	1.37392	2.1
7876	1.35504	2.1
7877	1.30347	2.1
7878	1.50877	2.1
7879	1.11193	2.1
7880	1.40984	2.1
7881	1.67682	2.1
7882	1.00371	2.1
7883	1.15412	2.1
7884	1.41638	2.1
7885	1.03796	2.1

7886	0.946117	2.1
7887	1.50823	2.1
7888	1.50822	2.1
7889	0.966923	2.1
7890	0.946131	2.1
7891	2.07593	2.1
7892	1.9339	2.1
7893	1.1817	0.847526
7894	0.729178	0.851973
7895	1.5079	0.825217
7896	1.26991	0.897962
7897	0.994049	0.858032
7898	1.17452	0.819556
7899	1.05711	0.867682
7900	1.04071	0.853473
7901	1.80299	0.853461
7902	1.60417	0.831957
7903	0.943405	0.470715
7904	1.01129	0.327836
7905	1.42972	0.382212
7906	1.15904	0.385889
7907	0.680409	0.348148
7908	1.2816	0.193738
7909	0.806176	0.358417
7910	1.18954	0.321913
7911	1.26055	0.35517
7912	1.06313	2.1
7913	1.19352	2.1
7914	1.55766	2.1
7915	1.53729	2.1
7916	0.993021	0.366683
7917	1.09056	0.854158
7918	1.03408	2.1
7919	0.993023	0.847962
7920	0.659901	2.1
7921	0.632905	2.1
7922	1.34072	0.466885
7923	1.17793	0.695865
7924	2.24885	0.658994
7925	0.946132	0.650984
7926	1.12443	0.581127
7927	0.904207	0.691997
7928	1.51237	0.534272
7929	1.51237	0.533116
7930	1.12443	0.472195
7931	1.29215	0.398678
7932	1.6943	1.79325
7933	2.43858	2.00438
7934	2.0589	1.30136
7935	1.67204	2.1
7936	1.67204	1.71785
7937	0.555875	1.60846
7938	0.91951	2.1
7939	0.946131	1.26674
7940	0.555904	1.50672
7941	0.946131	1.48812
7942	1.6943	2.1
7943	1.04744	1.37239

7944	1.50938	2.1
7945	1.60861	2.1
7946	1.92406	0.426711
7947	1.18716	0.447431
7948	0.962705	0.860541
7949	1.35382	0.985134
7950	1.43632	0.125365
7951	1.09386	0.170877
7952	1.92547	0.157043
7953	0.994164	0.124964
7954	0.903974	2.07562
7955	0.928829	1.79232
7956	0.810958	1.53638
7957	1.92853	1.81566
7958	1.598	1.74763
7959	1.28967	1.89103
7960	1.44993	1.7134
7961	0.946132	0.942938
7962	0.882984	0.892405
7963	0.882955	0.970093
7964	1.56071	0.81675
7965	1.56071	0.831872
7966	1.35946	0.905019
7967	1.08421	0.939863
7968	1.41943	0.188542
7969	1.26265	0.124239
7970	1.03235	0.806377
7971	1.50778	2.1
7972	1.70387	2.1
7973	1.65608	2.1
7974	1.39076	2.1
7975	1.6104	2.1
7976	1.37456	2.1
7977	1.51898	0.739208
7978	1.9726	0.706095
7979	2.13757	1.53677
7980	1.74856	1.62834
7981	0.635949	0.907441
7982	2.46448	1.04885
7983	1.23224	1.01479
7984	1.12786	1.1275
7985	1.82721	1.00449
7986	1.82721	0.90206
7987	0.532892	1.48765
7988	0.946131	1.15808
7989	0.53292	1.11105
7990	1.41394	2.1
7991	1.45506	2.1
7992	1.39523	2.1
7993	1.09006	2.1
7994	1.80264	0.24175
7995	1.80264	0.858451
7996	0.946131	0.727396
7997	1.34268	0.688632
7998	0.946132	0.825306
7999	1.23732	0.672368
8000	1.34268	0.772748
8001	1.2064	0.912964

8002	1.58164	2.1
8003	1.73853	2.1
8004	1.01243	1.03287
8005	0.910672	1.41115
8006	1.47793	1.28646
8007	1.47793	2.1
8008	0.910702	1.36373
8009	1.30382	1.02647
8010	1.08478	1.04597
8011	0.910702	1.69666
8012	2.60709	1.14044
8013	1.01243	1.06445
8014	1.85516	1.28571
8015	2.52823	0.887926
8016	1.24001	0.821585
8017	1.89793	1.32659
8018	1.73584	0.10171
8019	1.24613	2.08364
8020	1.37074	1.78423
8021	1.23763	1.50208
8022	1.04072	1.94281
8023	0.75499	1.34572
8024	1.72329	1.63877
8025	1.46195	1.5204
8026	0.946132	1.37053
8027	0.747438	1.87541
8028	1.24613	1.43209
8029	2.59886	1.33081
8030	1.87279	2.1
8031	1.07977	2.1
8032	1.29606	2.1
8033	1.45178	2.1
8034	1.45177	2.1
8035	0.92406	2.1
8036	0.946131	2.1
8037	0.924047	2.1
8038	2.04529	2.1
8039	1.04544	2.1
8040	1.08276	2.1
8041	1.67491	2.1
8042	0.961826	0.843615
8043	0.810942	0.889791
8044	0.826622	1.83226
8045	0.914378	1.48944
8046	0.89022	0.87233
8047	0.922383	1.03958
8048	0.988165	0.818901
8049	1.31357	0.231254
8050	0.946131	0.460747
8051	1.31357	0.348491
8052	1.10564	0.345854
8053	1.6797	0.251149
8054	1.6797	0.458696
8055	0.739506	0.382162
8056	1.33957	0.379523
8057	1.33957	0.26932
8058	1.33957	0.214525
8059	2.34223	0.319071

8060	1.5515	0.987655
8061	2.22137	0.914838
8062	0.94612	0.863038
8063	1.13908	0.949582
8064	1.45423	0.873973
8065	1.45422	0.961627
8066	0.795519	1.03959
8067	1.1391	0.917226
8068	1.11065	0.89423
8069	1.12266	2.08375
8070	1.24925	2.1
8071	1.20242	1.22841
8072	2.18297	1.39873
8073	3.00107	1.08672
8074	2.58297	0.780708
8075	0.884783	1.98048
8076	0.851538	0.854495
8077	0.713587	1.43508
8078	1.47949	0.889071
8079	1.66673	1.84426
8080	0.696199	1.48789
8081	0.9383	0.960318
8082	1.29149	0.965383
8083	1.32266	1.06588
8084	2.11428	0.0927723
8085	2.17439	0.0818793
8086	1.40489	0.083416
8087	1.21071	0.0736107
8088	0.946118	0.118405
8089	1.0746	0.106001
8090	0.973119	0.107509
8091	1.31333	0.102507
8092	1.08723	0.0972326
8093	0.994292	2.1
8094	1.4964	2.1
8095	1.15096	2.1
8096	1.10101	2.1
8097	1.50438	2.1
8098	1.67825	2.1
8099	2.02103	2.1
8100	2.01978	2.1
8101	1.50931	0.179061
8102	1.50931	0.181722
8103	0.65023	0.152505
8104	1.34831	0.172432
8105	0.811233	0.203894
8106	1.34831	0.206488
8107	0.94613	0.182102
8108	1.00754	1.1471
8109	0.714132	1.79877
8110	1.28877	1.34548
8111	1.39905	1.28192
8112	1.03282	1.24204
8113	0.997769	1.14631
8114	1.11743	1.17803
8115	1.86863	1.12759
8116	3.30723	1.3043
8117	1.66635	1.9073

8118	1.35609	0.110401
8119	1.38212	0.132573
8120	1.95597	2.1
8121	1.64257	2.1
8122	2.93316	2.1
8123	3.13417	2.1
8124	0.733947	2.1
8125	1.15259	2.1
8126	1.07976	2.1
8127	1.30237	2.1
8128	1.99943	0.159664
8129	0.907431	0.214544
8130	0.878161	0.2183
8131	1.07544	0.125785
8132	1.03401	0.171555
8133	0.991975	0.119983
8134	1.03635	0.187418
8135	0.788549	0.156242
8136	1.44984	0.13297
8137	1.84007	0.201743
8138	2.31127	0.158708
8139	1.27362	2.1
8140	1.27737	0.965776
8141	1.87783	0.862648
8142	2.35499	0.125616
8143	2.69662	0.20309
8144	2.24065	0.0980111
8145	0.996082	2.1
8146	0.996083	2.1
8147	1.44859	2.1
8148	1.03968	2.1
8149	1.60018	1.12002
8150	1.73082	1.70458
8151	0.946132	1.25305
8152	1.49742	1.79555
8153	1.49742	0.917403
8154	1.00501	1.10999
8155	1.41585	2.07252
8156	1.41585	1.18615
8157	0.946131	1.49806
8158	1.03697	1.37818
8159	2.95786	2.1
8160	2.32185	2.02098
8161	1.65783	0.8991
8162	1.772	1.70944
8163	0.946131	0.914499
8164	1.40404	0.632969
8165	1.13537	2.02285
8166	1.04559	1.02129
8167	1.16091	1.05759
8168	2.63751	2.1
8169	1.68455	0.132008
8170	1.9682	0.12662
8171	2.16834	0.722233
8172	2.45569	0.751102
8173	0.946128	0.714898
8174	1.36428	0.754877
8175	0.663978	0.781607

8176	1.58218	0.721181
8177	1.58219	0.719934
8178	1.36428	0.743555
8179	0.881886	0.754308
8180	1.73849	2.1
8181	1.35668	2.1
8182	1.1233	2.1
8183	1.15009	2.1
8184	1.16482	0.763694
8185	1.29313	0.772579
8186	1.47608	0.708994
8187	2.05949	0.646
8188	0.946119	0.532128
8189	1.02974	0.456477
8190	0.946119	0.501894
8191	1.66758	0.55659
8192	1.66757	0.438867
8193	1.32082	0.395539
8194	1.29287	0.241683
8195	1.36005	1.95682
8196	1.47109	2.1
8197	2.45958	2.1
8198	2.2975	2.1
8199	0.953521	1.05916
8200	0.953521	0.97831
8201	1.67054	2.1
8202	1.67054	2.1
8203	0.946131	2.1
8204	1.38154	2.1
8205	1.13326	2.1
8206	1.13326	2.1
8207	1.23513	2.1
8208	1.50998	2.1
8209	2.47982	0.185582
8210	1.45091	0.160598
8211	0.958953	0.236879
8212	0.79239	0.244371
8213	2.11929	0.203293
8214	1.26511	0.327389
8215	1.27733	0.173971
8216	2.18127	0.170846
8217	1.30169	0.165687
8218	1.19259	0.215642
8219	0.963395	1.09187
8220	0.999947	1.83362
8221	1.62241	2.1
8222	1.62027	2.1
8223	0.981586	0.0934008
8224	0.981585	0.0644172
8225	0.721961	2.1
8226	0.891321	2.1
8227	1.04442	2.1
8228	1.66554	2.1
8229	2.43434	2.1
8230	1.73944	0.0851589
8231	1.88032	0.0839716
8232	0.711391	0.091618
8233	1.40837	0.088249

8234	0.946133	0.0918982
8235	0.92252	0.0914812
8236	1.13964	0.0917173
8237	1.83394	0.0934631
8238	2.81674	0.0904588
8239	1.10049	1.40858
8240	1.25092	0.895442
8241	0.760299	1.05037
8242	1.48017	1.15757
8243	1.48017	1.25326
8244	0.760271	0.985353
8245	1.35861	1.10201
8246	0.946129	1.01927
8247	0.760269	1.00143
8248	2.26155	2.1
8249	2.15634	2.1
8250	1.39824	2.1
8251	1.07817	2.1
8252	0.946132	2.1
8253	1.53028	2.1
8254	1.53028	2.1
8255	1.00807	2.1
8256	0.946133	2.1
8257	2.06126	0.716482
8258	1.97385	1.26914
8259	1.75754	0.7
8260	2.01613	2.1
8261	1.97995	2.1
8262	1.47696	2.1
8263	1.50172	2.1
8264	1.5177	0.163066
8265	1.44146	0.267875
8266	1.8807	0.241828
8267	2.07068	0.270604
8268	1.84267	0.289752
8269	1.08975	2.1
8270	1.21327	2.1
8271	1.10106	0.0928758
8272	1.34496	0.0801784
8273	2.08452	0.707431
8274	1.70537	0.700082
8275	2.62464	1.19891
8276	1.5726	1.02373
8277	1.5726	2.1
8278	0.94613	2.1
8279	1.15038	2.1
8280	0.946132	0.997388
8281	1.03974	2.08467
8282	1.15038	2.08038
8283	0.99747	0.991633
8284	1.03472	1.31553
8285	1.58407	0.940381
8286	1.21148	0.832122
8287	1.35025	0.953969
8288	1.3428	0.71827
8289	1.00775	2.1
8290	1.00775	2.1
8291	1.3879	2.1

8292	1.39958	2.1
8293	0.853042	2.1
8294	1.06997	2.1
8295	0.853042	2.1
8296	0.853042	2.1
8297	1.12491	2.1
8298	1.17879	2.1
8299	1.07162	2.04543
8300	1.61179	2.08303
8301	1.76016	2.1
8302	0.946132	0.904591
8303	1.23449	0.872735
8304	1.70392	0.878097
8305	1.70392	0.837765
8306	0.94613	0.91739
8307	1.23449	0.871021
8308	1.41556	0.875958
8309	1.14547	0.859534
8310	1.1721	0.742867
8311	0.96478	0.829883
8312	1.41843	0.829254
8313	1.2358	0.792899
8314	1.70678	0.772806
8315	1.70678	0.782662
8316	0.946118	0.871811
8317	1.23582	0.850617
8318	0.946105	0.887182
8319	0.964769	0.672375
8320	1.1357	0.742662
8321	0.964767	0.701789
8322	0.619856	0.107209
8323	1.72366	0.11892
8324	1.72367	0.0945252
8325	0.619831	0.106649
8326	1.19262	0.122399
8327	0.619829	0.0850863
8328	0.946129	0.129843
8329	1.57459	2.1
8330	1.86512	2.1
8331	0.93841	2.1
8332	1.57459	2.1
8333	1.10476	0.227831
8334	1.00013	0.949326
8335	2.13446	0.921446
8336	0.759781	2.1
8337	1.96011	1.2576
8338	0.946129	0.950754
8339	1.32056	1.58435
8340	1.75702	1.04997
8341	1.75702	1.97659
8342	0.946124	1.39179
8343	1.32056	1.10205
8344	1.38258	0.993978
8345	1.96011	1.28868
8346	2.31099	1.2295
8347	0.97356	2.1
8348	1.38885	2.1
8349	1.3771	2.1

8350	0.946131	2.1
8351	1.84843	2.1
8352	1.84843	2.1
8353	1.3771	2.1
8354	0.946131	2.1
8355	1.00278	2.1
8356	1.05447	0.822071
8357	1.05446	0.830991
8358	1.24913	0.720649
8359	1.05447	0.814972
8360	1.80882	0.82445
8361	1.80882	2.1
8362	0.946129	2.1
8363	1.33997	2.1
8364	0.946131	0.790683
8365	1.28831	0.935373
8366	1.33997	0.899462
8367	1.44391	0.100266
8368	1.44391	0.0944082
8369	0.850964	0.115105
8370	0.850963	0.116847
8371	0.946132	0.109817
8372	0.921299	0.120831
8373	1.37358	0.106297
8374	1.46297	0.0920681
8375	1.46298	0.0819052
8376	0.823988	0.124222
8377	0.921287	0.128509
8378	0.94612	0.142959
8379	0.823995	0.120882
8380	0.946107	0.102714
8381	1.44437	2.1
8382	1.06203	2.1
8383	2.21397	2.1
8384	0.637677	2.1
8385	1.8892	2.1
8386	2.075 2.1	
8387	1.32051	0.639665
8388	2.23637	0.709965
8389	1.10904	0.754021
8390	1.11819	0.7176
8391	0.94613	0.760905
8392	0.735845	0.711331
8393	1.4914	0.775751
8394	1.4914	0.614625
8395	0.735831	0.791892
8396	1.61683	2.1
8397	1.61683	2.1
8398	0.946131	2.1
8399	1.3281	2.1
8400	1.10441	2.1
8401	1.10442	2.1
8402	1.23487	2.1
8403	1.09914	2.1
8404	1.20691	0.155427
8405	2.27379	0.160344
8406	1.1369	0.2255
8407	1.26868	0.231063

8408	1.54422	0.202756
8409	1.54422	0.202259
8410	0.861357	0.234414
8411	0.94613	0.215943
8412	0.861359	0.218575
8413	1.2858	0.791479
8414	1.31011	0.783409
8415	1.294 0.753933	
8416	1.03079	0.803151
8417	1.81979	2.01227
8418	1.57829	2.1
8419	1.19023	1.8627
8420	1.19022	2.0339
8421	1.46458	1.83803
8422	1.46458	2.09997
8423	0.789173	2.1
8424	1.34386	2.09561
8425	0.909896	2.1
8426	0.946131	1.91688
8427	0.909893	1.93247
8428	1.27725	2.1
8429	1.68451	2.1
8430	2.26155	2.1
8431	2.34217	2.1
8432	1.55485	0.913397
8433	0.90854	0.864789
8434	0.858256	0.861334
8435	1.47042	0.825967
8436	1.62771	0.832617
8437	0.777398	0.883109
8438	0.982525	0.853907
8439	1.23865	0.853623
8440	2.09928	0.846294
8441	0.946128	0.699718
8442	0.474856	0.707097
8443	0.474855	0.714301
8444	1.58747	0.614022
8445	1.58747	0.620158
8446	0.963917	0.684765
8447	1.09841	0.705733
8448	2.23904	1.45227
8449	2.60055	0.939839
8450	1.02599	2.1
8451	2.17837	2.1
8452	2.07122	2.1
8453	2.7309	1.00899
8454	2.08482	2.1
8455	1.18322	0.86768
8456	1.35381	1.13107
8457	1.01139	0.907232
8458	0.946133	1.10377
8459	1.59089	1.15303
8460	1.59089	0.300261
8461	0.946133	0.941227
8462	1.09634	0.295766
8463	2.02279	0.614972
8464	1.03108	0.743667
8465	0.808978	1.34461

8466	1.07317	1.42421
8467	0.831695	1.40196
8468	1.22583	1.42286
8469	1.10215	1.29506
8470	1.07508	1.50658
8471	1.22204	1.33714
8472	1.04679	2.0524
8473	1.92535	0.246413
8474	1.11081	0.118015
8475	0.946131	0.612499
8476	0.714824	0.618058
8477	0.714795	0.661442
8478	1.4231	0.561179
8479	1.4231	0.459255
8480	0.828617	0.688675
8481	1.30928	0.420159
8482	1.49894	2.1
8483	1.55702	2.1
8484	0.946131	2.1
8485	1.10689	2.1
8486	0.946227	2.1
8487	0.946131	2.1
8488	1.21754	2.1
8489	1.504 2.1	
8490	1.504 2.1	
8491	0.723573	2.1
8492	1.24512	2.1
8493	0.723544	2.1
8494	0.946131	2.1
8495	1.24512	2.1
8496	1.29383	0.689857
8497	1.29383	0.440174
8498	1.61969	0.4414
8499	0.946132	0.325049
8500	1.12818	0.277765
8501	0.946132	0.507724
8502	1.80922	0.450325
8503	1.80922	0.232777
8504	1.41383	0.28972
8505	1.34153	0.164726
8506	1.47689	0.504253
8507	1.79375	0.536346
8508	1.24333	0.795739
8509	0.768989	0.776502
8510	1.24333	0.753617
8511	1.0027	0.825556
8512	1.47705	0.816515
8513	1.47705	0.727442
8514	0.768977	0.768245
8515	1.28436	0.852124
8516	0.928583	0.850421
8517	1.28436	0.77009
8518	1.24333	0.879203
8519	1.5991	0.885583
8520	1.5991	0.687004
8521	0.928583	0.843891
8522	1.85717	0.727715
8523	2.48665	0.763457

8524	1.18423	0.0862717
8525	1.9493	1.23672
8526	1.8519	1.1015
8527	1.68802	2.1
8528	1.0302	0.323501
8529	0.900473	0.264276
8530	1.99663	0.417711
8531	2.61301	0.311415
8532	0.745772	0.587734
8533	0.946131	0.421534
8534	1.48881	0.414766
8535	1.48881	0.380363
8536	0.745745	0.512434
8537	0.809786	0.51762
8538	1.3065	0.464124
8539	0.946132	0.514584
8540	0.768357	0.595869
8541	0.768387	0.628321
8542	1.76897	0.438577
8543	1.76896	0.417965
8544	1.40271	0.491734
8545	1.00777	0.526382
8546	1.37427	0.337986
8547	1.1967	1.63502
8548	0.946132	1.60832
8549	1.59397	2.1
8550	1.59397	2.1
8551	0.946131	2.1
8552	1.14989	2.1
8553	1.14989	2.1
8554	1.10046	2.1
8555	1.09506	0.362446
8556	2.53251	0.440581
8557	1.9212	1.01465
8558	1.99586	0.959366
8559	0.913967	0.759182
8560	1.55028	0.737586
8561	1.55028	0.798787
8562	0.913966	0.765914
8563	1.16318	0.720258
8564	1.16318	0.74074
8565	0.946132	0.727735
8566	1.20478	2.1
8567	1.09662	2.1
8568	1.03472	2.1
8569	1.84774	2.1
8570	1.7984	2.1
8571	2.1221	2.1
8572	1.68223	2.0336
8573	1.68224	1.83435
8574	0.514872	1.50965
8575	0.888991	1.82101
8576	0.946127	1.09551
8577	0.514845	1.49625
8578	0.946129	1.26495
8579	1.10127	1.49412
8580	2.61623	1.17177
8581	0.983581	0.849548

8582	0.869663	0.760051
8583	1.1818	1.27464
8584	1.10406	0.821778
8585	1.07133	1.48396
8586	1.09405	0.815584
8587	0.838014	0.774331
8588	0.842998	1.23793
8589	1.03818	0.95154
8590	1.46338	0.703546
8591	1.903 2.1	
8592	1.23942	2.1
8593	0.965933	2.1
8594	0.810075	2.1
8595	1.40571	2.1
8596	1.66219	2.1
8597	0.810047	2.1
8598	0.936678	2.1
8599	1.45808	2.1
8600	1.6384	2.1
8601	1.54699	2.1
8602	0.719877	2.1
8603	1.33256	2.1
8604	0.753547	2.1
8605	1.04073	2.1
8606	1.25822	2.1
8607	0.851542	2.1
8608	0.714102	2.1
8609	1.45886	2.1
8610	1.80171	2.1
8611	0.597337	2.1
8612	0.957713	2.1
8613	1.3033	2.1
8614	0.596613	0.725909
8615	1.1386	0.906829
8616	0.596612	0.838978
8617	0.946133	0.964936
8618	0.596652	0.996201
8619	1.64092	0.920933
8620	1.64092	0.890647
8621	1.991 2.1	
8622	1.23013	2.1
8623	2.77816	2.1
8624	2.74107	0.863801
8625	2.07681	0.83135
8626	1.47764	0.324938
8627	1.72677	0.438884
8628	1.52161	2.1
8629	1.28526	2.1
8630	1.90502	2.1
8631	1.87359	0.542758
8632	1.93489	0.611967
8633	2.70895	2.1
8634	2.83141	2.1
8635	1.41766	0.690957
8636	1.11337	0.687774
8637	1.41765	0.974734
8638	1.29172	0.697616
8639	1.76324	0.696177

8640	1.76324	0.791513
8641	0.946131	0.689296
8642	1.52428	2.1
8643	1.71459	2.1
8644	1.93852	0.802554
8645	1.07119	1.1783
8646	1.29698	0.91423
8647	1.42203	0.787128
8648	1.42203	1.13101
8649	0.946132	0.802227
8650	0.946132	0.844491
8651	0.96926	0.90361
8652	0.769913	1.51719
8653	0.946119	1.34871
8654	1.37867	0.968791
8655	1.94028	0.949477
8656	0.94612	0.841466
8657	0.946132	1.12685
8658	1.37865	0.922827
8659	2.14234	0.989879
8660	3.06459	1.15693
8661	1.44421	0.198572
8662	1.33924	0.139637
8663	1.33671	0.377557
8664	1.10733	0.334899
8665	0.68396	0.437803
8666	1.76008	0.256001
8667	1.76008	0.242414
8668	0.68393	0.279793
8669	0.946131	0.372515
8670	3.79242	2.1
8671	2.46293	2.1
8672	3.56947	2.1
8673	1.35487	1.51094
8674	1.38616	1.8414
8675	0.954048	0.449349
8676	1.36663	0.701505
8677	0.909487	0.70591
8678	0.887288	1.6289
8679	0.810947	1.12237
8680	0.810934	1.46717
8681	1.07046	1.64105
8682	1.13979	1.83289
8683	0.927838	1.17899
8684	0.919995	1.5069
8685	1.61447	2.1
8686	2.56305	2.1
8687	0.943414	1.37207
8688	1.17016	2.1
8689	1.7238	2.1
8690	1.89334	1.90065
8691	0.946128	0.988889
8692	1.25184	1.67543
8693	1.55639	1.91886
8694	3.12098	1.10656
8695	3.57307	0.953399
8696	2.81281	2.1
8697	0.851542	2.1

8698	1.27689	1.93043
8699	1.52568	2.1
8700	0.839689	2.1
8701	1.36471	2.07153
8702	0.827865	2.1
8703	0.93667	2.1
8704	0.900861	2.1
8705	0.943152	2.1
8706	1.00667	2.1
8707	1.18141	2.1
8708	1.15298	2.1
8709	1.20335	2.1
8710	1.00713	2.1
8711	0.597537	2.1
8712	1.0577	2.1
8713	0.967342	1.08656
8714	0.838027	1.42004
8715	0.893587	0.950361
8716	1.06436	1.19377
8717	0.850048	0.962311
8718	1.22026	1.2931
8719	0.988077	1.09796
8720	1.18769	0.771672
8721	1.3991	2.1
8722	1.52984	2.1
8723	1.35469	1.16835
8724	1.25083	1.05901
8725	1.6594	1.2659
8726	1.6594	1.13298
8727	0.946129	1.00411
8728	1.02602	1.00087
8729	0.94613	0.954039
8730	1.29724	0.663938
8731	1.14501	0.656051
8732	1.13078	0.545003
8733	1.48381	0.458643
8734	1.45921	0.427358
8735	0.851539	0.680007
8736	0.890836	0.671696
8737	0.946131	0.755273
8738	1.60428	2.1
8739	1.50097	2.1
8740	0.714507	0.7
8741	1.51396	0.700168
8742	1.51396	0.624561
8743	0.946132	0.7
8744	0.813862	0.684104
8745	1.41461	0.698573
8746	0.813862	0.698356
8747	0.921311	2.1
8748	1.25084	1.01093
8749	1.30165	0.89746
8750	1.60636	1.10364
8751	1.60636	0.954376
8752	0.94613	0.887404
8753	0.972232	0.891933
8754	0.946131	0.852534
8755	2.35003	2.1

8756	0.92546	2.09938
8757	1.00713	2.1
8758	0.810947	2.1
8759	0.810934	2.1
8760	1.056	2.1
8761	0.926811	2.1
8762	0.766408	2.1
8763	1.34292	2.1
8764	1.57932	0.193854
8765	2.67595	0.153229
8766	1.77737	0.138879
8767	1.77738	0.140586
8768	0.789633	0.219519
8769	1.22903	0.224682
8770	0.789663	0.25533
8771	0.789662	0.197152
8772	1.33797	0.179132
8773	0.946128	2.1
8774	1.47351	2.1
8775	1.47351	2.1
8776	0.94613	2.1
8777	1.30082	2.1
8778	1.30082	2.1
8779	0.996486	2.1
8780	0.946127	2.1
8781	1.72122	2.1
8782	1.72122	2.1
8783	0.94613	2.1
8784	1.36653	2.1
8785	1.36653	2.1
8786	1.02608	2.1
8787	0.709123	0.886309
8788	0.946127	0.957503
8789	1.41342	0.956991
8790	1.818	1.0523
8791	1.818	1.01051
8792	0.946127	0.761023
8793	1.41342	0.9744
8794	1.35071	0.947381
8795	1.8993	0.883193
8796	1.4748	2.1
8797	1.18711	0.147102
8798	0.946131	0.290801
8799	1.18711	0.258363
8800	1.20416	0.194385
8801	1.91358	0.314767
8802	1.91358	0.131335
8803	0.477691	0.251225
8804	2.16798	0.0959565
8805	2.22307	0.0968344
8806	0.758971	0.224284
8807	1.80694	0.249159
8808	1.80694	0.147787
8809	0.946132	0.24874
8810	0.75897	0.233885
8811	1.25124	0.212885
8812	0.946131	0.31151
8813	1.76837	2.1

8814	1.76837	2.1
8815	0.946131	2.1
8816	1.36422	2.1
8817	0.946131	2.1
8818	1.14184	2.1
8819	1.36422	2.1
8820	0.793303	0.115165
8821	1.79584	0.0843733
8822	1.35333	1.97625
8823	1.19967	1.23234
8824	1.55088	1.36562
8825	1.64246	2.1
8826	0.946132	1.0372
8827	0.886293	1.78217
8828	1.25018	1.75741
8829	1.5795	1.40538
8830	2.15414	1.11148
8831	0.94613	0.841597
8832	1.37758	1.3596
8833	1.71567	0.895601
8834	1.71567	1.95394
8835	0.946125	1.02596
8836	1.37759	0.899295
8837	1.28421	0.853646
8838	0.933959	1.10586
8839	0.946133	0.358137
8840	1.7813	0.355599
8841	1.7813	0.175625
8842	1.05704	0.31423
8843	1.37794	0.44173
8844	1.37794	0.34105
8845	0.946132	0.447986
8846	0.946132	0.159793
8847	1.68197	0.149135
8848	1.68197	0.112946
8849	1.01367	0.147084
8850	1.25017	0.170289
8851	1.25017	0.15474
8852	0.946132	0.180568
8853	0.838953	2.1
8854	1.4045	2.1
8855	1.56388	2.1
8856	1.3182	2.1
8857	0.928518	2.1
8858	1.04073	2.1
8859	0.835676	2.1
8860	1.47507	2.1
8861	1.47506	2.1
8862	0.797664	2.1
8863	1.34285	2.1
8864	0.929875	2.1
8865	0.797663	2.1
8866	0.946133	2.1
8867	1.83628	0.749346
8868	1.83627	0.769561
8869	0.946131	0.772581
8870	1.36748	0.731077
8871	0.946133	0.712053

8872	1.21139	0.742401
8873	1.36748	0.532169
8874	1.06795	2.1
8875	1.08192	2.1
8876	0.827866	2.1
8877	1.02548	2.1
8878	0.95006	2.1
8879	1.53933	2.1
8880	0.586511	2.1
8881	0.868145	2.1
8882	1.28401	2.1
8883	1.37719	2.1
8884	1.37719	2.1
8885	0.77497	2.1
8886	0.946132	2.1
8887	0.774968	2.1
8888	1.7053	0.748014
8889	1.7053	0.882992
8890	0.94613	1.46796
8891	1.25715	0.855419
8892	0.946132	0.74532
8893	1.19655	0.79554
8894	1.25716	0.797859
8895	0.851542	2.1
8896	0.80043	2.1
8897	1.27239	2.1
8898	1.02719	2.1
8899	0.946131	2.1
8900	0.981318	2.1
8901	1.06628	2.1
8902	1.26022	2.1
8903	0.946132	2.1
8904	0.946132	2.1
8905	1.76447	2.1
8906	1.76447	2.1
8907	1.35357	2.1
8908	1.35703	2.1
8909	0.946131	2.1
8910	1.50538	2.1
8911	1.50538	2.1
8912	1.10311	2.1
8913	1.3484	2.1
8914	1.10312	2.1
8915	0.966428	2.1
8916	0.946133	2.1
8917	1.4008	2.1
8918	1.71908	2.1
8919	1.71908	2.1
8920	0.946133	2.1
8921	1.40081	2.1
8922	1.26441	2.1
8923	0.851543	2.1
8924	0.961732	2.1
8925	1.54576	2.1
8926	1.35364	2.1
8927	0.946133	2.1
8928	1.02913	2.1
8929	1.2735	2.1

8930	1.32608	2.1
8931	0.967643	2.1
8932	0.94613	2.1
8933	1.44873	2.1
8934	1.44873	2.1
8935	0.967641	2.1
8936	0.946131	2.1
8937	1.40488	2.1
8938	1.24272	2.1
8939	0.946131	2.1
8940	1.78483	2.1
8941	1.78483	2.1
8942	1.24272	2.1
8943	0.946131	2.1
8944	0.94613	1.80041
8945	1.5627	0.885872
8946	1.5627	1.91547
8947	1.03656	1.77543
8948	1.31649	0.820764
8949	1.19233	1.10419
8950	1.03656	1.06326
8951	0.912059	1.90291
8952	1.47954	0.911501
8953	1.47954	2.07355
8954	0.912061	1.84559
8955	1.34647	0.861146
8956	1.04514	1.32062
8957	0.912061	1.04695
8958	0.761972	0.676245
8959	0.946132	0.673266
8960	0.761974	0.723429
8961	1.70225	0.850786
8962	1.70225	0.785676
8963	1.33728	0.769339
8964	1.01188	0.684197
8965	0.94613	1.0445
8966	0.746361	1.19816
8967	1.6369	1.37009
8968	1.62454	1.23552
8969	0.736738	0.979328
8970	0.958487	1.01165
8971	1.40641	1.12409
8972	1.2284	2.1
8973	0.92609	2.1
8974	0.595102	2.1
8975	1.80871	2.1
8976	1.1516	2.1
8977	0.962474	2.1
8978	2.24024	2.1
8979	0.946131	2.1
8980	1.73689	2.1
8981	1.73689	2.1
8982	0.946132	2.1
8983	1.30492	2.1
8984	1.30492	2.1
8985	1.17949	2.1
8986	0.946131	2.1
8987	1.8566	2.1

8988	1.8566	2.1
8989	1.22392	2.1
8990	1.39826	2.1
8991	1.40447	2.1
8992	1.22392	2.1
8993	0.946131	1.7005
8994	1.74134	2.1
8995	1.74134	2.1
8996	1.09148	1.53278
8997	1.40447	2.1
8998	1.283	2.1
8999	1.09148	2.1
9000	1.33607	2.1
9001	1.41022	2.1
9002	1.80016	2.1
9003	1.80015	2.1
9004	0.946127	1.74073
9005	0.946129	2.1
9006	1.20541	2.1
9007	1.13078	2.1
9008	1.33607	2.1
9009	1.54624	2.1
9010	1.85553	2.1
9011	0.920606	2.1
9012	0.946131	2.1
9013	1.01271	2.1
9014	1.32664	1.04788
9015	0.954692	1.07735
9016	0.946116	0.943111
9017	1.47771	0.95225
9018	1.4777	2.1
9019	0.95468	0.999492
9020	0.94613	0.98752
9021	1.31381	0.947519
9022	1.14005	0.818812
9023	0.946128	0.906939
9024	1.69433	0.773632
9025	1.69433	1.08556
9026	1.14005	0.87817
9027	0.94613	0.800314
9028	1.521	2.1
9029	1.411	2.1
9030	0.94612	2.1
9031	1.35115	2.1
9032	1.04127	1.94352
9033	1.05017	2.1
9034	1.03564	2.1
9035	0.607156	1.32376
9036	1.54549	2.1
9037	1.36715	2.1
9038	0.607127	1.27573
9039	1.2571	2.1
9040	0.998701	1.96524
9041	0.546436	2.06566
9042	0.946133	2.1
9043	1.75286	2.1
9044	1.75286	2.1
9045	1.12206	2.1

9046	1.34237	2.1
9047	1.35663	2.1
9048	1.12206	2.1
9049	0.919455	2.1
9050	1.66517	2.1
9051	1.99822	2.1
9052	1.228 2.1	
9053	1.35662	2.1
9054	1.228 2.1	
9055	1.01143	2.1
9056	0.851543	1.03062
9057	0.986856	2.1
9058	1.42762	1.2312
9059	1.23095	2.1
9060	0.946132	1.99276
9061	1.00659	1.5154
9062	1.1779	1.17422
9063	1.40025	2.1
9064	1.40025	2.1
9065	0.889308	2.1
9066	1.34652	2.1
9067	0.943042	2.1
9068	0.943041	2.1
9069	1.34652	2.1
9070	1.39514	1.07896
9071	1.30809	0.950378
9072	1.75711	0.930719
9073	1.7571	2.03031
9074	0.946125	0.809494
9075	0.946127	0.993133
9076	1.18396	1.02232
9077	1.4844	1.03153
9078	1.4844	1.24944
9079	0.803322	1.44103
9080	0.946133	0.974971
9081	0.803324	2.1
9082	0.803324	1.3552
9083	1.36183	1.38926
9084	0.946131	0.428963
9085	1.01462	0.420955
9086	0.946132	0.439904
9087	1.59749	0.624572
9088	1.59749	0.538911
9089	1.16009	0.443655
9090	1.38354	0.413532
9091	0.851539	1.03171
9092	1.36005	1.67138
9093	1.5532	1.17348
9094	1.80402	1.9408
9095	0.851537	1.59655
9096	0.902632	1.32356
9097	1.44437	1.15103
9098	0.633098	2.1
9099	1.00307	0.794925
9100	0.94613	0.875002
9101	1.16623	0.781337
9102	0.946128	0.887123
9103	1.64588	0.881804

9104	1.64588	0.816411
9105	1.35059	0.858862
9106	1.24142	0.872228
9107	0.946131	0.433786
9108	1.13343	0.396778
9109	0.946127	0.56479
9110	1.63367	0.544581
9111	1.63367	0.347219
9112	1.33035	0.380403
9113	1.24945	0.326616
9114	1.78733	1.1669
9115	1.70856	1.65537
9116	0.637995	1.09697
9117	0.712454	1.11828
9118	0.946132	1.06781
9119	0.637964	1.05545
9120	1.13021	1.08769
9121	0.94613	0.626839
9122	1.20509	0.621017
9123	0.946127	0.647563
9124	1.70545	0.63791
9125	1.70545	0.382128
9126	1.28508	0.501656
9127	1.3665	0.35613
9128	1.58612	2.1
9129	1.51845	2.1
9130	0.797374	2.1
9131	1.20057	2.1
9132	0.946132	2.1
9133	0.946132	2.1
9134	0.899819	2.1
9135	1.50471	0.98228
9136	1.50472	1.10533
9137	0.59387	1.08254
9138	0.593844	0.936236
9139	0.946126	0.97375
9140	0.966137	0.990344
9141	1.13245	0.940533
9142	1.20801	1.06456
9143	0.946131	1.26844
9144	1.47644	1.114
9145	0.733223	1.08359
9146	0.563609	1.00522
9147	1.69218	1.09847
9148	1.6173	1.2679
9149	1.8126	1.97593
9150	1.8126	2.1
9151	0.678938	1.99772
9152	0.678935	2.09538
9153	0.946133	2.1
9154	0.946132	1.94531
9155	0.678936	2.1
9156	1.21997	1.67954
9157	1.28439	1.70285
9158	1.65282	1.98033
9159	1.86735	1.95606
9160	0.851539	1.73954
9161	0.94613	1.36469

9162	0.87688	1.40011
9163	0.804877	0.796922
9164	1.40747	0.915159
9165	1.41312	0.73373
9166	0.885349	0.768678
9167	1.25405	0.898708
9168	1.34463	0.763646
9169	0.804878	0.79178
9170	0.810299	0.614678
9171	1.04072	0.718775
9172	1.25601	0.706282
9173	1.04943	0.574058
9174	0.900294	0.709583
9175	1.38777	0.70633
9176	1.33131	0.473226
9177	1.39168	0.18485
9178	0.946132	0.229314
9179	0.747712	0.186974
9180	1.61944	0.143923
9181	1.61944	0.136607
9182	0.946132	0.243175
9183	0.747712	0.223375
9184	1.41201	0.670977
9185	1.12082	0.645389
9186	0.946128	0.662763
9187	1.79415	0.666686
9188	1.79415	0.678483
9189	1.12082	0.656821
9190	0.94613	0.649796
9191	0.711721	0.411041
9192	1.12316	0.492013
9193	0.707038	0.44114
9194	1.04072	0.492004
9195	0.704409	0.586013
9196	1.49036	0.516009
9197	1.27689	0.43397
9198	0.793044	0.994704
9199	0.810886	1.1749
9200	0.885957	0.993548
9201	1.38254	1.00568
9202	1.35991	0.963023
9203	1.01375	1.06912
9204	1.03169	1.06411
9205	0.946131	1.00659
9206	1.3279	1.16016
9207	1.42284	1.07802
9208	1.42284	1.00827
9209	0.851192	1.31753
9210	0.946131	1.22215
9211	0.851165	1.08809
9212	0.748062	0.175341
9213	1.27511	0.170383
9214	0.74806	0.15205
9215	0.94613	0.155664
9216	0.748059	0.168574
9217	1.86293	0.174728
9218	1.86293	0.148595
9219	0.851542	0.430637

9220	1.38186	0.437326
9221	0.851542	0.615852
9222	1.67869	0.342133
9223	1.66579	0.27096
9224	1.25626	0.403414
9225	1.23046	0.364702
9226	1.46023	0.0829149
9227	1.27083	0.0773355
9228	0.946131	0.0960339
9229	0.999304	0.0915847
9230	0.946803	0.0970288
9231	0.851554	0.0906533
9232	0.999227	0.0872488
9233	0.750097	0.294418
9234	1.91815	0.288269
9235	0.946132	0.290631
9236	0.768117	0.276887
9237	1.78003	0.275292
9238	1.99572	0.217936
9239	0.650453	0.334231
9240	0.946132	0.216652
9241	1.23845	0.211089
9242	0.946131	0.270687
9243	1.75477	0.160647
9244	1.75477	0.163434
9245	1.33393	0.189237
9246	1.36697	0.203229
9247	0.749018	0.997341
9248	1.02366	1.1824
9249	1.43924	1.05398
9250	1.00018	1.00737
9251	1.06438	0.909782
9252	0.946127	0.94149
9253	0.74906	0.952531
9254	0.946131	0.946239
9255	1.26488	0.122079
9256	0.946131	0.158959
9257	0.94613	0.128595
9258	1.49051	0.103837
9259	1.59837	0.104266
9260	0.907797	0.11973
9261	1.04455	0.134539
9262	1.25214	0.0676944
9263	0.68452	0.0756209
9264	1.25215	0.0730153
9265	1.24677	0.0714215
9266	1.8144	0.0649008
9267	1.81441	0.0650957
9268	0.684518	0.0703332
9269	0.851542	0.818794
9270	1.52275	0.802877
9271	1.45223	0.81089
9272	1.0269	0.811088
9273	1.29689	0.816299
9274	1.32822	0.835394
9275	0.851553	0.853146
9276	1.21756	0.144883
9277	0.946131	0.140879

9278	0.946127	0.143983
9279	1.78014	0.114568
9280	1.78014	0.0941287
9281	1.32076	0.124883
9282	1.40551	0.135166
9283	0.711214	0.442696
9284	1.82057	0.400778
9285	1.82057	0.326482
9286	0.946129	0.529652
9287	1.22716	0.309407
9288	1.30463	0.502006
9289	0.94613	0.503225
9290	0.568927	0.142574
9291	0.946132	0.142484
9292	0.568927	0.146176
9293	1.99266	0.0935777
9294	1.99266	0.0967146
9295	1.26201	0.150324
9296	1.29957	0.126229
9297	1.21614	0.466209
9298	0.851484	0.476787
9299	0.851543	0.497481
9300	1.85815	0.264252
9301	1.81937	0.244006
9302	0.697705	0.235945
9303	0.946132	0.464169
9304	0.593295	0.631326
9305	1.53642	0.550143
9306	1.53642	0.480753
9307	0.908276	0.685634
9308	0.593296	0.47765
9309	0.593295	0.561135
9310	0.94613	0.662425
9311	0.867593	0.739986
9312	1.42107	0.640916
9313	1.42107	0.617554
9314	0.867621	0.737342
9315	1.05516	0.733601
9316	0.867624	0.623813
9317	0.946129	0.74113
9318	0.766681	0.256879
9319	1.18119	0.295483
9320	1.57018	0.214699
9321	1.57018	0.189638
9322	0.766652	0.276843
9323	1.18119	0.289491
9324	1.15564	0.240064
9325	0.946131	0.708514
9326	1.239	0.646312
9327	0.851541	0.713552
9328	0.851487	0.625795
9329	1.40021	0.709015
9330	1.38373	0.564308
9331	0.851554	0.716158
9332	1.12266	0.329685
9333	0.946131	0.736976
9334	1.08234	0.690921
9335	1.2882	0.580386

9336	1.49963	0.876555
9337	1.46188	0.358938
9338	0.851555	0.662867
9339	1.33007	0.78341
9340	1.08054	0.771224
9341	0.946129	0.81373
9342	0.91151	0.746397
9343	1.49914	0.801077
9344	1.49914	0.733239
9345	0.911471	0.83205
9346	0.946132	0.974608
9347	1.35943	1.73456
9348	1.68117	1.24859
9349	1.68117	2.1
9350	0.946132	1.3522
9351	1.35943	1.07917
9352	1.26787	0.968682
9353	1.81612	0.955461
9354	1.81612	0.775145
9355	0.946131	0.531471
9356	1.41492	0.331579
9357	0.946132	0.979295
9358	1.25037	0.845485
9359	1.41492	0.693012
9360	1.30541	0.174665
9361	1.23646	0.223542
9362	0.94613	0.197991
9363	1.77304	0.124244
9364	1.77304	0.120077
9365	1.23646	0.174222
9366	0.946132	0.184092
9367	0.946131	2.1
9368	1.26689	2.1
9369	1.66806	2.1
9370	1.66806	2.1
9371	0.94613	2.1
9372	1.26689	2.1
9373	1.3473	2.1
9374	1.39384	2.1
9375	1.4181	2.1
9376	1.86581	2.1
9377	1.86581	2.1
9378	0.946128	2.1
9379	0.946129	2.1
9380	1.32068	2.1
9381	1.20328	2.1
9382	1.39384	2.1
9383	1.651	2.1
9384	1.651	2.1
9385	0.946129	2.1
9386	0.946131	2.1
9387	1.12913	2.1
9388	1.21474	2.1
9389	0.946132	2.1
9390	0.94613	2.1
9391	1.79724	2.1
9392	1.79724	2.1
9393	1.40987	2.1

9394	1.3335	2.1
9395	1.65642	1.44464
9396	1.65642	2.1
9397	0.946132	2.1
9398	1.19063	2.1
9399	0.946133	0.951051
9400	1.15203	2.1
9401	1.19063	2.1
9402	0.946131	0.939279
9403	1.77815	1.80903
9404	1.77815	2.1
9405	0.946131	1.22632
9406	1.31745	2.1
9407	1.31745	1.34716
9408	1.2597	1.83595
9409	0.946131	0.86837
9410	1.85184	1.06104
9411	1.85184	2.1
9412	0.946128	1.20224
9413	1.40684	2.1
9414	1.40684	1.03589
9415	1.34049	1.15007
9416	0.946132	1.33134
9417	1.71333	1.10025
9418	1.71333	2.03291
9419	1.1848	1.3287
9420	1.34297	1.00957
9421	1.31649	1.07101
9422	1.1848	1.51288
9423	1.36604	0.693947
9424	0.954559	0.693975
9425	1.36604	0.698764
9426	1.41766	0.692976
9427	1.83756	0.704096
9428	1.83756	1.24996
9429	0.946131	0.699148
9430	0.946127	0.594007
9431	1.14874	0.409056
9432	0.946127	0.620366
9433	1.66393	0.559348
9434	1.66393	0.449703
9435	1.35559	0.542779
9436	1.25447	0.562015
9437	0.94613	0.52739
9438	1.20732	0.567051
9439	0.946129	0.666138
9440	1.75073	0.650446
9441	1.75073	0.342176
9442	1.3665	0.46233
9443	1.33035	0.286132
9444	1.73256	2.1
9445	1.73256	2.1
9446	0.946131	2.1
9447	1.34921	2.1
9448	1.19462	2.1
9449	1.19462	2.1
9450	1.32949	2.1
9451	1.75754	0.730371

9452	1.75754	0.745263
9453	0.946132	0.748366
9454	1.3201	0.733349
9455	0.946132	0.725369
9456	1.25479	0.713475
9457	1.3201	0.712995
9458	1.8087	0.898904
9459	1.8087	0.741292
9460	0.946131	0.742223
9461	1.38357	0.73578
9462	0.946133	0.884618
9463	1.31034	0.785972
9464	1.38357	0.799218
9465	1.12702	0.638773
9466	0.946131	0.765653
9467	1.23923	0.7491
9468	1.4117	0.63641
9469	0.946131	0.708466
9470	1.70479	0.729169
9471	1.70479	0.429548
9472	0.946119	2.1
9473	1.55309	2.1
9474	1.55309	2.1
9475	0.946119	2.1
9476	1.08006	2.1
9477	1.08005	2.1
9478	1.02681	2.1
9479	1.36581	2.1
9480	1.93075	2.1
9481	0.946133	2.1
9482	0.772538	2.1
9483	0.946131	2.1
9484	0.946131	2.1
9485	1.33748	2.1
9486	1.44991	2.1
9487	1.31183	2.1
9488	0.946132	2.1
9489	0.944104	2.1
9490	0.915236	2.1
9491	1.05017	2.1
9492	0.944148	2.1
9493	0.946132	0.7
9494	1.60171	0.7
9495	1.60171	0.629374
9496	0.946131	0.700301
9497	1.16234	0.622522
9498	1.3855	0.7
9499	0.946132	0.7
9500	0.946133	0.700072
9501	1.73285	0.7
9502	1.73285	0.697256
9503	0.946133	0.7003
9504	1.3855	0.699681
9505	1.29348	0.7
9506	0.946132	0.7
9507	1.34828	2.1
9508	1.20943	2.1
9509	0.946129	1.15032

9510	1.71013	2.1
9511	1.71013	2.1
9512	1.20943	2.1
9513	0.94613	2.1
9514	1.19613	0.123071
9515	0.946118	0.123577
9516	0.946117	0.123799
9517	1.70868	0.105075
9518	1.70869	0.092777
9519	1.40551	0.116991
9520	1.2493	0.123558
9521	0.946131	2.1
9522	1.73123	2.1
9523	1.73123	2.1
9524	0.946132	2.1
9525	1.37811	2.1
9526	1.37811	2.1
9527	1.25144	2.1
9528	0.946132	2.1
9529	1.75139	2.1
9530	1.75139	2.1
9531	1.23374	2.1
9532	1.29926	2.1
9533	1.39827	2.1
9534	1.23374	2.1
9535	1.41375	0.210352
9536	1.25697	0.272966
9537	0.946118	0.263839
9538	1.80377	0.133315
9539	1.80377	0.119459
9540	1.25699	0.219721
9541	0.946106	0.226517
9542	0.946133	1.36244
9543	1.81806	1.13635
9544	1.81806	2.02813
9545	0.946133	1.85121
9546	1.37089	2.0992
9547	1.37089	1.17278
9548	1.34999	1.88655
9549	0.946131	2.1
9550	1.80855	1.09499
9551	1.80855	2.1
9552	1.31184	2.02881
9553	1.3933	1.23
9554	1.36139	1.2643
9555	1.31184	1.41033
9556	1.01175	1.1248
9557	0.94613	0.865211
9558	0.919796	1.26372
9559	1.50484	0.803301
9560	1.80584	1.4402
9561	1.53512	0.915259
9562	1.14539	0.872519
9563	0.946133	2.1
9564	1.72797	1.22736
9565	1.72797	2.1
9566	1.2111	2.1
9567	1.39731	1.17949

9568	1.27679	2.06718
9569	1.21111	1.94659
9570	0.946119	0.860631
9571	1.67673	1.11325
9572	1.67673	2.1
9573	0.946118	1.12315
9574	1.21519	1.95801
9575	1.2152	1.54182
9576	1.19808	1.84198
9577	0.946127	2.1
9578	1.54442	2.1
9579	1.54442	2.1
9580	1.04806	2.1
9581	1.13485	2.1
9582	1.3557	2.1
9583	1.04805	2.1
9584	0.946129	2.1
9585	1.32282	2.1
9586	1.77749	2.1
9587	1.77749	2.1
9588	0.946131	2.1
9589	1.32282	2.1
9590	1.4008	2.1
9591	0.946128	0.653658
9592	1.02366	0.547218
9593	0.94613	0.60092
9594	1.66684	0.580207
9595	1.66684	0.441609
9596	1.29215	0.452251
9597	1.32082	0.283423
9598	0.946133	0.412884
9599	1.01451	0.388662
9600	0.946132	0.419035
9601	1.62374	0.54046
9602	1.62374	0.415243
9603	1.2929	0.377068
9604	1.27697	0.238678
9605	1.37869	2.1
9606	1.17215	2.1
9607	0.946132	2.1
9608	1.70508	2.1
9609	1.70508	2.1
9610	1.17215	2.1
9611	0.946132	2.1
9612	1.03784	2.1
9613	0.946132	2.1
9614	0.946132	2.1
9615	1.56718	2.1
9616	1.56717	2.1
9617	1.35703	2.1
9618	1.15628	2.1
9619	1.35356	2.1
9620	1.28976	2.1
9621	0.946131	2.1
9622	1.78612	2.1
9623	1.78612	2.1
9624	1.28976	2.1
9625	0.94613	2.1

9626	0.946132	2.1
9627	1.36373	2.1
9628	1.92382	2.1
9629	0.946131	2.1
9630	1.92382	2.1
9631	1.33471	2.1
9632	0.946132	2.1
9633	0.94613	0.786286
9634	1.29149	0.984898
9635	1.75878	0.841213
9636	1.75878	1.58325
9637	0.946128	0.935758
9638	1.29149	0.805894
9639	1.41342	0.817815
9640	1.41022	2.1
9641	1.37034	1.18246
9642	1.83443	1.32927
9643	1.83443	2.1
9644	0.946128	1.0803
9645	0.946131	1.52574
9646	1.26928	1.34806
9647	0.946128	0.880958
9648	1.38259	1.53382
9649	1.81405	0.963696
9650	1.81405	1.98749
9651	0.946126	1.13585
9652	1.38259	0.976625
9653	1.37759	0.912041
9654	1.68131	0.637774
9655	1.68131	0.811469
9656	0.946131	0.632598
9657	1.38896	0.492928
9658	1.17438	0.9195
9659	1.17438	0.645597
9660	1.23848	0.799901
9661	1.80088	0.658905
9662	1.80088	0.244726
9663	0.946132	0.49277
9664	1.40332	0.693688
9665	1.28185	0.741239
9666	1.28185	0.377679
9667	1.34369	0.452873
9668	1.71939	2.1
9669	1.71939	2.1
9670	0.946131	2.1
9671	1.28308	2.1
9672	0.946133	2.1
9673	1.24024	2.1
9674	1.28309	2.1
9675	1.8525	2.1
9676	1.8525	2.1
9677	0.946132	2.1
9678	1.38244	2.1
9679	0.946132	0.973917
9680	1.33469	2.1
9681	1.38244	2.1
9682	1.26654	1.04862
9683	1.39514	0.994526

9684	1.71556	0.984366
9685	1.71556	1.98111
9686	0.946126	0.86547
9687	0.946129	1.10672
9688	1.1908	1.05253
9689	1.49995	2.1
9690	1.63875	2.1
9691	0.93608	2.1
9692	0.937208	2.1
9693	0.943708	2.1
9694	0.936134	2.1
9695	1.14341	2.1
9696	1.17802	0.0778477
9697	1.3542	0.0902461
9698	1.73981	0.0905563
9699	1.73981	0.082357
9700	0.94613	0.0781652
9701	1.3542	0.0837087
9702	1.33174	0.0891066
9703	1.59452	2.1
9704	1.59452	2.1
9705	0.946132	2.1
9706	1.25	2.1
9707	0.975266	2.1
9708	0.946132	2.1
9709	1.25	2.1
9710	0.946131	2.1
9711	1.48685	2.1
9712	1.48685	2.1
9713	0.946132	2.1
9714	1.17273	2.1
9715	1.17273	2.1
9716	0.955602	2.1
9717	1.32827	0.611384
9718	1.11166	0.627878
9719	0.946127	0.644077
9720	1.6136	0.52454
9721	1.6136	0.636817
9722	1.11166	0.641504
9723	0.946129	0.636735
9724	0.851541	0.877032
9725	1.22107	0.896506
9726	1.64594	0.854644
9727	1.5271	0.8703
9728	0.94613	0.926166
9729	1.22112	0.900391
9730	1.25002	0.873064
9731	1.65795	0.760425
9732	1.65795	1.1043
9733	0.946132	0.965219
9734	1.2366	0.90324
9735	0.946132	0.746569
9736	1.10023	0.834035
9737	1.2366	0.791748
9738	1.35856	0.425789
9739	0.946132	0.393172
9740	1.35856	0.272708
9741	1.08019	0.39435

9742	1.49261	0.298517
9743	1.49261	0.37934
9744	0.94613	0.518652
9745	1.29322	0.695271
9746	0.946132	0.605573
9747	1.29322	0.384011
9748	1.35856	0.571752
9749	1.70565	0.373238
9750	1.70565	0.515029
9751	0.946132	0.683098
9752	1.66635	2.1
9753	1.66635	2.1
9754	0.946133	2.1
9755	1.39558	2.1
9756	1.14793	2.1
9757	1.14793	2.1
9758	1.21691	2.1
9759	1.35381	0.772118
9760	1.3365	1.07595
9761	1.09905	0.874999
9762	0.946132	0.981927
9763	1.74418	1.05477
9764	1.74418	0.410799
9765	0.946132	0.855144
9766	1.84759	0.85057
9767	1.84759	0.578451
9768	0.94613	0.801542
9769	1.3904	0.844672
9770	1.34522	0.527782
9771	1.34522	0.495493
9772	1.40331	0.209687
9773	1.86047	0.873882
9774	1.86047	2.1
9775	0.946132	1.08879
9776	1.41619	2.1
9777	0.946132	0.565441
9778	1.3659	0.916714
9779	1.41619	0.942811
9780	1.28464	1.95748
9781	1.14496	1.3233
9782	1.57806	1.40842
9783	1.7753	1.73894
9784	0.851541	1.42355
9785	0.946131	1.54415
9786	0.872786	1.48034
9787	0.85062	0.797957
9788	1.52635	0.913963
9789	1.63785	0.813352
9790	1.69972	0.992793
9791	0.946132	1.04836
9792	1.37493	0.823033
9793	1.23854	0.831477
9794	1.41134	2.00452
9795	1.27079	1.63488
9796	1.736	1.20222
9797	1.736	2.1
9798	0.946132	0.923293
9799	0.946133	1.46411

9800	1.21177	1.70449
9801	0.946131	0.318537
9802	0.946129	0.242124
9803	0.94613	0.332485
9804	0.946131	0.20326
9805	0.946131	0.224655
9806	1.35403	0.231516
9807	0.946131	0.348565
9808	1.37052	0.595862
9809	0.946132	0.92033
9810	0.94613	0.772304
9811	0.946133	0.638513
9812	0.946131	0.672391
9813	0.946131	0.785593
9814	0.946131	0.687068
9815	1.24249	0.507279
9816	0.946132	0.500219
9817	0.946133	0.400804
9818	0.946132	0.535374
9819	0.946132	0.431866
9820	0.946132	0.519224
9821	0.946132	0.533564
9822	0.946132	2.1
9823	0.946132	1.95096
9824	0.946131	2.1
9825	0.946132	2.1
9826	0.946132	2.1
9827	0.946132	2.1
9828	1.20608	2.1
9829	1.41232	2.1
9830	0.946128	2.1
9831	0.946129	2.1
9832	0.94613	2.1
9833	0.94613	2.1
9834	0.94613	2.1
9835	0.94613	2.1
9836	0.946132	0.359575
9837	0.946131	0.40498
9838	0.946131	0.460838
9839	0.946132	0.201271
9840	0.946132	0.281076
9841	0.946131	0.402382
9842	0.946132	0.330195
9843	0.946116	0.901246
9844	0.94612	1.35281
9845	0.946118	1.08755
9846	0.946124	2.1
9847	0.946118	1.0634
9848	1.41561	1.49113
9849	0.946131	1.05433
9850	0.945846	2.1
9851	1.03508	2.1
9852	0.77579	1.43466
9853	0.658908	2.1
9854	1.17474	2.1
9855	0.94303	2.1
9856	0.849189	2.1
9857	0.94298	2.1

9858	1.03963	2.1
9859	0.946131	1.36868
9860	0.946131	1.20903
9861	0.946131	2.1
9862	0.946131	1.03465
9863	0.946131	0.82349
9864	0.946131	1.12843
9865	1.32819	1.27985
9866	0.946133	1.05505
9867	0.946129	2.1
9868	0.946128	1.73782
9869	0.94613	1.61662
9870	0.946131	2.1
9871	0.946131	1.0349
9872	0.946131	1.47528
9873	0.954072	0.0530246
9874	1.28895	0.0519605
9875	0.946133	0.061469
9876	1.15803	0.0649443
9877	1.29689	0.0544678
9878	1.29689	0.0603309
9879	0.946132	0.061604
9880	0.946132	1.23691
9881	0.946132	1.25114
9882	0.946132	1.12747
9883	0.946132	1.32896
9884	0.946132	1.51531
9885	0.946131	1.56473
9886	1.0264	1.54751
9887	0.946126	0.727806
9888	0.946132	0.640882
9889	0.946131	0.640933
9890	0.94613	0.66322
9891	0.946129	0.801445
9892	0.94613	0.726894
9893	1.35977	0.728963
9894	0.946132	0.705864
9895	0.946133	0.703235
9896	0.876632	0.722867
9897	0.946132	0.700769
9898	1.01566	0.728899
9899	1.01566	0.702832
9900	0.876606	0.713786
9901	0.946131	0.18855
9902	0.946128	0.187645
9903	0.946128	0.22929
9904	0.94613	0.178336
9905	0.94613	0.253767
9906	1.24979	0.152109
9907	0.94613	0.243752
9908	0.946131	0.135067
9909	0.946131	0.160062
9910	0.921762	0.129701
9911	0.9705	0.109561
9912	0.9705	0.121425
9913	0.946131	0.148468
9914	0.921791	0.143329
9915	0.810944	0.566823

9916	0.810945	0.665827
9917	0.810959	0.611626
9918	2.42267	0.423189
9919	1.35168	0.55243
9920	1.08132	0.574388
9921	1.08132	0.474101
9922	1.08131	0.580194
9923	0.946133	0.385256
9924	0.946132	0.425377
9925	0.946132	0.453608
9926	0.946133	0.288053
9927	0.946133	0.478662
9928	0.946132	0.641487
9929	0.946133	0.47364
9930	0.946131	0.925869
9931	0.946131	1.09294
9932	0.94613	0.870228
9933	0.851514	0.882127
9934	0.946131	0.969215
9935	0.965268	1.0179
9936	0.851513	0.901576
9937	0.946122	2.1
9938	0.946123	2.1
9939	0.946126	2.1
9940	0.946124	2.1
9941	0.946124	2.1
9942	0.946118	2.1
9943	0.94613	2.1
9944	0.946131	0.695724
9945	1.30448	0.688415
9946	0.946132	0.7
9947	0.946129	0.690388
9948	0.946131	0.785515
9949	0.946131	0.692511
9950	0.94613	0.699464
9951	0.946133	1.04108
9952	0.946128	2.1
9953	0.946128	1.91235
9954	0.94613	1.17179
9955	1.36503	2.1
9956	0.946131	1.83322
9957	0.946131	2.1
9958	0.810947	0.0763326
9959	0.810947	0.0724876
9960	2.48233	0.0683914
9961	1.3517	0.0655766
9962	1.08132	0.0738563
9963	1.08132	0.0672068
9964	1.08133	0.07046
9965	0.810934	0.0735044
9966	1.48874	0.0544051
9967	2.48233	0.0545442
9968	2.2369	0.0577197
9969	0.946133	0.832547
9970	0.946132	0.746164
9971	0.946132	0.877014
9972	0.946133	0.867435
9973	0.946133	0.878859

9974	1.41851	0.812286
9975	0.946132	0.782606
9976	1.03925	2.1
9977	0.946131	2.1
9978	0.94613	2.1
9979	0.946128	2.1
9980	0.946133	2.1
9981	0.944743	2.1
9982	1.13703	2.1
9983	0.946132	2.1
9984	0.946128	2.1
9985	0.946128	2.1
9986	0.946131	2.1
9987	0.94613	2.1
9988	0.94613	2.1
9989	0.94613	2.1
9990	0.946131	0.140535
9991	0.946132	0.173447
9992	0.946131	0.138968
9993	0.946132	0.131295
9994	0.946132	0.128167
9995	0.946131	0.143957
9996	1.20057	0.137361
9997	0.946129	2.1
9998	0.94613	2.1
9999	0.94613	2.1
10000	0.946128	2.1
10001	0.946132	2.1
10002	0.94613	2.1
10003	1.38302	2.1
10004	0.856567	0.333834
10005	1.14319	0.353207
10006	0.716526	0.292191
10007	1.20631	0.298815
10008	0.626936	0.236984
10009	0.946131	0.329262
10010	0.94613	0.409504
10011	1.39502	0.253047
10012	0.881445	0.199757
10013	0.946128	0.33374
10014	0.94613	0.240726
10015	0.881447	0.274661
10016	0.946117	0.25448
10017	0.946116	0.248344
10018	0.88146	0.439964
10019	0.946132	2.1
10020	1.39053	2.1
10021	0.946132	2.1
10022	0.946132	2.1
10023	0.946132	2.1
10024	0.946132	2.1
10025	0.946131	2.1
10026	0.946131	2.1
10027	0.946131	2.1
10028	0.946131	2.1
10029	0.946132	2.1
10030	0.946132	2.1
10031	1.40571	2.1

10032	0.946132	2.1
10033	0.94613	0.708599
10034	0.946127	0.730848
10035	0.946128	0.709379
10036	0.946131	0.712029
10037	0.946127	0.706069
10038	1.25819	0.709505
10039	0.946129	0.708579
10040	0.946129	0.943133
10041	1.29054	0.967446
10042	0.94613	0.938797
10043	0.94613	0.937159
10044	0.946131	1.24676
10045	0.946131	0.995117
10046	0.94613	0.960969
10047	0.810944	0.085032
10048	0.810941	0.0945522
10049	1.61558	0.0782594
10050	1.3517	0.0831673
10051	1.08131	0.0946456
10052	1.08132	0.0904509
10053	1.08133	0.0828237
10054	0.810931	0.0841388
10055	1.02036	1.2922
10056	0.946132	0.94658
10057	1.02036	0.94173
10058	0.946132	0.828549
10059	1.1576	0.910241
10060	0.964656	1.02621
10061	0.927608	0.831463
10062	0.946128	2.1
10063	1.38813	2.1
10064	0.946129	2.1
10065	0.946131	2.1
10066	0.946131	2.1
10067	0.94613	2.1
10068	0.946131	2.1
10069	0.946131	2.1
10070	0.94613	0.841374
10071	0.94613	2.1
10072	1.41665	2.1
10073	0.946132	0.84678
10074	0.946131	1.95167
10075	0.94613	0.972024
10076	0.946118	0.69692
10077	0.946118	0.613691
10078	0.946116	0.64018
10079	0.946126	0.401327
10080	0.94612	0.558754
10081	0.946131	0.685121
10082	0.946105	0.570933
10083	0.946132	2.1
10084	0.946132	2.1
10085	0.946132	2.1
10086	0.946131	2.1
10087	0.946132	2.1
10088	0.946132	2.1
10089	0.946132	2.1

10090	0.864997	0.0990352
10091	0.786573	0.098636
10092	0.978561	0.101833
10093	1.47385	0.0941132
10094	1.68652	0.0914489
10095	1.27803	0.0884546
10096	1.19771	0.102185
10097	1.27595	0.0969175
10098	0.946127	0.800617
10099	1.3771	0.769171
10100	0.946127	0.762445
10101	0.946131	0.83667
10102	0.946129	0.752512
10103	0.946129	0.810078
10104	0.946129	0.77599
10105	0.95697	0.0650045
10106	1.48133	0.0737553
10107	1.24499	0.0703144
10108	1.5295	0.0735313
10109	0.938161	0.0664118
10110	0.808717	0.0684214
10111	1.3542	0.0728951
10112	0.946133	0.954731
10113	1.14444	0.92349
10114	0.946132	0.914631
10115	0.946132	0.936896
10116	0.946133	1.14597
10117	0.946132	1.01007
10118	0.946132	0.948248
10119	0.946131	0.22644
10120	0.946132	0.35343
10121	0.946131	0.215434
10122	0.946132	0.219837
10123	0.946131	0.313298
10124	0.946132	0.207168
10125	0.946132	0.321882
10126	0.946132	2.1
10127	0.946133	2.1
10128	0.946133	2.1
10129	0.946132	2.1
10130	1.29322	2.1
10131	0.946132	2.1
10132	0.946132	2.1
10133	0.946131	2.1
10134	0.946132	2.1
10135	0.946131	2.1
10136	0.946132	2.1
10137	1.28441	2.1
10138	0.946131	2.1
10139	0.946131	2.1
10140	0.946113	2.1
10141	0.946118	2.1
10142	0.946118	1.42273
10143	1.28073	2.1
10144	0.946117	1.96415
10145	0.946131	2.1
10146	0.946105	2.1
10147	0.810945	2.1

10148	0.810942	2.1
10149	2.75904	2.1
10150	1.3517	2.1
10151	1.08131	2.1
10152	1.08133	2.1
10153	1.08132	2.1
10154	0.81093	2.1
10155	0.946127	2.1
10156	1.37952	2.1
10157	1.86965	2.1
10158	0.946127	2.1
10159	1.86965	2.1
10160	1.28228	2.1
10161	1.37952	2.1
10162	0.946128	2.1
10163	0.946132	2.1
10164	1.14042	2.1
10165	0.946132	2.1
10166	1.14042	2.1
10167	1.09974	2.1
10168	1.09974	2.1
10169	0.946132	2.1
10170	0.946132	0.636957
10171	0.946132	0.578301
10172	0.946133	0.696204
10173	0.946132	0.55192
10174	0.946132	0.696232
10175	0.946132	0.575856
10176	0.946133	0.693245
10177	1.31817	1.30909
10178	0.946131	1.11512
10179	0.946131	1.26986
10180	0.946131	1.22555
10181	0.946131	1.01096
10182	0.946131	1.1422
10183	0.946131	1.13569
10184	0.946132	2.1
10185	0.946132	2.1
10186	0.946132	2.1
10187	0.946132	2.1
10188	0.946132	2.1
10189	0.946132	2.1
10190	1.35048	2.1
10191	0.946131	2.1
10192	0.946131	2.1
10193	0.94613	2.1
10194	0.946133	2.1
10195	0.946133	2.1
10196	1.35943	2.1
10197	0.946131	2.1
10198	0.946133	2.1
10199	0.946132	2.1
10200	0.946132	2.1
10201	0.946133	2.1
10202	0.946133	2.1
10203	0.946133	2.1
10204	0.990712	2.1
10205	0.965725	1.71749

10206	0.946129	0.964271
10207	0.946133	1.22665
10208	1.21721	1.05126
10209	0.877931	0.874747
10210	0.946131	1.16738
10211	0.946131	0.945414
10212	0.946132	0.810179
10213	0.946131	0.707948
10214	0.946131	0.925993
10215	0.946133	0.788442
10216	0.946128	1.06903
10217	0.946131	0.878736
10218	1.39575	0.878317
10219	0.810943	0.78868
10220	0.810942	0.792664
10221	2.58794	0.696679
10222	1.21652	0.744304
10223	0.946154	0.775291
10224	0.979952	0.773474
10225	1.0813	0.772427
10226	0.810959	0.776622
10227	0.924071	0.751723
10228	1.16453	0.566161
10229	1.28586	0.505428
10230	0.946131	0.759572
10231	1.1227	0.497363
10232	1.16462	0.610565
10233	0.851553	0.771357
10234	0.709578	0.707729
10235	0.810943	0.878157
10236	0.810959	0.799592
10237	1.82615	0.723846
10238	1.21651	0.725297
10239	1.08132	0.75966
10240	0.946155	0.749295
10241	0.979937	0.838358
10242	0.946128	1.21098
10243	0.946132	2.1
10244	0.946132	0.993003
10245	0.94613	1.92338
10246	0.94613	1.68345
10247	0.94613	0.977311
10248	0.94613	1.49716
10249	0.946131	1.62606
10250	1.29735	2.01014
10251	0.946131	2.1
10252	0.946131	0.988567
10253	0.946131	1.27734
10254	0.946131	0.944725
10255	0.946131	1.86241
10256	0.946128	2.07054
10257	1.33266	1.09928
10258	0.94613	1.15833
10259	0.946131	1.64952
10260	0.94613	0.865762
10261	0.946131	1.09522
10262	0.946131	1.05017
10263	0.946131	0.871259

10264	0.946131	2.1
10265	0.946131	0.837565
10266	1.40608	0.839527
10267	0.946131	2.1
10268	0.946131	0.928533
10269	0.946131	1.00774
10270	0.946131	1.13407
10271	0.946131	1.60205
10272	0.946131	1.19264
10273	1.2334	1.2354
10274	0.946129	1.28132
10275	0.946131	1.24086
10276	0.946131	1.51774
10277	0.946133	1.87016
10278	0.946133	2.1
10279	0.946132	1.27362
10280	1.29198	1.97889
10281	0.946132	1.71891
10282	0.946132	1.6791
10283	0.946132	2.02728
10284	0.946131	2.1
10285	0.946128	2.1
10286	0.946128	2.1
10287	0.946131	2.1
10288	0.946127	2.1
10289	0.94613	2.1
10290	0.946129	2.1
10291	0.946123	2.1
10292	0.946126	2.1
10293	0.946125	2.1
10294	1.38639	2.1
10295	0.946126	2.1
10296	0.946132	2.1
10297	0.946119	2.1
10298	0.946128	0.692246
10299	0.946132	0.677619
10300	0.946131	0.710868
10301	0.94613	0.554177
10302	0.94613	0.642962
10303	0.946131	0.672555
10304	0.94613	0.667274
10305	0.946131	0.628105
10306	0.946131	0.618868
10307	0.946131	0.700906
10308	0.946131	0.414242
10309	0.946131	0.54978
10310	0.946131	0.648697
10311	0.946131	0.585448
10312	0.810945	2.1
10313	0.810943	2.1
10314	0.810933	2.1
10315	2.24688	2.1
10316	1.3517	2.1
10317	1.08132	2.1
10318	1.08132	2.1
10319	1.08133	2.1
10320	1.25809	0.671596
10321	0.946131	0.851245

10322	1.25809	0.697858
10323	0.946129	0.829365
10324	0.94613	0.703727
10325	0.946131	0.829357
10326	0.946131	0.740551
10327	0.946131	0.430254
10328	0.946131	0.399519
10329	0.946129	0.418301
10330	1.28911	0.388352
10331	0.946131	0.322921
10332	0.946131	0.392059
10333	0.946131	0.432916
10334	0.94613	0.723768
10335	0.946131	0.720707
10336	0.94613	0.70587
10337	0.946128	0.724761
10338	0.946132	0.705385
10339	1.38954	0.716878
10340	0.946131	0.716989
10341	0.946131	0.458474
10342	0.946131	0.764889
10343	0.946131	0.808131
10344	1.37981	0.740617
10345	0.946131	0.915096
10346	0.946131	0.775035
10347	0.946131	0.79331
10348	0.946131	1.5811
10349	0.946127	2.1
10350	0.946127	2.1
10351	0.946131	1.99495
10352	0.946127	2.1
10353	0.946129	2.1
10354	0.946128	2.1
10355	0.94613	2.1
10356	0.946126	2.1
10357	0.946127	2.1
10358	0.946131	2.1
10359	0.946126	2.09414
10360	0.946128	2.1
10361	0.946128	2.1
10362	0.946133	0.761151
10363	0.946132	0.739563
10364	0.946132	0.74694
10365	0.946132	0.749305
10366	0.946132	0.759771
10367	1.13511	0.656281
10368	0.946132	0.736746
10369	0.946132	1.71558
10370	0.946129	1.75101
10371	0.94613	1.51181
10372	0.946131	1.85365
10373	0.946131	1.94036
10374	0.94613	2.1
10375	1.27575	2.1
10376	0.946129	2.1
10377	0.946131	2.1
10378	0.946131	2.1
10379	0.946128	2.1

10380	0.946133	2.1
10381	0.946131	2.1
10382	0.94613	2.1
10383	1.15581	0.106341
10384	0.946132	0.0985107
10385	0.946131	0.14191
10386	0.94613	0.109413
10387	1.15581	0.112048
10388	0.94613	0.121508
10389	0.946131	0.1433
10390	0.611407	1.03785
10391	0.946131	0.899176
10392	1.37335	0.991235
10393	1.15086	1.08155
10394	0.617549	0.945533
10395	0.851553	1.00645
10396	1.14592	1.07864
10397	1.22618	1.23213
10398	0.946132	0.904916
10399	0.946132	1.25738
10400	0.946132	0.939722
10401	0.946131	0.955855
10402	0.946132	1.06356
10403	0.946132	1.02954
10404	0.946131	2.1
10405	0.94613	2.1
10406	0.946131	2.1
10407	0.946133	2.1
10408	0.946129	2.1
10409	0.946131	2.1
10410	0.946131	2.1
10411	0.810945	0.813386
10412	0.810943	1.06033
10413	2.50715	0.980582
10414	1.3517	0.92188
10415	1.08133	0.893949
10416	1.08132	1.03972
10417	1.08132	0.722005
10418	0.810932	0.838543
10419	1.07811	1.09841
10420	0.94613	0.872168
10421	0.946128	1.14419
10422	1.25358	0.780159
10423	0.946131	0.929973
10424	1.25358	0.86151
10425	0.94613	0.882503
10426	0.946129	2.1
10427	1.09197	2.1
10428	0.946131	2.1
10429	1.09197	2.1
10430	1.09532	2.1
10431	1.09532	2.1
10432	0.946131	2.1
10433	1.58788	2.1
10434	1.34255	2.1
10435	0.594912	2.1
10436	0.58894	2.1
10437	0.946129	2.1

10438	1.16679	2.1
10439	1.04071	2.1
10440	0.535418	2.1
10441	0.946131	2.09981
10442	0.946128	2.1
10443	0.946131	1.97593
10444	1.20221	2.1
10445	0.946128	2.1
10446	0.94613	2.1
10447	1.20221	2.1
10448	0.946132	2.1
10449	0.946133	2.1
10450	0.946132	2.1
10451	0.946132	2.1
10452	0.946133	2.1
10453	0.946133	2.1
10454	0.946132	2.1
10455	0.946132	1.1425
10456	0.946132	1.29527
10457	0.946133	1.61976
10458	0.946132	1.18622
10459	1.411	1.38386
10460	0.946132	1.26889
10461	0.946133	1.35237
10462	1.05575	0.634646
10463	0.563734	0.604888
10464	1.05575	0.652141
10465	0.773833	0.475987
10466	0.751686	0.524563
10467	0.75884	0.629852
10468	0.815612	0.506805
10469	0.793465	0.559398
10470	0.768563	0.696909
10471	0.946129	0.533703
10472	0.946125	0.463479
10473	0.946126	0.67016
10474	0.94613	0.471192
10475	0.946124	0.689309
10476	1.31678	0.411463
10477	0.946128	0.511754
10478	0.946132	0.758838
10479	1.29914	0.689717
10480	0.946132	0.677014
10481	0.946132	0.718796
10482	0.946132	0.760806
10483	1.29914	0.575253
10484	0.946132	0.734159
10485	0.810946	0.753576
10486	0.810943	0.748353
10487	2.21082	0.607691
10488	1.35168	0.684255
10489	1.08132	0.718041
10490	1.08132	0.725941
10491	1.0813	0.746108
10492	0.81096	0.739445
10493	0.625813	0.162758
10494	0.946132	0.170908
10495	0.946131	0.225505

10496	0.946132	0.205733
10497	0.946131	0.22387
10498	1.39301	0.251684
10499	0.625812	0.193784
10500	0.810946	1.32483
10501	0.810945	2.1
10502	0.810931	1.95891
10503	2.70043	2.1
10504	1.3517	2.05061
10505	1.08131	1.39228
10506	1.08131	2.1
10507	1.08133	1.4749
10508	0.810943	2.1
10509	0.810944	2.1
10510	0.810958	2.1
10511	2.44802	2.1
10512	1.35168	2.1
10513	1.08132	2.1
10514	1.08132	2.1
10515	1.0813	2.1
10516	0.946131	0.705373
10517	0.946129	0.703449
10518	0.946128	0.702132
10519	0.946131	0.70746
10520	1.3492	0.705951
10521	1.3492	0.706412
10522	0.94613	0.703009
10523	0.946132	0.717211
10524	0.946132	0.707486
10525	0.946133	0.707793
10526	0.946133	0.702002
10527	0.946132	0.719598
10528	0.946132	0.701493
10529	0.946132	0.71771
10530	0.946129	0.420623
10531	0.946133	0.364618
10532	0.946131	0.445615
10533	0.94613	0.370767
10534	0.946131	0.485074
10535	0.946131	0.405221
10536	0.946131	0.402221
10537	0.946119	0.330394
10538	0.946119	0.312941
10539	0.946119	0.36008
10540	0.946125	0.249219
10541	0.94612	0.371715
10542	0.946132	0.335718
10543	0.946107	0.312966
10544	0.946133	2.1
10545	0.946132	2.1
10546	0.946132	2.1
10547	0.946132	2.1
10548	0.946132	2.1
10549	0.946133	2.1
10550	1.3868	2.1
10551	0.810947	2.1
10552	0.810946	2.1
10553	0.81096	2.1

10554	2.66094	2.1
10555	1.35168	2.1
10556	1.08132	2.1
10557	1.08132	2.1
10558	1.0813	2.1
10559	0.810946	2.1
10560	0.810946	2.1
10561	0.810933	2.1
10562	2.75737	2.1
10563	1.3517	2.1
10564	1.08132	2.1
10565	1.08132	2.1
10566	1.08133	2.1
10567	0.946133	2.1
10568	0.946132	2.1
10569	0.946132	1.67469
10570	0.946132	2.1
10571	0.946133	1.4699
10572	0.946132	2.1
10573	0.946133	2.1
10574	0.946133	2.1
10575	0.946133	2.1
10576	0.946132	2.1
10577	0.946132	2.1
10578	0.946133	2.1
10579	0.946132	2.1
10580	1.28569	2.1
10581	0.946132	1.42984
10582	0.946133	2.1
10583	0.946133	2.1
10584	0.946132	1.89016
10585	0.946132	2.1
10586	0.946132	2.06553
10587	0.946133	2.0433
10588	0.946132	0.445103
10589	0.946131	0.469933
10590	0.94613	0.409105
10591	0.946133	0.396252
10592	0.946128	0.48822
10593	0.946131	0.412894
10594	0.946131	0.516265
10595	0.946131	1.83036
10596	0.94613	1.73395
10597	0.946131	2.1
10598	0.94613	1.87068
10599	1.41833	1.1725
10600	0.946131	1.07585
10601	0.946131	1.85964
10602	1.26155	0.140825
10603	0.946131	0.210812
10604	0.946131	0.156484
10605	0.946132	0.127383
10606	0.946132	0.152408
10607	0.946131	0.154156
10608	0.946132	0.129979
10609	0.810946	2.1
10610	0.810946	2.1
10611	0.810959	2.1

10612	2.47938	2.1
10613	1.35168	2.1
10614	1.08132	2.1
10615	1.08132	2.1
10616	1.08131	2.1
10617	0.946131	0.750478
10618	0.94613	0.718901
10619	0.94613	0.891869
10620	0.946132	0.580912
10621	0.946128	1.02769
10622	0.94613	0.848628
10623	0.94613	0.874476
10624	0.94613	0.174581
10625	0.946131	0.184571
10626	0.94613	0.253547
10627	0.946129	0.229823
10628	0.946132	0.207864
10629	0.94613	0.229881
10630	0.946131	0.207191
10631	0.946132	2.1
10632	0.946131	2.1
10633	0.946128	2.1
10634	1.40756	2.1
10635	0.946131	2.1
10636	0.946129	2.1
10637	0.94613	2.1
10638	0.946131	1.29149
10639	0.946131	1.27723
10640	0.946131	1.77173
10641	0.946132	1.34353
10642	1.31947	1.35841
10643	0.946131	1.32047
10644	0.946131	1.45216
10645	0.946131	2.1
10646	1.25768	2.1
10647	0.946132	2.1
10648	0.946132	2.1
10649	0.946129	2.1
10650	0.946131	2.1
10651	0.94613	2.1
10652	0.946132	0.7
10653	0.946129	0.7
10654	0.946129	0.7
10655	0.94613	0.7
10656	1.31716	0.7
10657	1.31716	0.7
10658	0.946131	0.7
10659	0.810943	0.996386
10660	0.810942	2.1
10661	0.81093	1.64016
10662	2.37052	2.1
10663	1.3517	1.32504
10664	1.08132	0.944515
10665	1.08132	1.98219
10666	1.08133	1.04615
10667	0.946128	0.902622
10668	1.10335	0.79452
10669	0.94613	0.79435

10670	0.946131	0.92846
10671	0.946131	0.801056
10672	1.10335	0.916268
10673	0.94613	0.922379
10674	0.946132	1.02307
10675	1.18818	0.925829
10676	0.946132	0.917566
10677	0.946132	1.03534
10678	0.946132	0.910167
10679	1.18818	1.04007
10680	0.946132	1.0417
10681	0.946125	2.1
10682	0.94612	2.1
10683	0.946119	2.1
10684	0.946119	2.1
10685	0.946119	2.1
10686	0.946132	2.1
10687	1.31522	2.1
10688	1.28803	1.78703
10689	0.946132	0.973697
10690	0.946132	2.1
10691	0.946133	2.038
10692	0.946133	0.938276
10693	0.946133	1.73772
10694	0.946132	1.28958
10695	0.946133	0.849243
10696	0.946132	1.03997
10697	0.946132	0.904788
10698	0.946132	0.796756
10699	0.946133	0.782
10700	0.946132	0.781048
10701	1.37658	0.836593
10702	1.02239	1.54705
10703	0.946128	1.01903
10704	0.946132	1.28753
10705	1.02239	1.09686
10706	0.94613	0.946854
10707	0.96283	1.27586
10708	0.92943	1.00009
10709	0.946132	0.106058
10710	0.946131	0.110914
10711	0.946126	0.149769
10712	0.946129	0.119032
10713	0.946128	0.125128
10714	0.946128	0.118111
10715	0.946129	0.118834
10716	0.810946	0.178367
10717	0.810944	0.130836
10718	2.22493	0.100305
10719	1.21651	0.0967328
10720	1.0813	0.129171
10721	0.946154	0.10471
10722	0.979951	0.108674
10723	0.70959	0.107874
10724	0.946132	0.241333
10725	0.946131	0.35962
10726	0.946131	0.235771
10727	0.946133	0.229621

10728	0.946131	0.356674
10729	0.946132	0.249785
10730	0.946132	0.330107
10731	0.946132	1.77456
10732	0.946131	1.36783
10733	0.946132	2.07741
10734	0.946132	1.0339
10735	0.946132	0.932519
10736	0.946132	1.07707
10737	1.40924	1.45782
10738	0.946131	2.1
10739	0.946128	2.1
10740	0.946127	2.1
10741	0.94613	2.1
10742	1.41743	2.1
10743	0.946128	2.1
10744	0.946129	2.1
10745	1.37675	0.771422
10746	0.946128	0.793975
10747	0.94613	0.767495
10748	0.94613	0.853112
10749	0.94613	0.767779
10750	0.946129	0.797045
10751	0.94613	0.815335
10752	0.946132	0.726863
10753	0.946132	0.765215
10754	0.946132	0.812819
10755	1.35721	0.595513
10756	0.946132	0.926314
10757	0.946132	0.80418
10758	1.35721	0.779349
10759	0.946133	0.882858
10760	0.946133	0.832049
10761	0.946132	0.853124
10762	1.10003	0.79897
10763	0.946133	0.788386
10764	0.946132	0.835238
10765	0.946132	0.828771
10766	0.946132	0.567553
10767	0.946131	0.405736
10768	0.946131	0.484019
10769	0.946131	0.635133
10770	1.0616	0.431172
10771	1.0616	0.473196
10772	0.946132	0.638404
10773	0.946132	0.661274
10774	0.946131	0.507768
10775	0.946131	0.581114
10776	0.946131	0.695837
10777	1.09402	0.522428
10778	1.09402	0.56211
10779	0.946131	0.703248
10780	0.946132	0.18835
10781	0.946126	0.153299
10782	1.24093	0.177018
10783	0.946129	0.205289
10784	0.946129	0.185138
10785	0.946128	0.205843

10786	0.946129	0.193491
10787	0.946131	0.296688
10788	0.946131	0.254492
10789	1.2929	0.2833
10790	0.946131	0.354189
10791	0.946131	0.316
10792	0.946131	0.349809
10793	0.946131	0.320137
10794	0.810946	0.120255
10795	0.810945	0.0924682
10796	0.810959	0.108295
10797	2.41233	0.0989836
10798	1.35168	0.096978
10799	1.08132	0.10435
10800	1.08132	0.100601
10801	1.0813	0.112371
10802	0.810946	1.36695
10803	0.810943	2.00274
10804	0.750103	1.70017
10805	2.22778	2.08419
10806	1.27059	1.93072
10807	1.00021	1.53066
10808	1.02049	2.04999
10809	1.08133	1.50608
10810	0.946128	1.66219
10811	0.946132	2.1
10812	0.946132	2.1
10813	0.94613	2.09042
10814	0.94613	2.1
10815	0.94613	2.1
10816	0.946131	2.1
10817	0.946126	0.769217
10818	0.946131	0.75623
10819	0.946127	0.791462
10820	1.3324	0.744073
10821	0.946127	0.74724
10822	0.946128	0.772941
10823	0.946128	0.810394
10824	0.94613	2.1
10825	0.946131	2.1
10826	0.94613	2.1
10827	0.946128	2.1
10828	0.946133	2.1
10829	0.946131	2.1
10830	0.946131	2.1
10831	0.946133	2.1
10832	0.946132	2.1
10833	0.946133	2.1
10834	0.946133	2.1
10835	0.946132	2.1
10836	0.946132	2.1
10837	1.36541	2.1
10838	0.946131	0.877539
10839	0.94613	2.1
10840	0.946131	1.02511
10841	0.94613	0.981192
10842	1.39509	1.38799
10843	0.946131	0.881822

10844	0.946131	1.60013
10845	0.946133	2.1
10846	0.946133	2.1
10847	0.946132	2.1
10848	0.946132	2.1
10849	0.946133	2.1
10850	0.946132	2.1
10851	1.29319	2.1
10852	1.01196	0.278341
10853	0.946131	0.451787
10854	0.946132	0.243998
10855	0.946132	0.309004
10856	0.946132	0.410295
10857	0.946132	0.412074
10858	0.946132	0.324421
10859	0.946112	2.1
10860	0.946118	2.1
10861	0.946116	2.1
10862	0.946117	2.1
10863	0.946118	2.1
10864	0.94613	2.1
10865	0.946105	2.1
10866	1.28684	1.00768
10867	0.946131	1.05352
10868	0.946132	1.03414
10869	0.946132	1.19626
10870	0.946132	1.27529
10871	0.946132	1.0722
10872	0.946132	1.2624
10873	0.94613	1.95373
10874	0.94613	2.06543
10875	0.946128	1.62629
10876	0.946131	1.57947
10877	0.946131	1.77159
10878	0.94613	1.63476
10879	1.10185	1.9815
10880	0.810947	0.0798317
10881	0.810945	0.0917649
10882	1.57348	0.0789941
10883	1.3517	0.0814416
10884	1.08131	0.093244
10885	1.08132	0.0875832
10886	1.08133	0.0769713
10887	0.810932	0.0817249
10888	0.946119	0.74182
10889	0.946116	0.725682
10890	0.946117	0.739585
10891	0.946121	0.71465
10892	0.946118	0.71912
10893	0.94613	0.72359
10894	0.946105	0.721591
10895	0.94612	0.784663
10896	0.946122	0.725619
10897	0.946119	0.74865
10898	0.946119	0.782852
10899	1.20884	0.730368
10900	0.946132	0.751697
10901	0.946107	0.747453

10902	2.67849	1.16382
10903	1.17709	1.133
10904	0.605418	1.24716
10905	0.286984	1.17508
10906	0.172654	1.19435
10907	0.810946	0.994943
10908	0.940878	0.954448
10909	0.941235	0.942422
10910	1.26696	0.962074
10911	1.56837	0.981755
10912	1.13737	0.968734
10913	1.34115	0.972863
10914	1.24889	0.946392
10915	0.946132	1.30848
10916	0.946129	1.07631
10917	0.946128	1.20157
10918	0.94613	1.13293
10919	0.94613	1.32094
10920	0.946131	1.24347
10921	0.94613	1.20545
10922	0.946116	2.1
10923	0.94612	2.1
10924	0.946118	2.1
10925	0.946123	2.1
10926	0.946118	2.1
10927	0.946131	2.1
10928	1.23836	2.1
10929	0.810946	0.112518
10930	0.810946	0.107171
10931	0.810959	0.113687
10932	2.80677	0.0990436
10933	1.35168	0.106568
10934	1.08132	0.0977037
10935	1.08132	0.108433
10936	1.08131	0.116325
10937	0.810947	0.706212
10938	0.810947	0.756957
10939	2.41574	0.755842
10940	1.21653	0.749531
10941	1.08133	0.727481
10942	0.946153	0.7521
10943	0.979949	0.759898
10944	0.709567	0.71257
10945	0.94613	2.1
10946	0.946131	2.1
10947	0.946128	2.1
10948	1.34782	2.1
10949	0.946128	2.1
10950	0.946129	2.1
10951	1.34782	2.1
10952	0.946132	2.02061
10953	0.946132	2.1
10954	0.94613	2.1
10955	1.16023	2.1
10956	0.946131	2.1
10957	0.946131	2.1
10958	1.16023	2.1
10959	0.946131	1.01656

10960	0.946127	2.1
10961	0.946127	1.73137
10962	0.946131	1.3444
10963	0.946128	2.1
10964	0.94613	1.09912
10965	0.946129	1.34461
10966	0.946133	0.715986
10967	0.946132	0.696646
10968	0.946132	0.710772
10969	0.946133	0.720696
10970	1.20045	0.693931
10971	1.20045	0.707014
10972	0.946132	0.720108
10973	0.918871	0.318819
10974	0.971961	0.299811
10975	1.37324	0.166909
10976	1.78255	0.151404
10977	1.25155	0.300472
10978	1.42172	0.152537
10979	1.40554	0.211866
10980	1.08862	0.228275
10981	1.03803	0.229696
10982	0.946132	0.236041
10983	0.946126	0.261013
10984	0.943647	0.257516
10985	0.948611	0.246816
10986	0.946129	0.277624
10987	0.946129	0.272985
10988	1.1699	0.897337
10989	0.946132	0.784834
10990	0.946133	0.947588
10991	0.946133	0.797123
10992	0.946132	0.825187
10993	0.946133	0.85746
10994	0.946132	0.876351
10995	0.946132	2.1
10996	0.946131	2.1
10997	0.946131	2.1
10998	0.946132	2.1
10999	0.946131	2.1
11000	0.946131	2.1
11001	0.946132	2.1
11002	0.946132	0.935942
11003	1.38623	0.895667
11004	0.946132	0.877415
11005	0.946132	0.862001
11006	0.946133	0.896906
11007	0.946132	0.834109
11008	0.946133	0.986417
11009	0.946132	2.1
11010	1.40098	2.1
11011	0.946131	2.1
11012	0.946131	2.1
11013	0.946132	2.1
11014	0.946132	2.1
11015	0.946132	2.1
11016	1.01777	0.753281
11017	0.946132	0.714692

11018	0.946133	0.813417
11019	1.68068	0.744541
11020	1.68068	0.840935
11021	1.41894	0.734486
11022	1.20787	0.7
11023	0.946132	0.717117
11024	0.946133	2.1
11025	0.946133	2.1
11026	0.946132	2.1
11027	1.40199	2.1
11028	0.946133	2.1
11029	0.946132	2.1
11030	0.946132	2.1
11031	0.810946	1.11734
11032	0.810947	0.947219
11033	2.37827	1.29617
11034	1.3517	1.15791
11035	1.08132	1.16508
11036	1.08132	1.17654
11037	1.08133	1.10292
11038	0.810934	1.09389
11039	0.946124	1.69923
11040	0.94613	1.25342
11041	1.40248	1.10104
11042	0.946131	1.07329
11043	0.946125	0.89457
11044	0.946127	1.22178
11045	0.946128	0.964764
11046	1.25703	1.81242
11047	0.87821	1.14224
11048	1.18912	1.7543
11049	0.946119	1.04905
11050	0.87825	1.17629
11051	1.1891	1.25647
11052	0.946107	1.43589
11053	0.946132	2.04137
11054	1.28543	1.67369
11055	0.946132	1.45348
11056	0.946132	1.18729
11057	0.946131	0.850086
11058	0.946132	0.853446
11059	0.946132	0.906877
11060	0.946115	2.1
11061	0.946119	2.1
11062	0.946116	2.1
11063	0.946123	2.1
11064	0.946118	2.1
11065	1.41636	2.1
11066	0.946129	2.1
11067	0.946132	0.973576
11068	0.946132	2.07701
11069	0.946132	1.15374
11070	0.946131	2.1
11071	0.946131	1.75645
11072	1.41432	2.09434
11073	0.946132	1.49985
11074	0.810947	0.284853
11075	0.810947	0.434111

11076	2.44433	0.204653
11077	1.21654	0.202514
11078	0.946154	0.372832
11079	0.97995	0.264918
11080	1.08133	0.236273
11081	0.810934	0.25324
11082	0.851542	0.180354
11083	0.835698	0.242271
11084	1.22216	0.18086
11085	0.946133	0.230434
11086	1.01217	0.217599
11087	1.09996	0.239004
11088	0.851529	0.280002
11089	0.946132	1.6248
11090	0.946132	1.9664
11091	0.946129	2.1
11092	0.94613	1.66384
11093	0.946131	2.07926
11094	0.946131	1.92902
11095	1.2254	2.1
11096	0.800897	1.26187
11097	0.838029	2.01986
11098	0.838013	1.49431
11099	0.93679	1.19067
11100	1.20217	1.28284
11101	1.16923	1.36576
11102	0.94948	1.32039
11103	0.958118	1.47454
11104	0.36751	1.40219
11105	0.800535	1.69797
11106	0.272087	1.07124
11107	0.428084	1.22101
11108	0.986335	1.68485
11109	0.946132	0.781276
11110	0.946133	0.843189
11111	1.19601	0.801643
11112	0.946132	0.790905
11113	0.946132	0.8145
11114	0.946133	0.877028
11115	0.946133	0.774682
11116	0.946132	1.27031
11117	1.36956	2.1
11118	0.946132	2.1
11119	0.946132	1.23146
11120	0.946132	2.1
11121	0.946133	1.41968
11122	0.946132	1.72902
11123	0.946132	0.197787
11124	0.946131	0.855218
11125	0.946131	0.43695
11126	1.34268	0.201669
11127	0.946133	0.811174
11128	0.946132	0.345814
11129	0.946131	0.772813
11130	0.946132	1.91261
11131	0.946131	2.03812
11132	0.946131	2.1
11133	0.946132	2.09928

11134	0.946132	2.02866
11135	0.946132	2.1
11136	0.946132	2.01098
11137	0.810946	1.08329
11138	0.810943	0.997569
11139	2.51472	1.0857
11140	1.3517	1.05236
11141	1.08132	0.992815
11142	1.08132	1.08317
11143	1.08133	1.05323
11144	0.810933	1.0612
11145	0.902316	0.956119
11146	1.04517	1.00401
11147	0.974924	0.871378
11148	0.530063	1.10956
11149	1.1272	1.02709
11150	0.68207	1.00535
11151	0.477061	1.08458
11152	0.810946	0.276868
11153	0.810943	0.458292
11154	0.810959	0.385223
11155	1.99211	0.359907
11156	1.35168	0.319064
11157	1.08132	0.341543
11158	1.08132	0.326597
11159	1.08131	0.462452
11160	0.94613	0.482074
11161	0.946131	0.403358
11162	0.946132	0.448093
11163	0.946132	0.434932
11164	0.946129	0.61284
11165	0.946131	0.476046
11166	1.16699	0.502913
11167	0.810943	1.19545
11168	0.810942	1.12121
11169	1.6943	1.4508
11170	1.27059	1.47397
11171	1.00021	1.42544
11172	1.02049	1.29423
11173	1.08133	1.20233
11174	0.750103	1.17342
11175	0.957813	1.22777
11176	0.946128	1.30917
11177	0.946132	1.09028
11178	1.04832	1.36629
11179	0.870714	1.08391
11180	0.94613	1.1437
11181	0.94613	1.20109
11182	1.86374	1.98794
11183	2.0815	1.16848
11184	1.15918	0.7
11185	0.946129	0.7
11186	1.15918	0.7001
11187	0.946131	0.7
11188	0.946131	0.7
11189	0.946131	0.7
11190	0.94613	0.7
11191	0.946119	0.9329

11192	1.3612	1.00058
11193	0.946125	0.975594
11194	0.946119	0.929746
11195	0.94612	1.23377
11196	0.946132	0.972908
11197	0.946107	0.965568
11198	0.810947	0.641077
11199	0.810947	0.647332
11200	2.50398	0.600264
11201	1.3517	0.55597
11202	1.08132	0.619691
11203	1.08133	0.644473
11204	1.08132	0.526642
11205	0.810934	0.639466
11206	0.946132	2.1
11207	0.946132	2.1
11208	0.946132	2.1
11209	1.39754	2.1
11210	0.946132	2.1
11211	0.946132	2.1
11212	0.946131	2.1
11213	0.946129	2.1
11214	1.41732	2.1
11215	0.946129	2.1
11216	0.946131	2.1
11217	0.946131	2.1
11218	0.946131	2.1
11219	0.946131	2.1
11220	0.946132	1.06052
11221	1.25276	1.55039
11222	0.946131	1.92072
11223	0.94613	1.07837
11224	0.94613	1.35433
11225	0.94613	1.11756
11226	0.946131	1.19289
11227	1.21261	1.10794
11228	0.94613	1.23107
11229	0.94613	1.58184
11230	0.946128	1.06003
11231	0.946131	1.34088
11232	0.94613	1.29166
11233	0.946129	1.41443
11234	0.946128	2.1
11235	0.946133	2.1
11236	0.946132	2.1
11237	0.946131	2.1
11238	0.94613	2.1
11239	0.946131	2.1
11240	1.39407	2.1
11241	0.946116	1.21979
11242	0.946119	1.23861
11243	0.946119	1.05091
11244	1.15369	1.23059
11245	0.946119	0.973519
11246	0.946131	1.12374
11247	0.946106	1.12011
11248	0.946131	2.08727
11249	0.946131	2.1

11250	0.946131	2.1
11251	0.946133	2.1
11252	0.946129	2.1
11253	0.946131	2.1
11254	0.946131	2.1
11255	1.19564	0.0804083
11256	0.498881	0.0918318
11257	1.33964	0.0799492
11258	0.498878	0.0895563
11259	0.923583	0.0836281
11260	0.831259	0.0898618
11261	0.553735	0.0793154
11262	0.946132	0.0930389
11263	0.946132	0.0988415
11264	0.946131	0.116834
11265	0.946131	0.0938476
11266	0.946132	0.1037
11267	0.946131	0.104189
11268	1.28673	0.10009
11269	0.946132	0.32557
11270	0.946132	0.309927
11271	1.33178	0.297706
11272	0.946132	0.332389
11273	0.946132	0.298033
11274	0.946131	0.339484
11275	0.946131	0.306045
11276	0.946128	0.743752
11277	0.946132	0.720269
11278	0.946129	0.75046
11279	0.946131	0.66612
11280	0.946131	0.703216
11281	0.946129	0.718137
11282	0.94613	0.710159
11283	0.946131	2.1
11284	0.946131	2.1
11285	0.946129	2.1
11286	1.40525	2.1
11287	0.94613	2.1
11288	0.94613	2.1
11289	1.40525	2.1
11290	0.946131	2.1
11291	0.946126	2.1
11292	0.946127	2.1
11293	0.946127	2.1
11294	1.4073	2.1
11295	0.946129	2.1
11296	0.946129	2.1
11297	0.946131	0.516617
11298	0.946131	0.407898
11299	0.946132	0.583257
11300	0.946131	0.364746
11301	0.946131	0.374401
11302	1.05016	0.420737
11303	0.946132	0.580539
11304	0.946128	0.549919
11305	0.946132	0.438927
11306	0.946131	0.597991
11307	0.946129	0.401265

11308	0.94613	0.410294
11309	1.13398	0.457952
11310	0.94613	0.633615
11311	0.946132	0.109691
11312	0.946132	0.12495
11313	0.946131	0.189527
11314	0.946131	0.151127
11315	0.946132	0.136275
11316	0.946131	0.139813
11317	0.946132	0.140131
11318	0.946131	0.678289
11319	0.94613	0.500876
11320	0.946131	0.631027
11321	0.94613	0.7
11322	1.38645	0.542693
11323	1.38645	0.57845
11324	0.946131	0.677793
11325	0.946131	0.750227
11326	0.946131	0.708211
11327	0.94613	0.763052
11328	1.23923	0.704866
11329	0.94613	0.701325
11330	0.946131	0.751574
11331	0.946131	0.776503
11332	0.946132	0.125852
11333	0.946131	0.152934
11334	0.946131	0.119101
11335	0.946132	0.114804
11336	0.946132	0.114659
11337	0.946132	0.121818
11338	1.40511	0.119902
11339	0.946132	2.1
11340	0.946132	1.70766
11341	0.946132	2.1
11342	0.946132	1.73757
11343	0.946132	1.46156
11344	0.946131	2.1
11345	1.12968	2.1
11346	0.946132	1.31181
11347	0.946128	2.1
11348	0.946128	1.93101
11349	0.94613	1.46739
11350	0.94613	2.1
11351	0.94613	1.35296
11352	0.94613	1.81807
11353	0.946133	2.1
11354	0.946133	2.1
11355	0.946132	2.1
11356	0.946132	2.1
11357	0.946132	2.1
11358	0.946133	2.1
11359	1.31323	2.1
11360	0.94613	2.0568
11361	0.946131	0.940619
11362	0.946131	0.940881
11363	0.94613	0.903556
11364	0.946131	2.08802
11365	0.946131	1.09933

11366	0.946131	1.05341
11367	0.946132	0.82095
11368	0.946132	0.71873
11369	0.946132	0.757322
11370	1.36899	0.457444
11371	0.946132	0.702483
11372	0.946132	0.29603
11373	0.946133	0.715485
11374	1.03543	0.427119
11375	0.946131	0.546654
11376	0.946131	0.262163
11377	0.946132	0.384629
11378	0.94613	0.461905
11379	0.950987	0.333131
11380	0.941275	0.610428
11381	0.946131	0.41703
11382	0.94613	0.443161
11383	1.28952	0.466807
11384	0.946132	0.385601
11385	0.94613	0.536724
11386	0.946131	0.514872
11387	0.946131	0.416014
11388	0.94613	2.1
11389	1.35966	2.1
11390	0.946131	2.1
11391	0.946131	2.1
11392	0.946129	2.1
11393	0.94613	2.1
11394	0.94613	2.1
11395	0.810946	2.1
11396	0.810942	0.980251
11397	0.810932	2.05191
11398	2.6655	1.82353
11399	1.3517	1.3307
11400	1.08132	2.1
11401	1.08131	0.955222
11402	1.08133	1.82306
11403	0.94613	2.1
11404	0.946131	2.1
11405	0.94613	1.99527
11406	0.946128	2.1
11407	0.946131	2.1
11408	0.94613	2.1
11409	0.94613	2.1
11410	0.946128	2.1
11411	0.946128	2.1
11412	0.946133	2.1
11413	1.25375	2.1
11414	0.94613	2.1
11415	0.946131	2.1
11416	1.25375	2.1
11417	0.94613	2.1
11418	0.94613	2.1
11419	0.946132	2.1
11420	0.946132	2.1
11421	0.946128	2.1
11422	0.94613	2.1
11423	1.31453	2.1

11424	0.946131	0.485317
11425	0.946129	0.321182
11426	0.94613	0.493471
11427	0.94613	0.29175
11428	0.94613	0.322535
11429	1.12018	0.328782
11430	0.94613	0.509593
11431	0.946131	0.678118
11432	0.946129	0.532225
11433	0.946131	0.678581
11434	1.16373	0.490048
11435	0.94613	0.489784
11436	0.94613	0.666201
11437	0.946131	0.701655
11438	1.36811	0.772087
11439	0.946132	0.807201
11440	1.36811	0.754618
11441	0.946129	0.788329
11442	0.94613	0.799589
11443	0.946129	0.793146
11444	0.946129	0.781384
11445	0.946132	0.707869
11446	0.946133	0.656285
11447	0.946132	0.702291
11448	0.946133	0.709594
11449	1.35732	0.678371
11450	1.35732	0.696432
11451	0.946133	0.703792
11452	1.36399	0.831433
11453	0.946129	0.939163
11454	0.94613	0.844313
11455	0.946131	0.948576
11456	0.94613	1.13872
11457	0.946131	0.910813
11458	0.946131	0.982046
11459	0.946133	2.1
11460	0.946132	2.1
11461	0.946132	2.1
11462	1.38302	2.1
11463	0.946133	2.1
11464	0.946132	2.1
11465	0.946133	2.1
11466	0.810943	2.1
11467	0.810944	2.1
11468	0.810945	2.1
11469	2.80542	2.1
11470	1.35169	2.1
11471	1.08132	2.1
11472	1.08132	2.1
11473	1.08132	2.1
11474	1.30189	2.1
11475	0.946131	2.1
11476	0.946132	2.1
11477	0.946131	2.1
11478	0.946131	2.1
11479	0.946131	2.1
11480	0.946132	2.1
11481	0.810946	0.905868

11482	0.810947	0.844662
11483	2.33293	0.886784
11484	1.24357	0.865783
11485	1.08132	0.86895
11486	0.973183	0.880102
11487	1.00023	0.891397
11488	0.810934	0.872847
11489	1.06584	0.840702
11490	0.946129	0.869393
11491	0.84731	0.843986
11492	1.16646	0.871968
11493	0.946126	0.884744
11494	1.2831	0.854221
11495	0.851527	0.860583
11496	0.946131	2.1
11497	0.946128	2.1
11498	0.946128	2.1
11499	0.94613	2.1
11500	1.31961	2.1
11501	0.94613	2.1
11502	0.94613	2.1
11503	0.946131	2.1
11504	1.3605	2.1
11505	0.946133	2.1
11506	0.946132	2.1
11507	0.94613	2.1
11508	0.946131	2.1
11509	0.946131	2.1
11510	0.946128	1.8638
11511	0.946133	1.26739
11512	0.946132	2.1
11513	0.946131	1.13957
11514	0.946131	0.921939
11515	0.946131	1.28366
11516	1.30116	1.57
11517	0.946132	0.170583
11518	0.946129	0.21167
11519	0.94613	0.18057
11520	1.3035	0.172939
11521	0.946131	0.130547
11522	0.94613	0.171096
11523	0.946131	0.275863
11524	0.946132	2.1
11525	1.2051	2.1
11526	1.2051	2.1
11527	0.946132	2.1
11528	1.37869	2.1
11529	0.946132	2.1
11530	0.946131	2.1
11531	0.810946	2.1
11532	0.810945	2.1
11533	2.69247	2.1
11534	1.35169	2.1
11535	1.08132	2.1
11536	1.08132	2.1
11537	1.08132	2.1
11538	0.810946	2.1
11539	1.22993	2.1

11540	0.946131	2.1
11541	0.946131	2.1
11542	1.34623	2.1
11543	0.946131	2.1
11544	1.34623	2.1
11545	0.946131	2.1
11546	0.946131	0.198647
11547	0.946132	0.286585
11548	0.946132	0.22138
11549	0.946132	0.182629
11550	0.946131	0.188557
11551	0.946132	0.265636
11552	1.32299	0.167642
11553	0.94613	0.334917
11554	0.946131	0.38616
11555	0.946128	0.331537
11556	0.946132	0.290586
11557	0.946133	0.461817
11558	0.946131	0.463994
11559	0.946131	0.360837
11560	0.946132	2.1
11561	0.946129	2.1
11562	0.946128	2.1
11563	0.94613	2.1
11564	0.94613	2.1
11565	0.946131	2.1
11566	0.94613	2.1
11567	0.94613	2.1
11568	0.94613	2.1
11569	0.946131	2.1
11570	0.946132	2.1
11571	0.946129	2.1
11572	0.946131	2.1
11573	0.946131	2.1
11574	0.946132	1.48536
11575	0.946131	1.52
11576	1.13731	1.36873
11577	0.94775	1.62549
11578	0.944514	1.77315
11579	0.946132	1.99657
11580	1.039	1.96115
11581	0.946131	1.40762
11582	0.946128	1.33774
11583	0.94613	1.20283
11584	0.946131	1.41349
11585	0.946128	1.52225
11586	0.946129	1.79879
11587	1.23726	1.73568
11588	0.946132	2.1
11589	0.946132	2.1
11590	0.946129	2.1
11591	1.39214	2.1
11592	0.946131	2.1
11593	0.946131	2.1
11594	0.94613	2.1
11595	0.946125	0.774768
11596	0.946119	0.335407
11597	0.946118	0.363638

11598	0.946118	0.386514
11599	0.94612	0.633255
11600	0.946106	0.37823
11601	0.946131	0.409518
11602	1.26893	0.700633
11603	0.946131	0.7
11604	1.26893	0.701926
11605	0.94613	0.701094
11606	0.946131	0.700929
11607	0.946131	0.700737
11608	0.946131	0.7
11609	0.946133	0.126977
11610	0.946133	0.12588
11611	0.946132	0.183769
11612	1.28924	0.114228
11613	0.946133	0.167044
11614	0.946132	0.168875
11615	0.946132	0.144961
11616	0.946133	0.853656
11617	0.946132	0.859971
11618	0.946132	0.846417
11619	0.946133	0.822815
11620	0.946133	0.830718
11621	0.946132	0.846669
11622	0.946133	0.837614
11623	0.946132	0.974952
11624	0.946133	0.855557
11625	0.946133	0.960467
11626	0.946132	0.979812
11627	1.15388	0.92247
11628	0.946132	0.965591
11629	0.946133	0.946039
11630	0.946132	0.822872
11631	0.946133	0.808128
11632	0.946132	0.816064
11633	0.946133	0.780381
11634	0.946133	0.79176
11635	0.946132	0.804118
11636	0.946132	0.795508
11637	1.0644	1.17275
11638	1.12486	1.63255
11639	1.08674	1.76297
11640	0.946132	1.25142
11641	0.946132	1.54924
11642	0.946133	1.34107
11643	0.946132	1.32859
11644	0.808978	1.14718
11645	0.911537	1.43738
11646	0.932832	1.31783
11647	1.14604	1.76774
11648	1.56953	1.46482
11649	1.25866	1.27441
11650	1.18488	1.59486
11651	1.341	1.18866
11652	0.946131	0.688144
11653	0.946131	0.629233
11654	0.946131	0.672929
11655	0.94613	0.705059

11656	1.33922	0.509383
11657	0.946131	0.701224
11658	0.946131	0.677107
11659	0.946132	0.258679
11660	0.946133	0.22812
11661	0.946132	0.241447
11662	0.946132	0.218747
11663	0.946132	0.266837
11664	0.946133	0.239092
11665	0.946132	0.227878
11666	0.946129	0.931521
11667	0.946133	2.1
11668	0.946133	1.03898
11669	0.946131	1.1313
11670	1.40496	1.94401
11671	0.946131	0.954087
11672	0.946131	2.1
11673	0.94613	1.05122
11674	0.946127	0.883963
11675	0.946127	1.0071
11676	0.94613	1.06517
11677	1.38913	0.94204
11678	0.946128	1.01529
11679	0.946128	0.974808
11680	1.98863	0.0937931
11681	2.59915	0.138438
11682	1.40338	0.0997983
11683	0.946131	0.114736
11684	1.22266	0.11285
11685	1.40338	0.104415
11686	0.946132	0.104882
11687	0.946132	0.116574
11688	1.22266	0.0991743
11689	0.94613	0.165952
11690	0.94613	0.196287
11691	0.946124	0.357829
11692	1.21428	0.170934
11693	0.946126	0.339292
11694	0.946127	0.336658
11695	0.946127	0.222495
11696	1.03411	0.284309
11697	0.946131	0.558008
11698	1.03411	0.285494
11699	0.946129	0.539832
11700	1.14258	0.327137
11701	0.952143	0.525393
11702	0.940118	0.340998
11703	0.810943	2.1
11704	0.810945	2.1
11705	0.810958	2.1
11706	2.82603	2.1
11707	1.35168	2.1
11708	1.08132	2.1
11709	1.08132	2.1
11710	1.0813	2.1
11711	0.946133	0.493929
11712	0.946132	0.406638
11713	0.946132	0.445272

11714	0.946132	0.367583
11715	0.946132	0.330326
11716	1.11539	0.338888
11717	0.946133	0.444224
11718	0.946133	2.1
11719	0.946132	2.1
11720	0.946133	2.1
11721	0.946133	2.1
11722	0.946132	2.1
11723	1.41195	2.1
11724	0.946133	2.1
11725	0.946131	0.120835
11726	0.946131	0.138812
11727	0.946131	0.113434
11728	0.946131	0.116652
11729	0.946131	0.116812
11730	0.946131	0.124323
11731	1.15968	0.125143
11732	0.946131	1.38628
11733	0.94613	1.43097
11734	0.94613	2.1
11735	1.34386	1.53047
11736	0.946132	1.87051
11737	0.94613	2.05206
11738	1.34386	1.7441
11739	0.946131	1.30311
11740	0.935202	1.29292
11741	0.946131	1.86223
11742	1.25054	1.39897
11743	0.850155	1.65963
11744	0.946131	1.69998
11745	0.935205	1.55981
11746	0.946131	0.757173
11747	1.40193	0.689622
11748	0.946132	0.696238
11749	0.946132	0.720567
11750	0.946129	0.760021
11751	1.40193	0.627295
11752	0.946131	0.741221
11753	0.808779	0.676245
11754	0.561937	0.451472
11755	1.40193	0.536521
11756	1.40193	0.42793
11757	0.561936	0.601716
11758	0.561937	0.418634
11759	1.40193	0.495322
11760	0.946132	0.110863
11761	0.946132	0.115136
11762	0.946132	0.146567
11763	0.946132	0.121756
11764	0.946133	0.155802
11765	0.946132	0.129975
11766	0.946133	0.130241
11767	0.946127	0.70913
11768	0.946131	0.706171
11769	0.946131	0.700755
11770	0.946127	0.702321
11771	1.19583	0.7

11772	1.19583	0.700607
11773	0.946129	0.704378
11774	0.589428	0.715373
11775	1.08737	0.709411
11776	1.10961	0.721676
11777	0.872901	0.705104
11778	0.541327	0.701589
11779	0.541312	0.704244
11780	0.85153	0.711359
11781	0.946132	1.38106
11782	0.946131	2.1
11783	0.946131	2.1
11784	0.946132	1.69601
11785	0.946132	2.1
11786	0.946132	2.04817
11787	0.946132	2.0763
11788	0.946132	1.16547
11789	1.2778	1.98692
11790	0.946131	2.1
11791	0.946131	1.19784
11792	0.94613	1.90131
11793	0.94613	1.24683
11794	0.94613	1.38378
11795	0.946118	0.910251
11796	0.946112	0.928257
11797	0.946118	0.875837
11798	0.946119	0.89353
11799	0.946117	0.893003
11800	1.35631	0.849502
11801	0.946105	0.852856
11802	0.946133	1.02771
11803	0.946133	2.1
11804	0.946132	1.12523
11805	0.946132	2.1
11806	0.946133	0.797821
11807	0.946132	1.01562
11808	0.946132	0.97019
11809	0.94613	2.1
11810	0.946131	0.921282
11811	0.946128	0.927581
11812	0.946128	0.894388
11813	0.94613	2.1
11814	0.946129	1.17489
11815	0.946129	1.03007
11816	0.946132	1.25795
11817	1.15139	1.26333
11818	0.946132	1.20121
11819	0.946132	1.3467
11820	0.946132	1.35829
11821	0.946132	1.43485
11822	0.946132	1.27942
11823	0.946133	2.1
11824	1.36151	2.1
11825	0.946132	2.1
11826	0.946132	2.1
11827	0.946132	2.1
11828	0.946132	2.1
11829	0.946133	2.1

11830	0.946127	2.1
11831	1.38397	2.1
11832	0.94613	2.1
11833	0.946131	2.1
11834	0.946128	2.1
11835	0.94613	2.1
11836	0.946129	2.1
11837	0.946131	0.521582
11838	0.946127	0.555987
11839	0.946127	0.515719
11840	0.946132	0.662773
11841	0.946131	0.744936
11842	0.946128	0.544577
11843	0.946129	0.619287
11844	1.0268	0.05
11845	1.23823	0.0511962
11846	1.2171	0.0579756
11847	0.883446	0.0554047
11848	1.23827	0.0526368
11849	1.32614	0.0514108
11850	0.94613	0.0591117
11851	0.946132	2.1
11852	0.946129	2.1
11853	0.946129	2.1
11854	0.94613	2.1
11855	1.36523	2.1
11856	0.946131	2.1
11857	0.94613	2.1
11858	0.946132	0.611451
11859	0.946131	0.466548
11860	0.946132	0.645182
11861	0.946131	0.425007
11862	0.946131	0.447981
11863	1.01653	0.467394
11864	0.946131	0.680576
11865	0.94612	2.1
11866	0.946121	1.82403
11867	0.946119	2.07865
11868	0.946119	1.99669
11869	0.946119	2.1
11870	0.946132	2.1
11871	0.946107	2.1
11872	0.810942	1.03939
11873	0.810944	0.919518
11874	2.60324	1.10039
11875	1.21651	1.04726
11876	1.0813	1.02236
11877	0.946153	0.919143
11878	0.979948	2.1
11879	0.709588	1.13955
11880	1.3492	0.591352
11881	0.946132	0.597612
11882	0.946131	0.484092
11883	0.94613	0.602718
11884	0.946129	0.692562
11885	0.946129	0.606709
11886	0.94613	0.641767
11887	0.946131	0.330542

11888	0.94613	0.397997
11889	0.946128	0.33723
11890	0.946133	0.429336
11891	0.946133	0.660958
11892	0.946131	0.44611
11893	0.946131	0.44171
11894	1.23351	0.106218
11895	0.946131	0.144398
11896	0.946131	0.114388
11897	0.946132	0.0924289
11898	0.946132	0.106574
11899	0.946131	0.108862
11900	0.946132	0.106708
11901	1.35657	0.119331
11902	0.946132	0.197141
11903	0.946131	0.135216
11904	0.946132	0.106457
11905	0.946132	0.120739
11906	0.946131	0.129468
11907	0.946131	0.119904
11908	0.946132	0.770763
11909	0.946132	0.731578
11910	0.946132	0.770793
11911	0.946131	0.719297
11912	0.946131	0.752588
11913	0.946132	0.728593
11914	0.946132	0.727049
11915	0.946133	0.71713
11916	0.946132	0.65456
11917	0.946133	0.717333
11918	0.946132	0.61175
11919	0.946132	0.706091
11920	0.946132	0.692167
11921	0.946133	0.673743
11922	0.946131	0.927777
11923	1.4101	1.55467
11924	0.946131	2.1
11925	0.946131	0.823106
11926	0.946131	1.03193
11927	0.946131	0.822519
11928	0.946131	1.64155
11929	0.810944	1.31846
11930	0.810939	1.20678
11931	0.709586	1.05474
11932	2.71261	1.09713
11933	1.21651	1.15037
11934	0.946151	1.02996
11935	0.979943	1.15673
11936	1.0813	1.37591
11937	0.946132	2.1
11938	0.946131	2.1
11939	0.946132	2.1
11940	0.946131	2.1
11941	0.946131	2.1
11942	1.37733	2.1
11943	0.946131	2.1
11944	0.946133	2.1
11945	0.946132	1.2586

11946	0.946132	2.1
11947	0.946133	2.1
11948	0.946133	2.1
11949	0.946132	1.81514
11950	1.32582	2.1
11951	0.946132	2.1
11952	0.946132	2.1
11953	0.946132	2.1
11954	0.946132	2.1
11955	0.946132	2.07393
11956	0.946132	2.1
11957	0.946131	2.1
11958	0.946133	1.47146
11959	0.946132	1.11006
11960	0.946132	1.13949
11961	0.946132	1.0711
11962	0.946132	1.12609
11963	0.946133	1.08587
11964	1.3469	1.08704
11965	0.946126	1.01279
11966	0.94613	2.1
11967	0.946131	1.8018
11968	0.946127	1.15606
11969	1.41206	2.1
11970	0.946128	2.07871
11971	0.946128	2.1
11972	0.946127	2.1
11973	0.94613	1.94149
11974	0.946131	0.988635
11975	0.94613	2.1
11976	0.946128	1.04399
11977	0.946129	1.77921
11978	0.946129	1.72402
11979	0.946131	0.492482
11980	0.94613	0.39846
11981	1.24448	0.452786
11982	0.946129	0.605101
11983	0.946131	0.514368
11984	0.94613	0.570291
11985	0.946131	0.522832
11986	0.946132	0.663203
11987	0.946133	0.586138
11988	1.11015	0.628268
11989	0.946133	0.811905
11990	0.946132	0.729933
11991	0.946132	0.768932
11992	0.946132	0.719695
11993	1.38775	2.1
11994	0.946131	2.1
11995	0.946131	2.1
11996	0.946132	2.1
11997	0.946132	2.1
11998	0.946131	2.1
11999	0.946131	2.1
12000	0.946129	1.40179
12001	0.946129	2.1
12002	0.946133	1.02689
12003	0.946131	2.1

12004	0.946131	0.846111
12005	0.946131	1.159
12006	0.946131	1.07833
12007	0.946132	2.1
12008	0.946133	2.1
12009	0.946132	2.1
12010	1.40774	2.1
12011	0.946132	2.1
12012	0.946133	2.1
12013	0.946132	2.1
12014	0.610968	0.0798193
12015	0.688233	0.0932966
12016	0.715226	0.0901501
12017	0.792409	0.0885086
12018	1.13418	0.0816198
12019	0.956667	0.0907618
12020	0.757321	0.0879456
12021	0.968845	0.0791867
12022	0.595679	0.0802758
12023	0.946133	1.02903
12024	1.28917	1.22776
12025	0.946131	1.62984
12026	0.94613	0.834052
12027	0.946131	1.04948
12028	0.946131	0.876446
12029	0.946131	1.30662
12030	0.946132	0.671895
12031	0.946128	0.65594
12032	0.94613	0.684026
12033	1.40668	0.678675
12034	0.946131	0.666232
12035	0.946131	0.677878
12036	0.94613	0.672084
12037	0.810946	2.1
12038	0.810945	2.1
12039	2.78163	2.1
12040	1.3517	2.1
12041	1.08132	2.1
12042	1.08133	2.1
12043	1.08132	2.1
12044	0.810933	2.1
12045	0.946131	2.1
12046	1.39081	2.1
12047	0.94613	2.1
12048	1.30339	2.1
12049	1.30339	2.1
12050	1.39081	2.1
12051	0.946131	2.1
12052	0.946127	0.954134
12053	0.821631	0.987063
12054	0.946131	0.889216
12055	0.946131	0.956514
12056	0.946128	0.888444
12057	0.996109	0.928028
12058	0.827876	0.918514
12059	0.946131	2.1
12060	0.946129	2.1
12061	0.946129	2.1

12062	0.94613	2.1
12063	0.94613	2.1
12064	0.94613	2.1
12065	0.94613	2.1
12066	0.946132	0.829229
12067	1.41723	0.871479
12068	0.946132	0.863069
12069	0.946133	0.795184
12070	0.946132	0.95692
12071	0.946132	0.876568
12072	0.946133	0.880684
12073	0.946133	0.82616
12074	0.946132	0.779729
12075	0.946133	0.771605
12076	0.946133	0.809067
12077	0.946132	0.887175
12078	0.946132	0.84146
12079	1.34254	0.828905
12080	1.26501	1.0621
12081	0.946132	1.11808
12082	0.946133	1.12969
12083	0.946133	1.30433
12084	0.946132	1.22128
12085	0.946133	1.12603
12086	0.946132	1.42839
12087	0.946131	0.36762
12088	0.946131	0.404858
12089	0.946131	0.44028
12090	0.946131	0.23433
12091	0.946131	0.313465
12092	0.946131	0.383413
12093	0.946131	0.342614
12094	0.878843	0.217347
12095	0.82561	0.218154
12096	0.586974	0.234837
12097	1.05343	0.215848
12098	0.80631	0.143153
12099	0.782663	0.243968
12100	0.789405	0.14739
12101	0.902491	0.134448
12102	1.05344	0.18952
12103	0.94613	0.196034
12104	0.946131	0.221062
12105	0.946131	0.376986
12106	0.94613	0.317234
12107	0.946131	0.245017
12108	0.946131	0.322649
12109	0.946131	0.260616
12110	0.946128	2.03195
12111	0.946133	2.1
12112	0.946133	0.945777
12113	0.94613	2.1
12114	0.946131	1.10609
12115	0.946131	1.15635
12116	0.946131	1.30791
12117	0.946131	0.754063
12118	0.946131	0.747949
12119	0.94613	0.743542

12120	0.946128	0.745285
12121	0.946133	0.752678
12122	1.2996	0.732471
12123	0.946131	0.742463
12124	0.946132	2.1
12125	0.946132	2.1
12126	0.946132	1.64253
12127	0.946132	2.1
12128	0.946131	2.1
12129	0.946132	2.1
12130	0.946132	2.1
12131	1.99211	0.232573
12132	2.55024	0.192332
12133	0.946131	0.251948
12134	0.946131	0.213528
12135	0.946131	0.267267
12136	0.946131	0.233812
12137	0.946131	0.290239
12138	0.946131	0.252936
12139	1.35726	0.272303
12140	0.946128	2.1
12141	0.946132	1.52525
12142	0.946131	1.65668
12143	0.94613	1.58735
12144	0.94613	1.59956
12145	0.94613	2.09853
12146	1.39852	2.05954
12147	1.31666	0.222655
12148	0.946131	0.400692
12149	0.946128	0.24851
12150	0.94613	0.180131
12151	0.946128	0.259273
12152	0.946128	0.255746
12153	0.946128	0.234452
12154	1.32275	0.366478
12155	0.946131	0.602864
12156	0.946129	0.416124
12157	0.946128	0.424049
12158	0.946129	0.43351
12159	0.946128	0.428804
12160	0.946129	0.393647
12161	0.946132	2.1
12162	0.946131	2.1
12163	0.946131	2.1
12164	0.946132	2.1
12165	0.94613	2.1
12166	0.946131	2.1
12167	1.41125	2.1
12168	0.946132	2.1
12169	0.946132	2.1
12170	0.946131	2.1
12171	0.946132	2.1
12172	0.946132	2.1
12173	0.946132	2.1
12174	1.31904	2.1
12175	1.02425	0.40537
12176	0.946132	0.326439
12177	0.94613	0.322057

12178	0.946131	0.547018
12179	1.1534	0.332892
12180	0.961161	0.310469
12181	0.9311	0.684187
12182	1.40092	1.07687
12183	0.946133	1.10742
12184	0.946132	1.11867
12185	0.946131	1.27359
12186	0.946131	1.0837
12187	0.94613	1.11759
12188	0.946131	1.31582
12189	0.946113	2.1
12190	0.946119	0.807012
12191	0.946117	2.1
12192	1.40015	2.04724
12193	0.946118	0.812338
12194	0.946131	1.40757
12195	0.946105	0.862943
12196	0.946132	0.696432
12197	0.946133	0.681037
12198	0.946132	0.70766
12199	1.03547	0.710238
12200	0.946133	0.68845
12201	0.946133	0.700838
12202	0.946132	0.680475
12203	0.946133	2.1
12204	1.36694	2.1
12205	0.946133	2.1
12206	0.946132	2.1
12207	0.946133	2.1
12208	0.946133	2.1
12209	0.946132	2.1
12210	0.946132	0.540006
12211	0.946133	0.521016
12212	0.946132	0.619567
12213	0.946133	0.359132
12214	0.946133	0.553885
12215	0.946132	0.65301
12216	0.946132	0.553148
12217	0.491552	0.468426
12218	0.913078	0.64015
12219	0.946132	0.728354
12220	0.491552	0.413629
12221	0.946132	0.639242
12222	0.49154	0.695649
12223	1.53168	0.649093
12224	1.53168	0.467829
12225	0.946127	2.1
12226	1.38752	2.1
12227	0.946129	2.1
12228	0.94613	2.1
12229	0.946128	2.1
12230	0.946129	2.1
12231	0.946129	2.1
12232	0.946132	2.1
12233	0.946132	2.1
12234	0.946132	2.1
12235	0.946132	2.1

12236	0.946132	2.1
12237	0.946131	2.1
12238	0.946132	2.1
12239	0.94613	0.75713
12240	0.94613	0.714523
12241	0.94613	0.732146
12242	0.946131	0.725622
12243	0.946129	0.756817
12244	1.16834	0.592558
12245	0.94613	0.733822
12246	0.946127	0.764696
12247	1.35594	0.731387
12248	0.946131	0.737347
12249	0.946128	0.77064
12250	0.946127	0.636325
12251	0.946129	0.756971
12252	0.94613	0.740655
12253	0.94613	0.927266
12254	0.946131	0.744164
12255	0.946128	0.760544
12256	0.946128	0.724504
12257	0.946131	0.894118
12258	0.94613	0.804864
12259	0.94613	0.798012
12260	0.946132	0.703899
12261	0.946132	0.728596
12262	0.946129	0.723849
12263	1.39751	0.686058
12264	0.94613	0.741883
12265	0.946131	0.693255
12266	0.946131	0.717478
12267	0.946132	0.560196
12268	0.700041	0.627486
12269	0.700013	0.693238
12270	1.7697	0.468608
12271	1.7697	0.450221
12272	1.21134	0.518127
12273	0.946131	0.513792
12274	1.25837	0.531925
12275	0.946133	2.1
12276	0.946132	1.52563
12277	0.946132	1.38061
12278	0.946132	1.62266
12279	0.946132	2.08179
12280	0.946133	2.01818
12281	1.40583	1.86785
12282	0.946131	1.21408
12283	0.946131	1.56613
12284	0.946131	1.22073
12285	0.946131	2.09573
12286	0.946131	1.64561
12287	0.827865	1.55403
12288	0.946131	1.51641
12289	0.946132	0.126588
12290	0.946129	0.113096
12291	0.946129	0.141504
12292	0.946131	0.122376
12293	0.94613	0.152848

12294	1.21087	0.112891
12295	0.946131	0.123073
12296	0.94613	0.813928
12297	0.946131	0.834173
12298	0.94613	0.774411
12299	0.946128	0.811894
12300	0.946132	0.776172
12301	1.17452	0.787307
12302	0.94613	0.78437
12303	0.946119	0.772258
12304	0.946114	0.777528
12305	0.946119	0.742997
12306	0.946119	0.770375
12307	0.946119	0.744676
12308	1.27376	0.753715
12309	0.946106	0.751634
12310	0.946133	2.1
12311	1.39621	2.1
12312	0.946132	2.1
12313	0.946132	2.1
12314	0.946133	2.1
12315	0.946132	2.1
12316	0.946132	2.1
12317	0.946132	2.1
12318	0.946132	2.1
12319	0.946131	2.1
12320	0.946131	2.1
12321	0.946132	2.1
12322	0.946132	2.1
12323	1.34352	2.1
12324	0.946132	1.85959
12325	1.41272	2.09558
12326	0.946131	2.1
12327	0.94613	1.3343
12328	0.94613	1.95313
12329	0.94613	1.40185
12330	0.94613	1.94855
12331	0.946132	2.1
12332	0.946131	1.39895
12333	0.946132	2.1
12334	0.946132	1.31155
12335	0.946132	1.1592
12336	0.946132	1.90631
12337	1.23536	1.92725
12338	0.946132	2.1
12339	0.946131	2.1
12340	0.946131	2.1
12341	0.946132	2.1
12342	0.946132	2.1
12343	1.41131	2.1
12344	0.946131	2.1
12345	1.41894	0.842072
12346	0.946132	0.790182
12347	0.946133	0.761549
12348	0.946132	0.821696
12349	0.946132	0.872778
12350	0.946133	0.783835
12351	0.946132	0.776553

12352	0.946128	0.70749
12353	0.946133	0.940194
12354	0.946131	0.841199
12355	0.946129	1.94193
12356	0.94613	0.875375
12357	1.4099	0.971961
12358	0.946131	0.866651
12359	0.810947	2.1
12360	0.810945	1.04635
12361	0.810933	2.06466
12362	2.62883	2.09236
12363	1.3517	1.94309
12364	1.08132	2.1
12365	1.08132	1.00452
12366	1.08133	1.7604
12367	0.946131	0.966743
12368	0.946126	1.81124
12369	0.946127	0.956281
12370	0.94613	0.978452
12371	0.946128	1.47288
12372	0.946129	0.856802
12373	0.946129	0.937566
12374	1.21347	2.1
12375	0.946129	2.1
12376	0.94613	2.1
12377	1.21347	2.1
12378	1.33064	2.1
12379	0.94613	2.1
12380	1.33064	2.1
12381	0.94613	0.0601391
12382	0.946129	0.0875047
12383	0.94613	0.0828377
12384	0.946132	0.0858715
12385	0.946126	0.0794318
12386	0.946129	0.0829108
12387	0.94613	0.0836842
12388	0.946132	0.910077
12389	1.37938	1.05609
12390	0.946131	1.202
12391	0.94613	0.882773
12392	0.946131	1.32709
12393	0.94613	0.919079
12394	0.94613	0.985052
12395	0.946133	0.132742
12396	0.946133	0.120521
12397	1.2968	0.128387
12398	0.946132	0.139351
12399	0.946133	0.167573
12400	1.2968	0.117049
12401	0.946133	0.137181
12402	1.43643	0.135898
12403	0.946129	0.867466
12404	0.946133	0.808523
12405	0.946131	0.871773
12406	0.94613	0.781681
12407	0.946131	0.835849
12408	0.946131	0.810629
12409	0.946131	0.803534

12410	0.872447	1.25653
12411	0.946132	1.16372
12412	0.872447	1.12215
12413	0.827867	1.22048
12414	0.946132	1.30982
12415	0.827867	1.42203
12416	0.872447	1.40323
12417	0.946117	1.04527
12418	1.27213	1.02641
12419	0.946124	0.976924
12420	0.946119	1.03576
12421	0.946113	1.27914
12422	0.946129	1.11212
12423	0.946104	1.06035
12424	0.94613	0.849599
12425	0.946131	0.908381
12426	0.94613	0.819202
12427	0.946129	0.904554
12428	0.946132	0.375886
12429	0.946131	0.818685
12430	0.94613	0.638971
12431	0.810942	2.1
12432	0.810943	2.1
12433	2.80212	2.1
12434	1.3517	2.1
12435	1.08133	2.1
12436	1.08132	2.1
12437	1.08132	2.1
12438	0.810932	2.1
12439	1.29521	2.1
12440	0.946129	2.1
12441	0.946131	2.1
12442	1.40106	2.1
12443	0.94613	2.1
12444	1.40106	2.1
12445	0.946131	2.1
12446	0.946132	0.813902
12447	1.24596	0.756828
12448	0.94613	0.770996
12449	0.946131	0.802483
12450	0.946131	0.731563
12451	0.946131	0.803509
12452	0.946131	0.777399
12453	0.946131	2.1
12454	0.946131	2.1
12455	0.946131	2.1
12456	0.946128	2.1
12457	0.946133	2.1
12458	0.94613	2.1
12459	0.946131	2.1
12460	0.879548	0.717599
12461	1.21527	0.702787
12462	0.967506	0.708538
12463	0.946132	0.777469
12464	0.946133	0.70497
12465	0.967511	0.836995
12466	0.946132	0.782044
12467	0.946132	0.867334

12468	0.946132	0.746532
12469	0.974253	0.761041
12470	0.974253	0.894022
12471	0.91801	0.767505
12472	0.918009	0.781416
12473	0.946132	0.83555
12474	0.946132	0.32757
12475	0.946131	0.260658
12476	0.946131	0.382626
12477	0.946133	0.221744
12478	0.946132	0.239779
12479	1.28644	0.261237
12480	0.946132	0.365714
12481	0.946131	0.357699
12482	0.946131	0.271953
12483	0.946131	0.411494
12484	0.946131	0.227637
12485	0.946131	0.254932
12486	1.40103	0.261227
12487	0.946131	0.39173
12488	0.946128	0.710614
12489	0.946132	0.658658
12490	0.946129	0.717749
12491	1.03327	0.619343
12492	0.946129	0.628369
12493	0.946129	0.710351
12494	0.94613	0.718461
12495	0.946134	0.764275
12496	0.946133	0.595691
12497	0.946132	0.394141
12498	0.946133	0.648355
12499	0.946132	0.276312
12500	0.946132	0.480493
12501	0.946133	0.148454
12502	0.856654	0.14468
12503	0.946131	0.181116
12504	0.946131	0.12869
12505	0.946132	0.138095
12506	0.856616	0.162719
12507	1.03565	0.11145
12508	1.03565	0.165977
12509	1.15844	0.723517
12510	0.946132	0.729866
12511	0.946132	0.711178
12512	0.946132	0.724395
12513	0.946132	0.708558
12514	0.946132	0.718964
12515	0.946131	0.724475
12516	1.4582	2.1
12517	1.35067	2.1
12518	0.946131	2.1
12519	1.04106	2.1
12520	0.938748	2.1
12521	0.943015	2.1
12522	1.05017	2.1
12523	0.938793	2.1
12524	0.946131	0.114684
12525	0.946132	0.134031

12526	0.946131	0.222085
12527	0.94613	0.184694
12528	0.946133	0.147181
12529	0.946131	0.175589
12530	0.946131	0.159821
12531	0.946132	0.913254
12532	0.946133	0.980903
12533	0.946132	0.911281
12534	0.946133	0.981224
12535	0.946133	0.953039
12536	0.946132	0.948203
12537	0.946133	0.971078
12538	0.946128	1.09617
12539	0.946132	1.25258
12540	0.946132	1.10306
12541	0.94613	1.12459
12542	1.23426	1.17811
12543	0.94613	1.16496
12544	0.946131	1.184
12545	0.810943	0.714608
12546	0.810946	0.751879
12547	2.60388	0.744197
12548	1.3517	0.729347
12549	1.08133	0.718636
12550	1.08132	0.752884
12551	1.08132	0.720989
12552	0.810933	0.7
12553	0.810945	0.96883
12554	0.810945	0.875059
12555	2.72377	1.2005
12556	1.3517	1.03895
12557	1.08133	0.984755
12558	1.08132	0.890569
12559	1.08132	2.1
12560	0.810932	1.05638
12561	1.25104	0.570778
12562	0.946131	0.940953
12563	0.946131	0.852425
12564	1.36188	2.1
12565	0.946131	0.985027
12566	1.36188	1.11775
12567	0.946132	1.14257
12568	0.946132	2.06618
12569	0.946132	2.09518
12570	0.946132	2.1
12571	0.946132	2.1
12572	0.946132	2.00305
12573	0.946132	2.1
12574	1.25488	2.1
12575	1.32132	0.178535
12576	0.946126	0.221164
12577	0.946127	0.128895
12578	0.946128	0.19007
12579	0.946128	0.196429
12580	0.946128	0.195552
12581	0.946128	0.191567
12582	0.946133	2.1
12583	1.41123	1.29697

12584	0.946134	1.40671
12585	0.946133	2.1
12586	0.946132	1.02759
12587	0.946133	1.54916
12588	0.946132	1.36717
12589	1.32163	0.435735
12590	0.946133	0.437191
12591	0.946133	0.464708
12592	0.946132	0.408054
12593	0.946132	0.253983
12594	0.946132	0.393428
12595	0.946133	0.449063
12596	0.946133	2.1
12597	0.946133	2.1
12598	0.946132	2.1
12599	0.946132	2.1
12600	0.946132	1.00934
12601	0.946133	2.1
12602	0.946132	2.1
12603	0.94613	0.703579
12604	0.946131	0.648638
12605	0.946128	0.664719
12606	0.946133	0.457003
12607	0.946133	0.589314
12608	0.946131	0.703482
12609	0.946131	0.669856
12610	0.946133	0.911214
12611	0.946132	0.806648
12612	0.946132	0.922316
12613	0.946134	0.820109
12614	0.946133	0.986789
12615	0.946133	0.879787
12616	0.946132	0.862299
12617	0.94613	1.67668
12618	0.946128	2.00326
12619	0.946127	1.4245
12620	0.94613	1.35957
12621	0.946131	1.60658
12622	0.946128	1.54069
12623	1.21099	1.81783
12624	0.946132	0.861912
12625	0.946131	0.830604
12626	0.946131	0.8815
12627	0.946133	0.761266
12628	0.946133	0.785677
12629	0.946131	0.825026
12630	0.946132	0.79472
12631	1.34794	0.99574
12632	0.517994	0.986638
12633	0.517965	0.904824
12634	0.946132	1.0455
12635	0.9198	1.49078
12636	0.946132	1.13061
12637	0.9198	1.18928
12638	0.946132	1.03921
12639	0.94613	1.05915
12640	0.946131	0.853449
12641	0.946131	1.05473

12642	0.946131	1.28867
12643	0.946131	1.06582
12644	1.23224	0.976968
12645	0.946132	1.49218
12646	0.946132	1.66149
12647	0.946133	1.33019
12648	0.946132	1.09007
12649	0.946132	1.23455
12650	0.946133	1.32818
12651	1.37511	1.40942
12652	0.946133	0.774951
12653	0.946129	0.780523
12654	0.851104	0.803332
12655	0.946131	0.772661
12656	1.0412	0.867526
12657	1.0412	0.805443
12658	0.851066	0.787584
12659	1.31357	0.496157
12660	0.946131	0.786034
12661	0.94613	0.644118
12662	0.946131	0.675421
12663	0.94613	0.513367
12664	0.946131	0.710332
12665	0.946131	0.597879
12666	0.946133	0.913544
12667	1.38149	1.03261
12668	0.946132	1.04071
12669	0.946132	0.90463
12670	0.946133	1.38505
12671	0.946132	0.950023
12672	0.946133	0.970728
12673	0.810944	1.03088
12674	0.810945	2.1
12675	0.810958	1.94716
12676	2.65782	2.06476
12677	1.35168	1.57197
12678	1.08132	1.04089
12679	1.08132	2.00541
12680	1.0813	1.03114
12681	0.946113	0.10237
12682	0.946119	0.105215
12683	0.946118	0.14471
12684	0.946118	0.113868
12685	0.946119	0.117512
12686	0.946131	0.109336
12687	0.946106	0.113489
12688	0.946132	0.0848256
12689	0.946132	0.147023
12690	0.946128	0.149509
12691	0.946131	0.146794
12692	0.94613	0.156934
12693	0.94613	0.149494
12694	0.94613	0.174094
12695	1.00099	0.219726
12696	0.946132	0.349014
12697	1.17876	0.189516
12698	0.982282	0.235771
12699	0.909981	0.316837

12700	0.946132	0.321566
12701	0.946131	0.251349
12702	0.946132	0.895864
12703	1.35328	1.09527
12704	0.946132	1.44658
12705	0.946132	0.847119
12706	0.946133	1.2398
12707	0.946132	0.873172
12708	0.946133	1.12818
12709	0.946133	0.0853351
12710	0.946133	0.117113
12711	0.946132	0.100008
12712	0.946132	0.119978
12713	0.946132	0.108964
12714	0.946132	0.118672
12715	0.946133	0.116855
12716	0.900195	0.0851343
12717	0.946132	0.0781198
12718	0.900167	0.0869886
12719	1.41963	0.104889
12720	0.900195	0.0799306
12721	0.946133	0.0860399
12722	1.35385	0.105394
12723	0.946132	1.17457
12724	0.946129	1.80819
12725	0.946129	1.55844
12726	0.946131	1.16128
12727	0.946131	1.62201
12728	1.41585	1.27594
12729	0.94613	1.83925
12730	0.810946	2.1
12731	0.810946	2.1
12732	2.68895	2.1
12733	1.3517	2.1
12734	1.08133	2.1
12735	1.08132	2.1
12736	1.08132	2.1
12737	0.810933	2.1
12738	1.19886	2.1
12739	0.946131	2.1
12740	0.946131	2.1
12741	1.34447	2.1
12742	0.946131	2.1
12743	1.34447	2.1
12744	0.946131	2.1
12745	0.946133	0.147442
12746	0.946133	0.166896
12747	0.946132	0.271243
12748	1.35065	0.149739
12749	0.946132	0.279371
12750	0.946133	0.264748
12751	0.946132	0.17982
12752	0.946131	0.820608
12753	0.946131	0.802843
12754	0.94613	0.838544
12755	0.946131	0.737238
12756	0.946131	0.749361
12757	0.946131	0.78593

12758	0.946131	0.761701
12759	0.946127	0.478794
12760	0.946131	0.507205
12761	0.946127	0.477221
12762	0.94613	0.336663
12763	0.946131	0.511447
12764	0.946128	0.570736
12765	0.946129	0.452099
12766	0.810946	0.1169
12767	0.810947	0.132003
12768	0.810959	0.126913
12769	2.4391	0.123761
12770	1.35168	0.115979
12771	1.08132	0.114279
12772	1.08132	0.122784
12773	1.08131	0.137195
12774	0.946132	0.0916444
12775	0.946132	0.0947846
12776	0.946132	0.10691
12777	1.40837	0.101889
12778	0.946132	0.104163
12779	0.946132	0.103679
12780	1.40837	0.100229
12781	0.946127	1.51377
12782	0.946132	2.1
12783	0.946131	0.931446
12784	0.946127	1.99078
12785	0.946131	1.35156
12786	0.946129	1.0208
12787	0.94613	1.4169
12788	0.946133	1.8022
12789	0.946132	1.4909
12790	0.946132	1.46421
12791	0.946132	1.25634
12792	0.946132	1.54603
12793	0.946132	1.0981
12794	0.946133	2.1
12795	0.946132	0.773433
12796	1.24344	0.705755
12797	0.946132	0.705608
12798	0.946132	0.826411
12799	0.946132	0.723846
12800	1.24344	0.741846
12801	0.946132	0.804555
12802	0.946131	0.709866
12803	0.946128	0.705612
12804	0.946128	0.701193
12805	0.946131	0.71038
12806	1.41232	0.7014
12807	0.946129	0.700954
12808	0.94613	0.703833
12809	0.946128	2.1
12810	0.946133	1.8657
12811	0.946133	0.94883
12812	0.946131	2.1
12813	0.946131	1.06486
12814	0.946131	1.38369
12815	0.94613	1.27307

12816	0.946133	0.839465
12817	1.41556	0.851079
12818	0.946133	0.819231
12819	0.946133	0.800699
12820	0.946132	0.9157
12821	0.946132	0.855095
12822	0.946132	0.879052
12823	0.94612	0.814886
12824	1.41843	0.81048
12825	0.946114	0.812602
12826	0.94612	0.789614
12827	0.946119	0.872975
12828	1.41845	0.805797
12829	0.946107	0.852389
12830	0.946129	0.138881
12831	0.946129	0.121768
12832	0.94613	0.171683
12833	0.946132	0.142016
12834	0.946126	0.18205
12835	1.1509	0.118328
12836	0.94613	0.137224
12837	0.946131	0.105176
12838	0.946132	0.0988642
12839	0.946132	0.119092
12840	0.946132	0.105451
12841	0.946132	0.12308
12842	1.19263	0.0921524
12843	0.946132	0.108746
12844	0.94613	0.835984
12845	0.946131	0.909284
12846	0.946128	0.83937
12847	0.946133	0.926758
12848	0.946133	0.876986
12849	0.946131	0.867388
12850	0.946131	0.903372
12851	0.946133	1.09602
12852	1.22848	1.28749
12853	1.00336	1.29337
12854	0.827866	1.13053
12855	0.946132	1.42054
12856	0.827865	1.20791
12857	0.946133	1.18011
12858	0.946131	0.85001
12859	0.94613	0.726833
12860	0.946127	0.803801
12861	1.41498	0.801574
12862	0.94613	0.722208
12863	0.946128	0.769617
12864	0.946128	0.743748
12865	0.94613	0.0903136
12866	0.94613	0.108723
12867	0.94613	0.12999
12868	1.37358	0.121789
12869	0.946129	0.126114
12870	0.94613	0.13046
12871	1.37357	0.113752
12872	0.946111	0.106442
12873	0.946118	0.104362

12874	0.946118	0.141503
12875	1.36568	0.0971342
12876	0.946116	0.12838
12877	0.94613	0.126313
12878	0.946105	0.114622
12879	0.946132	0.756616
12880	0.946131	0.718033
12881	0.94613	0.784565
12882	1.23175	0.711473
12883	0.946131	0.707271
12884	0.946131	0.763246
12885	0.946131	0.779527
12886	0.946132	0.172299
12887	0.946126	0.157953
12888	0.946128	0.189258
12889	0.946129	0.167492
12890	0.946129	0.190878
12891	0.946128	0.170626
12892	1.25698	0.180264
12893	0.810943	2.1
12894	0.810944	2.1
12895	2.81171	2.1
12896	1.3517	2.1
12897	1.08132	2.1
12898	1.08133	2.1
12899	1.08132	2.1
12900	0.810932	2.1
12901	0.946132	0.71543
12902	0.946131	0.711394
12903	0.946131	0.720335
12904	0.946132	0.597468
12905	0.946132	0.675036
12906	0.946132	0.705916
12907	0.946131	0.70138
12908	0.810936	0.990499
12909	0.810935	2.04914
12910	2.80914	1.355
12911	1.35167	1.23687
12912	1.0813	1.1651
12913	1.0813	2.02679
12914	1.0813	0.920322
12915	0.810934	1.14018
12916	1.23851	2.1
12917	0.946132	1.06158
12918	0.946131	2.1
12919	1.40459	1.00883
12920	0.946132	1.41746
12921	1.40459	1.2681
12922	0.946132	1.30422
12923	1.19974	0.533035
12924	0.946131	0.796088
12925	1.19974	0.549909
12926	0.946132	0.790827
12927	0.946132	0.557425
12928	0.946132	0.794312
12929	0.946132	0.621142
12930	0.939141	1.0643
12931	1.14375	1.31665

12932	1.03306	1.34817
12933	0.946132	1.1277
12934	0.946126	1.41708
12935	0.946129	1.19996
12936	0.94613	1.14544
12937	0.946132	0.620954
12938	0.946131	0.596963
12939	0.946132	0.665336
12940	0.946132	0.410499
12941	0.946132	0.567219
12942	0.946132	0.687489
12943	0.946132	0.6044
12944	0.946131	2.1
12945	0.94613	2.1
12946	0.94613	2.1
12947	0.946131	2.1
12948	0.946129	2.1
12949	0.94613	2.1
12950	1.24512	2.1
12951	0.946133	0.268774
12952	0.946129	0.261725
12953	0.946131	0.384445
12954	0.946132	0.166557
12955	0.946131	0.292377
12956	0.946131	0.377944
12957	0.946131	0.265705
12958	0.946132	0.290679
12959	0.946133	0.307382
12960	0.946132	0.315119
12961	0.946132	0.194795
12962	0.946133	0.36171
12963	0.946132	0.3902
12964	0.946132	0.326841
12965	0.946132	0.839036
12966	1.41392	0.780223
12967	0.946132	0.778266
12968	0.946132	0.872581
12969	0.946132	0.74728
12970	0.946132	0.845203
12971	0.946132	0.796535
12972	0.946132	0.975764
12973	1.24333	0.899952
12974	0.946132	0.863292
12975	0.946133	1.05845
12976	0.946132	0.810244
12977	0.946132	1.03776
12978	0.946133	0.886832
12979	1.30649	0.558073
12980	0.94613	0.617147
12981	0.94613	0.530944
12982	0.946128	0.569372
12983	0.946126	0.644574
12984	0.946127	0.625447
12985	0.946127	0.604363
12986	0.709578	0.743712
12987	0.810943	0.733994
12988	2.53303	0.514672
12989	1.21651	0.545235

12990	0.979951	0.617566
12991	1.08132	0.559575
12992	0.946141	0.737577
12993	0.81096	0.677968
12994	0.946131	0.533883
12995	0.946131	0.603535
12996	0.946131	0.588218
12997	0.946131	0.400808
12998	0.946131	0.488504
12999	0.946131	0.623037
13000	0.946131	0.561843
13001	0.946132	0.524701
13002	0.946129	0.444941
13003	0.946129	0.519585
13004	0.94613	0.478894
13005	1.20634	0.467864
13006	1.20634	0.410395
13007	0.946131	0.588467
13008	0.946133	0.747195
13009	0.946132	0.713021
13010	0.946132	0.701052
13011	0.946132	0.753855
13012	0.946132	0.7
13013	1.30107	0.742058
13014	0.946133	0.744924
13015	0.810946	2.1
13016	0.810944	2.1
13017	2.73936	2.1
13018	1.35168	2.1
13019	1.0813	2.1
13020	1.08132	2.1
13021	1.08132	2.1
13022	0.810958	2.1
13023	0.946129	1.77701
13024	0.946129	2.1
13025	0.946133	1.06148
13026	1.30812	1.77455
13027	0.946131	1.15085
13028	0.946131	1.37074
13029	0.946131	1.31682
13030	1.00901	1.11613
13031	0.94613	1.32126
13032	0.94613	0.910905
13033	1.17001	1.15578
13034	0.917259	0.916778
13035	0.94613	1.02486
13036	0.94613	1.04828
13037	0.946126	2.1
13038	0.946127	2.1
13039	0.946131	2.1
13040	0.946127	2.1
13041	0.946131	2.1
13042	0.946129	2.1
13043	1.398	2.1
13044	0.946133	2.1
13045	1.3033	2.1
13046	0.946132	2.1
13047	0.946132	2.1

13048	0.946132	2.1
13049	0.946132	2.1
13050	0.946133	2.1
13051	0.946131	0.894891
13052	0.946131	0.940811
13053	1.09897	0.863823
13054	0.946129	1.04391
13055	0.946131	0.971865
13056	0.94613	1.01636
13057	0.94613	0.915705
13058	0.946132	2.1
13059	0.946131	2.1
13060	0.946131	2.1
13061	0.946132	2.1
13062	0.94613	2.1
13063	0.946131	2.1
13064	1.39858	2.1
13065	1.07117	0.827346
13066	1.08878	0.908745
13067	0.946119	0.927209
13068	1.08878	1.13764
13069	0.946119	0.772673
13070	0.946132	0.880532
13071	1.07116	0.833924
13072	0.946132	0.401674
13073	0.946132	0.34867
13074	0.946132	0.486691
13075	0.946132	0.292341
13076	0.946132	0.310141
13077	1.10733	0.359479
13078	0.946132	0.443668
13079	0.946128	0.441649
13080	0.946132	0.349139
13081	0.94613	0.529761
13082	0.946129	0.307219
13083	0.94613	0.321638
13084	1.3367	0.362976
13085	0.94613	0.483118
13086	0.810943	2.1
13087	0.810944	2.1
13088	2.70234	2.1
13089	1.21653	2.1
13090	1.08132	2.1
13091	0.946155	2.1
13092	0.979951	2.1
13093	0.709578	2.1
13094	1.27174	2.1
13095	0.946129	2.1
13096	0.94613	2.1
13097	1.35117	2.1
13098	0.94613	2.1
13099	1.35117	2.1
13100	0.94613	2.1
13101	0.946133	1.16015
13102	0.946133	1.13133
13103	0.946132	1.51319
13104	0.946133	1.07522
13105	0.946132	2.07816

13106	0.946133	1.47491
13107	0.997296	1.4585
13108	0.810943	1.12112
13109	0.810944	2.04316
13110	0.810945	2.1
13111	2.78833	2.1
13112	1.35169	2.1
13113	1.08132	1.5866
13114	1.08132	2.1
13115	1.08132	2.1
13116	0.810946	2.1
13117	0.810943	1.00678
13118	0.810933	1.34651
13119	2.70049	1.92154
13120	1.3517	1.1346
13121	1.08132	1.89457
13122	1.08132	0.851177
13123	1.08133	1.02732
13124	0.946127	1.21153
13125	1.35469	1.17756
13126	0.94613	1.15667
13127	0.946131	1.23245
13128	0.946128	1.09524
13129	1.35469	1.2998
13130	0.946129	1.32652
13131	0.946131	0.749073
13132	1.2722	0.647029
13133	0.946132	0.631944
13134	0.946132	0.672747
13135	0.946129	0.758487
13136	1.2722	0.565377
13137	0.94613	0.690031
13138	0.946133	0.7
13139	0.946132	0.7
13140	0.946132	0.7
13141	0.946132	0.7
13142	1.41461	0.7
13143	1.41461	0.7
13144	0.946133	0.7
13145	0.946131	0.959099
13146	1.30165	0.827119
13147	0.946132	0.808753
13148	0.946132	0.985951
13149	0.946129	0.850533
13150	1.30165	0.952093
13151	0.946131	0.995107
13152	0.946132	1.08075
13153	0.946129	1.37906
13154	0.94613	1.0744
13155	1.39356	1.69746
13156	0.946131	1.12382
13157	0.946131	1.1548
13158	0.94613	1.45422
13159	0.810946	1.94428
13160	0.810947	2.1
13161	0.810934	2.1
13162	2.35003	2.1
13163	1.3517	2.1

13164	1.08132	2.1
13165	1.08132	2.1
13166	1.08133	2.1
13167	0.946131	0.145076
13168	0.946131	0.157363
13169	0.946125	0.236114
13170	0.946127	0.199553
13171	0.946129	0.186862
13172	0.946128	0.204639
13173	0.946128	0.188463
13174	0.946131	0.150106
13175	0.946131	0.162666
13176	0.946131	0.271337
13177	0.946131	0.218098
13178	0.946132	0.173305
13179	0.946132	0.225051
13180	0.946131	0.182833
13181	0.946129	2.1
13182	0.946132	2.1
13183	0.946132	2.1
13184	0.94613	2.1
13185	1.30082	2.1
13186	0.94613	2.1
13187	0.946131	2.1
13188	1.20416	0.282995
13189	0.946132	0.554532
13190	0.946131	0.398895
13191	0.946132	0.372429
13192	0.946131	0.316757
13193	0.946131	0.442586
13194	0.946132	0.309488
13195	1.18711	0.268609
13196	0.946131	0.531369
13197	0.946131	0.41945
13198	0.946132	0.33393
13199	0.946131	0.306595
13200	0.946131	0.46062
13201	0.946132	0.364724
13202	0.946133	0.119483
13203	0.946133	0.134326
13204	0.946132	0.126509
13205	1.30212	0.138751
13206	0.946133	0.150683
13207	0.946132	0.127926
13208	1.30212	0.129692
13209	0.946131	0.394545
13210	0.946131	0.29413
13211	0.946131	0.50109
13212	0.946132	0.383195
13213	1.31467	0.382185
13214	1.31467	0.327077
13215	0.946131	0.613832
13216	0.946133	0.254265
13217	0.946132	0.201917
13218	0.946132	0.312737
13219	0.946132	0.250334
13220	1.25124	0.251331
13221	1.25124	0.214551

13222	0.946132	0.376582
13223	0.946132	2.1
13224	0.946132	2.1
13225	0.946132	2.1
13226	1.36422	2.1
13227	0.946132	2.1
13228	0.946131	2.1
13229	0.946132	2.1
13230	0.946131	0.991978
13231	1.41851	1.49456
13232	0.946132	1.91643
13233	0.946132	0.968206
13234	0.946132	1.23932
13235	0.946132	0.998311
13236	0.946132	1.24495
13237	0.946133	0.361793
13238	0.946132	0.378019
13239	0.946132	0.33175
13240	0.946132	0.325223
13241	0.946132	0.365348
13242	1.37794	0.270419
13243	0.946132	0.324424
13244	0.946132	2.1
13245	0.946132	2.1
13246	0.946131	2.1
13247	0.946131	2.1
13248	0.946132	2.1
13249	0.946132	2.1
13250	1.4045	2.1
13251	0.946133	2.1
13252	0.946132	2.1
13253	0.946131	2.1
13254	0.946132	2.1
13255	0.946131	2.1
13256	0.946131	2.1
13257	0.946132	2.1
13258	0.946132	2.1
13259	0.946132	2.1
13260	0.946131	2.1
13261	0.946132	2.1
13262	0.946131	2.1
13263	0.946131	2.1
13264	0.946131	2.1
13265	0.946128	2.1
13266	1.31641	2.1
13267	0.946129	2.1
13268	0.94613	2.1
13269	0.94613	2.1
13270	0.94613	2.1
13271	0.94613	2.1
13272	0.946132	2.1
13273	0.946131	2.1
13274	0.946131	1.12285
13275	0.946132	2.1
13276	0.946132	1.33303
13277	0.946132	2.01741
13278	0.946132	2.1
13279	0.946132	2.1

13280	1.41507	2.1
13281	0.946132	2.1
13282	0.946132	2.1
13283	0.946133	2.1
13284	0.946133	2.1
13285	0.946132	2.1
13286	0.946132	2.1
13287	0.946131	2.1
13288	0.946131	2.1
13289	0.946133	2.1
13290	0.946132	2.1
13291	1.32608	2.1
13292	0.946132	2.1
13293	0.94613	1.53707
13294	0.94613	2.1
13295	0.94613	0.904153
13296	0.946131	1.80801
13297	0.946129	1.21734
13298	0.94613	0.936014
13299	0.94613	1.16503
13300	1.33728	0.959763
13301	0.946131	0.919182
13302	0.94613	0.807874
13303	0.946132	0.958745
13304	0.946132	1.12604
13305	0.946131	0.869189
13306	0.946131	0.883987
13307	0.946127	2.1
13308	1.40641	1.45973
13309	0.946129	1.91757
13310	0.94613	2.1
13311	0.946128	1.11219
13312	0.946129	1.63594
13313	0.946129	1.46885
13314	0.946129	2.1
13315	0.946128	2.1
13316	0.946133	2.1
13317	1.37467	2.1
13318	0.946131	2.1
13319	0.946131	2.1
13320	1.37467	2.1
13321	0.946131	2.1
13322	0.946131	2.1
13323	0.946132	2.1
13324	0.946132	2.1
13325	0.946129	2.1
13326	0.946131	2.1
13327	1.34912	2.1
13328	0.946132	2.1
13329	0.946133	2.1
13330	0.946133	2.1
13331	0.946132	2.1
13332	0.946132	2.1
13333	0.946133	2.1
13334	0.946132	2.1
13335	0.946127	2.1
13336	0.946131	1.69067
13337	0.946132	2.1

13338	0.946131	1.72509
13339	0.946127	1.3139
13340	0.94613	2.1
13341	1.33607	2.1
13342	0.946117	0.747343
13343	0.946117	0.842646
13344	0.946115	0.946416
13345	0.946124	1.82016
13346	0.946119	0.766009
13347	1.32662	0.867425
13348	0.94613	0.757204
13349	0.946119	2.1
13350	0.946114	2.1
13351	0.946118	2.1
13352	0.946114	2.1
13353	0.946118	2.1
13354	0.946104	2.1
13355	0.946129	2.1
13356	0.946132	1.36721
13357	0.946133	2.1
13358	0.946133	2.1
13359	0.946133	2.02491
13360	0.946132	2.1
13361	0.946132	2.1
13362	0.946133	2.1
13363	0.946133	2.1
13364	0.946132	2.1
13365	0.946132	2.1
13366	0.946133	2.1
13367	0.946132	2.1
13368	0.946132	2.1
13369	0.946132	2.1
13370	0.946133	1.38025
13371	1.30881	2.00315
13372	0.946132	2.1
13373	0.946131	1.10028
13374	0.946131	1.28136
13375	0.946131	1.04895
13376	0.946131	1.68128
13377	0.946128	2.1
13378	0.946128	2.1
13379	0.946132	2.1
13380	0.94613	2.1
13381	0.94613	2.1
13382	0.94613	2.1
13383	1.34652	2.1
13384	0.946133	1.07051
13385	0.946133	0.996505
13386	0.946132	1.51799
13387	0.946133	0.997243
13388	0.946132	1.91922
13389	0.946132	1.14147
13390	0.946132	1.30912
13391	0.946132	0.362943
13392	0.946128	0.404122
13393	0.94613	0.34013
13394	0.946131	0.36442
13395	0.94613	0.462672

13396	0.94613	0.313717
13397	0.94613	0.389778
13398	0.946128	0.856208
13399	0.946132	0.879579
13400	0.94613	0.848924
13401	0.94613	0.855853
13402	0.946131	0.851837
13403	0.94613	0.86172
13404	0.94613	0.859415
13405	0.946129	0.953296
13406	1.32056	1.22698
13407	0.946131	1.63214
13408	0.94613	0.959986
13409	0.946124	1.22979
13410	0.946128	0.993605
13411	0.946127	1.05551
13412	0.946129	1.0117
13413	1.20351	1.27241
13414	0.946132	1.49351
13415	0.946132	1.04449
13416	0.946126	1.40675
13417	0.946129	1.09871
13418	0.946129	1.09872
13419	0.946127	1.28475
13420	0.946127	1.6397
13421	0.946131	1.05683
13422	1.25592	1.3257
13423	0.946127	1.08805
13424	0.946129	1.18873
13425	0.94613	1.23668
13426	1.02332	1.5983
13427	0.946133	1.12035
13428	0.946132	1.13145
13429	1.02332	1.22864
13430	0.946132	0.949519
13431	0.96196	1.19391
13432	0.930306	1.05594
13433	0.946133	1.93808
13434	0.946133	1.96216
13435	0.946132	2.1
13436	1.20057	2.08216
13437	0.946132	2.1
13438	0.946133	2.1
13439	1.20057	2.1
13440	0.946128	1.47493
13441	0.946129	0.932691
13442	0.946129	1.1722
13443	1.13245	0.938232
13444	0.946126	0.848924
13445	0.946128	1.017
13446	0.946129	0.924064
13447	0.946131	1.5728
13448	0.946131	1.82002
13449	0.94613	2.1
13450	1.25	1.79046
13451	0.946129	2.1
13452	0.946131	2.1
13453	1.25	2.1

13454	0.540425	1.89225
13455	0.748493	1.97314
13456	1.46266	2.1
13457	1.20046	2.1
13458	0.600454	1.94082
13459	1.41283	2.1
13460	0.582567	1.95033
13461	0.946131	1.58695
13462	0.946131	1.46902
13463	0.946132	1.50161
13464	0.946132	1.52371
13465	0.94613	1.34985
13466	0.946131	2.08736
13467	1.28439	2.02136
13468	0.946132	0.858771
13469	0.946131	0.842491
13470	0.946131	0.959219
13471	0.946132	0.831635
13472	0.946132	0.983036
13473	1.39335	0.813686
13474	0.946132	0.880123
13475	0.946131	0.736788
13476	0.946131	0.847919
13477	0.94613	0.731889
13478	0.946132	0.72904
13479	0.946131	0.783719
13480	0.946131	0.761163
13481	0.946131	0.770433
13482	0.946131	0.71763
13483	0.94613	0.7119
13484	0.946126	0.713709
13485	0.946131	0.702902
13486	0.946125	0.73045
13487	0.946127	0.703517
13488	0.946128	0.72002
13489	0.946131	0.210835
13490	0.946131	0.315316
13491	0.946131	0.220001
13492	0.946131	0.192068
13493	0.946131	0.201209
13494	0.946131	0.282899
13495	1.39168	0.191619
13496	0.946131	0.68755
13497	0.946126	0.666457
13498	0.946127	0.689069
13499	0.946131	0.691662
13500	0.94613	0.683241
13501	1.412	0.68174
13502	0.946128	0.670036
13503	0.948901	0.767538
13504	0.943364	0.755615
13505	1.03768	0.765939
13506	0.946132	0.864958
13507	0.946133	0.836907
13508	1.03767	0.866906
13509	0.946132	0.813621
13510	1.00902	0.938058
13511	0.88324	0.92146

13512	0.971584	0.858626
13513	0.946132	0.955054
13514	0.946129	1.1076
13515	0.946131	1.03958
13516	0.971572	1.05641
13517	0.946131	0.190208
13518	0.946125	0.156145
13519	1.33587	0.184656
13520	0.946126	0.219414
13521	0.946127	0.180843
13522	0.946126	0.20682
13523	0.946128	0.199072
13524	0.946132	0.252203
13525	0.946132	0.213328
13526	1.27512	0.207127
13527	0.946133	0.325385
13528	0.946132	0.266277
13529	0.946133	0.274465
13530	0.946133	0.307644
13531	0.946128	0.505392
13532	0.946133	0.528998
13533	0.946131	0.577534
13534	0.94613	0.292878
13535	0.946131	0.44012
13536	0.94613	0.524196
13537	0.946131	0.469372
13538	0.946131	0.443568
13539	0.946131	0.457836
13540	0.946131	0.545751
13541	0.946131	0.230172
13542	0.946131	0.367459
13543	0.946131	0.474261
13544	0.946131	0.398783
13545	0.94613	0.0848562
13546	0.946131	0.0878707
13547	0.946131	0.100501
13548	0.94613	0.0879323
13549	0.946132	0.095264
13550	0.946131	0.0973961
13551	1.35316	0.0907589
13552	0.94613	0.706394
13553	0.94613	0.701356
13554	0.946128	0.70341
13555	0.946132	0.702461
13556	0.946132	0.704385
13557	1.28727	0.703315
13558	0.94613	0.698723
13559	0.946131	0.293708
13560	0.946132	0.330329
13561	0.946132	0.299718
13562	1.39502	0.275497
13563	0.946132	0.21011
13564	0.946132	0.290957
13565	0.946132	0.460854
13566	0.946125	0.828803
13567	0.946131	0.821356
13568	0.94613	0.778143
13569	0.946129	0.837142

13570	0.946126	0.933898
13571	0.946127	0.871314
13572	1.36173	0.853141
13573	0.946129	0.952847
13574	1.02363	0.886208
13575	0.94613	0.885929
13576	0.946131	0.964752
13577	0.946131	0.846279
13578	1.02363	0.94991
13579	0.946131	0.941009
13580	0.946128	0.0641904
13581	1.24678	0.0654878
13582	0.94613	0.0660297
13583	0.94613	0.0706241
13584	0.94613	0.0621581
13585	0.946131	0.0646199
13586	0.94613	0.0710786
13587	0.946129	0.0662365
13588	1.25215	0.0670752
13589	0.946132	0.0685935
13590	0.946126	0.071619
13591	0.946129	0.0646023
13592	0.94613	0.0655445
13593	0.946129	0.0713616
13594	0.946129	0.826981
13595	0.946133	0.838081
13596	0.946133	0.792604
13597	0.946131	0.81067
13598	0.946131	0.809548
13599	1.29689	0.77638
13600	0.946131	0.786663
13601	0.946128	0.545535
13602	0.946132	0.33162
13603	0.946132	0.468892
13604	0.94613	0.644685
13605	1.22716	0.389919
13606	1.22716	0.446632
13607	0.946131	0.619003
13608	0.946127	0.645494
13609	0.946131	0.477221
13610	0.946131	0.586414
13611	0.946128	0.69584
13612	1.30463	0.510694
13613	1.30463	0.547451
13614	0.946129	0.690239
13615	1.29958	0.147266
13616	0.946132	0.248561
13617	0.946132	0.159161
13618	0.946132	0.120612
13619	0.946132	0.145108
13620	0.946132	0.163816
13621	0.946133	0.144242
13622	1.26201	0.130416
13623	0.946133	0.273754
13624	0.946132	0.1684
13625	0.946132	0.125078
13626	0.946132	0.154734
13627	0.946133	0.183583

13628	0.946132	0.14414
13629	0.946133	0.497462
13630	0.946132	0.369106
13631	0.946132	0.560857
13632	0.946133	0.323754
13633	0.946132	0.343165
13634	1.40427	0.370314
13635	0.946133	0.546672
13636	0.946132	0.519381
13637	0.946132	0.361129
13638	0.946132	0.558575
13639	0.946132	0.323647
13640	0.946132	0.349435
13641	1.35123	0.352516
13642	0.946131	0.533498
13643	0.946128	0.705109
13644	0.946132	0.576549
13645	0.946132	0.65594
13646	0.946129	0.709377
13647	1.22144	0.586495
13648	1.22144	0.619179
13649	0.946131	0.703827
13650	0.946127	0.762543
13651	0.946132	0.752838
13652	0.946131	0.7446
13653	0.946127	0.754399
13654	0.946131	0.752942
13655	1.23351	0.73701
13656	0.94613	0.746064
13657	1.18119	0.321909
13658	0.946131	0.457873
13659	0.946132	0.272927
13660	0.946132	0.362515
13661	0.94613	0.431485
13662	0.946131	0.42279
13663	0.946131	0.384372
13664	0.946132	0.726966
13665	0.946132	0.718068
13666	0.946133	0.740352
13667	1.30563	0.71079
13668	0.946132	0.713399
13669	0.946132	0.727143
13670	0.946133	0.743574
13671	1.20256	0.875317
13672	0.946132	1.37521
13673	0.946133	1.02396
13674	0.946132	0.871954
13675	0.946132	0.946933
13676	0.946132	1.12128
13677	0.946133	0.903038
13678	0.94613	0.98014
13679	0.94613	0.899502
13680	0.946128	1.11561
13681	1.33007	0.87567
13682	0.946129	0.890113
13683	0.94613	1.02377
13684	0.946131	1.0855
13685	0.946127	2.1

13686	0.946127	2.1
13687	0.946131	2.1
13688	1.30136	2.1
13689	0.946128	2.1
13690	0.946129	2.1
13691	1.30136	2.1
13692	0.810942	2.1
13693	0.810942	2.1
13694	0.81093	2.1
13695	2.3208	2.1
13696	1.3517	2.1
13697	1.08132	2.1
13698	1.08132	2.1
13699	1.08133	2.1
13700	0.946132	2.1
13701	0.946132	2.1
13702	0.946132	2.1
13703	0.946132	2.1
13704	0.946132	2.1
13705	1.39823	2.1
13706	0.946132	2.1
13707	1.37713	2.1
13708	0.946131	2.1
13709	0.946131	2.1
13710	0.946131	2.1
13711	0.946131	2.1
13712	0.946131	2.1
13713	0.946131	2.1
13714	0.946128	2.1
13715	0.946128	2.1
13716	0.946133	2.1
13717	0.946131	2.1
13718	0.946131	1.18285
13719	0.946131	2.1
13720	0.94613	2.1
13721	0.946129	2.1
13722	0.946129	2.1
13723	0.946133	2.1
13724	0.946131	2.1
13725	0.946131	2.1
13726	0.946131	2.1
13727	0.946131	2.1
13728	0.946132	1.06191
13729	1.33694	1.90855
13730	0.946133	2.1
13731	0.946133	1.04716
13732	0.946132	1.67291
13733	0.946132	1.09572
13734	0.946133	1.32351
13735	0.946131	1.83211
13736	0.94613	2.1
13737	0.946131	2.1
13738	0.946133	2.1
13739	0.946129	2.1
13740	0.946131	2.1
13741	0.946131	2.1
13742	0.946132	1.59368
13743	1.35943	1.93975

13744	0.946132	2.1
13745	0.946132	1.07198
13746	0.946131	1.24977
13747	0.946132	1.01027
13748	0.946132	1.84776
13749	0.946132	0.167114
13750	0.946132	0.231964
13751	0.946133	0.135303
13752	0.946133	0.209995
13753	0.946132	0.159166
13754	0.946132	0.20335
13755	0.946132	0.194224
13756	1.29322	0.654106
13757	0.946131	0.7
13758	0.946132	0.387507
13759	0.946129	0.699727
13760	0.946131	0.54219
13761	0.946131	0.443987
13762	0.946131	0.697934
13763	0.946132	0.778021
13764	0.946132	1.1376
13765	0.946131	0.620566
13766	1.41492	0.332795
13767	0.946132	0.912416
13768	0.946132	0.813119
13769	0.946131	0.8586
13770	0.946132	2.1
13771	0.946132	2.1
13772	0.946132	2.1
13773	1.35028	2.1
13774	0.946132	2.1
13775	0.946132	2.1
13776	0.946132	2.1
13777	0.946132	1.33316
13778	0.946132	2.1
13779	0.946132	1.71397
13780	1.32461	1.52532
13781	0.946131	2.1
13782	0.946132	1.66685
13783	0.946132	2.1
13784	0.946131	2.1
13785	0.946131	2.1
13786	0.946131	2.1
13787	0.946132	2.1
13788	0.946129	2.1
13789	0.94613	2.1
13790	1.40369	2.1
13791	0.946132	0.702961
13792	0.946131	0.594433
13793	0.946131	0.707581
13794	0.979598	0.546178
13795	0.946131	0.56462
13796	0.946131	0.700663
13797	0.946132	0.707203
13798	0.946131	2.1
13799	1.41897	2.1
13800	0.946132	2.1
13801	0.946132	2.1

13802	0.94613	1.54915
13803	0.946131	2.1
13804	0.946131	2.1
13805	0.946131	2.1
13806	0.94613	2.1
13807	0.946132	2.1
13808	0.946132	2.1
13809	0.946129	2.1
13810	0.946131	2.1
13811	1.37791	2.1
13812	0.946127	2.1
13813	0.946131	2.1
13814	0.946131	2.1
13815	0.94613	2.1
13816	0.946128	2.1
13817	0.946129	2.1
13818	1.39384	2.1
13819	0.810945	2.05054
13820	0.810944	1.2882
13821	0.810958	1.59233
13822	2.70852	1.93746
13823	1.35168	1.43804
13824	1.08132	1.70628
13825	1.08132	1.09957
13826	1.0813	1.34286
13827	1.3335	2.1
13828	0.946132	2.1
13829	0.94613	2.1
13830	0.946132	2.1
13831	0.946131	2.1
13832	0.94613	2.1
13833	0.946131	2.1
13834	0.946132	1.72816
13835	0.946131	0.765664
13836	0.94613	0.991239
13837	1.41192	0.904472
13838	0.946132	0.765066
13839	0.946131	0.859725
13840	0.94613	0.79065
13841	0.946133	0.77555
13842	0.946133	0.74487
13843	0.946132	0.751016
13844	1.39428	0.724156
13845	0.946132	0.716628
13846	0.946132	0.719113
13847	0.946133	0.723744
13848	0.94613	0.908516
13849	0.946129	2.1
13850	0.94613	1.04439
13851	0.946128	1.02231
13852	1.40684	2.00793
13853	0.94613	1.006
13854	0.94613	2.1
13855	0.946131	1.01607
13856	0.946131	2.1
13857	0.946131	1.549
13858	0.946131	1.16403
13859	1.39021	2.1

13860	0.946131	1.5986
13861	0.946131	2.1
13862	0.946131	1.58488
13863	0.946131	2.07197
13864	0.946131	1.0868
13865	0.946131	1.98969
13866	0.946131	2.093
13867	0.946131	1.04263
13868	0.946131	1.27238
13869	0.810946	2.1
13870	0.810946	2.1
13871	2.69264	2.1
13872	1.35168	2.1
13873	1.0813	2.1
13874	1.08132	2.1
13875	1.08132	2.1
13876	0.810958	2.1
13877	1.16687	2.0715
13878	0.946131	2.1
13879	0.946131	2.1
13880	1.34632	2.1
13881	0.946131	2.1
13882	1.34632	2.1
13883	0.946131	2.1
13884	0.946128	0.921656
13885	0.946133	0.933607
13886	0.946133	0.851654
13887	0.946131	0.909632
13888	0.946131	0.861598
13889	1.3564	0.870387
13890	0.94613	0.868394
13891	0.946128	2.1
13892	0.946132	2.1
13893	0.94613	2.1
13894	0.94613	2.1
13895	0.946131	2.1
13896	1.35827	2.1
13897	0.94613	2.1
13898	0.946133	0.924793
13899	0.946132	1.01904
13900	0.946132	0.922899
13901	0.946133	1.01306
13902	0.946133	0.950466
13903	0.946132	0.958453
13904	0.946133	0.997247
13905	0.946132	2.1
13906	0.946132	2.07176
13907	0.946132	0.907672
13908	0.946131	2.03128
13909	0.946132	1.16772
13910	0.946132	1.10244
13911	0.946132	1.1358
13912	0.94613	0.850443
13913	0.94613	0.783207
13914	0.946128	0.854374
13915	0.946132	0.779934
13916	0.946132	0.878189
13917	0.94613	0.804489

13918	0.946131	0.801774
13919	0.946126	0.582422
13920	0.946131	0.424869
13921	0.946127	0.602244
13922	0.946129	0.401662
13923	0.94613	0.574298
13924	0.946128	0.475266
13925	0.946128	0.485043
13926	0.946131	0.359199
13927	0.946127	0.326413
13928	0.946127	0.48736
13929	0.946131	0.275299
13930	0.946131	0.466713
13931	0.946128	0.462845
13932	0.946129	0.395934
13933	0.94613	0.589701
13934	0.946131	0.493658
13935	0.946129	0.652025
13936	0.946132	0.340951
13937	0.946132	0.649777
13938	0.946131	0.648927
13939	0.94613	0.54575
13940	0.94613	0.651747
13941	0.946131	0.641783
13942	0.946129	0.661227
13943	0.946132	0.654516
13944	0.946132	0.652189
13945	1.32827	0.645028
13946	0.946131	0.64957
13947	0.946132	2.1
13948	0.946132	2.1
13949	0.946131	2.1
13950	0.946131	2.1
13951	0.946132	2.1
13952	0.946131	2.1
13953	0.946131	2.1
13954	0.946132	0.593119
13955	0.946132	0.916224
13956	0.946131	0.737634
13957	1.38356	0.732851
13958	0.946133	0.885371
13959	0.946131	0.773323
13960	0.946131	0.82388
13961	0.94613	2.1
13962	0.946131	2.1
13963	0.946131	2.1
13964	0.946128	2.1
13965	0.946133	2.1
13966	0.946131	2.1
13967	1.36918	2.1
13968	0.946131	0.913892
13969	0.94613	1.78463
13970	0.946129	1.12614
13971	0.946133	2.1
13972	0.946132	1.08078
13973	1.3771	1.66253
13974	0.946131	1.07005
13975	1.30029	0.997074

13976	0.94613	0.809456
13977	0.946129	1.15914
13978	0.946132	1.0936
13979	0.946132	0.716024
13980	0.946131	0.94932
13981	0.94613	0.84512
13982	0.946133	0.14275
13983	0.946132	0.185805
13984	0.946132	0.142113
13985	0.946132	0.133465
13986	0.946132	0.135705
13987	0.946132	0.16414
13988	1.21174	0.138736
13989	0.810946	2.1
13990	0.810945	2.1
13991	2.63045	2.1
13992	1.35169	2.1
13993	1.08132	2.1
13994	1.08132	2.1
13995	1.08132	2.1
13996	0.810946	2.1
13997	1.1796	2.1
13998	0.946131	2.1
13999	0.946132	2.1
14000	1.31522	2.1
14001	0.946132	2.1
14002	1.31522	2.1
14003	0.946132	2.1
14004	0.946131	2.1
14005	1.40615	2.1
14006	0.946129	2.1
14007	0.946128	2.1
14008	0.946133	2.1
14009	0.946131	2.1
14010	0.94613	2.1
14011	0.810945	2.1
14012	0.810944	2.1
14013	2.80479	2.1
14014	1.35169	2.1
14015	1.08132	2.1
14016	1.08132	2.1
14017	1.08132	2.1
14018	0.810945	2.1
14019	1.35562	2.1
14020	0.94613	2.1
14021	1.35561	2.1
14022	0.946133	2.1
14023	0.946131	2.1
14024	1.40239	2.1
14025	0.946131	2.1
14026	0.946125	0.749653
14027	0.946131	0.845374
14028	0.946127	0.683802
14029	0.946129	0.746896
14030	0.946128	0.814376
14031	0.946129	0.814848
14032	0.946128	0.779459
14033	0.946119	2.1

14034	0.946113	2.1
14035	0.94612	2.1
14036	0.946119	2.1
14037	1.41915	2.1
14038	0.946107	2.1
14039	0.946132	2.1
14040	0.810946	1.2945
14041	0.810943	1.99815
14042	0.810934	1.5739
14043	2.31411	2.08648
14044	1.35171	1.89756
14045	1.08132	1.40358
14046	1.08132	2.05507
14047	1.08133	1.37322
14048	0.946127	2.04895
14049	0.94613	2.1
14050	0.94613	2.1
14051	0.946131	2.1
14052	0.946126	2.1
14053	0.946128	2.1
14054	0.946128	2.1
14055	0.810944	2.1
14056	0.810947	2.1
14057	0.81096	2.1
14058	2.78019	2.1
14059	1.35168	2.1
14060	1.08132	2.1
14061	1.08132	2.1
14062	1.08131	2.1
14063	0.946133	0.656152
14064	0.946132	0.657478
14065	0.946132	0.65389
14066	0.946133	0.616188
14067	0.946132	0.675897
14068	1.3495	0.55133
14069	0.946133	0.680614
14070	0.946133	2.1
14071	0.946132	2.1
14072	0.946132	2.1
14073	1.39529	2.1
14074	0.946133	2.1
14075	0.946132	2.1
14076	0.946133	2.1
14077	0.946133	2.1
14078	0.946132	2.1
14079	0.946132	2.1
14080	0.946132	2.1
14081	0.946132	2.1
14082	0.946133	2.1
14083	0.946132	2.1
14084	0.810946	2.1
14085	0.810945	2.1
14086	2.67861	2.1
14087	1.35169	2.1
14088	1.08132	2.1
14089	1.08132	2.1
14090	1.08132	2.1
14091	0.810946	2.1

14092	1.24616	2.1
14093	0.946132	2.1
14094	0.946132	2.1
14095	1.33931	2.1
14096	0.946132	2.1
14097	1.33931	2.1
14098	0.946133	2.1
14099	0.946133	0.700316
14100	0.946133	0.7
14101	0.946132	0.7
14102	0.946133	0.70011
14103	1.3855	0.7
14104	1.3855	0.7
14105	0.946133	0.7
14106	0.946128	1.09957
14107	0.946132	2.1
14108	0.946128	1.63718
14109	0.94613	2.1
14110	0.946131	2.1
14111	1.40225	2.1
14112	0.94613	2.1
14113	1.40548	0.137009
14114	0.946118	0.199507
14115	0.946114	0.151387
14116	0.946112	0.103651
14117	0.946117	0.122817
14118	0.946128	0.133912
14119	0.946103	0.146106
14120	0.94613	2.1
14121	0.946132	2.1
14122	0.946131	2.1
14123	0.946132	2.1
14124	1.37811	2.1
14125	0.946131	2.1
14126	0.946131	2.1
14127	0.946132	2.1
14128	0.946133	2.1
14129	0.946133	2.1
14130	0.946132	2.1
14131	0.946133	2.1
14132	0.946132	2.1
14133	0.946132	2.1
14134	0.946118	1.38542
14135	0.946113	2.1
14136	0.946118	2.1
14137	0.946119	2.1
14138	0.946117	2.1
14139	0.946105	2.1
14140	0.946131	2.1
14141	0.946118	0.196296
14142	0.946118	0.174152
14143	0.946117	0.219296
14144	0.946113	0.131469
14145	0.94612	0.150738
14146	1.41377	0.160391
14147	0.946106	0.197103
14148	0.946132	1.02273
14149	1.28436	0.915004

14150	0.946131	0.907348
14151	0.946131	1.03495
14152	0.946131	0.80577
14153	0.94613	1.06829
14154	0.946131	0.933066
14155	0.810947	0.757791
14156	0.810946	0.77396
14157	2.68675	0.723291
14158	1.35168	0.734634
14159	1.08132	0.752372
14160	1.08132	0.742155
14161	1.0813	0.760744
14162	0.81096	0.745837
14163	0.946132	0.73138
14164	1.34338	0.708145
14165	0.946132	0.718397
14166	1.34338	0.739314
14167	0.946132	0.741744
14168	1.34337	0.732512
14169	0.946132	0.722641
14170	0.946127	1.95297
14171	0.946131	1.73384
14172	0.946127	1.61693
14173	0.946131	1.55796
14174	0.946131	1.80761
14175	0.946128	1.53506
14176	1.30281	1.96006
14177	0.946132	0.835985
14178	0.946132	0.813381
14179	0.946132	0.809417
14180	0.946131	0.812223
14181	0.946132	0.834494
14182	1.37973	0.734545
14183	0.946132	0.795249
14184	0.946131	0.944052
14185	1.41798	0.916793
14186	0.946132	0.903619
14187	0.946132	0.908606
14188	0.946129	0.979849
14189	0.946131	0.907196
14190	0.946131	0.97875
14191	0.946118	0.893097
14192	1.41776	0.872612
14193	0.946113	0.877544
14194	0.946119	0.861362
14195	0.946117	0.927516
14196	1.41778	0.853876
14197	0.946105	0.933225
14198	0.946131	0.993143
14199	1.41798	1.04008
14200	0.946132	1.07148
14201	0.946132	0.974734
14202	0.94613	1.00553
14203	1.41799	0.954578
14204	0.946131	0.961455
14205	0.946133	2.1
14206	0.946132	2.1
14207	0.946132	1.04694

14208	0.946132	2.1
14209	0.946133	1.39421
14210	0.946132	1.90385
14211	0.946132	2.1
14212	0.946131	1.99799
14213	0.946131	2.1
14214	0.94613	0.969565
14215	0.946129	2.1
14216	0.946132	1.21479
14217	0.94613	1.10991
14218	0.946131	1.87109
14219	0.94612	1.23289
14220	0.946126	2.1
14221	0.946119	1.14381
14222	0.94612	1.37154
14223	1.40766	1.88818
14224	0.946132	1.07602
14225	0.946107	2.1
14226	0.946128	2.1
14227	1.31213	2.1
14228	0.946129	2.1
14229	0.94613	2.1
14230	0.94613	2.1
14231	0.94613	2.1
14232	0.94613	2.1
14233	0.946127	1.53403
14234	0.946131	2.1
14235	0.94613	2.1
14236	0.94613	1.67879
14237	1.36653	2.07391
14238	0.946128	1.72058
14239	0.946128	2.05097
14240	0.946127	2.1
14241	0.94613	2.1
14242	0.946131	2.1
14243	0.94613	2.1
14244	0.946128	2.1
14245	0.946128	2.1
14246	0.946129	2.1
14247	0.946133	2.1
14248	1.40081	2.1
14249	0.946132	2.1
14250	0.946132	2.1
14251	0.946133	2.1
14252	0.946132	2.1
14253	0.946133	2.1
14254	0.946128	2.1
14255	1.32282	2.1
14256	0.946129	2.1
14257	0.94613	2.1
14258	0.946131	2.1
14259	0.946131	2.1
14260	0.946131	2.1
14261	0.810945	1.35549
14262	0.810943	2.1
14263	0.810933	2.1
14264	2.44097	2.1
14265	1.3517	2.1

14266	1.08132	1.42559
14267	1.08132	2.1
14268	1.08133	1.82205
14269	0.946131	2.1
14270	0.94613	2.1
14271	0.946133	2.1
14272	0.946133	2.1
14273	0.946128	2.1
14274	0.946131	2.1
14275	1.27676	2.1
14276	0.946126	0.851107
14277	0.946126	0.903806
14278	0.946131	0.799029
14279	0.946127	0.899654
14280	0.94613	0.713604
14281	0.946128	0.821104
14282	0.946128	0.783588
14283	0.946132	0.348182
14284	0.946133	0.370051
14285	0.946132	0.432589
14286	0.946132	0.238239
14287	0.946132	0.395266
14288	0.946133	0.608463
14289	0.946132	0.389145
14290	0.946128	0.515137
14291	0.946131	0.526078
14292	0.94613	0.582559
14293	0.946129	0.340727
14294	0.94613	0.488743
14295	0.94613	0.687801
14296	0.94613	0.528689
14297	0.946132	0.251299
14298	0.946128	0.267532
14299	0.94613	0.317017
14300	0.946131	0.170883
14301	0.94613	0.353229
14302	0.94613	0.39204
14303	0.946131	0.270931
14304	0.94612	0.272166
14305	0.946119	0.286431
14306	0.946119	0.352826
14307	0.946124	0.186437
14308	0.946119	0.331944
14309	0.946132	0.438778
14310	0.946107	0.284439
14311	0.946128	2.1
14312	0.946133	2.1
14313	0.946131	2.1
14314	0.94613	2.1
14315	0.946131	2.1
14316	1.37869	2.1
14317	0.946131	2.1
14318	1.35703	2.1
14319	0.946132	2.1
14320	0.946132	2.1
14321	0.946132	2.1
14322	0.946132	2.1
14323	0.946132	2.1

14324	0.946133	2.1
14325	0.946133	2.1
14326	0.946133	2.02425
14327	0.946132	2.1
14328	1.37017	2.1
14329	0.946133	2.1
14330	0.946132	2.1
14331	0.946133	2.1
14332	0.946114	0.0911419
14333	0.946119	0.102247
14334	0.946119	0.139271
14335	0.94612	0.114799
14336	0.946119	0.125386
14337	0.946132	0.111934
14338	0.946107	0.111537
14339	0.946131	0.186067
14340	0.946131	0.193981
14341	0.946126	0.279651
14342	0.946127	0.263852
14343	0.94613	0.197426
14344	0.946129	0.27267
14345	0.946128	0.234549
14346	0.946131	1.22926
14347	0.946131	1.17672
14348	0.946132	1.03998
14349	0.946132	1.23768
14350	0.94613	1.39511
14351	0.946131	1.4844
14352	1.3279	1.41705
14353	0.946132	2.1
14354	1.40932	2.1
14355	0.946132	2.1
14356	0.946132	2.1
14357	0.946132	2.1
14358	0.946131	2.1
14359	0.946132	2.1
14360	0.946131	0.350187
14361	0.946131	0.312425
14362	0.946132	0.37257
14363	0.946132	0.344046
14364	0.94613	0.406495
14365	0.946131	0.401573
14366	1.26868	0.397496
14367	0.810943	2.1
14368	0.810945	2.1
14369	0.810932	2.1
14370	2.82321	2.1
14371	1.3517	2.1
14372	1.08132	2.1
14373	1.08132	2.1
14374	1.08133	2.1
14375	0.810943	0.809953
14376	0.810944	0.747377
14377	2.63079	0.833777
14378	1.35168	0.760163
14379	1.0813	0.787091
14380	1.08132	0.562013
14381	1.08132	0.924481

14382	0.810957	0.811937
14383	1.17856	0.34239
14384	0.946132	0.776712
14385	0.946128	0.735802
14386	1.3154	0.935718
14387	0.94613	0.773294
14388	1.31539	0.789841
14389	0.94613	0.870895
14390	0.94613	1.53341
14391	0.94613	1.46421
14392	0.946131	1.35899
14393	0.946131	1.55253
14394	0.946129	1.55574
14395	0.94613	2.04956
14396	1.21997	1.99895
14397	0.946131	1.15847
14398	1.25083	1.00941
14399	0.946132	0.988285
14400	0.946132	1.1865
14401	0.946129	0.972574
14402	1.25083	1.19882
14403	0.946131	1.21437
14404	0.946127	1.20078
14405	1.41342	0.868976
14406	0.94613	1.14153
14407	0.946131	0.990537
14408	0.946127	0.791051
14409	0.946129	0.869376
14410	0.946129	0.919972
14411	0.946131	1.53781
14412	1.29149	1.62717
14413	0.946133	2.1
14414	0.946133	1.0383
14415	0.946128	1.26015
14416	0.946131	0.87196
14417	0.94613	1.22706
14418	0.94613	2.00196
14419	0.94613	1.20757
14420	0.946132	2.1
14421	0.946132	1.12257
14422	0.946128	0.972152
14423	0.94613	1.82556
14424	1.41022	1.95428
14425	0.946132	0.7
14426	0.946132	0.7
14427	0.946128	0.7
14428	0.94613	0.7
14429	0.94613	0.7
14430	0.94613	0.7
14431	0.946131	0.7
14432	0.946129	0.889997
14433	1.37758	1.38077
14434	0.946131	2.04722
14435	0.94613	0.831821
14436	0.946124	1.15428
14437	0.946127	0.85544
14438	0.946127	1.44652
14439	0.946129	0.908861

14440	1.38259	1.29676
14441	0.946132	1.83395
14442	0.946132	0.889387
14443	0.946126	1.23335
14444	0.946129	0.91886
14445	0.94613	1.17717
14446	0.946131	0.238041
14447	0.946132	0.294758
14448	0.946132	0.275957
14449	0.946132	0.212026
14450	0.946132	0.302147
14451	0.946132	0.350739
14452	0.946132	0.26742
14453	0.946131	0.171376
14454	0.94613	0.184837
14455	0.94613	0.151746
14456	0.946133	0.173705
14457	0.946128	0.148725
14458	1.25017	0.155489
14459	0.946131	0.1846
14460	0.946131	2.1
14461	0.946131	2.1
14462	0.946131	2.1
14463	1.3937	2.1
14464	0.946133	2.1
14465	0.946131	2.1
14466	0.94613	2.1
14467	0.946131	0.83566
14468	0.946131	0.748299
14469	0.946131	0.774413
14470	0.94613	0.735714
14471	0.946133	0.890733
14472	0.946132	0.794137
14473	0.946131	0.781427
14474	0.946132	0.674907
14475	0.946132	0.746478
14476	0.946132	0.623953
14477	0.946132	0.708461
14478	0.946131	0.696657
14479	0.946132	0.4491
14480	0.946132	0.690742
14481	0.946129	0.474898
14482	0.94613	0.852882
14483	0.94613	0.799076
14484	0.946128	0.852306
14485	0.946132	0.135842
14486	0.946131	0.787916
14487	0.94613	0.780326
14488	0.946132	2.0364
14489	0.946132	0.853191
14490	0.946132	0.970325
14491	1.41619	1.61585
14492	0.946133	0.784602
14493	0.946133	0.965044
14494	0.946132	0.850587
14495	0.946132	2.1
14496	0.946132	2.02632
14497	0.946132	2.1

14498	1.38244	2.1
14499	0.946132	1.00246
14500	0.946131	2.1
14501	0.946132	2.1
14502	0.810946	0.862583
14503	0.810943	1.01158
14504	0.810934	0.85606
14505	2.42741	0.765974
14506	1.35171	0.799808
14507	1.08132	0.8303
14508	1.08132	0.857101
14509	1.08133	0.859842
14510	0.810947	0.826773
14511	0.810943	0.980733
14512	2.44179	0.764428
14513	1.35171	0.801821
14514	1.08132	0.807305
14515	1.08132	0.803202
14516	1.08133	0.824646
14517	0.810934	0.809762
14518	0.946132	1.32434
14519	0.946132	1.28005
14520	0.946133	1.01365
14521	0.946133	1.28109
14522	0.946132	1.49672
14523	0.946132	1.32616
14524	1.12786	1.25436
14525	1.27806	0.923442
14526	0.946132	1.03793
14527	0.946133	0.963164
14528	0.946133	1.11316
14529	0.946132	1.33631
14530	0.946132	1.02892
14531	0.946133	1.22356
14532	0.946132	2.1
14533	0.946132	2.1
14534	0.946132	2.1
14535	1.37098	2.1
14536	0.946133	2.1
14537	0.946132	2.1
14538	1.37098	2.1
14539	0.946125	1.00224
14540	0.946129	0.925752
14541	0.946131	1.64938
14542	0.94613	0.866578
14543	0.946124	0.818479
14544	0.946127	0.867849
14545	1.39514	0.994298
14546	0.946132	2.1
14547	0.946131	2.1
14548	0.94613	2.1
14549	1.39909	2.1
14550	0.946132	1.06711
14551	0.946131	2.1
14552	0.94613	2.1
14553	0.946133	1.50189
14554	0.946133	0.788025
14555	0.946132	1.83098

14556	1.36835	1.12109
14557	0.946132	0.815794
14558	0.946133	1.00384
14559	0.946132	0.819728
14560	0.946132	2.1
14561	0.946133	2.1
14562	0.946133	2.1
14563	0.946132	2.1
14564	1.39047	2.1
14565	0.946132	2.1
14566	0.946132	2.1
14567	1.33174	0.134437
14568	0.946128	0.141326
14569	0.94613	0.164103
14570	0.94613	0.135871
14571	0.94613	0.126389
14572	0.94613	0.140591
14573	0.946131	0.138683
14574	0.946128	0.180167
14575	1.36004	0.160379
14576	0.946129	0.165995
14577	0.94613	0.186355
14578	0.94613	0.212382
14579	1.36003	0.131281
14580	0.94613	0.177096
14581	0.946124	2.1
14582	0.946119	2.1
14583	0.946119	2.1
14584	0.946119	2.1
14585	0.94612	2.1
14586	0.946132	2.1
14587	1.29062	2.1
14588	0.946128	2.1
14589	0.946128	2.1
14590	0.946133	2.1
14591	0.94613	2.1
14592	0.946131	2.1
14593	0.946131	2.1
14594	1.30584	2.1
14595	0.946131	2.1
14596	0.946131	2.1
14597	0.946131	2.1
14598	0.946132	2.1
14599	1.39549	2.1
14600	0.946131	2.1
14601	0.946131	2.1
14602	0.913386	1.04405
14603	1.17466	0.985416
14604	1.00473	0.956383
14605	0.946132	1.00472
14606	0.946126	1.26675
14607	0.94613	1.10185
14608	0.946129	1.05034
14609	0.946132	0.855537
14610	1.38894	0.871162
14611	0.946132	0.854685
14612	0.946132	0.838304
14613	0.946132	0.929532

14614	0.946132	0.858196
14615	0.946133	0.874522
14616	0.946128	0.848684
14617	1.23865	0.854631
14618	0.946129	0.837145
14619	0.94613	0.82785
14620	0.94613	0.867517
14621	0.94613	0.832326
14622	0.94613	0.861286
14623	0.946126	1.02604
14624	1.40248	1.11516
14625	0.946129	1.08041
14626	0.94613	1.03882
14627	0.946124	1.49037
14628	0.946127	1.11151
14629	0.946128	1.0794
14630	0.946133	0.860318
14631	1.1391	0.919197
14632	0.946132	0.909394
14633	0.946132	0.847535
14634	0.946132	1.01817
14635	0.946133	0.876665
14636	0.946132	0.887036
14637	1.17156	0.989397
14638	0.946133	0.911305
14639	1.33893	0.862082
14640	0.827864	0.857474
14641	0.995232	0.846055
14642	0.827864	0.949778
14643	0.94613	0.799281
14644	1.21387	0.77009
14645	0.946132	0.774904
14646	0.946131	0.740033
14647	0.94613	0.759337
14648	0.94613	0.748569
14649	0.94613	0.761214
14650	0.94613	0.761413
14651	0.810943	0.703774
14652	0.810945	0.7
14653	2.42463	0.700124
14654	1.3517	0.700183
14655	1.08132	0.70276
14656	1.08132	0.703769
14657	1.08133	0.700028
14658	0.810932	0.704543
14659	0.946133	0.795759
14660	0.946132	0.75143
14661	0.946131	0.77184
14662	1.36748	0.763334
14663	0.946133	0.767676
14664	0.946131	0.753819
14665	0.946132	0.715627
14666	1.35856	0.462711
14667	0.946131	0.587659
14668	0.94613	0.288301
14669	0.94613	0.692751
14670	0.946131	0.447088
14671	0.94613	0.403993

14672	0.94613	0.688408
14673	0.946132	2.01825
14674	0.946131	2.1
14675	0.946131	2.1
14676	0.946131	2.1
14677	1.41149	2.1
14678	1.41149	2.1
14679	0.946131	2.1
14680	0.946128	2.1
14681	0.946128	2.1
14682	0.946133	2.1
14683	0.946131	2.1
14684	0.946131	2.1
14685	0.946131	2.1
14686	0.94613	2.1
14687	1.41388	0.269587
14688	0.94613	0.36402
14689	0.946132	0.219855
14690	0.946132	0.306316
14691	0.946129	0.360041
14692	0.946131	0.336637
14693	0.946131	0.33201
14694	1.02798	0.308882
14695	0.946132	0.621074
14696	1.02798	0.301705
14697	0.946133	0.571821
14698	1.14931	0.360568
14699	0.957746	0.573238
14700	0.93452	0.388632
14701	0.946129	2.1
14702	0.946134	2.1
14703	0.946133	2.1
14704	0.946131	2.1
14705	0.946131	2.1
14706	0.946131	2.1
14707	0.946131	2.1
14708	0.946133	1.18599
14709	0.946132	1.01343
14710	0.946132	1.15141
14711	0.946132	1.35589
14712	1.38773	1.02117
14713	0.946132	1.15083
14714	0.946133	1.06859
14715	0.946131	0.565868
14716	0.946131	0.539804
14717	1.41512	0.558402
14718	0.946133	0.653316
14719	0.946131	0.668742
14720	0.946131	0.680715
14721	0.946131	0.597909
14722	1.35381	1.05133
14723	0.946132	2.1
14724	1.35381	1.04468
14725	0.946133	2.1
14726	0.946132	1.16672
14727	0.946132	1.8242
14728	0.946133	1.21512
14729	1.3365	1.02813

14730	0.946132	1.3093
14731	1.3365	1.00579
14732	0.946129	1.15525
14733	0.94613	1.14341
14734	0.946128	1.24408
14735	0.946129	1.02145
14736	0.939895	1.45397
14737	1.14285	2.08539
14738	0.952366	1.99118
14739	0.946131	1.76075
14740	0.94613	1.87694
14741	0.946131	1.79385
14742	0.94613	1.81397
14743	0.946131	2.1
14744	0.946131	2.1
14745	0.946131	1.20035
14746	0.946129	2.1
14747	0.946133	1.25241
14748	0.946131	2.1
14749	0.946131	2.1
14750	0.946133	2.1
14751	0.946132	2.1
14752	0.946132	2.1
14753	0.946133	2.1
14754	0.946132	2.1
14755	0.946132	2.1
14756	0.946133	2.1
14757	0.946133	2.08459
14758	0.946132	1.30731
14759	0.946132	1.79046
14760	0.946133	1.56233
14761	0.946132	2.1
14762	0.946132	2.03382
14763	0.946132	1.50215
14764	0.946132	1.00386
14765	0.946131	2.1
14766	0.946131	1.05943
14767	0.946131	1.06458
14768	1.38969	2.1
14769	0.946131	1.80787
14770	0.946132	2.1
14771	0.946128	1.99483
14772	0.946133	1.39508
14773	0.946132	1.75767
14774	0.946131	1.36536
14775	0.94613	1.44862
14776	0.946131	1.705
14777	1.28464	1.63714
14778	0.946119	0.828055
14779	1.37492	0.880346
14780	0.946125	0.907196
14781	0.946119	0.788626
14782	0.946119	0.968361
14783	0.946132	0.802692
14784	0.946107	0.868493
14785	0.810942	2.1
14786	0.810943	2.1
14787	0.709587	2.1

14788	2.64488	2.1
14789	1.21651	2.1
14790	0.946151	2.1
14791	0.979948	2.1
14792	1.0813	2.1
14793	0.946133	1.36605
14794	0.946132	1.92527
14795	0.946132	2.1
14796	0.946133	1.03567
14797	0.946132	0.873526
14798	0.946132	0.978746
14799	1.41134	1.27203
14800	0.946133	1.90845
14801	0.946132	1.43402
14802	0.946133	2.1
14803	0.946133	1.13166
14804	0.946132	0.979445
14805	0.946133	1.30678
14806	1.31338	1.52315
14807	2.07612	1.55878
14808	0.946102	0.0985409
14809	0.752448	0.110498
14810	1.20617	0.103092
14811	0.94613	0.101432
14812	0.75242	0.101573
14813	0.946131	0.105258
14814	0.752419	0.0829071
14815	0.946132	1.06907
14816	1.25736	1.08604
14817	1.25736	1.05495
14818	0.946132	1.01131
14819	1.08054	1.00383
14820	1.25736	1.03527
14821	0.946132	1.05658
14822	0.946128	2.1
14823	1.1896	2.1
14824	1.18959	2.1
14825	0.946131	1.04226
14826	1.33271	1.93568
14827	0.946105	2.08592
14828	0.946156	2.02608
14829	0.741946	0.115452
14830	1.33587	0.123845
14831	0.741947	0.11919
14832	1.21954	0.120494
14833	0.94613	0.116872
14834	0.741946	0.125935
14835	1.81347	0.1213
14836	1.81347	0.109713
14837	0.653322	0.0908876
14838	1.36935	0.0887775
14839	0.661882	0.08899
14840	1.04753	0.0910963
14841	0.507464	0.0958109
14842	0.945844	1.049
14843	1.59507	1.89455
14844	1.04043	0.989917
14845	0.946131	1.70766

14846	1.32891	1.35189
14847	0.946131	1.01743
14848	0.946131	0.971734
14849	1.28866	1.18861
14850	1.12682	0.385384
14851	0.851543	0.482914
14852	1.04072	0.473796
14853	0.946132	0.61401
14854	1.13437	0.363369
14855	1.04167	0.495519
14856	0.936652	0.572187
14857	1.258 2.1	
14858	0.946131	2.1
14859	0.946132	2.1
14860	1.36968	2.1
14861	0.946132	2.1
14862	1.36968	2.1
14863	0.946131	2.1
14864	2.30046	0.669997
14865	2.82969	0.720082
14866	0.946132	1.43439
14867	1.35022	2.1
14868	1.12356	1.38576
14869	0.94613	2.1
14870	1.35022	2.1
14871	0.946131	2.05508
14872	0.94613	1.90876
14873	0.946131	2.1
14874	1.22401	2.1
14875	0.952456	2.1
14876	0.94613	2.1
14877	1.22401	2.1
14878	0.946131	2.1
14879	0.946131	2.1
14880	1.33047	2.1
14881	0.946132	2.1
14882	1.33047	2.1
14883	0.946131	2.1
14884	1.33047	2.1
14885	0.946132	2.1
14886	1.2107	2.1
14887	0.61957	1.78458
14888	1.80666	2.1
14889	1.53743	2.1
14890	0.619572	1.52059
14891	1.32547	2.1
14892	1.16166	2.1
14893	1.04074	2.1
14894	0.557621	2.1
14895	0.946128	1.00633
14896	1.36607	2.1
14897	0.94613	1.92738
14898	1.36607	2.1
14899	1.39416	2.1
14900	0.94613	2.1
14901	0.94613	2.1
14902	1.23969	2.1
14903	0.946131	2.1

14904	0.946131	2.1
14905	1.13537	2.1
14906	1.05719	2.1
14907	0.806848	2.1
14908	1.30251	2.1
14909	0.851526	1.64455
14910	1.14176	2.1
14911	0.93666	1.14892
14912	0.946114	2.02508
14913	1.15832	1.44528
14914	0.924624	1.42002
14915	0.967609	1.09077
14916	0.771764	0.529414
14917	1.26652	0.525897
14918	0.946133	0.556131
14919	1.17957	0.343149
14920	0.771735	0.448316
14921	0.946122	0.555981
14922	0.946133	0.408642
14923	0.924325	1.15801
14924	0.656739	1.21161
14925	0.637876	1.20444
14926	0.530092	1.17982
14927	0.914415	1.13225
14928	0.821311	1.41575
14929	0.630589	1.31753
14930	0.738823	1.29169
14931	0.839109	1.22998
14932	1.55029	0.0805671
14933	1.13537	0.870925
14934	0.946131	0.994819
14935	0.946128	0.914977
14936	1.30162	2.1
14937	0.952476	1.04584
14938	1.29289	1.17926
14939	0.939783	1.24016
14940	0.94612	2.1
14941	0.946131	2.1
14942	1.07998	2.1
14943	0.836985	2.1
14944	1.1604	2.1
14945	0.94613	2.1
14946	0.837016	2.1
14947	0.712461	0.833577
14948	1.36399	0.84836
14949	1.8724	0.792247
14950	1.8724	0.760164
14951	0.623426	0.996079
14952	1.36399	0.825031
14953	1.22089	0.808395
14954	0.946132	0.806158
14955	0.946131	2.1
14956	1.32241	2.1
14957	1.32241	2.1
14958	0.946133	2.1
14959	1.40271	2.1
14960	0.946131	2.1
14961	0.946131	2.1

14962	1.1989	1.31398
14963	0.954106	1.49322
14964	0.820068	1.19027
14965	0.563139	1.40111
14966	1.06448	1.45872
14967	0.892472	1.92373
14968	0.862598	1.30071
14969	0.745851	1.94355
14970	1.13336	1.43027
14971	0.946129	2.1
14972	1.28254	2.1
14973	1.28254	2.1
14974	0.946131	2.1
14975	1.41302	2.1
14976	0.946131	2.1
14977	0.946131	2.1
14978	0.851543	1.48514
14979	1.21961	2.1
14980	0.851484	1.41581
14981	0.851543	2.1
14982	1.15705	2.00586
14983	0.946133	1.85413
14984	0.909417	1.80946
14985	1.13808	0.756278
14986	0.946133	0.708598
14987	0.946131	0.761105
14988	1.30194	0.71149
14989	0.946131	0.718859
14990	1.30194	0.70331
14991	0.946131	0.711524
14992	0.930941	1.19861
14993	1.20185	1.00941
14994	1.02404	1.05196
14995	0.946124	1.26109
14996	1.3563	1.11437
14997	0.946127	1.34269
14998	0.946128	1.16633
14999	0.946116	2.1
15000	1.3144	2.09617
15001	1.13088	2.1
15002	0.946118	1.01196
15003	1.31438	1.85281
15004	0.946105	1.86026
15005	0.946131	1.80134
15006	1.76972	0.666508
15007	1.42091	0.423694
15008	0.946128	0.624993
15009	0.946101	0.691726
15010	0.863552	0.700135
15011	0.94613	0.603284
15012	1.21232	0.692702
15013	0.94613	0.700325
15014	1.2137	0.785807
15015	1.23224	0.918235
15016	1.86512	0.824049
15017	1.86512	0.766017
15018	0.580825	1.121
15019	1.2137	0.930021

15020	0.946133	0.925981
15021	0.580822	0.908496
15022	0.946128	2.1
15023	1.40586	2.1
15024	0.94613	2.1
15025	1.3719	2.1
15026	1.3719	2.1
15027	1.40586	2.1
15028	0.94613	2.1
15029	0.946132	2.03707
15030	1.35025	1.87078
15031	1.1794	1.82412
15032	0.946132	0.924443
15033	1.35025	1.08151
15034	0.946131	1.09529
15035	0.946132	1.06683
15036	0.946133	2.1
15037	1.60149	2.1
15038	1.67058	2.1
15039	0.851542	2.1
15040	1.20296	2.1
15041	1.17501	2.1
15042	0.946132	2.1
15043	1.04993	2.1
15044	0.851541	1.20973
15045	1.48219	2.1
15046	0.874565	1.19547
15047	0.946129	2.1
15048	1.22049	2.1
15049	0.946131	2.1
15050	0.946131	1.88062
15051	0.94613	2.1
15052	0.848719	2.1
15053	1.17195	2.08566
15054	0.946129	1.2123
15055	1.35426	1.25583
15056	0.94613	1.44528
15057	0.94613	1.60961
15058	0.946133	2.1
15059	1.14342	2.1
15060	1.14342	2.1
15061	0.946133	2.1
15062	1.39009	2.1
15063	0.946132	2.1
15064	0.946132	2.1
15065	1.38819	2.1
15066	0.946133	2.1
15067	1.38819	2.1
15068	0.946131	2.1
15069	1.4116	2.1
15070	0.946131	2.1
15071	1.38819	2.1
15072	0.946127	2.1
15073	1.32244	2.1
15074	1.2176	2.1
15075	0.946131	2.1
15076	1.32244	2.1
15077	0.94613	2.1

15078	0.946129	2.1
15079	1.32415	2.1
15080	1.11239	2.1
15081	0.984099	2.1
15082	1.65532	2.1
15083	1.53082	2.1
15084	1.32068	2.1
15085	1.32068	2.1
15086	1.08172	2.1
15087	3.76293	0.193279
15088	1.36335	0.112204
15089	0.953925	2.1
15090	0.989497	2.1
15091	0.98949	2.1
15092	1.06341	2.1
15093	1.27875	2.1
15094	1.04598	2.1
15095	0.603154	2.1
15096	0.582457	2.1
15097	0.582457	2.1
15098	0.568994	2.1
15099	1.5791	2.1
15100	1.09506	2.1
15101	1.45327	2.1
15102	1.88406	2.1
15103	1.88404	2.1
15104	1.27389	1.93229
15105	1.14206	2.1
15106	1.00232	2.1
15107	1.00231	2.1
15108	1.68436	2.1
15109	1.4677	0.130672
15110	1.28828	0.208811
15111	1.28828	0.109587
15112	1.46932	0.129546
15113	1.28228	2.1
15114	1.64268	1.1222
15115	0.959473	2.1
15116	1.10101	2.1
15117	1.07976	2.1
15118	1.2873	1.31297
15119	1.70303	0.387078
15120	1.40066	0.341365
15121	1.5563	0.256743
15122	1.13727	0.180814
15123	1.03529	2.1
15124	1.11285	2.1
15125	1.03529	2.1
15126	1.28667	2.1
15127	1.32119	2.1
15128	1.44777	2.1
15129	1.44778	2.1
15130	1.61121	2.1
15131	1.09372	0.17852
15132	1.17664	0.20893
15133	1.17664	0.161313
15134	0.872931	0.134746
15135	1.02415	2.1

15136	1.28228	2.1
15137	2.14692	2.1
15138	3.90469	2.1
15139	1.00509	2.1
15140	1.28228	2.1
15141	3.32936	0.122667
15142	1.1333	0.112859
15143	1.11048	2.1
15144	0.979854	2.1
15145	1.65815	2.1
15146	1.00185	2.1
15147	1.84887	2.1
15148	1.59597	2.1
15149	1.78468	2.1
15150	1.87508	2.1
15151	1.82716	2.1
15152	1.33429	2.1
15153	1.34626	2.1
15154	0.797142	2.1
15155	1.00007	1.16177
15156	1.02051	2.1
15157	1.02051	1.24088
15158	1.03373	1.94293
15159	1.05786	2.1
15160	0.887631	2.1
15161	0.992773	2.1
15162	1.28462	2.1
15163	2.35274	1.8475
15164	1.82533	1.10855
15165	1.82533	1.7033
15166	1.19496	0.947852
15167	1.28597	0.05
15168	1.09725	2.1
15169	1.60948	2.1
15170	1.20421	2.1
15171	1.16566	2.1
15172	1.88522	2.1
15173	1.81948	0.759078
15174	1.14042	0.726288
15175	1.21558	0.897882
15176	1.56876	0.754832
15177	1.6697	2.1
15178	1.81655	2.1
15179	1.81655	2.1
15180	1.5956	2.1
15181	0.906059	2.1
15182	1.63045	2.1
15183	0.9533	1.11799
15184	1.1475	1.03677
15185	1.72231	1.96918
15186	1.94936	1.28667
15187	1.06826	2.1
15188	1.08576	2.1
15189	1.06826	2.1
15190	0.973559	2.1
15191	1.62737	0.102572
15192	0.965676	0.099339
15193	1.45055	0.1208

15194	1.17631	0.118824
15195	1.92535	0.185186
15196	1.9503	0.359099
15197	1.99828	0.173651
15198	1.70302	0.357149
15199	1.65139	0.36318
15200	1.26784	0.408296
15201	1.35923	0.500085
15202	1.70302	0.510566
15203	1.33033	2.1
15204	1.12481	2.1
15205	1.29452	2.1
15206	1.25399	2.1
15207	1.12481	2.1
15208	0.918358	2.1
15209	1.39332	2.1
15210	1.32368	2.1
15211	1.11712	0.937225
15212	1.07999	0.939531
15213	1.07999	0.854837
15214	1.05619	0.904285
15215	1.1766	2.1
15216	1.35735	2.1
15217	1.42456	0.177203
15218	1.089	0.261696
15219	1.11081	0.0808778
15220	2.88521	0.0865851
15221	1.09754	0.074679
15222	1.85479	0.0755926
15223	1.27922	0.950287
15224	1.03556	0.925552
15225	1.07508	1.3154
15226	1.09795	1.8398
15227	0.967672	1.00084
15228	1.07508	1.21324
15229	1.03933	1.43788
15230	1.02975	2.1
15231	1.02975	1.60726
15232	1.01121	2.1
15233	1.24526	0.101145
15234	1.72368	0.088313
15235	1.89607	0.0905912
15236	2.13512	0.0846606
15237	1.34591	0.744587
15238	1.14721	0.820148
15239	1.15539	1.18551
15240	1.90938	1.00334
15241	1.90941	2.1
15242	1.89242	1.35508
15243	1.18907	2.1
15244	1.20408	2.1
15245	1.20409	2.1
15246	1.22655	2.1
15247	0.701447	2.1
15248	1.57567	2.1
15249	1.38806	2.1
15250	2.09407	2.1
15251	1.07612	1.06841

15252	0.990019	1.10994
15253	1.15564	1.42864
15254	1.01243	1.26197
15255	1.28956	0.698439
15256	1.35146	0.60222
15257	1.35145	0.683994
15258	1.22811	0.671531
15259	1.09662	2.1
15260	1.42486	2.1
15261	1.10106	0.0919764
15262	1.90814	0.0724404
15263	1.3946	0.0740421
15264	1.11926	0.0766538
15265	0.725839	2.1
15266	0.925068	2.1
15267	0.925074	2.1
15268	1.43282	2.1
15269	1.91756	1.10433
15270	1.74321	1.11956
15271	1.74321	1.04002
15272	1.63413	1.0575
15273	1.9194	0.663704
15274	1.62246	0.532857
15275	1.90456	0.436053
15276	1.06578	0.487506
15277	1.46019	0.541023
15278	1.17036	0.337854
15279	1.70233	1.39728
15280	1.53515	1.34048
15281	2.03494	2.1
15282	1.78441	2.1
15283	1.75992	2.1
15284	1.94351	2.1
15285	1.51057	0.196992
15286	0.991435	0.198048
15287	1.98068	0.283482
15288	1.64283	0.274128
15289	2.96126	0.228473
15290	2.20692	0.241042
15291	1.95054	0.148672
15292	1.21325	0.14356
15293	0.996828	0.643882
15294	1.01976	0.552957
15295	1.17129	2.1
15296	1.13664	2.1
15297	1.13664	2.1
15298	1.09627	2.1
15299	2.65123	2.1
15300	3.23062	2.1
15301	3.23062	2.1
15302	3.92038	2.1
15303	1.89019	0.665416
15304	1.79109	0.73339
15305	1.79109	0.536739
15306	1.54197	0.595912
15307	3.41824	1.15495
15308	1.93553	0.545006
15309	2.26215	1.10449

15310	2.0103	0.517525
15311	1.51569	1.27078
15312	1.01683	1.21535
15313	1.78595	2.1
15314	1.66537	2.1
15315	3.73426	2.1
15316	3.53905	2.1
15317	1.66537	2.1
15318	1.52903	2.1
15319	3.53905	2.1
15320	3.3837	2.1
15321	1.19901	0.926538
15322	1.35002	1.48082
15323	1.35002	0.786074
15324	1.11683	0.785258
15325	1.75326	1.24429
15326	0.712404	1.25244
15327	2.97768	2.1
15328	2.98949	2.1
15329	1.2832	2.1
15330	1.39518	2.1
15331	2.98949	2.1
15332	2.99339	2.1
15333	1.39517	2.1
15334	1.46124	2.1
15335	1.28534	2.1
15336	1.03391	2.1
15337	1.75366	1.14347
15338	1.26247	1.3308
15339	1.13621	1.86427
15340	2.43994	2.1
15341	1.47904	0.295949
15342	1.80085	0.261976
15343	1.80085	0.282664
15344	1.64656	0.336984
15345	2.87331	2.1
15346	2.82863	2.1
15347	2.82859	2.1
15348	2.84673	2.1
15349	1.0961	0.494556
15350	1.39448	0.701523
15351	1.21366	0.676024
15352	1.20143	0.433439
15353	1.85795	0.336989
15354	1.36768	0.273401
15355	1.36769	0.246154
15356	1.45789	0.2219
15357	1.32605	0.101146
15358	1.27148	0.120047
15359	1.70218	0.276334
15360	1.3063	0.474391
15361	1.45603	0.317438
15362	1.89226	0.484528
15363	1.2742	0.382283
15364	2.10112	0.235105
15365	1.10748	0.629816
15366	1.1893	0.62971
15367	1.04921	2.1

15368	1.22927	2.1
15369	1.22927	2.1
15370	1.46568	2.1
15371	0.811186	2.1
15372	0.792162	2.1
15373	0.792162	2.1
15374	0.758749	2.1
15375	1.03581	2.1
15376	0.737481	2.1
15377	2.24071	2.1
15378	1.34868	2.1
15379	1.47203	0.939579
15380	1.40967	0.984319
15381	1.40967	1.03342
15382	1.27916	1.05594
15383	1.17418	1.08376
15384	1.04921	2.1
15385	1.28291	1.3851
15386	1.3213	0.930054
15387	1.46812	1.1768
15388	2.42522	1.49982
15389	1.17879	0.891319
15390	1.28291	0.910923
15391	1.28291	0.89257
15392	1.13438	0.920354
15393	1.75004	2.1
15394	1.78247	2.1
15395	1.78247	2.1
15396	1.76421	2.1
15397	2.25975	0.0943375
15398	3.66111	0.0771075
15399	3.6611	0.0657313
15400	3.44043	0.0631796
15401	1.13339	2.1
15402	1.13322	2.1
15403	1.13323	2.1
15404	1.20761	2.1
15405	1.34337	0.694473
15406	1.90912	1.59863
15407	1.04399	0.934351
15408	2.00578	1.56292
15409	1.04973	2.1
15410	1.22615	1.9092
15411	1.93384	2.1
15412	2.62816	0.905015
15413	1.13127	2.1
15414	1.47642	1.43792
15415	2.62816	2.1
15416	3.31755	0.897758
15417	1.47642	2.1
15418	1.80454	1.18792
15419	0.964782	1.94442
15420	0.949135	2.1
15421	0.949134	2.02892
15422	0.949134	2.1
15423	1.10476	0.837536
15424	0.982858	0.154993
15425	1.10938	0.748389

15426	0.982858	0.68136
15427	1.01012	0.0666499
15428	0.974081	0.0675213
15429	1.82643	0.0662343
15430	1.29585	0.0690997
15431	1.00763	0.886584
15432	1.3294	0.853768
15433	1.3294	0.766605
15434	1.30001	1.00095
15435	1.59151	2.1
15436	1.99443	2.1
15437	1.07369	2.1
15438	1.30584	2.1
15439	1.05071	2.1
15440	0.948676	2.1
15441	1.13842	2.1
15442	1.42884	2.1
15443	1.59019	0.0988623
15444	1.38212	0.136319
15445	1.34102	0.106917
15446	1.02663	0.121772
15447	0.797011	2.1
15448	0.81301	2.1
15449	0.81301	2.1
15450	0.811187	2.1
15451	0.991869	0.655488
15452	1.23868	0.65913
15453	1.67201	2.1
15454	1.61201	2.1
15455	1.61202	2.1
15456	1.57627	2.1
15457	0.94994	2.1
15458	1.77867	2.1
15459	0.854945	2.1
15460	0.973905	2.1
15461	1.64193	2.1
15462	1.68893	2.1
15463	1.68893	2.1
15464	1.70002	2.1
15465	2.08149	0.742275
15466	1.69456	0.688741
15467	1.67399	0.05
15468	1.83922	0.97264
15469	1.91703	2.1
15470	0.967492	2.1
15471	1.13317	2.1
15472	0.980766	2.1
15473	2.06158	0.314517
15474	0.986498	0.22027
15475	1.76289	0.309908
15476	1.17248	0.252833
15477	1.51794	0.215526
15478	1.30266	0.260951
15479	1.03165	0.602647
15480	0.947692	0.737056
15481	1.20473	0.585965
15482	0.947693	0.455485
15483	1.54357	2.1

15484	0.980841	2.1
15485	1.34124	0.730552
15486	1.39818	0.803337
15487	1.39818	0.690581
15488	1.48035	0.767997
15489	1.03932	2.1
15490	1.2065	2.1
15491	1.20651	2.1
15492	1.12717	2.1
15493	1.09483	2.1
15494	0.994293	2.1
15495	0.994293	2.1
15496	0.97356	2.1
15497	0.995252	1.00151
15498	1.66294	0.987347
15499	1.82775	0.866647
15500	1.42762	0.845312
15501	1.04713	0.701214
15502	1.05475	0.702496
15503	1.29002	0.23723
15504	1.05475	0.261279
15505	1.17585	2.1
15506	1.39223	2.1
15507	1.39223	2.1
15508	1.71255	2.1
15509	1.56166	0.959182
15510	1.37762	0.914971
15511	1.16858	2.1
15512	1.0361	2.1
15513	1.23966	0.0939605
15514	1.10718	0.0979709
15515	1.99957	0.0713385
15516	0.658392	0.0634037
15517	1.36904	0.0829335
15518	1.12524	0.070739
15519	1.04321	1.24099
15520	1.04627	1.2428
15521	1.91351	0.833377
15522	1.63379	0.92539
15523	1.79718	0.98842
15524	0.59634	1.08831
15525	0.958257	2.1
15526	1.06457	2.1
15527	0.958255	2.1
15528	1.03933	2.1
15529	1.14195	0.828821
15530	1.49438	0.809742
15531	1.49438	0.802828
15532	1.31259	2.1
15533	1.23036	2.1
15534	1.2016	2.1
15535	1.2016	2.1
15536	1.17129	2.1
15537	1.05724	0.930729
15538	1.04291	1.09395
15539	1.12341	2.1
15540	2.32525	2.1
15541	3.39038	2.1

15542	2.09374	2.1
15543	1.76185	0.740625
15544	1.7934	0.746124
15545	1.65809	0.735076
15546	1.54852	0.967443
15547	1.38766	2.1
15548	1.28765	2.1
15549	1.26599	2.1
15550	1.21397	2.1
15551	1.28765	2.1
15552	1.20045	2.1
15553	1.21398	2.1
15554	1.15234	2.1
15555	0.953634	0.469891
15556	1.39132	0.658455
15557	1.17285	2.1
15558	1.21408	2.1
15559	1.21408	2.1
15560	0.982848	2.1
15561	2.00101	2.1
15562	2.00973	2.1
15563	2.20314	2.1
15564	1.89318	2.1
15565	1.23267	1.38761
15566	0.947921	2.1
15567	1.18872	2.1
15568	0.740752	2.1
15569	1.92269	0.704851
15570	1.53584	0.7
15571	1.53584	0.700075
15572	1.21067	0.699589
15573	1.40707	0.181311
15574	1.50437	0.167687
15575	1.50435	0.165925
15576	1.45446	0.172756
15577	1.47954	0.861108
15578	2.08687	1.06752
15579	2.08687	1.82808
15580	1.1233	2.09036
15581	1.04025	0.894443
15582	1.36005	1.44303
15583	1.47606	0.704821
15584	1.3407	0.707942
15585	0.758743	2.1
15586	0.716917	2.1
15587	0.716922	2.1
15588	0.672964	2.1
15589	2.40014	2.1
15590	1.96848	1.53339
15591	1.96848	2.1
15592	1.53555	1.10814
15593	1.06191	2.1
15594	1.29579	2.1
15595	1.29579	2.1
15596	1.71289	2.1
15597	1.78584	2.1
15598	2.15211	2.1
15599	2.00427	2.1

15600	3.41615	2.1
15601	1.66408	2.1
15602	1.73954	2.1
15603	2.91663	2.1
15604	3.65818	2.1
15605	1.93286	2.1
15606	1.08831	2.1
15607	1.31242	0.127374
15608	1.11712	0.145027
15609	1.26311	0.136719
15610	1.31242	0.152168
15611	1.10378	2.1
15612	0.968594	2.1
15613	1.48389	0.0987592
15614	1.49613	0.120553
15615	1.04349	0.75263
15616	1.26409	0.736597
15617	1.26409	0.73329
15618	1.05724	0.741164
15619	1.65723	0.523105
15620	1.83116	0.618984
15621	2.06284	0.635956
15622	2.00512	0.702126
15623	1.83116	0.513924
15624	2.03139	0.572874
15625	2.20564	0.670416
15626	1.90048	0.666655
15627	0.963392	2.1
15628	1.01346	0.949325
15629	2.98092	2.1
15630	2.70295	2.1
15631	2.70295	2.1
15632	2.48828	2.1
15633	1.63661	2.1
15634	1.64158	2.1
15635	1.64158	2.1
15636	1.6488	2.1
15637	1.17506	0.663989
15638	1.09667	0.582755
15639	2.00539	0.781179
15640	1.09667	0.729164
15641	1.54946	2.1
15642	1.43577	2.1
15643	1.26103	2.1
15644	1.49808	2.1
15645	1.089	0.20097
15646	1.22586	0.204105
15647	2.35027	0.739033
15648	1.37549	0.654261
15649	3.33706	0.618299
15650	1.69894	0.541194
15651	2.00539	0.724408
15652	1.53798	0.636417
15653	1.28938	2.1
15654	1.20969	2.1
15655	1.20969	2.1
15656	1.15864	2.1
15657	1.7986	2.1

15658	1.75869	2.1
15659	1.58281	2.1
15660	1.7392	2.1
15661	1.30733	0.884731
15662	1.5419	1.53361
15663	1.50883	1.06052
15664	1.85439	1.03974
15665	1.73531	1.01924
15666	1.70225	0.941675
15667	1.22176	0.7
15668	1.59492	0.700265
15669	1.59492	0.7
15670	2.15542	0.702913
15671	3.24379	2.1
15672	2.64775	2.1
15673	3.16707	2.1
15674	1.93032	2.1
15675	1.50738	2.1
15676	1.24128	2.1
15677	1.24128	2.1
15678	0.994293	2.1
15679	2.90808	0.0549555
15680	2.45797	0.0540629
15681	1.85501	0.0578614
15682	2.90809	0.0560503
15683	0.892835	1.14171
15684	1.55871	1.90585
15685	1.04615	1.05928
15686	1.66643	0.813472
15687	1.70044	1.92673
15688	1.80224	2.1
15689	1.70186	0.864651
15690	3.30123	0.851092
15691	1.74789	2.1
15692	1.77446	2.1
15693	1.77901	2.1
15694	1.57995	2.1
15695	1.81518	2.1
15696	2.17716	2.1
15697	2.17716	2.1
15698	2.65123	2.1
15699	2.01899	1.56512
15700	1.77479	1.43393
15701	1.19122	1.06825
15702	1.46724	0.94712
15703	1.70944	0.092575
15704	1.08929	0.104789
15705	1.68454	0.0936133
15706	1.08929	0.0906506
15707	1.51067	1.06993
15708	1.06729	0.969066
15709	1.37762	0.95161
15710	1.13992	0.917506
15711	1.26397	2.1
15712	1.04083	2.1
15713	1.00278	2.1
15714	1.04084	2.1
15715	1.1016	0.212553

15716	1.23477	0.259959
15717	1.23477	0.121353
15718	1.64985	0.139061
15719	1.11117	2.1
15720	1.13006	2.1
15721	1.13006	2.1
15722	1.14721	2.1
15723	1.21946	2.1
15724	1.0186	2.1
15725	1.13178	2.1
15726	1.00133	2.1
15727	1.67046	0.7
15728	0.986036	0.714468
15729	1.67046	0.723675
15730	1.34857	1.25254
15731	2.46016	2.1
15732	2.07161	2.1
15733	2.10732	2.1
15734	2.08371	2.1
15735	1.35602	2.1
15736	0.829065	2.1
15737	1.81667	2.1
15738	0.99406	2.1
15739	1.81667	2.1
15740	1.68073	2.1
15741	2.05736	2.1
15742	2.97311	2.03386
15743	2.14011	2.1
15744	1.23411	2.1
15745	1.13632	0.446387
15746	1.40862	0.400022
15747	1.28055	0.392306
15748	1.14305	0.363723
15749	1.12035	0.0864686
15750	1.15483	0.0838024
15751	1.94099	0.0849902
15752	1.82126	0.0834774
15753	1.15481	0.0807905
15754	0.998957	0.0796355
15755	2.10078	0.0828179
15756	1.81617	0.0849284
15757	1.27581	2.1
15758	0.968536	2.1
15759	1.33916	2.1
15760	1.27581	2.1
15761	1.32784	2.1
15762	1.4259	2.1
15763	1.70377	2.1
15764	1.54231	2.1
15765	3.77569	2.1
15766	3.42809	2.1
15767	1.54231	2.1
15768	1.51298	2.1
15769	3.55866	2.1
15770	2.86136	2.1
15771	1.83301	1.11066
15772	1.26371	0.987429
15773	1.98301	1.10469

15774	2.0609	1.013
15775	2.26047	0.85176
15776	2.00537	0.80264
15777	1.7913	0.806462
15778	1.48864	0.759112
15779	1.09504	2.1
15780	1.39827	2.1
15781	2.28084	2.1
15782	1.05983	2.1
15783	1.33593	0.102594
15784	1.31508	0.0835789
15785	1.31508	0.0950053
15786	1.09599	0.086797
15787	1.30647	2.1
15788	1.28846	2.1
15789	1.28846	2.1
15790	1.26923	2.1
15791	1.2955	2.1
15792	1.09044	2.1
15793	2.12209	2.1
15794	1.26438	2.1
15795	0.911819	2.1
15796	1.38752	2.1
15797	2.57704	2.1
15798	2.71736	2.1
15799	1.42284	2.1
15800	1.41562	2.1
15801	2.69131	2.1
15802	2.36178	2.1
15803	1.41562	2.1
15804	1.39564	2.1
15805	1.32368	2.1
15806	0.908053	2.1
15807	0.908053	2.1
15808	0.629213	2.1
15809	1.8758	1.50294
15810	1.27362	1.16619
15811	1.3756	0.873113
15812	2.28749	1.0933
15813	1.84577	1.02964
15814	2.17765	1.00961
15815	1.38969	0.906307
15816	1.84578	0.987183
15817	1.32801	0.807762
15818	1.76377	0.818186
15819	1.22845	1.34292
15820	1.57766	0.996239
15821	1.57766	2.1
15822	1.39067	1.71894
15823	1.15743	0.262815
15824	1.37721	0.204487
15825	1.69055	0.551194
15826	1.14858	0.41182
15827	1.38333	1.15107
15828	1.10575	0.832137
15829	1.22418	2.1
15830	1.10574	1.19655
15831	1.91509	0.540253

15832	1.02001	0.440386
15833	1.03074	0.86853
15834	1.19122	0.665554
15835	1.67887	2.1
15836	1.67597	2.1
15837	1.84984	2.1
15838	1.77775	2.1
15839	1.73571	2.1
15840	1.27921	2.03644
15841	2.11851	1.36815
15842	1.27921	2.1
15843	1.42486	2.1
15844	1.12503	2.1
15845	1.12503	2.1
15846	1.30116	2.1
15847	0.568995	2.1
15848	0.557348	2.1
15849	0.557348	2.1
15850	0.53907	2.1
15851	0.633575	2.1
15852	1.33756	1.02382
15853	1.51074	2.1
15854	1.93384	0.924819
15855	0.655202	2.1
15856	0.731934	2.1
15857	0.731934	2.1
15858	1.09061	2.1
15859	0.966979	2.1
15860	1.31502	2.1
15861	1.31502	2.1
15862	1.72116	2.1
15863	1.70002	2.1
15864	1.67969	2.1
15865	1.67969	2.1
15866	1.62408	2.1
15867	1.01531	2.1
15868	1.66018	2.1
15869	1.01531	2.1
15870	0.886968	2.1
15871	1.22585	0.204759
15872	1.20674	0.207148
15873	1.20672	0.182491
15874	1.02962	0.202284
15875	0.956467	1.57444
15876	2.00551	1.03453
15877	0.553801	2.1
15878	0.617006	2.1
15879	1.26923	2.1
15880	1.23614	2.1
15881	1.23614	2.1
15882	1.16817	2.1
15883	2.14482	2.1
15884	1.72637	2.06761
15885	1.09368	2.1
15886	1.26195	2.1
15887	0.760546	2.1
15888	1.84094	2.1
15889	2.62868	2.1

15890	2.79784	2.1
15891	2.79784	2.1
15892	3.00129	2.1
15893	1.27593	0.961509
15894	1.38877	1.49885
15895	1.06486	0.646667
15896	1.2474	0.407403
15897	1.76234	0.0807714
15898	1.8685	0.106658
15899	1.69863	0.0979598
15900	1.29666	0.0963467
15901	1.91646	2.1
15902	1.8582	2.1
15903	1.8582	2.1
15904	1.7986	2.1
15905	3.44043	0.0623577
15906	1.94764	0.0586066
15907	1.94764	0.0743793
15908	1.36307	0.0687515
15909	1.32075	0.123027
15910	1.12971	0.10633
15911	2.06319	0.147363
15912	1.94982	0.112089
15913	1.77255	0.18138
15914	0.979297	0.111384
15915	1.59997	2.1
15916	1.93506	2.1
15917	1.94409	2.1
15918	1.9597	2.1
15919	0.57899	2.1
15920	0.953036	2.1
15921	0.953049	2.1
15922	1.53503	2.1
15923	1.39615	0.262614
15924	1.06474	0.347838
15925	0.947564	0.33223
15926	1.45506	0.305616
15927	1.17799	0.7
15928	1.21067	0.7
15929	1.35945	0.564005
15930	2.05648	0.742504
15931	1.36208	0.64425
15932	2.15229	0.559644
15933	2.89288	0.504353
15934	1.95719	0.550944
15935	2.25169	0.406854
15936	1.52756	0.485716
15937	1.19322	0.895894
15938	1.23146	0.765121
15939	2.45024	0.9994
15940	0.794122	0.810688
15941	1.6343	0.75409
15942	1.34161	0.608213
15943	1.05353	0.892251
15944	1.50795	0.609871
15945	1.05353	0.503249
15946	1.20669	0.622798
15947	1.59289	2.1

15948	0.989543	2.1
15949	1.40588	2.1
15950	0.991427	2.1
15951	0.921822	1.34948
15952	1.46903	0.866251
15953	1.46903	2.1
15954	1.8214	0.802753
15955	1.39702	2.1
15956	1.25103	2.1
15957	1.25103	2.1
15958	1.10378	2.1
15959	1.71813	2.1
15960	1.39595	2.1
15961	1.386 2.1	
15962	1.13008	2.1
15963	1.31711	0.104695
15964	1.70191	0.0985262
15965	0.893249	0.114972
15966	1.42669	0.110944
15967	1.23943	1.16814
15968	1.1483	0.940381
15969	1.04598	2.1
15970	1.1483	1.09776
15971	1.00381	0.750294
15972	1.11433	0.720308
15973	0.973501	1.01779
15974	1.0756	1.00226
15975	1.20669	0.615543
15976	1.18508	0.613299
15977	1.18508	0.651157
15978	0.983027	0.649428
15979	1.43687	2.1
15980	1.71059	2.1
15981	1.71059	2.1
15982	1.66762	2.1
15983	2.8415	2.1
15984	2.40555	2.1
15985	1.91546	2.1
15986	1.61385	2.1
15987	2.01569	2.1
15988	1.33183	2.1
15989	1.23218	2.1
15990	1.23999	2.1
15991	1.01335	0.847726
15992	1.12088	0.919065
15993	1.25346	1.36498
15994	1.15644	2.0721
15995	1.3764	1.01752
15996	1.27558	0.926113
15997	1.2064	2.1
15998	1.15067	2.1
15999	1.01595	2.1
16000	1.15067	2.1
16001	1.41902	0.995527
16002	1.30272	2.1
16003	1.30272	0.87468
16004	1.19548	2.1
16005	2.99339	2.1

16006	2.98041	2.1
16007	1.46124	2.1
16008	1.42105	2.1
16009	2.98041	2.1
16010	2.86339	2.1
16011	1.42105	2.1
16012	1.42284	2.1
16013	2.31301	1.04892
16014	1.80273	1.03255
16015	2.17765	1.08956
16016	2.31301	0.976496
16017	1.76378	0.836458
16018	2.10275	0.806969
16019	2.10275	0.842875
16020	2.26047	0.82519
16021	0.97541	2.1
16022	1.40863	2.1
16023	1.40863	2.1
16024	1.30751	2.1
16025	1.20143	0.631127
16026	1.19756	0.564508
16027	1.43707	0.642108
16028	1.28954	0.510487
16029	3.34254	0.246625
16030	1.92916	0.107397
16031	1.80089	0.21103
16032	1.1323	0.125042
16033	1.47321	0.273179
16034	1.23756	0.223262
16035	1.87291	2.1
16036	1.53642	2.1
16037	1.53642	2.1
16038	1.5791	2.1
16039	1.0446	2.1
16040	0.992674	2.1
16041	1.12324	0.0606907
16042	1.29163	0.0544194
16043	1.29163	0.0542979
16044	1.15371	0.0544764
16045	0.996831	0.371196
16046	1.00192	0.37796
16047	1.0019	0.326288
16048	0.958937	0.357168
16049	1.07283	2.1
16050	1.25294	1.07493
16051	1.25294	1.11183
16052	1.04788	1.10706
16053	1.14496	2.1
16054	1.07652	2.1
16055	1.07652	2.1
16056	0.991406	2.1
16057	1.60976	2.1
16058	1.37	2.1
16059	1.37	2.1
16060	1.14195	2.1
16061	1.26593	1.39549
16062	1.75366	2.1
16063	1.57621	0.705301

16064	1.24538	0.7086
16065	0.969118	2.1
16066	1.04147	2.1
16067	0.969118	2.1
16068	1.22615	2.1
16069	0.98084	2.1
16070	1.01843	2.1
16071	1.09435	2.1
16072	1.50876	2.1
16073	1.5976	2.1
16074	1.84322	2.1
16075	1.84324	2.1
16076	1.70377	2.1
16077	1.20709	2.1
16078	2.54729	2.1
16079	0.974716	0.376094
16080	1.54468	0.543478
16081	1.39021	0.327948
16082	1.70303	0.498
16083	1.22852	1.48449
16084	1.578	0.958482
16085	1.578	1.54256
16086	1.93852	0.85329
16087	2.2506	2.1
16088	1.39302	1.99648
16089	1.5478	2.1
16090	1.2269	2.1
16091	1.70048	2.1
16092	1.45477	1.24198
16093	1.30929	2.1
16094	1.15069	1.19346
16095	1.00255	1.63605
16096	1.05481	0.829406
16097	1.05481	2.01965
16098	1.1032	0.819973
16099	0.993888	2.1
16100	1.19294	2.1
16101	1.01121	0.556504
16102	0.991866	0.631524
16103	2.20052	0.993481
16104	1.7409	2.1
16105	1.75142	2.1
16106	1.46811	2.1
16107	1.7409	1.14426
16108	1.23267	2.1
16109	1.46811	2.1
16110	1.18872	2.1
16111	1.12665	2.1
16112	1.50045	2.1
16113	1.50045	2.1
16114	1.37704	2.1
16115	1.35308	0.847247
16116	1.29983	0.736726
16117	1.29983	0.821385
16118	0.970212	0.745266
16119	1.82272	2.1
16120	1.62784	2.1
16121	1.62784	2.1

16122	1.87691	2.1
16123	3.65294	2.1
16124	2.33374	2.1
16125	2.12888	2.1
16126	1.62229	2.1
16127	1.41496	2.1
16128	1.32707	0.945237
16129	2.18871	0.893212
16130	1.4235	0.484392
16131	1.46015	0.377799
16132	1.25635	0.370927
16133	1.45501	0.755435
16134	1.3543	0.672994
16135	1.38139	2.1
16136	1.98159	2.1
16137	2.15868	2.1
16138	4.04308	2.1
16139	1.5362	2.1
16140	1.56328	2.1
16141	1.56327	2.1
16142	1.58163	2.1
16143	1.88987	0.333473
16144	1.08761	0.22679
16145	0.862154	0.268956
16146	1.77688	0.258009
16147	1.14238	1.93413
16148	1.11691	2.1
16149	1.01537	1.73416
16150	1.02923	2.1
16151	0.977425	0.245939
16152	1.15211	0.131647
16153	2.0815	2.1
16154	1.18621	2.1
16155	1.01485	2.1
16156	1.17054	2.1
16157	1.17054	2.1
16158	1.12665	2.1
16159	1.87213	0.103255
16160	1.82244	0.093677
16161	1.45039	0.118435
16162	1.7226	0.118763
16163	1.841	0.0999791
16164	1.79011	0.0956012
16165	1.8792	0.121683
16166	1.45053	0.120677
16167	1.08947	2.1
16168	1.5008	2.1
16169	1.5008	2.1
16170	1.83186	2.1
16171	1.04159	0.0936415
16172	1.2489	0.0882773
16173	1.2489	0.100742
16174	1.33592	0.0882609
16175	1.51973	0.655762
16176	0.980547	0.69101
16177	1.51974	0.697554
16178	1.15991	0.724686
16179	1.34965	2.1

16180	1.2724	2.1
16181	1.2724	2.1
16182	1.38438	2.1
16183	1.02923	1.82994
16184	1.06521	2.1
16185	1.06521	1.97582
16186	1.0752	2.1
16187	2.03139	0.510473
16188	1.92951	0.487746
16189	1.74852	0.666449
16190	1.62772	0.555669
16191	1.73655	0.451902
16192	1.76809	0.43433
16193	1.62772	0.528694
16194	1.1432	0.452442
16195	1.09235	2.1
16196	1.48081	2.1
16197	1.46712	2.1
16198	1.89321	2.1
16199	1.94548	2.1
16200	1.5636	2.1
16201	1.5636	2.1
16202	1.21124	2.1
16203	1.52314	2.1
16204	2.0219	2.1
16205	1.51113	2.1
16206	2.0219	2.1
16207	0.987247	0.761592
16208	1.18347	0.450205
16209	1.89226	0.788577
16210	1.61441	0.605752
16211	1.42884	2.1
16212	1.10961	2.1
16213	1.56991	2.1
16214	1.05337	2.1
16215	1.05337	2.1
16216	1.91547	2.1
16217	1.05931	2.1
16218	1.1729	2.1
16219	1.34526	2.1
16220	1.0593	2.1
16221	1.0856	2.1
16222	1.90854	2.1
16223	1.91674	2.1
16224	1.11345	2.1
16225	1.75067	2.1
16226	1.50022	2.1
16227	1.50022	2.1
16228	1.53619	2.1
16229	1.4748	2.1
16230	1.74431	1.98244
16231	2.02581	1.91508
16232	1.25288	1.87048
16233	1.01814	0.920255
16234	1.2881	0.789107
16235	1.2881	0.816285
16236	1.54433	0.765099
16237	1.19999	0.801368

16238	1.44056	0.73083
16239	1.02011	0.469308
16240	1.17129	0.723683
16241	1.13775	2.1
16242	3.34815	2.1
16243	1.60428	1.88699
16244	1.85928	2.03723
16245	1.75592	2.1
16246	1.25989	2.1
16247	1.75592	2.1
16248	1.71549	2.1
16249	1.37634	2.1
16250	1.64693	2.1
16251	1.64693	2.1
16252	1.64193	2.1
16253	1.18351	0.14509
16254	2.19398	0.141528
16255	2.16272	1.29151
16256	1.19837	1.26578
16257	1.04973	2.1
16258	1.20149	2.1
16259	1.60574	2.1
16260	1.11164	0.957455
16261	1.87823	0.924796
16262	1.11164	1.02591
16263	0.772882	2.1
16264	1.4714	2.1
16265	2.14242	0.963691
16266	1.12336	2.1
16267	2.8386	0.900114
16268	2.13346	2.1
16269	1.06788	0.336114
16270	0.947662	0.330261
16271	1.05297	0.228407
16272	1.42907	0.275823
16273	2.17465	2.1
16274	2.18394	2.1
16275	1.14808	2.1
16276	1.21292	2.1
16277	1.21292	2.1
16278	1.27229	2.1
16279	1.20859	0.880238
16280	1.21073	0.890198
16281	1.21073	0.823817
16282	1.22857	0.877976
16283	1.79158	2.1
16284	1.73899	2.1
16285	1.34012	2.1
16286	1.39991	2.1
16287	1.73898	2.1
16288	1.61245	2.1
16289	1.40136	2.1
16290	1.57234	2.1
16291	1.82162	2.1
16292	3.09875	2.1
16293	1.10264	2.1
16294	1.02339	2.1
16295	1.02339	2.1

16296	1.23042	2.1
16297	2.82266	0.833585
16298	1.05698	0.75959
16299	1.96384	0.859039
16300	1.10244	0.831149
16301	0.929061	2.1
16302	1.47007	2.1
16303	0.985303	2.1
16304	1.24554	2.1
16305	1.05993	2.1
16306	2.02074	2.1
16307	2.10106	0.907861
16308	1.18171	0.885828
16309	1.68111	0.926396
16310	1.84226	0.851039
16311	1.82131	0.574344
16312	1.32371	0.429114
16313	1.41902	0.294675
16314	1.00205	0.259685
16315	1.65532	2.1
16316	1.18977	2.1
16317	1.15471	2.1
16318	0.993889	2.1
16319	2.08521	2.1
16320	2.09885	2.1
16321	2.09497	2.1
16322	2.10604	2.1
16323	0.881028	1.1357
16324	1.52828	0.920395
16325	1.40574	0.0873064
16326	1.09599	0.0873775
16327	1.45506	2.1
16328	1.50524	2.1
16329	1.50524	2.1
16330	1.54711	2.1
16331	1.41469	2.1
16332	2.40442	2.1
16333	1.40794	2.1
16334	1.89226	2.1
16335	1.31346	2.1
16336	1.51067	2.1
16337	1.31363	2.1
16338	1.11833	2.1
16339	2.3373	2.1
16340	2.19994	2.1
16341	2.19994	2.1
16342	1.09616	2.1
16343	1.63719	2.1
16344	1.52204	2.1
16345	1.52205	2.1
16346	1.40118	2.1
16347	2.5946	2.1
16348	4.07065	2.1
16349	1.33916	2.1
16350	2.05217	2.1
16351	1.67023	2.1
16352	2.05217	2.1
16353	1.59458	0.807128

16354	1.02247	0.778845
16355	1.68108	0.749265
16356	1.03079	0.735017
16357	1.1643	0.664088
16358	1.85795	0.674558
16359	1.70302	0.555781
16360	1.42076	0.462908
16361	1.91351	0.501955
16362	1.01148	0.455245
16363	2.52402	0.102146
16364	0.752625	0.0847152
16365	0.886968	2.1
16366	1.28845	2.1
16367	1.28845	2.1
16368	1.27766	2.1
16369	1.82268	2.1
16370	1.87118	2.1
16371	1.87119	2.1
16372	1.91704	2.1
16373	3.13938	2.1
16374	2.97839	2.1
16375	2.97839	2.1
16376	2.87331	2.1
16377	1.73443	0.954787
16378	1.59457	0.868756
16379	2.01827	2.1
16380	2.04586	2.1
16381	2.04586	2.1
16382	2.00865	2.1
16383	1.19901	1.55648
16384	1.16661	2.1
16385	1.13215	0.731364
16386	1.96256	0.623679
16387	2.34043	0.0862056
16388	2.04154	0.0836307
16389	1.58991	0.0786198
16390	1.45893	0.0813194
16391	2.56818	0.0821387
16392	1.70532	0.0835836
16393	1.17798	0.7
16394	1.25796	0.702116
16395	1.25797	0.703532
16396	1.10719	0.759691
16397	1.51067	2.1
16398	1.60707	2.1
16399	2.06946	1.58375
16400	3.19218	2.1
16401	2.19965	2.1
16402	1.30521	2.1
16403	2.23684	1.24449
16404	1.43363	1.54885
16405	1.51007	2.1
16406	2.46356	2.1
16407	1.90428	2.1
16408	1.13618	2.1
16409	2.13276	0.407057
16410	0.998345	0.341589
16411	1.2861	0.439721

16412	1.20457	0.408386
16413	1.51789	2.1
16414	1.49872	2.1
16415	1.49872	2.1
16416	1.47712	2.1
16417	1.87129	0.700002
16418	0.949364	0.701445
16419	1.83142	0.700971
16420	1.55063	0.703179
16421	1.03298	0.700565
16422	0.980664	0.767877
16423	1.51696	0.7
16424	1.34927	0.707923
16425	1.70302	0.840317
16426	1.65952	0.729626
16427	1.76658	0.635502
16428	0.986848	0.511663
16429	2.13757	1.31323
16430	1.98142	0.893505
16431	1.98142	1.14979
16432	1.65411	0.905971
16433	1.1323	0.345349
16434	1.46015	0.313034
16435	2.15376	2.00964
16436	1.64622	2.0071
16437	0.991867	0.7
16438	1.58074	0.7
16439	1.69368	2.1
16440	1.76403	2.1
16441	1.76403	2.1
16442	1.83318	2.1
16443	1.28418	0.817109
16444	1.16851	0.574051
16445	1.1685	0.606301
16446	1.1726	0.483953
16447	1.06455	2.1
16448	1.18773	2.1
16449	0.98511	2.1
16450	1.18774	2.1
16451	1.76476	2.1
16452	1.79825	2.1
16453	1.79825	2.1
16454	0.961895	2.1
16455	1.59589	0.103205
16456	2.25975	0.105182
16457	2.76339	2.1
16458	3.0551	2.1
16459	1.68761	2.1
16460	1.59561	2.1
16461	2.17257	2.1
16462	1.05845	2.1
16463	1.03512	2.1
16464	2.19011	2.1
16465	1.44859	2.02296
16466	1.35897	0.801238
16467	1.35897	1.86379
16468	1.17822	0.975711
16469	1.13641	2.1

16470	1.18301	2.1
16471	1.18301	2.1
16472	1.25173	2.1
16473	2.41297	2.1
16474	1.36463	2.1
16475	1.54711	2.1
16476	1.5735	2.1
16477	1.5735	2.1
16478	1.58713	2.1
16479	1.69044	0.0761914
16480	1.57979	0.0799303
16481	1.57979	0.0778721
16482	1.14387	0.0837623
16483	1.04199	2.1
16484	1.96491	2.1
16485	1.04199	2.1
16486	1.46892	2.1
16487	0.983724	2.1
16488	1.70579	2.1
16489	1.88522	2.1
16490	1.23914	2.1
16491	1.79424	2.1
16492	1.52851	2.1
16493	1.04921	2.1
16494	1.11285	2.1
16495	1.25334	0.90138
16496	1.07496	0.830888
16497	1.07497	1.99298
16498	1.34852	0.870406
16499	1.27391	2.1
16500	2.20307	2.1
16501	1.81311	2.1
16502	2.10305	2.1
16503	1.21059	0.7
16504	1.44253	0.700327
16505	1.9194	0.68475
16506	1.21159	0.7
16507	0.947979	2.1
16508	1.01225	0.862639
16509	0.947979	2.1
16510	1.01225	1.74363
16511	1.00255	2.1
16512	1.01225	2.1
16513	1.01225	2.1
16514	1.06561	1.78293
16515	3.50562	2.1
16516	3.61336	2.1
16517	3.61336	2.1
16518	3.73635	2.1
16519	1.61245	2.1
16520	1.52301	2.1
16521	1.57612	2.1
16522	1.83035	2.1
16523	1.52301	2.1
16524	1.24789	2.1
16525	1.85156	2.1
16526	1.89072	2.1
16527	0.995735	2.1

16528	1.57738	2.1
16529	1.87141	0.232559
16530	1.73388	0.286342
16531	1.61344	0.299213
16532	0.922676	0.369943
16533	1.56614	2.1
16534	0.956294	2.1
16535	1.82589	2.1
16536	1.63719	2.1
16537	1.12399	0.0883664
16538	1.50103	0.107707
16539	1.56976	0.087808
16540	1.03567	0.0933888
16541	1.21776	0.0917826
16542	1.89708	0.0880467
16543	1.16323	0.0860574
16544	1.63533	0.0792048
16545	2.29397	0.0897637
16546	3.01596	0.0877244
16547	1.80145	0.0810702
16548	1.7666	0.0789043
16549	1.91621	0.758416
16550	1.10719	0.704556
16551	1.57828	0.966649
16552	1.17105	1.03844
16553	1.15806	2.1
16554	1.31565	2.1
16555	1.31565	2.1
16556	1.46643	2.1
16557	1.47507	2.1
16558	1.87858	2.1
16559	0.958661	2.1
16560	1.87858	2.1
16561	1.44293	0.232826
16562	1.43213	0.370137
16563	1.43213	0.233636
16564	1.10476	0.330439
16565	1.03235	1.83511
16566	1.31	0.790235
16567	1.31	1.97518
16568	1.50108	0.890869
16569	1.32215	2.1
16570	1.10611	2.1
16571	1.10611	2.1
16572	0.904978	2.1
16573	1.06486	0.636346
16574	1.18347	0.467317
16575	1.70535	2.1
16576	1.94779	2.1
16577	1.77071	2.1
16578	1.82268	2.1
16579	0.61701	2.1
16580	0.672495	2.1
16581	0.672494	2.1
16582	0.713174	2.1
16583	1.40395	2.1
16584	1.34245	2.1
16585	1.34243	2.1

16586	1.30646	2.1
16587	1.06204	0.615641
16588	0.632691	0.737096
16589	1.75766	0.751879
16590	1.14287	0.836996
16591	1.52101	0.701251
16592	1.71414	0.702678
16593	1.86411	0.704668
16594	1.6253	0.704106
16595	1.04355	0.702106
16596	1.0443	0.703583
16597	1.34852	1.95237
16598	1.56016	0.789228
16599	1.56016	0.808658
16600	1.03235	0.807425
16601	1.2966	0.159683
16602	1.82354	0.119531
16603	1.2966	0.0932025
16604	1.67687	0.102491
16605	1.19669	1.01382
16606	1.82979	1.07827
16607	1.58972	1.13568
16608	1.09219	1.08092
16609	1.42077	1.08167
16610	1.89226	0.991772
16611	1.19547	0.870859
16612	1.10187	2.1
16613	1.10188	0.876965
16614	1.01479	2.1
16615	1.81663	2.1
16616	1.81533	2.1
16617	1.83247	2.1
16618	1.51511	2.1
16619	0.983148	2.1
16620	1.7513	2.1
16621	1.85017	2.1
16622	1.26103	2.1
16623	1.23152	2.1
16624	1.65508	2.1
16625	1.23152	2.1
16626	2.15082	2.1
16627	1.57974	2.1
16628	1.07289	2.1
16629	0.81534	2.1
16630	1.51007	2.1
16631	1.00898	0.121043
16632	1.18959	0.111896
16633	1.32178	0.0955934
16634	1.25929	0.0922212
16635	2.17892	0.720639
16636	1.22938	0.348187
16637	3.02933	0.816273
16638	2.34619	0.734238
16639	1.47679	0.7
16640	1.0819	0.7
16641	3.10558	2.1
16642	3.31457	2.1
16643	0.985109	2.1

16644	1.10667	2.1
16645	3.31457	2.1
16646	3.54443	2.1
16647	1.10667	2.1
16648	1.25987	2.1
16649	1.77063	1.12661
16650	1.51065	1.00792
16651	1.24688	2.04205
16652	1.37652	2.1
16653	1.37652	1.48338
16654	1.54168	2.1
16655	1.17042	0.969538
16656	1.13092	1.13461
16657	1.13092	1.33151
16658	1.52938	2.1
16659	1.11787	0.715247
16660	1.00613	0.824705
16661	0.781373	0.674
16662	1.6885	0.672558
16663	2.49439	0.513861
16664	1.42657	0.611242
16665	1.77903	1.62968
16666	2.63606	2.1
16667	2.10425	2.1
16668	1.21425	1.22511
16669	1.31253	0.987566
16670	1.28418	1.00577
16671	1.28418	1.07349
16672	1.58972	1.10571
16673	0.858948	2.1
16674	1.90196	2.1
16675	1.81024	2.1
16676	1.90194	2.1
16677	1.4263	0.957269
16678	0.947921	0.940195
16679	1.22852	0.829628
16680	1.09194	0.777566
16681	1.60578	0.207482
16682	1.14721	0.22214
16683	1.06336	0.306566
16684	1.14721	0.228029
16685	1.11924	0.0780586
16686	1.53814	0.0787941
16687	1.18421	0.102938
16688	1.8458	0.0854416
16689	1.7565	2.06339
16690	1.09344	1.37825
16691	1.09344	1.08651
16692	1.16971	1.98308
16693	1.71465	0.100391
16694	1.19412	0.090315
16695	1.64985	0.245481
16696	1.05417	0.246531
16697	1.2127	0.0780171
16698	1.48867	0.0748916
16699	1.27806	0.0819697
16700	1.21269	0.0750186
16701	1.37813	0.998467

16702	1.0417	1.01531
16703	1.88626	0.728467
16704	1.14977	0.866448
16705	1.78803	1.67375
16706	1.33793	1.11171
16707	1.42266	1.32715
16708	1.26865	1.16748
16709	1.09518	2.1
16710	1.36844	2.1
16711	1.20455	2.1
16712	1.36844	2.1
16713	1.22297	1.17044
16714	1.11256	1.22954
16715	1.11256	1.13168
16716	1.03217	1.18019
16717	1.18422	0.0860028
16718	1.4282	0.0901715
16719	1.04159	0.113147
16720	1.4282	0.087375
16721	0.988805	2.1
16722	1.05481	2.1
16723	1.52938	0.98506
16724	1.26593	1.19592
16725	1.53082	2.1
16726	1.09847	2.1
16727	1.75211	0.880004
16728	1.22222	1.89861
16729	1.75211	0.820449
16730	1.51592	1.84365
16731	1.63413	0.963522
16732	1.26662	0.966192
16733	1.42495	0.852161
16734	1.4356	0.883436
16735	1.29389	1.31492
16736	1.04164	2.05309
16737	1.40574	1.30437
16738	1.04164	1.13332
16739	2.22344	0.844989
16740	1.45945	0.587051
16741	0.987248	0.764714
16742	1.2474	0.547359
16743	0.974702	0.902755
16744	1.43777	0.80657
16745	1.71289	2.1
16746	1.91529	2.1
16747	1.91529	2.1
16748	2.01827	2.1
16749	1.87111	1.26994
16750	1.00864	0.807066
16751	1.26865	0.865926
16752	1.18769	1.24003
16753	1.24167	1.95374
16754	1.36133	0.914053
16755	2.28168	2.1
16756	3.98524	2.1
16757	1.36133	1.91947
16758	1.69436	0.925386
16759	2.21926	1.37501

16760	1.70302	2.1
16761	1.50732	0.422307
16762	1.42237	0.415686
16763	1.18659	0.490006
16764	1.50732	0.494797
16765	1.19785	0.185315
16766	1.40969	0.141036
16767	1.28339	0.239063
16768	1.40882	0.223007
16769	0.968595	2.0009
16770	1.41903	0.945146
16771	1.12567	2.1
16772	1.15898	2.1
16773	1.27241	2.1
16774	0.995796	2.1
16775	1.89983	0.087815
16776	1.05417	0.0904113
16777	0.571999	0.0772361
16778	0.743597	0.0754859
16779	1.89353	0.0860993
16780	0.924068	0.0790175
16781	1.52851	2.1
16782	1.26969	2.1
16783	1.07763	0.740965
16784	1.82301	0.717485
16785	1.07763	0.583962
16786	1.17506	0.659115
16787	1.20045	2.1
16788	1.12486	2.1
16789	1.15234	2.1
16790	1.11598	2.1
16791	1.12486	2.1
16792	1.0491	2.1
16793	1.11598	2.1
16794	1.1047	2.1
16795	1.05902	2.1
16796	1.14361	2.1
16797	1.14361	2.1
16798	1.26851	2.1
16799	1.29389	1.09866
16800	0.988456	1.06894
16801	1.83688	0.0968668
16802	1.70302	0.113647
16803	1.49386	0.102018
16804	1.83151	0.112213
16805	1.01346	0.937687
16806	0.983607	0.964593
16807	0.983596	2.1
16808	1.08332	1.00903
16809	2.36848	0.460487
16810	2.97066	0.340645
16811	1.12352	0.325029
16812	2.5311	0.259004
16813	1.09062	2.1
16814	0.962182	2.1
16815	0.962181	2.1
16816	1.2258	2.1
16817	0.974888	2.1

16818	1.06312	2.1
16819	1.73583	0.155024
16820	1.72399	0.150233
16821	1.724	0.234157
16822	1.50951	0.223072
16823	1.26755	2.1
16824	1.03824	2.1
16825	1.27875	2.1
16826	1.03824	2.1
16827	1.18902	2.1
16828	1.534	2.1
16829	1.11764	2.1
16830	1.534	2.1
16831	1.52008	2.1
16832	1.34406	2.1
16833	1.34406	2.1
16834	1.2187	2.1
16835	0.963571	1.00151
16836	1.19612	0.93141
16837	0.963571	1.68461
16838	1.00007	1.56989
16839	0.903333	2.1
16840	1.12724	2.1
16841	1.23771	2.1
16842	1.17683	2.1
16843	1.20059	0.907608
16844	0.772023	0.762186
16845	1.14749	0.970326
16846	1.2802	0.945143
16847	1.85091	1.30914
16848	2.11193	1.12889
16849	1.2802	0.917776
16850	1.42913	0.908738
16851	2.11193	1.06887
16852	1.27737	0.94876
16853	1.30584	2.1
16854	1.60936	2.1
16855	1.3413	0.35
16856	1.53336	0.180053
16857	2.60679	0.239168
16858	1.3413	0.18931
16859	1.35877	2.1
16860	1.11024	2.1
16861	1.27839	2.1
16862	1.08171	2.1
16863	1.27841	2.1
16864	1.0577	2.1
16865	1.71255	2.1
16866	1.10611	2.1
16867	0.960106	2.1
16868	1.40921	2.1
16869	1.90705	0.710956
16870	2.79564	0.70619
16871	1.45321	0.714796
16872	1.90705	0.709632
16873	0.974718	0.609858
16874	1.53671	0.580382
16875	1.69039	0.576221

16876	1.78229	0.536728
16877	1.0101	2.1
16878	1.3167	2.1
16879	1.19699	2.1
16880	1.3212	2.1
16881	1.60811	0.7
16882	1.43224	0.7
16883	1.35401	0.7
16884	1.17464	0.674162
16885	1.27696	2.1
16886	1.05025	2.1
16887	1.05025	2.1
16888	1.18076	2.1
16889	1.37224	1.28682
16890	1.13438	1.20479
16891	1.76788	1.13847
16892	1.73444	1.13187
16893	1.37704	2.1
16894	1.77042	2.1
16895	1.77042	2.1
16896	1.09778	2.1
16897	1.28667	2.1
16898	1.28228	2.1
16899	1.30646	0.70397
16900	1.68867	0.850745
16901	1.30645	0.986701
16902	0.986494	1.86961
16903	1.6253	0.704423
16904	1.48676	0.704843
16905	1.48676	0.70437
16906	1.32974	0.703487
16907	0.634347	0.05
16908	1.15142	0.0901043
16909	1.11395	0.075621
16910	1.18068	0.0759742
16911	1.36904	0.0691936
16912	0.764563	0.0626832
16913	2.09213	0.064067
16914	1.56631	2.1
16915	1.08011	2.1
16916	1.80283	2.1
16917	1.50738	2.1
16918	1.44951	0.406708
16919	1.50102	0.475859
16920	1.50102	0.292492
16921	1.39615	0.350478
16922	1.02653	0.754704
16923	2.98258	0.759691
16924	2.0815	0.108699
16925	1.39525	0.0845966
16926	1.82176	0.0905665
16927	0.977607	0.0987267
16928	1.96133	1.43318
16929	1.27189	1.21564
16930	0.826032	1.08603
16931	2.12831	1.05232
16932	1.77798	1.8205
16933	1.02969	1.29298

16934	1.06463	1.21715
16935	1.49219	2.01966
16936	1.10003	2.1
16937	1.21223	2.1
16938	1.00513	1.01662
16939	1.43788	1.40399
16940	1.47134	0.800462
16941	0.968249	0.821885
16942	3.22585	0.200775
16943	3.36933	0.171021
16944	3.36932	0.236833
16945	3.03865	0.138181
16946	1.66295	0.251413
16947	1.65583	0.179229
16948	1.65583	0.337842
16949	1.4732	0.190124
16950	1.73704	0.05
16951	1.76476	1.07255
16952	2.13345	0.977208
16953	1.55816	2.1
16954	1.55816	1.05031
16955	1.02601	2.1
16956	1.08039	1.54862
16957	1.05498	1.03236
16958	1.08039	2.1
16959	1.2873	2.1
16960	1.01121	2.1
16961	0.987703	2.1
16962	0.98771	2.02936
16963	0.964782	2.1
16964	1.35719	2.1
16965	2.61092	2.1
16966	2.6607	2.1
16967	3.41693	2.1
16968	1.23125	2.1
16969	1.73772	2.1
16970	0.99845	0.838059
16971	1.5176	0.879486
16972	1.13271	0.185532
16973	1.332 0.139435	
16974	1.63159	2.1
16975	1.61342	2.1
16976	1.03679	2.1
16977	1.19087	2.1
16978	1.97172	2.1
16979	2.68634	2.1
16980	1.19086	2.1
16981	1.50091	2.1
16982	2.84677	2.1
16983	2.77894	2.1
16984	2.77894	2.1
16985	2.74206	2.1
16986	1.3284	2.1
16987	1.83524	2.1
16988	1.65853	2.1
16989	1.56741	2.0957
16990	1.00509	2.1
16991	1.45246	2.1

16992	1.26193	2.1
16993	1.45244	2.1
16994	1.61963	0.364787
16995	1.68189	0.738092
16996	1.68189	0.995171
16997	1.49155	0.777898
16998	1.58054	2.1
16999	1.59169	2.1
17000	1.59169	2.1
17001	1.60497	2.1
17002	0.743595	0.0771381
17003	0.702359	0.077202
17004	1.15317	0.0963839
17005	2.24367	0.0917266
17006	1.15252	0.0892478
17007	2.60354	0.0802077
17008	1.45158	2.1
17009	1.56004	2.1
17010	1.64527	2.1
17011	1.00371	2.1
17012	1.38287	0.735012
17013	0.893024	0.758095
17014	1.23527	0.83346
17015	1.23706	0.76514
17016	2.20993	0.732746
17017	1.54858	0.887073
17018	0.798666	0.704851
17019	1.22487	0.705463
17020	1.33158	0.702466
17021	1.85902	0.702829
17022	1.08334	2.1
17023	1.17885	1.11587
17024	1.17885	0.951483
17025	1.07283	1.04979
17026	1.30723	0.0853774
17027	1.62507	0.0801881
17028	1.62507	0.0767531
17029	1.08854	0.0861143
17030	1.59475	2.1
17031	1.58874	2.1
17032	1.58875	2.1
17033	1.35787	2.1
17034	1.04147	2.1
17035	1.15564	1.60427
17036	1.78301	2.1
17037	1.90618	2.1
17038	1.71555	2.1
17039	2.13143	2.1
17040	1.10817	2.1
17041	1.032	2.1
17042	1.032	2.1
17043	0.953636	2.1
17044	1.27916	1.13456
17045	1.11938	1.10233
17046	1.11937	1.11126
17047	1.9186	1.27228
17048	1.19967	0.45137
17049	1.63489	0.515604

17050	1.00763	0.580065
17051	1.81656	0.607423
17052	1.31814	0.450775
17053	2.98183	0.557047
17054	1.20149	2.1
17055	1.29129	2.1
17056	1.29129	2.1
17057	1.37138	2.1
17058	1.23104	1.26981
17059	1.75236	1.57548
17060	1.6	0.89376
17061	1.60565	1.10106
17062	1.60565	1.03298
17063	1.40013	1.14894
17064	1.23042	2.1
17065	1.18278	2.1
17066	1.10853	2.1
17067	1.04724	2.1
17068	1.1766	2.1
17069	1.00319	2.1
17070	1.29435	2.1
17071	1.00319	2.1
17072	1.05655	1.59787
17073	1.01606	1.70153
17074	1.80391	2.1
17075	2.7699	2.1
17076	1.52073	2.1
17077	2.06835	2.1
17078	2.00202	2.1
17079	2.04906	2.1
17080	0.968536	2.1
17081	1.02461	2.1
17082	1.59784	2.1
17083	1.78249	2.1
17084	1.81409	2.1
17085	1.79844	2.1
17086	1.77093	1.86871
17087	2.02356	1.54401
17088	1.32449	2.1
17089	1.10003	2.1
17090	1.16915	0.802654
17091	1.05937	0.764914
17092	1.28096	2.09704
17093	1.34696	1.67812
17094	1.34695	2.1
17095	1.36457	1.79334
17096	1.06478	2.1
17097	1.06239	2.1
17098	1.06239	2.1
17099	1.09629	2.1
17100	0.798209	2.07158
17101	1.8532	2.1
17102	2.10112	0.305814
17103	2.8704	0.263245
17104	2.8704	0.273342
17105	2.92457	0.247854
17106	1.0752	2.0912
17107	1.07745	2.1

17108	1.07745	2.1
17109	1.08911	2.1
17110	1.40182	2.1
17111	1.00133	2.1
17112	1.40182	2.1
17113	1.24509	2.1
17114	2.22243	0.0820877
17115	1.07303	0.0952771
17116	1.29736	0.107152
17117	1.73584	0.10723
17118	2.0815	0.808181
17119	1.07999	0.780638
17120	2.1692	2.1
17121	1.99992	2.1
17122	1.99992	2.1
17123	1.78327	2.1
17124	1.52661	0.101279
17125	0.956003	0.085776
17126	1.53172	0.0984695
17127	0.955991	0.0874465
17128	1.35025	1.01584
17129	1.51084	2.1
17130	1.51084	1.09857
17131	1.67115	2.1
17132	2.00977	2.1
17133	1.63632	2.1
17134	1.63632	2.1
17135	2.66128	2.1
17136	1.13525	2.1
17137	2.37566	2.1
17138	1.25987	2.1
17139	1.4472	2.1
17140	1.4472	2.1
17141	1.66018	2.1
17142	0.994001	1.50373
17143	1.83151	0.952339
17144	1.83151	0.762267
17145	1.57621	0.800062
17146	1.40915	0.349129
17147	1.24653	0.818925
17148	1.24653	0.208134
17149	1.10609	0.893476
17150	1.61121	2.1
17151	1.74472	2.1
17152	1.74472	2.1
17153	1.0368	2.1
17154	1.04788	2.1
17155	1.28522	2.1
17156	1.28522	2.1
17157	1.54196	1.52237
17158	1.23387	2.1
17159	0.896519	2.1
17160	1.60428	2.1
17161	1.08467	2.1
17162	1.56875	0.915013
17163	1.39072	0.744253
17164	1.39073	0.741668
17165	1.6	0.97609

17166	1.01346	1.9752
17167	1.06838	1.08165
17168	0.968463	1.13754
17169	1.06837	1.10138
17170	3.22433	0.700468
17171	2.05993	1.11292
17172	2.01836	0.712071
17173	1.66154	1.17867
17174	1.20871	1.25941
17175	1.10586	2.1
17176	1.10586	1.1981
17177	1.03853	2.1
17178	1.12076	2.1
17179	1.03899	2.1
17180	1.03899	2.1
17181	0.963973	2.1
17182	0.987132	2.1
17183	1.0894	2.1
17184	2.37823	2.03663
17185	1.75647	1.51618
17186	1.64625	2.1
17187	1.56195	2.1
17188	1.41995	2.1
17189	1.15171	2.1
17190	1.36457	2.1
17191	1.33847	1.61566
17192	1.33847	2.1
17193	0.869601	1.42573
17194	1.42255	2.1
17195	1.3868	2.1
17196	1.3868	2.1
17197	1.33962	2.1
17198	1.05481	2.1
17199	1.08074	2.1
17200	1.08074	2.1
17201	0.857557	2.1
17202	1.13537	1.57391
17203	1.09194	0.75936
17204	1.01179	1.6491
17205	1.13537	0.757672
17206	1.15645	1.78416
17207	1.56409	0.645302
17208	1.56409	0.877501
17209	1.16239	0.965678
17210	1.10746	0.741101
17211	1.47952	0.784155
17212	1.14733	2.01977
17213	3.77068	2.1
17214	1.38497	2.1
17215	1.54599	2.1
17216	1.54601	2.1
17217	1.27697	2.1
17218	1.01393	1.33272
17219	1.10558	1.56016
17220	0.995012	0.763413
17221	1.17624	1.15226
17222	1.60497	2.1
17223	1.61588	2.1

17224	1.61587	2.1
17225	1.62182	2.1
17226	1.48866	0.0756009
17227	1.65977	0.0743119
17228	1.65977	0.0767441
17229	1.69044	0.0768565
17230	0.961892	2.1
17231	1.00659	2.1
17232	1.0066	2.1
17233	1.06226	2.1
17234	1.15171	2.1
17235	1.50853	1.24447
17236	1.50853	2.1
17237	1.2955	2.1
17238	1.3204	0.350544
17239	2.13295	0.207688
17240	2.75524	0.174512
17241	1.18156	0.118125
17242	1.73729	2.1
17243	1.20356	2.1
17244	1.62483	2.1
17245	1.52008	2.1
17246	1.19964	2.1
17247	1.0577	2.1
17248	1.19964	2.1
17249	1.07156	2.1
17250	1.98241	2.1
17251	1.43687	2.1
17252	1.27863	1.8938
17253	1.19915	1.17556
17254	0.97269	1.22932
17255	1.0739	1.04956
17256	1.1427	1.98503
17257	1.22476	0.930827
17258	1.06584	1.38625
17259	1.22626	1.23143
17260	1.31388	2.1
17261	1.16932	2.1
17262	1.28819	2.1
17263	1.0499	2.1
17264	1.55894	2.1
17265	1.78709	2.1
17266	1.75986	2.1
17267	1.764 2.1	
17268	1.44801	2.1
17269	1.28186	2.1
17270	1.20797	2.01153
17271	1.06821	1.98908
17272	1.87287	0.790838
17273	2.08014	0.857892
17274	1.63225	0.884026
17275	1.39501	1.03658
17276	1.20132	0.106072
17277	1.16182	0.109245
17278	1.08848	0.125327
17279	1.20132	0.138167
17280	1.23475	1.17515
17281	1.16661	1.55725

17282	2.31317	2.1
17283	2.50145	2.1
17284	1.04367	2.1
17285	2.23875	2.1
17286	0.953632	0.502049
17287	1.38646	0.514501
17288	1.38646	0.501351
17289	1.61963	0.419291
17290	1.62934	1.13246
17291	1.88626	0.895739
17292	1.0775	1.00509
17293	1.119	0.923548
17294	1.08133	0.830208
17295	1.48312	0.751018
17296	1.57125	0.50888
17297	1.74098	0.49155
17298	1.94372	1.12437
17299	1.22237	1.1603
17300	1.18549	0.730886
17301	1.01294	0.594258
17302	1.01294	0.856468
17303	1.2064	0.898327
17304	1.14599	2.1
17305	1.00065	0.881603
17306	1.76357	2.1
17307	2.23468	2.1
17308	2.21735	2.1
17309	3.32769	2.1
17310	1.19647	0.860278
17311	1.35307	0.757974
17312	1.19645	0.78043
17313	1.43778	0.766754
17314	1.34412	0.759229
17315	1.1315	1.17846
17316	1.1315	0.754604
17317	1.23943	0.744181
17318	1.57829	2.1
17319	1.22537	2.1
17320	1.14357	2.1
17321	1.32772	2.1
17322	1.4259	2.1
17323	1.34245	2.1
17324	1.47953	2.1
17325	1.70303	2.1
17326	2.21127	0.201383
17327	1.47904	0.154289
17328	1.18793	0.24892
17329	2.21126	0.237188
17330	1.1032	1.51877
17331	1.14986	0.86916
17332	1.14986	2.06036
17333	1.19611	0.882706
17334	1.91883	2.1
17335	1.83019	2.1
17336	1.83019	2.1
17337	1.82462	2.1
17338	1.27362	0.676777
17339	1.27421	0.579587

17340	1.27421	0.365919
17341	1.10736	0.286477
17342	1.3003	0.820409
17343	1.31415	1.05933
17344	1.31415	0.845352
17345	1.32201	1.0911
17346	1.21124	2.1
17347	0.81568	2.1
17348	0.81568	2.1
17349	0.551816	2.1
17350	1.67119	2.1
17351	1.63384	2.1
17352	1.63384	2.1
17353	1.63661	2.1
17354	1.48734	2.1
17355	1.52245	2.1
17356	1.52245	2.1
17357	1.50714	2.1
17358	0.996778	0.101834
17359	1.56091	0.115452
17360	1.56091	0.120099
17361	1.90457	0.125284
17362	1.1181	2.1
17363	1.06543	2.1
17364	2.66828	2.1
17365	2.76932	2.1
17366	1.06543	2.1
17367	1.01474	2.1
17368	2.76932	2.1
17369	2.86704	2.1
17370	1.29724	0.670012
17371	1.96938	0.663933
17372	1.82462	2.1
17373	1.84553	2.1
17374	1.05556	2.1
17375	1.25363	2.1
17376	1.84552	2.1
17377	1.53208	2.1
17378	1.12826	2.1
17379	1.39075	2.1
17380	0.9702	0.899783
17381	1.16481	0.790109
17382	1.08894	1.07968
17383	1.91756	1.15694
17384	1.50865	0.966978
17385	1.06012	0.891026
17386	1.5456	0.374846
17387	1.37427	0.433087
17388	1.1211	0.191589
17389	1.5456	0.241272
17390	1.33135	0.302968
17391	1.07956	0.325331
17392	2.23255	0.333595
17393	1.21031	0.301013
17394	1.52903	2.1
17395	1.35977	2.1
17396	3.3837	2.1
17397	3.20581	2.1

17398	1.35977	2.1
17399	1.15414	2.1
17400	3.20582	2.1
17401	2.07596	2.1
17402	1.6812	0.306118
17403	1.03939	0.28217
17404	1.30047	2.1
17405	1.5586	2.1
17406	1.5586	2.1
17407	1.84214	2.1
17408	1.31825	0.857489
17409	1.31923	1.09918
17410	1.31924	0.856698
17411	1.33026	1.04883
17412	1.15818	0.867825
17413	1.17822	0.952988
17414	1.15818	1.99762
17415	1.28096	1.13871
17416	1.06848	0.7
17417	1.89639	0.7
17418	1.9186	1.08266
17419	1.77065	1.03797
17420	1.59103	0.942852
17421	1.56164	0.907412
17422	1.09697	0.0608417
17423	2.38719	0.0598818
17424	2.22123	0.0526518
17425	2.46059	0.0516527
17426	1.45506	0.580061
17427	1.03645	0.575091
17428	1.30347	2.1
17429	1.2627	2.1
17430	1.2627	2.1
17431	1.23036	2.1
17432	1.41469	2.1
17433	1.45257	2.1
17434	1.46907	0.159658
17435	1.70302	0.1788
17436	1.49196	0.108353
17437	1.36307	0.125601
17438	1.05677	2.1
17439	0.978698	2.1
17440	0.978699	2.1
17441	2.01822	2.1
17442	1.47446	2.1
17443	1.55432	2.1
17444	1.55432	2.1
17445	1.06053	2.1
17446	1.86813	1.32458
17447	1.21892	1.73533
17448	1.53064	1.05833
17449	1.30664	1.13864
17450	1.09629	2.1
17451	1.19244	2.1
17452	1.19244	2.1
17453	1.37635	2.1
17454	1.49358	0.706834
17455	1.57702	0.692002

17456	2.09374	2.1
17457	1.88175	2.1
17458	1.88175	2.1
17459	1.51085	2.1
17460	1.10938	0.606864
17461	1.02478	0.520029
17462	1.33061	0.240763
17463	1.13272	0.132349
17464	1.75505	2.1
17465	1.78529	2.1
17466	1.6353	2.1
17467	1.59307	2.1
17468	1.60017	0.930628
17469	1.35608	1.10429
17470	1.35608	0.791217
17471	1.04713	0.79706
17472	1.01479	0.761268
17473	1.3868	0.742877
17474	1.3868	0.738658
17475	1.28418	0.910938
17476	2.15949	2.1
17477	2.90429	2.1
17478	1.51902	2.1
17479	1.54755	2.1
17480	2.90429	2.1
17481	3.49966	2.1
17482	1.70842	2.1
17483	1.97526	2.1
17484	0.949712	0.699775
17485	1.92784	0.596208
17486	1.34093	0.696628
17487	2.53426	0.69709
17488	1.92783	0.616803
17489	3.89373	0.490164
17490	2.53426	0.698337
17491	2.35961	0.690506
17492	1.68108	0.735797
17493	1.15558	0.728462
17494	1.67265	0.720079
17495	1.27608	0.718229
17496	1.42249	2.1
17497	1.39708	2.1
17498	1.39708	2.1
17499	1.39327	2.1
17500	1.97046	0.335437
17501	1.11688	0.237354
17502	1.28418	0.735226
17503	1.17302	0.8767
17504	1.17302	0.713643
17505	1.04349	0.877127
17506	0.979853	2.1
17507	1.26016	2.1
17508	1.18901	2.1
17509	1.26016	2.1
17510	1.22857	0.798451
17511	1.25848	0.864691
17512	1.25848	0.786619
17513	1.2858	0.843068

17514	1.09154	2.1
17515	2.01915	2.1
17516	1.23013	2.1
17517	1.55698	2.1
17518	1.55698	2.1
17519	1.8575	2.1
17520	2.01854	2.1
17521	1.20686	2.06951
17522	1.20684	1.65257
17523	1.77796	2.03882
17524	1.46129	2.1
17525	1.04193	2.1
17526	1.10051	2.1
17527	0.983723	2.1
17528	1.1729	2.1
17529	0.98505	2.1
17530	0.985052	2.1
17531	1.03951	2.1
17532	1.03949	2.1
17533	1.17583	2.1
17534	1.42866	2.1
17535	1.97261	2.1
17536	1.73274	2.1
17537	1.53629	2.1
17538	1.98182	2.1
17539	1.72511	2.1
17540	1.42174	2.1
17541	1.44472	2.1
17542	1.72511	2.1
17543	1.45411	2.1
17544	1.44472	2.1
17545	1.43836	2.1
17546	0.962703	0.778635
17547	1.0171	0.875859
17548	1.53336	0.218275
17549	1.40274	0.223045
17550	1.40272	0.228032
17551	1.00213	0.228444
17552	1.50892	0.197358
17553	1.64984	0.198249
17554	1.67464	0.210385
17555	1.67481	0.209931
17556	1.08212	2.1
17557	1.28095	2.1
17558	1.46956	2.1
17559	1.70303	2.1
17560	1.2925	0.0906827
17561	1.84716	0.0817156
17562	1.48676	0.426822
17563	1.48797	0.444823
17564	1.33917	0.412985
17565	1.22691	0.412626
17566	0.528744	2.1
17567	1.13728	2.1
17568	1.13728	2.1
17569	1.99843	2.1
17570	1.17464	0.568251
17571	1.0372	0.404785

17572	2.0815	0.213996
17573	0.933462	0.224763
17574	1.54931	2.1
17575	1.5485	2.1
17576	1.4077	2.1
17577	1.29931	2.1
17578	1.14931	0.719492
17579	2.02223	0.720604
17580	1.04482	0.699318
17581	0.94971	0.707981
17582	1.54632	1.23419
17583	1.15693	1.28173
17584	1.66763	2.1
17585	1.41238	2.1
17586	1.41238	2.1
17587	1.48068	2.1
17588	1.0171	0.571959
17589	1.1211	0.861717
17590	1.1211	0.514138
17591	1.28418	0.831615
17592	0.993021	2.1
17593	1.764 2.1	
17594	0.993023	2.1
17595	1.05787	2.1
17596	1.90914	0.724501
17597	1.12229	1.13165
17598	1.02025	0.782808
17599	0.585368	1.37405
17600	1.99323	2.1
17601	2.34988	2.1
17602	2.34988	2.1
17603	2.36987	2.1
17604	1.23107	0.257332
17605	1.90664	0.403081
17606	1.53496	0.241101
17607	1.70302	0.451509
17608	1.98859	2.1
17609	1.61057	2.1
17610	1.79844	2.1
17611	2.10811	2.1
17612	1.4618	2.1
17613	1.96722	2.1
17614	1.95191	2.1
17615	2.13418	2.1
17616	1.50368	0.138283
17617	1.61417	0.0881572
17618	1.15008	0.0944431
17619	1.19685	0.100809
17620	1.90456	0.129695
17621	1.90899	0.117688
17622	2.08864	0.147557
17623	2.10447	0.0969703
17624	1.16653	2.1
17625	1.23017	2.1
17626	1.0752	0.783331
17627	1.34412	0.753041
17628	1.06226	2.1
17629	1.09252	2.1

17630	1.09252	2.1
17631	0.88579	2.1
17632	1.56178	0.566945
17633	1.14305	0.553105
17634	1.56175	0.479672
17635	1.08211	0.398466
17636	1.32813	2.1
17637	1.32461	2.1
17638	1.32461	2.1
17639	1.32455	2.1
17640	1.42729	0.645463
17641	1.18716	0.601018
17642	1.47169	0.71848
17643	1.24687	0.674007
17644	1.09627	2.1
17645	1.04759	2.1
17646	1.04759	2.1
17647	0.987132	2.1
17648	1.11181	1.39606
17649	1.80662	2.1
17650	1.8066	2.1
17651	1.06461	1.74718
17652	1.56091	2.1
17653	1.9842	2.1
17654	1.73525	0.49067
17655	1.40557	0.389684
17656	2.26906	0.514851
17657	1.31248	0.72401
17658	1.66832	0.476372
17659	1.32634	0.346207
17660	1.03314	0.0865515
17661	1.24682	0.082818
17662	1.38536	0.0878336
17663	1.52661	0.07791
17664	1.177	1.16047
17665	1.36405	1.02829
17666	1.19837	1.08445
17667	1.17701	0.835248
17668	0.993831	0.685515
17669	1.0823	0.529509
17670	1.0823	0.660033
17671	1.01121	0.653862
17672	1.49653	0.333943
17673	1.89583	0.541201
17674	0.67242	0.291928
17675	1.36047	0.492983
17676	1.00366	0.543059
17677	1.24728	0.65422
17678	1.13387	0.0963969
17679	1.60867	0.113597
17680	1.30572	0.214349
17681	1.54982	0.13136
17682	0.788592	0.104978
17683	1.82961	0.110006
17684	1.54982	0.1818
17685	1.82961	0.113806
17686	1.26062	0.0973546
17687	1.64002	0.0945265

17688	1.12035	0.0927469
17689	1.64002	0.102833
17690	1.1673	2.1
17691	1.75367	2.1
17692	0.964034	1.88453
17693	1.0305	1.48969
17694	1.30896	1.60336
17695	1.52776	1.44024
17696	1.52776	2.1
17697	1.17989	1.84489
17698	1.16759	2.1
17699	1.8575	2.1
17700	1.16759	2.1
17701	1.60936	2.1
17702	1.10747	0.653917
17703	1.47688	0.454078
17704	1.40794	2.1
17705	1.95729	1.37044
17706	1.95729	2.1
17707	3.18156	1.55302
17708	1.87598	1.1307
17709	1.94343	2.1
17710	1.94343	1.103
17711	2.00551	2.1
17712	1.20067	1.02269
17713	1.70744	1.27389
17714	1.87244	1.51328
17715	1.32075	1.24953
17716	1.64955	2.1
17717	1.44221	2.1
17718	1.31109	2.1
17719	1.14206	2.1
17720	1.60452	1.69564
17721	1.12111	0.88659
17722	1.00832	2.1
17723	0.974137	2.1
17724	0.974137	2.1
17725	0.98505	2.1
17726	1.02368	2.1
17727	0.985051	2.1
17728	0.995734	0.721283
17729	1.31993	0.713509
17730	1.31993	0.695235
17731	1.18549	0.463722
17732	1.5176	0.833581
17733	1.33622	0.813079
17734	1.33621	0.818183
17735	1.02782	0.806572
17736	1.66774	2.1
17737	1.85305	2.1
17738	1.85305	2.1
17739	2.07088	2.1
17740	1.78873	2.1
17741	1.47533	2.1
17742	1.23152	2.1
17743	0.99922	2.1
17744	1.48493	2.1
17745	1.1394	2.1

17746	1.19179	2.1
17747	1.64289	2.1
17748	1.47858	2.1
17749	1.89687	2.1
17750	0.972464	2.1
17751	1.79473	2.1
17752	1.06971	2.1
17753	1.21089	2.1
17754	1.12572	2.1
17755	1.30566	2.1
17756	1.30566	2.1
17757	1.52724	2.1
17758	2.32237	0.704764
17759	2.20248	0.696346
17760	2.79564	0.710207
17761	2.32237	0.705033
17762	1.59354	0.51078
17763	3.098	0.506602
17764	3.09801	0.483527
17765	3.45132	0.497449
17766	1.24001	1.82637
17767	1.41822	0.780539
17768	1.41821	2.1
17769	1.44859	0.800458
17770	1.34539	2.1
17771	1.06991	2.1
17772	1.18076	2.1
17773	0.978586	2.1
17774	0.999189	0.318731
17775	2.41076	0.625442
17776	0.968475	2.02644
17777	1.02074	2.1
17778	1.02074	1.94732
17779	1.07866	2.1
17780	0.817702	0.282623
17781	1.5082	0.324441
17782	1.48376	0.373099
17783	1.18947	0.336362
17784	1.10638	2.1
17785	1.86766	2.1
17786	1.19785	2.1
17787	1.34141	2.1
17788	1.34141	2.1
17789	0.991985	2.1
17790	2.35516	0.857926
17791	1.24815	0.927787
17792	1.80454	2.1
17793	1.22633	2.1
17794	0.696255	2.1
17795	1.19379	0.974542
17796	1.14721	2.1
17797	1.16153	2.1
17798	1.16153	2.1
17799	1.17261	2.1
17800	1.20814	2.1
17801	1.54791	2.1
17802	1.54791	2.1
17803	1.60477	2.1

17804	1.29931	2.1
17805	1.19098	2.1
17806	1.19098	2.1
17807	1.13341	2.1
17808	1.00503	1.87176
17809	1.25334	0.806132
17810	1.22655	2.1
17811	1.26282	2.1
17812	1.26282	2.1
17813	0.999489	2.01526
17814	1.54197	0.435164
17815	1.17152	0.510881
17816	1.17152	0.494118
17817	1.49627	0.469226
17818	1.36405	1.03097
17819	0.976334	0.911212
17820	1.12495	2.1
17821	1.54096	2.1
17822	1.75263	2.1
17823	1.49114	2.1
17824	0.842998	1.74918
17825	1.60244	0.935408
17826	1.48911	1.36417
17827	2.18685	0.941558
17828	1.75236	1.47681
17829	1.54143	1.74962
17830	1.40013	1.09837
17831	0.946646	1.21567
17832	1.50714	2.1
17833	1.46055	2.1
17834	1.46055	2.1
17835	1.42249	2.1
17836	2.12839	0.455464
17837	1.75211	0.366037
17838	1.76297	0.301435
17839	1.70736	0.246306
17840	1.19952	0.363579
17841	1.56475	0.263852
17842	1.74312	0.452184
17843	1.48973	0.312163
17844	1.91518	0.624716
17845	1.16534	0.599889
17846	1.53556	2.1
17847	1.56253	2.1
17848	1.56253	2.1
17849	1.58054	2.1
17850	1.27806	0.0729644
17851	1.44593	0.0741593
17852	1.44593	0.0779326
17853	1.3946	0.0748063
17854	1.34857	0.736753
17855	1.03818	1.39083
17856	1.38969	0.774886
17857	1.85548	0.797388
17858	1.03108	0.748925
17859	1.85548	0.779172
17860	1.49346	1.92953
17861	1.83192	2.1

17862	1.75291	1.62542
17863	1.35435	2.03461
17864	0.921851	0.794358
17865	1.09646	0.776802
17866	1.2183	0.768056
17867	1.36717	0.741031
17868	2.14179	0.70202
17869	1.79805	0.70769
17870	1.3428	0.70793
17871	2.30493	0.707797
17872	1.81545	0.709075
17873	1.92269	0.703616
17874	2.30492	0.716231
17875	1.3663	0.715477
17876	1.22418	1.82214
17877	0.956698	0.956331
17878	1.4706	2.1
17879	0.953295	2.1
17880	1.47059	1.30743
17881	1.26929	2.1
17882	1.24538	0.648366
17883	1.4468	0.566465
17884	2.86762	2.1
17885	2.32911	2.1
17886	2.10864	2.1
17887	1.94548	2.1
17888	1.83134	2.1
17889	2.19519	2.1
17890	2.19853	2.1
17891	2.63855	2.1
17892	1.08213	0.397936
17893	1.46389	0.53294
17894	1.62235	0.640745
17895	0.980549	0.682435
17896	1.08195	1.8746
17897	0.99002	1.90547
17898	1.2269	2.1
17899	1.0838	2.1
17900	0.889491	2.1
17901	1.19587	2.1
17902	1.18801	0.221185
17903	1.57125	0.226261
17904	1.47712	2.1
17905	1.45182	2.1
17906	1.45182	2.1
17907	1.42255	2.1
17908	1.17816	0.111869
17909	1.00982	0.122788
17910	1.21056	0.0862344
17911	1.17816	0.0897527
17912	3.80902	0.880187
17913	0.976991	0.852893
17914	1.09962	2.1
17915	1.60976	2.1
17916	1.87029	2.1
17917	2.33806	2.1
17918	2.12549	2.1
17919	2.21472	2.1

17920	2.16221	0.390294
17921	1.99528	0.305572
17922	1.99528	0.313439
17923	1.97969	0.281217
17924	3.60854	2.1
17925	4.00847	2.1
17926	2.70372	2.1
17927	1.253 2.1	
17928	1.65739	2.1
17929	0.994294	2.1
17930	1.36203	2.1
17931	1.00965	2.1
17932	2.33374	2.1
17933	1.36209	2.1
17934	1.64983	0.151506
17935	1.36416	0.155712
17936	2.19766	0.147408
17937	1.36416	0.201039
17938	2.15871	0.839247
17939	1.81454	0.789216
17940	1.54607	0.705217
17941	1.57702	0.733445
17942	0.948748	0.0948024
17943	1.04522	0.102292
17944	1.60994	0.0887272
17945	1.90382	0.094888
17946	1.90382	0.093302
17947	1.57999	0.0799326
17948	1.73905	0.861487
17949	1.26535	1.11198
17950	2.12552	1.0999
17951	1.06891	0.991914
17952	1.13999	0.930706
17953	2.29812	1.15255
17954	2.83329	0.9978
17955	2.16272	0.935683
17956	1.71277	2.1
17957	1.68401	2.1
17958	1.68401	2.1
17959	1.69368	2.1
17960	1.06472	2.1
17961	1.56017	2.1
17962	1.2765	2.1
17963	1.99541	2.1
17964	1.46041	0.0503087
17965	2.04817	0.05
17966	2.04817	0.05
17967	2.33181	0.05
17968	2.06353	2.1
17969	1.35449	2.1
17970	1.52045	2.1
17971	1.45437	2.1
17972	1.04405	0.103715
17973	1.85945	0.0817366
17974	1.85945	0.0685255
17975	2.22679	0.0625204
17976	1.33339	2.1
17977	1.19376	2.1

17978	1.33266	2.1
17979	1.44244	2.1
17980	1.20455	2.1
17981	1.52852	2.1
17982	1.03882	2.1
17983	1.52852	2.1
17984	1.44801	2.09394
17985	1.00775	1.96118
17986	0.53907	2.1
17987	0.506124	2.1
17988	2.39045	2.1
17989	0.973794	2.1
17990	1.11193	2.1
17991	1.32784	2.1
17992	1.31595	2.1
17993	0.966115	2.1
17994	1.25953	2.1
17995	1.38086	2.1
17996	1.38086	2.1
17997	1.04101	2.1
17998	1.99319	0.267913
17999	1.23107	0.317509
18000	3.3606	0.308301
18001	1.23112	0.363744
18002	1.40239	2.1
18003	1.26091	2.1
18004	1.26091	2.1
18005	1.05776	2.1
18006	1.23768	0.717775
18007	1.0142	1.03241
18008	1.11435	0.868734
18009	1.01422	0.796955
18010	1.24001	0.79648
18011	1.42186	0.904045
18012	1.42186	0.861816
18013	1.47204	0.910959
18014	1.27361	2.1
18015	1.56642	2.1
18016	1.71676	2.1
18017	2.16815	2.1
18018	1.07855	1.16322
18019	1.07612	1.16883
18020	1.703	0.185093
18021	1.46853	0.18543
18022	1.45448	0.176015
18023	1.27778	0.177799
18024	0.991986	2.1
18025	1.1192	2.1
18026	1.1192	2.1
18027	1.25953	2.1
18028	2.08506	2.1
18029	1.75162	0.94601
18030	1.94523	2.1
18031	1.88405	1.99272
18032	1.15506	0.0995421
18033	1.1233	0.103775
18034	2.09736	2.1
18035	2.98303	2.1

18036	2.71184	2.1
18037	3.51614	2.1
18038	1.18769	2.06588
18039	1.24053	2.08921
18040	1.24053	2.1
18041	1.45327	2.1
18042	1.26865	1.2024
18043	1.1744	1.05825
18044	1.1744	0.970695
18045	1.11712	0.967253
18046	1.15841	0.716522
18047	1.02247	0.729249
18048	1.93649	2.1
18049	1.81191	2.1
18050	1.64718	2.1
18051	1.47521	2.1
18052	1.59654	2.1
18053	1.57823	2.1
18054	1.57823	2.1
18055	1.55768	2.1
18056	1.00665	0.485019
18057	1.28239	0.580557
18058	1.28239	0.390441
18059	1.44951	0.493972
18060	1.22083	2.1
18061	0.957622	2.1
18062	0.887896	2.1
18063	1.27387	2.1
18064	1.00521	0.991529
18065	1.00503	1.01133
18066	1.95844	2.1
18067	1.05042	2.1
18068	2.78662	2.1
18069	2.22399	2.1
18070	2.3356	0.13248
18071	1.49543	0.189389
18072	1.96854	0.121246
18073	1.21681	0.115508
18074	2.17803	1.74486
18075	1.50998	1.93602
18076	2.35239	1.59859
18077	2.26987	1.30072
18078	1.03824	0.681923
18079	1.51396	0.741191
18080	1.51396	0.711814
18081	1.48693	0.607985
18082	1.08784	1.57953
18083	1.36865	1.60398
18084	0.712405	1.81479
18085	1.08784	1.79201
18086	2.02522	2.1
18087	1.6738	2.1
18088	1.35175	2.1
18089	1.50094	2.1
18090	1.6738	2.1
18091	1.62871	2.1
18092	1.35084	2.1
18093	1.34276	2.1

18094	1.18041	2.1
18095	1.43652	2.1
18096	0.974196	2.1
18097	1.28037	2.1
18098	1.28037	2.1
18099	1.05071	2.1
18100	2.44737	0.0927917
18101	1.4672	0.103061
18102	1.22883	0.114306
18103	2.08149	0.138634
18104	1.82208	1.44643
18105	1.59826	1.5264
18106	1.59826	1.28263
18107	1.01393	1.77073
18108	1.42757	2.1
18109	0.996775	2.1
18110	1.42757	2.1
18111	1.26397	2.1
18112	1.02461	2.1
18113	1.46129	2.1
18114	1.11833	2.1
18115	1.335 2.1	
18116	2.07087	2.1
18117	1.24041	2.1
18118	1.335 2.1	
18119	1.55398	2.1
18120	1.21171	0.209578
18121	1.48589	0.227471
18122	1.32886	0.171884
18123	1.8027	0.178695
18124	1.53515	1.69592
18125	1.60428	1.74817
18126	1.00035	0.05
18127	1.03534	0.21771
18128	1.03535	0.90997
18129	1.09962	2.06456
18130	0.62132	1.12371
18131	1.21033	2.02039
18132	1.30326	1.34806
18133	2.6823	2.1
18134	0.991407	2.1
18135	1.29729	2.1
18136	1.29729	2.1
18137	1.16858	2.1
18138	1.66137	0.500216
18139	1.23012	0.184181
18140	1.66139	0.560835
18141	1.41821	0.445175
18142	1.56043	0.702259
18143	1.06848	0.85365
18144	1.56043	1.32328
18145	1.90911	1.02409
18146	0.982847	2.1
18147	1.05863	2.1
18148	1.05863	2.1
18149	1.22352	2.1
18150	1.23019	1.41144
18151	1.60509	1.95468

18152	1.19122	2.1
18153	1.47769	2.1
18154	2.18868	2.1
18155	1.40984	1.68734
18156	3.55197	2.1
18157	3.57227	2.1
18158	3.57227	2.1
18159	3.48926	2.1
18160	1.4375	0.701787
18161	1.11314	0.703113
18162	1.62408	2.1
18163	1.74438	2.1
18164	1.72387	2.1
18165	1.50018	2.1
18166	1.1486	2.1
18167	1.18474	2.1
18168	1.18474	2.1
18169	1.2295	2.1
18170	1.28754	2.1
18171	1.36838	2.1
18172	1.36838	2.1
18173	1.21703	2.1
18174	2.74206	2.1
18175	2.70795	2.1
18176	2.70795	2.1
18177	2.55918	2.1
18178	1.35065	1.00987
18179	1.65411	0.834179
18180	1.48572	0.971234
18181	1.28393	0.901031
18182	1.25265	0.129984
18183	1.70944	0.104461
18184	1.31259	0.811334
18185	1.14945	2.1
18186	1.14945	0.780337
18187	0.995725	2.1
18188	1.00307	0.723271
18189	2.05786	0.729319
18190	1.56357	0.70707
18191	1.10907	0.718377
18192	1.27558	1.22844
18193	1.05175	1.02607
18194	1.2685	2.1
18195	1.41369	2.1
18196	1.4137	2.1
18197	1.49654	2.1
18198	1.21489	2.00876
18199	1.14605	1.40365
18200	1.22476	2.1
18201	1.21489	1.03107
18202	2.51472	1.11904
18203	2.52091	1.08769
18204	2.52091	1.06399
18205	1.79171	1.01373
18206	1.03373	1.93106
18207	1.04037	2.1
18208	1.04038	1.87895
18209	1.03933	2.06386

18210	1.74123	0.345171
18211	1.41902	0.308379
18212	1.89777	0.355694
18213	1.89899	0.481564
18214	1.24728	0.51375
18215	1.30394	0.546747
18216	1.30394	0.40118
18217	1.16535	0.327243
18218	1.09914	2.1
18219	1.54259	2.1
18220	1.40233	2.1
18221	1.69858	2.1
18222	1.53555	2.1
18223	1.09859	1.06991
18224	1.09859	2.1
18225	0.725879	1.01896
18226	1.07213	2.1
18227	1.31589	2.1
18228	1.36904	2.1
18229	1.69711	2.1
18230	1.51028	0.148543
18231	0.993598	0.298491
18232	0.61563	0.14631
18233	1.26311	0.16262
18234	1.19496	2.1
18235	1.65925	2.1
18236	1.65925	2.1
18237	1.48162	2.1
18238	2.75738	1.20955
18239	1.24699	1.26256
18240	4.08729	1.79582
18241	2.35271	1.52369
18242	1.55063	0.700648
18243	1.34563	0.701098
18244	1.34563	0.7
18245	1.08189	0.7
18246	1.09662	0.658662
18247	2.35961	0.679516
18248	0.986952	0.518513
18249	0.813251	0.416727
18250	1.17897	0.1427
18251	1.67685	0.103971
18252	1.00573	1.27728
18253	1.08542	1.01788
18254	1.00573	2.1
18255	1.12572	2.1
18256	1.05499	2.04342
18257	1.00521	1.05348
18258	2.22679	0.0615016
18259	2.86746	0.0570989
18260	2.86746	0.0641349
18261	1.99958	0.0595256
18262	0.996517	2.1
18263	1.01169	2.1
18264	1.01169	2.1
18265	1.26346	2.1
18266	1.68274	2.1
18267	1.75505	2.1

18268	2.28748	1.70461
18269	1.36555	1.36897
18270	3.47888	0.100654
18271	0.692788	0.125389
18272	1.0685	0.836566
18273	1.14547	0.876626
18274	1.19017	2.1
18275	1.24976	2.1
18276	1.24976	2.1
18277	1.30682	2.1
18278	2.2153	2.1
18279	1.85457	2.1
18280	2.03267	2.1
18281	1.51042	2.1
18282	1.05619	0.796591
18283	1.05793	0.854424
18284	1.05793	0.770504
18285	1.00763	0.847918
18286	1.77677	2.1
18287	1.02524	2.1
18288	1.02524	2.1
18289	1.18803	2.1
18290	1.68669	2.1
18291	1.71162	2.1
18292	1.556	2.1
18293	1.39523	2.1
18294	0.960288	0.861103
18295	1.24201	0.863416
18296	1.24202	2.1
18297	1.3592	2.1
18298	2.14045	0.402227
18299	1.62054	0.398279
18300	2.20248	0.645161
18301	2.34699	0.644829
18302	3.45132	0.367036
18303	3.38241	0.407163
18304	3.38241	0.297331
18305	2.96127	0.314482
18306	2.02612	2.1
18307	2.09351	2.1
18308	2.09351	2.1
18309	2.17465	2.1
18310	1.07445	2.1
18311	1.14808	2.1
18312	1.39067	2.0877
18313	1.20958	1.25115
18314	1.20958	1.65758
18315	1.00382	1.11118
18316	0.986496	1.8173
18317	1.27142	1.74977
18318	1.39857	1.29997
18319	2.26482	1.51327
18320	2.24367	0.0877813
18321	1.9234	0.0907642
18322	1.59076	0.0925278
18323	1.185	0.0989558
18324	3.86179	0.0795607
18325	3.0638	0.0832283

18326	2.7574	0.0819924
18327	1.83266	0.0859962
18328	2.59418	2.1
18329	2.23048	2.1
18330	1.39564	2.1
18331	1.36763	2.1
18332	2.23048	2.1
18333	2.02522	2.1
18334	1.36763	2.1
18335	1.35175	2.1
18336	1.16181	0.104269
18337	0.97096	0.113137
18338	2.05665	0.365944
18339	0.483583	0.430054
18340	0.601928	0.225132
18341	3.88545	0.491901
18342	1.04909	2.1
18343	0.983143	2.1
18344	1.10469	2.1
18345	1.10516	2.1
18346	1.10517	2.1
18347	1.09951	2.1
18348	2.33985	2.1
18349	2.35026	2.1
18350	2.35025	2.1
18351	1.19462	2.1
18352	1.57627	2.1
18353	1.5493	2.1
18354	1.5493	2.1
18355	1.53556	2.1
18356	1.42763	0.819068
18357	1.24682	0.827269
18358	1.24682	0.772293
18359	1.00307	0.790202
18360	1.37392	2.1
18361	1.45408	2.1
18362	1.45408	2.1
18363	1.54249	2.1
18364	1.24474	1.1529
18365	1.07467	1.15168
18366	1.37813	1.36054
18367	1.24474	1.35861
18368	1.06341	2.1
18369	1.09194	2.1
18370	1.09194	2.1
18371	1.11117	2.1
18372	1.1658	2.1
18373	1.93707	2.1
18374	1.93707	2.1
18375	1.47729	2.1
18376	1.25669	0.937356
18377	1.07519	2.1
18378	1.65417	2.1
18379	1.50956	2.1
18380	1.37231	2.1
18381	1.27285	2.1
18382	1.23612	2.1
18383	1.1899	2.1

18384	1.08172	2.1
18385	1.20357	2.1
18386	1.5664	2.1
18387	1.7472	2.1
18388	1.48434	2.1
18389	1.23989	2.1
18390	1.58836	2.1
18391	1.38766	2.1
18392	1.23989	2.1
18393	1.26599	2.1
18394	2.56248	2.1
18395	2.08673	1.01361
18396	1.87216	1.05153
18397	1.60476	0.438258
18398	1.84355	2.1
18399	1.25971	0.990903
18400	1.44141	0.973607
18401	1.25521	0.293528
18402	1.19837	0.103656
18403	2.31936	0.0953094
18404	1.38153	2.1
18405	2.41734	2.1
18406	2.70856	2.1
18407	3.48385	2.1
18408	1.89226	0.821219
18409	1.64617	1.54866
18410	1.89112	1.15882
18411	1.23135	0.734783
18412	1.23134	0.728154
18413	1.34927	0.720894
18414	3.60096	0.132264
18415	1.68103	0.142085
18416	2.25644	2.1
18417	1.91955	2.1
18418	1.91955	2.1
18419	1.47688	2.1
18420	1.67375	2.1
18421	1.50033	2.1
18422	1.5098	2.1
18423	1.46908	2.1
18424	1.69055	0.371831
18425	1.53463	0.435121
18426	1.53463	0.266625
18427	1.41943	0.272545
18428	1.19022	1.83514
18429	1.95981	1.56766
18430	1.15009	1.23662
18431	1.31612	1.2549
18432	1.31611	0.895971
18433	1.10735	0.865233
18434	1.18601	0.387504
18435	1.3499	0.436001
18436	1.3499	0.516749
18437	1.29001	0.471892
18438	1.22083	2.1
18439	1.06191	2.1
18440	1.348	1.05171
18441	1.05758	1.39044

18442	1.34799	1.95252
18443	1.18647	1.49746
18444	2.92457	0.257427
18445	1.99182	0.242997
18446	2.25313	0.258312
18447	1.30859	0.261793
18448	1.30682	2.1
18449	1.38957	2.1
18450	1.38957	2.1
18451	1.47446	2.1
18452	0.953638	2.01124
18453	1.03408	2.1
18454	1.17885	0.718817
18455	1.3663	0.709352
18456	1.08265	0.726021
18457	1.71948	0.779206
18458	1.29501	0.989839
18459	0.955093	0.953608
18460	0.955081	0.881666
18461	1.88345	0.8616
18462	1.2243	2.1
18463	1.23169	2.1
18464	2.04726	1.08291
18465	0.842869	1.34429
18466	3.18156	2.1
18467	3.60786	1.37763
18468	3.60786	2.1
18469	2.38566	1.20352
18470	1.24526	0.0974864
18471	1.59019	0.112493
18472	1.16776	0.720365
18473	1.05042	1.5
18474	1.17418	2.1
18475	1.05042	2.1
18476	0.949135	2.1
18477	0.973448	2.1
18478	0.973447	2.1
18479	1.02958	2.1
18480	3.57768	2.1
18481	3.04246	2.1
18482	3.04246	2.1
18483	2.47824	2.1
18484	1.35879	2.1
18485	1.10407	2.07177
18486	1.14599	0.906575
18487	1.10407	0.923109
18488	1.87794	1.62053
18489	1.53429	1.4754
18490	1.12717	2.1
18491	1.38634	2.1
18492	1.38634	2.1
18493	1.13127	2.1
18494	1.04546	2.1
18495	1.06007	2.1
18496	1.06007	2.1
18497	1.19785	2.1
18498	1.43652	2.1
18499	1.71741	2.1

18500	1.70921	1.14283
18501	1.06994	1.42766
18502	0.960106	2.1
18503	1.48077	2.1
18504	1.48077	2.1
18505	2.5946	2.1
18506	2.03045	1.36849
18507	1.76788	1.70762
18508	2.03045	2.1
18509	0.633098	2.1
18510	1.06756	2.1
18511	1.38667	2.1
18512	0.966979	2.1
18513	1.38669	2.1
18514	1.07156	2.1
18515	0.986095	2.1
18516	1.32813	2.1
18517	0.986095	2.1
18518	1.98697	0.86921
18519	1.12016	1.41658
18520	1.24463	1.00856
18521	1.1967	1.44465
18522	1.30001	0.793816
18523	1.29175	1.05726
18524	1.29175	0.80737
18525	1.3003	1.06729
18526	1.5606	0.114702
18527	2.32098	0.105242
18528	2.57888	0.0964702
18529	2.97463	0.0915379
18530	1.24555	2.1
18531	1.46101	2.1
18532	1.90723	2.1
18533	2.29126	2.1
18534	1.46101	2.1
18535	1.84089	2.1
18536	2.5519	2.1
18537	2.91663	2.1
18538	1.10476	0.255813
18539	1.44103	0.225969
18540	1.44103	0.229374
18541	1.53336	0.224798
18542	0.672964	2.1
18543	0.633884	2.1
18544	0.633884	2.1
18545	0.603154	2.1
18546	2.29663	2.1
18547	2.06892	2.1
18548	1.3685	2.1
18549	1.39067	2.1
18550	2.06892	2.1
18551	1.78208	2.1
18552	1.39067	2.1
18553	1.42174	2.1
18554	1.01335	1.74817
18555	1.08195	1.02066
18556	1.86463	1.68325
18557	1.39827	1.88603

18558	0.861515	2.1
18559	2.10074	2.1
18560	2.09511	0.814239
18561	2.04853	0.744113
18562	2.21488	0.773983
18563	1.38287	0.780661
18564	0.974717	0.843846
18565	0.998451	0.825766
18566	1.42281	1.18139
18567	1.82595	1.11846
18568	1.50458	1.01378
18569	1.09408	0.945754
18570	1.23498	1.46297
18571	1.05654	1.43904
18572	1.17261	2.1
18573	1.18058	2.1
18574	1.18059	2.1
18575	1.18907	2.1
18576	2.38998	2.1
18577	2.77437	2.1
18578	1.04546	2.1
18579	1.16817	2.1
18580	2.77437	2.1
18581	2.97768	2.1
18582	1.16817	2.1
18583	1.2832	2.1
18584	1.75766	0.703207
18585	1.4934	0.881081
18586	1.4934	0.714803
18587	1.20324	0.951245
18588	1.07236	0.847993
18589	1.37641	0.807709
18590	1.42913	0.847302
18591	1.93373	0.846004
18592	1.42629	0.806024
18593	1.00636	0.768408
18594	0.933959	1.92334
18595	2.22873	0.995042
18596	2.22873	1.22129
18597	2.13757	0.884979
18598	1.10517	2.1
18599	1.86737	2.1
18600	0.811 2.1	
18601	1.19179	2.1
18602	1.18647	2.03048
18603	0.988453	2.1
18604	1.12457	0.320615
18605	1.23851	0.279195
18606	0.92492	2.1
18607	1.45656	2.1
18608	1.61251	0.978143
18609	1.56696	0.97954
18610	1.90245	0.88714
18611	1.46046	0.877792
18612	1.60329	0.140036
18613	1.38247	0.137998
18614	1.38247	0.119691
18615	1.23198	0.127539

18616	1.09518	2.1
18617	1.28159	2.1
18618	0.977777	2.1
18619	1.80778	2.1
18620	1.80777	2.1
18621	1.16655	2.1
18622	1.3515	2.1
18623	0.956573	2.1
18624	1.08022	2.1
18625	1.03437	2.1
18626	1.03437	2.1
18627	0.975409	2.1
18628	1.47369	0.235714
18629	1.31167	0.482243
18630	1.01847	0.261494
18631	2.75562	0.266735
18632	1.13443	2.1
18633	1.32709	2.1
18634	1.01051	0.549027
18635	0.958947	0.414947
18636	1.7211	2.1
18637	1.922 2.1	
18638	1.922 2.1	
18639	0.991113	2.1
18640	1.00636	0.804395
18641	1.31658	0.803197
18642	1.44627	0.758057
18643	1.03824	0.696954
18644	1.21223	2.1
18645	1.01242	2.1
18646	1.01243	2.1
18647	0.956698	1.63374
18648	1.23167	2.1
18649	1.06935	2.1
18650	1.29215	0.0862681
18651	0.998971	0.0936734
18652	1.50103	0.0911336
18653	1.44836	0.0957634
18654	3.02513	2.1
18655	3.7335	2.1
18656	2.49988	2.1
18657	1.24948	2.1
18658	0.963972	2.1
18659	1.1822	2.1
18660	1.1822	2.1
18661	1.10817	2.1
18662	1.30237	2.1
18663	1.41908	2.1
18664	1.41908	2.1
18665	1.48734	2.1
18666	1.31127	0.776055
18667	1.40822	0.760456
18668	1.40822	0.710519
18669	1.22176	0.707564
18670	1.82525	2.1
18671	1.59394	2.1
18672	1.22731	0.104343
18673	0.981584	0.0946017

18674	1.1427	0.779952
18675	1.44091	0.919396
18676	1.49154	0.8224
18677	1.07526	0.807319
18678	1.07527	0.837325
18679	0.96029	0.892465
18680	2.40568	1.15313
18681	3.46486	1.0452
18682	2.417 1.12503	
18683	1.52054	0.948942
18684	1.51638	0.692781
18685	1.04786	0.568403
18686	1.51404	0.610255
18687	1.3461	0.448522
18688	1.27696	0.400993
18689	0.736457	0.232305
18690	1.16817	2.1
18691	1.04263	2.1
18692	1.13449	2.1
18693	1.50524	2.1
18694	2.1182	2.1
18695	2.49001	2.1
18696	1.11764	2.1
18697	1.39292	2.1
18698	2.49001	2.1
18699	2.76339	2.1
18700	1.39292	2.1
18701	1.68761	2.1
18702	1.25173	2.1
18703	1.34701	2.1
18704	1.02415	2.1
18705	1.34701	2.06702
18706	1.332 0.117266	
18707	0.989764	0.0924884
18708	0.963167	0.0920676
18709	1.21056	0.0836211
18710	1.67375	2.1
18711	1.17965	2.1
18712	1.09154	2.1
18713	1.52314	2.1
18714	1.54433	0.754853
18715	1.81969	0.72652
18716	1.3598	0.726196
18717	1.71041	0.708533
18718	1.70934	0.707989
18719	1.33871	0.70316
18720	1.79485	0.703695
18721	1.51404	0.677373
18722	1.30751	2.1
18723	1.20969	2.1
18724	1.20969	2.1
18725	1.12076	2.1
18726	1.49808	2.1
18727	1.50201	2.1
18728	1.50201	2.1
18729	1.31594	2.1
18730	1.2187	2.1
18731	1.11545	2.1

18732	1.227	2.1
18733	1.09645	2.1
18734	1.05902	0.05
18735	1.41595	0.631258
18736	1.35696	2.1
18737	1.29446	0.893576
18738	0.857957	2.1
18739	1.21125	0.05
18740	2.43115	2.1
18741	2.1151	0.754889
18742	2.0815	1.12945
18743	1.1723	1.80825
18744	1.48035	0.704267
18745	1.59971	0.77342
18746	1.33668	0.681499
18747	1.0961	0.590303
18748	1.46235	2.1
18749	0.882195	2.1
18750	2.46624	2.1
18751	2.72161	2.1
18752	2.96094	2.1
18753	2.25644	2.1
18754	1.25923	0.115337
18755	0.976218	0.119865
18756	1.10319	0.0973427
18757	1.82583	0.0964039
18758	0.888955	2.1
18759	1.30817	2.1
18760	1.30817	2.1
18761	1.60787	2.1
18762	0.988805	1.85587
18763	1.54168	0.998847
18764	1.19496	0.932476
18765	1.12224	1.72227
18766	1.93597	1.25245
18767	1.31113	1.2171
18768	1.17879	1.02794
18769	1.07468	1.0148
18770	2.0523	2.1
18771	2.41385	2.1
18772	2.41385	2.1
18773	3.0355	2.1
18774	1.36717	0.732148
18775	1.45535	0.589885
18776	1.45535	0.556216
18777	1.49906	0.403756
18778	1.37785	1.07072
18779	1.04413	2.1
18780	1.06729	0.946551
18781	1.54305	0.889246
18782	1.07444	2.1
18783	1.52724	1.23253
18784	1.49151	2.1
18785	1.36814	2.1
18786	1.36814	2.1
18787	1.15541	2.1
18788	1.19379	2.1
18789	1.78657	0.889518

18790	1.6079	2.1
18791	2.59469	0.892382
18792	1.35545	0.810019
18793	1.23602	0.934889
18794	1.23452	2.1
18795	1.56842	0.995289
18796	1.15864	2.1
18797	1.11955	2.1
18798	1.11955	2.1
18799	1.08022	2.1
18800	1.64268	2.05375
18801	1.26247	2.1
18802	1.16912	2.1
18803	2.1095	0.995806
18804	1.75549	0.148239
18805	1.7491	0.182036
18806	1.59833	0.222004
18807	1.68338	0.237298
18808	1.14846	0.158791
18809	1.06515	0.158815
18810	1.2721	0.463512
18811	1.32639	0.389802
18812	1.32639	0.751287
18813	1.19323	0.551709
18814	1.81034	2.1
18815	1.9034	2.1
18816	2.08245	2.1
18817	1.84887	2.1
18818	1.41448	2.1
18819	1.77326	2.1
18820	1.76872	2.1
18821	1.86766	2.1
18822	1.46643	2.1
18823	1.58995	2.1
18824	1.58995	2.1
18825	1.13854	2.1
18826	1.23654	2.1
18827	1.04442	2.1
18828	1.23654	2.1
18829	1.08576	2.1
18830	0.966979	2.1
18831	1.41758	2.1
18832	1.03108	0.704276
18833	1.94412	0.71391
18834	1.32455	2.1
18835	1.32328	2.1
18836	1.32328	2.1
18837	1.34008	2.1
18838	1.60861	2.1
18839	1.69586	2.1
18840	1.69586	2.1
18841	1.78301	2.1
18842	1.7513	2.1
18843	1.67906	2.1
18844	1.67906	2.1
18845	1.60635	2.1
18846	1.68073	2.1
18847	1.57234	2.1

18848	1.57233	2.1
18849	1.47012	2.1
18850	1.22811	0.445804
18851	1.79683	0.625321
18852	1.53417	0.615716
18853	1.08732	0.662762
18854	1.8903	1.17617
18855	1.20247	1.16436
18856	1.12087	1.4128
18857	1.54699	1.79212
18858	1.43635	2.1
18859	1.02368	2.1
18860	1.43635	2.1
18861	1.23007	2.1
18862	1.035 2.1	
18863	1.07889	2.1
18864	3.0355	2.1
18865	3.78805	1.96416
18866	2.57183	1.35605
18867	1.27835	1.16758
18868	1.29813	0.460158
18869	1.42965	0.687005
18870	1.69832	0.593035
18871	2.18359	0.703466
18872	1.42964	0.50854
18873	1.65723	0.628547
18874	1.98508	0.591746
18875	2.06284	0.700342
18876	1.50705	0.7
18877	1.51153	0.7
18878	1.36037	0.7
18879	1.09767	0.700865
18880	1.70048	2.1
18881	1.15177	1.85716
18882	0.713174	2.1
18883	0.763575	2.1
18884	0.763575	2.1
18885	0.797011	2.1
18886	1.01179	0.794422
18887	1.27737	0.808934
18888	3.16531	0.29762
18889	1.0141	0.209991
18890	1.47354	0.601983
18891	1.62495	0.413644
18892	1.62495	0.667098
18893	1.39296	0.717186
18894	0.998682	2.1
18895	1.93528	2.1
18896	2.71383	2.1
18897	1.13987	2.1
18898	1.93528	2.1
18899	1.78595	2.1
18900	3.98933	2.1
18901	3.73426	2.1
18902	1.02438	2.1
18903	1.12376	2.1
18904	1.12375	2.1
18905	1.19016	2.1

18906	1.37157	0.334229
18907	1.60814	0.334745
18908	1.70303	0.456208
18909	1.01219	0.415646
18910	1.06757	1.86281
18911	1.23475	2.1
18912	1.2295	2.1
18913	1.28014	2.1
18914	1.28014	2.1
18915	1.33339	2.1
18916	1.3201	2.1
18917	1.2679	2.1
18918	1.2679	2.1
18919	1.23504	2.1
18920	1.50108	2.1
18921	1.3715	2.1
18922	1.3715	2.1
18923	1.25075	2.1
18924	1.99222	1.03263
18925	1.4155	1.00594
18926	1.4777	2.1
18927	1.52613	2.1
18928	1.91031	2.1
18929	1.03968	2.1
18930	2.04289	0.712505
18931	0.967192	0.636926
18932	1.03043	0.486181
18933	1.65452	0.731782
18934	1.21564	2.1
18935	1.16938	2.1
18936	2.47056	2.1
18937	2.56853	2.1
18938	1.16938	2.1
18939	1.1181	2.1
18940	2.56853	2.1
18941	2.66828	2.1
18942	1.0465	2.1
18943	1.44709	2.1
18944	1.44709	2.1
18945	1.96491	2.1
18946	1.01976	0.806369
18947	0.961779	0.755309
18948	1.11764	0.793706
18949	0.961779	0.756514
18950	1.21171	0.17454
18951	1.35007	0.266504
18952	1.03102	0.781371
18953	1.42446	0.781302
18954	1.42446	0.762666
18955	1.03166	0.745039
18956	1.2951	0.522661
18957	1.33575	0.589952
18958	1.33575	0.603608
18959	1.20473	0.566965
18960	1.10049	0.897625
18961	0.99747	0.897428
18962	1.54249	2.1
18963	1.65677	2.1

18964	1.65677	2.1
18965	1.77677	2.1
18966	1.73525	0.525433
18967	1.05689	0.718295
18968	1.26051	0.529917
18969	1.16259	0.389758
18970	1.34009	2.1
18971	1.398	2.1
18972	1.398	2.1
18973	1.50437	2.1
18974	2.3245	2.1
18975	1.18237	2.1
18976	1.24509	2.1
18977	1.01595	2.1
18978	3.29047	2.1
18979	2.79168	2.1
18980	1.06561	2.1
18981	1.11077	2.0305
18982	1.11077	1.1442
18983	0.953927	1.69532
18984	1.30509	0.678857
18985	1.04685	0.741894
18986	1.04685	0.543448
18987	0.834593	0.612908
18988	1.0775	0.74702
18989	1.27362	0.825896
18990	1.71762	0.914187
18991	2.5736	0.841426
18992	2.04717	0.936765
18993	1.45521	0.864156
18994	1.33962	2.1
18995	1.27956	2.1
18996	1.27956	2.1
18997	1.20478	2.1
18998	3.65841	0.156322
18999	1.18656	0.124074
19000	1.72272	0.216723
19001	1.6812	0.191069
19002	1.09951	2.1
19003	1.08231	2.1
19004	1.08231	2.1
19005	1.06478	2.1
19006	2.16196	1.48098
19007	1.00387	1.07734
19008	1.00387	1.03145
19009	0.993999	0.900685
19010	1.04101	2.1
19011	1.27119	2.1
19012	1.27119	2.1
19013	1.51748	2.1
19014	3.00129	2.1
19015	3.27303	2.1
19016	3.27303	2.1
19017	3.60855	2.1
19018	2.22399	2.1
19019	1.71995	2.1
19020	1.54793	2.1
19021	1.227	2.1

19022	1.07624	2.1
19023	1.264 2.1	
19024	1.19323	1.0882
19025	1.21286	1.00147
19026	1.21286	0.957952
19027	1.20859	0.921899
19028	1.60578	0.311316
19029	1.27778	0.218718
19030	1.0525	2.1
19031	1.05758	1.12217
19032	1.0525	0.946673
19033	1.17042	1.25892
19034	1.62536	0.0924842
19035	3.71662	0.0902665
19036	1.78169	0.266481
19037	1.91434	0.282622
19038	2.12331	0.32597
19039	1.33406	0.298343
19040	1.38437	2.1
19041	1.51829	2.1
19042	1.51829	2.1
19043	1.6697	2.1
19044	0.947114	2.1
19045	1.13335	2.1
19046	1.13335	2.1
19047	1.00965	2.1
19048	1.47521	2.1
19049	1.27679	2.1
19050	1.14911	2.1
19051	0.93841	2.1
19052	1.68437	2.1
19053	1.53822	2.1
19054	1.53822	2.1
19055	1.39702	2.1
19056	0.726453	2.1
19057	0.754101	2.1
19058	0.754101	2.1
19059	0.69776	2.1
19060	0.964033	2.1
19061	1.62397	1.93064
19062	1.70302	1.66655
19063	1.78637	1.43473
19064	3.68158	0.130335
19065	2.11685	0.126382
19066	1.33125	2.1
19067	1.15471	2.1
19068	1.0465	2.1
19069	1.33125	2.1
19070	1.17683	2.1
19071	1.25889	2.1
19072	1.25889	2.1
19073	1.34239	2.1
19074	1.40591	0.496366
19075	0.79348	0.409783
19076	1.04019	0.354588
19077	0.999187	0.338189
19078	1.30218	0.297485
19079	1.83339	0.34005

19080	1.88574	0.187409
19081	1.5177	0.162546
19082	1.23504	2.1
19083	1.19583	2.1
19084	1.19583	2.1
19085	1.14496	2.1
19086	1.62183	2.1
19087	1.62316	2.1
19088	1.62316	2.1
19089	1.62241	2.1
19090	1.02305	0.723871
19091	1.21136	0.63186
19092	0.993832	0.515497
19093	1.21137	0.591335
19094	1.12064	2.1
19095	1.24965	2.1
19096	1.24965	2.1
19097	1.41077	2.1
19098	1.00052	0.383008
19099	1.49842	0.287434
19100	1.49843	0.24678
19101	1.10801	0.175775
19102	1.54196	1.01101
19103	1.18041	1.17421
19104	1.73571	1.35391
19105	1.37224	1.67846
19106	1.4375	0.543843
19107	1.09529	0.519653
19108	1.44421	0.350499
19109	1.0953	0.438028
19110	1.0753	2.1
19111	0.815365	2.1
19112	0.815365	2.1
19113	0.597537	2.1
19114	1.03609	0.812863
19115	1.18952	0.813975
19116	1.54283	0.701676
19117	1.16719	0.709904
19118	1.16719	0.734576
19119	1.89226	0.726841
19120	2.55919	2.1
19121	2.37492	2.1
19122	2.37492	2.1
19123	2.07435	2.1
19124	1.36016	0.689227
19125	1.70302	0.692291
19126	1.85252	0.69458
19127	1.2111	0.714152
19128	1.62905	2.1
19129	1.49692	2.1
19130	1.49692	2.1
19131	1.40395	2.1
19132	2.19083	2.1
19133	1.30542	2.1
19134	1.58736	2.1
19135	2.16579	2.1
19136	2.0815	2.1
19137	1.17935	2.1

19138	1.53803	1.26665
19139	1.09148	0.933865
19140	1.81301	1.17809
19141	1.38333	0.87096
19142	1.04412	2.1
19143	1.44437	2.1
19144	1.4675	0.586277
19145	1.00665	0.639642
19146	1.50951	0.373356
19147	0.983105	0.317302
19148	1.80252	0.485606
19149	0.540005	0.335369
19150	1.2545	0.713196
19151	2.14514	0.674043
19152	1.32051	0.603879
19153	1.82302	0.694419
19154	2.71057	2.1
19155	2.51107	2.1
19156	1.34817	2.1
19157	1.3551	2.1
19158	2.51107	2.1
19159	2.29663	2.1
19160	1.3551	2.1
19161	1.3685	2.1
19162	1.51748	2.1
19163	1.23389	2.1
19164	0.901928	0.21845
19165	1.70302	0.311904
19166	1.66787	0.188794
19167	1.92535	0.336021
19168	1.13571	0.613183
19169	1.02836	0.667509
19170	1.55398	2.1
19171	1.54179	2.1
19172	1.89608	2.1
19173	1.07167	2.1
19174	3.3488	0.430648
19175	0.550044	0.344734
19176	0.962877	0.729357
19177	1.00065	0.754538
19178	1.00613	0.734061
19179	1.07497	0.741161
19180	1.00486	0.05
19181	1.00486	0.05
19182	0.726453	2.1
19183	1.32634	0.523603
19184	1.40008	0.631045
19185	1.40008	0.534442
19186	2.42267	0.425317
19187	1.9216	0.173115
19188	1.28066	0.123714
19189	1.79102	1.01053
19190	1.18219	0.705244
19191	1.18219	0.950649
19192	0.709123	0.73325
19193	1.07866	2.1
19194	1.15038	2.1
19195	1.15038	2.01494

19196	1.24687	2.1
19197	1.83613	1.78407
19198	1.99157	1.70889
19199	1.45789	0.130708
19200	1.1706	0.137653
19201	1.74746	0.0657811
19202	1.1706	0.0690221
19203	1.96383	0.176542
19204	1.67727	0.186711
19205	1.67727	0.15202
19206	1.60329	0.144527
19207	1.18803	2.1
19208	1.3227	2.1
19209	1.3227	2.1
19210	1.40844	2.1
19211	1.07889	2.1
19212	1.07721	2.1
19213	1.07721	2.1
19214	1.00832	2.1
19215	0.953523	0.96545
19216	1.62875	0.954532
19217	1.07427	0.787317
19218	1.19722	0.769877
19219	1.1972	0.73145
19220	1.1599	0.738931
19221	2.97463	0.078086
19222	1.9477	0.0698197
19223	1.95222	0.0594565
19224	1.75083	0.0675728
19225	3.46787	2.1
19226	3.02896	2.1
19227	3.02896	2.1
19228	2.46145	2.1
19229	0.970962	0.097991
19230	1.0227	0.0940453
19231	1.12328	0.0968346
19232	1.02269	0.11467
19233	0.904978	2.1
19234	0.725887	2.1
19235	0.725887	2.1
19236	0.601592	2.1
19237	0.956294	2.1
19238	1.13179	2.1
19239	0.956294	2.1
19240	1.13179	2.1
19241	1.05556	2.1
19242	1.13179	2.1
19243	1.38859	2.1
19244	1.13179	2.1
19245	1.06659	2.1
19246	1.05776	2.1
19247	1.06659	2.1
19248	1.04291	0.975243
19249	1.54699	1.01532
19250	1.42307	1.08311
19251	1.5654	1.39068
19252	1.97906	2.1
19253	1.15354	0.116985

19254	1.15505	0.15085
19255	1.26455	0.136071
19256	2.50042	0.162545
19257	2.50042	0.171411
19258	3.22585	0.193449
19259	1.19258	0.183754
19260	1.34562	0.145359
19261	1.49515	0.176545
19262	1.66295	0.197236
19263	1.02395	2.1
19264	1.51067	2.1
19265	1.35821	1.78214
19266	2.10671	2.1
19267	1.0305	1.3407
19268	1.07855	1.27508
19269	1.20761	2.1
19270	0.98488	2.1
19271	1.4162	2.1
19272	1.14825	2.1
19273	1.29415	2.1
19274	1.6067	2.1
19275	1.23851	0.42446
19276	1.09651	0.369318
19277	1.60636	2.1
19278	1.53521	2.1
19279	1.53521	2.1
19280	1.46279	2.1
19281	1.28044	0.58377
19282	1.64147	0.7
19283	1.18422	0.677258
19284	1.68208	0.7
19285	1.09726	2.1
19286	1.234	2.1
19287	1.234	2.1
19288	1.09483	2.1
19289	1.89318	2.1
19290	1.98448	2.1
19291	1.98448	2.1
19292	2.02612	2.1
19293	1.22026	1.0023
19294	1.23527	0.893332
19295	1.09044	0.974412
19296	0.993366	0.842585
19297	1.22026	0.971704
19298	0.993368	0.858827
19299	2.61357	2.1
19300	2.48208	2.1
19301	2.48208	2.1
19302	2.62868	2.1
19303	1.85439	0.993631
19304	1.81776	0.926216
19305	1.81776	0.878367
19306	2.1587	0.898582
19307	1.8914	0.958561
19308	2.23961	0.919323
19309	1.49357	0.706903
19310	2.23961	0.853946
19311	1.78014	0.827332

19312	1.67635	0.809115
19313	1.66219	0.787863
19314	1.56036	0.776261
19315	1.14721	0.721579
19316	2.22495	0.799252
19317	1.88509	0.7
19318	1.95249	0.495125
19319	2.11959	0.494047
19320	1.37427	0.346799
19321	1.39379	0.915392
19322	1.17614	0.885209
19323	1.39379	1.75959
19324	1.14397	0.596784
19325	1.09651	2.1
19326	1.42843	2.1
19327	1.7069	2.1
19328	2.31293	2.1
19329	1.56839	2.1
19330	1.78252	0.885691
19331	1.78254	0.84412
19332	1.09142	0.95545
19333	0.99829	0.210415
19334	1.09372	0.230731
19335	1.88175	0.58233
19336	0.898452	0.391517
19337	1.06936	2.1
19338	1.4162	2.1
19339	1.17989	2.1
19340	1.47411	1.38159
19341	1.25376	1.91384
19342	1.00757	0.932113
19343	1.4185	0.579121
19344	1.02011	0.670885
19345	2.22495	0.687366
19346	1.79542	0.66587
19347	1.79542	0.490991
19348	1.19352	0.524265
19349	2.38566	2.1
19350	1.83255	1.36551
19351	1.62667	2.1
19352	1.17879	1.53319
19353	2.79168	2.1
19354	2.28162	2.1
19355	2.28162	2.1
19356	1.90905	2.1
19357	1.40117	2.1
19358	1.28268	2.1
19359	1.41096	2.1
19360	1.22978	2.1
19361	3.20034	2.1
19362	3.42052	2.1
19363	3.42052	2.1
19364	3.50562	2.1
19365	1.46279	2.1
19366	1.3864	2.1
19367	1.3864	2.1
19368	1.3201	2.1
19369	1.35561	0.094163

19370	0.974077	0.093423
19371	0.967673	0.968797
19372	1.22482	0.89689
19373	1.02305	0.742152
19374	1.22482	0.818153
19375	0.999492	2.1
19376	1.14657	2.1
19377	1.14657	2.1
19378	1.31346	2.1
19379	1.84417	0.14867
19380	1.31777	0.1099
19381	1.19796	0.0925391
19382	0.691742	0.0813228
19383	1.16725	2.1
19384	1.9679	2.1
19385	1.08848	0.147316
19386	1.15356	0.134595
19387	1.58713	2.1
19388	1.58742	2.1
19389	1.58742	2.1
19390	1.49662	2.1
19391	1.32201	0.851297
19392	1.32097	1.05999
19393	1.32097	0.85457
19394	1.31825	1.0716
19395	1.12028	2.1
19396	1.15852	2.06509
19397	1.15852	2.1
19398	1.16239	1.52153
19399	1.07624	2.1
19400	1.30047	2.1
19401	1.44847	0.866705
19402	1.55292	0.8722
19403	1.21125	0.05
19404	1.15262	2.1
19405	1.05025	2.1
19406	1.55351	2.1
19407	1.5329	0.16433
19408	2.26758	0.119722
19409	1.94968	0.250443
19410	1.65747	0.240417
19411	1.42549	0.274284
19412	1.57656	0.294291
19413	2.11157	0.717955
19414	1.56531	0.745379
19415	1.69279	0.600375
19416	1.06204	0.753389
19417	0.947863	1.0816
19418	1.19854	1.34562
19419	0.947862	0.941258
19420	1.31126	0.863482
19421	1.3425	1.51664
19422	1.29976	1.22532
19423	1.05129	0.534131
19424	1.51026	0.562856
19425	1.05129	0.484625
19426	1.2951	0.515635
19427	1.7155	2.1

19428	1.72196	2.1
19429	1.72196	2.1
19430	1.77867	2.1
19431	0.964755	2.1
19432	1.14142	2.1
19433	1.03766	2.1
19434	0.986723	2.1
19435	1.28072	0.900815
19436	1.10609	1.05662
19437	1.28072	0.807774
19438	1.16776	1.72592
19439	1.61687	2.1
19440	1.60965	2.1
19441	1.60965	2.1
19442	1.59654	2.1
19443	1.26512	2.1
19444	1.23964	2.1
19445	2.07997	2.1
19446	2.60048	2.1
19447	1.23732	2.1
19448	1.3372	2.1
19449	2.62253	2.1
19450	2.47056	2.1
19451	0.92782	0.457362
19452	1.40335	0.565141
19453	1.26301	0.544413
19454	1.52668	0.527931
19455	0.99818	0.70191
19456	1.51165	0.703637
19457	1.66213	0.689613
19458	1.55246	0.702537
19459	2.31255	2.1
19460	1.34366	2.1
19461	1.90577	2.1
19462	0.969739	2.1
19463	3.4245	2.1
19464	2.8415	2.1
19465	1.97559	2.1
19466	1.82362	2.1
19467	1.48162	2.1
19468	1.23204	2.1
19469	1.23204	2.1
19470	0.95947	2.1
19471	1.05267	2.1
19472	1.44091	0.973087
19473	1.05267	1.19806
19474	1.5795	1.9623
19475	0.738711	2.1
19476	1.28549	2.1
19477	1.15694	2.1
19478	1.72061	2.1
19479	1.69858	2.1
19480	0.954563	2.1
19481	0.859087	2.1
19482	0.788886	2.1
19483	1.08732	0.489609
19484	1.3357	0.562515
19485	1.3357	0.47231

19486	1.48676	0.546359
19487	2.15079	2.1
19488	2.12414	2.1
19489	2.12414	2.1
19490	2.04664	2.1
19491	1.98859	2.1
19492	0.992159	2.1
19493	1.48064	2.1
19494	1.2163	2.1
19495	1.26337	2.1
19496	1.63159	2.1
19497	1.00366	0.785286
19498	1.65452	0.7588
19499	1.10408	2.1
19500	1.37704	2.1
19501	1.50224	2.1
19502	1.42866	2.1
19503	1.43939	2.1
19504	1.02784	2.1
19505	1.00763	0.494722
19506	1.23746	0.482086
19507	1.23747	0.482639
19508	1.13642	0.521568
19509	1.49906	0.403297
19510	1.46506	0.304216
19511	1.46506	0.34154
19512	1.33061	0.216379
19513	1.08166	0.41311
19514	1.01052	0.36301
19515	1.02836	0.418559
19516	1.08166	0.398203
19517	1.25334	0.252634
19518	1.23749	0.241674
19519	1.37501	0.347958
19520	1.47354	0.265635
19521	1.30167	2.1
19522	1.29897	2.1
19523	1.51042	2.1
19524	1.07684	2.1
19525	1.07684	2.1
19526	0.765121	2.1
19527	1.5535	0.979387
19528	1.40602	2.1
19529	1.40603	1.33509
19530	1.25669	2.1
19531	1.08276	2.1
19532	1.60948	2.1
19533	1.26929	1.53304
19534	1.09847	2.1
19535	0.827082	0.717731
19536	1.15841	0.717541
19537	1.25063	0.733228
19538	1.14397	0.662643
19539	1.25063	0.849812
19540	1.10476	0.219425
19541	1.14872	0.196563
19542	1.3659	0.242109
19543	1.36589	0.171535

19544	1.4677	0.25091
19545	0.590354	2.1
19546	0.655202	2.1
19547	3.48926	2.1
19548	3.32709	2.1
19549	3.32709	2.1
19550	3.13938	2.1
19551	1.24458	0.294545
19552	1.29681	0.573797
19553	1.40915	0.581786
19554	1.17891	0.769439
19555	1.33171	0.05
19556	1.37681	0.770299
19557	1.37681	2.1
19558	1.4516	2.1
19559	2.68241	0.232492
19560	1.61031	0.158141
19561	1.6488	2.1
19562	1.6585	2.1
19563	1.65848	2.1
19564	1.67061	2.1
19565	1.15152	2.1
19566	1.32548	2.1
19567	1.43677	2.1
19568	1.10264	2.1
19569	1.01472	2.1
19570	0.966964	2.1
19571	2.867	2.1
19572	2.94897	2.1
19573	1.65815	2.1
19574	1.4502	2.1
19575	1.4502	2.1
19576	1.26755	2.1
19577	1.37138	2.1
19578	1.45026	2.1
19579	1.45026	2.1
19580	1.53446	2.1
19581	0.973502	1.90996
19582	1.08542	1.74955
19583	1.13387	0.137747
19584	1.71031	0.11032
19585	1.08911	2.1
19586	1.10748	2.1
19587	1.21823	2.1
19588	1.25005	2.1
19589	0.657014	2.05445
19590	1.38139	2.1
19591	1.01485	2.1
19592	1.26045	2.1
19593	1.12468	2.1
19594	1.26045	2.1
19595	1.89278	0.902691
19596	1.06433	0.863522
19597	1.87861	0.853718
19598	0.967544	0.751458
19599	1.50319	2.1
19600	3.47245	1.84652
19601	2.2354	0.812999

19602	1.16701	0.727009
19603	1.3592	2.1
19604	1.3305	1.01862
19605	1.3305	1.55818
19606	1.15008	0.881924
19607	1.12457	0.192569
19608	1.40707	0.173843
19609	1.01778	2.1
19610	1.35733	2.1
19611	1.01779	2.1
19612	1.12064	2.1
19613	1.30694	0.830872
19614	1.42716	0.988062
19615	1.42717	1.08397
19616	1.04321	1.12067
19617	1.19352	0.408565
19618	1.32634	0.700589
19619	1.14605	2.01308
19620	1.0417	1.65341
19621	1.19676	0.702701
19622	1.03804	0.702031
19623	2.09455	0.7
19624	1.15338	0.7
19625	2.21119	2.1
19626	1.26097	2.1
19627	1.88394	0.86727
19628	1.24106	0.495734
19629	2.98143	2.1
19630	2.56248	1.18696
19631	1.88394	1.3068
19632	1.68703	0.377602
19633	1.5846	2.1
19634	1.94799	2.1
19635	1.20091	2.1
19636	1.40393	2.1
19637	3.09031	2.1
19638	2.90243	2.1
19639	1.27227	2.1
19640	1.32592	2.1
19641	2.90247	2.1
19642	2.71057	2.1
19643	1.32594	2.1
19644	1.34817	2.1
19645	1.4092	2.1
19646	1.44859	2.1
19647	1.52008	2.1
19648	1.401	2.1
19649	1.40099	2.1
19650	1.28936	2.1
19651	1.56002	0.0597258
19652	1.08811	0.0525147
19653	1.46041	0.0594664
19654	1.56002	0.052082
19655	1.50524	2.1
19656	1.21304	2.1
19657	1.21304	2.1
19658	0.947115	2.1
19659	1.60665	2.03796

19660	1.85179	1.63405
19661	1.85178	1.6623
19662	2.06277	1.12875
19663	2.17534	2.1
19664	1.23007	2.1
19665	2.17533	2.1
19666	1.66635	1.90563
19667	2.00869	2.1
19668	0.986724	2.1
19669	2.00475	2.1
19670	1.99394	2.1
19671	1.67244	0.573813
19672	1.39604	0.516335
19673	1.39604	0.554818
19674	0.954046	0.394966
19675	0.988863	2.1
19676	1.9864	2.1
19677	1.9864	2.1
19678	3.38645	2.1
19679	1.46932	0.107069
19680	1.50847	0.113665
19681	1.50848	0.102193
19682	1.42705	0.110013
19683	1.50108	2.1
19684	1.51621	0.848258
19685	1.94317	0.869579
19686	1.18382	0.762475
19687	2.05091	0.149757
19688	1.88593	0.110247
19689	1.54299	0.0897007
19690	0.987265	0.0792755
19691	1.27766	2.1
19692	1.5178	2.1
19693	1.5178	2.1
19694	1.81518	2.1
19695	1.04626	1.23455
19696	1.31057	1.25248
19697	1.31057	1.2384
19698	1.22297	1.24497
19699	1.67844	1.14305
19700	1.62862	2.1
19701	1.62112	1.12548
19702	1.87598	2.1
19703	1.17844	2.1
19704	1.37063	2.1
19705	1.37063	2.1
19706	1.68723	2.1
19707	1.99943	0.215754
19708	1.17631	0.143668
19709	1.4263	1.07777
19710	1.31023	2.1
19711	1.31023	1.11477
19712	1.20871	2.1
19713	1.50356	2.1
19714	1.69311	2.1
19715	1.69599	2.1
19716	1.6801	2.1
19717	1.5843	0.0792902

19718	0.840233	0.0917996
19719	1.95358	0.0859591
19720	1.40216	0.0983009
19721	1.39078	2.1
19722	1.18948	1.99841
19723	1.01606	1.52704
19724	1.18948	1.91399
19725	1.63823	2.1
19726	1.67687	2.1
19727	1.67687	2.1
19728	1.52855	2.1
19729	1.11048	2.1
19730	1.25346	2.1
19731	2.42984	1.65716
19732	3.41731	0.918038
19733	1.2258	2.1
19734	1.2966	2.1
19735	1.2966	2.1
19736	1.37392	2.1
19737	1.41758	2.1
19738	1.35504	2.1
19739	1.35504	2.1
19740	1.30347	2.1
19741	1.50877	2.1
19742	1.11193	2.1
19743	1.40984	2.1
19744	1.54537	1.8143
19745	1.71638	2.1
19746	1.77795	1.44131
19747	2.07593	2.1
19748	1.93387	2.1
19749	1.9339	2.1
19750	1.75669	2.1
19751	1.80299	0.925429
19752	1.31251	0.894849
19753	1.31249	0.892372
19754	1.13411	0.845903
19755	1.06313	2.1
19756	1.04921	2.1
19757	1.55766	2.1
19758	1.53728	2.1
19759	1.53729	2.1
19760	1.51789	2.1
19761	0.993021	0.847086
19762	1.02478	0.860022
19763	1.03408	0.852494
19764	1.09056	0.854876
19765	0.697755	2.1
19766	0.632901	2.1
19767	1.34072	0.531862
19768	1.80841	0.702714
19769	1.80841	0.694645
19770	1.17793	0.702759
19771	1.75032	1.68358
19772	1.13076	2.1
19773	2.11851	2.1
19774	1.73255	2.07531
19775	1.73255	2.1

19776	1.11181	2.06393
19777	1.43772	2.1
19778	1.67381	2.1
19779	1.52164	2.1
19780	1.60861	2.1
19781	1.30588	0.381414
19782	1.75241	0.403331
19783	1.33026	1.05746
19784	0.962705	0.989858
19785	0.99678	0.177821
19786	1.30572	0.125665
19787	1.41943	0.192574
19788	1.2683	0.179254
19789	1.14145	0.129576
19790	1.01361	0.121009
19791	1.29943	2.1
19792	1.3483	2.1
19793	1.34832	2.1
19794	1.83488	2.1
19795	1.71065	2.1
19796	1.66178	2.1
19797	1.39076	2.1
19798	1.37456	2.1
19799	1.66126	2.1
19800	1.562	2.1
19801	1.37456	2.1
19802	1.36065	2.1
19803	1.04026	0.739231
19804	1.51898	0.734642
19805	1.67088	0.70822
19806	1.9726	0.704698
19807	1.74852	1.5391
19808	1.95512	1.63139
19809	1.16165	1.02663
19810	1.06584	0.929117
19811	1.39327	2.1
19812	1.41394	2.1
19813	1.41394	2.1
19814	1.45506	2.1
19815	1.39523	2.1
19816	1.21119	2.1
19817	1.09005	2.1
19818	0.921311	2.1
19819	1.58164	2.1
19820	1.77574	2.1
19821	1.73184	2.1
19822	1.59423	2.1
19823	1.8214	2.0935
19824	3.10544	0.979115
19825	1.01243	1.65356
19826	1.30896	0.998886
19827	2.16956	1.05138
19828	1.29608	0.892115
19829	1.38617	1.97535
19830	1.49536	1.65249
19831	2.35239	1.3512
19832	1.24613	1.53707
19833	1.94351	2.1

19834	2.05361	2.1
19835	2.05358	2.1
19836	2.38994	2.1
19837	1.13533	2.1
19838	1.08276	2.1
19839	1.55611	0.302704
19840	1.33957	0.270711
19841	1.33957	0.41318
19842	1.69011	0.409589
19843	1.5515	0.895828
19844	1.59106	0.900453
19845	1.02958	2.1
19846	1.12266	2.1
19847	1.12266	2.1
19848	1.24925	2.1
19849	1.80223	2.1
19850	1.20242	1.17585
19851	2.13665	0.78228
19852	1.3924	0.782753
19853	0.904894	1.04375
19854	1.82633	2.1
19855	1.94138	0.0996424
19856	1.34009	0.0819378
19857	1.15096	2.1
19858	0.994292	2.1
19859	1.10101	2.1
19860	1.15096	2.1
19861	1.50438	2.1
19862	1.67825	2.1
19863	1.67825	2.1
19864	1.91894	2.1
19865	2.04664	2.1
19866	1.92063	2.1
19867	1.72857	2.1
19868	1.89757	2.1
19869	1.61843	0.128403
19870	1.49431	0.125005
19871	2.33925	2.1
19872	1.80683	2.1
19873	1.64257	2.1
19874	1.50108	2.1
19875	2.82561	2.1
19876	2.97847	2.1
19877	2.97848	2.1
19878	3.20034	2.1
19879	0.66055	2.1
19880	1.95919	2.1
19881	1.07976	2.1
19882	1.30237	2.1
19883	1.99943	0.140096
19884	1.31802	0.143848
19885	1.53336	0.248002
19886	1.31802	0.150657
19887	1.27737	0.825305
19888	1.44848	0.840528
19889	2.35499	0.170417
19890	1.30041	0.186594
19891	2.35499	0.127123

19892	1.65381	0.0856399
19893	0.996083	2.1
19894	1.28754	2.1
19895	0.996081	2.1
19896	1.44859	2.1
19897	1.24763	1.9855
19898	1.03697	2.1
19899	0.951273	1.69457
19900	1.60018	1.23267
19901	2.0815	2.07825
19902	1.20488	2.1
19903	2.95785	2.1
19904	2.52245	1.75455
19905	1.68455	0.14073
19906	1.12971	0.117235
19907	1.1952	0.719187
19908	2.0815	0.73983
19909	1.3963	2.1
19910	1.4934	2.1
19911	1.47973	2.1
19912	1.25559	2.1
19913	1.1233	2.1
19914	1.15009	2.1
19915	1.15009	2.1
19916	0.977775	2.1
19917	1.16482	0.964072
19918	1.29313	0.768424
19919	1.29313	0.813815
19920	1.294	0.767493
19921	1.48693	0.708155
19922	1.47608	0.687537
19923	1.36005	1.76464
19924	1.47109	2.1
19925	1.47109	2.1
19926	1.3515	2.1
19927	2.48828	2.1
19928	2.32378	2.1
19929	2.32378	2.1
19930	2.1692	2.1
19931	1.03217	1.06544
19932	0.953521	1.10869
19933	1.4515	0.189449
19934	3.72406	0.176311
19935	0.963395	1.59447
19936	1.01346	1.09321
19937	1.62241	2.1
19938	1.62027	2.1
19939	1.62027	2.1
19940	1.61687	2.1
19941	1.2593	0.0755724
19942	0.981586	0.0813749
19943	0.721961	2.1
19944	0.888955	2.1
19945	1.22031	0.05
19946	1.27566	2.1
19947	2.50066	2.1
19948	1.19814	2.1
19949	1.42278	0.098485

19950	2.02657	0.0853523
19951	1.0756	0.906688
19952	1.10049	0.901691
19953	2.26155	2.1
19954	2.15634	2.1
19955	2.15634	2.1
19956	2.01613	2.1
19957	1.94233	0.92878
19958	1.18127	1.35175
19959	1.36865	1.12556
19960	1.75755	1.23565
19961	2.01613	2.1
19962	1.78958	2.1
19963	1.97995	2.1
19964	1.79198	2.1
19965	1.20841	2.1
19966	1.6519	2.1
19967	1.80207	2.1
19968	1.66774	2.1
19969	1.17935	0.211044
19970	1.88071	0.374291
19971	1.58723	0.28921
19972	1.7361	0.282367
19973	0.794165	2.1
19974	1.21327	2.1
19975	1.21327	2.1
19976	1.34268	2.1
19977	1.34497	0.0909107
19978	0.793303	0.0839448
19979	1.12323	0.0803747
19980	1.34496	0.0745456
19981	0.928226	0.7
19982	1.89804	0.702191
19983	2.60141	0.05
19984	2.60142	2.1
19985	2.60772	0.822232
19986	0.99747	2.03668
19987	1.27096	0.921006
19988	1.27096	0.849028
19989	1.03472	0.919491
19990	1.09962	0.831224
19991	1.21148	1.33019
19992	1.21148	0.951133
19993	1.35025	2.1
19994	1.09795	2.06909
19995	1.00775	2.1
19996	1.07162	2.1
19997	1.60665	2.01501
19998	1.52984	2.1
19999	1.61179	2.1
20000	1.70057	2.1
20001	1.76063	2.1
20002	1.14547	0.787929
20003	0.964782	0.735477
20004	1.17208	0.740468
20005	0.964769	0.668834
20006	1.04268	2.1
20007	1.57459	2.1

20008	1.57459	2.1
20009	1.32449	2.1
20010	2.07527	0.942666
20011	1.00013	0.896285
20012	0.813711	0.05
20013	1.05677	2.07934
20014	1.30664	1.31909
20015	1.96011	1.94187
20016	1.7151	1.1206
20017	1.61309	1.20527
20018	1.18954	0.821615
20019	1.05447	2.1
20020	1.05446	0.731375
20021	1.11787	0.723696
20022	1.44437	2.1
20023	1.84739	2.1
20024	1.84739	2.03405
20025	1.06203	2.1
20026	1.78391	2.1
20027	1.89267	2.1
20028	1.9212	2.1
20029	2.02997	2.1
20030	1.32051	0.735554
20031	1.47169	0.708409
20032	1.20691	0.148276
20033	1.72271	0.14409
20034	1.2858	0.756197
20035	1.31011	0.836527
20036	1.31011	0.733891
20037	1.34124	0.830364
20038	1.294	0.833514
20039	1.03079	0.767209
20040	1.83192	2.1
20041	1.81979	2.1
20042	1.81979	2.1
20043	1.57829	2.1
20044	1.35435	1.94119
20045	1.19023	2.08179
20046	0.991115	2.1
20047	1.27725	2.1
20048	1.38204	2.1
20049	1.48013	2.1
20050	0.983493	2.1
20051	2.26155	2.1
20052	1.02311	0.897838
20053	1.55485	0.848473
20054	1.71033	0.84817
20055	2.07319	0.800254
20056	2.29074	0.830564
20057	1.50822	0.927681
20058	2.08147	1.95435
20059	1.1585	2.1
20060	3.74092	1.03135
20061	2.40799	2.1
20062	2.05349	1.62381
20063	1.92656	2.1
20064	1.09634	0.633668
20065	1.45502	0.661212

20066	1.17261	0.515143
20067	1.29383	0.428068
20068	1.29383	0.618877
20069	1.47689	0.527924
20070	1.85717	0.82142
20071	1.53798	0.756572
20072	1.9493	2.1
20073	2.0371	1.30439
20074	1.8519	1.69982
20075	1.92119	1.15066
20076	1.00764	0.302229
20077	1.0302	0.293213
20078	1.0302	0.238329
20079	0.90047	0.263768
20080	1.49155	0.56492
20081	2.26403	0.295983
20082	2.06158	0.492693
20083	1.09506	0.454261
20084	1.9212	1.17614
20085	1.99586	1.02977
20086	1.99586	1.09027
20087	2.07527	0.992107
20088	1.20478	2.1
20089	1.09662	2.1
20090	1.55961	2.1
20091	1.03472	2.1
20092	1.54983	2.1
20093	2.02953	2.1
20094	1.84502	2.1
20095	2.1221	2.1
20096	1.02969	1.62698
20097	2.23523	2.08327
20098	1.2377	0.7
20099	1.03818	0.708945
20100	1.978 2.1	
20101	1.78159	2.1
20102	1.45762	2.1
20103	1.34234	2.1
20104	2.01624	2.1
20105	2.52072	2.1
20106	1.23013	2.1
20107	2.5593	2.1
20108	3.06464	1.25967
20109	2.63697	0.874977
20110	2.63697	1.05024
20111	1.87111	0.828777
20112	1.04574	0.413387
20113	2.13428	0.334519
20114	1.61715	2.1
20115	1.44137	2.1
20116	1.44867	2.1
20117	1.18283	2.1
20118	1.50584	0.494254
20119	1.45466	0.501227
20120	1.4959	0.591109
20121	1.93489	0.600302
20122	2.60772	2.1
20123	2.68868	2.1

20124	2.68868	2.1
20125	2.82561	2.1
20126	1.49654	2.1
20127	1.38672	2.1
20128	1.36813	2.1
20129	1.98396	2.1
20130	1.93852	1.26084
20131	2.14237	0.92896
20132	1.44421	0.276122
20133	1.21749	0.212322
20134	1.09572	0.246269
20135	1.11081	0.136395
20136	2.32363	2.1
20137	1.24375	2.1
20138	3.79242	2.1
20139	3.46787	2.1
20140	1.19854	1.48298
20141	1.35487	1.4604
20142	1.35486	1.68434
20143	1.38616	1.63041
20144	1.67265	0.710927
20145	0.909487	0.702811
20146	2.53562	2.1
20147	1.09564	2.1
20148	2.9068	2.1
20149	3.57307	0.847238
20150	2.50369	1.14249
20151	1.47348	2.1
20152	1.24925	2.1
20153	1.3991	2.1
20154	1.3991	2.1
20155	1.52984	2.1
20156	1.60428	2.1
20157	1.50097	2.1
20158	1.50097	2.1
20159	1.40239	2.1
20160	2.19621	2.1
20161	2.35003	2.1
20162	1.57932	0.220111
20163	1.61194	0.125452
20164	2.0815	0.111292
20165	1.18539	0.094002
20166	1.5795	0.926882
20167	1.53064	1.04191
20168	1.89227	0.061944
20169	3.01037	0.0568225
20170	2.08149	1.56687
20171	2.06678	1.4814
20172	1.89226	0.101643
20173	3.47888	0.117524
20174	1.89226	0.322542
20175	3.65842	0.19825
20176	3.88546	0.426855
20177	1.89226	0.724415
20178	1.11239	2.1
20179	1.29133	0.16474
20180	0.889309	0.134851
20181	0.881934	0.197743

20182	1.88406	2.1
20183	0.636946	2.0729
20184	0.636945	1.50691
20185	1.28073	2.1
20186	1.28075	2.1
20187	0.942007	2.1
20188	1.68436	2.1
20189	1.4677	0.123362
20190	0.851542	0.315775
20191	1.39063	0.214346
20192	0.851539	0.257335
20193	1.11597	0.291839
20194	1.35403	0.21773
20195	0.959473	2.1
20196	1.07976	2.1
20197	0.946129	1.00183
20198	0.946129	0.738554
20199	1.38464	0.876694
20200	1.36991	0.795728
20201	0.946131	0.817146
20202	0.94613	0.790147
20203	1.34493	0.718932
20204	1.36991	0.73108
20205	1.36991	0.666028
20206	0.946131	0.706562
20207	0.78392	0.760204
20208	0.80282	0.763657
20209	1.10351	0.731324
20210	0.80846	0.824249
20211	0.838026	0.779966
20212	1.08558	0.782535
20213	0.718203	0.82555
20214	0.501866	0.785797
20215	0.85426	0.829454
20216	1.24249	0.433262
20217	1.37052	0.611768
20218	0.851541	0.601544
20219	1.0092	0.391328
20220	0.851554	0.575702
20221	1.49826	0.278682
20222	1.13727	0.184773
20223	0.778149	0.535662
20224	1.20257	0.823351
20225	0.946133	0.835318
20226	0.778149	0.512906
20227	0.77815	0.677884
20228	0.946132	2.1
20229	1.17602	2.1
20230	0.946132	2.1
20231	1.31257	2.1
20232	1.17602	2.1
20233	1.35821	0.691055
20234	1.19993	0.633247
20235	0.946131	0.620176
20236	0.946131	0.676416
20237	1.08848	0.610913
20238	1.00509	2.1
20239	1.19629	2.1

20240	0.867593	2.1
20241	0.757921	2.1
20242	0.729116	0.165439
20243	0.89162	0.193243
20244	1.16811	0.144804
20245	1.3676	2.1
20246	1.2638	1.06414
20247	0.946132	2.1
20248	0.946132	2.1
20249	1.2638	2.1
20250	1.00185	2.1
20251	1.60948	2.1
20252	0.602075	2.1
20253	0.942611	2.1
20254	0.946132	2.1
20255	0.942611	2.1
20256	1.11822	2.1
20257	1.21558	0.708768
20258	0.607788	0.760471
20259	0.827029	0.749784
20260	1.00691	0.888022
20261	0.607789	0.748189
20262	0.607775	0.825533
20263	1.23248	1.06368
20264	0.902653	1.21729
20265	0.639143	1.23918
20266	0.851545	1.10914
20267	0.605153	1.10359
20268	1.06826	2.1
20269	1.29365	2.1
20270	1.41232	2.1
20271	0.946131	2.1
20272	1.20854	2.1
20273	0.946131	2.1
20274	0.909005	0.152804
20275	1.16672	0.136023
20276	0.810959	0.150385
20277	0.946131	0.369164
20278	1.40104	0.253752
20279	0.946131	0.320779
20280	1.35403	0.322353
20281	1.40104	0.250787
20282	1.92535	0.184569
20283	2.14534	0.168638
20284	1.77532	0.330512
20285	0.851541	0.501246
20286	1.05585	0.600347
20287	1.27098	0.437408
20288	0.870925	0.544776
20289	1.15547	0.531686
20290	1.47583	0.436829
20291	1.35007	0.186251
20292	0.740095	0.0920176
20293	0.644912	0.0950515
20294	0.819489	0.0888158
20295	0.983028	0.708369
20296	0.861849	0.0951765
20297	1.40511	0.103

20298	0.86182	0.123525
20299	0.9705	0.102563
20300	1.40511	0.0974717
20301	1.15539	2.1
20302	0.946119	1.93034
20303	0.736382	2.1
20304	0.736384	2.1
20305	1.41561	2.1
20306	0.785651	2.1
20307	1.34479	2.1
20308	1.38885	2.1
20309	0.94613	2.1
20310	1.29461	2.1
20311	0.94613	2.1
20312	0.776179	2.00977
20313	0.858611	2.1
20314	0.946128	1.98245
20315	1.04073	2.1
20316	1.16399	2.1
20317	0.990019	2.1
20318	1.15564	1.9856
20319	1.28956	0.516431
20320	1.35145	0.547795
20321	1.09662	2.1
20322	1.8155	0.0707105
20323	0.811459	0.0709384
20324	0.731982	0.0607031
20325	0.900554	0.0769252
20326	0.946127	0.974827
20327	0.817066	0.990418
20328	0.817064	0.977586
20329	1.41798	1.01109
20330	0.871606	1.00517
20331	1.30265	0.332353
20332	0.849402	1.75972
20333	0.868582	1.41445
20334	0.909137	1.53371
20335	0.807016	1.44234
20336	0.83393	0.999289
20337	0.9034	1.2208
20338	1.53515	1.23008
20339	1.17665	0.776405
20340	0.946132	0.841466
20341	0.946129	0.764763
20342	1.35977	0.724879
20343	1.34254	0.710774
20344	1.14241	0.631799
20345	0.946132	0.772452
20346	0.946132	0.606156
20347	1.27376	0.568686
20348	1.35978	0.542475
20349	1.01976	0.51711
20350	1.23498	1.10445
20351	1.35002	0.993179
20352	1.11683	0.981663
20353	0.88359	0.716817
20354	0.751374	0.748968
20355	0.791714	0.739077

20356	1.6067	2.1
20357	0.923782	2.1
20358	1.44689	2.1
20359	0.94613	2.1
20360	0.946131	2.1
20361	1.5261	2.1
20362	1.26247	2.1
20363	1.80085	0.17439
20364	1.7693	0.139344
20365	0.900422	0.301549
20366	1.31357	0.492056
20367	0.94613	0.433418
20368	0.900424	0.406804
20369	0.900397	0.458044
20370	1.39448	0.437581
20371	1.36769	0.122761
20372	0.713524	0.310151
20373	0.713554	0.161751
20374	0.946124	0.234474
20375	1.24979	0.171769
20376	1.04765	0.266619
20377	0.70054	0.0930852
20378	0.776991	0.112531
20379	0.738905	0.112338
20380	1.32605	0.0928902
20381	0.784957	0.0891725
20382	0.803955	0.108167
20383	0.819486	0.0978137
20384	1.79306	0.296743
20385	1.18275	0.371854
20386	1.25626	0.291849
20387	0.936806	0.217244
20388	0.653124	0.390681
20389	0.946132	0.351329
20390	0.653151	0.401367
20391	1.10748	0.431547
20392	1.95244	2.1
20393	1.40967	1.84943
20394	1.27916	1.72271
20395	0.946132	0.834758
20396	0.946132	0.985293
20397	1.30555	1.02388
20398	1.38651	1.03343
20399	0.946132	0.948893
20400	1.23571	1.03393
20401	1.21261	1.35041
20402	1.34225	1.20319
20403	0.946132	1.46545
20404	0.734059	0.946841
20405	0.734084	1.16489
20406	0.78438	0.798347
20407	1.13748	0.932757
20408	0.946131	1.04175
20409	0.78438	0.799546
20410	0.784392	0.901877
20411	0.626495	2.1
20412	0.694635	2.1
20413	0.946125	2.1

20414	0.694632	2.1
20415	1.38521	2.1
20416	0.795469	2.1
20417	0.850877	2.1
20418	1.37098	2.1
20419	0.946131	2.1
20420	0.79547	2.1
20421	0.946132	0.714878
20422	1.30448	0.730938
20423	0.628361	0.715914
20424	0.686964	0.700111
20425	0.493261	0.697775
20426	1.90912	1.25296
20427	0.946127	0.875851
20428	1.36604	0.699092
20429	0.671687	0.708702
20430	0.946128	0.775934
20431	0.671699	0.708827
20432	1.04973	2.1
20433	0.982858	0.174268
20434	0.974081	0.0650619
20435	1.69993	0.0621384
20436	1.00763	0.795526
20437	0.755566	2.1
20438	0.946129	2.1
20439	1.04184	2.1
20440	1.13509	2.1
20441	0.755566	2.1
20442	1.114 2.1	
20443	0.810946	2.1
20444	0.786259	2.1
20445	1.43455	2.1
20446	1.05071	2.1
20447	0.746095	2.1
20448	0.82737	2.1
20449	0.946126	2.09736
20450	1.04071	2.1
20451	1.0852	2.1
20452	1.59019	0.104696
20453	0.621972	0.168067
20454	1.14475	0.168996
20455	0.691062	0.175875
20456	1.20057	0.13595
20457	0.846868	0.158893
20458	1.26062	0.108277
20459	0.795093	0.139707
20460	1.20056	0.135454
20461	0.795065	0.150979
20462	1.18203	0.114733
20463	1.20057	0.132327
20464	0.991869	0.656301
20465	0.841088	2.1
20466	0.946131	2.1
20467	0.869097	2.1
20468	0.946131	2.1
20469	0.841089	2.1
20470	1.77867	2.1
20471	1.99296	0.401046

20472	1.91703	2.1
20473	0.651334	0.360867
20474	1.31467	0.372407
20475	0.651332	0.306704
20476	0.651332	0.339164
20477	1.03079	0.373807
20478	0.808519	0.197808
20479	1.41417	0.157126
20480	1.03165	0.553329
20481	1.43577	2.1
20482	1.39053	2.1
20483	1.07006	2.1
20484	0.946132	2.1
20485	0.946133	2.1
20486	1.07006	2.1
20487	1.12717	2.1
20488	0.994293	2.1
20489	0.946132	2.1
20490	1.31912	2.1
20491	0.946131	2.1
20492	1.40571	2.1
20493	1.31912	2.1
20494	1.12292	0.77246
20495	1.07755	0.815667
20496	0.851539	0.675551
20497	0.946131	0.560767
20498	0.929834	0.616515
20499	1.83919	0.864649
20500	1.04713	0.704062
20501	0.929726	0.588873
20502	1.50985	0.498931
20503	0.946127	0.699562
20504	1.0227	0.624455
20505	1.2582	0.544119
20506	1.1391	0.872355
20507	0.780831	0.965815
20508	0.78086	0.890731
20509	0.946133	0.951235
20510	0.780831	0.885277
20511	1.37762	0.945203
20512	0.946116	2.1
20513	1.22882	2.1
20514	1.40015	2.07646
20515	1.40017	2.1
20516	0.946105	2.1
20517	0.553567	0.0975116
20518	1.19263	0.0778627
20519	0.946132	0.0863959
20520	0.553594	0.0830668
20521	1.19263	0.0958968
20522	1.18237	0.0828605
20523	0.946133	0.0722904
20524	0.978467	0.0693797
20525	0.56262	0.0724515
20526	0.946132	0.0708781
20527	0.562633	0.0675391
20528	0.900175	0.0695037
20529	1.04627	1.16619

20530	0.549928	0.846568
20531	1.17765	0.946773
20532	1.11479	0.953044
20533	0.398261	0.91334
20534	0.927608	0.972182
20535	1.38467	0.827468
20536	1.04824	0.789773
20537	1.33893	0.948665
20538	1.55775	0.84004
20539	0.816895	0.842323
20540	1.96057	0.960372
20541	1.35798	2.1
20542	1.29262	2.1
20543	0.94613	2.1
20544	0.94613	2.1
20545	1.29261	2.1
20546	1.18067	2.1
20547	0.946131	2.1
20548	0.946132	2.1
20549	1.27809	2.1
20550	1.35799	2.1
20551	1.03933	2.1
20552	1.49438	2.1
20553	0.946132	2.1
20554	1.13537	2.1
20555	1.41666	2.1
20556	1.74237	2.1
20557	0.946132	2.1
20558	1.04291	0.759342
20559	1.63802	2.1
20560	1.24714	1.57491
20561	0.842013	2.1
20562	0.810959	2.1
20563	0.767923	0.703689
20564	1.19583	0.702996
20565	0.767921	0.701619
20566	0.767921	0.7
20567	0.961348	0.703457
20568	2.08687	1.42185
20569	0.739729	0.816834
20570	1.04342	0.797936
20571	0.739757	0.800876
20572	0.946129	0.824146
20573	1.04341	0.924379
20574	1.38683	0.722787
20575	1.34153	0.715878
20576	0.946129	0.64166
20577	0.946131	0.619396
20578	1.02028	0.561409
20579	1.47606	0.631179
20580	1.20739	0.711254
20581	0.957684	0.660353
20582	0.946116	0.715509
20583	0.851528	0.676343
20584	0.957685	0.715571
20585	1.04504	2.1
20586	0.902938	2.1
20587	0.946132	2.1

20588	1.07566	2.1
20589	1.4558	2.1
20590	1.93286	2.1
20591	1.27241	2.1
20592	0.828671	2.1
20593	0.810933	2.1
20594	1.31335	0.100369
20595	1.40305	0.0874924
20596	0.912698	0.131522
20597	0.810946	0.13449
20598	1.11608	0.126134
20599	1.26409	0.737794
20600	0.946132	0.741626
20601	1.27376	0.753101
20602	0.946132	0.741146
20603	1.03333	0.753355
20604	1.27376	0.75051
20605	0.94613	0.735128
20606	0.812384	0.730731
20607	0.687744	0.653789
20608	0.946132	0.73764
20609	0.687758	0.730222
20610	1.09667	0.681895
20611	1.56977	2.1
20612	0.851544	2.1
20613	1.01136	2.1
20614	0.851481	2.00766
20615	0.946131	2.1
20616	0.705409	2.1
20617	1.32449	0.240325
20618	1.13508	0.192217
20619	0.94613	0.330884
20620	0.94613	0.298431
20621	0.965822	0.31779
20622	1.95301	0.466333
20623	0.851538	0.761922
20624	1.29262	0.745675
20625	0.95417	0.721567
20626	0.851541	0.770534
20627	0.94613	0.747572
20628	1.02727	0.869615
20629	1.37675	1.06247
20630	0.955646	0.83094
20631	0.95114	0.848575
20632	0.851528	0.855625
20633	1.76513	1.19131
20634	0.87155	2.1
20635	0.94613	2.1
20636	0.871519	2.1
20637	0.966631	2.1
20638	1.37791	2.1
20639	1.19122	0.947257
20640	0.818971	1.27532
20641	0.628799	0.877401
20642	0.861027	1.12907
20643	0.800034	0.169276
20644	0.91846	0.160831
20645	0.765645	0.165397

20646	1.13709	0.94316
20647	1.29054	0.972196
20648	0.755336	1.02736
20649	0.755334	0.977178
20650	0.946133	1.05418
20651	0.755336	0.902701
20652	1.14444	0.976419
20653	0.872823	0.977945
20654	0.533645	0.953452
20655	0.946132	0.916763
20656	0.533645	0.83324
20657	1.02311	0.90679
20658	1.00278	2.1
20659	1.1016	0.122001
20660	0.629615	0.138788
20661	1.12628	0.206558
20662	0.946131	0.187942
20663	0.629615	0.13624
20664	0.629641	0.174502
20665	1.67046	0.968317
20666	1.81667	2.1
20667	1.7145	2.1
20668	0.94613	2.1
20669	1.29322	2.1
20670	1.00365	2.1
20671	0.946131	2.1
20672	1.29322	2.1
20673	1.27581	2.1
20674	1.33916	2.1
20675	1.32784	2.1
20676	1.74787	2.1
20677	2.28084	2.1
20678	2.98092	2.1
20679	0.946132	1.00345
20680	1.2435	2.1
20681	0.946132	2.09999
20682	1.4099	1.06618
20683	1.2435	1.56493
20684	1.26438	2.1
20685	0.745287	2.1
20686	0.696827	2.1
20687	0.696781	2.1
20688	1.41195	2.1
20689	0.946131	2.1
20690	1.8758	1.10097
20691	0.832979	0.847074
20692	0.75054	0.798048
20693	0.810958	0.803525
20694	0.922889	0.932999
20695	0.694844	0.783818
20696	0.694814	0.856957
20697	0.946131	0.823601
20698	0.694844	0.817409
20699	1.02369	2.1
20700	1.4468	0.152255
20701	0.946125	0.700391
20702	0.93487	0.7
20703	1.23608	0.710144

20704	1.34257	0.7
20705	0.851525	0.70008
20706	1.03298	0.580082
20707	1.22418	1.03672
20708	0.946131	1.50242
20709	1.41833	1.10136
20710	1.23452	0.994707
20711	0.946131	0.889791
20712	1.41833	0.985173
20713	1.13077	0.715443
20714	0.942874	1.35947
20715	0.946132	1.23462
20716	0.504366	1.32072
20717	0.593647	2.1
20718	0.898829	1.40316
20719	1.27921	2.1
20720	1.12503	2.1
20721	1.22539	2.1
20722	1.10332	1.09486
20723	0.851541	2.1
20724	0.94613	2.1
20725	0.945109	2.1
20726	0.607962	2.1
20727	0.946133	2.1
20728	0.607961	2.1
20729	0.661956	2.1
20730	1.35048	2.1
20731	1.66018	2.1
20732	0.956467	2.1
20733	0.851542	2.1
20734	0.890734	2.1
20735	0.802455	2.1
20736	1.35943	2.1
20737	1.04798	2.1
20738	1.26426	0.784027
20739	1.3689	0.729838
20740	0.946133	0.74296
20741	0.946133	0.790896
20742	1.09729	0.773571
20743	0.765259	2.1
20744	0.810945	2.1
20745	0.810957	2.1
20746	1.10284	1.93909
20747	1.09368	2.1
20748	0.772601	2.1
20749	0.788011	2.1
20750	0.810934	2.1
20751	0.694357	0.976989
20752	1.22641	1.03934
20753	0.946132	1.17152
20754	0.946132	0.842326
20755	0.946133	0.888431
20756	1.42754	1.56316
20757	0.776414	0.882211
20758	0.806525	0.934264
20759	0.575339	0.998323
20760	0.97154	0.874234
20761	0.8217	0.98375

20762	0.838039	1.17244
20763	0.94613	1.04054
20764	0.946132	0.908785
20765	1.39575	0.77553
20766	1.39575	0.858822
20767	1.12818	0.885098
20768	0.946132	1.19416
20769	0.946133	1.04
20770	1.06879	0.835648
20771	1.06879	0.964454
20772	0.970358	0.955504
20773	1.2474	0.683194
20774	1.01919	0.173604
20775	0.94613	0.226937
20776	1.28952	0.20769
20777	1.00809	0.175081
20778	0.849344	0.175637
20779	0.640304	0.255192
20780	1.35065	0.164225
20781	0.946132	0.170194
20782	0.640333	0.15204
20783	0.946132	0.253747
20784	0.685489	2.1
20785	0.94613	2.1
20786	0.68546	2.1
20787	0.874264	2.1
20788	1.27676	2.1
20789	0.946129	0.399336
20790	1.09024	0.305189
20791	0.714563	0.391846
20792	1.12335	0.445883
20793	1.07931	0.398571
20794	1.17799	0.7
20795	1.8795	0.460419
20796	2.15229	0.530017
20797	1.13859	0.911466
20798	0.946132	1.18862
20799	1.11374	1.00131
20800	1.1386	0.990644
20801	0.615713	1.01829
20802	1.19322	0.742219
20803	1.3377	0.623937
20804	0.670801	0.707164
20805	0.94613	0.989243
20806	0.66617	0.873947
20807	1.36173	0.868512
20808	0.980473	0.855979
20809	1.39132	0.359717
20810	0.864653	0.140962
20811	1.23837	0.122775
20812	0.810959	0.114368
20813	1.1483	0.690991
20814	1.00381	1.12758
20815	0.946128	1.67507
20816	1.39696	1.2329
20817	0.946131	1.04489
20818	0.946131	1.16104
20819	1.38613	1.20527

20820	0.973501	1.72494
20821	0.946127	1.15066
20822	1.35968	1.11311
20823	0.946131	0.953999
20824	0.946129	1.19002
20825	1.39696	1.15358
20826	1.12088	2.1
20827	1.41122	1.75888
20828	1.14464	0.905367
20829	0.946131	1.96664
20830	0.946131	1.43994
20831	1.14464	1.66101
20832	1.33266	1.27781
20833	1.07341	0.967657
20834	0.827287	1.38506
20835	0.946132	1.15082
20836	0.827287	1.83536
20837	1.15644	1.21162
20838	1.27558	0.811802
20839	1.15067	0.906264
20840	0.946131	2.1
20841	1.29143	0.844006
20842	1.36281	2.1
20843	1.36281	0.973839
20844	0.946131	0.986471
20845	1.40863	2.1
20846	1.20143	0.451168
20847	1.19755	0.511673
20848	0.566153	0.259974
20849	0.946131	0.523371
20850	1.07979	0.239529
20851	1.00463	0.488141
20852	0.566153	0.671637
20853	0.768212	2.1
20854	1.32461	2.1
20855	1.13268	2.1
20856	1.13268	1.76934
20857	0.946133	1.68317
20858	1.53642	2.1
20859	1.5791	2.1
20860	0.726635	2.0139
20861	1.2334	1.53172
20862	1.29198	1.88552
20863	1.29198	1.26142
20864	0.946131	1.14531
20865	0.956585	2.1
20866	1.25294	2.1
20867	0.946133	2.1
20868	1.35287	2.1
20869	1.23877	2.1
20870	0.946131	2.1
20871	1.35287	2.1
20872	1.01843	2.1
20873	3.77569	2.1
20874	0.86707	2.1
20875	0.867066	2.1
20876	1.38639	2.1
20877	1.38639	2.1

20878	0.896168	2.1
20879	0.974716	0.489216
20880	1.09411	0.608884
20881	0.663171	0.59726
20882	0.663171	0.649056
20883	0.946132	0.627145
20884	0.66317	0.638383
20885	1.47104	0.397217
20886	1.13629	0.438058
20887	0.772342	0.452263
20888	0.772312	0.497618
20889	0.94613	0.447118
20890	0.77234	0.48563
20891	1.43154	2.1
20892	1.37704	2.1
20893	1.40025	2.1
20894	1.3063	2.1
20895	0.946132	2.1
20896	1.04075	2.1
20897	0.946131	2.1
20898	0.946118	1.59045
20899	0.750011	0.771996
20900	1.0971	0.943266
20901	0.889667	0.801153
20902	0.709598	0.969502
20903	2.39737	0.99558
20904	1.19974	0.355572
20905	0.94613	0.691181
20906	1.25809	0.426225
20907	1.19974	0.622533
20908	0.73005	0.744312
20909	1.50764	0.188326
20910	1.25809	0.346882
20911	0.946133	0.708049
20912	1.10357	0.499547
20913	1.25809	0.607717
20914	0.628177	0.740447
20915	1.3543	0.296211
20916	0.793711	0.264988
20917	1.18455	0.316264
20918	1.03966	0.280165
20919	0.946131	0.353276
20920	0.767453	0.222681
20921	0.612292	0.351557
20922	0.946131	0.357627
20923	1.24128	0.403386
20924	1.08761	0.202499
20925	0.762059	0.132231
20926	1.005 2.1	
20927	1.17054	2.1
20928	1.12665	2.1
20929	0.750368	2.1
20930	0.851542	2.1
20931	0.975436	2.1
20932	0.975442	2.1
20933	0.675367	2.1
20934	1.51973	0.689817
20935	0.94613	0.695761

20936	1.38954	0.699701
20937	0.946131	0.706761
20938	1.07912	0.712844
20939	1.38954	0.703009
20940	2.0219	2.1
20941	1.18347	0.45325
20942	0.946132	0.817883
20943	0.94613	0.752046
20944	1.00202	0.667107
20945	1.00201	0.7135
20946	0.946132	0.757436
20947	0.946132	0.875465
20948	1.37981	0.78449
20949	1.26874	0.762746
20950	1.26874	0.763991
20951	0.946132	0.78672
20952	2.8687	0.67619
20953	1.42884	2.1
20954	1.05188	2.1
20955	0.625307	2.1
20956	0.810956	2.1
20957	0.526656	2.1
20958	0.526684	2.1
20959	0.724122	2.1
20960	0.946132	2.1
20961	0.526684	2.1
20962	1.05337	2.1
20963	1.0593	2.1
20964	1.47981	2.1
20965	1.74431	2.1
20966	0.885612	2.08976
20967	0.810946	1.57609
20968	0.845185	1.79425
20969	1.38622	0.881599
20970	0.723194	0.851426
20971	0.554302	0.926943
20972	0.709597	0.811901
20973	0.909507	0.84111
20974	0.912754	0.756501
20975	1.15174	0.676191
20976	0.946131	0.758874
20977	0.912753	0.756147
20978	1.15174	0.757116
20979	1.02011	0.414382
20980	0.792754	1.95455
20981	0.946129	2.1
20982	0.792754	1.87997
20983	1.27575	1.82817
20984	0.94613	1.80402
20985	1.60132	2.1
20986	0.922455	2.07223
20987	1.18713	1.58925
20988	0.926957	1.77522
20989	1.66525	1.93631
20990	0.946132	2.1
20991	0.851541	2.1
20992	0.961191	2.1
20993	1.27534	2.1

20994	0.851554	2.1
20995	1.0499	2.1
20996	1.01776	0.178565
20997	0.97622	0.137328
20998	0.810934	0.203547
20999	2.6802	0.137295
21000	1.31485	0.15095
21001	1.09699	0.113973
21002	0.681649	0.0898365
21003	0.790369	0.0876663
21004	0.946131	0.123198
21005	0.838026	1.50727
21006	0.567833	1.00524
21007	0.891492	1.3557
21008	0.986984	0.911123
21009	0.946132	1.2586
21010	1.19028	0.994036
21011	0.986984	0.975068
21012	0.595623	1.08514
21013	0.946131	2.1
21014	1.36502	2.1
21015	1.08109	2.1
21016	0.946129	2.1
21017	1.36503	2.1
21018	0.773897	2.1
21019	0.946133	1.24445
21020	0.613447	2.1
21021	0.613449	1.77281
21022	0.946131	2.04707
21023	1.11163	2.00847
21024	0.966114	2.1
21025	0.735699	2.1
21026	0.662147	2.1
21027	1.04072	2.1
21028	0.946131	2.1
21029	0.862978	2.1
21030	1.48839	1.00516
21031	0.936141	0.976339
21032	0.946131	1.16478
21033	0.936142	2.1
21034	1.40595	1.74406
21035	0.946131	1.19946
21036	1.05297	0.264782
21037	3.09035	2.1
21038	1.19952	2.1
21039	0.907198	2.1
21040	0.849144	2.1
21041	0.612469	0.789489
21042	0.551237	0.800497
21043	0.985256	0.856065
21044	0.551209	0.806379
21045	0.85153	0.875316
21046	0.624712	2.1
21047	0.900018	2.1
21048	0.851542	2.1
21049	0.864787	2.1
21050	1.27669	2.1
21051	0.946711	0.799848

21052	0.810947	0.811657
21053	0.841547	0.839305
21054	0.764114	0.853865
21055	1.26692	1.14973
21056	0.946132	0.857281
21057	1.37675	0.873803
21058	1.26692	0.875134
21059	1.37545	1.15641
21060	1.49637	0.537927
21061	0.709598	0.673176
21062	0.751252	0.595608
21063	1.36811	0.60051
21064	0.635625	0.630295
21065	1.31713	0.319591
21066	1.18977	2.1
21067	0.893377	1.35026
21068	0.707344	2.1
21069	0.946131	2.1
21070	1.20221	2.1
21071	1.20221	2.1
21072	0.707345	2.1
21073	1.26786	2.1
21074	0.988048	2.08786
21075	0.751293	2.04301
21076	0.632008	1.80803
21077	1.51067	2.1
21078	1.13534	2.1
21079	0.946133	2.1
21080	0.946131	2.1
21081	1.25716	2.1
21082	1.3199	2.1
21083	1.09616	2.1
21084	1.36677	1.30648
21085	1.34752	1.52163
21086	0.838041	1.21537
21087	1.67023	2.1
21088	1.02247	0.75853
21089	1.03079	0.724605
21090	0.928982	0.697748
21091	1.13635	0.470093
21092	0.946132	0.699418
21093	0.92898	0.570035
21094	1.13635	0.659337
21095	1.1643	0.276835
21096	0.851541	0.691646
21097	1.39939	0.571696
21098	0.946129	0.705271
21099	1.07775	0.630975
21100	1.14503	0.614872
21101	1.78573	0.439041
21102	1.29827	0.462929
21103	0.872373	0.182049
21104	1.01162	0.179497
21105	0.83804	0.152269
21106	1.48851	0.14661
21107	1.47385	0.13899
21108	0.843858	0.262574
21109	0.938626	0.24856

21110	0.89567	0.206799
21111	1.59457	0.831076
21112	2.09666	0.430611
21113	0.775132	0.0915771
21114	0.927005	0.117582
21115	0.854224	0.0822743
21116	0.775142	0.0844835
21117	0.946106	0.0974273
21118	1.80784	2.1
21119	2.04593	2.1
21120	1.32282	2.1
21121	0.952138	2.1
21122	0.946132	2.1
21123	0.946132	2.1
21124	0.952138	2.1
21125	1.46988	2.1
21126	0.80465	0.375667
21127	1.14155	0.394702
21128	0.810931	0.349683
21129	1.66203	0.704513
21130	0.674605	0.715038
21131	0.946132	0.716565
21132	1.26706	0.76111
21133	0.674633	0.719313
21134	0.946132	0.735227
21135	0.980664	0.755268
21136	1.34927	0.705894
21137	0.758454	0.7
21138	0.946133	0.663015
21139	0.985371	0.522747
21140	1.28632	0.635507
21141	0.946133	0.7
21142	1.46868	0.710134
21143	1.7522	0.168035
21144	0.737431	2.1
21145	0.663679	2.1
21146	1.16541	1.82153
21147	1.3179	1.99439
21148	1.04081	1.9926
21149	0.887866	1.72547
21150	0.806961	1.4135
21151	0.929061	1.67255
21152	1.28418	0.54854
21153	1.38752	0.451789
21154	1.33038	0.544207
21155	0.946132	0.366538
21156	0.946132	0.361985
21157	1.14897	0.358329
21158	1.1685	0.403021
21159	1.18774	2.1
21160	0.961899	2.1
21161	1.48841	2.1
21162	1.79105	2.1
21163	3.51614	2.1
21164	0.682342	2.1
21165	0.783184	2.1
21166	0.946132	2.1
21167	0.868396	2.1

21168	1.21813	2.1
21169	0.848305	0.811782
21170	0.94559	0.81425
21171	0.805691	0.822788
21172	0.752217	2.1
21173	0.946132	2.1
21174	0.839123	2.1
21175	0.946131	2.1
21176	0.752187	2.1
21177	1.96491	2.1
21178	0.520997	2.1
21179	0.946132	2.1
21180	0.73446	2.1
21181	0.946133	2.1
21182	0.520995	2.1
21183	1.88522	2.1
21184	1.8361	2.1
21185	1.11285	2.1
21186	1.25334	1.1255
21187	2.00756	2.1
21188	1.44252	0.7
21189	1.00255	1.10933
21190	1.64516	2.1
21191	1.12399	0.0915741
21192	1.13542	0.117209
21193	1.09038	0.151669
21194	0.746903	0.173639
21195	0.672241	0.117349
21196	0.871774	0.139917
21197	1.73513	0.95475
21198	0.949995	2.04785
21199	1.17244	2.00522
21200	0.946127	2.01183
21201	1.3028	1.88628
21202	1.17244	1.79403
21203	0.774101	2.1
21204	0.946127	2.1
21205	0.939289	2.1
21206	0.946129	2.1
21207	0.774101	2.1
21208	1.87858	2.1
21209	1.44293	0.252046
21210	1.43213	0.2284
21211	0.946131	2.1
21212	0.716614	2.1
21213	0.716644	2.1
21214	1.35828	2.1
21215	0.790935	2.1
21216	0.730157	2.1
21217	1.41149	2.1
21218	0.730186	2.1
21219	0.730186	2.1
21220	0.946131	2.1
21221	0.946132	1.19126
21222	0.946132	1.03943
21223	0.990713	0.850166
21224	1.40779	0.929085
21225	0.946132	0.990782

21226	1.06486	0.732952
21227	0.946131	1.02591
21228	0.946133	0.855763
21229	1.40779	0.81904
21230	1.26874	0.849535
21231	0.946131	0.855523
21232	1.02588	0.758205
21233	0.826073	0.702122
21234	1.41233	0.702447
21235	0.861269	0.701867
21236	1.04073	0.70851
21237	1.27111	0.707198
21238	1.56016	1.33845
21239	1.60866	0.109243
21240	0.94613	0.193444
21241	0.946129	0.214097
21242	1.36376	0.145863
21243	1.22187	0.222504
21244	0.94613	0.217279
21245	1.82581	1.00699
21246	1.19705	1.14903
21247	1.7513	2.1
21248	1.85017	2.1
21249	1.72116	2.1
21250	1.12058	2.1
21251	0.899991	2.1
21252	0.616288	2.1
21253	1.07289	2.1
21254	1.7092	0.788425
21255	0.946132	0.746571
21256	0.946133	0.249307
21257	1.18717	0.323773
21258	1.40029	0.767998
21259	0.946132	0.516492
21260	0.94613	0.7
21261	1.18547	0.7
21262	0.960326	0.7
21263	0.960345	0.7
21264	0.942557	0.7
21265	1.18601	0.671216
21266	0.946132	0.7
21267	1.30307	0.7
21268	1.08169	0.7
21269	1.08169	0.7
21270	1.31716	0.7
21271	1.51065	0.995406
21272	0.877642	0.113458
21273	0.617384	0.086347
21274	0.575741	0.105649
21275	0.972448	0.0821658
21276	1.11334	0.106268
21277	1.13092	2.1
21278	0.946127	0.767708
21279	1.31724	0.815454
21280	1.39751	0.705555
21281	1.39751	0.763807
21282	0.946128	0.759542
21283	0.946132	0.664904

21284	0.713284	0.652833
21285	1.40668	0.666191
21286	1.03547	0.624781
21287	0.713298	0.661067
21288	1.88774	0.420452
21289	0.830164	1.90249
21290	1.05211	2.1
21291	0.946119	1.21824
21292	0.946107	2.1
21293	1.283	2.1
21294	2.63609	2.1
21295	1.28418	0.855133
21296	0.946128	0.93638
21297	1.10335	0.858914
21298	0.598377	0.973146
21299	1.18818	0.86266
21300	1.10335	0.853836
21301	1.58972	1.05222
21302	1.90194	2.1
21303	0.946118	2.1
21304	0.946115	2.1
21305	0.950969	2.1
21306	1.36918	2.1
21307	0.950955	2.1
21308	1.4263	2.05859
21309	1.11978	0.797624
21310	0.946132	0.912914
21311	0.946133	1.01461
21312	1.37658	0.796252
21313	1.2364	0.844213
21314	1.05496	0.860541
21315	0.946132	0.97546
21316	0.946132	0.922694
21317	1.21858	0.825324
21318	1.37658	0.912495
21319	1.09194	1.13524
21320	1.14721	0.175859
21321	1.53814	0.0821105
21322	0.546751	0.967625
21323	0.492063	1.19032
21324	1.16889	1.64704
21325	1.00349	1.69204
21326	0.863559	1.07407
21327	1.38102	1.56947
21328	0.597449	1.0106
21329	0.537748	0.91822
21330	1.16297	1.32909
21331	0.999366	1.24776
21332	0.896554	0.994986
21333	0.566962	0.0939557
21334	0.946129	0.121423
21335	0.452729	0.0776413
21336	0.696552	0.0908872
21337	0.852524	0.098847
21338	1.57992	0.0772112
21339	1.64985	0.118424
21340	0.946128	2.1
21341	1.21847	2.1

21342	0.946129	2.1
21343	1.41131	2.1
21344	1.21847	2.1
21345	1.21269	0.0730767
21346	1.0417	1.88745
21347	1.15821	1.05023
21348	0.946132	0.854937
21349	0.946132	1.16605
21350	1.37967	0.848426
21351	0.851529	0.864487
21352	1.20455	2.1
21353	1.4282	0.0916402
21354	1.26593	2.1
21355	0.838026	0.295339
21356	0.985096	0.261982
21357	0.83804	0.285952
21358	0.822395	0.889275
21359	0.420272	0.742514
21360	0.946131	2.1
21361	1.17461	2.1
21362	0.94613	2.1
21363	1.40225	2.1
21364	1.17461	2.1
21365	1.09847	2.1
21366	1.22222	0.970795
21367	1.41798	0.907908
21368	0.791658	0.942089
21369	0.791656	0.943808
21370	0.946129	0.959209
21371	0.817064	0.948125
21372	0.946114	0.887423
21373	0.75824	0.886317
21374	0.75821	0.891809
21375	1.41778	0.871814
21376	0.791635	0.882491
21377	1.04164	1.58645
21378	0.946131	0.954117
21379	1.11172	0.869256
21380	1.30076	0.813753
21381	1.35721	0.826592
21382	1.11172	0.838702
21383	1.87218	0.610286
21384	0.987248	0.709332
21385	0.729695	0.82988
21386	0.974389	0.749534
21387	1.18002	0.742532
21388	0.822612	0.73107
21389	1.35721	0.74175
21390	0.634295	0.843778
21391	0.634326	0.844327
21392	1.13245	1.12415
21393	1.13245	0.991114
21394	0.937961	0.832067
21395	1.18769	0.946656
21396	0.854949	0.783458
21397	0.967357	0.817777
21398	0.810934	0.775138
21399	3.91082	2.1

21400	0.946132	2.1
21401	0.946132	2.1
21402	1.05383	2.1
21403	1.05383	2.1
21404	0.986498	2.1
21405	1.50732	0.38897
21406	0.753659	0.523757
21407	1.22144	0.591102
21408	1.30463	0.475522
21409	0.753659	0.549296
21410	1.30463	0.525391
21411	0.886189	0.5442
21412	1.04321	0.532077
21413	0.909928	0.454983
21414	1.18659	0.456781
21415	1.19785	0.139463
21416	1.40969	0.225146
21417	1.24094	0.157224
21418	0.946126	0.197653
21419	1.2929	0.154337
21420	1.24093	0.172773
21421	0.867944	0.148635
21422	1.41903	2.1
21423	2.47881	0.0846654
21424	1.12943	0.0758173
21425	0.764229	2.1
21426	0.764256	2.1
21427	0.94613	2.1
21428	0.94613	2.1
21429	1.1995	2.1
21430	1.83423	2.1
21431	0.634816	2.06301
21432	0.877837	2.1
21433	0.94613	2.08753
21434	1.04073	2.1
21435	1.06485	2.1
21436	0.94613	0.719934
21437	0.62725	0.713815
21438	0.798212	0.720647
21439	1.33241	0.638247
21440	0.62725	0.714385
21441	1.82301	0.63954
21442	1.40615	2.1
21443	0.782504	2.1
21444	0.748635	2.1
21445	0.946131	2.1
21446	0.748607	2.1
21447	0.988456	2.07899
21448	1.64833	0.103946
21449	1.35657	0.124487
21450	1.26155	0.128218
21451	0.915755	0.139151
21452	0.915755	0.120511
21453	0.946132	0.131483
21454	1.49386	0.10146
21455	1.01346	2.1
21456	0.946131	0.998227
21457	0.946132	2.1

21458	1.38651	2.1
21459	1.39523	2.1
21460	0.946132	2.1
21461	1.06312	2.1
21462	0.946131	2.1
21463	0.946129	2.1
21464	1.39523	2.1
21465	1.37372	2.1
21466	0.946131	2.1
21467	1.2929	0.244468
21468	0.94613	0.357459
21469	1.24448	0.229903
21470	1.2929	0.283064
21471	0.862011	0.250993
21472	1.73583	0.140125
21473	1.724	0.171521
21474	1.03824	2.1
21475	1.534	2.1
21476	0.725492	1.76676
21477	0.771967	1.9255
21478	0.61731	1.22693
21479	0.475743	1.55016
21480	0.719694	1.62692
21481	1.14878	1.16164
21482	0.946117	1.70996
21483	0.94613	1.78798
21484	0.946105	1.78051
21485	0.482666	1.18551
21486	0.609172	1.19267
21487	0.946131	2.1
21488	1.39509	0.921561
21489	1.07439	2.1
21490	0.946131	1.0311
21491	1.39509	1.17116
21492	0.963571	1.23691
21493	0.591011	0.793487
21494	1.1357	0.724004
21495	2.11193	1.12494
21496	1.60936	2.1
21497	1.34118	0.418535
21498	1.27696	0.54897
21499	0.94613	0.35752
21500	0.94613	0.310158
21501	1.04257	0.248674
21502	1.53336	0.145012
21503	1.0631	0.209448
21504	1.04072	0.377244
21505	0.670678	0.269186
21506	0.709531	0.32729
21507	0.891275	0.346113
21508	0.683583	0.487791
21509	0.902478	0.330715
21510	0.807671	0.349658
21511	1.11024	2.1
21512	1.08171	2.1
21513	1.16725	2.1
21514	0.946128	2.1
21515	1.13537	2.1

21516	1.40525	2.1
21517	1.28365	2.1
21518	1.04523	2.1
21519	0.960106	2.1
21520	0.946128	2.1
21521	1.23452	2.1
21522	0.946129	2.1
21523	1.3704	2.1
21524	1.23452	2.1
21525	1.16906	0.907195
21526	0.946131	1.20385
21527	0.867246	0.95
21528	0.867217	1.50468
21529	0.946131	0.996534
21530	1.13438	1.15504
21531	1.28684	1.22458
21532	0.946132	1.36067
21533	0.883935	1.09143
21534	0.883938	2.02771
21535	0.946133	1.17641
21536	1.73444	1.03475
21537	1.21835	2.1
21538	1.28228	2.1
21539	1.8643	1.21612
21540	0.743379	0.704615
21541	1.34921	0.703124
21542	0.743381	0.704676
21543	0.743381	0.704762
21544	0.946131	0.705638
21545	0.915528	2.1
21546	0.982742	2.1
21547	0.975965	2.1
21548	0.891132	0.0740826
21549	1.27533	0.071466
21550	0.946132	0.067601
21551	1.25215	0.0633551
21552	0.590343	0.0679298
21553	0.946133	0.0672549
21554	0.590331	0.0674676
21555	1.62375	2.1
21556	0.687769	0.833245
21557	1.14374	0.823833
21558	1.20885	0.793557
21559	0.946132	0.842395
21560	1.14374	0.887023
21561	1.09308	0.723297
21562	1.02055	0.721036
21563	0.810934	0.709688
21564	0.917609	1.26751
21565	1.13094	1.27222
21566	0.70959	1.2256
21567	1.11223	2.1
21568	0.83051	1.25745
21569	0.810946	1.43801
21570	0.810959	1.21441
21571	1.5773	0.0815788
21572	1.96133	1.08972
21573	1.0214	1.11304

21574	0.762783	1.46558
21575	0.838036	1.02688
21576	0.63591	1.55151
21577	0.967411	1.05733
21578	0.946132	2.0302
21579	0.946132	1.96247
21580	1.39699	1.54025
21581	1.61642	1.8593
21582	0.860954	1.02058
21583	0.957411	1.18202
21584	0.660856	1.1846
21585	1.10003	2.1
21586	0.968249	2.00937
21587	0.910184	1.15004
21588	1.30029	0.81633
21589	0.754119	0.873939
21590	0.754121	1.02167
21591	0.94613	1.0479
21592	1.08039	2.1
21593	0.810942	0.531869
21594	1.15153	0.532764
21595	1.06968	0.444121
21596	1.38456	2.1
21597	1.04118	2.1
21598	0.838027	2.1
21599	1.04073	2.1
21600	0.754209	2.1
21601	1.332	0.118415
21602	1.26193	2.1
21603	1.15317	0.089444
21604	3.86179	0.0802847
21605	1.56005	2.1
21606	0.683035	0.712673
21607	0.931463	0.712235
21608	0.556019	0.780571
21609	0.929172	0.757329
21610	0.756874	0.704013
21611	1.23527	0.760961
21612	2.05961	0.778918
21613	0.946132	2.1
21614	1.16684	2.1
21615	1.3937	2.1
21616	1.3937	2.1
21617	0.946132	2.1
21618	1.17885	2.1
21619	1.62507	0.0808929
21620	1.58874	2.1
21621	0.819953	2.1
21622	1.16023	2.1
21623	1.34782	2.1
21624	1.16023	2.1
21625	0.819955	2.1
21626	0.79437	2.1
21627	0.794373	2.1
21628	1.20056	2.06275
21629	1.25	2.1
21630	1.20056	2.1
21631	1.35787	2.1

21632	0.946131	1.95821
21633	1.38585	1.54385
21634	0.946128	0.955135
21635	0.946129	1.22935
21636	1.35714	1.3116
21637	1.04147	2.1
21638	0.946133	1.37274
21639	1.30382	1.0751
21640	0.946131	0.830041
21641	0.946131	1.0588
21642	1.38585	1.22342
21643	1.22305	1.4611
21644	1.00763	0.536131
21645	0.813199	0.687578
21646	0.810948	0.682941
21647	1.15928	0.634976
21648	0.944166	0.268827
21649	0.94613	0.351192
21650	0.946133	0.286022
21651	1.4179	0.329329
21652	0.946132	0.28557
21653	1.36965	0.133936
21654	1.05444	0.226924
21655	0.690302	0.300497
21656	0.838038	0.228181
21657	0.802821	0.978901
21658	1.40944	1.06719
21659	0.946126	1.27071
21660	0.799998	0.83367
21661	0.800035	0.955214
21662	1.1766	2.1
21663	1.00319	2.1
21664	1.01606	1.68229
21665	0.872158	2.1
21666	1.04884	2.1
21667	0.679494	2.1
21668	0.946131	2.1
21669	1.37609	2.1
21670	0.94613	2.1
21671	0.946131	2.1
21672	1.18486	2.1
21673	0.726287	2.1
21674	0.946131	2.1
21675	0.726286	2.1
21676	1.30318	2.1
21677	1.01712	2.1
21678	0.946132	2.1
21679	1.04404	2.1
21680	1.37017	2.1
21681	1.37017	2.1
21682	0.946133	2.1
21683	0.992084	1.03698
21684	0.946131	0.868941
21685	0.529655	1.25435
21686	0.529686	1.06059
21687	0.946131	0.903752
21688	1.05937	1.10644
21689	1.14622	0.796053

21690	0.946131	0.791381
21691	0.584575	0.873115
21692	0.584575	0.737442
21693	0.946129	0.742533
21694	0.709714	2.1
21695	0.946132	2.1
21696	0.709714	2.1
21697	1.37199	2.1
21698	0.82899	2.1
21699	1.00133	2.1
21700	0.778424	0.10047
21701	0.946132	0.126893
21702	1.24094	0.107503
21703	1.00691	0.116219
21704	0.64868	0.105625
21705	1.29736	0.0991331
21706	1.24767	0.799229
21707	1.28953	0.761335
21708	1.34612	0.817548
21709	0.946133	0.767883
21710	1.14187	0.881745
21711	0.946132	0.766509
21712	0.946129	0.810473
21713	1.4099	1.8638
21714	0.946131	0.888575
21715	1.34612	0.804362
21716	1.4099	0.852734
21717	0.956003	0.0815512
21718	1.40572	0.088163
21719	3.54443	2.1
21720	0.994001	0.772297
21721	1.03679	2.1
21722	1.08467	2.1
21723	1.56875	0.743084
21724	0.968463	1.99713
21725	0.974889	2.1
21726	0.946131	2.1
21727	1.30203	2.1
21728	1.40199	2.1
21729	1.40199	2.1
21730	0.946132	2.1
21731	0.946132	2.1
21732	1.36994	2.1
21733	1.37372	2.1
21734	1.37372	2.1
21735	0.946132	2.1
21736	0.928979	1.06704
21737	0.565509	1.19468
21738	1.60196	0.738975
21739	1.16239	0.859247
21740	1.10746	0.732574
21741	1.15412	2.1
21742	1.15171	2.1
21743	0.946133	2.1
21744	0.940877	2.1
21745	0.940878	2.1
21746	1.41432	2.1
21747	1.04687	2.1

21748	2.75524	0.169792
21749	0.946132	0.214636
21750	0.766452	0.145304
21751	0.766427	0.202914
21752	1.36004	0.126481
21753	1.06648	0.190355
21754	1.62483	2.1
21755	1.0577	2.1
21756	1.39158	2.1
21757	0.94255	2.1
21758	1.05029	2.1
21759	1.03735	1.96676
21760	0.931055	2.1
21761	1.03797	2.03332
21762	1.43687	2.01892
21763	1.28765	1.287
21764	1.1811	1.00989
21765	0.946132	1.13369
21766	0.946132	1.08188
21767	1.1811	1.1021
21768	0.67805	0.99861
21769	1.50998	1.97141
21770	1.16661	2.1
21771	1.36749	2.1
21772	0.810945	0.853006
21773	1.14745	1.07881
21774	0.865588	0.755282
21775	1.36665	0.74457
21776	0.506964	0.809023
21777	0.506966	0.768421
21778	0.552786	0.734612
21779	0.946133	0.704467
21780	1.21336	0.724342
21781	0.552786	0.711401
21782	0.552757	1.19352
21783	0.653468	0.727474
21784	1.36665	1.62258
21785	1.27922	0.746984
21786	1.08133	0.794627
21787	1.05348	0.427606
21788	0.946132	0.628604
21789	1.19601	0.472681
21790	1.13276	0.48556
21791	0.7856	0.471303
21792	1.57125	0.463946
21793	1.19601	0.716977
21794	0.946131	0.953152
21795	1.34534	0.91984
21796	1.19601	0.790511
21797	0.870489	0.799042
21798	1.49404	2.1
21799	1.35943	1.06486
21800	1.10427	1.36106
21801	0.94613	1.06047
21802	0.946131	1.13082
21803	1.10427	1.14384
21804	1.2064	0.220636
21805	0.946132	0.737323

21806	1.11288	0.78334
21807	1.34268	0.673961
21808	1.34268	0.373099
21809	0.946132	0.687327
21810	0.946128	2.1
21811	0.946128	0.901059
21812	1.30835	2.1
21813	1.38463	1.02891
21814	0.946129	1.05878
21815	1.43778	0.867992
21816	1.23943	0.77236
21817	1.4748	2.1
21818	0.71295	2.1
21819	0.712951	2.1
21820	0.946132	2.1
21821	0.946133	2.1
21822	1.13976	2.1
21823	1.71109	2.1
21824	1.32426	2.1
21825	0.671255	2.1
21826	1.29123	2.1
21827	0.946132	2.1
21828	0.946132	2.07141
21829	1.25603	2.1
21830	2.21126	0.191996
21831	0.892889	2.1
21832	0.946129	2.1
21833	0.892888	2.1
21834	0.92872	2.1
21835	1.34352	2.1
21836	0.622784	0.730748
21837	1.02947	0.705609
21838	0.810957	0.674887
21839	1.91756	1.16888
21840	1.06012	1.12482
21841	1.09569	0.846026
21842	1.5456	0.202084
21843	1.1211	0.134776
21844	1.21031	0.206767
21845	1.03939	0.300623
21846	0.519697	0.332144
21847	0.933889	0.591711
21848	0.519726	0.580917
21849	1.16699	0.490025
21850	0.933889	0.57091
21851	0.840599	0.222099
21852	0.94613	0.456746
21853	0.840568	0.375964
21854	1.26868	0.348569
21855	1.16699	0.331694
21856	1.17822	1.83813
21857	0.922044	0.7
21858	1.29322	0.694937
21859	0.946132	0.661487
21860	0.995178	0.7
21861	0.922056	0.7
21862	1.76232	0.7
21863	1.77065	1.1675

21864	1.38149	0.933168
21865	0.959301	1.10282
21866	0.946133	0.976686
21867	0.946132	1.02835
21868	0.959301	0.989443
21869	1.3612	0.951465
21870	0.885314	1.09047
21871	0.885309	0.976556
21872	0.94613	1.05584
21873	0.8853	0.954936
21874	1.56164	0.994669
21875	1.45506	0.551512
21876	1.45257	2.1
21877	0.851541	0.204285
21878	1.29018	0.11868
21879	0.851541	0.155078
21880	1.21087	0.13136
21881	1.24979	0.164642
21882	1.56983	0.106327
21883	1.60151	0.0879243
21884	1.78952	2.1
21885	0.638164	2.1
21886	0.638163	2.1
21887	1.39754	2.1
21888	1.39754	2.1
21889	0.681589	2.1
21890	1.34993	2.1
21891	1.04525	2.1
21892	0.946133	2.1
21893	1.04072	2.1
21894	0.857805	2.1
21895	1.06053	2.1
21896	1.49066	2.1
21897	1.4185	1.06902
21898	0.934059	1.45099
21899	0.93406	1.08834
21900	0.94613	1.2466
21901	0.93406	1.16183
21902	1.25277	1.24743
21903	1.00792	1.99798
21904	0.609466	1.26797
21905	0.946132	1.5151
21906	0.609463	1.66528
21907	1.30664	2.1
21908	0.810943	1.70095
21909	1.05741	1.4522
21910	1.3868	0.943557
21911	1.15558	0.70738
21912	1.13537	2.1
21913	0.946132	2.1
21914	0.946132	2.1
21915	1.08266	2.1
21916	1.54934	2.1
21917	1.26016	2.1
21918	2.01915	2.1
21919	0.60346	1.21712
21920	0.651529	1.28247
21921	1.15581	1.35306

21922	1.13037	1.14807
21923	1.04071	1.12422
21924	1.77796	1.62584
21925	0.532308	1.20042
21926	0.532308	1.15483
21927	1.15369	1.55214
21928	1.1537	1.42458
21929	0.903288	1.32908
21930	0.804766	2.1
21931	0.804739	2.1
21932	0.946128	2.1
21933	0.946131	2.1
21934	1.16745	2.1
21935	1.04193	2.1
21936	0.98505	2.1
21937	1.40724	0.852586
21938	1.14216	0.790594
21939	0.946131	0.753541
21940	0.946131	0.797424
21941	1.10941	0.791298
21942	0.81268	0.107955
21943	0.946131	0.116108
21944	1.28673	0.100472
21945	1.28673	0.101565
21946	0.812681	0.106362
21947	1.84716	0.0775605
21948	1.84716	0.0780384
21949	0.92361	0.0894504
21950	0.94613	0.0894587
21951	0.923582	0.0880845
21952	1.26227	0.0920974
21953	0.923581	0.0920686
21954	0.747394	2.1
21955	1.32582	2.1
21956	0.747365	2.1
21957	0.97587	2.1
21958	1.32582	2.1
21959	1.04182	0.1974
21960	0.961743	0.126488
21961	0.946126	0.190471
21962	1.47463	0.145224
21963	1.12183	0.156179
21964	0.851552	0.145727
21965	1.33177	0.240823
21966	0.946131	0.374399
21967	1.41512	0.329608
21968	1.33178	0.287587
21969	0.946131	0.272855
21970	0.927735	0.762009
21971	0.97206	0.713237
21972	0.515566	0.740988
21973	0.946129	0.729474
21974	0.515538	0.717719
21975	1.04482	0.700644
21976	0.975535	0.712696
21977	0.522411	0.700615
21978	0.522382	0.71777
21979	0.925346	0.717551

21980	0.575817	0.704157
21981	0.946129	2.1
21982	1.14256	2.1
21983	1.32054	2.1
21984	1.40525	2.1
21985	1.14256	2.1
21986	1.59475	2.1
21987	0.773813	2.1
21988	1.4073	2.1
21989	0.773813	2.1
21990	0.773813	2.1
21991	1.4073	2.1
21992	0.993021	2.1
21993	1.50466	0.249402
21994	0.851539	0.477292
21995	1.20299	0.347404
21996	0.946131	0.394315
21997	1.54464	0.423749
21998	1.20309	0.35223
21999	2.00701	0.254607
22000	0.810946	0.506116
22001	1.07678	0.498079
22002	0.900128	0.411702
22003	1.26842	2.1
22004	1.11206	2.1
22005	0.946132	2.1
22006	1.04074	2.1
22007	0.948809	2.1
22008	0.946131	0.103147
22009	0.929617	0.145159
22010	1.24057	0.111887
22011	1.36485	0.108595
22012	0.851554	0.110331
22013	1.96823	0.0855048
22014	0.914836	0.125621
22015	0.94613	0.185285
22016	1.38911	0.125059
22017	0.914805	0.111935
22018	0.946131	0.131309
22019	0.946131	0.217863
22020	1.04072	0.192461
22021	1.26837	0.136334
22022	0.970054	0.212424
22023	0.923958	0.196396
22024	1.16653	2.1
22025	1.34412	1.52785
22026	0.851541	0.397907
22027	1.38645	0.520784
22028	0.891637	0.420772
22029	0.851487	0.440593
22030	0.890871	0.476648
22031	1.56178	0.413303
22032	0.946128	0.05
22033	1.26868	0.05
22034	0.946131	0.0796715
22035	1.18789	0.226308
22036	1.26868	0.0788644
22037	1.26868	0.079731

22038	0.946128	0.079731
22039	1.18716	0.401021
22040	0.946131	0.694687
22041	0.59358	0.548297
22042	1.23923	0.697215
22043	1.00024	0.576686
22044	0.593593	0.652431
22045	0.946131	0.707597
22046	0.713645	0.675844
22047	1.23175	0.714621
22048	1.23923	0.607393
22049	0.713633	0.693445
22050	1.24687	0.495737
22051	1.80662	2.1
22052	2.15762	2.1
22053	0.780455	2.1
22054	0.946131	2.1
22055	0.780455	2.1
22056	1.40583	2.1
22057	1.39852	2.1
22058	0.565619	2.1
22059	1.1428	2.1
22060	0.663168	0.722936
22061	1.02163	0.586062
22062	1.04072	0.740751
22063	0.663169	0.677489
22064	1.13511	0.66531
22065	1.85277	0.432478
22066	1.17701	1.02414
22067	0.993831	0.448327
22068	1.0823	0.522536
22069	0.795761	0.721862
22070	1.24596	0.598152
22071	0.566075	0.713247
22072	0.94613	0.734467
22073	0.56605	0.72049
22074	0.774911	0.144
22075	0.946131	0.202284
22076	1.24417	0.131924
22077	0.774911	0.124678
22078	0.946132	0.169768
22079	1.54982	0.115676
22080	1.82961	0.104012
22081	1.12035	0.0916825
22082	0.876835	2.1
22083	0.946131	2.0308
22084	0.876836	2.1
22085	1.12968	2.1
22086	1.23536	2.1
22087	2.15082	2.1
22088	0.58365	2.1
22089	0.946133	2.1
22090	0.583653	2.1
22091	0.992097	2.1
22092	1.12968	2.1
22093	1.0305	2.1
22094	0.82613	0.616226
22095	0.879644	0.580434

22096	1.00688	0.531124
22097	1.52776	2.05958
22098	0.851537	1.48929
22099	1.00851	1.25538
22100	0.946128	1.00859
22101	0.851552	1.23702
22102	1.4895	1.50829
22103	0.761574	2.1
22104	0.946132	2.1
22105	1.01095	2.1
22106	1.01095	2.1
22107	0.761572	2.1
22108	1.8575	2.1
22109	1.19984	0.500406
22110	1.25797	0.599933
22111	0.946132	0.449313
22112	0.946132	0.377554
22113	0.95332	0.346039
22114	0.946131	2.1
22115	0.851542	2.1
22116	1.03172	2.1
22117	1.22889	2.1
22118	1.0313	2.1
22119	1.15977	1.39339
22120	0.918875	1.0035
22121	0.8654	1.42898
22122	0.865374	1.06117
22123	0.946132	1.08172
22124	0.985051	2.1
22125	1.31993	0.498977
22126	1.33622	0.850429
22127	0.959992	0.820745
22128	0.926499	2.1
22129	1.03544	2.1
22130	0.946132	2.1
22131	0.946132	2.1
22132	1.40335	2.1
22133	0.772299	2.1
22134	0.851529	2.1
22135	0.857243	2.1
22136	1.38155	2.1
22137	1.05462	2.1
22138	0.832138	2.1
22139	0.946132	2.1
22140	0.832137	2.1
22141	0.959414	2.1
22142	1.36541	2.1
22143	1.91894	2.1
22144	1.04661	1.11028
22145	1.28355	2.1
22146	0.877353	1.03078
22147	0.877353	1.11175
22148	0.94613	1.06493
22149	1.20537	0.465893
22150	1.09914	0.249544
22151	0.94613	0.278978
22152	0.499593	0.371418
22153	0.499606	0.340729

22154	1.0959	0.262192
22155	0.838027	0.2301
22156	0.883644	0.336039
22157	0.95019	0.324395
22158	1.10638	2.1
22159	0.946132	2.1
22160	1.41744	2.1
22161	1.05842	2.1
22162	0.94613	2.1
22163	1.41744	2.1
22164	0.991985	2.1
22165	3.31755	1.18062
22166	1.05556	2.1
22167	0.624075	1.09198
22168	0.814554	1.03613
22169	0.946131	1.12418
22170	0.814554	1.183
22171	1.31857	1.48025
22172	1.26282	2.1
22173	0.946132	2.1
22174	1.14302	2.1
22175	0.946132	2.1
22176	0.946132	2.1
22177	1.40372	2.1
22178	1.24688	0.440881
22179	0.89889	2.1
22180	1.17286	2.1
22181	0.71741	2.1
22182	0.491096	0.875989
22183	1.05432	0.778491
22184	0.719531	2.1
22185	1.25145	2.1
22186	0.864686	2.1
22187	0.867974	2.1
22188	1.21734	2.1
22189	0.405517	1.05888
22190	0.818878	1.42988
22191	0.502457	0.251165
22192	1.05868	0.357021
22193	0.912018	0.268371
22194	1.40591	0.416903
22195	0.957584	0.783163
22196	0.946131	0.861939
22197	1.11612	0.827004
22198	1.3365	0.809867
22199	0.946128	0.865018
22200	1.16534	0.599476
22201	1.38969	0.775198
22202	0.891597	1.58796
22203	0.640032	1.95436
22204	0.956758	1.64286
22205	1.3428	0.712029
22206	0.953295	2.1
22207	0.946131	0.708736
22208	1.16167	0.70797
22209	0.94613	0.711293
22210	0.951675	0.708406
22211	1.16167	0.71366

22212	1.24538	0.301003
22213	1.13482	2.1
22214	1.38775	2.1
22215	0.934061	2.1
22216	0.91408	2.1
22217	1.06099	2.1
22218	0.980549	0.553225
22219	0.946132	0.637985
22220	1.10321	0.6898
22221	0.99126	0.653727
22222	0.991261	0.670772
22223	1.35732	0.648368
22224	1.1952	1.87949
22225	0.946132	1.14784
22226	0.946132	2.1
22227	1.32819	1.19042
22228	1.30171	1.06574
22229	1.29667	0.228031
22230	1.00982	0.113975
22231	0.407589	0.843747
22232	1.18266	1.15474
22233	1.04037	0.852799
22234	0.956694	0.938869
22235	0.552639	0.866209
22236	0.810943	1.64235
22237	1.43144	0.942134
22238	0.918074	0.925964
22239	1.60976	2.1
22240	4.00847	2.1
22241	2.3995	0.189927
22242	1.36203	2.1
22243	0.919619	2.1
22244	1.30188	2.1
22245	0.681073	2.1
22246	0.681043	2.1
22247	1.04687	2.1
22248	3.32085	2.1
22249	1.80396	0.138695
22250	0.773007	0.713299
22251	0.773034	0.704827
22252	1.22141	0.718806
22253	1.22141	0.712587
22254	0.946131	0.727877
22255	2.02071	0.701632
22256	0.951912	0.127087
22257	1.39027	0.161453
22258	0.94613	0.163911
22259	0.829922	0.131593
22260	0.829946	0.134391
22261	1.24902	1.43521
22262	2.8333	1.18281
22263	0.946131	0.082794
22264	0.929725	0.0773422
22265	1.1134	0.0856233
22266	1.17056	0.089195
22267	0.929713	0.0774461
22268	0.749096	2.1
22269	1.31961	2.1

22270	0.870191	2.1
22271	1.04071	2.1
22272	1.1877	2.1
22273	1.03882	2.1
22274	1.44801	2.07849
22275	3.55198	2.1
22276	1.11193	2.1
22277	0.663922	2.1
22278	0.663922	2.1
22279	0.946129	2.1
22280	0.946131	2.1
22281	0.97079	2.1
22282	1.36049	2.1
22283	1.06105	2.1
22284	0.92251	2.1
22285	0.94613	2.1
22286	0.922511	2.1
22287	1.04101	2.1
22288	0.810944	0.377629
22289	0.994109	0.353702
22290	0.623143	0.326643
22291	0.864244	0.420472
22292	1.14627	0.383241
22293	0.64586	0.367148
22294	0.721422	0.188203
22295	0.870885	0.13118
22296	0.855175	0.201733
22297	0.946131	0.996522
22298	1.38157	2.1
22299	1.33266	0.890891
22300	0.946131	1.64035
22301	1.38158	1.26056
22302	0.946132	2.09179
22303	1.3268	2.1
22304	0.946132	1.66742
22305	0.946132	2.1
22306	1.38158	2.1
22307	1.42186	1.85859
22308	1.47204	1.80667
22309	1.14665	2.08241
22310	0.946132	1.1514
22311	0.94613	2.1
22312	1.30116	1.73149
22313	1.32819	1.23901
22314	1.25388	2.1
22315	0.887808	1.25254
22316	0.946132	2.1
22317	1.23536	1.99966
22318	1.34309	1.95286
22319	1.07612	2.1
22320	0.728982	0.185857
22321	1.3157	0.161887
22322	0.946131	0.254424
22323	0.686176	0.2156
22324	1.3035	0.230768
22325	0.686139	0.223563
22326	0.906806	0.209201
22327	0.794122	0.166582

22328	1.17173	0.191259
22329	1.8194	0.155019
22330	0.851543	2.1
22331	1.19345	2.1
22332	0.946133	2.1
22333	0.946132	2.1
22334	1.56179	2.1
22335	0.620263	1.96082
22336	1.30226	1.67911
22337	1.2334	1.24539
22338	1.2334	1.12057
22339	0.946133	1.11819
22340	1.24053	2.1
22341	1.4828	2.05652
22342	0.957622	2.1
22343	1.00521	1.38812
22344	1.49463	2.1
22345	1.39621	2.1
22346	1.09861	2.1
22347	0.946131	2.1
22348	0.946131	2.1
22349	1.09861	2.1
22350	2.16987	0.120898
22351	0.572182	0.14654
22352	1.15802	0.15814
22353	0.63574	0.154496
22354	1.18897	0.129671
22355	0.996733	0.146527
22356	1.82402	1.71324
22357	0.969602	1.4507
22358	0.794777	1.77598
22359	1.02626	1.54829
22360	1.51396	0.564127
22361	1.40574	0.725182
22362	1.38683	0.727967
22363	0.946126	0.531676
22364	0.946128	0.510948
22365	1.07138	0.400798
22366	0.946131	2.1
22367	1.41331	2.1
22368	0.94613	2.1
22369	0.94613	2.1
22370	1.27451	2.1
22371	0.974196	2.1
22372	1.2049	0.109805
22373	1.42757	2.1
22374	1.02461	2.1
22375	2.07087	2.1
22376	1.92515	1.57585
22377	0.802138	1.53639
22378	0.946128	1.78991
22379	0.802107	1.45411
22380	1.23726	1.43224
22381	1.21997	1.40883
22382	0.767577	1.19924
22383	0.946131	1.63351
22384	0.767577	1.29281
22385	1.3279	1.30233

22386	1.23726	1.24958
22387	1.29729	2.1
22388	0.946128	2.1
22389	1.14186	2.1
22390	1.39214	2.1
22391	1.39214	2.1
22392	0.946129	2.1
22393	1.23012	0.297028
22394	0.653228	0.7
22395	0.946133	0.731561
22396	1.15918	0.855657
22397	1.26893	0.700109
22398	0.653228	0.7
22399	1.06848	1.4469
22400	0.615121	2.1
22401	0.841488	1.35506
22402	0.946131	2.1
22403	1.04071	2.1
22404	0.965717	2.1
22405	1.77893	1.3749
22406	1.11314	0.693249
22407	1.65411	0.982593
22408	0.85472	0.159724
22409	1.1604	0.120754
22410	1.19141	0.146704
22411	1.19141	0.119777
22412	0.94613	0.15381
22413	1.70944	0.0933358
22414	1.89082	0.705072
22415	1.05175	0.949175
22416	0.815276	2.1
22417	0.843283	2.1
22418	0.946131	2.1
22419	0.843284	2.1
22420	1.38897	2.1
22421	1.01019	0.913904
22422	1.05197	0.895374
22423	0.810959	0.87665
22424	1.33037	0.386825
22425	1.3415	0.508478
22426	0.946118	0.352437
22427	0.946131	0.328152
22428	1.09803	0.302484
22429	0.930281	2.1
22430	1.3335	2.1
22431	0.784151	2.1
22432	0.78415	2.1
22433	0.946132	2.1
22434	1.6299	0.148391
22435	0.995795	2.1
22436	1.84846	1.34601
22437	0.929873	1.81011
22438	0.889245	1.75193
22439	0.808072	1.93589
22440	0.851541	1.55599
22441	1.51956	1.45944
22442	0.94613	1.39583
22443	0.851553	1.44125

22444	0.946787	1.42404
22445	1.15544	1.78823
22446	1.30289	0.536918
22447	1.28323	0.501208
22448	0.85024	0.540857
22449	1.09662	0.671249
22450	1.67687	0.105534
22451	0.946127	2.1
22452	1.40496	2.1
22453	1.22442	1.79306
22454	0.946128	1.76843
22455	1.40496	1.86097
22456	1.00573	2.1
22457	0.636837	1.613
22458	0.988642	1.77907
22459	0.573141	1.27013
22460	0.946128	1.67971
22461	0.786538	2.03084
22462	2.28748	1.28637
22463	0.682805	2.1
22464	0.946132	2.07767
22465	0.682776	1.99184
22466	0.946132	2.1
22467	0.79775	2.1
22468	1.05403	0.151189
22469	0.888273	0.138119
22470	0.876373	0.128187
22471	0.576083	0.12617
22472	0.946132	0.191269
22473	0.576057	0.110199
22474	0.576058	0.129334
22475	0.946131	0.158236
22476	1.41724	0.85228
22477	1.31678	0.987886
22478	0.946132	0.96262
22479	0.946132	0.934637
22480	1.31678	0.918652
22481	0.935738	2.1
22482	0.946132	2.1
22483	0.935765	2.1
22484	1.32949	2.1
22485	0.946131	2.1
22486	1.02525	2.1
22487	0.946128	2.1
22488	1.41207	2.1
22489	1.04675	2.1
22490	0.946131	2.1
22491	1.41207	2.1
22492	0.946131	2.1
22493	0.946132	2.1
22494	1.41125	2.1
22495	1.41125	2.1
22496	1.27087	2.1
22497	0.924071	0.681171
22498	0.608893	0.533992
22499	0.528907	0.671058
22500	1.78249	0.65944
22501	0.720094	0.571705

22502	0.781667	2.1
22503	0.781638	2.1
22504	1.40756	2.1
22505	1.40756	2.1
22506	0.797635	2.1
22507	1.11946	0.05
22508	1.32068	0.05
22509	0.88757	0.251037
22510	1.27891	0.23184
22511	0.810957	0.235508
22512	0.914656	0.301435
22513	0.640819	0.203344
22514	0.745643	0.307031
22515	0.865108	0.359844
22516	1.06883	0.325751
22517	0.806001	0.371835
22518	1.19462	2.1
22519	1.42763	0.810663
22520	1.24682	0.761673
22521	0.78392	0.354412
22522	1.02874	0.359703
22523	0.884512	0.294629
22524	1.1658	2.1
22525	0.663026	0.102222
22526	1.21174	0.1331
22527	0.663056	0.139564
22528	1.15968	0.115926
22529	1.21174	0.126689
22530	0.993976	0.104428
22531	0.945495	0.107153
22532	0.671202	0.0986605
22533	1.37312	2.1
22534	1.28499	2.1
22535	0.946133	2.1
22536	0.946132	2.1
22537	1.28499	2.1
22538	1.25081	2.1
22539	0.946132	2.1
22540	0.946132	2.1
22541	1.27985	2.1
22542	1.37312	2.1
22543	0.830638	2.1
22544	1.05358	2.1
22545	1.22209	2.1
22546	1.09874	2.1
22547	0.74758	2.1
22548	2.50011	1.58584
22549	0.6157	0.691065
22550	0.674634	0.713079
22551	0.946128	0.82648
22552	0.674633	0.72305
22553	0.946131	0.78983
22554	1.96192	2.00978
22555	1.31612	0.481211
22556	1.10735	0.613023
22557	0.936676	0.635824
22558	0.808779	0.704243
22559	0.808808	0.71328

22560	1.00245	0.703705
22561	1.26179	0.568986
22562	1.05758	2.07191
22563	1.03408	2.1
22564	0.946132	0.157045
22565	0.914368	0.136209
22566	1.20524	0.107117
22567	1.12743	0.141807
22568	0.851555	0.136256
22569	1.17885	0.713231
22570	0.867881	0.700893
22571	1.00122	0.700372
22572	0.867883	0.7
22573	0.867881	0.7
22574	1.19583	0.701441
22575	1.17743	1.71993
22576	0.878684	1.43192
22577	0.687087	2.1
22578	0.955079	0.857769
22579	0.672977	0.87739
22580	0.735937	0.900313
22581	0.851531	0.88476
22582	0.672957	0.891002
22583	0.923974	0.877437
22584	1.2243	2.1
22585	0.808463	0.859536
22586	0.971078	0.883194
22587	1.06161	0.873831
22588	0.810947	0.90516
22589	0.886074	0.924932
22590	0.786829	0.945353
22591	0.55062	0.876526
22592	0.574458	1.25774
22593	0.946129	0.886131
22594	1.42526	0.890484
22595	1.15962	0.90318
22596	1.24526	0.0980137
22597	0.94613	0.732342
22598	0.946128	0.779243
22599	1.23279	0.818344
22600	1.38645	0.836371
22601	0.94613	0.76937
22602	1.05042	1.71068
22603	0.946128	2.1
22604	0.946129	2.1
22605	1.24286	2.1
22606	1.39659	2.1
22607	0.94613	2.1
22608	1.10407	2.02573
22609	1.32809	1.76851
22610	0.993999	2.02635
22611	0.810959	1.51063
22612	0.983643	1.73726
22613	0.869552	2.01715
22614	0.747801	1.57093
22615	1.53429	1.26739
22616	1.13127	2.1
22617	1.38397	2.1

22618	1.08366	2.1
22619	0.946129	2.1
22620	0.946131	2.1
22621	1.08366	2.1
22622	1.06007	2.1
22623	1.79641	2.1
22624	0.758799	2.1
22625	1.28442	2.1
22626	1.06957	2.1
22627	0.946133	2.1
22628	1.28442	2.1
22629	1.76788	1.60944
22630	1.38669	2.1
22631	0.986095	2.1
22632	1.38478	1.19464
22633	0.946132	0.822318
22634	1.24344	0.727676
22635	0.622283	1.02025
22636	1.10335	0.755622
22637	1.24344	0.772458
22638	1.1967	0.837604
22639	0.946131	0.153406
22640	0.901005	0.141734
22641	1.05751	0.143194
22642	1.37771	0.146954
22643	0.851527	0.144396
22644	1.26643	1.04475
22645	1.08992	1.408
22646	0.946132	1.36305
22647	0.946132	1.00693
22648	1.08992	1.4733
22649	1.53534	2.1
22650	1.58825	1.36155
22651	1.0447	1.92874
22652	0.593818	1.42265
22653	0.89883	1.57911
22654	0.734088	1.84213
22655	0.998451	0.903296
22656	1.64156	1.04357
22657	1.05654	1.35649
22658	1.37641	0.739566
22659	1.74553	0.802966
22660	1.31112	0.833074
22661	0.74518	0.892621
22662	0.851539	0.843472
22663	0.851554	0.751091
22664	0.851475	0.766304
22665	0.946132	0.0579793
22666	1.28895	0.0553737
22667	1.11356	0.0536628
22668	0.946132	0.0524849
22669	0.946132	0.0599953
22670	1.84214	2.1
22671	1.23851	0.27768
22672	1.25866	0.822562
22673	1.28655	0.996349
22674	0.946131	0.934705
22675	0.946132	0.910205

22676	1.28655	0.896345
22677	1.11929	0.84385
22678	0.946132	0.948603
22679	0.946132	0.842903
22680	1.34254	0.841754
22681	1.25866	0.837793
22682	0.946132	2.1
22683	1.27451	2.1
22684	1.19803	2.1
22685	0.94613	2.1
22686	1.27451	2.1
22687	0.946131	2.1
22688	1.36523	2.1
22689	1.14599	2.1
22690	0.946129	2.1
22691	1.36523	2.1
22692	1.09518	2.1
22693	0.977777	2.1
22694	0.583237	2.1
22695	0.583267	2.1
22696	0.903872	2.1
22697	0.946129	2.1
22698	0.583258	2.1
22699	1.58179	0.245853
22700	0.946131	0.429706
22701	1.35123	0.363548
22702	0.655837	0.403587
22703	1.12018	0.405376
22704	1.35123	0.342604
22705	0.62967	0.397231
22706	0.860497	0.540161
22707	0.816639	0.416277
22708	0.746716	2.1
22709	0.810947	2.1
22710	0.810959	2.1
22711	0.653422	2.09368
22712	0.289127	1.396
22713	0.475716	1.9846
22714	0.980651	1.38704
22715	0.946119	2.1
22716	0.946132	2.1
22717	1.08299	2.1
22718	1.34539	2.1
22719	1.28753	0.279163
22720	0.946132	0.517218
22721	0.946132	0.483006
22722	1.19719	0.464212
22723	0.946133	0.476754
22724	0.958947	0.318389
22725	1.3492	0.348591
22726	0.946132	0.590105
22727	0.946128	0.598453
22728	1.22588	0.566067
22729	0.946129	0.562976
22730	0.991113	2.1
22731	1.35966	2.1
22732	0.961 2.1	
22733	0.946129	2.1

22734	0.946131	2.1
22735	0.961001	2.1
22736	0.727096	1.84231
22737	0.810946	1.3093
22738	0.810959	1.54544
22739	1.31658	0.73366
22740	1.23167	2.1
22741	1.44836	0.0792901
22742	1.50103	0.0881691
22743	1.23351	0.0987217
22744	1.35657	0.108141
22745	0.851541	0.125547
22746	0.93668	0.100458
22747	0.946131	0.10622
22748	3.7335	2.1
22749	1.1822	2.1
22750	1.31127	0.737347
22751	1.3689	0.718605
22752	1.35822	0.676696
22753	0.946133	0.688348
22754	0.946132	0.721748
22755	1.14926	0.703241
22756	1.40822	0.7
22757	1.04935	0.733552
22758	0.704112	0.708521
22759	0.704112	0.721919
22760	0.946131	0.744637
22761	0.704112	0.731648
22762	1.37721	0.106741
22763	0.85154	2.1
22764	1.06949	2.1
22765	0.851541	2.1
22766	0.946128	2.1
22767	1.1175	2.1
22768	1.44091	2.1
22769	0.96029	0.823306
22770	3.46486	1.1702
22771	0.603286	0.672156
22772	0.432283	0.305094
22773	0.851541	0.570983
22774	1.09355	0.306937
22775	0.851534	0.639816
22776	1.04263	2.1
22777	0.851541	2.1
22778	0.965604	2.1
22779	0.94613	2.1
22780	1.23953	2.1
22781	0.869615	2.1
22782	1.02415	2.1
22783	0.983032	2.1
22784	1.35385	1.43763
22785	1.59971	0.57918
22786	0.915178	2.1
22787	0.962435	2.1
22788	1.3116	2.1
22789	1.38302	2.1
22790	1.13227	2.1
22791	1.25923	0.0898593

22792	0.988805	2.1
22793	0.611486	0.981901
22794	1.1019	1.06901
22795	1.11124	1.31651
22796	0.716713	0.991096
22797	0.946131	1.16322
22798	1.22297	1.21386
22799	1.12873	1.19353
22800	0.946131	1.2396
22801	0.946132	1.20727
22802	1.3469	1.38204
22803	1.25366	1.5597
22804	1.1611	0.980069
22805	0.946132	1.1173
22806	0.946132	1.0011
22807	1.2854	1.06994
22808	1.3469	1.14498
22809	1.07468	0.993056
22810	1.79317	2.1
22811	1.07444	2.1
22812	1.69436	1.56013
22813	0.851539	2.1
22814	0.870925	2.1
22815	0.851541	2.1
22816	0.870951	2.1
22817	1.26097	2.1
22818	1.26247	2.1
22819	0.946132	2.1
22820	1.24041	2.1
22821	0.946132	1.81819
22822	0.946132	2.1
22823	1.40274	2.1
22824	0.946131	0.124505
22825	0.797945	0.125355
22826	1.12988	0.124435
22827	1.00681	0.125104
22828	0.797932	0.129405
22829	0.977483	0.679553
22830	0.943362	0.865071
22831	0.9891	0.681746
22832	1.1703	0.77641
22833	0.663171	0.776169
22834	1.40882	0.363429
22835	1.32639	0.56449
22836	1.24448	0.370037
22837	0.946132	0.548702
22838	1.11015	0.350957
22839	1.24448	0.454458
22840	0.754768	0.414586
22841	1.13854	2.1
22842	1.23654	2.1
22843	0.714101	0.768657
22844	0.851538	0.837534
22845	0.79261	1.08336
22846	1.30598	0.945456
22847	1.05338	0.828208
22848	1.41758	2.1
22849	0.83317	0.704031

22850	1.00058	0.70853
22851	0.810958	0.706265
22852	1.63791	0.46502
22853	1.55116	2.1
22854	1.29735	1.07905
22855	0.945147	1.87032
22856	0.94515	1.10848
22857	0.94613	1.46601
22858	0.945149	1.28153
22859	1.02368	2.1
22860	1.12787	2.1
22861	1.04007	2.1
22862	3.78805	2.09309
22863	0.816891	0.423136
22864	0.943996	0.431853
22865	0.870002	0.404708
22866	0.564533	0.7
22867	0.564536	0.7
22868	0.946128	0.708427
22869	0.564535	0.702038
22870	0.946128	0.700285
22871	1.15177	2.1
22872	1.16806	0.836048
22873	0.999083	0.969127
22874	0.946131	0.874706
22875	0.946131	0.884176
22876	0.999084	0.932361
22877	1.28918	0.847216
22878	1.0383	1.01257
22879	0.946133	0.893497
22880	0.946132	0.911251
22881	1.0383	0.910886
22882	1.27737	1.35059
22883	1.39296	0.698452
22884	0.998682	2.1
22885	3.98933	2.1
22886	1.05328	0.404745
22887	1.23475	2.1
22888	0.900856	1.15747
22889	0.94001	0.953993
22890	0.707748	0.904893
22891	0.707751	1.03343
22892	0.946128	0.909189
22893	0.70775	0.9613
22894	0.996109	0.952123
22895	1.68551	2.1
22896	0.851539	2.1
22897	0.962688	1.86001
22898	0.94613	1.18687
22899	1.04073	2.1
22900	1.44824	2.1
22901	0.794313	0.742157
22902	0.838031	0.744294
22903	1.11076	0.723565
22904	1.38813	2.1
22905	1.17204	2.1
22906	0.946132	2.1
22907	0.946131	2.1

22908	1.17204	2.1
22909	0.961779	0.708285
22910	1.0685	0.857554
22911	0.810947	1.70469
22912	1.2899	1.06027
22913	0.890404	1.03914
22914	0.909981	1.98918
22915	0.810946	1.24284
22916	0.890402	1.14015
22917	0.522844	1.54613
22918	0.593846	1.49443
22919	1.30226	1.14287
22920	1.30226	1.14784
22921	0.946132	1.22296
22922	1.03537	0.302161
22923	1.50035	0.212552
22924	0.940356	0.396843
22925	0.946131	0.322094
22926	1.03441	0.375779
22927	1.21171	0.184606
22928	1.35169	0.27003
22929	1.32449	0.202229
22930	0.946131	0.337389
22931	0.946131	0.324257
22932	1.06627	0.323717
22933	1.03102	0.789244
22934	0.488712	0.160571
22935	0.946131	0.271249
22936	1.23146	0.212254
22937	1.13242	0.258613
22938	0.946131	0.239665
22939	0.409199	0.116841
22940	0.429959	0.120382
22941	1.08357	0.269357
22942	0.80926	0.273217
22943	0.879791	0.261794
22944	0.946129	1.15441
22945	1.28828	1.40966
22946	0.946131	1.05332
22947	0.946131	1.60843
22948	1.35969	1.51824
22949	1.10049	1.51951
22950	0.923724	1.21637
22951	0.923695	2.07922
22952	0.94613	1.1414
22953	0.94613	2.1
22954	1.28828	2.02518
22955	1.73525	0.462747
22956	0.656239	0.702751
22957	1.2996	0.587241
22958	0.812939	0.72977
22959	0.65624	0.675156
22960	1.2996	0.671502
22961	1.67372	2.1
22962	0.946131	2.1
22963	0.946132	2.1
22964	1.05978	2.1
22965	1.31049	2.1

22966	0.946132	2.1
22967	1.11077	2.1
22968	0.579725	0.791364
22969	0.373532	0.619242
22970	1.13537	0.804192
22971	1.20726	0.737958
22972	0.946131	0.727106
22973	1.45694	0.847462
22974	0.750367	0.169026
22975	0.946126	0.197135
22976	0.750365	0.171195
22977	1.25698	0.161595
22978	1.35726	0.165312
22979	1.6812	0.187555
22980	1.78665	1.65632
22981	1.46568	2.1
22982	1.29606	2.1
22983	1.25298	2.1
22984	0.946115	2.1
22985	0.946129	2.1
22986	1.25296	2.1
22987	1.27778	0.179052
22988	1.17042	2.1
22989	1.25858	0.093573
22990	0.93106	0.0953046
22991	0.810959	0.0891176
22992	1.38905	2.1
22993	1.19216	2.1
22994	0.946131	2.1
22995	0.946131	2.1
22996	1.19216	2.1
22997	1.58339	1.97197
22998	0.81094	0.151726
22999	0.845129	0.19539
23000	0.918535	0.158568
23001	0.946132	2.1
23002	0.946132	2.1
23003	1.31904	2.1
23004	1.31904	2.1
23005	1.21275	2.1
23006	1.15471	2.1
23007	1.33125	2.1
23008	0.702954	0.378993
23009	0.942973	0.330156
23010	0.94613	0.530468
23011	0.641772	0.284995
23012	0.702942	0.481597
23013	0.991865	0.641572
23014	0.639952	0.688811
23015	1.17333	0.383654
23016	0.946132	0.7
23017	0.490975	0.541738
23018	0.495934	0.698326
23019	1.19904	0.46613
23020	1.30488	0.177035
23021	1.02305	0.614404
23022	1.49842	0.241258
23023	1.54196	2.1

23024	1.37224	1.66199
23025	1.0953	0.357662
23026	1.18952	2.1
23027	1.68851	0.725219
23028	0.946131	0.70298
23029	1.12491	0.682373
23030	0.583591	0.690606
23031	1.28727	0.713966
23032	1.12491	0.69562
23033	0.884641	0.741105
23034	0.810944	0.720715
23035	0.917973	0.71197
23036	0.846671	0.707864
23037	0.596968	0.706523
23038	0.716347	0.709072
23039	1.36255	0.689051
23040	1.11077	2.1
23041	0.946132	2.1
23042	0.946132	2.1
23043	1.26267	2.1
23044	1.26441	2.1
23045	1.85238	2.1
23046	1.36693	2.1
23047	0.616123	2.1
23048	0.481844	2.1
23049	0.709599	2.1
23050	0.815889	2.1
23051	1.1502	2.1
23052	1.63937	0.924936
23053	1.25797	0.578293
23054	1.22878	0.64045
23055	0.946132	0.508107
23056	0.946132	0.454841
23057	0.965737	0.404702
23058	1.1893	0.49421
23059	1.22877	0.536427
23060	0.735217	0.473276
23061	0.85154	0.565739
23062	0.851553	0.448552
23063	0.851473	0.446845
23064	1.58916	0.295207
23065	1.25545	0.462688
23066	0.810942	0.711931
23067	0.919314	0.715911
23068	0.928633	0.701971
23069	2.14514	0.569384
23070	1.23389	2.1
23071	0.946128	0.350152
23072	0.969504	0.243677
23073	0.851539	0.288983
23074	1.41503	0.298733
23075	0.958517	0.25235
23076	0.990615	0.212015
23077	1.7943	0.194061
23078	0.946133	0.792597
23079	1.27581	0.799012
23080	0.946132	0.79489
23081	1.36362	0.77309

23082	1.36936	0.762746
23083	1.71903	2.1
23084	0.751639	2.1
23085	0.77464	2.1
23086	0.946133	2.1
23087	1.04073	2.1
23088	1.23551	2.1
23089	0.94613	2.1
23090	1.18682	2.1
23091	0.946131	2.1
23092	0.94613	2.1
23093	1.40638	2.1
23094	0.483346	0.565726
23095	0.432527	0.418511
23096	0.946132	0.680632
23097	0.483312	0.555379
23098	0.946132	0.584796
23099	0.878798	0.718008
23100	0.810944	0.722535
23101	1.14604	0.598137
23102	0.946129	0.685198
23103	1.35594	0.5798
23104	0.538378	0.57271
23105	0.946131	0.70549
23106	0.538378	0.670852
23107	1.00065	0.872295
23108	0.962878	0.685584
23109	1.32634	0.571214
23110	1.28066	0.13492
23111	0.785651	2.1
23112	1.20327	1.84552
23113	1.47058	1.63572
23114	0.551834	1.14333
23115	1.04072	1.86358
23116	0.506719	1.3182
23117	1.12786	1.42061
23118	1.45789	0.0915529
23119	0.681532	0.109928
23120	1.21087	0.081593
23121	0.94613	0.0952099
23122	0.681534	0.0986095
23123	1.21087	0.103605
23124	1.98618	0.984406
23125	1.71742	1.01403
23126	1.07427	0.839581
23127	1.1972	0.759036
23128	0.933474	0.771574
23129	1.1745	0.804871
23130	0.94612	0.771972
23131	0.933485	0.796733
23132	1.17449	0.791988
23133	0.851541	0.0976525
23134	1.12774	0.089281
23135	0.946129	0.0898263
23136	0.851486	0.109333
23137	1.12517	0.0931142
23138	1.12328	0.0904275
23139	1.13179	2.1

23140	1.02438	2.1
23141	1.05776	2.1
23142	1.41273	1.15718
23143	1.1229	2.1
23144	0.875912	1.21715
23145	0.946132	2.00554
23146	0.875912	1.4092
23147	1.42307	2.1
23148	1.15354	0.114409
23149	0.848382	0.115469
23150	1.2685	1.58885
23151	0.938104	1.42235
23152	0.793166	2.1
23153	1.07855	2.1
23154	0.98488	2.1
23155	1.4162	2.1
23156	1.09651	0.377818
23157	1.98502	0.597391
23158	1.41168	0.676372
23159	1.33879	0.731996
23160	0.946127	0.7567
23161	1.13528	0.674002
23162	0.946128	0.685298
23163	1.09726	2.1
23164	1.22026	0.891963
23165	1.09044	2.09096
23166	2.23961	0.898374
23167	1.14721	0.590875
23168	1.17614	1.19628
23169	0.615065	2.1
23170	0.946131	2.1
23171	0.778488	2.1
23172	0.94613	2.1
23173	0.615066	2.1
23174	1.78254	1.91605
23175	0.898452	0.264683
23176	1.47411	2.1
23177	0.59676	0.718442
23178	0.59676	0.512149
23179	0.94613	0.752139
23180	1.13511	0.708122
23181	0.946131	0.673452
23182	0.916275	2.1
23183	0.946132	1.94971
23184	0.916274	1.31385
23185	1.36182	1.69307
23186	0.946131	1.7777
23187	0.857202	0.0939589
23188	0.941918	0.0885374
23189	0.943349	0.0858655
23190	1.2493	0.0777011
23191	0.946133	0.0946437
23192	1.01814	0.0869578
23193	0.927881	0.0623958
23194	1.11845	0.0618904
23195	1.2493	0.0700954
23196	1.11845	0.0540361
23197	0.946129	0.0676584

23198	0.967673	0.828781
23199	1.01332	0.119214
23200	0.946133	0.130988
23201	0.81938	0.122938
23202	0.819377	0.117134
23203	0.946133	0.138384
23204	1.22474	0.706416
23205	1.15321	0.701767
23206	0.797489	0.700976
23207	0.946131	0.709685
23208	0.797461	0.703428
23209	1.35328	0.879076
23210	1.03318	1.09395
23211	0.946131	0.923465
23212	0.94613	0.95242
23213	1.03319	0.940625
23214	1.37938	0.912482
23215	1.00512	1.13558
23216	0.946133	0.957209
23217	0.946132	0.994258
23218	1.00512	0.971487
23219	1.5515	1.21322
23220	1.55351	2.1
23221	0.94261	0.173022
23222	0.828156	0.155681
23223	0.916495	0.16181
23224	3.06309	0.109502
23225	0.619832	0.109427
23226	0.705903	0.127503
23227	1.2968	0.105535
23228	0.946133	0.114268
23229	0.705904	0.104976
23230	1.2968	0.125218
23231	1.01971	0.103777
23232	0.60302	0.092564
23233	1.07242	0.890157
23234	1.26426	0.826181
23235	0.946131	0.839799
23236	0.946131	0.895994
23237	0.963109	0.883721
23238	0.947862	0.839588
23239	1.40248	1.14185
23240	0.767143	1.21608
23241	0.767114	1.18183
23242	0.946129	1.22526
23243	0.767143	1.10937
23244	1.22637	1.15934
23245	1.2281	1.13287
23246	0.590909	1.24475
23247	0.490832	1.14816
23248	0.804174	1.24523
23249	0.747368	1.0769
23250	1.05129	0.490406
23251	1.10609	0.863925
23252	1.3639	0.703745
23253	0.776117	0.702585
23254	0.752329	0.704338
23255	1.04073	0.708025

23256	0.67713	0.706558
23257	3.4245	2.1
23258	1.18526	2.1
23259	1.2161	1.03857
23260	0.876834	1.38233
23261	0.876831	1.03838
23262	0.946132	1.33478
23263	0.876834	1.11124
23264	0.954563	2.1
23265	0.924132	2.1
23266	1.25075	2.1
23267	1.01652	2.1
23268	0.92413	2.1
23269	1.37579	2.1
23270	0.992159	2.1
23271	1.26092	2.1
23272	0.929435	2.1
23273	0.844958	2.1
23274	0.79598	2.1
23275	0.852559	2.1
23276	0.946131	0.843761
23277	1.28435	0.771367
23278	0.827261	0.816245
23279	0.946128	0.839232
23280	0.827298	0.818283
23281	1.65452	0.651656
23282	1.79929	2.1
23283	0.791947	0.900609
23284	0.902072	0.925103
23285	0.882672	0.898546
23286	1.21703	2.1
23287	0.900338	2.1
23288	1.37712	2.1
23289	0.782591	2.1
23290	0.782562	2.1
23291	0.94613	2.1
23292	1.34749	2.1
23293	0.61359	2.1
23294	1.14397	0.576967
23295	1.02963	0.172682
23296	1.36589	0.152957
23297	0.946132	0.377144
23298	1.28644	0.260069
23299	0.946131	0.297465
23300	1.40104	0.313592
23301	1.28644	0.256915
23302	0.810946	0.618871
23303	1.09178	0.609878
23304	1.26956	0.512316
23305	0.810943	0.626006
23306	0.885633	0.629541
23307	1.00844	0.564873
23308	0.825671	0.649731
23309	0.792093	0.620644
23310	0.925037	0.594305
23311	0.946131	0.88825
23312	0.946131	0.160843
23313	1.18052	0.61823

23314	1.40265	0.668151
23315	0.946132	0.785582
23316	1.17891	0.603159
23317	0.734248	0.10025
23318	0.810943	0.110903
23319	0.731621	0.104557
23320	0.799857	0.738779
23321	0.834507	0.775055
23322	0.810932	0.738912
23323	1.01316	0.183492
23324	1.23884	0.229325
23325	1.88438	2.1
23326	0.946129	1.96612
23327	1.38614	1.96726
23328	1.34245	1.15662
23329	0.946131	1.15032
23330	1.38614	1.18091
23331	0.946132	0.128219
23332	0.910733	0.139675
23333	1.24726	0.101494
23334	1.13742	0.125625
23335	0.851555	0.119483
23336	1.50368	0.0949792
23337	0.94613	0.82577
23338	1.08998	2.1
23339	1.36899	0.494019
23340	1.36898	0.888498
23341	0.946131	0.866528
23342	1.26045	2.1
23343	1.06432	0.961699
23344	1.89016	0.845748
23345	0.83514	1.56023
23346	1.36653	2.07866
23347	1.23426	1.79778
23348	0.94613	1.44549
23349	0.835112	1.9995
23350	1.14567	1.20345
23351	1.12096	1.1643
23352	0.881106	1.28388
23353	1.17787	0.756659
23354	1.29738	0.996131
23355	0.940472	0.824455
23356	1.03452	0.894514
23357	0.946131	0.837333
23358	2.7489	0.759564
23359	1.01778	2.1
23360	1.04321	0.948198
23361	1.19352	0.374942
23362	1.16912	1.31768
23363	0.946132	1.08802
23364	0.946132	1.59716
23365	1.40924	1.50004
23366	1.22927	1.75429
23367	2.09455	0.692412
23368	3.00575	2.1
23369	1.08928	0.998696
23370	1.94799	2.1
23371	1.4092	2.1

23372	0.946132	2.1
23373	1.16	2.1
23374	1.01985	2.1
23375	0.946133	2.1
23376	1.16	2.1
23377	1.15371	0.0528633
23378	1.56002	0.0514539
23379	1.23007	2.0784
23380	1.9992	2.1
23381	0.652605	2.0348
23382	0.937903	2.1
23383	0.946132	1.97639
23384	0.937902	2.1
23385	1.228	2.1
23386	1.33694	1.264
23387	1.00191	2.1
23388	0.716814	1.2956
23389	0.94613	1.8201
23390	0.716816	1.74976
23391	1.70388	1.47033
23392	0.946129	0.76391
23393	1.09362	0.754949
23394	0.946131	0.766272
23395	1.4185	0.799805
23396	1.09362	0.747226
23397	1.31057	1.25783
23398	1.17631	0.116582
23399	1.19404	0.139079
23400	0.94613	0.241906
23401	0.946129	0.195367
23402	0.99972	0.206111
23403	0.94613	0.216689
23404	0.835199	0.0852903
23405	1.23351	0.0965075
23406	0.582785	0.113672
23407	0.701079	0.102669
23408	0.946132	0.09553
23409	2.17385	0.0798966
23410	1.18948	2.05612
23411	1.25346	2.1
23412	1.18586	0.804683
23413	0.880876	0.745336
23414	0.68632	0.768324
23415	0.946118	2.1
23416	0.966936	2.1
23417	0.946117	2.1
23418	1.41636	2.1
23419	1.03798	2.1
23420	0.729178	0.809716
23421	0.721888	0.89398
23422	0.946132	0.814399
23423	0.656286	0.85926
23424	1.13898	0.843249
23425	1.02351	0.377339
23426	1.48803	0.412627
23427	0.946131	0.408668
23428	1.04921	2.1
23429	1.02478	0.379684

23430	0.993023	2.1
23431	1.80841	0.526044
23432	1.29215	0.573852
23433	0.904206	0.502444
23434	0.904207	0.597885
23435	0.946132	0.491552
23436	0.904207	0.509399
23437	1.07347	2.1
23438	0.555875	1.21169
23439	0.555904	1.33474
23440	1.30841	1.50887
23441	1.30841	1.69852
23442	0.91951	1.69644
23443	1.33026	0.853064
23444	1.43289	0.110853
23445	1.15703	1.68746
23446	0.886594	2.02501
23447	1.05129	1.84843
23448	1.08421	0.843234
23449	1.35946	0.825467
23450	0.882955	0.887094
23451	0.946131	0.897754
23452	0.882985	0.87942
23453	0.583501	0.88346
23454	0.532921	0.884157
23455	0.94613	1.36726
23456	0.532892	1.1267
23457	1.23224	1.16044
23458	1.12786	1.12264
23459	0.946131	0.85864
23460	1.23732	0.712922
23461	1.40609	0.875486
23462	1.40609	0.350637
23463	0.946131	0.76917
23464	0.910672	1.26117
23465	1.08478	1.11684
23466	0.94613	0.834786
23467	0.94613	1.10613
23468	1.30382	1.10171
23469	3.10544	1.80243
23470	0.679509	1.80777
23471	1.15966	1.53244
23472	0.75499	1.91125
23473	1.37511	1.63822
23474	1.01776	1.50614
23475	2.35239	1.35565
23476	0.92406	2.1
23477	0.946118	2.1
23478	0.92406	2.1
23479	1.07979	2.1
23480	1.29604	2.1
23481	1.29897	2.1
23482	0.902645	0.76461
23483	0.729493	0.898075
23484	0.906844	0.838247
23485	0.739518	0.264085
23486	1.10564	0.490744
23487	0.946131	0.441897

23488	0.739519	0.368975
23489	0.739506	0.428868
23490	1.79907	0.240292
23491	1.59106	1.04349
23492	1.11067	0.854884
23493	0.795516	0.972893
23494	0.795519	0.88034
23495	0.946132	0.940138
23496	0.795506	0.89036
23497	1.80223	2.1
23498	1.95572	1.42136
23499	1.16237	0.889031
23500	1.07518	1.33683
23501	0.696199	0.926147
23502	0.94613	0.967025
23503	0.696199	0.997088
23504	1.6379	0.0831773
23505	0.946118	0.0960883
23506	0.851529	0.121107
23507	1.08716	0.0891614
23508	0.973118	0.0834267
23509	0.851528	0.094454
23510	1.15096	2.1
23511	0.65023	0.188087
23512	0.94613	0.178673
23513	0.650205	0.240029
23514	0.650205	0.192279
23515	0.811233	0.31486
23516	0.778721	1.75297
23517	0.810945	1.0646
23518	0.997518	1.07247
23519	0.838028	0.211223
23520	0.952893	0.178338
23521	0.838037	0.206848
23522	1.58166	0.137112
23523	1.44848	1.32597
23524	1.30041	0.140894
23525	1.03697	0.99901
23526	0.946132	1.39396
23527	1.0277	1.75243
23528	0.946131	1.52752
23529	1.00501	1.41706
23530	1.0277	1.3008
23531	1.64988	2.1
23532	0.946131	2.1
23533	0.94613	0.904108
23534	1.27702	1.93872
23535	1.55163	1.01854
23536	0.897905	1.04598
23537	1.12971	0.129029
23538	1.35715	0.715448
23539	0.881884	0.824824
23540	0.664007	0.721191
23541	0.663978	0.761442
23542	0.94613	0.742759
23543	0.664006	0.746686
23544	1.48693	0.601555
23545	1.29289	0.559092

23546	1.32081	0.690439
23547	0.946119	0.399859
23548	0.946132	0.358684
23549	1.02972	0.285704
23550	0.946131	2.1
23551	0.946127	2.1
23552	1.23513	2.1
23553	1.38154	2.1
23554	0.94613	2.1
23555	1.25933	0.234371
23556	0.917954	0.244678
23557	0.868589	0.305647
23558	3.0957	2.1
23559	0.711391	0.0930204
23560	0.711391	0.0956848
23561	0.991303	0.0921976
23562	1.37322	0.0899931
23563	0.729145	0.101946
23564	2.55648	0.0934857
23565	1.0756	1.76965
23566	0.760299	1.28325
23567	0.763592	0.87514
23568	0.946126	0.936978
23569	0.76359	0.856487
23570	1.35862	1.2257
23571	0.946133	2.1
23572	1.00807	2.1
23573	0.946132	2.1
23574	1.39823	2.1
23575	1.07817	2.1
23576	1.44379	0.79998
23577	1.17934	0.268128
23578	1.13451	0.701833
23579	0.94613	0.96044
23580	1.03974	2.1
23581	1.36835	0.966558
23582	1.36835	1.86282
23583	0.94613	2.1
23584	1.27096	1.79965
23585	0.853042	2.1
23586	0.946131	2.1
23587	1.13658	2.1
23588	1.06997	2.1
23589	0.94613	2.1
23590	1.41556	0.834984
23591	1.21708	0.909243
23592	0.94613	0.911205
23593	0.946131	0.880664
23594	1.21708	0.877489
23595	0.964782	0.84741
23596	0.946119	0.866724
23597	1.23447	0.866162
23598	0.946118	0.869278
23599	1.41844	0.853575
23600	1.23446	0.875819
23601	1.1357	0.754736
23602	0.619856	0.142606
23603	1.1509	0.102778

23604	0.946126	0.115818
23605	1.19262	0.102103
23606	1.1509	0.128619
23607	1.04268	2.1
23608	2.07527	0.973249
23609	1.38259	0.961334
23610	0.980054	1.2652
23611	0.946124	0.997196
23612	0.946127	1.07003
23613	0.980056	1.02758
23614	1.62916	1.92779
23615	0.946131	2.1
23616	1.40571	2.1
23617	0.946131	2.1
23618	1.38885	2.1
23619	1.40571	2.1
23620	1.11787	0.828955
23621	0.946129	0.829693
23622	1.28831	2.1
23623	1.41498	0.786872
23624	1.41498	0.951889
23625	0.946131	0.900123
23626	0.850964	0.124745
23627	0.921299	0.117704
23628	1.37358	0.101383
23629	0.946132	0.0981612
23630	0.850964	0.125171
23631	0.823988	0.129502
23632	0.823984	0.13136
23633	1.36568	0.109435
23634	1.36569	0.0931271
23635	0.921275	0.128332
23636	1.84739	2.1
23637	1.47169	0.604326
23638	0.946131	0.748253
23639	0.735844	0.724814
23640	1.11819	0.768798
23641	1.10904	0.69313
23642	0.735831	0.718166
23643	0.946131	2.1
23644	0.946132	2.1
23645	1.23487	2.1
23646	1.3281	2.1
23647	0.946131	2.1
23648	1.72271	0.140649
23649	0.861359	0.170112
23650	0.946131	0.308666
23651	0.861357	0.240065
23652	1.1369	0.236892
23653	1.26868	0.225763
23654	0.789173	2.06704
23655	0.789143	2.1
23656	0.946133	1.72262
23657	1.34386	1.96407
23658	0.789144	1.97045
23659	0.983493	2.1
23660	1.18944	0.863713
23661	1.05121	0.861037

23662	0.777398	0.878733
23663	0.946132	0.894143
23664	0.777423	0.928136
23665	1.09841	0.671806
23666	0.963919	0.566587
23667	0.474855	0.704919
23668	0.946131	0.684402
23669	0.474855	0.705941
23670	3.40074	1.18907
23671	1.29453	2.1
23672	1.01139	0.85022
23673	0.946133	1.10003
23674	1.35381	0.835761
23675	1.18322	1.01343
23676	0.946133	1.14161
23677	1.45502	0.252014
23678	0.970088	1.26266
23679	0.960333	1.34289
23680	0.838036	1.45496
23681	1.30927	0.612544
23682	0.828617	0.684777
23683	0.714795	0.627582
23684	0.946132	0.589928
23685	0.714824	0.57534
23686	0.946131	2.1
23687	0.851542	2.1
23688	1.38204	2.1
23689	1.24512	2.1
23690	1.04086	2.1
23691	0.723573	2.1
23692	0.946131	2.1
23693	0.982454	2.1
23694	0.982454	2.1
23695	0.723544	2.1
23696	1.17261	0.364457
23697	1.34153	0.367709
23698	1.41383	0.469565
23699	0.946132	0.357309
23700	0.946133	0.292534
23701	1.12818	0.29199
23702	1.29383	0.450122
23703	0.946133	0.79943
23704	1.0027	0.789261
23705	0.768989	0.751075
23706	0.946133	0.821975
23707	0.768977	0.793666
23708	0.946132	0.85517
23709	1.24333	0.785283
23710	0.928583	0.833923
23711	0.946132	0.868157
23712	0.928583	0.856482
23713	1.53798	0.687529
23714	2.95436	0.263483
23715	1.30649	0.332642
23716	0.809789	0.607039
23717	0.745744	0.551166
23718	0.745774	0.522316
23719	0.946128	0.504482

23720	1.00777	0.474773
23721	1.40271	0.351016
23722	0.768387	0.539112
23723	0.946132	0.520738
23724	0.922045	0.52826
23725	0.946132	2.1
23726	1.39021	2.1
23727	1.10046	2.1
23728	0.946132	2.1
23729	1.39021	2.1
23730	2.06158	0.328717
23731	0.913967	0.735965
23732	1.30107	0.800983
23733	0.946132	0.735178
23734	0.913966	0.744876
23735	1.30107	0.738551
23736	1.64117	2.1
23737	0.514872	1.37218
23738	0.514846	1.11587
23739	1.30811	1.6047
23740	1.30812	1.13559
23741	0.888989	1.20726
23742	1.99279	1.63689
23743	0.910271	0.833949
23744	0.838027	0.851668
23745	0.928444	0.857346
23746	1.2377	0.961185
23747	0.851537	2.09407
23748	0.899142	2.1
23749	0.810075	2.1
23750	1.3771	2.1
23751	1.03281	2.1
23752	0.719877	2.1
23753	0.901838	2.1
23754	1.01748	2.1
23755	1.12315	2.1
23756	0.64787	2.1
23757	1.173 2.1	
23758	0.876903	2.1
23759	0.597337	2.1
23760	1.00291	2.1
23761	0.597309	2.1
23762	1.09897	0.83097
23763	0.946132	1.04969
23764	1.1386	0.843119
23765	1.09897	0.895075
23766	0.596652	0.931307
23767	2.5593	2.1
23768	0.946133	0.695904
23769	1.29172	0.68869
23770	1.11337	0.686135
23771	0.946131	0.696855
23772	0.946131	0.681457
23773	0.96926	0.837041
23774	0.946132	0.850376
23775	0.946132	0.918438
23776	1.07118	0.784365
23777	1.29698	0.794269

23778	1.37867	0.936602
23779	0.946119	1.08312
23780	0.94612	1.44827
23781	1.33154	0.92173
23782	0.946107	1.0098
23783	2.17757	1.32109
23784	0.946131	0.412821
23785	0.68393	0.232196
23786	0.68396	0.328295
23787	1.33671	0.359183
23788	1.10733	0.334803
23789	2.31367	2.1
23790	0.989326	1.59351
23791	0.689952	2.1
23792	0.854787	1.47611
23793	3.73635	2.1
23794	1.41489	2.1
23795	1.25544	0.992742
23796	0.946128	1.95224
23797	0.946129	1.79472
23798	1.13536	2.035
23799	2.10284	1.13392
23800	0.851542	2.1
23801	0.828523	2.1
23802	0.946132	2.1
23803	0.946132	2.1
23804	1.50873	2.1
23805	0.989048	2.1
23806	0.925055	2.1
23807	0.838041	2.1
23808	0.807714	1.14194
23809	0.656831	0.822656
23810	0.997603	1.05143
23811	0.946131	1.10363
23812	1.02602	1.0067
23813	0.946129	1.10945
23814	1.35469	1.00322
23815	1.25083	0.995369
23816	0.851541	0.69472
23817	0.851483	0.694963
23818	0.851539	0.711294
23819	1.19785	0.631244
23820	1.07615	0.59289
23821	0.714507	0.7
23822	1.41461	0.7
23823	0.714507	0.7
23824	0.714507	0.7
23825	0.946133	0.70032
23826	0.946131	0.98667
23827	0.972232	0.88756
23828	0.94613	1.03242
23829	1.25083	0.893342
23830	1.30165	0.890491
23831	1.05332	2.1
23832	0.574768	2.1
23833	1.94541	0.118287
23834	0.789633	0.173483
23835	0.946127	0.215925

23836	1.33798	0.181066
23837	1.22903	0.205274
23838	0.946128	0.201933
23839	0.946128	2.1
23840	1.11882	2.1
23841	0.996487	2.1
23842	0.94613	2.1
23843	1.11883	2.1
23844	0.946127	2.1
23845	1.30082	2.1
23846	1.02608	2.1
23847	0.946128	2.1
23848	1.30081	2.1
23849	1.3507	1.22133
23850	1.19583	0.756882
23851	0.946127	1.51882
23852	0.94613	1.06288
23853	1.19583	1.19997
23854	0.477652	0.143146
23855	1.20416	0.313207
23856	0.946131	0.251815
23857	0.477652	0.188163
23858	0.477691	0.291936
23859	1.34536	0.0942363
23860	0.758971	0.281035
23861	1.25124	0.264947
23862	1.31467	0.230083
23863	0.758971	0.241603
23864	1.31467	0.277452
23865	0.946131	2.1
23866	1.14184	2.1
23867	1.35028	2.1
23868	1.35028	2.1
23869	0.946131	2.1
23870	1.13537	1.97364
23871	0.946132	1.15122
23872	0.946132	2.1
23873	1.23031	1.70927
23874	1.27555	1.63521
23875	1.53064	2.07831
23876	1.28421	0.908808
23877	1.07707	0.971361
23878	0.946125	0.905804
23879	0.946127	0.936389
23880	1.07707	0.931955
23881	0.946133	0.418479
23882	1.3495	0.248907
23883	0.946132	0.448477
23884	1.05704	0.359574
23885	1.3495	0.368084
23886	0.946132	0.158747
23887	1.37794	0.129268
23888	0.946132	0.162501
23889	1.01367	0.18111
23890	1.37794	0.157092
23891	0.928516	2.1
23892	0.946133	2.1
23893	0.928518	2.1

23894	1.14105	2.1
23895	1.26406	2.1
23896	0.797664	2.1
23897	0.946132	2.1
23898	0.797663	2.1
23899	1.34285	2.1
23900	0.929875	2.1
23901	0.946131	0.845875
23902	1.21139	0.742719
23903	1.41492	0.705736
23904	1.41492	0.732426
23905	0.946131	0.722712
23906	0.946131	2.1
23907	0.689388	2.1
23908	0.94365	2.1
23909	0.777345	2.1
23910	0.709599	2.1
23911	0.774968	2.1
23912	0.946132	2.1
23913	0.77497	2.1
23914	0.868145	2.1
23915	1.28401	2.1
23916	0.94613	0.789055
23917	1.19655	1.08401
23918	1.39428	0.731271
23919	1.39428	0.79455
23920	0.946131	0.795569
23921	1.1848	2.1
23922	1.07591	2.1
23923	0.946131	2.1
23924	0.946131	2.1
23925	0.874788	2.1
23926	1.35703	2.1
23927	1.35357	2.1
23928	0.946132	2.1
23929	1.26022	2.1
23930	0.946132	2.1
23931	0.946131	2.1
23932	0.966426	2.1
23933	0.946133	2.1
23934	0.946131	2.1
23935	1.3484	2.1
23936	1.26441	2.1
23937	1.2079	2.1
23938	0.946133	2.1
23939	0.946132	2.1
23940	1.2079	2.1
23941	1.2736	2.1
23942	1.25774	2.1
23943	0.946133	2.1
23944	0.851555	2.1
23945	0.96172	2.1
23946	0.946128	2.1
23947	1.06879	2.1
23948	0.94613	2.1
23949	1.32608	2.1
23950	1.06879	2.1
23951	0.946132	2.1

23952	1.32608	2.1
23953	0.946131	2.1
23954	1.40488	2.1
23955	1.32608	2.1
23956	0.94613	1.06372
23957	1.19234	0.876119
23958	0.946129	0.911936
23959	0.94613	1.08815
23960	1.31649	1.17665
23961	0.912059	0.996541
23962	1.04514	1.01388
23963	0.946132	0.940463
23964	0.946132	1.34582
23965	1.34646	1.43495
23966	1.01187	0.739477
23967	1.33728	0.847973
23968	0.761974	0.559013
23969	0.761974	0.775191
23970	0.94613	0.758541
23971	1.40641	1.38747
23972	0.955752	0.933928
23973	0.736738	1.5048
23974	0.94613	1.00448
23975	0.736739	1.07567
23976	0.861471	2.1
23977	1.34556	2.1
23978	0.810957	2.1
23979	0.946131	2.1
23980	1.37811	2.1
23981	1.17949	2.1
23982	0.946132	2.1
23983	1.37811	2.1
23984	0.946131	2.1
23985	1.40447	2.1
23986	0.946132	2.1
23987	0.946132	2.1
23988	1.39826	2.1
23989	0.946131	2.1
23990	1.283 2.1	
23991	0.94613	2.1
23992	0.946131	2.1
23993	1.40447	2.1
23994	1.20541	2.1
23995	0.946131	1.94883
23996	0.946127	2.1
23997	1.33607	2.1
23998	1.41021	2.1
23999	1.01271	2.1
24000	0.946131	2.1
24001	0.920606	2.1
24002	1.13078	2.1
24003	1.60331	2.1
24004	0.946118	0.896281
24005	1.09718	1.96217
24006	0.946116	1.07366
24007	1.32662	0.945063
24008	1.0972	0.99006
24009	0.94613	0.847141

24010	1.32666	1.04462
24011	0.946128	0.811097
24012	1.31381	0.800089
24013	1.32666	0.780835
24014	0.94612	1.89185
24015	0.940748	2.1
24016	1.03561	2.1
24017	1.35111	2.1
24018	0.851527	2.1
24019	0.607156	1.36918
24020	0.789158	2.1
24021	0.946129	1.15265
24022	1.04071	1.97923
24023	1.13144	2.04927
24024	0.946133	2.1
24025	1.35663	2.1
24026	0.946132	2.1
24027	0.946133	2.1
24028	1.34237	2.1
24029	0.919455	2.1
24030	1.01143	2.1
24031	0.946128	2.1
24032	0.946131	2.1
24033	1.62796	2.1
24034	1.17796	1.13468
24035	1.23024	1.94452
24036	0.946132	1.10647
24037	0.85153	1.49781
24038	0.986867	1.23737
24039	0.889308	2.1
24040	0.94613	2.1
24041	0.889338	2.1
24042	0.946131	2.1
24043	0.889337	2.1
24044	1.18396	0.978923
24045	0.94613	0.870366
24046	0.946125	1.2677
24047	1.39514	0.94675
24048	1.30809	1.07962
24049	0.803322	2.07181
24050	0.925893	1.61663
24051	1.36183	1.35366
24052	0.925892	1.4367
24053	0.946133	1.38908
24054	1.38354	0.482204
24055	1.16009	0.612602
24056	0.946132	0.474414
24057	0.946132	0.401616
24058	1.01462	0.34914
24059	1.18854	1.03996
24060	0.888265	1.583
24061	0.745095	1.08672
24062	0.709597	1.37181
24063	0.936541	1.24151
24064	1.24142	0.891537
24065	1.35059	0.824059
24066	0.946128	0.800667
24067	0.94613	0.860855

24068	1.16623	0.854483
24069	1.24945	0.441295
24070	1.33035	0.444984
24071	0.946127	0.415904
24072	0.946128	0.38174
24073	1.13343	0.38397
24074	0.637995	0.991346
24075	1.10455	1.08367
24076	1.22641	1.16708
24077	1.22641	1.61066
24078	0.946132	1.18119
24079	1.3665	0.577919
24080	1.28507	0.578186
24081	0.946127	0.574957
24082	0.946129	0.48077
24083	1.2051	0.465799
24084	0.797374	2.1
24085	0.797376	2.1
24086	1.13113	2.1
24087	1.20057	2.1
24088	1.12049	2.1
24089	0.59387	0.894987
24090	1.13245	0.933123
24091	0.966134	1.02148
24092	0.593842	0.89281
24093	0.946128	0.991081
24094	0.641455	1.01305
24095	0.916507	0.917738
24096	1.04072	1.25739
24097	0.415585	0.911848
24098	0.507244	1.18905
24099	0.678938	2.1
24100	1.24154	2.1
24101	1.25	1.78453
24102	1.24154	2.00092
24103	1.25	1.97362
24104	0.851009	1.74987
24105	0.946131	1.88574
24106	0.851539	1.59638
24107	1.21997	1.45979
24108	1.45109	1.49999
24109	0.804877	0.964371
24110	1.00062	0.751275
24111	0.851536	0.903049
24112	0.909673	0.753067
24113	0.946129	0.917007
24114	1.20494	0.625856
24115	1.09088	0.714652
24116	0.887634	0.720538
24117	0.81027	0.565354
24118	0.900294	0.59539
24119	0.747713	0.220921
24120	0.975467	0.242637
24121	0.747712	0.227976
24122	1.39168	0.174867
24123	0.975467	0.210947
24124	0.94613	0.659465
24125	1.32827	0.63838

24126	0.946128	0.636163
24127	1.41201	0.686241
24128	1.32827	0.649392
24129	0.981121	0.424291
24130	0.936311	0.643607
24131	1.00715	0.422743
24132	1.15914	0.518062
24133	0.704409	0.494056
24134	0.713444	1.17139
24135	0.954538	1.02535
24136	0.944508	1.04065
24137	0.851165	1.19112
24138	0.946131	1.44058
24139	0.851192	1.14244
24140	0.946131	1.15889
24141	1.3279	1.11235
24142	1.33587	0.145722
24143	0.946126	0.180172
24144	1.27512	0.169406
24145	1.33587	0.170159
24146	0.748059	0.151021
24147	1.17451	0.369524
24148	1.04299	0.37684
24149	0.851542	0.45654
24150	0.946131	0.408963
24151	0.892109	0.427795
24152	0.946131	0.0913317
24153	0.851541	0.0933665
24154	1.2178	0.0877691
24155	1.2179	0.0902492
24156	0.946793	0.0920509
24157	0.709599	0.308641
24158	0.987426	0.234897
24159	1.39502	0.319278
24160	1.13624	0.223328
24161	0.73176	0.289969
24162	1.36697	0.159956
24163	1.33393	0.137365
24164	0.946131	0.248049
24165	0.946132	0.218599
24166	1.23845	0.219785
24167	0.50702	0.937782
24168	1.06442	0.89404
24169	0.946131	0.942867
24170	1.02363	0.936882
24171	1.13537	0.160176
24172	1.25049	0.1262
24173	0.94613	0.158989
24174	1.14993	0.121289
24175	1.38056	0.120886
24176	0.946126	0.0744063
24177	1.24678	0.0663924
24178	0.684521	0.0705997
24179	0.946124	0.0705824
24180	0.684518	0.0701609
24181	0.851542	0.8077
24182	0.925459	0.807659
24183	0.946131	0.811892

24184	0.851474	0.830768
24185	0.923305	0.832282
24186	1.4055	0.0933136
24187	1.32077	0.12058
24188	0.946127	0.171595
24189	1.21757	0.134207
24190	0.946129	0.134196
24191	0.711214	0.372093
24192	1.30463	0.508829
24193	0.711185	0.423949
24194	0.711185	0.445696
24195	1.22716	0.421135
24196	1.29958	0.102955
24197	1.26201	0.148271
24198	0.568927	0.153231
24199	0.568927	0.0923417
24200	0.946132	0.140323
24201	0.851542	0.488605
24202	1.44968	0.360421
24203	0.851543	0.411267
24204	1.35123	0.453315
24205	1.40427	0.355113
24206	0.593295	0.588587
24207	0.946129	0.636918
24208	1.22144	0.516065
24209	0.908278	0.649954
24210	1.22144	0.571961
24211	0.867593	0.738529
24212	1.23351	0.627867
24213	0.946131	0.738438
24214	1.05516	0.734869
24215	1.23351	0.701554
24216	1.15564	0.166753
24217	0.946131	0.313136
24218	0.766652	0.235257
24219	0.766681	0.205952
24220	0.946131	0.27542
24221	0.946131	0.707159
24222	0.897851	0.672509
24223	1.1751	0.713223
24224	1.00273	0.663848
24225	0.851554	0.672609
24226	0.883272	0.499446
24227	1.17111	0.800979
24228	0.851541	0.703886
24229	1.01665	0.660404
24230	0.851555	0.629953
24231	0.946131	0.798004
24232	0.911508	0.769299
24233	1.08054	0.821765
24234	1.33007	0.734796
24235	0.911471	0.764014
24236	1.26787	1.69411
24237	1.16641	1.35785
24238	0.946132	1.35773
24239	0.946133	1.14732
24240	1.16641	1.54933
24241	0.946131	2.1

24242	1.25037	0.178762
24243	1.34733	0.98988
24244	1.34733	0.841901
24245	0.946131	0.854347
24246	0.946131	0.206816
24247	1.41377	0.166134
24248	0.94613	0.195928
24249	1.30541	0.163285
24250	1.41377	0.157742
24251	1.3473	2.1
24252	1.11804	2.08972
24253	0.94613	2.1
24254	0.946131	2.1
24255	1.11805	2.1
24256	1.32068	2.1
24257	0.946131	2.1
24258	0.946128	2.1
24259	1.39384	2.1
24260	1.4181	2.1
24261	1.12913	2.1
24262	0.94613	2.1
24263	0.946129	2.1
24264	1.20328	2.1
24265	1.39385	2.1
24266	1.3335	2.1
24267	1.40987	2.1
24268	0.94613	2.1
24269	1.21474	2.1
24270	0.94613	2.1
24271	0.946132	1.2118
24272	1.15203	2.1
24273	1.41192	0.911065
24274	1.41192	2.1
24275	0.946132	2.1
24276	0.946131	2.1
24277	1.40684	2.1
24278	1.2597	2.03121
24279	0.946131	1.81424
24280	1.40684	1.19845
24281	0.946131	1.77594
24282	1.39113	2.05969
24283	1.34049	1.10417
24284	0.946131	1.15075
24285	1.39113	1.03377
24286	0.946132	1.64004
24287	1.31649	0.996363
24288	0.946132	0.918577
24289	0.946132	1.07515
24290	1.34297	1.10651
24291	0.946131	0.702054
24292	1.41766	0.688038
24293	0.954559	1.0345
24294	0.946131	0.70208
24295	0.946131	0.687248
24296	1.25447	0.609117
24297	1.35559	0.490912
24298	0.946127	0.513715
24299	0.946128	0.614703

24300	1.14874	0.555945
24301	1.33035	0.574969
24302	1.3665	0.599895
24303	0.946129	0.502095
24304	0.94613	0.428074
24305	1.20732	0.425488
24306	0.946131	2.1
24307	0.946131	2.08801
24308	1.32949	2.1
24309	1.34921	2.1
24310	0.946131	2.1
24311	0.946132	0.753994
24312	1.25479	0.739631
24313	1.38357	0.737695
24314	1.38357	0.706486
24315	0.946132	0.704104
24316	0.946131	0.973575
24317	1.31034	0.727394
24318	1.37127	0.917141
24319	1.37127	0.778198
24320	0.946131	0.800374
24321	1.23923	0.595217
24322	1.41169	0.720422
24323	0.94613	0.757731
24324	1.12703	0.592363
24325	0.946131	0.62547
24326	0.946119	2.1
24327	1.41915	2.1
24328	1.02679	2.1
24329	0.946107	2.1
24330	1.41917	2.1
24331	0.946133	2.1
24332	1.36581	2.1
24333	0.946131	2.1
24334	1.36581	2.1
24335	0.946131	2.1
24336	0.946132	2.1
24337	1.04384	2.1
24338	1.34973	2.1
24339	1.34967	2.1
24340	0.851555	2.1
24341	0.946132	0.699052
24342	1.3855	0.7
24343	1.08603	0.69639
24344	1.08602	0.7
24345	1.16234	0.7
24346	0.946133	0.7
24347	1.29348	0.7
24348	1.20111	0.7
24349	1.20112	0.7
24350	1.3855	0.7
24351	0.946128	1.17294
24352	1.30798	2.1
24353	0.946129	2.1
24354	1.34828	2.07611
24355	1.30797	2.1
24356	1.24932	0.0897404
24357	1.40549	0.10466

24358	0.946117	0.143225
24359	1.19615	0.119433
24360	0.946106	0.120919
24361	0.946131	2.1
24362	1.29926	2.1
24363	1.25144	2.1
24364	0.946131	2.1
24365	1.29925	2.1
24366	0.946132	2.1
24367	1.39826	2.1
24368	0.946133	2.1
24369	0.946133	2.1
24370	1.29926	2.1
24371	0.946119	0.267191
24372	1.33614	0.201372
24373	0.946118	0.225495
24374	1.41377	0.184604
24375	1.33612	0.192696
24376	0.946133	1.42132
24377	1.3933	1.95569
24378	1.34999	1.07656
24379	0.946133	1.3745
24380	1.3933	1.26903
24381	0.946131	1.5389
24382	1.36139	2.1
24383	0.946132	1.18763
24384	0.946131	1.79462
24385	1.39329	1.1933
24386	1.37449	1.17302
24387	1.27925	0.793697
24388	0.919796	0.837826
24389	1.01176	0.95357
24390	0.94613	0.912619
24391	0.946133	1.75331
24392	1.27679	2.1
24393	0.946133	1.23103
24394	0.946132	2.1
24395	1.39731	1.58328
24396	0.946119	2.1
24397	1.40765	1.50036
24398	1.1981	1.77062
24399	0.946131	1.20895
24400	1.40764	1.84706
24401	0.946127	2.1
24402	1.3557	2.1
24403	0.946127	2.1
24404	0.946128	2.1
24405	1.13485	2.1
24406	1.4008	2.1
24407	1.1587	2.1
24408	0.946131	2.1
24409	0.946131	2.1
24410	1.1587	2.1
24411	1.32082	0.634148
24412	1.29215	0.693135
24413	0.94613	0.4559
24414	0.946131	0.421173
24415	1.02366	0.35931

24416	1.27697	0.500546
24417	1.2929	0.610714
24418	0.946132	0.382655
24419	0.946132	0.342518
24420	1.01451	0.259278
24421	0.946132	2.1
24422	1.27252	2.1
24423	0.946132	2.1
24424	1.37869	2.1
24425	1.27252	2.1
24426	1.15627	2.1
24427	1.35703	2.1
24428	0.946132	2.1
24429	1.03783	2.1
24430	0.946132	2.1
24431	0.946128	2.1
24432	1.37869	2.1
24433	0.946131	2.1
24434	1.35357	2.1
24435	1.37869	2.1
24436	0.946132	2.1
24437	0.946131	2.1
24438	1.33471	2.1
24439	0.946131	2.1
24440	1.36373	2.1
24441	1.41342	0.87587
24442	1.06832	0.904711
24443	0.946128	1.31561
24444	0.94613	0.807761
24445	1.06832	1.15799
24446	1.26929	2.1
24447	0.946131	1.13665
24448	0.946128	2.1
24449	1.41022	1.49537
24450	1.37034	1.17828
24451	1.37759	0.901518
24452	1.07612	1.07513
24453	0.946126	0.934675
24454	0.946129	0.97467
24455	1.07612	0.958947
24456	0.946131	0.955887
24457	0.94613	0.736963
24458	1.23848	0.800089
24459	1.38897	0.765045
24460	0.946131	0.815383
24461	0.946132	0.764566
24462	0.946133	0.47931
24463	1.34369	0.555513
24464	1.40332	0.265739
24465	0.946132	0.666462
24466	0.946131	2.1
24467	1.24023	2.1
24468	1.38244	2.1
24469	1.38244	2.1
24470	0.946131	2.1
24471	0.946132	1.10524
24472	1.33468	2.1
24473	1.41619	1.82477

24474	1.41619	2.1
24475	0.946131	2.1
24476	1.1908	1.03391
24477	0.946128	0.948916
24478	0.946126	1.12852
24479	1.26654	1.15227
24480	1.39514	1.51719
24481	0.93608	2.1
24482	1.04105	2.1
24483	1.39551	2.1
24484	1.52471	2.1
24485	1.04072	2.1
24486	1.33174	0.0959161
24487	0.946128	0.091031
24488	0.94613	0.0864233
24489	1.17801	0.0800543
24490	0.94613	0.097628
24491	0.946132	2.1
24492	0.946132	2.1
24493	1.29065	2.1
24494	1.29065	2.1
24495	0.975266	2.1
24496	0.946131	2.1
24497	1.26025	2.1
24498	0.955601	2.1
24499	0.946131	2.1
24500	1.26025	2.1
24501	0.94613	0.637252
24502	1.23146	0.531357
24503	0.946127	0.601156
24504	1.32827	0.656287
24505	1.23146	0.632446
24506	1.25008	0.867634
24507	1.05018	0.899093
24508	0.94613	0.873827
24509	0.932744	0.929407
24510	1.04207	0.898716
24511	0.946132	0.767938
24512	1.10023	0.910521
24513	1.36748	0.745765
24514	1.36748	0.817162
24515	0.946133	0.800953
24516	1.06023	0.330136
24517	1.08019	0.340043
24518	0.94613	0.581375
24519	1.06023	0.347721
24520	0.94613	0.37636
24521	1.16727	0.457192
24522	1.35856	0.566542
24523	0.946132	0.699117
24524	1.16727	0.543248
24525	0.946132	0.550184
24526	0.946133	2.1
24527	0.946131	2.1
24528	1.21691	2.1
24529	1.39558	2.1
24530	0.946131	2.1
24531	1.09905	0.781097

24532	0.946132	1.00255
24533	1.3365	0.864869
24534	1.35381	0.910569
24535	0.946132	1.04354
24536	0.94613	0.733352
24537	0.946128	0.794699
24538	1.40332	0.62281
24539	1.39041	0.497287
24540	0.94613	0.72901
24541	0.946132	0.826462
24542	1.3659	1.031
24543	1.39041	0.862378
24544	1.39041	0.94502
24545	0.946132	0.941179
24546	0.840292	1.81266
24547	0.946133	1.44873
24548	0.851541	1.89006
24549	1.28464	1.57625
24550	1.28806	1.44983
24551	1.12591	0.80818
24552	1.05033	0.889838
24553	0.946132	0.837295
24554	0.946132	0.864943
24555	1.13537	0.857435
24556	1.21177	1.11536
24557	0.946132	1.15333
24558	0.946132	2.02544
24559	1.41134	1.14372
24560	1.27079	1.24467
24561	0.946131	0.222518
24562	1.35403	0.218352
24563	0.94613	0.343093
24564	0.94613	0.313216
24565	0.946131	0.244181
24566	0.946131	0.655695
24567	0.946132	0.392886
24568	0.94613	0.816412
24569	1.37052	0.595501
24570	0.946131	0.6852
24571	0.946133	0.453754
24572	0.946132	0.740315
24573	0.946133	0.508491
24574	1.24249	0.476217
24575	0.946132	0.570763
24576	1.20608	2.1
24577	0.946132	2.1
24578	0.946131	1.86709
24579	0.946132	2.1
24580	0.946132	2.1
24581	0.946131	2.1
24582	0.946128	2.1
24583	0.946129	2.1
24584	1.41232	2.1
24585	0.94613	2.1
24586	0.946132	0.277904
24587	1.32449	0.321029
24588	0.946131	0.388584
24589	0.946131	0.343412

24590	1.32449	0.315446
24591	0.946116	1.07966
24592	1.41563	2.05799
24593	0.946118	1.54503
24594	0.946105	1.63277
24595	0.946131	1.05909
24596	1.26563	2.1
24597	1.32819	1.70443
24598	0.946131	0.85278
24599	0.946131	2.01687
24600	0.946131	1.07655
24601	0.946131	1.78761
24602	0.946133	1.19134
24603	1.30382	2.1
24604	0.946131	1.1632
24605	0.946131	1.11801
24606	1.30382	1.66749
24607	0.946133	0.0608369
24608	1.15803	0.0674673
24609	1.28895	0.0634853
24610	0.954071	0.0672625
24611	0.946132	0.0754138
24612	1.0264	1.06256
24613	0.946132	1.54443
24614	0.946132	1.25466
24615	0.946131	1.44193
24616	0.946132	1.30312
24617	1.35977	0.515419
24618	0.946132	0.761689
24619	0.946129	0.764959
24620	0.94613	0.738848
24621	0.946129	0.706894
24622	0.876631	0.705961
24623	0.946133	0.701425
24624	0.946132	0.723364
24625	0.946132	0.704763
24626	0.876606	0.702953
24627	0.946131	0.366127
24628	1.24979	0.174965
24629	0.94613	0.263264
24630	0.94613	0.175944
24631	0.94613	0.238473
24632	0.921791	0.162731
24633	0.946131	0.14608
24634	0.921762	0.153955
24635	0.946131	0.128713
24636	0.946131	0.13178
24637	0.810944	0.488659
24638	0.810945	0.588549
24639	0.810959	0.560531
24640	0.946133	0.445922
24641	1.25797	0.408242
24642	0.946132	0.421212
24643	0.946132	0.540646
24644	1.25797	0.505307
24645	1.13748	0.831836
24646	0.851512	0.931911
24647	0.94613	1.11053

24648	1.02373	0.827387
24649	0.946131	0.975675
24650	0.946122	2.1
24651	1.3852	2.1
24652	0.946124	2.1
24653	0.946118	2.1
24654	1.38521	2.1
24655	0.946131	0.699691
24656	0.946131	0.694968
24657	1.30448	0.698734
24658	0.946129	0.7
24659	0.94613	0.689711
24660	0.946133	2.1
24661	0.94613	2.1
24662	0.946131	2.04852
24663	0.946131	1.9528
24664	1.36503	2.1
24665	0.810947	0.0771624
24666	0.810947	0.0737197
24667	0.810934	0.0812501
24668	3.34066	0.0527475
24669	0.946133	0.877654
24670	1.4185	0.772901
24671	0.946132	0.753315
24672	0.946133	0.818702
24673	0.946132	0.793536
24674	0.94613	2.1
24675	0.946128	2.1
24676	1.03925	2.1
24677	0.944743	2.1
24678	0.947518	2.1
24679	0.946132	2.1
24680	1.20573	2.1
24681	0.94613	2.1
24682	0.94613	2.1
24683	1.20573	2.1
24684	1.20056	0.124891
24685	0.946132	0.148319
24686	0.946131	0.174298
24687	0.946131	0.140698
24688	0.946131	0.146566
24689	0.94613	2.1
24690	0.946128	2.1
24691	1.38302	2.1
24692	0.94613	2.1
24693	0.94613	2.1
24694	0.827866	0.325473
24695	0.913552	0.238485
24696	0.716526	0.373576
24697	0.82784	0.258792
24698	1.20634	0.37797
24699	0.946131	0.338952
24700	0.94613	0.344585
24701	1.39502	0.238179
24702	0.88146	0.35775
24703	0.946132	2.1
24704	0.946132	2.1
24705	0.946132	2.1

24706	0.946132	2.1
24707	1.39053	2.1
24708	0.946131	2.1
24709	1.40571	2.1
24710	0.946131	2.1
24711	0.946131	2.1
24712	0.946132	2.1
24713	0.94613	0.703773
24714	1.2582	0.719584
24715	0.946127	0.703618
24716	0.946129	0.710744
24717	0.946129	0.707698
24718	0.946129	0.98362
24719	0.946132	1.02778
24720	0.946131	0.949309
24721	0.946131	1.07355
24722	1.29054	0.942067
24723	0.810944	0.0987942
24724	0.810941	0.0932853
24725	0.810931	0.0929976
24726	0.946132	0.844476
24727	0.946132	0.817303
24728	0.946132	1.01778
24729	0.946132	0.836264
24730	0.927608	0.821139
24731	0.946128	2.1
24732	0.946133	2.1
24733	0.946131	2.1
24734	0.94613	2.1
24735	1.38813	2.1
24736	0.94613	0.844503
24737	0.946128	2.1
24738	0.946132	0.836992
24739	1.41666	1.71326
24740	0.94613	0.967862
24741	0.946118	0.632751
24742	1.34151	0.40518
24743	0.946116	0.600331
24744	0.946131	0.609278
24745	1.34149	0.585247
24746	0.946132	2.1
24747	1.31889	2.1
24748	0.946132	2.1
24749	0.946132	2.1
24750	1.31889	2.1
24751	0.864997	0.100995
24752	0.928981	0.094804
24753	0.872219	0.0888738
24754	0.946127	0.82935
24755	0.946131	0.790458
24756	1.37709	0.764682
24757	0.946131	0.833988
24758	0.946129	0.790582
24759	1.21882	0.0727227
24760	0.938168	0.0645303
24761	0.938161	0.0693066
24762	1.06327	0.0655676
24763	1.33457	0.0749424

24764	0.946133	0.981333
24765	0.946132	0.987805
24766	0.946133	0.956196
24767	0.946132	1.1401
24768	1.14444	0.915873
24769	1.12628	0.217269
24770	0.946132	0.325231
24771	0.946131	0.360154
24772	1.12628	0.212077
24773	0.946131	0.237251
24774	0.946132	2.1
24775	0.946132	2.1
24776	0.946133	2.1
24777	0.946132	2.1
24778	1.29322	2.1
24779	0.946131	2.1
24780	0.946132	2.1
24781	0.94613	2.1
24782	0.946131	2.1
24783	1.28441	2.1
24784	0.946118	1.94685
24785	0.946119	1.97683
24786	0.946117	2.1
24787	1.28075	2.1
24788	0.946105	2.1
24789	0.810945	2.1
24790	0.810942	2.1
24791	0.81093	2.1
24792	0.946127	2.1
24793	0.946127	2.1
24794	1.28228	2.1
24795	0.946129	2.1
24796	0.946132	2.1
24797	1.14042	2.1
24798	0.946132	2.1
24799	0.946133	2.1
24800	1.14042	2.1
24801	0.946133	0.7
24802	0.946132	0.6677
24803	1.28632	0.583069
24804	1.28632	0.306152
24805	0.946133	0.656688
24806	0.946131	1.16915
24807	0.946131	1.05926
24808	0.946131	1.10875
24809	1.31817	1.20835
24810	0.946131	1.0626
24811	1.35048	2.1
24812	0.946132	2.1
24813	0.946132	2.1
24814	0.946132	2.1
24815	0.946131	2.1
24816	0.946131	2.1
24817	1.35943	2.1
24818	0.94613	2.1
24819	0.946132	2.1
24820	0.946131	2.1
24821	0.990713	2.1

24822	0.946132	2.1
24823	0.946132	2.1
24824	0.946133	2.1
24825	0.946132	2.1
24826	0.946133	0.961382
24827	1.01433	1.07873
24828	0.877931	1.02173
24829	0.96573	0.956703
24830	0.946131	0.942948
24831	0.946131	1.0698
24832	0.946133	0.953862
24833	1.39575	0.848088
24834	0.946131	0.873615
24835	0.94613	0.881971
24836	0.810943	0.806652
24837	0.810942	0.809
24838	0.709589	0.773823
24839	0.924071	0.759876
24840	1.04293	0.678623
24841	1.15747	0.478541
24842	0.946131	0.762809
24843	1.04071	0.747364
24844	0.810946	0.871536
24845	0.810943	0.835926
24846	0.810959	0.802198
24847	0.946128	1.2854
24848	1.39696	1.75821
24849	0.94613	1.01218
24850	0.94613	1.02845
24851	1.39696	1.3962
24852	0.946131	0.911233
24853	0.946131	1.47928
24854	0.946131	1.384
24855	0.946131	1.0066
24856	1.29735	1.51812
24857	0.946128	1.20054
24858	0.946133	0.87789
24859	0.94613	2.0483
24860	0.946131	1.14099
24861	1.33266	1.0151
24862	0.946131	2.1
24863	0.94613	0.563608
24864	0.946131	2.1
24865	1.40609	0.924691
24866	0.946131	1.00101
24867	0.946131	1.31434
24868	0.946133	1.36935
24869	0.946129	1.42824
24870	1.23339	1.60367
24871	0.946131	1.30997
24872	0.946132	1.91913
24873	0.946133	1.72208
24874	0.946132	2.1
24875	1.29198	2.1
24876	0.946132	1.47442
24877	0.946131	2.1
24878	1.07297	2.1
24879	0.946127	2.1

24880	0.94613	2.1
24881	1.07297	2.1
24882	0.946125	2.1
24883	0.946126	2.1
24884	0.946126	2.1
24885	1.3864	2.1
24886	0.946119	2.1
24887	0.946128	0.67031
24888	1.09411	0.655262
24889	0.946131	0.691979
24890	0.946131	0.674627
24891	1.09411	0.666579
24892	0.946131	0.578008
24893	1.25837	0.581901
24894	0.946131	0.630793
24895	0.946131	0.613352
24896	1.25837	0.598397
24897	0.810945	2.1
24898	0.810943	2.1
24899	0.810933	2.1
24900	0.94613	0.695586
24901	0.946131	0.85138
24902	0.946132	0.736835
24903	0.946129	0.658958
24904	0.946131	0.804964
24905	0.946131	0.291493
24906	1.28911	0.335948
24907	0.946129	0.556358
24908	0.946133	0.333325
24909	0.946131	0.371285
24910	0.94613	0.71314
24911	1.38954	0.720795
24912	0.946132	0.710758
24913	0.94613	0.718763
24914	0.946131	0.713624
24915	0.946131	0.897839
24916	0.946131	0.80507
24917	0.946131	0.774417
24918	1.37981	0.773796
24919	0.946131	0.794747
24920	0.946131	1.62416
24921	1.26958	2.1
24922	0.946127	2.00873
24923	0.946129	2.1
24924	1.26957	2.1
24925	0.94613	2.1
24926	1.16889	2.1
24927	0.946126	2.1
24928	0.946128	2.1
24929	1.16889	2.1
24930	0.946133	0.764634
24931	1.13511	0.715335
24932	0.946132	0.767696
24933	0.946133	0.745897
24934	0.946132	0.745235
24935	1.27575	1.50384
24936	0.946129	2.00942
24937	0.946131	1.64479

24938	0.94613	2.08885
24939	0.946131	1.89657
24940	0.946131	2.1
24941	0.946128	2.1
24942	1.41712	2.1
24943	1.41712	2.1
24944	0.94613	2.1
24945	0.946129	0.144236
24946	0.946132	0.126577
24947	0.946131	0.130501
24948	0.946131	0.178756
24949	0.946131	0.136498
24950	0.979045	1.47685
24951	0.946131	0.990096
24952	0.617549	1.42624
24953	0.555844	1.83184
24954	1.04071	1.05853
24955	0.946132	1.28329
24956	0.946132	1.00405
24957	0.946131	1.5028
24958	1.22618	1.0653
24959	0.946132	0.9786
24960	0.946131	2.1
24961	1.13976	2.1
24962	0.946129	2.1
24963	0.946131	2.1
24964	1.13976	2.1
24965	0.810945	0.766828
24966	0.810943	0.772571
24967	0.810932	0.823542
24968	1.25358	1.00376
24969	0.94613	0.806625
24970	0.946128	0.831844
24971	1.25358	1.26122
24972	1.07811	0.919255
24973	0.946129	2.1
24974	1.09197	2.1
24975	0.946131	2.1
24976	0.946131	2.1
24977	1.09197	2.1
24978	0.594912	2.1
24979	0.993703	2.1
24980	1.10173	2.1
24981	0.9916	2.1
24982	0.946131	2.1
24983	0.946128	2.1
24984	0.946131	2.1
24985	0.94613	2.1
24986	0.946129	2.1
24987	0.946132	2.1
24988	1.40335	2.1
24989	0.946133	2.1
24990	0.946133	2.1
24991	1.40335	2.1
24992	0.946132	1.18129
24993	0.946132	1.46697
24994	0.946132	1.2186
24995	0.946132	1.31149

24996	1.411	1.47068
24997	0.611675	0.659958
24998	0.946129	0.7
24999	1.31678	0.412886
25000	0.946124	0.699813
25001	0.946127	0.50486
25002	0.946128	0.698934
25003	0.946132	0.772974
25004	0.946131	0.727756
25005	0.946132	0.774484
25006	0.946132	0.74582
25007	0.946132	0.742016
25008	0.810946	0.757653
25009	0.810943	0.759576
25010	0.81096	0.739386
25011	1.39301	0.244833
25012	0.946132	0.203303
25013	0.625812	0.189624
25014	0.946132	0.205202
25015	0.625812	0.2203
25016	0.810946	1.21843
25017	0.810945	1.34173
25018	0.810931	1.95857
25019	0.810943	2.1
25020	0.810944	2.1
25021	0.810958	2.1
25022	0.946131	0.708137
25023	0.946131	0.704042
25024	0.946128	0.709252
25025	0.946129	0.706183
25026	0.94613	0.702769
25027	0.946133	0.708862
25028	0.946133	0.710323
25029	0.98537	0.706995
25030	0.985371	0.7
25031	0.946132	0.720432
25032	0.946129	0.489456
25033	1.38752	0.385965
25034	0.946131	0.39351
25035	0.946131	0.387912
25036	1.38752	0.389195
25037	0.946119	0.373842
25038	1.33036	0.293978
25039	0.946119	0.343446
25040	0.946132	0.320349
25041	1.33035	0.305578
25042	1.3868	2.1
25043	0.946132	2.1
25044	0.946132	2.1
25045	0.946133	2.1
25046	0.946132	2.1
25047	0.810947	2.1
25048	0.810946	2.1
25049	0.81096	2.1
25050	0.810946	2.1
25051	0.810946	2.1
25052	0.810933	2.1
25053	0.946133	2.1

25054	1.35342	2.1
25055	0.946133	2.1
25056	0.946132	2.1
25057	1.35342	2.1
25058	0.946132	2.1
25059	0.946132	2.1
25060	1.28569	2.1
25061	0.946132	2.1
25062	0.946133	2.1
25063	0.946132	1.52172
25064	1.32547	2.1
25065	0.946132	1.65662
25066	0.946132	1.7231
25067	1.32547	2.1
25068	0.94613	0.45752
25069	0.946133	0.459818
25070	1.39664	0.457105
25071	1.39664	0.357734
25072	0.946131	0.506103
25073	0.946131	1.18898
25074	0.94613	2.1
25075	0.946132	1.00614
25076	0.946131	1.19216
25077	1.41833	1.28094
25078	0.946131	0.151507
25079	0.946131	0.144312
25080	0.946131	0.156886
25081	1.26155	0.142122
25082	0.946132	0.150676
25083	0.810946	2.1
25084	0.810946	2.1
25085	0.810959	2.1
25086	0.94613	1.05826
25087	0.946132	0.887787
25088	1.40779	0.765607
25089	1.40779	0.843422
25090	0.94613	0.883299
25091	0.94613	0.212617
25092	0.946129	0.249795
25093	1.36376	0.187686
25094	1.36376	0.194116
25095	0.946131	0.214982
25096	0.946128	2.1
25097	0.946127	2.1
25098	0.946131	2.1
25099	1.40756	2.1
25100	0.94613	2.1
25101	0.946131	1.30144
25102	0.946132	1.72909
25103	0.94613	1.28612
25104	0.946131	1.27204
25105	1.31947	1.51231
25106	0.946131	2.1
25107	0.946131	2.1
25108	0.946129	2.1
25109	0.946131	2.1
25110	1.25768	2.1
25111	0.946132	0.7

25112	0.94613	0.7
25113	0.946131	0.7
25114	0.946131	0.7
25115	0.946131	0.7
25116	0.810943	0.910022
25117	0.810942	1.03273
25118	0.81093	1.5801
25119	0.946128	0.893386
25120	0.946133	0.803794
25121	0.946131	0.90847
25122	0.946131	0.859159
25123	0.94613	0.9126
25124	0.946132	1.00364
25125	0.946131	0.919301
25126	0.946132	1.00966
25127	0.946132	0.998235
25128	0.946132	1.04831
25129	0.946119	2.1
25130	0.946119	2.1
25131	1.31523	2.1
25132	0.946132	2.1
25133	0.946107	2.1
25134	0.946133	2.03384
25135	0.946132	0.96668
25136	0.946132	0.967221
25137	1.28803	1.40698
25138	0.946132	1.28102
25139	1.37658	0.872764
25140	0.946132	0.809974
25141	0.946133	1.01991
25142	0.946132	0.84194
25143	0.946132	0.981857
25144	0.946132	0.989685
25145	0.94613	1.0856
25146	0.94613	1.18244
25147	1.15541	1.01156
25148	0.92943	1.10855
25149	0.946126	0.125095
25150	0.946129	0.143271
25151	1.33849	0.0968938
25152	1.33849	0.121853
25153	0.946129	0.125147
25154	0.810946	0.10173
25155	0.810944	0.143016
25156	0.810959	0.115364
25157	1.39027	0.220996
25158	0.946131	0.335887
25159	0.946131	0.382093
25160	1.39027	0.227209
25161	0.946131	0.250899
25162	1.40924	1.66825
25163	0.946131	1.09125
25164	0.946132	1.70719
25165	0.946132	1.01638
25166	0.946132	1.30838
25167	0.946131	2.1
25168	0.94613	2.1
25169	0.946128	2.1

25170	0.946128	2.1
25171	1.41744	2.1
25172	0.946132	0.765049
25173	0.946128	0.761687
25174	0.94613	0.823558
25175	1.37675	0.772966
25176	0.94613	0.856203
25177	0.946132	0.947802
25178	0.946132	0.80812
25179	0.946132	0.764897
25180	0.946132	0.800385
25181	0.946131	0.794751
25182	0.946132	0.847472
25183	0.946132	0.809824
25184	0.946133	1.09293
25185	1.10003	0.791236
25186	0.946132	0.90876
25187	0.946132	0.519744
25188	0.946131	0.588979
25189	0.946132	0.448095
25190	0.946131	0.577941
25191	0.946132	0.498048
25192	0.946132	0.634507
25193	0.946131	0.677241
25194	0.946132	0.54053
25195	0.946132	0.657943
25196	0.946131	0.584443
25197	1.24094	0.149933
25198	0.946126	0.196083
25199	0.946128	0.164742
25200	0.946129	0.218684
25201	0.946129	0.174141
25202	1.2929	0.240084
25203	0.946131	0.336612
25204	0.946131	0.275025
25205	0.946131	0.346365
25206	0.946131	0.3033
25207	0.810946	0.115334
25208	0.810945	0.121874
25209	0.810959	0.107404
25210	0.810946	1.38989
25211	0.810943	1.41694
25212	0.810933	1.61293
25213	0.946128	2.00161
25214	1.18315	2.1
25215	0.94613	2.02558
25216	0.94613	1.97985
25217	1.18316	2.1
25218	0.946126	0.772513
25219	1.3324	0.741998
25220	0.946127	0.779954
25221	0.94613	0.741225
25222	0.946128	0.754993
25223	0.94613	2.1
25224	0.946128	2.1
25225	1.39523	2.1
25226	1.39523	2.1
25227	0.946131	2.1

25228	1.36541	2.1
25229	0.946132	2.1
25230	0.946132	2.1
25231	0.946132	2.1
25232	0.946132	2.1
25233	0.946131	1.87795
25234	0.94613	1.09349
25235	0.946132	1.26564
25236	0.946131	0.878269
25237	1.39509	1.27814
25238	0.946132	2.1
25239	0.946132	2.1
25240	1.29319	2.1
25241	0.946132	2.1
25242	0.946133	2.1
25243	0.946132	0.284305
25244	0.946131	0.476008
25245	0.946132	0.328522
25246	1.01196	0.317137
25247	0.946132	0.437613
25248	0.946116	2.1
25249	0.946117	2.01326
25250	1.38153	2.1
25251	1.38155	2.1
25252	0.946105	2.1
25253	0.946132	1.40739
25254	0.946131	1.49041
25255	0.946132	1.1888
25256	1.28684	1.32359
25257	0.946132	1.08475
25258	1.10185	1.58582
25259	0.94613	1.62884
25260	0.946128	2.0638
25261	0.94613	1.98742
25262	0.94613	1.61933
25263	0.810947	0.0928133
25264	0.810945	0.090467
25265	0.810932	0.0926919
25266	0.946119	0.726403
25267	1.16717	0.712368
25268	0.946117	0.731291
25269	0.94613	0.716463
25270	1.16715	0.714106
25271	0.94612	0.758839
25272	0.946119	0.759074
25273	0.946119	0.744997
25274	0.946132	0.724568
25275	1.20882	0.730648
25276	1.07043	0.947934
25277	0.823268	0.957837
25278	0.810959	0.91922
25279	0.946132	1.58902
25280	1.14879	1.0148
25281	0.94613	1.37306
25282	0.946131	1.54611
25283	1.14879	1.05798
25284	1.23837	2.1
25285	0.94612	2.1

25286	0.946118	2.1
25287	0.946131	2.1
25288	0.946105	2.1
25289	0.810946	0.113318
25290	0.810946	0.107391
25291	0.810959	0.102505
25292	0.810947	0.856281
25293	0.810947	0.713113
25294	0.810934	0.713485
25295	0.946128	2.1
25296	0.946131	2.1
25297	0.946128	2.1
25298	0.946129	2.1
25299	0.94613	2.1
25300	0.94613	2.1
25301	0.946131	2.1
25302	0.946131	2.07758
25303	0.946131	2.1
25304	0.946131	2.1
25305	0.946131	1.41632
25306	1.38585	2.1
25307	0.946128	1.13211
25308	0.94613	1.27559
25309	1.38585	1.39988
25310	0.946133	0.713647
25311	0.946133	0.715399
25312	0.946132	0.707099
25313	0.946132	0.714905
25314	0.946132	0.711826
25315	0.918871	0.267145
25316	0.810248	0.313854
25317	0.838037	0.313417
25318	0.946126	0.325373
25319	0.943647	0.270124
25320	1.13836	0.230933
25321	1.03802	0.246322
25322	0.946129	0.254856
25323	0.946133	0.998105
25324	0.946132	0.843
25325	0.946132	1.12418
25326	1.1699	0.868769
25327	0.946132	0.849502
25328	0.946131	2.1
25329	0.946132	2.1
25330	1.37199	2.1
25331	1.37199	2.1
25332	0.946132	2.1
25333	0.946132	0.959322
25334	0.946133	0.868908
25335	0.946133	1.04203
25336	0.946132	0.875687
25337	1.38623	0.893074
25338	0.946132	2.1
25339	0.946132	2.1
25340	0.946132	2.1
25341	0.946132	2.1
25342	1.40098	2.1
25343	1.20787	0.725706

25344	1.41894	0.74439
25345	0.946133	0.718056
25346	1.01777	0.770015
25347	0.946132	2.1
25348	0.946132	2.1
25349	0.946133	2.1
25350	1.40199	2.1
25351	0.946132	2.1
25352	0.810946	1.03886
25353	0.810947	1.22008
25354	0.810934	1.10228
25355	0.946126	0.987465
25356	0.94613	1.03072
25357	0.946125	1.08381
25358	1.40247	1.01762
25359	0.946128	0.98785
25360	1.25703	1.26114
25361	0.87821	1.04836
25362	0.878238	1.05026
25363	0.946119	1.37215
25364	0.946132	1.83932
25365	0.946132	0.850282
25366	0.946132	0.813975
25367	0.946131	1.04603
25368	0.946132	0.879048
25369	1.28543	0.859715
25370	0.946115	2.1
25371	1.41638	2.1
25372	0.946116	2.1
25373	0.946104	2.1
25374	0.946129	2.1
25375	0.946132	1.79564
25376	1.41432	2.1
25377	0.946132	2.06315
25378	0.946132	2.1
25379	0.946132	1.60481
25380	0.810947	0.405513
25381	0.810947	0.382034
25382	0.709567	0.41276
25383	0.851542	0.288259
25384	1.01217	0.187352
25385	0.697993	0.163303
25386	0.946133	0.219659
25387	0.851488	0.160612
25388	0.946129	1.99415
25389	0.94613	2.1
25390	1.2254	1.71742
25391	0.946131	2.1
25392	0.946131	1.77306
25393	0.700785	2.0612
25394	0.915893	1.56124
25395	0.838013	1.48472
25396	0.760188	1.33257
25397	1.19601	0.675089
25398	0.946133	0.807113
25399	0.946132	0.832736
25400	0.946132	0.768467
25401	0.946132	0.810816

25402	0.946132	1.07535
25403	0.946133	2.0863
25404	0.946132	1.21279
25405	0.946133	1.511
25406	1.36956	1.71275
25407	0.946131	0.873686
25408	0.94613	0.34057
25409	0.946133	0.859921
25410	1.34268	0.343831
25411	0.946131	0.766801
25412	0.946132	2.04179
25413	1.25603	2.03957
25414	0.946132	2.04842
25415	0.946132	2.02305
25416	1.25603	2.1
25417	0.810946	1.05471
25418	0.810943	1.05271
25419	0.810933	1.05866
25420	0.496545	0.866944
25421	0.805767	1.11222
25422	0.936671	1.04589
25423	0.530063	0.991321
25424	1.05954	1.00214
25425	0.810946	0.421489
25426	0.810943	0.463587
25427	0.810959	0.368104
25428	1.16699	0.355284
25429	0.946131	0.549669
25430	0.946129	0.581863
25431	0.946131	0.49535
25432	0.946131	0.511913
25433	0.810943	1.16882
25434	0.810942	1.22104
25435	0.810933	1.15831
25436	0.946132	1.15055
25437	1.04832	1.15732
25438	0.870714	1.22929
25439	0.957819	1.3466
25440	0.94613	1.11153
25441	1.82931	2.1
25442	0.946132	0.7
25443	0.946129	0.7
25444	0.94613	0.7
25445	0.946131	0.7
25446	0.94613	0.7
25447	0.946119	0.949094
25448	0.94612	1.04364
25449	0.94612	0.931606
25450	0.946132	1.00917
25451	1.36118	0.95622
25452	0.810947	0.636273
25453	0.810947	0.627341
25454	0.810934	0.629001
25455	0.946132	2.1
25456	0.946132	2.1
25457	0.946132	2.1
25458	1.39754	2.1
25459	0.946131	2.1

25460	0.946129	2.1
25461	0.946133	2.1
25462	0.946131	2.1
25463	0.946131	2.1
25464	1.41732	2.1
25465	0.946132	0.977333
25466	0.946128	1.33302
25467	0.94613	1.04007
25468	0.94613	1.09235
25469	1.25277	1.20949
25470	0.94613	1.35883
25471	0.946128	1.75323
25472	1.21261	1.13476
25473	0.94613	1.3918
25474	0.94613	1.2137
25475	1.39407	2.1
25476	0.946133	2.1
25477	0.94613	2.1
25478	0.946131	2.1
25479	0.946131	2.1
25480	0.946119	1.057
25481	0.946119	1.19649
25482	0.946119	1.20941
25483	1.1537	1.22659
25484	0.946106	1.04402
25485	0.946131	2.1
25486	1.11822	2.1
25487	0.946129	2.1
25488	0.946131	2.1
25489	1.11822	2.1
25490	1.46806	0.0785426
25491	0.498881	0.0872085
25492	0.996658	0.0887375
25493	1.2179	0.079193
25494	0.991519	0.0843092
25495	0.946131	0.105797
25496	0.946131	0.118397
25497	1.28673	0.100537
25498	0.946131	0.103544
25499	0.946131	0.102291
25500	1.33178	0.250732
25501	0.946132	0.322746
25502	0.946132	0.296655
25503	0.946132	0.336958
25504	0.946132	0.307408
25505	0.946128	0.71491
25506	0.972061	0.701804
25507	0.946129	0.725814
25508	0.946129	0.718924
25509	0.972059	0.705562
25510	0.946129	2.1
25511	0.94613	2.1
25512	0.94613	2.1
25513	0.94613	2.1
25514	0.94613	2.1
25515	0.946131	2.1
25516	0.946127	2.1
25517	0.946131	2.1

25518	0.946129	2.1
25519	1.4073	2.1
25520	0.946131	0.446824
25521	1.05016	0.341895
25522	0.946132	0.517717
25523	0.946131	0.510727
25524	0.946132	0.415161
25525	0.946128	0.483294
25526	1.13398	0.385493
25527	0.946131	0.574747
25528	0.946131	0.549401
25529	0.94613	0.450076
25530	0.946131	0.128456
25531	0.946131	0.182023
25532	1.38911	0.109139
25533	1.38911	0.133366
25534	0.946132	0.140624
25535	0.946131	0.612207
25536	0.94613	0.674154
25537	0.946131	0.576539
25538	0.946131	0.668319
25539	0.946131	0.633213
25540	0.946131	0.729289
25541	1.23923	0.706115
25542	0.94613	0.740003
25543	0.946131	0.705672
25544	0.946131	0.70937
25545	1.40511	0.10642
25546	0.946131	0.120232
25547	0.946131	0.154216
25548	0.946132	0.125278
25549	0.946131	0.119509
25550	1.12968	2.1
25551	0.946132	1.67624
25552	0.946132	2.1
25553	0.946131	2.07888
25554	0.946132	2.1
25555	0.946132	1.36846
25556	1.4172	2.1
25557	0.94613	1.18491
25558	0.94613	1.4908
25559	1.4172	1.78695
25560	0.946132	2.1
25561	0.946132	2.1
25562	1.31323	2.1
25563	0.946133	2.1
25564	0.946132	2.1
25565	0.946131	2.1
25566	0.94613	0.91979
25567	1.36546	1.65959
25568	1.36546	1.11185
25569	0.946131	1.05514
25570	0.946132	0.766689
25571	0.946133	0.803073
25572	0.946132	0.696985
25573	1.36899	0.353221
25574	0.946133	0.720517
25575	0.946131	0.396115

25576	0.946132	0.34095
25577	1.03543	0.396276
25578	1.1412	0.336452
25579	0.941275	0.413852
25580	1.28952	0.372007
25581	0.94613	0.452348
25582	0.946129	0.455781
25583	0.946132	0.379035
25584	0.94613	0.397832
25585	0.94613	2.1
25586	0.94613	2.1
25587	0.946129	2.1
25588	0.94613	2.1
25589	1.35966	2.1
25590	0.810946	2.08187
25591	0.810942	2.1
25592	0.810932	1.8429
25593	0.94613	2.1
25594	1.40372	2.1
25595	0.946131	1.86332
25596	0.94613	2.1
25597	1.40372	2.1
25598	0.946133	2.1
25599	0.946131	2.1
25600	0.94613	2.1
25601	0.946131	2.1
25602	0.946131	2.1
25603	1.31453	2.1
25604	0.94613	2.1
25605	0.946128	2.1
25606	0.94613	2.1
25607	0.94613	2.1
25608	0.946131	0.351837
25609	1.12018	0.305616
25610	0.94613	0.408364
25611	0.94613	0.466471
25612	0.94613	0.331811
25613	0.946131	0.55921
25614	1.16373	0.500878
25615	0.946131	0.666428
25616	0.94613	0.521344
25617	0.946131	0.537578
25618	0.946126	0.766603
25619	0.946132	0.803034
25620	0.946128	0.781648
25621	0.946129	0.797868
25622	0.946129	0.829583
25623	0.946132	0.694543
25624	0.946133	0.704398
25625	0.946132	0.692341
25626	0.946132	0.702265
25627	0.946133	0.703178
25628	0.946132	0.955293
25629	0.946129	1.1078
25630	0.94613	0.919041
25631	1.36399	0.990837
25632	0.946131	0.943377
25633	0.946132	2.1

25634	0.946132	2.1
25635	0.946133	2.1
25636	1.38302	2.1
25637	0.946133	2.1
25638	0.810943	2.1
25639	0.810944	2.1
25640	0.810945	2.1
25641	0.946132	2.1
25642	0.946131	2.1
25643	0.946132	2.1
25644	1.30188	2.1
25645	0.946132	2.1
25646	0.810946	0.863515
25647	0.72985	1.03306
25648	0.810934	0.919434
25649	0.856243	0.86667
25650	0.946129	0.833749
25651	0.946132	0.85784
25652	1.19686	0.828794
25653	0.851527	0.896214
25654	0.946131	2.1
25655	0.94613	2.1
25656	0.94613	2.1
25657	0.94613	2.1
25658	1.31961	2.1
25659	0.946131	2.1
25660	0.946131	2.1
25661	0.94613	2.1
25662	0.946131	2.1
25663	1.3605	2.1
25664	1.30116	2.1
25665	0.946133	0.956745
25666	0.946131	2.03343
25667	0.946131	1.24035
25668	0.94613	1.67987
25669	0.946132	0.262378
25670	1.3035	0.148868
25671	0.94613	0.211569
25672	0.946131	0.178999
25673	0.946131	0.220964
25674	0.946132	2.1
25675	1.37869	2.1
25676	1.2051	2.1
25677	0.946132	2.1
25678	1.2051	2.1
25679	0.810946	2.1
25680	0.810945	2.1
25681	0.810946	2.1
25682	1.34623	2.1
25683	0.946131	2.1
25684	0.946131	2.1
25685	1.34623	2.1
25686	1.22993	2.1
25687	1.32299	0.184869
25688	0.946132	0.262258
25689	0.946132	0.282919
25690	0.946132	0.206946
25691	0.946131	0.25641

25692	0.94613	0.411315
25693	1.40574	0.280508
25694	0.946128	0.363532
25695	0.946131	0.509625
25696	1.40574	0.459424
25697	0.946132	2.1
25698	1.41331	2.1
25699	0.94613	2.1
25700	0.946131	2.1
25701	1.41331	2.1
25702	0.94613	2.1
25703	1.37609	2.1
25704	0.946129	2.1
25705	0.946131	2.1
25706	1.37609	2.1
25707	1.039	1.25993
25708	0.946131	1.8457
25709	0.944514	1.45829
25710	0.946132	1.79931
25711	0.946132	1.60589
25712	1.23726	1.20377
25713	0.946128	1.55357
25714	0.946128	1.37459
25715	0.946129	1.64027
25716	0.94613	1.46216
25717	0.946129	2.1
25718	0.946131	2.1
25719	0.946131	2.1
25720	1.39214	2.1
25721	0.94613	2.1
25722	0.946118	0.906353
25723	0.946118	0.546442
25724	1.41078	0.560235
25725	1.41076	0.23232
25726	0.946131	0.715349
25727	0.946131	0.701137
25728	0.946131	0.7
25729	0.946132	0.7
25730	0.94613	0.701338
25731	0.946131	0.7
25732	0.946132	0.200423
25733	0.946132	0.142574
25734	0.946133	0.123224
25735	1.28924	0.13417
25736	0.946132	0.161867
25737	0.946133	0.832503
25738	1.09812	0.791772
25739	0.946132	0.856198
25740	0.946132	0.857319
25741	1.09812	0.866117
25742	0.946132	0.968568
25743	0.946132	0.961036
25744	0.946132	0.956625
25745	0.946132	0.949865
25746	1.15388	0.97416
25747	0.946132	0.800893
25748	0.974688	0.754962
25749	0.946132	0.811213

25750	0.946132	0.800983
25751	0.974687	0.810226
25752	1.0644	1.19501
25753	0.946133	1.59697
25754	0.946132	1.21655
25755	0.946133	1.24303
25756	1.26547	1.37185
25757	1.02905	1.13506
25758	0.911537	1.16105
25759	0.838038	1.26644
25760	0.946131	0.66927
25761	0.94613	0.674358
25762	0.946132	0.666988
25763	0.946131	0.68581
25764	1.33922	0.67655
25765	0.946132	0.289516
25766	0.946132	0.221015
25767	1.40926	0.24746
25768	1.40927	0.240877
25769	0.946132	0.220171
25770	0.946129	1.85427
25771	0.946131	1.73969
25772	0.946131	1.17414
25773	0.946131	1.01947
25774	1.40496	1.61543
25775	0.94613	1.01842
25776	0.94613	1.01473
25777	0.946127	0.991936
25778	0.946128	0.948772
25779	1.38914	0.970727
25780	2.675	0.0861732
25781	1.22266	0.091707
25782	0.946131	0.115915
25783	0.946132	0.111767
25784	0.946132	0.0999496
25785	1.22266	0.11708
25786	0.946124	0.381779
25787	0.946128	0.204348
25788	0.946126	0.200229
25789	1.21428	0.202415
25790	0.946127	0.355999
25791	0.946131	0.30179
25792	0.946131	0.598318
25793	0.946132	0.337101
25794	0.946129	0.328142
25795	0.940118	0.545295
25796	0.810943	2.1
25797	0.810945	2.1
25798	0.810958	2.1
25799	0.946133	0.344882
25800	1.11539	0.361582
25801	0.946132	0.488506
25802	0.946132	0.394253
25803	0.946133	0.386541
25804	0.946133	2.1
25805	1.41195	2.1
25806	0.946133	2.1
25807	0.946132	2.1

25808	0.946133	2.1
25809	1.15968	0.110787
25810	0.946131	0.124178
25811	0.946131	0.140465
25812	0.946131	0.122225
25813	0.946131	0.121426
25814	0.94613	2.0462
25815	0.946128	2.1
25816	0.946132	1.39837
25817	0.94613	1.9354
25818	0.94613	1.6255
25819	0.946131	1.80611
25820	1.04211	1.83174
25821	0.850155	1.29168
25822	0.946131	1.61425
25823	0.946131	1.44412
25824	0.946131	0.77049
25825	0.94613	0.729146
25826	0.946129	0.773055
25827	0.946131	0.748178
25828	0.946131	0.742393
25829	1.40193	0.487522
25830	1.19756	0.552618
25831	0.561936	0.637919
25832	0.808778	0.651567
25833	1.19756	0.583065
25834	0.946132	0.159247
25835	0.946132	0.140609
25836	1.33916	0.130142
25837	1.33916	0.127438
25838	0.946133	0.133954
25839	0.946127	0.708779
25840	0.946127	0.702514
25841	0.946131	0.707942
25842	0.946128	0.70163
25843	0.946129	0.703078
25844	0.589428	0.713677
25845	0.851501	0.706604
25846	0.868924	0.71249
25847	0.877703	0.707699
25848	0.85153	0.710858
25849	0.946132	1.33914
25850	1.25372	2.1
25851	0.946132	1.4678
25852	0.946132	1.83499
25853	1.25372	2.1
25854	0.946132	1.04127
25855	0.946128	1.7674
25856	0.94613	1.12685
25857	0.94613	1.22171
25858	1.2778	1.42495
25859	0.946118	0.887955
25860	1.35629	0.89906
25861	0.946117	0.89105
25862	0.946131	0.878372
25863	0.946105	0.853076
25864	0.946132	0.81985
25865	0.946132	0.988195

25866	1.38646	0.978897
25867	1.38646	1.01845
25868	0.946132	1.01723
25869	0.946128	2.1
25870	0.946128	0.914663
25871	1.39659	1.98121
25872	1.39659	1.17469
25873	0.946129	1.03593
25874	0.946132	1.61002
25875	0.946131	1.37939
25876	0.946132	1.41299
25877	0.946132	1.40055
25878	1.15139	1.34922
25879	0.946133	2.1
25880	0.946132	2.1
25881	0.946132	2.1
25882	0.946132	2.1
25883	1.36151	2.1
25884	0.946127	2.1
25885	0.946131	2.1
25886	0.946128	2.1
25887	0.94613	2.1
25888	1.38397	2.1
25889	0.946131	0.670161
25890	1.31112	0.659512
25891	0.946127	0.547726
25892	0.946128	0.714749
25893	1.31112	0.681196
25894	1.29778	0.0597502
25895	0.883417	0.0571769
25896	1.32613	0.0555951
25897	0.94613	0.0577511
25898	0.94613	0.0619672
25899	0.946132	2.1
25900	0.94613	2.1
25901	0.946131	2.1
25902	0.946131	2.1
25903	1.36523	2.1
25904	0.946132	0.520556
25905	1.01653	0.426966
25906	0.946132	0.618575
25907	0.946131	0.611585
25908	0.946131	0.480158
25909	0.94612	2.1
25910	1.08301	1.57377
25911	0.946119	2.1
25912	0.946132	2.1
25913	1.08299	1.9679
25914	0.810942	2.1
25915	0.810944	1.04698
25916	0.810957	1.15568
25917	0.946126	0.652518
25918	0.946132	0.675019
25919	0.946129	0.655293
25920	1.3492	0.607614
25921	0.94613	0.646618
25922	0.946131	0.542669
25923	1.24766	0.404999

25924	0.946128	0.3872
25925	0.946131	0.581007
25926	1.24766	0.552759
25927	0.946132	0.11652
25928	0.946131	0.105669
25929	0.946131	0.128268
25930	1.23351	0.105523
25931	0.946132	0.109242
25932	0.946131	0.12821
25933	0.946132	0.115363
25934	0.946131	0.158305
25935	1.35657	0.116743
25936	0.946131	0.123785
25937	0.946132	0.758545
25938	1.3689	0.720468
25939	0.946132	0.735531
25940	0.946132	0.740125
25941	1.3689	0.721296
25942	0.946133	0.710074
25943	1.35822	0.642964
25944	0.946133	0.674999
25945	0.946132	0.703167
25946	1.35822	0.67053
25947	0.946131	0.778188
25948	0.946131	1.05041
25949	0.946131	0.997396
25950	0.946131	0.848019
25951	1.4101	1.20086
25952	0.810944	1.46039
25953	0.810939	1.55987
25954	0.810955	1.19886
25955	0.946132	2.1
25956	1.37733	2.1
25957	0.946132	2.1
25958	0.946131	2.1
25959	0.946131	2.1
25960	1.32582	2.1
25961	0.946132	1.84572
25962	0.946132	1.25155
25963	0.946132	2.00831
25964	0.946132	2.1
25965	0.946132	2.09521
25966	0.946132	2.1
25967	1.31401	2.1
25968	1.31401	2.1
25969	0.946131	2.1
25970	1.3469	1.11
25971	0.946132	1.3679
25972	0.946132	1.37417
25973	0.946133	1.02474
25974	0.946132	1.0782
25975	0.946126	2.1
25976	0.946127	2.1
25977	0.94613	2.1
25978	0.946128	2.1
25979	1.41206	2.1
25980	0.946127	1.24114
25981	1.40109	1.90642

25982	0.946128	1.23389
25983	0.946129	1.90626
25984	1.40109	1.67165
25985	1.24448	0.368019
25986	0.94613	0.564756
25987	0.946132	0.435318
25988	0.946129	0.586201
25989	0.946131	0.508623
25990	1.11015	0.513199
25991	0.946133	0.790139
25992	0.946132	0.637936
25993	0.946133	0.755081
25994	0.946132	0.713083
25995	0.946132	2.1
25996	0.946131	2.1
25997	0.946131	2.1
25998	1.38775	2.1
25999	0.946131	2.1
26000	0.946133	0.873908
26001	0.946131	0.966123
26002	1.30598	1.24195
26003	1.30598	1.14868
26004	0.946131	1.09505
26005	0.946132	2.1
26006	0.946132	2.1
26007	0.946132	2.1
26008	1.40774	2.1
26009	0.946132	2.1
26010	0.798396	0.0896662
26011	0.946133	0.802663
26012	0.946128	1.14498
26013	0.946131	0.900653
26014	0.946131	0.898674
26015	1.28918	0.938242
26016	0.946132	0.670656
26017	1.40668	0.651316
26018	0.94613	0.664874
26019	0.94613	0.658734
26020	0.94613	0.651813
26021	0.810946	2.1
26022	0.810945	2.1
26023	0.810933	2.1
26024	0.946131	2.1
26025	1.30338	2.1
26026	0.94613	2.1
26027	1.30339	2.1
26028	0.946131	2.1
26029	0.946127	0.907325
26030	0.87161	0.971352
26031	0.946128	0.910309
26032	0.946129	0.938905
26033	0.827876	0.922517
26034	0.946131	2.1
26035	1.40796	2.1
26036	0.94613	2.1
26037	0.94613	2.1
26038	1.40796	2.1
26039	0.946132	0.90865

26040	0.946132	0.980405
26041	0.946132	0.903105
26042	0.946132	0.881373
26043	1.41724	0.875631
26044	1.34254	0.689178
26045	0.946132	0.883916
26046	0.946132	0.840379
26047	0.946132	0.838296
26048	0.946133	0.825107
26049	0.946133	1.48554
26050	0.946132	1.24976
26051	0.946132	1.20334
26052	1.26501	1.53474
26053	0.946132	1.1638
26054	0.946131	0.318455
26055	1.35169	0.337521
26056	0.946131	0.397919
26057	0.946131	0.350368
26058	1.35169	0.331665
26059	0.676844	0.192305
26060	0.946131	0.250518
26061	0.94613	0.33082
26062	1.13242	0.200757
26063	1.13242	0.233653
26064	0.946131	0.286179
26065	0.946128	1.13442
26066	1.28828	1.76503
26067	0.946131	1.13113
26068	0.946131	1.16231
26069	1.28828	1.34206
26070	0.946131	0.758366
26071	1.2996	0.742386
26072	0.946133	0.757974
26073	0.946131	0.748609
26074	0.946131	0.745161
26075	0.946132	1.14691
26076	1.40638	2.1
26077	0.946131	1.02655
26078	0.946132	2.1
26079	1.40638	2.1
26080	2.84909	0.182731
26081	1.35726	0.208538
26082	0.946131	0.264629
26083	0.946131	0.285023
26084	0.946131	0.243631
26085	0.946131	0.261802
26086	1.39852	1.93248
26087	0.946132	1.78552
26088	0.94613	2.1
26089	0.94613	2.08652
26090	0.94613	1.96412
26091	0.946126	0.227594
26092	0.946131	0.218144
26093	0.946128	0.290929
26094	1.31666	0.250089
26095	0.946128	0.256257
26096	0.946126	0.382965
26097	0.946131	0.367487

26098	0.946129	0.469093
26099	1.32275	0.332042
26100	0.946129	0.429278
26101	0.946131	2.1
26102	0.946132	2.1
26103	1.41125	2.1
26104	0.946131	2.1
26105	0.946131	2.1
26106	0.946131	2.1
26107	0.946132	2.1
26108	1.31904	2.1
26109	0.946132	2.1
26110	0.946132	2.1
26111	0.946129	0.496642
26112	0.946132	0.416649
26113	0.94613	0.429788
26114	1.02426	0.309866
26115	0.9311	0.489525
26116	0.946128	1.43029
26117	0.946133	1.12588
26118	0.946131	1.21974
26119	1.40092	1.35014
26120	0.946131	1.16016
26121	0.946117	0.821397
26122	0.946118	2.1
26123	0.946118	0.80546
26124	1.40017	1.28185
26125	0.946105	0.859879
26126	0.946132	0.689801
26127	1.03547	0.679805
26128	0.946132	0.671179
26129	0.946133	0.701661
26130	0.946132	0.677301
26131	0.946133	2.1
26132	0.946132	2.1
26133	0.946133	2.1
26134	0.946133	2.1
26135	1.36694	2.1
26136	0.946132	0.534716
26137	1.22878	0.487346
26138	0.946132	0.515695
26139	0.946132	0.63678
26140	1.22878	0.581773
26141	1.11014	0.491603
26142	0.913078	0.511586
26143	1.11015	0.656522
26144	0.49154	0.642631
26145	0.946127	2.1
26146	0.946131	2.1
26147	0.946128	2.1
26148	0.946129	2.1
26149	1.38752	2.1
26150	0.946132	2.1
26151	1.37277	2.1
26152	0.946132	2.1
26153	0.946131	2.1
26154	1.37277	2.1
26155	0.94613	0.768284

26156	1.16834	0.688473
26157	0.946129	0.769962
26158	0.94613	0.743424
26159	0.94613	0.738729
26160	0.946127	0.776695
26161	0.946131	0.741232
26162	1.35594	0.730591
26163	0.946128	0.780817
26164	0.94613	0.745298
26165	0.946128	0.960812
26166	0.946128	0.743451
26167	1.38464	0.866609
26168	1.38463	0.80305
26169	0.94613	0.801779
26170	0.946129	0.777881
26171	0.946131	0.720032
26172	0.94613	0.728867
26173	1.39751	0.683107
26174	0.946131	0.717911
26175	1.25837	0.502923
26176	1.21134	0.396369
26177	0.700013	0.558517
26178	0.700041	0.563272
26179	1.40583	1.59213
26180	0.946132	2.1
26181	0.946132	2.1
26182	0.946133	1.98351
26183	0.946132	1.75698
26184	0.827865	1.99238
26185	0.946131	2.1
26186	0.946131	1.59794
26187	0.946131	1.78673
26188	0.946131	1.58643
26189	0.946132	0.159364
26190	1.21087	0.126464
26191	0.94613	0.143777
26192	0.94613	0.124738
26193	0.946131	0.133723
26194	0.94613	0.784988
26195	1.17452	0.817365
26196	0.946132	0.78524
26197	0.94613	0.800196
26198	0.94613	0.783629
26199	0.946119	0.753478
26200	1.27374	0.769848
26201	0.946119	0.75262
26202	0.946131	0.762015
26203	0.946106	0.74993
26204	0.946133	2.1
26205	0.946132	2.1
26206	0.946133	2.1
26207	0.946132	2.1
26208	1.39621	2.1
26209	1.34352	2.1
26210	0.946132	2.1
26211	0.946132	2.1
26212	0.946132	2.1
26213	0.946132	2.1

26214	0.946132	0.914833
26215	0.946128	1.95907
26216	0.94613	1.10859
26217	0.94613	1.43939
26218	1.41273	1.77241
26219	1.23536	2.1
26220	0.946131	1.2348
26221	0.946132	2.1
26222	0.946132	1.76914
26223	0.946132	1.95454
26224	0.946132	2.1
26225	1.41131	2.1
26226	0.946131	2.1
26227	0.946131	2.1
26228	0.946131	2.1
26229	0.946133	0.757694
26230	0.946132	1.09231
26231	0.946133	0.778212
26232	1.41894	0.753438
26233	0.946132	0.784094
26234	0.946128	0.885699
26235	1.4099	1.10201
26236	0.946131	0.937788
26237	0.946131	1.39886
26238	0.946131	0.864813
26239	0.810947	1.2734
26240	0.810945	1.27424
26241	0.810933	1.94494
26242	0.946131	0.846078
26243	1.25765	1.76629
26244	0.946128	0.999316
26245	0.946129	0.839832
26246	1.25765	0.953262
26247	1.21347	2.1
26248	0.946129	2.1
26249	0.94613	2.1
26250	1.21347	2.1
26251	0.94613	2.1
26252	0.94613	0.086927
26253	0.946132	0.0844858
26254	1.2493	0.0784622
26255	1.2493	0.0749867
26256	0.94613	0.109432
26257	0.946132	0.871903
26258	0.946128	1.09077
26259	0.946131	0.896722
26260	0.94613	0.949475
26261	1.37938	0.946738
26262	0.946133	0.180511
26263	0.946132	0.16407
26264	0.946133	0.167322
26265	0.946132	0.121828
26266	0.946133	0.173841
26267	0.946129	0.843622
26268	1.26426	0.787718
26269	0.946131	0.814644
26270	0.946131	0.824131
26271	1.26426	0.79727

26272	0.872447	1.83301
26273	0.946132	1.33687
26274	0.946132	1.61815
26275	0.946132	1.72379
26276	0.946133	1.42509
26277	0.946117	1.19392
26278	0.946116	1.12897
26279	0.946113	1.0919
26280	0.946129	1.21152
26281	1.27211	1.03634
26282	0.94613	0.748904
26283	0.946129	0.597677
26284	1.40029	0.793664
26285	1.40028	0.815007
26286	0.94613	0.750682
26287	0.810942	2.1
26288	0.810943	2.1
26289	0.810932	2.1
26290	1.29521	2.1
26291	0.946129	2.1
26292	0.946131	2.1
26293	1.29521	2.1
26294	1.29521	2.1
26295	0.946132	0.822052
26296	0.946129	0.759597
26297	1.24596	0.758359
26298	0.946131	0.821937
26299	0.946131	0.770927
26300	0.946131	2.1
26301	1.35616	2.1
26302	0.946133	2.1
26303	0.94613	2.1
26304	1.35616	2.1
26305	0.879548	0.744088
26306	1.01272	0.707324
26307	0.946133	0.765158
26308	0.946133	0.704074
26309	0.946132	0.713192
26310	0.946132	0.862883
26311	0.918009	0.75371
26312	0.91801	0.891879
26313	0.946132	0.787842
26314	0.946132	0.820705
26315	0.946132	0.260146
26316	1.28644	0.215935
26317	0.946131	0.34032
26318	0.946131	0.32527
26319	0.946132	0.267931
26320	0.946131	0.252032
26321	1.40103	0.234787
26322	0.946131	0.366759
26323	0.946131	0.362063
26324	0.946131	0.285148
26325	0.946128	0.705432
26326	1.03327	0.627391
26327	0.946129	0.710673
26328	0.946131	0.631458
26329	0.94613	0.655069

26330	0.946132	0.744246
26331	0.946133	0.114527
26332	1.40265	0.567014
26333	1.40265	0.449051
26334	0.946133	0.249144
26335	0.946131	0.151451
26336	0.946131	0.171709
26337	0.946131	0.178294
26338	0.856655	0.13716
26339	0.856616	0.138799
26340	0.946132	0.720258
26341	0.946132	0.723678
26342	0.946132	0.749151
26343	1.15844	0.711239
26344	0.946131	0.738809
26345	0.946131	2.1
26346	1.4031	2.1
26347	1.40304	2.1
26348	0.851554	2.1
26349	0.946131	0.14108
26350	0.94613	0.218469
26351	1.24417	0.125382
26352	1.24416	0.141299
26353	0.946131	0.161283
26354	0.946132	0.930035
26355	1.20992	0.961457
26356	0.946132	0.963113
26357	0.946132	1.00019
26358	1.20992	1.00399
26359	0.946128	1.13358
26360	0.94613	1.22239
26361	0.94613	1.15254
26362	0.94613	1.18205
26363	1.23426	1.23957
26364	0.810943	0.747541
26365	0.810946	0.676162
26366	0.810933	0.70078
26367	0.810945	2.1
26368	0.810945	0.950578
26369	0.810932	1.05549
26370	1.25104	1.19052
26371	0.946131	2.1
26372	0.946131	0.929681
26373	1.25104	1.202
26374	1.25104	0.97191
26375	1.25488	2.1
26376	0.946132	2.07789
26377	0.946132	2.01231
26378	0.946132	2.1
26379	0.946131	2.1
26380	0.946131	0.166619
26381	0.946126	0.232139
26382	0.946128	0.166754
26383	1.32131	0.189331
26384	0.946128	0.210378
26385	0.946133	2.08043
26386	0.946132	1.0451
26387	0.946132	2.1

26388	0.946133	1.90615
26389	1.41123	1.25218
26390	0.946132	0.432009
26391	0.946133	0.440681
26392	0.946132	0.560931
26393	1.32163	0.353508
26394	0.946133	0.496974
26395	0.946132	1.01719
26396	0.946132	2.1
26397	1.38651	2.1
26398	1.38651	2.1
26399	0.946132	2.1
26400	0.94613	0.691331
26401	1.3639	0.50782
26402	0.946128	0.663334
26403	0.946131	0.588505
26404	1.3639	0.588572
26405	0.946133	0.971685
26406	1.40724	0.840718
26407	0.946132	0.808735
26408	0.946133	0.881314
26409	1.40724	0.862009
26410	1.21099	1.3679
26411	0.946128	1.51788
26412	0.946127	2.02912
26413	0.946128	1.82834
26414	0.946129	1.4519
26415	0.946132	0.795948
26416	1.35946	0.774166
26417	0.946131	0.835809
26418	0.946131	0.824627
26419	1.35946	0.792535
26420	1.34794	0.915543
26421	0.517994	1.31708
26422	0.946132	0.973048
26423	0.946132	1.05694
26424	0.517965	1.14782
26425	1.23224	0.906995
26426	0.94613	1.4368
26427	0.946131	1.09395
26428	0.946131	1.02761
26429	0.946131	0.991942
26430	1.37511	1.13463
26431	0.946132	1.18408
26432	0.946133	1.6572
26433	0.946133	1.50156
26434	0.946132	1.18445
26435	0.851106	0.771064
26436	0.946129	0.752041
26437	0.946131	0.852489
26438	0.946131	0.777164
26439	0.851066	0.768707
26440	0.946131	0.549231
26441	0.946131	0.726297
26442	0.94613	0.708162
26443	1.31357	0.554676
26444	0.946131	0.693442
26445	0.946133	0.91252

26446	0.946132	1.10161
26447	0.946133	0.91433
26448	0.946132	0.981229
26449	1.38149	0.957753
26450	0.810944	0.89404
26451	0.810945	1.06208
26452	0.810958	1.87442
26453	0.946118	0.111153
26454	0.946118	0.14094
26455	1.37835	0.0943936
26456	1.37837	0.111547
26457	0.946106	0.116288
26458	0.946128	0.172433
26459	0.946131	0.161829
26460	1.34831	0.163499
26461	1.34831	0.132017
26462	0.94613	0.240904
26463	0.946132	0.21813
26464	0.946132	0.366271
26465	0.909981	0.23838
26466	1.00099	0.254121
26467	0.946131	0.319416
26468	0.946132	0.819972
26469	0.946133	1.07765
26470	0.946133	0.871167
26471	0.946132	0.898773
26472	1.35328	0.9189
26473	0.946132	0.122101
26474	0.946132	0.11299
26475	1.35386	0.147714
26476	1.35385	0.105577
26477	0.946133	0.155552
26478	0.946121	0.116141
26479	0.946132	0.111694
26480	0.900167	0.085451
26481	1.01189	0.102092
26482	0.946132	0.116851
26483	0.946132	2.08104
26484	1.41585	1.42847
26485	0.946131	1.38896
26486	0.946131	1.38195
26487	0.94613	1.32543
26488	0.810946	2.1
26489	0.810946	2.1
26490	0.810933	2.1
26491	1.34447	2.1
26492	0.946131	2.1
26493	0.946131	2.1
26494	1.34447	2.1
26495	1.19886	2.1
26496	0.946132	0.294783
26497	0.946132	0.161293
26498	0.946132	0.181659
26499	1.35065	0.16359
26500	0.946132	0.285089
26501	0.946131	0.759418
26502	1.3022	0.746832
26503	0.94613	0.81166

26504	0.946131	0.787428
26505	1.3022	0.757571
26506	0.946127	0.526102
26507	1.38683	0.345989
26508	0.946127	0.475078
26509	0.946128	0.520657
26510	1.38683	0.515219
26511	0.810946	0.133074
26512	0.810947	0.134041
26513	0.810959	0.114425
26514	0.946132	0.109491
26515	0.946131	0.105897
26516	0.946132	0.0987316
26517	0.946132	0.0967598
26518	0.946132	0.101584
26519	0.946127	1.17674
26520	1.35968	1.80417
26521	0.946131	1.07598
26522	0.946129	1.02586
26523	1.35969	1.44722
26524	0.946133	2.04891
26525	1.35862	1.76226
26526	0.946132	2.1
26527	0.946132	1.18422
26528	1.35862	1.55497
26529	0.946132	0.806504
26530	0.946132	0.717151
26531	0.946132	0.83446
26532	0.946131	0.725547
26533	0.946132	0.753754
26534	0.946131	0.707865
26535	0.946131	0.704059
26536	0.946128	0.707996
26537	0.946129	0.700703
26538	1.41232	0.700615
26539	0.946128	1.11905
26540	1.29331	1.70617
26541	0.946131	1.1428
26542	0.946131	1.39307
26543	1.29331	1.26932
26544	0.946133	0.883644
26545	0.946132	0.919197
26546	0.946132	0.885882
26547	0.946132	0.868067
26548	1.41556	0.863894
26549	0.94612	0.857998
26550	0.946119	0.858778
26551	0.946119	0.85932
26552	0.946132	0.829931
26553	0.946107	0.839663
26554	0.946129	0.199753
26555	1.1509	0.135521
26556	0.946126	0.175328
26557	0.946129	0.130456
26558	0.94613	0.1737
26559	0.946131	0.140829
26560	1.19263	0.10886
26561	0.946132	0.121451

26562	0.946132	0.102261
26563	0.946132	0.116751
26564	0.94613	0.852882
26565	1.36939	0.940298
26566	0.946128	0.906558
26567	0.946131	0.919625
26568	1.36939	0.910823
26569	0.946133	1.03223
26570	0.827865	1.36878
26571	0.946132	1.06813
26572	0.946132	1.17714
26573	1.40398	1.22218
26574	0.946127	0.734172
26575	0.946127	0.795827
26576	0.94613	0.72388
26577	1.41498	0.769192
26578	0.946128	0.741584
26579	0.94613	0.135642
26580	0.946131	0.130349
26581	0.946129	0.10762
26582	0.94613	0.110228
26583	0.94613	0.114167
26584	0.946118	0.151381
26585	0.946119	0.129138
26586	0.946116	0.100066
26587	1.36569	0.112732
26588	0.946105	0.137797
26589	0.946132	0.743041
26590	1.23175	0.714753
26591	0.94613	0.744339
26592	0.946133	0.711584
26593	0.946131	0.717931
26594	1.25698	0.145255
26595	0.946126	0.181747
26596	0.946129	0.186081
26597	0.946128	0.161836
26598	0.946129	0.175073
26599	0.810943	2.1
26600	0.810944	2.1
26601	0.810932	2.1
26602	0.946132	0.696891
26603	1.09841	0.694931
26604	0.946131	0.714394
26605	0.946132	0.702841
26606	1.09841	0.70241
26607	0.810936	0.944359
26608	0.810935	0.970489
26609	0.810934	1.15525
26610	1.23851	2.1
26611	0.946132	1.01177
26612	0.946131	1.05991
26613	1.23851	2.1
26614	1.23851	1.42636
26615	0.946132	0.584516
26616	0.946131	0.793062
26617	0.946132	0.620893
26618	0.946132	0.748349
26619	0.946132	0.804737

26620	0.939141	1.09028
26621	0.953118	1.43705
26622	0.946126	1.08444
26623	0.946129	1.13478
26624	1.03306	1.20908
26625	0.946132	0.600697
26626	1.30927	0.524778
26627	0.946132	0.612965
26628	0.946132	0.644519
26629	1.30928	0.588837
26630	0.94613	2.1
26631	0.946131	2.1
26632	1.24511	2.1
26633	0.94613	2.1
26634	0.94613	2.1
26635	0.946133	0.28698
26636	1.34152	0.234574
26637	0.946131	0.313067
26638	0.946131	0.288217
26639	1.34152	0.257832
26640	0.946132	0.340336
26641	1.41383	0.289827
26642	0.946132	0.331912
26643	0.946132	0.37644
26644	1.41383	0.328812
26645	0.946132	0.872654
26646	0.946131	0.802868
26647	1.41392	0.77553
26648	0.946132	0.879311
26649	0.946132	0.808134
26650	0.946132	0.994198
26651	0.946132	0.921464
26652	1.24333	0.87458
26653	0.946133	1.05041
26654	0.946133	0.945081
26655	0.946124	0.609737
26656	0.94613	0.657031
26657	0.946126	0.67009
26658	1.3065	0.576163
26659	0.946127	0.667475
26660	0.810946	0.74423
26661	0.810943	0.708959
26662	0.81096	0.739236
26663	0.946131	0.50347
26664	1.40271	0.550358
26665	0.946131	0.61565
26666	0.946131	0.544481
26667	1.40271	0.559192
26668	0.946132	0.458252
26669	0.94613	0.575624
26670	0.946131	0.44506
26671	0.94613	0.495258
26672	0.946131	0.522008
26673	0.946133	0.728478
26674	1.30107	0.719966
26675	0.946132	0.726618
26676	0.946132	0.72828
26677	0.946133	0.737195

26678	0.810946	2.1
26679	0.810944	2.1
26680	0.810958	2.1
26681	0.946133	1.21358
26682	0.946131	1.17754
26683	0.946131	2.1
26684	1.30812	1.49463
26685	0.946131	1.2164
26686	0.94613	0.981147
26687	0.975002	1.03633
26688	0.917259	1.35108
26689	1.00902	1.20687
26690	0.94613	0.964581
26691	0.946131	2.1
26692	0.946127	2.1
26693	1.398 2.1	
26694	0.946129	2.1
26695	0.946129	2.1
26696	0.946133	2.1
26697	0.946132	2.1
26698	0.946132	2.1
26699	0.946132	2.1
26700	1.3033	2.1
26701	1.09897	0.779821
26702	0.946131	1.03306
26703	0.946132	0.949951
26704	0.946129	0.933564
26705	0.946131	0.967174
26706	0.946131	2.1
26707	0.946132	2.1
26708	1.39858	2.1
26709	0.946131	2.1
26710	0.946131	2.1
26711	0.946119	0.793589
26712	1.08878	0.813417
26713	0.946119	1.03926
26714	1.0888	0.813183
26715	0.946107	0.996281
26716	0.946132	0.322724
26717	1.10733	0.261298
26718	0.946132	0.427247
26719	0.946131	0.405865
26720	0.946132	0.356772
26721	0.946128	0.348995
26722	1.33671	0.288861
26723	0.94613	0.42247
26724	0.94613	0.443552
26725	0.94613	0.35919
26726	0.810943	2.1
26727	0.810944	2.1
26728	0.810945	2.1
26729	1.35117	2.1
26730	0.946129	2.1
26731	0.94613	2.1
26732	1.35117	2.1
26733	1.27174	2.1
26734	0.946132	2.06689
26735	0.946133	1.92692

26736	0.997296	1.25656
26737	0.946133	1.50342
26738	0.946132	1.37187
26739	0.810943	2.1
26740	0.810944	1.5343
26741	0.810945	2.1
26742	0.810946	1.01419
26743	0.810943	1.5932
26744	0.810933	1.20364
26745	0.946127	1.19751
26746	0.946131	1.1154
26747	0.946128	1.18739
26748	0.94613	1.25363
26749	0.946129	1.30677
26750	0.946131	0.765906
26751	0.946131	0.696044
26752	0.946129	0.767406
26753	0.946131	0.73894
26754	0.94613	0.71797
26755	0.946133	0.7
26756	0.946132	0.7
26757	0.946132	0.7
26758	0.946132	0.7
26759	0.946133	0.7
26760	0.946131	0.967958
26761	0.94613	0.858516
26762	0.946129	0.980615
26763	0.946131	0.901524
26764	0.946131	0.974672
26765	0.946132	1.09067
26766	0.946129	1.89711
26767	0.94613	1.36125
26768	0.946131	1.29861
26769	1.39356	1.57109
26770	0.810946	2.1
26771	0.810947	2.1
26772	0.810934	2.1
26773	0.946125	0.208016
26774	0.946127	0.227229
26775	1.33798	0.150298
26776	1.33797	0.173245
26777	0.946128	0.197797
26778	0.946131	0.163264
26779	0.946131	0.22372
26780	1.23146	0.145238
26781	1.23146	0.187423
26782	0.946131	0.201492
26783	0.946129	2.1
26784	0.94613	2.1
26785	0.946131	2.1
26786	0.94613	2.1
26787	1.30082	2.1
26788	0.946131	0.291456
26789	0.946132	0.396273
26790	0.946131	0.425434
26791	1.20416	0.291345
26792	0.946132	0.419212
26793	0.946132	0.294415

26794	0.946131	0.349782
26795	0.946131	0.467736
26796	1.18711	0.276507
26797	0.946132	0.408372
26798	0.946132	0.148478
26799	0.946132	0.130944
26800	0.946133	0.146032
26801	0.946132	0.124174
26802	0.946133	0.139465
26803	0.946131	0.484498
26804	0.946132	0.519377
26805	0.94613	0.375994
26806	0.946131	0.393918
26807	0.946131	0.489935
26808	0.946133	0.328766
26809	0.946132	0.309871
26810	0.946133	0.25147
26811	0.946132	0.256665
26812	0.946132	0.311233
26813	0.946132	2.1
26814	0.946132	2.1
26815	0.946132	2.1
26816	1.36422	2.1
26817	0.946132	2.1
26818	0.946131	0.8786
26819	0.946132	1.22706
26820	0.946132	0.961052
26821	0.946132	0.990366
26822	1.4185	1.15301
26823	0.946133	0.443528
26824	1.37794	0.307186
26825	0.946132	0.449953
26826	0.946133	0.340456
26827	0.946132	0.342405
26828	1.4045	2.1
26829	0.946132	2.1
26830	0.946132	2.1
26831	0.946132	2.1
26832	0.946132	2.1
26833	0.946131	2.1
26834	0.946132	2.1
26835	1.34285	2.1
26836	1.34285	2.1
26837	0.946132	2.1
26838	0.946131	2.1
26839	0.946132	2.1
26840	1.40739	2.1
26841	1.40739	2.1
26842	0.946131	2.1
26843	0.946128	2.1
26844	0.946132	2.1
26845	0.94613	2.1
26846	0.94613	2.1
26847	1.3164	2.1
26848	0.946132	1.25443
26849	1.3484	2.1
26850	0.946132	1.14012
26851	0.946132	2.04257

26852	1.3484	2.1
26853	0.946132	2.1
26854	0.946132	2.1
26855	0.946133	2.1
26856	0.946133	2.1
26857	1.41507	2.1
26858	0.946132	2.1
26859	1.32608	2.1
26860	0.946131	2.1
26861	0.946131	2.1
26862	0.946132	2.1
26863	0.94613	0.993366
26864	1.34646	1.87555
26865	0.946129	1.00016
26866	0.94613	0.945487
26867	1.34646	1.19696
26868	0.946131	0.800425
26869	0.946131	1.93936
26870	0.94613	0.916407
26871	1.33728	0.836433
26872	0.946131	0.882351
26873	0.946127	1.59077
26874	0.946131	1.08509
26875	0.946128	1.94037
26876	0.946129	1.92008
26877	1.4064	1.34099
26878	0.946133	2.1
26879	0.946131	2.1
26880	0.946131	2.1
26881	0.946131	2.1
26882	0.94613	2.1
26883	1.34912	2.1
26884	0.946131	2.1
26885	0.946129	2.1
26886	0.946131	2.1
26887	0.94613	2.1
26888	0.946132	2.1
26889	1.40447	2.1
26890	0.946132	2.1
26891	0.946133	2.1
26892	1.40447	2.1
26893	1.33607	2.1
26894	0.946131	1.33217
26895	0.946127	2.1
26896	0.94613	2.1
26897	0.946129	2.1
26898	0.946117	0.789596
26899	1.32664	0.919825
26900	0.946115	0.845791
26901	0.946105	0.952613
26902	0.94613	0.763248
26903	0.946118	2.1
26904	0.946114	2.1
26905	1.35886	2.1
26906	1.35884	2.1
26907	0.946129	2.1
26908	0.946132	2.0156
26909	1.2571	2.1

26910	0.946132	2.1
26911	0.946132	2.1
26912	1.2571	2.1
26913	0.946133	2.1
26914	1.35663	2.1
26915	0.946132	2.1
26916	0.946132	2.1
26917	1.35663	2.1
26918	0.946133	0.909235
26919	0.946129	1.36308
26920	0.946131	1.10976
26921	0.946131	1.04164
26922	1.30881	1.63242
26923	0.946132	2.1
26924	0.94613	2.1
26925	1.34652	2.1
26926	0.94613	2.1
26927	0.94613	2.1
26928	0.946132	2.00905
26929	0.946133	1.39629
26930	1.36183	1.09738
26931	1.36183	1.14114
26932	0.946132	1.29667
26933	0.946132	0.420071
26934	1.38353	0.362292
26935	0.94613	0.433303
26936	0.94613	0.413432
26937	1.38354	0.389428
26938	0.946128	0.84524
26939	1.24887	0.8318
26940	0.94613	0.871179
26941	0.94613	0.886714
26942	1.24887	0.884848
26943	0.946129	0.912061
26944	0.946126	1.17414
26945	0.946124	0.951423
26946	0.946128	0.979887
26947	1.32056	1.05635
26948	0.946129	0.989198
26949	0.946128	1.33007
26950	0.946126	1.01254
26951	0.946129	1.06532
26952	1.20351	1.12912
26953	0.946131	1.1749
26954	0.946131	1.21982
26955	0.946127	1.40087
26956	1.25591	1.41371
26957	0.94613	1.08749
26958	0.946132	1.09656
26959	0.946133	1.14389
26960	0.946132	1.185
26961	1.15436	1.08916
26962	0.930306	1.03181
26963	0.946132	2.1
26964	0.946132	2.1
26965	0.946132	1.89974
26966	0.946133	2.1
26967	0.946132	2.1

26968	0.946129	1.01852
26969	0.946132	0.953913
26970	0.946126	1.13932
26971	1.13245	0.896719
26972	0.946129	0.958725
26973	0.94613	2.1
26974	0.946132	2.1
26975	0.946129	1.75332
26976	0.946131	2.1
26977	0.946131	2.03389
26978	1.41279	2.01299
26979	1.76254	2.1
26980	0.600454	1.91811
26981	0.858991	1.93273
26982	0.940722	2.01591
26983	1.28439	1.52706
26984	0.946131	1.40232
26985	0.94613	1.52359
26986	0.946131	1.98029
26987	0.946131	1.91936
26988	0.946132	1.07027
26989	1.39335	0.813297
26990	0.946132	1.00078
26991	0.946132	0.83157
26992	0.946132	0.970381
26993	1.33879	0.718654
26994	0.946131	0.78327
26995	0.94613	0.86006
26996	1.33879	0.717055
26997	0.946131	0.755068
26998	0.946126	0.714518
26999	0.946131	0.71147
27000	1.37342	0.712036
27001	1.37343	0.705737
27002	0.946128	0.733745
27003	1.39168	0.194804
27004	0.946131	0.290911
27005	0.946131	0.323533
27006	0.946131	0.222967
27007	0.946131	0.279506
27008	0.946131	0.682907
27009	1.412	0.670112
27010	0.946127	0.662863
27011	0.946128	0.680206
27012	0.946128	0.670362
27013	1.1387	0.619867
27014	0.943364	0.869457
27015	0.946132	0.790953
27016	0.946132	0.816112
27017	0.946133	0.811403
27018	1.21085	0.843738
27019	0.88324	1.09656
27020	0.946129	0.974356
27021	0.946131	1.02309
27022	0.946131	0.956967
27023	1.33587	0.157545
27024	0.946125	0.202775
27025	0.946129	0.170048

27026	0.946126	0.222794
27027	0.946127	0.180399
27028	1.27512	0.228373
27029	0.946132	0.315722
27030	0.946132	0.345541
27031	0.946133	0.322409
27032	0.946132	0.255821
27033	0.946128	0.455878
27034	1.30498	0.469811
27035	0.946131	0.531624
27036	0.94613	0.496649
27037	1.30497	0.478277
27038	0.946131	0.372605
27039	1.25626	0.385938
27040	0.946131	0.451141
27041	0.946131	0.421945
27042	1.25626	0.403527
27043	0.946131	0.0960162
27044	0.94613	0.10297
27045	1.35316	0.0912368
27046	0.946131	0.0916309
27047	0.946131	0.0930653
27048	0.94613	0.706216
27049	1.28727	0.706661
27050	0.946128	0.695612
27051	0.946131	0.704211
27052	0.94613	0.700312
27053	0.946131	0.319297
27054	1.39502	0.245267
27055	0.946132	0.40571
27056	0.946132	0.273009
27057	0.946132	0.345448
27058	1.36173	0.716288
27059	0.946131	0.925727
27060	0.946126	0.854339
27061	0.946127	0.866617
27062	0.946128	0.833974
27063	0.946129	0.912105
27064	0.946133	0.854565
27065	0.946131	0.915115
27066	0.946131	0.924891
27067	0.946131	0.944288
27068	0.946128	0.0725182
27069	0.946132	0.0713368
27070	1.24678	0.0689526
27071	0.94613	0.068655
27072	0.94613	0.0698613
27073	0.946129	0.0725723
27074	0.94613	0.0729925
27075	1.25215	0.0693639
27076	0.946126	0.0700389
27077	0.946129	0.0721475
27078	0.946129	0.821218
27079	1.29689	0.815031
27080	0.946131	0.823107
27081	0.946131	0.804462
27082	0.946131	0.786778
27083	0.946128	0.495794

27084	0.94613	0.576624
27085	0.946131	0.422678
27086	0.94613	0.557555
27087	0.946131	0.488384
27088	0.946127	0.583514
27089	0.946128	0.679823
27090	0.94613	0.532462
27091	0.946129	0.647803
27092	0.946129	0.595331
27093	0.946133	0.11193
27094	0.946132	0.137483
27095	0.946132	0.212231
27096	1.29957	0.126044
27097	0.946133	0.150939
27098	0.946132	0.141422
27099	0.946133	0.134307
27100	0.946132	0.193892
27101	1.26201	0.144024
27102	0.946132	0.161497
27103	0.946133	0.382863
27104	1.40427	0.321513
27105	0.946132	0.465943
27106	0.946132	0.492156
27107	0.946133	0.37894
27108	0.946132	0.372581
27109	1.35123	0.334786
27110	0.946132	0.456083
27111	0.946132	0.509655
27112	0.946131	0.370427
27113	0.946128	0.665985
27114	0.946129	0.703013
27115	0.946131	0.617593
27116	0.94613	0.702595
27117	0.946131	0.661346
27118	0.946127	0.761806
27119	1.23351	0.748182
27120	0.946131	0.762609
27121	0.946129	0.750577
27122	0.94613	0.746839
27123	0.946131	0.347003
27124	0.946131	0.478932
27125	0.94613	0.369545
27126	1.18119	0.359442
27127	0.946131	0.427471
27128	0.946132	0.722632
27129	1.30563	0.710511
27130	0.946133	0.730249
27131	0.946132	0.711637
27132	0.946133	0.719389
27133	0.946132	0.861494
27134	0.946132	0.653075
27135	0.946133	1.0884
27136	1.20256	0.829691
27137	0.946133	0.905935
27138	0.94613	0.943656
27139	1.33007	0.880669
27140	0.946128	0.974713
27141	0.946132	0.852751

27142	0.946131	0.878581
27143	0.946131	2.1
27144	0.946131	2.1
27145	0.946128	2.1
27146	0.946129	2.1
27147	0.94613	2.1
27148	0.810942	2.1
27149	0.810942	2.1
27150	0.81093	2.1
27151	0.946132	2.1
27152	1.39823	2.1
27153	0.946132	2.1
27154	0.946131	2.1
27155	0.946132	2.1
27156	0.946131	2.1
27157	0.946131	2.1
27158	0.946131	2.1
27159	1.37713	2.1
27160	0.946131	2.1
27161	0.946133	1.2075
27162	0.946131	2.1
27163	1.40594	2.1
27164	1.40594	2.1
27165	0.94613	2.1
27166	0.946133	2.1
27167	0.946131	2.1
27168	1.38155	2.1
27169	1.38155	2.1
27170	0.946131	2.1
27171	0.946132	0.971159
27172	0.946133	1.55548
27173	0.946132	1.05348
27174	0.946132	1.08623
27175	1.33694	1.32175
27176	0.946131	2.04439
27177	1.228 2.1	
27178	0.946129	2.1
27179	0.946131	2.1
27180	1.228 2.1	
27181	0.946132	1.01925
27182	0.946132	1.37074
27183	0.946131	1.73739
27184	0.946132	1.06845
27185	1.35943	1.6676
27186	0.946133	0.193551
27187	0.946133	0.167745
27188	1.02377	0.277653
27189	1.02377	0.178191
27190	0.946132	0.225206
27191	0.94613	0.630354
27192	0.946131	0.698651
27193	0.946132	0.7
27194	1.29322	0.445481
27195	0.946131	0.7
27196	0.946131	0.946097
27197	0.946131	0.270406
27198	0.946132	1.04332
27199	1.41492	0.49257

27200	0.946131	0.868205
27201	0.946132	2.1
27202	0.946131	2.1
27203	0.946132	2.1
27204	1.35028	2.1
27205	0.946132	2.1
27206	0.946132	2.1
27207	0.946132	2.04742
27208	0.946131	1.94482
27209	1.32461	2.1
27210	0.946132	1.88529
27211	0.946131	2.1
27212	0.946132	2.1
27213	1.40369	2.1
27214	0.94613	2.1
27215	0.946131	2.1
27216	0.946132	0.653915
27217	0.979597	0.557679
27218	0.946131	0.690943
27219	0.946132	0.568378
27220	0.946132	0.588971
27221	0.946131	2.1
27222	0.946131	1.51602
27223	0.94613	2.1
27224	0.946131	2.1
27225	1.41897	2.1
27226	1.37791	2.1
27227	0.94613	2.1
27228	0.946129	2.1
27229	0.946131	2.1
27230	0.946131	2.1
27231	1.39384	2.1
27232	0.946131	2.1
27233	0.946128	2.1
27234	0.946129	2.1
27235	0.946129	2.1
27236	0.810945	1.32742
27237	0.810944	2.09474
27238	0.810958	1.46585
27239	0.946128	2.1
27240	0.946132	2.1
27241	0.94613	2.1
27242	1.3335	2.1
27243	0.946131	2.1
27244	0.94613	0.771881
27245	0.946129	0.939944
27246	0.946132	0.752836
27247	1.41192	0.84521
27248	0.94613	0.785203
27249	0.946132	0.755012
27250	0.946133	0.743253
27251	0.946132	0.735047
27252	1.39428	0.720563
27253	0.946133	0.713438
27254	0.94613	1.47287
27255	0.946128	1.60789
27256	0.946132	1.07618
27257	0.94613	1.3659

27258	1.40684	1.79408
27259	0.946131	2.1
27260	0.946131	2.1
27261	0.946131	2.1
27262	0.946131	1.76627
27263	1.39021	2.1
27264	0.946131	1.11887
27265	1.31649	1.9431
27266	0.946131	0.992031
27267	0.946131	1.00965
27268	1.31649	1.27205
27269	0.810946	2.1
27270	0.810946	2.1
27271	0.810958	2.1
27272	1.16687	2.1
27273	0.946131	2.1
27274	0.946131	2.1
27275	1.16687	2.1
27276	1.16687	2.1
27277	0.946128	0.890413
27278	1.3564	0.908354
27279	0.946131	0.891306
27280	0.946131	0.893759
27281	0.94613	0.869288
27282	0.946128	2.1
27283	1.35828	2.1
27284	0.94613	2.1
27285	0.946131	2.1
27286	0.94613	2.1
27287	0.946133	0.930303
27288	1.36477	1.01092
27289	0.946132	1.00712
27290	0.946132	1.00956
27291	1.36477	1.01509
27292	0.946132	1.03835
27293	1.31857	1.69971
27294	0.946132	1.12279
27295	0.946132	1.15526
27296	1.31857	1.13222
27297	0.94613	0.877474
27298	1.35059	0.791075
27299	0.946128	0.785957
27300	0.94613	0.814728
27301	1.35059	0.798208
27302	0.946126	0.614116
27303	1.35559	0.423319
27304	0.946127	0.503442
27305	0.946128	0.525607
27306	1.35559	0.439486
27307	0.946131	0.420825
27308	1.33035	0.335538
27309	0.946127	0.346663
27310	0.946128	0.500283
27311	1.33035	0.429288
27312	0.94613	0.596461
27313	1.3665	0.45992
27314	0.946129	0.463071
27315	0.946131	0.654352

27316	1.3665	0.615427
27317	0.94613	0.655074
27318	1.32828	0.640904
27319	0.946129	0.606575
27320	0.94613	0.648857
27321	0.946131	0.647944
27322	0.946131	2.1
27323	0.946131	2.1
27324	1.32949	2.1
27325	1.32949	2.1
27326	0.946131	2.1
27327	0.946131	0.995014
27328	0.94613	0.718045
27329	0.946133	0.903358
27330	1.38357	0.77014
27331	0.946131	0.818061
27332	0.946131	2.1
27333	0.946128	2.1
27334	1.36919	2.1
27335	0.946131	2.1
27336	0.94613	2.1
27337	0.946131	1.08746
27338	1.3771	2.1
27339	0.946129	1.96394
27340	0.94613	1.68809
27341	0.946131	1.07378
27342	0.946131	1.05966
27343	0.94613	0.826406
27344	0.946129	0.762857
27345	1.30029	0.902442
27346	0.94613	0.810938
27347	1.21174	0.129199
27348	0.946132	0.165347
27349	0.946132	0.184904
27350	0.946132	0.147782
27351	0.946133	0.159234
27352	0.810946	2.1
27353	0.810945	2.1
27354	0.810946	2.1
27355	1.1796	2.1
27356	0.946131	2.1
27357	0.946132	2.1
27358	1.1796	2.1
27359	1.1796	2.1
27360	0.946131	2.1
27361	0.946131	2.1
27362	0.946133	2.1
27363	0.946131	2.1
27364	1.40615	2.1
27365	0.810945	2.1
27366	0.810944	2.1
27367	0.810945	2.1
27368	1.40239	2.1
27369	0.94613	2.1
27370	1.35561	2.1
27371	0.946133	2.1
27372	1.35562	2.1
27373	1.41169	0.720978

27374	0.946131	0.797294
27375	0.946127	0.880945
27376	1.41169	0.729073
27377	0.946128	0.776372
27378	0.946119	2.1
27379	0.946119	2.1
27380	0.946119	2.1
27381	0.946107	2.1
27382	1.41917	2.1
27383	0.810946	1.21267
27384	0.810943	1.27055
27385	0.810934	1.55256
27386	0.946127	2.1
27387	1.1995	2.1
27388	0.946126	2.1
27389	0.946128	2.1
27390	1.1995	2.1
27391	0.810944	2.1
27392	0.810947	2.1
27393	0.81096	2.1
27394	0.946133	0.700712
27395	1.3495	0.563232
27396	0.946132	0.7
27397	0.946132	0.644046
27398	0.946133	0.685509
27399	0.946132	2.1
27400	0.946132	2.1
27401	0.946133	2.1
27402	1.39529	2.1
27403	0.946133	2.1
27404	0.946133	2.1
27405	1.28525	2.1
27406	0.946132	2.1
27407	0.946133	2.1
27408	1.28525	2.1
27409	0.810946	2.1
27410	0.810945	2.1
27411	0.810946	2.1
27412	1.33931	2.1
27413	0.946132	2.1
27414	0.946132	2.1
27415	1.33931	2.1
27416	1.24616	2.1
27417	0.946133	0.7
27418	0.946133	0.7
27419	0.946132	0.700023
27420	0.946132	0.7
27421	0.946133	0.7
27422	0.946128	2.1
27423	1.40225	2.1
27424	0.946128	2.1
27425	0.94613	2.1
27426	0.94613	2.1
27427	0.946113	0.169438
27428	0.946118	0.135541
27429	0.946114	0.171687
27430	1.40551	0.134201
27431	0.946103	0.133836

27432	0.94613	2.1
27433	0.946132	2.1
27434	0.946129	2.1
27435	0.946131	2.1
27436	1.37811	2.1
27437	0.946132	2.1
27438	1.39826	2.1
27439	0.946133	2.1
27440	0.946132	2.1
27441	1.39826	2.1
27442	0.946118	2.1
27443	1.28298	2.1
27444	0.946117	2.1
27445	0.946105	2.1
27446	1.283 2.1	
27447	0.946118	0.130382
27448	1.41375	0.138183
27449	0.946117	0.224977
27450	0.946131	0.190379
27451	0.946106	0.17043
27452	0.946132	1.02824
27453	0.946129	0.914309
27454	1.28436	0.914332
27455	0.946131	1.03293
27456	0.946131	0.933908
27457	0.810947	0.765
27458	0.810946	0.77435
27459	0.81096	0.754298
27460	0.946132	0.737078
27461	1.34338	0.729739
27462	0.961435	0.691121
27463	0.961435	0.72834
27464	0.946132	0.733396
27465	1.3028	1.60136
27466	0.946131	1.48363
27467	0.946127	1.71685
27468	0.946128	1.93651
27469	0.946129	1.52345
27470	0.946132	0.852521
27471	1.37973	0.798995
27472	0.946132	0.857363
27473	0.946132	0.803016
27474	0.946132	0.798431
27475	0.946131	0.98613
27476	0.94613	0.949453
27477	0.946129	0.988211
27478	0.946131	0.942807
27479	1.41798	0.942433
27480	0.946118	0.931186
27481	0.946118	0.892071
27482	0.946117	0.93271
27483	0.946131	0.8886
27484	0.946105	0.896054
27485	0.946131	1.03568
27486	0.946131	0.985881
27487	0.94613	1.03278
27488	0.946131	0.986819
27489	0.946131	0.963423

27490	0.946133	1.14482
27491	1.40274	2.1
27492	0.946133	0.985856
27493	0.946132	1.77185
27494	1.40274	1.82275
27495	0.946131	1.33778
27496	1.39731	2.04437
27497	0.946132	1.06577
27498	0.94613	1.15105
27499	1.39731	1.56211
27500	0.94612	1.95152
27501	0.94612	1.42919
27502	0.946119	1.35379
27503	0.946132	1.46358
27504	1.40764	1.87777
27505	0.946128	2.1
27506	0.946132	2.1
27507	0.94613	2.1
27508	0.94613	2.1
27509	1.31213	2.1
27510	0.946127	1.52811
27511	0.94613	2.1
27512	0.946127	1.54125
27513	0.946128	1.59153
27514	1.36653	2.04927
27515	0.946127	2.1
27516	1.3557	2.1
27517	0.946128	2.1
27518	0.946128	2.1
27519	1.3557	2.1
27520	0.946133	2.1
27521	0.946132	2.1
27522	0.946133	2.1
27523	0.946132	2.1
27524	1.40081	2.1
27525	0.946128	2.1
27526	0.946133	2.1
27527	0.946131	2.1
27528	0.946131	2.1
27529	1.32282	2.1
27530	0.810945	1.39176
27531	0.810943	1.55369
27532	0.810933	2.1
27533	1.27676	2.1
27534	0.94613	2.1
27535	0.946128	2.1
27536	0.946131	2.1
27537	0.946131	2.1
27538	0.946131	0.753146
27539	0.946127	0.775897
27540	1.41328	0.813407
27541	1.41327	0.818196
27542	0.946128	0.783498
27543	0.946132	0.391325
27544	1.32083	0.304661
27545	0.946132	0.353226
27546	0.946133	0.442913
27547	1.32082	0.444263

27548	0.946128	0.510705
27549	1.29215	0.414182
27550	0.94613	0.513131
27551	0.94613	0.578046
27552	1.29215	0.572609
27553	0.946132	0.311269
27554	1.27696	0.233726
27555	0.94613	0.267459
27556	0.94613	0.350062
27557	1.27696	0.319359
27558	0.94612	0.305676
27559	1.29289	0.236776
27560	0.946119	0.270173
27561	0.946132	0.353769
27562	1.29287	0.342496
27563	0.946128	2.1
27564	1.37869	2.1
27565	0.946131	2.1
27566	0.94613	2.1
27567	0.946131	2.1
27568	0.946133	2.1
27569	0.946132	2.1
27570	0.946132	2.1
27571	1.35703	2.1
27572	0.946133	2.1
27573	0.946132	2.1
27574	0.946132	2.1
27575	0.946133	2.00121
27576	1.37017	2.1
27577	0.946133	2.1
27578	0.946119	0.123081
27579	0.94612	0.138898
27580	1.38583	0.0996535
27581	1.38585	0.108836
27582	0.946107	0.118184
27583	0.946126	0.21996
27584	0.946127	0.281463
27585	1.22904	0.192027
27586	1.22904	0.191405
27587	0.946128	0.227255
27588	1.3279	1.04352
27589	0.946131	1.4021
27590	0.94613	1.22529
27591	0.946131	1.39728
27592	0.946131	1.23603
27593	0.946132	2.1
27594	0.946132	2.1
27595	0.946132	2.1
27596	0.946131	2.1
27597	1.40932	2.1
27598	1.26868	0.259752
27599	0.946131	0.369285
27600	0.94613	0.392262
27601	0.946131	0.35691
27602	0.946131	0.362277
27603	0.810943	2.1
27604	0.810945	2.1
27605	0.810932	2.1

27606	0.810943	0.991379
27607	0.810944	0.790774
27608	0.810957	0.791066
27609	1.31539	0.795515
27610	0.946132	1.05814
27611	0.946128	0.47421
27612	1.3154	0.883298
27613	1.17856	0.640285
27614	1.21997	1.36407
27615	0.94613	1.6306
27616	0.946129	1.46874
27617	0.94613	1.90444
27618	0.94613	1.72809
27619	0.946131	1.12105
27620	0.946131	0.987693
27621	0.946129	1.13495
27622	0.94613	1.1288
27623	0.946131	1.21543
27624	0.946127	0.908338
27625	0.946131	0.780348
27626	0.946127	1.34974
27627	0.946129	0.920518
27628	1.41342	0.859
27629	0.946131	0.844031
27630	0.946131	1.18054
27631	0.946128	1.58911
27632	0.946131	0.882907
27633	1.29149	1.12422
27634	1.41022	2.1
27635	0.94613	0.984732
27636	0.946128	2.06491
27637	0.94613	1.86876
27638	0.94613	2.08973
27639	0.946128	0.7
27640	0.94613	0.7
27641	1.27864	0.7
27642	1.27864	0.606962
27643	0.946131	0.7
27644	0.946129	0.795392
27645	0.946125	1.17793
27646	0.946124	0.910957
27647	0.946127	0.864711
27648	1.37759	1.05017
27649	0.946129	0.848767
27650	0.94613	1.17495
27651	0.946126	0.904947
27652	0.946129	0.917662
27653	1.38259	1.01724
27654	0.946131	0.281175
27655	1.34118	0.246137
27656	0.946132	0.304398
27657	0.946132	0.302836
27658	1.34118	0.286606
27659	0.946131	0.178932
27660	1.25017	0.178162
27661	0.946128	0.15947
27662	0.946131	0.172378
27663	0.946131	0.145827

27664	0.946131	2.1
27665	0.946128	2.1
27666	0.946133	2.1
27667	1.3937	2.1
27668	0.94613	2.1
27669	0.946131	0.952591
27670	0.94613	0.760711
27671	1.40404	0.847053
27672	1.40404	0.477013
27673	0.946131	0.789855
27674	0.946132	0.801612
27675	0.946132	0.678211
27676	1.38897	0.612913
27677	1.38897	0.25392
27678	0.946132	0.714868
27679	0.94613	0.739241
27680	0.946128	0.79124
27681	1.40332	0.343324
27682	1.40331	0.596764
27683	0.94613	0.79512
27684	0.946132	0.795705
27685	0.946132	0.965994
27686	0.946133	0.864477
27687	1.41619	0.974594
27688	0.946132	0.85515
27689	0.946132	1.01188
27690	0.946132	2.1
27691	0.946132	2.02929
27692	1.38244	2.1
27693	0.946132	2.1
27694	0.810946	1.04483
27695	0.810943	0.874873
27696	0.810934	0.84585
27697	0.810947	0.960105
27698	0.810943	0.806289
27699	0.810934	0.820062
27700	1.12786	1.12151
27701	0.946132	1.75333
27702	0.946132	1.35636
27703	0.946132	1.26859
27704	0.946133	1.22493
27705	0.946132	1.22433
27706	0.946132	1.32822
27707	0.946132	1.08998
27708	1.27806	1.32757
27709	0.946133	1.08681
27710	0.946132	2.1
27711	0.946132	2.1
27712	0.946133	2.1
27713	0.946132	2.1
27714	0.946133	2.1
27715	1.39514	1.18325
27716	0.946129	0.890845
27717	0.946124	1.05424
27718	0.946127	0.842677
27719	0.946127	1.0537
27720	0.94613	2.1
27721	0.946129	2.1

27722	0.946132	2.1
27723	1.3991	2.1
27724	0.94613	2.1
27725	0.946132	0.782361
27726	0.946133	1.30606
27727	0.946132	1.18352
27728	1.36835	0.901877
27729	0.946132	0.837223
27730	0.946132	2.1
27731	0.946132	2.1
27732	0.946133	2.1
27733	0.946132	2.1
27734	1.39047	2.1
27735	0.946132	0.140895
27736	0.946128	0.137425
27737	0.94613	0.157824
27738	1.33174	0.127241
27739	0.946131	0.1603
27740	0.946128	0.224293
27741	0.946131	0.194744
27742	0.94613	0.238125
27743	0.94613	0.158177
27744	0.94613	0.239549
27745	0.946119	2.1
27746	0.946119	2.1
27747	1.29064	2.1
27748	0.946132	2.1
27749	0.946107	2.1
27750	0.946133	2.1
27751	0.94613	2.1
27752	1.30584	2.1
27753	0.946131	2.1
27754	0.946131	2.1
27755	0.946131	2.1
27756	0.946132	2.1
27757	0.94613	2.1
27758	0.946131	2.1
27759	1.39549	2.1
27760	0.913386	1.16633
27761	0.978873	1.09415
27762	0.946126	1.09247
27763	0.94613	1.25432
27764	1.00473	0.983564
27765	0.946132	0.860904
27766	0.946133	0.880753
27767	0.946132	0.863616
27768	0.946132	0.896813
27769	1.38894	0.849204
27770	0.946128	0.85762
27771	0.946131	0.852339
27772	0.94613	0.85923
27773	0.94613	0.853355
27774	1.23865	0.840396
27775	0.946126	1.1466
27776	0.946129	1.22416
27777	0.946124	1.06904
27778	0.946127	1.16474
27779	1.40248	1.06947

27780	0.946133	0.870495
27781	0.946132	0.934963
27782	0.946132	0.864488
27783	0.946133	0.911921
27784	1.1391	0.872322
27785	0.827862	0.814945
27786	0.946133	0.807487
27787	0.946131	0.879304
27788	0.946131	0.810824
27789	0.94613	0.809577
27790	0.946128	0.757136
27791	0.946132	0.778798
27792	0.94613	0.793135
27793	1.21388	0.739945
27794	0.94613	0.781072
27795	0.810943	0.703158
27796	0.810945	0.71007
27797	0.810932	0.706806
27798	0.946131	0.7824
27799	0.946131	0.760893
27800	0.946133	0.775158
27801	1.36748	0.731083
27802	0.946132	0.716159
27803	0.946128	0.466582
27804	0.946131	0.466772
27805	0.94613	0.627301
27806	1.35856	0.308873
27807	0.94613	0.575852
27808	0.946132	2.1
27809	0.946131	2.1
27810	0.946133	2.1
27811	0.946132	2.1
27812	0.946131	2.1
27813	0.946133	2.1
27814	0.946131	2.1
27815	1.39558	2.1
27816	1.39558	2.1
27817	0.94613	2.1
27818	0.946131	0.297628
27819	0.94613	0.399326
27820	0.946129	0.316175
27821	1.41388	0.283649
27822	0.946131	0.358849
27823	0.946133	0.332182
27824	0.946132	0.653273
27825	0.946132	0.380659
27826	0.946133	0.397478
27827	0.93452	0.612499
27828	0.946129	2.1
27829	1.38897	2.1
27830	0.946131	2.1
27831	0.946131	2.1
27832	1.38897	2.1
27833	0.946133	1.18061
27834	0.946132	1.15495
27835	0.946133	1.12213
27836	0.946132	0.921667
27837	1.38773	1.03369

27838	1.41512	0.456915
27839	0.946131	0.663378
27840	0.946129	0.566335
27841	0.946133	0.616055
27842	0.946131	0.599245
27843	0.946132	1.02621
27844	0.946132	2.1
27845	0.946132	1.14291
27846	0.946133	1.19488
27847	0.946133	2.1
27848	0.946126	0.967934
27849	0.946132	1.22177
27850	0.946129	1.06581
27851	0.946129	1.07274
27852	0.946129	1.24014
27853	0.939895	1.69347
27854	1.03386	2.1
27855	0.94613	1.69817
27856	0.946131	1.45654
27857	1.03386	1.8187
27858	0.946131	1.26975
27859	1.39309	2.1
27860	0.946133	1.14718
27861	0.946131	2.1
27862	1.39309	2.1
27863	0.946133	2.1
27864	1.24853	2.1
27865	0.946132	2.1
27866	0.946132	2.1
27867	1.24853	2.1
27868	0.946133	1.91787
27869	1.39699	1.09367
27870	0.946132	1.57777
27871	0.946132	2.1
27872	1.39699	1.27001
27873	0.946132	2.1
27874	0.946131	1.10842
27875	0.946133	2.1
27876	0.946131	2.1
27877	1.38969	2.1
27878	1.28464	1.80081
27879	0.946133	1.83106
27880	0.94613	1.92437
27881	0.946131	1.65265
27882	0.946131	1.58882
27883	0.946119	0.814805
27884	0.94612	0.870394
27885	0.946119	0.832498
27886	0.946132	0.840906
27887	1.3749	0.822398
27888	0.810942	2.1
27889	0.810943	2.1
27890	0.810957	2.1
27891	1.41134	1.90256
27892	0.946132	0.954089
27893	0.946132	1.61798
27894	0.946132	1.02401
27895	0.946132	1.64043

27896	1.31338	1.93318
27897	0.946132	1.09828
27898	0.946132	1.91164
27899	0.946133	1.28196
27900	0.946132	1.62699
27901	0.946103	0.090094
27902	0.752448	0.103319
27903	0.946132	0.0959424
27904	0.946131	0.0973249
27905	1.20617	0.099336
27906	0.946132	1.02597
27907	1.08054	0.981079
27908	1.25736	1.0915
27909	0.946132	1.02645
27910	0.946131	1.03055
27911	0.946128	1.92356
27912	1.33275	1.03753
27913	1.1896	1.82097
27914	0.946131	1.95873
27915	1.18962	1.9546
27916	1.21955	0.114476
27917	0.946131	0.137464
27918	1.33587	0.118977
27919	0.741946	0.107012
27920	1.07387	0.0898184
27921	0.945844	0.867968
27922	1.3292	1.86054
27923	1.59472	1.26633
27924	0.946131	0.959359
27925	1.04043	1.00273
27926	1.13529	0.298668
27927	0.946132	0.501245
27928	1.24893	0.302147
27929	1.12675	0.524037
27930	0.936652	0.546993
27931	1.36968	2.1
27932	0.946131	2.1
27933	0.946132	2.1
27934	1.36968	2.1
27935	1.258 2.1	
27936	3.17458	0.357589
27937	0.946132	1.32929
27938	1.35022	2.1
27939	1.35022	2.1
27940	0.94613	1.41182
27941	1.12356	1.94012
27942	0.946131	2.1
27943	1.22401	2.1
27944	1.22401	2.1
27945	0.94613	2.1
27946	0.952455	2.1
27947	1.2107	2.1
27948	0.946132	2.1
27949	1.33047	2.1
27950	0.946131	2.1
27951	0.946132	2.1
27952	0.61957	1.81932
27953	0.8636	2.1

27954	0.946132	1.79586
27955	1.19294	2.1
27956	0.946128	2.1
27957	1.36607	2.1
27958	0.94613	1.92332
27959	1.39416	2.1
27960	1.36607	2.1
27961	1.21474	2.1
27962	0.946131	2.1
27963	0.946131	2.1
27964	1.16288	2.1
27965	1.08542	2.1
27966	0.851526	0.942241
27967	1.30374	1.98274
27968	1.01708	1.86377
27969	0.946114	1.00345
27970	0.96976	1.12704
27971	0.946132	0.284602
27972	1.49997	0.398629
27973	0.771735	0.356307
27974	0.771764	0.444626
27975	0.946133	0.523791
27976	0.700863	1.23253
27977	1.42222	1.25552
27978	0.946131	2.1
27979	0.946128	0.995458
27980	1.42509	2.03675
27981	1.14298	1.04522
27982	0.837014	2.1
27983	0.946131	2.1
27984	1.29426	2.1
27985	0.836985	2.1
27986	0.946129	2.1
27987	1.22089	0.766936
27988	0.946132	0.969345
27989	0.712486	0.803065
27990	0.6234	0.812038
27991	0.946131	2.1
27992	1.40271	2.1
27993	1.32241	2.1
27994	0.946133	2.1
27995	1.32241	2.1
27996	0.769373	1.23266
27997	0.946129	2.1
27998	1.41302	2.1
27999	1.28254	2.1
28000	0.946131	2.1
28001	1.28254	2.1
28002	0.851543	1.38335
28003	1.23056	2.1
28004	1.24356	2.1
28005	0.851543	1.48648
28006	0.909417	1.81615
28007	1.13808	0.70395
28008	0.946133	0.75308
28009	0.946131	0.712968
28010	1.13808	0.792605
28011	1.13808	0.715759

28012	0.930941	1.22066
28013	1.37808	1.11085
28014	1.17573	0.984532
28015	0.946124	1.24903
28016	1.02404	1.04037
28017	0.946116	2.0809
28018	1.3144	0.997826
28019	1.3144	1.96883
28020	0.946118	2.1
28021	1.13089	1.75246
28022	0.946128	0.633973
28023	0.946101	0.575819
28024	1.2606	0.688208
28025	1.26055	0.565149
28026	0.580823	0.888449
28027	0.946132	1.06604
28028	0.580825	0.906112
28029	1.23224	0.925341
28030	0.946128	2.1
28031	1.3719	2.1
28032	0.94613	2.1
28033	1.3719	2.1
28034	0.94613	2.1
28035	0.946132	1.16341
28036	1.35025	0.895362
28037	1.35025	1.02028
28038	0.946132	1.32389
28039	1.1794	1.06884
28040	0.946133	2.1
28041	1.25598	2.1
28042	1.29249	2.1
28043	1.38154	2.1
28044	0.851541	1.45098
28045	1.31508	2.1
28046	1.37558	2.1
28047	0.946129	1.58295
28048	0.867663	1.83998
28049	0.94613	1.26075
28050	1.35426	1.12902
28051	0.84872	1.13154
28052	0.946129	1.06464
28053	1.17195	1.65051
28054	0.946133	2.1
28055	1.39009	2.1
28056	1.14342	2.1
28057	0.946133	2.1
28058	1.14343	2.1
28059	1.38819	2.1
28060	0.946133	2.1
28061	1.4116	2.1
28062	0.946131	2.1
28063	0.946131	2.1
28064	0.946127	2.1
28065	1.32244	2.1
28066	1.32244	2.1
28067	0.946131	2.1
28068	1.2176	2.1
28069	1.75823	2.1

28070	1.11239	2.1
28071	2.17979	2.1
28072	1.65533	2.1
28073	3.41356	2.1
28074	1.94841	2.1
28075	1.91767	2.1
28076	1.76726	2.1
28077	1.70302	2.1
28078	3.76137	2.1
28079	1.94841	2.1
28080	1.33962	2.1
28081	1.31365	2.1
28082	1.70302	2.1
28083	3.2123	2.1
28084	1.76726	2.1
28085	1.70302	2.1
28086	1.47581	2.1
28087	1.7457	2.1
28088	3.22764	2.1
28089	1.20478	2.1
28090	1.70302	2.1
28091	1.27956	2.1
28092	1.18569	2.1
28093	1.63979	2.1
28094	1.51949	2.1
28095	1.53082	2.1
28096	1.92911	2.1
28097	1.32068	2.1
28098	1.74454	2.1
28099	3.27536	2.1
28100	1.08172	2.1
28101	1.74454	2.1
28102	1.52603	2.1
28103	1.52603	2.1
28104	1.32068	2.1
28105	1.32068	2.1
28106	1.89226	2.1
28107	2.37364	2.1
28108	1.89226	2.1
28109	2.42769	2.1
28110	4.31995	2.1
28111	2.37365	2.1
28112	1.5362	2.1
28113	2.4277	2.1
28114	1.56328	2.1
28115	3.9639	2.1
28116	1.89225	2.1
28117	2.42768	2.1
28118	1.89213	2.1
28119	1.89225	2.1
28120	2.42756	2.1
28121	2.42768	2.1
28122	2.42768	2.1
28123	1.58163	2.1
28124	1.56315	2.1
28125	1.56327	2.1
28126	2.42757	2.1
28127	2.42768	2.1

28128	1.01641	2.1
28129	2.15005	2.1
28130	1.00099	2.1
28131	2.15114	2.1
28132	3.15104	2.1
28133	0.994813	2.1
28134	2.15114	2.1
28135	2.17159	2.1
28136	2.17159	2.1
28137	1.00099	2.1
28138	1.00099	2.1
28139	1.29608	2.1
28140	1.29608	2.1
28141	0.942035	1.98463
28142	0.942035	1.40195
28143	1.29198	1.96199
28144	0.942033	2.1
28145	0.946132	2.1
28146	0.946132	1.81435
28147	0.946132	1.47535
28148	0.942033	1.81615
28149	0.946133	1.248
28150	1.29198	1.74157
28151	1.99985	2.1
28152	1.14207	2.1
28153	1.83584	2.1
28154	1.00232	2.1
28155	3.00217	2.1
28156	1.99867	0.123655
28157	2.0815	0.180606
28158	1.56806	0.139402
28159	1.28828	0.173972
28160	3.58519	0.184465
28161	1.89226	0.245431
28162	1.89226	0.345847
28163	1.89226	0.294153
28164	1.89226	0.324886
28165	1.89226	0.269664
28166	1.89226	0.313623
28167	1.89226	0.190277
28168	1.89226	0.274574
28169	2.23193	0.204357
28170	2.48978	0.380358
28171	4.38204	0.208878
28172	1.85293	0.884801
28173	1.64279	0.731532
28174	1.76268	0.90354
28175	1.54214	0.620478
28176	3.39507	0.788449
28177	1.6428	0.731364
28178	1.17302	0.732184
28179	1.17302	0.822783
28180	1.28419	0.732224
28181	1.54214	0.720876
28182	1.65331	0.741841
28183	1.76268	0.919183
28184	1.54214	0.736199
28185	1.65324	0.874955

28186	1.42491	0.68105
28187	3.19538	0.785942
28188	0.651043	0.788985
28189	0.651043	0.776594
28190	0.647925	0.734117
28191	0.647925	0.755883
28192	0.521747	0.720882
28193	0.58651	0.730873
28194	0.77107	0.715715
28195	0.712457	0.735481
28196	0.712457	0.702914
28197	0.521747	0.708276
28198	0.58651	0.710516
28199	0.77107	0.719539
28200	1.2873	2.1
28201	1.48751	2.1
28202	1.59659	2.1
28203	1.77596	1.30047
28204	3.06326	2.1
28205	1.07976	2.1
28206	1.59659	2.1
28207	1.30237	2.1
28208	1.77723	2.1
28209	2.89897	2.1
28210	1.77723	2.1
28211	1.77596	1.345
28212	1.82614	2.1
28213	1.59659	2.1
28214	2.0055	2.1
28215	1.77596	2.1
28216	2.64523	0.919237
28217	2.73982	0.73637
28218	2.50057	0.917669
28219	2.61606	0.739324
28220	5.26129	0.802003
28221	2.73982	0.723077
28222	2.68987	0.721212
28223	2.61607	0.741533
28224	2.57611	0.674743
28225	5.30593	0.686434
28226	1.89226	0.703222
28227	1.89226	0.732946
28228	1.89226	0.713379
28229	2.02178	0.71186
28230	3.78452	0.719702
28231	1.89226	0.834794
28232	1.89226	0.852696
28233	1.89226	1.24672
28234	1.89226	0.74024
28235	1.89226	1.34392
28236	1.89226	0.696042
28237	2.35204	2.1
28238	1.50108	2.1
28239	1.3715	2.1
28240	2.18469	2.1
28241	3.68577	2.1
28242	2.62514	2.1
28243	2.1847	2.1

28244	2.0487	2.1
28245	2.45213	2.1
28246	4.63682	2.1
28247	1.25075	2.1
28248	2.18469	2.1
28249	1.3715	2.1
28250	1.3715	2.1
28251	2.0487	2.1
28252	2.0487	2.1
28253	1.28667	2.1
28254	1.11285	2.1
28255	1.03529	2.1
28256	1.03529	2.1
28257	1.30711	2.1
28258	1.30711	2.1
28259	2.35053	2.1
28260	1.89225	2.1
28261	1.89225	2.1
28262	2.51216	2.1
28263	4.24278	2.1
28264	1.89225	2.1
28265	1.89225	2.1
28266	1.89225	2.1
28267	1.89225	2.1
28268	3.7845	2.1
28269	1.92025	2.1
28270	1.32119	2.1
28271	1.44777	2.1
28272	1.33834	2.1
28273	1.92025	2.1
28274	1.81082	2.1
28275	1.89226	2.1
28276	2.67272	2.1
28277	2.51218	2.1
28278	2.51218	2.1
28279	1.89226	2.1
28280	1.89226	2.1
28281	1.89226	2.1
28282	1.89226	2.1
28283	1.89226	2.1
28284	1.89226	2.1
28285	3.78452	2.1
28286	1.92027	2.1
28287	1.44778	2.1
28288	1.61121	2.1
28289	2.01555	2.1
28290	3.53147	2.1
28291	1.90727	0.605222
28292	2.29851	0.655776
28293	2.71643	0.7
28294	2.17695	0.563504
28295	4.47547	0.592102
28296	1.90728	0.682935
28297	1.56339	0.433486
28298	1.58143	0.642095
28299	2.17696	0.671867
28300	3.74035	0.65113
28301	1.91368	0.625758

28302	2.17696	0.671931
28303	2.39986	0.7
28304	1.75904	0.63687
28305	3.936	0.636589
28306	1.18793	0.281509
28307	2.74744	0.357818
28308	1.38716	0.342697
28309	2.57465	0.272899
28310	3.93537	0.349053
28311	1.3148	0.32693
28312	2.74744	0.482098
28313	2.70805	0.357856
28314	1.38716	0.392805
28315	2.70805	0.494983
28316	1.38716	0.411649
28317	1.20608	2.1
28318	0.975871	2.1
28319	0.946132	2.1
28320	0.946132	2.1
28321	0.946132	2.1
28322	0.946132	2.1
28323	0.975871	2.1
28324	0.975871	2.1
28325	1.20608	2.1
28326	0.946132	2.1
28327	0.946133	2.1
28328	0.975871	2.1
28329	2.8198	1.08724
28330	2.29967	1.76048
28331	2.09542	2.1
28332	2.63784	0.96111
28333	4.93751	1.77561
28334	2.09542	2.1
28335	1.39523	2.1
28336	1.21119	2.1
28337	1.39523	2.1
28338	2.11563	2.1
28339	2.29967	1.88424
28340	1.88584	2.1
28341	2.63784	0.890699
28342	2.11262	1.15879
28343	1.91132	1.0963
28344	4.02153	1.15396
28345	1.88584	2.1
28346	1.21119	2.1
28347	1.12606	2.1
28348	1.91132	1.15074
28349	3.1225	2.1
28350	2.18077	1.8475
28351	1.78167	1.80778
28352	2.2947	0.94048
28353	1.93139	1.22152
28354	4.07637	1.01322
28355	1.5709	1.03949
28356	1.03235	1.84822
28357	2.05933	1.58666
28358	1.78168	1.0412
28359	1.31	0.867748

28360	1.03235	0.883363
28361	2.2947	1.10705
28362	2.31785	1.86985
28363	2.40627	1.86716
28364	2.08288	0.983691
28365	4.37758	1.92724
28366	1.95737	1.3446
28367	1.31	1.81237
28368	2.08289	0.839859
28369	1.50108	0.935898
28370	3.39289	0.914057
28371	2.49676	2.1
28372	2.24781	1.99784
28373	2.5276	1.16614
28374	2.7352	2.1
28375	5.02436	2.0844
28376	2.49676	2.1
28377	2.43549	2.1
28378	2.6398	2.1
28379	2.7352	2.1
28380	5.13656	2.1
28381	1.89226	2.1
28382	2.7352	2.1
28383	1.89226	1.99741
28384	2.6398	2.1
28385	1.89226	2.1
28386	2.6398	2.1
28387	2.16843	0.05
28388	1.00185	0.05
28389	1.90039	2.1
28390	3.1547	0.05
28391	2.54064	0.05
28392	2.46752	2.1
28393	1.90039	2.1
28394	2.26044	2.1
28395	4.36791	0.805276
28396	1.65815	2.1
28397	2.48652	2.1
28398	1.65815	2.1
28399	1.9004	2.1
28400	2.21711	2.1
28401	2.5034	2.1
28402	2.37167	2.1
28403	2.59965	2.1
28404	2.62812	2.1
28405	2.40308	2.1
28406	4.99979	2.1
28407	2.50137	2.1
28408	2.59965	2.1
28409	2.62812	2.1
28410	2.5214	2.1
28411	5.12948	2.1
28412	2.62814	2.1
28413	2.4031	2.1
28414	2.43509	2.1
28415	2.65066	2.1
28416	5.06323	2.1
28417	2.62814	2.1

28418	2.52142	2.1
28419	2.53008	2.1
28420	2.65066	2.1
28421	5.15822	2.1
28422	2.0815	2.1
28423	1.92067	2.1
28424	1.59611	2.1
28425	1.89226	2.1
28426	1.62453	2.1
28427	1.92067	2.1
28428	1.89226	2.1
28429	1.89226	2.1
28430	1.89226	2.1
28431	1.89226	2.1
28432	3.78453	2.1
28433	0.946131	2.1
28434	0.946131	2.1
28435	0.929501	2.1
28436	0.910761	2.1
28437	0.9461	2.1
28438	0.9461	2.1
28439	0.910792	2.1
28440	0.910792	2.1
28441	0.946131	2.1
28442	0.929501	2.1
28443	0.910761	2.1
28444	0.946131	2.1
28445	2.0815	2.1
28446	1.81661	2.1
28447	2.1341	2.1
28448	2.0815	2.1
28449	1.86921	2.1
28450	1.81661	2.1
28451	1.89226	2.1
28452	1.89226	2.1
28453	1.89226	2.1
28454	1.89226	2.1
28455	1.89226	2.1
28456	1.89226	2.1
28457	0.946131	2.1
28458	0.946131	2.1
28459	0.949104	2.1
28460	0.9295	2.1
28461	0.946132	2.1
28462	0.946132	2.1
28463	0.929499	2.1
28464	0.929499	2.1
28465	0.946131	2.1
28466	0.949105	2.1
28467	0.946131	2.1
28468	0.929501	2.1
28469	1.66104	2.1
28470	2.34044	2.1
28471	2.08889	2.1
28472	1.33264	2.1
28473	3.67308	2.1
28474	2.0889	2.1
28475	1.08011	2.1

28476	1.08011	2.1
28477	1.33264	2.1
28478	1.87598	2.1
28479	2.12851	2.1
28480	2.27472	2.1
28481	1.89226	1.28687
28482	2.27703	1.72533
28483	1.89226	0.941767
28484	4.16699	0.998382
28485	1.89226	1.89856
28486	1.89226	1.38115
28487	1.89226	1.21831
28488	1.89226	1.10358
28489	3.78452	1.70607
28490	1.00007	1.54663
28491	1.32316	1.05269
28492	1.29417	0.976134
28493	1.29417	1.4142
28494	1.02051	0.970872
28495	1.02051	1.25512
28496	2.2846	1.97402
28497	1.89226	1.10978
28498	2.27703	1.07019
28499	2.27703	2.1
28500	1.89226	0.888171
28501	1.89226	2.1
28502	1.89226	1.72783
28503	1.89226	1.77796
28504	1.89226	1.03011
28505	1.89226	0.900873
28506	3.78452	1.07022
28507	1.02051	2.1
28508	1.32316	1.63774
28509	1.03373	1.01489
28510	1.34066	1.09478
28511	2.35689	1.87797
28512	1.703	2.1
28513	1.89224	2.1
28514	1.52266	2.1
28515	1.89224	2.1
28516	1.23344	2.1
28517	1.53282	2.1
28518	1.89223	2.1
28519	1.89223	2.1
28520	1.89223	2.1
28521	1.89223	2.1
28522	3.78447	2.1
28523	0.796845	2.1
28524	0.796845	2.1
28525	0.812146	2.1
28526	0.812146	2.1
28527	1.08006	2.1
28528	1.02679	2.1
28529	0.551538	2.1
28530	0.528929	2.1
28531	0.551529	2.1
28532	1.08005	2.1
28533	0.528935	2.1

28534	1.0268	2.1
28535	1.5001	2.1
28536	1.70302	2.1
28537	2.0726	2.1
28538	1.70302	2.1
28539	1.99601	2.1
28540	1.70303	2.1
28541	1.89226	2.1
28542	1.89226	2.1
28543	1.89226	2.1
28544	1.89226	2.1
28545	1.89226	2.1
28546	1.89226	2.1
28547	1.17285	2.1
28548	2.16015	2.1
28549	2.23563	2.1
28550	2.23563	2.1
28551	1.10309	2.1
28552	1.10309	2.1
28553	0.946128	2.1
28554	0.946128	2.1
28555	1.21847	0.819159
28556	1.2884	2.1
28557	0.94613	2.1
28558	0.94613	2.1
28559	1.21847	2.1
28560	1.21847	0.825182
28561	0.946131	2.1
28562	1.21847	2.1
28563	0.94613	2.1
28564	1.2884	2.1
28565	1.83922	0.88478
28566	2.5768	2.1
28567	1.83488	2.1
28568	1.82846	0.05
28569	2.43693	2.1
28570	2.43693	1.98767
28571	2.36134	2.1
28572	1.81656	2.1
28573	1.86454	2.1
28574	1.6697	2.1
28575	2.55618	2.1
28576	2.36134	2.1
28577	2.55618	2.1
28578	1.9323	2.1
28579	1.9302	2.1
28580	1.81656	2.1
28581	2.66983	2.1
28582	2.55618	2.1
28583	1.89226	0.993344
28584	1.70302	1.01141
28585	1.94935	1.12326
28586	1.70302	0.993078
28587	1.95569	1.01053
28588	1.70302	0.886766
28589	2.82465	2.1
28590	2.41707	2.1
28591	2.24481	2.1

28592	2.68958	2.1
28593	5.10665	2.1
28594	1.41232	2.1
28595	1.34479	2.1
28596	0.946129	2.1
28597	0.946129	2.1
28598	0.94613	2.1
28599	0.94613	2.1
28600	1.41232	2.1
28601	1.41232	2.1
28602	1.34479	2.1
28603	0.94613	2.1
28604	0.946131	2.1
28605	1.41232	2.1
28606	1.86859	0.122226
28607	1.89226	0.159912
28608	1.89226	0.160807
28609	1.89226	0.13702
28610	1.45875	0.148422
28611	1.45875	0.120213
28612	2.1769	0.711851
28613	2.50259	0.723669
28614	1.94955	0.702533
28615	2.25959	0.872588
28616	4.45214	0.825115
28617	1.89226	0.741018
28618	1.89226	0.758515
28619	1.89226	0.70719
28620	1.89226	0.731754
28621	3.78452	0.715463
28622	1.89226	0.81129
28623	1.89226	0.877809
28624	1.89226	0.748692
28625	1.89226	0.880134
28626	3.78452	1.30702
28627	2.50259	0.76187
28628	2.49445	0.91402
28629	2.25959	0.70255
28630	2.20843	0.779105
28631	4.71102	0.741297
28632	1.94955	0.704051
28633	2.25959	0.700993
28634	1.24689	0.7
28635	1.79906	0.701228
28636	3.74861	0.700415
28637	1.89226	0.708862
28638	1.89226	0.715097
28639	1.89226	0.700447
28640	1.89226	0.703783
28641	3.78452	0.713942
28642	1.89226	0.728559
28643	1.89226	0.770501
28644	1.89226	0.703499
28645	1.89226	0.72231
28646	3.78452	0.710121
28647	2.25959	0.701593
28648	2.20843	0.72972
28649	1.79906	0.701

28650	1.98391	0.70592
28651	4.00748	0.704911
28652	1.89226	2.1
28653	1.89226	2.1
28654	1.89226	2.1
28655	1.89226	2.1
28656	3.78452	2.1
28657	1.89226	2.1
28658	1.89226	2.1
28659	1.89226	2.1
28660	1.89226	2.1
28661	3.78452	2.1
28662	1.64718	2.1
28663	1.23085	2.1
28664	1.46028	2.1
28665	1.39564	2.1
28666	1.68372	2.1
28667	1.64718	2.1
28668	1.64718	2.1
28669	1.64981	2.1
28670	1.70303	2.1
28671	1.6991	2.1
28672	3.3199	2.1
28673	1.89226	2.1
28674	1.89226	2.1
28675	1.89226	2.1
28676	1.89226	2.1
28677	3.78452	2.1
28678	1.89226	2.1
28679	1.89226	2.1
28680	1.89226	2.1
28681	1.89226	2.1
28682	3.78452	2.1
28683	0.666701	2.1
28684	0.65896	2.1
28685	0.920797	2.1
28686	0.895441	2.1
28687	0.775871	2.1
28688	0.687493	2.1
28689	0.777393	2.1
28690	0.777393	2.1
28691	0.677856	2.1
28692	0.90095	2.1
28693	0.621435	2.1
28694	0.858124	2.1
28695	1.70303	2.1
28696	1.70731	2.1
28697	1.79663	2.1
28698	1.68783	2.1
28699	3.17337	2.1
28700	1.22724	2.1
28701	2.0021	2.1
28702	1.90427	2.1
28703	1.08698	2.1
28704	3.08907	2.1
28705	2.41689	2.1
28706	2.00209	2.1
28707	1.90427	2.1

28708	2.35245	2.1
28709	4.35454	2.1
28710	1.90427	2.1
28711	0.956294	2.1
28712	0.956294	2.1
28713	1.08698	2.1
28714	1.81488	2.1
28715	1.94556	2.1
28716	1.90427	2.1
28717	2.35245	2.1
28718	2.28979	2.1
28719	1.81488	2.1
28720	4.16733	2.1
28721	1.6272	2.1
28722	1.70302	2.1
28723	1.70303	2.1
28724	1.70302	2.1
28725	1.91167	2.1
28726	1.91167	2.1
28727	1.49063	2.1
28728	1.89226	2.1
28729	1.89226	2.1
28730	1.89226	2.1
28731	1.6272	2.1
28732	1.6272	2.1
28733	2.25988	0.977566
28734	1.89226	0.746485
28735	1.89226	0.926587
28736	2.23152	0.892948
28737	4.12379	0.830336
28738	2.25988	0.962541
28739	1.16361	1.12988
28740	1.15853	1.89893
28741	2.23153	0.902578
28742	3.39513	1.12766
28743	1.89226	0.898784
28744	2.23153	0.813679
28745	2.21022	0.986321
28746	2.23153	0.86334
28747	1.87095	0.804334
28748	1.89226	0.734929
28749	1.16239	1.31244
28750	2.23153	0.821352
28751	1.15853	1.14312
28752	1.15853	0.857785
28753	2.21022	1.16644
28754	2.21022	0.857689
28755	1.78381	0.185944
28756	1.70302	0.243746
28757	1.15453	0.291102
28758	1.35007	0.316764
28759	1.82021	0.170559
28760	2.12851	0.200954
28761	1.89226	0.305944
28762	1.89226	0.408363
28763	1.89226	0.459645
28764	1.89226	0.498786
28765	1.89226	0.236209

28766	1.89226	0.288745
28767	1.89258	0.192543
28768	1.089	0.275002
28769	1.22586	0.18759
28770	1.79877	0.263238
28771	2.88777	0.193659
28772	1.91564	0.182714
28773	1.79877	0.274894
28774	1.96356	0.291345
28775	1.70302	0.300702
28776	2.37828	0.18332
28777	2.04859	0.192866
28778	1.89226	0.288838
28779	1.89226	0.400997
28780	1.89226	0.276997
28781	1.89226	0.453127
28782	3.78453	0.297495
28783	1.89226	0.264765
28784	1.89226	0.405607
28785	1.89226	0.285682
28786	1.89226	0.466133
28787	1.89226	0.239174
28788	1.89226	0.378263
28789	2.28593	2.1
28790	1.89226	1.52871
28791	2.2731	2.1
28792	1.89226	0.942068
28793	4.17819	1.12277
28794	1.89226	2.08996
28795	1.89226	1.99225
28796	1.89226	1.12522
28797	1.89226	1.1368
28798	3.78452	1.45945
28799	1.03933	1.94785
28800	1.34355	1.08782
28801	1.35048	1.04293
28802	1.35048	1.81485
28803	1.02975	0.9781
28804	1.02975	1.3976
28805	2.25105	2.1
28806	1.89226	1.22278
28807	2.27311	1.57612
28808	2.27311	2.1
28809	1.89226	1.02699
28810	1.89226	2.1
28811	1.89226	2.01039
28812	1.89226	1.88895
28813	1.89226	1.20083
28814	1.89226	0.888115
28815	3.78452	1.12442
28816	1.02975	2.1
28817	1.34355	1.76075
28818	1.01121	1.10709
28819	1.33079	1.082
28820	2.35476	1.9905
28821	1.24526	0.104348
28822	2.36406	0.120605
28823	2.81021	0.0941852

28824	1.7237	0.102025
28825	4.08776	0.0973204
28826	1.89226	2.1
28827	1.89226	2.09608
28828	1.89226	2.1
28829	1.89226	1.8452
28830	3.78453	2.1
28831	1.79028	2.1
28832	1.89238	2.1
28833	1.65533	2.1
28834	1.77821	2.1
28835	3.5477	2.1
28836	1.89238	2.1
28837	1.7465	0.827999
28838	1.77821	2.1
28839	1.63557	0.812517
28840	3.52795	0.976157
28841	1.89226	2.06949
28842	1.89226	0.799749
28843	1.89226	1.80637
28844	1.89226	0.529274
28845	3.78452	0.910332
28846	1.89226	2.1
28847	1.89226	1.98991
28848	1.89226	2.1
28849	1.89226	2.1
28850	3.78452	2.1
28851	1.65532	2.1
28852	1.77821	2.1
28853	1.53278	2.1
28854	1.67571	2.1
28855	3.33103	2.1
28856	1.77821	2.1
28857	1.63557	0.846364
28858	1.67571	2.1
28859	1.52615	0.668189
28860	3.31128	0.976422
28861	1.89226	1.95427
28862	1.89226	0.744087
28863	1.89226	2.1
28864	1.89226	0.666147
28865	3.78452	0.974678
28866	2.83129	2.1
28867	1.89226	0.858847
28868	1.89226	1.03303
28869	2.6491	1.24227
28870	4.54136	1.01236
28871	2.83129	2.1
28872	1.47278	2.1
28873	1.3909	2.1
28874	2.6491	1.53764
28875	4.12188	2.1
28876	1.89225	2.1
28877	2.65393	2.1
28878	1.89225	2.1
28879	2.69591	2.1
28880	4.58816	2.1
28881	1.91703	2.1

28882	2.00209	2.1
28883	2.65393	2.1
28884	2.65393	2.1
28885	2.69591	2.1
28886	2.00209	2.1
28887	1.89226	2.1
28888	2.69593	2.1
28889	1.93967	2.1
28890	1.89226	2.1
28891	2.74334	2.1
28892	2.69593	2.1
28893	2.74335	2.1
28894	2.0021	2.1
28895	2.76367	2.1
28896	2.69593	2.1
28897	2.06984	2.1
28898	2.0021	2.1
28899	2.24481	2.1
28900	2.58921	2.1
28901	2.68958	2.1
28902	2.68958	2.1
28903	2.11984	2.1
28904	2.11984	2.1
28905	2.11984	2.1
28906	2.58921	2.1
28907	2.46488	2.1
28908	1.98339	2.1
28909	4.5726	2.1
28910	2.7777	2.1
28911	2.58921	2.1
28912	2.46488	2.1
28913	2.70401	2.1
28914	5.29322	2.1
28915	2.17741	2.1
28916	2.54197	2.1
28917	2.54018	2.1
28918	2.18608	2.1
28919	4.72805	2.1
28920	2.17742	2.1
28921	1.58054	2.1
28922	1.59169	2.1
28923	2.18608	2.1
28924	3.76662	2.1
28925	2.54018	2.1
28926	2.18608	2.1
28927	2.19104	2.1
28928	2.5392	2.1
28929	4.72528	2.1
28930	1.60497	2.1
28931	2.18608	2.1
28932	1.59169	2.1
28933	1.59169	2.1
28934	2.19105	2.1
28935	2.19105	2.1
28936	2.58661	1.1899
28937	1.06203	2.1
28938	2.7043	2.1
28939	2.21397	2.1

28940	1.55236	2.1
28941	1.06203	2.1
28942	1.89226	1.25715
28943	1.89226	1.79473
28944	1.89226	0.878616
28945	1.89226	1.77774
28946	3.78453	1.30648
28947	1.08195	1.50018
28948	0.990019	2.1
28949	1.31976	1.70829
28950	1.4539	0.97316
28951	2.40171	2.00261
28952	1.89226	1.06878
28953	1.89226	2.06749
28954	1.89226	1.05573
28955	1.89226	0.857613
28956	3.78453	1.16132
28957	1.89226	1.4373
28958	1.89226	1.25651
28959	1.89226	0.951858
28960	1.89226	2.00856
28961	3.78453	1.18569
28962	1.43895	1.88011
28963	1.31976	1.87669
28964	1.70066	0.936437
28965	1.33783	2.1
28966	1.43895	2.08008
28967	1.07612	2.1
28968	0.65988	1.76282
28969	0.850329	1.3404
28970	0.919598	1.01497
28971	0.726952	1.50681
28972	0.788106	0.923011
28973	0.788106	2.1
28974	0.789175	1.96714
28975	0.789175	0.83683
28976	0.850329	1.03827
28977	0.919598	1.00111
28978	0.659879	1.5336
28979	0.726952	1.29942
28980	1.34389	2.1
28981	1.41279	1.49255
28982	1.15564	2.1
28983	1.15564	2.1
28984	1.55998	1.2619
28985	1.55998	1.84784
28986	1.89226	1.23357
28987	1.89226	1.74521
28988	1.89226	2.1
28989	1.89226	1.07968
28990	1.89226	2.1
28991	1.89226	1.08301
28992	1.55998	2.1
28993	1.41279	1.82988
28994	1.72901	1.41592
28995	1.62414	2.04709
28996	3.1418	1.82084
28997	1.89226	1.25285

28998	1.89226	2.08077
28999	1.89226	1.44456
29000	1.89226	1.00156
29001	3.78452	1.61389
29002	1.89226	0.934349
29003	1.89226	2.1
29004	1.89226	1.21469
29005	1.89226	1.82745
29006	1.89226	1.00614
29007	1.89226	1.17244
29008	1.62414	1.8615
29009	1.01243	2.02239
29010	1.70932	2.01647
29011	1.41279	1.31528
29012	1.30896	1.35415
29013	1.01243	1.17193
29014	1.9719	2.1
29015	1.10725	2.1
29016	1.12411	2.1
29017	1.98691	2.1
29018	3.09601	2.1
29019	1.97189	2.1
29020	2.47932	2.1
29021	2.49092	2.1
29022	1.98691	2.1
29023	4.46282	2.1
29024	1.1486	2.1
29025	1.98691	2.1
29026	1.12411	2.1
29027	1.12411	2.1
29028	2.01602	2.1
29029	2.01602	2.1
29030	2.49093	2.1
29031	1.98691	2.1
29032	2.01601	2.1
29033	2.51651	2.1
29034	4.50694	2.1
29035	1.93147	0.629124
29036	2.51593	0.667367
29037	1.35146	0.433919
29038	1.35146	0.407606
29039	2.48665	0.469616
29040	1.90664	0.401144
29041	1.63979	2.1
29042	1.7457	2.1
29043	1.66046	2.1
29044	1.56558	2.1
29045	3.31128	2.1
29046	0.819895	2.1
29047	0.782792	2.1
29048	0.60239	2.1
29049	0.54831	2.1
29050	0.688347	2.1
29051	0.688347	2.1
29052	0.679857	2.1
29053	0.679857	2.1
29054	0.782792	2.1
29055	0.54831	2.1

29056	0.60239	2.1
29057	0.819895	2.1
29058	1.66046	2.1
29059	1.42485	2.1
29060	1.56558	2.1
29061	1.56558	2.1
29062	1.52995	2.1
29063	1.52995	2.1
29064	2.58101	0.779263
29065	2.09803	1.9298
29066	2.408	0.578692
29067	1.86893	2.1
29068	4.44994	0.855274
29069	2.36062	1.68084
29070	1.25104	2.1
29071	1.25104	2.1
29072	1.39703	2.1
29073	1.86893	2.1
29074	2.01492	2.1
29075	2.408	0.760881
29076	1.86893	1.99845
29077	2.22575	0.596902
29078	1.70677	1.37922
29079	4.09468	0.847452
29080	0.737605	2.1
29081	0.737605	1.86114
29082	0.741302	2.1
29083	0.741302	2.1
29084	0.55189	2.1
29085	0.625517	2.1
29086	0.934468	1.7113
29087	0.853389	1.15192
29088	0.853389	2.1
29089	0.55189	2.1
29090	0.625517	2.1
29091	0.934467	2.1
29092	2.35053	2.1
29093	1.09615	2.1
29094	1.25998	2.1
29095	2.51216	2.1
29096	3.61051	2.1
29097	1.33636	2.1
29098	1.25609	2.1
29099	0.698455	2.1
29100	0.629995	2.1
29101	0.986092	2.1
29102	0.986092	2.1
29103	0.980263	2.1
29104	0.980263	2.1
29105	1.33636	2.1
29106	0.698455	2.1
29107	0.629995	2.1
29108	1.25609	2.1
29109	1.89226	1.02731
29110	1.89226	1.0721
29111	1.89226	1.09786
29112	1.89226	1.06692
29113	3.78452	1.04701

29114	1.74321	1.04095
29115	1.89226	1.06817
29116	1.91755	1.09249
29117	1.91755	1.04187
29118	1.89226	1.10231
29119	1.89226	1.04993
29120	0.946132	1.02115
29121	0.946132	0.989204
29122	0.949798	0.954832
29123	1.08054	1.04401
29124	1.08054	1.00271
29125	1.08054	1.04611
29126	0.946132	0.991134
29127	0.946132	0.987949
29128	0.946132	0.994486
29129	0.949798	0.962139
29130	1.08054	0.99709
29131	0.946131	1.02607
29132	1.89226	1.06646
29133	1.89226	1.01507
29134	1.89226	0.992013
29135	1.89226	0.99941
29136	1.89226	1.02764
29137	1.89226	1.03716
29138	2.39378	0.635256
29139	2.29234	0.378581
29140	2.29234	0.583443
29141	2.7933	0.481954
29142	1.18422	0.462183
29143	1.68518	0.435923
29144	2.79329	0.291549
29145	1.89226	0.414885
29146	2.29233	0.316671
29147	1.89226	0.227
29148	4.18459	0.264981
29149	0.857046	0.155552
29150	0.857037	0.255104
29151	0.94613	0.21248
29152	0.94613	0.375793
29153	0.851539	0.243069
29154	0.851539	0.199835
29155	1.0315	0.181571
29156	0.952867	0.209865
29157	0.851486	0.140294
29158	0.952869	0.186768
29159	1.03156	0.292781
29160	0.851528	0.203319
29161	1.61981	2.1
29162	1.93096	2.1
29163	2.00776	2.1
29164	1.62218	2.1
29165	3.62757	2.1
29166	2.00776	2.1
29167	1.68541	2.1
29168	1.83215	2.1
29169	1.62218	2.1
29170	2.21773	2.1
29171	2.00776	2.1

29172	1.89188	0.234185
29173	1.70303	0.295164
29174	1.89226	0.276364
29175	1.72465	0.267883
29176	1.89226	0.284731
29177	1.70552	0.291554
29178	2.48659	0.340756
29179	2.0815	0.452169
29180	1.89226	0.43509
29181	2.08111	0.398038
29182	1.89226	0.424945
29183	2.08111	0.426078
29184	2.35332	0.747036
29185	1.83284	0.490799
29186	2.28483	0.556566
29187	2.71955	0.461332
29188	4.63815	0.474182
29189	1.89227	2.1
29190	1.89227	2.1
29191	1.89227	2.1
29192	1.89227	2.1
29193	3.78453	2.1
29194	1.33506	2.1
29195	1.17129	2.1
29196	1.33506	2.1
29197	1.38415	2.1
29198	1.13664	2.1
29199	1.18573	2.1
29200	1.89226	2.1
29201	2.68785	2.1
29202	1.89226	2.1
29203	2.6692	2.1
29204	4.58011	2.1
29205	1.89226	2.1
29206	1.89226	2.1
29207	1.89226	2.1
29208	1.89226	2.1
29209	3.78453	2.1
29210	1.33506	2.1
29211	1.13664	2.1
29212	1.27575	2.1
29213	1.09627	2.1
29214	2.43133	2.1
29215	1.89226	2.1
29216	2.65349	2.1
29217	2.66919	2.1
29218	2.66919	2.1
29219	1.89226	2.1
29220	1.89226	2.1
29221	1.89226	2.1
29222	1.89226	2.1
29223	1.89226	2.1
29224	1.89226	2.1
29225	3.78453	2.1
29226	1.16834	2.1
29227	1.16834	2.1
29228	0.946132	2.1
29229	0.946132	2.1

29230	1.26978	2.1
29231	1.16834	2.1
29232	0.94613	2.1
29233	0.94613	2.1
29234	0.94613	2.1
29235	1.26978	2.1
29236	0.94613	2.1
29237	1.16834	2.1
29238	2.14698	2.1
29239	1.89226	2.1
29240	1.89226	2.1
29241	1.89226	2.1
29242	2.40719	2.1
29243	2.40719	2.1
29244	1.89226	2.1
29245	1.89226	2.1
29246	1.89226	2.1
29247	1.89226	2.1
29248	3.78453	2.1
29249	1.26977	2.1
29250	1.26977	2.1
29251	0.946133	2.1
29252	0.946133	2.1
29253	1.37467	2.1
29254	1.26978	2.1
29255	0.946131	2.1
29256	0.946131	2.1
29257	1.37467	2.1
29258	0.94613	2.1
29259	0.946131	2.1
29260	1.26978	2.1
29261	1.2036	2.1
29262	1.34912	2.1
29263	0.946131	2.1
29264	0.946131	2.1
29265	1.34912	2.1
29266	1.34912	2.1
29267	0.946129	2.1
29268	0.946129	2.1
29269	1.34912	2.1
29270	0.94613	2.1
29271	0.946131	2.1
29272	1.2036	2.1
29273	2.47164	0.829265
29274	1.89019	0.62004
29275	1.79109	0.824528
29276	2.41632	0.666742
29277	4.26273	0.822093
29278	1.54197	0.782583
29279	2.41632	0.533356
29280	1.79109	0.736612
29281	1.79109	0.781989
29282	2.20929	0.608865
29283	2.20929	0.704253
29284	1.70302	1.1798
29285	1.48104	1.32736
29286	1.99365	1.3544
29287	1.32897	1.18718

29288	1.70302	1.34506
29289	1.14034	1.17328
29290	1.70302	1.14947
29291	1.99365	1.20882
29292	1.99297	1.22688
29293	2.36337	1.05546
29294	4.04804	1.09797
29295	1.51569	1.07913
29296	2.49093	1.09619
29297	1.6026	1.1632
29298	1.43229	1.00259
29299	2.0815	1.17275
29300	1.83402	1.01038
29301	2.54665	2.1
29302	2.34044	2.1
29303	2.08889	2.1
29304	2.37653	2.1
29305	4.71697	2.1
29306	2.54665	2.1
29307	2.45212	2.1
29308	2.31012	2.1
29309	2.37653	2.1
29310	4.82865	2.1
29311	2.08889	2.1
29312	2.37653	2.1
29313	2.23366	2.1
29314	1.87598	2.1
29315	4.25251	2.1
29316	2.31012	2.1
29317	2.37653	2.1
29318	2.23367	2.1
29319	2.17384	2.1
29320	4.55037	2.1
29321	0.946131	2.1
29322	0.946131	2.1
29323	1.30338	2.1
29324	1.23186	2.1
29325	0.94613	2.1
29326	0.94613	2.1
29327	1.23187	2.1
29328	1.23187	2.1
29329	0.946131	2.1
29330	1.23186	2.1
29331	1.30338	2.1
29332	0.946131	2.1
29333	1.89226	2.1
29334	2.46373	2.1
29335	2.34725	2.1
29336	2.46373	2.1
29337	1.77579	2.1
29338	1.89226	2.1
29339	2.61901	2.1
29340	2.45899	2.1
29341	2.64067	2.1
29342	2.52708	2.1
29343	5.14609	2.1
29344	2.41795	2.1
29345	2.0815	2.1

29346	2.52708	2.1
29347	1.92277	2.1
29348	4.31368	2.1
29349	2.64067	2.1
29350	2.52708	2.1
29351	2.58638	2.1
29352	2.51039	2.1
29353	5.11346	2.1
29354	2.52708	2.1
29355	2.13642	2.1
29356	2.51039	2.1
29357	2.16633	2.1
29358	4.69341	2.1
29359	1.61028	1.25058
29360	1.65712	0.814979
29361	1.35002	0.772005
29362	1.35002	1.26227
29363	1.88637	0.836145
29364	1.88637	1.89533
29365	2.57663	2.07107
29366	2.24227	2.1
29367	2.48354	2.03768
29368	2.14317	2.1
29369	4.7198	2.1
29370	2.24227	2.1
29371	1.77353	2.1
29372	2.14317	2.1
29373	1.69956	2.1
29374	3.9167	2.1
29375	2.48353	1.92175
29376	2.14317	2.1
29377	2.39282	2.1
29378	1.79824	2.1
29379	4.53599	2.1
29380	2.14317	2.1
29381	1.69956	2.1
29382	1.91427	2.1
29383	1.6347	2.1
29384	3.77787	2.1
29385	1.89226	0.704222
29386	1.89226	0.71476
29387	1.89226	0.701118
29388	1.89226	0.709828
29389	3.78453	0.708085
29390	1.89226	0.704321
29391	1.89226	0.717719
29392	1.89226	0.701516
29393	1.89226	0.741659
29394	3.78452	0.707492
29395	1.89226	0.720732
29396	1.89652	1.16256
29397	1.89226	0.763164
29398	1.89226	0.783714
29399	1.19352	0.741084
29400	1.19352	0.749887
29401	1.89226	0.717031
29402	1.89653	0.729309
29403	1.89226	0.770769

29404	1.89226	0.737749
29405	1.89226	0.721753
29406	1.89226	0.711789
29407	1.89226	0.755369
29408	1.75326	0.763098
29409	1.89226	1.46466
29410	1.12948	1.54592
29411	1.89226	0.700404
29412	1.12948	0.700823
29413	2.61064	2.1
29414	1.89226	2.1
29415	1.89226	2.1
29416	1.89226	2.1
29417	2.6085	2.1
29418	2.6085	2.1
29419	2.6085	2.1
29420	1.89226	2.1
29421	1.89226	2.1
29422	1.89226	2.1
29423	2.62161	2.1
29424	2.62161	2.1
29425	2.16535	2.1
29426	1.44603	2.1
29427	2.79784	2.1
29428	1.84756	2.1
29429	4.01292	2.1
29430	2.65262	2.1
29431	1.03391	2.1
29432	2.20239	2.1
29433	1.74416	2.1
29434	1.49213	2.1
29435	1.03391	2.1
29436	2.29066	2.1
29437	2.49174	2.1
29438	2.56502	2.1
29439	2.33802	2.1
29440	4.85568	2.1
29441	2.39663	2.1
29442	2.49174	2.1
29443	2.56502	2.1
29444	2.45929	2.1
29445	4.96165	2.1
29446	2.56502	2.1
29447	2.33802	2.1
29448	2.37168	2.1
29449	2.59967	2.1
29450	4.9367	2.1
29451	2.56502	2.1
29452	2.45929	2.1
29453	2.50138	2.1
29454	2.59967	2.1
29455	5.0664	2.1
29456	2.27009	1.09311
29457	1.26247	2.1
29458	1.86812	1.96137
29459	2.837	1.2754
29460	4.13822	1.88533
29461	1.89226	0.897639

29462	1.89226	2.1
29463	1.89226	1.78287
29464	1.89226	2.1
29465	3.78453	2.1
29466	1.75835	2.1
29467	1.72866	2.1
29468	1.65313	1.65864
29469	1.64822	2.1
29470	3.3818	2.1
29471	1.72866	2.1
29472	1.58401	2.1
29473	1.64822	2.1
29474	1.52441	2.1
29475	3.25307	2.1
29476	1.89226	2.1
29477	1.89226	2.1
29478	1.89226	2.1
29479	1.89226	2.1
29480	3.78452	2.1
29481	1.89226	1.90616
29482	1.89226	2.1
29483	1.89226	0.92293
29484	1.89226	2.1
29485	3.78453	2.00853
29486	1.65313	1.93484
29487	1.64822	2.1
29488	1.55779	2.1
29489	1.57968	2.1
29490	3.23281	2.1
29491	1.64822	2.1
29492	1.52441	2.1
29493	1.57967	2.1
29494	1.474 2.1	
29495	3.10408	2.1
29496	1.89226	2.1
29497	1.89226	2.1
29498	1.89226	2.1
29499	1.89226	2.1
29500	3.78452	2.1
29501	1.86067	1.19614
29502	1.58407	2.1
29503	2.07965	0.930869
29504	1.83123	1.44022
29505	3.66372	1.05357
29506	1.89226	1.00047
29507	1.89226	2.1
29508	1.89226	1.01479
29509	1.89226	1.3495
29510	3.78452	1.2475
29511	1.89226	0.98751
29512	1.89226	1.78288
29513	1.89226	0.944508
29514	1.89226	2.1
29515	1.89226	0.89345
29516	1.89226	2.1
29517	1.83123	0.934879
29518	1.03472	1.93854
29519	1.91068	1.51966

29520	1.58406	1.11753
29521	1.36133	1.09569
29522	1.03472	0.974244
29523	2.07965	1.07477
29524	1.83123	1.61142
29525	2.28385	1.00794
29526	2.09114	1.20674
29527	4.1708	1.54235
29528	1.89226	1.23797
29529	1.89226	1.83241
29530	1.89226	0.909891
29531	1.89226	1.50456
29532	3.78452	1.02358
29533	1.89226	1.03247
29534	1.89226	2.0481
29535	1.89226	0.95675
29536	1.89226	1.89992
29537	3.78452	1.1604
29538	1.83123	0.985361
29539	1.36133	2.0849
29540	2.09114	0.906156
29541	1.69436	1.44665
29542	3.45248	1.06148
29543	1.89226	2.1
29544	1.89226	2.1
29545	1.89226	2.1
29546	1.89226	1.51336
29547	3.78453	2.1
29548	1.53278	2.1
29549	1.67571	2.1
29550	1.40182	2.1
29551	1.55686	2.1
29552	3.07752	2.1
29553	1.67571	2.1
29554	1.52615	0.775231
29555	1.55686	2.1
29556	1.38963	0.435324
29557	3.06534	0.961281
29558	1.89226	2.1
29559	1.89226	0.823138
29560	1.89226	1.50055
29561	1.89226	0.483031
29562	3.78452	0.922193
29563	1.89226	2.1
29564	1.89226	2.0318
29565	1.89226	2.1
29566	1.89226	2.1
29567	3.78453	2.1
29568	1.40182	2.1
29569	1.55686	2.1
29570	1.24509	2.1
29571	1.38426	2.1
29572	2.78608	2.1
29573	1.55686	2.1
29574	1.38963	0.857158
29575	1.2064	2.1
29576	1.2064	0.886638
29577	1.38426	2.1

29578	1.56749	0.917024
29579	0.810946	1.45733
29580	0.810946	0.840807
29581	0.810945	1.68121
29582	0.810945	2.1
29583	0.810945	1.1143
29584	0.810945	1.01178
29585	1.89226	0.861017
29586	1.35169	0.931363
29587	1.08132	2.1
29588	1.08132	0.599859
29589	1.08132	0.964882
29590	1.28418	0.732515
29591	1.59382	0.780799
29592	1.78289	0.906497
29593	1.51021	0.645389
29594	3.10403	0.701184
29595	1.76897	1.10384
29596	1.59382	0.785913
29597	1.78289	0.923815
29598	1.94302	0.980719
29599	3.53684	0.987294
29600	1.89226	0.738744
29601	1.89226	0.887448
29602	1.89226	0.96904
29603	1.89226	0.609175
29604	3.78453	0.707387
29605	1.89227	0.736345
29606	1.89227	0.45148
29607	1.89227	0.451589
29608	1.89227	0.567545
29609	1.89227	0.578825
29610	1.89227	0.648371
29611	1.78286	0.877387
29612	1.51019	0.56955
29613	1.79979	0.721068
29614	2.02511	0.786244
29615	3.5353	0.796427
29616	1.78286	0.889346
29617	1.94299	0.940287
29618	2.17831	1.05992
29619	2.02511	0.81492
29620	3.9681	0.864853
29621	1.89224	0.929463
29622	1.89224	0.663372
29623	1.89224	0.737258
29624	1.89224	0.901896
29625	3.78448	0.870826
29626	1.89224	0.707857
29627	1.89224	0.456482
29628	1.89224	0.515867
29629	1.89224	0.634773
29630	1.89224	0.530222
29631	1.89224	0.62899
29632	1.83198	0.575576
29633	1.0961	0.316588
29634	1.39448	0.593014
29635	1.99568	0.498325

29636	3.09178	0.543043
29637	1.25503	0.381328
29638	1.99568	0.351771
29639	2.3661	0.61161
29640	1.70302	0.466303
29641	3.55833	0.377546
29642	2.69899	0.370983
29643	1.85796	0.321086
29644	2.11407	0.326628
29645	1.36769	0.236115
29646	3.97203	0.220627
29647	1.89226	0.521307
29648	1.89226	0.473922
29649	1.89226	0.455462
29650	1.89226	0.293596
29651	3.78452	0.594067
29652	1.89225	0.335285
29653	2.63355	0.337461
29654	2.09529	0.306047
29655	2.09529	0.402515
29656	1.89225	0.333579
29657	1.89225	0.402626
29658	1.89226	0.491122
29659	1.89226	0.540382
29660	1.89226	0.414426
29661	1.89226	0.699916
29662	1.89226	0.311379
29663	1.89226	0.580877
29664	2.12204	0.370539
29665	1.89226	0.527305
29666	2.05798	0.314538
29667	2.01954	0.365392
29668	1.70302	0.467541
29669	1.3865	0.540778
29670	1.28326	0.660612
29671	1.33326	0.859741
29672	1.19052	0.153262
29673	1.25369	0.83003
29674	2.53694	0.8015
29675	1.33327	0.863163
29676	1.27339	1.612
29677	1.25369	0.834532
29678	1.19733	2.1
29679	2.52708	0.906587
29680	1.19052	0.227861
29681	1.25369	0.828994
29682	1.10938	0.214231
29683	1.17614	0.891791
29684	2.36307	0.538014
29685	1.25369	0.830502
29686	1.19733	2.1
29687	1.17614	0.89809
29688	1.11868	2.04515
29689	2.37237	1.15469
29690	1.10747	0.368684
29691	1.47688	0.360013
29692	1.97732	0.565054
29693	1.62206	0.586611

29694	3.09894	0.476879
29695	1.89226	0.316713
29696	1.89226	0.497295
29697	1.89226	0.375418
29698	1.89226	0.330983
29699	1.89226	0.520284
29700	1.89226	0.383592
29701	1.62206	0.409762
29702	1.70302	0.595542
29703	1.22962	0.455344
29704	1.1893	0.444637
29705	2.15177	0.582341
29706	2.08121	0.442652
29707	1.97732	0.542716
29708	2.08121	0.521987
29709	1.62206	0.471586
29710	1.62206	0.413081
29711	2.41464	0.642695
29712	2.41464	0.462006
29713	1.89226	0.358238
29714	1.89226	0.45047
29715	1.89226	0.572734
29716	1.89226	0.465394
29717	3.78452	0.389832
29718	0.946133	0.323925
29719	0.946133	0.42814
29720	0.946132	0.505076
29721	0.946132	0.485051
29722	0.946132	0.53484
29723	0.946132	0.44646
29724	0.946133	0.552727
29725	0.946133	0.436123
29726	0.946132	0.62033
29727	0.946132	0.649772
29728	0.946132	0.437721
29729	0.946132	0.32471
29730	1.83157	1.11776
29731	2.19989	1.0182
29732	2.20664	1.0764
29733	1.81487	1.05398
29734	4.03146	1.10698
29735	2.41055	2.1
29736	2.19989	1.7836
29737	2.20665	1.64119
29738	2.38023	1.51961
29739	4.61044	1.41102
29740	2.19989	2.03444
29741	2.22558	1.85554
29742	2.38023	1.67834
29743	2.07065	1.18753
29744	4.42547	1.4042
29745	2.19988	1.10224
29746	1.70303	0.88479
29747	1.89939	1.10485
29748	1.86357	1.02969
29749	3.78536	1.02132
29750	1.04921	2.1
29751	1.30711	2.1

29752	1.53076	2.1
29753	1.22927	2.1
29754	2.57997	2.1
29755	1.53076	2.1
29756	1.46568	2.1
29757	1.56056	2.1
29758	1.22927	2.1
29759	1.86206	2.1
29760	1.53076	2.1
29761	1.18353	2.1
29762	2.28593	2.1
29763	1.16979	2.1
29764	2.2731	2.1
29765	3.45571	2.1
29766	0.854776	2.0946
29767	0.854776	2.1
29768	0.855641	2.1
29769	0.855641	2.1
29770	1.12552	2.1
29771	1.13655	2.1
29772	0.57216	2.1
29773	0.584893	2.1
29774	0.57216	2.1
29775	1.12552	2.1
29776	0.584893	2.1
29777	1.13655	2.1
29778	1.54462	2.1
29779	2.76309	2.1
29780	1.34869	2.1
29781	2.48573	2.1
29782	4.03035	2.1
29783	1.35328	0.961979
29784	1.0383	0.908236
29785	1.03318	1.60757
29786	0.704834	1.30533
29787	1.0257	1.42877
29788	1.0257	0.970803
29789	1.03241	0.893994
29790	1.03241	1.4521
29791	1.35328	1.12209
29792	1.03319	1.5431
29793	1.0383	1.24598
29794	0.704835	1.15807
29795	2.27126	0.660461
29796	2.77291	1.54899
29797	1.81771	0.889752
29798	2.6111	0.843144
29799	4.88236	0.904463
29800	2.77291	1.20967
29801	1.89227	0.964199
29802	1.89227	0.8824
29803	1.89227	2.0815
29804	2.6111	0.854102
29805	2.6111	2.1
29806	2.22061	0.862107
29807	2.41522	0.851903
29808	2.41522	0.789286
29809	2.6111	1.08136

29810	1.75765	0.787956
29811	1.95353	1.63158
29812	2.27502	1.00254
29813	1.89227	1.54327
29814	1.89227	0.963998
29815	2.8189	0.888895
29816	4.71116	0.911772
29817	1.89226	0.872631
29818	1.89226	1.39031
29819	1.89226	0.990266
29820	1.89226	0.941288
29821	1.89226	1.23171
29822	1.89226	1.08786
29823	1.89226	1.34177
29824	1.89226	1.31255
29825	1.89226	1.18553
29826	1.89226	0.986408
29827	3.78453	1.3609
29828	1.89226	1.2303
29829	2.57079	0.877039
29830	2.5932	1.01309
29831	1.89226	0.99175
29832	4.48546	0.96355
29833	0.589396	1.00036
29834	0.641454	0.906434
29835	0.814062	1.19279
29836	0.726518	1.01241
29837	0.675126	1.09964
29838	0.675125	0.966491
29839	0.692847	1.11005
29840	0.692847	1.00435
29841	0.641454	0.945906
29842	0.814062	1.03141
29843	0.726518	1.16542
29844	0.589396	1.02589
29845	1.89226	1.2349
29846	1.89226	0.987489
29847	1.89226	1.18448
29848	1.89226	1.38123
29849	1.89226	1.08404
29850	1.89226	1.29057
29851	1.16906	0.822413
29852	1.2966	0.974583
29853	0.946132	0.967232
29854	0.946132	1.36915
29855	1.16906	0.979481
29856	1.16906	0.847262
29857	0.946132	0.985691
29858	0.946132	1.07983
29859	1.16906	1.10861
29860	0.946131	1.00492
29861	1.2966	1.01409
29862	0.946132	1.431
29863	0.641455	1.00252
29864	0.567191	0.918298
29865	0.807594	1.2528
29866	0.814062	1.05453
29867	0.673735	1.26947

29868	0.673734	0.954764
29869	0.707519	1.00941
29870	0.707519	1.21402
29871	0.807594	1.10685
29872	0.56719	1.44457
29873	0.641455	1.12294
29874	0.814063	1.49415
29875	1.89225	2.1
29876	1.89225	2.1
29877	1.89225	2.1
29878	1.89225	2.1
29879	3.7845	2.1
29880	1.89225	2.1
29881	1.89225	2.1
29882	1.89225	2.1
29883	1.89225	2.1
29884	1.89225	2.1
29885	1.89225	2.1
29886	1.89226	2.1
29887	1.89226	2.1
29888	1.89226	2.1
29889	1.89226	2.1
29890	3.78452	2.1
29891	1.89226	2.1
29892	1.89226	2.1
29893	1.89226	2.1
29894	1.89226	2.1
29895	1.89226	2.1
29896	1.89226	2.1
29897	1.89226	2.1
29898	1.89226	2.1
29899	1.89226	2.1
29900	1.89226	2.1
29901	3.78452	2.1
29902	1.89226	2.1
29903	1.38928	2.1
29904	1.89226	2.1
29905	1.51378	2.1
29906	1.89061	2.1
29907	1.89226	2.1
29908	1.89226	2.1
29909	1.89226	1.85759
29910	1.89226	1.73776
29911	1.89226	2.1
29912	3.78453	2.1
29913	1.04572	2.1
29914	0.994213	2.1
29915	0.912466	1.94113
29916	0.912466	2.1
29917	0.947743	2.07958
29918	1.00388	2.1
29919	0.900913	1.84094
29920	0.900913	2.1
29921	0.900915	2.1
29922	0.946131	2.1
29923	0.946131	2.1
29924	0.999196	2.1
29925	1.89226	1.57153

29926	1.89226	2.1
29927	1.89226	2.1
29928	1.89226	1.90351
29929	3.78453	2.1
29930	0.946161	2.1
29931	1.1354	2.1
29932	0.850847	2.1
29933	0.850847	2.1
29934	1.01445	2.1
29935	1.02106	2.1
29936	0.946131	2.1
29937	0.946131	2.1
29938	1.13537	2.1
29939	1.01444	2.1
29940	0.850877	2.1
29941	0.946131	2.1
29942	1.80183	2.1
29943	1.89226	2.05452
29944	1.89226	1.94995
29945	1.89226	2.1
29946	1.79606	1.91383
29947	1.79606	2.1
29948	1.24001	1.75731
29949	1.41822	1.41651
29950	1.95665	0.777993
29951	1.77342	1.25606
29952	3.19666	0.971483
29953	1.95665	0.968838
29954	1.44859	1.67664
29955	1.41821	1.52545
29956	1.41821	1.47173
29957	1.95543	0.966228
29958	1.95543	1.20248
29959	1.95665	0.844038
29960	1.77342	1.32658
29961	2.33611	1.08143
29962	2.49694	0.780951
29963	4.29276	1.36663
29964	1.95665	1.20922
29965	1.95544	1.2658
29966	2.4728	0.743312
29967	2.49693	1.08893
29968	4.42945	0.888273
29969	2.24937	2.1
29970	2.49053	2.1
29971	2.16323	2.1
29972	2.36377	2.1
29973	4.61314	2.1
29974	2.49053	2.1
29975	2.58639	2.1
29976	2.36377	2.1
29977	2.47395	2.1
29978	4.95016	2.1
29979	2.45054	2.1
29980	2.36377	2.1
29981	1.92911	2.1
29982	2.181	2.1
29983	4.29288	2.1

29984	2.36377	2.1
29985	2.47395	2.1
29986	2.181 2.1	
29987	2.29881	2.1
29988	4.66258	2.1
29989	1.57885	2.1
29990	1.13339	2.1
29991	1.48594	2.1
29992	1.13322	2.1
29993	2.71207	2.1
29994	1.48596	2.1
29995	1.20761	2.1
29996	1.20761	2.1
29997	1.13323	2.1
29998	1.46568	2.1
29999	1.3913	2.1
30000	1.89226	0.69762
30001	1.89226	0.798706
30002	1.89226	0.7
30003	1.89226	0.720608
30004	1.89226	0.699362
30005	1.89226	0.70074
30006	1.11712	0.728858
30007	0.946129	0.769879
30008	0.686934	0.696393
30009	0.946133	0.690847
30010	0.9461	0.72715
30011	1.10729	1.44674
30012	0.686963	0.806063
30013	0.686963	0.692456
30014	1.10945	0.758194
30015	0.80397	1.52638
30016	0.946131	0.696671
30017	0.946131	0.804594
30018	1.89226	2.1
30019	1.89226	1.21005
30020	1.89226	1.11224
30021	1.89226	1.22242
30022	3.78452	1.23737
30023	1.22615	2.1
30024	1.3439	2.1
30025	1.47868	2.1
30026	1.55998	1.20817
30027	2.78613	2.1
30028	1.04973	2.1
30029	1.47868	2.1
30030	1.20149	2.1
30031	1.61721	2.1
30032	2.68017	2.1
30033	1.89226	2.1
30034	1.89226	2.1
30035	1.89226	2.1
30036	1.89226	1.03025
30037	3.78452	2.1
30038	0.946133	0.948599
30039	0.946133	1.71469
30040	0.946128	2.1
30041	0.946128	2.09273

30042	0.946131	0.924141
30043	0.946131	1.26347
30044	0.946131	2.1
30045	0.946131	1.04649
30046	0.94613	1.60074
30047	0.94613	1.23103
30048	0.946131	1.36808
30049	0.946131	1.21556
30050	0.798126	2.1
30051	0.798126	1.97025
30052	0.805719	2.1
30053	0.805719	2.1
30054	0.779992	2.1
30055	0.864507	1.95173
30056	0.739338	2.1
30057	0.808607	2.1
30058	0.808606	2.1
30059	0.864506	2.1
30060	0.739339	2.1
30061	0.779993	2.1
30062	0.946128	2.1
30063	0.946128	2.1
30064	1.18959	1.11604
30065	1.00304	2.1
30066	1.00304	2.1
30067	1.00304	2.05088
30068	0.94613	2.1
30069	0.94613	1.13593
30070	0.94613	2.1
30071	1.18959	2.1
30072	1.00304	2.08955
30073	0.94613	2.1
30074	2.19163	2.1
30075	1.89226	1.95228
30076	2.16541	2.1
30077	1.89226	1.13097
30078	4.08389	1.75086
30079	1.89226	2.1
30080	1.89226	1.51141
30081	1.89226	1.76491
30082	1.89226	1.17756
30083	3.78452	1.7671
30084	0.964782	2.1
30085	1.30151	1.13063
30086	1.30318	1.1125
30087	1.30318	2.1
30088	0.949134	1.04445
30089	0.949134	2.02309
30090	2.15005	2.1
30091	1.89226	1.26411
30092	2.16541	2.00114
30093	2.16541	2.1
30094	1.89226	1.08071
30095	1.89226	2.1
30096	1.89226	2.0066
30097	1.89226	2.02197
30098	1.89226	1.45822
30099	1.89226	0.920094

30100	3.78452	1.26696
30101	0.949134	2.1
30102	1.30151	1.92768
30103	0.949134	1.36701
30104	1.32143	1.17625
30105	2.25064	2.1
30106	0.982859	0.176713
30107	1.28326	0.675977
30108	1.19052	0.257534
30109	1.19052	0.701602
30110	1.10476	0.152629
30111	1.10476	0.677652
30112	1.37265	0.834958
30113	1.38247	0.976717
30114	1.36636	1.09184
30115	1.3294	0.84898
30116	2.71187	0.90969
30117	1.89226	0.987284
30118	1.89226	0.822133
30119	1.89226	0.862339
30120	1.89226	0.94299
30121	1.89226	0.863098
30122	1.89226	0.951578
30123	1.36636	1.1756
30124	1.3294	0.85258
30125	1.30001	0.943106
30126	1.36416	1.10555
30127	2.69356	1.01237
30128	1.89226	1.1222
30129	1.89226	0.82117
30130	1.89226	0.909295
30131	1.89226	1.03038
30132	1.89226	0.853452
30133	1.89226	0.96203
30134	2.83701	0.794149
30135	1.89226	0.847653
30136	1.89226	1.00158
30137	2.79312	0.774089
30138	4.68539	0.821017
30139	1.44288	2.1
30140	0.948677	2.1
30141	1.84076	2.1
30142	1.42884	2.1
30143	2.78943	2.1
30144	1.89226	2.06977
30145	1.89226	2.1
30146	1.89226	2.1
30147	1.89226	2.05422
30148	3.78452	2.1
30149	0.909402	1.86446
30150	0.909402	2.0777
30151	1.3063	2.1
30152	1.56757	2.1
30153	1.00038	1.62165
30154	1.5235	2.1
30155	0.946127	2.1
30156	0.946127	1.82207
30157	0.946128	2.1

30158	1.26957	2.1
30159	1.00038	2.1
30160	0.946129	2.1
30161	1.17256	2.1
30162	2.2846	2.1
30163	1.1844	2.1
30164	2.28904	1.41173
30165	3.469 2.1	
30166	0.867886	1.62437
30167	0.867886	2.1
30168	0.867277	2.1
30169	0.867277	2.1
30170	1.14296	1.77216
30171	1.14452	2.1
30172	0.591766	2.1
30173	0.592199	2.1
30174	0.591766	2.1
30175	1.14296	2.1
30176	0.5922	2.1
30177	1.14452	2.1
30178	1.27408	0.53837
30179	1.56339	0.641035
30180	1.52522	0.685363
30181	1.34447	0.638673
30182	2.90786	0.646029
30183	1.27408	0.601507
30184	1.01121	0.464085
30185	0.991868	0.651275
30186	1.34447	0.662186
30187	2.35569	0.648953
30188	0.944818	0.685732
30189	0.883008	0.689449
30190	0.66957	0.657616
30191	0.616883	0.660156
30192	0.755679	0.668566
30193	0.755679	0.659048
30194	0.791912	0.690573
30195	0.701943	0.615879
30196	0.901541	0.684346
30197	0.634847	0.685821
30198	0.82781	0.674196
30199	0.655405	0.608343
30200	1.73819	2.1
30201	1.70303	2.1
30202	1.70303	2.1
30203	1.70303	2.1
30204	1.94516	2.1
30205	1.94517	2.1
30206	0.94994	2.1
30207	2.07469	2.1
30208	1.08506	2.1
30209	2.0815	2.1
30210	3.12902	2.1
30211	0.946131	2.1
30212	0.946131	2.1
30213	0.946131	2.1
30214	0.946131	2.1
30215	0.946128	2.1

30216	0.946128	2.1
30217	0.946133	2.1
30218	0.946133	2.1
30219	0.946131	2.1
30220	0.946131	2.1
30221	0.94613	2.1
30222	0.94613	2.1
30223	2.51796	2.1
30224	1.64193	2.1
30225	2.51172	2.1
30226	1.68893	2.1
30227	4.20689	2.1
30228	2.51172	2.1
30229	1.70002	2.1
30230	1.70002	2.1
30231	1.68893	2.1
30232	2.48689	2.1
30233	2.4758	2.1
30234	2.27963	0.0946268
30235	2.21582	0.0787077
30236	2.60486	0.077221
30237	2.5105	0.0935193
30238	4.79013	0.087244
30239	2.21582	0.0777865
30240	2.02069	0.0992964
30241	2.5105	0.0964738
30242	2.28615	0.076685
30243	4.53119	0.0826665
30244	2.60486	0.0776065
30245	2.5105	0.0984729
30246	2.55791	0.104166
30247	2.47141	0.0770238
30248	5.06842	0.0836105
30249	2.5105	0.101912
30250	2.28615	0.080042
30251	2.47141	0.0788169
30252	2.23337	0.0985946
30253	4.74387	0.0921209
30254	2.06984	2.1
30255	0.967499	2.1
30256	2.12435	2.1
30257	2.0021	2.1
30258	1.08975	2.1
30259	0.967499	2.1
30260	1.89226	0.489953
30261	1.89226	0.45012
30262	1.89226	0.482979
30263	1.89226	0.498742
30264	1.89226	0.39953
30265	1.89226	0.411641
30266	1.89226	2.1
30267	1.89226	2.1
30268	1.89226	2.1
30269	1.89226	2.1
30270	3.78452	2.1
30271	1.89227	2.1
30272	1.89227	2.1
30273	1.89227	2.1

30274	1.89227	2.1
30275	3.78453	2.1
30276	0.55882	2.1
30277	0.696462	2.1
30278	1.07003	2.1
30279	0.937179	2.1
30280	0.818077	2.1
30281	0.818077	2.1
30282	0.815565	2.1
30283	0.815565	2.1
30284	0.696462	2.1
30285	1.07003	2.1
30286	0.93718	2.1
30287	0.55882	2.1
30288	2.53857	2.1
30289	1.87436	2.1
30290	2.27541	2.1
30291	2.27541	2.1
30292	2.14005	2.1
30293	2.14005	2.1
30294	1.89226	2.1
30295	1.89226	2.1
30296	1.89226	1.48981
30297	1.89226	2.1
30298	3.78452	2.1
30299	1.89227	2.1
30300	1.89227	2.1
30301	1.89227	2.1
30302	1.89227	2.1
30303	3.78453	2.1
30304	1.39292	2.1
30305	2.14006	2.1
30306	2.41083	2.1
30307	1.68761	2.1
30308	3.80376	2.1
30309	2.53857	2.1
30310	2.14005	2.1
30311	2.41083	2.1
30312	2.82043	2.1
30313	4.9494	2.1
30314	1.90439	0.704296
30315	1.51835	0.681673
30316	1.20473	0.652599
30317	0.947693	0.728105
30318	2.85208	0.703441
30319	2.586	2.1
30320	1.46262	2.1
30321	2.14009	2.1
30322	2.78101	2.1
30323	4.72609	2.1
30324	1.89224	2.1
30325	2.78101	2.1
30326	2.586	2.1
30327	2.586	2.1
30328	1.89224	2.1
30329	1.89224	2.1
30330	0.994293	2.1
30331	1.19	2.1

30332	1.09483	2.1
30333	1.09483	2.1
30334	1.06827	2.1
30335	1.06827	2.1
30336	1.89226	2.1
30337	1.89226	2.1
30338	1.89226	2.1
30339	1.89226	2.1
30340	3.78452	2.1
30341	0.946132	2.1
30342	0.946132	2.1
30343	1.31912	2.1
30344	1.3572	2.1
30345	0.946131	2.1
30346	0.946131	2.1
30347	1.31912	2.1
30348	1.31912	2.1
30349	0.946131	2.1
30350	1.31912	2.1
30351	1.35721	2.1
30352	0.946131	2.1
30353	2.24577	0.855061
30354	1.09408	0.851968
30355	1.60239	0.871526
30356	1.09408	0.852226
30357	2.39404	0.863117
30358	2.07382	0.833108
30359	1.70303	0.700068
30360	1.3216	0.704498
30361	1.23104	0.7
30362	1.05475	0.703448
30363	2.60408	0.7
30364	1.89226	0.702314
30365	1.89226	0.713202
30366	1.89226	0.7
30367	1.89226	0.709076
30368	3.78452	0.708083
30369	1.89226	0.7
30370	2.3845	0.691175
30371	2.6875	0.7
30372	1.89226	0.7
30373	2.6875	0.7
30374	1.89226	0.7
30375	1.46759	2.1
30376	1.17585	2.1
30377	1.85189	2.1
30378	1.39223	2.1
30379	2.85981	2.1
30380	1.85189	2.1
30381	1.71255	2.1
30382	1.71255	2.1
30383	1.39223	2.1
30384	2.28511	2.1
30385	1.96479	2.1
30386	1.89227	0.905699
30387	1.89227	0.97903
30388	1.89227	1.02304
30389	1.89227	0.883047

30390	1.89227	1.25684
30391	1.89227	0.918297
30392	1.89226	0.944427
30393	1.89226	0.969837
30394	1.89226	1.0567
30395	1.89226	0.893971
30396	1.89226	1.291
30397	1.89226	0.931762
30398	1.89226	0.903438
30399	1.89226	0.971245
30400	1.89226	0.994451
30401	1.89226	0.870467
30402	1.89226	1.31108
30403	1.89226	0.919523
30404	1.89226	0.87395
30405	2.77788	0.908789
30406	2.21062	0.935723
30407	2.21062	0.854908
30408	1.89226	0.986116
30409	1.89226	0.876073
30410	0.868548	0.904009
30411	0.610104	0.938045
30412	0.796678	0.894556
30413	0.865021	0.869531
30414	1.0883	0.886637
30415	0.904938	0.919427
30416	2.21062	0.901072
30417	1.49548	0.88573
30418	1.2068	0.971577
30419	1.54764	0.887856
30420	0.790005	0.869275
30421	1.89226	2.06255
30422	2.67457	2.1
30423	1.89226	2.1
30424	2.56352	2.1
30425	4.56683	2.1
30426	2.67456	2.1
30427	2.20011	2.1
30428	2.56351	2.1
30429	2.11083	2.1
30430	4.78539	2.1
30431	2.56348	2.1
30432	2.1108	2.1
30433	2.45763	2.1
30434	2.00986	2.1
30435	4.56843	2.1
30436	0.946133	0.0874353
30437	0.946133	0.0985074
30438	0.946132	0.0811898
30439	0.946132	0.0950617
30440	0.946132	0.0890713
30441	0.946132	0.0887863
30442	0.946132	0.0947707
30443	0.946132	0.0935912
30444	0.946132	0.0887672
30445	0.946132	0.0903528
30446	0.946133	0.094026
30447	0.946133	0.0891047

30448	1.45927	0.984779
30449	1.25133	1.26587
30450	1.00082	1.12906
30451	1.41689	1.2078
30452	1.0432	1.05553
30453	1.45927	1.18611
30454	1.41689	1.15339
30455	1.29579	1.49904
30456	1.34874	1.31244
30457	1.25133	1.30138
30458	2.76563	1.59936
30459	0.675555	1.39754
30460	0.674371	1.33812
30461	0.625664	1.22776
30462	0.523133	1.25296
30463	0.676509	1.31693
30464	0.676509	1.47769
30465	0.62471	1.33056
30466	0.62471	1.16283
30467	0.675556	1.19174
30468	0.523132	1.12689
30469	0.674373	1.12769
30470	0.625663	1.17423
30471	1.89226	0.827174
30472	1.89226	0.844177
30473	1.89226	0.8233
30474	1.89226	0.857708
30475	3.78453	0.888816
30476	0.851517	1.2201
30477	0.946133	0.888469
30478	0.946132	0.906123
30479	0.946132	1.09682
30480	0.946133	0.855701
30481	0.851517	1.41471
30482	0.946132	0.876873
30483	0.946132	0.864808
30484	0.851516	0.919649
30485	0.851516	0.860778
30486	0.946133	0.96285
30487	0.946133	0.876266
30488	0.569995	0.938763
30489	0.816895	1.00185
30490	1.33893	1.07103
30491	1.18795	1.02208
30492	1.18795	0.96156
30493	0.569995	0.983545
30494	0.816895	0.940064
30495	1.33893	1.1367
30496	0.859572	1.16148
30497	0.859572	1.26765
30498	1.04936	0.969272
30499	1.04936	0.883307
30500	1.89226	2.1
30501	1.89226	2.1
30502	2.55618	2.1
30503	2.55618	2.1
30504	2.66983	2.1
30505	2.00591	2.1

30506	1.89226	0.842223
30507	2.64586	2.1
30508	2.83331	2.1
30509	2.83331	0.824278
30510	1.89226	2.1
30511	1.89226	0.816333
30512	2.64586	2.1
30513	2.0815	2.1
30514	1.99985	2.1
30515	1.94005	2.1
30516	2.83332	2.1
30517	2.74859	2.1
30518	1.98061	1.5238
30519	1.68356	2.1
30520	1.83879	2.04835
30521	1.55987	2.1
30522	3.54048	2.1
30523	1.68356	2.1
30524	1.20969	2.1
30525	1.20969	2.1
30526	1.30751	2.1
30527	1.55987	2.1
30528	1.65769	2.1
30529	1.83879	2.04131
30530	1.55987	2.1
30531	1.68443	1.45339
30532	1.4449	2.1
30533	3.2443	2.1
30534	0.660368	2.1
30535	0.660368	2.1
30536	0.666922	2.1
30537	0.666922	2.1
30538	0.560377	2.1
30539	0.604842	2.1
30540	0.779935	2.1
30541	0.722448	2.1
30542	0.722446	2.1
30543	0.560377	2.1
30544	0.604844	2.1
30545	0.779934	2.1
30546	1.89227	2.1
30547	1.89227	2.1
30548	1.89227	2.1
30549	1.89227	2.1
30550	3.78453	2.1
30551	1.42896	2.1
30552	1.23037	2.1
30553	1.42896	2.1
30554	1.474 2.1	
30555	1.2016	2.1
30556	1.24665	2.1
30557	1.89226	2.1
30558	2.7188	2.1
30559	1.89226	2.1
30560	2.70702	2.1
30561	4.61106	2.1
30562	1.89226	2.1
30563	1.89226	2.1

30564	1.89226	2.1
30565	1.89226	2.1
30566	3.78453	2.1
30567	0.644603	2.1
30568	0.644603	2.1
30569	0.648272	2.1
30570	0.648272	2.1
30571	0.585644	2.1
30572	0.600802	2.1
30573	0.692073	2.1
30574	0.714478	2.1
30575	0.585644	2.1
30576	0.692073	2.1
30577	0.714478	2.1
30578	0.600802	2.1
30579	1.35351	2.1
30580	1.35351	2.1
30581	0.946131	2.1
30582	0.946131	2.1
30583	1.34392	2.1
30584	1.35351	2.1
30585	0.946132	2.1
30586	0.946132	2.1
30587	0.946132	2.1
30588	1.34392	2.1
30589	0.946132	2.1
30590	1.35351	2.1
30591	1.87609	2.1
30592	1.79155	2.1
30593	1.79155	2.1
30594	1.89226	2.1
30595	1.89226	2.1
30596	1.99211	2.1
30597	1.89226	2.1
30598	1.71706	2.1
30599	1.71706	2.1
30600	1.79155	2.1
30601	1.89226	2.1
30602	1.96675	2.1
30603	1.46869	2.1
30604	1.89226	2.1
30605	1.89226	2.1
30606	1.89226	2.1
30607	1.5001	2.1
30608	1.5001	2.1
30609	1.89226	2.1
30610	1.89226	2.1
30611	1.89226	2.1
30612	1.89226	2.1
30613	1.89226	2.1
30614	1.89226	1.91274
30615	1.21408	2.1
30616	2.23563	2.1
30617	2.34021	2.1
30618	2.34021	2.1
30619	1.17285	2.1
30620	1.17285	2.1
30621	1.89226	2.1

30622	1.89226	2.1
30623	1.47937	2.1
30624	1.89226	2.1
30625	1.14822	2.1
30626	1.46869	2.03562
30627	1.89226	2.1
30628	1.89226	2.1
30629	1.89226	2.1
30630	1.89226	2.1
30631	1.89226	2.1
30632	1.89226	1.52386
30633	1.25716	2.1
30634	2.34021	2.1
30635	2.4542	2.1
30636	2.4542	2.1
30637	1.21408	2.1
30638	1.21408	2.1
30639	1.66824	2.1
30640	2.44883	2.1
30641	1.93003	2.1
30642	2.24795	2.1
30643	3.88907	2.1
30644	1.89319	2.1
30645	2.24795	2.1
30646	2.82724	2.1
30647	2.31318	2.1
30648	2.30868	2.1
30649	1.93003	2.1
30650	1.89226	1.20955
30651	1.42589	2.1
30652	1.42589	1.96786
30653	1.80501	2.1
30654	1.89226	1.82174
30655	2.27137	2.1
30656	1.21067	0.7
30657	2.39166	0.702121
30658	1.73577	0.7
30659	1.73577	0.7
30660	1.53584	0.700523
30661	1.53584	0.7
30662	1.35025	1.13938
30663	2.0599	1.03014
30664	1.51084	0.948194
30665	2.27218	0.814821
30666	3.57074	0.997172
30667	2.0599	0.952428
30668	2.51818	1.0845
30669	2.27218	0.784328
30670	2.66532	0.961912
30671	4.72522	0.896453
30672	1.67115	1.01093
30673	2.27218	0.981065
30674	2.39605	0.922579
30675	2.39605	0.923135
30676	1.51084	1.00885
30677	1.51084	1.11043
30678	2.27218	0.898177
30679	2.66532	1.24693

30680	2.39605	0.822279
30681	2.76829	0.94429
30682	5.06137	0.927056
30683	2.08684	0.933288
30684	2.36033	1.10877
30685	1.89224	0.880323
30686	1.89224	1.10237
30687	4.25257	0.917548
30688	2.41473	0.715611
30689	1.00307	0.732497
30690	2.01365	0.720547
30691	1.00307	0.737769
30692	3.05113	0.707502
30693	2.04056	0.742055
30694	1.11099	2.1
30695	2.25103	2.1
30696	2.22227	2.1
30697	2.22227	2.1
30698	1.14431	2.1
30699	1.14431	2.1
30700	1.07485	2.1
30701	2.22229	2.1
30702	2.19163	2.1
30703	2.19163	2.1
30704	1.111 2.1	
30705	1.111 2.1	
30706	1.0547	2.02986
30707	1.19886	2.1
30708	0.946131	2.1
30709	0.946131	2.1
30710	0.946131	2.1
30711	0.946131	2.08908
30712	1.19886	2.1
30713	1.19886	2.1
30714	1.0547	2.1
30715	0.946131	2.1
30716	0.946132	2.1
30717	1.19886	2.1
30718	1.89417	2.1
30719	2.64424	0.816732
30720	1.76585	1.5115
30721	2.47695	0.930702
30722	4.37112	1.04192
30723	1.89226	2.1
30724	1.89226	1.0009
30725	1.89226	2.1
30726	1.89226	0.937938
30727	3.78453	1.13846
30728	0.930281	1.62384
30729	1.0547	2.1
30730	0.946131	2.1
30731	0.946131	2.1
30732	0.930309	2.1
30733	0.930309	1.76486
30734	1.07052	2.1
30735	1.07052	2.1
30736	0.93028	2.1
30737	0.946131	2.1

30738	1.0547	2.1
30739	0.946131	2.1
30740	1.00015	0.411218
30741	1.00015	1.10621
30742	1.01918	1.46205
30743	1.01918	0.899671
30744	1.17437	0.911963
30745	1.23847	0.44396
30746	0.882926	1.47695
30747	0.780859	1.10299
30748	0.780859	0.921745
30749	1.17438	0.950247
30750	0.882925	0.986778
30751	1.23848	0.987773
30752	1.89226	2.1
30753	1.89226	0.912205
30754	1.89226	1.01465
30755	1.89226	1.94229
30756	1.89226	1.00113
30757	1.89226	2.1
30758	1.22083	2.1
30759	1.29579	2.1
30760	1.26236	2.1
30761	1.06191	2.1
30762	1.42128	2.1
30763	1.22083	2.1
30764	1.42128	2.1
30765	1.45806	2.1
30766	1.43502	2.1
30767	1.29579	2.1
30768	1.5605	2.1
30769	1.42128	2.1
30770	1.02813	2.1
30771	1.12121	2.1
30772	1.40557	2.1
30773	1.35298	2.1
30774	1.1314	2.1
30775	1.28679	2.1
30776	1.1715	2.1
30777	1.1715	2.1
30778	0.975656	2.1
30779	1.24908	2.1
30780	0.98956	2.1
30781	1.2681	2.1
30782	0.946132	2.1
30783	0.946132	2.1
30784	1.31889	2.1
30785	1.31889	2.1
30786	1.16546	2.1
30787	1.31889	2.1
30788	0.946133	2.1
30789	0.946133	2.1
30790	0.946133	2.1
30791	1.31889	2.1
30792	0.946132	2.1
30793	1.16546	2.1
30794	2.51518	0.127331
30795	2.37584	0.164301

30796	1.11712	0.137627
30797	1.31242	0.193694
30798	3.68826	0.166988
30799	2.51518	0.147257
30800	1.26311	0.217424
30801	2.48977	0.158325
30802	2.48977	0.179662
30803	1.31242	0.1586
30804	1.31242	0.179824
30805	1.89226	0.738177
30806	1.89226	0.841747
30807	1.89226	0.729507
30808	1.89226	0.834182
30809	3.78452	0.776093
30810	2.04737	0.721639
30811	1.77122	0.84272
30812	1.94498	0.633754
30813	1.65174	0.847355
30814	3.71621	0.775285
30815	1.41077	2.1
30816	1.29729	2.1
30817	1.77123	0.854531
30818	1.77123	1.87149
30819	1.65175	0.844655
30820	1.29129	0.855218
30821	1.89226	0.836849
30822	1.89226	2.1
30823	1.89226	2.1
30824	1.89226	0.840179
30825	1.89226	2.1
30826	1.89226	0.911459
30827	1.89226	0.726846
30828	1.89226	0.833287
30829	1.89226	0.71317
30830	1.89226	0.826623
30831	3.78451	0.758299
30832	1.94498	0.708157
30833	1.65174	0.836127
30834	1.7968	0.65873
30835	1.49831	0.822232
30836	3.44329	0.78061
30837	1.49831	0.825283
30838	1.29729	2.1
30839	1.49831	2.1
30840	1.65175	0.832664
30841	1.16858	2.1
30842	1.32201	0.881783
30843	1.89226	0.828552
30844	1.89226	2.1
30845	1.89226	0.825762
30846	1.89226	2.1
30847	3.78452	0.928599
30848	1.89226	0.135789
30849	1.89226	0.145648
30850	1.89226	0.142681
30851	1.89226	0.147398
30852	1.89226	0.13585
30853	1.89226	0.140688

30854	1.65324	0.877759
30855	1.26409	0.731036
30856	1.42491	0.731702
30857	1.42491	0.892823
30858	1.52268	0.751691
30859	1.52268	0.889924
30860	1.52268	0.951617
30861	1.26409	0.744739
30862	1.32697	0.882368
30863	1.05724	0.687023
30864	2.57991	0.811673
30865	1.89226	0.68359
30866	1.96548	0.703825
30867	2.0178	0.704228
30868	1.96548	0.70318
30869	1.94458	0.682558
30870	1.89226	0.507714
30871	1.99366	0.671622
30872	2.0178	0.702658
30873	1.89226	0.702615
30874	2.0178	0.701601
30875	1.75999	0.673625
30876	1.89226	0.590263
30877	0.946126	2.1
30878	0.946126	2.1
30879	1.28228	2.1
30880	1.19444	2.1
30881	0.946127	2.1
30882	0.946127	2.1
30883	1.19444	2.1
30884	1.19444	2.1
30885	0.946128	2.1
30886	1.19444	2.1
30887	1.28228	2.1
30888	0.946128	2.1
30889	1.89226	2.1
30890	1.89226	2.1
30891	1.89226	2.1
30892	1.89226	1.98736
30893	3.78452	2.1
30894	2.14104	2.1
30895	1.89226	2.1
30896	2.19948	2.1
30897	1.89226	2.1
30898	1.89226	2.1
30899	2.19948	2.1
30900	1.89226	2.1
30901	2.38888	2.1
30902	2.23932	2.1
30903	2.38888	2.1
30904	1.74269	2.1
30905	1.89226	2.1
30906	1.89226	2.1
30907	1.89226	1.81997
30908	1.89226	2.1
30909	1.89226	2.1
30910	3.78452	2.1
30911	0.946132	2.1

30912	0.946132	2.1
30913	1.07052	2.1
30914	1.07052	2.1
30915	0.946132	2.1
30916	0.946132	2.1
30917	1.05132	2.1
30918	1.07052	2.1
30919	1.05132	2.1
30920	0.946132	2.1
30921	1.07052	2.1
30922	0.946133	2.1
30923	2.40171	2.1
30924	1.89226	2.1
30925	1.89226	2.1
30926	1.89226	2.1
30927	2.42723	2.1
30928	2.42723	2.1
30929	1.89226	2.1
30930	1.89226	1.2513
30931	1.89226	2.1
30932	1.89226	1.65073
30933	3.78452	2.1
30934	1.63661	2.1
30935	2.3532	1.15576
30936	2.33287	1.09246
30937	2.33287	2.1
30938	1.64158	1.03864
30939	1.64158	2.1
30940	2.38918	2.1
30941	1.89226	2.1
30942	1.89226	2.1
30943	1.89226	2.1
30944	2.40171	2.1
30945	2.40171	2.1
30946	1.89226	2.1
30947	1.89226	1.92768
30948	1.89226	2.1
30949	1.89226	1.76727
30950	1.89226	2.1
30951	1.89226	2.06521
30952	1.64158	2.1
30953	2.3532	1.38373
30954	1.6488	2.1
30955	2.37364	1.77836
30956	4.002 2.1	
30957	2.54752	0.754373
30958	1.42445	0.776979
30959	1.86697	0.781844
30960	1.86697	0.758881
30961	2.06666	0.78093
30962	2.06666	0.760769
30963	2.50872	0.724469
30964	2.06666	0.747107
30965	1.90439	0.734403
30966	1.51835	0.735379
30967	3.97105	0.732598
30968	0.755161	0.754798
30969	0.755161	0.744683

30970	0.793994	0.735248
30971	0.793994	0.747266
30972	0.712225	0.7517
30973	0.515826	0.740116
30974	1.03333	0.747715
30975	0.759175	0.740325
30976	0.759173	0.742947
30977	0.515827	0.671947
30978	0.712226	0.730226
30979	1.03333	0.743911
30980	1.69633	0.733636
30981	1.89226	0.730142
30982	1.62477	0.72601
30983	1.89226	0.732488
30984	1.62477	0.732706
30985	1.89226	0.737701
30986	1.89226	0.793875
30987	1.89226	0.819108
30988	1.89226	0.80835
30989	1.89226	0.811093
30990	3.78452	0.810687
30991	2.28622	0.738771
30992	1.09667	0.691875
30993	1.17506	0.686228
30994	2.24411	0.72969
30995	1.17506	0.734806
30996	2.24411	0.753389
30997	2.24741	2.1
30998	1.70303	2.1
30999	2.49168	2.1
31000	1.57936	2.1
31001	2.53729	2.1
31002	1.70303	2.1
31003	2.24741	2.1
31004	2.47557	2.1
31005	1.89227	2.1
31006	1.89227	2.1
31007	4.36784	2.1
31008	2.27016	0.225232
31009	1.10476	0.341815
31010	1.44103	0.238983
31011	1.10476	0.491145
31012	2.26791	0.24143
31013	1.93165	0.369953
31014	2.27016	0.212845
31015	1.89226	0.385435
31016	1.89226	0.262009
31017	2.4065	0.336609
31018	4.29876	0.343181
31019	2.27016	0.210141
31020	1.44103	0.301796
31021	1.53336	0.21841
31022	2.4065	0.313843
31023	3.84753	0.221179
31024	2.7542	0.762057
31025	1.89226	0.775265
31026	2.35027	0.774221
31027	1.89226	0.773648

31028	2.35027	0.762845
31029	1.89226	0.762697
31030	2.3301	2.1
31031	2.36071	1.62668
31032	2.07629	2.1
31033	2.01105	2.1
31034	4.37176	2.1
31035	1.86512	2.1
31036	2.01105	2.1
31037	2.01105	2.1
31038	2.3301	2.1
31039	1.57459	2.1
31040	1.89364	2.1
31041	2.52671	2.1
31042	2.01105	2.1
31043	1.78251	2.1
31044	1.70302	1.44841
31045	3.98362	2.1
31046	1.227 2.1	
31047	2.01105	2.1
31048	1.45869	2.1
31049	1.41713	2.1
31050	1.70302	2.1
31051	1.64994	2.1
31052	2.00989	2.1
31053	1.28938	2.1
31054	1.8769	2.1
31055	1.20969	2.1
31056	3.21958	2.1
31057	1.8769	2.1
31058	1.15864	2.1
31059	1.15864	2.1
31060	1.20969	2.1
31061	1.77353	2.1
31062	1.82458	2.1
31063	1.7986	2.1
31064	2.48717	2.1
31065	2.50161	2.1
31066	1.75869	2.1
31067	4.30021	2.1
31068	2.50161	2.1
31069	1.75251	2.1
31070	1.81678	2.1
31071	1.75869	2.1
31072	2.5597	2.1
31073	2.50161	2.1
31074	1.91334	0.808637
31075	1.85439	0.872184
31076	1.91334	0.832973
31077	1.70303	0.851146
31078	1.70303	0.780583
31079	1.49692	0.772242
31080	2.39986	0.645872
31081	1.89226	0.690377
31082	1.89226	0.700252
31083	2.50756	0.665226
31084	4.39982	0.681548
31085	2.39986	0.7

31086	1.70303	0.687274
31087	1.75564	0.657422
31088	1.75904	0.675182
31089	2.50272	0.7
31090	2.50756	0.7
31091	1.89226	0.701698
31092	2.50756	0.683873
31093	2.47857	0.700473
31094	2.50756	0.701049
31095	1.86327	0.696283
31096	1.89226	0.699827
31097	1.22241	0.7
31098	1.20827	0.70322
31099	0.772416	0.698656
31100	0.555208	0.702395
31101	1.004	0.699225
31102	1.004	0.699946
31103	0.93504	0.704976
31104	0.744364	0.701191
31105	1.2089	0.703276
31106	0.654935	0.704487
31107	0.773927	0.697985
31108	1.03618	0.7
31109	1.89227	0.0891613
31110	1.89227	0.101168
31111	1.89227	0.0701307
31112	1.89227	0.0759626
31113	3.78453	0.0820538
31114	1.89226	0.0781644
31115	1.89226	0.0954928
31116	1.89226	0.0664367
31117	1.89226	0.0781938
31118	3.78452	0.077635
31119	1.82643	0.0668389
31120	2.7084	0.066576
31121	2.12654	0.0613669
31122	2.65614	0.0713833
31123	1.82643	0.0636665
31124	2.35603	0.0704391
31125	1.29385	0.0583836
31126	0.946133	0.0621491
31127	1.05492	0.0602048
31128	0.946132	0.0637233
31129	1.05492	0.0620272
31130	1.29385	0.060496
31131	0.946133	0.0599373
31132	0.946133	0.0650815
31133	1.29385	0.05735
31134	1.29385	0.0569181
31135	0.946132	0.0686429
31136	0.946132	0.0644765
31137	0.946132	0.0580244
31138	1.27329	0.0569541
31139	1.29385	0.0584277
31140	0.946129	0.0608385
31141	1.29385	0.0585857
31142	1.27328	0.0567158
31143	0.946131	0.0579701

31144	0.946131	0.0570836
31145	1.27328	0.0568042
31146	1.27328	0.05601
31147	0.94613	0.0631056
31148	0.94613	0.0595828
31149	1.89226	1.39505
31150	1.89226	1.6169
31151	1.89226	0.879531
31152	1.89226	1.06025
31153	3.78453	1.15303
31154	1.89226	1.23953
31155	1.89226	1.58234
31156	1.89226	0.859664
31157	1.89226	1.24433
31158	3.78453	0.994336
31159	1.21488	1.56488
31160	1.14605	2.07005
31161	1.62974	1.16195
31162	1.52399	1.02839
31163	2.84463	1.6236
31164	0.577078	1.0843
31165	0.81251	1.96819
31166	0.821003	1.29923
31167	0.543413	1.7914
31168	0.683929	1.1172
31169	0.722433	2.1
31170	0.730674	1.72837
31171	0.730674	1.03111
31172	0.857766	1.30459
31173	0.862119	1.09274
31174	0.600938	1.68114
31175	0.596094	1.32377
31176	1.89226	0.964258
31177	1.89226	1.36284
31178	1.89226	0.828269
31179	1.89226	1.09086
31180	3.78453	0.958612
31181	1.89226	0.924662
31182	1.89226	1.75654
31183	1.89226	0.916931
31184	1.89226	0.996385
31185	3.78453	1.13925
31186	1.62974	1.66008
31187	1.56958	1.87695
31188	2.2362	0.926946
31189	2.11424	1.2621
31190	3.86595	1.26205
31191	2.2362	0.99263
31192	1.85414	1.91704
31193	2.28211	0.832824
31194	1.78367	0.900222
31195	2.2362	1.52868
31196	1.73256	1.82331
31197	0.851539	1.07849
31198	0.851539	0.793271
31199	1.14454	0.962756
31200	0.938988	1.45139
31201	0.851485	1.09147

31202	0.920593	0.832654
31203	0.851541	0.730323
31204	0.851541	0.887401
31205	1.04073	0.789953
31206	1.16342	0.818986
31207	0.825315	0.94632
31208	0.825316	0.819037
31209	1.89226	1.95969
31210	1.89226	2.1
31211	1.89226	2.1
31212	1.89226	2.03395
31213	3.78453	2.1
31214	2.57016	2.1
31215	1.74789	2.1
31216	2.57016	2.1
31217	2.522	2.1
31218	1.61727	2.1
31219	1.56911	2.1
31220	1.28075	2.1
31221	1.406	2.1
31222	0.946131	2.09541
31223	0.946131	1.98728
31224	1.28075	2.1
31225	1.00333	2.1
31226	0.94613	2.1
31227	0.94613	2.1
31228	1.041	2.1
31229	1.041	2.1
31230	0.943484	2.1
31231	1.27717	2.1
31232	1.89226	2.1
31233	1.89226	1.92813
31234	1.89226	1.89717
31235	1.89226	2.1
31236	1.89226	1.96096
31237	1.89226	2.1
31238	2.57016	2.1
31239	1.61727	2.1
31240	2.74196	2.1
31241	1.59094	2.1
31242	4.16109	2.1
31243	1.89226	1.95394
31244	2.0815	2.1
31245	2.2034	2.1
31246	1.82493	1.81507
31247	1.87847	2.1
31248	2.16142	2.1
31249	1.89226	2.1
31250	1.89226	2.1
31251	1.89226	2.1
31252	1.89226	2.1
31253	3.78453	2.1
31254	0.985252	2.1
31255	0.985252	2.1
31256	0.946132	2.1
31257	0.946132	2.1
31258	1.0655	2.1
31259	0.985255	2.1

31260	0.94613	2.1
31261	0.94613	2.1
31262	0.94613	2.1
31263	1.0655	2.1
31264	0.94613	2.1
31265	0.985255	2.1
31266	1.89226	2.1
31267	1.89226	2.1
31268	1.89226	2.1
31269	1.89226	2.1
31270	3.78453	2.1
31271	1.06549	2.1
31272	1.06549	2.1
31273	0.946133	2.1
31274	0.946133	2.1
31275	1.16834	2.1
31276	1.0655	2.1
31277	0.946131	2.1
31278	0.946131	2.1
31279	0.946131	2.1
31280	1.16834	2.1
31281	0.94613	2.1
31282	1.0655	2.1
31283	0.966631	2.1
31284	1.07349	2.1
31285	0.946131	2.1
31286	0.946131	2.1
31287	1.07349	2.1
31288	1.07349	2.1
31289	0.946129	2.1
31290	0.946129	2.1
31291	1.07349	2.1
31292	0.946131	2.1
31293	0.94613	2.1
31294	0.966631	2.1
31295	2.38055	1.07279
31296	1.89226	1.53977
31297	1.89226	1.33077
31298	1.46724	0.867679
31299	3.35949	1.12693
31300	1.42249	2.1
31301	2.05499	2.1
31302	1.39708	2.1
31303	2.09033	2.1
31304	3.45207	2.1
31305	2.05499	2.1
31306	2.57425	2.1
31307	2.51287	2.1
31308	2.47753	2.1
31309	2.09033	2.1
31310	2.05499	1.94205
31311	1.39327	2.1
31312	2.09033	2.1
31313	2.16217	2.1
31314	2.16217	2.1
31315	1.39708	2.1
31316	1.39708	2.1
31317	2.16217	2.1

31318	2.57426	2.1
31319	2.73006	2.1
31320	2.73006	2.1
31321	2.09033	2.1
31322	2.09033	2.1
31323	1.89226	0.24679
31324	1.89226	0.130275
31325	1.89226	0.175067
31326	1.89226	0.242314
31327	3.78452	0.24178
31328	0.838749	0.093592
31329	0.838749	0.0970827
31330	0.85824	0.123312
31331	0.85824	0.145655
31332	0.544644	0.138754
31333	0.854718	0.113345
31334	0.842271	0.128535
31335	1.19141	0.136256
31336	0.842272	0.131167
31337	0.544643	0.0905176
31338	0.854718	0.126583
31339	1.19141	0.102086
31340	1.34214	0.111277
31341	1.34214	0.103239
31342	0.893236	0.183525
31343	0.893236	0.1578
31344	0.893265	0.171261
31345	1.28924	0.131821
31346	0.946133	0.119355
31347	0.946133	0.151078
31348	0.946132	0.138241
31349	0.893265	0.16708
31350	0.946133	0.154247
31351	1.28924	0.130591
31352	2.7013	0.153097
31353	1.89226	0.213854
31354	1.9216	0.23154
31355	1.9216	0.131657
31356	1.89226	0.226369
31357	1.89226	0.130005
31358	1.26397	2.1
31359	1.52995	2.1
31360	1.30116	2.1
31361	1.04084	2.1
31362	2.57079	2.1
31363	1.47013	2.1
31364	2.17459	2.1
31365	2.10668	2.1
31366	1.38328	2.1
31367	3.55787	2.1
31368	2.55006	2.1
31369	2.17459	2.1
31370	2.10668	2.1
31371	2.49168	2.1
31372	4.66626	2.1
31373	0.613622	2.1
31374	0.691639	2.1
31375	1.00105	2.1

31376	1.05334	2.1
31377	0.839874	2.1
31378	0.839874	2.1
31379	0.852814	2.1
31380	0.852814	2.1
31381	0.613622	2.1
31382	1.00105	2.1
31383	1.05334	2.1
31384	0.691639	2.1
31385	1.00105	2.1
31386	1.05334	2.1
31387	1.20844	2.1
31388	1.24584	2.1
31389	1.12717	2.1
31390	1.12717	2.1
31391	1.13462	2.1
31392	1.13462	2.1
31393	1.20845	2.1
31394	1.00105	2.1
31395	1.05334	2.1
31396	1.24584	2.1
31397	2.22558	1.64921
31398	1.85981	1.49748
31399	2.18943	1.52376
31400	1.89142	1.01486
31401	4.0854	1.33937
31402	2.02641	2.1
31403	1.85981	1.34283
31404	2.18943	1.48418
31405	1.67346	1.44884
31406	3.88622	1.33063
31407	1.85981	0.880409
31408	1.10736	0.745689
31409	1.27421	0.933162
31410	1.67346	0.645201
31411	1.27421	0.6683
31412	1.67346	1.12554
31413	1.85981	0.992053
31414	1.27362	0.920116
31415	1.89142	1.05246
31416	1.27421	0.947666
31417	3.13343	0.946483
31418	1.81893	0.255442
31419	1.64985	0.30556
31420	1.23477	0.189642
31421	2.25405	0.381517
31422	3.48882	0.33326
31423	2.23933	0.155372
31424	1.89226	0.255797
31425	1.89226	0.272325
31426	2.78053	0.245767
31427	1.89226	0.176379
31428	2.78053	0.152348
31429	0.946131	0.319423
31430	0.946131	0.311681
31431	0.946132	0.391837
31432	0.946132	0.488053
31433	0.946131	0.509944

31434	0.946131	0.49448
31435	0.946132	0.325427
31436	0.946132	0.492167
31437	0.946131	0.33476
31438	0.946131	0.45432
31439	0.946132	0.283228
31440	0.946132	0.492332
31441	1.89226	2.1
31442	2.48596	2.1
31443	1.89226	2.1
31444	2.51506	2.1
31445	4.40732	2.1
31446	2.48596	2.1
31447	1.63823	2.1
31448	2.51507	2.1
31449	1.67687	2.1
31450	4.1533	2.1
31451	1.89226	2.1
31452	2.51506	2.1
31453	1.92044	2.1
31454	1.89226	2.1
31455	2.54325	2.1
31456	2.51506	2.1
31457	2.51507	2.1
31458	1.5704	2.1
31459	1.70505	2.1
31460	1.67687	2.1
31461	2.54325	2.1
31462	2.51507	2.1
31463	2.0815	2.1
31464	1.70303	2.1
31465	1.70303	2.1
31466	1.80354	2.1
31467	1.98172	2.1
31468	2.09868	2.1
31469	1.27944	2.1
31470	1.79028	2.1
31471	1.13179	2.1
31472	1.65533	2.1
31473	2.93477	2.1
31474	1.89226	2.1
31475	1.89226	2.1
31476	1.89226	2.1
31477	1.89226	2.1
31478	1.89226	2.1
31479	1.89226	2.1
31480	1.98171	2.1
31481	1.89226	2.1
31482	1.89226	2.1
31483	1.89226	2.1
31484	1.65798	2.1
31485	1.65797	2.1
31486	1.00133	2.1
31487	1.65532	2.1
31488	1.53278	2.1
31489	1.53278	2.1
31490	1.13179	2.1
31491	1.13179	2.1

31492	0.810946	2.1
31493	0.810946	2.1
31494	0.810944	2.1
31495	0.810944	2.1
31496	0.810946	2.1
31497	0.810946	2.1
31498	1.89226	2.1
31499	1.35169	2.1
31500	1.08132	2.1
31501	1.08132	2.1
31502	1.08132	2.1
31503	1.99528	0.45294
31504	1.70303	0.562272
31505	2.08152	0.470009
31506	1.70303	0.7
31507	2.33877	0.359658
31508	1.93967	0.562457
31509	1.89226	0.561507
31510	1.89226	0.701507
31511	1.89226	0.565555
31512	1.89226	0.7
31513	3.78452	0.7
31514	1.26259	0.560552
31515	2.17205	0.669097
31516	2.32468	0.586173
31517	2.32468	0.582591
31518	1.28055	0.584833
31519	1.28055	0.593917
31520	0.946131	0.511917
31521	0.946131	0.437683
31522	0.997642	0.509533
31523	0.997642	0.32517
31524	0.946131	0.546462
31525	0.946131	0.594155
31526	0.989846	0.445279
31527	0.997642	0.323002
31528	0.946132	0.470182
31529	0.989846	0.436122
31530	0.997641	0.404117
31531	0.946131	0.507861
31532	1.89226	0.595683
31533	1.89226	0.7
31534	1.89226	0.543327
31535	1.89226	0.7
31536	3.78452	0.598133
31537	1.28055	0.474701
31538	2.17205	0.661506
31539	1.14305	0.471141
31540	1.92737	0.584291
31541	3.3151	0.604417
31542	0.954908	0.104766
31543	0.9705	0.0933886
31544	0.946131	0.0954661
31545	0.946131	0.105384
31546	0.946131	0.10449
31547	0.946131	0.102467
31548	0.9705	0.102051
31549	0.9705	0.0901373

31550	0.954908	0.0979627
31551	0.946131	0.0972189
31552	0.946131	0.102067
31553	0.9705	0.102134
31554	0.835187	0.102742
31555	0.954896	0.0874586
31556	0.946118	0.0942292
31557	0.946118	0.101781
31558	0.835186	0.0995342
31559	0.835186	0.0953345
31560	1.06583	0.0937546
31561	1.06583	0.0843219
31562	0.835198	0.0994451
31563	0.946131	0.101031
31564	0.954883	0.101928
31565	0.946106	0.0989861
31566	1.89227	2.1
31567	1.89227	2.1
31568	1.89227	2.1
31569	1.89227	2.1
31570	1.89227	2.1
31571	1.89227	2.07735
31572	0.885265	2.1
31573	0.885265	2.1
31574	0.871467	2.1
31575	0.871467	2.1
31576	1.11883	2.1
31577	0.787875	2.1
31578	0.996487	2.1
31579	0.637907	2.1
31580	0.996487	2.1
31581	1.11883	2.1
31582	0.787875	2.1
31583	0.637906	2.1
31584	1.89226	2.1
31585	2.39258	2.1
31586	2.39258	2.1
31587	2.54729	2.1
31588	1.89226	2.1
31589	2.04697	2.1
31590	1.89226	2.1
31591	2.0815	2.1
31592	2.0815	2.1
31593	2.48371	2.1
31594	2.0815	2.1
31595	2.51469	2.1
31596	1.00035	0.05
31597	1.55755	0.120941
31598	1.03534	0.585332
31599	1.61333	0.20315
31600	2.59289	0.0999822
31601	1.55755	0.120941
31602	1.85217	1.11032
31603	1.61333	0.676999
31604	1.92875	0.903907
31605	3.48629	0.650707
31606	1.09962	0.742006
31607	1.61334	0.674957

31608	1.70868	0.789934
31609	1.70868	0.790744
31610	1.03535	0.919916
31611	1.03535	0.77643
31612	1.61334	0.388991
31613	1.92876	0.906455
31614	1.70868	0.354447
31615	2.05274	0.998289
31616	3.63744	0.65291
31617	1.82233	0.788457
31618	2.05768	0.950816
31619	1.89226	1.00139
31620	1.65406	0.821841
31621	1.89226	0.947545
31622	1.65406	0.816499
31623	2.26047	0.80817
31624	2.0815	1.04117
31625	1.89226	1.02112
31626	2.01156	0.834056
31627	1.89226	0.962349
31628	2.01156	0.822138
31629	1.89226	2.1
31630	1.89226	2.1
31631	1.89226	2.1
31632	1.89226	1.91433
31633	3.78453	2.02606
31634	2.70055	2.1
31635	2.28367	2.1
31636	2.56456	2.1
31637	2.33565	2.1
31638	4.84823	2.1
31639	2.28367	2.1
31640	1.5791	2.1
31641	1.96966	2.1
31642	1.09506	2.1
31643	3.37873	2.1
31644	1.89226	2.1
31645	1.89226	1.78588
31646	1.89226	1.88286
31647	1.89226	2.1
31648	3.78452	2.1
31649	0.94612	2.1
31650	0.94612	2.1
31651	0.946119	2.07389
31652	0.946119	1.90688
31653	0.946119	1.82176
31654	0.946119	2.1
31655	0.946119	2.1
31656	0.946119	2.0281
31657	0.946132	2.1
31658	0.946132	2.1
31659	0.946107	2.1
31660	0.946107	2.1
31661	2.56452	2.1
31662	1.57307	2.1
31663	2.10299	2.1
31664	2.1115	2.1
31665	2.52196	2.1

31666	2.52196	2.1
31667	1.89226	2.1
31668	1.89226	1.20513
31669	1.89226	2.1
31670	1.89226	2.1
31671	3.78452	2.1
31672	2.37665	2.1
31673	1.89226	2.1
31674	2.28084	2.1
31675	1.89226	2.1
31676	4.26891	2.1
31677	0.946129	1.22131
31678	0.946129	2.1
31679	0.946133	2.1
31680	0.946133	2.1
31681	0.946131	2.1
31682	0.946131	1.17998
31683	0.946131	2.1
31684	0.946131	2.1
31685	0.946131	2.1
31686	0.946131	2.1
31687	0.946131	2.1
31688	0.946131	2.1
31689	1.7207	0.0862336
31690	1.65815	0.105058
31691	1.33593	0.117115
31692	1.31508	0.0833153
31693	2.99408	0.085188
31694	0.676047	0.0787622
31695	0.676047	0.0837651
31696	0.684362	0.121296
31697	0.684362	0.0909538
31698	0.547994	0.10217
31699	0.702871	0.0904702
31700	0.829076	0.12211
31701	0.657538	0.105174
31702	0.657538	0.101643
31703	0.547994	0.0873396
31704	0.829077	0.0920788
31705	0.702871	0.0906485
31706	1.9518	2.1
31707	1.30648	2.1
31708	1.28846	2.1
31709	1.92033	2.1
31710	3.22681	2.1
31711	1.89226	2.1
31712	1.89226	2.1
31713	1.89226	2.1
31714	1.89226	2.1
31715	3.78453	2.1
31716	0.946128	2.1
31717	0.946128	2.1
31718	1.10113	2.1
31719	1.09587	2.1
31720	0.946131	2.1
31721	0.946131	2.1
31722	1.09587	2.1
31723	1.09587	2.1

31724	0.946131	2.1
31725	1.09587	2.1
31726	1.10112	2.1
31727	0.94613	2.1
31728	1.26923	2.1
31729	1.92033	2.1
31730	1.28846	2.1
31731	1.28846	2.1
31732	1.89452	2.1
31733	1.89452	2.1
31734	1.89226	2.1
31735	1.89226	2.1
31736	1.89226	2.1
31737	1.89226	2.1
31738	3.78453	2.1
31739	1.89226	2.1
31740	2.19174	2.1
31741	2.15616	2.1
31742	2.19174	2.1
31743	1.85669	2.1
31744	1.89226	2.1
31745	1.89226	2.1
31746	1.89226	2.1
31747	1.89226	2.1
31748	1.89226	2.1
31749	3.78452	2.1
31750	1.89226	2.1
31751	1.89226	2.1
31752	1.89226	2.1
31753	1.89226	2.1
31754	3.78452	2.1
31755	1.51223	2.1
31756	1.41562	2.1
31757	1.5139	2.1
31758	1.42284	2.1
31759	1.6033	2.1
31760	1.51223	2.1
31761	1.51223	2.1
31762	1.25953	2.1
31763	1.38085	2.1
31764	1.6033	2.1
31765	2.89309	2.1
31766	0.946131	2.1
31767	0.946131	2.1
31768	0.946131	2.1
31769	0.946131	2.1
31770	0.946132	2.1
31771	0.946132	2.1
31772	0.946129	2.1
31773	0.946129	2.1
31774	0.946131	2.1
31775	0.946131	2.1
31776	0.94613	2.1
31777	0.94613	2.1
31778	1.89226	2.1
31779	1.89226	2.1
31780	1.89226	2.1
31781	1.89226	2.1

31782	3.78452	2.1
31783	0.707806	2.1
31784	0.697816	2.1
31785	0.823593	2.1
31786	0.801649	2.1
31787	0.74182	2.1
31788	0.74182	2.1
31789	0.757645	2.1
31790	0.757645	2.1
31791	0.823592	2.1
31792	0.697818	2.1
31793	0.707807	2.1
31794	0.801647	2.1
31795	1.52534	2.1
31796	1.6033	2.1
31797	1.64719	2.1
31798	1.38085	2.1
31799	1.64719	2.1
31800	1.38085	2.1
31801	2.67041	2.1
31802	1.89226	0.988693
31803	1.89226	2.07552
31804	1.89226	1.39817
31805	2.487	1.95968
31806	2.487	1.86913
31807	2.70097	2.1
31808	1.70303	2.1
31809	1.71163	2.1
31810	2.487	2.1
31811	4.46723	2.1
31812	1.14984	2.02481
31813	1.2435	2.1
31814	0.778	2.1
31815	0.697616	2.1
31816	0.967211	2.1
31817	0.967211	1.64142
31818	0.973907	2.1
31819	0.973907	2.1
31820	0.697616	2.1
31821	1.14984	2.1
31822	0.778	2.1
31823	1.2435	2.1
31824	2.4158	2.1
31825	1.26438	2.1
31826	1.676	2.1
31827	2.77505	2.1
31828	4.0918	2.1
31829	2.58258	2.1
31830	1.89226	2.1
31831	1.89226	2.1
31832	1.89226	2.1
31833	2.47032	2.1
31834	2.47032	2.1
31835	1.23516	2.1
31836	1.18734	2.1
31837	0.946131	2.1
31838	0.946131	2.1
31839	1.18734	2.1

31840	1.18734	2.1
31841	0.94613	2.1
31842	0.94613	2.1
31843	1.18734	2.1
31844	0.946131	2.1
31845	0.946131	2.1
31846	1.23516	2.1
31847	2.8239	2.1
31848	1.39361	2.1
31849	1.12342	2.1
31850	2.57275	2.1
31851	3.96637	2.1
31852	0.937929	0.980773
31853	0.937929	0.927647
31854	1.39734	0.863721
31855	1.39734	0.849721
31856	1.38914	0.897232
31857	0.94613	0.938879
31858	0.937902	0.945743
31859	0.94613	0.78796
31860	0.94613	0.856381
31861	0.94613	0.882303
31862	1.38914	0.903378
31863	0.937902	0.876062
31864	1.89226	0.774151
31865	2.17765	0.967941
31866	1.84577	0.91826
31867	1.84577	0.818466
31868	1.89226	0.86905
31869	1.89226	0.785075
31870	2.11262	1.00837
31871	1.57766	1.10501
31872	2.22689	2.1
31873	1.91132	0.849088
31874	2.19624	1.77961
31875	1.79689	0.720011
31876	1.99656	0.782075
31877	1.57766	1.02067
31878	1.39067	1.99131
31879	1.73802	0.856979
31880	3.31568	1.0228
31881	1.89226	0.707287
31882	1.89226	0.7
31883	1.89226	0.70061
31884	1.89226	0.725835
31885	1.89226	0.656865
31886	1.89226	0.707042
31887	1.84936	0.778719
31888	2.0815	0.7
31889	2.0815	0.701294
31890	2.47217	0.718199
31891	1.04528	0.682422
31892	1.62774	0.712255
31893	1.89226	0.701653
31894	1.89226	0.7
31895	1.89226	0.7
31896	1.89226	0.707252
31897	1.89226	0.64756

31898	1.89226	0.701286
31899	1.69055	0.369038
31900	2.68518	0.652591
31901	1.69055	0.439324
31902	2.25055	0.665901
31903	2.39587	0.287647
31904	3.01801	0.642214
31905	1.89226	0.692678
31906	1.89226	0.7
31907	1.89226	0.437246
31908	1.89226	0.406975
31909	3.78452	0.7
31910	1.24499	1.10155
31911	1.70302	1.16945
31912	1.41207	2.1
31913	1.10574	0.921126
31914	2.66056	1.17825
31915	1.70303	1.16292
31916	2.39605	1.14851
31917	2.72674	0.97919
31918	2.46904	0.95563
31919	1.84557	1.13194
31920	1.67115	1.17434
31921	2.46904	0.837964
31922	2.76829	1.55261
31923	2.83666	0.806677
31924	2.83666	0.990402
31925	2.39605	0.929049
31926	2.39605	1.3766
31927	1.07323	0.786656
31928	1.07323	2.06465
31929	1.06715	1.219
31930	1.18052	1.06248
31931	1.30067	0.898131
31932	1.26542	0.933329
31933	0.914516	1.12321
31934	0.839633	1.40464
31935	0.936958	0.984685
31936	1.26751	0.930216
31937	0.847764	1.03458
31938	1.35395	1.05114
31939	2.69069	0.914624
31940	1.03074	0.854872
31941	1.74098	0.602298
31942	1.97397	0.805405
31943	3.71495	0.803937
31944	2.44554	2.1
31945	1.89226	2.1
31946	1.89226	2.1
31947	1.89226	2.1
31948	2.40645	2.1
31949	2.40645	2.1
31950	1.89226	2.1
31951	1.89226	1.34306
31952	1.89226	2.1
31953	1.89226	1.22002
31954	3.78452	2.1
31955	1.82117	2.1

31956	2.55786	1.18718
31957	2.51431	2.1
31958	2.51431	2.1
31959	1.85054	2.1
31960	1.84375	2.1
31961	2.38467	2.1
31962	1.89226	2.1
31963	1.89226	2.1
31964	1.89226	2.1
31965	2.44554	2.1
31966	2.44554	2.1
31967	1.89226	2.1
31968	1.89226	2.1
31969	1.89226	2.1
31970	1.89226	2.08416
31971	1.89226	2.1
31972	1.89226	2.1
31973	2.02397	2.1
31974	2.55786	2.07444
31975	1.77775	2.1
31976	2.61404	2.1
31977	4.33561	2.1
31978	1.89226	1.17213
31979	1.89226	1.14959
31980	1.89226	0.994616
31981	1.89226	1.07783
31982	3.78453	1.15139
31983	1.89226	1.25677
31984	2.80184	1.39304
31985	1.89226	1.12474
31986	1.89226	1.25926
31987	2.03045	1.39488
31988	2.03045	1.7045
31989	2.52263	1.16766
31990	1.27921	1.96329
31991	1.58429	2.03925
31992	1.73571	1.81431
31993	2.37121	1.34325
31994	2.52263	1.20469
31995	0.764974	2.1
31996	0.650577	2.1
31997	0.712428	2.1
31998	0.562516	2.1
31999	0.67264	2.1
32000	0.67264	2.1
32001	0.654851	2.1
32002	0.654851	2.1
32003	0.562515	2.1
32004	0.650579	2.1
32005	0.712427	2.1
32006	0.764976	2.1
32007	1.89227	2.1
32008	1.89227	2.1
32009	1.89227	2.1
32010	1.89227	2.1
32011	3.78453	2.1
32012	1.89226	2.1
32013	1.89226	2.1

32014	1.89226	2.1
32015	1.89226	2.1
32016	3.78453	2.1
32017	1.49248	2.1
32018	1.24959	2.1
32019	1.28211	2.1
32020	1.26432	2.1
32021	1.51027	2.1
32022	1.49248	2.1
32023	1.49248	2.1
32024	1.19017	2.1
32025	1.24977	2.1
32026	1.51027	2.1
32027	2.74225	2.1
32028	1.89226	2.1
32029	1.89226	2.1
32030	1.89226	2.1
32031	1.89226	2.1
32032	3.78453	2.1
32033	1.89226	2.1
32034	1.89226	2.1
32035	1.89226	2.1
32036	1.89226	2.1
32037	3.78453	2.1
32038	0.624796	2.1
32039	0.618472	2.1
32040	0.770147	2.1
32041	0.755132	2.1
32042	0.681445	2.1
32043	0.681445	2.1
32044	0.69216	2.1
32045	0.69216	2.1
32046	0.618472	2.1
32047	0.770147	2.1
32048	0.624796	2.1
32049	0.755132	2.1
32050	1.54029	2.1
32051	1.51027	2.1
32052	1.54029	2.1
32053	1.30682	2.1
32054	1.24977	2.1
32055	1.24977	2.1
32056	0.994293	2.1
32057	2.17159	2.1
32058	2.21149	2.1
32059	2.21149	2.1
32060	0.994814	2.1
32061	0.994814	2.1
32062	0.990481	2.1
32063	2.21149	2.1
32064	2.2671	2.1
32065	2.2671	2.1
32066	0.994293	2.1
32067	0.994293	2.1
32068	2.41579	2.1
32069	1.09847	2.1
32070	1.70302	1.15171
32071	2.45069	2.1

32072	3.61169	2.1
32073	2.48689	2.1
32074	1.67969	2.1
32075	1.67969	2.1
32076	1.70002	2.1
32077	2.43826	2.1
32078	2.45859	2.1
32079	2.43827	2.1
32080	1.62408	2.1
32081	1.62408	2.1
32082	1.67969	2.1
32083	2.36729	2.1
32084	2.4229	2.1
32085	2.47199	2.1
32086	1.0823	2.1
32087	1.0607	2.1
32088	2.44276	2.1
32089	3.52506	2.1
32090	2.47199	2.1
32091	1.89226	2.1
32092	1.89226	2.1
32093	2.44277	2.1
32094	4.33503	2.1
32095	1.89226	2.1
32096	1.89226	2.1
32097	1.89226	2.1
32098	1.89226	2.1
32099	3.78453	2.1
32100	1.89226	2.1
32101	1.89226	2.1
32102	1.89226	2.1
32103	1.89226	2.1
32104	1.89226	2.1
32105	1.89226	2.1
32106	1.02986	2.1
32107	2.44276	2.1
32108	1.0607	2.1
32109	1.0607	2.1
32110	2.40055	2.1
32111	2.40055	2.1
32112	1.89226	2.1
32113	2.44277	2.1
32114	2.40056	2.1
32115	2.44277	2.1
32116	1.85005	2.1
32117	1.89226	2.1
32118	1.89226	2.1
32119	1.89226	2.1
32120	1.89226	2.1
32121	1.89226	2.1
32122	3.78453	2.1
32123	1.89226	2.1
32124	1.89226	2.1
32125	1.89226	2.1
32126	1.89226	2.1
32127	3.78452	2.1
32128	2.79502	0.706618
32129	2.63448	0.808496

32130	2.68986	0.686968
32131	2.53868	0.81516
32132	5.32434	0.726671
32133	2.63449	0.805755
32134	2.39282	2.1
32135	2.53869	0.823252
32136	2.3085	1.81398
32137	4.94299	0.907057
32138	2.68986	0.714116
32139	2.53868	0.809019
32140	2.5761	0.592692
32141	2.43404	0.79975
32142	5.1239	0.755622
32143	2.53869	0.811538
32144	2.3085	1.84119
32145	2.43405	0.805002
32146	2.22575	1.95338
32147	4.74255	0.876059
32148	1.89226	2.1
32149	1.89226	2.1
32150	1.89226	2.1
32151	1.89226	2.1
32152	3.78453	2.1
32153	1.32391	2.1
32154	1.44951	2.1
32155	1.44951	2.1
32156	1.89226	2.1
32157	1.89226	2.1
32158	2.20203	2.1
32159	0.507656	2.1
32160	0.578945	2.1
32161	1.05568	2.1
32162	0.959007	2.1
32163	0.76264	2.1
32164	0.76264	2.1
32165	0.775313	2.1
32166	0.775313	2.1
32167	0.578945	2.1
32168	1.05568	2.1
32169	0.959007	2.1
32170	0.507656	2.1
32171	1.89226	0.246899
32172	2.4065	0.334703
32173	2.36561	0.247502
32174	2.4065	0.399049
32175	1.85138	0.190917
32176	1.89226	0.324149
32177	1.40274	0.216445
32178	2.4065	0.314653
32179	1.53336	0.21101
32180	1.53336	0.232011
32181	2.36562	0.285378
32182	2.36562	0.316316
32183	0.946115	0.292118
32184	0.946115	0.214272
32185	1.06496	0.191519
32186	1.18279	0.256256
32187	0.946117	0.315037

32188	0.946117	0.348175
32189	1.06496	0.243177
32190	1.06496	0.186046
32191	0.94613	0.283313
32192	1.06497	0.229699
32193	0.946105	0.227307
32194	1.18278	0.276704
32195	1.00213	0.221546
32196	2.36559	0.298862
32197	1.40272	0.207994
32198	1.40272	0.222634
32199	2.12992	0.279962
32200	2.12992	0.303814
32201	0.95646	2.1
32202	2.07201	1.75725
32203	1.01808	2.1
32204	2.14876	1.04499
32205	3.09009	2.1
32206	1.89226	2.1
32207	1.89226	1.14479
32208	1.89226	2.1
32209	1.89226	2.1
32210	3.78453	2.1
32211	1.89227	2.1
32212	1.89227	2.1
32213	1.89227	2.1
32214	1.89227	2.1
32215	1.89227	2.1
32216	1.89227	2.1
32217	2.71886	2.1
32218	1.60497	2.1
32219	1.43935	2.1
32220	2.55151	2.1
32221	4.15648	2.1
32222	2.71886	2.1
32223	1.89226	2.1
32224	1.89226	2.1
32225	2.55151	2.1
32226	4.44377	2.1
32227	1.89226	2.1
32228	1.89226	2.1
32229	1.89226	2.1
32230	1.89226	1.17176
32231	3.78453	2.1
32232	1.89226	2.1
32233	1.89226	2.1
32234	1.89226	2.1
32235	1.89226	2.1
32236	3.78453	2.1
32237	1.58942	0.762782
32238	1.92622	0.821673
32239	2.52852	0.833665
32240	2.19457	0.75299
32241	4.12079	0.751453
32242	1.92649	0.748271
32243	1.44426	0.681603
32244	1.87529	0.705984
32245	2.19457	0.749701

32246	3.63883	0.740785
32247	1.87529	0.681988
32248	2.19457	0.733782
32249	2.7378	0.73326
32250	2.29852	0.672503
32251	4.49309	0.677646
32252	1.35976	2.1
32253	1.89452	2.1
32254	1.39616	2.1
32255	1.1423	2.1
32256	1.8996	2.1
32257	1.52068	2.1
32258	1.89226	2.1
32259	1.89226	2.1
32260	1.89226	2.1
32261	1.89226	2.1
32262	1.89226	2.1
32263	1.89226	2.1
32264	1.89226	2.1
32265	2.15617	2.1
32266	2.0953	2.1
32267	2.15617	2.1
32268	1.83139	2.1
32269	1.89226	2.1
32270	1.16817	2.1
32271	1.8996	2.1
32272	1.23614	2.1
32273	1.48338	2.1
32274	1.80218	2.1
32275	2.16263	2.1
32276	1.89226	2.1
32277	1.89226	2.1
32278	1.89226	2.1
32279	1.89226	2.1
32280	1.89226	2.1
32281	1.89226	2.1
32282	1.89226	2.1
32283	2.0953	2.1
32284	2.03074	2.1
32285	2.0953	2.1
32286	1.8277	2.1
32287	1.89226	2.1
32288	1.74455	2.1
32289	2.181	2.1
32290	1.74455	2.1
32291	1.92911	2.1
32292	1.98195	2.1
32293	2.16651	2.1
32294	2.181	2.1
32295	2.29881	2.1
32296	1.98195	2.1
32297	1.80729	2.1
32298	4.28075	2.1
32299	1.74455	2.1
32300	1.98195	2.1
32301	1.52603	2.1
32302	1.76603	2.1
32303	3.51058	2.1

32304	1.76603	2.1
32305	1.94506	2.1
32306	1.76603	2.1
32307	1.98195	2.1
32308	1.86795	2.1
32309	2.08387	2.1
32310	0.984305	2.1
32311	1.9	2.1
32312	2.86619	2.1
32313	1.86189	2.1
32314	3.76189	2.1
32315	1.4621	2.1
32316	1.89226	2.1
32317	2.81374	2.1
32318	1.89226	2.1
32319	3.35437	2.1
32320	0.946133	2.1
32321	0.946133	2.1
32322	0.946132	1.98631
32323	0.946132	2.1
32324	0.946132	2.1
32325	0.946132	2.1
32326	0.946133	2.1
32327	0.946133	2.1
32328	0.946132	2.1
32329	0.946132	1.97291
32330	0.946132	2.1
32331	0.946132	2.1
32332	1.64268	1.82143
32333	1.93673	2.1
32334	2.25284	2.1
32335	1.97548	2.1
32336	3.89552	2.1
32337	1.86206	2.1
32338	1.93673	2.1
32339	2.25284	2.1
32340	2.20075	2.1
32341	4.1149	2.1
32342	2.25284	2.1
32343	2.24781	2.1
32344	1.97548	1.53465
32345	1.97548	2.1
32346	2.49676	1.83265
32347	2.49676	2.1
32348	2.25284	2.1
32349	2.43549	2.1
32350	2.44467	2.1
32351	2.20075	2.1
32352	2.49676	2.1
32353	2.25284	2.1
32354	2.57928	2.1
32355	2.32606	2.1
32356	2.59522	2.1
32357	2.39022	2.1
32358	4.9695	2.1
32359	2.32606	2.1
32360	1.77677	2.1
32361	2.39022	2.1

32362	2.28669	2.1
32363	4.16699	2.1
32364	2.59522	2.1
32365	2.39022	2.1
32366	2.61901	2.1
32367	2.45899	2.1
32368	5.00923	2.1
32369	2.39022	2.1
32370	2.11575	2.1
32371	2.41795	2.1
32372	2.0815	2.1
32373	4.67085	2.1
32374	1.89226	2.1
32375	2.24723	2.1
32376	1.95405	2.1
32377	1.89226	2.1
32378	2.30902	2.1
32379	2.24723	2.1
32380	1.89226	2.1
32381	1.89226	2.1
32382	1.89226	2.1
32383	1.89226	2.1
32384	3.78453	2.1
32385	0.946131	2.1
32386	0.946131	2.1
32387	1.14643	2.1
32388	1.10673	2.1
32389	1.10673	2.1
32390	1.10673	2.1
32391	0.946133	2.1
32392	0.946133	2.1
32393	0.946131	2.1
32394	1.14643	2.1
32395	1.10673	2.1
32396	0.946131	2.1
32397	1.89226	2.1
32398	2.30902	2.1
32399	1.96635	2.1
32400	1.89226	2.1
32401	2.38311	2.1
32402	2.30902	2.1
32403	1.89226	2.1
32404	1.89226	2.1
32405	1.89226	2.1
32406	1.89226	2.1
32407	1.89226	2.1
32408	1.89226	2.1
32409	0.946131	2.1
32410	0.946131	2.1
32411	1.19156	2.1
32412	1.14643	2.1
32413	1.14643	2.1
32414	1.14643	2.1
32415	0.946133	2.1
32416	0.946133	2.1
32417	0.946131	2.1
32418	1.19156	2.1
32419	0.946131	2.1

32420	1.14643	2.1
32421	1.89226	0.963045
32422	1.89226	0.876906
32423	1.89226	0.861006
32424	1.89226	1.00759
32425	1.89226	0.841909
32426	1.89226	1.26665
32427	1.89226	0.863262
32428	1.89226	0.892746
32429	1.89226	0.837726
32430	1.9645	0.915834
32431	3.78452	0.974058
32432	2.39489	2.1
32433	2.15715	2.1
32434	2.07821	2.1
32435	2.33541	2.1
32436	4.49256	2.1
32437	2.39489	2.1
32438	2.34898	2.1
32439	2.30729	2.1
32440	2.33541	2.1
32441	4.6844	2.1
32442	2.07821	2.1
32443	2.33541	2.1
32444	2.26415	2.1
32445	1.99632	2.1
32446	4.33174	2.1
32447	2.30729	2.1
32448	2.33542	2.1
32449	2.26416	2.1
32450	2.26618	2.1
32451	4.60159	2.1
32452	1.19837	1.35778
32453	2.79151	0.776109
32454	2.25636	1.09182
32455	2.25636	0.953309
32456	1.94072	1.02341
32457	1.94072	0.979843
32458	2.53725	0.200218
32459	1.16699	0.211235
32460	1.69864	0.164525
32461	2.0815	0.223531
32462	3.58273	0.197812
32463	1.91646	2.1
32464	2.51686	2.1
32465	2.49872	2.1
32466	1.85819	2.1
32467	4.41518	2.1
32468	0.929098	2.1
32469	0.8993	2.1
32470	1.24359	2.1
32471	1.24936	2.1
32472	1.06832	2.1
32473	1.06832	2.1
32474	1.08034	2.1
32475	1.08034	2.1
32476	0.899298	2.1
32477	1.24359	2.1

32478	1.24936	2.1
32479	0.929097	2.1
32480	2.0815	0.267406
32481	1.89226	0.127378
32482	2.0815	0.202902
32483	1.82811	0.165153
32484	1.94982	0.202861
32485	1.71246	0.157556
32486	2.3256	2.1
32487	1.89226	2.1
32488	1.89226	2.1
32489	1.89226	2.1
32490	2.38467	2.1
32491	2.38467	2.1
32492	1.89226	2.1
32493	1.89226	2.1
32494	1.89226	2.1
32495	1.89226	2.1
32496	1.89226	2.1
32497	1.89226	2.1
32498	1.89763	2.1
32499	2.61405	2.1
32500	2.15035	2.1
32501	2.68796	2.1
32502	1.42219	2.1
32503	1.77775	2.1
32504	2.26306	2.1
32505	1.89226	2.1
32506	1.89226	2.1
32507	1.89226	2.1
32508	2.3256	2.1
32509	2.3256	2.1
32510	1.89226	2.1
32511	1.89226	2.1
32512	1.89226	2.1
32513	1.89226	2.1
32514	1.89226	2.1
32515	1.89226	2.1
32516	1.92887	2.1
32517	2.41915	2.1
32518	2.50142	2.1
32519	2.50142	2.1
32520	1.90388	2.1
32521	1.89763	2.1
32522	1.89224	2.1
32523	1.89224	2.1
32524	1.89224	2.1
32525	1.89224	2.1
32526	3.78448	2.1
32527	2.25315	2.1
32528	1.05843	2.1
32529	2.38995	2.1
32530	1.31541	2.1
32531	3.56856	2.1
32532	0.517552	2.1
32533	0.68548	2.1
32534	1.27675	2.1
32535	1.13076	2.1

32536	0.913611	2.1
32537	0.913612	2.1
32538	0.902633	2.1
32539	0.902633	2.1
32540	0.68547	2.1
32541	1.27673	2.1
32542	1.13078	2.1
32543	0.517559	2.1
32544	1.89226	2.1
32545	1.89226	2.1
32546	1.89226	2.1
32547	1.89226	2.1
32548	3.78453	2.1
32549	0.962936	2.1
32550	0.962936	2.1
32551	0.996141	2.1
32552	0.996141	2.1
32553	0.830609	2.1
32554	0.657714	2.1
32555	1.30136	2.1
32556	1.19499	2.1
32557	1.30136	2.1
32558	0.830609	2.1
32559	1.19499	2.1
32560	0.657713	2.1
32561	1.60057	0.471369
32562	1.39615	0.428419
32563	1.06474	0.481555
32564	1.39615	0.619794
32565	1.64655	0.328438
32566	2.04424	0.42892
32567	1.89226	0.508419
32568	1.89226	0.621177
32569	1.89226	0.620502
32570	1.89226	0.549421
32571	1.89226	0.605473
32572	1.89226	0.581531
32573	2.56322	0.393393
32574	1.89226	0.521561
32575	1.89226	0.520664
32576	1.89226	0.434002
32577	2.18047	0.479645
32578	2.18047	0.42079
32579	1.89226	0.91682
32580	1.89226	0.92625
32581	1.89226	0.989144
32582	1.65573	0.857653
32583	1.89226	0.998616
32584	1.65573	0.864338
32585	1.05353	0.783193
32586	1.83158	0.709598
32587	1.50282	0.511991
32588	1.20669	0.653627
32589	2.88511	0.570341
32590	1.44807	2.1
32591	1.93592	2.1
32592	1.80703	2.1
32593	1.27806	2.1

32594	3.21398	2.1
32595	2.0815	2.1
32596	1.87465	2.1
32597	1.87465	2.1
32598	1.50566	2.1
32599	2.0815	2.1
32600	1.69263	2.1
32601	1.89226	2.1
32602	1.89226	2.1
32603	1.89226	2.1
32604	1.89226	2.1
32605	1.89226	2.1
32606	1.89226	2.1
32607	1.62632	2.1
32608	0.994294	2.1
32609	0.994294	2.1
32610	1.27806	2.1
32611	1.81921	2.1
32612	2.10776	2.1
32613	2.0815	2.1
32614	1.70303	2.1
32615	1.70303	2.1
32616	1.85749	2.1
32617	1.90451	2.1
32618	2.07724	2.1
32619	1.89226	2.1
32620	1.89226	2.1
32621	1.89226	2.1
32622	1.89226	2.1
32623	1.89226	2.1
32624	1.89226	2.1
32625	2.69773	2.1
32626	1.88129	2.1
32627	2.55196	2.1
32628	2.26469	2.1
32629	4.94367	2.1
32630	1.88129	2.1
32631	1.25889	2.1
32632	1.26614	2.1
32633	1.05914	2.1
32634	2.26469	2.1
32635	1.88129	2.1
32636	2.55196	2.1
32637	2.0588	2.1
32638	2.6536	2.1
32639	2.15287	2.1
32640	4.70484	2.1
32641	0.8516	2.1
32642	0.8516	2.1
32643	0.849002	2.1
32644	0.849002	2.1
32645	0.629445	2.1
32646	0.671197	2.1
32647	1.0294	2.1
32648	1.07644	2.1
32649	1.07644	2.1
32650	0.671197	2.1
32651	0.629446	2.1

32652	1.0294	2.1
32653	2.02115	1.75612
32654	1.18382	1.9288
32655	2.29107	0.80872
32656	1.46903	1.17393
32657	3.47488	0.953032
32658	2.60763	1.0538
32659	2.29107	1.31312
32660	1.82141	1.11294
32661	1.82141	1.92914
32662	1.46903	1.6997
32663	2.29107	1.72047
32664	1.64007	2.1
32665	2.2024	2.1
32666	1.44044	2.1
32667	2.02634	2.1
32668	3.6664	2.1
32669	1.25565	2.1
32670	2.02635	2.1
32671	1.87228	2.1
32672	1.87228	2.1
32673	1.44045	2.1
32674	1.44045	2.1
32675	1.89226	0.129073
32676	1.78503	0.137329
32677	1.78503	0.134524
32678	1.40048	0.130482
32679	2.0815	0.132271
32680	1.62972	0.131001
32681	1.27875	2.1
32682	1.1483	0.93068
32683	1.40014	0.954494
32684	1.40014	2.1
32685	1.04598	1.02923
32686	1.04598	2.1
32687	2.63443	1.22897
32688	2.26444	2.03714
32689	2.79392	0.897868
32690	2.4863	1.99007
32691	5.12073	1.49054
32692	1.83158	0.899059
32693	1.18508	0.790765
32694	1.20669	0.773421
32695	1.81488	0.846131
32696	1.20669	0.781875
32697	1.81488	0.905045
32698	1.08134	0.798492
32699	1.70303	0.832685
32700	1.24027	0.871286
32701	1.30359	0.738188
32702	1.89939	0.920272
32703	1.70303	0.865061
32704	1.89226	2.1
32705	2.69027	2.1
32706	1.89226	2.1
32707	2.2078	2.1
32708	1.89226	2.1
32709	2.2078	2.1

32710	1.89226	2.1
32711	2.78977	2.1
32712	1.89226	2.1
32713	2.69027	2.1
32714	1.89226	2.1
32715	2.69027	2.1
32716	1.89226	2.1
32717	1.89226	2.1
32718	1.89226	1.6512
32719	1.89226	2.1
32720	3.78452	2.07408
32721	1.43687	2.1
32722	1.71059	2.1
32723	2.53014	2.1
32724	2.6521	2.1
32725	4.24073	2.1
32726	2.6521	2.1
32727	1.66762	2.1
32728	2.50299	2.1
32729	2.50299	2.1
32730	1.71059	2.1
32731	1.71059	2.1
32732	1.89226	2.1
32733	1.89226	2.1
32734	1.89226	2.1
32735	1.89226	2.05276
32736	1.89226	2.1
32737	1.89226	2.07796
32738	2.10662	0.775304
32739	2.77417	0.800466
32740	2.78873	0.929355
32741	2.11661	0.777755
32742	4.89078	0.799866
32743	2.10662	0.811741
32744	1.22857	0.729023
32745	1.25849	0.845446
32746	2.11661	0.808354
32747	3.34518	0.799356
32748	2.78873	0.878455
32749	2.11661	0.747375
32750	2.12475	0.810806
32751	2.79029	0.838331
32752	4.9069	0.806586
32753	1.2858	0.804546
32754	2.11661	0.766693
32755	1.25849	0.788863
32756	1.25849	0.790581
32757	2.12475	0.784763
32758	2.12475	0.782974
32759	1.13537	2.1
32760	1.20714	2.1
32761	0.946129	2.1
32762	0.946129	2.1
32763	0.946131	2.1
32764	0.946131	2.1
32765	1.29521	2.1
32766	1.20713	2.1
32767	1.13537	2.1

32768	0.946131	2.1
32769	0.94613	2.1
32770	1.29521	2.1
32771	1.00533	2.1
32772	0.876055	2.1
32773	0.946128	2.1
32774	0.946128	2.1
32775	0.913908	2.1
32776	0.913908	2.1
32777	1.17081	2.1
32778	0.896413	2.1
32779	0.955905	2.1
32780	0.893715	2.1
32781	1.20327	2.1
32782	0.998544	2.1
32783	1.89226	1.53032
32784	1.89226	1.82978
32785	1.89226	0.866619
32786	1.89226	1.18963
32787	3.78452	1.01799
32788	0.540975	1.20774
32789	0.726949	2.1
32790	0.712198	1.94149
32791	0.506675	1.10462
32792	0.611916	1.13668
32793	0.611916	2.1
32794	0.621708	1.70013
32795	0.621708	1.07337
32796	0.726951	1.31824
32797	0.712196	1.15454
32798	0.506674	1.53178
32799	0.540976	1.34221
32800	1.89226	1.12532
32801	1.89226	2.1
32802	1.89226	1.12047
32803	1.89226	1.12564
32804	3.78453	1.47685
32805	1.89226	1.05171
32806	1.89226	1.12767
32807	1.89226	0.874974
32808	1.89226	1.89196
32809	1.89226	0.898904
32810	1.89226	2.1
32811	0.919594	1.10747
32812	0.726949	1.70056
32813	0.898318	1.12004
32814	0.712198	1.6519
32815	0.817559	0.96364
32816	0.817559	2.1
32817	0.814234	0.975273
32818	0.814234	1.70931
32819	0.919597	1.02739
32820	0.898316	0.910453
32821	0.726951	1.76854
32822	0.712196	1.34738
32823	1.79663	0.935224
32824	1.12088	2.02769
32825	1.54699	1.12027

32826	1.17475	1.17826
32827	1.79663	1.82739
32828	1.42439	2.01489
32829	2.28928	1.12075
32830	1.23077	1.3809
32831	1.65458	1.00961
32832	2.66533	1.49522
32833	3.94385	1.21878
32834	1.89226	2.1
32835	1.89226	2.1
32836	1.89226	2.1
32837	1.89226	2.0713
32838	3.78452	2.1
32839	1.38426	2.1
32840	1.15067	2.1
32841	1.38426	2.1
32842	1.01595	2.1
32843	1.24509	2.1
32844	1.01595	2.1
32845	2.62097	2.1
32846	1.89226	2.1
32847	2.72561	2.1
32848	2.72561	2.1
32849	1.89226	2.1
32850	1.89226	2.1
32851	1.90103	0.739393
32852	1.41902	1.10991
32853	1.77249	0.651072
32854	1.30272	1.90753
32855	3.20375	0.833013
32856	1.77249	0.756123
32857	1.19548	1.22089
32858	1.19548	0.802103
32859	1.30272	0.869891
32860	1.65815	0.473433
32861	1.76539	0.890582
32862	2.62161	2.1
32863	1.89226	2.1
32864	1.89226	2.1
32865	1.89226	2.1
32866	2.6446	2.1
32867	2.6446	2.1
32868	2.64459	2.1
32869	1.89226	2.1
32870	1.89226	2.1
32871	1.89226	2.1
32872	2.58257	2.1
32873	2.58257	2.1
32874	1.89226	0.79088
32875	1.89226	1.0594
32876	2.31301	1.03297
32877	2.76309	0.84363
32878	1.89226	0.970965
32879	2.26047	0.821837
32880	1.89226	0.783443
32881	2.31301	1.03601
32882	2.17765	0.968507
32883	2.17765	0.827697

32884	1.89226	0.923334
32885	1.89226	0.799516
32886	2.18516	2.1
32887	1.85196	2.1
32888	1.78595	2.1
32889	2.14606	2.1
32890	3.99802	2.1
32891	2.18516	2.1
32892	2.21189	2.1
32893	2.18937	2.1
32894	2.14606	2.1
32895	4.35796	2.1
32896	1.60735	2.1
32897	2.14606	2.1
32898	2.15714	2.1
32899	1.70302	2.1
32900	3.70055	2.1
32901	2.18937	2.1
32902	2.14606	2.1
32903	2.0927	2.1
32904	2.162 2.1	
32905	4.30806	2.1
32906	2.17326	2.1
32907	2.58517	2.1
32908	2.55318	2.1
32909	2.1665	2.1
32910	4.75167	2.1
32911	2.17326	2.1
32912	1.53556	2.1
32913	1.56253	2.1
32914	2.1665	2.1
32915	3.70206	2.1
32916	2.55317	2.1
32917	2.1665	2.1
32918	2.17741	2.1
32919	2.54197	2.1
32920	4.70847	2.1
32921	2.17742	2.1
32922	2.1665	2.1
32923	2.18452	2.1
32924	1.58054	2.1
32925	1.56253	2.1
32926	1.56253	2.1
32927	1.71051	0.500864
32928	1.89807	0.47433
32929	1.19755	0.306858
32930	1.19755	0.296615
32931	2.25632	0.569614
32932	2.25632	0.443869
32933	0.876046	0.40254
32934	1.27293	0.338622
32935	0.946132	0.511232
32936	0.946132	0.5255
32937	0.946131	0.387895
32938	0.869312	0.374735
32939	1.12767	0.469757
32940	0.946132	0.495607
32941	1.22225	0.496601

32942	1.37968	0.313697
32943	0.851555	0.595639
32944	0.851555	0.375329
32945	0.946133	1.39213
32946	0.946133	2.1
32947	0.946132	1.57257
32948	0.946132	1.16437
32949	0.946133	1.33537
32950	0.946133	1.4502
32951	0.946132	2.1
32952	0.946132	1.48758
32953	0.946132	2.01928
32954	0.946132	1.4997
32955	0.946133	1.45956
32956	0.946133	1.85006
32957	1.35152	2.1
32958	0.948936	2.1
32959	1.90166	2.1
32960	1.70303	2.1
32961	2.90858	2.1
32962	1.89226	2.1
32963	1.89226	2.1
32964	1.89226	1.98188
32965	1.89226	2.1
32966	3.78452	2.0983
32967	1.90166	2.1
32968	1.70303	2.1
32969	2.52454	1.46604
32970	2.13919	2.1
32971	3.99529	2.1
32972	1.89226	1.0554
32973	1.89226	1.9221
32974	1.89226	2.1
32975	1.89226	2.1
32976	3.78452	2.1
32977	1.89226	2.1
32978	1.89226	2.1
32979	1.89226	2.1
32980	1.89226	2.1
32981	1.89226	1.14516
32982	1.89226	2.09513
32983	2.13918	2.1
32984	0.992674	2.1
32985	2.3397	2.1
32986	1.8023	2.1
32987	1.47769	2.1
32988	0.992674	2.1
32989	1.89226	0.728164
32990	1.89226	0.695397
32991	1.89226	0.804069
32992	1.89226	0.560783
32993	3.78452	0.737605
32994	1.89226	0.703603
32995	2.54752	0.432267
32996	2.63974	0.588054
32997	1.89226	0.543145
32998	4.53201	0.528752
32999	0.498412	0.56562

33000	0.500955	0.511324
33001	0.57955	0.667237
33002	0.563873	0.568359
33003	0.529107	0.52046
33004	0.529107	0.494227
33005	0.53572	0.700452
33006	0.53572	0.515601
33007	0.500956	0.529149
33008	0.579548	0.51392
33009	0.563871	0.548676
33010	0.498414	0.553057
33011	1.89223	0.761539
33012	1.89223	0.553271
33013	1.89223	0.667737
33014	1.89223	0.717831
33015	1.89223	0.644917
33016	1.89223	0.694909
33017	1.28752	0.457536
33018	1.31985	0.54469
33019	0.946119	0.500224
33020	0.946119	0.592381
33021	1.28752	0.510712
33022	1.28752	0.458799
33023	0.946119	0.495423
33024	0.946119	0.766139
33025	1.28753	0.508901
33026	0.946132	0.492162
33027	1.31984	0.551149
33028	0.946107	0.55632
33029	0.500948	0.497779
33030	0.479465	0.463275
33031	0.562335	0.616862
33032	0.579542	0.506985
33033	0.528436	0.469301
33034	0.528436	0.456255
33035	0.530571	0.476821
33036	0.530571	0.639756
33037	0.56234	0.482948
33038	0.479473	0.48975
33039	0.500943	0.514445
33040	0.579533	0.499089
33041	1.89226	2.1
33042	1.89226	2.1
33043	1.89226	2.1
33044	1.89226	2.1
33045	3.78452	2.1
33046	1.50767	2.1
33047	1.14496	2.1
33048	1.50767	2.1
33049	1.58539	2.1
33050	1.07653	2.1
33051	1.15425	2.1
33052	1.89226	2.1
33053	2.43826	2.1
33054	2.43826	2.1
33055	2.48688	2.1
33056	1.89226	2.1
33057	1.94088	2.1

33058	1.89226	2.1
33059	1.89226	2.1
33060	1.89226	2.1
33061	1.89226	2.1
33062	3.78452	2.1
33063	1.50767	2.1
33064	1.07653	2.1
33065	1.41077	2.1
33066	0.991407	2.1
33067	2.49907	2.1
33068	1.21913	2.1
33069	1.21913	2.1
33070	0.946128	2.1
33071	0.946128	2.1
33072	1.21913	2.1
33073	1.18364	2.1
33074	0.946131	2.1
33075	0.946131	2.1
33076	1.18364	2.1
33077	0.946129	2.1
33078	1.21913	2.1
33079	0.94613	2.1
33080	1.89226	0.905806
33081	1.89226	2.1
33082	1.89226	0.910642
33083	1.89226	2.1
33084	3.78452	1.07799
33085	1.93823	0.906125
33086	1.60976	2.1
33087	1.93823	2.1
33088	2.20045	0.896727
33089	1.36999	2.1
33090	1.63222	0.990562
33091	1.38302	2.1
33092	1.38302	2.1
33093	0.946132	2.1
33094	0.946132	2.1
33095	1.27258	2.1
33096	1.38302	2.1
33097	0.946133	2.1
33098	0.946133	2.1
33099	0.946133	2.1
33100	1.27258	2.1
33101	0.946132	2.1
33102	1.38302	2.1
33103	1.89226	0.85104
33104	1.89226	2.1
33105	1.89226	0.89538
33106	1.89226	2.1
33107	3.78452	1.83977
33108	1.93823	0.847783
33109	1.36999	2.1
33110	1.70423	0.858184
33111	1.14195	2.1
33112	3.08018	0.996731
33113	1.27258	2.1
33114	1.27258	2.1
33115	0.946132	2.1

33116	0.946132	2.1
33117	1.17233	2.1
33118	1.27258	2.1
33119	0.946132	2.1
33120	0.946132	2.1
33121	1.17233	2.1
33122	0.946132	2.1
33123	0.946132	2.1
33124	1.27258	2.1
33125	2.70575	2.1
33126	1.89226	1.00346
33127	2.71429	2.1
33128	2.71429	1.72656
33129	1.89226	1.47398
33130	1.89226	1.05956
33131	0.946133	1.03947
33132	0.946133	2.1
33133	1.28713	2.1
33134	1.28713	2.1
33135	0.946131	2.1
33136	0.946131	1.02642
33137	1.28713	2.1
33138	1.36503	1.8894
33139	0.946131	2.1
33140	1.36503	2.1
33141	1.28713	2.1
33142	0.946131	2.1
33143	1.89226	2.1
33144	1.89226	1.24626
33145	1.89226	2.1
33146	1.89226	1.21394
33147	1.89226	2.00841
33148	1.89226	1.20205
33149	1.22615	2.1
33150	1.04147	2.1
33151	1.3439	2.1
33152	1.3439	2.1
33153	0.969118	2.1
33154	0.969118	2.07148
33155	0.980841	2.1
33156	0.992505	2.1
33157	1.34289	2.1
33158	1.70303	2.1
33159	2.56081	2.1
33160	1.89225	2.1
33161	2.66785	2.1
33162	2.79506	2.1
33163	2.79506	2.1
33164	1.89225	2.1
33165	1.89225	2.1
33166	0.978778	2.1
33167	0.978778	2.1
33168	0.993303	2.1
33169	0.993303	2.1
33170	0.603538	2.1
33171	0.638158	2.1
33172	1.33393	2.1
33173	1.39753	2.1

33174	1.33391	2.1
33175	0.603535	2.1
33176	0.638164	2.1
33177	1.39754	2.1
33178	1.33393	2.1
33179	1.33393	2.1
33180	0.946132	2.1
33181	0.946132	2.1
33182	1.33393	2.1
33183	1.27365	2.1
33184	0.946132	2.1
33185	0.946132	2.1
33186	0.946132	2.1
33187	1.27365	2.1
33188	1.33393	2.1
33189	0.946131	2.1
33190	1.89226	2.1
33191	1.89226	2.1
33192	1.89226	2.1
33193	1.89226	2.1
33194	3.78452	2.1
33195	1.89226	2.1
33196	1.89226	2.1
33197	1.89226	2.1
33198	1.89226	2.1
33199	3.78453	2.1
33200	0.979364	2.1
33201	0.979364	2.1
33202	0.89618	2.1
33203	0.89618	2.1
33204	0.896178	2.1
33205	0.929411	2.1
33206	0.946133	2.1
33207	0.946133	2.1
33208	0.946131	2.1
33209	0.89618	2.1
33210	0.946131	2.1
33211	0.929413	2.1
33212	1.89226	2.1
33213	1.89226	2.1
33214	1.89226	2.1
33215	1.89226	2.1
33216	1.89226	2.1
33217	1.89226	2.1
33218	1.89225	2.1
33219	1.89225	2.1
33220	1.89225	2.1
33221	1.89225	2.1
33222	3.7845	2.1
33223	1.89225	2.1
33224	1.89225	2.1
33225	1.89225	2.1
33226	1.89225	2.1
33227	1.89225	2.1
33228	1.89225	2.1
33229	0.946128	0.697248
33230	0.946128	0.649607
33231	0.946132	0.686608

33232	0.946132	0.641726
33233	0.94613	0.687635
33234	0.94613	0.709669
33235	0.946129	0.62258
33236	0.946129	0.50812
33237	0.94613	0.663271
33238	0.94613	0.66127
33239	0.94613	0.660095
33240	0.94613	0.662575
33241	1.89227	0.907996
33242	1.89227	0.937515
33243	1.89227	0.830227
33244	1.89227	0.82409
33245	3.78453	0.84649
33246	1.89226	0.926163
33247	2.43717	1.49626
33248	2.63259	0.944494
33249	1.89226	0.827632
33250	4.52485	0.997552
33251	0.616808	0.884847
33252	0.792277	1.15375
33253	1.04986	0.814472
33254	0.72303	1.08989
33255	0.702675	0.74997
33256	0.76862	1.78916
33257	0.809154	1.25038
33258	0.809154	0.853852
33259	0.808757	1.00054
33260	1.07789	0.777309
33261	0.772568	0.851372
33262	0.598878	1.18134
33263	1.89226	0.914507
33264	1.89226	0.952616
33265	1.89226	0.862099
33266	1.89226	0.83314
33267	3.78453	0.89469
33268	1.36428	1.67608
33269	1.31629	0.987496
33270	0.946132	0.969137
33271	0.946132	0.917571
33272	1.36428	0.863159
33273	1.36428	1.67046
33274	0.946132	1.00545
33275	0.946132	1.14202
33276	1.36428	1.4171
33277	0.946132	0.898431
33278	1.31629	1.05482
33279	0.946132	0.859934
33280	1.578	1.15965
33281	2.85336	0.787574
33282	2.11368	0.755358
33283	2.14375	0.880511
33284	1.93852	0.863177
33285	1.93852	1.36547
33286	2.34806	0.753105
33287	1.42913	0.674626
33288	1.61539	0.794716
33289	2.45322	0.754336

33290	3.88235	0.761558
33291	2.34806	0.722393
33292	1.89226	0.617243
33293	1.89226	0.78127
33294	2.45322	0.710923
33295	4.34548	0.695467
33296	1.89226	0.605239
33297	1.89226	0.37612
33298	1.89226	0.653826
33299	1.89226	0.557458
33300	3.78452	0.615842
33301	1.89226	0.634277
33302	1.89226	0.429621
33303	1.89226	0.733829
33304	1.89226	0.630166
33305	3.78452	0.580941
33306	1.25972	0.721013
33307	2.45322	0.591931
33308	2.58823	0.766452
33309	1.79398	0.694861
33310	4.2472	0.695898
33311	1.89226	0.74677
33312	2.45322	0.504582
33313	2.58823	0.679048
33314	2.45322	0.555043
33315	2.02727	0.688292
33316	1.89226	0.581762
33317	1.89226	0.425204
33318	1.89226	0.359504
33319	1.89226	0.646337
33320	1.89226	0.364496
33321	3.78452	0.356858
33322	1.89226	0.527343
33323	1.89226	0.366924
33324	1.89226	0.358406
33325	1.89226	0.216232
33326	1.89226	0.702906
33327	1.89226	0.51443
33328	1.70048	2.1
33329	2.1572	0.829092
33330	1.45477	1.19265
33331	1.93973	0.873484
33332	3.64021	1.0217
33333	1.17614	1.19554
33334	1.93973	0.879641
33335	1.69661	1.82471
33336	1.69661	0.880592
33337	1.45477	1.8207
33338	1.45477	0.888538
33339	1.94343	1.81542
33340	2.53782	1.34056
33341	2.59903	1.2292
33342	2.59903	0.962054
33343	1.87598	1.26142
33344	1.87598	1.06457
33345	0.946131	0.890761
33346	0.946131	1.1653
33347	1.26891	1.8583

33348	1.26891	0.848763
33349	0.94613	1.29897
33350	0.94613	1.09064
33351	1.26891	1.28277
33352	1.29952	0.971239
33353	1.29952	0.884864
33354	0.946131	0.850133
33355	1.26891	1.08286
33356	0.946131	1.0934
33357	2.00551	1.82682
33358	2.59903	1.63569
33359	2.65811	1.43269
33360	2.65811	1.06041
33361	1.94343	1.19891
33362	1.94343	1.13625
33363	2.65811	0.921152
33364	1.89226	1.6012
33365	1.89226	0.926378
33366	1.89226	1.22352
33367	2.59903	1.05519
33368	2.59903	1.80477
33369	1.89227	2.1
33370	1.89227	2.1
33371	1.89227	2.1
33372	1.89227	2.1
33373	3.78453	2.1
33374	1.15471	2.1
33375	0.993889	2.1
33376	1.78919	2.1
33377	1.77325	2.1
33378	2.78308	2.1
33379	1.89226	2.1
33380	1.89226	2.1
33381	1.89226	2.1
33382	1.89226	2.1
33383	3.78453	2.1
33384	1.78919	2.1
33385	1.77325	2.1
33386	2.45969	2.1
33387	2.70835	2.1
33388	4.49753	2.1
33389	0.939314	2.1
33390	0.939315	2.1
33391	1.01133	2.1
33392	1.01133	2.1
33393	0.596471	2.1
33394	1.20813	2.1
33395	0.886619	2.1
33396	1.35417	2.1
33397	1.35417	2.1
33398	1.20813	2.1
33399	0.886621	2.1
33400	0.59647	2.1
33401	0.946131	2.1
33402	0.946131	2.1
33403	0.94613	2.1
33404	0.94613	2.1
33405	0.946133	2.1

33406	0.946133	2.1
33407	0.946128	2.1
33408	0.946128	2.1
33409	0.946131	2.1
33410	0.946131	2.1
33411	0.946131	2.1
33412	0.946131	2.1
33413	1.03939	2.1
33414	1.03939	2.1
33415	0.946132	1.1097
33416	0.946132	1.20504
33417	0.946132	1.29578
33418	0.946132	2.1
33419	1.23851	1.13442
33420	1.03939	2.1
33421	1.03939	1.91044
33422	0.946132	1.92663
33423	0.946133	1.80448
33424	1.23851	1.83316
33425	0.902503	2.1
33426	0.946129	2.1
33427	0.946132	1.14131
33428	0.946132	1.72553
33429	0.902472	1.61114
33430	0.902472	2.1
33431	1.08736	1.16593
33432	0.994093	2.1
33433	0.946133	1.74744
33434	0.902504	1.87157
33435	1.03939	1.86467
33436	0.946132	1.84194
33437	1.35306	0.855603
33438	1.29982	0.783472
33439	1.7216	0.759971
33440	1.89183	0.766118
33441	3.07466	0.769428
33442	0.485098	0.776828
33443	0.582401	0.792546
33444	0.649908	0.813269
33445	0.8608	0.779716
33446	0.587739	0.744682
33447	0.58774	0.812479
33448	0.644567	0.77878
33449	0.644567	0.989264
33450	0.58241	0.803489
33451	0.86081	0.744588
33452	0.649898	0.869952
33453	0.485093	0.785952
33454	2.25936	2.1
33455	2.71441	2.1
33456	2.63824	2.1
33457	2.20652	2.1
33458	4.92092	2.1
33459	2.25936	2.1
33460	1.57627	2.1
33461	1.5493	2.1
33462	2.20652	2.1
33463	3.78279	2.1

33464	2.63824	2.1
33465	2.20652	2.1
33466	2.17326	2.1
33467	2.58517	2.1
33468	4.79169	2.1
33469	2.17326	2.1
33470	2.20652	2.1
33471	2.19278	2.1
33472	1.53556	2.1
33473	1.5493	2.1
33474	1.5493	2.1
33475	1.97465	2.1
33476	2.13925	2.1
33477	2.04483	2.1
33478	1.62784	2.1
33479	3.76709	2.1
33480	1.89226	2.1
33481	1.89226	2.1
33482	1.89226	2.1
33483	1.89226	2.1
33484	1.89226	2.1
33485	1.89226	2.1
33486	0.724037	2.1
33487	0.81392	2.1
33488	0.96796	2.1
33489	1.02242	2.1
33490	0.882091	2.1
33491	0.882091	2.1
33492	0.899789	2.1
33493	0.899789	2.1
33494	0.724036	2.1
33495	0.96796	2.1
33496	1.02242	2.1
33497	0.81392	2.1
33498	1.13537	2.1
33499	1.63054	2.1
33500	0.946131	2.1
33501	0.946131	2.1
33502	0.946131	2.1
33503	0.946131	2.1
33504	1.258	2.1
33505	1.63054	2.1
33506	1.22632	2.1
33507	1.22632	2.1
33508	0.946131	2.1
33509	1.258	2.1
33510	1.89226	2.1
33511	1.89226	2.1
33512	1.89226	2.1
33513	1.89226	2.1
33514	1.89226	2.1
33515	1.89226	2.1
33516	0.809739	2.1
33517	0.862674	2.1
33518	1.1995	2.1
33519	1.33899	2.1
33520	1.06324	2.1
33521	1.06324	2.1

33522	0.981932	2.1
33523	1.19563	2.1
33524	0.809727	2.1
33525	1.14092	2.1
33526	1.33902	2.1
33527	0.863494	2.1
33528	2.23742	2.1
33529	1.84902	2.1
33530	1.88088	2.1
33531	2.20392	2.1
33532	4.08644	2.1
33533	1.3592	2.1
33534	1.84902	2.1
33535	1.88088	2.1
33536	1.3305	2.1
33537	1.88088	2.1
33538	1.3305	2.1
33539	1.84902	2.1
33540	1.15008	0.810884
33541	1.3305	1.95793
33542	1.70539	2.1
33543	2.9991	2.1
33544	1.84901	2.1
33545	2.0264	2.1
33546	2.20391	2.1
33547	1.70539	2.1
33548	3.87541	2.1
33549	1.03852	1.99459
33550	1.38247	0.948165
33551	1.36635	1.0395
33552	1.01537	1.38274
33553	2.39784	1.17434
33554	2.0815	1.45931
33555	1.91744	1.50705
33556	1.91744	1.15704
33557	2.06496	2.1
33558	1.89227	1.14907
33559	2.03474	2.1
33560	1.89226	0.957875
33561	1.89226	1.16748
33562	1.89226	0.952415
33563	1.89226	1.53702
33564	1.89226	0.950806
33565	1.89226	1.41263
33566	1.36635	1.10895
33567	1.02923	1.83906
33568	1.02923	1.09687
33569	1.01537	1.84294
33570	1.36416	1.0467
33571	1.3503	2.04252
33572	1.89226	1.53061
33573	1.89359	2.1
33574	1.89359	1.30804
33575	1.91744	2.1
33576	1.89226	1.27829
33577	1.91611	2.1
33578	0.946128	1.76506
33579	0.946128	1.30108

33580	0.946133	1.05128
33581	0.946133	0.962325
33582	0.94613	0.971047
33583	0.94613	1.25608
33584	0.946131	1.88772
33585	0.946131	1.01211
33586	0.946131	1.14532
33587	0.946131	1.14636
33588	0.946131	1.15324
33589	0.946131	1.15343
33590	2.689	2.1
33591	2.0815	2.1
33592	2.72786	2.1
33593	2.48295	2.1
33594	5.02397	2.1
33595	2.72786	2.1
33596	1.86424	2.1
33597	2.7188	2.1
33598	2.08981	2.1
33599	4.81768	2.1
33600	2.08982	2.1
33601	0.986987	2.1
33602	2.08982	2.1
33603	1.87249	2.1
33604	1.01964	2.1
33605	0.906115	2.1
33606	1.44247	2.1
33607	1.43872	2.1
33608	1.17054	2.1
33609	1.17054	2.1
33610	1.43872	2.1
33611	1.26045	2.1
33612	2.0815	0.128789
33613	2.0815	0.14404
33614	2.0815	0.147613
33615	1.81468	0.130354
33616	1.89065	0.139435
33617	1.55625	0.131232
33618	1.89062	0.123761
33619	1.89224	0.151712
33620	1.89224	0.146795
33621	2.27073	0.133916
33622	1.86856	0.141257
33623	2.24232	0.128688
33624	1.21419	2.1
33625	2.79716	2.1
33626	2.50005	2.1
33627	2.50005	2.1
33628	1.5008	2.1
33629	1.5008	2.1
33630	1.60139	0.0915261
33631	1.7103	0.101232
33632	1.04159	0.117733
33633	1.2489	0.0843252
33634	2.75189	0.0893259
33635	0.732812	0.0840933
33636	0.732812	0.0893167
33637	0.751986	0.114399

33638	0.751986	0.104095
33639	0.860346	0.0939248
33640	0.667961	0.114139
33641	0.85515	0.120135
33642	0.624452	0.110045
33643	0.62445	0.104361
33644	0.667962	0.0929169
33645	0.855148	0.100107
33646	0.860348	0.0947319
33647	1.89226	0.728824
33648	2.50872	0.733292
33649	2.77909	0.725975
33650	1.89226	0.738005
33651	2.77909	0.724346
33652	1.89226	0.737309
33653	2.15825	0.722292
33654	2.50872	0.717721
33655	1.90439	0.665542
33656	1.90439	0.731339
33657	2.77909	0.698788
33658	2.77909	0.733463
33659	1.12468	2.1
33660	1.43872	2.1
33661	1.64926	2.1
33662	1.2724	2.1
33663	2.77395	2.1
33664	1.64926	2.1
33665	1.38438	2.1
33666	1.47244	2.1
33667	1.2724	2.1
33668	1.8493	2.1
33669	1.64926	2.1
33670	1.36416	1.10967
33671	1.0652	1.976
33672	1.0652	1.14346
33673	1.02923	1.98198
33674	1.37496	1.09363
33675	1.33898	2.1
33676	1.89227	1.72394
33677	1.90433	2.1
33678	1.90433	1.64038
33679	1.89359	2.1
33680	1.89227	1.54164
33681	1.88153	2.1
33682	0.946129	1.93461
33683	0.946129	1.60244
33684	0.946133	1.10713
33685	0.946133	1.02402
33686	0.946131	1.03405
33687	0.946131	1.51039
33688	0.946131	2.00514
33689	0.946131	1.05333
33690	0.946131	1.2207
33691	0.946131	1.22889
33692	0.946131	1.22788
33693	0.946131	1.23176
33694	1.37496	1.13492
33695	1.07519	2.06628

33696	1.07519	1.17431
33697	1.0652	2.06705
33698	1.39725	1.12574
33699	1.38726	2.1
33700	1.89227	1.8931
33701	1.9052	2.1
33702	1.9052	1.81631
33703	1.90433	2.1
33704	1.89227	1.67406
33705	1.8914	2.1
33706	0.946129	2.03616
33707	0.946129	1.7224
33708	0.946133	1.15282
33709	0.946133	1.04218
33710	0.946131	1.05338
33711	0.946131	1.66844
33712	0.946131	2.07949
33713	0.946131	1.08805
33714	0.946131	1.26732
33715	0.946131	1.2905
33716	0.946131	1.27679
33717	0.946131	1.28945
33718	1.91316	0.628903
33719	1.89226	0.700997
33720	1.89226	0.696502
33721	1.89226	0.700355
33722	1.99366	0.61497
33723	1.99366	0.603754
33724	1.93083	0.464145
33725	1.89226	0.690877
33726	1.51379	0.615075
33727	1.89226	0.672348
33728	1.53051	0.518453
33729	1.91316	0.593278
33730	2.16731	2.1
33731	1.21373	2.1
33732	1.47804	2.1
33733	2.37601	2.1
33734	3.64535	2.1
33735	1.7211	2.1
33736	2.37601	2.1
33737	1.47804	2.1
33738	1.47804	2.1
33739	2.56843	2.1
33740	2.56843	2.1
33741	2.58731	2.1
33742	1.59094	2.1
33743	1.59094	2.1
33744	1.76816	2.1
33745	2.41707	2.1
33746	2.5943	2.1
33747	0.718346	2.1
33748	0.795468	2.1
33749	1.20854	2.1
33750	1.1224	2.1
33751	0.961203	2.1
33752	0.961203	2.1
33753	0.95667	2.1

33754	0.95667	2.1
33755	0.718346	2.1
33756	1.1224	2.1
33757	0.795469	2.1
33758	1.20854	2.1
33759	1.84076	2.1
33760	1.42884	2.1
33761	2.24274	2.1
33762	1.85415	2.1
33763	3.69491	2.1
33764	1.89226	2.1
33765	1.89226	1.59532
33766	1.89226	1.96468
33767	1.89226	2.1
33768	3.78452	2.1
33769	2.24273	2.1
33770	2.24412	2.1
33771	1.85415	2.1
33772	1.85415	2.1
33773	2.68473	2.1
33774	2.68473	2.1
33775	1.89226	1.52514
33776	1.89226	2.1
33777	1.89226	2.1
33778	1.89226	1.34407
33779	1.89226	2.1
33780	1.89226	1.33378
33781	1.89226	1.33095
33782	1.89226	2.1
33783	1.89226	2.1
33784	1.89226	1.52665
33785	3.78451	2.1
33786	1.85415	2.1
33787	1.10961	2.1
33788	2.24411	2.1
33789	1.51067	2.1
33790	3.35373	2.1
33791	1.70302	2.1
33792	1.89226	2.1
33793	1.76399	2.1
33794	1.43577	2.1
33795	2.08149	2.1
33796	1.70302	2.1
33797	1.89226	2.1
33798	1.89226	2.1
33799	1.89226	2.1
33800	1.89226	2.1
33801	1.89226	2.07492
33802	1.89226	2.1
33803	1.0593	2.1
33804	1.17289	2.1
33805	1.5379	2.1
33806	1.46757	2.1
33807	2.71079	2.1
33808	0.931235	2.1
33809	1.1151	2.1
33810	0.975236	2.1
33811	0.975236	2.1

33812	1.31147	2.1
33813	0.762882	2.1
33814	1.13152	2.1
33815	0.505799	2.1
33816	1.31149	2.1
33817	1.07629	2.1
33818	0.884817	2.1
33819	0.505793	2.1
33820	2.69027	2.1
33821	1.60441	2.1
33822	2.69027	2.1
33823	1.64771	2.1
33824	2.78977	2.1
33825	1.72731	2.1
33826	0.77878	0.05
33827	0.843226	0.05
33828	0.845997	0.05
33829	0.926091	0.05
33830	0.77878	0.05
33831	0.926093	0.05
33832	1.3922	1.56468
33833	1.3922	1.64177
33834	0.872156	2.05481
33835	0.872156	1.98073
33836	0.872157	2.1
33837	0.946132	1.57134
33838	0.946129	1.29911
33839	1.31822	2.1
33840	0.94613	1.88759
33841	0.94613	1.96146
33842	1.31823	1.82056
33843	0.872157	1.97042
33844	1.81534	0.807273
33845	1.89226	0.821549
33846	1.59602	0.886583
33847	1.59602	0.938202
33848	1.89226	0.742065
33849	1.89226	0.767966
33850	1.89227	0.789062
33851	1.89227	0.809326
33852	1.89227	0.827671
33853	1.89227	0.778974
33854	3.78453	0.777741
33855	1.89226	0.809103
33856	1.89226	0.758122
33857	1.89226	0.779771
33858	1.89226	0.790368
33859	3.78453	0.813947
33860	2.30348	0.647052
33861	1.4185	0.690628
33862	1.82551	0.758807
33863	1.02011	0.607053
33864	3.24401	0.62777
33865	2.46702	0.632961
33866	1.17129	0.712273
33867	1.82551	0.607224
33868	1.82551	0.654707
33869	1.73525	0.65609

33870	1.73525	0.733013
33871	1.89227	0.78821
33872	1.89227	0.751337
33873	1.89227	0.751749
33874	1.89227	0.761426
33875	1.89227	0.776593
33876	1.89227	0.786512
33877	2.80034	2.1
33878	2.45767	2.1
33879	2.67994	2.06271
33880	2.35395	2.1
33881	5.1376	2.1
33882	2.45766	2.1
33883	1.8769	2.1
33884	2.00989	2.1
33885	2.00989	2.1
33886	2.35395	2.1
33887	2.35395	2.1
33888	2.57662	2.1
33889	2.35395	2.1
33890	2.57662	2.1
33891	2.67993	2.1
33892	2.24227	2.1
33893	2.34558	2.1
33894	1.02874	2.1
33895	1.02874	2.1
33896	1.03084	2.1
33897	1.03084	2.1
33898	0.938448	2.1
33899	0.886762	2.1
33900	1.17698	2.1
33901	1.12114	2.1
33902	1.12113	2.1
33903	0.886764	2.1
33904	0.93845	2.1
33905	1.17697	2.1
33906	1.91969	2.1
33907	2.5508	2.1
33908	1.88273	2.04667
33909	2.76609	2.1
33910	4.68578	2.1
33911	2.83423	2.1
33912	1.89226	2.1
33913	2.76609	2.1
33914	1.89226	2.1
33915	4.65835	2.1
33916	1.56685	0.785954
33917	1.11712	0.765185
33918	1.07999	0.93517
33919	1.51448	0.83797
33920	2.6316	0.826609
33921	1.56685	0.736377
33922	1.67652	0.874123
33923	1.5955	1.00488
33924	1.51448	0.800096
33925	3.19099	0.847495
33926	1.89226	0.88564
33927	1.89226	0.73458

33928	1.89226	0.817178
33929	1.89226	0.889698
33930	3.78453	0.836257
33931	1.89226	0.85674
33932	1.89226	0.744308
33933	1.89226	0.89246
33934	1.89226	0.908141
33935	1.89226	0.741859
33936	1.89226	0.752772
33937	1.0562	0.861367
33938	1.51448	0.770392
33939	1.07999	0.821006
33940	1.07999	0.781518
33941	1.46441	0.845062
33942	1.46441	0.785716
33943	1.5955	0.966657
33944	1.51448	0.754033
33945	1.46441	0.845883
33946	1.52909	0.9454
33947	3.04357	0.858705
33948	1.89226	0.768198
33949	1.89226	0.873131
33950	1.89226	0.956275
33951	1.89226	0.778525
33952	3.78453	0.812281
33953	1.89227	0.785981
33954	1.89227	0.723606
33955	1.89227	0.862125
33956	1.89227	0.806679
33957	3.78453	0.796828
33958	1.89994	0.868652
33959	1.70303	0.994279
33960	1.68576	0.56167
33961	1.89608	0.691772
33962	3.8237	0.785843
33963	1.89994	0.87772
33964	2.31116	1.137
33965	1.9386	1.06687
33966	1.88653	1.33793
33967	3.74885	1.09692
33968	2.31117	1.15927
33969	2.20665	1.52858
33970	2.09617	1.29318
33971	2.41055	2.1
33972	2.09617	1.44293
33973	2.41055	2.1
33974	1.10777	0.707162
33975	1.0523	0.879152
33976	0.912098	0.854201
33977	0.757533	0.940463
33978	0.89287	0.986408
33979	1.07571	0.446981
33980	0.77485	0.695095
33981	0.98645	0.440924
33982	0.989171	0.520264
33983	0.875089	0.593995
33984	0.979686	0.697119
33985	0.979686	0.911611

33986	2.52442	2.1
33987	1.49998	2.1
33988	2.51692	2.1
33989	1.56957	2.1
33990	4.09399	2.1
33991	1.01298	2.1
33992	1.01298	2.1
33993	1.03079	2.1
33994	1.03079	2.1
33995	0.820964	2.1
33996	0.784786	2.1
33997	1.25898	2.1
33998	1.25846	2.1
33999	1.25898	2.1
34000	0.820964	2.1
34001	1.25846	2.1
34002	0.784786	2.1
34003	1.89226	0.142318
34004	1.89226	0.12149
34005	2.6297	0.116418
34006	1.89226	0.125702
34007	3.78452	0.128389
34008	1.89226	0.110426
34009	1.89226	0.0977952
34010	1.89226	0.0880719
34011	1.89226	0.120454
34012	1.89226	0.0957648
34013	1.89226	0.136571
34014	2.19397	0.0923699
34015	1.89226	0.0960685
34016	2.34112	0.0836699
34017	2.34112	0.0804961
34018	1.89226	0.103646
34019	1.89226	0.0947565
34020	2.23366	2.1
34021	1.87598	2.1
34022	1.64716	2.1
34023	2.06048	2.1
34024	3.93645	2.1
34025	2.23367	2.1
34026	2.17384	2.1
34027	2.04818	2.1
34028	2.06048	2.1
34029	4.23432	2.1
34030	1.9855	2.1
34031	2.06048	2.1
34032	1.93163	2.1
34033	1.50737	2.1
34034	3.56785	2.1
34035	2.04818	2.1
34036	2.06048	2.1
34037	2.27465	2.1
34038	1.92264	2.1
34039	3.98312	2.1
34040	2.08149	0.966951
34041	1.89226	1.69596
34042	1.89226	1.35247
34043	1.33153	0.9859

34044	1.68038	1.81476
34045	1.18244	1.10951
34046	1.89226	1.09203
34047	1.89226	1.6837
34048	1.89226	1.49937
34049	1.89226	1.0101
34050	3.78452	1.14536
34051	2.69069	0.927381
34052	1.89226	1.07414
34053	1.89226	1.06729
34054	1.97397	0.958022
34055	1.89226	1.03196
34056	1.97397	0.923461
34057	1.89226	1.68064
34058	2.38056	1.06669
34059	1.89226	1.0103
34060	1.89226	1.3358
34061	3.78452	1.23678
34062	0.904435	2.1
34063	0.904435	2.1
34064	0.925315	2.1
34065	0.925315	2.1
34066	1.13312	2.1
34067	1.08109	2.1
34068	0.70697	2.1
34069	0.696633	2.1
34070	0.70697	2.1
34071	1.13312	2.1
34072	0.696633	2.1
34073	1.08109	2.1
34074	1.45506	2.1
34075	2.26623	2.1
34076	2.38363	2.1
34077	2.38363	2.1
34078	1.41394	2.1
34079	1.41394	2.1
34080	0.946131	2.07591
34081	0.946131	2.1
34082	1.13311	2.1
34083	1.13311	2.1
34084	0.946127	1.76106
34085	0.946127	2.1
34086	1.13312	2.1
34087	1.19182	2.1
34088	1.19181	2.1
34089	0.946129	2.1
34090	1.13312	2.1
34091	0.94613	2.1
34092	1.89226	0.921413
34093	1.89226	1.37207
34094	1.89226	0.929613
34095	1.89226	1.29544
34096	1.89226	0.963045
34097	1.89226	1.47183
34098	1.05168	2.1
34099	0.696527	2.08837
34100	0.907442	1.10911
34101	0.907442	1.20008

34102	0.953145	1.07838
34103	0.782285	2.1
34104	0.773928	2.1
34105	0.773928	1.14421
34106	0.696538	1.21702
34107	0.851554	1.22868
34108	1.04071	1.31555
34109	1.12335	1.32819
34110	1.89226	0.859831
34111	1.89226	0.766386
34112	1.89226	0.744235
34113	1.89226	0.726176
34114	3.78453	0.732295
34115	1.89226	0.800723
34116	1.89226	0.759706
34117	1.89226	0.740731
34118	1.89226	0.728757
34119	3.78453	0.781547
34120	2.50912	0.743302
34121	1.89226	0.732855
34122	1.89226	0.722897
34123	2.63195	0.721055
34124	4.40139	0.719511
34125	2.50912	0.74829
34126	1.3598	0.732796
34127	1.70303	0.71622
34128	2.36874	0.69426
34129	3.82055	0.73622
34130	1.89227	0.737419
34131	1.89227	0.722156
34132	1.89227	0.709848
34133	1.89227	0.703643
34134	3.78453	0.73184
34135	1.89227	0.735126
34136	1.89227	0.72082
34137	1.89227	0.706733
34138	1.89227	0.70379
34139	3.78453	0.705893
34140	1.89226	0.71543
34141	2.67088	0.6237
34142	2.63195	0.700611
34143	2.63195	0.711643
34144	1.89226	0.701821
34145	1.89226	0.718945
34146	1.30202	0.707054
34147	1.28304	0.70824
34148	0.809902	0.70507
34149	0.888801	0.705599
34150	1.13262	0.484291
34151	0.968276	0.590297
34152	1.0733	0.706603
34153	1.0733	0.702305
34154	1.35419	0.679433
34155	0.801222	0.530019
34156	0.899732	0.710467
34157	1.23967	0.682277
34158	1.31595	2.1
34159	0.869501	2.1

34160	2.65473	2.1
34161	1.88782	2.1
34162	3.20377	2.1
34163	1.89227	2.1
34164	1.89227	2.1
34165	1.89227	2.1
34166	1.89227	2.1
34167	3.78453	2.1
34168	2.65473	2.1
34169	1.89226	2.1
34170	1.88781	2.1
34171	1.88782	2.1
34172	2.0815	2.1
34173	2.0815	2.1
34174	1.89226	1.63874
34175	1.89226	2.1
34176	1.89226	2.07201
34177	1.89226	1.93541
34178	1.89226	1.80052
34179	1.89226	1.569
34180	1.89226	1.75144
34181	1.89226	2.0508
34182	1.89226	2.1
34183	1.89226	2.08465
34184	1.89226	2.00031
34185	1.89226	1.78969
34186	2.39057	1.97967
34187	1.89226	1.10437
34188	1.89226	0.981996
34189	1.89226	2.1
34190	2.14243	0.962774
34191	2.14243	2.1
34192	1.89226	0.772464
34193	1.89226	1.31931
34194	1.89226	0.889834
34195	1.89226	0.959832
34196	1.89226	0.933815
34197	1.89226	1.17231
34198	1.89226	0.823772
34199	1.89226	1.65433
34200	1.89226	0.91082
34201	1.89226	0.997855
34202	1.89226	0.932413
34203	1.89226	1.27715
34204	2.56323	0.302132
34205	1.36324	0.356146
34206	1.05296	0.398114
34207	2.18048	0.383528
34208	3.54372	0.340035
34209	0.946128	2.1
34210	0.946128	2.1
34211	1.08733	2.1
34212	1.08733	2.1
34213	0.946131	2.1
34214	0.946131	2.1
34215	1.09197	2.1
34216	1.08732	2.1
34217	0.946131	2.1

34218	1.09197	2.1
34219	1.08732	2.1
34220	0.946131	2.1
34221	1.35111	1.23805
34222	1.51327	1.25068
34223	1.64493	1.22289
34224	1.31058	1.2853
34225	2.99605	1.42263
34226	1.8124	1.21573
34227	1.51327	1.33909
34228	1.64493	1.35855
34229	1.31358	1.23948
34230	1.8124	1.31799
34231	1.48105	1.19133
34232	1.64493	1.20374
34233	1.31058	1.27155
34234	1.22297	1.19687
34235	1.70101	1.20906
34236	2.8679	1.12172
34237	1.70101	1.26003
34238	1.99297	1.17229
34239	1.64493	1.28737
34240	1.64493	1.15685
34241	1.99297	1.27042
34242	1.8124	1.1113
34243	2.0815	2.1
34244	1.80258	2.1
34245	1.80258	2.1
34246	1.83506	2.1
34247	2.0815	2.1
34248	2.1212	2.1
34249	2.0815	2.1
34250	1.82129	2.1
34251	1.82129	2.1
34252	1.80445	2.1
34253	2.0815	2.1
34254	2.06092	2.1
34255	1.89224	2.1
34256	1.703 2.1	
34257	1.97839	2.1
34258	1.703 2.1	
34259	1.97402	2.1
34260	1.703 2.1	
34261	1.89226	0.775077
34262	1.89226	0.854257
34263	1.89226	0.819198
34264	2.75351	0.813307
34265	3.78453	0.822828
34266	1.89226	0.819097
34267	3.02887	0.96632
34268	2.03149	0.797935
34269	2.03149	0.846971
34270	1.89226	0.810712
34271	1.89226	0.864161
34272	2.17979	2.1
34273	1.65533	2.1
34274	2.57212	2.1
34275	2.20346	2.1

34276	4.22745	2.1
34277	1.89227	2.1
34278	2.20347	2.1
34279	1.89227	2.1
34280	2.57212	2.1
34281	1.89227	2.1
34282	2.57212	2.1
34283	2.05274	0.953074
34284	2.16783	2.1
34285	2.24429	0.905564
34286	2.38404	2.1
34287	4.41212	1.26644
34288	2.16783	2.1
34289	2.05967	2.1
34290	2.38404	2.1
34291	2.24695	2.1
34292	4.41478	2.1
34293	2.24429	0.929823
34294	2.38404	2.1
34295	2.51819	0.932779
34296	2.60723	2.1
34297	4.85152	1.49113
34298	2.38404	2.1
34299	2.24695	2.1
34300	2.60723	2.1
34301	2.45247	2.1
34302	4.85418	2.1
34303	1.89227	2.1
34304	1.89227	2.1
34305	1.89227	2.1
34306	1.89227	2.1
34307	3.78453	2.1
34308	1.89226	2.1
34309	1.89226	2.1
34310	1.89226	2.1
34311	1.89226	2.1
34312	3.78453	2.1
34313	0.618473	2.1
34314	0.619945	2.1
34315	0.797692	2.1
34316	0.770147	2.1
34317	0.68852	2.1
34318	0.68852	2.1
34319	0.701573	2.1
34320	0.701573	2.1
34321	0.619945	2.1
34322	0.797691	2.1
34323	0.618473	2.1
34324	0.770147	2.1
34325	0.797692	2.1
34326	0.770148	2.1
34327	0.694787	2.1
34328	0.653411	2.1
34329	0.722102	2.1
34330	0.722102	2.1
34331	0.729001	2.1
34332	0.729001	2.1
34333	0.797692	2.1

34334	0.694787	2.1
34335	0.770148	2.1
34336	0.653412	2.1
34337	1.89227	2.1
34338	1.89227	2.1
34339	1.89227	2.1
34340	1.89227	2.1
34341	3.78453	2.1
34342	1.89226	2.1
34343	1.89226	2.1
34344	1.89226	2.1
34345	1.89226	2.1
34346	3.78453	2.1
34347	1.23989	2.1
34348	1.59538	2.1
34349	1.66647	2.1
34350	1.26599	2.1
34351	2.90636	2.1
34352	1.66647	2.1
34353	1.38957	2.1
34354	1.47446	2.1
34355	1.40337	2.1
34356	1.66647	2.1
34357	1.59538	2.1
34358	2.80619	2.1
34359	2.0815	2.1
34360	2.0815	2.1
34361	2.0815	2.1
34362	2.73721	2.1
34363	2.74192	2.1
34364	2.73721	2.1
34365	2.0815	2.1
34366	2.0815	2.1
34367	2.0815	2.1
34368	2.689 2.1	
34369	2.69166	2.1
34370	2.62369	2.1
34371	2.19717	2.1
34372	2.60405	2.1
34373	2.20566	2.1
34374	4.80122	2.1
34375	2.19717	2.1
34376	1.37392	2.1
34377	2.20566	2.1
34378	1.45408	2.1
34379	3.65125	2.1
34380	2.60405	2.1
34381	2.20566	2.1
34382	2.57466	2.1
34383	2.21715	2.1
34384	4.8212	2.1
34385	2.20566	2.1
34386	1.54249	2.1
34387	1.54249	2.1
34388	1.45408	2.1
34389	2.21715	2.1
34390	2.12874	2.1
34391	1.60647	2.02955

34392	1.31346	2.1
34393	1.82649	1.70016
34394	1.51067	2.1
34395	3.13995	2.1
34396	1.89226	1.24669
34397	1.89226	2.1
34398	1.89226	1.72466
34399	1.89226	2.1
34400	3.78452	2.1
34401	1.89226	2.1
34402	2.6096	2.1
34403	2.8067	2.1
34404	2.8067	2.1
34405	1.89226	2.1
34406	1.89226	2.1
34407	1.82649	1.30344
34408	1.3596	2.1
34409	2.17708	1.74576
34410	1.70302	2.1
34411	3.40243	2.1
34412	1.89226	1.3134
34413	1.89226	2.1
34414	1.89226	1.10581
34415	1.89226	2.1
34416	3.78452	2.1
34417	2.27067	2.1
34418	2.43549	2.1
34419	2.63979	2.1
34420	2.41828	2.1
34421	2.27067	2.1
34422	2.04916	2.1
34423	1.30047	2.1
34424	2.04916	2.1
34425	2.27068	2.1
34426	1.5586	2.1
34427	3.57115	2.1
34428	2.27068	2.1
34429	1.84214	2.1
34430	1.80224	2.1
34431	1.5586	2.1
34432	2.51432	2.1
34433	2.27068	2.1
34434	1.63719	2.1
34435	2.15714	2.1
34436	2.07821	2.1
34437	1.52204	2.1
34438	3.67919	2.1
34439	2.07821	2.1
34440	1.40118	2.1
34441	1.40118	2.1
34442	1.52204	2.1
34443	1.99632	2.1
34444	2.11718	2.1
34445	0.946131	1.87996
34446	0.946131	1.73184
34447	1.06957	2.1
34448	1.06957	2.1
34449	0.946132	2.1

34450	0.946132	1.29705
34451	1.411	1.9429
34452	1.06957	2.1
34453	0.946132	1.96033
34454	1.411	2.06271
34455	1.06957	2.1
34456	0.946132	2.07871
34457	1.99875	0.866945
34458	2.57079	0.847982
34459	2.59319	1.01205
34460	1.9105	0.792834
34461	4.59194	0.872711
34462	1.99875	0.85084
34463	1.02247	0.748383
34464	1.07017	0.769233
34465	1.15841	0.7694
34466	1.91051	0.764901
34467	1.99875	0.770889
34468	2.59319	0.967065
34469	1.9105	0.805991
34470	2.33812	0.861721
34471	2.59319	0.853801
34472	1.59457	0.884868
34473	1.59457	0.881542
34474	2.50906	0.757412
34475	2.0467	0.723448
34476	2.03357	0.73437
34477	2.53176	0.727092
34478	4.54263	0.728939
34479	1.294	0.783514
34480	1.66636	0.747967
34481	2.03357	0.734953
34482	1.03079	0.728904
34483	3.32757	0.740439
34484	1.68108	0.726199
34485	2.53176	0.727849
34486	2.36417	0.713325
34487	2.71666	0.714597
34488	1.68108	0.764205
34489	2.03357	0.754766
34490	1.89226	0.707328
34491	2.59302	0.710635
34492	2.2727	0.710449
34493	2.2727	0.706078
34494	1.89226	0.716206
34495	1.89226	0.707359
34496	1.89226	0.72656
34497	1.89226	0.722509
34498	1.89226	0.715651
34499	1.89226	0.70076
34500	3.78452	0.728989
34501	1.89225	0.736883
34502	1.89225	0.627273
34503	1.89225	0.701282
34504	1.89225	0.705865
34505	1.89225	0.700718
34506	1.89225	0.704505
34507	0.934114	0.712219

34508	0.934114	0.683062
34509	0.944544	0.704275
34510	0.944544	0.703725
34511	0.721493	0.677686
34512	0.58215	0.680649
34513	1.2965	0.704635
34514	1.13634	0.700377
34515	1.13635	0.696151
34516	0.582148	0.676872
34517	1.29651	0.704218
34518	0.721491	0.701768
34519	1.89226	0.700848
34520	1.89226	0.661604
34521	1.89226	0.681624
34522	1.89226	0.531235
34523	3.78452	0.553223
34524	0.946131	0.779481
34525	0.946131	0.786228
34526	0.946131	0.763634
34527	0.946131	0.778359
34528	0.946131	0.763169
34529	0.946131	0.770632
34530	0.946131	0.783279
34531	0.946131	0.794659
34532	0.946131	0.777997
34533	0.946131	0.776482
34534	0.946131	0.775542
34535	0.946131	0.774148
34536	1.89226	0.250594
34537	1.89226	0.231641
34538	2.78602	0.222991
34539	1.89226	0.351926
34540	3.78453	0.231746
34541	1.52601	2.1
34542	1.76601	2.1
34543	1.27839	2.1
34544	1.56095	2.1
34545	3.0444	2.1
34546	1.76601	2.1
34547	1.86793	2.1
34548	1.56095	2.1
34549	1.68152	2.1
34550	3.44753	2.1
34551	1.27841	2.1
34552	1.56097	2.1
34553	1.0577	2.1
34554	1.36861	2.1
34555	2.64702	2.1
34556	1.56097	2.1
34557	1.68154	2.1
34558	1.36861	2.1
34559	1.53296	2.1
34560	3.05015	2.1
34561	1.89226	2.1
34562	1.89226	2.1
34563	1.89226	2.1
34564	1.89226	2.1
34565	3.78453	2.1

34566	0.983075	2.1
34567	0.788361	2.1
34568	0.724726	2.1
34569	0.724726	2.1
34570	0.983027	2.1
34571	0.724734	2.1
34572	0.788364	2.1
34573	0.788364	2.1
34574	0.788335	2.1
34575	0.94613	2.1
34576	0.724757	2.1
34577	0.946131	2.1
34578	1.15789	2.1
34579	2.11135	2.1
34580	2.25826	2.1
34581	1.28845	2.1
34582	3.41615	2.1
34583	1.89226	2.1
34584	1.89226	2.1
34585	1.89226	2.1
34586	1.89226	2.1
34587	3.78453	2.1
34588	1.57667	2.1
34589	1.70002	2.1
34590	1.70002	2.1
34591	1.89226	2.1
34592	1.89226	2.1
34593	2.05244	2.1
34594	2.25826	2.1
34595	1.42214	2.1
34596	1.43675	2.1
34597	1.28845	2.1
34598	2.40656	2.1
34599	2.25826	2.1
34600	1.58066	2.1
34601	1.74247	2.1
34602	1.85097	2.1
34603	1.61155	2.1
34604	3.43163	2.1
34605	0.785612	2.1
34606	0.789638	2.1
34607	0.882561	2.1
34608	0.763529	2.1
34609	0.686921	2.1
34610	0.86048	2.1
34611	0.871812	2.1
34612	0.871812	2.1
34613	0.830183	2.1
34614	0.882572	2.1
34615	0.902315	2.1
34616	0.785602	2.1
34617	1.01972	0.0971805
34618	0.936657	0.0851987
34619	0.946131	0.0976688
34620	0.946131	0.109463
34621	0.927018	0.10434
34622	0.927018	0.0921158
34623	1.1376	0.104239

34624	0.953663	0.0854599
34625	1.1257	0.0924038
34626	1.04073	0.0947347
34627	1.00358	0.101542
34628	0.851528	0.0910702
34629	1.73576	0.7
34630	2.00244	0.716783
34631	1.17798	0.699618
34632	1.25796	0.7
34633	2.99373	0.7
34634	2.00244	0.70953
34635	1.10719	0.703699
34636	2.03236	0.737779
34637	2.03236	0.704687
34638	1.25797	0.738963
34639	1.25797	0.7
34640	1.89226	2.1
34641	1.89226	2.1
34642	1.89226	2.1
34643	1.89226	2.1
34644	3.78452	2.1
34645	2.3174	2.1
34646	1.50005	2.1
34647	1.90428	2.1
34648	2.64564	2.1
34649	4.22168	2.1
34650	1.89226	2.1
34651	1.89226	2.1
34652	1.89226	2.1
34653	1.89226	2.1
34654	1.89226	2.1
34655	1.89226	2.1
34656	1.32749	0.389712
34657	1.70302	0.553686
34658	2.08149	0.459246
34659	1.70302	0.490372
34660	2.19634	0.439528
34661	1.93083	0.443744
34662	1.89226	1.24549
34663	1.89226	1.86238
34664	1.89226	0.854038
34665	1.89226	2.0484
34666	3.78453	1.04919
34667	1.89226	1.25006
34668	1.89226	1.78372
34669	1.89226	0.98814
34670	1.89226	1.29968
34671	3.78452	1.4863
34672	2.17499	1.04985
34673	1.79663	1.78722
34674	2.1538	0.872214
34675	1.818	1.0862
34676	2.17499	1.47528
34677	1.83919	2.1
34678	0.898316	1.93865
34679	1.0769	1.1406
34680	0.968276	2.00581
34681	0.773495	1.32632

34682	0.921126	1.61265
34683	0.921126	1.9929
34684	0.92927	1.60422
34685	0.92927	1.49409
34686	1.0769	1.46619
34687	0.968276	1.15648
34688	0.898316	1.89675
34689	0.773495	1.33861
34690	1.89226	0.976609
34691	1.89226	2.1
34692	1.89226	1.13305
34693	1.89226	1.08041
34694	3.78453	1.76332
34695	1.89226	1.19738
34696	1.89226	2.03506
34697	1.89226	0.921759
34698	1.89226	1.39928
34699	3.78452	1.11325
34700	2.17499	1.73031
34701	2.1538	2.1
34702	2.53574	1.08384
34703	2.54157	0.998875
34704	4.71073	1.62369
34705	1.0769	1.37415
34706	1.26787	1.07253
34707	1.16641	1.23008
34708	0.968276	1.8927
34709	1.11625	1.24448
34710	1.11625	1.63221
34711	1.11989	1.69493
34712	1.11989	0.91111
34713	1.26787	1.10973
34714	1.16641	2.0289
34715	1.0769	1.99759
34716	0.968277	1.55137
34717	1.89226	0.713955
34718	1.89226	0.722283
34719	1.89226	0.7163
34720	1.89226	0.70646
34721	3.78452	0.71077
34722	1.91739	0.707466
34723	2.20045	0.7
34724	1.79005	0.700999
34725	2.33802	0.701434
34726	4.25539	0.70347
34727	2.52754	0.703813
34728	1.89226	0.704429
34729	2.69841	0.702432
34730	2.69841	0.706309
34731	1.89226	0.701428
34732	1.89226	0.704315
34733	1.89226	0.717894
34734	1.89226	0.714096
34735	1.89226	0.706579
34736	1.89226	0.711347
34737	3.78452	0.712406
34738	1.93544	0.703394
34739	2.33802	0.70425

34740	1.55063	0.704297
34741	2.1769	0.7
34742	3.88865	0.701106
34743	2.20929	0.683382
34744	2.55162	0.49832
34745	2.27807	0.73887
34746	1.48831	0.525868
34747	4.48736	0.533609
34748	2.27807	0.751372
34749	2.72723	0.519363
34750	2.55162	0.674706
34751	2.55162	0.719137
34752	2.43157	0.556589
34753	2.43157	0.648127
34754	2.27807	0.627542
34755	1.51612	0.469028
34756	1.96048	0.413258
34757	1.73022	0.429764
34758	1.70302	0.699129
34759	1.61016	0.701999
34760	2.27807	0.640975
34761	2.08048	0.438949
34762	1.98522	0.481123
34763	2.43157	0.562056
34764	1.83798	0.489515
34765	2.27807	0.579041
34766	1.70302	0.785584
34767	2.68507	0.646383
34768	2.23856	0.714444
34769	2.52174	0.648369
34770	2.35331	0.812796
34771	2.65099	0.757546
34772	1.70303	0.77377
34773	0.986848	0.570543
34774	2.41566	0.524442
34775	1.70303	0.515462
34776	1.88139	0.516707
34777	1.64955	0.533563
34778	1.89226	2.08342
34779	2.34978	1.82442
34780	1.89226	2.05419
34781	2.68773	1.50644
34782	1.89226	2.1
34783	2.68773	1.7244
34784	1.89226	2.06641
34785	1.89226	1.4823
34786	1.89226	1.45926
34787	1.89226	1.97398
34788	3.78453	1.8661
34789	1.00825	1.30199
34790	1.09847	1.53076
34791	0.939987	1.35265
34792	0.783516	1.75746
34793	0.838041	1.54219
34794	0.838041	1.86913
34795	1.49693	1.90932
34796	1.26602	1.54079
34797	1.10875	1.4067

34798	1.05187	2.06733
34799	1.28743	1.60285
34800	0.946132	1.36163
34801	0.946132	1.51852
34802	0.946132	2.1
34803	0.946132	1.91956
34804	0.946132	1.29672
34805	1.31823	2.1
34806	0.946132	1.47426
34807	0.946132	1.91467
34808	0.946131	1.9973
34809	0.946131	2.1
34810	0.946132	1.77293
34811	1.31823	1.97098
34812	1.51688	0.458634
34813	1.79978	0.711992
34814	1.1685	0.460183
34815	1.1685	0.508829
34816	1.85857	0.512324
34817	1.51018	0.548482
34818	0.946116	0.38469
34819	0.946116	0.515523
34820	0.94612	0.333792
34821	0.94612	0.33917
34822	0.946118	0.297277
34823	0.946118	0.433981
34824	0.946118	0.367067
34825	0.946118	0.509823
34826	0.946131	0.3728
34827	0.946131	0.445558
34828	0.946105	0.416279
34829	0.946105	0.383715
34830	1.89224	2.1
34831	1.89224	2.1
34832	1.89224	2.1
34833	1.89224	2.1
34834	1.89224	2.1
34835	1.89224	2.1
34836	1.38678	2.1
34837	1.3349	2.1
34838	0.946119	2.1
34839	0.946119	2.1
34840	1.38678	2.1
34841	1.38678	2.1
34842	0.946119	2.1
34843	0.946119	2.1
34844	1.38676	2.1
34845	0.946107	2.1
34846	0.946132	2.1
34847	1.33491	2.1
34848	1.06455	2.1
34849	1.03932	2.1
34850	1.20649	2.1
34851	1.18773	2.1
34852	2.27105	2.1
34853	1.89226	2.1
34854	1.89226	2.1
34855	1.89226	2.1

34856	1.89226	2.1
34857	1.89226	2.1
34858	1.89226	2.1
34859	1.36918	2.1
34860	1.18774	2.1
34861	1.31144	2.1
34862	1.20651	2.1
34863	1.31144	2.1
34864	1.20651	2.1
34865	2.29066	2.1
34866	1.76476	2.1
34867	1.79825	2.1
34868	2.33801	2.1
34869	4.08891	2.1
34870	1.79825	2.1
34871	2.33801	2.1
34872	2.37168	2.1
34873	1.49325	2.1
34874	4.16993	2.1
34875	1.13641	2.1
34876	1.43213	1.11775
34877	1.44085	1.29884
34878	1.18301	2.1
34879	2.61513	1.87613
34880	1.89226	2.00097
34881	2.23875	2.1
34882	2.23875	1.77247
34883	2.10287	2.1
34884	1.89226	1.82199
34885	1.75639	2.1
34886	1.89226	1.12575
34887	1.89226	1.90878
34888	1.89226	1.13533
34889	1.89226	2.09291
34890	1.89226	1.12938
34891	1.89226	2.1
34892	1.44085	1.36317
34893	1.25173	2.09564
34894	1.25173	1.20818
34895	1.18301	2.1
34896	1.4617	1.19339
34897	1.39298	2.1
34898	1.89227	1.90326
34899	2.23875	2.1
34900	2.42504	2.1
34901	1.89227	1.42786
34902	4.13101	2.1
34903	1.89226	1.14056
34904	1.89226	1.34852
34905	1.89226	1.09404
34906	1.89226	1.97974
34907	1.89226	1.10028
34908	1.89226	2.08826
34909	1.89226	2.1
34910	2.41297	2.1
34911	2.70685	2.1
34912	2.70685	2.1
34913	1.89226	2.1

34914	1.89226	2.1
34915	1.89226	2.1
34916	1.89226	2.1
34917	1.89226	2.1
34918	1.89226	2.1
34919	1.89226	2.1
34920	1.89226	2.1
34921	1.89226	2.1
34922	1.89226	2.1
34923	1.89226	2.1
34924	1.89226	2.1
34925	3.78453	2.1
34926	1.89226	2.1
34927	1.89226	2.1
34928	1.89226	2.1
34929	1.89226	2.1
34930	1.89226	2.1
34931	1.89226	2.1
34932	1.69044	0.0771383
34933	1.57979	0.0858494
34934	2.27963	0.0858228
34935	2.21582	0.0770317
34936	3.85942	0.0789834
34937	2.21582	0.0761786
34938	1.30722	0.0853994
34939	2.02069	0.0841844
34940	2.02069	0.0801245
34941	1.57979	0.0882632
34942	1.57979	0.0778974
34943	1.67825	2.1
34944	1.89226	2.1
34945	1.89226	2.1
34946	1.89226	2.1
34947	1.91894	2.1
34948	1.91894	2.1
34949	0.946133	2.1
34950	0.946133	2.1
34951	0.946132	2.1
34952	0.946132	2.1
34953	0.946132	2.1
34954	0.946132	2.1
34955	0.946133	2.1
34956	0.946133	2.1
34957	0.946133	2.1
34958	0.946133	2.1
34959	0.946132	2.1
34960	0.946132	2.1
34961	0.942609	1.99662
34962	0.942609	2.1
34963	1.329	2.1
34964	1.329	2.1
34965	1.32547	2.1
34966	0.94261	2.1
34967	0.946132	2.095
34968	0.946132	2.1
34969	0.946132	2.1
34970	1.32547	2.1
34971	0.946132	2.1

34972	0.94261	2.1
34973	1.55831	1.81319
34974	1.07496	2.00402
34975	1.80327	0.882523
34976	1.34851	0.957924
34977	2.87824	1.02872
34978	2.14346	0.792476
34979	2.77937	0.81841
34980	2.79173	0.912367
34981	2.17129	0.774961
34982	4.95066	0.805613
34983	2.14346	0.810027
34984	1.34124	0.703674
34985	1.39818	0.771353
34986	2.1713	0.785572
34987	3.51253	0.765738
34988	2.79173	0.86957
34989	2.21882	0.768681
34990	2.17129	0.785068
34991	2.17129	0.808876
34992	2.81448	0.798968
34993	2.81448	0.818064
34994	1.10941	0.771891
34995	1.08565	0.801254
34996	0.699089	0.721392
34997	0.740176	0.739888
34998	0.908594	0.719232
34999	0.908594	0.745209
35000	0.91723	0.825872
35001	0.91723	0.726266
35002	1.10941	0.762422
35003	0.740176	0.756332
35004	0.699089	0.744155
35005	1.08565	0.743425
35006	1.89227	0.7
35007	1.89227	0.7
35008	1.89227	0.7
35009	1.89227	0.7
35010	3.78453	0.7
35011	2.40032	0.712317
35012	2.19526	0.7
35013	1.12907	0.688017
35014	1.44253	0.700915
35015	3.32433	0.7
35016	2.19526	0.7
35017	2.71093	0.70031
35018	1.29826	0.7
35019	2.0815	0.7
35020	4.18911	0.7
35021	1.89226	0.7
35022	1.89226	0.7
35023	1.89226	0.542926
35024	1.89226	0.631
35025	3.78452	0.502292
35026	2.30822	0.696708
35027	1.89226	0.7
35028	2.79329	0.551589
35029	2.79329	0.408012

35030	1.89226	0.7
35031	1.89226	0.7
35032	0.946131	1.81055
35033	0.946131	1.0104
35034	1.38414	2.1
35035	1.38414	0.877331
35036	0.94613	2.1
35037	0.94613	2.09672
35038	1.38415	1.04555
35039	1.41833	0.879961
35040	0.946132	1.04627
35041	1.41833	1.03529
35042	1.38414	1.10966
35043	0.946131	1.22526
35044	1.89226	1.78489
35045	2.6536	2.1
35046	1.89226	1.55934
35047	2.72238	2.1
35048	4.61465	2.1
35049	0.946132	2.1
35050	0.946132	1.94543
35051	1.38657	1.7384
35052	1.38657	2.1
35053	1.38657	2.1
35054	1.36119	2.1
35055	0.946131	1.11567
35056	0.946131	2.07797
35057	0.946132	2.1
35058	1.38657	2.1
35059	0.946132	2.1
35060	1.36119	2.1
35061	1.89226	2.1
35062	2.4717	2.1
35063	2.5026	2.1
35064	2.4717	2.1
35065	1.92316	2.1
35066	1.89226	2.1
35067	1.89226	2.1
35068	1.89226	2.1
35069	1.89226	2.1
35070	1.89226	0.995551
35071	3.78453	2.1
35072	0.946128	2.1
35073	0.946128	0.982137
35074	1.3419	1.91659
35075	1.3419	2.1
35076	0.946131	1.49232
35077	0.946131	1.64829
35078	1.36607	2.1
35079	1.3419	2.1
35080	0.946131	2.1
35081	1.36607	2.1
35082	1.3419	2.1
35083	0.94613	2.1
35084	1.89226	2.1
35085	2.50259	2.1
35086	2.53563	2.1
35087	2.50259	2.1

35088	1.92529	2.1
35089	1.89226	2.1
35090	0.946132	1.63516
35091	0.946132	2.1
35092	0.946131	1.99149
35093	0.946131	2.1
35094	0.946132	1.67558
35095	0.946132	2.1
35096	0.946131	2.1
35097	0.946131	2.1
35098	0.946131	2.1
35099	0.946131	2.1
35100	0.946131	2.1
35101	0.946131	2.1
35102	2.0815	2.1
35103	1.86506	2.1
35104	1.86506	2.1
35105	1.82567	2.1
35106	2.0815	2.1
35107	2.03335	2.1
35108	2.0815	2.1
35109	2.0815	2.1
35110	2.0815	2.1
35111	1.88668	2.1
35112	1.83221	2.1
35113	1.57275	2.1
35114	2.34061	0.211077
35115	1.89226	0.327582
35116	2.23254	0.19776
35117	1.89226	0.212398
35118	2.23254	0.292553
35119	1.89226	0.32107
35120	1.8619	0.248353
35121	1.89226	0.463842
35122	2.34061	0.261318
35123	1.89226	0.264293
35124	2.34061	0.377839
35125	1.89226	0.381327
35126	1.89227	0.382714
35127	1.89227	0.275483
35128	1.89227	0.300477
35129	1.89227	0.499778
35130	1.89227	0.212146
35131	1.89227	0.375097
35132	1.89227	0.309119
35133	1.89227	0.222533
35134	1.89227	0.232302
35135	1.89227	0.380879
35136	1.89227	0.186423
35137	1.89227	0.314383
35138	1.06365	0.311349
35139	0.503305	0.287552
35140	0.794912	0.223418
35141	1.42752	0.265726
35142	1.34826	0.311853
35143	1.07163	0.345352
35144	0.533113	0.301871
35145	0.761277	0.280139

35146	0.848403	0.315763
35147	0.876191	0.25643
35148	0.963372	0.438869
35149	0.963372	0.322437
35150	1.55571	0.1715
35151	2.76574	0.281435
35152	2.92453	0.200042
35153	1.70301	0.223287
35154	2.33089	0.237938
35155	1.06905	0.195087
35156	2.28979	2.1
35157	1.81488	2.1
35158	1.63722	2.1
35159	2.22627	2.1
35160	4.04115	2.1
35161	1.98246	2.1
35162	2.15715	2.1
35163	2.22627	2.1
35164	2.22627	2.1
35165	1.63719	2.1
35166	1.63719	2.1
35167	2.1644	0.0884162
35168	1.903	0.111085
35169	1.23639	0.0988412
35170	1.42704	0.136834
35171	3.61546	0.109558
35172	1.89226	0.155994
35173	1.89226	0.210353
35174	1.89226	0.194877
35175	1.89226	0.210584
35176	3.78451	0.190492
35177	2.61343	0.158536
35178	1.89226	0.170477
35179	2.08149	0.149066
35180	1.74357	0.117871
35181	2.08149	0.21507
35182	1.74357	0.197324
35183	1.63483	0.106484
35184	1.8631	0.0920569
35185	1.8631	0.116756
35186	1.70301	0.0937682
35187	2.0815	0.108014
35188	1.94319	0.0941472
35189	2.0815	0.104067
35190	1.89226	0.0895977
35191	1.89226	0.120302
35192	2.05236	0.0932911
35193	2.23014	0.107643
35194	2.41882	0.0898961
35195	2.14993	2.1
35196	1.87217	1.84192
35197	2.34488	2.1
35198	2.5254	1.46241
35199	4.39757	1.5729
35200	0.949995	2.1
35201	0.630972	2.1
35202	0.936084	2.1
35203	1.17244	2.1

35204	0.922394	2.1
35205	0.922394	2.1
35206	0.88102	2.1
35207	0.88102	2.1
35208	0.949998	2.1
35209	1.17244	2.1
35210	0.936081	2.1
35211	0.630973	2.1
35212	1.89226	2.1
35213	1.89226	1.93865
35214	1.89226	2.1
35215	1.89226	2.1
35216	3.78452	2.1
35217	1.89226	1.33581
35218	1.89226	1.50591
35219	1.89226	1.2648
35220	1.89226	1.57593
35221	1.89226	1.24722
35222	1.89226	1.54823
35223	2.34488	1.69715
35224	2.5254	1.30475
35225	1.89226	1.41299
35226	1.89226	1.61099
35227	4.41766	1.89328
35228	1.89226	1.7396
35229	1.89226	2.00127
35230	1.89226	1.7751
35231	1.89226	1.81711
35232	3.78452	1.44857
35233	1.89226	1.23031
35234	1.89226	1.49729
35235	1.89226	1.1908
35236	1.89226	1.38183
35237	1.89226	1.18317
35238	1.89226	1.3789
35239	1.5605	2.1
35240	1.15806	2.1
35241	1.31565	2.1
35242	1.65642	2.1
35243	2.87615	2.1
35244	1.46643	2.1
35245	1.65642	2.1
35246	1.31565	2.1
35247	1.31565	2.1
35248	1.74246	2.1
35249	1.74246	2.1
35250	1.89226	2.1
35251	1.89226	2.1
35252	1.89226	2.1
35253	1.89226	2.1
35254	1.89226	2.1
35255	1.89226	2.1
35256	1.15099	2.1
35257	1.15099	2.1
35258	0.939316	2.1
35259	0.939316	2.1
35260	0.946127	2.1
35261	0.946127	2.1

35262	1.14418	2.1
35263	0.939289	2.1
35264	1.14417	2.1
35265	0.946129	2.1
35266	0.939287	2.1
35267	0.94613	2.1
35268	1.89226	2.1
35269	1.89226	2.1
35270	1.89226	2.1
35271	1.89226	2.0865
35272	3.78452	2.1
35273	1.93164	0.317137
35274	2.64898	0.220671
35275	1.43213	0.375283
35276	1.43213	0.436418
35277	2.63206	0.223903
35278	2.13254	0.358045
35279	1.89226	2.1
35280	1.89226	2.1
35281	1.89226	2.1
35282	1.89226	2.1
35283	3.78452	2.1
35284	2.71655	2.1
35285	1.43323	2.1
35286	1.28413	2.1
35287	2.53614	2.1
35288	3.96937	2.1
35289	1.89226	2.1
35290	1.89226	2.1
35291	1.89226	2.1
35292	1.89226	2.1
35293	1.89226	2.1
35294	1.89226	2.1
35295	1.33356	2.1
35296	2.82297	2.1
35297	2.771	2.1
35298	2.771	2.1
35299	1.46037	2.1
35300	1.46037	2.1
35301	2.56034	2.1
35302	1.18474	2.1
35303	1.15113	2.1
35304	2.53724	2.1
35305	3.72198	2.1
35306	2.56034	2.1
35307	1.89226	2.1
35308	1.89226	2.1
35309	2.53725	2.1
35310	4.42951	2.1
35311	1.89226	2.1
35312	1.89226	2.1
35313	1.89226	2.1
35314	1.89226	2.1
35315	3.78453	2.1
35316	1.89226	2.1
35317	1.89226	2.1
35318	1.89226	2.1
35319	1.89226	2.1

35320	3.78452	2.1
35321	1.1229	2.1
35322	2.53724	2.1
35323	1.15113	2.1
35324	1.15113	2.1
35325	2.51559	2.1
35326	2.51559	2.1
35327	1.89226	2.1
35328	2.53724	2.1
35329	2.51559	2.1
35330	2.53724	2.1
35331	1.87061	2.1
35332	1.89226	2.1
35333	1.89226	2.1
35334	1.89226	2.1
35335	1.89226	2.1
35336	1.89226	2.1
35337	3.78453	2.1
35338	1.89226	2.1
35339	1.89226	2.1
35340	1.89226	2.1
35341	1.89226	2.1
35342	3.78452	2.1
35343	1.98143	1.18896
35344	2.81559	0.741425
35345	1.65411	0.881395
35346	2.26675	1.05097
35347	4.24818	0.940878
35348	0.810656	0.975068
35349	0.810656	2.1
35350	0.821888	2.1
35351	0.821888	1.64583
35352	1.1235	1.02585
35353	1.07439	1.9255
35354	0.535693	2.1
35355	0.509042	2.1
35356	0.535693	2.1
35357	1.1235	2.1
35358	0.509043	2.1
35359	1.07439	2.1
35360	1.10378	2.1
35361	2.247 1.88997	
35362	2.27472	2.1
35363	2.27472	2.05109
35364	1.07139	2.1
35365	1.07139	2.1
35366	2.08081	2.1
35367	1.40395	2.1
35368	1.34245	2.1
35369	2.00239	2.1
35370	3.40634	2.1
35371	1.89226	2.1
35372	1.89226	2.1
35373	1.89226	2.1
35374	1.89226	2.1
35375	3.78453	2.1
35376	0.946128	2.1
35377	0.946128	2.1

35378	1.13785	2.1
35379	1.11496	2.1
35380	0.946131	2.1
35381	0.946131	2.1
35382	1.11495	2.1
35383	1.11495	2.1
35384	0.94613	2.1
35385	1.11495	2.1
35386	1.13785	2.1
35387	0.946131	2.1
35388	0.975888	2.1
35389	1.00118	2.1
35390	0.671218	2.1
35391	0.653229	2.1
35392	0.825372	2.1
35393	0.825372	2.1
35394	0.829038	2.1
35395	0.829038	2.1
35396	0.975901	2.1
35397	0.653238	2.1
35398	0.671209	2.1
35399	1.00117	2.1
35400	1.89224	2.1
35401	1.89224	2.1
35402	1.89224	2.1
35403	1.89224	2.1
35404	3.78448	2.1
35405	0.946115	2.1
35406	0.946115	2.1
35407	1.11494	2.1
35408	1.10111	2.1
35409	0.946118	2.1
35410	0.946118	2.1
35411	1.10111	2.1
35412	1.10111	2.1
35413	1.10112	2.1
35414	0.94613	2.1
35415	0.946105	2.1
35416	1.11492	2.1
35417	1.04028	0.74578
35418	2.27126	0.680006
35419	2.0815	0.732165
35420	1.82249	0.810192
35421	1.33723	0.732863
35422	1.18684	0.795784
35423	0.812616	0.703457
35424	0.812616	0.703685
35425	0.997777	0.702334
35426	1.08122	0.703684
35427	0.94613	0.702641
35428	0.94613	0.703609
35429	0.812648	0.703836
35430	0.932779	0.703615
35431	0.812648	0.702352
35432	0.94613	0.701755
35433	0.849335	0.700293
35434	0.94613	0.701217
35435	1.80327	1.02273

35436	1.56016	0.94316
35437	1.57731	1.61805
35438	1.34851	1.6147
35439	2.03207	1.01052
35440	1.80327	1.76164
35441	2.03207	1.49789
35442	1.56016	1.40177
35443	2.18076	1.05335
35444	1.78167	1.03827
35445	3.81374	1.41383
35446	1.37597	1.69081
35447	1.60058	2.04986
35448	1.70302	1.02634
35449	1.85448	1.21928
35450	3.15028	1.18905
35451	1.60058	1.14892
35452	1.4054	2.02607
35453	1.66202	1.06537
35454	1.70302	1.73867
35455	3.09979	1.35111
35456	1.70302	1.42341
35457	1.85448	1.59379
35458	2.21317	1.11451
35459	2.0961	1.15718
35460	4.01757	1.56482
35461	1.66202	1.30736
35462	1.70302	1.82096
35463	2.09611	0.973141
35464	2.00892	1.3221
35465	3.6468	1.17367
35466	1.2966	0.158848
35467	2.25495	0.114136
35468	1.2966	0.198317
35469	2.21117	0.120507
35470	1.67687	0.179737
35471	2.6167	0.115243
35472	1.89227	0.23756
35473	1.89227	0.159051
35474	1.89227	0.215019
35475	1.89227	0.312405
35476	3.78453	0.227784
35477	2.44375	0.202827
35478	1.89227	0.248668
35479	2.81853	0.271432
35480	2.81853	0.238965
35481	1.89227	0.217048
35482	1.89227	0.14599
35483	1.89226	0.995928
35484	1.75853	1.04485
35485	2.37636	0.989396
35486	2.37636	1.10087
35487	1.89226	0.93023
35488	1.89226	1.03317
35489	1.65814	0.752122
35490	1.10187	0.928368
35491	1.10187	0.804581
35492	1.19547	0.871676
35493	1.56263	0.492872

35494	1.65624	0.909181
35495	1.56264	0.748542
35496	1.01479	0.923814
35497	1.01479	0.830864
35498	1.10187	0.880722
35499	1.47561	0.438157
35500	1.5627	0.936523
35501	1.89225	2.1
35502	1.89225	2.1
35503	1.89225	2.1
35504	1.89225	2.1
35505	3.7845	2.1
35506	2.54386	2.1
35507	1.91546	2.1
35508	2.46008	2.1
35509	1.82815	2.1
35510	4.37554	2.1
35511	1.91546	2.1
35512	1.04909	2.1
35513	1.82815	2.1
35514	0.983141	2.1
35515	2.8986	2.1
35516	1.89225	2.1
35517	1.89225	2.1
35518	1.89225	2.1
35519	1.89225	2.1
35520	3.78449	2.1
35521	1.89226	2.1
35522	1.89226	2.1
35523	1.89226	2.1
35524	1.89226	2.1
35525	1.89226	2.1
35526	1.89226	2.1
35527	2.46009	2.1
35528	1.7513	2.1
35529	1.82817	2.1
35530	1.82817	2.1
35531	2.38127	2.1
35532	2.38127	2.1
35533	2.55006	2.1
35534	2.64569	2.1
35535	2.60128	2.1
35536	2.49168	2.1
35537	5.13737	2.1
35538	2.51605	2.1
35539	2.64569	2.1
35540	2.60128	2.1
35541	2.48891	2.1
35542	5.1346	2.1
35543	2.60128	2.1
35544	2.49168	2.1
35545	2.41689	2.1
35546	2.55248	2.1
35547	5.04416	2.1
35548	2.60128	2.1
35549	2.48891	2.1
35550	2.45922	2.1
35551	2.55249	2.1

35552	5.04139	2.1
35553	1.89227	1.55262
35554	1.89227	2.04066
35555	1.89227	2.01093
35556	1.89227	1.86057
35557	1.89227	1.72743
35558	1.89227	1.55827
35559	0.96063	2.1
35560	0.96063	2.1
35561	0.958806	2.1
35562	0.958806	2.1
35563	1.28892	2.1
35564	0.749042	2.1
35565	1.17409	2.1
35566	0.630516	2.1
35567	1.17409	2.1
35568	1.28892	2.1
35569	0.630515	2.1
35570	0.749042	2.1
35571	0.92511	1.63272
35572	0.92511	1.77137
35573	1.19511	1.75216
35574	1.19511	2.00381
35575	0.946132	1.89392
35576	1.17409	1.7129
35577	0.925081	1.53137
35578	0.946129	1.89937
35579	0.946131	1.36448
35580	0.946131	1.55645
35581	1.17409	1.73538
35582	0.925083	1.425
35583	1.89226	1.01801
35584	1.89226	1.14361
35585	1.89226	1.06544
35586	1.89226	1.0634
35587	1.89226	1.07232
35588	1.89226	1.07252
35589	1.89226	1.09328
35590	1.89226	1.19644
35591	1.89226	1.16616
35592	1.89226	1.13525
35593	3.78453	1.14521
35594	0.946132	2.1
35595	0.946132	2.00091
35596	1.28892	2.1
35597	1.28892	2.1
35598	0.946132	2.1
35599	1.28892	2.1
35600	0.946133	2.06787
35601	1.17409	1.94543
35602	0.946132	1.83461
35603	0.946132	2.1
35604	1.17409	2.1
35605	1.28892	2.1
35606	0.625229	2.1
35607	0.890321	2.1
35608	1.21636	2.1
35609	1.13022	2.1

35610	1.07094	2.1
35611	1.03541	2.1
35612	0.942207	2.1
35613	0.942207	2.1
35614	0.898766	2.1
35615	1.26626	2.1
35616	1.0853	2.1
35617	0.600358	2.1
35618	0.719012	2.1
35619	0.719012	2.1
35620	1.17957	2.1
35621	1.17957	2.1
35622	0.719012	2.1
35623	0.952456	2.1
35624	0.946129	2.1
35625	0.946129	2.1
35626	0.946131	2.1
35627	0.952454	2.1
35628	0.946131	2.1
35629	0.719011	2.1
35630	0.94613	2.1
35631	0.94613	2.1
35632	0.94613	2.1
35633	0.94613	2.1
35634	0.946131	2.1
35635	0.946131	2.1
35636	0.946129	2.1
35637	0.946129	2.1
35638	0.94613	2.1
35639	0.94613	2.1
35640	0.94613	2.1
35641	0.94613	2.1
35642	1.1587	2.1
35643	0.973158	2.1
35644	0.750023	2.1
35645	0.536443	2.1
35646	0.84054	2.1
35647	0.84054	2.1
35648	0.854602	2.1
35649	0.854602	2.1
35650	1.1587	2.1
35651	0.750023	2.1
35652	0.973158	2.1
35653	0.536443	2.1
35654	1.89227	0.925331
35655	1.89227	2.08534
35656	1.89227	1.26798
35657	1.89227	0.97528
35658	3.78453	1.13004
35659	1.89226	2.1
35660	1.89226	1.67089
35661	1.89226	1.17444
35662	1.89226	2.1
35663	3.78453	2.03928
35664	1.7632	1.80081
35665	1.61149	2.1
35666	1.98749	2.1
35667	2.11615	2.1

35668	3.75068	2.1
35669	1.7632	2.1
35670	1.61149	1.57547
35671	1.97733	0.96363
35672	2.11615	2.1
35673	3.74052	1.4261
35674	1.89226	1.18124
35675	1.89226	1.02045
35676	1.89226	0.876659
35677	1.89226	1.82499
35678	3.78453	1.06518
35679	1.89226	1.09307
35680	1.89226	2.1
35681	1.89226	2.1
35682	1.89226	1.3637
35683	3.78453	2.1
35684	1.98749	2.1
35685	2.11615	2.1
35686	2.43151	1.48606
35687	2.27541	2.1
35688	4.41899	2.1
35689	1.97733	0.975019
35690	2.11615	2.1
35691	2.43151	1.93333
35692	2.33986	0.969758
35693	4.40883	1.64565
35694	2.17892	0.421589
35695	2.80057	0.819146
35696	1.88349	0.764543
35697	2.63062	0.733688
35698	4.80954	0.750042
35699	1.89226	0.928932
35700	1.89226	0.74571
35701	1.89226	0.900544
35702	1.89226	0.724531
35703	3.78452	0.807781
35704	2.0155	0.948097
35705	1.97652	0.745303
35706	1.8247	0.893759
35707	1.80645	0.687161
35708	3.80122	0.801784
35709	1.97652	0.73016
35710	1.79681	0.730599
35711	1.80645	0.730239
35712	1.59244	0.71132
35713	3.56896	0.707184
35714	1.89226	0.73468
35715	1.89226	0.748587
35716	1.89226	0.721295
35717	1.89226	0.726099
35718	3.78452	0.704619
35719	1.89223	0.902286
35720	1.89223	0.741273
35721	1.89223	0.886658
35722	1.89223	0.727551
35723	3.78447	0.790585
35724	1.82467	0.896157
35725	1.80643	0.733528

35726	1.55159	0.887839
35727	1.53969	0.72349
35728	3.36437	0.791422
35729	1.80643	0.731919
35730	1.59242	0.729106
35731	1.5397	0.725375
35732	1.40538	0.707645
35733	3.13211	0.708904
35734	1.89224	0.732253
35735	1.89224	0.730498
35736	1.89224	0.728965
35737	1.89224	0.732293
35738	3.78447	0.710847
35739	1.37427	0.37422
35740	2.37086	0.553172
35741	2.37972	0.334974
35742	1.94703	0.625987
35743	2.19299	0.476115
35744	1.91813	0.7
35745	1.07566	2.1
35746	1.12954	2.1
35747	0.946131	2.1
35748	0.946131	2.1
35749	1.12954	2.1
35750	1.12954	2.1
35751	0.946131	2.1
35752	0.946131	2.1
35753	1.12954	2.1
35754	0.946131	2.1
35755	0.946131	2.1
35756	1.07566	2.1
35757	2.25908	2.1
35758	1.89226	2.1
35759	1.89226	2.1
35760	1.89226	2.1
35761	2.37567	2.1
35762	2.37567	2.1
35763	1.37597	1.71887
35764	1.70302	0.963271
35765	1.24162	1.5336
35766	1.12219	1.87733
35767	1.37072	1.11049
35768	1.70302	2.1
35769	1.37072	2.1
35770	1.70302	1.22769
35771	1.54168	1.19923
35772	2.21318	1.11278
35773	3.39238	1.38061
35774	1.89226	0.202611
35775	1.89226	0.197729
35776	1.89226	0.137216
35777	1.89226	0.128527
35778	3.78452	0.139119
35779	1.89227	0.194829
35780	1.89227	0.18388
35781	1.89227	0.129708
35782	1.89227	0.161294
35783	3.78453	0.182603

35784	1.89226	0.137589
35785	1.89226	0.163384
35786	1.89226	0.12535
35787	1.89226	0.114792
35788	3.78453	0.125804
35789	1.89226	0.148473
35790	2.21654	0.112573
35791	2.72007	0.121231
35792	2.72007	0.14236
35793	1.89226	0.108557
35794	1.89226	0.129177
35795	0.946128	0.106354
35796	0.946128	0.122237
35797	0.946132	0.102852
35798	0.946132	0.121365
35799	0.94613	0.10752
35800	0.94613	0.114766
35801	0.946131	0.111244
35802	0.946131	0.121161
35803	0.94613	0.104163
35804	0.94613	0.119642
35805	0.94613	0.1429
35806	0.94613	0.12792
35807	1.89226	0.122621
35808	1.89226	0.132165
35809	1.89226	0.0989355
35810	1.89226	0.100753
35811	3.78453	0.108766
35812	1.33457	0.0966616
35813	0.946131	0.101021
35814	1.08045	0.0945281
35815	0.946132	0.102322
35816	1.08045	0.0985774
35817	1.08045	0.0994669
35818	0.946132	0.106535
35819	0.946132	0.100465
35820	1.33457	0.0923443
35821	1.08045	0.0879931
35822	0.946132	0.11133
35823	0.946132	0.106185
35824	1.53347	1.04371
35825	1.13092	2.1
35826	1.52938	1.61362
35827	1.93568	1.50319
35828	3.06285	1.88006
35829	1.89226	0.729679
35830	2.72959	0.818502
35831	2.82996	0.817843
35832	2.82996	0.735312
35833	1.89226	0.821344
35834	1.89226	0.729527
35835	2.7296	0.828973
35836	2.57663	2.1
35837	2.48354	0.830024
35838	2.48354	1.91601
35839	2.82996	0.821663
35840	2.82996	1.99203
35841	2.63449	0.823031

35842	2.7296	0.821756
35843	2.39282	0.867053
35844	2.39282	2.1
35845	2.48354	2.00169
35846	2.72521	2.00606
35847	1.89224	2.1
35848	1.89224	1.50013
35849	1.89224	0.962423
35850	1.89224	1.92672
35851	3.78447	1.10784
35852	1.89226	1.95584
35853	2.10425	2.1
35854	2.5142	2.1
35855	2.5142	2.1
35856	1.89226	2.1
35857	1.89226	1.81978
35858	0.946133	0.954866
35859	0.946133	1.14712
35860	0.946128	2.1
35861	0.946128	2.1
35862	0.946131	1.76598
35863	0.946131	0.957746
35864	0.94613	0.874304
35865	0.94613	2.07437
35866	0.946131	1.26949
35867	0.946131	1.32611
35868	0.946131	1.20626
35869	0.946131	1.21737
35870	1.89226	0.865976
35871	1.89226	1.01971
35872	1.89226	0.887784
35873	1.89226	0.994304
35874	1.89226	0.884605
35875	1.89226	0.987252
35876	1.94446	0.898686
35877	2.50167	1.10256
35878	1.28418	0.930371
35879	1.94446	1.0323
35880	2.05205	0.99373
35881	2.05205	1.04649
35882	1.27835	2.1
35883	1.08341	2.1
35884	0.946132	2.1
35885	0.946132	2.1
35886	0.851542	2.1
35887	0.851542	2.1
35888	0.977356	2.1
35889	1.18368	2.1
35890	1.18378	2.1
35891	0.851555	2.1
35892	0.977346	2.1
35893	0.851476	2.1
35894	1.07369	2.1
35895	2.5813	2.1
35896	1.95053	2.1
35897	1.95053	2.1
35898	1.90196	2.1
35899	1.90196	2.1

35900	1.89226	2.1
35901	1.89226	2.1
35902	1.89226	2.1
35903	1.89226	2.1
35904	3.78452	2.1
35905	1.89224	2.1
35906	1.89224	2.1
35907	1.89224	2.1
35908	1.89224	2.1
35909	1.89224	2.1
35910	1.89224	2.1
35911	1.97004	0.756878
35912	1.8072	1.09225
35913	1.5866	1.09016
35914	1.79478	0.828767
35915	3.60198	0.961397
35916	1.23267	1.89599
35917	1.8072	0.972912
35918	1.58661	0.936824
35919	0.947921	1.63566
35920	2.75512	1.18436
35921	1.89226	0.971665
35922	1.89226	2.1
35923	1.89226	0.989964
35924	1.89226	1.35258
35925	1.89226	0.980388
35926	1.89226	1.7978
35927	1.89226	1.09107
35928	1.89226	0.795351
35929	1.89226	0.844647
35930	1.89226	1.16367
35931	3.78452	0.914654
35932	1.4263	1.23995
35933	1.79478	0.828243
35934	1.67651	0.900616
35935	1.79478	1.23981
35936	1.46834	0.814976
35937	1.5866	0.993257
35938	1.89226	0.842398
35939	1.89226	1.09364
35940	1.89226	0.853899
35941	1.89226	1.0187
35942	1.89226	0.862976
35943	1.89226	1.25244
35944	2.23956	1.14096
35945	1.81847	1.45065
35946	2.10991	0.878449
35947	2.75317	0.930893
35948	4.34947	1.01333
35949	1.60578	0.264733
35950	1.94974	0.269311
35951	1.68062	0.393482
35952	1.14721	0.24339
35953	3.2864	0.267998
35954	1.3946	0.0888095
35955	1.692	0.0785851
35956	2.0154	0.10081
35957	2.0154	0.0825381

35958	1.11926	0.0932477
35959	1.11926	0.07918
35960	1.90347	0.0952649
35961	1.83626	0.0755223
35962	1.84583	0.0807129
35963	1.89434	0.0943835
35964	3.79781	0.0834419
35965	1.89226	1.09828
35966	1.89226	1.01785
35967	1.89226	0.922702
35968	1.89226	1.13297
35969	3.78452	1.00472
35970	1.20986	1.28133
35971	1.20986	1.33567
35972	0.912635	1.09612
35973	0.912635	1.49073
35974	0.912667	0.997026
35975	1.17637	1.67403
35976	0.946125	1.2458
35977	0.946125	1.39617
35978	0.946128	1.56595
35979	0.912664	1.11636
35980	0.946127	1.77773
35981	1.17637	1.21321
35982	1.89226	0.116129
35983	1.65573	0.135959
35984	1.65573	0.170265
35985	1.89226	0.103012
35986	1.89226	0.16055
35987	2.12879	0.0947723
35988	1.89226	0.153752
35989	1.89226	0.154639
35990	1.89226	0.239082
35991	1.89226	0.134199
35992	1.89226	0.22868
35993	1.89226	0.126496
35994	2.40928	0.099349
35995	1.89226	0.132491
35996	1.95959	0.131544
35997	1.70302	0.116152
35998	3.47338	0.13393
35999	1.89226	0.14855
36000	1.89226	0.143956
36001	1.89226	0.133529
36002	1.89226	0.214738
36003	3.78452	0.12709
36004	1.64985	0.336061
36005	2.47846	0.483992
36006	2.25405	0.398153
36007	1.80768	0.361002
36008	4.06173	0.34228
36009	1.98846	0.218693
36010	0.912729	0.261048
36011	1.05417	0.179331
36012	1.70303	0.296999
36013	2.82107	0.284952
36014	2.47846	0.511792
36015	1.70303	0.506182

36016	2.86918	0.579984
36017	2.05973	0.579219
36018	2.51808	0.488124
36019	1.80768	0.478892
36020	0.946131	0.483423
36021	0.946131	0.455646
36022	0.946132	0.440983
36023	0.946132	0.60407
36024	0.946131	0.6344
36025	0.946131	0.608695
36026	0.946133	0.475667
36027	0.946133	0.460202
36028	0.946131	0.501621
36029	0.946131	0.594651
36030	0.946132	0.569798
36031	0.946132	0.599456
36032	0.946133	0.296414
36033	0.946133	0.293455
36034	0.946132	0.392098
36035	0.946132	0.400359
36036	0.946133	0.412421
36037	0.946133	0.405651
36038	0.946132	0.282234
36039	0.946132	0.438163
36040	0.946132	0.303229
36041	0.946132	0.408605
36042	0.946133	0.284794
36043	0.946133	0.406877
36044	1.89227	0.629949
36045	1.89227	0.695382
36046	1.89227	0.658312
36047	1.89227	0.624667
36048	1.89227	0.715292
36049	1.89227	0.7021
36050	1.1656	2.1
36051	1.21847	2.1
36052	0.836006	2.1
36053	0.806007	2.1
36054	1.00653	2.1
36055	1.00653	2.1
36056	1.01794	2.1
36057	1.01794	2.1
36058	1.1656	2.1
36059	0.806007	2.1
36060	0.836006	2.1
36061	1.21847	2.1
36062	2.82263	2.1
36063	2.3312	2.1
36064	2.25936	2.1
36065	2.71441	2.1
36066	5.0456	2.1
36067	2.25936	2.1
36068	1.61201	2.1
36069	1.57627	2.1
36070	1.61201	2.1
36071	2.29545	2.1
36072	2.3312	2.1
36073	1.21269	0.0783

36074	1.48867	0.0841586
36075	1.68264	0.0836056
36076	1.9898	0.0755707
36077	3.17131	0.078242
36078	2.0487	2.1
36079	2.45212	2.1
36080	2.31012	2.1
36081	1.94095	2.1
36082	4.39307	2.1
36083	1.02435	2.1
36084	0.970469	2.1
36085	0.625375	2.1
36086	0.567166	2.1
36087	0.796854	2.1
36088	0.796854	2.1
36089	0.79466	2.1
36090	0.79466	2.1
36091	0.970471	2.1
36092	0.567165	2.1
36093	0.625374	2.1
36094	1.02435	2.1
36095	2.31012	2.1
36096	1.94095	2.1
36097	1.4825	2.1
36098	2.17384	2.1
36099	4.11479	2.1
36100	0.946187	2.1
36101	0.803763	2.1
36102	0.581357	2.1
36103	0.510356	2.1
36104	0.745632	2.1
36105	0.745632	2.1
36106	0.734566	2.1
36107	0.734563	2.1
36108	0.896142	2.1
36109	0.510366	2.1
36110	0.946173	2.1
36111	0.552965	2.1
36112	1.89226	0.966156
36113	1.89226	1.19009
36114	1.89226	1.4111
36115	1.89226	1.56862
36116	1.89226	0.978145
36117	1.89226	1.29881
36118	1.50743	1.17266
36119	1.0417	1.98033
36120	1.50743	1.72321
36121	1.62974	0.980307
36122	1.14605	0.97527
36123	1.14605	1.94114
36124	1.89226	0.890616
36125	1.89226	1.49577
36126	1.89226	0.839352
36127	1.89226	1.23015
36128	1.89226	0.866886
36129	1.89226	1.45856
36130	1.89226	1.44516
36131	1.89226	1.10947

36132	1.89226	0.887874
36133	1.89226	1.45142
36134	3.78453	1.0221
36135	1.77186	1.26786
36136	1.35668	1.62935
36137	1.70254	0.969633
36138	1.38756	1.53679
36139	1.77186	1.38398
36140	1.37814	2.05217
36141	0.734857	1.74723
36142	1.00549	1.32518
36143	0.835261	0.979101
36144	1.08357	1.67681
36145	0.97665	1.09766
36146	0.795559	2.1
36147	0.891385	1.29809
36148	0.891385	0.852821
36149	0.874231	0.977075
36150	1.08356	1.06319
36151	0.734863	1.13463
36152	0.79449	1.28186
36153	1.89226	0.873003
36154	1.89226	0.715083
36155	2.42671	0.752798
36156	1.98783	0.92004
36157	4.31897	0.790984
36158	0.579854	0.837982
36159	0.965582	0.864333
36160	0.634403	0.779379
36161	1.17796	0.731187
36162	1.00592	0.734475
36163	0.797259	1.94065
36164	0.905246	0.880822
36165	0.905246	0.738118
36166	0.695715	0.783492
36167	0.637106	0.823912
36168	1.24374	0.790281
36169	0.95617	0.874812
36170	2.0815	0.847124
36171	1.60521	1.12048
36172	1.29556	0.893761
36173	2.32205	1.20307
36174	1.28445	0.830251
36175	2.0815	1.01633
36176	2.3085	1.89094
36177	2.21147	2.1
36178	2.3085	2.1
36179	2.39282	2.1
36180	1.98899	2.1
36181	2.0733	2.1
36182	2.3275	2.1
36183	1.63471	2.1
36184	1.98899	2.1
36185	1.56801	2.1
36186	3.62369	2.1
36187	2.3085	1.71646
36188	1.98899	2.1
36189	2.22575	2.08485

36190	1.90502	2.1
36191	4.21353	2.1
36192	1.90502	2.1
36193	1.56801	2.1
36194	1.90502	2.1
36195	1.98899	2.1
36196	1.49733	2.1
36197	1.58129	2.1
36198	1.89226	1.04252
36199	2.6491	1.1119
36200	2.49266	2.1
36201	2.6491	1.04406
36202	1.73582	1.09905
36203	1.89226	0.859102
36204	1.31854	2.1
36205	2.6491	1.15926
36206	1.3909	2.1
36207	1.3909	1.93069
36208	2.49266	2.1
36209	2.49266	2.09539
36210	1.89226	1.03359
36211	2.49266	1.04559
36212	2.38634	2.1
36213	2.49266	0.992604
36214	1.78595	1.03899
36215	1.89226	0.848746
36216	1.23042	2.1
36217	2.49266	1.05961
36218	1.31854	2.1
36219	1.31854	1.34842
36220	2.38635	2.1
36221	2.38635	1.60372
36222	2.83487	2.1
36223	1.89226	2.1
36224	2.594	2.1
36225	2.594	2.1
36226	1.89226	2.1
36227	1.89226	2.1
36228	1.89226	2.1
36229	1.89226	2.1
36230	1.89226	2.1
36231	1.89226	2.1
36232	3.78452	2.1
36233	1.09518	2.1
36234	1.28158	2.1
36235	1.36844	2.1
36236	1.59972	2.1
36237	2.65003	2.1
36238	1.89226	2.1
36239	1.89226	2.1
36240	1.89226	2.1
36241	1.89226	2.1
36242	3.78452	2.1
36243	1.36844	2.1
36244	1.59972	2.1
36245	1.67352	2.1
36246	1.94291	2.1
36247	3.31135	2.1

36248	1.52938	1.86241
36249	1.93568	1.79623
36250	2.36221	0.900212
36251	1.94723	2.1
36252	3.89158	1.49012
36253	1.94724	1.03912
36254	1.26593	2.1
36255	1.75367	1.8528
36256	2.4322	1.15906
36257	3.7009	1.91234
36258	2.36221	0.949345
36259	2.43219	1.86732
36260	1.94723	1.22569
36261	1.94723	1.34352
36262	2.8202	1.37481
36263	2.8202	1.58835
36264	2.51604	2.1
36265	2.18533	2.1
36266	2.17228	2.1
36267	2.4889	2.1
36268	4.67423	2.1
36269	2.17228	2.1
36270	1.62183	2.1
36271	1.62316	2.1
36272	1.62183	2.1
36273	2.18666	2.1
36274	2.18533	2.1
36275	2.17227	2.1
36276	2.4889	2.1
36277	2.45922	2.1
36278	2.15686	2.1
36279	4.64576	2.1
36280	2.17228	2.1
36281	1.62316	2.1
36282	1.62241	2.1
36283	2.15686	2.1
36284	3.78002	2.1
36285	1.89226	0.832217
36286	1.89226	0.822329
36287	1.89226	0.765273
36288	1.89226	0.75637
36289	3.78453	0.870774
36290	1.89226	0.806668
36291	1.89226	0.807654
36292	1.89226	0.77976
36293	1.89226	0.730299
36294	3.78452	0.77255
36295	1.89226	0.808098
36296	1.89226	0.732056
36297	1.89226	0.773294
36298	1.89226	0.784828
36299	1.89226	0.739114
36300	1.89226	0.749157
36301	1.89226	0.782845
36302	1.89226	0.817237
36303	2.53413	0.729394
36304	1.89226	0.714082
36305	3.78453	0.740632

36306	1.38048	0.800469
36307	1.38048	0.80656
36308	0.490332	0.745687
36309	0.490332	0.74482
36310	0.924679	0.709421
36311	0.946131	1.28279
36312	0.946131	0.905829
36313	0.490332	0.705158
36314	0.924678	0.711795
36315	0.490332	0.715046
36316	0.946131	0.818709
36317	0.946131	0.763437
36318	1.89227	0.709623
36319	1.89227	0.766533
36320	1.89227	0.714263
36321	1.89227	0.793961
36322	1.89227	0.710724
36323	1.89227	0.759621
36324	1.30803	2.1
36325	2.55151	2.1
36326	1.43935	2.1
36327	1.43935	2.1
36328	2.34922	2.1
36329	2.34922	2.1
36330	0.946131	2.1
36331	0.946131	2.1
36332	1.27575	2.1
36333	1.17461	2.1
36334	0.94613	2.1
36335	0.94613	2.1
36336	1.17461	2.1
36337	1.17461	2.1
36338	0.946131	2.1
36339	1.17461	2.1
36340	0.946132	2.1
36341	1.27575	2.1
36342	1.89226	2.1
36343	1.89226	1.12155
36344	1.89226	2.1
36345	1.89226	2.1
36346	1.89226	2.1
36347	1.89226	2.1
36348	0.946133	2.1
36349	0.946133	2.1
36350	0.946132	2.1
36351	0.946132	2.1
36352	0.946133	2.1
36353	0.946133	2.06325
36354	0.946132	2.1
36355	0.946132	2.1
36356	0.946132	2.1
36357	0.946132	2.1
36358	0.946133	2.1
36359	0.946133	2.1
36360	1.07043	2.1
36361	1.17461	2.1
36362	0.654016	2.1
36363	0.5829	2.1

36364	0.870481	2.1
36365	0.870481	2.1
36366	0.887027	2.1
36367	0.887027	2.1
36368	1.07043	2.1
36369	0.5829	2.1
36370	0.654016	2.1
36371	1.17461	2.1
36372	1.89226	2.1
36373	1.89226	1.09547
36374	1.89226	2.1
36375	1.89226	2.0178
36376	3.78453	2.1
36377	1.89226	2.1
36378	1.89226	2.1
36379	1.89226	2.1
36380	1.89226	2.1
36381	3.78453	2.1
36382	1.26929	2.1
36383	2.19313	2.1
36384	1.09847	2.1
36385	1.69916	2.1
36386	2.96844	2.1
36387	1.84124	2.1
36388	2.24937	2.1
36389	1.69915	2.1
36390	1.75691	2.1
36391	3.94852	2.1
36392	1.53082	2.1
36393	2.04422	2.1
36394	1.53082	2.1
36395	1.69915	2.1
36396	1.92911	2.1
36397	2.09744	2.1
36398	1.39263	0.948844
36399	1.0171	0.651159
36400	1.1211	0.855425
36401	1.46482	0.913185
36402	2.48192	0.879606
36403	1.39263	0.952196
36404	1.57962	1.05331
36405	1.65238	1.19627
36406	1.46482	0.923112
36407	3.04443	0.985892
36408	1.89226	1.05022
36409	1.89226	0.726639
36410	1.89226	0.919408
36411	1.89226	0.98654
36412	3.78453	0.96128
36413	1.89227	0.638649
36414	1.89227	0.725464
36415	1.89227	0.910255
36416	1.89227	0.581608
36417	3.78453	0.613368
36418	1.28418	0.804627
36419	1.46482	0.856526
36420	1.1211	0.70754
36421	1.1211	0.932464

36422	1.59382	0.721449
36423	1.59382	0.926828
36424	1.76897	1.11762
36425	1.46482	0.883495
36426	1.59382	0.956925
36427	1.46482	1.03141
36428	1.78138	0.936094
36429	1.65238	1.00041
36430	1.89226	0.833602
36431	1.89226	0.92654
36432	1.89226	0.806756
36433	1.89226	1.00143
36434	1.89226	0.699253
36435	1.89226	1.01671
36436	1.89226	0.808496
36437	1.89226	0.469005
36438	1.89226	0.4996
36439	1.89226	0.651359
36440	1.89226	0.66069
36441	1.89226	0.72694
36442	1.96832	1.43525
36443	2.40858	1.27846
36444	1.75211	0.900096
36445	2.20797	2.1
36446	4.1607	1.19482
36447	1.75211	0.907517
36448	2.20797	2.1
36449	1.51592	1.80145
36450	2.02064	1.34442
36451	3.77275	1.92682
36452	2.71793	0.752104
36453	2.67099	0.725401
36454	2.55105	0.721675
36455	2.71297	0.724937
36456	5.26898	0.726596
36457	2.36417	0.739496
36458	2.67098	0.730527
36459	2.55104	0.724099
36460	1.83418	0.707984
36461	4.91522	0.723661
36462	2.55105	0.722095
36463	2.71297	0.724751
36464	2.58032	0.718161
36465	2.28922	0.706801
36466	5.13137	0.722481
36467	2.55104	0.724512
36468	2.23787	0.709181
36469	1.67265	0.707077
36470	2.28922	0.710167
36471	4.22369	0.709918
36472	0.851542	0.967965
36473	0.851542	0.95385
36474	0.981419	0.926777
36475	0.854774	0.975818
36476	0.949431	0.944484
36477	0.753763	0.978716
36478	0.946131	0.955142
36479	0.946131	0.957845

36480	1.04073	0.953314
36481	0.981433	0.911155
36482	0.851528	0.984186
36483	0.854828	0.960785
36484	1.89226	1.02077
36485	1.89226	0.962423
36486	1.89226	0.952426
36487	1.89226	0.959624
36488	1.89226	1.00263
36489	1.89226	1.01202
36490	0.814227	0.89937
36491	0.814227	0.899588
36492	0.946117	0.876888
36493	1.07285	0.887885
36494	0.879132	0.87832
36495	0.923293	0.885144
36496	0.946119	0.900434
36497	0.946119	0.899197
36498	0.946131	0.888138
36499	0.814236	0.841029
36500	0.946106	0.917397
36501	0.870405	0.900824
36502	1.89224	0.965644
36503	1.89224	0.897143
36504	1.89224	0.897927
36505	1.89224	0.905079
36506	1.89224	0.948535
36507	1.89224	0.957188
36508	1.29388	1.17843
36509	1.04164	1.93091
36510	1.64834	1.66114
36511	1.82955	1.08977
36512	2.94222	1.66024
36513	1.4934	0.876536
36514	2.41522	0.817008
36515	2.20387	0.784132
36516	2.20387	1.08162
36517	1.75765	0.815252
36518	1.75765	1.07534
36519	2.41522	0.818043
36520	2.77303	2.1
36521	2.20387	1.58485
36522	2.58627	1.0506
36523	5.00149	1.416
36524	1.22222	0.905034
36525	2.20387	0.889786
36526	1.96831	0.810838
36527	1.96831	1.2675
36528	1.4934	0.837785
36529	1.4934	1.33929
36530	1.96832	1.76105
36531	2.58627	1.0532
36532	2.40858	0.930193
36533	2.40858	2.1
36534	2.20387	1.02945
36535	2.20387	2.1
36536	1.5605	2.1
36537	1.53775	2.1

36538	1.55398	2.1
36539	1.45806	2.1
36540	1.65642	2.1
36541	1.5605	2.1
36542	0.768876	2.1
36543	0.79033	2.1
36544	0.828211	2.1
36545	0.871233	2.1
36546	0.803843	2.1
36547	0.803843	2.1
36548	0.814698	2.1
36549	0.814698	2.1
36550	0.79033	2.1
36551	0.871233	2.1
36552	0.768876	2.1
36553	0.828212	2.1
36554	1.80252	2.1
36555	2.09715	0.969734
36556	1.9394	1.897
36557	1.50853	1.86553
36558	3.60567	2.04746
36559	1.96416	0.899521
36560	2.09715	0.957097
36561	1.88484	1.42609
36562	1.57916	0.825735
36563	3.67631	0.974413
36564	1.89226	0.937542
36565	1.89226	0.725575
36566	1.89226	0.829356
36567	1.89226	0.869164
36568	3.78453	0.810962
36569	1.89226	0.946911
36570	1.89226	1.02982
36571	1.89226	2.1
36572	1.89226	0.836517
36573	1.89226	1.9031
36574	1.89226	0.512435
36575	1.5852	1.53459
36576	1.50853	1.52735
36577	1.2955	2.1
36578	1.44472	0.962186
36579	2.95325	1.44173
36580	1.53065	1.20808
36581	1.57916	0.787167
36582	1.44472	0.93464
36583	1.78861	0.931534
36584	1.23527	0.943356
36585	1.23527	0.922247
36586	1.89227	0.786705
36587	1.89227	0.809418
36588	1.89227	0.956396
36589	1.89227	0.758051
36590	3.78453	0.820001
36591	1.89226	0.968122
36592	1.89226	0.982866
36593	1.89226	2.05184
36594	1.89226	0.944765
36595	1.89226	1.12038

36596	1.89226	0.80903
36597	1.89227	0.933618
36598	1.89227	0.826962
36599	1.89227	0.820149
36600	1.89227	0.832003
36601	3.78453	0.818099
36602	0.943708	0.834963
36603	0.752219	0.807405
36604	0.946132	0.799888
36605	0.946132	0.805953
36606	0.946133	0.765747
36607	0.946133	1.17289
36608	0.752218	0.88255
36609	0.752218	0.842367
36610	0.752218	0.827357
36611	0.946132	0.786104
36612	0.946133	0.880539
36613	0.943708	0.836278
36614	0.943992	2.1
36615	0.943992	1.71039
36616	1.23507	1.03765
36617	1.48209	1.96089
36618	1.0384	2.1
36619	1.47953	2.03545
36620	0.946128	1.02168
36621	0.946128	1.40438
36622	0.946129	2.03543
36623	1.23293	1.93788
36624	1.0384	1.89186
36625	0.946129	1.89997
36626	1.89226	1.49033
36627	2.46586	1.78451
36628	2.80218	1.00541
36629	2.80218	2.1
36630	1.89226	1.00598
36631	1.89226	2.1
36632	1.32455	2.1
36633	1.89128	2.1
36634	1.32328	2.1
36635	1.97299	2.1
36636	3.29754	2.1
36637	1.89128	2.1
36638	2.4255	2.1
36639	1.973	2.1
36640	2.63807	2.1
36641	4.3985	2.1
36642	1.34008	2.1
36643	1.97299	2.1
36644	2.10766	2.1
36645	2.10766	2.1
36646	1.32328	2.1
36647	1.32328	2.1
36648	2.41055	2.1
36649	2.37491	2.1
36650	2.40754	2.1
36651	2.38023	2.1
36652	4.78546	2.1
36653	2.23743	2.1

36654	2.37491	2.1
36655	2.40754	2.1
36656	2.20392	2.1
36657	4.61234	2.1
36658	2.37491	2.1
36659	2.02641	2.1
36660	2.20392	2.1
36661	2.18943	2.1
36662	4.40132	2.1
36663	2.37491	2.1
36664	2.22558	2.1
36665	2.38023	2.1
36666	2.18943	2.1
36667	4.60049	2.1
36668	1.89226	0.588786
36669	1.89226	0.709933
36670	1.89226	0.702456
36671	1.89226	0.701935
36672	1.89226	0.646079
36673	1.89226	0.638894
36674	1.03599	0.292608
36675	0.706698	0.267351
36676	1.41141	0.337517
36677	1.17573	0.303398
36678	1.11957	0.235101
36679	0.765034	0.247415
36680	1.05913	0.229214
36681	1.45054	0.325861
36682	1.04847	0.252233
36683	1.04847	0.294104
36684	1.15323	0.299057
36685	1.05825	0.238498
36686	1.89226	0.386069
36687	1.89226	0.363679
36688	1.89226	0.362617
36689	1.89226	0.348983
36690	1.89226	0.390363
36691	1.89226	0.38145
36692	2.22575	0.75724
36693	1.70677	1.50573
36694	1.90157	0.532579
36695	1.5534	2.05044
36696	3.77914	0.850184
36697	1.70678	1.39168
36698	0.968594	2.1
36699	0.968594	2.1
36700	1.10378	2.1
36701	1.5534	2.09845
36702	1.68859	2.1
36703	2.31429	0.740122
36704	1.5534	2.02819
36705	1.90103	0.442363
36706	1.41902	1.0175
36707	3.45443	0.846687
36708	2.53709	0.0884296
36709	1.209	0.0930561
36710	1.05417	0.0845244
36711	2.6513	0.0975179

36712	3.70547	0.0972451
36713	1.02675	0.0793274
36714	1.16384	0.08661
36715	2.22062	0.0876852
36716	2.48862	0.0793144
36717	3.38446	0.0786484
36718	1.89226	0.0983219
36719	1.89226	0.121174
36720	1.89226	0.119961
36721	1.89226	0.0909749
36722	1.89226	0.140109
36723	1.89226	0.0997056
36724	1.89226	1.1674
36725	1.89226	1.8853
36726	1.89226	1.90764
36727	1.89226	1.3085
36728	1.89226	1.59525
36729	1.89226	1.26949
36730	1.89226	1.08568
36731	1.89226	1.41739
36732	1.89226	1.45544
36733	1.89226	1.16409
36734	1.89226	1.3971
36735	1.89226	1.14998
36736	1.89227	1.18012
36737	1.89227	1.62571
36738	1.75032	1.59151
36739	1.75032	1.27662
36740	1.89227	1.47481
36741	1.89227	1.23948
36742	1.89226	0.785108
36743	1.89226	0.80087
36744	1.89226	0.776876
36745	1.89226	0.781751
36746	1.89226	0.789547
36747	1.89226	0.799235
36748	0.946132	0.729789
36749	0.946132	0.724972
36750	0.798215	0.723904
36751	0.848163	0.720987
36752	0.848163	0.729152
36753	0.946131	0.731185
36754	0.946132	0.719344
36755	0.798216	0.720502
36756	0.996105	0.725809
36757	0.996105	0.716064
36758	0.79819	0.7238
36759	0.79819	0.734907
36760	1.07763	0.737694
36761	2.28622	0.733433
36762	2.16109	0.714552
36763	1.17506	0.72407
36764	3.36385	0.725895
36765	1.89226	2.1
36766	1.6663	2.1
36767	1.6663	2.1
36768	1.71706	2.1
36769	1.89226	2.1

36770	1.94302	2.1
36771	1.89226	2.1
36772	1.62801	2.1
36773	1.62801	2.1
36774	1.6663	2.1
36775	1.89226	2.1
36776	1.93055	2.1
36777	1.48734	2.1
36778	1.99609	2.1
36779	1.52245	2.1
36780	2.04137	2.1
36781	3.51854	2.1
36782	1.99609	2.1
36783	2.28563	2.1
36784	2.04136	2.1
36785	2.35019	1.69556
36786	4.34628	2.1
36787	1.50714	2.1
36788	2.04137	2.1
36789	2.04945	2.1
36790	2.04945	2.1
36791	1.52245	2.1
36792	1.52245	2.1
36793	2.04136	2.1
36794	2.35019	1.83882
36795	2.04945	2.1
36796	2.38681	2.1
36797	4.39964	2.1
36798	2.50924	0.05
36799	1.38305	2.1
36800	2.56918	2.1
36801	1.44535	2.1
36802	3.95222	2.1
36803	1.89226	2.1
36804	1.89226	2.1
36805	1.89226	2.1
36806	1.89226	2.1
36807	3.78453	2.1
36808	1.40615	2.1
36809	1.38851	2.1
36810	0.748607	2.1
36811	0.724468	2.1
36812	1.06362	2.1
36813	1.06362	2.1
36814	1.067 2.1	
36815	1.067 2.1	
36816	1.40615	2.1
36817	0.748607	2.1
36818	0.724468	2.1
36819	1.38851	2.1
36820	2.56918	2.1
36821	1.53377	2.1
36822	1.53706	2.1
36823	1.44535	2.1
36824	2.66088	2.1
36825	2.56918	2.1
36826	1.89226	2.1
36827	1.89226	2.1

36828	1.89226	2.1
36829	1.89226	2.1
36830	3.78453	2.1
36831	1.40239	2.1
36832	1.68466	2.1
36833	1.26091	2.1
36834	1.54641	2.1
36835	2.94881	2.1
36836	1.68466	2.1
36837	1.65324	0.88347
36838	1.54641	2.1
36839	1.52268	0.895189
36840	3.19965	1.0379
36841	1.05776	2.1
36842	1.54641	2.1
36843	1.36595	2.1
36844	1.36595	2.1
36845	1.26091	2.1
36846	1.26091	2.1
36847	1.54641	2.1
36848	1.32697	0.885121
36849	1.52268	0.895876
36850	1.52268	2.1
36851	1.36595	0.896663
36852	1.36595	2.1
36853	1.18647	1.34044
36854	0.988454	2.1
36855	1.4867	1.70154
36856	1.63892	1.43968
36857	2.67317	2.05295
36858	1.4867	2.00996
36859	1.63892	1.5613
36860	2.09605	0.913799
36861	1.97645	1.85871
36862	3.58275	1.22856
36863	1.29389	1.86365
36864	1.97645	1.33359
36865	1.4867	1.23702
36866	1.4867	2.09102
36867	1.82955	1.06212
36868	1.82955	2.00028
36869	1.13454	1.15315
36870	0.983594	2.1
36871	1.16809	2.1
36872	1.06836	1.74323
36873	1.08332	1.65139
36874	0.983594	1.22313
36875	1.48751	2.1
36876	1.48047	1.56181
36877	1.77596	1.44826
36878	1.75506	1.4845
36879	3.25643	1.72154
36880	1.48047	1.42169
36881	1.25334	2.09415
36882	1.75505	1.22006
36883	1.55831	0.907078
36884	3.03878	1.60051
36885	1.77596	1.45803

36886	1.75506	1.94582
36887	2.00551	2.0934
36888	2.00083	1.01935
36889	3.77679	1.85035
36890	1.75506	1.65228
36891	1.80328	0.940788
36892	1.55831	1.63943
36893	1.55831	1.96566
36894	2.00083	0.984153
36895	2.00083	1.16011
36896	2.58627	1.03905
36897	2.79047	2.1
36898	2.40858	2.1
36899	2.64748	2.1
36900	5.23375	2.1
36901	1.26755	2.1
36902	1.71717	2.1
36903	1.03824	2.1
36904	1.48901	2.1
36905	2.75656	2.1
36906	1.89226	2.1
36907	1.89226	2.1
36908	1.89226	2.1
36909	1.89226	2.1
36910	1.89226	2.1
36911	1.89226	2.1
36912	1.36541	2.1
36913	1.22156	2.1
36914	0.946132	2.1
36915	0.946132	2.1
36916	1.36541	2.1
36917	1.36541	2.1
36918	0.946132	2.1
36919	0.946132	2.1
36920	1.36541	2.1
36921	0.946133	2.1
36922	0.946132	2.1
36923	1.22156	2.1
36924	1.18901	2.1
36925	1.61149	2.1
36926	1.98748	2.1
36927	1.534 2.1	
36928	3.1765	2.1
36929	1.89226	2.1
36930	1.89226	2.1
36931	1.89226	2.1
36932	1.89226	2.1
36933	3.78452	2.1
36934	1.98748	2.1
36935	1.534 2.1	
36936	2.27541	2.1
36937	1.98748	2.1
36938	1.87436	2.1
36939	1.87436	2.1
36940	2.2988	2.1
36941	2.2604	2.1
36942	2.20831	2.1
36943	2.02802	2.1

36944	4.32683	2.1
36945	2.26041	2.1
36946	1.98957	2.1
36947	2.02803	2.1
36948	1.75021	2.1
36949	4.0176	2.1
36950	2.34607	2.1
36951	2.02803	2.1
36952	1.86795	2.1
36953	1.81448	2.1
36954	3.89597	2.1
36955	2.02803	2.1
36956	1.75021	2.1
36957	1.81448	2.1
36958	1.57887	2.1
36959	3.6069	2.1
36960	0.946131	0.805091
36961	0.946131	2.1
36962	1.1235	1.68456
36963	1.1235	1.80645
36964	0.946131	1.24695
36965	0.946131	0.972849
36966	1.1235	2.09311
36967	1.13736	2.1
36968	1.13736	0.937949
36969	0.946131	1.0521
36970	1.1235	1.25253
36971	0.946131	1.18492
36972	1.89226	1.89678
36973	1.89226	2.1
36974	1.89226	1.02427
36975	1.89226	0.967206
36976	3.78452	0.991913
36977	0.563266	2.1
36978	0.563266	1.06242
36979	0.565606	0.899593
36980	0.565606	2.06803
36981	0.647086	1.6708
36982	0.624219	1.45979
36983	0.500035	1.10536
36984	0.481785	1.14023
36985	0.500034	0.923456
36986	0.647087	0.951801
36987	0.624219	1.38938
36988	0.481785	1.16482
36989	0.915788	0.707268
36990	0.915788	0.757122
36991	1.02732	0.869993
36992	1.02732	0.797135
36993	0.660831	0.824397
36994	1.11155	0.723372
36995	0.713295	0.751523
36996	1.26874	0.857973
36997	0.713295	0.788443
36998	0.660831	0.778133
36999	1.11155	0.778697
37000	1.26874	0.775632
37001	1.89226	0.926612

37002	1.85091	0.943396
37003	2.11193	0.993546
37004	1.85091	0.925823
37005	2.15328	0.908888
37006	1.89226	0.856881
37007	0.946131	0.820552
37008	0.946131	0.847862
37009	1.18275	0.862451
37010	1.05596	0.905042
37011	0.946127	0.879883
37012	0.946127	0.604354
37013	1.18275	0.869981
37014	1.18275	0.893511
37015	0.946128	0.899886
37016	1.18275	0.843566
37017	0.946129	0.857316
37018	1.05596	0.83003
37019	1.60936	2.1
37020	2.35846	2.1
37021	2.06424	2.1
37022	2.57651	2.1
37023	4.42269	2.1
37024	1.89226	2.1
37025	1.89226	2.1
37026	1.89226	2.1
37027	1.89226	2.1
37028	3.78453	2.1
37029	1.89226	2.1
37030	1.89226	2.1
37031	1.89226	2.1
37032	1.89226	2.1
37033	1.89226	2.1
37034	1.89226	2.1
37035	1.95053	2.1
37036	2.06424	2.1
37037	1.95053	2.1
37038	1.30584	2.1
37039	2.5	2.1
37040	1.8553	2.1
37041	2.49999	2.1
37042	2.57651	2.1
37043	2.49999	2.1
37044	2.06423	2.1
37045	2.80738	2.1
37046	2.37162	2.1
37047	1.89226	2.1
37048	1.89226	2.1
37049	1.89226	2.1
37050	1.89226	2.1
37051	1.89226	2.1
37052	1.89226	2.1
37053	1.49295	0.659396
37054	1.03824	0.31547
37055	1.51396	0.67782
37056	2.08514	0.556555
37057	3.12338	0.545503
37058	1.49798	0.475523
37059	2.32017	0.241081

37060	2.68236	0.673522
37061	2.08514	0.463043
37062	4.4053	0.435667
37063	1.89226	0.230865
37064	1.89226	0.176329
37065	1.89226	0.34833
37066	1.89226	0.255183
37067	3.78452	0.252226
37068	1.89226	0.476972
37069	1.89226	0.214817
37070	1.89226	0.406654
37071	1.89226	0.312365
37072	1.89226	0.312671
37073	1.89226	0.258183
37074	1.51396	0.464846
37075	2.08514	0.295636
37076	2.55393	0.689974
37077	2.02901	0.478679
37078	4.11415	0.447656
37079	1.89226	0.181217
37080	1.89226	0.146035
37081	1.89226	0.331636
37082	1.89226	0.282937
37083	3.78451	0.187005
37084	1.89226	0.250653
37085	1.89226	0.291089
37086	1.89226	0.169648
37087	1.89226	0.14931
37088	1.89226	0.363282
37089	1.89226	0.309515
37090	0.946131	0.442435
37091	0.946131	0.472542
37092	0.946132	0.72985
37093	0.946132	0.568437
37094	0.946132	0.603965
37095	0.946132	0.519001
37096	0.946132	0.62508
37097	0.946132	0.684998
37098	0.946132	0.512775
37099	0.946132	0.632876
37100	0.946132	0.646577
37101	0.946132	0.506007
37102	1.89226	2.1
37103	1.89226	2.1
37104	1.89226	2.1
37105	1.89226	1.84068
37106	3.78452	2.1
37107	1.28367	2.1
37108	1.82707	2.06333
37109	1.11025	2.1
37110	1.6365	1.61943
37111	2.92016	2.1
37112	1.82707	2.1
37113	1.8247	0.895115
37114	1.8247	2.1
37115	2.01549	0.939305
37116	1.6365	1.60706
37117	1.82729	0.94155

37118	1.89226	2.1
37119	1.89226	0.898109
37120	1.89226	0.849404
37121	1.89226	2.07424
37122	1.89226	0.878288
37123	1.89226	2.03303
37124	1.63648	2.1
37125	1.82467	0.900229
37126	1.35877	2.1
37127	1.55159	0.787662
37128	3.18345	1.05952
37129	1.89223	2.1
37130	1.89223	0.90247
37131	1.89223	2.1
37132	1.89223	0.885683
37133	3.78447	1.07331
37134	1.59972	2.1
37135	1.28037	2.1
37136	1.63702	2.1
37137	1.28158	2.1
37138	1.63581	2.1
37139	1.28037	2.1
37140	1.89226	2.1
37141	1.89226	2.1
37142	1.89226	2.1
37143	1.89226	2.1
37144	1.89226	2.1
37145	1.89226	2.1
37146	1.89226	2.1
37147	1.89226	2.1
37148	1.89226	2.1
37149	1.89226	2.1
37150	3.78451	2.1
37151	1.28037	2.1
37152	1.05071	2.1
37153	1.63581	2.1
37154	1.44287	2.1
37155	2.72324	2.1
37156	1.59972	2.1
37157	1.63581	2.1
37158	1.94291	2.1
37159	2.00118	2.1
37160	3.57872	2.1
37161	1.89226	2.1
37162	1.89226	2.1
37163	1.89226	2.1
37164	1.89226	2.1
37165	3.78452	2.1
37166	0.946131	2.0007
37167	0.946131	2.1
37168	0.946127	2.1
37169	0.946127	2.1
37170	0.94613	1.79009
37171	0.94613	2.1
37172	0.946128	2.1
37173	0.946128	2.1
37174	0.946128	2.1
37175	0.946128	2.1

37176	0.946129	2.1
37177	0.946129	2.1
37178	0.865056	2.1
37179	0.865056	2.1
37180	0.873224	2.1
37181	0.873224	2.1
37182	0.721437	2.1
37183	0.920378	2.1
37184	0.817903	2.1
37185	1.00059	2.1
37186	1.00059	2.1
37187	0.920376	2.1
37188	0.817904	2.1
37189	0.721437	2.1
37190	3.09157	2.1
37191	1.71255	2.1
37192	2.0815	2.1
37193	1.9918	2.1
37194	2.51364	2.1
37195	2.39396	2.1
37196	2.0815	2.1
37197	1.05051	2.1
37198	2.56724	2.1
37199	1.63991	2.1
37200	3.47929	2.1
37201	1.89226	0.643433
37202	2.2973	0.724692
37203	1.90705	0.724489
37204	1.90705	0.704662
37205	1.89226	0.710883
37206	1.89226	0.698842
37207	1.89226	0.649576
37208	1.90705	0.723519
37209	1.45321	0.729154
37210	1.45321	0.705317
37211	1.89226	0.711944
37212	1.89226	0.697352
37213	0.901146	2.1
37214	0.901146	2.1
37215	1.15227	2.1
37216	1.21279	2.1
37217	0.991265	2.1
37218	1.15003	2.1
37219	0.946132	2.1
37220	0.946132	2.1
37221	0.946132	2.1
37222	1.09997	2.1
37223	0.991266	2.1
37224	0.946132	2.1
37225	1.89226	2.1
37226	1.89226	2.1
37227	1.89226	2.1
37228	1.89226	2.1
37229	1.89226	2.1
37230	1.89226	2.1
37231	1.62229	2.1
37232	1.06053	2.1
37233	1.19698	2.1

37234	1.70106	2.1
37235	2.81928	2.1
37236	0.946132	2.1
37237	0.946132	2.1
37238	1.17527	2.1
37239	1.09997	2.1
37240	1.09997	2.1
37241	1.09997	2.1
37242	0.946133	2.1
37243	0.946133	2.1
37244	1.17527	2.1
37245	0.946132	2.1
37246	0.946133	2.1
37247	1.09997	2.1
37248	1.89226	2.1
37249	1.89226	2.1
37250	1.89226	2.1
37251	1.89226	2.1
37252	1.89226	2.1
37253	1.89226	2.1
37254	0.905416	2.1
37255	0.850527	2.1
37256	0.660601	2.1
37257	0.598494	2.1
37258	0.750137	2.1
37259	0.750137	2.1
37260	0.753774	2.1
37261	0.753774	2.1
37262	0.660599	2.1
37263	0.905418	2.1
37264	0.598493	2.1
37265	0.850529	2.1
37266	2.43509	2.1
37267	2.65066	2.1
37268	2.66602	2.1
37269	2.46339	2.1
37270	5.10111	2.1
37271	2.53008	2.1
37272	2.65066	2.1
37273	2.66602	2.1
37274	2.52852	2.1
37275	5.1961	2.1
37276	2.66602	2.1
37277	2.46339	2.1
37278	2.47932	2.1
37279	2.67393	2.1
37280	5.14535	2.1
37281	2.66602	2.1
37282	2.52853	2.1
37283	2.51686	2.1
37284	2.67393	2.1
37285	5.18288	2.1
37286	1.89226	0.910775
37287	1.89226	0.742545
37288	1.89226	0.902557
37289	1.89226	0.735542
37290	3.78452	0.80619
37291	1.55161	0.915702

37292	1.53972	0.738603
37293	1.32259	0.899032
37294	1.35082	0.730198
37295	2.8623	0.803025
37296	1.53972	0.733385
37297	1.4054	0.727442
37298	1.35083	0.736042
37299	1.24913	0.709267
37300	2.78885	0.713591
37301	1.89226	0.737305
37302	1.89226	0.741259
37303	1.89226	0.735077
37304	1.89226	0.712568
37305	3.78452	0.705991
37306	1.89226	0.88884
37307	1.89226	0.753007
37308	1.89226	0.913039
37309	1.89226	0.762275
37310	1.89226	0.90797
37311	1.89226	0.77429
37312	1.14599	0.886831
37313	1.35082	0.746414
37314	1.14599	0.794743
37315	1.32259	0.908127
37316	1.19993	0.742803
37317	1.37652	0.935739
37318	1.35083	0.74113
37319	1.24913	0.731201
37320	1.19993	0.763603
37321	1.11787	0.69522
37322	2.44906	0.712012
37323	1.89226	0.749134
37324	1.89226	0.731178
37325	1.89226	0.763367
37326	1.89226	0.718647
37327	3.78452	0.71536
37328	1.51697	0.511333
37329	2.57264	0.597359
37330	1.30203	0.306824
37331	2.41701	0.537631
37332	3.93398	0.512999
37333	1.17464	0.264485
37334	2.41701	0.482883
37335	2.32462	0.412614
37336	2.32462	0.424854
37337	1.30203	0.374276
37338	1.30203	0.360794
37339	2.08149	2.1
37340	1.9839	2.1
37341	1.71398	2.1
37342	1.99182	0.836341
37343	3.71121	2.04669
37344	2.16061	2.04071
37345	2.01399	0.73431
37346	1.99182	0.867512
37347	1.83895	0.829612
37348	4.00581	0.82884
37349	1.90444	2.1

37350	1.99182	0.865327
37351	1.71717	2.1
37352	1.805	0.959798
37353	3.70899	2.1
37354	1.99182	0.879807
37355	1.83896	0.751786
37356	1.805	1.38856
37357	1.65243	0.762056
37358	3.64425	0.811821
37359	1.18076	2.1
37360	1.27697	2.1
37361	1.05025	2.1
37362	1.27697	2.1
37363	1.25	2.1
37364	1.47671	2.1
37365	1.89226	2.1
37366	1.89226	1.91933
37367	1.89226	2.1
37368	1.89226	2.1
37369	3.78452	2.1
37370	2.64124	2.1
37371	1.89226	2.1
37372	1.89226	2.1
37373	1.89226	2.1
37374	2.46904	2.1
37375	2.46904	2.1
37376	1.89226	2.1
37377	1.89226	2.1
37378	2.7408	2.1
37379	2.82858	2.06743
37380	4.72084	2.1
37381	1.87875	2.1
37382	1.37704	2.1
37383	1.77042	2.1
37384	2.27801	2.1
37385	3.64916	2.1
37386	1.77042	2.1
37387	2.27801	2.1
37388	2.58523	2.1
37389	1.90178	2.1
37390	4.35565	2.1
37391	1.28667	2.1
37392	1.30711	2.1
37393	1.53077	2.1
37394	1.55635	2.1
37395	2.81743	2.1
37396	1.55635	2.1
37397	1.28228	2.1
37398	1.64268	1.88138
37399	1.93673	2.1
37400	3.19903	2.1
37401	1.53077	2.1
37402	1.93673	2.1
37403	1.55635	2.1
37404	1.55635	2.1
37405	1.86206	2.1
37406	1.86206	2.1
37407	1.64892	2.1

37408	1.60532	2.1
37409	1.85282	2.1
37410	1.96716	2.1
37411	3.61608	2.1
37412	1.60531	2.1
37413	1.32414	2.1
37414	1.96716	2.1
37415	1.75823	2.1
37416	3.2913	2.1
37417	1.91219	2.1
37418	1.96716	2.1
37419	2.0815	2.1
37420	2.51396	2.1
37421	4.30843	2.1
37422	1.96716	2.1
37423	2.17979	2.1
37424	1.75823	2.1
37425	1.75823	2.1
37426	2.28541	2.1
37427	2.28541	2.1
37428	1.89226	1.11403
37429	1.89226	1.53235
37430	1.89226	1.13311
37431	1.89226	1.02149
37432	3.78453	1.12152
37433	1.89226	1.25144
37434	1.89226	1.1838
37435	1.89226	1.1557
37436	1.89226	1.28604
37437	1.89226	1.15551
37438	1.89226	1.34109
37439	1.61016	1.11946
37440	1.45303	1.41241
37441	1.62812	1.13252
37442	1.86379	1.10378
37443	3.23828	1.10976
37444	1.61016	1.30755
37445	1.78716	1.52138
37446	2.0815	1.00334
37447	1.6774	1.33321
37448	3.54454	1.27684
37449	1.89226	1.25898
37450	1.89226	1.12404
37451	1.89226	0.996614
37452	1.89226	1.22185
37453	3.78453	1.1205
37454	1.89226	1.33668
37455	1.89226	1.2908
37456	1.89226	1.22763
37457	1.89226	1.66673
37458	1.89226	1.02863
37459	1.89226	1.06256
37460	0.807591	1.347
37461	0.814059	1.09726
37462	1.00217	1.45583
37463	0.931896	1.17085
37464	0.850586	1.20979
37465	0.850586	1.51486

37466	0.8889	1.10105
37467	0.8889	1.295
37468	0.807592	0.988415
37469	1.00217	1.34684
37470	0.814061	1.1908
37471	0.931894	1.65203
37472	2.0815	1.08525
37473	1.6774	1.71946
37474	2.00435	1.11281
37475	2.36104	1.09644
37476	3.86809	1.09123
37477	1.00532	0.702019
37478	1.00532	0.704004
37479	1.00183	0.705497
37480	1.00183	0.702858
37481	1.26377	0.702923
37482	1.34921	0.701839
37483	0.664872	0.703541
37484	0.743381	0.704757
37485	0.664872	0.703151
37486	1.26377	0.703704
37487	1.34921	0.703912
37488	0.743381	0.705108
37489	1.26826	0.05
37490	0.946131	2.0049
37491	1.02404	1.86272
37492	0.94613	2.1
37493	1.30281	1.95487
37494	0.930941	2.1
37495	0.930941	1.9748
37496	1.318	1.99162
37497	1.58161	1.88801
37498	0.94613	1.94819
37499	0.94613	1.63433
37500	1.56338	1.81132
37501	1.02404	1.78121
37502	2.81374	1.87324
37503	1.89226	2.1
37504	1.89226	2.02622
37505	1.89226	2.08056
37506	3.78453	2.1
37507	0.946133	2.1
37508	0.946133	1.74623
37509	0.946132	1.69208
37510	0.946132	1.99344
37511	0.946132	1.98926
37512	0.946132	1.81193
37513	0.946133	2.04654
37514	0.946133	1.7503
37515	0.946132	2.04663
37516	0.946132	1.6819
37517	0.946133	2.1
37518	0.946133	1.80755
37519	1.89226	1.47264
37520	1.89226	1.99683
37521	1.89226	1.53031
37522	1.89226	1.65913
37523	3.78452	1.75826

37524	1.89226	2.00686
37525	1.89226	1.8908
37526	1.89226	2.1
37527	1.89226	1.57875
37528	1.89226	2.1
37529	1.89226	1.59632
37530	0.946133	1.52396
37531	0.946133	1.98007
37532	0.946132	1.40496
37533	0.946132	1.90225
37534	0.946132	1.89792
37535	0.946132	1.77225
37536	0.946133	1.88133
37537	0.946133	1.53788
37538	0.946132	1.91863
37539	0.946132	1.55688
37540	0.946132	1.99228
37541	0.946132	1.5941
37542	2.56953	2.1
37543	2.64148	2.1
37544	2.48631	2.1
37545	2.62213	2.1
37546	5.19166	2.1
37547	2.64148	2.1
37548	2.57929	2.1
37549	2.62213	2.1
37550	2.59522	2.1
37551	5.20142	2.1
37552	2.48631	2.1
37553	2.62213	2.1
37554	2.41176	2.1
37555	2.59262	2.1
37556	5.03389	2.1
37557	2.62213	2.1
37558	2.59522	2.1
37559	2.59262	2.1
37560	2.61901	2.1
37561	5.24114	2.1
37562	0.946132	0.089602
37563	0.946132	0.0790687
37564	0.946133	0.0778672
37565	0.946133	0.0878797
37566	0.946132	0.0808474
37567	0.946132	0.0848674
37568	0.946133	0.0830837
37569	0.946133	0.0865249
37570	0.946132	0.0767977
37571	0.946132	0.0805307
37572	0.946133	0.0971994
37573	0.946133	0.0904498
37574	1.5681	0.0785885
37575	1.01248	0.0686836
37576	1.10749	0.0722111
37577	0.570913	0.0704186
37578	1.15589	0.0709698
37579	1.15588	0.0759704
37580	0.977549	0.0714062
37581	0.977549	0.0743914

37582	1.01249	0.0681026
37583	0.654445	0.067898
37584	1.36795	0.0773634
37585	1.10748	0.0747812
37586	1.73883	0.05
37587	1.87638	0.05
37588	1.85219	0.05
37589	1.93113	2.1
37590	3.7407	0.05
37591	1.87638	0.05
37592	1.73883	2.1
37593	1.93113	2.1
37594	1.80426	2.1
37595	3.66996	2.1
37596	2.02912	0.403728
37597	1.44951	0.481998
37598	1.50103	0.575412
37599	2.02248	0.60016
37600	3.47199	0.422171
37601	1.89226	0.523918
37602	1.89226	0.561652
37603	1.89226	0.625396
37604	1.89226	0.64674
37605	3.78453	0.630446
37606	2.57881	0.518315
37607	1.89226	0.628382
37608	1.89226	0.611701
37609	1.89226	0.528586
37610	2.6428	0.584954
37611	2.6428	0.512911
37612	1.01379	0.500602
37613	0.985937	0.471093
37614	0.769295	0.472179
37615	0.715547	0.427853
37616	0.853042	0.519347
37617	0.853042	0.451382
37618	0.864117	0.588225
37619	0.864114	0.33116
37620	0.715556	0.623766
37621	1.03047	0.601668
37622	0.731721	0.576347
37623	0.985921	0.552598
37624	1.89227	0.479631
37625	1.89227	0.608771
37626	1.89227	0.620295
37627	1.89227	0.642015
37628	3.78453	0.537389
37629	0.946129	0.505095
37630	0.946129	0.464598
37631	1.28161	0.446993
37632	1.3214	0.531703
37633	0.94613	0.583679
37634	0.94613	0.485517
37635	1.28161	0.455755
37636	1.28161	0.35774
37637	1.28161	0.47413
37638	0.94613	0.510025
37639	0.946131	0.530967

37640	1.3214	0.501443
37641	0.946132	0.789578
37642	0.946132	0.766726
37643	0.706045	0.752535
37644	1.16718	0.739824
37645	0.706017	0.74765
37646	0.706017	0.806475
37647	1.4073	0.741608
37648	1.4073	0.733642
37649	0.94613	0.757483
37650	1.16719	0.756905
37651	0.706046	0.772806
37652	0.946131	0.767851
37653	1.89226	0.805747
37654	1.89226	0.789531
37655	1.89226	0.750913
37656	1.89226	0.842279
37657	1.89226	0.74849
37658	1.89226	0.837476
37659	0.946119	0.767012
37660	0.946119	0.751915
37661	0.946115	0.759363
37662	0.946115	0.736987
37663	0.946117	0.76139
37664	0.946117	0.786408
37665	0.946117	0.744205
37666	0.946117	0.736022
37667	0.94613	0.739121
37668	0.94613	0.756739
37669	0.946105	0.764226
37670	0.946105	0.750547
37671	0.946132	1.02026
37672	0.946132	0.980551
37673	0.946131	1.03421
37674	0.946131	0.947076
37675	0.946132	0.999129
37676	0.946132	1.04493
37677	0.946132	0.970745
37678	0.827865	0.969281
37679	0.946132	0.979551
37680	0.946132	0.996822
37681	0.946132	0.979946
37682	0.827865	0.988647
37683	1.89226	1.32747
37684	1.89226	1.16989
37685	1.89226	1.46332
37686	1.89226	1.09243
37687	1.89226	1.81542
37688	1.89226	1.24313
37689	0.93094	1.04817
37690	0.93094	1.1151
37691	1.04649	1.07071
37692	0.614468	0.953013
37693	0.950358	0.98649
37694	0.799012	0.957532
37695	0.682742	1.07738
37696	0.682742	1.07098
37697	1.04073	1.48064

37698	1.15596	1.25221
37699	0.614478	1.264
37700	0.851524	1.12442
37701	2.22575	2.0101
37702	1.90502	2.1
37703	2.10893	1.62173
37704	1.80258	2.1
37705	4.01395	2.1
37706	1.90502	2.1
37707	1.40863	2.1
37708	1.49733	2.1
37709	1.49733	2.1
37710	1.80258	2.1
37711	1.80258	2.1
37712	2.10893	1.40162
37713	1.68356	2.1
37714	1.80258	2.1
37715	1.80258	2.1
37716	1.98061	2.1
37717	1.98061	2.1
37718	1.80258	2.1
37719	1.40863	2.1
37720	1.68356	2.1
37721	1.30751	2.1
37722	3.11009	2.1
37723	1.35374	1.32326
37724	1.35374	1.54952
37725	0.746096	1.1549
37726	0.746096	1.11901
37727	1.1537	1.19343
37728	0.746095	1.42617
37729	0.946132	1.18614
37730	0.946132	1.05156
37731	0.946132	0.986036
37732	0.746095	1.10291
37733	0.946131	1.00594
37734	1.1537	1.06581
37735	2.15247	2.1
37736	1.70303	1.40586
37737	1.41207	2.1
37738	1.43052	2.1
37739	3.22793	2.1
37740	0.940246	2.1
37741	0.728442	2.1
37742	0.650037	2.1
37743	0.539874	2.1
37744	0.694756	2.1
37745	0.694756	2.1
37746	0.776346	2.1
37747	0.656772	2.1
37748	0.733146	2.1
37749	0.563769	2.1
37750	0.66316	2.1
37751	0.899692	2.1
37752	1.21223	2.1
37753	1.22418	2.1
37754	1.43052	2.1
37755	1.43052	1.38538

37756	1.24353	2.1
37757	1.46181	1.695
37758	1.89226	0.611719
37759	2.39894	0.423626
37760	1.89226	0.691395
37761	2.37417	0.516204
37762	1.89226	0.619821
37763	2.37417	0.393124
37764	1.12767	0.393837
37765	1.19947	0.477463
37766	0.94613	0.633649
37767	0.94613	0.476456
37768	1.12766	0.343188
37769	0.946128	0.362594
37770	1.19947	0.480029
37771	0.946132	0.656646
37772	1.19947	0.618694
37773	1.19947	0.333012
37774	0.94613	0.6348
37775	0.94613	0.366882
37776	2.22621	0.05
37777	1.14533	2.1
37778	1.1131	2.1
37779	0.88238	2.1
37780	0.868521	1.4117
37781	1.01153	2.1
37782	1.01153	2.1
37783	1.00232	2.1
37784	1.00232	2.1
37785	1.14533	2.1
37786	0.882378	2.1
37787	0.868519	2.1
37788	1.11311	2.1
37789	0.910183	1.26352
37790	1.07811	1.05405
37791	0.94613	0.786054
37792	0.94613	0.850022
37793	0.910212	0.882171
37794	0.910212	1.33162
37795	1.11403	0.790131
37796	1.11403	1.41766
37797	0.910183	0.929402
37798	0.94613	0.950028
37799	0.94613	0.984967
37800	1.07811	0.974196
37801	1.87228	1.03775
37802	2.81189	2.1
37803	1.6197	2.1
37804	2.56716	1.96379
37805	4.43944	2.1
37806	0.946132	1.10033
37807	0.946132	2.06491
37808	0.946132	0.847653
37809	0.946132	0.920291
37810	0.946131	0.993032
37811	0.946131	2.07277
37812	0.946132	0.867297
37813	0.946132	1.1207

37814	0.946132	1.03321
37815	0.946132	1.05881
37816	0.946132	1.11517
37817	0.946132	1.1258
37818	1.28724	2.1
37819	2.56716	1.80623
37820	2.3014	1.30901
37821	2.3014	2.1
37822	1.6197	1.73534
37823	1.6197	2.1
37824	0.946132	1.98699
37825	0.946132	1.54942
37826	0.946132	0.887673
37827	0.946132	0.984944
37828	0.946132	1.02714
37829	0.946132	2.04081
37830	0.946133	2.08702
37831	0.946133	0.894826
37832	0.946133	1.08534
37833	0.946133	1.06881
37834	0.946132	1.08045
37835	0.946132	1.09737
37836	1.08039	2.1
37837	1.24445	1.58514
37838	1.2873	2.0517
37839	1.48751	2.00738
37840	2.53175	2.1
37841	0.946125	0.935387
37842	0.946125	2.1
37843	1.12552	2.00792
37844	1.12552	2.1
37845	0.946124	1.47948
37846	0.946124	1.68487
37847	1.12552	2.1
37848	1.11114	2.1
37849	1.11113	1.64006
37850	0.946118	1.97287
37851	1.12552	2.05562
37852	0.946131	1.98151
37853	1.89225	1.09964
37854	1.89225	1.25324
37855	1.89225	1.84052
37856	1.89225	2.1
37857	1.89225	1.0124
37858	1.89225	2.03067
37859	0.987703	1.05938
37860	1.33078	1.63818
37861	1.31565	1.12427
37862	1.31565	1.71635
37863	1.01121	1.15281
37864	1.01121	1.99793
37865	2.19163	2.1
37866	1.89226	1.85877
37867	1.89226	2.1
37868	1.89226	1.33647
37869	2.22229	2.1
37870	2.22229	1.21946
37871	1.89226	2.1

37872	1.89226	0.950668
37873	1.89226	0.942026
37874	1.89226	2.02499
37875	1.89226	0.980048
37876	1.89226	2.02838
37877	0.57141	2.027
37878	0.57141	0.981163
37879	0.574036	1.10838
37880	0.574036	2.1
37881	0.651591	1.75921
37882	0.657827	1.18601
37883	0.482391	1.24569
37884	0.493855	1.50518
37885	0.482391	0.994583
37886	0.651591	1.05573
37887	0.493855	1.53066
37888	0.657828	1.46022
37889	1.88428	2.1
37890	2.03975	2.1
37891	1.97316	2.1
37892	1.8266	2.1
37893	3.86635	2.1
37894	2.23053	2.1
37895	1.42255	2.1
37896	1.3868	2.1
37897	1.8266	2.1
37898	3.24915	2.1
37899	1.97316	2.1
37900	1.8266	2.1
37901	1.77128	2.1
37902	1.91767	2.1
37903	3.74427	2.1
37904	1.77128	2.1
37905	1.8266	2.1
37906	1.77942	2.1
37907	1.33962	2.1
37908	1.3868	2.1
37909	1.3868	2.1
37910	0.944111	2.1
37911	0.944111	2.1
37912	1.26036	2.1
37913	1.16968	2.1
37914	1.03853	2.1
37915	1.16646	2.1
37916	0.946129	2.1
37917	0.946129	2.1
37918	0.94613	2.1
37919	1.25768	2.1
37920	1.03853	2.1
37921	0.946131	2.1
37922	1.82441	0.225525
37923	1.33061	0.21485
37924	1.13272	0.155393
37925	1.60249	0.192127
37926	2.95712	0.220227
37927	1.82441	0.232456
37928	2.08757	0.236076
37929	1.81292	0.187072

37930	1.60249	0.169506
37931	3.63733	0.203975
37932	1.81292	0.16647
37933	1.332	0.12213
37934	1.60249	0.134222
37935	1.60249	0.178374
37936	1.5344	0.117356
37937	1.5344	0.155432
37938	1.10107	2.1
37939	2.51559	2.1
37940	1.1229	2.1
37941	1.1229	2.1
37942	2.49456	2.1
37943	2.49456	2.1
37944	1.89226	2.1
37945	2.51559	2.1
37946	2.49457	2.1
37947	2.51559	2.1
37948	1.87124	2.1
37949	1.89226	2.1
37950	1.89226	2.1
37951	1.89226	2.1
37952	1.89226	2.1
37953	1.89226	2.1
37954	1.89226	2.1
37955	1.89226	2.1
37956	1.89226	2.1
37957	1.89226	2.1
37958	1.89226	2.1
37959	1.89226	2.1
37960	1.89226	2.1
37961	1.89226	2.1
37962	1.23599	2.1
37963	1.24728	2.1
37964	0.550534	2.1
37965	0.541151	2.1
37966	0.893754	2.1
37967	0.893754	2.1
37968	0.894678	2.1
37969	0.894678	2.1
37970	1.23599	2.1
37971	0.541149	2.1
37972	0.550533	2.1
37973	1.24728	2.1
37974	0.946132	2.1
37975	0.946132	2.1
37976	1.23599	2.1
37977	1.24728	2.1
37978	0.946132	2.1
37979	0.946132	2.1
37980	1.23599	2.1
37981	1.23599	2.1
37982	1.23599	2.1
37983	0.946132	2.1
37984	0.946132	2.1
37985	1.24728	2.1
37986	1.89226	2.1
37987	1.89226	2.1

37988	1.89226	2.1
37989	1.89226	2.1
37990	1.89226	2.1
37991	1.89226	2.1
37992	0.946128	2.1
37993	0.946128	2.1
37994	0.946132	2.1
37995	0.946132	2.1
37996	0.94613	2.1
37997	0.94613	2.1
37998	0.946129	2.1
37999	0.946129	2.1
38000	0.94613	2.1
38001	0.94613	2.1
38002	0.94613	2.1
38003	0.94613	2.1
38004	2.58696	0.7
38005	2.57073	0.711182
38006	2.12585	0.701042
38007	2.07607	0.704904
38008	4.69658	0.703442
38009	1.89226	0.712403
38010	1.89226	0.711242
38011	1.89226	0.701139
38012	1.89226	0.734585
38013	3.78452	0.708674
38014	1.89226	0.712091
38015	1.89226	0.75871
38016	1.89226	0.737264
38017	1.89226	0.737199
38018	3.78453	0.738827
38019	2.57074	0.71212
38020	2.32635	0.733026
38021	2.07608	0.707042
38022	1.73623	0.733651
38023	4.30697	0.713298
38024	2.12582	0.702698
38025	1.51971	0.704513
38026	1.57255	0.700904
38027	2.07604	0.710317
38028	1.62233	0.697098
38029	2.12582	0.70782
38030	1.89223	0.702892
38031	1.89223	0.737647
38032	1.89223	0.713127
38033	1.89223	0.714776
38034	1.89223	0.714367
38035	1.89223	0.715727
38036	0.946119	0.748879
38037	0.946119	0.735757
38038	0.946119	0.712981
38039	0.946119	0.735421
38040	0.946119	0.741361
38041	0.946119	0.74913
38042	0.94612	0.739786
38043	0.94612	0.715141
38044	0.946132	0.735016
38045	0.946132	0.729141

38046	0.946107	0.733993
38047	0.946107	0.73247
38048	0.811456	0.715454
38049	0.811456	0.738107
38050	0.806521	0.705962
38051	0.806521	0.732243
38052	0.868104	0.730155
38053	0.579949	0.735087
38054	0.759859	0.705697
38055	1.03802	0.710627
38056	0.579957	0.717382
38057	0.759868	0.716042
38058	1.03801	0.723865
38059	0.868093	0.725543
38060	2.7408	2.1
38061	2.82858	1.96236
38062	2.57368	2.1
38063	2.52633	2.1
38064	5.3549	2.1
38065	2.7408	2.1
38066	2.31353	2.1
38067	2.14063	2.1
38068	2.52632	2.1
38069	4.83985	2.1
38070	2.57368	2.1
38071	2.52633	2.1
38072	2.3301	2.1
38073	2.36071	1.96383
38074	4.88704	2.1
38075	1.86512	2.1
38076	2.52632	2.1
38077	2.14063	2.1
38078	2.14063	2.1
38079	2.3301	2.1
38080	2.3301	2.1
38081	1.86431	2.1
38082	1.89227	2.1
38083	1.68992	2.1
38084	1.68992	2.1
38085	1.89227	2.1
38086	1.89227	2.1
38087	2.0815	2.1
38088	1.89226	2.1
38089	2.04022	2.1
38090	1.86431	2.1
38091	2.07081	2.1
38092	1.89226	2.1
38093	1.91686	1.16063
38094	1.65683	1.39534
38095	1.86871	2.1
38096	1.65683	1.18894
38097	2.13075	1.38549
38098	1.89226	0.936693
38099	1.89226	1.44942
38100	1.89226	0.918377
38101	1.89226	1.02194
38102	1.89226	1.4132
38103	3.78452	1.08148

38104	0.851541	2.1
38105	0.851541	1.8731
38106	0.952803	2.1
38107	0.851573	2.1
38108	0.946129	2.1
38109	0.946129	1.24782
38110	0.929184	2.1
38111	0.757482	2.1
38112	0.836274	2.1
38113	0.851528	2.1
38114	0.952816	2.1
38115	1.04073	2.1
38116	1.70303	1.0507
38117	1.70302	1.08968
38118	1.5544	1.54228
38119	1.70302	1.0379
38120	1.74957	1.08295
38121	1.91686	0.889971
38122	1.89226	0.957465
38123	1.89226	1.13874
38124	1.89226	1.33529
38125	1.89226	1.01211
38126	1.89226	1.02867
38127	1.89226	0.859577
38128	1.89226	1.91381
38129	1.89226	2.1
38130	2.03472	2.1
38131	1.89226	2.084
38132	1.99827	2.1
38133	1.85836	1.29127
38134	1.00509	2.1
38135	1.55998	2.1
38136	1.83347	2.1
38137	1.45246	2.1
38138	3.01244	2.1
38139	1.23839	2.1
38140	0.884251	2.1
38141	0.946133	2.1
38142	0.946133	2.1
38143	0.884226	2.1
38144	0.884226	2.1
38145	0.946158	2.1
38146	0.946159	2.1
38147	1.23839	2.1
38148	0.946131	2.1
38149	0.946131	2.1
38150	0.884254	2.1
38151	1.89226	2.1
38152	1.89226	1.47795
38153	1.89226	2.03006
38154	1.89226	2.1
38155	1.89226	2.1
38156	1.89226	2.1
38157	2.1499	2.1
38158	1.45244	2.1
38159	1.83345	2.05658
38160	1.83345	2.1
38161	1.87214	2.00143

38162	1.87214	2.1
38163	1.89224	2.1
38164	1.89224	1.25166
38165	1.89224	1.78451
38166	1.89224	1.90872
38167	3.78448	2.1
38168	1.49155	1.06419
38169	2.0815	1.21231
38170	1.77515	1.35122
38171	1.72377	2.1
38172	3.50659	1.34448
38173	1.69876	0.385183
38174	2.0815	1.80823
38175	1.9133	1.02777
38176	1.68189	0.607866
38177	2.17628	2.00272
38178	1.94335	1.75194
38179	2.65129	0.100847
38180	1.15317	0.0931321
38181	2.57085	0.0893173
38182	1.209	0.0954316
38183	2.57085	0.090528
38184	1.209	0.0978833
38185	1.89226	0.124754
38186	1.89226	0.102907
38187	1.89226	0.0977299
38188	1.89226	0.116997
38189	3.78452	0.119534
38190	2.48861	0.0781342
38191	1.15252	0.0847879
38192	2.60515	0.0926545
38193	2.60515	0.0786343
38194	1.16384	0.0902963
38195	1.16384	0.0784624
38196	1.59903	0.823448
38197	1.26865	0.832163
38198	1.30486	1.05023
38199	1.70302	0.877213
38200	2.98573	0.880029
38201	1.59903	0.795276
38202	1.79478	0.896364
38203	1.79478	0.828568
38204	1.97005	0.852553
38205	1.50365	0.809321
38206	1.64192	1.0202
38207	1.89226	0.755799
38208	1.89226	0.818533
38209	1.89226	0.911678
38210	1.89226	0.786855
38211	3.78453	0.778488
38212	1.89226	0.871814
38213	1.89226	0.808632
38214	1.89226	0.872749
38215	1.89226	0.777849
38216	1.89226	0.871434
38217	1.89226	0.900221
38218	0.761672	0.784102
38219	0.896406	0.819559

38220	0.603509	0.825389
38221	0.574071	0.879402
38222	0.695159	0.896842
38223	0.695159	0.753461
38224	0.762564	1.05009
38225	0.770159	0.809112
38226	0.803021	0.889934
38227	0.572529	0.948223
38228	0.696466	0.774071
38229	0.899733	0.776642
38230	1.66034	0.720019
38231	1.79478	0.87585
38232	1.56685	0.803945
38233	1.68512	1.09989
38234	1.67652	0.831002
38235	1.67652	0.887924
38236	1.89226	0.888334
38237	1.89226	0.811295
38238	1.89226	0.860566
38239	1.89226	0.861391
38240	1.89226	0.871419
38241	1.89226	0.863409
38242	0.946133	0.770639
38243	0.946133	0.812478
38244	0.946132	0.859008
38245	0.946132	0.815164
38246	0.946132	0.835588
38247	0.946132	0.725663
38248	0.946133	1.10117
38249	0.946133	0.788432
38250	0.946133	0.840726
38251	0.946133	0.86463
38252	0.946132	0.754826
38253	0.946132	0.753379
38254	1.80424	2.1
38255	1.45158	2.1
38256	1.9098	2.1
38257	1.56003	2.1
38258	3.36138	2.1
38259	1.90982	2.1
38260	1.71606	2.1
38261	2.15314	2.1
38262	1.70302	2.1
38263	3.89051	2.1
38264	1.96416	0.892802
38265	1.23706	0.765468
38266	1.00053	0.808899
38267	1.38287	0.749187
38268	1.57916	0.785094
38269	1.9615	0.793738
38270	0.827865	0.724192
38271	0.946132	0.717699
38272	0.946132	0.73122
38273	0.946132	0.766087
38274	0.946132	0.758806
38275	0.946132	0.726144
38276	0.946131	0.764139
38277	0.946131	0.765329

38278	0.827865	0.795423
38279	0.946132	0.731228
38280	0.946132	0.714768
38281	0.946132	0.715716
38282	1.89227	2.1
38283	1.89227	2.1
38284	1.89227	2.1
38285	1.89227	2.1
38286	3.78453	2.1
38287	2.3819	2.1
38288	1.58089	2.1
38289	1.58089	2.1
38290	1.6287	2.1
38291	2.33368	2.1
38292	2.3815	2.1
38293	0.946133	2.1
38294	0.946133	2.1
38295	0.946132	2.1
38296	0.946132	2.1
38297	0.946132	2.1
38298	0.946132	2.1
38299	0.946132	2.1
38300	0.946132	2.1
38301	0.946133	2.1
38302	0.946133	2.1
38303	0.946132	2.1
38304	0.946132	2.1
38305	1.89226	2.1
38306	1.89226	2.1
38307	1.89226	2.1
38308	1.89226	2.1
38309	3.78453	2.1
38310	2.7874	2.1
38311	2.33368	2.1
38312	2.71897	2.1
38313	2.27495	2.1
38314	5.05266	2.1
38315	0.970991	2.1
38316	0.970991	2.1
38317	0.956929	2.1
38318	0.956929	2.1
38319	0.732928	2.1
38320	0.790444	2.1
38321	1.16684	2.1
38322	1.13748	2.1
38323	1.13748	2.1
38324	0.732928	2.1
38325	0.790444	2.1
38326	1.16684	2.1
38327	0.946132	2.1
38328	0.946132	2.1
38329	0.946131	2.1
38330	0.946131	2.1
38331	0.946132	2.1
38332	0.946132	2.1
38333	0.946132	2.1
38334	0.946132	2.1
38335	0.946132	2.1

38336	0.946132	2.1
38337	0.946132	2.1
38338	0.946132	2.1
38339	1.10747	0.709274
38340	2.30331	0.732799
38341	1.47954	0.708696
38342	2.63789	0.706714
38343	3.78285	0.71025
38344	1.65215	0.703151
38345	2.63789	0.704543
38346	2.82465	0.703921
38347	2.82465	0.708646
38348	1.47954	0.702145
38349	1.47954	0.705922
38350	1.13456	1.91076
38351	1.08334	2.1
38352	1.20674	1.05444
38353	1.17885	1.94155
38354	2.29008	1.61028
38355	1.20674	1.54028
38356	1.17885	2.1
38357	1.28649	1.12005
38358	1.28516	1.57554
38359	2.4919	1.61044
38360	1.89226	2.09904
38361	1.89226	2.1
38362	1.89226	2.1
38363	1.89226	2.1
38364	1.89226	2.1
38365	1.89226	2.05593
38366	2.50288	1.33831
38367	2.02115	1.90857
38368	2.7717	1.26666
38369	2.29107	0.930657
38370	4.79395	1.90588
38371	1.27339	1.61512
38372	1.10817	2.1
38373	1.19733	2.1
38374	1.032	2.1
38375	2.30539	2.1
38376	1.19733	2.1
38377	0.953637	2.1
38378	0.953637	2.1
38379	1.032	2.1
38380	1.11868	2.04727
38381	1.19704	2.1
38382	2.01024	1.01909
38383	1.11938	1.14846
38384	1.9186	1.3873
38385	2.76298	1.03992
38386	3.92884	1.1566
38387	0.851541	0.703778
38388	0.851541	0.679302
38389	0.957577	0.636828
38390	0.848952	0.650483
38391	1.08043	0.704518
38392	0.851541	0.708845
38393	0.851484	0.637982

38394	0.957578	0.58272
38395	1.20045	0.700924
38396	0.726289	0.565615
38397	0.946131	0.633343
38398	0.946131	0.705428
38399	1.50524	2.1
38400	2.38363	2.1
38401	2.49018	2.1
38402	2.49018	2.1
38403	1.45506	2.1
38404	1.45506	2.1
38405	2.49017	2.1
38406	1.89226	2.1
38407	1.89226	2.1
38408	1.89226	2.1
38409	2.38363	2.1
38410	2.38363	2.1
38411	1.54711	2.1
38412	2.49018	2.1
38413	2.56975	2.1
38414	2.56975	2.1
38415	1.50524	2.1
38416	1.50524	2.1
38417	2.56975	2.1
38418	1.89226	2.1
38419	1.89226	2.1
38420	1.89226	2.1
38421	2.49017	2.1
38422	2.49017	2.1
38423	1.89226	0.36669
38424	1.89226	0.422098
38425	1.89226	0.554574
38426	1.89226	0.31314
38427	1.89226	0.55472
38428	1.89226	0.29807
38429	1.89226	0.450832
38430	1.89226	0.46257
38431	1.89226	0.713683
38432	1.89226	0.348905
38433	1.89226	0.709664
38434	1.89226	0.351092
38435	1.89226	0.370242
38436	1.89226	0.35167
38437	2.8358	0.275976
38438	1.89226	0.643419
38439	3.78453	0.310068
38440	1.60565	0.776461
38441	1.89226	1.50245
38442	1.89226	1.18951
38443	1.40013	1.01642
38444	1.89226	1.33566
38445	1.40013	1.12356
38446	1.1766	2.1
38447	1.29435	2.1
38448	1.35735	2.1
38449	1.53509	2.1
38450	2.65169	2.1
38451	0.69701	2.1

38452	0.69701	2.1
38453	0.711303	2.1
38454	0.711303	2.1
38455	0.761138	2.1
38456	0.612149	2.1
38457	0.647174	2.1
38458	0.767548	2.1
38459	0.767548	2.1
38460	0.761139	2.1
38461	0.612149	2.1
38462	0.647174	2.1
38463	1.29764	1.40917
38464	1.29579	1.67695
38465	1.34875	1.47465
38466	1.06717	1.68459
38467	1.29764	1.38975
38468	1.01606	1.4461
38469	0.756635	1.35857
38470	0.648821	1.36247
38471	0.675557	1.5125
38472	0.674374	1.41295
38473	0.74214	1.56698
38474	0.74214	1.43076
38475	0.688868	1.31395
38476	0.688868	1.37605
38477	0.675557	1.21533
38478	0.756634	1.2832
38479	0.648821	1.19521
38480	0.674374	1.25734
38481	1.51327	1.50637
38482	1.48105	1.30902
38483	1.29764	1.51322
38484	1.29764	1.41794
38485	1.48105	1.48003
38486	1.05655	1.30348
38487	1.13537	2.1
38488	1.07326	2.1
38489	0.94613	2.1
38490	0.94613	2.1
38491	0.946132	2.1
38492	0.946132	2.1
38493	1.13537	2.1
38494	1.08927	2.1
38495	1.13537	2.1
38496	0.946131	2.1
38497	0.946131	2.1
38498	0.95947	2.1
38499	1.46892	2.1
38500	1.89226	2.1
38501	1.89226	2.1
38502	1.89226	2.1
38503	1.88031	2.1
38504	1.88031	2.1
38505	1.89226	2.1
38506	1.89226	2.1
38507	1.89226	2.1
38508	1.89226	2.1
38509	3.78453	2.1

38510	2.18254	2.1
38511	1.89226	2.1
38512	2.50005	2.1
38513	1.89226	2.1
38514	4.39231	2.1
38515	1.88031	2.1
38516	2.0815	2.1
38517	2.0815	2.1
38518	1.92078	2.1
38519	2.0815	2.1
38520	1.9218	2.1
38521	1.89227	2.1
38522	1.89227	2.1
38523	1.89227	2.1
38524	1.89227	2.1
38525	1.89227	2.1
38526	1.89227	2.1
38527	1.98693	2.1
38528	1.82857	2.1
38529	2.32	2.1
38530	2.36972	2.1
38531	4.14857	2.1
38532	1.82857	2.1
38533	1.58688	2.1
38534	2.36971	2.1
38535	2.19093	2.1
38536	4.0195	2.1
38537	0.946132	2.1
38538	0.946132	2.1
38539	1.18486	2.1
38540	1.18486	2.1
38541	1.18486	2.1
38542	0.946132	2.1
38543	0.946132	2.1
38544	1.16	2.1
38545	0.946133	2.1
38546	0.946133	2.1
38547	1.16	2.1
38548	1.18486	2.1
38549	1.07798	1.54907
38550	1.08101	1.36371
38551	0.910786	2.1
38552	0.910786	1.96654
38553	0.851537	1.74965
38554	0.901377	1.3638
38555	1.14652	1.74126
38556	1.04072	2.1
38557	0.946129	1.85767
38558	0.946129	1.89181
38559	1.0019	1.60256
38560	1.04263	1.65787
38561	1.89226	2.1
38562	1.89226	2.1
38563	1.89226	2.1
38564	1.89226	2.1
38565	3.78452	2.1
38566	1.89226	2.1
38567	2.30146	2.1

38568	1.89226	2.1
38569	2.18412	2.1
38570	4.19372	2.1
38571	2.30146	2.1
38572	1.39962	2.1
38573	2.18411	2.1
38574	1.28765	2.1
38575	3.5891	2.1
38576	1.89226	2.1
38577	1.89226	2.1
38578	1.89226	2.1
38579	1.89226	2.1
38580	1.89226	2.1
38581	1.89226	2.1
38582	1.89226	2.1
38583	1.89226	2.1
38584	1.89226	2.1
38585	1.89226	2.1
38586	3.78452	2.1
38587	2.18412	2.1
38588	1.20045	2.1
38589	1.20045	2.1
38590	1.28765	2.1
38591	2.08808	2.1
38592	2.17528	2.1
38593	0.946131	2.1
38594	0.946131	2.1
38595	0.946128	2.1
38596	0.946128	2.1
38597	0.946127	2.1
38598	0.946127	2.1
38599	0.946131	2.1
38600	0.946131	2.1
38601	0.946129	2.1
38602	0.946129	2.1
38603	0.94613	2.1
38604	0.94613	2.1
38605	1.89226	0.778841
38606	1.89226	0.912513
38607	1.89226	0.866228
38608	1.89226	0.762386
38609	3.78452	0.771687
38610	1.89227	0.817968
38611	1.89227	1.1537
38612	1.89227	0.791277
38613	1.89227	0.975969
38614	1.89227	0.808804
38615	1.89227	1.05509
38616	1.89226	0.958074
38617	1.89226	0.816063
38618	1.89226	0.766321
38619	2.29245	0.831757
38620	3.78452	0.911503
38621	1.89226	0.873527
38622	1.89226	0.727164
38623	1.89226	0.773642
38624	1.89226	0.841469
38625	1.89226	0.748463

38626	1.89226	0.780492
38627	1.28095	1.21172
38628	1.81846	1.73046
38629	2.10991	0.923776
38630	1.34695	1.39173
38631	3.39086	1.45054
38632	2.10991	1.00256
38633	1.36457	1.48328
38634	1.67421	1.78731
38635	1.34695	1.42283
38636	2.43717	1.11288
38637	2.10991	1.13704
38638	2.69541	2.1
38639	1.48572	2.1
38640	2.63553	2.1
38641	1.45385	2.1
38642	4.14926	2.1
38643	2.63553	2.1
38644	1.44207	2.1
38645	1.44207	2.1
38646	1.45385	2.1
38647	2.588	2.1
38648	2.59978	2.1
38649	1.89227	2.1
38650	1.89227	2.1
38651	1.89227	2.1
38652	1.89227	2.1
38653	3.78453	2.1
38654	1.89226	2.1
38655	1.89226	2.1
38656	1.89226	2.1
38657	1.89226	2.1
38658	3.78453	2.1
38659	2.15929	2.1
38660	1.38276	2.1
38661	1.43617	2.1
38662	1.35614	2.1
38663	2.23932	2.1
38664	2.15929	2.1
38665	2.15929	2.1
38666	2.25168	2.1
38667	2.3558	2.1
38668	2.23932	2.1
38669	4.51509	2.1
38670	1.89226	2.1
38671	1.89226	2.1
38672	1.89226	2.1
38673	1.89226	2.1
38674	3.78453	2.1
38675	1.89226	2.1
38676	1.89226	2.1
38677	1.89226	2.1
38678	1.89226	2.1
38679	1.89226	2.1
38680	1.89226	2.1
38681	2.23932	2.1
38682	1.43854	2.1
38683	1.49173	2.1

38684	1.38276	2.1
38685	2.34829	2.1
38686	2.23932	2.1
38687	2.3558	2.1
38688	2.34829	2.1
38689	2.40685	2.1
38690	2.23932	2.1
38691	2.52332	2.1
38692	2.3558	2.1
38693	1.89226	0.310529
38694	1.87361	0.395578
38695	2.25082	0.28717
38696	1.87361	0.508497
38697	2.26947	0.241404
38698	1.89226	0.391508
38699	1.89226	0.415202
38700	1.89226	0.560155
38701	1.89226	0.424186
38702	1.89226	0.697506
38703	1.89226	0.324245
38704	1.89226	0.595388
38705	2.41314	0.360341
38706	1.89226	0.504661
38707	1.89226	0.358866
38708	1.89226	0.645061
38709	2.12204	0.266916
38710	2.12204	0.517649
38711	1.89226	0.306974
38712	2.25082	0.367832
38713	2.28189	0.280387
38714	2.25082	0.518686
38715	1.92333	0.208749
38716	1.89226	0.398172
38717	1.89226	0.427202
38718	1.89226	0.518813
38719	1.89226	0.446868
38720	1.89226	0.7
38721	1.89226	0.317978
38722	1.89226	0.548364
38723	2.42521	0.345862
38724	1.89226	0.44858
38725	1.89226	0.331866
38726	1.89226	0.60046
38727	2.41314	0.256311
38728	2.41314	0.451546
38729	1.39725	1.20636
38730	1.07745	2.1
38731	1.07745	1.18657
38732	1.07519	2.1
38733	1.41336	1.13946
38734	1.41111	2.1
38735	1.89226	1.99977
38736	1.92039	2.1
38737	1.92039	1.7844
38738	1.9052	2.1
38739	1.89226	1.64225
38740	1.87708	2.1
38741	0.946128	2.09849

38742	0.946128	1.687
38743	0.946133	1.15935
38744	0.946133	1.07061
38745	0.94613	1.07979
38746	0.94613	1.61992
38747	0.946131	2.1
38748	0.946131	1.10493
38749	0.946131	1.26993
38750	0.946131	1.27342
38751	0.946131	1.27786
38752	0.946131	1.29581
38753	1.41336	1.1935
38754	1.08911	2.1
38755	1.08911	1.23404
38756	1.07745	2.1
38757	1.42381	1.19006
38758	1.41215	2.1
38759	1.89227	2.0799
38760	1.95833	2.1
38761	1.95833	1.92495
38762	1.92039	2.1
38763	1.89227	1.79259
38764	1.85433	2.1
38765	0.946129	2.1
38766	0.946129	1.80704
38767	0.946133	1.2252
38768	0.946133	1.09083
38769	0.946131	1.09763
38770	0.946131	1.73562
38771	0.946131	2.1
38772	0.946131	1.15063
38773	0.946131	1.33111
38774	0.946131	1.337
38775	0.946131	1.35217
38776	0.946131	1.36555
38777	1.41943	2.1
38778	2.74398	2.1
38779	1.18238	2.1
38780	2.53897	2.1
38781	3.9584	2.1
38782	2.74398	2.1
38783	1.89226	2.1
38784	1.89226	2.1
38785	1.89226	2.1
38786	2.53897	2.1
38787	2.53897	2.1
38788	1.89227	0.969428
38789	1.89227	0.866959
38790	1.89227	0.928706
38791	1.89227	0.833583
38792	3.78453	0.868495
38793	1.89226	0.851616
38794	2.08134	0.799273
38795	2.00632	0.788734
38796	2.00632	0.817729
38797	1.89226	0.812395
38798	1.89226	0.854179
38799	1.89227	0.88911

38800	1.89227	0.83231
38801	1.89227	0.830765
38802	1.89227	0.776198
38803	3.78453	0.857404
38804	1.89227	0.908818
38805	1.89227	0.768704
38806	1.89227	0.767136
38807	1.89227	0.821572
38808	1.89227	0.815224
38809	1.89227	0.939075
38810	2.50912	0.753108
38811	1.89226	0.795488
38812	1.89226	0.743203
38813	2.31261	0.774136
38814	2.31261	0.785583
38815	1.89226	0.816902
38816	2.50912	0.758934
38817	1.19999	0.762953
38818	1.44056	0.743002
38819	1.24405	0.760034
38820	2.50912	0.764378
38821	2.31261	0.78503
38822	2.69224	0.81738
38823	2.28373	0.718578
38824	1.94788	0.857337
38825	2.4106	0.757598
38826	4.69434	0.792652
38827	2.69223	0.798018
38828	2.63783	0.864187
38829	2.34737	1.04583
38830	2.4106	0.787121
38831	5.04843	0.877259
38832	1.89226	2.1
38833	1.99753	2.1
38834	1.87366	2.1
38835	1.99753	2.1
38836	1.76839	2.1
38837	1.89226	2.1
38838	1.89226	2.1
38839	1.89226	1.17771
38840	1.89226	2.1
38841	1.89226	2.1
38842	3.78452	2.1
38843	0.946132	2.1
38844	0.946132	2.1
38845	1.02744	2.1
38846	1.02744	2.1
38847	0.946132	2.1
38848	0.946132	2.1
38849	1.00989	2.1
38850	1.02744	2.1
38851	1.00989	2.1
38852	0.946133	2.1
38853	1.02744	2.1
38854	0.946132	2.1
38855	1.70302	2.1
38856	1.87366	2.1
38857	1.71618	2.1

38858	1.87366	2.1
38859	1.73105	2.1
38860	1.89226	2.1
38861	1.89226	2.1
38862	1.89226	2.1
38863	1.89226	2.1
38864	1.89226	2.1
38865	1.89226	2.1
38866	1.89226	1.15166
38867	1.96624	2.1
38868	1.89226	2.1
38869	1.89226	2.1
38870	1.89226	2.1
38871	2.01977	2.1
38872	2.01977	2.1
38873	1.1032	1.39898
38874	1.15114	2.1
38875	1.14986	0.85097
38876	1.19242	2.1
38877	2.301	1.67907
38878	1.89226	1.29459
38879	1.89226	2.1
38880	1.89226	0.868274
38881	1.89226	1.50436
38882	3.78453	1.15396
38883	0.946132	2.1
38884	0.946132	2.02109
38885	0.946132	1.10232
38886	0.946132	2.1
38887	0.946133	2.1
38888	0.946133	2.1
38889	0.946132	1.79706
38890	0.946132	1.35781
38891	0.946132	2.1
38892	0.946132	2.1
38893	0.946133	2.1
38894	0.946133	2.1
38895	1.19243	1.99989
38896	0.953928	2.1
38897	1.18671	2.1
38898	1.15114	1.96373
38899	0.9895	2.1
38900	0.953928	2.1
38901	1.19611	0.89471
38902	1.19242	2.1
38903	1.24693	0.916092
38904	1.24693	2.05714
38905	1.14986	0.991422
38906	1.14986	2.1
38907	1.89225	0.912112
38908	1.89225	2.1
38909	1.89225	2.1
38910	1.89225	1.14887
38911	1.89225	1.23177
38912	1.89225	1.08702
38913	1.89225	1.51563
38914	1.89225	2.1
38915	1.89225	2.1

38916	1.89225	2.1
38917	1.89225	1.01088
38918	1.89225	2.1
38919	0.561532	2.1
38920	0.561532	1.89401
38921	0.566382	1.52909
38922	0.566382	2.1
38923	0.494747	2.1
38924	0.531705	2.1
38925	0.596208	1.99068
38926	0.623464	1.11709
38927	0.623469	2.1
38928	0.531708	2.1
38929	0.596205	2.1
38930	0.494743	2.1
38931	1.25742	2.1
38932	1.18783	2.1
38933	0.946131	2.1
38934	0.946131	2.1
38935	1.25742	2.1
38936	1.25742	2.1
38937	0.946131	2.1
38938	0.946131	2.1
38939	1.25742	2.1
38940	0.946131	2.1
38941	0.946131	2.1
38942	1.18783	2.1
38943	1.35048	2.1
38944	1.25742	2.1
38945	0.946132	2.1
38946	0.946132	2.1
38947	1.35048	2.1
38948	1.35048	2.1
38949	0.946132	2.1
38950	0.946132	2.1
38951	1.35044	2.1
38952	0.946106	2.1
38953	1.25745	2.1
38954	0.946158	2.1
38955	1.13525	2.1
38956	2.70095	2.1
38957	2.51483	2.1
38958	2.51483	2.1
38959	1.21593	2.1
38960	1.21593	2.1
38961	1.93142	0.752089
38962	2.8053	0.241523
38963	1.559	0.777085
38964	2.60359	0.383815
38965	4.53501	0.632309
38966	2.80531	0.387231
38967	1.89226	0.2495
38968	2.60359	0.607359
38969	1.89226	0.786525
38970	4.49586	0.240228
38971	1.22938	0.765189
38972	2.60359	0.216833
38973	2.37434	0.825145

38974	2.37434	0.603801
38975	1.55901	0.819158
38976	1.55901	0.220337
38977	1.18717	0.77256
38978	1.18717	0.273714
38979	0.946132	0.49317
38980	0.946132	0.503268
38981	0.946133	0.725172
38982	0.946133	0.768371
38983	1.3018	0.484094
38984	1.18717	0.767473
38985	1.18714	0.637329
38986	0.946107	0.721295
38987	1.30183	0.411024
38988	0.946158	0.348592
38989	1.89226	2.1
38990	2.80196	2.1
38991	2.67272	2.1
38992	2.67272	2.1
38993	1.89226	2.1
38994	1.89226	2.1
38995	1.89226	2.1
38996	1.89226	2.1
38997	1.89226	2.1
38998	1.89226	2.1
38999	1.89226	2.1
39000	1.89226	2.1
39001	1.74471	2.1
39002	2.01555	2.1
39003	1.61121	2.1
39004	1.61121	2.1
39005	2.13878	2.1
39006	2.13878	2.1
39007	1.89226	2.1
39008	1.89226	2.1
39009	1.89226	2.1
39010	1.89226	2.1
39011	1.89226	2.1
39012	1.89226	2.1
39013	1.09766	2.1
39014	1.04263	2.1
39015	0.881794	2.1
39016	0.894188	2.1
39017	0.795402	2.1
39018	0.99524	2.1
39019	1.00647	2.1
39020	1.00647	2.1
39021	1.15401	2.1
39022	0.881789	2.1
39023	0.850526	2.1
39024	1.04263	2.1
39025	1.4054	1.15822
39026	1.04788	2.1
39027	1.70302	1.09864
39028	1.41375	2.1
39029	2.82706	1.55417
39030	1.70302	1.08516
39031	1.38776	2.1

39032	1.59457	1.57709
39033	1.41375	2.1
39034	1.77261	1.02994
39035	1.70302	2.1
39036	1.89226	2.1
39037	1.89226	2.1
39038	1.89226	2.1
39039	1.89226	2.1
39040	3.78453	2.1
39041	2.18042	2.1
39042	1.74738	2.1
39043	2.08433	2.1
39044	1.66347	2.1
39045	3.83171	2.1
39046	1.74738	2.1
39047	1.1181	2.1
39048	1.66347	2.1
39049	1.06543	2.1
39050	2.81281	2.1
39051	1.89226	2.1
39052	1.89226	2.1
39053	1.89226	2.1
39054	1.89226	2.1
39055	3.78453	2.1
39056	1.89227	2.1
39057	1.89227	2.1
39058	1.89227	2.1
39059	1.89227	2.1
39060	3.78453	2.1
39061	2.08433	2.1
39062	1.66347	2.1
39063	1.97092	2.1
39064	1.57431	2.1
39065	3.65864	2.1
39066	1.66347	2.1
39067	1.01474	2.1
39068	1.01474	2.1
39069	1.06544	2.1
39070	1.57431	2.1
39071	1.625 2.1	
39072	1.89226	2.1
39073	1.89226	2.1
39074	1.89226	2.1
39075	1.89226	2.1
39076	1.89226	2.1
39077	1.89226	2.1
39078	1.08467	2.1
39079	1.87228	2.1
39080	1.73305	2.1
39081	1.73305	2.1
39082	1.25565	2.1
39083	1.25565	2.1
39084	0.695361	0.845463
39085	0.799996	0.9232
39086	1.13751	1.05305
39087	1.40945	1.04765
39088	1.40944	1.13287
39089	0.799999	0.737568

39090	1.13751	0.943053
39091	0.695363	0.791376
39092	1.01059	0.843989
39093	1.01059	0.905908
39094	0.926908	1.23038
39095	0.926909	0.82235
39096	1.05481	2.1
39097	1.78069	2.09878
39098	1.08074	2.1
39099	1.83284	2.1
39100	2.86143	2.1
39101	1.78069	2.1
39102	2.22044	1.50011
39103	1.83284	2.1
39104	2.28696	1.02424
39105	4.06766	1.62325
39106	1.07196	2.1
39107	1.64955	2.1
39108	2.02665	2.1
39109	2.0284	2.1
39110	1.08074	2.1
39111	1.08074	2.1
39112	1.64955	2.1
39113	2.28696	1.24423
39114	1.92033	2.1
39115	2.1004	1.76359
39116	3.74988	2.04284
39117	2.83787	0.759981
39118	1.70303	0.875039
39119	1.70303	0.723516
39120	1.83195	0.711057
39121	2.64075	0.720022
39122	2.70231	0.786675
39123	1.2087	1.51348
39124	1.52909	0.908762
39125	1.43063	0.920472
39126	1.10586	1.31174
39127	2.63494	1.03923
39128	1.89227	1.11845
39129	2.10732	2.1
39130	2.10732	1.22989
39131	2.2365	2.1
39132	1.89227	1.1807
39133	2.02145	2.1
39134	1.89226	0.91661
39135	1.89226	1.27099
39136	1.89226	0.962805
39137	1.89226	1.20138
39138	1.89226	0.938193
39139	1.89226	1.43547
39140	0.51926	1.40439
39141	0.552927	1.41748
39142	0.691235	0.951159
39143	0.715314	0.910474
39144	0.619684	0.938747
39145	0.619684	1.71003
39146	0.624477	1.97315
39147	0.624477	0.914746

39148	0.519262	1.08887
39149	0.691234	1.09625
39150	0.715313	1.07619
39151	0.552928	1.07031
39152	1.89226	1.2145
39153	1.87321	2.1
39154	1.87321	1.80921
39155	1.89658	2.1
39156	2.0815	1.24532
39157	2.14789	2.1
39158	0.946128	1.54842
39159	0.946128	1.40564
39160	0.946133	0.918776
39161	0.946133	0.921836
39162	0.94613	0.916551
39163	0.94613	1.35224
39164	0.946131	0.960814
39165	0.946131	1.3489
39166	0.946131	1.0119
39167	0.946131	1.01961
39168	0.946131	1.07918
39169	0.946131	1.0699
39170	1.99297	1.18377
39171	2.19514	0.976537
39172	2.46153	1.07843
39173	2.187 1.09196	
39174	4.45451	1.1057
39175	2.0815	1.01388
39176	2.33402	0.998567
39177	2.46154	1.10512
39178	2.83046	0.913331
39179	4.94496	0.989314
39180	2.46153	1.03366
39181	2.187 1.02481	
39182	2.27351	1.0895
39183	2.63356	0.932844
39184	4.73504	1.01108
39185	2.46154	1.05829
39186	2.83447	0.873632
39187	2.74513	0.969808
39188	2.5731	1.01997
39189	2.63357	0.943179
39190	2.46154	0.978038
39191	2.80398	2.1
39192	2.60406	2.1
39193	2.73988	2.1
39194	2.57466	2.1
39195	5.34394	2.1
39196	1.89226	1.02703
39197	1.89226	0.903356
39198	1.89226	0.832039
39199	1.89226	1.09077
39200	1.89226	0.863518
39201	1.89226	1.28954
39202	1.89224	1.10962
39203	1.89224	0.925234
39204	1.89224	0.868741
39205	1.89224	1.26333

39206	1.89224	0.908706
39207	1.89224	1.54284
39208	1.40245	1.11515
39209	1.40246	1.70088
39210	0.946117	1.10165
39211	0.946117	1.47666
39212	1.17635	0.998039
39213	1.40246	1.77826
39214	0.946112	0.942174
39215	0.946112	1.36826
39216	0.946127	1.11217
39217	1.17637	1.00014
39218	0.946102	1.44984
39219	1.40244	1.11941
39220	2.53269	2.1
39221	1.70302	2.1
39222	1.56194	2.1
39223	2.17516	2.1
39224	4.19807	2.1
39225	1.89226	2.1
39226	1.89226	1.09154
39227	1.89226	2.1
39228	1.89226	2.1
39229	3.78453	2.1
39230	2.57276	2.1
39231	1.89226	2.1
39232	1.89226	2.1
39233	1.89226	2.1
39234	2.32525	2.1
39235	2.32525	2.1
39236	1.15171	2.1
39237	2.17517	2.1
39238	1.41995	2.1
39239	1.41995	2.1
39240	1.80252	2.1
39241	1.80252	2.1
39242	1.89226	2.1
39243	1.89226	2.1
39244	1.89226	2.1
39245	1.89226	1.0149
39246	3.78453	2.1
39247	0.946133	2.1
39248	0.946133	2.1
39249	1.16263	2.1
39250	1.04687	2.1
39251	0.946133	2.1
39252	0.946133	2.1
39253	1.04687	2.1
39254	1.04687	2.1
39255	0.946133	2.1
39256	1.04687	2.1
39257	0.946132	2.1
39258	1.16263	2.1
39259	0.682281	1.36001
39260	0.669233	1.86914
39261	1.21859	1.87704
39262	1.31629	1.15901
39263	0.916158	1.08509

39264	0.916158	2.03698
39265	0.971658	1.26053
39266	0.971658	1.33477
39267	0.669234	1.29985
39268	1.31629	1.1903
39269	0.682283	1.41747
39270	1.21858	1.48168
39271	0.669232	1.23502
39272	0.623495	1.66087
39273	1.36428	1.42744
39274	1.31629	1.0205
39275	0.946475	1.01367
39276	0.946476	1.97603
39277	0.99331	1.26994
39278	0.99331	1.2189
39279	1.36428	1.30473
39280	0.623497	1.12006
39281	0.669234	1.228
39282	1.31629	1.14897
39283	2.02069	0.0990942
39284	1.46253	0.0797691
39285	2.51479	0.0763078
39286	1.87136	0.0895021
39287	3.89203	0.0830882
39288	1.46253	0.0770753
39289	1.38536	0.0908829
39290	1.38536	0.0856646
39291	1.05579	0.0928693
39292	1.73281	0.0828513
39293	1.46916	0.0821106
39294	2.51479	0.0808049
39295	1.87136	0.108858
39296	2.23338	0.102354
39297	2.15603	0.0832671
39298	4.55146	0.0860474
39299	0.822553	0.0811392
39300	0.822563	0.087432
39301	0.842935	0.110553
39302	0.842935	0.0979271
39303	0.744203	0.105947
39304	0.675346	0.0969167
39305	0.874795	0.116058
39306	1.00453	0.0915831
39307	1.00454	0.0836026
39308	0.782417	0.0957787
39309	0.675337	0.100996
39310	0.858007	0.0871079
39311	0.946132	0.972492
39312	1.24847	0.770893
39313	0.946132	0.94245
39314	1.16806	1.10037
39315	1.24847	0.766638
39316	1.24847	1.51865
39317	0.946133	0.747337
39318	0.946133	1.32411
39319	0.946133	0.822149
39320	0.946133	0.764083
39321	1.16805	1.09248

39322	1.24847	0.804723
39323	1.2364	1.04294
39324	0.946129	0.817256
39325	0.946133	1.05489
39326	1.24847	0.835587
39327	1.2364	0.794553
39328	1.2364	1.31318
39329	0.946131	0.750415
39330	0.946131	1.54867
39331	0.946131	0.87704
39332	0.946131	0.786004
39333	1.2364	1.27965
39334	1.24847	0.867739
39335	1.89226	0.990912
39336	1.89226	1.04755
39337	1.89226	0.791642
39338	1.89226	0.813032
39339	1.89226	0.962305
39340	1.89226	1.20248
39341	1.89226	0.882911
39342	1.89226	0.814273
39343	1.89226	0.761959
39344	1.89226	0.995911
39345	1.89226	0.807685
39346	1.89226	1.1067
39347	1.69014	0.858984
39348	1.09194	1.02136
39349	1.13537	1.25125
39350	1.13537	1.0784
39351	1.58481	0.793432
39352	1.58481	0.810728
39353	1.58147	0.873776
39354	1.13537	0.865407
39355	1.69015	0.989081
39356	1.01179	0.816367
39357	1.69015	0.85679
39358	1.01179	1.10822
39359	0.946132	1.48494
39360	0.946132	1.0742
39361	1.28543	0.848769
39362	1.07341	1.47407
39363	1.07341	1.35201
39364	1.07341	0.795791
39365	0.946132	0.896528
39366	0.946132	0.87105
39367	0.946133	0.878857
39368	1.28543	0.869476
39369	1.07341	0.9287
39370	0.946132	1.01579
39371	1.89226	1.02113
39372	1.89226	0.848424
39373	1.89226	0.813392
39374	1.89226	1.46272
39375	1.89226	0.816603
39376	1.89226	2.1
39377	0.805744	1.97761
39378	0.988659	1.08856
39379	0.782044	0.839305

39380	0.578223	1.04894
39381	0.77823	0.881177
39382	0.778231	2.1
39383	0.788653	0.815954
39384	0.788653	1.25667
39385	0.988662	1.00366
39386	0.782042	0.982582
39387	0.805745	1.12635
39388	0.578222	1.0486
39389	1.89226	0.951535
39390	1.89226	0.939254
39391	1.89226	0.808155
39392	1.89226	1.62691
39393	1.89226	0.770792
39394	1.89226	2.02711
39395	1.97732	1.20495
39396	1.97819	0.884551
39397	1.56408	0.808349
39398	1.56408	1.49907
39399	2.33986	0.876981
39400	2.33986	1.9089
39401	1.84516	2.1
39402	2.21152	2.1
39403	1.90702	2.1
39404	1.54599	2.1
39405	3.75751	2.1
39406	1.89224	2.1
39407	1.89224	2.1
39408	1.89224	2.1
39409	1.89224	2.1
39410	1.89224	2.1
39411	1.89224	2.01357
39412	1.47672	2.1
39413	1.54601	2.1
39414	1.27697	2.1
39415	1.54601	2.1
39416	1.638 2.1	
39417	1.90705	2.1
39418	0.946132	1.75747
39419	0.946132	2.1
39420	0.946131	2.1
39421	0.946131	2.1
39422	0.946131	2.1
39423	0.946131	1.85308
39424	0.946132	2.1
39425	0.946132	2.1
39426	0.946132	2.1
39427	0.946132	2.1
39428	0.946131	2.1
39429	0.946131	2.1
39430	0.946128	2.1
39431	0.946128	2.1
39432	1.32062	2.1
39433	1.4164	2.1
39434	0.946129	2.1
39435	0.946129	2.1
39436	1.32062	2.1
39437	1.32062	2.1

39438	1.32062	2.1
39439	0.94613	2.1
39440	1.41639	2.1
39441	0.946129	2.1
39442	1.98979	0.0776433
39443	1.65977	0.0858569
39444	2.21772	0.0830316
39445	2.21772	0.0784698
39446	1.48866	0.0883742
39447	1.48866	0.0761499
39448	0.973129	0.0743114
39449	0.973129	0.0760799
39450	0.980954	0.0864821
39451	0.980954	0.0852595
39452	0.845218	0.0828152
39453	1.13981	0.0847121
39454	0.829886	0.0887081
39455	1.10886	0.0806182
39456	1.10886	0.076562
39457	1.13982	0.0821637
39458	0.845219	0.0829732
39459	0.829886	0.0769636
39460	0.946132	1.09405
39461	0.946132	2.02655
39462	1.3352	2.1
39463	1.41432	2.1
39464	0.946133	2.1
39465	0.946133	0.951845
39466	1.3352	2.04047
39467	1.3352	2.1
39468	1.3352	2.1
39469	0.946132	2.1
39470	1.41432	2.1
39471	0.946133	2.1
39472	2.70097	2.1
39473	1.88175	2.1
39474	1.51482	2.1
39475	1.88175	2.1
39476	2.37784	2.1
39477	2.82864	2.1
39478	1.89227	0.533856
39479	1.89227	0.397969
39480	1.89227	0.308764
39481	1.89227	0.257597
39482	3.78453	0.252639
39483	1.89226	0.307512
39484	2.13295	0.194741
39485	2.43203	0.248236
39486	2.43203	0.308685
39487	1.89226	0.209326
39488	1.89226	0.276284
39489	0.946133	0.196794
39490	0.946133	0.290245
39491	0.946132	0.164128
39492	0.946132	0.259009
39493	0.946132	0.180116
39494	0.946132	0.194969
39495	0.946133	0.187714

39496	0.946133	0.24951
39497	0.946132	0.183331
39498	0.946132	0.232986
39499	0.946132	0.321442
39500	0.946132	0.282453
39501	1.54987	2.1
39502	1.24128	2.1
39503	1.24128	2.1
39504	1.50738	2.1
39505	1.75702	2.1
39506	2.02312	2.1
39507	1.89289	2.1
39508	1.92264	2.1
39509	1.72994	2.1
39510	1.75702	2.1
39511	3.67966	2.1
39512	1.75702	2.1
39513	0.994291	2.1
39514	0.994291	2.1
39515	1.24128	2.1
39516	1.4964	2.1
39517	1.74339	2.1
39518	1.72993	2.1
39519	1.49641	2.1
39520	1.49641	2.1
39521	1.75702	2.1
39522	1.3939	2.1
39523	1.65452	2.1
39524	1.19964	2.1
39525	1.53296	2.1
39526	1.19964	2.1
39527	1.36861	2.1
39528	1.42624	2.1
39529	1.59521	2.1
39530	1.19964	2.1
39531	1.42624	2.1
39532	1.07156	2.1
39533	1.36226	2.1
39534	2.5619	2.1
39535	2.14058	1.0655
39536	1.93569	2.03682
39537	2.36221	1.20298
39538	2.5753	1.12475
39539	4.50279	1.78878
39540	2.14057	1.53859
39541	2.20831	2.03441
39542	2.64725	0.976586
39543	2.57529	1.55957
39544	4.78782	1.48123
39545	2.64725	1.08675
39546	2.57529	1.75879
39547	1.89226	0.970085
39548	1.89226	0.882351
39549	4.53951	1.28196
39550	2.0815	2.1
39551	1.86137	2.1
39552	1.86137	2.1
39553	1.86159	2.1

39554	2.0815	2.1
39555	2.10235	2.1
39556	1.33552	2.1
39557	2.32417	2.1
39558	1.18872	2.1
39559	1.93101	2.1
39560	3.26653	2.1
39561	1.89226	2.1
39562	1.89226	2.1
39563	1.89226	2.1
39564	1.89226	2.1
39565	1.89226	2.1
39566	1.89226	2.1
39567	0.955191	2.1
39568	1.03372	2.1
39569	0.946131	2.1
39570	0.946131	2.1
39571	0.930654	2.1
39572	0.930654	2.1
39573	1.04233	2.1
39574	1.05247	2.1
39575	0.955341	2.1
39576	0.946131	2.1
39577	1.02375	2.1
39578	1.13537	2.1
39579	1.1549	2.1
39580	1.93101	2.1
39581	1.91108	2.1
39582	1.52487	2.1
39583	1.3076	2.1
39584	1.06985	2.1
39585	1.89226	2.1
39586	1.89226	2.1
39587	1.89226	2.1
39588	1.89226	2.1
39589	1.89226	2.1
39590	1.89226	2.1
39591	1.58713	2.1
39592	2.6845	2.1
39593	1.58742	2.1
39594	2.74201	2.1
39595	4.27191	2.1
39596	2.68449	2.1
39597	1.89226	2.1
39598	2.74201	2.1
39599	1.89226	2.1
39600	4.57675	2.1
39601	1.57523	2.1
39602	2.74201	2.1
39603	2.8146	2.1
39604	2.8146	2.1
39605	1.58742	2.1
39606	1.58742	2.1
39607	0.946131	2.1
39608	0.946131	2.1
39609	1.371	2.1
39610	1.371	2.1
39611	0.946127	2.1

39612	0.946127	1.89981
39613	1.37101	2.1
39614	1.4073	2.1
39615	0.94613	2.1
39616	1.4073	2.1
39617	1.371	2.1
39618	0.946129	2.1
39619	1.44801	2.02585
39620	1.06821	1.68824
39621	2.00542	1.84775
39622	2.27703	1.9452
39623	3.34524	1.63781
39624	1.70301	0.702443
39625	1.89226	0.707212
39626	1.65809	0.706971
39627	1.89226	0.711192
39628	1.62758	0.705038
39629	1.85744	0.706448
39630	1.89226	0.702893
39631	1.89226	0.708066
39632	1.89226	0.7045
39633	1.89226	0.701704
39634	3.78452	0.702182
39635	1.89226	0.708364
39636	1.89226	0.702812
39637	1.89226	0.701223
39638	1.89226	0.711618
39639	3.78452	0.704265
39640	0.851541	0.707215
39641	0.851541	0.707781
39642	0.91193	0.695588
39643	0.824837	0.69613
39644	0.854559	0.695877
39645	0.851534	0.769954
39646	0.81851	0.706469
39647	0.717219	0.706926
39648	0.871743	0.702959
39649	0.837985	0.702322
39650	0.82902	0.701182
39651	0.82902	0.701922
39652	1.89226	0.706189
39653	1.92659	0.696694
39654	2.03235	0.699332
39655	1.89226	0.719212
39656	2.03235	0.696597
39657	1.89226	0.705416
39658	0.585557	0.768434
39659	0.585557	0.727062
39660	1.22612	0.705652
39661	1.09436	0.747545
39662	0.851537	0.782584
39663	0.526987	0.803078
39664	0.983879	0.702162
39665	1.04072	0.722503
39666	0.585526	0.751092
39667	0.792325	0.732842
39668	0.946129	0.729977
39669	0.946129	0.735035

39670	1.68152	2.1
39671	1.81445	2.1
39672	1.68152	2.1
39673	1.86792	2.1
39674	1.65888	2.1
39675	1.84529	2.1
39676	1.81445	2.1
39677	1.57885	2.1
39678	1.65889	2.1
39679	1.48594	2.1
39680	3.23773	2.1
39681	1.68154	2.1
39682	1.65891	2.1
39683	1.53296	2.1
39684	1.56299	2.1
39685	3.24453	2.1
39686	1.56299	2.1
39687	1.48596	2.1
39688	1.56299	2.1
39689	1.65891	2.1
39690	1.46568	2.1
39691	1.5616	2.1
39692	2.68785	2.1
39693	2.08831	2.1
39694	2.66919	2.1
39695	2.1024	2.1
39696	4.79025	2.1
39697	2.08832	2.1
39698	1.15478	2.1
39699	1.15478	2.1
39700	1.09062	2.1
39701	2.10241	2.1
39702	2.03825	2.1
39703	2.65348	2.1
39704	2.1024	2.1
39705	2.12313	2.1
39706	2.12313	2.1
39707	2.66919	2.1
39708	2.66919	2.1
39709	0.813169	2.1
39710	0.813169	2.1
39711	0.825788	2.1
39712	0.825788	2.1
39713	0.612902	2.1
39714	0.577387	2.1
39715	1.0512	2.1
39716	1.06157	2.1
39717	1.06157	2.1
39718	0.612902	2.1
39719	0.577387	2.1
39720	1.0512	2.1
39721	1.27227	2.1
39722	1.91724	2.1
39723	1.32591	2.1
39724	1.99837	2.1
39725	3.24315	2.1
39726	1.89223	2.1
39727	1.89223	2.1

39728	1.89223	2.1
39729	1.89223	1.87316
39730	3.78446	2.1
39731	1.89224	2.1
39732	1.89224	1.07932
39733	1.89224	1.97554
39734	1.89224	1.95464
39735	3.78447	1.87838
39736	1.91724	2.1
39737	2.321	2.1
39738	1.99838	2.1
39739	2.4149	1.88739
39740	4.33214	2.1
39741	1.34817	2.1
39742	1.9984	2.1
39743	2.06238	2.1
39744	2.06238	2.1
39745	1.32593	2.1
39746	1.32593	2.1
39747	1.89226	2.1
39748	1.89226	1.8553
39749	1.89226	2.1
39750	1.89226	2.1
39751	1.89226	2.1
39752	1.89226	2.1
39753	1.89226	1.96746
39754	1.89226	2.05889
39755	1.89226	2.1
39756	1.89226	1.05891
39757	3.78452	2.05959
39758	1.9984	2.1
39759	2.41494	2.00992
39760	2.06239	2.1
39761	2.5194	2.1
39762	4.47732	2.1
39763	1.19901	1.02915
39764	1.16661	2.1
39765	1.56859	1.995
39766	1.61029	1.13586
39767	2.7676	1.88873
39768	1.56859	2.02477
39769	1.88637	0.867177
39770	1.61029	0.839562
39771	1.61029	1.87676
39772	1.856	0.852982
39773	1.856	1.88674
39774	1.856	0.990307
39775	1.57916	2.1
39776	1.56859	1.05528
39777	1.56859	2.1
39778	1.57916	1.37067
39779	1.23475	2.1
39780	0.890924	0.725147
39781	1.07608	0.922078
39782	0.72877	0.915546
39783	0.910106	0.897373
39784	1.00196	1.00755
39785	1.09519	1.07691

39786	0.839967	0.864862
39787	0.719043	0.918932
39788	0.893859	0.689281
39789	0.942408	1.10127
39790	0.873497	0.875525
39791	0.873497	1.18492
39792	1.53046	0.686909
39793	1.89994	0.934886
39794	1.049	0.681392
39795	1.049	0.70326
39796	1.52512	0.703113
39797	1.89994	0.827516
39798	1.65573	0.75341
39799	1.89226	0.760957
39800	1.89226	0.725781
39801	2.73329	0.947364
39802	3.548	0.794443
39803	1.65573	0.727685
39804	2.42671	0.791778
39805	2.73329	0.731235
39806	1.89226	0.721068
39807	2.73329	0.935248
39808	1.89226	0.875399
39809	0.941078	0.933273
39810	1.01599	0.959664
39811	0.818936	0.908891
39812	0.651554	0.92776
39813	0.904729	0.928983
39814	0.665756	0.939676
39815	0.843423	1.09399
39816	0.918649	1.03154
39817	0.855806	1.09642
39818	0.814384	0.792454
39819	0.844437	0.986863
39820	0.844437	0.947231
39821	2.22558	1.3149
39822	1.95785	0.991787
39823	2.07065	1.07895
39824	1.89142	1.00625
39825	2.07065	1.83888
39826	1.89142	1.60371
39827	0.640813	1.03337
39828	0.951654	1.13434
39829	0.797442	0.902612
39830	0.535364	0.956475
39831	0.525269	0.995701
39832	0.620874	0.962909
39833	0.916953	1.08764
39834	0.969374	1.04813
39835	0.788925	1.37177
39836	0.69596	0.786417
39837	0.743154	0.974574
39838	0.743154	1.02519
39839	1.89226	1.61833
39840	1.89226	1.28248
39841	1.89226	0.911129
39842	1.89226	2.1
39843	3.78452	1.0719

39844	1.32541	1.34037
39845	1.13722	1.06123
39846	0.91103	2.1
39847	0.881123	1.8935
39848	1.03363	2.03884
39849	1.03364	1.61262
39850	1.1505	1.36121
39851	1.1505	1.93243
39852	1.32539	1.72917
39853	1.07662	2.1
39854	0.881121	1.7275
39855	0.962303	1.54856
39856	1.89226	1.07093
39857	1.89226	2.1
39858	1.89226	1.85506
39859	1.89226	1.11657
39860	3.78453	1.6046
39861	1.89226	0.900219
39862	1.89226	1.90047
39863	1.89226	1.20628
39864	1.89226	1.09864
39865	1.89226	1.22803
39866	1.89226	1.23097
39867	1.27606	1.32319
39868	1.07664	1.13727
39869	0.905895	2.1
39870	0.696099	2.1
39871	0.984902	2.09685
39872	0.984905	1.31001
39873	1.01878	2.07421
39874	1.01878	1.01751
39875	1.27604	1.84661
39876	1.06044	1.90572
39877	1.13192	1.73013
39878	0.696111	1.73831
39879	1.89226	0.81904
39880	1.89226	0.132062
39881	1.89226	0.210375
39882	1.89226	0.826937
39883	1.89226	0.217326
39884	1.89226	0.814642
39885	1.52615	0.847026
39886	1.01294	0.103931
39887	1.01294	0.880901
39888	1.18549	0.586768
39889	1.38963	0.577701
39890	1.56218	0.399717
39891	1.89226	0.740766
39892	2.40801	0.55112
39893	2.58101	0.7626
39894	2.58101	0.73547
39895	1.89226	0.627697
39896	1.89226	0.719559
39897	2.24071	2.1
39898	2.79317	2.00196
39899	1.99136	2.1
39900	2.6167	1.92536
39901	4.85741	2.1

39902	2.79317	2.06044
39903	1.89226	0.797309
39904	1.89226	2.1
39905	1.89226	0.949672
39906	2.6167	2.07365
39907	2.6167	0.923742
39908	1.08354	2.04869
39909	1.08354	2.1
39910	1.10222	2.1
39911	1.10222	2.1
39912	1.2275	2.1
39913	1.30835	2.02095
39914	0.995678	2.1
39915	0.877412	2.1
39916	0.877411	2.1
39917	1.2275	2.1
39918	0.995678	2.1
39919	1.30835	2.1
39920	1.05793	0.830472
39921	1.46441	0.772365
39922	1.0562	0.798156
39923	1.0562	0.796794
39924	1.41787	0.822171
39925	1.41787	0.796018
39926	1.41787	0.835773
39927	1.52909	0.944753
39928	1.46441	0.813954
39929	1.46441	0.925115
39930	1.43063	0.817098
39931	1.43063	0.915789
39932	1.89226	0.952486
39933	1.89226	0.766981
39934	1.89226	0.829077
39935	1.89226	1.00495
39936	3.78453	0.879246
39937	1.89227	0.831735
39938	1.89227	0.782663
39939	1.89227	0.783817
39940	1.89227	0.79614
39941	1.89227	0.785206
39942	1.89227	0.787058
39943	1.00763	0.790908
39944	1.41787	0.803437
39945	1.05793	0.785783
39946	1.05793	0.824104
39947	1.37265	0.797409
39948	1.37265	0.815324
39949	1.37265	0.8338
39950	1.43063	0.921621
39951	1.41787	0.826516
39952	1.41787	0.968753
39953	1.38247	0.827043
39954	1.38247	0.955432
39955	0.946132	0.940051
39956	0.946132	0.92138
39957	0.946132	0.812459
39958	0.946132	0.79194
39959	0.946131	0.806124

39960	0.946131	0.913099
39961	0.946132	0.940121
39962	0.946132	0.857396
39963	0.946132	0.877032
39964	0.946132	0.879722
39965	0.946132	0.860095
39966	0.946132	0.850025
39967	1.89226	0.806394
39968	1.89226	0.800786
39969	1.89226	0.774629
39970	1.89226	0.828742
39971	1.89226	0.777357
39972	1.89226	0.818403
39973	1.19645	0.860349
39974	1.89183	0.748996
39975	1.91106	0.820054
39976	1.35306	0.770232
39977	1.75446	0.821995
39978	1.19645	0.76704
39979	2.37576	0.837458
39980	1.90139	1.15154
39981	2.1845	2.1
39982	1.86568	0.937216
39983	4.24145	1.12277
39984	2.29435	0.988286
39985	1.40602	2.1
39986	1.40602	0.873667
39987	1.55351	1.01237
39988	1.86568	1.4189
39989	2.01317	2.1
39990	2.18452	1.96295
39991	1.8657	0.781686
39992	2.01399	0.841258
39993	1.70141	1.66647
39994	3.87969	0.850647
39995	0.774999	2.1
39996	0.774999	0.863276
39997	0.778721	1.02425
39998	0.778721	2.1
39999	0.628348	1.94212
40000	0.703016	1.50794
40001	0.932848	0.892999
40002	0.850704	0.98564
40003	0.850704	1.07453
40004	0.628348	1.1121
40005	0.703015	0.893523
40006	0.932848	0.922451
40007	1.65243	0.746062
40008	1.34412	0.734793
40009	1.45569	0.800214
40010	1.1315	0.797562
40011	2.78393	0.746826
40012	1.48901	2.1
40013	1.805	0.937118
40014	1.48901	0.948877
40015	1.71717	2.1
40016	1.60635	0.86913
40017	1.83451	2.1

40018	1.805	1.24773
40019	1.65243	0.741622
40020	1.60635	0.924208
40021	1.45569	0.776052
40022	3.25879	0.807098
40023	1.27875	2.1
40024	1.60635	0.920193
40025	1.27875	1.04771
40026	1.48901	2.1
40027	1.40014	0.934458
40028	1.6104	2.1
40029	0.715199	0.805832
40030	0.715199	0.985723
40031	0.712714	0.982685
40032	0.712714	0.57684
40033	0.727843	0.699101
40034	0.619713	0.755067
40035	0.803177	0.967938
40036	0.70007	0.934242
40037	0.700069	0.833363
40038	0.619713	0.823839
40039	0.803177	0.763966
40040	0.727844	0.800271
40041	1.14357	2.1
40042	2.3506	2.1
40043	1.14357	2.1
40044	2.68773	2.1
40045	1.57829	2.1
40046	2.68773	2.09011
40047	2.00551	2.1
40048	2.65811	1.48628
40049	2.07203	2.1
40050	2.72117	0.794705
40051	4.72667	1.32992
40052	0.946131	0.877277
40053	0.946131	1.77621
40054	1.32905	1.92241
40055	1.32905	0.876985
40056	0.94613	1.1077
40057	0.94613	1.3388
40058	1.32905	1.29293
40059	1.36058	1.13417
40060	1.36058	0.902335
40061	0.946131	0.864985
40062	1.32905	1.16077
40063	0.946131	1.43217
40064	1.21649	0.810314
40065	1.21649	2.1
40066	1.21459	1.95089
40067	1.21459	2.00537
40068	1.39508	1.12044
40069	1.36057	0.975571
40070	1.03601	1.78631
40071	1.07438	2.1
40072	1.07439	1.11051
40073	1.39509	1.12082
40074	1.036	1.17241
40075	1.36056	1.09707

40076	2.79015	0.953296
40077	2.72114	2.1
40078	1.89225	1.03743
40079	1.89225	1.94444
40080	1.89225	1.46163
40081	2.72114	0.910976
40082	0.876258	2.1
40083	0.892889	2.1
40084	1.34352	2.1
40085	1.27985	2.1
40086	1.07462	2.1
40087	1.07462	2.1
40088	1.09812	2.1
40089	1.09812	2.1
40090	0.892888	2.1
40091	1.34352	2.1
40092	0.876256	2.1
40093	1.27985	2.1
40094	1.39725	1.33894
40095	1.30029	0.91785
40096	1.31415	1.00519
40097	1.41336	1.22511
40098	2.71366	1.0854
40099	1.89226	0.924854
40100	1.89226	1.07019
40101	1.89226	1.24884
40102	1.89226	0.91549
40103	3.78453	0.999712
40104	0.946133	0.991391
40105	0.946133	0.924558
40106	1.38709	0.800822
40107	1.39437	0.880484
40108	0.946132	0.809258
40109	0.946132	0.89894
40110	1.39437	1.0118
40111	1.39437	0.824648
40112	0.946132	0.888635
40113	1.39437	0.87673
40114	1.38709	0.864641
40115	0.946132	0.864344
40116	0.706681	1.32854
40117	0.711908	1.23206
40118	0.657077	0.94736
40119	0.661003	1.03188
40120	0.684188	0.958911
40121	0.684188	1.20359
40122	0.683496	1.56417
40123	0.683496	0.981087
40124	0.711908	1.10628
40125	0.661003	1.11971
40126	0.657076	1.08917
40127	0.706681	1.08571
40128	0.946132	1.23561
40129	0.946132	1.09232
40130	0.946132	0.965675
40131	0.946132	0.900603
40132	0.946132	0.908708
40133	0.946132	1.09233

40134	0.946133	0.985674
40135	0.946133	1.23117
40136	0.946133	1.02553
40137	0.946133	1.02654
40138	0.946132	1.02482
40139	0.946132	1.03178
40140	2.79029	0.815332
40141	1.89227	1.01104
40142	2.78873	0.874671
40143	2.78873	0.893371
40144	1.89383	0.853594
40145	1.89227	0.898965
40146	1.43669	2.1
40147	2.11984	2.1
40148	2.24481	2.1
40149	2.24481	2.1
40150	1.20311	2.1
40151	1.20311	2.1
40152	0.49703	2.1
40153	0.601553	2.1
40154	1.05992	2.1
40155	0.991694	2.1
40156	0.787558	2.1
40157	0.787558	2.1
40158	0.805689	2.1
40159	0.805689	2.1
40160	0.991694	2.1
40161	0.49703	2.1
40162	1.05992	2.1
40163	0.601553	2.1
40164	2.45905	2.1
40165	1.89226	2.1
40166	1.89226	2.1
40167	1.89226	2.1
40168	2.47395	2.1
40169	2.47395	2.1
40170	1.89226	2.1
40171	1.89226	1.40277
40172	1.89226	2.1
40173	1.89226	1.1255
40174	3.78452	1.93395
40175	1.38427	2.1
40176	2.31203	1.06594
40177	2.28743	1.07533
40178	2.28743	2.1
40179	1.63384	1.0138
40180	1.63384	2.1
40181	2.42723	2.1
40182	1.89226	2.1
40183	1.89226	2.1
40184	1.89226	2.1
40185	2.45905	2.1
40186	2.45905	2.1
40187	0.946131	0.906991
40188	0.946131	2.1
40189	0.946131	2.1
40190	0.946131	1.39912
40191	0.94613	1.17356

40192	0.94613	1.38213
40193	0.946132	2.1
40194	0.946132	1.93402
40195	0.946131	1.62393
40196	0.946131	1.93815
40197	0.946131	1.85382
40198	0.946131	2.1
40199	0.989441	2.1
40200	0.989441	1.56606
40201	0.993918	2.1
40202	0.993918	1.99584
40203	1.16644	1.73841
40204	1.15601	1.13059
40205	0.818308	2.1
40206	0.816922	2.1
40207	0.818307	2.09671
40208	1.16644	2.1
40209	1.15601	2.09439
40210	0.816922	1.63216
40211	0.996778	0.152679
40212	2.44375	0.133307
40213	0.996778	0.226024
40214	1.92547	0.122103
40215	1.56091	0.254286
40216	2.4896	0.135917
40217	1.56091	0.246861
40218	2.44375	0.132938
40219	1.90456	0.224152
40220	1.90456	0.243305
40221	2.81853	0.140071
40222	2.47487	0.147982
40223	1.32143	1.89212
40224	1.43294	2.04676
40225	1.37444	1.04547
40226	1.51691	1.72465
40227	2.80738	1.59388
40228	1.89226	1.40215
40229	1.89226	2.09419
40230	1.89226	1.10377
40231	1.89226	1.26401
40232	3.78453	1.31507
40233	1.89226	1.38806
40234	1.89226	2.1
40235	1.89226	1.09536
40236	1.89226	2.1
40237	3.78453	1.47146
40238	1.43294	1.36856
40239	1.3268	2.1
40240	1.51691	1.3074
40241	1.44091	2.1
40242	2.87385	2.1
40243	1.37444	2.05514
40244	1.51691	2.07866
40245	1.4714	1.06644
40246	1.65434	1.36313
40247	3.02878	1.54735
40248	1.89226	2.1
40249	1.89226	1.72115

40250	1.89226	1.1806
40251	1.89226	1.41333
40252	3.78452	1.5272
40253	1.89226	1.33534
40254	1.89226	2.1
40255	1.89226	1.17522
40256	1.89226	2.1
40257	3.78453	1.88127
40258	1.51691	1.5695
40259	1.44091	2.1
40260	1.65434	1.17177
40261	1.60647	2.1
40262	3.09526	1.98793
40263	1.05013	2.1
40264	1.05013	2.1
40265	0.94613	2.1
40266	0.94613	2.1
40267	1.04959	2.1
40268	1.05014	2.1
40269	0.946129	2.1
40270	0.946129	2.1
40271	0.946131	2.1
40272	1.04958	2.1
40273	0.946131	2.1
40274	1.05013	2.1
40275	1.04959	2.1
40276	1.04959	2.1
40277	0.946131	2.1
40278	0.946131	2.1
40279	1.04765	2.1
40280	1.04959	2.1
40281	0.94613	2.1
40282	0.94613	2.1
40283	0.946131	2.1
40284	1.04765	2.1
40285	0.946131	2.1
40286	1.04959	2.1
40287	1.85744	2.1
40288	1.89226	2.1
40289	1.89226	2.1
40290	1.89226	2.1
40291	1.94839	2.1
40292	1.94839	2.1
40293	0.97419	2.1
40294	0.822054	2.1
40295	0.946123	2.1
40296	0.946123	2.1
40297	1.04202	2.1
40298	0.84898	2.1
40299	0.933307	2.1
40300	0.933307	2.1
40301	0.933285	2.1
40302	1.05018	2.1
40303	0.851528	2.1
40304	0.876787	2.1
40305	1.87528	0.702234
40306	1.21137	0.451453
40307	1.57893	0.561444

40308	1.21137	0.705512
40309	1.81182	0.546611
40310	1.44426	0.684523
40311	0.993833	0.401153
40312	1.21137	0.542148
40313	1.57893	0.70033
40314	1.31774	0.503117
40315	2.5291	0.468019
40316	1.87528	0.687188
40317	1.57893	0.44106
40318	1.90728	0.61426
40319	2.29851	0.681337
40320	3.87744	0.674565
40321	1.90728	0.681385
40322	1.31774	0.461084
40323	1.56339	0.597092
40324	1.31774	0.685795
40325	1.82458	0.523647
40326	1.57893	0.615063
40327	1.89226	0.987131
40328	1.89226	1.1013
40329	1.89226	1.04054
40330	1.89226	1.06866
40331	1.89226	1.04263
40332	1.89226	1.07156
40333	1.89226	1.05469
40334	1.89226	1.14561
40335	1.89226	1.17706
40336	1.89226	1.09933
40337	3.78452	1.1394
40338	1.89226	1.1159
40339	2.17251	1.17605
40340	2.45575	1.10494
40341	2.45575	1.15122
40342	1.89226	1.07363
40343	1.89226	1.12087
40344	1.08625	1.18072
40345	1.22788	1.09728
40346	0.54447	1.03313
40347	0.794861	1.1001
40348	0.967729	1.16874
40349	0.967729	1.1825
40350	0.913386	1.09126
40351	0.913386	1.02951
40352	1.08625	1.14871
40353	0.544469	1.07651
40354	0.794862	1.15042
40355	1.22788	1.12932
40356	0.946128	1.11573
40357	0.946128	1.13552
40358	0.946132	1.08911
40359	0.946132	1.05951
40360	0.94613	1.08962
40361	0.94613	1.11101
40362	0.94613	1.13819
40363	0.94613	1.03914
40364	0.94613	1.09218
40365	0.94613	1.09278

40366	0.94613	1.0825
40367	0.94613	1.0925
40368	0.946127	1.10757
40369	0.946127	1.08286
40370	1.08625	1.07433
40371	0.958779	1.16148
40372	1.08625	1.11841
40373	1.08625	1.14873
40374	0.946128	1.06272
40375	0.946128	1.08089
40376	0.946129	1.08558
40377	0.958777	1.08288
40378	1.08625	1.08155
40379	0.946129	1.06511
40380	2.16338	0.383352
40381	1.94002	0.255756
40382	1.98836	0.306161
40383	2.60614	0.36316
40384	1.5456	0.247703
40385	2.16338	0.258036
40386	1.19952	0.290065
40387	2.34061	0.345011
40388	1.21031	0.271764
40389	2.1711	0.305704
40390	3.54013	0.272718
40391	1.13624	2.1
40392	1.70302	2.1
40393	1.8267	2.1
40394	1.11545	2.1
40395	2.85455	2.1
40396	1.82671	2.1
40397	1.91767	2.1
40398	1.81563	2.1
40399	1.91767	2.1
40400	1.71362	2.1
40401	1.83277	2.1
40402	1.66064	2.1
40403	0.996776	2.1
40404	0.996776	2.1
40405	1.11545	2.1
40406	1.54278	2.1
40407	1.66145	2.1
40408	1.66064	2.1
40409	1.7457	2.1
40410	1.81563	2.1
40411	1.81563	2.1
40412	1.54278	2.1
40413	1.54278	2.1
40414	2.71672	2.1
40415	2.70402	2.1
40416	2.64996	2.1
40417	2.69443	2.1
40418	5.39845	2.1
40419	2.71672	2.1
40420	2.54198	2.1
40421	2.54019	2.1
40422	2.69443	2.1
40423	5.23641	2.1

40424	2.64996	2.1
40425	2.69443	2.1
40426	2.68213	2.1
40427	2.61243	2.1
40428	5.30686	2.1
40429	2.54019	2.1
40430	2.69443	2.1
40431	2.68213	2.1
40432	2.5392	2.1
40433	5.23363	2.1
40434	1.89226	2.1
40435	2.34725	2.1
40436	2.22414	2.1
40437	2.34725	2.1
40438	1.76914	2.1
40439	1.89226	2.1
40440	0.946131	2.1
40441	0.946131	2.1
40442	1.11207	2.1
40443	1.03798	2.1
40444	0.94613	2.1
40445	0.94613	2.1
40446	1.03798	2.1
40447	1.03798	2.1
40448	0.946131	2.1
40449	1.03798	2.1
40450	0.946131	2.1
40451	1.11207	2.1
40452	1.89226	0.662752
40453	1.89226	0.582528
40454	1.89226	0.600275
40455	1.89226	0.569805
40456	1.89226	0.683075
40457	1.89226	0.654437
40458	1.89226	0.669961
40459	1.89226	0.64456
40460	1.89226	0.666558
40461	1.89226	0.596311
40462	1.89226	0.732004
40463	1.89226	0.674186
40464	1.89226	0.464007
40465	1.89226	0.386937
40466	1.89226	0.404063
40467	1.89226	0.401877
40468	1.89226	0.472412
40469	1.89226	0.478116
40470	1.44085	1.3169
40471	1.31825	0.957217
40472	1.31923	1.06615
40473	1.31825	1.2101
40474	1.43311	1.02186
40475	1.43213	1.13384
40476	1.89226	0.960756
40477	1.89226	1.13487
40478	1.89226	0.937543
40479	1.89226	1.13683
40480	1.89226	0.929889
40481	1.89226	1.19306

40482	0.946133	0.99342
40483	0.946133	0.929838
40484	1.38969	0.823434
40485	1.39587	0.892753
40486	0.946132	0.826792
40487	0.946132	0.912237
40488	1.39587	1.00288
40489	1.39587	0.859303
40490	0.946132	0.888798
40491	1.39587	0.884492
40492	1.38969	0.881843
40493	0.946132	0.883082
40494	1.44085	1.40674
40495	1.31923	0.938996
40496	1.33026	1.04288
40497	1.4617	1.20264
40498	2.78093	1.11757
40499	1.89226	0.941861
40500	1.89226	1.10593
40501	1.89226	0.931798
40502	1.89226	1.14959
40503	1.89226	0.926123
40504	1.89226	1.21177
40505	0.946133	0.966156
40506	0.946133	0.919611
40507	1.39587	0.863401
40508	1.40724	0.810072
40509	0.946132	0.817377
40510	0.946132	0.909114
40511	1.40724	0.975457
40512	1.40724	0.858142
40513	0.946132	0.878228
40514	1.40724	0.878817
40515	0.946133	0.906501
40516	1.39587	0.890645
40517	1.15818	1.3461
40518	1.81846	1.38564
40519	1.52782	0.907688
40520	1.52782	1.96165
40521	1.28095	0.869802
40522	1.28095	1.59606
40523	2.56709	2.1
40524	1.4349	0.863058
40525	1.4349	0.979735
40526	1.75471	1.01731
40527	2.29909	0.973693
40528	2.61889	1.10793
40529	2.5671	2.1
40530	2.82858	1.82536
40531	2.57368	2.1
40532	2.29909	1.00405
40533	5.12767	2.1
40534	2.29909	1.96556
40535	1.09148	0.760382
40536	1.09148	0.976827
40537	1.4349	0.939579
40538	1.99776	0.938483
40539	2.34119	1.00157

40540	2.57368	2.1
40541	1.99777	0.975484
40542	1.99777	1.76962
40543	2.29909	2.06007
40544	2.36071	1.67471
40545	2.66203	2.1
40546	1.89226	0.959219
40547	1.89226	1.04953
40548	1.89226	1.01751
40549	1.89226	0.954699
40550	3.78453	0.951124
40551	0.946128	1.10473
40552	0.946128	1.30826
40553	0.946133	1.02929
40554	0.946133	1.08997
40555	0.946131	1.10313
40556	0.946131	1.1859
40557	0.946131	1.21852
40558	0.946131	1.10204
40559	0.94613	1.25935
40560	0.94613	1.12605
40561	0.946131	1.08049
40562	0.946131	1.16385
40563	1.89226	1.05854
40564	1.89226	1.11923
40565	1.89226	0.964317
40566	1.89226	1.13219
40567	3.78453	1.14223
40568	1.89226	1.08226
40569	1.89226	1.01792
40570	1.89226	0.942526
40571	1.89226	1.04517
40572	3.78453	1.06926
40573	1.89226	1.03176
40574	2.61682	1.27713
40575	1.89226	1.20345
40576	1.89226	1.22963
40577	1.89226	1.03685
40578	1.89226	1.14256
40579	1.89226	0.7
40580	1.89226	0.7
40581	1.89226	0.699698
40582	1.89226	0.7
40583	3.78452	0.7
40584	1.89226	0.7
40585	1.89226	0.7
40586	1.89226	0.7
40587	1.89226	0.7
40588	1.89226	0.697877
40589	1.89226	0.7
40590	1.89227	0.7
40591	1.89227	0.7
40592	1.89227	0.7
40593	1.89227	0.7
40594	1.89227	0.7
40595	1.89227	0.7
40596	0.80447	0.700446
40597	0.534241	0.701166

40598	0.995175	0.7
40599	0.92204	0.7
40600	0.922042	0.7
40601	0.534241	0.7
40602	0.804468	0.701086
40603	0.995176	0.699037
40604	0.71543	0.702261
40605	0.71543	0.701532
40606	0.813988	0.7
40607	0.813988	0.7
40608	0.922045	0.7
40609	0.942899	0.7
40610	0.946132	0.7
40611	0.946132	0.7
40612	1.05169	0.7
40613	0.936661	0.7
40614	0.922045	0.7
40615	0.946133	0.686487
40616	1.01665	0.672979
40617	1.13008	0.7
40618	0.851529	0.7
40619	0.851529	0.7
40620	1.41908	2.1
40621	1.77723	2.1
40622	1.90485	2.1
40623	1.90485	2.1
40624	1.30237	2.1
40625	1.30237	2.1
40626	1.77723	2.1
40627	2.0055	1.59816
40628	1.90485	2.1
40629	2.17158	2.1
40630	3.91035	2.1
40631	0.850906	2.09253
40632	0.850906	2.1
40633	0.85668	2.1
40634	0.85668	2.1
40635	0.998045	2.1
40636	0.952425	2.1
40637	0.74367	2.1
40638	0.709541	2.1
40639	0.74367	2.1
40640	0.998045	2.1
40641	0.709541	2.1
40642	0.952425	2.1
40643	1.90485	2.1
40644	2.28563	2.1
40645	2.26282	2.1
40646	2.17158	1.89093
40647	1.99609	2.1
40648	1.90485	1.22015
40649	2.00098	0.524691
40650	1.90462	0.504053
40651	1.60057	0.608442
40652	1.79224	0.646108
40653	3.69686	0.526239
40654	2.65654	0.625742
40655	1.79224	0.644258

40656	2.22332	0.637283
40657	2.22332	0.629786
40658	2.24163	0.665337
40659	2.24163	0.655778
40660	1.89226	0.641718
40661	1.89226	0.615104
40662	1.89226	0.64382
40663	1.89226	0.661253
40664	3.78452	0.637918
40665	1.89226	0.57805
40666	1.89226	0.631421
40667	1.89226	0.627932
40668	1.89226	0.633316
40669	1.89226	0.643992
40670	1.89226	0.635137
40671	2.24163	0.66724
40672	1.03645	0.652857
40673	1.79224	0.656824
40674	1.79224	0.644582
40675	1.54283	0.683899
40676	1.54283	0.67025
40677	0.946132	0.655935
40678	0.946132	0.65259
40679	0.946129	0.646672
40680	0.946129	0.643224
40681	0.94613	0.653301
40682	0.94613	0.646446
40683	0.946131	0.665671
40684	0.946131	0.650025
40685	0.94613	0.665136
40686	0.94613	0.65177
40687	0.946131	0.644975
40688	0.946131	0.651904
40689	1.89226	2.1
40690	1.89226	2.1
40691	1.89226	2.1
40692	1.89226	2.1
40693	3.78453	2.1
40694	1.52441	2.1
40695	1.30347	2.1
40696	1.52441	2.1
40697	1.58401	2.1
40698	1.2627	2.1
40699	1.3223	2.1
40700	1.89226	2.1
40701	2.72787	2.1
40702	2.72787	2.1
40703	2.74762	2.1
40704	1.89226	2.1
40705	1.91202	2.1
40706	1.89227	2.1
40707	1.89227	2.1
40708	1.89227	2.1
40709	1.89227	2.1
40710	3.78453	2.1
40711	0.56414	2.1
40712	0.561557	2.1
40713	0.527284	2.1

40714	0.541143	2.1
40715	0.527276	2.1
40716	0.631685	2.1
40717	1.52441	2.1
40718	1.00232	2.1
40719	0.784495	2.1
40720	0.842304	2.1
40721	0.721569	2.1
40722	1.36393	2.1
40723	1.36393	2.1
40724	0.946132	2.1
40725	0.946132	2.1
40726	1.36393	2.1
40727	1.3594	2.1
40728	0.946132	2.1
40729	0.946132	2.1
40730	0.946107	2.1
40731	1.35936	2.1
40732	1.36397	2.1
40733	0.946158	2.1
40734	1.89226	2.1
40735	1.89226	2.1
40736	1.89226	2.1
40737	1.89226	2.1
40738	3.78453	2.1
40739	2.20802	2.1
40740	1.05677	2.1
40741	2.30479	2.1
40742	0.978699	2.1
40743	3.18672	2.1
40744	0.94613	2.1
40745	0.94613	2.1
40746	0.669866	2.1
40747	0.669866	2.1
40748	0.681588	2.1
40749	0.669865	2.1
40750	0.946133	2.1
40751	0.946133	2.1
40752	0.94613	2.1
40753	0.681589	2.1
40754	0.946131	2.1
40755	0.669867	2.1
40756	2.73514	2.1
40757	1.78301	2.1
40758	1.90618	2.1
40759	2.83464	2.1
40760	4.64132	2.1
40761	2.73514	2.1
40762	1.89226	2.1
40763	1.89226	2.1
40764	2.83464	2.1
40765	4.6274	2.1
40766	2.03915	2.1
40767	2.26616	2.1
40768	2.23549	2.1
40769	2.01704	2.1
40770	4.2832	2.1
40771	2.03916	2.1

40772	1.55766	2.1
40773	1.53728	2.1
40774	2.01704	2.1
40775	3.5747	2.1
40776	2.23551	2.1
40777	2.01705	2.1
40778	1.99823	2.1
40779	2.21189	2.1
40780	4.22894	2.1
40781	1.51788	2.1
40782	2.01705	2.1
40783	1.53729	2.1
40784	1.53729	2.1
40785	1.99823	2.1
40786	1.99823	2.1
40787	2.588	2.1
40788	1.45581	2.1
40789	1.45581	2.1
40790	1.44207	2.1
40791	2.54949	2.1
40792	2.53574	2.1
40793	0.98641	2.1
40794	0.98641	2.1
40795	1.00371	2.1
40796	1.00371	2.1
40797	0.749992	2.1
40798	0.727904	2.1
40799	1.26221	2.1
40800	1.27474	2.1
40801	1.26221	2.1
40802	0.749993	2.1
40803	0.727904	2.1
40804	1.27474	2.1
40805	1.23105	1.09772
40806	1.35435	1.80567
40807	1.89226	1.39924
40808	1.89226	1.35256
40809	1.89226	1.68047
40810	1.7043	1.50738
40811	1.89226	1.87491
40812	1.89226	1.32832
40813	1.89226	1.0523
40814	1.89226	1.75006
40815	3.78453	1.46782
40816	1.89226	0.914453
40817	1.89226	1.71074
40818	1.89226	1.08036
40819	1.89226	1.14588
40820	1.89226	1.29788
40821	1.89226	1.40205
40822	0.946131	1.02015
40823	1.40945	0.954466
40824	0.94613	1.00006
40825	0.94613	1.44839
40826	0.946129	1.47272
40827	0.946129	1.15939
40828	0.946132	1.65966
40829	1.40945	0.93068

40830	0.94613	1.09915
40831	0.94613	1.06208
40832	0.946131	1.12331
40833	0.94613	1.08907
40834	0.946133	1.17589
40835	0.946133	1.07183
40836	0.946132	1.28615
40837	0.946132	1.63304
40838	0.946132	1.61569
40839	0.946132	1.18953
40840	0.946133	1.3185
40841	0.946133	1.07558
40842	0.946132	1.28679
40843	0.946132	1.24709
40844	0.946133	1.19757
40845	0.946133	1.20024
40846	1.89226	1.10313
40847	1.89226	1.87487
40848	1.89226	1.32359
40849	1.89226	1.26238
40850	1.89226	1.57474
40851	1.89226	1.44796
40852	1.89226	2.1
40853	1.89226	2.1
40854	1.89226	2.1
40855	1.89226	2.1
40856	1.89226	2.1
40857	1.89226	2.1
40858	1.89226	2.1
40859	1.89226	2.1
40860	1.89226	2.1
40861	1.89226	2.1
40862	3.78453	2.1
40863	1.66647	2.1
40864	1.15399	2.1
40865	1.25443	2.1
40866	1.26599	2.1
40867	1.58697	2.1
40868	1.66647	2.1
40869	1.66647	2.1
40870	1.47446	2.1
40871	1.55433	2.1
40872	1.74241	2.1
40873	3.2208	2.1
40874	1.89227	2.1
40875	1.89227	2.1
40876	1.89227	2.1
40877	1.89227	2.1
40878	3.78453	2.1
40879	0.946132	2.1
40880	0.946132	2.1
40881	0.946132	2.1
40882	0.946132	2.1
40883	0.946132	2.1
40884	0.946132	2.1
40885	0.946133	2.1
40886	0.946133	2.1
40887	0.946132	2.1

40888	0.946132	2.1
40889	0.946133	2.1
40890	0.946133	2.1
40891	0.625067	2.1
40892	0.61907	2.1
40893	0.896913	2.1
40894	0.946199	2.1
40895	0.806287	2.1
40896	0.677685	2.1
40897	0.750657	2.1
40898	0.750657	2.1
40899	0.646004	2.1
40900	0.890236	2.1
40901	0.61049	2.1
40902	0.888979	2.1
40903	1.6223	2.1
40904	1.98034	2.1
40905	1.75003	2.1
40906	1.55433	2.1
40907	1.75003	2.1
40908	1.55433	2.1
40909	1.88602	2.1
40910	2.08149	2.1
40911	2.07991	2.1
40912	1.88473	2.1
40913	2.08149	2.1
40914	1.88602	2.1
40915	1.88458	2.1
40916	2.08149	2.1
40917	1.7069	2.1
40918	1.56284	2.1
40919	2.08149	2.1
40920	1.88458	2.1
40921	1.5955	2.1
40922	1.89226	2.1
40923	1.91975	2.1
40924	1.62299	2.1
40925	1.89226	2.06155
40926	1.5955	2.1
40927	1.89226	1.69927
40928	1.89226	2.08617
40929	1.89226	1.67479
40930	1.89226	2.1
40931	1.89226	1.22274
40932	1.89226	1.93551
40933	1.89226	2.1
40934	1.89226	2.1
40935	1.89226	2.1
40936	1.89226	2.1
40937	1.89226	1.77007
40938	1.89226	2.1
40939	1.62299	2.1
40940	1.89226	2.1
40941	1.71752	2.1
40942	1.44824	2.1
40943	1.89226	2.1
40944	1.62299	2.1
40945	1.89226	0.985972

40946	2.8317	1.00696
40947	1.89226	1.03496
40948	2.64055	0.847343
40949	4.53281	1.00321
40950	2.8317	1.19232
40951	1.83151	0.825323
40952	2.01001	0.955746
40953	2.01001	1.10206
40954	2.64055	0.837403
40955	2.64055	0.904739
40956	0.946132	0.761877
40957	0.946132	0.784809
40958	1.16167	0.817484
40959	1.16167	0.82926
40960	1.16167	0.802408
40961	1.32027	0.763075
40962	0.946131	0.770642
40963	0.946131	0.845291
40964	0.94613	0.767935
40965	1.16167	0.759519
40966	0.946131	0.788759
40967	1.32027	0.783561
40968	2.32335	0.86609
40969	1.83151	0.773586
40970	2.38525	0.79318
40971	2.64055	0.797999
40972	1.57621	0.761941
40973	1.83151	0.765907
40974	2.25677	2.1
40975	1.88158	2.1
40976	1.86856	2.1
40977	2.0815	2.1
40978	1.70303	2.1
40979	1.88158	2.1
40980	2.50381	2.1
40981	1.89227	2.1
40982	2.25677	2.1
40983	2.25677	2.1
40984	1.89227	2.1
40985	1.89227	2.1
40986	1.89226	0.68278
40987	1.50899	0.703333
40988	2.13659	0.682664
40989	1.50899	0.712819
40990	2.51986	0.661007
40991	1.89226	0.712408
40992	0.946131	0.69548
40993	0.946131	0.661755
40994	1.06829	0.684515
40995	0.895375	0.56428
40996	0.94613	0.673358
40997	0.94613	0.714126
40998	1.33922	0.651138
40999	0.895376	0.572977
41000	0.946131	0.626058
41001	1.33922	0.643529
41002	0.946131	0.645997
41003	1.06829	0.691045

41004	0.841467	0.721541
41005	0.578428	0.726472
41006	1.18079	0.718032
41007	0.920237	0.727439
41008	0.772317	0.714763
41009	0.856555	0.733874
41010	0.910271	0.721121
41011	0.910271	0.74021
41012	0.975752	0.720708
41013	0.536929	0.716861
41014	0.81952	0.738181
41015	1.15252	0.71988
41016	1.89226	2.1
41017	2.51431	2.1
41018	1.89226	2.1
41019	1.89226	2.1
41020	2.78815	2.1
41021	2.78815	2.1
41022	2.0815	2.1
41023	2.0516	2.1
41024	2.0815	2.1
41025	3.06699	2.1
41026	2.76576	2.1
41027	2.8002	2.1
41028	1.94112	2.1
41029	1.89226	2.1
41030	2.20358	2.1
41031	1.89226	2.1
41032	4.09584	2.1
41033	1.09154	2.1
41034	2.62647	2.1
41035	2.20358	2.1
41036	2.20358	2.1
41037	1.52314	2.1
41038	1.52314	2.1
41039	1.31323	2.1
41040	1.31323	2.1
41041	0.946132	2.1
41042	0.946132	2.1
41043	0.946132	2.1
41044	0.946132	2.1
41045	1.31323	2.1
41046	1.10179	2.1
41047	1.31323	2.1
41048	0.946132	2.1
41049	1.10179	2.1
41050	0.946133	2.1
41051	1.89226	0.966484
41052	1.89226	1.16101
41053	1.89226	1.1072
41054	1.89226	1.19337
41055	1.89226	0.953148
41056	1.89226	1.07845
41057	1.35126	1.36935
41058	1.35126	2.07311
41059	0.903285	1.21479
41060	0.903285	1.31833
41061	1.30841	1.37174

41062	0.903313	1.87542
41063	0.946132	1.24073
41064	0.946132	1.13821
41065	0.946132	1.21071
41066	0.903312	1.27915
41067	1.30841	1.22404
41068	0.946133	1.15586
41069	2.62097	2.1
41070	2.72561	2.1
41071	2.39016	2.1
41072	2.51062	2.1
41073	5.11577	2.1
41074	2.51062	2.1
41075	2.58286	0.86197
41076	2.40107	2.1
41077	2.40107	0.921978
41078	2.72561	2.1
41079	2.72561	0.925989
41080	2.39016	2.1
41081	2.51062	2.1
41082	2.20242	2.1
41083	2.34107	2.1
41084	4.73124	2.1
41085	2.51062	2.1
41086	2.40107	0.853515
41087	2.34107	2.1
41088	2.25197	0.905101
41089	4.74215	0.993646
41090	2.75218	2.1
41091	1.89226	2.1
41092	2.3349	2.1
41093	2.3349	2.1
41094	1.89226	2.1
41095	1.89226	2.1
41096	1.46129	2.1
41097	0.996308	2.1
41098	1.88649	2.1
41099	1.70302	2.1
41100	2.95419	2.1
41101	1.89227	2.1
41102	1.89227	2.06929
41103	1.89227	2.1
41104	1.89227	2.1
41105	3.78453	2.1
41106	0.646558	2.1
41107	0.646557	2.1
41108	0.60697	2.1
41109	0.60697	2.1
41110	0.587917	2.1
41111	0.519747	2.1
41112	0.586443	2.1
41113	0.733782	2.1
41114	0.733793	2.1
41115	0.587924	2.1
41116	0.519739	2.1
41117	0.586436	2.1
41118	2.1642	2.1
41119	1.42867	2.1

41120	2.28847	2.1
41121	1.68299	2.1
41122	3.71714	2.1
41123	1.89226	2.1
41124	1.89226	2.1
41125	1.89226	2.1
41126	1.89226	2.1
41127	3.78453	2.1
41128	0.946131	2.1
41129	0.946131	2.1
41130	1.24994	2.1
41131	1.24994	2.1
41132	1.11582	2.1
41133	1.24994	2.1
41134	0.946133	2.1
41135	0.946133	2.1
41136	0.946131	2.1
41137	1.24994	2.1
41138	0.94613	2.1
41139	1.11582	2.1
41140	2.28847	2.1
41141	1.53629	2.1
41142	1.57498	2.1
41143	1.47417	2.1
41144	2.42221	2.1
41145	2.28847	2.1
41146	1.89226	2.1
41147	1.89226	2.1
41148	1.89226	2.1
41149	1.89226	2.1
41150	3.78453	2.1
41151	1.89226	2.1
41152	2.49988	2.1
41153	2.71233	2.1
41154	2.71233	2.1
41155	1.89226	2.1
41156	1.89226	2.1
41157	0.924534	2.1
41158	0.924534	2.1
41159	0.990395	2.1
41160	0.990395	2.1
41161	0.946131	2.1
41162	0.946131	2.1
41163	0.924505	2.1
41164	0.968797	2.1
41165	0.924505	2.1
41166	0.946131	2.1
41167	0.968797	2.1
41168	0.946131	2.1
41169	0.924533	2.1
41170	0.924533	2.1
41171	0.948264	2.1
41172	0.945345	2.1
41173	0.851541	2.1
41174	0.851541	2.1
41175	0.958934	2.1
41176	1.02617	2.1
41177	0.936668	2.1

41178	1.04829	2.1
41179	0.924505	2.1
41180	0.946131	2.1
41181	2.28431	0.801776
41182	1.48035	0.586517
41183	1.59971	0.750543
41184	1.48035	0.725475
41185	2.33818	0.735009
41186	2.21882	0.685053
41187	2.28431	0.757303
41188	1.89226	0.602168
41189	1.89226	0.850593
41190	2.3968	0.617761
41191	4.28906	0.621757
41192	2.28431	0.756098
41193	1.70303	0.494403
41194	1.5428	0.459339
41195	1.59971	0.438176
41196	2.31152	0.720562
41197	2.3968	0.704287
41198	1.99823	2.1
41199	2.21189	2.1
41200	2.18937	2.1
41201	1.979 2.1	
41202	4.19089	2.1
41203	0.999114	2.1
41204	0.9895	2.1
41205	0.758942	2.1
41206	0.749358	2.1
41207	0.874236	2.1
41208	0.874236	2.1
41209	0.874236	2.1
41210	0.874236	2.1
41211	0.9895	2.1
41212	0.749358	2.1
41213	0.758942	2.1
41214	0.999114	2.1
41215	2.18937	2.1
41216	1.979 2.1	
41217	1.95416	2.1
41218	2.162 2.1	
41219	4.14099	2.1
41220	1.95416	2.1
41221	1.49871	2.1
41222	1.47712	2.1
41223	1.49871	2.1
41224	1.9574	2.1
41225	1.979 2.1	
41226	1.68508	0.189679
41227	1.64984	0.307499
41228	2.67711	0.139757
41229	2.76407	0.290833
41230	4.41391	0.292908
41231	1.84227	0.208222
41232	2.76407	0.274795
41233	2.76407	0.31455
41234	1.85007	0.220356
41235	2.72769	0.286072

41236	1.80812	0.204614
41237	1.94516	2.1
41238	1.89226	2.1
41239	1.89226	2.1
41240	1.89226	2.1
41241	1.98853	2.1
41242	1.98852	2.1
41243	1.28095	2.1
41244	2.0815	2.1
41245	2.29123	2.1
41246	2.06833	2.1
41247	1.08212	2.1
41248	0.976848	2.1
41249	1.89226	2.1
41250	1.89226	2.1
41251	1.89226	2.1
41252	1.89226	2.1
41253	1.89226	2.1
41254	1.89226	2.1
41255	1.13537	2.1
41256	1.18233	2.1
41257	0.946132	2.1
41258	0.946132	2.1
41259	0.946132	2.1
41260	0.946132	2.1
41261	0.994264	2.1
41262	1.18233	2.1
41263	1.13537	2.1
41264	0.946131	2.1
41265	0.946132	2.1
41266	0.994264	2.1
41267	0.953204	2.1
41268	0.953204	2.1
41269	0.966515	2.1
41270	0.966515	2.1
41271	1.30583	2.1
41272	1.14561	2.1
41273	0.640475	2.1
41274	0.774103	2.1
41275	1.30584	2.1
41276	0.774101	2.1
41277	0.640474	2.1
41278	1.14562	2.1
41279	0.946131	2.1
41280	0.946131	2.1
41281	0.946131	2.1
41282	0.946131	2.1
41283	0.946128	2.1
41284	0.946128	2.1
41285	0.946133	2.1
41286	0.946133	2.1
41287	0.94613	2.1
41288	0.94613	2.1
41289	0.946131	2.1
41290	0.946131	2.1
41291	0.946132	0.10256
41292	0.946132	0.0957622
41293	0.946131	0.110991

41294	0.946131	0.119895
41295	0.946131	0.122571
41296	0.946131	0.120398
41297	0.946132	0.100659
41298	0.946132	0.132877
41299	0.946131	0.1079
41300	0.946131	0.115979
41301	0.946132	0.118597
41302	0.946132	0.104861
41303	0.946131	0.107156
41304	0.946131	0.100232
41305	0.946131	0.112368
41306	0.946131	0.123495
41307	0.94613	0.123319
41308	0.94613	0.118115
41309	0.946132	0.104168
41310	0.946132	0.1302
41311	0.946131	0.111605
41312	0.946131	0.112979
41313	0.946131	0.109012
41314	0.946131	0.118061
41315	2.57882	0.402252
41316	1.48676	0.453275
41317	1.48797	0.472719
41318	2.64281	0.500406
41319	4.12957	0.431773
41320	2.56323	0.365683
41321	2.64281	0.414527
41322	2.51807	0.36475
41323	1.36324	0.510014
41324	1.48797	0.504496
41325	1.48797	0.570886
41326	1.04367	2.1
41327	2.42504	2.1
41328	2.65164	2.1
41329	1.49479	2.1
41330	3.91983	2.1
41331	2.15622	1.20814
41332	1.70539	2.1
41333	2.02369	2.1
41334	2.39039	1.38633
41335	4.17992	2.1
41336	0.966979	2.1
41337	1.7054	2.1
41338	2.0237	2.1
41339	1.31502	2.1
41340	2.99067	2.1
41341	2.02369	2.1
41342	2.65637	1.43381
41343	2.39039	1.17027
41344	2.39039	2.1
41345	2.29332	1.17154
41346	2.29332	2.1
41347	2.0237	2.1
41348	1.72116	2.1
41349	1.58465	2.1
41350	1.31502	2.1
41351	2.29332	2.05211

41352	2.0237	2.1
41353	1.0372	0.203414
41354	2.32462	0.391309
41355	2.2291	0.280797
41356	2.2291	0.324283
41357	1.17464	0.25937
41358	1.17464	0.274677
41359	2.68289	0.147831
41360	1.07805	0.169281
41361	1.70302	0.137199
41362	2.06653	0.156116
41363	3.5779	0.156919
41364	1.89226	0.428695
41365	1.89226	0.570396
41366	1.89226	0.456354
41367	1.89226	0.297096
41368	3.78453	0.417461
41369	1.89226	0.221359
41370	1.89226	0.253485
41371	1.89226	0.253791
41372	1.89226	0.253683
41373	1.89226	0.227135
41374	1.89226	0.225256
41375	1.89225	0.236395
41376	1.89225	0.213704
41377	1.89225	0.227141
41378	1.89225	0.222943
41379	1.89225	0.239255
41380	1.89225	0.238091
41381	0.94613	0.34488
41382	0.94613	0.433758
41383	0.94613	0.554059
41384	0.94613	0.453597
41385	0.946128	0.407166
41386	0.946128	0.410173
41387	0.946132	0.593145
41388	0.946132	0.320003
41389	0.94613	0.360465
41390	0.94613	0.43982
41391	0.94613	0.440663
41392	0.94613	0.362296
41393	1.29417	1.81982
41394	1.32732	2.1
41395	1.32316	1.01785
41396	1.34973	2.1
41397	2.65048	1.72661
41398	1.89226	1.27034
41399	1.89226	2.1
41400	1.89226	1.04311
41401	1.89226	1.92137
41402	3.78453	1.25849
41403	1.89226	1.51851
41404	1.89226	2.1
41405	1.89226	1.03931
41406	1.89226	2.1
41407	3.78453	2.1
41408	1.32732	1.85328
41409	1.13006	2.1

41410	1.13358	2.1
41411	1.11117	2.1
41412	1.34973	1.8064
41413	1.32732	2.1
41414	1.32316	1.52209
41415	1.34973	2.1
41416	1.34066	1.09286
41417	1.36601	2.07982
41418	2.68918	1.84839
41419	1.89226	1.55228
41420	1.89226	2.1
41421	1.89226	0.889288
41422	1.89226	2.1
41423	3.78453	1.42474
41424	1.89226	1.18679
41425	1.89226	2.1
41426	1.89226	2.1
41427	1.89226	2.1
41428	1.89226	1.10436
41429	1.89226	2.1
41430	0.624134	2.1
41431	0.624134	2.1
41432	0.624334	1.11702
41433	0.624334	2.1
41434	0.56503	2.1
41435	0.573605	2.1
41436	0.674864	1.32162
41437	0.683007	1.55191
41438	0.683007	2.1
41439	0.573605	2.1
41440	0.565029	2.1
41441	0.674864	2.1
41442	2.16633	2.1
41443	1.40846	2.1
41444	2.13977	2.1
41445	1.40771	2.1
41446	3.57404	2.1
41447	2.13977	2.1
41448	1.29932	2.1
41449	1.29932	2.1
41450	1.40771	2.1
41451	1.98957	2.1
41452	2.09796	2.1
41453	2.6044	0.744397
41454	1.85548	0.761339
41455	1.89226	0.715029
41456	1.89226	0.796184
41457	3.74774	0.76454
41458	1.89226	0.713867
41459	1.89226	0.795215
41460	1.89226	0.723465
41461	1.89226	0.744391
41462	3.78453	0.726828
41463	1.89226	2.1
41464	2.64107	2.1
41465	1.89226	2.1
41466	2.78977	2.1
41467	1.89226	2.1

41468	2.78977	2.1
41469	1.89226	2.1
41470	1.89226	2.1
41471	1.89226	2.1
41472	1.89226	2.1
41473	1.89226	2.1
41474	1.89226	2.1
41475	2.43173	2.1
41476	1.41238	2.1
41477	2.0815	2.1
41478	1.98048	2.1
41479	1.62014	2.1
41480	1.50086	2.1
41481	0.946129	0.727809
41482	0.946129	0.620533
41483	1.27636	0.728093
41484	1.1984	0.426634
41485	0.946131	0.42462
41486	0.946131	0.53608
41487	1.27636	0.746375
41488	1.27636	0.55339
41489	0.946131	0.607822
41490	1.27636	0.526596
41491	0.946131	0.564031
41492	1.1984	0.526768
41493	1.99569	0.509792
41494	2.55272	0.745768
41495	1.77403	0.428904
41496	1.70303	0.372672
41497	3.05772	0.567513
41498	2.55121	0.444053
41499	0.946128	0.510346
41500	0.946128	0.59338
41501	1.38752	0.64459
41502	1.27636	0.334117
41503	0.94613	0.326089
41504	0.94613	0.456379
41505	1.38752	0.600614
41506	1.38752	0.495879
41507	0.946131	0.479043
41508	1.38752	0.471285
41509	0.946131	0.495906
41510	1.27636	0.450973
41511	2.77504	0.626218
41512	1.99569	0.365236
41513	2.29794	0.512247
41514	1.99569	0.40929
41515	2.85497	0.409611
41516	2.55272	0.320481
41517	1.53284	2.1
41518	1.89226	2.1
41519	1.89226	2.1
41520	1.89226	2.1
41521	1.54763	2.1
41522	1.54763	2.1
41523	0.946131	2.1
41524	0.946131	2.1
41525	0.946127	2.1

41526	0.946127	2.1
41527	0.946127	2.1
41528	0.946127	2.05556
41529	0.946131	2.1
41530	0.946131	2.1
41531	0.94613	2.1
41532	0.94613	2.1
41533	0.946129	2.1
41534	0.946129	2.1
41535	0.751754	2.1
41536	0.751754	2.1
41537	0.771562	2.1
41538	0.771561	2.1
41539	1.0268	2.1
41540	0.95482	2.1
41541	0.528937	2.1
41542	0.496512	2.1
41543	0.528936	2.1
41544	1.0268	2.1
41545	0.954821	2.1
41546	0.49651	2.1
41547	2.64075	0.720786
41548	1.35181	0.887105
41549	1.35181	0.716311
41550	1.83823	0.750299
41551	2.28373	0.756759
41552	2.88566	0.858187
41553	0.493018	0.888088
41554	0.675903	0.85983
41555	1.14187	0.715087
41556	0.973937	0.700406
41557	0.821166	0.752076
41558	0.821166	0.945368
41559	0.828674	1.35909
41560	0.828674	0.710165
41561	0.973937	0.781002
41562	0.493018	0.791622
41563	0.675903	0.750665
41564	1.14187	0.739805
41565	1.89226	2.1
41566	2.11667	2.1
41567	1.91184	2.1
41568	1.89226	2.1
41569	2.13625	2.1
41570	2.11667	2.1
41571	1.89226	2.1
41572	1.89226	2.1
41573	1.89226	2.1
41574	1.89226	2.1
41575	3.78453	2.1
41576	0.907729	2.1
41577	0.907729	2.1
41578	1.04459	2.1
41579	0.890955	2.1
41580	0.988181	2.1
41581	0.841856	2.1
41582	0.946133	2.1
41583	0.946133	2.1

41584	1.04072	2.1
41585	1.1064	2.1
41586	0.89099	2.1
41587	0.851541	2.1
41588	1.89225	2.1
41589	2.13623	2.1
41590	1.91044	2.1
41591	1.89225	2.1
41592	2.15442	2.1
41593	2.13623	2.1
41594	1.89225	2.1
41595	1.89225	2.1
41596	1.89225	2.1
41597	1.89225	2.1
41598	1.89225	2.1
41599	1.89225	2.1
41600	0.907723	2.1
41601	0.907723	2.1
41602	1.07536	2.1
41603	1.07814	2.1
41604	0.998499	2.1
41605	1.03575	2.1
41606	0.946126	2.1
41607	0.946126	2.1
41608	1.04072	2.1
41609	1.14025	2.1
41610	0.851528	2.1
41611	0.898284	2.1
41612	1.89226	0.397419
41613	1.89226	0.703539
41614	1.89226	0.611223
41615	1.89226	0.610467
41616	1.89226	0.512341
41617	1.89226	0.508811
41618	2.53008	2.1
41619	1.94534	2.1
41620	1.98702	2.1
41621	1.98858	2.1
41622	2.52852	2.1
41623	2.53008	2.1
41624	1.03267	2.1
41625	1.12591	2.1
41626	1.2959	2.1
41627	1.28992	2.1
41628	1.10851	2.1
41629	1.33078	2.1
41630	1.10661	2.1
41631	1.10661	2.1
41632	0.982208	2.1
41633	1.28992	2.1
41634	1.03267	2.1
41635	1.23262	2.1
41636	2.2712	2.1
41637	1.89226	2.1
41638	1.95954	2.1
41639	1.95954	2.1
41640	1.89226	2.1
41641	1.89226	2.1

41642	1.89226	2.1
41643	1.89226	2.1
41644	1.89226	2.1
41645	1.89226	2.1
41646	3.78453	2.1
41647	1.33887	2.1
41648	1.25395	2.1
41649	0.88327	2.1
41650	0.676665	2.1
41651	0.84881	2.1
41652	0.987503	2.1
41653	1.13243	2.1
41654	1.13243	2.1
41655	1.15338	2.1
41656	0.849966	2.1
41657	0.793074	2.1
41658	1.11957	2.1
41659	0.946129	2.1
41660	0.946129	2.1
41661	1.28254	2.1
41662	1.1356	2.1
41663	1.1356	2.1
41664	1.1356	2.1
41665	0.946131	2.1
41666	0.946131	2.1
41667	0.946131	2.1
41668	1.28254	2.1
41669	1.1356	2.1
41670	0.946131	2.1
41671	1.89227	2.1
41672	1.89227	2.1
41673	1.89227	2.1
41674	1.89227	2.1
41675	3.78453	2.1
41676	0.936287	0.117723
41677	1.01399	0.0914255
41678	0.959415	0.129041
41679	0.959415	0.136951
41680	0.910786	0.131717
41681	1.38301	0.0908749
41682	1.0286	0.103137
41683	0.535742	0.090936
41684	0.505202	0.117994
41685	0.937685	0.116882
41686	1.11066	0.115606
41687	1.46907	0.0966289
41688	1.00546	0.168591
41689	1.01524	0.153115
41690	0.94613	0.245375
41691	0.94613	0.214751
41692	1.05018	0.227903
41693	0.914025	0.145159
41694	0.913512	0.244364
41695	0.851542	0.246381
41696	1.05385	0.244991
41697	1.05494	0.140931
41698	0.91404	0.230984
41699	0.91404	0.170143

41700	2.03253	0.872794
41701	1.82325	0.733011
41702	1.93938	0.922145
41703	1.73756	0.553493
41704	3.77009	0.791277
41705	1.82325	0.730332
41706	1.47562	0.764237
41707	1.73756	0.603593
41708	1.3868	0.607645
41709	3.21317	0.641068
41710	1.93938	0.917727
41711	1.73756	0.731311
41712	1.85293	0.876932
41713	1.64279	0.680294
41714	3.59049	0.788515
41715	1.73756	0.721514
41716	1.3868	0.74412
41717	1.64279	0.714735
41718	1.28419	0.767165
41719	3.02174	0.721989
41720	1.35048	2.03599
41721	1.37999	2.1
41722	1.34355	1.07872
41723	1.37854	2.1
41724	2.72354	1.51512
41725	1.89226	1.28818
41726	1.89226	2.1
41727	1.89226	1.05806
41728	1.89226	1.26404
41729	3.78452	1.13783
41730	1.89226	1.39085
41731	1.89226	2.1
41732	1.89226	0.964161
41733	1.89226	2.1
41734	3.78453	2.1
41735	1.37999	1.48967
41736	1.18059	2.1
41737	1.17117	2.1
41738	1.17262	2.1
41739	1.37855	1.57778
41740	1.37999	2.1
41741	1.34355	1.72278
41742	1.37854	2.1
41743	1.33079	1.15168
41744	1.3741	2.05827
41745	2.71765	1.62691
41746	1.89226	1.56335
41747	1.89226	1.96807
41748	1.89226	1.00228
41749	1.89226	2.08573
41750	3.78453	1.51152
41751	1.89226	1.1348
41752	1.89226	2.1
41753	1.89226	1.18854
41754	1.89226	2.1
41755	3.78453	1.99675
41756	0.640305	2.1
41757	0.640305	1.56782

41758	0.643507	0.972536
41759	0.643507	2.1
41760	0.590294	2.1
41761	0.594539	2.1
41762	0.689273	1.21364
41763	0.687048	1.75122
41764	0.687048	2.1
41765	0.594538	1.59213
41766	0.590294	2.1
41767	0.689273	2.1
41768	2.01399	0.753652
41769	1.70141	0.788148
41770	1.83896	1.00892
41771	1.52482	0.857565
41772	3.53881	0.803219
41773	1.70141	0.842018
41774	1.0752	1.73417
41775	1.0752	0.818066
41776	1.25669	0.933145
41777	1.52482	0.885477
41778	1.70632	1.87031
41779	1.83896	0.787633
41780	1.52482	0.757704
41781	1.65244	0.778882
41782	1.34412	0.821189
41783	3.17725	0.778407
41784	1.26782	0.377634
41785	2.90353	0.42439
41786	2.54203	0.353984
41787	2.41979	0.535347
41788	1.77007	0.292132
41789	1.703	0.418453
41790	0.946118	0.63245
41791	0.946118	0.551626
41792	1.38643	0.554334
41793	1.38644	0.397695
41794	0.946117	0.665413
41795	0.946117	0.675779
41796	1.38644	0.413528
41797	1.15545	0.511175
41798	0.946131	0.626198
41799	1.15546	0.520343
41800	1.38642	0.552527
41801	0.946105	0.637619
41802	2.42256	0.72365
41803	2.63056	0.721185
41804	2.43174	0.709422
41805	2.25035	0.715108
41806	4.8543	0.71202
41807	2.58031	0.718258
41808	2.63056	0.721688
41809	2.43174	0.708413
41810	2.32127	0.706491
41811	5.01205	0.719246
41812	2.43174	0.707236
41813	2.25035	0.640618
41814	2.11465	0.580652
41815	2.13042	0.614255

41816	4.54639	0.625625
41817	2.43174	0.706438
41818	2.32127	0.694424
41819	1.92997	0.674519
41820	2.13041	0.62862
41821	4.36171	0.697314
41822	1.89227	2.1
41823	1.89227	2.1
41824	1.89227	2.1
41825	1.89227	2.1
41826	3.78453	2.1
41827	0.568724	2.1
41828	0.568724	2.1
41829	0.57678	2.1
41830	0.57678	2.1
41831	0.523796	2.1
41832	0.548137	2.1
41833	0.597368	2.1
41834	0.637877	2.1
41835	0.597367	2.1
41836	0.523796	2.1
41837	0.637877	2.1
41838	0.548137	2.1
41839	1.32674	2.1
41840	1.32674	2.1
41841	0.946132	2.1
41842	0.946132	2.1
41843	1.3195	2.1
41844	1.32674	2.1
41845	0.946132	2.1
41846	0.946132	2.1
41847	0.946132	2.1
41848	1.3195	2.1
41849	1.32674	2.1
41850	0.946133	2.1
41851	1.89227	2.1
41852	1.89227	2.1
41853	1.89227	2.1
41854	1.89227	2.1
41855	3.78453	2.1
41856	1.19473	2.1
41857	0.987132	2.1
41858	0.987132	2.1
41859	1.04759	2.1
41860	1.0894	2.1
41861	1.14986	2.1
41862	1.3195	2.1
41863	1.3195	2.1
41864	0.946132	2.1
41865	0.946132	2.1
41866	1.31185	2.1
41867	1.3195	2.1
41868	0.946132	2.1
41869	0.946132	2.1
41870	1.31185	2.1
41871	0.946133	2.1
41872	0.946132	2.1
41873	1.3195	2.1

41874	1.11256	1.13635
41875	1.70101	1.14126
41876	1.22297	1.15188
41877	1.22297	1.14214
41878	1.7252	1.18867
41879	1.7252	1.17
41880	1.99298	1.13156
41881	1.70101	1.18247
41882	1.7252	1.16939
41883	2.18701	1.06788
41884	3.71818	1.08379
41885	1.03218	1.10636
41886	1.7252	1.07894
41887	1.11256	1.12326
41888	1.11256	1.09027
41889	1.7457	1.14103
41890	1.7457	1.10417
41891	1.7457	1.103
41892	2.187 1.00442	
41893	1.7252	1.07897
41894	1.7252	1.0711
41895	2.27351	1.08319
41896	2.27351	1.07602
41897	1.95544	0.857088
41898	1.35897	1.81154
41899	1.44859	1.14076
41900	1.44859	1.38953
41901	1.80709	0.90914
41902	1.80709	1.33309
41903	1.80709	0.824656
41904	1.17822	1.58569
41905	1.35897	1.05118
41906	1.35897	1.55973
41907	1.52782	0.825813
41908	1.52782	1.25279
41909	1.11978	1.27134
41910	0.903546	0.868735
41911	0.977719	1.4381
41912	1.2364	0.88905
41913	1.03812	0.802558
41914	1.03812	1.73161
41915	1.05938	0.791922
41916	1.05938	1.48042
41917	1.11978	0.919924
41918	1.2364	0.777828
41919	0.97772	1.14471
41920	0.903546	0.95482
41921	1.80709	1.42007
41922	1.81847	1.07825
41923	1.96029	1.54527
41924	1.52782	1.20994
41925	2.23956	0.935666
41926	1.80709	1.02563
41927	1.89226	0.757282
41928	2.32962	0.690599
41929	2.5992	0.688711
41930	1.89226	0.718964
41931	2.5992	0.75158

41932	1.89226	0.754142
41933	2.55791	0.108693
41934	2.47141	0.0792545
41935	2.13515	0.0789358
41936	2.10939	0.103197
41937	4.66731	0.0949998
41938	2.47141	0.0811383
41939	2.23337	0.102578
41940	2.10939	0.106584
41941	2.03438	0.0810823
41942	4.34276	0.0845083
41943	2.13512	0.0826844
41944	1.71027	0.11128
41945	1.71027	0.110088
41946	2.10936	0.090075
41947	1.60137	0.108657
41948	2.00046	0.0833686
41949	1.71027	0.124343
41950	2.03435	0.0827175
41951	1.71027	0.100579
41952	2.10936	0.093032
41953	1.72067	0.102662
41954	2.11976	0.0913592
41955	1.177 0.835785	
41956	1.36405	1.02829
41957	2.25635	1.07789
41958	2.60151	0.791488
41959	3.6204	0.957922
41960	0.658869	0.629362
41961	0.781697	0.656964
41962	0.637037	0.475984
41963	0.541148	0.487123
41964	0.654682	0.480366
41965	0.654682	0.483135
41966	0.641224	0.682286
41967	0.641224	0.3432
41968	0.637039	0.625153
41969	0.781696	0.579916
41970	0.658867	0.613988
41971	0.541149	0.443535
41972	1.89226	0.756405
41973	1.59152	0.676406
41974	1.89226	0.689375
41975	1.82125	0.68036
41976	1.89226	0.750128
41977	1.82125	0.74789
41978	2.18967	0.160531
41979	1.89227	0.223451
41980	2.72752	0.252995
41981	2.72752	0.242634
41982	1.89227	0.154211
41983	1.89227	0.124326
41984	2.18967	0.136199
41985	1.92547	0.284368
41986	1.92547	0.201925
41987	2.72752	0.158496
41988	1.30572	0.178001
41989	2.10777	0.132355

41990	2.39663	2.1
41991	1.92293	2.1
41992	1.96889	2.1
41993	2.45928	2.1
41994	4.36552	2.1
41995	1.02605	2.1
41996	1.92293	2.1
41997	1.9689	2.1
41998	1.04679	2.1
41999	2.99495	2.1
42000	1.96889	2.1
42001	2.45928	2.1
42002	2.50138	2.1
42003	1.99782	2.1
42004	4.47027	2.1
42005	1.9689	2.1
42006	1.03449	2.1
42007	1.07572	2.1
42008	1.04679	2.1
42009	1.99783	2.1
42010	1.9689	2.1
42011	1.72162	0.803991
42012	1.89186	0.771796
42013	2.29771	0.769802
42014	1.80149	0.72479
42015	4.01933	0.764388
42016	1.16482	0.843822
42017	1.97003	0.740549
42018	1.72162	0.725884
42019	1.72162	0.764255
42020	1.29313	0.766091
42021	1.29313	0.848449
42022	2.29771	0.783091
42023	2.84159	0.712608
42024	2.19622	0.737719
42025	1.97366	0.732984
42026	2.50906	0.744417
42027	2.29771	0.74731
42028	1.29313	0.820841
42029	2.4483	0.734726
42030	2.14223	0.730935
42031	2.14228	0.761297
42032	1.294	0.75377
42033	1.294	0.79725
42034	1.21495	0.113414
42035	1.70303	0.137728
42036	1.7924	0.098409
42037	1.64002	0.118291
42038	3.11963	0.10471
42039	2.81021	0.106577
42040	1.89226	0.152417
42041	1.89226	0.111963
42042	1.89226	0.116049
42043	2.36406	0.121895
42044	2.36406	0.125845
42045	1.89226	0.128357
42046	1.89226	0.194386
42047	1.89226	0.128664

42048	1.89226	0.144022
42049	1.89226	0.143067
42050	1.89226	0.164217
42051	2.29976	0.0934682
42052	1.64002	0.131324
42053	1.8959	0.110209
42054	1.90873	0.102256
42055	1.85594	0.123095
42056	1.85594	0.121526
42057	1.89226	0.141886
42058	1.89226	0.197077
42059	1.89226	0.139226
42060	1.89226	0.154869
42061	3.78453	0.13815
42062	1.89226	0.995615
42063	2.52823	2.1
42064	2.83441	1.39197
42065	2.83441	2.06782
42066	1.89226	1.21875
42067	1.89226	1.56009
42068	1.04522	1.60368
42069	1.04522	1.36998
42070	1.12831	1.57848
42071	1.18364	2.1
42072	0.640525	1.55121
42073	0.924882	1.45374
42074	1.27877	1.64799
42075	1.43363	1.00338
42076	0.93474	1.48123
42077	1.58926	1.30302
42078	1.29573	1.6226
42079	0.664247	1.80217
42080	2.02191	2.1
42081	1.89226	2.1
42082	1.89226	2.1
42083	1.89226	2.1
42084	2.58639	2.1
42085	2.58639	2.1
42086	1.16759	2.1
42087	2.11828	2.1
42088	1.60936	2.1
42089	2.35846	2.1
42090	3.52605	2.1
42091	1.89226	2.1
42092	1.89226	2.1
42093	1.89226	2.1
42094	1.89226	2.1
42095	3.78453	2.1
42096	2.82765	0.556196
42097	1.89809	0.319007
42098	2.15971	0.516637
42099	1.89809	0.398032
42100	2.29233	0.342642
42101	2.03071	0.245406
42102	1.90664	0.588175
42103	2.15971	0.690361
42104	1.28956	0.39172
42105	1.28956	0.337034

42106	2.64414	0.420289
42107	2.02706	0.370893
42108	2.82765	0.312921
42109	1.89226	0.426952
42110	2.39968	0.336819
42111	2.39968	0.277698
42112	1.89226	0.407247
42113	1.89226	0.334212
42114	0.978643	2.1
42115	0.703968	2.1
42116	0.946129	2.1
42117	0.946129	2.1
42118	0.703939	2.1
42119	0.703939	2.1
42120	0.946159	2.1
42121	0.946159	2.1
42122	0.978642	2.1
42123	0.94613	2.1
42124	0.94613	2.1
42125	0.703968	2.1
42126	1.89226	2.1
42127	2.17309	1.1951
42128	2.17309	2.07189
42129	1.47475	1.66359
42130	1.89226	1.98241
42131	1.19393	1.30615
42132	1.89226	2.1
42133	1.89226	2.1
42134	1.89226	2.1
42135	1.89226	1.91052
42136	3.78452	2.1
42137	1.17426	2.1
42138	0.978644	2.1
42139	0.946129	2.1
42140	0.946129	2.1
42141	0.946131	2.1
42142	0.946131	2.1
42143	0.978643	2.1
42144	0.978642	2.1
42145	1.17426	2.1
42146	0.946131	2.1
42147	0.978642	2.1
42148	0.94613	2.1
42149	1.89226	2.1
42150	1.89226	1.18383
42151	1.89226	2.00073
42152	2.17309	1.26354
42153	2.56346	1.92712
42154	2.94389	1.4446
42155	0.946131	2.1
42156	0.946131	1.9427
42157	0.946128	2.1
42158	0.946128	2.1
42159	0.946131	2.1
42160	0.946131	1.93412
42161	0.946128	2.1
42162	0.946128	2.1
42163	0.94613	2.1

42164	0.94613	2.1
42165	0.946129	2.1
42166	0.946129	2.1
42167	1.89226	0.758344
42168	1.89226	0.774563
42169	1.89226	0.775136
42170	1.89226	0.850443
42171	3.78453	0.760859
42172	1.89226	0.791587
42173	1.89226	0.783529
42174	1.89226	0.896803
42175	1.89226	0.886527
42176	1.89226	0.749898
42177	1.89226	0.748242
42178	1.89226	0.793823
42179	1.30733	0.841231
42180	1.89226	0.910159
42181	1.92403	1.03527
42182	3.38635	0.800623
42183	1.89226	0.783799
42184	3.02887	1.07863
42185	2.0815	0.79064
42186	1.88422	0.810064
42187	2.0815	0.86965
42188	1.88422	1.27974
42189	1.89226	0.869861
42190	1.70221	0.870024
42191	1.89226	1.49457
42192	1.20068	1.34849
42193	1.89226	0.764788
42194	1.20068	0.755495
42195	1.01393	0.854949
42196	1.20067	0.831554
42197	1.89226	1.61436
42198	2.44958	1.16896
42199	1.01393	0.734812
42200	1.89226	0.728553
42201	1.89226	2.1
42202	1.89226	2.1
42203	1.89226	2.1
42204	1.89226	2.1
42205	1.89226	2.1
42206	1.89226	2.1
42207	1.89226	2.1
42208	1.89226	0.931986
42209	1.89226	0.977252
42210	1.89226	2.1
42211	1.89226	0.988872
42212	1.89226	2.1
42213	1.89226	2.1
42214	1.89226	2.00607
42215	1.89226	2.1
42216	1.89226	2.1
42217	1.89226	2.1
42218	1.89226	2.1
42219	1.91161	2.1
42220	2.45782	1.49235
42221	1.39653	2.1

42222	2.44652	1.82578
42223	4.35812	2.1
42224	2.73092	1.46013
42225	1.89226	0.949045
42226	1.89226	2.1
42227	1.89226	0.930366
42228	2.44652	1.97171
42229	2.44652	0.908149
42230	1.89226	2.1
42231	1.89226	0.966752
42232	1.89226	1.00891
42233	1.89226	2.1
42234	1.89226	1.00594
42235	1.89226	2.1
42236	2.64924	2.1
42237	1.31109	2.1
42238	1.31109	2.1
42239	1.49958	2.1
42240	1.95039	2.1
42241	2.14451	2.1
42242	0.825157	2.1
42243	0.634856	2.1
42244	0.816836	2.1
42245	0.816836	2.1
42246	0.556743	2.1
42247	0.639142	2.1
42248	0.985106	2.1
42249	0.795045	2.1
42250	0.967461	2.1
42251	0.552494	2.1
42252	0.676829	2.1
42253	0.98803	2.1
42254	2.28564	2.1
42255	2.34084	1.23559
42256	2.3502	1.44249
42257	2.42908	2.1
42258	4.69104	1.7151
42259	2.34084	1.67377
42260	2.18077	2.03579
42261	2.42908	2.1
42262	2.2947	0.994304
42263	4.63554	1.74319
42264	2.3502	1.61726
42265	2.42908	2.1
42266	2.38681	2.1
42267	2.50438	1.10744
42268	4.85458	2.1
42269	2.42908	2.1
42270	2.2947	1.23226
42271	2.50438	1.0687
42272	2.40627	2.05408
42273	4.79908	1.15189
42274	1.89226	0.917654
42275	2.20711	1.31348
42276	1.89226	0.924788
42277	1.89226	1.30142
42278	2.02693	0.941538
42279	2.02693	1.43253

42280	1.2269	2.1
42281	2.20711	1.29642
42282	2.0815	1.19536
42283	1.11506	2.1
42284	3.22109	1.91124
42285	0.545802	1.65983
42286	0.547489	2.06231
42287	0.85914	1.25675
42288	1.11641	1.36633
42289	0.899974	1.44464
42290	0.849893	2.1
42291	0.747885	1.70696
42292	0.747885	1.19133
42293	0.557184	2.03064
42294	0.887039	1.97935
42295	1.08948	1.92227
42296	0.59815	2.00941
42297	0.974139	2.1
42298	1.33385	2.1
42299	1.02368	2.1
42300	1.02368	2.1
42301	1.00833	2.1
42302	1.00833	2.1
42303	1.89226	0.799039
42304	1.89226	0.706897
42305	1.89226	0.83123
42306	1.89226	0.330749
42307	3.78453	0.707606
42308	1.74651	0.85346
42309	1.44605	0.625611
42310	1.63557	0.814366
42311	1.31993	0.069672
42312	3.08162	0.587671
42313	1.89226	0.842695
42314	1.89226	0.723253
42315	1.89226	0.809819
42316	1.89226	0.110327
42317	3.78452	0.568525
42318	1.63557	0.840758
42319	1.31992	0.384765
42320	1.52615	0.856525
42321	1.18549	0.0724889
42322	2.82106	0.474727
42323	1.89226	0.703393
42324	2.58101	0.403502
42325	2.73797	0.786515
42326	2.73797	0.71898
42327	1.89226	0.796543
42328	1.89226	0.71469
42329	1.91762	0.802346
42330	1.44354	0.800355
42331	1.33622	0.792822
42332	1.33622	0.790549
42333	2.20248	0.817191
42334	2.20248	0.812657
42335	1.11712	2.1
42336	1.11712	2.1
42337	1.11421	2.1

42338	1.11421	2.1
42339	0.833871	2.1
42340	0.926528	2.1
42341	1.3048	2.1
42342	1.40335	2.1
42343	0.926527	2.1
42344	1.40335	2.1
42345	1.3048	2.1
42346	0.833871	2.1
42347	0.879657	2.1
42348	0.937695	2.1
42349	0.946129	2.1
42350	0.946129	2.1
42351	0.851541	2.1
42352	0.851541	2.1
42353	1.1126	2.1
42354	1.04519	2.1
42355	0.851486	2.1
42356	0.980501	2.1
42357	0.898251	2.1
42358	0.851528	2.1
42359	1.37756	2.1
42360	2.73081	2.1
42361	2.44311	2.1
42362	2.44311	2.1
42363	1.66428	2.1
42364	1.66428	2.1
42365	1.89226	2.1
42366	1.89226	2.1
42367	1.89226	2.1
42368	1.89226	2.1
42369	3.78452	2.1
42370	1.65781	2.1
42371	1.89837	2.1
42372	1.46713	2.1
42373	1.98027	2.1
42374	3.63808	2.1
42375	2.29054	2.1
42376	2.26369	0.901114
42377	1.98027	2.1
42378	2.15617	0.570984
42379	4.24396	1.04507
42380	1.89226	2.1
42381	1.89226	0.903104
42382	1.89226	2.1
42383	1.89226	0.883455
42384	3.78452	1.089
42385	0.946131	2.1
42386	0.946131	2.1
42387	0.946128	2.1
42388	0.946128	2.1
42389	0.946129	2.1
42390	0.946129	2.1
42391	0.94613	2.1
42392	0.94613	2.1
42393	0.94613	2.1
42394	0.94613	2.1
42395	0.94613	2.1

42396	0.94613	2.1
42397	0.812186	2.1
42398	0.812186	2.1
42399	0.819781	2.1
42400	0.819781	2.1
42401	0.913532	2.1
42402	0.990134	2.1
42403	0.733564	2.1
42404	0.641833	2.1
42405	0.641832	2.1
42406	0.913533	2.1
42407	0.733564	2.1
42408	0.990134	2.1
42409	0.994262	0.844989
42410	0.994262	2.1
42411	0.997354	2.06032
42412	0.997354	0.898055
42413	1.00775	0.798471
42414	1.07808	0.874415
42415	0.913533	2.1
42416	0.990135	2.1
42417	0.913532	1.0208
42418	1.00775	1.01977
42419	0.990134	1.00859
42420	1.07808	1.01105
42421	0.946131	2.1
42422	0.946131	0.906124
42423	0.946128	2.06409
42424	0.946128	0.90286
42425	0.946128	0.878835
42426	0.946128	0.866036
42427	0.94613	2.1
42428	0.94613	2.1
42429	0.946129	1.07982
42430	0.946129	1.08573
42431	0.946129	1.00494
42432	0.946129	1.00528
42433	0.816232	2.1
42434	0.760038	2.1
42435	0.845999	2.1
42436	0.845999	2.1
42437	1.0631	2.1
42438	1.28131	2.1
42439	0.474065	2.1
42440	0.591905	2.1
42441	0.601756	2.1
42442	1.23903	2.1
42443	0.988592	2.1
42444	0.49051	2.1
42445	2.57021	2.1
42446	1.89226	2.1
42447	2.68172	2.1
42448	1.89226	1.11422
42449	4.46247	2.00062
42450	1.89226	2.1
42451	1.89226	1.57916
42452	1.89226	1.36849
42453	1.89226	1.24715

42454	3.78452	1.5044
42455	1.12573	2.1
42456	1.65504	1.20516
42457	1.38848	1.78315
42458	1.38848	2.1
42459	1.30567	1.11869
42460	1.30567	2.00117
42461	2.82413	2.1
42462	1.89226	1.18786
42463	2.68172	2.1
42464	2.68172	2.1
42465	1.89226	1.95549
42466	1.89226	2.1
42467	1.89226	1.45567
42468	1.89226	1.55093
42469	1.89226	2.1
42470	1.89226	0.984991
42471	3.78452	1.36795
42472	1.30567	2.1
42473	1.65504	1.43589
42474	1.52724	2.1
42475	1.89249	1.88284
42476	3.18228	2.1
42477	2.57386	0.577122
42478	1.89226	0.711463
42479	1.89226	0.718169
42480	2.66753	0.674598
42481	1.89226	0.694405
42482	2.66753	0.686615
42483	1.89226	0.575363
42484	1.89226	0.722675
42485	2.2973	0.722654
42486	3.12479	0.698885
42487	1.89226	0.711329
42488	2.57385	0.698951
42489	0.489293	2.1
42490	0.672698	2.1
42491	0.59038	2.1
42492	0.738357	2.1
42493	0.62269	2.1
42494	0.62269	2.1
42495	0.640388	2.1
42496	0.640388	2.1
42497	0.59038	2.1
42498	0.489293	2.1
42499	0.738357	2.1
42500	0.672698	2.1
42501	1.89226	2.1
42502	1.89226	1.64655
42503	1.89226	2.1
42504	1.89226	0.994141
42505	3.78452	2.041
42506	2.82858	1.93902
42507	1.89226	1.04076
42508	2.5671	2.1
42509	1.89226	2.1
42510	2.5671	2.1
42511	1.89226	1.5005

42512	2.06581	0.0809329
42513	2.47308	0.100469
42514	1.89433	0.0959519
42515	1.67571	0.0775837
42516	3.87274	0.0872643
42517	2.55791	0.102413
42518	2.10276	0.0755504
42519	2.47308	0.103334
42520	2.47308	0.0891721
42521	1.83415	0.0848707
42522	1.90922	0.0812949
42523	1.18421	0.10417
42524	2.03277	0.0795765
42525	1.18421	0.115889
42526	2.01275	0.0868552
42527	1.42818	0.109697
42528	2.25673	0.0817307
42529	0.882904	0.0780775
42530	0.958492	0.0800811
42531	0.905756	0.101799
42532	0.905756	0.104411
42533	1.06352	0.0815652
42534	0.797654	0.111365
42535	1.11319	0.0815714
42536	0.716795	0.110204
42537	0.820721	0.0948016
42538	0.731963	0.0953681
42539	1.13143	0.0842547
42540	1.10528	0.089429
42541	1.89226	0.414748
42542	1.89226	0.335477
42543	1.89226	0.320221
42544	1.89226	0.668156
42545	1.89226	0.21427
42546	1.89226	0.520036
42547	1.01241	0.691076
42548	0.703929	0.635268
42549	0.946133	0.363948
42550	0.946133	0.532351
42551	1.04072	0.575346
42552	1.163	0.435125
42553	0.639952	0.629407
42554	0.851543	0.294811
42555	0.946132	0.211339
42556	0.920761	0.583656
42557	0.711038	0.612558
42558	0.711038	0.7
42559	1.89226	0.327616
42560	1.89226	0.232017
42561	1.89226	0.215896
42562	1.89226	0.461287
42563	1.89226	0.178397
42564	1.89226	0.396794
42565	1.89226	0.196652
42566	1.89226	0.260151
42567	2.41075	0.208626
42568	1.89226	0.165477
42569	3.78451	0.233307

42570	0.968476	1.75778
42571	1.20674	1.10053
42572	1.13456	1.08236
42573	1.13456	2.0369
42574	1.02074	1.0347
42575	1.02074	1.68161
42576	1.02074	2.1
42577	1.20674	1.75075
42578	1.07866	1.15487
42579	1.28649	1.15916
42580	2.2854	1.70847
42581	1.94221	0.72838
42582	1.36717	0.727169
42583	1.45535	0.716577
42584	1.93771	0.655498
42585	3.39756	0.729267
42586	1.94222	0.732868
42587	2.25035	0.709658
42588	2.41806	0.726998
42589	2.42256	0.714002
42590	1.93772	0.721844
42591	1.94222	0.713955
42592	1.49906	0.55888
42593	1.93771	0.561326
42594	1.45535	0.681146
42595	1.45535	0.70742
42596	1.91484	0.418982
42597	1.91484	0.447606
42598	1.91484	0.542564
42599	2.25035	0.634034
42600	1.93772	0.663715
42601	1.93772	0.693221
42602	2.11465	0.45689
42603	2.11465	0.496862
42604	2.61735	0.641175
42605	1.5082	0.575036
42606	2.36481	0.722372
42607	1.58597	0.694713
42608	2.36481	0.626722
42609	1.46653	0.581922
42610	2.36482	0.750083
42611	1.08133	0.831486
42612	1.5082	0.546242
42613	2.01868	0.781135
42614	3.52688	0.799931
42615	0.94613	0.675612
42616	0.94613	0.681579
42617	0.946131	0.729045
42618	0.946131	0.773041
42619	0.946129	0.77525
42620	0.946129	0.741034
42621	0.946132	0.692498
42622	0.946132	0.712114
42623	0.94613	0.709758
42624	0.94613	0.725143
42625	0.946131	0.764133
42626	0.946131	0.739954
42627	2.53725	0.418017

42628	1.89227	0.491723
42629	1.89227	0.511036
42630	2.27073	0.513099
42631	1.89227	0.424642
42632	2.27073	0.4234
42633	1.89227	0.704446
42634	1.89227	0.803474
42635	1.89227	0.806149
42636	1.89227	0.773799
42637	1.89227	0.737383
42638	1.89227	0.692165
42639	1.86766	2.1
42640	2.83487	2.1
42641	2.84318	2.1
42642	2.59401	2.1
42643	2.11684	2.1
42644	1.86766	2.1
42645	1.10638	2.1
42646	2.11684	2.1
42647	1.29111	2.1
42648	2.37439	2.1
42649	3.40796	2.1
42650	2.56843	2.1
42651	1.89226	2.1
42652	1.89226	2.1
42653	2.71932	2.1
42654	4.46069	2.1
42655	1.89226	2.1
42656	1.89226	2.1
42657	1.89226	2.1
42658	1.89226	2.1
42659	3.78452	2.1
42660	0.6416	2.1
42661	0.697585	2.1
42662	0.670706	2.1
42663	0.598927	2.1
42664	0.644313	2.1
42665	0.644313	2.1
42666	0.652199	2.1
42667	0.652199	2.1
42668	0.697587	2.1
42669	0.670705	2.1
42670	0.598926	2.1
42671	0.641601	2.1
42672	1.89226	2.1
42673	1.89226	2.1
42674	1.89226	2.1
42675	1.89226	2.1
42676	1.89226	2.1
42677	1.89226	2.1
42678	0.697585	2.1
42679	0.730617	2.1
42680	0.712082	2.1
42681	0.670706	2.1
42682	0.69854	2.1
42683	0.69854	2.1
42684	0.702783	2.1
42685	0.702783	2.1

42686	0.730618	2.1
42687	0.712081	2.1
42688	0.697586	2.1
42689	0.670705	2.1
42690	1.89226	1.43174
42691	1.89226	1.41318
42692	1.89226	1.01198
42693	1.89226	2.1
42694	1.89226	0.979338
42695	1.89226	2.1
42696	2.0815	2.1
42697	1.12076	2.1
42698	1.38662	1.21187
42699	2.01244	2.1
42700	3.41508	2.1
42701	1.89226	2.1
42702	2.54324	2.1
42703	1.91981	2.1
42704	1.89226	2.1
42705	2.57079	2.1
42706	2.54324	2.1
42707	2.54325	2.1
42708	1.94779	2.1
42709	1.76879	2.1
42710	1.73811	2.1
42711	2.5708	2.1
42712	2.54325	2.1
42713	1.89226	2.1
42714	2.57079	2.1
42715	1.91894	2.1
42716	1.89226	2.1
42717	2.59747	2.1
42718	2.57079	2.1
42719	2.5708	2.1
42720	1.82268	2.1
42721	1.79738	2.1
42722	1.77071	2.1
42723	2.59747	2.1
42724	2.5708	2.1
42725	1.89226	0.748316
42726	1.89226	0.827252
42727	1.89226	0.731006
42728	1.89226	0.848614
42729	3.78451	0.777588
42730	2.21582	0.717776
42731	1.94412	0.826214
42732	2.13428	0.628466
42733	1.86292	0.815497
42734	4.07839	0.757536
42735	1.94412	0.824823
42736	1.5854	2.1
42737	1.86293	0.837901
42738	1.50767	2.1
42739	3.45179	1.1301
42740	1.89226	0.826904
42741	1.89226	2.1
42742	1.89226	0.854469
42743	1.89226	2.1

42744	3.78452	1.35994
42745	1.89226	0.731818
42746	1.89226	0.895912
42747	1.89226	0.724054
42748	1.89226	0.837931
42749	3.78452	0.775296
42750	2.13428	0.729081
42751	1.86293	0.800643
42752	2.04737	0.633301
42753	1.77122	0.838323
42754	3.9055	0.766329
42755	1.86293	0.679601
42756	1.50767	2.1
42757	1.41077	0.847914
42758	1.41077	1.86207
42759	1.77123	0.853631
42760	1.86813	2.05944
42761	0.810943	0.861466
42762	0.810943	2.1
42763	0.810945	0.850563
42764	0.810945	0.836044
42765	0.810958	1.01618
42766	0.810958	0.966525
42767	1.89226	2.1
42768	1.35168	1.09407
42769	1.08132	0.859888
42770	1.08132	2.1
42771	1.0813	0.974383
42772	0.745979	2.1
42773	0.745979	2.1
42774	0.778781	2.1
42775	0.778781	2.1
42776	0.59549	2.1
42777	0.649658	2.1
42778	0.994784	2.1
42779	0.875102	2.1
42780	0.875102	2.1
42781	0.59549	2.1
42782	0.649658	2.1
42783	0.994784	2.1
42784	1.57887	2.1
42785	1.75021	2.1
42786	1.13341	2.1
42787	1.13341	2.1
42788	1.19098	2.1
42789	1.63644	2.1
42790	2.5761	0.672776
42791	2.43404	0.79659
42792	2.44813	0.596449
42793	2.31751	0.815795
42794	4.88218	0.696552
42795	2.43405	0.796547
42796	2.22575	2.00298
42797	2.31751	0.824692
42798	2.10893	1.44204
42799	4.54298	0.897174
42800	2.44813	0.684872
42801	2.31751	0.807065

42802	2.30908	0.41277
42803	2.19815	0.7968
42804	4.64628	0.735101
42805	2.31751	0.808563
42806	2.10893	1.53645
42807	2.19815	0.778064
42808	1.98061	2.1
42809	4.30708	0.874032
42810	0.94613	2.1
42811	0.94613	2.08255
42812	1.40372	2.1
42813	1.40372	2.1
42814	1.40372	2.1
42815	1.37167	2.1
42816	0.946132	1.45401
42817	0.946132	2.1
42818	0.946131	2.1
42819	1.40372	2.1
42820	0.94613	2.1
42821	1.37167	2.1
42822	2.80744	2.1
42823	2.06984	2.1
42824	2.83418	2.1
42825	2.74335	2.1
42826	2.16067	2.1
42827	2.06984	2.1
42828	1.077	0.648992
42829	0.809116	0.581909
42830	0.600419	0.766948
42831	0.790277	0.591199
42832	0.927647	0.509527
42833	0.758598	0.516276
42834	0.85749	0.676557
42835	0.85749	0.709327
42836	1.02417	0.582733
42837	0.600411	0.629929
42838	0.751701	0.625057
42839	1.07702	0.557655
42840	1.89226	2.1
42841	1.89226	2.1
42842	1.89226	2.1
42843	1.89226	2.1
42844	1.89226	2.1
42845	1.89226	2.1
42846	1.89226	2.1
42847	1.89226	2.1
42848	1.89226	2.1
42849	1.89226	2.1
42850	3.78453	2.1
42851	1.89226	2.1
42852	1.89226	2.1
42853	1.89226	2.1
42854	1.89226	2.1
42855	1.89226	2.1
42856	1.89226	2.1
42857	0.946131	2.1
42858	0.946131	2.1
42859	0.946131	2.1

42860	0.946131	2.1
42861	0.946133	2.1
42862	0.946133	2.1
42863	0.946128	2.1
42864	0.946128	2.1
42865	0.946131	2.1
42866	0.946131	2.1
42867	0.94613	2.1
42868	0.94613	2.1
42869	2.17159	2.1
42870	2.00083	1.76453
42871	2.21305	2.1
42872	2.00551	1.38451
42873	2.20837	1.96151
42874	2.00083	1.34258
42875	2.20837	1.38978
42876	1.80328	1.11354
42877	2.22962	1.97322
42878	2.00083	1.31459
42879	2.03207	1.01804
42880	1.80328	0.989008
42881	2.28564	2.1
42882	2.20837	1.34656
42883	2.30406	2.1
42884	2.17159	2.00904
42885	2.34084	1.68493
42886	2.20837	1.89197
42887	1.09523	0.974242
42888	1.09523	1.67712
42889	1.09934	1.82148
42890	1.09934	2.1
42891	1.01603	0.9453
42892	1.09038	1.61371
42893	1.10419	1.08185
42894	1.17042	2.1
42895	1.17042	1.88552
42896	1.09038	1.4186
42897	1.10419	1.16371
42898	1.01603	1.8003
42899	2.60152	0.793151
42900	0.976334	0.894999
42901	2.22344	0.919889
42902	2.22344	0.802138
42903	1.36405	0.982165
42904	1.36405	0.751401
42905	1.89226	2.03875
42906	1.70302	2.1
42907	1.69019	2.1
42908	1.70302	2.04214
42909	1.73933	2.1
42910	1.70303	1.12457
42911	1.89226	2.1
42912	1.89226	0.998906
42913	1.89226	1.24821
42914	1.89226	2.01612
42915	3.78452	1.57119
42916	1.89226	1.28999
42917	1.59036	2.00637

42918	1.65683	2.1
42919	1.59036	1.39455
42920	1.95873	2.1
42921	1.89226	1.0164
42922	0.946131	0.959254
42923	0.946131	1.03565
42924	0.946127	1.9996
42925	0.946127	1.42621
42926	0.946127	1.40543
42927	0.946127	0.907495
42928	0.946131	1.88831
42929	0.946131	1.04682
42930	0.946129	1.13277
42931	0.946129	1.14285
42932	0.94613	1.15284
42933	0.94613	1.13656
42934	0.851541	2.1
42935	0.851541	2.1
42936	0.851483	2.1
42937	1.15713	2.1
42938	0.851539	2.1
42939	0.851539	1.4272
42940	0.942127	2.1
42941	1.14455	2.1
42942	0.838683	2.1
42943	0.946131	2.1
42944	0.957503	2.1
42945	0.957504	2.1
42946	1.89226	0.98678
42947	1.89226	0.731133
42948	1.89226	0.854114
42949	1.89226	1.64599
42950	3.78452	0.850984
42951	1.89226	0.39758
42952	1.89226	0.816904
42953	1.89226	0.856637
42954	1.89226	0.808778
42955	1.89226	0.469167
42956	1.89226	0.400576
42957	1.89226	0.764755
42958	1.89226	0.631375
42959	1.89226	0.676349
42960	1.89226	0.748422
42961	3.78453	0.702744
42962	1.89226	0.650021
42963	1.89226	2.1
42964	1.89226	1.8031
42965	1.89226	1.31422
42966	3.78453	1.67784
42967	1.22575	0.896443
42968	1.05438	0.827806
42969	0.946131	0.903592
42970	0.946131	0.905056
42971	0.946129	1.30106
42972	1.22575	0.603249
42973	1.05438	0.841535
42974	0.946132	0.834042
42975	1.05438	0.911019

42976	1.05438	1.07409
42977	0.946131	0.843402
42978	0.946131	0.941338
42979	2.4515	0.407921
42980	1.13726	0.167412
42981	2.10875	0.368957
42982	1.49515	0.392395
42983	2.10875	0.292315
42984	1.49515	0.227646
42985	0.831477	0.259095
42986	0.747575	0.258858
42987	1.22575	0.707595
42988	1.28174	0.395527
42989	1.28174	0.445064
42990	0.831476	0.406189
42991	1.22575	0.295395
42992	0.747575	0.250574
42993	1.03561	0.315395
42994	1.03561	0.670982
42995	1.02162	0.284918
42996	1.02162	0.282299
42997	1.89227	1.53535
42998	2.56348	1.02917
42999	1.89227	2.1
43000	2.45151	0.859183
43001	1.89227	2.1
43002	2.45151	0.976214
43003	1.40013	1.00698
43004	1.89226	1.70199
43005	1.89226	1.21622
43006	0.946643	1.01563
43007	1.89226	1.45817
43008	0.946643	1.19357
43009	1.89226	0.299975
43010	1.89226	0.466882
43011	1.89226	0.403851
43012	1.89226	0.493523
43013	3.78453	0.49333
43014	1.89226	0.643746
43015	1.89226	0.681428
43016	1.89226	0.643746
43017	1.89226	0.716757
43018	3.78453	0.638113
43019	0.946131	0.710856
43020	0.946131	0.70626
43021	0.946128	0.594245
43022	0.946128	0.61628
43023	0.94613	0.707371
43024	0.94613	0.711191
43025	0.946129	0.655991
43026	0.946129	0.604702
43027	0.94613	0.703316
43028	0.94613	0.55879
43029	0.94613	0.635223
43030	0.94613	0.708051
43031	1.89226	0.283437
43032	1.89226	0.343764
43033	1.17244	0.315154

43034	1.89226	0.338973
43035	0.975291	0.322886
43036	1.57408	0.35646
43037	0.946132	0.675629
43038	0.946132	0.521821
43039	0.946131	0.695906
43040	0.946131	0.57441
43041	0.946132	0.659275
43042	0.946132	0.677852
43043	0.946131	0.565831
43044	0.946131	0.585364
43045	0.946131	0.652807
43046	0.946131	0.651135
43047	0.946132	0.578782
43048	0.946132	0.648444
43049	2.74744	0.679425
43050	1.89226	0.72806
43051	2.70805	0.696988
43052	1.89226	0.709076
43053	2.70805	0.705436
43054	1.89226	0.721752
43055	2.74744	0.621046
43056	1.89226	0.647034
43057	2.57465	0.638522
43058	1.89226	0.727732
43059	4.6397	0.681437
43060	1.89226	0.74034
43061	1.89226	0.815379
43062	1.89226	0.82013
43063	1.89226	0.975292
43064	3.78452	0.796819
43065	1.89226	0.774951
43066	1.89226	0.875456
43067	1.89226	0.762377
43068	1.89226	0.736249
43069	1.89226	1.00163
43070	1.89226	0.93192
43071	2.16206	0.462373
43072	2.67861	0.470574
43073	3.11996	0.557173
43074	1.70303	0.535731
43075	2.89015	0.520011
43076	1.486	0.370306
43077	2.12839	0.60739
43078	2.83631	0.745234
43079	3.08096	0.662142
43080	1.84157	0.609244
43081	3.08096	0.725719
43082	1.84157	0.597477
43083	1.89225	1.12795
43084	1.89225	0.862655
43085	1.89225	0.970336
43086	1.89225	1.13091
43087	1.89225	0.942328
43088	1.89225	1.09969
43089	1.89226	0.835051
43090	1.89226	0.864125
43091	1.89226	1.17278

43092	1.89226	0.819368
43093	3.78452	0.825776
43094	1.89226	0.766101
43095	2.49041	0.770855
43096	1.89226	0.768671
43097	1.89226	0.722435
43098	2.27073	0.70982
43099	2.27073	0.76219
43100	1.09904	0.601658
43101	0.677147	0.543659
43102	1.3365	0.53627
43103	0.95759	0.813901
43104	0.957591	0.621666
43105	0.677148	0.685109
43106	1.09904	0.681901
43107	1.3365	0.591247
43108	0.996015	0.531716
43109	0.996015	0.590087
43110	1.01764	0.817144
43111	1.01764	0.617235
43112	2.77349	0.721541
43113	2.72723	0.461099
43114	2.43156	0.750245
43115	2.4564	0.491903
43116	5.20505	0.712666
43117	2.77349	0.666999
43118	2.68508	0.451519
43119	2.33761	0.643237
43120	2.4564	0.470444
43121	5.1111	0.500168
43122	2.43156	0.579759
43123	2.70208	0.435822
43124	1.85779	0.686673
43125	2.10695	0.419393
43126	4.77572	0.434926
43127	2.33761	0.516853
43128	2.70208	0.416492
43129	1.85779	0.596228
43130	1.61597	0.37948
43131	4.19542	0.517554
43132	0.946132	1.43389
43133	0.946132	1.77617
43134	0.746729	1.54959
43135	0.746729	1.89298
43136	0.946132	1.93778
43137	0.915958	1.71536
43138	0.946133	1.41359
43139	0.74673	2.01019
43140	0.946133	1.65005
43141	0.946133	1.6518
43142	0.915958	1.70046
43143	0.746729	1.75827
43144	1.50743	2.1
43145	2.45501	2.1
43146	2.29118	2.1
43147	2.29118	2.1
43148	1.75482	2.1
43149	1.75482	2.1

43150	2.45501	2.1
43151	2.76927	0.893747
43152	2.29118	2.1
43153	2.64523	0.90813
43154	5.10024	1.08382
43155	1.25559	2.1
43156	2.29118	2.1
43157	2.0815	2.1
43158	2.26387	2.1
43159	1.35668	2.1
43160	1.47667	2.1
43161	2.28481	1.98863
43162	2.64523	0.920936
43163	2.50057	2.07329
43164	2.50057	0.925434
43165	2.29118	2.08849
43166	2.29118	0.93084
43167	0.946129	0.706997
43168	0.946129	0.703264
43169	1.23929	0.7
43170	1.15246	0.701003
43171	0.946131	0.702113
43172	0.946131	0.706165
43173	1.15246	0.701869
43174	1.15246	0.702463
43175	0.946131	0.701784
43176	1.15246	0.702815
43177	0.946131	0.700137
43178	1.23928	0.701778
43179	0.946128	0.709535
43180	0.946128	0.706829
43181	1.15246	0.702797
43182	0.942814	0.713365
43183	0.946131	0.702863
43184	0.946131	0.70866
43185	0.961347	0.703601
43186	0.942811	0.714272
43187	0.946131	0.704353
43188	0.961347	0.703746
43189	0.946131	0.704271
43190	1.15246	0.70599
43191	2.32334	0.715739
43192	1.57621	0.707036
43193	1.90335	0.710774
43194	1.24538	0.703987
43195	3.47956	0.705003
43196	0.965762	0.443193
43197	0.965762	0.678347
43198	1.00245	0.4519
43199	1.00245	0.509053
43200	0.92786	0.498831
43201	0.705297	0.36273
43202	0.856414	0.626823
43203	1.28968	0.460901
43204	1.00306	0.453434
43205	0.705303	0.393826
43206	1.28967	0.523293
43207	0.975478	0.526006

43208	3.05308	2.1
43209	1.76816	2.1
43210	1.76816	2.1
43211	1.69129	2.1
43212	2.58731	2.1
43213	2.51043	2.1
43214	1.89226	0.626024
43215	2.71464	0.673003
43216	2.34627	0.684379
43217	1.89226	0.693442
43218	2.34627	0.596444
43219	1.89226	0.616039
43220	2.34627	0.544372
43221	2.71464	0.554484
43222	1.98252	0.496656
43223	1.98252	0.665209
43224	1.51026	0.498919
43225	2.34627	0.551597
43226	2.38698	1.10282
43227	2.35142	2.1
43228	2.60343	1.097
43229	2.6636	1.13523
43230	4.99041	1.20351
43231	1.07811	1.98162
43232	1.1952	2.1
43233	1.17571	1.47944
43234	1.30171	1.03872
43235	1.18322	1.00496
43236	1.18322	2.1
43237	1.18769	1.85132
43238	1.18769	1.08197
43239	1.1952	2.01493
43240	1.30171	1.75907
43241	1.17571	1.69411
43242	1.07811	1.52672
43243	1.89226	1.18432
43244	2.6636	1.67511
43245	1.89226	0.851758
43246	2.60343	1.1969
43247	1.89226	1.22232
43248	2.60343	2.1
43249	1.5344	0.108926
43250	1.4516	0.12884
43251	1.17816	0.109996
43252	1.21056	0.106839
43253	2.71257	0.126388
43254	1.89226	1.59454
43255	1.89226	0.903171
43256	1.89226	1.03254
43257	1.89226	1.09418
43258	3.78452	1.00641
43259	1.89226	1.0796
43260	1.89226	0.800194
43261	1.89226	0.913307
43262	1.89226	0.858709
43263	1.65573	0.989171
43264	1.65573	0.895364
43265	1.89226	1.02576

43266	1.89226	1.14126
43267	1.89226	1.83261
43268	2.64212	0.943754
43269	3.78452	1.17426
43270	1.89226	1.02223
43271	1.89226	2.1
43272	1.89226	1.09606
43273	1.89226	2.1
43274	3.78452	2.01872
43275	2.7367	0.915367
43276	2.07947	2.1
43277	2.47319	1.09273
43278	1.85057	2.1
43279	4.55267	2.1
43280	2.07948	2.1
43281	1.3294	2.1
43282	1.85057	2.1
43283	1.09962	2.1
43284	3.1791	2.1
43285	1.89226	2.1
43286	1.89226	2.1
43287	1.89226	2.1
43288	1.89226	2.1
43289	3.78453	2.1
43290	0.94613	2.1
43291	0.94613	0.938844
43292	0.94613	1.02553
43293	0.94613	2.1
43294	0.946128	2.1
43295	0.946128	2.1
43296	0.946132	0.933914
43297	0.946132	0.944639
43298	0.94613	2.1
43299	0.94613	2.1
43300	0.946131	2.1
43301	0.946131	2.1
43302	1.00875	2.1
43303	1.00875	0.898471
43304	1.01676	2.1
43305	1.01676	0.911168
43306	0.925282	2.1
43307	0.804877	2.1
43308	1.2366	0.969947
43309	1.10023	0.957114
43310	0.804878	1.89371
43311	1.10023	2.03481
43312	1.2366	1.93506
43313	0.925284	1.63614
43314	1.89226	2.1
43315	1.89226	2.1
43316	1.89226	2.1
43317	1.89226	2.1
43318	3.78453	2.1
43319	1.89226	2.1
43320	1.89226	2.1
43321	1.89226	2.1
43322	1.89226	2.1
43323	1.89226	2.1

43324	1.89226	2.1
43325	1.09766	2.1
43326	1.14842	2.1
43327	1.02909	2.1
43328	1.07659	2.1
43329	1.16718	2.1
43330	0.954189	2.1
43331	1.06653	2.1
43332	1.06653	2.1
43333	1.20738	2.1
43334	1.02909	2.1
43335	1.09767	2.1
43336	0.815951	2.1
43337	2.00793	2.1
43338	1.14048	2.1
43339	1.19086	2.1
43340	1.13723	2.1
43341	2.00793	2.1
43342	2.11772	2.1
43343	1.89226	2.1
43344	1.89226	2.1
43345	1.89226	2.1
43346	1.89226	2.1
43347	1.89226	2.1
43348	1.89226	2.1
43349	1.89226	2.1
43350	1.89226	2.1
43351	1.89226	2.1
43352	1.89226	2.1
43353	1.89226	2.1
43354	1.89226	2.1
43355	1.1779	2.1
43356	1.26166	2.1
43357	1.0875	2.1
43358	1.00397	2.1
43359	1.13286	2.1
43360	1.13286	2.1
43361	1.13277	2.1
43362	1.13277	2.1
43363	1.26166	2.1
43364	1.08749	2.1
43365	1.1779	2.1
43366	1.00396	2.1
43367	1.0875	2.1
43368	1.00397	2.1
43369	0.685749	2.1
43370	0.595432	2.1
43371	0.83976	2.1
43372	0.83976	2.1
43373	0.843169	2.1
43374	0.843169	2.1
43375	1.0875	2.1
43376	0.685749	2.1
43377	1.00397	2.1
43378	0.595432	2.1
43379	1.89226	2.1
43380	1.86443	2.1
43381	2.4988	2.1

43382	2.0815	2.1
43383	2.28173	2.1
43384	1.86443	2.1
43385	1.89226	2.1
43386	1.89226	2.1
43387	1.89226	2.1
43388	1.89226	2.1
43389	1.89226	2.1
43390	1.89226	2.1
43391	0.946131	2.1
43392	0.946131	2.1
43393	0.969491	2.1
43394	0.949105	2.1
43395	0.949103	2.1
43396	0.949103	2.1
43397	0.946133	2.1
43398	0.946133	2.1
43399	0.946131	2.1
43400	0.969492	2.1
43401	0.946131	2.1
43402	0.949105	2.1
43403	1.89226	2.1
43404	2.07428	2.1
43405	1.93464	2.1
43406	1.89226	2.1
43407	2.11667	2.1
43408	2.07428	2.1
43409	0.946132	2.1
43410	0.946132	2.1
43411	0.946131	2.1
43412	0.946131	2.1
43413	0.946132	2.1
43414	0.946132	2.1
43415	0.946132	2.1
43416	0.946132	2.1
43417	0.946132	2.1
43418	0.946132	2.1
43419	0.946132	2.1
43420	0.946132	2.1
43421	1.97992	2.1
43422	1.89226	2.1
43423	1.89226	2.1
43424	1.93898	2.1
43425	1.93898	2.1
43426	1.89226	2.1
43427	2.12475	0.781068
43428	2.79029	0.825462
43429	2.7803	0.941131
43430	2.13052	0.777517
43431	4.92081	0.805016
43432	1.06238	0.848411
43433	1.06526	0.784831
43434	0.642901	0.73933
43435	0.655055	0.769661
43436	0.856391	0.755175
43437	0.856391	0.751588
43438	0.86104	0.901058
43439	0.86104	0.734966

43440	1.06526	0.791825
43441	0.655055	0.784194
43442	0.642901	0.760696
43443	1.06238	0.75276
43444	2.7803	0.886219
43445	2.13053	0.747312
43446	2.14346	0.808636
43447	2.77937	0.835695
43448	4.9099	0.814613
43449	2.14346	0.834077
43450	1.31011	0.717227
43451	1.34124	0.785311
43452	1.31011	0.825583
43453	2.16166	0.741441
43454	2.13053	0.761462
43455	1.89226	2.1
43456	2.58176	2.1
43457	2.01422	2.1
43458	1.89226	2.1
43459	2.70373	2.1
43460	2.58176	2.1
43461	1.89226	2.1
43462	1.89226	2.1
43463	1.89226	2.1
43464	1.89226	2.1
43465	1.89226	2.1
43466	1.89226	2.1
43467	0.946128	2.1
43468	0.946128	2.1
43469	1.38521	2.1
43470	1.38521	2.1
43471	1.38521	2.1
43472	1.35186	2.1
43473	0.946131	2.1
43474	0.946131	2.1
43475	0.94613	2.1
43476	1.38521	2.1
43477	0.946131	2.1
43478	1.35186	2.1
43479	0.946133	2.1
43480	0.946133	2.1
43481	0.946132	2.1
43482	0.946132	2.1
43483	0.946132	2.1
43484	0.946132	2.1
43485	0.946132	2.1
43486	0.946132	2.1
43487	0.946132	2.1
43488	0.946132	2.1
43489	0.946133	2.1
43490	0.946133	2.1
43491	2.33374	2.1
43492	1.89226	2.1
43493	1.89226	2.1
43494	1.89226	2.1
43495	2.60377	2.1
43496	2.60377	2.1
43497	2.6771	0.156476

43498	1.36416	0.237589
43499	1.64983	0.178881
43500	1.64983	0.171153
43501	2.44131	0.207003
43502	2.44131	0.208092
43503	2.58321	0.730255
43504	1.89226	0.724775
43505	2.44283	0.703846
43506	2.44283	0.709263
43507	1.89226	0.776807
43508	1.89226	0.818591
43509	2.58321	0.783175
43510	2.44283	0.712275
43511	1.49358	0.701615
43512	1.57702	0.715426
43513	3.93641	0.706502
43514	0.951913	0.151788
43515	0.81556	0.134741
43516	0.946132	0.203129
43517	0.946132	0.192563
43518	1.04072	0.191666
43519	1.12182	0.194862
43520	0.856745	0.139051
43521	0.851542	0.140381
43522	0.923163	0.145411
43523	0.923163	0.217867
43524	1.04304	0.190644
43525	0.85098	0.119684
43526	2.70125	1.21936
43527	2.37734	1.21369
43528	2.63495	0.99755
43529	2.30539	2.1
43530	5.01229	1.75191
43531	1.89226	1.23666
43532	1.89226	1.51817
43533	1.89226	1.13965
43534	1.89226	1.62643
43535	3.78452	1.01487
43536	1.89226	1.84959
43537	1.89226	2.1
43538	1.89226	1.49857
43539	1.89226	2.1
43540	3.78452	2.1
43541	2.37734	1.25038
43542	1.79346	2.1
43543	2.53594	1.74933
43544	1.70302	2.04265
43545	4.27682	1.84072
43546	2.63495	0.938947
43547	2.30539	1.52689
43548	2.49445	1.05463
43549	2.12089	1.01602
43550	4.75584	0.985812
43551	1.89226	1.04812
43552	1.89226	1.29023
43553	1.89226	0.939005
43554	1.89226	1.55405
43555	3.78452	1.91364

43556	1.89226	1.26447
43557	1.89226	1.86921
43558	1.89226	1.68451
43559	1.89226	1.64678
43560	1.89226	1.16698
43561	1.89226	1.18691
43562	2.53594	1.22823
43563	1.60017	1.24131
43564	1.57079	1.37848
43565	1.70302	1.06598
43566	2.15772	1.29937
43567	2.33299	1.02033
43568	2.09114	2.1
43569	2.34898	2.1
43570	2.30729	2.1
43571	2.06556	2.1
43572	4.41454	2.1
43573	2.09114	2.1
43574	1.59654	2.1
43575	1.57823	2.1
43576	2.06556	2.1
43577	3.6621	2.1
43578	2.30729	2.1
43579	2.06556	2.1
43580	2.03917	2.1
43581	2.26617	2.1
43582	4.33173	2.1
43583	2.03917	2.1
43584	2.06556	2.1
43585	2.045 2.1	
43586	1.55767	2.1
43587	1.57823	2.1
43588	1.57823	2.1
43589	1.89225	1.90152
43590	1.09726	2.1
43591	1.61196	2.1
43592	1.28597	2.1
43593	1.28597	0.05
43594	1.45633	2.1
43595	1.45633	2.1
43596	1.9544	0.05
43597	1.61196	2.1
43598	1.45633	2.1
43599	1.53735	2.1
43600	3.14931	2.1
43601	1.89226	2.1
43602	1.89226	2.1
43603	1.89226	2.1
43604	1.89226	2.1
43605	3.78453	2.1
43606	0.810946	2.1
43607	0.810946	2.1
43608	0.810947	2.1
43609	0.810947	2.1
43610	0.810933	2.1
43611	0.810933	2.1
43612	1.89226	2.1
43613	1.3517	2.1

43614	1.08132	2.1
43615	1.08132	2.1
43616	1.08133	2.1
43617	0.851539	2.1
43618	0.851539	2.1
43619	0.972053	2.1
43620	1.05632	2.1
43621	0.94613	2.1
43622	0.94613	1.89266
43623	1.0279	2.1
43624	0.94991	2.1
43625	1.0279	2.1
43626	0.946131	2.1
43627	0.865643	2.1
43628	0.94613	2.1
43629	1.89226	2.1
43630	1.89226	2.1
43631	1.89226	2.1
43632	1.89226	2.09549
43633	3.78452	2.1
43634	1.03882	2.1
43635	1.91121	2.1
43636	1.41833	2.1
43637	2.34547	2.1
43638	3.32954	2.1
43639	2.3979	2.1
43640	1.89226	2.1
43641	1.89226	2.1
43642	1.89226	2.1
43643	2.0558	2.1
43644	2.0558	1.578
43645	1.89226	2.1
43646	1.89226	1.22421
43647	1.89226	2.06651
43648	1.89226	2.1
43649	3.78452	2.1
43650	1.05816	2.1
43651	1.05816	2.1
43652	0.842045	2.1
43653	1.05252	2.1
43654	1.36057	2.1
43655	1.14339	2.1
43656	0.873397	2.1
43657	0.585073	2.1
43658	0.873388	2.1
43659	1.15129	2.1
43660	0.69143	2.1
43661	1.1434	2.1
43662	1.93338	2.1
43663	1.85883	2.1
43664	1.51714	2.1
43665	1.85883	2.1
43666	1.54795	2.1
43667	1.89226	2.1
43668	1.89226	2.1
43669	1.89226	2.1
43670	1.89226	2.1
43671	1.89226	2.1

43672	3.78452	2.1
43673	0.844726	2.1
43674	0.844726	2.1
43675	0.960524	2.1
43676	1.16288	2.1
43677	0.851541	2.1
43678	0.851541	2.1
43679	0.929147	2.1
43680	1.15533	2.1
43681	0.844697	2.1
43682	0.946131	2.1
43683	0.851632	2.1
43684	0.946131	2.1
43685	1.89226	2.1
43686	1.70303	2.1
43687	1.74006	2.1
43688	1.70303	2.1
43689	1.99471	2.1
43690	1.93338	2.1
43691	0.946131	2.1
43692	0.946131	2.1
43693	0.946131	2.1
43694	0.946131	2.1
43695	0.94613	2.1
43696	0.94613	2.1
43697	0.946132	2.1
43698	0.946132	2.1
43699	0.946131	2.1
43700	0.946131	2.1
43701	0.946131	2.1
43702	0.946131	2.1
43703	1.58158	2.1
43704	1.89226	2.1
43705	1.89226	2.1
43706	1.89226	2.1
43707	1.68939	2.1
43708	1.68939	2.1
43709	0.946132	0.0860811
43710	0.946132	0.115819
43711	1.12988	0.0945415
43712	1.30783	0.0753951
43713	1.30783	0.0915495
43714	0.946133	0.0903801
43715	0.946132	0.0926262
43716	1.12988	0.0909134
43717	1.30783	0.0854981
43718	1.30783	0.0767648
43719	0.946133	0.0957053
43720	0.946133	0.10934
43721	1.89227	0.111172
43722	1.89227	0.098863
43723	1.89227	0.0901004
43724	1.89227	0.138151
43725	1.89227	0.0882495
43726	1.89227	0.136435
43727	1.89226	0.0945759
43728	1.89226	0.0901134
43729	1.89226	0.0847495

43730	1.89226	0.109446
43731	1.89226	0.0813969
43732	1.89226	0.10509
43733	0.946131	0.0968552
43734	0.946131	0.0766545
43735	1.30783	0.0766558
43736	1.37715	0.0660709
43737	1.37715	0.0770352
43738	0.946133	0.0777571
43739	1.30783	0.0763829
43740	0.946129	0.0763627
43741	1.37715	0.0757275
43742	1.37715	0.0660619
43743	0.946131	0.0758867
43744	0.946131	0.096541
43745	1.17056	0.0995259
43746	0.591757	0.148941
43747	0.522025	0.107439
43748	0.929727	0.0821782
43749	0.929728	0.0806758
43750	1.17056	0.0977914
43751	0.591756	0.106791
43752	0.522025	0.101063
43753	0.889054	0.0968742
43754	0.889054	0.100114
43755	0.803535	0.113706
43756	0.803535	0.109253
43757	1.49814	2.1
43758	2.37439	2.1
43759	2.63923	2.1
43760	2.63923	2.1
43761	1.29111	2.1
43762	1.29111	2.1
43763	0.946132	2.1
43764	0.946132	2.1
43765	1.18719	2.1
43766	1.18719	2.1
43767	0.94613	2.1
43768	0.94613	2.1
43769	1.1872	2.1
43770	1.31961	2.1
43771	0.946131	2.1
43772	1.31961	2.1
43773	1.1872	2.1
43774	0.94613	2.1
43775	2.49445	0.781727
43776	2.12089	0.893673
43777	2.20843	0.759587
43778	1.82805	0.997985
43779	4.32932	0.83778
43780	1.89226	0.880201
43781	1.89226	1.32646
43782	1.89226	0.82714
43783	1.89226	0.870537
43784	3.78452	0.816322
43785	0.94613	0.926553
43786	0.94613	0.886467
43787	0.946128	0.885816

43788	0.946128	1.07571
43789	0.946131	1.02125
43790	0.946131	1.0693
43791	0.946127	1.02774
43792	0.946127	0.861966
43793	0.946129	0.927908
43794	0.946129	0.903411
43795	0.946129	0.979449
43796	0.946129	0.976538
43797	1.82804	0.892586
43798	1.60017	0.985474
43799	1.87679	1.04603
43800	2.12088	0.840949
43801	1.35608	1.08069
43802	1.60017	0.893963
43803	2.20843	0.719184
43804	1.3216	0.721275
43805	1.44426	0.716672
43806	1.82805	0.746105
43807	1.80399	0.703504
43808	2.20843	0.733009
43809	1.89226	0.751066
43810	1.89226	0.748242
43811	1.89226	0.831124
43812	1.89226	0.754603
43813	1.89226	0.792481
43814	1.89226	0.7479
43815	1.89226	0.86943
43816	1.89226	0.775055
43817	1.89226	0.76348
43818	1.89226	0.780114
43819	1.89226	0.825595
43820	1.89226	0.746303
43821	0.627705	0.711527
43822	0.634941	0.792016
43823	0.629317	0.714789
43824	0.783422	0.729058
43825	0.629328	0.728933
43826	0.448764	0.730638
43827	1.35608	0.777639
43828	1.00805	0.776777
43829	0.814295	0.729071
43830	0.598375	0.792804
43831	1.04461	0.761209
43832	1.09795	1.78032
43833	1.00775	1.60525
43834	1.61202	1.97366
43835	1.63419	1.4291
43836	2.64194	1.39099
43837	1.44801	1.68067
43838	1.61201	1.69063
43839	2.35614	1.8438
43840	2.27703	1.66853
43841	3.88904	1.80683
43842	2.35615	1.62858
43843	1.63419	1.21951
43844	2.29852	1.40009
43845	1.63419	1.29396

43846	2.27634	1.45565
43847	1.61202	1.48074
43848	0.973793	2.1
43849	2.2671	2.1
43850	2.32981	2.1
43851	2.32981	2.1
43852	0.990481	2.1
43853	0.990481	2.1
43854	1.50877	2.1
43855	1.11193	2.1
43856	2.29667	2.1
43857	1.94158	2.1
43858	3.45035	2.1
43859	2.29667	2.1
43860	1.89227	2.1
43861	1.94158	2.1
43862	1.94158	2.1
43863	1.89227	2.1
43864	1.89227	2.1
43865	2.51379	2.1
43866	1.30026	2.1
43867	1.84502	2.1
43868	2.72099	2.1
43869	4.35882	2.1
43870	1.89226	2.1
43871	2.721	2.1
43872	2.5138	2.1
43873	2.5138	2.1
43874	1.89226	2.1
43875	1.89226	2.1
43876	1.89226	0.372554
43877	1.89226	0.487645
43878	2.59101	0.361004
43879	1.89226	0.602226
43880	3.78453	0.376934
43881	0.851541	0.403793
43882	0.851541	0.341102
43883	1.42502	0.377437
43884	0.948679	0.300713
43885	0.946132	0.464998
43886	0.946132	0.476898
43887	0.853617	0.38609
43888	0.853618	0.267462
43889	0.851475	0.405326
43890	0.948666	0.484511
43891	1.166	0.384257
43892	0.851554	0.437407
43893	2.51818	0.949611
43894	2.60723	2.1
43895	2.66532	0.891695
43896	2.76315	2.1
43897	5.27255	1.04815
43898	2.60723	2.06684
43899	2.45247	2.1
43900	2.76315	2.1
43901	2.55197	2.1
43902	5.1592	2.1
43903	1.23768	0.741713

43904	1.11433	0.811284
43905	1.32037	1.00576
43906	1.32037	0.801344
43907	1.0142	0.868437
43908	1.0142	0.756652
43909	0.886711	0.854454
43910	1.16806	0.824443
43911	0.999083	1.76314
43912	0.710926	1.08481
43913	0.937757	1.27839
43914	0.937757	0.933749
43915	0.941224	0.78413
43916	0.941224	1.64232
43917	1.16805	0.997884
43918	0.999084	1.7555
43919	0.886709	1.33161
43920	0.710928	1.12424
43921	2.29332	1.54066
43922	1.54904	2.1
43923	2.0815	2.1
43924	2.44199	2.03235
43925	4.00418	2.1
43926	1.53873	0.622101
43927	1.53422	0.847698
43928	1.39661	0.339083
43929	1.42387	0.821659
43930	2.9626	0.775684
43931	1.53423	0.847704
43932	1.44091	1.28933
43933	1.42387	0.828616
43934	1.35215	2.1
43935	2.86479	0.913487
43936	1.39661	0.505207
43937	1.42387	0.818564
43938	1.28325	0.473117
43939	1.33326	0.863835
43940	2.70713	0.729918
43941	1.42387	0.820571
43942	1.35215	2.1
43943	1.33327	0.873843
43944	1.27339	1.49811
43945	2.69726	0.998593
43946	2.58442	2.1
43947	1.1468	2.1
43948	1.18775	2.1
43949	2.60047	2.1
43950	3.74728	2.1
43951	2.58442	2.1
43952	1.89226	2.1
43953	1.89226	2.1
43954	2.60048	2.1
43955	4.49274	2.1
43956	1.89226	2.1
43957	1.89226	2.1
43958	1.89226	2.1
43959	1.89226	2.1
43960	3.78453	2.1
43961	1.89226	2.1

43962	1.89226	2.1
43963	1.89226	2.1
43964	1.89226	0.997787
43965	3.78452	2.1
43966	1.22262	2.1
43967	2.60047	2.1
43968	1.18775	2.1
43969	1.18775	2.1
43970	2.61433	2.1
43971	2.61433	2.1
43972	1.89226	2.1
43973	2.60048	2.1
43974	2.61434	2.1
43975	2.60048	2.1
43976	1.90612	2.1
43977	1.89226	2.1
43978	1.89226	2.1
43979	1.89226	2.1
43980	1.89226	2.1
43981	1.89226	2.1
43982	3.78453	2.1
43983	1.89226	2.1
43984	1.89226	2.1
43985	1.89226	2.1
43986	1.89226	2.09692
43987	3.78452	2.1
43988	2.12556	2.1
43989	0.991113	2.1
43990	1.27725	2.1
43991	2.31081	2.1
43992	3.40281	2.1
43993	1.89226	2.1
43994	2.31082	2.1
43995	2.12556	2.1
43996	2.12556	2.1
43997	1.89226	2.1
43998	1.89226	2.1
43999	1.22544	2.1
44000	1.12649	2.1
44001	0.775254	2.1
44002	0.643133	2.1
44003	0.917924	2.1
44004	0.934955	2.1
44005	0.975579	2.1
44006	0.975579	2.1
44007	1.23113	2.1
44008	0.685272	2.1
44009	0.651719	2.1
44010	1.1791	2.1
44011	1.89226	2.1
44012	2.5138	2.1
44013	2.31082	2.1
44014	2.31082	2.1
44015	1.89226	2.1
44016	1.89226	2.1
44017	1.89226	2.1
44018	1.89226	2.1
44019	1.89226	2.1

44020	1.89226	2.1
44021	3.78453	2.1
44022	0.922224	2.1
44023	0.922224	2.1
44024	1.23555	2.1
44025	1.19715	2.1
44026	1.01445	2.1
44027	1.16061	2.1
44028	0.946132	2.1
44029	0.946132	2.1
44030	0.946131	2.1
44031	1.2051	2.1
44032	1.13537	2.1
44033	1.01445	2.1
44034	0.946133	2.1
44035	0.946133	2.1
44036	1.20648	2.1
44037	1.20648	2.1
44038	1.06454	2.1
44039	1.20648	2.1
44040	0.946133	2.1
44041	0.946133	2.1
44042	1.20649	2.1
44043	0.946133	2.1
44044	1.06454	2.1
44045	0.946132	2.1
44046	0.946132	2.1
44047	0.946132	2.1
44048	0.946132	2.1
44049	0.946132	2.1
44050	0.946132	2.1
44051	0.946132	2.1
44052	0.946133	2.1
44053	0.946133	2.1
44054	0.946133	2.1
44055	0.946133	2.1
44056	0.946132	2.1
44057	0.946132	2.1
44058	1.33078	1.79892
44059	1.37409	2.1
44060	1.31565	0.974145
44061	1.37114	2.1
44062	2.68973	1.70888
44063	1.89225	1.79355
44064	1.89225	2.1
44065	1.89225	0.990618
44066	1.89225	1.34818
44067	3.7845	1.24309
44068	1.89225	1.25978
44069	1.89225	2.1
44070	1.89225	1.05554
44071	1.89225	2.1
44072	3.7845	1.77934
44073	1.37409	1.32212
44074	1.20408	2.1
44075	1.18612	2.1
44076	1.18907	2.1
44077	1.37114	1.15104

44078	1.37409	2.1
44079	1.31566	1.9874
44080	1.37115	2.1
44081	1.30318	0.991359
44082	1.37433	1.39692
44083	2.68998	1.48097
44084	1.89226	2.04809
44085	1.89226	1.90478
44086	1.89226	0.980338
44087	1.89226	1.43828
44088	3.78453	1.29084
44089	1.89226	1.20203
44090	1.89226	2.1
44091	1.89226	2.1
44092	1.89226	2.1
44093	1.89226	1.0809
44094	1.89226	2.1
44095	0.647001	2.1
44096	0.647001	2.06313
44097	0.651851	1.01918
44098	0.651851	2.1
44099	0.602046	2.1
44100	0.613277	2.1
44101	0.685576	1.20868
44102	0.687164	1.27596
44103	0.687164	2.1
44104	0.613276	2.1
44105	0.602046	2.1
44106	0.685576	2.1
44107	0.946128	0.928422
44108	0.946128	0.797337
44109	1.15307	1.28162
44110	1.19317	1.05286
44111	0.94613	1.09571
44112	0.94613	0.593507
44113	1.15307	1.01776
44114	1.15307	1.12509
44115	0.946131	0.984524
44116	1.15307	0.987089
44117	0.946131	0.930964
44118	1.19317	0.907475
44119	1.18278	2.1
44120	2.38635	1.00385
44121	1.23042	2.1
44122	1.23042	1.18732
44123	2.30614	2.1
44124	2.30614	1.47192
44125	0.946129	0.859126
44126	0.946129	0.815868
44127	1.12994	1.03842
44128	1.15307	0.95774
44129	0.946131	0.996213
44130	0.946131	0.745066
44131	1.12994	0.883519
44132	1.12994	0.953307
44133	1.12994	0.937902
44134	0.946131	0.953268
44135	0.946131	0.866935

44136	1.15307	0.868465
44137	2.25988	1.15324
44138	2.30614	0.905123
44139	2.28696	1.58695
44140	1.16361	2.00721
44141	1.18278	1.83473
44142	1.18278	1.06211
44143	2.3771	2.1
44144	1.90121	2.1
44145	1.81191	2.1
44146	2.46904	2.1
44147	4.37025	2.1
44148	1.15676	2.1
44149	1.23452	2.1
44150	0.823592	2.1
44151	0.737606	2.1
44152	0.988113	2.1
44153	0.988113	2.1
44154	0.984014	2.1
44155	0.984014	2.1
44156	1.15676	2.1
44157	0.737605	2.1
44158	0.823592	2.1
44159	1.23452	2.1
44160	1.30318	2.1
44161	1.37433	2.1
44162	1.30151	1.13071
44163	1.39067	1.9414
44164	2.67584	1.50595
44165	1.89226	1.60502
44166	1.89226	2.1
44167	1.89226	1.1294
44168	1.89226	1.25505
44169	3.78453	1.22552
44170	1.89226	1.01691
44171	1.89226	2.1
44172	1.89226	0.984989
44173	1.89226	2.1
44174	1.89226	1.12378
44175	1.89226	2.1
44176	0.656819	2.1
44177	0.656819	1.05881
44178	0.661755	1.0983
44179	0.661755	2.1
44180	0.613277	2.1
44181	0.63141	2.1
44182	0.687164	1.10851
44183	0.695336	1.46174
44184	0.695337	2.09963
44185	0.63141	1.98335
44186	0.687164	2.1
44187	0.613276	2.1
44188	1.30151	1.91197
44189	1.39067	2.1
44190	1.32143	1.15395
44191	1.43294	1.86631
44192	2.73445	1.85786
44193	1.89226	1.85446

44194	1.89226	1.80813
44195	1.89226	1.00074
44196	1.89226	1.98651
44197	3.78452	1.41566
44198	1.89226	1.26064
44199	1.89226	1.20751
44200	1.89226	1.09627
44201	1.89226	2.1
44202	1.89226	2.1
44203	1.89226	2.1
44204	1.43294	1.23588
44205	1.26282	2.1
44206	1.3268	1.06599
44207	1.3268	2.1
44208	1.39067	1.21078
44209	1.39067	2.1
44210	1.5511	0.432403
44211	1.00665	0.483202
44212	1.28239	0.58324
44213	1.88631	0.556056
44214	2.89296	0.434863
44215	1.89227	0.581782
44216	1.89227	0.502042
44217	1.89227	0.647252
44218	1.89227	0.642948
44219	1.89227	0.42633
44220	1.89227	0.415312
44221	2.08848	0.492353
44222	1.89226	0.438492
44223	1.89226	0.603411
44224	2.37947	0.650189
44225	4.27173	0.568033
44226	1.01456	0.54896
44227	0.943157	0.527595
44228	0.641198	0.500729
44229	0.724757	0.535851
44230	0.830923	0.516825
44231	0.830923	0.463678
44232	0.836991	0.631666
44233	0.836991	0.391187
44234	1.01456	0.641854
44235	0.724757	0.64861
44236	0.641197	0.625106
44237	0.943157	0.57393
44238	1.89227	0.479422
44239	1.89227	0.571758
44240	1.89227	0.624077
44241	1.89227	0.633179
44242	3.78453	0.545534
44243	0.946129	0.525666
44244	0.946129	0.520393
44245	1.28941	0.622427
44246	1.18974	0.556003
44247	0.94613	0.594789
44248	0.94613	0.483482
44249	1.28941	0.525414
44250	1.28941	0.440445
44251	1.28941	0.490703

44252	0.946131	0.512215
44253	0.94613	0.276843
44254	1.18974	0.423856
44255	0.710636	2.1
44256	0.780249	2.1
44257	0.579033	2.1
44258	0.478812	2.1
44259	0.621822	2.1
44260	0.621822	2.1
44261	0.63724	2.1
44262	0.63724	2.1
44263	0.780251	2.1
44264	0.579032	2.1
44265	0.710637	2.1
44266	0.478811	2.1
44267	1.05499	1.94462
44268	1.00521	1.98808
44269	1.24445	1.47748
44270	1.23082	1.02481
44271	2.28581	1.8728
44272	1.48751	1.83601
44273	1.23083	1.40657
44274	1.4941	1.89759
44275	1.24445	1.2819
44276	1.48047	1.1748
44277	1.23083	1.17334
44278	0.621185	1.56747
44279	0.621185	1.80901
44280	0.620899	1.0882
44281	0.620899	2.1
44282	0.502515	1.43504
44283	0.626672	1.14048
44284	0.615412	1.06105
44285	0.740234	1.91429
44286	0.740233	1.04417
44287	0.626672	0.996191
44288	0.615413	1.16907
44289	0.502516	1.32059
44290	1.95844	2.1
44291	2.6562	2.1
44292	1.95844	2.1
44293	2.20883	2.1
44294	2.38225	2.1
44295	2.63264	2.1
44296	1.89226	2.1
44297	1.89226	2.1
44298	1.89226	2.1
44299	1.89226	2.1
44300	3.78453	2.1
44301	1.95844	2.1
44302	2.38225	2.1
44303	1.71082	2.1
44304	2.14647	2.1
44305	4.1049	2.1
44306	0.946131	2.1
44307	0.946131	2.1
44308	0.946131	2.1
44309	0.946131	2.1

44310	0.946131	2.1
44311	0.946131	2.1
44312	0.946131	2.1
44313	0.946131	2.1
44314	0.946131	2.1
44315	0.946131	2.1
44316	0.946131	2.1
44317	0.946131	2.1
44318	2.12597	2.1
44319	1.2295	2.1
44320	1.28014	2.1
44321	2.19723	2.1
44322	3.40611	2.1
44323	2.12597	2.1
44324	2.79242	2.1
44325	2.73375	2.1
44326	2.66249	2.1
44327	2.19723	2.1
44328	2.12597	2.1
44329	1.13716	2.1
44330	1.09861	2.1
44331	0.666692	2.1
44332	0.640071	2.1
44333	0.891617	2.1
44334	0.891617	2.1
44335	0.885614	2.1
44336	0.885614	2.1
44337	1.13716	2.1
44338	0.666692	2.1
44339	0.640071	2.1
44340	1.09861	2.1
44341	1.89226	0.178388
44342	1.70303	0.182263
44343	1.89226	0.135735
44344	2.64598	0.224296
44345	1.81091	0.12241
44346	2.53222	0.196534
44347	2.32422	0.153865
44348	1.89226	0.191694
44349	2.78336	0.245556
44350	2.78336	0.224233
44351	1.89226	0.145593
44352	1.89226	0.123663
44353	0.946132	0.153577
44354	0.946132	0.138595
44355	0.946131	0.142884
44356	0.946131	0.187682
44357	0.946132	0.209899
44358	0.946132	0.198116
44359	0.946131	0.154155
44360	0.946131	0.207855
44361	0.946131	0.16541
44362	0.946131	0.176368
44363	0.946131	0.1904
44364	0.946131	0.171833
44365	0.946131	0.229389
44366	0.946131	0.295426
44367	0.946132	0.221856

44368	0.946132	0.2926
44369	0.946132	0.301064
44370	0.946132	0.303135
44371	0.946131	0.20405
44372	0.946131	0.211747
44373	0.946131	0.215713
44374	0.946131	0.284477
44375	0.946131	0.289086
44376	0.946131	0.225955
44377	2.24323	1.25683
44378	1.89226	1.69987
44379	2.75021	1.36555
44380	2.75021	1.51155
44381	1.89226	1.1357
44382	1.89226	1.25836
44383	2.30908	0.578079
44384	2.19815	0.795809
44385	2.1609	0.460871
44386	1.83796	0.853598
44387	4.35904	0.572221
44388	2.19815	0.783449
44389	2.02268	1.94759
44390	2.17869	1.71198
44391	1.9559	0.827465
44392	2.0815	1.66048
44393	1.83424	0.821102
44394	2.1609	0.672931
44395	1.88877	0.84232
44396	1.98413	0.360315
44397	1.87355	0.811925
44398	4.03445	0.775088
44399	1.87355	0.815947
44400	2.02268	2.03167
44401	1.87355	1.82849
44402	2.10488	0.838042
44403	1.68443	1.49764
44404	1.89448	0.878108
44405	0.946131	0.383059
44406	0.946131	0.519105
44407	1.40574	0.700821
44408	1.12373	0.502174
44409	0.946129	0.404236
44410	0.946129	0.372809
44411	1.40574	0.676104
44412	1.40574	0.610319
44413	0.946131	0.486394
44414	1.40574	0.594753
44415	1.12373	0.54692
44416	0.946131	0.400371
44417	2.14277	0.765081
44418	2.81148	0.769561
44419	1.42762	0.698603
44420	1.42762	0.69443
44421	2.96261	0.75474
44422	2.24746	0.689684
44423	1.89226	0.702183
44424	1.89226	0.722234
44425	1.89226	0.7

44426	1.89226	0.781004
44427	3.78453	0.712669
44428	1.89226	0.7
44429	1.89226	0.722404
44430	1.89226	0.703325
44431	1.89226	0.783403
44432	3.78452	0.7
44433	0.68433	0.700168
44434	0.946127	0.778107
44435	0.543921	0.746084
44436	0.946131	0.733162
44437	0.946131	0.798156
44438	0.946131	1.77378
44439	0.543918	0.752741
44440	0.543918	0.816919
44441	0.684332	0.779149
44442	0.54392	1.61157
44443	0.946129	0.700153
44444	0.946129	0.803734
44445	1.89226	0.727971
44446	1.08784	0.791952
44447	1.19352	0.720873
44448	1.89226	0.817622
44449	1.19352	1.68415
44450	1.89226	1.35439
44451	0.635711	0.7
44452	0.946132	0.760666
44453	0.946132	0.722942
44454	0.56474	0.713581
44455	0.750045	0.779323
44456	1.25657	1.56876
44457	0.635682	0.750422
44458	0.635682	0.7
44459	0.891954	0.729654
44460	1.32751	1.28913
44461	0.564766	0.7
44462	0.564766	0.761072
44463	0.858345	0.71829
44464	0.946128	0.793958
44465	0.635712	0.762889
44466	0.946133	0.725013
44467	0.977198	0.778082
44468	0.944521	1.62278
44469	0.635682	0.804473
44470	0.635682	0.7
44471	1.02924	0.762325
44472	0.705625	1.63918
44473	0.851528	0.7
44474	0.851528	0.956175
44475	2.18903	2.1
44476	2.10182	2.1
44477	2.10182	2.1
44478	1.89226	2.1
44479	1.89226	2.1
44480	1.64983	2.1
44481	1.89226	2.1
44482	1.83867	2.1
44483	1.83867	2.1

44484	1.89164	2.1
44485	2.0815	2.1
44486	2.17638	2.1
44487	2.57957	1.09074
44488	2.3506	1.48964
44489	2.69056	0.892908
44490	2.49439	1.42094
44491	5.04116	0.981963
44492	2.3506	1.05389
44493	1.8911	1.84885
44494	2.49439	1.05429
44495	2.06689	2.1
44496	4.41748	1.60158
44497	2.69056	1.076
44498	2.49439	1.55303
44499	2.77227	0.926449
44500	2.63443	1.23105
44501	5.32499	1.5877
44502	2.49439	1.14293
44503	2.06689	2.1
44504	2.63443	0.903859
44505	2.26444	2.1
44506	4.70131	1.35543
44507	2.46327	2.1
44508	2.54347	2.1
44509	2.52869	2.1
44510	2.16534	2.1
44511	2.82661	2.1
44512	2.46327	2.1
44513	0.946131	2.1
44514	0.946131	2.1
44515	1.27174	2.1
44516	1.27174	2.1
44517	1.27174	2.1
44518	0.94613	2.1
44519	0.946128	2.1
44520	1.4133	2.1
44521	0.946129	2.1
44522	0.946129	2.1
44523	1.27174	2.1
44524	1.4133	2.1
44525	1.42757	2.1
44526	1.7457	2.1
44527	1.66047	2.1
44528	1.7457	2.1
44529	1.45754	2.1
44530	1.54278	2.1
44531	1.42757	2.1
44532	1.52996	2.1
44533	1.66047	2.1
44534	1.66047	2.1
44535	1.26397	2.1
44536	1.26397	2.1
44537	0.968536	2.1
44538	1.02461	2.1
44539	1.57575	2.1
44540	1.62391	2.1
44541	2.59245	2.1

44542	0.946131	2.1
44543	0.946131	2.1
44544	0.946132	2.1
44545	0.946132	2.1
44546	0.946132	2.1
44547	0.946132	2.1
44548	0.946132	2.1
44549	0.946132	2.1
44550	0.946132	2.1
44551	0.946132	2.1
44552	0.946132	2.1
44553	0.946132	2.1
44554	2.23765	2.1
44555	1.62391	2.1
44556	2.22154	2.1
44557	1.57575	2.1
44558	2.2697	2.1
44559	1.62391	2.1
44560	1.89226	2.1
44561	1.89226	2.1
44562	1.89226	2.1
44563	1.89226	2.1
44564	1.89226	2.1
44565	1.89226	2.05316
44566	1.89226	2.1
44567	1.89226	2.1
44568	1.89226	2.1
44569	1.89226	2.1
44570	1.89226	2.1
44571	1.89226	1.96875
44572	2.2697	2.1
44573	1.46129	2.1
44574	2.25873	2.1
44575	1.62391	2.1
44576	2.09611	2.1
44577	1.46129	2.1
44578	1.89226	2.1
44579	2.07088	2.1
44580	1.89226	2.1
44581	2.31884	2.1
44582	4.21111	2.1
44583	1.89226	2.1
44584	2.31884	2.1
44585	2.11713	2.1
44586	1.89226	2.1
44587	2.54371	2.1
44588	2.31884	2.1
44589	2.54371	2.1
44590	1.24041	2.1
44591	2.55208	2.1
44592	2.31884	2.1
44593	1.23712	2.1
44594	1.00388	2.1
44595	2.72769	0.263407
44596	2.57507	0.41318
44597	1.89226	0.221952
44598	1.89226	0.396887
44599	4.46733	0.271763

44600	1.89226	0.368154
44601	2.57506	0.434065
44602	1.89226	0.241823
44603	2.30076	0.429637
44604	1.89226	0.250885
44605	2.30076	0.418204
44606	1.21171	0.18613
44607	1.48589	0.272232
44608	2.09166	0.176988
44609	1.78381	0.223277
44610	3.2697	0.231453
44611	2.09166	0.169592
44612	1.70302	0.258739
44613	1.48589	0.198523
44614	1.18027	0.173139
44615	2.26571	0.229403
44616	1.79969	0.218121
44617	1.89226	0.237302
44618	1.89226	0.433431
44619	1.89226	0.351152
44620	1.89226	0.310906
44621	1.89226	0.382891
44622	1.89226	0.398531
44623	0.946131	0.315175
44624	0.946131	0.585762
44625	0.946128	0.430599
44626	0.946128	0.373471
44627	0.946129	0.412035
44628	0.946129	0.42787
44629	0.94613	0.269434
44630	0.94613	0.260451
44631	0.94613	0.305381
44632	0.94613	0.353766
44633	0.94613	0.374336
44634	0.94613	0.340157
44635	1.89226	1.56369
44636	1.89226	1.67093
44637	1.89226	1.96826
44638	1.89226	1.4251
44639	1.89226	1.88523
44640	1.89226	1.37544
44641	1.89226	1.55504
44642	1.89226	1.56249
44643	1.89226	1.85395
44644	1.89226	1.37084
44645	1.89226	1.78051
44646	1.89226	1.33833
44647	1.89226	1.40973
44648	1.89226	1.48744
44649	1.89226	1.76242
44650	1.89226	1.24938
44651	1.89226	1.71649
44652	1.89226	1.227
44653	1.79103	0.92156
44654	0.968249	2.08802
44655	1.44807	1.25745
44656	2.17984	0.821112
44657	3.2391	1.68492

44658	1.29735	1.24182
44659	1.08992	1.22753
44660	0.945147	1.69987
44661	0.724033	2.1
44662	1.00723	2.01317
44663	1.00723	1.36807
44664	1.01416	0.87559
44665	1.01416	1.5358
44666	1.29735	1.37598
44667	0.94515	1.72445
44668	0.724034	1.48883
44669	1.08992	1.86626
44670	1.39214	2.1
44671	1.39214	2.1
44672	0.946129	2.1
44673	0.946129	2.1
44674	1.33728	2.1
44675	1.39214	2.1
44676	0.94613	2.1
44677	0.94613	2.1
44678	0.94613	2.1
44679	1.33728	2.1
44680	0.94613	2.1
44681	1.39214	2.1
44682	2.78428	2.1
44683	2.28373	2.1
44684	2.67456	2.1
44685	2.20011	2.1
44686	4.95829	2.1
44687	1.90165	0.162128
44688	2.24206	0.273646
44689	1.66137	0.275681
44690	2.07108	0.561541
44691	3.90343	0.11376
44692	1.89224	0.737992
44693	1.89224	0.278216
44694	1.89224	0.251548
44695	1.89224	0.490791
44696	3.78448	0.369142
44697	2.82159	0.482083
44698	1.89226	0.728416
44699	2.50138	0.877277
44700	1.89226	0.384927
44701	4.39365	0.380188
44702	1.66139	0.16002
44703	1.86399	0.597721
44704	1.56005	0.408753
44705	1.90324	0.365013
44706	3.56463	0.285096
44707	0.946133	0.148423
44708	0.946133	0.393792
44709	0.946132	0.235059
44710	0.946132	0.682823
44711	0.946133	0.437531
44712	0.946133	0.295467
44713	0.946132	0.175896
44714	0.946132	0.402622
44715	0.946133	0.180295

44716	0.946133	0.436476
44717	0.946132	0.14057
44718	0.946132	0.562594
44719	1.5176	0.847827
44720	1.97964	0.791392
44721	2.32889	0.799385
44722	1.91762	0.825363
44723	3.84649	0.799506
44724	2.3376	0.785388
44725	1.97963	0.801997
44726	2.32889	0.815535
44727	2.58303	0.761922
44728	4.66649	0.814394
44729	2.32889	0.787142
44730	2.20248	0.784551
44731	1.91762	0.775517
44732	1.91762	0.784529
44733	2.5615	0.798
44734	2.5615	0.812922
44735	2.32889	0.802205
44736	2.58303	0.755974
44737	2.71793	0.750935
44738	2.56149	0.764004
44739	5.04681	0.755879
44740	1.89226	0.699412
44741	2.53785	1.04856
44742	1.89226	0.707428
44743	2.60895	0.700895
44744	1.89226	0.692189
44745	2.60895	0.690993
44746	0.493248	0.748161
44747	1.30448	0.839717
44748	1.26893	0.876312
44749	0.653226	0.959498
44750	0.653227	0.757889
44751	0.493247	1.89005
44752	1.26893	1.56066
44753	1.30448	0.702269
44754	0.832232	1.4172
44755	0.832233	1.59317
44756	0.929944	0.7
44757	0.929944	0.712681
44758	1.89226	2.1
44759	1.47937	2.1
44760	1.50143	2.1
44761	1.47937	2.1
44762	1.91432	2.1
44763	1.89226	1.7264
44764	1.89226	2.1
44765	1.89226	2.1
44766	1.89226	2.1
44767	1.89226	2.1
44768	1.89226	2.1
44769	1.89226	1.19954
44770	1.33419	2.1
44771	2.4542	2.1
44772	2.59834	2.1
44773	2.59834	2.1

44774	1.25716	2.1
44775	1.25716	2.1
44776	1.70303	2.1
44777	1.50143	2.1
44778	1.60878	2.1
44779	1.50143	2.1
44780	1.93599	2.1
44781	1.89226	1.26498
44782	0.946131	1.13802
44783	0.946131	1.53029
44784	0.946127	2.1
44785	0.946127	2.1
44786	0.946127	2.1
44787	0.946127	1.03244
44788	0.946131	2.1
44789	0.946131	1.55365
44790	0.94613	2.05488
44791	0.94613	2.07691
44792	0.946129	2.1
44793	0.946129	2.1
44794	1.43912	2.1
44795	2.59834	2.1
44796	2.78093	2.1
44797	2.78093	2.1
44798	1.33419	2.1
44799	1.33419	2.1
44800	1.89226	2.1
44801	2.26322	2.03454
44802	2.55682	2.1
44803	2.55682	2.1
44804	1.89226	2.1
44805	1.89226	2.1
44806	2.58032	0.719118
44807	2.28922	0.708423
44808	1.88424	0.703746
44809	2.32127	0.707869
44810	4.46456	0.70673
44811	1.67265	0.715306
44812	2.28922	0.712221
44813	1.88424	0.701614
44814	1.11314	0.702323
44815	3.55689	0.704474
44816	1.43751	0.7
44817	2.32127	0.703146
44818	1.92998	0.603586
44819	2.37671	0.684432
44820	1.43751	0.705938
44821	1.88424	0.708111
44822	2.36729	2.1
44823	1.56714	2.1
44824	1.56714	2.1
44825	1.62408	2.1
44826	2.28373	2.1
44827	2.34067	2.1
44828	0.937268	2.1
44829	0.937268	2.1
44830	0.94636	2.1
44831	0.94636	2.1

44832	0.76004	2.1
44833	0.783572	2.1
44834	1.14187	2.1
44835	1.10006	2.1
44836	1.10006	2.1
44837	0.76004	2.1
44838	0.783572	2.1
44839	1.14187	2.1
44840	1.28753	2.1
44841	1.36838	2.1
44842	1.62617	2.1
44843	1.82857	2.1
44844	3.1161	2.1
44845	1.82856	2.1
44846	1.17996	2.1
44847	1.53417	2.1
44848	1.56618	2.1
44849	1.36838	2.1
44850	1.36838	2.1
44851	1.35065	0.999991
44852	1.42659	0.759228
44853	2.26675	0.859499
44854	2.26675	0.833984
44855	1.62137	0.826992
44856	2.53747	0.86415
44857	1.89226	0.239185
44858	1.89226	0.145843
44859	1.89226	0.179103
44860	1.89226	0.221487
44861	3.78452	0.220069
44862	1.36569	0.10506
44863	1.36569	0.0943846
44864	0.946131	0.126849
44865	0.946131	0.17532
44866	1.16041	0.13793
44867	1.36569	0.11511
44868	0.946129	0.145836
44869	0.946129	0.179557
44870	1.1604	0.109779
44871	0.94613	0.140289
44872	0.946131	0.128766
44873	1.36569	0.117654
44874	2.73139	0.0861548
44875	1.25265	0.131414
44876	1.25265	0.134812
44877	1.64799	0.119234
44878	2.32081	0.124229
44879	2.71615	0.0921788
44880	0.946133	0.145457
44881	0.946133	0.151674
44882	0.946132	0.223277
44883	0.946132	0.180925
44884	0.946132	0.167431
44885	0.946132	0.189762
44886	0.946133	0.153442
44887	0.946133	0.174544
44888	0.946132	0.163243
44889	0.946132	0.185851

44890	0.946133	0.17543
44891	0.946133	0.160152
44892	0.946131	0.149311
44893	0.946131	0.160593
44894	0.94613	0.313482
44895	0.94613	0.19528
44896	0.946132	0.267692
44897	0.946132	0.19744
44898	0.946129	0.227074
44899	0.946129	0.290655
44900	0.946131	0.226517
44901	0.946131	0.180218
44902	0.946131	0.171647
44903	0.946131	0.210174
44904	1.89226	0.833577
44905	2.48503	2.09299
44906	2.64586	2.1
44907	2.64586	0.828653
44908	1.89226	2.1
44909	1.89226	0.812919
44910	2.48504	2.1
44911	1.99985	2.1
44912	1.83584	2.1
44913	1.83584	2.1
44914	2.64586	2.1
44915	2.64586	2.1
44916	1.89225	0.821616
44917	2.33095	1.99406
44918	2.48501	2.1
44919	2.48501	0.808954
44920	1.89225	2.1
44921	1.89225	0.797685
44922	2.33096	2.1
44923	1.83583	2.1
44924	1.68436	2.1
44925	1.68436	2.1
44926	2.48502	2.1
44927	2.48502	2.1
44928	1.14807	2.1
44929	1.76545	2.1
44930	1.21292	2.1
44931	1.83936	2.1
44932	2.97837	2.1
44933	1.89226	2.1
44934	1.89226	2.1
44935	1.89226	2.1
44936	1.89226	1.56416
44937	3.78451	2.1
44938	1.89226	2.1
44939	1.89226	1.09832
44940	1.89226	1.65779
44941	1.89226	1.40293
44942	3.78452	1.61103
44943	1.76545	2.1
44944	2.12608	2.1
44945	1.83937	2.1
44946	2.22529	1.31564
44947	3.99074	2.1

44948	0.780222	2.1
44949	0.780222	2.1
44950	0.784871	2.1
44951	0.784871	2.1
44952	0.95863	2.1
44953	0.919681	2.1
44954	0.636145	2.1
44955	0.606461	2.1
44956	0.636144	2.1
44957	0.958631	2.1
44958	0.60646	2.1
44959	0.919683	2.1
44960	1.89226	2.1
44961	1.89226	1.73055
44962	1.89226	2.1
44963	1.89226	2.1
44964	3.78452	2.1
44965	1.89226	1.82324
44966	1.89226	1.62912
44967	1.89226	2.1
44968	1.89226	1.01422
44969	3.78452	1.54506
44970	1.03789	1.92289
44971	1.03789	2.1
44972	1.04231	2.1
44973	1.04231	2.1
44974	1.16052	2.1
44975	1.11265	1.09514
44976	0.958634	2.1
44977	0.919685	2.1
44978	0.958634	2.1
44979	1.16052	2.1
44980	0.919685	2.1
44981	1.11265	2.1
44982	1.43837	2.1
44983	2.57085	2.1
44984	1.39332	2.1
44985	2.58228	2.1
44986	3.96417	2.1
44987	1.89226	2.1
44988	1.89226	2.1
44989	1.89226	2.1
44990	1.89226	2.1
44991	1.89226	2.1
44992	1.89226	2.1
44993	1.89226	2.1
44994	1.89226	1.20503
44995	1.89226	2.1
44996	1.89226	2.1
44997	3.78452	2.1
44998	2.57085	2.1
44999	1.89226	2.1
45000	2.58229	2.1
45001	1.89226	2.1
45002	4.46312	2.1
45003	1.32368	2.1
45004	2.58228	2.1
45005	2.55352	2.1

45006	2.55352	2.1
45007	1.39332	2.1
45008	1.39332	2.1
45009	1.89226	2.1
45010	1.89226	2.1
45011	1.89226	2.1
45012	1.89226	2.1
45013	1.89226	2.1
45014	1.89226	2.1
45015	1.89226	2.1
45016	1.89226	2.1
45017	1.89226	2.1
45018	1.89226	2.1
45019	1.89226	2.1
45020	1.89226	1.85549
45021	2.55353	2.1
45022	1.89226	2.1
45023	2.58228	2.1
45024	1.89226	2.1
45025	1.89226	2.1
45026	2.58228	2.1
45027	1.40574	1.31346
45028	1.05175	1.08729
45029	1.71613	1.2887
45030	1.99961	1.11171
45031	3.12187	1.20501
45032	1.71613	1.26051
45033	2.50733	1.09699
45034	1.99961	1.47024
45035	1.99961	1.19135
45036	2.25746	1.13169
45037	2.25746	1.04761
45038	2.25746	1.03521
45039	1.89526	1.05427
45040	1.71613	1.24469
45041	1.71613	0.945004
45042	1.89526	1.08891
45043	1.27558	0.943888
45044	1.07536	2.1
45045	1.07536	2.1
45046	1.07034	2.1
45047	1.07034	2.1
45048	0.766879	2.1
45049	0.81527	2.1
45050	1.38896	2.1
45051	1.33043	2.1
45052	0.815264	2.1
45053	1.38895	2.1
45054	0.766884	2.1
45055	1.33044	2.1
45056	1.89225	2.1
45057	1.89225	2.1
45058	1.89225	2.1
45059	1.89225	2.1
45060	1.89225	2.1
45061	1.89225	2.1
45062	1.64059	2.1
45063	1.89225	2.1

45064	1.565	2.1
45065	1.565	2.1
45066	1.89225	2.1
45067	1.89225	2.1
45068	1.89226	2.1
45069	1.89226	2.1
45070	1.89226	2.1
45071	1.89226	2.1
45072	1.89226	2.1
45073	1.89226	2.1
45074	1.67906	2.1
45075	1.89226	2.1
45076	1.6406	2.1
45077	1.6406	2.1
45078	1.89226	2.1
45079	1.89226	2.1
45080	2.18106	1.35323
45081	2.20831	2.05731
45082	2.64725	0.996835
45083	2.63102	1.23315
45084	4.82831	1.40044
45085	2.18106	1.16697
45086	2.09605	2.1
45087	2.5601	1.26289
45088	2.63102	1.2631
45089	4.74116	1.84503
45090	2.64725	1.10604
45091	1.89226	1.1891
45092	1.87603	1.98426
45093	2.63102	1.12003
45094	1.89226	1.35269
45095	2.64725	0.959938
45096	0.946132	1.23622
45097	1.28005	1.07887
45098	0.946132	1.86259
45099	1.31551	1.00619
45100	1.28005	1.32
45101	1.28005	1.8099
45102	0.946132	1.00902
45103	0.946132	1.21835
45104	0.946132	1.03314
45105	0.946132	1.12659
45106	1.28005	1.33542
45107	1.31551	1.53306
45108	2.0815	2.1
45109	1.59318	2.1
45110	2.0815	2.1
45111	1.40161	2.1
45112	2.49782	2.1
45113	1.68553	2.1
45114	0.819885	2.1
45115	0.935082	2.1
45116	0.867363	2.1
45117	0.867363	2.1
45118	0.715431	2.1
45119	0.902777	2.1
45120	0.738933	2.1
45121	0.980053	2.1

45122	0.936716	2.1
45123	0.917651	2.1
45124	0.775853	2.1
45125	0.74037	2.1
45126	2.78977	2.1
45127	2.38239	2.1
45128	2.78977	2.1
45129	1.96468	2.1
45130	2.64107	2.1
45131	1.80733	2.1
45132	1.80141	2.1
45133	2.28512	2.1
45134	2.28512	2.1
45135	1.8519	2.1
45136	2.64107	2.1
45137	2.20785	2.1
45138	0.946133	0.895955
45139	0.946133	0.856389
45140	0.946132	0.884863
45141	0.946132	0.862333
45142	0.946132	0.885792
45143	0.946132	0.882217
45144	0.946132	0.852242
45145	0.946132	0.859812
45146	0.946132	0.880341
45147	0.946132	0.873268
45148	0.946133	0.869098
45149	0.946133	0.877055
45150	1.89226	0.771007
45151	1.89226	0.776368
45152	1.89226	0.56234
45153	1.89226	0.743679
45154	1.89226	0.757246
45155	1.89226	0.795948
45156	1.89226	0.770019
45157	1.89226	0.749878
45158	1.89226	0.777608
45159	1.89226	0.815105
45160	3.78453	0.779856
45161	1.89226	0.86418
45162	1.89226	0.824107
45163	1.89226	0.802434
45164	1.89226	0.867676
45165	1.89226	0.822805
45166	1.89226	0.860273
45167	2.12925	0.149369
45168	2.51397	0.241455
45169	2.22488	0.154838
45170	1.86113	0.22929
45171	4.3751	0.232193
45172	2.82754	0.139091
45173	2.51397	0.227779
45174	2.22489	0.132751
45175	2.47291	0.247441
45176	4.98688	0.138779
45177	1.89226	0.250069
45178	1.89226	0.401114
45179	1.89226	0.295939

45180	1.89226	0.402507
45181	3.78453	0.401739
45182	1.89226	0.264323
45183	1.89226	0.404976
45184	1.89226	0.257089
45185	1.89226	0.423844
45186	3.78452	0.268305
45187	0.90848	0.194881
45188	1.11244	0.144985
45189	0.930566	0.165093
45190	0.73385	0.215736
45191	0.921298	0.209055
45192	0.921298	0.232409
45193	0.924994	0.148661
45194	0.924994	0.144895
45195	0.908478	0.197042
45196	0.733851	0.146302
45197	1.11244	0.156954
45198	0.930567	0.190503
45199	2.22489	0.131349
45200	2.0815	0.198709
45201	2.0815	0.200701
45202	2.30674	0.159467
45203	1.99867	0.185181
45204	2.33358	0.135511
45205	1.89226	0.274369
45206	1.89226	0.375123
45207	1.89226	0.284398
45208	1.89226	0.338448
45209	1.89226	0.277627
45210	1.89226	0.316728
45211	0.946131	0.434659
45212	0.946131	0.36815
45213	0.946128	0.421449
45214	0.946128	0.298555
45215	0.946129	0.3834
45216	0.946129	0.422522
45217	0.94613	0.240809
45218	0.94613	0.260369
45219	0.94613	0.355957
45220	0.94613	0.251143
45221	0.94613	0.290549
45222	0.94613	0.336365
45223	2.28904	2.1
45224	1.89226	1.16418
45225	2.2846	1.307
45226	2.2846	2.1
45227	1.89226	0.940372
45228	1.89226	2.1
45229	1.89226	1.16179
45230	1.89226	1.27784
45231	1.89226	2.1
45232	1.89226	2.06485
45233	1.89226	1.01884
45234	1.89226	1.34134
45235	1.04037	1.03237
45236	1.34066	1.63595
45237	1.34955	1.04139

45238	1.34955	1.75676
45239	1.03373	1.16261
45240	1.03373	2.1
45241	0.946131	0.859724
45242	0.946131	2.1
45243	1.14452	1.89027
45244	1.14452	2.1
45245	0.946131	1.17268
45246	0.946131	1.12979
45247	1.14296	1.91568
45248	1.14452	2.1
45249	1.14296	0.991212
45250	0.946131	1.14146
45251	1.14452	1.39557
45252	0.946132	1.21952
45253	1.89226	1.08706
45254	1.89226	1.79432
45255	1.89226	1.50671
45256	1.89226	2.1
45257	1.89226	0.957554
45258	1.89226	1.88657
45259	0.597454	1.06818
45260	0.597454	1.73693
45261	0.597972	1.02129
45262	0.597972	2.1
45263	0.675238	1.79067
45264	0.674776	1.19679
45265	0.519667	1.13756
45266	0.520188	1.24754
45267	0.519667	0.98837
45268	0.675238	1.05372
45269	0.520188	1.38864
45270	0.674777	1.35589
45271	1.89226	0.749605
45272	1.82125	0.689418
45273	1.89226	0.71525
45274	1.84314	0.661012
45275	1.89226	0.767829
45276	1.84314	0.753243
45277	1.89226	0.689358
45278	1.84314	0.721969
45279	1.89226	0.678137
45280	1.65794	0.653876
45281	1.89226	0.677457
45282	1.65794	0.646879
45283	1.348	1.85026
45284	1.43485	2.1
45285	1.79929	1.0073
45286	1.75309	1.64445
45287	3.14728	1.63303
45288	1.348	1.23792
45289	1.63892	1.79096
45290	1.59157	2.0927
45291	1.18647	1.85798
45292	1.75309	1.30354
45293	1.348	1.62627
45294	1.79929	1.43325
45295	1.75309	2.1

45296	2.18106	1.17344
45297	2.20831	1.21165
45298	3.98034	1.73138
45299	2.18106	1.0633
45300	1.63892	1.88081
45301	1.75309	1.07692
45302	1.75309	1.97656
45303	2.09605	1.05734
45304	2.09605	2.00673
45305	2.66076	0.549685
45306	1.70302	0.308491
45307	2.41575	0.494376
45308	1.70302	0.410349
45309	3.08598	0.342142
45310	2.3661	0.25821
45311	1.71053	0.437668
45312	2.41575	0.623659
45313	1.20143	0.327235
45314	1.20143	0.302027
45315	2.2123	0.330808
45316	1.66739	0.346407
45317	2.25633	0.486607
45318	2.68302	0.596602
45319	1.71051	0.387071
45320	1.71051	0.313349
45321	2.74192	0.390182
45322	2.1961	0.274257
45323	1.32553	1.06759
45324	2.34875	0.871157
45325	2.19139	1.38323
45326	2.19139	0.83789
45327	1.56172	1.19377
45328	1.56172	0.535941
45329	1.89226	2.1
45330	1.89226	0.875176
45331	1.89226	1.60429
45332	1.89226	0.97594
45333	3.78453	1.06902
45334	2.667 2.1	
45335	1.26097	1.17428
45336	1.26097	2.1
45337	1.5683	2.1
45338	2.42949	2.1
45339	2.73682	2.1
45340	1.13213	1.01725
45341	2.19139	0.855717
45342	2.0815	1.12893
45343	1.77615	0.807858
45344	1.37767	1.10844
45345	1.19297	0.524114
45346	0.946132	0.890015
45347	0.946132	1.23685
45348	0.946131	2.1
45349	0.946131	0.9762
45350	0.946131	0.959108
45351	0.946131	0.854018
45352	0.946132	1.1992
45353	0.946132	2.1

45354	0.946132	1.11552
45355	0.946132	1.10472
45356	0.946131	1.0455
45357	0.946131	1.07534
45358	2.02064	1.62009
45359	2.3606	2.1
45360	1.47478	2.1
45361	2.22997	2.1
45362	4.25061	2.1
45363	2.36059	2.1
45364	2.56952	2.1
45365	2.22997	2.1
45366	2.48631	2.1
45367	4.79949	2.1
45368	1.84261	2.1
45369	2.22997	2.1
45370	1.65706	1.99901
45371	1.78837	2.1
45372	3.88703	2.1
45373	2.22997	2.1
45374	2.48631	2.1
45375	2.19528	2.1
45376	2.41175	2.1
45377	4.64172	2.1
45378	1.6466	2.1
45379	1.89226	2.1
45380	1.35972	2.1
45381	1.35972	2.1
45382	1.89226	2.1
45383	1.89226	2.1
45384	1.89226	2.1
45385	1.89226	2.1
45386	1.89226	2.1
45387	1.89226	2.1
45388	3.78453	2.1
45389	2.44797	2.1
45390	1.07214	2.1
45391	1.31589	2.1
45392	1.1211	2.1
45393	2.44797	2.1
45394	2.25318	2.1
45395	1.95954	2.1
45396	1.89226	2.1
45397	1.6466	2.1
45398	1.6466	2.1
45399	1.89226	2.1
45400	1.89226	2.1
45401	0.946133	2.1
45402	0.946133	2.1
45403	0.946132	2.1
45404	0.946132	2.1
45405	0.946132	2.1
45406	0.946132	2.1
45407	0.946133	2.1
45408	0.946133	2.1
45409	0.946132	2.1
45410	0.946132	2.1
45411	0.946133	2.1

45412	0.946133	2.1
45413	2.44797	2.1
45414	1.36904	2.1
45415	1.70303	2.1
45416	2.37586	2.1
45417	4.00507	2.1
45418	0.631553	0.227751
45419	1.24489	0.15198
45420	1.11597	0.172265
45421	0.496799	0.25444
45422	0.872272	0.239105
45423	0.872272	0.221665
45424	0.869415	0.144687
45425	0.869415	0.163557
45426	0.4968	0.208604
45427	0.631553	0.2109
45428	1.11597	0.162845
45429	1.24489	0.221731
45430	1.84253	2.1
45431	2.17384	2.1
45432	2.04818	2.1
45433	1.65925	2.1
45434	3.83309	2.1
45435	2.04818	2.1
45436	1.65925	2.1
45437	1.48162	2.1
45438	1.92264	2.1
45439	3.58189	2.1
45440	2.3305	0.701548
45441	1.89226	0.701796
45442	2.52753	0.7009
45443	2.52753	0.703716
45444	1.89226	0.700252
45445	1.89226	0.702046
45446	1.89226	0.701764
45447	1.89226	0.711021
45448	1.89226	0.706023
45449	1.89226	0.712331
45450	1.89226	0.701101
45451	1.89226	0.70755
45452	1.34563	0.701033
45453	2.1769	0.700459
45454	1.94955	0.700889
45455	1.94955	0.70641
45456	1.55063	0.700628
45457	1.55063	0.707128
45458	2.09455	0.7
45459	1.89226	0.700272
45460	2.3305	0.7
45461	2.3305	0.700924
45462	1.89226	0.7
45463	1.89226	0.700163
45464	1.89226	0.700032
45465	1.89226	0.700162
45466	1.89226	0.702368
45467	1.89226	0.700612
45468	1.89226	0.701735
45469	1.89226	0.701472

45470	0.773392	0.7
45471	0.612765	0.700914
45472	0.79504	0.7
45473	0.575323	0.7
45474	1.0819	0.7
45475	1.06106	0.7
45476	1.04675	0.7
45477	0.86858	0.701691
45478	0.796873	0.700121
45479	0.557469	0.700988
45480	0.807663	0.700774
45481	2.2024	2.1
45482	2.34106	2.1
45483	2.02634	2.1
45484	2.20125	2.1
45485	4.36739	2.1
45486	2.34106	2.1
45487	2.25195	0.919618
45488	2.20125	2.1
45489	2.13132	0.864161
45490	4.47237	1.06542
45491	2.02635	2.1
45492	2.22465	2.1
45493	1.68504	2.1
45494	2.0815	2.1
45495	3.92673	2.1
45496	2.22465	2.1
45497	2.13133	0.871952
45498	2.0815	2.1
45499	2.23579	0.911633
45500	4.44862	1.03717
45501	1.89226	1.06654
45502	1.89226	1.32784
45503	1.89226	1.51192
45504	1.89226	1.13529
45505	1.89226	1.36393
45506	1.89226	1.10272
45507	1.93967	0.405311
45508	1.70302	0.577257
45509	1.21883	0.491935
45510	1.70302	0.670539
45511	1.19889	0.393748
45512	1.70302	0.618485
45513	0.946131	0.65207
45514	0.946131	0.61682
45515	0.946131	0.535276
45516	0.946131	0.675382
45517	0.94613	0.689556
45518	0.94613	0.7024
45519	0.946131	0.52
45520	0.946131	0.611635
45521	0.946131	0.593092
45522	0.946131	0.619498
45523	0.946131	0.672565
45524	0.946131	0.550557
45525	0.883128	0.6228
45526	0.883128	0.698757
45527	0.827519	0.569234

45528	0.82752	0.676749
45529	1.16234	0.655173
45530	1.19063	0.67705
45531	0.631294	0.619821
45532	0.548311	0.580461
45533	0.631293	0.592464
45534	1.16234	0.602174
45535	1.19063	0.662829
45536	0.54831	0.690309
45537	1.89226	0.168752
45538	1.89226	0.253937
45539	1.89226	0.334122
45540	1.89226	0.218402
45541	3.78453	0.215019
45542	2.33328	0.117362
45543	1.87991	0.152724
45544	1.87991	0.194453
45545	2.82938	0.107107
45546	1.84457	0.188915
45547	2.79403	0.105177
45548	2.82937	0.11199
45549	2.70881	0.141422
45550	1.8799	0.179185
45551	1.82739	0.104278
45552	4.58874	0.110525
45553	1.89226	0.295636
45554	1.89226	0.163562
45555	1.89226	0.249827
45556	1.89226	0.310333
45557	3.78452	0.297644
45558	1.17897	0.171671
45559	1.82739	0.103122
45560	1.17897	0.134454
45561	1.76199	0.110982
45562	1.23885	0.156265
45563	1.80989	0.112629
45564	0.946131	0.222224
45565	0.946131	0.205302
45566	0.946131	0.266488
45567	0.946131	0.247895
45568	0.946129	0.306236
45569	0.946129	0.18358
45570	0.946133	0.262767
45571	0.946133	0.270045
45572	0.946131	0.263617
45573	0.946131	0.187821
45574	0.946131	0.213556
45575	0.946131	0.20994
45576	0.946126	0.91258
45577	0.946126	2.1
45578	1.24887	2.1
45579	1.24887	2.1
45580	0.946127	1.94175
45581	0.946127	0.968744
45582	1.24887	2.1
45583	1.28511	1.91833
45584	1.2851	1.63716
45585	0.946128	1.95053

45586	1.24887	2.0144
45587	0.946128	2.04774
45588	1.89226	2.05965
45589	1.89226	1.65004
45590	1.89226	1.83374
45591	1.89226	0.927885
45592	3.78452	1.30274
45593	0.592142	1.8161
45594	0.592142	1.76173
45595	0.60496	2.1
45596	0.60496	2.1
45597	0.694237	1.94795
45598	0.608714	1.12533
45599	0.562863	1.78637
45600	0.502864	2.1
45601	0.562863	1.88069
45602	0.694237	1.88063
45603	0.608714	2.06342
45604	0.502864	1.72245
45605	1.89226	0.926046
45606	1.89226	2.1
45607	1.89226	1.89693
45608	1.89226	2.1
45609	3.78453	2.1
45610	1.55779	2.1
45611	1.57968	2.1
45612	1.47186	1.71861
45613	1.51725	2.1
45614	3.05154	2.1
45615	1.57967	2.1
45616	1.474 2.1	
45617	1.51725	2.1
45618	1.42895	2.1
45619	3.00863	2.1
45620	1.89226	2.1
45621	1.89226	2.1
45622	1.89226	2.1
45623	1.89226	2.1
45624	3.78452	2.1
45625	1.89226	1.67246
45626	1.89226	2.1
45627	1.89226	0.926447
45628	1.89226	2.1
45629	3.78453	1.92985
45630	1.47186	1.6214
45631	1.51725	2.1
45632	1.38859	2.1
45633	1.45332	2.1
45634	2.92519	2.1
45635	1.51725	2.1
45636	1.42895	2.1
45637	1.45332	2.1
45638	1.38414	2.1
45639	2.88228	2.1
45640	1.89226	2.1
45641	1.89226	2.1
45642	1.89226	2.1
45643	1.89226	2.1

45644	3.78452	2.1
45645	1.34497	0.0723738
45646	1.36747	0.0608586
45647	1.18919	0.0633383
45648	2.08149	0.064777
45649	1.23556	0.0576419
45650	2.13702	0.0603442
45651	1.10106	0.0791808
45652	2.08001	0.0685345
45653	1.34497	0.0712398
45654	2.31605	0.0740457
45655	1.34497	0.064778
45656	2.31605	0.0683777
45657	2.31605	0.0637684
45658	2.0815	0.0670293
45659	1.9419	0.0549906
45660	2.46665	0.0510119
45661	4.52922	0.0573996
45662	1.20349	0.0570516
45663	2.08149	0.0648517
45664	1.36948	0.0510619
45665	1.34737	0.0572314
45666	2.08149	0.0531985
45667	2.08149	0.0580802
45668	0.946132	0.0787026
45669	0.946132	0.0859922
45670	1.37715	0.0713695
45671	0.823199	0.0736204
45672	1.21598	0.0754901
45673	0.946132	0.0777543
45674	1.37715	0.0728025
45675	0.946132	0.0727005
45676	1.21598	0.0779158
45677	0.823198	0.0764568
45678	0.946133	0.0704759
45679	0.946133	0.0854177
45680	1.89226	0.0883288
45681	1.89226	0.103382
45682	1.89226	0.0810453
45683	1.89226	0.0890937
45684	3.78453	0.0861392
45685	1.89227	0.0967077
45686	1.89227	0.0961501
45687	1.89227	0.083781
45688	1.89227	0.0863149
45689	3.78453	0.0935038
45690	0.946132	0.0950578
45691	0.946132	0.0753782
45692	1.21599	0.0790339
45693	1.21599	0.0756735
45694	0.966749	0.0817418
45695	0.946133	0.0812118
45696	1.21598	0.0808853
45697	0.946132	0.0806563
45698	0.966748	0.0828496
45699	1.21598	0.083364
45700	0.946133	0.0790885
45701	0.946133	0.0807712

45702	0.946133	0.0639968
45703	0.946133	0.0788867
45704	1.1134	0.0683695
45705	1.11555	0.0596571
45706	1.11555	0.0668034
45707	0.946132	0.0650609
45708	0.946132	0.0655892
45709	1.1134	0.0672179
45710	1.11555	0.0642211
45711	1.11555	0.0592885
45712	0.946132	0.0775204
45713	0.946132	0.0700601
45714	0.946133	0.0636248
45715	0.946133	0.070199
45716	1.11555	0.0684249
45717	0.978467	0.063337
45718	0.978468	0.0671519
45719	0.946133	0.0651954
45720	1.11555	0.0669323
45721	0.946132	0.0657506
45722	0.978468	0.0643983
45723	0.978468	0.063163
45724	0.946132	0.0677014
45725	0.946132	0.0704331
45726	0.851542	2.1
45727	0.851542	2.1
45728	1.14168	2.1
45729	1.27897	2.1
45730	1.02817	2.1
45731	1.13536	2.1
45732	0.946131	2.08213
45733	0.861207	2.04534
45734	0.94613	1.86079
45735	0.94613	2.1
45736	0.868751	2.1
45737	1.15181	2.1
45738	0.946132	1.21336
45739	0.946132	1.1698
45740	0.946128	1.302
45741	0.946128	1.08354
45742	0.94613	1.98139
45743	0.94613	1.29682
45744	0.946131	1.12653
45745	0.946131	1.06254
45746	0.94613	1.024
45747	0.94613	1.24737
45748	0.94613	1.14869
45749	0.94613	1.60764
45750	2.53273	0.145358
45751	1.89226	0.192359
45752	1.89226	0.191759
45753	1.89226	0.206885
45754	1.89226	0.125808
45755	1.89226	0.149251
45756	1.89226	2.1
45757	1.89226	2.1
45758	1.89226	2.1
45759	1.89226	2.1

45760	3.78452	2.1
45761	1.89226	2.1
45762	2.54394	2.1
45763	1.89226	2.1
45764	2.42221	2.1
45765	4.43621	2.1
45766	2.54394	2.1
45767	1.62743	2.1
45768	2.42221	2.1
45769	1.5437	2.1
45770	4.08764	2.1
45771	1.89226	2.1
45772	1.89226	2.1
45773	1.89226	2.1
45774	1.89226	2.1
45775	3.78452	2.1
45776	1.89226	2.1
45777	1.89226	2.1
45778	1.89226	2.1
45779	1.89226	2.1
45780	3.78452	2.1
45781	1.89226	2.1
45782	2.30146	2.1
45783	2.42221	2.1
45784	1.89226	2.1
45785	1.89226	2.1
45786	2.42221	2.1
45787	2.30146	2.1
45788	2.42221	2.1
45789	1.39962	2.1
45790	1.39962	2.1
45791	1.5437	2.1
45792	2.44553	2.1
45793	1.89226	2.1
45794	1.89226	2.1
45795	1.89226	2.1
45796	1.89226	2.1
45797	3.78452	2.1
45798	1.62196	2.1
45799	2.00695	2.1
45800	1.95288	2.1
45801	1.70303	2.1
45802	3.53316	2.1
45803	1.80703	2.1
45804	2.00695	2.1
45805	1.92991	2.1
45806	2.00695	2.1
45807	1.85888	2.1
45808	1.93592	2.1
45809	1.89226	2.1
45810	1.89226	2.1
45811	1.89226	2.1
45812	1.89226	2.1
45813	1.89226	2.1
45814	1.89226	2.1
45815	1.89226	2.1
45816	1.89226	2.1
45817	1.89226	2.1

45818	1.89226	2.1
45819	3.78453	2.1
45820	1.95288	2.1
45821	1.50524	2.1
45822	1.83564	2.1
45823	1.70303	2.1
45824	2.00749	2.1
45825	1.84671	2.1
45826	1.62632	2.1
45827	1.82498	2.1
45828	1.92991	2.1
45829	1.92991	2.1
45830	1.47928	2.1
45831	1.47751	2.1
45832	0.946128	2.1
45833	0.946128	2.1
45834	0.946132	2.1
45835	0.946132	2.1
45836	0.94613	2.1
45837	0.94613	2.1
45838	0.946129	2.1
45839	0.946129	2.1
45840	0.94613	2.1
45841	0.94613	2.1
45842	0.94613	2.1
45843	0.94613	2.1
45844	1.89226	2.1
45845	1.89226	2.1
45846	1.89226	2.1
45847	1.89226	2.1
45848	1.89226	2.1
45849	1.89226	2.1
45850	2.27351	1.03869
45851	2.74068	0.907456
45852	2.72278	0.965482
45853	2.63356	0.996324
45854	2.36273	0.953751
45855	2.27351	0.98025
45856	2.36273	0.975292
45857	2.83112	0.873694
45858	2.81212	0.929123
45859	2.74068	0.940471
45860	2.43416	0.918661
45861	2.36273	0.92694
45862	1.89227	0.916213
45863	2.83112	0.874455
45864	2.74069	0.918944
45865	2.74069	0.855642
45866	1.89227	0.948381
45867	1.89227	0.87904
45868	2.40887	2.1
45869	1.4494	2.1
45870	1.52164	2.1
45871	2.47534	2.1
45872	3.93051	2.1
45873	1.89226	2.1
45874	2.47534	2.1
45875	2.40887	2.1

45876	2.40887	2.1
45877	1.89226	2.1
45878	1.89226	2.1
45879	1.60861	2.1
45880	2.47534	2.1
45881	1.52164	2.1
45882	1.52164	2.1
45883	2.55301	2.1
45884	2.55301	2.1
45885	0.946128	2.1
45886	0.946128	2.1
45887	1.23767	2.1
45888	1.2765	2.1
45889	1.23767	2.1
45890	1.23767	2.1
45891	0.946131	2.1
45892	0.946131	2.1
45893	0.946131	2.1
45894	1.2765	2.1
45895	1.23767	2.1
45896	0.946131	2.1
45897	1.89226	2.1
45898	1.87511	2.1
45899	1.87511	2.1
45900	1.95852	2.1
45901	2.0815	2.1
45902	2.22169	2.1
45903	1.89226	2.1
45904	1.89226	2.1
45905	1.89226	2.1
45906	1.89226	2.1
45907	1.89226	2.1
45908	1.89226	2.1
45909	2.0815	2.1
45910	1.80067	2.1
45911	1.80067	2.1
45912	2.05518	2.1
45913	1.89226	2.1
45914	2.16509	2.1
45915	1.87153	2.1
45916	2.65897	2.1
45917	1.58724	2.1
45918	2.38924	2.1
45919	4.26077	2.1
45920	1.89226	2.1
45921	1.89226	2.1
45922	1.89226	2.1
45923	1.89226	2.1
45924	1.89226	2.1
45925	1.89226	2.1
45926	1.89226	0.865848
45927	2.21022	0.775491
45928	2.19769	0.913101
45929	2.21022	0.83032
45930	1.87973	0.765616
45931	1.89226	0.724324
45932	1.18376	1.11914
45933	2.21022	0.781619

45934	1.16239	1.06359
45935	1.16239	0.802804
45936	2.19769	1.06877
45937	2.19769	0.797991
45938	0.946128	0.787756
45939	0.946128	0.765401
45940	1.09362	0.839466
45941	1.09884	0.777496
45942	0.94613	0.804682
45943	0.94613	0.714834
45944	1.09362	0.826205
45945	1.09362	0.76289
45946	0.946131	0.806748
45947	1.09362	0.809959
45948	0.946131	0.732774
45949	1.09884	0.719127
45950	1.19323	1.00854
45951	2.19769	0.750418
45952	1.18376	0.958123
45953	1.18376	0.789926
45954	2.18724	0.966286
45955	2.18724	0.79584
45956	2.0815	2.1
45957	0.922714	2.1
45958	2.0815	2.1
45959	1.84882	2.1
45960	1.20787	2.1
45961	1.08321	2.1
45962	2.6238	0.404837
45963	1.89226	0.606531
45964	1.89226	0.549254
45965	2.4866	0.481838
45966	1.89226	0.528553
45967	2.4866	0.519918
45968	2.66753	0.513613
45969	1.89226	0.696354
45970	1.89226	0.674658
45971	2.6238	0.615323
45972	1.89226	0.642289
45973	2.6238	0.645519
45974	0.946129	2.1
45975	0.946129	2.1
45976	1.04676	2.1
45977	1.04676	2.1
45978	0.946131	2.1
45979	0.946131	2.1
45980	1.08732	2.1
45981	1.04676	2.1
45982	1.08732	2.1
45983	0.946131	2.1
45984	1.04676	2.1
45985	0.946131	2.1
45986	1.20958	1.33809
45987	1.73802	0.827731
45988	1.39067	0.959738
45989	1.39067	0.862697
45990	1.50969	0.972803
45991	1.50969	0.972109

45992	1.00382	1.07074
45993	1.50969	0.748107
45994	1.20958	1.00904
45995	1.20958	0.788732
45996	1.32039	1.07969
45997	1.32039	0.830211
45998	2.0815	2.1
45999	2.51396	2.1
46000	2.19826	2.1
46001	2.54175	2.1
46002	4.79599	2.1
46003	2.54174	2.1
46004	2.17978	2.1
46005	2.54174	2.1
46006	2.2854	2.1
46007	2.57212	2.1
46008	2.31578	2.1
46009	2.27103	2.1
46010	2.54175	2.1
46011	2.4255	2.1
46012	2.82251	2.1
46013	4.96725	2.1
46014	2.60614	0.414038
46015	2.75374	0.259683
46016	1.94002	0.227834
46017	2.08392	0.362094
46018	4.69006	0.276644
46019	2.75373	0.273646
46020	2.6875	0.477938
46021	2.08392	0.252492
46022	1.99944	0.331097
46023	4.77142	0.370912
46024	1.1211	0.131789
46025	2.08392	0.239725
46026	1.17787	0.197262
46027	1.94002	0.173696
46028	1.32178	0.163932
46029	2.08392	0.166078
46030	2.08392	0.166086
46031	1.99944	0.164115
46032	1.32178	0.113314
46033	1.25929	0.155223
46034	3.34321	0.138158
46035	1.22266	0.104811
46036	0.779473	0.0989215
46037	0.94613	0.119431
46038	0.94613	0.112695
46039	1.04072	0.115453
46040	1.1407	0.117683
46041	1.10042	0.110645
46042	0.851542	0.109114
46043	0.942046	0.103126
46044	0.942046	0.121304
46045	1.04114	0.12373
46046	0.826602	0.109894
46047	2.37468	2.1
46048	1.89226	2.1
46049	1.89226	2.1

46050	1.89226	2.1
46051	2.27917	2.1
46052	2.27917	2.1
46053	1.13959	2.1
46054	1.09451	2.1
46055	0.946131	2.1
46056	0.946131	2.1
46057	1.09451	2.1
46058	1.09451	2.1
46059	0.94613	2.1
46060	0.94613	2.1
46061	1.09451	2.1
46062	0.946131	2.1
46063	0.946131	2.1
46064	1.13959	2.1
46065	2.52454	1.04039
46066	2.12221	2.1
46067	2.67624	1.15578
46068	2.33865	1.38734
46069	4.86319	1.49185
46070	1.89226	1.29033
46071	1.89226	2.1
46072	1.89226	1.27172
46073	1.89226	2.1
46074	3.78452	2.1
46075	1.89226	2.1
46076	1.89226	2.1
46077	1.89226	2.1
46078	1.89226	2.1
46079	3.78452	2.1
46080	2.12221	2.1
46081	1.47769	2.1
46082	2.33865	2.1
46083	1.62898	2.1
46084	3.81634	2.1
46085	2.67624	2.1
46086	2.37734	1.33722
46087	2.33865	2.1
46088	2.33865	1.77624
46089	2.70125	2.1
46090	2.70125	2.1
46091	1.89226	1.49893
46092	1.89226	2.08561
46093	1.89226	2.1
46094	1.89226	2.1
46095	1.89226	1.05605
46096	1.89226	1.0947
46097	1.89226	2.1
46098	1.89226	2.1
46099	1.89226	2.1
46100	1.89226	2.1
46101	1.89226	2.1
46102	1.89226	2.1
46103	2.37733	1.39601
46104	1.95927	2.1
46105	1.82516	1.36797
46106	1.82516	2.1
46107	2.33865	2.1

46108	2.33865	2.1
46109	2.57466	2.1
46110	2.21715	2.1
46111	2.55653	2.1
46112	2.24775	2.1
46113	4.77367	2.1
46114	0.937095	2.1
46115	0.937095	2.1
46116	0.958028	2.1
46117	0.958028	2.1
46118	0.828386	2.1
46119	0.771244	2.1
46120	1.10858	2.1
46121	1.12388	2.1
46122	1.12388	2.1
46123	0.828386	2.1
46124	0.771244	2.1
46125	1.10858	2.1
46126	2.55653	2.1
46127	2.32606	2.1
46128	2.24775	2.1
46129	2.24775	2.1
46130	2.57928	2.1
46131	2.57928	2.1
46132	0.990481	2.1
46133	0.990481	2.1
46134	1.00094	2.1
46135	1.00094	2.1
46136	0.888385	2.1
46137	0.828386	2.1
46138	1.16303	2.1
46139	1.12388	2.1
46140	1.16303	2.1
46141	0.888385	2.1
46142	1.12388	2.1
46143	0.828387	2.1
46144	0.962524	2.1
46145	0.962524	2.1
46146	0.7976	2.1
46147	0.7976	2.1
46148	0.797628	2.1
46149	0.814 2.1	
46150	0.946125	2.1
46151	0.946125	2.1
46152	0.946118	2.1
46153	0.797623	2.1
46154	0.814006	2.1
46155	0.946131	2.1
46156	1.8978	0.05
46157	2.23892	0.05
46158	1.71469	0.05
46159	4.13672	0.05
46160	2.64136	0.05
46161	2.23892	0.789807
46162	2.37578	0.825022
46163	1.77489	2.1
46164	4.6147	0.851396
46165	1.02284	2.1

46166	0.898796	0.845131
46167	0.948554	1.03264
46168	0.948554	2.1
46169	0.878797	2.1
46170	0.796197	2.1
46171	1.09145	0.89934
46172	0.963109	0.906451
46173	1.12114	1.61072
46174	0.782739	1.19069
46175	1.11084	1.48281
46176	0.850742	1.48281
46177	2.48024	2.1
46178	2.405	2.1
46179	2.24585	2.1
46180	2.34795	2.1
46181	4.75295	2.1
46182	1.93794	2.1
46183	2.405	2.1
46184	2.24585	2.1
46185	1.67594	1.9723
46186	4.08094	2.1
46187	1.89226	2.1
46188	1.89226	0.917795
46189	1.89226	1.96393
46190	1.89226	1.09088
46191	3.78452	1.45575
46192	1.89226	2.1
46193	1.89226	2.1
46194	1.89226	2.1
46195	1.89226	1.11456
46196	3.78452	2.1
46197	2.24585	2.1
46198	2.34795	2.1
46199	2.21155	2.1
46200	2.06626	2.1
46201	4.4142	2.1
46202	2.24585	2.1
46203	1.41498	1.69035
46204	1.41498	2.1
46205	1.67594	2.1
46206	2.06625	2.1
46207	2.32721	2.1
46208	1.89226	1.66709
46209	1.89226	1.08114
46210	1.89226	2.1
46211	1.89226	0.992559
46212	1.89226	2.1
46213	1.89226	0.933116
46214	1.89226	2.1
46215	1.89226	1.09739
46216	1.89226	2.1
46217	1.89226	1.90305
46218	3.78452	2.1
46219	1.89226	0.412738
46220	1.89226	0.599647
46221	1.89226	0.71194
46222	1.89226	0.356684
46223	3.78452	0.399292

46224	1.89226	0.448801
46225	2.53655	0.244393
46226	1.89226	0.208001
46227	1.89226	0.235154
46228	1.89226	0.484138
46229	1.89226	0.510829
46230	1.89227	2.1
46231	1.89227	2.1
46232	1.89227	2.1
46233	1.89227	2.1
46234	1.89227	2.1
46235	1.89227	2.1
46236	0.91339	2.1
46237	0.91339	2.1
46238	1.2158	2.1
46239	1.20629	2.1
46240	1.00473	2.1
46241	1.15749	2.1
46242	0.946132	2.1
46243	0.946132	2.1
46244	0.946133	2.1
46245	1.17513	2.1
46246	1.00473	2.1
46247	0.946132	2.1
46248	1.89226	2.1
46249	1.89226	2.1
46250	1.89226	2.1
46251	1.89226	2.1
46252	1.89226	2.1
46253	1.89226	2.1
46254	1.89226	2.1
46255	2.6066	2.1
46256	2.35026	2.1
46257	2.35026	2.1
46258	1.89226	2.1
46259	1.89226	2.1
46260	2.04056	0.758633
46261	2.77366	0.743021
46262	1.24682	0.659274
46263	1.24682	0.766541
46264	2.93651	0.738201
46265	2.14277	0.759509
46266	2.68524	1.42986
46267	2.81847	1.25414
46268	1.89226	1.04245
46269	1.89226	1.23346
46270	4.57751	1.09392
46271	2.68524	1.21665
46272	1.89226	1.56067
46273	1.89226	1.55222
46274	2.50732	1.21847
46275	1.89226	1.39285
46276	2.50732	1.4341
46277	1.89226	1.08287
46278	1.89226	1.46246
46279	1.89226	1.19589
46280	1.89226	1.56908
46281	1.89226	1.06456

46282	1.89226	1.45121
46283	1.89226	1.55288
46284	1.89226	1.15109
46285	1.89226	1.30594
46286	1.89226	1.6688
46287	1.89226	1.04257
46288	1.89226	1.05909
46289	1.61016	1.09344
46290	1.07467	1.16215
46291	1.24474	1.03056
46292	1.24474	1.53537
46293	1.45303	1.08963
46294	1.45303	1.54967
46295	1.61016	1.23904
46296	1.24474	1.79141
46297	1.61016	1.30923
46298	1.77186	1.1254
46299	1.37813	1.09207
46300	1.37813	1.21815
46301	1.73305	2.1
46302	2.0815	2.1
46303	1.97658	2.1
46304	1.75791	2.1
46305	1.87228	2.1
46306	1.66515	2.1
46307	2.0815	2.1
46308	1.93938	0.919518
46309	2.03253	0.872771
46310	2.2142	2.1
46311	1.97658	0.880213
46312	2.15324	2.1
46313	1.73305	2.1
46314	1.97658	2.1
46315	1.60428	2.1
46316	1.8589	2.1
46317	3.58085	2.1
46318	1.97657	2.1
46319	1.93938	0.916867
46320	1.51316	2.1
46321	1.85293	0.877368
46322	3.82951	1.07101
46323	1.70303	0.361404
46324	1.96087	0.482801
46325	1.42308	0.467596
46326	1.96087	0.491946
46327	1.25023	0.380322
46328	1.70303	0.415658
46329	0.946132	0.600059
46330	0.946132	0.417774
46331	0.946131	0.438207
46332	0.946131	0.510365
46333	0.946132	0.55392
46334	0.946132	0.512594
46335	0.946132	0.393629
46336	0.946132	0.382376
46337	0.946132	0.476926
46338	0.946132	0.420733
46339	0.946132	0.479281

46340	0.946132	0.519071
46341	1.89226	0.550675
46342	1.89226	0.695209
46343	1.89226	0.670519
46344	1.89226	0.674476
46345	3.78452	0.635158
46346	1.89226	0.484696
46347	1.89226	0.695367
46348	1.89226	0.647686
46349	1.89226	0.663035
46350	1.89226	0.46344
46351	1.89226	0.480765
46352	1.89226	0.550054
46353	1.89226	0.587652
46354	1.89226	0.67624
46355	1.89226	0.566453
46356	1.89226	0.609806
46357	1.89226	0.464902
46358	1.89226	0.357324
46359	1.89226	0.574503
46360	2.48255	0.438864
46361	1.89226	0.441214
46362	3.78452	0.37834
46363	1.89226	2.1
46364	2.42758	2.1
46365	1.9209	2.1
46366	1.89226	2.1
46367	2.45622	2.1
46368	2.42758	2.1
46369	2.42758	2.1
46370	1.49987	2.1
46371	1.61028	2.1
46372	1.58164	2.1
46373	2.45622	2.1
46374	2.42758	2.1
46375	0.94613	2.1
46376	0.94613	2.1
46377	1.24298	2.1
46378	1.24298	2.1
46379	1.24298	2.1
46380	1.22811	2.1
46381	0.946131	2.1
46382	0.946131	2.1
46383	0.94613	2.1
46384	1.24298	2.1
46385	0.94613	2.1
46386	1.22811	2.1
46387	2.48596	2.1
46388	2.45622	2.1
46389	1.63823	2.1
46390	1.4304	2.1
46391	1.45739	2.1
46392	2.45622	2.1
46393	2.4169	2.1
46394	2.55249	2.1
46395	2.51125	2.1
46396	2.35245	2.1
46397	4.90494	2.1

46398	2.45922	2.1
46399	2.55249	2.1
46400	2.51126	2.1
46401	2.43226	2.1
46402	4.98474	2.1
46403	2.51125	2.1
46404	2.35245	2.1
46405	2.28979	2.1
46406	2.47228	2.1
46407	4.82473	2.1
46408	2.51126	2.1
46409	2.43226	2.1
46410	2.40668	2.1
46411	2.47228	2.1
46412	4.90453	2.1
46413	2.8045	2.1
46414	1.93707	2.1
46415	2.14087	2.1
46416	2.14087	2.1
46417	2.61595	2.1
46418	2.61595	2.1
46419	1.89226	2.1
46420	1.89226	2.1
46421	1.89226	2.1
46422	1.89226	1.16888
46423	1.89226	2.1
46424	1.89226	1.06037
46425	1.22368	2.00068
46426	1.32339	2.1
46427	0.957125	2.1
46428	0.750074	2.1
46429	1.08438	2.1
46430	1.08438	2.08267
46431	1.13094	2.1
46432	0.970797	2.1
46433	1.17568	2.1
46434	0.764392	2.1
46435	0.944286	2.1
46436	1.27523	2.1
46437	1.89226	2.1
46438	1.89226	2.1
46439	1.89226	2.1
46440	1.89226	1.12956
46441	3.78453	2.1
46442	2.8239	2.1
46443	1.89227	2.1
46444	2.57276	2.1
46445	2.57276	2.1
46446	1.89227	2.1
46447	1.89227	2.1
46448	1.18139	2.1
46449	1.85195	2.1
46450	1.78595	2.1
46451	1.08172	2.1
46452	2.93367	2.1
46453	1.60735	2.1
46454	1.20356	2.1
46455	0.967034	2.1

46456	1.08172	2.1
46457	1.67324	2.1
46458	1.77293	2.1
46459	1.89226	2.1
46460	1.89226	2.1
46461	1.8213	2.1
46462	1.82129	2.1
46463	2.14171	2.1
46464	2.40192	2.1
46465	0.810943	2.1
46466	0.810943	2.1
46467	0.798469	2.1
46468	0.788336	2.1
46469	0.804015	2.1
46470	0.810945	2.1
46471	1.947	2.1
46472	1.36506	2.1
46473	1.08686	2.1
46474	1.07208	2.1
46475	1.08132	2.1
46476	1.13537	0.460064
46477	1.14754	1.04108
46478	0.946131	0.910056
46479	0.946131	1.29909
46480	0.94613	0.905707
46481	0.94613	0.57925
46482	1.25104	1.40968
46483	1.14754	1.01324
46484	1.13537	0.940122
46485	0.946131	0.949522
46486	0.946131	0.984416
46487	1.25104	0.969386
46488	0.96542	0.30548
46489	0.922284	0.957236
46490	0.946131	1.20748
46491	0.946131	0.925329
46492	0.946131	0.917983
46493	0.946131	0.358082
46494	1.13537	1.21292
46495	0.924824	1.00018
46496	0.859476	0.911985
46497	0.842305	0.924743
46498	1.25996	0.924156
46499	1.04996	0.934841
46500	1.71007	0.113361
46501	2.73283	0.103441
46502	2.73283	0.121775
46503	1.89226	0.108745
46504	1.89226	0.120108
46505	1.13262	0.0957207
46506	0.946131	0.118374
46507	0.946131	0.12827
46508	0.946131	0.128407
46509	0.946131	0.164539
46510	0.946132	0.17051
46511	0.946132	0.161121
46512	0.946131	0.125689
46513	0.946131	0.179513

46514	0.946131	0.135427
46515	0.946131	0.139448
46516	0.946132	0.157643
46517	0.946132	0.133507
46518	0.946131	0.117501
46519	1.36642	0.109162
46520	0.946125	0.108991
46521	0.946125	0.123446
46522	0.946128	0.127117
46523	0.946128	0.122892
46524	0.946126	0.1224
46525	0.946128	0.118349
46526	0.946128	0.120606
46527	0.946128	0.118095
46528	1.36641	0.118499
46529	0.946128	0.129718
46530	1.89226	0.123839
46531	1.89226	0.186275
46532	1.89226	0.138305
46533	1.89226	0.139202
46534	1.89226	0.153659
46535	1.89226	0.15492
46536	2.69784	2.1
46537	2.51651	2.1
46538	2.56999	2.1
46539	2.74623	2.1
46540	5.26783	2.1
46541	2.69784	2.1
46542	2.48718	2.1
46543	2.50161	2.1
46544	2.74623	2.1
46545	5.19945	2.1
46546	1.89226	2.1
46547	1.89226	2.1
46548	1.89226	2.1
46549	1.89226	2.1
46550	3.78453	2.1
46551	0.874264	2.1
46552	1.09879	2.1
46553	0.946131	2.1
46554	0.946131	2.1
46555	1.17062	2.1
46556	1.17062	2.1
46557	0.874293	2.1
46558	0.874293	2.1
46559	1.09879	2.1
46560	0.94613	2.1
46561	0.946131	2.1
46562	0.874265	2.1
46563	1.89226	2.1
46564	1.89226	2.1
46565	1.89226	2.1
46566	1.89226	2.1
46567	3.78453	2.1
46568	0.977382	2.1
46569	1.16424	2.1
46570	0.946133	2.1
46571	0.946133	2.1

46572	1.33012	2.1
46573	1.25589	2.1
46574	0.888498	2.1
46575	0.888498	2.1
46576	1.25375	2.1
46577	0.946131	2.1
46578	0.946131	2.1
46579	0.977384	2.1
46580	1.89226	2.1
46581	2.19757	2.1
46582	2.62906	2.1
46583	1.89226	2.1
46584	1.89226	2.1
46585	2.62906	2.1
46586	1.27944	0.800218
46587	1.27731	1.73557
46588	0.615701	1.4521
46589	0.615701	0.960301
46590	0.615673	0.706315
46591	0.946133	1.26614
46592	0.948264	0.734003
46593	0.946132	0.77202
46594	0.94752	0.740102
46595	0.61706	0.713907
46596	0.946132	0.765593
46597	0.946132	0.771493
46598	2.76604	2.1
46599	1.66728	2.1
46600	1.66728	2.1
46601	1.83036	2.1
46602	2.54515	2.1
46603	2.70823	2.1
46604	2.54515	2.1
46605	1.49958	2.1
46606	1.49958	2.1
46607	1.66728	2.1
46608	2.34465	2.1
46609	2.51235	2.1
46610	1.21128	2.1
46611	1.21128	2.1
46612	0.946131	2.1
46613	0.946131	2.1
46614	0.946131	2.1
46615	0.946131	2.1
46616	1.31401	2.1
46617	1.21128	2.1
46618	1.21128	2.1
46619	0.946131	2.1
46620	0.946131	2.1
46621	1.31401	2.1
46622	1.67374	2.1
46623	2.29586	2.1
46624	1.50033	2.1
46625	2.16598	2.1
46626	3.83972	2.1
46627	1.89226	2.1
46628	1.89226	2.1
46629	1.89226	2.1

46630	1.89226	2.1
46631	3.78452	2.1
46632	1.13537	2.1
46633	1.02188	2.1
46634	0.946132	2.1
46635	0.946132	2.1
46636	0.946132	2.1
46637	0.946132	2.1
46638	1.21128	2.1
46639	1.02188	2.1
46640	1.04672	2.1
46641	1.04672	2.1
46642	1.21128	2.1
46643	0.946132	2.1
46644	0.815411	2.1
46645	0.870464	2.1
46646	0.885437	2.1
46647	0.885437	2.1
46648	1.06395	2.1
46649	1.00483	2.1
46650	0.757125	2.1
46651	0.673956	2.1
46652	0.684461	2.1
46653	1.06979	2.1
46654	0.731403	2.1
46655	1.0559	2.1
46656	1.89226	2.1
46657	1.89226	2.1
46658	1.89226	2.1
46659	1.89226	2.1
46660	1.89226	2.1
46661	1.89226	2.1
46662	1.76897	1.1327
46663	1.76932	1.17014
46664	1.97455	1.67145
46665	1.94302	1.00885
46666	3.71233	1.11492
46667	1.83347	2.1
46668	1.76932	1.18761
46669	1.97455	1.77796
46670	1.76932	1.86744
46671	1.76522	1.86981
46672	1.55999	1.99422
46673	1.89227	1.18663
46674	1.89227	1.31028
46675	1.89227	1.88175
46676	1.89227	1.09479
46677	3.78453	1.17864
46678	1.89226	1.17022
46679	1.89226	0.908957
46680	1.89226	0.994773
46681	1.89226	1.06995
46682	3.78452	1.08763
46683	1.97452	1.44832
46684	1.94299	0.958509
46685	2.17831	1.07309
46686	2.23195	1.09473
46687	4.17495	1.14924

46688	2.23196	1.13409
46689	2.1499	2.04723
46690	1.97452	1.47403
46691	1.97452	1.37753
46692	2.09088	1.73329
46693	1.83345	1.5268
46694	0.94612	1.22608
46695	0.94612	1.4189
46696	0.946119	1.08761
46697	0.946119	1.05291
46698	0.946119	1.05459
46699	0.946119	1.2142
46700	0.94612	1.1273
46701	0.94612	1.42233
46702	0.946132	1.15134
46703	0.946132	1.11817
46704	0.946107	1.18667
46705	0.946107	1.18164
46706	1.89224	0.941939
46707	1.89224	1.01793
46708	1.89224	1.07273
46709	1.89224	0.888318
46710	3.78447	0.914224
46711	1.70302	0.791148
46712	1.056	0.787081
46713	1.21829	0.792644
46714	1.89906	0.774731
46715	3.07892	0.784989
46716	2.20248	0.780136
46717	1.56498	0.771538
46718	1.46539	0.763572
46719	1.48787	0.770371
46720	2.37625	0.781724
46721	2.37625	0.787828
46722	1.14104	0.759066
46723	1.00675	0.758319
46724	0.700689	0.757844
46725	0.624389	0.766258
46726	0.782149	0.742985
46727	0.940197	0.760289
46728	0.803437	0.742403
46729	0.803437	0.762144
46730	0.995423	0.745466
46731	0.700699	0.74064
46732	0.593895	0.780448
46733	1.00673	0.764698
46734	1.18097	0.748679
46735	1.15839	0.742607
46736	0.995407	0.756245
46737	0.865932	0.747508
46738	1.17299	0.742516
46739	0.957054	0.756426
46740	1.08016	0.739423
46741	1.08016	0.762293
46742	0.995425	0.746167
46743	1.24161	0.73485
46744	0.796771	0.750924
46745	1.15837	0.765624

46746	2.57264	0.289788
46747	1.89226	0.505835
46748	2.41701	0.392011
46749	1.89226	0.256334
46750	4.30928	0.285109
46751	1.69055	0.374191
46752	2.39587	0.398206
46753	1.53463	0.219978
46754	2.2053	0.226379
46755	3.89585	0.419488
46756	0.946129	0.335499
46757	0.946129	0.255789
46758	0.94613	0.287807
46759	0.94613	0.375995
46760	0.946132	0.288734
46761	0.946132	0.56125
46762	0.946126	0.343995
46763	0.946126	0.344623
46764	0.94613	0.250818
46765	0.94613	0.392038
46766	0.946129	0.310831
46767	0.946129	0.439756
46768	2.41701	0.351587
46769	1.89226	0.213326
46770	1.89226	0.178641
46771	1.89226	0.212365
46772	2.32462	0.305734
46773	2.32462	0.382557
46774	1.41942	0.159275
46775	2.2053	0.217737
46776	2.02097	0.185548
46777	2.02097	0.207207
46778	1.53463	0.166938
46779	1.53463	0.170808
46780	1.89226	0.179923
46781	1.89226	0.291971
46782	1.89226	0.212846
46783	1.89226	0.296317
46784	1.89226	0.197273
46785	1.89226	0.228425
46786	1.89226	1.2813
46787	1.89226	1.94175
46788	1.89226	1.86548
46789	1.89226	1.44818
46790	1.89226	1.66988
46791	1.89226	1.34895
46792	1.02816	1.3997
46793	1.02816	1.70052
46794	0.595141	1.61384
46795	0.595141	1.82858
46796	0.946132	1.56633
46797	0.595113	1.8614
46798	0.677174	1.76585
46799	0.946132	1.36165
46800	0.946132	1.61827
46801	0.946132	1.60676
46802	0.595113	1.602
46803	0.677174	1.69754

46804	1.89226	1.24124
46805	1.89226	2.04042
46806	1.89226	1.6165
46807	1.89226	1.34534
46808	1.89226	1.83809
46809	1.89226	1.44657
46810	0.946133	1.34765
46811	0.946133	1.1216
46812	0.946132	1.61975
46813	0.946132	1.7639
46814	0.946132	1.75132
46815	0.946132	1.33643
46816	0.946132	1.45493
46817	0.946132	1.11863
46818	0.946132	1.41936
46819	0.946132	1.54169
46820	0.946133	1.35531
46821	0.946133	1.34249
46822	0.946131	1.46976
46823	0.946131	1.3941
46824	0.946131	1.98542
46825	0.946131	1.99546
46826	0.946128	2.06151
46827	0.946128	1.47473
46828	0.946133	2.01631
46829	0.946133	1.35928
46830	0.94613	1.66657
46831	0.94613	1.84931
46832	0.946131	1.69555
46833	0.946131	1.57
46834	2.0264	1.36878
46835	1.67345	1.47327
46836	2.0264	1.16316
46837	1.70539	1.175
46838	1.31611	1.29607
46839	1.31611	1.19452
46840	1.89226	0.802383
46841	1.89226	0.797076
46842	1.89226	0.858855
46843	1.89226	0.773987
46844	3.78452	0.777322
46845	1.89226	0.844974
46846	1.89226	0.822044
46847	1.89226	0.818711
46848	1.89226	0.861221
46849	1.89226	0.830687
46850	1.89226	0.882131
46851	1.02645	0.663675
46852	0.810942	0.7225
46853	0.882781	0.745994
46854	0.882781	0.733194
46855	2.80386	0.687446
46856	1.56719	0.734766
46857	1.22499	0.670454
46858	1.08132	0.747377
46859	1.36865	0.721075
46860	0.810957	0.733104
46861	0.810957	0.714306

46862	0.946132	0.78072
46863	0.946132	0.79131
46864	0.946129	0.747313
46865	0.946129	0.755718
46866	0.946131	0.746268
46867	0.946131	0.754593
46868	0.946131	0.78547
46869	0.946131	0.771835
46870	0.94613	0.783795
46871	0.94613	0.77865
46872	0.946131	0.81679
46873	0.946131	0.769996
46874	1.89226	0.791024
46875	1.89226	0.764587
46876	1.89226	0.826619
46877	1.89226	0.75708
46878	3.78453	0.761048
46879	1.30462	0.7
46880	1.79906	0.7
46881	1.01299	0.7
46882	1.44185	0.7
46883	1.3499	0.7
46884	1.79906	0.7
46885	1.89226	0.7
46886	1.89226	0.700301
46887	1.89226	0.7
46888	1.89226	0.700194
46889	1.89226	0.7
46890	1.89226	0.701996
46891	1.89226	0.700105
46892	1.89226	0.700415
46893	1.89226	0.700229
46894	1.89226	0.700296
46895	1.89226	0.700346
46896	1.89226	0.700492
46897	1.3499	0.7
46898	1.79905	0.7
46899	1.29001	0.7
46900	1.36005	0.7
46901	1.88294	0.70027
46902	1.79905	0.7
46903	1.89226	0.7
46904	2.75374	0.685133
46905	2.60614	0.7
46906	1.89226	0.7
46907	2.60614	0.699047
46908	1.89226	0.7
46909	0.946131	0.7
46910	0.946131	0.7
46911	1.37687	0.7
46912	1.37687	0.7
46913	0.946131	0.7
46914	1.34375	0.7
46915	1.37686	0.7
46916	0.946128	0.7
46917	1.37687	0.7
46918	1.34375	0.7
46919	0.94613	0.7

46920	0.94613	0.7
46921	1.44247	2.1
46922	1.12665	2.1
46923	1.50044	2.1
46924	1.73651	2.1
46925	2.94291	2.1
46926	1.73652	2.1
46927	1.64926	2.1
46928	1.44247	2.1
46929	1.44247	2.1
46930	1.64926	2.1
46931	1.43872	2.1
46932	1.50044	2.1
46933	1.73651	2.1
46934	2.00955	2.1
46935	1.87875	2.1
46936	3.50999	2.1
46937	1.64926	2.1
46938	1.73652	2.1
46939	2.00955	2.1
46940	1.8493	2.1
46941	3.65881	2.1
46942	1.89226	0.279699
46943	2.28188	0.35477
46944	1.99182	0.28716
46945	2.28188	0.499865
46946	1.6022	0.197447
46947	1.89226	0.377514
46948	1.89226	0.433239
46949	1.89226	0.532409
46950	1.89226	0.462001
46951	1.89226	0.7
46952	1.89226	0.319206
46953	1.89226	0.526191
46954	1.89226	0.33826
46955	1.89226	0.40671
46956	2.19578	0.312899
46957	1.89226	0.633479
46958	2.81422	0.261579
46959	2.42521	0.437357
46960	1.89224	0.263955
46961	1.99179	0.332045
46962	1.454	0.296029
46963	1.99179	0.416794
46964	1.35444	0.211057
46965	1.89224	0.311355
46966	1.89224	0.380207
46967	1.89224	0.505545
46968	1.89224	0.450886
46969	1.89224	0.657539
46970	1.89224	0.318173
46971	1.89224	0.472899
46972	1.703	0.317705
46973	2.19575	0.3923
46974	1.9911	0.334873
46975	2.19575	0.537033
46976	1.67811	0.217115
46977	1.89223	0.381226

46978	1.69586	2.1
46979	2.55301	2.1
46980	1.60861	2.1
46981	1.60861	2.1
46982	2.65233	2.1
46983	2.65233	2.1
46984	0.946129	2.1
46985	0.946129	2.1
46986	1.2765	2.1
46987	1.32617	2.1
46988	1.2765	2.1
46989	1.2765	2.1
46990	0.946131	2.1
46991	0.946131	2.1
46992	0.946131	2.1
46993	1.32616	2.1
46994	0.946131	2.1
46995	1.2765	2.1
46996	1.36757	2.1
46997	1.32617	2.1
46998	0.891503	2.1
46999	0.847932	2.1
47000	1.10722	2.1
47001	1.10722	2.1
47002	1.10829	2.1
47003	1.10829	2.1
47004	1.36757	2.1
47005	0.891504	2.1
47006	0.847932	2.1
47007	1.32617	2.1
47008	0.946129	2.1
47009	0.946129	2.1
47010	1.36757	2.1
47011	1.32617	2.1
47012	1.32616	2.1
47013	1.32616	2.1
47014	0.946131	2.1
47015	0.946131	2.1
47016	1.36757	2.1
47017	0.946131	2.1
47018	0.946131	2.1
47019	1.32616	2.1
47020	1.89226	0.21378
47021	1.89226	0.153796
47022	1.89226	0.162568
47023	1.89226	0.238537
47024	3.78452	0.182377
47025	2.36336	0.114363
47026	1.89226	0.15062
47027	2.67831	0.157612
47028	2.67831	0.138705
47029	1.89226	0.144545
47030	1.89226	0.095602
47031	1.89227	0.157073
47032	1.89227	0.255025
47033	1.89227	0.243931
47034	1.89227	0.175071
47035	3.78453	0.201747

47036	1.89226	0.143323
47037	1.89226	0.238412
47038	1.89226	0.271739
47039	1.89226	0.192831
47040	3.78452	0.21073
47041	2.36336	0.111575
47042	2.0815	0.182637
47043	2.0815	0.15918
47044	2.41048	0.112013
47045	1.66267	0.170207
47046	2.13452	0.107195
47047	1.89226	0.243381
47048	1.89226	0.190134
47049	1.89226	0.224011
47050	1.89226	0.153085
47051	1.89226	0.267615
47052	1.89226	0.216955
47053	1.89226	0.70329
47054	1.89226	0.704703
47055	1.89226	0.703288
47056	1.89226	0.70747
47057	1.89226	0.700751
47058	1.89226	0.704675
47059	0.946128	0.704908
47060	0.946128	0.703608
47061	1.00122	0.698988
47062	1.00122	0.699329
47063	0.94613	0.707533
47064	1.01618	0.7
47065	0.946131	0.703373
47066	1.00122	0.69814
47067	1.00122	0.7
47068	1.01618	0.7
47069	0.946131	0.704965
47070	0.946131	0.702803
47071	1.89226	1.33151
47072	1.89226	1.05829
47073	1.89226	1.04754
47074	1.89226	1.45111
47075	3.78452	1.13817
47076	1.89226	1.21528
47077	1.89226	2.1
47078	1.89226	2.1
47079	1.89226	1.33092
47080	1.89226	1.94433
47081	1.89226	1.31784
47082	1.89226	0.979304
47083	1.89226	1.36418
47084	1.89226	1.25065
47085	1.89226	1.01864
47086	1.89226	1.25597
47087	1.89226	1.02663
47088	1.89226	1.02178
47089	1.89226	1.74648
47090	1.89226	1.55929
47091	1.89226	1.08975
47092	1.89226	1.44344
47093	1.89226	1.10254

47094	2.71279	0.920762
47095	1.4155	0.933266
47096	2.28488	0.884588
47097	1.06122	0.948811
47098	3.70038	0.904577
47099	1.89226	0.945385
47100	1.89226	0.940461
47101	1.89226	0.90452
47102	1.89226	0.938365
47103	3.78452	0.982176
47104	0.851542	0.945033
47105	0.851542	0.911876
47106	0.851484	0.913772
47107	0.865567	0.987712
47108	0.981616	0.950173
47109	0.864767	0.97853
47110	0.851542	0.929447
47111	0.851542	0.916472
47112	0.851477	0.952172
47113	0.851477	0.959375
47114	0.865635	0.937472
47115	0.816472	0.931112
47116	1.68443	1.45438
47117	1.44489	2.1
47118	1.54924	2.1
47119	1.34389	2.1
47120	3.02832	2.1
47121	1.44489	2.1
47122	1.03899	2.1
47123	1.03899	2.1
47124	1.12075	2.1
47125	1.34389	2.1
47126	1.42566	2.1
47127	1.54924	2.1
47128	1.34389	2.1
47129	1.44091	1.6958
47130	1.25866	2.1
47131	2.7848	2.1
47132	0.573141	2.1
47133	0.573141	2.1
47134	0.575681	2.1
47135	0.575681	2.1
47136	0.481985	2.1
47137	0.519492	2.1
47138	0.671948	2.1
47139	0.629331	2.1
47140	0.629329	2.1
47141	0.481986	2.1
47142	0.519494	2.1
47143	0.671947	2.1
47144	1.89226	0.762037
47145	1.89226	0.871357
47146	1.89226	0.851228
47147	2.31925	0.800504
47148	3.78452	0.857728
47149	1.30916	2.1
47150	1.17426	2.1
47151	0.946129	2.1

47152	0.946129	2.1
47153	0.94613	2.1
47154	0.94613	2.1
47155	1.17426	2.1
47156	1.17426	2.1
47157	1.30916	2.1
47158	0.94613	2.1
47159	1.17426	2.1
47160	0.94613	2.1
47161	2.56346	2.1
47162	1.89226	1.19582
47163	1.89226	1.95237
47164	1.89226	1.29971
47165	2.6074	1.92994
47166	2.6074	1.31142
47167	1.89226	2.1
47168	1.89226	2.1
47169	1.89226	2.1
47170	1.89226	1.92542
47171	3.78452	2.1
47172	1.33064	2.1
47173	1.84121	2.1
47174	0.946129	2.1
47175	0.946129	2.1
47176	0.946131	2.1
47177	0.946131	2.1
47178	1.30916	2.1
47179	1.8412	2.1
47180	1.33064	2.1
47181	0.94613	2.1
47182	1.30916	2.1
47183	0.946131	2.1
47184	1.19283	1.19928
47185	1.19283	1.13018
47186	0.946131	2.1
47187	0.946131	1.94649
47188	0.946132	2.00576
47189	0.946132	1.18665
47190	1.3037	2.1
47191	1.19283	1.15448
47192	1.19283	1.49611
47193	0.946131	1.49491
47194	0.946131	1.4561
47195	1.3037	1.55057
47196	1.89226	2.1
47197	1.89226	1.93895
47198	1.89226	2.1
47199	1.89226	2.1
47200	1.89226	2.1
47201	1.89226	2.1
47202	2.17708	1.38546
47203	1.70302	2.1
47204	2.28188	1.3008
47205	2.13621	2.1
47206	4.28253	2.1
47207	1.89226	1.12092
47208	1.89226	2.1
47209	1.89226	1.33803

47210	1.89226	2.1
47211	3.78452	2.1
47212	0.946132	2.1
47213	0.946132	2.1
47214	0.946132	2.1
47215	0.946132	2.1
47216	0.946131	2.1
47217	0.946131	2.1
47218	0.946132	2.1
47219	0.946132	2.1
47220	0.946132	2.1
47221	0.946132	2.1
47222	0.946132	2.1
47223	0.946132	2.1
47224	1.74933	2.1
47225	1.11833	2.1
47226	1.90074	2.1
47227	1.669 2.1	
47228	1.335 2.1	
47229	1.11833	2.1
47230	2.28188	1.20816
47231	2.31804	2.1
47232	2.42758	1.34029
47233	2.14895	2.1
47234	4.43083	2.1
47235	1.89226	1.22779
47236	1.89226	2.1
47237	1.89226	1.09405
47238	1.89226	2.1
47239	3.78452	1.74979
47240	1.89226	2.1
47241	1.89226	2.1
47242	1.89226	2.1
47243	1.89226	2.1
47244	1.89226	2.1
47245	1.89226	2.1
47246	0.864133	2.1
47247	0.864133	2.1
47248	0.967939	2.1
47249	0.793739	2.1
47250	0.650797	2.1
47251	0.757546	2.1
47252	1.08941	2.1
47253	1.10136	2.1
47254	1.10138	2.1
47255	0.796445	2.1
47256	0.866907	2.1
47257	0.650788	2.1
47258	2.63062	0.818528
47259	1.89226	0.798949
47260	1.89226	0.783834
47261	1.89226	0.902998
47262	2.46557	0.753641
47263	2.46557	0.93136
47264	1.16777	0.869416
47265	1.38859	2.1
47266	1.05042	1.22935
47267	1.28909	2.03987

47268	2.45686	1.8169
47269	1.89227	0.881893
47270	1.89227	0.929378
47271	1.89227	0.801736
47272	1.89227	2.1
47273	3.78453	0.878595
47274	2.15114	2.1
47275	1.89226	2.05822
47276	2.15005	2.1
47277	2.15005	2.1
47278	1.89226	1.16994
47279	1.89226	2.1
47280	1.89226	2.07804
47281	1.89226	1.17616
47282	1.89226	2.1
47283	1.89226	2.1
47284	1.89226	1.0498
47285	1.89226	1.99927
47286	0.973447	1.68347
47287	1.32143	2.01145
47288	1.37444	1.76442
47289	1.37444	1.89845
47290	0.949134	1.97952
47291	0.949134	2.1
47292	0.946131	1.0221
47293	0.946131	2.1
47294	1.07557	2.09965
47295	1.07557	2.1
47296	0.946131	1.63892
47297	0.946131	2.00189
47298	1.07557	2.1
47299	1.08579	2.1
47300	1.08579	2.1
47301	0.946131	2.1
47302	1.07557	2.1
47303	0.946131	2.1
47304	0.946131	0.940394
47305	0.946131	2.1
47306	0.946131	1.31231
47307	0.946131	1.84534
47308	0.94613	1.18103
47309	0.94613	1.31285
47310	0.946131	1.84023
47311	0.946131	2.09791
47312	0.946131	1.29657
47313	0.946131	1.26107
47314	0.946131	1.42161
47315	0.946131	1.55812
47316	0.606115	1.56149
47317	0.606115	1.84834
47318	0.616308	2.01849
47319	0.616308	2.1
47320	0.68722	1.17057
47321	0.735699	1.92035
47322	0.514789	1.50802
47323	0.486724	2.04753
47324	0.514788	1.2752
47325	0.735699	1.33658

47326	0.486723	1.90119
47327	0.687221	1.66977
47328	2.33761	0.797384
47329	2.51749	0.741567
47330	2.68092	0.74471
47331	2.58304	0.763931
47332	5.01853	0.743828
47333	2.50906	0.756317
47334	2.51749	0.752055
47335	2.68091	0.753235
47336	2.53176	0.720678
47337	5.18997	0.754244
47338	2.68092	0.745849
47339	2.58304	0.757769
47340	2.71793	0.757074
47341	2.67098	0.726346
47342	5.39885	0.756458
47343	2.68091	0.755107
47344	2.53175	0.721446
47345	2.36417	0.727563
47346	2.67098	0.731668
47347	5.04508	0.72638
47348	2.76309	2.1
47349	1.89226	2.1
47350	1.89226	2.1
47351	1.89226	2.1
47352	2.48573	2.1
47353	2.48573	2.1
47354	1.35879	2.1
47355	1.55161	0.917275
47356	1.10407	2.09181
47357	1.32259	0.897436
47358	2.68138	1.12983
47359	1.89226	2.1
47360	1.89226	0.907964
47361	1.89226	2.1
47362	1.89226	0.899493
47363	3.78452	1.04914
47364	1.19612	1.69371
47365	1.24694	2.1
47366	1.24844	0.888007
47367	1.29747	2.1
47368	2.49358	1.66055
47369	1.89226	1.84074
47370	1.89226	2.1
47371	1.89226	0.839743
47372	1.89226	2.1
47373	3.78453	1.30373
47374	1.89226	1.31693
47375	1.89226	2.1
47376	1.89226	2.1
47377	1.89226	2.1
47378	1.89226	1.41181
47379	1.89226	2.1
47380	0.587463	2.1
47381	0.587463	2.1
47382	0.581977	1.53967
47383	0.581977	2.1

47384	0.531709	2.1
47385	0.545972	2.1
47386	0.623469	2.03869
47387	0.648733	2.0301
47388	0.648733	2.1
47389	0.545971	2.1
47390	0.623469	2.1
47391	0.531709	2.1
47392	1.29417	0.944968
47393	1.29747	2.1
47394	1.27829	2.1
47395	1.24844	1.18687
47396	1.32732	1.55826
47397	1.29747	1.05403
47398	1.89226	0.934899
47399	1.89226	2.1
47400	1.89226	0.890767
47401	1.89226	1.1083
47402	1.89226	1.2118
47403	1.89226	2.1
47404	1.89226	2.1
47405	1.89226	2.1
47406	1.89226	1.06991
47407	1.89226	2.1
47408	1.89226	2.1
47409	1.89226	1.08421
47410	0.60346	2.1
47411	0.60346	1.68987
47412	0.60086	0.978853
47413	0.60086	2.1
47414	0.545971	2.1
47415	0.555587	2.1
47416	0.648733	1.42152
47417	0.663661	1.3533
47418	0.663661	2.1
47419	0.555587	2.06345
47420	0.648733	2.1
47421	0.545971	2.1
47422	1.34066	1.56002
47423	1.36601	2.1
47424	1.34955	0.92573
47425	1.37623	2.1
47426	2.71556	1.59118
47427	1.89226	1.2601
47428	1.89226	2.1
47429	1.89226	0.935209
47430	1.89226	1.17561
47431	3.78452	1.13397
47432	0.946132	2.1
47433	0.946132	1.40527
47434	0.946132	0.936805
47435	0.946132	2.1
47436	0.946132	2.1
47437	0.946132	2.1
47438	0.946131	1.15417
47439	0.946131	1.10143
47440	0.946132	2.1
47441	0.946132	2.1

47442	0.946132	2.1
47443	0.946132	2.1
47444	0.631351	0.995438
47445	0.631351	2.1
47446	0.632421	1.22008
47447	0.632421	2.1
47448	0.573604	2.1
47449	0.580765	2.1
47450	0.683006	1.00961
47451	0.688118	1.55767
47452	0.688118	2.1
47453	0.580765	2.1
47454	0.683007	2.1
47455	0.573604	2.1
47456	1.34955	1.6372
47457	1.37624	2.1
47458	1.35048	0.956362
47459	1.37999	2.1
47460	2.72954	1.44191
47461	0.946131	1.16635
47462	0.946131	1.21574
47463	0.946131	2.05414
47464	0.946131	2.1
47465	0.94613	2.1
47466	0.94613	1.27439
47467	0.946133	1.11353
47468	0.946133	1.35228
47469	0.946131	1.28976
47470	0.946131	1.07758
47471	0.946132	1.26718
47472	0.946132	1.19465
47473	1.89226	1.37224
47474	1.89226	1.41045
47475	1.89226	1.66776
47476	1.89226	2.1
47477	1.89226	2.1
47478	1.89226	2.1
47479	0.532189	1.01223
47480	0.531141	2.1
47481	0.497784	2.1
47482	0.502536	2.1
47483	1.37999	1.4632
47484	0.92433	2.1
47485	0.670081	2.1
47486	0.727155	2.1
47487	0.786438	1.10549
47488	0.589798	2.1
47489	0.497784	2.1
47490	1.89226	2.1
47491	2.72301	2.1
47492	2.4158	2.1
47493	2.4158	2.1
47494	1.89226	2.1
47495	1.89226	2.1
47496	1.89226	2.1
47497	1.89226	2.1
47498	1.89226	2.1
47499	1.89226	2.1

47500	1.89226	2.1
47501	1.89226	2.1
47502	1.36919	2.1
47503	1.12717	2.1
47504	1.38634	2.1
47505	1.56541	2.1
47506	2.75552	2.1
47507	1.89226	2.1
47508	1.89226	2.1
47509	1.89226	2.1
47510	1.89226	2.1
47511	1.89226	2.1
47512	1.89226	2.1
47513	0.907036	2.1
47514	0.782706	2.1
47515	0.838808	2.1
47516	0.693168	2.1
47517	0.794747	2.1
47518	0.794747	2.1
47519	0.805458	2.1
47520	0.805458	2.1
47521	0.907036	2.1
47522	0.838808	2.1
47523	0.693169	2.1
47524	0.782707	2.1
47525	0.94613	2.1
47526	0.94613	2.1
47527	1.188	2.1
47528	1.28421	2.1
47529	1.18801	2.1
47530	1.18801	2.1
47531	0.946129	2.1
47532	0.946129	2.1
47533	1.28421	2.1
47534	0.94613	2.1
47535	1.188	2.1
47536	0.94613	2.1
47537	1.89226	2.1
47538	1.89226	2.1
47539	1.89226	2.1
47540	1.89226	2.1
47541	1.89226	2.1
47542	1.89226	2.1
47543	1.16817	2.1
47544	1.06007	2.1
47545	1.19785	2.1
47546	1.2832	2.1
47547	2.36602	2.1
47548	0.960106	2.1
47549	2.0073	2.1
47550	1.51754	2.1
47551	2.56883	2.1
47552	3.52484	2.1
47553	1.06756	2.1
47554	1.57913	2.057
47555	1.87532	1.65521
47556	1.38666	2.1
47557	2.94287	2.1

47558	2.15622	1.18703
47559	1.38668	2.1
47560	1.87534	2.1
47561	1.87534	2.1
47562	1.70539	2.03933
47563	1.70539	2.1
47564	0.986095	2.1
47565	1.36226	2.1
47566	1.3331	2.1
47567	1.3331	2.1
47568	1.07156	2.1
47569	1.07156	2.1
47570	0.682081	1.23014
47571	0.68748	1.12592
47572	0.650003	0.888515
47573	0.645873	0.967607
47574	0.666344	0.901376
47575	0.666344	1.09931
47576	0.66161	1.41041
47577	0.66161	0.922559
47578	0.68748	1.02482
47579	0.645873	1.02317
47580	0.650004	0.99804
47581	0.682082	0.993628
47582	0.946132	1.12118
47583	0.946132	1.02967
47584	0.946132	0.907263
47585	0.946132	0.845621
47586	0.946132	0.852733
47587	0.946132	1.02113
47588	0.946133	0.932595
47589	0.946133	1.11808
47590	0.946132	0.965367
47591	0.946132	0.96849
47592	0.946133	0.960078
47593	0.946133	0.962106
47594	1.89227	0.988735
47595	2.79312	0.754368
47596	2.77591	0.867637
47597	2.79312	0.899332
47598	1.87505	0.800846
47599	1.89227	0.853506
47600	0.68748	1.25893
47601	0.698626	1.16017
47602	0.645873	0.918
47603	0.650148	0.975792
47604	0.670561	0.930379
47605	0.670561	1.14391
47606	0.667067	1.42644
47607	0.667067	0.928108
47608	0.698627	1.0612
47609	0.650148	1.06777
47610	0.68748	1.03669
47611	0.645873	1.0396
47612	0.946132	1.16604
47613	0.946132	1.04589
47614	0.946132	0.938895
47615	0.946132	0.886609

47616	0.946132	0.893022
47617	0.946132	1.04306
47618	0.946133	0.962925
47619	0.946133	1.16576
47620	0.946133	0.985091
47621	0.946133	0.989033
47622	0.946132	0.983237
47623	0.946132	0.988451
47624	0.946133	0.957317
47625	0.946133	0.898751
47626	1.38709	0.87018
47627	1.38795	0.787822
47628	0.946132	0.798016
47629	0.946132	0.884714
47630	1.38709	0.981526
47631	1.38709	0.812569
47632	1.38709	0.885303
47633	0.946133	0.884889
47634	0.946132	0.856804
47635	1.38795	0.845994
47636	1.70301	0.154969
47637	2.07659	0.132787
47638	1.89226	0.139647
47639	1.89226	0.18777
47640	1.76332	0.120282
47641	1.76331	0.158194
47642	1.89226	0.21163
47643	1.89226	0.164732
47644	1.89226	0.181731
47645	1.89226	0.256879
47646	1.89226	0.149716
47647	1.89226	0.202368
47648	1.89226	0.189577
47649	1.89226	0.22695
47650	1.89226	0.134326
47651	1.89226	0.160671
47652	3.78452	0.195921
47653	1.89226	0.13157
47654	2.12492	0.120343
47655	1.89226	0.133658
47656	1.89226	0.163145
47657	2.07659	0.110755
47658	2.07659	0.126675
47659	0.94613	0.103131
47660	0.94613	0.146388
47661	1.17498	0.113143
47662	1.32446	0.0926424
47663	1.32446	0.105446
47664	0.94613	0.108126
47665	0.946127	0.113654
47666	1.17498	0.109745
47667	1.32446	0.101678
47668	1.32446	0.0880654
47669	0.946128	0.121471
47670	0.946128	0.11917
47671	1.89226	2.1
47672	1.86472	2.1
47673	2.11049	2.1

47674	1.89226	2.1
47675	2.08295	2.1
47676	1.86472	2.1
47677	1.89226	2.1
47678	2.08295	2.1
47679	2.14023	2.1
47680	1.89226	2.1
47681	2.33091	2.1
47682	2.08295	2.1
47683	0.809215	2.1
47684	0.809215	2.1
47685	0.810915	2.1
47686	0.810915	2.1
47687	1.09581	2.1
47688	1.08271	2.1
47689	0.520996	2.1
47690	0.537426	2.1
47691	0.520997	2.1
47692	1.08271	2.1
47693	0.537426	2.1
47694	1.09581	2.1
47695	0.79674	2.1
47696	0.79674	2.1
47697	0.799279	2.1
47698	0.799279	2.1
47699	1.07502	2.1
47700	1.0827	2.1
47701	0.508206	2.1
47702	0.520996	2.1
47703	0.508206	2.1
47704	1.07502	2.1
47705	0.520996	2.1
47706	1.0827	2.1
47707	2.15714	2.1
47708	1.70302	2.1
47709	1.78732	2.1
47710	2.02219	2.1
47711	3.98971	2.1
47712	2.0927	2.1
47713	2.162 2.1	
47714	2.11563	2.1
47715	2.02219	2.1
47716	4.18419	2.1
47717	1.62483	2.1
47718	2.02219	2.1
47719	1.93617	2.1
47720	1.52008	2.1
47721	3.54227	2.1
47722	2.11563	2.1
47723	2.02219	2.1
47724	2.29218	2.1
47725	2.03975	2.1
47726	4.06194	2.1
47727	2.007 2.1	
47728	1.89226	2.1
47729	1.89226	2.1
47730	1.89226	2.1
47731	2.06776	2.1

47732	2.06776	2.1
47733	0.946129	2.1
47734	0.946129	2.1
47735	1.0035	2.1
47736	1.0035	2.1
47737	0.946131	2.1
47738	0.946131	2.1
47739	0.968797	2.1
47740	1.0035	2.1
47741	0.968771	2.1
47742	0.946105	2.1
47743	1.00353	2.1
47744	0.946157	2.1
47745	1.79103	1.86884
47746	2.53286	0.839679
47747	2.60781	1.93968
47748	2.219	0.933493
47749	2.17985	1.31582
47750	1.79103	0.774071
47751	2.219	1.4356
47752	2.66359	1.37993
47753	2.70084	2.04194
47754	2.38698	1.13933
47755	2.53285	1.03216
47756	2.219	0.870447
47757	1.33179	1.26064
47758	0.946128	0.943067
47759	0.946133	1.12709
47760	1.26643	1.72962
47761	1.3318	1.02248
47762	1.3318	1.71981
47763	0.946131	0.893271
47764	0.946131	1.25686
47765	0.946131	0.911182
47766	0.946131	0.846238
47767	1.33179	1.11584
47768	1.26643	1.8558
47769	2.28979	2.1
47770	2.47227	2.1
47771	2.43451	2.1
47772	2.22627	2.1
47773	4.69855	2.1
47774	2.40667	2.1
47775	2.47228	2.1
47776	2.43451	2.1
47777	2.38069	2.1
47778	4.85296	2.1
47779	2.43451	2.1
47780	2.15715	2.1
47781	2.15715	2.1
47782	2.22627	2.1
47783	2.39489	2.1
47784	2.46402	2.1
47785	2.43451	2.1
47786	2.34899	2.1
47787	2.38069	2.1
47788	2.38069	2.1
47789	2.3949	2.1

47790	2.3949	2.1
47791	2.85425	1.03297
47792	1.70302	0.756378
47793	1.70302	0.819499
47794	1.52254	0.805444
47795	2.34181	0.835711
47796	2.08499	0.860923
47797	1.89226	0.822879
47798	1.89226	0.738934
47799	1.89226	0.743549
47800	1.89226	0.729362
47801	1.89226	0.735105
47802	1.89226	0.779437
47803	2.34181	0.905306
47804	1.38287	0.770929
47805	1.38287	0.836568
47806	1.84132	0.77795
47807	1.96416	0.8124
47808	2.4907	0.825398
47809	0.931432	0.806378
47810	0.946135	0.706634
47811	0.946133	0.700717
47812	0.946133	0.709566
47813	0.931464	0.739507
47814	0.931464	0.806966
47815	1.15573	0.704162
47816	0.96378	0.816963
47817	0.931434	0.762427
47818	0.946131	0.71822
47819	0.946131	0.706466
47820	1.13808	0.704193
47821	0.946132	0.783555
47822	0.946132	0.730673
47823	0.946132	0.797211
47824	0.946132	0.733248
47825	0.946133	0.771027
47826	0.946133	0.712832
47827	0.946132	0.780148
47828	0.946132	0.787283
47829	0.946133	0.761598
47830	0.946133	0.795079
47831	0.946132	0.715406
47832	0.946132	0.71405
47833	1.68074	2.1
47834	2.29459	2.1
47835	2.23361	2.1
47836	1.57234	2.1
47837	3.86693	2.1
47838	2.61243	2.1
47839	2.29458	2.1
47840	2.2336	2.1
47841	2.58234	2.1
47842	4.87692	2.1
47843	0.73506	2.1
47844	0.786166	2.1
47845	1.08729	2.1
47846	1.11679	2.1
47847	0.93134	2.1

47848	0.93134	2.1
47849	0.942113	2.1
47850	0.942113	2.1
47851	0.735065	2.1
47852	1.08729	2.1
47853	1.11679	2.1
47854	0.78616	2.1
47855	1.08729	2.1
47856	1.11679	2.1
47857	1.27502	2.1
47858	1.29116	2.1
47859	1.19259	2.1
47860	1.19259	2.1
47861	1.19923	2.1
47862	1.19923	2.1
47863	1.27503	2.1
47864	1.08729	2.1
47865	1.11679	2.1
47866	1.29115	2.1
47867	0.974715	0.898204
47868	1.43779	0.806759
47869	1.93915	0.805295
47870	1.53908	0.838174
47871	2.91387	0.817428
47872	1.53908	0.822451
47873	0.99845	0.845127
47874	1.5176	0.890056
47875	1.97963	0.802116
47876	3.05668	0.848058
47877	1.93915	0.794319
47878	1.97963	0.8114
47879	1.53908	0.803781
47880	1.53908	0.774624
47881	2.3376	0.839133
47882	2.3376	0.799787
47883	1.89226	2.1
47884	2.59747	2.1
47885	1.91802	2.1
47886	1.89226	2.1
47887	2.62322	2.1
47888	2.59747	2.1
47889	2.59747	2.1
47890	1.87119	2.1
47891	1.84844	2.1
47892	1.82268	2.1
47893	2.62323	2.1
47894	2.59747	2.1
47895	0.94613	2.1
47896	0.94613	2.1
47897	1.32697	2.1
47898	1.32697	2.1
47899	1.32697	2.1
47900	1.31161	2.1
47901	0.946132	2.1
47902	0.946132	2.1
47903	0.94613	2.1
47904	1.32697	2.1
47905	0.946131	2.1

47906	1.31161	2.1
47907	2.62323	2.1
47908	1.91704	2.1
47909	1.90191	2.1
47910	1.87119	2.1
47911	2.65395	2.1
47912	2.62323	2.1
47913	1.89226	2.1
47914	1.89226	2.1
47915	2.15958	2.1
47916	2.15958	2.1
47917	2.46558	2.1
47918	2.19826	2.1
47919	2.46558	2.1
47920	1.89226	2.1
47921	1.89226	2.1
47922	1.89226	2.1
47923	2.61064	2.1
47924	2.61064	2.1
47925	1.81759	0.872328
47926	1.89526	0.89826
47927	2.32219	1.00633
47928	1.881	0.836055
47929	1.81759	0.949923
47930	1.3764	0.820559
47931	1.81759	0.87748
47932	2.57079	0.853215
47933	2.32219	1.0556
47934	2.32219	0.83457
47935	1.99875	0.933023
47936	1.99875	0.79195
47937	0.536182	0.76941
47938	0.579206	0.736233
47939	0.908796	0.871317
47940	0.999375	0.801906
47941	0.732091	0.81359
47942	0.732091	0.742278
47943	0.755911	0.765179
47944	0.755911	0.836733
47945	0.999376	0.78886
47946	0.579206	0.807043
47947	0.908796	0.809936
47948	0.536182	0.804075
47949	0.94613	0.790769
47950	0.94613	0.78497
47951	1.31112	0.827838
47952	1.18275	0.785962
47953	0.946126	0.805489
47954	0.946126	0.514138
47955	1.31112	0.807326
47956	1.31112	0.816223
47957	0.946128	0.781327
47958	1.31112	0.807313
47959	0.946128	0.77997
47960	1.18275	0.636951
47961	2.64617	0.935474
47962	1.27737	0.913414
47963	1.83076	0.960151

47964	1.27737	0.91433
47965	3.03764	0.942858
47966	2.3655	0.864916
47967	1.12991	0.0530191
47968	1.22375	0.05
47969	1.35931	0.051452
47970	1.04971	0.0599761
47971	1.0419	0.05149
47972	1.15237	0.0517337
47973	1.19593	0.0517849
47974	1.30306	0.0504636
47975	1.12937	0.05
47976	1.31934	0.05
47977	1.15203	0.058392
47978	1.15203	0.0522885
47979	2.65227	0.05
47980	1.46041	0.0578021
47981	2.22712	0.05
47982	2.04816	0.0543471
47983	2.22712	0.0513581
47984	2.04816	0.0576707
47985	1.89226	0.276997
47986	1.89226	0.867221
47987	1.89226	0.779668
47988	1.89226	0.77569
47989	3.78452	0.796489
47990	1.24653	0.832767
47991	1.66554	0.863224
47992	1.53538	0.727143
47993	1.53538	0.939406
47994	1.40915	0.768171
47995	1.40915	0.959161
47996	1.66555	0.866819
47997	1.75835	2.1
47998	1.53538	1.0653
47999	1.65313	1.72993
48000	3.31868	1.35454
48001	1.89226	0.868257
48002	1.89226	0.897043
48003	1.89226	0.785881
48004	1.89226	1.89467
48005	3.78453	0.857855
48006	1.89226	0.543702
48007	1.89226	0.782636
48008	1.89226	0.71852
48009	1.89226	0.928958
48010	3.78452	0.418382
48011	0.658778	1.66997
48012	0.658778	0.782994
48013	0.661956	0.774409
48014	0.661956	0.909719
48015	0.703822	0.903251
48016	0.767689	1.3412
48017	0.623266	0.708215
48018	0.553046	0.878814
48019	0.703804	0.895753
48020	0.55303	0.89542
48021	0.623282	0.817312

48022	0.767711	0.826567
48023	1.53538	1.05436
48024	1.65313	1.92617
48025	1.40765	0.907863
48026	1.55779	2.1
48027	3.06078	1.09305
48028	1.89227	0.777485
48029	1.89227	1.94068
48030	1.89227	0.924502
48031	1.89227	0.92141
48032	3.78453	0.957755
48033	1.10517	2.1
48034	2.16532	2.1
48035	1.94374	2.1
48036	1.94001	2.1
48037	1.37756	2.1
48038	1.37756	2.1
48039	1.7453	0.315906
48040	1.83284	0.409345
48041	2.28483	0.461926
48042	1.77804	0.32694
48043	1.7453	0.590029
48044	1.23851	0.368193
48045	1.7453	0.415508
48046	2.54752	0.333922
48047	2.28483	0.354874
48048	2.28483	0.336917
48049	2.0189	0.430418
48050	2.0189	0.419192
48051	1.12457	0.31218
48052	2.0189	0.233184
48053	1.7453	0.263205
48054	1.7453	0.264291
48055	1.40707	0.317837
48056	1.40707	0.327392
48057	2.41176	2.1
48058	2.59262	2.1
48059	2.34085	2.1
48060	2.55827	2.1
48061	4.97003	2.1
48062	2.59262	2.1
48063	2.61901	2.1
48064	2.55827	2.1
48065	2.64067	2.1
48066	5.17728	2.1
48067	2.34084	2.1
48068	2.55827	2.1
48069	2.24937	2.1
48070	2.49053	2.1
48071	4.80764	2.1
48072	2.55826	2.1
48073	2.64067	2.1
48074	2.49053	2.1
48075	2.58639	2.1
48076	5.14465	2.1
48077	2.27918	0.172824
48078	2.44132	0.240964
48079	1.89226	0.13829

48080	1.89226	0.171737
48081	4.33359	0.148713
48082	1.89226	0.160045
48083	2.44132	0.248614
48084	2.6771	0.213299
48085	2.6771	0.193456
48086	1.89226	0.234235
48087	1.89226	0.242354
48088	1.60329	0.13442
48089	1.38246	0.183823
48090	1.98182	0.127653
48091	2.2217	0.164491
48092	3.60417	0.169121
48093	2.05362	0.111832
48094	1.02663	0.144306
48095	1.43255	0.122296
48096	1.52072	0.123321
48097	1.70302	0.130741
48098	1.7685	0.138244
48099	0.946131	0.16506
48100	0.946131	0.143242
48101	0.946131	0.123815
48102	0.946131	0.195244
48103	0.946131	0.210529
48104	0.946131	0.206195
48105	0.946131	0.151433
48106	0.946131	0.146376
48107	0.946131	0.165087
48108	0.946131	0.179308
48109	0.946131	0.175434
48110	0.946131	0.184784
48111	1.89226	0.171145
48112	1.89226	0.283777
48113	1.89226	0.218624
48114	1.89226	0.199314
48115	1.89226	0.252266
48116	1.89226	0.26589
48117	1.92645	0.92805
48118	2.83045	0.93082
48119	1.92225	0.992408
48120	2.01873	0.873685
48121	2.51732	1.00705
48122	2.51732	0.881641
48123	2.51732	0.855119
48124	2.23857	0.834346
48125	1.92645	0.899099
48126	1.92645	0.809559
48127	2.23857	0.947899
48128	1.50865	0.80273
48129	2.02687	2.1
48130	2.39604	2.1
48131	2.29198	2.1
48132	2.73046	2.1
48133	4.68803	2.1
48134	1.21148	0.783114
48135	1.87957	0.854712
48136	2.03555	0.77679
48137	2.03555	0.79461

48138	1.09962	0.768686
48139	1.09962	0.853401
48140	1.92558	0.761544
48141	2.05274	0.994615
48142	2.2443	0.761639
48143	2.24429	0.933052
48144	1.70869	0.73061
48145	1.70868	1.02389
48146	0.818397	0.771424
48147	0.818397	0.89656
48148	0.827911	0.813756
48149	0.827908	0.897184
48150	1.00418	0.841418
48151	1.02054	0.817424
48152	0.692018	0.823175
48153	0.620899	0.813742
48154	0.65823	0.770992
48155	1.00418	0.772034
48156	0.620899	0.825535
48157	1.0262	0.776139
48158	1.92558	0.869427
48159	2.51818	0.986295
48160	1.94816	0.803197
48161	2.01985	0.950719
48162	1.85389	0.789035
48163	1.92558	0.959851
48164	0.903844	2.1
48165	0.903844	2.1
48166	0.946148	2.1
48167	0.946151	2.1
48168	0.946118	2.1
48169	0.903876	2.1
48170	0.946118	2.1
48171	1.10235	2.1
48172	0.946131	2.1
48173	0.946131	2.1
48174	1.10234	2.1
48175	0.903863	2.1
48176	1.63471	2.1
48177	1.08022	2.1
48178	1.56801	2.1
48179	1.03437	2.1
48180	2.66908	2.1
48181	1.56801	2.1
48182	0.97541	2.1
48183	0.97541	2.1
48184	1.03437	2.1
48185	1.49733	2.1
48186	1.55629	2.1
48187	1.89226	0.455257
48188	1.89226	0.695078
48189	1.89226	0.610595
48190	1.89226	0.673373
48191	3.78452	0.682107
48192	0.946131	0.556657
48193	1.16373	0.548219
48194	0.946131	0.619344
48195	0.734637	0.521201

48196	0.946132	0.567378
48197	0.946132	0.590051
48198	1.16373	0.467966
48199	0.734636	0.447437
48200	0.946093	0.565144
48201	0.946093	0.538029
48202	0.734675	0.508194
48203	0.734675	0.54595
48204	1.98413	0.489612
48205	1.87355	0.811778
48206	1.72774	0.280607
48207	1.67865	0.856187
48208	3.60128	0.482892
48209	1.87355	0.812972
48210	1.54924	2.06364
48211	1.68443	1.48748
48212	1.68443	0.820207
48213	1.67866	1.64129
48214	1.67866	0.829062
48215	1.72774	0.643736
48216	1.53422	0.843375
48217	1.67865	0.804875
48218	1.67865	0.58908
48219	1.53873	0.802783
48220	1.53873	0.677516
48221	1.67866	0.799159
48222	1.54924	2.1
48223	1.53423	0.845746
48224	1.44091	1.65189
48225	3.11957	0.925363
48226	1.89226	2.1
48227	1.89226	1.57685
48228	1.65573	1.84149
48229	1.65573	2.02735
48230	1.89226	1.92639
48231	1.89226	2.09257
48232	1.89226	2.09338
48233	1.89614	1.38441
48234	1.7508	1.57422
48235	1.72666	1.74165
48236	1.89226	1.69706
48237	1.89226	1.94756
48238	1.89223	1.94804
48239	1.89223	1.39493
48240	1.89223	1.39246
48241	1.89223	1.72612
48242	1.89223	1.48965
48243	1.89223	1.99008
48244	1.89224	1.8803
48245	1.89224	1.1639
48246	1.89224	1.39197
48247	1.89224	1.43172
48248	1.89224	1.51776
48249	1.89224	1.78442
48250	2.09773	2.1
48251	2.06623	2.1
48252	2.43268	2.1
48253	2.33713	2.1

48254	1.703	2.1
48255	1.61372	2.1
48256	0.646929	1.32502
48257	0.689776	2.1
48258	1.05897	2.1
48259	0.956364	2.1
48260	0.905594	2.1
48261	0.904315	1.41129
48262	0.727014	2.1
48263	0.850757	2.1
48264	0.798445	2.1
48265	0.654101	2.1
48266	0.717539	2.1
48267	1.0478	2.1
48268	1.89223	1.27017
48269	1.89223	1.07375
48270	1.89223	2.1
48271	1.6557	0.88875
48272	3.54794	1.11085
48273	0.946118	1.18693
48274	0.946118	2.1
48275	0.946118	2.1
48276	0.946118	2.1
48277	0.946117	2.1
48278	0.946117	1.05463
48279	0.946119	2.1
48280	0.946119	2.1
48281	0.946105	2.1
48282	0.946105	2.1
48283	0.946131	2.1
48284	0.946131	2.1
48285	1.47671	2.1
48286	1.70303	2.1
48287	1.90705	2.1
48288	1.98945	2.1
48289	1.3454	2.1
48290	1.40353	2.1
48291	1.89226	2.1
48292	1.89226	1.90175
48293	1.89226	2.1
48294	1.89226	1.10111
48295	1.89226	2.1
48296	1.89226	1.01674
48297	2.39438	0.409337
48298	2.69842	0.367043
48299	2.45177	0.536898
48300	2.28563	0.331947
48301	4.84615	0.425211
48302	1.28421	2.1
48303	1.35966	2.1
48304	0.961001	2.1
48305	0.860549	2.1
48306	1.1039	2.1
48307	1.1039	2.1
48308	1.11631	2.1
48309	1.11631	2.1
48310	1.35966	2.1
48311	0.961001	2.1

48312	0.860549	2.1
48313	1.28421	2.1
48314	1.89226	1.44635
48315	1.89226	1.91722
48316	1.89226	2.05476
48317	1.89226	1.31153
48318	3.78452	1.47227
48319	1.48702	1.22488
48320	1.40656	1.3211
48321	0.626442	1.6851
48322	0.626442	1.57761
48323	0.626442	1.85019
48324	0.946132	1.30424
48325	1.05489	1.08053
48326	1.05012	1.91363
48327	0.946131	1.52708
48328	0.946131	1.55895
48329	0.626442	1.57856
48330	1.05012	1.49267
48331	1.89226	1.91042
48332	1.97212	1.3889
48333	1.89226	1.31757
48334	1.89226	1.61887
48335	3.78453	1.67546
48336	1.89226	2.00589
48337	1.89226	1.27065
48338	1.89226	1.31415
48339	1.89226	1.72798
48340	3.78452	1.67927
48341	1.89226	1.21296
48342	1.89226	1.54555
48343	1.89226	1.74274
48344	1.89226	1.10501
48345	1.89226	1.75598
48346	1.89226	1.1252
48347	0.946161	1.06845
48348	0.946161	1.27495
48349	0.932156	1.5511
48350	0.932156	1.35884
48351	0.932184	1.55989
48352	0.946132	1.01602
48353	0.946133	1.54469
48354	0.946133	1.09876
48355	0.946132	1.20363
48356	0.946132	1.25221
48357	0.932185	1.24633
48358	0.946133	1.15588
48359	0.946132	1.23731
48360	0.946132	1.12889
48361	0.946132	2.05139
48362	0.946132	1.56215
48363	0.946132	1.50471
48364	0.946132	1.23401
48365	0.946133	1.27363
48366	0.946133	1.67304
48367	0.946132	1.44681
48368	0.946132	1.61206
48369	0.946133	1.23967

48370	0.946133	1.34031
48371	1.79397	0.730081
48372	1.00636	0.450256
48373	1.31658	0.780391
48374	2.02924	0.661806
48375	3.0356	0.693503
48376	1.89226	0.599311
48377	1.89226	0.2741
48378	1.89226	0.481239
48379	1.89226	0.367512
48380	1.89226	0.443648
48381	1.89226	0.34717
48382	0.946131	0.544807
48383	0.946131	0.652113
48384	1.24766	0.785215
48385	1.03691	0.65451
48386	0.946129	0.593855
48387	0.946129	0.445839
48388	1.24766	0.777879
48389	1.24766	0.749274
48390	0.946131	0.619709
48391	1.24766	0.731384
48392	1.03691	0.685839
48393	0.946131	0.556393
48394	1.44824	0.578395
48395	2.02924	0.435358
48396	1.7065	0.601625
48397	1.38604	0.736537
48398	2.32017	0.49959
48399	2.32017	0.578117
48400	1.89226	0.367653
48401	1.89226	0.278722
48402	1.89226	0.22361
48403	1.89226	0.155316
48404	1.89226	0.48042
48405	1.89226	0.355818
48406	0.478349	0.981609
48407	0.612092	1.95137
48408	0.606115	2.1
48409	0.506213	2.1
48410	0.550677	2.1
48411	0.550677	0.979914
48412	0.567628	2.1
48413	0.567628	2.1
48414	0.506213	2.0415
48415	0.478349	2.08575
48416	0.606116	1.98855
48417	0.612092	2.1
48418	1.52226	2.1
48419	1.54241	2.1
48420	1.23167	2.1
48421	1.23167	2.1
48422	1.75555	2.1
48423	1.75555	2.1
48424	1.75557	2.1
48425	1.54243	2.1
48426	1.9943	2.1
48427	1.85871	2.1

48428	3.53673	2.1
48429	1.85871	2.1
48430	1.06936	2.1
48431	1.88926	2.1
48432	1.54243	2.1
48433	1.41619	2.1
48434	1.06936	2.1
48435	1.28649	1.7147
48436	1.28516	2.1
48437	1.38986	0.938142
48438	1.42399	1.474
48439	2.67502	1.40712
48440	1.42399	1.043
48441	1.07283	2.1
48442	1.46528	2.1
48443	1.28517	1.19906
48444	1.25294	1.99861
48445	1.07283	1.03005
48446	1.38986	1.93784
48447	1.42399	2.1
48448	1.52885	1.04563
48449	1.60058	1.18703
48450	2.99043	1.40756
48451	0.715316	2.06264
48452	0.715316	1.53883
48453	0.719126	0.891133
48454	0.719126	2.1
48455	0.722447	1.95651
48456	0.626469	2.09144
48457	0.711995	1.09392
48458	0.800291	1.12681
48459	0.80029	1.33411
48460	0.722447	1.17255
48461	0.711995	1.49441
48462	0.62647	1.54332
48463	2.05632	0.0869933
48464	1.70303	0.10553
48465	2.3923	0.099188
48466	2.61277	0.0854848
48467	1.44836	0.111535
48468	1.58184	0.100036
48469	1.89226	0.116246
48470	1.89226	0.167921
48471	1.89226	0.137405
48472	1.89226	0.141718
48473	3.78453	0.143381
48474	1.44091	1.26487
48475	1.1822	2.1
48476	1.25866	2.1
48477	1.25866	2.1
48478	1.35215	2.1
48479	1.35215	2.1
48480	1.27338	1.57078
48481	1.1822	2.1
48482	1.27338	2.1
48483	1.35215	2.1
48484	1.10817	2.1
48485	1.18694	2.1

48486	0.946132	0.754213
48487	0.946132	0.742221
48488	0.946132	0.71588
48489	0.946132	0.705389
48490	0.946133	0.709942
48491	0.946133	0.744441
48492	0.946132	0.738385
48493	0.946132	0.697938
48494	0.946132	0.732439
48495	0.946132	0.722048
48496	0.946133	0.727937
48497	0.946133	0.734957
48498	2.6875	0.264972
48499	2.40558	0.216518
48500	2.1994	0.214772
48501	1.70303	0.297484
48502	4.68305	0.236443
48503	0.915523	0.205135
48504	0.915523	0.237852
48505	0.861057	0.155273
48506	0.946796	0.201074
48507	0.838652	0.21158
48508	0.654182	0.196356
48509	1.14044	0.147644
48510	0.80252	0.228797
48511	0.836435	0.206075
48512	0.608659	0.208776
48513	1.22198	0.194642
48514	0.863472	0.237961
48515	2.1994	0.105539
48516	0.981586	0.119855
48517	1.59619	0.117188
48518	1.70303	0.126744
48519	1.24816	0.111389
48520	1.38523	0.10777
48521	2.71141	2.1
48522	1.70302	2.1
48523	2.56527	2.1
48524	1.88649	2.1
48525	2.41279	2.1
48526	1.70302	2.1
48527	1.89226	1.73441
48528	1.89226	2.1
48529	1.89226	2.1
48530	1.89226	1.5656
48531	3.78453	2.1
48532	1.89226	1.84763
48533	1.89226	2.1
48534	1.89226	2.1
48535	1.89226	2.1
48536	1.89226	2.1
48537	1.89226	1.63695
48538	2.18867	2.1
48539	0.983723	2.1
48540	2.42263	2.1
48541	1.8014	2.1
48542	1.62992	2.1
48543	0.983723	2.1

48544	2.71141	2.1
48545	1.89226	2.1
48546	2.31157	2.1
48547	2.31157	2.1
48548	1.89226	2.1
48549	1.89226	2.1
48550	1.89227	2.09322
48551	1.89227	1.28587
48552	1.89227	1.28196
48553	1.89227	1.98146
48554	3.78453	1.43687
48555	1.89226	1.36651
48556	1.89226	2.1
48557	1.89226	2.1
48558	1.89226	1.37554
48559	1.89226	2.0561
48560	1.89226	1.35808
48561	1.89226	1.20297
48562	1.89226	1.04141
48563	1.89226	1.93185
48564	1.89226	0.891287
48565	1.89226	1.10968
48566	1.89226	0.779881
48567	1.1427	1.60837
48568	1.14956	2.09733
48569	1.70302	1.04056
48570	1.81267	1.762
48571	2.88983	1.69578
48572	1.89226	0.843618
48573	1.89226	1.71831
48574	1.89226	0.89721
48575	1.89226	0.986323
48576	3.78453	1.02705
48577	1.89226	1.14363
48578	1.89226	1.3755
48579	1.89226	0.780179
48580	1.89226	1.0926
48581	3.78452	0.951697
48582	0.884197	1.90195
48583	1.1125	1.11658
48584	1.13273	1.15771
48585	0.844557	1.4913
48586	0.981137	0.994925
48587	1.13971	1.63557
48588	0.980432	1.56126
48589	0.980432	1.02768
48590	1.15859	1.03691
48591	1.12206	0.85667
48592	0.898619	1.84408
48593	0.94426	1.23911
48594	2.21016	0.972822
48595	1.44091	2.06634
48596	1.98408	1.05128
48597	1.4218	0.943124
48598	2.21016	1.81295
48599	1.64788	1.85961
48600	1.07526	0.757889
48601	1.91531	2.1

48602	1.49155	2.0838
48603	1.58551	1.06476
48604	1.49155	0.908505
48605	1.58551	1.30725
48606	1.98979	0.0778816
48607	2.21773	0.0906961
48608	2.30533	0.0888152
48609	2.55895	0.0739369
48610	4.52305	0.0794852
48611	2.55896	0.075745
48612	2.27963	0.0935505
48613	2.60486	0.0893737
48614	2.60486	0.079938
48615	2.21773	0.0955525
48616	2.21773	0.0779645
48617	2.30533	0.0923555
48618	2.55895	0.0739065
48619	2.29539	0.0791227
48620	2.47308	0.0933883
48621	4.7784	0.0820112
48622	2.55896	0.0756488
48623	2.55792	0.0984061
48624	2.55792	0.0959962
48625	2.60486	0.0841384
48626	2.47308	0.091717
48627	2.52003	0.0786206
48628	0.946133	1.67458
48629	0.946133	1.81336
48630	1.20851	1.37701
48631	1.20851	1.30197
48632	0.967411	1.32785
48633	1.20851	1.16087
48634	0.946132	1.91299
48635	0.946132	1.56139
48636	0.946132	1.58641
48637	1.20851	1.32712
48638	0.967412	1.37454
48639	0.946133	1.60796
48640	1.89226	1.66172
48641	1.89226	1.21808
48642	1.89226	1.22871
48643	1.89226	1.23055
48644	1.89226	1.61314
48645	1.89226	1.76757
48646	1.89226	1.52048
48647	2.41701	1.60851
48648	2.71724	1.26693
48649	2.71724	1.52165
48650	1.89226	1.8176
48651	1.89226	2.05168
48652	0.946131	1.77619
48653	0.946131	2.01731
48654	0.946128	1.40242
48655	0.946128	1.48102
48656	0.94613	1.11955
48657	0.94613	1.26472
48658	0.94613	1.74694
48659	0.94613	1.91273

48660	0.94613	1.53846
48661	0.94613	1.195
48662	0.94613	1.37147
48663	0.94613	1.96359
48664	0.851541	0.643785
48665	0.851541	0.700939
48666	0.851481	0.700655
48667	0.997219	0.702295
48668	0.997112	0.585192
48669	0.992853	0.576845
48670	0.85154	0.699999
48671	0.85154	0.700684
48672	0.894824	0.575251
48673	0.894824	0.7
48674	0.94613	0.702348
48675	0.906485	0.621151
48676	0.802947	2.1
48677	0.845556	2.1
48678	0.582339	2.1
48679	0.519756	2.1
48680	0.714508	2.1
48681	0.714508	2.1
48682	0.628932	2.1
48683	0.708518	2.1
48684	0.801358	2.1
48685	0.476016	2.1
48686	0.569477	2.1
48687	0.878553	2.1
48688	1.89226	2.1
48689	1.89226	2.1
48690	1.89226	2.1
48691	1.89226	2.1
48692	1.89226	2.1
48693	1.89226	2.1
48694	0.946128	2.1
48695	0.946128	2.1
48696	1.01537	2.1
48697	0.9662	2.1
48698	0.946131	2.1
48699	0.946131	2.1
48700	0.966198	2.1
48701	0.966198	2.1
48702	0.94613	2.1
48703	0.966198	2.1
48704	0.946131	2.1
48705	1.01537	2.1
48706	1.89226	2.1
48707	1.89226	2.1
48708	1.91883	2.1
48709	1.91883	2.1
48710	2.15905	2.1
48711	2.13249	2.1
48712	1.07953	2.1
48713	1.2107	2.1
48714	0.946132	2.1
48715	0.946132	2.1
48716	1.2107	2.1
48717	1.2107	2.1

48718	0.946132	2.1
48719	0.946132	2.1
48720	1.2107	2.1
48721	0.946133	2.1
48722	0.946132	2.1
48723	1.07953	2.1
48724	2.46752	2.1
48725	2.53733	2.1
48726	2.26044	2.1
48727	2.33222	1.15661
48728	4.79974	2.1
48729	2.53733	2.1
48730	2.37576	0.832411
48731	2.33222	1.49875
48732	2.1845	2.1
48733	4.70798	1.72348
48734	2.48652	2.1
48735	2.33224	1.08059
48736	2.08149	2.1
48737	1.9839	2.1
48738	4.45623	2.1
48739	2.33223	1.13045
48740	2.18452	1.19707
48741	2.16061	2.1
48742	2.01399	0.798892
48743	4.34623	1.02983
48744	1.4617	1.18792
48745	1.34701	2.08522
48746	1.34701	1.19189
48747	1.25173	2.1
48748	1.5117	1.17282
48749	1.41642	2.1
48750	2.65164	2.1
48751	1.89227	1.39232
48752	1.89227	2.1
48753	1.89227	2.1
48754	2.42504	2.0046
48755	2.42504	2.1
48756	1.89226	1.11621
48757	1.89226	1.34261
48758	1.89226	2.09183
48759	1.89226	1.03704
48760	3.78452	1.18026
48761	1.5117	1.21629
48762	1.34701	1.66372
48763	1.47001	2.1
48764	1.57961	1.1131
48765	2.92663	1.34266
48766	1.89226	1.67295
48767	1.89226	1.019
48768	1.89226	1.10793
48769	1.89226	1.26829
48770	3.78452	1.21609
48771	0.412773	0.0879279
48772	0.570839	0.107119
48773	0.50721	0.0939473
48774	0.513638	0.128064
48775	0.518805	0.0875337

48776	0.41278	0.0900058
48777	1.5344	0.12372
48778	0.939282	0.11118
48779	0.720051	0.0919005
48780	0.691767	0.112491
48781	0.761152	0.115974
48782	1.89226	1.9481
48783	1.89226	2.1
48784	1.89226	2.1
48785	1.89226	2.1
48786	1.89226	2.08136
48787	1.89226	2.1
48788	1.24618	2.1
48789	2.10585	2.1
48790	1.08134	2.1
48791	1.93032	2.1
48792	3.1765	2.1
48793	1.98474	2.1
48794	2.57777	2.1
48795	1.93031	2.1
48796	2.19041	2.1
48797	3.99251	2.1
48798	1.89226	2.1
48799	1.89226	2.1
48800	1.89226	2.1
48801	1.89226	2.1
48802	3.78452	2.1
48803	1.67374	2.1
48804	2.19042	2.1
48805	1.67374	2.1
48806	2.09769	2.1
48807	2.29586	2.1
48808	2.7198	2.1
48809	1.89226	2.1
48810	1.89226	2.1
48811	1.89226	2.1
48812	1.89226	2.1
48813	1.89226	2.1
48814	1.89226	2.1
48815	0.946132	0.772661
48816	0.946132	0.743051
48817	0.947763	0.754576
48818	0.909304	0.769927
48819	0.946127	0.735884
48820	0.947831	0.723707
48821	0.907673	0.725366
48822	0.907673	0.763011
48823	0.94613	0.727982
48824	0.947834	0.72521
48825	0.907671	0.794658
48826	0.94613	0.739515
48827	0.906519	0.710512
48828	0.906519	0.703801
48829	0.946097	0.710367
48830	0.946097	0.715763
48831	0.947834	0.709669
48832	0.906485	0.703989
48833	0.946131	0.704068

48834	0.946131	0.713274
48835	0.94613	0.710201
48836	0.906485	0.701578
48837	0.946131	0.715777
48838	0.947835	0.714918
48839	2.58638	2.1
48840	2.51039	2.1
48841	2.47395	2.1
48842	2.41539	2.1
48843	5.00178	2.1
48844	2.51039	2.1
48845	2.16633	2.1
48846	2.4154	2.1
48847	2.13977	2.1
48848	4.58173	2.1
48849	2.47395	2.1
48850	2.41539	2.1
48851	2.2988	2.1
48852	2.2604	2.1
48853	4.7142	2.1
48854	2.4154	2.1
48855	2.13977	2.1
48856	2.26041	2.1
48857	1.98957	2.1
48858	4.40497	2.1
48859	2.15721	0.839419
48860	2.31843	0.74789
48861	1.93973	0.891048
48862	2.13243	0.207874
48863	4.25816	0.765909
48864	2.31844	0.737591
48865	2.30908	0.684728
48866	2.13243	0.2977
48867	2.1609	0.616366
48868	4.47934	0.617788
48869	1.93973	0.883344
48870	2.13243	0.680818
48871	1.69661	0.878845
48872	1.9339	0.486942
48873	3.87363	0.78361
48874	2.13243	0.652634
48875	2.1609	0.701729
48876	1.9339	0.638737
48877	1.98414	0.536305
48878	4.0948	0.589348
48879	1.88857	0.05
48880	2.34107	0.05
48881	0.502431	0.05
48882	0.71416	0.05
48883	0.50243	0.05
48884	0.944284	0.05
48885	1.88856	2.1
48886	2.34107	2.1
48887	2.39662	2.1
48888	1.92293	2.1
48889	4.28519	2.1
48890	1.00486	2.1
48891	1.92293	2.1

48892	1.88857	2.1
48893	1.88857	2.1
48894	1.02605	2.1
48895	1.02605	2.1
48896	2.53728	2.1
48897	1.70303	2.1
48898	2.03734	2.1
48899	1.3624	2.1
48900	2.54706	2.1
48901	1.70303	2.1
48902	2.54706	2.1
48903	1.50876	2.1
48904	2.74067	2.1
48905	1.85391	2.1
48906	2.29667	2.1
48907	1.50876	2.1
48908	0.946133	2.1
48909	0.946133	2.1
48910	1.24191	2.1
48911	1.24191	2.1
48912	0.946132	2.1
48913	1.24191	2.1
48914	1.23779	2.1
48915	0.946132	2.1
48916	0.946132	2.1
48917	0.946132	2.1
48918	1.23779	2.1
48919	1.24191	2.1
48920	0.946131	2.1
48921	0.946131	2.1
48922	1.14833	2.1
48923	1.14833	2.1
48924	1.14834	2.1
48925	0.946132	2.1
48926	0.94613	2.1
48927	1.24191	2.1
48928	0.946131	2.1
48929	0.946131	2.1
48930	1.24191	2.1
48931	1.14833	2.1
48932	2.08149	2.1
48933	2.31116	2.1
48934	1.9701	1.45758
48935	2.18328	2.1
48936	4.49563	2.1
48937	2.08149	2.1
48938	2.14059	2.1
48939	1.72377	2.1
48940	2.18328	2.1
48941	3.88931	2.1
48942	2.14059	2.1
48943	2.40755	2.1
48944	2.31145	2.1
48945	2.23743	2.1
48946	2.31145	2.1
48947	2.23743	2.1
48948	2.31117	2.1
48949	2.40754	2.1

48950	2.41055	2.1
48951	2.31145	2.1
48952	2.41055	2.1
48953	2.31145	2.1
48954	2.5615	0.763598
48955	2.37625	0.755097
48956	2.20248	0.74897
48957	2.20248	0.756695
48958	2.65967	0.764571
48959	2.65967	0.777935
48960	2.71793	0.746217
48961	2.65966	0.740449
48962	2.56149	0.730783
48963	2.56149	0.739883
48964	2.71296	0.743914
48965	2.71296	0.75988
48966	1.31528	0.733373
48967	1.32983	0.726869
48968	1.21128	0.742974
48969	1.18812	0.735706
48970	1.24232	0.732239
48971	1.24232	0.737339
48972	1.26109	0.725649
48973	1.26109	0.749861
48974	1.21128	0.735132
48975	1.31528	0.726824
48976	1.18812	0.738289
48977	1.32983	0.752278
48978	2.63056	0.724679
48979	2.58032	0.717513
48980	2.65966	0.713381
48981	2.65966	0.728266
48982	2.58032	0.737694
48983	2.71296	0.742707
48984	1.92039	2.1
48985	1.06226	2.1
48986	1.09252	2.1
48987	1.96052	2.1
48988	3.01291	2.1
48989	1.92039	2.1
48990	2.46338	2.1
48991	2.47522	2.1
48992	2.43508	2.1
48993	1.96052	2.1
48994	1.92039	2.1
48995	0.985949	2.1
48996	0.980261	2.1
48997	0.553625	2.1
48998	0.546262	2.1
48999	0.765671	2.1
49000	0.765671	2.1
49001	0.766539	2.1
49002	0.766539	2.1
49003	0.985949	2.1
49004	0.553625	2.1
49005	0.546261	2.1
49006	0.980261	2.1
49007	1.96052	2.1

49008	2.46338	2.1
49009	1.97189	2.1
49010	1.96052	2.1
49011	2.47932	2.1
49012	2.47932	2.1
49013	0.964608	0.05
49014	0.691524	0.05
49015	1.25462	0.05
49016	0.691524	0.05
49017	1.89226	0.05
49018	1.89226	0.05
49019	1.44894	0.05
49020	2.77655	0.05
49021	1.4237	0.05
49022	2.77701	0.05
49023	2.77701	0.05
49024	1.38847	2.1
49025	1.46031	1.77716
49026	1.65504	1.19908
49027	1.75396	1.21965
49028	3.11535	1.39864
49029	1.89226	1.50678
49030	1.89226	1.90796
49031	1.89226	1.26096
49032	1.89226	1.03354
49033	3.78452	1.56689
49034	1.89226	1.37017
49035	1.89226	1.93723
49036	1.89226	0.916955
49037	1.89226	1.54426
49038	3.78452	1.03793
49039	1.46031	1.22974
49040	1.34851	1.97324
49041	1.75395	1.07514
49042	1.62287	1.24255
49043	3.08318	1.69834
49044	1.65504	1.45147
49045	1.75396	1.77883
49046	1.89249	1.59167
49047	1.9857	1.13574
49048	3.64073	1.69853
49049	1.89226	1.61563
49050	1.89226	1.50269
49051	1.89226	1.02421
49052	1.89226	1.20872
49053	3.78452	1.11583
49054	1.89226	1.12274
49055	1.89226	1.98797
49056	1.89226	1.10958
49057	1.89226	1.33959
49058	3.78452	1.79803
49059	1.75395	1.47581
49060	1.62287	1.67138
49061	1.98569	1.02549
49062	1.86067	1.22354
49063	3.60856	1.15553
49064	1.35697	1.85217
49065	2.02064	1.69155

49066	1.89989	1.04533
49067	1.49571	2.1
49068	1.66753	1.15021
49069	1.36431	2.1
49070	0.744004	2.1
49071	0.743997	2.00874
49072	0.717972	1.05425
49073	0.717972	2.02721
49074	0.997982	2.1
49075	0.807796	2.07221
49076	0.632235	1.10406
49077	0.488575	2.1
49078	0.849275	2.1
49079	0.488581	2.1
49080	0.724729	2.1
49081	0.997967	2.1
49082	2.5276	1.42
49083	1.70791	1.39547
49084	1.82872	0.996482
49085	1.56362	2.1
49086	2.5276	1.13556
49087	2.24781	2.1
49088	1.37591	2.1
49089	1.23217	1.25645
49090	1.00442	1.54479
49091	0.945282	1.02054
49092	1.15446	1.14262
49093	1.09123	2.1
49094	1.21506	1.33104
49095	1.21506	1.11175
49096	1.39452	1.22117
49097	1.13108	1.52866
49098	1.27876	1.15116
49099	0.929006	1.08313
49100	1.46505	0.42327
49101	1.91484	0.436106
49102	1.49906	0.505123
49103	1.49906	0.564098
49104	1.90196	0.301319
49105	1.90196	0.34669
49106	1.90196	0.4146
49107	2.11465	0.501235
49108	1.91484	0.53461
49109	1.91484	0.621177
49110	2.04016	0.33583
49111	2.04016	0.412467
49112	0.912201	0.300224
49113	0.950978	0.332387
49114	0.665307	0.24941
49115	0.732528	0.348451
49116	0.829512	0.192982
49117	0.829512	0.301908
49118	0.815217	0.271563
49119	0.815217	0.281588
49120	0.912202	0.244142
49121	0.665305	0.260738
49122	0.732527	0.448387
49123	0.95098	0.287151

49124	1.90196	0.381766
49125	2.08757	0.280642
49126	1.9626	0.30288
49127	2.04016	0.291224
49128	1.82441	0.299415
49129	1.90196	0.311254
49130	1.13048	2.1
49131	0.821162	2.1
49132	0.946131	2.1
49133	0.946131	2.1
49134	1.04567	2.1
49135	0.882347	2.1
49136	0.897276	2.1
49137	0.897276	2.1
49138	0.863109	2.1
49139	1.01055	2.1
49140	0.940305	2.1
49141	0.942166	2.1
49142	2.23933	0.10173
49143	0.976217	0.108129
49144	2.78053	0.122334
49145	2.78053	0.105173
49146	0.976217	0.14941
49147	1.65984	0.112872
49148	1.54168	2.1
49149	2.01197	1.50349
49150	1.69136	2.1
49151	2.13284	0.988734
49152	3.67452	1.61411
49153	0.988805	2.1
49154	1.78069	1.70684
49155	1.69136	1.87768
49156	1.69136	2.1
49157	1.05481	1.74608
49158	1.05481	2.1
49159	1.69136	2.1
49160	2.13284	1.20449
49161	1.78069	1.98796
49162	2.22044	1.17645
49163	3.91353	1.85069
49164	2.69379	0.970303
49165	2.32219	1.0184
49166	2.25746	1.4226
49167	2.25746	0.936126
49168	2.32219	1.04941
49169	1.89526	0.918997
49170	1.4556	2.1
49171	1.85028	2.1
49172	2.4909	0.93918
49173	2.03799	2.1
49174	2.73495	1.13932
49175	1.85028	1.63797
49176	2.71938	0.948934
49177	1.85028	2.1
49178	3.36051	2.08351
49179	2.73495	1.47646
49180	2.47584	1.59471
49181	1.85028	1.08692

49182	2.47584	1.34292
49183	1.04413	2.1
49184	2.1461	2.1
49185	1.74586	2.1
49186	1.44437	2.1
49187	1.04413	2.1
49188	1.89226	2.1
49189	1.89226	1.14181
49190	1.89226	1.29332
49191	1.89226	1.22499
49192	3.78452	1.35587
49193	1.52724	2.1
49194	1.89249	2.1
49195	1.68143	2.1
49196	2.02433	1.18405
49197	3.55157	2.1
49198	1.07444	2.1
49199	1.76545	2.1
49200	1.68142	2.1
49201	1.68142	2.1
49202	1.14807	2.1
49203	1.14807	2.1
49204	1.89226	2.1
49205	1.89226	2.1
49206	1.89226	2.1
49207	1.89226	1.32222
49208	1.89226	2.1
49209	1.89226	1.08509
49210	1.89226	2.1
49211	1.89226	1.34822
49212	1.89226	2.1
49213	1.89226	0.984922
49214	3.78452	1.29674
49215	1.68143	2.1
49216	2.02433	1.30457
49217	1.76545	2.1
49218	2.12608	2.1
49219	3.78978	2.1
49220	2.62871	2.1
49221	1.49149	2.1
49222	1.36814	2.1
49223	2.44761	1.48669
49224	3.9391	2.1
49225	1.89226	2.1
49226	1.89226	0.966928
49227	1.89226	1.59133
49228	1.89226	2.07974
49229	3.78452	1.66522
49230	0.851542	2.1
49231	0.851542	1.89708
49232	1.00897	2.1
49233	0.931224	2.1
49234	0.838952	2.1
49235	0.838952	1.61381
49236	0.956848	2.1
49237	0.942474	2.1
49238	0.946132	2.1
49239	0.838952	2.1

49240	0.878743	2.1
49241	0.946132	2.1
49242	1.15541	2.1
49243	2.44761	1.06865
49244	1.36814	2.1
49245	1.36814	1.88261
49246	2.19439	2.1
49247	2.19439	2.1
49248	1.89226	1.07255
49249	1.89226	1.92593
49250	1.89226	2.1
49251	1.89226	1.01821
49252	3.78452	1.54735
49253	1.89226	2.1
49254	1.67791	2.1
49255	1.5713	2.1
49256	1.67791	2.1
49257	1.78566	2.1
49258	1.89226	2.0994
49259	1.89226	0.845072
49260	1.62911	1.80536
49261	2.24602	1.54964
49262	1.89226	1.57141
49263	1.98287	1.00921
49264	1.62911	0.99216
49265	1.89226	1.46048
49266	1.89226	1.5344
49267	1.89226	0.980956
49268	1.89226	2.1
49269	3.78452	1.20516
49270	1.26688	2.1
49271	1.11804	2.1
49272	0.852467	1.13819
49273	0.693314	2.1
49274	0.977459	1.97538
49275	0.977459	2.1
49276	0.982741	1.10671
49277	0.982741	2.1
49278	1.26689	2.1
49279	0.852464	2.1
49280	0.693312	2.1
49281	1.11804	2.1
49282	0.946131	2.1
49283	0.946131	0.946066
49284	1.1794	0.937706
49285	1.1794	1.91614
49286	0.991434	2.01767
49287	1.1794	1.0144
49288	0.946131	0.895719
49289	0.946131	0.916667
49290	0.946131	1.51929
49291	1.1794	1.26151
49292	0.946131	1.34122
49293	0.991434	1.11073
49294	1.89226	0.937313
49295	1.89226	2.1
49296	1.89226	1.03735
49297	1.89226	1.18685

49298	3.78452	1.21492
49299	2.53377	2.1
49300	1.53443	2.08943
49301	2.0815	1.0501
49302	2.82979	2.1
49303	4.35462	2.1
49304	1.96192	0.911442
49305	1.23602	1.86881
49306	2.16506	0.941262
49307	1.56841	1.28759
49308	3.40108	1.11875
49309	1.89226	1.19813
49310	1.89226	1.83338
49311	1.89226	0.958218
49312	1.89226	2.1
49313	3.78452	1.18679
49314	0.946129	2.1
49315	0.946129	2.1
49316	1.13067	1.09721
49317	1.12952	2.1
49318	0.946129	2.1
49319	1.13089	2.1
49320	0.944978	1.08909
49321	0.944978	2.1
49322	0.946131	2.1
49323	1.13089	2.1
49324	0.944977	2.1
49325	0.946131	2.1
49326	0.710208	2.1
49327	0.710208	2.1
49328	0.718894	2.1
49329	0.718894	2.1
49330	0.559773	2.1
49331	0.579321	2.1
49332	0.886767	2.1
49333	0.849781	2.1
49334	0.849781	2.1
49335	0.559774	2.1
49336	0.579321	2.1
49337	0.886766	2.1
49338	1.63471	2.1
49339	1.69956	2.1
49340	1.08022	2.1
49341	1.08022	2.1
49342	1.11955	2.1
49343	1.67403	2.1
49344	1.03766	2.1
49345	2.02825	2.1
49346	2.02794	2.1
49347	2.0348	2.1
49348	1.07196	2.1
49349	1.07196	2.1
49350	1.9276	2.1
49351	2.1004	2.1
49352	2.1306	2.1
49353	2.1306	2.09106
49354	1.92178	2.1
49355	1.92032	1.69

49356	0.986724	2.1
49357	2.03064	2.1
49358	2.01385	2.1
49359	2.02404	2.1
49360	1.03766	2.1
49361	1.03766	2.1
49362	1.92027	2.1
49363	2.1306	2.1
49364	2.15388	2.1
49365	2.15388	2.1
49366	1.92614	2.1
49367	1.9276	2.1
49368	1.09578	2.1
49369	0.963018	2.1
49370	0.782956	1.17434
49371	0.529693	2.1
49372	0.6994	1.18481
49373	0.862532	2.1
49374	0.898605	2.1
49375	0.898605	1.26102
49376	0.776756	1.97056
49377	1.14805	2.02434
49378	0.966521	1.99771
49379	0.617676	1.41119
49380	2.67185	1.77247
49381	2.099 2.1	
49382	2.80548	1.62489
49383	2.2727	2.1
49384	4.94455	2.1
49385	1.89226	1.76889
49386	1.89226	2.1
49387	1.89226	1.91073
49388	1.89226	2.1
49389	3.78453	2.1
49390	1.89226	2.1
49391	1.89226	2.1
49392	1.89226	2.1
49393	1.89226	2.1
49394	1.89226	2.1
49395	1.89226	2.1
49396	0.884801	2.1
49397	0.884801	2.1
49398	0.895199	2.1
49399	0.895199	2.1
49400	0.622774	2.1
49401	0.730501	2.1
49402	1.0495	2.1
49403	1.13635	2.1
49404	1.13635	2.1
49405	0.730503	2.1
49406	1.0495	2.1
49407	0.622775	2.1
49408	1.89226	1.23746
49409	1.89226	2.1
49410	1.89226	2.07695
49411	1.89226	2.1
49412	1.89226	1.64324
49413	1.89226	2.1

49414	1.89226	2.1
49415	1.89226	2.1
49416	1.89226	2.1
49417	1.89226	2.1
49418	1.89226	2.1
49419	1.89226	2.1
49420	2.2727	2.1
49421	1.89586	2.1
49422	1.66913	2.1
49423	1.46101	2.1
49424	2.48082	2.1
49425	2.2727	2.1
49426	1.89226	1.75233
49427	1.89226	2.1
49428	1.84641	2.1
49429	1.89226	2.04594
49430	1.30868	2.1
49431	1.34118	1.25626
49432	1.89226	1.04452
49433	1.89226	1.15584
49434	1.89226	1.22104
49435	1.89226	1.06397
49436	1.89226	1.10375
49437	1.89226	0.975365
49438	1.89226	1.17387
49439	1.89226	0.912307
49440	1.89226	1.02675
49441	1.89226	1.16219
49442	3.78452	1.07588
49443	0.946132	1.60949
49444	0.946132	1.31325
49445	0.946131	2.1
49446	0.946131	2.1
49447	0.946132	2.1
49448	0.946132	1.16773
49449	0.946132	2.1
49450	0.946132	1.73414
49451	0.946132	1.99519
49452	0.946132	2.0028
49453	0.946132	2.1
49454	0.946132	2.0707
49455	1.89227	1.29045
49456	1.84641	1.38055
49457	2.29517	2.01036
49458	1.84641	1.25671
49459	2.34102	1.3433
49460	1.89227	1.11281
49461	1.89226	0.963925
49462	1.89226	1.00937
49463	1.89226	1.08363
49464	1.89226	0.9623
49465	1.89226	0.991277
49466	1.89226	0.888399
49467	1.89226	0.930252
49468	1.89226	1.02579
49469	1.89226	1.06173
49470	1.89226	0.953087
49471	1.89226	0.984476

49472	1.89226	0.889921
49473	1.89226	1.48904
49474	1.89226	1.18934
49475	1.89226	1.85949
49476	1.89226	1.13237
49477	1.89226	1.30767
49478	1.89226	1.12594
49479	0.851544	0.130275
49480	0.757542	0.20899
49481	1.13421	0.128584
49482	1.04072	0.147537
49483	0.946132	0.121608
49484	0.946132	0.13122
49485	0.841686	0.146187
49486	0.834428	0.130186
49487	1.13649	0.128762
49488	0.937687	0.1216
49489	0.841713	0.153532
49490	0.841713	0.166354
49491	1.89226	0.785571
49492	1.89226	0.808288
49493	1.89226	0.815027
49494	1.89226	0.754461
49495	1.89226	0.816828
49496	1.89226	0.76875
49497	2.31988	1.31772
49498	2.00082	1.89758
49499	2.4594	0.941386
49500	2.17638	1.79882
49501	4.46022	1.14368
49502	2.00082	1.08784
49503	1.41829	2.07673
49504	1.61207	1.3172
49505	1.43652	2.1
49506	2.17637	1.03455
49507	2.00082	2.1
49508	2.4594	1.00606
49509	2.17638	2.1
49510	2.57957	0.964978
49511	2.3506	1.62818
49512	4.81	1.2962
49513	2.3506	1.09307
49514	1.57368	2.1
49515	1.89111	0.909895
49516	1.89111	2.1
49517	2.17638	0.934302
49518	2.17638	2.1
49519	1.32813	2.1
49520	1.7237	2.1
49521	1.32461	2.1
49522	1.81656	2.1
49523	3.14468	2.1
49524	1.55132	2.1
49525	2.0815	2.1
49526	1.81656	2.1
49527	2.19826	2.1
49528	3.65818	2.1
49529	0.775951	2.1

49530	0.775951	2.1
49531	0.794601	2.1
49532	0.794601	2.1
49533	0.945641	2.1
49534	0.90828	2.1
49535	0.662302	2.1
49536	0.662273	2.1
49537	0.662274	2.1
49538	0.945639	2.1
49539	0.662304	2.1
49540	0.908279	2.1
49541	1.03062	2.1
49542	1.03062	2.1
49543	1.05054	2.1
49544	1.05054	2.1
49545	1.13551	2.1
49546	1.21275	2.1
49547	0.945641	2.1
49548	0.90828	2.1
49549	0.945641	2.1
49550	1.21275	2.1
49551	0.90828	2.1
49552	1.13552	2.1
49553	1.89226	2.1
49554	1.79883	2.1
49555	1.95341	2.1
49556	1.89226	2.1
49557	1.85998	2.1
49558	1.79883	2.1
49559	1.89226	2.1
49560	1.89226	2.1
49561	1.89226	2.1
49562	1.89226	2.1
49563	3.78453	2.1
49564	0.851541	2.1
49565	0.851541	2.1
49566	0.970398	2.1
49567	0.954365	2.1
49568	0.855356	2.1
49569	0.855357	2.1
49570	0.946133	2.1
49571	0.946133	2.1
49572	1.04072	2.1
49573	0.970397	2.1
49574	0.950003	2.1
49575	0.851538	2.1
49576	1.89226	2.1
49577	1.85998	2.1
49578	1.95296	2.1
49579	1.89226	2.1
49580	1.92067	2.1
49581	1.85998	2.1
49582	0.946133	2.1
49583	0.946133	2.1
49584	0.946132	2.1
49585	0.946132	2.1
49586	0.946132	2.1
49587	0.946132	2.1

49588	0.946132	2.1
49589	0.946132	2.1
49590	0.946132	2.1
49591	0.946132	2.1
49592	0.946133	2.1
49593	0.946133	2.1
49594	1.82152	2.1
49595	1.89226	2.1
49596	1.89226	2.1
49597	2.11152	2.1
49598	1.7472	2.1
49599	1.89226	2.1
49600	2.02687	2.1
49601	2.29199	2.1
49602	1.2734	2.1
49603	1.41077	2.1
49604	1.47381	2.1
49605	2.29199	2.1
49606	1.46889	2.1
49607	2.29199	2.1
49608	1.86766	2.1
49609	2.59401	2.1
49610	4.15965	2.1
49611	1.58995	2.1
49612	1.74246	2.1
49613	1.46643	2.1
49614	1.46643	2.1
49615	1.85097	2.1
49616	1.85097	2.1
49617	1.58995	2.1
49618	1.66584	2.1
49619	1.89214	2.1
49620	1.91787	2.1
49621	3.48208	2.1
49622	1.18474	2.1
49623	1.8144	2.1
49624	1.1486	2.1
49625	1.1486	2.1
49626	2.13071	2.1
49627	2.1257	2.1
49628	2.0815	2.1
49629	2.51651	2.1
49630	1.73681	2.1
49631	2.01601	2.1
49632	2.21406	2.1
49633	2.56998	2.1
49634	1.19995	2.1
49635	1.06256	2.1
49636	0.620677	2.1
49637	0.598084	2.1
49638	0.831426	2.1
49639	0.946056	2.1
49640	0.827692	2.1
49641	0.827692	2.1
49642	1.08958	2.1
49643	0.630132	2.1
49644	0.577547	2.1
49645	1.06104	2.1

49646	2.0815	2.1
49647	2.66249	2.1
49648	2.78966	2.1
49649	2.47108	2.1
49650	2.33858	2.1
49651	2.0815	2.1
49652	1.53735	2.1
49653	1.42278	2.1
49654	1.23654	2.1
49655	1.34678	2.1
49656	2.76955	2.1
49657	1.89226	2.1
49658	1.89226	2.1
49659	1.89226	2.1
49660	1.89226	2.1
49661	3.78452	2.1
49662	1.23654	2.1
49663	1.34678	2.1
49664	1.19	2.1
49665	1.08576	2.1
49666	2.43254	2.1
49667	1.38776	2.1
49668	1.29366	2.1
49669	0.946131	2.1
49670	0.946131	2.1
49671	0.946131	2.1
49672	0.946131	2.1
49673	1.38775	2.1
49674	1.38775	2.1
49675	1.29366	2.1
49676	0.946132	2.1
49677	0.946131	2.1
49678	1.38775	2.1
49679	0.946131	2.1
49680	0.946131	2.1
49681	0.946129	2.1
49682	0.946129	2.1
49683	0.94613	2.1
49684	0.94613	2.1
49685	0.946131	2.1
49686	0.946131	2.1
49687	0.946131	2.1
49688	0.946131	2.1
49689	0.94613	2.1
49690	0.94613	2.1
49691	1.89226	0.707796
49692	1.89226	0.97152
49693	1.89226	0.76928
49694	1.89226	0.781575
49695	1.89226	0.787197
49696	1.89226	0.871719
49697	1.89226	0.757341
49698	1.89226	1.16245
49699	1.89226	0.816738
49700	1.89226	0.857912
49701	1.89226	0.82333
49702	1.89226	0.972534
49703	1.89226	0.741551

49704	1.89226	1.00115
49705	1.89226	0.773045
49706	1.89226	0.8199
49707	1.89226	0.788087
49708	1.89226	1.00218
49709	0.92338	2.1
49710	0.92338	0.955613
49711	0.94428	0.94063
49712	0.94428	0.822402
49713	1.19528	1.02563
49714	1.30598	2.1
49715	0.714073	0.844696
49716	0.561679	1.20747
49717	1.19528	1.04282
49718	0.561678	1.05437
49719	0.714072	1.0915
49720	1.30598	1.09406
49721	2.61197	1.84304
49722	1.89226	1.1277
49723	1.89226	1.07236
49724	1.89226	2.1
49725	2.39056	1.00068
49726	2.39056	2.1
49727	0.946132	1.22091
49728	0.946132	0.982607
49729	0.946132	0.754643
49730	0.946132	0.829188
49731	0.946131	0.862343
49732	0.946131	1.23627
49733	0.946132	0.955134
49734	0.946132	0.79371
49735	0.946132	0.870358
49736	0.946132	0.892569
49737	0.946132	0.934118
49738	0.946132	0.919765
49739	1.89224	2.1
49740	1.89224	2.1
49741	1.89224	2.1
49742	1.89224	2.1
49743	3.78448	2.1
49744	1.97089	2.1
49745	1.57428	2.1
49746	1.84991	2.1
49747	1.49078	2.1
49748	3.42419	2.1
49749	0.621927	2.1
49750	0.621927	2.1
49751	0.630824	2.1
49752	0.630824	2.1
49753	0.483483	2.1
49754	0.507361	2.1
49755	0.787142	2.1
49756	0.745391	2.1
49757	0.745381	2.1
49758	0.483476	2.1
49759	0.507368	2.1
49760	0.787152	2.1
49761	1.89224	2.1

49762	1.89224	2.1
49763	1.89224	2.1
49764	1.89224	2.1
49765	1.89224	2.1
49766	1.89224	2.1
49767	1.89227	2.1
49768	1.89227	2.1
49769	1.89227	2.1
49770	1.89227	2.1
49771	1.89227	2.1
49772	1.89227	2.1
49773	1.84994	2.1
49774	1.41758	2.1
49775	1.4908	2.1
49776	1.4908	2.1
49777	1.74391	2.1
49778	1.74391	2.1
49779	1.89226	2.1
49780	1.89226	2.1
49781	1.89226	2.1
49782	1.89226	2.1
49783	3.78452	2.1
49784	1.01323	2.1
49785	1.01323	2.1
49786	1.01445	2.1
49787	1.01445	2.1
49788	0.839531	2.1
49789	0.875652	2.1
49790	1.19063	2.1
49791	1.15203	2.1
49792	1.15203	2.1
49793	0.839531	2.1
49794	0.875652	2.1
49795	1.19063	2.1
49796	1.89226	2.1
49797	2.75587	2.1
49798	2.81512	2.1
49799	2.81512	2.1
49800	1.89226	2.1
49801	1.89226	2.1
49802	1.89226	2.1
49803	1.89226	2.1
49804	1.89226	2.1
49805	1.89226	2.1
49806	3.78452	2.1
49807	0.974831	2.1
49808	0.974831	2.1
49809	0.976681	2.1
49810	0.976681	2.1
49811	0.803179	2.1
49812	0.83953	2.1
49813	1.11198	2.1
49814	1.15203	2.1
49815	1.11198	2.1
49816	0.803179	2.1
49817	1.15203	2.1
49818	0.83953	2.1
49819	1.37794	2.1

49820	1.37794	2.1
49821	0.946128	2.1
49822	0.946128	2.1
49823	1.3477	2.1
49824	1.37793	2.1
49825	0.946131	2.1
49826	0.946131	2.1
49827	0.946129	2.1
49828	1.3477	2.1
49829	1.37793	2.1
49830	0.946129	2.1
49831	2.38577	1.18598
49832	1.20247	2.1
49833	1.75182	2.1
49834	2.82546	1.21998
49835	4.1376	2.1
49836	2.82546	2.06381
49837	1.89226	0.921703
49838	2.38577	1.4424
49839	2.38577	1.35985
49840	1.89226	1.66233
49841	1.89226	1.23161
49842	1.035 2.1	
49843	2.15429	2.1
49844	0.970998	2.1
49845	2.0815	2.1
49846	2.89672	2.1
49847	0.946131	0.704993
49848	0.946131	0.703078
49849	0.853249	0.699302
49850	1.02715	0.704525
49851	0.851537	0.693647
49852	0.851537	0.705306
49853	1.02663	0.686122
49854	1.13139	0.574163
49855	0.946128	0.652509
49856	0.932039	0.70288
49857	0.843985	0.669979
49858	0.946129	0.697078
49859	1.89226	0.63634
49860	1.86408	0.70246
49861	1.96548	0.704798
49862	1.86408	0.704251
49863	1.99366	0.638525
49864	1.89226	0.487773
49865	2.70702	2.1
49866	2.08981	2.1
49867	2.08693	2.1
49868	2.08693	2.1
49869	2.7188	2.1
49870	2.7188	2.1
49871	2.08982	2.1
49872	1.01964	2.1
49873	2.08693	2.1
49874	1.04615	2.1
49875	3.13597	2.1
49876	2.70702	2.1
49877	2.08693	2.1

49878	2.68784	2.1
49879	2.08831	2.1
49880	4.77477	2.1
49881	0.778233	2.1
49882	0.778233	2.1
49883	0.789001	2.1
49884	0.789001	2.1
49885	0.545307	2.1
49886	0.523075	2.1
49887	1.04347	2.1
49888	1.04416	2.1
49889	1.04416	2.1
49890	0.545308	2.1
49891	1.04347	2.1
49892	0.523075	2.1
49893	1.89226	0.7
49894	1.89226	0.7
49895	1.89226	0.7
49896	1.89226	0.7
49897	3.78453	0.7
49898	1.39237	0.7
49899	1.17933	1.15441
49900	0.755796	0.760962
49901	0.755796	0.803583
49902	0.755767	0.7
49903	0.94613	0.711087
49904	1.15918	0.7
49905	0.94613	0.7
49906	1.11632	0.7
49907	0.92595	0.7
49908	0.94613	0.7
49909	0.94613	0.7
49910	1.89226	0.7
49911	1.89226	0.7
49912	1.89226	0.7
49913	1.89226	0.7
49914	3.78452	0.7
49915	0.950067	0.7
49916	0.75071	0.7
49917	0.906516	0.7
49918	0.906516	0.7
49919	0.81586	0.7
49920	0.851541	0.7
49921	0.962101	0.545162
49922	0.851476	0.7
49923	0.962105	0.466796
49924	0.815808	0.7
49925	0.851529	0.7
49926	0.851529	0.7
49927	1.05032	0.7
49928	0.972326	0.7
49929	0.906515	0.694458
49930	0.906515	0.7
49931	1.12245	0.7
49932	1.04072	0.7
49933	0.815857	0.520978
49934	0.851539	0.7
49935	1.04451	0.642076

49936	0.815853	0.7
49937	0.919795	0.7
49938	0.919795	0.7
49939	1.5855	2.1
49940	2.14058	2.1
49941	1.9153	2.1
49942	1.79922	2.1
49943	3.72608	2.1
49944	1.58551	0.939699
49945	1.24201	1.37666
49946	0.960287	0.95981
49947	1.79922	2.1
49948	2.82752	2.1
49949	0.679602	2.1
49950	0.621009	2.1
49951	0.899614	2.1
49952	0.940441	2.1
49953	0.679602	2.1
49954	0.94044	2.1
49955	0.621009	2.1
49956	0.899615	2.1
49957	0.794027	2.1
49958	0.794027	2.1
49959	0.785189	2.1
49960	0.785189	1.34636
49961	2.14058	2.1
49962	1.79922	2.1
49963	2.23742	2.1
49964	2.23742	2.1
49965	1.78404	2.1
49966	1.88088	2.1
49967	1.15177	2.1
49968	2.0815	2.05822
49969	1.93765	2.1
49970	1.78342	1.98032
49971	1.39511	2.1
49972	1.28406	2.0139
49973	1.97719	2.03367
49974	2.50057	0.90573
49975	2.35349	1.87973
49976	2.35349	0.905112
49977	2.31686	1.98796
49978	2.32857	0.917313
49979	1.93765	1.9056
49980	2.35349	0.919091
49981	1.70048	2.0206
49982	2.15721	0.600989
49983	4.05397	1.03018
49984	0.850388	1.19275
49985	0.850388	2.1
49986	0.840019	2.1
49987	0.840019	2.1
49988	1.13736	2.1
49989	1.13852	1.79556
49990	0.573661	2.1
49991	0.551891	2.1
49992	0.573661	2.1
49993	1.13852	2.1

49994	0.551892	2.1
49995	1.13736	2.1
49996	0.860203	1.48367
49997	0.860203	2.1
49998	0.855758	2.1
49999	0.855758	2.1
50000	1.1423	1.84454
50001	1.13852	2.1
50002	0.58628	2.1
50003	0.573661	2.1
50004	0.586281	2.1
50005	1.1423	2.1
50006	0.573661	2.1
50007	1.13852	2.1
50008	1.28918	0.905025
50009	0.999086	0.855936
50010	1.0383	1.70154
50011	0.736019	1.17845
50012	1.00954	1.32597
50013	1.00954	0.924322
50014	1.01566	0.836732
50015	1.01566	1.49169
50016	1.28918	1.05222
50017	1.0383	1.64639
50018	0.999086	1.25458
50019	0.736019	1.10628
50020	2.20797	2.1
50021	2.64748	2.1
50022	2.50375	1.43062
50023	2.50375	2.1
50024	2.40858	2.07577
50025	2.40858	2.1
50026	2.64748	2.1
50027	2.74744	2.1
50028	2.50375	2.1
50029	2.6521	2.1
50030	5.29958	2.1
50031	2.20797	2.1
50032	2.50375	2.1
50033	2.02064	1.19759
50034	2.3606	2.1
50035	4.52439	2.1
50036	2.36059	2.1
50037	2.6521	2.1
50038	2.56952	2.1
50039	2.56952	2.1
50040	2.50375	2.1
50041	2.50375	2.1
50042	2.49439	0.479586
50043	1.89226	0.630668
50044	2.81337	0.63347
50045	1.89226	0.63721
50046	4.38665	0.627984
50047	1.89226	0.646532
50048	1.89226	0.67386
50049	1.89226	0.680801
50050	1.89226	0.670711
50051	3.78452	0.662209

50052	1.89226	0.629903
50053	1.89226	0.652333
50054	1.89226	0.642986
50055	1.89226	0.665697
50056	3.78452	0.65236
50057	1.93948	0.713781
50058	1.98874	0.660084
50059	1.62494	0.675618
50060	2.14218	0.701827
50061	1.62494	0.639026
50062	2.14218	0.670646
50063	1.47354	0.585782
50064	1.98874	0.527717
50065	2.01865	0.609132
50066	1.62495	0.471482
50067	2.01865	0.674231
50068	1.62495	0.50076
50069	1.89226	0.907296
50070	1.89226	2.1
50071	1.89226	1.53589
50072	1.89226	2.1
50073	3.78453	2.0924
50074	2.05257	2.1
50075	1.94441	2.1
50076	1.88487	1.44293
50077	1.82441	2.1
50078	3.82928	2.1
50079	1.94441	2.1
50080	1.74391	2.1
50081	1.82441	2.1
50082	1.65636	2.1
50083	3.60077	2.1
50084	1.89226	2.1
50085	1.89226	2.1
50086	1.89226	2.1
50087	1.89226	2.1
50088	3.78452	2.1
50089	1.89226	1.64966
50090	1.89226	2.1
50091	1.89226	0.907929
50092	1.89226	2.1
50093	3.78453	1.8844
50094	1.88487	1.69125
50095	1.82441	2.1
50096	1.75835	2.1
50097	1.72866	2.1
50098	3.61354	2.1
50099	1.82441	2.1
50100	1.65636	2.1
50101	1.72866	2.1
50102	1.58401	2.1
50103	3.38503	2.1
50104	1.89226	2.1
50105	1.89226	2.1
50106	1.89226	2.1
50107	1.89226	2.1
50108	3.78452	2.1
50109	2.27432	2.1

50110	1.33339	2.1
50111	1.38905	2.1
50112	2.34471	2.1
50113	3.66337	2.1
50114	1.89226	2.1
50115	2.34471	2.1
50116	2.27432	2.1
50117	2.27432	2.1
50118	1.89226	2.1
50119	1.89226	2.1
50120	1.20443	2.1
50121	1.17235	2.1
50122	0.724695	2.1
50123	0.694522	2.1
50124	0.949994	2.1
50125	0.949993	2.1
50126	0.948955	2.1
50127	0.948955	2.1
50128	1.20443	2.1
50129	0.7247	2.1
50130	0.694517	2.1
50131	1.17234	2.1
50132	1.89225	2.1
50133	2.40885	2.1
50134	2.3447	2.1
50135	2.3447	2.1
50136	1.89225	2.1
50137	1.89225	2.1
50138	1.37138	2.1
50139	1.82597	2.1
50140	1.45027	2.1
50141	1.92772	2.1
50142	3.27624	2.1
50143	1.89226	2.1
50144	1.89226	2.1
50145	1.89226	2.1
50146	1.89226	2.01369
50147	3.78452	2.1
50148	1.89226	2.1
50149	1.89226	1.12041
50150	1.89226	1.73206
50151	1.89226	1.28743
50152	3.78452	2.06214
50153	1.82597	2.1
50154	2.02312	0.994307
50155	1.92772	2.1
50156	2.15888	2.1
50157	3.98485	1.26296
50158	1.53446	2.1
50159	1.92772	2.1
50160	2.03975	2.1
50161	2.03975	2.1
50162	1.45027	2.1
50163	1.45027	2.1
50164	1.89226	2.1
50165	1.89226	2.1
50166	1.89226	2.1
50167	1.89226	2.1

50168	1.89226	2.1
50169	1.89226	2.1
50170	1.89226	1.88409
50171	1.89226	2.1
50172	1.89226	2.1
50173	1.89226	1.20522
50174	3.78452	1.97713
50175	1.92772	2.1
50176	2.15888	2.1
50177	2.03975	2.1
50178	2.3111	2.1
50179	4.19863	2.1
50180	1.89226	2.1
50181	1.89226	2.1
50182	1.89226	2.1
50183	1.89226	2.1
50184	3.78452	2.1
50185	1.78376	2.1
50186	1.3201	2.1
50187	1.78376	2.1
50188	1.86102	2.1
50189	1.2679	2.1
50190	1.34517	2.1
50191	1.89226	2.1
50192	2.52442	2.1
50193	1.89226	2.1
50194	2.51691	2.1
50195	4.41667	2.1
50196	1.89226	2.1
50197	1.89226	2.1
50198	1.89226	2.1
50199	1.89226	2.1
50200	3.78452	2.1
50201	1.78376	2.1
50202	1.2679	2.1
50203	1.71839	2.1
50204	1.23504	2.1
50205	3.0188	2.1
50206	1.89226	2.1
50207	2.51795	2.1
50208	2.51691	2.1
50209	2.51691	2.1
50210	1.89226	2.1
50211	1.89226	2.1
50212	1.89226	0.897256
50213	1.89226	0.995045
50214	1.89226	0.975387
50215	1.89226	0.945015
50216	3.78452	0.940533
50217	1.89227	0.934982
50218	1.89227	1.01525
50219	3.26088	1.01012
50220	2.79543	0.981152
50221	2.20733	1.02369
50222	1.89227	0.984155
50223	1.89226	0.969403
50224	1.89226	0.956017
50225	1.89226	0.920656

50226	1.89226	0.964264
50227	1.89226	0.932637
50228	1.89226	0.982325
50229	1.89227	0.94521
50230	2.20733	0.993793
50231	1.46973	1.04313
50232	1.89227	0.953129
50233	1.7848	1.03938
50234	2.20733	0.953387
50235	1.89226	2.1
50236	2.55682	2.1
50237	2.81593	2.1
50238	2.81593	2.1
50239	1.89226	2.1
50240	1.89226	2.1
50241	1.0607	2.1
50242	1.0607	2.1
50243	1.12617	2.1
50244	0.986957	2.1
50245	0.699328	2.1
50246	0.785567	2.01896
50247	1.28855	2.1
50248	1.41913	2.1
50249	0.766737	1.35051
50250	1.34711	2.1
50251	1.24643	2.1
50252	0.687289	1.84609
50253	3.11063	0.749171
50254	1.89226	0.771493
50255	1.96536	0.762041
50256	1.89226	0.763499
50257	1.96536	0.752672
50258	1.89226	0.752692
50259	1.89226	0.902847
50260	1.89226	0.786249
50261	1.89226	0.827796
50262	1.89226	0.823117
50263	3.78452	0.894152
50264	1.89226	0.764562
50265	1.96256	0.700318
50266	1.89226	0.733972
50267	1.89226	0.77436
50268	2.49192	0.771134
50269	2.49192	0.727403
50270	1.85716	0.769214
50271	2.56871	0.765
50272	1.03043	0.743413
50273	1.03043	0.750542
50274	2.48125	0.705626
50275	1.65452	0.760301
50276	1.89226	2.1
50277	2.09622	2.1
50278	2.09622	2.1
50279	2.08508	2.1
50280	1.89226	2.1
50281	1.88112	2.1
50282	1.04811	2.1
50283	1.04811	2.1

50284	0.946131	2.1
50285	0.946131	2.1
50286	1.05014	2.1
50287	1.04811	2.1
50288	0.94613	2.1
50289	0.94613	2.1
50290	0.946131	2.1
50291	1.05013	2.1
50292	1.04811	2.1
50293	0.946131	2.1
50294	1.398	2.1
50295	2.10766	2.1
50296	2.3018	2.1
50297	2.3018	2.1
50298	1.34008	2.1
50299	1.34008	2.1
50300	1.1509	2.1
50301	1.1509	2.1
50302	0.946132	2.1
50303	0.946132	2.1
50304	0.946132	2.1
50305	0.946132	2.1
50306	1.05383	2.1
50307	1.1509	2.1
50308	1.1509	2.1
50309	0.946133	2.1
50310	0.946132	2.1
50311	1.05383	2.1
50312	0.931141	2.1
50313	0.931141	2.1
50314	0.971946	2.1
50315	0.971946	2.1
50316	1.28569	2.1
50317	1.1509	2.1
50318	0.699	2.1
50319	0.752185	2.1
50320	1.28568	2.1
50321	0.752186	2.1
50322	0.699001	2.1
50323	1.1509	2.1
50324	2.30181	2.1
50325	1.89226	2.1
50326	1.89226	2.1
50327	1.89226	2.1
50328	2.57137	2.1
50329	2.57137	2.1
50330	2.0815	2.1
50331	1.21962	2.1
50332	1.55173	2.1
50333	2.57849	2.1
50334	3.79001	2.1
50335	1.38779	2.1
50336	1.2923	2.1
50337	1.03974	2.1
50338	1.28832	2.1
50339	1.33566	2.1
50340	1.21116	2.1
50341	1.22343	2.1

50342	1.22343	2.1
50343	1.42288	2.1
50344	1.20138	2.1
50345	1.26025	2.1
50346	1.00644	2.1
50347	1.26066	2.1
50348	1.14271	2.1
50349	0.886635	2.1
50350	0.795277	2.1
50351	1.02966	2.1
50352	1.02967	2.1
50353	1.05216	2.1
50354	1.05216	2.1
50355	1.26068	2.1
50356	0.790042	2.1
50357	0.795269	2.1
50358	1.20135	2.1
50359	1.89226	0.872419
50360	1.89226	0.854221
50361	1.89226	0.890937
50362	1.89226	0.822815
50363	1.89226	0.910957
50364	1.89226	0.838882
50365	1.89227	0.819975
50366	1.89227	0.812031
50367	1.89227	0.847873
50368	1.89227	0.765003
50369	1.89227	0.859246
50370	1.89227	0.796061
50371	0.563873	0.712232
50372	0.50988	0.623852
50373	0.598693	0.809164
50374	0.558819	0.733659
50375	0.564855	0.677839
50376	0.564855	0.65468
50377	0.557838	0.785415
50378	0.557838	0.754095
50379	0.563873	0.734173
50380	0.598694	0.711024
50381	0.558819	0.709477
50382	0.50988	0.732535
50383	0.598693	0.796174
50384	0.558819	0.704306
50385	0.572737	0.857878
50386	0.534248	0.828906
50387	0.566788	0.802152
50388	0.566788	0.758446
50389	0.566153	0.853139
50390	0.566153	0.818769
50391	0.598694	0.820133
50392	0.572737	0.812497
50393	0.534248	0.796943
50394	0.558819	0.79574
50395	1.85217	1.11032
50396	1.93111	2.1
50397	1.92875	0.924213
50398	2.02391	2.1
50399	3.85986	2.1

50400	1.93112	2.1
50401	1.80425	2.1
50402	2.02392	2.1
50403	1.90981	2.1
50404	3.84092	2.1
50405	1.92876	0.894868
50406	2.02393	2.1
50407	2.05274	1.00423
50408	2.16783	2.1
50409	4.09659	2.1
50410	2.02393	2.1
50411	1.90982	2.1
50412	2.16784	2.1
50413	2.05967	2.1
50414	4.07766	2.1
50415	1.89226	1.25693
50416	1.89226	1.13765
50417	1.89226	1.25511
50418	1.89226	1.18829
50419	3.78453	1.07149
50420	1.89226	1.08428
50421	1.89226	1.27438
50422	1.89226	1.7486
50423	2.44829	1.0549
50424	3.78453	1.55619
50425	1.89226	1.24283
50426	1.89226	1.31023
50427	1.89226	1.15948
50428	2.60452	1.11882
50429	3.78453	1.70077
50430	1.58723	0.253803
50431	1.88071	0.468282
50432	2.97376	0.237276
50433	2.13255	0.327515
50434	4.43982	0.340814
50435	2.20641	0.588707
50436	1.33575	0.716309
50437	2.15824	0.643405
50438	2.15824	0.649833
50439	1.2951	0.65584
50440	1.2951	0.680733
50441	0.825383	0.715484
50442	0.825383	0.678543
50443	0.856104	0.642155
50444	0.856104	0.632745
50445	0.952196	0.673169
50446	0.602363	0.641429
50447	0.667877	0.718088
50448	1.07913	0.649913
50449	0.602363	0.699863
50450	0.667876	0.711759
50451	1.07913	0.715838
50452	0.952197	0.664952
50453	2.57657	1.07798
50454	1.44437	2.1
50455	2.65378	2.1
50456	2.25076	1.79808
50457	1.84739	2.1

50458	1.44437	1.35829
50459	2.0189	0.392625
50460	2.54752	0.38505
50461	2.63975	0.481347
50462	2.31049	0.292775
50463	4.65865	0.403357
50464	1.40707	0.276665
50465	1.89605	0.237605
50466	2.0189	0.245943
50467	2.0189	0.236355
50468	1.50438	0.287084
50469	1.50438	0.281813
50470	2.63971	0.43881
50471	2.38246	0.307521
50472	2.13402	0.344601
50473	2.57503	0.293575
50474	4.77373	0.319636
50475	1.50436	0.255668
50476	1.96803	0.244953
50477	2.13402	0.305789
50478	1.45446	0.203166
50479	3.63838	0.260776
50480	2.30793	0.686206
50481	2.11032	0.587879
50482	1.24082	0.61318
50483	1.05689	0.679782
50484	3.36482	0.677781
50485	1.89226	0.737357
50486	1.89226	0.754572
50487	1.89226	0.755585
50488	1.89226	0.742046
50489	3.78453	0.744667
50490	0.812967	0.722353
50491	0.812967	0.726968
50492	1.00181	0.717973
50493	1.08141	0.705956
50494	0.946132	0.683417
50495	0.812938	0.726509
50496	0.932815	0.729976
50497	1.05577	0.714571
50498	0.812938	0.721716
50499	0.932812	0.725255
50500	0.946133	0.727782
50501	0.946133	0.720812
50502	2.53897	2.1
50503	1.89226	2.1
50504	1.89226	2.1
50505	1.89226	2.1
50506	2.3245	2.1
50507	2.3245	2.1
50508	1.00255	1.18137
50509	1.06561	2.1
50510	1.05481	0.858277
50511	1.11077	2.1
50512	2.12042	1.54101
50513	1.89226	1.39826
50514	1.89226	2.1
50515	1.89226	0.882339

50516	1.89226	2.05464
50517	3.78453	1.16433
50518	1.89226	1.28225
50519	2.77314	2.1
50520	2.81276	1.60635
50521	2.81276	2.1
50522	1.89226	1.90299
50523	1.89226	2.1
50524	1.11077	2.1
50525	1.15114	2.1
50526	1.10274	0.99252
50527	1.1032	1.57348
50528	1.05481	1.44259
50529	1.1032	1.1006
50530	1.89226	1.20216
50531	1.89226	2.1
50532	1.89226	0.818023
50533	1.89226	2.1
50534	3.78452	1.14804
50535	0.721957	0.83199
50536	0.721957	0.832716
50537	0.946103	0.832366
50538	0.946103	0.803295
50539	0.814237	0.787769
50540	0.721929	0.752212
50541	0.946132	0.835808
50542	0.946132	0.838613
50543	0.721928	0.848056
50544	0.946131	0.855999
50545	0.946132	0.86738
50546	0.814237	0.861465
50547	1.89226	0.913709
50548	1.89226	0.838004
50549	1.89226	0.839463
50550	1.89226	0.846673
50551	1.89226	0.889636
50552	1.89226	0.89846
50553	1.37196	0.830097
50554	2.83556	0.708308
50555	1.5165	0.813868
50556	1.5165	0.836699
50557	2.74923	0.767684
50558	2.74923	0.825948
50559	0.946128	0.816214
50560	0.946128	0.831697
50561	0.827866	0.791417
50562	0.946132	0.811754
50563	0.94613	0.786118
50564	0.94613	0.796329
50565	0.94613	0.832929
50566	0.94613	0.833386
50567	0.94613	0.837646
50568	0.827864	0.853781
50569	0.94613	0.841068
50570	0.94613	0.892373
50571	1.36208	0.750214
50572	2.74923	0.585941
50573	1.37196	0.7062

50574	1.37196	0.742956
50575	2.53256	0.628436
50576	2.53256	0.684329
50577	1.07705	2.1
50578	1.07705	2.1
50579	1.08253	2.1
50580	1.08253	2.1
50581	0.762985	2.1
50582	0.781638	2.1
50583	1.37794	2.1
50584	1.40756	2.1
50585	1.37794	2.1
50586	0.762986	2.1
50587	0.781638	2.1
50588	1.40756	2.1
50589	2.69541	2.1
50590	2.75587	2.1
50591	1.48572	2.1
50592	1.48572	2.1
50593	1.52597	2.1
50594	2.73566	2.1
50595	2.0554	1.73314
50596	1.00387	1.0353
50597	2.01001	0.968171
50598	0.994002	0.926581
50599	3.01389	0.887189
50600	1.89226	2.1
50601	2.41579	2.1
50602	2.12209	2.1
50603	2.12209	2.1
50604	1.89226	2.1
50605	1.89226	2.1
50606	1.89226	2.1
50607	1.89226	2.1
50608	1.89226	2.1
50609	1.89226	2.1
50610	1.89226	2.1
50611	1.89226	2.1
50612	0.88209	2.1
50613	0.899471	2.1
50614	0.651501	2.1
50615	0.533531	2.1
50616	0.724105	2.1
50617	0.711243	2.1
50618	0.692622	2.1
50619	0.692622	2.1
50620	0.890668	2.1
50621	0.738066	2.1
50622	0.778223	2.1
50623	0.531121	2.1
50624	1.89226	2.1
50625	2.77504	2.1
50626	2.4158	2.1
50627	2.4158	2.1
50628	1.89226	2.1
50629	1.89226	2.1
50630	1.89226	2.1
50631	1.89226	2.1

50632	1.89226	2.1
50633	1.89226	2.1
50634	1.89226	2.1
50635	1.89226	2.1
50636	1.70303	2.1
50637	1.39832	2.1
50638	1.51749	2.1
50639	1.68783	2.1
50640	3.24844	2.1
50641	1.89226	2.1
50642	2.38311	2.1
50643	1.98321	2.1
50644	1.89226	2.1
50645	2.47406	2.1
50646	2.38311	2.1
50647	1.89226	2.1
50648	1.89226	2.1
50649	1.89226	2.1
50650	1.89226	2.1
50651	1.89226	2.1
50652	1.89226	2.1
50653	0.946131	2.1
50654	0.946131	2.1
50655	1.25167	2.1
50656	1.19156	2.1
50657	1.19156	2.1
50658	1.19156	2.1
50659	0.946133	2.1
50660	0.946133	2.1
50661	0.946131	2.1
50662	1.25167	2.1
50663	0.946131	2.1
50664	1.19156	2.1
50665	1.89226	2.1
50666	2.47406	2.1
50667	1.99996	2.1
50668	1.89226	2.1
50669	2.58176	2.1
50670	2.47406	2.1
50671	1.89226	2.1
50672	1.89226	2.1
50673	1.89226	2.1
50674	1.89226	2.1
50675	1.89226	2.1
50676	1.89226	2.1
50677	0.946131	2.1
50678	0.946131	2.1
50679	1.32241	2.1
50680	1.25167	2.1
50681	1.25167	2.1
50682	1.25167	2.1
50683	0.946133	2.1
50684	0.946133	2.1
50685	0.946131	2.1
50686	1.32241	2.1
50687	0.946131	2.1
50688	1.25167	2.1
50689	1.65151	2.1

50690	1.92471	2.1
50691	1.81834	2.1
50692	2.13272	2.1
50693	3.74306	2.1
50694	1.92472	2.1
50695	1.9943	2.1
50696	2.13273	2.1
50697	2.24475	2.1
50698	4.16947	2.1
50699	1.81834	2.1
50700	2.13272	2.1
50701	2.02687	2.1
50702	2.39605	2.1
50703	4.21439	2.1
50704	2.13273	2.1
50705	2.24475	2.1
50706	2.39605	2.1
50707	2.54903	2.1
50708	4.6408	2.1
50709	1.10534	2.1
50710	1.22993	2.1
50711	0.946132	2.1
50712	0.946132	2.1
50713	0.946132	2.1
50714	0.946132	2.1
50715	1.22993	2.1
50716	1.22993	2.1
50717	1.10534	2.1
50718	0.946131	2.1
50719	0.946132	2.1
50720	1.22993	2.1
50721	1.47221	2.1
50722	2.14647	2.1
50723	1.95567	2.1
50724	1.95567	2.1
50725	1.71082	2.1
50726	1.71082	2.1
50727	0.946131	2.1
50728	0.946131	2.1
50729	0.946131	2.1
50730	0.946131	2.1
50731	0.946132	2.1
50732	0.946132	2.1
50733	0.946131	2.1
50734	0.946131	2.1
50735	0.946131	2.1
50736	0.946131	2.1
50737	0.946132	2.1
50738	0.946132	2.1
50739	0.998086	2.1
50740	1.09429	2.1
50741	0.946125	2.1
50742	0.946125	2.1
50743	0.90732	2.1
50744	0.90732	2.1
50745	1.14414	2.1
50746	1.16553	2.1
50747	1.05018	2.1

50748	0.907332	2.1
50749	0.99482	2.1
50750	0.851529	2.1
50751	0.80534	2.1
50752	0.80534	2.1
50753	0.812206	2.1
50754	0.812206	2.1
50755	0.895134	2.1
50756	0.977828	2.1
50757	0.736099	2.1
50758	0.639718	2.1
50759	0.639722	2.1
50760	0.89514	2.1
50761	0.736095	2.1
50762	0.977821	2.1
50763	0.946125	2.1
50764	0.946125	2.1
50765	0.946125	2.1
50766	0.946125	2.1
50767	0.946125	2.1
50768	0.946125	2.1
50769	0.946124	2.1
50770	0.946124	2.1
50771	0.946131	2.1
50772	0.946131	2.1
50773	0.946118	2.1
50774	0.946118	2.1
50775	2.22622	0.05
50776	2.43347	0.05
50777	2.34108	0.05
50778	1.24587	2.1
50779	1.21674	2.1
50780	1.14533	2.1
50781	1.11311	2.1
50782	1.17874	2.1
50783	1.17874	2.1
50784	1.18024	2.1
50785	1.18024	2.1
50786	1.14533	2.1
50787	1.24587	2.1
50788	1.21674	2.1
50789	1.11311	2.1
50790	2.43347	2.1
50791	2.34107	2.1
50792	2.39663	2.1
50793	2.49174	2.1
50794	4.8301	2.1
50795	1.86206	2.1
50796	1.41058	2.1
50797	1.70302	2.1
50798	2.42083	2.1
50799	3.68129	2.1
50800	1.70302	2.1
50801	2.43549	2.1
50802	2.20075	2.1
50803	2.50358	2.1
50804	2.04916	2.1
50805	2.33113	2.1

50806	1.07624	2.1
50807	2.12621	2.1
50808	1.57297	2.1
50809	1.60383	2.1
50810	1.30047	2.1
50811	1.30047	2.1
50812	2.33379	2.1
50813	2.56149	1.20741
50814	2.36734	1.13335
50815	2.62796	1.13561
50816	4.92883	1.25658
50817	2.5615	1.23188
50818	2.57957	1.47386
50819	2.62796	1.11788
50820	2.69056	1.09609
50821	5.25206	1.53336
50822	2.39322	1.27664
50823	2.62796	1.72187
50824	2.6849	2.04335
50825	2.6849	1.70757
50826	2.36734	1.94022
50827	2.36734	1.65989
50828	2.62796	1.52005
50829	2.69056	1.64279
50830	2.6849	0.956756
50831	2.77227	0.977125
50832	5.37546	1.08503
50833	1.89226	0.100956
50834	1.89226	0.138352
50835	1.89226	0.108254
50836	1.89226	0.104598
50837	1.89226	0.118032
50838	1.89226	0.112247
50839	1.06974	0.0857292
50840	1.06974	0.0843671
50841	1.02299	0.0868746
50842	1.02299	0.0896717
50843	1.35299	0.0871721
50844	1.26227	0.0852054
50845	0.646252	0.0986227
50846	0.830464	0.084006
50847	0.830463	0.0840905
50848	1.35299	0.0857695
50849	1.26227	0.0860815
50850	0.646252	0.0945017
50851	2.52454	0.0919122
50852	1.89226	0.114111
50853	2.70598	0.0977682
50854	1.89226	0.0837513
50855	4.59824	0.092106
50856	1.89226	0.109377
50857	1.89226	0.163965
50858	1.89226	0.127282
50859	1.89226	0.117841
50860	1.89226	0.142537
50861	1.89226	0.128588
50862	1.89226	0.103341
50863	1.89226	0.136597

50864	1.89226	0.105655
50865	1.89226	0.107809
50866	1.89226	0.114159
50867	1.89226	0.110281
50868	1.7666	0.0828238
50869	2.70598	0.0952635
50870	2.65378	0.0902251
50871	2.65378	0.0948298
50872	1.66093	0.0853764
50873	1.66093	0.0851189
50874	2.70598	0.101399
50875	1.89226	0.0866802
50876	1.89226	0.115093
50877	1.89226	0.0857698
50878	2.65377	0.105559
50879	2.65377	0.0847176
50880	0.946132	0.10765
50881	0.946132	0.115958
50882	0.946131	0.124908
50883	0.946131	0.145455
50884	0.946131	0.141518
50885	0.946131	0.119719
50886	0.946132	0.146821
50887	0.946132	0.110352
50888	0.946132	0.123114
50889	0.946132	0.119526
50890	0.946131	0.123154
50891	0.946131	0.124632
50892	1.69858	2.1
50893	2.18238	2.1
50894	2.506	2.1
50895	1.93586	2.1
50896	4.20458	2.1
50897	2.21772	2.1
50898	2.18238	2.1
50899	2.506	2.1
50900	2.59215	2.1
50901	4.72372	2.1
50902	2.50596	2.1
50903	1.93584	2.1
50904	2.16728	2.1
50905	2.7679	2.1
50906	4.67325	2.1
50907	1.53296	2.1
50908	1.56299	2.1
50909	1.42624	2.1
50910	1.52961	2.1
50911	2.98923	2.1
50912	1.56299	2.1
50913	1.46568	2.1
50914	1.52961	2.1
50915	1.51916	2.1
50916	3.08215	2.1
50917	1.36226	2.1
50918	1.52961	2.1
50919	1.36226	2.1
50920	1.42624	2.1
50921	1.55369	2.1

50922	1.61768	2.1
50923	1.52961	2.1
50924	1.51916	2.1
50925	1.55369	2.1
50926	1.64892	2.1
50927	3.07285	2.1
50928	2.13405	0.314077
50929	2.57507	0.324078
50930	2.39438	0.417096
50931	1.93408	0.270109
50932	4.52843	0.335098
50933	2.13405	0.27859
50934	1.27778	0.205128
50935	1.2545	0.235808
50936	1.45448	0.260799
50937	1.93408	0.219495
50938	2.13405	0.239492
50939	2.39438	0.365583
50940	1.93407	0.267019
50941	1.60578	0.296905
50942	1.87985	0.284929
50943	4.00016	0.296236
50944	1.0525	2.07905
50945	1.05758	2.09914
50946	1.43484	0.993594
50947	1.38506	1.71934
50948	2.48734	1.83147
50949	1.20455	2.1
50950	1.67352	2.1
50951	1.52852	2.1
50952	2.05164	2.1
50953	3.20203	2.1
50954	1.89226	2.1
50955	1.89226	2.1
50956	1.89226	2.1
50957	1.89226	2.1
50958	3.78452	2.1
50959	1.89226	2.1
50960	1.89226	2.1
50961	1.89226	2.1
50962	1.89226	2.1
50963	1.89226	2.1
50964	1.89226	2.1
50965	2.05165	2.1
50966	1.94291	2.1
50967	2.09398	2.1
50968	1.67352	2.1
50969	2.36337	2.1
50970	1.94291	2.1
50971	1.52851	2.1
50972	2.52049	2.1
50973	2.43433	2.1
50974	2.05164	2.1
50975	1.9112	2.1
50976	1.52851	2.1
50977	1.89226	2.1
50978	1.89226	1.93902
50979	1.89226	2.1

50980	1.89226	2.1
50981	3.78451	2.1
50982	1.89226	2.06085
50983	1.89226	2.1
50984	1.89226	2.1
50985	1.89226	1.88768
50986	3.78452	2.1
50987	2.5205	2.1
50988	2.36337	2.1
50989	2.4605	2.1
50990	2.05165	2.1
50991	2.77222	2.1
50992	2.36337	2.1
50993	1.89226	2.1
50994	1.89226	2.1
50995	1.89226	2.1
50996	1.89226	2.1
50997	1.89226	2.1
50998	1.89226	2.1
50999	1.89226	2.1
51000	1.89226	2.1
51001	1.89226	2.1
51002	1.89226	2.1
51003	3.78452	2.1
51004	1.38905	2.1
51005	1.26166	2.1
51006	1.19216	2.1
51007	1.0875	2.1
51008	1.24393	2.1
51009	1.24393	2.1
51010	1.23261	2.1
51011	1.23261	2.1
51012	1.38905	2.1
51013	1.19216	2.1
51014	1.26166	2.1
51015	1.0875	2.1
51016	1.16233	2.1
51017	1.06028	2.1
51018	0.777395	2.1
51019	0.705508	2.1
51020	0.940018	2.1
51021	0.936465	2.1
51022	0.954216	2.1
51023	0.954216	2.1
51024	1.16125	2.1
51025	0.685505	2.1
51026	1.1157	2.1
51027	0.703534	2.1
51028	1.89226	2.1
51029	1.89226	2.1
51030	1.89226	2.1
51031	1.89226	2.1
51032	1.89226	2.1
51033	1.89226	2.1
51034	1.89226	2.1
51035	1.89226	2.1
51036	1.89226	2.1
51037	1.89226	2.1

51038	1.89226	2.1
51039	1.89226	2.1
51040	2.38433	2.1
51041	1.43456	2.1
51042	1.93881	2.1
51043	2.36952	2.1
51044	4.32313	2.1
51045	1.8493	2.1
51046	1.5927	2.1
51047	1.45535	2.1
51048	1.38438	2.1
51049	1.89657	2.1
51050	1.8493	2.1
51051	2.0815	2.1
51052	1.6697	2.1
51053	2.0657	2.1
51054	1.5927	2.1
51055	2.59749	2.1
51056	2.0815	2.1
51057	2.76597	2.1
51058	1.89226	2.1
51059	1.89226	2.1
51060	1.89226	2.1
51061	2.6126	2.1
51062	2.6126	2.1
51063	2.62513	2.1
51064	1.89226	2.1
51065	1.89226	2.1
51066	1.89226	2.1
51067	2.76598	2.1
51068	2.76598	2.1
51069	2.54665	2.1
51070	2.6126	2.1
51071	2.76598	2.1
51072	2.76598	2.1
51073	2.34044	2.1
51074	2.34044	2.1
51075	2.62514	2.1
51076	2.76598	2.1
51077	2.54666	2.1
51078	2.45212	2.1
51079	5.2181	2.1
51080	2.14063	2.1
51081	1.47521	2.1
51082	1.27679	2.1
51083	1.47521	2.1
51084	2.11511	2.1
51085	2.31353	2.1
51086	0.93256	2.1
51087	1.07032	2.1
51088	0.638396	2.1
51089	0.521342	2.1
51090	0.790674	2.1
51091	0.790674	2.1
51092	0.80098	2.1
51093	0.800981	2.1
51094	0.932535	2.1
51095	0.521328	2.1

51096	1.07034	2.1
51097	0.638414	2.1
51098	1.89226	2.1
51099	1.89226	1.31888
51100	1.89226	1.46913
51101	1.89226	2.1
51102	3.78453	1.59357
51103	1.89226	2.1
51104	1.9842	2.1
51105	1.89226	1.91163
51106	1.89226	2.1
51107	2.79705	2.05309
51108	2.79705	2.1
51109	0.469955	2.1
51110	0.791665	2.1
51111	0.989115	1.40331
51112	0.763235	1.86956
51113	0.776356	1.55307
51114	0.805461	2.1
51115	0.776759	1.55041
51116	0.776759	2.1
51117	0.799151	1.90858
51118	0.940869	2.01184
51119	0.78449	1.7672
51120	0.489635	1.79669
51121	1.89226	1.34434
51122	1.89226	1.83649
51123	1.89226	1.3086
51124	1.89226	2.05266
51125	1.89226	1.27644
51126	1.89226	2.08042
51127	2.46058	1.54482
51128	2.28991	2.01487
51129	1.8525	1.73338
51130	1.87697	2.08367
51131	2.28991	1.56919
51132	1.62397	2.00535
51133	0.946131	0.171759
51134	0.974033	0.175558
51135	1.31667	0.208465
51136	0.946131	0.201465
51137	0.885497	0.189004
51138	0.885497	0.183359
51139	1.0468	0.190336
51140	1.04678	0.177289
51141	1.31667	0.210428
51142	0.974019	0.164037
51143	0.946131	0.187163
51144	0.946131	0.165237
51145	1.89226	0.251214
51146	1.89226	0.428454
51147	1.89226	0.444554
51148	1.89226	0.348388
51149	3.78452	0.418293
51150	0.946125	0.450591
51151	0.946125	0.465013
51152	0.946131	0.522877
51153	0.946131	0.541307

51154	0.946128	0.511834
51155	0.946128	0.482223
51156	0.946128	0.442809
51157	0.946128	0.495087
51158	0.946128	0.499045
51159	0.946128	0.512502
51160	0.946128	0.50996
51161	0.946128	0.442798
51162	0.946131	0.224027
51163	0.98453	0.239971
51164	1.32276	0.362058
51165	0.946131	0.369254
51166	0.946132	0.312252
51167	0.946132	0.297099
51168	0.98453	0.300202
51169	1.32276	0.280071
51170	1.32276	0.343866
51171	1.32276	0.207701
51172	0.946132	0.315364
51173	0.946132	0.202876
51174	1.89227	2.1
51175	1.89227	2.1
51176	1.89227	2.1
51177	1.89227	2.1
51178	1.89227	2.1
51179	1.89227	2.1
51180	1.89226	2.1
51181	1.89226	2.1
51182	1.89226	2.1
51183	1.89226	2.1
51184	1.89226	2.1
51185	1.89226	2.1
51186	1.05556	2.1
51187	1.38859	2.1
51188	1.46996	2.1
51189	1.25364	2.1
51190	2.52552	2.1
51191	0.694296	2.1
51192	0.73498	2.1
51193	0.561881	2.1
51194	0.512189	2.1
51195	0.621304	2.1
51196	0.621304	2.1
51197	0.625865	2.1
51198	0.625865	2.1
51199	0.734979	2.1
51200	0.561881	2.1
51201	0.51219	2.1
51202	0.694296	2.1
51203	0.946126	2.1
51204	0.946126	2.1
51205	0.946126	2.1
51206	0.946126	2.1
51207	0.946126	2.1
51208	0.946126	2.1
51209	0.946126	2.1
51210	0.946126	2.1
51211	0.946132	2.1

51212	0.946132	2.1
51213	0.94612	2.1
51214	0.94612	2.1
51215	1.89225	2.1
51216	1.89225	2.1
51217	1.89225	2.1
51218	1.89225	2.1
51219	1.89225	2.1
51220	1.89225	2.1
51221	0.626814	2.1
51222	0.632155	2.1
51223	0.746234	2.1
51224	0.734974	2.1
51225	0.68202	2.1
51226	0.68202	2.1
51227	0.685108	2.1
51228	0.685108	2.1
51229	0.746239	2.1
51230	0.632159	2.1
51231	0.734969	2.1
51232	0.62681	2.1
51233	0.746235	2.1
51234	0.734975	2.1
51235	0.595081	2.1
51236	0.561877	2.1
51237	0.648584	2.1
51238	0.648584	2.1
51239	0.659528	2.1
51240	0.659528	2.1
51241	0.746239	2.1
51242	0.595085	2.1
51243	0.734969	2.1
51244	0.561874	2.1
51245	1.89249	2.1
51246	2.2137	2.1
51247	1.33125	2.1
51248	1.06499	2.1
51249	2.12571	2.1
51250	1.78918	2.1
51251	0.946131	2.1
51252	0.946131	2.1
51253	0.94613	2.1
51254	0.94613	2.1
51255	0.946132	2.1
51256	0.946132	2.1
51257	0.946129	2.1
51258	0.946129	2.1
51259	0.946131	2.1
51260	0.946131	2.1
51261	0.946131	2.1
51262	0.946131	2.1
51263	1.89227	2.1
51264	1.89227	2.1
51265	1.89227	2.1
51266	1.89227	2.1
51267	3.78453	2.1
51268	1.89226	2.1
51269	1.89226	2.1

51270	1.89226	2.1
51271	1.89226	2.1
51272	1.89226	2.1
51273	1.89226	2.1
51274	2.01388	2.1
51275	1.38757	2.1
51276	1.24229	2.1
51277	1.3309	2.1
51278	1.88011	2.1
51279	2.01388	2.1
51280	2.01388	2.1
51281	2.01555	2.1
51282	2.13879	2.1
51283	1.80766	2.1
51284	4.15267	2.1
51285	0.946133	2.1
51286	0.946133	2.1
51287	0.946132	2.1
51288	0.946132	2.1
51289	0.946132	2.1
51290	0.946132	2.1
51291	0.946133	2.1
51292	0.946133	2.1
51293	0.946132	2.1
51294	0.946132	2.1
51295	0.946132	2.1
51296	0.946132	2.1
51297	1.89226	2.1
51298	1.89226	2.1
51299	1.89226	2.1
51300	1.89226	2.1
51301	1.89226	2.1
51302	1.89226	2.1
51303	0.676745	2.1
51304	0.68382	2.1
51305	1.07048	2.1
51306	1.1327	2.1
51307	0.929324	2.1
51308	0.871438	2.1
51309	0.867362	2.1
51310	0.867362	2.1
51311	1.10666	2.1
51312	0.695036	2.1
51313	0.744098	2.1
51314	1.10144	2.1
51315	2.25168	2.1
51316	2.22499	2.1
51317	2.15929	2.1
51318	2.13879	2.1
51319	2.15929	2.1
51320	2.13879	2.1
51321	2.15314	2.1
51322	1.70302	2.1
51323	2.24694	2.1
51324	1.65245	2.1
51325	3.82722	2.1
51326	1.65245	2.1
51327	1.00371	2.1

51328	1.7437	2.1
51329	1.65748	2.1
51330	1.10459	2.1
51331	1.00371	2.1
51332	2.24694	2.1
51333	1.65246	2.1
51334	2.69773	2.1
51335	1.88129	2.1
51336	4.12824	2.1
51337	1.65246	2.1
51338	1.10459	2.1
51339	1.88129	2.1
51340	1.05914	2.1
51341	2.76496	2.1
51342	1.89226	2.1
51343	1.89226	2.1
51344	1.89226	2.1
51345	1.89226	2.1
51346	3.78452	2.1
51347	0.718577	2.1
51348	0.718577	2.1
51349	0.725249	2.1
51350	0.725249	2.1
51351	0.597916	2.1
51352	0.61752	2.1
51353	0.826306	2.1
51354	0.859194	2.1
51355	0.826306	2.1
51356	0.597916	2.1
51357	0.859194	2.1
51358	0.61752	2.1
51359	1.25898	2.1
51360	1.25898	2.1
51361	0.946128	2.1
51362	0.946128	2.1
51363	1.25897	2.1
51364	1.25585	2.1
51365	0.946131	2.1
51366	0.946131	2.1
51367	0.946129	2.1
51368	1.25586	2.1
51369	1.25898	2.1
51370	0.946129	2.1
51371	1.89226	2.1
51372	1.89226	2.1
51373	1.89226	2.1
51374	1.89226	2.1
51375	3.78452	2.1
51376	0.693258	2.1
51377	0.693258	2.1
51378	0.697356	2.1
51379	0.697356	2.1
51380	0.572479	2.1
51381	0.597916	2.1
51382	0.792698	2.1
51383	0.826306	2.1
51384	0.572478	2.1
51385	0.792697	2.1

51386	0.826307	2.1
51387	0.597917	2.1
51388	1.25586	2.1
51389	1.25586	2.1
51390	0.946128	2.1
51391	0.946128	2.1
51392	1.25585	2.1
51393	1.24344	2.1
51394	0.946131	2.1
51395	0.946131	2.1
51396	0.946129	2.1
51397	1.24344	2.1
51398	0.94613	2.1
51399	1.25586	2.1
51400	1.65151	2.1
51401	1.81835	2.1
51402	1.12064	2.1
51403	1.12064	2.1
51404	1.24965	2.1
51405	1.81835	2.1
51406	1.24965	2.1
51407	1.81835	2.1
51408	1.41077	2.1
51409	2.02687	2.1
51410	3.22911	2.1
51411	1.89122	1.22434
51412	1.38776	2.05747
51413	2.37014	0.994769
51414	1.80818	2.1
51415	3.6994	1.64883
51416	2.15466	1.04737
51417	2.00083	1.98538
51418	1.80818	1.42805
51419	1.80818	1.09575
51420	2.31988	1.48692
51421	2.31988	1.02681
51422	1.80818	1.01182
51423	1.18041	2.1
51424	2.00082	1.21009
51425	1.43652	2.1
51426	3.18123	2.04239
51427	1.89226	1.0301
51428	1.89226	1.2233
51429	1.89226	1.06596
51430	1.89226	1.00724
51431	3.78453	1.03791
51432	1.89226	1.27392
51433	1.89226	1.27731
51434	1.89226	1.08182
51435	1.89226	1.04551
51436	3.78452	1.42032
51437	0.80759	1.09759
51438	0.686119	1.26801
51439	0.972147	1.20714
51440	1.00217	1.25346
51441	0.821334	1.57105
51442	0.821334	1.21342
51443	0.866957	1.15627

51444	0.866957	1.09989
51445	0.972145	1.39866
51446	0.686121	2.01083
51447	0.807592	1.46798
51448	1.00217	1.96748
51449	0.972146	1.15369
51450	1.00217	1.1863
51451	1.26654	1.3402
51452	1.1908	1.20895
51453	1.05501	1.1953
51454	1.05501	1.90337
51455	1.10794	1.13305
51456	1.10794	0.977635
51457	0.972146	0.960597
51458	1.26654	1.20604
51459	1.00217	1.16267
51460	1.1908	1.62744
51461	1.89226	1.13778
51462	1.89226	0.997176
51463	1.89226	1.03464
51464	1.89226	1.41759
51465	1.89226	0.94634
51466	1.89226	1.08054
51467	0.972147	1.21141
51468	0.867854	1.32433
51469	1.26132	1.10367
51470	1.26654	1.22811
51471	1.0424	1.39506
51472	1.0424	1.70057
51473	1.092	0.980361
51474	1.092	1.22168
51475	1.26132	1.27246
51476	0.867854	2.1
51477	0.972147	1.53168
51478	1.26654	2.00811
51479	1.4375	0.686402
51480	1.92998	0.576843
51481	1.66953	0.355107
51482	1.0953	0.596688
51483	3.10703	0.476766
51484	1.89226	2.1
51485	1.89226	2.1
51486	1.89226	2.1
51487	1.89226	2.1
51488	3.78452	2.1
51489	2.74034	2.1
51490	2.08808	2.1
51491	2.63859	2.1
51492	2.00343	2.1
51493	4.72667	2.1
51494	0.799825	2.1
51495	0.799825	2.1
51496	0.80211	2.1
51497	0.80211	2.1
51498	0.562428	2.1
51499	0.600223	2.1
51500	1.04404	2.1
51501	1.00171	2.1

51502	1.00171	2.1
51503	0.56243	2.1
51504	0.600225	2.1
51505	1.04404	2.1
51506	0.94613	2.1
51507	0.94613	2.1
51508	0.946128	2.1
51509	0.946128	2.1
51510	0.946127	2.1
51511	0.946127	2.1
51512	0.946131	2.1
51513	0.946131	2.1
51514	0.946129	2.1
51515	0.946129	2.1
51516	0.946129	2.1
51517	0.946129	2.1
51518	1.89226	2.1
51519	1.89226	2.1
51520	1.89226	2.1
51521	1.89226	2.1
51522	3.78452	2.1
51523	2.63859	2.1
51524	2.00343	2.1
51525	2.54388	2.1
51526	1.91548	2.1
51527	4.55407	2.1
51528	1.91548	2.1
51529	2.00342	2.1
51530	1.04909	2.1
51531	1.04909	2.1
51532	1.12486	2.1
51533	1.99124	2.1
51534	0.810942	2.1
51535	0.810942	2.1
51536	0.810942	2.1
51537	0.810942	2.1
51538	1.89226	2.1
51539	1.35169	2.1
51540	1.08131	2.1
51541	1.08132	2.1
51542	1.08132	2.1
51543	0.810944	2.1
51544	0.810944	2.1
51545	1.89226	0.732604
51546	1.89226	0.82623
51547	1.89226	0.729645
51548	1.89226	0.844858
51549	3.78452	0.76963
51550	1.7968	0.72557
51551	1.49831	0.821564
51552	1.59244	0.664175
51553	1.3395	0.804361
51554	3.09074	0.767279
51555	1.49831	0.82234
51556	1.16858	2.1
51557	1.33951	0.700309
51558	1.0361	2.04775
51559	2.53442	0.93009

51560	1.89226	0.843366
51561	1.89226	0.822611
51562	1.89226	0.854798
51563	1.89226	2.1
51564	1.89226	2.0981
51565	1.89226	2.1
51566	1.89223	0.728786
51567	1.89223	0.815726
51568	1.89223	0.766869
51569	1.89223	0.803453
51570	1.89223	0.723361
51571	1.89223	0.816984
51572	1.59241	0.70411
51573	1.18952	0.825843
51574	1.33949	0.805246
51575	1.33949	0.729498
51576	1.40538	0.809404
51577	1.40538	0.706265
51578	1.89226	0.673696
51579	1.89226	0.700741
51580	1.89226	0.699609
51581	1.89226	0.693376
51582	1.89226	0.705904
51583	1.89226	0.706162
51584	1.89226	2.1
51585	1.89226	2.1
51586	1.89226	2.1
51587	1.89226	2.1
51588	3.78452	2.1
51589	1.89226	2.1
51590	1.89226	2.1
51591	1.89226	2.1
51592	1.89226	2.1
51593	3.78452	2.1
51594	2.01867	2.1
51595	1.46955	2.1
51596	1.58476	2.1
51597	1.38189	2.1
51598	2.22153	2.1
51599	2.01867	2.1
51600	2.01867	2.1
51601	2.52881	2.1
51602	2.21732	2.1
51603	2.21732	2.1
51604	2.22154	2.1
51605	2.22154	2.1
51606	1.89226	2.1
51607	1.89226	2.1
51608	1.89226	2.1
51609	1.89226	2.1
51610	1.89226	2.1
51611	1.89226	2.1
51612	1.89226	2.1
51613	1.89226	2.1
51614	1.89226	2.1
51615	1.89226	2.1
51616	1.89226	2.1
51617	1.89226	2.1

51618	2.22153	2.1
51619	1.63361	2.1
51620	1.77336	2.1
51621	1.46955	2.1
51622	2.52534	2.1
51623	2.22153	2.1
51624	2.34431	2.1
51625	1.62905	2.1
51626	1.49693	2.1
51627	2.19197	2.1
51628	3.82103	2.1
51629	1.89226	2.1
51630	1.89226	2.1
51631	1.89226	2.1
51632	1.89226	2.1
51633	3.78453	2.1
51634	0.946128	2.1
51635	0.946128	2.1
51636	1.22372	2.1
51637	1.17331	2.1
51638	0.946131	2.1
51639	0.946131	2.1
51640	1.17331	2.1
51641	1.17331	2.1
51642	0.946131	2.1
51643	1.17331	2.1
51644	1.22372	2.1
51645	0.94613	2.1
51646	1.04041	2.1
51647	1.09599	2.1
51648	0.748462	2.1
51649	0.701976	2.1
51650	0.896701	2.1
51651	0.896701	2.1
51652	0.901262	2.1
51653	0.901262	2.1
51654	1.04041	2.1
51655	0.701977	2.1
51656	0.748463	2.1
51657	1.09599	2.1
51658	1.89226	2.1
51659	1.89226	2.1
51660	1.89226	2.1
51661	1.89226	2.1
51662	3.78453	2.1
51663	0.946128	2.1
51664	0.946128	2.1
51665	1.17331	2.1
51666	1.13785	2.1
51667	0.94613	2.1
51668	0.94613	2.1
51669	1.13785	2.1
51670	1.13785	2.1
51671	1.13785	2.1
51672	0.94613	2.1
51673	0.94613	2.1
51674	1.17331	2.1
51675	2.50381	2.1

51676	1.89226	2.1
51677	1.89226	2.1
51678	2.73387	2.1
51679	4.39607	2.1
51680	1.80045	2.1
51681	1.99777	0.961321
51682	2.45134	1.38906
51683	2.12463	1.75847
51684	1.70302	1.44151
51685	1.42655	2.1
51686	2.20231	2.1
51687	1.70302	1.16993
51688	1.93128	2.1
51689	1.66768	1.6834
51690	1.24499	0.965551
51691	1.70302	0.959992
51692	2.55572	0.640934
51693	1.22811	0.463973
51694	1.82584	0.652655
51695	1.22811	0.660766
51696	2.62764	0.477573
51697	1.93147	0.438988
51698	2.14513	0.710633
51699	1.89226	0.750078
51700	2.66481	0.712744
51701	1.89226	0.714717
51702	2.66481	0.756891
51703	1.89226	0.755648
51704	2.11649	2.1
51705	1.89226	2.1
51706	1.89226	2.1
51707	1.89226	2.1
51708	2.15426	2.1
51709	2.15426	2.1
51710	2.06776	2.1
51711	1.89226	2.1
51712	1.89226	2.1
51713	1.89226	2.1
51714	2.11649	2.1
51715	2.11649	2.1
51716	1.89226	2.1
51717	1.89226	2.1
51718	1.89226	2.1
51719	1.89226	2.1
51720	1.89226	2.1
51721	1.89226	2.1
51722	0.998134	2.1
51723	0.918904	2.1
51724	0.894708	2.1
51725	0.758742	2.1
51726	0.864276	2.1
51727	0.864276	2.1
51728	0.8926	2.1
51729	0.8926	2.1
51730	0.998134	2.1
51731	0.894709	2.1
51732	0.758742	2.1
51733	0.918904	2.1

51734	1.89226	2.1
51735	1.89226	2.1
51736	1.89226	2.1
51737	1.89226	2.1
51738	1.89226	2.1
51739	1.89226	2.1
51740	1.89226	2.1
51741	1.89226	2.1
51742	1.89226	2.1
51743	1.89226	2.1
51744	1.89226	2.1
51745	1.89226	2.1
51746	1.99627	2.1
51747	1.61047	2.1
51748	2.0815	2.1
51749	2.07569	2.1
51750	3.75063	2.1
51751	0.848077	2.1
51752	1.07748	2.1
51753	0.802498	2.1
51754	0.626808	2.1
51755	0.875552	2.1
51756	0.737527	2.1
51757	0.835602	2.1
51758	0.835602	2.1
51759	0.983245	2.1
51760	0.781108	2.1
51761	0.872306	2.1
51762	0.601497	2.1
51763	2.74745	2.1
51764	2.73988	2.1
51765	2.6521	2.1
51766	2.67792	2.1
51767	5.39198	2.1
51768	2.73988	2.1
51769	2.57466	2.1
51770	2.67791	2.1
51771	2.55653	2.1
51772	5.29641	2.1
51773	2.6521	2.1
51774	2.64148	2.1
51775	2.67792	2.1
51776	2.67792	2.1
51777	2.56953	2.1
51778	2.56953	2.1
51779	2.64148	2.1
51780	2.55653	2.1
51781	2.57929	2.1
51782	2.57929	2.1
51783	2.67792	2.1
51784	2.67792	2.1
51785	1.89226	0.827036
51786	1.89226	0.775191
51787	1.89226	0.903613
51788	1.89226	0.181066
51789	3.78453	0.756776
51790	1.68703	0.823483
51791	2.50385	0.212738

51792	1.45958	0.347655
51793	2.0815	0.230912
51794	3.93806	0.337719
51795	2.50385	0.205849
51796	2.57414	0.436161
51797	2.0815	0.371255
51798	2.39921	0.480485
51799	4.91181	0.159855
51800	1.89226	0.775559
51801	1.89226	0.440508
51802	1.89226	0.208207
51803	1.89226	0.552769
51804	3.78453	0.602748
51805	1.89226	0.91786
51806	1.89226	0.249028
51807	1.89226	0.779024
51808	1.89226	0.785066
51809	1.89226	0.332689
51810	1.89226	0.836076
51811	1.23013	0.319139
51812	2.0815	0.306395
51813	1.90167	0.572577
51814	1.71243	0.38925
51815	1.45887	0.379659
51816	1.31369	0.187294
51817	2.0815	0.548488
51818	2.39921	0.540386
51819	1.7115	0.218066
51820	2.2421	0.301353
51821	4.09829	0.358897
51822	1.89227	0.312934
51823	1.89227	0.639757
51824	1.89227	0.528112
51825	1.89227	0.308148
51826	3.78453	0.258657
51827	2.39697	0.0993848
51828	1.85594	0.117752
51829	1.91161	0.0953044
51830	2.49867	0.120337
51831	4.35461	0.113845
51832	1.12035	0.0939656
51833	1.85594	0.106034
51834	1.91161	0.0842879
51835	1.15483	0.0935676
51836	3.01077	0.0902786
51837	1.89226	0.104945
51838	1.89226	0.145231
51839	1.89226	0.111042
51840	1.89226	0.124168
51841	1.89226	0.112568
51842	1.89226	0.132085
51843	1.89226	0.131826
51844	1.89226	0.163437
51845	1.89226	0.147158
51846	1.89226	0.129533
51847	1.89226	0.165014
51848	1.89226	0.157287
51849	0.936516	0.103502

51850	1.03496	0.0902685
51851	1.22607	0.130728
51852	1.28058	0.0961215
51853	1.06317	0.108537
51854	1.06317	0.109329
51855	1.19735	0.0882512
51856	1.0813	0.0937274
51857	0.858168	0.107998
51858	1.32278	0.0991452
51859	0.957674	0.102887
51860	1.25178	0.100668
51861	1.92659	0.0850963
51862	0.998957	0.0891684
51863	0.998957	0.112296
51864	1.15481	0.0878539
51865	1.6031	0.0977705
51866	1.74459	0.0854708
51867	0.946119	0.109219
51868	0.946119	0.121156
51869	0.946118	0.108705
51870	0.946118	0.126349
51871	0.946118	0.125807
51872	0.946118	0.114197
51873	0.946119	0.09651
51874	0.946119	0.109584
51875	0.946131	0.113952
51876	0.946131	0.118881
51877	0.946106	0.119708
51878	0.946106	0.115537
51879	0.946118	0.137828
51880	0.946118	0.161971
51881	0.946119	0.130283
51882	0.946119	0.15128
51883	0.946118	0.15499
51884	0.946118	0.151669
51885	0.946119	0.134089
51886	0.946119	0.132174
51887	0.946131	0.138356
51888	0.946131	0.134529
51889	0.946106	0.141383
51890	0.946106	0.137724
51891	2.47164	0.834027
51892	2.83689	0.68118
51893	2.73872	0.834022
51894	2.41632	0.689323
51895	5.21036	0.697498
51896	0.946133	0.797508
51897	0.946133	0.828114
51898	1.41845	0.754933
51899	1.36936	0.832503
51900	1.41845	0.688423
51901	1.41845	0.670481
51902	0.946132	0.82627
51903	0.946132	0.838798
51904	0.946132	0.806189
51905	1.36936	0.754016
51906	1.41845	0.787633
51907	0.946133	0.820526

51908	1.36936	0.496289
51909	1.27581	0.761194
51910	1.20816	0.654324
51911	1.10464	0.802185
51912	1.24454	0.556178
51913	1.24454	0.58093
51914	1.23943	0.786755
51915	1.23943	0.799145
51916	1.10465	0.761091
51917	1.27581	0.728302
51918	1.36936	0.761375
51919	1.20816	0.67446
51920	0.946131	2.1
51921	0.946131	2.1
51922	1.37277	2.1
51923	1.37277	2.1
51924	1.37277	2.1
51925	1.27186	2.1
51926	0.946131	2.1
51927	0.946131	2.1
51928	0.946131	2.1
51929	1.37277	2.1
51930	0.946131	2.1
51931	1.27186	2.1
51932	2.74554	2.1
51933	1.23712	2.1
51934	2.80992	2.1
51935	2.54371	2.1
51936	1.50333	2.1
51937	1.23712	2.1
51938	2.77314	2.1
51939	2.33316	2.1
51940	2.28748	2.1
51941	2.28748	2.1
51942	2.81275	2.1
51943	2.81275	2.1
51944	2.28749	2.1
51945	1.45887	2.1
51946	2.33317	2.1
51947	1.50022	2.1
51948	3.79204	2.1
51949	2.37364	2.1
51950	2.33317	2.1
51951	1.5362	2.1
51952	1.5362	2.1
51953	1.50022	2.1
51954	2.33317	2.1
51955	1.89226	0.803044
51956	1.89226	0.797856
51957	1.89226	0.794121
51958	1.89226	0.814979
51959	1.89226	0.797735
51960	1.89226	0.825316
51961	0.946128	0.751664
51962	0.946128	0.758728
51963	1.0644	0.736179
51964	0.946133	0.749302
51965	1.0644	0.729575

51966	0.94613	0.742504
51967	0.946131	0.761902
51968	0.946131	0.753887
51969	1.0644	0.747897
51970	1.0644	0.750415
51971	0.946131	0.749505
51972	0.946131	0.744642
51973	1.89226	0.964438
51974	1.89226	0.759549
51975	1.89226	0.893691
51976	1.89226	0.724383
51977	3.78452	0.797793
51978	1.14599	0.95934
51979	1.19993	0.740056
51980	1.00065	0.905033
51981	1.07497	0.719016
51982	2.22096	0.836054
51983	0.546551	0.762569
51984	0.546551	0.750392
51985	0.54987	0.740132
51986	0.54987	0.753846
51987	0.503067	0.696413
51988	0.558937	0.697694
51989	0.599966	0.7156
51990	0.537484	0.723798
51991	0.503067	0.675796
51992	0.537485	0.6743
51993	0.599966	0.709778
51994	0.558936	0.700926
51995	0.946132	0.742151
51996	0.946132	0.735627
51997	0.94613	0.722072
51998	0.94613	0.743126
51999	0.946131	0.723702
52000	0.946131	0.724906
52001	0.946131	0.72425
52002	0.946131	0.72202
52003	0.946131	0.721546
52004	0.946131	0.705637
52005	0.946131	0.717169
52006	0.946131	0.724503
52007	2.19104	2.1
52008	2.5392	2.1
52009	2.53302	2.1
52010	2.19226	2.1
52011	4.73146	2.1
52012	1.09552	2.1
52013	1.09613	2.1
52014	0.802484	2.1
52015	0.807941	2.1
52016	0.95052	2.1
52017	0.95052	2.1
52018	0.952944	2.1
52019	0.952944	2.1
52020	1.09613	2.1
52021	0.807941	2.1
52022	0.802484	2.1
52023	1.09552	2.1

52024	2.533	2.1
52025	2.18531	2.1
52026	2.19224	2.1
52027	2.19224	2.1
52028	2.51603	2.1
52029	2.51603	2.1
52030	1.09266	2.1
52031	1.09612	2.1
52032	0.807935	2.1
52033	0.81091	2.1
52034	0.951897	2.1
52035	0.951897	2.1
52036	0.955134	2.1
52037	0.955134	2.1
52038	0.810915	2.1
52039	1.09266	2.1
52040	1.09611	2.1
52041	0.80793	2.1
52042	2.12741	0.763027
52043	2.79312	0.781028
52044	2.77591	0.942066
52045	2.10714	0.784954
52046	4.90026	0.773966
52047	2.12741	0.783048
52048	1.20859	0.754551
52049	1.21073	0.922097
52050	2.10714	0.80626
52051	3.31573	0.81327
52052	1.38708	0.883461
52053	1.38795	0.804392
52054	1.05331	0.79455
52055	1.05357	0.759348
52056	1.22049	0.776057
52057	1.22049	0.792997
52058	1.22078	0.958192
52059	1.22078	0.751733
52060	1.05331	0.827241
52061	1.38709	0.833295
52062	1.38795	0.76682
52063	1.05357	0.768646
52064	2.10662	0.87406
52065	2.10714	0.742144
52066	2.12499	0.758963
52067	1.22857	0.844624
52068	1.21073	0.79467
52069	1.21073	0.748116
52070	1.82014	0.251296
52071	2.08757	0.235806
52072	1.81292	0.175471
52073	1.67485	0.239839
52074	3.63306	0.196426
52075	1.82013	0.232381
52076	1.21748	0.283618
52077	1.29891	0.261615
52078	1.4442	0.316336
52079	1.67484	0.222166
52080	1.82013	0.290351
52081	1.81292	0.15678

52082	1.4516	0.149417
52083	1.39633	0.140432
52084	1.67485	0.157432
52085	1.5344	0.136411
52086	1.81292	0.160609
52087	1.67484	0.146626
52088	1.00982	0.161203
52089	0.994235	0.160332
52090	1.21748	0.186604
52091	1.45159	0.143578
52092	1.67484	0.183896
52093	1.55216	2.1
52094	1.34407	2.1
52095	1.34407	2.1
52096	1.52008	2.1
52097	1.81783	2.1
52098	1.99384	2.1
52099	1.90817	2.1
52100	2.03975	2.1
52101	1.97317	2.1
52102	1.81783	2.1
52103	3.85758	2.1
52104	0.599049	2.1
52105	0.660673	2.1
52106	0.785063	2.1
52107	0.924278	2.1
52108	0.760416	2.1
52109	0.760416	2.1
52110	0.719088	2.1
52111	0.694532	2.1
52112	0.522441	2.1
52113	0.796789	2.1
52114	0.655207	2.1
52115	0.886155	2.1
52116	1.97317	2.1
52117	1.81783	2.1
52118	1.65098	2.1
52119	1.91767	2.1
52120	3.7355	2.1
52121	1.15038	1.16443
52122	1.28649	1.85671
52123	1.38986	1.2597
52124	1.38986	1.97712
52125	1.07866	1.35358
52126	1.07866	2.08475
52127	0.664498	0.90744
52128	0.664498	2.1
52129	0.675122	1.55346
52130	0.675122	2.1
52131	0.69493	1.06881
52132	0.764429	1.48061
52133	0.623438	1.29136
52134	0.575191	1.91423
52135	0.764429	1.14173
52136	0.623438	1.0822
52137	0.575191	1.86988
52138	0.69493	1.69991
52139	1.89226	0.783058

52140	1.89226	0.414511
52141	1.89226	0.788458
52142	1.89226	0.520623
52143	3.78453	0.318175
52144	2.50075	0.738405
52145	2.15276	0.477116
52146	2.24902	0.788063
52147	2.30422	0.328291
52148	4.40178	0.271764
52149	1.70424	0.817066
52150	1.34494	0.841551
52151	2.15276	0.613981
52152	2.15276	0.819306
52153	2.0793	0.573272
52154	1.63078	0.768619
52155	1.89226	0.350209
52156	1.89226	0.813498
52157	1.89226	0.871558
52158	1.89226	0.631716
52159	1.89226	0.839842
52160	1.89226	0.779345
52161	1.89226	0.792068
52162	1.89226	0.736381
52163	1.89226	0.548127
52164	1.89226	0.265624
52165	3.78452	0.584674
52166	2.24902	0.793145
52167	1.91497	0.253742
52168	1.97135	0.839416
52169	1.73957	0.384734
52170	3.98859	0.202652
52171	1.73957	0.201859
52172	1.34494	0.802929
52173	1.73957	0.878471
52174	1.69006	0.504286
52175	1.3126	0.821025
52176	1.34848	0.751736
52177	0.946132	0.471141
52178	0.946132	0.819082
52179	0.946132	0.38054
52180	0.946132	0.856883
52181	0.946132	0.840607
52182	0.946132	0.818769
52183	0.946131	0.507045
52184	0.946131	0.535724
52185	0.946132	0.602613
52186	0.946132	0.465976
52187	0.946132	0.767479
52188	0.946132	0.772658
52189	1.89226	2.08422
52190	1.89226	1.4928
52191	1.89226	1.99037
52192	1.89226	1.57051
52193	3.78453	1.75858
52194	1.89226	1.81977
52195	2.82624	1.35205
52196	1.89226	1.68455
52197	2.02536	1.37134

52198	1.89226	1.6194
52199	2.02536	1.33291
52200	2.25027	0.0869581
52201	1.1706	0.102105
52202	2.02733	0.0935355
52203	2.02733	0.10291
52204	1.61462	0.0874144
52205	1.69832	0.0987444
52206	1.89226	0.147915
52207	1.89226	0.141249
52208	1.89226	0.127394
52209	1.89226	0.12647
52210	3.78452	0.112822
52211	0.946131	0.0891865
52212	0.946131	0.0944155
52213	1.18182	0.0802144
52214	1.18182	0.0751386
52215	0.973819	0.0830645
52216	0.94613	0.0874406
52217	1.18182	0.0875847
52218	0.94613	0.0860452
52219	0.973819	0.0931863
52220	1.18182	0.0912783
52221	0.94613	0.100777
52222	0.94613	0.0915677
52223	2.23944	2.1
52224	1.52903	2.1
52225	1.35977	2.1
52226	2.04847	2.1
52227	3.5775	2.1
52228	2.23944	2.1
52229	2.34795	2.1
52230	2.34795	2.1
52231	2.48025	2.1
52232	2.04847	2.1
52233	2.18077	2.1
52234	1.89226	2.1
52235	1.89226	2.1
52236	1.89226	2.1
52237	1.89226	2.1
52238	3.78452	2.1
52239	0.946127	2.1
52240	0.946127	2.1
52241	0.946131	2.1
52242	0.946131	2.1
52243	0.946128	2.1
52244	0.946128	2.1
52245	0.94613	2.1
52246	0.94613	2.1
52247	0.946129	2.1
52248	0.946129	2.1
52249	0.946129	2.1
52250	0.946129	2.1
52251	1.26956	2.1
52252	2.04847	2.1
52253	1.49576	2.1
52254	1.22377	2.1
52255	1.91172	2.1

52256	1.50198	2.1
52257	1.48568	2.1
52258	2.34795	2.1
52259	2.04847	2.1
52260	2.04847	2.1
52261	2.21155	2.1
52262	2.21155	2.1
52263	1.89226	2.1
52264	1.89226	2.1
52265	1.89226	2.1
52266	1.89226	2.1
52267	1.89226	2.1
52268	1.89226	1.79278
52269	0.810943	2.1
52270	0.810943	2.1
52271	0.810944	2.1
52272	0.810944	2.1
52273	0.810957	2.1
52274	0.810957	2.1
52275	1.89226	2.1
52276	1.35168	2.1
52277	1.08132	2.1
52278	1.08132	2.1
52279	1.0813	2.1
52280	0.946132	0.220141
52281	1.33855	0.32209
52282	0.946131	0.160998
52283	1.38204	0.308821
52284	0.946131	0.321382
52285	0.946131	0.322479
52286	1.38204	0.201592
52287	1.38204	0.185688
52288	0.946131	0.215335
52289	0.946131	0.273409
52290	1.33855	0.197426
52291	1.38204	0.301901
52292	1.89226	0.333597
52293	2.76407	0.38235
52294	1.89226	0.213894
52295	2.72769	0.38986
52296	1.89226	0.215555
52297	2.72769	0.36033
52298	2.03914	0.143342
52299	1.67727	0.232895
52300	1.94831	0.179524
52301	1.81591	0.158874
52302	2.32098	0.202962
52303	2.32098	0.200441
52304	0.801647	0.123322
52305	1.11086	0.15441
52306	0.838634	0.184891
52307	1.16049	0.136367
52308	0.977891	0.196758
52309	0.977891	0.195274
52310	0.984246	0.123123
52311	0.984246	0.154204
52312	1.11086	0.147734
52313	1.16049	0.175138

52314	0.801647	0.16996
52315	0.838634	0.16863
52316	0.946132	0.238005
52317	0.946132	0.368596
52318	0.946131	0.17608
52319	0.946131	0.320719
52320	0.946131	0.342905
52321	0.946131	0.334154
52322	0.946132	0.207577
52323	0.946132	0.205701
52324	0.946131	0.234086
52325	0.946131	0.258696
52326	0.946131	0.23885
52327	0.946131	0.300824
52328	0.946131	0.257571
52329	0.946131	0.462741
52330	0.946131	0.315115
52331	0.946131	0.206345
52332	0.946131	0.335388
52333	0.946131	0.348439
52334	0.946131	0.22673
52335	0.946131	0.221818
52336	0.946131	0.246992
52337	0.946131	0.291194
52338	0.946131	0.318225
52339	0.946131	0.318965
52340	2.0815	2.1
52341	1.20787	2.1
52342	1.92277	2.1
52343	1.3227	2.1
52344	3.29401	2.1
52345	2.16633	2.1
52346	2.13642	2.1
52347	1.40846	2.1
52348	1.40846	2.1
52349	1.3227	2.1
52350	2.08058	2.1
52351	0.938106	2.1
52352	0.790258	2.1
52353	0.742975	2.1
52354	0.742975	2.1
52355	0.874389	2.1
52356	1.17364	2.1
52357	0.525948	2.1
52358	0.52513	2.1
52359	0.553732	2.1
52360	0.876996	2.1
52361	0.530954	2.1
52362	1.095	2.1
52363	1.00833	2.1
52364	1.70608	2.1
52365	1.33385	2.1
52366	1.33385	2.1
52367	1.07722	2.1
52368	1.07722	2.1
52369	0.953523	1.05918
52370	1.7457	1.02981
52371	1.03218	1.06425

52372	1.03218	1.02896
52373	1.79675	1.07625
52374	1.79675	1.03832
52375	1.13676	1.02389
52376	1.18136	0.952989
52377	0.87285	1.03069
52378	0.898375	1.03901
52379	1.03191	1.02628
52380	1.03191	0.956089
52381	1.0223	1.01305
52382	1.0223	1.04305
52383	1.18136	1.00394
52384	0.898374	0.990646
52385	0.87285	1.01335
52386	1.13676	1.01452
52387	2.36273	0.963001
52388	1.46965	0.947887
52389	1.79675	0.95769
52390	1.79675	0.908938
52391	2.43417	0.975928
52392	2.43417	0.925663
52393	1.4714	2.09931
52394	1.65434	1.79797
52395	1.62322	1.08133
52396	1.86068	1.55955
52397	3.27756	1.52904
52398	1.89226	2.1
52399	1.89226	1.85463
52400	1.89226	1.10587
52401	1.89226	1.17603
52402	3.78453	1.32364
52403	1.89226	1.3895
52404	1.89226	2.1
52405	1.89226	1.07623
52406	1.89226	2.1
52407	3.78453	1.42451
52408	1.65434	1.3886
52409	1.60647	2.1
52410	1.86068	1.21759
52411	1.82649	2.1
52412	3.48083	1.99862
52413	1.62322	2.05925
52414	1.86068	1.83576
52415	1.84368	1.0492
52416	2.18862	1.28914
52417	3.81184	1.48772
52418	1.89226	2.09533
52419	1.89226	1.71264
52420	1.89226	0.989583
52421	1.89226	1.34112
52422	3.78453	1.40566
52423	1.89226	1.4094
52424	1.89226	2.1
52425	1.89226	1.17297
52426	1.89226	2.07384
52427	3.78453	1.80413
52428	1.86068	1.31421
52429	1.82649	2.1

52430	2.18862	1.14748
52431	2.1128	2.1
52432	4.01511	1.42201
52433	1.74351	2.1
52434	1.13854	2.1
52435	1.40233	2.1
52436	1.922 2.1	
52437	3.14584	2.1
52438	1.922 2.1	
52439	2.00775	2.1
52440	1.7435	2.1
52441	2.09224	2.1
52442	2.00775	2.1
52443	2.09295	2.1
52444	1.69858	2.1
52445	1.922 2.1	
52446	1.40233	2.1
52447	1.40233	2.1
52448	2.18238	2.1
52449	2.18238	2.1
52450	1.00387	2.1
52451	1.10886	2.1
52452	0.961001	2.1
52453	1.09119	2.1
52454	1.02868	2.1
52455	1.02868	2.1
52456	1.04118	2.1
52457	1.04118	2.1
52458	1.10886	2.1
52459	1.09119	2.1
52460	0.960999	2.1
52461	1.00388	2.1
52462	0.885958	0.760868
52463	0.885958	0.736222
52464	0.875826	0.730923
52465	0.875826	0.786945
52466	0.913965	0.782786
52467	0.598609	0.762993
52468	1.16317	0.732425
52469	0.868115	0.736451
52470	0.868115	0.753161
52471	0.598609	0.749696
52472	1.16317	0.754127
52473	0.913965	0.75726
52474	0.946131	0.778699
52475	0.946131	0.760846
52476	0.946131	0.771761
52477	0.946131	0.743646
52478	0.946131	0.770982
52479	0.946131	0.779984
52480	0.946131	0.75949
52481	0.946131	0.742787
52482	0.946131	0.767759
52483	0.946131	0.767105
52484	0.946131	0.762411
52485	0.946131	0.764423
52486	0.946133	0.108233
52487	0.946133	0.114813

52488	1.06246	0.10891
52489	1.06246	0.105864
52490	0.973851	0.114396
52491	0.946133	0.110992
52492	1.06246	0.102117
52493	0.946132	0.112055
52494	0.97385	0.114344
52495	1.06246	0.101796
52496	0.946133	0.105772
52497	0.946133	0.124284
52498	1.89226	0.154243
52499	1.89226	0.150968
52500	1.89226	0.168024
52501	1.89226	0.176818
52502	1.89226	0.132079
52503	1.89226	0.13685
52504	0.946131	0.13454
52505	0.946131	0.134863
52506	0.946127	0.135191
52507	0.946127	0.135654
52508	0.946131	0.157377
52509	0.946131	0.12688
52510	0.946127	0.127584
52511	0.946127	0.124792
52512	0.946128	0.129069
52513	0.946128	0.144359
52514	0.946129	0.12622
52515	0.946129	0.154232
52516	0.946132	0.091892
52517	0.946132	0.111949
52518	1.32446	0.0919501
52519	0.794912	0.0808516
52520	1.32429	0.0940041
52521	0.946131	0.0935984
52522	1.32446	0.0901076
52523	0.94613	0.0929345
52524	1.32429	0.0952736
52525	0.794915	0.0769615
52526	0.94613	0.0989307
52527	0.94613	0.0996168
52528	1.89226	0.0864467
52529	2.36365	0.0885489
52530	2.64857	0.0885799
52531	1.89226	0.0892191
52532	2.64857	0.080605
52533	1.89226	0.0848982
52534	1.18463	2.1
52535	1.27174	2.1
52536	0.946129	2.1
52537	0.946129	2.1
52538	0.946131	2.1
52539	0.946131	2.1
52540	1.27174	2.1
52541	1.27174	2.1
52542	1.18463	2.1
52543	0.94613	2.1
52544	0.946131	2.1
52545	1.27174	2.1

52546	2.57997	2.1
52547	1.89226	2.1
52548	1.89226	2.1
52549	1.89226	2.1
52550	2.41915	2.1
52551	2.41915	2.1
52552	1.89226	2.1
52553	1.89226	2.1
52554	1.89226	2.1
52555	1.89226	2.1
52556	3.78452	2.1
52557	1.08807	2.1
52558	1.18463	2.1
52559	0.946129	2.1
52560	0.946129	2.1
52561	0.946131	2.1
52562	0.946131	2.1
52563	1.18463	2.1
52564	1.18463	2.1
52565	0.946131	2.1
52566	1.08807	2.1
52567	1.18463	2.1
52568	0.94613	2.1
52569	1.07017	2.1
52570	1.07017	2.1
52571	0.946131	2.1
52572	0.946131	2.1
52573	0.946132	2.1
52574	0.946132	2.1
52575	1.20957	2.1
52576	1.07017	2.1
52577	1.07017	2.1
52578	0.946132	2.1
52579	0.946131	2.1
52580	1.20957	2.1
52581	1.89226	2.1
52582	1.89226	2.1
52583	1.89226	2.1
52584	1.89226	2.1
52585	1.89226	2.1
52586	1.89226	2.1
52587	1.16181	0.102078
52588	1.0227	0.112845
52589	1.0227	0.131417
52590	0.970959	0.109199
52591	1.24295	0.109063
52592	1.19121	0.0987882
52593	1.12076	2.1
52594	2.53614	2.1
52595	1.28413	2.1
52596	1.28413	2.1
52597	2.36423	2.1
52598	2.36423	2.1
52599	1.89226	2.1
52600	1.89226	2.1
52601	1.89226	2.1
52602	1.89226	2.1
52603	3.78452	2.1

52604	1.22557	2.1
52605	2.771	2.1
52606	2.73012	2.1
52607	2.73012	2.1
52608	1.33356	2.1
52609	1.33356	2.1
52610	0.998682	2.1
52611	2.36423	2.1
52612	1.12076	2.1
52613	1.12076	2.1
52614	2.13757	2.1
52615	2.13757	2.1
52616	1.89226	2.1
52617	1.89226	2.1
52618	1.89226	2.1
52619	1.89226	2.1
52620	1.89226	2.1
52621	1.89226	2.1
52622	1.13987	2.1
52623	2.73011	2.1
52624	2.70875	2.1
52625	2.70875	2.1
52626	1.22557	2.1
52627	1.22557	2.1
52628	1.89227	2.1
52629	2.79243	2.1
52630	2.6625	2.1
52631	2.6625	2.1
52632	1.89227	2.1
52633	1.89227	2.1
52634	1.89226	2.1
52635	2.55971	2.1
52636	1.89226	2.1
52637	1.89226	2.1
52638	2.68704	2.1
52639	2.68704	2.1
52640	2.22043	1.64169
52641	2.38975	1.62385
52642	2.28696	1.09197
52643	2.48682	1.00837
52644	4.67671	1.13344
52645	2.38976	1.1696
52646	2.31988	1.55481
52647	2.48683	1.01164
52648	2.4594	1.22805
52649	4.84915	1.37452
52650	2.28696	1.42206
52651	2.48682	1.41449
52652	2.33379	1.63555
52653	2.56149	0.953351
52654	4.84845	1.3071
52655	2.48683	1.47554
52656	2.4594	1.38699
52657	2.5615	0.966706
52658	2.57957	1.18188
52659	5.0209	1.07852
52660	1.06659	2.1
52661	1.04291	0.954329

52662	1.36595	0.917064
52663	1.36595	2.1
52664	1.02761	2.1
52665	1.32697	0.915258
52666	0.946132	1.2702
52667	0.946132	1.22139
52668	1.36956	2.1
52669	1.1229	2.1
52670	1.1229	1.19988
52671	1.1229	2.1
52672	0.946132	1.0699
52673	0.946132	2.02292
52674	0.946132	1.6656
52675	1.36956	1.73358
52676	1.1229	1.60289
52677	0.946133	1.56135
52678	0.946133	1.03174
52679	0.946133	0.996815
52680	0.946128	2.03585
52681	0.946128	1.72598
52682	0.946131	1.8108
52683	0.946131	1.25081
52684	0.946131	0.873763
52685	0.946131	1.94109
52686	0.94613	1.46241
52687	0.94613	1.45708
52688	0.946131	1.25796
52689	0.946131	1.23359
52690	0.966427	1.14065
52691	0.802265	1.94116
52692	0.803714	1.87795
52693	0.808476	1.45056
52694	1.94371	1.87186
52695	1.36851	1.95301
52696	0.853864	2.1
52697	1.26635	1.35747
52698	1.09085	1.21991
52699	0.857653	1.90545
52700	0.630243	1.45942
52701	1.20132	0.108122
52702	1.24295	0.125192
52703	1.24295	0.137353
52704	1.16182	0.117063
52705	1.28465	0.124126
52706	1.20351	0.108596
52707	0.600658	0.112442
52708	0.642323	0.13254
52709	0.54424	0.133437
52710	0.576779	0.117778
52711	0.590985	0.1414
52712	0.590985	0.153934
52713	0.586452	0.113365
52714	0.586452	0.127069
52715	0.642323	0.13652
52716	0.576779	0.127832
52717	0.600658	0.138335
52718	0.54424	0.133469
52719	1.28463	0.107054

52720	1.24294	0.12472
52721	1.12328	0.103907
52722	1.15505	0.122468
52723	2.39798	0.118678
52724	1.98431	0.232823
52725	2.18735	0.29682
52726	1.98431	0.301737
52727	1.70303	0.223537
52728	1.70302	0.403972
52729	1.37788	0.242638
52730	1.89226	0.390531
52731	2.37417	0.470033
52732	1.89226	0.488376
52733	2.18735	0.371469
52734	1.89226	0.562484
52735	2.18735	0.419273
52736	1.89226	0.282591
52737	1.89226	0.836789
52738	1.89226	0.64087
52739	1.89226	0.773327
52740	3.78452	0.776482
52741	1.8613	0.54073
52742	2.03576	0.837357
52743	1.61652	0.640119
52744	1.83082	1.01385
52745	3.65228	0.510766
52746	2.03576	0.837192
52747	2.05257	2.1
52748	1.83082	1.00121
52749	1.88487	1.43758
52750	3.92064	1.13336
52751	1.89226	0.834112
52752	1.89226	0.90997
52753	1.89226	0.780456
52754	1.89226	1.58452
52755	3.78453	0.849788
52756	0.946131	0.869045
52757	0.946131	0.301833
52758	0.94613	0.153156
52759	0.94613	0.78884
52760	0.946129	0.696124
52761	0.946129	0.85669
52762	0.946132	0.788943
52763	0.946132	0.138318
52764	0.94613	0.586653
52765	0.94613	0.732952
52766	0.946131	0.231128
52767	0.946131	0.620131
52768	0.804708	1.51065
52769	0.804708	0.572636
52770	0.815274	0.863411
52771	0.815274	0.639627
52772	0.83277	0.859971
52773	0.915407	1.16674
52774	0.80826	0.733552
52775	0.704574	0.533929
52776	0.832771	0.852692
52777	0.704573	0.864148

52778	0.808259	0.816572
52779	0.915409	0.79702
52780	1.83082	1.01375
52781	1.88487	1.64215
52782	1.66555	0.867315
52783	1.75835	2.1
52784	3.55042	1.01496
52785	0.810947	0.630794
52786	0.810947	0.91256
52787	0.810947	0.765545
52788	0.810947	0.843187
52789	0.810934	0.869954
52790	0.810934	0.868899
52791	1.89227	1.65085
52792	1.3517	0.937771
52793	1.08132	0.875216
52794	1.08132	0.905115
52795	1.08133	0.88022
52796	1.89226	1.44168
52797	1.70303	1.59848
52798	1.89226	2.1
52799	2.24712	2.1
52800	2.07363	2.1
52801	1.74617	1.57115
52802	1.89226	2.1
52803	1.89226	1.23232
52804	1.89226	1.08911
52805	1.89226	2.1
52806	3.78452	1.2713
52807	1.50108	2.1
52808	1.43894	1.92841
52809	1.8322	1.02398
52810	1.40967	2.1
52811	1.50108	2.02801
52812	1.07854	2.1
52813	1.89226	1.06108
52814	1.89226	2.1
52815	1.89226	1.91201
52816	1.89226	0.930155
52817	3.78452	1.63341
52818	1.89226	2.1
52819	1.89226	1.22523
52820	1.89226	1.2386
52821	1.89226	2.1
52822	3.78453	1.31941
52823	1.54457	2.1
52824	1.35097	1.42308
52825	1.70754	1.11555
52826	1.29629	2.1
52827	1.54457	1.86123
52828	1.0305	2.1
52829	0.731763	2.1
52830	1.00043	1.72192
52831	1.13883	1.14285
52832	0.939026	1.74948
52833	1.04003	1.12359
52834	0.853868	2.1
52835	0.927188	2.02836

52836	0.927188	0.921987
52837	0.861565	1.3223
52838	1.13882	1.67758
52839	0.731769	1.57256
52840	0.89319	1.8456
52841	1.46568	2.1
52842	1.20761	2.1
52843	1.51916	2.1
52844	1.36261	2.1
52845	2.72677	2.1
52846	1.51916	2.1
52847	1.60532	2.1
52848	1.36261	2.1
52849	1.36261	2.1
52850	1.64892	2.1
52851	1.64892	2.1
52852	1.3626	2.1
52853	0.98488	2.1
52854	1.60532	2.1
52855	1.32414	2.1
52856	2.68675	2.1
52857	1.78711	2.1
52858	1.14825	2.1
52859	2.16535	2.1
52860	1.6067	2.1
52861	3.3136	2.1
52862	1.89226	2.1
52863	1.89226	2.1
52864	1.89226	2.1
52865	1.89226	2.1
52866	3.78452	2.1
52867	2.34552	2.1
52868	1.53521	2.1
52869	1.53521	2.1
52870	1.60636	2.1
52871	1.92797	2.1
52872	1.99589	2.1
52873	1.34771	2.1
52874	1.34771	2.1
52875	0.946128	2.1
52876	0.946128	2.1
52877	1.3477	2.1
52878	1.31776	2.1
52879	0.946131	2.1
52880	0.946131	2.1
52881	0.94613	2.1
52882	1.31776	2.1
52883	0.946129	2.1
52884	1.3477	2.1
52885	1.89226	2.1
52886	1.89226	2.1
52887	1.89226	2.1
52888	1.89226	2.1
52889	3.78452	2.1
52890	0.92843	2.1
52891	0.672114	2.1
52892	0.89514	2.1
52893	0.89514	2.1

52894	0.720104	2.1
52895	0.755753	2.1
52896	0.789158	2.1
52897	0.967302	2.1
52898	1.06565	2.1
52899	0.7397	2.1
52900	0.758893	2.1
52901	0.94672	2.1
52902	1.89226	2.1
52903	2.588	2.1
52904	2.588	2.1
52905	2.63552	2.1
52906	1.89226	2.1
52907	1.93978	2.1
52908	1.49347	0.35819
52909	2.67757	0.559406
52910	1.80061	0.44621
52911	2.27056	0.498608
52912	4.07117	0.3931
52913	1.1016	0.242642
52914	2.27057	0.358102
52915	1.49348	0.448976
52916	1.81893	0.458159
52917	1.49348	0.259093
52918	1.81893	0.230444
52919	1.81893	0.399109
52920	2.82339	0.589794
52921	2.27057	0.445625
52922	2.25405	0.444804
52923	4.52462	0.363791
52924	1.89226	0.464375
52925	1.89226	0.662261
52926	1.89226	0.615408
52927	1.89226	0.572631
52928	1.89226	0.628427
52929	1.89226	0.63967
52930	1.89226	0.365365
52931	1.89226	0.555573
52932	1.89226	0.353338
52933	1.89226	0.412373
52934	3.78453	0.323988
52935	1.89226	0.616551
52936	1.89226	0.674396
52937	1.89226	0.443916
52938	1.89226	0.673375
52939	3.78452	0.701091
52940	1.89226	0.693014
52941	1.89226	0.794003
52942	1.89226	0.659271
52943	1.89226	0.707329
52944	3.78451	0.648953
52945	1.45633	2.1
52946	1.34678	2.1
52947	1.34678	2.1
52948	1.53735	2.1
52949	1.234	2.1
52950	1.42457	2.1
52951	1.89226	2.1

52952	1.89226	2.1
52953	1.89226	2.1
52954	1.89226	2.1
52955	1.89226	2.1
52956	1.89226	2.1
52957	1.09483	2.1
52958	1.34678	2.1
52959	1.234	2.1
52960	1.234	2.1
52961	1.19	2.1
52962	1.19	2.1
52963	0.946131	2.1
52964	0.946131	2.1
52965	0.946131	2.1
52966	0.946131	2.1
52967	0.946131	2.1
52968	0.946131	2.1
52969	0.946132	2.1
52970	0.946132	2.1
52971	0.946132	2.1
52972	0.946132	2.1
52973	0.946131	2.1
52974	0.946131	2.1
52975	2.82263	2.1
52976	1.89226	2.1
52977	2.71441	2.1
52978	2.71441	2.1
52979	1.89226	2.1
52980	1.89226	2.1
52981	1.1412	1.72711
52982	1.1412	2.1
52983	1.14626	2.1
52984	1.14626	2.1
52985	1.28511	2.05861
52986	1.34086	2.1
52987	0.992242	2.1
52988	0.946593	2.1
52989	0.992242	2.1
52990	1.34086	2.1
52991	0.946594	2.1
52992	1.28511	2.1
52993	1.18954	2.1
52994	1.18954	2.1
52995	1.21477	2.1
52996	1.21477	2.1
52997	1.41207	2.1
52998	1.34086	2.1
52999	1.01306	2.1
53000	0.992242	2.1
53001	1.01306	2.1
53002	1.41207	2.1
53003	0.992242	2.1
53004	1.34086	2.1
53005	1.09044	2.1
53006	1.44472	0.910269
53007	1.2955	1.42291
53008	1.2955	1.04261
53009	1.22025	1.41032

53010	1.22025	1.12872
53011	0.810944	0.955225
53012	0.810944	2.1
53013	0.810946	0.971157
53014	0.810946	0.717628
53015	0.810959	0.954184
53016	0.810959	0.942517
53017	1.89226	0.916505
53018	1.35167	0.962933
53019	1.08132	2.04505
53020	1.08132	0.755703
53021	1.0813	0.96092
53022	1.41894	0.759463
53023	1.28953	0.704423
53024	0.946132	0.763759
53025	0.946132	1.03456
53026	0.946133	0.787416
53027	0.946133	0.716494
53028	1.41894	0.859793
53029	1.41894	0.875312
53030	1.28953	0.744704
53031	0.946132	0.763967
53032	0.946132	0.804149
53033	1.41894	0.76444
53034	1.89226	2.1
53035	2.15444	2.1
53036	1.93239	2.1
53037	1.89226	2.1
53038	2.19457	2.1
53039	2.15444	2.1
53040	1.89226	2.1
53041	1.89226	2.1
53042	1.89226	2.1
53043	1.89226	2.1
53044	1.89226	2.1
53045	1.89226	2.1
53046	0.938507	2.1
53047	0.938507	2.1
53048	1.07871	2.1
53049	1.15801	2.1
53050	1.03236	2.1
53051	1.14766	2.1
53052	0.946133	2.1
53053	0.946133	2.1
53054	0.946131	2.1
53055	1.07009	2.1
53056	0.946131	2.1
53057	1.03237	2.1
53058	1.89226	2.1
53059	2.19457	2.1
53060	1.94492	2.1
53061	1.89226	2.1
53062	2.24723	2.1
53063	2.19457	2.1
53064	0.946132	2.1
53065	0.946132	2.1
53066	0.946131	2.1
53067	0.946131	2.1

53068	0.946132	2.1
53069	0.946132	2.1
53070	0.946132	2.1
53071	0.946132	2.1
53072	0.946132	2.1
53073	0.946132	2.1
53074	0.946132	2.1
53075	0.946132	2.1
53076	2.21345	2.1
53077	1.89226	2.1
53078	1.89226	2.1
53079	2.14017	2.1
53080	2.14017	2.1
53081	1.89226	2.1
53082	1.89226	0.751525
53083	1.89226	0.863756
53084	1.89226	0.776798
53085	1.85439	0.842692
53086	2.23961	0.741083
53087	2.19479	0.771814
53088	1.2916	0.761206
53089	1.2916	0.753045
53090	0.946128	0.734146
53091	0.946128	0.737663
53092	1.2916	0.727424
53093	0.946131	0.738355
53094	0.946126	0.826739
53095	1.1198	0.721798
53096	1.2916	0.725717
53097	1.1198	0.786841
53098	0.946128	0.79754
53099	0.946128	0.768965
53100	1.89226	0.858411
53101	2.71262	0.828934
53102	1.89226	0.825889
53103	2.49959	0.845596
53104	4.39185	0.882523
53105	2.71262	0.82654
53106	1.34592	0.832354
53107	2.49959	0.838753
53108	1.14721	0.811895
53109	3.84551	0.809054
53110	0.946131	0.811482
53111	0.946131	0.81748
53112	1.11247	0.789817
53113	1.11247	0.789502
53114	1.24979	0.794108
53115	1.11247	0.800546
53116	0.946129	0.821366
53117	0.946129	0.799508
53118	0.946131	0.784725
53119	1.11247	0.689769
53120	0.946131	0.770359
53121	1.24979	0.784711
53122	0.946131	2.1
53123	0.946131	2.1
53124	0.94613	2.1
53125	0.94613	2.1

53126	0.946132	2.1
53127	0.946132	2.1
53128	0.946129	2.1
53129	0.946129	2.1
53130	0.946131	2.1
53131	0.946131	2.1
53132	0.946131	2.1
53133	0.946131	2.1
53134	2.77816	2.1
53135	1.89226	2.1
53136	1.89226	2.1
53137	1.89226	2.1
53138	3.78453	2.1
53139	1.89226	2.1
53140	1.89226	2.1
53141	1.89226	2.1
53142	1.89226	2.1
53143	1.89226	2.1
53144	1.89226	2.1
53145	1.89226	2.1
53146	1.89226	2.1
53147	1.89226	2.1
53148	1.89226	2.1
53149	3.78452	2.1
53150	1.55698	2.1
53151	1.89226	2.1
53152	1.89226	2.1
53153	1.89226	2.1
53154	1.8575	2.1
53155	1.8575	2.1
53156	1.89226	2.1
53157	1.89226	2.1
53158	1.89226	2.1
53159	1.89226	2.1
53160	1.89226	2.1
53161	1.89226	2.1
53162	1.89227	2.1
53163	1.89227	2.1
53164	1.89227	2.1
53165	1.89227	2.1
53166	1.89227	2.1
53167	1.89227	2.1
53168	1.35509	2.1
53169	2.06238	2.1
53170	2.12244	2.1
53171	2.12244	2.1
53172	1.34817	2.1
53173	1.34817	2.1
53174	0.946126	1.63524
53175	0.946126	2.1
53176	0.946131	2.1
53177	0.946131	2.1
53178	0.946127	2.1
53179	0.946127	1.85619
53180	0.94613	2.1
53181	0.94613	2.1
53182	0.946128	2.1
53183	0.946128	2.1

53184	0.946128	2.1
53185	0.946128	2.1
53186	1.89226	2.1
53187	1.89226	1.12197
53188	1.89226	2.03502
53189	1.89226	2.1
53190	1.89226	1.21574
53191	1.89226	2.1
53192	1.16742	2.1
53193	1.16742	2.09904
53194	1.18122	2.1
53195	1.18122	2.1
53196	1.31745	2.1
53197	1.2597	1.18734
53198	1.03119	2.1
53199	1.06122	2.1
53200	1.06122	2.1
53201	1.31745	2.1
53202	1.03119	2.1
53203	1.2597	2.1
53204	1.36849	2.1
53205	2.12244	2.1
53206	2.20092	2.1
53207	2.20092	2.1
53208	1.35509	2.1
53209	1.35509	2.1
53210	1.89226	2.1
53211	1.89226	2.1
53212	1.89226	2.1
53213	1.89226	2.1
53214	1.89226	2.1
53215	1.89226	2.1
53216	1.89226	2.1
53217	1.89226	1.11613
53218	1.89226	2.1
53219	1.89226	1.90206
53220	3.78452	2.1
53221	1.21731	2.1
53222	1.21731	1.62421
53223	1.23412	2.1
53224	1.23412	2.1
53225	1.39021	2.1
53226	1.31745	1.82409
53227	1.06122	2.1
53228	1.10046	2.1
53229	1.10046	2.1
53230	1.39021	2.1
53231	1.06122	2.1
53232	1.31745	2.1
53233	2.16503	0.921848
53234	1.78252	1.05757
53235	1.77293	1.01707
53236	1.56839	1.9422
53237	2.36957	0.811538
53238	2.16503	2.04931
53239	1.89223	0.935008
53240	1.89223	2.1
53241	1.89223	0.935552

53242	1.89223	1.11429
53243	3.78447	1.28994
53244	2.3696	1.13978
53245	1.78254	1.43134
53246	2.63298	0.824523
53247	2.07312	1.57703
53248	4.44272	1.25378
53249	1.89226	1.13296
53250	1.89226	1.27469
53251	1.89226	1.11265
53252	1.89226	2.1
53253	1.89226	0.850142
53254	1.89226	2.1
53255	1.89226	0.904141
53256	1.89226	0.751051
53257	1.89226	0.885334
53258	1.89226	0.700559
53259	3.78452	0.78468
53260	2.26369	0.900885
53261	2.2168	0.737696
53262	2.15617	0.878573
53263	2.10864	0.66232
53264	4.37234	0.790225
53265	2.2168	0.728628
53266	1.94499	0.72918
53267	1.94499	0.743267
53268	2.04737	0.725907
53269	2.10864	0.70376
53270	2.21103	0.739454
53271	1.89226	0.754079
53272	1.89226	0.725521
53273	1.89226	0.729691
53274	1.89226	0.729839
53275	1.89226	0.734888
53276	1.89226	0.723272
53277	1.89226	0.87123
53278	1.89226	0.755435
53279	1.89226	0.777865
53280	1.89226	0.884996
53281	1.89226	0.736187
53282	1.89226	0.923538
53283	1.02995	0.731628
53284	1.02995	0.898293
53285	1.03212	0.749585
53286	1.03212	0.863004
53287	0.988256	0.733749
53288	1.05432	0.728401
53289	1.00775	0.860283
53290	1.07809	0.878705
53291	1.00775	0.786779
53292	0.988257	0.789166
53293	1.07808	0.802348
53294	1.05432	0.796412
53295	2.10864	0.7428
53296	1.94499	0.731751
53297	1.97652	0.736089
53298	1.79681	0.713283
53299	3.9215	0.709044

53300	0.810943	0.744031
53301	0.810943	0.742509
53302	0.810943	0.739765
53303	0.810943	0.737436
53304	0.810958	0.707345
53305	0.810958	0.719813
53306	1.89226	0.728124
53307	1.35168	0.711306
53308	1.08132	0.760261
53309	1.08132	0.723599
53310	1.0813	0.709401
53311	0.657403	0.414174
53312	1.35403	0.443494
53313	1.24038	0.421843
53314	0.499144	0.406905
53315	1.24039	0.468907
53316	1.35403	0.446908
53317	0.657402	0.426373
53318	0.499144	0.416289
53319	0.915415	0.449199
53320	0.915415	0.433669
53321	0.937755	0.429766
53322	0.937755	0.437908
53323	1.72526	2.1
53324	1.17989	1.8393
53325	1.96035	2.1
53326	1.47412	2.1
53327	3.14024	2.1
53328	1.89226	1.24856
53329	1.89226	1.96364
53330	1.89226	1.3008
53331	1.89226	0.954949
53332	3.78452	1.49747
53333	0.946131	1.3483
53334	0.946131	1.16453
53335	1.25764	1.25191
53336	1.25764	2.07064
53337	1.07087	1.08507
53338	1.25765	1.03196
53339	0.946128	1.0533
53340	0.946128	0.854324
53341	0.94613	0.972602
53342	1.25765	1.02421
53343	1.07086	1.14173
53344	0.946129	1.14801
53345	1.96035	2.1
53346	1.32668	2.1
53347	2.29301	2.1
53348	1.70303	1.57746
53349	3.56428	2.1
53350	1.89226	1.25561
53351	1.89226	1.0878
53352	1.89226	0.862478
53353	1.89226	1.67378
53354	3.78452	0.8597
53355	0.897738	0.775102
53356	0.897738	0.776766
53357	0.946102	0.75605

53358	0.946102	0.666032
53359	1.11247	0.703453
53360	0.89771	0.62188
53361	0.94613	0.757928
53362	0.94613	0.778791
53363	0.897709	0.77828
53364	0.946131	0.782638
53365	0.946131	0.800282
53366	1.11247	0.770732
53367	1.89226	2.1
53368	1.89226	1.90696
53369	1.89226	2.1
53370	1.89226	2.09048
53371	1.89226	2.1
53372	1.89226	2.1
53373	2.15429	2.1
53374	1.89226	2.1
53375	1.89226	2.1
53376	1.89226	2.1
53377	2.27074	2.1
53378	2.27074	2.1
53379	0.993311	1.34758
53380	0.993311	1.58085
53381	0.904322	2.1
53382	0.904322	1.87348
53383	0.803324	2.1
53384	1.36183	1.34877
53385	0.916274	2.1
53386	0.535808	1.49861
53387	0.535808	1.63646
53388	0.803322	1.66509
53389	0.916276	1.56881
53390	1.36183	1.72717
53391	1.89226	2.1
53392	1.89226	1.57722
53393	1.89226	2.1
53394	1.89226	2.1
53395	1.89226	2.1
53396	1.89226	2.1
53397	2.11955	2.1
53398	2.62097	2.1
53399	1.85542	2.1
53400	2.39016	2.1
53401	4.50971	2.1
53402	2.39016	2.1
53403	2.20242	2.1
53404	2.39016	2.1
53405	1.64008	2.1
53406	1.85542	2.1
53407	1.64008	2.1
53408	1.79667	2.1
53409	1.28267	2.1
53410	1.28267	2.1
53411	1.40117	2.1
53412	1.74514	2.1
53413	1.83994	2.1
53414	0.575914	2.1
53415	0.625292	2.1

53416	0.949144	2.1
53417	0.882782	2.1
53418	0.773697	2.1
53419	0.773697	2.1
53420	0.786497	2.1
53421	0.786494	2.1
53422	0.605477	2.1
53423	0.949148	2.1
53424	0.625291	2.1
53425	0.862374	2.1
53426	1.89226	2.1
53427	2.44918	2.1
53428	2.4616	2.1
53429	2.44918	2.1
53430	1.90468	2.1
53431	1.89226	2.1
53432	1.89226	2.1
53433	1.89226	1.90805
53434	1.89226	2.1
53435	1.89226	0.972485
53436	3.78453	2.1
53437	0.946128	2.1
53438	0.946128	0.946355
53439	1.23631	1.66305
53440	1.23631	2.1
53441	0.946131	1.25676
53442	0.946131	1.53521
53443	1.3147	2.1
53444	1.23631	2.1
53445	1.3147	2.1
53446	0.946131	2.1
53447	1.23631	2.1
53448	0.94613	2.1
53449	1.89226	2.1
53450	2.4616	2.1
53451	2.4717	2.1
53452	2.4616	2.1
53453	1.90237	2.1
53454	1.89226	2.1
53455	0.946132	1.03041
53456	0.946132	2.1
53457	0.946131	2.1
53458	0.946131	1.69747
53459	0.946132	1.11595
53460	0.946132	2.1
53461	0.946131	2.1
53462	0.946131	2.1
53463	0.946131	2.1
53464	0.946131	2.1
53465	0.946131	2.1
53466	0.946131	2.1
53467	0.946128	2.1
53468	0.946128	2.1
53469	1.3147	1.06493
53470	1.3147	2.1
53471	0.94613	1.03809
53472	0.94613	1.90834
53473	1.3419	2.1

53474	1.3147	2.1
53475	1.3419	2.1
53476	0.94613	2.1
53477	1.3147	2.1
53478	0.94613	2.1
53479	1.89226	2.1
53480	1.89226	2.1
53481	1.89226	2.1
53482	1.89226	2.1
53483	3.78452	2.1
53484	2.3333	2.1
53485	1.3864	2.1
53486	1.3864	2.1
53487	1.4628	2.1
53488	1.95763	2.1
53489	2.03403	2.1
53490	1.294	2.1
53491	1.294	2.1
53492	0.946128	2.1
53493	0.946128	2.1
53494	1.294	2.1
53495	1.27474	2.1
53496	0.946131	2.1
53497	0.946131	2.1
53498	0.946129	2.1
53499	1.27474	2.1
53500	1.294	2.1
53501	0.94613	2.1
53502	1.89226	2.1
53503	1.89226	2.1
53504	1.89226	2.1
53505	1.89226	2.1
53506	1.89226	2.1
53507	1.89226	2.1
53508	0.808088	2.1
53509	0.808088	2.1
53510	0.815623	2.1
53511	0.815623	2.1
53512	0.660053	2.1
53513	0.693199	2.1
53514	0.930512	2.1
53515	0.978817	2.1
53516	0.660053	2.1
53517	0.930513	2.1
53518	0.693198	2.1
53519	0.978817	2.1
53520	1.27474	2.1
53521	1.27474	2.1
53522	0.946128	2.1
53523	0.946128	2.1
53524	1.26221	2.1
53525	1.27474	2.1
53526	0.946131	2.1
53527	0.946131	2.1
53528	0.94613	2.1
53529	1.26221	2.1
53530	0.946129	2.1
53531	1.27474	2.1

53532	0.90207	0.0743707
53533	0.90207	0.0749259
53534	0.895719	0.075549
53535	0.895719	0.0844468
53536	0.927855	0.0621929
53537	1.2493	0.0756561
53538	0.85723	0.0740956
53539	0.548484	0.0697917
53540	0.548485	0.070672
53541	0.927855	0.0616872
53542	0.85723	0.0880586
53543	1.2493	0.0768042
53544	1.41319	0.875364
53545	1.22482	0.761741
53546	1.46629	0.782313
53547	1.92622	0.862713
53548	3.15104	0.849811
53549	1.02305	0.716314
53550	1.22482	0.765203
53551	1.80336	0.776227
53552	1.44426	0.712418
53553	2.66908	0.71509
53554	1.3268	2.1
53555	0.999489	2.1
53556	1.44091	2.1
53557	1.14657	2.1
53558	2.4404	2.1
53559	1.89226	1.67013
53560	1.89226	2.1
53561	1.89226	2.1
53562	1.89226	2.1
53563	3.78452	2.1
53564	1.89226	2.1
53565	2.28605	2.1
53566	2.43157	2.1
53567	2.43157	2.1
53568	1.89226	2.1
53569	1.89226	2.1
53570	1.60647	2.1
53571	1.44091	1.97171
53572	1.31346	2.1
53573	1.31346	2.1
53574	1.14657	2.1
53575	1.44091	2.1
53576	1.89226	1.95154
53577	1.89226	2.1
53578	1.89226	1.65573
53579	1.89226	2.1
53580	3.78452	2.1
53581	0.946132	2.1
53582	0.946132	2.1
53583	1.3048	2.1
53584	1.3048	2.1
53585	1.3048	2.1
53586	1.21578	2.1
53587	0.946132	2.1
53588	0.946132	2.1
53589	1.3048	2.1

53590	0.946132	2.1
53591	0.946132	2.1
53592	1.21578	2.1
53593	1.89227	0.148771
53594	1.89227	0.153983
53595	1.89227	0.0986886
53596	1.89227	0.135786
53597	3.78453	0.152398
53598	1.6671	0.0941444
53599	1.87338	0.133945
53600	1.6671	0.136532
53601	2.02098	0.191487
53602	1.38328	0.0918946
53603	1.73715	0.125202
53604	1.89226	0.100214
53605	1.89226	0.121831
53606	1.89226	0.0744901
53607	1.89226	0.097914
53608	3.78453	0.091822
53609	1.6671	0.0981141
53610	1.38328	0.0960528
53611	1.35562	0.0770867
53612	1.01012	0.0924905
53613	2.67722	0.0987446
53614	0.819377	0.0899292
53615	0.647925	0.0861228
53616	1.17802	0.0708524
53617	1.33174	0.0816394
53618	0.98543	0.0826821
53619	0.98543	0.10094
53620	0.994237	0.0746263
53621	0.994237	0.0861767
53622	1.17802	0.0810629
53623	0.647926	0.0768956
53624	1.33174	0.0831821
53625	0.819377	0.0821044
53626	1.27806	0.0863566
53627	1.68264	0.0748603
53628	1.44593	0.0780352
53629	1.878	0.0832719
53630	3.15606	0.0788293
53631	1.878	0.0848666
53632	1.9898	0.0757844
53633	1.878	0.0862146
53634	1.68264	0.0828164
53635	2.30533	0.0842178
53636	2.10997	0.0806976
53637	0.814587	0.0813331
53638	0.814596	0.0762787
53639	0.82775	0.0873516
53640	0.82775	0.0885145
53641	1.0107	0.0869609
53642	0.91549	0.0793259
53643	0.741058	0.0899482
53644	0.714748	0.0821508
53645	0.714758	0.0866068
53646	1.03225	0.0832931
53647	0.704864	0.0798949

53648	0.915488	0.0812594
53649	1.90347	0.0902283
53650	2.06584	0.0742194
53651	1.878	0.0900742
53652	1.50236	0.0829137
53653	2.05749	0.0810994
53654	2.30533	0.0881203
53655	2.24475	2.1
53656	1.85871	2.1
53657	2.13711	2.1
53658	2.13711	2.1
53659	1.9943	2.1
53660	1.9943	2.1
53661	1.85871	2.1
53662	1.7871	2.1
53663	1.41619	2.1
53664	1.41619	2.1
53665	2.13711	2.1
53666	2.13711	2.1
53667	2.24475	2.1
53668	2.13711	2.1
53669	2.54902	2.1
53670	2.46327	2.1
53671	4.70802	2.1
53672	1.06913	2.1
53673	1.06913	2.1
53674	1.0821	2.1
53675	1.0821	2.1
53676	0.893552	2.1
53677	1.08267	2.1
53678	1.06855	2.1
53679	1.23163	2.1
53680	1.23163	2.1
53681	1.08267	2.1
53682	0.893551	2.1
53683	1.06855	2.1
53684	1.67046	0.812854
53685	2.4106	0.715205
53686	2.05835	0.899982
53687	2.4106	0.90569
53688	1.59562	0.750046
53689	1.94788	0.728952
53690	2.34737	0.947734
53691	2.4106	0.737779
53692	2.05834	0.892395
53693	1.99655	0.97792
53694	4.40715	0.835218
53695	1.67046	0.743943
53696	2.05835	0.71084
53697	1.588	0.836223
53698	1.34857	1.11574
53699	3.40692	0.761218
53700	1.73802	0.810273
53701	1.89668	0.876881
53702	1.99655	1.41221
53703	1.99655	0.752331
53704	1.79981	0.866577
53705	2.05834	0.729892

53706	1.89226	1.14651
53707	1.89226	0.984757
53708	1.89226	0.822714
53709	1.89226	1.66863
53710	1.89226	0.839209
53711	1.89226	2.1
53712	1.89226	1.00386
53713	1.89226	2.1
53714	1.89226	1.45136
53715	1.89226	0.858425
53716	3.78453	1.39238
53717	0.719473	2.05047
53718	0.916103	1.43709
53719	1.02744	1.07329
53720	0.850328	1.90018
53721	0.888094	1.01754
53722	0.888094	2.1
53723	0.878337	1.97876
53724	0.878337	0.843125
53725	0.916103	1.17474
53726	1.02744	1.24069
53727	0.719473	1.5505
53728	0.850328	1.77076
53729	1.02744	1.20899
53730	0.850329	1.94378
53731	1.0875	1.07425
53732	0.919597	1.27061
53733	0.975841	0.928574
53734	0.975841	2.1
53735	0.971196	0.910041
53736	0.971196	1.7632
53737	1.02744	1.42956
53738	1.0875	1.04174
53739	0.850328	1.77844
53740	0.919598	1.49843
53741	1.89226	1.63354
53742	1.89226	1.34157
53743	1.89226	0.892233
53744	1.89226	1.39416
53745	3.78453	1.19444
53746	1.89226	1.29679
53747	1.89226	0.976495
53748	1.89226	0.862699
53749	1.89226	1.80408
53750	1.89226	0.843289
53751	1.89226	2.07077
53752	1.11103	1.40884
53753	0.916103	2.05255
53754	1.21177	1.14672
53755	1.02744	1.22738
53756	1.07188	0.958153
53757	1.07188	2.1
53758	1.06659	0.937023
53759	1.06659	1.91468
53760	1.11103	1.80641
53761	1.21177	1.36717
53762	0.916104	1.74233
53763	1.02744	1.86992

53764	2.54158	1.0426
53765	2.42354	1.23577
53766	2.17499	0.900659
53767	2.17499	2.09518
53768	2.42354	1.57137
53769	2.05488	2.1
53770	1.89226	0.30289
53771	1.89226	0.42627
53772	1.89226	0.361768
53773	1.89226	0.492377
53774	3.78453	0.34084
53775	1.89226	0.242103
53776	1.89226	0.411999
53777	1.89226	0.290841
53778	1.89226	0.349369
53779	1.89226	0.312973
53780	1.89226	0.385051
53781	1.89226	0.601791
53782	1.89226	0.600171
53783	1.89226	0.56969
53784	1.89226	0.477635
53785	1.89226	0.687677
53786	1.89226	0.629938
53787	1.89226	0.631298
53788	1.89226	0.685344
53789	1.89226	0.645031
53790	1.89226	0.710247
53791	3.78452	0.687414
53792	0.802544	0.330998
53793	0.946132	0.214039
53794	0.946132	0.26815
53795	0.787038	0.395397
53796	0.787039	0.301351
53797	0.787039	0.319493
53798	0.961637	0.283899
53799	0.961637	0.275963
53800	0.787038	0.313453
53801	0.802544	0.316051
53802	0.946132	0.263467
53803	0.946132	0.277425
53804	1.89226	0.196706
53805	1.60509	0.304543
53806	1.89226	0.213521
53807	1.89226	0.254078
53808	1.3905	0.252777
53809	1.3905	0.289991
53810	0.946132	0.595449
53811	0.946132	0.361683
53812	0.946131	0.533192
53813	0.946131	0.563258
53814	0.946132	0.559949
53815	0.946132	0.557752
53816	0.946131	0.500467
53817	0.946131	0.527314
53818	0.946131	0.516704
53819	0.946131	0.551395
53820	0.946132	0.52104
53821	0.946132	0.463004

53822	0.946131	0.630845
53823	0.946131	0.652907
53824	0.946131	0.685011
53825	0.946131	0.531404
53826	0.946132	0.641391
53827	0.946132	0.660026
53828	0.946131	0.561546
53829	0.946131	0.559463
53830	0.946131	0.64189
53831	0.946131	0.519049
53832	0.946132	0.573102
53833	0.946132	0.577894
53834	1.42382	1.48985
53835	1.32201	0.953828
53836	1.32096	1.03754
53837	1.42866	1.29802
53838	2.75067	1.12034
53839	1.89226	1.34844
53840	1.89226	0.893438
53841	1.89226	1.00872
53842	1.89226	1.11516
53843	1.89226	0.953786
53844	1.89226	1.0525
53845	1.89226	1.05614
53846	2.79029	0.810318
53847	2.7803	0.894053
53848	2.79029	0.938713
53849	1.88227	0.849633
53850	1.89226	0.90264
53851	0.716065	1.20216
53852	0.714333	1.46905
53853	0.660483	0.971539
53854	0.659126	1.04576
53855	0.687538	0.978775
53856	0.687538	1.17362
53857	0.685921	1.7579
53858	0.685921	1.00196
53859	0.659126	1.09853
53860	0.716065	1.10371
53861	0.660483	1.10009
53862	0.714333	1.09825
53863	1.89226	1.17163
53864	1.89226	0.901092
53865	1.89226	0.992614
53866	1.89226	1.0973
53867	3.78453	1.04726
53868	0.946133	0.914025
53869	0.946133	0.976929
53870	1.39015	0.867682
53871	1.38969	0.80692
53872	0.946132	0.814181
53873	0.946132	0.905134
53874	1.38969	0.990701
53875	1.38969	0.861704
53876	1.38969	0.887703
53877	0.946132	0.888492
53878	0.946133	0.907266
53879	1.39015	0.88657

53880	2.72157	0.706016
53881	1.22176	0.697698
53882	1.59492	0.7
53883	1.22176	0.715434
53884	2.40743	0.7
53885	2.03426	0.713646
53886	0.946131	0.740053
53887	0.946131	0.722623
53888	1.36079	0.706795
53889	1.01713	0.715309
53890	0.94613	0.713858
53891	0.94613	0.733988
53892	1.36079	0.714356
53893	1.36079	0.703841
53894	0.946131	0.711511
53895	1.36079	0.711669
53896	1.01713	0.706991
53897	0.946131	0.71139
53898	1.89226	0.734216
53899	1.89226	0.686837
53900	1.89226	0.683302
53901	1.89226	0.751074
53902	3.78453	0.737284
53903	1.89226	0.709439
53904	1.89226	0.636559
53905	1.89226	0.678461
53906	1.89226	0.71148
53907	1.89226	0.641377
53908	1.89226	0.70864
53909	0.946131	0.733527
53910	0.946131	0.720227
53911	0.946131	0.710301
53912	1.36079	0.697692
53913	0.94613	0.709196
53914	0.94613	0.727353
53915	0.946133	0.715204
53916	0.946133	0.603608
53917	0.946131	0.717928
53918	0.946131	0.714107
53919	0.946132	0.688357
53920	1.36079	0.709192
53921	1.89226	0.681125
53922	1.89226	0.745489
53923	1.89226	0.687604
53924	1.89226	0.724921
53925	1.89226	0.687639
53926	1.89226	0.724998
53927	1.89227	0.708484
53928	1.89227	0.627889
53929	1.89227	0.689544
53930	1.89227	0.716586
53931	1.89227	0.639525
53932	1.89227	0.701754
53933	1.60428	2.1
53934	2.2367	2.1
53935	1.50097	2.1
53936	1.80125	2.00629
53937	3.40553	2.1

53938	1.89095	2.1
53939	1.85293	0.884865
53940	1.80125	2.05045
53941	1.76268	0.902893
53942	3.65418	1.05622
53943	0.803177	2.1
53944	0.803177	2.1
53945	0.798644	2.1
53946	0.798644	2.1
53947	0.84233	2.1
53948	0.900624	2.1
53949	0.750483	2.1
53950	0.701197	2.1
53951	0.701197	2.1
53952	0.84233	2.1
53953	0.750483	2.1
53954	0.900625	2.1
53955	0.860925	0.928216
53956	0.860925	2.1
53957	0.862743	2.1
53958	0.862743	0.930132
53959	0.881338	0.906237
53960	0.826621	0.883357
53961	0.84233	2.1
53962	0.900625	2.1
53963	0.84233	1.02349
53964	0.826621	1.02494
53965	0.900624	1.05291
53966	0.881339	1.03199
53967	1.37938	1.0159
53968	1.03319	0.973377
53969	1.00512	1.37485
53970	0.639582	1.36291
53971	1.00463	1.36868
53972	1.00463	1.0104
53973	1.01434	0.963218
53974	1.01434	1.35252
53975	1.37938	1.14368
53976	1.00512	1.36396
53977	1.03319	1.18132
53978	0.639582	1.18356
53979	0.728713	0.336993
53980	0.728713	0.330106
53981	0.73425	0.798324
53982	0.73425	0.892901
53983	0.525122	2.1
53984	0.605622	0.284274
53985	0.948901	0.284274
53986	0.857342	0.284274
53987	0.85734	0.299145
53988	0.525123	0.883242
53989	0.948899	0.299145
53990	0.605624	0.299145
53991	1.75449	0.785304
53992	1.4378	0.826137
53993	1.93916	0.849941
53994	2.22512	0.757084
53995	3.69364	0.824417

53996	1.75448	0.829233
53997	1.89186	0.752829
53998	2.29771	0.755454
53999	2.22512	0.757353
54000	4.05219	0.759019
54001	1.93916	0.837737
54002	2.51749	0.738194
54003	2.62357	0.784257
54004	2.22512	0.76063
54005	2.33761	0.798889
54006	1.93916	0.76804
54007	2.29771	0.766072
54008	2.51749	0.747276
54009	2.22512	0.742633
54010	2.22512	0.734916
54011	2.50905	0.773083
54012	2.50905	0.762644
54013	0.87382	0.160801
54014	1.33662	0.247298
54015	1.20756	0.203476
54016	0.890467	0.368475
54017	1.08859	0.369535
54018	1.08859	0.367504
54019	1.13084	0.161503
54020	1.08793	0.255218
54021	1.34722	0.234608
54022	1.27184	0.305055
54023	0.917678	0.290677
54024	0.97821	0.291735
54025	1.28396	0.243299
54026	1.70302	0.465457
54027	2.30076	0.33063
54028	1.20417	0.271715
54029	2.57506	0.345456
54030	2.2656	0.331114
54031	1.03642	0.931342
54032	1.03642	0.689135
54033	1.07029	0.695608
54034	1.07029	0.61552
54035	1.31531	0.876776
54036	1.23279	0.742692
54037	0.941743	0.710074
54038	0.791399	0.793566
54039	0.791399	0.741868
54040	1.23279	0.740561
54041	0.941744	0.727569
54042	1.31531	0.728784
54043	0.937354	0.9493
54044	0.937354	0.659581
54045	0.954794	0.706313
54046	0.954794	0.654269
54047	1.13563	0.739181
54048	1.23279	0.880729
54049	0.791398	0.713665
54050	0.659359	0.77757
54051	1.13563	0.739047
54052	0.659359	0.740798
54053	0.791399	0.723083

54054	1.23279	0.724212
54055	2.29852	1.02238
54056	1.89227	0.937283
54057	2.14485	0.908047
54058	2.14485	1.03943
54059	1.89227	0.949784
54060	1.89227	1.07883
54061	0.947863	0.953074
54062	1.19854	1.05336
54063	2.16841	1.0557
54064	1.76597	0.943366
54065	2.96451	0.924642
54066	1.89227	0.922149
54067	1.89227	0.876429
54068	1.89227	0.856424
54069	1.89227	0.976979
54070	1.89227	0.869845
54071	1.89227	0.98804
54072	2.46489	2.1
54073	1.98339	2.1
54074	1.81667	2.1
54075	2.36914	2.1
54076	4.35253	2.1
54077	2.46488	2.1
54078	2.70401	2.1
54079	2.64996	2.1
54080	2.36914	2.1
54081	5.07315	2.1
54082	1.81667	2.1
54083	2.29459	2.1
54084	2.36914	2.1
54085	2.36914	2.1
54086	1.68073	2.1
54087	1.68073	2.1
54088	2.64996	2.1
54089	2.29458	2.1
54090	2.29458	2.1
54091	2.36914	2.1
54092	2.61243	2.1
54093	2.68698	2.1
54094	1.89226	1.22303
54095	1.89226	1.13053
54096	1.89226	1.41148
54097	1.89226	1.16351
54098	1.89226	1.47527
54099	1.89226	1.15431
54100	1.89224	1.50196
54101	1.89224	1.10515
54102	1.65571	1.26321
54103	1.65571	1.1037
54104	1.89224	1.37893
54105	1.89224	1.19751
54106	1.89224	1.1756
54107	1.89224	1.13354
54108	1.89224	1.32352
54109	1.89224	1.0562
54110	1.89224	1.56751
54111	1.89224	1.10481

54112	1.98252	0.558371
54113	2.20641	0.575263
54114	1.05129	0.536941
54115	1.2951	0.676312
54116	3.27762	0.551963
54117	1.85598	1.92215
54118	1.88635	1.12495
54119	2.12346	0.949652
54120	2.10838	2.02173
54121	3.97943	1.18736
54122	2.10838	1.22708
54123	1.87532	2.1
54124	1.85597	0.97292
54125	1.85597	2.1
54126	1.87532	1.26868
54127	1.57914	2.1
54128	2.12348	1.09434
54129	2.35141	1.75757
54130	2.37191	1.23714
54131	2.10841	1.39192
54132	2.38698	1.02684
54133	2.12348	1.34114
54134	1.07811	1.92068
54135	0.937672	2.1
54136	1.17571	1.6139
54137	1.05421	0.957083
54138	1.07087	1.10599
54139	1.07087	2.1
54140	1.06145	1.97933
54141	1.06145	1.0766
54142	1.17571	1.92381
54143	1.07811	1.82086
54144	1.05421	1.73097
54145	0.937672	1.78263
54146	1.67334	2.1
54147	2.76609	2.1
54148	2.74144	2.1
54149	2.74144	2.1
54150	1.71157	2.1
54151	1.71157	2.1
54152	2.76609	2.1
54153	1.89226	2.1
54154	1.89226	2.1
54155	1.89226	2.1
54156	2.74143	2.1
54157	2.74143	2.1
54158	1.10393	2.1
54159	1.10393	2.1
54160	1.10788	2.1
54161	1.10788	2.1
54162	1.38302	2.1
54163	1.37072	2.1
54164	0.836672	2.1
54165	0.841089	2.1
54166	1.38302	2.1
54167	0.84109	2.1
54168	0.836671	2.1
54169	1.37072	2.1

54170	1.38302	2.1
54171	1.38302	2.1
54172	0.946131	2.1
54173	0.946131	2.1
54174	0.946128	2.1
54175	0.946128	2.1
54176	1.38302	2.1
54177	1.37072	2.1
54178	1.38302	2.1
54179	0.946131	2.1
54180	0.94613	2.1
54181	1.37072	2.1
54182	1.28072	0.927014
54183	1.55779	2.1
54184	1.28072	1.91302
54185	1.40765	1.02285
54186	1.47186	1.94133
54187	1.59879	1.47817
54188	1.89227	0.939323
54189	1.89227	0.922317
54190	1.89227	0.822879
54191	1.89227	1.6548
54192	1.89227	0.840836
54193	1.89227	1.6156
54194	2.80057	0.816392
54195	1.89226	0.822842
54196	1.89226	0.796233
54197	1.89226	0.898533
54198	2.63061	0.746377
54199	2.63061	0.93676
54200	1.28072	0.887888
54201	1.38859	2.1
54202	1.47186	1.83489
54203	1.47186	1.03999
54204	1.16777	1.99815
54205	1.16777	1.16204
54206	1.89226	0.890896
54207	1.89226	0.919652
54208	1.89226	0.847016
54209	1.89226	1.83915
54210	1.89226	0.821553
54211	1.89226	2.08699
54212	1.83221	2.1
54213	2.0815	2.1
54214	2.0815	2.1
54215	2.08153	2.1
54216	1.86703	2.1
54217	1.87402	2.1
54218	1.86703	2.1
54219	2.08508	2.1
54220	2.08508	2.1
54221	2.29001	2.1
54222	1.89226	2.1
54223	2.08048	2.1
54224	2.7278	0.703053
54225	1.10909	0.706716
54226	1.50472	0.702982
54227	1.10909	0.710399

54228	2.69881	0.603854
54229	2.30319	0.706234
54230	1.23204	2.1
54231	1.92264	2.1
54232	1.48162	2.1
54233	1.48162	2.1
54234	1.72994	2.1
54235	1.72994	2.1
54236	0.864968	2.1
54237	0.696952	2.1
54238	0.61602	2.1
54239	0.479736	2.1
54240	0.66444	2.1
54241	0.66444	2.1
54242	0.680264	2.1
54243	0.680264	2.1
54244	0.696952	2.1
54245	0.479736	2.1
54246	0.61602	2.1
54247	0.864968	2.1
54248	1.89226	0.814526
54249	2.43219	1.72267
54250	1.89226	1.08553
54251	2.82019	1.05428
54252	1.89226	1.37203
54253	2.82019	1.08374
54254	1.89226	0.904344
54255	2.83701	1.96882
54256	2.2701	1.16751
54257	2.2701	1.00962
54258	1.89226	1.17677
54259	1.89226	1.05575
54260	1.89226	1.28091
54261	1.89226	0.812213
54262	1.89226	0.93688
54263	1.89226	1.0079
54264	1.89226	1.02826
54265	1.89226	0.978885
54266	1.5795	1.4586
54267	1.44091	1.45291
54268	1.05267	1.77403
54269	1.05267	1.09372
54270	1.98408	1.56801
54271	1.98408	1.0446
54272	2.20624	2.1
54273	1.08831	2.1
54274	2.49053	2.1
54275	1.42486	2.1
54276	3.57883	2.1
54277	1.89226	2.1
54278	1.89226	2.1
54279	1.89226	2.1
54280	1.89226	2.1
54281	3.78453	2.1
54282	0.946132	2.1
54283	0.946132	2.1
54284	0.853591	2.1
54285	0.642207	2.1

54286	0.94613	2.1
54287	0.94613	2.1
54288	0.642209	2.1
54289	0.642209	2.1
54290	0.946131	2.1
54291	0.853591	2.1
54292	0.642207	2.1
54293	0.946132	2.1
54294	1.0506	2.1
54295	1.0506	2.1
54296	1.0879	2.1
54297	0.960424	2.1
54298	0.708687	2.1
54299	0.782927	2.1
54300	1.40041	2.1
54301	1.2518	2.1
54302	0.767748	2.1
54303	1.31801	2.1
54304	1.21412	2.1
54305	0.69461	2.1
54306	1.89226	2.1
54307	1.89226	2.1
54308	1.89226	2.1
54309	1.89226	2.1
54310	1.89226	2.1
54311	1.89226	2.1
54312	0.922223	2.1
54313	0.922223	2.1
54314	1.04548	2.1
54315	0.768229	2.1
54316	0.948441	2.1
54317	0.806483	2.1
54318	0.85362	2.1
54319	0.85362	2.1
54320	1.05942	2.1
54321	0.922187	2.1
54322	0.851554	2.1
54323	0.768264	2.1
54324	2.16728	2.1
54325	0.95455	2.1
54326	1.21372	2.1
54327	0.98227	2.1
54328	2.16728	2.1
54329	1.93584	2.1
54330	2.08849	0.446279
54331	1.08732	0.544124
54332	1.3357	0.561736
54333	2.37948	0.584836
54334	3.4668	0.512987
54335	2.57882	0.464271
54336	2.37948	0.435943
54337	2.53055	0.423069
54338	1.48676	0.632038
54339	1.3357	0.573537
54340	1.3357	0.620327
54341	1.89226	2.1
54342	1.89226	2.1
54343	2.17471	2.1

54344	1.89226	2.1
54345	2.60085	2.1
54346	2.26306	2.1
54347	0.946131	1.91548
54348	0.946131	2.1
54349	0.946131	2.1
54350	0.946131	2.1
54351	0.94613	2.1
54352	0.94613	2.1
54353	0.946132	2.1
54354	0.946132	2.1
54355	0.946131	2.1
54356	0.946131	2.1
54357	0.946131	2.1
54358	0.946131	2.1
54359	1.89226	2.1
54360	2.17471	2.1
54361	2.08196	2.1
54362	2.17471	2.1
54363	1.79952	2.1
54364	1.89226	2.1
54365	1.89226	2.1
54366	1.89226	2.1
54367	1.89226	2.1
54368	1.89226	2.1
54369	1.89226	2.1
54370	1.89226	2.1
54371	1.86016	2.1
54372	1.89226	2.1
54373	1.89226	2.1
54374	1.89226	2.1
54375	1.84826	2.1
54376	1.84826	2.1
54377	2.50136	2.1
54378	1.99781	2.1
54379	1.99729	2.1
54380	2.5214	2.1
54381	4.49865	2.1
54382	0.51724	2.1
54383	0.496076	2.1
54384	0.998906	2.1
54385	0.998645	2.1
54386	0.742249	2.1
54387	0.742249	2.1
54388	0.752734	2.1
54389	0.752734	2.1
54390	0.998639	2.1
54391	0.496073	2.1
54392	0.517243	2.1
54393	0.998913	2.1
54394	0.998653	2.1
54395	0.994293	2.1
54396	1.26071	2.1
54397	1.26504	2.1
54398	1.12529	2.1
54399	1.12529	2.1
54400	1.12971	2.1
54401	1.12971	2.1

54402	1.26504	2.1
54403	0.994292	2.1
54404	0.998651	2.1
54405	1.26071	2.1
54406	1.89226	2.1
54407	1.89226	2.1
54408	1.89226	2.1
54409	1.89226	2.1
54410	3.78452	2.1
54411	1.89226	2.1
54412	1.89226	2.1
54413	1.89226	2.1
54414	1.89226	2.1
54415	3.78452	2.1
54416	0.675872	2.1
54417	0.675641	2.1
54418	0.943072	2.1
54419	0.898317	2.1
54420	0.775717	2.1
54421	0.775717	2.1
54422	0.79824	2.1
54423	0.79824	2.1
54424	0.675642	2.1
54425	0.94307	2.1
54426	0.675874	2.1
54427	0.898315	2.1
54428	0.943071	2.1
54429	0.898316	2.1
54430	0.998133	2.1
54431	0.918903	2.1
54432	0.922369	2.1
54433	0.922369	2.1
54434	0.939606	2.1
54435	0.939606	2.1
54436	0.943071	2.1
54437	0.998133	2.1
54438	0.898316	2.1
54439	0.918903	2.1
54440	0.946131	2.1
54441	0.946131	2.1
54442	0.946131	2.1
54443	0.946131	2.1
54444	0.946132	2.1
54445	0.946132	2.1
54446	0.946129	2.1
54447	0.946129	2.1
54448	0.94613	2.1
54449	0.94613	2.1
54450	0.946131	2.1
54451	0.946131	2.1
54452	1.89226	2.1
54453	1.89226	2.1
54454	1.89226	2.1
54455	1.89226	2.1
54456	1.89226	2.1
54457	1.89226	2.1
54458	1.35129	2.1
54459	1.88614	2.1

54460	2.01867	2.1
54461	1.38189	2.1
54462	3.36995	2.1
54463	0.943071	2.1
54464	1.00934	2.1
54465	0.998134	2.1
54466	1.10866	2.1
54467	0.992617	2.1
54468	0.992617	2.1
54469	1.01485	2.1
54470	1.01485	2.1
54471	1.00934	2.1
54472	1.10866	2.1
54473	0.943072	2.1
54474	0.998134	2.1
54475	2.67272	2.1
54476	1.61859	2.1
54477	1.70303	2.1
54478	2.52175	2.1
54479	4.5326	2.1
54480	2.00954	2.1
54481	2.27801	2.1
54482	1.87875	2.1
54483	1.87875	2.1
54484	2.37837	2.1
54485	2.37837	2.1
54486	2.00955	2.1
54487	1.78 2.1	
54488	2.21813	2.1
54489	1.8493	2.1
54490	2.37838	2.1
54491	2.00955	2.1
54492	2.37838	2.1
54493	2.27801	2.1
54494	2.58523	2.1
54495	2.71597	2.1
54496	4.9636	2.1
54497	2.37838	2.1
54498	2.71597	2.1
54499	1.72709	2.1
54500	2.0815	2.1
54501	2.59749	2.1
54502	2.78034	2.1
54503	0.94613	0.992476
54504	0.94613	1.01791
54505	0.946128	0.900801
54506	0.946128	0.881766
54507	0.94613	0.890501
54508	0.94613	0.916494
54509	0.946128	1.01293
54510	0.946128	0.854862
54511	0.946129	0.980548
54512	0.946129	0.961398
54513	0.946129	0.918889
54514	0.946129	0.930221
54515	1.89226	1.10131
54516	1.89226	1.0486
54517	1.89226	1.12419

54518	1.89226	0.961471
54519	3.78452	0.979639
54520	0.946133	0.876598
54521	0.946133	0.883019
54522	0.946132	0.795076
54523	0.946132	0.80255
54524	0.946132	0.821681
54525	0.946132	0.817501
54526	0.946133	0.87499
54527	0.946133	0.85984
54528	0.946132	0.85893
54529	0.946132	0.751029
54530	0.946132	0.80688
54531	0.946132	0.818781
54532	1.89226	0.944168
54533	2.10495	0.806619
54534	1.89226	0.805161
54535	2.30932	0.803322
54536	1.89226	1.02015
54537	2.30932	1.01092
54538	2.30932	0.812034
54539	1.00366	0.733054
54540	2.10496	0.74155
54541	1.24728	0.763834
54542	3.31298	0.76533
54543	1.87557	2.1
54544	1.07167	2.1
54545	2.03045	2.1
54546	1.25185	2.1
54547	3.10212	2.1
54548	1.89226	2.1
54549	1.89226	2.1
54550	1.89226	2.1
54551	1.89226	2.1
54552	3.78453	2.1
54553	0.851541	2.1
54554	0.851541	2.1
54555	1.12236	2.1
54556	1.05783	2.1
54557	0.870663	2.1
54558	0.951856	2.1
54559	0.946132	2.1
54560	0.946132	2.1
54561	1.04073	2.1
54562	1.1296	2.1
54563	0.776062	2.1
54564	0.851527	2.1
54565	0.859395	2.1
54566	0.859395	2.1
54567	0.870165	2.1
54568	0.870165	2.1
54569	0.625924	2.1
54570	0.714333	2.1
54571	1.0821	2.1
54572	1.01523	2.1
54573	1.0821	2.1
54574	0.714333	2.1
54575	1.01523	2.1

54576	0.625924	2.1
54577	1.89226	2.1
54578	1.89226	2.1
54579	1.89226	2.1
54580	1.89226	2.1
54581	3.78453	2.1
54582	1.89226	2.1
54583	1.89226	2.1
54584	2.23164	2.1
54585	2.67802	2.1
54586	2.03663	2.1
54587	1.89226	2.1
54588	2.42221	2.1
54589	1.61933	2.1
54590	2.80896	2.1
54591	1.87043	2.1
54592	4.29262	2.1
54593	1.89226	2.1
54594	1.89226	2.1
54595	1.89226	2.1
54596	1.89226	2.1
54597	1.89226	2.1
54598	1.89226	2.1
54599	2.80892	2.1
54600	1.87041	2.1
54601	2.67182	2.02178
54602	2.30891	2.1
54603	5.07665	2.1
54604	1.89224	2.1
54605	1.89224	2.1
54606	1.89224	2.00355
54607	1.89224	2.1
54608	3.78448	2.1
54609	0.946118	2.1
54610	0.946118	2.1
54611	0.946118	2.1
54612	0.946118	2.1
54613	0.946115	2.1
54614	0.946115	2.1
54615	0.94612	2.1
54616	0.94612	2.1
54617	0.94613	2.1
54618	0.94613	2.1
54619	0.946105	2.1
54620	0.946105	2.1
54621	2.3089	2.1
54622	1.02783	2.1
54623	1.98533	2.1
54624	1.76762	2.1
54625	1.24553	2.1
54626	1.02783	2.1
54627	2.2049	0.610683
54628	2.34673	0.57041
54629	1.00763	0.571359
54630	1.23746	0.692187
54631	3.44236	0.597319
54632	1.89226	0.717974
54633	1.89226	0.736264

54634	1.89226	0.722076
54635	1.89226	0.730844
54636	3.78452	0.733627
54637	1.89226	0.739329
54638	1.89226	0.732449
54639	1.89226	0.732835
54640	1.89226	0.750411
54641	3.78453	0.736071
54642	0.891618	0.667561
54643	0.891618	0.66243
54644	0.902158	0.575961
54645	0.902158	0.591613
54646	1.15396	0.657033
54647	0.620408	0.609228
54648	1.17337	0.56649
54649	0.618733	0.691333
54650	0.620408	0.648359
54651	0.618733	0.610976
54652	1.17337	0.633402
54653	1.15396	0.648
54654	0.69978	0.703929
54655	0.69978	0.691295
54656	0.946102	0.704536
54657	0.946102	0.708592
54658	0.831702	0.712043
54659	0.946129	0.715135
54660	0.699752	0.685967
54661	0.946132	0.673302
54662	0.831703	0.70942
54663	0.699751	0.672186
54664	0.946131	0.681247
54665	0.946131	0.707819
54666	0.831675	0.717737
54667	0.831675	0.718863
54668	0.946163	0.71604
54669	0.864397	0.716024
54670	0.853471	0.725471
54671	0.962623	0.712411
54672	0.748555	0.709375
54673	0.851543	0.71623
54674	0.864714	0.718155
54675	0.760411	0.713275
54676	0.946133	0.717681
54677	0.946133	0.721264
54678	1.89226	0.700789
54679	1.89226	0.738254
54680	1.89226	0.734998
54681	1.89226	0.701798
54682	3.78452	0.704737
54683	1.89226	0.796919
54684	1.89226	0.721858
54685	1.89226	0.732875
54686	1.89226	0.825393
54687	3.78452	0.756369
54688	0.946132	0.7956
54689	0.946132	0.827687
54690	0.946132	0.711442
54691	0.946132	0.728722

54692	0.946132	0.721656
54693	0.946132	0.826742
54694	0.946133	0.720947
54695	0.946133	0.826664
54696	0.946133	0.751841
54697	0.946133	0.750302
54698	0.946132	0.756907
54699	0.946132	0.76893
54700	1.89226	0.76629
54701	2.39715	0.720834
54702	1.89226	0.746216
54703	1.89226	0.828379
54704	1.89226	0.711686
54705	1.89226	0.739118
54706	0.94613	0.825997
54707	0.94613	0.817525
54708	0.94613	0.749395
54709	0.94613	0.730433
54710	0.946131	0.744586
54711	0.946131	0.828164
54712	0.946129	0.818905
54713	0.946129	0.739204
54714	0.94613	0.76278
54715	0.94613	0.770729
54716	0.94613	0.779932
54717	0.94613	0.77729
54718	2.54204	2.1
54719	2.3148	2.1
54720	2.55328	2.1
54721	2.39646	2.1
54722	4.71126	2.1
54723	1.62611	2.1
54724	2.3148	2.1
54725	2.16986	2.1
54726	1.43745	2.1
54727	3.75225	2.1
54728	1.89226	2.1
54729	1.89226	2.1
54730	1.89226	2.1
54731	1.89226	2.1
54732	3.78453	2.1
54733	1.89226	2.1
54734	1.89226	2.1
54735	1.89226	2.1
54736	1.89226	2.1
54737	3.78453	2.1
54738	2.12125	2.1
54739	2.39642	2.1
54740	2.2941	2.1
54741	1.9933	2.1
54742	4.38972	2.1
54743	1.73784	2.1
54744	1.26892	2.1
54745	1.26892	2.1
54746	1.43743	2.1
54747	1.9933	2.1
54748	2.1618	2.1
54749	0.946118	2.1

54750	0.946118	2.1
54751	0.946118	2.1
54752	0.946118	2.1
54753	0.946119	2.1
54754	0.946119	2.1
54755	0.946118	2.1
54756	0.946118	2.1
54757	0.946131	2.1
54758	0.946131	2.1
54759	0.946106	2.1
54760	0.946106	2.1
54761	1.89224	2.1
54762	1.89224	2.1
54763	1.89224	2.1
54764	1.89224	2.1
54765	3.78448	2.1
54766	2.45922	2.1
54767	2.15686	2.1
54768	2.14259	2.1
54769	2.43225	2.1
54770	4.58911	2.1
54771	1.62027	2.1
54772	2.15686	2.1
54773	1.62241	2.1
54774	1.62241	2.1
54775	2.1426	2.1
54776	2.1426	2.1
54777	2.14259	2.1
54778	2.43225	2.1
54779	2.40667	2.1
54780	2.1281	2.1
54781	4.56035	2.1
54782	1.0713	2.1
54783	1.06405	2.1
54784	0.810136	2.1
54785	0.808433	2.1
54786	0.938508	2.1
54787	0.938508	2.1
54788	0.941223	2.1
54789	0.941223	2.1
54790	1.06405	2.1
54791	0.808433	2.1
54792	0.810135	2.1
54793	1.0713	2.1
54794	1.89226	0.826341
54795	1.89226	0.745037
54796	1.89226	0.805926
54797	1.89226	0.78234
54798	3.78453	0.782635
54799	1.89227	0.776653
54800	1.89227	0.731611
54801	1.89227	0.799607
54802	1.89227	0.672927
54803	3.78453	0.77411
54804	1.19	0.635292
54805	1.12469	0.470792
54806	1.01052	0.578787
54807	1.08166	0.504158

54808	2.20051	0.501683
54809	0.594999	0.655113
54810	0.540832	0.607401
54811	0.514182	0.730553
54812	0.567857	0.588766
54813	0.554259	0.541182
54814	0.554259	0.535758
54815	0.55443	0.658432
54816	0.55443	0.663002
54817	0.540832	0.619778
54818	0.514182	0.539408
54819	0.567857	0.591857
54820	0.594999	0.571507
54821	1.38674	0.738142
54822	0.946132	0.776442
54823	1.36361	0.710552
54824	0.946132	0.767378
54825	1.36362	0.707001
54826	1.36362	0.586944
54827	0.946131	0.776096
54828	0.946131	0.795452
54829	1.38674	0.750386
54830	1.36361	0.656165
54831	0.946132	0.781372
54832	0.946132	0.751032
54833	1.89226	0.756986
54834	2.68508	0.582812
54835	2.77349	0.657103
54836	1.89226	0.70063
54837	2.77349	0.664644
54838	1.89226	0.703241
54839	2.15315	0.299463
54840	1.96254	0.46187
54841	1.51377	0.338246
54842	1.89226	0.327517
54843	1.47799	0.460285
54844	1.89226	0.377372
54845	2.49439	0.385442
54846	1.89226	0.585457
54847	2.15315	0.475085
54848	1.89226	0.452501
54849	2.15315	0.608562
54850	1.89226	0.492018
54851	1.89226	0.611289
54852	1.89226	0.627448
54853	1.89226	0.629361
54854	1.89226	0.495825
54855	1.89226	0.658736
54856	1.89226	0.619543
54857	1.89226	0.589752
54858	1.89226	0.471434
54859	1.89226	0.521876
54860	1.89226	0.488239
54861	1.89226	0.400609
54862	1.89226	0.58251
54863	1.375	0.359962
54864	2.01865	0.389354
54865	2.12047	0.384386

54866	1.47354	0.290706
54867	2.12047	0.510762
54868	1.47354	0.355074
54869	0.687499	0.2689
54870	1.06023	0.293082
54871	1.08019	0.278159
54872	0.604229	0.266442
54873	1.08018	0.29623
54874	1.06023	0.256264
54875	0.687502	0.246456
54876	0.60423	0.272113
54877	0.80646	0.308792
54878	0.806461	0.300542
54879	0.858001	0.236071
54880	0.858001	0.25322
54881	2.26414	2.1
54882	1.87342	2.1
54883	1.87342	2.1
54884	1.79667	2.1
54885	2.44045	2.1
54886	2.31924	2.1
54887	2.26414	2.1
54888	2.26616	2.1
54889	2.2355	2.1
54890	2.21858	2.1
54891	4.48474	2.1
54892	2.18516	2.1
54893	1.87344	2.1
54894	2.21859	2.1
54895	2.66233	2.1
54896	1.85196	2.1
54897	2.22236	2.1
54898	2.23551	2.1
54899	2.21859	2.1
54900	2.18516	2.1
54901	2.21189	2.1
54902	4.43049	2.1
54903	1.17171	2.1
54904	2.38924	2.1
54905	2.0815	2.1
54906	2.33348	2.1
54907	1.42851	2.1
54908	1.5959	2.1
54909	1.89226	2.1
54910	1.89226	2.1
54911	1.89226	2.1
54912	1.89226	2.1
54913	3.78452	2.1
54914	2.75425	2.1
54915	1.34366	2.1
54916	1.34366	2.1
54917	1.56512	2.1
54918	2.54186	2.1
54919	2.76332	2.1
54920	1.07751	2.1
54921	2.0815	2.1
54922	1.97259	2.1
54923	1.76455	2.1

54924	1.30191	2.1
54925	1.16461	2.1
54926	0.946131	2.1
54927	0.946131	2.1
54928	0.946129	2.1
54929	0.946129	2.1
54930	0.946131	2.1
54931	0.946131	2.1
54932	0.94613	2.1
54933	0.94613	2.1
54934	0.94613	2.1
54935	0.94613	2.1
54936	0.946131	2.1
54937	0.946131	2.1
54938	1.89226	0.705525
54939	1.89226	0.701366
54940	1.89226	0.7
54941	1.89226	0.550997
54942	3.78453	0.597836
54943	1.89226	0.703762
54944	1.89226	0.70176
54945	1.89226	0.7
54946	1.89226	0.546059
54947	3.78453	0.70201
54948	0.946132	0.593372
54949	0.946132	0.7003
54950	1.30327	0.508313
54951	1.33544	0.633507
54952	1.33544	0.501086
54953	1.33544	0.4504
54954	0.946132	0.559343
54955	0.946132	0.554258
54956	0.946132	0.474256
54957	1.30327	0.406956
54958	1.33544	0.621106
54959	0.946132	0.69507
54960	1.30327	0.518568
54961	1.33544	0.611689
54962	0.709426	0.484661
54963	0.790129	0.58536
54964	1.05886	0.348417
54965	1.05886	0.423534
54966	1.03454	0.667842
54967	1.03454	0.577049
54968	1.30327	0.464933
54969	0.709426	0.354876
54970	1.33544	0.664523
54971	0.790128	0.56849
54972	0.946132	0.400896
54973	0.946132	0.617498
54974	0.827866	0.317236
54975	0.946133	0.579961
54976	0.946132	0.315607
54977	0.946132	0.35378
54978	0.946133	0.412335
54979	0.946133	0.584178
54980	0.946132	0.333724
54981	0.827866	0.571002

54982	0.946133	0.65691
54983	0.946133	0.590515
54984	1.89227	0.687737
54985	1.89227	0.374474
54986	1.89227	0.489716
54987	1.89227	0.555526
54988	1.89227	0.466666
54989	1.89227	0.539055
54990	0.946133	0.370228
54991	0.946133	0.551811
54992	1.21602	0.319903
54993	1.30327	0.45274
54994	1.30327	0.333561
54995	1.30327	0.306747
54996	0.946132	0.34849
54997	0.946132	0.381129
54998	0.946132	0.309989
54999	1.21602	0.280355
55000	0.946133	0.486006
55001	1.30327	0.417937
55002	1.04227	0.283974
55003	1.11706	0.435753
55004	0.968413	0.233521
55005	0.945482	0.302662
55006	1.3204	0.341843
55007	1.23215	0.36714
55008	0.810791	0.39309
55009	1.38978	0.260425
55010	1.07728	0.372265
55011	1.11704	0.405997
55012	0.608052	0.387929
55013	1.89226	0.377706
55014	1.89226	0.247915
55015	1.89226	0.276384
55016	1.89226	0.332734
55017	3.78453	0.303101
55018	1.89226	0.195675
55019	1.89226	0.208263
55020	1.89226	0.247626
55021	1.89226	0.159667
55022	3.78451	0.199674
55023	2.58476	0.191043
55024	1.56598	0.138999
55025	1.55045	0.164081
55026	1.5124	0.172707
55027	2.62281	0.14532
55028	2.58476	0.151862
55029	2.58477	0.228411
55030	1.89226	0.261125
55031	1.89226	0.24128
55032	1.89226	0.22463
55033	2.62282	0.25311
55034	2.62282	0.241364
55035	1.89224	0.285241
55036	1.89224	0.332328
55037	1.89224	0.420268
55038	1.89224	0.254198
55039	3.78448	0.293193

55040	1.89223	0.256447
55041	1.89223	0.166205
55042	1.89223	0.20228
55043	1.89223	0.20469
55044	1.89223	0.216832
55045	1.89223	0.221663
55046	2.62278	0.199383
55047	1.45053	0.140768
55048	1.49678	0.147608
55049	1.56596	0.170639
55050	2.5536	0.138442
55051	2.62278	0.155062
55052	1.31139	0.220897
55053	1.2768	0.192792
55054	0.946118	0.362736
55055	0.946118	0.252918
55056	1.2768	0.254754
55057	1.2768	0.249141
55058	0.946116	0.303566
55059	0.946116	0.210158
55060	1.27682	0.193376
55061	0.94613	0.27196
55062	0.946105	0.26152
55063	1.31138	0.204434
55064	1.38506	1.54303
55065	1.53348	2.03838
55066	1.17042	1.52212
55067	1.17042	1.89076
55068	1.73669	1.52269
55069	1.73669	1.87777
55070	1.73669	1.2697
55071	1.79928	0.965231
55072	1.38506	1.17316
55073	1.38506	1.87339
55074	1.79928	1.77714
55075	1.43484	1.9063
55076	1.73669	1.13374
55077	1.93568	1.80171
55078	1.53347	1.1066
55079	1.53347	1.76639
55080	2.14058	1.12827
55081	2.14058	1.86639
55082	0.89964	0.949259
55083	1.10415	2.09377
55084	0.868346	1.21076
55085	1.07029	1.98348
55086	0.98687	1.32244
55087	0.98687	2.1
55088	0.985627	0.831638
55089	0.985627	1.82757
55090	1.07029	1.56218
55091	1.10415	1.31275
55092	0.868344	1.70444
55093	0.899642	1.72099
55094	2.40667	2.1
55095	2.1281	2.1
55096	2.11176	2.1
55097	2.38069	2.1

55098	4.50878	2.1
55099	2.11176	2.1
55100	1.61687	2.1
55101	1.60965	2.1
55102	1.61687	2.1
55103	2.12088	2.1
55104	2.1281	2.1
55105	2.11176	2.1
55106	2.34898	2.1
55107	2.34898	2.1
55108	2.38069	2.1
55109	2.09114	2.1
55110	2.12285	2.1
55111	1.05588	2.1
55112	1.04557	2.1
55113	0.804824	2.1
55114	0.798269	2.1
55115	0.92615	2.1
55116	0.92615	2.1
55117	0.927999	2.1
55118	0.927999	2.1
55119	1.04557	2.1
55120	0.798269	2.1
55121	1.05588	2.1
55122	0.804824	2.1
55123	1.43548	0.688514
55124	1.53873	0.738485
55125	1.25063	0.442103
55126	1.39662	0.440126
55127	2.78937	0.561768
55128	1.25063	0.722256
55129	1.39662	0.696088
55130	1.10476	0.143886
55131	1.28326	0.554369
55132	2.53389	0.670379
55133	1.51147	0.166403
55134	1.86114	0.272382
55135	1.86114	0.243853
55136	2.12926	0.215723
55137	1.3659	0.205403
55138	1.63401	0.170906
55139	1.89226	0.240875
55140	1.89226	0.40471
55141	1.89226	0.312756
55142	1.89226	0.26745
55143	1.89226	0.423672
55144	1.89226	0.453344
55145	1.89226	0.597808
55146	1.89226	0.689427
55147	2.46286	0.545169
55148	1.89226	0.708996
55149	3.78452	0.573575
55150	1.89226	0.703066
55151	1.89226	0.740335
55152	1.89226	0.724398
55153	1.89226	0.72402
55154	1.89226	0.707495
55155	1.89226	0.709062

55156	1.89226	0.642094
55157	1.89226	0.700755
55158	2.28992	0.606576
55159	1.89226	0.711589
55160	3.78452	0.633751
55161	1.13537	0.242332
55162	0.886595	0.349673
55163	0.946131	0.549688
55164	0.946131	0.494333
55165	0.94613	0.67182
55166	0.94613	0.270778
55167	1.25069	0.571609
55168	0.841855	0.822398
55169	1.27436	0.342157
55170	1.06196	0.504457
55171	1.09758	0.33797
55172	0.830306	0.126514
55173	0.826931	0.839068
55174	0.826933	0.159642
55175	0.764631	0.305162
55176	0.764631	0.339742
55177	1.00427	0.776203
55178	0.788034	0.556843
55179	0.726852	0.301016
55180	0.604206	0.743786
55181	0.828475	0.450245
55182	0.604198	0.441932
55183	0.8332	0.289298
55184	1.00428	0.304654
55185	1.89226	0.590494
55186	1.89226	0.405931
55187	1.89226	0.275896
55188	1.89226	0.775689
55189	1.89226	0.207731
55190	1.89226	0.604503
55191	0.768789	1.41515
55192	0.849132	0.05
55193	0.666423	1.34192
55194	0.688993	0.787053
55195	0.868671	0.05
55196	0.89117	1.19856
55197	0.705632	2.1
55198	1.80425	2.1
55199	1.73883	2.1
55200	1.45159	2.1
55201	1.45159	2.1
55202	1.37681	2.1
55203	1.73883	2.1
55204	1.39679	0.102338
55205	2.08149	0.168849
55206	1.89226	0.135233
55207	2.32763	0.10968
55208	1.89226	0.117024
55209	2.31274	0.0989375
55210	1.89226	0.141106
55211	1.89226	0.274015
55212	1.89226	0.191906
55213	1.89226	0.147006

55214	1.89226	0.223817
55215	1.89226	0.168445
55216	1.89226	0.147211
55217	1.89226	0.218184
55218	1.89226	0.165739
55219	1.89226	0.151021
55220	1.89226	0.17724
55221	1.89226	0.160481
55222	1.95181	0.113844
55223	1.89226	0.148644
55224	1.89226	0.129109
55225	1.89226	0.108644
55226	3.78451	0.107937
55227	0.946131	0.122954
55228	0.946131	0.115356
55229	0.946125	0.130484
55230	0.946125	0.153538
55231	0.946127	0.162038
55232	0.946127	0.161072
55233	0.946128	0.157501
55234	0.946128	0.12514
55235	0.946128	0.131474
55236	0.946128	0.146262
55237	0.946128	0.118786
55238	0.946128	0.154591
55239	1.89226	0.155644
55240	1.89226	0.283306
55241	1.89226	0.198935
55242	1.89226	0.189564
55243	1.89226	0.214942
55244	1.89226	0.20556
55245	1.89226	0.76274
55246	1.89226	0.763961
55247	1.89226	0.740318
55248	1.89226	0.727105
55249	3.78453	0.749282
55250	1.89226	0.818684
55251	1.89226	0.774621
55252	1.89226	0.769106
55253	1.89226	0.775398
55254	3.78452	0.769895
55255	1.21387	0.77126
55256	0.8164	0.748505
55257	0.946133	0.749192
55258	0.946133	0.761018
55259	0.946102	0.731436
55260	0.946102	0.794223
55261	0.816431	0.794755
55262	0.816431	0.772824
55263	0.816401	0.759242
55264	0.94613	0.738114
55265	0.946131	0.82117
55266	1.21388	0.772519
55267	0.946132	0.723551
55268	0.946132	0.728991
55269	0.946132	0.735201
55270	0.946132	0.738843
55271	0.946132	0.71733

55272	0.946132	0.737411
55273	0.946132	0.751191
55274	0.946132	0.722054
55275	0.946131	0.716751
55276	0.946131	0.726747
55277	0.946132	0.749036
55278	0.946132	0.737386
55279	1.89226	0.761711
55280	1.89226	0.712089
55281	1.89226	0.713564
55282	1.89226	0.762858
55283	1.89226	0.725217
55284	1.89226	0.804126
55285	1.89226	0.799931
55286	1.89226	0.80536
55287	2.15871	0.751113
55288	2.15871	0.816932
55289	1.81454	0.740872
55290	1.54809	0.83544
55291	1.89226	0.799635
55292	1.89226	1.16191
55293	1.89226	0.844892
55294	1.89226	0.785028
55295	3.78453	0.906556
55296	1.89226	0.894068
55297	1.89226	0.77517
55298	1.89226	0.751735
55299	1.89226	0.849372
55300	1.89226	1.08349
55301	1.89226	0.941346
55302	2.29205	0.876634
55303	1.42633	0.886571
55304	1.69015	1.06531
55305	1.69015	0.944166
55306	2.10364	0.778133
55307	2.05344	0.771155
55308	2.29205	0.782439
55309	1.58147	0.8852
55310	2.16529	0.788095
55311	1.56339	0.768627
55312	2.29205	1.10596
55313	1.69015	0.958174
55314	1.89226	0.925019
55315	1.89226	0.825693
55316	1.89226	0.775035
55317	1.89226	0.845009
55318	3.78453	0.799974
55319	1.89226	0.826153
55320	1.89226	1.10163
55321	1.89226	0.859355
55322	1.89226	0.800023
55323	3.78452	1.02666
55324	2.29205	1.09095
55325	2.85335	0.748321
55326	2.55409	0.831782
55327	2.04345	0.782498
55328	2.76338	0.840498
55329	2.29205	0.801424

55330	2.29205	0.823801
55331	2.16529	0.983937
55332	2.72418	0.83961
55333	2.76338	0.737807
55334	5.01623	0.852723
55335	1.89227	0.436778
55336	1.89227	0.347084
55337	1.89227	0.213037
55338	1.89227	0.33527
55339	3.78453	0.247455
55340	2.39588	0.229611
55341	2.14918	0.262387
55342	2.14918	0.312231
55343	2.37907	0.411855
55344	2.20531	0.16122
55345	2.4352	0.220812
55346	1.89226	0.236425
55347	2.64327	0.371453
55348	2.36175	0.233375
55349	1.89226	0.220061
55350	4.25402	0.337515
55351	1.89226	0.238649
55352	1.89226	0.179195
55353	1.89226	0.163916
55354	1.89226	0.2318
55355	1.89226	0.195791
55356	1.89226	0.289012
55357	1.05395	0.138859
55358	1.05395	0.168618
55359	1.03114	0.167208
55360	1.03114	0.198019
55361	0.936689	0.141303
55362	1.07459	0.202714
55363	1.10265	0.162606
55364	1.01049	0.173267
55365	0.93669	0.117836
55366	1.01049	0.158143
55367	1.07459	0.212193
55368	1.10265	0.160187
55369	1.18088	0.189024
55370	0.776509	0.168058
55371	0.946132	0.15021
55372	0.946132	0.182251
55373	1.04225	0.164679
55374	0.830392	0.292156
55375	0.931352	0.186502
55376	0.931352	0.179857
55377	1.13006	0.151874
55378	1.04074	0.161905
55379	1.0628	0.180677
55380	0.851529	0.233176
55381	2.40032	2.1
55382	1.89226	2.1
55383	1.89226	2.1
55384	1.89226	2.1
55385	2.38918	2.1
55386	2.38918	2.1
55387	1.89226	2.1

55388	1.89226	2.04733
55389	1.89226	2.1
55390	1.89226	1.76605
55391	1.89226	2.1
55392	1.89226	1.99915
55393	1.6585	2.1
55394	2.37364	2.08159
55395	2.39622	2.1
55396	2.39622	2.09585
55397	1.6488	2.1
55398	1.6488	2.1
55399	2.44603	2.1
55400	1.89224	2.1
55401	1.89224	2.1
55402	1.89224	2.1
55403	2.40029	2.1
55404	2.40029	2.1
55405	1.89224	2.1
55406	1.89224	1.74921
55407	1.89224	2.1
55408	1.89224	1.38132
55409	1.89224	2.1
55410	1.89224	1.47156
55411	1.01946	1.95211
55412	1.01946	2.1
55413	1.02497	2.1
55414	1.02497	2.1
55415	1.21519	1.55093
55416	1.19809	1.315
55417	0.835303	2.1
55418	0.82924	2.1
55419	0.835314	2.1
55420	1.2152	2.1
55421	0.829228	2.1
55422	1.19808	2.1
55423	1.91027	2.1
55424	1.05809	2.1
55425	1.00659	2.1
55426	2.06727	2.1
55427	3.11819	2.1
55428	2.37166	2.1
55429	2.06726	2.1
55430	1.66994	2.1
55431	1.67623	2.1
55432	2.40308	2.1
55433	2.40308	2.1
55434	0.960194	2.1
55435	0.939664	2.1
55436	0.531131	2.1
55437	0.503298	2.1
55438	0.729927	2.1
55439	0.729927	2.1
55440	0.733565	2.1
55441	0.733565	2.1
55442	0.960195	2.1
55443	0.531132	2.1
55444	0.503297	2.1
55445	0.939663	2.1

55446	1.20154	2.1
55447	1.21754	2.1
55448	0.960195	2.1
55449	0.939665	2.1
55450	1.07745	2.1
55451	1.07745	2.1
55452	1.07976	2.1
55453	1.07976	2.1
55454	0.960194	2.1
55455	1.21754	2.1
55456	0.939662	2.1
55457	1.20155	2.1
55458	1.89224	2.1
55459	1.89224	2.1
55460	2.08609	2.1
55461	2.08609	2.1
55462	2.09527	2.1
55463	1.90142	2.1
55464	1.65815	2.1
55465	1.71398	2.1
55466	2.2001	2.1
55467	2.23791	2.1
55468	1.4502	2.1
55469	1.4502	2.1
55470	0.793587	2.1
55471	0.793587	2.1
55472	0.792408	2.1
55473	0.792408	2.1
55474	0.952221	2.1
55475	0.858585	2.1
55476	0.725101	2.1
55477	0.633774	2.1
55478	0.633775	2.1
55479	0.858585	2.1
55480	0.952221	2.1
55481	0.725101	2.1
55482	1.5735	2.1
55483	2.56975	2.1
55484	2.63166	2.1
55485	2.63166	2.1
55486	1.54711	2.1
55487	1.54711	2.1
55488	0.946131	2.1
55489	0.946131	2.1
55490	1.28487	2.1
55491	1.28487	2.1
55492	0.946127	2.1
55493	0.946127	2.1
55494	1.28488	2.1
55495	1.31583	2.1
55496	1.31583	2.1
55497	0.94613	2.1
55498	1.28487	2.1
55499	0.946129	2.1
55500	1.0596	2.1
55501	1.0596	2.1
55502	1.06939	2.1
55503	1.06939	2.1

55504	1.34225	2.1
55505	1.31583	2.1
55506	0.793563	2.1
55507	0.786748	2.1
55508	0.793563	2.1
55509	1.34225	2.1
55510	0.786748	2.1
55511	1.31583	2.1
55512	0.946131	2.1
55513	0.946131	2.1
55514	1.31582	2.1
55515	1.31582	2.1
55516	0.946127	2.1
55517	0.946127	2.1
55518	1.34225	2.1
55519	1.31583	2.1
55520	1.34225	2.1
55521	0.946129	2.1
55522	1.31583	2.1
55523	0.946129	2.1
55524	1.89226	2.1
55525	1.89226	2.1
55526	1.89226	2.1
55527	1.89226	2.1
55528	1.89226	2.1
55529	1.89226	2.1
55530	1.89226	2.1
55531	1.89226	2.1
55532	1.89226	2.1
55533	1.89226	2.1
55534	1.89226	2.1
55535	1.89226	2.1
55536	1.26992	2.1
55537	1.75003	2.1
55538	1.79883	2.1
55539	1.26732	2.1
55540	3.06875	2.1
55541	0.875017	2.1
55542	0.899414	2.1
55543	0.811147	2.1
55544	0.85053	2.1
55545	0.851513	2.1
55546	0.851513	2.1
55547	0.859048	2.1
55548	0.859048	2.1
55549	0.899413	2.1
55550	0.85053	2.1
55551	0.811147	2.1
55552	0.875016	2.1
55553	1.89227	2.1
55554	1.89227	2.1
55555	1.89227	2.1
55556	1.89227	2.1
55557	1.89227	2.1
55558	1.89227	2.1
55559	1.89226	2.1
55560	1.89226	2.1
55561	1.89226	2.1

55562	1.89226	2.1
55563	1.89226	2.1
55564	1.89226	2.1
55565	0.633661	2.1
55566	0.642005	2.1
55567	0.93285	2.1
55568	0.899413	2.1
55569	0.764487	2.1
55570	0.764487	2.1
55571	0.776931	2.1
55572	0.776931	2.1
55573	0.642004	2.1
55574	0.93285	2.1
55575	0.899414	2.1
55576	0.633661	2.1
55577	0.932851	2.1
55578	0.899414	2.1
55579	0.905419	2.1
55580	0.85053	2.1
55581	0.886336	2.1
55582	0.886336	2.1
55583	0.897045	2.1
55584	0.897045	2.1
55585	0.93285	2.1
55586	0.905419	2.1
55587	0.899414	2.1
55588	0.850531	2.1
55589	2.39322	2.1
55590	2.74149	1.02285
55591	2.70944	2.1
55592	2.6849	1.06097
55593	2.41776	2.0957
55594	2.39322	0.899382
55595	2.41776	2.1
55596	2.80992	1.83977
55597	2.77256	2.1
55598	2.74149	1.29185
55599	2.44882	2.1
55600	2.41776	0.959847
55601	2.80992	2.00755
55602	1.89226	1.61266
55603	2.74149	1.67073
55604	2.74149	2.00764
55605	1.89226	1.05069
55606	1.89226	1.24527
55607	1.89226	0.1684
55608	1.89226	0.153809
55609	1.89226	0.249982
55610	1.89226	0.117567
55611	1.89226	0.220464
55612	1.89226	0.122514
55613	2.38796	0.0883878
55614	2.0815	0.153006
55615	2.0815	0.120878
55616	2.49452	0.0941494
55617	1.55994	0.134287
55618	2.14721	0.0929344
55619	1.89227	0.1895

55620	1.89227	0.163743
55621	1.89227	0.256749
55622	1.89227	0.130004
55623	1.89227	0.239282
55624	1.89227	0.147556
55625	1.13387	0.13536
55626	2.27494	0.110029
55627	1.13387	0.205616
55628	2.19489	0.108393
55629	1.60867	0.176268
55630	2.6994	0.101535
55631	1.89226	0.193513
55632	1.89226	0.142079
55633	1.89226	0.161282
55634	1.89226	0.288911
55635	3.78453	0.162153
55636	1.42381	1.26979
55637	1.10747	2.1
55638	1.10747	1.2097
55639	1.08911	2.1
55640	1.42866	1.18752
55641	1.4103	2.1
55642	1.89226	2.1
55643	1.88181	2.1
55644	1.88181	1.97441
55645	1.76249	2.1
55646	2.0815	1.85052
55647	1.97539	2.1
55648	0.946128	2.1
55649	0.946128	1.85336
55650	0.946133	1.23104
55651	0.946133	1.06029
55652	0.94613	1.07085
55653	0.94613	1.77651
55654	0.946131	2.1
55655	0.946131	1.15576
55656	0.946131	1.32824
55657	0.946131	1.35131
55658	0.946131	1.35271
55659	0.946131	1.36218
55660	1.42866	1.23135
55661	1.43213	1.20351
55662	1.10748	1.91853
55663	1.10748	2.1
55664	1.13641	2.1
55665	1.43213	2.1
55666	2.0815	2.1
55667	2.10287	2.1
55668	2.10287	1.95459
55669	2.0921	2.1
55670	1.89227	1.90971
55671	1.83938	2.1
55672	0.810944	1.16555
55673	0.810944	1.06992
55674	0.810945	1.08587
55675	0.810945	1.78294
55676	0.810933	1.23735
55677	0.810933	1.24879

55678	1.89226	2.1
55679	1.3517	1.44415
55680	1.08132	1.22558
55681	1.08132	1.95458
55682	1.08133	1.27474
55683	2.33097	2.07625
55684	1.53822	2.1
55685	1.53822	2.1
55686	1.68437	2.1
55687	2.17996	2.1
55688	2.32612	2.1
55689	0.99189	2.1
55690	1.18948	0.782236
55691	1.19756	0.73664
55692	1.19756	2.1
55693	0.978851	2.1
55694	0.926995	1.8192
55695	1.26924	0.407304
55696	1.34642	0.782776
55697	1.3228	0.84993
55698	0.990899	0.851588
55699	1.0627	0.867848
55700	1.33473	0.860298
55701	1.96196	1.94718
55702	1.39702	2.1
55703	1.39702	2.1
55704	1.53822	2.1
55705	2.17372	2.1
55706	2.31011	2.1
55707	1.89226	0.8085
55708	1.89226	0.704795
55709	1.89226	0.805339
55710	1.89226	0.287269
55711	3.78453	0.727694
55712	2.38475	0.861256
55713	1.73957	0.723307
55714	1.88452	0.804242
55715	1.58695	0.156744
55716	3.6241	0.595583
55717	1.73958	0.706405
55718	1.3126	0.811623
55719	1.58695	0.244103
55720	1.14946	0.791413
55721	2.88903	0.516435
55722	1.89226	0.708107
55723	1.89226	0.827981
55724	1.89226	0.222095
55725	1.89226	0.834744
55726	3.78453	0.716202
55727	1.89225	0.811428
55728	1.89225	0.720926
55729	1.89225	0.552188
55730	1.89225	0.266315
55731	3.7845	0.326253
55732	1.88451	0.809927
55733	1.58694	0.216041
55734	1.74649	0.845066
55735	1.44604	0.189645

55736	3.33055	0.238174
55737	1.58694	0.49051
55738	0.995727	0.681079
55739	0.995727	0.777588
55740	1.14945	0.7651
55741	1.44604	0.199219
55742	1.59976	0.797835
55743	1.89225	0.706648
55744	1.89225	0.64553
55745	1.89225	0.823856
55746	1.89225	0.726162
55747	1.89225	0.828012
55748	1.89225	0.715846
55749	2.01197	1.54083
55750	2.0961	1.55429
55751	2.13283	1.06429
55752	2.25399	0.991139
55753	4.22894	1.2614
55754	2.09611	1.14188
55755	2.37014	1.1214
55756	2.29031	1.51498
55757	2.10084	1.77863
55758	2.25399	1.00816
55759	2.09611	1.56882
55760	2.13283	1.39892
55761	2.25399	1.99186
55762	2.22044	1.2395
55763	2.38975	1.00528
55764	4.52258	1.33398
55765	2.38976	0.900868
55766	2.15466	1.25609
55767	2.31988	0.853439
55768	2.31988	1.62814
55769	2.25399	0.919032
55770	2.25399	2.07256
55771	2.58286	0.858539
55772	2.29193	0.729037
55773	2.40107	0.926184
55774	2.13815	0.435768
55775	4.72101	0.777062
55776	2.29193	0.727782
55777	1.90104	0.762555
55778	2.13815	0.493387
55779	1.77249	0.748063
55780	4.03919	0.680909
55781	2.25197	0.907407
55782	2.13815	0.723183
55783	2.00625	0.837104
55784	2.00625	0.731545
55785	2.40107	0.833557
55786	2.40107	0.757308
55787	2.13815	0.715082
55788	1.77249	0.752139
55789	2.00625	0.407338
55790	1.65815	0.760878
55791	3.7963	0.721839
55792	2.43417	0.912794
55793	2.06653	0.882729

55794	1.64587	0.89801
55795	1.65284	0.864406
55796	2.46898	0.921049
55797	2.46898	0.883401
55798	2.46895	0.866701
55799	1.89016	0.789293
55800	1.87861	0.808349
55801	1.87861	0.74342
55802	2.47161	0.87076
55803	2.47161	0.833029
55804	0.946132	1.04443
55805	0.946132	1.0205
55806	0.863311	1.14401
55807	1.34768	1.11776
55808	0.851542	1.1363
55809	0.851542	0.971439
55810	1.30451	1.06537
55811	1.45304	1.1039
55812	0.946132	1.04947
55813	1.20992	1.07839
55814	0.855162	1.11714
55815	0.946132	1.06132
55816	1.89226	0.985571
55817	1.89226	1.15388
55818	1.89226	1.13205
55819	1.89226	0.986179
55820	1.89226	1.16621
55821	1.89226	1.00967
55822	0.946132	0.935136
55823	0.946132	0.901104
55824	0.946133	0.956302
55825	0.946133	0.956298
55826	0.946132	0.961748
55827	0.946132	0.799653
55828	0.946132	0.909933
55829	0.946132	0.955208
55830	0.946132	0.974713
55831	0.946132	0.92434
55832	0.946133	0.922169
55833	0.946133	0.936686
55834	2.35574	0.976655
55835	1.89226	0.842691
55836	1.89226	1.10827
55837	1.89226	0.827891
55838	2.67456	1.00924
55839	2.67456	0.428578
55840	0.583505	0.704023
55841	0.761972	0.726432
55842	1.17787	0.899766
55843	1.33728	0.731423
55844	0.965128	0.78813
55845	0.965128	0.709318
55846	0.974714	0.786491
55847	0.974715	0.894095
55848	1.17787	0.769772
55849	0.583506	0.801165
55850	1.33728	0.675993
55851	0.761974	0.747159

55852	1.89226	0.790754
55853	1.89226	0.729772
55854	1.89226	0.564484
55855	1.89226	0.621175
55856	1.89226	0.706185
55857	1.89226	0.758389
55858	1.89226	0.829693
55859	1.89226	0.466276
55860	1.89226	0.778425
55861	1.89226	0.761436
55862	3.78453	0.75095
55863	0.946132	0.705164
55864	0.946132	0.726034
55865	0.946132	0.80438
55866	0.946132	0.708158
55867	0.946133	0.757363
55868	0.946133	0.712386
55869	0.946132	0.785385
55870	0.946132	0.797487
55871	0.946133	0.76625
55872	0.946133	0.794049
55873	0.946132	0.681681
55874	0.946132	0.773798
55875	1.01779	2.1
55876	1.50685	2.1
55877	1.12064	2.1
55878	1.65151	2.1
55879	2.6275	2.1
55880	1.35733	2.1
55881	1.53507	2.1
55882	1.50683	2.1
55883	1.73626	2.1
55884	3.09359	2.1
55885	1.73626	2.1
55886	1.52226	2.1
55887	1.75555	2.1
55888	1.75555	2.1
55889	1.53507	2.1
55890	1.53507	2.1
55891	1.65151	2.1
55892	1.73628	2.1
55893	1.69528	2.1
55894	1.50685	2.1
55895	1.92471	2.1
55896	1.73628	2.1
55897	1.73629	2.1
55898	1.75558	2.1
55899	1.9943	2.1
55900	1.9943	2.1
55901	1.92471	2.1
55902	1.73629	2.1
55903	2.5601	1.02209
55904	1.97645	1.8022
55905	2.09605	0.95352
55906	2.09605	1.6317
55907	2.45853	1.03008
55908	2.45853	1.94174
55909	2.33824	1.4337

55910	1.97646	1.27223
55911	2.45853	1.01798
55912	1.94984	1.79792
55913	2.33824	1.40625
55914	1.82955	2.1
55915	1.22926	1.87674
55916	0.946128	1.79303
55917	0.946133	1.96727
55918	1.28005	1.18987
55919	1.22927	1.1969
55920	1.22926	1.52726
55921	0.946131	1.45262
55922	0.946131	1.22121
55923	0.946131	1.24034
55924	0.946131	1.15257
55925	1.28005	1.29704
55926	1.22926	1.22817
55927	2.35349	0.907049
55928	2.61606	0.750085
55929	2.48209	0.892216
55930	2.48209	0.751866
55931	2.50057	0.888354
55932	2.50057	0.76083
55933	2.61607	0.745612
55934	2.57611	0.701112
55935	2.4821	0.696839
55936	2.44814	0.663361
55937	5.06421	0.689859
55938	2.15721	0.830412
55939	2.48209	0.752122
55940	2.31844	0.913234
55941	2.31844	0.739041
55942	2.35349	0.909104
55943	2.35349	0.734084
55944	2.44814	0.711636
55945	2.30908	0.568297
55946	2.44814	0.694801
55947	2.31844	0.468627
55948	2.4821	0.732539
55949	2.31844	0.693075
55950	0.91766	0.700297
55951	0.91766	0.702636
55952	0.912463	0.703053
55953	0.912463	0.701491
55954	1.16525	0.70119
55955	1.26377	0.700537
55956	0.576692	0.702224
55957	0.664871	0.70305
55958	1.16525	0.701165
55959	0.576692	0.700999
55960	0.664871	0.703347
55961	1.26377	0.702838
55962	1.72901	1.94303
55963	1.62414	2.1
55964	1.88971	2.1
55965	1.8411	1.24203
55966	3.57011	2.03519
55967	1.89226	2.04159

55968	1.89226	1.24926
55969	1.89226	1.01513
55970	1.89226	1.49069
55971	3.78452	1.05023
55972	1.89226	1.08539
55973	1.89226	2.1
55974	1.89226	1.36647
55975	1.89226	1.11031
55976	3.78452	2.08811
55977	1.62414	2.05124
55978	1.30896	2.09813
55979	1.8411	1.15268
55980	1.52776	1.9038
55981	3.15006	1.31419
55982	1.51448	1.94736
55983	1.8411	1.71287
55984	2.02311	1.86675
55985	1.99309	0.965182
55986	3.86421	1.33284
55987	1.89226	1.0465
55988	1.89226	2.08774
55989	1.89226	1.56405
55990	1.89226	1.09565
55991	3.78452	1.94786
55992	1.89226	2.07067
55993	1.89226	1.36867
55994	1.89226	1.25304
55995	1.89226	2.07558
55996	3.78452	1.64212
55997	1.8411	1.4213
55998	1.52776	2.1
55999	1.99309	2.1
56000	1.72526	1.88486
56001	3.56636	2.1
56002	1.89226	2.1
56003	1.89226	1.58451
56004	1.89226	1.59996
56005	1.89226	2.1
56006	3.78453	1.81634
56007	1.0424	2.1
56008	1.12935	2.1
56009	0.929383	2.1
56010	0.929383	2.1
56011	1.24941	2.1
56012	1.12686	2.1
56013	0.946132	2.1
56014	0.946132	2.1
56015	1.12944	2.1
56016	0.851554	2.1
56017	1.05017	2.1
56018	0.929315	2.1
56019	0.898029	2.1
56020	0.973994	2.1
56021	0.946131	1.64276
56022	0.946131	2.04285
56023	1.0221	2.0573
56024	1.0221	2.1
56025	0.898028	1.53167

56026	0.898028	2.1
56027	0.973994	2.1
56028	0.946131	2.1
56029	0.946131	2.1
56030	0.898029	2.1
56031	1.89226	1.56062
56032	1.89226	1.9728
56033	1.89226	1.4651
56034	1.89226	1.88252
56035	1.89226	1.49599
56036	1.89226	1.91368
56037	1.13537	1.9303
56038	0.879308	2.1
56039	0.946131	1.59893
56040	0.946131	1.81161
56041	1.13537	1.86258
56042	0.85187	2.1
56043	0.94613	1.93997
56044	0.94613	1.4352
56045	1.05777	2.04654
56046	1.05777	2.1
56047	0.799318	2.08372
56048	0.776452	2.1
56049	0.946116	2.1
56050	0.946116	2.1
56051	1.09531	2.1
56052	1.09531	2.1
56053	0.946118	2.1
56054	0.946118	2.1
56055	1.09135	2.1
56056	1.09531	2.1
56057	1.09134	2.1
56058	0.946105	2.1
56059	1.09532	2.1
56060	0.946131	2.1
56061	0.946129	2.1
56062	0.946129	2.1
56063	1.09137	2.1
56064	1.09137	2.1
56065	0.946131	2.1
56066	0.946131	2.1
56067	1.07713	2.1
56068	1.09137	2.1
56069	0.946131	2.1
56070	1.07713	2.1
56071	1.09137	2.1
56072	0.946131	2.1
56073	0.938916	2.1
56074	0.938916	2.1
56075	0.936995	2.1
56076	1.00447	2.1
56077	0.979819	2.1
56078	1.16821	2.1
56079	1.01263	2.1
56080	0.719167	2.1
56081	1.04536	2.1
56082	1.18901	2.1
56083	0.758057	2.1

56084	0.924111	2.1
56085	1.00532	2.1
56086	1.00532	2.1
56087	0.9925	2.1
56088	0.9925	2.1
56089	1.29322	2.1
56090	1.01985	2.1
56091	1.00365	2.1
56092	0.704601	2.1
56093	1.00365	2.1
56094	1.29322	2.1
56095	1.01985	2.1
56096	0.704602	2.1
56097	2.20011	2.1
56098	1.401	2.1
56099	1.401	2.1
56100	1.52008	2.1
56101	2.11084	2.1
56102	2.22991	2.1
56103	0.854068	2.1
56104	0.854068	2.1
56105	0.851357	2.1
56106	0.851357	2.1
56107	0.644681	2.1
56108	0.700493	2.1
56109	1.0554	2.1
56110	1.00493	2.1
56111	1.00495	2.1
56112	0.64469	2.1
56113	0.700483	2.1
56114	1.05539	2.1
56115	2.46665	0.05
56116	2.22713	0.0604206
56117	1.85877	0.052946
56118	1.8967	0.050927
56119	1.56002	0.0586793
56120	2.22713	0.0573892
56121	1.89225	2.1
56122	1.89225	2.1
56123	1.89225	2.1
56124	1.89225	2.1
56125	1.89225	2.1
56126	1.89225	2.1
56127	1.89225	2.1
56128	1.89225	2.1
56129	1.89225	2.1
56130	1.89225	2.1
56131	1.89225	2.1
56132	1.89225	2.1
56133	1.284	2.1
56134	1.86569	2.1
56135	1.95398	2.1
56136	1.31091	2.1
56137	3.23798	2.1
56138	0.932844	2.1
56139	0.976992	2.1
56140	0.960128	2.1
56141	0.905413	2.1

56142	0.938531	2.1
56143	0.938531	2.1
56144	0.943874	2.1
56145	0.943874	2.1
56146	0.976985	2.1
56147	0.960122	2.1
56148	0.93285	2.1
56149	0.905419	2.1
56150	1.89227	2.1
56151	1.89227	2.1
56152	1.89227	2.1
56153	1.89227	2.1
56154	3.78453	2.1
56155	1.89226	2.1
56156	1.89226	2.1
56157	1.89226	2.1
56158	1.89226	2.1
56159	1.89226	2.1
56160	1.89226	2.1
56161	0.65546	2.1
56162	0.665451	2.1
56163	1.00694	2.1
56164	0.976998	2.1
56165	0.816261	2.1
56166	0.816261	2.1
56167	0.826189	2.1
56168	0.826189	2.1
56169	1.00694	2.1
56170	0.665451	2.1
56171	0.976998	2.1
56172	0.65546	2.1
56173	2.01555	2.1
56174	2.01388	2.1
56175	1.92027	2.1
56176	1.92027	2.1
56177	2.01388	2.1
56178	1.954 2.1	
56179	2.75466	2.1
56180	1.89226	2.1
56181	1.89226	2.1
56182	2.54931	2.1
56183	4.44158	2.1
56184	2.47917	2.1
56185	1.92772	2.1
56186	1.80682	2.1
56187	2.54931	2.1
56188	4.47702	2.1
56189	0.946132	2.1
56190	0.946132	2.1
56191	1.17602	2.1
56192	1.27466	2.1
56193	0.946132	2.1
56194	0.946132	2.1
56195	1.17602	2.1
56196	1.17602	2.1
56197	0.946132	2.1
56198	1.17602	2.1
56199	0.946131	2.1

56200	1.27466	2.1
56201	2.35204	2.1
56202	2.54931	2.1
56203	2.40783	2.1
56204	1.50108	2.1
56205	1.64256	2.1
56206	1.64256	2.1
56207	1.89226	2.1
56208	1.89226	1.15276
56209	2.31012	1.68285
56210	2.17534	1.97656
56211	4.0676	1.77054
56212	2.17533	1.93473
56213	1.89226	1.06293
56214	2.17533	1.19228
56215	1.89226	1.14189
56216	1.66635	1.77698
56217	1.38328	1.64497
56218	1.92027	2.1
56219	2.17597	2.1
56220	2.14619	2.1
56221	2.15388	2.1
56222	1.91087	2.1
56223	1.92027	1.12575
56224	1.08622	1.15236
56225	1.08622	2.1
56226	0.971184	2.1
56227	0.972761	2.1
56228	1.14845	1.59966
56229	1.16321	2.1
56230	0.894769	2.1
56231	0.894967	2.1
56232	0.894775	2.1
56233	1.28563	2.1
56234	1.14844	2.1
56235	1.0167	2.1
56236	1.89226	1.1706
56237	1.89226	2.1
56238	1.89226	2.01096
56239	1.89226	1.23268
56240	1.89226	2.00072
56241	1.89226	1.25752
56242	0.937933	1.82539
56243	0.937933	2.00885
56244	1.26192	2.1
56245	1.26192	2.1
56246	0.937903	1.5905
56247	1.25372	2.1
56248	0.946132	1.8677
56249	0.946132	2.1
56250	0.946133	2.1
56251	1.25372	2.1
56252	0.946132	2.1
56253	0.937902	2.1
56254	0.946132	1.15601
56255	0.946132	1.06864
56256	0.946128	1.9895
56257	0.946128	1.96046

56258	0.94613	1.81065
56259	0.94613	1.13204
56260	0.94613	1.0346
56261	0.94613	1.67101
56262	0.946131	1.29894
56263	0.946131	1.33326
56264	0.94613	1.33288
56265	0.94613	1.32201
56266	0.946132	1.2432
56267	0.946132	1.12437
56268	1.2778	2.0956
56269	1.00191	2.1
56270	1.00191	1.21778
56271	1.00191	2.1
56272	0.94613	2.09253
56273	0.94613	1.21089
56274	0.946131	1.51565
56275	1.2778	1.69268
56276	1.00191	1.42593
56277	0.94613	1.39164
56278	1.50847	0.120075
56279	2.0815	0.157416
56280	1.56806	0.118987
56281	2.0815	0.138524
56282	1.52043	0.120875
56283	1.9224	0.134792
56284	1.06882	0.0978318
56285	0.69567	0.14712
56286	0.773112	0.101349
56287	1.09473	0.115542
56288	0.8941	0.134913
56289	0.8941	0.148399
56290	0.937064	0.119572
56291	0.981131	0.102229
56292	0.763762	0.126799
56293	0.722535	0.127055
56294	1.2153	0.119313
56295	1.07279	0.12461
56296	1.89226	0.23533
56297	1.89226	0.250998
56298	1.89226	0.191326
56299	1.89226	0.178589
56300	1.89226	0.313291
56301	1.89226	0.30952
56302	1.89226	0.218523
56303	1.89226	0.311428
56304	1.89226	0.264281
56305	1.89226	0.344248
56306	3.78453	0.308204
56307	2.51518	0.195906
56308	1.89226	0.216281
56309	1.89226	0.172913
56310	1.89226	0.157612
56311	2.37584	0.253471
56312	2.37584	0.24553
56313	1.25759	0.24033
56314	0.946131	0.248213
56315	0.946131	0.202967

56316	1.24489	0.316226
56317	0.946131	0.229401
56318	0.946131	0.26367
56319	1.25759	0.214489
56320	1.25759	0.20103
56321	1.25759	0.235092
56322	1.24489	0.22949
56323	0.946131	0.217406
56324	0.946131	0.228079
56325	1.08028	0.787165
56326	1.09362	0.80025
56327	0.596616	0.849903
56328	0.606432	0.87131
56329	0.844265	0.882822
56330	0.844265	0.711892
56331	0.855786	0.907349
56332	0.855786	0.835032
56333	1.08028	0.872934
56334	0.606433	0.835088
56335	0.596616	0.805092
56336	1.09362	0.794829
56337	2.837	0.829078
56338	2.16055	0.72026
56339	2.12741	0.812867
56340	2.79312	0.815922
56341	4.95367	0.795831
56342	2.12741	0.845326
56343	1.21286	0.775714
56344	1.20859	0.890231
56345	1.21286	0.916481
56346	2.15628	0.79551
56347	2.16056	0.72721
56348	1.89226	2.1
56349	1.89226	2.1
56350	1.89226	2.1
56351	1.89226	2.1
56352	3.78453	2.1
56353	0.946102	2.1
56354	0.946102	2.1
56355	0.850039	2.1
56356	0.850039	2.1
56357	0.914891	2.1
56358	0.85001	2.1
56359	0.946131	2.1
56360	0.946131	2.1
56361	0.94613	2.1
56362	0.91489	2.1
56363	0.850011	2.1
56364	0.946131	2.1
56365	0.71107	2.1
56366	0.784813	2.1
56367	1.28453	2.1
56368	1.20328	2.1
56369	0.992128	2.1
56370	0.992128	2.1
56371	0.995965	2.1
56372	0.995965	2.1
56373	0.784813	2.1

56374	1.28453	2.1
56375	0.711071	2.1
56376	1.20328	2.1
56377	1.89226	2.1
56378	1.89226	2.1
56379	1.89226	2.1
56380	1.89226	2.1
56381	3.78453	2.1
56382	0.946132	2.1
56383	0.946132	2.1
56384	0.914889	2.1
56385	0.914889	2.1
56386	0.985255	2.1
56387	0.914891	2.1
56388	0.946131	2.1
56389	0.946131	2.1
56390	0.946131	2.1
56391	0.985255	2.1
56392	0.94613	2.1
56393	0.91489	2.1
56394	0.784813	2.1
56395	0.871551	2.1
56396	1.37791	2.1
56397	1.28453	2.1
56398	1.07644	2.1
56399	1.07644	2.1
56400	1.07964	2.1
56401	1.07964	2.1
56402	0.871551	2.1
56403	1.37791	2.1
56404	0.784813	2.1
56405	1.28453	2.1
56406	1.73075	1.25086
56407	2.10582	1.02099
56408	1.89319	0.990376
56409	1.38766	1.6203
56410	3.27777	1.03981
56411	1.08715	1.61916
56412	1.54185	1.47115
56413	1.73074	1.32756
56414	1.73074	1.98145
56415	1.18948	1.17566
56416	1.18948	1.60239
56417	1.89319	0.989278
56418	1.41689	1.51277
56419	1.11619	1.0936
56420	1.38766	1.24696
56421	1.6052	1.0404
56422	1.89319	1.20705
56423	0.647894	1.46302
56424	0.594738	1.69105
56425	0.708445	1.43891
56426	0.770926	1.22377
56427	0.738326	1.46744
56428	0.738326	1.85974
56429	0.680494	1.42907
56430	0.680494	1.12405
56431	0.708443	1.22127

56432	0.647895	1.24169
56433	0.770925	1.26271
56434	0.59474	1.14679
56435	1.89111	1.05612
56436	1.06994	2.1
56437	2.06689	1.88556
56438	1.37063	2.1
56439	3.13683	2.1
56440	2.06689	1.20724
56441	1.39295	2.1
56442	1.56818	1.74634
56443	1.37063	2.1
56444	2.26444	1.23183
56445	2.06689	2.1
56446	1.49808	2.1
56447	1.50201	2.1
56448	2.57784	2.1
56449	2.58615	2.1
56450	4.08424	2.1
56451	0.946133	1.78714
56452	0.946133	1.79431
56453	0.946132	2.1
56454	0.946132	2.1
56455	0.946132	2.1
56456	0.946132	1.82243
56457	0.946132	2.0957
56458	0.946132	1.80882
56459	0.946133	1.71665
56460	0.946133	2.1
56461	0.946132	2.1
56462	0.946132	2.1
56463	1.89226	1.87973
56464	1.89226	2.1
56465	1.89226	2.1
56466	1.89226	2.1
56467	3.78452	2.1
56468	0.977201	2.1
56469	0.977201	2.1
56470	0.980489	2.1
56471	0.980489	2.1
56472	0.657973	2.1
56473	1.20669	2.1
56474	0.751002	2.1
56475	1.29307	2.1
56476	1.29308	2.1
56477	1.20669	2.1
56478	0.751003	2.1
56479	0.657972	2.1
56480	0.946131	2.1
56481	0.946131	2.1
56482	1.29308	2.1
56483	1.29308	2.1
56484	0.946132	2.1
56485	1.29308	2.1
56486	0.946132	2.1
56487	1.28892	2.1
56488	0.946132	2.1
56489	0.946132	2.1

56490	1.28892	2.1
56491	1.29308	2.1
56492	0.946132	1.21139
56493	0.946132	1.13926
56494	0.946131	1.39784
56495	0.946131	1.32219
56496	0.946132	1.40175
56497	0.946132	1.2505
56498	0.946132	1.21975
56499	0.946132	1.13971
56500	0.946132	1.18337
56501	0.946132	1.26533
56502	0.946132	1.2421
56503	0.946132	1.54463
56504	1.89226	1.43554
56505	1.89226	1.55405
56506	1.89226	1.90742
56507	1.89226	1.22771
56508	1.89226	1.83991
56509	1.89226	1.21576
56510	0.94613	2.1
56511	0.94613	2.1
56512	1.20669	2.1
56513	1.20669	2.1
56514	1.20669	2.1
56515	0.946132	2.1
56516	0.946129	2.1
56517	1.29307	2.1
56518	0.946131	2.1
56519	0.946131	2.1
56520	1.29308	2.1
56521	1.20669	2.1
56522	1.89226	0.416887
56523	1.89226	0.245807
56524	1.89226	0.284455
56525	1.89226	0.393702
56526	3.78453	0.366492
56527	0.946132	0.184862
56528	0.946132	0.203539
56529	0.946126	0.202456
56530	0.946126	0.307495
56531	0.946129	0.217298
56532	0.946129	0.205439
56533	0.946129	0.233042
56534	0.946129	0.292558
56535	0.946128	0.200912
56536	0.946128	0.222969
56537	0.946129	0.207429
56538	0.946129	0.216839
56539	0.725277	0.149615
56540	0.588155	0.142604
56541	1.19403	0.200178
56542	1.27681	0.145179
56543	0.918903	0.147385
56544	0.918903	0.146052
56545	0.946068	0.154721
56546	0.946068	0.145956
56547	1.19404	0.154079

56548	0.588153	0.154634
56549	0.725275	0.15309
56550	1.27682	0.147534
56551	1.19404	0.220807
56552	1.27682	0.156314
56553	0.946131	0.307508
56554	0.946131	0.231037
56555	1.19404	0.237375
56556	1.19404	0.23206
56557	0.946129	0.215138
56558	0.946129	0.21895
56559	1.19404	0.224006
56560	0.94613	0.22911
56561	1.27682	0.188529
56562	0.946131	0.261839
56563	1.89226	0.242072
56564	1.89226	0.393249
56565	1.89226	0.40671
56566	1.89226	0.301715
56567	1.89226	0.334906
56568	1.89226	0.234048
56569	1.4263	1.23661
56570	1.67651	0.815303
56571	1.5955	0.911453
56572	1.31022	1.42663
56573	2.98674	0.998599
56574	1.18824	2.1
56575	1.28802	2.1
56576	0.946132	1.13711
56577	0.946132	1.14686
56578	0.946132	1.15756
56579	0.946132	2.1
56580	1.28802	1.08419
56581	1.28802	2.1
56582	1.18824	1.6071
56583	0.946132	1.82106
56584	0.946132	1.79379
56585	1.28802	1.56324
56586	1.89226	0.826679
56587	1.89226	1.13994
56588	1.89226	1.41487
56589	1.89226	0.856986
56590	3.78452	0.97463
56591	1.5955	0.906148
56592	1.52909	0.9011
56593	1.31022	0.974854
56594	1.31022	1.42902
56595	1.2087	1.32238
56596	1.52909	1.32182
56597	1.11825	2.1
56598	1.18824	2.1
56599	0.946132	1.20118
56600	0.946132	1.14502
56601	0.946132	1.20465
56602	0.946132	2.1
56603	1.18824	1.10555
56604	1.18824	2.1
56605	0.946132	1.23186

56606	1.11825	1.80422
56607	1.18824	1.72055
56608	0.946133	1.81321
56609	0.810943	1.25019
56610	0.810943	1.14845
56611	0.810945	0.894123
56612	0.810945	1.15889
56613	1.89226	0.910733
56614	1.35168	0.969083
56615	1.08132	0.869194
56616	1.0813	1.03904
56617	1.08132	1.20229
56618	0.810959	0.993024
56619	0.810959	1.00334
56620	1.89226	0.791116
56621	1.89226	0.523636
56622	1.89226	0.661437
56623	1.89226	0.762569
56624	3.78453	0.779466
56625	1.89226	0.359538
56626	1.89226	0.407083
56627	1.89226	0.447585
56628	1.89226	0.247113
56629	1.89226	0.441113
56630	1.89226	0.257947
56631	0.946131	0.261595
56632	0.946131	0.271129
56633	0.946126	0.447284
56634	0.946126	0.315185
56635	0.946128	0.518783
56636	0.946128	0.327572
56637	0.946128	0.433125
56638	0.946128	0.376174
56639	0.946128	0.348311
56640	0.946128	0.370953
56641	0.946128	0.349111
56642	0.946128	0.286855
56643	1.89226	0.771332
56644	1.89226	0.355384
56645	1.89226	0.679021
56646	1.89226	0.658602
56647	1.89226	0.554122
56648	1.89226	0.534453
56649	1.89226	0.548329
56650	1.89226	0.714416
56651	1.89226	0.859605
56652	1.89226	0.542797
56653	3.78453	0.549522
56654	1.07176	0.27232
56655	1.07176	0.29779
56656	0.946126	0.410435
56657	0.946126	0.379039
56658	1.07176	0.352996
56659	0.946129	0.235923
56660	1.27474	0.384871
56661	0.94613	0.408172
56662	1.27474	0.397993
56663	1.07176	0.169824

56664	0.94613	0.30154
56665	0.94613	0.286051
56666	0.723486	0.215746
56667	0.94613	0.220226
56668	0.946126	0.308081
56669	0.946126	0.274015
56670	0.946129	0.310664
56671	0.723484	0.228401
56672	1.07176	0.29282
56673	0.946129	0.193924
56674	1.32398	0.317047
56675	1.19835	0.201509
56676	0.723471	0.241587
56677	0.723471	0.269675
56678	0.810946	0.761686
56679	0.810946	0.607314
56680	0.810946	0.760853
56681	0.810946	0.722897
56682	0.810933	0.372963
56683	0.810933	0.728008
56684	1.89226	0.275711
56685	1.3517	0.636603
56686	1.08132	0.413551
56687	1.08132	0.634777
56688	1.08133	0.636372
56689	2.39923	2.1
56690	1.89226	2.1
56691	1.89226	2.1
56692	1.89226	2.1
56693	2.44606	2.1
56694	2.44606	2.1
56695	1.89226	2.1
56696	1.89226	2.1
56697	1.89226	2.1
56698	1.89226	2.1
56699	1.89226	2.1
56700	1.89226	2.1
56701	1.70302	2.1
56702	2.43041	2.1
56703	2.25275	2.1
56704	2.4747	2.0831
56705	1.52079	2.1
56706	1.67063	2.1
56707	2.40644	2.1
56708	1.89226	2.1
56709	1.89226	2.1
56710	1.89226	2.1
56711	2.39923	2.1
56712	2.39923	2.1
56713	0.946131	1.06321
56714	0.946131	2.1
56715	0.946131	2.1
56716	0.946131	2.07649
56717	0.94613	1.59544
56718	0.94613	1.46731
56719	0.946131	2.1
56720	0.946131	2.1
56721	0.946131	2.1

56722	0.946131	2.1
56723	0.946131	2.1
56724	0.946131	2.1
56725	1.04938	2.1
56726	1.04938	2.1
56727	1.111	2.1
56728	1.06978	2.1
56729	1.2748	2.1
56730	1.25472	1.35953
56731	0.88782	2.1
56732	0.882987	2.1
56733	0.973779	2.1
56734	1.31997	2.1
56735	0.913905	2.1
56736	1.31312	2.1
56737	2.6536	2.1
56738	2.15287	2.1
56739	2.72238	2.1
56740	2.22887	2.1
56741	4.87525	2.1
56742	2.15288	2.1
56743	1.40719	2.1
56744	1.41839	2.1
56745	1.34239	2.1
56746	2.22887	2.1
56747	2.15288	2.1
56748	2.72237	2.1
56749	2.22887	2.1
56750	2.77314	2.1
56751	2.28748	2.1
56752	5.00985	2.1
56753	0.922802	2.1
56754	0.922802	2.1
56755	0.92107	2.1
56756	0.92107	2.1
56757	0.703593	2.1
56758	0.729436	2.1
56759	1.11444	2.1
56760	1.14374	2.1
56761	1.14374	2.1
56762	0.729436	2.1
56763	0.703593	2.1
56764	1.11444	2.1
56765	1.89226	2.1
56766	1.89226	2.1
56767	1.89226	1.81531
56768	1.89226	2.1
56769	1.89226	2.04922
56770	1.89226	2.1
56771	0.489927	2.1
56772	0.63008	2.1
56773	0.697385	2.1
56774	0.55524	2.1
56775	0.592141	2.1
56776	0.592141	2.1
56777	0.593179	2.1
56778	0.593179	2.1
56779	0.63008	2.1

56780	0.697385	2.1
56781	0.55524	2.1
56782	0.489927	2.1
56783	0.946133	2.1
56784	0.946133	1.89955
56785	0.946132	1.2421
56786	0.946132	1.00168
56787	0.946133	1.11732
56788	0.946133	2.1
56789	0.946132	1.99152
56790	0.946132	1.06162
56791	0.946132	1.31125
56792	0.946132	1.22446
56793	0.946132	1.14802
56794	0.946132	1.19674
56795	1.89226	2.1
56796	1.89226	1.97749
56797	1.89226	1.38724
56798	1.89226	2.1
56799	1.89226	1.41304
56800	1.89226	2.1
56801	0.805746	2.1
56802	0.63008	2.1
56803	0.881598	2.1
56804	0.697385	2.1
56805	0.749417	2.1
56806	0.749417	2.1
56807	0.753714	2.03218
56808	0.753714	2.1
56809	0.881598	2.1
56810	0.805746	2.1
56811	0.63008	2.1
56812	0.697385	2.1
56813	0.881599	2.1
56814	0.697385	2.1
56815	0.805746	1.49679
56816	0.626731	1.12227
56817	0.755479	1.32739
56818	0.755479	2.1
56819	0.752851	1.11085
56820	0.752851	2.1
56821	0.881598	2.1
56822	0.805747	2.09347
56823	0.697385	2.1
56824	0.626731	2.1
56825	2.65348	2.1
56826	2.12314	2.1
56827	2.63899	2.1
56828	2.13898	2.1
56829	4.76213	2.1
56830	0.763518	2.1
56831	0.765889	2.1
56832	0.84086	2.1
56833	0.84086	2.1
56834	0.632078	2.1
56835	0.597565	2.1
56836	1.08813	2.1
56837	0.985643	2.1

56838	0.982464	2.1
56839	0.66404	2.1
56840	1.0358	2.1
56841	0.598023	2.1
56842	1.29293	2.1
56843	1.29628	2.1
56844	1.19266	2.1
56845	1.19266	2.1
56846	1.07109	2.1
56847	1.13093	2.1
56848	1.34467	2.1
56849	1.35251	2.1
56850	1.34369	2.1
56851	1.12525	2.1
56852	1.28746	2.1
56853	1.13511	2.1
56854	2.19717	2.1
56855	2.16547	2.1
56856	1.37392	2.1
56857	1.37392	2.1
56858	1.2966	2.1
56859	2.11985	2.1
56860	1.89226	2.1
56861	1.89226	2.1
56862	1.89226	2.1
56863	1.89226	2.1
56864	1.89226	2.1
56865	1.89226	2.1
56866	1.65637	2.1
56867	1.41758	2.1
56868	1.65637	2.1
56869	1.74391	2.1
56870	1.35504	2.1
56871	1.44258	2.1
56872	1.40774	2.1
56873	1.40774	2.1
56874	0.946131	2.1
56875	0.946131	2.1
56876	1.40774	2.1
56877	1.38882	2.1
56878	0.946132	2.1
56879	0.946132	2.1
56880	0.946132	2.1
56881	1.38882	2.1
56882	0.946132	2.1
56883	1.40774	2.1
56884	1.89227	2.1
56885	1.89227	2.1
56886	1.89227	2.1
56887	1.89227	2.1
56888	3.78453	2.1
56889	0.603148	2.1
56890	0.600177	2.1
56891	0.558615	2.1
56892	0.580714	2.1
56893	0.558606	2.1
56894	0.67883	2.1
56895	1.65637	2.1

56896	1.07933	2.1
56897	0.842649	2.1
56898	0.905168	2.1
56899	0.774334	2.1
56900	1.38882	2.1
56901	1.38882	2.1
56902	0.946132	2.1
56903	0.946132	2.1
56904	1.38882	2.1
56905	1.37381	2.1
56906	0.946132	2.1
56907	0.946132	2.1
56908	0.946132	2.1
56909	1.37381	2.1
56910	0.946133	2.1
56911	1.38882	2.1
56912	2.3771	2.1
56913	1.9339	2.1
56914	1.70782	2.1
56915	1.9339	2.1
56916	2.42693	2.1
56917	2.83279	2.1
56918	2.34901	0.807191
56919	1.45833	0.840819
56920	1.86695	0.799895
56921	1.03101	0.841332
56922	3.32528	0.816866
56923	1.89226	0.700206
56924	1.89226	0.701026
56925	1.89226	0.7
56926	1.89226	0.584584
56927	3.78453	0.7
56928	2.71753	0.676445
56929	1.89226	0.700375
56930	2.76228	0.524757
56931	2.76228	0.60385
56932	1.89226	0.700654
56933	1.89226	0.70319
56934	0.946131	0.631613
56935	0.946131	0.642735
56936	0.946132	0.602628
56937	0.946132	0.606443
56938	0.946132	0.700248
56939	0.946132	0.533683
56940	0.946132	0.7
56941	0.946132	0.46167
56942	0.946132	0.530326
56943	0.946132	0.57744
56944	0.946132	0.685008
56945	0.946132	0.659011
56946	1.89227	0.7
56947	1.89227	0.336504
56948	1.89227	0.518062
56949	1.89227	0.689351
56950	1.89227	0.274087
56951	1.89227	0.453126
56952	2.71753	0.434955
56953	2.37907	0.254602

56954	2.37907	0.468304
56955	2.76228	0.59608
56956	2.39588	0.178758
56957	2.77909	0.316611
56958	1.6571	0.926301
56959	1.11682	1.44062
56960	1.47132	0.913478
56961	1.93763	0.742951
56962	3.12842	1.12889
56963	1.93762	0.828227
56964	2.12345	0.999762
56965	1.65709	0.764784
56966	1.65709	1.46174
56967	2.12345	1.00784
56968	1.88635	1.94585
56969	1.47134	1.6712
56970	2.219	0.827689
56971	2.25734	1.9457
56972	1.93765	0.890831
56973	1.79103	1.12676
56974	1.47134	0.800511
56975	1.06174	0.969261
56976	1.19349	1.77595
56977	1.1095	1.50186
56978	0.968827	0.900642
56979	1.07892	0.884549
56980	1.07892	2.1
56981	1.0834	0.876519
56982	1.0834	1.05655
56983	1.1095	1.10399
56984	1.19349	0.987954
56985	0.968825	1.06825
56986	1.06174	1.06604
56987	2.40627	2.1
56988	2.08288	1.21216
56989	2.5444	0.938321
56990	2.29285	1.27091
56991	4.62729	1.03165
56992	2.08289	0.964278
56993	1.65703	1.01696
56994	1.71105	2.1
56995	1.50108	1.54522
56996	2.29286	1.47458
56997	2.08289	1.67424
56998	2.5444	1.06264
56999	2.29285	2.1
57000	2.71429	1.87931
57001	2.50288	0.915779
57002	5.04728	1.93247
57003	2.50288	0.914152
57004	2.00927	1.73065
57005	2.02116	1.05689
57006	2.02116	1.9953
57007	2.29286	0.961638
57008	2.29286	1.48333
57009	1.89226	1.9628
57010	1.89226	2.1
57011	1.89226	0.956457

57012	1.89226	2.1
57013	3.78453	2.00801
57014	1.17418	1.95893
57015	1.29741	2.1
57016	1.04921	2.1
57017	1.19352	2.1
57018	2.3677	2.1
57019	1.29741	2.1
57020	1.27575	2.1
57021	1.19352	2.1
57022	1.19473	2.1
57023	2.46927	2.1
57024	1.89226	2.1
57025	1.89226	2.1
57026	1.89226	2.1
57027	1.89226	2.1
57028	1.89226	2.1
57029	1.89226	2.1
57030	0.561332	2.1
57031	0.561332	2.1
57032	0.567597	2.1
57033	0.567597	2.1
57034	0.597365	2.1
57035	0.544699	2.1
57036	0.596761	2.1
57037	0.531564	2.1
57038	0.5447	2.1
57039	0.531563	2.1
57040	0.596759	2.1
57041	0.597366	2.1
57042	0.946131	2.1
57043	0.946131	2.1
57044	0.946131	2.1
57045	0.946131	2.1
57046	0.946129	2.1
57047	0.946129	2.1
57048	0.946133	2.1
57049	0.946133	2.1
57050	0.946131	2.1
57051	0.946131	2.1
57052	0.946131	2.1
57053	0.946131	2.1
57054	1.10938	0.584124
57055	1.17614	0.88536
57056	1.02478	0.361922
57057	1.09056	0.853326
57058	2.19994	0.826865
57059	1.17614	0.886725
57060	1.11868	1.98511
57061	1.03408	0.858948
57062	1.03408	2.1
57063	1.09056	0.854453
57064	1.17516	2.1
57065	1.89226	0.849405
57066	1.89226	0.82938
57067	1.89226	0.860502
57068	1.89226	0.801187
57069	1.89226	0.879462

57070	1.89226	0.824293
57071	1.89226	0.790523
57072	1.89226	0.814155
57073	1.89226	0.810116
57074	1.89226	0.735439
57075	1.89226	0.855357
57076	1.89226	0.732524
57077	0.579551	0.675005
57078	0.563873	0.572446
57079	0.611341	0.773661
57080	0.598693	0.68369
57081	0.589856	0.635307
57082	0.589856	0.613446
57083	0.588387	0.732949
57084	0.588387	0.707814
57085	0.57955	0.682996
57086	0.611341	0.660208
57087	0.563873	0.68578
57088	0.598693	0.660808
57089	0.611341	0.760834
57090	0.598693	0.661952
57091	0.586048	0.741703
57092	0.572737	0.785353
57093	0.591824	0.742204
57094	0.591824	0.713348
57095	0.592254	0.812451
57096	0.592254	0.786853
57097	0.611341	0.774395
57098	0.586048	0.739011
57099	0.598693	0.765749
57100	0.572737	0.756832
57101	0.946119	0.793696
57102	0.946119	0.834551
57103	0.946119	0.807729
57104	0.946119	0.848396
57105	0.94612	0.803721
57106	0.94612	0.787209
57107	0.946119	0.832082
57108	0.946119	0.862219
57109	0.946132	0.817412
57110	0.946132	0.815097
57111	0.946107	0.829543
57112	0.946107	0.833036
57113	1.89224	0.792722
57114	1.89224	0.767057
57115	1.89224	0.779944
57116	1.89224	0.680992
57117	1.89224	0.829118
57118	1.89224	0.779021
57119	0.562335	0.614745
57120	0.579543	0.504659
57121	0.59499	0.701923
57122	0.611333	0.603849
57123	0.586619	0.563414
57124	0.586619	0.555729
57125	0.587049	0.661448
57126	0.587049	0.638579
57127	0.594999	0.635848

57128	0.562342	0.625613
57129	0.579535	0.627501
57130	0.611325	0.604831
57131	0.59499	0.679856
57132	0.611333	0.579404
57133	0.567849	0.647498
57134	0.58604	0.700316
57135	0.590978	0.636671
57136	0.590978	0.631419
57137	0.590052	0.73256
57138	0.590052	0.718503
57139	0.567857	0.706329
57140	0.594998	0.688436
57141	0.611325	0.714455
57142	0.586032	0.67733
57143	1.69661	0.875005
57144	1.9339	0.737629
57145	1.39379	0.890355
57146	1.69107	0.212127
57147	3.32768	0.77934
57148	1.9339	0.723397
57149	1.98414	0.683564
57150	1.69107	0.229801
57151	1.72774	0.514175
57152	3.66164	0.610805
57153	1.39379	0.91965
57154	1.69107	0.62693
57155	1.14397	0.669207
57156	1.43548	0.204166
57157	2.82927	0.796632
57158	1.69107	0.646113
57159	1.72774	0.678973
57160	1.53873	0.673417
57161	1.53873	0.715183
57162	1.43548	0.493762
57163	1.62449	0.704071
57164	2.58431	0.684694
57165	1.34072	0.463694
57166	1.80841	0.697636
57167	1.34072	0.653616
57168	2.51501	0.490308
57169	2.04732	0.467555
57170	1.89226	0.634461
57171	1.89226	0.443491
57172	1.89226	0.70388
57173	1.89226	0.590952
57174	1.89226	0.477544
57175	1.89226	0.415428
57176	0.946131	0.508836
57177	0.946131	0.705123
57178	1.3639	0.703293
57179	1.1516	0.594698
57180	0.946129	0.552967
57181	0.946129	0.698762
57182	1.36391	0.698739
57183	1.36391	0.688772
57184	0.946131	0.705241
57185	1.3639	0.689015

57186	1.1516	0.595065
57187	0.946131	0.623707
57188	0.946131	0.615355
57189	0.946131	0.584518
57190	0.94613	0.607701
57191	0.94613	0.467192
57192	0.946128	0.591264
57193	0.946128	0.57424
57194	0.946133	0.532824
57195	0.946133	0.41015
57196	0.946131	0.540168
57197	0.946131	0.705094
57198	0.946131	0.393313
57199	0.946131	0.524123
57200	1.89226	1.14163
57201	2.63634	1.71497
57202	1.89226	1.17497
57203	1.83902	1.25781
57204	3.73128	1.15079
57205	1.47059	2.1
57206	2.02928	2.1
57207	1.47059	2.1
57208	1.65706	2.1
57209	1.98628	2.1
57210	2.17274	2.1
57211	2.43618	2.1
57212	2.41176	2.1
57213	1.98627	2.1
57214	2.34084	2.1
57215	4.39803	2.1
57216	1.47059	2.1
57217	1.82453	2.1
57218	1.98627	2.1
57219	1.98627	2.1
57220	1.26929	2.1
57221	1.26929	2.1
57222	1.98627	2.1
57223	2.34084	2.1
57224	1.47264	2.1
57225	2.24937	2.1
57226	4.23564	2.1
57227	1.33026	1.00194
57228	1.51171	1.16701
57229	1.51171	0.973789
57230	1.4617	1.19916
57231	1.35382	0.974134
57232	1.30382	1.33421
57233	1.89226	0.944528
57234	1.89226	1.02275
57235	1.89226	0.915104
57236	1.89226	1.12459
57237	1.89226	0.913909
57238	1.89226	1.16651
57239	1.39263	0.98502
57240	0.962704	0.717786
57241	1.0171	0.869538
57242	0.962704	0.938703
57243	1.40822	0.845109

57244	1.35382	0.900666
57245	0.755853	1.26099
57246	0.789808	1.08286
57247	0.696315	0.983779
57248	0.676912	0.916682
57249	0.729751	0.918211
57250	0.729751	1.06508
57251	0.722417	1.33935
57252	0.722417	0.950786
57253	0.696315	1.04132
57254	0.789808	1.03472
57255	0.755853	1.03138
57256	0.676912	1.03015
57257	0.946132	1.01136
57258	0.946132	1.06399
57259	0.946132	0.923859
57260	0.946132	0.876742
57261	0.946131	0.880027
57262	0.946131	1.00831
57263	0.946132	0.928036
57264	0.946132	1.06305
57265	0.946132	0.949732
57266	0.946132	0.954756
57267	0.946132	0.96281
57268	0.946132	0.962541
57269	0.946133	0.908984
57270	0.946133	0.781036
57271	0.946132	0.658495
57272	0.946132	0.845034
57273	0.946132	0.654018
57274	0.946132	0.809651
57275	0.946133	0.76037
57276	0.946133	0.918073
57277	0.946132	0.856844
57278	0.946132	0.85796
57279	0.946132	0.827879
57280	0.946132	0.849877
57281	1.89227	1.73323
57282	2.53014	1.85856
57283	1.89227	2.09385
57284	1.98241	1.63822
57285	1.89227	2.1
57286	1.98241	1.6984
57287	1.89226	1.40219
57288	1.89226	2.1
57289	1.89226	1.58705
57290	1.89226	1.60379
57291	1.89226	2.09015
57292	1.89226	2.04625
57293	1.89226	1.28944
57294	1.89226	2.01896
57295	1.89226	1.42869
57296	1.89226	1.46445
57297	3.78452	1.4924
57298	2.45304	1.30757
57299	1.89226	1.81358
57300	1.89226	1.5223
57301	1.89226	1.52112

57302	1.89226	1.37294
57303	1.89226	1.38702
57304	1.89227	1.68276
57305	1.89227	1.6232
57306	1.89227	2.03355
57307	1.89227	1.39783
57308	1.89227	2.05141
57309	1.89227	1.4298
57310	1.89226	1.39138
57311	1.89226	2.1
57312	1.89226	1.51479
57313	1.89226	1.60152
57314	1.89226	1.94443
57315	1.89226	1.97596
57316	2.09871	0.77878
57317	2.71891	0.832893
57318	1.31126	0.774756
57319	1.31126	0.828179
57320	2.55341	0.776088
57321	1.76597	0.894748
57322	1.89226	0.873079
57323	1.89226	0.848642
57324	1.89226	0.804003
57325	1.89226	0.952193
57326	1.89226	0.80932
57327	1.89226	0.956614
57328	0.946128	0.849767
57329	0.946128	0.802346
57330	0.946133	0.77411
57331	0.946133	0.760383
57332	0.946131	0.771066
57333	0.946131	0.825483
57334	0.94613	0.7709
57335	0.94613	0.809766
57336	0.946131	0.791497
57337	0.946131	0.782919
57338	0.946131	0.79616
57339	0.946131	0.805576
57340	2.71891	0.772948
57341	1.89226	0.752765
57342	2.09871	0.735436
57343	2.09871	0.819265
57344	1.89226	0.738818
57345	1.89226	0.821432
57346	1.89227	0.834181
57347	1.89227	0.823102
57348	1.89227	0.789205
57349	1.89227	0.906941
57350	1.89227	0.792251
57351	1.89227	0.9087
57352	0.946129	0.825163
57353	0.946129	0.788054
57354	0.946133	0.756146
57355	0.946133	0.74311
57356	0.946131	0.754026
57357	0.946131	0.806546
57358	0.946131	0.752097
57359	0.946131	0.796341

57360	0.946131	0.772846
57361	0.946131	0.766642
57362	0.946131	0.782991
57363	0.946131	0.776925
57364	2.32462	0.235261
57365	1.89226	0.141631
57366	1.89226	0.129835
57367	1.89226	0.142524
57368	2.2291	0.218055
57369	2.2291	0.247169
57370	0.847669	0.148701
57371	0.847669	0.152099
57372	0.796966	0.136622
57373	0.796966	0.183379
57374	0.833552	0.108633
57375	1.01049	0.167566
57376	0.709709	0.13074
57377	0.634147	0.131399
57378	0.634148	0.121381
57379	0.833549	0.156365
57380	0.709711	0.147837
57381	1.01049	0.171515
57382	1.89226	0.117855
57383	1.89226	0.230929
57384	1.89226	0.143075
57385	1.89226	0.21159
57386	1.89226	0.13215
57387	1.89226	0.171448
57388	1.03611	0.130677
57389	0.936655	0.1351
57390	0.946132	0.137018
57391	0.946132	0.164725
57392	0.941918	0.0947552
57393	0.941918	0.131504
57394	1.00308	0.145281
57395	1.04117	0.116426
57396	1.1406	0.1023
57397	1.04074	0.111817
57398	0.851529	0.138785
57399	1.00311	0.202631
57400	1.01814	0.0954102
57401	1.6671	0.140096
57402	1.35561	0.0937416
57403	1.35561	0.120662
57404	1.2683	0.0932948
57405	1.2683	0.0998264
57406	0.946128	0.13864
57407	0.946128	0.0896761
57408	0.946129	0.0953519
57409	0.946129	0.141229
57410	0.946132	0.136913
57411	0.946132	0.0965835
57412	0.946126	0.0975291
57413	0.946126	0.132
57414	0.946129	0.0743878
57415	0.946129	0.0949582
57416	0.946129	0.115618
57417	0.946129	0.141304

57418	2.23338	0.106555
57419	1.96	0.085646
57420	2.03438	0.0838898
57421	1.87835	0.099061
57422	4.11172	0.100176
57423	1.96	0.084436
57424	1.52661	0.0986839
57425	1.87834	0.100776
57426	1.53175	0.0818393
57427	3.40495	0.0826837
57428	2.03435	0.0860548
57429	1.65813	0.104392
57430	1.65813	0.112951
57431	1.87832	0.0938829
57432	1.72067	0.104006
57433	1.94086	0.0823796
57434	1.65813	0.108888
57435	1.40572	0.0814225
57436	1.87832	0.0931541
57437	1.87832	0.0858263
57438	1.31154	0.0858848
57439	1.53173	0.0897474
57440	1.86662	2.1
57441	2.0815	2.1
57442	2.0815	2.1
57443	2.0815	2.1
57444	1.87246	2.1
57445	1.87362	2.1
57446	1.0273	2.1
57447	0.996347	2.1
57448	0.946129	2.1
57449	0.946129	2.1
57450	1.04427	2.1
57451	1.04196	2.1
57452	0.910674	2.1
57453	0.910674	2.1
57454	1.04072	2.1
57455	1.10931	2.1
57456	1.03132	2.1
57457	0.933698	2.1
57458	2.63789	0.719211
57459	1.89226	0.77346
57460	2.3033	0.72591
57461	2.3033	0.763168
57462	1.89226	0.727029
57463	1.89226	0.767261
57464	1.89226	0.799603
57465	1.89226	0.841749
57466	1.89226	0.773057
57467	1.89226	0.797798
57468	3.78452	0.823073
57469	1.51898	0.765215
57470	1.36006	0.839243
57471	2.03143	0.831602
57472	2.03143	0.834414
57473	1.04026	0.788977
57474	1.04026	0.795891
57475	0.94613	0.710503

57476	0.94613	0.722101
57477	1.31894	0.70453
57478	1.31894	0.703857
57479	0.94613	0.704491
57480	0.94613	0.73121
57481	1.31894	0.708988
57482	1.41232	0.718718
57483	0.946129	0.712387
57484	1.41232	0.711684
57485	1.31895	0.709402
57486	0.946129	0.716548
57487	1.89226	0.714844
57488	1.89226	0.76678
57489	1.89226	0.714411
57490	1.89226	0.763852
57491	1.89226	0.710821
57492	1.89226	0.760506
57493	1.89804	0.709711
57494	2.03143	0.745493
57495	2.46286	0.733789
57496	2.46287	0.737617
57497	1.51898	0.714945
57498	1.51898	0.7215
57499	1.89226	2.1
57500	1.89226	2.1
57501	1.89226	2.1
57502	1.89226	2.1
57503	3.78452	2.1
57504	1.3864	2.1
57505	1.3864	2.1
57506	0.946132	2.1
57507	0.946132	2.1
57508	1.32905	2.1
57509	1.3864	2.1
57510	0.946132	2.1
57511	0.946132	2.1
57512	0.946132	2.1
57513	1.32905	2.1
57514	0.946133	2.1
57515	1.3864	2.1
57516	2.77279	2.1
57517	1.67883	2.1
57518	1.67883	2.1
57519	1.73415	2.1
57520	2.65811	2.1
57521	2.71343	2.1
57522	0.946131	2.1
57523	0.946131	2.1
57524	0.946128	2.1
57525	0.946128	2.1
57526	0.946127	2.1
57527	0.946127	2.1
57528	0.946131	2.1
57529	0.946131	2.1
57530	0.946129	2.1
57531	0.946129	2.1
57532	0.94613	2.1
57533	0.94613	2.1

57534	1.89226	2.1
57535	1.89226	2.1
57536	1.89226	2.1
57537	1.89226	2.1
57538	3.78452	2.1
57539	1.89226	2.1
57540	2.54394	2.1
57541	2.54394	2.1
57542	2.65811	2.1
57543	1.89226	2.1
57544	2.00643	2.1
57545	2.54394	2.1
57546	2.6581	2.1
57547	1.62743	2.1
57548	1.62743	2.1
57549	1.67883	2.1
57550	2.59533	2.1
57551	1.89226	2.1
57552	1.89226	2.1
57553	1.89226	2.1
57554	1.89226	2.1
57555	1.89226	2.1
57556	1.89226	2.1
57557	1.89226	1.33404
57558	1.89226	1.23584
57559	1.89226	1.86949
57560	1.89226	1.06169
57561	1.89226	1.67424
57562	1.89226	1.04701
57563	1.89226	1.27677
57564	1.89226	1.20672
57565	1.89226	1.79148
57566	1.89226	1.08978
57567	1.89226	1.56105
57568	1.89226	1.05383
57569	1.89226	1.03386
57570	1.89226	0.918858
57571	1.89226	1.25998
57572	1.89226	0.893741
57573	1.89226	1.23849
57574	1.89226	0.889283
57575	2.68537	0.723335
57576	2.22575	0.778326
57577	2.47464	0.361746
57578	1.79093	0.634018
57579	4.70039	0.654992
57580	2.81217	0.889015
57581	2.47464	0.720181
57582	2.58286	0.839087
57583	2.29193	0.367324
57584	5.0575	0.760218
57585	2.47464	0.713511
57586	2.20365	0.748939
57587	2.29193	0.569258
57588	1.90104	0.752134
57589	4.37568	0.701214
57590	1.89226	0.863834
57591	2.16957	2.1

57592	2.52823	1.07907
57593	2.52823	2.05041
57594	1.89226	0.968275
57595	1.89226	1.39375
57596	1.36226	2.1
57597	1.55369	2.1
57598	1.33309	2.1
57599	1.62593	2.1
57600	2.98819	2.1
57601	0.802281	2.1
57602	0.802281	2.1
57603	0.835143	2.1
57604	0.835143	2.1
57605	0.824458	2.1
57606	0.92641	2.1
57607	0.776847	2.1
57608	0.812967	2.1
57609	0.812967	2.1
57610	0.926411	2.1
57611	0.776847	2.1
57612	0.824458	2.1
57613	1.33309	2.1
57614	1.62593	2.1
57615	1.32813	2.1
57616	1.7237	2.1
57617	2.95406	2.1
57618	0.814544	2.1
57619	0.875312	2.1
57620	0.910502	2.1
57621	0.910502	2.1
57622	0.886633	2.1
57623	0.975739	2.1
57624	0.792623	2.1
57625	0.840283	2.1
57626	0.796372	2.1
57627	1.06159	2.1
57628	0.81948	2.1
57629	0.933833	2.1
57630	2.63997	2.1
57631	1.29694	2.1
57632	1.28141	2.1
57633	2.62623	2.1
57634	3.92317	2.1
57635	2.63998	2.1
57636	1.89226	2.1
57637	1.89226	2.1
57638	2.62623	2.1
57639	4.5185	2.1
57640	1.89226	2.1
57641	1.89226	2.1
57642	1.89226	2.1
57643	1.89226	2.1
57644	3.78453	2.1
57645	1.89226	2.1
57646	1.89226	2.1
57647	1.89226	2.1
57648	1.89226	2.1
57649	1.89226	2.1

57650	1.89226	2.1
57651	1.25468	2.1
57652	2.62623	2.1
57653	1.28141	2.1
57654	1.28141	2.1
57655	2.60665	2.1
57656	2.60665	2.1
57657	1.89226	2.1
57658	2.62623	2.1
57659	2.60666	2.1
57660	2.62623	2.1
57661	1.87269	2.1
57662	1.89226	2.1
57663	1.89226	2.1
57664	1.89226	2.1
57665	1.89226	2.1
57666	1.89226	2.1
57667	3.78453	2.1
57668	1.89226	2.1
57669	1.89226	2.1
57670	1.89226	2.1
57671	1.89226	2.1
57672	1.89226	2.1
57673	1.89226	2.1
57674	2.30147	1.36431
57675	2.27703	1.73356
57676	1.89227	1.25914
57677	1.89227	1.51281
57678	4.1693	1.49428
57679	1.38617	1.45206
57680	1.24613	1.3811
57681	2.59886	1.24968
57682	2.6238	1.42314
57683	3.86993	1.22092
57684	0.946133	1.33885
57685	0.946133	1.09157
57686	0.946132	1.04851
57687	0.946132	1.68983
57688	0.946133	1.63482
57689	0.946133	1.42253
57690	0.946132	1.07748
57691	0.946132	1.13448
57692	0.946133	1.23744
57693	0.946133	1.19556
57694	0.946132	1.13715
57695	0.946132	1.15012
57696	2.21773	2.1
57697	2.59216	2.1
57698	1.68541	2.1
57699	1.68541	2.1
57700	1.84814	2.1
57701	2.38046	2.1
57702	1.89966	2.1
57703	1.70303	2.1
57704	1.2028	2.1
57705	1.29897	2.1
57706	2.02873	2.1
57707	2.19093	2.1

57708	2.75218	2.1
57709	1.70303	2.1
57710	3.10254	2.1
57711	2.24783	2.1
57712	2.56839	2.1
57713	1.70303	2.1
57714	2.56839	2.1
57715	1.08276	2.1
57716	2.24061	2.1
57717	1.67491	2.1
57718	1.60948	2.1
57719	1.08276	2.1
57720	1.89226	0.771122
57721	1.89226	0.924433
57722	1.89226	0.74304
57723	1.89226	0.772688
57724	3.78453	0.748469
57725	1.89226	0.754307
57726	1.89226	0.889769
57727	1.89226	0.752438
57728	1.89226	0.78129
57729	3.78452	0.829273
57730	1.89226	0.81815
57731	2.7535	0.783807
57732	1.89226	0.948785
57733	1.89226	1.00339
57734	1.89226	0.782908
57735	1.89226	0.780373
57736	1.89226	0.831735
57737	1.89226	0.778612
57738	1.89226	0.747767
57739	1.89226	0.918935
57740	1.89226	0.767571
57741	1.89226	1.1251
57742	1.89227	0.752125
57743	1.89227	0.782632
57744	1.89227	0.736991
57745	1.89227	0.755614
57746	3.78453	0.865711
57747	1.89226	0.799903
57748	2.53412	0.740859
57749	1.89226	1.25975
57750	1.89226	0.752124
57751	1.89226	0.777446
57752	1.89226	0.740804
57753	2.21126	0.469592
57754	1.89226	0.610541
57755	2.57464	0.622582
57756	1.89226	0.592401
57757	2.57464	0.47709
57758	1.89226	0.461307
57759	1.89226	0.734043
57760	1.89226	0.77852
57761	1.89226	0.945202
57762	1.89226	0.724649
57763	1.89226	0.980815
57764	1.89226	0.733227
57765	1.1418	0.280774

57766	0.653021	0.326905
57767	1.37282	0.377165
57768	0.931386	0.430573
57769	1.34568	0.476632
57770	1.17669	0.35908
57771	0.666595	0.441601
57772	0.786895	0.440466
57773	0.933982	0.347879
57774	1.05351	0.469424
57775	1.00792	0.30311
57776	1.00792	0.455216
57777	1.89226	2.1
57778	1.89226	2.1
57779	1.89226	2.1
57780	1.89226	2.1
57781	3.78452	2.1
57782	1.89226	2.1
57783	1.89226	2.1
57784	1.89226	2.1
57785	1.89226	2.1
57786	1.89226	2.1
57787	1.89226	2.1
57788	1.65642	2.1
57789	1.30248	2.1
57790	1.38888	2.1
57791	1.25987	2.1
57792	1.70611	2.1
57793	1.65642	2.1
57794	1.65642	2.1
57795	1.81407	2.1
57796	2.19428	2.1
57797	1.92555	2.1
57798	3.8507	2.1
57799	1.89226	2.1
57800	1.89226	2.1
57801	1.89226	2.1
57802	1.89226	2.1
57803	3.78452	2.1
57804	1.89226	2.1
57805	1.89226	2.1
57806	1.89226	2.1
57807	1.89226	2.1
57808	1.89226	2.1
57809	1.89226	2.1
57810	0.705495	2.1
57811	0.809317	2.1
57812	1.1655	2.1
57813	1.09605	2.1
57814	0.983375	2.1
57815	0.797301	2.1
57816	0.906633	2.1
57817	0.906633	2.1
57818	0.85085	2.1
57819	1.16548	2.1
57820	0.705501	2.1
57821	1.00314	2.1
57822	2.19428	2.1
57823	2.31862	2.1

57824	2.27408	2.1
57825	2.45893	2.1
57826	4.46836	2.1
57827	1.89226	0.847924
57828	1.89226	0.979367
57829	1.89226	0.919256
57830	1.89226	0.8498
57831	1.89226	1.13512
57832	1.89226	0.907687
57833	2.0815	0.850622
57834	2.22136	0.953758
57835	1.5515	0.912216
57836	1.86323	0.841815
57837	2.74986	1.01556
57838	3.30238	0.870774
57839	1.89224	0.873008
57840	1.89224	0.979473
57841	1.89224	0.968184
57842	1.89224	0.867347
57843	1.89224	1.21504
57844	1.89224	0.907967
57845	1.89224	0.845779
57846	1.89224	0.961656
57847	1.89224	0.895822
57848	1.89224	0.839685
57849	1.89224	1.115
57850	1.89224	0.893728
57851	1.17846	0.821687
57852	0.810935	0.821491
57853	0.93347	1.00404
57854	0.93347	0.845267
57855	0.810932	0.895472
57856	0.810932	0.859013
57857	2.22133	0.895081
57858	1.71919	0.890199
57859	1.32629	0.836747
57860	1.0813	0.925019
57861	1.57137	0.861894
57862	2.21149	2.1
57863	1.89226	2.1
57864	1.89226	2.1
57865	1.89226	1.86986
57866	2.17159	2.1
57867	2.17159	2.03795
57868	0.946131	0.914183
57869	0.946131	2.1
57870	0.946131	1.55657
57871	0.946131	2.1
57872	0.94613	1.18615
57873	0.94613	1.7519
57874	0.946132	2.1
57875	0.946132	1.98741
57876	0.946131	1.29097
57877	0.946131	1.25026
57878	0.946131	1.6589
57879	0.946131	1.52709
57880	1.12266	1.4913
57881	1.4714	1.93153

57882	1.62322	1.4589
57883	1.62322	1.70187
57884	1.02958	1.8053
57885	1.02958	2.1
57886	2.2671	2.1
57887	1.89226	2.1
57888	1.89226	2.1
57889	1.89226	2.05904
57890	2.21149	2.1
57891	2.21149	2.1
57892	1.89226	1.7742
57893	1.89226	1.656
57894	1.89226	1.63909
57895	1.89226	1.30851
57896	1.89226	2.00353
57897	1.89226	1.82192
57898	0.736914	1.8604
57899	0.736914	1.88059
57900	0.822839	1.96072
57901	0.822836	2.1
57902	0.791298	1.1165
57903	1.00003	1.90195
57904	0.640254	1.93274
57905	0.575379	2.08806
57906	0.609001	1.37346
57907	1.00005	1.35563
57908	0.575371	1.87621
57909	0.831908	1.44835
57910	1.89226	2.1
57911	1.89226	2.1
57912	1.89226	2.1
57913	1.89226	2.1
57914	1.89226	2.1
57915	1.89226	2.1
57916	1.89226	2.1
57917	1.89226	2.1
57918	1.89226	2.1
57919	1.89226	2.1
57920	1.89226	2.1
57921	1.89226	2.1
57922	0.730617	2.1
57923	0.71052	2.1
57924	0.7329	2.1
57925	0.712083	2.1
57926	0.701051	2.1
57927	0.701051	2.1
57928	0.721552	2.1
57929	0.721552	2.1
57930	0.710521	2.1
57931	0.732898	2.1
57932	0.730619	2.1
57933	0.712081	2.1
57934	0.7329	2.1
57935	0.712082	2.1
57936	0.559601	2.1
57937	0.495993	2.1
57938	0.603718	2.1
57939	0.603718	2.1

57940	0.625174	2.1
57941	0.625174	2.1
57942	0.732899	2.1
57943	0.559601	2.1
57944	0.712082	2.1
57945	0.495993	2.1
57946	0.94613	2.1
57947	0.94613	2.1
57948	0.94613	2.1
57949	0.94613	2.1
57950	0.946131	2.1
57951	0.946131	2.1
57952	0.946129	2.1
57953	0.946129	2.1
57954	0.94613	2.1
57955	0.94613	2.1
57956	0.94613	2.1
57957	0.94613	2.1
57958	1.89226	2.1
57959	1.89226	2.1
57960	1.89226	2.1
57961	1.89226	2.1
57962	1.89226	2.1
57963	1.89226	2.1
57964	1.42104	2.1
57965	1.4658	2.1
57966	1.51223	2.1
57967	1.42283	2.1
57968	2.93327	2.1
57969	0.732899	2.1
57970	0.756115	2.1
57971	0.629763	2.1
57972	0.559601	2.1
57973	0.646134	2.1
57974	0.646134	2.1
57975	0.669582	2.1
57976	0.669582	2.1
57977	0.756115	2.1
57978	0.629763	2.1
57979	0.732899	2.1
57980	0.559601	2.1
57981	2.566 2.1	
57982	1.20242	1.44355
57983	2.64084	2.1
57984	2.18297	2.1
57985	1.66029	2.1
57986	1.20242	1.62572
57987	0.946133	1.06444
57988	0.946133	1.2435
57989	0.946129	2.09493
57990	0.946129	2.07484
57991	0.946131	1.93678
57992	0.946131	1.03601
57993	0.946131	0.837916
57994	0.946131	1.90275
57995	0.946131	1.00862
57996	0.946131	1.32681
57997	0.946131	1.16116

57998	0.946131	1.21245
57999	1.89226	0.103868
58000	1.89226	0.136704
58001	1.89226	0.118248
58002	1.89226	0.108991
58003	3.78453	0.11757
58004	1.03073	0.0838491
58005	1.03073	0.0829665
58006	1.04066	0.0873522
58007	1.04066	0.0997071
58008	1.20802	0.0891859
58009	1.32689	0.0794604
58010	0.883301	0.089579
58011	0.744506	0.0813948
58012	1.20802	0.085532
58013	0.744506	0.0871191
58014	0.883301	0.0821035
58015	1.32689	0.0870478
58016	1.20802	0.0938039
58017	1.20802	0.085243
58018	0.946131	0.0972952
58019	0.946131	0.110015
58020	0.94613	0.107335
58021	0.94613	0.0883949
58022	1.20802	0.101385
58023	1.32689	0.0986956
58024	1.20802	0.0916994
58025	0.946131	0.0931548
58026	0.946131	0.0870242
58027	1.32689	0.0971287
58028	1.89226	0.111272
58029	1.89226	0.159784
58030	1.89226	0.135154
58031	1.89226	0.133662
58032	1.89226	0.135834
58033	1.89226	0.132116
58034	0.946119	0.135191
58035	0.946119	0.110397
58036	0.946118	0.144481
58037	0.946118	0.173006
58038	0.946118	0.166625
58039	0.946118	0.115504
58040	0.946119	0.10963
58041	0.946119	0.143383
58042	0.946131	0.138483
58043	0.946131	0.139186
58044	0.946106	0.13613
58045	0.946106	0.133845
58046	1.3939	2.1
58047	1.49641	2.1
58048	1.15096	2.1
58049	1.10101	2.1
58050	2.59742	2.1
58051	1.22106	2.1
58052	2.60663	2.1
58053	1.25467	2.1
58054	1.25467	2.1
58055	2.58377	2.1

58056	2.58377	2.1
58057	1.89225	2.1
58058	2.60664	2.1
58059	2.58377	2.1
58060	2.60664	2.1
58061	1.86938	2.1
58062	1.89225	2.1
58063	1.89225	2.1
58064	1.89225	2.1
58065	1.89225	2.1
58066	1.89225	2.1
58067	1.89225	2.1
58068	1.89225	2.1
58069	0.946121	2.1
58070	0.946121	2.1
58071	0.946126	2.1
58072	0.946126	2.1
58073	0.946124	2.1
58074	0.946124	2.1
58075	0.946123	2.1
58076	0.946123	2.1
58077	0.946117	2.1
58078	0.946117	2.1
58079	0.94613	2.1
58080	0.94613	2.1
58081	1.28017	2.1
58082	1.29189	2.1
58083	0.610534	2.1
58084	0.592373	2.1
58085	0.943764	2.1
58086	0.943764	2.1
58087	0.940501	2.1
58088	0.940501	2.1
58089	1.28017	2.1
58090	0.592371	2.1
58091	0.610533	2.1
58092	1.29189	2.1
58093	0.946133	2.1
58094	0.946133	2.1
58095	1.28017	2.1
58096	1.29189	2.1
58097	0.946132	2.1
58098	0.946132	2.1
58099	1.28017	2.1
58100	1.28017	2.1
58101	1.28017	2.1
58102	0.946132	2.1
58103	0.946133	2.1
58104	1.2919	2.1
58105	1.89226	2.1
58106	1.89226	2.1
58107	1.89226	2.1
58108	1.89226	2.1
58109	1.89226	2.1
58110	1.89226	2.1
58111	1.89226	2.1
58112	1.89226	2.1
58113	1.89226	2.1

58114	1.89226	2.1
58115	3.78452	2.1
58116	1.89226	2.1
58117	2.08196	2.1
58118	1.97854	2.1
58119	2.08196	2.1
58120	1.78884	2.1
58121	1.89226	2.1
58122	1.89226	2.1
58123	1.89226	2.1
58124	1.89226	2.1
58125	1.89226	2.1
58126	1.89226	2.1
58127	1.89226	2.1
58128	1.83688	2.1
58129	1.89226	2.1
58130	1.89226	2.1
58131	1.89226	2.1
58132	1.86015	2.1
58133	1.86015	2.1
58134	1.89226	2.1
58135	1.97854	2.1
58136	1.85883	2.1
58137	1.97854	2.1
58138	1.77255	2.1
58139	1.89226	2.1
58140	0.946131	2.1
58141	0.946131	2.1
58142	0.946131	2.1
58143	0.946131	2.1
58144	0.94613	2.1
58145	0.94613	2.1
58146	0.946132	2.1
58147	0.946132	2.1
58148	0.946131	2.1
58149	0.946131	2.1
58150	0.946131	2.1
58151	0.946131	2.1
58152	0.918412	2.1
58153	0.918412	2.1
58154	0.94893	2.1
58155	0.750211	2.1
58156	0.851541	2.1
58157	0.851541	2.1
58158	0.970566	2.1
58159	0.826567	2.1
58160	0.970554	2.1
58161	1.04071	2.1
58162	0.826632	2.1
58163	0.851554	2.1
58164	1.89226	0.276461
58165	1.89226	0.221784
58166	1.89226	0.174698
58167	1.89226	0.253213
58168	3.78453	0.248697
58169	0.946131	0.20983
58170	0.946131	0.272568
58171	0.811232	0.347815

58172	0.811232	0.272497
58173	0.94613	0.313163
58174	0.94613	0.267966
58175	0.959701	0.266891
58176	0.811233	0.338988
58177	0.94613	0.161012
58178	0.811233	0.282767
58179	0.959702	0.278881
58180	0.946131	0.261713
58181	1.89226	0.227998
58182	1.89226	0.222897
58183	1.89226	0.317414
58184	1.89226	0.218772
58185	1.89226	0.171931
58186	1.89226	0.13714
58187	1.89227	0.212026
58188	1.89227	0.237256
58189	1.89227	0.291567
58190	1.89227	0.214829
58191	1.89227	0.21905
58192	1.89227	0.175999
58193	1.89226	0.149447
58194	1.89226	0.204191
58195	1.89226	0.151321
58196	1.89226	0.120302
58197	3.78453	0.146636
58198	1.89226	0.164499
58199	1.89226	0.132906
58200	1.89226	0.136641
58201	1.89226	0.15629
58202	1.89226	0.13627
58203	1.89226	0.15858
58204	1.89226	2.1
58205	2.36204	2.1
58206	2.39853	2.1
58207	2.36204	2.1
58208	1.92876	2.1
58209	1.89226	2.1
58210	1.89226	2.1
58211	1.89226	1.60638
58212	1.89226	2.1
58213	1.89226	0.906727
58214	3.78453	2.1
58215	0.946128	2.1
58216	0.946128	0.896712
58217	1.13182	1.11399
58218	1.13182	2.1
58219	0.94613	1.07044
58220	0.94613	1.22948
58221	1.1749	2.1
58222	1.13182	2.1
58223	1.1749	2.1
58224	0.94613	2.1
58225	1.13182	2.1
58226	0.94613	2.1
58227	1.89226	2.1
58228	2.39853	2.1
58229	2.44918	2.1

58230	2.39853	2.1
58231	1.94291	2.1
58232	1.89226	2.1
58233	0.946132	0.953061
58234	0.946132	2.1
58235	0.946131	2.1
58236	0.946131	1.72586
58237	0.946132	1.179
58238	0.946132	2.1
58239	0.946131	2.1
58240	0.946131	2.1
58241	0.946131	2.1
58242	0.946131	2.1
58243	0.946132	2.1
58244	0.946132	2.1
58245	0.946128	2.1
58246	0.946128	2.1
58247	1.1749	1.15233
58248	1.1749	2.1
58249	0.946131	1.13147
58250	0.946131	1.32951
58251	1.23631	2.1
58252	1.1749	2.1
58253	1.23631	2.1
58254	0.94613	2.1
58255	1.1749	2.1
58256	0.946131	1.6705
58257	1.89226	0.933322
58258	1.89226	2.1
58259	1.89226	2.07693
58260	1.89226	2.1
58261	3.78453	2.1
58262	1.38859	2.1
58263	1.45333	2.1
58264	1.28909	1.9909
58265	1.38091	2.1
58266	2.74242	2.1
58267	1.45332	2.1
58268	1.38414	2.1
58269	1.38091	2.1
58270	1.33506	2.1
58271	2.78838	2.1
58272	1.89226	2.1
58273	1.89226	2.1
58274	1.89226	2.1
58275	1.89226	2.1
58276	3.78452	2.1
58277	1.89226	2.1
58278	1.89226	2.1
58279	1.89226	1.0321
58280	1.89226	2.1
58281	1.89226	1.0895
58282	1.89226	2.1
58283	1.17418	2.1
58284	1.38091	2.1
58285	1.17418	2.1
58286	1.28909	2.1
58287	1.29741	2.1

58288	1.41232	2.1
58289	1.38091	2.1
58290	1.33506	2.1
58291	1.27575	2.1
58292	1.27575	2.1
58293	1.29741	2.1
58294	1.35671	2.1
58295	1.89226	2.1
58296	1.89226	2.1
58297	1.89226	2.1
58298	1.89226	2.1
58299	3.78452	2.1
58300	1.92737	0.586952
58301	2.40223	0.677209
58302	1.56178	0.524909
58303	1.97605	0.7
58304	3.90343	0.574269
58305	1.89226	0.695878
58306	1.89226	0.701898
58307	1.89226	0.7
58308	1.89226	0.7
58309	3.78452	0.7
58310	1.89226	0.702324
58311	1.89226	0.700979
58312	1.89226	0.7
58313	1.89226	0.711145
58314	1.89226	0.7
58315	1.89226	0.708479
58316	1.13641	0.700223
58317	1.13641	0.7
58318	1.12763	0.66561
58319	1.12763	0.7
58320	1.29348	0.7
58321	1.06293	0.701307
58322	1.20111	0.656996
58323	0.988028	0.7
58324	0.988028	0.7
58325	1.06293	0.7
58326	1.29348	0.7
58327	1.20111	0.697749
58328	1.56176	0.489044
58329	1.46387	0.67963
58330	1.97603	0.594341
58331	1.97603	0.565242
58332	1.08211	0.574554
58333	1.08211	0.53899
58334	1.89224	0.683889
58335	1.89224	0.7
58336	1.89224	0.7
58337	1.89224	0.70096
58338	1.89224	0.660173
58339	1.89224	0.682847
58340	1.89224	0.70162
58341	1.89224	0.702667
58342	1.89224	0.7
58343	1.89224	0.71389
58344	3.78448	0.701421
58345	0.820185	0.561026

58346	0.818947	0.69103
58347	0.911041	0.70219
58348	0.695265	0.695701
58349	1.46388	0.685515
58350	1.23607	0.689159
58351	0.927068	0.678146
58352	1.02691	0.702877
58353	1.12918	0.602869
58354	0.846843	0.695398
58355	0.911039	0.693668
58356	1.29129	2.1
58357	1.45547	2.1
58358	1.67327	2.1
58359	1.66698	2.1
58360	1.20149	2.1
58361	1.20149	2.1
58362	0.946133	1.0977
58363	0.946133	2.1
58364	0.946129	2.1
58365	0.946129	2.1
58366	0.946131	2.1
58367	0.946131	1.09042
58368	0.946131	2.1
58369	0.946131	2.08596
58370	0.946131	2.1
58371	0.946131	2.1
58372	0.946131	2.1
58373	0.946131	2.1
58374	1.89226	1.88418
58375	1.89226	1.52823
58376	1.89226	2.1
58377	1.89226	1.04749
58378	3.78452	1.36593
58379	0.86875	1.43503
58380	0.86875	2.1
58381	0.899002	2.1
58382	0.966245	2.1
58383	0.849364	1.13762
58384	1.01918	2.06955
58385	0.764737	2.1
58386	0.89249	2.1
58387	0.764624	2.1
58388	0.967869	2.1
58389	0.808606	2.04764
58390	0.864506	1.84877
58391	0.773929	2.1
58392	0.773929	2.1
58393	0.781991	2.1
58394	0.836559	2.1
58395	0.983761	2.1
58396	0.834687	2.1
58397	0.680545	2.1
58398	0.640799	2.1
58399	0.70286	2.1
58400	0.935844	2.1
58401	0.629479	2.1
58402	0.823655	2.1
58403	0.946133	1.20375

58404	0.946133	2.1
58405	0.946128	2.1
58406	0.946128	2.1
58407	0.94613	2.1
58408	0.94613	1.15377
58409	0.946131	2.1
58410	0.946131	2.1
58411	0.946131	2.1
58412	0.946131	2.1
58413	0.946131	2.1
58414	0.946131	2.1
58415	1.89226	2.1
58416	1.89226	1.08109
58417	1.89226	1.76634
58418	1.89226	1.74165
58419	1.89226	1.25402
58420	1.89226	1.56973
58421	0.931638	2.1
58422	0.931638	1.31588
58423	1.03559	2.1
58424	0.977442	1.74016
58425	0.826487	1.32961
58426	0.993902	1.28988
58427	1.04922	2.1
58428	0.894324	2.1
58429	0.912984	2.1
58430	1.01156	2.1
58431	0.944692	1.89085
58432	1.15456	1.94557
58433	1.89226	0.228157
58434	1.89226	0.349684
58435	1.89226	0.461173
58436	1.89226	0.225392
58437	3.78453	0.234394
58438	1.89226	0.222295
58439	1.89226	0.222666
58440	2.64262	0.21495
58441	2.64262	0.168624
58442	1.78052	0.137575
58443	1.03015	0.25777
58444	0.999721	0.172061
58445	0.659011	0.151848
58446	1.15564	0.318284
58447	1.41388	0.190537
58448	1.01583	0.214862
58449	1.01583	0.17638
58450	1.05706	0.295138
58451	1.05706	0.207419
58452	1.15564	0.200225
58453	0.65901	0.183077
58454	1.41388	0.198742
58455	0.999721	0.178828
58456	1.89226	2.1
58457	1.89226	2.1
58458	1.89226	2.1
58459	1.89226	2.1
58460	3.78453	2.1
58461	2.39022	2.1

58462	1.90734	2.1
58463	2.27634	2.1
58464	1.82666	2.1
58465	4.18368	2.1
58466	1.90734	2.1
58467	1.21564	2.1
58468	1.82666	2.1
58469	1.16938	2.1
58470	3.07672	2.1
58471	1.89226	2.1
58472	1.89226	2.1
58473	1.89226	2.1
58474	1.89226	2.1
58475	1.89226	2.1
58476	1.89226	2.1
58477	1.89227	2.1
58478	1.89227	2.1
58479	1.89227	2.1
58480	1.89227	2.1
58481	3.78453	2.1
58482	2.27634	2.1
58483	1.82666	2.1
58484	2.18042	2.1
58485	1.74738	2.1
58486	4.02372	2.1
58487	1.74738	2.1
58488	1.82666	2.1
58489	1.1181	2.1
58490	1.1181	2.1
58491	1.16938	2.1
58492	1.79865	2.1
58493	1.89226	2.1
58494	1.89226	2.1
58495	1.89226	2.1
58496	1.89226	2.1
58497	1.89226	2.1
58498	1.89226	2.1
58499	1.89226	1.13398
58500	1.89226	0.778193
58501	1.89226	0.830429
58502	1.89226	0.82876
58503	1.89226	1.08497
58504	1.89226	0.85878
58505	0.790736	0.826438
58506	1.08265	0.800064
58507	0.938909	1.1388
58508	0.638683	0.88559
58509	0.858588	0.911364
58510	0.858588	0.810562
58511	0.862743	1.16487
58512	0.862743	0.849433
58513	1.08265	1.01142
58514	0.938911	0.820003
58515	0.790735	0.950331
58516	0.638685	0.820001
58517	1.89226	0.818734
58518	1.89226	1.02527
58519	1.89226	0.872055

58520	1.89226	0.829697
58521	1.89226	1.07068
58522	1.89226	0.907683
58523	1.89226	0.794938
58524	1.89226	1.02408
58525	1.89226	0.818549
58526	1.89226	0.827245
58527	1.89226	0.890106
58528	1.89226	0.96558
58529	1.08265	0.812903
58530	1.36209	0.790327
58531	1.25233	0.837974
58532	0.938909	0.877108
58533	1.14195	0.82269
58534	1.14195	0.794326
58535	1.15904	1.01512
58536	1.15904	0.829562
58537	1.36209	0.862111
58538	1.25233	0.776058
58539	1.08265	0.883574
58540	0.938911	0.807486
58541	1.22077	0.812134
58542	0.915244	0.832076
58543	0.967835	1.01761
58544	0.742495	0.948157
58545	0.78648	0.954804
58546	0.985041	0.839427
58547	0.989098	1.24475
58548	0.989098	0.849011
58549	1.03206	0.889589
58550	0.967897	0.998965
58551	0.915404	0.922615
58552	0.706105	0.87423
58553	2.69662	0.118316
58554	1.89226	0.148721
58555	2.35499	0.10319
58556	1.89226	0.0773568
58557	4.24725	0.11894
58558	1.89227	0.110034
58559	1.89227	0.107393
58560	1.89227	0.0778846
58561	1.89227	0.10223
58562	3.78453	0.101707
58563	2.35499	0.102197
58564	1.70302	0.0643258
58565	1.70302	0.0687836
58566	1.703	0.0718063
58567	1.97374	0.0778556
58568	1.97372	0.0833655
58569	0.946133	0.0818935
58570	0.946133	0.0704462
58571	0.946132	0.0783056
58572	0.946132	0.095982
58573	0.946133	0.0789495
58574	0.946133	0.0767144
58575	0.946132	0.0785434
58576	0.946132	0.0775545
58577	0.946133	0.0617444

58578	0.946133	0.0749922
58579	0.946132	0.0769891
58580	0.946132	0.0832208
58581	0.6794	2.1
58582	0.6794	2.1
58583	0.670812	2.1
58584	0.62182	2.1
58585	0.648326	2.1
58586	0.792154	2.1
58587	0.719167	2.1
58588	0.494515	2.1
58589	0.649421	2.1
58590	0.804869	2.1
58591	0.485585	2.1
58592	0.627666	2.1
58593	1.79347	2.1
58594	1.03968	1.74263
58595	1.70303	2.07258
58596	0.933265	1.61305
58597	2.62068	1.44772
58598	1.89226	2.1
58599	1.89226	2.1
58600	1.89226	2.1
58601	1.89226	1.29627
58602	3.78452	2.1
58603	1.89226	1.49248
58604	2.24604	2.1
58605	2.0554	2.1
58606	2.0554	1.95889
58607	1.89226	2.1
58608	1.89226	2.1
58609	1.89226	2.1
58610	1.89226	0.918258
58611	1.89226	0.995516
58612	1.89226	2.1
58613	1.89226	1.00387
58614	1.89226	2.1
58615	2.0815	2.1
58616	2.64424	0.470345
58617	2.80807	0.892184
58618	3.232 1.50303	
58619	1.89417	0.901042
58620	2.18012	1.61105
58621	0.946131	2.1
58622	0.946131	0.876064
58623	0.946131	2.1
58624	0.946131	1.03983
58625	0.94613	1.00496
58626	0.94613	0.916766
58627	0.946132	2.1
58628	0.946132	2.1
58629	0.946131	1.15959
58630	0.946131	1.29132
58631	0.946131	1.15365
58632	0.946131	1.14249
58633	1.89227	0.176144
58634	1.89227	0.241495
58635	1.89227	0.33463

58636	1.89227	0.186019
58637	3.78453	0.185336
58638	2.70881	0.155988
58639	2.68687	0.0812583
58640	2.03046	0.113923
58641	1.9682	0.153704
58642	4.67701	0.14718
58643	1.08518	0.0848287
58644	1.08518	0.0917124
58645	1.09033	0.154524
58646	1.09033	0.113894
58647	0.842273	0.0874515
58648	1.19141	0.0990043
58649	1.34343	0.0910078
58650	0.9841	0.129072
58651	0.9841	0.112794
58652	0.842273	0.122337
58653	1.34344	0.120948
58654	1.19141	0.0996019
58655	0.946133	0.185959
58656	0.946133	0.152351
58657	0.946132	0.329415
58658	0.946132	0.212663
58659	0.946132	0.175114
58660	0.946132	0.152814
58661	0.946132	0.31294
58662	0.946132	0.254671
58663	0.946132	0.157775
58664	0.946132	0.24602
58665	0.946133	0.248193
58666	0.946133	0.181627
58667	1.89227	0.309282
58668	1.89227	0.156158
58669	1.89227	0.257281
58670	1.89227	0.185827
58671	1.89227	0.25108
58672	1.89227	0.18081
58673	2.03046	0.111892
58674	1.9682	0.127686
58675	1.23886	0.191111
58676	1.12971	0.109578
58677	3.20706	0.108526
58678	1.89226	0.762138
58679	1.89226	0.935189
58680	1.89226	0.749436
58681	1.89226	0.832189
58682	3.78452	0.796865
58683	2.45569	0.717845
58684	1.89226	0.729251
58685	2.22922	0.711935
58686	2.0815	0.762938
58687	1.85074	0.681854
58688	1.70303	0.74492
58689	2.72856	0.718763
58690	1.89226	0.786312
58691	1.89226	0.71097
58692	1.89226	0.745957
58693	3.78452	0.733754

58694	1.89226	0.765082
58695	1.89226	0.846423
58696	1.89226	0.733964
58697	1.89226	0.843745
58698	3.78452	0.788807
58699	0.946131	0.847014
58700	0.946131	0.731972
58701	0.946131	0.796562
58702	0.946131	0.725408
58703	0.94613	0.780254
58704	0.94613	0.809531
58705	0.946132	0.732792
58706	0.946132	0.740578
58707	0.946131	0.754831
58708	0.946131	0.756989
58709	0.946131	0.768586
58710	0.946131	0.770771
58711	2.0595	0.562025
58712	2.58581	0.693743
58713	1.48694	0.360457
58714	1.48694	0.38568
58715	2.60157	0.507646
58716	2.02901	0.421466
58717	1.89226	0.330451
58718	1.89226	0.41773
58719	1.89226	0.208316
58720	1.89226	0.218574
58721	1.89226	0.42075
58722	1.89226	0.454558
58723	2.04728	0.652993
58724	2.64161	0.699796
58725	1.47606	0.383711
58726	1.47606	0.475565
58727	2.63069	0.539657
58728	2.05947	0.446238
58729	1.89224	0.478934
58730	1.89224	0.305452
58731	1.89224	0.547439
58732	1.89224	0.360615
58733	1.89224	0.300661
58734	1.89224	0.219924
58735	0.946114	0.307443
58736	0.946114	0.43521
58737	0.946118	0.457238
58738	0.946118	0.295536
58739	0.946114	0.42138
58740	0.946114	0.441165
58741	0.946118	0.460533
58742	0.946118	0.481169
58743	0.946128	0.532751
58744	0.946128	0.64419
58745	0.946103	0.375194
58746	0.946103	0.343002
58747	1.1233	2.1
58748	1.15009	2.1
58749	2.36036	2.1
58750	2.38225	2.1
58751	3.50554	2.1

58752	2.38224	2.1
58753	0.977775	2.1
58754	2.20473	2.1
58755	2.20473	2.1
58756	1.15009	2.1
58757	1.15009	2.1
58758	0.946119	1.88122
58759	0.946119	1.89069
58760	1.19111	2.06504
58761	1.19111	1.92657
58762	0.946118	1.98396
58763	1.19111	2.09592
58764	1.18016	1.86297
58765	0.946118	1.12439
58766	0.94613	0.884388
58767	0.94613	1.70057
58768	1.18015	1.76614
58769	1.19109	1.82712
58770	0.946118	2.1
58771	0.946118	2.1
58772	1.10235	2.1
58773	1.10235	2.1
58774	0.946118	2.1
58775	1.10235	2.1
58776	0.946115	2.05247
58777	1.1911	2.1
58778	0.946129	1.88753
58779	0.946129	2.1
58780	1.19109	2.1
58781	1.10234	2.1
58782	1.89226	2.1
58783	2.23932	2.1
58784	2.11568	2.1
58785	2.23932	2.1
58786	1.76862	2.1
58787	1.89226	2.1
58788	1.89226	2.1
58789	1.89226	2.1
58790	1.89226	2.1
58791	1.89226	2.1
58792	1.89226	2.1
58793	1.89226	1.70299
58794	2.07838	2.1
58795	1.89226	2.1
58796	1.89226	2.1
58797	1.89226	2.1
58798	2.10264	2.1
58799	2.10264	2.1
58800	1.89226	2.1
58801	2.11568	2.1
58802	1.99753	2.1
58803	2.11568	2.1
58804	1.77411	2.1
58805	1.89226	2.1
58806	0.946128	1.16412
58807	0.946128	2.1
58808	0.946133	2.1
58809	0.946133	2.1

58810	0.94613	2.1
58811	0.94613	1.14183
58812	0.946131	2.1
58813	0.946131	2.1
58814	0.946131	2.1
58815	0.946131	2.1
58816	0.946131	2.1
58817	0.946131	2.1
58818	0.946132	2.1
58819	0.946132	2.1
58820	1.03919	2.1
58821	1.03919	2.1
58822	0.946131	2.1
58823	0.946131	2.1
58824	1.02744	2.1
58825	1.03919	2.1
58826	1.02744	2.1
58827	0.946132	2.1
58828	1.03919	2.1
58829	0.946132	2.1
58830	1.64834	1.72462
58831	1.82955	1.32973
58832	2.33824	1.11061
58833	2.19301	1.78812
58834	3.98658	1.2995
58835	1.40574	1.80755
58836	2.19301	1.21059
58837	1.64834	1.41764
58838	1.64834	1.44309
58839	1.99961	1.18231
58840	1.99961	1.213
58841	2.33824	1.14024
58842	2.19301	1.72625
58843	2.68524	1.41144
58844	2.81847	1.1545
58845	5.02349	1.44054
58846	1.99961	1.72542
58847	2.19301	1.13412
58848	2.68524	1.22294
58849	2.50732	1.37012
58850	4.68485	1.21222
58851	1.89226	2.1
58852	1.89226	1.422
58853	1.89226	1.27067
58854	1.89226	2.1
58855	3.78453	2.03527
58856	1.75142	2.1
58857	2.47026	2.1
58858	1.46811	2.1
58859	2.26652	2.1
58860	4.01794	2.1
58861	1.89226	1.61407
58862	1.89226	2.1
58863	1.89226	2.08667
58864	1.89226	2.1
58865	1.89226	1.99734
58866	1.89226	2.1
58867	1.89226	1.65966

58868	1.89226	2.1
58869	1.89226	2.06015
58870	1.89226	2.1
58871	1.89226	1.68523
58872	1.89226	2.1
58873	0.812082	2.1
58874	0.878759	2.1
58875	0.882782	2.1
58876	0.882782	2.1
58877	1.05572	2.1
58878	1.04037	2.1
58879	0.73849	2.1
58880	0.626857	2.1
58881	0.638677	2.1
58882	1.05581	2.1
58883	0.715695	2.1
58884	1.10492	2.1
58885	1.17058	2.1
58886	1.07863	2.1
58887	1.21113	2.1
58888	1.21113	2.1
58889	1.2811	2.1
58890	1.37319	2.1
58891	1.01788	2.1
58892	0.999492	2.1
58893	1.04352	2.1
58894	1.32112	2.1
58895	1.10492	2.1
58896	1.34699	2.1
58897	1.89226	1.91502
58898	1.89226	2.1
58899	1.89226	2.1
58900	1.89226	2.1
58901	1.89226	2.08912
58902	1.89226	2.1
58903	1.19813	0.05
58904	1.19813	0.05
58905	2.43432	0.05
58906	1.21716	0.05
58907	0.946124	0.05
58908	1.21715	0.05
58909	0.946118	0.05
58910	2.56627	2.1
58911	1.89226	2.1
58912	1.89226	2.1
58913	1.89226	2.1
58914	2.43434	2.1
58915	2.43434	2.1
58916	1.89227	0.128785
58917	1.89227	0.137885
58918	1.89227	0.131082
58919	1.89227	0.134308
58920	1.89227	0.126024
58921	1.89227	0.129345
58922	1.89226	0.160491
58923	1.89226	0.216861
58924	1.89226	0.186183
58925	1.89226	0.126043

58926	3.78453	0.166507
58927	1.89226	0.108947
58928	2.0764	0.0890655
58929	1.89226	0.106302
58930	2.81674	0.102641
58931	1.89226	0.0971124
58932	2.81674	0.0884945
58933	0.946132	0.144351
58934	0.946132	0.121884
58935	0.946131	0.208029
58936	0.946131	0.154785
58937	0.946132	0.147982
58938	0.946132	0.146727
58939	0.946132	0.224929
58940	0.946132	0.13488
58941	0.946132	0.14404
58942	0.946132	0.161788
58943	0.946132	0.157702
58944	0.946132	0.146845
58945	0.578684	1.31176
58946	0.578684	2.1
58947	0.589487	1.37469
58948	0.589487	2.1
58949	0.625461	1.99228
58950	0.5378	1.97683
58951	0.608714	2.1
58952	0.54271	1.42267
58953	0.608714	1.43811
58954	0.625461	1.16545
58955	0.54271	1.75032
58956	0.537801	1.70159
58957	0.946128	1.08562
58958	0.946128	1.86454
58959	0.946133	1.09544
58960	0.946133	2.1
58961	0.946131	1.26789
58962	0.946131	1.07742
58963	0.946131	1.06415
58964	0.946131	1.2746
58965	0.94613	1.24033
58966	0.94613	1.06101
58967	0.946131	1.25724
58968	0.946131	1.37497
58969	1.21743	1.77648
58970	1.25092	2.1
58971	1.38847	1.1282
58972	1.46031	1.13081
58973	2.67774	1.47118
58974	0.946128	1.04337
58975	0.946128	1.95391
58976	0.946132	1.94959
58977	0.946132	1.1572
58978	0.94613	1.4891
58979	0.94613	1.05352
58980	0.94613	1.25107
58981	0.94613	1.09822
58982	0.94613	1.08629
58983	0.94613	1.05167

58984	0.94613	1.40533
58985	0.94613	1.14648
58986	1.89226	1.01764
58987	1.89226	1.19025
58988	1.89226	0.929499
58989	1.89226	2.05772
58990	1.89226	2.08865
58991	1.89226	1.762
58992	0.539566	1.04182
58993	0.534046	2.1
58994	0.577914	1.63158
58995	0.471623	1.67401
58996	1.46031	0.961066
58997	0.945865	1.11799
58998	0.770603	0.995955
58999	0.737201	1.95765
59000	0.714829	1.15054
59001	0.536085	1.39818
59002	0.471617	1.34883
59003	1.89226	1.98134
59004	1.89226	1.06154
59005	1.89226	1.05958
59006	1.89226	1.31918
59007	1.89226	1.29351
59008	1.89226	1.98738
59009	1.89225	1.51764
59010	1.89225	0.936404
59011	1.65572	0.950659
59012	1.65572	1.11111
59013	1.89225	1.07535
59014	1.89225	1.65476
59015	1.89226	1.36849
59016	1.89226	1.20603
59017	1.89226	1.18006
59018	1.89226	0.838669
59019	3.78453	1.5371
59020	1.89226	1.69853
59021	1.89226	0.922701
59022	1.89226	0.947966
59023	1.89226	1.179
59024	1.89226	1.10179
59025	1.89226	1.86827
59026	1.89225	1.26756
59027	1.89225	0.814712
59028	1.89225	0.856725
59029	1.89225	0.997198
59030	1.89225	0.914195
59031	1.89225	1.43439
59032	0.763592	0.920443
59033	0.763592	1.27502
59034	0.987417	1.04228
59035	0.987417	1.06892
59036	0.763592	0.86579
59037	0.804881	0.828655
59038	0.946126	1.05873
59039	0.946126	0.893718
59040	0.804879	0.815523
59041	0.94613	0.956592

59042	0.946129	1.12284
59043	0.76359	0.886657
59044	1.46055	2.1
59045	2.04945	2.1
59046	2.04587	2.1
59047	2.04587	2.1
59048	1.50714	2.1
59049	1.50714	2.1
59050	2.04587	2.1
59051	2.38681	2.1
59052	2.42082	2.1
59053	2.42082	2.1
59054	2.04945	2.1
59055	2.04945	2.1
59056	0.872964	2.1
59057	0.872964	2.1
59058	0.884806	2.1
59059	0.884806	2.1
59060	1.0275	2.1
59061	1.02293	2.1
59062	0.711243	2.1
59063	0.730273	2.1
59064	0.711244	2.1
59065	1.0275	2.1
59066	0.730273	2.1
59067	1.02293	2.1
59068	2.04587	2.1
59069	2.47753	2.1
59070	2.42994	2.1
59071	2.42082	2.1
59072	2.05499	2.1
59073	2.04587	1.8744
59074	0.946132	2.1
59075	0.946132	2.1
59076	1.13078	2.1
59077	1.07817	2.1
59078	0.946132	2.1
59079	0.946132	2.1
59080	1.07817	2.1
59081	1.07817	2.1
59082	0.946132	2.1
59083	1.07817	2.1
59084	1.13078	2.1
59085	0.946132	2.1
59086	1.89226	2.1
59087	1.89226	2.1
59088	1.89226	2.1
59089	1.89226	2.1
59090	1.89226	2.1
59091	1.89226	2.1
59092	1.89226	2.1
59093	1.89226	2.1
59094	1.89226	2.1
59095	1.89226	2.1
59096	1.89226	2.1
59097	1.89226	2.1
59098	1.89226	2.1
59099	1.89226	2.1

59100	1.8173	2.1
59101	2.66885	2.1
59102	2.7438	2.1
59103	2.7438	2.1
59104	1.89226	0.722874
59105	1.89226	0.7
59106	1.89226	0.70079
59107	1.89226	0.774544
59108	3.78453	0.707607
59109	1.89226	0.714189
59110	1.89226	0.757198
59111	1.89226	0.726632
59112	1.89226	0.852906
59113	1.89226	0.710157
59114	1.89226	0.763214
59115	1.89226	0.715318
59116	1.97385	0.772124
59117	1.7531	0.727519
59118	1.89226	1.01141
59119	1.7531	0.703371
59120	1.89226	0.75122
59121	0.946131	0.832304
59122	0.840904	0.703648
59123	0.684333	0.737001
59124	0.94613	0.733065
59125	0.972337	0.748247
59126	0.946278	1.40112
59127	0.684304	0.7
59128	0.684304	0.75911
59129	0.999838	0.707364
59130	0.759605	1.44579
59131	0.851528	0.7
59132	0.851528	0.847485
59133	1.89226	0.7
59134	2.23818	0.812746
59135	1.79771	0.71073
59136	1.89226	0.90644
59137	1.79771	0.7
59138	1.89226	0.721909
59139	2.79647	2.1
59140	2.01613	2.1
59141	1.46826	2.1
59142	2.54504	2.1
59143	4.56117	2.1
59144	1.89226	2.1
59145	1.89226	2.1
59146	1.89226	2.1
59147	1.89226	2.1
59148	1.89226	2.1
59149	1.89226	2.1
59150	1.89226	2.1
59151	2.66884	2.1
59152	2.56022	2.1
59153	2.66884	2.1
59154	1.78364	2.1
59155	1.89226	2.1
59156	1.14282	2.1
59157	1.24068	2.1

59158	0.880748	2.1
59159	0.834916	2.1
59160	1.05539	2.1
59161	1.05539	2.1
59162	1.04312	2.1
59163	1.04313	2.1
59164	1.2015	2.1
59165	0.83492	2.1
59166	0.789149	2.1
59167	1.24066	2.1
59168	1.89226	2.1
59169	1.89226	2.1
59170	1.89226	2.1
59171	1.89226	2.1
59172	3.78453	2.1
59173	0.946128	2.1
59174	0.946128	2.1
59175	1.28011	2.1
59176	1.22372	2.1
59177	0.946131	2.1
59178	0.946131	2.1
59179	1.22372	2.1
59180	1.22372	2.1
59181	1.22372	2.1
59182	0.94613	2.1
59183	0.946131	2.1
59184	1.28011	2.1
59185	2.28604	2.1
59186	1.34268	2.1
59187	2.43157	2.1
59188	1.50172	2.1
59189	3.77425	2.1
59190	2.6096	2.1
59191	2.43157	2.1
59192	1.66774	2.1
59193	1.66774	2.1
59194	1.50172	2.1
59195	2.43157	2.1
59196	2.16067	2.1
59197	1.08975	2.1
59198	2.19336	2.1
59199	2.06984	2.1
59200	1.21327	2.1
59201	1.08975	2.1
59202	2.28604	2.1
59203	1.21327	2.1
59204	2.29008	2.1
59205	2.16067	2.1
59206	1.34268	2.1
59207	1.21327	2.1
59208	1.89226	0.713306
59209	1.89226	0.719247
59210	1.89226	0.715454
59211	1.89226	0.704966
59212	3.78452	0.702987
59213	1.89804	0.708931
59214	2.21656	0.710343
59215	1.70537	0.7

59216	2.50023	0.700998
59217	3.8982	0.701121
59218	1.03136	0.70137
59219	1.89486	0.701881
59220	0.949364	0.702437
59221	1.82283	0.700974
59222	2.84422	0.700818
59223	1.89226	0.704031
59224	1.89226	0.702505
59225	1.89226	0.702871
59226	1.89226	0.705619
59227	3.78452	0.709024
59228	0.946132	0.71995
59229	0.946132	0.714963
59230	0.946129	0.711269
59231	0.946129	0.703025
59232	0.94613	0.703455
59233	0.94613	0.74161
59234	0.946131	0.703905
59235	0.946131	0.717026
59236	0.946131	0.724509
59237	0.946131	0.72388
59238	0.946131	0.715285
59239	0.946131	0.74531
59240	1.09466	0.705161
59241	1.09466	0.700181
59242	0.866512	0.7
59243	1.08352	0.7
59244	1.19189	0.7
59245	1.21883	0.700666
59246	0.971139	0.704189
59247	0.873213	0.7
59248	0.873202	0.700921
59249	1.00855	0.7
59250	0.923735	0.7
59251	1.21885	0.7
59252	1.89225	0.05
59253	2.40019	0.05
59254	1.89225	0.193811
59255	1.89225	0.147527
59256	1.89225	0.144869
59257	1.89225	0.258418
59258	1.89225	0.193811
59259	1.89225	0.188552
59260	2.05088	0.146302
59261	1.89225	0.0818161
59262	1.89225	0.103115
59263	1.89225	0.202015
59264	2.03956	0.146302
59265	2.03956	0.147527
59266	1.89226	2.1
59267	2.40021	1.86493
59268	2.38473	0.810957
59269	2.40021	2.1
59270	1.87679	0.836143
59271	1.89226	2.04413
59272	1.89226	2.1
59273	1.89226	0.792599

59274	1.89226	1.963
59275	1.89226	0.614001
59276	1.89226	0.960801
59277	1.89226	0.723157
59278	2.0692	1.55188
59279	1.89226	0.810696
59280	1.89226	2.1
59281	1.89226	0.80167
59282	2.05089	2.1
59283	2.05089	0.79226
59284	1.89226	1.80533
59285	1.89226	2.1
59286	1.89226	2.04544
59287	1.89226	2.1
59288	3.78452	2.1
59289	1.89226	0.936475
59290	2.50051	2.1
59291	1.89226	1.99769
59292	2.30076	2.1
59293	4.39277	2.1
59294	2.50052	2.1
59295	1.70377	2.1
59296	2.30076	2.1
59297	1.54231	2.1
59298	4.04283	2.1
59299	1.89226	2.1
59300	1.89226	2.1
59301	1.89226	2.1
59302	1.89226	2.1
59303	1.89226	2.1
59304	1.89226	2.1
59305	1.89226	0.974535
59306	1.89226	2.1
59307	1.89226	1.41197
59308	1.89226	2.1
59309	3.78452	2.1
59310	2.07948	2.1
59311	2.30076	2.1
59312	1.3294	2.1
59313	1.3294	2.1
59314	1.54232	2.1
59315	2.29239	2.1
59316	0.810947	2.1
59317	0.810947	2.1
59318	0.810946	2.1
59319	0.810946	2.1
59320	1.89226	2.1
59321	1.35169	2.1
59322	1.08132	2.1
59323	1.08132	2.1
59324	1.08132	2.1
59325	0.810946	2.1
59326	0.810946	2.1
59327	1.34851	1.19382
59328	0.997471	2.1
59329	1.62287	0.955575
59330	1.27096	1.46761
59331	2.62034	1.13336

59332	1.89226	1.07963
59333	1.89226	2.1
59334	1.89226	1.04914
59335	1.89226	1.95562
59336	3.78452	1.27491
59337	0.946128	2.1
59338	0.946128	1.60541
59339	1.29331	0.970104
59340	1.29331	1.97839
59341	1.29331	1.32685
59342	1.10699	2.1
59343	0.946131	0.952064
59344	0.946131	1.24884
59345	0.94613	1.48342
59346	1.29331	1.4714
59347	1.10699	1.31416
59348	0.946131	1.27973
59349	1.62287	1.08464
59350	1.27096	1.81105
59351	1.86067	0.973487
59352	1.58407	1.65512
59353	3.20694	1.53124
59354	1.89226	1.24118
59355	1.89226	1.84016
59356	1.89226	0.964619
59357	1.89226	1.68346
59358	3.78452	1.16277
59359	2.62761	0.729827
59360	2.55849	0.892777
59361	2.12261	1.05512
59362	2.22939	0.833495
59363	4.78788	0.871137
59364	0.772522	1.33817
59365	0.839588	1.19901
59366	1.31126	0.845788
59367	1.08787	0.824659
59368	1.01318	0.907625
59369	1.01318	1.44358
59370	0.900815	1.75718
59371	1.10799	0.786448
59372	0.772511	1.03354
59373	1.03473	1.05254
59374	1.31126	1.00059
59375	0.841464	1.05175
59376	1.89227	0.889787
59377	1.89227	1.47802
59378	1.89227	0.924952
59379	1.89227	1.19894
59380	1.89227	0.923118
59381	1.89227	1.25671
59382	1.89226	0.836603
59383	1.89226	0.934397
59384	1.89226	0.803768
59385	1.89226	0.900194
59386	1.89226	0.811348
59387	1.89226	0.916434
59388	2.12261	0.95452
59389	2.22939	0.761186

59390	1.97004	0.818257
59391	1.8072	1.04355
59392	4.03658	0.841163
59393	1.5847	1.44045
59394	1.8072	0.923857
59395	2.12262	1.00193
59396	2.12262	1.373
59397	1.23267	0.952104
59398	1.23267	1.35292
59399	1.89227	0.924771
59400	1.89227	1.79743
59401	1.89227	0.946484
59402	1.89227	1.30248
59403	1.89227	0.948294
59404	1.89227	1.44142
59405	1.89226	0.767988
59406	1.89226	0.941419
59407	1.89226	1.04574
59408	1.89226	0.807554
59409	3.78452	0.867915
59410	2.49093	2.1
59411	2.67393	2.1
59412	2.47932	2.1
59413	2.47932	2.1
59414	2.67994	2.1
59415	2.67994	2.1
59416	2.49873	2.1
59417	2.67393	2.1
59418	2.67993	2.1
59419	2.69807	2.1
59420	2.49873	2.1
59421	2.51686	2.1
59422	2.49093	2.1
59423	2.67994	2.1
59424	2.69784	2.1
59425	2.51651	2.1
59426	5.18877	2.1
59427	1.24359	2.1
59428	1.24936	2.1
59429	1.33997	2.1
59430	1.34892	2.1
59431	1.28805	2.1
59432	1.28805	2.1
59433	1.2955	2.1
59434	1.2955	2.1
59435	1.34892	2.1
59436	1.24359	2.1
59437	1.24936	2.1
59438	1.33997	2.1
59439	1.15506	1.56836
59440	1.15506	2.1
59441	0.946128	2.1
59442	0.946128	2.1
59443	0.946131	2.1
59444	1.15506	1.62977
59445	1.06997	2.1
59446	0.946128	2.1
59447	1.06997	2.03126

59448	1.15506	2.1
59449	0.946129	2.1
59450	0.946129	2.1
59451	1.70608	2.1
59452	2.13994	2.1
59453	1.33384	2.1
59454	1.43635	2.1
59455	3.14243	2.1
59456	2.13994	2.1
59457	2.31012	2.1
59458	1.43635	2.1
59459	1.23007	2.1
59460	3.74646	2.1
59461	2.46361	2.1
59462	1.89226	2.1
59463	1.89226	2.1
59464	1.89226	2.09648
59465	2.39045	2.1
59466	2.39045	2.1
59467	1.89226	2.1
59468	1.89226	1.46756
59469	1.89226	2.053
59470	1.89226	1.10451
59471	3.78452	1.85318
59472	1.52984	2.1
59473	2.25682	1.06207
59474	2.20831	1.0399
59475	2.20831	2.1
59476	1.61178	0.977399
59477	1.61178	2.1
59478	2.47395	2.1
59479	1.89226	2.1
59480	1.89226	2.1
59481	1.89226	2.1
59482	2.46362	2.1
59483	2.46362	2.1
59484	0.946131	0.912245
59485	0.946131	2.1
59486	0.946131	2.1
59487	0.946131	1.39066
59488	0.94613	1.1942
59489	0.94613	1.59564
59490	0.946132	2.1
59491	0.946132	1.99595
59492	0.946131	1.26675
59493	0.946131	1.64251
59494	0.946131	1.90013
59495	0.946131	2.1
59496	0.982398	2.1
59497	0.982398	1.47578
59498	0.931703	2.1
59499	0.97549	1.84925
59500	1.11509	1.69106
59501	1.10017	1.14391
59502	0.774576	2.1
59503	0.789859	2.1
59504	0.716502	1.77201
59505	1.12793	1.98167

59506	1.14398	1.92283
59507	0.817011	1.33572
59508	1.82499	2.1
59509	1.50524	2.1
59510	1.21304	2.1
59511	1.54191	2.1
59512	3.04715	2.1
59513	1.54191	2.1
59514	1.49823	2.1
59515	1.82499	2.1
59516	1.82499	2.1
59517	1.21147	2.1
59518	1.48977	2.1
59519	1.89226	2.1
59520	1.89226	2.1
59521	1.89226	2.1
59522	1.89226	2.1
59523	3.78452	2.1
59524	1.89226	2.1
59525	1.89226	2.1
59526	1.89226	2.1
59527	1.89226	2.1
59528	1.89226	2.1
59529	1.89226	2.1
59530	0.566674	2.1
59531	0.770957	2.1
59532	0.60652	2.1
59533	0.473557	2.1
59534	0.604441	2.1
59535	0.604441	2.1
59536	0.640073	2.1
59537	0.640073	2.1
59538	0.473557	2.1
59539	0.566674	2.1
59540	0.60652	2.1
59541	0.770956	2.1
59542	1.36203	2.1
59543	1.13335	2.1
59544	1.54191	2.1
59545	1.54191	2.1
59546	1.00965	2.1
59547	1.00965	2.1
59548	1.89226	2.1
59549	1.89226	2.1
59550	1.89226	2.1
59551	1.89226	2.1
59552	1.89226	2.1
59553	1.89226	2.1
59554	0.946131	2.1
59555	0.946131	2.1
59556	0.946131	2.1
59557	0.946131	2.1
59558	0.946132	2.1
59559	0.946132	2.1
59560	0.946131	2.1
59561	0.946131	2.1
59562	0.946132	2.1
59563	0.946132	2.1

59564	0.946131	2.1
59565	0.946131	2.1
59566	0.946132	0.150338
59567	0.946132	0.195427
59568	0.946133	0.129104
59569	0.946133	0.191763
59570	0.946132	0.148172
59571	0.946132	0.148807
59572	0.946132	0.157243
59573	0.946132	0.187672
59574	0.946132	0.151813
59575	0.946132	0.165217
59576	0.946133	0.189078
59577	0.946133	0.151798
59578	1.89226	0.239408
59579	1.89226	0.203038
59580	1.89226	0.176018
59581	1.89226	0.151296
59582	3.78453	0.143586
59583	1.89226	0.205848
59584	1.89226	0.136008
59585	1.89226	0.176143
59586	1.89226	0.191192
59587	1.89226	0.163804
59588	1.89226	0.179735
59589	0.946132	0.113501
59590	0.946132	0.132918
59591	0.946131	0.101755
59592	0.946131	0.134274
59593	0.946132	0.121127
59594	0.946132	0.117332
59595	0.946132	0.114017
59596	0.946132	0.138451
59597	0.946132	0.128591
59598	0.946132	0.117729
59599	0.946132	0.155111
59600	0.946132	0.138125
59601	0.946129	0.10994
59602	0.946129	0.143286
59603	0.946128	0.0980974
59604	0.946128	0.125187
59605	0.946132	0.108512
59606	0.946132	0.113344
59607	0.946126	0.131896
59608	0.946126	0.127024
59609	0.946129	0.110395
59610	0.946129	0.123077
59611	0.946129	0.132904
59612	0.946129	0.157352
59613	1.20674	0.192684
59614	1.79877	0.259396
59615	1.22586	0.187096
59616	1.22586	0.197315
59617	1.7919	0.244971
59618	1.7919	0.267794
59619	2.27622	0.187146
59620	1.79877	0.270467
59621	1.7919	0.179978

59622	2.27102	0.281899
59623	4.06979	0.187196
59624	1.89226	0.272633
59625	1.89226	0.448603
59626	1.89226	0.28069
59627	1.89226	0.424354
59628	3.78452	0.426333
59629	1.89226	0.260998
59630	1.89226	0.349653
59631	1.89226	0.248903
59632	1.89226	0.427339
59633	3.78453	0.259785
59634	1.13259	0.186647
59635	1.79187	0.245811
59636	1.32741	0.176149
59637	1.18784	0.191063
59638	1.703	0.227839
59639	1.49575	0.253912
59640	1.46201	0.168749
59641	2.27099	0.268696
59642	1.79187	0.169942
59643	1.79187	0.181028
59644	2.12923	0.260867
59645	2.12923	0.288493
59646	1.89224	0.264437
59647	1.89224	0.411756
59648	1.89224	0.266228
59649	1.89224	0.424423
59650	3.78447	0.277268
59651	0.946119	0.481328
59652	0.946119	0.355703
59653	0.946118	0.396442
59654	0.946118	0.297358
59655	0.946118	0.401778
59656	0.946118	0.423201
59657	0.946119	0.24834
59658	0.946119	0.224433
59659	0.946131	0.345043
59660	0.946131	0.257371
59661	0.946106	0.299817
59662	0.946106	0.342953
59663	2.07526	0.793835
59664	1.89226	0.736275
59665	1.89226	0.842592
59666	2.13445	0.852531
59667	4.02671	0.774326
59668	1.89226	0.740574
59669	1.89226	0.804712
59670	1.89226	0.81739
59671	1.89226	0.546133
59672	1.89226	0.861895
59673	1.89226	0.734517
59674	2.13446	0.816571
59675	1.00013	0.865679
59676	1.1475	1.0057
59677	2.20046	0.850975
59678	3.20059	0.895807
59679	0.946133	0.767309

59680	0.946133	0.788017
59681	1.06722	0.788147
59682	1.10023	0.767622
59683	0.946131	0.78478
59684	0.946131	0.698908
59685	1.10023	0.809244
59686	1.10023	0.766162
59687	0.946131	0.817455
59688	1.10023	0.769181
59689	0.94613	0.757487
59690	1.06723	0.744009
59691	1.89226	0.714341
59692	1.89226	0.726391
59693	1.89226	0.80489
59694	1.89226	0.747915
59695	3.78452	0.760795
59696	1.89226	0.855019
59697	1.89226	0.844295
59698	1.89226	0.887405
59699	1.89226	0.837046
59700	1.89226	0.842557
59701	1.89226	0.793643
59702	1.89226	2.1
59703	1.89226	0.753169
59704	1.89226	0.879005
59705	1.89226	0.761638
59706	1.89226	2.1
59707	0.828588	0.05
59708	0.828588	0.05
59709	0.841316	0.05
59710	0.841316	2.1
59711	0.528387	2.1
59712	0.565893	0.05
59713	1.10401	0.05
59714	1.10401	2.1
59715	0.528387	2.1
59716	1.89226	2.1
59717	1.36047	0.879005
59718	1.33973	2.1
59719	1.33973	2.1
59720	1.89226	0.05
59721	2.28951	0.756785
59722	1.89226	1.10151
59723	2.80796	1.88719
59724	2.01584	1.59816
59725	2.01584	1.22079
59726	1.89226	1.44416
59727	1.89226	1.19509
59728	1.89226	0.931117
59729	1.89226	1.34049
59730	1.89226	1.15578
59731	1.89226	0.98967
59732	1.89226	1.15934
59733	1.89226	0.994113
59734	2.15223	1.00307
59735	1.30664	1.98949
59736	1.96011	1.68317
59737	2.76516	1.06848

59738	4.11234	1.67774
59739	1.89226	0.893871
59740	1.89226	1.25821
59741	1.89226	1.06635
59742	1.89226	0.953407
59743	1.89226	1.07441
59744	1.89226	0.962221
59745	2.81143	2.1
59746	2.58517	2.1
59747	2.55318	2.1
59748	2.7542	2.1
59749	5.33937	2.1
59750	2.7777	2.1
59751	2.7542	2.1
59752	2.71672	2.1
59753	2.70401	2.1
59754	5.45821	2.1
59755	2.55318	2.1
59756	2.7542	2.1
59757	2.71672	2.1
59758	2.54198	2.1
59759	5.29618	2.1
59760	1.95416	2.1
59761	2.162 2.1	
59762	2.11563	2.1
59763	1.91813	2.1
59764	4.08013	2.1
59765	1.95417	2.1
59766	1.47712	2.1
59767	1.45182	2.1
59768	1.91813	2.1
59769	3.39525	2.1
59770	2.11562	2.1
59771	1.91813	2.1
59772	1.50979	2.1
59773	2.03974	2.1
59774	3.95787	2.1
59775	1.85605	2.1
59776	1.91813	2.1
59777	1.88885	2.1
59778	1.42255	2.1
59779	1.45182	2.1
59780	1.45182	2.1
59781	0.94613	0.845248
59782	0.94613	0.736156
59783	0.946128	0.710384
59784	0.946128	0.821755
59785	0.946127	0.804766
59786	0.946127	0.818979
59787	0.946131	0.722248
59788	0.946131	0.718557
59789	0.946129	0.758435
59790	0.946129	0.755918
59791	0.946128	0.763214
59792	0.946128	0.764927
59793	0.543052	0.847485
59794	0.54608	0.736282
59795	0.602293	0.69973

59796	0.535325	0.725929
59797	1.05446	0.829206
59798	0.850496	0.781614
59799	0.699838	0.739947
59800	0.679751	0.821213
59801	0.713797	0.761879
59802	0.509794	0.738948
59803	0.602304	0.730658
59804	1.40017	0.821146
59805	1.40017	2.1
59806	0.94613	0.767803
59807	0.94613	2.1
59808	1.40017	2.1
59809	1.33997	1.97215
59810	0.946131	0.805608
59811	0.946131	0.817462
59812	1.33997	0.902368
59813	0.946131	0.905476
59814	0.946131	0.910481
59815	1.40017	0.906419
59816	1.89226	0.158829
59817	1.89226	0.225231
59818	1.89226	0.222465
59819	1.89226	0.1432
59820	3.78452	0.164421
59821	0.946133	0.137092
59822	0.946133	0.153664
59823	0.946132	0.264478
59824	0.946132	0.164301
59825	0.946132	0.164491
59826	0.946132	0.178804
59827	0.946133	0.251808
59828	0.946133	0.157471
59829	0.946132	0.142662
59830	0.946132	0.184479
59831	0.946132	0.162268
59832	0.946132	0.167912
59833	1.89224	0.22194
59834	1.89224	0.152103
59835	1.89224	0.166346
59836	1.89224	0.180837
59837	1.89224	0.196877
59838	1.89224	0.213078
59839	0.94612	0.141187
59840	0.94612	0.150138
59841	0.946119	0.238806
59842	0.946119	0.166737
59843	0.946119	0.170636
59844	0.946119	0.182395
59845	0.94612	0.167494
59846	0.94612	0.22019
59847	0.946132	0.169318
59848	0.946132	0.153057
59849	0.946107	0.140093
59850	0.946107	0.184256
59851	2.63939	2.1
59852	1.2862	2.1
59853	1.29891	2.1

59854	2.64419	2.1
59855	3.93039	2.1
59856	2.6394	2.1
59857	1.89226	2.1
59858	1.89226	2.1
59859	2.64419	2.1
59860	4.53646	2.1
59861	1.89226	2.1
59862	1.89226	2.1
59863	1.89226	2.1
59864	1.89226	2.1
59865	3.78453	2.1
59866	1.89226	2.1
59867	1.89226	2.1
59868	1.89226	2.1
59869	1.89226	2.1
59870	1.89226	2.1
59871	1.89226	2.1
59872	1.31998	2.1
59873	1.32209	2.1
59874	0.649456	2.1
59875	0.648474	2.1
59876	0.984995	2.1
59877	0.984995	2.1
59878	0.985573	2.1
59879	0.985573	2.1
59880	1.31999	2.1
59881	0.648472	2.1
59882	0.649455	2.1
59883	1.32209	2.1
59884	0.946133	2.1
59885	0.946133	2.1
59886	1.31999	2.1
59887	1.3221	2.1
59888	0.946132	2.1
59889	0.946132	2.1
59890	1.31999	2.1
59891	1.31999	2.1
59892	1.31999	2.1
59893	0.946132	2.1
59894	0.946133	2.1
59895	1.3221	2.1
59896	0.946132	2.1
59897	0.946132	2.1
59898	0.946131	2.1
59899	0.946131	2.1
59900	0.946132	2.1
59901	0.946132	2.1
59902	0.946131	2.1
59903	0.946131	2.1
59904	0.946131	2.1
59905	0.946131	2.1
59906	0.946132	2.1
59907	0.946132	2.1
59908	0.946128	2.1
59909	0.946128	2.1
59910	0.946132	2.1
59911	0.946132	2.1

59912	0.946131	2.1
59913	0.946131	2.1
59914	0.94613	2.1
59915	0.94613	2.1
59916	0.94613	2.1
59917	0.94613	2.1
59918	0.946131	2.1
59919	0.946131	2.1
59920	1.89226	0.732983
59921	1.89226	0.876331
59922	1.89226	0.842316
59923	1.89226	0.836688
59924	1.89226	0.753426
59925	1.89226	0.758738
59926	1.89226	0.80026
59927	1.89226	1.02601
59928	1.89226	0.892895
59929	1.89226	0.997748
59930	3.78453	1.04207
59931	0.946133	0.800192
59932	0.946133	0.810744
59933	0.946132	0.787453
59934	0.946132	0.776291
59935	0.946133	0.848862
59936	0.946133	0.847315
59937	0.946132	0.79118
59938	0.946132	0.75343
59939	0.946132	0.809822
59940	0.946132	0.868843
59941	0.946132	0.787108
59942	0.946132	0.918804
59943	2.23638	0.750718
59944	1.89226	0.782658
59945	2.66014	0.864856
59946	2.66014	0.886363
59947	1.89226	0.862758
59948	1.89226	0.796665
59949	1.11819	0.710215
59950	1.33007	0.715525
59951	0.660255	0.737921
59952	0.91151	0.724975
59953	0.911511	0.7534
59954	1.33007	0.736487
59955	1.11819	0.69113
59956	0.660255	0.724806
59957	0.985327	0.709745
59958	0.985327	0.742221
59959	1.005	0.724465
59960	1.005	0.745192
59961	1.89226	2.1
59962	1.89226	2.1
59963	1.89226	2.1
59964	1.89226	2.1
59965	3.78453	2.1
59966	1.59533	2.1
59967	2.68571	2.1
59968	1.28291	2.1
59969	2.46974	2.1

59970	4.06506	2.1
59971	2.68571	2.1
59972	1.89226	2.1
59973	1.89226	2.1
59974	1.89226	2.1
59975	2.46973	2.1
59976	2.46973	2.1
59977	1.89226	2.1
59978	1.89226	2.1
59979	1.89226	2.1
59980	1.89226	2.1
59981	1.89226	2.1
59982	1.89226	2.1
59983	1.89226	2.1
59984	1.89226	2.1
59985	1.89226	2.1
59986	1.89226	2.1
59987	1.89226	2.1
59988	1.89226	2.1
59989	0.883594	2.1
59990	0.883594	2.1
59991	0.876484	2.1
59992	0.876484	2.1
59993	1.10442	2.1
59994	1.23487	2.1
59995	0.641457	2.1
59996	0.52521	2.1
59997	1.10442	2.1
59998	0.52521	2.1
59999	0.641457	2.1
60000	1.23487	2.1
60001	0.946131	2.1
60002	0.946131	2.1
60003	0.946131	2.1
60004	0.946131	2.1
60005	0.946131	2.1
60006	0.946131	2.1
60007	0.946131	2.1
60008	0.946131	2.1
60009	0.946131	2.1
60010	0.946131	2.1
60011	0.946131	2.1
60012	0.946131	2.1
60013	2.04016	0.420548
60014	2.13042	0.513114
60015	2.11465	0.527694
60016	2.11465	0.693686
60017	1.95908	0.336772
60018	1.95908	0.471887
60019	1.66953	0.433449
60020	2.13041	0.529077
60021	1.95908	0.331492
60022	2.21952	0.47089
60023	1.66953	0.587912
60024	1.92997	0.651361
60025	2.04016	0.359937
60026	1.95908	0.393018
60027	1.82014	0.247006

60028	2.08757	0.280648
60029	3.86029	0.334222
60030	0.834764	0.459292
60031	0.722102	0.331785
60032	0.979537	0.342581
60033	0.910065	0.252907
60034	0.840053	0.270455
60035	0.840052	0.58032
60036	0.861587	0.364272
60037	0.861587	0.321145
60038	0.910067	0.304027
60039	0.722101	0.26242
60040	0.834763	0.379187
60041	0.979539	0.360832
60042	1.89226	0.298232
60043	1.89226	0.260321
60044	1.89226	0.26185
60045	1.89226	0.258762
60046	1.89226	0.307928
60047	1.89226	0.314691
60048	1.89226	0.307502
60049	1.89226	0.333267
60050	1.89226	0.400409
60051	1.89226	0.232511
60052	3.78453	0.311763
60053	1.89226	0.198799
60054	1.89226	0.195163
60055	1.89226	0.192456
60056	1.89226	0.180711
60057	1.89226	0.216607
60058	1.89226	0.211176
60059	1.20691	0.159741
60060	2.27379	0.159484
60061	2.58476	0.177399
60062	1.5124	0.133811
60063	3.79167	0.168896
60064	1.89226	0.324443
60065	2.2738	0.211769
60066	2.58477	0.212431
60067	1.89226	0.243369
60068	4.47703	0.236753
60069	0.952225	1.62277
60070	0.952225	1.9311
60071	0.909865	1.74643
60072	0.909865	2.00513
60073	0.909895	2.09933
60074	0.946132	1.71316
60075	0.946132	1.81674
60076	0.915958	1.61459
60077	0.946132	1.82109
60078	0.946132	1.78009
60079	0.909894	1.81656
60080	0.915958	1.92555
60081	0.983493	2.1
60082	2.40055	2.1
60083	1.02986	2.1
60084	1.02986	2.1
60085	2.34217	2.1

60086	2.34217	2.1
60087	1.89226	2.1
60088	2.40056	2.1
60089	2.34217	2.1
60090	2.40056	2.1
60091	1.83388	2.1
60092	1.89226	2.1
60093	1.89226	2.1
60094	1.89226	2.1
60095	1.89226	2.1
60096	1.89226	2.1
60097	1.89226	2.1
60098	1.89226	2.1
60099	1.89226	2.1
60100	1.89226	2.1
60101	1.89226	2.1
60102	1.89226	2.1
60103	1.89226	2.1
60104	1.89226	2.1
60105	0.946133	2.1
60106	0.946133	2.1
60107	1.17109	2.1
60108	1.13078	2.1
60109	0.946132	2.1
60110	0.946132	2.1
60111	1.13078	2.1
60112	1.13078	2.1
60113	1.13078	2.1
60114	0.946133	2.1
60115	0.946132	2.1
60116	1.17109	2.1
60117	1.89226	2.1
60118	1.89226	2.1
60119	1.89226	2.1
60120	1.89226	2.1
60121	1.89226	2.1
60122	1.89226	2.1
60123	2.33963	1.12049
60124	1.89227	0.91543
60125	1.74565	0.944366
60126	1.74565	0.999067
60127	1.89227	0.869532
60128	1.89227	0.94229
60129	1.89227	0.988001
60130	1.89227	0.920681
60131	1.89227	1.23057
60132	1.89227	0.942195
60133	3.78453	0.930104
60134	1.31398	0.883375
60135	1.10392	0.898309
60136	0.796878	0.893427
60137	0.524356	0.878836
60138	0.870614	0.875467
60139	0.945739	0.862496
60140	0.89208	0.910956
60141	0.89208	0.951411
60142	1.23425	0.86648
60143	0.774658	0.933629

60144	1.03182	0.87371
60145	0.557289	0.845762
60146	2.33963	1.06042
60147	1.89226	0.863375
60148	1.89226	1.01943
60149	2.77245	0.896588
60150	4.23189	0.977355
60151	1.89226	0.99501
60152	1.89226	0.892138
60153	1.89226	1.0955
60154	1.89226	1.06318
60155	1.89226	0.893494
60156	1.89226	0.886102
60157	1.89226	0.716276
60158	1.89226	0.728332
60159	1.89226	0.708582
60160	1.89226	0.761719
60161	1.89226	0.659441
60162	1.89226	0.758217
60163	1.89226	0.698753
60164	1.89226	0.71339
60165	1.89226	0.688626
60166	1.89226	0.731039
60167	1.89226	0.597731
60168	1.89226	0.727362
60169	0.946128	0.612226
60170	0.946128	0.57479
60171	0.963918	0.638353
60172	1.37543	0.439894
60173	0.946129	0.608021
60174	0.946129	0.677494
60175	1.37542	0.518953
60176	1.37542	0.45279
60177	0.94613	0.580106
60178	1.37542	0.627233
60179	0.963916	0.634049
60180	0.94613	0.632512
60181	0.946132	0.711497
60182	0.946132	0.682633
60183	0.946131	0.678253
60184	0.946131	0.644865
60185	0.946131	0.680487
60186	0.946131	0.715269
60187	0.946132	0.658276
60188	0.946132	0.482027
60189	0.946131	0.676141
60190	0.946131	0.675367
60191	0.946131	0.672696
60192	0.946131	0.653398
60193	1.89226	0.720213
60194	1.89226	0.728895
60195	1.89226	0.666401
60196	1.89226	0.748441
60197	3.78452	0.72617
60198	0.946127	0.727656
60199	0.946127	0.682502
60200	0.946131	0.709819
60201	0.946131	0.694069

60202	0.946128	0.710353
60203	0.946128	0.724292
60204	0.94613	0.621107
60205	0.94613	0.696691
60206	0.946129	0.688408
60207	0.946129	0.706462
60208	0.946129	0.686884
60209	0.946129	0.712809
60210	1.89226	0.981504
60211	1.89226	1.06187
60212	1.89226	2.04347
60213	1.89226	0.879225
60214	3.78453	0.886906
60215	1.89226	0.920127
60216	1.89226	0.650313
60217	1.89226	0.834582
60218	1.89226	0.860583
60219	3.78453	0.925202
60220	0.946131	0.79453
60221	0.946131	0.791861
60222	0.946132	1.20022
60223	0.946132	0.87874
60224	0.946132	1.07261
60225	0.946132	0.850309
60226	0.946132	1.07837
60227	0.946132	0.929695
60228	0.946132	0.952684
60229	0.946132	1.02707
60230	0.946132	1.05326
60231	0.946132	0.889228
60232	0.548169	0.687769
60233	0.736603	0.509054
60234	1.18322	0.652625
60235	1.01139	0.685909
60236	1.01139	0.552193
60237	0.548168	0.564639
60238	0.736603	0.449229
60239	1.18322	0.468212
60240	0.861589	0.452782
60241	0.861589	0.648641
60242	0.869798	0.611399
60243	0.869798	0.647246
60244	1.16037	0.646513
60245	0.942012	0.593715
60246	0.942012	0.566901
60247	0.62356	0.695994
60248	0.891932	0.460188
60249	0.900255	0.601694
60250	2.02279	0.713094
60251	1.48677	0.591967
60252	1.54724	0.499962
60253	0.831456	0.536009
60254	1.19775	0.748861
60255	1.89226	1.22588
60256	2.76291	1.3329
60257	1.89226	1.48579
60258	1.89226	1.19257
60259	3.78452	1.39816

60260	1.89226	0.996672
60261	1.89226	1.23272
60262	1.89226	1.36246
60263	1.89226	1.05129
60264	1.89226	1.35774
60265	1.89226	1.04628
60266	1.89226	1.39328
60267	1.89226	1.08819
60268	1.89226	1.16794
60269	1.89226	1.20206
60270	3.78452	1.12364
60271	1.89226	0.94851
60272	1.89226	1.25142
60273	1.89226	1.30945
60274	1.89226	0.977593
60275	3.78453	1.12916
60276	0.946128	1.00017
60277	0.946128	1.01326
60278	0.946129	1.37304
60279	0.946129	1.21615
60280	0.946132	0.985716
60281	0.946132	1.18266
60282	0.946126	1.20565
60283	0.946126	0.984096
60284	0.946129	1.11341
60285	0.946129	1.11373
60286	0.946129	1.09366
60287	0.946129	1.06781
60288	2.80495	1.27105
60289	1.89225	1.10693
60290	1.89225	1.36259
60291	1.87794	1.3457
60292	1.89225	1.16441
60293	1.87794	1.15004
60294	2.25195	0.919136
60295	2.00624	0.739024
60296	2.13132	0.862617
60297	1.90239	0.229677
60298	4.13756	0.778666
60299	0.804661	0.730193
60300	0.804663	0.722367
60301	0.889591	0.538817
60302	0.889591	0.769173
60303	0.808324	0.742458
60304	0.761765	0.749781
60305	1.02822	0.705714
60306	0.879624	0.59167
60307	0.879617	0.414331
60308	0.800864	0.691636
60309	0.978023	0.730177
60310	0.808329	0.727132
60311	2.13133	0.868257
60312	2.00558	0.328451
60313	2.09316	0.33831
60314	2.09316	0.909382
60315	2.03253	0.63644
60316	2.03253	0.908014
60317	0.84577	0.779489

60318	0.845767	0.749944
60319	0.843917	0.636133
60320	0.843917	0.774991
60321	0.76177	0.733641
60322	0.719345	0.754266
60323	0.934435	0.694981
60324	0.884197	0.703495
60325	0.934439	0.469373
60326	0.75627	0.693199
60327	0.849424	0.70619
60328	0.76177	0.722737
60329	2.61855	0.633539
60330	1.17793	0.5722
60331	1.42965	0.691021
60332	1.17793	0.668921
60333	2.50057	0.517074
60334	2.24885	0.46879
60335	0.946133	0.653322
60336	0.946133	0.652402
60337	1.30928	0.675707
60338	1.12443	0.562293
60339	0.946132	0.54088
60340	0.946132	0.502127
60341	1.30928	0.570035
60342	1.30928	0.501571
60343	0.946132	0.584861
60344	1.30928	0.573021
60345	1.12443	0.531636
60346	0.946133	0.544295
60347	1.89226	0.445168
60348	1.89226	0.656617
60349	1.89226	0.683991
60350	1.89226	0.69038
60351	3.78452	0.652303
60352	1.89226	0.705483
60353	1.89226	0.671935
60354	1.89226	0.570535
60355	1.89226	0.678018
60356	1.89226	0.609875
60357	1.89226	0.465091
60358	1.89226	0.577145
60359	1.89226	0.580975
60360	1.89226	0.64733
60361	1.89226	0.704634
60362	3.78452	0.656697
60363	0.94613	0.668687
60364	0.94613	0.69266
60365	0.946131	0.70278
60366	0.946131	0.702433
60367	0.946128	0.701554
60368	0.946128	0.699223
60369	0.946133	0.619939
60370	0.946133	0.581984
60371	0.946131	0.672922
60372	0.946131	0.703245
60373	0.946131	0.687553
60374	0.946131	0.411616
60375	1.89226	2.1

60376	1.89226	2.1
60377	1.89226	2.1
60378	1.89226	2.1
60379	3.78453	2.1
60380	1.0465	2.1
60381	2.73926	2.1
60382	2.03721	2.1
60383	2.03721	2.1
60384	1.44709	2.1
60385	1.44709	2.1
60386	0.946131	2.1
60387	0.946131	2.1
60388	0.946131	2.1
60389	0.946131	2.1
60390	0.946132	2.1
60391	0.946132	2.1
60392	0.94613	2.1
60393	0.94613	2.1
60394	0.946131	2.1
60395	0.946131	2.1
60396	0.946131	2.1
60397	0.946131	2.1
60398	0.946132	2.1
60399	0.946132	2.1
60400	0.946131	2.1
60401	0.946131	2.1
60402	0.946132	2.1
60403	0.946132	2.1
60404	0.946132	2.1
60405	0.946132	2.1
60406	0.946132	2.1
60407	0.946132	2.1
60408	0.946132	2.1
60409	0.946132	2.1
60410	1.89226	2.1
60411	1.89226	2.1
60412	1.89226	2.1
60413	1.89226	2.1
60414	3.78452	2.1
60415	1.61969	0.417347
60416	1.79374	0.359213
60417	1.29382	0.31158
60418	1.29382	0.340763
60419	2.05759	0.529575
60420	2.05759	0.403244
60421	1.89226	0.272925
60422	1.89226	0.330691
60423	1.89226	0.260661
60424	1.89226	0.321879
60425	1.89226	0.292034
60426	1.89226	0.285179
60427	1.89226	0.886086
60428	1.89226	0.91098
60429	1.89226	0.896873
60430	1.89226	0.901421
60431	1.89226	0.86088
60432	1.89226	0.864658
60433	1.89226	1.26153

60434	1.89226	0.992227
60435	1.89226	1.11
60436	1.89226	1.15913
60437	3.78453	1.26391
60438	0.946131	0.967287
60439	0.946131	0.98948
60440	0.946131	0.913483
60441	0.946131	0.959919
60442	0.946131	0.913251
60443	0.946131	0.897416
60444	0.946131	0.979065
60445	0.946131	1.04395
60446	0.946131	0.958457
60447	0.946131	0.98793
60448	0.946131	0.963405
60449	0.946131	0.926293
60450	2.45177	0.499805
60451	2.68507	0.405056
60452	2.3376	0.590684
60453	2.22086	0.388651
60454	4.78937	0.500859
60455	2.45178	0.420435
60456	2.35552	0.315417
60457	1.68062	0.446705
60458	1.81902	0.340036
60459	4.13239	0.355583
60460	2.3376	0.464545
60461	1.55612	0.337027
60462	1.69811	0.335349
60463	2.31527	0.403827
60464	1.79553	0.33884
60465	2.3376	0.401746
60466	1.55613	0.309663
60467	1.06336	0.326874
60468	1.97516	0.298254
60469	1.88966	0.286784
60470	1.06336	0.475555
60471	1.68062	0.270476
60472	1.89226	0.99264
60473	1.91634	1.00607
60474	2.03709	1.20865
60475	1.91634	0.983696
60476	1.83185	1.02357
60477	1.70303	0.838716
60478	1.89226	0.916854
60479	1.89226	0.970178
60480	1.89226	1.12856
60481	1.89226	0.941648
60482	1.89226	0.960242
60483	1.89226	0.827422
60484	1.89226	1.31395
60485	2.03472	1.91362
60486	2.22183	2.1
60487	2.03472	1.20458
60488	2.07936	1.8624
60489	1.89226	1.01642
60490	1.89226	0.937879
60491	1.8519	0.927812

60492	1.92119	1.07478
60493	1.8519	0.929331
60494	1.96156	0.919159
60495	1.89226	0.81035
60496	1.89226	0.877235
60497	1.89226	0.930049
60498	1.89226	1.05165
60499	1.89226	0.908587
60500	1.89226	0.897284
60501	1.89226	0.792436
60502	1.89226	1.14689
60503	2.22183	1.23242
60504	2.39541	1.90303
60505	2.22183	1.09476
60506	2.06585	1.21539
60507	1.89226	0.976531
60508	1.47001	2.09785
60509	1.57962	1.06461
60510	1.65238	1.2124
60511	1.60502	1.36763
60512	3.18464	1.32574
60513	1.02415	2.1
60514	1.60503	1.67055
60515	1.47001	2.1
60516	1.47001	2.1
60517	1.28228	2.1
60518	1.28228	2.1
60519	0.946133	2.1
60520	0.946133	2.04069
60521	0.946132	1.3917
60522	0.946132	1.21064
60523	0.946132	1.21136
60524	0.946132	1.9685
60525	0.946133	1.74682
60526	0.946133	2.1
60527	0.946132	1.81055
60528	0.946132	1.45378
60529	0.946133	1.87063
60530	0.946133	1.98607
60531	1.89226	1.0606
60532	1.89226	1.1579
60533	1.89226	1.3587
60534	1.89226	0.991036
60535	3.78452	1.07171
60536	1.76897	1.153
60537	1.60502	1.24778
60538	1.65238	1.06753
60539	1.65238	1.71981
60540	1.76932	1.05069
60541	1.76932	1.55015
60542	1.55998	2.1
60543	1.60503	1.30487
60544	1.76932	1.97738
60545	1.60503	2.1
60546	1.44657	2.0175
60547	1.28228	2.1
60548	0.946133	1.6175
60549	0.946133	2.09621

60550	0.946132	1.41757
60551	0.946132	1.12847
60552	0.946132	1.11804
60553	0.946132	1.43058
60554	0.946133	1.30166
60555	0.946133	2.1
60556	0.946132	1.67586
60557	0.946132	1.62805
60558	0.946132	1.88812
60559	0.946132	1.64983
60560	0.946128	1.25898
60561	0.946128	1.07111
60562	0.946133	0.932849
60563	0.946133	1.01019
60564	0.94613	0.939241
60565	0.94613	1.07642
60566	0.946131	1.26122
60567	0.946131	1.05012
60568	0.946131	1.04387
60569	0.946131	1.03566
60570	0.946131	1.0778
60571	0.946131	1.08061
60572	1.85779	0.484645
60573	1.00763	0.392116
60574	1.74874	0.366356
60575	1.74873	0.35591
60576	1.14467	0.551637
60577	1.14466	0.526861
60578	1.85779	0.386967
60579	1.61597	0.327153
60580	1.00053	0.431363
60581	1.03018	0.310631
60582	2.65819	0.320492
60583	1.89226	0.805628
60584	1.89226	0.74966
60585	1.89226	0.805549
60586	1.89226	0.788794
60587	3.78451	0.797229
60588	1.89226	0.799543
60589	1.89226	0.609851
60590	1.89226	0.738282
60591	1.89226	0.751913
60592	1.89226	0.72184
60593	1.89226	0.742947
60594	1.11983	0.489951
60595	0.810945	0.544978
60596	0.913906	0.461763
60597	0.913906	0.506204
60598	2.23303	0.386752
60599	1.66057	0.405769
60600	1.28724	0.412391
60601	1.08132	0.418313
60602	1.49316	0.497805
60603	0.810958	0.499047
60604	0.810958	0.453126
60605	0.827861	0.743113
60606	0.946126	0.74802
60607	0.946132	0.783864

60608	0.946132	0.741017
60609	0.827863	0.748311
60610	0.827863	0.749763
60611	0.946129	0.756179
60612	0.946129	0.758929
60613	0.827863	0.744123
60614	0.946128	0.752742
60615	0.946129	0.737095
60616	0.946129	0.749682
60617	0.974718	0.426834
60618	1.53671	0.634169
60619	2.80542	0.379869
60620	2.27259	0.498808
60621	3.8093	0.527369
60622	1.89226	0.686474
60623	1.89226	0.726256
60624	1.89226	0.606258
60625	1.89226	0.716776
60626	3.78453	0.708586
60627	0.946132	0.662768
60628	0.946132	0.706055
60629	0.946131	0.71036
60630	0.946131	0.699885
60631	0.946132	0.710821
60632	0.946132	0.716765
60633	0.946131	0.635254
60634	0.946131	0.640266
60635	0.946132	0.689709
60636	0.946132	0.708862
60637	0.946131	0.699621
60638	0.946131	0.702163
60639	2.80542	0.510454
60640	1.89226	0.518117
60641	2.27259	0.618151
60642	2.27259	0.682144
60643	1.89226	0.306772
60644	1.89226	0.454594
60645	1.89226	0.571367
60646	1.89226	0.721793
60647	1.89226	0.595072
60648	1.89226	0.72161
60649	1.89226	0.602779
60650	1.89226	0.720669
60651	0.946132	0.72054
60652	0.946132	0.643456
60653	0.946131	0.71327
60654	0.946131	0.701341
60655	0.946132	0.714708
60656	0.946132	0.716026
60657	0.946131	0.59323
60658	0.946131	0.632843
60659	0.946131	0.663183
60660	0.946131	0.698143
60661	0.946131	0.664637
60662	0.946131	0.706984
60663	2.68213	2.1
60664	2.58234	2.1
60665	2.58234	2.1

60666	2.61243	2.1
60667	2.67035	2.1
60668	2.70044	2.1
60669	2.68213	2.1
60670	2.53921	2.1
60671	2.53303	2.1
60672	2.67035	2.1
60673	5.20956	2.1
60674	2.67033	2.1
60675	2.58232	2.1
60676	2.55004	2.1
60677	2.64567	2.1
60678	5.228 2.1	
60679	2.53301	2.1
60680	2.64567	2.1
60681	2.64567	2.1
60682	2.67033	2.1
60683	2.51603	2.1
60684	2.54069	2.1
60685	0.907497	2.1
60686	0.907497	2.1
60687	0.926641	2.1
60688	0.926641	2.1
60689	1.14989	2.1
60690	1.10046	2.1
60691	0.695334	2.1
60692	0.684247	2.1
60693	0.695333	2.1
60694	1.14989	2.1
60695	0.684246	2.1
60696	1.10046	2.1
60697	0.946126	1.20224
60698	0.946126	2.1
60699	0.946131	2.1
60700	0.946131	2.1
60701	0.946127	2.1
60702	0.946127	1.60247
60703	0.94613	2.1
60704	0.94613	2.1
60705	0.946128	2.1
60706	0.946128	2.1
60707	0.946128	2.1
60708	0.946128	2.1
60709	1.89226	2.1
60710	1.89226	1.8512
60711	1.89226	2.1
60712	1.89226	1.14952
60713	1.89226	2.1
60714	1.89226	1.03298
60715	1.42174	2.1
60716	2.29978	2.1
60717	2.41233	2.1
60718	2.41233	2.1
60719	1.39067	2.1
60720	1.39067	2.1
60721	1.89226	2.1
60722	1.89226	2.1
60723	1.89226	2.1

60724	1.89226	2.1
60725	3.78452	2.1
60726	1.89226	2.1
60727	1.89226	2.1
60728	1.89226	2.1
60729	1.89226	2.09843
60730	1.89226	2.1
60731	1.89226	1.28473
60732	2.41234	2.1
60733	1.89226	2.1
60734	2.29979	2.1
60735	1.89226	2.1
60736	1.89226	2.1
60737	2.29979	2.1
60738	1.09506	0.458245
60739	2.82921	0.635266
60740	2.53251	0.469096
60741	2.53251	0.621043
60742	1.42901	0.504971
60743	1.42901	0.661946
60744	0.946132	0.7
60745	0.946132	0.654173
60746	0.946128	0.58298
60747	0.946128	0.7
60748	0.94613	0.7
60749	0.94613	0.7
60750	0.946131	0.683469
60751	0.946131	0.617225
60752	0.94613	0.67689
60753	0.94613	0.614162
60754	0.94613	0.7
60755	0.94613	0.7
60756	1.89226	0.7
60757	1.89226	0.7
60758	1.89226	0.7
60759	1.89226	0.7
60760	3.78452	0.7
60761	2.53251	0.654431
60762	1.89226	0.7
60763	2.82921	0.66415
60764	1.89226	0.678315
60765	1.89226	0.7
60766	2.82921	0.7
60767	1.89226	0.7
60768	1.89226	0.695207
60769	1.89226	0.7
60770	1.89226	0.7
60771	1.89226	0.7
60772	1.89226	0.7
60773	2.53251	0.554272
60774	1.89226	0.698804
60775	2.06158	0.445543
60776	1.89226	0.581158
60777	4.42477	0.437018
60778	1.23296	2.1
60779	2.55352	2.1
60780	2.47476	2.1
60781	2.47476	2.1

60782	1.32368	2.1
60783	1.32368	2.1
60784	1.89226	2.1
60785	1.89226	2.1
60786	1.89226	2.1
60787	1.89226	2.1
60788	1.89226	2.1
60789	1.89226	2.1
60790	1.89226	2.1
60791	1.89226	2.1
60792	1.89226	2.1
60793	1.89226	2.1
60794	1.89226	2.1
60795	1.89226	1.51414
60796	2.47476	2.1
60797	1.89226	2.1
60798	1.89226	2.1
60799	1.89226	2.1
60800	2.55353	2.1
60801	2.55353	2.1
60802	1.05983	2.1
60803	2.47476	2.1
60804	2.37664	2.1
60805	2.37664	2.1
60806	1.23296	2.1
60807	1.23296	2.1
60808	0.946127	2.1
60809	0.946127	2.1
60810	0.946131	2.1
60811	0.946131	2.1
60812	0.946127	2.1
60813	0.946127	2.1
60814	0.946131	2.1
60815	0.946131	2.1
60816	0.946129	2.1
60817	0.946129	2.1
60818	0.946128	2.1
60819	0.946128	2.1
60820	1.89226	2.1
60821	1.89226	2.1
60822	1.89226	2.1
60823	1.89226	1.1946
60824	3.78452	2.1
60825	2.37665	2.1
60826	1.89226	2.1
60827	1.89226	2.1
60828	1.89226	2.1
60829	2.47476	2.1
60830	2.47476	2.1
60831	1.89226	0.884474
60832	1.92119	0.875835
60833	1.99586	0.988335
60834	1.92119	0.895033
60835	1.96693	0.838839
60836	1.89226	0.77264
60837	1.89226	0.846735
60838	1.89226	0.870576
60839	1.89226	0.978905

60840	1.89226	0.867211
60841	1.89226	0.849772
60842	1.89226	0.765604
60843	1.89226	1.03614
60844	2.39541	1.12805
60845	2.57183	1.38887
60846	2.39541	1.01872
60847	2.06868	1.0334
60848	1.89226	0.904391
60849	0.946133	0.800191
60850	0.946133	0.801723
60851	0.997928	0.854467
60852	1.03763	0.867983
60853	0.946131	0.880861
60854	0.946131	0.722422
60855	1.03763	0.818726
60856	1.03763	0.817719
60857	1.03763	0.836206
60858	0.946131	0.830944
60859	0.94613	0.796971
60860	0.99793	0.785575
60861	1.89226	0.747484
60862	1.89226	0.867429
60863	1.89226	0.880283
60864	1.89226	0.723687
60865	1.89226	0.945242
60866	1.89226	0.763095
60867	0.946131	0.881126
60868	0.946131	0.888066
60869	1.36939	0.999537
60870	1.28592	1.07197
60871	0.946129	1.02637
60872	0.946129	0.751672
60873	1.36939	0.908134
60874	1.36939	1.00154
60875	0.946131	0.910937
60876	1.36939	0.918546
60877	0.946131	0.930433
60878	1.28592	0.916737
60879	1.89226	0.722331
60880	1.89226	0.79462
60881	1.89226	0.762928
60882	1.89226	0.745168
60883	3.78453	0.756689
60884	1.89226	0.794443
60885	1.89226	0.77163
60886	1.89226	0.748319
60887	1.89226	0.815569
60888	3.78452	0.771111
60889	1.91005	0.763993
60890	1.89226	0.801336
60891	2.60215	0.721681
60892	2.60215	0.753771
60893	1.89226	0.756652
60894	1.89226	0.825449
60895	0.946132	0.785979
60896	0.946132	0.762577
60897	0.946132	0.762169

60898	0.946132	0.73811
60899	0.946132	0.763697
60900	0.946132	0.787078
60901	0.946132	0.76483
60902	0.946132	0.731087
60903	0.946132	0.760807
60904	0.946132	0.763133
60905	0.946131	0.754512
60906	0.946131	0.755533
60907	1.89226	0.764716
60908	1.89226	0.817583
60909	1.89226	0.812769
60910	1.89226	0.793387
60911	1.89226	0.791991
60912	1.89226	0.813109
60913	0.780483	0.799617
60914	0.78928	0.9244
60915	0.818566	0.832607
60916	0.460387	0.913359
60917	1.82793	0.765946
60918	1.32039	0.817493
60919	0.613889	0.80209
60920	1.0592	0.834017
60921	1.48698	0.811843
60922	0.818555	0.820098
60923	1.11515	0.817172
60924	1.2802	0.931896
60925	2.20046	0.774343
60926	1.1475	0.898802
60927	1.1475	0.778356
60928	2.27062	0.88172
60929	2.27062	0.763669
60930	1.89226	0.811438
60931	2.20046	0.740777
60932	2.27062	0.805679
60933	2.20046	0.786739
60934	1.96242	0.737863
60935	1.89226	0.732211
60936	1.89226	0.743625
60937	1.89226	0.636796
60938	1.89226	0.766075
60939	1.89226	0.748194
60940	3.78452	0.713008
60941	1.89226	0.779588
60942	1.89226	0.729383
60943	1.89226	0.861856
60944	1.89226	0.784153
60945	3.78452	0.801436
60946	1.17403	0.773739
60947	1.13531	0.799308
60948	0.6401	0.801602
60949	0.714566	0.810646
60950	0.915987	0.817882
60951	0.915987	0.664612
60952	0.933891	0.844209
60953	0.933891	0.801953
60954	1.17403	0.835269
60955	0.714566	0.807047

60956	0.6401	0.810017
60957	1.13531	0.778699
60958	0.946133	0.740577
60959	0.946133	0.762366
60960	1.17403	0.734701
60961	1.13531	0.74136
60962	0.946131	0.745957
60963	0.946131	0.661777
60964	1.17403	0.779235
60965	1.17403	0.727535
60966	1.17403	0.746431
60967	0.94613	0.743885
60968	0.946131	0.735539
60969	1.13531	0.723853
60970	1.89226	0.671688
60971	1.89226	0.642459
60972	1.89226	0.58022
60973	1.89226	0.366502
60974	1.89226	0.762552
60975	1.89226	0.712004
60976	0.946131	0.709099
60977	0.946131	0.723361
60978	0.946131	0.750867
60979	0.946131	0.795666
60980	0.946129	0.747922
60981	0.946129	0.538003
60982	0.946133	0.852193
60983	0.946133	0.701536
60984	0.946131	0.745929
60985	0.946131	0.737062
60986	0.946131	0.68829
60987	0.946131	0.698745
60988	2.29413	2.1
60989	1.99332	2.1
60990	1.84774	2.1
60991	2.21582	2.1
60992	4.20915	2.1
60993	0.517359	2.1
60994	0.634469	2.1
60995	0.996661	2.1
60996	0.92387	2.1
60997	0.768098	2.1
60998	0.768098	2.1
60999	0.790242	2.1
61000	0.790242	2.1
61001	0.923871	2.1
61002	0.517359	2.1
61003	0.634469	2.1
61004	0.996661	2.1
61005	1.89226	2.1
61006	1.89226	2.1
61007	1.89226	2.1
61008	1.89226	2.1
61009	3.78453	2.1
61010	0.946132	2.1
61011	0.946132	2.1
61012	0.946131	2.1
61013	0.946131	2.1

61014	0.946132	2.1
61015	0.946132	2.1
61016	0.946131	2.1
61017	0.946131	2.1
61018	0.946132	2.1
61019	0.946132	2.1
61020	0.946131	2.1
61021	0.946131	2.1
61022	1.62708	2.1
61023	2.21582	2.1
61024	2.13925	2.1
61025	2.21582	2.1
61026	1.77117	2.1
61027	1.84774	2.1
61028	0.946132	2.1
61029	0.946132	2.1
61030	0.946131	2.1
61031	0.946131	2.1
61032	0.946132	2.1
61033	0.946132	2.1
61034	0.946131	2.1
61035	0.946131	2.1
61036	0.946131	2.1
61037	0.946131	2.1
61038	0.946131	2.1
61039	0.946131	2.1
61040	1.89226	1.25955
61041	1.89226	0.941714
61042	1.89226	0.934283
61043	1.89226	1.07646
61044	3.78453	1.12064
61045	1.89226	0.93696
61046	1.89226	1.17335
61047	1.89226	1.09472
61048	1.89226	1.40661
61049	1.89226	0.896841
61050	1.89226	1.077
61051	1.89226	0.934163
61052	1.89226	1.34091
61053	1.89226	0.892635
61054	1.89226	1.23321
61055	1.89226	0.932189
61056	1.89226	1.4183
61057	0.914369	1.12782
61058	0.914369	1.18019
61059	0.810945	2.01375
61060	0.810945	1.14857
61061	2.26581	2.1
61062	1.66199	1.87295
61063	1.49503	1.2261
61064	1.28816	2.1
61065	1.08133	1.35021
61066	0.810933	1.36538
61067	1.1212	1.37539
61068	1.89227	0.7456
61069	1.89227	0.704588
61070	1.89227	0.708208
61071	1.89227	0.775766

61072	3.78453	0.718334
61073	1.89226	0.737493
61074	1.89226	0.815583
61075	1.89226	0.803421
61076	1.89226	0.752223
61077	3.78453	0.782295
61078	1.89226	0.818886
61079	1.89226	0.751397
61080	1.89226	0.755356
61081	1.89226	0.848433
61082	3.78453	0.778243
61083	1.89226	0.705923
61084	1.89226	0.815451
61085	1.89226	0.779499
61086	2.25318	0.72831
61087	3.78453	0.778678
61088	1.89226	0.723104
61089	1.89226	0.821726
61090	1.89226	0.769978
61091	1.9637	0.7434
61092	3.78453	0.785951
61093	1.89226	0.812987
61094	1.89226	0.774079
61095	1.89226	0.75275
61096	1.89226	0.836147
61097	3.78453	0.78093
61098	1.89226	0.778839
61099	1.89226	0.881648
61100	1.89226	0.837361
61101	2.53384	0.802742
61102	3.78453	0.84457
61103	1.55405	0.735138
61104	1.03818	1.05933
61105	1.03818	0.751702
61106	1.34857	0.793454
61107	1.46338	0.743751
61108	1.77377	0.906455
61109	1.86273	0.753034
61110	1.73802	0.788212
61111	1.50969	1.25249
61112	1.46337	0.871934
61113	3.20139	0.816556
61114	1.2377	0.897801
61115	1.46337	0.748008
61116	1.27408	0.818146
61117	1.32039	1.07321
61118	1.50969	0.872716
61119	1.50969	0.776956
61120	1.89226	2.1
61121	1.89226	2.1
61122	1.89226	2.1
61123	1.89226	2.1
61124	1.89226	2.1
61125	1.89226	1.15697
61126	1.89169	2.1
61127	1.89226	2.1
61128	1.89226	2.1
61129	1.89226	2.1

61130	1.96624	2.1
61131	1.96624	2.1
61132	2.62871	2.1
61133	1.6201	2.1
61134	1.49149	2.1
61135	1.6201	2.1
61136	2.62559	2.1
61137	2.75419	1.57775
61138	0.946129	0.997589
61139	0.946129	2.05614
61140	0.946133	2.1
61141	0.946133	2.1
61142	0.946131	2.1
61143	0.946131	0.967338
61144	0.946131	2.1
61145	0.946131	2.1
61146	0.946131	2.05705
61147	0.946131	2.09022
61148	0.946131	2.1
61149	0.946131	2.05005
61150	0.851543	2.1
61151	0.851543	2.1
61152	1.04043	2.1
61153	0.997939	2.1
61154	0.946132	2.1
61155	0.946132	1.81346
61156	0.894185	2.1
61157	0.893901	2.1
61158	0.998267	2.1
61159	1.04074	2.1
61160	0.85127	2.1
61161	0.85153	2.1
61162	1.89226	2.1
61163	1.89226	2.1
61164	1.89226	2.1
61165	1.89226	2.1
61166	1.89226	2.1
61167	1.89226	2.1
61168	0.928691	2.1
61169	0.928691	2.1
61170	0.935332	2.1
61171	0.935332	2.1
61172	1.398	2.1
61173	1.14418	2.1
61174	0.479331	2.1
61175	0.719848	2.1
61176	1.398	2.1
61177	0.719848	2.1
61178	1.14417	2.1
61179	0.47933	2.1
61180	2.28835	2.1
61181	1.89226	2.1
61182	1.89226	2.1
61183	1.89226	2.1
61184	2.796	2.1
61185	2.796	2.1
61186	0.946131	2.1
61187	0.946131	2.1

61188	0.946131	2.1
61189	0.946131	1.94505
61190	0.946128	1.97016
61191	0.946128	2.1
61192	0.946133	2.1
61193	0.946133	2.1
61194	0.946131	2.1
61195	0.946131	2.1
61196	0.94613	2.1
61197	0.94613	2.1
61198	0.946131	2.1
61199	0.946131	2.1
61200	0.946131	2.1
61201	0.946131	2.04053
61202	0.946131	2.0585
61203	0.946131	2.1
61204	0.946131	2.1
61205	0.946131	2.1
61206	0.946131	2.1
61207	0.946131	2.1
61208	0.946131	2.1
61209	0.946131	2.1
61210	1.89226	2.1
61211	1.89226	1.64922
61212	1.89226	2.08445
61213	1.89226	2.1
61214	3.78452	2.1
61215	2.05207	0.784149
61216	1.70302	0.89287
61217	1.33794	1.25714
61218	1.98194	0.852183
61219	3.36873	0.900378
61220	2.62761	0.770925
61221	1.98194	0.846182
61222	2.28009	1.04083
61223	2.28009	0.764966
61224	2.22939	0.889239
61225	2.22939	0.754511
61226	1.89226	0.85331
61227	1.89226	0.762334
61228	1.89226	0.802382
61229	1.89226	0.764514
61230	1.89226	0.834222
61231	1.89226	0.807012
61232	1.89227	0.857734
61233	1.89227	0.870266
61234	1.89227	1.15199
61235	1.89227	0.955999
61236	1.89227	0.832129
61237	1.89227	0.741707
61238	0.888355	0.778829
61239	0.990973	0.897291
61240	0.71133	0.914001
61241	0.634324	1.00231
61242	0.806209	0.954237
61243	0.806209	0.73249
61244	0.819087	1.13214
61245	0.819087	0.979967

61246	0.888355	0.990787
61247	0.634324	0.978471
61248	0.71133	0.893792
61249	0.990972	0.880675
61250	1.11469	0.869546
61251	0.985023	0.842213
61252	0.888354	0.778563
61253	0.990972	0.917795
61254	0.994754	0.857749
61255	0.994754	0.807669
61256	1.0083	0.96961
61257	1.0083	0.761533
61258	0.888354	0.79343
61259	0.985024	0.839546
61260	0.990971	0.778139
61261	1.11469	0.734398
61262	0.946132	0.891076
61263	0.946132	0.793541
61264	0.946132	0.798467
61265	0.946132	0.78159
61266	0.946132	0.803318
61267	0.946132	0.76722
61268	0.946133	1.00864
61269	0.946133	0.815728
61270	0.946133	0.833795
61271	0.946133	0.833393
61272	0.946132	0.76463
61273	0.946132	0.767789
61274	0.810944	0.821685
61275	0.810944	0.771277
61276	0.810947	0.897002
61277	0.810947	0.721036
61278	1.89227	1.07043
61279	1.35168	0.883615
61280	1.08132	0.880402
61281	1.08131	0.892242
61282	1.08132	0.775432
61283	0.810959	0.766201
61284	0.810959	0.779877
61285	1.70303	2.1
61286	2.03975	2.1
61287	1.99324	2.1
61288	2.17615	2.1
61289	1.40549	2.1
61290	1.53446	2.1
61291	0.946133	2.1
61292	0.946133	2.1
61293	0.946129	2.1
61294	0.946129	2.1
61295	0.946131	2.1
61296	0.946131	1.33102
61297	0.946131	2.1
61298	0.946131	2.1
61299	0.946131	2.1
61300	0.946131	2.1
61301	0.946131	2.1
61302	0.946131	2.1
61303	1.89226	2.1

61304	1.89226	1.79949
61305	1.89226	2.1
61306	1.89226	1.61488
61307	1.89226	2.1
61308	1.89226	2.01379
61309	2.17615	2.1
61310	2.3111	2.1
61311	2.50288	2.1
61312	2.50288	2.1
61313	2.03975	2.1
61314	2.03975	2.1
61315	1.76401	2.1
61316	1.95852	2.1
61317	2.32134	2.1
61318	2.12306	2.1
61319	1.8513	2.1
61320	1.70303	2.1
61321	1.89226	2.1
61322	1.89226	2.1
61323	1.89226	2.1
61324	1.89226	2.01955
61325	3.78452	2.1
61326	1.89226	2.1
61327	1.89226	1.04089
61328	1.89226	2.00448
61329	1.89226	2.1
61330	3.78452	2.1
61331	1.2243	2.1
61332	1.2243	2.1
61333	1.24189	2.1
61334	1.24189	2.1
61335	1.37811	2.1
61336	1.25144	2.1
61337	1.08807	2.1
61338	1.17949	2.1
61339	1.17949	2.1
61340	1.37811	2.1
61341	1.08807	2.1
61342	1.25144	2.1
61343	1.89226	2.1
61344	1.89226	2.1
61345	1.89226	2.1
61346	1.89226	2.1
61347	1.89226	2.1
61348	1.89226	2.1
61349	1.89227	2.1
61350	1.89227	2.1
61351	1.89227	2.1
61352	1.89227	2.1
61353	1.89227	2.1
61354	1.89227	2.1
61355	1.89224	2.1
61356	1.89224	2.1
61357	1.89224	2.1
61358	1.89224	2.1
61359	3.78447	2.1
61360	1.89224	2.1
61361	1.89224	2.1

61362	1.89224	2.1
61363	1.89224	2.1
61364	1.89224	2.1
61365	1.89224	2.1
61366	1.89224	2.1
61367	1.89224	2.1
61368	1.89224	2.1
61369	1.89224	2.1
61370	3.78448	2.1
61371	1.89224	2.1
61372	1.703 2.1	
61373	1.89224	2.1
61374	1.72991	2.1
61375	1.07643	2.1
61376	1.36657	2.1
61377	1.44472	2.1
61378	2.41233	2.1
61379	2.51628	2.1
61380	2.51628	2.1
61381	1.42174	2.1
61382	1.42174	2.1
61383	1.89226	2.1
61384	1.89226	2.1
61385	1.89226	2.1
61386	1.89226	2.1
61387	1.89226	2.1
61388	1.89226	2.1
61389	1.89226	2.1
61390	1.89226	2.1
61391	1.89226	2.1
61392	1.89226	1.11562
61393	3.78452	2.1
61394	2.51628	2.1
61395	1.89226	2.1
61396	1.89226	2.1
61397	1.89226	2.1
61398	2.41234	2.1
61399	2.41234	2.1
61400	0.996254	2.1
61401	0.996254	2.1
61402	1.01153	2.1
61403	1.01153	2.1
61404	1.28542	2.1
61405	1.25814	2.1
61406	0.719184	2.1
61407	0.72236	2.1
61408	0.719183	2.1
61409	1.28542	2.1
61410	0.722359	2.1
61411	1.25814	2.1
61412	0.946126	2.1
61413	0.946126	2.1
61414	0.946131	2.1
61415	0.946131	2.1
61416	0.946127	2.1
61417	0.946127	2.1
61418	0.94613	2.1
61419	0.94613	2.1

61420	0.946128	2.1
61421	0.946128	2.1
61422	0.946128	2.1
61423	0.946128	2.1
61424	1.89226	2.1
61425	1.89226	1.24746
61426	1.89226	2.1
61427	1.89226	2.1
61428	1.89226	2.01197
61429	1.89226	2.1
61430	0.946132	2.1
61431	0.946132	2.1
61432	1.25814	2.1
61433	1.25814	2.1
61434	0.946131	2.1
61435	0.946131	1.97105
61436	1.28543	2.1
61437	1.25814	2.1
61438	1.28543	2.1
61439	0.946132	2.1
61440	1.25814	2.1
61441	0.946132	2.1
61442	1.89226	1.34513
61443	1.89226	0.948573
61444	1.89226	1.29502
61445	1.89226	1.15621
61446	1.89226	1.05268
61447	1.89226	0.96558
61448	1.89226	1.28514
61449	2.27719	0.969174
61450	1.89226	0.94803
61451	1.89226	1.08518
61452	3.78452	1.04583
61453	1.89225	1.37155
61454	1.89225	0.967087
61455	1.89225	1.04232
61456	1.89225	1.18987
61457	3.78451	1.1555
61458	1.89226	0.878395
61459	1.89226	0.970132
61460	1.89226	1.0438
61461	1.89226	0.814898
61462	3.78452	0.899149
61463	0.946132	0.799053
61464	0.946132	0.901112
61465	0.946132	0.968558
61466	0.946132	0.9026
61467	0.946131	0.865592
61468	0.946131	0.81893
61469	0.946132	0.966019
61470	0.946132	0.813781
61471	0.946132	0.840739
61472	0.946132	0.858889
61473	0.946132	0.868517
61474	0.946132	0.825502
61475	0.946126	0.928406
61476	0.946126	0.87478
61477	0.946132	1.34982

61478	0.946132	1.03757
61479	0.946128	0.904835
61480	0.946128	0.996339
61481	0.946129	0.965622
61482	0.946129	1.17817
61483	0.946129	1.04881
61484	0.946129	1.10503
61485	0.946129	0.967742
61486	0.946129	0.973263
61487	1.39858	2.1
61488	1.39858	2.1
61489	0.94613	2.1
61490	0.94613	2.1
61491	0.946132	2.1
61492	0.946132	2.1
61493	1.39858	2.1
61494	1.25002	2.1
61495	1.39858	2.1
61496	0.946131	2.1
61497	1.25002	2.1
61498	0.946131	2.1
61499	2.0815	2.1
61500	1.89226	2.1
61501	1.89226	2.1
61502	2.05298	2.1
61503	2.5593	2.1
61504	2.77667	2.1
61505	0.946133	2.1
61506	0.946133	2.1
61507	0.946132	2.1
61508	0.946132	2.1
61509	0.946132	2.1
61510	0.946132	2.1
61511	0.946133	2.1
61512	0.946133	2.1
61513	0.946132	2.1
61514	0.946132	2.1
61515	0.946132	2.1
61516	0.946132	2.1
61517	1.38908	2.1
61518	1.38908	2.1
61519	0.946132	2.1
61520	0.946132	2.1
61521	0.946132	2.1
61522	0.946132	2.1
61523	1.38908	2.1
61524	1.27965	2.1
61525	1.38908	2.1
61526	0.946132	2.1
61527	0.946133	2.1
61528	1.27965	2.1
61529	0.946133	2.1
61530	0.946133	2.1
61531	0.946132	2.1
61532	0.946132	2.1
61533	0.946132	2.1
61534	0.946132	2.1
61535	0.946133	2.1

61536	0.946133	2.1
61537	0.946132	2.1
61538	0.946132	2.1
61539	0.946133	2.1
61540	0.946133	2.1
61541	1.89227	0.908004
61542	1.89227	1.25627
61543	1.89227	0.877484
61544	1.89227	0.794879
61545	3.78453	0.88568
61546	1.33154	0.994023
61547	1.33154	1.14016
61548	0.946132	1.05514
61549	0.946132	0.923386
61550	1.19095	0.87762
61551	1.33154	1.24746
61552	0.946133	1.02672
61553	0.946133	1.06084
61554	0.946133	1.54327
61555	1.19095	0.911747
61556	0.946132	1.37209
61557	1.33154	0.991072
61558	1.0888	0.884015
61559	1.04745	0.897984
61560	0.946132	0.837782
61561	0.946132	0.855966
61562	1.04745	0.791876
61563	1.04745	1.27659
61564	0.946132	1.32294
61565	0.946132	0.792479
61566	1.04745	1.12128
61567	0.946133	0.801653
61568	0.946132	1.15471
61569	1.0888	0.886576
61570	1.89226	1.58479
61571	1.89226	0.78821
61572	1.89226	0.778936
61573	1.89226	0.871194
61574	1.89226	0.819272
61575	1.89226	1.01602
61576	1.89226	1.39584
61577	1.87592	0.810008
61578	1.87592	0.842497
61579	2.3819	0.944313
61580	1.89226	0.993657
61581	2.39825	1.36639
61582	1.89226	0.794811
61583	2.0949	0.994776
61584	1.88741	0.767396
61585	1.89226	0.770941
61586	1.89226	0.807591
61587	1.88741	1.089
61588	1.07508	1.13797
61589	1.63419	1.31473
61590	1.63419	1.18802
61591	1.09795	1.49845
61592	1.56137	1.13233
61593	1.02513	1.80594

61594	0.967672	0.962814
61595	1.56137	1.07415
61596	1.56137	0.967523
61597	1.07508	1.09476
61598	1.41319	0.937239
61599	0.926902	1.1847
61600	1.56137	1.11682
61601	1.63419	1.163
61602	2.29852	1.40759
61603	2.14485	1.01702
61604	3.77904	1.0223
61605	1.41319	0.882856
61606	1.92622	0.870711
61607	1.56137	0.848832
61608	1.56137	1.11061
61609	2.14485	1.01955
61610	1.92622	0.935615
61611	1.89227	2.1
61612	1.89227	2.1
61613	1.89227	2.1
61614	1.89227	2.1
61615	3.78453	2.1
61616	2.56618	2.1
61617	2.08612	2.1
61618	2.48048	2.1
61619	1.99644	2.1
61620	4.5666	2.1
61621	2.08612	2.1
61622	1.30659	2.1
61623	1.99644	2.1
61624	1.25404	2.1
61625	3.34016	2.1
61626	1.89226	2.1
61627	1.89226	2.1
61628	1.89226	2.1
61629	1.89226	2.1
61630	1.89226	2.1
61631	1.89226	2.1
61632	1.89227	2.1
61633	1.89227	2.1
61634	1.89227	2.1
61635	1.89227	2.1
61636	3.78453	2.1
61637	2.48048	2.1
61638	1.99644	2.1
61639	2.39022	2.1
61640	1.90734	2.1
61641	4.38782	2.1
61642	1.90734	2.1
61643	1.99644	2.1
61644	1.21564	2.1
61645	1.21564	2.1
61646	1.25404	2.1
61647	1.94574	2.1
61648	0.810946	2.1
61649	0.810946	2.1
61650	0.810947	2.1
61651	0.810947	2.1

61652	1.89226	2.1
61653	1.3517	2.1
61654	1.08133	2.1
61655	1.08132	2.1
61656	1.08132	2.1
61657	0.810934	2.1
61658	0.810934	2.1
61659	1.89226	2.1
61660	1.89226	0.972941
61661	1.89226	1.32591
61662	1.89226	2.1
61663	3.78453	2.02648
61664	1.89226	0.762117
61665	1.89226	0.556743
61666	1.89226	1.2839
61667	1.89226	0.809975
61668	1.89226	1.08352
61669	1.89226	0.472138
61670	0.946131	0.863663
61671	0.946131	0.809477
61672	0.94613	0.985182
61673	0.94613	0.898152
61674	0.946128	0.838637
61675	0.946128	0.796261
61676	0.946133	0.532738
61677	0.946133	1.07492
61678	0.946131	0.952725
61679	0.946131	0.900725
61680	0.946131	0.958829
61681	0.946131	0.862103
61682	1.89226	2.1
61683	1.89226	0.923943
61684	1.89226	1.23767
61685	1.89226	1.12313
61686	1.89226	1.10618
61687	1.89226	1.02629
61688	2.56348	1.02839
61689	1.89226	1.53375
61690	1.89226	2.1
61691	2.53234	1.26339
61692	4.45574	1.06953
61693	2.53234	0.503674
61694	1.65583	0.314855
61695	2.56348	0.445037
61696	2.56348	0.674571
61697	1.66295	0.286582
61698	1.65583	0.270601
61699	0.736602	0.636272
61700	0.827916	0.532344
61701	1.26617	0.589926
61702	1.18322	0.369006
61703	0.7366	0.527439
61704	1.18321	0.537347
61705	1.26617	0.446111
61706	0.827918	0.495076
61707	0.99929	0.457865
61708	0.99929	0.523532
61709	1.00348	0.551633

61710	1.00348	0.613648
61711	1.26617	1.07701
61712	1.18322	0.958382
61713	0.946131	2.1
61714	0.946131	0.987251
61715	0.946129	0.982262
61716	1.18321	0.918723
61717	0.946133	1.95463
61718	1.26617	1.3833
61719	1.26617	1.39845
61720	1.26617	0.566809
61721	0.946131	1.49342
61722	0.946131	1.08824
61723	2.23267	0.613008
61724	1.89226	0.701603
61725	2.07099	0.67707
61726	1.89226	0.692602
61727	2.07099	0.688255
61728	1.89226	0.696699
61729	0.946132	0.722336
61730	1.35403	0.690715
61731	1.24038	0.674831
61732	0.946131	0.692261
61733	0.946131	0.713877
61734	0.946131	0.715904
61735	1.24039	0.676916
61736	1.35403	0.687308
61737	1.24038	0.714649
61738	1.24038	0.724654
61739	0.946131	0.687584
61740	0.946131	0.731907
61741	1.89226	0.74583
61742	1.89226	0.847704
61743	1.89226	0.732801
61744	1.89226	0.726615
61745	1.89226	0.916007
61746	1.89226	0.894337
61747	1.89226	0.711791
61748	1.89226	0.815036
61749	1.89226	0.751276
61750	1.89226	0.779296
61751	3.78452	0.803297
61752	1.56971	0.659931
61753	2.80086	0.756826
61754	2.59806	0.705106
61755	1.93489	0.703051
61756	2.59806	0.781793
61757	1.93489	0.743382
61758	0.827467	0.688146
61759	1.26652	0.701867
61760	1.13679	0.702382
61761	0.639035	0.700376
61762	1.11547	0.724891
61763	1.30656	0.716505
61764	0.869062	0.691724
61765	0.696608	0.691098
61766	1.07205	0.699004
61767	0.953656	0.730322

61768	0.970744	0.701682
61769	0.970744	0.710928
61770	2.38681	2.1
61771	2.50438	1.19641
61772	2.42082	1.84228
61773	2.59106	2.1
61774	4.92521	2.05395
61775	2.50438	1.14556
61776	2.40627	2.1
61777	2.59106	2.1
61778	2.5444	1.03591
61779	5.04879	2.1
61780	2.42082	2.1
61781	2.70575	2.1
61782	2.64778	2.1
61783	2.59106	1.66504
61784	2.47753	2.1
61785	2.42082	1.12828
61786	2.70575	2.1
61787	2.5444	1.23752
61788	2.76095	2.1
61789	2.59106	1.68878
61790	2.71429	2.1
61791	2.5444	1.34289
61792	1.89226	2.1
61793	2.38473	2.1
61794	2.36689	2.1
61795	2.38473	2.1
61796	1.87442	2.1
61797	1.89226	2.1
61798	1.89226	2.1
61799	1.89226	0.895605
61800	1.89226	1.72942
61801	1.89226	0.889166
61802	1.89226	2.1
61803	1.89226	0.915832
61804	2.14952	1.88751
61805	1.89226	0.834852
61806	1.89226	1.80572
61807	1.89226	0.824475
61808	2.0692	2.1
61809	2.0692	0.934951
61810	1.89226	2.1
61811	2.36689	2.1
61812	2.36204	2.1
61813	2.36689	2.1
61814	1.88742	2.1
61815	1.89226	2.1
61816	0.946132	0.862618
61817	0.946132	2.1
61818	0.946131	2.1
61819	0.946131	1.96272
61820	0.946132	1.09823
61821	0.946132	1.932
61822	0.946131	2.1
61823	0.946131	2.1
61824	0.946132	2.1
61825	0.946132	2.1

61826	0.946131	2.1
61827	0.946131	2.1
61828	0.946128	2.1
61829	0.946128	2.1
61830	1.07476	1.13502
61831	1.07476	2.1
61832	0.946131	1.19984
61833	0.946131	1.90698
61834	1.13182	2.1
61835	1.07476	2.1
61836	1.13182	1.9338
61837	0.946131	2.01366
61838	1.07476	1.9091
61839	0.94613	0.974099
61840	1.89227	0.674203
61841	1.89227	0.688797
61842	1.89227	0.688664
61843	1.89227	0.684859
61844	3.78453	0.681376
61845	1.89226	0.683983
61846	1.89226	0.689428
61847	1.89226	0.675873
61848	1.89226	0.690923
61849	3.78452	0.68179
61850	1.89226	0.687698
61851	1.89226	0.699499
61852	1.89226	0.691466
61853	1.89226	0.691945
61854	3.78452	0.690084
61855	1.89226	0.688543
61856	1.89226	0.69974
61857	1.89226	0.699289
61858	1.89226	0.700672
61859	1.89226	0.68788
61860	1.89226	0.688667
61861	2.58344	0.724103
61862	2.00267	0.685219
61863	2.14218	0.698341
61864	2.22674	0.795269
61865	2.14218	0.66542
61866	2.22674	0.685861
61867	2.83531	0.933672
61868	1.56043	0.692939
61869	2.22674	0.857637
61870	1.90912	1.26405
61871	2.22674	0.676762
61872	1.90912	0.689127
61873	0.780217	0.698835
61874	1.11337	0.706528
61875	1.00133	0.705706
61876	0.804472	0.700666
61877	0.804472	0.735518
61878	0.780217	0.701114
61879	1.00133	0.68195
61880	1.11337	0.701685
61881	0.856668	0.713512
61882	0.856668	1.11516
61883	0.924883	0.700476

61884	0.924883	0.686896
61885	1.93107	2.1
61886	1.68657	2.1
61887	1.7237	2.1
61888	1.89226	2.1
61889	1.53436	2.1
61890	1.68657	2.1
61891	1.89226	2.1
61892	1.89226	2.1
61893	1.89226	2.1
61894	1.89226	2.1
61895	3.78453	2.1
61896	0.946131	2.1
61897	0.946131	2.1
61898	0.855092	2.1
61899	0.83953	2.1
61900	0.946131	2.1
61901	0.946131	2.1
61902	0.839531	2.1
61903	0.839531	2.1
61904	0.946131	2.1
61905	0.855093	2.1
61906	0.839531	2.1
61907	0.946131	2.1
61908	1.89226	2.1
61909	1.70302	2.1
61910	2.21419	2.1
61911	1.93107	2.1
61912	1.97872	2.1
61913	1.70302	2.1
61914	0.946133	2.1
61915	0.946133	2.1
61916	0.946132	2.1
61917	0.946132	2.1
61918	0.946132	2.1
61919	0.946132	2.1
61920	0.946132	2.1
61921	0.946132	2.1
61922	0.946133	2.1
61923	0.946133	2.1
61924	0.946132	2.1
61925	0.946132	2.1
61926	1.90081	2.1
61927	1.89226	2.1
61928	1.51379	2.1
61929	1.36813	2.1
61930	1.71019	2.1
61931	1.89226	2.1
61932	1.89227	0.974242
61933	1.89227	0.805756
61934	1.89227	0.789499
61935	1.89227	0.917768
61936	1.89227	0.96371
61937	1.89227	1.53737
61938	1.36428	1.02234
61939	1.37869	0.964967
61940	0.946132	0.866889
61941	0.946132	0.954498

61942	1.37869	0.864495
61943	1.37869	1.75056
61944	0.946133	1.01506
61945	0.946133	1.06448
61946	1.37869	0.867982
61947	0.946132	0.922277
61948	1.36428	1.04541
61949	0.946133	0.817571
61950	0.94612	0.92176
61951	0.94612	0.893164
61952	0.946119	0.870784
61953	0.946119	0.905757
61954	0.946119	0.798806
61955	0.946119	1.41938
61956	0.94612	0.949334
61957	0.94612	1.24442
61958	0.946132	0.859683
61959	0.946132	0.807608
61960	0.946107	0.825331
61961	0.946107	1.02242
61962	1.89225	0.05
61963	1.89225	2.1
61964	1.63564	0.05
61965	1.42278	2.1
61966	1.42278	2.1
61967	1.57534	0.05
61968	1.53735	2.1
61969	1.68991	0.05
61970	1.57535	0.05
61971	1.04442	2.1
61972	1.04442	2.1
61973	1.22031	0.05
61974	1.42278	2.1
61975	1.59868	0.05
61976	0.946132	2.1
61977	0.946132	2.1
61978	0.946131	2.1
61979	0.946131	2.1
61980	0.946131	2.1
61981	0.946131	2.1
61982	0.946132	2.1
61983	0.946132	2.1
61984	0.946132	2.1
61985	0.946132	2.1
61986	0.946131	2.1
61987	0.946131	2.1
61988	1.89226	2.1
61989	1.89226	2.1
61990	1.89226	2.1
61991	1.89226	2.1
61992	1.89226	2.1
61993	1.89226	2.1
61994	2.03143	0.910514
61995	1.47111	1.25514
61996	2.28685	1.24065
61997	2.28685	1.14542
61998	1.36006	0.970258
61999	1.36006	0.973226

62000	1.89226	0.840431
62001	1.89226	1.1235
62002	1.89226	1.0444
62003	1.89226	1.06577
62004	1.89226	0.856268
62005	1.89226	0.90445
62006	1.89226	1.04393
62007	1.89226	2.1
62008	1.89226	2.1
62009	1.89226	2.1
62010	1.89226	1.08919
62011	1.89226	1.55828
62012	0.902821	2.1
62013	0.902821	1.6397
62014	0.889218	2.1
62015	0.889218	2.1
62016	0.675759	2.1
62017	1.05649	2.1
62018	0.735554	2.05217
62019	1.14342	1.95662
62020	1.14343	1.18907
62021	1.05649	2.1
62022	0.735554	2.1
62023	0.675758	2.1
62024	2.46286	0.772975
62025	2.28685	0.893734
62026	2.67843	0.86768
62027	2.67843	0.838992
62028	2.03143	0.806242
62029	2.03143	0.795964
62030	1.89226	0.794873
62031	1.89226	0.849371
62032	1.89226	0.788006
62033	1.89226	0.784353
62034	3.78452	0.846892
62035	0.94613	0.970763
62036	0.94613	0.961645
62037	0.946128	1.25196
62038	0.946128	0.9827
62039	0.94613	2.1
62040	0.94613	0.980586
62041	0.946128	0.831878
62042	0.946128	0.936442
62043	0.946129	0.745968
62044	0.946129	1.0277
62045	0.946129	0.843674
62046	0.946129	1.84539
62047	2.67843	0.914422
62048	2.11297	1.32232
62049	2.52453	1.19338
62050	2.52453	1.38645
62051	2.28685	1.02391
62052	2.28685	0.971538
62053	1.89226	0.747464
62054	1.89226	0.880439
62055	1.89226	0.740405
62056	1.89226	0.886522
62057	3.78451	0.793029

62058	2.39524	0.730841
62059	2.10188	0.865541
62060	2.29839	0.709724
62061	2.02242	0.895072
62062	4.40028	0.783341
62063	1.71839	2.1
62064	1.65261	2.1
62065	2.10189	0.863428
62066	2.10189	2.1
62067	2.02243	0.881802
62068	1.63893	0.867799
62069	0.946132	2.1
62070	0.946132	0.892051
62071	0.946129	0.873163
62072	0.946129	2.1
62073	0.94613	2.1
62074	0.94613	2.1
62075	0.946131	0.858985
62076	0.946131	0.867641
62077	0.946131	1.41768
62078	0.946131	1.42888
62079	0.946131	1.72843
62080	0.946131	1.79905
62081	1.89226	0.734699
62082	1.89226	0.934347
62083	1.89226	0.666017
62084	1.89226	0.835928
62085	3.78452	0.778062
62086	2.2984	0.728698
62087	2.02242	0.848498
62088	2.21582	0.257977
62089	1.94412	0.834855
62090	4.24251	0.786358
62091	1.94412	0.840463
62092	1.5854	2.1
62093	2.02243	2.1
62094	2.02243	0.874778
62095	1.57431	0.853653
62096	1.65261	2.1
62097	0.810943	0.876349
62098	0.810943	2.1
62099	0.810945	2.1
62100	0.810945	2.1
62101	1.89226	0.842919
62102	1.3517	1.07232
62103	1.08133	1.68803
62104	1.08132	2.1
62105	1.08132	0.860136
62106	0.810933	1.75556
62107	0.810933	1.48298
62108	1.89226	0.29794
62109	1.89226	0.586653
62110	1.89226	0.490006
62111	1.89226	0.451727
62112	1.89226	0.411086
62113	1.89226	0.377037
62114	1.89226	0.484864
62115	1.89226	0.698515

62116	1.89226	0.599916
62117	1.89226	0.689929
62118	3.78452	0.694277
62119	1.89226	0.434041
62120	1.89226	0.664664
62121	1.89226	0.594795
62122	1.89226	0.572842
62123	1.89226	0.522074
62124	1.89226	0.497002
62125	0.946128	0.64444
62126	0.946128	0.515936
62127	0.946132	0.508689
62128	0.946132	0.588088
62129	0.94613	0.693705
62130	0.94613	0.700604
62131	0.946129	0.577206
62132	0.946129	0.473721
62133	0.94613	0.498528
62134	0.94613	0.652503
62135	0.94613	0.527743
62136	0.94613	0.630461
62137	1.89226	0.339691
62138	1.89226	0.614941
62139	1.89226	0.461594
62140	1.89226	0.455616
62141	1.89226	0.628797
62142	1.89226	0.645455
62143	2.81478	2.1
62144	1.89226	2.1
62145	2.65557	2.1
62146	1.89226	2.1
62147	4.54783	2.1
62148	1.89226	2.1
62149	1.89226	2.1
62150	1.89226	2.1
62151	1.89226	2.1
62152	3.78452	2.1
62153	2.65557	2.1
62154	1.89226	2.1
62155	1.89226	2.1
62156	1.89226	2.1
62157	2.57997	2.1
62158	2.57997	2.1
62159	0.946131	2.1
62160	0.946131	2.1
62161	0.946128	2.1
62162	0.946128	2.1
62163	0.94613	2.1
62164	0.94613	2.1
62165	0.946129	2.1
62166	0.946129	2.1
62167	0.94613	2.1
62168	0.94613	2.1
62169	0.94613	2.1
62170	0.94613	2.1
62171	1.89226	1.12526
62172	2.27703	1.45081
62173	2.35615	1.51904

62174	2.35615	1.18876
62175	1.89226	1.45509
62176	1.89226	1.17916
62177	0.946132	1.09056
62178	1.17807	1.49241
62179	1.14926	1.03148
62180	0.946132	1.10891
62181	0.946133	1.10182
62182	0.946133	1.1834
62183	1.14926	1.12097
62184	1.14926	1.11285
62185	0.946132	1.1843
62186	0.946132	1.09094
62187	1.17807	1.27386
62188	1.14926	1.09401
62189	2.16841	1.0793
62190	1.35487	1.18376
62191	1.19854	1.0884
62192	1.19854	1.1542
62193	2.46078	1.10001
62194	2.46078	1.14764
62195	0.69308	1.18415
62196	1.3119	1.29028
62197	0.677433	1.25423
62198	1.23039	1.21771
62199	0.978178	1.26625
62200	0.978178	1.25639
62201	0.945294	1.23131
62202	0.945294	1.22421
62203	1.3119	1.21262
62204	1.23039	1.15557
62205	0.693081	1.15794
62206	0.677432	1.15997
62207	1.89226	1.01521
62208	1.89226	1.55366
62209	1.89226	1.35718
62210	1.89226	1.06096
62211	1.89226	1.28533
62212	1.89226	1.05058
62213	0.946132	0.9965
62214	0.946132	1.39629
62215	0.946132	0.930675
62216	0.946132	1.02138
62217	0.946133	1.01779
62218	0.946133	1.11984
62219	0.946132	1.01207
62220	0.946132	0.96447
62221	0.946133	1.03757
62222	0.946133	0.980314
62223	0.946132	1.07217
62224	0.946132	1.00926
62225	2.60637	1.83138
62226	1.89226	2.1
62227	1.89226	2.1
62228	1.89226	1.36674
62229	3.78452	2.1
62230	1.89226	1.0926
62231	1.8396	1.48

62232	1.89226	1.63786
62233	1.89226	1.08932
62234	1.60993	2.04772
62235	1.60993	1.13893
62236	0.946131	1.21769
62237	0.946131	1.44405
62238	0.946128	2.1
62239	0.946128	1.71191
62240	0.946131	1.20978
62241	0.946131	1.53966
62242	0.946127	2.1
62243	0.946127	1.39043
62244	0.946129	1.64966
62245	0.946129	1.63425
62246	0.94613	1.51854
62247	0.94613	1.51223
62248	1.89226	2.1
62249	1.89226	1.39317
62250	1.89226	2.1
62251	1.89226	1.98815
62252	1.89226	2.07368
62253	1.89226	1.93847
62254	1.89226	1.04547
62255	1.89226	1.3092
62256	1.89226	1.54675
62257	1.89226	1.00194
62258	1.89226	1.78917
62259	1.89226	1.00009
62260	0.946131	1.18504
62261	0.946131	1.44484
62262	0.946128	2.1
62263	0.946128	1.53087
62264	0.946131	1.17165
62265	0.946131	1.43241
62266	0.946127	1.43145
62267	0.946127	2.1
62268	0.94613	1.55763
62269	0.94613	1.56445
62270	0.946129	1.46725
62271	0.946129	1.48842
62272	0.946133	2.1
62273	0.946133	2.1
62274	1.28153	2.1
62275	1.26781	2.1
62276	0.946132	2.1
62277	0.946132	2.1
62278	1.28153	2.1
62279	1.28153	2.1
62280	0.946132	2.1
62281	1.28153	2.1
62282	0.946133	2.1
62283	1.26781	2.1
62284	1.89226	2.1
62285	1.89226	1.21688
62286	1.89226	2.1
62287	1.89226	2.1
62288	1.89226	2.1
62289	1.89226	2.05608

62290	2.5844	2.1
62291	1.09563	2.1
62292	1.1468	2.1
62293	1.09563	2.1
62294	2.6142	2.1
62295	2.56304	2.1
62296	0.946126	2.1
62297	0.946126	2.1
62298	1.2922	2.1
62299	1.28152	2.1
62300	0.946126	2.1
62301	0.946126	2.1
62302	1.2922	2.1
62303	1.2922	2.1
62304	1.29221	2.1
62305	0.946132	2.1
62306	0.946119	2.1
62307	1.28151	2.1
62308	1.89225	2.1
62309	1.89225	2.05212
62310	1.89225	2.1
62311	1.89225	2.1
62312	1.89225	2.1
62313	1.89225	2.1
62314	0.946121	1.67527
62315	0.946121	2.1
62316	0.946126	1.43917
62317	0.946126	2.1
62318	0.946124	1.12617
62319	0.946124	2.1
62320	0.946123	2.1
62321	0.946123	2.1
62322	0.94613	2.1
62323	0.94613	2.1
62324	0.946117	2.1
62325	0.946117	2.1
62326	1.89226	1.078
62327	1.89226	1.05474
62328	1.89226	0.985588
62329	1.89226	2.1
62330	1.89226	0.860318
62331	1.89226	2.1
62332	1.89226	1.50483
62333	1.89226	1.27991
62334	1.89226	0.934165
62335	1.89226	2.1
62336	1.89226	0.856863
62337	1.89226	2.1
62338	1.89226	1.74404
62339	2.81281	2.0356
62340	2.50369	1.03606
62341	2.50369	2.1
62342	1.89226	1.07225
62343	1.89226	2.1
62344	1.89226	2.1
62345	1.89226	2.1
62346	1.89226	2.07257
62347	1.89226	2.1

62348	1.89226	2.09282
62349	1.89226	2.1
62350	1.89226	2.1
62351	1.89226	2.1
62352	1.89226	2.1
62353	1.89226	2.1
62354	1.89226	2.1
62355	1.89226	2.1
62356	0.907036	2.1
62357	1.09714	2.1
62358	0.994609	2.1
62359	0.838808	2.1
62360	0.976536	2.1
62361	0.976536	2.1
62362	0.959412	2.1
62363	0.959412	2.1
62364	1.09714	2.1
62365	0.994609	2.1
62366	0.907036	2.1
62367	0.838808	2.1
62368	0.99461	2.1
62369	0.838808	2.1
62370	0.738212	2.1
62371	0.565634	2.1
62372	0.775921	2.1
62373	0.775921	2.1
62374	0.784322	2.1
62375	0.784322	2.1
62376	0.994609	2.1
62377	0.738212	2.1
62378	0.838808	2.1
62379	0.565635	2.1
62380	1.89226	2.1
62381	1.89226	2.1
62382	1.89226	1.85684
62383	1.89226	2.1
62384	1.89226	1.41894
62385	1.89226	2.1
62386	1.89226	2.1
62387	1.89226	2.1
62388	1.89226	2.1
62389	1.89226	2.1
62390	1.89226	2.1
62391	1.89226	2.1
62392	2.19428	2.1
62393	1.98922	2.1
62394	2.27322	2.1
62395	2.45893	2.1
62396	4.4675	2.1
62397	0.99461	2.1
62398	1.13661	2.1
62399	0.902272	2.1
62400	0.738212	2.1
62401	0.931874	2.1
62402	0.931874	2.1
62403	0.942953	2.1
62404	0.942953	2.1
62405	1.13658	2.1

62406	0.902248	2.1
62407	0.994637	2.1
62408	0.738233	2.1
62409	1.89226	1.54294
62410	1.89226	1.45556
62411	1.89226	1.85732
62412	1.89226	1.75029
62413	1.89226	1.57651
62414	1.89226	1.44369
62415	1.89227	1.83598
62416	2.58246	1.29074
62417	1.89227	1.47358
62418	1.89227	1.66494
62419	3.78453	1.41773
62420	1.89226	2.03216
62421	1.89226	1.60762
62422	1.65573	1.88015
62423	1.65573	1.93077
62424	1.89226	1.88226
62425	1.89226	1.94054
62426	1.89226	1.19023
62427	1.89226	1.3102
62428	1.89226	1.28588
62429	1.89226	1.28947
62430	1.89226	1.26553
62431	1.89226	1.25394
62432	1.89226	1.30643
62433	1.89226	1.3126
62434	1.89226	1.29617
62435	1.89226	1.3099
62436	1.89226	1.28665
62437	1.89226	1.33192
62438	0.946132	1.88836
62439	0.946132	1.80909
62440	1.01023	1.35888
62441	1.13651	1.76474
62442	1.01023	1.33824
62443	0.946133	1.59383
62444	1.0644	1.63372
62445	1.0644	1.87277
62446	0.946132	1.91661
62447	0.946132	1.71523
62448	1.13651	1.57105
62449	0.946132	1.61785
62450	1.89226	1.01109
62451	1.89226	1.64232
62452	1.89226	1.23869
62453	1.89226	0.961516
62454	3.78451	1.05225
62455	1.00934	0.883587
62456	0.741561	0.871632
62457	0.946131	1.01993
62458	0.946131	1.11497
62459	0.946131	1.34656
62460	0.741561	0.710758
62461	1.00934	0.796202
62462	0.946131	1.15794
62463	0.741586	0.929882

62464	0.741586	0.945961
62465	0.946106	1.02978
62466	0.946106	0.908815
62467	1.14895	1.05232
62468	0.957449	1.10435
62469	0.934806	1.36893
62470	0.934806	1.32356
62471	1.13536	0.967599
62472	1.02829	1.6686
62473	0.946129	1.70544
62474	0.946129	0.989491
62475	0.946127	1.22958
62476	0.946127	1.29799
62477	0.946127	1.2122
62478	1.0283	1.31231
62479	1.89226	1.48996
62480	1.89226	1.02186
62481	1.89226	0.968773
62482	1.89226	1.36528
62483	3.78452	1.21388
62484	1.89226	0.884541
62485	1.89226	1.13428
62486	1.89226	1.02514
62487	1.89226	0.8525
62488	3.78453	0.900742
62489	1.89226	1.12913
62490	2.69068	0.927085
62491	1.89226	0.853197
62492	1.89226	1.04469
62493	3.78452	1.00557
62494	1.89226	1.01833
62495	1.89226	1.51265
62496	1.89226	1.47019
62497	1.89226	1.12164
62498	1.89226	1.32994
62499	1.89226	1.03461
62500	1.89226	2.1
62501	1.89226	2.1
62502	1.89226	2.1
62503	1.89226	2.1
62504	1.89226	2.1
62505	1.89226	2.1
62506	1.89226	2.1
62507	1.89226	2.1
62508	1.89226	2.1
62509	1.89226	2.1
62510	1.89226	2.1
62511	1.89226	2.1
62512	0.985109	2.1
62513	1.31144	2.1
62514	1.45864	2.1
62515	1.10667	2.1
62516	2.44375	2.1
62517	0.655718	2.1
62518	0.729316	2.1
62519	0.684594	2.1
62520	0.782707	2.1
62521	0.700849	2.1

62522	0.700849	2.1
62523	0.71306	2.1
62524	0.71306	2.1
62525	0.729318	2.1
62526	0.782705	2.1
62527	0.684592	2.1
62528	0.655719	2.1
62529	0.946128	2.1
62530	0.946128	2.1
62531	0.946133	2.1
62532	0.946133	2.1
62533	0.946131	2.1
62534	0.946131	2.1
62535	0.946131	2.1
62536	0.946131	2.1
62537	0.94613	2.1
62538	0.94613	2.1
62539	0.946131	2.1
62540	0.946131	2.1
62541	1.89226	2.1
62542	1.89226	2.1
62543	1.89226	2.1
62544	1.89226	2.1
62545	1.89226	2.1
62546	1.89226	2.1
62547	0.553334	2.1
62548	0.629936	2.1
62549	0.828211	2.1
62550	0.729319	2.1
62551	0.674025	2.1
62552	0.674025	2.1
62553	0.68523	2.1
62554	0.68523	2.1
62555	0.828211	2.1
62556	0.629935	2.1
62557	0.72932	2.1
62558	0.553334	2.1
62559	0.828208	2.1
62560	0.729316	2.1
62561	0.907037	2.1
62562	0.782707	2.1
62563	0.799048	2.1
62564	0.799048	2.1
62565	0.811867	2.1
62566	0.811867	2.1
62567	0.828209	2.1
62568	0.907035	2.1
62569	0.729318	2.1
62570	0.782706	2.1
62571	2.32981	2.1
62572	1.89226	2.1
62573	1.89226	2.1
62574	1.89226	2.1
62575	2.2671	2.1
62576	2.2671	2.1
62577	1.89226	1.97072
62578	1.89226	2.02895
62579	1.89226	1.99338

62580	1.89226	1.44448
62581	1.89226	2.05124
62582	1.89226	1.89896
62583	1.3991	1.84166
62584	1.81793	1.80821
62585	2.07226	1.50151
62586	2.07226	1.45903
62587	1.24924	2.02388
62588	1.24924	2.09543
62589	0.946131	1.04806
62590	0.946131	2.1
62591	1.16491	2.1
62592	1.16491	2.1
62593	0.946131	2.1
62594	0.946131	1.26553
62595	1.16491	2.1
62596	1.19523	2.1
62597	0.946132	2.1
62598	1.19523	2.1
62599	1.16491	2.1
62600	0.946131	2.1
62601	1.89226	2.1
62602	1.89226	1.46043
62603	1.89226	2.1
62604	1.89226	0.950689
62605	3.78452	1.24254
62606	0.901204	1.54634
62607	0.901204	2.1
62608	0.902504	2.00744
62609	0.902504	2.1
62610	1.10416	1.68733
62611	1.03613	1.15939
62612	0.764922	2.1
62613	0.699552	2.1
62614	0.764921	1.54151
62615	1.10416	1.74854
62616	0.699551	1.77022
62617	1.03613	1.36503
62618	1.89226	0.971705
62619	1.89226	1.09932
62620	1.89226	1.09151
62621	1.89226	1.00497
62622	3.78453	1.0723
62623	1.89226	1.09097
62624	1.89226	1.19791
62625	1.89226	1.09984
62626	1.89226	1.26186
62627	1.89226	1.10702
62628	1.89226	1.26498
62629	0.946131	1.10805
62630	0.946131	1.09689
62631	0.946131	1.01817
62632	0.946131	1.00032
62633	0.946131	1.02221
62634	0.946131	1.10823
62635	0.946131	1.09908
62636	0.946131	0.974824
62637	0.946131	1.01499

62638	0.946131	1.01778
62639	0.946131	1.00286
62640	0.946131	1.02276
62641	1.89226	1.08607
62642	1.89226	1.19759
62643	1.89226	1.11108
62644	1.89226	1.19899
62645	1.89226	1.11016
62646	1.89226	1.19873
62647	0.946127	1.17642
62648	0.946127	1.15736
62649	1.22788	1.1165
62650	1.35469	1.1638
62651	1.35469	1.20689
62652	1.35469	1.17694
62653	0.946128	1.16186
62654	0.946128	1.08106
62655	0.946129	1.14228
62656	1.22788	1.17564
62657	1.35469	1.15386
62658	0.946129	1.11629
62659	1.22788	1.13872
62660	1.35469	1.18727
62661	1.02602	1.03302
62662	0.794861	1.07661
62663	1.15301	1.13941
62664	1.15301	1.18583
62665	1.10089	1.02729
62666	1.10089	1.11632
62667	0.794861	1.05775
62668	1.22788	1.13886
62669	1.02602	1.14093
62670	1.35469	1.13713
62671	1.89226	0.714592
62672	1.89226	0.80375
62673	1.89226	0.84306
62674	1.89226	0.740091
62675	3.78452	0.766652
62676	1.13536	1.45205
62677	0.942524	0.838944
62678	0.946131	0.809298
62679	0.946131	0.73601
62680	0.946127	0.839768
62681	0.946127	1.48797
62682	1.26282	0.735146
62683	0.917471	1.20134
62684	1.21023	0.890839
62685	1.00851	0.888366
62686	1.17955	0.830027
62687	0.883746	0.848811
62688	1.89226	0.823494
62689	1.89226	1.81482
62690	1.89226	0.935619
62691	1.89226	1.00899
62692	1.89226	0.959933
62693	1.89226	1.17291
62694	1.89226	0.745583
62695	1.89226	0.944964

62696	1.89226	0.793735
62697	1.89226	0.801906
62698	1.89226	0.808662
62699	1.89226	0.897078
62700	0.94613	0.866244
62701	0.94613	0.761968
62702	0.94613	0.701923
62703	0.94613	0.74215
62704	0.946128	0.763597
62705	0.946128	0.878541
62706	0.946131	0.867525
62707	0.946131	0.7
62708	0.94613	0.758885
62709	0.94613	0.794356
62710	0.94613	0.733037
62711	0.94613	0.739692
62712	0.883758	1.20234
62713	1.03041	0.944709
62714	0.946131	0.841844
62715	0.946131	0.764127
62716	0.851537	0.862743
62717	0.851537	1.21068
62718	1.23942	0.760484
62719	1.14906	1.19913
62720	0.851484	0.862985
62721	0.985518	0.824232
62722	1.0156	0.844127
62723	0.851526	0.849563
62724	1.89226	0.842938
62725	1.89226	1.99671
62726	1.89226	0.934171
62727	1.89226	1.04335
62728	1.89226	0.96429
62729	1.89226	1.20766
62730	1.89226	0.764963
62731	1.89226	0.946066
62732	1.89226	0.808119
62733	1.89226	0.836883
62734	1.89226	0.814201
62735	1.89226	0.91056
62736	1.89227	0.656115
62737	1.89227	0.676452
62738	1.89227	0.663076
62739	1.89227	0.681687
62740	3.78453	0.660942
62741	1.89226	0.672532
62742	1.89226	0.682334
62743	1.89226	0.670784
62744	1.89226	0.69199
62745	3.78452	0.683414
62746	0.946132	0.694839
62747	0.946132	0.695041
62748	0.946128	0.69553
62749	0.946128	0.695134
62750	0.94613	0.699759
62751	0.94613	0.696033
62752	0.94613	0.682611
62753	0.94613	0.68659

62754	0.946131	0.684518
62755	0.946131	0.695637
62756	0.94613	0.687239
62757	0.94613	0.697287
62758	1.89226	0.686231
62759	1.89226	0.697079
62760	1.89226	0.689857
62761	1.89226	0.686834
62762	1.89226	0.69709
62763	1.89226	0.695194
62764	1.98874	0.512376
62765	2.33455	0.669712
62766	2.01865	0.653694
62767	2.07024	0.639615
62768	4.32329	0.679767
62769	1.98874	0.658868
62770	2.00266	0.691626
62771	2.14218	0.70076
62772	2.07024	0.695609
62773	3.9914	0.672051
62774	1.00133	0.700321
62775	0.804471	0.696611
62776	1.03512	0.69614
62777	0.995176	0.6994
62778	0.80447	0.703542
62779	0.995178	0.7
62780	1.00133	0.69656
62781	1.03512	0.704406
62782	0.880587	0.705893
62783	0.880587	0.696996
62784	0.959002	0.689099
62785	0.959002	0.7
62786	2.33455	0.676522
62787	1.99036	0.699254
62788	2.07024	0.626371
62789	2.58645	0.697988
62790	4.32491	0.545053
62791	1.89226	0.834832
62792	1.89226	0.815741
62793	1.89226	0.854266
62794	1.89226	0.792311
62795	3.78453	0.792016
62796	1.89226	0.923895
62797	1.89226	0.824672
62798	1.89226	0.873489
62799	1.89226	0.856819
62800	3.78453	0.923954
62801	1.89226	0.802949
62802	1.89226	0.775114
62803	1.89226	0.784524
62804	1.89226	0.799384
62805	1.89226	0.782464
62806	1.89226	0.798757
62807	1.89226	0.794811
62808	2.1523	0.527359
62809	2.53257	0.557506
62810	2.53257	0.557093
62811	1.89226	0.788339

62812	1.89226	0.785686
62813	0.946133	0.780577
62814	0.946133	0.802346
62815	0.946132	0.756631
62816	0.946132	0.765615
62817	0.946132	0.755001
62818	0.946132	0.762385
62819	0.946132	0.802598
62820	0.946132	0.768472
62821	0.946133	0.788674
62822	0.946133	0.791561
62823	0.946132	0.811714
62824	0.946132	0.7858
62825	0.946132	0.823884
62826	0.946132	0.855173
62827	0.946132	0.793201
62828	0.946132	0.817319
62829	0.946132	0.792889
62830	0.946132	0.803206
62831	0.946132	0.855046
62832	0.946132	0.819942
62833	0.946132	0.827082
62834	0.946132	0.828076
62835	0.946131	0.82037
62836	0.946131	0.817815
62837	1.62772	0.700762
62838	1.70303	0.701667
62839	1.70303	0.702235
62840	1.70303	0.701348
62841	1.90116	0.701478
62842	1.90116	0.70094
62843	1.89226	0.700088
62844	1.89226	0.701043
62845	1.89226	0.700872
62846	1.89226	0.700906
62847	1.89226	0.700825
62848	1.89226	0.70041
62849	1.89226	0.701641
62850	1.89226	0.700708
62851	1.89226	0.700256
62852	1.89226	0.700494
62853	3.78452	0.700335
62854	1.89226	0.702896
62855	1.89226	0.700395
62856	1.89226	0.700504
62857	1.89226	0.701471
62858	1.89226	0.700549
62859	1.89226	0.701718
62860	0.946132	0.7
62861	0.946132	0.7
62862	0.946129	0.7
62863	0.946129	0.7
62864	0.94613	0.7
62865	0.94613	0.7
62866	0.946131	0.7
62867	0.946131	0.7
62868	0.94613	0.7
62869	0.94613	0.7

62870	0.946131	0.671467
62871	0.946131	0.700105
62872	1.89226	0.7
62873	1.89226	0.7
62874	1.89226	0.7
62875	1.89226	0.7
62876	1.89226	0.7
62877	1.89226	0.7
62878	1.89226	0.758635
62879	1.89226	0.867151
62880	1.89226	0.864574
62881	1.89226	0.747528
62882	3.78452	0.795574
62883	1.89226	0.849072
62884	1.89226	0.954627
62885	1.89226	0.833812
62886	1.89226	0.975283
62887	1.89226	0.836519
62888	1.89226	0.983704
62889	1.30165	0.825762
62890	0.946127	0.861743
62891	1.09659	0.74545
62892	0.946131	0.759298
62893	1.09659	0.749173
62894	1.09659	0.933922
62895	0.946128	0.928666
62896	0.946128	0.765488
62897	1.30165	0.800592
62898	1.09659	0.84989
62899	0.946129	0.809621
62900	0.946129	0.83311
62901	0.946128	0.869596
62902	0.946128	0.887006
62903	0.946133	0.764403
62904	0.946133	0.788437
62905	0.946131	0.767322
62906	0.946131	0.895579
62907	0.946131	0.886194
62908	0.946131	0.769898
62909	0.94613	0.799664
62910	0.94613	0.798799
62911	0.946131	0.788593
62912	0.946131	0.805714
62913	0.946133	0.970559
62914	0.946133	0.970785
62915	0.946132	0.859635
62916	0.946132	0.87673
62917	0.946132	0.857183
62918	0.946132	0.97697
62919	0.946132	0.973169
62920	0.946132	0.862666
62921	0.946132	0.902179
62922	0.946132	0.899541
62923	0.946133	0.917326
62924	0.946133	0.916897
62925	1.30165	0.924457
62926	0.972228	0.908434
62927	1.09659	0.795965

62928	0.656268	0.820248
62929	1.06217	0.805562
62930	1.06217	1.35862
62931	1.00665	0.794016
62932	1.00665	0.971273
62933	0.97223	0.808596
62934	0.656267	0.793686
62935	1.30165	0.81465
62936	1.09659	0.832231
62937	0.946132	1.17829
62938	0.946132	1.47178
62939	1.19895	2.06812
62940	1.19895	2.1
62941	0.94613	2.1
62942	0.94613	1.05919
62943	1.39356	1.70156
62944	1.19895	2.1
62945	0.94613	1.5668
62946	1.39356	1.69066
62947	1.19895	1.89448
62948	0.946131	1.68411
62949	1.89226	1.11493
62950	1.89226	2.0676
62951	1.89226	2.1
62952	1.89226	1.09573
62953	1.89226	2.1
62954	1.89226	1.127
62955	0.946132	1.04321
62956	0.946132	1.04025
62957	0.946128	1.27157
62958	0.946128	1.38661
62959	0.94613	1.27189
62960	0.94613	0.959235
62961	0.94613	1.06414
62962	0.94613	1.28538
62963	0.946131	1.0815
62964	0.946131	1.07261
62965	0.94613	1.1083
62966	0.94613	1.13789
62967	1.89226	0.215456
62968	1.89226	0.255552
62969	1.89226	0.331041
62970	1.89226	0.158739
62971	1.89226	0.347324
62972	1.89226	0.158609
62973	1.29881	0.126495
62974	1.09985	0.124726
62975	0.946126	0.223346
62976	0.946126	0.1408
62977	0.851539	0.218807
62978	1.20421	0.183005
62979	0.851483	0.150378
62980	0.999426	0.221402
62981	1.47172	0.181155
62982	0.999413	0.122792
62983	0.851552	0.161786
62984	0.851552	0.158302
62985	1.89226	0.282138

62986	1.89226	0.295117
62987	1.89226	0.453356
62988	1.89226	0.229925
62989	1.89226	0.439554
62990	1.89226	0.224095
62991	1.89226	0.246171
62992	1.89226	0.280869
62993	1.89226	0.381201
62994	1.89226	0.206372
62995	1.89226	0.389264
62996	1.89226	0.199534
62997	1.33915	2.1
62998	1.99297	2.1
62999	2.05216	2.1
63000	2.60163	2.1
63001	4.04513	2.1
63002	1.89226	1.42206
63003	1.89226	2.1
63004	1.89226	2.1
63005	1.89226	1.65308
63006	3.78452	2.09672
63007	0.946132	1.80061
63008	0.946132	1.61493
63009	0.946133	2.1
63010	0.946133	2.1
63011	0.946132	2.1
63012	0.946132	1.27762
63013	0.946133	2.1
63014	0.946133	1.70508
63015	0.946132	2.1
63016	0.946132	2.04067
63017	0.946132	2.1
63018	0.946132	2.1
63019	1.89226	1.43416
63020	1.89226	1.13055
63021	1.89226	1.20457
63022	1.89226	1.567
63023	3.78452	1.27988
63024	1.89227	1.15995
63025	1.89227	1.49019
63026	1.89227	1.62952
63027	1.89227	1.13051
63028	1.89227	1.74706
63029	1.89227	1.16753
63030	2.29301	2.1
63031	1.70303	2.1
63032	2.46749	2.1
63033	2.30728	2.1
63034	4.53937	2.1
63035	1.89226	0.979878
63036	1.89226	2.1
63037	1.89226	2.1
63038	1.89226	0.951538
63039	3.78452	2.1
63040	1.89226	0.843995
63041	1.89226	1.55787
63042	1.89226	1.0544
63043	1.89226	1.56726

63044	1.89226	0.90592
63045	1.89226	1.05998
63046	2.30728	2.1
63047	1.00757	1.09198
63048	2.0833	2.1
63049	1.65318	1.71283
63050	1.417	1.54345
63051	1.00757	1.73876
63052	2.46749	2.1
63053	2.0975	2.1
63054	2.79653	2.1
63055	2.44785	2.1
63056	4.91534	2.1
63057	1.89226	2.1
63058	1.89226	1.6
63059	1.89226	0.977196
63060	1.89226	2.1
63061	3.78452	1.06806
63062	1.89226	1.03999
63063	1.89226	1.53996
63064	1.89226	2.1
63065	1.89226	1.2216
63066	1.89226	2.1
63067	1.89226	1.18153
63068	0.970559	1.44436
63069	0.970559	2.1
63070	0.979305	2.1
63071	0.979305	2.1
63072	0.708502	1.39183
63073	0.901117	2.1
63074	1.04875	2.1
63075	1.22392	1.5778
63076	1.22392	2.1
63077	0.901117	2.1
63078	1.04875	2.1
63079	0.7085	2.1
63080	1.89226	1.31317
63081	1.89226	0.994831
63082	1.89226	1.11142
63083	1.89226	1.18193
63084	3.78452	1.41752
63085	1.89226	1.19588
63086	1.89226	0.989161
63087	1.89226	1.17726
63088	1.89226	1.06891
63089	3.78452	1.04845
63090	1.89226	0.824871
63091	1.89226	0.789052
63092	1.89226	0.856725
63093	1.89226	0.819718
63094	3.78453	0.823045
63095	1.89226	0.870579
63096	1.89226	0.921314
63097	1.89226	0.83337
63098	1.89226	0.832833
63099	1.89226	0.81574
63100	1.89226	0.815849
63101	1.89226	0.929013

63102	2.35441	0.926817
63103	1.89226	1.13768
63104	2.30931	0.848984
63105	4.24667	0.884933
63106	1.11612	0.905844
63107	0.946131	0.893626
63108	1.1772	0.874133
63109	0.946131	0.943334
63110	1.17721	0.956363
63111	1.11612	0.890723
63112	0.94613	0.917408
63113	0.94613	0.90605
63114	1.11612	0.923377
63115	1.11612	0.89017
63116	0.946131	0.884615
63117	0.946131	0.878945
63118	1.11612	0.638812
63119	0.582675	0.606981
63120	1.17721	0.713512
63121	0.651969	0.70259
63122	0.651969	0.71873
63123	0.582674	0.61284
63124	1.17721	0.659799
63125	1.11612	0.632144
63126	0.877955	0.704132
63127	0.877955	0.726779
63128	0.881925	0.664775
63129	0.881925	0.67251
63130	1.17721	0.768705
63131	0.651969	0.77364
63132	1.15466	0.753128
63133	0.623642	0.749376
63134	0.623642	0.75544
63135	0.651969	0.733932
63136	1.17721	0.765344
63137	1.15466	0.762336
63138	0.904714	0.774354
63139	0.904714	0.737785
63140	0.901914	0.796361
63141	0.901914	0.753243
63142	1.89226	1.03872
63143	1.89226	0.850493
63144	1.89226	0.811036
63145	1.89226	1.14465
63146	1.89226	0.769168
63147	1.89226	1.84522
63148	1.89226	1.06131
63149	1.89226	0.805436
63150	1.89226	0.789352
63151	1.89226	1.96642
63152	1.89226	0.782317
63153	1.89226	2.03936
63154	2.33986	1.56772
63155	1.97819	0.951342
63156	2.39166	0.75255
63157	2.70141	1.68373
63158	4.73151	0.938615
63159	0.989095	0.960866

63160	1.19583	0.809057
63161	0.851194	0.756944
63162	0.581197	0.824428
63163	0.872704	0.716961
63164	0.872704	1.04616
63165	0.904322	0.766416
63166	0.904322	0.914603
63167	1.19583	0.745382
63168	0.851193	0.745441
63169	0.989095	0.877359
63170	0.581197	0.808858
63171	1.89226	0.908177
63172	1.89226	0.896039
63173	1.89226	0.883824
63174	1.89226	1.24778
63175	1.89226	0.825151
63176	1.89226	1.87087
63177	1.89226	1.05616
63178	1.89226	0.794325
63179	1.89226	0.802263
63180	1.89226	1.89291
63181	1.89226	0.766337
63182	1.89226	2.08111
63183	2.39166	0.879455
63184	1.70239	0.954812
63185	2.13665	0.771202
63186	2.82684	0.848774
63187	4.52831	0.860866
63188	1.89226	0.276921
63189	1.89226	0.549641
63190	1.89226	0.502145
63191	1.89226	0.489081
63192	3.78453	0.532461
63193	1.89226	0.286299
63194	1.89226	0.346222
63195	1.89226	0.435953
63196	1.89226	0.208768
63197	1.89226	0.468804
63198	1.89226	0.225529
63199	1.89226	0.41498
63200	1.89226	0.469953
63201	1.89226	0.57246
63202	1.89226	0.329828
63203	1.89226	0.600206
63204	1.89226	0.336536
63205	1.89226	0.635368
63206	1.89226	0.668494
63207	1.89226	0.786829
63208	1.89226	0.464604
63209	1.89226	0.802797
63210	1.89226	0.489871
63211	1.89226	0.576353
63212	1.89226	0.640973
63213	1.89226	0.732712
63214	1.89226	0.460248
63215	1.89226	0.770503
63216	1.89226	0.512947
63217	1.5169	0.515014

63218	1.61969	0.384547
63219	1.17262	0.348548
63220	1.17262	0.401325
63221	1.88868	0.488909
63222	1.88868	0.488873
63223	1.79981	0.683243
63224	1.88868	0.460634
63225	1.5169	0.481348
63226	1.5169	0.571532
63227	2.12891	0.579972
63228	2.12891	0.627796
63229	1.89226	0.494851
63230	1.89226	0.341281
63231	1.89226	0.381033
63232	1.89226	0.446522
63233	3.78452	0.438097
63234	0.946132	0.276226
63235	0.946132	0.390768
63236	0.946132	0.263484
63237	0.946132	0.249454
63238	0.946132	0.238099
63239	0.946132	0.33118
63240	0.946133	0.293898
63241	0.946133	0.384931
63242	0.946133	0.298136
63243	0.946133	0.331273
63244	0.946132	0.321007
63245	0.946132	0.28091
63246	1.88868	0.510878
63247	2.05759	0.375655
63248	1.61969	0.366085
63249	1.61969	0.413624
63250	2.29747	0.512068
63251	2.29747	0.469501
63252	2.12891	0.678616
63253	2.29747	0.441932
63254	1.88868	0.470652
63255	1.88868	0.532918
63256	2.50894	0.562961
63257	2.50894	0.59322
63258	1.89226	0.364498
63259	1.89226	0.444434
63260	1.89226	0.270312
63261	1.89226	0.478798
63262	1.89226	0.321196
63263	1.89226	0.557375
63264	0.946132	0.378073
63265	0.946132	0.26563
63266	0.946132	0.271012
63267	0.946132	0.273511
63268	0.946131	0.246663
63269	0.946131	0.362848
63270	0.946132	0.315053
63271	0.946132	0.370811
63272	0.946132	0.317492
63273	0.946132	0.34326
63274	0.946132	0.272788
63275	0.946132	0.351226

63276	2.42758	1.27141
63277	2.14895	2.1
63278	2.56975	1.16368
63279	2.28142	2.1
63280	4.71869	1.89516
63281	1.89226	1.1364
63282	1.89226	2.1
63283	1.89226	1.18382
63284	1.89226	2.1
63285	3.78452	2.04969
63286	1.89226	2.1
63287	1.89226	2.1
63288	1.89226	2.1
63289	1.89226	2.1
63290	1.89226	2.1
63291	1.89226	2.1
63292	0.960916	2.1
63293	0.960916	2.1
63294	0.978118	2.1
63295	0.875674	2.1
63296	0.775661	2.1
63297	0.773613	2.1
63298	1.07631	2.1
63299	1.14266	2.1
63300	1.16927	2.1
63301	0.898612	2.1
63302	1.04757	2.1
63303	0.757536	2.1
63304	2.56975	1.1506
63305	2.28142	2.1
63306	2.64384	1.22895
63307	2.40621	2.1
63308	4.97596	2.00164
63309	1.89226	1.16999
63310	1.89226	2.1
63311	1.89226	0.928989
63312	1.89226	2.1
63313	3.78452	1.29353
63314	1.89226	1.9384
63315	1.89226	2.1
63316	1.89226	2.1
63317	1.89226	2.1
63318	1.89226	2.07523
63319	1.89226	2.1
63320	1.03328	2.1
63321	1.03328	2.1
63322	1.04321	1.95128
63323	1.15424	2.1
63324	0.936183	2.1
63325	0.944491	2.1
63326	1.14266	2.0573
63327	1.20517	2.1
63328	1.23323	2.1
63329	0.961268	2.1
63330	1.11215	2.1
63331	0.827247	2.1
63332	1.89226	2.1
63333	1.89226	1.0924

63334	1.89226	2.1
63335	1.89226	1.26218
63336	3.78453	2.1
63337	2.14647	2.1
63338	2.20589	2.1
63339	1.95567	2.1
63340	2.03224	1.32538
63341	4.17871	2.1
63342	2.06035	2.1
63343	1.88452	0.807396
63344	1.88452	2.1
63345	2.26997	0.772516
63346	2.03224	1.30172
63347	2.42272	0.887928
63348	1.89226	1.36342
63349	1.89226	0.443277
63350	1.89226	0.269732
63351	1.89226	1.64701
63352	1.89226	0.423538
63353	1.89226	1.85535
63354	1.89225	2.1
63355	1.89225	2.1
63356	1.89225	2.0942
63357	1.89225	2.1
63358	1.89225	1.59262
63359	1.89225	2.1
63360	0.952418	2.1
63361	0.952418	2.1
63362	0.95883	2.1
63363	0.95883	2.1
63364	0.946182	2.04426
63365	1.01611	2.1
63366	0.895134	2.1
63367	0.977828	2.1
63368	0.89514	2.1
63369	0.946189	2.1
63370	0.977821	2.1
63371	1.01611	2.1
63372	2.03223	2.1
63373	1.88451	0.817541
63374	1.89236	2.06062
63375	1.74649	0.577007
63376	3.77687	0.929893
63377	0.81094	1.96167
63378	0.81094	0.799405
63379	0.810939	2.03578
63380	0.810939	1.65862
63381	0.810945	0.955232
63382	0.810945	0.974696
63383	1.89225	0.452072
63384	1.35167	0.893369
63385	1.08131	2.1
63386	1.08131	0.521183
63387	1.0813	0.925877
63388	1.89226	0.147161
63389	1.89226	0.180508
63390	1.89226	0.163016
63391	1.89226	0.158675

63392	1.89226	0.159155
63393	1.89226	0.155095
63394	1.01842	0.100422
63395	0.936656	0.106263
63396	0.946131	0.113249
63397	0.946131	0.10921
63398	1.11157	0.0985641
63399	1.04281	0.11309
63400	0.925803	0.0985949
63401	0.925803	0.123273
63402	0.851553	0.0953278
63403	1.11158	0.109744
63404	1.05017	0.121238
63405	0.925798	0.10537
63406	1.89226	0.154732
63407	1.89226	0.196248
63408	1.89226	0.205499
63409	1.89226	0.177855
63410	1.89226	0.18981
63411	1.89226	0.161084
63412	1.23504	0.118559
63413	1.23504	0.0981298
63414	0.946131	0.122984
63415	0.946131	0.115089
63416	1.37358	0.118062
63417	1.23504	0.115083
63418	0.946129	0.11638
63419	0.946129	0.145682
63420	1.37358	0.110142
63421	0.946131	0.112466
63422	0.94613	0.110817
63423	1.23504	0.127548
63424	1.00261	0.09424
63425	1.00261	0.0923446
63426	1.02952	0.124592
63427	1.02952	0.102799
63428	0.850964	0.10588
63429	0.658553	0.106671
63430	1.37358	0.1001
63431	1.23504	0.090968
63432	1.37358	0.0940436
63433	0.850965	0.10951
63434	1.23504	0.0980662
63435	0.658553	0.0943317
63436	0.946133	0.147382
63437	0.946133	0.123676
63438	0.946132	0.22
63439	0.946132	0.145079
63440	0.946132	0.143879
63441	0.946132	0.145474
63442	0.946133	0.160909
63443	0.946133	0.151811
63444	0.946133	0.151088
63445	0.946133	0.155025
63446	0.946132	0.150517
63447	0.946132	0.150445
63448	1.89226	0.682134
63449	1.89226	0.7

63450	1.89226	0.459327
63451	1.89226	0.60191
63452	3.78452	0.7
63453	1.89226	0.7
63454	1.89226	0.7
63455	1.89226	0.587996
63456	1.89226	0.682918
63457	3.78452	0.610418
63458	1.89227	0.686871
63459	1.89227	0.7
63460	1.89227	0.530795
63461	1.89227	0.640207
63462	1.89227	0.611033
63463	1.89227	0.652051
63464	0.94613	0.285907
63465	0.94613	0.378946
63466	0.946127	0.298625
63467	0.946127	0.361356
63468	0.94613	0.346087
63469	0.94613	0.350016
63470	0.946127	0.320117
63471	0.946127	0.4633
63472	0.946128	0.283805
63473	0.946128	0.227858
63474	0.946128	0.368057
63475	0.946128	0.354426
63476	0.946132	0.38155
63477	0.946132	0.465777
63478	0.946128	0.354331
63479	0.946128	0.431424
63480	0.94613	0.414896
63481	0.94613	0.442385
63482	0.946131	0.373954
63483	0.946131	0.382798
63484	0.94613	0.364172
63485	0.94613	0.303743
63486	0.94613	0.432922
63487	0.94613	0.529072
63488	1.89226	2.1
63489	1.89226	1.97509
63490	1.89226	2.1
63491	1.89226	2.1
63492	3.78453	2.1
63493	2.72844	2.1
63494	1.89226	2.1
63495	2.26536	2.1
63496	2.26536	2.1
63497	1.89226	2.1
63498	1.89226	2.1
63499	1.89226	2.1
63500	1.89226	1.52458
63501	1.89226	2.0053
63502	1.89226	2.0961
63503	1.89226	1.9936
63504	1.89226	2.07646
63505	1.89226	2.1
63506	1.89226	2.1
63507	1.89226	2.1

63508	1.89226	2.1
63509	1.89226	1.96688
63510	1.89226	2.1
63511	2.72844	2.1
63512	2.26536	2.1
63513	2.28367	2.1
63514	1.5791	2.1
63515	4.54903	2.1
63516	0.810945	2.09667
63517	0.810945	1.72435
63518	0.810943	1.6785
63519	0.810943	2.1
63520	1.89226	2.1
63521	1.3517	1.96733
63522	1.08133	2.1
63523	1.08132	2.1
63524	1.08132	2.03011
63525	0.810933	2.1
63526	0.810933	2.06722
63527	1.89227	2.1
63528	1.89227	2.1
63529	1.89227	2.1
63530	1.89227	2.1
63531	3.78453	2.1
63532	2.71897	2.1
63533	2.27495	2.1
63534	2.648	2.1
63535	2.20832	2.1
63536	4.92729	2.1
63537	2.27495	2.1
63538	1.38455	2.1
63539	1.38455	2.1
63540	1.46586	2.1
63541	2.20831	2.1
63542	2.28962	2.1
63543	0.946133	2.1
63544	0.946133	2.1
63545	0.946132	2.1
63546	0.946132	2.1
63547	0.946132	2.1
63548	0.946132	2.1
63549	0.946132	2.1
63550	0.946132	2.1
63551	0.946132	2.1
63552	0.946132	2.1
63553	0.946133	2.1
63554	0.946133	2.1
63555	1.89226	2.1
63556	1.89226	2.1
63557	1.89226	2.1
63558	1.89226	2.1
63559	1.89226	2.1
63560	1.89226	2.1
63561	2.648	2.1
63562	2.08612	2.1
63563	2.08612	2.1
63564	2.20831	2.1
63565	2.56618	2.1

63566	2.68837	2.1
63567	2.08612	2.1
63568	2.20831	2.1
63569	1.30659	2.1
63570	1.30659	2.1
63571	1.38455	2.1
63572	2.16408	2.1
63573	0.810946	2.1
63574	0.810946	2.1
63575	0.810946	2.1
63576	0.810946	2.1
63577	1.89226	2.1
63578	1.3517	2.1
63579	1.08133	2.1
63580	1.08132	2.1
63581	1.08132	2.1
63582	0.810934	2.1
63583	0.810934	2.1
63584	1.89226	1.46173
63585	1.89226	1.24768
63586	1.89226	0.991751
63587	1.89226	1.93579
63588	3.78452	1.15664
63589	1.89226	1.11981
63590	1.89226	2.06906
63591	1.89226	1.99953
63592	1.89226	1.17494
63593	3.78453	1.68962
63594	1.08733	1.90024
63595	0.856615	2.1
63596	1.25964	1.27317
63597	1.12378	1.24841
63598	1.23551	1.11153
63599	0.969894	2.1
63600	0.985977	2.01087
63601	0.985977	1.14753
63602	1.16169	1.36244
63603	1.21072	1.60719
63604	0.765175	1.46803
63605	0.863419	1.41403
63606	2.39934	1.85526
63607	2.44427	1.32241
63608	2.64003	1.00278
63609	2.39077	1.90007
63610	2.39934	1.6122
63611	2.10194	2.1
63612	1.89226	1.0164
63613	1.89226	2.02794
63614	1.89226	1.37526
63615	1.89226	0.921218
63616	3.78452	1.40114
63617	0.946132	1.6368
63618	0.946132	1.81303
63619	0.946131	1.09546
63620	0.946131	1.13519
63621	0.946132	1.09115
63622	0.946132	2.1
63623	0.946132	1.69044

63624	0.946132	1.19028
63625	0.946132	1.33184
63626	0.946132	1.40981
63627	0.946132	1.3332
63628	0.946132	1.58783
63629	1.19967	1.88757
63630	0.946128	1.4605
63631	0.946133	1.06594
63632	1.32002	1.34772
63633	1.19967	1.79847
63634	1.19967	1.76091
63635	0.94613	1.8698
63636	0.94613	0.909966
63637	0.946131	1.16338
63638	0.946131	1.26317
63639	1.19967	1.3858
63640	1.32001	1.42154
63641	1.89226	0.770832
63642	1.89226	0.873102
63643	1.89226	0.743183
63644	1.89226	1.04938
63645	3.78451	0.808683
63646	2.6402	0.736039
63647	2.28552	0.874037
63648	2.50958	0.731963
63649	2.18948	0.844095
63650	4.79509	0.787351
63651	2.28552	0.856569
63652	1.86102	2.1
63653	2.18949	0.868008
63654	1.78376	2.1
63655	4.06928	2.07765
63656	1.89226	0.857835
63657	1.89226	2.1
63658	1.89226	0.927942
63659	1.89226	2.1
63660	3.78452	2.1
63661	1.89226	0.741162
63662	1.89226	1.27232
63663	1.89226	0.734304
63664	1.89226	0.888618
63665	3.78452	0.792306
63666	2.50958	0.73876
63667	2.18948	0.823357
63668	2.39524	0.726236
63669	2.10188	0.899952
63670	4.61146	0.776794
63671	2.18949	0.804713
63672	1.78376	2.1
63673	1.71839	0.887336
63674	1.71839	2.1
63675	2.10189	0.924765
63676	2.16726	2.1
63677	0.810943	0.911662
63678	0.810943	2.1
63679	0.810945	0.901627
63680	0.810945	0.87642
63681	0.810959	1.94565

63682	0.810959	2.05948
63683	1.89226	2.1
63684	1.35168	2.1
63685	1.08132	0.903117
63686	1.08132	2.1
63687	1.0813	1.9228
63688	1.89226	0.80202
63689	1.89226	1.3313
63690	1.89226	0.949994
63691	1.89226	0.921278
63692	1.89226	0.987611
63693	1.89226	1.17958
63694	1.28421	1.23379
63695	0.992041	0.840006
63696	1.07707	2.00701
63697	0.789746	1.3553
63698	1.03815	1.76473
63699	1.03815	1.43276
63700	1.03581	1.44498
63701	1.03581	1.02653
63702	1.28421	1.26907
63703	1.07707	1.69901
63704	0.789748	1.36828
63705	0.992038	1.05956
63706	1.89226	0.862911
63707	1.89226	1.35028
63708	1.89226	1.03471
63709	1.89226	0.913218
63710	1.89226	1.04914
63711	1.89226	0.92775
63712	1.89226	0.800566
63713	1.89226	1.02072
63714	1.89226	0.892309
63715	1.89226	0.884262
63716	1.89226	0.912226
63717	1.89226	0.98063
63718	0.92236	2.00773
63719	0.655971	1.56104
63720	0.916772	1.84966
63721	0.913834	1.09614
63722	1.18074	1.49251
63723	0.922346	1.85811
63724	2.15414	0.918507
63725	1.53528	1.37861
63726	1.22748	1.62165
63727	1.57442	0.983392
63728	0.874683	1.49044
63729	1.25935	2.1
63730	2.61433	2.1
63731	1.22262	2.1
63732	1.22262	2.1
63733	2.62813	2.1
63734	2.62813	2.1
63735	1.89226	2.1
63736	2.61434	2.1
63737	2.62814	2.1
63738	2.61434	2.1
63739	1.90607	2.1

63740	1.89226	2.1
63741	1.89226	2.1
63742	1.89226	2.1
63743	1.89226	2.1
63744	1.89226	2.1
63745	1.89226	2.1
63746	1.89226	2.1
63747	1.89226	1.2348
63748	1.89226	2.1
63749	1.89226	2.1
63750	1.89226	2.1
63751	1.89226	2.1
63752	1.89226	2.1
63753	1.31969	2.1
63754	1.31406	2.1
63755	0.629677	2.1
63756	0.643104	2.1
63757	0.976621	2.1
63758	0.976621	2.1
63759	0.98055	2.1
63760	0.98055	2.1
63761	1.3197	2.1
63762	0.643102	2.1
63763	0.629675	2.1
63764	1.31407	2.1
63765	0.946133	2.1
63766	0.946133	2.1
63767	1.3197	2.1
63768	1.31407	2.1
63769	0.946132	2.1
63770	0.946132	2.1
63771	1.3197	2.1
63772	1.3197	2.1
63773	1.3197	2.1
63774	0.946133	2.1
63775	0.946132	2.1
63776	1.31407	2.1
63777	1.89226	2.1
63778	1.89226	2.1
63779	1.89226	2.1
63780	1.89226	2.1
63781	1.89226	2.1
63782	1.89226	2.1
63783	1.89226	2.1
63784	1.89226	1.82253
63785	1.89226	2.1
63786	1.89226	2.1
63787	1.89226	2.1
63788	1.89226	2.00269
63789	1.89226	0.680956
63790	1.89226	0.499353
63791	1.89226	0.420428
63792	1.89226	0.419219
63793	3.78452	0.409501
63794	1.89226	0.544257
63795	1.89226	0.366773
63796	1.89226	0.365945
63797	1.89226	0.383408

63798	1.89226	0.469646
63799	1.89226	0.495575
63800	0.946131	0.223077
63801	0.946131	0.227477
63802	0.946128	0.230872
63803	0.946128	0.227993
63804	0.94613	0.233724
63805	0.94613	0.217489
63806	0.94613	0.176049
63807	0.94613	0.303952
63808	0.94613	0.261268
63809	0.94613	0.264167
63810	0.94613	0.270755
63811	0.94613	0.243742
63812	1.89226	0.25489
63813	1.89226	0.278526
63814	1.89226	0.272004
63815	1.89226	0.200962
63816	1.89226	0.219794
63817	1.89226	0.264516
63818	0.924614	0.163939
63819	0.914275	0.156277
63820	0.906003	0.152158
63821	0.62479	0.14846
63822	2.02733	0.173851
63823	1.48332	0.140077
63824	0.833106	0.144847
63825	1.19364	0.157709
63826	1.57482	0.159469
63827	1.18103	0.139033
63828	0.905988	0.144582
63829	1.89226	2.1
63830	1.89226	2.1
63831	1.89226	2.1
63832	1.89226	2.1
63833	3.78452	2.1
63834	1.89226	2.1
63835	1.89226	2.1
63836	1.89226	2.1
63837	1.89226	2.1
63838	1.89226	2.1
63839	1.89226	2.1
63840	0.816805	2.1
63841	0.928516	2.1
63842	1.4045	2.1
63843	1.26268	2.1
63844	1.0881	2.1
63845	1.0881	2.1
63846	1.10309	2.1
63847	1.10309	2.1
63848	0.928518	2.1
63849	1.4045	2.1
63850	0.816807	2.1
63851	1.26267	2.1
63852	1.89226	2.1
63853	2.52535	2.1
63854	1.89226	2.1
63855	1.89226	2.1

63856	2.809	2.1
63857	2.809	2.1
63858	1.89226	2.1
63859	1.89226	2.1
63860	1.89226	2.1
63861	1.89226	2.1
63862	3.78452	2.1
63863	0.946127	2.1
63864	0.946127	2.1
63865	0.946131	2.1
63866	0.946131	2.1
63867	0.94613	2.1
63868	0.94613	2.1
63869	0.946128	2.1
63870	0.946128	2.1
63871	0.946129	2.1
63872	0.946129	2.1
63873	0.946129	2.1
63874	0.946129	2.1
63875	1.89224	0.900498
63876	1.89224	2.1
63877	1.89224	1.29557
63878	1.89224	2.1
63879	3.78448	2.0671
63880	2.39244	2.1
63881	2.27036	2.1
63882	2.25945	1.14553
63883	2.27398	2.1
63884	4.52981	2.1
63885	2.27036	2.1
63886	1.97088	2.1
63887	1.86302	2.1
63888	1.84991	2.1
63889	4.12027	2.1
63890	1.89224	2.1
63891	1.89224	2.1
63892	1.89224	2.1
63893	1.89224	2.1
63894	3.78447	2.1
63895	1.89226	1.31704
63896	1.89226	2.1
63897	1.89226	0.904972
63898	1.89226	2.1
63899	3.78453	1.95752
63900	2.25948	1.35839
63901	2.33611	2.1
63902	2.05257	2.1
63903	1.94441	2.1
63904	4.20389	2.1
63905	1.92514	2.1
63906	1.84993	2.1
63907	1.94441	2.1
63908	1.74391	2.1
63909	3.79434	2.1
63910	1.89226	2.1
63911	1.89226	2.1
63912	1.89226	2.1
63913	1.89226	2.1

63914	3.78452	2.1
63915	15.1381	0.735191
63916	15.1381	0.724344
63917	15.1381	0.711561
63918	15.1381	0.830387
63919	30.2762	0.72391
63920	15.1381	0.708948
63921	15.1381	0.773925
63922	15.1381	0.716469
63923	15.1381	0.726137
63924	30.2762	0.72865
63925	15.1381	1.54989
63926	15.1381	1.81728
63927	15.1381	1.21469
63928	15.1381	2.01557
63929	30.2762	1.81397
63930	15.1381	0.704461
63931	15.1381	0.739067
63932	15.1381	0.729917
63933	15.1381	0.705334
63934	30.2762	0.705883
63935	1.89225	2.05272
63936	1.89225	2.1
63937	1.89225	2.1
63938	1.89225	2.1
63939	3.78449	2.1
63940	1.39909	2.1
63941	1.39909	2.1
63942	0.946123	2.1
63943	0.946123	2.1
63944	1.3291	2.1
63945	1.39908	2.1
63946	0.946126	2.1
63947	0.946126	2.1
63948	0.946118	2.1
63949	1.32909	2.1
63950	0.94613	2.1
63951	1.3991	2.1
63952	3.07801	2.1
63953	1.84322	2.1
63954	1.84322	2.1
63955	1.68788	2.1
63956	2.6582	2.1
63957	2.50287	2.1
63958	1.89225	2.1
63959	1.89225	2.1
63960	1.89225	2.1
63961	1.89225	2.1
63962	3.7845	2.1
63963	1.89226	2.01339
63964	1.89226	2.1
63965	1.89226	2.04201
63966	1.89226	2.1
63967	3.78452	2.1
63968	1.89226	2.0058
63969	2.50051	2.1
63970	2.50051	1.45069
63971	2.65822	2.1

63972	1.89226	2.1
63973	2.04997	2.1
63974	2.50052	2.1
63975	2.65822	2.1
63976	1.70377	2.1
63977	1.70377	2.1
63978	1.84324	2.1
63979	2.63998	2.1
63980	0.810947	2.1
63981	0.810947	2.1
63982	0.810946	2.1
63983	0.810946	2.1
63984	1.89226	2.1
63985	1.35169	2.1
63986	1.08132	2.1
63987	1.08132	2.1
63988	1.08132	2.1
63989	0.810946	2.1
63990	0.810946	2.1
63991	1.89226	2.1
63992	1.89226	2.1
63993	1.89226	2.1
63994	1.89226	2.1
63995	3.78453	2.1
63996	0.946164	2.1
63997	0.946164	2.1
63998	0.929842	2.1
63999	0.929842	2.1
64000	0.946132	2.1
64001	0.946132	2.1
64002	1.04101	2.1
64003	0.929874	2.1
64004	0.946132	2.1
64005	0.929874	2.1
64006	1.04101	2.1
64007	0.946132	2.1
64008	1.89226	2.1
64009	1.89226	2.1
64010	1.89226	2.1
64011	1.89226	2.1
64012	3.78453	2.1
64013	1.89226	2.1
64014	1.89226	2.1
64015	1.89226	2.1
64016	1.89226	2.1
64017	1.89226	2.1
64018	1.89226	2.1
64019	1.89226	2.1
64020	1.89226	2.1
64021	1.89226	2.1
64022	1.89226	2.1
64023	1.89226	2.1
64024	1.89226	2.1
64025	0.946131	2.1
64026	0.946131	2.1
64027	0.946131	2.1
64028	0.946131	2.1
64029	0.946132	2.1

64030	0.946132	2.1
64031	0.946131	2.1
64032	0.946131	2.1
64033	0.946131	2.1
64034	0.946131	2.1
64035	0.946132	2.1
64036	0.946132	2.1
64037	1.89226	0.799982
64038	1.89226	0.763481
64039	1.89226	0.649035
64040	1.89226	0.776415
64041	3.78453	0.747489
64042	2.73497	0.73801
64043	1.93823	0.891697
64044	1.93823	0.75941
64045	2.20046	0.869006
64046	2.42279	0.775092
64047	2.68501	1.13563
64048	1.89226	0.760769
64049	1.89226	0.891488
64050	1.89226	0.954145
64051	1.89226	0.757052
64052	1.89226	0.954666
64053	1.89226	0.572061
64054	1.89226	0.735915
64055	1.89226	0.721773
64056	1.89226	0.73218
64057	1.89226	0.763927
64058	1.89226	0.55685
64059	1.89226	0.738134
64060	2.82984	0.768953
64061	2.15276	0.648506
64062	2.15276	0.75984
64063	2.42278	0.491161
64064	2.50074	0.48705
64065	2.77077	0.704111
64066	2.42279	0.714174
64067	1.93823	0.850231
64068	1.70423	0.51033
64069	1.70423	0.823403
64070	2.15276	0.63874
64071	2.38676	0.858418
64072	0.810946	0.758557
64073	0.810946	0.906439
64074	0.810944	0.691286
64075	0.810944	0.724983
64076	0.810946	0.7582
64077	0.810946	0.770114
64078	1.89226	0.849551
64079	1.35169	0.781385
64080	1.08132	0.746886
64081	1.08132	0.86202
64082	1.08132	0.790602
64083	1.89226	2.1
64084	1.89226	2.1
64085	1.89226	2.1
64086	1.89226	2.1
64087	3.78452	2.1

64088	1.89226	2.1
64089	1.89226	2.1
64090	1.89226	2.1
64091	1.89226	2.1
64092	3.78453	2.1
64093	1.04144	2.1
64094	1.04144	2.1
64095	0.938972	2.1
64096	0.938972	2.1
64097	0.969661	2.1
64098	1.03285	2.1
64099	0.946133	2.1
64100	0.946133	2.1
64101	0.946131	2.1
64102	0.969663	2.1
64103	0.946131	2.1
64104	1.03284	2.1
64105	0.946131	2.1
64106	0.946131	2.1
64107	0.946128	2.1
64108	0.946128	2.1
64109	0.946127	2.1
64110	0.946127	2.1
64111	0.946131	2.1
64112	0.946131	2.1
64113	0.94613	2.1
64114	0.94613	2.1
64115	0.946129	2.1
64116	0.946129	2.1
64117	1.89226	2.1
64118	1.89226	2.1
64119	1.89226	2.1
64120	1.89226	2.1
64121	3.78452	2.1
64122	1.89226	2.1
64123	1.89226	2.1
64124	1.89226	2.1
64125	1.89226	2.1
64126	3.78453	2.1
64127	1.89226	2.1
64128	1.89226	2.1
64129	1.85882	2.1
64130	1.85882	2.1
64131	1.93932	2.1
64132	1.97276	2.1
64133	0.94613	2.1
64134	0.94613	2.1
64135	0.946128	2.1
64136	0.946128	2.1
64137	0.946127	2.1
64138	0.946127	2.1
64139	0.946131	2.1
64140	0.946131	2.1
64141	0.946129	2.1
64142	0.946129	2.1
64143	0.946129	2.1
64144	0.946129	2.1
64145	1.89225	2.1

64146	1.89225	2.1
64147	1.89225	2.1
64148	1.89225	2.1
64149	3.7845	2.1
64150	1.0086	2.1
64151	1.09028	2.1
64152	0.946125	2.1
64153	0.946125	2.1
64154	0.916906	2.1
64155	0.916906	2.1
64156	1.13051	2.1
64157	1.1505	2.1
64158	1.11557	2.1
64159	1.04071	2.1
64160	0.851542	2.1
64161	0.991196	2.1
64162	1.89225	2.1
64163	1.89225	2.1
64164	1.89225	2.1
64165	1.89225	2.1
64166	3.7845	2.1
64167	1.89225	2.1
64168	1.89225	2.1
64169	1.89225	2.1
64170	1.89225	2.1
64171	1.89225	2.1
64172	1.89225	2.1
64173	1.89226	2.1
64174	1.89226	2.1
64175	1.89226	2.1
64176	1.89226	2.1
64177	3.78452	2.1
64178	0.890962	2.1
64179	0.952547	2.1
64180	0.946132	2.1
64181	0.946132	2.1
64182	0.851543	2.1
64183	0.851543	2.1
64184	1.11558	2.1
64185	1.06304	2.1
64186	0.994315	2.1
64187	1.04072	2.1
64188	0.900943	2.1
64189	0.851542	2.1
64190	1.89226	2.1
64191	1.89226	2.1
64192	1.89226	2.1
64193	1.89226	2.1
64194	1.89226	2.1
64195	1.89226	2.1
64196	1.89226	2.1
64197	1.89226	2.1
64198	1.89226	2.1
64199	1.89226	2.1
64200	3.78452	2.1
64201	1.89226	2.1
64202	1.89226	2.1
64203	1.89226	2.1

64204	1.89226	2.1
64205	3.78452	2.1
64206	0.782015	2.1
64207	0.929718	2.1
64208	0.946132	2.1
64209	0.946132	2.1
64210	0.782016	2.1
64211	0.782016	2.1
64212	1.06706	2.1
64213	1.11785	2.1
64214	0.782015	2.1
64215	0.946133	2.1
64216	0.878931	2.1
64217	0.946132	2.1
64218	1.89226	2.1
64219	1.89226	2.1
64220	1.89226	2.1
64221	1.89226	2.1
64222	1.89226	2.1
64223	1.89226	2.1
64224	0.946131	2.1
64225	0.946131	2.1
64226	0.946129	2.1
64227	0.946129	2.1
64228	0.946131	2.1
64229	0.946131	2.1
64230	0.94613	2.1
64231	0.94613	2.1
64232	0.946131	2.1
64233	0.946131	2.1
64234	0.94613	2.1
64235	0.94613	2.1
64236	0.827866	2.1
64237	0.946131	2.1
64238	0.946129	2.1
64239	0.946129	2.1
64240	0.94613	2.1
64241	0.94613	2.1
64242	0.94613	2.1
64243	0.94613	2.1
64244	0.827865	2.1
64245	0.94613	2.1
64246	0.94613	2.1
64247	0.94613	2.1
64248	1.89226	2.1
64249	1.89226	2.1
64250	1.89226	2.1
64251	1.89226	2.1
64252	3.78452	2.1
64253	1.89226	2.1
64254	1.89226	2.1
64255	1.89226	2.1
64256	1.89226	2.1
64257	1.89226	2.1
64258	1.89226	2.1
64259	1.89226	2.1
64260	1.89226	2.1
64261	1.89226	2.1

64262	1.89226	2.1
64263	3.78453	2.1
64264	1.89226	2.1
64265	1.89226	2.1
64266	1.89226	2.1
64267	1.89226	2.1
64268	3.78452	2.1
64269	0.744377	2.1
64270	0.541919	2.1
64271	0.652022	2.1
64272	0.544198	2.1
64273	0.764633	2.1
64274	0.673254	2.1
64275	1.89226	2.1
64276	1.31149	2.1
64277	1.05423	2.1
64278	1.02189	2.1
64279	0.870602	2.1
64280	1.89226	2.1
64281	1.89226	2.1
64282	1.89226	2.1
64283	1.89226	2.1
64284	1.89226	2.1
64285	1.89226	2.1
64286	1.89226	2.1
64287	1.89226	2.1
64288	1.89226	2.1
64289	1.89226	2.1
64290	1.89226	2.1
64291	1.89226	2.1
64292	1.89226	2.1
64293	1.89226	2.1
64294	1.89226	2.1
64295	1.89226	2.1
64296	1.89226	2.1
64297	1.89226	2.1
64298	0.815314	2.1
64299	0.815313	2.1
64300	0.714029	2.1
64301	0.782951	2.1
64302	0.810944	2.1
64303	0.782954	2.1
64304	1.89226	2.1
64305	1.30999	2.1
64306	1.08132	2.1
64307	1.03962	2.1
64308	0.95209	2.1
64309	1.89226	2.1
64310	1.89226	2.1
64311	1.89226	2.1
64312	1.89226	2.1
64313	1.89226	2.1
64314	1.89226	2.1
64315	1.89226	2.1
64316	1.89226	2.1
64317	1.89226	2.1
64318	1.89226	2.1
64319	1.89226	2.1

64320	1.89226	2.1
64321	1.89226	2.1
64322	1.89226	2.1
64323	1.89226	2.1
64324	1.89226	2.1
64325	1.89226	2.1
64326	1.89226	2.1
64327	0.871558	2.1
64328	0.713078	2.1
64329	0.756858	2.1
64330	0.703674	2.1
64331	0.838025	2.1
64332	0.862196	2.1
64333	1.89226	2.1
64334	1.33194	2.1
64335	1.10834	2.1
64336	1.06163	2.1
64337	1.01785	2.1
64338	1.89226	2.1
64339	1.89226	2.1
64340	1.89226	2.1
64341	1.89226	2.1
64342	3.78452	2.1
64343	1.89226	2.1
64344	1.89226	2.1
64345	1.89226	2.1
64346	1.89226	2.1
64347	1.89226	2.1
64348	1.89226	2.1
64349	1.89226	2.1
64350	1.89226	2.1
64351	1.89226	2.1
64352	1.89226	2.1
64353	1.89226	2.1
64354	1.89226	2.1
64355	0.958406	2.1
64356	1.1383	2.1
64357	0.865636	2.1
64358	1.10093	2.1
64359	0.838024	2.1
64360	0.975154	2.1
64361	1.89226	2.1
64362	1.38553	2.1
64363	1.10834	2.1
64364	1.11522	2.1
64365	1.15271	2.1
64366	1.89227	2.1
64367	1.89227	2.1
64368	1.89227	2.1
64369	1.89227	2.1
64370	3.78453	2.1
64371	0.946132	2.1
64372	0.946132	2.1
64373	0.946132	2.1
64374	0.946132	2.1
64375	0.946132	2.1
64376	0.946132	2.1
64377	0.946133	2.1

64378	0.946133	2.1
64379	0.946132	2.1
64380	0.946132	2.1
64381	0.946133	2.1
64382	0.946133	2.1
64383	0.719271	2.1
64384	0.774968	2.1
64385	1.28401	2.1
64386	1.17415	2.1
64387	0.960999	2.1
64388	0.960999	2.1
64389	0.988115	2.1
64390	0.988115	2.1
64391	0.774968	2.1
64392	1.28401	2.1
64393	0.719271	2.1
64394	1.17415	2.1
64395	2.77811	2.1
64396	2.34829	2.1
64397	2.56802	2.1
64398	2.52333	2.1
64399	2.56802	2.1
64400	2.52333	2.1
64401	1.89227	2.1
64402	1.89227	2.1
64403	1.89227	2.1
64404	1.89227	2.1
64405	3.78453	2.1
64406	1.89226	2.1
64407	1.89226	2.1
64408	1.89226	2.1
64409	1.89226	2.1
64410	1.89226	2.1
64411	1.89226	2.1
64412	1.89226	2.1
64413	2.77811	2.1
64414	1.89226	2.1
64415	2.56802	2.1
64416	1.89226	2.1
64417	2.56802	2.1
64418	1.89226	0.75733
64419	1.89226	1.83372
64420	1.89226	0.756645
64421	1.89226	1.31196
64422	3.78452	0.803599
64423	2.41802	0.855767
64424	1.95764	2.1
64425	1.95764	1.02213
64426	2.27727	2.1
64427	2.39311	0.909207
64428	2.73961	2.1
64429	0.946132	2.1
64430	0.946132	0.923446
64431	0.94613	0.948528
64432	0.94613	2.1
64433	0.946131	2.1
64434	0.946131	2.1
64435	0.946131	0.958296

64436	0.946131	1.006
64437	0.946131	2.04322
64438	0.946131	2.1
64439	0.946131	2.00986
64440	0.946131	2.08841
64441	1.89226	0.764436
64442	1.89226	0.870302
64443	1.89226	1.01531
64444	1.89226	0.767736
64445	1.89226	1.27805
64446	1.89226	0.758692
64447	2.78856	0.737075
64448	2.28552	0.867942
64449	2.28552	0.760179
64450	2.3931	0.826695
64451	2.6402	0.704094
64452	2.74778	0.835422
64453	2.39311	0.823856
64454	1.95764	2.1
64455	2.28552	0.868427
64456	1.86103	2.1
64457	4.24316	2.1
64458	1.89226	0.872758
64459	1.89226	2.1
64460	1.89226	0.927875
64461	1.89226	2.1
64462	1.89226	0.933864
64463	1.89226	2.1
64464	1.47412	1.92715
64465	2.18862	1.56434
64466	2.07226	1.09925
64467	2.33645	1.27241
64468	4.26088	1.32052
64469	1.89226	1.0388
64470	1.89226	1.64215
64471	1.89226	1.12236
64472	1.89226	1.68866
64473	1.89226	1.31842
64474	1.89226	2.1
64475	1.89226	1.33402
64476	1.89226	2.1
64477	1.89226	0.879879
64478	1.89226	2.1
64479	3.78453	1.28184
64480	2.18862	1.30678
64481	2.1128	2.1
64482	2.33645	1.10931
64483	2.28188	2.1
64484	4.47051	1.77672
64485	2.07226	2.1
64486	2.33645	1.65297
64487	2.20831	1.02078
64488	2.4646	1.14681
64489	4.53686	1.26226
64490	0.946131	1.00165
64491	0.946131	2.1
64492	0.946131	2.1
64493	0.946131	1.26738

64494	0.94613	2.08888
64495	0.94613	1.16784
64496	0.946133	1.91615
64497	0.946133	2.04253
64498	0.946132	1.0564
64499	0.946132	1.02156
64500	0.946131	1.85025
64501	0.946131	1.44037
64502	1.89226	1.68994
64503	1.89226	2.1
64504	1.89226	1.08533
64505	1.89226	1.87638
64506	3.78453	1.44174
64507	2.33646	1.30434
64508	2.28189	2.08217
64509	2.4646	1.0597
64510	2.42758	2.1
64511	4.74648	1.32596
64512	1.89226	2.1
64513	1.89226	2.1
64514	1.89226	2.1
64515	1.89226	2.1
64516	1.89226	2.1
64517	1.89226	2.1
64518	1.89226	2.1
64519	1.89226	2.1
64520	1.89226	2.1
64521	1.89226	2.1
64522	1.89226	2.1
64523	1.89226	2.1
64524	1.89226	2.1
64525	1.89226	2.1
64526	1.89226	2.1
64527	1.89226	2.1
64528	3.78453	2.1
64529	1.89226	2.1
64530	1.89226	2.1
64531	1.89226	2.1
64532	1.89226	2.1
64533	1.89226	2.1
64534	1.89226	2.1
64535	1.89226	2.1
64536	1.89226	2.1
64537	1.89226	2.1
64538	1.89226	2.1
64539	3.78452	2.1
64540	0.877238	2.1
64541	0.877238	2.1
64542	1.24746	2.1
64543	1.04764	2.1
64544	0.964969	2.1
64545	1.1521	2.1
64546	0.946132	2.1
64547	0.946132	2.1
64548	0.946132	2.1
64549	1.12659	2.1
64550	0.964969	2.1
64551	0.946131	2.1

64552	1.89226	2.1
64553	1.89226	2.1
64554	1.89226	2.1
64555	1.89226	2.1
64556	1.89226	2.1
64557	1.89226	2.1
64558	1.89226	2.1
64559	1.89226	2.1
64560	1.89226	2.1
64561	1.89226	2.1
64562	3.78453	2.1
64563	0.868144	2.1
64564	0.992762	2.1
64565	0.946131	2.1
64566	0.946131	2.1
64567	1.07072	2.1
64568	1.07072	2.1
64569	0.868171	2.1
64570	0.868171	2.1
64571	0.992762	2.1
64572	0.946131	2.1
64573	0.946131	2.1
64574	0.868144	2.1
64575	0.946132	2.1
64576	0.946132	2.1
64577	0.946131	2.1
64578	0.946131	2.1
64579	0.946132	2.1
64580	0.946132	2.1
64581	0.946132	2.1
64582	0.946132	2.1
64583	0.946132	2.1
64584	0.946132	2.1
64585	0.946132	2.1
64586	0.946132	2.1
64587	1.89227	2.1
64588	1.89227	2.1
64589	1.89227	2.1
64590	1.89227	2.1
64591	1.89227	2.1
64592	1.89227	2.1
64593	1.89226	2.1
64594	1.89226	2.1
64595	1.89226	2.1
64596	1.89226	2.1
64597	1.89226	2.1
64598	1.89226	2.1
64599	1.98552	2.1
64600	2.22298	2.1
64601	2.22298	2.1
64602	1.89226	2.1
64603	1.89226	2.1
64604	1.54525	2.1
64605	0.810946	2.1
64606	0.810946	2.1
64607	0.810947	2.1
64608	0.810947	2.1
64609	0.81096	2.1

64610	0.81096	2.1
64611	1.89226	2.1
64612	1.35168	2.1
64613	1.08132	2.1
64614	1.08132	2.1
64615	1.0813	2.1
64616	1.89226	2.1
64617	1.89226	2.1
64618	1.89226	2.1
64619	1.89226	2.1
64620	3.78452	2.1
64621	1.7753	2.1
64622	2.39703	2.1
64623	1.90577	2.1
64624	2.03445	2.1
64625	3.82335	2.1
64626	2.39703	2.1
64627	2.47949	0.916613
64628	2.26051	2.1
64629	2.36891	0.769543
64630	4.76594	1.08469
64631	1.89226	2.1
64632	1.89226	0.88546
64633	1.89226	2.1
64634	1.89226	0.808526
64635	3.78452	1.00873
64636	1.89226	2.1
64637	1.89226	2.1
64638	1.89226	2.1
64639	1.89226	2.1
64640	1.89226	2.1
64641	1.89226	2.1
64642	0.883745	2.1
64643	0.973143	2.1
64644	0.988173	2.1
64645	0.988173	2.1
64646	0.941927	1.90281
64647	1.02971	2.1
64648	0.854065	2.1
64649	0.82702	2.1
64650	0.847835	2.1
64651	0.981126	2.1
64652	1.02996	2.1
64653	1.10704	2.1
64654	2.28031	2.1
64655	2.26369	0.892879
64656	2.26369	2.1
64657	2.36891	0.928199
64658	2.30788	1.83726
64659	2.42455	0.926751
64660	1.89226	2.1
64661	1.89226	0.894692
64662	1.89226	0.874359
64663	1.89226	2.1
64664	1.89226	0.882269
64665	1.89226	2.1
64666	2.71406	2.1
64667	1.83244	2.1

64668	1.83244	2.1
64669	2.07567	2.1
64670	2.52044	2.1
64671	2.76367	2.1
64672	1.89226	2.1
64673	1.89226	2.1
64674	1.89226	2.1
64675	1.89226	2.1
64676	3.78453	2.1
64677	1.89226	2.1
64678	1.89226	2.1
64679	1.89226	2.1
64680	1.89226	2.1
64681	1.89226	2.1
64682	1.89226	2.1
64683	2.70714	2.1
64684	2.3148	2.1
64685	2.3148	2.1
64686	2.52044	2.1
64687	2.54204	2.1
64688	2.74768	2.1
64689	2.52044	2.1
64690	2.3148	2.1
64691	1.83244	2.1
64692	1.83244	2.1
64693	1.62611	2.1
64694	2.3148	2.1
64695	0.946131	2.1
64696	0.946131	2.1
64697	0.946131	2.1
64698	0.946131	2.1
64699	0.946131	2.1
64700	0.946131	2.1
64701	0.946131	2.1
64702	0.946131	2.1
64703	0.946131	2.1
64704	0.946131	2.1
64705	0.946131	2.1
64706	0.946131	2.1
64707	1.89226	2.1
64708	1.89226	2.1
64709	1.89226	2.1
64710	1.89226	2.1
64711	3.78453	2.1
64712	1.89226	2.1
64713	1.89226	2.1
64714	1.89226	2.1
64715	1.89226	2.1
64716	1.89226	2.1
64717	1.89226	2.1
64718	1.89227	2.1
64719	1.89227	2.1
64720	1.89227	2.1
64721	1.89227	2.1
64722	3.78453	2.1
64723	1.89226	2.1
64724	1.89226	2.1
64725	1.89226	2.1

64726	1.89226	2.1
64727	3.78453	2.1
64728	0.915781	2.1
64729	0.863524	2.1
64730	0.717056	2.1
64731	0.830182	2.1
64732	0.865094	2.1
64733	0.918003	2.1
64734	1.89226	2.1
64735	1.29933	2.1
64736	1.11215	2.1
64737	1.05227	2.1
64738	0.966269	2.1
64739	1.89226	2.1
64740	1.89226	2.1
64741	1.89226	2.1
64742	1.89226	2.1
64743	1.89226	2.1
64744	1.89226	2.1
64745	1.89226	2.1
64746	1.89226	2.1
64747	1.89226	2.1
64748	1.89226	2.1
64749	1.89226	2.1
64750	1.89226	2.1
64751	1.89226	2.1
64752	1.89226	2.1
64753	1.89226	2.1
64754	1.89226	2.1
64755	1.89226	2.1
64756	1.89226	2.1
64757	1.01725	2.1
64758	0.997048	2.1
64759	0.818657	2.1
64760	0.960724	2.1
64761	0.838013	2.1
64762	1.04859	2.1
64763	1.89226	2.1
64764	1.41754	2.1
64765	1.17625	2.1
64766	1.0793	2.1
64767	1.18822	2.1
64768	2.16402	2.1
64769	1.89452	2.1
64770	1.50084	2.1
64771	2.09288	2.1
64772	3.9874	2.1
64773	2.16402	2.1
64774	2.13925	2.1
64775	2.04483	2.1
64776	2.09288	2.1
64777	4.23212	2.1
64778	1.89226	2.1
64779	1.89226	2.1
64780	1.89226	2.1
64781	1.89226	2.1
64782	3.78452	2.1
64783	1.89226	2.1

64784	1.89226	2.1
64785	1.89226	2.1
64786	1.89226	2.1
64787	1.89226	2.1
64788	1.89226	2.1
64789	1.9824	2.1
64790	2.09288	2.1
64791	1.69889	2.1
64792	1.6817	2.1
64793	2.00695	2.1
64794	2.00695	2.1
64795	1.93592	2.1
64796	2.09288	2.1
64797	2.00695	2.1
64798	2.09288	2.1
64799	1.9589	2.1
64800	2.04483	2.1
64801	0.946128	2.1
64802	0.946128	2.1
64803	0.946132	2.1
64804	0.946132	2.1
64805	0.94613	2.1
64806	0.94613	2.1
64807	0.946129	2.1
64808	0.946129	2.1
64809	0.94613	2.1
64810	0.94613	2.1
64811	0.94613	2.1
64812	0.94613	2.1
64813	0.946131	2.1
64814	0.946131	2.1
64815	0.946131	2.1
64816	0.946131	2.1
64817	0.946131	2.1
64818	0.946131	2.1
64819	0.946131	2.1
64820	0.946131	2.1
64821	0.946131	2.1
64822	0.946131	2.1
64823	0.946131	2.1
64824	0.946131	2.1
64825	1.89226	1.22837
64826	2.48083	2.1
64827	2.6968	1.16832
64828	2.6968	2.1
64829	1.89226	1.21286
64830	1.89226	2.1
64831	1.89226	1.34101
64832	1.89226	2.1
64833	1.89226	1.24581
64834	1.89226	2.1
64835	3.78453	2.1
64836	1.89226	2.1
64837	1.89226	2.1
64838	1.89226	2.1
64839	1.89226	2.1
64840	1.89226	2.1
64841	1.89226	2.1

64842	1.1017	2.1
64843	1.1017	2.1
64844	1.10605	2.1
64845	1.01647	2.1
64846	0.868255	2.1
64847	0.883424	2.1
64848	1.24624	2.1
64849	1.35473	2.1
64850	0.942231	2.1
64851	1.31464	2.1
64852	0.910064	2.1
64853	1.27147	2.1
64854	1.89226	1.23216
64855	1.89226	2.1
64856	1.89226	0.978807
64857	1.89226	2.1
64858	3.78453	2.1
64859	1.89226	2.1
64860	1.89226	2.1
64861	1.89226	2.1
64862	1.89226	2.1
64863	1.89226	2.1
64864	1.89226	2.1
64865	1.89226	2.1
64866	1.89226	2.1
64867	1.89226	2.1
64868	1.89226	2.1
64869	3.78453	2.1
64870	1.89226	2.1
64871	2.70015	2.1
64872	1.89226	2.1
64873	2.61064	2.1
64874	4.59241	2.1
64875	2.70015	2.1
64876	2.3372	2.1
64877	2.61064	2.1
64878	1.85184	2.1
64879	4.55198	2.1
64880	1.89226	2.1
64881	1.89226	2.1
64882	1.89226	2.1
64883	1.89226	2.1
64884	1.89226	2.1
64885	1.89226	2.1
64886	1.89227	2.1
64887	1.89227	2.1
64888	1.89227	2.1
64889	1.89227	2.1
64890	3.78453	2.1
64891	1.89226	2.1
64892	2.52194	2.1
64893	2.61064	2.1
64894	2.61064	2.1
64895	1.89226	2.1
64896	1.89226	2.1
64897	1.095 2.1	
64898	1.095 2.1	
64899	1.09189	2.1

64900	1.09189	2.1
64901	0.875334	2.1
64902	0.925919	2.1
64903	1.26097	2.1
64904	1.30532	2.1
64905	1.26097	2.1
64906	0.875334	2.1
64907	1.30532	2.1
64908	0.92592	2.1
64909	1.89226	2.1
64910	1.89226	2.1
64911	1.89226	2.1
64912	1.89226	2.1
64913	1.89226	2.1
64914	1.89226	2.1
64915	1.89226	2.1
64916	1.89226	2.1
64917	1.89226	2.1
64918	1.89226	2.1
64919	3.78453	2.1
64920	1.26097	2.1
64921	1.26097	2.1
64922	0.946132	2.1
64923	0.946132	2.1
64924	1.22176	2.1
64925	1.26097	2.1
64926	0.946132	2.1
64927	0.946132	2.1
64928	0.946132	2.1
64929	1.22176	2.1
64930	1.26097	2.1
64931	0.946132	2.1
64932	2.52194	2.1
64933	1.67381	2.1
64934	1.67381	2.1
64935	1.75067	2.1
64936	2.44352	2.1
64937	2.52038	2.1
64938	1.89226	2.1
64939	1.89226	2.1
64940	1.89226	2.1
64941	1.89226	2.1
64942	1.89226	2.1
64943	1.89226	2.1
64944	1.89227	2.1
64945	1.89227	2.1
64946	1.89227	2.1
64947	1.89227	2.1
64948	1.89227	2.1
64949	1.89227	2.1
64950	1.22176	2.1
64951	1.22176	2.1
64952	0.946132	2.1
64953	0.946132	2.1
64954	1.19095	2.1
64955	1.22176	2.1
64956	0.946133	2.1
64957	0.946133	2.1

64958	1.19095	2.1
64959	0.946132	2.1
64960	0.946133	2.1
64961	1.22176	2.1
64962	2.44352	2.1
64963	1.6287	2.1
64964	1.6287	2.1
64965	1.67381	2.1
64966	2.3819	2.1
64967	2.427	2.1
64968	1.89226	2.1
64969	1.89226	2.1
64970	1.89226	2.1
64971	1.89226	2.1
64972	1.89226	2.1
64973	1.89226	2.1
64974	1.89226	2.1
64975	1.89226	2.1
64976	1.89226	1.89321
64977	1.89226	2.1
64978	3.78453	2.1
64979	1.89226	2.1
64980	1.89226	2.1
64981	1.89226	2.1
64982	1.89226	2.1
64983	1.89226	1.97404
64984	1.89226	2.1
64985	1.89226	2.1
64986	1.89226	2.1
64987	1.89226	2.1
64988	1.89226	2.1
64989	1.89226	2.1
64990	1.89226	2.1
64991	0.851542	2.1
64992	0.851542	2.1
64993	1.1736	2.1
64994	1.29261	2.1
64995	0.852708	2.1
64996	1.18843	2.1
64997	0.946132	2.1
64998	0.946132	2.1
64999	0.946132	2.1
65000	1.07901	2.1
65001	1.04208	2.1
65002	0.936669	2.1
65003	1.89227	1.20523
65004	1.89227	2.1
65005	1.89227	1.62273
65006	1.89227	2.1
65007	3.78453	2.1
65008	1.89226	2.1
65009	1.89226	2.1
65010	1.89226	1.73507
65011	1.89226	2.1
65012	3.78453	2.1
65013	1.89226	2.1
65014	1.89226	2.1
65015	1.89226	2.1

65016	1.89226	2.1
65017	1.89226	2.1
65018	1.89226	2.1
65019	0.946133	2.1
65020	0.946133	2.1
65021	1.28525	2.1
65022	1.28525	2.1
65023	1.07901	2.1
65024	1.28525	2.1
65025	0.946132	2.1
65026	0.946132	2.1
65027	0.946132	2.1
65028	1.28525	2.1
65029	0.946133	2.1
65030	1.07901	2.1
65031	1.89226	2.1
65032	1.89226	2.1
65033	1.89226	2.1
65034	1.89226	2.1
65035	1.89226	2.1
65036	1.89226	2.1
65037	1.89226	2.1
65038	1.89226	2.1
65039	1.89226	2.1
65040	1.89226	2.1
65041	1.89226	2.1
65042	1.89226	2.1
65043	1.24419	2.1
65044	1.09093	2.1
65045	1.22722	2.1
65046	1.18154	2.1
65047	1.27455	2.1
65048	1.08473	2.1
65049	1.15541	2.1
65050	1.15541	2.1
65051	1.29607	2.1
65052	1.23814	2.1
65053	1.13024	2.1
65054	1.12096	2.1
65055	1.18858	2.1
65056	1.13181	2.1
65057	0.988718	2.1
65058	0.802498	2.1
65059	0.994034	2.1
65060	1.15773	2.1
65061	1.0029	2.1
65062	1.0029	2.1
65063	1.23814	2.1
65064	0.997523	2.1
65065	1.11498	2.1
65066	0.899532	2.1
65067	1.89226	2.1
65068	1.89226	2.1
65069	1.89226	2.1
65070	1.89226	2.1
65071	1.89226	2.1
65072	1.89226	2.1
65073	1.89226	2.1

65074	1.89226	2.1
65075	1.89226	2.1
65076	1.89226	2.1
65077	1.89226	2.1
65078	1.89226	2.1
65079	0.993139	2.1
65080	0.834108	2.1
65081	1.01147	2.1
65082	1.01358	2.1
65083	1.20067	2.1
65084	0.993156	2.1
65085	2.4158	2.1
65086	1.69956	2.1
65087	1.3544	2.1
65088	1.60095	2.1
65089	1.11219	2.1
65090	1.89226	2.1
65091	1.89226	2.1
65092	1.89226	2.1
65093	1.89226	2.1
65094	3.78452	2.1
65095	1.89226	2.1
65096	1.89226	2.1
65097	1.89226	2.1
65098	1.89226	2.1
65099	1.89226	2.1
65100	1.89226	2.1
65101	1.89226	2.1
65102	2.83014	2.1
65103	2.32404	2.1
65104	2.32404	2.1
65105	1.89226	2.1
65106	1.89226	2.1
65107	1.37966	2.1
65108	1.13294	2.1
65109	0.881723	2.1
65110	0.582304	2.1
65111	0.977944	2.1
65112	0.977948	2.1
65113	1.0264	2.1
65114	1.0264	2.1
65115	1.37967	2.1
65116	1.03896	2.1
65117	1.19108	2.1
65118	0.582298	2.1
65119	1.89226	1.99544
65120	1.89226	2.1
65121	1.89226	2.1
65122	1.89226	2.1
65123	3.78452	2.1
65124	0.946131	2.1
65125	0.946131	2.1
65126	0.946131	2.1
65127	0.946131	2.1
65128	0.946132	2.1
65129	0.946132	2.1
65130	0.946129	2.1
65131	0.946129	2.1

65132	0.946131	2.1
65133	0.946131	2.1
65134	0.94613	2.1
65135	0.94613	2.1
65136	1.89226	2.1
65137	1.89226	2.1
65138	1.89226	2.1
65139	1.89226	2.1
65140	3.78453	2.1
65141	1.89226	2.1
65142	1.89226	2.1
65143	1.89226	2.1
65144	1.89226	2.1
65145	1.89226	2.1
65146	1.89226	2.1
65147	2.65216	2.1
65148	1.93528	2.1
65149	1.78595	2.1
65150	2.48544	2.1
65151	4.42072	2.1
65152	1.89226	2.1
65153	1.89226	2.1
65154	1.89226	2.1
65155	1.89226	2.1
65156	1.89226	2.1
65157	1.89226	2.1
65158	1.89226	2.1
65159	1.89226	2.1
65160	1.89226	2.1
65161	1.89226	2.1
65162	3.78452	2.1
65163	1.66537	2.1
65164	2.48544	2.1
65165	1.78595	2.1
65166	1.78595	2.1
65167	2.36197	2.1
65168	2.36197	2.1
65169	2.80975	2.1
65170	2.48544	2.1
65171	2.36198	2.1
65172	2.65135	2.1
65173	5.13679	2.1
65174	1.89226	2.1
65175	1.89226	2.1
65176	1.89226	2.1
65177	1.89226	2.1
65178	1.89226	2.1
65179	1.89226	2.1
65180	0.946127	2.1
65181	0.946127	2.1
65182	0.946131	2.1
65183	0.946131	2.1
65184	0.946128	2.1
65185	0.946128	2.1
65186	0.94613	2.1
65187	0.94613	2.1
65188	0.946129	2.1
65189	0.946129	2.1

65190	0.946129	2.1
65191	0.946129	2.1
65192	1.11972	2.1
65193	1.18099	2.1
65194	0.832687	2.1
65195	0.764517	2.1
65196	0.974426	2.1
65197	0.974426	2.1
65198	0.971078	2.1
65199	0.971078	2.1
65200	1.11972	2.1
65201	0.764515	2.1
65202	0.832686	2.1
65203	1.18099	2.1
65204	2.36198	2.1
65205	2.48025	2.1
65206	2.48025	2.1
65207	2.65135	2.1
65208	2.23944	2.1
65209	2.41055	2.1
65210	0.946132	1.86422
65211	0.946132	2.1
65212	0.946131	2.1
65213	0.946131	2.1
65214	0.946131	2.1
65215	0.946131	2.1
65216	0.946132	2.1
65217	0.946132	2.1
65218	0.946131	2.1
65219	0.946131	2.1
65220	0.946132	2.1
65221	0.946132	2.1
65222	0.810942	2.1
65223	0.810942	2.1
65224	0.810944	2.1
65225	0.810944	2.1
65226	1.89226	2.1
65227	1.3517	2.1
65228	1.08132	2.1
65229	1.08133	2.1
65230	1.08132	2.1
65231	0.810932	2.1
65232	0.810932	2.1
65233	1.89249	2.07934
65234	1.9857	1.59331
65235	2.02433	1.33119
65236	2.15807	1.28734
65237	4.01003	1.44068
65238	1.89226	1.22248
65239	1.89226	1.82192
65240	1.89226	1.58226
65241	1.89226	1.01886
65242	3.78452	1.71366
65243	1.89226	1.58898
65244	1.89226	1.53577
65245	1.89226	0.916226
65246	1.89226	1.3752
65247	3.78452	1.04137

65248	1.98569	1.29974
65249	1.86067	1.7106
65250	2.15807	1.17366
65251	2.07965	1.17087
65252	4.06534	1.77467
65253	2.02433	1.5119
65254	2.15807	1.88681
65255	2.12608	2.1
65256	2.3025	1.06127
65257	4.32683	1.83645
65258	1.89226	1.73397
65259	1.89226	1.35395
65260	1.89226	1.04031
65261	1.89226	1.09566
65262	3.78452	1.11968
65263	1.89226	1.09032
65264	1.89226	1.89596
65265	1.89226	1.08399
65266	1.89226	1.08442
65267	3.78452	1.62715
65268	2.15807	1.68684
65269	2.07965	1.58426
65270	2.3025	1.02462
65271	2.28384	1.21121
65272	4.38215	1.10628
65273	1.89226	0.97717
65274	1.89226	0.740507
65275	1.89226	0.873482
65276	1.89226	0.761289
65277	3.78453	0.820235
65278	2.16905	0.557574
65279	2.56369	0.735506
65280	1.88394	0.852427
65281	2.41938	0.153655
65282	4.44763	0.547826
65283	2.56369	0.71156
65284	2.80663	0.582425
65285	2.41938	0.141188
65286	2.69045	0.292879
65287	5.25414	0.271966
65288	1.89226	0.710791
65289	1.89226	0.71784
65290	1.89226	0.75779
65291	1.89226	0.297811
65292	3.78453	0.673337
65293	1.89226	0.846056
65294	1.89226	0.757773
65295	1.89226	0.946349
65296	1.89226	0.754756
65297	1.89226	0.913099
65298	1.89226	0.727646
65299	1.01988	0.228438
65300	1.01988	0.195567
65301	1.03333	0.580628
65302	1.03333	0.744771
65303	1.13811	0.221668
65304	1.20969	0.184149
65305	0.941974	0.63722

65306	0.843515	0.337916
65307	0.843514	0.294796
65308	1.13811	0.237866
65309	0.941973	0.729698
65310	1.20969	0.263208
65311	2.41938	0.132529
65312	2.57414	0.383406
65313	2.57414	0.762641
65314	2.69045	0.724265
65315	2.27623	0.164665
65316	2.39253	0.675452
65317	1.89227	0.746982
65318	1.89227	0.390392
65319	1.89227	0.602972
65320	1.89227	0.652931
65321	1.89227	0.562105
65322	1.89227	0.691366
65323	2.28385	1.25654
65324	2.09114	1.43974
65325	2.46968	0.930619
65326	2.32438	1.28504
65327	4.56082	0.980336
65328	1.89226	1.00219
65329	1.89226	1.77448
65330	1.89226	1.01036
65331	1.89226	1.26271
65332	3.78452	1.38424
65333	1.89226	1.01003
65334	1.89226	1.89153
65335	1.89226	0.981089
65336	1.89226	1.19254
65337	3.78452	1.07646
65338	2.09114	0.952758
65339	1.5249	1.7806
65340	2.55684	0.926159
65341	1.86899	1.25401
65342	3.96011	1.26059
65343	2.46968	1.09051
65344	2.32439	1.66808
65345	2.62768	1.02103
65346	2.506	1.19316
65347	4.97568	1.77248
65348	1.89226	1.25909
65349	1.89226	1.34162
65350	1.89226	0.826986
65351	1.89226	1.22001
65352	3.78452	0.963927
65353	1.89226	0.997306
65354	1.89226	1.23203
65355	1.89226	1.09388
65356	1.89226	2.1
65357	1.89226	0.877354
65358	1.89226	2.1
65359	2.55684	1.08554
65360	2.16506	1.18514
65361	1.86901	1.65629
65362	1.86899	1.12567
65363	2.75664	1.06399

65364	2.75662	0.949161
65365	1.89224	0.210064
65366	1.89224	0.765235
65367	1.89224	0.350614
65368	1.89224	0.858185
65369	3.78447	0.276248
65370	2.24206	0.324859
65371	2.38862	0.948073
65372	2.07107	0.524154
65373	2.23097	0.849629
65374	4.47303	0.787007
65375	2.38863	0.954222
65376	2.25945	1.08929
65377	2.25945	0.849763
65378	2.39244	2.1
65379	2.23098	0.852721
65380	2.36397	2.1
65381	1.89224	0.684629
65382	1.89224	1.28269
65383	1.89224	0.906697
65384	1.89224	0.846845
65385	1.89224	0.895873
65386	1.89224	0.853437
65387	1.89226	0.409609
65388	1.89226	0.8237
65389	1.89226	0.794128
65390	1.89226	0.444953
65391	1.89226	0.650546
65392	1.89226	0.787651
65393	0.920975	1.07645
65394	0.920979	0.576254
65395	1.03015	0.824094
65396	1.03015	0.274751
65397	0.992407	0.831112
65398	1.08759	0.990792
65399	0.854346	0.608595
65400	0.87241	0.302654
65401	0.872397	0.744555
65402	1.04334	0.741695
65403	1.00965	0.738221
65404	1.0876	0.719666
65405	2.23101	0.940143
65406	2.25948	1.29154
65407	2.03577	0.84377
65408	2.05257	2.1
65409	4.29524	0.975087
65410	0.810947	0.592291
65411	0.810947	0.895432
65412	0.810947	0.824784
65413	0.810947	0.755595
65414	0.810934	0.844205
65415	0.810934	0.848071
65416	1.89227	1.30497
65417	1.3517	0.916393
65418	1.08132	0.858311
65419	1.08132	0.898426
65420	1.08133	0.858102
65421	1.89226	1.17059

65422	1.89226	1.45766
65423	1.89226	1.03615
65424	1.89226	2.1
65425	1.89226	0.866086
65426	1.89226	2.1
65427	1.89226	1.51865
65428	1.89226	1.44582
65429	1.89226	1.05604
65430	1.89226	2.1
65431	1.89226	1.02004
65432	1.89226	2.1
65433	0.879432	1.93948
65434	0.879432	1.14412
65435	0.900223	0.843031
65436	0.900223	2.00004
65437	0.54571	1.88338
65438	0.743092	1.25866
65439	1.03656	1.89134
65440	1.19234	0.948055
65441	1.19234	1.25112
65442	0.743092	1.59595
65443	1.03656	1.60385
65444	0.545711	1.32883
65445	1.89226	0.874312
65446	2.38467	1.63393
65447	2.69293	0.889718
65448	2.69293	1.90368
65449	1.89226	0.830548
65450	1.89226	1.43597
65451	1.89226	1.07497
65452	1.89226	1.45615
65453	1.89226	0.891053
65454	1.89226	1.72908
65455	3.78452	1.15401
65456	0.946129	2.1
65457	0.946129	1.70882
65458	0.946133	0.9107
65459	0.946133	2.0972
65460	0.946131	1.15703
65461	0.946131	2.1
65462	0.946131	0.901439
65463	0.946131	1.23026
65464	0.946131	1.29696
65465	0.946131	1.30098
65466	0.946131	1.17704
65467	0.946131	1.16254
65468	1.04846	0.867455
65469	1.04846	1.68036
65470	1.05594	0.865163
65471	1.05594	1.92454
65472	0.743092	1.53239
65473	0.912061	1.10946
65474	1.19234	0.870289
65475	1.34647	0.936204
65476	0.912061	0.900958
65477	1.34646	1.0239
65478	1.19234	1.01208
65479	0.743093	1.01051

65480	1.89226	0.992342
65481	1.89226	1.74116
65482	1.89226	0.843656
65483	1.89226	1.01597
65484	3.78452	1.00035
65485	0.946128	2.1
65486	0.946128	1.15786
65487	0.946133	1.30795
65488	0.946133	0.947864
65489	0.94613	2.1
65490	0.94613	1.91499
65491	0.946131	1.23423
65492	0.946131	0.99982
65493	0.946131	1.65967
65494	0.946131	1.588
65495	0.946131	1.14976
65496	0.946131	1.15318
65497	1.89226	1.19649
65498	1.89226	2.1
65499	1.89226	0.939194
65500	1.89226	1.1935
65501	3.78452	1.40826
65502	1.89226	1.08224
65503	1.89226	1.15457
65504	1.89226	1.05837
65505	1.89226	2.1
65506	1.89226	0.834577
65507	1.89226	2.1
65508	1.89226	1.18234
65509	1.89226	1.26778
65510	1.89226	1.00255
65511	1.89226	2.1
65512	1.89226	0.931068
65513	1.89226	2.1
65514	0.946132	1.96466
65515	0.946132	1.15623
65516	1.17758	0.9242
65517	1.17759	2.06937
65518	1.04514	1.89976
65519	1.17758	1.0817
65520	0.946133	0.901202
65521	0.946133	0.976907
65522	0.946107	1.10658
65523	1.17755	1.09756
65524	1.04517	1.14473
65525	0.946158	1.10481
65526	1.89226	2.1
65527	2.10171	2.1
65528	1.95064	2.1
65529	2.10171	2.1
65530	1.7412	2.1
65531	1.89226	2.1
65532	1.89226	2.1
65533	1.89226	2.1
65534	1.89226	2.1
65535	1.89226	2.1
65536	1.89226	2.1
65537	1.89226	2.1

65538	1.89226	2.1
65539	1.89226	1.10834
65540	1.89226	2.1
65541	1.89226	2.1
65542	3.78453	2.1
65543	0.946133	2.1
65544	0.946133	2.01884
65545	0.946132	2.1
65546	0.946132	2.1
65547	0.946133	2.1
65548	0.946133	2.1
65549	0.946132	2.1
65550	0.946132	2.1
65551	0.946132	2.1
65552	0.946132	2.1
65553	0.946132	2.1
65554	0.946132	2.1
65555	1.70303	2.1
65556	1.95064	2.1
65557	1.74039	2.1
65558	1.95064	2.1
65559	1.69368	2.1
65560	1.89226	2.1
65561	1.89226	2.1
65562	1.89226	2.1
65563	1.89226	2.1
65564	1.89226	2.1
65565	3.78453	2.1
65566	0.946132	1.05357
65567	0.946132	2.1
65568	0.946132	2.1
65569	0.946132	2.1
65570	0.946133	2.07896
65571	0.946133	1.09892
65572	0.946132	2.1
65573	0.946132	2.1
65574	0.946107	2.1
65575	0.946107	2.1
65576	0.946158	2.1
65577	0.946158	2.1
65578	0.946133	2.1
65579	0.946133	2.1
65580	0.946132	2.1
65581	0.946132	2.1
65582	0.946133	2.1
65583	0.946133	1.99295
65584	0.946132	2.1
65585	0.946132	2.1
65586	0.946107	2.1
65587	0.946107	2.1
65588	0.946158	2.1
65589	0.946158	2.1
65590	1.89226	2.1
65591	1.89226	2.1
65592	1.89226	1.76308
65593	1.89226	2.1
65594	1.89226	1.61092
65595	1.89226	2.1

65596	1.89226	2.1
65597	1.89226	2.1
65598	1.89226	2.1
65599	1.89226	2.1
65600	1.89226	2.1
65601	1.89226	2.1
65602	2.5198	2.1
65603	2.45893	2.1
65604	2.27322	2.1
65605	2.27322	2.1
65606	2.63576	2.1
65607	2.63576	2.1
65608	1.24045	2.1
65609	1.11906	2.1
65610	1.13578	2.1
65611	0.916205	2.1
65612	1.12539	2.1
65613	1.08404	2.1
65614	1.08484	2.1
65615	1.08484	2.1
65616	1.22836	2.1
65617	0.919363	2.1
65618	1.16507	2.1
65619	0.924864	2.1
65620	1.89226	2.1
65621	1.89226	2.08437
65622	1.89226	1.19585
65623	1.89226	2.1
65624	1.89226	1.12453
65625	1.89226	2.1
65626	1.89226	2.1
65627	1.89226	2.1
65628	1.89226	2.1
65629	1.89226	2.1
65630	1.89226	2.1
65631	1.89226	2.1
65632	2.6946	2.1
65633	2.63576	2.1
65634	2.5198	2.1
65635	2.5198	2.1
65636	2.8362	2.1
65637	2.8362	2.1
65638	1.3265	2.1
65639	1.24045	2.1
65640	0.983223	2.1
65641	0.954429	2.1
65642	1.06046	2.1
65643	1.10594	2.1
65644	1.1915	2.08092
65645	1.1915	2.1
65646	1.31357	2.1
65647	1.09005	2.1
65648	1.29145	2.1
65649	0.995825	2.1
65650	1.89226	2.1
65651	1.89226	2.1
65652	1.89226	2.1
65653	1.89226	2.1

65654	3.78452	2.1
65655	1.89226	2.1
65656	1.89226	2.1
65657	1.89226	2.1
65658	1.89226	2.1
65659	3.78453	2.1
65660	1.91802	2.1
65661	2.27409	2.1
65662	2.48048	2.1
65663	2.22264	2.1
65664	1.91802	2.1
65665	1.66018	2.1
65666	1.13704	2.1
65667	1.24023	2.1
65668	1.22947	2.1
65669	1.31788	2.1
65670	1.23859	2.1
65671	1.23859	2.1
65672	1.23111	2.1
65673	1.23111	2.1
65674	1.24024	2.1
65675	1.31788	2.1
65676	1.13704	2.1
65677	1.22947	2.1
65678	1.89226	2.1
65679	1.89226	2.1
65680	1.89226	2.1
65681	1.89226	2.1
65682	1.89226	2.1
65683	1.89226	2.1
65684	1.89226	2.1
65685	1.89226	2.1
65686	1.89226	2.1
65687	1.89226	2.1
65688	3.78453	2.1
65689	1.05568	2.1
65690	0.959008	2.1
65691	1.32068	2.1
65692	1.24024	2.1
65693	1.15206	2.1
65694	1.15206	2.1
65695	1.14386	2.1
65696	1.14386	2.1
65697	1.05568	2.1
65698	1.32068	2.1
65699	0.959008	2.1
65700	1.24024	2.1
65701	1.32068	2.1
65702	1.24023	2.1
65703	1.31788	2.1
65704	1.4181	2.1
65705	1.31436	2.1
65706	1.31436	2.1
65707	1.3242	2.1
65708	1.3242	2.1
65709	1.32068	2.1
65710	1.4181	2.1
65711	1.24024	2.1

65712	1.31788	2.1
65713	1.89226	1.12551
65714	1.89226	0.773201
65715	1.89226	0.903018
65716	1.89226	0.903617
65717	1.89226	0.900302
65718	1.89226	0.94651
65719	1.02925	0.392724
65720	1.16678	0.651456
65721	0.946132	0.759934
65722	0.946132	0.96917
65723	0.935677	0.765896
65724	0.935677	0.44775
65725	1.18902	0.893286
65726	1.21562	0.839964
65727	1.13433	0.685977
65728	1.04071	0.754106
65729	0.851553	0.786563
65730	1.06075	0.708769
65731	1.89226	0.772329
65732	1.89226	0.613488
65733	1.89226	0.499919
65734	1.89226	0.316463
65735	1.89226	0.722292
65736	1.89226	0.793445
65737	1.89226	0.920687
65738	1.89226	0.225376
65739	1.89226	0.772891
65740	1.89226	0.766936
65741	3.78453	0.721377
65742	0.946128	0.782806
65743	0.946128	0.737159
65744	0.946133	0.734793
65745	0.946133	0.529789
65746	0.946131	0.712069
65747	0.946131	0.729841
65748	0.94613	0.737254
65749	0.94613	0.723133
65750	0.946131	0.748945
65751	0.946131	0.714765
65752	0.946131	0.36641
65753	0.946131	0.621666
65754	0.946132	0.815696
65755	0.946132	0.732693
65756	0.946132	0.885057
65757	0.946132	0.763721
65758	0.946132	0.768357
65759	0.946132	0.408216
65760	0.946131	0.807218
65761	0.946131	0.873096
65762	0.946132	0.798551
65763	0.946132	0.823589
65764	0.946132	0.516225
65765	0.946132	0.773392
65766	0.946131	1.89027
65767	0.946131	1.14255
65768	0.946131	2.1
65769	0.946131	0.924637

65770	0.946131	0.988619
65771	0.946131	1.6657
65772	0.946131	0.915587
65773	0.946131	0.897552
65774	0.946131	1.05226
65775	0.946131	1.04249
65776	0.946131	1.10143
65777	0.946131	1.11471
65778	1.89226	1.10954
65779	1.89226	1.15803
65780	1.89226	0.956448
65781	1.89226	2.1
65782	1.89226	0.90193
65783	1.89226	2.1
65784	1.89226	1.04482
65785	1.89226	1.00686
65786	1.89226	1.02455
65787	1.89226	1.81227
65788	1.89226	0.919523
65789	1.89226	2.1
65790	1.89226	1.16712
65791	1.89226	1.03408
65792	1.89226	0.922523
65793	1.89226	2.09359
65794	1.89226	0.844919
65795	1.89226	2.1
65796	1.89226	1.52611
65797	1.89226	2.00278
65798	1.89226	0.932505
65799	1.89226	2.1
65800	3.78452	1.37109
65801	0.946131	1.82967
65802	0.946131	0.954459
65803	1.17195	0.923903
65804	1.17195	2.06982
65805	0.958489	2.04597
65806	1.17195	1.27429
65807	0.946129	0.933528
65808	0.946129	1.20966
65809	0.946131	1.10286
65810	1.17195	1.10741
65811	0.958487	1.17514
65812	0.94613	1.17718
65813	2.69656	2.1
65814	2.17516	2.1
65815	2.41885	2.1
65816	2.41885	1.48886
65817	2.46476	2.1
65818	2.46476	1.06606
65819	2.69657	2.06256
65820	2.30233	0.8895
65821	2.30233	1.28737
65822	2.59476	1.03465
65823	2.46477	1.32163
65824	2.7572	1.08565
65825	1.89226	1.60209
65826	1.89226	0.762083
65827	1.89226	0.828577

65828	1.89226	0.854307
65829	1.89226	0.819691
65830	1.89226	0.909879
65831	1.89226	1.60632
65832	1.89226	1.05346
65833	1.89226	2.1
65834	1.89226	0.83333
65835	3.78452	1.09198
65836	2.46477	2.03847
65837	2.17516	2.1
65838	1.80252	2.1
65839	2.09715	0.971008
65840	4.27231	2.1
65841	2.46477	1.97597
65842	2.09715	0.953557
65843	2.30233	1.04184
65844	2.30233	0.997518
65845	1.96416	0.825314
65846	2.09715	0.820144
65847	1.89227	0.941857
65848	1.89227	0.735818
65849	1.89227	0.820131
65850	1.89227	0.821777
65851	1.89227	0.847358
65852	1.89227	0.897532
65853	1.89226	2.04524
65854	1.89226	0.824073
65855	1.89226	0.955868
65856	1.89226	0.987925
65857	3.78452	0.961389
65858	0.946132	2.1
65859	0.946132	2.1
65860	0.946132	2.1
65861	0.946132	2.1
65862	0.946132	2.1
65863	0.946132	2.1
65864	0.946132	2.1
65865	0.946132	2.1
65866	0.946132	2.1
65867	0.946132	2.1
65868	0.946131	2.1
65869	0.946131	2.1
65870	1.89226	2.1
65871	1.89226	2.1
65872	1.89226	2.1
65873	1.89226	2.1
65874	3.78453	2.1
65875	1.89226	2.1
65876	1.89226	2.1
65877	1.89226	2.1
65878	1.89226	2.1
65879	1.89226	2.1
65880	1.89226	2.1
65881	1.89226	2.1
65882	1.63707	2.1
65883	1.63707	2.1
65884	1.38859	2.1
65885	1.89226	2.1

65886	1.64377	2.1
65887	0.94613	2.1
65888	0.94613	2.1
65889	0.94613	2.1
65890	0.94613	2.1
65891	0.946131	2.1
65892	0.946131	2.1
65893	0.946129	2.1
65894	0.946129	2.1
65895	0.94613	2.1
65896	0.94613	2.1
65897	0.94613	2.1
65898	0.94613	2.1
65899	2.15888	2.1
65900	1.99309	2.1
65901	2.16841	2.1
65902	2.16841	2.1
65903	2.02311	1.05262
65904	2.02311	0.974614
65905	1.89226	1.12747
65906	1.89226	1.17878
65907	1.89226	2.1
65908	1.89226	1.15511
65909	1.89226	1.88578
65910	1.89226	0.98342
65911	1.89226	1.55764
65912	1.89226	2.1
65913	1.89226	2.1
65914	1.89226	1.31522
65915	1.89226	2.1
65916	1.89226	1.6294
65917	1.99309	2.1
65918	1.96035	2.1
65919	1.72526	2.1
65920	1.72526	1.72642
65921	2.16841	2.1
65922	2.16841	2.1
65923	2.15888	2.1
65924	2.16841	2.1
65925	2.3111	2.1
65926	2.35938	2.1
65927	4.51826	2.1
65928	1.89226	2.1
65929	1.89226	1.08703
65930	1.89226	1.03145
65931	1.89226	0.601611
65932	3.78452	2.1
65933	1.89226	1.45997
65934	1.89226	2.1
65935	1.89226	2.1
65936	1.89226	0.842595
65937	3.78452	2.1
65938	2.16841	2.1
65939	1.96035	2.1
65940	2.35938	2.1
65941	2.18493	2.1
65942	4.31973	2.1
65943	1.89226	2.1

65944	1.89226	2.1
65945	1.89226	2.1
65946	1.89226	2.1
65947	3.78453	2.1
65948	1.89226	2.1
65949	1.89226	2.1
65950	1.89226	2.1
65951	1.89226	2.1
65952	3.78452	2.1
65953	0.946158	2.1
65954	0.946158	2.1
65955	0.818509	2.1
65956	0.818509	2.1
65957	0.933975	2.1
65958	0.818539	2.1
65959	0.946128	2.1
65960	0.946128	2.1
65961	0.94613	2.1
65962	0.933974	2.1
65963	0.818537	2.1
65964	0.946129	2.1
65965	0.946131	2.1
65966	0.946131	2.1
65967	0.946131	2.1
65968	0.946131	2.1
65969	0.946132	2.1
65970	0.946132	2.1
65971	0.946129	2.1
65972	0.946129	2.1
65973	0.946131	2.1
65974	0.946131	2.1
65975	0.94613	2.1
65976	0.94613	2.1
65977	1.89226	2.1
65978	1.89226	2.1
65979	1.89226	2.1
65980	1.89226	2.1
65981	3.78453	2.1
65982	1.89226	2.1
65983	1.89226	2.1
65984	1.89226	2.1
65985	1.89226	2.1
65986	3.78452	2.1
65987	1.89226	2.1
65988	1.8706	2.1
65989	1.8706	2.1
65990	1.68112	2.1
65991	2.0815	2.1
65992	1.88715	2.1
65993	0.94613	2.1
65994	0.94613	2.1
65995	0.94613	2.1
65996	0.94613	2.1
65997	0.946131	2.1
65998	0.946131	2.1
65999	0.946129	2.1
66000	0.946129	2.1
66001	0.94613	2.1

66002	0.94613	2.1
66003	0.94613	2.1
66004	0.94613	2.1
66005	1.76401	2.1
66006	2.60983	2.1
66007	2.50461	2.1
66008	2.35897	2.1
66009	1.90965	2.1
66010	1.76401	2.1
66011	1.89226	2.1
66012	1.89226	1.83126
66013	1.89226	2.1
66014	1.89226	2.1
66015	3.78452	2.1
66016	1.89226	1.78584
66017	1.89226	2.1
66018	1.89226	2.1
66019	1.89226	1.07641
66020	1.89226	2.1
66021	1.89226	1.14293
66022	1.90965	2.1
66023	2.83834	2.1
66024	2.7538	2.1
66025	2.60983	2.1
66026	2.05361	2.1
66027	1.90965	2.1
66028	1.89226	2.1
66029	1.89226	2.1
66030	1.89226	2.1
66031	1.89226	1.76982
66032	1.89226	2.1
66033	1.89226	1.75837
66034	1.89226	2.01622
66035	1.89226	2.1
66036	1.89226	2.1
66037	1.89226	1.10033
66038	3.78452	2.1
66039	2.83834	2.1
66040	1.89226	2.1
66041	2.60983	2.1
66042	2.60983	2.1
66043	1.89226	2.1
66044	1.89226	2.1
66045	1.89226	2.1
66046	1.89226	2.1
66047	1.89226	2.1
66048	1.89226	1.55499
66049	1.89226	2.1
66050	1.89226	1.22388
66051	1.89226	1.18545
66052	1.89226	2.1
66053	1.89226	2.1
66054	1.89226	1.11592
66055	1.89226	2.1
66056	1.89226	1.14459
66057	2.44785	2.1
66058	1.80223	2.1
66059	2.80894	2.1

66060	2.18296	1.84904
66061	4.63081	2.1
66062	1.89226	1.76712
66063	1.89226	2.1
66064	1.89226	2.1
66065	1.89226	1.23918
66066	1.89226	2.1
66067	1.89226	1.2316
66068	0.946131	1.04373
66069	0.946131	1.30604
66070	0.946127	1.90436
66071	0.946127	2.1
66072	0.946131	1.16871
66073	0.946131	2.1
66074	0.946128	2.1
66075	0.946128	1.51948
66076	0.94613	1.8488
66077	0.94613	1.86264
66078	0.946129	1.8469
66079	0.946129	1.73525
66080	1.89226	1.37153
66081	1.89226	2.1
66082	1.89226	2.1
66083	1.89226	1.66561
66084	3.78452	2.1
66085	1.89226	2.1
66086	1.89226	2.1
66087	1.89226	2.1
66088	1.89226	2.1
66089	3.78453	2.1
66090	2.41083	2.1
66091	2.93937	2.1
66092	1.68761	2.1
66093	1.68761	2.1
66094	1.67248	2.1
66095	2.3957	2.1
66096	1.89226	2.1
66097	1.89226	1.31112
66098	1.89226	1.19006
66099	1.89226	2.1
66100	1.89226	1.1803
66101	1.89226	2.1
66102	1.89227	2.1
66103	1.89227	2.1
66104	1.89227	2.1
66105	1.89227	2.1
66106	1.89227	2.1
66107	1.89227	2.1
66108	1.89224	0.816694
66109	1.89224	0.74209
66110	1.89224	0.882164
66111	1.89224	0.817118
66112	3.78448	0.770193
66113	1.89224	1.06453
66114	1.89224	0.826307
66115	1.89224	1.01262
66116	1.89224	0.823271
66117	1.89224	1.02711

66118	1.89224	0.894354
66119	1.11152	0.785604
66120	1.29346	0.909206
66121	0.978596	0.976251
66122	0.918324	1.09296
66123	1.06821	1.03024
66124	1.06821	0.76924
66125	0.985654	1.1242
66126	1.15278	1.05112
66127	1.07693	1.00162
66128	0.902432	1.03621
66129	0.968939	0.974741
66130	1.34644	0.918417
66131	0.946132	0.769633
66132	0.946132	0.809213
66133	0.946132	0.811567
66134	0.946132	0.776694
66135	0.946131	0.784532
66136	0.946131	0.737934
66137	0.946132	0.90934
66138	0.946132	0.761692
66139	0.946132	0.80582
66140	0.946132	0.79654
66141	0.946132	0.738562
66142	0.946132	0.741549
66143	0.946133	0.864047
66144	0.946133	0.789218
66145	0.946132	0.900274
66146	0.946132	0.975324
66147	0.946132	0.918516
66148	0.946132	0.729831
66149	0.946133	1.20425
66150	0.946133	0.810273
66151	0.946132	0.936625
66152	0.946132	0.904861
66153	0.946132	0.802354
66154	0.946132	0.802827
66155	1.89224	0.969979
66156	1.89224	2.1
66157	1.89224	1.11684
66158	1.89224	1.94939
66159	1.89224	1.13402
66160	1.89224	2.1
66161	1.89224	1.11373
66162	1.89224	2.1
66163	1.89224	1.87675
66164	1.89224	2.1
66165	1.89224	1.67438
66166	1.89224	2.1
66167	1.89226	1.05618
66168	1.89226	2.1
66169	1.89226	2.1
66170	1.89226	1.18988
66171	3.78453	2.1
66172	1.11056	2.1
66173	1.21595	2.1
66174	1.12763	2.1
66175	1.12763	2.1

66176	1.23339	2.1
66177	1.35697	2.1
66178	1.03581	2.1
66179	0.95954	2.1
66180	0.89761	2.1
66181	1.26602	2.1
66182	1.12749	2.1
66183	1.45307	2.1
66184	2.71775	2.1
66185	1.89226	2.1
66186	1.89226	2.1
66187	1.89226	2.1
66188	2.47025	2.1
66189	2.47025	2.1
66190	0.94613	2.1
66191	0.94613	2.1
66192	0.94613	1.19409
66193	0.94613	2.07112
66194	0.946128	1.86978
66195	0.946128	2.1
66196	0.946132	2.1
66197	0.946132	1.54998
66198	0.94613	2.1
66199	0.94613	2.1
66200	0.94613	2.1
66201	0.94613	2.1
66202	1.89226	1.00047
66203	1.89226	1.94077
66204	1.89226	2.1
66205	1.89226	1.07564
66206	1.89226	2.0868
66207	1.89226	1.1405
66208	1.89226	1.0086
66209	1.89226	1.92654
66210	1.89226	1.81683
66211	1.89226	0.992267
66212	1.89226	1.74298
66213	1.89226	1.07937
66214	0.946132	1.27411
66215	0.946132	1.59108
66216	0.946133	2.1
66217	0.946133	2.1
66218	0.946133	2.1
66219	0.946133	1.22995
66220	0.946132	1.1906
66221	0.946132	2.1
66222	0.946133	2.1
66223	0.946133	2.1
66224	0.946132	2.1
66225	0.946132	2.1
66226	1.89226	1.03207
66227	1.89226	1.96319
66228	1.89226	2.07703
66229	1.89226	1.15399
66230	1.89226	2.09696
66231	1.89226	1.17621
66232	0.946131	1.20477
66233	0.946131	1.01604

66234	0.946127	1.77908
66235	0.946127	2.02638
66236	0.946131	1.15512
66237	0.946131	2.1
66238	0.946127	2.1
66239	0.946127	1.16486
66240	0.94613	1.47666
66241	0.94613	1.46065
66242	0.946129	1.37497
66243	0.946129	1.37837
66244	1.94291	2.1
66245	2.00118	2.1
66246	2.36337	2.1
66247	2.41217	2.1
66248	4.35508	2.1
66249	1.89226	2.1
66250	1.89226	1.96727
66251	1.89226	2.1
66252	1.89226	2.1
66253	3.78452	2.1
66254	1.89226	2.00641
66255	1.89226	2.1
66256	1.89226	2.1
66257	1.89226	1.90515
66258	3.78452	2.1
66259	1.06209	2.1
66260	1.06209	2.1
66261	1.05987	2.1
66262	1.05987	2.1
66263	0.920378	2.1
66264	1.12137	2.1
66265	1.00059	2.1
66266	1.20608	2.1
66267	1.20608	2.1
66268	1.12137	2.1
66269	1.00059	2.1
66270	0.920376	2.1
66271	2.77222	2.1
66272	2.41216	2.1
66273	2.77002	2.1
66274	2.36337	2.1
66275	2.81882	2.1
66276	2.41216	2.1
66277	1.89226	2.01074
66278	1.89226	2.1
66279	1.89226	2.1
66280	1.89226	2.1
66281	1.89226	2.1
66282	1.89226	1.63067
66283	1.89226	2.1
66284	1.89226	1.46297
66285	1.89226	1.87211
66286	1.89226	2.1
66287	3.78451	2.1
66288	2.41216	2.1
66289	2.24273	2.1
66290	2.81882	2.1
66291	2.68473	2.1

66292	5.09689	2.1
66293	1.89226	2.1
66294	1.89226	2.1
66295	1.89226	2.1
66296	1.89226	2.1
66297	3.78453	2.1
66298	2.17614	2.1
66299	2.65326	2.1
66300	1.93101	2.1
66301	2.58552	2.1
66302	4.58427	2.1
66303	2.65326	2.1
66304	1.89226	2.1
66305	1.89226	2.1
66306	1.89226	2.1
66307	2.58552	2.1
66308	2.58552	2.1
66309	1.89226	2.1
66310	1.89226	2.1
66311	1.89226	2.1
66312	1.89226	2.1
66313	3.78453	2.1
66314	1.89226	2.1
66315	1.89226	2.1
66316	1.89226	2.1
66317	1.89226	2.1
66318	3.78453	2.1
66319	1.93101	2.1
66320	2.58552	2.1
66321	1.46507	2.1
66322	2.52893	2.1
66323	4.45994	2.1
66324	1.26446	2.1
66325	1.26446	2.1
66326	0.946132	2.1
66327	0.946132	2.1
66328	0.94613	2.1
66329	0.94613	2.1
66330	1.29276	2.1
66331	1.26446	2.1
66332	1.26446	2.1
66333	0.94613	2.1
66334	0.94613	2.1
66335	1.29276	2.1
66336	1.89226	2.1
66337	1.89226	2.1
66338	1.89226	2.1
66339	1.89226	2.1
66340	3.78453	2.1
66341	1.89226	2.1
66342	1.89226	2.1
66343	1.89226	2.1
66344	1.89226	2.1
66345	3.78453	2.1
66346	1.83378	2.1
66347	2.52893	2.1
66348	1.75592	2.1
66349	2.49844	2.1

66350	4.28485	2.1
66351	1.24922	2.1
66352	1.24922	2.1
66353	0.946133	2.1
66354	0.946133	2.1
66355	0.946131	2.1
66356	0.946131	2.1
66357	1.26446	2.1
66358	1.24922	2.1
66359	1.24922	2.1
66360	0.946131	2.1
66361	1.26446	2.1
66362	0.94613	2.1
66363	0.946132	2.1
66364	0.946132	2.1
66365	0.946132	2.1
66366	0.946132	2.1
66367	0.946132	2.1
66368	0.946132	2.1
66369	0.946132	2.1
66370	0.946132	2.1
66371	0.946132	2.1
66372	0.946132	2.1
66373	0.946131	2.1
66374	0.946131	2.1
66375	1.89226	2.1
66376	1.89226	2.1
66377	1.89226	2.1
66378	1.89226	2.1
66379	1.89226	2.1
66380	1.89226	2.1
66381	1.0465	2.1
66382	1.0465	2.1
66383	1.06047	2.1
66384	1.06047	2.1
66385	1.25687	2.1
66386	1.24922	2.1
66387	0.87796	2.1
66388	0.857749	2.1
66389	0.857749	2.1
66390	1.25687	2.1
66391	0.877959	2.1
66392	1.24922	2.1
66393	2.49843	2.1
66394	1.89226	2.1
66395	2.51374	2.1
66396	1.89226	2.1
66397	4.406 2.1	
66398	1.89226	2.1
66399	1.89226	2.1
66400	1.89226	2.1
66401	1.89226	2.1
66402	1.89226	2.1
66403	1.89226	2.1
66404	1.89226	1.25664
66405	1.89226	2.1
66406	1.89226	2.1
66407	1.89226	1.33975

66408	1.89226	2.1
66409	1.89226	1.37997
66410	1.89226	2.1
66411	1.89226	1.35017
66412	1.89226	2.1
66413	1.89226	1.35152
66414	1.89226	2.1
66415	1.89226	1.37333
66416	1.03835	2.1
66417	1.03835	2.1
66418	1.15388	2.1
66419	0.94267	2.1
66420	0.736433	2.1
66421	0.849239	2.1
66422	1.15014	2.1
66423	1.39058	2.1
66424	0.838639	2.1
66425	1.39056	2.1
66426	1.09399	2.1
66427	0.736444	2.1
66428	1.89226	1.18966
66429	1.89226	2.09247
66430	1.89226	2.1
66431	1.89226	1.20887
66432	1.89226	2.08753
66433	1.89226	1.23114
66434	0.94613	1.22494
66435	0.94613	1.21757
66436	0.946127	2.1
66437	0.946127	2.05273
66438	0.946131	2.07906
66439	0.946131	1.19461
66440	0.946126	2.1
66441	0.946126	1.2641
66442	0.946128	1.69823
66443	0.946128	1.69547
66444	0.946128	1.44788
66445	0.946128	1.60062
66446	1.89226	1.61774
66447	1.89226	1.09137
66448	1.89226	0.999734
66449	1.89226	2.02333
66450	3.78453	1.14368
66451	0.946133	1.19849
66452	0.946133	0.874386
66453	0.946128	1.52789
66454	0.946128	1.21283
66455	0.946131	1.23248
66456	0.946131	1.1767
66457	0.946131	0.826147
66458	0.946131	1.20902
66459	0.946131	1.05705
66460	0.946131	1.05104
66461	0.94613	1.10702
66462	0.94613	1.12002
66463	1.89226	0.994356
66464	1.89226	1.82043
66465	1.89226	1.38287

66466	1.89226	1.05961
66467	1.89226	1.3686
66468	1.89226	1.0964
66469	1.89226	0.878698
66470	1.89226	1.37849
66471	1.89226	1.10217
66472	1.89226	0.967122
66473	1.89226	1.10095
66474	1.89226	1.05286
66475	1.89226	0.900635
66476	1.89226	1.75615
66477	1.89226	1.13089
66478	1.89226	1.12346
66479	1.89226	1.14892
66480	1.89226	1.38242
66481	1.89226	1.01812
66482	2.67388	2.1
66483	2.23684	1.66491
66484	2.23684	1.17318
66485	1.89226	1.71799
66486	1.89226	1.23673
66487	1.89226	2.1
66488	1.89226	2.1
66489	1.89226	2.1
66490	1.89226	2.1
66491	1.89226	2.1
66492	1.89226	2.1
66493	1.72197	2.1
66494	2.51374	2.1
66495	2.58004	2.1
66496	2.58004	2.1
66497	1.7155	2.1
66498	1.7155	2.1
66499	2.51374	2.1
66500	1.89226	2.1
66501	1.89226	2.1
66502	1.89226	2.1
66503	2.58003	2.1
66504	2.58003	2.1
66505	0.946132	2.1
66506	0.946132	2.1
66507	0.946132	2.1
66508	0.946132	2.1
66509	0.946132	2.1
66510	0.946132	2.1
66511	0.946132	2.1
66512	0.946132	2.1
66513	0.946131	2.1
66514	0.946131	2.1
66515	0.946132	2.1
66516	0.946132	2.1
66517	1.89226	2.1
66518	1.89226	2.1
66519	1.89226	2.1
66520	1.89226	2.1
66521	3.78453	2.1
66522	1.08262	2.1
66523	1.08262	2.1

66524	1.09674	2.1
66525	1.09674	2.1
66526	1.34652	2.1
66527	1.29002	2.1
66528	0.860984	2.1
66529	0.889338	2.1
66530	1.34652	2.1
66531	0.889338	2.1
66532	0.860983	2.1
66533	1.29002	2.1
66534	1.34652	2.1
66535	1.34652	2.1
66536	0.946133	2.1
66537	0.946133	2.1
66538	0.946131	2.1
66539	0.946131	2.1
66540	1.34652	2.1
66541	1.29002	2.1
66542	1.34652	2.1
66543	0.946131	2.1
66544	0.94613	2.1
66545	1.29002	2.1
66546	1.89226	2.1
66547	1.89226	2.1
66548	1.89226	2.1
66549	1.89226	2.1
66550	1.89226	2.1
66551	1.89226	2.1
66552	1.89226	2.1
66553	1.89226	2.1
66554	1.89226	2.1
66555	1.89226	2.1
66556	1.89226	2.1
66557	1.89226	2.1
66558	0.946131	2.1
66559	0.946131	2.1
66560	0.946131	2.1
66561	0.946131	2.00292
66562	0.946131	2.00259
66563	0.946131	2.1
66564	0.946131	2.1
66565	0.946131	2.1
66566	0.946131	2.1
66567	0.946131	2.1
66568	0.946131	2.1
66569	0.946131	2.1
66570	1.89226	2.1
66571	1.89226	2.1
66572	1.89226	2.1
66573	1.89226	2.1
66574	1.89226	2.1
66575	1.89226	2.1
66576	1.04103	2.1
66577	1.13849	2.1
66578	0.943014	2.1
66579	0.943014	2.1
66580	0.943591	2.1
66581	0.943591	2.1

66582	1.03757	2.1
66583	1.04098	2.1
66584	1.03761	2.1
66585	0.946131	2.1
66586	0.943041	2.1
66587	0.946131	2.1
66588	0.946128	2.1
66589	0.946128	2.1
66590	0.946133	2.1
66591	0.946133	2.1
66592	0.946131	2.1
66593	0.946131	2.1
66594	0.946131	2.1
66595	0.946131	2.1
66596	0.946131	2.1
66597	0.946131	2.1
66598	0.94613	2.1
66599	0.94613	2.1
66600	0.946132	2.1
66601	0.946132	2.1
66602	0.946132	2.1
66603	0.946132	2.0406
66604	0.946132	2.05045
66605	0.946132	2.1
66606	0.946132	2.1
66607	0.946132	2.1
66608	0.946132	2.1
66609	0.946132	2.1
66610	0.946131	2.1
66611	0.946131	2.1
66612	1.89226	0.90449
66613	1.89226	1.66577
66614	1.89226	0.953945
66615	1.89226	0.90457
66616	3.78452	1.09294
66617	0.946132	1.07894
66618	0.946132	1.08874
66619	0.946131	0.94437
66620	0.946131	0.94321
66621	0.946132	0.951256
66622	0.946132	1.92948
66623	0.946132	1.0812
66624	0.946132	1.02977
66625	0.946132	0.991506
66626	0.946132	1.01134
66627	0.946132	1.04007
66628	0.946132	1.10173
66629	0.935482	1.33306
66630	1.08999	1.22751
66631	1.24315	1.06336
66632	1.12559	1.21007
66633	1.21343	1.02748
66634	0.889397	2.09231
66635	1.01265	0.909191
66636	1.01265	1.46672
66637	1.07332	0.967314
66638	1.34292	1.12065
66639	0.865649	1.23523

66640	0.840024	1.54701
66641	1.15433	1.18685
66642	1.02858	1.48125
66643	1.34082	1.07225
66644	1.08357	1.06709
66645	1.10938	0.910101
66646	1.33349	2.03263
66647	1.12232	1.49288
66648	1.12232	0.866446
66649	1.21357	1.06941
66650	1.3408	1.18641
66651	1.02859	1.28672
66652	1.24211	1.54489
66653	1.89226	1.04831
66654	1.89226	0.822638
66655	1.89226	0.879401
66656	1.89226	1.68313
66657	1.89226	0.817206
66658	1.89226	1.05087
66659	1.89226	0.906341
66660	1.89226	1.0131
66661	1.89226	0.932851
66662	1.89226	1.34018
66663	1.89226	1.01568
66664	1.89226	1.33914
66665	2.0815	1.19134
66666	2.79029	1.04652
66667	2.36793	1.03205
66668	2.58428	1.83713
66669	2.38161	0.892739
66670	2.59922	1.2136
66671	1.89226	2.1
66672	1.89226	1.36929
66673	1.89226	1.80534
66674	1.89226	1.84872
66675	1.89226	1.79845
66676	1.89226	1.87934
66677	1.89226	1.38433
66678	1.89226	0.86023
66679	1.89226	0.986449
66680	1.89226	1.07576
66681	3.78452	1.02985
66682	1.89226	1.81655
66683	1.89226	1.03041
66684	1.89226	1.4035
66685	1.89226	1.35824
66686	1.89226	1.355
66687	1.89226	1.35847
66688	0.946132	1.14189
66689	0.946132	1.61837
66690	0.94613	2.1
66691	0.94613	1.53928
66692	0.946131	1.94857
66693	0.946131	1.139
66694	0.946131	1.57904
66695	0.946131	2.1
66696	0.946131	1.58684
66697	0.946131	1.74686

66698	0.946131	1.7619
66699	0.946131	1.68448
66700	1.85178	1.59685
66701	1.89226	1.06879
66702	2.0815	0.965194
66703	2.27054	1.36913
66704	1.70303	0.865583
66705	1.5217	0.936569
66706	1.89226	1.90925
66707	1.89226	1.02193
66708	1.89226	1.2472
66709	1.89226	1.13441
66710	1.89226	1.78185
66711	1.89226	1.27622
66712	2.58823	0.726497
66713	2.02924	0.530562
66714	1.79398	0.53691
66715	1.79398	0.358493
66716	2.76708	0.733141
66717	2.76708	0.624195
66718	0.946133	0.536969
66719	0.946133	0.614311
66720	1.29411	0.579214
66721	1.38353	0.467847
66722	0.946131	0.426896
66723	0.946131	0.420307
66724	1.38354	0.66248
66725	1.38354	0.610186
66726	0.946131	0.470022
66727	1.38354	0.483871
66728	0.94613	0.62915
66729	1.29411	0.483126
66730	1.89226	0.342547
66731	1.89226	0.213837
66732	1.89226	0.421188
66733	1.89226	0.3353
66734	3.78452	0.346025
66735	0.946131	0.249857
66736	0.946131	0.411252
66737	0.946131	0.370371
66738	0.946131	0.240042
66739	0.946129	0.243754
66740	0.946129	0.19677
66741	0.946133	0.447626
66742	0.946133	0.416812
66743	0.946131	0.468985
66744	0.946131	0.314502
66745	0.946131	0.189187
66746	0.946131	0.293395
66747	1.89226	0.232326
66748	1.89226	0.203453
66749	1.89226	0.304781
66750	1.89226	0.291816
66751	3.78452	0.208405
66752	0.94613	0.280655
66753	0.94613	0.272738
66754	0.946131	0.214527
66755	0.946131	0.267514

66756	0.946128	0.208488
66757	0.946128	0.199275
66758	0.946133	0.305059
66759	0.946133	0.268222
66760	0.946131	0.318046
66761	0.946131	0.226527
66762	0.946131	0.282879
66763	0.946131	0.190016
66764	0.946129	0.996528
66765	0.946129	0.959244
66766	1.13486	0.978252
66767	0.859753	1.02464
66768	0.851541	1.01329
66769	0.851541	0.926304
66770	1.34346	0.974749
66771	1.26212	0.988252
66772	0.851553	0.934933
66773	1.12403	0.958829
66774	0.851474	0.904815
66775	0.956163	0.964896
66776	0.946132	0.799715
66777	0.946132	0.843573
66778	0.946133	0.878709
66779	0.946133	0.806404
66780	0.946132	0.892376
66781	0.946132	0.539623
66782	0.946133	0.861414
66783	0.946133	0.849345
66784	0.946133	0.855594
66785	0.946133	0.845997
66786	0.946132	0.716531
66787	0.946132	0.851853
66788	1.89226	0.698963
66789	1.89226	0.751686
66790	1.89226	0.902859
66791	1.89226	0.761369
66792	3.78453	0.781869
66793	0.946128	0.980877
66794	0.946128	0.952416
66795	0.946132	1.01641
66796	0.946132	0.999324
66797	0.94613	1.01525
66798	0.94613	0.95773
66799	0.94613	1.04214
66800	0.94613	0.945802
66801	0.946131	1.0212
66802	0.946131	0.993537
66803	0.94613	0.973243
66804	0.94613	0.98464
66805	0.946132	0.558552
66806	0.946132	0.618956
66807	0.946133	0.478321
66808	0.946133	0.477099
66809	0.946132	0.448732
66810	0.946132	0.356364
66811	0.946133	0.626543
66812	0.946133	0.507621
66813	0.946132	0.578907

66814	0.946132	0.495633
66815	0.946132	0.503305
66816	0.946132	0.453943
66817	1.89226	0.5269
66818	1.89226	0.395451
66819	1.89226	0.646971
66820	1.89226	0.36289
66821	1.89226	0.719445
66822	1.89226	0.363222
66823	1.89226	0.911006
66824	1.89226	0.854918
66825	1.89226	0.861959
66826	1.89226	0.743506
66827	1.89226	0.867531
66828	1.89226	0.848914
66829	1.89226	2.1
66830	1.89226	2.1
66831	1.89226	2.1
66832	1.89226	2.1
66833	3.78452	2.1
66834	1.07652	2.1
66835	1.07652	2.1
66836	0.928749	2.1
66837	0.928749	2.1
66838	0.946132	2.1
66839	0.946132	2.1
66840	1.05914	2.1
66841	0.928746	2.1
66842	1.05914	2.1
66843	0.946131	2.1
66844	0.94613	2.1
66845	0.928747	2.1
66846	1.89226	2.1
66847	1.89226	2.1
66848	1.89226	2.1
66849	1.89226	2.1
66850	1.89226	2.1
66851	1.89226	2.1
66852	1.89227	2.1
66853	1.89227	2.1
66854	1.89227	2.1
66855	1.89227	2.1
66856	1.89227	2.1
66857	1.89227	2.1
66858	1.89226	2.1
66859	1.89226	2.1
66860	1.89226	2.1
66861	1.89226	2.1
66862	1.89226	2.1
66863	1.89226	2.1
66864	2.11828	2.1
66865	1.89226	2.1
66866	2.35845	2.1
66867	1.89226	2.1
66868	4.25071	2.1
66869	1.89226	2.1
66870	1.89226	2.1
66871	1.89226	2.1

66872	1.89226	2.1
66873	3.78453	2.1
66874	1.89226	2.1
66875	1.89226	2.1
66876	1.89226	2.1
66877	1.89226	2.1
66878	1.89226	2.1
66879	1.89226	2.1
66880	1.89226	0.966149
66881	1.89226	1.49609
66882	1.65573	1.21696
66883	1.65573	1.01752
66884	1.89226	1.21619
66885	1.89226	1.02115
66886	1.89226	0.864674
66887	1.89226	1.12273
66888	1.89226	1.00442
66889	1.89226	0.920452
66890	1.89226	1.01794
66891	1.89226	0.928128
66892	1.89226	0.924172
66893	1.89226	1.24751
66894	1.89226	1.12092
66895	1.89226	0.983368
66896	1.89226	1.13582
66897	1.89226	0.986747
66898	1.89226	0.841103
66899	1.89226	1.12294
66900	1.89226	0.959308
66901	1.89226	0.882232
66902	1.89226	0.978509
66903	1.89226	0.893758
66904	0.946128	0.910815
66905	0.946128	0.861646
66906	0.946125	1.16225
66907	0.946125	1.08198
66908	0.94613	0.920094
66909	0.94613	1.7058
66910	0.946124	1.20982
66911	0.946124	0.902825
66912	0.946127	0.972209
66913	0.946127	0.974403
66914	0.946127	0.977531
66915	0.946127	0.980872
66916	1.89226	0.863013
66917	1.89226	0.999518
66918	1.89226	0.873017
66919	1.89226	0.837673
66920	3.78452	0.879867
66921	1.89226	1.0415
66922	1.89226	1.05429
66923	1.89226	0.926494
66924	1.89226	1.0726
66925	3.78453	1.03712
66926	1.05926	1.23602
66927	0.891358	2.06282
66928	0.946129	1.12581
66929	0.946129	1.29003

66930	0.946101	1.13975
66931	0.946101	2.1
66932	0.891386	1.11996
66933	0.891386	1.18439
66934	0.891357	1.17201
66935	0.946129	1.16908
66936	0.94613	1.18449
66937	1.05926	1.20556
66938	0.946126	0.957646
66939	0.946126	1.03504
66940	0.94613	0.92012
66941	0.94613	0.999636
66942	0.946131	1.00081
66943	0.946131	1.03343
66944	0.946125	1.01701
66945	0.946125	0.839137
66946	0.946127	0.921222
66947	0.946127	0.929316
66948	0.946128	1.01242
66949	0.946128	1.0034
66950	1.89226	0.934431
66951	1.89226	0.866355
66952	1.89226	0.822857
66953	1.89226	1.02868
66954	1.89226	0.848102
66955	1.89226	1.09462
66956	1.89226	0.996841
66957	1.89226	1.1825
66958	1.89226	1.0936
66959	1.89226	1.16226
66960	1.89226	0.996397
66961	1.89226	1.10756
66962	1.16109	1.44927
66963	1.23243	2.08729
66964	0.659819	1.20843
66965	0.659819	1.53597
66966	0.697965	1.0944
66967	1.00087	2.1
66968	0.891386	1.38574
66969	0.891386	1.21644
66970	0.946129	1.37335
66971	0.65979	1.1745
66972	0.891357	1.34702
66973	0.946129	1.32355
66974	1.89225	0.886294
66975	1.89225	1.02547
66976	1.89225	1.10469
66977	1.89225	0.8645
66978	1.89225	1.30563
66979	1.89225	0.967316
66980	1.79981	0.703493
66981	2.02514	0.77597
66982	2.33247	0.863785
66983	2.12891	0.628026
66984	4.15405	0.682687
66985	2.48284	0.980728
66986	2.02514	0.808235
66987	2.33247	0.861595

66988	2.02514	0.93032
66989	2.48567	0.853303
66990	2.17834	0.914819
66991	1.89226	0.744429
66992	1.89226	0.873094
66993	1.89226	0.917018
66994	1.89226	0.651985
66995	3.78453	0.793247
66996	1.89227	0.70804
66997	1.89227	0.454656
66998	1.89227	0.506636
66999	1.89227	0.67477
67000	1.89227	0.545452
67001	1.89227	0.653592
67002	2.50894	0.600748
67003	2.70118	0.820877
67004	2.12891	0.695413
67005	2.12891	0.781323
67006	2.7125	0.697665
67007	2.33247	0.776287
67008	1.89226	0.872727
67009	1.89226	0.680461
67010	1.89226	0.72535
67011	1.89226	0.847235
67012	1.89226	0.707782
67013	1.89226	0.833218
67014	0.946133	0.632052
67015	0.946133	0.740444
67016	0.946132	0.432963
67017	0.946132	0.530194
67018	0.946132	0.404046
67019	0.946132	0.690019
67020	0.946133	0.537602
67021	0.946133	0.773935
67022	0.946132	0.573463
67023	0.946132	0.583352
67024	0.946132	0.601917
67025	0.946132	0.571245
67026	1.90098	0.706127
67027	1.36463	0.711371
67028	1.56656	0.704915
67029	1.36463	0.714257
67030	2.04386	0.7032
67031	1.89227	0.715751
67032	1.89226	0.710542
67033	1.89226	0.715486
67034	1.89226	0.707981
67035	1.89226	0.719679
67036	1.89226	0.708067
67037	1.89226	0.719852
67038	1.89226	0.720684
67039	1.89226	0.73183
67040	1.89226	0.719453
67041	1.89226	0.740595
67042	1.89226	0.717587
67043	1.89226	0.73909
67044	1.89226	0.716403
67045	1.89226	0.724931

67046	1.89226	0.714843
67047	1.89226	0.7311
67048	1.89226	0.713551
67049	1.89226	0.731123
67050	1.89226	0.703051
67051	1.70303	0.705269
67052	2.1292	0.702472
67053	1.70303	0.708113
67054	2.32302	0.702041
67055	1.90098	0.70836
67056	1.89226	0.706456
67057	1.89226	0.709274
67058	1.89226	0.704208
67059	1.89226	0.712855
67060	1.89226	0.70448
67061	1.89226	0.635132
67062	1.89226	0.712656
67063	1.89226	0.715721
67064	1.89226	0.710603
67065	1.89226	0.722518
67066	1.89226	0.709526
67067	1.89226	0.721645
67068	1.89226	0.708242
67069	1.89226	0.709278
67070	1.89226	0.705343
67071	1.89226	0.714229
67072	1.89226	0.705114
67073	1.89226	0.714348
67074	1.89226	0.951307
67075	1.89226	1.13296
67076	1.89226	1.11589
67077	1.89226	0.947554
67078	3.78453	0.942802
67079	1.89226	1.10984
67080	1.89226	1.41089
67081	1.89226	1.02197
67082	1.89226	1.57105
67083	1.89226	1.1199
67084	1.89226	2.01936
67085	1.89226	1.1304
67086	1.89226	0.969742
67087	1.89226	0.943885
67088	1.89226	1.03724
67089	3.78452	1.0867
67090	1.89224	1.127
67091	1.89224	0.919106
67092	1.89224	0.977318
67093	1.89224	1.1573
67094	1.89224	0.944766
67095	1.89224	1.07468
67096	1.89224	1.12204
67097	1.89224	1.58005
67098	1.89224	1.22735
67099	1.89224	2.0977
67100	1.89224	1.02024
67101	1.89224	1.52786
67102	1.31307	1.98274
67103	1.31307	1.96061

67104	0.888948	1.23152
67105	0.888948	1.1517
67106	1.2559	1.28647
67107	0.888979	1.91613
67108	0.946114	1.51962
67109	0.946114	1.14336
67110	0.946129	1.0854
67111	0.888989	1.19132
67112	0.946104	1.03982
67113	1.25588	1.15121
67114	1.89224	0.91294
67115	1.89224	1.18903
67116	1.89224	0.921506
67117	1.89224	1.15552
67118	1.89224	0.991827
67119	1.89224	1.30521
67120	1.79375	0.431683
67121	1.47689	0.27031
67122	1.97732	0.479776
67123	2.26687	0.449686
67124	3.74375	0.378358
67125	2.05759	0.53064
67126	2.26686	0.407709
67127	1.79374	0.369674
67128	1.79374	0.368777
67129	2.49889	0.630705
67130	2.49889	0.45088
67131	1.89226	0.298148
67132	1.89226	0.304441
67133	1.89226	0.31164
67134	1.89226	0.349799
67135	3.78452	0.332118
67136	1.89227	0.376525
67137	1.89227	0.347292
67138	1.89227	0.299477
67139	1.89227	0.306804
67140	1.89227	0.193681
67141	1.89227	0.248782
67142	1.97732	0.445282
67143	2.26687	0.364007
67144	2.6607	0.661833
67145	2.41464	0.463299
67146	4.68151	0.497146
67147	1.89226	0.276791
67148	1.89226	0.3309
67149	1.89226	0.427713
67150	1.89226	0.373829
67151	3.78452	0.295639
67152	1.89226	0.301065
67153	1.89226	0.448026
67154	1.89226	0.366064
67155	1.89226	0.366753
67156	1.89226	0.416159
67157	1.89226	0.352145
67158	1.89226	0.863678
67159	1.89226	1.01198
67160	1.89226	0.909798
67161	1.89226	0.87786

67162	3.78452	0.881945
67163	1.89226	0.909975
67164	1.89226	1.13844
67165	1.89226	0.897422
67166	1.89226	1.05811
67167	1.89226	0.946278
67168	1.89226	1.13534
67169	1.89226	1.0468
67170	1.89226	0.906332
67171	1.89226	0.865916
67172	1.89226	1.05453
67173	1.89226	0.880644
67174	1.89226	1.10494
67175	1.89226	0.909804
67176	1.89226	0.874257
67177	1.89226	0.848385
67178	1.89226	1.00943
67179	1.89226	0.833445
67180	1.89226	1.05265
67181	1.89226	0.911902
67182	2.45282	1.05857
67183	1.89226	1.04847
67184	1.89226	1.10588
67185	1.89226	0.888717
67186	1.89226	0.985054
67187	1.89226	0.889977
67188	1.89226	1.03336
67189	1.89226	0.901773
67190	1.89226	0.929656
67191	1.89226	1.14156
67192	1.89226	1.54633
67193	1.87308	0.527233
67194	1.88631	0.593542
67195	1.63908	0.524351
67196	1.70302	0.447502
67197	2.08745	0.62604
67198	2.16916	0.519181
67199	2.41019	0.575796
67200	2.733	0.644834
67201	2.08121	0.501689
67202	2.08121	0.440553
67203	2.74362	0.480777
67204	2.41464	0.47515
67205	1.89226	0.585187
67206	1.89226	0.425866
67207	1.89226	0.593307
67208	1.89226	0.494636
67209	1.89226	0.571849
67210	1.89226	0.458673
67211	0.946133	0.489703
67212	0.946133	0.454756
67213	0.946132	0.620524
67214	0.946132	0.589398
67215	0.946132	0.608737
67216	0.946132	0.498108
67217	0.946133	0.575471
67218	0.946133	0.474047
67219	0.946132	0.633976

67220	0.946132	0.6538
67221	0.946133	0.356597
67222	0.946133	0.471162
67223	2.02912	0.634462
67224	2.57016	0.598236
67225	1.88631	0.60467
67226	1.88631	0.560966
67227	2.55301	0.449215
67228	2.4102	0.51098
67229	1.89226	0.515316
67230	1.89226	0.501852
67231	1.89226	0.644493
67232	1.89226	0.64325
67233	3.78452	0.623235
67234	1.89227	0.51781
67235	1.89227	0.647728
67236	1.89227	0.628754
67237	1.89227	0.600607
67238	1.89227	0.634022
67239	1.89227	0.610561
67240	1.89226	2.1
67241	1.89226	2.1
67242	1.89226	2.02023
67243	1.89226	2.1
67244	3.78452	2.1
67245	1.2515	2.08656
67246	1.34788	2.1
67247	0.946132	2.1
67248	0.946132	2.1
67249	0.973043	2.1
67250	1.16766	2.1
67251	0.919222	1.91317
67252	1.2159	2.1
67253	0.946132	2.1
67254	0.946132	2.1
67255	1.33746	2.1
67256	1.13537	2.1
67257	1.89226	2.1
67258	1.89226	2.1
67259	1.89226	2.1
67260	1.89226	2.1
67261	1.89226	2.1
67262	1.89226	2.1
67263	1.89226	1.97438
67264	1.89226	2.1
67265	1.89226	2.1
67266	1.89226	2.03204
67267	3.78452	2.08379
67268	1.89227	2.1
67269	1.89227	1.66224
67270	1.89227	2.1
67271	1.89227	1.95201
67272	1.89227	2.1
67273	1.89227	1.92384
67274	0.946132	2.09159
67275	0.946132	2.03736
67276	0.946129	2.1
67277	0.946129	2.1

67278	0.946131	2.1
67279	0.946131	2.1
67280	0.946131	2.1
67281	0.946131	2.03813
67282	0.946131	2.1
67283	0.946131	2.1
67284	0.94613	2.1
67285	0.94613	2.1
67286	1.89226	0.884798
67287	1.89226	0.921018
67288	1.89226	0.895623
67289	1.89226	0.844837
67290	3.78453	0.876472
67291	0.946132	1.47677
67292	0.946132	0.953355
67293	0.946132	0.995474
67294	0.946132	0.934134
67295	0.946132	0.872983
67296	0.946132	1.46365
67297	0.946132	0.950193
67298	0.946132	0.955493
67299	0.946131	1.51829
67300	0.946131	0.913381
67301	0.946132	1.00464
67302	0.946132	0.948594
67303	1.89226	1.02396
67304	1.89226	0.815156
67305	1.89226	0.793313
67306	1.89226	0.867008
67307	1.89226	0.875291
67308	1.89226	1.32393
67309	1.89226	1.1367
67310	1.89226	0.856758
67311	1.89226	0.833922
67312	1.89226	0.874743
67313	3.78453	0.865057
67314	0.96614	1.05392
67315	0.96614	0.94116
67316	0.946131	0.833437
67317	0.946131	0.905215
67318	0.946131	0.93333
67319	0.96614	0.924292
67320	1.37736	1.00386
67321	0.946131	1.03668
67322	1.37736	1.33758
67323	0.96614	0.899829
67324	0.946131	1.16904
67325	0.946131	0.877609
67326	0.946129	1.40183
67327	0.946129	0.901491
67328	0.946128	0.89768
67329	0.946128	1.22825
67330	0.946132	0.824157
67331	0.946132	1.62579
67332	0.946126	0.909277
67333	0.946126	0.886009
67334	0.946129	0.834875
67335	0.946129	0.861174

67336	0.946129	0.830224
67337	0.946129	0.929675
67338	1.89226	0.592584
67339	1.65723	0.621586
67340	1.83117	0.689289
67341	1.65723	0.683171
67342	2.0662	0.520124
67343	1.89226	0.45975
67344	1.89226	0.597395
67345	1.89226	0.660864
67346	1.89226	0.682699
67347	1.89226	0.679985
67348	1.89226	0.485781
67349	1.89226	0.452503
67350	1.89226	0.672342
67351	1.89226	0.703646
67352	1.89226	0.702192
67353	1.89226	0.702375
67354	1.89226	0.597609
67355	1.89226	0.557227
67356	1.89226	0.678128
67357	1.89226	0.701996
67358	1.89226	0.702872
67359	1.89226	0.703139
67360	1.89226	0.610355
67361	1.89226	0.55467
67362	1.89226	0.607607
67363	1.83117	0.640192
67364	1.92807	0.685178
67365	1.83117	0.670884
67366	1.98916	0.510556
67367	1.89226	0.449764
67368	1.89226	0.447593
67369	1.89226	0.668152
67370	1.89226	0.661279
67371	1.89226	0.661367
67372	3.78453	0.568661
67373	1.89226	0.671948
67374	1.89226	0.701988
67375	1.89226	0.701031
67376	1.89226	0.700917
67377	1.89226	0.600999
67378	1.89226	0.60953
67379	1.89226	0.651752
67380	1.89226	0.702151
67381	1.89226	0.701456
67382	1.89226	0.701243
67383	1.89226	0.61691
67384	1.89226	0.583225
67385	1.88037	0.703629
67386	1.89227	0.704136
67387	1.89227	0.705045
67388	1.89227	0.704456
67389	1.98599	0.705461
67390	1.98599	0.70405
67391	1.89226	0.702943
67392	1.89226	0.704172
67393	1.89226	0.703896

67394	1.89226	0.705421
67395	1.89226	0.703883
67396	1.89226	0.704175
67397	1.89226	0.704741
67398	1.89226	0.706119
67399	1.89226	0.704432
67400	1.89226	0.708246
67401	3.78452	0.70299
67402	1.89226	0.70487
67403	1.89226	0.706459
67404	1.89226	0.706774
67405	1.89226	0.704355
67406	1.89226	0.705566
67407	1.89226	0.704629
67408	1.90116	0.702271
67409	1.89227	0.703003
67410	1.89226	0.703484
67411	1.89227	0.703077
67412	1.88036	0.704346
67413	1.88037	0.70248
67414	1.89226	0.701319
67415	1.89226	0.702656
67416	1.89226	0.702478
67417	1.89226	0.702966
67418	1.89226	0.702481
67419	1.89226	0.702305
67420	0.946132	0.702714
67421	0.946132	0.702595
67422	0.946128	0.702785
67423	0.946128	0.703524
67424	0.94613	0.703494
67425	0.94613	0.703771
67426	0.94613	0.702724
67427	0.94613	0.702909
67428	0.946131	0.704946
67429	0.946131	0.704665
67430	0.94613	0.705507
67431	0.94613	0.707777
67432	1.89226	0.704256
67433	1.89226	0.70309
67434	1.89226	0.702595
67435	1.89226	0.703491
67436	1.89226	0.702448
67437	1.89226	0.702929
67438	1.89226	0.918817
67439	1.89226	1.35535
67440	2.35239	1.33513
67441	2.27873	1.00286
67442	1.89226	1.25932
67443	1.83301	0.991872
67444	2.68449	1.08811
67445	1.89226	1.84571
67446	1.89226	1.10316
67447	1.89226	1.20115
67448	3.78453	1.13198
67449	0.946132	1.63597
67450	0.946132	1.08712
67451	0.946129	1.00359

67452	0.946129	1.52568
67453	0.946131	1.51259
67454	0.946131	1.24594
67455	0.94613	1.08711
67456	0.94613	1.16992
67457	0.946131	1.25726
67458	0.946131	1.09942
67459	0.946131	1.16899
67460	0.946131	1.34854
67461	1.89226	0.873879
67462	1.89226	1.11566
67463	1.89226	1.25335
67464	1.89226	0.912426
67465	1.89226	1.18443
67466	1.89226	0.903682
67467	1.70303	0.913084
67468	1.89226	1.22544
67469	1.89226	0.877268
67470	1.89226	0.982292
67471	3.78453	0.928908
67472	1.89227	1.10659
67473	1.89227	1.20528
67474	1.89227	0.941801
67475	1.89227	1.43927
67476	3.78453	1.16773
67477	1.89226	1.02683
67478	1.89226	1.53517
67479	1.89226	1.04468
67480	1.89226	1.29386
67481	3.78452	1.1813
67482	1.89226	2.00001
67483	1.89226	2.1
67484	1.89226	2.1
67485	1.89226	1.98144
67486	3.78452	1.92344
67487	0.946132	1.75019
67488	0.946132	2.00921
67489	0.946129	2.1
67490	0.946129	2.1
67491	0.94613	2.1
67492	0.94613	1.92654
67493	0.946131	2.1
67494	0.946131	1.95352
67495	0.946131	1.98529
67496	0.946131	2.1
67497	0.946131	2.06168
67498	0.946131	1.80972
67499	1.89226	2.1
67500	1.89226	1.71648
67501	1.89226	1.86555
67502	1.89226	2.08908
67503	3.78452	2.09775
67504	1.89226	1.49007
67505	1.89226	2.07789
67506	1.89226	2.1
67507	1.89226	1.61676
67508	1.89226	2.1
67509	1.89226	1.5663

67510	1.89227	2.05875
67511	2.48308	1.57526
67512	1.89227	1.62052
67513	1.89227	1.95327
67514	3.78453	2.06657
67515	1.89226	1.6845
67516	1.89226	2.01945
67517	1.89226	2.1
67518	1.89226	1.67289
67519	1.89226	2.0526
67520	1.89226	1.57316
67521	1.89226	1.44479
67522	1.89226	1.64115
67523	1.89226	1.81101
67524	1.89226	1.50562
67525	3.78453	1.95143
67526	0.946131	1.79316
67527	0.946131	1.93063
67528	0.946131	1.95645
67529	0.946131	2.09137
67530	0.946131	1.97563
67531	0.946131	1.94524
67532	0.946131	1.93985
67533	0.946131	1.92064
67534	0.946131	1.87546
67535	0.946131	2.05541
67536	0.946131	1.97614
67537	0.946131	1.76295
67538	1.23719	2.07231
67539	1.28439	1.71526
67540	0.94613	1.48689
67541	0.94613	1.7498
67542	1.28439	1.90171
67543	1.6431	2.09912
67544	0.946129	1.64139
67545	0.946129	1.45433
67546	1.28439	1.41581
67547	0.946131	1.34693
67548	1.21038	1.36293
67549	1.21038	1.4386
67550	1.89226	1.72359
67551	1.89226	1.39557
67552	1.89226	1.44939
67553	1.89226	1.57635
67554	1.89226	1.37497
67555	1.89226	1.51746
67556	1.89226	1.65684
67557	1.89226	1.83772
67558	1.89226	2.00946
67559	1.89226	1.61266
67560	1.89226	1.87545
67561	1.89226	1.51113
67562	1.89226	1.08842
67563	1.89226	0.804
67564	1.89226	0.839455
67565	1.89226	0.916025
67566	1.89226	0.907622
67567	1.89226	1.12422

67568	0.946128	0.79682
67569	0.946128	0.898605
67570	0.946125	0.737402
67571	0.946125	0.825689
67572	0.94613	0.765156
67573	0.94613	0.824848
67574	0.946124	0.850228
67575	0.946124	0.913009
67576	0.946127	0.758551
67577	0.946127	0.746015
67578	0.946127	0.856845
67579	0.946127	0.905096
67580	1.89226	0.733889
67581	2.78671	0.76206
67582	2.59302	0.759278
67583	2.59302	0.749752
67584	1.89226	0.787724
67585	1.89226	0.753821
67586	1.89226	0.758725
67587	1.89226	0.796731
67588	1.89226	0.766089
67589	1.89226	0.754676
67590	1.89226	0.82802
67591	1.89226	0.78536
67592	1.89225	0.741863
67593	1.89225	0.705996
67594	1.89225	0.796224
67595	1.89225	0.722519
67596	1.89225	0.791493
67597	1.89225	0.726747
67598	0.979536	0.727798
67599	0.816597	0.731629
67600	1.15446	0.726436
67601	1.07423	0.711176
67602	1.08452	0.716833
67603	0.603522	0.714647
67604	1.70302	0.717083
67605	1.29344	0.70961
67606	1.21806	0.712262
67607	0.723758	0.709771
67608	1.37562	0.732137
67609	1.89226	0.495569
67610	1.92807	0.629744
67611	1.90843	0.65523
67612	1.92807	0.657071
67613	1.87263	0.477798
67614	1.89226	0.479096
67615	0.946132	0.647507
67616	0.946132	0.571784
67617	0.946131	0.62727
67618	0.946131	0.626076
67619	0.946132	0.650313
67620	0.946132	0.597537
67621	0.946132	0.474304
67622	0.946132	0.517268
67623	0.946132	0.657828
67624	0.946132	0.678295
67625	0.946132	0.627897

67626	0.946132	0.652484
67627	1.89226	0.590587
67628	1.89226	0.700017
67629	1.89226	0.687922
67630	1.89226	0.691268
67631	3.78452	0.679107
67632	1.89226	0.640973
67633	1.89226	0.700663
67634	1.89226	0.700063
67635	1.89226	0.698703
67636	1.89226	0.608318
67637	1.89226	0.539994
67638	1.70303	0.500284
67639	1.90843	0.530378
67640	1.77002	0.557988
67641	1.90843	0.539692
67642	1.75709	0.454305
67643	1.89226	0.417979
67644	1.89226	0.436665
67645	1.89226	0.647342
67646	1.89226	0.622256
67647	1.89226	0.645554
67648	3.78453	0.510688
67649	1.89226	0.67645
67650	1.89226	0.685067
67651	1.89226	0.657265
67652	1.89226	0.698831
67653	3.78452	0.690081
67654	0.946131	0.591136
67655	0.946131	0.685126
67656	0.946131	0.638403
67657	0.946131	0.696018
67658	0.946129	0.696701
67659	0.946129	0.694433
67660	0.946133	0.602498
67661	0.946133	0.565948
67662	0.946131	0.697956
67663	0.946131	0.683263
67664	0.946131	0.473003
67665	0.946131	0.695574
67666	1.89226	0.716096
67667	2.10275	0.832278
67668	1.76377	0.812915
67669	1.76377	0.743827
67670	1.89226	0.771977
67671	1.89226	0.721694
67672	1.89226	0.718683
67673	1.89226	0.850664
67674	2.10275	0.827509
67675	2.55889	0.739891
67676	1.89226	0.789575
67677	2.30274	0.725894
67678	1.89226	0.771387
67679	1.89226	1.00303
67680	1.89226	0.974057
67681	1.89226	0.800407
67682	1.89226	0.911715
67683	1.89226	0.780983

67684	1.89226	0.755431
67685	1.89226	0.988598
67686	1.89226	0.929611
67687	1.89226	0.796352
67688	1.89226	0.879021
67689	1.89226	0.776548
67690	1.89226	0.712813
67691	1.89226	0.803462
67692	1.89226	0.78551
67693	1.89226	0.726142
67694	1.89226	0.75668
67695	1.89226	0.714856
67696	1.89226	0.714085
67697	1.89226	0.819194
67698	1.89226	0.799551
67699	1.89226	0.732506
67700	1.89226	0.770967
67701	1.89226	0.721097
67702	1.89226	0.763399
67703	1.89226	0.950275
67704	1.89226	0.911534
67705	1.89226	0.793076
67706	1.89226	0.874809
67707	1.89226	0.778993
67708	1.89226	0.850479
67709	1.89226	0.919254
67710	1.89226	0.752878
67711	1.89226	0.78472
67712	1.89226	0.755661
67713	1.89226	0.875953
67714	0.946131	0.712497
67715	0.976399	0.709137
67716	1.10124	0.675627
67717	0.946131	0.792635
67718	0.887633	0.770865
67719	0.887633	0.737805
67720	1.16933	0.714203
67721	1.23163	0.706252
67722	0.946131	0.722942
67723	0.946131	0.729373
67724	0.976399	0.734941
67725	1.14992	0.735957
67726	1.89226	0.765256
67727	1.89226	0.988539
67728	1.89226	0.754793
67729	1.89226	0.796277
67730	3.78452	0.758829
67731	1.89226	0.709359
67732	1.89226	0.786201
67733	1.89226	0.68373
67734	1.89226	0.7257
67735	3.78453	0.69954
67736	0.946132	0.642429
67737	0.946132	0.722702
67738	0.946131	0.717935
67739	0.946131	0.737629
67740	0.946131	0.739219
67741	0.946131	0.722382

67742	0.946132	0.583835
67743	0.946132	0.601452
67744	0.946131	0.642242
67745	0.946131	0.708979
67746	0.946132	0.715107
67747	0.946132	0.632543
67748	0.946132	0.77982
67749	0.946132	0.719809
67750	0.946131	0.735251
67751	0.946131	0.822215
67752	0.946131	0.822367
67753	0.946131	0.781431
67754	0.946132	0.731419
67755	0.946132	0.717337
67756	0.946131	0.743481
67757	0.946131	0.765396
67758	0.946132	0.767756
67759	0.946132	0.741022
67760	1.89226	0.785085
67761	1.89226	0.843603
67762	1.89226	0.730708
67763	1.89226	0.866543
67764	3.78452	0.831802
67765	1.89226	0.768933
67766	1.89226	0.759168
67767	1.89226	0.72988
67768	1.89226	0.716852
67769	3.78453	0.734851
67770	1.89226	0.727667
67771	1.89226	0.788355
67772	1.89226	0.73848
67773	1.89226	0.791623
67774	1.89226	0.723553
67775	1.89226	0.751489
67776	1.89225	0.712306
67777	1.89225	0.742386
67778	1.89225	0.724234
67779	1.89225	0.774334
67780	1.89225	0.708804
67781	1.89225	0.731884
67782	1.89226	0.713318
67783	1.89226	0.744709
67784	1.89226	0.721818
67785	1.89226	0.750812
67786	1.89226	0.710948
67787	1.89226	0.724315
67788	1.89226	0.764824
67789	1.89226	0.719784
67790	1.89226	0.704432
67791	1.89226	0.704503
67792	3.78453	0.707907
67793	1.39491	0.740575
67794	1.39491	0.738291
67795	0.924649	0.727171
67796	0.924649	0.722694
67797	0.946131	0.726379
67798	1.37343	0.709485
67799	0.924678	0.702212

67800	0.946131	0.86078
67801	0.924678	0.736275
67802	1.37343	0.708675
67803	0.946131	0.779108
67804	0.946131	0.757838
67805	1.89226	0.71655
67806	1.89226	0.701528
67807	1.89226	0.707997
67808	1.89226	0.719729
67809	1.89226	0.700884
67810	1.89226	0.706685
67811	0.946126	0.246953
67812	0.975461	0.246933
67813	0.946132	0.182344
67814	1.12674	0.315609
67815	0.946129	0.332737
67816	0.946129	0.326054
67817	1.12673	0.253409
67818	1.12673	0.245593
67819	0.946129	0.254405
67820	0.946129	0.302671
67821	0.975464	0.303812
67822	1.12673	0.287797
67823	1.89226	0.344376
67824	1.89226	0.288156
67825	1.89226	0.389328
67826	1.89226	0.328533
67827	1.89226	0.33274
67828	1.89226	0.315402
67829	0.946126	0.357352
67830	0.946126	0.386774
67831	0.946131	0.295334
67832	0.946131	0.523352
67833	0.946129	0.543529
67834	0.946129	0.523486
67835	0.946128	0.370111
67836	0.946128	0.364407
67837	0.946129	0.411664
67838	0.946129	0.5173
67839	0.946128	0.480326
67840	0.946128	0.480412
67841	1.89226	0.288197
67842	1.89226	0.490097
67843	1.89226	0.242541
67844	1.89226	0.298863
67845	3.78451	0.247922
67846	1.89226	0.40235
67847	1.89226	0.407959
67848	1.89226	0.246123
67849	1.89226	0.388024
67850	3.78452	0.434786
67851	0.946125	0.369034
67852	0.946125	0.412765
67853	0.946131	0.349494
67854	0.946131	0.554065
67855	0.946128	0.579672
67856	0.946128	0.575106
67857	0.946128	0.457333

67858	0.946128	0.362399
67859	0.946128	0.417305
67860	0.946128	0.517967
67861	0.946128	0.425314
67862	0.946128	0.529889
67863	1.89226	0.635234
67864	1.89226	0.677896
67865	1.89226	0.691721
67866	1.89226	0.656758
67867	3.78452	0.664464
67868	1.89226	0.6324
67869	1.89226	0.667131
67870	1.89226	0.665996
67871	1.89226	0.665926
67872	1.89226	0.651941
67873	1.89226	0.666224
67874	1.12492	0.692182
67875	1.41201	0.68866
67876	1.12082	0.669322
67877	0.771416	0.6738
67878	1.10725	0.675329
67879	1.10725	0.668262
67880	1.07618	0.707077
67881	1.07618	0.672227
67882	1.12492	0.688555
67883	0.771415	0.695815
67884	1.12082	0.653322
67885	1.41201	0.681641
67886	2.82401	0.675194
67887	1.89226	0.697643
67888	1.89226	0.692668
67889	1.89226	0.686003
67890	2.24983	0.695664
67891	2.24983	0.690898
67892	0.946133	0.694123
67893	0.946133	0.687362
67894	0.946129	0.670349
67895	0.946129	0.670491
67896	0.946131	0.681204
67897	0.946131	0.685139
67898	0.946131	0.691454
67899	0.946131	0.668101
67900	0.946131	0.684174
67901	0.946131	0.686914
67902	0.946131	0.681385
67903	0.946131	0.673079
67904	0.946132	0.674911
67905	0.946132	0.666934
67906	0.946133	0.660013
67907	0.946133	0.656271
67908	0.946132	0.662802
67909	0.946132	0.664737
67910	0.946133	0.683289
67911	0.946133	0.670186
67912	0.946132	0.677474
67913	0.946132	0.666004
67914	0.946133	0.66477
67915	0.946133	0.659909

67916	1.89227	0.813789
67917	1.89227	0.863736
67918	1.89227	0.868081
67919	1.89227	0.815022
67920	1.89227	0.863119
67921	1.89227	0.810823
67922	1.42982	0.469704
67923	1.19944	0.33379
67924	0.936311	0.583134
67925	0.936311	0.468445
67926	0.946132	0.496106
67927	1.02993	0.525876
67928	1.41513	0.550444
67929	0.946133	0.558843
67930	0.946133	0.490489
67931	0.946133	0.566846
67932	1.02993	0.575441
67933	1.18181	0.462918
67934	1.89226	0.808732
67935	1.89226	0.903358
67936	1.89226	0.91817
67937	1.89226	0.746796
67938	3.78453	0.82063
67939	1.89226	0.595603
67940	1.89226	0.669985
67941	1.89226	0.666652
67942	1.89226	0.614484
67943	1.89226	0.653015
67944	1.89226	0.601191
67945	1.89226	0.601765
67946	1.89226	0.62282
67947	1.89226	0.647525
67948	1.89226	0.591238
67949	1.89226	0.651771
67950	1.89226	0.598218
67951	0.946131	0.766257
67952	0.946131	0.801266
67953	0.946132	0.882288
67954	0.946132	0.84241
67955	0.946132	0.788996
67956	0.946132	0.820877
67957	0.946132	0.903461
67958	0.946132	0.757421
67959	0.946132	0.796646
67960	0.946132	0.814262
67961	0.946132	0.818699
67962	0.946132	0.792622
67963	1.89226	1.19921
67964	1.89226	1.2624
67965	1.89226	1.43052
67966	1.89226	1.09969
67967	1.89226	1.4143
67968	1.89226	1.08892
67969	1.89226	1.41208
67970	2.22749	1.00331
67971	1.89226	1.02475
67972	1.89226	1.11306
67973	3.78453	1.1174

67974	1.89226	1.20125
67975	1.89226	1.3016
67976	1.89226	1.41462
67977	1.89226	1.03731
67978	3.78453	1.1343
67979	1.89226	1.03472
67980	1.89226	1.08606
67981	1.89226	1.20774
67982	1.89226	0.959318
67983	1.89226	1.22508
67984	1.89226	0.972129
67985	0.94613	0.948967
67986	0.94613	1.15626
67987	0.94613	1.35351
67988	0.94613	1.11318
67989	0.946131	1.06069
67990	0.946131	0.961501
67991	0.946129	1.30175
67992	0.946129	1.03927
67993	0.94613	1.0707
67994	0.94613	1.0951
67995	0.94613	1.06158
67996	0.94613	1.03035
67997	1.89226	0.352191
67998	1.89226	0.330572
67999	1.89226	0.327803
68000	1.89226	0.323818
68001	1.89226	0.347215
68002	1.89226	0.346979
68003	1.89226	0.388761
68004	1.89226	0.510213
68005	1.89226	0.459786
68006	1.89226	0.282746
68007	3.78452	0.396478
68008	1.89225	0.23688
68009	1.89225	0.218375
68010	1.89225	0.210666
68011	1.89225	0.204246
68012	1.89225	0.247697
68013	1.89225	0.248977
68014	1.89226	0.192897
68015	1.89226	0.17859
68016	1.89226	0.197418
68017	1.89226	0.191621
68018	1.89226	0.202691
68019	1.89226	0.201391
68020	0.946132	0.301348
68021	0.946132	0.404502
68022	0.946132	0.502786
68023	0.946132	0.356322
68024	0.946131	0.302628
68025	0.946131	0.296091
68026	0.946132	0.524232
68027	0.946132	0.28634
68028	0.946132	0.304932
68029	0.946132	0.337895
68030	0.946132	0.34317
68031	0.946132	0.328217

68032	1.89226	0.566758
68033	1.89226	0.689831
68034	1.89226	0.578711
68035	1.89226	0.710759
68036	1.89226	0.514779
68037	1.89226	0.708375
68038	1.89226	0.609535
68039	1.89226	0.553386
68040	1.89226	0.656856
68041	1.89226	0.696467
68042	1.89226	0.356222
68043	1.89226	0.667809
68044	1.89226	0.393564
68045	1.89226	0.495416
68046	1.89226	0.549197
68047	1.89226	0.59308
68048	1.89226	0.292405
68049	1.89226	0.366904
68050	1.89226	0.603251
68051	1.89226	0.704985
68052	1.89226	0.525488
68053	1.89226	0.706562
68054	3.78452	0.589328
68055	1.89226	0.535042
68056	1.89226	0.598984
68057	1.89226	0.520015
68058	1.89226	0.704471
68059	1.89226	0.427312
68060	1.89226	0.702533
68061	1.89226	0.117815
68062	1.89226	0.140051
68063	1.89226	0.132446
68064	1.89226	0.11117
68065	3.78453	0.113316
68066	1.89226	0.0982812
68067	2.48862	0.0889846
68068	1.89226	0.092719
68069	2.22062	0.0918507
68070	1.89226	0.0947887
68071	2.22062	0.0961004
68072	1.89227	0.116412
68073	1.89227	0.164615
68074	1.89227	0.125413
68075	1.89227	0.105811
68076	1.89227	0.148496
68077	1.89227	0.121586
68078	0.946132	0.121578
68079	0.946132	0.116731
68080	0.946131	0.121879
68081	0.946131	0.145678
68082	0.94613	0.146876
68083	0.94613	0.13577
68084	0.946133	0.128396
68085	0.946133	0.123939
68086	0.946131	0.131176
68087	0.946131	0.130141
68088	0.946131	0.139514
68089	0.946131	0.137911

68090	1.89226	0.108468
68091	1.89226	0.0994717
68092	1.89226	0.0899303
68093	1.89226	0.106621
68094	3.78453	0.104782
68095	1.89226	0.133036
68096	1.89226	0.18946
68097	1.89226	0.148783
68098	1.89226	0.122623
68099	3.78453	0.129859
68100	1.89226	0.704219
68101	1.89226	0.700415
68102	1.89226	0.7
68103	1.89226	0.703178
68104	1.89226	0.701258
68105	1.89226	0.709072
68106	1.89226	0.703572
68107	1.89226	0.698136
68108	1.89226	0.700231
68109	1.89226	0.704805
68110	3.78452	0.702136
68111	1.89226	0.682797
68112	1.89226	0.698838
68113	1.89226	0.70381
68114	1.89226	0.697875
68115	3.78453	0.694413
68116	2.57454	0.710643
68117	1.89226	0.707699
68118	1.3663	0.706866
68119	1.89226	0.70982
68120	3.25856	0.705883
68121	1.89226	0.70566
68122	1.89226	0.701348
68123	1.89226	0.702007
68124	1.89226	0.705216
68125	3.78451	0.705633
68126	1.89226	0.701158
68127	1.89226	0.705712
68128	1.89226	0.701633
68129	1.89226	0.705318
68130	1.89226	0.700823
68131	1.89226	0.703395
68132	0.810946	0.703187
68133	0.810946	0.695078
68134	0.810946	0.704303
68135	0.810946	0.705821
68136	0.810959	0.704639
68137	0.810959	0.704406
68138	1.89226	0.707279
68139	1.35168	0.706661
68140	1.08132	0.704996
68141	1.08132	0.706224
68142	1.0813	0.701381
68143	1.89226	0.64979
68144	1.89226	0.67678
68145	1.89226	0.709767
68146	1.89226	0.671662
68147	3.78453	0.672065

68148	1.89226	0.698151
68149	1.89226	0.670081
68150	1.89226	0.67968
68151	1.89226	0.688314
68152	1.89226	0.676844
68153	1.89226	0.684606
68154	1.89226	0.668142
68155	1.89226	0.683718
68156	1.89226	0.69424
68157	1.89226	0.674674
68158	3.78452	0.677952
68159	1.89226	0.676294
68160	1.89226	0.675021
68161	1.89226	0.670684
68162	1.89226	0.689619
68163	3.78452	0.675945
68164	2.142	0.751772
68165	1.93949	0.670575
68166	1.39296	0.716688
68167	2.55711	0.729589
68168	1.39296	0.680927
68169	2.55711	0.708003
68170	1.89226	0.701353
68171	2.5571	0.675896
68172	2.142	0.712805
68173	1.89226	0.716608
68174	4.44936	0.710062
68175	0.946131	0.699263
68176	0.946131	0.696451
68177	1.27855	0.691365
68178	1.29171	0.686425
68179	1.29171	0.693749
68180	0.946127	0.69047
68181	1.27855	0.679822
68182	0.946131	0.705252
68183	1.29172	0.700351
68184	1.29172	0.696978
68185	0.946129	0.687179
68186	0.946129	0.685238
68187	1.27855	0.714972
68188	1.29172	0.702766
68189	0.969743	0.690985
68190	1.07109	0.683103
68191	1.29172	0.720631
68192	1.07109	0.682145
68193	0.969745	0.671484
68194	1.27855	0.72007
68195	1.10873	0.734074
68196	1.10873	0.712313
68197	1.15273	0.676538
68198	1.15273	0.685223
68199	1.89227	0.419009
68200	1.89227	0.456568
68201	1.89227	0.50427
68202	1.89227	0.432073
68203	2.1288	0.386503
68204	2.1288	0.334584
68205	1.89227	0.55456

68206	1.89227	0.541263
68207	1.89227	0.479545
68208	1.89227	0.591089
68209	3.78453	0.630685
68210	1.89226	0.694304
68211	1.89226	0.7
68212	1.89226	0.6168
68213	1.89226	0.655828
68214	3.78453	0.5798
68215	1.89226	0.592113
68216	1.89226	0.688324
68217	1.89226	0.550773
68218	1.89226	0.552839
68219	1.89226	0.667345
68220	1.89226	0.684036
68221	1.94432	0.277552
68222	1.89226	0.384163
68223	2.07911	0.286192
68224	1.89226	0.291317
68225	2.07911	0.340904
68226	1.89226	0.35464
68227	1.89226	0.406029
68228	1.89226	0.339612
68229	1.89226	0.661645
68230	1.89226	0.289912
68231	1.89226	0.401179
68232	1.89226	0.512748
68233	1.89226	0.913866
68234	1.89226	0.927304
68235	1.89226	0.99457
68236	1.89226	0.852472
68237	1.89226	1.01019
68238	1.89226	0.864209
68239	1.89226	0.806822
68240	1.89226	0.796243
68241	1.89226	0.850348
68242	1.89226	0.749112
68243	1.89226	0.86792
68244	1.89226	0.789123
68245	1.86778	0.750316
68246	2.72348	0.728334
68247	1.89226	0.788244
68248	1.89226	0.476914
68249	3.76004	0.754723
68250	1.89226	0.894984
68251	1.89226	0.930592
68252	1.89226	1.03046
68253	1.89226	0.822773
68254	3.78451	0.889195
68255	1.89226	0.799796
68256	1.89226	0.812556
68257	1.89226	0.84613
68258	1.89226	0.761795
68259	1.89226	0.857237
68260	1.89226	0.779954
68261	0.94613	0.680674
68262	0.94613	0.771196
68263	0.94613	0.844114

68264	0.94613	0.75551
68265	0.946131	0.775467
68266	0.946131	0.538036
68267	0.946129	0.811909
68268	0.946129	0.75288
68269	0.94613	0.735206
68270	0.94613	0.771276
68271	0.94613	0.743772
68272	0.94613	0.760487
68273	0.946124	0.825366
68274	0.946124	0.704449
68275	0.94613	0.923735
68276	0.94613	0.836182
68277	0.946128	0.784631
68278	0.946128	0.814702
68279	0.946126	0.927568
68280	0.946126	0.821428
68281	0.946127	0.83027
68282	0.946127	0.832569
68283	0.946127	0.822964
68284	0.946127	0.820027
68285	2.09166	0.16874
68286	2.1285	0.239397
68287	1.78381	0.257991
68288	1.78381	0.279618
68289	2.47689	0.167349
68290	2.47689	0.192342
68291	2.09167	0.16165
68292	2.26572	0.270843
68293	2.73393	0.151924
68294	2.4769	0.243215
68295	4.74261	0.237196
68296	1.89226	0.23989
68297	1.89226	0.433898
68298	1.89226	0.31536
68299	1.89226	0.282284
68300	1.89226	0.383519
68301	1.89226	0.397371
68302	0.946131	0.316808
68303	0.946131	0.534499
68304	0.946129	0.354207
68305	0.946129	0.452618
68306	0.94613	0.419214
68307	0.94613	0.430548
68308	0.946131	0.250976
68309	0.946131	0.262942
68310	0.94613	0.304909
68311	0.94613	0.35051
68312	0.946131	0.316292
68313	0.946131	0.365196
68314	2.27622	0.242754
68315	2.66786	0.16569
68316	2.1285	0.262976
68317	2.1285	0.271345
68318	2.62461	0.166372
68319	2.47689	0.246969
68320	1.89226	0.255221
68321	1.89226	0.424692

68322	1.89226	0.268536
68323	1.89226	0.393152
68324	3.78453	0.387986
68325	1.89226	0.262279
68326	1.89226	0.407311
68327	1.89226	0.442533
68328	1.89226	0.456907
68329	1.89226	0.24206
68330	1.89226	0.279707
68331	1.89226	0.830698
68332	1.89226	0.900408
68333	1.89226	0.833225
68334	1.89226	0.891127
68335	1.89226	0.835516
68336	1.89226	0.887369
68337	1.89226	0.984902
68338	1.89226	0.883387
68339	1.89226	0.88172
68340	1.89226	0.995648
68341	3.78452	0.927537
68342	1.89227	0.886129
68343	1.89227	0.99818
68344	1.89227	0.988197
68345	1.89227	0.938762
68346	3.78453	0.967653
68347	0.946127	0.940271
68348	0.946127	0.913272
68349	1.06438	0.907442
68350	0.946131	0.862735
68351	1.06438	0.902392
68352	0.94613	0.938844
68353	0.946128	0.915285
68354	0.946128	0.847345
68355	1.06438	0.911158
68356	1.06438	0.916872
68357	0.946129	0.906759
68358	0.946129	0.902355
68359	1.89226	0.892752
68360	1.89226	0.984481
68361	1.89226	0.927968
68362	1.89226	0.966391
68363	1.89226	0.93139
68364	1.89226	0.972917
68365	0.946133	0.994471
68366	0.946133	0.970423
68367	1.39771	0.984922
68368	0.946132	0.93078
68369	1.39771	0.991418
68370	1.39772	0.99375
68371	0.946133	0.972599
68372	0.946133	0.913706
68373	0.946132	0.981749
68374	1.39772	0.977855
68375	0.946132	0.945294
68376	0.946132	0.951962
68377	1.98182	0.133934
68378	2.2217	0.187944
68379	2.79889	0.122219

68380	2.50097	0.160232
68381	4.72267	0.134723
68382	2.05362	0.116598
68383	1.70302	0.141121
68384	2.75109	0.131581
68385	2.138	0.113954
68386	2.0815	0.147802
68387	1.84751	0.14624
68388	0.946131	0.220545
68389	0.946131	0.16725
68390	0.946131	0.139852
68391	0.946131	0.198405
68392	0.946131	0.216076
68393	0.946131	0.20329
68394	0.946132	0.14317
68395	0.946132	0.154889
68396	0.946131	0.168963
68397	0.946131	0.171136
68398	0.946132	0.171637
68399	0.946132	0.188081
68400	1.89226	0.195321
68401	1.89226	0.283474
68402	1.89226	0.167854
68403	1.89226	0.255742
68404	3.78451	0.261404
68405	0.946126	0.148424
68406	1.25048	0.119251
68407	0.946131	0.101355
68408	1.39945	0.155307
68409	0.946127	0.172384
68410	0.946127	0.171829
68411	1.39944	0.121521
68412	1.39944	0.122689
68413	0.946129	0.142677
68414	0.946129	0.150659
68415	1.39944	0.13916
68416	1.25048	0.149648
68417	1.89226	0.145157
68418	1.89226	0.238001
68419	1.89226	0.150983
68420	1.89226	0.184444
68421	1.89226	0.164006
68422	1.89226	0.209474
68423	0.946126	0.291525
68424	0.946126	0.214236
68425	0.946131	0.167416
68426	0.946131	0.233046
68427	0.946128	0.256267
68428	0.946128	0.254066
68429	0.946129	0.18081
68430	0.946129	0.181516
68431	0.946129	0.205088
68432	0.946129	0.205343
68433	0.946128	0.225782
68434	0.946128	0.227526
68435	1.89226	0.113918
68436	1.89226	0.165963
68437	1.89226	0.118695

68438	1.89226	0.119833
68439	1.89226	0.132193
68440	1.89226	0.134853
68441	1.01393	0.105304
68442	0.775245	0.0971039
68443	0.946126	0.0993622
68444	0.946126	0.11038
68445	0.78305	0.108116
68446	0.78305	0.109961
68447	0.946159	0.11525
68448	0.929267	0.0970179
68449	1.14956	0.105231
68450	1.04073	0.107272
68451	0.851526	0.112532
68452	0.704781	0.113151
68453	1.89226	0.12168
68454	1.89226	0.15286
68455	1.89226	0.122594
68456	1.89226	0.115858
68457	3.78451	0.117833
68458	1.89226	0.12973
68459	1.89226	0.219402
68460	1.89226	0.1459
68461	1.89226	0.136939
68462	1.89226	0.175339
68463	1.89226	0.161676
68464	1.89226	0.113038
68465	1.89226	0.164515
68466	1.89226	0.117942
68467	1.89226	0.125132
68468	1.89226	0.130972
68469	1.89226	0.141348
68470	1.16317	0.104562
68471	1.0292	0.0954931
68472	0.946126	0.100352
68473	0.946126	0.112736
68474	0.93562	0.110285
68475	0.93562	0.105758
68476	1.0418	0.115257
68477	1.04181	0.0932933
68478	1.16317	0.100921
68479	0.946129	0.101105
68480	1.02919	0.104599
68481	0.946128	0.107308
68482	0.946131	0.109175
68483	0.946131	0.122348
68484	0.946126	0.117299
68485	0.946126	0.138552
68486	0.946129	0.139445
68487	0.946129	0.123396
68488	0.946128	0.125874
68489	0.946128	0.122561
68490	0.946129	0.124725
68491	0.946129	0.120554
68492	0.946128	0.126957
68493	0.946128	0.123459
68494	1.89226	0.131874
68495	1.89226	0.218736

68496	1.89226	0.150034
68497	1.89226	0.153584
68498	1.89226	0.170743
68499	1.89226	0.176822
68500	1.89226	0.105073
68501	1.89226	0.0870668
68502	1.89226	0.0912597
68503	1.89226	0.0986647
68504	1.89226	0.0906152
68505	1.89226	0.0974575
68506	1.89226	0.109289
68507	1.89226	0.1047
68508	1.89226	0.0861343
68509	1.89226	0.0896683
68510	3.78453	0.0922105
68511	1.89226	0.101802
68512	1.89226	0.107214
68513	1.89226	0.0857032
68514	1.89226	0.0860931
68515	3.78452	0.0961157
68516	1.89225	0.105892
68517	1.89225	0.107741
68518	1.89225	0.0872596
68519	1.89225	0.0965584
68520	1.89225	0.0882873
68521	1.89225	0.0811471
68522	0.946132	0.0644853
68523	0.946132	0.0736253
68524	0.946133	0.0649176
68525	0.946133	0.0733761
68526	0.946132	0.0718687
68527	0.946132	0.0682735
68528	0.946132	0.0693787
68529	0.946132	0.0760786
68530	0.946133	0.0642632
68531	0.946133	0.0628742
68532	0.946132	0.0825491
68533	0.946132	0.0739326
68534	1.89226	0.116859
68535	1.89226	0.152968
68536	1.89226	0.126986
68537	1.89226	0.126458
68538	3.78453	0.129044
68539	1.13537	0.103886
68540	1.09222	0.0931572
68541	0.946126	0.0998101
68542	0.946126	0.125917
68543	0.946128	0.10694
68544	0.946128	0.100286
68545	1.16317	0.104947
68546	1.13034	0.0911138
68547	1.30155	0.102399
68548	1.08462	0.101811
68549	0.992915	0.101263
68550	0.807642	0.101352
68551	0.946131	0.114295
68552	0.946131	0.130431
68553	0.946125	0.117603

68554	0.946125	0.136809
68555	0.946128	0.13485
68556	0.946128	0.123053
68557	0.946128	0.124535
68558	0.946128	0.116908
68559	0.946128	0.122826
68560	0.946128	0.117397
68561	0.946128	0.129319
68562	0.946128	0.123917
68563	1.89226	0.131151
68564	1.89226	0.205463
68565	1.89226	0.155743
68566	1.89226	0.165889
68567	1.89226	0.152919
68568	1.89226	0.17162
68569	1.89224	0.123641
68570	1.89224	0.120372
68571	1.89224	0.142771
68572	1.89224	0.110681
68573	1.89224	0.142068
68574	1.89224	0.112956
68575	0.946086	0.103362
68576	1.17919	0.0907277
68577	0.89738	0.105831
68578	0.89738	0.098259
68579	0.946116	0.0994039
68580	0.946116	0.0953618
68581	1.13048	0.0996405
68582	1.11844	0.0838235
68583	0.946128	0.099154
68584	0.897362	0.0986605
68585	1.1282	0.103772
68586	0.946103	0.100761
68587	1.89223	0.106797
[truncated: 613,203 more chars]
