# Supplementary material for: On the Use of Bone Remodelling Models to Estimate the Density Distribution of Bones. Uniqueness of the Solution
Source: PLoS One. 2016 Feb 9;11(2):e0148603. doi: 10.1371/journal.pone.0148603 (PMC4747586; doi:10.1371/journal.pone.0148603)
Supplement: S9 File — (PDF) [file pone.0148603.s009.pdf]

| N° Elem | Volume (mm3) | Density (g/cm3) |
|---|---|---|
| 1 | 1.32415 | 2.1 |
| 2 | 1.75823 | 2.1 |
| 3 | 0.984099 | 2.1 |
| 4 | 1.46442 | 2.1 |
| 5 | 1.53082 | 2.1 |
| 6 | 1.32068 | 2.1 |
| 7 | 1.91404 | 0.05 |
| 8 | 1.17036 | 0.05 |
| 9 | 0.929209 | 0.05 |
| 10 | 1.27254 | 0.05 |
| 11 | 2.0926 | 0.05 |
| 12 | 2.20407 | 0.05 |
| 13 | 1.21488 | 0.05 |
| 14 | 1.26024 | 0.05 |
| 15 | 0.794454 | 0.05 |
| 16 | 1.95087 | 0.05 |
| 17 | 0.953925 | 2.1 |
| 18 | 0.98949 | 2.1 |
| 19 | 1.27875 | 2.1 |
| 20 | 1.04598 | 2.1 |
| 21 | 0.594005 | 2.1 |
| 22 | 0.578861 | 2.1 |
| 23 | 1.09506 | 2.1 |
| 24 | 1.45327 | 2.1 |
| 25 | 2.58396 | 2.1 |
| 26 | 1.58581 | 2.1 |
| 27 | 1.5858 | 2.1 |
| 28 | 0.636946 | 2.1 |
| 29 | 0.94202 | 2.1 |
| 30 | 0.946119 | 2.1 |
| 31 | 0.636953 | 2.1 |
| 32 | 0.946107 | 2.1 |
| 33 | 1.09505 | 2.1 |
| 34 | 1.27389 | 2.1 |
| 35 | 1.00232 | 2.1 |
| 36 | 1.00231 | 2.1 |
| 37 | 1.83583 | 2.1 |
| 38 | 1.00231 | 2.1 |
| 39 | 1.28828 | 0.05 |
| 40 | 1.81696 | 0.05 |
| 41 | 1.28828 | 0.05 |
| 42 | 1.28828 | 0.05 |
| 43 | 1.0044 | 0.05 |
| 44 | 0.85148 | 0.05 |
| 45 | 0.851539 | 0.05 |
| 46 | 1.57767 | 0.05 |
| 47 | 1.52227 | 0.05 |
| 48 | 0.730657 | 0.05 |
| 49 | 0.94613 | 0.05 |
| 50 | 1.28228 | 2.1 |
| 51 | 1.64268 | 2.1 |
| 52 | 1.10101 | 2.1 |
| 53 | 1.3939 | 2.1 |
| 54 | 0.959471 | 2.1 |
| 55 | 1.10101 | 2.1 |

| | | |
|---|---|---|
| 56 | 1.2873 | 2.1 |
| 57 | 1.59659 | 2.1 |
| 58 | 1.80841 | 0.136762 |
| 59 | 1.80841 | 0.492758 |
| 60 | 0.946129 | 0.05 |
| 61 | 1.36991 | 0.05 |
| 62 | 1.32262 | 0.67083 |
| 63 | 1.32261 | 0.05 |
| 64 | 1.38464 | 0.476625 |
| 65 | 0.962877 | 0.333815 |
| 66 | 0.962878 | 0.308117 |
| 67 | 1.82129 | 0.322944 |
| 68 | 1.82129 | 0.173965 |
| 69 | 0.94613 | 0.359184 |
| 70 | 1.39751 | 0.232191 |
| 71 | 0.946131 | 0.468908 |
| 72 | 1.34493 | 0.11102 |
| 73 | 1.39751 | 0.203653 |
| 74 | 0.837577 | 0.84294 |
| 75 | 0.823478 | 0.390236 |
| 76 | 1.10889 | 1.83329 |
| 77 | 1.01686 | 0.874275 |
| 78 | 1.1942 | 1.84203 |
| 79 | 0.836129 | 0.807328 |
| 80 | 0.937165 | 0.267095 |
| 81 | 0.969438 | 0.98188 |
| 82 | 0.895386 | 0.719387 |
| 83 | 1.30554 | 2.1 |
| 84 | 1.15798 | 0.785913 |
| 85 | 1.22578 | 2.1 |
| 86 | 1.15255 | 0.94683 |
| 87 | 0.9765 | 0.204661 |
| 88 | 2.02178 | 2.03255 |
| 89 | 1.63755 | 1.87728 |
| 90 | 0.727713 | 1.01718 |
| 91 | 0.810945 | 1.01435 |
| 92 | 1.00559 | 2.07038 |
| 93 | 0.879098 | 1.07716 |
| 94 | 1.07852 | 2.1 |
| 95 | 0.657764 | 0.885619 |
| 96 | 0.829406 | 0.765032 |
| 97 | 0.720201 | 0.05 |
| 98 | 0.946131 | 0.05 |
| 99 | 1.11827 | 0.05 |
| 100 | 1.50754 | 0.05 |
| 101 | 1.64835 | 0.05 |
| 102 | 1.71903 | 0.05 |
| 103 | 0.851554 | 0.05 |
| 104 | 1.75626 | 0.05 |
| 105 | 2.1646 | 0.05 |
| 106 | 1.5563 | 0.05 |
| 107 | 1.5563 | 0.05 |
| 108 | 2.10876 | 0.05 |
| 109 | 1.05438 | 0.0899719 |
| 110 | 0.946133 | 0.05 |
| 111 | 1.05438 | 0.57464 |
| 112 | 1.20256 | 0.05 |
| 113 | 1.47879 | 0.17096 |

| | | |
|---|---|---|
| 114 | 1.47879 | 1.6762 |
| 115 | 0.77815 | 1.52499 |
| 116 | 0.947114 | 2.1 |
| 117 | 1.31257 | 2.1 |
| 118 | 1.09235 | 2.1 |
| 119 | 0.946132 | 2.1 |
| 120 | 1.54246 | 2.1 |
| 121 | 1.54246 | 2.1 |
| 122 | 1.09235 | 2.1 |
| 123 | 0.946131 | 2.1 |
| 124 | 1.29507 | 1.92226 |
| 125 | 1.0353 | 2.1 |
| 126 | 1.03529 | 2.1 |
| 127 | 1.28667 | 2.1 |
| 128 | 1.44777 | 2.1 |
| 129 | 1.61121 | 2.1 |
| 130 | 1.22176 | 0.05 |
| 131 | 0.946129 | 0.05 |
| 132 | 1.08848 | 0.05 |
| 133 | 0.946131 | 0.05 |
| 134 | 1.61201 | 0.05 |
| 135 | 1.61201 | 0.05 |
| 136 | 1.19993 | 0.05 |
| 137 | 1.35822 | 0.05 |
| 138 | 1.1539 | 0.05 |
| 139 | 1.00765 | 0.05 |
| 140 | 1.28228 | 2.1 |
| 141 | 2.65339 | 2.1 |
| 142 | 1.28228 | 2.1 |
| 143 | 1.55998 | 2.1 |
| 144 | 1.00509 | 2.1 |
| 145 | 0.93845 | 2.1 |
| 146 | 1.27674 | 2.1 |
| 147 | 0.810958 | 2.1 |
| 148 | 1.91381 | 2.1 |
| 149 | 1.1861 | 2.1 |
| 150 | 1.24886 | 2.1 |
| 151 | 2.02842 | 2.1 |
| 152 | 0.991436 | 0.05 |
| 153 | 1.34588 | 0.05 |
| 154 | 2.02729 | 0.05 |
| 155 | 0.72904 | 0.05 |
| 156 | 1.06139 | 0.05 |
| 157 | 0.835368 | 0.05 |
| 158 | 1.63819 | 0.05 |
| 159 | 1.10745 | 0.05 |
| 160 | 1.79925 | 0.05 |
| 161 | 1.00085 | 0.05 |
| 162 | 0.946133 | 2.1 |
| 163 | 1.41123 | 2.1 |
| 164 | 1.8327 | 2.1 |
| 165 | 1.8327 | 2.1 |
| 166 | 0.946132 | 0.234728 |
| 167 | 1.41123 | 2.1 |
| 168 | 1.3676 | 2.1 |
| 169 | 1.11048 | 2.1 |
| 170 | 1.11048 | 2.1 |
| 171 | 1.65815 | 2.1 |

| | | |
|---|---|---|
| 172 | 1.9004 | 2.1 |
| 173 | 1.88691 | 2.1 |
| 174 | 1.82232 | 2.1 |
| 175 | 1.46591 | 2.1 |
| 176 | 1.09115 | 2.1 |
| 177 | 1.02051 | 0.939497 |
| 178 | 1.03373 | 1.02153 |
| 179 | 1.10307 | 2.1 |
| 180 | 1.05556 | 2.1 |
| 181 | 1.82533 | 0.991936 |
| 182 | 1.19496 | 0.811781 |
| 183 | 1.28597 | 2.1 |
| 184 | 1.20421 | 2.1 |
| 185 | 2.23644 | 2.1 |
| 186 | 1.08378 | 2.1 |
| 187 | 0.983724 | 2.1 |
| 188 | 0.602075 | 2.1 |
| 189 | 1.45876 | 2.1 |
| 190 | 1.45875 | 2.1 |
| 191 | 0.602103 | 2.1 |
| 192 | 1.11822 | 2.1 |
| 193 | 0.946133 | 2.1 |
| 194 | 0.602103 | 2.1 |
| 195 | 1.20421 | 2.1 |
| 196 | 1.26371 | 0.05 |
| 197 | 1.55179 | 0.05 |
| 198 | 1.14042 | 0.05 |
| 199 | 1.56876 | 0.05 |
| 200 | 2.27496 | 0.05 |
| 201 | 1.13748 | 0.05 |
| 202 | 1.02884 | 0.05 |
| 203 | 1.25124 | 0.05 |
| 204 | 0.82703 | 0.05 |
| 205 | 0.607775 | 0.05 |
| 206 | 1.49242 | 0.05 |
| 207 | 1.35674 | 0.075956 |
| 208 | 1.6697 | 2.1 |
| 209 | 1.81655 | 2.1 |
| 210 | 0.900108 | 1.11744 |
| 211 | 0.851537 | 0.538326 |
| 212 | 0.703076 | 1.0611 |
| 213 | 0.639143 | 0.865717 |
| 214 | 1.42358 | 1.14598 |
| 215 | 1.4976 | 1.54168 |
| 216 | 0.86284 | 0.893361 |
| 217 | 1.2965 | 1.05146 |
| 218 | 1.27835 | 2.1 |
| 219 | 1.00012 | 0.926731 |
| 220 | 1.74332 | 1.41194 |
| 221 | 1.08576 | 2.1 |
| 222 | 1.19 2.1 | |
| 223 | 1.08576 | 2.1 |
| 224 | 1.20854 | 2.1 |
| 225 | 0.946131 | 2.1 |
| 226 | 0.946131 | 2.1 |
| 227 | 1.75985 | 2.1 |
| 228 | 1.75985 | 2.1 |
| 229 | 1.41232 | 2.1 |

| | | |
|---|---|---|
| 230 | 1.29365 | 2.1 |
| 231 | 0.973559 | 2.1 |
| 232 | 1.23698 | 0.05 |
| 233 | 1.45055 | 0.05 |
| 234 | 1.26947 | 0.05 |
| 235 | 0.856218 | 0.05 |
| 236 | 1.75303 | 0.05 |
| 237 | 1.14938 | 0.05 |
| 238 | 0.984711 | 0.05 |
| 239 | 2.05771 | 0.05 |
| 240 | 0.625171 | 0.05 |
| 241 | 0.965675 | 0.05 |
| 242 | 1.17631 | 0.05 |
| 243 | 1.4677 | 0.05 |
| 244 | 1.35403 | 0.05 |
| 245 | 0.975149 | 0.05 |
| 246 | 0.946131 | 0.05 |
| 247 | 1.80893 | 0.05 |
| 248 | 1.80893 | 0.05 |
| 249 | 0.975149 | 0.05 |
| 250 | 0.946132 | 0.05 |
| 251 | 1.9503 | 0.05 |
| 252 | 2.80207 | 0.05 |
| 253 | 1.76411 | 0.05 |
| 254 | 2.77469 | 0.05 |
| 255 | 1.9503 | 0.05 |
| 256 | 1.26784 | 0.05 |
| 257 | 2.31093 | 0.05 |
| 258 | 0.851541 | 0.05 |
| 259 | 1.39521 | 0.05 |
| 260 | 1.45613 | 0.05 |
| 261 | 0.851541 | 0.05 |
| 262 | 0.996013 | 0.05 |
| 263 | 1.07989 | 0.05 |
| 264 | 0.946131 | 0.05 |
| 265 | 1.08213 | 0.05 |
| 266 | 1.74289 | 0.05 |
| 267 | 1.75838 | 0.05 |
| 268 | 2.11163 | 0.05 |
| 269 | 1.23773 | 2.1 |
| 270 | 1.25398 | 2.1 |
| 271 | 1.0304 | 2.1 |
| 272 | 1.32368 | 2.1 |
| 273 | 1.11712 | 0.972466 |
| 274 | 1.07999 | 0.875448 |
| 275 | 1.1766 | 2.1 |
| 276 | 1.35735 | 2.1 |
| 277 | 1.089 0.05 | |
| 278 | 1.81193 | 0.05 |
| 279 | 1.11081 | 0.05 |
| 280 | 1.78069 | 0.05 |
| 281 | 0.717512 | 0.05 |
| 282 | 0.810946 | 0.05 |
| 283 | 0.703901 | 0.05 |
| 284 | 1.09028 | 0.05 |
| 285 | 0.958031 | 0.05 |
| 286 | 0.892585 | 0.05 |
| 287 | 0.873431 | 0.05 |

| | | |
|---|---|---|
| 288 | 0.997755 | 0.05 |
| 289 | 1.85479 | 0.05 |
| 290 | 0.983029 | 0.05 |
| 291 | 1.27922 | 0.05 |
| 292 | 1.64346 | 0.109333 |
| 293 | 1.27922 | 0.05 |
| 294 | 1.09795 | 2.05105 |
| 295 | 1.07508 | 0.325237 |
| 296 | 1.02975 | 1.29343 |
| 297 | 1.01121 | 2.1 |
| 298 | 1.72369 | 0.05 |
| 299 | 1.12035 | 0.05 |
| 300 | 2.13511 | 0.05 |
| 301 | 0.9705 | 0.05 |
| 302 | 0.946131 | 0.05 |
| 303 | 0.86182 | 0.05 |
| 304 | 1.51379 | 0.05 |
| 305 | 1.51379 | 0.05 |
| 306 | 0.946132 | 0.05 |
| 307 | 0.861849 | 0.05 |
| 308 | 1.34591 | 0.216174 |
| 309 | 1.14721 | 0.05 |
| 310 | 1.15539 | 2.1 |
| 311 | 1.90938 | 2.1 |
| 312 | 2.19436 | 2.09658 |
| 313 | 1.41563 | 2.1 |
| 314 | 0.785641 | 2.1 |
| 315 | 0.736384 | 2.1 |
| 316 | 1.46489 | 2.1 |
| 317 | 1.46488 | 2.1 |
| 318 | 0.736372 | 2.1 |
| 319 | 0.946132 | 2.1 |
| 320 | 1.90941 | 1.10158 |
| 321 | 1.18907 | 2.1 |
| 322 | 1.20409 | 2.1 |
| 323 | 0.973559 | 2.1 |
| 324 | 1.29461 | 2.1 |
| 325 | 0.946129 | 2.1 |
| 326 | 0.94613 | 2.1 |
| 327 | 1.78751 | 2.1 |
| 328 | 1.78751 | 2.1 |
| 329 | 1.38885 | 2.1 |
| 330 | 1.34479 | 2.1 |
| 331 | 1.07283 | 1.84271 |
| 332 | 1.6869 | 2.1 |
| 333 | 1.52544 | 2.1 |
| 334 | 1.07797 | 2.1 |
| 335 | 1.13819 | 0.251683 |
| 336 | 0.776179 | 2.1 |
| 337 | 1.61092 | 1.0247 |
| 338 | 1.36155 | 2.1 |
| 339 | 0.77618 | 2.1 |
| 340 | 1.2933 | 0.781129 |
| 341 | 1.15946 | 2.1 |
| 342 | 0.69857 | 2.1 |
| 343 | 1.87607 | 2.1 |
| 344 | 1.07612 | 2.1 |
| 345 | 1.31976 | 1.17595 |

| | | |
|---|---|---|
| 346 | 1.01243 | 2.1 |
| 347 | 1.41279 | 1.3115 |
| 348 | 1.35146 | 0.05 |
| 349 | 1.90664 | 0.05 |
| 350 | 1.35145 | 0.05 |
| 351 | 1.22811 | 0.05 |
| 352 | 1.93147 | 0.05 |
| 353 | 1.42486 | 2.1 |
| 354 | 1.56558 | 2.1 |
| 355 | 1.0476 | 0.05 |
| 356 | 2.31605 | 0.05 |
| 357 | 1.89367 | 0.05 |
| 358 | 0.804706 | 0.05 |
| 359 | 0.783919 | 0.05 |
| 360 | 1.3116 | 0.05 |
| 361 | 1.03891 | 0.05 |
| 362 | 0.960473 | 0.05 |
| 363 | 1.08462 | 0.05 |
| 364 | 0.785994 | 0.05 |
| 365 | 1.1318 | 0.05 |
| 366 | 1.29576 | 0.05 |
| 367 | 1.3946 | 0.05 |
| 368 | 1.11924 | 0.05 |
| 369 | 0.834322 | 2.1 |
| 370 | 1.0256 | 2.1 |
| 371 | 1.74321 | 0.893912 |
| 372 | 1.41798 | 0.690892 |
| 373 | 0.871608 | 0.870974 |
| 374 | 1.47253 | 0.546089 |
| 375 | 1.47252 | 0.536502 |
| 376 | 0.817064 | 0.805413 |
| 377 | 0.817064 | 0.832433 |
| 378 | 0.946129 | 0.390224 |
| 379 | 1.63413 | 0.644435 |
| 380 | 1.9194 | 0.05 |
| 381 | 1.93562 | 0.05 |
| 382 | 2.06307 | 0.05 |
| 383 | 1.46021 | 0.05 |
| 384 | 0.955002 | 0.756055 |
| 385 | 0.833409 | 1.616 |
| 386 | 0.838039 | 0.70585 |
| 387 | 1.48702 | 0.789321 |
| 388 | 1.1697 | 0.81193 |
| 389 | 1.23549 | 0.930856 |
| 390 | 1.18373 | 0.646647 |
| 391 | 2.02254 | 0.316131 |
| 392 | 1.68867 | 1.04162 |
| 393 | 0.875481 | 0.0923572 |
| 394 | 0.821635 | 1.29897 |
| 395 | 0.858878 | 0.0777049 |
| 396 | 1.30245 | 0.243071 |
| 397 | 1.07476 | 0.267903 |
| 398 | 1.11907 | 0.231629 |
| 399 | 1.10453 | 0.0920311 |
| 400 | 1.70233 | 0.282366 |
| 401 | 1.70233 | 0.164605 |
| 402 | 2.6558 | 1.22587 |
| 403 | 1.7818 | 2.1 |

| | | |
|---|---|---|
| 404 | 1.87059 | 2.1 |
| 405 | 1.34412 | 0.05 |
| 406 | 1.98068 | 0.05 |
| 407 | 2.56369 | 0.05 |
| 408 | 1.49512 | 0.05 |
| 409 | 1.35977 | 0.05 |
| 410 | 1.34254 | 0.05 |
| 411 | 1.75618 | 0.05 |
| 412 | 1.75618 | 0.05 |
| 413 | 0.946129 | 0.05 |
| 414 | 0.946129 | 0.05 |
| 415 | 1.17665 | 0.05 |
| 416 | 1.01976 | 0.05 |
| 417 | 1.27376 | 0.05 |
| 418 | 1.35978 | 0.05 |
| 419 | 1.68741 | 0.05 |
| 420 | 1.68741 | 0.05 |
| 421 | 0.946132 | 0.05 |
| 422 | 0.946133 | 0.05 |
| 423 | 1.14241 | 0.05 |
| 424 | 0.996831 | 0.05 |
| 425 | 0.996828 | 0.05 |
| 426 | 1.12774 | 0.05 |
| 427 | 1.13664 | 2.1 |
| 428 | 1.09627 | 2.1 |
| 429 | 2.8814 | 2.1 |
| 430 | 3.49751 | 2.1 |
| 431 | 1.79109 | 0.05 |
| 432 | 1.54197 | 0.05 |
| 433 | 2.75153 | 0.05 |
| 434 | 1.93019 | 0.05 |
| 435 | 1.51569 | 0.170308 |
| 436 | 1.55145 | 0.716648 |
| 437 | 1.78595 | 2.1 |
| 438 | 3.74452 | 2.1 |
| 439 | 1.66537 | 2.1 |
| 440 | 3.55161 | 2.1 |
| 441 | 1.35002 | 0.249621 |
| 442 | 1.19901 | 0.514691 |
| 443 | 1.61028 | 2.1 |
| 444 | 1.35002 | 0.906025 |
| 445 | 1.65712 | 0.827446 |
| 446 | 1.10585 | 0.883832 |
| 447 | 0.961347 | 0.897627 |
| 448 | 0.810944 | 1.20597 |
| 449 | 1.16148 | 2.03275 |
| 450 | 1.08066 | 1.73274 |
| 451 | 0.934577 | 0.656488 |
| 452 | 1.05164 | 1.29437 |
| 453 | 0.79216 | 0.818524 |
| 454 | 2.99194 | 2.1 |
| 455 | 1.2832 | 2.1 |
| 456 | 2.97842 | 2.1 |
| 457 | 1.39517 | 2.1 |
| 458 | 1.03391 | 2.1 |
| 459 | 1.74416 | 2.1 |
| 460 | 1.05071 | 2.1 |
| 461 | 0.923782 | 2.1 |

| | | |
|---|---|---|
| 462 | 1.55369 | 2.1 |
| 463 | 1.86444 | 2.1 |
| 464 | 1.01617 | 2.1 |
| 465 | 1.27174 | 2.1 |
| 466 | 1.20573 | 2.1 |
| 467 | 1.01616 | 2.1 |
| 468 | 1.13621 | 2.1 |
| 469 | 1.75366 | 2.1 |
| 470 | 2.27009 | 1.95524 |
| 471 | 1.6813 | 2.08531 |
| 472 | 1.47904 | 0.05 |
| 473 | 2.62714 | 0.05 |
| 474 | 1.80085 | 0.05 |
| 475 | 1.80085 | 0.05 |
| 476 | 2.74104 | 0.05 |
| 477 | 1.37052 | 0.05 |
| 478 | 0.946131 | 0.05 |
| 479 | 1.37052 | 0.05 |
| 480 | 1.31357 | 0.05 |
| 481 | 1.7837 | 0.05 |
| 482 | 1.7837 | 0.05 |
| 483 | 0.900397 | 0.05 |
| 484 | 1.74487 | 0.05 |
| 485 | 2.88672 | 2.1 |
| 486 | 2.88249 | 2.1 |
| 487 | 1.0961 | 0.05 |
| 488 | 1.20143 | 0.0871351 |
| 489 | 1.66739 | 0.05 |
| 490 | 1.85795 | 0.05 |
| 491 | 1.45789 | 0.05 |
| 492 | 2.11407 | 0.05 |
| 493 | 0.713524 | 0.05 |
| 494 | 1.58391 | 0.05 |
| 495 | 1.58391 | 0.05 |
| 496 | 1.04765 | 0.05 |
| 497 | 1.24979 | 0.05 |
| 498 | 0.713551 | 0.05 |
| 499 | 0.946127 | 0.05 |
| 500 | 1.36768 | 0.05 |
| 501 | 1.14433 | 0.05 |
| 502 | 1.66923 | 0.05 |
| 503 | 0.699479 | 0.05 |
| 504 | 0.70351 | 0.05 |
| 505 | 0.758416 | 0.05 |
| 506 | 1.07678 | 0.05 |
| 507 | 0.909874 | 0.05 |
| 508 | 0.940218 | 0.05 |
| 509 | 0.876604 | 0.05 |
| 510 | 1.27148 | 0.05 |
| 511 | 2.42348 | 0.05 |
| 512 | 1.19837 | 0.05 |
| 513 | 0.764764 | 0.05 |
| 514 | 0.829259 | 0.05 |
| 515 | 0.740584 | 0.05 |
| 516 | 1.2772 | 0.05 |
| 517 | 0.964465 | 0.05 |
| 518 | 1.06231 | 0.05 |
| 519 | 1.06924 | 0.05 |

| | | |
|---|---|---|
| 520 | 1.17566 | 0.05 |
| 521 | 1.3063 | 0.05 |
| 522 | 2.51253 | 0.05 |
| 523 | 2.42267 | 0.05 |
| 524 | 1.89226 | 0.05 |
| 525 | 1.46492 | 0.05 |
| 526 | 0.946132 | 0.05 |
| 527 | 0.653151 | 0.05 |
| 528 | 0.653124 | 0.05 |
| 529 | 1.53995 | 0.05 |
| 530 | 1.53995 | 0.05 |
| 531 | 0.936806 | 0.05 |
| 532 | 1.25626 | 0.05 |
| 533 | 1.1893 | 0.05 |
| 534 | 1.62206 | 0.05 |
| 535 | 1.04921 | 2.1 |
| 536 | 1.22927 | 2.1 |
| 537 | 0.801765 | 2.1 |
| 538 | 0.774917 | 2.1 |
| 539 | 0.844623 | 2.1 |
| 540 | 0.737481 | 2.1 |
| 541 | 3.24379 | 2.1 |
| 542 | 2.48573 | 2.1 |
| 543 | 1.40967 | 1.05926 |
| 544 | 1.47203 | 1.56217 |
| 545 | 2.07659 | 1.03991 |
| 546 | 1.40967 | 1.5161 |
| 547 | 2.06638 | 1.05498 |
| 548 | 1.17418 | 2.1 |
| 549 | 1.04921 | 2.1 |
| 550 | 1.74593 | 2.1 |
| 551 | 1.74593 | 2.1 |
| 552 | 0.946132 | 1.19434 |
| 553 | 1.38651 | 2.1 |
| 554 | 1.20761 | 0.05 |
| 555 | 1.20761 | 1.16142 |
| 556 | 1.30555 | 0.19242 |
| 557 | 1.46812 | 0.060172 |
| 558 | 2.41602 | 0.266557 |
| 559 | 1.46812 | 0.301728 |
| 560 | 1.46812 | 0.05 |
| 561 | 0.734059 | 0.05 |
| 562 | 0.946132 | 1.61746 |
| 563 | 1.21261 | 0.504413 |
| 564 | 1.34225 | 0.40534 |
| 565 | 0.734084 | 0.40207 |
| 566 | 1.82077 | 0.248137 |
| 567 | 1.82077 | 0.134455 |
| 568 | 1.13751 | 0.05 |
| 569 | 0.946131 | 0.260479 |
| 570 | 1.13751 | 0.0704152 |
| 571 | 1.13748 | 0.0559101 |
| 572 | 0.784392 | 0.0608374 |
| 573 | 1.4906 | 0.05 |
| 574 | 1.4906 | 0.05 |
| 575 | 1.17879 | 1.29768 |
| 576 | 1.28291 | 1.21954 |
| 577 | 0.626495 | 2.1 |

| | | |
|---|---|---|
| 578 | 1.45334 | 2.1 |
| 579 | 1.45334 | 2.1 |
| 580 | 0.626494 | 2.1 |
| 581 | 1.3852 | 2.1 |
| 582 | 0.946116 | 2.1 |
| 583 | 0.626499 | 2.1 |
| 584 | 1.47203 | 0.933316 |
| 585 | 1.42639 | 2.1 |
| 586 | 1.42639 | 2.1 |
| 587 | 0.795469 | 2.1 |
| 588 | 0.795469 | 2.1 |
| 589 | 0.946132 | 2.1 |
| 590 | 0.850877 | 2.1 |
| 591 | 1.37098 | 2.1 |
| 592 | 1.87194 | 2.1 |
| 593 | 1.76988 | 2.1 |
| 594 | 3.16295 | 0.05 |
| 595 | 3.26725 | 0.05 |
| 596 | 1.13322 | 2.1 |
| 597 | 1.20761 | 2.1 |
| 598 | 0.686933 | 1.36245 |
| 599 | 0.493248 | 0.855841 |
| 600 | 1.2053 | 2.1 |
| 601 | 0.947149 | 0.995602 |
| 602 | 1.27591 | 1.07865 |
| 603 | 1.75732 | 2.1 |
| 604 | 0.493261 | 1.21095 |
| 605 | 1.34337 | 2.1 |
| 606 | 2.73207 | 0.815656 |
| 607 | 1.19857 | 1.03994 |
| 608 | 0.671688 | 0.711263 |
| 609 | 0.946134 | 2.1 |
| 610 | 1.36603 | 0.794399 |
| 611 | 2.07817 | 1.04594 |
| 612 | 1.82573 | 2.1 |
| 613 | 0.671699 | 1.29517 |
| 614 | 1.47772 | 0.794527 |
| 615 | 0.975349 | 2.1 |
| 616 | 1.22615 | 2.1 |
| 617 | 1.47868 | 2.1 |
| 618 | 2.29351 | 2.05567 |
| 619 | 1.47642 | 2.1 |
| 620 | 2.94443 | 1.77301 |
| 621 | 1.80454 | 2.1 |
| 622 | 0.949135 | 2.1 |
| 623 | 0.949134 | 2.1 |
| 624 | 1.10476 | 0.05 |
| 625 | 0.982857 | 0.231873 |
| 626 | 1.10938 | 0.05 |
| 627 | 1.19052 | 0.393727 |
| 628 | 1.01012 | 0.05 |
| 629 | 1.35562 | 0.05 |
| 630 | 0.691742 | 0.05 |
| 631 | 2.39596 | 0.05 |
| 632 | 2.35604 | 0.05 |
| 633 | 0.974081 | 0.05 |
| 634 | 3.22444 | 0.05 |
| 635 | 1.3294 | 0.34371 |

| | | |
|---|---|---|
| 636 | 1.37265 | 1.00877 |
| 637 | 1.3294 | 0.858068 |
| 638 | 1.51113 | 2.1 |
| 639 | 1.43643 | 2.1 |
| 640 | 1.72726 | 2.1 |
| 641 | 1.634 2.1 | |
| 642 | 0.755566 | 2.1 |
| 643 | 1.29319 | 2.1 |
| 644 | 0.755567 | 2.1 |
| 645 | 0.946131 | 2.1 |
| 646 | 1.29319 | 2.1 |
| 647 | 0.973563 | 2.1 |
| 648 | 0.904398 | 2.1 |
| 649 | 0.904406 | 2.1 |
| 650 | 1.1538 | 2.1 |
| 651 | 1.10437 | 2.1 |
| 652 | 0.935418 | 2.1 |
| 653 | 1.16649 | 2.1 |
| 654 | 0.975667 | 2.1 |
| 655 | 1.17952 | 2.1 |
| 656 | 1.95053 | 2.1 |
| 657 | 0.948676 | 2.1 |
| 658 | 1.44287 | 2.1 |
| 659 | 0.746095 | 2.1 |
| 660 | 1.51224 | 2.1 |
| 661 | 1.27354 | 2.1 |
| 662 | 0.746066 | 2.1 |
| 663 | 1.20573 | 2.1 |
| 664 | 1.12442 | 2.1 |
| 665 | 0.671487 | 2.1 |
| 666 | 1.04355 | 2.1 |
| 667 | 0.948675 | 2.1 |
| 668 | 0.967673 | 0.252901 |
| 669 | 1.12931 | 0.05 |
| 670 | 1.38212 | 0.05 |
| 671 | 2.40113 | 0.05 |
| 672 | 1.08054 | 0.05 |
| 673 | 1.04072 | 0.05 |
| 674 | 0.691062 | 0.05 |
| 675 | 1.58052 | 0.05 |
| 676 | 1.33502 | 0.05 |
| 677 | 0.946131 | 0.05 |
| 678 | 0.691062 | 0.05 |
| 679 | 1.02663 | 0.05 |
| 680 | 1.80569 | 0.05 |
| 681 | 1.26062 | 0.05 |
| 682 | 1.18203 | 0.05 |
| 683 | 0.946131 | 0.05 |
| 684 | 0.795065 | 0.05 |
| 685 | 1.58753 | 0.05 |
| 686 | 1.58753 | 0.05 |
| 687 | 0.946131 | 0.05 |
| 688 | 0.795093 | 0.05 |
| 689 | 0.805057 | 2.1 |
| 690 | 0.812415 | 2.1 |
| 691 | 1.23868 | 0.0680674 |
| 692 | 1.34448 | 0.0629554 |
| 693 | 1.67201 | 2.1 |

| | | |
|---|---|---|
| 694 | 1.61202 | 2.1 |
| 695 | 1.41103 | 2.1 |
| 696 | 1.41103 | 2.1 |
| 697 | 0.841088 | 2.1 |
| 698 | 1.38302 | 2.1 |
| 699 | 0.841089 | 2.1 |
| 700 | 0.869097 | 2.1 |
| 701 | 1.38302 | 2.1 |
| 702 | 0.949941 | 2.1 |
| 703 | 0.94994 | 2.1 |
| 704 | 1.88608 | 2.1 |
| 705 | 0.854942 | 2.1 |
| 706 | 1.68893 | 2.1 |
| 707 | 1.70002 | 2.1 |
| 708 | 2.02107 | 0.05 |
| 709 | 2.04982 | 0.05 |
| 710 | 3.11063 | 0.05 |
| 711 | 2.03805 | 0.05 |
| 712 | 1.67638 | 2.1 |
| 713 | 0.967492 | 2.1 |
| 714 | 2.00209 | 2.1 |
| 715 | 0.967491 | 2.1 |
| 716 | 0.870742 | 2.1 |
| 717 | 1.30266 | 0.05 |
| 718 | 0.651334 | 0.05 |
| 719 | 1.69413 | 0.05 |
| 720 | 1.69413 | 0.05 |
| 721 | 0.946132 | 0.05 |
| 722 | 1.03079 | 0.05 |
| 723 | 1.31468 | 0.05 |
| 724 | 0.946132 | 0.05 |
| 725 | 0.919055 | 0.05 |
| 726 | 1.16815 | 0.05 |
| 727 | 2.79004 | 0.05 |
| 728 | 1.2136 | 0.05 |
| 729 | 2.62935 | 0.05 |
| 730 | 0.947692 | 0.05 |
| 731 | 1.51835 | 0.0557127 |
| 732 | 0.947693 | 0.05 |
| 733 | 0.947692 | 0.05 |
| 734 | 0.980841 | 2.1 |
| 735 | 1.43577 | 2.1 |
| 736 | 0.980841 | 2.1 |
| 737 | 1.67858 | 2.1 |
| 738 | 1.34124 | 0.535125 |
| 739 | 1.39818 | 0.108791 |
| 740 | 1.2065 | 2.1 |
| 741 | 0.946133 | 2.1 |
| 742 | 1.2079 | 2.1 |
| 743 | 1.65229 | 2.1 |
| 744 | 1.65229 | 2.1 |
| 745 | 0.946132 | 2.1 |
| 746 | 1.2079 | 2.1 |
| 747 | 1.39053 | 2.1 |
| 748 | 1.20651 | 2.1 |
| 749 | 1.36919 | 2.1 |
| 750 | 1.12717 | 2.1 |
| 751 | 1.09483 | 2.1 |

| | | |
|---|---|---|
| 752 | 0.994294 | 2.1 |
| 753 | 0.97356 | 2.1 |
| 754 | 1.06827 | 2.1 |
| 755 | 1.40571 | 2.1 |
| 756 | 1.29259 | 2.1 |
| 757 | 0.946131 | 2.1 |
| 758 | 1.7787 | 2.1 |
| 759 | 1.7787 | 2.1 |
| 760 | 1.29259 | 2.1 |
| 761 | 0.946132 | 2.1 |
| 762 | 1.09408 | 0.232057 |
| 763 | 1.03824 | 0.257047 |
| 764 | 0.851541 | 0.05 |
| 765 | 1.23608 | 0.05 |
| 766 | 0.851539 | 0.0785055 |
| 767 | 1.5021 | 0.069153 |
| 768 | 1.54839 | 0.05 |
| 769 | 1.12373 | 0.05 |
| 770 | 1.22617 | 0.05 |
| 771 | 1.42762 | 0.05 |
| 772 | 2.24746 | 0.05 |
| 773 | 1.05475 | 0.05 |
| 774 | 1.46845 | 0.05 |
| 775 | 1.05475 | 0.05 |
| 776 | 0.929726 | 0.05 |
| 777 | 1.52072 | 0.05 |
| 778 | 1.82488 | 0.05 |
| 779 | 1.02271 | 0.05 |
| 780 | 1.19225 | 0.05 |
| 781 | 1.43071 | 0.05 |
| 782 | 0.94613 | 0.05 |
| 783 | 1.05475 | 0.05 |
| 784 | 1.39223 | 2.1 |
| 785 | 1.71255 | 2.1 |
| 786 | 0.946132 | 0.829653 |
| 787 | 1.10531 | 0.811918 |
| 788 | 1.46355 | 0.843813 |
| 789 | 1.46355 | 0.586689 |
| 790 | 0.78086 | 1.05817 |
| 791 | 1.10531 | 0.831294 |
| 792 | 1.1391 | 0.831274 |
| 793 | 1.56166 | 0.944156 |
| 794 | 2.21062 | 0.632667 |
| 795 | 1.0361 | 2.1 |
| 796 | 1.73577 | 2.1 |
| 797 | 1.73577 | 2.1 |
| 798 | 0.946116 | 2.1 |
| 799 | 1.28174 | 2.1 |
| 800 | 0.946118 | 2.1 |
| 801 | 1.22883 | 2.1 |
| 802 | 1.28172 | 2.1 |
| 803 | 1.03609 | 2.1 |
| 804 | 0.553567 | 0.05 |
| 805 | 1.60581 | 0.05 |
| 806 | 1.60581 | 0.05 |
| 807 | 0.553594 | 0.05 |
| 808 | 0.966748 | 0.05 |
| 809 | 0.966748 | 0.05 |

| | | |
|---|---|---|
| 810 | 0.946133 | 0.05 |
| 811 | 1.05602 | 0.05 |
| 812 | 2.38525 | 0.05 |
| 813 | 1.14503 | 0.05 |
| 814 | 1.24678 | 0.05 |
| 815 | 0.56262 | 0.05 |
| 816 | 1.24678 | 0.05 |
| 817 | 0.978467 | 0.05 |
| 818 | 1.66262 | 0.05 |
| 819 | 1.66262 | 0.05 |
| 820 | 0.562633 | 0.05 |
| 821 | 1.09522 | 0.05 |
| 822 | 2.49356 | 0.05 |
| 823 | 0.592547 | 0.05 |
| 824 | 1.04321 | 0.338909 |
| 825 | 1.25133 | 1.03394 |
| 826 | 1.70508 | 1.2058 |
| 827 | 1.81043 | 0.883124 |
| 828 | 0.494934 | 0.982331 |
| 829 | 0.601047 | 0.896683 |
| 830 | 0.993428 | 0.94962 |
| 831 | 0.946573 | 1.04142 |
| 832 | 1.35039 | 0.841853 |
| 833 | 0.816895 | 1.51423 |
| 834 | 1.5063 | 0.836861 |
| 835 | 0.816895 | 0.730714 |
| 836 | 0.946132 | 0.805083 |
| 837 | 1.9067 | 1.12438 |
| 838 | 2.14504 | 1.43336 |
| 839 | 0.816895 | 0.702254 |
| 840 | 2.29624 | 0.716199 |
| 841 | 2.76933 | 0.765186 |
| 842 | 1.254 1.20761 | |
| 843 | 0.946128 | 2.1 |
| 844 | 1.38813 | 2.1 |
| 845 | 1.79998 | 2.1 |
| 846 | 1.79998 | 2.1 |
| 847 | 0.94613 | 2.1 |
| 848 | 1.38813 | 2.1 |
| 849 | 1.35798 | 2.1 |
| 850 | 1.27809 | 2.1 |
| 851 | 1.35798 | 2.1 |
| 852 | 1.68994 | 2.1 |
| 853 | 1.68994 | 2.1 |
| 854 | 0.946132 | 2.1 |
| 855 | 0.946132 | 2.1 |
| 856 | 1.18067 | 2.1 |
| 857 | 0.958256 | 2.1 |
| 858 | 0.958255 | 2.1 |
| 859 | 0.958257 | 2.1 |
| 860 | 0.958255 | 2.1 |
| 861 | 1.06457 | 2.1 |
| 862 | 1.14195 | 2.1 |
| 863 | 1.70423 | 0.05 |
| 864 | 1.14195 | 2.1 |
| 865 | 1.64285 | 2.1 |
| 866 | 2.02057 | 2.1 |
| 867 | 0.946132 | 2.1 |

| | | |
|---|---|---|
| 868 | 1.44186 | 2.1 |
| 869 | 0.946133 | 2.1 |
| 870 | 1.16366 | 2.1 |
| 871 | 1.17233 | 2.1 |
| 872 | 1.31259 | 0.205683 |
| 873 | 1.2016 | 2.1 |
| 874 | 1.17129 | 2.1 |
| 875 | 1.05724 | 0.05 |
| 876 | 1.32697 | 0.897435 |
| 877 | 1.05724 | 0.594166 |
| 878 | 1.04292 | 1.19507 |
| 879 | 3.39037 | 2.1 |
| 880 | 2.57275 | 2.1 |
| 881 | 0.629212 | 2.1 |
| 882 | 2.32525 | 2.1 |
| 883 | 1.76949 | 0.831603 |
| 884 | 1.65809 | 1.18782 |
| 885 | 1.27665 | 2.1 |
| 886 | 1.19861 | 2.1 |
| 887 | 1.20045 | 2.1 |
| 888 | 1.1635 | 2.1 |
| 889 | 0.953633 | 0.05 |
| 890 | 0.953634 | 0.05 |
| 891 | 1.21408 | 2.1 |
| 892 | 1.25716 | 2.1 |
| 893 | 2.01791 | 2.1 |
| 894 | 1.89318 | 2.1 |
| 895 | 0.947921 | 1.12427 |
| 896 | 0.939049 | 2.1 |
| 897 | 0.947921 | 2.1 |
| 898 | 0.740753 | 2.1 |
| 899 | 0.916104 | 2.1 |
| 900 | 1.16941 | 2.1 |
| 901 | 1.72038 | 2.1 |
| 902 | 2.04441 | 2.1 |
| 903 | 0.973182 | 2.1 |
| 904 | 1.11751 | 2.1 |
| 905 | 0.611089 | 2.1 |
| 906 | 1.53584 | 0.05 |
| 907 | 0.767923 | 0.086717 |
| 908 | 1.38925 | 0.0876907 |
| 909 | 1.38926 | 0.0714208 |
| 910 | 0.946131 | 0.177422 |
| 911 | 0.961347 | 0.0696033 |
| 912 | 1.19583 | 0.351107 |
| 913 | 0.946131 | 0.354854 |
| 914 | 1.3663 | 0.373352 |
| 915 | 1.21067 | 0.0626002 |
| 916 | 1.00166 | 0.05 |
| 917 | 1.40707 | 0.05 |
| 918 | 1.50435 | 0.05 |
| 919 | 1.47954 | 0.587346 |
| 920 | 1.1233 | 2.1 |
| 921 | 2.36036 | 1.44176 |
| 922 | 1.04024 | 0.636915 |
| 923 | 0.739729 | 0.712036 |
| 924 | 1.45533 | 0.585998 |
| 925 | 1.45532 | 0.496328 |

| | | |
|---|---|---|
| 926 | 0.946118 | 0.653476 |
| 927 | 1.15163 | 0.117283 |
| 928 | 1.15165 | 0.724009 |
| 929 | 0.739748 | 0.585839 |
| 930 | 1.04025 | 0.924751 |
| 931 | 1.47608 | 0.05 |
| 932 | 0.946131 | 0.101632 |
| 933 | 1.02028 | 0.05 |
| 934 | 0.946129 | 0.05 |
| 935 | 1.78223 | 0.05 |
| 936 | 1.78223 | 0.05 |
| 937 | 1.34153 | 0.05 |
| 938 | 1.38683 | 0.05 |
| 939 | 1.3407 | 0.05 |
| 940 | 1.3407 | 0.0578267 |
| 941 | 2.04729 | 0.05 |
| 942 | 0.851529 | 0.101171 |
| 943 | 1.26671 | 0.0575673 |
| 944 | 0.946116 | 0.0697152 |
| 945 | 1.4869 | 0.05 |
| 946 | 1.29553 | 0.05 |
| 947 | 1.03645 | 0.05 |
| 948 | 1.20731 | 0.05 |
| 949 | 0.73665 | 2.1 |
| 950 | 0.693596 | 2.1 |
| 951 | 2.11152 | 2.1 |
| 952 | 1.73627 | 2.1 |
| 953 | 1.06191 | 2.1 |
| 954 | 1.52226 | 2.1 |
| 955 | 1.76869 | 2.1 |
| 956 | 1.1881 | 2.1 |
| 957 | 1.32349 | 2.1 |
| 958 | 1.51041 | 2.1 |
| 959 | 1.61591 | 2.1 |
| 960 | 0.946132 | 2.1 |
| 961 | 0.946132 | 2.1 |
| 962 | 1.13537 | 2.1 |
| 963 | 3.0048 | 2.1 |
| 964 | 0.985109 | 2.1 |
| 965 | 1.68899 | 2.1 |
| 966 | 3.23261 | 2.1 |
| 967 | 1.08831 | 2.1 |
| 968 | 2.20623 | 2.1 |
| 969 | 0.91749 | 2.1 |
| 970 | 1.15515 | 2.1 |
| 971 | 0.550438 | 2.1 |
| 972 | 1.66294 | 2.1 |
| 973 | 1.08257 | 2.1 |
| 974 | 0.87851 | 2.1 |
| 975 | 2.09046 | 2.1 |
| 976 | 0.738711 | 2.1 |
| 977 | 1.31242 | 0.05 |
| 978 | 1.26311 | 0.05 |
| 979 | 1.67652 | 0.05 |
| 980 | 0.968594 | 2.1 |
| 981 | 0.968594 | 2.1 |
| 982 | 0.981099 | 0.05 |
| 983 | 1.32418 | 0.05 |

| | | |
|---|---|---|
| 984 | 2.67175 | 0.05 |
| 985 | 2.09678 | 0.05 |
| 986 | 1.2511 | 0.05 |
| 987 | 0.912698 | 0.05 |
| 988 | 0.810946 | 0.05 |
| 989 | 0.810959 | 0.05 |
| 990 | 1.67479 | 0.05 |
| 991 | 1.65677 | 0.05 |
| 992 | 1.28469 | 0.05 |
| 993 | 1.48817 | 0.05 |
| 994 | 1.08131 | 0.05 |
| 995 | 1.04349 | 0.546973 |
| 996 | 1.42492 | 0.480507 |
| 997 | 1.05724 | 0.384155 |
| 998 | 1.65723 | 0.05 |
| 999 | 1.95313 | 0.05 |
| 1000 | 1.9293 | 0.05 |
| 1001 | 2.02729 | 0.05 |
| 1002 | 0.963393 | 0.893272 |
| 1003 | 0.963392 | 0.457763 |
| 1004 | 2.90171 | 2.1 |
| 1005 | 2.65447 | 2.1 |
| 1006 | 1.64158 | 2.1 |
| 1007 | 1.6488 | 2.1 |
| 1008 | 1.15991 | 0.0833704 |
| 1009 | 0.946132 | 0.508555 |
| 1010 | 1.58199 | 0.254827 |
| 1011 | 1.58199 | 0.297728 |
| 1012 | 1.03333 | 0.509871 |
| 1013 | 1.25436 | 0.42238 |
| 1014 | 1.25436 | 0.547556 |
| 1015 | 0.946132 | 0.577817 |
| 1016 | 1.3771 | 0.05 |
| 1017 | 0.687745 | 0.05 |
| 1018 | 1.37709 | 0.05 |
| 1019 | 0.812386 | 0.05 |
| 1020 | 1.50172 | 0.05 |
| 1021 | 1.50172 | 0.05 |
| 1022 | 0.687758 | 0.05 |
| 1023 | 1.17506 | 0.05 |
| 1024 | 2.00539 | 0.05 |
| 1025 | 2.2441 | 0.05 |
| 1026 | 1.09667 | 0.05 |
| 1027 | 1.43577 | 2.1 |
| 1028 | 2.24741 | 2.1 |
| 1029 | 1.26103 | 2.1 |
| 1030 | 0.851544 | 2.1 |
| 1031 | 1.5543 | 2.1 |
| 1032 | 1.59756 | 2.1 |
| 1033 | 0.851543 | 2.1 |
| 1034 | 1.44673 | 2.1 |
| 1035 | 1.31947 | 2.1 |
| 1036 | 1.1237 | 2.1 |
| 1037 | 1.26103 | 2.1 |
| 1038 | 1.089 | 0.05 |
| 1039 | 0.946128 | 0.05 |
| 1040 | 0.965824 | 0.05 |
| 1041 | 0.94613 | 0.05 |

| | | |
|---|---|---|
| 1042 | 1.51344 | 0.05 |
| 1043 | 1.51344 | 0.05 |
| 1044 | 1.13508 | 0.05 |
| 1045 | 1.32449 | 0.05 |
| 1046 | 1.089 | 0.05 |
| 1047 | 3.33706 | 0.05 |
| 1048 | 2.7542 | 0.05 |
| 1049 | 1.27256 | 0.05 |
| 1050 | 0.954172 | 0.05 |
| 1051 | 1.55527 | 0.05 |
| 1052 | 1.05765 | 0.0708492 |
| 1053 | 1.57326 | 0.05 |
| 1054 | 1.38251 | 0.05 |
| 1055 | 0.94613 | 0.05 |
| 1056 | 2.38829 | 0.05 |
| 1057 | 2.00539 | 0.05 |
| 1058 | 1.53798 | 0.05 |
| 1059 | 1.20969 | 2.1 |
| 1060 | 1.15864 | 2.1 |
| 1061 | 1.7986 | 2.1 |
| 1062 | 1.74533 | 2.1 |
| 1063 | 0.944827 | 1.98429 |
| 1064 | 0.946131 | 1.06757 |
| 1065 | 0.851543 | 0.981963 |
| 1066 | 1.52816 | 0.267253 |
| 1067 | 1.5314 | 1.30536 |
| 1068 | 1.39266 | 0.0895613 |
| 1069 | 0.851528 | 0.256275 |
| 1070 | 1.39589 | 0.655568 |
| 1071 | 1.7348 | 0.582672 |
| 1072 | 1.85439 | 1.30697 |
| 1073 | 1.86367 | 0.894189 |
| 1074 | 1.90905 | 0.340889 |
| 1075 | 1.22176 | 0.0568127 |
| 1076 | 1.59492 | 0.0631161 |
| 1077 | 2.97929 | 2.1 |
| 1078 | 2.1905 | 2.1 |
| 1079 | 1.24128 | 2.1 |
| 1080 | 0.994293 | 2.1 |
| 1081 | 2.49767 | 0.05 |
| 1082 | 2.37105 | 0.05 |
| 1083 | 1.10834 | 0.997158 |
| 1084 | 1.53214 | 1.01705 |
| 1085 | 1.417 | 2.1 |
| 1086 | 2.13599 | 0.751828 |
| 1087 | 1.61727 | 2.1 |
| 1088 | 1.75004 | 2.1 |
| 1089 | 1.96301 | 2.1 |
| 1090 | 0.966631 | 2.1 |
| 1091 | 1.37791 | 2.1 |
| 1092 | 1.47302 | 2.1 |
| 1093 | 1.47302 | 2.1 |
| 1094 | 0.871519 | 2.1 |
| 1095 | 0.94613 | 2.1 |
| 1096 | 0.87155 | 2.1 |
| 1097 | 2.38731 | 2.1 |
| 1098 | 2.12399 | 0.752008 |
| 1099 | 1.74865 | 0.609391 |

| | | |
|---|---|---|
| 1100 | 1.46724 | 0.0535535 |
| 1101 | 2.38056 | 0.05 |
| 1102 | 1.46724 | 0.0707122 |
| 1103 | 0.818887 | 0.05 |
| 1104 | 0.810944 | 1.31455 |
| 1105 | 0.805227 | 0.05 |
| 1106 | 1.06558 | 0.250573 |
| 1107 | 0.841487 | 0.0691195 |
| 1108 | 1.08456 | 0.310926 |
| 1109 | 0.903083 | 0.203398 |
| 1110 | 1.08929 | 0.05 |
| 1111 | 1.68454 | 0.05 |
| 1112 | 1.08929 | 0.05 |
| 1113 | 1.07658 | 0.05 |
| 1114 | 0.904439 | 0.05 |
| 1115 | 1.58501 | 0.05 |
| 1116 | 1.19729 | 0.05 |
| 1117 | 1.05427 | 0.05 |
| 1118 | 1.47764 | 0.05 |
| 1119 | 0.810959 | 0.05 |
| 1120 | 1.60946 | 0.898948 |
| 1121 | 2.28887 | 0.91338 |
| 1122 | 0.946133 | 0.979902 |
| 1123 | 1.14444 | 0.682243 |
| 1124 | 1.67964 | 0.373437 |
| 1125 | 1.67964 | 0.441519 |
| 1126 | 0.755334 | 1.07219 |
| 1127 | 1.14444 | 0.925185 |
| 1128 | 1.29054 | 0.854581 |
| 1129 | 1.37762 | 0.60457 |
| 1130 | 0.946132 | 0.968306 |
| 1131 | 0.533645 | 0.648926 |
| 1132 | 1.48361 | 0.311436 |
| 1133 | 1.48361 | 0.674189 |
| 1134 | 0.533645 | 0.946924 |
| 1135 | 0.872823 | 0.991962 |
| 1136 | 1.14444 | 0.599435 |
| 1137 | 1.37762 | 0.74527 |
| 1138 | 2.10101 | 0.803161 |
| 1139 | 1.02311 | 0.863822 |
| 1140 | 1.04083 | 2.1 |
| 1141 | 1.04084 | 2.1 |
| 1142 | 1.04084 | 2.1 |
| 1143 | 1.30116 | 2.1 |
| 1144 | 1.23477 | 0.05 |
| 1145 | 1.81893 | 0.05 |
| 1146 | 1.23477 | 0.05 |
| 1147 | 1.23477 | 0.05 |
| 1148 | 1.11967 | 0.05 |
| 1149 | 0.946131 | 0.05 |
| 1150 | 1.11966 | 0.05 |
| 1151 | 1.12628 | 0.05 |
| 1152 | 0.629641 | 0.05 |
| 1153 | 1.6163 | 0.05 |
| 1154 | 1.6163 | 0.05 |
| 1155 | 1.11117 | 2.1 |
| 1156 | 1.13006 | 2.1 |
| 1157 | 1.15149 | 2.1 |

| | | |
|---|---|---|
| 1158 | 1.13179 | 2.1 |
| 1159 | 0.585368 | 0.05 |
| 1160 | 0.986036 | 1.24788 |
| 1161 | 1.94788 | 0.200346 |
| 1162 | 1.34857 | 0.05 |
| 1163 | 2.31806 | 2.1 |
| 1164 | 2.06363 | 2.1 |
| 1165 | 1.00423 | 2.1 |
| 1166 | 0.829065 | 2.1 |
| 1167 | 0.551816 | 2.1 |
| 1168 | 0.99406 | 2.1 |
| 1169 | 1.98339 | 2.1 |
| 1170 | 1.68073 | 2.1 |
| 1171 | 2.558 2.1 | |
| 1172 | 0.711814 | 1.07457 |
| 1173 | 2.97316 | 2.1 |
| 1174 | 2.41579 | 2.1 |
| 1175 | 1.15248 | 0.05 |
| 1176 | 1.14305 | 0.05 |
| 1177 | 1.15483 | 0.05 |
| 1178 | 1.90981 | 0.05 |
| 1179 | 0.998957 | 0.05 |
| 1180 | 1.83742 | 0.05 |
| 1181 | 1.27581 | 2.1 |
| 1182 | 0.94613 | 2.1 |
| 1183 | 1.6315 | 2.1 |
| 1184 | 1.6315 | 2.1 |
| 1185 | 0.946132 | 2.1 |
| 1186 | 1.28441 | 2.1 |
| 1187 | 1.28441 | 2.1 |
| 1188 | 1.00365 | 2.1 |
| 1189 | 0.968536 | 2.1 |
| 1190 | 1.57575 | 2.1 |
| 1191 | 1.27581 | 2.1 |
| 1192 | 1.99297 | 2.1 |
| 1193 | 1.32784 | 2.1 |
| 1194 | 1.4259 | 2.1 |
| 1195 | 2.27952 | 2.1 |
| 1196 | 1.5685 | 2.1 |
| 1197 | 1.54231 | 2.1 |
| 1198 | 3.54638 | 2.1 |
| 1199 | 1.3294 | 2.1 |
| 1200 | 2.98237 | 2.1 |
| 1201 | 2.01633 | 0.256336 |
| 1202 | 2.0985 | 0.477183 |
| 1203 | 2.26047 | 0.0938606 |
| 1204 | 1.7913 | 0.0515785 |
| 1205 | 1.09504 | 2.1 |
| 1206 | 1.74126 | 2.1 |
| 1207 | 1.05983 | 2.1 |
| 1208 | 2.37664 | 2.1 |
| 1209 | 2.28084 | 2.1 |
| 1210 | 2.19948 | 2.1 |
| 1211 | 1.33593 | 0.05 |
| 1212 | 1.31508 | 0.05 |
| 1213 | 1.30647 | 2.1 |
| 1214 | 1.28846 | 2.1 |
| 1215 | 1.2955 | 2.1 |

| | | |
|---|---|---|
| 1216 | 1.09044 | 2.1 |
| 1217 | 1.4099 | 2.1 |
| 1218 | 1.14984 | 2.1 |
| 1219 | 0.946132 | 0.866075 |
| 1220 | 1.70727 | 2.1 |
| 1221 | 1.70727 | 2.1 |
| 1222 | 1.14984 | 2.1 |
| 1223 | 0.946132 | 2.1 |
| 1224 | 0.911818 | 2.1 |
| 1225 | 2.12209 | 2.1 |
| 1226 | 2.4158 | 2.1 |
| 1227 | 1.20867 | 2.1 |
| 1228 | 2.84085 | 2.1 |
| 1229 | 1.42284 | 2.1 |
| 1230 | 2.45689 | 2.1 |
| 1231 | 1.41562 | 2.1 |
| 1232 | 0.918358 | 2.1 |
| 1233 | 1.11222 | 2.1 |
| 1234 | 0.999083 | 2.1 |
| 1235 | 0.696781 | 2.1 |
| 1236 | 1.30869 | 2.1 |
| 1237 | 1.66132 | 2.1 |
| 1238 | 0.696807 | 2.1 |
| 1239 | 0.745315 | 2.1 |
| 1240 | 1.23296 | 2.1 |
| 1241 | 0.780548 | 2.1 |
| 1242 | 1.93242 | 0.944439 |
| 1243 | 1.27362 | 1.0629 |
| 1244 | 2.77828 | 1.46835 |
| 1245 | 1.02653 | 0.4013 |
| 1246 | 0.808576 | 0.419561 |
| 1247 | 0.744012 | 0.961473 |
| 1248 | 0.605174 | 0.189955 |
| 1249 | 1.16501 | 0.0905253 |
| 1250 | 0.803432 | 0.0670843 |
| 1251 | 0.961016 | 0.120798 |
| 1252 | 1.10425 | 0.349783 |
| 1253 | 1.3756 | 0.706064 |
| 1254 | 2.17765 | 0.501979 |
| 1255 | 1.84578 | 0.232854 |
| 1256 | 1.32801 | 0.0971508 |
| 1257 | 1.76377 | 0.0885499 |
| 1258 | 0.946131 | 0.05 |
| 1259 | 1.3022 | 0.232929 |
| 1260 | 0.694814 | 0.262018 |
| 1261 | 1.53028 | 0.05 |
| 1262 | 1.53028 | 0.05 |
| 1263 | 1.3022 | 0.0662773 |
| 1264 | 0.922889 | 0.15266 |
| 1265 | 1.57766 | 2.1 |
| 1266 | 2.06911 | 2.1 |
| 1267 | 1.57766 | 2.1 |
| 1268 | 1.37721 | 0.05 |
| 1269 | 1.72174 | 0.05 |
| 1270 | 1.4468 | 0.05 |
| 1271 | 1.37721 | 0.05 |
| 1272 | 1.73972 | 0.05 |
| 1273 | 1.62324 | 0.05 |

| | | |
|---|---|---|
| 1274 | 0.946125 | 0.05 |
| 1275 | 1.34259 | 0.05 |
| 1276 | 1.04187 | 0.05 |
| 1277 | 1.05019 | 0.05 |
| 1278 | 1.2361 | 0.05 |
| 1279 | 1.69055 | 0.05 |
| 1280 | 2.25056 | 0.05 |
| 1281 | 1.10574 | 0.05 |
| 1282 | 1.21632 | 0.827201 |
| 1283 | 1.10574 | 0.812807 |
| 1284 | 1.46181 | 2.1 |
| 1285 | 0.946131 | 0.190278 |
| 1286 | 1.74111 | 1.43773 |
| 1287 | 1.74111 | 0.954044 |
| 1288 | 0.94613 | 0.877406 |
| 1289 | 1.26891 | 0.767725 |
| 1290 | 1.26891 | 0.468602 |
| 1291 | 1.23452 | 1.10389 |
| 1292 | 1.00255 | 0.660354 |
| 1293 | 1.72286 | 0.05 |
| 1294 | 1.12233 | 0.05 |
| 1295 | 1.30682 | 0.05 |
| 1296 | 1.97397 | 0.05 |
| 1297 | 1.67425 | 2.1 |
| 1298 | 1.77775 | 2.1 |
| 1299 | 1.73571 | 2.1 |
| 1300 | 0.426265 | 2.1 |
| 1301 | 1.31817 | 1.94895 |
| 1302 | 1.81042 | 2.1 |
| 1303 | 1.85456 | 2.1 |
| 1304 | 0.466532 | 1.95508 |
| 1305 | 1.28025 | 1.79458 |
| 1306 | 0.964467 | 1.60443 |
| 1307 | 1.27921 | 2.1 |
| 1308 | 2.11851 | 2.1 |
| 1309 | 2.37121 | 2.1 |
| 1310 | 1.49357 | 0.119 |
| 1311 | 1.42486 | 2.1 |
| 1312 | 1.00278 | 2.1 |
| 1313 | 1.00278 | 2.1 |
| 1314 | 1.12503 | 2.1 |
| 1315 | 0.568697 | 2.1 |
| 1316 | 0.555211 | 2.1 |
| 1317 | 0.982272 | 2.1 |
| 1318 | 1.57558 | 2.00814 |
| 1319 | 0.851539 | 2.1 |
| 1320 | 1.07307 | 2.1 |
| 1321 | 1.58292 | 2.1 |
| 1322 | 1.50731 | 2.1 |
| 1323 | 0.851541 | 0.778786 |
| 1324 | 1.00304 | 2.1 |
| 1325 | 1.36003 | 2.1 |
| 1326 | 1.13127 | 2.1 |
| 1327 | 0.672234 | 2.1 |
| 1328 | 0.763044 | 2.1 |
| 1329 | 0.966979 | 2.1 |
| 1330 | 1.31502 | 2.1 |
| 1331 | 1.67969 | 2.1 |

| | | |
|---|---|---|
| 1332 | 1.62408 | 2.1 |
| 1333 | 0.661955 | 2.1 |
| 1334 | 1.35048 | 2.1 |
| 1335 | 1.40447 | 2.1 |
| 1336 | 1.40447 | 2.1 |
| 1337 | 0.607961 | 2.1 |
| 1338 | 0.946131 | 2.1 |
| 1339 | 0.607963 | 2.1 |
| 1340 | 1.01531 | 2.1 |
| 1341 | 1.01531 | 2.1 |
| 1342 | 1.91802 | 2.1 |
| 1343 | 0.777749 | 2.1 |
| 1344 | 1.22585 | 0.05 |
| 1345 | 1.20672 | 0.05 |
| 1346 | 1.03166 | 0.05 |
| 1347 | 0.553805 | 2.1 |
| 1348 | 2.00551 | 2.1 |
| 1349 | 2.07203 | 2.1 |
| 1350 | 0.589479 | 2.1 |
| 1351 | 1.22352 | 2.1 |
| 1352 | 0.984227 | 2.1 |
| 1353 | 0.802455 | 2.1 |
| 1354 | 1.40942 | 2.1 |
| 1355 | 1.6662 | 2.1 |
| 1356 | 0.802485 | 2.1 |
| 1357 | 0.936669 | 2.1 |
| 1358 | 1.31127 | 0.0995079 |
| 1359 | 0.946132 | 0.0902646 |
| 1360 | 1.09729 | 0.05 |
| 1361 | 0.946133 | 0.141566 |
| 1362 | 1.68703 | 0.05 |
| 1363 | 1.68703 | 0.05 |
| 1364 | 1.3689 | 0.05 |
| 1365 | 1.26426 | 0.05 |
| 1366 | 1.26923 | 2.1 |
| 1367 | 1.23614 | 2.1 |
| 1368 | 0.828371 | 2.1 |
| 1369 | 2.04556 | 2.1 |
| 1370 | 0.772648 | 2.1 |
| 1371 | 0.791059 | 2.1 |
| 1372 | 0.809958 | 2.1 |
| 1373 | 1.70012 | 2.1 |
| 1374 | 0.267032 | 2.1 |
| 1375 | 1.67604 | 2.1 |
| 1376 | 0.803823 | 2.1 |
| 1377 | 0.267305 | 2.1 |
| 1378 | 0.80246 | 2.1 |
| 1379 | 0.874582 | 2.1 |
| 1380 | 1.00188 | 2.1 |
| 1381 | 0.709588 | 2.1 |
| 1382 | 1.50679 | 2.1 |
| 1383 | 1.22456 | 2.1 |
| 1384 | 0.870622 | 2.1 |
| 1385 | 1.05754 | 2.1 |
| 1386 | 1.1689 | 2.1 |
| 1387 | 1.54556 | 2.1 |
| 1388 | 1.89226 | 2.1 |
| 1389 | 0.995311 | 2.1 |

| | | |
|---|---|---|
| 1390 | 1.26195 | 2.1 |
| 1391 | 1.9 | 2.1 |
| 1392 | 0.845055 | 2.1 |
| 1393 | 0.737836 | 2.1 |
| 1394 | 0.823707 | 2.1 |
| 1395 | 0.626587 | 2.1 |
| 1396 | 1.16109 | 2.1 |
| 1397 | 0.85517 | 2.1 |
| 1398 | 0.932036 | 2.1 |
| 1399 | 1.08321 | 2.1 |
| 1400 | 1.43863 | 2.1 |
| 1401 | 2.72297 | 2.1 |
| 1402 | 2.90798 | 2.1 |
| 1403 | 1.47818 | 1.32091 |
| 1404 | 1.47819 | 1.17091 |
| 1405 | 0.694357 | 1.05686 |
| 1406 | 0.694382 | 1.07329 |
| 1407 | 0.946132 | 0.780493 |
| 1408 | 0.694382 | 0.94791 |
| 1409 | 1.22641 | 0.844358 |
| 1410 | 1.31155 | 1.39457 |
| 1411 | 2.45282 | 1.16261 |
| 1412 | 0.787843 | 0.915949 |
| 1413 | 0.808984 | 0.70634 |
| 1414 | 1.14232 | 1.58057 |
| 1415 | 0.795037 | 1.1251 |
| 1416 | 0.887382 | 1.2859 |
| 1417 | 1.14493 | 1.09419 |
| 1418 | 0.78393 | 0.812883 |
| 1419 | 1.09129 | 1.07938 |
| 1420 | 0.791482 | 0.988339 |
| 1421 | 1.1137 | 1.42632 |
| 1422 | 1.11819 | 1.08229 |
| 1423 | 0.960173 | 1.00669 |
| 1424 | 1.09447 | 1.61019 |
| 1425 | 0.874365 | 0.933747 |
| 1426 | 1.2474 | 0.05 |
| 1427 | 1.75038 | 0.05 |
| 1428 | 1.75038 | 0.05 |
| 1429 | 0.94613 | 0.0992922 |
| 1430 | 1.30076 | 0.05 |
| 1431 | 1.12817 | 0.435685 |
| 1432 | 0.94613 | 0.0613299 |
| 1433 | 1.30076 | 0.05 |
| 1434 | 1.51841 | 0.05 |
| 1435 | 1.51841 | 0.05 |
| 1436 | 0.946132 | 0.292794 |
| 1437 | 1.39575 | 0.05 |
| 1438 | 0.970358 | 1.18913 |
| 1439 | 0.946133 | 0.127281 |
| 1440 | 1.39575 | 0.069419 |
| 1441 | 1.06486 | 0.05 |
| 1442 | 1.06486 | 0.05 |
| 1443 | 1.38622 | 0.05 |
| 1444 | 1.04521 | 0.05 |
| 1445 | 1.00809 | 0.05 |
| 1446 | 1.28952 | 0.05 |
| 1447 | 0.849316 | 0.05 |

| | | |
|---|---|---|
| 1448 | 0.946132 | 0.05 |
| 1449 | 0.849344 | 0.05 |
| 1450 | 1.53631 | 0.05 |
| 1451 | 1.53631 | 0.05 |
| 1452 | 1.77843 | 0.05 |
| 1453 | 1.69863 | 0.05 |
| 1454 | 1.91646 | 2.1 |
| 1455 | 1.8582 | 2.1 |
| 1456 | 2.8349 | 0.05 |
| 1457 | 1.36307 | 0.05 |
| 1458 | 1.23886 | 0.05 |
| 1459 | 1.12971 | 0.05 |
| 1460 | 1.59662 | 0.05 |
| 1461 | 1.59662 | 0.05 |
| 1462 | 0.640304 | 0.05 |
| 1463 | 0.640333 | 0.05 |
| 1464 | 0.886276 | 0.05 |
| 1465 | 0.886276 | 0.05 |
| 1466 | 1.35065 | 0.05 |
| 1467 | 2.09002 | 0.05 |
| 1468 | 1.35543 | 0.05 |
| 1469 | 1.94688 | 2.1 |
| 1470 | 1.97221 | 2.1 |
| 1471 | 0.719563 | 2.1 |
| 1472 | 1.20358 | 2.1 |
| 1473 | 0.874264 | 2.1 |
| 1474 | 1.27676 | 2.1 |
| 1475 | 1.46557 | 2.1 |
| 1476 | 1.46557 | 2.1 |
| 1477 | 0.68546 | 2.1 |
| 1478 | 0.94613 | 2.1 |
| 1479 | 0.685489 | 2.1 |
| 1480 | 1.39615 | 0.05 |
| 1481 | 1.05404 | 0.05 |
| 1482 | 1.06474 | 0.05 |
| 1483 | 1.00713 | 0.05 |
| 1484 | 0.714535 | 0.05 |
| 1485 | 0.714563 | 0.122561 |
| 1486 | 1.41646 | 0.05 |
| 1487 | 1.52582 | 0.05 |
| 1488 | 0.721694 | 0.05 |
| 1489 | 0.93665 | 0.05 |
| 1490 | 1.21067 | 0.0729783 |
| 1491 | 1.21067 | 0.0607381 |
| 1492 | 1.73577 | 0.207155 |
| 1493 | 1.17799 | 0.0557718 |
| 1494 | 3.21432 | 0.05 |
| 1495 | 2.41452 | 0.0542231 |
| 1496 | 1.36208 | 0.05 |
| 1497 | 2.53256 | 0.05 |
| 1498 | 0.834593 | 0.05 |
| 1499 | 2.5853 | 0.05 |
| 1500 | 1.77259 | 0.05 |
| 1501 | 0.615724 | 0.05 |
| 1502 | 1.11374 | 0.05 |
| 1503 | 0.615726 | 0.05 |
| 1504 | 0.946129 | 0.149657 |
| 1505 | 0.615714 | 0.102653 |

| | | |
|---|---|---|
| 1506 | 1.75489 | 0.05 |
| 1507 | 1.75489 | 0.05 |
| 1508 | 1.23146 | 0.05 |
| 1509 | 2.2772 | 0.05 |
| 1510 | 0.74463 | 0.05 |
| 1511 | 1.3546 | 0.05 |
| 1512 | 1.80252 | 0.05 |
| 1513 | 2.38304 | 0.05 |
| 1514 | 1.19151 | 0.05 |
| 1515 | 1.1231 | 0.05 |
| 1516 | 1.82345 | 0.05 |
| 1517 | 1.79805 | 0.05 |
| 1518 | 0.761337 | 0.0775781 |
| 1519 | 0.946127 | 0.05 |
| 1520 | 0.557726 | 0.05 |
| 1521 | 1.05353 | 0.05 |
| 1522 | 1.05353 | 0.05 |
| 1523 | 1.68599 | 0.05 |
| 1524 | 1.05353 | 0.05 |
| 1525 | 1.40588 | 2.1 |
| 1526 | 1.09373 | 2.1 |
| 1527 | 1.18382 | 1.04774 |
| 1528 | 1.46903 | 0.963463 |
| 1529 | 1.25103 | 2.1 |
| 1530 | 1.10378 | 2.1 |
| 1531 | 1.58941 | 2.1 |
| 1532 | 1.29639 | 2.1 |
| 1533 | 1.14874 | 0.05 |
| 1534 | 0.899817 | 0.05 |
| 1535 | 0.666898 | 0.05 |
| 1536 | 1.76519 | 0.05 |
| 1537 | 1.17289 | 0.05 |
| 1538 | 1.03526 | 0.05 |
| 1539 | 1.97708 | 0.05 |
| 1540 | 1.31711 | 0.05 |
| 1541 | 0.992517 | 0.05 |
| 1542 | 1.70193 | 0.05 |
| 1543 | 1.13852 | 0.05 |
| 1544 | 1.23943 | 0.05 |
| 1545 | 1.40014 | 1.09256 |
| 1546 | 1.04598 | 2.1 |
| 1547 | 1.1483 | 1.70554 |
| 1548 | 1.11433 | 1.02091 |
| 1549 | 1.32037 | 0.19038 |
| 1550 | 1.00381 | 0.507681 |
| 1551 | 1.11435 | 0.0837662 |
| 1552 | 0.973502 | 0.888773 |
| 1553 | 0.946128 | 1.87153 |
| 1554 | 1.83697 | 1.13402 |
| 1555 | 1.83697 | 2.1 |
| 1556 | 1.31721 | 1.77993 |
| 1557 | 1.38614 | 1.25681 |
| 1558 | 1.39696 | 1.11985 |
| 1559 | 1.31721 | 1.54685 |
| 1560 | 1.0756 | 1.83582 |
| 1561 | 1.08542 | 0.958259 |
| 1562 | 0.946127 | 2.1 |
| 1563 | 1.81052 | 0.40198 |

| | | |
|---|---|---|
| 1564 | 1.81052 | 2.1 |
| 1565 | 1.24315 | 2.1 |
| 1566 | 1.39696 | 0.350694 |
| 1567 | 1.35969 | 1.34436 |
| 1568 | 1.24315 | 1.20046 |
| 1569 | 1.0756 | 0.05 |
| 1570 | 1.20669 | 0.05 |
| 1571 | 1.18508 | 0.05 |
| 1572 | 1.71059 | 2.1 |
| 1573 | 1.66762 | 2.1 |
| 1574 | 2.50853 | 2.1 |
| 1575 | 1.8236 | 2.1 |
| 1576 | 1.78872 | 2.1 |
| 1577 | 1.41721 | 2.1 |
| 1578 | 1.01335 | 1.74853 |
| 1579 | 1.42439 | 1.05962 |
| 1580 | 0.946129 | 0.864059 |
| 1581 | 1.33266 | 1.30518 |
| 1582 | 1.79776 | 1.41548 |
| 1583 | 1.79776 | 2.1 |
| 1584 | 0.946131 | 0.196001 |
| 1585 | 1.33266 | 1.52349 |
| 1586 | 1.41123 | 1.3055 |
| 1587 | 1.25346 | 2.1 |
| 1588 | 0.946132 | 0.793771 |
| 1589 | 0.827287 | 2.09436 |
| 1590 | 1.57878 | 1.15328 |
| 1591 | 1.57878 | 1.33142 |
| 1592 | 0.827287 | 1.12023 |
| 1593 | 1.07341 | 1.1938 |
| 1594 | 1.33266 | 1.1427 |
| 1595 | 1.25346 | 1.16281 |
| 1596 | 1.61149 | 2.1 |
| 1597 | 1.15644 | 2.1 |
| 1598 | 1.27558 | 0.758389 |
| 1599 | 1.3764 | 0.178328 |
| 1600 | 1.89526 | 1.09245 |
| 1601 | 1.37641 | 0.158457 |
| 1602 | 1.2064 | 0.05 |
| 1603 | 1.38426 | 2.1 |
| 1604 | 1.01595 | 2.1 |
| 1605 | 1.15067 | 2.1 |
| 1606 | 1.82276 | 2.1 |
| 1607 | 1.82276 | 0.05 |
| 1608 | 0.946131 | 0.0904081 |
| 1609 | 1.40609 | 0.05 |
| 1610 | 0.946132 | 2.1 |
| 1611 | 1.29143 | 1.11065 |
| 1612 | 1.40609 | 0.162457 |
| 1613 | 1.30272 | 2.1 |
| 1614 | 1.19548 | 2.1 |
| 1615 | 2.95451 | 2.1 |
| 1616 | 1.46124 | 2.1 |
| 1617 | 2.93215 | 2.1 |
| 1618 | 1.42105 | 2.1 |
| 1619 | 1.75477 | 0.579156 |
| 1620 | 2.31301 | 1.00365 |
| 1621 | 2.10275 | 0.143353 |

| | | |
|---|---|---|
| 1622 | 1.80099 | 0.208373 |
| 1623 | 0.97541 | 2.1 |
| 1624 | 1.49733 | 2.1 |
| 1625 | 0.97541 | 2.1 |
| 1626 | 1.30751 | 2.1 |
| 1627 | 1.28614 | 0.05 |
| 1628 | 1.74759 | 0.05 |
| 1629 | 1.19756 | 0.05 |
| 1630 | 1.71053 | 0.05 |
| 1631 | 1.19757 | 0.05 |
| 1632 | 1.28954 | 0.05 |
| 1633 | 2.02703 | 0.05 |
| 1634 | 2.29453 | 0.05 |
| 1635 | 1.28265 | 0.05 |
| 1636 | 0.793267 | 0.05 |
| 1637 | 1.19974 | 0.05 |
| 1638 | 0.509551 | 0.05 |
| 1639 | 1.04072 | 0.05 |
| 1640 | 1.35302 | 0.05 |
| 1641 | 1.53133 | 0.05 |
| 1642 | 0.566153 | 0.05 |
| 1643 | 1.09634 | 0.05 |
| 1644 | 1.12792 | 0.05 |
| 1645 | 1.68908 | 2.1 |
| 1646 | 1.68908 | 2.1 |
| 1647 | 0.768212 | 2.1 |
| 1648 | 0.768213 | 2.1 |
| 1649 | 0.946132 | 2.1 |
| 1650 | 0.768212 | 2.1 |
| 1651 | 1.32461 | 2.1 |
| 1652 | 1.87291 | 2.1 |
| 1653 | 2.64921 | 2.1 |
| 1654 | 1.53642 | 2.1 |
| 1655 | 2.26537 | 2.1 |
| 1656 | 1.53642 | 2.1 |
| 1657 | 1.79874 | 2.1 |
| 1658 | 1.79874 | 2.1 |
| 1659 | 0.726635 | 1.80199 |
| 1660 | 0.726636 | 2.1 |
| 1661 | 0.946128 | 2.1 |
| 1662 | 0.726634 | 1.93058 |
| 1663 | 1.23339 | 1.11331 |
| 1664 | 0.992674 | 2.1 |
| 1665 | 1.8023 | 2.1 |
| 1666 | 1.12324 | 0.05 |
| 1667 | 1.29163 | 0.05 |
| 1668 | 0.996831 | 0.05 |
| 1669 | 1.0019 | 0.05 |
| 1670 | 0.993831 | 0.05 |
| 1671 | 1.07283 | 2.1 |
| 1672 | 1.04788 | 2.1 |
| 1673 | 1.44489 | 0.455078 |
| 1674 | 1.04788 | 2.1 |
| 1675 | 1.07652 | 2.1 |
| 1676 | 0.991406 | 2.1 |
| 1677 | 1.37 | 2.1 |
| 1678 | 1.14195 | 2.1 |
| 1679 | 1.47904 | 0.05 |

| | | |
|---|---|---|
| 1680 | 1.26593 | 2.05873 |
| 1681 | 1.75366 | 1.40897 |
| 1682 | 1.57621 | 0.05 |
| 1683 | 1.24538 | 0.05 |
| 1684 | 0.969118 | 2.1 |
| 1685 | 0.946133 | 0.893137 |
| 1686 | 1.69387 | 2.1 |
| 1687 | 1.69387 | 2.1 |
| 1688 | 0.94613 | 2.1 |
| 1689 | 1.28713 | 2.1 |
| 1690 | 1.28713 | 2.1 |
| 1691 | 1.23877 | 2.1 |
| 1692 | 0.969118 | 2.1 |
| 1693 | 1.22615 | 2.1 |
| 1694 | 0.98084 | 2.1 |
| 1695 | 1.01843 | 2.1 |
| 1696 | 1.50876 | 2.1 |
| 1697 | 1.85391 | 2.1 |
| 1698 | 1.84322 | 2.1 |
| 1699 | 1.70377 | 2.1 |
| 1700 | 1.78919 | 2.1 |
| 1701 | 2.66787 | 2.1 |
| 1702 | 1.41549 | 2.1 |
| 1703 | 1.41549 | 2.1 |
| 1704 | 0.86707 | 2.1 |
| 1705 | 0.896172 | 2.1 |
| 1706 | 0.946126 | 2.1 |
| 1707 | 0.867074 | 2.1 |
| 1708 | 0.946118 | 2.1 |
| 1709 | 1.54468 | 0.05 |
| 1710 | 1.54468 | 0.05 |
| 1711 | 2.27259 | 0.05 |
| 1712 | 0.946131 | 0.05 |
| 1713 | 1.25837 | 0.05 |
| 1714 | 0.663171 | 0.05 |
| 1715 | 1.68931 | 0.05 |
| 1716 | 1.68931 | 0.05 |
| 1717 | 1.25837 | 0.05 |
| 1718 | 1.09411 | 0.05 |
| 1719 | 1.73571 | 0.05 |
| 1720 | 2.34894 | 0.05 |
| 1721 | 0.946128 | 0.05 |
| 1722 | 1.30497 | 0.05 |
| 1723 | 0.772312 | 0.05 |
| 1724 | 1.66895 | 0.05 |
| 1725 | 1.66895 | 0.05 |
| 1726 | 1.30497 | 0.05 |
| 1727 | 1.13629 | 0.05 |
| 1728 | 1.22852 | 0.904745 |
| 1729 | 1.578 | 0.839683 |
| 1730 | 2.07781 | 2.1 |
| 1731 | 1.5478 | 2.1 |
| 1732 | 1.70048 | 2.1 |
| 1733 | 1.42329 | 2.1 |
| 1734 | 1.05481 | 2.1 |
| 1735 | 1.1032 | 1.03155 |
| 1736 | 1.19267 | 2.1 |
| 1737 | 1.77324 | 2.1 |

| | | |
|---|---|---|
| 1738 | 1.01121 | 0.05 |
| 1739 | 0.991867 | 0.05 |
| 1740 | 2.0744 | 0.866847 |
| 1741 | 1.75142 | 2.1 |
| 1742 | 1.35418 | 1.07619 |
| 1743 | 1.40041 | 2.1 |
| 1744 | 1.50045 | 2.1 |
| 1745 | 1.37704 | 2.1 |
| 1746 | 1.50045 | 2.1 |
| 1747 | 1.87875 | 2.1 |
| 1748 | 1.35308 | 0.726218 |
| 1749 | 1.29983 | 0.740768 |
| 1750 | 1.62784 | 2.1 |
| 1751 | 1.44807 | 2.1 |
| 1752 | 1.00965 | 2.1 |
| 1753 | 2.69264 | 2.1 |
| 1754 | 1.04075 | 2.1 |
| 1755 | 0.946131 | 2.1 |
| 1756 | 0.946132 | 2.1 |
| 1757 | 1.52704 | 2.1 |
| 1758 | 1.83246 | 2.1 |
| 1759 | 1.56757 | 2.1 |
| 1760 | 1.16687 | 2.1 |
| 1761 | 2.04857 | 2.1 |
| 1762 | 1.32707 | 1.94337 |
| 1763 | 1.25519 | 0.05 |
| 1764 | 1.32101 | 0.05 |
| 1765 | 1.25055 | 0.206905 |
| 1766 | 0.827853 | 0.05 |
| 1767 | 1.11835 | 0.05 |
| 1768 | 1.07281 | 1.8888 |
| 1769 | 1.54302 | 0.05 |
| 1770 | 0.724064 | 0.439077 |
| 1771 | 1.32709 | 2.1 |
| 1772 | 3.44123 | 0.679518 |
| 1773 | 2.46889 | 0.05 |
| 1774 | 0.730076 | 0.05 |
| 1775 | 1.25809 | 0.05 |
| 1776 | 0.730077 | 0.05 |
| 1777 | 0.946129 | 0.05 |
| 1778 | 1.72777 | 0.05 |
| 1779 | 1.72777 | 0.05 |
| 1780 | 0.73005 | 0.05 |
| 1781 | 1.7522 | 0.05 |
| 1782 | 2.51618 | 0.05 |
| 1783 | 0.628177 | 0.05 |
| 1784 | 1.1068 | 0.05 |
| 1785 | 0.747435 | 0.05 |
| 1786 | 0.946133 | 0.05 |
| 1787 | 1.84668 | 0.05 |
| 1788 | 1.8563 | 0.05 |
| 1789 | 0.628177 | 0.05 |
| 1790 | 1.45501 | 0.05 |
| 1791 | 2.19809 | 0.05 |
| 1792 | 1.45501 | 0.05 |
| 1793 | 1.48974 | 0.05 |
| 1794 | 1.7709 | 2.1 |
| 1795 | 2.63288 | 2.1 |

| | | |
|---|---|---|
| 1796 | 1.5362 | 2.1 |
| 1797 | 1.56327 | 2.1 |
| 1798 | 3.03945 | 0.05 |
| 1799 | 0.899979 | 0.05 |
| 1800 | 0.869386 | 0.05 |
| 1801 | 1.85864 | 0.05 |
| 1802 | 1.78028 | 0.05 |
| 1803 | 1.28719 | 0.05 |
| 1804 | 1.09532 | 0.05 |
| 1805 | 0.881898 | 0.05 |
| 1806 | 1.28911 | 0.05 |
| 1807 | 1.39644 | 0.05 |
| 1808 | 0.688829 | 0.05 |
| 1809 | 2.14611 | 0.05 |
| 1810 | 1.80313 | 0.05 |
| 1811 | 0.68883 | 0.05 |
| 1812 | 0.946131 | 0.05 |
| 1813 | 1.25334 | 0.05 |
| 1814 | 1.88987 | 0.05 |
| 1815 | 1.94432 | 0.05 |
| 1816 | 1.37592 | 0.05 |
| 1817 | 1.11691 | 1.90176 |
| 1818 | 1.02923 | 2.1 |
| 1819 | 0.45824 | 0.05 |
| 1820 | 0.646512 | 0.05 |
| 1821 | 2.46292 | 0.05 |
| 1822 | 0.493451 | 0.05 |
| 1823 | 2.00826 | 2.1 |
| 1824 | 2.49784 | 2.1 |
| 1825 | 1.17054 | 2.1 |
| 1826 | 1.01485 | 2.1 |
| 1827 | 1.26045 | 2.1 |
| 1828 | 1.17054 | 2.1 |
| 1829 | 1.44247 | 2.1 |
| 1830 | 2.02687 | 0.05 |
| 1831 | 1.66365 | 0.05 |
| 1832 | 1.64656 | 0.05 |
| 1833 | 1.56597 | 0.05 |
| 1834 | 1.21419 | 2.1 |
| 1835 | 1.5008 | 2.1 |
| 1836 | 1.10711 | 2.1 |
| 1837 | 1.44287 | 2.1 |
| 1838 | 1.44287 | 2.1 |
| 1839 | 0.750368 | 2.1 |
| 1840 | 1.25876 | 2.1 |
| 1841 | 0.82542 | 2.1 |
| 1842 | 1.04074 | 2.1 |
| 1843 | 1.25874 | 2.1 |
| 1844 | 1.04159 | 0.05 |
| 1845 | 1.2489 | 0.05 |
| 1846 | 0.980547 | 0.05 |
| 1847 | 1.62235 | 0.05 |
| 1848 | 1.15991 | 0.05 |
| 1849 | 0.980547 | 0.05 |
| 1850 | 0.94613 | 0.05 |
| 1851 | 1.54662 | 0.05 |
| 1852 | 1.54661 | 0.05 |
| 1853 | 1.07912 | 0.05 |

| | | |
|---|---|---|
| 1854 | 1.1032 | 0.05 |
| 1855 | 1.1032 | 0.05 |
| 1856 | 0.94613 | 0.05 |
| 1857 | 1.12468 | 2.1 |
| 1858 | 1.2724 | 2.1 |
| 1859 | 1.06521 | 2.1 |
| 1860 | 1.0752 | 2.1 |
| 1861 | 1.94936 | 0.05 |
| 1862 | 1.62772 | 0.05 |
| 1863 | 1.94733 | 0.05 |
| 1864 | 1.42901 | 0.0533847 |
| 1865 | 1.33022 | 2.1 |
| 1866 | 1.70839 | 2.1 |
| 1867 | 1.75048 | 2.1 |
| 1868 | 1.41201 | 2.1 |
| 1869 | 1.52314 | 2.1 |
| 1870 | 1.44039 | 2.1 |
| 1871 | 1.51113 | 2.1 |
| 1872 | 2.58638 | 2.1 |
| 1873 | 0.987247 | 0.05 |
| 1874 | 1.38622 | 0.05 |
| 1875 | 0.858359 | 0.05 |
| 1876 | 1.19967 | 0.05 |
| 1877 | 1.06414 | 0.05 |
| 1878 | 0.946132 | 0.05 |
| 1879 | 0.94613 | 0.05 |
| 1880 | 0.687079 | 0.05 |
| 1881 | 0.946132 | 0.05 |
| 1882 | 0.822611 | 0.05 |
| 1883 | 1.70242 | 0.05 |
| 1884 | 1.70242 | 0.05 |
| 1885 | 0.946132 | 0.0862746 |
| 1886 | 1.11155 | 0.05 |
| 1887 | 0.946132 | 0.172163 |
| 1888 | 1.11155 | 0.0558769 |
| 1889 | 1.37981 | 0.05 |
| 1890 | 1.18348 | 0.05 |
| 1891 | 2.44179 | 0.05 |
| 1892 | 2.00403 | 0.05 |
| 1893 | 1.10961 | 2.1 |
| 1894 | 1.85415 | 2.1 |
| 1895 | 1.44825 | 2.1 |
| 1896 | 0.932272 | 2.1 |
| 1897 | 0.810942 | 2.1 |
| 1898 | 1.00189 | 2.1 |
| 1899 | 1.11779 | 2.1 |
| 1900 | 1.3359 | 2.1 |
| 1901 | 0.965279 | 2.1 |
| 1902 | 0.833779 | 2.1 |
| 1903 | 1.05337 | 2.1 |
| 1904 | 0.526656 | 2.1 |
| 1905 | 1.44599 | 2.1 |
| 1906 | 1.44599 | 2.1 |
| 1907 | 1.24853 | 2.1 |
| 1908 | 0.946132 | 2.1 |
| 1909 | 1.24853 | 2.1 |
| 1910 | 0.724122 | 2.1 |
| 1911 | 1.91547 | 2.1 |

| | | |
|---|---|---|
| 1912 | 2.49705 | 2.1 |
| 1913 | 1.05931 | 2.1 |
| 1914 | 1.1729 | 2.1 |
| 1915 | 1.34526 | 2.1 |
| 1916 | 1.5379 | 2.1 |
| 1917 | 1.90855 | 2.1 |
| 1918 | 2.08597 | 2.1 |
| 1919 | 1.68165 | 2.1 |
| 1920 | 1.45887 | 2.1 |
| 1921 | 1.50022 | 2.1 |
| 1922 | 1.4748 | 2.1 |
| 1923 | 2.63645 | 1.97817 |
| 1924 | 1.74431 | 2.08745 |
| 1925 | 1.25288 | 2.01982 |
| 1926 | 1.25289 | 1.44197 |
| 1927 | 0.970025 | 1.51613 |
| 1928 | 0.747542 | 2.1 |
| 1929 | 0.810959 | 1.30717 |
| 1930 | 1.15369 | 1.47136 |
| 1931 | 0.977472 | 1.59433 |
| 1932 | 1.12695 | 1.50858 |
| 1933 | 0.996762 | 1.4691 |
| 1934 | 1.28249 | 0.463412 |
| 1935 | 0.946132 | 0.633544 |
| 1936 | 0.528621 | 0.843319 |
| 1937 | 1.59186 | 0.351056 |
| 1938 | 1.44072 | 0.382424 |
| 1939 | 0.633488 | 0.28551 |
| 1940 | 0.598506 | 0.508867 |
| 1941 | 1.51618 | 0.792466 |
| 1942 | 1.19999 | 0.272405 |
| 1943 | 1.46511 | 0.363692 |
| 1944 | 1.44056 | 0.245127 |
| 1945 | 0.912754 | 0.17651 |
| 1946 | 1.4725 | 0.05 |
| 1947 | 1.4725 | 0.05 |
| 1948 | 0.912753 | 0.17386 |
| 1949 | 1.23351 | 0.247137 |
| 1950 | 1.23351 | 0.357456 |
| 1951 | 0.946131 | 0.555037 |
| 1952 | 1.17129 | 0.05 |
| 1953 | 1.82551 | 0.05 |
| 1954 | 1.27575 | 1.88898 |
| 1955 | 0.946129 | 2.1 |
| 1956 | 0.946129 | 2.1 |
| 1957 | 0.946128 | 2.00185 |
| 1958 | 0.792754 | 2.1 |
| 1959 | 0.94613 | 1.66978 |
| 1960 | 0.792752 | 1.5834 |
| 1961 | 2.08054 | 2.1 |
| 1962 | 1.20091 | 2.1 |
| 1963 | 2.1669 | 2.0657 |
| 1964 | 1.24383 | 1.70352 |
| 1965 | 0.904669 | 2.1 |
| 1966 | 0.83804 | 1.35708 |
| 1967 | 1.90486 | 1.70778 |
| 1968 | 1.29162 | 1.57725 |
| 1969 | 1.37383 | 1.90881 |

| | | |
|---|---|---|
| 1970 | 1.86533 | 1.53722 |
| 1971 | 1.76472 | 1.94986 |
| 1972 | 1.60428 | 1.57584 |
| 1973 | 2.43993 | 2.08003 |
| 1974 | 1.37833 | 2.1 |
| 1975 | 1.18632 | 2.1 |
| 1976 | 0.946132 | 2.1 |
| 1977 | 1.27544 | 2.1 |
| 1978 | 1.05085 | 2.1 |
| 1979 | 0.961178 | 2.1 |
| 1980 | 0.859844 | 2.1 |
| 1981 | 1.0499 | 2.1 |
| 1982 | 1.75592 | 2.1 |
| 1983 | 2.08668 | 2.1 |
| 1984 | 1.75592 | 2.1 |
| 1985 | 1.4402 | 2.1 |
| 1986 | 1.72285 | 2.1 |
| 1987 | 0.83312 | 0.05 |
| 1988 | 1.07295 | 0.05 |
| 1989 | 1.37607 | 0.05 |
| 1990 | 1.32097 | 0.05 |
| 1991 | 1.11332 | 0.05 |
| 1992 | 1.18568 | 0.05 |
| 1993 | 0.877482 | 0.05 |
| 1994 | 1.4458 | 0.05 |
| 1995 | 2.34112 | 0.05 |
| 1996 | 2.68021 | 0.05 |
| 1997 | 0.681677 | 0.05 |
| 1998 | 0.946131 | 0.05 |
| 1999 | 0.681649 | 0.05 |
| 2000 | 1.83115 | 0.05 |
| 2001 | 1.68029 | 0.05 |
| 2002 | 1.05482 | 0.05 |
| 2003 | 1.31485 | 0.05 |
| 2004 | 2.08247 | 0.350024 |
| 2005 | 1.33153 | 0.19824 |
| 2006 | 0.935062 | 0.292849 |
| 2007 | 0.806958 | 1.73872 |
| 2008 | 0.827683 | 0.203698 |
| 2009 | 1.01041 | 0.528754 |
| 2010 | 0.741832 | 0.144825 |
| 2011 | 1.15668 | 0.83767 |
| 2012 | 0.908649 | 0.282682 |
| 2013 | 0.59561 | 0.05 |
| 2014 | 1.19028 | 0.0722835 |
| 2015 | 0.59561 | 0.0806794 |
| 2016 | 0.946132 | 0.0967391 |
| 2017 | 0.595623 | 0.178436 |
| 2018 | 1.58164 | 0.0808478 |
| 2019 | 1.58164 | 0.134685 |
| 2020 | 0.946131 | 2.1 |
| 2021 | 1.55201 | 2.1 |
| 2022 | 1.55201 | 2.1 |
| 2023 | 0.946127 | 2.1 |
| 2024 | 1.13312 | 2.1 |
| 2025 | 1.13312 | 2.1 |
| 2026 | 1.08108 | 2.1 |
| 2027 | 1.04973 | 2.1 |

| | | |
|---|---|---|
| 2028 | 1.20149 | 2.1 |
| 2029 | 1.86902 | 0.139446 |
| 2030 | 0.613447 | 2.1 |
| 2031 | 1.22618 | 0.719576 |
| 2032 | 1.38663 | 1.33465 |
| 2033 | 1.38662 | 2.1 |
| 2034 | 0.613447 | 0.879211 |
| 2035 | 1.22617 | 2.1 |
| 2036 | 0.773899 | 2.1 |
| 2037 | 1.23515 | 1.43483 |
| 2038 | 1.87823 | 1.23524 |
| 2039 | 1.70303 | 0.484341 |
| 2040 | 1.4714 | 2.1 |
| 2041 | 1.74067 | 2.1 |
| 2042 | 0.735699 | 2.1 |
| 2043 | 1.73461 | 2.1 |
| 2044 | 1.47224 | 2.1 |
| 2045 | 1.22827 | 2.1 |
| 2046 | 1.1634 | 2.1 |
| 2047 | 1.36471 | 2.1 |
| 2048 | 0.735701 | 2.1 |
| 2049 | 2.8386 | 0.129953 |
| 2050 | 2.39056 | 2.1 |
| 2051 | 2.44128 | 0.434747 |
| 2052 | 1.54102 | 2.1 |
| 2053 | 1.54102 | 2.1 |
| 2054 | 0.936141 | 1.53783 |
| 2055 | 1.40594 | 2.1 |
| 2056 | 1.07121 | 0.0531162 |
| 2057 | 0.936142 | 2.1 |
| 2058 | 1.07121 | 2.01518 |
| 2059 | 1.22691 | 0.05 |
| 2060 | 1.42907 | 0.05 |
| 2061 | 2.18048 | 0.05 |
| 2062 | 0.947662 | 0.05 |
| 2063 | 2.18394 | 2.1 |
| 2064 | 1.21292 | 2.1 |
| 2065 | 2.18394 | 2.1 |
| 2066 | 2.19064 | 2.1 |
| 2067 | 1.27229 | 2.1 |
| 2068 | 1.20859 | 0.785194 |
| 2069 | 1.21073 | 0.725558 |
| 2070 | 1.73899 | 2.1 |
| 2071 | 1.3164 | 2.1 |
| 2072 | 1.61245 | 2.1 |
| 2073 | 1.41444 | 2.1 |
| 2074 | 2.55116 | 2.1 |
| 2075 | 1.26193 | 2.1 |
| 2076 | 1.89553 | 2.1 |
| 2077 | 0.916438 | 2.1 |
| 2078 | 1.26113 | 2.1 |
| 2079 | 0.810934 | 2.1 |
| 2080 | 2.00419 | 2.1 |
| 2081 | 1.27872 | 2.1 |
| 2082 | 1.35306 | 2.1 |
| 2083 | 1.96749 | 2.1 |
| 2084 | 1.07955 | 2.1 |
| 2085 | 1.31854 | 2.1 |

| | | |
|---|---|---|
| 2086 | 1.29505 | 0.502059 |
| 2087 | 1.29504 | 0.545981 |
| 2088 | 0.612469 | 0.820767 |
| 2089 | 0.990053 | 0.778013 |
| 2090 | 1.04072 | 0.752745 |
| 2091 | 0.985266 | 0.667621 |
| 2092 | 0.990039 | 0.508673 |
| 2093 | 1.06892 | 0.811839 |
| 2094 | 1.57158 | 0.36252 |
| 2095 | 1.895 | 0.44776 |
| 2096 | 1.05698 | 0.321217 |
| 2097 | 0.925053 | 2.1 |
| 2098 | 0.624712 | 2.1 |
| 2099 | 1.42465 | 2.1 |
| 2100 | 1.49014 | 2.1 |
| 2101 | 0.687198 | 2.1 |
| 2102 | 1.22058 | 2.1 |
| 2103 | 0.851488 | 2.1 |
| 2104 | 0.588128 | 2.1 |
| 2105 | 1.12452 | 2.1 |
| 2106 | 1.02783 | 2.1 |
| 2107 | 1.7083 | 2.1 |
| 2108 | 1.06977 | 1.29901 |
| 2109 | 0.818569 | 0.860391 |
| 2110 | 1.14386 | 1.48186 |
| 2111 | 0.983791 | 0.980963 |
| 2112 | 1.12214 | 1.80064 |
| 2113 | 1.0915 | 1.02714 |
| 2114 | 0.810934 | 0.954289 |
| 2115 | 0.764114 | 1.43597 |
| 2116 | 1.87956 | 0.985795 |
| 2117 | 1.87956 | 1.55696 |
| 2118 | 0.764141 | 0.943836 |
| 2119 | 1.37675 | 0.881282 |
| 2120 | 0.764141 | 0.922943 |
| 2121 | 0.946132 | 1.25126 |
| 2122 | 1.9637 | 2.01128 |
| 2123 | 2.10107 | 0.774892 |
| 2124 | 1.67814 | 1.37771 |
| 2125 | 3.02886 | 1.31519 |
| 2126 | 0.908982 | 0.05 |
| 2127 | 0.621727 | 0.05 |
| 2128 | 0.525963 | 0.05 |
| 2129 | 0.946128 | 0.05 |
| 2130 | 1.60979 | 0.05 |
| 2131 | 1.45596 | 0.05 |
| 2132 | 0.556172 | 0.05 |
| 2133 | 2.06779 | 0.05 |
| 2134 | 2.17911 | 0.05 |
| 2135 | 1.00205 | 0.05 |
| 2136 | 1.30685 | 0.05 |
| 2137 | 1.16534 | 0.05 |
| 2138 | 1.65532 | 2.1 |
| 2139 | 2.20346 | 2.1 |
| 2140 | 0.993889 | 2.1 |
| 2141 | 1.09328 | 2.1 |
| 2142 | 2.09372 | 2.1 |
| 2143 | 2.10036 | 2.1 |

| | | |
|---|---|---|
| 2144 | 1.33727 | 1.96676 |
| 2145 | 2.53383 | 1.93616 |
| 2146 | 0.893379 | 1.6918 |
| 2147 | 1.52828 | 1.4916 |
| 2148 | 1.09599 | 0.05 |
| 2149 | 1.09599 | 0.05 |
| 2150 | 1.50524 | 2.1 |
| 2151 | 1.54711 | 2.1 |
| 2152 | 1.41469 | 2.1 |
| 2153 | 1.99459 | 1.9442 |
| 2154 | 1.79805 | 2.1 |
| 2155 | 1.79805 | 2.1 |
| 2156 | 0.707344 | 2.1 |
| 2157 | 1.30318 | 2.1 |
| 2158 | 0.707344 | 2.1 |
| 2159 | 0.946129 | 2.1 |
| 2160 | 1.30318 | 2.1 |
| 2161 | 2.40442 | 2.1 |
| 2162 | 1.89226 | 2.1 |
| 2163 | 1.40794 | 2.1 |
| 2164 | 0.898721 | 2.1 |
| 2165 | 0.791921 | 0.810946 |
| 2166 | 0.810933 | 2.1 |
| 2167 | 1.10904 | 2.04025 |
| 2168 | 0.991102 | 2.1 |
| 2169 | 0.808502 | 0.48547 |
| 2170 | 1.1398 | 2.1 |
| 2171 | 1.31346 | 2.1 |
| 2172 | 1.11833 | 2.1 |
| 2173 | 1.669 | 2.1 |
| 2174 | 1.25715 | 2.1 |
| 2175 | 1.3199 | 2.1 |
| 2176 | 1.63092 | 2.1 |
| 2177 | 1.63092 | 2.1 |
| 2178 | 0.946131 | 2.1 |
| 2179 | 0.946131 | 2.1 |
| 2180 | 1.13534 | 2.1 |
| 2181 | 0.979853 | 2.1 |
| 2182 | 2.19994 | 2.1 |
| 2183 | 0.725844 | 2.1 |
| 2184 | 2.19994 | 2.1 |
| 2185 | 2.35055 | 2.1 |
| 2186 | 1.52204 | 2.1 |
| 2187 | 1.40118 | 2.1 |
| 2188 | 3.42285 | 2.1 |
| 2189 | 2.05217 | 2.1 |
| 2190 | 1.50319 | 2.1 |
| 2191 | 0.947516 | 1.18079 |
| 2192 | 0.903572 | 1.10054 |
| 2193 | 0.76996 | 1.23484 |
| 2194 | 1.90959 | 2.1 |
| 2195 | 1.18897 | 2.1 |
| 2196 | 1.24166 | 2.08244 |
| 2197 | 2.26785 | 1.78841 |
| 2198 | 2.05217 | 2.1 |
| 2199 | 2.73306 | 2.1 |
| 2200 | 1.42729 | 0.05 |
| 2201 | 1.387 | 0.05 |

| | | |
|---|---|---|
| 2202 | 1.59458 | 0.146581 |
| 2203 | 1.91051 | 0.129792 |
| 2204 | 1.68108 | 0.37184 |
| 2205 | 2.03357 | 0.556354 |
| 2206 | 0.928982 | 0.05 |
| 2207 | 1.55687 | 0.05 |
| 2208 | 1.55687 | 0.05 |
| 2209 | 0.92898 | 0.05 |
| 2210 | 1.3495 | 0.05 |
| 2211 | 1.3495 | 0.05 |
| 2212 | 0.946133 | 0.05 |
| 2213 | 1.85795 | 0.05 |
| 2214 | 2.2727 | 0.05 |
| 2215 | 1.20324 | 1.85333 |
| 2216 | 1.07784 | 0.05 |
| 2217 | 0.957792 | 0.05 |
| 2218 | 1.2745 | 0.05 |
| 2219 | 1.4659 | 0.05 |
| 2220 | 0.946129 | 0.05 |
| 2221 | 0.95778 | 0.05 |
| 2222 | 0.851553 | 0.05 |
| 2223 | 1.74103 | 0.05 |
| 2224 | 2.28994 | 0.05 |
| 2225 | 2.08213 | 0.05 |
| 2226 | 2.1556 | 0.05 |
| 2227 | 2.20372 | 0.05 |
| 2228 | 0.838027 | 0.05 |
| 2229 | 1.11373 | 0.05 |
| 2230 | 1.2909 | 0.05 |
| 2231 | 0.998424 | 0.05 |
| 2232 | 1.40621 | 0.05 |
| 2233 | 1.19478 | 0.05 |
| 2234 | 1.11783 | 0.05 |
| 2235 | 1.6695 | 0.05 |
| 2236 | 1.7498 | 0.05 |
| 2237 | 0.477652 | 0.05 |
| 2238 | 1.77291 | 0.05 |
| 2239 | 0.590448 | 0.05 |
| 2240 | 1.07668 | 0.05 |
| 2241 | 0.810946 | 0.05 |
| 2242 | 1.1946 | 0.05 |
| 2243 | 2.03914 | 0.05 |
| 2244 | 1.25585 | 0.05 |
| 2245 | 2.02622 | 0.05 |
| 2246 | 1.39091 | 0.05 |
| 2247 | 1.31871 | 0.05 |
| 2248 | 1.15789 | 2.1 |
| 2249 | 1.15755 | 2.1 |
| 2250 | 1.82268 | 2.1 |
| 2251 | 1.87119 | 2.1 |
| 2252 | 3.10636 | 2.1 |
| 2253 | 2.96285 | 2.1 |
| 2254 | 1.73443 | 0.661141 |
| 2255 | 2.33812 | 0.731282 |
| 2256 | 1.59457 | 0.296937 |
| 2257 | 1.73443 | 1.08437 |
| 2258 | 2.00665 | 2.1 |
| 2259 | 1.99841 | 2.1 |

| | | |
|---|---|---|
| 2260 | 1.19901 | 2.1 |
| 2261 | 1.16661 | 2.1 |
| 2262 | 2.15883 | 0.05 |
| 2263 | 1.20951 | 0.05 |
| 2264 | 2.49192 | 0.05 |
| 2265 | 0.65112 | 0.05 |
| 2266 | 2.45317 | 0.05 |
| 2267 | 1.73091 | 0.05 |
| 2268 | 2.0795 | 0.05 |
| 2269 | 1.45891 | 0.05 |
| 2270 | 1.6531 | 0.05 |
| 2271 | 1.78304 | 0.05 |
| 2272 | 0.775132 | 0.05 |
| 2273 | 1.35051 | 0.05 |
| 2274 | 1.0461 | 0.05 |
| 2275 | 1.37837 | 0.05 |
| 2276 | 1.00259 | 0.05 |
| 2277 | 1.25796 | 0.339652 |
| 2278 | 1.10719 | 0.601329 |
| 2279 | 1.51067 | 2.1 |
| 2280 | 2.65632 | 2.1 |
| 2281 | 1.30521 | 2.1 |
| 2282 | 2.456 2.1 | |
| 2283 | 3.19218 | 2.1 |
| 2284 | 2.67387 | 2.1 |
| 2285 | 0.988862 | 2.1 |
| 2286 | 1.917 2.1 | |
| 2287 | 0.946132 | 2.1 |
| 2288 | 1.16202 | 2.1 |
| 2289 | 1.53871 | 2.1 |
| 2290 | 1.53871 | 2.1 |
| 2291 | 0.946132 | 2.1 |
| 2292 | 1.16202 | 2.1 |
| 2293 | 1.32282 | 2.1 |
| 2294 | 2.46356 | 2.1 |
| 2295 | 2.32404 | 2.1 |
| 2296 | 1.37562 | 0.05 |
| 2297 | 0.985546 | 0.05 |
| 2298 | 1.28983 | 0.05 |
| 2299 | 1.05566 | 0.05 |
| 2300 | 0.895312 | 0.05 |
| 2301 | 1.53325 | 0.05 |
| 2302 | 1.04342 | 0.05 |
| 2303 | 1.80881 | 0.05 |
| 2304 | 0.876865 | 0.05 |
| 2305 | 0.5689 | 0.05 |
| 2306 | 1.09506 | 0.05 |
| 2307 | 1.51789 | 2.1 |
| 2308 | 1.49872 | 2.1 |
| 2309 | 0.949364 | 0.204617 |
| 2310 | 2.01233 | 0.265416 |
| 2311 | 1.55063 | 0.247808 |
| 2312 | 1.57783 | 0.05 |
| 2313 | 1.57783 | 0.33344 |
| 2314 | 0.674605 | 0.206604 |
| 2315 | 0.98537 | 0.05 |
| 2316 | 0.674633 | 0.05 |
| 2317 | 0.98537 | 0.05 |

| | | |
|---|---|---|
| 2318 | 1.26706 | 0.0797595 |
| 2319 | 0.980664 | 0.193561 |
| 2320 | 1.03298 | 0.05 |
| 2321 | 1.84936 | 0.238348 |
| 2322 | 1.51696 | 0.05 |
| 2323 | 1.97074 | 0.05 |
| 2324 | 1.03298 | 0.0598894 |
| 2325 | 1.51324 | 0.05 |
| 2326 | 1.51324 | 0.05 |
| 2327 | 0.758454 | 0.05 |
| 2328 | 1.28632 | 0.05 |
| 2329 | 0.758482 | 0.05 |
| 2330 | 0.758482 | 0.05 |
| 2331 | 0.985372 | 0.05 |
| 2332 | 1.14105 | 0.05 |
| 2333 | 1.59787 | 0.05 |
| 2334 | 2.46244 | 0.05 |
| 2335 | 1.70367 | 0.05 |
| 2336 | 2.13757 | 1.05598 |
| 2337 | 1.98142 | 1.11351 |
| 2338 | 1.35878 | 0.05 |
| 2339 | 2.39947 | 0.05 |
| 2340 | 1.24554 | 0.05 |
| 2341 | 1.7522 | 0.05 |
| 2342 | 1.49693 | 1.82338 |
| 2343 | 1.49652 | 1.83792 |
| 2344 | 1.6211 | 1.82955 |
| 2345 | 1.45938 | 1.95181 |
| 2346 | 0.737431 | 2.1 |
| 2347 | 1.18643 | 1.955 |
| 2348 | 1.04072 | 2.1 |
| 2349 | 0.9459 | 1.93513 |
| 2350 | 0.66364 | 2.1 |
| 2351 | 0.985109 | 1.37363 |
| 2352 | 0.638368 | 2.08943 |
| 2353 | 0.83804 | 1.24782 |
| 2354 | 0.998024 | 1.1811 |
| 2355 | 0.914471 | 0.811401 |
| 2356 | 1.14281 | 1.36629 |
| 2357 | 0.833972 | 1.37662 |
| 2358 | 0.991865 | 0.129018 |
| 2359 | 1.42208 | 0.05 |
| 2360 | 1.70718 | 2.1 |
| 2361 | 1.79888 | 2.1 |
| 2362 | 1.16851 | 0.0975911 |
| 2363 | 1.16851 | 0.0661778 |
| 2364 | 1.51021 | 0.0513312 |
| 2365 | 0.946132 | 0.0729971 |
| 2366 | 1.14897 | 0.05 |
| 2367 | 0.946132 | 0.05 |
| 2368 | 1.77177 | 0.05 |
| 2369 | 1.77177 | 0.05 |
| 2370 | 1.33038 | 0.05 |
| 2371 | 1.38752 | 0.05 |
| 2372 | 1.1726 | 0.05 |
| 2373 | 1.51688 | 0.05 |
| 2374 | 1.06455 | 2.1 |
| 2375 | 0.98511 | 2.1 |

| | | |
|---|---|---|
| 2376 | 1.31144 | 2.1 |
| 2377 | 1.79825 | 2.1 |
| 2378 | 1.79825 | 2.1 |
| 2379 | 1.73044 | 2.1 |
| 2380 | 1.59589 | 0.05 |
| 2381 | 1.59589 | 0.05 |
| 2382 | 2.78009 | 2.1 |
| 2383 | 1.68761 | 2.1 |
| 2384 | 3.05511 | 2.1 |
| 2385 | 2.25318 | 2.1 |
| 2386 | 0.578998 | 2.1 |
| 2387 | 1.03512 | 2.1 |
| 2388 | 2.26156 | 2.1 |
| 2389 | 1.44859 | 1.1887 |
| 2390 | 1.35897 | 1.74799 |
| 2391 | 1.18301 | 2.1 |
| 2392 | 1.25173 | 2.1 |
| 2393 | 1.98851 | 2.1 |
| 2394 | 2.70685 | 2.1 |
| 2395 | 0.682342 | 2.1 |
| 2396 | 1.52491 | 2.1 |
| 2397 | 1.37119 | 2.1 |
| 2398 | 0.682313 | 2.1 |
| 2399 | 1.35342 | 2.1 |
| 2400 | 0.947555 | 2.1 |
| 2401 | 0.614107 | 2.1 |
| 2402 | 1.23007 | 2.1 |
| 2403 | 1.5735 | 2.1 |
| 2404 | 1.58713 | 2.1 |
| 2405 | 1.57979 | 0.05 |
| 2406 | 1.30723 | 0.05 |
| 2407 | 0.935658 | 0.801427 |
| 2408 | 0.8952 | 0.767277 |
| 2409 | 1.27352 | 1.31604 |
| 2410 | 1.0985 | 0.909684 |
| 2411 | 1.07163 | 0.753498 |
| 2412 | 1.15823 | 0.894436 |
| 2413 | 0.836451 | 0.719696 |
| 2414 | 2.08051 | 0.571406 |
| 2415 | 1.92601 | 1.17148 |
| 2416 | 2.0644 | 0.680034 |
| 2417 | 1.37259 | 2.1 |
| 2418 | 1.37259 | 2.1 |
| 2419 | 0.752217 | 2.1 |
| 2420 | 1.28569 | 2.1 |
| 2421 | 0.752186 | 2.1 |
| 2422 | 0.839124 | 2.1 |
| 2423 | 1.28568 | 2.1 |
| 2424 | 1.04199 | 2.1 |
| 2425 | 1.04199 | 2.1 |
| 2426 | 2.59268 | 2.1 |
| 2427 | 1.04199 | 2.1 |
| 2428 | 1.5098 | 2.1 |
| 2429 | 1.5098 | 2.1 |
| 2430 | 0.520997 | 2.1 |
| 2431 | 1.29634 | 2.1 |
| 2432 | 0.520996 | 2.1 |
| 2433 | 0.73446 | 2.1 |

| | | |
|---|---|---|
| 2434 | 1.29634 | 2.1 |
| 2435 | 0.983724 | 2.1 |
| 2436 | 1.23914 | 2.1 |
| 2437 | 2.65095 | 2.1 |
| 2438 | 1.52851 | 2.1 |
| 2439 | 2.399 2.1 | |
| 2440 | 1.68137 | 2.1 |
| 2441 | 1.11285 | 2.1 |
| 2442 | 1.04921 | 2.1 |
| 2443 | 1.30711 | 2.1 |
| 2444 | 1.04921 | 2.1 |
| 2445 | 1.07496 | 1.05687 |
| 2446 | 1.55831 | 0.573425 |
| 2447 | 1.07497 | 0.05 |
| 2448 | 1.07497 | 0.860757 |
| 2449 | 1.27391 | 2.1 |
| 2450 | 2.5615 | 2.1 |
| 2451 | 1.2739 | 2.1 |
| 2452 | 1.75253 | 2.1 |
| 2453 | 1.12907 | 0.05 |
| 2454 | 1.9194 | 0.05 |
| 2455 | 1.96617 | 0.05 |
| 2456 | 1.01225 | 2.1 |
| 2457 | 0.947979 | 2.1 |
| 2458 | 1.01225 | 0.956895 |
| 2459 | 1.06561 | 2.1 |
| 2460 | 1.01225 | 2.1 |
| 2461 | 3.56876 | 2.1 |
| 2462 | 3.68767 | 2.1 |
| 2463 | 1.52301 | 2.1 |
| 2464 | 1.61013 | 2.1 |
| 2465 | 1.43726 | 2.1 |
| 2466 | 2.04275 | 2.1 |
| 2467 | 0.995734 | 2.1 |
| 2468 | 1.16199 | 2.1 |
| 2469 | 1.73704 | 0.05 |
| 2470 | 1.04019 | 0.05 |
| 2471 | 0.956294 | 2.1 |
| 2472 | 1.81488 | 2.1 |
| 2473 | 1.63719 | 2.1 |
| 2474 | 1.50103 | 0.05 |
| 2475 | 1.75289 | 0.05 |
| 2476 | 1.31491 | 0.05 |
| 2477 | 1.03567 | 0.05 |
| 2478 | 0.672257 | 0.05 |
| 2479 | 1.04072 | 0.05 |
| 2480 | 0.746903 | 0.05 |
| 2481 | 1.43317 | 0.05 |
| 2482 | 1.39986 | 0.05 |
| 2483 | 1.06385 | 0.05 |
| 2484 | 1.1624 | 0.05 |
| 2485 | 1.61667 | 0.05 |
| 2486 | 1.27258 | 0.05 |
| 2487 | 2.559 0.05 | |
| 2488 | 1.66093 | 0.05 |
| 2489 | 1.10719 | 0.64365 |
| 2490 | 2.03235 | 0.774269 |
| 2491 | 1.17105 | 1.07174 |

| | | |
|---|---|---|
| 2492 | 1.10719 | 0.792197 |
| 2493 | 1.3028 | 2.1 |
| 2494 | 0.946131 | 2.1 |
| 2495 | 0.946127 | 2.1 |
| 2496 | 1.52912 | 2.1 |
| 2497 | 1.52912 | 2.1 |
| 2498 | 0.946128 | 1.958 |
| 2499 | 0.949997 | 2.1 |
| 2500 | 1.31565 | 2.1 |
| 2501 | 1.46643 | 2.1 |
| 2502 | 1.5482 | 2.1 |
| 2503 | 1.47102 | 2.1 |
| 2504 | 1.47102 | 2.1 |
| 2505 | 0.774101 | 2.1 |
| 2506 | 1.30583 | 2.1 |
| 2507 | 0.774103 | 2.1 |
| 2508 | 0.939287 | 2.1 |
| 2509 | 1.30584 | 2.1 |
| 2510 | 0.958661 | 2.1 |
| 2511 | 2.28835 | 2.1 |
| 2512 | 1.43213 | 0.05 |
| 2513 | 2.13255 | 0.05 |
| 2514 | 1.43213 | 0.05 |
| 2515 | 1.10476 | 0.05 |
| 2516 | 1.93165 | 0.05 |
| 2517 | 1.03235 | 0.963063 |
| 2518 | 1.31 | 1.01493 |
| 2519 | 1.22082 | 2.1 |
| 2520 | 1.35827 | 2.1 |
| 2521 | 0.790936 | 2.1 |
| 2522 | 0.716644 | 2.1 |
| 2523 | 1.43257 | 2.1 |
| 2524 | 1.43257 | 2.1 |
| 2525 | 0.716615 | 2.1 |
| 2526 | 0.946131 | 2.1 |
| 2527 | 0.730157 | 2.1 |
| 2528 | 1.47212 | 2.1 |
| 2529 | 1.47212 | 2.1 |
| 2530 | 0.946131 | 2.1 |
| 2531 | 0.790792 | 2.1 |
| 2532 | 1.41149 | 2.1 |
| 2533 | 0.790791 | 2.1 |
| 2534 | 1.01006 | 2.1 |
| 2535 | 1.53045 | 0.05 |
| 2536 | 1.53045 | 0.05 |
| 2537 | 0.946132 | 0.301253 |
| 2538 | 1.40779 | 0.05 |
| 2539 | 1.06879 | 0.991726 |
| 2540 | 0.990713 | 0.0879203 |
| 2541 | 1.06879 | 0.131501 |
| 2542 | 1.06486 | 0.05 |
| 2543 | 1.18347 | 0.05 |
| 2544 | 1.38622 | 0.05 |
| 2545 | 1.18347 | 0.05 |
| 2546 | 1.7304 | 0.05 |
| 2547 | 1.7304 | 0.05 |
| 2548 | 0.946131 | 0.159856 |
| 2549 | 1.26874 | 0.05 |

| | | |
|---|---|---|
| 2550 | 1.13337 | 0.74043 |
| 2551 | 1.13337 | 0.0894586 |
| 2552 | 1.40779 | 0.0799149 |
| 2553 | 1.695 2.1 | |
| 2554 | 1.77071 | 2.1 |
| 2555 | 0.649313 | 2.1 |
| 2556 | 0.692829 | 2.1 |
| 2557 | 1.40395 | 2.1 |
| 2558 | 1.34243 | 2.1 |
| 2559 | 0.813292 | 0.05 |
| 2560 | 0.632691 | 0.0649869 |
| 2561 | 1.75766 | 0.471807 |
| 2562 | 2.25727 | 0.786448 |
| 2563 | 1.74206 | 0.05 |
| 2564 | 0.826073 | 0.180997 |
| 2565 | 1.30022 | 0.218426 |
| 2566 | 1.45641 | 0.136723 |
| 2567 | 0.946131 | 0.0990727 |
| 2568 | 0.752245 | 0.183297 |
| 2569 | 0.960952 | 0.191722 |
| 2570 | 0.743477 | 0.0579459 |
| 2571 | 1.04355 | 0.0736559 |
| 2572 | 1.6253 | 0.05 |
| 2573 | 0.960577 | 0.05 |
| 2574 | 1.34852 | 0.949411 |
| 2575 | 1.03235 | 1.00166 |
| 2576 | 1.78168 | 0.611857 |
| 2577 | 1.2966 | 0.05 |
| 2578 | 2.21116 | 0.05 |
| 2579 | 1.6395 | 0.05 |
| 2580 | 1.6395 | 0.05 |
| 2581 | 0.94613 | 0.05 |
| 2582 | 1.22187 | 0.05 |
| 2583 | 0.962737 | 0.05 |
| 2584 | 0.962735 | 0.05 |
| 2585 | 1.36376 | 0.05 |
| 2586 | 1.2966 | 0.05 |
| 2587 | 1.2966 | 0.05 |
| 2588 | 1.19669 | 1.09612 |
| 2589 | 2.37636 | 1.16233 |
| 2590 | 1.08894 | 1.12321 |
| 2591 | 1.62551 | 1.50088 |
| 2592 | 2.51472 | 1.28364 |
| 2593 | 1.89226 | 1.04378 |
| 2594 | 0.980038 | 1.15194 |
| 2595 | 1.10187 | 0.825814 |
| 2596 | 1.01479 | 1.72464 |
| 2597 | 1.81663 | 2.1 |
| 2598 | 1.83247 | 2.1 |
| 2599 | 0.921851 | 0.190874 |
| 2600 | 0.983148 | 2.1 |
| 2601 | 1.82816 | 2.1 |
| 2602 | 1.26103 | 2.1 |
| 2603 | 2.34818 | 2.1 |
| 2604 | 1.23152 | 2.1 |
| 2605 | 2.15738 | 2.1 |
| 2606 | 1.23152 | 2.1 |
| 2607 | 1.51042 | 2.1 |

| | | |
|---|---|---|
| 2608 | 0.842995 | 2.1 |
| 2609 | 1.22257 | 2.1 |
| 2610 | 0.810959 | 2.1 |
| 2611 | 1.68242 | 2.1 |
| 2612 | 0.95701 | 2.1 |
| 2613 | 1.12389 | 2.1 |
| 2614 | 1.95255 | 2.1 |
| 2615 | 1.57974 | 2.1 |
| 2616 | 1.94632 | 2.1 |
| 2617 | 0.81534 | 2.1 |
| 2618 | 1.13996 | 2.1 |
| 2619 | 1.18958 | 0.05 |
| 2620 | 1.25929 | 0.05 |
| 2621 | 3.02933 | 0.608135 |
| 2622 | 2.37434 | 0.05 |
| 2623 | 1.10609 | 0.162028 |
| 2624 | 1.64132 | 0.224351 |
| 2625 | 1.64132 | 0.380617 |
| 2626 | 0.946132 | 0.348463 |
| 2627 | 1.40029 | 0.10713 |
| 2628 | 1.08946 | 0.33253 |
| 2629 | 1.08946 | 0.456349 |
| 2630 | 1.18717 | 0.05 |
| 2631 | 2.71421 | 0.05 |
| 2632 | 1.08189 | 0.220065 |
| 2633 | 0.94613 | 0.217126 |
| 2634 | 1.37109 | 0.20838 |
| 2635 | 1.17925 | 0.05 |
| 2636 | 0.851541 | 0.231419 |
| 2637 | 0.942512 | 0.168531 |
| 2638 | 1.18541 | 0.12884 |
| 2639 | 0.851526 | 0.262042 |
| 2640 | 1.0819 | 0.09299 |
| 2641 | 1.86009 | 0.239775 |
| 2642 | 0.946132 | 0.0755399 |
| 2643 | 1.67409 | 0.05 |
| 2644 | 1.67409 | 0.0685883 |
| 2645 | 0.946131 | 0.0995459 |
| 2646 | 1.31716 | 0.0562757 |
| 2647 | 1.30307 | 0.05 |
| 2648 | 0.946131 | 0.114407 |
| 2649 | 1.18601 | 0.05 |
| 2650 | 3.15646 | 2.1 |
| 2651 | 0.985109 | 2.1 |
| 2652 | 3.37048 | 2.1 |
| 2653 | 1.10667 | 2.1 |
| 2654 | 1.77065 | 1.1539 |
| 2655 | 1.45837 | 0.96765 |
| 2656 | 1.77063 | 1.05162 |
| 2657 | 2.58104 | 0.957967 |
| 2658 | 1.37652 | 2.1 |
| 2659 | 1.54168 | 2.1 |
| 2660 | 1.00224 | 0.05 |
| 2661 | 1.05851 | 0.05 |
| 2662 | 1.39093 | 0.05 |
| 2663 | 1.43358 | 0.05 |
| 2664 | 0.575741 | 0.05 |
| 2665 | 0.518161 | 0.05 |

| | | |
|---|---|---|
| 2666 | 1.04071 | 0.05 |
| 2667 | 1.13092 | 1.3099 |
| 2668 | 1.17042 | 2.1 |
| 2669 | 1.53347 | 1.92521 |
| 2670 | 1.52938 | 1.35933 |
| 2671 | 1.00613 | 0.240576 |
| 2672 | 1.81618 | 0.306058 |
| 2673 | 1.81618 | 0.234833 |
| 2674 | 0.946127 | 0.682659 |
| 2675 | 1.36479 | 0.441817 |
| 2676 | 0.94613 | 0.39844 |
| 2677 | 1.31724 | 0.308138 |
| 2678 | 1.3648 | 0.423545 |
| 2679 | 1.00613 | 0.2753 |
| 2680 | 0.781375 | 0.05 |
| 2681 | 1.13431 | 0.0574766 |
| 2682 | 1.03547 | 0.0856864 |
| 2683 | 1.40668 | 0.347663 |
| 2684 | 0.94613 | 0.329096 |
| 2685 | 0.713285 | 0.283172 |
| 2686 | 1.72885 | 0.444848 |
| 2687 | 1.72885 | 0.230864 |
| 2688 | 0.713298 | 0.269558 |
| 2689 | 3.30075 | 0.0796534 |
| 2690 | 2.81337 | 0.206122 |
| 2691 | 1.39296 | 0.345804 |
| 2692 | 0.830164 | 2.1 |
| 2693 | 1.50493 | 2.1 |
| 2694 | 1.50494 | 2.1 |
| 2695 | 0.830136 | 1.53175 |
| 2696 | 1.28298 | 2.1 |
| 2697 | 1.0521 | 2.1 |
| 2698 | 0.830147 | 2.1 |
| 2699 | 2.0799 | 2.1 |
| 2700 | 1.52115 | 1.40586 |
| 2701 | 2.5142 | 2.04751 |
| 2702 | 1.31253 | 0.864919 |
| 2703 | 1.94446 | 1.24502 |
| 2704 | 1.18817 | 1.23586 |
| 2705 | 0.598348 | 1.99504 |
| 2706 | 1.69315 | 1.32239 |
| 2707 | 1.69315 | 1.7333 |
| 2708 | 0.598377 | 0.882858 |
| 2709 | 0.598347 | 1.00245 |
| 2710 | 0.94613 | 0.882858 |
| 2711 | 1.28418 | 0.937473 |
| 2712 | 2.05205 | 1.06494 |
| 2713 | 1.28418 | 1.6423 |
| 2714 | 1.0737 | 2.1 |
| 2715 | 1.81024 | 2.1 |
| 2716 | 2.73834 | 2.1 |
| 2717 | 1.71369 | 2.1 |
| 2718 | 1.71368 | 2.1 |
| 2719 | 0.946118 | 2.1 |
| 2720 | 1.29063 | 2.1 |
| 2721 | 1.36917 | 2.1 |
| 2722 | 0.94613 | 2.1 |
| 2723 | 1.29062 | 2.1 |

| | | |
|---|---|---|
| 2724 | 0.947921 | 2.1 |
| 2725 | 1.58661 | 0.05 |
| 2726 | 1.37658 | 1.98849 |
| 2727 | 1.2364 | 1.94266 |
| 2728 | 1.66685 | 0.998068 |
| 2729 | 1.66685 | 2.1 |
| 2730 | 0.946133 | 0.921727 |
| 2731 | 0.946133 | 1.32857 |
| 2732 | 1.11978 | 1.74558 |
| 2733 | 1.09194 | 1.13412 |
| 2734 | 1.21858 | 2.1 |
| 2735 | 1.37658 | 1.70799 |
| 2736 | 1.64903 | 0.828223 |
| 2737 | 1.64903 | 2.1 |
| 2738 | 0.946132 | 0.919504 |
| 2739 | 0.946132 | 1.4544 |
| 2740 | 1.05496 | 1.1714 |
| 2741 | 1.22851 | 0.839299 |
| 2742 | 1.22852 | 2.1 |
| 2743 | 1.58482 | 0.779092 |
| 2744 | 1.60578 | 0.05 |
| 2745 | 1.06336 | 0.05 |
| 2746 | 1.68062 | 0.05 |
| 2747 | 1.14721 | 0.05 |
| 2748 | 1.11924 | 0.05 |
| 2749 | 1.18421 | 0.05 |
| 2750 | 2.01276 | 0.05 |
| 2751 | 1.23502 | 2.1 |
| 2752 | 1.34926 | 2.08014 |
| 2753 | 0.546751 | 1.43358 |
| 2754 | 0.790446 | 2.02626 |
| 2755 | 1.04072 | 1.4173 |
| 2756 | 0.555868 | 1.32684 |
| 2757 | 0.930305 | 1.31079 |
| 2758 | 1.09344 | 1.67705 |
| 2759 | 1.18351 | 1.80767 |
| 2760 | 1.97215 | 1.39537 |
| 2761 | 1.07416 | 2.0405 |
| 2762 | 1.33204 | 1.34175 |
| 2763 | 1.22038 | 1.23256 |
| 2764 | 0.597449 | 1.00082 |
| 2765 | 0.821422 | 1.45276 |
| 2766 | 1.04071 | 1.2027 |
| 2767 | 0.608026 | 0.882686 |
| 2768 | 0.929427 | 1.11662 |
| 2769 | 1.64929 | 0.05 |
| 2770 | 1.68541 | 0.05 |
| 2771 | 0.496093 | 0.05 |
| 2772 | 1.3385 | 0.05 |
| 2773 | 0.790798 | 0.05 |
| 2774 | 1.22971 | 0.05 |
| 2775 | 0.898853 | 0.05 |
| 2776 | 2.14361 | 0.05 |
| 2777 | 2.10813 | 0.05 |
| 2778 | 1.05417 | 0.05 |
| 2779 | 1.80768 | 0.05 |
| 2780 | 1.09725 | 2.1 |
| 2781 | 1.41131 | 2.1 |

| | | |
|---|---|---|
| 2782 | 1.1656 | 2.1 |
| 2783 | 0.946129 | 2.1 |
| 2784 | 1.68365 | 2.1 |
| 2785 | 1.68365 | 2.1 |
| 2786 | 1.1656 | 2.1 |
| 2787 | 0.94613 | 2.1 |
| 2788 | 1.21269 | 0.05 |
| 2789 | 1.48867 | 0.05 |
| 2790 | 1.27806 | 0.05 |
| 2791 | 1.68264 | 0.05 |
| 2792 | 1.37813 | 2.09845 |
| 2793 | 1.50743 | 1.31882 |
| 2794 | 1.04121 | 0.381279 |
| 2795 | 1.12434 | 0.0556237 |
| 2796 | 1.39553 | 0.05 |
| 2797 | 1.44721 | 1.33339 |
| 2798 | 0.946132 | 0.0582902 |
| 2799 | 1.23943 | 0.0881293 |
| 2800 | 1.05292 | 0.160594 |
| 2801 | 1.28607 | 0.0506473 |
| 2802 | 1.16915 | 0.208189 |
| 2803 | 1.76306 | 0.977024 |
| 2804 | 1.42266 | 1.08248 |
| 2805 | 1.36844 | 2.1 |
| 2806 | 1.36844 | 2.1 |
| 2807 | 1.67352 | 2.1 |
| 2808 | 1.11256 | 0.821482 |
| 2809 | 1.03217 | 0.504136 |
| 2810 | 1.18422 | 0.05 |
| 2811 | 1.04159 | 0.05 |
| 2812 | 1.60139 | 0.05 |
| 2813 | 1.04159 | 0.05 |
| 2814 | 0.988805 | 2.1 |
| 2815 | 1.05481 | 2.1 |
| 2816 | 1.52938 | 2.1 |
| 2817 | 1.94724 | 1.9729 |
| 2818 | 0.896336 | 0.05 |
| 2819 | 0.911484 | 0.05 |
| 2820 | 1.18315 | 0.05 |
| 2821 | 1.10515 | 0.05 |
| 2822 | 1.04067 | 0.05 |
| 2823 | 1.12758 | 0.05 |
| 2824 | 0.904106 | 0.05 |
| 2825 | 1.6901 | 0.05 |
| 2826 | 1.68671 | 0.05 |
| 2827 | 1.99908 | 0.05 |
| 2828 | 0.772426 | 0.324717 |
| 2829 | 0.810945 | 2.1 |
| 2830 | 0.667562 | 0.951166 |
| 2831 | 0.496617 | 0.05 |
| 2832 | 0.847689 | 0.569464 |
| 2833 | 0.914378 | 1.62887 |
| 2834 | 0.934003 | 0.158227 |
| 2835 | 1.07459 | 1.03066 |
| 2836 | 1.40225 | 2.1 |
| 2837 | 1.07043 | 2.1 |
| 2838 | 0.94613 | 2.1 |
| 2839 | 1.63072 | 2.1 |

| | | |
|---|---|---|
| 2840 | 1.63072 | 2.1 |
| 2841 | 1.07043 | 2.1 |
| 2842 | 0.946131 | 2.1 |
| 2843 | 1.53082 | 2.1 |
| 2844 | 1.69916 | 2.1 |
| 2845 | 1.75211 | 0.0768023 |
| 2846 | 1.96831 | 2.1 |
| 2847 | 1.75211 | 0.127816 |
| 2848 | 0.946127 | 0.3209 |
| 2849 | 0.817065 | 0.65043 |
| 2850 | 1.41798 | 0.34256 |
| 2851 | 1.41798 | 0.115632 |
| 2852 | 0.791656 | 0.585015 |
| 2853 | 0.791656 | 0.689033 |
| 2854 | 1.41798 | 0.251064 |
| 2855 | 1.58331 | 0.376196 |
| 2856 | 1.41776 | 0.0985104 |
| 2857 | 0.791647 | 0.168607 |
| 2858 | 1.4512 | 0.0777504 |
| 2859 | 1.45119 | 0.0782429 |
| 2860 | 0.75821 | 0.22757 |
| 2861 | 0.758248 | 0.194477 |
| 2862 | 0.946104 | 0.110051 |
| 2863 | 1.36482 | 0.10816 |
| 2864 | 1.29389 | 2.1 |
| 2865 | 1.04164 | 1.89901 |
| 2866 | 1.40574 | 1.95674 |
| 2867 | 1.64833 | 1.57949 |
| 2868 | 1.71183 | 0.05 |
| 2869 | 1.71183 | 0.05 |
| 2870 | 0.946131 | 0.104781 |
| 2871 | 1.35721 | 0.05 |
| 2872 | 0.946131 | 0.526436 |
| 2873 | 0.946131 | 0.157803 |
| 2874 | 1.30076 | 0.0625508 |
| 2875 | 1.2474 | 0.05 |
| 2876 | 2.85225 | 0.0506304 |
| 2877 | 2.71441 | 0.05 |
| 2878 | 1.2474 | 0.05 |
| 2879 | 1.38622 | 0.05 |
| 2880 | 0.858359 | 0.05 |
| 2881 | 1.3682 | 0.05 |
| 2882 | 1.57364 | 0.05 |
| 2883 | 0.729695 | 0.05 |
| 2884 | 0.729723 | 0.05 |
| 2885 | 0.822611 | 0.160249 |
| 2886 | 0.946133 | 0.05 |
| 2887 | 0.729723 | 0.05 |
| 2888 | 0.974704 | 0.559328 |
| 2889 | 0.974702 | 0.732783 |
| 2890 | 1.81603 | 2.1 |
| 2891 | 1.96348 | 2.1 |
| 2892 | 1.43612 | 0.939693 |
| 2893 | 1.43612 | 1.07365 |
| 2894 | 0.634295 | 0.984055 |
| 2895 | 0.93796 | 1.28224 |
| 2896 | 0.946133 | 1.11649 |
| 2897 | 0.634326 | 0.81247 |

| | | |
|---|---|---|
| 2898 | 0.946133 | 0.938055 |
| 2899 | 1.2654 | 0.700483 |
| 2900 | 1.26865 | 0.976237 |
| 2901 | 2.2649 | 0.868658 |
| 2902 | 1.00864 | 0.918944 |
| 2903 | 1.09689 | 0.706847 |
| 2904 | 0.832021 | 0.712736 |
| 2905 | 1.53229 | 0.630409 |
| 2906 | 1.1145 | 0.719883 |
| 2907 | 0.929583 | 0.686098 |
| 2908 | 1.613 | 0.534257 |
| 2909 | 0.644729 | 0.677788 |
| 2910 | 1.03471 | 1.09603 |
| 2911 | 2.7739 | 2.1 |
| 2912 | 1.36133 | 1.12775 |
| 2913 | 2.50215 | 2.1 |
| 2914 | 2.52195 | 2.1 |
| 2915 | 1.0465 | 2.1 |
| 2916 | 1.42673 | 2.1 |
| 2917 | 1.42673 | 2.1 |
| 2918 | 0.946132 | 2.1 |
| 2919 | 1.31904 | 2.1 |
| 2920 | 0.986499 | 2.1 |
| 2921 | 0.946132 | 2.1 |
| 2922 | 1.31904 | 2.1 |
| 2923 | 0.966978 | 2.1 |
| 2924 | 1.42237 | 0.05 |
| 2925 | 2.60925 | 0.05 |
| 2926 | 0.753659 | 0.05 |
| 2927 | 1.77241 | 0.05 |
| 2928 | 1.77241 | 0.05 |
| 2929 | 0.946129 | 0.05 |
| 2930 | 0.75366 | 0.05 |
| 2931 | 1.22144 | 0.05 |
| 2932 | 0.94613 | 0.05 |
| 2933 | 1.50732 | 0.05 |
| 2934 | 1.18514 | 0.05 |
| 2935 | 0.902187 | 0.05 |
| 2936 | 1.89858 | 0.05 |
| 2937 | 1.24294 | 0.05 |
| 2938 | 1.67211 | 0.05 |
| 2939 | 1.41786 | 0.05 |
| 2940 | 0.810959 | 0.05 |
| 2941 | 1.50732 | 0.05 |
| 2942 | 2.44288 | 0.05 |
| 2943 | 1.40969 | 0.05 |
| 2944 | 2.06654 | 0.05 |
| 2945 | 1.40969 | 0.05 |
| 2946 | 1.40882 | 0.05 |
| 2947 | 1.94063 | 0.05 |
| 2948 | 0.867921 | 0.05 |
| 2949 | 1.29289 | 0.05 |
| 2950 | 0.867918 | 0.05 |
| 2951 | 0.946128 | 0.05 |
| 2952 | 0.867944 | 0.05 |
| 2953 | 1.66589 | 0.05 |
| 2954 | 1.66589 | 0.05 |
| 2955 | 0.968595 | 2.1 |

| | | |
|---|---|---|
| 2956 | 1.5534 | 2.1 |
| 2957 | 1.27793 | 2.1 |
| 2958 | 1.13433 | 2.1 |
| 2959 | 1.3439 | 0.05 |
| 2960 | 2.53709 | 0.05 |
| 2961 | 0.571998 | 0.05 |
| 2962 | 0.64837 | 0.05 |
| 2963 | 1.99755 | 0.05 |
| 2964 | 1.99854 | 0.05 |
| 2965 | 0.764229 | 2.1 |
| 2966 | 1.61842 | 2.1 |
| 2967 | 1.61842 | 2.1 |
| 2968 | 1.18315 | 2.1 |
| 2969 | 1.1995 | 2.1 |
| 2970 | 1.18315 | 2.1 |
| 2971 | 0.764256 | 2.1 |
| 2972 | 1.52364 | 2.1 |
| 2973 | 2.36631 | 2.1 |
| 2974 | 0.641912 | 1.8874 |
| 2975 | 1.7181 | 2.1 |
| 2976 | 1.459 2.1 | |
| 2977 | 0.634845 | 1.55609 |
| 2978 | 1.18315 | 2.1 |
| 2979 | 1.17615 | 2.1 |
| 2980 | 0.571367 | 2.1 |
| 2981 | 1.3324 | 0.05 |
| 2982 | 0.798214 | 0.05 |
| 2983 | 0.946128 | 0.05 |
| 2984 | 0.627252 | 0.05 |
| 2985 | 1.50337 | 0.05 |
| 2986 | 1.50337 | 0.05 |
| 2987 | 0.62725 | 0.05 |
| 2988 | 1.07763 | 0.05 |
| 2989 | 1.07763 | 0.05 |
| 2990 | 2.16109 | 0.05 |
| 2991 | 1.07763 | 0.05 |
| 2992 | 1.12486 | 2.1 |
| 2993 | 1.11827 | 2.1 |
| 2994 | 1.0491 | 2.1 |
| 2995 | 1.09034 | 2.1 |
| 2996 | 1.09431 | 2.1 |
| 2997 | 0.946131 | 2.1 |
| 2998 | 0.748607 | 2.1 |
| 2999 | 1.44002 | 2.1 |
| 3000 | 1.44002 | 2.1 |
| 3001 | 0.748635 | 2.1 |
| 3002 | 0.782505 | 2.1 |
| 3003 | 1.40615 | 2.1 |
| 3004 | 1.19539 | 2.1 |
| 3005 | 1.29389 | 2.1 |
| 3006 | 1.4867 | 1.23738 |
| 3007 | 1.50103 | 0.05 |
| 3008 | 1.63025 | 0.05 |
| 3009 | 2.72109 | 0.05 |
| 3010 | 0.915755 | 0.05 |
| 3011 | 0.946132 | 0.05 |
| 3012 | 0.915755 | 0.05 |
| 3013 | 1.70236 | 0.05 |

| | | |
|---|---|---|
| 3014 | 1.70236 | 0.05 |
| 3015 | 1.26155 | 0.05 |
| 3016 | 1.35657 | 0.05 |
| 3017 | 1.83151 | 0.05 |
| 3018 | 1.83151 | 0.05 |
| 3019 | 2.52309 | 0.05 |
| 3020 | 0.983607 | 2.1 |
| 3021 | 1.06838 | 0.0554413 |
| 3022 | 0.983609 | 0.445485 |
| 3023 | 0.983596 | 0.212137 |
| 3024 | 2.74384 | 0.05 |
| 3025 | 1.6261 | 0.05 |
| 3026 | 1.15477 | 2.1 |
| 3027 | 1.02136 | 2.1 |
| 3028 | 1.83561 | 2.1 |
| 3029 | 1.83561 | 2.1 |
| 3030 | 0.946131 | 2.1 |
| 3031 | 1.39523 | 2.1 |
| 3032 | 1.29313 | 0.817959 |
| 3033 | 1.29313 | 2.1 |
| 3034 | 1.38651 | 2.1 |
| 3035 | 1.06313 | 2.1 |
| 3036 | 0.974888 | 2.1 |
| 3037 | 1.0894 | 2.1 |
| 3038 | 0.974888 | 2.1 |
| 3039 | 1.82282 | 2.1 |
| 3040 | 1.82282 | 2.1 |
| 3041 | 0.946131 | 2.1 |
| 3042 | 1.37372 | 2.1 |
| 3043 | 1.32374 | 2.1 |
| 3044 | 1.32374 | 2.1 |
| 3045 | 1.39523 | 2.1 |
| 3046 | 1.40882 | 0.05 |
| 3047 | 0.861998 | 0.05 |
| 3048 | 1.24448 | 0.05 |
| 3049 | 0.861999 | 0.05 |
| 3050 | 0.946129 | 0.05 |
| 3051 | 0.862011 | 0.05 |
| 3052 | 1.67537 | 0.05 |
| 3053 | 1.67537 | 0.05 |
| 3054 | 1.72399 | 0.05 |
| 3055 | 2.58579 | 0.05 |
| 3056 | 1.72399 | 0.05 |
| 3057 | 1.50951 | 0.05 |
| 3058 | 2.48897 | 0.05 |
| 3059 | 1.26755 | 2.1 |
| 3060 | 1.03824 | 2.1 |
| 3061 | 1.27875 | 2.1 |
| 3062 | 1.48901 | 2.1 |
| 3063 | 1.18902 | 2.1 |
| 3064 | 1.11764 | 2.1 |
| 3065 | 1.87436 | 2.1 |
| 3066 | 0.95345 | 2.1 |
| 3067 | 0.724191 | 1.52541 |
| 3068 | 0.86668 | 1.49677 |
| 3069 | 0.811523 | 1.73382 |
| 3070 | 1.04074 | 1.02977 |
| 3071 | 0.843097 | 1.32097 |

| | | |
|---|---|---|
| 3072 | 0.833434 | 1.62394 |
| 3073 | 0.965727 | 1.33402 |
| 3074 | 0.85346 | 1.59868 |
| 3075 | 1.00185 | 0.469363 |
| 3076 | 1.00184 | 1.36156 |
| 3077 | 0.980651 | 1.63514 |
| 3078 | 0.980664 | 1.33879 |
| 3079 | 0.475737 | 0.995552 |
| 3080 | 0.719696 | 1.12143 |
| 3081 | 0.946109 | 0.904822 |
| 3082 | 0.946116 | 1.17363 |
| 3083 | 0.719693 | 1.01673 |
| 3084 | 0.946117 | 1.17754 |
| 3085 | 1.14879 | 1.60994 |
| 3086 | 0.719683 | 1.51463 |
| 3087 | 0.900977 | 1.10088 |
| 3088 | 0.972364 | 1.24738 |
| 3089 | 1.22961 | 1.22728 |
| 3090 | 0.405384 | 1.10044 |
| 3091 | 1.34406 | 2.1 |
| 3092 | 1.2187 | 2.1 |
| 3093 | 0.963571 | 0.05 |
| 3094 | 0.946131 | 0.05 |
| 3095 | 1.57246 | 0.633379 |
| 3096 | 1.57246 | 2.1 |
| 3097 | 0.946131 | 0.74222 |
| 3098 | 1.1235 | 2.1 |
| 3099 | 1.1235 | 1.48537 |
| 3100 | 1.07439 | 1.5348 |
| 3101 | 1.19612 | 1.11085 |
| 3102 | 1.24844 | 1.11548 |
| 3103 | 1.00007 | 1.41714 |
| 3104 | 1.02429 | 2.1 |
| 3105 | 1.10459 | 2.1 |
| 3106 | 1.06724 | 0.05 |
| 3107 | 0.67519 | 0.152831 |
| 3108 | 0.49762 | 0.05 |
| 3109 | 1.43435 | 0.05 |
| 3110 | 0.833315 | 0.05 |
| 3111 | 0.58135 | 0.05 |
| 3112 | 1.14749 | 0.406921 |
| 3113 | 1.85091 | 1.07483 |
| 3114 | 1.2802 | 0.41108 |
| 3115 | 1.27737 | 0.0701414 |
| 3116 | 2.3655 | 0.19768 |
| 3117 | 1.30584 | 2.1 |
| 3118 | 2.06424 | 2.1 |
| 3119 | 0.946131 | 0.0540113 |
| 3120 | 1.04257 | 0.05 |
| 3121 | 0.94613 | 0.05 |
| 3122 | 1.67201 | 0.05 |
| 3123 | 1.67201 | 0.05 |
| 3124 | 1.27696 | 0.05 |
| 3125 | 1.34118 | 0.05 |
| 3126 | 1.3413 | 0.05 |
| 3127 | 2.36238 | 0.05 |
| 3128 | 0.6036 | 0.05 |
| 3129 | 1.11745 | 0.05 |

| | | |
|---|---|---|
| 3130 | 1.46126 | 0.05 |
| 3131 | 1.39612 | 0.05 |
| 3132 | 0.670678 | 0.05 |
| 3133 | 1.03402 | 0.05 |
| 3134 | 1.11271 | 0.05 |
| 3135 | 0.838028 | 0.05 |
| 3136 | 0.807019 | 0.05 |
| 3137 | 1.27787 | 0.05 |
| 3138 | 0.994705 | 0.05 |
| 3139 | 1.20014 | 0.05 |
| 3140 | 0.964793 | 0.05 |
| 3141 | 0.863868 | 0.05 |
| 3142 | 1.3413 | 0.05 |
| 3143 | 1.79549 | 0.05 |
| 3144 | 1.35877 | 2.1 |
| 3145 | 1.63648 | 2.1 |
| 3146 | 1.08171 | 2.1 |
| 3147 | 1.27839 | 2.1 |
| 3148 | 1.52601 | 2.1 |
| 3149 | 1.27841 | 2.1 |
| 3150 | 1.71255 | 2.1 |
| 3151 | 2.16307 | 2.1 |
| 3152 | 1.91592 | 2.1 |
| 3153 | 1.78508 | 2.1 |
| 3154 | 0.946128 | 2.1 |
| 3155 | 1.41199 | 2.1 |
| 3156 | 0.946128 | 2.1 |
| 3157 | 0.901088 | 2.1 |
| 3158 | 1.55244 | 2.1 |
| 3159 | 1.59475 | 2.1 |
| 3160 | 1.40921 | 2.1 |
| 3161 | 0.960106 | 2.1 |
| 3162 | 1.40921 | 2.1 |
| 3163 | 2.0073 | 2.1 |
| 3164 | 2.2973 | 0.05 |
| 3165 | 1.90705 | 0.05 |
| 3166 | 1.53671 | 0.05 |
| 3167 | 1.86035 | 0.05 |
| 3168 | 1.33747 | 2.1 |
| 3169 | 1.14007 | 2.1 |
| 3170 | 1.3212 | 2.1 |
| 3171 | 1.66867 | 0.05 |
| 3172 | 1.30203 | 0.05 |
| 3173 | 1.27696 | 2.1 |
| 3174 | 1.05025 | 2.1 |
| 3175 | 1.05025 | 2.1 |
| 3176 | 1.3704 | 2.1 |
| 3177 | 1.15677 | 2.1 |
| 3178 | 0.946129 | 2.1 |
| 3179 | 1.65879 | 2.1 |
| 3180 | 1.65879 | 2.1 |
| 3181 | 1.15676 | 2.1 |
| 3182 | 0.94613 | 2.1 |
| 3183 | 0.867217 | 1.32263 |
| 3184 | 1.28684 | 1.06973 |
| 3185 | 1.58866 | 1.19122 |
| 3186 | 1.58866 | 0.852316 |
| 3187 | 0.867246 | 2.02658 |

| | | |
|---|---|---|
| 3188 | 1.28684 | 0.941474 |
| 3189 | 1.16906 | 1.42236 |
| 3190 | 1.13438 | 1.18725 |
| 3191 | 1.37224 | 1.31249 |
| 3192 | 1.61519 | 1.37536 |
| 3193 | 0.883939 | 2.1 |
| 3194 | 1.40092 | 1.17526 |
| 3195 | 1.80383 | 2.07096 |
| 3196 | 1.80383 | 1.80801 |
| 3197 | 0.883935 | 1.9528 |
| 3198 | 1.40092 | 1.08491 |
| 3199 | 1.28684 | 1.93672 |
| 3200 | 1.76788 | 0.914445 |
| 3201 | 2.57368 | 1.1513 |
| 3202 | 1.37224 | 2.1 |
| 3203 | 1.65411 | 0.104286 |
| 3204 | 1.77042 | 2.1 |
| 3205 | 1.77042 | 2.1 |
| 3206 | 1.90446 | 2.1 |
| 3207 | 1.28667 | 2.1 |
| 3208 | 1.55635 | 2.1 |
| 3209 | 1.30646 | 1.06965 |
| 3210 | 2.31836 | 1.5597 |
| 3211 | 1.30645 | 0.948961 |
| 3212 | 1.86429 | 1.17364 |
| 3213 | 1.48676 | 0.05 |
| 3214 | 0.743379 | 0.157449 |
| 3215 | 1.41848 | 0.219379 |
| 3216 | 1.41848 | 0.0858309 |
| 3217 | 0.946131 | 0.152739 |
| 3218 | 0.812649 | 0.126091 |
| 3219 | 1.34921 | 0.137932 |
| 3220 | 0.812649 | 0.0767189 |
| 3221 | 0.949366 | 0.05 |
| 3222 | 1.32974 | 0.05 |
| 3223 | 0.794204 | 0.05 |
| 3224 | 0.634352 | 2.1 |
| 3225 | 0.838025 | 2.1 |
| 3226 | 1.08802 | 2.1 |
| 3227 | 0.838038 | 2.1 |
| 3228 | 1.18314 | 2.1 |
| 3229 | 1.12495 | 2.1 |
| 3230 | 1.22774 | 2.1 |
| 3231 | 1.04343 | 2.1 |
| 3232 | 0.921111 | 0.05 |
| 3233 | 2.1609 | 0.05 |
| 3234 | 1.09987 | 0.05 |
| 3235 | 2.5043 | 0.05 |
| 3236 | 1.05492 | 0.05 |
| 3237 | 0.590344 | 0.05 |
| 3238 | 1.05492 | 0.05 |
| 3239 | 1.25215 | 0.05 |
| 3240 | 1.71674 | 0.05 |
| 3241 | 1.71674 | 0.05 |
| 3242 | 0.590331 | 0.05 |
| 3243 | 1.17078 | 0.05 |
| 3244 | 0.625542 | 0.05 |
| 3245 | 0.724673 | 2.1 |

| | | |
|---|---|---|
| 3246 | 1.08011 | 2.1 |
| 3247 | 1.87598 | 2.1 |
| 3248 | 1.50738 | 2.1 |
| 3249 | 1.44951 | 0.05 |
| 3250 | 1.50102 | 0.05 |
| 3251 | 1.8042 | 0.112952 |
| 3252 | 0.687769 | 0.363453 |
| 3253 | 1.66483 | 0.235194 |
| 3254 | 1.66483 | 0.304393 |
| 3255 | 0.946132 | 0.493853 |
| 3256 | 0.687798 | 0.461843 |
| 3257 | 1.20885 | 0.647192 |
| 3258 | 0.687799 | 0.55065 |
| 3259 | 0.886063 | 0.175305 |
| 3260 | 0.798751 | 0.157915 |
| 3261 | 0.584813 | 0.186058 |
| 3262 | 1.49483 | 0.05 |
| 3263 | 0.888005 | 0.0657689 |
| 3264 | 1.03589 | 0.0507194 |
| 3265 | 1.70435 | 0.12184 |
| 3266 | 0.710414 | 2.1 |
| 3267 | 0.917609 | 1.10586 |
| 3268 | 0.810947 | 1.31191 |
| 3269 | 0.81096 | 1.30286 |
| 3270 | 1.48658 | 1.31621 |
| 3271 | 1.53653 | 1.35306 |
| 3272 | 1.19329 | 1.72149 |
| 3273 | 0.946153 | 1.39075 |
| 3274 | 1.50798 | 1.37891 |
| 3275 | 1.85017 | 2.1 |
| 3276 | 1.19768 | 2.1 |
| 3277 | 0.864304 | 2.1 |
| 3278 | 0.792906 | 1.42441 |
| 3279 | 0.989025 | 1.25295 |
| 3280 | 1.19768 | 1.72944 |
| 3281 | 1.54548 | 1.47375 |
| 3282 | 1.18798 | 1.96874 |
| 3283 | 1.31875 | 1.1529 |
| 3284 | 1.05725 | 1.30904 |
| 3285 | 2.17365 | 0.05 |
| 3286 | 2.48106 | 0.05 |
| 3287 | 1.87451 | 0.05 |
| 3288 | 1.27189 | 1.15181 |
| 3289 | 2.79399 | 1.84998 |
| 3290 | 0.859565 | 1.26615 |
| 3291 | 0.926174 | 1.12262 |
| 3292 | 0.604744 | 1.06915 |
| 3293 | 1.41832 | 0.232382 |
| 3294 | 1.05494 | 0.939428 |
| 3295 | 0.874749 | 0.906585 |
| 3296 | 1.50067 | 0.692666 |
| 3297 | 0.917817 | 1.34295 |
| 3298 | 0.63591 | 1.34929 |
| 3299 | 1.72849 | 1.21508 |
| 3300 | 1.72849 | 0.35682 |
| 3301 | 0.635943 | 0.638019 |
| 3302 | 1.39699 | 1.32644 |
| 3303 | 0.967411 | 1.88024 |

| | | |
|---|---|---|
| 3304 | 0.635943 | 1.20164 |
| 3305 | 1.39908 | 1.08705 |
| 3306 | 1.26971 | 1.21819 |
| 3307 | 1.02969 | 1.09008 |
| 3308 | 1.15326 | 2.1 |
| 3309 | 2.30741 | 1.76942 |
| 3310 | 0.93545 | 1.17801 |
| 3311 | 0.802747 | 0.871909 |
| 3312 | 1.15466 | 1.37645 |
| 3313 | 0.859164 | 1.59922 |
| 3314 | 1.02616 | 1.84958 |
| 3315 | 1.23149 | 1.54939 |
| 3316 | 0.83804 | 0.944322 |
| 3317 | 1.13266 | 2.1 |
| 3318 | 1.64142 | 2.1 |
| 3319 | 1.21223 | 2.1 |
| 3320 | 1.43051 | 2.1 |
| 3321 | 1.32418 | 0.991245 |
| 3322 | 0.621319 | 0.878215 |
| 3323 | 1.47134 | 2.1 |
| 3324 | 1.79103 | 0.362785 |
| 3325 | 3.25951 | 0.05 |
| 3326 | 3.16764 | 0.05 |
| 3327 | 1.65583 | 0.05 |
| 3328 | 1.4732 | 0.05 |
| 3329 | 1.76476 | 2.1 |
| 3330 | 1.80114 | 1.06577 |
| 3331 | 0.754121 | 2.1 |
| 3332 | 0.94613 | 0.220391 |
| 3333 | 0.754119 | 2.1 |
| 3334 | 1.45636 | 2.1 |
| 3335 | 1.45636 | 0.181028 |
| 3336 | 1.30029 | 1.03696 |
| 3337 | 0.910183 | 0.634233 |
| 3338 | 1.29099 | 2.04967 |
| 3339 | 1.08039 | 2.1 |
| 3340 | 1.05498 | 2.1 |
| 3341 | 1.24445 | 2.1 |
| 3342 | 1.2873 | 2.1 |
| 3343 | 0.987703 | 2.1 |
| 3344 | 0.964782 | 2.05114 |
| 3345 | 1.73645 | 0.05 |
| 3346 | 0.871279 | 0.05 |
| 3347 | 0.884744 | 0.05 |
| 3348 | 1.85193 | 0.05 |
| 3349 | 1.68681 | 0.05 |
| 3350 | 1.33689 | 0.05 |
| 3351 | 1.23493 | 0.05 |
| 3352 | 0.892482 | 0.05 |
| 3353 | 2.95185 | 0.05 |
| 3354 | 2.10496 | 0.05 |
| 3355 | 1.87334 | 2.1 |
| 3356 | 0.94411 | 2.1 |
| 3357 | 0.754196 | 2.1 |
| 3358 | 1.43122 | 2.1 |
| 3359 | 1.58684 | 2.1 |
| 3360 | 0.838027 | 2.1 |
| 3361 | 1.1536 | 2.1 |

| | | |
|---|---|---|
| 3362 | 1.24878 | 2.1 |
| 3363 | 1.23125 | 2.1 |
| 3364 | 2.86862 | 2.1 |
| 3365 | 1.57636 | 2.1 |
| 3366 | 0.998451 | 0.515575 |
| 3367 | 1.5176 | 0.344247 |
| 3368 | 1.13271 | 0.05 |
| 3369 | 1.60248 | 0.05 |
| 3370 | 1.22083 | 2.1 |
| 3371 | 1.79996 | 2.1 |
| 3372 | 1.19087 | 2.1 |
| 3373 | 2.00342 | 2.1 |
| 3374 | 1.3715 | 2.1 |
| 3375 | 2.79227 | 2.1 |
| 3376 | 2.7873 | 2.1 |
| 3377 | 0.987265 | 0.05 |
| 3378 | 1.62715 | 2.1 |
| 3379 | 1.70117 | 2.1 |
| 3380 | 1.27806 | 0.05 |
| 3381 | 1.45246 | 2.1 |
| 3382 | 1.45244 | 2.1 |
| 3383 | 1.87214 | 2.1 |
| 3384 | 0.995796 | 2.1 |
| 3385 | 1.68189 | 0.05 |
| 3386 | 1.49155 | 2.1 |
| 3387 | 1.58054 | 2.1 |
| 3388 | 1.59169 | 2.1 |
| 3389 | 0.736364 | 0.05 |
| 3390 | 0.70236 | 0.05 |
| 3391 | 2.24367 | 0.05 |
| 3392 | 2.57085 | 0.05 |
| 3393 | 1.70952 | 0.05 |
| 3394 | 2.60516 | 0.05 |
| 3395 | 1.45158 | 2.1 |
| 3396 | 1.00371 | 2.1 |
| 3397 | 1.65748 | 2.1 |
| 3398 | 1.23706 | 0.43515 |
| 3399 | 1.55834 | 0.758796 |
| 3400 | 1.72671 | 0.686708 |
| 3401 | 0.597656 | 0.580795 |
| 3402 | 1.39137 | 0.679951 |
| 3403 | 0.855578 | 0.350827 |
| 3404 | 1.48398 | 0.572629 |
| 3405 | 0.745117 | 0.498812 |
| 3406 | 1.23706 | 1.14324 |
| 3407 | 1.57916 | 0.52498 |
| 3408 | 0.893025 | 0.444711 |
| 3409 | 3.22433 | 0.534126 |
| 3410 | 2.47324 | 1.46795 |
| 3411 | 1.63852 | 2.1 |
| 3412 | 1.63852 | 2.1 |
| 3413 | 0.946132 | 2.1 |
| 3414 | 1.19095 | 2.1 |
| 3415 | 0.946133 | 2.1 |
| 3416 | 1.16684 | 2.1 |
| 3417 | 1.19095 | 2.1 |
| 3418 | 1.06698 | 0.05 |
| 3419 | 1.48692 | 0.0579807 |

| | | |
|---|---|---|
| 3420 | 1.08334 | 0.774253 |
| 3421 | 1.07283 | 2.1 |
| 3422 | 1.28517 | 0.190719 |
| 3423 | 1.2746 | 0.05 |
| 3424 | 1.30723 | 0.05 |
| 3425 | 2.02069 | 0.05 |
| 3426 | 1.05579 | 0.05 |
| 3427 | 1.59475 | 2.1 |
| 3428 | 2.40113 | 2.1 |
| 3429 | 1.58875 | 2.1 |
| 3430 | 1.6881 | 2.1 |
| 3431 | 1.6881 | 2.1 |
| 3432 | 0.819953 | 2.1 |
| 3433 | 0.819954 | 2.1 |
| 3434 | 0.946131 | 2.1 |
| 3435 | 0.946131 | 2.1 |
| 3436 | 1.34782 | 2.1 |
| 3437 | 1.65619 | 2.1 |
| 3438 | 1.65619 | 2.1 |
| 3439 | 0.79437 | 2.1 |
| 3440 | 1.25 | 2.1 |
| 3441 | 0.94613 | 2.1 |
| 3442 | 0.946131 | 2.1 |
| 3443 | 0.794371 | 2.1 |
| 3444 | 1.58875 | 2.1 |
| 3445 | 2.5 | 2.1 |
| 3446 | 1.04147 | 2.1 |
| 3447 | 0.946131 | 0.812514 |
| 3448 | 1.79686 | 2.1 |
| 3449 | 1.79686 | 2.1 |
| 3450 | 1.25144 | 0.828553 |
| 3451 | 1.35714 | 1.84808 |
| 3452 | 1.38585 | 1.82683 |
| 3453 | 1.25144 | 2.1 |
| 3454 | 1.15564 | 2.1 |
| 3455 | 1.34389 | 2.1 |
| 3456 | 0.946133 | 1.16292 |
| 3457 | 1.74353 | 2.1 |
| 3458 | 1.74353 | 2.1 |
| 3459 | 1.14553 | 1.08848 |
| 3460 | 1.38585 | 1.0426 |
| 3461 | 1.30382 | 1.28075 |
| 3462 | 1.14553 | 2.1 |
| 3463 | 1.15564 | 2.1 |
| 3464 | 1.90618 | 2.1 |
| 3465 | 2.11056 | 2.1 |
| 3466 | 1.032 | 2.1 |
| 3467 | 0.953636 | 2.1 |
| 3468 | 1.23131 | 1.29734 |
| 3469 | 1.39763 | 1.45668 |
| 3470 | 2.01024 | 1.05246 |
| 3471 | 1.9186 | 1.07076 |
| 3472 | 1.83658 | 0.05 |
| 3473 | 1.81656 | 0.05 |
| 3474 | 2.2049 | 0.05 |
| 3475 | 2.16081 | 0.05 |
| 3476 | 1.12219 | 0.05 |
| 3477 | 0.599771 | 0.05 |

| | | |
|---|---|---|
| 3478 | 1.61401 | 0.05 |
| 3479 | 1.05912 | 0.05 |
| 3480 | 0.951648 | 0.05 |
| 3481 | 1.89328 | 0.05 |
| 3482 | 0.913682 | 0.05 |
| 3483 | 1.00763 | 0.05 |
| 3484 | 1.29129 | 2.1 |
| 3485 | 1.37138 | 2.1 |
| 3486 | 1.41986 | 0.05 |
| 3487 | 1.41986 | 0.05 |
| 3488 | 0.944166 | 0.05 |
| 3489 | 1.41789 | 0.05 |
| 3490 | 0.944167 | 0.05 |
| 3491 | 0.944166 | 0.05 |
| 3492 | 0.946132 | 0.05 |
| 3493 | 1.98713 | 0.05 |
| 3494 | 1.23626 | 0.05 |
| 3495 | 0.932791 | 0.05 |
| 3496 | 0.810251 | 0.05 |
| 3497 | 0.986748 | 0.05 |
| 3498 | 0.898155 | 0.05 |
| 3499 | 0.92624 | 0.05 |
| 3500 | 1.1349 | 0.05 |
| 3501 | 0.934572 | 0.05 |
| 3502 | 1.23104 | 0.165637 |
| 3503 | 1.23104 | 0.247701 |
| 3504 | 0.799994 | 0.05 |
| 3505 | 0.94613 | 0.0871044 |
| 3506 | 0.802822 | 0.05 |
| 3507 | 1.40944 | 0.501273 |
| 3508 | 0.800035 | 0.05 |
| 3509 | 1.41223 | 0.0613824 |
| 3510 | 1.41223 | 0.0842985 |
| 3511 | 1.6 | 0.05 |
| 3512 | 1.60565 | 0.05 |
| 3513 | 1.23042 | 2.1 |
| 3514 | 1.0645 | 2.1 |
| 3515 | 1.00319 | 2.1 |
| 3516 | 1.29435 | 2.1 |
| 3517 | 1.2243 | 2.1 |
| 3518 | 1.2243 | 2.1 |
| 3519 | 1.05655 | 1.28834 |
| 3520 | 1.29764 | 1.61378 |
| 3521 | 2.55252 | 2.1 |
| 3522 | 1.15676 | 2.1 |
| 3523 | 0.855295 | 2.1 |
| 3524 | 0.810959 | 2.1 |
| 3525 | 1.65857 | 2.1 |
| 3526 | 1.00849 | 2.1 |
| 3527 | 1.16021 | 2.1 |
| 3528 | 1.75869 | 2.1 |
| 3529 | 1.13775 | 2.1 |
| 3530 | 1.20091 | 2.1 |
| 3531 | 1.61582 | 2.1 |
| 3532 | 1.88031 | 2.1 |
| 3533 | 0.968536 | 2.1 |
| 3534 | 0.946131 | 2.1 |
| 3535 | 1.61481 | 2.1 |

| | | |
|---|---|---|
| 3536 | 1.61481 | 2.1 |
| 3537 | 1.09547 | 2.1 |
| 3538 | 1.18486 | 2.1 |
| 3539 | 1.37609 | 2.1 |
| 3540 | 1.09546 | 2.1 |
| 3541 | 1.02461 | 2.1 |
| 3542 | 1.83223 | 2.1 |
| 3543 | 1.74002 | 2.1 |
| 3544 | 1.70939 | 2.1 |
| 3545 | 1.7094 | 2.1 |
| 3546 | 0.726287 | 2.1 |
| 3547 | 1.01712 | 1.97334 |
| 3548 | 1.30318 | 2.1 |
| 3549 | 0.946131 | 2.1 |
| 3550 | 0.87155 | 2.1 |
| 3551 | 1.77093 | 1.76952 |
| 3552 | 2.02356 | 1.83935 |
| 3553 | 1.32449 | 2.1 |
| 3554 | 1.10003 | 2.1 |
| 3555 | 1.5161 | 2.1 |
| 3556 | 1.5161 | 2.1 |
| 3557 | 0.946132 | 2.1 |
| 3558 | 1.09206 | 2.1 |
| 3559 | 0.946132 | 2.1 |
| 3560 | 1.04404 | 2.1 |
| 3561 | 1.09206 | 2.1 |
| 3562 | 0.635628 | 1.07977 |
| 3563 | 1.14623 | 0.24054 |
| 3564 | 1.73197 | 0.675025 |
| 3565 | 1.73197 | 0.973213 |
| 3566 | 0.529655 | 0.179914 |
| 3567 | 1.14623 | 0.675519 |
| 3568 | 0.992085 | 0.812788 |
| 3569 | 1.16915 | 1.20359 |
| 3570 | 2.29245 | 0.05 |
| 3571 | 0.584573 | 0.889779 |
| 3572 | 0.956756 | 0.329661 |
| 3573 | 1.52471 | 0.517594 |
| 3574 | 1.51409 | 1.15924 |
| 3575 | 0.584575 | 0.390459 |
| 3576 | 0.94613 | 0.790052 |
| 3577 | 1.14622 | 0.198859 |
| 3578 | 1.28096 | 1.17804 |
| 3579 | 1.34695 | 1.99773 |
| 3580 | 1.04193 | 2.1 |
| 3581 | 1.05378 | 2.1 |
| 3582 | 0.798209 | 2.1 |
| 3583 | 1.32007 | 2.1 |
| 3584 | 1.64327 | 0.05 |
| 3585 | 2.64674 | 2.1 |
| 3586 | 2.38936 | 0.05 |
| 3587 | 2.88475 | 0.05 |
| 3588 | 1.07745 | 2.1 |
| 3589 | 1.08911 | 2.1 |
| 3590 | 1.49127 | 2.1 |
| 3591 | 1.49127 | 2.1 |
| 3592 | 0.709714 | 2.1 |
| 3593 | 1.37199 | 2.1 |

| | | |
|---|---|---|
| 3594 | 0.82899 | 2.1 |
| 3595 | 0.709714 | 2.1 |
| 3596 | 0.946132 | 2.1 |
| 3597 | 1.00133 | 2.1 |
| 3598 | 1.40182 | 2.1 |
| 3599 | 1.53278 | 2.1 |
| 3600 | 1.40182 | 2.1 |
| 3601 | 1.31764 | 0.05 |
| 3602 | 1.00691 | 0.05 |
| 3603 | 1.24094 | 0.05 |
| 3604 | 0.64868 | 0.05 |
| 3605 | 0.946133 | 0.05 |
| 3606 | 0.64868 | 0.05 |
| 3607 | 1.71762 | 0.05 |
| 3608 | 1.71762 | 0.05 |
| 3609 | 1.41662 | 0.05 |
| 3610 | 0.975477 | 0.05 |
| 3611 | 1.73584 | 0.05 |
| 3612 | 2.48188 | 0.05 |
| 3613 | 2.2045 | 0.245371 |
| 3614 | 2.08134 | 0.21507 |
| 3615 | 0.993366 | 0.05 |
| 3616 | 0.993368 | 0.758052 |
| 3617 | 1.14187 | 0.656002 |
| 3618 | 0.946132 | 0.575032 |
| 3619 | 0.946133 | 1.05594 |
| 3620 | 1.68951 | 0.753605 |
| 3621 | 1.68951 | 1.87406 |
| 3622 | 1.34612 | 0.652874 |
| 3623 | 1.28953 | 0.60101 |
| 3624 | 1.34611 | 1.11618 |
| 3625 | 1.31892 | 0.05 |
| 3626 | 0.946131 | 0.678008 |
| 3627 | 1.80988 | 0.0722428 |
| 3628 | 1.80988 | 2.1 |
| 3629 | 1.31892 | 0.794736 |
| 3630 | 0.946131 | 0.706763 |
| 3631 | 2.13214 | 2.1 |
| 3632 | 1.94691 | 2.1 |
| 3633 | 1.52661 | 0.05 |
| 3634 | 1.53175 | 0.05 |
| 3635 | 0.956005 | 0.05 |
| 3636 | 0.955991 | 0.05 |
| 3637 | 1.40572 | 0.05 |
| 3638 | 0.955992 | 0.05 |
| 3639 | 1.51084 | 0.838779 |
| 3640 | 1.67115 | 1.9642 |
| 3641 | 1.73143 | 2.1 |
| 3642 | 1.55234 | 2.1 |
| 3643 | 1.69377 | 2.1 |
| 3644 | 2.51483 | 2.1 |
| 3645 | 1.25987 | 2.1 |
| 3646 | 1.4472 | 2.1 |
| 3647 | 1.83151 | 0.366317 |
| 3648 | 2.01001 | 0.959605 |
| 3649 | 0.993998 | 0.127571 |
| 3650 | 1.83151 | 0.183822 |
| 3651 | 1.40915 | 0.05 |

| | | |
|---|---|---|
| 3652 | 1.24653 | 0.176861 |
| 3653 | 1.74472 | 2.1 |
| 3654 | 1.74472 | 2.1 |
| 3655 | 1.74244 | 2.1 |
| 3656 | 1.04788 | 2.1 |
| 3657 | 1.28522 | 2.1 |
| 3658 | 1.03785 | 2.1 |
| 3659 | 0.896519 | 2.1 |
| 3660 | 1.60428 | 2.1 |
| 3661 | 1.73305 | 2.1 |
| 3662 | 1.39072 | 0.05 |
| 3663 | 2.27501 | 0.05 |
| 3664 | 1.39073 | 0.05 |
| 3665 | 1.39073 | 0.05 |
| 3666 | 1.06838 | 2.1 |
| 3667 | 0.968462 | 0.710321 |
| 3668 | 1.06837 | 1.38651 |
| 3669 | 1.13455 | 1.29436 |
| 3670 | 1.23527 | 0.661801 |
| 3671 | 2.71686 | 0.0954019 |
| 3672 | 1.86881 | 0.0701983 |
| 3673 | 1.10586 | 0.302604 |
| 3674 | 1.03852 | 1.71117 |
| 3675 | 1.03899 | 2.1 |
| 3676 | 0.963973 | 2.1 |
| 3677 | 1.00319 | 2.1 |
| 3678 | 1.00319 | 2.1 |
| 3679 | 0.987132 | 2.1 |
| 3680 | 0.974889 | 2.1 |
| 3681 | 0.987131 | 2.1 |
| 3682 | 1.7677 | 2.1 |
| 3683 | 1.7677 | 2.1 |
| 3684 | 0.946131 | 2.1 |
| 3685 | 1.31185 | 2.1 |
| 3686 | 0.946132 | 2.1 |
| 3687 | 1.30203 | 2.1 |
| 3688 | 1.31185 | 2.1 |
| 3689 | 1.82958 | 2.1 |
| 3690 | 1.82958 | 2.1 |
| 3691 | 0.946132 | 2.1 |
| 3692 | 1.40199 | 2.1 |
| 3693 | 0.946133 | 2.1 |
| 3694 | 1.36994 | 2.1 |
| 3695 | 1.40199 | 2.1 |
| 3696 | 0.97489 | 2.1 |
| 3697 | 1.05939 | 2.1 |
| 3698 | 1.01922 | 2.1 |
| 3699 | 0.810941 | 1.97529 |
| 3700 | 1.05372 | 2.1 |
| 3701 | 0.754065 | 2.1 |
| 3702 | 0.926131 | 2.1 |
| 3703 | 1.35905 | 2.1 |
| 3704 | 0.810931 | 2.1 |
| 3705 | 1.75647 | 1.05493 |
| 3706 | 1.68265 | 2.1 |
| 3707 | 1.41995 | 2.1 |
| 3708 | 1.36457 | 1.84166 |
| 3709 | 0.933392 | 1.30287 |

| | | |
|---|---|---|
| 3710 | 1.42255 | 2.1 |
| 3711 | 1.3868 | 2.1 |
| 3712 | 1.08074 | 2.1 |
| 3713 | 1.07196 | 2.1 |
| 3714 | 1.13537 | 1.14243 |
| 3715 | 1.01179 | 0.851197 |
| 3716 | 1.60196 | 0.671566 |
| 3717 | 1.72798 | 0.777281 |
| 3718 | 1.3715 | 0.826687 |
| 3719 | 1.56409 | 1.0755 |
| 3720 | 1.56409 | 0.491827 |
| 3721 | 1.97819 | 1.27433 |
| 3722 | 1.47954 | 0.456488 |
| 3723 | 0.798655 | 0.0705513 |
| 3724 | 1.47952 | 0.507285 |
| 3725 | 2.30327 | 0.0948486 |
| 3726 | 1.49111 | 2.1 |
| 3727 | 1.96013 | 2.1 |
| 3728 | 1.54599 | 2.1 |
| 3729 | 2.06632 | 2.1 |
| 3730 | 1.54601 | 2.1 |
| 3731 | 1.10557 | 0.57302 |
| 3732 | 1.17624 | 0.0602985 |
| 3733 | 1.60497 | 2.1 |
| 3734 | 1.61587 | 2.1 |
| 3735 | 1.65977 | 0.05 |
| 3736 | 1.69044 | 0.05 |
| 3737 | 1.00659 | 2.1 |
| 3738 | 1.06226 | 2.1 |
| 3739 | 1.15171 | 2.1 |
| 3740 | 1.50853 | 2.1 |
| 3741 | 1.80252 | 2.1 |
| 3742 | 1.41432 | 2.1 |
| 3743 | 1.04687 | 2.1 |
| 3744 | 0.940878 | 2.1 |
| 3745 | 1.52031 | 2.1 |
| 3746 | 1.52031 | 2.1 |
| 3747 | 0.940877 | 2.1 |
| 3748 | 0.946132 | 2.1 |
| 3749 | 1.50853 | 2.1 |
| 3750 | 0.963973 | 2.1 |
| 3751 | 1.70562 | 0.05 |
| 3752 | 2.43203 | 0.05 |
| 3753 | 1.36004 | 0.05 |
| 3754 | 1.06648 | 0.05 |
| 3755 | 1.66009 | 0.05 |
| 3756 | 1.66009 | 0.05 |
| 3757 | 0.766427 | 0.05 |
| 3758 | 0.766452 | 0.05 |
| 3759 | 0.946132 | 0.05 |
| 3760 | 1.98012 | 0.05 |
| 3761 | 1.20357 | 2.1 |
| 3762 | 1.20356 | 2.1 |
| 3763 | 1.67324 | 2.1 |
| 3764 | 1.52008 | 2.1 |
| 3765 | 1.19964 | 2.1 |
| 3766 | 1.36861 | 2.1 |
| 3767 | 1.19964 | 2.1 |

| | | |
|---|---|---|
| 3768 | 2.85415 | 2.1 |
| 3769 | 1.43687 | 2.1 |
| 3770 | 0.935852 | 2.1 |
| 3771 | 1.09972 | 2.1 |
| 3772 | 0.810933 | 2.1 |
| 3773 | 2.25974 | 2.1 |
| 3774 | 1.74149 | 2.1 |
| 3775 | 1.36984 | 2.1 |
| 3776 | 1.87602 | 2.1 |
| 3777 | 0.890748 | 2.1 |
| 3778 | 1.20587 | 1.95687 |
| 3779 | 0.810956 | 1.70777 |
| 3780 | 2.03174 | 2.01903 |
| 3781 | 1.34403 | 2.08936 |
| 3782 | 1.58825 | 1.99621 |
| 3783 | 1.63096 | 1.95828 |
| 3784 | 2.53735 | 2.1 |
| 3785 | 1.98241 | 2.05832 |
| 3786 | 2.53014 | 2.1 |
| 3787 | 1.1775 | 1.20025 |
| 3788 | 1.06538 | 1.02329 |
| 3789 | 0.946132 | 1.2348 |
| 3790 | 1.4101 | 2.1 |
| 3791 | 1.75162 | 2.1 |
| 3792 | 1.75162 | 1.14848 |
| 3793 | 0.946132 | 1.24055 |
| 3794 | 1.4101 | 1.10189 |
| 3795 | 1.28765 | 1.1563 |
| 3796 | 1.1427 | 1.02459 |
| 3797 | 1.1427 | 0.81452 |
| 3798 | 1.3109 | 2.1 |
| 3799 | 1.02721 | 1.24845 |
| 3800 | 0.846447 | 1.52243 |
| 3801 | 0.862076 | 2.1 |
| 3802 | 0.644098 | 1.6299 |
| 3803 | 0.581308 | 1.6134 |
| 3804 | 1.02306 | 1.40553 |
| 3805 | 0.322641 | 0.456481 |
| 3806 | 0.589346 | 0.441066 |
| 3807 | 2.25572 | 1.5209 |
| 3808 | 1.31372 | 2.1 |
| 3809 | 1.18873 | 2.1 |
| 3810 | 1.78225 | 2.1 |
| 3811 | 1.764 | 2.1 |
| 3812 | 1.17503 | 2.1 |
| 3813 | 1.06821 | 2.1 |
| 3814 | 1.06821 | 1.85602 |
| 3815 | 1.7777 | 2.00218 |
| 3816 | 1.9096 | 1.09729 |
| 3817 | 1.70239 | 1.30554 |
| 3818 | 1.16182 | 0.05 |
| 3819 | 1.20132 | 0.05 |
| 3820 | 1.23475 | 2.1 |
| 3821 | 1.56859 | 1.93675 |
| 3822 | 2.46263 | 2.1 |
| 3823 | 0.528744 | 2.1 |
| 3824 | 2.93337 | 2.1 |
| 3825 | 2.42504 | 2.1 |

| | | |
|---|---|---|
| 3826 | 1.38646 | 0.05 |
| 3827 | 1.61963 | 0.05 |
| 3828 | 1.88016 | 0.0804428 |
| 3829 | 0.622283 | 0.314592 |
| 3830 | 1.03243 | 0.5225 |
| 3831 | 0.710307 | 1.13268 |
| 3832 | 0.798124 | 0.278764 |
| 3833 | 0.931073 | 0.991877 |
| 3834 | 0.892862 | 1.33093 |
| 3835 | 1.06791 | 0.475899 |
| 3836 | 0.552787 | 1.41413 |
| 3837 | 0.946132 | 0.440485 |
| 3838 | 1.36665 | 0.278352 |
| 3839 | 1.36665 | 1.17885 |
| 3840 | 0.506966 | 0.774601 |
| 3841 | 0.506964 | 1.54934 |
| 3842 | 1.36665 | 0.261168 |
| 3843 | 0.653469 | 0.461984 |
| 3844 | 1.36665 | 0.0530413 |
| 3845 | 1.21339 | 0.05 |
| 3846 | 1.21339 | 0.9802 |
| 3847 | 0.552757 | 0.315468 |
| 3848 | 0.552786 | 1.51679 |
| 3849 | 1.21336 | 0.05 |
| 3850 | 1.70744 | 1.02224 |
| 3851 | 1.0775 | 0.05 |
| 3852 | 1.59527 | 0.0892662 |
| 3853 | 1.48312 | 0.05 |
| 3854 | 2.01868 | 0.05 |
| 3855 | 1.18947 | 0.05 |
| 3856 | 1.48312 | 0.05 |
| 3857 | 0.785626 | 0.05 |
| 3858 | 1.19601 | 0.05 |
| 3859 | 0.785626 | 0.05 |
| 3860 | 0.946132 | 0.05 |
| 3861 | 0.7856 | 0.05 |
| 3862 | 1.59087 | 0.05 |
| 3863 | 1.51158 | 0.05 |
| 3864 | 1.74098 | 0.05 |
| 3865 | 2.39202 | 0.05 |
| 3866 | 0.870489 | 0.05 |
| 3867 | 1.34534 | 0.05 |
| 3868 | 0.870488 | 0.05 |
| 3869 | 0.946133 | 0.05 |
| 3870 | 0.870489 | 0.05 |
| 3871 | 1.67086 | 0.05 |
| 3872 | 1.67086 | 0.05 |
| 3873 | 2.11311 | 2.1 |
| 3874 | 1.98765 | 1.59119 |
| 3875 | 0.946133 | 0.924669 |
| 3876 | 1.30881 | 2.1 |
| 3877 | 1.72211 | 2.1 |
| 3878 | 1.72211 | 1.60559 |
| 3879 | 0.94613 | 2.1 |
| 3880 | 1.30881 | 1.4329 |
| 3881 | 1.35943 | 0.996112 |
| 3882 | 1.01294 | 0.267305 |
| 3883 | 1.01294 | 0.05 |

| | | |
|---|---|---|
| 3884 | 1.38963 | 0.576494 |
| 3885 | 1.01294 | 0.0520713 |
| 3886 | 1.60056 | 0.0720768 |
| 3887 | 1.60056 | 0.0710637 |
| 3888 | 0.946132 | 0.064581 |
| 3889 | 1.204 | 0.05 |
| 3890 | 0.946132 | 0.347437 |
| 3891 | 1.11288 | 0.05 |
| 3892 | 1.204 | 0.0751497 |
| 3893 | 1.14599 | 0.05 |
| 3894 | 1.00065 | 0.0793415 |
| 3895 | 1.74685 | 0.12463 |
| 3896 | 1.74685 | 2.1 |
| 3897 | 0.946128 | 0.0538841 |
| 3898 | 1.38463 | 0.05 |
| 3899 | 1.2275 | 2.1 |
| 3900 | 1.2275 | 0.3858 |
| 3901 | 1.30835 | 0.830326 |
| 3902 | 2.04407 | 2.1 |
| 3903 | 2.50425 | 2.1 |
| 3904 | 1.19647 | 0.65835 |
| 3905 | 1.19647 | 0.754692 |
| 3906 | 1.19645 | 0.834436 |
| 3907 | 1.75446 | 0.715531 |
| 3908 | 1.1315 | 0.05 |
| 3909 | 1.1315 | 0.599649 |
| 3910 | 1.45569 | 0.0755366 |
| 3911 | 1.1315 | 0.05 |
| 3912 | 1.14357 | 2.1 |
| 3913 | 1.25793 | 2.1 |
| 3914 | 1.14357 | 2.1 |
| 3915 | 2.08181 | 2.1 |
| 3916 | 1.4714 | 2.1 |
| 3917 | 0.71295 | 2.1 |
| 3918 | 1.68285 | 2.1 |
| 3919 | 1.68285 | 2.1 |
| 3920 | 1.25603 | 2.1 |
| 3921 | 1.13976 | 2.1 |
| 3922 | 1.25603 | 2.1 |
| 3923 | 0.71295 | 2.1 |
| 3924 | 1.61095 | 2.1 |
| 3925 | 2.51207 | 2.1 |
| 3926 | 1.63762 | 2.1 |
| 3927 | 2.74451 | 2.1 |
| 3928 | 0.671255 | 1.91586 |
| 3929 | 1.87601 | 2.1 |
| 3930 | 1.87601 | 2.1 |
| 3931 | 0.671226 | 2.04201 |
| 3932 | 1.25603 | 2.1 |
| 3933 | 1.29123 | 2.1 |
| 3934 | 0.671226 | 2.1 |
| 3935 | 1.63723 | 2.1 |
| 3936 | 0.872928 | 0.05 |
| 3937 | 1.47904 | 0.05 |
| 3938 | 1.18793 | 0.05 |
| 3939 | 2.57464 | 0.05 |
| 3940 | 1.14986 | 2.1 |
| 3941 | 1.19611 | 2.1 |

| | | |
|---|---|---|
| 3942 | 0.96029 | 0.610545 |
| 3943 | 1.7961 | 2.1 |
| 3944 | 0.928721 | 2.1 |
| 3945 | 1.34352 | 2.1 |
| 3946 | 1.37935 | 2.1 |
| 3947 | 1.37935 | 2.1 |
| 3948 | 0.892888 | 2.1 |
| 3949 | 0.946131 | 2.1 |
| 3950 | 0.892888 | 2.1 |
| 3951 | 1.75422 | 2.1 |
| 3952 | 1.05556 | 2.1 |
| 3953 | 1.27362 | 0.05 |
| 3954 | 1.27421 | 0.05 |
| 3955 | 1.3003 | 0.992842 |
| 3956 | 1.31415 | 0.391365 |
| 3957 | 1.01431 | 2.1 |
| 3958 | 0.673951 | 2.1 |
| 3959 | 1.63384 | 2.1 |
| 3960 | 1.63661 | 2.1 |
| 3961 | 1.52245 | 2.1 |
| 3962 | 1.50714 | 2.1 |
| 3963 | 0.996778 | 0.05 |
| 3964 | 1.56091 | 0.05 |
| 3965 | 1.06543 | 2.1 |
| 3966 | 2.66689 | 2.1 |
| 3967 | 1.01474 | 2.1 |
| 3968 | 2.76422 | 2.1 |
| 3969 | 1.45321 | 0.05 |
| 3970 | 0.974716 | 0.05 |
| 3971 | 0.810943 | 0.05 |
| 3972 | 0.909982 | 0.05 |
| 3973 | 0.937793 | 0.05 |
| 3974 | 1.05138 | 0.05 |
| 3975 | 1.25044 | 0.05 |
| 3976 | 0.92095 | 0.05 |
| 3977 | 0.830414 | 0.05 |
| 3978 | 2.1402 | 0.05 |
| 3979 | 1.75938 | 2.1 |
| 3980 | 1.05556 | 2.1 |
| 3981 | 1.78558 | 2.1 |
| 3982 | 1.379 2.1 | |
| 3983 | 0.970214 | 0.765223 |
| 3984 | 0.9702 | 0.703859 |
| 3985 | 1.08894 | 1.0302 |
| 3986 | 2.17251 | 0.949324 |
| 3987 | 1.51569 | 0.141486 |
| 3988 | 1.06012 | 0.108002 |
| 3989 | 0.98966 | 0.0615867 |
| 3990 | 1.55926 | 0.05 |
| 3991 | 1.92645 | 0.0594376 |
| 3992 | 1.37427 | 0.05 |
| 3993 | 2.16338 | 0.05 |
| 3994 | 1.5456 | 0.05 |
| 3995 | 1.5456 | 0.05 |
| 3996 | 1.94002 | 0.05 |
| 3997 | 1.33135 | 0.05 |
| 3998 | 1.31948 | 0.05 |
| 3999 | 2.23255 | 0.05 |

| | | |
|---|---|---|
| 4000 | 2.34061 | 0.05 |
| 4001 | 1.52903 | 2.1 |
| 4002 | 3.38327 | 2.1 |
| 4003 | 1.35977 | 2.1 |
| 4004 | 2.22414 | 2.1 |
| 4005 | 1.6812 | 0.05 |
| 4006 | 2.33397 | 0.05 |
| 4007 | 1.16699 | 0.05 |
| 4008 | 0.946131 | 0.05 |
| 4009 | 1.58115 | 0.05 |
| 4010 | 1.58115 | 0.05 |
| 4011 | 0.519726 | 0.05 |
| 4012 | 0.946131 | 0.05 |
| 4013 | 0.519697 | 0.05 |
| 4014 | 1.26868 | 0.05 |
| 4015 | 1.16699 | 0.05 |
| 4016 | 1.5951 | 0.05 |
| 4017 | 1.5951 | 0.05 |
| 4018 | 0.840568 | 0.05 |
| 4019 | 0.946131 | 0.05 |
| 4020 | 0.840598 | 0.05 |
| 4021 | 1.30047 | 2.1 |
| 4022 | 1.5586 | 2.1 |
| 4023 | 1.31825 | 0.801901 |
| 4024 | 1.31924 | 0.632028 |
| 4025 | 1.15818 | 2.1 |
| 4026 | 1.52782 | 1.11235 |
| 4027 | 1.15818 | 1.62999 |
| 4028 | 1.15818 | 1.38714 |
| 4029 | 0.99518 | 0.475304 |
| 4030 | 0.94613 | 0.45666 |
| 4031 | 0.922045 | 0.506008 |
| 4032 | 1.29322 | 0.587366 |
| 4033 | 1.29321 | 0.0758391 |
| 4034 | 1.29321 | 0.586192 |
| 4035 | 0.922056 | 0.501936 |
| 4036 | 1.06848 | 0.0912376 |
| 4037 | 1.84409 | 0.126525 |
| 4038 | 1.9186 | 1.33476 |
| 4039 | 2.72244 | 0.959125 |
| 4040 | 0.946132 | 0.931741 |
| 4041 | 1.36122 | 1.04828 |
| 4042 | 1.79658 | 1.11359 |
| 4043 | 1.79658 | 0.859884 |
| 4044 | 0.946133 | 1.32883 |
| 4045 | 1.36122 | 1.01222 |
| 4046 | 1.38149 | 0.950919 |
| 4047 | 1.59106 | 0.731359 |
| 4048 | 0.946116 | 0.849255 |
| 4049 | 1.29052 | 0.754531 |
| 4050 | 1.76641 | 0.868919 |
| 4051 | 1.76641 | 0.601031 |
| 4052 | 0.885309 | 1.25329 |
| 4053 | 1.29054 | 0.879405 |
| 4054 | 1.36118 | 0.926528 |
| 4055 | 1.59103 | 1.03265 |
| 4056 | 2.27816 | 0.716521 |
| 4057 | 1.56164 | 0.522451 |

| | | |
|---|---|---|
| 4058 | 1.41114 | 0.05 |
| 4059 | 2.04128 | 0.05 |
| 4060 | 0.986723 | 2.1 |
| 4061 | 1.03645 | 0.104033 |
| 4062 | 1.79224 | 0.05 |
| 4063 | 1.2627 | 2.1 |
| 4064 | 1.23036 | 2.1 |
| 4065 | 1.45257 | 2.1 |
| 4066 | 1.41469 | 2.1 |
| 4067 | 1.41469 | 2.1 |
| 4068 | 2.60636 | 2.1 |
| 4069 | 1.45789 | 0.05 |
| 4070 | 0.851541 | 0.05 |
| 4071 | 1.57877 | 0.05 |
| 4072 | 1.53134 | 0.05 |
| 4073 | 0.851479 | 0.05 |
| 4074 | 1.08982 | 0.05 |
| 4075 | 0.716284 | 0.05 |
| 4076 | 0.946131 | 0.05 |
| 4077 | 1.73193 | 0.05 |
| 4078 | 2.57309 | 0.05 |
| 4079 | 1.36307 | 0.05 |
| 4080 | 2.42175 | 0.05 |
| 4081 | 1.72948 | 0.05 |
| 4082 | 0.978698 | 2.1 |
| 4083 | 0.978699 | 2.1 |
| 4084 | 2.08398 | 2.1 |
| 4085 | 0.978698 | 2.1 |
| 4086 | 1.44096 | 2.1 |
| 4087 | 1.44096 | 2.1 |
| 4088 | 0.638164 | 2.1 |
| 4089 | 0.681588 | 2.1 |
| 4090 | 0.946133 | 2.1 |
| 4091 | 0.638165 | 2.1 |
| 4092 | 0.94613 | 2.1 |
| 4093 | 1.55432 | 2.1 |
| 4094 | 0.901147 | 2.1 |
| 4095 | 0.85777 | 2.1 |
| 4096 | 1.36073 | 2.1 |
| 4097 | 1.44004 | 2.1 |
| 4098 | 0.946133 | 2.1 |
| 4099 | 1.09982 | 2.1 |
| 4100 | 1.27562 | 2.1 |
| 4101 | 1.55432 | 2.1 |
| 4102 | 1.62229 | 2.1 |
| 4103 | 1.0101 | 2.1 |
| 4104 | 2.28459 | 2.1 |
| 4105 | 2.50554 | 2.1 |
| 4106 | 0.946131 | 0.956345 |
| 4107 | 1.25276 | 2.1 |
| 4108 | 1.73721 | 2.1 |
| 4109 | 1.73721 | 1.3702 |
| 4110 | 0.93406 | 2.1 |
| 4111 | 1.25277 | 1.83449 |
| 4112 | 1.4185 | 1.1481 |
| 4113 | 1.53064 | 1.12815 |
| 4114 | 0.946133 | 1.15223 |
| 4115 | 0.609462 | 2.1 |

| | | |
|---|---|---|
| 4116 | 1.65123 | 2.1 |
| 4117 | 1.65123 | 1.74254 |
| 4118 | 0.609466 | 2.1 |
| 4119 | 1.00792 | 1.88435 |
| 4120 | 1.25277 | 1.41078 |
| 4121 | 1.53064 | 2.1 |
| 4122 | 2.15223 | 1.2592 |
| 4123 | 1.30664 | 1.74557 |
| 4124 | 1.10674 | 2.1 |
| 4125 | 1.22862 | 2.1 |
| 4126 | 1.49358 | 0.0788896 |
| 4127 | 2.0207 | 0.05 |
| 4128 | 2.09374 | 2.1 |
| 4129 | 1.88175 | 2.1 |
| 4130 | 1.10938 | 0.05 |
| 4131 | 1.02478 | 0.0756998 |
| 4132 | 1.13272 | 0.05 |
| 4133 | 1.13272 | 0.05 |
| 4134 | 1.03935 | 0.8536 |
| 4135 | 0.810945 | 1.50645 |
| 4136 | 0.527573 | 0.0712764 |
| 4137 | 0.925011 | 0.735544 |
| 4138 | 1.03193 | 0.0603311 |
| 4139 | 1.38587 | 0.159213 |
| 4140 | 0.703469 | 0.131063 |
| 4141 | 0.912282 | 0.730239 |
| 4142 | 1.75505 | 2.1 |
| 4143 | 1.78529 | 2.1 |
| 4144 | 1.60017 | 0.693282 |
| 4145 | 1.35608 | 0.05 |
| 4146 | 1.01479 | 0.597682 |
| 4147 | 1.47561 | 0.0962569 |
| 4148 | 1.01479 | 0.309061 |
| 4149 | 1.28418 | 0.0516666 |
| 4150 | 2.62605 | 2.1 |
| 4151 | 1.71233 | 2.1 |
| 4152 | 3.22222 | 2.1 |
| 4153 | 1.97526 | 2.1 |
| 4154 | 0.949712 | 0.05 |
| 4155 | 1.78986 | 0.05 |
| 4156 | 2.72879 | 0.05 |
| 4157 | 2.66428 | 0.05 |
| 4158 | 1.68108 | 0.0836014 |
| 4159 | 1.67265 | 0.0510999 |
| 4160 | 2.24877 | 0.148548 |
| 4161 | 1.15558 | 0.15672 |
| 4162 | 1.39708 | 2.1 |
| 4163 | 1.39327 | 2.1 |
| 4164 | 1.69788 | 0.05 |
| 4165 | 1.1273 | 0.05 |
| 4166 | 1.17302 | 0.05 |
| 4167 | 1.04349 | 0.05 |
| 4168 | 0.979853 | 2.1 |
| 4169 | 1.19094 | 2.1 |
| 4170 | 1.39408 | 2.1 |
| 4171 | 1.61857 | 2.1 |
| 4172 | 1.66523 | 2.1 |
| 4173 | 0.946132 | 2.1 |

| | | |
|---|---|---|
| 4174 | 0.838554 | 2.1 |
| 4175 | 1.05151 | 2.1 |
| 4176 | 1.18901 | 2.1 |
| 4177 | 1.18901 | 2.1 |
| 4178 | 1.61149 | 2.1 |
| 4179 | 1.22857 | 0.733009 |
| 4180 | 1.25848 | 0.714434 |
| 4181 | 1.09154 | 2.1 |
| 4182 | 2.20358 | 2.1 |
| 4183 | 1.23013 | 2.1 |
| 4184 | 1.55698 | 2.1 |
| 4185 | 1.55828 | 2.1 |
| 4186 | 1.52391 | 2.1 |
| 4187 | 0.60346 | 1.88815 |
| 4188 | 1.06336 | 2.1 |
| 4189 | 0.870714 | 1.09823 |
| 4190 | 0.543109 | 1.86123 |
| 4191 | 1.10367 | 1.08662 |
| 4192 | 1.20686 | 1.93631 |
| 4193 | 1.20684 | 2.1 |
| 4194 | 2.5118 | 1.32716 |
| 4195 | 1.20684 | 2.1 |
| 4196 | 1.52468 | 2.1 |
| 4197 | 1.52469 | 2.1 |
| 4198 | 0.532308 | 1.54798 |
| 4199 | 0.903301 | 2.1 |
| 4200 | 0.946119 | 1.33934 |
| 4201 | 0.532315 | 1.36612 |
| 4202 | 0.946107 | 1.43195 |
| 4203 | 1.46129 | 2.1 |
| 4204 | 0.804766 | 2.1 |
| 4205 | 1.4809 | 2.1 |
| 4206 | 1.4809 | 2.1 |
| 4207 | 1.11822 | 2.1 |
| 4208 | 1.16744 | 2.1 |
| 4209 | 1.11822 | 2.1 |
| 4210 | 0.804737 | 2.1 |
| 4211 | 1.04193 | 2.1 |
| 4212 | 0.983723 | 2.1 |
| 4213 | 1.8014 | 2.1 |
| 4214 | 1.1729 | 2.1 |
| 4215 | 1.03951 | 2.1 |
| 4216 | 1.03951 | 2.1 |
| 4217 | 1.17583 | 2.1 |
| 4218 | 1.42866 | 2.1 |
| 4219 | 1.78503 | 2.1 |
| 4220 | 1.17822 | 1.66914 |
| 4221 | 1.89121 | 2.1 |
| 4222 | 1.44472 | 2.1 |
| 4223 | 1.62143 | 2.1 |
| 4224 | 1.43837 | 2.1 |
| 4225 | 1.01121 | 0.05 |
| 4226 | 0.962703 | 0.782026 |
| 4227 | 0.946129 | 0.527014 |
| 4228 | 1.10941 | 0.366087 |
| 4229 | 0.946131 | 0.543157 |
| 4230 | 1.60326 | 0.608885 |
| 4231 | 1.60326 | 0.336354 |

| | | |
|---|---|---|
| 4232 | 1.14215 | 0.512335 |
| 4233 | 1.40724 | 0.5239 |
| 4234 | 0.962703 | 0.448332 |
| 4235 | 1.53336 | 0.05 |
| 4236 | 1.40272 | 0.05 |
| 4237 | 1.64984 | 0.05 |
| 4238 | 1.67345 | 0.05 |
| 4239 | 1.08212 | 2.1 |
| 4240 | 1.40905 | 2.1 |
| 4241 | 0.946132 | 0.05 |
| 4242 | 0.946132 | 0.05 |
| 4243 | 0.81268 | 0.05 |
| 4244 | 0.946131 | 0.05 |
| 4245 | 0.812681 | 0.05 |
| 4246 | 0.946131 | 0.05 |
| 4247 | 0.946132 | 0.05 |
| 4248 | 0.997755 | 0.05 |
| 4249 | 1.94585 | 0.05 |
| 4250 | 1.2925 | 0.05 |
| 4251 | 2.52454 | 0.05 |
| 4252 | 1.69182 | 0.05 |
| 4253 | 1.69182 | 0.05 |
| 4254 | 0.92361 | 0.05 |
| 4255 | 1.35316 | 0.05 |
| 4256 | 1.26227 | 0.05 |
| 4257 | 0.946131 | 0.05 |
| 4258 | 1.35316 | 0.05 |
| 4259 | 1.48676 | 0.05 |
| 4260 | 1.33917 | 0.05 |
| 4261 | 0.757292 | 2.1 |
| 4262 | 1.02415 | 2.1 |
| 4263 | 1.48495 | 2.1 |
| 4264 | 0.97587 | 2.1 |
| 4265 | 0.946132 | 2.1 |
| 4266 | 0.747365 | 2.1 |
| 4267 | 1.55432 | 2.1 |
| 4268 | 1.55432 | 2.1 |
| 4269 | 0.946132 | 2.1 |
| 4270 | 0.747394 | 2.1 |
| 4271 | 1.17464 | 0.05 |
| 4272 | 1.0372 | 0.05 |
| 4273 | 2.16553 | 0.05 |
| 4274 | 2.00616 | 0.05 |
| 4275 | 1.12184 | 0.05 |
| 4276 | 1.33177 | 0.05 |
| 4277 | 0.936674 | 0.05 |
| 4278 | 0.946129 | 0.05 |
| 4279 | 0.851552 | 0.05 |
| 4280 | 1.60205 | 0.05 |
| 4281 | 1.77391 | 0.05 |
| 4282 | 1.03327 | 0.05 |
| 4283 | 1.41512 | 0.05 |
| 4284 | 1.03327 | 0.05 |
| 4285 | 0.946133 | 0.05 |
| 4286 | 0.946131 | 0.05 |
| 4287 | 1.80077 | 0.05 |
| 4288 | 1.80077 | 0.05 |
| 4289 | 1.54848 | 2.1 |

| | | |
|---|---|---|
| 4290 | 1.29931 | 2.1 |
| 4291 | 1.04482 | 0.05 |
| 4292 | 2.08069 | 0.05 |
| 4293 | 0.946127 | 0.05 |
| 4294 | 0.515539 | 0.05 |
| 4295 | 0.515566 | 0.0586993 |
| 4296 | 1.38423 | 0.05 |
| 4297 | 1.38423 | 0.05 |
| 4298 | 0.972058 | 0.05 |
| 4299 | 0.927737 | 0.05 |
| 4300 | 0.94971 | 0.05 |
| 4301 | 2.19682 | 0.05 |
| 4302 | 0.946132 | 0.05 |
| 4303 | 1.09841 | 0.05 |
| 4304 | 0.522382 | 0.05 |
| 4305 | 1.5962 | 0.05 |
| 4306 | 1.72791 | 0.05 |
| 4307 | 1.07428 | 0.05 |
| 4308 | 1.05215 | 0.05 |
| 4309 | 1.54632 | 1.52667 |
| 4310 | 1.15693 | 1.67687 |
| 4311 | 1.41238 | 2.1 |
| 4312 | 1.41238 | 2.1 |
| 4313 | 1.77966 | 2.1 |
| 4314 | 1.77966 | 2.1 |
| 4315 | 0.946129 | 2.1 |
| 4316 | 1.40525 | 2.1 |
| 4317 | 0.946131 | 2.1 |
| 4318 | 0.94613 | 2.1 |
| 4319 | 1.32054 | 2.1 |
| 4320 | 1.41238 | 2.1 |
| 4321 | 1.94026 | 2.1 |
| 4322 | 1.0171 | 0.527408 |
| 4323 | 1.1211 | 0.05 |
| 4324 | 0.993022 | 2.1 |
| 4325 | 0.773813 | 2.1 |
| 4326 | 1.4073 | 2.1 |
| 4327 | 1.4073 | 2.1 |
| 4328 | 0.94613 | 2.1 |
| 4329 | 0.787615 | 2.1 |
| 4330 | 0.94613 | 2.1 |
| 4331 | 0.787615 | 2.1 |
| 4332 | 1.764 | 2.1 |
| 4333 | 1.90964 | 2.1 |
| 4334 | 1.05787 | 2.1 |
| 4335 | 1.403 | 0.05 |
| 4336 | 0.744189 | 0.194671 |
| 4337 | 2.32834 | 2.1 |
| 4338 | 2.34986 | 2.1 |
| 4339 | 1.17106 | 1.69945 |
| 4340 | 1.94826 | 0.05 |
| 4341 | 2.40606 | 0.05 |
| 4342 | 1.26387 | 0.05 |
| 4343 | 0.954013 | 0.05 |
| 4344 | 0.946131 | 0.05 |
| 4345 | 1.71 | 0.05 |
| 4346 | 1.51926 | 0.05 |
| 4347 | 0.953997 | 0.05 |

| | | |
|---|---|---|
| 4348 | 0.851554 | 0.05 |
| 4349 | 1.96502 | 0.05 |
| 4350 | 1.72954 | 0.05 |
| 4351 | 2.89117 | 0.05 |
| 4352 | 0.971958 | 0.05 |
| 4353 | 0.985575 | 0.05 |
| 4354 | 1.41943 | 0.05 |
| 4355 | 1.20021 | 0.05 |
| 4356 | 1.29599 | 0.05 |
| 4357 | 1.15687 | 0.05 |
| 4358 | 0.810958 | 0.05 |
| 4359 | 1.12919 | 0.05 |
| 4360 | 1.98859 | 2.1 |
| 4361 | 1.82044 | 2.1 |
| 4362 | 1.90488 | 2.1 |
| 4363 | 0.851542 | 2.1 |
| 4364 | 1.02973 | 2.1 |
| 4365 | 1.39286 | 2.1 |
| 4366 | 1.44873 | 2.1 |
| 4367 | 0.946132 | 2.1 |
| 4368 | 1.11826 | 2.1 |
| 4369 | 1.26842 | 2.1 |
| 4370 | 2.09461 | 2.1 |
| 4371 | 1.76952 | 0.05 |
| 4372 | 1.6473 | 0.05 |
| 4373 | 0.946131 | 0.05 |
| 4374 | 1.36486 | 0.05 |
| 4375 | 1.04243 | 0.05 |
| 4376 | 1.05017 | 0.05 |
| 4377 | 1.24059 | 0.05 |
| 4378 | 2.11646 | 0.05 |
| 4379 | 1.50368 | 0.05 |
| 4380 | 2.38796 | 0.05 |
| 4381 | 1.50368 | 0.05 |
| 4382 | 1.71844 | 0.05 |
| 4383 | 1.71844 | 0.05 |
| 4384 | 0.914836 | 0.05 |
| 4385 | 1.24416 | 0.05 |
| 4386 | 0.914805 | 0.05 |
| 4387 | 1.24416 | 0.05 |
| 4388 | 1.38911 | 0.05 |
| 4389 | 1.46859 | 0.05 |
| 4390 | 1.35348 | 0.05 |
| 4391 | 0.946131 | 0.05 |
| 4392 | 1.1226 | 0.05 |
| 4393 | 0.857078 | 0.05 |
| 4394 | 0.9746 | 0.05 |
| 4395 | 1.37624 | 0.05 |
| 4396 | 1.15131 | 0.05 |
| 4397 | 1.139 0.05 | |
| 4398 | 1.90456 | 0.05 |
| 4399 | 2.09692 | 0.05 |
| 4400 | 1.16655 | 2.1 |
| 4401 | 1.23017 | 2.1 |
| 4402 | 2.14592 | 2.1 |
| 4403 | 1.23017 | 2.1 |
| 4404 | 1.0752 | 1.46052 |
| 4405 | 1.52482 | 0.05 |

| | | |
|---|---|---|
| 4406 | 1.09252 | 2.1 |
| 4407 | 1.10725 | 2.1 |
| 4408 | 1.14305 | 0.05 |
| 4409 | 0.851541 | 0.05 |
| 4410 | 1.48073 | 0.05 |
| 4411 | 1.45607 | 0.05 |
| 4412 | 0.946131 | 0.05 |
| 4413 | 0.932823 | 0.05 |
| 4414 | 1.45828 | 0.05 |
| 4415 | 0.851528 | 0.05 |
| 4416 | 1.14305 | 0.05 |
| 4417 | 1.92737 | 0.05 |
| 4418 | 1.08211 | 0.05 |
| 4419 | 1.69831 | 0.05 |
| 4420 | 1.69831 | 0.05 |
| 4421 | 0.946131 | 0.05 |
| 4422 | 1.26868 | 0.05 |
| 4423 | 1.23377 | 0.05 |
| 4424 | 1.23377 | 0.05 |
| 4425 | 0.94723 | 0.05 |
| 4426 | 1.64322 | 0.05 |
| 4427 | 1.64322 | 0.05 |
| 4428 | 0.946131 | 0.05 |
| 4429 | 1.32068 | 0.05 |
| 4430 | 0.946129 | 0.05 |
| 4431 | 1.18789 | 0.05 |
| 4432 | 1.32068 | 0.05 |
| 4433 | 1.32813 | 2.1 |
| 4434 | 1.32461 | 2.1 |
| 4435 | 1.42729 | 0.05 |
| 4436 | 2.47846 | 0.05 |
| 4437 | 1.00024 | 0.05 |
| 4438 | 1.23923 | 0.05 |
| 4439 | 0.946131 | 0.05 |
| 4440 | 0.712304 | 0.05 |
| 4441 | 1.77501 | 0.05 |
| 4442 | 1.77501 | 0.05 |
| 4443 | 0.593593 | 0.05 |
| 4444 | 1.23923 | 0.05 |
| 4445 | 1.23175 | 0.05 |
| 4446 | 0.94613 | 0.05 |
| 4447 | 0.713646 | 0.05 |
| 4448 | 1.75735 | 0.05 |
| 4449 | 1.75735 | 0.05 |
| 4450 | 0.713633 | 0.05 |
| 4451 | 1.37157 | 0.05 |
| 4452 | 1.47169 | 0.05 |
| 4453 | 2.21808 | 0.05 |
| 4454 | 1.47169 | 0.05 |
| 4455 | 1.04759 | 2.1 |
| 4456 | 0.987132 | 2.1 |
| 4457 | 1.11181 | 2.1 |
| 4458 | 2.61682 | 2.1 |
| 4459 | 1.11181 | 2.1 |
| 4460 | 1.06461 | 1.26894 |
| 4461 | 1.69733 | 2.1 |
| 4462 | 2.79705 | 2.1 |
| 4463 | 1.08245 | 2.1 |

| | | |
|---|---|---|
| 4464 | 1.40583 | 2.1 |
| 4465 | 1.39852 | 2.1 |
| 4466 | 2.0239 | 2.1 |
| 4467 | 2.0239 | 2.1 |
| 4468 | 0.780455 | 2.1 |
| 4469 | 0.946132 | 2.1 |
| 4470 | 0.780455 | 2.1 |
| 4471 | 1.01885 | 2.1 |
| 4472 | 0.564217 | 2.1 |
| 4473 | 0.733461 | 2.1 |
| 4474 | 0.537126 | 2.1 |
| 4475 | 0.683838 | 2.1 |
| 4476 | 0.779662 | 2.1 |
| 4477 | 0.634141 | 2.1 |
| 4478 | 0.685337 | 2.1 |
| 4479 | 1.58848 | 2.1 |
| 4480 | 1.17129 | 0.05 |
| 4481 | 0.663168 | 0.05 |
| 4482 | 1.55587 | 0.05 |
| 4483 | 1.31342 | 0.05 |
| 4484 | 0.596868 | 0.05 |
| 4485 | 1.1666 | 0.05 |
| 4486 | 0.867261 | 0.05 |
| 4487 | 0.946132 | 0.0621039 |
| 4488 | 1.28843 | 0.05 |
| 4489 | 1.31248 | 0.05 |
| 4490 | 2.59921 | 0.05 |
| 4491 | 1.32634 | 0.05 |
| 4492 | 1.35564 | 0.05 |
| 4493 | 1.52661 | 0.05 |
| 4494 | 1.12323 | 0.05 |
| 4495 | 1.17701 | 0.410668 |
| 4496 | 1.36405 | 0.431263 |
| 4497 | 1.19837 | 0.197434 |
| 4498 | 2.25636 | 0.133506 |
| 4499 | 1.0823 | 0.05 |
| 4500 | 1.31774 | 0.05 |
| 4501 | 1.0823 | 0.05 |
| 4502 | 1.01121 | 0.05 |
| 4503 | 1.27408 | 0.05 |
| 4504 | 0.946131 | 0.05 |
| 4505 | 0.566074 | 0.05 |
| 4506 | 0.79576 | 0.05 |
| 4507 | 1.24596 | 0.05 |
| 4508 | 1.47567 | 0.05 |
| 4509 | 1.47567 | 0.05 |
| 4510 | 0.56605 | 0.05 |
| 4511 | 1.6567 | 0.05 |
| 4512 | 0.967792 | 0.05 |
| 4513 | 1.00366 | 0.05 |
| 4514 | 1.00366 | 0.05 |
| 4515 | 1.56409 | 0.05 |
| 4516 | 1.56409 | 0.05 |
| 4517 | 0.774911 | 0.05 |
| 4518 | 1.09483 | 0.05 |
| 4519 | 0.774912 | 0.05 |
| 4520 | 1.09483 | 0.05 |
| 4521 | 1.24417 | 0.05 |

| | | |
|---|---|---|
| 4522 | 1.13387 | 0.05 |
| 4523 | 1.13387 | 0.05 |
| 4524 | 1.30572 | 0.05 |
| 4525 | 2.18966 | 0.05 |
| 4526 | 0.98575 | 0.05 |
| 4527 | 1.54982 | 0.05 |
| 4528 | 2.48833 | 0.05 |
| 4529 | 1.54982 | 0.05 |
| 4530 | 1.28954 | 0.05 |
| 4531 | 1.64002 | 0.05 |
| 4532 | 1.64002 | 0.05 |
| 4533 | 1.85594 | 0.05 |
| 4534 | 1.12968 | 2.1 |
| 4535 | 1.23536 | 2.1 |
| 4536 | 1.4882 | 2.1 |
| 4537 | 1.4882 | 2.1 |
| 4538 | 0.876836 | 2.1 |
| 4539 | 0.946131 | 2.1 |
| 4540 | 0.876835 | 2.1 |
| 4541 | 1.43166 | 2.1 |
| 4542 | 2.25936 | 2.1 |
| 4543 | 1.0305 | 2.1 |
| 4544 | 0.992095 | 2.1 |
| 4545 | 1.12968 | 2.1 |
| 4546 | 1.53813 | 2.1 |
| 4547 | 1.53812 | 2.1 |
| 4548 | 0.583653 | 2.1 |
| 4549 | 0.94613 | 2.1 |
| 4550 | 0.583651 | 2.1 |
| 4551 | 0.964033 | 2.1 |
| 4552 | 0.964034 | 2.1 |
| 4553 | 1.54457 | 2.1 |
| 4554 | 3.54109 | 0.05 |
| 4555 | 0.99974 | 0.05 |
| 4556 | 0.78814 | 0.05 |
| 4557 | 1.65729 | 0.05 |
| 4558 | 1.1283 | 0.05 |
| 4559 | 1.09407 | 0.05 |
| 4560 | 1.55091 | 0.05 |
| 4561 | 0.870773 | 0.05 |
| 4562 | 1.19967 | 0.05 |
| 4563 | 1.56521 | 0.05 |
| 4564 | 1.30896 | 1.30089 |
| 4565 | 1.17989 | 2.1 |
| 4566 | 1.72526 | 2.1 |
| 4567 | 0.851537 | 1.86764 |
| 4568 | 1.58944 | 2.1 |
| 4569 | 1.56915 | 2.1 |
| 4570 | 0.891039 | 1.66014 |
| 4571 | 1.4172 | 1.87073 |
| 4572 | 0.963818 | 1.47094 |
| 4573 | 0.851474 | 2.08626 |
| 4574 | 1.17989 | 2.1 |
| 4575 | 1.56261 | 2.1 |
| 4576 | 1.56261 | 2.1 |
| 4577 | 0.761574 | 2.1 |
| 4578 | 1.31323 | 2.1 |
| 4579 | 0.761573 | 2.1 |

| | | |
|---|---|---|
| 4580 | 0.946133 | 2.1 |
| 4581 | 1.31323 | 2.1 |
| 4582 | 1.16759 | 2.1 |
| 4583 | 1.16759 | 2.1 |
| 4584 | 2.11829 | 2.1 |
| 4585 | 1.16759 | 2.1 |
| 4586 | 1.10748 | 0.05 |
| 4587 | 1.47688 | 0.05 |
| 4588 | 0.946133 | 0.05 |
| 4589 | 0.953319 | 0.05 |
| 4590 | 0.946132 | 0.05 |
| 4591 | 1.51168 | 0.05 |
| 4592 | 1.51168 | 0.05 |
| 4593 | 1.25797 | 0.05 |
| 4594 | 1.19984 | 0.05 |
| 4595 | 1.95729 | 2.1 |
| 4596 | 2.34853 | 2.1 |
| 4597 | 1.94343 | 2.1 |
| 4598 | 2.00551 | 2.1 |
| 4599 | 1.20067 | 0.379972 |
| 4600 | 1.87244 | 0.599562 |
| 4601 | 1.4178 | 2.1 |
| 4602 | 1.33538 | 2.1 |
| 4603 | 0.946131 | 2.1 |
| 4604 | 1.22895 | 2.1 |
| 4605 | 1.12433 | 2.1 |
| 4606 | 0.956757 | 2.1 |
| 4607 | 0.91826 | 2.1 |
| 4608 | 1.44221 | 2.1 |
| 4609 | 1.14206 | 2.1 |
| 4610 | 1.80514 | 0.121147 |
| 4611 | 0.890304 | 0.91006 |
| 4612 | 1.1362 | 0.0714474 |
| 4613 | 1.32819 | 0.0802316 |
| 4614 | 1.46311 | 2.05854 |
| 4615 | 0.8654 | 0.0743536 |
| 4616 | 1.1699 | 1.04645 |
| 4617 | 1.00267 | 0.99813 |
| 4618 | 1.02882 | 0.106108 |
| 4619 | 1.00832 | 2.1 |
| 4620 | 0.974137 | 2.1 |
| 4621 | 1.02368 | 2.1 |
| 4622 | 0.974138 | 2.1 |
| 4623 | 0.995734 | 0.05 |
| 4624 | 1.44605 | 0.121207 |
| 4625 | 1.18549 | 0.05 |
| 4626 | 1.33621 | 0.345396 |
| 4627 | 1.5176 | 0.516326 |
| 4628 | 1.91762 | 0.515991 |
| 4629 | 1.33621 | 0.245545 |
| 4630 | 1.6638 | 0.380778 |
| 4631 | 1.66774 | 2.1 |
| 4632 | 1.11833 | 2.1 |
| 4633 | 0.926499 | 2.1 |
| 4634 | 1.51229 | 2.1 |
| 4635 | 1.51229 | 2.1 |
| 4636 | 0.926528 | 2.1 |
| 4637 | 1.40335 | 2.1 |

| | | |
|---|---|---|
| 4638 | 1.03544 | 2.1 |
| 4639 | 0.926527 | 2.1 |
| 4640 | 1.85305 | 2.1 |
| 4641 | 1.5812 | 2.1 |
| 4642 | 1.15529 | 2.1 |
| 4643 | 1.31086 | 2.1 |
| 4644 | 0.999206 | 2.1 |
| 4645 | 1.46714 | 2.1 |
| 4646 | 1.73087 | 2.1 |
| 4647 | 0.772299 | 2.1 |
| 4648 | 1.24341 | 2.1 |
| 4649 | 0.997867 | 2.1 |
| 4650 | 0.772311 | 2.1 |
| 4651 | 0.936657 | 2.1 |
| 4652 | 1.35986 | 2.1 |
| 4653 | 0.959414 | 2.1 |
| 4654 | 1.36541 | 2.1 |
| 4655 | 1.49268 | 2.1 |
| 4656 | 1.49268 | 2.1 |
| 4657 | 0.832137 | 2.1 |
| 4658 | 0.946132 | 2.1 |
| 4659 | 0.832138 | 2.1 |
| 4660 | 1.64523 | 2.1 |
| 4661 | 1.11764 | 2.1 |
| 4662 | 1.05776 | 2.1 |
| 4663 | 0.972465 | 2.1 |
| 4664 | 0.972464 | 2.1 |
| 4665 | 1.98425 | 2.1 |
| 4666 | 0.969822 | 2.1 |
| 4667 | 1.30566 | 2.1 |
| 4668 | 1.52724 | 2.1 |
| 4669 | 2.32237 | 0.05 |
| 4670 | 2.30274 | 0.05 |
| 4671 | 2.27761 | 0.05 |
| 4672 | 3.33012 | 0.05 |
| 4673 | 1.24001 | 0.913463 |
| 4674 | 1.41821 | 1.24107 |
| 4675 | 0.978584 | 2.1 |
| 4676 | 1.18076 | 2.1 |
| 4677 | 1.07645 | 2.1 |
| 4678 | 0.877351 | 0.05 |
| 4679 | 0.946132 | 0.815896 |
| 4680 | 0.877353 | 0.05 |
| 4681 | 1.58444 | 2.1 |
| 4682 | 1.49614 | 2.1 |
| 4683 | 1.28355 | 1.4028 |
| 4684 | 1.13491 | 0.854806 |
| 4685 | 1.56875 | 0.05 |
| 4686 | 0.499592 | 0.05 |
| 4687 | 0.946131 | 0.05 |
| 4688 | 1.20538 | 0.05 |
| 4689 | 1.09913 | 0.05 |
| 4690 | 1.8049 | 0.05 |
| 4691 | 1.8049 | 0.05 |
| 4692 | 0.499606 | 0.05 |
| 4693 | 2.41076 | 0.05 |
| 4694 | 0.860202 | 0.05 |
| 4695 | 0.906798 | 0.05 |

| | | |
|---|---|---|
| 4696 | 1.8626 | 0.05 |
| 4697 | 1.33986 | 0.05 |
| 4698 | 1.2992 | 0.05 |
| 4699 | 1.58702 | 0.05 |
| 4700 | 1.05939 | 0.05 |
| 4701 | 1.02074 | 2.1 |
| 4702 | 1.07866 | 2.1 |
| 4703 | 1.5082 | 0.05 |
| 4704 | 1.5522 | 0.05 |
| 4705 | 1.65903 | 0.05 |
| 4706 | 1.86766 | 2.1 |
| 4707 | 2.11684 | 2.1 |
| 4708 | 0.946132 | 2.1 |
| 4709 | 1.6585 | 2.1 |
| 4710 | 1.6585 | 2.1 |
| 4711 | 0.94613 | 2.1 |
| 4712 | 1.1872 | 2.1 |
| 4713 | 1.1872 | 2.1 |
| 4714 | 1.05842 | 2.1 |
| 4715 | 1.20455 | 2.1 |
| 4716 | 1.34141 | 2.1 |
| 4717 | 1.34141 | 2.1 |
| 4718 | 1.42416 | 2.1 |
| 4719 | 1.75818 | 2.1 |
| 4720 | 2.63715 | 1.06128 |
| 4721 | 1.80454 | 2.1 |
| 4722 | 2.26198 | 2.1 |
| 4723 | 0.696255 | 2.1 |
| 4724 | 0.624075 | 2.1 |
| 4725 | 1.50905 | 0.708681 |
| 4726 | 1.50905 | 2.1 |
| 4727 | 0.624075 | 2.1 |
| 4728 | 1.31857 | 0.381531 |
| 4729 | 0.946131 | 2.1 |
| 4730 | 0.624075 | 1.06143 |
| 4731 | 0.914628 | 2.1 |
| 4732 | 1.14721 | 2.1 |
| 4733 | 1.16153 | 2.1 |
| 4734 | 1.47664 | 2.1 |
| 4735 | 1.54791 | 2.1 |
| 4736 | 1.19098 | 2.1 |
| 4737 | 1.13341 | 2.1 |
| 4738 | 1.00503 | 0.113338 |
| 4739 | 1.00503 | 1.11417 |
| 4740 | 1.22655 | 2.1 |
| 4741 | 0.999489 | 2.1 |
| 4742 | 1.3268 | 2.1 |
| 4743 | 0.946132 | 2.1 |
| 4744 | 1.60061 | 2.1 |
| 4745 | 1.60061 | 2.1 |
| 4746 | 1.08034 | 2.1 |
| 4747 | 1.40372 | 2.1 |
| 4748 | 1.14302 | 2.1 |
| 4749 | 1.08034 | 2.1 |
| 4750 | 0.999491 | 2.1 |
| 4751 | 1.17152 | 0.05 |
| 4752 | 1.17152 | 0.05 |
| 4753 | 1.17152 | 0.05 |

| | | |
|---|---|---|
| 4754 | 2.05423 | 0.05 |
| 4755 | 1.40984 | 2.1 |
| 4756 | 1.2489 | 2.1 |
| 4757 | 0.885089 | 2.1 |
| 4758 | 0.810931 | 2.1 |
| 4759 | 1.84128 | 2.1 |
| 4760 | 1.11206 | 2.1 |
| 4761 | 1.21926 | 2.1 |
| 4762 | 1.99084 | 2.1 |
| 4763 | 0.976334 | 0.318459 |
| 4764 | 0.599363 | 0.141167 |
| 4765 | 1.02621 | 0.075301 |
| 4766 | 0.462959 | 0.253024 |
| 4767 | 1.42612 | 0.0660128 |
| 4768 | 0.299682 | 0.05997 |
| 4769 | 1.51379 | 2.1 |
| 4770 | 0.719531 | 2.1 |
| 4771 | 1.3702 | 2.1 |
| 4772 | 1.33293 | 2.1 |
| 4773 | 0.851539 | 2.1 |
| 4774 | 0.62998 | 2.1 |
| 4775 | 0.851543 | 2.1 |
| 4776 | 0.629966 | 2.1 |
| 4777 | 1.43132 | 2.1 |
| 4778 | 1.13328 | 1.2519 |
| 4779 | 1.93212 | 0.950742 |
| 4780 | 1.62345 | 0.675339 |
| 4781 | 1.38511 | 1.15591 |
| 4782 | 1.40013 | 0.12242 |
| 4783 | 1.04132 | 0.05 |
| 4784 | 0.83473 | 0.663721 |
| 4785 | 0.868732 | 0.443836 |
| 4786 | 0.810595 | 1.67866 |
| 4787 | 0.729919 | 0.239206 |
| 4788 | 0.992428 | 0.418369 |
| 4789 | 0.577107 | 0.05 |
| 4790 | 1.18464 | 0.591547 |
| 4791 | 0.791199 | 0.500897 |
| 4792 | 0.810943 | 0.05 |
| 4793 | 0.860723 | 0.05 |
| 4794 | 1.33348 | 0.05 |
| 4795 | 1.57467 | 0.05 |
| 4796 | 0.981032 | 0.05 |
| 4797 | 0.731569 | 0.05 |
| 4798 | 0.770312 | 0.05 |
| 4799 | 1.46055 | 2.1 |
| 4800 | 1.42249 | 2.1 |
| 4801 | 1.75999 | 0.05 |
| 4802 | 1.6868 | 0.05 |
| 4803 | 1.17042 | 0.860512 |
| 4804 | 1.01485 | 2.1 |
| 4805 | 1.19952 | 0.05 |
| 4806 | 1.40592 | 0.0751242 |
| 4807 | 1.19952 | 0.115457 |
| 4808 | 2.20145 | 0.05 |
| 4809 | 1.33649 | 0.05 |
| 4810 | 1.11612 | 0.05 |
| 4811 | 0.957588 | 0.05 |

| | | |
|---|---|---|
| 4812 | 0.946126 | 0.05 |
| 4813 | 1.50648 | 0.05 |
| 4814 | 1.50648 | 0.05 |
| 4815 | 0.946128 | 0.05 |
| 4816 | 2.10671 | 0.05 |
| 4817 | 1.91518 | 0.05 |
| 4818 | 2.23223 | 0.05 |
| 4819 | 1.53556 | 2.1 |
| 4820 | 1.56253 | 2.1 |
| 4821 | 1.27806 | 0.05 |
| 4822 | 1.44593 | 0.05 |
| 4823 | 1.03818 | 0.05 |
| 4824 | 1.03818 | 0.05 |
| 4825 | 1.38969 | 0.171402 |
| 4826 | 1.85548 | 0.0530454 |
| 4827 | 2.6044 | 0.145048 |
| 4828 | 1.85548 | 0.05 |
| 4829 | 1.49346 | 2.00601 |
| 4830 | 1.83192 | 1.84846 |
| 4831 | 0.834043 | 2.03539 |
| 4832 | 0.810943 | 0.250938 |
| 4833 | 0.887113 | 0.591545 |
| 4834 | 0.893827 | 0.631861 |
| 4835 | 0.991061 | 0.778804 |
| 4836 | 0.91939 | 1.12383 |
| 4837 | 0.793307 | 1.12444 |
| 4838 | 1.75291 | 1.33282 |
| 4839 | 1.35435 | 0.954954 |
| 4840 | 1.16989 | 0.05 |
| 4841 | 1.36717 | 0.05 |
| 4842 | 2.12276 | 0.0581226 |
| 4843 | 2.30493 | 0.114632 |
| 4844 | 2.47857 | 0.131559 |
| 4845 | 1.92269 | 0.0861543 |
| 4846 | 1.66426 | 0.139167 |
| 4847 | 0.956698 | 0.355318 |
| 4848 | 0.956698 | 2.1 |
| 4849 | 0.857957 | 2.1 |
| 4850 | 1.4706 | 2.1 |
| 4851 | 1.65706 | 2.1 |
| 4852 | 1.47059 | 2.1 |
| 4853 | 1.04713 | 0.05 |
| 4854 | 0.946131 | 0.05 |
| 4855 | 1.47374 | 0.05 |
| 4856 | 1.47374 | 0.05 |
| 4857 | 0.951675 | 0.05 |
| 4858 | 1.2582 | 0.05 |
| 4859 | 1.2582 | 0.05 |
| 4860 | 0.94613 | 0.0519184 |
| 4861 | 1.4468 | 0.05 |
| 4862 | 1.90335 | 0.05 |
| 4863 | 1.14886 | 2.1 |
| 4864 | 2.59689 | 2.1 |
| 4865 | 1.02747 | 2.1 |
| 4866 | 0.946131 | 2.1 |
| 4867 | 0.934061 | 2.1 |
| 4868 | 1.63979 | 2.1 |
| 4869 | 1.65206 | 2.1 |

| | | |
|---|---|---|
| 4870 | 1.48 | 2.1 |
| 4871 | 1.27467 | 2.1 |
| 4872 | 2.20449 | 2.1 |
| 4873 | 1.1333 | 0.05 |
| 4874 | 1.95334 | 2.1 |
| 4875 | 2.20984 | 2.1 |
| 4876 | 1.46389 | 0.05 |
| 4877 | 1.4639 | 0.05 |
| 4878 | 1.4639 | 0.05 |
| 4879 | 0.946132 | 0.05 |
| 4880 | 1.5144 | 0.05 |
| 4881 | 1.5144 | 0.05 |
| 4882 | 0.946132 | 0.05 |
| 4883 | 1.35732 | 0.05 |
| 4884 | 1.10321 | 0.05 |
| 4885 | 0.946132 | 0.05 |
| 4886 | 0.980549 | 0.05 |
| 4887 | 1.32819 | 2.1 |
| 4888 | 1.30171 | 1.25104 |
| 4889 | 1.68377 | 1.02701 |
| 4890 | 1.68377 | 2.1 |
| 4891 | 0.946132 | 0.600298 |
| 4892 | 0.946132 | 2.1 |
| 4893 | 1.1952 | 1.50538 |
| 4894 | 0.990019 | 1.36322 |
| 4895 | 1.08195 | 1.99313 |
| 4896 | 1.2269 | 2.1 |
| 4897 | 1.19219 | 2.1 |
| 4898 | 1.01485 | 2.1 |
| 4899 | 1.57125 | 0.05 |
| 4900 | 2.10696 | 0.05 |
| 4901 | 1.29666 | 0.05 |
| 4902 | 1.57125 | 0.05 |
| 4903 | 1.47712 | 2.1 |
| 4904 | 1.45182 | 2.1 |
| 4905 | 1.17816 | 0.05 |
| 4906 | 1.4516 | 0.05 |
| 4907 | 1.17816 | 0.05 |
| 4908 | 1.17816 | 0.05 |
| 4909 | 0.596954 | 0.993481 |
| 4910 | 1.65133 | 0.188163 |
| 4911 | 0.583859 | 0.293525 |
| 4912 | 1.04072 | 1.02079 |
| 4913 | 1.28795 | 0.261166 |
| 4914 | 1.59423 | 0.172049 |
| 4915 | 0.483559 | 0.389388 |
| 4916 | 1.74088 | 0.156394 |
| 4917 | 1.09545 | 0.86777 |
| 4918 | 1.02519 | 1.62784 |
| 4919 | 2.47579 | 0.866927 |
| 4920 | 1.54732 | 1.01616 |
| 4921 | 2.17663 | 0.797856 |
| 4922 | 1.75006 | 0.816682 |
| 4923 | 1.32354 | 0.971357 |
| 4924 | 1.09962 | 2.1 |
| 4925 | 1.85057 | 2.1 |
| 4926 | 1.91046 | 2.1 |
| 4927 | 2.17037 | 2.1 |

| | | |
|---|---|---|
| 4928 | 1.99528 | 0.05 |
| 4929 | 1.97969 | 0.05 |
| 4930 | 3.82765 | 2.1 |
| 4931 | 1.85767 | 2.1 |
| 4932 | 2.77042 | 2.1 |
| 4933 | 1.37236 | 0.05 |
| 4934 | 2.23254 | 0.05 |
| 4935 | 1.10089 | 0.05 |
| 4936 | 0.994294 | 2.1 |
| 4937 | 1.81921 | 2.1 |
| 4938 | 1.00073 | 2.1 |
| 4939 | 0.681043 | 2.1 |
| 4940 | 0.946131 | 2.1 |
| 4941 | 0.681073 | 2.1 |
| 4942 | 1.51804 | 2.1 |
| 4943 | 1.60653 | 2.1 |
| 4944 | 1.30188 | 2.1 |
| 4945 | 0.919622 | 2.1 |
| 4946 | 1.00965 | 2.1 |
| 4947 | 1.93821 | 2.1 |
| 4948 | 2.60377 | 2.1 |
| 4949 | 1.36416 | 0.05 |
| 4950 | 1.36416 | 0.05 |
| 4951 | 1.36416 | 0.05 |
| 4952 | 2.44132 | 0.05 |
| 4953 | 2.15871 | 0.191909 |
| 4954 | 2.22171 | 0.05 |
| 4955 | 1.70747 | 0.05 |
| 4956 | 1.49515 | 0.05 |
| 4957 | 0.773007 | 0.0667245 |
| 4958 | 0.946125 | 0.086385 |
| 4959 | 0.946131 | 0.198497 |
| 4960 | 0.773034 | 0.05 |
| 4961 | 1.15844 | 0.0561349 |
| 4962 | 1.98104 | 0.0525413 |
| 4963 | 2.44283 | 0.05 |
| 4964 | 1.05417 | 0.05 |
| 4965 | 1.1596 | 0.05 |
| 4966 | 0.829921 | 0.05 |
| 4967 | 0.946131 | 0.05 |
| 4968 | 0.951911 | 0.05 |
| 4969 | 1.39027 | 0.05 |
| 4970 | 0.829946 | 0.05 |
| 4971 | 1.51223 | 0.05 |
| 4972 | 1.51223 | 0.05 |
| 4973 | 1.65984 | 0.05 |
| 4974 | 1.90382 | 0.05 |
| 4975 | 1.93228 | 1.03007 |
| 4976 | 2.47924 | 0.990959 |
| 4977 | 1.73904 | 1.10625 |
| 4978 | 1.40547 | 0.698806 |
| 4979 | 2.37589 | 0.809854 |
| 4980 | 0.662605 | 1.09573 |
| 4981 | 2.33293 | 0.881601 |
| 4982 | 1.72249 | 2.1 |
| 4983 | 1.68478 | 2.1 |
| 4984 | 1.00514 | 0.105177 |
| 4985 | 1.4537 | 2.1 |

| | | |
|---|---|---|
| 4986 | 1.92698 | 2.1 |
| 4987 | 1.46041 | 0.05 |
| 4988 | 2.04817 | 0.05 |
| 4989 | 1.65954 | 2.1 |
| 4990 | 1.42342 | 2.1 |
| 4991 | 1.04405 | 0.05 |
| 4992 | 1.17056 | 0.05 |
| 4993 | 1.11339 | 0.05 |
| 4994 | 0.946133 | 0.05 |
| 4995 | 0.929724 | 0.05 |
| 4996 | 1.35424 | 0.05 |
| 4997 | 1.35424 | 0.05 |
| 4998 | 0.929713 | 0.05 |
| 4999 | 1.85945 | 0.05 |
| 5000 | 1.38905 | 2.1 |
| 5001 | 1.39056 | 2.1 |
| 5002 | 1.52852 | 2.1 |
| 5003 | 1.06472 | 2.1 |
| 5004 | 0.749096 | 2.1 |
| 5005 | 1.46076 | 2.1 |
| 5006 | 1.29263 | 2.1 |
| 5007 | 0.94613 | 2.1 |
| 5008 | 0.69097 | 2.1 |
| 5009 | 0.982284 | 2.1 |
| 5010 | 0.67419 | 2.1 |
| 5011 | 1.52852 | 2.1 |
| 5012 | 1.91121 | 2.1 |
| 5013 | 1.00775 | 2.1 |
| 5014 | 1.61201 | 2.1 |
| 5015 | 0.529987 | 2.1 |
| 5016 | 0.506125 | 2.1 |
| 5017 | 1.44696 | 2.1 |
| 5018 | 2.32981 | 2.1 |
| 5019 | 1.32784 | 2.1 |
| 5020 | 1.94158 | 2.1 |
| 5021 | 1.31595 | 2.1 |
| 5022 | 0.663922 | 2.1 |
| 5023 | 1.44663 | 2.1 |
| 5024 | 1.44663 | 2.1 |
| 5025 | 1.13976 | 2.1 |
| 5026 | 0.970788 | 2.1 |
| 5027 | 1.13976 | 2.1 |
| 5028 | 0.663921 | 2.1 |
| 5029 | 0.966114 | 2.1 |
| 5030 | 1.05498 | 0.979184 |
| 5031 | 1.38086 | 2.1 |
| 5032 | 0.946127 | 2.1 |
| 5033 | 0.922513 | 2.1 |
| 5034 | 1.49903 | 2.1 |
| 5035 | 1.49903 | 2.1 |
| 5036 | 0.92251 | 2.1 |
| 5037 | 1.06105 | 2.1 |
| 5038 | 1.36049 | 2.1 |
| 5039 | 1.38086 | 2.1 |
| 5040 | 1.52534 | 2.1 |
| 5041 | 1.04101 | 2.1 |
| 5042 | 1.30861 | 0.05 |
| 5043 | 0.949112 | 0.05 |

| | | |
|---|---|---|
| 5044 | 0.894275 | 0.05 |
| 5045 | 1.10232 | 0.05 |
| 5046 | 0.830889 | 0.05 |
| 5047 | 1.26554 | 0.05 |
| 5048 | 0.948347 | 0.05 |
| 5049 | 0.81096 | 0.05 |
| 5050 | 1.18338 | 0.05 |
| 5051 | 0.883764 | 0.05 |
| 5052 | 1.54793 | 0.05 |
| 5053 | 1.02867 | 0.05 |
| 5054 | 1.8573 | 0.05 |
| 5055 | 1.06065 | 0.05 |
| 5056 | 0.64842 | 0.05 |
| 5057 | 1.99319 | 0.05 |
| 5058 | 1.9476 | 0.05 |
| 5059 | 2.51436 | 0.05 |
| 5060 | 1.68337 | 0.05 |
| 5061 | 1.92321 | 0.05 |
| 5062 | 0.810942 | 0.05 |
| 5063 | 0.921001 | 0.05 |
| 5064 | 0.99758 | 0.05 |
| 5065 | 0.960785 | 0.05 |
| 5066 | 1.01565 | 0.05 |
| 5067 | 0.908624 | 0.05 |
| 5068 | 0.912835 | 0.05 |
| 5069 | 1.18351 | 0.05 |
| 5070 | 1.40239 | 2.1 |
| 5071 | 1.26091 | 2.1 |
| 5072 | 0.947979 | 2.1 |
| 5073 | 0.946131 | 2.1 |
| 5074 | 1.81959 | 2.1 |
| 5075 | 1.81959 | 0.809652 |
| 5076 | 0.94613 | 2.1 |
| 5077 | 1.38415 | 0.527537 |
| 5078 | 1.38414 | 2.1 |
| 5079 | 1.33266 | 2.1 |
| 5080 | 0.946132 | 2.1 |
| 5081 | 1.76225 | 2.1 |
| 5082 | 1.76225 | 2.1 |
| 5083 | 1.27599 | 2.1 |
| 5084 | 1.38158 | 2.1 |
| 5085 | 1.3268 | 2.1 |
| 5086 | 1.27599 | 2.1 |
| 5087 | 0.947979 | 2.1 |
| 5088 | 1.0142 | 0.0720197 |
| 5089 | 1.01422 | 0.0649952 |
| 5090 | 1.11435 | 0.857764 |
| 5091 | 1.42186 | 0.870466 |
| 5092 | 1.24001 | 1.17719 |
| 5093 | 1.77342 | 1.34048 |
| 5094 | 1.42186 | 1.42293 |
| 5095 | 1.99817 | 1.01184 |
| 5096 | 1.27361 | 2.1 |
| 5097 | 1.68914 | 2.1 |
| 5098 | 1.30116 | 2.1 |
| 5099 | 1.32819 | 1.6875 |
| 5100 | 1.68322 | 1.50412 |
| 5101 | 1.68322 | 2.1 |

| | | |
|---|---|---|
| 5102 | 0.94613 | 0.124609 |
| 5103 | 0.94613 | 2.1 |
| 5104 | 1.14666 | 2.04314 |
| 5105 | 1.07612 | 1.21437 |
| 5106 | 1.31151 | 2.1 |
| 5107 | 1.22095 | 2.1 |
| 5108 | 1.57256 | 2.1 |
| 5109 | 1.6679 | 2.1 |
| 5110 | 0.946132 | 0.419884 |
| 5111 | 0.946133 | 2.1 |
| 5112 | 1.13537 | 2.1 |
| 5113 | 1.07855 | 2.1 |
| 5114 | 1.07855 | 2.1 |
| 5115 | 1.43895 | 1.18777 |
| 5116 | 1.38481 | 0.05 |
| 5117 | 1.72385 | 0.05 |
| 5118 | 0.550441 | 0.05 |
| 5119 | 0.79807 | 0.05 |
| 5120 | 1.11627 | 0.05 |
| 5121 | 1.30349 | 0.05 |
| 5122 | 0.94613 | 0.05 |
| 5123 | 0.686176 | 0.05 |
| 5124 | 1.11627 | 0.05 |
| 5125 | 1.73363 | 0.05 |
| 5126 | 1.73363 | 0.05 |
| 5127 | 0.686139 | 0.05 |
| 5128 | 1.24932 | 0.05 |
| 5129 | 0.899582 | 0.05 |
| 5130 | 2.00472 | 0.05 |
| 5131 | 1.17121 | 0.05 |
| 5132 | 1.2874 | 0.05 |
| 5133 | 2.0194 | 0.05 |
| 5134 | 0.88601 | 0.05 |
| 5135 | 1.77409 | 0.05 |
| 5136 | 1.73293 | 0.05 |
| 5137 | 2.74045 | 0.05 |
| 5138 | 1.83787 | 0.05 |
| 5139 | 1.45448 | 0.05 |
| 5140 | 1.27778 | 0.05 |
| 5141 | 1.1192 | 2.1 |
| 5142 | 1.25953 | 2.1 |
| 5143 | 1.93611 | 2.1 |
| 5144 | 1.92104 | 2.1 |
| 5145 | 1.15506 | 0.05 |
| 5146 | 1.15506 | 0.05 |
| 5147 | 1.23943 | 0.521597 |
| 5148 | 0.851543 | 2.1 |
| 5149 | 1.6061 | 2.1 |
| 5150 | 1.80058 | 2.1 |
| 5151 | 0.869471 | 2.1 |
| 5152 | 1.3931 | 2.1 |
| 5153 | 1.06454 | 2.1 |
| 5154 | 0.93667 | 2.1 |
| 5155 | 2.16916 | 2.1 |
| 5156 | 3.10246 | 2.1 |
| 5157 | 1.91539 | 2.1 |
| 5158 | 1.91539 | 2.1 |
| 5159 | 0.620263 | 1.32422 |

| | | |
|---|---|---|
| 5160 | 0.620265 | 2.1 |
| 5161 | 0.946132 | 1.67191 |
| 5162 | 0.620265 | 1.23896 |
| 5163 | 1.30226 | 1.13736 |
| 5164 | 1.87292 | 2.1 |
| 5165 | 1.18769 | 2.1 |
| 5166 | 2.60452 | 2.1 |
| 5167 | 1.26574 | 2.1 |
| 5168 | 2.46679 | 2.1 |
| 5169 | 1.36459 | 2.1 |
| 5170 | 1.26865 | 1.15869 |
| 5171 | 1.1744 | 0.871111 |
| 5172 | 1.15841 | 0.0511068 |
| 5173 | 1.02247 | 0.05 |
| 5174 | 1.93649 | 2.1 |
| 5175 | 1.64718 | 2.1 |
| 5176 | 1.59654 | 2.1 |
| 5177 | 1.57823 | 2.1 |
| 5178 | 1.00665 | 0.05 |
| 5179 | 1.28239 | 0.05 |
| 5180 | 0.887895 | 2.1 |
| 5181 | 1.22083 | 2.1 |
| 5182 | 1.42127 | 2.1 |
| 5183 | 1.07374 | 2.1 |
| 5184 | 1.00503 | 1.44644 |
| 5185 | 1.23082 | 0.961083 |
| 5186 | 2.78663 | 2.1 |
| 5187 | 2.20883 | 2.1 |
| 5188 | 2.54588 | 2.1 |
| 5189 | 0.946128 | 2.1 |
| 5190 | 1.13716 | 2.1 |
| 5191 | 1.58724 | 2.1 |
| 5192 | 1.58724 | 2.1 |
| 5193 | 0.946131 | 2.1 |
| 5194 | 1.13716 | 2.1 |
| 5195 | 1.39621 | 2.1 |
| 5196 | 1.02438 | 2.1 |
| 5197 | 1.49543 | 0.05 |
| 5198 | 2.78336 | 0.05 |
| 5199 | 1.27148 | 0.05 |
| 5200 | 1.0906 | 0.05 |
| 5201 | 1.04072 | 0.05 |
| 5202 | 0.63574 | 0.05 |
| 5203 | 1.45506 | 0.05 |
| 5204 | 1.5863 | 0.05 |
| 5205 | 0.928352 | 0.05 |
| 5206 | 0.647687 | 0.05 |
| 5207 | 2.2375 | 1.89717 |
| 5208 | 1.50998 | 1.61724 |
| 5209 | 2.75021 | 1.44952 |
| 5210 | 1.91912 | 1.34502 |
| 5211 | 1.99872 | 0.913282 |
| 5212 | 0.918127 | 2.01602 |
| 5213 | 1.03031 | 1.06646 |
| 5214 | 0.951169 | 0.391676 |
| 5215 | 1.27975 | 0.549956 |
| 5216 | 1.12735 | 0.946271 |
| 5217 | 1.15741 | 0.975829 |

| | | |
|---|---|---|
| 5218 | 1.24998 | 0.870352 |
| 5219 | 1.65953 | 0.05 |
| 5220 | 0.967555 | 0.198144 |
| 5221 | 0.967542 | 0.05 |
| 5222 | 1.03824 | 0.05 |
| 5223 | 1.48693 | 0.0582769 |
| 5224 | 1.48693 | 0.0587886 |
| 5225 | 2.02901 | 0.05 |
| 5226 | 0.946127 | 0.070251 |
| 5227 | 1.07139 | 0.05 |
| 5228 | 0.946126 | 0.05 |
| 5229 | 1.84644 | 0.05 |
| 5230 | 1.84644 | 0.05 |
| 5231 | 1.38683 | 0.05 |
| 5232 | 1.40574 | 0.05 |
| 5233 | 1.36866 | 1.11227 |
| 5234 | 0.649325 | 0.965515 |
| 5235 | 1.93631 | 2.1 |
| 5236 | 1.50145 | 2.1 |
| 5237 | 1.6287 | 2.1 |
| 5238 | 1.31302 | 2.1 |
| 5239 | 1.18041 | 2.1 |
| 5240 | 1.18041 | 2.1 |
| 5241 | 0.974196 | 2.1 |
| 5242 | 0.946131 | 2.1 |
| 5243 | 1.74169 | 2.1 |
| 5244 | 1.74169 | 2.1 |
| 5245 | 1.23163 | 2.1 |
| 5246 | 1.27451 | 2.1 |
| 5247 | 1.41331 | 2.1 |
| 5248 | 1.23163 | 2.1 |
| 5249 | 1.28037 | 2.1 |
| 5250 | 1.28158 | 2.1 |
| 5251 | 1.28037 | 2.1 |
| 5252 | 2.28015 | 0.05 |
| 5253 | 2.06561 | 0.05 |
| 5254 | 2.61343 | 0.05 |
| 5255 | 1.11712 | 0.05 |
| 5256 | 1.63689 | 1.23285 |
| 5257 | 1.01393 | 0.681671 |
| 5258 | 0.996775 | 2.1 |
| 5259 | 1.54277 | 2.1 |
| 5260 | 1.26397 | 2.1 |
| 5261 | 1.46129 | 2.1 |
| 5262 | 1.62391 | 2.1 |
| 5263 | 1.08848 | 0.05 |
| 5264 | 1.11833 | 2.1 |
| 5265 | 1.24041 | 2.1 |
| 5266 | 2.31884 | 2.1 |
| 5267 | 1.335 | 2.1 |
| 5268 | 1.48589 | 0.05 |
| 5269 | 1.77131 | 0.05 |
| 5270 | 1.8422 | 0.859766 |
| 5271 | 2.47453 | 1.73665 |
| 5272 | 1.23726 | 1.43689 |
| 5273 | 1.21996 | 1.38932 |
| 5274 | 1.65512 | 1.55703 |
| 5275 | 1.65512 | 1.51452 |

| | | |
|---|---|---|
| 5276 | 0.802107 | 1.71966 |
| 5277 | 0.946129 | 1.00478 |
| 5278 | 0.802137 | 0.719174 |
| 5279 | 1.3279 | 1.01763 |
| 5280 | 1.23726 | 0.924313 |
| 5281 | 1.79759 | 0.962849 |
| 5282 | 1.79759 | 0.711493 |
| 5283 | 0.767577 | 1.49894 |
| 5284 | 0.946131 | 0.501583 |
| 5285 | 0.767577 | 0.341376 |
| 5286 | 1.03534 | 0.05 |
| 5287 | 1.09962 | 0.0542969 |
| 5288 | 0.929217 | 1.1738 |
| 5289 | 1.70126 | 1.21445 |
| 5290 | 0.991407 | 2.1 |
| 5291 | 1.41077 | 2.1 |
| 5292 | 0.991406 | 2.1 |
| 5293 | 1.62966 | 2.1 |
| 5294 | 1.62966 | 2.1 |
| 5295 | 0.946128 | 2.1 |
| 5296 | 1.18364 | 2.1 |
| 5297 | 0.946131 | 2.1 |
| 5298 | 1.14186 | 2.1 |
| 5299 | 1.18364 | 2.1 |
| 5300 | 1.16858 | 2.1 |
| 5301 | 1.66137 | 0.05 |
| 5302 | 1.90165 | 0.05 |
| 5303 | 1.66139 | 0.05 |
| 5304 | 1.26893 | 0.181293 |
| 5305 | 1.15918 | 0.616899 |
| 5306 | 0.653228 | 0.75163 |
| 5307 | 0.946133 | 0.679254 |
| 5308 | 1.77488 | 2.1 |
| 5309 | 1.77489 | 1.08605 |
| 5310 | 0.653228 | 1.14118 |
| 5311 | 1.56043 | 0.723524 |
| 5312 | 1.60894 | 1.07536 |
| 5313 | 1.56043 | 0.613895 |
| 5314 | 1.06848 | 1.36368 |
| 5315 | 1.04307 | 2.1 |
| 5316 | 1.14188 | 2.1 |
| 5317 | 1.09195 | 2.1 |
| 5318 | 0.615121 | 2.1 |
| 5319 | 1.50867 | 2.1 |
| 5320 | 1.27152 | 2.1 |
| 5321 | 0.615094 | 2.1 |
| 5322 | 1.07297 | 2.1 |
| 5323 | 1.1372 | 2.1 |
| 5324 | 0.553608 | 2.1 |
| 5325 | 1.23019 | 2.1 |
| 5326 | 0.992675 | 2.1 |
| 5327 | 1.40984 | 1.22523 |
| 5328 | 2.55682 | 2.1 |
| 5329 | 3.66069 | 2.1 |
| 5330 | 3.58726 | 2.1 |
| 5331 | 1.4375 | 0.05 |
| 5332 | 1.88424 | 0.05 |
| 5333 | 1.56714 | 2.1 |

| | | |
|---|---|---|
| 5334 | 1.6721 | 2.1 |
| 5335 | 1.18474 | 2.1 |
| 5336 | 1.2295 | 2.1 |
| 5337 | 1.28754 | 2.1 |
| 5338 | 1.36838 | 2.1 |
| 5339 | 2.78661 | 2.1 |
| 5340 | 2.64055 | 2.1 |
| 5341 | 1.35065 | 0.901887 |
| 5342 | 2.26675 | 0.0510571 |
| 5343 | 1.35065 | 0.168072 |
| 5344 | 1.21558 | 0.175797 |
| 5345 | 1.49709 | 0.05 |
| 5346 | 1.4971 | 0.05 |
| 5347 | 0.85472 | 0.05 |
| 5348 | 0.854718 | 0.05 |
| 5349 | 0.946129 | 0.05 |
| 5350 | 0.854717 | 0.05 |
| 5351 | 1.1604 | 0.05 |
| 5352 | 1.25265 | 0.05 |
| 5353 | 2.32081 | 0.05 |
| 5354 | 1.15371 | 0.05 |
| 5355 | 1.14945 | 0.05 |
| 5356 | 0.995725 | 0.0685694 |
| 5357 | 1.00307 | 0.05 |
| 5358 | 1.10907 | 0.144236 |
| 5359 | 2.30315 | 0.0595354 |
| 5360 | 1.10908 | 0.0946862 |
| 5361 | 1.27558 | 0.793538 |
| 5362 | 1.71613 | 1.5037 |
| 5363 | 1.32977 | 2.1 |
| 5364 | 0.815276 | 2.1 |
| 5365 | 1.41697 | 2.1 |
| 5366 | 1.41697 | 2.1 |
| 5367 | 0.815276 | 2.1 |
| 5368 | 1.38897 | 2.1 |
| 5369 | 0.946131 | 2.1 |
| 5370 | 0.815275 | 2.1 |
| 5371 | 1.44684 | 2.1 |
| 5372 | 1.21489 | 1.97946 |
| 5373 | 1.22476 | 1.97111 |
| 5374 | 0.870366 | 0.51444 |
| 5375 | 0.862572 | 0.326987 |
| 5376 | 0.835435 | 0.288031 |
| 5377 | 1.6993 | 0.204448 |
| 5378 | 1.23618 | 0.0941533 |
| 5379 | 1.18624 | 0.338192 |
| 5380 | 1.53058 | 0.139534 |
| 5381 | 2.16108 | 0.934067 |
| 5382 | 2.21589 | 0.830095 |
| 5383 | 1.04037 | 2.1 |
| 5384 | 1.03933 | 2.0672 |
| 5385 | 1.57753 | 0.05 |
| 5386 | 1.87644 | 0.05 |
| 5387 | 1.24728 | 0.05 |
| 5388 | 1.30394 | 0.05 |
| 5389 | 1.3538 | 2.1 |
| 5390 | 1.69858 | 2.1 |
| 5391 | 1.1726 | 0.0737901 |

| | | |
|---|---|---|
| 5392 | 0.94612 | 0.05 |
| 5393 | 1.09805 | 0.05 |
| 5394 | 0.946118 | 0.05 |
| 5395 | 1.72576 | 0.05 |
| 5396 | 1.72575 | 0.05 |
| 5397 | 1.34152 | 0.05 |
| 5398 | 1.33034 | 0.05 |
| 5399 | 0.784151 | 2.1 |
| 5400 | 0.946131 | 2.1 |
| 5401 | 0.784151 | 2.1 |
| 5402 | 1.47963 | 2.1 |
| 5403 | 1.47963 | 2.1 |
| 5404 | 1.3335 | 2.1 |
| 5405 | 0.930281 | 2.1 |
| 5406 | 1.29435 | 1.7877 |
| 5407 | 0.883308 | 0.570325 |
| 5408 | 1.31589 | 2.1 |
| 5409 | 1.67846 | 2.1 |
| 5410 | 0.993598 | 0.05 |
| 5411 | 2.23193 | 0.05 |
| 5412 | 0.9936 | 0.05 |
| 5413 | 0.615629 | 0.05 |
| 5414 | 1.65925 | 2.1 |
| 5415 | 2.05992 | 2.1 |
| 5416 | 1.65925 | 2.1 |
| 5417 | 4.08735 | 1.87954 |
| 5418 | 2.72856 | 1.91903 |
| 5419 | 0.869602 | 2.1 |
| 5420 | 3.42833 | 1.02214 |
| 5421 | 1.14397 | 0.05 |
| 5422 | 1.34563 | 0.374253 |
| 5423 | 1.08189 | 0.313093 |
| 5424 | 0.782128 | 2.08258 |
| 5425 | 1.0022 | 2.1 |
| 5426 | 0.900427 | 1.85407 |
| 5427 | 1.51499 | 1.90455 |
| 5428 | 1.05588 | 1.92183 |
| 5429 | 1.13739 | 2.1 |
| 5430 | 1.37004 | 1.97836 |
| 5431 | 1.14603 | 2.1 |
| 5432 | 0.851541 | 1.2736 |
| 5433 | 0.946799 | 1.82959 |
| 5434 | 1.14854 | 1.74122 |
| 5435 | 1.33793 | 1.38447 |
| 5436 | 0.94613 | 1.39273 |
| 5437 | 1.24336 | 1.68583 |
| 5438 | 0.851473 | 1.80557 |
| 5439 | 2.08792 | 1.764 |
| 5440 | 1.01869 | 1.78612 |
| 5441 | 0.986953 | 0.05 |
| 5442 | 0.896238 | 0.05 |
| 5443 | 0.906574 | 0.05 |
| 5444 | 0.852145 | 0.05 |
| 5445 | 2.14191 | 0.05 |
| 5446 | 1.15979 | 0.05 |
| 5447 | 1.18327 | 0.05 |
| 5448 | 2.32742 | 0.05 |
| 5449 | 2.35961 | 0.05 |

| | | |
|---|---|---|
| 5450 | 2.38126 | 0.05 |
| 5451 | 1.13632 | 0.05 |
| 5452 | 1.17897 | 0.05 |
| 5453 | 1.76199 | 0.05 |
| 5454 | 1.00573 | 2.1 |
| 5455 | 0.946127 | 0.467969 |
| 5456 | 1.7077 | 2.1 |
| 5457 | 1.7077 | 2.1 |
| 5458 | 0.946127 | 1.62682 |
| 5459 | 1.24887 | 2.1 |
| 5460 | 1.24887 | 2.1 |
| 5461 | 1.22441 | 2.1 |
| 5462 | 1.08542 | 2.1 |
| 5463 | 1.21743 | 2.09169 |
| 5464 | 1.12572 | 2.1 |
| 5465 | 1.05499 | 0.940252 |
| 5466 | 1.00521 | 1.01249 |
| 5467 | 2.86191 | 0.05 |
| 5468 | 2.27972 | 0.05 |
| 5469 | 1.01169 | 2.1 |
| 5470 | 1.03373 | 2.1 |
| 5471 | 1.39319 | 2.1 |
| 5472 | 0.636837 | 2.1 |
| 5473 | 1.63908 | 2.1 |
| 5474 | 1.45713 | 2.06959 |
| 5475 | 1.04072 | 2.1 |
| 5476 | 1.25025 | 2.1 |
| 5477 | 1.38914 | 2.1 |
| 5478 | 0.636807 | 2.1 |
| 5479 | 1.50211 | 2.1 |
| 5480 | 1.36555 | 2.1 |
| 5481 | 2.77545 | 1.43777 |
| 5482 | 0.682805 | 2.1 |
| 5483 | 1.50267 | 2.1 |
| 5484 | 1.50267 | 2.1 |
| 5485 | 0.797749 | 2.1 |
| 5486 | 1.38773 | 2.1 |
| 5487 | 1.38773 | 2.1 |
| 5488 | 0.682776 | 2.1 |
| 5489 | 2.01245 | 0.05 |
| 5490 | 0.925918 | 0.05 |
| 5491 | 0.75689 | 0.05 |
| 5492 | 1.25823 | 0.05 |
| 5493 | 1.16505 | 0.05 |
| 5494 | 0.838779 | 0.05 |
| 5495 | 1.14722 | 0.05 |
| 5496 | 0.756849 | 0.05 |
| 5497 | 1.52298 | 0.05 |
| 5498 | 2.11034 | 0.05 |
| 5499 | 1.92174 | 0.05 |
| 5500 | 1.92174 | 0.05 |
| 5501 | 0.576083 | 0.05 |
| 5502 | 1.23146 | 0.05 |
| 5503 | 1.26637 | 0.05 |
| 5504 | 1.23146 | 0.05 |
| 5505 | 1.26637 | 0.05 |
| 5506 | 0.946133 | 0.133671 |
| 5507 | 1.37034 | 0.05 |

| | | |
|---|---|---|
| 5508 | 1.84145 | 0.0635981 |
| 5509 | 1.84145 | 0.05 |
| 5510 | 0.946132 | 0.267125 |
| 5511 | 1.37034 | 0.0989992 |
| 5512 | 1.41724 | 0.0903935 |
| 5513 | 1.0685 | 0.05 |
| 5514 | 1.14547 | 0.05 |
| 5515 | 1.24976 | 2.1 |
| 5516 | 1.30682 | 2.1 |
| 5517 | 2.12095 | 2.1 |
| 5518 | 1.46392 | 2.1 |
| 5519 | 1.46392 | 2.1 |
| 5520 | 0.935738 | 2.1 |
| 5521 | 1.32949 | 2.1 |
| 5522 | 1.07017 | 2.1 |
| 5523 | 0.935766 | 2.1 |
| 5524 | 1.07017 | 2.1 |
| 5525 | 1.78095 | 2.1 |
| 5526 | 1.05619 | 0.775739 |
| 5527 | 1.05793 | 0.790371 |
| 5528 | 1.77677 | 2.1 |
| 5529 | 2.04766 | 2.1 |
| 5530 | 1.02524 | 2.1 |
| 5531 | 1.18803 | 2.1 |
| 5532 | 1.70047 | 2.1 |
| 5533 | 1.556 2.1 | |
| 5534 | 1.24201 | 2.1 |
| 5535 | 1.3592 | 2.1 |
| 5536 | 2.11824 | 0.05 |
| 5537 | 2.20248 | 0.05 |
| 5538 | 3.43878 | 0.05 |
| 5539 | 3.12466 | 0.05 |
| 5540 | 2.09351 | 2.1 |
| 5541 | 0.946128 | 2.1 |
| 5542 | 1.51269 | 2.1 |
| 5543 | 1.51269 | 2.1 |
| 5544 | 0.946131 | 2.1 |
| 5545 | 1.01306 | 2.1 |
| 5546 | 1.04676 | 2.1 |
| 5547 | 1.01306 | 2.1 |
| 5548 | 1.07444 | 2.1 |
| 5549 | 2.17465 | 2.1 |
| 5550 | 1.14808 | 2.1 |
| 5551 | 1.39067 | 1.1675 |
| 5552 | 1.20958 | 1.16351 |
| 5553 | 0.662832 | 0.700928 |
| 5554 | 1.93512 | 0.97226 |
| 5555 | 1.15471 | 2.1 |
| 5556 | 1.75119 | 2.1 |
| 5557 | 1.75118 | 2.1 |
| 5558 | 0.946131 | 2.1 |
| 5559 | 1.28606 | 2.1 |
| 5560 | 1.27087 | 2.1 |
| 5561 | 0.946131 | 2.1 |
| 5562 | 1.28606 | 2.1 |
| 5563 | 1.58916 | 0.05 |
| 5564 | 0.927822 | 0.05 |
| 5565 | 2.24367 | 0.05 |

| | | |
|---|---|---|
| 5566 | 1.8734 | 0.05 |
| 5567 | 3.51668 | 0.05 |
| 5568 | 2.68986 | 0.05 |
| 5569 | 2.32395 | 2.1 |
| 5570 | 1.39564 | 2.1 |
| 5571 | 2.10862 | 2.1 |
| 5572 | 1.36763 | 2.1 |
| 5573 | 0.970963 | 0.05 |
| 5574 | 0.97096 | 0.05 |
| 5575 | 1.11897 | 0.05 |
| 5576 | 1.26885 | 0.05 |
| 5577 | 1.11605 | 0.05 |
| 5578 | 2.06612 | 0.05 |
| 5579 | 1.717 0.05 | |
| 5580 | 0.59502 | 0.05 |
| 5581 | 0.825755 | 0.05 |
| 5582 | 0.709624 | 0.05 |
| 5583 | 1.65741 | 0.05 |
| 5584 | 0.307486 | 0.05 |
| 5585 | 1.4785 | 0.05 |
| 5586 | 0.983143 | 2.1 |
| 5587 | 1.08247 | 2.1 |
| 5588 | 1.42353 | 2.1 |
| 5589 | 1.42353 | 2.1 |
| 5590 | 0.781667 | 2.1 |
| 5591 | 0.797634 | 2.1 |
| 5592 | 0.946131 | 2.1 |
| 5593 | 0.781638 | 2.1 |
| 5594 | 0.946131 | 2.1 |
| 5595 | 0.98315 | 2.1 |
| 5596 | 1.083 2.1 | |
| 5597 | 1.61975 | 0.05 |
| 5598 | 1.11946 | 0.05 |
| 5599 | 1.18977 | 0.05 |
| 5600 | 0.996484 | 0.05 |
| 5601 | 2.24343 | 0.05 |
| 5602 | 1.4787 | 0.05 |
| 5603 | 1.66127 | 0.05 |
| 5604 | 1.86028 | 0.05 |
| 5605 | 1.09128 | 0.05 |
| 5606 | 1.03115 | 0.05 |
| 5607 | 0.838025 | 0.05 |
| 5608 | 1.3736 | 0.05 |
| 5609 | 1.44009 | 0.05 |
| 5610 | 0.92917 | 0.05 |
| 5611 | 1.03889 | 0.05 |
| 5612 | 0.956974 | 0.05 |
| 5613 | 3.23468 | 0.05 |
| 5614 | 0.953922 | 0.05 |
| 5615 | 0.957104 | 0.05 |
| 5616 | 1.66231 | 0.05 |
| 5617 | 1.26778 | 0.05 |
| 5618 | 1.47615 | 0.05 |
| 5619 | 1.28621 | 0.05 |
| 5620 | 1.08043 | 0.05 |
| 5621 | 2.35026 | 2.1 |
| 5622 | 2.35025 | 2.1 |
| 5623 | 2.60659 | 2.1 |

| | | |
|---|---|---|
| 5624 | 1.57627 | 2.1 |
| 5625 | 1.5493 | 2.1 |
| 5626 | 1.24682 | 0.05 |
| 5627 | 2.14277 | 0.0805526 |
| 5628 | 1.24682 | 0.492928 |
| 5629 | 1.00307 | 0.0583088 |
| 5630 | 2.04056 | 0.0962433 |
| 5631 | 1.45408 | 2.1 |
| 5632 | 1.54249 | 2.1 |
| 5633 | 1.24474 | 1.70799 |
| 5634 | 1.37813 | 2.04976 |
| 5635 | 0.924158 | 0.05 |
| 5636 | 0.916623 | 0.05 |
| 5637 | 1.35344 | 0.05 |
| 5638 | 1.00859 | 0.05 |
| 5639 | 1.34786 | 0.05 |
| 5640 | 0.952457 | 0.05 |
| 5641 | 0.85404 | 0.05 |
| 5642 | 1.06341 | 2.1 |
| 5643 | 1.09194 | 2.1 |
| 5644 | 1.93707 | 2.1 |
| 5645 | 2.14087 | 2.1 |
| 5646 | 1.93707 | 2.1 |
| 5647 | 1.07519 | 0.924851 |
| 5648 | 1.07519 | 0.479548 |
| 5649 | 1.62518 | 2.1 |
| 5650 | 1.3592 | 2.1 |
| 5651 | 1.13183 | 2.1 |
| 5652 | 1.20357 | 2.1 |
| 5653 | 1.4858 | 2.1 |
| 5654 | 1.23694 | 2.1 |
| 5655 | 1.44011 | 2.1 |
| 5656 | 1.23989 | 2.1 |
| 5657 | 2.21604 | 2.1 |
| 5658 | 1.85574 | 0.05 |
| 5659 | 1.55649 | 2.1 |
| 5660 | 1.48861 | 0.05 |
| 5661 | 1.15968 | 0.05 |
| 5662 | 0.946132 | 0.05 |
| 5663 | 0.663056 | 0.05 |
| 5664 | 1.70837 | 0.05 |
| 5665 | 1.70837 | 0.05 |
| 5666 | 0.946131 | 0.05 |
| 5667 | 0.663026 | 0.05 |
| 5668 | 2.09604 | 0.05 |
| 5669 | 0.81094 | 0.05 |
| 5670 | 1.06559 | 0.05 |
| 5671 | 0.810956 | 0.05 |
| 5672 | 1.15474 | 0.05 |
| 5673 | 0.990051 | 0.05 |
| 5674 | 0.894971 | 0.05 |
| 5675 | 1.32536 | 0.05 |
| 5676 | 0.946133 | 2.1 |
| 5677 | 1.33125 | 2.1 |
| 5678 | 1.75823 | 2.1 |
| 5679 | 1.75823 | 2.1 |
| 5680 | 0.946133 | 2.1 |
| 5681 | 1.33125 | 2.1 |

| | | |
|---|---|---|
| 5682 | 1.37312 | 2.1 |
| 5683 | 1.27985 | 2.1 |
| 5684 | 1.37312 | 2.1 |
| 5685 | 1.70684 | 2.1 |
| 5686 | 1.70684 | 2.1 |
| 5687 | 0.946132 | 2.1 |
| 5688 | 0.946132 | 2.1 |
| 5689 | 1.25081 | 2.1 |
| 5690 | 0.956293 | 2.1 |
| 5691 | 0.956294 | 2.1 |
| 5692 | 1.36035 | 2.1 |
| 5693 | 1.41944 | 2.1 |
| 5694 | 0.830638 | 2.1 |
| 5695 | 0.747568 | 2.1 |
| 5696 | 0.888498 | 2.1 |
| 5697 | 1.04071 | 2.1 |
| 5698 | 1.17128 | 2.1 |
| 5699 | 1.92339 | 2.1 |
| 5700 | 2.91208 | 2.1 |
| 5701 | 1.82208 | 0.999122 |
| 5702 | 1.89653 | 2.1 |
| 5703 | 1.82588 | 1.33622 |
| 5704 | 1.26703 | 0.0647562 |
| 5705 | 1.26703 | 0.922322 |
| 5706 | 0.6157 | 0.05 |
| 5707 | 1.26706 | 1.55343 |
| 5708 | 0.61567 | 0.05 |
| 5709 | 0.615671 | 0.05 |
| 5710 | 1.26706 | 0.0879771 |
| 5711 | 1.89158 | 0.418859 |
| 5712 | 1.23134 | 0.05 |
| 5713 | 2.49497 | 0.05 |
| 5714 | 1.68103 | 0.05 |
| 5715 | 2.0554 | 2.1 |
| 5716 | 1.72647 | 2.1 |
| 5717 | 1.67375 | 2.1 |
| 5718 | 1.65037 | 2.1 |
| 5719 | 1.04349 | 0.05 |
| 5720 | 1.69055 | 0.05 |
| 5721 | 1.53463 | 0.05 |
| 5722 | 1.19022 | 2.06971 |
| 5723 | 1.89226 | 0.874229 |
| 5724 | 1.15009 | 1.36633 |
| 5725 | 1.7054 | 0.05 |
| 5726 | 1.31612 | 0.05 |
| 5727 | 1.31611 | 0.133798 |
| 5728 | 1.67345 | 0.05 |
| 5729 | 1.06577 | 0.05 |
| 5730 | 1.40193 | 0.05 |
| 5731 | 1.72411 | 0.05 |
| 5732 | 1.46123 | 0.05 |
| 5733 | 0.808808 | 0.05 |
| 5734 | 0.897714 | 0.05 |
| 5735 | 0.851554 | 0.05 |
| 5736 | 1.3499 | 0.05 |
| 5737 | 1.29001 | 0.05 |
| 5738 | 1.22083 | 2.1 |
| 5739 | 1.06191 | 2.1 |

| | | |
|---|---|---|
| 5740 | 1.05758 | 1.41859 |
| 5741 | 1.348 | 2.08338 |
| 5742 | 1.43485 | 1.35713 |
| 5743 | 1.34799 | 1.94685 |
| 5744 | 1.36307 | 0.05 |
| 5745 | 2.28188 | 0.05 |
| 5746 | 1.7926 | 0.05 |
| 5747 | 1.38957 | 2.1 |
| 5748 | 1.47446 | 2.1 |
| 5749 | 0.953638 | 2.1 |
| 5750 | 1.11868 | 2.1 |
| 5751 | 0.953636 | 2.1 |
| 5752 | 1.03408 | 2.1 |
| 5753 | 1.52443 | 0.05 |
| 5754 | 1.38539 | 0.05 |
| 5755 | 0.946132 | 0.05 |
| 5756 | 1.12747 | 0.05 |
| 5757 | 1.04393 | 0.05 |
| 5758 | 1.05017 | 0.05 |
| 5759 | 1.20529 | 0.05 |
| 5760 | 1.3663 | 0.446486 |
| 5761 | 1.89226 | 0.47266 |
| 5762 | 1.17885 | 0.743201 |
| 5763 | 0.867881 | 0.217062 |
| 5764 | 1.32917 | 0.179348 |
| 5765 | 1.32917 | 0.247038 |
| 5766 | 0.946127 | 0.337274 |
| 5767 | 1.19583 | 0.263204 |
| 5768 | 1.00122 | 0.574269 |
| 5769 | 0.946128 | 0.561464 |
| 5770 | 1.71405 | 0.856911 |
| 5771 | 0.871815 | 2.1 |
| 5772 | 1.16435 | 2.1 |
| 5773 | 0.686619 | 2.1 |
| 5774 | 1.63102 | 2.1 |
| 5775 | 1.06589 | 2.1 |
| 5776 | 1.05375 | 2.1 |
| 5777 | 1.9023 | 2.1 |
| 5778 | 1.27394 | 0.791114 |
| 5779 | 1.88345 | 0.756011 |
| 5780 | 2.05636 | 0.880317 |
| 5781 | 0.672977 | 0.696943 |
| 5782 | 1.41274 | 0.719153 |
| 5783 | 1.65606 | 0.675444 |
| 5784 | 0.869329 | 0.696645 |
| 5785 | 1.2207 | 0.744882 |
| 5786 | 1.35631 | 0.724347 |
| 5787 | 0.908776 | 0.693986 |
| 5788 | 0.955081 | 0.215183 |
| 5789 | 1.2243 | 2.1 |
| 5790 | 1.23169 | 2.1 |
| 5791 | 1.52228 | 2.1 |
| 5792 | 1.23169 | 2.1 |
| 5793 | 0.990892 | 1.30871 |
| 5794 | 1.08376 | 0.806913 |
| 5795 | 1.79348 | 2.1 |
| 5796 | 1.50043 | 1.00538 |
| 5797 | 1.32599 | 2.1 |

| | | |
|---|---|---|
| 5798 | 1.37924 | 1.1118 |
| 5799 | 0.838014 | 0.805836 |
| 5800 | 0.87737 | 1.87152 |
| 5801 | 0.947086 | 0.933733 |
| 5802 | 1.27199 | 2.1 |
| 5803 | 1.04918 | 1.01787 |
| 5804 | 1.04851 | 2.1 |
| 5805 | 1.1699 | 1.45927 |
| 5806 | 0.810934 | 1.27053 |
| 5807 | 1.83602 | 2.1 |
| 5808 | 2.21909 | 2.1 |
| 5809 | 0.619441 | 1.71414 |
| 5810 | 2.03755 | 0.987517 |
| 5811 | 1.83472 | 2.1 |
| 5812 | 1.20416 | 1.69883 |
| 5813 | 1.26691 | 0.852148 |
| 5814 | 0.655795 | 1.00226 |
| 5815 | 0.94613 | 1.38481 |
| 5816 | 2.35169 | 1.62025 |
| 5817 | 3.54705 | 2.1 |
| 5818 | 2.60741 | 2.1 |
| 5819 | 1.59019 | 0.05 |
| 5820 | 1.24526 | 0.05 |
| 5821 | 1.59019 | 0.05 |
| 5822 | 2.36406 | 0.05 |
| 5823 | 1.16776 | 0.05 |
| 5824 | 1.67311 | 0.849985 |
| 5825 | 1.67311 | 0.27255 |
| 5826 | 0.94613 | 0.498443 |
| 5827 | 1.38645 | 0.403479 |
| 5828 | 1.13563 | 0.05 |
| 5829 | 1.13563 | 0.528657 |
| 5830 | 1.23279 | 0.112072 |
| 5831 | 1.05042 | 2.1 |
| 5832 | 1.17418 | 1.66472 |
| 5833 | 1.28909 | 2.1 |
| 5834 | 0.973448 | 2.1 |
| 5835 | 1.02958 | 2.1 |
| 5836 | 3.3005 | 2.1 |
| 5837 | 2.86325 | 2.1 |
| 5838 | 1.35879 | 2.1 |
| 5839 | 1.69332 | 2.1 |
| 5840 | 1.69332 | 2.1 |
| 5841 | 0.946128 | 2.1 |
| 5842 | 1.39659 | 2.1 |
| 5843 | 1.12035 | 2.1 |
| 5844 | 1.12035 | 2.1 |
| 5845 | 1.24286 | 2.1 |
| 5846 | 1.10407 | 2.1 |
| 5847 | 1.14599 | 2.02084 |
| 5848 | 1.32259 | 0.0578193 |
| 5849 | 0.788592 | 0.05 |
| 5850 | 2.4588 | 2.1 |
| 5851 | 0.909581 | 2.1 |
| 5852 | 1.35656 | 1.36549 |
| 5853 | 0.986895 | 1.78396 |
| 5854 | 2.30602 | 1.26426 |
| 5855 | 1.46873 | 1.66393 |

| | | |
|---|---|---|
| 5856 | 1.55584 | 1.23966 |
| 5857 | 2.1547 | 1.40472 |
| 5858 | 1.87794 | 2.045 |
| 5859 | 0.808286 | 2.1 |
| 5860 | 0.928464 | 1.77892 |
| 5861 | 0.810959 | 1.63822 |
| 5862 | 1.50962 | 1.11528 |
| 5863 | 1.1053 | 1.46149 |
| 5864 | 1.0712 | 1.40342 |
| 5865 | 1.40504 | 1.37081 |
| 5866 | 1.87794 | 1.27893 |
| 5867 | 2.80496 | 1.4524 |
| 5868 | 1.41479 | 1.82486 |
| 5869 | 1.38634 | 2.1 |
| 5870 | 1.38634 | 2.1 |
| 5871 | 1.67762 | 2.1 |
| 5872 | 0.946131 | 2.1 |
| 5873 | 1.188 | 2.1 |
| 5874 | 1.62585 | 2.1 |
| 5875 | 1.62585 | 2.1 |
| 5876 | 0.946129 | 2.1 |
| 5877 | 1.18801 | 2.1 |
| 5878 | 1.38397 | 2.1 |
| 5879 | 1.04546 | 2.1 |
| 5880 | 1.16817 | 2.1 |
| 5881 | 1.06007 | 2.1 |
| 5882 | 1.19785 | 2.1 |
| 5883 | 1.43652 | 2.1 |
| 5884 | 1.06994 | 2.1 |
| 5885 | 1.89111 | 2.1 |
| 5886 | 1.07144 | 2.1 |
| 5887 | 1.51754 | 2.1 |
| 5888 | 2.0873 | 2.1 |
| 5889 | 0.758799 | 2.1 |
| 5890 | 1.59518 | 2.1 |
| 5891 | 1.59518 | 2.1 |
| 5892 | 0.946132 | 2.1 |
| 5893 | 0.758771 | 2.1 |
| 5894 | 1.06957 | 2.1 |
| 5895 | 0.758771 | 2.1 |
| 5896 | 1.33916 | 2.1 |
| 5897 | 2.03045 | 1.23653 |
| 5898 | 2.80184 | 1.62547 |
| 5899 | 1.76788 | 2.1 |
| 5900 | 1.27442 | 2.1 |
| 5901 | 1.06756 | 2.1 |
| 5902 | 0.966979 | 2.1 |
| 5903 | 1.7054 | 2.1 |
| 5904 | 1.07156 | 2.1 |
| 5905 | 0.986096 | 2.1 |
| 5906 | 1.32813 | 2.1 |
| 5907 | 1.3331 | 2.1 |
| 5908 | 2.08369 | 0.934885 |
| 5909 | 2.23818 | 2.1 |
| 5910 | 1.10335 | 1.05229 |
| 5911 | 0.622314 | 1.84139 |
| 5912 | 1.72451 | 1.18291 |
| 5913 | 1.72451 | 2.09609 |

| | | |
|---|---|---|
| 5914 | 0.622283 | 0.823531 |
| 5915 | 0.622314 | 0.876799 |
| 5916 | 0.946131 | 0.837471 |
| 5917 | 1.31254 | 2.1 |
| 5918 | 1.15704 | 2.1 |
| 5919 | 1.24463 | 0.985246 |
| 5920 | 2.2067 | 1.58587 |
| 5921 | 1.30001 | 0.780895 |
| 5922 | 1.29175 | 0.881978 |
| 5923 | 1.1233 | 0.05 |
| 5924 | 1.8689 | 0.05 |
| 5925 | 1.29494 | 0.05 |
| 5926 | 1.11895 | 0.05 |
| 5927 | 0.851539 | 0.05 |
| 5928 | 0.851474 | 0.05 |
| 5929 | 1.58352 | 0.05 |
| 5930 | 1.58228 | 0.05 |
| 5931 | 0.851527 | 0.05 |
| 5932 | 2.6389 | 0.05 |
| 5933 | 1.77548 | 0.05 |
| 5934 | 1.24555 | 2.1 |
| 5935 | 2.0512 | 2.1 |
| 5936 | 1.46101 | 2.1 |
| 5937 | 2.60635 | 2.1 |
| 5938 | 1.10476 | 0.05 |
| 5939 | 1.44103 | 0.05 |
| 5940 | 0.652391 | 2.1 |
| 5941 | 0.618322 | 2.1 |
| 5942 | 2.22915 | 2.1 |
| 5943 | 1.39067 | 2.1 |
| 5944 | 1.99737 | 2.1 |
| 5945 | 1.42174 | 2.1 |
| 5946 | 0.946132 | 0.825601 |
| 5947 | 1.29735 | 1.57321 |
| 5948 | 1.61765 | 2.1 |
| 5949 | 1.61765 | 1.06189 |
| 5950 | 0.946132 | 1.95654 |
| 5951 | 1.29735 | 1.02744 |
| 5952 | 1.26643 | 0.972254 |
| 5953 | 1.01335 | 1.28936 |
| 5954 | 1.01335 | 0.182626 |
| 5955 | 0.947669 | 2.1 |
| 5956 | 2.17167 | 2.1 |
| 5957 | 2.59733 | 2.03587 |
| 5958 | 1.50954 | 2.1 |
| 5959 | 1.02638 | 1.50245 |
| 5960 | 0.555785 | 2.1 |
| 5961 | 1.91101 | 2.1 |
| 5962 | 2.09809 | 1.92697 |
| 5963 | 0.641974 | 2.1 |
| 5964 | 1.2022 | 2.00752 |
| 5965 | 1.36359 | 1.92765 |
| 5966 | 1.8128 | 2.1 |
| 5967 | 2.34348 | 0.42372 |
| 5968 | 1.66343 | 0.429519 |
| 5969 | 0.974717 | 0.835481 |
| 5970 | 1.53908 | 0.599207 |
| 5971 | 1.27737 | 0.920475 |

| | | |
|---|---|---|
| 5972 | 1.09408 | 0.0578991 |
| 5973 | 2.07382 | 0.147819 |
| 5974 | 1.10466 | 1.25099 |
| 5975 | 1.05654 | 1.58619 |
| 5976 | 1.23498 | 0.735462 |
| 5977 | 1.48104 | 1.38387 |
| 5978 | 1.23498 | 0.815153 |
| 5979 | 1.17261 | 2.1 |
| 5980 | 1.18059 | 2.1 |
| 5981 | 2.43005 | 2.1 |
| 5982 | 1.04546 | 2.1 |
| 5983 | 2.81222 | 2.1 |
| 5984 | 1.16817 | 2.1 |
| 5985 | 1.75766 | 0.191239 |
| 5986 | 1.47021 | 0.50906 |
| 5987 | 1.07236 | 0.05 |
| 5988 | 1.81759 | 0.531529 |
| 5989 | 1.42913 | 0.274134 |
| 5990 | 1.00636 | 0.672359 |
| 5991 | 1.00636 | 0.398387 |
| 5992 | 1.79397 | 0.373509 |
| 5993 | 0.946131 | 0.05 |
| 5994 | 1.03691 | 0.05 |
| 5995 | 0.851539 | 0.322714 |
| 5996 | 1.46001 | 0.408561 |
| 5997 | 1.37878 | 0.05 |
| 5998 | 0.933258 | 0.05 |
| 5999 | 1.32423 | 0.05 |
| 6000 | 1.32614 | 0.05 |
| 6001 | 1.11356 | 0.05 |
| 6002 | 1.32614 | 0.05 |
| 6003 | 1.28895 | 0.05 |
| 6004 | 1.66895 | 0.05 |
| 6005 | 1.66895 | 0.05 |
| 6006 | 0.946132 | 0.05 |
| 6007 | 1.29576 | 0.05 |
| 6008 | 1.1318 | 0.05 |
| 6009 | 1.37621 | 1.53264 |
| 6010 | 1.94071 | 1.32119 |
| 6011 | 1.10517 | 2.1 |
| 6012 | 1.97098 | 2.1 |
| 6013 | 0.811 2.1 | |
| 6014 | 0.956127 | 2.1 |
| 6015 | 1.18647 | 2.1 |
| 6016 | 0.988455 | 1.52853 |
| 6017 | 1.12457 | 0.05 |
| 6018 | 1.7453 | 0.05 |
| 6019 | 0.92492 | 2.1 |
| 6020 | 1.24353 | 2.1 |
| 6021 | 1.84536 | 0.755479 |
| 6022 | 1.67662 | 0.326871 |
| 6023 | 1.38247 | 0.05 |
| 6024 | 1.02663 | 0.05 |
| 6025 | 0.946132 | 0.0537371 |
| 6026 | 1.41723 | 0.05 |
| 6027 | 1.72976 | 0.05 |
| 6028 | 1.72976 | 0.05 |
| 6029 | 0.946131 | 0.191042 |

| | | |
|---|---|---|
| 6030 | 1.41723 | 0.0572571 |
| 6031 | 1.25866 | 0.05 |
| 6032 | 1.34254 | 0.05 |
| 6033 | 1.25866 | 0.05 |
| 6034 | 1.65507 | 0.05 |
| 6035 | 1.65507 | 0.05 |
| 6036 | 0.946132 | 0.0540149 |
| 6037 | 0.946132 | 0.05 |
| 6038 | 1.11929 | 0.05 |
| 6039 | 0.961779 | 0.05 |
| 6040 | 0.961779 | 0.05 |
| 6041 | 0.946132 | 2.1 |
| 6042 | 1.69361 | 2.1 |
| 6043 | 1.69361 | 2.1 |
| 6044 | 0.94613 | 2.1 |
| 6045 | 1.36523 | 2.1 |
| 6046 | 1.36523 | 2.1 |
| 6047 | 1.19802 | 2.1 |
| 6048 | 0.974196 | 2.1 |
| 6049 | 1.09518 | 2.1 |
| 6050 | 0.946131 | 2.1 |
| 6051 | 1.7161 | 2.1 |
| 6052 | 1.7161 | 2.1 |
| 6053 | 0.94613 | 2.1 |
| 6054 | 1.297 2.1 | |
| 6055 | 1.297 2.1 | |
| 6056 | 1.14599 | 2.1 |
| 6057 | 0.974197 | 2.1 |
| 6058 | 0.974197 | 2.1 |
| 6059 | 1.80778 | 2.1 |
| 6060 | 2.20473 | 2.1 |
| 6061 | 1.80777 | 2.1 |
| 6062 | 1.3515 | 2.1 |
| 6063 | 0.583237 | 2.1 |
| 6064 | 1.39359 | 2.1 |
| 6065 | 1.39359 | 2.1 |
| 6066 | 1.07296 | 2.1 |
| 6067 | 0.946114 | 2.1 |
| 6068 | 1.07297 | 2.1 |
| 6069 | 0.903862 | 2.1 |
| 6070 | 0.956572 | 2.1 |
| 6071 | 1.03437 | 2.1 |
| 6072 | 0.975409 | 2.1 |
| 6073 | 1.31167 | 0.05 |
| 6074 | 2.70246 | 0.05 |
| 6075 | 1.12018 | 0.05 |
| 6076 | 0.655836 | 0.05 |
| 6077 | 0.655837 | 0.05 |
| 6078 | 1.81557 | 0.05 |
| 6079 | 1.81557 | 0.05 |
| 6080 | 0.655836 | 0.05 |
| 6081 | 0.946131 | 0.05 |
| 6082 | 1.01847 | 0.05 |
| 6083 | 1.61334 | 0.05 |
| 6084 | 0.810946 | 0.05 |
| 6085 | 0.8311 | 0.05 |
| 6086 | 1.2466 | 0.05 |
| 6087 | 1.20286 | 0.05 |

| | | |
|---|---|---|
| 6088 | 0.97508 | 0.05 |
| 6089 | 0.870805 | 0.05 |
| 6090 | 0.760087 | 0.05 |
| 6091 | 0.853389 | 2.1 |
| 6092 | 0.820894 | 2.1 |
| 6093 | 0.928291 | 2.1 |
| 6094 | 1.06922 | 2.1 |
| 6095 | 1.32829 | 2.1 |
| 6096 | 1.12173 | 2.1 |
| 6097 | 1.16615 | 2.1 |
| 6098 | 0.957748 | 2.1 |
| 6099 | 0.612491 | 2.02646 |
| 6100 | 0.548141 | 2.1 |
| 6101 | 0.979614 | 2.1 |
| 6102 | 0.658184 | 2.1 |
| 6103 | 0.678456 | 1.56999 |
| 6104 | 0.529544 | 1.80197 |
| 6105 | 0.739013 | 2.1 |
| 6106 | 0.694037 | 2.1 |
| 6107 | 0.475716 | 2.04558 |
| 6108 | 1.58794 | 1.60366 |
| 6109 | 1.58794 | 1.20224 |
| 6110 | 0.475743 | 1.39812 |
| 6111 | 1.08301 | 2.1 |
| 6112 | 0.980664 | 2.1 |
| 6113 | 0.475737 | 2.1 |
| 6114 | 1.32709 | 2.1 |
| 6115 | 1.59396 | 2.1 |
| 6116 | 1.19719 | 0.05 |
| 6117 | 1.34921 | 0.05 |
| 6118 | 1.69061 | 0.05 |
| 6119 | 1.69061 | 0.05 |
| 6120 | 0.946132 | 0.05 |
| 6121 | 1.34921 | 0.05 |
| 6122 | 1.28753 | 0.05 |
| 6123 | 0.95895 | 0.05 |
| 6124 | 1.01051 | 0.05 |
| 6125 | 1.12468 | 0.05 |
| 6126 | 1.22588 | 0.05 |
| 6127 | 1.34254 | 0.05 |
| 6128 | 1.74561 | 0.05 |
| 6129 | 1.74561 | 0.05 |
| 6130 | 0.946128 | 0.05 |
| 6131 | 1.34253 | 0.05 |
| 6132 | 1.3492 | 0.05 |
| 6133 | 1.01051 | 0.05 |
| 6134 | 1.922 | 2.1 |
| 6135 | 1.922 | 2.1 |
| 6136 | 2.12556 | 2.1 |
| 6137 | 0.946131 | 2.1 |
| 6138 | 1.06278 | 2.1 |
| 6139 | 1.47631 | 2.1 |
| 6140 | 1.47631 | 2.1 |
| 6141 | 0.946129 | 2.1 |
| 6142 | 1.06278 | 2.1 |
| 6143 | 1.35966 | 2.1 |
| 6144 | 0.991985 | 2.1 |
| 6145 | 0.738507 | 0.924733 |

| | | |
|---|---|---|
| 6146 | 0.536935 | 1.9144 |
| 6147 | 0.799004 | 1.03666 |
| 6148 | 1.04281 | 0.375442 |
| 6149 | 0.879197 | 0.35434 |
| 6150 | 1.06538 | 0.285664 |
| 6151 | 0.715942 | 0.929557 |
| 6152 | 1.00636 | 0.0696411 |
| 6153 | 1.03824 | 0.0673397 |
| 6154 | 1.70147 | 0.0766451 |
| 6155 | 1.21223 | 2.1 |
| 6156 | 1.01243 | 2.1 |
| 6157 | 1.01242 | 2.1 |
| 6158 | 1.06935 | 2.1 |
| 6159 | 1.54241 | 2.1 |
| 6160 | 0.998971 | 0.05 |
| 6161 | 1.60312 | 0.05 |
| 6162 | 1.44836 | 0.05 |
| 6163 | 2.05632 | 0.05 |
| 6164 | 1.44836 | 0.05 |
| 6165 | 0.973765 | 0.05 |
| 6166 | 0.946131 | 0.05 |
| 6167 | 0.851541 | 0.05 |
| 6168 | 1.73854 | 0.05 |
| 6169 | 1.91752 | 0.05 |
| 6170 | 1.49622 | 0.05 |
| 6171 | 1.23351 | 0.05 |
| 6172 | 3.33289 | 2.1 |
| 6173 | 1.86606 | 2.1 |
| 6174 | 2.71233 | 2.1 |
| 6175 | 0.788816 | 2.1 |
| 6176 | 0.963972 | 2.1 |
| 6177 | 1.25866 | 2.1 |
| 6178 | 1.10817 | 2.1 |
| 6179 | 1.41908 | 2.1 |
| 6180 | 1.48734 | 2.1 |
| 6181 | 1.40822 | 0.05 |
| 6182 | 1.40822 | 0.05 |
| 6183 | 2.09871 | 0.0585304 |
| 6184 | 0.946133 | 0.05 |
| 6185 | 1.14926 | 0.05 |
| 6186 | 0.946133 | 0.0961169 |
| 6187 | 1.78098 | 0.05 |
| 6188 | 1.78098 | 0.05 |
| 6189 | 1.35822 | 0.05 |
| 6190 | 1.3689 | 0.05 |
| 6191 | 1.22176 | 0.05 |
| 6192 | 2.03426 | 0.05 |
| 6193 | 0.946131 | 0.05 |
| 6194 | 1.01713 | 0.05 |
| 6195 | 0.704112 | 0.0550761 |
| 6196 | 1.36237 | 0.05 |
| 6197 | 1.36237 | 0.05 |
| 6198 | 1.01713 | 0.05 |
| 6199 | 1.04936 | 0.05 |
| 6200 | 1.87377 | 2.1 |
| 6201 | 1.60501 | 2.1 |
| 6202 | 0.981584 | 0.05 |
| 6203 | 1.69435 | 0.05 |

| | | |
|---|---|---|
| 6204 | 0.85154 | 2.1 |
| 6205 | 1.47841 | 2.1 |
| 6206 | 1.4952 | 2.1 |
| 6207 | 1.31941 | 2.1 |
| 6208 | 1.04023 | 2.1 |
| 6209 | 1.1995 | 2.1 |
| 6210 | 0.914791 | 2.1 |
| 6211 | 1.1427 | 2.00008 |
| 6212 | 1.64787 | 1.27725 |
| 6213 | 1.07526 | 0.896452 |
| 6214 | 1.07527 | 0.294605 |
| 6215 | 1.07527 | 1.66127 |
| 6216 | 1.58551 | 0.468846 |
| 6217 | 2.77362 | 1.20965 |
| 6218 | 2.17974 | 0.713731 |
| 6219 | 2.71723 | 0.985761 |
| 6220 | 1.29868 | 0.05 |
| 6221 | 1.3594 | 0.05 |
| 6222 | 1.04786 | 0.05 |
| 6223 | 0.527876 | 0.05 |
| 6224 | 1.4085 | 0.05 |
| 6225 | 1.48538 | 0.05 |
| 6226 | 0.76829 | 0.05 |
| 6227 | 1.18514 | 0.05 |
| 6228 | 0.617954 | 0.05 |
| 6229 | 0.864161 | 0.05 |
| 6230 | 1.18835 | 0.05 |
| 6231 | 1.16817 | 2.1 |
| 6232 | 1.04263 | 2.1 |
| 6233 | 1.50524 | 2.1 |
| 6234 | 1.85355 | 2.1 |
| 6235 | 1.23963 | 2.1 |
| 6236 | 1.06279 | 2.1 |
| 6237 | 0.94613 | 2.1 |
| 6238 | 1.35229 | 2.1 |
| 6239 | 1.15947 | 2.1 |
| 6240 | 0.965593 | 2.1 |
| 6241 | 0.851553 | 2.1 |
| 6242 | 2.21296 | 2.1 |
| 6243 | 1.11764 | 2.1 |
| 6244 | 2.46399 | 2.1 |
| 6245 | 1.39292 | 2.1 |
| 6246 | 1.34701 | 2.1 |
| 6247 | 1.34701 | 2.1 |
| 6248 | 1.47001 | 2.1 |
| 6249 | 0.963166 | 0.05 |
| 6250 | 0.893445 | 0.05 |
| 6251 | 1.21056 | 0.05 |
| 6252 | 1.67375 | 2.1 |
| 6253 | 2.0977 | 2.1 |
| 6254 | 1.09154 | 2.1 |
| 6255 | 1.09154 | 2.1 |
| 6256 | 1.57122 | 0.931691 |
| 6257 | 1.60976 | 0.144188 |
| 6258 | 1.69533 | 0.0532697 |
| 6259 | 1.6951 | 0.05 |
| 6260 | 1.5831 | 0.05 |
| 6261 | 1.20969 | 2.1 |

| | | |
|---|---|---|
| 6262 | 1.12076 | 2.1 |
| 6263 | 1.49808 | 2.1 |
| 6264 | 1.50201 | 2.1 |
| 6265 | 1.00278 | 2.1 |
| 6266 | 1.22811 | 0.05 |
| 6267 | 1.11545 | 2.1 |
| 6268 | 1.09646 | 2.1 |
| 6269 | 1.03107 | 0.05 |
| 6270 | 1.44293 | 0.05 |
| 6271 | 1.66752 | 1.42761 |
| 6272 | 1.11023 | 1.20656 |
| 6273 | 2.57831 | 2.1 |
| 6274 | 2.20191 | 1.22582 |
| 6275 | 2.18051 | 2.1 |
| 6276 | 1.48035 | 0.492711 |
| 6277 | 1.0961 | 0.273319 |
| 6278 | 1.63241 | 0.0866133 |
| 6279 | 1.0961 | 0.303355 |
| 6280 | 1.20959 | 2.1 |
| 6281 | 2.86136 | 2.1 |
| 6282 | 1.64933 | 2.1 |
| 6283 | 1.47217 | 2.1 |
| 6284 | 0.915178 | 2.1 |
| 6285 | 1.19074 | 2.1 |
| 6286 | 0.897276 | 2.1 |
| 6287 | 0.915177 | 2.1 |
| 6288 | 0.987037 | 2.1 |
| 6289 | 1.04288 | 2.1 |
| 6290 | 2.39323 | 2.1 |
| 6291 | 0.976218 | 0.05 |
| 6292 | 2.23933 | 0.05 |
| 6293 | 0.976215 | 0.05 |
| 6294 | 1.07384 | 0.05 |
| 6295 | 1.07652 | 2.1 |
| 6296 | 1.55678 | 2.1 |
| 6297 | 1.54168 | 2.1 |
| 6298 | 1.69136 | 2.1 |
| 6299 | 1.64391 | 1.46106 |
| 6300 | 1.76477 | 1.32611 |
| 6301 | 0.611486 | 1.76638 |
| 6302 | 0.683421 | 1.02689 |
| 6303 | 0.946131 | 1.26393 |
| 6304 | 1.1019 | 1.22758 |
| 6305 | 1.12946 | 1.04497 |
| 6306 | 1.54226 | 2.03928 |
| 6307 | 1.19496 | 1.28391 |
| 6308 | 1.96199 | 1.13948 |
| 6309 | 2.0775 | 1.76676 |
| 6310 | 1.22297 | 1.17961 |
| 6311 | 1.3469 | 1.00969 |
| 6312 | 1.25366 | 1.27221 |
| 6313 | 1.65443 | 1.32878 |
| 6314 | 1.65443 | 1.53567 |
| 6315 | 0.946132 | 1.55437 |
| 6316 | 0.946132 | 2.028 |
| 6317 | 1.12873 | 1.54766 |
| 6318 | 1.07468 | 1.14927 |
| 6319 | 1.2854 | 0.502485 |

| | | |
|---|---|---|
| 6320 | 1.3469 | 1.05504 |
| 6321 | 1.68616 | 1.01067 |
| 6322 | 1.68616 | 0.835512 |
| 6323 | 0.946132 | 1.61789 |
| 6324 | 0.946132 | 1.41389 |
| 6325 | 1.16109 | 1.12027 |
| 6326 | 1.17879 | 0.93556 |
| 6327 | 1.17879 | 0.982058 |
| 6328 | 1.45304 | 1.31964 |
| 6329 | 2.21058 | 2.1 |
| 6330 | 2.81552 | 2.1 |
| 6331 | 1.45535 | 0.05 |
| 6332 | 1.49906 | 0.05 |
| 6333 | 1.04413 | 2.1 |
| 6334 | 1.74586 | 2.1 |
| 6335 | 1.34526 | 2.1 |
| 6336 | 1.17402 | 0.981132 |
| 6337 | 1.74564 | 0.80688 |
| 6338 | 1.52724 | 2.1 |
| 6339 | 1.68142 | 2.1 |
| 6340 | 1.09651 | 2.1 |
| 6341 | 1.49149 | 2.1 |
| 6342 | 1.36814 | 2.1 |
| 6343 | 1.46783 | 2.02558 |
| 6344 | 2.18116 | 2.1 |
| 6345 | 1.23602 | 1.13417 |
| 6346 | 1.74538 | 0.286482 |
| 6347 | 0.851539 | 2.1 |
| 6348 | 1.45775 | 2.1 |
| 6349 | 1.28919 | 2.1 |
| 6350 | 0.851483 | 2.1 |
| 6351 | 1.26102 | 1.9961 |
| 6352 | 0.869889 | 2.1 |
| 6353 | 0.85151 | 2.1 |
| 6354 | 1.23451 | 0.248139 |
| 6355 | 1.23602 | 1.51297 |
| 6356 | 1.11955 | 2.1 |
| 6357 | 1.08022 | 2.1 |
| 6358 | 0.977776 | 2.1 |
| 6359 | 1.27896 | 2.1 |
| 6360 | 1.64268 | 2.1 |
| 6361 | 1.97548 | 2.1 |
| 6362 | 1.72527 | 2.1 |
| 6363 | 0.946132 | 2.1 |
| 6364 | 1.69702 | 2.1 |
| 6365 | 1.69702 | 2.1 |
| 6366 | 1.13635 | 2.1 |
| 6367 | 1.40274 | 1.86564 |
| 6368 | 1.24041 | 2.1 |
| 6369 | 1.13635 | 2.1 |
| 6370 | 1.78427 | 0.05 |
| 6371 | 1.68338 | 0.05 |
| 6372 | 1.00681 | 0.05 |
| 6373 | 1.12988 | 0.05 |
| 6374 | 0.946132 | 0.05 |
| 6375 | 0.957543 | 0.05 |
| 6376 | 1.40515 | 0.05 |
| 6377 | 1.40515 | 0.05 |

| | | |
|---|---|---|
| 6378 | 0.797932 | 0.05 |
| 6379 | 1.30187 | 0.05 |
| 6380 | 1.14846 | 0.05 |
| 6381 | 0.665137 | 0.05 |
| 6382 | 1.09575 | 0.05 |
| 6383 | 0.596894 | 0.05 |
| 6384 | 1.04072 | 0.05 |
| 6385 | 0.663171 | 0.05 |
| 6386 | 1.47218 | 0.05 |
| 6387 | 1.32889 | 0.05 |
| 6388 | 1.32639 | 0.05 |
| 6389 | 1.94887 | 0.05 |
| 6390 | 1.32639 | 0.05 |
| 6391 | 1.19323 | 0.05 |
| 6392 | 2.19794 | 0.05 |
| 6393 | 0.754756 | 0.05 |
| 6394 | 1.11015 | 0.05 |
| 6395 | 0.754755 | 0.05 |
| 6396 | 0.946133 | 0.05 |
| 6397 | 0.754768 | 0.05 |
| 6398 | 1.59986 | 0.05 |
| 6399 | 1.59986 | 0.05 |
| 6400 | 1.84082 | 2.1 |
| 6401 | 1.79044 | 2.1 |
| 6402 | 1.75922 | 2.1 |
| 6403 | 1.86766 | 2.1 |
| 6404 | 1.58995 | 2.1 |
| 6405 | 1.58995 | 2.1 |
| 6406 | 1.74351 | 2.1 |
| 6407 | 1.04442 | 2.1 |
| 6408 | 1.42278 | 2.1 |
| 6409 | 1.08576 | 2.1 |
| 6410 | 0.994294 | 2.1 |
| 6411 | 1.44884 | 2.1 |
| 6412 | 1.71036 | 1.09323 |
| 6413 | 0.714101 | 0.217489 |
| 6414 | 1.17541 | 1.71685 |
| 6415 | 1.00872 | 0.05 |
| 6416 | 0.714072 | 0.489277 |
| 6417 | 0.936677 | 0.0513173 |
| 6418 | 0.966979 | 2.1 |
| 6419 | 1.4908 | 2.1 |
| 6420 | 1.03108 | 0.05 |
| 6421 | 0.949712 | 0.05 |
| 6422 | 0.810946 | 0.05 |
| 6423 | 0.88209 | 0.05 |
| 6424 | 0.646702 | 0.05 |
| 6425 | 0.972663 | 0.05 |
| 6426 | 0.862301 | 0.05 |
| 6427 | 0.868143 | 0.05 |
| 6428 | 1.11094 | 0.05 |
| 6429 | 1.35809 | 0.05 |
| 6430 | 1.32455 | 2.1 |
| 6431 | 1.32328 | 2.1 |
| 6432 | 1.69586 | 2.1 |
| 6433 | 1.78301 | 2.1 |
| 6434 | 1.67906 | 2.1 |
| 6435 | 1.60635 | 2.1 |

| | | |
|---|---|---|
| 6436 | 1.57234 | 2.1 |
| 6437 | 1.47012 | 2.1 |
| 6438 | 1.22811 | 0.05 |
| 6439 | 1.08732 | 0.05 |
| 6440 | 2.08849 | 0.05 |
| 6441 | 1.08732 | 0.05 |
| 6442 | 1.30635 | 0.05 |
| 6443 | 0.77315 | 0.05 |
| 6444 | 1.25732 | 0.05 |
| 6445 | 2.43845 | 2.1 |
| 6446 | 2.38577 | 1.90892 |
| 6447 | 0.946132 | 0.96752 |
| 6448 | 1.19288 | 2.1 |
| 6449 | 1.54508 | 2.1 |
| 6450 | 1.54508 | 1.92581 |
| 6451 | 0.94515 | 2.1 |
| 6452 | 1.19288 | 2.1 |
| 6453 | 1.29735 | 1.41525 |
| 6454 | 1.12087 | 0.870041 |
| 6455 | 1.54699 | 1.20323 |
| 6456 | 1.43635 | 2.1 |
| 6457 | 1.33385 | 2.1 |
| 6458 | 1.43635 | 2.1 |
| 6459 | 1.02368 | 2.1 |
| 6460 | 1.035 2.1 | |
| 6461 | 1.00128 | 2.1 |
| 6462 | 1.04009 | 2.1 |
| 6463 | 1.14052 | 2.1 |
| 6464 | 0.443552 | 2.1 |
| 6465 | 0.984351 | 2.1 |
| 6466 | 0.591443 | 2.1 |
| 6467 | 0.929561 | 2.1 |
| 6468 | 1.52079 | 2.1 |
| 6469 | 1.035 2.1 | |
| 6470 | 3.5282 | 2.1 |
| 6471 | 1.88288 | 2.1 |
| 6472 | 2.73877 | 1.16204 |
| 6473 | 0.906059 | 2.1 |
| 6474 | 1.23107 | 0.05 |
| 6475 | 0.906712 | 0.05 |
| 6476 | 0.841322 | 0.05 |
| 6477 | 1.53005 | 0.05 |
| 6478 | 1.10007 | 0.05 |
| 6479 | 1.38033 | 0.05 |
| 6480 | 1.03442 | 0.05 |
| 6481 | 0.787223 | 0.05 |
| 6482 | 1.42589 | 0.05 |
| 6483 | 2.35665 | 0.05 |
| 6484 | 1.17793 | 0.05 |
| 6485 | 1.81356 | 0.05 |
| 6486 | 1.42964 | 0.05 |
| 6487 | 1.95641 | 0.05 |
| 6488 | 1.39139 | 0.05 |
| 6489 | 1.39139 | 0.0798113 |
| 6490 | 0.564533 | 0.05 |
| 6491 | 1.20016 | 0.104462 |
| 6492 | 0.755765 | 0.05 |
| 6493 | 0.755766 | 0.05 |

| | | |
|---|---|---|
| 6494 | 1.20016 | 0.05 |
| 6495 | 1.91011 | 0.224155 |
| 6496 | 1.4695 | 0.05 |
| 6497 | 1.70048 | 2.1 |
| 6498 | 1.93765 | 2.1 |
| 6499 | 0.741307 | 2.1 |
| 6500 | 0.780371 | 2.1 |
| 6501 | 0.946133 | 0.864137 |
| 6502 | 1.28918 | 1.25362 |
| 6503 | 1.5111 | 2.1 |
| 6504 | 1.5111 | 0.885711 |
| 6505 | 0.946131 | 1.66167 |
| 6506 | 1.28918 | 0.938166 |
| 6507 | 1.16805 | 0.810143 |
| 6508 | 1.01179 | 0.689304 |
| 6509 | 0.946132 | 0.869639 |
| 6510 | 1.35328 | 1.28585 |
| 6511 | 1.69633 | 2.07088 |
| 6512 | 1.69633 | 0.910631 |
| 6513 | 0.946133 | 1.76872 |
| 6514 | 1.35328 | 0.955458 |
| 6515 | 1.28918 | 0.806586 |
| 6516 | 1.01179 | 1.12767 |
| 6517 | 1.58147 | 1.27091 |
| 6518 | 1.27737 | 0.767132 |
| 6519 | 2.17223 | 0.05 |
| 6520 | 0.697168 | 0.05 |
| 6521 | 1.62495 | 0.0902165 |
| 6522 | 1.62495 | 0.150807 |
| 6523 | 1.93948 | 0.607166 |
| 6524 | 0.601592 | 2.1 |
| 6525 | 1.93528 | 2.1 |
| 6526 | 2.13757 | 2.1 |
| 6527 | 1.67561 | 2.1 |
| 6528 | 2.70875 | 2.1 |
| 6529 | 1.93528 | 2.1 |
| 6530 | 3.98475 | 2.1 |
| 6531 | 1.12376 | 2.1 |
| 6532 | 1.19016 | 2.1 |
| 6533 | 1.67323 | 0.05 |
| 6534 | 1.74019 | 0.05 |
| 6535 | 1.84719 | 0.05 |
| 6536 | 1.00764 | 0.05 |
| 6537 | 1.23475 | 2.1 |
| 6538 | 1.06757 | 2.1 |
| 6539 | 1.57916 | 2.1 |
| 6540 | 1.06758 | 2.1 |
| 6541 | 1.28014 | 2.1 |
| 6542 | 1.33339 | 2.1 |
| 6543 | 1.2679 | 2.1 |
| 6544 | 1.23504 | 2.1 |
| 6545 | 1.50108 | 2.1 |
| 6546 | 1.3715 | 2.1 |
| 6547 | 0.58831 | 0.986131 |
| 6548 | 0.865971 | 1.13237 |
| 6549 | 0.813704 | 1.41653 |
| 6550 | 0.658856 | 1.40797 |
| 6551 | 0.495869 | 1.33219 |

| | | |
|---|---|---|
| 6552 | 1.13845 | 1.10355 |
| 6553 | 0.780804 | 1.49239 |
| 6554 | 0.642023 | 1.12248 |
| 6555 | 0.707748 | 0.973304 |
| 6556 | 1.64476 | 0.919383 |
| 6557 | 1.64476 | 1.1884 |
| 6558 | 0.99611 | 0.936987 |
| 6559 | 1.35639 | 0.9525 |
| 6560 | 1.3564 | 1.70745 |
| 6561 | 0.946129 | 1.80619 |
| 6562 | 1.99222 | 0.884847 |
| 6563 | 1.4777 | 2.1 |
| 6564 | 1.03968 | 2.1 |
| 6565 | 1.82516 | 2.1 |
| 6566 | 0.851539 | 2.1 |
| 6567 | 1.63693 | 2.1 |
| 6568 | 1.59416 | 2.1 |
| 6569 | 0.878566 | 2.1 |
| 6570 | 1.40796 | 2.1 |
| 6571 | 1.13172 | 2.1 |
| 6572 | 0.851527 | 1.01761 |
| 6573 | 1.14366 | 2.1 |
| 6574 | 0.823822 | 0.05 |
| 6575 | 0.882001 | 0.05 |
| 6576 | 1.09756 | 0.05 |
| 6577 | 1.11857 | 0.05 |
| 6578 | 1.0934 | 0.05 |
| 6579 | 0.960104 | 0.05 |
| 6580 | 0.982001 | 0.05 |
| 6581 | 1.74319 | 0.05 |
| 6582 | 1.03043 | 0.05 |
| 6583 | 0.967194 | 0.05 |
| 6584 | 1.16938 | 2.1 |
| 6585 | 2.48377 | 2.1 |
| 6586 | 1.1181 | 2.1 |
| 6587 | 2.57349 | 2.1 |
| 6588 | 1.0465 | 2.1 |
| 6589 | 1.44709 | 2.1 |
| 6590 | 0.946132 | 2.1 |
| 6591 | 1.29302 | 2.1 |
| 6592 | 1.73502 | 2.1 |
| 6593 | 1.73502 | 2.1 |
| 6594 | 0.946132 | 2.1 |
| 6595 | 1.29302 | 2.1 |
| 6596 | 1.38813 | 2.1 |
| 6597 | 1.03933 | 2.1 |
| 6598 | 1.01976 | 0.05 |
| 6599 | 1.11764 | 0.05 |
| 6600 | 0.961779 | 0.05 |
| 6601 | 1.0685 | 0.05 |
| 6602 | 1.08426 | 1.1398 |
| 6603 | 0.99695 | 2.00512 |
| 6604 | 2.25593 | 1.80784 |
| 6605 | 1.47992 | 1.94346 |
| 6606 | 1.88989 | 1.20749 |
| 6607 | 1.65813 | 1.06475 |
| 6608 | 1.20278 | 1.55985 |
| 6609 | 0.981842 | 1.12601 |

| | | |
|---|---|---|
| 6610 | 0.802655 | 2.1 |
| 6611 | 0.953489 | 2.1 |
| 6612 | 0.961719 | 2.1 |
| 6613 | 0.975614 | 2.1 |
| 6614 | 0.991617 | 1.15646 |
| 6615 | 0.940748 | 1.50821 |
| 6616 | 2.25019 | 2.1 |
| 6617 | 2.30712 | 1.74664 |
| 6618 | 1.98823 | 2.1 |
| 6619 | 0.522844 | 1.33452 |
| 6620 | 0.946122 | 2.1 |
| 6621 | 0.946132 | 2.00451 |
| 6622 | 0.593846 | 1.23324 |
| 6623 | 1.26501 | 1.32844 |
| 6624 | 1.21171 | 0.05 |
| 6625 | 0.851542 | 0.05 |
| 6626 | 0.940327 | 0.05 |
| 6627 | 0.940356 | 0.05 |
| 6628 | 1.52117 | 0.05 |
| 6629 | 1.58072 | 0.05 |
| 6630 | 1.35169 | 0.05 |
| 6631 | 1.13889 | 0.05 |
| 6632 | 1.35007 | 0.05 |
| 6633 | 1.35007 | 0.05 |
| 6634 | 1.78381 | 0.05 |
| 6635 | 0.946132 | 0.05 |
| 6636 | 1.06627 | 0.05 |
| 6637 | 0.946131 | 0.05 |
| 6638 | 1.73005 | 0.05 |
| 6639 | 1.73005 | 0.05 |
| 6640 | 1.32449 | 0.05 |
| 6641 | 1.35169 | 0.05 |
| 6642 | 1.42446 | 0.624531 |
| 6643 | 1.86697 | 0.605997 |
| 6644 | 1.13413 | 0.138779 |
| 6645 | 1.42446 | 0.0991641 |
| 6646 | 1.33575 | 0.05 |
| 6647 | 1.20473 | 0.05 |
| 6648 | 1.87517 | 0.05 |
| 6649 | 1.87517 | 0.05 |
| 6650 | 0.488712 | 0.05 |
| 6651 | 1.13242 | 0.05 |
| 6652 | 0.488713 | 0.05 |
| 6653 | 0.488713 | 0.05 |
| 6654 | 1.23146 | 0.05 |
| 6655 | 1.57932 | 0.05 |
| 6656 | 1.01698 | 0.05 |
| 6657 | 1.42085 | 0.05 |
| 6658 | 0.246725 | 0.05 |
| 6659 | 0.996283 | 0.05 |
| 6660 | 1.36965 | 0.05 |
| 6661 | 0.86361 | 0.05 |
| 6662 | 0.810945 | 0.05 |
| 6663 | 1.72938 | 0.05 |
| 6664 | 1.21649 | 0.05 |
| 6665 | 1.1865 | 0.05 |
| 6666 | 1.62387 | 0.05 |
| 6667 | 1.05302 | 0.05 |

| | | |
|---|---|---|
| 6668 | 1.10049 | 0.05 |
| 6669 | 0.946129 | 2.1 |
| 6670 | 1.70184 | 0.355585 |
| 6671 | 1.70184 | 2.1 |
| 6672 | 1.12538 | 2.1 |
| 6673 | 1.35969 | 0.17205 |
| 6674 | 1.28828 | 2.1 |
| 6675 | 1.12538 | 1.35637 |
| 6676 | 0.99747 | 1.49538 |
| 6677 | 1.34851 | 0.593693 |
| 6678 | 0.923724 | 2.1 |
| 6679 | 1.47154 | 0.241191 |
| 6680 | 1.47154 | 2.1 |
| 6681 | 1.10698 | 2.1 |
| 6682 | 1.28828 | 0.117677 |
| 6683 | 1.10698 | 2.1 |
| 6684 | 0.923695 | 2.1 |
| 6685 | 0.99747 | 0.05 |
| 6686 | 1.65677 | 2.1 |
| 6687 | 1.77677 | 2.1 |
| 6688 | 1.05689 | 0.05 |
| 6689 | 2.11031 | 0.05 |
| 6690 | 1.07367 | 0.05 |
| 6691 | 0.656239 | 0.05 |
| 6692 | 1.4563 | 0.05 |
| 6693 | 1.4563 | 0.05 |
| 6694 | 0.656238 | 0.05 |
| 6695 | 0.946133 | 0.05 |
| 6696 | 0.812937 | 0.05 |
| 6697 | 0.946131 | 0.05 |
| 6698 | 1.05689 | 0.05 |
| 6699 | 1.34009 | 2.1 |
| 6700 | 1.398 2.1 | |
| 6701 | 3.29047 | 2.1 |
| 6702 | 2.53897 | 2.1 |
| 6703 | 1.24509 | 2.1 |
| 6704 | 3.06162 | 2.1 |
| 6705 | 1.01595 | 2.1 |
| 6706 | 1.52661 | 2.1 |
| 6707 | 1.5266 | 2.1 |
| 6708 | 0.946131 | 2.1 |
| 6709 | 1.31049 | 2.1 |
| 6710 | 1.16225 | 2.1 |
| 6711 | 1.05978 | 2.1 |
| 6712 | 1.16225 | 2.1 |
| 6713 | 1.06561 | 2.1 |
| 6714 | 0.953927 | 2.1 |
| 6715 | 1.15114 | 2.1 |
| 6716 | 1.1807 | 0.05 |
| 6717 | 0.507259 | 0.05 |
| 6718 | 1.47709 | 0.05 |
| 6719 | 1.36192 | 0.05 |
| 6720 | 0.487293 | 0.05 |
| 6721 | 1.65569 | 0.0540792 |
| 6722 | 0.679725 | 0.05 |
| 6723 | 0.721928 | 0.05 |
| 6724 | 1.03931 | 0.05 |
| 6725 | 1.0775 | 0.0517157 |

| | | |
|---|---|---|
| 6726 | 1.0775 | 0.05 |
| 6727 | 2.30207 | 0.701757 |
| 6728 | 1.01814 | 0.624659 |
| 6729 | 2.33963 | 0.391353 |
| 6730 | 2.42589 | 0.778075 |
| 6731 | 1.33962 | 2.1 |
| 6732 | 1.27956 | 2.1 |
| 6733 | 2.14624 | 0.05 |
| 6734 | 1.6812 | 0.05 |
| 6735 | 1.25698 | 0.05 |
| 6736 | 1.35725 | 0.05 |
| 6737 | 1.86387 | 0.05 |
| 6738 | 1.86387 | 0.05 |
| 6739 | 0.750365 | 0.05 |
| 6740 | 0.946128 | 0.05 |
| 6741 | 0.750365 | 0.05 |
| 6742 | 1.72271 | 0.05 |
| 6743 | 1.72272 | 0.05 |
| 6744 | 2.53736 | 0.05 |
| 6745 | 1.07327 | 2.1 |
| 6746 | 1.05376 | 2.1 |
| 6747 | 1.00387 | 0.95217 |
| 6748 | 2.0554 | 0.834183 |
| 6749 | 1.00387 | 0.920486 |
| 6750 | 1.00387 | 0.905997 |
| 6751 | 1.27119 | 2.1 |
| 6752 | 1.51748 | 2.1 |
| 6753 | 3.15271 | 2.1 |
| 6754 | 3.45801 | 2.1 |
| 6755 | 1.99871 | 2.1 |
| 6756 | 1.41186 | 2.1 |
| 6757 | 1.07624 | 2.1 |
| 6758 | 1.5954 | 2.1 |
| 6759 | 1.19323 | 0.930596 |
| 6760 | 1.21286 | 0.844923 |
| 6761 | 0.946114 | 2.1 |
| 6762 | 1.38395 | 2.1 |
| 6763 | 1.73389 | 2.1 |
| 6764 | 1.7339 | 2.1 |
| 6765 | 0.946115 | 2.1 |
| 6766 | 1.38397 | 2.1 |
| 6767 | 1.29604 | 2.1 |
| 6768 | 1.04544 | 2.1 |
| 6769 | 1.60578 | 0.05 |
| 6770 | 1.93407 | 0.05 |
| 6771 | 1.0525 | 0.941508 |
| 6772 | 1.0525 | 1.30309 |
| 6773 | 1.0525 | 2.06532 |
| 6774 | 1.38506 | 1.89095 |
| 6775 | 2.34737 | 0.05 |
| 6776 | 1.3531 | 0.05 |
| 6777 | 1.2925 | 0.05 |
| 6778 | 0.871466 | 0.05 |
| 6779 | 1.26623 | 0.05 |
| 6780 | 0.700631 | 0.05 |
| 6781 | 1.88774 | 0.05 |
| 6782 | 1.14167 | 0.05 |
| 6783 | 1.18606 | 0.05 |

| | | |
|---|---|---|
| 6784 | 2.1238 | 0.05 |
| 6785 | 1.42762 | 0.620798 |
| 6786 | 1.67791 | 0.05 |
| 6787 | 1.47118 | 0.05 |
| 6788 | 0.946128 | 2.1 |
| 6789 | 1.31641 | 2.1 |
| 6790 | 1.75933 | 2.1 |
| 6791 | 1.75933 | 2.1 |
| 6792 | 0.946131 | 2.1 |
| 6793 | 1.31641 | 2.1 |
| 6794 | 1.38905 | 2.1 |
| 6795 | 1.38437 | 2.1 |
| 6796 | 1.51829 | 2.1 |
| 6797 | 0.947114 | 2.1 |
| 6798 | 1.13335 | 2.1 |
| 6799 | 1.47521 | 2.1 |
| 6800 | 1.14911 | 2.1 |
| 6801 | 1.53822 | 2.1 |
| 6802 | 1.39702 | 2.1 |
| 6803 | 0.739166 | 2.1 |
| 6804 | 0.706056 | 2.1 |
| 6805 | 0.964033 | 2.1 |
| 6806 | 1.58993 | 2.1 |
| 6807 | 2.51892 | 2.1 |
| 6808 | 0.595905 | 0.05 |
| 6809 | 1.18155 | 0.05 |
| 6810 | 1.73364 | 0.05 |
| 6811 | 0.952735 | 0.05 |
| 6812 | 2.10381 | 0.05 |
| 6813 | 1.12115 | 0.05 |
| 6814 | 1.27826 | 0.05 |
| 6815 | 2.74021 | 0.05 |
| 6816 | 1.78416 | 2.1 |
| 6817 | 1.78416 | 2.1 |
| 6818 | 0.946132 | 2.1 |
| 6819 | 1.41126 | 2.1 |
| 6820 | 1.21275 | 2.1 |
| 6821 | 0.946133 | 2.1 |
| 6822 | 1.41126 | 2.1 |
| 6823 | 1.33125 | 2.1 |
| 6824 | 1.33125 | 2.1 |
| 6825 | 1.78918 | 2.1 |
| 6826 | 1.0465 | 2.1 |
| 6827 | 1.99715 | 2.1 |
| 6828 | 1.17683 | 2.1 |
| 6829 | 1.25889 | 2.1 |
| 6830 | 0.860834 | 0.05 |
| 6831 | 0.946131 | 0.05 |
| 6832 | 1.05008 | 0.05 |
| 6833 | 0.970444 | 0.05 |
| 6834 | 1.19401 | 0.05 |
| 6835 | 1.25989 | 0.05 |
| 6836 | 0.702942 | 0.05 |
| 6837 | 1.40591 | 0.363136 |
| 6838 | 1.72168 | 0.0969044 |
| 6839 | 0.446352 | 0.05 |
| 6840 | 1.04072 | 0.05 |
| 6841 | 0.711038 | 0.0671697 |

| | | |
|---|---|---|
| 6842 | 0.980737 | 0.05 |
| 6843 | 1.05099 | 0.05 |
| 6844 | 1.26003 | 0.05 |
| 6845 | 0.495934 | 0.05 |
| 6846 | 1.03114 | 0.05 |
| 6847 | 1.09911 | 0.05 |
| 6848 | 1.24825 | 0.05 |
| 6849 | 2.19827 | 0.05 |
| 6850 | 2.0953 | 0.05 |
| 6851 | 2.30437 | 0.05 |
| 6852 | 0.775923 | 0.05 |
| 6853 | 2.2283 | 0.05 |
| 6854 | 1.19583 | 2.1 |
| 6855 | 1.14496 | 2.1 |
| 6856 | 1.62183 | 2.1 |
| 6857 | 1.62316 | 2.1 |
| 6858 | 1.02305 | 0.05 |
| 6859 | 1.21136 | 0.05 |
| 6860 | 1.44426 | 0.05 |
| 6861 | 1.21137 | 0.05 |
| 6862 | 1.20143 | 0.0619427 |
| 6863 | 1.15009 | 0.05 |
| 6864 | 1.24965 | 2.1 |
| 6865 | 1.41077 | 2.1 |
| 6866 | 1.00052 | 0.05 |
| 6867 | 1.79553 | 0.05 |
| 6868 | 1.06336 | 0.05 |
| 6869 | 1.00053 | 0.05 |
| 6870 | 1.18041 | 2.1 |
| 6871 | 1.80818 | 2.1 |
| 6872 | 1.73571 | 1.41353 |
| 6873 | 1.94429 | 1.75523 |
| 6874 | 1.4375 | 0.05 |
| 6875 | 1.44421 | 0.05 |
| 6876 | 1.66953 | 0.05 |
| 6877 | 1.09529 | 0.05 |
| 6878 | 0.952395 | 2.1 |
| 6879 | 0.717337 | 2.1 |
| 6880 | 1.03609 | 2.1 |
| 6881 | 1.33949 | 0.05 |
| 6882 | 1.2774 | 0.481173 |
| 6883 | 2.24983 | 0.179012 |
| 6884 | 1.28727 | 0.144979 |
| 6885 | 0.583592 | 0.279204 |
| 6886 | 0.583591 | 0.41377 |
| 6887 | 1.82859 | 0.237853 |
| 6888 | 1.82859 | 0.279344 |
| 6889 | 0.583593 | 0.490705 |
| 6890 | 0.946128 | 0.219491 |
| 6891 | 0.692233 | 0.460128 |
| 6892 | 1.12226 | 0.512667 |
| 6893 | 2.57454 | 0.434746 |
| 6894 | 0.810944 | 0.396144 |
| 6895 | 0.810945 | 0.349965 |
| 6896 | 1.12226 | 0.495637 |
| 6897 | 1.45867 | 0.45124 |
| 6898 | 1.22403 | 0.321887 |
| 6899 | 1.15262 | 0.562409 |

| | | |
|---|---|---|
| 6900 | 1.08132 | 0.371476 |
| 6901 | 0.846671 | 0.499018 |
| 6902 | 0.480954 | 0.31844 |
| 6903 | 0.917975 | 0.374188 |
| 6904 | 0.917975 | 0.672309 |
| 6905 | 0.720957 | 0.282953 |
| 6906 | 0.795998 | 0.404343 |
| 6907 | 0.69316 | 0.625842 |
| 6908 | 0.641306 | 0.451878 |
| 6909 | 0.690817 | 0.265712 |
| 6910 | 0.961899 | 2.1 |
| 6911 | 2.49579 | 2.1 |
| 6912 | 2.31106 | 2.1 |
| 6913 | 1.70482 | 0.0968085 |
| 6914 | 1.93156 | 0.0704959 |
| 6915 | 1.23868 | 0.0729483 |
| 6916 | 1.73878 | 0.136346 |
| 6917 | 1.26267 | 2.1 |
| 6918 | 1.26441 | 2.1 |
| 6919 | 1.58095 | 2.1 |
| 6920 | 1.58095 | 2.1 |
| 6921 | 0.946132 | 2.1 |
| 6922 | 0.946132 | 2.1 |
| 6923 | 1.11077 | 2.1 |
| 6924 | 1.62905 | 2.1 |
| 6925 | 1.49692 | 2.1 |
| 6926 | 3.49966 | 2.1 |
| 6927 | 2.39213 | 2.1 |
| 6928 | 2.12603 | 2.1 |
| 6929 | 0.946129 | 2.1 |
| 6930 | 0.461661 | 2.1 |
| 6931 | 1.52841 | 2.1 |
| 6932 | 1.39091 | 2.1 |
| 6933 | 0.550679 | 2.1 |
| 6934 | 0.509894 | 2.1 |
| 6935 | 1.49508 | 2.1 |
| 6936 | 2.05862 | 2.1 |
| 6937 | 2.47851 | 2.1 |
| 6938 | 1.07213 | 2.1 |
| 6939 | 1.09148 | 0.376261 |
| 6940 | 1.99776 | 0.105597 |
| 6941 | 1.38333 | 0.0662658 |
| 6942 | 1.04412 | 2.1 |
| 6943 | 1.04412 | 2.1 |
| 6944 | 1.1893 | 0.05 |
| 6945 | 0.946132 | 0.05 |
| 6946 | 0.965737 | 0.05 |
| 6947 | 0.946132 | 0.05 |
| 6948 | 1.54061 | 0.05 |
| 6949 | 1.54061 | 0.05 |
| 6950 | 1.22878 | 0.05 |
| 6951 | 1.25797 | 0.05 |
| 6952 | 1.00665 | 0.05 |
| 6953 | 1.00665 | 0.05 |
| 6954 | 1.80369 | 0.05 |
| 6955 | 0.946128 | 0.05 |
| 6956 | 1.04424 | 0.05 |
| 6957 | 0.85154 | 0.05 |

| | | |
|---|---|---|
| 6958 | 1.39322 | 0.05 |
| 6959 | 1.35464 | 0.05 |
| 6960 | 0.939857 | 0.05 |
| 6961 | 1.28272 | 0.05 |
| 6962 | 1.19323 | 0.05 |
| 6963 | 1.03498 | 0.05 |
| 6964 | 2.22029 | 0.05 |
| 6965 | 0.540005 | 0.05 |
| 6966 | 0.970382 | 0.05 |
| 6967 | 2.14514 | 0.05 |
| 6968 | 1.31542 | 0.05 |
| 6969 | 1.05575 | 0.05 |
| 6970 | 0.810943 | 0.05 |
| 6971 | 1.31542 | 0.05 |
| 6972 | 1.70493 | 0.05 |
| 6973 | 1.22582 | 0.05 |
| 6974 | 1.31681 | 0.05 |
| 6975 | 1.40775 | 0.05 |
| 6976 | 0.928633 | 0.05 |
| 6977 | 1.2545 | 0.05 |
| 6978 | 2.66481 | 0.05 |
| 6979 | 1.32051 | 0.05 |
| 6980 | 1.32051 | 0.05 |
| 6981 | 2.66305 | 2.1 |
| 6982 | 1.3551 | 2.1 |
| 6983 | 2.45325 | 2.1 |
| 6984 | 1.3685 | 2.1 |
| 6985 | 1.51748 | 2.1 |
| 6986 | 1.78941 | 2.1 |
| 6987 | 1.02963 | 0.05 |
| 6988 | 1.28643 | 0.05 |
| 6989 | 0.86131 | 0.05 |
| 6990 | 0.851539 | 0.05 |
| 6991 | 1.46585 | 0.05 |
| 6992 | 1.38822 | 0.05 |
| 6993 | 0.996402 | 0.05 |
| 6994 | 0.851553 | 0.05 |
| 6995 | 1.73156 | 0.05 |
| 6996 | 1.91695 | 0.05 |
| 6997 | 1.92535 | 0.05 |
| 6998 | 2.57287 | 0.05 |
| 6999 | 1.77743 | 0.05 |
| 7000 | 1.02836 | 0.05 |
| 7001 | 1.36362 | 0.05 |
| 7002 | 1.36936 | 0.05 |
| 7003 | 1.78684 | 0.05 |
| 7004 | 1.78685 | 0.05 |
| 7005 | 0.946132 | 0.05 |
| 7006 | 1.27581 | 0.05 |
| 7007 | 0.946132 | 0.05 |
| 7008 | 1.02836 | 0.05 |
| 7009 | 1.55398 | 2.1 |
| 7010 | 1.07167 | 2.1 |
| 7011 | 1.87557 | 2.1 |
| 7012 | 0.751639 | 2.1 |
| 7013 | 1.50884 | 2.1 |
| 7014 | 1.33793 | 2.1 |
| 7015 | 0.751664 | 2.1 |

| | | |
|---|---|---|
| 7016 | 1.37277 | 2.1 |
| 7017 | 0.979515 | 2.1 |
| 7018 | 0.676508 | 2.1 |
| 7019 | 1.09578 | 2.1 |
| 7020 | 0.953925 | 2.1 |
| 7021 | 0.94613 | 2.1 |
| 7022 | 1.64707 | 2.1 |
| 7023 | 1.64707 | 2.1 |
| 7024 | 1.16658 | 2.1 |
| 7025 | 1.40638 | 2.1 |
| 7026 | 1.18682 | 2.1 |
| 7027 | 1.16658 | 2.1 |
| 7028 | 0.543765 | 0.05 |
| 7029 | 1.57462 | 0.05 |
| 7030 | 1.57462 | 0.05 |
| 7031 | 1.00083 | 0.05 |
| 7032 | 1.16834 | 0.05 |
| 7033 | 1.16834 | 0.05 |
| 7034 | 0.889626 | 0.05 |
| 7035 | 1.25944 | 0.05 |
| 7036 | 0.824061 | 0.05 |
| 7037 | 1.9397 | 0.05 |
| 7038 | 1.21893 | 0.05 |
| 7039 | 1.91364 | 0.05 |
| 7040 | 1.33672 | 0.05 |
| 7041 | 0.928069 | 0.05 |
| 7042 | 1.41484 | 0.05 |
| 7043 | 0.538379 | 0.05 |
| 7044 | 1.41484 | 0.05 |
| 7045 | 1.35594 | 0.05 |
| 7046 | 2.11414 | 0.05 |
| 7047 | 2.11414 | 0.05 |
| 7048 | 0.538378 | 0.05 |
| 7049 | 1.38532 | 0.05 |
| 7050 | 0.962877 | 0.465614 |
| 7051 | 1.07497 | 0.317448 |
| 7052 | 1.00613 | 0.19337 |
| 7053 | 0.962878 | 0.203978 |
| 7054 | 0.711447 | 2.1 |
| 7055 | 1.40008 | 0.05 |
| 7056 | 2.51674 | 0.05 |
| 7057 | 1.40008 | 0.05 |
| 7058 | 1.7263 | 0.05 |
| 7059 | 1.40008 | 0.05 |
| 7060 | 1.9216 | 0.05 |
| 7061 | 2.7013 | 0.05 |
| 7062 | 2.58584 | 0.05 |
| 7063 | 0.979296 | 0.05 |
| 7064 | 1.46459 | 0.05 |
| 7065 | 0.899254 | 0.05 |
| 7066 | 1.15038 | 2.1 |
| 7067 | 1.24687 | 2.1 |
| 7068 | 1.16738 | 2.1 |
| 7069 | 0.942323 | 2.1 |
| 7070 | 1.65573 | 1.47536 |
| 7071 | 0.844791 | 2.1 |
| 7072 | 0.54674 | 2.05937 |
| 7073 | 0.795811 | 2.1 |

| | | |
|---|---|---|
| 7074 | 1.08131 | 2.1 |
| 7075 | 1.03553 | 2.1 |
| 7076 | 0.758334 | 2.1 |
| 7077 | 0.708665 | 2.01334 |
| 7078 | 1.22626 | 1.97201 |
| 7079 | 0.835439 | 1.96474 |
| 7080 | 0.861131 | 1.65696 |
| 7081 | 0.68296 | 2.09798 |
| 7082 | 1.65224 | 2.09935 |
| 7083 | 2.82624 | 2.1 |
| 7084 | 1.0151 | 1.30486 |
| 7085 | 1.15905 | 1.93494 |
| 7086 | 1.71076 | 1.52163 |
| 7087 | 1.4529 | 1.04863 |
| 7088 | 0.494895 | 2.1 |
| 7089 | 0.946132 | 1.88017 |
| 7090 | 0.613132 | 1.63704 |
| 7091 | 0.863631 | 1.91513 |
| 7092 | 1.78391 | 2.1 |
| 7093 | 1.1706 | 0.05 |
| 7094 | 2.02734 | 0.05 |
| 7095 | 1.1706 | 0.05 |
| 7096 | 0.681532 | 0.05 |
| 7097 | 1.50316 | 0.05 |
| 7098 | 1.50316 | 0.05 |
| 7099 | 0.681534 | 0.05 |
| 7100 | 0.973819 | 0.05 |
| 7101 | 0.97382 | 0.05 |
| 7102 | 0.94613 | 0.05 |
| 7103 | 1.1706 | 0.05 |
| 7104 | 1.67727 | 0.05 |
| 7105 | 1.60329 | 0.05 |
| 7106 | 1.3227 | 2.1 |
| 7107 | 1.40846 | 2.1 |
| 7108 | 1.07889 | 2.1 |
| 7109 | 1.07721 | 2.1 |
| 7110 | 0.953523 | 0.161619 |
| 7111 | 1.79675 | 0.165494 |
| 7112 | 2.09859 | 0.81227 |
| 7113 | 1.21309 | 2.1 |
| 7114 | 1.54567 | 0.67964 |
| 7115 | 1.19722 | 0.775741 |
| 7116 | 1.82793 | 0.809163 |
| 7117 | 1.1599 | 0.577324 |
| 7118 | 1.73621 | 0.524501 |
| 7119 | 0.933474 | 0.647891 |
| 7120 | 1.51477 | 0.626646 |
| 7121 | 1.51477 | 0.670262 |
| 7122 | 0.93347 | 0.626448 |
| 7123 | 1.27374 | 0.637888 |
| 7124 | 1.27376 | 0.68943 |
| 7125 | 0.946105 | 0.735701 |
| 7126 | 1.1972 | 0.444444 |
| 7127 | 0.851541 | 0.05 |
| 7128 | 1.36971 | 0.05 |
| 7129 | 1.27832 | 0.05 |
| 7130 | 0.853489 | 0.05 |
| 7131 | 0.973847 | 0.05 |

| | | |
|---|---|---|
| 7132 | 0.976308 | 0.05 |
| 7133 | 0.851528 | 0.05 |
| 7134 | 2.72654 | 0.05 |
| 7135 | 1.77867 | 0.05 |
| 7136 | 3.23032 | 2.1 |
| 7137 | 2.78208 | 2.1 |
| 7138 | 1.0227 | 0.05 |
| 7139 | 1.02269 | 0.05 |
| 7140 | 1.02269 | 0.05 |
| 7141 | 1.24293 | 0.05 |
| 7142 | 0.831695 | 2.1 |
| 7143 | 0.67513 | 2.1 |
| 7144 | 0.956294 | 2.1 |
| 7145 | 1.08623 | 2.1 |
| 7146 | 1.05556 | 2.1 |
| 7147 | 1.38859 | 2.1 |
| 7148 | 1.13179 | 2.1 |
| 7149 | 1.02438 | 2.1 |
| 7150 | 1.06659 | 2.1 |
| 7151 | 1.36595 | 2.1 |
| 7152 | 1.06659 | 2.1 |
| 7153 | 1.06659 | 0.724579 |
| 7154 | 0.946131 | 1.13776 |
| 7155 | 0.875912 | 2.1 |
| 7156 | 1.65971 | 2.1 |
| 7157 | 1.65971 | 2.1 |
| 7158 | 0.875912 | 2.1 |
| 7159 | 1.1229 | 2.1 |
| 7160 | 1.41273 | 2.1 |
| 7161 | 1.54699 | 1.98503 |
| 7162 | 1.93655 | 1.81327 |
| 7163 | 1.42307 | 1.06955 |
| 7164 | 1.97907 | 1.54433 |
| 7165 | 0.996774 | 2.1 |
| 7166 | 1.15505 | 0.05 |
| 7167 | 1.28463 | 0.05 |
| 7168 | 1.86353 | 0.05 |
| 7169 | 2.71398 | 0.05 |
| 7170 | 1.56787 | 0.05 |
| 7171 | 1.66295 | 0.05 |
| 7172 | 1.73432 | 1.16677 |
| 7173 | 1.05019 | 0.05 |
| 7174 | 1.42117 | 0.05 |
| 7175 | 0.888299 | 0.05 |
| 7176 | 1.38127 | 0.05 |
| 7177 | 1.72993 | 0.05 |
| 7178 | 0.664129 | 0.05 |
| 7179 | 0.99302 | 0.654031 |
| 7180 | 0.993023 | 0.0798954 |
| 7181 | 1.13491 | 2.1 |
| 7182 | 0.876316 | 2.1 |
| 7183 | 1.2588 | 2.1 |
| 7184 | 0.895613 | 2.1 |
| 7185 | 2.07526 | 2.1 |
| 7186 | 1.23369 | 2.1 |
| 7187 | 1.25146 | 2.1 |
| 7188 | 2.15282 | 2.1 |
| 7189 | 1.22239 | 2.1 |

| | | |
|---|---|---|
| 7190 | 1.10962 | 2.1 |
| 7191 | 1.77402 | 2.1 |
| 7192 | 1.0305 | 2.1 |
| 7193 | 1.50108 | 1.609 |
| 7194 | 1.20761 | 2.1 |
| 7195 | 1.3626 | 2.1 |
| 7196 | 1.14825 | 2.1 |
| 7197 | 1.78711 | 2.1 |
| 7198 | 1.17933 | 2.1 |
| 7199 | 1.14824 | 2.1 |
| 7200 | 1.09651 | 0.05 |
| 7201 | 1.23851 | 0.05 |
| 7202 | 1.83284 | 0.05 |
| 7203 | 1.23851 | 0.05 |
| 7204 | 1.53521 | 2.1 |
| 7205 | 1.46279 | 2.1 |
| 7206 | 1.9233 | 0.05 |
| 7207 | 1.2966 | 0.05 |
| 7208 | 2.11443 | 0.05 |
| 7209 | 1.30265 | 0.05 |
| 7210 | 1.13528 | 0.05 |
| 7211 | 0.946131 | 0.05 |
| 7212 | 1.41169 | 0.05 |
| 7213 | 1.33879 | 0.05 |
| 7214 | 0.946128 | 0.05 |
| 7215 | 1.80435 | 0.05 |
| 7216 | 1.80435 | 0.05 |
| 7217 | 1.234 2.1 | |
| 7218 | 1.45633 | 2.1 |
| 7219 | 1.234 2.1 | |
| 7220 | 1.98448 | 2.1 |
| 7221 | 2.02612 | 2.1 |
| 7222 | 1.23527 | 0.798161 |
| 7223 | 1.44472 | 0.614039 |
| 7224 | 0.993366 | 0.689124 |
| 7225 | 1.22025 | 0.354164 |
| 7226 | 0.993368 | 0.722119 |
| 7227 | 2.43669 | 2.1 |
| 7228 | 2.56707 | 2.1 |
| 7229 | 1.80348 | 1.17958 |
| 7230 | 1.74406 | 0.463919 |
| 7231 | 1.90937 | 1.03664 |
| 7232 | 1.49357 | 0.05 |
| 7233 | 2.5832 | 0.221145 |
| 7234 | 1.82274 | 0.567073 |
| 7235 | 1.6957 | 0.247052 |
| 7236 | 2.22495 | 0.0921366 |
| 7237 | 2.49959 | 0.2351 |
| 7238 | 2.01183 | 0.128567 |
| 7239 | 1.37427 | 0.05 |
| 7240 | 1.15069 | 2.1 |
| 7241 | 1.39379 | 1.02846 |
| 7242 | 1.69661 | 0.05 |
| 7243 | 1.39379 | 0.05 |
| 7244 | 1.39654 | 2.1 |
| 7245 | 1.93505 | 2.1 |
| 7246 | 1.5525 | 2.1 |
| 7247 | 1.5525 | 2.1 |

| | | |
|---|---|---|
| 7248 | 0.615065 | 2.1 |
| 7249 | 1.38908 | 2.1 |
| 7250 | 0.615065 | 2.1 |
| 7251 | 0.77849 | 2.1 |
| 7252 | 1.38908 | 2.1 |
| 7253 | 1.56839 | 1.13689 |
| 7254 | 1.09142 | 1.99864 |
| 7255 | 2.07313 | 0.975043 |
| 7256 | 0.998288 | 0.05 |
| 7257 | 1.31481 | 0.05 |
| 7258 | 1.88175 | 0.05 |
| 7259 | 2.23267 | 0.05 |
| 7260 | 1.06935 | 2.1 |
| 7261 | 1.06936 | 2.1 |
| 7262 | 1.17989 | 2.1 |
| 7263 | 1.00757 | 1.47941 |
| 7264 | 1.65318 | 1.74114 |
| 7265 | 1.4185 | 0.0861675 |
| 7266 | 2.22495 | 0.05 |
| 7267 | 1.02011 | 0.05 |
| 7268 | 0.59676 | 0.05 |
| 7269 | 1.43606 | 0.05 |
| 7270 | 1.43606 | 0.05 |
| 7271 | 0.89771 | 0.05 |
| 7272 | 1.13511 | 0.05 |
| 7273 | 0.59676 | 0.05 |
| 7274 | 0.897709 | 0.05 |
| 7275 | 1.79542 | 0.05 |
| 7276 | 2.38566 | 2.1 |
| 7277 | 1.63838 | 0.298765 |
| 7278 | 1.63837 | 1.46222 |
| 7279 | 0.916275 | 2.1 |
| 7280 | 1.36183 | 0.679372 |
| 7281 | 1.19282 | 2.1 |
| 7282 | 0.916275 | 2.1 |
| 7283 | 1.19283 | 2.1 |
| 7284 | 2.01582 | 2.1 |
| 7285 | 1.00215 | 0.05 |
| 7286 | 2.60643 | 2.1 |
| 7287 | 2.15971 | 2.1 |
| 7288 | 1.28267 | 2.1 |
| 7289 | 1.29954 | 2.1 |
| 7290 | 3.40327 | 2.1 |
| 7291 | 3.49128 | 2.1 |
| 7292 | 1.3864 | 2.1 |
| 7293 | 1.3201 | 2.1 |
| 7294 | 1.45666 | 0.05 |
| 7295 | 1.55798 | 0.05 |
| 7296 | 0.857202 | 0.05 |
| 7297 | 1.24374 | 0.05 |
| 7298 | 1.04157 | 0.05 |
| 7299 | 0.85723 | 0.05 |
| 7300 | 1.02722 | 0.05 |
| 7301 | 1.01361 | 0.05 |
| 7302 | 0.974077 | 0.05 |
| 7303 | 1.01814 | 0.05 |
| 7304 | 0.974077 | 0.05 |
| 7305 | 1.43987 | 0.05 |

| | | |
|---|---|---|
| 7306 | 1.43986 | 0.05 |
| 7307 | 0.927881 | 0.05 |
| 7308 | 0.946132 | 0.05 |
| 7309 | 0.927849 | 0.05 |
| 7310 | 0.927852 | 0.05 |
| 7311 | 1.2493 | 0.05 |
| 7312 | 1.22482 | 0.05 |
| 7313 | 1.41319 | 0.145541 |
| 7314 | 1.22482 | 0.0516386 |
| 7315 | 0.999492 | 2.1 |
| 7316 | 1.14657 | 2.1 |
| 7317 | 1.3617 | 0.05 |
| 7318 | 0.983269 | 0.05 |
| 7319 | 1.33174 | 0.05 |
| 7320 | 1.62816 | 0.05 |
| 7321 | 1.62817 | 0.05 |
| 7322 | 0.81938 | 0.05 |
| 7323 | 1.33174 | 0.05 |
| 7324 | 1.01332 | 0.05 |
| 7325 | 0.874788 | 0.05 |
| 7326 | 1.16725 | 2.1 |
| 7327 | 1.63991 | 2.1 |
| 7328 | 1.08848 | 0.05 |
| 7329 | 1.15354 | 0.05 |
| 7330 | 1.12665 | 2.1 |
| 7331 | 1.58742 | 2.1 |
| 7332 | 1.57523 | 2.1 |
| 7333 | 1.32201 | 0.664848 |
| 7334 | 1.32097 | 0.583591 |
| 7335 | 0.851542 | 0.05 |
| 7336 | 0.877188 | 0.05 |
| 7337 | 0.797489 | 0.0573676 |
| 7338 | 1.44721 | 0.05 |
| 7339 | 1.61761 | 0.05 |
| 7340 | 0.943799 | 0.05 |
| 7341 | 1.36079 | 0.05 |
| 7342 | 1.16361 | 2.1 |
| 7343 | 1.15852 | 1.83564 |
| 7344 | 1.07624 | 2.1 |
| 7345 | 1.07624 | 2.1 |
| 7346 | 0.946132 | 0.867572 |
| 7347 | 1.37938 | 1.24232 |
| 7348 | 1.78653 | 1.89503 |
| 7349 | 1.78653 | 0.929099 |
| 7350 | 0.946131 | 1.83677 |
| 7351 | 1.37938 | 1.00106 |
| 7352 | 1.35328 | 0.844755 |
| 7353 | 1.44847 | 0.749361 |
| 7354 | 0.946133 | 0.902167 |
| 7355 | 1.38149 | 1.16185 |
| 7356 | 1.81474 | 1.4021 |
| 7357 | 1.81474 | 0.933034 |
| 7358 | 0.946133 | 1.8716 |
| 7359 | 1.38149 | 1.02666 |
| 7360 | 1.37938 | 0.894545 |
| 7361 | 1.44847 | 1.09913 |
| 7362 | 1.93665 | 0.905575 |
| 7363 | 1.5515 | 0.773174 |

| | | |
|---|---|---|
| 7364 | 1.05025 | 2.1 |
| 7365 | 1.05025 | 2.1 |
| 7366 | 1.71468 | 2.1 |
| 7367 | 1.04759 | 2.1 |
| 7368 | 1.04646 | 0.05 |
| 7369 | 1.00739 | 0.05 |
| 7370 | 1.37086 | 0.05 |
| 7371 | 1.12547 | 0.05 |
| 7372 | 1.03853 | 0.05 |
| 7373 | 1.34824 | 0.05 |
| 7374 | 0.799786 | 0.05 |
| 7375 | 2.10105 | 0.05 |
| 7376 | 2.72007 | 0.05 |
| 7377 | 0.589918 | 0.05 |
| 7378 | 0.589918 | 0.05 |
| 7379 | 1.1509 | 0.05 |
| 7380 | 0.619832 | 0.05 |
| 7381 | 1.1509 | 0.05 |
| 7382 | 0.705903 | 0.05 |
| 7383 | 1.53703 | 0.05 |
| 7384 | 1.53703 | 0.05 |
| 7385 | 0.705904 | 0.05 |
| 7386 | 0.946133 | 0.05 |
| 7387 | 0.946132 | 0.05 |
| 7388 | 0.946132 | 0.05 |
| 7389 | 0.588467 | 0.05 |
| 7390 | 1.1078 | 0.05 |
| 7391 | 0.408061 | 0.05 |
| 7392 | 1.38703 | 0.05 |
| 7393 | 1.68328 | 0.05 |
| 7394 | 1.87031 | 0.05 |
| 7395 | 1.86268 | 0.05 |
| 7396 | 1.27472 | 0.05 |
| 7397 | 0.947862 | 0.359347 |
| 7398 | 1.19854 | 0.802442 |
| 7399 | 0.946129 | 0.181403 |
| 7400 | 0.963111 | 0.0754768 |
| 7401 | 0.946131 | 0.267543 |
| 7402 | 1.39055 | 0.05 |
| 7403 | 1.39055 | 0.0510406 |
| 7404 | 1.26426 | 0.0894492 |
| 7405 | 1.07242 | 0.087565 |
| 7406 | 1.31126 | 0.0511153 |
| 7407 | 1.76597 | 0.284085 |
| 7408 | 0.94613 | 1.31397 |
| 7409 | 1.27214 | 0.960824 |
| 7410 | 1.90751 | 0.817499 |
| 7411 | 1.90751 | 0.6003 |
| 7412 | 0.767114 | 1.20738 |
| 7413 | 1.27214 | 1.17608 |
| 7414 | 1.40248 | 1.10866 |
| 7415 | 1.41479 | 1.20438 |
| 7416 | 2.22625 | 1.23156 |
| 7417 | 0.913373 | 1.33557 |
| 7418 | 1.0158 | 0.867403 |
| 7419 | 1.61259 | 0.600113 |
| 7420 | 1.69051 | 0.262838 |
| 7421 | 0.560949 | 1.20449 |

| | | |
|---|---|---|
| 7422 | 0.898559 | 1.23393 |
| 7423 | 1.28011 | 1.10884 |
| 7424 | 1.51026 | 0.05 |
| 7425 | 1.98252 | 0.05 |
| 7426 | 1.05129 | 0.05 |
| 7427 | 1.2951 | 0.05 |
| 7428 | 1.46041 | 0.05 |
| 7429 | 1.7155 | 2.1 |
| 7430 | 1.72196 | 2.1 |
| 7431 | 0.933885 | 2.1 |
| 7432 | 0.986723 | 2.1 |
| 7433 | 1.28072 | 0.05 |
| 7434 | 1.40765 | 1.76611 |
| 7435 | 1.28072 | 0.106034 |
| 7436 | 1.61687 | 2.1 |
| 7437 | 1.60965 | 2.1 |
| 7438 | 1.21424 | 2.1 |
| 7439 | 2.07297 | 2.1 |
| 7440 | 1.19943 | 2.1 |
| 7441 | 2.38784 | 2.1 |
| 7442 | 1.10278 | 0.05 |
| 7443 | 1.43678 | 0.05 |
| 7444 | 1.1142 | 0.05 |
| 7445 | 1.18034 | 0.05 |
| 7446 | 0.94613 | 0.111491 |
| 7447 | 0.752356 | 0.0531933 |
| 7448 | 0.752329 | 0.05 |
| 7449 | 1.46634 | 0.05 |
| 7450 | 1.31789 | 0.05 |
| 7451 | 0.958159 | 0.0629109 |
| 7452 | 1.22752 | 0.05 |
| 7453 | 1.50472 | 0.05 |
| 7454 | 1.98974 | 2.1 |
| 7455 | 2.54186 | 2.1 |
| 7456 | 0.76512 | 2.1 |
| 7457 | 2.08777 | 2.1 |
| 7458 | 1.7753 | 2.1 |
| 7459 | 3.07372 | 2.1 |
| 7460 | 1.9756 | 2.1 |
| 7461 | 1.48162 | 2.1 |
| 7462 | 1.23204 | 2.1 |
| 7463 | 0.946131 | 1.01178 |
| 7464 | 1.13505 | 2.1 |
| 7465 | 1.47432 | 2.1 |
| 7466 | 1.47432 | 1.48752 |
| 7467 | 0.876831 | 2.1 |
| 7468 | 1.13505 | 1.97214 |
| 7469 | 1.2161 | 1.22962 |
| 7470 | 1.05267 | 1.86883 |
| 7471 | 1.05267 | 1.04145 |
| 7472 | 1.5795 | 1.2123 |
| 7473 | 0.98186 | 2.1 |
| 7474 | 1.56735 | 2.1 |
| 7475 | 0.859099 | 2.1 |
| 7476 | 1.69858 | 2.1 |
| 7477 | 1.93586 | 2.1 |
| 7478 | 1.09233 | 2.1 |
| 7479 | 1.08732 | 0.05 |

| | | |
|---|---|---|
| 7480 | 1.3357 | 0.05 |
| 7481 | 1.84826 | 2.1 |
| 7482 | 0.924132 | 2.1 |
| 7483 | 1.39188 | 2.1 |
| 7484 | 1.43685 | 2.1 |
| 7485 | 0.851541 | 2.1 |
| 7486 | 1.16251 | 2.1 |
| 7487 | 0.946131 | 2.1 |
| 7488 | 1.05685 | 2.1 |
| 7489 | 2.13781 | 2.1 |
| 7490 | 1.98859 | 2.1 |
| 7491 | 1.99731 | 2.1 |
| 7492 | 1.51379 | 2.1 |
| 7493 | 1.48796 | 2.1 |
| 7494 | 0.851542 | 2.1 |
| 7495 | 0.802558 | 2.1 |
| 7496 | 1.09244 | 2.1 |
| 7497 | 1.33891 | 2.1 |
| 7498 | 0.844958 | 2.1 |
| 7499 | 0.710864 | 2.1 |
| 7500 | 1.26087 | 2.1 |
| 7501 | 1.03679 | 2.1 |
| 7502 | 1.51026 | 0.05 |
| 7503 | 1.05248 | 0.10969 |
| 7504 | 0.827258 | 0.0891923 |
| 7505 | 1.05248 | 0.112644 |
| 7506 | 1.28435 | 0.118208 |
| 7507 | 1.50953 | 0.132282 |
| 7508 | 1.50953 | 0.05 |
| 7509 | 0.827298 | 0.147831 |
| 7510 | 1.00366 | 0.05 |
| 7511 | 2.10495 | 0.05 |
| 7512 | 1.17885 | 2.1 |
| 7513 | 1.25185 | 2.1 |
| 7514 | 1.02784 | 2.1 |
| 7515 | 1.76764 | 2.1 |
| 7516 | 1.23746 | 0.05 |
| 7517 | 1.24082 | 0.05 |
| 7518 | 1.46506 | 0.05 |
| 7519 | 1.33061 | 0.05 |
| 7520 | 0.768407 | 1.29279 |
| 7521 | 0.972058 | 0.864266 |
| 7522 | 1.16536 | 2.1 |
| 7523 | 1.13325 | 1.17508 |
| 7524 | 0.978906 | 2.1 |
| 7525 | 1.02291 | 1.0648 |
| 7526 | 0.838015 | 0.86088 |
| 7527 | 1.01052 | 0.05 |
| 7528 | 1.08166 | 0.05 |
| 7529 | 1.42607 | 0.05 |
| 7530 | 1.47354 | 0.05 |
| 7531 | 1.21703 | 2.1 |
| 7532 | 1.29897 | 2.1 |
| 7533 | 1.83293 | 2.1 |
| 7534 | 1.29897 | 2.1 |
| 7535 | 1.24688 | 0.05 |
| 7536 | 0.782563 | 2.1 |
| 7537 | 0.946129 | 2.1 |

| | | |
|---|---|---|
| 7538 | 0.782591 | 2.1 |
| 7539 | 1.49487 | 2.1 |
| 7540 | 1.49487 | 2.1 |
| 7541 | 1.37713 | 2.1 |
| 7542 | 0.900337 | 2.1 |
| 7543 | 1.31284 | 2.1 |
| 7544 | 0.924468 | 2.1 |
| 7545 | 1.40602 | 2.1 |
| 7546 | 1.25669 | 2.1 |
| 7547 | 0.986901 | 2.1 |
| 7548 | 0.958565 | 2.1 |
| 7549 | 1.37266 | 2.1 |
| 7550 | 1.64146 | 2.1 |
| 7551 | 0.49345 | 2.1 |
| 7552 | 1.08276 | 2.1 |
| 7553 | 1.08276 | 2.1 |
| 7554 | 1.26929 | 2.1 |
| 7555 | 1.09847 | 2.1 |
| 7556 | 0.827081 | 0.05 |
| 7557 | 1.07236 | 0.0899572 |
| 7558 | 1.25063 | 0.072759 |
| 7559 | 1.43548 | 0.05 |
| 7560 | 1.25063 | 0.05 |
| 7561 | 1.3659 | 0.05 |
| 7562 | 1.72441 | 0.05 |
| 7563 | 1.3659 | 0.05 |
| 7564 | 1.4677 | 0.05 |
| 7565 | 1.86114 | 0.05 |
| 7566 | 1.40104 | 0.05 |
| 7567 | 0.962675 | 0.05 |
| 7568 | 0.946131 | 0.05 |
| 7569 | 1.74134 | 0.05 |
| 7570 | 1.74134 | 0.05 |
| 7571 | 0.962676 | 0.05 |
| 7572 | 0.946131 | 0.05 |
| 7573 | 1.05548 | 0.05 |
| 7574 | 0.902187 | 0.05 |
| 7575 | 2.14178 | 0.05 |
| 7576 | 1.74998 | 0.05 |
| 7577 | 1.38541 | 0.05 |
| 7578 | 1.64468 | 0.05 |
| 7579 | 0.949317 | 0.05 |
| 7580 | 2.06654 | 0.05 |
| 7581 | 0.839626 | 0.05 |
| 7582 | 0.810945 | 0.05 |
| 7583 | 1.29203 | 0.05 |
| 7584 | 1.18091 | 0.05 |
| 7585 | 1.06872 | 0.05 |
| 7586 | 1.11956 | 0.05 |
| 7587 | 0.926911 | 0.05 |
| 7588 | 0.901716 | 0.05 |
| 7589 | 0.909146 | 0.05 |
| 7590 | 1.33776 | 0.05 |
| 7591 | 1.02048 | 0.05 |
| 7592 | 1.08365 | 0.05 |
| 7593 | 1.21042 | 0.05 |
| 7594 | 0.865634 | 0.05 |
| 7595 | 0.590354 | 2.1 |

| | | |
|---|---|---|
| 7596 | 0.61173 | 2.1 |
| 7597 | 3.46825 | 2.1 |
| 7598 | 3.28404 | 2.1 |
| 7599 | 1.56004 | 0.05 |
| 7600 | 1.69344 | 0.05 |
| 7601 | 1.7151 | 0.115548 |
| 7602 | 0.946131 | 0.05 |
| 7603 | 1.40265 | 0.05 |
| 7604 | 1.13537 | 0.05 |
| 7605 | 0.965708 | 0.05 |
| 7606 | 1.13537 | 0.05 |
| 7607 | 1.29681 | 0.05 |
| 7608 | 1.40915 | 0.05 |
| 7609 | 1.61652 | 0.105088 |
| 7610 | 1.33171 | 0.16221 |
| 7611 | 1.37681 | 2.1 |
| 7612 | 0.81094 | 0.05 |
| 7613 | 0.598606 | 0.05 |
| 7614 | 0.723111 | 0.05 |
| 7615 | 1.04562 | 0.05 |
| 7616 | 0.979052 | 0.05 |
| 7617 | 0.798185 | 0.05 |
| 7618 | 0.880117 | 0.05 |
| 7619 | 0.979341 | 0.121618 |
| 7620 | 0.800229 | 0.05 |
| 7621 | 1.39126 | 0.126398 |
| 7622 | 1.13005 | 0.210748 |
| 7623 | 0.913877 | 0.159348 |
| 7624 | 1.26959 | 0.0626575 |
| 7625 | 0.699741 | 0.17902 |
| 7626 | 1.19496 | 1.3568 |
| 7627 | 1.13392 | 0.05 |
| 7628 | 1.11913 | 0.05 |
| 7629 | 1.06866 | 0.05 |
| 7630 | 0.615546 | 0.05 |
| 7631 | 0.831006 | 0.05 |
| 7632 | 1.53286 | 0.05 |
| 7633 | 0.690977 | 0.05 |
| 7634 | 1.01956 | 0.05 |
| 7635 | 2.33818 | 0.05 |
| 7636 | 1.6585 | 2.1 |
| 7637 | 1.67061 | 2.1 |
| 7638 | 1.41414 | 2.1 |
| 7639 | 1.16756 | 2.1 |
| 7640 | 1.00898 | 0.05 |
| 7641 | 0.966964 | 2.1 |
| 7642 | 2.85443 | 2.1 |
| 7643 | 0.966977 | 2.1 |
| 7644 | 2.08427 | 2.1 |
| 7645 | 2.94901 | 2.1 |
| 7646 | 1.10735 | 0.05 |
| 7647 | 1.65815 | 2.1 |
| 7648 | 1.4502 | 2.1 |
| 7649 | 1.45026 | 2.1 |
| 7650 | 1.53446 | 2.1 |
| 7651 | 0.946129 | 0.938693 |
| 7652 | 1.81075 | 2.1 |
| 7653 | 1.81075 | 0.891255 |

| | | |
|---|---|---|
| 7654 | 0.946131 | 1.51201 |
| 7655 | 1.37075 | 2.1 |
| 7656 | 1.37075 | 0.924205 |
| 7657 | 1.34245 | 2.00662 |
| 7658 | 0.973502 | 0.937343 |
| 7659 | 1.08542 | 2.1 |
| 7660 | 1.5814 | 0.05 |
| 7661 | 1.43836 | 0.05 |
| 7662 | 0.946132 | 0.05 |
| 7663 | 1.13747 | 0.05 |
| 7664 | 1.04429 | 0.05 |
| 7665 | 1.05017 | 0.05 |
| 7666 | 1.24732 | 0.05 |
| 7667 | 1.13387 | 0.05 |
| 7668 | 2.19489 | 0.05 |
| 7669 | 1.10748 | 2.1 |
| 7670 | 1.25006 | 2.1 |
| 7671 | 1.58834 | 2.1 |
| 7672 | 1.58834 | 0.05 |
| 7673 | 0.94613 | 2.1 |
| 7674 | 1.16548 | 2.1 |
| 7675 | 0.946132 | 0.05 |
| 7676 | 1.08998 | 0.675525 |
| 7677 | 1.16548 | 0.0974401 |
| 7678 | 0.995732 | 0.05 |
| 7679 | 0.657014 | 2.1 |
| 7680 | 0.957173 | 2.1 |
| 7681 | 1.01485 | 2.1 |
| 7682 | 1.12468 | 2.1 |
| 7683 | 1.43871 | 2.1 |
| 7684 | 1.23716 | 2.1 |
| 7685 | 1.89277 | 0.474797 |
| 7686 | 2.06653 | 0.100205 |
| 7687 | 0.967544 | 0.05 |
| 7688 | 1.89016 | 0.05 |
| 7689 | 2.22165 | 2.1 |
| 7690 | 0.83514 | 2.1 |
| 7691 | 1.76565 | 2.1 |
| 7692 | 1.76565 | 2.1 |
| 7693 | 0.94613 | 2.02466 |
| 7694 | 0.835112 | 2.1 |
| 7695 | 1.23426 | 2.1 |
| 7696 | 1.36653 | 2.1 |
| 7697 | 0.895892 | 0.944922 |
| 7698 | 0.859278 | 0.910142 |
| 7699 | 0.791461 | 0.989901 |
| 7700 | 1.86249 | 1.29527 |
| 7701 | 1.14607 | 1.00367 |
| 7702 | 1.19904 | 1.23912 |
| 7703 | 1.84898 | 1.07249 |
| 7704 | 1.03452 | 0.875348 |
| 7705 | 0.946131 | 0.0721891 |
| 7706 | 0.940472 | 0.453403 |
| 7707 | 1.53478 | 0.394287 |
| 7708 | 1.84175 | 0.405798 |
| 7709 | 1.55687 | 0.285308 |
| 7710 | 1.17787 | 0.05 |
| 7711 | 1.60589 | 0.510471 |

| | | |
|---|---|---|
| 7712 | 2.35574 | 0.289763 |
| 7713 | 1.3305 | 0.05 |
| 7714 | 1.15008 | 0.0712014 |
| 7715 | 1.12457 | 0.05 |
| 7716 | 1.12457 | 0.05 |
| 7717 | 1.01778 | 2.1 |
| 7718 | 1.35733 | 2.1 |
| 7719 | 1.50683 | 2.1 |
| 7720 | 1.12064 | 2.1 |
| 7721 | 1.42716 | 0.0649904 |
| 7722 | 1.42717 | 0.191361 |
| 7723 | 1.45927 | 0.867359 |
| 7724 | 1.04321 | 1.67424 |
| 7725 | 1.32634 | 0.05 |
| 7726 | 2.27022 | 0.05 |
| 7727 | 1.19352 | 0.05 |
| 7728 | 1.32634 | 0.05 |
| 7729 | 1.40924 | 2.1 |
| 7730 | 1.22927 | 1.38166 |
| 7731 | 1.69237 | 2.0452 |
| 7732 | 1.69237 | 2.1 |
| 7733 | 0.946132 | 1.02623 |
| 7734 | 0.946133 | 1.869 |
| 7735 | 1.16912 | 1.53097 |
| 7736 | 1.0417 | 1.42157 |
| 7737 | 1.14605 | 2.09971 |
| 7738 | 1.1233 | 2.1 |
| 7739 | 1.96882 | 1.69971 |
| 7740 | 1.03804 | 0.0533768 |
| 7741 | 1.15338 | 0.0627909 |
| 7742 | 1.15338 | 0.0570203 |
| 7743 | 2.33051 | 0.242782 |
| 7744 | 1.81507 | 2.1 |
| 7745 | 2.42949 | 2.1 |
| 7746 | 0.725879 | 0.109663 |
| 7747 | 1.88394 | 0.203951 |
| 7748 | 2.2699 | 0.05 |
| 7749 | 2.78952 | 2.1 |
| 7750 | 1.88394 | 0.05 |
| 7751 | 1.79606 | 2.1 |
| 7752 | 1.20091 | 2.1 |
| 7753 | 1.9614 | 2.1 |
| 7754 | 1.19901 | 2.1 |
| 7755 | 3.07915 | 2.1 |
| 7756 | 1.32592 | 2.1 |
| 7757 | 2.86461 | 2.1 |
| 7758 | 1.34817 | 2.1 |
| 7759 | 1.44859 | 2.1 |
| 7760 | 2.03969 | 2.1 |
| 7761 | 0.946132 | 2.1 |
| 7762 | 1.50709 | 2.1 |
| 7763 | 1.50709 | 2.1 |
| 7764 | 0.946132 | 2.1 |
| 7765 | 1.29322 | 2.1 |
| 7766 | 1.29322 | 2.1 |
| 7767 | 1.01985 | 2.1 |
| 7768 | 0.968537 | 2.1 |
| 7769 | 1.401 | 2.1 |

| | | |
|---|---|---|
| 7770 | 1.28936 | 2.1 |
| 7771 | 1.56002 | 0.05 |
| 7772 | 1.92548 | 0.05 |
| 7773 | 1.46041 | 0.05 |
| 7774 | 2.22712 | 0.05 |
| 7775 | 1.56001 | 0.05 |
| 7776 | 1.50524 | 2.1 |
| 7777 | 1.21304 | 2.1 |
| 7778 | 1.60665 | 1.73943 |
| 7779 | 1.85178 | 0.901797 |
| 7780 | 2.17534 | 2.1 |
| 7781 | 2.31012 | 2.1 |
| 7782 | 2.17533 | 1.63222 |
| 7783 | 0.986724 | 2.1 |
| 7784 | 2.01895 | 2.1 |
| 7785 | 2.00433 | 2.1 |
| 7786 | 1.45752 | 0.05 |
| 7787 | 1.12695 | 0.05 |
| 7788 | 0.652605 | 2.1 |
| 7789 | 1.5133 | 2.1 |
| 7790 | 1.5133 | 2.1 |
| 7791 | 0.652603 | 2.1 |
| 7792 | 1.228 2.1 | |
| 7793 | 0.946132 | 2.1 |
| 7794 | 0.652604 | 2.1 |
| 7795 | 0.946132 | 1.13644 |
| 7796 | 0.716815 | 2.1 |
| 7797 | 1.62203 | 2.1 |
| 7798 | 1.62203 | 2.1 |
| 7799 | 0.716814 | 2.1 |
| 7800 | 1.00191 | 2.1 |
| 7801 | 1.33694 | 2.1 |
| 7802 | 1.38216 | 2.1 |
| 7803 | 2.53338 | 2.1 |
| 7804 | 1.46932 | 0.05 |
| 7805 | 1.50848 | 0.05 |
| 7806 | 1.50108 | 1.27259 |
| 7807 | 1.18382 | 0.766289 |
| 7808 | 2.02115 | 0.59156 |
| 7809 | 0.921823 | 0.05 |
| 7810 | 2.08356 | 0.05 |
| 7811 | 1.543 0.05 | |
| 7812 | 1.00763 | 0.259126 |
| 7813 | 1.4185 | 0.602361 |
| 7814 | 1.08028 | 0.452549 |
| 7815 | 0.946131 | 0.803091 |
| 7816 | 1.56599 | 0.488024 |
| 7817 | 1.56599 | 0.864627 |
| 7818 | 1.08028 | 0.696835 |
| 7819 | 0.946131 | 0.34979 |
| 7820 | 1.37518 | 2.1 |
| 7821 | 1.63453 | 2.1 |
| 7822 | 1.04627 | 0.992324 |
| 7823 | 1.04626 | 0.470102 |
| 7824 | 1.35111 | 1.05656 |
| 7825 | 1.22297 | 0.966197 |
| 7826 | 1.64499 | 2.1 |
| 7827 | 1.87598 | 1.96327 |

| | | |
|---|---|---|
| 7828 | 1.06994 | 2.1 |
| 7829 | 1.37063 | 2.1 |
| 7830 | 1.99943 | 0.05 |
| 7831 | 2.38807 | 0.05 |
| 7832 | 0.99972 | 0.05 |
| 7833 | 1.41388 | 0.05 |
| 7834 | 1.66179 | 0.05 |
| 7835 | 1.66179 | 0.05 |
| 7836 | 0.946129 | 0.05 |
| 7837 | 1.41388 | 0.05 |
| 7838 | 1.19404 | 0.05 |
| 7839 | 1.31023 | 0.078258 |
| 7840 | 1.20871 | 0.236093 |
| 7841 | 1.69731 | 2.1 |
| 7842 | 1.67971 | 2.1 |
| 7843 | 0.998971 | 0.05 |
| 7844 | 1.32988 | 0.05 |
| 7845 | 0.701079 | 0.05 |
| 7846 | 0.946131 | 0.05 |
| 7847 | 0.582785 | 0.05 |
| 7848 | 1.48592 | 0.05 |
| 7849 | 1.48592 | 0.05 |
| 7850 | 1.23351 | 0.05 |
| 7851 | 0.835199 | 0.05 |
| 7852 | 0.92427 | 0.05 |
| 7853 | 1.68262 | 0.05 |
| 7854 | 2.46702 | 0.05 |
| 7855 | 1.08715 | 2.1 |
| 7856 | 1.01606 | 1.68223 |
| 7857 | 1.29579 | 1.75954 |
| 7858 | 1.01606 | 2.0752 |
| 7859 | 1.63823 | 2.1 |
| 7860 | 1.67687 | 2.1 |
| 7861 | 1.11048 | 2.1 |
| 7862 | 1.39477 | 2.1 |
| 7863 | 0.991114 | 2.1 |
| 7864 | 2.90978 | 0.84711 |
| 7865 | 0.906811 | 1.00291 |
| 7866 | 0.872849 | 0.178217 |
| 7867 | 1.17091 | 0.838546 |
| 7868 | 0.685763 | 0.82923 |
| 7869 | 1.6386 | 2.04083 |
| 7870 | 1.06907 | 0.917246 |
| 7871 | 1.05516 | 1.04112 |
| 7872 | 1.9189 | 1.05547 |
| 7873 | 1.04615 | 0.741799 |
| 7874 | 1.2966 | 2.1 |
| 7875 | 1.37392 | 2.1 |
| 7876 | 1.35504 | 2.1 |
| 7877 | 1.30347 | 2.1 |
| 7878 | 1.50877 | 2.1 |
| 7879 | 1.11193 | 2.1 |
| 7880 | 1.40984 | 2.1 |
| 7881 | 1.67682 | 2.1 |
| 7882 | 1.00371 | 2.1 |
| 7883 | 1.15412 | 2.1 |
| 7884 | 1.41638 | 2.1 |
| 7885 | 1.03796 | 2.1 |

| | | |
|---|---|---|
| 7886 | 0.946117 | 2.1 |
| 7887 | 1.50823 | 2.1 |
| 7888 | 1.50822 | 2.1 |
| 7889 | 0.966923 | 2.1 |
| 7890 | 0.946131 | 2.1 |
| 7891 | 2.07593 | 2.1 |
| 7892 | 1.9339 | 2.1 |
| 7893 | 1.1817 | 0.786617 |
| 7894 | 0.729178 | 0.792789 |
| 7895 | 1.5079 | 0.833187 |
| 7896 | 1.26991 | 0.971491 |
| 7897 | 0.994049 | 0.777406 |
| 7898 | 1.17452 | 0.829396 |
| 7899 | 1.05711 | 0.930204 |
| 7900 | 1.04071 | 0.925253 |
| 7901 | 1.80299 | 0.76281 |
| 7902 | 1.60417 | 0.684805 |
| 7903 | 0.943405 | 0.05 |
| 7904 | 1.01129 | 0.05 |
| 7905 | 1.42972 | 0.05 |
| 7906 | 1.15904 | 0.05 |
| 7907 | 0.680409 | 0.05 |
| 7908 | 1.2816 | 0.05 |
| 7909 | 0.806176 | 0.05 |
| 7910 | 1.18954 | 0.05 |
| 7911 | 1.26055 | 0.05 |
| 7912 | 1.06313 | 2.1 |
| 7913 | 1.19352 | 2.1 |
| 7914 | 1.55766 | 2.1 |
| 7915 | 1.53729 | 2.1 |
| 7916 | 0.993021 | 0.05 |
| 7917 | 1.09056 | 0.18111 |
| 7918 | 1.03408 | 2.1 |
| 7919 | 0.993023 | 0.05 |
| 7920 | 0.659901 | 2.1 |
| 7921 | 0.632905 | 2.1 |
| 7922 | 1.34072 | 0.05 |
| 7923 | 1.17793 | 0.074312 |
| 7924 | 2.24885 | 0.05 |
| 7925 | 0.946132 | 0.05 |
| 7926 | 1.12443 | 0.05 |
| 7927 | 0.904207 | 0.05 |
| 7928 | 1.51237 | 0.05 |
| 7929 | 1.51237 | 0.05 |
| 7930 | 1.12443 | 0.05 |
| 7931 | 1.29215 | 0.05 |
| 7932 | 1.6943 | 2.1 |
| 7933 | 2.43858 | 2.1 |
| 7934 | 2.0589 | 2.06595 |
| 7935 | 1.67204 | 2.1 |
| 7936 | 1.67204 | 2.1 |
| 7937 | 0.555875 | 2.1 |
| 7938 | 0.91951 | 2.1 |
| 7939 | 0.946131 | 1.44054 |
| 7940 | 0.555904 | 2.1 |
| 7941 | 0.946131 | 2.1 |
| 7942 | 1.6943 | 2.1 |
| 7943 | 1.04744 | 1.99818 |

| | | |
|---|---|---|
| 7944 | 1.50938 | 2.1 |
| 7945 | 1.60861 | 2.1 |
| 7946 | 1.92406 | 0.05 |
| 7947 | 1.18716 | 0.05 |
| 7948 | 0.962705 | 0.608663 |
| 7949 | 1.35382 | 0.764065 |
| 7950 | 1.43632 | 0.05 |
| 7951 | 1.09386 | 0.05 |
| 7952 | 1.92547 | 0.05 |
| 7953 | 0.994164 | 0.05 |
| 7954 | 0.903974 | 2.1 |
| 7955 | 0.928829 | 1.79725 |
| 7956 | 0.810958 | 1.44019 |
| 7957 | 1.92853 | 1.59191 |
| 7958 | 1.598 1.64873 | |
| 7959 | 1.28967 | 1.85164 |
| 7960 | 1.44993 | 1.67619 |
| 7961 | 0.946132 | 0.397957 |
| 7962 | 0.882984 | 0.274639 |
| 7963 | 0.882955 | 0.61588 |
| 7964 | 1.56071 | 0.05 |
| 7965 | 1.56071 | 0.0905498 |
| 7966 | 1.35946 | 0.208725 |
| 7967 | 1.08421 | 0.256721 |
| 7968 | 1.41943 | 0.05 |
| 7969 | 1.26265 | 0.05 |
| 7970 | 1.03235 | 0.05 |
| 7971 | 1.50778 | 2.1 |
| 7972 | 1.70387 | 2.1 |
| 7973 | 1.65608 | 2.1 |
| 7974 | 1.39076 | 2.1 |
| 7975 | 1.6104 | 2.1 |
| 7976 | 1.37456 | 2.1 |
| 7977 | 1.51898 | 0.151708 |
| 7978 | 1.9726 | 0.0786796 |
| 7979 | 2.13757 | 0.651033 |
| 7980 | 1.74856 | 0.836305 |
| 7981 | 0.635949 | 0.246898 |
| 7982 | 2.46448 | 0.961419 |
| 7983 | 1.23224 | 0.860923 |
| 7984 | 1.12786 | 1.12719 |
| 7985 | 1.82721 | 0.59895 |
| 7986 | 1.82721 | 0.153686 |
| 7987 | 0.532892 | 2.07678 |
| 7988 | 0.946131 | 1.19924 |
| 7989 | 0.53292 | 1.12816 |
| 7990 | 1.41394 | 2.1 |
| 7991 | 1.45506 | 2.1 |
| 7992 | 1.39523 | 2.1 |
| 7993 | 1.09006 | 2.1 |
| 7994 | 1.80264 | 0.05 |
| 7995 | 1.80264 | 0.180299 |
| 7996 | 0.946131 | 0.05 |
| 7997 | 1.34268 | 0.05 |
| 7998 | 0.946132 | 0.196465 |
| 7999 | 1.23732 | 0.05 |
| 8000 | 1.34268 | 0.05 |
| 8001 | 1.2064 | 0.685511 |

| | | |
|---|---|---|
| 8002 | 1.58164 | 2.1 |
| 8003 | 1.73853 | 2.1 |
| 8004 | 1.01243 | 1.0872 |
| 8005 | 0.910672 | 1.78606 |
| 8006 | 1.47793 | 2.1 |
| 8007 | 1.47793 | 2.1 |
| 8008 | 0.910702 | 1.66972 |
| 8009 | 1.30382 | 1.08745 |
| 8010 | 1.08478 | 1.12504 |
| 8011 | 0.910702 | 2.1 |
| 8012 | 2.60709 | 1.27125 |
| 8013 | 1.01243 | 1.08992 |
| 8014 | 1.85516 | 1.53941 |
| 8015 | 2.52823 | 0.825239 |
| 8016 | 1.24001 | 0.762942 |
| 8017 | 1.89793 | 0.278299 |
| 8018 | 1.73584 | 0.05 |
| 8019 | 1.24613 | 2.1 |
| 8020 | 1.37074 | 1.76406 |
| 8021 | 1.23763 | 1.51986 |
| 8022 | 1.04072 | 2.1 |
| 8023 | 0.75499 | 1.21164 |
| 8024 | 1.72329 | 1.592 |
| 8025 | 1.46195 | 1.34201 |
| 8026 | 0.946132 | 1.33729 |
| 8027 | 0.747438 | 1.98494 |
| 8028 | 1.24613 | 1.51962 |
| 8029 | 2.59886 | 1.04987 |
| 8030 | 1.87279 | 2.1 |
| 8031 | 1.07977 | 2.1 |
| 8032 | 1.29606 | 2.1 |
| 8033 | 1.45178 | 2.1 |
| 8034 | 1.45177 | 2.1 |
| 8035 | 0.92406 | 2.1 |
| 8036 | 0.946131 | 2.1 |
| 8037 | 0.924047 | 2.1 |
| 8038 | 2.04529 | 2.1 |
| 8039 | 1.04544 | 2.1 |
| 8040 | 1.08276 | 2.1 |
| 8041 | 1.67491 | 2.1 |
| 8042 | 0.961826 | 0.961455 |
| 8043 | 0.810942 | 0.68937 |
| 8044 | 0.826622 | 1.59761 |
| 8045 | 0.914378 | 1.86906 |
| 8046 | 0.89022 | 0.127109 |
| 8047 | 0.922383 | 1.07196 |
| 8048 | 0.988165 | 0.150712 |
| 8049 | 1.31357 | 0.05 |
| 8050 | 0.946131 | 0.05 |
| 8051 | 1.31357 | 0.05 |
| 8052 | 1.10564 | 0.05 |
| 8053 | 1.6797 | 0.05 |
| 8054 | 1.6797 | 0.05 |
| 8055 | 0.739506 | 0.05 |
| 8056 | 1.33957 | 0.05 |
| 8057 | 1.33957 | 0.05 |
| 8058 | 1.33957 | 0.05 |
| 8059 | 2.34223 | 0.05 |

| | | |
|---|---|---|
| 8060 | 1.5515 | 1.00812 |
| 8061 | 2.22137 | 0.875542 |
| 8062 | 0.94612 | 0.813018 |
| 8063 | 1.13908 | 0.906469 |
| 8064 | 1.45423 | 0.754923 |
| 8065 | 1.45422 | 0.975916 |
| 8066 | 0.795519 | 1.15342 |
| 8067 | 1.1391 | 0.876239 |
| 8068 | 1.11065 | 0.875732 |
| 8069 | 1.12266 | 2.1 |
| 8070 | 1.24925 | 2.1 |
| 8071 | 1.20242 | 2.1 |
| 8072 | 2.18297 | 2.1 |
| 8073 | 3.00107 | 1.97748 |
| 8074 | 2.58297 | 0.263739 |
| 8075 | 0.884783 | 2.1 |
| 8076 | 0.851538 | 0.868183 |
| 8077 | 0.713587 | 2.1 |
| 8078 | 1.47949 | 0.925954 |
| 8079 | 1.66673 | 2.1 |
| 8080 | 0.696199 | 2.1 |
| 8081 | 0.9383 | 1.44024 |
| 8082 | 1.29149 | 1.10515 |
| 8083 | 1.32266 | 1.91365 |
| 8084 | 2.11428 | 0.05 |
| 8085 | 2.17439 | 0.05 |
| 8086 | 1.40489 | 0.05 |
| 8087 | 1.21071 | 0.05 |
| 8088 | 0.946118 | 0.05 |
| 8089 | 1.0746 | 0.05 |
| 8090 | 0.973119 | 0.05 |
| 8091 | 1.31333 | 0.05 |
| 8092 | 1.08723 | 0.05 |
| 8093 | 0.994292 | 2.1 |
| 8094 | 1.4964 | 2.1 |
| 8095 | 1.15096 | 2.1 |
| 8096 | 1.10101 | 2.1 |
| 8097 | 1.50438 | 2.1 |
| 8098 | 1.67825 | 2.1 |
| 8099 | 2.02103 | 2.1 |
| 8100 | 2.01978 | 2.1 |
| 8101 | 1.50931 | 0.05 |
| 8102 | 1.50931 | 0.05 |
| 8103 | 0.65023 | 0.05 |
| 8104 | 1.34831 | 0.05 |
| 8105 | 0.811233 | 0.05 |
| 8106 | 1.34831 | 0.05 |
| 8107 | 0.94613 | 0.05 |
| 8108 | 1.00754 | 1.02138 |
| 8109 | 0.714132 | 2.1 |
| 8110 | 1.28877 | 1.44184 |
| 8111 | 1.39905 | 1.31529 |
| 8112 | 1.03282 | 1.4575 |
| 8113 | 0.997769 | 1.0219 |
| 8114 | 1.11743 | 1.17292 |
| 8115 | 1.86863 | 1.02421 |
| 8116 | 3.30723 | 1.68222 |
| 8117 | 1.66635 | 2.1 |

| | | |
|---|---|---|
| 8118 | 1.35609 | 0.05 |
| 8119 | 1.38212 | 0.05 |
| 8120 | 1.95597 | 2.1 |
| 8121 | 1.64257 | 2.1 |
| 8122 | 2.93316 | 2.1 |
| 8123 | 3.13417 | 2.1 |
| 8124 | 0.733947 | 2.1 |
| 8125 | 1.15259 | 2.1 |
| 8126 | 1.07976 | 2.1 |
| 8127 | 1.30237 | 2.1 |
| 8128 | 1.99943 | 0.05 |
| 8129 | 0.907431 | 0.05 |
| 8130 | 0.878161 | 0.05 |
| 8131 | 1.07544 | 0.05 |
| 8132 | 1.03401 | 0.05 |
| 8133 | 0.991975 | 0.05 |
| 8134 | 1.03635 | 0.05 |
| 8135 | 0.788549 | 0.05 |
| 8136 | 1.44984 | 0.05 |
| 8137 | 1.84007 | 0.05 |
| 8138 | 2.31127 | 0.05 |
| 8139 | 1.27362 | 2.1 |
| 8140 | 1.27737 | 1.16465 |
| 8141 | 1.87783 | 0.937272 |
| 8142 | 2.35499 | 0.05 |
| 8143 | 2.69662 | 0.05 |
| 8144 | 2.24065 | 0.05 |
| 8145 | 0.996082 | 2.1 |
| 8146 | 0.996083 | 2.1 |
| 8147 | 1.44859 | 2.1 |
| 8148 | 1.03968 | 2.1 |
| 8149 | 1.60018 | 0.526864 |
| 8150 | 1.73082 | 1.57724 |
| 8151 | 0.946132 | 1.21132 |
| 8152 | 1.49742 | 1.56411 |
| 8153 | 1.49742 | 0.05 |
| 8154 | 1.00501 | 0.911623 |
| 8155 | 1.41585 | 1.99392 |
| 8156 | 1.41585 | 0.911335 |
| 8157 | 0.946131 | 1.4945 |
| 8158 | 1.03697 | 0.613761 |
| 8159 | 2.95786 | 2.1 |
| 8160 | 2.32185 | 2.1 |
| 8161 | 1.65783 | 0.05 |
| 8162 | 1.772 2.1 | |
| 8163 | 0.946131 | 0.05 |
| 8164 | 1.40404 | 0.05 |
| 8165 | 1.13537 | 2.1 |
| 8166 | 1.04559 | 0.0803583 |
| 8167 | 1.16091 | 0.186458 |
| 8168 | 2.63751 | 2.1 |
| 8169 | 1.68455 | 0.05 |
| 8170 | 1.9682 | 0.05 |
| 8171 | 2.16834 | 0.05 |
| 8172 | 2.45569 | 0.0602768 |
| 8173 | 0.946128 | 0.05 |
| 8174 | 1.36428 | 0.0694387 |
| 8175 | 0.663978 | 0.0517867 |

| | | |
|---|---|---|
| 8176 | 1.58218 | 0.05 |
| 8177 | 1.58219 | 0.05 |
| 8178 | 1.36428 | 0.05 |
| 8179 | 0.881886 | 0.05 |
| 8180 | 1.73849 | 2.1 |
| 8181 | 1.35668 | 2.1 |
| 8182 | 1.1233 | 2.1 |
| 8183 | 1.15009 | 2.1 |
| 8184 | 1.16482 | 0.644593 |
| 8185 | 1.29313 | 0.671822 |
| 8186 | 1.47608 | 0.0567695 |
| 8187 | 2.05949 | 0.05 |
| 8188 | 0.946119 | 0.05 |
| 8189 | 1.02974 | 0.05 |
| 8190 | 0.946119 | 0.05 |
| 8191 | 1.66758 | 0.05 |
| 8192 | 1.66757 | 0.05 |
| 8193 | 1.32082 | 0.05 |
| 8194 | 1.29287 | 0.05 |
| 8195 | 1.36005 | 2.1 |
| 8196 | 1.47109 | 2.1 |
| 8197 | 2.45958 | 2.1 |
| 8198 | 2.2975 | 2.1 |
| 8199 | 0.953521 | 0.3493 |
| 8200 | 0.953521 | 0.0505886 |
| 8201 | 1.67054 | 2.1 |
| 8202 | 1.67054 | 2.1 |
| 8203 | 0.946131 | 2.1 |
| 8204 | 1.38154 | 2.1 |
| 8205 | 1.13326 | 2.1 |
| 8206 | 1.13326 | 2.1 |
| 8207 | 1.23513 | 2.1 |
| 8208 | 1.50998 | 2.1 |
| 8209 | 2.47982 | 0.05 |
| 8210 | 1.45091 | 0.05 |
| 8211 | 0.958953 | 0.05 |
| 8212 | 0.79239 | 0.05 |
| 8213 | 2.11929 | 0.05 |
| 8214 | 1.26511 | 0.05 |
| 8215 | 1.27733 | 0.05 |
| 8216 | 2.18127 | 0.05 |
| 8217 | 1.30169 | 0.05 |
| 8218 | 1.19259 | 0.05 |
| 8219 | 0.963395 | 1.13129 |
| 8220 | 0.999947 | 2.09495 |
| 8221 | 1.62241 | 2.1 |
| 8222 | 1.62027 | 2.1 |
| 8223 | 0.981586 | 0.05 |
| 8224 | 0.981585 | 0.05 |
| 8225 | 0.721961 | 2.1 |
| 8226 | 0.891321 | 2.1 |
| 8227 | 1.04442 | 2.1 |
| 8228 | 1.66554 | 2.1 |
| 8229 | 2.43434 | 2.1 |
| 8230 | 1.73944 | 0.05 |
| 8231 | 1.88032 | 0.05 |
| 8232 | 0.711391 | 0.05 |
| 8233 | 1.40837 | 0.05 |

| | | |
|---|---|---|
| 8234 | 0.946133 | 0.05 |
| 8235 | 0.92252 | 0.05 |
| 8236 | 1.13964 | 0.05 |
| 8237 | 1.83394 | 0.05 |
| 8238 | 2.81674 | 0.05 |
| 8239 | 1.10049 | 1.53128 |
| 8240 | 1.25092 | 0.439145 |
| 8241 | 0.760299 | 0.994718 |
| 8242 | 1.48017 | 1.09456 |
| 8243 | 1.48017 | 1.22914 |
| 8244 | 0.760271 | 0.805427 |
| 8245 | 1.35861 | 1.02307 |
| 8246 | 0.946129 | 0.881987 |
| 8247 | 0.760269 | 0.844407 |
| 8248 | 2.26155 | 2.1 |
| 8249 | 2.15634 | 2.1 |
| 8250 | 1.39824 | 2.1 |
| 8251 | 1.07817 | 2.1 |
| 8252 | 0.946132 | 2.1 |
| 8253 | 1.53028 | 2.1 |
| 8254 | 1.53028 | 2.1 |
| 8255 | 1.00807 | 2.1 |
| 8256 | 0.946133 | 2.1 |
| 8257 | 2.06126 | 0.476827 |
| 8258 | 1.97385 | 1.72813 |
| 8259 | 1.75754 | 0.587425 |
| 8260 | 2.01613 | 2.1 |
| 8261 | 1.97995 | 2.1 |
| 8262 | 1.47696 | 2.1 |
| 8263 | 1.50172 | 2.1 |
| 8264 | 1.5177 | 0.05 |
| 8265 | 1.44146 | 0.05 |
| 8266 | 1.8807 | 0.05 |
| 8267 | 2.07068 | 0.05 |
| 8268 | 1.84267 | 0.05 |
| 8269 | 1.08975 | 2.1 |
| 8270 | 1.21327 | 2.1 |
| 8271 | 1.10106 | 0.05 |
| 8272 | 1.34496 | 0.05 |
| 8273 | 2.08452 | 0.374775 |
| 8274 | 1.70537 | 0.0606001 |
| 8275 | 2.62464 | 2.1 |
| 8276 | 1.5726 | 0.81477 |
| 8277 | 1.5726 | 2.1 |
| 8278 | 0.94613 | 2.1 |
| 8279 | 1.15038 | 2.1 |
| 8280 | 0.946132 | 0.748867 |
| 8281 | 1.03974 | 2.1 |
| 8282 | 1.15038 | 2.1 |
| 8283 | 0.99747 | 0.218966 |
| 8284 | 1.03472 | 1.9287 |
| 8285 | 1.58407 | 0.161211 |
| 8286 | 1.21148 | 0.600076 |
| 8287 | 1.35025 | 0.796629 |
| 8288 | 1.3428 | 0.213148 |
| 8289 | 1.00775 | 2.1 |
| 8290 | 1.00775 | 2.1 |
| 8291 | 1.3879 | 2.1 |

| | | |
|---|---|---|
| 8292 | 1.39958 | 2.1 |
| 8293 | 0.853042 | 2.1 |
| 8294 | 1.06997 | 2.1 |
| 8295 | 0.853042 | 2.1 |
| 8296 | 0.853042 | 2.1 |
| 8297 | 1.12491 | 2.1 |
| 8298 | 1.17879 | 2.1 |
| 8299 | 1.07162 | 2.1 |
| 8300 | 1.61179 | 2.1 |
| 8301 | 1.76016 | 2.1 |
| 8302 | 0.946132 | 0.111505 |
| 8303 | 1.23449 | 0.05 |
| 8304 | 1.70392 | 0.05 |
| 8305 | 1.70392 | 0.05 |
| 8306 | 0.94613 | 0.147706 |
| 8307 | 1.23449 | 0.0676968 |
| 8308 | 1.41556 | 0.0625561 |
| 8309 | 1.14547 | 0.05 |
| 8310 | 1.1721 | 0.05 |
| 8311 | 0.96478 | 0.05 |
| 8312 | 1.41843 | 0.05 |
| 8313 | 1.2358 | 0.05 |
| 8314 | 1.70678 | 0.05 |
| 8315 | 1.70678 | 0.05 |
| 8316 | 0.946118 | 0.0868771 |
| 8317 | 1.23582 | 0.0569426 |
| 8318 | 0.946105 | 0.082359 |
| 8319 | 0.964769 | 0.05 |
| 8320 | 1.1357 | 0.05 |
| 8321 | 0.964767 | 0.05 |
| 8322 | 0.619856 | 0.05 |
| 8323 | 1.72366 | 0.05 |
| 8324 | 1.72367 | 0.05 |
| 8325 | 0.619831 | 0.05 |
| 8326 | 1.19262 | 0.05 |
| 8327 | 0.619829 | 0.05 |
| 8328 | 0.946129 | 0.05 |
| 8329 | 1.57459 | 2.1 |
| 8330 | 1.86512 | 2.1 |
| 8331 | 0.93841 | 2.1 |
| 8332 | 1.57459 | 2.1 |
| 8333 | 1.10476 | 0.05 |
| 8334 | 1.00013 | 0.37786 |
| 8335 | 2.13446 | 0.806825 |
| 8336 | 0.759781 | 2.1 |
| 8337 | 1.96011 | 1.26628 |
| 8338 | 0.946129 | 0.925848 |
| 8339 | 1.32056 | 1.97142 |
| 8340 | 1.75702 | 1.13435 |
| 8341 | 1.75702 | 2.1 |
| 8342 | 0.946124 | 1.93596 |
| 8343 | 1.32056 | 1.29255 |
| 8344 | 1.38258 | 0.971238 |
| 8345 | 1.96011 | 1.37959 |
| 8346 | 2.31099 | 1.28692 |
| 8347 | 0.97356 | 2.1 |
| 8348 | 1.38885 | 2.1 |
| 8349 | 1.3771 | 2.1 |

| | | |
|---|---|---|
| 8350 | 0.946131 | 2.1 |
| 8351 | 1.84843 | 2.1 |
| 8352 | 1.84843 | 2.1 |
| 8353 | 1.3771 | 2.1 |
| 8354 | 0.946131 | 2.1 |
| 8355 | 1.00278 | 2.1 |
| 8356 | 1.05447 | 0.05 |
| 8357 | 1.05446 | 0.375155 |
| 8358 | 1.24913 | 0.194579 |
| 8359 | 1.05447 | 0.05 |
| 8360 | 1.80882 | 0.05 |
| 8361 | 1.80882 | 2.1 |
| 8362 | 0.946129 | 2.1 |
| 8363 | 1.33997 | 2.1 |
| 8364 | 0.946131 | 0.05 |
| 8365 | 1.28831 | 0.342948 |
| 8366 | 1.33997 | 0.232386 |
| 8367 | 1.44391 | 0.05 |
| 8368 | 1.44391 | 0.05 |
| 8369 | 0.850964 | 0.05 |
| 8370 | 0.850963 | 0.05 |
| 8371 | 0.946132 | 0.05 |
| 8372 | 0.921299 | 0.05 |
| 8373 | 1.37358 | 0.05 |
| 8374 | 1.46297 | 0.05 |
| 8375 | 1.46298 | 0.05 |
| 8376 | 0.823988 | 0.05 |
| 8377 | 0.921287 | 0.05 |
| 8378 | 0.94612 | 0.05 |
| 8379 | 0.823995 | 0.05 |
| 8380 | 0.946107 | 0.05 |
| 8381 | 1.44437 | 2.1 |
| 8382 | 1.06203 | 2.1 |
| 8383 | 2.21397 | 2.1 |
| 8384 | 0.637677 | 2.1 |
| 8385 | 1.8892 | 2.1 |
| 8386 | 2.075 | 2.1 |
| 8387 | 1.32051 | 0.05 |
| 8388 | 2.23637 | 0.05 |
| 8389 | 1.10904 | 0.05 |
| 8390 | 1.11819 | 0.05 |
| 8391 | 0.94613 | 0.05 |
| 8392 | 0.735845 | 0.05 |
| 8393 | 1.4914 | 0.05 |
| 8394 | 1.4914 | 0.05 |
| 8395 | 0.735831 | 0.05 |
| 8396 | 1.61683 | 2.1 |
| 8397 | 1.61683 | 2.1 |
| 8398 | 0.946131 | 2.1 |
| 8399 | 1.3281 | 2.1 |
| 8400 | 1.10441 | 2.1 |
| 8401 | 1.10442 | 2.1 |
| 8402 | 1.23487 | 2.1 |
| 8403 | 1.09914 | 2.1 |
| 8404 | 1.20691 | 0.05 |
| 8405 | 2.27379 | 0.05 |
| 8406 | 1.1369 | 0.05 |
| 8407 | 1.26868 | 0.05 |

| | | |
|---|---|---|
| 8408 | 1.54422 | 0.05 |
| 8409 | 1.54422 | 0.05 |
| 8410 | 0.861357 | 0.05 |
| 8411 | 0.94613 | 0.05 |
| 8412 | 0.861359 | 0.05 |
| 8413 | 1.2858 | 0.625933 |
| 8414 | 1.31011 | 0.63087 |
| 8415 | 1.294 | 0.50209 |
| 8416 | 1.03079 | 0.70607 |
| 8417 | 1.81979 | 2.0239 |
| 8418 | 1.57829 | 2.1 |
| 8419 | 1.19023 | 1.64635 |
| 8420 | 1.19022 | 1.99051 |
| 8421 | 1.46458 | 1.8004 |
| 8422 | 1.46458 | 2.1 |
| 8423 | 0.789173 | 2.1 |
| 8424 | 1.34386 | 2.1 |
| 8425 | 0.909896 | 2.1 |
| 8426 | 0.946131 | 1.94932 |
| 8427 | 0.909893 | 1.96901 |
| 8428 | 1.27725 | 2.1 |
| 8429 | 1.68451 | 2.1 |
| 8430 | 2.26155 | 2.1 |
| 8431 | 2.34217 | 2.1 |
| 8432 | 1.55485 | 0.80783 |
| 8433 | 0.90854 | 0.467967 |
| 8434 | 0.858256 | 0.427787 |
| 8435 | 1.47042 | 0.180981 |
| 8436 | 1.62771 | 0.214547 |
| 8437 | 0.777398 | 0.768267 |
| 8438 | 0.982525 | 0.406601 |
| 8439 | 1.23865 | 0.372964 |
| 8440 | 2.09928 | 0.41924 |
| 8441 | 0.946128 | 0.05 |
| 8442 | 0.474856 | 0.05 |
| 8443 | 0.474855 | 0.05 |
| 8444 | 1.58747 | 0.05 |
| 8445 | 1.58747 | 0.05 |
| 8446 | 0.963917 | 0.05 |
| 8447 | 1.09841 | 0.05 |
| 8448 | 2.23904 | 2.1 |
| 8449 | 2.60055 | 0.263187 |
| 8450 | 1.02599 | 2.1 |
| 8451 | 2.17837 | 2.1 |
| 8452 | 2.07122 | 2.1 |
| 8453 | 2.7309 | 0.108764 |
| 8454 | 2.08482 | 2.1 |
| 8455 | 1.18322 | 0.05 |
| 8456 | 1.35381 | 0.401785 |
| 8457 | 1.01139 | 0.05 |
| 8458 | 0.946133 | 0.71527 |
| 8459 | 1.59089 | 0.110735 |
| 8460 | 1.59089 | 0.05 |
| 8461 | 0.946133 | 0.0628159 |
| 8462 | 1.09634 | 0.05 |
| 8463 | 2.02279 | 0.05 |
| 8464 | 1.03108 | 0.05 |
| 8465 | 0.808978 | 1.43503 |

| | | |
|---|---|---|
| 8466 | 1.07317 | 1.44351 |
| 8467 | 0.831695 | 1.39643 |
| 8468 | 1.22583 | 1.39557 |
| 8469 | 1.10215 | 1.26532 |
| 8470 | 1.07508 | 1.48373 |
| 8471 | 1.22204 | 1.33109 |
| 8472 | 1.04679 | 2.1 |
| 8473 | 1.92535 | 0.05 |
| 8474 | 1.11081 | 0.05 |
| 8475 | 0.946131 | 0.0542521 |
| 8476 | 0.714824 | 0.05 |
| 8477 | 0.714795 | 0.05 |
| 8478 | 1.4231 | 0.05 |
| 8479 | 1.4231 | 0.05 |
| 8480 | 0.828617 | 0.0747946 |
| 8481 | 1.30928 | 0.05 |
| 8482 | 1.49894 | 2.1 |
| 8483 | 1.55702 | 2.1 |
| 8484 | 0.946131 | 2.1 |
| 8485 | 1.10689 | 2.1 |
| 8486 | 0.946227 | 2.1 |
| 8487 | 0.946131 | 2.1 |
| 8488 | 1.21754 | 2.1 |
| 8489 | 1.504 2.1 | |
| 8490 | 1.504 2.1 | |
| 8491 | 0.723573 | 2.1 |
| 8492 | 1.24512 | 2.1 |
| 8493 | 0.723544 | 2.1 |
| 8494 | 0.946131 | 2.1 |
| 8495 | 1.24512 | 2.1 |
| 8496 | 1.29383 | 0.05 |
| 8497 | 1.29383 | 0.05 |
| 8498 | 1.61969 | 0.05 |
| 8499 | 0.946132 | 0.05 |
| 8500 | 1.12818 | 0.05 |
| 8501 | 0.946132 | 0.05 |
| 8502 | 1.80922 | 0.05 |
| 8503 | 1.80922 | 0.05 |
| 8504 | 1.41383 | 0.05 |
| 8505 | 1.34153 | 0.05 |
| 8506 | 1.47689 | 0.05 |
| 8507 | 1.79375 | 0.05 |
| 8508 | 1.24333 | 0.0532591 |
| 8509 | 0.768989 | 0.05 |
| 8510 | 1.24333 | 0.05 |
| 8511 | 1.0027 | 0.11309 |
| 8512 | 1.47705 | 0.07991 |
| 8513 | 1.47705 | 0.05 |
| 8514 | 0.768977 | 0.0680435 |
| 8515 | 1.28436 | 0.0907975 |
| 8516 | 0.928583 | 0.132231 |
| 8517 | 1.28436 | 0.05 |
| 8518 | 1.24333 | 0.126961 |
| 8519 | 1.5991 | 0.113705 |
| 8520 | 1.5991 | 0.05 |
| 8521 | 0.928583 | 0.172623 |
| 8522 | 1.85717 | 0.05 |
| 8523 | 2.48665 | 0.05 |

| | | |
|---|---|---|
| 8524 | 1.18423 | 0.05 |
| 8525 | 1.9493 | 1.78497 |
| 8526 | 1.8519 | 1.10413 |
| 8527 | 1.68802 | 2.1 |
| 8528 | 1.0302 | 0.05 |
| 8529 | 0.900473 | 0.05 |
| 8530 | 1.99663 | 0.05 |
| 8531 | 2.61301 | 0.05 |
| 8532 | 0.745772 | 0.05 |
| 8533 | 0.946131 | 0.05 |
| 8534 | 1.48881 | 0.05 |
| 8535 | 1.48881 | 0.05 |
| 8536 | 0.745745 | 0.05 |
| 8537 | 0.809786 | 0.05 |
| 8538 | 1.3065 | 0.05 |
| 8539 | 0.946132 | 0.05 |
| 8540 | 0.768357 | 0.05 |
| 8541 | 0.768387 | 0.05 |
| 8542 | 1.76897 | 0.05 |
| 8543 | 1.76896 | 0.05 |
| 8544 | 1.40271 | 0.05 |
| 8545 | 1.00777 | 0.05 |
| 8546 | 1.37427 | 0.05 |
| 8547 | 1.1967 | 2.1 |
| 8548 | 0.946132 | 2.1 |
| 8549 | 1.59397 | 2.1 |
| 8550 | 1.59397 | 2.1 |
| 8551 | 0.946131 | 2.1 |
| 8552 | 1.14989 | 2.1 |
| 8553 | 1.14989 | 2.1 |
| 8554 | 1.10046 | 2.1 |
| 8555 | 1.09506 | 0.05 |
| 8556 | 2.53251 | 0.05 |
| 8557 | 1.9212 | 0.960655 |
| 8558 | 1.99586 | 0.877066 |
| 8559 | 0.913967 | 0.665667 |
| 8560 | 1.55028 | 0.696929 |
| 8561 | 1.55028 | 0.814001 |
| 8562 | 0.913966 | 0.642927 |
| 8563 | 1.16318 | 0.623452 |
| 8564 | 1.16318 | 0.635913 |
| 8565 | 0.946132 | 0.613028 |
| 8566 | 1.20478 | 2.1 |
| 8567 | 1.09662 | 2.1 |
| 8568 | 1.03472 | 2.1 |
| 8569 | 1.84774 | 2.1 |
| 8570 | 1.7984 | 2.1 |
| 8571 | 2.1221 | 2.1 |
| 8572 | 1.68223 | 2.1 |
| 8573 | 1.68224 | 2.1 |
| 8574 | 0.514872 | 1.9911 |
| 8575 | 0.888991 | 2.1 |
| 8576 | 0.946127 | 0.911836 |
| 8577 | 0.514845 | 1.94892 |
| 8578 | 0.946129 | 1.1748 |
| 8579 | 1.10127 | 1.73125 |
| 8580 | 2.61623 | 0.849793 |
| 8581 | 0.983581 | 1.09787 |

| | | |
|---|---|---|
| 8582 | 0.869663 | 0.785796 |
| 8583 | 1.1818 | 2.1 |
| 8584 | 1.10406 | 0.930723 |
| 8585 | 1.07133 | 2.1 |
| 8586 | 1.09405 | 0.992617 |
| 8587 | 0.838014 | 0.852543 |
| 8588 | 0.842998 | 1.54149 |
| 8589 | 1.03818 | 1.05435 |
| 8590 | 1.46338 | 0.512028 |
| 8591 | 1.903 2.1 | |
| 8592 | 1.23942 | 2.1 |
| 8593 | 0.965933 | 2.1 |
| 8594 | 0.810075 | 2.1 |
| 8595 | 1.40571 | 2.1 |
| 8596 | 1.66219 | 2.1 |
| 8597 | 0.810047 | 2.1 |
| 8598 | 0.936678 | 2.1 |
| 8599 | 1.45808 | 2.1 |
| 8600 | 1.6384 | 2.1 |
| 8601 | 1.54699 | 2.1 |
| 8602 | 0.719877 | 2.1 |
| 8603 | 1.33256 | 2.1 |
| 8604 | 0.753547 | 2.1 |
| 8605 | 1.04073 | 2.1 |
| 8606 | 1.25822 | 2.1 |
| 8607 | 0.851542 | 2.1 |
| 8608 | 0.714102 | 2.1 |
| 8609 | 1.45886 | 2.1 |
| 8610 | 1.80171 | 2.1 |
| 8611 | 0.597337 | 2.1 |
| 8612 | 0.957713 | 2.1 |
| 8613 | 1.3033 | 2.1 |
| 8614 | 0.596613 | 0.05 |
| 8615 | 1.1386 | 0.05 |
| 8616 | 0.596612 | 0.05 |
| 8617 | 0.946133 | 0.05 |
| 8618 | 0.596652 | 0.0715043 |
| 8619 | 1.64092 | 0.05 |
| 8620 | 1.64092 | 0.05 |
| 8621 | 1.991 2.1 | |
| 8622 | 1.23013 | 2.1 |
| 8623 | 2.77816 | 2.1 |
| 8624 | 2.74107 | 0.83877 |
| 8625 | 2.07681 | 0.715902 |
| 8626 | 1.47764 | 0.05 |
| 8627 | 1.72677 | 0.05 |
| 8628 | 1.52161 | 2.1 |
| 8629 | 1.28526 | 2.1 |
| 8630 | 1.90502 | 2.1 |
| 8631 | 1.87359 | 0.05 |
| 8632 | 1.93489 | 0.05 |
| 8633 | 2.70895 | 2.1 |
| 8634 | 2.83141 | 2.1 |
| 8635 | 1.41766 | 0.612356 |
| 8636 | 1.11337 | 0.603752 |
| 8637 | 1.41765 | 1.48677 |
| 8638 | 1.29172 | 0.61666 |
| 8639 | 1.76324 | 0.679496 |

| | | |
|---|---|---|
| 8640 | 1.76324 | 1.0738 |
| 8641 | 0.946131 | 0.626611 |
| 8642 | 1.52428 | 2.1 |
| 8643 | 1.71459 | 2.1 |
| 8644 | 1.93852 | 0.692055 |
| 8645 | 1.07119 | 1.55232 |
| 8646 | 1.29698 | 1.27458 |
| 8647 | 1.42203 | 0.723686 |
| 8648 | 1.42203 | 1.61025 |
| 8649 | 0.946132 | 0.828964 |
| 8650 | 0.946132 | 0.995897 |
| 8651 | 0.96926 | 1.06804 |
| 8652 | 0.769913 | 2.06045 |
| 8653 | 0.946119 | 1.93106 |
| 8654 | 1.37867 | 1.06725 |
| 8655 | 1.94028 | 1.00925 |
| 8656 | 0.94612 | 0.77334 |
| 8657 | 0.946132 | 1.63017 |
| 8658 | 1.37865 | 0.919025 |
| 8659 | 2.14234 | 1.13671 |
| 8660 | 3.06459 | 1.35764 |
| 8661 | 1.44421 | 0.05 |
| 8662 | 1.33924 | 0.05 |
| 8663 | 1.33671 | 0.05 |
| 8664 | 1.10733 | 0.05 |
| 8665 | 0.68396 | 0.05 |
| 8666 | 1.76008 | 0.05 |
| 8667 | 1.76008 | 0.05 |
| 8668 | 0.68393 | 0.05 |
| 8669 | 0.946131 | 0.05 |
| 8670 | 3.79242 | 2.1 |
| 8671 | 2.46293 | 2.1 |
| 8672 | 3.56947 | 2.1 |
| 8673 | 1.35487 | 1.571 |
| 8674 | 1.38616 | 1.9112 |
| 8675 | 0.954048 | 0.05 |
| 8676 | 1.36663 | 0.05 |
| 8677 | 0.909487 | 0.0526617 |
| 8678 | 0.887288 | 2.1 |
| 8679 | 0.810947 | 0.192436 |
| 8680 | 0.810934 | 1.54302 |
| 8681 | 1.07046 | 1.71536 |
| 8682 | 1.13979 | 1.96693 |
| 8683 | 0.927838 | 0.225216 |
| 8684 | 0.919995 | 1.1361 |
| 8685 | 1.61447 | 2.1 |
| 8686 | 2.56305 | 2.1 |
| 8687 | 0.943414 | 2.1 |
| 8688 | 1.17016 | 2.1 |
| 8689 | 1.7238 | 2.1 |
| 8690 | 1.89334 | 2.1 |
| 8691 | 0.946128 | 0.22042 |
| 8692 | 1.25184 | 2.1 |
| 8693 | 1.55639 | 2.1 |
| 8694 | 3.12098 | 1.36808 |
| 8695 | 3.57307 | 0.880173 |
| 8696 | 2.81281 | 2.1 |
| 8697 | 0.851542 | 2.1 |

| | | |
|---|---|---|
| 8698 | 1.27689 | 2.1 |
| 8699 | 1.52568 | 2.1 |
| 8700 | 0.839689 | 2.1 |
| 8701 | 1.36471 | 2.1 |
| 8702 | 0.827865 | 2.1 |
| 8703 | 0.93667 | 2.1 |
| 8704 | 0.900861 | 2.1 |
| 8705 | 0.943152 | 2.1 |
| 8706 | 1.00667 | 2.1 |
| 8707 | 1.18141 | 2.1 |
| 8708 | 1.15298 | 2.1 |
| 8709 | 1.20335 | 2.1 |
| 8710 | 1.00713 | 2.1 |
| 8711 | 0.597537 | 2.1 |
| 8712 | 1.0577 | 2.1 |
| 8713 | 0.967342 | 0.0567198 |
| 8714 | 0.838027 | 0.688764 |
| 8715 | 0.893587 | 0.05 |
| 8716 | 1.06436 | 0.111591 |
| 8717 | 0.850048 | 0.05 |
| 8718 | 1.22026 | 0.257688 |
| 8719 | 0.988077 | 0.0551869 |
| 8720 | 1.18769 | 0.05 |
| 8721 | 1.3991 | 2.1 |
| 8722 | 1.52984 | 2.1 |
| 8723 | 1.35469 | 1.14147 |
| 8724 | 1.25083 | 0.638931 |
| 8725 | 1.6594 | 1.34109 |
| 8726 | 1.6594 | 0.983952 |
| 8727 | 0.946129 | 0.539909 |
| 8728 | 1.02602 | 0.416792 |
| 8729 | 0.94613 | 0.336805 |
| 8730 | 1.29724 | 0.05 |
| 8731 | 1.14501 | 0.05 |
| 8732 | 1.13078 | 0.05 |
| 8733 | 1.48381 | 0.05 |
| 8734 | 1.45921 | 0.05 |
| 8735 | 0.851539 | 0.05 |
| 8736 | 0.890836 | 0.05 |
| 8737 | 0.946131 | 0.05 |
| 8738 | 1.60428 | 2.1 |
| 8739 | 1.50097 | 2.1 |
| 8740 | 0.714507 | 0.152669 |
| 8741 | 1.51396 | 0.125579 |
| 8742 | 1.51396 | 0.0754731 |
| 8743 | 0.946132 | 0.153441 |
| 8744 | 0.813862 | 0.102558 |
| 8745 | 1.41461 | 0.115553 |
| 8746 | 0.813862 | 0.124476 |
| 8747 | 0.921311 | 2.1 |
| 8748 | 1.25084 | 0.921669 |
| 8749 | 1.30165 | 0.512975 |
| 8750 | 1.60636 | 1.38709 |
| 8751 | 1.60636 | 0.768197 |
| 8752 | 0.94613 | 0.607363 |
| 8753 | 0.972232 | 0.30477 |
| 8754 | 0.946131 | 0.361087 |
| 8755 | 2.35003 | 2.1 |

| | | |
|---|---|---|
| 8756 | 0.92546 | 2.1 |
| 8757 | 1.00713 | 2.1 |
| 8758 | 0.810947 | 2.1 |
| 8759 | 0.810934 | 2.1 |
| 8760 | 1.056 | 2.1 |
| 8761 | 0.926811 | 2.1 |
| 8762 | 0.766408 | 2.1 |
| 8763 | 1.34292 | 2.1 |
| 8764 | 1.57932 | 0.05 |
| 8765 | 2.67595 | 0.05 |
| 8766 | 1.77737 | 0.05 |
| 8767 | 1.77738 | 0.05 |
| 8768 | 0.789633 | 0.05 |
| 8769 | 1.22903 | 0.05 |
| 8770 | 0.789663 | 0.05 |
| 8771 | 0.789662 | 0.05 |
| 8772 | 1.33797 | 0.05 |
| 8773 | 0.946128 | 2.1 |
| 8774 | 1.47351 | 2.1 |
| 8775 | 1.47351 | 2.1 |
| 8776 | 0.94613 | 2.1 |
| 8777 | 1.30082 | 2.1 |
| 8778 | 1.30082 | 2.1 |
| 8779 | 0.996486 | 2.1 |
| 8780 | 0.946127 | 2.1 |
| 8781 | 1.72122 | 2.1 |
| 8782 | 1.72122 | 2.1 |
| 8783 | 0.94613 | 2.1 |
| 8784 | 1.36653 | 2.1 |
| 8785 | 1.36653 | 2.1 |
| 8786 | 1.02608 | 2.1 |
| 8787 | 0.709123 | 0.666891 |
| 8788 | 0.946127 | 0.763866 |
| 8789 | 1.41342 | 1.05374 |
| 8790 | 1.818 | 1.30507 |
| 8791 | 1.818 | 1.03563 |
| 8792 | 0.946127 | 0.141298 |
| 8793 | 1.41342 | 1.12522 |
| 8794 | 1.35071 | 0.796932 |
| 8795 | 1.8993 | 1.24659 |
| 8796 | 1.4748 | 2.1 |
| 8797 | 1.18711 | 0.05 |
| 8798 | 0.946131 | 0.05 |
| 8799 | 1.18711 | 0.05 |
| 8800 | 1.20416 | 0.05 |
| 8801 | 1.91358 | 0.05 |
| 8802 | 1.91358 | 0.05 |
| 8803 | 0.477691 | 0.05 |
| 8804 | 2.16798 | 0.05 |
| 8805 | 2.22307 | 0.05 |
| 8806 | 0.758971 | 0.05 |
| 8807 | 1.80694 | 0.05 |
| 8808 | 1.80694 | 0.05 |
| 8809 | 0.946132 | 0.05 |
| 8810 | 0.75897 | 0.05 |
| 8811 | 1.25124 | 0.05 |
| 8812 | 0.946131 | 0.05 |
| 8813 | 1.76837 | 2.1 |

| | | |
|---|---|---|
| 8814 | 1.76837 | 2.1 |
| 8815 | 0.946131 | 2.1 |
| 8816 | 1.36422 | 2.1 |
| 8817 | 0.946131 | 2.1 |
| 8818 | 1.14184 | 2.1 |
| 8819 | 1.36422 | 2.1 |
| 8820 | 0.793303 | 0.05 |
| 8821 | 1.79584 | 0.05 |
| 8822 | 1.35333 | 2.1 |
| 8823 | 1.19967 | 1.49392 |
| 8824 | 1.55088 | 1.9249 |
| 8825 | 1.64246 | 2.1 |
| 8826 | 0.946132 | 0.553071 |
| 8827 | 0.886293 | 2.1 |
| 8828 | 1.25018 | 2.1 |
| 8829 | 1.5795 | 1.80664 |
| 8830 | 2.15414 | 1.40361 |
| 8831 | 0.94613 | 0.968063 |
| 8832 | 1.37758 | 1.78243 |
| 8833 | 1.71567 | 1.04069 |
| 8834 | 1.71567 | 2.1 |
| 8835 | 0.946125 | 1.27694 |
| 8836 | 1.37759 | 1.0106 |
| 8837 | 1.28421 | 0.900599 |
| 8838 | 0.933959 | 0.150384 |
| 8839 | 0.946133 | 0.05 |
| 8840 | 1.7813 | 0.05 |
| 8841 | 1.7813 | 0.05 |
| 8842 | 1.05704 | 0.05 |
| 8843 | 1.37794 | 0.05 |
| 8844 | 1.37794 | 0.05 |
| 8845 | 0.946132 | 0.05 |
| 8846 | 0.946132 | 0.05 |
| 8847 | 1.68197 | 0.05 |
| 8848 | 1.68197 | 0.05 |
| 8849 | 1.01367 | 0.05 |
| 8850 | 1.25017 | 0.05 |
| 8851 | 1.25017 | 0.05 |
| 8852 | 0.946132 | 0.05 |
| 8853 | 0.838953 | 2.1 |
| 8854 | 1.4045 | 2.1 |
| 8855 | 1.56388 | 2.1 |
| 8856 | 1.3182 | 2.1 |
| 8857 | 0.928518 | 2.1 |
| 8858 | 1.04073 | 2.1 |
| 8859 | 0.835676 | 2.1 |
| 8860 | 1.47507 | 2.1 |
| 8861 | 1.47506 | 2.1 |
| 8862 | 0.797664 | 2.1 |
| 8863 | 1.34285 | 2.1 |
| 8864 | 0.929875 | 2.1 |
| 8865 | 0.797663 | 2.1 |
| 8866 | 0.946133 | 2.1 |
| 8867 | 1.83628 | 0.289406 |
| 8868 | 1.83627 | 0.470087 |
| 8869 | 0.946131 | 0.320701 |
| 8870 | 1.36748 | 0.420854 |
| 8871 | 0.946133 | 0.05 |

| | | |
|---|---|---|
| 8872 | 1.21139 | 0.429929 |
| 8873 | 1.36748 | 0.05 |
| 8874 | 1.06795 | 2.1 |
| 8875 | 1.08192 | 2.1 |
| 8876 | 0.827866 | 2.1 |
| 8877 | 1.02548 | 2.1 |
| 8878 | 0.95006 | 2.1 |
| 8879 | 1.53933 | 2.1 |
| 8880 | 0.586511 | 2.1 |
| 8881 | 0.868145 | 2.1 |
| 8882 | 1.28401 | 2.1 |
| 8883 | 1.37719 | 2.1 |
| 8884 | 1.37719 | 2.1 |
| 8885 | 0.77497 | 2.1 |
| 8886 | 0.946132 | 2.1 |
| 8887 | 0.774968 | 2.1 |
| 8888 | 1.7053 | 0.0643648 |
| 8889 | 1.7053 | 0.675553 |
| 8890 | 0.94613 | 2.1 |
| 8891 | 1.25715 | 0.198143 |
| 8892 | 0.946132 | 0.183483 |
| 8893 | 1.19655 | 0.0623656 |
| 8894 | 1.25716 | 0.0510383 |
| 8895 | 0.851542 | 2.1 |
| 8896 | 0.80043 | 2.1 |
| 8897 | 1.27239 | 2.1 |
| 8898 | 1.02719 | 2.1 |
| 8899 | 0.946131 | 2.1 |
| 8900 | 0.981318 | 2.1 |
| 8901 | 1.06628 | 2.1 |
| 8902 | 1.26022 | 2.1 |
| 8903 | 0.946132 | 2.1 |
| 8904 | 0.946132 | 2.1 |
| 8905 | 1.76447 | 2.1 |
| 8906 | 1.76447 | 2.1 |
| 8907 | 1.35357 | 2.1 |
| 8908 | 1.35703 | 2.1 |
| 8909 | 0.946131 | 2.1 |
| 8910 | 1.50538 | 2.1 |
| 8911 | 1.50538 | 2.1 |
| 8912 | 1.10311 | 2.1 |
| 8913 | 1.3484 | 2.1 |
| 8914 | 1.10312 | 2.1 |
| 8915 | 0.966428 | 2.1 |
| 8916 | 0.946133 | 2.1 |
| 8917 | 1.4008 | 2.1 |
| 8918 | 1.71908 | 2.1 |
| 8919 | 1.71908 | 2.1 |
| 8920 | 0.946133 | 2.1 |
| 8921 | 1.40081 | 2.1 |
| 8922 | 1.26441 | 2.1 |
| 8923 | 0.851543 | 2.1 |
| 8924 | 0.961732 | 2.1 |
| 8925 | 1.54576 | 2.1 |
| 8926 | 1.35364 | 2.1 |
| 8927 | 0.946133 | 2.1 |
| 8928 | 1.02913 | 2.1 |
| 8929 | 1.2735 | 2.1 |

| | | |
|---|---|---|
| 8930 | 1.32608 | 2.1 |
| 8931 | 0.967643 | 2.1 |
| 8932 | 0.94613 | 2.1 |
| 8933 | 1.44873 | 2.1 |
| 8934 | 1.44873 | 2.1 |
| 8935 | 0.967641 | 2.1 |
| 8936 | 0.946131 | 2.1 |
| 8937 | 1.40488 | 2.1 |
| 8938 | 1.24272 | 2.1 |
| 8939 | 0.946131 | 2.1 |
| 8940 | 1.78483 | 2.1 |
| 8941 | 1.78483 | 2.1 |
| 8942 | 1.24272 | 2.1 |
| 8943 | 0.946131 | 2.1 |
| 8944 | 0.94613 | 2.1 |
| 8945 | 1.5627 | 0.553327 |
| 8946 | 1.5627 | 2.1 |
| 8947 | 1.03656 | 2.1 |
| 8948 | 1.31649 | 0.167568 |
| 8949 | 1.19233 | 1.31805 |
| 8950 | 1.03656 | 1.04417 |
| 8951 | 0.912059 | 2.1 |
| 8952 | 1.47954 | 0.57283 |
| 8953 | 1.47954 | 2.1 |
| 8954 | 0.912061 | 2.1 |
| 8955 | 1.34647 | 0.30167 |
| 8956 | 1.04514 | 1.95018 |
| 8957 | 0.912061 | 1.06511 |
| 8958 | 0.761972 | 0.0820449 |
| 8959 | 0.946132 | 0.05 |
| 8960 | 0.761974 | 0.438304 |
| 8961 | 1.70225 | 0.262112 |
| 8962 | 1.70225 | 0.525595 |
| 8963 | 1.33728 | 0.288985 |
| 8964 | 1.01188 | 0.05 |
| 8965 | 0.94613 | 1.07807 |
| 8966 | 0.746361 | 1.49748 |
| 8967 | 1.6369 | 2.1 |
| 8968 | 1.62454 | 1.55647 |
| 8969 | 0.736738 | 0.96804 |
| 8970 | 0.958487 | 1.06844 |
| 8971 | 1.40641 | 1.23291 |
| 8972 | 1.2284 | 2.1 |
| 8973 | 0.92609 | 2.1 |
| 8974 | 0.595102 | 2.1 |
| 8975 | 1.80871 | 2.1 |
| 8976 | 1.1516 | 2.1 |
| 8977 | 0.962474 | 2.1 |
| 8978 | 2.24024 | 2.1 |
| 8979 | 0.946131 | 2.1 |
| 8980 | 1.73689 | 2.1 |
| 8981 | 1.73689 | 2.1 |
| 8982 | 0.946132 | 2.1 |
| 8983 | 1.30492 | 2.1 |
| 8984 | 1.30492 | 2.1 |
| 8985 | 1.17949 | 2.1 |
| 8986 | 0.946131 | 2.1 |
| 8987 | 1.8566 | 2.1 |

| | | |
|---|---|---|
| 8988 | 1.8566 | 2.1 |
| 8989 | 1.22392 | 2.1 |
| 8990 | 1.39826 | 2.1 |
| 8991 | 1.40447 | 2.1 |
| 8992 | 1.22392 | 2.1 |
| 8993 | 0.946131 | 2.1 |
| 8994 | 1.74134 | 2.1 |
| 8995 | 1.74134 | 2.1 |
| 8996 | 1.09148 | 2.1 |
| 8997 | 1.40447 | 2.1 |
| 8998 | 1.283 | 2.1 |
| 8999 | 1.09148 | 2.1 |
| 9000 | 1.33607 | 2.1 |
| 9001 | 1.41022 | 2.1 |
| 9002 | 1.80016 | 2.1 |
| 9003 | 1.80015 | 2.1 |
| 9004 | 0.946127 | 2.1 |
| 9005 | 0.946129 | 2.1 |
| 9006 | 1.20541 | 2.1 |
| 9007 | 1.13078 | 2.1 |
| 9008 | 1.33607 | 2.1 |
| 9009 | 1.54624 | 2.1 |
| 9010 | 1.85553 | 2.1 |
| 9011 | 0.920606 | 2.1 |
| 9012 | 0.946131 | 2.1 |
| 9013 | 1.01271 | 2.1 |
| 9014 | 1.32664 | 1.91311 |
| 9015 | 0.954692 | 1.29068 |
| 9016 | 0.946116 | 0.939358 |
| 9017 | 1.47771 | 0.900304 |
| 9018 | 1.4777 | 2.1 |
| 9019 | 0.95468 | 1.11239 |
| 9020 | 0.94613 | 0.953367 |
| 9021 | 1.31381 | 1.19736 |
| 9022 | 1.14005 | 0.740088 |
| 9023 | 0.946128 | 0.998034 |
| 9024 | 1.69433 | 0.490461 |
| 9025 | 1.69433 | 1.4633 |
| 9026 | 1.14005 | 0.962093 |
| 9027 | 0.94613 | 0.770752 |
| 9028 | 1.521 | 2.1 |
| 9029 | 1.411 | 2.1 |
| 9030 | 0.94612 | 2.1 |
| 9031 | 1.35115 | 2.1 |
| 9032 | 1.04127 | 2.1 |
| 9033 | 1.05017 | 2.1 |
| 9034 | 1.03564 | 2.1 |
| 9035 | 0.607156 | 2.1 |
| 9036 | 1.54549 | 2.1 |
| 9037 | 1.36715 | 2.1 |
| 9038 | 0.607127 | 2.07301 |
| 9039 | 1.2571 | 2.1 |
| 9040 | 0.998701 | 2.1 |
| 9041 | 0.546436 | 2.1 |
| 9042 | 0.946133 | 2.1 |
| 9043 | 1.75286 | 2.1 |
| 9044 | 1.75286 | 2.1 |
| 9045 | 1.12206 | 2.1 |

| | | |
|---|---|---|
| 9046 | 1.34237 | 2.1 |
| 9047 | 1.35663 | 2.1 |
| 9048 | 1.12206 | 2.1 |
| 9049 | 0.919455 | 2.1 |
| 9050 | 1.66517 | 2.1 |
| 9051 | 1.99822 | 2.1 |
| 9052 | 1.228 2.1 | |
| 9053 | 1.35662 | 2.1 |
| 9054 | 1.228 2.1 | |
| 9055 | 1.01143 | 2.1 |
| 9056 | 0.851543 | 1.00674 |
| 9057 | 0.986856 | 2.1 |
| 9058 | 1.42762 | 2.05166 |
| 9059 | 1.23095 | 2.1 |
| 9060 | 0.946132 | 2.1 |
| 9061 | 1.00659 | 2.05668 |
| 9062 | 1.1779 | 1.48886 |
| 9063 | 1.40025 | 2.1 |
| 9064 | 1.40025 | 2.1 |
| 9065 | 0.889308 | 2.1 |
| 9066 | 1.34652 | 2.1 |
| 9067 | 0.943042 | 2.1 |
| 9068 | 0.943041 | 2.1 |
| 9069 | 1.34652 | 2.1 |
| 9070 | 1.39514 | 1.88808 |
| 9071 | 1.30809 | 1.39993 |
| 9072 | 1.75711 | 1.23016 |
| 9073 | 1.7571 | 2.1 |
| 9074 | 0.946125 | 0.751829 |
| 9075 | 0.946127 | 1.45664 |
| 9076 | 1.18396 | 1.30992 |
| 9077 | 1.4844 | 0.269459 |
| 9078 | 1.4844 | 1.05242 |
| 9079 | 0.803322 | 1.98811 |
| 9080 | 0.946133 | 0.106392 |
| 9081 | 0.803324 | 2.1 |
| 9082 | 0.803324 | 1.44094 |
| 9083 | 1.36183 | 1.5211 |
| 9084 | 0.946131 | 0.222734 |
| 9085 | 1.01462 | 0.05 |
| 9086 | 0.946132 | 0.05 |
| 9087 | 1.59749 | 0.178756 |
| 9088 | 1.59749 | 0.05 |
| 9089 | 1.16009 | 0.0715064 |
| 9090 | 1.38354 | 0.05 |
| 9091 | 0.851539 | 1.00123 |
| 9092 | 1.36005 | 1.7437 |
| 9093 | 1.5532 | 1.21788 |
| 9094 | 1.80402 | 2.1 |
| 9095 | 0.851537 | 2.00902 |
| 9096 | 0.902632 | 1.53684 |
| 9097 | 1.44437 | 1.26982 |
| 9098 | 0.633098 | 2.1 |
| 9099 | 1.00307 | 0.165119 |
| 9100 | 0.94613 | 0.0706376 |
| 9101 | 1.16623 | 0.05 |
| 9102 | 0.946128 | 0.102635 |
| 9103 | 1.64588 | 0.05 |

| | | |
|---|---|---|
| 9104 | 1.64588 | 0.05 |
| 9105 | 1.35059 | 0.0565805 |
| 9106 | 1.24142 | 0.0584221 |
| 9107 | 0.946131 | 0.05 |
| 9108 | 1.13343 | 0.05 |
| 9109 | 0.946127 | 0.05 |
| 9110 | 1.63367 | 0.05 |
| 9111 | 1.63367 | 0.05 |
| 9112 | 1.33035 | 0.05 |
| 9113 | 1.24945 | 0.05 |
| 9114 | 1.78733 | 1.51321 |
| 9115 | 1.70856 | 1.99031 |
| 9116 | 0.637995 | 1.11915 |
| 9117 | 0.712454 | 1.11197 |
| 9118 | 0.946132 | 1.15355 |
| 9119 | 0.637964 | 1.01715 |
| 9120 | 1.13021 | 1.12303 |
| 9121 | 0.94613 | 0.05 |
| 9122 | 1.20509 | 0.05 |
| 9123 | 0.946127 | 0.05 |
| 9124 | 1.70545 | 0.05 |
| 9125 | 1.70545 | 0.05 |
| 9126 | 1.28508 | 0.05 |
| 9127 | 1.3665 | 0.05 |
| 9128 | 1.58612 | 2.1 |
| 9129 | 1.51845 | 2.1 |
| 9130 | 0.797374 | 2.1 |
| 9131 | 1.20057 | 2.1 |
| 9132 | 0.946132 | 2.1 |
| 9133 | 0.946132 | 2.1 |
| 9134 | 0.899819 | 2.1 |
| 9135 | 1.50471 | 0.951403 |
| 9136 | 1.50472 | 1.02968 |
| 9137 | 0.59387 | 1.07322 |
| 9138 | 0.593844 | 0.812225 |
| 9139 | 0.946126 | 0.984823 |
| 9140 | 0.966137 | 0.920397 |
| 9141 | 1.13245 | 0.858707 |
| 9142 | 1.20801 | 0.190534 |
| 9143 | 0.946131 | 0.978662 |
| 9144 | 1.47644 | 0.360652 |
| 9145 | 0.733223 | 0.235139 |
| 9146 | 0.563609 | 0.183904 |
| 9147 | 1.69218 | 0.231477 |
| 9148 | 1.6173 | 0.897872 |
| 9149 | 1.8126 | 1.95498 |
| 9150 | 1.8126 | 2.07883 |
| 9151 | 0.678938 | 2.05012 |
| 9152 | 0.678935 | 2.06521 |
| 9153 | 0.946133 | 2.1 |
| 9154 | 0.946132 | 2.04681 |
| 9155 | 0.678936 | 2.1 |
| 9156 | 1.21997 | 1.68978 |
| 9157 | 1.28439 | 1.64461 |
| 9158 | 1.65282 | 1.95986 |
| 9159 | 1.86735 | 1.92501 |
| 9160 | 0.851539 | 1.98824 |
| 9161 | 0.94613 | 1.05402 |

| | | |
|---|---|---|
| 9162 | 0.87688 | 0.577091 |
| 9163 | 0.804877 | 0.262744 |
| 9164 | 1.40747 | 0.605927 |
| 9165 | 1.41312 | 0.05 |
| 9166 | 0.885349 | 0.0845609 |
| 9167 | 1.25405 | 0.587703 |
| 9168 | 1.34463 | 0.116562 |
| 9169 | 0.804878 | 0.106763 |
| 9170 | 0.810299 | 0.05 |
| 9171 | 1.04072 | 0.05 |
| 9172 | 1.25601 | 0.05 |
| 9173 | 1.04943 | 0.05 |
| 9174 | 0.900294 | 0.05 |
| 9175 | 1.38777 | 0.05 |
| 9176 | 1.33131 | 0.05 |
| 9177 | 1.39168 | 0.05 |
| 9178 | 0.946132 | 0.05 |
| 9179 | 0.747712 | 0.05 |
| 9180 | 1.61944 | 0.05 |
| 9181 | 1.61944 | 0.05 |
| 9182 | 0.946132 | 0.05 |
| 9183 | 0.747712 | 0.05 |
| 9184 | 1.41201 | 0.0773016 |
| 9185 | 1.12082 | 0.121576 |
| 9186 | 0.946128 | 0.116796 |
| 9187 | 1.79415 | 0.0758774 |
| 9188 | 1.79415 | 0.0965446 |
| 9189 | 1.12082 | 0.0567576 |
| 9190 | 0.94613 | 0.103232 |
| 9191 | 0.711721 | 0.05 |
| 9192 | 1.12316 | 0.05 |
| 9193 | 0.707038 | 0.05 |
| 9194 | 1.04072 | 0.05 |
| 9195 | 0.704409 | 0.05 |
| 9196 | 1.49036 | 0.05 |
| 9197 | 1.27689 | 0.05 |
| 9198 | 0.793044 | 0.05 |
| 9199 | 0.810886 | 0.333015 |
| 9200 | 0.885957 | 0.0510564 |
| 9201 | 1.38254 | 0.0562356 |
| 9202 | 1.35991 | 0.05 |
| 9203 | 1.01375 | 0.104546 |
| 9204 | 1.03169 | 0.0989864 |
| 9205 | 0.946131 | 0.0688205 |
| 9206 | 1.3279 | 0.3535 |
| 9207 | 1.42284 | 0.0957413 |
| 9208 | 1.42284 | 0.0597293 |
| 9209 | 0.851192 | 1.1699 |
| 9210 | 0.946131 | 0.613904 |
| 9211 | 0.851165 | 0.152149 |
| 9212 | 0.748062 | 0.05 |
| 9213 | 1.27511 | 0.05 |
| 9214 | 0.74806 | 0.05 |
| 9215 | 0.94613 | 0.05 |
| 9216 | 0.748059 | 0.05 |
| 9217 | 1.86293 | 0.05 |
| 9218 | 1.86293 | 0.05 |
| 9219 | 0.851542 | 0.05 |

| | | |
|---|---|---|
| 9220 | 1.38186 | 0.05 |
| 9221 | 0.851542 | 0.05 |
| 9222 | 1.67869 | 0.05 |
| 9223 | 1.66579 | 0.05 |
| 9224 | 1.25626 | 0.05 |
| 9225 | 1.23046 | 0.05 |
| 9226 | 1.46023 | 0.05 |
| 9227 | 1.27083 | 0.05 |
| 9228 | 0.946131 | 0.05 |
| 9229 | 0.999304 | 0.05 |
| 9230 | 0.946803 | 0.05 |
| 9231 | 0.851554 | 0.05 |
| 9232 | 0.999227 | 0.05 |
| 9233 | 0.750097 | 0.05 |
| 9234 | 1.91815 | 0.05 |
| 9235 | 0.946132 | 0.05 |
| 9236 | 0.768117 | 0.05 |
| 9237 | 1.78003 | 0.05 |
| 9238 | 1.99572 | 0.05 |
| 9239 | 0.650453 | 0.05 |
| 9240 | 0.946132 | 0.05 |
| 9241 | 1.23845 | 0.05 |
| 9242 | 0.946131 | 0.05 |
| 9243 | 1.75477 | 0.05 |
| 9244 | 1.75477 | 0.05 |
| 9245 | 1.33393 | 0.05 |
| 9246 | 1.36697 | 0.05 |
| 9247 | 0.749018 | 1.25752 |
| 9248 | 1.02366 | 1.7825 |
| 9249 | 1.43924 | 1.37736 |
| 9250 | 1.00018 | 1.36832 |
| 9251 | 1.06438 | 0.979055 |
| 9252 | 0.946127 | 1.29856 |
| 9253 | 0.74906 | 1.06441 |
| 9254 | 0.946131 | 1.25998 |
| 9255 | 1.26488 | 0.05 |
| 9256 | 0.946131 | 0.05 |
| 9257 | 0.94613 | 0.05 |
| 9258 | 1.49051 | 0.05 |
| 9259 | 1.59837 | 0.05 |
| 9260 | 0.907797 | 0.05 |
| 9261 | 1.04455 | 0.05 |
| 9262 | 1.25214 | 0.05 |
| 9263 | 0.68452 | 0.05 |
| 9264 | 1.25215 | 0.05 |
| 9265 | 1.24677 | 0.05 |
| 9266 | 1.8144 | 0.05 |
| 9267 | 1.81441 | 0.05 |
| 9268 | 0.684518 | 0.05 |
| 9269 | 0.851542 | 0.619063 |
| 9270 | 1.52275 | 0.610628 |
| 9271 | 1.45223 | 0.542953 |
| 9272 | 1.0269 | 0.547728 |
| 9273 | 1.29689 | 0.655466 |
| 9274 | 1.32822 | 0.691868 |
| 9275 | 0.851553 | 0.751628 |
| 9276 | 1.21756 | 0.05 |
| 9277 | 0.946131 | 0.05 |

| | | |
|---|---|---|
| 9278 | 0.946127 | 0.05 |
| 9279 | 1.78014 | 0.05 |
| 9280 | 1.78014 | 0.05 |
| 9281 | 1.32076 | 0.05 |
| 9282 | 1.40551 | 0.05 |
| 9283 | 0.711214 | 0.05 |
| 9284 | 1.82057 | 0.05 |
| 9285 | 1.82057 | 0.05 |
| 9286 | 0.946129 | 0.05 |
| 9287 | 1.22716 | 0.05 |
| 9288 | 1.30463 | 0.05 |
| 9289 | 0.94613 | 0.05 |
| 9290 | 0.568927 | 0.05 |
| 9291 | 0.946132 | 0.05 |
| 9292 | 0.568927 | 0.05 |
| 9293 | 1.99266 | 0.05 |
| 9294 | 1.99266 | 0.05 |
| 9295 | 1.26201 | 0.05 |
| 9296 | 1.29957 | 0.05 |
| 9297 | 1.21614 | 0.05 |
| 9298 | 0.851484 | 0.05 |
| 9299 | 0.851543 | 0.05 |
| 9300 | 1.85815 | 0.05 |
| 9301 | 1.81937 | 0.05 |
| 9302 | 0.697705 | 0.05 |
| 9303 | 0.946132 | 0.05 |
| 9304 | 0.593295 | 0.05 |
| 9305 | 1.53642 | 0.05 |
| 9306 | 1.53642 | 0.05 |
| 9307 | 0.908276 | 0.05 |
| 9308 | 0.593296 | 0.05 |
| 9309 | 0.593295 | 0.05 |
| 9310 | 0.94613 | 0.05 |
| 9311 | 0.867593 | 0.0637563 |
| 9312 | 1.42107 | 0.05 |
| 9313 | 1.42107 | 0.05 |
| 9314 | 0.867621 | 0.05 |
| 9315 | 1.05516 | 0.0539694 |
| 9316 | 0.867624 | 0.05 |
| 9317 | 0.946129 | 0.09477 |
| 9318 | 0.766681 | 0.05 |
| 9319 | 1.18119 | 0.05 |
| 9320 | 1.57018 | 0.05 |
| 9321 | 1.57018 | 0.05 |
| 9322 | 0.766652 | 0.05 |
| 9323 | 1.18119 | 0.05 |
| 9324 | 1.15564 | 0.05 |
| 9325 | 0.946131 | 0.05 |
| 9326 | 1.239 0.05 | |
| 9327 | 0.851541 | 0.05 |
| 9328 | 0.851487 | 0.05 |
| 9329 | 1.40021 | 0.05 |
| 9330 | 1.38373 | 0.05 |
| 9331 | 0.851554 | 0.05 |
| 9332 | 1.12266 | 0.05 |
| 9333 | 0.946131 | 0.05 |
| 9334 | 1.08234 | 0.05 |
| 9335 | 1.2882 | 0.05 |

| | | |
|---|---|---|
| 9336 | 1.49963 | 0.067102 |
| 9337 | 1.46188 | 0.05 |
| 9338 | 0.851555 | 0.05 |
| 9339 | 1.33007 | 0.05 |
| 9340 | 1.08054 | 0.05 |
| 9341 | 0.946129 | 0.0801703 |
| 9342 | 0.91151 | 0.05 |
| 9343 | 1.49914 | 0.05 |
| 9344 | 1.49914 | 0.05 |
| 9345 | 0.911471 | 0.083979 |
| 9346 | 0.946132 | 1.02734 |
| 9347 | 1.35943 | 2.1 |
| 9348 | 1.68117 | 1.60548 |
| 9349 | 1.68117 | 2.1 |
| 9350 | 0.946132 | 1.80442 |
| 9351 | 1.35943 | 1.21362 |
| 9352 | 1.26787 | 0.981533 |
| 9353 | 1.81612 | 0.611817 |
| 9354 | 1.81612 | 0.05 |
| 9355 | 0.946131 | 0.05 |
| 9356 | 1.41492 | 0.05 |
| 9357 | 0.946132 | 0.176116 |
| 9358 | 1.25037 | 0.157324 |
| 9359 | 1.41492 | 0.05 |
| 9360 | 1.30541 | 0.05 |
| 9361 | 1.23646 | 0.05 |
| 9362 | 0.94613 | 0.05 |
| 9363 | 1.77304 | 0.05 |
| 9364 | 1.77304 | 0.05 |
| 9365 | 1.23646 | 0.05 |
| 9366 | 0.946132 | 0.05 |
| 9367 | 0.946131 | 2.1 |
| 9368 | 1.26689 | 2.1 |
| 9369 | 1.66806 | 2.1 |
| 9370 | 1.66806 | 2.1 |
| 9371 | 0.94613 | 2.1 |
| 9372 | 1.26689 | 2.1 |
| 9373 | 1.3473 | 2.1 |
| 9374 | 1.39384 | 2.1 |
| 9375 | 1.4181 | 2.1 |
| 9376 | 1.86581 | 2.1 |
| 9377 | 1.86581 | 2.1 |
| 9378 | 0.946128 | 2.1 |
| 9379 | 0.946129 | 2.1 |
| 9380 | 1.32068 | 2.1 |
| 9381 | 1.20328 | 2.1 |
| 9382 | 1.39384 | 2.1 |
| 9383 | 1.651 | 2.1 |
| 9384 | 1.651 | 2.1 |
| 9385 | 0.946129 | 2.1 |
| 9386 | 0.946131 | 2.1 |
| 9387 | 1.12913 | 2.1 |
| 9388 | 1.21474 | 2.1 |
| 9389 | 0.946132 | 2.1 |
| 9390 | 0.94613 | 2.1 |
| 9391 | 1.79724 | 2.1 |
| 9392 | 1.79724 | 2.1 |
| 9393 | 1.40987 | 2.1 |

| | | |
|---|---|---|
| 9394 | 1.3335 | 2.1 |
| 9395 | 1.65642 | 2.1 |
| 9396 | 1.65642 | 2.1 |
| 9397 | 0.946132 | 2.1 |
| 9398 | 1.19063 | 2.1 |
| 9399 | 0.946133 | 0.816321 |
| 9400 | 1.15203 | 2.1 |
| 9401 | 1.19063 | 2.1 |
| 9402 | 0.946131 | 0.694532 |
| 9403 | 1.77815 | 2.1 |
| 9404 | 1.77815 | 2.1 |
| 9405 | 0.946131 | 1.79259 |
| 9406 | 1.31745 | 2.1 |
| 9407 | 1.31745 | 2.1 |
| 9408 | 1.2597 | 2.1 |
| 9409 | 0.946131 | 0.469423 |
| 9410 | 1.85184 | 1.08127 |
| 9411 | 1.85184 | 2.1 |
| 9412 | 0.946128 | 1.33414 |
| 9413 | 1.40684 | 2.1 |
| 9414 | 1.40684 | 1.01036 |
| 9415 | 1.34049 | 1.97717 |
| 9416 | 0.946132 | 1.90381 |
| 9417 | 1.71333 | 1.22716 |
| 9418 | 1.71333 | 2.1 |
| 9419 | 1.1848 | 1.86666 |
| 9420 | 1.34297 | 1.09611 |
| 9421 | 1.31649 | 1.17071 |
| 9422 | 1.1848 | 1.87553 |
| 9423 | 1.36604 | 0.715302 |
| 9424 | 0.954559 | 0.765841 |
| 9425 | 1.36604 | 0.766715 |
| 9426 | 1.41766 | 0.687103 |
| 9427 | 1.83756 | 0.861327 |
| 9428 | 1.83756 | 2.1 |
| 9429 | 0.946131 | 0.829203 |
| 9430 | 0.946127 | 0.05 |
| 9431 | 1.14874 | 0.05 |
| 9432 | 0.946127 | 0.05 |
| 9433 | 1.66393 | 0.05 |
| 9434 | 1.66393 | 0.05 |
| 9435 | 1.35559 | 0.05 |
| 9436 | 1.25447 | 0.05 |
| 9437 | 0.94613 | 0.05 |
| 9438 | 1.20732 | 0.05 |
| 9439 | 0.946129 | 0.05 |
| 9440 | 1.75073 | 0.05 |
| 9441 | 1.75073 | 0.05 |
| 9442 | 1.3665 | 0.05 |
| 9443 | 1.33035 | 0.05 |
| 9444 | 1.73256 | 2.1 |
| 9445 | 1.73256 | 2.1 |
| 9446 | 0.946131 | 2.1 |
| 9447 | 1.34921 | 2.1 |
| 9448 | 1.19462 | 2.1 |
| 9449 | 1.19462 | 2.1 |
| 9450 | 1.32949 | 2.1 |
| 9451 | 1.75754 | 0.459368 |

| | | |
|---|---|---|
| 9452 | 1.75754 | 0.213204 |
| 9453 | 0.946132 | 0.65532 |
| 9454 | 1.3201 | 0.450571 |
| 9455 | 0.946132 | 0.546378 |
| 9456 | 1.25479 | 0.121038 |
| 9457 | 1.3201 | 0.459415 |
| 9458 | 1.8087 | 0.703973 |
| 9459 | 1.8087 | 0.05 |
| 9460 | 0.946131 | 0.05 |
| 9461 | 1.38357 | 0.05 |
| 9462 | 0.946133 | 0.637026 |
| 9463 | 1.31034 | 0.05 |
| 9464 | 1.38357 | 0.299786 |
| 9465 | 1.12702 | 0.05 |
| 9466 | 0.946131 | 0.05 |
| 9467 | 1.23923 | 0.05 |
| 9468 | 1.4117 | 0.05 |
| 9469 | 0.946131 | 0.05 |
| 9470 | 1.70479 | 0.05 |
| 9471 | 1.70479 | 0.05 |
| 9472 | 0.946119 | 2.1 |
| 9473 | 1.55309 | 2.1 |
| 9474 | 1.55309 | 2.1 |
| 9475 | 0.946119 | 2.1 |
| 9476 | 1.08006 | 2.1 |
| 9477 | 1.08005 | 2.1 |
| 9478 | 1.02681 | 2.1 |
| 9479 | 1.36581 | 2.1 |
| 9480 | 1.93075 | 2.1 |
| 9481 | 0.946133 | 2.1 |
| 9482 | 0.772538 | 2.1 |
| 9483 | 0.946131 | 2.1 |
| 9484 | 0.946131 | 2.1 |
| 9485 | 1.33748 | 2.1 |
| 9486 | 1.44991 | 2.1 |
| 9487 | 1.31183 | 2.1 |
| 9488 | 0.946132 | 2.1 |
| 9489 | 0.944104 | 2.1 |
| 9490 | 0.915236 | 2.1 |
| 9491 | 1.05017 | 2.1 |
| 9492 | 0.944148 | 2.1 |
| 9493 | 0.946132 | 0.05 |
| 9494 | 1.60171 | 0.05 |
| 9495 | 1.60171 | 0.05 |
| 9496 | 0.946131 | 0.05 |
| 9497 | 1.16234 | 0.05 |
| 9498 | 1.3855 | 0.0542153 |
| 9499 | 0.946132 | 0.0527709 |
| 9500 | 0.946133 | 0.0799763 |
| 9501 | 1.73285 | 0.0661889 |
| 9502 | 1.73285 | 0.05 |
| 9503 | 0.946133 | 0.119578 |
| 9504 | 1.3855 | 0.05 |
| 9505 | 1.29348 | 0.136827 |
| 9506 | 0.946132 | 0.211629 |
| 9507 | 1.34828 | 2.1 |
| 9508 | 1.20943 | 2.1 |
| 9509 | 0.946129 | 1.33648 |

| | | |
|---|---|---|
| 9510 | 1.71013 | 2.1 |
| 9511 | 1.71013 | 2.1 |
| 9512 | 1.20943 | 2.1 |
| 9513 | 0.94613 | 2.1 |
| 9514 | 1.19613 | 0.05 |
| 9515 | 0.946118 | 0.05 |
| 9516 | 0.946117 | 0.05 |
| 9517 | 1.70868 | 0.05 |
| 9518 | 1.70869 | 0.05 |
| 9519 | 1.40551 | 0.05 |
| 9520 | 1.2493 | 0.05 |
| 9521 | 0.946131 | 2.1 |
| 9522 | 1.73123 | 2.1 |
| 9523 | 1.73123 | 2.1 |
| 9524 | 0.946132 | 2.1 |
| 9525 | 1.37811 | 2.1 |
| 9526 | 1.37811 | 2.1 |
| 9527 | 1.25144 | 2.1 |
| 9528 | 0.946132 | 2.1 |
| 9529 | 1.75139 | 2.1 |
| 9530 | 1.75139 | 2.1 |
| 9531 | 1.23374 | 2.1 |
| 9532 | 1.29926 | 2.1 |
| 9533 | 1.39827 | 2.1 |
| 9534 | 1.23374 | 2.1 |
| 9535 | 1.41375 | 0.05 |
| 9536 | 1.25697 | 0.05 |
| 9537 | 0.946118 | 0.05 |
| 9538 | 1.80377 | 0.05 |
| 9539 | 1.80377 | 0.05 |
| 9540 | 1.25699 | 0.05 |
| 9541 | 0.946106 | 0.05 |
| 9542 | 0.946133 | 2.1 |
| 9543 | 1.81806 | 1.10134 |
| 9544 | 1.81806 | 2.1 |
| 9545 | 0.946133 | 2.1 |
| 9546 | 1.37089 | 2.1 |
| 9547 | 1.37089 | 1.17082 |
| 9548 | 1.34999 | 2.1 |
| 9549 | 0.946131 | 2.1 |
| 9550 | 1.80855 | 1.0228 |
| 9551 | 1.80855 | 2.1 |
| 9552 | 1.31184 | 2.1 |
| 9553 | 1.3933 | 1.18099 |
| 9554 | 1.36139 | 1.97943 |
| 9555 | 1.31184 | 2.09192 |
| 9556 | 1.01175 | 1.2729 |
| 9557 | 0.94613 | 0.610615 |
| 9558 | 0.919796 | 1.9793 |
| 9559 | 1.50484 | 0.291802 |
| 9560 | 1.80584 | 2.08494 |
| 9561 | 1.53512 | 0.545225 |
| 9562 | 1.14539 | 0.774982 |
| 9563 | 0.946133 | 2.1 |
| 9564 | 1.72797 | 1.49603 |
| 9565 | 1.72797 | 2.1 |
| 9566 | 1.2111 | 2.1 |
| 9567 | 1.39731 | 1.14051 |

| | | |
|---|---|---|
| 9568 | 1.27679 | 2.1 |
| 9569 | 1.21111 | 2.1 |
| 9570 | 0.946119 | 0.106656 |
| 9571 | 1.67673 | 1.05987 |
| 9572 | 1.67673 | 2.1 |
| 9573 | 0.946118 | 1.06775 |
| 9574 | 1.21519 | 2.1 |
| 9575 | 1.2152 | 2.1 |
| 9576 | 1.19808 | 2.1 |
| 9577 | 0.946127 | 2.1 |
| 9578 | 1.54442 | 2.1 |
| 9579 | 1.54442 | 2.1 |
| 9580 | 1.04806 | 2.1 |
| 9581 | 1.13485 | 2.1 |
| 9582 | 1.3557 | 2.1 |
| 9583 | 1.04805 | 2.1 |
| 9584 | 0.946129 | 2.1 |
| 9585 | 1.32282 | 2.1 |
| 9586 | 1.77749 | 2.1 |
| 9587 | 1.77749 | 2.1 |
| 9588 | 0.946131 | 2.1 |
| 9589 | 1.32282 | 2.1 |
| 9590 | 1.4008 | 2.1 |
| 9591 | 0.946128 | 0.05 |
| 9592 | 1.02366 | 0.05 |
| 9593 | 0.94613 | 0.05 |
| 9594 | 1.66684 | 0.05 |
| 9595 | 1.66684 | 0.05 |
| 9596 | 1.29215 | 0.05 |
| 9597 | 1.32082 | 0.05 |
| 9598 | 0.946133 | 0.05 |
| 9599 | 1.01451 | 0.05 |
| 9600 | 0.946132 | 0.05 |
| 9601 | 1.62374 | 0.05 |
| 9602 | 1.62374 | 0.05 |
| 9603 | 1.2929 | 0.05 |
| 9604 | 1.27697 | 0.05 |
| 9605 | 1.37869 | 2.1 |
| 9606 | 1.17215 | 2.1 |
| 9607 | 0.946132 | 2.1 |
| 9608 | 1.70508 | 2.1 |
| 9609 | 1.70508 | 2.1 |
| 9610 | 1.17215 | 2.1 |
| 9611 | 0.946132 | 2.1 |
| 9612 | 1.03784 | 2.1 |
| 9613 | 0.946132 | 2.1 |
| 9614 | 0.946132 | 2.1 |
| 9615 | 1.56718 | 2.1 |
| 9616 | 1.56717 | 2.1 |
| 9617 | 1.35703 | 2.1 |
| 9618 | 1.15628 | 2.1 |
| 9619 | 1.35356 | 2.1 |
| 9620 | 1.28976 | 2.1 |
| 9621 | 0.946131 | 2.1 |
| 9622 | 1.78612 | 2.1 |
| 9623 | 1.78612 | 2.1 |
| 9624 | 1.28976 | 2.1 |
| 9625 | 0.94613 | 2.1 |

| | | |
|---|---|---|
| 9626 | 0.946132 | 2.1 |
| 9627 | 1.36373 | 2.1 |
| 9628 | 1.92382 | 2.1 |
| 9629 | 0.946131 | 2.1 |
| 9630 | 1.92382 | 2.1 |
| 9631 | 1.33471 | 2.1 |
| 9632 | 0.946132 | 2.1 |
| 9633 | 0.94613 | 0.715827 |
| 9634 | 1.29149 | 1.11999 |
| 9635 | 1.75878 | 0.884927 |
| 9636 | 1.75878 | 2.1 |
| 9637 | 0.946128 | 1.13175 |
| 9638 | 1.29149 | 0.804968 |
| 9639 | 1.41342 | 0.240322 |
| 9640 | 1.41022 | 2.1 |
| 9641 | 1.37034 | 1.91764 |
| 9642 | 1.83443 | 2.1 |
| 9643 | 1.83443 | 2.1 |
| 9644 | 0.946128 | 0.67028 |
| 9645 | 0.946131 | 2.1 |
| 9646 | 1.26928 | 2.1 |
| 9647 | 0.946128 | 0.919233 |
| 9648 | 1.38259 | 2.01178 |
| 9649 | 1.81405 | 1.0659 |
| 9650 | 1.81405 | 2.1 |
| 9651 | 0.946126 | 1.65016 |
| 9652 | 1.38259 | 1.09492 |
| 9653 | 1.37759 | 0.882073 |
| 9654 | 1.68131 | 0.05 |
| 9655 | 1.68131 | 0.05 |
| 9656 | 0.946131 | 0.05 |
| 9657 | 1.38896 | 0.05 |
| 9658 | 1.17438 | 0.438095 |
| 9659 | 1.17438 | 0.05 |
| 9660 | 1.23848 | 0.477947 |
| 9661 | 1.80088 | 0.120271 |
| 9662 | 1.80088 | 0.05 |
| 9663 | 0.946132 | 0.05 |
| 9664 | 1.40332 | 0.123839 |
| 9665 | 1.28185 | 0.243212 |
| 9666 | 1.28185 | 0.05 |
| 9667 | 1.34369 | 0.0762232 |
| 9668 | 1.71939 | 2.1 |
| 9669 | 1.71939 | 2.1 |
| 9670 | 0.946131 | 2.1 |
| 9671 | 1.28308 | 2.1 |
| 9672 | 0.946133 | 2.1 |
| 9673 | 1.24024 | 2.1 |
| 9674 | 1.28309 | 2.1 |
| 9675 | 1.8525 | 2.1 |
| 9676 | 1.8525 | 2.1 |
| 9677 | 0.946132 | 2.1 |
| 9678 | 1.38244 | 2.1 |
| 9679 | 0.946132 | 0.477932 |
| 9680 | 1.33469 | 2.1 |
| 9681 | 1.38244 | 2.1 |
| 9682 | 1.26654 | 1.69818 |
| 9683 | 1.39514 | 1.29063 |

| | | |
|---|---|---|
| 9684 | 1.71556 | 1.21284 |
| 9685 | 1.71556 | 2.1 |
| 9686 | 0.946126 | 0.78208 |
| 9687 | 0.946129 | 1.90357 |
| 9688 | 1.1908 | 1.30424 |
| 9689 | 1.49995 | 2.1 |
| 9690 | 1.63875 | 2.1 |
| 9691 | 0.93608 | 2.1 |
| 9692 | 0.937208 | 2.1 |
| 9693 | 0.943708 | 2.1 |
| 9694 | 0.936134 | 2.1 |
| 9695 | 1.14341 | 2.1 |
| 9696 | 1.17802 | 0.05 |
| 9697 | 1.3542 | 0.05 |
| 9698 | 1.73981 | 0.05 |
| 9699 | 1.73981 | 0.05 |
| 9700 | 0.94613 | 0.05 |
| 9701 | 1.3542 | 0.05 |
| 9702 | 1.33174 | 0.05 |
| 9703 | 1.59452 | 2.1 |
| 9704 | 1.59452 | 2.1 |
| 9705 | 0.946132 | 2.1 |
| 9706 | 1.25 | 2.1 |
| 9707 | 0.975266 | 2.1 |
| 9708 | 0.946132 | 2.1 |
| 9709 | 1.25 | 2.1 |
| 9710 | 0.946131 | 2.1 |
| 9711 | 1.48685 | 2.1 |
| 9712 | 1.48685 | 2.1 |
| 9713 | 0.946132 | 2.1 |
| 9714 | 1.17273 | 2.1 |
| 9715 | 1.17273 | 2.1 |
| 9716 | 0.955602 | 2.1 |
| 9717 | 1.32827 | 0.0612912 |
| 9718 | 1.11166 | 0.0967881 |
| 9719 | 0.946127 | 0.110786 |
| 9720 | 1.6136 | 0.05 |
| 9721 | 1.6136 | 0.0577525 |
| 9722 | 1.11166 | 0.0627425 |
| 9723 | 0.946129 | 0.0900171 |
| 9724 | 0.851541 | 0.744248 |
| 9725 | 1.22107 | 0.687215 |
| 9726 | 1.64594 | 0.488302 |
| 9727 | 1.5271 | 0.565017 |
| 9728 | 0.94613 | 0.894591 |
| 9729 | 1.22112 | 0.823019 |
| 9730 | 1.25002 | 0.669128 |
| 9731 | 1.65795 | 0.05 |
| 9732 | 1.65795 | 1.67208 |
| 9733 | 0.946132 | 2.06907 |
| 9734 | 1.2366 | 0.15783 |
| 9735 | 0.946132 | 0.05 |
| 9736 | 1.10023 | 0.6604 |
| 9737 | 1.2366 | 0.05 |
| 9738 | 1.35856 | 0.0509985 |
| 9739 | 0.946132 | 0.05 |
| 9740 | 1.35856 | 0.05 |
| 9741 | 1.08019 | 0.0638531 |

| | | |
|---|---|---|
| 9742 | 1.49261 | 0.05 |
| 9743 | 1.49261 | 0.0514419 |
| 9744 | 0.94613 | 0.0673596 |
| 9745 | 1.29322 | 0.380816 |
| 9746 | 0.946132 | 0.315874 |
| 9747 | 1.29322 | 0.0512648 |
| 9748 | 1.35856 | 0.403321 |
| 9749 | 1.70565 | 0.05 |
| 9750 | 1.70565 | 0.260946 |
| 9751 | 0.946132 | 0.370023 |
| 9752 | 1.66635 | 2.1 |
| 9753 | 1.66635 | 2.1 |
| 9754 | 0.946133 | 2.1 |
| 9755 | 1.39558 | 2.1 |
| 9756 | 1.14793 | 2.1 |
| 9757 | 1.14793 | 2.1 |
| 9758 | 1.21691 | 2.1 |
| 9759 | 1.35381 | 0.05 |
| 9760 | 1.3365 | 0.05 |
| 9761 | 1.09905 | 0.05 |
| 9762 | 0.946132 | 0.0570052 |
| 9763 | 1.74418 | 0.05 |
| 9764 | 1.74418 | 0.05 |
| 9765 | 0.946132 | 0.05 |
| 9766 | 1.84759 | 0.05 |
| 9767 | 1.84759 | 0.05 |
| 9768 | 0.94613 | 0.05 |
| 9769 | 1.3904 | 0.05 |
| 9770 | 1.34522 | 0.05 |
| 9771 | 1.34522 | 0.05 |
| 9772 | 1.40331 | 0.05 |
| 9773 | 1.86047 | 0.05 |
| 9774 | 1.86047 | 2.1 |
| 9775 | 0.946132 | 2.1 |
| 9776 | 1.41619 | 2.1 |
| 9777 | 0.946132 | 0.05 |
| 9778 | 1.3659 | 0.05 |
| 9779 | 1.41619 | 0.136878 |
| 9780 | 1.28464 | 2.1 |
| 9781 | 1.14496 | 1.5788 |
| 9782 | 1.57806 | 1.89662 |
| 9783 | 1.7753 | 2.1 |
| 9784 | 0.851541 | 1.64611 |
| 9785 | 0.946131 | 2.1 |
| 9786 | 0.872786 | 2.08566 |
| 9787 | 0.85062 | 0.791178 |
| 9788 | 1.52635 | 0.947516 |
| 9789 | 1.63785 | 0.750912 |
| 9790 | 1.69972 | 1.1933 |
| 9791 | 0.946132 | 1.49542 |
| 9792 | 1.37493 | 0.813837 |
| 9793 | 1.23854 | 0.819177 |
| 9794 | 1.41134 | 2.1 |
| 9795 | 1.27079 | 2.06736 |
| 9796 | 1.736 | 1.38448 |
| 9797 | 1.736 | 2.1 |
| 9798 | 0.946132 | 0.901105 |
| 9799 | 0.946133 | 1.94842 |

| | | |
|---|---|---|
| 9800 | 1.21177 | 2.1 |
| 9801 | 0.946131 | 0.05 |
| 9802 | 0.946129 | 0.05 |
| 9803 | 0.94613 | 0.05 |
| 9804 | 0.946131 | 0.05 |
| 9805 | 0.946131 | 0.05 |
| 9806 | 1.35403 | 0.05 |
| 9807 | 0.946131 | 0.05 |
| 9808 | 1.37052 | 0.05 |
| 9809 | 0.946132 | 0.05 |
| 9810 | 0.94613 | 0.05 |
| 9811 | 0.946133 | 0.05 |
| 9812 | 0.946131 | 0.05 |
| 9813 | 0.946131 | 0.05 |
| 9814 | 0.946131 | 0.05 |
| 9815 | 1.24249 | 0.05 |
| 9816 | 0.946132 | 0.05 |
| 9817 | 0.946133 | 0.05 |
| 9818 | 0.946132 | 0.05 |
| 9819 | 0.946132 | 0.05 |
| 9820 | 0.946132 | 0.05 |
| 9821 | 0.946132 | 0.05 |
| 9822 | 0.946132 | 2.1 |
| 9823 | 0.946132 | 2.1 |
| 9824 | 0.946131 | 2.1 |
| 9825 | 0.946132 | 2.1 |
| 9826 | 0.946132 | 2.1 |
| 9827 | 0.946132 | 2.1 |
| 9828 | 1.20608 | 2.1 |
| 9829 | 1.41232 | 2.1 |
| 9830 | 0.946128 | 2.1 |
| 9831 | 0.946129 | 2.1 |
| 9832 | 0.94613 | 2.1 |
| 9833 | 0.94613 | 2.1 |
| 9834 | 0.94613 | 2.1 |
| 9835 | 0.94613 | 2.1 |
| 9836 | 0.946132 | 0.05 |
| 9837 | 0.946131 | 0.05 |
| 9838 | 0.946131 | 0.05 |
| 9839 | 0.946132 | 0.05 |
| 9840 | 0.946132 | 0.05 |
| 9841 | 0.946131 | 0.05 |
| 9842 | 0.946132 | 0.05 |
| 9843 | 0.946116 | 0.626558 |
| 9844 | 0.94612 | 2.05533 |
| 9845 | 0.946118 | 0.946643 |
| 9846 | 0.946124 | 2.1 |
| 9847 | 0.946118 | 0.856904 |
| 9848 | 1.41561 | 1.96046 |
| 9849 | 0.946131 | 1.34141 |
| 9850 | 0.945846 | 2.1 |
| 9851 | 1.03508 | 2.1 |
| 9852 | 0.77579 | 2.1 |
| 9853 | 0.658908 | 2.1 |
| 9854 | 1.17474 | 2.1 |
| 9855 | 0.94303 | 2.1 |
| 9856 | 0.849189 | 2.1 |
| 9857 | 0.94298 | 2.1 |

| | | |
|---|---|---|
| 9858 | 1.03963 | 2.1 |
| 9859 | 0.946131 | 1.86738 |
| 9860 | 0.946131 | 1.43895 |
| 9861 | 0.946131 | 2.1 |
| 9862 | 0.946131 | 1.09832 |
| 9863 | 0.946131 | 0.05 |
| 9864 | 0.946131 | 1.22392 |
| 9865 | 1.32819 | 1.5089 |
| 9866 | 0.946133 | 1.12099 |
| 9867 | 0.946129 | 2.1 |
| 9868 | 0.946128 | 2.1 |
| 9869 | 0.94613 | 2.1 |
| 9870 | 0.946131 | 2.1 |
| 9871 | 0.946131 | 1.07153 |
| 9872 | 0.946131 | 2.06254 |
| 9873 | 0.954072 | 0.05 |
| 9874 | 1.28895 | 0.05 |
| 9875 | 0.946133 | 0.05 |
| 9876 | 1.15803 | 0.05 |
| 9877 | 1.29689 | 0.05 |
| 9878 | 1.29689 | 0.05 |
| 9879 | 0.946132 | 0.05 |
| 9880 | 0.946132 | 0.603744 |
| 9881 | 0.946132 | 0.699306 |
| 9882 | 0.946132 | 0.162781 |
| 9883 | 0.946132 | 0.982394 |
| 9884 | 0.946132 | 1.51033 |
| 9885 | 0.946131 | 1.40514 |
| 9886 | 1.0264 | 1.49068 |
| 9887 | 0.946126 | 0.05 |
| 9888 | 0.946132 | 0.05 |
| 9889 | 0.946131 | 0.05 |
| 9890 | 0.94613 | 0.05 |
| 9891 | 0.946129 | 0.05 |
| 9892 | 0.94613 | 0.05 |
| 9893 | 1.35977 | 0.05 |
| 9894 | 0.946132 | 0.695788 |
| 9895 | 0.946133 | 0.681581 |
| 9896 | 0.876632 | 1.0417 |
| 9897 | 0.946132 | 0.961997 |
| 9898 | 1.01566 | 1.57727 |
| 9899 | 1.01566 | 0.898483 |
| 9900 | 0.876606 | 1.13206 |
| 9901 | 0.946131 | 0.05 |
| 9902 | 0.946128 | 0.05 |
| 9903 | 0.946128 | 0.05 |
| 9904 | 0.94613 | 0.05 |
| 9905 | 0.94613 | 0.05 |
| 9906 | 1.24979 | 0.05 |
| 9907 | 0.94613 | 0.05 |
| 9908 | 0.946131 | 0.05 |
| 9909 | 0.946131 | 0.05 |
| 9910 | 0.921762 | 0.05 |
| 9911 | 0.9705 | 0.05 |
| 9912 | 0.9705 | 0.05 |
| 9913 | 0.946131 | 0.05 |
| 9914 | 0.921791 | 0.05 |
| 9915 | 0.810944 | 0.05 |

| | | |
|---|---|---|
| 9916 | 0.810945 | 0.05 |
| 9917 | 0.810959 | 0.05 |
| 9918 | 2.42267 | 0.05 |
| 9919 | 1.35168 | 0.05 |
| 9920 | 1.08132 | 0.05 |
| 9921 | 1.08132 | 0.05 |
| 9922 | 1.08131 | 0.05 |
| 9923 | 0.946133 | 0.05 |
| 9924 | 0.946132 | 0.05 |
| 9925 | 0.946132 | 0.05 |
| 9926 | 0.946133 | 0.05 |
| 9927 | 0.946133 | 0.05 |
| 9928 | 0.946132 | 0.05 |
| 9929 | 0.946133 | 0.05 |
| 9930 | 0.946131 | 0.10846 |
| 9931 | 0.946131 | 0.370031 |
| 9932 | 0.94613 | 0.05 |
| 9933 | 0.851514 | 0.0542632 |
| 9934 | 0.946131 | 0.153339 |
| 9935 | 0.965268 | 0.371469 |
| 9936 | 0.851513 | 0.05 |
| 9937 | 0.946122 | 2.1 |
| 9938 | 0.946123 | 2.1 |
| 9939 | 0.946126 | 2.1 |
| 9940 | 0.946124 | 2.1 |
| 9941 | 0.946124 | 2.1 |
| 9942 | 0.946118 | 2.1 |
| 9943 | 0.94613 | 2.1 |
| 9944 | 0.946131 | 0.608718 |
| 9945 | 1.30448 | 0.733674 |
| 9946 | 0.946132 | 0.809926 |
| 9947 | 0.946129 | 0.68651 |
| 9948 | 0.946131 | 1.9302 |
| 9949 | 0.946131 | 0.631147 |
| 9950 | 0.94613 | 1.80138 |
| 9951 | 0.946133 | 0.791323 |
| 9952 | 0.946128 | 2.1 |
| 9953 | 0.946128 | 2.1 |
| 9954 | 0.94613 | 1.30587 |
| 9955 | 1.36503 | 2.1 |
| 9956 | 0.946131 | 2.1 |
| 9957 | 0.946131 | 2.1 |
| 9958 | 0.810947 | 0.05 |
| 9959 | 0.810947 | 0.05 |
| 9960 | 2.48233 | 0.05 |
| 9961 | 1.3517 | 0.05 |
| 9962 | 1.08132 | 0.05 |
| 9963 | 1.08132 | 0.05 |
| 9964 | 1.08133 | 0.05 |
| 9965 | 0.810934 | 0.05 |
| 9966 | 1.48874 | 0.05 |
| 9967 | 2.48233 | 0.05 |
| 9968 | 2.2369 | 0.05 |
| 9969 | 0.946133 | 0.355645 |
| 9970 | 0.946132 | 0.113589 |
| 9971 | 0.946132 | 0.772356 |
| 9972 | 0.946133 | 0.803999 |
| 9973 | 0.946133 | 0.899789 |

| | | |
|---|---|---|
| 9974 | 1.41851 | 0.276876 |
| 9975 | 0.946132 | 0.550641 |
| 9976 | 1.03925 | 2.1 |
| 9977 | 0.946131 | 2.1 |
| 9978 | 0.94613 | 2.1 |
| 9979 | 0.946128 | 2.1 |
| 9980 | 0.946133 | 2.1 |
| 9981 | 0.944743 | 2.1 |
| 9982 | 1.13703 | 2.1 |
| 9983 | 0.946132 | 2.1 |
| 9984 | 0.946128 | 2.1 |
| 9985 | 0.946128 | 2.1 |
| 9986 | 0.946131 | 2.1 |
| 9987 | 0.94613 | 2.1 |
| 9988 | 0.94613 | 2.1 |
| 9989 | 0.94613 | 2.1 |
| 9990 | 0.946131 | 0.05 |
| 9991 | 0.946132 | 0.05 |
| 9992 | 0.946131 | 0.05 |
| 9993 | 0.946132 | 0.05 |
| 9994 | 0.946132 | 0.05 |
| 9995 | 0.946131 | 0.05 |
| 9996 | 1.20057 | 0.05 |
| 9997 | 0.946129 | 2.1 |
| 9998 | 0.94613 | 2.1 |
| 9999 | 0.94613 | 2.1 |
| 10000 | 0.946128 | 2.1 |
| 10001 | 0.946132 | 2.1 |
| 10002 | 0.94613 | 2.1 |
| 10003 | 1.38302 | 2.1 |
| 10004 | 0.856567 | 0.05 |
| 10005 | 1.14319 | 0.05 |
| 10006 | 0.716526 | 0.05 |
| 10007 | 1.20631 | 0.05 |
| 10008 | 0.626936 | 0.05 |
| 10009 | 0.946131 | 0.05 |
| 10010 | 0.94613 | 0.05 |
| 10011 | 1.39502 | 0.05 |
| 10012 | 0.881445 | 0.05 |
| 10013 | 0.946128 | 0.05 |
| 10014 | 0.94613 | 0.05 |
| 10015 | 0.881447 | 0.05 |
| 10016 | 0.946117 | 0.05 |
| 10017 | 0.946116 | 0.05 |
| 10018 | 0.88146 | 0.05 |
| 10019 | 0.946132 | 2.1 |
| 10020 | 1.39053 | 2.1 |
| 10021 | 0.946132 | 2.1 |
| 10022 | 0.946132 | 2.1 |
| 10023 | 0.946132 | 2.1 |
| 10024 | 0.946132 | 2.1 |
| 10025 | 0.946131 | 2.1 |
| 10026 | 0.946131 | 2.1 |
| 10027 | 0.946131 | 2.1 |
| 10028 | 0.946131 | 2.1 |
| 10029 | 0.946132 | 2.1 |
| 10030 | 0.946132 | 2.1 |
| 10031 | 1.40571 | 2.1 |

| | | |
|---|---|---|
| 10032 | 0.946132 | 2.1 |
| 10033 | 0.94613 | 0.0815439 |
| 10034 | 0.946127 | 0.114089 |
| 10035 | 0.946128 | 0.077616 |
| 10036 | 0.946131 | 0.089089 |
| 10037 | 0.946127 | 0.063482 |
| 10038 | 1.25819 | 0.0655527 |
| 10039 | 0.946129 | 0.0544773 |
| 10040 | 0.946129 | 0.861889 |
| 10041 | 1.29054 | 0.911749 |
| 10042 | 0.94613 | 0.751077 |
| 10043 | 0.94613 | 0.751152 |
| 10044 | 0.946131 | 1.5708 |
| 10045 | 0.946131 | 0.960736 |
| 10046 | 0.94613 | 0.918818 |
| 10047 | 0.810944 | 0.05 |
| 10048 | 0.810941 | 0.05 |
| 10049 | 1.61558 | 0.05 |
| 10050 | 1.3517 | 0.05 |
| 10051 | 1.08131 | 0.05 |
| 10052 | 1.08132 | 0.05 |
| 10053 | 1.08133 | 0.05 |
| 10054 | 0.810931 | 0.05 |
| 10055 | 1.02036 | 1.58536 |
| 10056 | 0.946132 | 1.06342 |
| 10057 | 1.02036 | 0.958252 |
| 10058 | 0.946132 | 0.587949 |
| 10059 | 1.1576 | 0.892259 |
| 10060 | 0.964656 | 1.03818 |
| 10061 | 0.927608 | 0.662158 |
| 10062 | 0.946128 | 2.1 |
| 10063 | 1.38813 | 2.1 |
| 10064 | 0.946129 | 2.1 |
| 10065 | 0.946131 | 2.1 |
| 10066 | 0.946131 | 2.1 |
| 10067 | 0.94613 | 2.1 |
| 10068 | 0.946131 | 2.1 |
| 10069 | 0.946131 | 2.1 |
| 10070 | 0.94613 | 0.05 |
| 10071 | 0.94613 | 2.1 |
| 10072 | 1.41665 | 2.1 |
| 10073 | 0.946132 | 0.05 |
| 10074 | 0.946131 | 2.1 |
| 10075 | 0.94613 | 2.1 |
| 10076 | 0.946118 | 0.0701209 |
| 10077 | 0.946118 | 0.05 |
| 10078 | 0.946116 | 0.05 |
| 10079 | 0.946126 | 0.05 |
| 10080 | 0.94612 | 0.05 |
| 10081 | 0.946131 | 0.0511326 |
| 10082 | 0.946105 | 0.05 |
| 10083 | 0.946132 | 2.1 |
| 10084 | 0.946132 | 2.1 |
| 10085 | 0.946132 | 2.1 |
| 10086 | 0.946131 | 2.1 |
| 10087 | 0.946132 | 2.1 |
| 10088 | 0.946132 | 2.1 |
| 10089 | 0.946132 | 2.1 |

| | | |
|---|---|---|
| 10090 | 0.864997 | 0.05 |
| 10091 | 0.786573 | 0.05 |
| 10092 | 0.978561 | 0.05 |
| 10093 | 1.47385 | 0.05 |
| 10094 | 1.68652 | 0.05 |
| 10095 | 1.27803 | 0.05 |
| 10096 | 1.19771 | 0.05 |
| 10097 | 1.27595 | 0.05 |
| 10098 | 0.946127 | 0.0634359 |
| 10099 | 1.3771 | 0.05 |
| 10100 | 0.946127 | 0.05 |
| 10101 | 0.946131 | 0.19346 |
| 10102 | 0.946129 | 0.05 |
| 10103 | 0.946129 | 0.0769072 |
| 10104 | 0.946129 | 0.0760008 |
| 10105 | 0.95697 | 0.05 |
| 10106 | 1.48133 | 0.05 |
| 10107 | 1.24499 | 0.05 |
| 10108 | 1.5295 | 0.05 |
| 10109 | 0.938161 | 0.05 |
| 10110 | 0.808717 | 0.05 |
| 10111 | 1.3542 | 0.05 |
| 10112 | 0.946133 | 0.893099 |
| 10113 | 1.14444 | 0.747517 |
| 10114 | 0.946132 | 0.505744 |
| 10115 | 0.946132 | 0.755567 |
| 10116 | 0.946133 | 1.39617 |
| 10117 | 0.946132 | 0.998748 |
| 10118 | 0.946132 | 0.868822 |
| 10119 | 0.946131 | 0.05 |
| 10120 | 0.946132 | 0.05 |
| 10121 | 0.946131 | 0.05 |
| 10122 | 0.946132 | 0.05 |
| 10123 | 0.946131 | 0.05 |
| 10124 | 0.946132 | 0.05 |
| 10125 | 0.946132 | 0.05 |
| 10126 | 0.946132 | 2.1 |
| 10127 | 0.946133 | 2.1 |
| 10128 | 0.946133 | 2.1 |
| 10129 | 0.946132 | 2.1 |
| 10130 | 1.29322 | 2.1 |
| 10131 | 0.946132 | 2.1 |
| 10132 | 0.946132 | 2.1 |
| 10133 | 0.946131 | 2.1 |
| 10134 | 0.946132 | 2.1 |
| 10135 | 0.946131 | 2.1 |
| 10136 | 0.946132 | 2.1 |
| 10137 | 1.28441 | 2.1 |
| 10138 | 0.946131 | 2.1 |
| 10139 | 0.946131 | 2.1 |
| 10140 | 0.946113 | 2.1 |
| 10141 | 0.946118 | 2.1 |
| 10142 | 0.946118 | 1.90509 |
| 10143 | 1.28073 | 2.1 |
| 10144 | 0.946117 | 2.1 |
| 10145 | 0.946131 | 2.1 |
| 10146 | 0.946105 | 2.1 |
| 10147 | 0.810945 | 2.1 |

| | | |
|---|---|---|
| 10148 | 0.810942 | 2.1 |
| 10149 | 2.75904 | 2.1 |
| 10150 | 1.3517 | 2.1 |
| 10151 | 1.08131 | 2.1 |
| 10152 | 1.08133 | 2.1 |
| 10153 | 1.08132 | 2.1 |
| 10154 | 0.81093 | 2.1 |
| 10155 | 0.946127 | 2.1 |
| 10156 | 1.37952 | 2.1 |
| 10157 | 1.86965 | 2.1 |
| 10158 | 0.946127 | 2.1 |
| 10159 | 1.86965 | 2.1 |
| 10160 | 1.28228 | 2.1 |
| 10161 | 1.37952 | 2.1 |
| 10162 | 0.946128 | 2.1 |
| 10163 | 0.946132 | 2.1 |
| 10164 | 1.14042 | 2.1 |
| 10165 | 0.946132 | 2.1 |
| 10166 | 1.14042 | 2.1 |
| 10167 | 1.09974 | 2.1 |
| 10168 | 1.09974 | 2.1 |
| 10169 | 0.946132 | 2.1 |
| 10170 | 0.946132 | 0.05 |
| 10171 | 0.946132 | 0.05 |
| 10172 | 0.946133 | 0.05 |
| 10173 | 0.946132 | 0.05 |
| 10174 | 0.946132 | 0.05 |
| 10175 | 0.946132 | 0.05 |
| 10176 | 0.946133 | 0.05 |
| 10177 | 1.31817 | 1.94671 |
| 10178 | 0.946131 | 1.17941 |
| 10179 | 0.946131 | 1.77028 |
| 10180 | 0.946131 | 1.52954 |
| 10181 | 0.946131 | 0.96497 |
| 10182 | 0.946131 | 1.27034 |
| 10183 | 0.946131 | 1.26854 |
| 10184 | 0.946132 | 2.1 |
| 10185 | 0.946132 | 2.1 |
| 10186 | 0.946132 | 2.1 |
| 10187 | 0.946132 | 2.1 |
| 10188 | 0.946132 | 2.1 |
| 10189 | 0.946132 | 2.1 |
| 10190 | 1.35048 | 2.1 |
| 10191 | 0.946131 | 2.1 |
| 10192 | 0.946131 | 2.1 |
| 10193 | 0.94613 | 2.1 |
| 10194 | 0.946133 | 2.1 |
| 10195 | 0.946133 | 2.1 |
| 10196 | 1.35943 | 2.1 |
| 10197 | 0.946131 | 2.1 |
| 10198 | 0.946133 | 2.1 |
| 10199 | 0.946132 | 2.1 |
| 10200 | 0.946132 | 2.1 |
| 10201 | 0.946133 | 2.1 |
| 10202 | 0.946133 | 2.1 |
| 10203 | 0.946133 | 2.1 |
| 10204 | 0.990712 | 2.1 |
| 10205 | 0.965725 | 2.1 |

| | | |
|---|---|---|
| 10206 | 0.946129 | 0.916 |
| 10207 | 0.946133 | 1.73213 |
| 10208 | 1.21721 | 1.0862 |
| 10209 | 0.877931 | 0.794196 |
| 10210 | 0.946131 | 1.69173 |
| 10211 | 0.946131 | 0.872971 |
| 10212 | 0.946132 | 0.05 |
| 10213 | 0.946131 | 0.05 |
| 10214 | 0.946131 | 0.113817 |
| 10215 | 0.946133 | 0.05 |
| 10216 | 0.946128 | 0.617901 |
| 10217 | 0.946131 | 0.0701492 |
| 10218 | 1.39575 | 0.0536874 |
| 10219 | 0.810943 | 0.0876021 |
| 10220 | 0.810942 | 0.126029 |
| 10221 | 2.58794 | 0.05 |
| 10222 | 1.21652 | 0.05 |
| 10223 | 0.946154 | 0.0977791 |
| 10224 | 0.979952 | 0.0879701 |
| 10225 | 1.0813 | 0.0655548 |
| 10226 | 0.810959 | 0.121077 |
| 10227 | 0.924071 | 0.05 |
| 10228 | 1.16453 | 0.05 |
| 10229 | 1.28586 | 0.05 |
| 10230 | 0.946131 | 0.0509661 |
| 10231 | 1.1227 | 0.05 |
| 10232 | 1.16462 | 0.05 |
| 10233 | 0.851553 | 0.0812158 |
| 10234 | 0.709578 | 0.05 |
| 10235 | 0.810943 | 0.05 |
| 10236 | 0.810959 | 0.05 |
| 10237 | 1.82615 | 0.05 |
| 10238 | 1.21651 | 0.05 |
| 10239 | 1.08132 | 0.05 |
| 10240 | 0.946155 | 0.05 |
| 10241 | 0.979937 | 0.05 |
| 10242 | 0.946128 | 1.48871 |
| 10243 | 0.946132 | 2.1 |
| 10244 | 0.946132 | 0.984989 |
| 10245 | 0.94613 | 2.1 |
| 10246 | 0.94613 | 2.1 |
| 10247 | 0.94613 | 0.889597 |
| 10248 | 0.94613 | 1.53652 |
| 10249 | 0.946131 | 2.1 |
| 10250 | 1.29735 | 2.1 |
| 10251 | 0.946131 | 2.1 |
| 10252 | 0.946131 | 1.03652 |
| 10253 | 0.946131 | 1.90872 |
| 10254 | 0.946131 | 0.962638 |
| 10255 | 0.946131 | 2.1 |
| 10256 | 0.946128 | 2.1 |
| 10257 | 1.33266 | 1.19492 |
| 10258 | 0.94613 | 1.22996 |
| 10259 | 0.946131 | 2.1 |
| 10260 | 0.94613 | 0.0815536 |
| 10261 | 0.946131 | 1.19451 |
| 10262 | 0.946131 | 1.05038 |
| 10263 | 0.946131 | 0.05 |

| | | |
|---|---|---|
| 10264 | 0.946131 | 2.1 |
| 10265 | 0.946131 | 0.05 |
| 10266 | 1.40608 | 0.05 |
| 10267 | 0.946131 | 2.1 |
| 10268 | 0.946131 | 0.05 |
| 10269 | 0.946131 | 0.566222 |
| 10270 | 0.946131 | 0.584723 |
| 10271 | 0.946131 | 2.1 |
| 10272 | 0.946131 | 1.12931 |
| 10273 | 1.2334 | 1.12172 |
| 10274 | 0.946129 | 1.52774 |
| 10275 | 0.946131 | 1.15155 |
| 10276 | 0.946131 | 2.09636 |
| 10277 | 0.946133 | 2.1 |
| 10278 | 0.946133 | 2.1 |
| 10279 | 0.946132 | 1.25885 |
| 10280 | 1.29198 | 2.1 |
| 10281 | 0.946132 | 2.1 |
| 10282 | 0.946132 | 2.1 |
| 10283 | 0.946132 | 2.1 |
| 10284 | 0.946131 | 2.1 |
| 10285 | 0.946128 | 2.1 |
| 10286 | 0.946128 | 2.1 |
| 10287 | 0.946131 | 2.1 |
| 10288 | 0.946127 | 2.1 |
| 10289 | 0.94613 | 2.1 |
| 10290 | 0.946129 | 2.1 |
| 10291 | 0.946123 | 2.1 |
| 10292 | 0.946126 | 2.1 |
| 10293 | 0.946125 | 2.1 |
| 10294 | 1.38639 | 2.1 |
| 10295 | 0.946126 | 2.1 |
| 10296 | 0.946132 | 2.1 |
| 10297 | 0.946119 | 2.1 |
| 10298 | 0.946128 | 0.05 |
| 10299 | 0.946132 | 0.05 |
| 10300 | 0.946131 | 0.05 |
| 10301 | 0.94613 | 0.05 |
| 10302 | 0.94613 | 0.05 |
| 10303 | 0.946131 | 0.05 |
| 10304 | 0.94613 | 0.05 |
| 10305 | 0.946131 | 0.05 |
| 10306 | 0.946131 | 0.05 |
| 10307 | 0.946131 | 0.05 |
| 10308 | 0.946131 | 0.05 |
| 10309 | 0.946131 | 0.05 |
| 10310 | 0.946131 | 0.05 |
| 10311 | 0.946131 | 0.05 |
| 10312 | 0.810945 | 2.1 |
| 10313 | 0.810943 | 2.1 |
| 10314 | 0.810933 | 2.1 |
| 10315 | 2.24688 | 2.1 |
| 10316 | 1.3517 | 2.1 |
| 10317 | 1.08132 | 2.1 |
| 10318 | 1.08132 | 2.1 |
| 10319 | 1.08133 | 2.1 |
| 10320 | 1.25809 | 0.05 |
| 10321 | 0.946131 | 0.0978157 |

| | | |
|---|---|---|
| 10322 | 1.25809 | 0.05 |
| 10323 | 0.946129 | 0.0545193 |
| 10324 | 0.94613 | 0.05 |
| 10325 | 0.946131 | 0.05 |
| 10326 | 0.946131 | 0.05 |
| 10327 | 0.946131 | 0.0565155 |
| 10328 | 0.946131 | 0.05 |
| 10329 | 0.946129 | 0.0904054 |
| 10330 | 1.28911 | 0.05 |
| 10331 | 0.946131 | 0.05 |
| 10332 | 0.946131 | 0.0527591 |
| 10333 | 0.946131 | 0.05 |
| 10334 | 0.94613 | 0.106958 |
| 10335 | 0.946131 | 0.153026 |
| 10336 | 0.94613 | 0.05 |
| 10337 | 0.946128 | 0.139618 |
| 10338 | 0.946132 | 0.05 |
| 10339 | 1.38954 | 0.113036 |
| 10340 | 0.946131 | 0.0902086 |
| 10341 | 0.946131 | 0.05 |
| 10342 | 0.946131 | 0.05 |
| 10343 | 0.946131 | 0.0515433 |
| 10344 | 1.37981 | 0.05 |
| 10345 | 0.946131 | 0.324304 |
| 10346 | 0.946131 | 0.05 |
| 10347 | 0.946131 | 0.05 |
| 10348 | 0.946131 | 2.1 |
| 10349 | 0.946127 | 2.1 |
| 10350 | 0.946127 | 2.1 |
| 10351 | 0.946131 | 2.1 |
| 10352 | 0.946127 | 2.1 |
| 10353 | 0.946129 | 2.1 |
| 10354 | 0.946128 | 2.1 |
| 10355 | 0.94613 | 2.1 |
| 10356 | 0.946126 | 2.1 |
| 10357 | 0.946127 | 2.1 |
| 10358 | 0.946131 | 2.1 |
| 10359 | 0.946126 | 2.1 |
| 10360 | 0.946128 | 2.1 |
| 10361 | 0.946128 | 2.1 |
| 10362 | 0.946133 | 0.0792422 |
| 10363 | 0.946132 | 0.0550662 |
| 10364 | 0.946132 | 0.103168 |
| 10365 | 0.946132 | 0.0541276 |
| 10366 | 0.946132 | 0.166677 |
| 10367 | 1.13511 | 0.05 |
| 10368 | 0.946132 | 0.05 |
| 10369 | 0.946132 | 1.84931 |
| 10370 | 0.946129 | 1.81398 |
| 10371 | 0.94613 | 1.29747 |
| 10372 | 0.946131 | 1.96618 |
| 10373 | 0.946131 | 2.1 |
| 10374 | 0.94613 | 2.1 |
| 10375 | 1.27575 | 2.1 |
| 10376 | 0.946129 | 2.1 |
| 10377 | 0.946131 | 2.1 |
| 10378 | 0.946131 | 2.1 |
| 10379 | 0.946128 | 2.1 |

| | | |
|---|---|---|
| 10380 | 0.946133 | 2.1 |
| 10381 | 0.946131 | 2.1 |
| 10382 | 0.94613 | 2.1 |
| 10383 | 1.15581 | 0.05 |
| 10384 | 0.946132 | 0.05 |
| 10385 | 0.946131 | 0.05 |
| 10386 | 0.94613 | 0.05 |
| 10387 | 1.15581 | 0.05 |
| 10388 | 0.94613 | 0.05 |
| 10389 | 0.946131 | 0.05 |
| 10390 | 0.611407 | 0.990773 |
| 10391 | 0.946131 | 0.295528 |
| 10392 | 1.37335 | 0.809537 |
| 10393 | 1.15086 | 1.01575 |
| 10394 | 0.617549 | 0.621079 |
| 10395 | 0.851553 | 0.929185 |
| 10396 | 1.14592 | 1.02799 |
| 10397 | 1.22618 | 1.66909 |
| 10398 | 0.946132 | 0.359383 |
| 10399 | 0.946132 | 1.84406 |
| 10400 | 0.946132 | 0.463951 |
| 10401 | 0.946131 | 0.375064 |
| 10402 | 0.946132 | 1.10165 |
| 10403 | 0.946132 | 0.654275 |
| 10404 | 0.946131 | 2.1 |
| 10405 | 0.94613 | 2.1 |
| 10406 | 0.946131 | 2.1 |
| 10407 | 0.946133 | 2.1 |
| 10408 | 0.946129 | 2.1 |
| 10409 | 0.946131 | 2.1 |
| 10410 | 0.946131 | 2.1 |
| 10411 | 0.810945 | 0.774713 |
| 10412 | 0.810943 | 1.50033 |
| 10413 | 2.50715 | 1.24678 |
| 10414 | 1.3517 | 0.873493 |
| 10415 | 1.08133 | 0.812802 |
| 10416 | 1.08132 | 1.12904 |
| 10417 | 1.08132 | 0.105081 |
| 10418 | 0.810932 | 0.759229 |
| 10419 | 1.07811 | 1.218 |
| 10420 | 0.94613 | 0.484212 |
| 10421 | 0.946128 | 1.37844 |
| 10422 | 1.25358 | 0.0723779 |
| 10423 | 0.946131 | 0.701289 |
| 10424 | 1.25358 | 0.281606 |
| 10425 | 0.94613 | 0.421166 |
| 10426 | 0.946129 | 2.1 |
| 10427 | 1.09197 | 2.1 |
| 10428 | 0.946131 | 2.1 |
| 10429 | 1.09197 | 2.1 |
| 10430 | 1.09532 | 2.1 |
| 10431 | 1.09532 | 2.1 |
| 10432 | 0.946131 | 2.1 |
| 10433 | 1.58788 | 2.1 |
| 10434 | 1.34255 | 2.1 |
| 10435 | 0.594912 | 2.1 |
| 10436 | 0.58894 | 2.1 |
| 10437 | 0.946129 | 2.1 |

| | | |
|---|---|---|
| 10438 | 1.16679 | 2.1 |
| 10439 | 1.04071 | 2.1 |
| 10440 | 0.535418 | 2.1 |
| 10441 | 0.946131 | 2.1 |
| 10442 | 0.946128 | 2.1 |
| 10443 | 0.946131 | 2.1 |
| 10444 | 1.20221 | 2.1 |
| 10445 | 0.946128 | 2.1 |
| 10446 | 0.94613 | 2.1 |
| 10447 | 1.20221 | 2.1 |
| 10448 | 0.946132 | 2.1 |
| 10449 | 0.946133 | 2.1 |
| 10450 | 0.946132 | 2.1 |
| 10451 | 0.946132 | 2.1 |
| 10452 | 0.946133 | 2.1 |
| 10453 | 0.946133 | 2.1 |
| 10454 | 0.946132 | 2.1 |
| 10455 | 0.946132 | 0.889646 |
| 10456 | 0.946132 | 1.3286 |
| 10457 | 0.946133 | 2.1 |
| 10458 | 0.946132 | 0.968617 |
| 10459 | 1.411 | 1.6906 |
| 10460 | 0.946132 | 1.11978 |
| 10461 | 0.946133 | 1.37074 |
| 10462 | 1.05575 | 0.05 |
| 10463 | 0.563734 | 0.05 |
| 10464 | 1.05575 | 0.05 |
| 10465 | 0.773833 | 0.05 |
| 10466 | 0.751686 | 0.05 |
| 10467 | 0.75884 | 0.05 |
| 10468 | 0.815612 | 0.05 |
| 10469 | 0.793465 | 0.05 |
| 10470 | 0.768563 | 0.05 |
| 10471 | 0.946129 | 0.05 |
| 10472 | 0.946125 | 0.05 |
| 10473 | 0.946126 | 0.05 |
| 10474 | 0.94613 | 0.05 |
| 10475 | 0.946124 | 0.05 |
| 10476 | 1.31678 | 0.05 |
| 10477 | 0.946128 | 0.05 |
| 10478 | 0.946132 | 0.05 |
| 10479 | 1.29914 | 0.05 |
| 10480 | 0.946132 | 0.05 |
| 10481 | 0.946132 | 0.05 |
| 10482 | 0.946132 | 0.05 |
| 10483 | 1.29914 | 0.05 |
| 10484 | 0.946132 | 0.05 |
| 10485 | 0.810946 | 0.05 |
| 10486 | 0.810943 | 0.05 |
| 10487 | 2.21082 | 0.05 |
| 10488 | 1.35168 | 0.05 |
| 10489 | 1.08132 | 0.05 |
| 10490 | 1.08132 | 0.05 |
| 10491 | 1.0813 | 0.05 |
| 10492 | 0.81096 | 0.05 |
| 10493 | 0.625813 | 0.05 |
| 10494 | 0.946132 | 0.05 |
| 10495 | 0.946131 | 0.05 |

| | | |
|---|---|---|
| 10496 | 0.946132 | 0.05 |
| 10497 | 0.946131 | 0.05 |
| 10498 | 1.39301 | 0.05 |
| 10499 | 0.625812 | 0.05 |
| 10500 | 0.810946 | 1.81509 |
| 10501 | 0.810945 | 2.1 |
| 10502 | 0.810931 | 2.1 |
| 10503 | 2.70043 | 2.1 |
| 10504 | 1.3517 | 2.1 |
| 10505 | 1.08131 | 2.0465 |
| 10506 | 1.08131 | 2.1 |
| 10507 | 1.08133 | 2.1 |
| 10508 | 0.810943 | 2.1 |
| 10509 | 0.810944 | 2.1 |
| 10510 | 0.810958 | 2.1 |
| 10511 | 2.44802 | 2.1 |
| 10512 | 1.35168 | 2.1 |
| 10513 | 1.08132 | 2.1 |
| 10514 | 1.08132 | 2.1 |
| 10515 | 1.0813 | 2.1 |
| 10516 | 0.946131 | 0.295872 |
| 10517 | 0.946129 | 0.435109 |
| 10518 | 0.946128 | 0.185155 |
| 10519 | 0.946131 | 0.264747 |
| 10520 | 1.3492 | 0.265272 |
| 10521 | 1.3492 | 0.316147 |
| 10522 | 0.94613 | 0.20997 |
| 10523 | 0.946132 | 0.115954 |
| 10524 | 0.946132 | 0.05 |
| 10525 | 0.946133 | 0.0715264 |
| 10526 | 0.946133 | 0.05 |
| 10527 | 0.946132 | 0.239475 |
| 10528 | 0.946132 | 0.05 |
| 10529 | 0.946132 | 0.151442 |
| 10530 | 0.946129 | 0.05 |
| 10531 | 0.946133 | 0.05 |
| 10532 | 0.946131 | 0.0930811 |
| 10533 | 0.94613 | 0.0602043 |
| 10534 | 0.946131 | 0.05 |
| 10535 | 0.946131 | 0.05 |
| 10536 | 0.946131 | 0.05 |
| 10537 | 0.946119 | 0.05 |
| 10538 | 0.946119 | 0.05 |
| 10539 | 0.946119 | 0.0507122 |
| 10540 | 0.946125 | 0.05 |
| 10541 | 0.94612 | 0.05 |
| 10542 | 0.946132 | 0.05 |
| 10543 | 0.946107 | 0.05 |
| 10544 | 0.946133 | 2.1 |
| 10545 | 0.946132 | 2.1 |
| 10546 | 0.946132 | 2.1 |
| 10547 | 0.946132 | 2.1 |
| 10548 | 0.946132 | 2.1 |
| 10549 | 0.946133 | 2.1 |
| 10550 | 1.3868 | 2.1 |
| 10551 | 0.810947 | 2.1 |
| 10552 | 0.810946 | 2.1 |
| 10553 | 0.81096 | 2.1 |

| | | |
|---|---|---|
| 10554 | 2.66094 | 2.1 |
| 10555 | 1.35168 | 2.1 |
| 10556 | 1.08132 | 2.1 |
| 10557 | 1.08132 | 2.1 |
| 10558 | 1.0813 | 2.1 |
| 10559 | 0.810946 | 2.1 |
| 10560 | 0.810946 | 2.1 |
| 10561 | 0.810933 | 2.1 |
| 10562 | 2.75737 | 2.1 |
| 10563 | 1.3517 | 2.1 |
| 10564 | 1.08132 | 2.1 |
| 10565 | 1.08132 | 2.1 |
| 10566 | 1.08133 | 2.1 |
| 10567 | 0.946133 | 2.1 |
| 10568 | 0.946132 | 2.1 |
| 10569 | 0.946132 | 2.1 |
| 10570 | 0.946132 | 2.1 |
| 10571 | 0.946133 | 2.1 |
| 10572 | 0.946132 | 2.1 |
| 10573 | 0.946133 | 2.1 |
| 10574 | 0.946133 | 2.1 |
| 10575 | 0.946133 | 2.1 |
| 10576 | 0.946132 | 2.1 |
| 10577 | 0.946132 | 2.1 |
| 10578 | 0.946133 | 2.1 |
| 10579 | 0.946132 | 2.1 |
| 10580 | 1.28569 | 2.1 |
| 10581 | 0.946132 | 1.81916 |
| 10582 | 0.946133 | 2.1 |
| 10583 | 0.946133 | 2.1 |
| 10584 | 0.946132 | 2.1 |
| 10585 | 0.946132 | 2.1 |
| 10586 | 0.946132 | 2.1 |
| 10587 | 0.946133 | 2.1 |
| 10588 | 0.946132 | 0.05 |
| 10589 | 0.946131 | 0.05 |
| 10590 | 0.94613 | 0.05 |
| 10591 | 0.946133 | 0.05 |
| 10592 | 0.946128 | 0.05 |
| 10593 | 0.946131 | 0.05 |
| 10594 | 0.946131 | 0.05 |
| 10595 | 0.946131 | 2.1 |
| 10596 | 0.94613 | 2.1 |
| 10597 | 0.946131 | 2.1 |
| 10598 | 0.94613 | 2.1 |
| 10599 | 1.41833 | 1.24517 |
| 10600 | 0.946131 | 1.506 |
| 10601 | 0.946131 | 2.1 |
| 10602 | 1.26155 | 0.05 |
| 10603 | 0.946131 | 0.05 |
| 10604 | 0.946131 | 0.05 |
| 10605 | 0.946132 | 0.05 |
| 10606 | 0.946132 | 0.05 |
| 10607 | 0.946131 | 0.05 |
| 10608 | 0.946132 | 0.05 |
| 10609 | 0.810946 | 2.1 |
| 10610 | 0.810946 | 2.1 |
| 10611 | 0.810959 | 2.1 |

| | | |
|---|---|---|
| 10612 | 2.47938 | 2.1 |
| 10613 | 1.35168 | 2.1 |
| 10614 | 1.08132 | 2.1 |
| 10615 | 1.08132 | 2.1 |
| 10616 | 1.08131 | 2.1 |
| 10617 | 0.946131 | 0.05 |
| 10618 | 0.94613 | 0.05 |
| 10619 | 0.94613 | 0.106724 |
| 10620 | 0.946132 | 0.05 |
| 10621 | 0.946128 | 0.43397 |
| 10622 | 0.94613 | 0.0759864 |
| 10623 | 0.94613 | 0.0615563 |
| 10624 | 0.94613 | 0.05 |
| 10625 | 0.946131 | 0.05 |
| 10626 | 0.94613 | 0.05 |
| 10627 | 0.946129 | 0.05 |
| 10628 | 0.946132 | 0.05 |
| 10629 | 0.94613 | 0.05 |
| 10630 | 0.946131 | 0.05 |
| 10631 | 0.946132 | 2.1 |
| 10632 | 0.946131 | 2.1 |
| 10633 | 0.946128 | 2.1 |
| 10634 | 1.40756 | 2.1 |
| 10635 | 0.946131 | 2.1 |
| 10636 | 0.946129 | 2.1 |
| 10637 | 0.94613 | 2.1 |
| 10638 | 0.946131 | 1.36646 |
| 10639 | 0.946131 | 1.54852 |
| 10640 | 0.946131 | 2.1 |
| 10641 | 0.946132 | 1.99189 |
| 10642 | 1.31947 | 2.0425 |
| 10643 | 0.946131 | 2.1 |
| 10644 | 0.946131 | 2.09396 |
| 10645 | 0.946131 | 2.1 |
| 10646 | 1.25768 | 2.1 |
| 10647 | 0.946132 | 2.1 |
| 10648 | 0.946132 | 2.1 |
| 10649 | 0.946129 | 2.1 |
| 10650 | 0.946131 | 2.1 |
| 10651 | 0.94613 | 2.1 |
| 10652 | 0.946132 | 0.294845 |
| 10653 | 0.946129 | 0.145577 |
| 10654 | 0.946129 | 0.354077 |
| 10655 | 0.94613 | 0.334766 |
| 10656 | 1.31716 | 0.254502 |
| 10657 | 1.31716 | 0.229298 |
| 10658 | 0.946131 | 0.395093 |
| 10659 | 0.810943 | 1.13773 |
| 10660 | 0.810942 | 2.1 |
| 10661 | 0.81093 | 2.1 |
| 10662 | 2.37052 | 2.1 |
| 10663 | 1.3517 | 2.1 |
| 10664 | 1.08132 | 0.970336 |
| 10665 | 1.08132 | 2.1 |
| 10666 | 1.08133 | 1.19125 |
| 10667 | 0.946128 | 1.04168 |
| 10668 | 1.10335 | 0.876057 |
| 10669 | 0.94613 | 0.794236 |

| | | |
|---|---|---|
| 10670 | 0.946131 | 1.28965 |
| 10671 | 0.946131 | 0.713075 |
| 10672 | 1.10335 | 1.03822 |
| 10673 | 0.94613 | 1.16954 |
| 10674 | 0.946132 | 1.07208 |
| 10675 | 1.18818 | 0.927018 |
| 10676 | 0.946132 | 0.873345 |
| 10677 | 0.946132 | 1.10831 |
| 10678 | 0.946132 | 0.812034 |
| 10679 | 1.18818 | 1.003 |
| 10680 | 0.946132 | 1.06602 |
| 10681 | 0.946125 | 2.1 |
| 10682 | 0.94612 | 2.1 |
| 10683 | 0.946119 | 2.1 |
| 10684 | 0.946119 | 2.1 |
| 10685 | 0.946119 | 2.1 |
| 10686 | 0.946132 | 2.1 |
| 10687 | 1.31522 | 2.1 |
| 10688 | 1.28803 | 2.1 |
| 10689 | 0.946132 | 0.0795922 |
| 10690 | 0.946132 | 2.1 |
| 10691 | 0.946133 | 2.1 |
| 10692 | 0.946133 | 0.05 |
| 10693 | 0.946133 | 2.1 |
| 10694 | 0.946132 | 1.86872 |
| 10695 | 0.946133 | 1.15354 |
| 10696 | 0.946132 | 1.73783 |
| 10697 | 0.946132 | 1.12722 |
| 10698 | 0.946132 | 0.89342 |
| 10699 | 0.946133 | 0.879132 |
| 10700 | 0.946132 | 0.871192 |
| 10701 | 1.37658 | 0.984894 |
| 10702 | 1.02239 | 1.93108 |
| 10703 | 0.946128 | 0.991186 |
| 10704 | 0.946132 | 1.6693 |
| 10705 | 1.02239 | 1.11619 |
| 10706 | 0.94613 | 0.934376 |
| 10707 | 0.96283 | 1.741 |
| 10708 | 0.92943 | 0.942264 |
| 10709 | 0.946132 | 0.05 |
| 10710 | 0.946131 | 0.05 |
| 10711 | 0.946126 | 0.05 |
| 10712 | 0.946129 | 0.05 |
| 10713 | 0.946128 | 0.05 |
| 10714 | 0.946128 | 0.05 |
| 10715 | 0.946129 | 0.05 |
| 10716 | 0.810946 | 0.05 |
| 10717 | 0.810944 | 0.05 |
| 10718 | 2.22493 | 0.05 |
| 10719 | 1.21651 | 0.05 |
| 10720 | 1.0813 | 0.05 |
| 10721 | 0.946154 | 0.05 |
| 10722 | 0.979951 | 0.05 |
| 10723 | 0.70959 | 0.05 |
| 10724 | 0.946132 | 0.05 |
| 10725 | 0.946131 | 0.05 |
| 10726 | 0.946131 | 0.05 |
| 10727 | 0.946133 | 0.05 |

| | | |
|---|---|---|
| 10728 | 0.946131 | 0.05 |
| 10729 | 0.946132 | 0.05 |
| 10730 | 0.946132 | 0.05 |
| 10731 | 0.946132 | 2.1 |
| 10732 | 0.946131 | 2.1 |
| 10733 | 0.946132 | 2.1 |
| 10734 | 0.946132 | 1.2548 |
| 10735 | 0.946132 | 0.84613 |
| 10736 | 0.946132 | 1.31659 |
| 10737 | 1.40924 | 2.07417 |
| 10738 | 0.946131 | 2.1 |
| 10739 | 0.946128 | 2.1 |
| 10740 | 0.946127 | 2.1 |
| 10741 | 0.94613 | 2.1 |
| 10742 | 1.41743 | 2.1 |
| 10743 | 0.946128 | 2.1 |
| 10744 | 0.946129 | 2.1 |
| 10745 | 1.37675 | 0.0926964 |
| 10746 | 0.946128 | 0.159559 |
| 10747 | 0.94613 | 0.149354 |
| 10748 | 0.94613 | 0.972582 |
| 10749 | 0.94613 | 0.214202 |
| 10750 | 0.946129 | 0.193033 |
| 10751 | 0.94613 | 0.683344 |
| 10752 | 0.946132 | 0.05 |
| 10753 | 0.946132 | 0.05 |
| 10754 | 0.946132 | 0.0598156 |
| 10755 | 1.35721 | 0.05 |
| 10756 | 0.946132 | 0.389643 |
| 10757 | 0.946132 | 0.0826592 |
| 10758 | 1.35721 | 0.05 |
| 10759 | 0.946133 | 0.862216 |
| 10760 | 0.946133 | 0.66108 |
| 10761 | 0.946132 | 0.853282 |
| 10762 | 1.10003 | 0.36753 |
| 10763 | 0.946133 | 0.637893 |
| 10764 | 0.946132 | 0.789106 |
| 10765 | 0.946132 | 0.714941 |
| 10766 | 0.946132 | 0.05 |
| 10767 | 0.946131 | 0.05 |
| 10768 | 0.946131 | 0.05 |
| 10769 | 0.946131 | 0.05 |
| 10770 | 1.0616 | 0.05 |
| 10771 | 1.0616 | 0.05 |
| 10772 | 0.946132 | 0.05 |
| 10773 | 0.946132 | 0.05 |
| 10774 | 0.946131 | 0.05 |
| 10775 | 0.946131 | 0.05 |
| 10776 | 0.946131 | 0.05 |
| 10777 | 1.09402 | 0.05 |
| 10778 | 1.09402 | 0.05 |
| 10779 | 0.946131 | 0.05 |
| 10780 | 0.946132 | 0.05 |
| 10781 | 0.946126 | 0.05 |
| 10782 | 1.24093 | 0.05 |
| 10783 | 0.946129 | 0.05 |
| 10784 | 0.946129 | 0.05 |
| 10785 | 0.946128 | 0.05 |

| | | |
|---|---|---|
| 10786 | 0.946129 | 0.05 |
| 10787 | 0.946131 | 0.05 |
| 10788 | 0.946131 | 0.05 |
| 10789 | 1.2929 | 0.05 |
| 10790 | 0.946131 | 0.05 |
| 10791 | 0.946131 | 0.05 |
| 10792 | 0.946131 | 0.05 |
| 10793 | 0.946131 | 0.05 |
| 10794 | 0.810946 | 0.05 |
| 10795 | 0.810945 | 0.05 |
| 10796 | 0.810959 | 0.05 |
| 10797 | 2.41233 | 0.05 |
| 10798 | 1.35168 | 0.05 |
| 10799 | 1.08132 | 0.05 |
| 10800 | 1.08132 | 0.05 |
| 10801 | 1.0813 | 0.05 |
| 10802 | 0.810946 | 1.3809 |
| 10803 | 0.810943 | 2.1 |
| 10804 | 0.750103 | 2.1 |
| 10805 | 2.22778 | 2.1 |
| 10806 | 1.27059 | 2.1 |
| 10807 | 1.00021 | 1.84954 |
| 10808 | 1.02049 | 2.1 |
| 10809 | 1.08133 | 2.09033 |
| 10810 | 0.946128 | 2.1 |
| 10811 | 0.946132 | 2.1 |
| 10812 | 0.946132 | 2.1 |
| 10813 | 0.94613 | 2.1 |
| 10814 | 0.94613 | 2.1 |
| 10815 | 0.94613 | 2.1 |
| 10816 | 0.946131 | 2.1 |
| 10817 | 0.946126 | 0.0594637 |
| 10818 | 0.946131 | 0.05 |
| 10819 | 0.946127 | 0.0841638 |
| 10820 | 1.3324 | 0.05 |
| 10821 | 0.946127 | 0.05 |
| 10822 | 0.946128 | 0.0601574 |
| 10823 | 0.946128 | 0.138358 |
| 10824 | 0.94613 | 2.1 |
| 10825 | 0.946131 | 2.1 |
| 10826 | 0.94613 | 2.1 |
| 10827 | 0.946128 | 2.1 |
| 10828 | 0.946133 | 2.1 |
| 10829 | 0.946131 | 2.1 |
| 10830 | 0.946131 | 2.1 |
| 10831 | 0.946133 | 2.1 |
| 10832 | 0.946132 | 2.1 |
| 10833 | 0.946133 | 2.1 |
| 10834 | 0.946133 | 2.1 |
| 10835 | 0.946132 | 2.1 |
| 10836 | 0.946132 | 2.1 |
| 10837 | 1.36541 | 2.1 |
| 10838 | 0.946131 | 0.183228 |
| 10839 | 0.94613 | 2.1 |
| 10840 | 0.946131 | 1.03588 |
| 10841 | 0.94613 | 0.87635 |
| 10842 | 1.39509 | 1.89611 |
| 10843 | 0.946131 | 0.192324 |

| | | |
|---|---|---|
| 10844 | 0.946131 | 2.09469 |
| 10845 | 0.946133 | 2.1 |
| 10846 | 0.946133 | 2.1 |
| 10847 | 0.946132 | 2.1 |
| 10848 | 0.946132 | 2.1 |
| 10849 | 0.946133 | 2.1 |
| 10850 | 0.946132 | 2.1 |
| 10851 | 1.29319 | 2.1 |
| 10852 | 1.01196 | 0.05 |
| 10853 | 0.946131 | 0.05 |
| 10854 | 0.946132 | 0.05 |
| 10855 | 0.946132 | 0.05 |
| 10856 | 0.946132 | 0.05 |
| 10857 | 0.946132 | 0.05 |
| 10858 | 0.946132 | 0.05 |
| 10859 | 0.946112 | 2.1 |
| 10860 | 0.946118 | 2.1 |
| 10861 | 0.946116 | 2.1 |
| 10862 | 0.946117 | 2.1 |
| 10863 | 0.946118 | 2.1 |
| 10864 | 0.94613 | 2.1 |
| 10865 | 0.946105 | 2.1 |
| 10866 | 1.28684 | 1.17183 |
| 10867 | 0.946131 | 1.19707 |
| 10868 | 0.946132 | 1.23867 |
| 10869 | 0.946132 | 1.57957 |
| 10870 | 0.946132 | 1.68385 |
| 10871 | 0.946132 | 1.27601 |
| 10872 | 0.946132 | 1.86453 |
| 10873 | 0.94613 | 2.1 |
| 10874 | 0.94613 | 2.1 |
| 10875 | 0.946128 | 2.07096 |
| 10876 | 0.946131 | 1.56985 |
| 10877 | 0.946131 | 2.02229 |
| 10878 | 0.94613 | 2.06805 |
| 10879 | 1.10185 | 2.1 |
| 10880 | 0.810947 | 0.05 |
| 10881 | 0.810945 | 0.05 |
| 10882 | 1.57348 | 0.05 |
| 10883 | 1.3517 | 0.05 |
| 10884 | 1.08131 | 0.05 |
| 10885 | 1.08132 | 0.05 |
| 10886 | 1.08133 | 0.05 |
| 10887 | 0.810932 | 0.05 |
| 10888 | 0.946119 | 0.221243 |
| 10889 | 0.946116 | 0.122555 |
| 10890 | 0.946117 | 0.148172 |
| 10891 | 0.946121 | 0.05 |
| 10892 | 0.946118 | 0.05 |
| 10893 | 0.94613 | 0.127282 |
| 10894 | 0.946105 | 0.0929815 |
| 10895 | 0.94612 | 0.489421 |
| 10896 | 0.946122 | 0.072297 |
| 10897 | 0.946119 | 0.223873 |
| 10898 | 0.946119 | 0.557674 |
| 10899 | 1.20884 | 0.128588 |
| 10900 | 0.946132 | 0.421681 |
| 10901 | 0.946107 | 0.323232 |

| | | |
|---|---|---|
| 10902 | 2.67849 | 1.04878 |
| 10903 | 1.17709 | 0.999557 |
| 10904 | 0.605418 | 1.33517 |
| 10905 | 0.286984 | 1.05867 |
| 10906 | 0.172654 | 1.10378 |
| 10907 | 0.810946 | 0.386973 |
| 10908 | 0.940878 | 0.320135 |
| 10909 | 0.941235 | 0.199205 |
| 10910 | 1.26696 | 0.178074 |
| 10911 | 1.56837 | 0.314238 |
| 10912 | 1.13737 | 0.133038 |
| 10913 | 1.34115 | 0.39778 |
| 10914 | 1.24889 | 0.0829784 |
| 10915 | 0.946132 | 1.38512 |
| 10916 | 0.946129 | 1.04861 |
| 10917 | 0.946128 | 1.19465 |
| 10918 | 0.94613 | 1.08317 |
| 10919 | 0.94613 | 1.38061 |
| 10920 | 0.946131 | 1.24227 |
| 10921 | 0.94613 | 1.24228 |
| 10922 | 0.946116 | 2.1 |
| 10923 | 0.94612 | 2.1 |
| 10924 | 0.946118 | 2.1 |
| 10925 | 0.946123 | 2.1 |
| 10926 | 0.946118 | 2.1 |
| 10927 | 0.946131 | 2.1 |
| 10928 | 1.23836 | 2.1 |
| 10929 | 0.810946 | 0.05 |
| 10930 | 0.810946 | 0.05 |
| 10931 | 0.810959 | 0.05 |
| 10932 | 2.80677 | 0.05 |
| 10933 | 1.35168 | 0.05 |
| 10934 | 1.08132 | 0.05 |
| 10935 | 1.08132 | 0.05 |
| 10936 | 1.08131 | 0.05 |
| 10937 | 0.810947 | 0.27509 |
| 10938 | 0.810947 | 0.731112 |
| 10939 | 2.41574 | 0.725814 |
| 10940 | 1.21653 | 0.686574 |
| 10941 | 1.08133 | 0.566561 |
| 10942 | 0.946153 | 0.762285 |
| 10943 | 0.979949 | 0.597049 |
| 10944 | 0.709567 | 0.567722 |
| 10945 | 0.94613 | 2.1 |
| 10946 | 0.946131 | 2.1 |
| 10947 | 0.946128 | 2.1 |
| 10948 | 1.34782 | 2.1 |
| 10949 | 0.946128 | 2.1 |
| 10950 | 0.946129 | 2.1 |
| 10951 | 1.34782 | 2.1 |
| 10952 | 0.946132 | 2.06631 |
| 10953 | 0.946132 | 2.1 |
| 10954 | 0.94613 | 2.1 |
| 10955 | 1.16023 | 2.1 |
| 10956 | 0.946131 | 2.1 |
| 10957 | 0.946131 | 2.1 |
| 10958 | 1.16023 | 2.1 |
| 10959 | 0.946131 | 1.04317 |

| | | |
|---|---|---|
| 10960 | 0.946127 | 2.1 |
| 10961 | 0.946127 | 2.1 |
| 10962 | 0.946131 | 1.90978 |
| 10963 | 0.946128 | 2.1 |
| 10964 | 0.94613 | 1.18258 |
| 10965 | 0.946129 | 2.05913 |
| 10966 | 0.946133 | 0.05 |
| 10967 | 0.946132 | 0.05 |
| 10968 | 0.946132 | 0.05 |
| 10969 | 0.946133 | 0.05 |
| 10970 | 1.20045 | 0.05 |
| 10971 | 1.20045 | 0.05 |
| 10972 | 0.946132 | 0.05 |
| 10973 | 0.918871 | 0.05 |
| 10974 | 0.971961 | 0.05 |
| 10975 | 1.37324 | 0.05 |
| 10976 | 1.78255 | 0.05 |
| 10977 | 1.25155 | 0.05 |
| 10978 | 1.42172 | 0.05 |
| 10979 | 1.40554 | 0.05 |
| 10980 | 1.08862 | 0.05 |
| 10981 | 1.03803 | 0.05 |
| 10982 | 0.946132 | 0.05 |
| 10983 | 0.946126 | 0.05 |
| 10984 | 0.943647 | 0.05 |
| 10985 | 0.948611 | 0.05 |
| 10986 | 0.946129 | 0.05 |
| 10987 | 0.946129 | 0.05 |
| 10988 | 1.1699 | 0.167924 |
| 10989 | 0.946132 | 0.0666288 |
| 10990 | 0.946133 | 0.640195 |
| 10991 | 0.946133 | 0.0835274 |
| 10992 | 0.946132 | 0.0547653 |
| 10993 | 0.946133 | 0.147934 |
| 10994 | 0.946132 | 0.406 |
| 10995 | 0.946132 | 2.1 |
| 10996 | 0.946131 | 2.1 |
| 10997 | 0.946131 | 2.1 |
| 10998 | 0.946132 | 2.1 |
| 10999 | 0.946131 | 2.1 |
| 11000 | 0.946131 | 2.1 |
| 11001 | 0.946132 | 2.1 |
| 11002 | 0.946132 | 0.900747 |
| 11003 | 1.38623 | 0.792296 |
| 11004 | 0.946132 | 0.439995 |
| 11005 | 0.946132 | 0.509659 |
| 11006 | 0.946133 | 0.783231 |
| 11007 | 0.946132 | 0.281773 |
| 11008 | 0.946133 | 1.01699 |
| 11009 | 0.946132 | 2.1 |
| 11010 | 1.40098 | 2.1 |
| 11011 | 0.946131 | 2.1 |
| 11012 | 0.946131 | 2.1 |
| 11013 | 0.946132 | 2.1 |
| 11014 | 0.946132 | 2.1 |
| 11015 | 0.946132 | 2.1 |
| 11016 | 1.01777 | 0.734378 |
| 11017 | 0.946132 | 0.487512 |

| | | |
|---|---|---|
| 11018 | 0.946133 | 1.09226 |
| 11019 | 1.68068 | 0.624378 |
| 11020 | 1.68068 | 1.61679 |
| 11021 | 1.41894 | 0.526555 |
| 11022 | 1.20787 | 0.388626 |
| 11023 | 0.946132 | 0.560436 |
| 11024 | 0.946133 | 2.1 |
| 11025 | 0.946133 | 2.1 |
| 11026 | 0.946132 | 2.1 |
| 11027 | 1.40199 | 2.1 |
| 11028 | 0.946133 | 2.1 |
| 11029 | 0.946132 | 2.1 |
| 11030 | 0.946132 | 2.1 |
| 11031 | 0.810946 | 1.25814 |
| 11032 | 0.810947 | 0.847749 |
| 11033 | 2.37827 | 1.69478 |
| 11034 | 1.3517 | 1.20304 |
| 11035 | 1.08132 | 1.21993 |
| 11036 | 1.08132 | 1.3269 |
| 11037 | 1.08133 | 1.18964 |
| 11038 | 0.810934 | 1.10119 |
| 11039 | 0.946124 | 2.1 |
| 11040 | 0.94613 | 2.05265 |
| 11041 | 1.40248 | 1.22818 |
| 11042 | 0.946131 | 1.19833 |
| 11043 | 0.946125 | 0.825374 |
| 11044 | 0.946127 | 1.98814 |
| 11045 | 0.946128 | 0.887424 |
| 11046 | 1.25703 | 1.96134 |
| 11047 | 0.87821 | 1.16691 |
| 11048 | 1.18912 | 1.85454 |
| 11049 | 0.946119 | 1.05799 |
| 11050 | 0.87825 | 1.22235 |
| 11051 | 1.1891 | 1.38644 |
| 11052 | 0.946107 | 2.06246 |
| 11053 | 0.946132 | 2.1 |
| 11054 | 1.28543 | 2.1 |
| 11055 | 0.946132 | 1.97778 |
| 11056 | 0.946132 | 1.78668 |
| 11057 | 0.946131 | 1.07145 |
| 11058 | 0.946132 | 0.942886 |
| 11059 | 0.946132 | 0.943189 |
| 11060 | 0.946115 | 2.1 |
| 11061 | 0.946119 | 2.1 |
| 11062 | 0.946116 | 2.1 |
| 11063 | 0.946123 | 2.1 |
| 11064 | 0.946118 | 2.1 |
| 11065 | 1.41636 | 2.1 |
| 11066 | 0.946129 | 2.1 |
| 11067 | 0.946132 | 0.366124 |
| 11068 | 0.946132 | 2.1 |
| 11069 | 0.946132 | 1.02981 |
| 11070 | 0.946131 | 2.1 |
| 11071 | 0.946131 | 2.1 |
| 11072 | 1.41432 | 2.1 |
| 11073 | 0.946132 | 2.1 |
| 11074 | 0.810947 | 0.05 |
| 11075 | 0.810947 | 0.05 |

| | | |
|---|---|---|
| 11076 | 2.44433 | 0.05 |
| 11077 | 1.21654 | 0.05 |
| 11078 | 0.946154 | 0.05 |
| 11079 | 0.97995 | 0.05 |
| 11080 | 1.08133 | 0.05 |
| 11081 | 0.810934 | 0.05 |
| 11082 | 0.851542 | 0.05 |
| 11083 | 0.835698 | 0.05 |
| 11084 | 1.22216 | 0.05 |
| 11085 | 0.946133 | 0.05 |
| 11086 | 1.01217 | 0.05 |
| 11087 | 1.09996 | 0.05 |
| 11088 | 0.851529 | 0.05 |
| 11089 | 0.946132 | 1.54046 |
| 11090 | 0.946132 | 2.1 |
| 11091 | 0.946129 | 2.1 |
| 11092 | 0.94613 | 1.68442 |
| 11093 | 0.946131 | 2.1 |
| 11094 | 0.946131 | 2.1 |
| 11095 | 1.2254 | 2.1 |
| 11096 | 0.800897 | 1.18852 |
| 11097 | 0.838029 | 2.1 |
| 11098 | 0.838013 | 1.61417 |
| 11099 | 0.93679 | 0.499366 |
| 11100 | 1.20217 | 0.59214 |
| 11101 | 1.16923 | 1.21234 |
| 11102 | 0.94948 | 1.31862 |
| 11103 | 0.958118 | 1.49137 |
| 11104 | 0.36751 | 1.29647 |
| 11105 | 0.800535 | 1.75359 |
| 11106 | 0.272087 | 0.115664 |
| 11107 | 0.428084 | 0.416904 |
| 11108 | 0.986335 | 1.69069 |
| 11109 | 0.946132 | 0.05 |
| 11110 | 0.946133 | 0.05 |
| 11111 | 1.19601 | 0.05 |
| 11112 | 0.946132 | 0.05 |
| 11113 | 0.946132 | 0.05 |
| 11114 | 0.946133 | 0.05 |
| 11115 | 0.946133 | 0.05 |
| 11116 | 0.946132 | 2.09617 |
| 11117 | 1.36956 | 2.1 |
| 11118 | 0.946132 | 2.1 |
| 11119 | 0.946132 | 1.90333 |
| 11120 | 0.946132 | 2.1 |
| 11121 | 0.946133 | 2.1 |
| 11122 | 0.946132 | 2.1 |
| 11123 | 0.946132 | 0.05 |
| 11124 | 0.946131 | 0.567231 |
| 11125 | 0.946131 | 0.05 |
| 11126 | 1.34268 | 0.05 |
| 11127 | 0.946133 | 0.05 |
| 11128 | 0.946132 | 0.05 |
| 11129 | 0.946131 | 0.05 |
| 11130 | 0.946132 | 2.1 |
| 11131 | 0.946131 | 2.1 |
| 11132 | 0.946131 | 2.1 |
| 11133 | 0.946132 | 2.1 |

| | | |
|---|---|---|
| 11134 | 0.946132 | 2.1 |
| 11135 | 0.946132 | 2.1 |
| 11136 | 0.946132 | 2.1 |
| 11137 | 0.810946 | 1.04126 |
| 11138 | 0.810943 | 0.921361 |
| 11139 | 2.51472 | 1.02413 |
| 11140 | 1.3517 | 0.917783 |
| 11141 | 1.08132 | 0.888599 |
| 11142 | 1.08132 | 1.01039 |
| 11143 | 1.08133 | 0.961982 |
| 11144 | 0.810933 | 0.952163 |
| 11145 | 0.902316 | 0.0593731 |
| 11146 | 1.04517 | 0.0616179 |
| 11147 | 0.974924 | 0.05 |
| 11148 | 0.530063 | 0.311865 |
| 11149 | 1.1272 | 0.0950005 |
| 11150 | 0.68207 | 0.0643916 |
| 11151 | 0.477061 | 0.213093 |
| 11152 | 0.810946 | 0.05 |
| 11153 | 0.810943 | 0.05 |
| 11154 | 0.810959 | 0.05 |
| 11155 | 1.99211 | 0.05 |
| 11156 | 1.35168 | 0.05 |
| 11157 | 1.08132 | 0.05 |
| 11158 | 1.08132 | 0.05 |
| 11159 | 1.08131 | 0.05 |
| 11160 | 0.94613 | 0.05 |
| 11161 | 0.946131 | 0.05 |
| 11162 | 0.946132 | 0.05 |
| 11163 | 0.946132 | 0.05 |
| 11164 | 0.946129 | 0.05 |
| 11165 | 0.946131 | 0.05 |
| 11166 | 1.16699 | 0.05 |
| 11167 | 0.810943 | 1.20108 |
| 11168 | 0.810942 | 1.12707 |
| 11169 | 1.6943 | 2.1 |
| 11170 | 1.27059 | 2.1 |
| 11171 | 1.00021 | 2.1 |
| 11172 | 1.02049 | 1.97735 |
| 11173 | 1.08133 | 1.2004 |
| 11174 | 0.750103 | 1.20126 |
| 11175 | 0.957813 | 1.20812 |
| 11176 | 0.946128 | 1.53032 |
| 11177 | 0.946132 | 0.975161 |
| 11178 | 1.04832 | 1.68851 |
| 11179 | 0.870714 | 1.01797 |
| 11180 | 0.94613 | 1.04139 |
| 11181 | 0.94613 | 1.16641 |
| 11182 | 1.86374 | 2.1 |
| 11183 | 2.0815 | 1.1972 |
| 11184 | 1.15918 | 0.773059 |
| 11185 | 0.946129 | 0.740085 |
| 11186 | 1.15918 | 0.534345 |
| 11187 | 0.946131 | 1.0783 |
| 11188 | 0.946131 | 0.520627 |
| 11189 | 0.946131 | 0.733751 |
| 11190 | 0.94613 | 0.791886 |
| 11191 | 0.946119 | 0.901097 |

| | | |
|---|---|---|
| 11192 | 1.3612 | 0.997175 |
| 11193 | 0.946125 | 0.946641 |
| 11194 | 0.946119 | 0.841042 |
| 11195 | 0.94612 | 1.49877 |
| 11196 | 0.946132 | 0.979009 |
| 11197 | 0.946107 | 0.959541 |
| 11198 | 0.810947 | 0.371892 |
| 11199 | 0.810947 | 0.417786 |
| 11200 | 2.50398 | 0.131604 |
| 11201 | 1.3517 | 0.153117 |
| 11202 | 1.08132 | 0.232682 |
| 11203 | 1.08133 | 0.391706 |
| 11204 | 1.08132 | 0.099773 |
| 11205 | 0.810934 | 0.347887 |
| 11206 | 0.946132 | 2.1 |
| 11207 | 0.946132 | 2.1 |
| 11208 | 0.946132 | 2.1 |
| 11209 | 1.39754 | 2.1 |
| 11210 | 0.946132 | 2.1 |
| 11211 | 0.946132 | 2.1 |
| 11212 | 0.946131 | 2.1 |
| 11213 | 0.946129 | 2.1 |
| 11214 | 1.41732 | 2.1 |
| 11215 | 0.946129 | 2.1 |
| 11216 | 0.946131 | 2.1 |
| 11217 | 0.946131 | 2.1 |
| 11218 | 0.946131 | 2.1 |
| 11219 | 0.946131 | 2.1 |
| 11220 | 0.946132 | 1.17349 |
| 11221 | 1.25276 | 2.1 |
| 11222 | 0.946131 | 2.1 |
| 11223 | 0.94613 | 1.16136 |
| 11224 | 0.94613 | 2.09061 |
| 11225 | 0.94613 | 1.27914 |
| 11226 | 0.946131 | 1.54937 |
| 11227 | 1.21261 | 0.15148 |
| 11228 | 0.94613 | 0.59022 |
| 11229 | 0.94613 | 1.73673 |
| 11230 | 0.946128 | 0.053444 |
| 11231 | 0.946131 | 1.38609 |
| 11232 | 0.94613 | 1.01616 |
| 11233 | 0.946129 | 1.43 |
| 11234 | 0.946128 | 2.1 |
| 11235 | 0.946133 | 2.1 |
| 11236 | 0.946132 | 2.1 |
| 11237 | 0.946131 | 2.1 |
| 11238 | 0.94613 | 2.1 |
| 11239 | 0.946131 | 2.1 |
| 11240 | 1.39407 | 2.1 |
| 11241 | 0.946116 | 1.37773 |
| 11242 | 0.946119 | 1.4768 |
| 11243 | 0.946119 | 1.02391 |
| 11244 | 1.15369 | 1.35746 |
| 11245 | 0.946119 | 0.894151 |
| 11246 | 0.946131 | 1.18518 |
| 11247 | 0.946106 | 1.09004 |
| 11248 | 0.946131 | 2.1 |
| 11249 | 0.946131 | 2.1 |

| | | |
|---|---|---|
| 11250 | 0.946131 | 2.1 |
| 11251 | 0.946133 | 2.1 |
| 11252 | 0.946129 | 2.1 |
| 11253 | 0.946131 | 2.1 |
| 11254 | 0.946131 | 2.1 |
| 11255 | 1.19564 | 0.05 |
| 11256 | 0.498881 | 0.05 |
| 11257 | 1.33964 | 0.05 |
| 11258 | 0.498878 | 0.05 |
| 11259 | 0.923583 | 0.05 |
| 11260 | 0.831259 | 0.05 |
| 11261 | 0.553735 | 0.05 |
| 11262 | 0.946132 | 0.05 |
| 11263 | 0.946132 | 0.05 |
| 11264 | 0.946131 | 0.05 |
| 11265 | 0.946131 | 0.05 |
| 11266 | 0.946132 | 0.05 |
| 11267 | 0.946131 | 0.05 |
| 11268 | 1.28673 | 0.05 |
| 11269 | 0.946132 | 0.05 |
| 11270 | 0.946132 | 0.05 |
| 11271 | 1.33178 | 0.05 |
| 11272 | 0.946132 | 0.05 |
| 11273 | 0.946132 | 0.05 |
| 11274 | 0.946131 | 0.05 |
| 11275 | 0.946131 | 0.05 |
| 11276 | 0.946128 | 0.05 |
| 11277 | 0.946132 | 0.05 |
| 11278 | 0.946129 | 0.0622845 |
| 11279 | 0.946131 | 0.05 |
| 11280 | 0.946131 | 0.05 |
| 11281 | 0.946129 | 0.05 |
| 11282 | 0.94613 | 0.05 |
| 11283 | 0.946131 | 2.1 |
| 11284 | 0.946131 | 2.1 |
| 11285 | 0.946129 | 2.1 |
| 11286 | 1.40525 | 2.1 |
| 11287 | 0.94613 | 2.1 |
| 11288 | 0.94613 | 2.1 |
| 11289 | 1.40525 | 2.1 |
| 11290 | 0.946131 | 2.1 |
| 11291 | 0.946126 | 2.1 |
| 11292 | 0.946127 | 2.1 |
| 11293 | 0.946127 | 2.1 |
| 11294 | 1.4073 | 2.1 |
| 11295 | 0.946129 | 2.1 |
| 11296 | 0.946129 | 2.1 |
| 11297 | 0.946131 | 0.05 |
| 11298 | 0.946131 | 0.05 |
| 11299 | 0.946132 | 0.05 |
| 11300 | 0.946131 | 0.05 |
| 11301 | 0.946131 | 0.05 |
| 11302 | 1.05016 | 0.05 |
| 11303 | 0.946132 | 0.05 |
| 11304 | 0.946128 | 0.05 |
| 11305 | 0.946132 | 0.05 |
| 11306 | 0.946131 | 0.05 |
| 11307 | 0.946129 | 0.05 |

| | | |
|---|---|---|
| 11308 | 0.94613 | 0.05 |
| 11309 | 1.13398 | 0.05 |
| 11310 | 0.94613 | 0.05 |
| 11311 | 0.946132 | 0.05 |
| 11312 | 0.946132 | 0.05 |
| 11313 | 0.946131 | 0.05 |
| 11314 | 0.946131 | 0.05 |
| 11315 | 0.946132 | 0.05 |
| 11316 | 0.946131 | 0.05 |
| 11317 | 0.946132 | 0.05 |
| 11318 | 0.946131 | 0.05 |
| 11319 | 0.94613 | 0.05 |
| 11320 | 0.946131 | 0.05 |
| 11321 | 0.94613 | 0.05 |
| 11322 | 1.38645 | 0.05 |
| 11323 | 1.38645 | 0.05 |
| 11324 | 0.946131 | 0.05 |
| 11325 | 0.946131 | 0.05 |
| 11326 | 0.946131 | 0.05 |
| 11327 | 0.94613 | 0.05 |
| 11328 | 1.23923 | 0.05 |
| 11329 | 0.94613 | 0.05 |
| 11330 | 0.946131 | 0.05 |
| 11331 | 0.946131 | 0.05 |
| 11332 | 0.946132 | 0.05 |
| 11333 | 0.946131 | 0.05 |
| 11334 | 0.946131 | 0.05 |
| 11335 | 0.946132 | 0.05 |
| 11336 | 0.946132 | 0.05 |
| 11337 | 0.946132 | 0.05 |
| 11338 | 1.40511 | 0.05 |
| 11339 | 0.946132 | 2.1 |
| 11340 | 0.946132 | 2.1 |
| 11341 | 0.946132 | 2.1 |
| 11342 | 0.946132 | 2.1 |
| 11343 | 0.946132 | 2.1 |
| 11344 | 0.946131 | 2.1 |
| 11345 | 1.12968 | 2.1 |
| 11346 | 0.946132 | 1.623 |
| 11347 | 0.946128 | 2.1 |
| 11348 | 0.946128 | 2.1 |
| 11349 | 0.94613 | 2.1 |
| 11350 | 0.94613 | 2.1 |
| 11351 | 0.94613 | 1.84078 |
| 11352 | 0.94613 | 2.1 |
| 11353 | 0.946133 | 2.1 |
| 11354 | 0.946133 | 2.1 |
| 11355 | 0.946132 | 2.1 |
| 11356 | 0.946132 | 2.1 |
| 11357 | 0.946132 | 2.1 |
| 11358 | 0.946133 | 2.1 |
| 11359 | 1.31323 | 2.1 |
| 11360 | 0.94613 | 2.1 |
| 11361 | 0.946131 | 0.102746 |
| 11362 | 0.946131 | 0.05 |
| 11363 | 0.94613 | 0.05 |
| 11364 | 0.946131 | 2.1 |
| 11365 | 0.946131 | 1.19768 |

| | | |
|---|---|---|
| 11366 | 0.946131 | 0.824228 |
| 11367 | 0.946132 | 0.0536032 |
| 11368 | 0.946132 | 0.095638 |
| 11369 | 0.946132 | 0.05 |
| 11370 | 1.36899 | 0.05 |
| 11371 | 0.946132 | 0.185313 |
| 11372 | 0.946132 | 0.05 |
| 11373 | 0.946133 | 0.313164 |
| 11374 | 1.03543 | 0.05 |
| 11375 | 0.946131 | 0.05 |
| 11376 | 0.946131 | 0.05 |
| 11377 | 0.946132 | 0.05 |
| 11378 | 0.94613 | 0.05 |
| 11379 | 0.950987 | 0.05 |
| 11380 | 0.941275 | 0.05 |
| 11381 | 0.946131 | 0.05 |
| 11382 | 0.94613 | 0.05 |
| 11383 | 1.28952 | 0.05 |
| 11384 | 0.946132 | 0.05 |
| 11385 | 0.94613 | 0.05 |
| 11386 | 0.946131 | 0.05 |
| 11387 | 0.946131 | 0.05 |
| 11388 | 0.94613 | 2.1 |
| 11389 | 1.35966 | 2.1 |
| 11390 | 0.946131 | 2.1 |
| 11391 | 0.946131 | 2.1 |
| 11392 | 0.946129 | 2.1 |
| 11393 | 0.94613 | 2.1 |
| 11394 | 0.94613 | 2.1 |
| 11395 | 0.810946 | 2.1 |
| 11396 | 0.810942 | 0.154846 |
| 11397 | 0.810932 | 2.1 |
| 11398 | 2.6655 | 2.1 |
| 11399 | 1.3517 | 2.1 |
| 11400 | 1.08132 | 2.1 |
| 11401 | 1.08131 | 0.124396 |
| 11402 | 1.08133 | 2.1 |
| 11403 | 0.94613 | 2.1 |
| 11404 | 0.946131 | 2.1 |
| 11405 | 0.94613 | 2.1 |
| 11406 | 0.946128 | 2.1 |
| 11407 | 0.946131 | 2.1 |
| 11408 | 0.94613 | 2.1 |
| 11409 | 0.94613 | 2.1 |
| 11410 | 0.946128 | 2.1 |
| 11411 | 0.946128 | 2.1 |
| 11412 | 0.946133 | 2.1 |
| 11413 | 1.25375 | 2.1 |
| 11414 | 0.94613 | 2.1 |
| 11415 | 0.946131 | 2.1 |
| 11416 | 1.25375 | 2.1 |
| 11417 | 0.94613 | 2.1 |
| 11418 | 0.94613 | 2.1 |
| 11419 | 0.946132 | 2.1 |
| 11420 | 0.946132 | 2.1 |
| 11421 | 0.946128 | 2.1 |
| 11422 | 0.94613 | 2.1 |
| 11423 | 1.31453 | 2.1 |

| | | |
|---|---|---|
| 11424 | 0.946131 | 0.05 |
| 11425 | 0.946129 | 0.05 |
| 11426 | 0.94613 | 0.05 |
| 11427 | 0.94613 | 0.05 |
| 11428 | 0.94613 | 0.05 |
| 11429 | 1.12018 | 0.05 |
| 11430 | 0.94613 | 0.05 |
| 11431 | 0.946131 | 0.05 |
| 11432 | 0.946129 | 0.05 |
| 11433 | 0.946131 | 0.05 |
| 11434 | 1.16373 | 0.05 |
| 11435 | 0.94613 | 0.05 |
| 11436 | 0.94613 | 0.05 |
| 11437 | 0.946131 | 0.05 |
| 11438 | 1.36811 | 0.05 |
| 11439 | 0.946132 | 0.05 |
| 11440 | 1.36811 | 0.05 |
| 11441 | 0.946129 | 0.0520505 |
| 11442 | 0.94613 | 0.0670407 |
| 11443 | 0.946129 | 0.05 |
| 11444 | 0.946129 | 0.05 |
| 11445 | 0.946132 | 0.05 |
| 11446 | 0.946133 | 0.05 |
| 11447 | 0.946132 | 0.05 |
| 11448 | 0.946133 | 0.05 |
| 11449 | 1.35732 | 0.05 |
| 11450 | 1.35732 | 0.05 |
| 11451 | 0.946133 | 0.05 |
| 11452 | 1.36399 | 0.218348 |
| 11453 | 0.946129 | 0.790271 |
| 11454 | 0.94613 | 0.530496 |
| 11455 | 0.946131 | 0.916911 |
| 11456 | 0.94613 | 1.92547 |
| 11457 | 0.946131 | 0.728496 |
| 11458 | 0.946131 | 1.02478 |
| 11459 | 0.946133 | 2.1 |
| 11460 | 0.946132 | 2.1 |
| 11461 | 0.946132 | 2.1 |
| 11462 | 1.38302 | 2.1 |
| 11463 | 0.946133 | 2.1 |
| 11464 | 0.946132 | 2.1 |
| 11465 | 0.946133 | 2.1 |
| 11466 | 0.810943 | 2.1 |
| 11467 | 0.810944 | 2.1 |
| 11468 | 0.810945 | 2.1 |
| 11469 | 2.80542 | 2.1 |
| 11470 | 1.35169 | 2.1 |
| 11471 | 1.08132 | 2.1 |
| 11472 | 1.08132 | 2.1 |
| 11473 | 1.08132 | 2.1 |
| 11474 | 1.30189 | 2.1 |
| 11475 | 0.946131 | 2.1 |
| 11476 | 0.946132 | 2.1 |
| 11477 | 0.946131 | 2.1 |
| 11478 | 0.946131 | 2.1 |
| 11479 | 0.946131 | 2.1 |
| 11480 | 0.946132 | 2.1 |
| 11481 | 0.810946 | 0.900718 |

| | | |
|---|---|---|
| 11482 | 0.810947 | 0.678272 |
| 11483 | 2.33293 | 0.728224 |
| 11484 | 1.24357 | 0.744516 |
| 11485 | 1.08132 | 0.786487 |
| 11486 | 0.973183 | 0.84864 |
| 11487 | 1.00023 | 0.810705 |
| 11488 | 0.810934 | 0.829063 |
| 11489 | 1.06584 | 0.696095 |
| 11490 | 0.946129 | 0.785848 |
| 11491 | 0.84731 | 0.299397 |
| 11492 | 1.16646 | 0.757374 |
| 11493 | 0.946126 | 0.848748 |
| 11494 | 1.2831 | 0.562795 |
| 11495 | 0.851527 | 0.765365 |
| 11496 | 0.946131 | 2.1 |
| 11497 | 0.946128 | 2.1 |
| 11498 | 0.946128 | 2.1 |
| 11499 | 0.94613 | 2.1 |
| 11500 | 1.31961 | 2.1 |
| 11501 | 0.94613 | 2.1 |
| 11502 | 0.94613 | 2.1 |
| 11503 | 0.946131 | 2.1 |
| 11504 | 1.3605 | 2.1 |
| 11505 | 0.946133 | 2.1 |
| 11506 | 0.946132 | 2.1 |
| 11507 | 0.94613 | 2.1 |
| 11508 | 0.946131 | 2.1 |
| 11509 | 0.946131 | 2.1 |
| 11510 | 0.946128 | 2.1 |
| 11511 | 0.946133 | 1.9524 |
| 11512 | 0.946132 | 2.1 |
| 11513 | 0.946131 | 1.2847 |
| 11514 | 0.946131 | 0.05 |
| 11515 | 0.946131 | 2.1 |
| 11516 | 1.30116 | 2.1 |
| 11517 | 0.946132 | 0.05 |
| 11518 | 0.946129 | 0.05 |
| 11519 | 0.94613 | 0.05 |
| 11520 | 1.3035 | 0.05 |
| 11521 | 0.946131 | 0.05 |
| 11522 | 0.94613 | 0.05 |
| 11523 | 0.946131 | 0.05 |
| 11524 | 0.946132 | 2.1 |
| 11525 | 1.2051 | 2.1 |
| 11526 | 1.2051 | 2.1 |
| 11527 | 0.946132 | 2.1 |
| 11528 | 1.37869 | 2.1 |
| 11529 | 0.946132 | 2.1 |
| 11530 | 0.946131 | 2.1 |
| 11531 | 0.810946 | 2.1 |
| 11532 | 0.810945 | 2.1 |
| 11533 | 2.69247 | 2.1 |
| 11534 | 1.35169 | 2.1 |
| 11535 | 1.08132 | 2.1 |
| 11536 | 1.08132 | 2.1 |
| 11537 | 1.08132 | 2.1 |
| 11538 | 0.810946 | 2.1 |
| 11539 | 1.22993 | 2.1 |

| | | |
|---|---|---|
| 11540 | 0.946131 | 2.1 |
| 11541 | 0.946131 | 2.1 |
| 11542 | 1.34623 | 2.1 |
| 11543 | 0.946131 | 2.1 |
| 11544 | 1.34623 | 2.1 |
| 11545 | 0.946131 | 2.1 |
| 11546 | 0.946131 | 0.05 |
| 11547 | 0.946132 | 0.05 |
| 11548 | 0.946132 | 0.05 |
| 11549 | 0.946132 | 0.05 |
| 11550 | 0.946131 | 0.05 |
| 11551 | 0.946132 | 0.05 |
| 11552 | 1.32299 | 0.05 |
| 11553 | 0.94613 | 0.05 |
| 11554 | 0.946131 | 0.05 |
| 11555 | 0.946128 | 0.05 |
| 11556 | 0.946132 | 0.05 |
| 11557 | 0.946133 | 0.05 |
| 11558 | 0.946131 | 0.05 |
| 11559 | 0.946131 | 0.05 |
| 11560 | 0.946132 | 2.1 |
| 11561 | 0.946129 | 2.1 |
| 11562 | 0.946128 | 2.1 |
| 11563 | 0.94613 | 2.1 |
| 11564 | 0.94613 | 2.1 |
| 11565 | 0.946131 | 2.1 |
| 11566 | 0.94613 | 2.1 |
| 11567 | 0.94613 | 2.1 |
| 11568 | 0.94613 | 2.1 |
| 11569 | 0.946131 | 2.1 |
| 11570 | 0.946132 | 2.1 |
| 11571 | 0.946129 | 2.1 |
| 11572 | 0.946131 | 2.1 |
| 11573 | 0.946131 | 2.1 |
| 11574 | 0.946132 | 1.4165 |
| 11575 | 0.946131 | 1.45914 |
| 11576 | 1.13731 | 0.825559 |
| 11577 | 0.94775 | 1.58399 |
| 11578 | 0.944514 | 2.01006 |
| 11579 | 0.946132 | 2.07767 |
| 11580 | 1.039 | 2.03758 |
| 11581 | 0.946131 | 1.35593 |
| 11582 | 0.946128 | 1.22547 |
| 11583 | 0.94613 | 0.623172 |
| 11584 | 0.946131 | 1.31867 |
| 11585 | 0.946128 | 1.575 |
| 11586 | 0.946129 | 1.87959 |
| 11587 | 1.23726 | 1.76532 |
| 11588 | 0.946132 | 2.1 |
| 11589 | 0.946132 | 2.1 |
| 11590 | 0.946129 | 2.1 |
| 11591 | 1.39214 | 2.1 |
| 11592 | 0.946131 | 2.1 |
| 11593 | 0.946131 | 2.1 |
| 11594 | 0.94613 | 2.1 |
| 11595 | 0.946125 | 0.05 |
| 11596 | 0.946119 | 0.05 |
| 11597 | 0.946118 | 0.05 |

| | | |
|---|---|---|
| 11598 | 0.946118 | 0.05 |
| 11599 | 0.94612 | 0.05 |
| 11600 | 0.946106 | 0.05 |
| 11601 | 0.946131 | 0.05 |
| 11602 | 1.26893 | 0.659963 |
| 11603 | 0.946131 | 0.642837 |
| 11604 | 1.26893 | 0.740248 |
| 11605 | 0.94613 | 0.712592 |
| 11606 | 0.946131 | 0.776887 |
| 11607 | 0.946131 | 0.621399 |
| 11608 | 0.946131 | 0.93062 |
| 11609 | 0.946133 | 0.05 |
| 11610 | 0.946133 | 0.05 |
| 11611 | 0.946132 | 0.05 |
| 11612 | 1.28924 | 0.05 |
| 11613 | 0.946133 | 0.05 |
| 11614 | 0.946132 | 0.05 |
| 11615 | 0.946132 | 0.05 |
| 11616 | 0.946133 | 0.0714665 |
| 11617 | 0.946132 | 0.05 |
| 11618 | 0.946132 | 0.0526859 |
| 11619 | 0.946133 | 0.05 |
| 11620 | 0.946133 | 0.0991033 |
| 11621 | 0.946132 | 0.0709803 |
| 11622 | 0.946133 | 0.05 |
| 11623 | 0.946132 | 0.651503 |
| 11624 | 0.946133 | 0.05 |
| 11625 | 0.946133 | 0.132013 |
| 11626 | 0.946132 | 0.497948 |
| 11627 | 1.15388 | 0.218123 |
| 11628 | 0.946132 | 0.521539 |
| 11629 | 0.946133 | 0.29891 |
| 11630 | 0.946132 | 0.155122 |
| 11631 | 0.946133 | 0.05 |
| 11632 | 0.946132 | 0.0868327 |
| 11633 | 0.946133 | 0.05 |
| 11634 | 0.946133 | 0.106675 |
| 11635 | 0.946132 | 0.106606 |
| 11636 | 0.946132 | 0.0555172 |
| 11637 | 1.0644 | 1.01213 |
| 11638 | 1.12486 | 1.78086 |
| 11639 | 1.08674 | 1.92211 |
| 11640 | 0.946132 | 1.25157 |
| 11641 | 0.946132 | 1.91199 |
| 11642 | 0.946133 | 1.48409 |
| 11643 | 0.946132 | 1.397 |
| 11644 | 0.808978 | 1.16229 |
| 11645 | 0.911537 | 1.60168 |
| 11646 | 0.932832 | 1.39593 |
| 11647 | 1.14604 | 1.87523 |
| 11648 | 1.56953 | 1.52108 |
| 11649 | 1.25866 | 1.27785 |
| 11650 | 1.18488 | 1.73989 |
| 11651 | 1.341 | 1.18246 |
| 11652 | 0.946131 | 0.05 |
| 11653 | 0.946131 | 0.05 |
| 11654 | 0.946131 | 0.05 |
| 11655 | 0.94613 | 0.05 |

| | | |
|---|---|---|
| 11656 | 1.33922 | 0.05 |
| 11657 | 0.946131 | 0.05 |
| 11658 | 0.946131 | 0.05 |
| 11659 | 0.946132 | 0.05 |
| 11660 | 0.946133 | 0.05 |
| 11661 | 0.946132 | 0.05 |
| 11662 | 0.946132 | 0.05 |
| 11663 | 0.946132 | 0.05 |
| 11664 | 0.946133 | 0.05 |
| 11665 | 0.946132 | 0.05 |
| 11666 | 0.946129 | 0.758875 |
| 11667 | 0.946133 | 2.1 |
| 11668 | 0.946133 | 0.972712 |
| 11669 | 0.946131 | 1.19415 |
| 11670 | 1.40496 | 2.1 |
| 11671 | 0.946131 | 0.851316 |
| 11672 | 0.946131 | 2.1 |
| 11673 | 0.94613 | 0.983238 |
| 11674 | 0.946127 | 0.197697 |
| 11675 | 0.946127 | 0.82809 |
| 11676 | 0.94613 | 1.04656 |
| 11677 | 1.38913 | 0.718815 |
| 11678 | 0.946128 | 0.909898 |
| 11679 | 0.946128 | 0.773633 |
| 11680 | 1.98863 | 0.05 |
| 11681 | 2.59915 | 0.05 |
| 11682 | 1.40338 | 0.05 |
| 11683 | 0.946131 | 0.05 |
| 11684 | 1.22266 | 0.05 |
| 11685 | 1.40338 | 0.05 |
| 11686 | 0.946132 | 0.05 |
| 11687 | 0.946132 | 0.05 |
| 11688 | 1.22266 | 0.05 |
| 11689 | 0.94613 | 0.05 |
| 11690 | 0.94613 | 0.05 |
| 11691 | 0.946124 | 0.05 |
| 11692 | 1.21428 | 0.05 |
| 11693 | 0.946126 | 0.05 |
| 11694 | 0.946127 | 0.05 |
| 11695 | 0.946127 | 0.05 |
| 11696 | 1.03411 | 0.05 |
| 11697 | 0.946131 | 0.05 |
| 11698 | 1.03411 | 0.05 |
| 11699 | 0.946129 | 0.05 |
| 11700 | 1.14258 | 0.05 |
| 11701 | 0.952143 | 0.05 |
| 11702 | 0.940118 | 0.05 |
| 11703 | 0.810943 | 2.1 |
| 11704 | 0.810945 | 2.1 |
| 11705 | 0.810958 | 2.1 |
| 11706 | 2.82603 | 2.1 |
| 11707 | 1.35168 | 2.1 |
| 11708 | 1.08132 | 2.1 |
| 11709 | 1.08132 | 2.1 |
| 11710 | 1.0813 | 2.1 |
| 11711 | 0.946133 | 0.05 |
| 11712 | 0.946132 | 0.05 |
| 11713 | 0.946132 | 0.0517435 |

| | | |
|---|---|---|
| 11714 | 0.946132 | 0.05 |
| 11715 | 0.946132 | 0.05 |
| 11716 | 1.11539 | 0.05 |
| 11717 | 0.946133 | 0.05 |
| 11718 | 0.946133 | 2.1 |
| 11719 | 0.946132 | 2.1 |
| 11720 | 0.946133 | 2.1 |
| 11721 | 0.946133 | 2.1 |
| 11722 | 0.946132 | 2.1 |
| 11723 | 1.41195 | 2.1 |
| 11724 | 0.946133 | 2.1 |
| 11725 | 0.946131 | 0.05 |
| 11726 | 0.946131 | 0.05 |
| 11727 | 0.946131 | 0.05 |
| 11728 | 0.946131 | 0.05 |
| 11729 | 0.946131 | 0.05 |
| 11730 | 0.946131 | 0.05 |
| 11731 | 1.15968 | 0.05 |
| 11732 | 0.946131 | 0.665535 |
| 11733 | 0.94613 | 1.383 |
| 11734 | 0.94613 | 2.1 |
| 11735 | 1.34386 | 1.48187 |
| 11736 | 0.946132 | 2.1 |
| 11737 | 0.94613 | 2.1 |
| 11738 | 1.34386 | 2.036 |
| 11739 | 0.946131 | 0.538145 |
| 11740 | 0.935202 | 0.258635 |
| 11741 | 0.946131 | 2.06828 |
| 11742 | 1.25054 | 0.778553 |
| 11743 | 0.850155 | 1.95266 |
| 11744 | 0.946131 | 1.77016 |
| 11745 | 0.935205 | 1.53714 |
| 11746 | 0.946131 | 0.05 |
| 11747 | 1.40193 | 0.05 |
| 11748 | 0.946132 | 0.05 |
| 11749 | 0.946132 | 0.05 |
| 11750 | 0.946129 | 0.05 |
| 11751 | 1.40193 | 0.05 |
| 11752 | 0.946131 | 0.05 |
| 11753 | 0.808779 | 0.05 |
| 11754 | 0.561937 | 0.05 |
| 11755 | 1.40193 | 0.05 |
| 11756 | 1.40193 | 0.05 |
| 11757 | 0.561936 | 0.05 |
| 11758 | 0.561937 | 0.05 |
| 11759 | 1.40193 | 0.05 |
| 11760 | 0.946132 | 0.05 |
| 11761 | 0.946132 | 0.05 |
| 11762 | 0.946132 | 0.05 |
| 11763 | 0.946132 | 0.05 |
| 11764 | 0.946133 | 0.05 |
| 11765 | 0.946132 | 0.05 |
| 11766 | 0.946133 | 0.05 |
| 11767 | 0.946127 | 0.281644 |
| 11768 | 0.946131 | 0.310716 |
| 11769 | 0.946131 | 0.0550576 |
| 11770 | 0.946127 | 0.194846 |
| 11771 | 1.19583 | 0.0732548 |

| | | |
|---|---|---|
| 11772 | 1.19583 | 0.0514987 |
| 11773 | 0.946129 | 0.125479 |
| 11774 | 0.589428 | 0.406572 |
| 11775 | 1.08737 | 0.116031 |
| 11776 | 1.10961 | 0.570046 |
| 11777 | 0.872901 | 0.519363 |
| 11778 | 0.541327 | 0.43146 |
| 11779 | 0.541312 | 0.124733 |
| 11780 | 0.85153 | 0.55905 |
| 11781 | 0.946132 | 1.73941 |
| 11782 | 0.946131 | 2.1 |
| 11783 | 0.946131 | 2.1 |
| 11784 | 0.946132 | 2.1 |
| 11785 | 0.946132 | 2.1 |
| 11786 | 0.946132 | 2.1 |
| 11787 | 0.946132 | 2.1 |
| 11788 | 0.946132 | 1.18083 |
| 11789 | 1.2778 | 2.1 |
| 11790 | 0.946131 | 2.1 |
| 11791 | 0.946131 | 1.25592 |
| 11792 | 0.94613 | 2.1 |
| 11793 | 0.94613 | 1.82545 |
| 11794 | 0.94613 | 1.86486 |
| 11795 | 0.946118 | 0.810531 |
| 11796 | 0.946112 | 0.889029 |
| 11797 | 0.946118 | 0.749354 |
| 11798 | 0.946119 | 0.746968 |
| 11799 | 0.946117 | 0.819628 |
| 11800 | 1.35631 | 0.649496 |
| 11801 | 0.946105 | 0.652538 |
| 11802 | 0.946133 | 0.853976 |
| 11803 | 0.946133 | 2.1 |
| 11804 | 0.946132 | 1.73013 |
| 11805 | 0.946132 | 2.1 |
| 11806 | 0.946133 | 0.05 |
| 11807 | 0.946132 | 0.338129 |
| 11808 | 0.946132 | 0.174811 |
| 11809 | 0.94613 | 2.1 |
| 11810 | 0.946131 | 0.0850025 |
| 11811 | 0.946128 | 0.0784409 |
| 11812 | 0.946128 | 0.05 |
| 11813 | 0.94613 | 2.1 |
| 11814 | 0.946129 | 1.51814 |
| 11815 | 0.946129 | 0.518333 |
| 11816 | 0.946132 | 1.2576 |
| 11817 | 1.15139 | 1.19928 |
| 11818 | 0.946132 | 0.632213 |
| 11819 | 0.946132 | 1.35501 |
| 11820 | 0.946132 | 1.46236 |
| 11821 | 0.946132 | 1.51161 |
| 11822 | 0.946132 | 1.29783 |
| 11823 | 0.946133 | 2.1 |
| 11824 | 1.36151 | 2.1 |
| 11825 | 0.946132 | 2.1 |
| 11826 | 0.946132 | 2.1 |
| 11827 | 0.946132 | 2.1 |
| 11828 | 0.946132 | 2.1 |
| 11829 | 0.946133 | 2.1 |

| | | |
|---|---|---|
| 11830 | 0.946127 | 2.1 |
| 11831 | 1.38397 | 2.1 |
| 11832 | 0.94613 | 2.1 |
| 11833 | 0.946131 | 2.1 |
| 11834 | 0.946128 | 2.1 |
| 11835 | 0.94613 | 2.1 |
| 11836 | 0.946129 | 2.1 |
| 11837 | 0.946131 | 0.0503905 |
| 11838 | 0.946127 | 0.237422 |
| 11839 | 0.946127 | 0.0955963 |
| 11840 | 0.946132 | 0.187916 |
| 11841 | 0.946131 | 0.094814 |
| 11842 | 0.946128 | 0.05 |
| 11843 | 0.946129 | 0.119459 |
| 11844 | 1.0268 | 0.05 |
| 11845 | 1.23823 | 0.05 |
| 11846 | 1.2171 | 0.05 |
| 11847 | 0.883446 | 0.05 |
| 11848 | 1.23827 | 0.05 |
| 11849 | 1.32614 | 0.05 |
| 11850 | 0.94613 | 0.05 |
| 11851 | 0.946132 | 2.1 |
| 11852 | 0.946129 | 2.1 |
| 11853 | 0.946129 | 2.1 |
| 11854 | 0.94613 | 2.1 |
| 11855 | 1.36523 | 2.1 |
| 11856 | 0.946131 | 2.1 |
| 11857 | 0.94613 | 2.1 |
| 11858 | 0.946132 | 0.05 |
| 11859 | 0.946131 | 0.05 |
| 11860 | 0.946132 | 0.05 |
| 11861 | 0.946131 | 0.05 |
| 11862 | 0.946131 | 0.05 |
| 11863 | 1.01653 | 0.05 |
| 11864 | 0.946131 | 0.05 |
| 11865 | 0.94612 | 2.1 |
| 11866 | 0.946121 | 2.1 |
| 11867 | 0.946119 | 2.1 |
| 11868 | 0.946119 | 2.1 |
| 11869 | 0.946119 | 2.1 |
| 11870 | 0.946132 | 2.1 |
| 11871 | 0.946107 | 2.1 |
| 11872 | 0.810942 | 0.861775 |
| 11873 | 0.810944 | 0.333857 |
| 11874 | 2.60324 | 1.93775 |
| 11875 | 1.21651 | 1.79613 |
| 11876 | 1.0813 | 1.14672 |
| 11877 | 0.946153 | 0.167353 |
| 11878 | 0.979948 | 2.1 |
| 11879 | 0.709588 | 1.76756 |
| 11880 | 1.3492 | 0.05 |
| 11881 | 0.946132 | 0.05 |
| 11882 | 0.946131 | 0.05 |
| 11883 | 0.94613 | 0.05 |
| 11884 | 0.946129 | 0.05 |
| 11885 | 0.946129 | 0.05 |
| 11886 | 0.94613 | 0.05 |
| 11887 | 0.946131 | 0.05 |

| | | |
|---|---|---|
| 11888 | 0.94613 | 0.0674778 |
| 11889 | 0.946128 | 0.05 |
| 11890 | 0.946133 | 0.05 |
| 11891 | 0.946133 | 0.05 |
| 11892 | 0.946131 | 0.05 |
| 11893 | 0.946131 | 0.120272 |
| 11894 | 1.23351 | 0.05 |
| 11895 | 0.946131 | 0.05 |
| 11896 | 0.946131 | 0.05 |
| 11897 | 0.946132 | 0.05 |
| 11898 | 0.946132 | 0.05 |
| 11899 | 0.946131 | 0.05 |
| 11900 | 0.946132 | 0.05 |
| 11901 | 1.35657 | 0.05 |
| 11902 | 0.946132 | 0.05 |
| 11903 | 0.946131 | 0.05 |
| 11904 | 0.946132 | 0.05 |
| 11905 | 0.946132 | 0.05 |
| 11906 | 0.946131 | 0.05 |
| 11907 | 0.946131 | 0.05 |
| 11908 | 0.946132 | 0.0529195 |
| 11909 | 0.946132 | 0.05 |
| 11910 | 0.946132 | 0.0803233 |
| 11911 | 0.946131 | 0.05 |
| 11912 | 0.946131 | 0.05 |
| 11913 | 0.946132 | 0.05 |
| 11914 | 0.946132 | 0.05 |
| 11915 | 0.946133 | 0.05 |
| 11916 | 0.946132 | 0.05 |
| 11917 | 0.946133 | 0.075462 |
| 11918 | 0.946132 | 0.05 |
| 11919 | 0.946132 | 0.05 |
| 11920 | 0.946132 | 0.05 |
| 11921 | 0.946133 | 0.05 |
| 11922 | 0.946131 | 1.13322 |
| 11923 | 1.4101 | 2.1 |
| 11924 | 0.946131 | 2.1 |
| 11925 | 0.946131 | 0.816824 |
| 11926 | 0.946131 | 1.30964 |
| 11927 | 0.946131 | 0.78962 |
| 11928 | 0.946131 | 2.1 |
| 11929 | 0.810944 | 1.37975 |
| 11930 | 0.810939 | 1.32091 |
| 11931 | 0.709586 | 0.711319 |
| 11932 | 2.71261 | 1.03506 |
| 11933 | 1.21651 | 1.16506 |
| 11934 | 0.946151 | 0.455784 |
| 11935 | 0.979943 | 1.19552 |
| 11936 | 1.0813 | 1.5716 |
| 11937 | 0.946132 | 2.1 |
| 11938 | 0.946131 | 2.1 |
| 11939 | 0.946132 | 2.1 |
| 11940 | 0.946131 | 2.1 |
| 11941 | 0.946131 | 2.1 |
| 11942 | 1.37733 | 2.1 |
| 11943 | 0.946131 | 2.1 |
| 11944 | 0.946133 | 2.1 |
| 11945 | 0.946132 | 1.69991 |

| | | |
|---|---|---|
| 11946 | 0.946132 | 2.1 |
| 11947 | 0.946133 | 2.1 |
| 11948 | 0.946133 | 2.1 |
| 11949 | 0.946132 | 2.1 |
| 11950 | 1.32582 | 2.1 |
| 11951 | 0.946132 | 2.1 |
| 11952 | 0.946132 | 2.1 |
| 11953 | 0.946132 | 2.1 |
| 11954 | 0.946132 | 2.1 |
| 11955 | 0.946132 | 2.1 |
| 11956 | 0.946132 | 2.1 |
| 11957 | 0.946131 | 2.1 |
| 11958 | 0.946133 | 2.1 |
| 11959 | 0.946132 | 1.35923 |
| 11960 | 0.946132 | 1.95492 |
| 11961 | 0.946132 | 1.25548 |
| 11962 | 0.946132 | 1.2136 |
| 11963 | 0.946133 | 1.26695 |
| 11964 | 1.3469 | 1.30509 |
| 11965 | 0.946126 | 0.784648 |
| 11966 | 0.94613 | 2.1 |
| 11967 | 0.946131 | 2.1 |
| 11968 | 0.946127 | 1.94052 |
| 11969 | 1.41206 | 2.1 |
| 11970 | 0.946128 | 2.1 |
| 11971 | 0.946128 | 2.1 |
| 11972 | 0.946127 | 2.1 |
| 11973 | 0.94613 | 2.1 |
| 11974 | 0.946131 | 0.273432 |
| 11975 | 0.94613 | 2.1 |
| 11976 | 0.946128 | 0.379075 |
| 11977 | 0.946129 | 2.1 |
| 11978 | 0.946129 | 2.1 |
| 11979 | 0.946131 | 0.05 |
| 11980 | 0.94613 | 0.05 |
| 11981 | 1.24448 | 0.05 |
| 11982 | 0.946129 | 0.05 |
| 11983 | 0.946131 | 0.05 |
| 11984 | 0.94613 | 0.05 |
| 11985 | 0.946131 | 0.05 |
| 11986 | 0.946132 | 0.05 |
| 11987 | 0.946133 | 0.05 |
| 11988 | 1.11015 | 0.05 |
| 11989 | 0.946133 | 0.05 |
| 11990 | 0.946132 | 0.05 |
| 11991 | 0.946132 | 0.05 |
| 11992 | 0.946132 | 0.05 |
| 11993 | 1.38775 | 2.1 |
| 11994 | 0.946131 | 2.1 |
| 11995 | 0.946131 | 2.1 |
| 11996 | 0.946132 | 2.1 |
| 11997 | 0.946132 | 2.1 |
| 11998 | 0.946131 | 2.1 |
| 11999 | 0.946131 | 2.1 |
| 12000 | 0.946129 | 2.03545 |
| 12001 | 0.946129 | 2.1 |
| 12002 | 0.946133 | 0.718774 |
| 12003 | 0.946131 | 2.1 |

| | | |
|---|---|---|
| 12004 | 0.946131 | 0.05 |
| 12005 | 0.946131 | 0.708658 |
| 12006 | 0.946131 | 0.330323 |
| 12007 | 0.946132 | 2.1 |
| 12008 | 0.946133 | 2.1 |
| 12009 | 0.946132 | 2.1 |
| 12010 | 1.40774 | 2.1 |
| 12011 | 0.946132 | 2.1 |
| 12012 | 0.946133 | 2.1 |
| 12013 | 0.946132 | 2.1 |
| 12014 | 0.610968 | 0.05 |
| 12015 | 0.688233 | 0.05 |
| 12016 | 0.715226 | 0.05 |
| 12017 | 0.792409 | 0.05 |
| 12018 | 1.13418 | 0.05 |
| 12019 | 0.956667 | 0.05 |
| 12020 | 0.757321 | 0.05 |
| 12021 | 0.968845 | 0.05 |
| 12022 | 0.595679 | 0.05 |
| 12023 | 0.946133 | 1.53675 |
| 12024 | 1.28917 | 1.68676 |
| 12025 | 0.946131 | 2.1 |
| 12026 | 0.94613 | 1.02404 |
| 12027 | 0.946131 | 1.39639 |
| 12028 | 0.946131 | 1.3056 |
| 12029 | 0.946131 | 1.97204 |
| 12030 | 0.946132 | 0.426802 |
| 12031 | 0.946128 | 0.447781 |
| 12032 | 0.94613 | 0.562317 |
| 12033 | 1.40668 | 0.458842 |
| 12034 | 0.946131 | 0.514831 |
| 12035 | 0.946131 | 0.463245 |
| 12036 | 0.94613 | 0.477551 |
| 12037 | 0.810946 | 2.1 |
| 12038 | 0.810945 | 2.1 |
| 12039 | 2.78163 | 2.1 |
| 12040 | 1.3517 | 2.1 |
| 12041 | 1.08132 | 2.1 |
| 12042 | 1.08133 | 2.1 |
| 12043 | 1.08132 | 2.1 |
| 12044 | 0.810933 | 2.1 |
| 12045 | 0.946131 | 2.1 |
| 12046 | 1.39081 | 2.1 |
| 12047 | 0.94613 | 2.1 |
| 12048 | 1.30339 | 2.1 |
| 12049 | 1.30339 | 2.1 |
| 12050 | 1.39081 | 2.1 |
| 12051 | 0.946131 | 2.1 |
| 12052 | 0.946127 | 1.38368 |
| 12053 | 0.821631 | 1.86977 |
| 12054 | 0.946131 | 0.91463 |
| 12055 | 0.946131 | 1.05165 |
| 12056 | 0.946128 | 0.975797 |
| 12057 | 0.996109 | 0.855686 |
| 12058 | 0.827876 | 0.836528 |
| 12059 | 0.946131 | 2.1 |
| 12060 | 0.946129 | 2.1 |
| 12061 | 0.946129 | 2.1 |

| | | |
|---|---|---|
| 12062 | 0.94613 | 2.1 |
| 12063 | 0.94613 | 2.1 |
| 12064 | 0.94613 | 2.1 |
| 12065 | 0.94613 | 2.1 |
| 12066 | 0.946132 | 0.05 |
| 12067 | 1.41723 | 0.05 |
| 12068 | 0.946132 | 0.05 |
| 12069 | 0.946133 | 0.05 |
| 12070 | 0.946132 | 0.133327 |
| 12071 | 0.946132 | 0.05 |
| 12072 | 0.946133 | 0.05 |
| 12073 | 0.946133 | 0.05 |
| 12074 | 0.946132 | 0.05 |
| 12075 | 0.946133 | 0.05 |
| 12076 | 0.946133 | 0.05 |
| 12077 | 0.946132 | 0.05 |
| 12078 | 0.946132 | 0.05 |
| 12079 | 1.34254 | 0.05 |
| 12080 | 1.26501 | 1.01812 |
| 12081 | 0.946132 | 1.16423 |
| 12082 | 0.946133 | 1.17898 |
| 12083 | 0.946133 | 1.67206 |
| 12084 | 0.946132 | 1.52102 |
| 12085 | 0.946133 | 1.18069 |
| 12086 | 0.946132 | 1.99984 |
| 12087 | 0.946131 | 0.05 |
| 12088 | 0.946131 | 0.05 |
| 12089 | 0.946131 | 0.05 |
| 12090 | 0.946131 | 0.05 |
| 12091 | 0.946131 | 0.05 |
| 12092 | 0.946131 | 0.05 |
| 12093 | 0.946131 | 0.05 |
| 12094 | 0.878843 | 0.05 |
| 12095 | 0.82561 | 0.05 |
| 12096 | 0.586974 | 0.05 |
| 12097 | 1.05343 | 0.05 |
| 12098 | 0.80631 | 0.05 |
| 12099 | 0.782663 | 0.05 |
| 12100 | 0.789405 | 0.05 |
| 12101 | 0.902491 | 0.05 |
| 12102 | 1.05344 | 0.05 |
| 12103 | 0.94613 | 0.05 |
| 12104 | 0.946131 | 0.05 |
| 12105 | 0.946131 | 0.05 |
| 12106 | 0.94613 | 0.05 |
| 12107 | 0.946131 | 0.05 |
| 12108 | 0.946131 | 0.05 |
| 12109 | 0.946131 | 0.05 |
| 12110 | 0.946128 | 2.1 |
| 12111 | 0.946133 | 2.1 |
| 12112 | 0.946133 | 0.479642 |
| 12113 | 0.94613 | 2.1 |
| 12114 | 0.946131 | 1.07476 |
| 12115 | 0.946131 | 1.36376 |
| 12116 | 0.946131 | 1.47298 |
| 12117 | 0.946131 | 0.10338 |
| 12118 | 0.946131 | 0.100679 |
| 12119 | 0.94613 | 0.100106 |

| | | |
|---|---|---|
| 12120 | 0.946128 | 0.0909075 |
| 12121 | 0.946133 | 0.117374 |
| 12122 | 1.2996 | 0.0530086 |
| 12123 | 0.946131 | 0.0856077 |
| 12124 | 0.946132 | 2.1 |
| 12125 | 0.946132 | 2.1 |
| 12126 | 0.946132 | 2.1 |
| 12127 | 0.946132 | 2.1 |
| 12128 | 0.946131 | 2.1 |
| 12129 | 0.946132 | 2.1 |
| 12130 | 0.946132 | 2.1 |
| 12131 | 1.99211 | 0.05 |
| 12132 | 2.55024 | 0.05 |
| 12133 | 0.946131 | 0.05 |
| 12134 | 0.946131 | 0.05 |
| 12135 | 0.946131 | 0.05 |
| 12136 | 0.946131 | 0.05 |
| 12137 | 0.946131 | 0.05 |
| 12138 | 0.946131 | 0.05 |
| 12139 | 1.35726 | 0.05 |
| 12140 | 0.946128 | 2.1 |
| 12141 | 0.946132 | 2.05792 |
| 12142 | 0.946131 | 2.1 |
| 12143 | 0.94613 | 2.1 |
| 12144 | 0.94613 | 2.1 |
| 12145 | 0.94613 | 2.1 |
| 12146 | 1.39852 | 2.1 |
| 12147 | 1.31666 | 0.05 |
| 12148 | 0.946131 | 0.05 |
| 12149 | 0.946128 | 0.05 |
| 12150 | 0.94613 | 0.05 |
| 12151 | 0.946128 | 0.05 |
| 12152 | 0.946128 | 0.05 |
| 12153 | 0.946128 | 0.05 |
| 12154 | 1.32275 | 0.05 |
| 12155 | 0.946131 | 0.05 |
| 12156 | 0.946129 | 0.05 |
| 12157 | 0.946128 | 0.05 |
| 12158 | 0.946129 | 0.05 |
| 12159 | 0.946128 | 0.05 |
| 12160 | 0.946129 | 0.05 |
| 12161 | 0.946132 | 2.1 |
| 12162 | 0.946131 | 2.1 |
| 12163 | 0.946131 | 2.1 |
| 12164 | 0.946132 | 2.1 |
| 12165 | 0.94613 | 2.1 |
| 12166 | 0.946131 | 2.1 |
| 12167 | 1.41125 | 2.1 |
| 12168 | 0.946132 | 2.1 |
| 12169 | 0.946132 | 2.1 |
| 12170 | 0.946131 | 2.1 |
| 12171 | 0.946132 | 2.1 |
| 12172 | 0.946132 | 2.1 |
| 12173 | 0.946132 | 2.1 |
| 12174 | 1.31904 | 2.1 |
| 12175 | 1.02425 | 0.05 |
| 12176 | 0.946132 | 0.05 |
| 12177 | 0.94613 | 0.05 |

| | | |
|---|---|---|
| 12178 | 0.946131 | 0.05 |
| 12179 | 1.1534 | 0.05 |
| 12180 | 0.961161 | 0.05 |
| 12181 | 0.9311 | 0.05 |
| 12182 | 1.40092 | 1.19007 |
| 12183 | 0.946133 | 1.22124 |
| 12184 | 0.946132 | 1.27895 |
| 12185 | 0.946131 | 1.67508 |
| 12186 | 0.946131 | 1.13171 |
| 12187 | 0.94613 | 1.28381 |
| 12188 | 0.946131 | 1.8693 |
| 12189 | 0.946113 | 2.1 |
| 12190 | 0.946119 | 0.05 |
| 12191 | 0.946117 | 2.1 |
| 12192 | 1.40015 | 2.1 |
| 12193 | 0.946118 | 0.05 |
| 12194 | 0.946131 | 2.04648 |
| 12195 | 0.946105 | 0.280042 |
| 12196 | 0.946132 | 0.436922 |
| 12197 | 0.946133 | 0.44326 |
| 12198 | 0.946132 | 0.620258 |
| 12199 | 1.03547 | 0.464584 |
| 12200 | 0.946133 | 0.327043 |
| 12201 | 0.946133 | 0.499439 |
| 12202 | 0.946132 | 0.450463 |
| 12203 | 0.946133 | 2.1 |
| 12204 | 1.36694 | 2.1 |
| 12205 | 0.946133 | 2.1 |
| 12206 | 0.946132 | 2.1 |
| 12207 | 0.946133 | 2.1 |
| 12208 | 0.946133 | 2.1 |
| 12209 | 0.946132 | 2.1 |
| 12210 | 0.946132 | 0.05 |
| 12211 | 0.946133 | 0.05 |
| 12212 | 0.946132 | 0.05 |
| 12213 | 0.946133 | 0.05 |
| 12214 | 0.946133 | 0.05 |
| 12215 | 0.946132 | 0.0502344 |
| 12216 | 0.946132 | 0.05 |
| 12217 | 0.491552 | 0.05 |
| 12218 | 0.913078 | 0.05 |
| 12219 | 0.946132 | 0.05 |
| 12220 | 0.491552 | 0.05 |
| 12221 | 0.946132 | 0.05 |
| 12222 | 0.49154 | 0.05 |
| 12223 | 1.53168 | 0.05 |
| 12224 | 1.53168 | 0.05 |
| 12225 | 0.946127 | 2.1 |
| 12226 | 1.38752 | 2.1 |
| 12227 | 0.946129 | 2.1 |
| 12228 | 0.94613 | 2.1 |
| 12229 | 0.946128 | 2.1 |
| 12230 | 0.946129 | 2.1 |
| 12231 | 0.946129 | 2.1 |
| 12232 | 0.946132 | 2.1 |
| 12233 | 0.946132 | 2.1 |
| 12234 | 0.946132 | 2.1 |
| 12235 | 0.946132 | 2.1 |

| | | |
|---|---|---|
| 12236 | 0.946132 | 2.1 |
| 12237 | 0.946131 | 2.1 |
| 12238 | 0.946132 | 2.1 |
| 12239 | 0.94613 | 0.0627294 |
| 12240 | 0.94613 | 0.0551678 |
| 12241 | 0.94613 | 0.0586908 |
| 12242 | 0.946131 | 0.0501912 |
| 12243 | 0.946129 | 0.074947 |
| 12244 | 1.16834 | 0.05 |
| 12245 | 0.94613 | 0.0662072 |
| 12246 | 0.946127 | 0.05 |
| 12247 | 1.35594 | 0.05 |
| 12248 | 0.946131 | 0.05 |
| 12249 | 0.946128 | 0.0633208 |
| 12250 | 0.946127 | 0.05 |
| 12251 | 0.946129 | 0.05 |
| 12252 | 0.94613 | 0.05 |
| 12253 | 0.94613 | 0.627188 |
| 12254 | 0.946131 | 0.219246 |
| 12255 | 0.946128 | 0.05 |
| 12256 | 0.946128 | 0.05 |
| 12257 | 0.946131 | 0.644596 |
| 12258 | 0.94613 | 0.05 |
| 12259 | 0.94613 | 0.511479 |
| 12260 | 0.946132 | 0.157087 |
| 12261 | 0.946132 | 0.381178 |
| 12262 | 0.946129 | 0.368224 |
| 12263 | 1.39751 | 0.177177 |
| 12264 | 0.94613 | 0.520159 |
| 12265 | 0.946131 | 0.254786 |
| 12266 | 0.946131 | 0.322747 |
| 12267 | 0.946132 | 0.05 |
| 12268 | 0.700041 | 0.05 |
| 12269 | 0.700013 | 0.05 |
| 12270 | 1.7697 | 0.05 |
| 12271 | 1.7697 | 0.05 |
| 12272 | 1.21134 | 0.05 |
| 12273 | 0.946131 | 0.05 |
| 12274 | 1.25837 | 0.05 |
| 12275 | 0.946133 | 2.1 |
| 12276 | 0.946132 | 2.00227 |
| 12277 | 0.946132 | 1.67529 |
| 12278 | 0.946132 | 2.1 |
| 12279 | 0.946132 | 2.1 |
| 12280 | 0.946133 | 2.1 |
| 12281 | 1.40583 | 2.1 |
| 12282 | 0.946131 | 1.02019 |
| 12283 | 0.946131 | 2.06078 |
| 12284 | 0.946131 | 1.19266 |
| 12285 | 0.946131 | 2.1 |
| 12286 | 0.946131 | 2.08603 |
| 12287 | 0.827865 | 1.85597 |
| 12288 | 0.946131 | 2.01314 |
| 12289 | 0.946132 | 0.05 |
| 12290 | 0.946129 | 0.05 |
| 12291 | 0.946129 | 0.05 |
| 12292 | 0.946131 | 0.05 |
| 12293 | 0.94613 | 0.05 |

| | | |
|---|---|---|
| 12294 | 1.21087 | 0.05 |
| 12295 | 0.946131 | 0.05 |
| 12296 | 0.94613 | 0.745937 |
| 12297 | 0.946131 | 0.996369 |
| 12298 | 0.94613 | 0.678807 |
| 12299 | 0.946128 | 0.724342 |
| 12300 | 0.946132 | 0.695654 |
| 12301 | 1.17452 | 0.663876 |
| 12302 | 0.94613 | 0.649349 |
| 12303 | 0.946119 | 0.610228 |
| 12304 | 0.946114 | 0.678899 |
| 12305 | 0.946119 | 0.574817 |
| 12306 | 0.946119 | 0.601834 |
| 12307 | 0.946119 | 0.582633 |
| 12308 | 1.27376 | 0.528975 |
| 12309 | 0.946106 | 0.505843 |
| 12310 | 0.946133 | 2.1 |
| 12311 | 1.39621 | 2.1 |
| 12312 | 0.946132 | 2.1 |
| 12313 | 0.946132 | 2.1 |
| 12314 | 0.946133 | 2.1 |
| 12315 | 0.946132 | 2.1 |
| 12316 | 0.946132 | 2.1 |
| 12317 | 0.946132 | 2.1 |
| 12318 | 0.946132 | 2.1 |
| 12319 | 0.946131 | 2.1 |
| 12320 | 0.946131 | 2.1 |
| 12321 | 0.946132 | 2.1 |
| 12322 | 0.946132 | 2.1 |
| 12323 | 1.34352 | 2.1 |
| 12324 | 0.946132 | 2.1 |
| 12325 | 1.41272 | 2.1 |
| 12326 | 0.946131 | 2.1 |
| 12327 | 0.94613 | 1.98069 |
| 12328 | 0.94613 | 2.1 |
| 12329 | 0.94613 | 2.0559 |
| 12330 | 0.94613 | 2.1 |
| 12331 | 0.946132 | 2.1 |
| 12332 | 0.946131 | 2.1 |
| 12333 | 0.946132 | 2.1 |
| 12334 | 0.946132 | 2.0103 |
| 12335 | 0.946132 | 0.981514 |
| 12336 | 0.946132 | 2.1 |
| 12337 | 1.23536 | 2.1 |
| 12338 | 0.946132 | 2.1 |
| 12339 | 0.946131 | 2.1 |
| 12340 | 0.946131 | 2.1 |
| 12341 | 0.946132 | 2.1 |
| 12342 | 0.946132 | 2.1 |
| 12343 | 1.41131 | 2.1 |
| 12344 | 0.946131 | 2.1 |
| 12345 | 1.41894 | 1.51065 |
| 12346 | 0.946132 | 0.40804 |
| 12347 | 0.946133 | 0.632816 |
| 12348 | 0.946132 | 0.715358 |
| 12349 | 0.946132 | 0.371815 |
| 12350 | 0.946133 | 0.699424 |
| 12351 | 0.946132 | 0.629405 |

| | | |
|---|---|---|
| 12352 | 0.946128 | 0.05 |
| 12353 | 0.946133 | 0.72385 |
| 12354 | 0.946131 | 0.657515 |
| 12355 | 0.946129 | 2.1 |
| 12356 | 0.94613 | 0.05 |
| 12357 | 1.4099 | 0.81359 |
| 12358 | 0.946131 | 0.304935 |
| 12359 | 0.810947 | 2.1 |
| 12360 | 0.810945 | 0.581363 |
| 12361 | 0.810933 | 2.1 |
| 12362 | 2.62883 | 2.1 |
| 12363 | 1.3517 | 2.1 |
| 12364 | 1.08132 | 2.1 |
| 12365 | 1.08132 | 0.38732 |
| 12366 | 1.08133 | 2.1 |
| 12367 | 0.946131 | 1.11875 |
| 12368 | 0.946126 | 2.1 |
| 12369 | 0.946127 | 1.09392 |
| 12370 | 0.94613 | 1.19433 |
| 12371 | 0.946128 | 2.1 |
| 12372 | 0.946129 | 0.765092 |
| 12373 | 0.946129 | 1.09564 |
| 12374 | 1.21347 | 2.1 |
| 12375 | 0.946129 | 2.1 |
| 12376 | 0.94613 | 2.1 |
| 12377 | 1.21347 | 2.1 |
| 12378 | 1.33064 | 2.1 |
| 12379 | 0.94613 | 2.1 |
| 12380 | 1.33064 | 2.1 |
| 12381 | 0.94613 | 0.05 |
| 12382 | 0.946129 | 0.05 |
| 12383 | 0.94613 | 0.05 |
| 12384 | 0.946132 | 0.05 |
| 12385 | 0.946126 | 0.05 |
| 12386 | 0.946129 | 0.05 |
| 12387 | 0.94613 | 0.05 |
| 12388 | 0.946132 | 1.02951 |
| 12389 | 1.37938 | 1.23298 |
| 12390 | 0.946131 | 1.54813 |
| 12391 | 0.94613 | 0.899714 |
| 12392 | 0.946131 | 1.91794 |
| 12393 | 0.94613 | 1.03843 |
| 12394 | 0.94613 | 1.19305 |
| 12395 | 0.946133 | 0.05 |
| 12396 | 0.946133 | 0.05 |
| 12397 | 1.2968 | 0.05 |
| 12398 | 0.946132 | 0.05 |
| 12399 | 0.946133 | 0.05 |
| 12400 | 1.2968 | 0.05 |
| 12401 | 0.946133 | 0.05 |
| 12402 | 1.43643 | 0.05 |
| 12403 | 0.946129 | 0.120264 |
| 12404 | 0.946133 | 0.0554273 |
| 12405 | 0.946131 | 0.179151 |
| 12406 | 0.94613 | 0.05 |
| 12407 | 0.946131 | 0.05 |
| 12408 | 0.946131 | 0.0707073 |
| 12409 | 0.946131 | 0.05 |

| | | |
|---|---|---|
| 12410 | 0.872447 | 1.23253 |
| 12411 | 0.946132 | 0.94953 |
| 12412 | 0.872447 | 0.547105 |
| 12413 | 0.827867 | 1.10893 |
| 12414 | 0.946132 | 1.32953 |
| 12415 | 0.827867 | 1.48695 |
| 12416 | 0.872447 | 1.49271 |
| 12417 | 0.946117 | 1.00678 |
| 12418 | 1.27213 | 0.907855 |
| 12419 | 0.946124 | 0.319756 |
| 12420 | 0.946119 | 0.863325 |
| 12421 | 0.946113 | 1.41787 |
| 12422 | 0.946129 | 1.11679 |
| 12423 | 0.946104 | 1.04512 |
| 12424 | 0.94613 | 0.118749 |
| 12425 | 0.946131 | 0.687356 |
| 12426 | 0.94613 | 0.36688 |
| 12427 | 0.946129 | 0.467113 |
| 12428 | 0.946132 | 0.05 |
| 12429 | 0.946131 | 0.05 |
| 12430 | 0.94613 | 0.05 |
| 12431 | 0.810942 | 2.1 |
| 12432 | 0.810943 | 2.1 |
| 12433 | 2.80212 | 2.1 |
| 12434 | 1.3517 | 2.1 |
| 12435 | 1.08133 | 2.1 |
| 12436 | 1.08132 | 2.1 |
| 12437 | 1.08132 | 2.1 |
| 12438 | 0.810932 | 2.1 |
| 12439 | 1.29521 | 2.1 |
| 12440 | 0.946129 | 2.1 |
| 12441 | 0.946131 | 2.1 |
| 12442 | 1.40106 | 2.1 |
| 12443 | 0.94613 | 2.1 |
| 12444 | 1.40106 | 2.1 |
| 12445 | 0.946131 | 2.1 |
| 12446 | 0.946132 | 0.05 |
| 12447 | 1.24596 | 0.05 |
| 12448 | 0.94613 | 0.05 |
| 12449 | 0.946131 | 0.05 |
| 12450 | 0.946131 | 0.05 |
| 12451 | 0.946131 | 0.05 |
| 12452 | 0.946131 | 0.05 |
| 12453 | 0.946131 | 2.1 |
| 12454 | 0.946131 | 2.1 |
| 12455 | 0.946131 | 2.1 |
| 12456 | 0.946128 | 2.1 |
| 12457 | 0.946133 | 2.1 |
| 12458 | 0.94613 | 2.1 |
| 12459 | 0.946131 | 2.1 |
| 12460 | 0.879548 | 0.789578 |
| 12461 | 1.21527 | 0.776607 |
| 12462 | 0.967506 | 0.843519 |
| 12463 | 0.946132 | 1.08399 |
| 12464 | 0.946133 | 0.488041 |
| 12465 | 0.967511 | 1.32487 |
| 12466 | 0.946132 | 1.11129 |
| 12467 | 0.946132 | 1.11408 |

| | | |
|---|---|---|
| 12468 | 0.946132 | 0.826456 |
| 12469 | 0.974253 | 0.929079 |
| 12470 | 0.974253 | 1.54112 |
| 12471 | 0.91801 | 0.798029 |
| 12472 | 0.918009 | 0.877343 |
| 12473 | 0.946132 | 1.12003 |
| 12474 | 0.946132 | 0.05 |
| 12475 | 0.946131 | 0.05 |
| 12476 | 0.946131 | 0.05 |
| 12477 | 0.946133 | 0.05 |
| 12478 | 0.946132 | 0.05 |
| 12479 | 1.28644 | 0.05 |
| 12480 | 0.946132 | 0.05 |
| 12481 | 0.946131 | 0.05 |
| 12482 | 0.946131 | 0.05 |
| 12483 | 0.946131 | 0.05 |
| 12484 | 0.946131 | 0.05 |
| 12485 | 0.946131 | 0.05 |
| 12486 | 1.40103 | 0.05 |
| 12487 | 0.946131 | 0.05 |
| 12488 | 0.946128 | 0.05 |
| 12489 | 0.946132 | 0.05 |
| 12490 | 0.946129 | 0.05 |
| 12491 | 1.03327 | 0.05 |
| 12492 | 0.946129 | 0.05 |
| 12493 | 0.946129 | 0.05 |
| 12494 | 0.94613 | 0.05 |
| 12495 | 0.946134 | 0.251459 |
| 12496 | 0.946133 | 0.0726405 |
| 12497 | 0.946132 | 0.05 |
| 12498 | 0.946133 | 0.0829478 |
| 12499 | 0.946132 | 0.05 |
| 12500 | 0.946132 | 0.05 |
| 12501 | 0.946133 | 0.05 |
| 12502 | 0.856654 | 0.05 |
| 12503 | 0.946131 | 0.05 |
| 12504 | 0.946131 | 0.05 |
| 12505 | 0.946132 | 0.05 |
| 12506 | 0.856616 | 0.05 |
| 12507 | 1.03565 | 0.05 |
| 12508 | 1.03565 | 0.05 |
| 12509 | 1.15844 | 0.277627 |
| 12510 | 0.946132 | 0.38607 |
| 12511 | 0.946132 | 0.0628126 |
| 12512 | 0.946132 | 0.16992 |
| 12513 | 0.946132 | 0.0753982 |
| 12514 | 0.946132 | 0.272352 |
| 12515 | 0.946131 | 0.13433 |
| 12516 | 1.4582 | 2.1 |
| 12517 | 1.35067 | 2.1 |
| 12518 | 0.946131 | 2.1 |
| 12519 | 1.04106 | 2.1 |
| 12520 | 0.938748 | 2.1 |
| 12521 | 0.943015 | 2.1 |
| 12522 | 1.05017 | 2.1 |
| 12523 | 0.938793 | 2.1 |
| 12524 | 0.946131 | 0.05 |
| 12525 | 0.946132 | 0.05 |

| | | |
|---|---|---|
| 12526 | 0.946131 | 0.05 |
| 12527 | 0.94613 | 0.05 |
| 12528 | 0.946133 | 0.05 |
| 12529 | 0.946131 | 0.05 |
| 12530 | 0.946131 | 0.05 |
| 12531 | 0.946132 | 0.0517295 |
| 12532 | 0.946133 | 0.05 |
| 12533 | 0.946132 | 0.05 |
| 12534 | 0.946133 | 0.0834576 |
| 12535 | 0.946133 | 0.382695 |
| 12536 | 0.946132 | 0.05 |
| 12537 | 0.946133 | 0.145783 |
| 12538 | 0.946128 | 0.856611 |
| 12539 | 0.946132 | 1.08325 |
| 12540 | 0.946132 | 0.641809 |
| 12541 | 0.94613 | 0.898591 |
| 12542 | 1.23426 | 0.977575 |
| 12543 | 0.94613 | 0.954411 |
| 12544 | 0.946131 | 0.981549 |
| 12545 | 0.810943 | 0.503567 |
| 12546 | 0.810946 | 0.585114 |
| 12547 | 2.60388 | 0.590753 |
| 12548 | 1.3517 | 0.52729 |
| 12549 | 1.08133 | 0.489717 |
| 12550 | 1.08132 | 0.598152 |
| 12551 | 1.08132 | 0.50053 |
| 12552 | 0.810933 | 0.422657 |
| 12553 | 0.810945 | 0.05 |
| 12554 | 0.810945 | 0.05 |
| 12555 | 2.72377 | 1.67725 |
| 12556 | 1.3517 | 0.780971 |
| 12557 | 1.08133 | 0.05 |
| 12558 | 1.08132 | 0.0523391 |
| 12559 | 1.08132 | 2.1 |
| 12560 | 0.810932 | 0.898204 |
| 12561 | 1.25104 | 0.05 |
| 12562 | 0.946131 | 0.12312 |
| 12563 | 0.946131 | 0.05 |
| 12564 | 1.36188 | 2.1 |
| 12565 | 0.946131 | 0.151387 |
| 12566 | 1.36188 | 0.955565 |
| 12567 | 0.946132 | 0.740784 |
| 12568 | 0.946132 | 2.1 |
| 12569 | 0.946132 | 2.1 |
| 12570 | 0.946132 | 2.1 |
| 12571 | 0.946132 | 2.1 |
| 12572 | 0.946132 | 2.1 |
| 12573 | 0.946132 | 2.1 |
| 12574 | 1.25488 | 2.1 |
| 12575 | 1.32132 | 0.05 |
| 12576 | 0.946126 | 0.05 |
| 12577 | 0.946127 | 0.05 |
| 12578 | 0.946128 | 0.05 |
| 12579 | 0.946128 | 0.05 |
| 12580 | 0.946128 | 0.05 |
| 12581 | 0.946128 | 0.05 |
| 12582 | 0.946133 | 2.1 |
| 12583 | 1.41123 | 1.85889 |

| | | |
|---|---|---|
| 12584 | 0.946134 | 2.1 |
| 12585 | 0.946133 | 2.1 |
| 12586 | 0.946132 | 0.0728724 |
| 12587 | 0.946133 | 2.1 |
| 12588 | 0.946132 | 2.1 |
| 12589 | 1.32163 | 0.05 |
| 12590 | 0.946133 | 0.05 |
| 12591 | 0.946133 | 0.05 |
| 12592 | 0.946132 | 0.05 |
| 12593 | 0.946132 | 0.05 |
| 12594 | 0.946132 | 0.05 |
| 12595 | 0.946133 | 0.05 |
| 12596 | 0.946133 | 2.1 |
| 12597 | 0.946133 | 2.1 |
| 12598 | 0.946132 | 2.1 |
| 12599 | 0.946132 | 2.1 |
| 12600 | 0.946132 | 0.266832 |
| 12601 | 0.946133 | 2.1 |
| 12602 | 0.946132 | 2.1 |
| 12603 | 0.94613 | 0.0706179 |
| 12604 | 0.946131 | 0.0628067 |
| 12605 | 0.946128 | 0.05 |
| 12606 | 0.946133 | 0.05 |
| 12607 | 0.946133 | 0.05 |
| 12608 | 0.946131 | 0.0796185 |
| 12609 | 0.946131 | 0.05 |
| 12610 | 0.946133 | 0.627882 |
| 12611 | 0.946132 | 0.170864 |
| 12612 | 0.946132 | 0.6403 |
| 12613 | 0.946134 | 0.101622 |
| 12614 | 0.946133 | 0.777542 |
| 12615 | 0.946133 | 0.0788485 |
| 12616 | 0.946132 | 0.588175 |
| 12617 | 0.94613 | 1.96893 |
| 12618 | 0.946128 | 2.1 |
| 12619 | 0.946127 | 1.42476 |
| 12620 | 0.94613 | 1.0631 |
| 12621 | 0.946131 | 1.74638 |
| 12622 | 0.946128 | 1.84134 |
| 12623 | 1.21099 | 2.1 |
| 12624 | 0.946132 | 0.213593 |
| 12625 | 0.946131 | 0.210797 |
| 12626 | 0.946131 | 0.429223 |
| 12627 | 0.946133 | 0.05 |
| 12628 | 0.946133 | 0.05 |
| 12629 | 0.946131 | 0.131002 |
| 12630 | 0.946132 | 0.0771805 |
| 12631 | 1.34794 | 0.165982 |
| 12632 | 0.517994 | 0.153878 |
| 12633 | 0.517965 | 0.062223 |
| 12634 | 0.946132 | 0.412665 |
| 12635 | 0.9198 | 1.82031 |
| 12636 | 0.946132 | 0.993986 |
| 12637 | 0.9198 | 0.608979 |
| 12638 | 0.946132 | 1.07546 |
| 12639 | 0.94613 | 1.10383 |
| 12640 | 0.946131 | 0.238843 |
| 12641 | 0.946131 | 1.09818 |

| | | |
|---|---|---|
| 12642 | 0.946131 | 1.71367 |
| 12643 | 0.946131 | 1.11445 |
| 12644 | 1.23224 | 0.649362 |
| 12645 | 0.946132 | 2.01785 |
| 12646 | 0.946132 | 2.1 |
| 12647 | 0.946133 | 1.60762 |
| 12648 | 0.946132 | 0.399072 |
| 12649 | 0.946132 | 1.25844 |
| 12650 | 0.946133 | 1.51761 |
| 12651 | 1.37511 | 1.72931 |
| 12652 | 0.946133 | 0.183613 |
| 12653 | 0.946129 | 0.253048 |
| 12654 | 0.851104 | 1.01723 |
| 12655 | 0.946131 | 0.522759 |
| 12656 | 1.0412 | 0.591072 |
| 12657 | 1.0412 | 0.76697 |
| 12658 | 0.851066 | 0.601194 |
| 12659 | 1.31357 | 0.05 |
| 12660 | 0.946131 | 0.05 |
| 12661 | 0.94613 | 0.05 |
| 12662 | 0.946131 | 0.05 |
| 12663 | 0.94613 | 0.05 |
| 12664 | 0.946131 | 0.05 |
| 12665 | 0.946131 | 0.05 |
| 12666 | 0.946133 | 0.934917 |
| 12667 | 1.38149 | 1.10607 |
| 12668 | 0.946132 | 1.15614 |
| 12669 | 0.946132 | 0.87229 |
| 12670 | 0.946133 | 1.97689 |
| 12671 | 0.946132 | 1.00667 |
| 12672 | 0.946133 | 1.04663 |
| 12673 | 0.810944 | 1.20832 |
| 12674 | 0.810945 | 2.1 |
| 12675 | 0.810958 | 2.1 |
| 12676 | 2.65782 | 2.1 |
| 12677 | 1.35168 | 2.1 |
| 12678 | 1.08132 | 1.20524 |
| 12679 | 1.08132 | 2.1 |
| 12680 | 1.0813 | 1.24669 |
| 12681 | 0.946113 | 0.05 |
| 12682 | 0.946119 | 0.05 |
| 12683 | 0.946118 | 0.05 |
| 12684 | 0.946118 | 0.05 |
| 12685 | 0.946119 | 0.05 |
| 12686 | 0.946131 | 0.05 |
| 12687 | 0.946106 | 0.05 |
| 12688 | 0.946132 | 0.05 |
| 12689 | 0.946132 | 0.05 |
| 12690 | 0.946128 | 0.05 |
| 12691 | 0.946131 | 0.05 |
| 12692 | 0.94613 | 0.05 |
| 12693 | 0.94613 | 0.05 |
| 12694 | 0.94613 | 0.05 |
| 12695 | 1.00099 | 0.05 |
| 12696 | 0.946132 | 0.05 |
| 12697 | 1.17876 | 0.05 |
| 12698 | 0.982282 | 0.05 |
| 12699 | 0.909981 | 0.05 |

| | | |
|---|---|---|
| 12700 | 0.946132 | 0.05 |
| 12701 | 0.946131 | 0.05 |
| 12702 | 0.946132 | 1.12205 |
| 12703 | 1.35328 | 1.38536 |
| 12704 | 0.946132 | 2.06523 |
| 12705 | 0.946132 | 0.896712 |
| 12706 | 0.946133 | 1.87906 |
| 12707 | 0.946132 | 1.02568 |
| 12708 | 0.946133 | 1.682 |
| 12709 | 0.946133 | 0.05 |
| 12710 | 0.946133 | 0.05 |
| 12711 | 0.946132 | 0.05 |
| 12712 | 0.946132 | 0.05 |
| 12713 | 0.946132 | 0.05 |
| 12714 | 0.946132 | 0.05 |
| 12715 | 0.946133 | 0.05 |
| 12716 | 0.900195 | 0.05 |
| 12717 | 0.946132 | 0.05 |
| 12718 | 0.900167 | 0.05 |
| 12719 | 1.41963 | 0.05 |
| 12720 | 0.900195 | 0.05 |
| 12721 | 0.946133 | 0.05 |
| 12722 | 1.35385 | 0.05 |
| 12723 | 0.946132 | 1.05754 |
| 12724 | 0.946129 | 1.64754 |
| 12725 | 0.946129 | 1.47172 |
| 12726 | 0.946131 | 1.00478 |
| 12727 | 0.946131 | 1.64828 |
| 12728 | 1.41585 | 1.07193 |
| 12729 | 0.94613 | 2.1 |
| 12730 | 0.810946 | 2.1 |
| 12731 | 0.810946 | 2.1 |
| 12732 | 2.68895 | 2.1 |
| 12733 | 1.3517 | 2.1 |
| 12734 | 1.08133 | 2.1 |
| 12735 | 1.08132 | 2.1 |
| 12736 | 1.08132 | 2.1 |
| 12737 | 0.810933 | 2.1 |
| 12738 | 1.19886 | 2.1 |
| 12739 | 0.946131 | 2.1 |
| 12740 | 0.946131 | 2.1 |
| 12741 | 1.34447 | 2.1 |
| 12742 | 0.946131 | 2.1 |
| 12743 | 1.34447 | 2.1 |
| 12744 | 0.946131 | 2.1 |
| 12745 | 0.946133 | 0.05 |
| 12746 | 0.946133 | 0.05 |
| 12747 | 0.946132 | 0.05 |
| 12748 | 1.35065 | 0.05 |
| 12749 | 0.946132 | 0.05 |
| 12750 | 0.946133 | 0.05 |
| 12751 | 0.946132 | 0.05 |
| 12752 | 0.946131 | 0.126109 |
| 12753 | 0.946131 | 0.186796 |
| 12754 | 0.94613 | 0.31488 |
| 12755 | 0.946131 | 0.05 |
| 12756 | 0.946131 | 0.05 |
| 12757 | 0.946131 | 0.117395 |

| | | |
|---|---|---|
| 12758 | 0.946131 | 0.0652225 |
| 12759 | 0.946127 | 0.05 |
| 12760 | 0.946131 | 0.05 |
| 12761 | 0.946127 | 0.05 |
| 12762 | 0.94613 | 0.05 |
| 12763 | 0.946131 | 0.05 |
| 12764 | 0.946128 | 0.05 |
| 12765 | 0.946129 | 0.05 |
| 12766 | 0.810946 | 0.05 |
| 12767 | 0.810947 | 0.05 |
| 12768 | 0.810959 | 0.05 |
| 12769 | 2.4391 | 0.05 |
| 12770 | 1.35168 | 0.05 |
| 12771 | 1.08132 | 0.05 |
| 12772 | 1.08132 | 0.05 |
| 12773 | 1.08131 | 0.05 |
| 12774 | 0.946132 | 0.05 |
| 12775 | 0.946132 | 0.05 |
| 12776 | 0.946132 | 0.05 |
| 12777 | 1.40837 | 0.05 |
| 12778 | 0.946132 | 0.05 |
| 12779 | 0.946132 | 0.05 |
| 12780 | 1.40837 | 0.05 |
| 12781 | 0.946127 | 2.1 |
| 12782 | 0.946132 | 2.1 |
| 12783 | 0.946131 | 0.656491 |
| 12784 | 0.946127 | 2.1 |
| 12785 | 0.946131 | 1.52302 |
| 12786 | 0.946129 | 1.05992 |
| 12787 | 0.94613 | 1.55241 |
| 12788 | 0.946133 | 2.1 |
| 12789 | 0.946132 | 1.5405 |
| 12790 | 0.946132 | 1.46737 |
| 12791 | 0.946132 | 1.15005 |
| 12792 | 0.946132 | 1.935 |
| 12793 | 0.946132 | 0.937104 |
| 12794 | 0.946133 | 2.1 |
| 12795 | 0.946132 | 0.862255 |
| 12796 | 1.24344 | 0.581318 |
| 12797 | 0.946132 | 0.641536 |
| 12798 | 0.946132 | 1.10166 |
| 12799 | 0.946132 | 0.471133 |
| 12800 | 1.24344 | 0.584237 |
| 12801 | 0.946132 | 1.07516 |
| 12802 | 0.946131 | 0.210977 |
| 12803 | 0.946128 | 0.191778 |
| 12804 | 0.946128 | 0.169876 |
| 12805 | 0.946131 | 0.178376 |
| 12806 | 1.41232 | 0.16389 |
| 12807 | 0.946129 | 0.263068 |
| 12808 | 0.94613 | 0.192424 |
| 12809 | 0.946128 | 2.1 |
| 12810 | 0.946133 | 2.1 |
| 12811 | 0.946133 | 0.162577 |
| 12812 | 0.946131 | 2.1 |
| 12813 | 0.946131 | 0.351139 |
| 12814 | 0.946131 | 2.08483 |
| 12815 | 0.94613 | 2.0815 |

| | | |
|---|---|---|
| 12816 | 0.946133 | 0.05 |
| 12817 | 1.41556 | 0.05 |
| 12818 | 0.946133 | 0.05 |
| 12819 | 0.946133 | 0.05 |
| 12820 | 0.946132 | 0.141232 |
| 12821 | 0.946132 | 0.05 |
| 12822 | 0.946132 | 0.0664045 |
| 12823 | 0.94612 | 0.05 |
| 12824 | 1.41843 | 0.05 |
| 12825 | 0.946114 | 0.05 |
| 12826 | 0.94612 | 0.05 |
| 12827 | 0.946119 | 0.0949894 |
| 12828 | 1.41845 | 0.05 |
| 12829 | 0.946107 | 0.0659718 |
| 12830 | 0.946129 | 0.05 |
| 12831 | 0.946129 | 0.05 |
| 12832 | 0.94613 | 0.05 |
| 12833 | 0.946132 | 0.05 |
| 12834 | 0.946126 | 0.05 |
| 12835 | 1.1509 | 0.05 |
| 12836 | 0.94613 | 0.05 |
| 12837 | 0.946131 | 0.05 |
| 12838 | 0.946132 | 0.05 |
| 12839 | 0.946132 | 0.05 |
| 12840 | 0.946132 | 0.05 |
| 12841 | 0.946132 | 0.05 |
| 12842 | 1.19263 | 0.05 |
| 12843 | 0.946132 | 0.05 |
| 12844 | 0.94613 | 0.281056 |
| 12845 | 0.946131 | 0.198106 |
| 12846 | 0.946128 | 0.559604 |
| 12847 | 0.946133 | 0.610102 |
| 12848 | 0.946133 | 0.688629 |
| 12849 | 0.946131 | 0.229716 |
| 12850 | 0.946131 | 0.611334 |
| 12851 | 0.946133 | 1.12959 |
| 12852 | 1.22848 | 1.53671 |
| 12853 | 1.00336 | 1.50924 |
| 12854 | 0.827866 | 1.18546 |
| 12855 | 0.946132 | 2.1 |
| 12856 | 0.827865 | 1.57743 |
| 12857 | 0.946133 | 1.28134 |
| 12858 | 0.946131 | 0.639357 |
| 12859 | 0.94613 | 0.207157 |
| 12860 | 0.946127 | 0.329634 |
| 12861 | 1.41498 | 0.399138 |
| 12862 | 0.94613 | 0.282116 |
| 12863 | 0.946128 | 0.249493 |
| 12864 | 0.946128 | 0.232703 |
| 12865 | 0.94613 | 0.05 |
| 12866 | 0.94613 | 0.05 |
| 12867 | 0.94613 | 0.05 |
| 12868 | 1.37358 | 0.05 |
| 12869 | 0.946129 | 0.05 |
| 12870 | 0.94613 | 0.05 |
| 12871 | 1.37357 | 0.05 |
| 12872 | 0.946111 | 0.05 |
| 12873 | 0.946118 | 0.05 |

| | | |
|---|---|---|
| 12874 | 0.946118 | 0.05 |
| 12875 | 1.36568 | 0.05 |
| 12876 | 0.946116 | 0.05 |
| 12877 | 0.94613 | 0.05 |
| 12878 | 0.946105 | 0.05 |
| 12879 | 0.946132 | 0.05 |
| 12880 | 0.946131 | 0.05 |
| 12881 | 0.94613 | 0.0838037 |
| 12882 | 1.23175 | 0.05 |
| 12883 | 0.946131 | 0.05 |
| 12884 | 0.946131 | 0.05 |
| 12885 | 0.946131 | 0.05 |
| 12886 | 0.946132 | 0.05 |
| 12887 | 0.946126 | 0.05 |
| 12888 | 0.946128 | 0.05 |
| 12889 | 0.946129 | 0.05 |
| 12890 | 0.946129 | 0.05 |
| 12891 | 0.946128 | 0.05 |
| 12892 | 1.25698 | 0.05 |
| 12893 | 0.810943 | 2.1 |
| 12894 | 0.810944 | 2.1 |
| 12895 | 2.81171 | 2.1 |
| 12896 | 1.3517 | 2.1 |
| 12897 | 1.08132 | 2.1 |
| 12898 | 1.08133 | 2.1 |
| 12899 | 1.08132 | 2.1 |
| 12900 | 0.810932 | 2.1 |
| 12901 | 0.946132 | 0.05 |
| 12902 | 0.946131 | 0.05 |
| 12903 | 0.946131 | 0.05 |
| 12904 | 0.946132 | 0.05 |
| 12905 | 0.946132 | 0.05 |
| 12906 | 0.946132 | 0.05 |
| 12907 | 0.946131 | 0.05 |
| 12908 | 0.810936 | 0.0729321 |
| 12909 | 0.810935 | 2.1 |
| 12910 | 2.80914 | 1.95891 |
| 12911 | 1.35167 | 0.838378 |
| 12912 | 1.0813 | 0.427763 |
| 12913 | 1.0813 | 2.1 |
| 12914 | 1.0813 | 0.0840925 |
| 12915 | 0.810934 | 0.411617 |
| 12916 | 1.23851 | 2.1 |
| 12917 | 0.946132 | 0.13795 |
| 12918 | 0.946131 | 2.1 |
| 12919 | 1.40459 | 0.0655339 |
| 12920 | 0.946132 | 2.07651 |
| 12921 | 1.40459 | 1.84947 |
| 12922 | 0.946132 | 2.06582 |
| 12923 | 1.19974 | 0.05 |
| 12924 | 0.946131 | 0.05 |
| 12925 | 1.19974 | 0.05 |
| 12926 | 0.946132 | 0.05 |
| 12927 | 0.946132 | 0.05 |
| 12928 | 0.946132 | 0.05 |
| 12929 | 0.946132 | 0.05 |
| 12930 | 0.939141 | 1.03876 |
| 12931 | 1.14375 | 1.39509 |

| | | |
|---|---|---|
| 12932 | 1.03306 | 1.43175 |
| 12933 | 0.946132 | 1.13592 |
| 12934 | 0.946126 | 1.76134 |
| 12935 | 0.946129 | 1.30662 |
| 12936 | 0.94613 | 1.18049 |
| 12937 | 0.946132 | 0.0519871 |
| 12938 | 0.946131 | 0.0613413 |
| 12939 | 0.946132 | 0.0550161 |
| 12940 | 0.946132 | 0.05 |
| 12941 | 0.946132 | 0.05 |
| 12942 | 0.946132 | 0.105885 |
| 12943 | 0.946132 | 0.0556858 |
| 12944 | 0.946131 | 2.1 |
| 12945 | 0.94613 | 2.1 |
| 12946 | 0.94613 | 2.1 |
| 12947 | 0.946131 | 2.1 |
| 12948 | 0.946129 | 2.1 |
| 12949 | 0.94613 | 2.1 |
| 12950 | 1.24512 | 2.1 |
| 12951 | 0.946133 | 0.05 |
| 12952 | 0.946129 | 0.05 |
| 12953 | 0.946131 | 0.05 |
| 12954 | 0.946132 | 0.05 |
| 12955 | 0.946131 | 0.05 |
| 12956 | 0.946131 | 0.05 |
| 12957 | 0.946131 | 0.05 |
| 12958 | 0.946132 | 0.05 |
| 12959 | 0.946133 | 0.05 |
| 12960 | 0.946132 | 0.05 |
| 12961 | 0.946132 | 0.05 |
| 12962 | 0.946133 | 0.05 |
| 12963 | 0.946132 | 0.05 |
| 12964 | 0.946132 | 0.05 |
| 12965 | 0.946132 | 0.159766 |
| 12966 | 1.41392 | 0.0798739 |
| 12967 | 0.946132 | 0.0826773 |
| 12968 | 0.946132 | 0.393931 |
| 12969 | 0.946132 | 0.05 |
| 12970 | 0.946132 | 0.182853 |
| 12971 | 0.946132 | 0.219008 |
| 12972 | 0.946132 | 0.741841 |
| 12973 | 1.24333 | 0.400216 |
| 12974 | 0.946132 | 0.277006 |
| 12975 | 0.946133 | 0.873282 |
| 12976 | 0.946132 | 0.05 |
| 12977 | 0.946132 | 0.822367 |
| 12978 | 0.946133 | 0.628276 |
| 12979 | 1.30649 | 0.05 |
| 12980 | 0.94613 | 0.05 |
| 12981 | 0.94613 | 0.05 |
| 12982 | 0.946128 | 0.05 |
| 12983 | 0.946126 | 0.05 |
| 12984 | 0.946127 | 0.05 |
| 12985 | 0.946127 | 0.05 |
| 12986 | 0.709578 | 0.05 |
| 12987 | 0.810943 | 0.05 |
| 12988 | 2.53303 | 0.05 |
| 12989 | 1.21651 | 0.05 |

| | | |
|---|---|---|
| 12990 | 0.979951 | 0.05 |
| 12991 | 1.08132 | 0.05 |
| 12992 | 0.946141 | 0.05 |
| 12993 | 0.81096 | 0.05 |
| 12994 | 0.946131 | 0.05 |
| 12995 | 0.946131 | 0.05 |
| 12996 | 0.946131 | 0.05 |
| 12997 | 0.946131 | 0.05 |
| 12998 | 0.946131 | 0.05 |
| 12999 | 0.946131 | 0.05 |
| 13000 | 0.946131 | 0.05 |
| 13001 | 0.946132 | 0.0792592 |
| 13002 | 0.946129 | 0.05 |
| 13003 | 0.946129 | 0.0520121 |
| 13004 | 0.94613 | 0.0557812 |
| 13005 | 1.20634 | 0.05 |
| 13006 | 1.20634 | 0.05 |
| 13007 | 0.946131 | 0.0789341 |
| 13008 | 0.946133 | 0.655764 |
| 13009 | 0.946132 | 0.642147 |
| 13010 | 0.946132 | 0.592575 |
| 13011 | 0.946132 | 0.689804 |
| 13012 | 0.946132 | 0.566941 |
| 13013 | 1.30107 | 0.669294 |
| 13014 | 0.946133 | 0.684301 |
| 13015 | 0.810946 | 2.1 |
| 13016 | 0.810944 | 2.1 |
| 13017 | 2.73936 | 2.1 |
| 13018 | 1.35168 | 2.1 |
| 13019 | 1.0813 | 2.1 |
| 13020 | 1.08132 | 2.1 |
| 13021 | 1.08132 | 2.1 |
| 13022 | 0.810958 | 2.1 |
| 13023 | 0.946129 | 2.1 |
| 13024 | 0.946129 | 2.1 |
| 13025 | 0.946133 | 0.535259 |
| 13026 | 1.30812 | 2.1 |
| 13027 | 0.946131 | 1.05242 |
| 13028 | 0.946131 | 1.73378 |
| 13029 | 0.946131 | 1.3027 |
| 13030 | 1.00901 | 1.17143 |
| 13031 | 0.94613 | 1.66587 |
| 13032 | 0.94613 | 0.598781 |
| 13033 | 1.17001 | 1.33782 |
| 13034 | 0.917259 | 0.139612 |
| 13035 | 0.94613 | 0.985008 |
| 13036 | 0.94613 | 0.940767 |
| 13037 | 0.946126 | 2.1 |
| 13038 | 0.946127 | 2.1 |
| 13039 | 0.946131 | 2.1 |
| 13040 | 0.946127 | 2.1 |
| 13041 | 0.946131 | 2.1 |
| 13042 | 0.946129 | 2.1 |
| 13043 | 1.398 | 2.1 |
| 13044 | 0.946133 | 2.1 |
| 13045 | 1.3033 | 2.1 |
| 13046 | 0.946132 | 2.1 |
| 13047 | 0.946132 | 2.1 |

| | | |
|---|---|---|
| 13048 | 0.946132 | 2.1 |
| 13049 | 0.946132 | 2.1 |
| 13050 | 0.946133 | 2.1 |
| 13051 | 0.946131 | 0.05 |
| 13052 | 0.946131 | 0.05 |
| 13053 | 1.09897 | 0.05 |
| 13054 | 0.946129 | 0.117205 |
| 13055 | 0.946131 | 0.0620074 |
| 13056 | 0.94613 | 0.0886836 |
| 13057 | 0.94613 | 0.05 |
| 13058 | 0.946132 | 2.1 |
| 13059 | 0.946131 | 2.1 |
| 13060 | 0.946131 | 2.1 |
| 13061 | 0.946132 | 2.1 |
| 13062 | 0.94613 | 2.1 |
| 13063 | 0.946131 | 2.1 |
| 13064 | 1.39858 | 2.1 |
| 13065 | 1.07117 | 0.795438 |
| 13066 | 1.08878 | 1.02855 |
| 13067 | 0.946119 | 1.24665 |
| 13068 | 1.08878 | 1.32636 |
| 13069 | 0.946119 | 0.672562 |
| 13070 | 0.946132 | 1.08541 |
| 13071 | 1.07116 | 0.815777 |
| 13072 | 0.946132 | 0.05 |
| 13073 | 0.946132 | 0.05 |
| 13074 | 0.946132 | 0.05 |
| 13075 | 0.946132 | 0.05 |
| 13076 | 0.946132 | 0.05 |
| 13077 | 1.10733 | 0.05 |
| 13078 | 0.946132 | 0.05 |
| 13079 | 0.946128 | 0.05 |
| 13080 | 0.946132 | 0.05 |
| 13081 | 0.94613 | 0.05 |
| 13082 | 0.946129 | 0.05 |
| 13083 | 0.94613 | 0.05 |
| 13084 | 1.3367 | 0.05 |
| 13085 | 0.94613 | 0.05 |
| 13086 | 0.810943 | 2.1 |
| 13087 | 0.810944 | 2.1 |
| 13088 | 2.70234 | 2.1 |
| 13089 | 1.21653 | 2.1 |
| 13090 | 1.08132 | 2.1 |
| 13091 | 0.946155 | 2.1 |
| 13092 | 0.979951 | 2.1 |
| 13093 | 0.709578 | 2.1 |
| 13094 | 1.27174 | 2.1 |
| 13095 | 0.946129 | 2.1 |
| 13096 | 0.94613 | 2.1 |
| 13097 | 1.35117 | 2.1 |
| 13098 | 0.94613 | 2.1 |
| 13099 | 1.35117 | 2.1 |
| 13100 | 0.94613 | 2.1 |
| 13101 | 0.946133 | 0.485559 |
| 13102 | 0.946133 | 0.24944 |
| 13103 | 0.946132 | 2.02645 |
| 13104 | 0.946133 | 0.185197 |
| 13105 | 0.946132 | 2.1 |

| | | |
|---|---|---|
| 13106 | 0.946133 | 1.86832 |
| 13107 | 0.997296 | 1.96485 |
| 13108 | 0.810943 | 2.1 |
| 13109 | 0.810944 | 2.1 |
| 13110 | 0.810945 | 2.1 |
| 13111 | 2.78833 | 2.1 |
| 13112 | 1.35169 | 2.1 |
| 13113 | 1.08132 | 2.1 |
| 13114 | 1.08132 | 2.1 |
| 13115 | 1.08132 | 2.1 |
| 13116 | 0.810946 | 2.1 |
| 13117 | 0.810943 | 1.04369 |
| 13118 | 0.810933 | 1.83895 |
| 13119 | 2.70049 | 2.1 |
| 13120 | 1.3517 | 1.26185 |
| 13121 | 1.08132 | 2.1 |
| 13122 | 1.08132 | 0.568976 |
| 13123 | 1.08133 | 1.0613 |
| 13124 | 0.946127 | 1.15703 |
| 13125 | 1.35469 | 0.814587 |
| 13126 | 0.94613 | 0.742778 |
| 13127 | 0.946131 | 1.15704 |
| 13128 | 0.946128 | 0.59867 |
| 13129 | 1.35469 | 1.12464 |
| 13130 | 0.946129 | 1.30131 |
| 13131 | 0.946131 | 0.05 |
| 13132 | 1.2722 | 0.05 |
| 13133 | 0.946132 | 0.05 |
| 13134 | 0.946132 | 0.05 |
| 13135 | 0.946129 | 0.05 |
| 13136 | 1.2722 | 0.05 |
| 13137 | 0.94613 | 0.05 |
| 13138 | 0.946133 | 0.338207 |
| 13139 | 0.946132 | 0.146081 |
| 13140 | 0.946132 | 0.392931 |
| 13141 | 0.946132 | 0.388472 |
| 13142 | 1.41461 | 0.332824 |
| 13143 | 1.41461 | 0.303266 |
| 13144 | 0.946133 | 0.406443 |
| 13145 | 0.946131 | 0.468007 |
| 13146 | 1.30165 | 0.0863258 |
| 13147 | 0.946132 | 0.05 |
| 13148 | 0.946132 | 0.934596 |
| 13149 | 0.946129 | 0.131382 |
| 13150 | 1.30165 | 0.572803 |
| 13151 | 0.946131 | 0.918524 |
| 13152 | 0.946132 | 0.749722 |
| 13153 | 0.946129 | 1.95453 |
| 13154 | 0.94613 | 0.825687 |
| 13155 | 1.39356 | 2.1 |
| 13156 | 0.946131 | 0.986643 |
| 13157 | 0.946131 | 0.949627 |
| 13158 | 0.94613 | 1.72285 |
| 13159 | 0.810946 | 2.1 |
| 13160 | 0.810947 | 2.1 |
| 13161 | 0.810934 | 2.1 |
| 13162 | 2.35003 | 2.1 |
| 13163 | 1.3517 | 2.1 |

| | | |
|---|---|---|
| 13164 | 1.08132 | 2.1 |
| 13165 | 1.08132 | 2.1 |
| 13166 | 1.08133 | 2.1 |
| 13167 | 0.946131 | 0.05 |
| 13168 | 0.946131 | 0.05 |
| 13169 | 0.946125 | 0.05 |
| 13170 | 0.946127 | 0.05 |
| 13171 | 0.946129 | 0.05 |
| 13172 | 0.946128 | 0.05 |
| 13173 | 0.946128 | 0.05 |
| 13174 | 0.946131 | 0.05 |
| 13175 | 0.946131 | 0.05 |
| 13176 | 0.946131 | 0.05 |
| 13177 | 0.946131 | 0.05 |
| 13178 | 0.946132 | 0.05 |
| 13179 | 0.946132 | 0.05 |
| 13180 | 0.946131 | 0.05 |
| 13181 | 0.946129 | 2.1 |
| 13182 | 0.946132 | 2.1 |
| 13183 | 0.946132 | 2.1 |
| 13184 | 0.94613 | 2.1 |
| 13185 | 1.30082 | 2.1 |
| 13186 | 0.94613 | 2.1 |
| 13187 | 0.946131 | 2.1 |
| 13188 | 1.20416 | 0.05 |
| 13189 | 0.946132 | 0.05 |
| 13190 | 0.946131 | 0.05 |
| 13191 | 0.946132 | 0.05 |
| 13192 | 0.946131 | 0.05 |
| 13193 | 0.946131 | 0.05 |
| 13194 | 0.946132 | 0.05 |
| 13195 | 1.18711 | 0.05 |
| 13196 | 0.946131 | 0.05 |
| 13197 | 0.946131 | 0.05 |
| 13198 | 0.946132 | 0.05 |
| 13199 | 0.946131 | 0.05 |
| 13200 | 0.946131 | 0.05 |
| 13201 | 0.946132 | 0.05 |
| 13202 | 0.946133 | 0.05 |
| 13203 | 0.946133 | 0.05 |
| 13204 | 0.946132 | 0.05 |
| 13205 | 1.30212 | 0.05 |
| 13206 | 0.946133 | 0.05 |
| 13207 | 0.946132 | 0.05 |
| 13208 | 1.30212 | 0.05 |
| 13209 | 0.946131 | 0.05 |
| 13210 | 0.946131 | 0.05 |
| 13211 | 0.946131 | 0.05 |
| 13212 | 0.946132 | 0.05 |
| 13213 | 1.31467 | 0.05 |
| 13214 | 1.31467 | 0.05 |
| 13215 | 0.946131 | 0.05 |
| 13216 | 0.946133 | 0.05 |
| 13217 | 0.946132 | 0.05 |
| 13218 | 0.946132 | 0.05 |
| 13219 | 0.946132 | 0.05 |
| 13220 | 1.25124 | 0.05 |
| 13221 | 1.25124 | 0.05 |

| | | |
|---|---|---|
| 13222 | 0.946132 | 0.05 |
| 13223 | 0.946132 | 2.1 |
| 13224 | 0.946132 | 2.1 |
| 13225 | 0.946132 | 2.1 |
| 13226 | 1.36422 | 2.1 |
| 13227 | 0.946132 | 2.1 |
| 13228 | 0.946131 | 2.1 |
| 13229 | 0.946132 | 2.1 |
| 13230 | 0.946131 | 1.47583 |
| 13231 | 1.41851 | 2.1 |
| 13232 | 0.946132 | 2.1 |
| 13233 | 0.946132 | 1.13297 |
| 13234 | 0.946132 | 1.98791 |
| 13235 | 0.946132 | 1.16744 |
| 13236 | 0.946132 | 1.92884 |
| 13237 | 0.946133 | 0.05 |
| 13238 | 0.946132 | 0.05 |
| 13239 | 0.946132 | 0.05 |
| 13240 | 0.946132 | 0.05 |
| 13241 | 0.946132 | 0.05 |
| 13242 | 1.37794 | 0.05 |
| 13243 | 0.946132 | 0.05 |
| 13244 | 0.946132 | 2.1 |
| 13245 | 0.946132 | 2.1 |
| 13246 | 0.946131 | 2.1 |
| 13247 | 0.946131 | 2.1 |
| 13248 | 0.946132 | 2.1 |
| 13249 | 0.946132 | 2.1 |
| 13250 | 1.4045 | 2.1 |
| 13251 | 0.946133 | 2.1 |
| 13252 | 0.946132 | 2.1 |
| 13253 | 0.946131 | 2.1 |
| 13254 | 0.946132 | 2.1 |
| 13255 | 0.946131 | 2.1 |
| 13256 | 0.946131 | 2.1 |
| 13257 | 0.946132 | 2.1 |
| 13258 | 0.946132 | 2.1 |
| 13259 | 0.946132 | 2.1 |
| 13260 | 0.946131 | 2.1 |
| 13261 | 0.946132 | 2.1 |
| 13262 | 0.946131 | 2.1 |
| 13263 | 0.946131 | 2.1 |
| 13264 | 0.946131 | 2.1 |
| 13265 | 0.946128 | 2.1 |
| 13266 | 1.31641 | 2.1 |
| 13267 | 0.946129 | 2.1 |
| 13268 | 0.94613 | 2.1 |
| 13269 | 0.94613 | 2.1 |
| 13270 | 0.94613 | 2.1 |
| 13271 | 0.94613 | 2.1 |
| 13272 | 0.946132 | 2.1 |
| 13273 | 0.946131 | 2.1 |
| 13274 | 0.946131 | 1.1139 |
| 13275 | 0.946132 | 2.1 |
| 13276 | 0.946132 | 2.04546 |
| 13277 | 0.946132 | 2.1 |
| 13278 | 0.946132 | 2.1 |
| 13279 | 0.946132 | 2.1 |

| | | |
|---|---|---|
| 13280 | 1.41507 | 2.1 |
| 13281 | 0.946132 | 2.1 |
| 13282 | 0.946132 | 2.1 |
| 13283 | 0.946133 | 2.1 |
| 13284 | 0.946133 | 2.1 |
| 13285 | 0.946132 | 2.1 |
| 13286 | 0.946132 | 2.1 |
| 13287 | 0.946131 | 2.1 |
| 13288 | 0.946131 | 2.1 |
| 13289 | 0.946133 | 2.1 |
| 13290 | 0.946132 | 2.1 |
| 13291 | 1.32608 | 2.1 |
| 13292 | 0.946132 | 2.1 |
| 13293 | 0.94613 | 2.1 |
| 13294 | 0.94613 | 2.1 |
| 13295 | 0.94613 | 0.854291 |
| 13296 | 0.946131 | 2.1 |
| 13297 | 0.946129 | 1.8137 |
| 13298 | 0.94613 | 0.956692 |
| 13299 | 0.94613 | 1.31414 |
| 13300 | 1.33728 | 0.798372 |
| 13301 | 0.946131 | 0.195954 |
| 13302 | 0.94613 | 0.354468 |
| 13303 | 0.946132 | 0.749278 |
| 13304 | 0.946132 | 1.17399 |
| 13305 | 0.946131 | 0.61031 |
| 13306 | 0.946131 | 0.621235 |
| 13307 | 0.946127 | 2.1 |
| 13308 | 1.40641 | 2.1 |
| 13309 | 0.946129 | 2.1 |
| 13310 | 0.94613 | 2.1 |
| 13311 | 0.946128 | 1.17755 |
| 13312 | 0.946129 | 2.1 |
| 13313 | 0.946129 | 2.1 |
| 13314 | 0.946129 | 2.1 |
| 13315 | 0.946128 | 2.1 |
| 13316 | 0.946133 | 2.1 |
| 13317 | 1.37467 | 2.1 |
| 13318 | 0.946131 | 2.1 |
| 13319 | 0.946131 | 2.1 |
| 13320 | 1.37467 | 2.1 |
| 13321 | 0.946131 | 2.1 |
| 13322 | 0.946131 | 2.1 |
| 13323 | 0.946132 | 2.1 |
| 13324 | 0.946132 | 2.1 |
| 13325 | 0.946129 | 2.1 |
| 13326 | 0.946131 | 2.1 |
| 13327 | 1.34912 | 2.1 |
| 13328 | 0.946132 | 2.1 |
| 13329 | 0.946133 | 2.1 |
| 13330 | 0.946133 | 2.1 |
| 13331 | 0.946132 | 2.1 |
| 13332 | 0.946132 | 2.1 |
| 13333 | 0.946133 | 2.1 |
| 13334 | 0.946132 | 2.1 |
| 13335 | 0.946127 | 2.1 |
| 13336 | 0.946131 | 2.1 |
| 13337 | 0.946132 | 2.1 |

| | | |
|---|---|---|
| 13338 | 0.946131 | 2.1 |
| 13339 | 0.946127 | 2.1 |
| 13340 | 0.94613 | 2.1 |
| 13341 | 1.33607 | 2.1 |
| 13342 | 0.946117 | 0.132485 |
| 13343 | 0.946117 | 0.78825 |
| 13344 | 0.946115 | 1.24743 |
| 13345 | 0.946124 | 2.1 |
| 13346 | 0.946119 | 0.115143 |
| 13347 | 1.32662 | 0.883427 |
| 13348 | 0.94613 | 0.560292 |
| 13349 | 0.946119 | 2.1 |
| 13350 | 0.946114 | 2.1 |
| 13351 | 0.946118 | 2.1 |
| 13352 | 0.946114 | 2.1 |
| 13353 | 0.946118 | 2.1 |
| 13354 | 0.946104 | 2.1 |
| 13355 | 0.946129 | 2.1 |
| 13356 | 0.946132 | 2.1 |
| 13357 | 0.946133 | 2.1 |
| 13358 | 0.946133 | 2.1 |
| 13359 | 0.946133 | 2.1 |
| 13360 | 0.946132 | 2.1 |
| 13361 | 0.946132 | 2.1 |
| 13362 | 0.946133 | 2.1 |
| 13363 | 0.946133 | 2.1 |
| 13364 | 0.946132 | 2.1 |
| 13365 | 0.946132 | 2.1 |
| 13366 | 0.946133 | 2.1 |
| 13367 | 0.946132 | 2.1 |
| 13368 | 0.946132 | 2.1 |
| 13369 | 0.946132 | 2.1 |
| 13370 | 0.946133 | 2.1 |
| 13371 | 1.30881 | 2.1 |
| 13372 | 0.946132 | 2.1 |
| 13373 | 0.946131 | 1.4068 |
| 13374 | 0.946131 | 1.73551 |
| 13375 | 0.946131 | 1.16623 |
| 13376 | 0.946131 | 2.0906 |
| 13377 | 0.946128 | 2.1 |
| 13378 | 0.946128 | 2.1 |
| 13379 | 0.946132 | 2.1 |
| 13380 | 0.94613 | 2.1 |
| 13381 | 0.94613 | 2.1 |
| 13382 | 0.94613 | 2.1 |
| 13383 | 1.34652 | 2.1 |
| 13384 | 0.946133 | 0.492009 |
| 13385 | 0.946133 | 0.329818 |
| 13386 | 0.946132 | 2.03224 |
| 13387 | 0.946133 | 0.232735 |
| 13388 | 0.946132 | 2.1 |
| 13389 | 0.946132 | 1.22062 |
| 13390 | 0.946132 | 1.70683 |
| 13391 | 0.946132 | 0.0888799 |
| 13392 | 0.946128 | 0.169683 |
| 13393 | 0.94613 | 0.0570143 |
| 13394 | 0.946131 | 0.151096 |
| 13395 | 0.94613 | 0.132629 |

| | | |
|---|---|---|
| 13396 | 0.94613 | 0.05 |
| 13397 | 0.94613 | 0.0588754 |
| 13398 | 0.946128 | 0.0728448 |
| 13399 | 0.946132 | 0.05 |
| 13400 | 0.94613 | 0.05 |
| 13401 | 0.94613 | 0.05 |
| 13402 | 0.946131 | 0.0747565 |
| 13403 | 0.94613 | 0.05 |
| 13404 | 0.94613 | 0.05 |
| 13405 | 0.946129 | 1.0232 |
| 13406 | 1.32056 | 1.56279 |
| 13407 | 0.946131 | 2.1 |
| 13408 | 0.94613 | 0.965541 |
| 13409 | 0.946124 | 1.89643 |
| 13410 | 0.946128 | 1.05197 |
| 13411 | 0.946127 | 1.26482 |
| 13412 | 0.946129 | 1.0204 |
| 13413 | 1.20351 | 1.44842 |
| 13414 | 0.946132 | 1.89747 |
| 13415 | 0.946132 | 1.05371 |
| 13416 | 0.946126 | 1.94376 |
| 13417 | 0.946129 | 1.21299 |
| 13418 | 0.946129 | 1.18411 |
| 13419 | 0.946127 | 1.25363 |
| 13420 | 0.946127 | 2.1 |
| 13421 | 0.946131 | 0.894641 |
| 13422 | 1.25592 | 1.55634 |
| 13423 | 0.946127 | 0.965969 |
| 13424 | 0.946129 | 1.08777 |
| 13425 | 0.94613 | 1.23777 |
| 13426 | 1.02332 | 2.1 |
| 13427 | 0.946133 | 1.19384 |
| 13428 | 0.946132 | 1.29717 |
| 13429 | 1.02332 | 1.35956 |
| 13430 | 0.946132 | 0.912432 |
| 13431 | 0.96196 | 1.63162 |
| 13432 | 0.930306 | 1.02889 |
| 13433 | 0.946133 | 1.98483 |
| 13434 | 0.946133 | 2.1 |
| 13435 | 0.946132 | 2.1 |
| 13436 | 1.20057 | 2.1 |
| 13437 | 0.946132 | 2.1 |
| 13438 | 0.946133 | 2.1 |
| 13439 | 1.20057 | 2.1 |
| 13440 | 0.946128 | 1.92765 |
| 13441 | 0.946129 | 0.880132 |
| 13442 | 0.946129 | 1.55663 |
| 13443 | 1.13245 | 0.88355 |
| 13444 | 0.946126 | 0.73148 |
| 13445 | 0.946128 | 1.12087 |
| 13446 | 0.946129 | 0.878348 |
| 13447 | 0.946131 | 1.06958 |
| 13448 | 0.946131 | 1.9896 |
| 13449 | 0.94613 | 2.1 |
| 13450 | 1.25 | 1.74655 |
| 13451 | 0.946129 | 2.1 |
| 13452 | 0.946131 | 2.1 |
| 13453 | 1.25 | 2.1 |

| | | |
|---|---|---|
| 13454 | 0.540425 | 1.99944 |
| 13455 | 0.748493 | 1.92573 |
| 13456 | 1.46266 | 2.1 |
| 13457 | 1.20046 | 2.1 |
| 13458 | 0.600454 | 2.1 |
| 13459 | 1.41283 | 2.1 |
| 13460 | 0.582567 | 1.95391 |
| 13461 | 0.946131 | 1.75397 |
| 13462 | 0.946131 | 1.4207 |
| 13463 | 0.946132 | 1.38546 |
| 13464 | 0.946132 | 1.48954 |
| 13465 | 0.94613 | 1.36015 |
| 13466 | 0.946131 | 2.1 |
| 13467 | 1.28439 | 2.1 |
| 13468 | 0.946132 | 0.477417 |
| 13469 | 0.946131 | 0.44692 |
| 13470 | 0.946131 | 0.708275 |
| 13471 | 0.946132 | 0.26254 |
| 13472 | 0.946132 | 0.82712 |
| 13473 | 1.39335 | 0.136895 |
| 13474 | 0.946132 | 0.263705 |
| 13475 | 0.946131 | 0.05 |
| 13476 | 0.946131 | 0.05 |
| 13477 | 0.94613 | 0.05 |
| 13478 | 0.946132 | 0.05 |
| 13479 | 0.946131 | 0.05 |
| 13480 | 0.946131 | 0.05 |
| 13481 | 0.946131 | 0.05 |
| 13482 | 0.946131 | 0.05 |
| 13483 | 0.94613 | 0.05 |
| 13484 | 0.946126 | 0.088568 |
| 13485 | 0.946131 | 0.05 |
| 13486 | 0.946125 | 0.474067 |
| 13487 | 0.946127 | 0.05 |
| 13488 | 0.946128 | 0.185781 |
| 13489 | 0.946131 | 0.05 |
| 13490 | 0.946131 | 0.05 |
| 13491 | 0.946131 | 0.05 |
| 13492 | 0.946131 | 0.05 |
| 13493 | 0.946131 | 0.05 |
| 13494 | 0.946131 | 0.05 |
| 13495 | 1.39168 | 0.05 |
| 13496 | 0.946131 | 0.0822602 |
| 13497 | 0.946126 | 0.0725512 |
| 13498 | 0.946127 | 0.135284 |
| 13499 | 0.946131 | 0.0889741 |
| 13500 | 0.94613 | 0.0765079 |
| 13501 | 1.412 | 0.0594527 |
| 13502 | 0.946128 | 0.119449 |
| 13503 | 0.948901 | 0.05 |
| 13504 | 0.943364 | 0.05 |
| 13505 | 1.03768 | 0.05 |
| 13506 | 0.946132 | 0.05 |
| 13507 | 0.946133 | 0.05 |
| 13508 | 1.03767 | 0.05 |
| 13509 | 0.946132 | 0.05 |
| 13510 | 1.00902 | 0.0620317 |
| 13511 | 0.88324 | 0.05 |

| | | |
|---|---|---|
| 13512 | 0.971584 | 0.05 |
| 13513 | 0.946132 | 0.0545993 |
| 13514 | 0.946129 | 0.471184 |
| 13515 | 0.946131 | 0.191708 |
| 13516 | 0.971572 | 0.290654 |
| 13517 | 0.946131 | 0.05 |
| 13518 | 0.946125 | 0.05 |
| 13519 | 1.33587 | 0.05 |
| 13520 | 0.946126 | 0.05 |
| 13521 | 0.946127 | 0.05 |
| 13522 | 0.946126 | 0.05 |
| 13523 | 0.946128 | 0.05 |
| 13524 | 0.946132 | 0.05 |
| 13525 | 0.946132 | 0.05 |
| 13526 | 1.27512 | 0.05 |
| 13527 | 0.946133 | 0.05 |
| 13528 | 0.946132 | 0.05 |
| 13529 | 0.946133 | 0.05 |
| 13530 | 0.946133 | 0.05 |
| 13531 | 0.946128 | 0.05 |
| 13532 | 0.946133 | 0.05 |
| 13533 | 0.946131 | 0.05 |
| 13534 | 0.94613 | 0.05 |
| 13535 | 0.946131 | 0.05 |
| 13536 | 0.94613 | 0.05 |
| 13537 | 0.946131 | 0.05 |
| 13538 | 0.946131 | 0.05 |
| 13539 | 0.946131 | 0.05 |
| 13540 | 0.946131 | 0.05 |
| 13541 | 0.946131 | 0.05 |
| 13542 | 0.946131 | 0.05 |
| 13543 | 0.946131 | 0.05 |
| 13544 | 0.946131 | 0.05 |
| 13545 | 0.94613 | 0.05 |
| 13546 | 0.946131 | 0.05 |
| 13547 | 0.946131 | 0.05 |
| 13548 | 0.94613 | 0.05 |
| 13549 | 0.946132 | 0.05 |
| 13550 | 0.946131 | 0.05 |
| 13551 | 1.35316 | 0.05 |
| 13552 | 0.94613 | 0.089868 |
| 13553 | 0.94613 | 0.0657908 |
| 13554 | 0.946128 | 0.11099 |
| 13555 | 0.946132 | 0.0848223 |
| 13556 | 0.946132 | 0.0961778 |
| 13557 | 1.28727 | 0.0532501 |
| 13558 | 0.94613 | 0.11155 |
| 13559 | 0.946131 | 0.05 |
| 13560 | 0.946132 | 0.05 |
| 13561 | 0.946132 | 0.05 |
| 13562 | 1.39502 | 0.05 |
| 13563 | 0.946132 | 0.05 |
| 13564 | 0.946132 | 0.05 |
| 13565 | 0.946132 | 0.05 |
| 13566 | 0.946125 | 0.05 |
| 13567 | 0.946131 | 0.05 |
| 13568 | 0.94613 | 0.05 |
| 13569 | 0.946129 | 0.05 |

| | | |
|---|---|---|
| 13570 | 0.946126 | 0.102116 |
| 13571 | 0.946127 | 0.0570986 |
| 13572 | 1.36173 | 0.0629087 |
| 13573 | 0.946129 | 1.28413 |
| 13574 | 1.02363 | 1.28303 |
| 13575 | 0.94613 | 1.01297 |
| 13576 | 0.946131 | 1.2493 |
| 13577 | 0.946131 | 0.793412 |
| 13578 | 1.02363 | 0.953759 |
| 13579 | 0.946131 | 1.00325 |
| 13580 | 0.946128 | 0.05 |
| 13581 | 1.24678 | 0.05 |
| 13582 | 0.94613 | 0.05 |
| 13583 | 0.94613 | 0.05 |
| 13584 | 0.94613 | 0.05 |
| 13585 | 0.946131 | 0.05 |
| 13586 | 0.94613 | 0.05 |
| 13587 | 0.946129 | 0.05 |
| 13588 | 1.25215 | 0.05 |
| 13589 | 0.946132 | 0.05 |
| 13590 | 0.946126 | 0.05 |
| 13591 | 0.946129 | 0.05 |
| 13592 | 0.94613 | 0.05 |
| 13593 | 0.946129 | 0.05 |
| 13594 | 0.946129 | 0.675554 |
| 13595 | 0.946133 | 0.758565 |
| 13596 | 0.946133 | 0.621975 |
| 13597 | 0.946131 | 0.623135 |
| 13598 | 0.946131 | 0.679632 |
| 13599 | 1.29689 | 0.549046 |
| 13600 | 0.946131 | 0.571469 |
| 13601 | 0.946128 | 0.05 |
| 13602 | 0.946132 | 0.05 |
| 13603 | 0.946132 | 0.05 |
| 13604 | 0.94613 | 0.05 |
| 13605 | 1.22716 | 0.05 |
| 13606 | 1.22716 | 0.05 |
| 13607 | 0.946131 | 0.05 |
| 13608 | 0.946127 | 0.05 |
| 13609 | 0.946131 | 0.05 |
| 13610 | 0.946131 | 0.05 |
| 13611 | 0.946128 | 0.05 |
| 13612 | 1.30463 | 0.05 |
| 13613 | 1.30463 | 0.05 |
| 13614 | 0.946129 | 0.05 |
| 13615 | 1.29958 | 0.05 |
| 13616 | 0.946132 | 0.05 |
| 13617 | 0.946132 | 0.05 |
| 13618 | 0.946132 | 0.05 |
| 13619 | 0.946132 | 0.05 |
| 13620 | 0.946132 | 0.05 |
| 13621 | 0.946133 | 0.05 |
| 13622 | 1.26201 | 0.05 |
| 13623 | 0.946133 | 0.05 |
| 13624 | 0.946132 | 0.05 |
| 13625 | 0.946132 | 0.05 |
| 13626 | 0.946132 | 0.05 |
| 13627 | 0.946133 | 0.05 |

| | | |
|---|---|---|
| 13628 | 0.946132 | 0.05 |
| 13629 | 0.946133 | 0.05 |
| 13630 | 0.946132 | 0.05 |
| 13631 | 0.946132 | 0.05 |
| 13632 | 0.946133 | 0.05 |
| 13633 | 0.946132 | 0.05 |
| 13634 | 1.40427 | 0.05 |
| 13635 | 0.946133 | 0.05 |
| 13636 | 0.946132 | 0.05 |
| 13637 | 0.946132 | 0.05 |
| 13638 | 0.946132 | 0.05 |
| 13639 | 0.946132 | 0.05 |
| 13640 | 0.946132 | 0.05 |
| 13641 | 1.35123 | 0.05 |
| 13642 | 0.946131 | 0.05 |
| 13643 | 0.946128 | 0.05 |
| 13644 | 0.946132 | 0.05 |
| 13645 | 0.946132 | 0.05 |
| 13646 | 0.946129 | 0.05 |
| 13647 | 1.22144 | 0.05 |
| 13648 | 1.22144 | 0.05 |
| 13649 | 0.946131 | 0.05 |
| 13650 | 0.946127 | 0.312402 |
| 13651 | 0.946132 | 0.32051 |
| 13652 | 0.946131 | 0.265499 |
| 13653 | 0.946127 | 0.265332 |
| 13654 | 0.946131 | 0.328208 |
| 13655 | 1.23351 | 0.0840461 |
| 13656 | 0.94613 | 0.176773 |
| 13657 | 1.18119 | 0.05 |
| 13658 | 0.946131 | 0.05 |
| 13659 | 0.946132 | 0.05 |
| 13660 | 0.946132 | 0.05 |
| 13661 | 0.94613 | 0.05 |
| 13662 | 0.946131 | 0.05 |
| 13663 | 0.946131 | 0.05 |
| 13664 | 0.946132 | 0.05 |
| 13665 | 0.946132 | 0.05 |
| 13666 | 0.946133 | 0.05 |
| 13667 | 1.30563 | 0.05 |
| 13668 | 0.946132 | 0.05 |
| 13669 | 0.946132 | 0.05 |
| 13670 | 0.946133 | 0.0571254 |
| 13671 | 1.20256 | 0.05 |
| 13672 | 0.946132 | 1.09918 |
| 13673 | 0.946133 | 1.09111 |
| 13674 | 0.946132 | 0.05 |
| 13675 | 0.946132 | 0.731575 |
| 13676 | 0.946132 | 0.856092 |
| 13677 | 0.946133 | 0.176821 |
| 13678 | 0.94613 | 0.371815 |
| 13679 | 0.94613 | 0.112443 |
| 13680 | 0.946128 | 0.713032 |
| 13681 | 1.33007 | 0.0661768 |
| 13682 | 0.946129 | 0.165003 |
| 13683 | 0.94613 | 0.381539 |
| 13684 | 0.946131 | 0.798726 |
| 13685 | 0.946127 | 2.1 |

| | | |
|---|---|---|
| 13686 | 0.946127 | 2.1 |
| 13687 | 0.946131 | 2.1 |
| 13688 | 1.30136 | 2.1 |
| 13689 | 0.946128 | 2.1 |
| 13690 | 0.946129 | 2.1 |
| 13691 | 1.30136 | 2.1 |
| 13692 | 0.810942 | 2.1 |
| 13693 | 0.810942 | 2.1 |
| 13694 | 0.81093 | 2.1 |
| 13695 | 2.3208 | 2.1 |
| 13696 | 1.3517 | 2.1 |
| 13697 | 1.08132 | 2.1 |
| 13698 | 1.08132 | 2.1 |
| 13699 | 1.08133 | 2.1 |
| 13700 | 0.946132 | 2.1 |
| 13701 | 0.946132 | 2.1 |
| 13702 | 0.946132 | 2.1 |
| 13703 | 0.946132 | 2.1 |
| 13704 | 0.946132 | 2.1 |
| 13705 | 1.39823 | 2.1 |
| 13706 | 0.946132 | 2.1 |
| 13707 | 1.37713 | 2.1 |
| 13708 | 0.946131 | 2.1 |
| 13709 | 0.946131 | 2.1 |
| 13710 | 0.946131 | 2.1 |
| 13711 | 0.946131 | 2.1 |
| 13712 | 0.946131 | 2.1 |
| 13713 | 0.946131 | 2.1 |
| 13714 | 0.946128 | 2.1 |
| 13715 | 0.946128 | 2.1 |
| 13716 | 0.946133 | 2.1 |
| 13717 | 0.946131 | 2.1 |
| 13718 | 0.946131 | 1.07801 |
| 13719 | 0.946131 | 2.1 |
| 13720 | 0.94613 | 2.1 |
| 13721 | 0.946129 | 2.1 |
| 13722 | 0.946129 | 2.1 |
| 13723 | 0.946133 | 2.1 |
| 13724 | 0.946131 | 2.1 |
| 13725 | 0.946131 | 2.1 |
| 13726 | 0.946131 | 2.1 |
| 13727 | 0.946131 | 2.1 |
| 13728 | 0.946132 | 1.22285 |
| 13729 | 1.33694 | 2.1 |
| 13730 | 0.946133 | 2.1 |
| 13731 | 0.946133 | 1.02753 |
| 13732 | 0.946132 | 2.1 |
| 13733 | 0.946132 | 1.18027 |
| 13734 | 0.946133 | 1.83938 |
| 13735 | 0.946131 | 2.1 |
| 13736 | 0.94613 | 2.1 |
| 13737 | 0.946131 | 2.1 |
| 13738 | 0.946133 | 2.1 |
| 13739 | 0.946129 | 2.1 |
| 13740 | 0.946131 | 2.1 |
| 13741 | 0.946131 | 2.1 |
| 13742 | 0.946132 | 2.1 |
| 13743 | 1.35943 | 2.1 |

| | | |
|---|---|---|
| 13744 | 0.946132 | 2.1 |
| 13745 | 0.946132 | 1.22075 |
| 13746 | 0.946131 | 1.50748 |
| 13747 | 0.946132 | 1.08314 |
| 13748 | 0.946132 | 2.1 |
| 13749 | 0.946132 | 0.05 |
| 13750 | 0.946132 | 0.05 |
| 13751 | 0.946133 | 0.05 |
| 13752 | 0.946133 | 0.05 |
| 13753 | 0.946132 | 0.05 |
| 13754 | 0.946132 | 0.05 |
| 13755 | 0.946132 | 0.05 |
| 13756 | 1.29322 | 0.305607 |
| 13757 | 0.946131 | 0.259174 |
| 13758 | 0.946132 | 0.0715652 |
| 13759 | 0.946129 | 0.457447 |
| 13760 | 0.946131 | 0.332671 |
| 13761 | 0.946131 | 0.17596 |
| 13762 | 0.946131 | 0.401384 |
| 13763 | 0.946132 | 0.120694 |
| 13764 | 0.946132 | 2.1 |
| 13765 | 0.946131 | 0.05 |
| 13766 | 1.41492 | 0.05 |
| 13767 | 0.946132 | 0.0537574 |
| 13768 | 0.946132 | 0.05 |
| 13769 | 0.946131 | 0.05 |
| 13770 | 0.946132 | 2.1 |
| 13771 | 0.946132 | 2.1 |
| 13772 | 0.946132 | 2.1 |
| 13773 | 1.35028 | 2.1 |
| 13774 | 0.946132 | 2.1 |
| 13775 | 0.946132 | 2.1 |
| 13776 | 0.946132 | 2.1 |
| 13777 | 0.946132 | 1.37191 |
| 13778 | 0.946132 | 2.1 |
| 13779 | 0.946132 | 2.1 |
| 13780 | 1.32461 | 2.1 |
| 13781 | 0.946131 | 2.1 |
| 13782 | 0.946132 | 2.1 |
| 13783 | 0.946132 | 2.1 |
| 13784 | 0.946131 | 2.1 |
| 13785 | 0.946131 | 2.1 |
| 13786 | 0.946131 | 2.1 |
| 13787 | 0.946132 | 2.1 |
| 13788 | 0.946129 | 2.1 |
| 13789 | 0.94613 | 2.1 |
| 13790 | 1.40369 | 2.1 |
| 13791 | 0.946132 | 0.05 |
| 13792 | 0.946131 | 0.05 |
| 13793 | 0.946131 | 0.05 |
| 13794 | 0.979598 | 0.05 |
| 13795 | 0.946131 | 0.05 |
| 13796 | 0.946131 | 0.05 |
| 13797 | 0.946132 | 0.05 |
| 13798 | 0.946131 | 2.1 |
| 13799 | 1.41897 | 2.1 |
| 13800 | 0.946132 | 2.1 |
| 13801 | 0.946132 | 2.1 |

| | | |
|---|---|---|
| 13802 | 0.94613 | 2.1 |
| 13803 | 0.946131 | 2.1 |
| 13804 | 0.946131 | 2.1 |
| 13805 | 0.946131 | 2.1 |
| 13806 | 0.94613 | 2.1 |
| 13807 | 0.946132 | 2.1 |
| 13808 | 0.946132 | 2.1 |
| 13809 | 0.946129 | 2.1 |
| 13810 | 0.946131 | 2.1 |
| 13811 | 1.37791 | 2.1 |
| 13812 | 0.946127 | 2.1 |
| 13813 | 0.946131 | 2.1 |
| 13814 | 0.946131 | 2.1 |
| 13815 | 0.94613 | 2.1 |
| 13816 | 0.946128 | 2.1 |
| 13817 | 0.946129 | 2.1 |
| 13818 | 1.39384 | 2.1 |
| 13819 | 0.810945 | 2.1 |
| 13820 | 0.810944 | 1.531 |
| 13821 | 0.810958 | 2.00098 |
| 13822 | 2.70852 | 2.1 |
| 13823 | 1.35168 | 1.74033 |
| 13824 | 1.08132 | 2.1 |
| 13825 | 1.08132 | 1.12995 |
| 13826 | 1.0813 | 1.60014 |
| 13827 | 1.3335 | 2.1 |
| 13828 | 0.946132 | 2.1 |
| 13829 | 0.94613 | 2.1 |
| 13830 | 0.946132 | 2.1 |
| 13831 | 0.946131 | 2.1 |
| 13832 | 0.94613 | 2.1 |
| 13833 | 0.946131 | 2.1 |
| 13834 | 0.946132 | 2.1 |
| 13835 | 0.946131 | 0.05 |
| 13836 | 0.94613 | 2.1 |
| 13837 | 1.41192 | 1.49989 |
| 13838 | 0.946132 | 0.0668172 |
| 13839 | 0.946131 | 1.4383 |
| 13840 | 0.94613 | 0.166136 |
| 13841 | 0.946133 | 0.701962 |
| 13842 | 0.946133 | 0.579482 |
| 13843 | 0.946132 | 0.574604 |
| 13844 | 1.39428 | 0.388995 |
| 13845 | 0.946132 | 0.468216 |
| 13846 | 0.946132 | 0.229944 |
| 13847 | 0.946133 | 0.52953 |
| 13848 | 0.94613 | 0.827802 |
| 13849 | 0.946129 | 2.1 |
| 13850 | 0.94613 | 1.00975 |
| 13851 | 0.946128 | 1.03713 |
| 13852 | 1.40684 | 2.1 |
| 13853 | 0.94613 | 0.989607 |
| 13854 | 0.94613 | 2.1 |
| 13855 | 0.946131 | 0.800637 |
| 13856 | 0.946131 | 2.1 |
| 13857 | 0.946131 | 2.1 |
| 13858 | 0.946131 | 1.63031 |
| 13859 | 1.39021 | 2.1 |

| | | |
|---|---|---|
| 13860 | 0.946131 | 2.1 |
| 13861 | 0.946131 | 2.1 |
| 13862 | 0.946131 | 2.1 |
| 13863 | 0.946131 | 2.1 |
| 13864 | 0.946131 | 1.21167 |
| 13865 | 0.946131 | 2.1 |
| 13866 | 0.946131 | 2.1 |
| 13867 | 0.946131 | 1.08292 |
| 13868 | 0.946131 | 1.37795 |
| 13869 | 0.810946 | 2.1 |
| 13870 | 0.810946 | 2.1 |
| 13871 | 2.69264 | 2.1 |
| 13872 | 1.35168 | 2.1 |
| 13873 | 1.0813 | 2.1 |
| 13874 | 1.08132 | 2.1 |
| 13875 | 1.08132 | 2.1 |
| 13876 | 0.810958 | 2.1 |
| 13877 | 1.16687 | 2.1 |
| 13878 | 0.946131 | 2.1 |
| 13879 | 0.946131 | 2.1 |
| 13880 | 1.34632 | 2.1 |
| 13881 | 0.946131 | 2.1 |
| 13882 | 1.34632 | 2.1 |
| 13883 | 0.946131 | 2.1 |
| 13884 | 0.946128 | 1.41732 |
| 13885 | 0.946133 | 1.64833 |
| 13886 | 0.946133 | 0.821226 |
| 13887 | 0.946131 | 0.901537 |
| 13888 | 0.946131 | 0.927811 |
| 13889 | 1.3564 | 0.762993 |
| 13890 | 0.94613 | 0.772697 |
| 13891 | 0.946128 | 2.1 |
| 13892 | 0.946132 | 2.1 |
| 13893 | 0.94613 | 2.1 |
| 13894 | 0.94613 | 2.1 |
| 13895 | 0.946131 | 2.1 |
| 13896 | 1.35827 | 2.1 |
| 13897 | 0.94613 | 2.1 |
| 13898 | 0.946133 | 0.05 |
| 13899 | 0.946132 | 0.127762 |
| 13900 | 0.946132 | 0.0973485 |
| 13901 | 0.946133 | 0.0920414 |
| 13902 | 0.946133 | 0.257334 |
| 13903 | 0.946132 | 0.0570519 |
| 13904 | 0.946133 | 0.274586 |
| 13905 | 0.946132 | 2.1 |
| 13906 | 0.946132 | 2.1 |
| 13907 | 0.946132 | 0.874839 |
| 13908 | 0.946131 | 2.1 |
| 13909 | 0.946132 | 1.44664 |
| 13910 | 0.946132 | 1.32948 |
| 13911 | 0.946132 | 1.28556 |
| 13912 | 0.94613 | 0.0613919 |
| 13913 | 0.94613 | 0.05 |
| 13914 | 0.946128 | 0.090777 |
| 13915 | 0.946132 | 0.05 |
| 13916 | 0.946132 | 0.05 |
| 13917 | 0.94613 | 0.05 |

| | | |
|---|---|---|
| 13918 | 0.946131 | 0.05 |
| 13919 | 0.946126 | 0.05 |
| 13920 | 0.946131 | 0.05 |
| 13921 | 0.946127 | 0.05 |
| 13922 | 0.946129 | 0.05 |
| 13923 | 0.94613 | 0.05 |
| 13924 | 0.946128 | 0.05 |
| 13925 | 0.946128 | 0.05 |
| 13926 | 0.946131 | 0.05 |
| 13927 | 0.946127 | 0.05 |
| 13928 | 0.946127 | 0.05 |
| 13929 | 0.946131 | 0.05 |
| 13930 | 0.946131 | 0.05 |
| 13931 | 0.946128 | 0.05 |
| 13932 | 0.946129 | 0.05 |
| 13933 | 0.94613 | 0.05 |
| 13934 | 0.946131 | 0.05 |
| 13935 | 0.946129 | 0.05 |
| 13936 | 0.946132 | 0.05 |
| 13937 | 0.946132 | 0.05 |
| 13938 | 0.946131 | 0.05 |
| 13939 | 0.94613 | 0.05 |
| 13940 | 0.94613 | 0.0804858 |
| 13941 | 0.946131 | 0.0802394 |
| 13942 | 0.946129 | 0.145197 |
| 13943 | 0.946132 | 0.0681761 |
| 13944 | 0.946132 | 0.0739063 |
| 13945 | 1.32827 | 0.0662026 |
| 13946 | 0.946131 | 0.0948664 |
| 13947 | 0.946132 | 2.1 |
| 13948 | 0.946132 | 2.1 |
| 13949 | 0.946131 | 2.1 |
| 13950 | 0.946131 | 2.1 |
| 13951 | 0.946132 | 2.1 |
| 13952 | 0.946131 | 2.1 |
| 13953 | 0.946131 | 2.1 |
| 13954 | 0.946132 | 0.05 |
| 13955 | 0.946132 | 0.813287 |
| 13956 | 0.946131 | 0.05 |
| 13957 | 1.38356 | 0.05 |
| 13958 | 0.946133 | 0.350962 |
| 13959 | 0.946131 | 0.05 |
| 13960 | 0.946131 | 0.445519 |
| 13961 | 0.94613 | 2.1 |
| 13962 | 0.946131 | 2.1 |
| 13963 | 0.946131 | 2.1 |
| 13964 | 0.946128 | 2.1 |
| 13965 | 0.946133 | 2.1 |
| 13966 | 0.946131 | 2.1 |
| 13967 | 1.36918 | 2.1 |
| 13968 | 0.946131 | 0.629519 |
| 13969 | 0.94613 | 2.1 |
| 13970 | 0.946129 | 1.00679 |
| 13971 | 0.946133 | 2.1 |
| 13972 | 0.946132 | 0.833777 |
| 13973 | 1.3771 | 2.1 |
| 13974 | 0.946131 | 1.64206 |
| 13975 | 1.30029 | 1.17151 |

| | | |
|---|---|---|
| 13976 | 0.94613 | 0.761596 |
| 13977 | 0.946129 | 1.91263 |
| 13978 | 0.946132 | 1.83634 |
| 13979 | 0.946132 | 0.161414 |
| 13980 | 0.946131 | 1.0385 |
| 13981 | 0.94613 | 0.887058 |
| 13982 | 0.946133 | 0.05 |
| 13983 | 0.946132 | 0.05 |
| 13984 | 0.946132 | 0.05 |
| 13985 | 0.946132 | 0.05 |
| 13986 | 0.946132 | 0.05 |
| 13987 | 0.946132 | 0.05 |
| 13988 | 1.21174 | 0.05 |
| 13989 | 0.810946 | 2.1 |
| 13990 | 0.810945 | 2.1 |
| 13991 | 2.63045 | 2.1 |
| 13992 | 1.35169 | 2.1 |
| 13993 | 1.08132 | 2.1 |
| 13994 | 1.08132 | 2.1 |
| 13995 | 1.08132 | 2.1 |
| 13996 | 0.810946 | 2.1 |
| 13997 | 1.1796 | 2.1 |
| 13998 | 0.946131 | 2.1 |
| 13999 | 0.946132 | 2.1 |
| 14000 | 1.31522 | 2.1 |
| 14001 | 0.946132 | 2.1 |
| 14002 | 1.31522 | 2.1 |
| 14003 | 0.946132 | 2.1 |
| 14004 | 0.946131 | 2.1 |
| 14005 | 1.40615 | 2.1 |
| 14006 | 0.946129 | 2.1 |
| 14007 | 0.946128 | 2.1 |
| 14008 | 0.946133 | 2.1 |
| 14009 | 0.946131 | 2.1 |
| 14010 | 0.94613 | 2.1 |
| 14011 | 0.810945 | 2.1 |
| 14012 | 0.810944 | 2.1 |
| 14013 | 2.80479 | 2.1 |
| 14014 | 1.35169 | 2.1 |
| 14015 | 1.08132 | 2.1 |
| 14016 | 1.08132 | 2.1 |
| 14017 | 1.08132 | 2.1 |
| 14018 | 0.810945 | 2.1 |
| 14019 | 1.35562 | 2.1 |
| 14020 | 0.94613 | 2.1 |
| 14021 | 1.35561 | 2.1 |
| 14022 | 0.946133 | 2.1 |
| 14023 | 0.946131 | 2.1 |
| 14024 | 1.40239 | 2.1 |
| 14025 | 0.946131 | 2.1 |
| 14026 | 0.946125 | 0.05 |
| 14027 | 0.946131 | 0.05 |
| 14028 | 0.946127 | 0.05 |
| 14029 | 0.946129 | 0.05 |
| 14030 | 0.946128 | 0.05 |
| 14031 | 0.946129 | 0.0550148 |
| 14032 | 0.946128 | 0.05 |
| 14033 | 0.946119 | 2.1 |

| | | |
|---|---|---|
| 14034 | 0.946113 | 2.1 |
| 14035 | 0.94612 | 2.1 |
| 14036 | 0.946119 | 2.1 |
| 14037 | 1.41915 | 2.1 |
| 14038 | 0.946107 | 2.1 |
| 14039 | 0.946132 | 2.1 |
| 14040 | 0.810946 | 1.33327 |
| 14041 | 0.810943 | 2.1 |
| 14042 | 0.810934 | 2.08451 |
| 14043 | 2.31411 | 2.1 |
| 14044 | 1.35171 | 2.1 |
| 14045 | 1.08132 | 1.74159 |
| 14046 | 1.08132 | 2.1 |
| 14047 | 1.08133 | 1.87822 |
| 14048 | 0.946127 | 2.1 |
| 14049 | 0.94613 | 2.1 |
| 14050 | 0.94613 | 2.1 |
| 14051 | 0.946131 | 2.1 |
| 14052 | 0.946126 | 2.1 |
| 14053 | 0.946128 | 2.1 |
| 14054 | 0.946128 | 2.1 |
| 14055 | 0.810944 | 2.1 |
| 14056 | 0.810947 | 2.1 |
| 14057 | 0.81096 | 2.1 |
| 14058 | 2.78019 | 2.1 |
| 14059 | 1.35168 | 2.1 |
| 14060 | 1.08132 | 2.1 |
| 14061 | 1.08132 | 2.1 |
| 14062 | 1.08131 | 2.1 |
| 14063 | 0.946133 | 0.05 |
| 14064 | 0.946132 | 0.05 |
| 14065 | 0.946132 | 0.05 |
| 14066 | 0.946133 | 0.05 |
| 14067 | 0.946132 | 0.05 |
| 14068 | 1.3495 | 0.05 |
| 14069 | 0.946133 | 0.05 |
| 14070 | 0.946133 | 2.1 |
| 14071 | 0.946132 | 2.1 |
| 14072 | 0.946132 | 2.1 |
| 14073 | 1.39529 | 2.1 |
| 14074 | 0.946133 | 2.1 |
| 14075 | 0.946132 | 2.1 |
| 14076 | 0.946133 | 2.1 |
| 14077 | 0.946133 | 2.1 |
| 14078 | 0.946132 | 2.1 |
| 14079 | 0.946132 | 2.1 |
| 14080 | 0.946132 | 2.1 |
| 14081 | 0.946132 | 2.1 |
| 14082 | 0.946133 | 2.1 |
| 14083 | 0.946132 | 2.1 |
| 14084 | 0.810946 | 2.1 |
| 14085 | 0.810945 | 2.1 |
| 14086 | 2.67861 | 2.1 |
| 14087 | 1.35169 | 2.1 |
| 14088 | 1.08132 | 2.1 |
| 14089 | 1.08132 | 2.1 |
| 14090 | 1.08132 | 2.1 |
| 14091 | 0.810946 | 2.1 |

| | | |
|---|---|---|
| 14092 | 1.24616 | 2.1 |
| 14093 | 0.946132 | 2.1 |
| 14094 | 0.946132 | 2.1 |
| 14095 | 1.33931 | 2.1 |
| 14096 | 0.946132 | 2.1 |
| 14097 | 1.33931 | 2.1 |
| 14098 | 0.946133 | 2.1 |
| 14099 | 0.946133 | 0.189958 |
| 14100 | 0.946133 | 0.0834212 |
| 14101 | 0.946132 | 0.0632388 |
| 14102 | 0.946133 | 0.171343 |
| 14103 | 1.3855 | 0.05 |
| 14104 | 1.3855 | 0.05 |
| 14105 | 0.946133 | 0.169388 |
| 14106 | 0.946128 | 0.995153 |
| 14107 | 0.946132 | 2.1 |
| 14108 | 0.946128 | 2.1 |
| 14109 | 0.94613 | 2.1 |
| 14110 | 0.946131 | 2.1 |
| 14111 | 1.40225 | 2.1 |
| 14112 | 0.94613 | 2.1 |
| 14113 | 1.40548 | 0.05 |
| 14114 | 0.946118 | 0.05 |
| 14115 | 0.946114 | 0.05 |
| 14116 | 0.946112 | 0.05 |
| 14117 | 0.946117 | 0.05 |
| 14118 | 0.946128 | 0.05 |
| 14119 | 0.946103 | 0.05 |
| 14120 | 0.94613 | 2.1 |
| 14121 | 0.946132 | 2.1 |
| 14122 | 0.946131 | 2.1 |
| 14123 | 0.946132 | 2.1 |
| 14124 | 1.37811 | 2.1 |
| 14125 | 0.946131 | 2.1 |
| 14126 | 0.946131 | 2.1 |
| 14127 | 0.946132 | 2.1 |
| 14128 | 0.946133 | 2.1 |
| 14129 | 0.946133 | 2.1 |
| 14130 | 0.946132 | 2.1 |
| 14131 | 0.946133 | 2.1 |
| 14132 | 0.946132 | 2.1 |
| 14133 | 0.946132 | 2.1 |
| 14134 | 0.946118 | 2.1 |
| 14135 | 0.946113 | 2.1 |
| 14136 | 0.946118 | 2.1 |
| 14137 | 0.946119 | 2.1 |
| 14138 | 0.946117 | 2.1 |
| 14139 | 0.946105 | 2.1 |
| 14140 | 0.946131 | 2.1 |
| 14141 | 0.946118 | 0.05 |
| 14142 | 0.946118 | 0.05 |
| 14143 | 0.946117 | 0.05 |
| 14144 | 0.946113 | 0.05 |
| 14145 | 0.94612 | 0.05 |
| 14146 | 1.41377 | 0.05 |
| 14147 | 0.946106 | 0.05 |
| 14148 | 0.946132 | 0.422727 |
| 14149 | 1.28436 | 0.16649 |

| | | |
|---|---|---|
| 14150 | 0.946131 | 0.164218 |
| 14151 | 0.946131 | 0.557456 |
| 14152 | 0.946131 | 0.05 |
| 14153 | 0.94613 | 0.641395 |
| 14154 | 0.946131 | 0.292795 |
| 14155 | 0.810947 | 0.0507397 |
| 14156 | 0.810946 | 0.113254 |
| 14157 | 2.68675 | 0.05 |
| 14158 | 1.35168 | 0.05 |
| 14159 | 1.08132 | 0.0578671 |
| 14160 | 1.08132 | 0.05 |
| 14161 | 1.0813 | 0.0781137 |
| 14162 | 0.81096 | 0.05 |
| 14163 | 0.946132 | 0.05 |
| 14164 | 1.34338 | 0.05 |
| 14165 | 0.946132 | 0.05 |
| 14166 | 1.34338 | 0.05 |
| 14167 | 0.946132 | 0.05 |
| 14168 | 1.34337 | 0.05 |
| 14169 | 0.946132 | 0.05 |
| 14170 | 0.946127 | 2.1 |
| 14171 | 0.946131 | 2.1 |
| 14172 | 0.946127 | 2.1 |
| 14173 | 0.946131 | 1.81169 |
| 14174 | 0.946131 | 2.1 |
| 14175 | 0.946128 | 2.1 |
| 14176 | 1.30281 | 2.1 |
| 14177 | 0.946132 | 0.41835 |
| 14178 | 0.946132 | 0.326671 |
| 14179 | 0.946132 | 0.527755 |
| 14180 | 0.946131 | 0.241873 |
| 14181 | 0.946132 | 0.640024 |
| 14182 | 1.37973 | 0.05 |
| 14183 | 0.946132 | 0.152908 |
| 14184 | 0.946131 | 0.635086 |
| 14185 | 1.41798 | 0.231701 |
| 14186 | 0.946132 | 0.0821762 |
| 14187 | 0.946132 | 0.360487 |
| 14188 | 0.946129 | 0.826891 |
| 14189 | 0.946131 | 0.219442 |
| 14190 | 0.946131 | 0.748454 |
| 14191 | 0.946118 | 0.381143 |
| 14192 | 1.41776 | 0.131676 |
| 14193 | 0.946113 | 0.147138 |
| 14194 | 0.946119 | 0.123088 |
| 14195 | 0.946117 | 0.607709 |
| 14196 | 1.41778 | 0.126315 |
| 14197 | 0.946105 | 0.668666 |
| 14198 | 0.946131 | 0.673331 |
| 14199 | 1.41798 | 0.870132 |
| 14200 | 0.946132 | 0.944607 |
| 14201 | 0.946132 | 0.460222 |
| 14202 | 0.94613 | 0.799029 |
| 14203 | 1.41799 | 0.287787 |
| 14204 | 0.946131 | 0.329902 |
| 14205 | 0.946133 | 2.1 |
| 14206 | 0.946132 | 2.1 |
| 14207 | 0.946132 | 0.653561 |

| | | |
|---|---|---|
| 14208 | 0.946132 | 2.1 |
| 14209 | 0.946133 | 1.39064 |
| 14210 | 0.946132 | 2.1 |
| 14211 | 0.946132 | 2.1 |
| 14212 | 0.946131 | 2.1 |
| 14213 | 0.946131 | 2.1 |
| 14214 | 0.94613 | 0.371873 |
| 14215 | 0.946129 | 2.1 |
| 14216 | 0.946132 | 1.09634 |
| 14217 | 0.94613 | 1.06809 |
| 14218 | 0.946131 | 2.1 |
| 14219 | 0.94612 | 1.28625 |
| 14220 | 0.946126 | 2.1 |
| 14221 | 0.946119 | 1.15379 |
| 14222 | 0.94612 | 1.7106 |
| 14223 | 1.40766 | 2.1 |
| 14224 | 0.946132 | 0.983513 |
| 14225 | 0.946107 | 2.1 |
| 14226 | 0.946128 | 2.1 |
| 14227 | 1.31213 | 2.1 |
| 14228 | 0.946129 | 2.1 |
| 14229 | 0.94613 | 2.1 |
| 14230 | 0.94613 | 2.1 |
| 14231 | 0.94613 | 2.1 |
| 14232 | 0.94613 | 2.1 |
| 14233 | 0.946127 | 2.1 |
| 14234 | 0.946131 | 2.1 |
| 14235 | 0.94613 | 2.1 |
| 14236 | 0.94613 | 2.1 |
| 14237 | 1.36653 | 2.1 |
| 14238 | 0.946128 | 2.1 |
| 14239 | 0.946128 | 2.1 |
| 14240 | 0.946127 | 2.1 |
| 14241 | 0.94613 | 2.1 |
| 14242 | 0.946131 | 2.1 |
| 14243 | 0.94613 | 2.1 |
| 14244 | 0.946128 | 2.1 |
| 14245 | 0.946128 | 2.1 |
| 14246 | 0.946129 | 2.1 |
| 14247 | 0.946133 | 2.1 |
| 14248 | 1.40081 | 2.1 |
| 14249 | 0.946132 | 2.1 |
| 14250 | 0.946132 | 2.1 |
| 14251 | 0.946133 | 2.1 |
| 14252 | 0.946132 | 2.1 |
| 14253 | 0.946133 | 2.1 |
| 14254 | 0.946128 | 2.1 |
| 14255 | 1.32282 | 2.1 |
| 14256 | 0.946129 | 2.1 |
| 14257 | 0.94613 | 2.1 |
| 14258 | 0.946131 | 2.1 |
| 14259 | 0.946131 | 2.1 |
| 14260 | 0.946131 | 2.1 |
| 14261 | 0.810945 | 2.1 |
| 14262 | 0.810943 | 2.1 |
| 14263 | 0.810933 | 2.1 |
| 14264 | 2.44097 | 2.1 |
| 14265 | 1.3517 | 2.1 |

| | | |
|---|---|---|
| 14266 | 1.08132 | 2.1 |
| 14267 | 1.08132 | 2.1 |
| 14268 | 1.08133 | 2.1 |
| 14269 | 0.946131 | 2.1 |
| 14270 | 0.94613 | 2.1 |
| 14271 | 0.946133 | 2.1 |
| 14272 | 0.946133 | 2.1 |
| 14273 | 0.946128 | 2.1 |
| 14274 | 0.946131 | 2.1 |
| 14275 | 1.27676 | 2.1 |
| 14276 | 0.946126 | 0.736737 |
| 14277 | 0.946126 | 0.806593 |
| 14278 | 0.946131 | 0.658472 |
| 14279 | 0.946127 | 0.795426 |
| 14280 | 0.94613 | 0.447819 |
| 14281 | 0.946128 | 0.594014 |
| 14282 | 0.946128 | 0.0560647 |
| 14283 | 0.946132 | 0.05 |
| 14284 | 0.946133 | 0.05 |
| 14285 | 0.946132 | 0.05 |
| 14286 | 0.946132 | 0.05 |
| 14287 | 0.946132 | 0.05 |
| 14288 | 0.946133 | 0.05 |
| 14289 | 0.946132 | 0.05 |
| 14290 | 0.946128 | 0.05 |
| 14291 | 0.946131 | 0.05 |
| 14292 | 0.94613 | 0.05 |
| 14293 | 0.946129 | 0.05 |
| 14294 | 0.94613 | 0.05 |
| 14295 | 0.94613 | 0.061771 |
| 14296 | 0.94613 | 0.05 |
| 14297 | 0.946132 | 0.05 |
| 14298 | 0.946128 | 0.05 |
| 14299 | 0.94613 | 0.05 |
| 14300 | 0.946131 | 0.05 |
| 14301 | 0.94613 | 0.05 |
| 14302 | 0.94613 | 0.05 |
| 14303 | 0.946131 | 0.05 |
| 14304 | 0.94612 | 0.05 |
| 14305 | 0.946119 | 0.05 |
| 14306 | 0.946119 | 0.05 |
| 14307 | 0.946124 | 0.05 |
| 14308 | 0.946119 | 0.05 |
| 14309 | 0.946132 | 0.05 |
| 14310 | 0.946107 | 0.05 |
| 14311 | 0.946128 | 2.1 |
| 14312 | 0.946133 | 2.1 |
| 14313 | 0.946131 | 2.1 |
| 14314 | 0.94613 | 2.1 |
| 14315 | 0.946131 | 2.1 |
| 14316 | 1.37869 | 2.1 |
| 14317 | 0.946131 | 2.1 |
| 14318 | 1.35703 | 2.1 |
| 14319 | 0.946132 | 2.1 |
| 14320 | 0.946132 | 2.1 |
| 14321 | 0.946132 | 2.1 |
| 14322 | 0.946132 | 2.1 |
| 14323 | 0.946132 | 2.1 |

| | | |
|---|---|---|
| 14324 | 0.946133 | 2.1 |
| 14325 | 0.946133 | 2.1 |
| 14326 | 0.946133 | 2.1 |
| 14327 | 0.946132 | 2.1 |
| 14328 | 1.37017 | 2.1 |
| 14329 | 0.946133 | 2.1 |
| 14330 | 0.946132 | 2.1 |
| 14331 | 0.946133 | 2.1 |
| 14332 | 0.946114 | 0.05 |
| 14333 | 0.946119 | 0.05 |
| 14334 | 0.946119 | 0.05 |
| 14335 | 0.94612 | 0.05 |
| 14336 | 0.946119 | 0.05 |
| 14337 | 0.946132 | 0.05 |
| 14338 | 0.946107 | 0.05 |
| 14339 | 0.946131 | 0.05 |
| 14340 | 0.946131 | 0.05 |
| 14341 | 0.946126 | 0.05 |
| 14342 | 0.946127 | 0.05 |
| 14343 | 0.94613 | 0.05 |
| 14344 | 0.946129 | 0.05 |
| 14345 | 0.946128 | 0.05 |
| 14346 | 0.946131 | 1.02441 |
| 14347 | 0.946131 | 0.77518 |
| 14348 | 0.946132 | 0.173441 |
| 14349 | 0.946132 | 1.04452 |
| 14350 | 0.94613 | 1.37836 |
| 14351 | 0.946131 | 1.43886 |
| 14352 | 1.3279 | 1.35546 |
| 14353 | 0.946132 | 2.1 |
| 14354 | 1.40932 | 2.1 |
| 14355 | 0.946132 | 2.1 |
| 14356 | 0.946132 | 2.1 |
| 14357 | 0.946132 | 2.1 |
| 14358 | 0.946131 | 2.1 |
| 14359 | 0.946132 | 2.1 |
| 14360 | 0.946131 | 0.05 |
| 14361 | 0.946131 | 0.05 |
| 14362 | 0.946132 | 0.05 |
| 14363 | 0.946132 | 0.05 |
| 14364 | 0.94613 | 0.05 |
| 14365 | 0.946131 | 0.05 |
| 14366 | 1.26868 | 0.05 |
| 14367 | 0.810943 | 2.1 |
| 14368 | 0.810945 | 2.1 |
| 14369 | 0.810932 | 2.1 |
| 14370 | 2.82321 | 2.1 |
| 14371 | 1.3517 | 2.1 |
| 14372 | 1.08132 | 2.1 |
| 14373 | 1.08132 | 2.1 |
| 14374 | 1.08133 | 2.1 |
| 14375 | 0.810943 | 0.05 |
| 14376 | 0.810944 | 0.0765444 |
| 14377 | 2.63079 | 0.05 |
| 14378 | 1.35168 | 0.0675844 |
| 14379 | 1.0813 | 0.0944924 |
| 14380 | 1.08132 | 0.05 |
| 14381 | 1.08132 | 0.134187 |

| | | |
|---|---|---|
| 14382 | 0.810957 | 0.121038 |
| 14383 | 1.17856 | 0.05 |
| 14384 | 0.946132 | 0.05 |
| 14385 | 0.946128 | 0.096213 |
| 14386 | 1.3154 | 0.12164 |
| 14387 | 0.94613 | 0.245046 |
| 14388 | 1.31539 | 0.0564978 |
| 14389 | 0.94613 | 0.809447 |
| 14390 | 0.94613 | 1.54219 |
| 14391 | 0.94613 | 1.39235 |
| 14392 | 0.946131 | 1.04979 |
| 14393 | 0.946131 | 1.50038 |
| 14394 | 0.946129 | 1.77248 |
| 14395 | 0.94613 | 2.1 |
| 14396 | 1.21997 | 2.1 |
| 14397 | 0.946131 | 1.11046 |
| 14398 | 1.25083 | 0.311478 |
| 14399 | 0.946132 | 0.221284 |
| 14400 | 0.946132 | 1.17559 |
| 14401 | 0.946129 | 0.226353 |
| 14402 | 1.25083 | 1.06579 |
| 14403 | 0.946131 | 1.23047 |
| 14404 | 0.946127 | 1.55477 |
| 14405 | 1.41342 | 0.832768 |
| 14406 | 0.94613 | 1.23922 |
| 14407 | 0.946131 | 1.11906 |
| 14408 | 0.946127 | 0.445299 |
| 14409 | 0.946129 | 0.687276 |
| 14410 | 0.946129 | 0.935414 |
| 14411 | 0.946131 | 2.1 |
| 14412 | 1.29149 | 2.0745 |
| 14413 | 0.946133 | 2.1 |
| 14414 | 0.946133 | 1.19988 |
| 14415 | 0.946128 | 1.86487 |
| 14416 | 0.946131 | 0.899017 |
| 14417 | 0.94613 | 1.47649 |
| 14418 | 0.94613 | 2.1 |
| 14419 | 0.94613 | 1.87933 |
| 14420 | 0.946132 | 2.1 |
| 14421 | 0.946132 | 1.10935 |
| 14422 | 0.946128 | 0.0682353 |
| 14423 | 0.94613 | 2.1 |
| 14424 | 1.41022 | 2.1 |
| 14425 | 0.946132 | 0.05 |
| 14426 | 0.946132 | 0.05 |
| 14427 | 0.946128 | 0.05 |
| 14428 | 0.94613 | 0.05 |
| 14429 | 0.94613 | 0.05 |
| 14430 | 0.94613 | 0.05 |
| 14431 | 0.946131 | 0.05 |
| 14432 | 0.946129 | 1.11023 |
| 14433 | 1.37758 | 1.99501 |
| 14434 | 0.946131 | 2.1 |
| 14435 | 0.94613 | 0.862512 |
| 14436 | 0.946124 | 1.81742 |
| 14437 | 0.946127 | 0.907213 |
| 14438 | 0.946127 | 2.06062 |
| 14439 | 0.946129 | 1.09036 |

| | | |
|---|---|---|
| 14440 | 1.38259 | 1.81483 |
| 14441 | 0.946132 | 2.1 |
| 14442 | 0.946132 | 0.900516 |
| 14443 | 0.946126 | 1.93489 |
| 14444 | 0.946129 | 0.966801 |
| 14445 | 0.94613 | 1.74162 |
| 14446 | 0.946131 | 0.05 |
| 14447 | 0.946132 | 0.0536796 |
| 14448 | 0.946132 | 0.05 |
| 14449 | 0.946132 | 0.0508435 |
| 14450 | 0.946132 | 0.05 |
| 14451 | 0.946132 | 0.05 |
| 14452 | 0.946132 | 0.05 |
| 14453 | 0.946131 | 0.05 |
| 14454 | 0.94613 | 0.05 |
| 14455 | 0.94613 | 0.05 |
| 14456 | 0.946133 | 0.05 |
| 14457 | 0.946128 | 0.05 |
| 14458 | 1.25017 | 0.05 |
| 14459 | 0.946131 | 0.05 |
| 14460 | 0.946131 | 2.1 |
| 14461 | 0.946131 | 2.1 |
| 14462 | 0.946131 | 2.1 |
| 14463 | 1.3937 | 2.1 |
| 14464 | 0.946133 | 2.1 |
| 14465 | 0.946131 | 2.1 |
| 14466 | 0.94613 | 2.1 |
| 14467 | 0.946131 | 0.05 |
| 14468 | 0.946131 | 0.125007 |
| 14469 | 0.946131 | 0.05 |
| 14470 | 0.94613 | 0.0897454 |
| 14471 | 0.946133 | 0.524218 |
| 14472 | 0.946132 | 0.05 |
| 14473 | 0.946131 | 0.169689 |
| 14474 | 0.946132 | 0.05 |
| 14475 | 0.946132 | 0.428129 |
| 14476 | 0.946132 | 0.05 |
| 14477 | 0.946132 | 0.0842862 |
| 14478 | 0.946131 | 0.216665 |
| 14479 | 0.946132 | 0.05 |
| 14480 | 0.946132 | 0.144982 |
| 14481 | 0.946129 | 0.05 |
| 14482 | 0.94613 | 0.187658 |
| 14483 | 0.94613 | 0.0846164 |
| 14484 | 0.946128 | 0.0896847 |
| 14485 | 0.946132 | 0.05 |
| 14486 | 0.946131 | 0.370634 |
| 14487 | 0.94613 | 0.05 |
| 14488 | 0.946132 | 2.1 |
| 14489 | 0.946132 | 0.05 |
| 14490 | 0.946132 | 0.490995 |
| 14491 | 1.41619 | 2.1 |
| 14492 | 0.946133 | 0.05 |
| 14493 | 0.946133 | 1.45831 |
| 14494 | 0.946132 | 0.05 |
| 14495 | 0.946132 | 2.1 |
| 14496 | 0.946132 | 2.1 |
| 14497 | 0.946132 | 2.1 |

| | | |
|---|---|---|
| 14498 | 1.38244 | 2.1 |
| 14499 | 0.946132 | 0.995311 |
| 14500 | 0.946131 | 2.1 |
| 14501 | 0.946132 | 2.1 |
| 14502 | 0.810946 | 0.381311 |
| 14503 | 0.810943 | 0.969825 |
| 14504 | 0.810934 | 0.472513 |
| 14505 | 2.42741 | 0.0530387 |
| 14506 | 1.35171 | 0.139278 |
| 14507 | 1.08132 | 0.393715 |
| 14508 | 1.08132 | 0.4096 |
| 14509 | 1.08133 | 0.418451 |
| 14510 | 0.810947 | 0.201884 |
| 14511 | 0.810943 | 0.937788 |
| 14512 | 2.44179 | 0.0521578 |
| 14513 | 1.35171 | 0.148327 |
| 14514 | 1.08132 | 0.178037 |
| 14515 | 1.08132 | 0.257405 |
| 14516 | 1.08133 | 0.162283 |
| 14517 | 0.810934 | 0.170699 |
| 14518 | 0.946132 | 1.89028 |
| 14519 | 0.946132 | 1.78284 |
| 14520 | 0.946133 | 0.898196 |
| 14521 | 0.946133 | 1.73228 |
| 14522 | 0.946132 | 2.03655 |
| 14523 | 0.946132 | 1.70831 |
| 14524 | 1.12786 | 1.38336 |
| 14525 | 1.27806 | 0.767206 |
| 14526 | 0.946132 | 1.04939 |
| 14527 | 0.946133 | 0.895746 |
| 14528 | 0.946133 | 1.2965 |
| 14529 | 0.946132 | 2.01541 |
| 14530 | 0.946132 | 1.04181 |
| 14531 | 0.946133 | 1.92955 |
| 14532 | 0.946132 | 2.1 |
| 14533 | 0.946132 | 2.1 |
| 14534 | 0.946132 | 2.1 |
| 14535 | 1.37098 | 2.1 |
| 14536 | 0.946133 | 2.1 |
| 14537 | 0.946132 | 2.1 |
| 14538 | 1.37098 | 2.1 |
| 14539 | 0.946125 | 1.29745 |
| 14540 | 0.946129 | 1.14377 |
| 14541 | 0.946131 | 2.1 |
| 14542 | 0.94613 | 0.943713 |
| 14543 | 0.946124 | 0.824732 |
| 14544 | 0.946127 | 0.96205 |
| 14545 | 1.39514 | 1.44464 |
| 14546 | 0.946132 | 2.1 |
| 14547 | 0.946131 | 2.1 |
| 14548 | 0.94613 | 2.1 |
| 14549 | 1.39909 | 2.1 |
| 14550 | 0.946132 | 0.843573 |
| 14551 | 0.946131 | 2.1 |
| 14552 | 0.94613 | 2.1 |
| 14553 | 0.946133 | 2.1 |
| 14554 | 0.946133 | 0.05 |
| 14555 | 0.946132 | 2.1 |

| | | |
|---|---|---|
| 14556 | 1.36835 | 2.01877 |
| 14557 | 0.946132 | 0.592114 |
| 14558 | 0.946133 | 2.1 |
| 14559 | 0.946132 | 0.0880919 |
| 14560 | 0.946132 | 2.1 |
| 14561 | 0.946133 | 2.1 |
| 14562 | 0.946133 | 2.1 |
| 14563 | 0.946132 | 2.1 |
| 14564 | 1.39047 | 2.1 |
| 14565 | 0.946132 | 2.1 |
| 14566 | 0.946132 | 2.1 |
| 14567 | 1.33174 | 0.05 |
| 14568 | 0.946128 | 0.05 |
| 14569 | 0.94613 | 0.05 |
| 14570 | 0.94613 | 0.05 |
| 14571 | 0.94613 | 0.05 |
| 14572 | 0.94613 | 0.05 |
| 14573 | 0.946131 | 0.05 |
| 14574 | 0.946128 | 0.05 |
| 14575 | 1.36004 | 0.05 |
| 14576 | 0.946129 | 0.05 |
| 14577 | 0.94613 | 0.05 |
| 14578 | 0.94613 | 0.05 |
| 14579 | 1.36003 | 0.05 |
| 14580 | 0.94613 | 0.05 |
| 14581 | 0.946124 | 2.1 |
| 14582 | 0.946119 | 2.1 |
| 14583 | 0.946119 | 2.1 |
| 14584 | 0.946119 | 2.1 |
| 14585 | 0.94612 | 2.1 |
| 14586 | 0.946132 | 2.1 |
| 14587 | 1.29062 | 2.1 |
| 14588 | 0.946128 | 2.1 |
| 14589 | 0.946128 | 2.1 |
| 14590 | 0.946133 | 2.1 |
| 14591 | 0.94613 | 2.1 |
| 14592 | 0.946131 | 2.1 |
| 14593 | 0.946131 | 2.1 |
| 14594 | 1.30584 | 2.1 |
| 14595 | 0.946131 | 2.1 |
| 14596 | 0.946131 | 2.1 |
| 14597 | 0.946131 | 2.1 |
| 14598 | 0.946132 | 2.1 |
| 14599 | 1.39549 | 2.1 |
| 14600 | 0.946131 | 2.1 |
| 14601 | 0.946131 | 2.1 |
| 14602 | 0.913386 | 0.998161 |
| 14603 | 1.17466 | 0.860615 |
| 14604 | 1.00473 | 0.354221 |
| 14605 | 0.946132 | 0.81553 |
| 14606 | 0.946126 | 1.39352 |
| 14607 | 0.94613 | 1.08033 |
| 14608 | 0.946129 | 1.02214 |
| 14609 | 0.946132 | 0.785344 |
| 14610 | 1.38894 | 0.774339 |
| 14611 | 0.946132 | 0.578748 |
| 14612 | 0.946132 | 0.623646 |
| 14613 | 0.946132 | 1.02615 |

| | | |
|---|---|---|
| 14614 | 0.946132 | 0.789338 |
| 14615 | 0.946133 | 0.80653 |
| 14616 | 0.946128 | 0.620733 |
| 14617 | 1.23865 | 0.704964 |
| 14618 | 0.946129 | 0.250225 |
| 14619 | 0.94613 | 0.40355 |
| 14620 | 0.94613 | 0.665064 |
| 14621 | 0.94613 | 0.412069 |
| 14622 | 0.94613 | 0.542788 |
| 14623 | 0.946126 | 0.972223 |
| 14624 | 1.40248 | 1.12914 |
| 14625 | 0.946129 | 1.05843 |
| 14626 | 0.94613 | 0.940472 |
| 14627 | 0.946124 | 1.93366 |
| 14628 | 0.946127 | 1.15012 |
| 14629 | 0.946128 | 1.08415 |
| 14630 | 0.946133 | 0.810431 |
| 14631 | 1.1391 | 0.882327 |
| 14632 | 0.946132 | 0.87517 |
| 14633 | 0.946132 | 0.689049 |
| 14634 | 0.946132 | 1.24536 |
| 14635 | 0.946133 | 0.830412 |
| 14636 | 0.946132 | 0.854619 |
| 14637 | 1.17156 | 1.18194 |
| 14638 | 0.946133 | 1.28488 |
| 14639 | 1.33893 | 0.670677 |
| 14640 | 0.827864 | 0.862704 |
| 14641 | 0.995232 | 0.470098 |
| 14642 | 0.827864 | 1.10243 |
| 14643 | 0.94613 | 0.705358 |
| 14644 | 1.21387 | 0.72224 |
| 14645 | 0.946132 | 0.744225 |
| 14646 | 0.946131 | 0.375954 |
| 14647 | 0.94613 | 0.639855 |
| 14648 | 0.94613 | 0.612715 |
| 14649 | 0.94613 | 0.683402 |
| 14650 | 0.94613 | 0.626073 |
| 14651 | 0.810943 | 0.05 |
| 14652 | 0.810945 | 0.05 |
| 14653 | 2.42463 | 0.05 |
| 14654 | 1.3517 | 0.05 |
| 14655 | 1.08132 | 0.05 |
| 14656 | 1.08132 | 0.05 |
| 14657 | 1.08133 | 0.05 |
| 14658 | 0.810932 | 0.05 |
| 14659 | 0.946133 | 1.52286 |
| 14660 | 0.946132 | 0.377926 |
| 14661 | 0.946131 | 0.326281 |
| 14662 | 1.36748 | 1.07893 |
| 14663 | 0.946133 | 0.890292 |
| 14664 | 0.946131 | 0.25923 |
| 14665 | 0.946132 | 0.05 |
| 14666 | 1.35856 | 0.10802 |
| 14667 | 0.946131 | 0.100913 |
| 14668 | 0.94613 | 0.05 |
| 14669 | 0.94613 | 0.159301 |
| 14670 | 0.946131 | 0.106912 |
| 14671 | 0.94613 | 0.126023 |

| | | |
|---|---|---|
| 14672 | 0.94613 | 0.170907 |
| 14673 | 0.946132 | 2.1 |
| 14674 | 0.946131 | 2.1 |
| 14675 | 0.946131 | 2.1 |
| 14676 | 0.946131 | 2.1 |
| 14677 | 1.41149 | 2.1 |
| 14678 | 1.41149 | 2.1 |
| 14679 | 0.946131 | 2.1 |
| 14680 | 0.946128 | 2.1 |
| 14681 | 0.946128 | 2.1 |
| 14682 | 0.946133 | 2.1 |
| 14683 | 0.946131 | 2.1 |
| 14684 | 0.946131 | 2.1 |
| 14685 | 0.946131 | 2.1 |
| 14686 | 0.94613 | 2.1 |
| 14687 | 1.41388 | 0.05 |
| 14688 | 0.94613 | 0.05 |
| 14689 | 0.946132 | 0.05 |
| 14690 | 0.946132 | 0.05 |
| 14691 | 0.946129 | 0.05 |
| 14692 | 0.946131 | 0.05 |
| 14693 | 0.946131 | 0.05 |
| 14694 | 1.02798 | 0.05 |
| 14695 | 0.946132 | 0.05 |
| 14696 | 1.02798 | 0.05 |
| 14697 | 0.946133 | 0.05 |
| 14698 | 1.14931 | 0.05 |
| 14699 | 0.957746 | 0.05 |
| 14700 | 0.93452 | 0.05 |
| 14701 | 0.946129 | 2.1 |
| 14702 | 0.946134 | 2.1 |
| 14703 | 0.946133 | 2.1 |
| 14704 | 0.946131 | 2.1 |
| 14705 | 0.946131 | 2.1 |
| 14706 | 0.946131 | 2.1 |
| 14707 | 0.946131 | 2.1 |
| 14708 | 0.946133 | 2.1 |
| 14709 | 0.946132 | 0.863357 |
| 14710 | 0.946132 | 1.46193 |
| 14711 | 0.946132 | 2.1 |
| 14712 | 1.38773 | 0.944202 |
| 14713 | 0.946132 | 2.0252 |
| 14714 | 0.946133 | 1.12766 |
| 14715 | 0.946131 | 0.05 |
| 14716 | 0.946131 | 0.05 |
| 14717 | 1.41512 | 0.05 |
| 14718 | 0.946133 | 0.05 |
| 14719 | 0.946131 | 0.05 |
| 14720 | 0.946131 | 0.05 |
| 14721 | 0.946131 | 0.05 |
| 14722 | 1.35381 | 0.792762 |
| 14723 | 0.946132 | 2.1 |
| 14724 | 1.35381 | 0.05 |
| 14725 | 0.946133 | 2.1 |
| 14726 | 0.946132 | 0.979087 |
| 14727 | 0.946132 | 2.08854 |
| 14728 | 0.946133 | 1.04255 |
| 14729 | 1.3365 | 0.285359 |

| | | |
|---|---|---|
| 14730 | 0.946132 | 1.12581 |
| 14731 | 1.3365 | 0.05 |
| 14732 | 0.946129 | 0.944993 |
| 14733 | 0.94613 | 0.156006 |
| 14734 | 0.946128 | 1.03726 |
| 14735 | 0.946129 | 0.778163 |
| 14736 | 0.939895 | 1.61247 |
| 14737 | 1.14285 | 2.1 |
| 14738 | 0.952366 | 2.1 |
| 14739 | 0.946131 | 2.0457 |
| 14740 | 0.94613 | 2.1 |
| 14741 | 0.946131 | 2.1 |
| 14742 | 0.94613 | 2.1 |
| 14743 | 0.946131 | 2.1 |
| 14744 | 0.946131 | 2.1 |
| 14745 | 0.946131 | 2.1 |
| 14746 | 0.946129 | 2.1 |
| 14747 | 0.946133 | 2.0059 |
| 14748 | 0.946131 | 2.1 |
| 14749 | 0.946131 | 2.1 |
| 14750 | 0.946133 | 2.1 |
| 14751 | 0.946132 | 2.1 |
| 14752 | 0.946132 | 2.1 |
| 14753 | 0.946133 | 2.1 |
| 14754 | 0.946132 | 2.1 |
| 14755 | 0.946132 | 2.1 |
| 14756 | 0.946133 | 2.1 |
| 14757 | 0.946133 | 2.1 |
| 14758 | 0.946132 | 1.41388 |
| 14759 | 0.946132 | 2.1 |
| 14760 | 0.946133 | 1.92644 |
| 14761 | 0.946132 | 2.1 |
| 14762 | 0.946132 | 2.1 |
| 14763 | 0.946132 | 1.72882 |
| 14764 | 0.946132 | 0.813403 |
| 14765 | 0.946131 | 2.1 |
| 14766 | 0.946131 | 0.938634 |
| 14767 | 0.946131 | 1.05854 |
| 14768 | 1.38969 | 2.1 |
| 14769 | 0.946131 | 2.1 |
| 14770 | 0.946132 | 2.1 |
| 14771 | 0.946128 | 2.1 |
| 14772 | 0.946133 | 1.95277 |
| 14773 | 0.946132 | 2.1 |
| 14774 | 0.946131 | 1.82445 |
| 14775 | 0.94613 | 1.92072 |
| 14776 | 0.946131 | 2.1 |
| 14777 | 1.28464 | 2.1 |
| 14778 | 0.946119 | 0.878431 |
| 14779 | 1.37492 | 0.977401 |
| 14780 | 0.946125 | 1.10867 |
| 14781 | 0.946119 | 0.719115 |
| 14782 | 0.946119 | 1.5238 |
| 14783 | 0.946132 | 0.779119 |
| 14784 | 0.946107 | 1.00944 |
| 14785 | 0.810942 | 2.1 |
| 14786 | 0.810943 | 2.1 |
| 14787 | 0.709587 | 2.1 |

| | | |
|---|---|---|
| 14788 | 2.64488 | 2.1 |
| 14789 | 1.21651 | 2.1 |
| 14790 | 0.946151 | 2.1 |
| 14791 | 0.979948 | 2.1 |
| 14792 | 1.0813 | 2.1 |
| 14793 | 0.946133 | 1.69009 |
| 14794 | 0.946132 | 2.1 |
| 14795 | 0.946132 | 2.1 |
| 14796 | 0.946133 | 1.08709 |
| 14797 | 0.946132 | 0.815978 |
| 14798 | 0.946132 | 0.98533 |
| 14799 | 1.41134 | 1.46402 |
| 14800 | 0.946133 | 2.1 |
| 14801 | 0.946132 | 2.07932 |
| 14802 | 0.946133 | 2.1 |
| 14803 | 0.946133 | 1.2676 |
| 14804 | 0.946132 | 0.211054 |
| 14805 | 0.946133 | 1.79074 |
| 14806 | 1.31338 | 2.02452 |
| 14807 | 2.07612 | 2.1 |
| 14808 | 0.946102 | 0.05 |
| 14809 | 0.752448 | 0.05 |
| 14810 | 1.20617 | 0.05 |
| 14811 | 0.94613 | 0.05 |
| 14812 | 0.75242 | 0.05 |
| 14813 | 0.946131 | 0.05 |
| 14814 | 0.752419 | 0.05 |
| 14815 | 0.946132 | 1.05234 |
| 14816 | 1.25736 | 0.981588 |
| 14817 | 1.25736 | 0.904083 |
| 14818 | 0.946132 | 0.968992 |
| 14819 | 1.08054 | 0.470847 |
| 14820 | 1.25736 | 0.921027 |
| 14821 | 0.946132 | 1.11947 |
| 14822 | 0.946128 | 2.1 |
| 14823 | 1.1896 | 2.1 |
| 14824 | 1.18959 | 2.1 |
| 14825 | 0.946131 | 0.334219 |
| 14826 | 1.33271 | 2.1 |
| 14827 | 0.946105 | 2.1 |
| 14828 | 0.946156 | 2.1 |
| 14829 | 0.741946 | 0.05 |
| 14830 | 1.33587 | 0.05 |
| 14831 | 0.741947 | 0.05 |
| 14832 | 1.21954 | 0.05 |
| 14833 | 0.94613 | 0.05 |
| 14834 | 0.741946 | 0.05 |
| 14835 | 1.81347 | 0.05 |
| 14836 | 1.81347 | 0.05 |
| 14837 | 0.653322 | 0.05 |
| 14838 | 1.36935 | 0.05 |
| 14839 | 0.661882 | 0.05 |
| 14840 | 1.04753 | 0.05 |
| 14841 | 0.507464 | 0.05 |
| 14842 | 0.945844 | 1.31069 |
| 14843 | 1.59507 | 2.1 |
| 14844 | 1.04043 | 1.15173 |
| 14845 | 0.946131 | 2.1 |

| | | |
|---|---|---|
| 14846 | 1.32891 | 2.1 |
| 14847 | 0.946131 | 1.38073 |
| 14848 | 0.946131 | 1.15226 |
| 14849 | 1.28866 | 1.69814 |
| 14850 | 1.12682 | 0.05 |
| 14851 | 0.851543 | 0.228376 |
| 14852 | 1.04072 | 0.062981 |
| 14853 | 0.946132 | 0.35357 |
| 14854 | 1.13437 | 0.05 |
| 14855 | 1.04167 | 0.05 |
| 14856 | 0.936652 | 0.0900439 |
| 14857 | 1.258 | 2.1 |
| 14858 | 0.946131 | 2.1 |
| 14859 | 0.946132 | 2.1 |
| 14860 | 1.36968 | 2.1 |
| 14861 | 0.946132 | 2.1 |
| 14862 | 1.36968 | 2.1 |
| 14863 | 0.946131 | 2.1 |
| 14864 | 2.30046 | 0.05 |
| 14865 | 2.82969 | 0.05 |
| 14866 | 0.946132 | 2.1 |
| 14867 | 1.35022 | 2.1 |
| 14868 | 1.12356 | 2.00796 |
| 14869 | 0.94613 | 2.1 |
| 14870 | 1.35022 | 2.1 |
| 14871 | 0.946131 | 2.1 |
| 14872 | 0.94613 | 2.1 |
| 14873 | 0.946131 | 2.1 |
| 14874 | 1.22401 | 2.1 |
| 14875 | 0.952456 | 2.1 |
| 14876 | 0.94613 | 2.1 |
| 14877 | 1.22401 | 2.1 |
| 14878 | 0.946131 | 2.1 |
| 14879 | 0.946131 | 2.1 |
| 14880 | 1.33047 | 2.1 |
| 14881 | 0.946132 | 2.1 |
| 14882 | 1.33047 | 2.1 |
| 14883 | 0.946131 | 2.1 |
| 14884 | 1.33047 | 2.1 |
| 14885 | 0.946132 | 2.1 |
| 14886 | 1.2107 | 2.1 |
| 14887 | 0.61957 | 2.1 |
| 14888 | 1.80666 | 2.1 |
| 14889 | 1.53743 | 2.1 |
| 14890 | 0.619572 | 1.65311 |
| 14891 | 1.32547 | 2.1 |
| 14892 | 1.16166 | 2.1 |
| 14893 | 1.04074 | 2.1 |
| 14894 | 0.557621 | 2.1 |
| 14895 | 0.946128 | 1.26937 |
| 14896 | 1.36607 | 2.1 |
| 14897 | 0.94613 | 2.1 |
| 14898 | 1.36607 | 2.1 |
| 14899 | 1.39416 | 2.1 |
| 14900 | 0.94613 | 2.1 |
| 14901 | 0.94613 | 2.1 |
| 14902 | 1.23969 | 2.1 |
| 14903 | 0.946131 | 2.1 |

| | | |
|---|---|---|
| 14904 | 0.946131 | 2.1 |
| 14905 | 1.13537 | 2.1 |
| 14906 | 1.05719 | 2.1 |
| 14907 | 0.806848 | 2.1 |
| 14908 | 1.30251 | 2.1 |
| 14909 | 0.851526 | 2.06299 |
| 14910 | 1.14176 | 2.1 |
| 14911 | 0.93666 | 1.39275 |
| 14912 | 0.946114 | 2.1 |
| 14913 | 1.15832 | 2.1 |
| 14914 | 0.924624 | 2.08859 |
| 14915 | 0.967609 | 1.7463 |
| 14916 | 0.771764 | 0.05 |
| 14917 | 1.26652 | 0.05 |
| 14918 | 0.946133 | 0.05 |
| 14919 | 1.17957 | 0.05 |
| 14920 | 0.771735 | 0.05 |
| 14921 | 0.946122 | 0.05 |
| 14922 | 0.946133 | 0.05 |
| 14923 | 0.924325 | 0.908222 |
| 14924 | 0.656739 | 1.08696 |
| 14925 | 0.637876 | 1.09387 |
| 14926 | 0.530092 | 1.03944 |
| 14927 | 0.914415 | 0.947107 |
| 14928 | 0.821311 | 1.61887 |
| 14929 | 0.630589 | 1.32797 |
| 14930 | 0.738823 | 1.30956 |
| 14931 | 0.839109 | 1.12152 |
| 14932 | 1.55029 | 0.05 |
| 14933 | 1.13537 | 0.05 |
| 14934 | 0.946131 | 0.749541 |
| 14935 | 0.946128 | 0.05 |
| 14936 | 1.30162 | 2.1 |
| 14937 | 0.952476 | 0.867049 |
| 14938 | 1.29289 | 1.48305 |
| 14939 | 0.939783 | 1.49302 |
| 14940 | 0.94612 | 2.1 |
| 14941 | 0.946131 | 2.1 |
| 14942 | 1.07998 | 2.1 |
| 14943 | 0.836985 | 2.1 |
| 14944 | 1.1604 | 2.1 |
| 14945 | 0.94613 | 2.1 |
| 14946 | 0.837016 | 2.1 |
| 14947 | 0.712461 | 0.230938 |
| 14948 | 1.36399 | 0.362245 |
| 14949 | 1.8724 | 0.213133 |
| 14950 | 1.8724 | 0.127821 |
| 14951 | 0.623426 | 1.05144 |
| 14952 | 1.36399 | 0.145709 |
| 14953 | 1.22089 | 0.165917 |
| 14954 | 0.946132 | 0.1927 |
| 14955 | 0.946131 | 2.1 |
| 14956 | 1.32241 | 2.1 |
| 14957 | 1.32241 | 2.1 |
| 14958 | 0.946133 | 2.1 |
| 14959 | 1.40271 | 2.1 |
| 14960 | 0.946131 | 2.1 |
| 14961 | 0.946131 | 2.1 |

| | | |
|---|---|---|
| 14962 | 1.1989 | 1.29174 |
| 14963 | 0.954106 | 1.55372 |
| 14964 | 0.820068 | 1.11488 |
| 14965 | 0.563139 | 1.43946 |
| 14966 | 1.06448 | 1.51607 |
| 14967 | 0.892472 | 1.9817 |
| 14968 | 0.862598 | 1.24223 |
| 14969 | 0.745851 | 2.0058 |
| 14970 | 1.13336 | 1.4645 |
| 14971 | 0.946129 | 2.1 |
| 14972 | 1.28254 | 2.1 |
| 14973 | 1.28254 | 2.1 |
| 14974 | 0.946131 | 2.1 |
| 14975 | 1.41302 | 2.1 |
| 14976 | 0.946131 | 2.1 |
| 14977 | 0.946131 | 2.1 |
| 14978 | 0.851543 | 1.95479 |
| 14979 | 1.21961 | 2.1 |
| 14980 | 0.851484 | 1.48965 |
| 14981 | 0.851543 | 2.1 |
| 14982 | 1.15705 | 2.1 |
| 14983 | 0.946133 | 2.1 |
| 14984 | 0.909417 | 2.1 |
| 14985 | 1.13808 | 0.684463 |
| 14986 | 0.946133 | 0.452193 |
| 14987 | 0.946131 | 0.697185 |
| 14988 | 1.30194 | 0.456899 |
| 14989 | 0.946131 | 0.523652 |
| 14990 | 1.30194 | 0.436115 |
| 14991 | 0.946131 | 0.468472 |
| 14992 | 0.930941 | 1.24064 |
| 14993 | 1.20185 | 0.943223 |
| 14994 | 1.02404 | 0.931572 |
| 14995 | 0.946124 | 1.39221 |
| 14996 | 1.3563 | 1.05581 |
| 14997 | 0.946127 | 1.4985 |
| 14998 | 0.946128 | 1.19544 |
| 14999 | 0.946116 | 2.1 |
| 15000 | 1.3144 | 2.1 |
| 15001 | 1.13088 | 2.1 |
| 15002 | 0.946118 | 0.0898786 |
| 15003 | 1.31438 | 2.1 |
| 15004 | 0.946105 | 2.1 |
| 15005 | 0.946131 | 2.1 |
| 15006 | 1.76972 | 0.05 |
| 15007 | 1.42091 | 0.05 |
| 15008 | 0.946128 | 0.05 |
| 15009 | 0.946101 | 0.05 |
| 15010 | 0.863552 | 0.05 |
| 15011 | 0.94613 | 0.05 |
| 15012 | 1.21232 | 0.05 |
| 15013 | 0.94613 | 0.05 |
| 15014 | 1.2137 | 0.0688649 |
| 15015 | 1.23224 | 0.481313 |
| 15016 | 1.86512 | 0.115989 |
| 15017 | 1.86512 | 0.05 |
| 15018 | 0.580825 | 1.30016 |
| 15019 | 1.2137 | 0.778715 |

| | | |
|---|---|---|
| 15020 | 0.946133 | 0.71832 |
| 15021 | 0.580822 | 0.44691 |
| 15022 | 0.946128 | 2.1 |
| 15023 | 1.40586 | 2.1 |
| 15024 | 0.94613 | 2.1 |
| 15025 | 1.3719 | 2.1 |
| 15026 | 1.3719 | 2.1 |
| 15027 | 1.40586 | 2.1 |
| 15028 | 0.94613 | 2.1 |
| 15029 | 0.946132 | 2.1 |
| 15030 | 1.35025 | 2.1 |
| 15031 | 1.1794 | 2.1 |
| 15032 | 0.946132 | 0.85348 |
| 15033 | 1.35025 | 1.26134 |
| 15034 | 0.946131 | 1.32877 |
| 15035 | 0.946132 | 1.12255 |
| 15036 | 0.946133 | 2.1 |
| 15037 | 1.60149 | 2.1 |
| 15038 | 1.67058 | 2.1 |
| 15039 | 0.851542 | 2.1 |
| 15040 | 1.20296 | 2.1 |
| 15041 | 1.17501 | 2.1 |
| 15042 | 0.946132 | 2.1 |
| 15043 | 1.04993 | 2.1 |
| 15044 | 0.851541 | 1.98363 |
| 15045 | 1.48219 | 2.1 |
| 15046 | 0.874565 | 1.66565 |
| 15047 | 0.946129 | 2.1 |
| 15048 | 1.22049 | 2.1 |
| 15049 | 0.946131 | 2.1 |
| 15050 | 0.946131 | 2.1 |
| 15051 | 0.94613 | 2.1 |
| 15052 | 0.848719 | 2.1 |
| 15053 | 1.17195 | 2.1 |
| 15054 | 0.946129 | 1.37662 |
| 15055 | 1.35426 | 1.49657 |
| 15056 | 0.94613 | 1.93794 |
| 15057 | 0.94613 | 2.1 |
| 15058 | 0.946133 | 2.1 |
| 15059 | 1.14342 | 2.1 |
| 15060 | 1.14342 | 2.1 |
| 15061 | 0.946133 | 2.1 |
| 15062 | 1.39009 | 2.1 |
| 15063 | 0.946132 | 2.1 |
| 15064 | 0.946132 | 2.1 |
| 15065 | 1.38819 | 2.1 |
| 15066 | 0.946133 | 2.1 |
| 15067 | 1.38819 | 2.1 |
| 15068 | 0.946131 | 2.1 |
| 15069 | 1.4116 | 2.1 |
| 15070 | 0.946131 | 2.1 |
| 15071 | 1.38819 | 2.1 |
| 15072 | 0.946127 | 2.1 |
| 15073 | 1.32244 | 2.1 |
| 15074 | 1.2176 | 2.1 |
| 15075 | 0.946131 | 2.1 |
| 15076 | 1.32244 | 2.1 |
| 15077 | 0.94613 | 2.1 |

| | | |
|---|---|---|
| 15078 | 0.946129 | 2.1 |
| 15079 | 1.32415 | 2.1 |
| 15080 | 1.11239 | 2.1 |
| 15081 | 0.984099 | 2.1 |
| 15082 | 1.65532 | 2.1 |
| 15083 | 1.53082 | 2.1 |
| 15084 | 1.32068 | 2.1 |
| 15085 | 1.32068 | 2.1 |
| 15086 | 1.08172 | 2.1 |
| 15087 | 3.76293 | 0.05 |
| 15088 | 1.36335 | 0.05 |
| 15089 | 0.953925 | 2.1 |
| 15090 | 0.989497 | 2.1 |
| 15091 | 0.98949 | 2.1 |
| 15092 | 1.06341 | 2.1 |
| 15093 | 1.27875 | 2.1 |
| 15094 | 1.04598 | 2.1 |
| 15095 | 0.603154 | 2.1 |
| 15096 | 0.582457 | 2.1 |
| 15097 | 0.582457 | 2.1 |
| 15098 | 0.568994 | 2.1 |
| 15099 | 1.5791 | 2.1 |
| 15100 | 1.09506 | 2.1 |
| 15101 | 1.45327 | 2.1 |
| 15102 | 1.88406 | 2.1 |
| 15103 | 1.88404 | 2.1 |
| 15104 | 1.27389 | 2.1 |
| 15105 | 1.14206 | 2.1 |
| 15106 | 1.00232 | 2.1 |
| 15107 | 1.00231 | 2.1 |
| 15108 | 1.68436 | 2.1 |
| 15109 | 1.4677 | 0.05 |
| 15110 | 1.28828 | 0.05 |
| 15111 | 1.28828 | 0.05 |
| 15112 | 1.46932 | 0.05 |
| 15113 | 1.28228 | 2.1 |
| 15114 | 1.64268 | 1.05187 |
| 15115 | 0.959473 | 2.1 |
| 15116 | 1.10101 | 2.1 |
| 15117 | 1.07976 | 2.1 |
| 15118 | 1.2873 | 2.1 |
| 15119 | 1.70303 | 0.05 |
| 15120 | 1.40066 | 0.05 |
| 15121 | 1.5563 | 0.05 |
| 15122 | 1.13727 | 0.05 |
| 15123 | 1.03529 | 2.1 |
| 15124 | 1.11285 | 2.1 |
| 15125 | 1.03529 | 2.1 |
| 15126 | 1.28667 | 2.1 |
| 15127 | 1.32119 | 2.1 |
| 15128 | 1.44777 | 2.1 |
| 15129 | 1.44778 | 2.1 |
| 15130 | 1.61121 | 2.1 |
| 15131 | 1.09372 | 0.05 |
| 15132 | 1.17664 | 0.05 |
| 15133 | 1.17664 | 0.05 |
| 15134 | 0.872931 | 0.05 |
| 15135 | 1.02415 | 2.1 |

| | | |
|---|---|---|
| 15136 | 1.28228 | 2.1 |
| 15137 | 2.14692 | 2.1 |
| 15138 | 3.90469 | 2.1 |
| 15139 | 1.00509 | 2.1 |
| 15140 | 1.28228 | 2.1 |
| 15141 | 3.32936 | 0.05 |
| 15142 | 1.1333 | 0.05 |
| 15143 | 1.11048 | 2.1 |
| 15144 | 0.979854 | 2.1 |
| 15145 | 1.65815 | 2.1 |
| 15146 | 1.00185 | 2.1 |
| 15147 | 1.84887 | 2.1 |
| 15148 | 1.59597 | 2.1 |
| 15149 | 1.78468 | 2.1 |
| 15150 | 1.87508 | 2.1 |
| 15151 | 1.82716 | 2.1 |
| 15152 | 1.33429 | 2.1 |
| 15153 | 1.34626 | 2.1 |
| 15154 | 0.797142 | 2.1 |
| 15155 | 1.00007 | 1.05459 |
| 15156 | 1.02051 | 2.1 |
| 15157 | 1.02051 | 1.12622 |
| 15158 | 1.03373 | 2.1 |
| 15159 | 1.05786 | 2.1 |
| 15160 | 0.887631 | 2.1 |
| 15161 | 0.992773 | 2.1 |
| 15162 | 1.28462 | 2.1 |
| 15163 | 2.35274 | 1.96537 |
| 15164 | 1.82533 | 1.12395 |
| 15165 | 1.82533 | 1.66511 |
| 15166 | 1.19496 | 0.86657 |
| 15167 | 1.28597 | 0.05 |
| 15168 | 1.09725 | 2.1 |
| 15169 | 1.60948 | 2.1 |
| 15170 | 1.20421 | 2.1 |
| 15171 | 1.16566 | 2.1 |
| 15172 | 1.88522 | 2.1 |
| 15173 | 1.81948 | 0.05 |
| 15174 | 1.14042 | 0.05 |
| 15175 | 1.21558 | 0.05 |
| 15176 | 1.56876 | 0.05 |
| 15177 | 1.6697 | 2.1 |
| 15178 | 1.81655 | 2.1 |
| 15179 | 1.81655 | 2.1 |
| 15180 | 1.5956 | 2.1 |
| 15181 | 0.906059 | 2.1 |
| 15182 | 1.63045 | 2.1 |
| 15183 | 0.9533 | 1.03267 |
| 15184 | 1.1475 | 0.981315 |
| 15185 | 1.72231 | 2.1 |
| 15186 | 1.94936 | 1.49033 |
| 15187 | 1.06826 | 2.1 |
| 15188 | 1.08576 | 2.1 |
| 15189 | 1.06826 | 2.1 |
| 15190 | 0.973559 | 2.1 |
| 15191 | 1.62737 | 0.05 |
| 15192 | 0.965676 | 0.05 |
| 15193 | 1.45055 | 0.05 |

| | | |
|---|---|---|
| 15194 | 1.17631 | 0.05 |
| 15195 | 1.92535 | 0.05 |
| 15196 | 1.9503 | 0.05 |
| 15197 | 1.99828 | 0.05 |
| 15198 | 1.70302 | 0.05 |
| 15199 | 1.65139 | 0.05 |
| 15200 | 1.26784 | 0.05 |
| 15201 | 1.35923 | 0.05 |
| 15202 | 1.70302 | 0.05 |
| 15203 | 1.33033 | 2.1 |
| 15204 | 1.12481 | 2.1 |
| 15205 | 1.29452 | 2.1 |
| 15206 | 1.25399 | 2.1 |
| 15207 | 1.12481 | 2.1 |
| 15208 | 0.918358 | 2.1 |
| 15209 | 1.39332 | 2.1 |
| 15210 | 1.32368 | 2.1 |
| 15211 | 1.11712 | 0.985815 |
| 15212 | 1.07999 | 1.07258 |
| 15213 | 1.07999 | 0.624132 |
| 15214 | 1.05619 | 1.01354 |
| 15215 | 1.1766 | 2.1 |
| 15216 | 1.35735 | 2.1 |
| 15217 | 1.42456 | 0.05 |
| 15218 | 1.089 | 0.05 |
| 15219 | 1.11081 | 0.05 |
| 15220 | 2.88521 | 0.05 |
| 15221 | 1.09754 | 0.05 |
| 15222 | 1.85479 | 0.05 |
| 15223 | 1.27922 | 0.315985 |
| 15224 | 1.03556 | 0.0995661 |
| 15225 | 1.07508 | 1.63586 |
| 15226 | 1.09795 | 2.1 |
| 15227 | 0.967672 | 0.201115 |
| 15228 | 1.07508 | 1.21611 |
| 15229 | 1.03933 | 1.99553 |
| 15230 | 1.02975 | 2.1 |
| 15231 | 1.02975 | 2.1 |
| 15232 | 1.01121 | 2.1 |
| 15233 | 1.24526 | 0.05 |
| 15234 | 1.72368 | 0.05 |
| 15235 | 1.89607 | 0.05 |
| 15236 | 2.13512 | 0.05 |
| 15237 | 1.34591 | 0.05 |
| 15238 | 1.14721 | 0.166449 |
| 15239 | 1.15539 | 1.14041 |
| 15240 | 1.90938 | 0.782096 |
| 15241 | 1.90941 | 2.1 |
| 15242 | 1.89242 | 2.1 |
| 15243 | 1.18907 | 2.1 |
| 15244 | 1.20408 | 2.1 |
| 15245 | 1.20409 | 2.1 |
| 15246 | 1.22655 | 2.1 |
| 15247 | 0.701447 | 2.1 |
| 15248 | 1.57567 | 2.1 |
| 15249 | 1.38806 | 2.1 |
| 15250 | 2.09407 | 2.1 |
| 15251 | 1.07612 | 1.1484 |

| | | |
|---|---|---|
| 15252 | 0.990019 | 1.19695 |
| 15253 | 1.15564 | 2.1 |
| 15254 | 1.01243 | 1.89879 |
| 15255 | 1.28956 | 0.05 |
| 15256 | 1.35146 | 0.05 |
| 15257 | 1.35145 | 0.05 |
| 15258 | 1.22811 | 0.05 |
| 15259 | 1.09662 | 2.1 |
| 15260 | 1.42486 | 2.1 |
| 15261 | 1.10106 | 0.05 |
| 15262 | 1.90814 | 0.05 |
| 15263 | 1.3946 | 0.05 |
| 15264 | 1.11926 | 0.05 |
| 15265 | 0.725839 | 2.1 |
| 15266 | 0.925068 | 2.1 |
| 15267 | 0.925074 | 2.1 |
| 15268 | 1.43282 | 2.1 |
| 15269 | 1.91756 | 0.984896 |
| 15270 | 1.74321 | 1.02271 |
| 15271 | 1.74321 | 0.671661 |
| 15272 | 1.63413 | 0.918747 |
| 15273 | 1.9194 | 0.05 |
| 15274 | 1.62246 | 0.05 |
| 15275 | 1.90456 | 0.05 |
| 15276 | 1.06578 | 0.05 |
| 15277 | 1.46019 | 0.05 |
| 15278 | 1.17036 | 0.05 |
| 15279 | 1.70233 | 1.00085 |
| 15280 | 1.53515 | 0.407877 |
| 15281 | 2.03494 | 2.1 |
| 15282 | 1.78441 | 2.1 |
| 15283 | 1.75992 | 2.1 |
| 15284 | 1.94351 | 2.1 |
| 15285 | 1.51057 | 0.05 |
| 15286 | 0.991435 | 0.05 |
| 15287 | 1.98068 | 0.05 |
| 15288 | 1.64283 | 0.05 |
| 15289 | 2.96126 | 0.05 |
| 15290 | 2.20692 | 0.05 |
| 15291 | 1.95054 | 0.05 |
| 15292 | 1.21325 | 0.05 |
| 15293 | 0.996828 | 0.05 |
| 15294 | 1.01976 | 0.05 |
| 15295 | 1.17129 | 2.1 |
| 15296 | 1.13664 | 2.1 |
| 15297 | 1.13664 | 2.1 |
| 15298 | 1.09627 | 2.1 |
| 15299 | 2.65123 | 2.1 |
| 15300 | 3.23062 | 2.1 |
| 15301 | 3.23062 | 2.1 |
| 15302 | 3.92038 | 2.1 |
| 15303 | 1.89019 | 0.05 |
| 15304 | 1.79109 | 0.05 |
| 15305 | 1.79109 | 0.05 |
| 15306 | 1.54197 | 0.05 |
| 15307 | 3.41824 | 1.13227 |
| 15308 | 1.93553 | 0.05 |
| 15309 | 2.26215 | 1.25064 |

| | | |
|---|---|---|
| 15310 | 2.0103 | 0.05 |
| 15311 | 1.51569 | 0.710036 |
| 15312 | 1.01683 | 0.389358 |
| 15313 | 1.78595 | 2.1 |
| 15314 | 1.66537 | 2.1 |
| 15315 | 3.73426 | 2.1 |
| 15316 | 3.53905 | 2.1 |
| 15317 | 1.66537 | 2.1 |
| 15318 | 1.52903 | 2.1 |
| 15319 | 3.53905 | 2.1 |
| 15320 | 3.3837 | 2.1 |
| 15321 | 1.19901 | 0.819014 |
| 15322 | 1.35002 | 2.07295 |
| 15323 | 1.35002 | 0.5214 |
| 15324 | 1.11683 | 0.399314 |
| 15325 | 1.75326 | 2.0002 |
| 15326 | 0.712404 | 1.47493 |
| 15327 | 2.97768 | 2.1 |
| 15328 | 2.98949 | 2.1 |
| 15329 | 1.2832 | 2.1 |
| 15330 | 1.39518 | 2.1 |
| 15331 | 2.98949 | 2.1 |
| 15332 | 2.99339 | 2.1 |
| 15333 | 1.39517 | 2.1 |
| 15334 | 1.46124 | 2.1 |
| 15335 | 1.28534 | 2.1 |
| 15336 | 1.03391 | 2.1 |
| 15337 | 1.75366 | 1.07721 |
| 15338 | 1.26247 | 1.86247 |
| 15339 | 1.13621 | 2.1 |
| 15340 | 2.43994 | 2.1 |
| 15341 | 1.47904 | 0.05 |
| 15342 | 1.80085 | 0.05 |
| 15343 | 1.80085 | 0.05 |
| 15344 | 1.64656 | 0.05 |
| 15345 | 2.87331 | 2.1 |
| 15346 | 2.82863 | 2.1 |
| 15347 | 2.82859 | 2.1 |
| 15348 | 2.84673 | 2.1 |
| 15349 | 1.0961 | 0.05 |
| 15350 | 1.39448 | 0.092261 |
| 15351 | 1.21366 | 0.0978404 |
| 15352 | 1.20143 | 0.05 |
| 15353 | 1.85795 | 0.05 |
| 15354 | 1.36768 | 0.05 |
| 15355 | 1.36769 | 0.05 |
| 15356 | 1.45789 | 0.05 |
| 15357 | 1.32605 | 0.05 |
| 15358 | 1.27148 | 0.05 |
| 15359 | 1.70218 | 0.05 |
| 15360 | 1.3063 | 0.05 |
| 15361 | 1.45603 | 0.05 |
| 15362 | 1.89226 | 0.05 |
| 15363 | 1.2742 | 0.05 |
| 15364 | 2.10112 | 0.05 |
| 15365 | 1.10748 | 0.05 |
| 15366 | 1.1893 | 0.05 |
| 15367 | 1.04921 | 2.1 |

| | | |
|---|---|---|
| 15368 | 1.22927 | 2.1 |
| 15369 | 1.22927 | 2.1 |
| 15370 | 1.46568 | 2.1 |
| 15371 | 0.811186 | 2.1 |
| 15372 | 0.792162 | 2.1 |
| 15373 | 0.792162 | 2.1 |
| 15374 | 0.758749 | 2.1 |
| 15375 | 1.03581 | 2.1 |
| 15376 | 0.737481 | 2.1 |
| 15377 | 2.24071 | 2.1 |
| 15378 | 1.34868 | 2.1 |
| 15379 | 1.47203 | 0.907233 |
| 15380 | 1.40967 | 0.969837 |
| 15381 | 1.40967 | 1.0204 |
| 15382 | 1.27916 | 1.01378 |
| 15383 | 1.17418 | 0.833845 |
| 15384 | 1.04921 | 2.1 |
| 15385 | 1.28291 | 0.389195 |
| 15386 | 1.3213 | 0.05 |
| 15387 | 1.46812 | 0.151764 |
| 15388 | 2.42522 | 0.340307 |
| 15389 | 1.17879 | 0.866257 |
| 15390 | 1.28291 | 0.897725 |
| 15391 | 1.28291 | 0.859731 |
| 15392 | 1.13438 | 0.848225 |
| 15393 | 1.75004 | 2.1 |
| 15394 | 1.78247 | 2.1 |
| 15395 | 1.78247 | 2.1 |
| 15396 | 1.76421 | 2.1 |
| 15397 | 2.25975 | 0.05 |
| 15398 | 3.66111 | 0.05 |
| 15399 | 3.6611 | 0.05 |
| 15400 | 3.44043 | 0.05 |
| 15401 | 1.13339 | 2.1 |
| 15402 | 1.13322 | 2.1 |
| 15403 | 1.13323 | 2.1 |
| 15404 | 1.20761 | 2.1 |
| 15405 | 1.34337 | 0.795498 |
| 15406 | 1.90912 | 2.1 |
| 15407 | 1.04399 | 2.1 |
| 15408 | 2.00578 | 2.1 |
| 15409 | 1.04973 | 2.1 |
| 15410 | 1.22615 | 2.1 |
| 15411 | 1.93384 | 2.1 |
| 15412 | 2.62816 | 0.0909319 |
| 15413 | 1.13127 | 2.1 |
| 15414 | 1.47642 | 2.1 |
| 15415 | 2.62816 | 2.1 |
| 15416 | 3.31755 | 0.291377 |
| 15417 | 1.47642 | 2.1 |
| 15418 | 1.80454 | 1.83514 |
| 15419 | 0.964782 | 2.1 |
| 15420 | 0.949135 | 2.1 |
| 15421 | 0.949134 | 2.1 |
| 15422 | 0.949134 | 2.1 |
| 15423 | 1.10476 | 0.532794 |
| 15424 | 0.982858 | 0.05 |
| 15425 | 1.10938 | 0.42359 |

| | | |
|---|---|---|
| 15426 | 0.982858 | 0.274269 |
| 15427 | 1.01012 | 0.05 |
| 15428 | 0.974081 | 0.05 |
| 15429 | 1.82643 | 0.05 |
| 15430 | 1.29585 | 0.05 |
| 15431 | 1.00763 | 1.00881 |
| 15432 | 1.3294 | 0.861446 |
| 15433 | 1.3294 | 0.57434 |
| 15434 | 1.30001 | 1.0834 |
| 15435 | 1.59151 | 2.1 |
| 15436 | 1.99443 | 2.1 |
| 15437 | 1.07369 | 2.1 |
| 15438 | 1.30584 | 2.1 |
| 15439 | 1.05071 | 2.1 |
| 15440 | 0.948676 | 2.1 |
| 15441 | 1.13842 | 2.1 |
| 15442 | 1.42884 | 2.1 |
| 15443 | 1.59019 | 0.05 |
| 15444 | 1.38212 | 0.05 |
| 15445 | 1.34102 | 0.05 |
| 15446 | 1.02663 | 0.05 |
| 15447 | 0.797011 | 2.1 |
| 15448 | 0.81301 | 2.1 |
| 15449 | 0.81301 | 2.1 |
| 15450 | 0.811187 | 2.1 |
| 15451 | 0.991869 | 0.0773332 |
| 15452 | 1.23868 | 0.0628682 |
| 15453 | 1.67201 | 2.1 |
| 15454 | 1.61201 | 2.1 |
| 15455 | 1.61202 | 2.1 |
| 15456 | 1.57627 | 2.1 |
| 15457 | 0.94994 | 2.1 |
| 15458 | 1.77867 | 2.1 |
| 15459 | 0.854945 | 2.1 |
| 15460 | 0.973905 | 2.1 |
| 15461 | 1.64193 | 2.1 |
| 15462 | 1.68893 | 2.1 |
| 15463 | 1.68893 | 2.1 |
| 15464 | 1.70002 | 2.1 |
| 15465 | 2.08149 | 0.05 |
| 15466 | 1.69456 | 0.05 |
| 15467 | 1.67399 | 0.05 |
| 15468 | 1.83922 | 0.824253 |
| 15469 | 1.91703 | 2.1 |
| 15470 | 0.967492 | 2.1 |
| 15471 | 1.13317 | 2.1 |
| 15472 | 0.980766 | 2.1 |
| 15473 | 2.06158 | 0.05 |
| 15474 | 0.986498 | 0.05 |
| 15475 | 1.76289 | 0.05 |
| 15476 | 1.17248 | 0.05 |
| 15477 | 1.51794 | 0.05 |
| 15478 | 1.30266 | 0.05 |
| 15479 | 1.03165 | 0.05 |
| 15480 | 0.947692 | 0.05 |
| 15481 | 1.20473 | 0.05 |
| 15482 | 0.947693 | 0.05 |
| 15483 | 1.54357 | 2.1 |

| | | |
|---|---|---|
| 15484 | 0.980841 | 2.1 |
| 15485 | 1.34124 | 0.510573 |
| 15486 | 1.39818 | 0.638747 |
| 15487 | 1.39818 | 0.0607569 |
| 15488 | 1.48035 | 0.574831 |
| 15489 | 1.03932 | 2.1 |
| 15490 | 1.2065 | 2.1 |
| 15491 | 1.20651 | 2.1 |
| 15492 | 1.12717 | 2.1 |
| 15493 | 1.09483 | 2.1 |
| 15494 | 0.994293 | 2.1 |
| 15495 | 0.994293 | 2.1 |
| 15496 | 0.97356 | 2.1 |
| 15497 | 0.995252 | 0.0505126 |
| 15498 | 1.66294 | 0.428641 |
| 15499 | 1.82775 | 0.196135 |
| 15500 | 1.42762 | 0.0633343 |
| 15501 | 1.04713 | 0.05 |
| 15502 | 1.05475 | 0.05 |
| 15503 | 1.29002 | 0.05 |
| 15504 | 1.05475 | 0.05 |
| 15505 | 1.17585 | 2.1 |
| 15506 | 1.39223 | 2.1 |
| 15507 | 1.39223 | 2.1 |
| 15508 | 1.71255 | 2.1 |
| 15509 | 1.56166 | 0.851435 |
| 15510 | 1.37762 | 0.516255 |
| 15511 | 1.16858 | 2.1 |
| 15512 | 1.0361 | 2.1 |
| 15513 | 1.23966 | 0.05 |
| 15514 | 1.10718 | 0.05 |
| 15515 | 1.99957 | 0.05 |
| 15516 | 0.658392 | 0.05 |
| 15517 | 1.36904 | 0.05 |
| 15518 | 1.12524 | 0.05 |
| 15519 | 1.04321 | 1.23363 |
| 15520 | 1.04627 | 1.12585 |
| 15521 | 1.91351 | 0.786873 |
| 15522 | 1.63379 | 0.886363 |
| 15523 | 1.79718 | 0.995958 |
| 15524 | 0.59634 | 1.08049 |
| 15525 | 0.958257 | 2.1 |
| 15526 | 1.06457 | 2.1 |
| 15527 | 0.958255 | 2.1 |
| 15528 | 1.03933 | 2.1 |
| 15529 | 1.14195 | 0.05 |
| 15530 | 1.49438 | 0.05 |
| 15531 | 1.49438 | 0.05 |
| 15532 | 1.31259 | 2.1 |
| 15533 | 1.23036 | 2.1 |
| 15534 | 1.2016 | 2.1 |
| 15535 | 1.2016 | 2.1 |
| 15536 | 1.17129 | 2.1 |
| 15537 | 1.05724 | 1.00648 |
| 15538 | 1.04291 | 1.61423 |
| 15539 | 1.12341 | 2.1 |
| 15540 | 2.32525 | 2.1 |
| 15541 | 3.39038 | 2.1 |

| | | |
|---|---|---|
| 15542 | 2.09374 | 2.1 |
| 15543 | 1.76185 | 0.762254 |
| 15544 | 1.7934 | 0.872107 |
| 15545 | 1.65809 | 0.864983 |
| 15546 | 1.54852 | 1.28069 |
| 15547 | 1.38766 | 2.1 |
| 15548 | 1.28765 | 2.1 |
| 15549 | 1.26599 | 2.1 |
| 15550 | 1.21397 | 2.1 |
| 15551 | 1.28765 | 2.1 |
| 15552 | 1.20045 | 2.1 |
| 15553 | 1.21398 | 2.1 |
| 15554 | 1.15234 | 2.1 |
| 15555 | 0.953634 | 0.05 |
| 15556 | 1.39132 | 0.05 |
| 15557 | 1.17285 | 2.1 |
| 15558 | 1.21408 | 2.1 |
| 15559 | 1.21408 | 2.1 |
| 15560 | 0.982848 | 2.1 |
| 15561 | 2.00101 | 2.1 |
| 15562 | 2.00973 | 2.1 |
| 15563 | 2.20314 | 2.1 |
| 15564 | 1.89318 | 2.1 |
| 15565 | 1.23267 | 1.88198 |
| 15566 | 0.947921 | 2.1 |
| 15567 | 1.18872 | 2.1 |
| 15568 | 0.740752 | 2.1 |
| 15569 | 1.92269 | 0.0656187 |
| 15570 | 1.53584 | 0.0630587 |
| 15571 | 1.53584 | 0.100619 |
| 15572 | 1.21067 | 0.05 |
| 15573 | 1.40707 | 0.05 |
| 15574 | 1.50437 | 0.05 |
| 15575 | 1.50435 | 0.05 |
| 15576 | 1.45446 | 0.05 |
| 15577 | 1.47954 | 0.692315 |
| 15578 | 2.08687 | 1.05481 |
| 15579 | 2.08687 | 2.1 |
| 15580 | 1.1233 | 2.1 |
| 15581 | 1.04025 | 0.755307 |
| 15582 | 1.36005 | 1.65153 |
| 15583 | 1.47606 | 0.05 |
| 15584 | 1.3407 | 0.05 |
| 15585 | 0.758743 | 2.1 |
| 15586 | 0.716917 | 2.1 |
| 15587 | 0.716922 | 2.1 |
| 15588 | 0.672964 | 2.1 |
| 15589 | 2.40014 | 2.1 |
| 15590 | 1.96848 | 2.1 |
| 15591 | 1.96848 | 2.1 |
| 15592 | 1.53555 | 1.49418 |
| 15593 | 1.06191 | 2.1 |
| 15594 | 1.29579 | 2.1 |
| 15595 | 1.29579 | 2.1 |
| 15596 | 1.71289 | 2.1 |
| 15597 | 1.78584 | 2.1 |
| 15598 | 2.15211 | 2.1 |
| 15599 | 2.00427 | 2.1 |

| | | |
|---|---|---|
| 15600 | 3.41615 | 2.1 |
| 15601 | 1.66408 | 2.1 |
| 15602 | 1.73954 | 2.1 |
| 15603 | 2.91663 | 2.1 |
| 15604 | 3.65818 | 2.1 |
| 15605 | 1.93286 | 2.1 |
| 15606 | 1.08831 | 2.1 |
| 15607 | 1.31242 | 0.05 |
| 15608 | 1.11712 | 0.05 |
| 15609 | 1.26311 | 0.05 |
| 15610 | 1.31242 | 0.05 |
| 15611 | 1.10378 | 2.1 |
| 15612 | 0.968594 | 2.1 |
| 15613 | 1.48389 | 0.05 |
| 15614 | 1.49613 | 0.05 |
| 15615 | 1.04349 | 0.605871 |
| 15616 | 1.26409 | 0.469509 |
| 15617 | 1.26409 | 0.252983 |
| 15618 | 1.05724 | 0.553695 |
| 15619 | 1.65723 | 0.05 |
| 15620 | 1.83116 | 0.05 |
| 15621 | 2.06284 | 0.05 |
| 15622 | 2.00512 | 0.0547737 |
| 15623 | 1.83116 | 0.05 |
| 15624 | 2.03139 | 0.05 |
| 15625 | 2.20564 | 0.05 |
| 15626 | 1.90048 | 0.0500715 |
| 15627 | 0.963392 | 2.1 |
| 15628 | 1.01346 | 0.283971 |
| 15629 | 2.98092 | 2.1 |
| 15630 | 2.70295 | 2.1 |
| 15631 | 2.70295 | 2.1 |
| 15632 | 2.48828 | 2.1 |
| 15633 | 1.63661 | 2.1 |
| 15634 | 1.64158 | 2.1 |
| 15635 | 1.64158 | 2.1 |
| 15636 | 1.6488 | 2.1 |
| 15637 | 1.17506 | 0.05 |
| 15638 | 1.09667 | 0.05 |
| 15639 | 2.00539 | 0.05 |
| 15640 | 1.09667 | 0.05 |
| 15641 | 1.54946 | 2.1 |
| 15642 | 1.43577 | 2.1 |
| 15643 | 1.26103 | 2.1 |
| 15644 | 1.49808 | 2.1 |
| 15645 | 1.089 | 0.05 |
| 15646 | 1.22586 | 0.05 |
| 15647 | 2.35027 | 0.05 |
| 15648 | 1.37549 | 0.05 |
| 15649 | 3.33706 | 0.05 |
| 15650 | 1.69894 | 0.05 |
| 15651 | 2.00539 | 0.05 |
| 15652 | 1.53798 | 0.05 |
| 15653 | 1.28938 | 2.1 |
| 15654 | 1.20969 | 2.1 |
| 15655 | 1.20969 | 2.1 |
| 15656 | 1.15864 | 2.1 |
| 15657 | 1.7986 | 2.1 |

| | | |
|---|---|---|
| 15658 | 1.75869 | 2.1 |
| 15659 | 1.58281 | 2.1 |
| 15660 | 1.7392 | 2.1 |
| 15661 | 1.30733 | 0.945895 |
| 15662 | 1.5419 | 1.28839 |
| 15663 | 1.50883 | 1.09102 |
| 15664 | 1.85439 | 0.992257 |
| 15665 | 1.73531 | 0.475583 |
| 15666 | 1.70225 | 0.476097 |
| 15667 | 1.22176 | 0.05 |
| 15668 | 1.59492 | 0.060257 |
| 15669 | 1.59492 | 0.05 |
| 15670 | 2.15542 | 0.0635722 |
| 15671 | 3.24379 | 2.1 |
| 15672 | 2.64775 | 2.1 |
| 15673 | 3.16707 | 2.1 |
| 15674 | 1.93032 | 2.1 |
| 15675 | 1.50738 | 2.1 |
| 15676 | 1.24128 | 2.1 |
| 15677 | 1.24128 | 2.1 |
| 15678 | 0.994293 | 2.1 |
| 15679 | 2.90808 | 0.05 |
| 15680 | 2.45797 | 0.05 |
| 15681 | 1.85501 | 0.05 |
| 15682 | 2.90809 | 0.05 |
| 15683 | 0.892835 | 1.98985 |
| 15684 | 1.55871 | 2.1 |
| 15685 | 1.04615 | 1.36834 |
| 15686 | 1.66643 | 0.559738 |
| 15687 | 1.70044 | 2.1 |
| 15688 | 1.80224 | 2.1 |
| 15689 | 1.70186 | 0.928117 |
| 15690 | 3.30123 | 0.860258 |
| 15691 | 1.74789 | 2.1 |
| 15692 | 1.77446 | 2.1 |
| 15693 | 1.77901 | 2.1 |
| 15694 | 1.57995 | 2.1 |
| 15695 | 1.81518 | 2.1 |
| 15696 | 2.17716 | 2.1 |
| 15697 | 2.17716 | 2.1 |
| 15698 | 2.65123 | 2.1 |
| 15699 | 2.01899 | 0.644496 |
| 15700 | 1.77479 | 0.432195 |
| 15701 | 1.19122 | 0.05 |
| 15702 | 1.46724 | 0.05 |
| 15703 | 1.70944 | 0.05 |
| 15704 | 1.08929 | 0.05 |
| 15705 | 1.68454 | 0.05 |
| 15706 | 1.08929 | 0.05 |
| 15707 | 1.51067 | 1.0381 |
| 15708 | 1.06729 | 0.675771 |
| 15709 | 1.37762 | 0.8255 |
| 15710 | 1.13992 | 0.626711 |
| 15711 | 1.26397 | 2.1 |
| 15712 | 1.04083 | 2.1 |
| 15713 | 1.00278 | 2.1 |
| 15714 | 1.04084 | 2.1 |
| 15715 | 1.1016 | 0.05 |

| | | |
|---|---|---|
| 15716 | 1.23477 | 0.05 |
| 15717 | 1.23477 | 0.05 |
| 15718 | 1.64985 | 0.05 |
| 15719 | 1.11117 | 2.1 |
| 15720 | 1.13006 | 2.1 |
| 15721 | 1.13006 | 2.1 |
| 15722 | 1.14721 | 2.1 |
| 15723 | 1.21946 | 2.1 |
| 15724 | 1.0186 | 2.1 |
| 15725 | 1.13178 | 2.1 |
| 15726 | 1.00133 | 2.1 |
| 15727 | 1.67046 | 0.208651 |
| 15728 | 0.986036 | 0.0761112 |
| 15729 | 1.67046 | 0.281682 |
| 15730 | 1.34857 | 1.94492 |
| 15731 | 2.46016 | 2.1 |
| 15732 | 2.07161 | 2.1 |
| 15733 | 2.10732 | 2.1 |
| 15734 | 2.08371 | 2.1 |
| 15735 | 1.35602 | 2.1 |
| 15736 | 0.829065 | 2.1 |
| 15737 | 1.81667 | 2.1 |
| 15738 | 0.99406 | 2.1 |
| 15739 | 1.81667 | 2.1 |
| 15740 | 1.68073 | 2.1 |
| 15741 | 2.05736 | 2.1 |
| 15742 | 2.97311 | 2.1 |
| 15743 | 2.14011 | 2.1 |
| 15744 | 1.23411 | 2.1 |
| 15745 | 1.13632 | 0.05 |
| 15746 | 1.40862 | 0.05 |
| 15747 | 1.28055 | 0.05 |
| 15748 | 1.14305 | 0.05 |
| 15749 | 1.12035 | 0.05 |
| 15750 | 1.15483 | 0.05 |
| 15751 | 1.94099 | 0.05 |
| 15752 | 1.82126 | 0.05 |
| 15753 | 1.15481 | 0.05 |
| 15754 | 0.998957 | 0.05 |
| 15755 | 2.10078 | 0.05 |
| 15756 | 1.81617 | 0.05 |
| 15757 | 1.27581 | 2.1 |
| 15758 | 0.968536 | 2.1 |
| 15759 | 1.33916 | 2.1 |
| 15760 | 1.27581 | 2.1 |
| 15761 | 1.32784 | 2.1 |
| 15762 | 1.4259 | 2.1 |
| 15763 | 1.70377 | 2.1 |
| 15764 | 1.54231 | 2.1 |
| 15765 | 3.77569 | 2.1 |
| 15766 | 3.42809 | 2.1 |
| 15767 | 1.54231 | 2.1 |
| 15768 | 1.51298 | 2.1 |
| 15769 | 3.55866 | 2.1 |
| 15770 | 2.86136 | 2.1 |
| 15771 | 1.83301 | 0.269637 |
| 15772 | 1.26371 | 0.0991735 |
| 15773 | 1.98301 | 0.434081 |

| | | |
|---|---|---|
| 15774 | 2.0609 | 0.146795 |
| 15775 | 2.26047 | 0.104244 |
| 15776 | 2.00537 | 0.0518503 |
| 15777 | 1.7913 | 0.0589149 |
| 15778 | 1.48864 | 0.05 |
| 15779 | 1.09504 | 2.1 |
| 15780 | 1.39827 | 2.1 |
| 15781 | 2.28084 | 2.1 |
| 15782 | 1.05983 | 2.1 |
| 15783 | 1.33593 | 0.05 |
| 15784 | 1.31508 | 0.05 |
| 15785 | 1.31508 | 0.05 |
| 15786 | 1.09599 | 0.05 |
| 15787 | 1.30647 | 2.1 |
| 15788 | 1.28846 | 2.1 |
| 15789 | 1.28846 | 2.1 |
| 15790 | 1.26923 | 2.1 |
| 15791 | 1.2955 | 2.1 |
| 15792 | 1.09044 | 2.1 |
| 15793 | 2.12209 | 2.1 |
| 15794 | 1.26438 | 2.1 |
| 15795 | 0.911819 | 2.1 |
| 15796 | 1.38752 | 2.1 |
| 15797 | 2.57704 | 2.1 |
| 15798 | 2.71736 | 2.1 |
| 15799 | 1.42284 | 2.1 |
| 15800 | 1.41562 | 2.1 |
| 15801 | 2.69131 | 2.1 |
| 15802 | 2.36178 | 2.1 |
| 15803 | 1.41562 | 2.1 |
| 15804 | 1.39564 | 2.1 |
| 15805 | 1.32368 | 2.1 |
| 15806 | 0.908053 | 2.1 |
| 15807 | 0.908053 | 2.1 |
| 15808 | 0.629213 | 2.1 |
| 15809 | 1.8758 | 1.65994 |
| 15810 | 1.27362 | 1.09853 |
| 15811 | 1.3756 | 0.623091 |
| 15812 | 2.28749 | 1.07427 |
| 15813 | 1.84577 | 0.572996 |
| 15814 | 2.17765 | 0.50325 |
| 15815 | 1.38969 | 0.240922 |
| 15816 | 1.84578 | 0.464464 |
| 15817 | 1.32801 | 0.0941052 |
| 15818 | 1.76377 | 0.130962 |
| 15819 | 1.22845 | 2.1 |
| 15820 | 1.57766 | 0.737604 |
| 15821 | 1.57766 | 2.1 |
| 15822 | 1.39067 | 2.1 |
| 15823 | 1.15743 | 0.05 |
| 15824 | 1.37721 | 0.05 |
| 15825 | 1.69055 | 0.05 |
| 15826 | 1.14858 | 0.05 |
| 15827 | 1.38333 | 1.9913 |
| 15828 | 1.10575 | 0.109978 |
| 15829 | 1.22418 | 2.1 |
| 15830 | 1.10574 | 2.1 |
| 15831 | 1.91509 | 0.05 |

| | | |
|---|---|---|
| 15832 | 1.02001 | 0.05 |
| 15833 | 1.03074 | 0.05 |
| 15834 | 1.19122 | 0.05 |
| 15835 | 1.67887 | 2.1 |
| 15836 | 1.67597 | 2.1 |
| 15837 | 1.84984 | 2.1 |
| 15838 | 1.77775 | 2.1 |
| 15839 | 1.73571 | 2.1 |
| 15840 | 1.27921 | 2.1 |
| 15841 | 2.11851 | 2.1 |
| 15842 | 1.27921 | 2.1 |
| 15843 | 1.42486 | 2.1 |
| 15844 | 1.12503 | 2.1 |
| 15845 | 1.12503 | 2.1 |
| 15846 | 1.30116 | 2.1 |
| 15847 | 0.568995 | 2.1 |
| 15848 | 0.557348 | 2.1 |
| 15849 | 0.557348 | 2.1 |
| 15850 | 0.53907 | 2.1 |
| 15851 | 0.633575 | 2.1 |
| 15852 | 1.33756 | 0.648803 |
| 15853 | 1.51074 | 2.1 |
| 15854 | 1.93384 | 0.19726 |
| 15855 | 0.655202 | 2.1 |
| 15856 | 0.731934 | 2.1 |
| 15857 | 0.731934 | 2.1 |
| 15858 | 1.09061 | 2.1 |
| 15859 | 0.966979 | 2.1 |
| 15860 | 1.31502 | 2.1 |
| 15861 | 1.31502 | 2.1 |
| 15862 | 1.72116 | 2.1 |
| 15863 | 1.70002 | 2.1 |
| 15864 | 1.67969 | 2.1 |
| 15865 | 1.67969 | 2.1 |
| 15866 | 1.62408 | 2.1 |
| 15867 | 1.01531 | 2.1 |
| 15868 | 1.66018 | 2.1 |
| 15869 | 1.01531 | 2.1 |
| 15870 | 0.886968 | 2.1 |
| 15871 | 1.22585 | 0.05 |
| 15872 | 1.20674 | 0.05 |
| 15873 | 1.20672 | 0.05 |
| 15874 | 1.02962 | 0.05 |
| 15875 | 0.956467 | 2.1 |
| 15876 | 2.00551 | 1.8942 |
| 15877 | 0.553801 | 2.1 |
| 15878 | 0.617006 | 2.1 |
| 15879 | 1.26923 | 2.1 |
| 15880 | 1.23614 | 2.1 |
| 15881 | 1.23614 | 2.1 |
| 15882 | 1.16817 | 2.1 |
| 15883 | 2.14482 | 2.1 |
| 15884 | 1.72637 | 2.1 |
| 15885 | 1.09368 | 2.1 |
| 15886 | 1.26195 | 2.1 |
| 15887 | 0.760546 | 2.1 |
| 15888 | 1.84094 | 2.1 |
| 15889 | 2.62868 | 2.1 |

| | | |
|---|---|---|
| 15890 | 2.79784 | 2.1 |
| 15891 | 2.79784 | 2.1 |
| 15892 | 3.00129 | 2.1 |
| 15893 | 1.27593 | 0.780625 |
| 15894 | 1.38877 | 1.96241 |
| 15895 | 1.06486 | 0.05 |
| 15896 | 1.2474 | 0.05 |
| 15897 | 1.76234 | 0.05 |
| 15898 | 1.8685 | 0.05 |
| 15899 | 1.69863 | 0.05 |
| 15900 | 1.29666 | 0.05 |
| 15901 | 1.91646 | 2.1 |
| 15902 | 1.8582 | 2.1 |
| 15903 | 1.8582 | 2.1 |
| 15904 | 1.7986 | 2.1 |
| 15905 | 3.44043 | 0.05 |
| 15906 | 1.94764 | 0.05 |
| 15907 | 1.94764 | 0.05 |
| 15908 | 1.36307 | 0.05 |
| 15909 | 1.32075 | 0.05 |
| 15910 | 1.12971 | 0.05 |
| 15911 | 2.06319 | 0.05 |
| 15912 | 1.94982 | 0.05 |
| 15913 | 1.77255 | 0.05 |
| 15914 | 0.979297 | 0.05 |
| 15915 | 1.59997 | 2.1 |
| 15916 | 1.93506 | 2.1 |
| 15917 | 1.94409 | 2.1 |
| 15918 | 1.9597 | 2.1 |
| 15919 | 0.57899 | 2.1 |
| 15920 | 0.953036 | 2.1 |
| 15921 | 0.953049 | 2.1 |
| 15922 | 1.53503 | 2.1 |
| 15923 | 1.39615 | 0.05 |
| 15924 | 1.06474 | 0.05 |
| 15925 | 0.947564 | 0.05 |
| 15926 | 1.45506 | 0.05 |
| 15927 | 1.17799 | 0.459051 |
| 15928 | 1.21067 | 0.0511135 |
| 15929 | 1.35945 | 0.05 |
| 15930 | 2.05648 | 0.05 |
| 15931 | 1.36208 | 0.05 |
| 15932 | 2.15229 | 0.05 |
| 15933 | 2.89288 | 0.05 |
| 15934 | 1.95719 | 0.05 |
| 15935 | 2.25169 | 0.05 |
| 15936 | 1.52756 | 0.05 |
| 15937 | 1.19322 | 0.05 |
| 15938 | 1.23146 | 0.05 |
| 15939 | 2.45024 | 0.05 |
| 15940 | 0.794122 | 0.05 |
| 15941 | 1.6343 | 0.05 |
| 15942 | 1.34161 | 0.05 |
| 15943 | 1.05353 | 0.05 |
| 15944 | 1.50795 | 0.05 |
| 15945 | 1.05353 | 0.05 |
| 15946 | 1.20669 | 0.05 |
| 15947 | 1.59289 | 2.1 |

| | | |
|---|---|---|
| 15948 | 0.989543 | 2.1 |
| 15949 | 1.40588 | 2.1 |
| 15950 | 0.991427 | 2.1 |
| 15951 | 0.921822 | 1.98219 |
| 15952 | 1.46903 | 0.839236 |
| 15953 | 1.46903 | 2.1 |
| 15954 | 1.8214 | 0.308172 |
| 15955 | 1.39702 | 2.1 |
| 15956 | 1.25103 | 2.1 |
| 15957 | 1.25103 | 2.1 |
| 15958 | 1.10378 | 2.1 |
| 15959 | 1.71813 | 2.1 |
| 15960 | 1.39595 | 2.1 |
| 15961 | 1.386 2.1 | |
| 15962 | 1.13008 | 2.1 |
| 15963 | 1.31711 | 0.05 |
| 15964 | 1.70191 | 0.05 |
| 15965 | 0.893249 | 0.05 |
| 15966 | 1.42669 | 0.05 |
| 15967 | 1.23943 | 2.1 |
| 15968 | 1.1483 | 1.26323 |
| 15969 | 1.04598 | 2.1 |
| 15970 | 1.1483 | 1.35056 |
| 15971 | 1.00381 | 0.05 |
| 15972 | 1.11433 | 0.05 |
| 15973 | 0.973501 | 0.978845 |
| 15974 | 1.0756 | 1.02558 |
| 15975 | 1.20669 | 0.05 |
| 15976 | 1.18508 | 0.05 |
| 15977 | 1.18508 | 0.05 |
| 15978 | 0.983027 | 0.05 |
| 15979 | 1.43687 | 2.1 |
| 15980 | 1.71059 | 2.1 |
| 15981 | 1.71059 | 2.1 |
| 15982 | 1.66762 | 2.1 |
| 15983 | 2.8415 | 2.1 |
| 15984 | 2.40555 | 2.1 |
| 15985 | 1.91546 | 2.1 |
| 15986 | 1.61385 | 2.1 |
| 15987 | 2.01569 | 2.1 |
| 15988 | 1.33183 | 2.1 |
| 15989 | 1.23218 | 2.1 |
| 15990 | 1.23999 | 2.1 |
| 15991 | 1.01335 | 0.778354 |
| 15992 | 1.12088 | 0.924265 |
| 15993 | 1.25346 | 2.1 |
| 15994 | 1.15644 | 2.1 |
| 15995 | 1.3764 | 1.02914 |
| 15996 | 1.27558 | 0.688126 |
| 15997 | 1.2064 | 2.1 |
| 15998 | 1.15067 | 2.1 |
| 15999 | 1.01595 | 2.1 |
| 16000 | 1.15067 | 2.1 |
| 16001 | 1.41902 | 1.73838 |
| 16002 | 1.30272 | 2.1 |
| 16003 | 1.30272 | 0.894226 |
| 16004 | 1.19548 | 2.1 |
| 16005 | 2.99339 | 2.1 |

| | | |
|---|---|---|
| 16006 | 2.98041 | 2.1 |
| 16007 | 1.46124 | 2.1 |
| 16008 | 1.42105 | 2.1 |
| 16009 | 2.98041 | 2.1 |
| 16010 | 2.86339 | 2.1 |
| 16011 | 1.42105 | 2.1 |
| 16012 | 1.42284 | 2.1 |
| 16013 | 2.31301 | 0.432312 |
| 16014 | 1.80273 | 0.305449 |
| 16015 | 2.17765 | 0.650394 |
| 16016 | 2.31301 | 0.333161 |
| 16017 | 1.76378 | 0.143073 |
| 16018 | 2.10275 | 0.101807 |
| 16019 | 2.10275 | 0.14187 |
| 16020 | 2.26047 | 0.0809962 |
| 16021 | 0.97541 | 2.1 |
| 16022 | 1.40863 | 2.1 |
| 16023 | 1.40863 | 2.1 |
| 16024 | 1.30751 | 2.1 |
| 16025 | 1.20143 | 0.05 |
| 16026 | 1.19756 | 0.05 |
| 16027 | 1.43707 | 0.05 |
| 16028 | 1.28954 | 0.05 |
| 16029 | 3.34254 | 0.05 |
| 16030 | 1.92916 | 0.05 |
| 16031 | 1.80089 | 0.05 |
| 16032 | 1.1323 | 0.05 |
| 16033 | 1.47321 | 0.05 |
| 16034 | 1.23756 | 0.05 |
| 16035 | 1.87291 | 2.1 |
| 16036 | 1.53642 | 2.1 |
| 16037 | 1.53642 | 2.1 |
| 16038 | 1.5791 | 2.1 |
| 16039 | 1.0446 | 2.1 |
| 16040 | 0.992674 | 2.1 |
| 16041 | 1.12324 | 0.05 |
| 16042 | 1.29163 | 0.05 |
| 16043 | 1.29163 | 0.05 |
| 16044 | 1.15371 | 0.05 |
| 16045 | 0.996831 | 0.05 |
| 16046 | 1.00192 | 0.05 |
| 16047 | 1.0019 | 0.05 |
| 16048 | 0.958937 | 0.05 |
| 16049 | 1.07283 | 2.1 |
| 16050 | 1.25294 | 0.325246 |
| 16051 | 1.25294 | 0.840139 |
| 16052 | 1.04788 | 0.794699 |
| 16053 | 1.14496 | 2.1 |
| 16054 | 1.07652 | 2.1 |
| 16055 | 1.07652 | 2.1 |
| 16056 | 0.991406 | 2.1 |
| 16057 | 1.60976 | 2.1 |
| 16058 | 1.37 2.1 | |
| 16059 | 1.37 2.1 | |
| 16060 | 1.14195 | 2.1 |
| 16061 | 1.26593 | 1.98687 |
| 16062 | 1.75366 | 2.1 |
| 16063 | 1.57621 | 0.05 |

| | | |
|---|---|---|
| 16064 | 1.24538 | 0.05 |
| 16065 | 0.969118 | 2.1 |
| 16066 | 1.04147 | 2.1 |
| 16067 | 0.969118 | 2.1 |
| 16068 | 1.22615 | 2.1 |
| 16069 | 0.98084 | 2.1 |
| 16070 | 1.01843 | 2.1 |
| 16071 | 1.09435 | 2.1 |
| 16072 | 1.50876 | 2.1 |
| 16073 | 1.5976 | 2.1 |
| 16074 | 1.84322 | 2.1 |
| 16075 | 1.84324 | 2.1 |
| 16076 | 1.70377 | 2.1 |
| 16077 | 1.20709 | 2.1 |
| 16078 | 2.54729 | 2.1 |
| 16079 | 0.974716 | 0.05 |
| 16080 | 1.54468 | 0.05 |
| 16081 | 1.39021 | 0.05 |
| 16082 | 1.70303 | 0.05 |
| 16083 | 1.22852 | 2.07258 |
| 16084 | 1.578 | 1.21639 |
| 16085 | 1.578 | 2.05506 |
| 16086 | 1.93852 | 0.910274 |
| 16087 | 2.2506 | 2.1 |
| 16088 | 1.39302 | 2.1 |
| 16089 | 1.5478 | 2.1 |
| 16090 | 1.2269 | 2.1 |
| 16091 | 1.70048 | 2.1 |
| 16092 | 1.45477 | 2.1 |
| 16093 | 1.30929 | 2.1 |
| 16094 | 1.15069 | 1.62758 |
| 16095 | 1.00255 | 2.09963 |
| 16096 | 1.05481 | 0.05 |
| 16097 | 1.05481 | 2.1 |
| 16098 | 1.1032 | 0.05 |
| 16099 | 0.993888 | 2.1 |
| 16100 | 1.19294 | 2.1 |
| 16101 | 1.01121 | 0.05 |
| 16102 | 0.991866 | 0.05 |
| 16103 | 2.20052 | 0.05 |
| 16104 | 1.7409 | 2.1 |
| 16105 | 1.75142 | 2.1 |
| 16106 | 1.46811 | 2.1 |
| 16107 | 1.7409 | 1.01205 |
| 16108 | 1.23267 | 2.1 |
| 16109 | 1.46811 | 2.1 |
| 16110 | 1.18872 | 2.1 |
| 16111 | 1.12665 | 2.1 |
| 16112 | 1.50045 | 2.1 |
| 16113 | 1.50045 | 2.1 |
| 16114 | 1.37704 | 2.1 |
| 16115 | 1.35308 | 1.09025 |
| 16116 | 1.29983 | 0.603925 |
| 16117 | 1.29983 | 1.21917 |
| 16118 | 0.970212 | 0.654924 |
| 16119 | 1.82272 | 2.1 |
| 16120 | 1.62784 | 2.1 |
| 16121 | 1.62784 | 2.1 |

| | | |
|---|---|---|
| 16122 | 1.87691 | 2.1 |
| 16123 | 3.65294 | 2.1 |
| 16124 | 2.33374 | 2.1 |
| 16125 | 2.12888 | 2.1 |
| 16126 | 1.62229 | 2.1 |
| 16127 | 1.41496 | 2.1 |
| 16128 | 1.32707 | 0.0740035 |
| 16129 | 2.18871 | 0.05 |
| 16130 | 1.4235 | 0.05 |
| 16131 | 1.46015 | 0.05 |
| 16132 | 1.25635 | 0.05 |
| 16133 | 1.45501 | 0.05 |
| 16134 | 1.3543 | 0.05 |
| 16135 | 1.38139 | 2.1 |
| 16136 | 1.98159 | 2.1 |
| 16137 | 2.15868 | 2.1 |
| 16138 | 4.04308 | 2.1 |
| 16139 | 1.5362 | 2.1 |
| 16140 | 1.56328 | 2.1 |
| 16141 | 1.56327 | 2.1 |
| 16142 | 1.58163 | 2.1 |
| 16143 | 1.88987 | 0.05 |
| 16144 | 1.08761 | 0.05 |
| 16145 | 0.862154 | 0.05 |
| 16146 | 1.77688 | 0.05 |
| 16147 | 1.14238 | 2.1 |
| 16148 | 1.11691 | 2.1 |
| 16149 | 1.01537 | 2.1 |
| 16150 | 1.02923 | 2.1 |
| 16151 | 0.977425 | 0.05 |
| 16152 | 1.15211 | 0.05 |
| 16153 | 2.0815 | 2.1 |
| 16154 | 1.18621 | 2.1 |
| 16155 | 1.01485 | 2.1 |
| 16156 | 1.17054 | 2.1 |
| 16157 | 1.17054 | 2.1 |
| 16158 | 1.12665 | 2.1 |
| 16159 | 1.87213 | 0.05 |
| 16160 | 1.82244 | 0.05 |
| 16161 | 1.45039 | 0.05 |
| 16162 | 1.7226 | 0.05 |
| 16163 | 1.841 | 0.05 |
| 16164 | 1.79011 | 0.05 |
| 16165 | 1.8792 | 0.05 |
| 16166 | 1.45053 | 0.05 |
| 16167 | 1.08947 | 2.1 |
| 16168 | 1.5008 | 2.1 |
| 16169 | 1.5008 | 2.1 |
| 16170 | 1.83186 | 2.1 |
| 16171 | 1.04159 | 0.05 |
| 16172 | 1.2489 | 0.05 |
| 16173 | 1.2489 | 0.05 |
| 16174 | 1.33592 | 0.05 |
| 16175 | 1.51973 | 0.05 |
| 16176 | 0.980547 | 0.05 |
| 16177 | 1.51974 | 0.05 |
| 16178 | 1.15991 | 0.05 |
| 16179 | 1.34965 | 2.1 |

| | | |
|---|---|---|
| 16180 | 1.2724 | 2.1 |
| 16181 | 1.2724 | 2.1 |
| 16182 | 1.38438 | 2.1 |
| 16183 | 1.02923 | 2.1 |
| 16184 | 1.06521 | 2.1 |
| 16185 | 1.06521 | 2.1 |
| 16186 | 1.0752 | 2.1 |
| 16187 | 2.03139 | 0.05 |
| 16188 | 1.92951 | 0.05 |
| 16189 | 1.74852 | 0.0544979 |
| 16190 | 1.62772 | 0.05 |
| 16191 | 1.73655 | 0.05 |
| 16192 | 1.76809 | 0.05 |
| 16193 | 1.62772 | 0.05 |
| 16194 | 1.1432 | 0.05 |
| 16195 | 1.09235 | 2.1 |
| 16196 | 1.48081 | 2.1 |
| 16197 | 1.46712 | 2.1 |
| 16198 | 1.89321 | 2.1 |
| 16199 | 1.94548 | 2.1 |
| 16200 | 1.5636 | 2.1 |
| 16201 | 1.5636 | 2.1 |
| 16202 | 1.21124 | 2.1 |
| 16203 | 1.52314 | 2.1 |
| 16204 | 2.0219 | 2.1 |
| 16205 | 1.51113 | 2.1 |
| 16206 | 2.0219 | 2.1 |
| 16207 | 0.987247 | 0.05 |
| 16208 | 1.18347 | 0.05 |
| 16209 | 1.89226 | 0.13408 |
| 16210 | 1.61441 | 0.05 |
| 16211 | 1.42884 | 2.1 |
| 16212 | 1.10961 | 2.1 |
| 16213 | 1.56991 | 2.1 |
| 16214 | 1.05337 | 2.1 |
| 16215 | 1.05337 | 2.1 |
| 16216 | 1.91547 | 2.1 |
| 16217 | 1.05931 | 2.1 |
| 16218 | 1.1729 | 2.1 |
| 16219 | 1.34526 | 2.1 |
| 16220 | 1.0593 | 2.1 |
| 16221 | 1.0856 | 2.1 |
| 16222 | 1.90854 | 2.1 |
| 16223 | 1.91674 | 2.1 |
| 16224 | 1.11345 | 2.1 |
| 16225 | 1.75067 | 2.1 |
| 16226 | 1.50022 | 2.1 |
| 16227 | 1.50022 | 2.1 |
| 16228 | 1.53619 | 2.1 |
| 16229 | 1.4748 | 2.1 |
| 16230 | 1.74431 | 1.9389 |
| 16231 | 2.02581 | 1.47342 |
| 16232 | 1.25288 | 1.55257 |
| 16233 | 1.01814 | 0.722628 |
| 16234 | 1.2881 | 0.153957 |
| 16235 | 1.2881 | 0.420752 |
| 16236 | 1.54433 | 0.153564 |
| 16237 | 1.19999 | 0.347719 |

| | | |
|---|---|---|
| 16238 | 1.44056 | 0.05 |
| 16239 | 1.02011 | 0.05 |
| 16240 | 1.17129 | 0.05 |
| 16241 | 1.13775 | 2.06966 |
| 16242 | 3.34815 | 2.1 |
| 16243 | 1.60428 | 1.98171 |
| 16244 | 1.85928 | 1.9967 |
| 16245 | 1.75592 | 2.1 |
| 16246 | 1.25989 | 2.1 |
| 16247 | 1.75592 | 2.1 |
| 16248 | 1.71549 | 2.1 |
| 16249 | 1.37634 | 2.1 |
| 16250 | 1.64693 | 2.1 |
| 16251 | 1.64693 | 2.1 |
| 16252 | 1.64193 | 2.1 |
| 16253 | 1.18351 | 0.05 |
| 16254 | 2.19398 | 0.05 |
| 16255 | 2.16272 | 0.244065 |
| 16256 | 1.19837 | 0.129489 |
| 16257 | 1.04973 | 2.1 |
| 16258 | 1.20149 | 2.1 |
| 16259 | 1.60574 | 2.1 |
| 16260 | 1.11164 | 0.11708 |
| 16261 | 1.87823 | 0.422961 |
| 16262 | 1.11164 | 0.942426 |
| 16263 | 0.772882 | 2.1 |
| 16264 | 1.4714 | 2.1 |
| 16265 | 2.14242 | 0.162411 |
| 16266 | 1.12336 | 2.1 |
| 16267 | 2.8386 | 0.0890511 |
| 16268 | 2.13346 | 2.1 |
| 16269 | 1.06788 | 0.05 |
| 16270 | 0.947662 | 0.05 |
| 16271 | 1.05297 | 0.05 |
| 16272 | 1.42907 | 0.05 |
| 16273 | 2.17465 | 2.1 |
| 16274 | 2.18394 | 2.1 |
| 16275 | 1.14808 | 2.1 |
| 16276 | 1.21292 | 2.1 |
| 16277 | 1.21292 | 2.1 |
| 16278 | 1.27229 | 2.1 |
| 16279 | 1.20859 | 0.368984 |
| 16280 | 1.21073 | 0.887755 |
| 16281 | 1.21073 | 0.380157 |
| 16282 | 1.22857 | 0.908434 |
| 16283 | 1.79158 | 2.1 |
| 16284 | 1.73899 | 2.1 |
| 16285 | 1.34012 | 2.1 |
| 16286 | 1.39991 | 2.1 |
| 16287 | 1.73898 | 2.1 |
| 16288 | 1.61245 | 2.1 |
| 16289 | 1.40136 | 2.1 |
| 16290 | 1.57234 | 2.1 |
| 16291 | 1.82162 | 2.1 |
| 16292 | 3.09875 | 2.1 |
| 16293 | 1.10264 | 2.1 |
| 16294 | 1.02339 | 2.1 |
| 16295 | 1.02339 | 2.1 |

| | | |
|---|---|---|
| 16296 | 1.23042 | 2.1 |
| 16297 | 2.82266 | 0.663719 |
| 16298 | 1.05698 | 0.0810053 |
| 16299 | 1.96384 | 0.519161 |
| 16300 | 1.10244 | 0.38878 |
| 16301 | 0.929061 | 2.1 |
| 16302 | 1.47007 | 2.1 |
| 16303 | 0.985303 | 2.1 |
| 16304 | 1.24554 | 2.1 |
| 16305 | 1.05993 | 2.1 |
| 16306 | 2.02074 | 2.1 |
| 16307 | 2.10106 | 1.08891 |
| 16308 | 1.18171 | 0.964788 |
| 16309 | 1.68111 | 1.05126 |
| 16310 | 1.84226 | 0.799605 |
| 16311 | 1.82131 | 0.05 |
| 16312 | 1.32371 | 0.05 |
| 16313 | 1.41902 | 0.05 |
| 16314 | 1.00205 | 0.05 |
| 16315 | 1.65532 | 2.1 |
| 16316 | 1.18977 | 2.1 |
| 16317 | 1.15471 | 2.1 |
| 16318 | 0.993889 | 2.1 |
| 16319 | 2.08521 | 2.1 |
| 16320 | 2.09885 | 2.1 |
| 16321 | 2.09497 | 2.1 |
| 16322 | 2.10604 | 2.1 |
| 16323 | 0.881028 | 1.43998 |
| 16324 | 1.52828 | 0.977308 |
| 16325 | 1.40574 | 0.05 |
| 16326 | 1.09599 | 0.05 |
| 16327 | 1.45506 | 2.1 |
| 16328 | 1.50524 | 2.1 |
| 16329 | 1.50524 | 2.1 |
| 16330 | 1.54711 | 2.1 |
| 16331 | 1.41469 | 2.1 |
| 16332 | 2.40442 | 2.1 |
| 16333 | 1.40794 | 2.1 |
| 16334 | 1.89226 | 2.1 |
| 16335 | 1.31346 | 2.1 |
| 16336 | 1.51067 | 2.1 |
| 16337 | 1.31363 | 2.1 |
| 16338 | 1.11833 | 2.1 |
| 16339 | 2.3373 | 2.1 |
| 16340 | 2.19994 | 2.1 |
| 16341 | 2.19994 | 2.1 |
| 16342 | 1.09616 | 2.1 |
| 16343 | 1.63719 | 2.1 |
| 16344 | 1.52204 | 2.1 |
| 16345 | 1.52205 | 2.1 |
| 16346 | 1.40118 | 2.1 |
| 16347 | 2.5946 | 2.1 |
| 16348 | 4.07065 | 2.1 |
| 16349 | 1.33916 | 2.1 |
| 16350 | 2.05217 | 2.1 |
| 16351 | 1.67023 | 2.1 |
| 16352 | 2.05217 | 2.1 |
| 16353 | 1.59458 | 0.255114 |

| | | |
|---|---|---|
| 16354 | 1.02247 | 0.16224 |
| 16355 | 1.68108 | 0.609826 |
| 16356 | 1.03079 | 0.260565 |
| 16357 | 1.1643 | 0.05 |
| 16358 | 1.85795 | 0.05 |
| 16359 | 1.70302 | 0.05 |
| 16360 | 1.42076 | 0.05 |
| 16361 | 1.91351 | 0.05 |
| 16362 | 1.01148 | 0.05 |
| 16363 | 2.52402 | 0.05 |
| 16364 | 0.752625 | 0.05 |
| 16365 | 0.886968 | 2.1 |
| 16366 | 1.28845 | 2.1 |
| 16367 | 1.28845 | 2.1 |
| 16368 | 1.27766 | 2.1 |
| 16369 | 1.82268 | 2.1 |
| 16370 | 1.87118 | 2.1 |
| 16371 | 1.87119 | 2.1 |
| 16372 | 1.91704 | 2.1 |
| 16373 | 3.13938 | 2.1 |
| 16374 | 2.97839 | 2.1 |
| 16375 | 2.97839 | 2.1 |
| 16376 | 2.87331 | 2.1 |
| 16377 | 1.73443 | 0.961161 |
| 16378 | 1.59457 | 0.767785 |
| 16379 | 2.01827 | 2.1 |
| 16380 | 2.04586 | 2.1 |
| 16381 | 2.04586 | 2.1 |
| 16382 | 2.00865 | 2.1 |
| 16383 | 1.19901 | 2.1 |
| 16384 | 1.16661 | 2.1 |
| 16385 | 1.13215 | 0.05 |
| 16386 | 1.96256 | 0.05 |
| 16387 | 2.34043 | 0.05 |
| 16388 | 2.04154 | 0.05 |
| 16389 | 1.58991 | 0.05 |
| 16390 | 1.45893 | 0.05 |
| 16391 | 2.56818 | 0.05 |
| 16392 | 1.70532 | 0.05 |
| 16393 | 1.17798 | 0.0717798 |
| 16394 | 1.25796 | 0.375956 |
| 16395 | 1.25797 | 0.461851 |
| 16396 | 1.10719 | 0.759934 |
| 16397 | 1.51067 | 2.1 |
| 16398 | 1.60707 | 2.1 |
| 16399 | 2.06946 | 2.1 |
| 16400 | 3.19218 | 2.1 |
| 16401 | 2.19965 | 2.1 |
| 16402 | 1.30521 | 2.1 |
| 16403 | 2.23684 | 1.21745 |
| 16404 | 1.43363 | 2.1 |
| 16405 | 1.51007 | 2.1 |
| 16406 | 2.46356 | 2.1 |
| 16407 | 1.90428 | 2.1 |
| 16408 | 1.13618 | 2.1 |
| 16409 | 2.13276 | 0.05 |
| 16410 | 0.998345 | 0.05 |
| 16411 | 1.2861 | 0.05 |

| | | |
|---|---|---|
| 16412 | 1.20457 | 0.05 |
| 16413 | 1.51789 | 2.1 |
| 16414 | 1.49872 | 2.1 |
| 16415 | 1.49872 | 2.1 |
| 16416 | 1.47712 | 2.1 |
| 16417 | 1.87129 | 0.220759 |
| 16418 | 0.949364 | 0.0919254 |
| 16419 | 1.83142 | 0.217376 |
| 16420 | 1.55063 | 0.259581 |
| 16421 | 1.03298 | 0.05 |
| 16422 | 0.980664 | 0.0749261 |
| 16423 | 1.51696 | 0.05 |
| 16424 | 1.34927 | 0.206314 |
| 16425 | 1.70302 | 0.05 |
| 16426 | 1.65952 | 0.05 |
| 16427 | 1.76658 | 0.05 |
| 16428 | 0.986848 | 0.05 |
| 16429 | 2.13757 | 1.53829 |
| 16430 | 1.98142 | 0.05 |
| 16431 | 1.98142 | 0.999322 |
| 16432 | 1.65411 | 0.0856203 |
| 16433 | 1.1323 | 0.05 |
| 16434 | 1.46015 | 0.05 |
| 16435 | 2.15376 | 1.60476 |
| 16436 | 1.64622 | 1.8455 |
| 16437 | 0.991867 | 0.05 |
| 16438 | 1.58074 | 0.05 |
| 16439 | 1.69368 | 2.1 |
| 16440 | 1.76403 | 2.1 |
| 16441 | 1.76403 | 2.1 |
| 16442 | 1.83318 | 2.1 |
| 16443 | 1.28418 | 0.146213 |
| 16444 | 1.16851 | 0.05 |
| 16445 | 1.1685 | 0.0673351 |
| 16446 | 1.1726 | 0.05 |
| 16447 | 1.06455 | 2.1 |
| 16448 | 1.18773 | 2.1 |
| 16449 | 0.98511 | 2.1 |
| 16450 | 1.18774 | 2.1 |
| 16451 | 1.76476 | 2.1 |
| 16452 | 1.79825 | 2.1 |
| 16453 | 1.79825 | 2.1 |
| 16454 | 0.961895 | 2.1 |
| 16455 | 1.59589 | 0.05 |
| 16456 | 2.25975 | 0.05 |
| 16457 | 2.76339 | 2.1 |
| 16458 | 3.0551 | 2.1 |
| 16459 | 1.68761 | 2.1 |
| 16460 | 1.59561 | 2.1 |
| 16461 | 2.17257 | 2.1 |
| 16462 | 1.05845 | 2.1 |
| 16463 | 1.03512 | 2.1 |
| 16464 | 2.19011 | 2.1 |
| 16465 | 1.44859 | 2.1 |
| 16466 | 1.35897 | 0.943113 |
| 16467 | 1.35897 | 2.1 |
| 16468 | 1.17822 | 1.43895 |
| 16469 | 1.13641 | 2.1 |

| | | |
|---|---|---|
| 16470 | 1.18301 | 2.1 |
| 16471 | 1.18301 | 2.1 |
| 16472 | 1.25173 | 2.1 |
| 16473 | 2.41297 | 2.1 |
| 16474 | 1.36463 | 2.1 |
| 16475 | 1.54711 | 2.1 |
| 16476 | 1.5735 | 2.1 |
| 16477 | 1.5735 | 2.1 |
| 16478 | 1.58713 | 2.1 |
| 16479 | 1.69044 | 0.05 |
| 16480 | 1.57979 | 0.05 |
| 16481 | 1.57979 | 0.05 |
| 16482 | 1.14387 | 0.05 |
| 16483 | 1.04199 | 2.1 |
| 16484 | 1.96491 | 2.1 |
| 16485 | 1.04199 | 2.1 |
| 16486 | 1.46892 | 2.1 |
| 16487 | 0.983724 | 2.1 |
| 16488 | 1.70579 | 2.1 |
| 16489 | 1.88522 | 2.1 |
| 16490 | 1.23914 | 2.1 |
| 16491 | 1.79424 | 2.1 |
| 16492 | 1.52851 | 2.1 |
| 16493 | 1.04921 | 2.1 |
| 16494 | 1.11285 | 2.1 |
| 16495 | 1.25334 | 0.910665 |
| 16496 | 1.07496 | 0.568304 |
| 16497 | 1.07497 | 2.1 |
| 16498 | 1.34852 | 0.799253 |
| 16499 | 1.27391 | 2.1 |
| 16500 | 2.20307 | 2.1 |
| 16501 | 1.81311 | 2.1 |
| 16502 | 2.10305 | 2.1 |
| 16503 | 1.21059 | 0.05 |
| 16504 | 1.44253 | 0.05 |
| 16505 | 1.9194 | 0.05 |
| 16506 | 1.21159 | 0.05 |
| 16507 | 0.947979 | 2.1 |
| 16508 | 1.01225 | 0.05 |
| 16509 | 0.947979 | 2.1 |
| 16510 | 1.01225 | 2.1 |
| 16511 | 1.00255 | 2.1 |
| 16512 | 1.01225 | 2.1 |
| 16513 | 1.01225 | 2.1 |
| 16514 | 1.06561 | 2.1 |
| 16515 | 3.50562 | 2.1 |
| 16516 | 3.61336 | 2.1 |
| 16517 | 3.61336 | 2.1 |
| 16518 | 3.73635 | 2.1 |
| 16519 | 1.61245 | 2.1 |
| 16520 | 1.52301 | 2.1 |
| 16521 | 1.57612 | 2.1 |
| 16522 | 1.83035 | 2.1 |
| 16523 | 1.52301 | 2.1 |
| 16524 | 1.24789 | 2.1 |
| 16525 | 1.85156 | 2.1 |
| 16526 | 1.89072 | 2.1 |
| 16527 | 0.995735 | 2.1 |

| | | |
|---|---|---|
| 16528 | 1.57738 | 2.1 |
| 16529 | 1.87141 | 0.05 |
| 16530 | 1.73388 | 0.05 |
| 16531 | 1.61344 | 0.05 |
| 16532 | 0.922676 | 0.05 |
| 16533 | 1.56614 | 2.1 |
| 16534 | 0.956294 | 2.1 |
| 16535 | 1.82589 | 2.1 |
| 16536 | 1.63719 | 2.1 |
| 16537 | 1.12399 | 0.05 |
| 16538 | 1.50103 | 0.05 |
| 16539 | 1.56976 | 0.05 |
| 16540 | 1.03567 | 0.05 |
| 16541 | 1.21776 | 0.05 |
| 16542 | 1.89708 | 0.05 |
| 16543 | 1.16323 | 0.05 |
| 16544 | 1.63533 | 0.05 |
| 16545 | 2.29397 | 0.05 |
| 16546 | 3.01596 | 0.05 |
| 16547 | 1.80145 | 0.05 |
| 16548 | 1.7666 | 0.05 |
| 16549 | 1.91621 | 0.808085 |
| 16550 | 1.10719 | 0.585214 |
| 16551 | 1.57828 | 1.16785 |
| 16552 | 1.17105 | 1.28803 |
| 16553 | 1.15806 | 2.1 |
| 16554 | 1.31565 | 2.1 |
| 16555 | 1.31565 | 2.1 |
| 16556 | 1.46643 | 2.1 |
| 16557 | 1.47507 | 2.1 |
| 16558 | 1.87858 | 2.1 |
| 16559 | 0.958661 | 2.1 |
| 16560 | 1.87858 | 2.1 |
| 16561 | 1.44293 | 0.05 |
| 16562 | 1.43213 | 0.05 |
| 16563 | 1.43213 | 0.05 |
| 16564 | 1.10476 | 0.05 |
| 16565 | 1.03235 | 2.1 |
| 16566 | 1.31 0.05 | |
| 16567 | 1.31 2.1 | |
| 16568 | 1.50108 | 0.908863 |
| 16569 | 1.32215 | 2.1 |
| 16570 | 1.10611 | 2.1 |
| 16571 | 1.10611 | 2.1 |
| 16572 | 0.904978 | 2.1 |
| 16573 | 1.06486 | 0.05 |
| 16574 | 1.18347 | 0.05 |
| 16575 | 1.70535 | 2.1 |
| 16576 | 1.94779 | 2.1 |
| 16577 | 1.77071 | 2.1 |
| 16578 | 1.82268 | 2.1 |
| 16579 | 0.61701 | 2.1 |
| 16580 | 0.672495 | 2.1 |
| 16581 | 0.672494 | 2.1 |
| 16582 | 0.713174 | 2.1 |
| 16583 | 1.40395 | 2.1 |
| 16584 | 1.34245 | 2.1 |
| 16585 | 1.34243 | 2.1 |

| | | |
|---|---|---|
| 16586 | 1.30646 | 2.1 |
| 16587 | 1.06204 | 0.05 |
| 16588 | 0.632691 | 0.409513 |
| 16589 | 1.75766 | 0.172035 |
| 16590 | 1.14287 | 0.749466 |
| 16591 | 1.52101 | 0.0705965 |
| 16592 | 1.71414 | 0.0692252 |
| 16593 | 1.86411 | 0.05 |
| 16594 | 1.6253 | 0.0831418 |
| 16595 | 1.04355 | 0.05 |
| 16596 | 1.0443 | 0.131668 |
| 16597 | 1.34852 | 2.1 |
| 16598 | 1.56016 | 0.314133 |
| 16599 | 1.56016 | 0.29176 |
| 16600 | 1.03235 | 0.336966 |
| 16601 | 1.2966 | 0.05 |
| 16602 | 1.82354 | 0.05 |
| 16603 | 1.2966 | 0.05 |
| 16604 | 1.67687 | 0.05 |
| 16605 | 1.19669 | 1.32177 |
| 16606 | 1.82979 | 1.83718 |
| 16607 | 1.58972 | 1.29638 |
| 16608 | 1.09219 | 1.29871 |
| 16609 | 1.42077 | 1.03241 |
| 16610 | 1.89226 | 0.896781 |
| 16611 | 1.19547 | 0.438428 |
| 16612 | 1.10187 | 2.1 |
| 16613 | 1.10188 | 0.372453 |
| 16614 | 1.01479 | 2.1 |
| 16615 | 1.81663 | 2.1 |
| 16616 | 1.81533 | 2.1 |
| 16617 | 1.83247 | 2.1 |
| 16618 | 1.51511 | 2.1 |
| 16619 | 0.983148 | 2.1 |
| 16620 | 1.7513 | 2.1 |
| 16621 | 1.85017 | 2.1 |
| 16622 | 1.26103 | 2.1 |
| 16623 | 1.23152 | 2.1 |
| 16624 | 1.65508 | 2.1 |
| 16625 | 1.23152 | 2.1 |
| 16626 | 2.15082 | 2.1 |
| 16627 | 1.57974 | 2.1 |
| 16628 | 1.07289 | 2.1 |
| 16629 | 0.81534 | 2.1 |
| 16630 | 1.51007 | 2.1 |
| 16631 | 1.00898 | 0.05 |
| 16632 | 1.18959 | 0.05 |
| 16633 | 1.32178 | 0.05 |
| 16634 | 1.25929 | 0.05 |
| 16635 | 2.17892 | 0.10278 |
| 16636 | 1.22938 | 0.05 |
| 16637 | 3.02933 | 0.58092 |
| 16638 | 2.34619 | 0.483255 |
| 16639 | 1.47679 | 0.23441 |
| 16640 | 1.0819 | 0.315937 |
| 16641 | 3.10558 | 2.1 |
| 16642 | 3.31457 | 2.1 |
| 16643 | 0.985109 | 2.1 |

| | | |
|---|---|---|
| 16644 | 1.10667 | 2.1 |
| 16645 | 3.31457 | 2.1 |
| 16646 | 3.54443 | 2.1 |
| 16647 | 1.10667 | 2.1 |
| 16648 | 1.25987 | 2.1 |
| 16649 | 1.77063 | 1.12658 |
| 16650 | 1.51065 | 0.765741 |
| 16651 | 1.24688 | 2.1 |
| 16652 | 1.37652 | 2.1 |
| 16653 | 1.37652 | 2.1 |
| 16654 | 1.54168 | 2.1 |
| 16655 | 1.17042 | 1.02953 |
| 16656 | 1.13092 | 1.30602 |
| 16657 | 1.13092 | 1.93394 |
| 16658 | 1.52938 | 2.1 |
| 16659 | 1.11787 | 0.266525 |
| 16660 | 1.00613 | 0.240656 |
| 16661 | 0.781373 | 0.194638 |
| 16662 | 1.6885 | 0.135933 |
| 16663 | 2.49439 | 0.06949 |
| 16664 | 1.42657 | 0.108886 |
| 16665 | 1.77903 | 2.1 |
| 16666 | 2.63606 | 2.1 |
| 16667 | 2.10425 | 2.1 |
| 16668 | 1.21425 | 2.1 |
| 16669 | 1.31253 | 1.37569 |
| 16670 | 1.28418 | 1.67862 |
| 16671 | 1.28418 | 1.14481 |
| 16672 | 1.58972 | 1.25256 |
| 16673 | 0.858948 | 2.1 |
| 16674 | 1.90196 | 2.1 |
| 16675 | 1.81024 | 2.1 |
| 16676 | 1.90194 | 2.1 |
| 16677 | 1.4263 | 0.05 |
| 16678 | 0.947921 | 0.05 |
| 16679 | 1.22852 | 1.00502 |
| 16680 | 1.09194 | 0.860739 |
| 16681 | 1.60578 | 0.05 |
| 16682 | 1.14721 | 0.05 |
| 16683 | 1.06336 | 0.05 |
| 16684 | 1.14721 | 0.05 |
| 16685 | 1.11924 | 0.05 |
| 16686 | 1.53814 | 0.05 |
| 16687 | 1.18421 | 0.05 |
| 16688 | 1.8458 | 0.05 |
| 16689 | 1.7565 | 2.1 |
| 16690 | 1.09344 | 1.42237 |
| 16691 | 1.09344 | 1.03744 |
| 16692 | 1.16971 | 2.1 |
| 16693 | 1.71465 | 0.05 |
| 16694 | 1.19412 | 0.05 |
| 16695 | 1.64985 | 0.05 |
| 16696 | 1.05417 | 0.05 |
| 16697 | 1.2127 | 0.05 |
| 16698 | 1.48867 | 0.05 |
| 16699 | 1.27806 | 0.05 |
| 16700 | 1.21269 | 0.05 |
| 16701 | 1.37813 | 1.15353 |

| | | |
|---|---|---|
| 16702 | 1.0417 | 1.2431 |
| 16703 | 1.88626 | 0.0875492 |
| 16704 | 1.14977 | 0.772825 |
| 16705 | 1.78803 | 2.1 |
| 16706 | 1.33793 | 1.23492 |
| 16707 | 1.42266 | 2.1 |
| 16708 | 1.26865 | 1.8407 |
| 16709 | 1.09518 | 2.1 |
| 16710 | 1.36844 | 2.1 |
| 16711 | 1.20455 | 2.1 |
| 16712 | 1.36844 | 2.1 |
| 16713 | 1.22297 | 0.76531 |
| 16714 | 1.11256 | 1.1717 |
| 16715 | 1.11256 | 0.53749 |
| 16716 | 1.03217 | 0.905541 |
| 16717 | 1.18422 | 0.05 |
| 16718 | 1.4282 | 0.05 |
| 16719 | 1.04159 | 0.05 |
| 16720 | 1.4282 | 0.05 |
| 16721 | 0.988805 | 2.1 |
| 16722 | 1.05481 | 2.1 |
| 16723 | 1.52938 | 0.865679 |
| 16724 | 1.26593 | 1.49443 |
| 16725 | 1.53082 | 2.1 |
| 16726 | 1.09847 | 2.1 |
| 16727 | 1.75211 | 0.05 |
| 16728 | 1.22222 | 2.1 |
| 16729 | 1.75211 | 0.05 |
| 16730 | 1.51592 | 2.1 |
| 16731 | 1.63413 | 0.311446 |
| 16732 | 1.26662 | 0.648661 |
| 16733 | 1.42495 | 0.0805775 |
| 16734 | 1.4356 | 0.133909 |
| 16735 | 1.29389 | 1.83619 |
| 16736 | 1.04164 | 2.1 |
| 16737 | 1.40574 | 1.52998 |
| 16738 | 1.04164 | 1.24577 |
| 16739 | 2.22344 | 0.482053 |
| 16740 | 1.45945 | 0.05 |
| 16741 | 0.987248 | 0.05 |
| 16742 | 1.2474 | 0.05 |
| 16743 | 0.974702 | 0.894904 |
| 16744 | 1.43777 | 0.670753 |
| 16745 | 1.71289 | 2.1 |
| 16746 | 1.91529 | 2.1 |
| 16747 | 1.91529 | 2.1 |
| 16748 | 2.01827 | 2.1 |
| 16749 | 1.87111 | 1.15651 |
| 16750 | 1.00864 | 0.600696 |
| 16751 | 1.26865 | 0.695235 |
| 16752 | 1.18769 | 1.05445 |
| 16753 | 1.24167 | 2.1 |
| 16754 | 1.36133 | 0.05 |
| 16755 | 2.28168 | 2.1 |
| 16756 | 3.98524 | 2.1 |
| 16757 | 1.36133 | 2.1 |
| 16758 | 1.69436 | 0.279261 |
| 16759 | 2.21926 | 2.1 |

| | | |
|---|---|---|
| 16760 | 1.70302 | 2.1 |
| 16761 | 1.50732 | 0.05 |
| 16762 | 1.42237 | 0.05 |
| 16763 | 1.18659 | 0.05 |
| 16764 | 1.50732 | 0.05 |
| 16765 | 1.19785 | 0.05 |
| 16766 | 1.40969 | 0.05 |
| 16767 | 1.28339 | 0.05 |
| 16768 | 1.40882 | 0.05 |
| 16769 | 0.968595 | 2.1 |
| 16770 | 1.41903 | 2.1 |
| 16771 | 1.12567 | 2.1 |
| 16772 | 1.15898 | 2.1 |
| 16773 | 1.27241 | 2.1 |
| 16774 | 0.995796 | 2.1 |
| 16775 | 1.89983 | 0.05 |
| 16776 | 1.05417 | 0.05 |
| 16777 | 0.571999 | 0.05 |
| 16778 | 0.743597 | 0.05 |
| 16779 | 1.89353 | 0.05 |
| 16780 | 0.924068 | 0.05 |
| 16781 | 1.52851 | 2.1 |
| 16782 | 1.26969 | 2.1 |
| 16783 | 1.07763 | 0.05 |
| 16784 | 1.82301 | 0.05 |
| 16785 | 1.07763 | 0.05 |
| 16786 | 1.17506 | 0.05 |
| 16787 | 1.20045 | 2.1 |
| 16788 | 1.12486 | 2.1 |
| 16789 | 1.15234 | 2.1 |
| 16790 | 1.11598 | 2.1 |
| 16791 | 1.12486 | 2.1 |
| 16792 | 1.0491 | 2.1 |
| 16793 | 1.11598 | 2.1 |
| 16794 | 1.1047 | 2.1 |
| 16795 | 1.05902 | 2.1 |
| 16796 | 1.14361 | 2.1 |
| 16797 | 1.14361 | 2.1 |
| 16798 | 1.26851 | 2.1 |
| 16799 | 1.29389 | 1.19721 |
| 16800 | 0.988456 | 1.20263 |
| 16801 | 1.83688 | 0.05 |
| 16802 | 1.70302 | 0.05 |
| 16803 | 1.49386 | 0.05 |
| 16804 | 1.83151 | 0.05 |
| 16805 | 1.01346 | 0.0541993 |
| 16806 | 0.983607 | 0.13797 |
| 16807 | 0.983596 | 2.1 |
| 16808 | 1.08332 | 0.138792 |
| 16809 | 2.36848 | 0.0757172 |
| 16810 | 2.97066 | 0.05 |
| 16811 | 1.12352 | 0.05 |
| 16812 | 2.5311 | 0.05 |
| 16813 | 1.09062 | 2.1 |
| 16814 | 0.962182 | 2.1 |
| 16815 | 0.962181 | 2.1 |
| 16816 | 1.2258 | 2.1 |
| 16817 | 0.974888 | 2.1 |

| | | |
|---|---|---|
| 16818 | 1.06312 | 2.1 |
| 16819 | 1.73583 | 0.05 |
| 16820 | 1.72399 | 0.05 |
| 16821 | 1.724 | 0.05 |
| 16822 | 1.50951 | 0.05 |
| 16823 | 1.26755 | 2.1 |
| 16824 | 1.03824 | 2.1 |
| 16825 | 1.27875 | 2.1 |
| 16826 | 1.03824 | 2.1 |
| 16827 | 1.18902 | 2.1 |
| 16828 | 1.534 | 2.1 |
| 16829 | 1.11764 | 2.1 |
| 16830 | 1.534 | 2.1 |
| 16831 | 1.52008 | 2.1 |
| 16832 | 1.34406 | 2.1 |
| 16833 | 1.34406 | 2.1 |
| 16834 | 1.2187 | 2.1 |
| 16835 | 0.963571 | 0.944728 |
| 16836 | 1.19612 | 0.80752 |
| 16837 | 0.963571 | 2.1 |
| 16838 | 1.00007 | 2.1 |
| 16839 | 0.903333 | 2.1 |
| 16840 | 1.12724 | 2.1 |
| 16841 | 1.23771 | 2.1 |
| 16842 | 1.17683 | 2.1 |
| 16843 | 1.20059 | 0.466805 |
| 16844 | 0.772023 | 0.05 |
| 16845 | 1.14749 | 0.18323 |
| 16846 | 1.2802 | 0.817681 |
| 16847 | 1.85091 | 1.38018 |
| 16848 | 2.11193 | 1.04666 |
| 16849 | 1.2802 | 0.0644375 |
| 16850 | 1.42913 | 0.766149 |
| 16851 | 2.11193 | 0.934235 |
| 16852 | 1.27737 | 0.165711 |
| 16853 | 1.30584 | 2.1 |
| 16854 | 1.60936 | 2.1 |
| 16855 | 1.3413 | 0.05 |
| 16856 | 1.53336 | 0.05 |
| 16857 | 2.60679 | 0.05 |
| 16858 | 1.3413 | 0.05 |
| 16859 | 1.35877 | 2.1 |
| 16860 | 1.11024 | 2.1 |
| 16861 | 1.27839 | 2.1 |
| 16862 | 1.08171 | 2.1 |
| 16863 | 1.27841 | 2.1 |
| 16864 | 1.0577 | 2.1 |
| 16865 | 1.71255 | 2.1 |
| 16866 | 1.10611 | 2.1 |
| 16867 | 0.960106 | 2.1 |
| 16868 | 1.40921 | 2.1 |
| 16869 | 1.90705 | 0.05 |
| 16870 | 2.79564 | 0.05 |
| 16871 | 1.45321 | 0.05 |
| 16872 | 1.90705 | 0.05 |
| 16873 | 0.974718 | 0.05 |
| 16874 | 1.53671 | 0.05 |
| 16875 | 1.69039 | 0.05 |

| | | |
|---|---|---|
| 16876 | 1.78229 | 0.05 |
| 16877 | 1.0101 | 2.1 |
| 16878 | 1.3167 | 2.1 |
| 16879 | 1.19699 | 2.1 |
| 16880 | 1.3212 | 2.1 |
| 16881 | 1.60811 | 0.05 |
| 16882 | 1.43224 | 0.05 |
| 16883 | 1.35401 | 0.05 |
| 16884 | 1.17464 | 0.05 |
| 16885 | 1.27696 | 2.1 |
| 16886 | 1.05025 | 2.1 |
| 16887 | 1.05025 | 2.1 |
| 16888 | 1.18076 | 2.1 |
| 16889 | 1.37224 | 1.7018 |
| 16890 | 1.13438 | 1.5792 |
| 16891 | 1.76788 | 1.43199 |
| 16892 | 1.73444 | 1.30841 |
| 16893 | 1.37704 | 2.1 |
| 16894 | 1.77042 | 2.1 |
| 16895 | 1.77042 | 2.1 |
| 16896 | 1.09778 | 2.1 |
| 16897 | 1.28667 | 2.1 |
| 16898 | 1.28228 | 2.1 |
| 16899 | 1.30646 | 0.809303 |
| 16900 | 1.68867 | 0.915347 |
| 16901 | 1.30645 | 1.16272 |
| 16902 | 0.986494 | 2.08762 |
| 16903 | 1.6253 | 0.05 |
| 16904 | 1.48676 | 0.073076 |
| 16905 | 1.48676 | 0.05 |
| 16906 | 1.32974 | 0.0659427 |
| 16907 | 0.634347 | 0.05 |
| 16908 | 1.15142 | 0.05 |
| 16909 | 1.11395 | 0.05 |
| 16910 | 1.18068 | 0.05 |
| 16911 | 1.36904 | 0.05 |
| 16912 | 0.764563 | 0.05 |
| 16913 | 2.09213 | 0.05 |
| 16914 | 1.56631 | 2.1 |
| 16915 | 1.08011 | 2.1 |
| 16916 | 1.80283 | 2.1 |
| 16917 | 1.50738 | 2.1 |
| 16918 | 1.44951 | 0.05 |
| 16919 | 1.50102 | 0.05 |
| 16920 | 1.50102 | 0.05 |
| 16921 | 1.39615 | 0.05 |
| 16922 | 1.02653 | 0.131934 |
| 16923 | 2.98258 | 0.162712 |
| 16924 | 2.0815 | 0.05 |
| 16925 | 1.39525 | 0.05 |
| 16926 | 1.82176 | 0.05 |
| 16927 | 0.977607 | 0.05 |
| 16928 | 1.96133 | 1.46127 |
| 16929 | 1.27189 | 1.19026 |
| 16930 | 0.826032 | 0.9918 |
| 16931 | 2.12831 | 0.971444 |
| 16932 | 1.77798 | 2.1 |
| 16933 | 1.02969 | 1.81574 |

| | | |
|---|---|---|
| 16934 | 1.06463 | 1.24362 |
| 16935 | 1.49219 | 2.1 |
| 16936 | 1.10003 | 2.1 |
| 16937 | 1.21223 | 2.1 |
| 16938 | 1.00513 | 1.23144 |
| 16939 | 1.43788 | 2.1 |
| 16940 | 1.47134 | 0.101244 |
| 16941 | 0.968249 | 0.418193 |
| 16942 | 3.22585 | 0.05 |
| 16943 | 3.36933 | 0.05 |
| 16944 | 3.36932 | 0.05 |
| 16945 | 3.03865 | 0.05 |
| 16946 | 1.66295 | 0.05 |
| 16947 | 1.65583 | 0.05 |
| 16948 | 1.65583 | 0.05 |
| 16949 | 1.4732 | 0.05 |
| 16950 | 1.73704 | 0.05 |
| 16951 | 1.76476 | 0.792002 |
| 16952 | 2.13345 | 0.12357 |
| 16953 | 1.55816 | 2.1 |
| 16954 | 1.55816 | 0.252329 |
| 16955 | 1.02601 | 2.1 |
| 16956 | 1.08039 | 2.1 |
| 16957 | 1.05498 | 0.982982 |
| 16958 | 1.08039 | 2.1 |
| 16959 | 1.2873 | 2.1 |
| 16960 | 1.01121 | 2.1 |
| 16961 | 0.987703 | 2.1 |
| 16962 | 0.98771 | 2.1 |
| 16963 | 0.964782 | 2.1 |
| 16964 | 1.35719 | 2.1 |
| 16965 | 2.61092 | 2.1 |
| 16966 | 2.6607 | 2.1 |
| 16967 | 3.41693 | 2.1 |
| 16968 | 1.23125 | 2.1 |
| 16969 | 1.73772 | 2.1 |
| 16970 | 0.99845 | 0.39981 |
| 16971 | 1.5176 | 0.5999 |
| 16972 | 1.13271 | 0.05 |
| 16973 | 1.332 | 0.05 |
| 16974 | 1.63159 | 2.1 |
| 16975 | 1.61342 | 2.1 |
| 16976 | 1.03679 | 2.1 |
| 16977 | 1.19087 | 2.1 |
| 16978 | 1.97172 | 2.1 |
| 16979 | 2.68634 | 2.1 |
| 16980 | 1.19086 | 2.1 |
| 16981 | 1.50091 | 2.1 |
| 16982 | 2.84677 | 2.1 |
| 16983 | 2.77894 | 2.1 |
| 16984 | 2.77894 | 2.1 |
| 16985 | 2.74206 | 2.1 |
| 16986 | 1.3284 | 2.1 |
| 16987 | 1.83524 | 2.1 |
| 16988 | 1.65853 | 2.1 |
| 16989 | 1.56741 | 2.1 |
| 16990 | 1.00509 | 2.1 |
| 16991 | 1.45246 | 2.1 |

| | | |
|---|---|---|
| 16992 | 1.26193 | 2.1 |
| 16993 | 1.45244 | 2.1 |
| 16994 | 1.61963 | 0.05 |
| 16995 | 1.68189 | 0.508383 |
| 16996 | 1.68189 | 1.53112 |
| 16997 | 1.49155 | 0.151876 |
| 16998 | 1.58054 | 2.1 |
| 16999 | 1.59169 | 2.1 |
| 17000 | 1.59169 | 2.1 |
| 17001 | 1.60497 | 2.1 |
| 17002 | 0.743595 | 0.05 |
| 17003 | 0.702359 | 0.05 |
| 17004 | 1.15317 | 0.05 |
| 17005 | 2.24367 | 0.05 |
| 17006 | 1.15252 | 0.05 |
| 17007 | 2.60354 | 0.05 |
| 17008 | 1.45158 | 2.1 |
| 17009 | 1.56004 | 2.1 |
| 17010 | 1.64527 | 2.1 |
| 17011 | 1.00371 | 2.1 |
| 17012 | 1.38287 | 0.525736 |
| 17013 | 0.893024 | 0.654519 |
| 17014 | 1.23527 | 0.665109 |
| 17015 | 1.23706 | 0.626784 |
| 17016 | 2.20993 | 0.508565 |
| 17017 | 1.54858 | 1.47117 |
| 17018 | 0.798666 | 0.0677588 |
| 17019 | 1.22487 | 0.111741 |
| 17020 | 1.33158 | 0.05 |
| 17021 | 1.85902 | 0.157155 |
| 17022 | 1.08334 | 2.1 |
| 17023 | 1.17885 | 1.04782 |
| 17024 | 1.17885 | 0.0785069 |
| 17025 | 1.07283 | 0.511451 |
| 17026 | 1.30723 | 0.05 |
| 17027 | 1.62507 | 0.05 |
| 17028 | 1.62507 | 0.05 |
| 17029 | 1.08854 | 0.05 |
| 17030 | 1.59475 | 2.1 |
| 17031 | 1.58874 | 2.1 |
| 17032 | 1.58875 | 2.1 |
| 17033 | 1.35787 | 2.1 |
| 17034 | 1.04147 | 2.1 |
| 17035 | 1.15564 | 2.1 |
| 17036 | 1.78301 | 2.1 |
| 17037 | 1.90618 | 2.1 |
| 17038 | 1.71555 | 2.1 |
| 17039 | 2.13143 | 2.1 |
| 17040 | 1.10817 | 2.1 |
| 17041 | 1.032 | 2.1 |
| 17042 | 1.032 | 2.1 |
| 17043 | 0.953636 | 2.1 |
| 17044 | 1.27916 | 1.1468 |
| 17045 | 1.11938 | 1.0175 |
| 17046 | 1.11937 | 1.02461 |
| 17047 | 1.9186 | 1.26381 |
| 17048 | 1.19967 | 0.05 |
| 17049 | 1.63489 | 0.05 |

| | | |
|---|---|---|
| 17050 | 1.00763 | 0.05 |
| 17051 | 1.81656 | 0.05 |
| 17052 | 1.31814 | 0.05 |
| 17053 | 2.98183 | 0.05 |
| 17054 | 1.20149 | 2.1 |
| 17055 | 1.29129 | 2.1 |
| 17056 | 1.29129 | 2.1 |
| 17057 | 1.37138 | 2.1 |
| 17058 | 1.23104 | 0.0971987 |
| 17059 | 1.75236 | 0.628084 |
| 17060 | 1.6 | 0.05 |
| 17061 | 1.60565 | 0.0769119 |
| 17062 | 1.60565 | 0.0588474 |
| 17063 | 1.40013 | 0.0614448 |
| 17064 | 1.23042 | 2.1 |
| 17065 | 1.18278 | 2.1 |
| 17066 | 1.10853 | 2.1 |
| 17067 | 1.04724 | 2.1 |
| 17068 | 1.1766 | 2.1 |
| 17069 | 1.00319 | 2.1 |
| 17070 | 1.29435 | 2.1 |
| 17071 | 1.00319 | 2.1 |
| 17072 | 1.05655 | 1.52653 |
| 17073 | 1.01606 | 1.60582 |
| 17074 | 1.80391 | 2.1 |
| 17075 | 2.7699 | 2.1 |
| 17076 | 1.52073 | 2.1 |
| 17077 | 2.06835 | 2.1 |
| 17078 | 2.00202 | 2.1 |
| 17079 | 2.04906 | 2.1 |
| 17080 | 0.968536 | 2.1 |
| 17081 | 1.02461 | 2.1 |
| 17082 | 1.59784 | 2.1 |
| 17083 | 1.78249 | 2.1 |
| 17084 | 1.81409 | 2.1 |
| 17085 | 1.79844 | 2.1 |
| 17086 | 1.77093 | 1.94848 |
| 17087 | 2.02356 | 1.14357 |
| 17088 | 1.32449 | 2.1 |
| 17089 | 1.10003 | 2.1 |
| 17090 | 1.16915 | 0.106246 |
| 17091 | 1.05937 | 0.0724593 |
| 17092 | 1.28096 | 2.1 |
| 17093 | 1.34696 | 2.1 |
| 17094 | 1.34695 | 2.1 |
| 17095 | 1.36457 | 2.1 |
| 17096 | 1.06478 | 2.1 |
| 17097 | 1.06239 | 2.1 |
| 17098 | 1.06239 | 2.1 |
| 17099 | 1.09629 | 2.1 |
| 17100 | 0.798209 | 2.1 |
| 17101 | 1.8532 | 2.1 |
| 17102 | 2.10112 | 0.05 |
| 17103 | 2.8704 | 0.05 |
| 17104 | 2.8704 | 0.05 |
| 17105 | 2.92457 | 0.05 |
| 17106 | 1.0752 | 2.1 |
| 17107 | 1.07745 | 2.1 |

| | | |
|---|---|---|
| 17108 | 1.07745 | 2.1 |
| 17109 | 1.08911 | 2.1 |
| 17110 | 1.40182 | 2.1 |
| 17111 | 1.00133 | 2.1 |
| 17112 | 1.40182 | 2.1 |
| 17113 | 1.24509 | 2.1 |
| 17114 | 2.22243 | 0.05 |
| 17115 | 1.07303 | 0.05 |
| 17116 | 1.29736 | 0.05 |
| 17117 | 1.73584 | 0.05 |
| 17118 | 2.0815 | 0.324914 |
| 17119 | 1.07999 | 0.078746 |
| 17120 | 2.1692 | 2.1 |
| 17121 | 1.99992 | 2.1 |
| 17122 | 1.99992 | 2.1 |
| 17123 | 1.78327 | 2.1 |
| 17124 | 1.52661 | 0.05 |
| 17125 | 0.956003 | 0.05 |
| 17126 | 1.53172 | 0.05 |
| 17127 | 0.955991 | 0.05 |
| 17128 | 1.35025 | 0.836957 |
| 17129 | 1.51084 | 2.1 |
| 17130 | 1.51084 | 1.76214 |
| 17131 | 1.67115 | 2.1 |
| 17132 | 2.00977 | 2.1 |
| 17133 | 1.63632 | 2.1 |
| 17134 | 1.63632 | 2.1 |
| 17135 | 2.66128 | 2.1 |
| 17136 | 1.13525 | 2.1 |
| 17137 | 2.37566 | 2.1 |
| 17138 | 1.25987 | 2.1 |
| 17139 | 1.4472 | 2.1 |
| 17140 | 1.4472 | 2.1 |
| 17141 | 1.66018 | 2.1 |
| 17142 | 0.994001 | 0.966119 |
| 17143 | 1.83151 | 0.656272 |
| 17144 | 1.83151 | 0.117331 |
| 17145 | 1.57621 | 0.107524 |
| 17146 | 1.40915 | 0.05 |
| 17147 | 1.24653 | 0.22797 |
| 17148 | 1.24653 | 0.05 |
| 17149 | 1.10609 | 0.618712 |
| 17150 | 1.61121 | 2.1 |
| 17151 | 1.74472 | 2.1 |
| 17152 | 1.74472 | 2.1 |
| 17153 | 1.0368 | 2.1 |
| 17154 | 1.04788 | 2.1 |
| 17155 | 1.28522 | 2.1 |
| 17156 | 1.28522 | 2.1 |
| 17157 | 1.54196 | 2.1 |
| 17158 | 1.23387 | 2.1 |
| 17159 | 0.896519 | 2.1 |
| 17160 | 1.60428 | 2.1 |
| 17161 | 1.08467 | 2.1 |
| 17162 | 1.56875 | 0.05 |
| 17163 | 1.39072 | 0.05 |
| 17164 | 1.39073 | 0.05 |
| 17165 | 1.6 | 0.0757323 |

| | | |
|---|---|---|
| 17166 | 1.01346 | 2.1 |
| 17167 | 1.06838 | 1.05771 |
| 17168 | 0.968463 | 1.0699 |
| 17169 | 1.06837 | 1.07392 |
| 17170 | 3.22433 | 0.05 |
| 17171 | 2.05993 | 1.83279 |
| 17172 | 2.01836 | 0.05 |
| 17173 | 1.66154 | 1.93296 |
| 17174 | 1.20871 | 1.97365 |
| 17175 | 1.10586 | 2.1 |
| 17176 | 1.10586 | 1.85089 |
| 17177 | 1.03853 | 2.1 |
| 17178 | 1.12076 | 2.1 |
| 17179 | 1.03899 | 2.1 |
| 17180 | 1.03899 | 2.1 |
| 17181 | 0.963973 | 2.1 |
| 17182 | 0.987132 | 2.1 |
| 17183 | 1.0894 | 2.1 |
| 17184 | 2.37823 | 2.1 |
| 17185 | 1.75647 | 1.6403 |
| 17186 | 1.64625 | 2.1 |
| 17187 | 1.56195 | 2.1 |
| 17188 | 1.41995 | 2.1 |
| 17189 | 1.15171 | 2.1 |
| 17190 | 1.36457 | 2.1 |
| 17191 | 1.33847 | 2.1 |
| 17192 | 1.33847 | 2.1 |
| 17193 | 0.869601 | 1.94113 |
| 17194 | 1.42255 | 2.1 |
| 17195 | 1.3868 | 2.1 |
| 17196 | 1.3868 | 2.1 |
| 17197 | 1.33962 | 2.1 |
| 17198 | 1.05481 | 2.1 |
| 17199 | 1.08074 | 2.1 |
| 17200 | 1.08074 | 2.1 |
| 17201 | 0.857557 | 2.1 |
| 17202 | 1.13537 | 2.07112 |
| 17203 | 1.09194 | 0.927066 |
| 17204 | 1.01179 | 2.1 |
| 17205 | 1.13537 | 0.756927 |
| 17206 | 1.15645 | 2.1 |
| 17207 | 1.56409 | 0.05 |
| 17208 | 1.56409 | 0.9228 |
| 17209 | 1.16239 | 1.15676 |
| 17210 | 1.10746 | 0.172893 |
| 17211 | 1.47952 | 0.571753 |
| 17212 | 1.14733 | 2.1 |
| 17213 | 3.77068 | 2.1 |
| 17214 | 1.38497 | 2.1 |
| 17215 | 1.54599 | 2.1 |
| 17216 | 1.54601 | 2.1 |
| 17217 | 1.27697 | 2.1 |
| 17218 | 1.01393 | 1.79309 |
| 17219 | 1.10558 | 1.80239 |
| 17220 | 0.995012 | 0.554663 |
| 17221 | 1.17624 | 1.42968 |
| 17222 | 1.60497 | 2.1 |
| 17223 | 1.61588 | 2.1 |

| | | |
|---|---|---|
| 17224 | 1.61587 | 2.1 |
| 17225 | 1.62182 | 2.1 |
| 17226 | 1.48866 | 0.05 |
| 17227 | 1.65977 | 0.05 |
| 17228 | 1.65977 | 0.05 |
| 17229 | 1.69044 | 0.05 |
| 17230 | 0.961892 | 2.1 |
| 17231 | 1.00659 | 2.1 |
| 17232 | 1.0066 | 2.1 |
| 17233 | 1.06226 | 2.1 |
| 17234 | 1.15171 | 2.1 |
| 17235 | 1.50853 | 1.96704 |
| 17236 | 1.50853 | 2.1 |
| 17237 | 1.2955 | 2.1 |
| 17238 | 1.3204 | 0.05 |
| 17239 | 2.13295 | 0.05 |
| 17240 | 2.75524 | 0.05 |
| 17241 | 1.18156 | 0.05 |
| 17242 | 1.73729 | 2.1 |
| 17243 | 1.20356 | 2.1 |
| 17244 | 1.62483 | 2.1 |
| 17245 | 1.52008 | 2.1 |
| 17246 | 1.19964 | 2.1 |
| 17247 | 1.0577 | 2.1 |
| 17248 | 1.19964 | 2.1 |
| 17249 | 1.07156 | 2.1 |
| 17250 | 1.98241 | 2.1 |
| 17251 | 1.43687 | 2.1 |
| 17252 | 1.27863 | 2.1 |
| 17253 | 1.19915 | 1.49843 |
| 17254 | 0.97269 | 1.66806 |
| 17255 | 1.0739 | 1.12092 |
| 17256 | 1.1427 | 2.1 |
| 17257 | 1.22476 | 1.02418 |
| 17258 | 1.06584 | 1.75964 |
| 17259 | 1.22626 | 1.20056 |
| 17260 | 1.31388 | 2.1 |
| 17261 | 1.16932 | 2.1 |
| 17262 | 1.28819 | 2.1 |
| 17263 | 1.0499 | 2.1 |
| 17264 | 1.55894 | 2.1 |
| 17265 | 1.78709 | 2.1 |
| 17266 | 1.75986 | 2.1 |
| 17267 | 1.764 | 2.1 |
| 17268 | 1.44801 | 2.1 |
| 17269 | 1.28186 | 2.1 |
| 17270 | 1.20797 | 2.0348 |
| 17271 | 1.06821 | 2.1 |
| 17272 | 1.87287 | 0.914089 |
| 17273 | 2.08014 | 1.07912 |
| 17274 | 1.63225 | 1.13364 |
| 17275 | 1.39501 | 1.38297 |
| 17276 | 1.20132 | 0.05 |
| 17277 | 1.16182 | 0.05 |
| 17278 | 1.08848 | 0.05 |
| 17279 | 1.20132 | 0.05 |
| 17280 | 1.23475 | 1.7746 |
| 17281 | 1.16661 | 2.1 |

| | | |
|---|---|---|
| 17282 | 2.31317 | 2.1 |
| 17283 | 2.50145 | 2.1 |
| 17284 | 1.04367 | 2.1 |
| 17285 | 2.23875 | 2.1 |
| 17286 | 0.953632 | 0.05 |
| 17287 | 1.38646 | 0.05 |
| 17288 | 1.38646 | 0.05 |
| 17289 | 1.61963 | 0.05 |
| 17290 | 1.62934 | 1.13672 |
| 17291 | 1.88626 | 0.723648 |
| 17292 | 1.0775 | 0.502448 |
| 17293 | 1.119 | 0.166254 |
| 17294 | 1.08133 | 0.05 |
| 17295 | 1.48312 | 0.05 |
| 17296 | 1.57125 | 0.05 |
| 17297 | 1.74098 | 0.05 |
| 17298 | 1.94372 | 1.16223 |
| 17299 | 1.22237 | 1.28843 |
| 17300 | 1.18549 | 0.301058 |
| 17301 | 1.01294 | 0.05 |
| 17302 | 1.01294 | 0.325159 |
| 17303 | 1.2064 | 0.503027 |
| 17304 | 1.14599 | 2.1 |
| 17305 | 1.00065 | 0.05 |
| 17306 | 1.76357 | 2.1 |
| 17307 | 2.23468 | 2.1 |
| 17308 | 2.21735 | 2.1 |
| 17309 | 3.32769 | 2.1 |
| 17310 | 1.19647 | 1.0352 |
| 17311 | 1.35307 | 0.639096 |
| 17312 | 1.19645 | 0.695986 |
| 17313 | 1.43778 | 0.587891 |
| 17314 | 1.34412 | 0.05 |
| 17315 | 1.1315 | 2.1 |
| 17316 | 1.1315 | 0.468132 |
| 17317 | 1.23943 | 0.0923839 |
| 17318 | 1.57829 | 2.1 |
| 17319 | 1.22537 | 2.1 |
| 17320 | 1.14357 | 2.1 |
| 17321 | 1.32772 | 2.1 |
| 17322 | 1.4259 | 2.1 |
| 17323 | 1.34245 | 2.1 |
| 17324 | 1.47953 | 2.1 |
| 17325 | 1.70303 | 2.1 |
| 17326 | 2.21127 | 0.05 |
| 17327 | 1.47904 | 0.05 |
| 17328 | 1.18793 | 0.05 |
| 17329 | 2.21126 | 0.05 |
| 17330 | 1.1032 | 2.03404 |
| 17331 | 1.14986 | 0.18095 |
| 17332 | 1.14986 | 2.1 |
| 17333 | 1.19611 | 0.579115 |
| 17334 | 1.91883 | 2.1 |
| 17335 | 1.83019 | 2.1 |
| 17336 | 1.83019 | 2.1 |
| 17337 | 1.82462 | 2.1 |
| 17338 | 1.27362 | 0.05 |
| 17339 | 1.27421 | 0.05 |

| | | |
|---|---|---|
| 17340 | 1.27421 | 0.05 |
| 17341 | 1.10736 | 0.05 |
| 17342 | 1.3003 | 0.149166 |
| 17343 | 1.31415 | 1.50309 |
| 17344 | 1.31415 | 0.241341 |
| 17345 | 1.32201 | 1.78272 |
| 17346 | 1.21124 | 2.1 |
| 17347 | 0.81568 | 2.1 |
| 17348 | 0.81568 | 2.1 |
| 17349 | 0.551816 | 2.1 |
| 17350 | 1.67119 | 2.1 |
| 17351 | 1.63384 | 2.1 |
| 17352 | 1.63384 | 2.1 |
| 17353 | 1.63661 | 2.1 |
| 17354 | 1.48734 | 2.1 |
| 17355 | 1.52245 | 2.1 |
| 17356 | 1.52245 | 2.1 |
| 17357 | 1.50714 | 2.1 |
| 17358 | 0.996778 | 0.05 |
| 17359 | 1.56091 | 0.05 |
| 17360 | 1.56091 | 0.05 |
| 17361 | 1.90457 | 0.05 |
| 17362 | 1.1181 | 2.1 |
| 17363 | 1.06543 | 2.1 |
| 17364 | 2.66828 | 2.1 |
| 17365 | 2.76932 | 2.1 |
| 17366 | 1.06543 | 2.1 |
| 17367 | 1.01474 | 2.1 |
| 17368 | 2.76932 | 2.1 |
| 17369 | 2.86704 | 2.1 |
| 17370 | 1.29724 | 0.05 |
| 17371 | 1.96938 | 0.05 |
| 17372 | 1.82462 | 2.1 |
| 17373 | 1.84553 | 2.1 |
| 17374 | 1.05556 | 2.1 |
| 17375 | 1.25363 | 2.1 |
| 17376 | 1.84552 | 2.1 |
| 17377 | 1.53208 | 2.1 |
| 17378 | 1.12826 | 2.1 |
| 17379 | 1.39075 | 2.1 |
| 17380 | 0.9702 | 1.13071 |
| 17381 | 1.16481 | 0.728922 |
| 17382 | 1.08894 | 0.968611 |
| 17383 | 1.91756 | 1.06781 |
| 17384 | 1.50865 | 0.05 |
| 17385 | 1.06012 | 0.05 |
| 17386 | 1.5456 | 0.05 |
| 17387 | 1.37427 | 0.05 |
| 17388 | 1.1211 | 0.05 |
| 17389 | 1.5456 | 0.05 |
| 17390 | 1.33135 | 0.05 |
| 17391 | 1.07956 | 0.05 |
| 17392 | 2.23255 | 0.05 |
| 17393 | 1.21031 | 0.05 |
| 17394 | 1.52903 | 2.1 |
| 17395 | 1.35977 | 2.1 |
| 17396 | 3.3837 | 2.1 |
| 17397 | 3.20581 | 2.1 |

| | | |
|---|---|---|
| 17398 | 1.35977 | 2.1 |
| 17399 | 1.15414 | 2.1 |
| 17400 | 3.20582 | 2.1 |
| 17401 | 2.07596 | 2.1 |
| 17402 | 1.6812 | 0.05 |
| 17403 | 1.03939 | 0.05 |
| 17404 | 1.30047 | 2.1 |
| 17405 | 1.5586 | 2.1 |
| 17406 | 1.5586 | 2.1 |
| 17407 | 1.84214 | 2.1 |
| 17408 | 1.31825 | 0.05 |
| 17409 | 1.31923 | 1.80136 |
| 17410 | 1.31924 | 0.05 |
| 17411 | 1.33026 | 0.953856 |
| 17412 | 1.15818 | 0.960477 |
| 17413 | 1.17822 | 1.25383 |
| 17414 | 1.15818 | 2.1 |
| 17415 | 1.28096 | 2.00436 |
| 17416 | 1.06848 | 0.204315 |
| 17417 | 1.89639 | 0.159874 |
| 17418 | 1.9186 | 1.0699 |
| 17419 | 1.77065 | 0.856657 |
| 17420 | 1.59103 | 0.88152 |
| 17421 | 1.56164 | 0.626448 |
| 17422 | 1.09697 | 0.05 |
| 17423 | 2.38719 | 0.05 |
| 17424 | 2.22123 | 0.05 |
| 17425 | 2.46059 | 0.05 |
| 17426 | 1.45506 | 0.05 |
| 17427 | 1.03645 | 0.0568696 |
| 17428 | 1.30347 | 2.1 |
| 17429 | 1.2627 | 2.1 |
| 17430 | 1.2627 | 2.1 |
| 17431 | 1.23036 | 2.1 |
| 17432 | 1.41469 | 2.1 |
| 17433 | 1.45257 | 2.1 |
| 17434 | 1.46907 | 0.05 |
| 17435 | 1.70302 | 0.05 |
| 17436 | 1.49196 | 0.05 |
| 17437 | 1.36307 | 0.05 |
| 17438 | 1.05677 | 2.1 |
| 17439 | 0.978698 | 2.1 |
| 17440 | 0.978699 | 2.1 |
| 17441 | 2.01822 | 2.1 |
| 17442 | 1.47446 | 2.1 |
| 17443 | 1.55432 | 2.1 |
| 17444 | 1.55432 | 2.1 |
| 17445 | 1.06053 | 2.1 |
| 17446 | 1.86813 | 1.37438 |
| 17447 | 1.21892 | 2.1 |
| 17448 | 1.53064 | 1.07514 |
| 17449 | 1.30664 | 1.1503 |
| 17450 | 1.09629 | 2.1 |
| 17451 | 1.19244 | 2.1 |
| 17452 | 1.19244 | 2.1 |
| 17453 | 1.37635 | 2.1 |
| 17454 | 1.49358 | 0.0595629 |
| 17455 | 1.57702 | 0.05 |

| | | |
|---|---|---|
| 17456 | 2.09374 | 2.1 |
| 17457 | 1.88175 | 2.1 |
| 17458 | 1.88175 | 2.1 |
| 17459 | 1.51085 | 2.1 |
| 17460 | 1.10938 | 0.343004 |
| 17461 | 1.02478 | 0.0828769 |
| 17462 | 1.33061 | 0.05 |
| 17463 | 1.13272 | 0.05 |
| 17464 | 1.75505 | 2.1 |
| 17465 | 1.78529 | 2.1 |
| 17466 | 1.6353 | 2.1 |
| 17467 | 1.59307 | 2.1 |
| 17468 | 1.60017 | 0.122387 |
| 17469 | 1.35608 | 0.734599 |
| 17470 | 1.35608 | 0.0929879 |
| 17471 | 1.04713 | 0.05 |
| 17472 | 1.01479 | 0.05 |
| 17473 | 1.3868 | 0.05 |
| 17474 | 1.3868 | 0.05 |
| 17475 | 1.28418 | 1.01064 |
| 17476 | 2.15949 | 2.1 |
| 17477 | 2.90429 | 2.1 |
| 17478 | 1.51902 | 2.1 |
| 17479 | 1.54755 | 2.1 |
| 17480 | 2.90429 | 2.1 |
| 17481 | 3.49966 | 2.1 |
| 17482 | 1.70842 | 2.1 |
| 17483 | 1.97526 | 2.1 |
| 17484 | 0.949712 | 0.05 |
| 17485 | 1.92784 | 0.05 |
| 17486 | 1.34093 | 0.05 |
| 17487 | 2.53426 | 0.05 |
| 17488 | 1.92783 | 0.05 |
| 17489 | 3.89373 | 0.05 |
| 17490 | 2.53426 | 0.05 |
| 17491 | 2.35961 | 0.05 |
| 17492 | 1.68108 | 0.18507 |
| 17493 | 1.15558 | 0.138467 |
| 17494 | 1.67265 | 0.139808 |
| 17495 | 1.27608 | 0.0824363 |
| 17496 | 1.42249 | 2.1 |
| 17497 | 1.39708 | 2.1 |
| 17498 | 1.39708 | 2.1 |
| 17499 | 1.39327 | 2.1 |
| 17500 | 1.97046 | 0.05 |
| 17501 | 1.11688 | 0.05 |
| 17502 | 1.28418 | 0.05 |
| 17503 | 1.17302 | 0.913828 |
| 17504 | 1.17302 | 0.05 |
| 17505 | 1.04349 | 0.831599 |
| 17506 | 0.979853 | 2.1 |
| 17507 | 1.26016 | 2.1 |
| 17508 | 1.18901 | 2.1 |
| 17509 | 1.26016 | 2.1 |
| 17510 | 1.22857 | 0.67332 |
| 17511 | 1.25848 | 0.839714 |
| 17512 | 1.25848 | 0.647832 |
| 17513 | 1.2858 | 0.777807 |

| | | |
|---|---|---|
| 17514 | 1.09154 | 2.1 |
| 17515 | 2.01915 | 2.1 |
| 17516 | 1.23013 | 2.1 |
| 17517 | 1.55698 | 2.1 |
| 17518 | 1.55698 | 2.1 |
| 17519 | 1.8575 | 2.1 |
| 17520 | 2.01854 | 2.1 |
| 17521 | 1.20686 | 2.1 |
| 17522 | 1.20684 | 2.1 |
| 17523 | 1.77796 | 2.1 |
| 17524 | 1.46129 | 2.1 |
| 17525 | 1.04193 | 2.1 |
| 17526 | 1.10051 | 2.1 |
| 17527 | 0.983723 | 2.1 |
| 17528 | 1.1729 | 2.1 |
| 17529 | 0.98505 | 2.1 |
| 17530 | 0.985052 | 2.1 |
| 17531 | 1.03951 | 2.1 |
| 17532 | 1.03949 | 2.1 |
| 17533 | 1.17583 | 2.1 |
| 17534 | 1.42866 | 2.1 |
| 17535 | 1.97261 | 2.1 |
| 17536 | 1.73274 | 2.1 |
| 17537 | 1.53629 | 2.1 |
| 17538 | 1.98182 | 2.1 |
| 17539 | 1.72511 | 2.1 |
| 17540 | 1.42174 | 2.1 |
| 17541 | 1.44472 | 2.1 |
| 17542 | 1.72511 | 2.1 |
| 17543 | 1.45411 | 2.1 |
| 17544 | 1.44472 | 2.1 |
| 17545 | 1.43836 | 2.1 |
| 17546 | 0.962703 | 0.50657 |
| 17547 | 1.0171 | 0.648446 |
| 17548 | 1.53336 | 0.05 |
| 17549 | 1.40274 | 0.05 |
| 17550 | 1.40272 | 0.05 |
| 17551 | 1.00213 | 0.05 |
| 17552 | 1.50892 | 0.05 |
| 17553 | 1.64984 | 0.05 |
| 17554 | 1.67464 | 0.05 |
| 17555 | 1.67481 | 0.05 |
| 17556 | 1.08212 | 2.1 |
| 17557 | 1.28095 | 2.1 |
| 17558 | 1.46956 | 2.1 |
| 17559 | 1.70303 | 2.1 |
| 17560 | 1.2925 | 0.05 |
| 17561 | 1.84716 | 0.05 |
| 17562 | 1.48676 | 0.05 |
| 17563 | 1.48797 | 0.05 |
| 17564 | 1.33917 | 0.05 |
| 17565 | 1.22691 | 0.05 |
| 17566 | 0.528744 | 2.1 |
| 17567 | 1.13728 | 2.1 |
| 17568 | 1.13728 | 2.1 |
| 17569 | 1.99843 | 2.1 |
| 17570 | 1.17464 | 0.05 |
| 17571 | 1.0372 | 0.05 |

| | | |
|---|---|---|
| 17572 | 2.0815 | 0.05 |
| 17573 | 0.933462 | 0.05 |
| 17574 | 1.54931 | 2.1 |
| 17575 | 1.5485 | 2.1 |
| 17576 | 1.4077 | 2.1 |
| 17577 | 1.29931 | 2.1 |
| 17578 | 1.14931 | 0.05 |
| 17579 | 2.02223 | 0.05 |
| 17580 | 1.04482 | 0.05 |
| 17581 | 0.94971 | 0.05 |
| 17582 | 1.54632 | 1.43429 |
| 17583 | 1.15693 | 1.59588 |
| 17584 | 1.66763 | 2.1 |
| 17585 | 1.41238 | 2.1 |
| 17586 | 1.41238 | 2.1 |
| 17587 | 1.48068 | 2.1 |
| 17588 | 1.0171 | 0.05 |
| 17589 | 1.1211 | 0.300437 |
| 17590 | 1.1211 | 0.05 |
| 17591 | 1.28418 | 0.0907383 |
| 17592 | 0.993021 | 2.1 |
| 17593 | 1.764 | 2.1 |
| 17594 | 0.993023 | 2.1 |
| 17595 | 1.05787 | 2.1 |
| 17596 | 1.90914 | 0.05 |
| 17597 | 1.12229 | 1.84583 |
| 17598 | 1.02025 | 0.05 |
| 17599 | 0.585368 | 2.1 |
| 17600 | 1.99323 | 2.1 |
| 17601 | 2.34988 | 2.1 |
| 17602 | 2.34988 | 2.1 |
| 17603 | 2.36987 | 2.1 |
| 17604 | 1.23107 | 0.05 |
| 17605 | 1.90664 | 0.05 |
| 17606 | 1.53496 | 0.05 |
| 17607 | 1.70302 | 0.05 |
| 17608 | 1.98859 | 2.1 |
| 17609 | 1.61057 | 2.1 |
| 17610 | 1.79844 | 2.1 |
| 17611 | 2.10811 | 2.1 |
| 17612 | 1.4618 | 2.1 |
| 17613 | 1.96722 | 2.1 |
| 17614 | 1.95191 | 2.1 |
| 17615 | 2.13418 | 2.1 |
| 17616 | 1.50368 | 0.05 |
| 17617 | 1.61417 | 0.05 |
| 17618 | 1.15008 | 0.05 |
| 17619 | 1.19685 | 0.05 |
| 17620 | 1.90456 | 0.05 |
| 17621 | 1.90899 | 0.05 |
| 17622 | 2.08864 | 0.05 |
| 17623 | 2.10447 | 0.05 |
| 17624 | 1.16653 | 2.1 |
| 17625 | 1.23017 | 2.1 |
| 17626 | 1.0752 | 0.05 |
| 17627 | 1.34412 | 0.05 |
| 17628 | 1.06226 | 2.1 |
| 17629 | 1.09252 | 2.1 |

| | | |
|---|---|---|
| 17630 | 1.09252 | 2.1 |
| 17631 | 0.88579 | 2.1 |
| 17632 | 1.56178 | 0.05 |
| 17633 | 1.14305 | 0.05 |
| 17634 | 1.56175 | 0.05 |
| 17635 | 1.08211 | 0.05 |
| 17636 | 1.32813 | 2.1 |
| 17637 | 1.32461 | 2.1 |
| 17638 | 1.32461 | 2.1 |
| 17639 | 1.32455 | 2.1 |
| 17640 | 1.42729 | 0.05 |
| 17641 | 1.18716 | 0.05 |
| 17642 | 1.47169 | 0.05 |
| 17643 | 1.24687 | 0.05 |
| 17644 | 1.09627 | 2.1 |
| 17645 | 1.04759 | 2.1 |
| 17646 | 1.04759 | 2.1 |
| 17647 | 0.987132 | 2.1 |
| 17648 | 1.11181 | 1.84798 |
| 17649 | 1.80662 | 2.1 |
| 17650 | 1.8066 | 2.1 |
| 17651 | 1.06461 | 1.99863 |
| 17652 | 1.56091 | 2.1 |
| 17653 | 1.9842 | 2.1 |
| 17654 | 1.73525 | 0.05 |
| 17655 | 1.40557 | 0.05 |
| 17656 | 2.26906 | 0.05 |
| 17657 | 1.31248 | 0.05 |
| 17658 | 1.66832 | 0.05 |
| 17659 | 1.32634 | 0.05 |
| 17660 | 1.03314 | 0.05 |
| 17661 | 1.24682 | 0.05 |
| 17662 | 1.38536 | 0.05 |
| 17663 | 1.52661 | 0.05 |
| 17664 | 1.177 | 0.563127 |
| 17665 | 1.36405 | 0.140941 |
| 17666 | 1.19837 | 0.134878 |
| 17667 | 1.17701 | 0.05 |
| 17668 | 0.993831 | 0.05 |
| 17669 | 1.0823 | 0.05 |
| 17670 | 1.0823 | 0.05 |
| 17671 | 1.01121 | 0.05 |
| 17672 | 1.49653 | 0.05 |
| 17673 | 1.89583 | 0.05 |
| 17674 | 0.67242 | 0.05 |
| 17675 | 1.36047 | 0.05 |
| 17676 | 1.00366 | 0.05 |
| 17677 | 1.24728 | 0.05 |
| 17678 | 1.13387 | 0.05 |
| 17679 | 1.60867 | 0.05 |
| 17680 | 1.30572 | 0.05 |
| 17681 | 1.54982 | 0.05 |
| 17682 | 0.788592 | 0.05 |
| 17683 | 1.82961 | 0.05 |
| 17684 | 1.54982 | 0.05 |
| 17685 | 1.82961 | 0.05 |
| 17686 | 1.26062 | 0.05 |
| 17687 | 1.64002 | 0.05 |

| | | |
|---|---|---|
| 17688 | 1.12035 | 0.05 |
| 17689 | 1.64002 | 0.05 |
| 17690 | 1.1673 | 2.1 |
| 17691 | 1.75367 | 2.1 |
| 17692 | 0.964034 | 2.1 |
| 17693 | 1.0305 | 2.1 |
| 17694 | 1.30896 | 2.1 |
| 17695 | 1.52776 | 2.1 |
| 17696 | 1.52776 | 2.1 |
| 17697 | 1.17989 | 2.1 |
| 17698 | 1.16759 | 2.1 |
| 17699 | 1.8575 | 2.1 |
| 17700 | 1.16759 | 2.1 |
| 17701 | 1.60936 | 2.1 |
| 17702 | 1.10747 | 0.05 |
| 17703 | 1.47688 | 0.05 |
| 17704 | 1.40794 | 2.1 |
| 17705 | 1.95729 | 1.62868 |
| 17706 | 1.95729 | 2.1 |
| 17707 | 3.18156 | 2.1 |
| 17708 | 1.87598 | 1.89778 |
| 17709 | 1.94343 | 2.1 |
| 17710 | 1.94343 | 1.87159 |
| 17711 | 2.00551 | 2.1 |
| 17712 | 1.20067 | 1.06246 |
| 17713 | 1.70744 | 1.98428 |
| 17714 | 1.87244 | 1.78749 |
| 17715 | 1.32075 | 1.30609 |
| 17716 | 1.64955 | 2.1 |
| 17717 | 1.44221 | 2.1 |
| 17718 | 1.31109 | 2.1 |
| 17719 | 1.14206 | 2.1 |
| 17720 | 1.60452 | 2.07406 |
| 17721 | 1.12111 | 0.191391 |
| 17722 | 1.00832 | 2.1 |
| 17723 | 0.974137 | 2.1 |
| 17724 | 0.974137 | 2.1 |
| 17725 | 0.98505 | 2.1 |
| 17726 | 1.02368 | 2.1 |
| 17727 | 0.985051 | 2.1 |
| 17728 | 0.995734 | 0.05 |
| 17729 | 1.31993 | 0.053789 |
| 17730 | 1.31993 | 0.0654964 |
| 17731 | 1.18549 | 0.05 |
| 17732 | 1.5176 | 0.483519 |
| 17733 | 1.33622 | 0.326696 |
| 17734 | 1.33621 | 0.294897 |
| 17735 | 1.02782 | 0.232129 |
| 17736 | 1.66774 | 2.1 |
| 17737 | 1.85305 | 2.1 |
| 17738 | 1.85305 | 2.1 |
| 17739 | 2.07088 | 2.1 |
| 17740 | 1.78873 | 2.1 |
| 17741 | 1.47533 | 2.1 |
| 17742 | 1.23152 | 2.1 |
| 17743 | 0.99922 | 2.1 |
| 17744 | 1.48493 | 2.1 |
| 17745 | 1.1394 | 2.1 |

| | | |
|---|---|---|
| 17746 | 1.19179 | 2.1 |
| 17747 | 1.64289 | 2.1 |
| 17748 | 1.47858 | 2.1 |
| 17749 | 1.89687 | 2.1 |
| 17750 | 0.972464 | 2.1 |
| 17751 | 1.79473 | 2.1 |
| 17752 | 1.06971 | 2.1 |
| 17753 | 1.21089 | 2.1 |
| 17754 | 1.12572 | 2.1 |
| 17755 | 1.30566 | 2.1 |
| 17756 | 1.30566 | 2.1 |
| 17757 | 1.52724 | 2.1 |
| 17758 | 2.32237 | 0.05 |
| 17759 | 2.20248 | 0.05 |
| 17760 | 2.79564 | 0.05 |
| 17761 | 2.32237 | 0.05 |
| 17762 | 1.59354 | 0.05 |
| 17763 | 3.098 | 0.05 |
| 17764 | 3.09801 | 0.05 |
| 17765 | 3.45132 | 0.05 |
| 17766 | 1.24001 | 2.1 |
| 17767 | 1.41822 | 0.79478 |
| 17768 | 1.41821 | 2.1 |
| 17769 | 1.44859 | 0.966394 |
| 17770 | 1.34539 | 2.1 |
| 17771 | 1.06991 | 2.1 |
| 17772 | 1.18076 | 2.1 |
| 17773 | 0.978586 | 2.1 |
| 17774 | 0.999189 | 0.05 |
| 17775 | 2.41076 | 0.05 |
| 17776 | 0.968475 | 2.1 |
| 17777 | 1.02074 | 2.1 |
| 17778 | 1.02074 | 2.1 |
| 17779 | 1.07866 | 2.1 |
| 17780 | 0.817702 | 0.05 |
| 17781 | 1.5082 | 0.05 |
| 17782 | 1.48376 | 0.05 |
| 17783 | 1.18947 | 0.05 |
| 17784 | 1.10638 | 2.1 |
| 17785 | 1.86766 | 2.1 |
| 17786 | 1.19785 | 2.1 |
| 17787 | 1.34141 | 2.1 |
| 17788 | 1.34141 | 2.1 |
| 17789 | 0.991985 | 2.1 |
| 17790 | 2.35516 | 0.216349 |
| 17791 | 1.24815 | 0.101653 |
| 17792 | 1.80454 | 2.1 |
| 17793 | 1.22633 | 2.1 |
| 17794 | 0.696255 | 2.1 |
| 17795 | 1.19379 | 0.233514 |
| 17796 | 1.14721 | 2.1 |
| 17797 | 1.16153 | 2.1 |
| 17798 | 1.16153 | 2.1 |
| 17799 | 1.17261 | 2.1 |
| 17800 | 1.20814 | 2.1 |
| 17801 | 1.54791 | 2.1 |
| 17802 | 1.54791 | 2.1 |
| 17803 | 1.60477 | 2.1 |

| | | |
|---|---|---|
| 17804 | 1.29931 | 2.1 |
| 17805 | 1.19098 | 2.1 |
| 17806 | 1.19098 | 2.1 |
| 17807 | 1.13341 | 2.1 |
| 17808 | 1.00503 | 2.1 |
| 17809 | 1.25334 | 0.284852 |
| 17810 | 1.22655 | 2.1 |
| 17811 | 1.26282 | 2.1 |
| 17812 | 1.26282 | 2.1 |
| 17813 | 0.999489 | 2.1 |
| 17814 | 1.54197 | 0.05 |
| 17815 | 1.17152 | 0.05 |
| 17816 | 1.17152 | 0.05 |
| 17817 | 1.49627 | 0.05 |
| 17818 | 1.36405 | 0.388095 |
| 17819 | 0.976334 | 0.0682369 |
| 17820 | 1.12495 | 2.1 |
| 17821 | 1.54096 | 2.1 |
| 17822 | 1.75263 | 2.1 |
| 17823 | 1.49114 | 2.1 |
| 17824 | 0.842998 | 2.1 |
| 17825 | 1.60244 | 0.990586 |
| 17826 | 1.48911 | 1.86136 |
| 17827 | 2.18685 | 1.06253 |
| 17828 | 1.75236 | 0.40983 |
| 17829 | 1.54143 | 1.2725 |
| 17830 | 1.40013 | 0.05 |
| 17831 | 0.946646 | 0.05 |
| 17832 | 1.50714 | 2.1 |
| 17833 | 1.46055 | 2.1 |
| 17834 | 1.46055 | 2.1 |
| 17835 | 1.42249 | 2.1 |
| 17836 | 2.12839 | 0.05 |
| 17837 | 1.75211 | 0.05 |
| 17838 | 1.76297 | 0.05 |
| 17839 | 1.70736 | 0.05 |
| 17840 | 1.19952 | 0.05 |
| 17841 | 1.56475 | 0.05 |
| 17842 | 1.74312 | 0.05 |
| 17843 | 1.48973 | 0.05 |
| 17844 | 1.91518 | 0.05 |
| 17845 | 1.16534 | 0.05 |
| 17846 | 1.53556 | 2.1 |
| 17847 | 1.56253 | 2.1 |
| 17848 | 1.56253 | 2.1 |
| 17849 | 1.58054 | 2.1 |
| 17850 | 1.27806 | 0.05 |
| 17851 | 1.44593 | 0.05 |
| 17852 | 1.44593 | 0.05 |
| 17853 | 1.3946 | 0.05 |
| 17854 | 1.34857 | 0.05 |
| 17855 | 1.03818 | 2.07586 |
| 17856 | 1.38969 | 0.05 |
| 17857 | 1.85548 | 0.0801466 |
| 17858 | 1.03108 | 0.05 |
| 17859 | 1.85548 | 0.05 |
| 17860 | 1.49346 | 1.68764 |
| 17861 | 1.83192 | 2.1 |

| | | |
|---|---|---|
| 17862 | 1.75291 | 0.749852 |
| 17863 | 1.35435 | 1.78285 |
| 17864 | 0.921851 | 0.0968676 |
| 17865 | 1.09646 | 0.0507846 |
| 17866 | 1.2183 | 0.05 |
| 17867 | 1.36717 | 0.05 |
| 17868 | 2.14179 | 0.05 |
| 17869 | 1.79805 | 0.0754445 |
| 17870 | 1.3428 | 0.132036 |
| 17871 | 2.30493 | 0.133202 |
| 17872 | 1.81545 | 0.0521377 |
| 17873 | 1.92269 | 0.0847777 |
| 17874 | 2.30492 | 0.183173 |
| 17875 | 1.3663 | 0.212457 |
| 17876 | 1.22418 | 2.1 |
| 17877 | 0.956698 | 0.338246 |
| 17878 | 1.4706 | 2.1 |
| 17879 | 0.953295 | 2.1 |
| 17880 | 1.47059 | 1.8281 |
| 17881 | 1.26929 | 2.1 |
| 17882 | 1.24538 | 0.05 |
| 17883 | 1.4468 | 0.05 |
| 17884 | 2.86762 | 2.1 |
| 17885 | 2.32911 | 2.1 |
| 17886 | 2.10864 | 2.1 |
| 17887 | 1.94548 | 2.1 |
| 17888 | 1.83134 | 2.1 |
| 17889 | 2.19519 | 2.1 |
| 17890 | 2.19853 | 2.1 |
| 17891 | 2.63855 | 2.1 |
| 17892 | 1.08213 | 0.05 |
| 17893 | 1.46389 | 0.05 |
| 17894 | 1.62235 | 0.05 |
| 17895 | 0.980549 | 0.05 |
| 17896 | 1.08195 | 2.08955 |
| 17897 | 0.99002 | 2.1 |
| 17898 | 1.2269 | 2.1 |
| 17899 | 1.0838 | 2.1 |
| 17900 | 0.889491 | 2.1 |
| 17901 | 1.19587 | 2.1 |
| 17902 | 1.18801 | 0.05 |
| 17903 | 1.57125 | 0.05 |
| 17904 | 1.47712 | 2.1 |
| 17905 | 1.45182 | 2.1 |
| 17906 | 1.45182 | 2.1 |
| 17907 | 1.42255 | 2.1 |
| 17908 | 1.17816 | 0.05 |
| 17909 | 1.00982 | 0.05 |
| 17910 | 1.21056 | 0.05 |
| 17911 | 1.17816 | 0.05 |
| 17912 | 3.80902 | 0.48065 |
| 17913 | 0.976991 | 0.424011 |
| 17914 | 1.09962 | 2.1 |
| 17915 | 1.60976 | 2.1 |
| 17916 | 1.87029 | 2.1 |
| 17917 | 2.33806 | 2.1 |
| 17918 | 2.12549 | 2.1 |
| 17919 | 2.21472 | 2.1 |

| | | |
|---|---|---|
| 17920 | 2.16221 | 0.05 |
| 17921 | 1.99528 | 0.05 |
| 17922 | 1.99528 | 0.05 |
| 17923 | 1.97969 | 0.05 |
| 17924 | 3.60854 | 2.1 |
| 17925 | 4.00847 | 2.1 |
| 17926 | 2.70372 | 2.1 |
| 17927 | 1.253 | 2.1 |
| 17928 | 1.65739 | 2.1 |
| 17929 | 0.994294 | 2.1 |
| 17930 | 1.36203 | 2.1 |
| 17931 | 1.00965 | 2.1 |
| 17932 | 2.33374 | 2.1 |
| 17933 | 1.36209 | 2.1 |
| 17934 | 1.64983 | 0.05 |
| 17935 | 1.36416 | 0.05 |
| 17936 | 2.19766 | 0.05 |
| 17937 | 1.36416 | 0.05 |
| 17938 | 2.15871 | 0.272408 |
| 17939 | 1.81454 | 0.05 |
| 17940 | 1.54607 | 0.05 |
| 17941 | 1.57702 | 0.119379 |
| 17942 | 0.948748 | 0.05 |
| 17943 | 1.04522 | 0.05 |
| 17944 | 1.60994 | 0.05 |
| 17945 | 1.90382 | 0.05 |
| 17946 | 1.90382 | 0.05 |
| 17947 | 1.57999 | 0.05 |
| 17948 | 1.73905 | 0.65687 |
| 17949 | 1.26535 | 1.1349 |
| 17950 | 2.12552 | 1.02077 |
| 17951 | 1.06891 | 0.879764 |
| 17952 | 1.13999 | 0.983919 |
| 17953 | 2.29812 | 1.01601 |
| 17954 | 2.83329 | 0.881096 |
| 17955 | 2.16272 | 0.604128 |
| 17956 | 1.71277 | 2.1 |
| 17957 | 1.68401 | 2.1 |
| 17958 | 1.68401 | 2.1 |
| 17959 | 1.69368 | 2.1 |
| 17960 | 1.06472 | 2.1 |
| 17961 | 1.56017 | 2.1 |
| 17962 | 1.2765 | 2.1 |
| 17963 | 1.99541 | 2.1 |
| 17964 | 1.46041 | 0.05 |
| 17965 | 2.04817 | 0.05 |
| 17966 | 2.04817 | 0.05 |
| 17967 | 2.33181 | 0.05 |
| 17968 | 2.06353 | 2.1 |
| 17969 | 1.35449 | 2.1 |
| 17970 | 1.52045 | 2.1 |
| 17971 | 1.45437 | 2.1 |
| 17972 | 1.04405 | 0.05 |
| 17973 | 1.85945 | 0.05 |
| 17974 | 1.85945 | 0.05 |
| 17975 | 2.22679 | 0.05 |
| 17976 | 1.33339 | 2.1 |
| 17977 | 1.19376 | 2.1 |

| | | |
|---|---|---|
| 17978 | 1.33266 | 2.1 |
| 17979 | 1.44244 | 2.1 |
| 17980 | 1.20455 | 2.1 |
| 17981 | 1.52852 | 2.1 |
| 17982 | 1.03882 | 2.1 |
| 17983 | 1.52852 | 2.1 |
| 17984 | 1.44801 | 2.1 |
| 17985 | 1.00775 | 2.1 |
| 17986 | 0.53907 | 2.1 |
| 17987 | 0.506124 | 2.1 |
| 17988 | 2.39045 | 2.1 |
| 17989 | 0.973794 | 2.1 |
| 17990 | 1.11193 | 2.1 |
| 17991 | 1.32784 | 2.1 |
| 17992 | 1.31595 | 2.1 |
| 17993 | 0.966115 | 2.1 |
| 17994 | 1.25953 | 2.1 |
| 17995 | 1.38086 | 2.1 |
| 17996 | 1.38086 | 2.1 |
| 17997 | 1.04101 | 2.1 |
| 17998 | 1.99319 | 0.05 |
| 17999 | 1.23107 | 0.05 |
| 18000 | 3.3606 | 0.05 |
| 18001 | 1.23112 | 0.05 |
| 18002 | 1.40239 | 2.1 |
| 18003 | 1.26091 | 2.1 |
| 18004 | 1.26091 | 2.1 |
| 18005 | 1.05776 | 2.1 |
| 18006 | 1.23768 | 0.225275 |
| 18007 | 1.0142 | 1.51922 |
| 18008 | 1.11435 | 1.06078 |
| 18009 | 1.01422 | 0.854685 |
| 18010 | 1.24001 | 0.749261 |
| 18011 | 1.42186 | 1.10902 |
| 18012 | 1.42186 | 0.818907 |
| 18013 | 1.47204 | 0.926759 |
| 18014 | 1.27361 | 2.1 |
| 18015 | 1.56642 | 2.1 |
| 18016 | 1.71676 | 2.1 |
| 18017 | 2.16815 | 2.1 |
| 18018 | 1.07855 | 1.28354 |
| 18019 | 1.07612 | 1.2903 |
| 18020 | 1.703 0.05 | |
| 18021 | 1.46853 | 0.05 |
| 18022 | 1.45448 | 0.05 |
| 18023 | 1.27778 | 0.05 |
| 18024 | 0.991986 | 2.1 |
| 18025 | 1.1192 | 2.1 |
| 18026 | 1.1192 | 2.1 |
| 18027 | 1.25953 | 2.1 |
| 18028 | 2.08506 | 2.1 |
| 18029 | 1.75162 | 2.1 |
| 18030 | 1.94523 | 2.1 |
| 18031 | 1.88405 | 2.09158 |
| 18032 | 1.15506 | 0.05 |
| 18033 | 1.1233 | 0.05 |
| 18034 | 2.09736 | 2.1 |
| 18035 | 2.98303 | 2.1 |

| | | |
|---|---|---|
| 18036 | 2.71184 | 2.1 |
| 18037 | 3.51614 | 2.1 |
| 18038 | 1.18769 | 2.1 |
| 18039 | 1.24053 | 2.1 |
| 18040 | 1.24053 | 2.1 |
| 18041 | 1.45327 | 2.1 |
| 18042 | 1.26865 | 1.8857 |
| 18043 | 1.1744 | 1.36921 |
| 18044 | 1.1744 | 0.990406 |
| 18045 | 1.11712 | 1.1493 |
| 18046 | 1.15841 | 0.05 |
| 18047 | 1.02247 | 0.05 |
| 18048 | 1.93649 | 2.1 |
| 18049 | 1.81191 | 2.1 |
| 18050 | 1.64718 | 2.1 |
| 18051 | 1.47521 | 2.1 |
| 18052 | 1.59654 | 2.1 |
| 18053 | 1.57823 | 2.1 |
| 18054 | 1.57823 | 2.1 |
| 18055 | 1.55768 | 2.1 |
| 18056 | 1.00665 | 0.05 |
| 18057 | 1.28239 | 0.05 |
| 18058 | 1.28239 | 0.05 |
| 18059 | 1.44951 | 0.05 |
| 18060 | 1.22083 | 2.1 |
| 18061 | 0.957622 | 2.1 |
| 18062 | 0.887896 | 2.1 |
| 18063 | 1.27387 | 2.1 |
| 18064 | 1.00521 | 1.051 |
| 18065 | 1.00503 | 1.10969 |
| 18066 | 1.95844 | 2.1 |
| 18067 | 1.05042 | 2.1 |
| 18068 | 2.78662 | 2.1 |
| 18069 | 2.22399 | 2.1 |
| 18070 | 2.3356 | 0.05 |
| 18071 | 1.49543 | 0.05 |
| 18072 | 1.96854 | 0.05 |
| 18073 | 1.21681 | 0.05 |
| 18074 | 2.17803 | 1.71846 |
| 18075 | 1.50998 | 2.1 |
| 18076 | 2.35239 | 1.32186 |
| 18077 | 2.26987 | 0.555336 |
| 18078 | 1.03824 | 0.05 |
| 18079 | 1.51396 | 0.082264 |
| 18080 | 1.51396 | 0.05 |
| 18081 | 1.48693 | 0.05 |
| 18082 | 1.08784 | 2.1 |
| 18083 | 1.36865 | 2.1 |
| 18084 | 0.712405 | 2.1 |
| 18085 | 1.08784 | 2.1 |
| 18086 | 2.02522 | 2.1 |
| 18087 | 1.6738 | 2.1 |
| 18088 | 1.35175 | 2.1 |
| 18089 | 1.50094 | 2.1 |
| 18090 | 1.6738 | 2.1 |
| 18091 | 1.62871 | 2.1 |
| 18092 | 1.35084 | 2.1 |
| 18093 | 1.34276 | 2.1 |

| | | |
|---|---|---|
| 18094 | 1.18041 | 2.1 |
| 18095 | 1.43652 | 2.1 |
| 18096 | 0.974196 | 2.1 |
| 18097 | 1.28037 | 2.1 |
| 18098 | 1.28037 | 2.1 |
| 18099 | 1.05071 | 2.1 |
| 18100 | 2.44737 | 0.05 |
| 18101 | 1.4672 | 0.05 |
| 18102 | 1.22883 | 0.05 |
| 18103 | 2.08149 | 0.05 |
| 18104 | 1.82208 | 2.00179 |
| 18105 | 1.59826 | 1.8583 |
| 18106 | 1.59826 | 0.943653 |
| 18107 | 1.01393 | 2.1 |
| 18108 | 1.42757 | 2.1 |
| 18109 | 0.996775 | 2.1 |
| 18110 | 1.42757 | 2.1 |
| 18111 | 1.26397 | 2.1 |
| 18112 | 1.02461 | 2.1 |
| 18113 | 1.46129 | 2.1 |
| 18114 | 1.11833 | 2.1 |
| 18115 | 1.335 | 2.1 |
| 18116 | 2.07087 | 2.1 |
| 18117 | 1.24041 | 2.1 |
| 18118 | 1.335 | 2.1 |
| 18119 | 1.55398 | 2.1 |
| 18120 | 1.21171 | 0.05 |
| 18121 | 1.48589 | 0.05 |
| 18122 | 1.32886 | 0.05 |
| 18123 | 1.8027 | 0.05 |
| 18124 | 1.53515 | 1.64327 |
| 18125 | 1.60428 | 1.45399 |
| 18126 | 1.00035 | 0.05 |
| 18127 | 1.03534 | 0.05 |
| 18128 | 1.03535 | 0.799696 |
| 18129 | 1.09962 | 2.1 |
| 18130 | 0.62132 | 1.26072 |
| 18131 | 1.21033 | 2.1 |
| 18132 | 1.30326 | 2.1 |
| 18133 | 2.6823 | 2.1 |
| 18134 | 0.991407 | 2.1 |
| 18135 | 1.29729 | 2.1 |
| 18136 | 1.29729 | 2.1 |
| 18137 | 1.16858 | 2.1 |
| 18138 | 1.66137 | 0.05 |
| 18139 | 1.23012 | 0.05 |
| 18140 | 1.66139 | 0.05 |
| 18141 | 1.41821 | 0.133512 |
| 18142 | 1.56043 | 0.638856 |
| 18143 | 1.06848 | 1.06447 |
| 18144 | 1.56043 | 1.98338 |
| 18145 | 1.90911 | 1.452 |
| 18146 | 0.982847 | 2.1 |
| 18147 | 1.05863 | 2.1 |
| 18148 | 1.05863 | 2.1 |
| 18149 | 1.22352 | 2.1 |
| 18150 | 1.23019 | 1.55892 |
| 18151 | 1.60509 | 2.1 |

| | | |
|---|---|---|
| 18152 | 1.19122 | 2.1 |
| 18153 | 1.47769 | 2.1 |
| 18154 | 2.18868 | 2.1 |
| 18155 | 1.40984 | 2.1 |
| 18156 | 3.55197 | 2.1 |
| 18157 | 3.57227 | 2.1 |
| 18158 | 3.57227 | 2.1 |
| 18159 | 3.48926 | 2.1 |
| 18160 | 1.4375 | 0.05 |
| 18161 | 1.11314 | 0.05 |
| 18162 | 1.62408 | 2.1 |
| 18163 | 1.74438 | 2.1 |
| 18164 | 1.72387 | 2.1 |
| 18165 | 1.50018 | 2.1 |
| 18166 | 1.1486 | 2.1 |
| 18167 | 1.18474 | 2.1 |
| 18168 | 1.18474 | 2.1 |
| 18169 | 1.2295 | 2.1 |
| 18170 | 1.28754 | 2.1 |
| 18171 | 1.36838 | 2.1 |
| 18172 | 1.36838 | 2.1 |
| 18173 | 1.21703 | 2.1 |
| 18174 | 2.74206 | 2.1 |
| 18175 | 2.70795 | 2.1 |
| 18176 | 2.70795 | 2.1 |
| 18177 | 2.55918 | 2.1 |
| 18178 | 1.35065 | 0.237994 |
| 18179 | 1.65411 | 0.05 |
| 18180 | 1.48572 | 0.281819 |
| 18181 | 1.28393 | 0.429712 |
| 18182 | 1.25265 | 0.05 |
| 18183 | 1.70944 | 0.05 |
| 18184 | 1.31259 | 0.0501135 |
| 18185 | 1.14945 | 2.1 |
| 18186 | 1.14945 | 0.05 |
| 18187 | 0.995725 | 2.1 |
| 18188 | 1.00307 | 0.05 |
| 18189 | 2.05786 | 0.0960208 |
| 18190 | 1.56357 | 0.0705321 |
| 18191 | 1.10907 | 0.089385 |
| 18192 | 1.27558 | 1.59122 |
| 18193 | 1.05175 | 0.998137 |
| 18194 | 1.2685 | 2.1 |
| 18195 | 1.41369 | 2.1 |
| 18196 | 1.4137 | 2.1 |
| 18197 | 1.49654 | 2.1 |
| 18198 | 1.21489 | 2.1 |
| 18199 | 1.14605 | 1.76894 |
| 18200 | 1.22476 | 2.1 |
| 18201 | 1.21489 | 1.22926 |
| 18202 | 2.51472 | 1.15835 |
| 18203 | 2.52091 | 1.13947 |
| 18204 | 2.52091 | 0.996531 |
| 18205 | 1.79171 | 0.960406 |
| 18206 | 1.03373 | 2.1 |
| 18207 | 1.04037 | 2.1 |
| 18208 | 1.04038 | 2.1 |
| 18209 | 1.03933 | 2.1 |

| | | |
|---|---|---|
| 18210 | 1.74123 | 0.05 |
| 18211 | 1.41902 | 0.05 |
| 18212 | 1.89777 | 0.05 |
| 18213 | 1.89899 | 0.05 |
| 18214 | 1.24728 | 0.05 |
| 18215 | 1.30394 | 0.05 |
| 18216 | 1.30394 | 0.05 |
| 18217 | 1.16535 | 0.05 |
| 18218 | 1.09914 | 2.1 |
| 18219 | 1.54259 | 2.1 |
| 18220 | 1.40233 | 2.1 |
| 18221 | 1.69858 | 2.1 |
| 18222 | 1.53555 | 2.1 |
| 18223 | 1.09859 | 0.613481 |
| 18224 | 1.09859 | 2.1 |
| 18225 | 0.725879 | 0.0568847 |
| 18226 | 1.07213 | 2.1 |
| 18227 | 1.31589 | 2.1 |
| 18228 | 1.36904 | 2.1 |
| 18229 | 1.69711 | 2.1 |
| 18230 | 1.51028 | 0.05 |
| 18231 | 0.993598 | 0.05 |
| 18232 | 0.61563 | 0.05 |
| 18233 | 1.26311 | 0.05 |
| 18234 | 1.19496 | 2.1 |
| 18235 | 1.65925 | 2.1 |
| 18236 | 1.65925 | 2.1 |
| 18237 | 1.48162 | 2.1 |
| 18238 | 2.75738 | 1.68002 |
| 18239 | 1.24699 | 1.41576 |
| 18240 | 4.08729 | 1.99331 |
| 18241 | 2.35271 | 1.68753 |
| 18242 | 1.55063 | 0.433933 |
| 18243 | 1.34563 | 0.248663 |
| 18244 | 1.34563 | 0.356214 |
| 18245 | 1.08189 | 0.116536 |
| 18246 | 1.09662 | 0.05 |
| 18247 | 2.35961 | 0.05 |
| 18248 | 0.986952 | 0.05 |
| 18249 | 0.813251 | 0.05 |
| 18250 | 1.17897 | 0.05 |
| 18251 | 1.67685 | 0.05 |
| 18252 | 1.00573 | 1.45364 |
| 18253 | 1.08542 | 0.926742 |
| 18254 | 1.00573 | 2.1 |
| 18255 | 1.12572 | 2.1 |
| 18256 | 1.05499 | 2.1 |
| 18257 | 1.00521 | 1.10915 |
| 18258 | 2.22679 | 0.05 |
| 18259 | 2.86746 | 0.05 |
| 18260 | 2.86746 | 0.05 |
| 18261 | 1.99958 | 0.05 |
| 18262 | 0.996517 | 2.1 |
| 18263 | 1.01169 | 2.1 |
| 18264 | 1.01169 | 2.1 |
| 18265 | 1.26346 | 2.1 |
| 18266 | 1.68274 | 2.1 |
| 18267 | 1.75505 | 2.1 |

| | | |
|---|---|---|
| 18268 | 2.28748 | 2.02872 |
| 18269 | 1.36555 | 2.1 |
| 18270 | 3.47888 | 0.05 |
| 18271 | 0.692788 | 0.05 |
| 18272 | 1.0685 | 0.05 |
| 18273 | 1.14547 | 0.05 |
| 18274 | 1.19017 | 2.1 |
| 18275 | 1.24976 | 2.1 |
| 18276 | 1.24976 | 2.1 |
| 18277 | 1.30682 | 2.1 |
| 18278 | 2.2153 | 2.1 |
| 18279 | 1.85457 | 2.1 |
| 18280 | 2.03267 | 2.1 |
| 18281 | 1.51042 | 2.1 |
| 18282 | 1.05619 | 0.39836 |
| 18283 | 1.05793 | 0.89466 |
| 18284 | 1.05793 | 0.235631 |
| 18285 | 1.00763 | 0.88941 |
| 18286 | 1.77677 | 2.1 |
| 18287 | 1.02524 | 2.1 |
| 18288 | 1.02524 | 2.1 |
| 18289 | 1.18803 | 2.1 |
| 18290 | 1.68669 | 2.1 |
| 18291 | 1.71162 | 2.1 |
| 18292 | 1.556 | 2.1 |
| 18293 | 1.39523 | 2.1 |
| 18294 | 0.960288 | 0.772412 |
| 18295 | 1.24201 | 1.14063 |
| 18296 | 1.24202 | 2.1 |
| 18297 | 1.3592 | 2.1 |
| 18298 | 2.14045 | 0.05 |
| 18299 | 1.62054 | 0.05 |
| 18300 | 2.20248 | 0.05 |
| 18301 | 2.34699 | 0.05 |
| 18302 | 3.45132 | 0.05 |
| 18303 | 3.38241 | 0.05 |
| 18304 | 3.38241 | 0.05 |
| 18305 | 2.96127 | 0.05 |
| 18306 | 2.02612 | 2.1 |
| 18307 | 2.09351 | 2.1 |
| 18308 | 2.09351 | 2.1 |
| 18309 | 2.17465 | 2.1 |
| 18310 | 1.07445 | 2.1 |
| 18311 | 1.14808 | 2.1 |
| 18312 | 1.39067 | 2.1 |
| 18313 | 1.20958 | 2.05503 |
| 18314 | 1.20958 | 2.1 |
| 18315 | 1.00382 | 1.71566 |
| 18316 | 0.986496 | 2.1 |
| 18317 | 1.27142 | 2.1 |
| 18318 | 1.39857 | 2.1 |
| 18319 | 2.26482 | 2.1 |
| 18320 | 2.24367 | 0.05 |
| 18321 | 1.9234 | 0.05 |
| 18322 | 1.59076 | 0.05 |
| 18323 | 1.185 | 0.05 |
| 18324 | 3.86179 | 0.05 |
| 18325 | 3.0638 | 0.05 |

| | | |
|---|---|---|
| 18326 | 2.7574 | 0.05 |
| 18327 | 1.83266 | 0.05 |
| 18328 | 2.59418 | 2.1 |
| 18329 | 2.23048 | 2.1 |
| 18330 | 1.39564 | 2.1 |
| 18331 | 1.36763 | 2.1 |
| 18332 | 2.23048 | 2.1 |
| 18333 | 2.02522 | 2.1 |
| 18334 | 1.36763 | 2.1 |
| 18335 | 1.35175 | 2.1 |
| 18336 | 1.16181 | 0.05 |
| 18337 | 0.97096 | 0.05 |
| 18338 | 2.05665 | 0.05 |
| 18339 | 0.483583 | 0.05 |
| 18340 | 0.601928 | 0.05 |
| 18341 | 3.88545 | 0.05 |
| 18342 | 1.04909 | 2.1 |
| 18343 | 0.983143 | 2.1 |
| 18344 | 1.10469 | 2.1 |
| 18345 | 1.10516 | 2.1 |
| 18346 | 1.10517 | 2.1 |
| 18347 | 1.09951 | 2.1 |
| 18348 | 2.33985 | 2.1 |
| 18349 | 2.35026 | 2.1 |
| 18350 | 2.35025 | 2.1 |
| 18351 | 1.19462 | 2.1 |
| 18352 | 1.57627 | 2.1 |
| 18353 | 1.5493 | 2.1 |
| 18354 | 1.5493 | 2.1 |
| 18355 | 1.53556 | 2.1 |
| 18356 | 1.42763 | 0.159442 |
| 18357 | 1.24682 | 0.0993741 |
| 18358 | 1.24682 | 0.16402 |
| 18359 | 1.00307 | 0.177539 |
| 18360 | 1.37392 | 2.1 |
| 18361 | 1.45408 | 2.1 |
| 18362 | 1.45408 | 2.1 |
| 18363 | 1.54249 | 2.1 |
| 18364 | 1.24474 | 1.53721 |
| 18365 | 1.07467 | 1.75515 |
| 18366 | 1.37813 | 2.01853 |
| 18367 | 1.24474 | 1.93221 |
| 18368 | 1.06341 | 2.1 |
| 18369 | 1.09194 | 2.1 |
| 18370 | 1.09194 | 2.1 |
| 18371 | 1.11117 | 2.1 |
| 18372 | 1.1658 | 2.1 |
| 18373 | 1.93707 | 2.1 |
| 18374 | 1.93707 | 2.1 |
| 18375 | 1.47729 | 2.1 |
| 18376 | 1.25669 | 0.6648 |
| 18377 | 1.07519 | 2.1 |
| 18378 | 1.65417 | 2.1 |
| 18379 | 1.50956 | 2.1 |
| 18380 | 1.37231 | 2.1 |
| 18381 | 1.27285 | 2.1 |
| 18382 | 1.23612 | 2.1 |
| 18383 | 1.1899 | 2.1 |

| | | |
|---|---|---|
| 18384 | 1.08172 | 2.1 |
| 18385 | 1.20357 | 2.1 |
| 18386 | 1.5664 | 2.1 |
| 18387 | 1.7472 | 2.1 |
| 18388 | 1.48434 | 2.1 |
| 18389 | 1.23989 | 2.1 |
| 18390 | 1.58836 | 2.1 |
| 18391 | 1.38766 | 2.1 |
| 18392 | 1.23989 | 2.1 |
| 18393 | 1.26599 | 2.1 |
| 18394 | 2.56248 | 2.1 |
| 18395 | 2.08673 | 0.588658 |
| 18396 | 1.87216 | 1.06635 |
| 18397 | 1.60476 | 0.05 |
| 18398 | 1.84355 | 2.1 |
| 18399 | 1.25971 | 0.36041 |
| 18400 | 1.44141 | 0.754389 |
| 18401 | 1.25521 | 0.05 |
| 18402 | 1.19837 | 0.05 |
| 18403 | 2.31936 | 0.05 |
| 18404 | 1.38153 | 2.1 |
| 18405 | 2.41734 | 2.1 |
| 18406 | 2.70856 | 2.1 |
| 18407 | 3.48385 | 2.1 |
| 18408 | 1.89226 | 1.2014 |
| 18409 | 1.64617 | 1.4853 |
| 18410 | 1.89112 | 1.21747 |
| 18411 | 1.23135 | 0.05 |
| 18412 | 1.23134 | 0.151836 |
| 18413 | 1.34927 | 0.0768962 |
| 18414 | 3.60096 | 0.05 |
| 18415 | 1.68103 | 0.05 |
| 18416 | 2.25644 | 2.1 |
| 18417 | 1.91955 | 2.1 |
| 18418 | 1.91955 | 2.1 |
| 18419 | 1.47688 | 2.1 |
| 18420 | 1.67375 | 2.1 |
| 18421 | 1.50033 | 2.1 |
| 18422 | 1.5098 | 2.1 |
| 18423 | 1.46908 | 2.1 |
| 18424 | 1.69055 | 0.05 |
| 18425 | 1.53463 | 0.05 |
| 18426 | 1.53463 | 0.05 |
| 18427 | 1.41943 | 0.05 |
| 18428 | 1.19022 | 1.98546 |
| 18429 | 1.95981 | 0.930586 |
| 18430 | 1.15009 | 0.950241 |
| 18431 | 1.31612 | 0.757647 |
| 18432 | 1.31611 | 0.05 |
| 18433 | 1.10735 | 0.05 |
| 18434 | 1.18601 | 0.05 |
| 18435 | 1.3499 | 0.05 |
| 18436 | 1.3499 | 0.05 |
| 18437 | 1.29001 | 0.05 |
| 18438 | 1.22083 | 2.1 |
| 18439 | 1.06191 | 2.1 |
| 18440 | 1.348 | 1.12124 |
| 18441 | 1.05758 | 1.65877 |

| | | |
|---|---|---|
| 18442 | 1.34799 | 2.1 |
| 18443 | 1.18647 | 1.78206 |
| 18444 | 2.92457 | 0.05 |
| 18445 | 1.99182 | 0.05 |
| 18446 | 2.25313 | 0.05 |
| 18447 | 1.30859 | 0.05 |
| 18448 | 1.30682 | 2.1 |
| 18449 | 1.38957 | 2.1 |
| 18450 | 1.38957 | 2.1 |
| 18451 | 1.47446 | 2.1 |
| 18452 | 0.953638 | 2.1 |
| 18453 | 1.03408 | 2.1 |
| 18454 | 1.17885 | 0.554494 |
| 18455 | 1.3663 | 0.109111 |
| 18456 | 1.08265 | 0.731942 |
| 18457 | 1.71948 | 0.871406 |
| 18458 | 1.29501 | 0.928337 |
| 18459 | 0.955093 | 0.916354 |
| 18460 | 0.955081 | 0.738104 |
| 18461 | 1.88345 | 0.71109 |
| 18462 | 1.2243 | 2.1 |
| 18463 | 1.23169 | 2.1 |
| 18464 | 2.04726 | 1.89559 |
| 18465 | 0.842869 | 1.98528 |
| 18466 | 3.18156 | 2.1 |
| 18467 | 3.60786 | 1.79526 |
| 18468 | 3.60786 | 2.1 |
| 18469 | 2.38566 | 1.20544 |
| 18470 | 1.24526 | 0.05 |
| 18471 | 1.59019 | 0.05 |
| 18472 | 1.16776 | 0.05 |
| 18473 | 1.05042 | 2.1 |
| 18474 | 1.17418 | 2.1 |
| 18475 | 1.05042 | 2.1 |
| 18476 | 0.949135 | 2.1 |
| 18477 | 0.973448 | 2.1 |
| 18478 | 0.973447 | 2.1 |
| 18479 | 1.02958 | 2.1 |
| 18480 | 3.57768 | 2.1 |
| 18481 | 3.04246 | 2.1 |
| 18482 | 3.04246 | 2.1 |
| 18483 | 2.47824 | 2.1 |
| 18484 | 1.35879 | 2.1 |
| 18485 | 1.10407 | 2.1 |
| 18486 | 1.14599 | 0.05 |
| 18487 | 1.10407 | 0.0734476 |
| 18488 | 1.87794 | 1.77762 |
| 18489 | 1.53429 | 1.40343 |
| 18490 | 1.12717 | 2.1 |
| 18491 | 1.38634 | 2.1 |
| 18492 | 1.38634 | 2.1 |
| 18493 | 1.13127 | 2.1 |
| 18494 | 1.04546 | 2.1 |
| 18495 | 1.06007 | 2.1 |
| 18496 | 1.06007 | 2.1 |
| 18497 | 1.19785 | 2.1 |
| 18498 | 1.43652 | 2.1 |
| 18499 | 1.71741 | 2.1 |

| | | |
|---|---|---|
| 18500 | 1.70921 | 2.05646 |
| 18501 | 1.06994 | 2.1 |
| 18502 | 0.960106 | 2.1 |
| 18503 | 1.48077 | 2.1 |
| 18504 | 1.48077 | 2.1 |
| 18505 | 2.5946 | 2.1 |
| 18506 | 2.03045 | 1.87693 |
| 18507 | 1.76788 | 2.1 |
| 18508 | 2.03045 | 2.1 |
| 18509 | 0.633098 | 2.1 |
| 18510 | 1.06756 | 2.1 |
| 18511 | 1.38667 | 2.1 |
| 18512 | 0.966979 | 2.1 |
| 18513 | 1.38669 | 2.1 |
| 18514 | 1.07156 | 2.1 |
| 18515 | 0.986095 | 2.1 |
| 18516 | 1.32813 | 2.1 |
| 18517 | 0.986095 | 2.1 |
| 18518 | 1.98697 | 1.26161 |
| 18519 | 1.12016 | 2.1 |
| 18520 | 1.24463 | 1.61671 |
| 18521 | 1.1967 | 2.1 |
| 18522 | 1.30001 | 0.05 |
| 18523 | 1.29175 | 1.45258 |
| 18524 | 1.29175 | 0.0905155 |
| 18525 | 1.3003 | 1.70411 |
| 18526 | 1.5606 | 0.05 |
| 18527 | 2.32098 | 0.05 |
| 18528 | 2.57888 | 0.05 |
| 18529 | 2.97463 | 0.05 |
| 18530 | 1.24555 | 2.1 |
| 18531 | 1.46101 | 2.1 |
| 18532 | 1.90723 | 2.1 |
| 18533 | 2.29126 | 2.1 |
| 18534 | 1.46101 | 2.1 |
| 18535 | 1.84089 | 2.1 |
| 18536 | 2.5519 | 2.1 |
| 18537 | 2.91663 | 2.1 |
| 18538 | 1.10476 | 0.05 |
| 18539 | 1.44103 | 0.05 |
| 18540 | 1.44103 | 0.05 |
| 18541 | 1.53336 | 0.05 |
| 18542 | 0.672964 | 2.1 |
| 18543 | 0.633884 | 2.1 |
| 18544 | 0.633884 | 2.1 |
| 18545 | 0.603154 | 2.1 |
| 18546 | 2.29663 | 2.1 |
| 18547 | 2.06892 | 2.1 |
| 18548 | 1.3685 | 2.1 |
| 18549 | 1.39067 | 2.1 |
| 18550 | 2.06892 | 2.1 |
| 18551 | 1.78208 | 2.1 |
| 18552 | 1.39067 | 2.1 |
| 18553 | 1.42174 | 2.1 |
| 18554 | 1.01335 | 1.89708 |
| 18555 | 1.08195 | 1.10467 |
| 18556 | 1.86463 | 2.08485 |
| 18557 | 1.39827 | 2.04172 |

| | | |
|---|---|---|
| 18558 | 0.861515 | 2.1 |
| 18559 | 2.10074 | 2.1 |
| 18560 | 2.09511 | 0.631974 |
| 18561 | 2.04853 | 0.587991 |
| 18562 | 2.21488 | 0.597313 |
| 18563 | 1.38287 | 0.764809 |
| 18564 | 0.974717 | 0.61197 |
| 18565 | 0.998451 | 0.500441 |
| 18566 | 1.42281 | 0.638246 |
| 18567 | 1.82595 | 0.981189 |
| 18568 | 1.50458 | 0.685261 |
| 18569 | 1.09408 | 0.180445 |
| 18570 | 1.23498 | 1.3608 |
| 18571 | 1.05654 | 0.940621 |
| 18572 | 1.17261 | 2.1 |
| 18573 | 1.18058 | 2.1 |
| 18574 | 1.18059 | 2.1 |
| 18575 | 1.18907 | 2.1 |
| 18576 | 2.38998 | 2.1 |
| 18577 | 2.77437 | 2.1 |
| 18578 | 1.04546 | 2.1 |
| 18579 | 1.16817 | 2.1 |
| 18580 | 2.77437 | 2.1 |
| 18581 | 2.97768 | 2.1 |
| 18582 | 1.16817 | 2.1 |
| 18583 | 1.2832 | 2.1 |
| 18584 | 1.75766 | 0.05 |
| 18585 | 1.4934 | 0.857225 |
| 18586 | 1.4934 | 0.05 |
| 18587 | 1.20324 | 1.4501 |
| 18588 | 1.07236 | 0.456158 |
| 18589 | 1.37641 | 0.174946 |
| 18590 | 1.42913 | 0.05 |
| 18591 | 1.93373 | 0.651762 |
| 18592 | 1.42629 | 0.599887 |
| 18593 | 1.00636 | 0.0869356 |
| 18594 | 0.933959 | 2.1 |
| 18595 | 2.22873 | 0.140433 |
| 18596 | 2.22873 | 0.75847 |
| 18597 | 2.13757 | 0.05 |
| 18598 | 1.10517 | 2.1 |
| 18599 | 1.86737 | 2.1 |
| 18600 | 0.811 2.1 | |
| 18601 | 1.19179 | 2.1 |
| 18602 | 1.18647 | 2.1 |
| 18603 | 0.988453 | 2.1 |
| 18604 | 1.12457 | 0.05 |
| 18605 | 1.23851 | 0.05 |
| 18606 | 0.92492 | 2.1 |
| 18607 | 1.45656 | 2.1 |
| 18608 | 1.61251 | 0.810797 |
| 18609 | 1.56696 | 0.837958 |
| 18610 | 1.90245 | 0.32963 |
| 18611 | 1.46046 | 0.349187 |
| 18612 | 1.60329 | 0.05 |
| 18613 | 1.38247 | 0.05 |
| 18614 | 1.38247 | 0.05 |
| 18615 | 1.23198 | 0.05 |

| | | |
|---|---|---|
| 18616 | 1.09518 | 2.1 |
| 18617 | 1.28159 | 2.1 |
| 18618 | 0.977777 | 2.1 |
| 18619 | 1.80778 | 2.1 |
| 18620 | 1.80777 | 2.1 |
| 18621 | 1.16655 | 2.1 |
| 18622 | 1.3515 | 2.1 |
| 18623 | 0.956573 | 2.1 |
| 18624 | 1.08022 | 2.1 |
| 18625 | 1.03437 | 2.1 |
| 18626 | 1.03437 | 2.1 |
| 18627 | 0.975409 | 2.1 |
| 18628 | 1.47369 | 0.05 |
| 18629 | 1.31167 | 0.05 |
| 18630 | 1.01847 | 0.05 |
| 18631 | 2.75562 | 0.05 |
| 18632 | 1.13443 | 2.1 |
| 18633 | 1.32709 | 2.1 |
| 18634 | 1.01051 | 0.05 |
| 18635 | 0.958947 | 0.05 |
| 18636 | 1.7211 | 2.1 |
| 18637 | 1.922 2.1 | |
| 18638 | 1.922 2.1 | |
| 18639 | 0.991113 | 2.1 |
| 18640 | 1.00636 | 0.05 |
| 18641 | 1.31658 | 0.408722 |
| 18642 | 1.44627 | 0.150057 |
| 18643 | 1.03824 | 0.05 |
| 18644 | 1.21223 | 2.1 |
| 18645 | 1.01242 | 2.1 |
| 18646 | 1.01243 | 2.1 |
| 18647 | 0.956698 | 2.1 |
| 18648 | 1.23167 | 2.1 |
| 18649 | 1.06935 | 2.1 |
| 18650 | 1.29215 | 0.05 |
| 18651 | 0.998971 | 0.05 |
| 18652 | 1.50103 | 0.05 |
| 18653 | 1.44836 | 0.05 |
| 18654 | 3.02513 | 2.1 |
| 18655 | 3.7335 | 2.1 |
| 18656 | 2.49988 | 2.1 |
| 18657 | 1.24948 | 2.1 |
| 18658 | 0.963972 | 2.1 |
| 18659 | 1.1822 | 2.1 |
| 18660 | 1.1822 | 2.1 |
| 18661 | 1.10817 | 2.1 |
| 18662 | 1.30237 | 2.1 |
| 18663 | 1.41908 | 2.1 |
| 18664 | 1.41908 | 2.1 |
| 18665 | 1.48734 | 2.1 |
| 18666 | 1.31127 | 0.05 |
| 18667 | 1.40822 | 0.05 |
| 18668 | 1.40822 | 0.05 |
| 18669 | 1.22176 | 0.05 |
| 18670 | 1.82525 | 2.1 |
| 18671 | 1.59394 | 2.1 |
| 18672 | 1.22731 | 0.05 |
| 18673 | 0.981584 | 0.05 |

| | | |
|---|---|---|
| 18674 | 1.1427 | 0.726989 |
| 18675 | 1.44091 | 1.02345 |
| 18676 | 1.49154 | 0.0688886 |
| 18677 | 1.07526 | 0.481604 |
| 18678 | 1.07527 | 0.191435 |
| 18679 | 0.96029 | 0.182411 |
| 18680 | 2.40568 | 1.10576 |
| 18681 | 3.46486 | 0.968422 |
| 18682 | 2.417 | 1.06429 |
| 18683 | 1.52054 | 0.503541 |
| 18684 | 1.51638 | 0.05 |
| 18685 | 1.04786 | 0.05 |
| 18686 | 1.51404 | 0.05 |
| 18687 | 1.3461 | 0.05 |
| 18688 | 1.27696 | 0.05 |
| 18689 | 0.736457 | 0.05 |
| 18690 | 1.16817 | 2.1 |
| 18691 | 1.04263 | 2.1 |
| 18692 | 1.13449 | 2.1 |
| 18693 | 1.50524 | 2.1 |
| 18694 | 2.1182 | 2.1 |
| 18695 | 2.49001 | 2.1 |
| 18696 | 1.11764 | 2.1 |
| 18697 | 1.39292 | 2.1 |
| 18698 | 2.49001 | 2.1 |
| 18699 | 2.76339 | 2.1 |
| 18700 | 1.39292 | 2.1 |
| 18701 | 1.68761 | 2.1 |
| 18702 | 1.25173 | 2.1 |
| 18703 | 1.34701 | 2.1 |
| 18704 | 1.02415 | 2.1 |
| 18705 | 1.34701 | 2.1 |
| 18706 | 1.332 | 0.05 |
| 18707 | 0.989764 | 0.05 |
| 18708 | 0.963167 | 0.05 |
| 18709 | 1.21056 | 0.05 |
| 18710 | 1.67375 | 2.1 |
| 18711 | 1.17965 | 2.1 |
| 18712 | 1.09154 | 2.1 |
| 18713 | 1.52314 | 2.1 |
| 18714 | 1.54433 | 0.187191 |
| 18715 | 1.81969 | 0.0719438 |
| 18716 | 1.3598 | 0.0816554 |
| 18717 | 1.71041 | 0.05 |
| 18718 | 1.70934 | 0.05 |
| 18719 | 1.33871 | 0.05 |
| 18720 | 1.79485 | 0.05 |
| 18721 | 1.51404 | 0.05 |
| 18722 | 1.30751 | 2.1 |
| 18723 | 1.20969 | 2.1 |
| 18724 | 1.20969 | 2.1 |
| 18725 | 1.12076 | 2.1 |
| 18726 | 1.49808 | 2.1 |
| 18727 | 1.50201 | 2.1 |
| 18728 | 1.50201 | 2.1 |
| 18729 | 1.31594 | 2.1 |
| 18730 | 1.2187 | 2.1 |
| 18731 | 1.11545 | 2.1 |

| | | |
|---|---|---|
| 18732 | 1.227 | 2.1 |
| 18733 | 1.09645 | 2.1 |
| 18734 | 1.05902 | 0.05 |
| 18735 | 1.41595 | 0.05 |
| 18736 | 1.35696 | 2.1 |
| 18737 | 1.29446 | 0.0531237 |
| 18738 | 0.857957 | 2.1 |
| 18739 | 1.21125 | 0.05 |
| 18740 | 2.43115 | 2.1 |
| 18741 | 2.1151 | 0.05 |
| 18742 | 2.0815 | 1.27684 |
| 18743 | 1.1723 | 2.1 |
| 18744 | 1.48035 | 0.295112 |
| 18745 | 1.59971 | 0.568221 |
| 18746 | 1.33668 | 0.320698 |
| 18747 | 1.0961 | 0.0940918 |
| 18748 | 1.46235 | 2.1 |
| 18749 | 0.882195 | 2.1 |
| 18750 | 2.46624 | 2.1 |
| 18751 | 2.72161 | 2.1 |
| 18752 | 2.96094 | 2.1 |
| 18753 | 2.25644 | 2.1 |
| 18754 | 1.25923 | 0.05 |
| 18755 | 0.976218 | 0.05 |
| 18756 | 1.10319 | 0.05 |
| 18757 | 1.82583 | 0.05 |
| 18758 | 0.888955 | 2.1 |
| 18759 | 1.30817 | 2.1 |
| 18760 | 1.30817 | 2.1 |
| 18761 | 1.60787 | 2.1 |
| 18762 | 0.988805 | 2.1 |
| 18763 | 1.54168 | 0.789568 |
| 18764 | 1.19496 | 0.826327 |
| 18765 | 1.12224 | 1.67241 |
| 18766 | 1.93597 | 1.30111 |
| 18767 | 1.31113 | 1.19203 |
| 18768 | 1.17879 | 1.09274 |
| 18769 | 1.07468 | 1.14041 |
| 18770 | 2.0523 | 2.1 |
| 18771 | 2.41385 | 2.1 |
| 18772 | 2.41385 | 2.1 |
| 18773 | 3.0355 | 2.1 |
| 18774 | 1.36717 | 0.05 |
| 18775 | 1.45535 | 0.05 |
| 18776 | 1.45535 | 0.05 |
| 18777 | 1.49906 | 0.05 |
| 18778 | 1.37785 | 1.7299 |
| 18779 | 1.04413 | 2.1 |
| 18780 | 1.06729 | 0.632923 |
| 18781 | 1.54305 | 0.488844 |
| 18782 | 1.07444 | 2.1 |
| 18783 | 1.52724 | 2.1 |
| 18784 | 1.49151 | 2.1 |
| 18785 | 1.36814 | 2.1 |
| 18786 | 1.36814 | 2.1 |
| 18787 | 1.15541 | 2.1 |
| 18788 | 1.19379 | 2.1 |
| 18789 | 1.78657 | 0.0830737 |

| | | |
|---|---|---|
| 18790 | 1.6079 | 2.1 |
| 18791 | 2.59469 | 0.168405 |
| 18792 | 1.35545 | 0.05 |
| 18793 | 1.23602 | 0.0940804 |
| 18794 | 1.23452 | 2.1 |
| 18795 | 1.56842 | 0.310726 |
| 18796 | 1.15864 | 2.1 |
| 18797 | 1.11955 | 2.1 |
| 18798 | 1.11955 | 2.1 |
| 18799 | 1.08022 | 2.1 |
| 18800 | 1.64268 | 2.1 |
| 18801 | 1.26247 | 2.1 |
| 18802 | 1.16912 | 2.1 |
| 18803 | 2.1095 | 1.01209 |
| 18804 | 1.75549 | 0.05 |
| 18805 | 1.7491 | 0.05 |
| 18806 | 1.59833 | 0.05 |
| 18807 | 1.68338 | 0.05 |
| 18808 | 1.14846 | 0.05 |
| 18809 | 1.06515 | 0.05 |
| 18810 | 1.2721 | 0.05 |
| 18811 | 1.32639 | 0.05 |
| 18812 | 1.32639 | 0.05 |
| 18813 | 1.19323 | 0.05 |
| 18814 | 1.81034 | 2.1 |
| 18815 | 1.9034 | 2.1 |
| 18816 | 2.08245 | 2.1 |
| 18817 | 1.84887 | 2.1 |
| 18818 | 1.41448 | 2.1 |
| 18819 | 1.77326 | 2.1 |
| 18820 | 1.76872 | 2.1 |
| 18821 | 1.86766 | 2.1 |
| 18822 | 1.46643 | 2.1 |
| 18823 | 1.58995 | 2.1 |
| 18824 | 1.58995 | 2.1 |
| 18825 | 1.13854 | 2.1 |
| 18826 | 1.23654 | 2.1 |
| 18827 | 1.04442 | 2.1 |
| 18828 | 1.23654 | 2.1 |
| 18829 | 1.08576 | 2.1 |
| 18830 | 0.966979 | 2.1 |
| 18831 | 1.41758 | 2.1 |
| 18832 | 1.03108 | 0.05 |
| 18833 | 1.94412 | 0.05 |
| 18834 | 1.32455 | 2.1 |
| 18835 | 1.32328 | 2.1 |
| 18836 | 1.32328 | 2.1 |
| 18837 | 1.34008 | 2.1 |
| 18838 | 1.60861 | 2.1 |
| 18839 | 1.69586 | 2.1 |
| 18840 | 1.69586 | 2.1 |
| 18841 | 1.78301 | 2.1 |
| 18842 | 1.7513 | 2.1 |
| 18843 | 1.67906 | 2.1 |
| 18844 | 1.67906 | 2.1 |
| 18845 | 1.60635 | 2.1 |
| 18846 | 1.68073 | 2.1 |
| 18847 | 1.57234 | 2.1 |

| | | |
|---|---|---|
| 18848 | 1.57233 | 2.1 |
| 18849 | 1.47012 | 2.1 |
| 18850 | 1.22811 | 0.05 |
| 18851 | 1.79683 | 0.05 |
| 18852 | 1.53417 | 0.05 |
| 18853 | 1.08732 | 0.0543399 |
| 18854 | 1.8903 | 1.28507 |
| 18855 | 1.20247 | 1.75828 |
| 18856 | 1.12087 | 1.82585 |
| 18857 | 1.54699 | 2.1 |
| 18858 | 1.43635 | 2.1 |
| 18859 | 1.02368 | 2.1 |
| 18860 | 1.43635 | 2.1 |
| 18861 | 1.23007 | 2.1 |
| 18862 | 1.035 | 2.1 |
| 18863 | 1.07889 | 2.1 |
| 18864 | 3.0355 | 2.1 |
| 18865 | 3.78805 | 2.1 |
| 18866 | 2.57183 | 1.9915 |
| 18867 | 1.27835 | 1.09514 |
| 18868 | 1.29813 | 0.05 |
| 18869 | 1.42965 | 0.0559253 |
| 18870 | 1.69832 | 0.0506475 |
| 18871 | 2.18359 | 0.0730742 |
| 18872 | 1.42964 | 0.05 |
| 18873 | 1.65723 | 0.05 |
| 18874 | 1.98508 | 0.05 |
| 18875 | 2.06284 | 0.0603153 |
| 18876 | 1.50705 | 0.489117 |
| 18877 | 1.51153 | 0.05 |
| 18878 | 1.36037 | 0.05 |
| 18879 | 1.09767 | 0.05 |
| 18880 | 1.70048 | 2.1 |
| 18881 | 1.15177 | 2.1 |
| 18882 | 0.713174 | 2.1 |
| 18883 | 0.763575 | 2.1 |
| 18884 | 0.763575 | 2.1 |
| 18885 | 0.797011 | 2.1 |
| 18886 | 1.01179 | 0.881618 |
| 18887 | 1.27737 | 0.868519 |
| 18888 | 3.16531 | 0.05 |
| 18889 | 1.0141 | 0.05 |
| 18890 | 1.47354 | 0.442434 |
| 18891 | 1.62495 | 0.105175 |
| 18892 | 1.62495 | 0.166386 |
| 18893 | 1.39296 | 0.594152 |
| 18894 | 0.998682 | 2.1 |
| 18895 | 1.93528 | 2.1 |
| 18896 | 2.71383 | 2.1 |
| 18897 | 1.13987 | 2.1 |
| 18898 | 1.93528 | 2.1 |
| 18899 | 1.78595 | 2.1 |
| 18900 | 3.98933 | 2.1 |
| 18901 | 3.73426 | 2.1 |
| 18902 | 1.02438 | 2.1 |
| 18903 | 1.12376 | 2.1 |
| 18904 | 1.12375 | 2.1 |
| 18905 | 1.19016 | 2.1 |

| | | |
|---|---|---|
| 18906 | 1.37157 | 0.05 |
| 18907 | 1.60814 | 0.05 |
| 18908 | 1.70303 | 0.05 |
| 18909 | 1.01219 | 0.05 |
| 18910 | 1.06757 | 2.1 |
| 18911 | 1.23475 | 2.1 |
| 18912 | 1.2295 | 2.1 |
| 18913 | 1.28014 | 2.1 |
| 18914 | 1.28014 | 2.1 |
| 18915 | 1.33339 | 2.1 |
| 18916 | 1.3201 | 2.1 |
| 18917 | 1.2679 | 2.1 |
| 18918 | 1.2679 | 2.1 |
| 18919 | 1.23504 | 2.1 |
| 18920 | 1.50108 | 2.1 |
| 18921 | 1.3715 | 2.1 |
| 18922 | 1.3715 | 2.1 |
| 18923 | 1.25075 | 2.1 |
| 18924 | 1.99222 | 1.19557 |
| 18925 | 1.4155 | 1.08265 |
| 18926 | 1.4777 | 2.1 |
| 18927 | 1.52613 | 2.1 |
| 18928 | 1.91031 | 2.1 |
| 18929 | 1.03968 | 2.1 |
| 18930 | 2.04289 | 0.05 |
| 18931 | 0.967192 | 0.05 |
| 18932 | 1.03043 | 0.05 |
| 18933 | 1.65452 | 0.05 |
| 18934 | 1.21564 | 2.1 |
| 18935 | 1.16938 | 2.1 |
| 18936 | 2.47056 | 2.1 |
| 18937 | 2.56853 | 2.1 |
| 18938 | 1.16938 | 2.1 |
| 18939 | 1.1181 | 2.1 |
| 18940 | 2.56853 | 2.1 |
| 18941 | 2.66828 | 2.1 |
| 18942 | 1.0465 | 2.1 |
| 18943 | 1.44709 | 2.1 |
| 18944 | 1.44709 | 2.1 |
| 18945 | 1.96491 | 2.1 |
| 18946 | 1.01976 | 0.05 |
| 18947 | 0.961779 | 0.05 |
| 18948 | 1.11764 | 0.05 |
| 18949 | 0.961779 | 0.05 |
| 18950 | 1.21171 | 0.05 |
| 18951 | 1.35007 | 0.05 |
| 18952 | 1.03102 | 0.16776 |
| 18953 | 1.42446 | 0.580585 |
| 18954 | 1.42446 | 0.13272 |
| 18955 | 1.03166 | 0.0979808 |
| 18956 | 1.2951 | 0.05 |
| 18957 | 1.33575 | 0.05 |
| 18958 | 1.33575 | 0.05 |
| 18959 | 1.20473 | 0.05 |
| 18960 | 1.10049 | 0.373751 |
| 18961 | 0.99747 | 0.0931928 |
| 18962 | 1.54249 | 2.1 |
| 18963 | 1.65677 | 2.1 |

| | | |
|---|---|---|
| 18964 | 1.65677 | 2.1 |
| 18965 | 1.77677 | 2.1 |
| 18966 | 1.73525 | 0.05 |
| 18967 | 1.05689 | 0.05 |
| 18968 | 1.26051 | 0.05 |
| 18969 | 1.16259 | 0.05 |
| 18970 | 1.34009 | 2.1 |
| 18971 | 1.398 | 2.1 |
| 18972 | 1.398 | 2.1 |
| 18973 | 1.50437 | 2.1 |
| 18974 | 2.3245 | 2.1 |
| 18975 | 1.18237 | 2.1 |
| 18976 | 1.24509 | 2.1 |
| 18977 | 1.01595 | 2.1 |
| 18978 | 3.29047 | 2.1 |
| 18979 | 2.79168 | 2.1 |
| 18980 | 1.06561 | 2.1 |
| 18981 | 1.11077 | 2.1 |
| 18982 | 1.11077 | 2.1 |
| 18983 | 0.953927 | 2.1 |
| 18984 | 1.30509 | 0.05 |
| 18985 | 1.04685 | 0.05 |
| 18986 | 1.04685 | 0.05 |
| 18987 | 0.834593 | 0.05 |
| 18988 | 1.0775 | 0.05 |
| 18989 | 1.27362 | 0.05 |
| 18990 | 1.71762 | 0.539705 |
| 18991 | 2.5736 | 0.194113 |
| 18992 | 2.04717 | 0.68034 |
| 18993 | 1.45521 | 0.376745 |
| 18994 | 1.33962 | 2.1 |
| 18995 | 1.27956 | 2.1 |
| 18996 | 1.27956 | 2.1 |
| 18997 | 1.20478 | 2.1 |
| 18998 | 3.65841 | 0.05 |
| 18999 | 1.18656 | 0.05 |
| 19000 | 1.72272 | 0.05 |
| 19001 | 1.6812 | 0.05 |
| 19002 | 1.09951 | 2.1 |
| 19003 | 1.08231 | 2.1 |
| 19004 | 1.08231 | 2.1 |
| 19005 | 1.06478 | 2.1 |
| 19006 | 2.16196 | 1.4209 |
| 19007 | 1.00387 | 0.92637 |
| 19008 | 1.00387 | 0.95999 |
| 19009 | 0.993999 | 0.428651 |
| 19010 | 1.04101 | 2.1 |
| 19011 | 1.27119 | 2.1 |
| 19012 | 1.27119 | 2.1 |
| 19013 | 1.51748 | 2.1 |
| 19014 | 3.00129 | 2.1 |
| 19015 | 3.27303 | 2.1 |
| 19016 | 3.27303 | 2.1 |
| 19017 | 3.60855 | 2.1 |
| 19018 | 2.22399 | 2.1 |
| 19019 | 1.71995 | 2.1 |
| 19020 | 1.54793 | 2.1 |
| 19021 | 1.227 | 2.1 |

| | | |
|---|---|---|
| 19022 | 1.07624 | 2.1 |
| 19023 | 1.264 | 2.1 |
| 19024 | 1.19323 | 1.1672 |
| 19025 | 1.21286 | 1.0619 |
| 19026 | 1.21286 | 0.623444 |
| 19027 | 1.20859 | 0.91929 |
| 19028 | 1.60578 | 0.05 |
| 19029 | 1.27778 | 0.05 |
| 19030 | 1.0525 | 2.1 |
| 19031 | 1.05758 | 1.23492 |
| 19032 | 1.0525 | 0.978308 |
| 19033 | 1.17042 | 1.43804 |
| 19034 | 1.62536 | 0.05 |
| 19035 | 3.71662 | 0.05 |
| 19036 | 1.78169 | 0.05 |
| 19037 | 1.91434 | 0.05 |
| 19038 | 2.12331 | 0.05 |
| 19039 | 1.33406 | 0.05 |
| 19040 | 1.38437 | 2.1 |
| 19041 | 1.51829 | 2.1 |
| 19042 | 1.51829 | 2.1 |
| 19043 | 1.6697 | 2.1 |
| 19044 | 0.947114 | 2.1 |
| 19045 | 1.13335 | 2.1 |
| 19046 | 1.13335 | 2.1 |
| 19047 | 1.00965 | 2.1 |
| 19048 | 1.47521 | 2.1 |
| 19049 | 1.27679 | 2.1 |
| 19050 | 1.14911 | 2.1 |
| 19051 | 0.93841 | 2.1 |
| 19052 | 1.68437 | 2.1 |
| 19053 | 1.53822 | 2.1 |
| 19054 | 1.53822 | 2.1 |
| 19055 | 1.39702 | 2.1 |
| 19056 | 0.726453 | 2.1 |
| 19057 | 0.754101 | 2.1 |
| 19058 | 0.754101 | 2.1 |
| 19059 | 0.69776 | 2.1 |
| 19060 | 0.964033 | 2.1 |
| 19061 | 1.62397 | 2.1 |
| 19062 | 1.70302 | 2.1 |
| 19063 | 1.78637 | 2.03833 |
| 19064 | 3.68158 | 0.05 |
| 19065 | 2.11685 | 0.05 |
| 19066 | 1.33125 | 2.1 |
| 19067 | 1.15471 | 2.1 |
| 19068 | 1.0465 | 2.1 |
| 19069 | 1.33125 | 2.1 |
| 19070 | 1.17683 | 2.1 |
| 19071 | 1.25889 | 2.1 |
| 19072 | 1.25889 | 2.1 |
| 19073 | 1.34239 | 2.1 |
| 19074 | 1.40591 | 0.05 |
| 19075 | 0.79348 | 0.05 |
| 19076 | 1.04019 | 0.05 |
| 19077 | 0.999187 | 0.05 |
| 19078 | 1.30218 | 0.05 |
| 19079 | 1.83339 | 0.05 |

| | | |
|---|---|---|
| 19080 | 1.88574 | 0.05 |
| 19081 | 1.5177 | 0.05 |
| 19082 | 1.23504 | 2.1 |
| 19083 | 1.19583 | 2.1 |
| 19084 | 1.19583 | 2.1 |
| 19085 | 1.14496 | 2.1 |
| 19086 | 1.62183 | 2.1 |
| 19087 | 1.62316 | 2.1 |
| 19088 | 1.62316 | 2.1 |
| 19089 | 1.62241 | 2.1 |
| 19090 | 1.02305 | 0.05 |
| 19091 | 1.21136 | 0.05 |
| 19092 | 0.993832 | 0.05 |
| 19093 | 1.21137 | 0.05 |
| 19094 | 1.12064 | 2.1 |
| 19095 | 1.24965 | 2.1 |
| 19096 | 1.24965 | 2.1 |
| 19097 | 1.41077 | 2.1 |
| 19098 | 1.00052 | 0.05 |
| 19099 | 1.49842 | 0.05 |
| 19100 | 1.49843 | 0.05 |
| 19101 | 1.10801 | 0.05 |
| 19102 | 1.54196 | 0.411465 |
| 19103 | 1.18041 | 2.04945 |
| 19104 | 1.73571 | 1.96932 |
| 19105 | 1.37224 | 2.1 |
| 19106 | 1.4375 | 0.05 |
| 19107 | 1.09529 | 0.05 |
| 19108 | 1.44421 | 0.05 |
| 19109 | 1.0953 | 0.05 |
| 19110 | 1.0753 | 2.1 |
| 19111 | 0.815365 | 2.1 |
| 19112 | 0.815365 | 2.1 |
| 19113 | 0.597537 | 2.1 |
| 19114 | 1.03609 | 0.05 |
| 19115 | 1.18952 | 0.05 |
| 19116 | 1.54283 | 0.255609 |
| 19117 | 1.16719 | 0.324787 |
| 19118 | 1.16719 | 0.527414 |
| 19119 | 1.89226 | 0.417102 |
| 19120 | 2.55919 | 2.1 |
| 19121 | 2.37492 | 2.1 |
| 19122 | 2.37492 | 2.1 |
| 19123 | 2.07435 | 2.1 |
| 19124 | 1.36016 | 0.090068 |
| 19125 | 1.70302 | 0.075624 |
| 19126 | 1.85252 | 0.0709602 |
| 19127 | 1.2111 | 0.186028 |
| 19128 | 1.62905 | 2.1 |
| 19129 | 1.49692 | 2.1 |
| 19130 | 1.49692 | 2.1 |
| 19131 | 1.40395 | 2.1 |
| 19132 | 2.19083 | 2.1 |
| 19133 | 1.30542 | 2.1 |
| 19134 | 1.58736 | 2.1 |
| 19135 | 2.16579 | 2.1 |
| 19136 | 2.0815 | 2.1 |
| 19137 | 1.17935 | 2.1 |

| | | |
|---|---|---|
| 19138 | 1.53803 | 2.1 |
| 19139 | 1.09148 | 0.269921 |
| 19140 | 1.81301 | 1.88889 |
| 19141 | 1.38333 | 0.05 |
| 19142 | 1.04412 | 2.1 |
| 19143 | 1.44437 | 2.1 |
| 19144 | 1.4675 | 0.05 |
| 19145 | 1.00665 | 0.05 |
| 19146 | 1.50951 | 0.05 |
| 19147 | 0.983105 | 0.05 |
| 19148 | 1.80252 | 0.05 |
| 19149 | 0.540005 | 0.05 |
| 19150 | 1.2545 | 0.05 |
| 19151 | 2.14514 | 0.05 |
| 19152 | 1.32051 | 0.05 |
| 19153 | 1.82302 | 0.05 |
| 19154 | 2.71057 | 2.1 |
| 19155 | 2.51107 | 2.1 |
| 19156 | 1.34817 | 2.1 |
| 19157 | 1.3551 | 2.1 |
| 19158 | 2.51107 | 2.1 |
| 19159 | 2.29663 | 2.1 |
| 19160 | 1.3551 | 2.1 |
| 19161 | 1.3685 | 2.1 |
| 19162 | 1.51748 | 2.1 |
| 19163 | 1.23389 | 2.1 |
| 19164 | 0.901928 | 0.05 |
| 19165 | 1.70302 | 0.05 |
| 19166 | 1.66787 | 0.05 |
| 19167 | 1.92535 | 0.05 |
| 19168 | 1.13571 | 0.05 |
| 19169 | 1.02836 | 0.05 |
| 19170 | 1.55398 | 2.1 |
| 19171 | 1.54179 | 2.1 |
| 19172 | 1.89608 | 2.1 |
| 19173 | 1.07167 | 2.1 |
| 19174 | 3.3488 | 0.05 |
| 19175 | 0.550044 | 0.05 |
| 19176 | 0.962877 | 0.0894995 |
| 19177 | 1.00065 | 0.148173 |
| 19178 | 1.00613 | 0.426256 |
| 19179 | 1.07497 | 0.442307 |
| 19180 | 1.00486 | 0.05 |
| 19181 | 1.00486 | 0.05 |
| 19182 | 0.726453 | 2.1 |
| 19183 | 1.32634 | 0.05 |
| 19184 | 1.40008 | 0.05 |
| 19185 | 1.40008 | 0.05 |
| 19186 | 2.42267 | 0.05 |
| 19187 | 1.9216 | 0.05 |
| 19188 | 1.28066 | 0.05 |
| 19189 | 1.79102 | 0.756679 |
| 19190 | 1.18219 | 0.05 |
| 19191 | 1.18219 | 0.807356 |
| 19192 | 0.709123 | 0.0927607 |
| 19193 | 1.07866 | 2.1 |
| 19194 | 1.15038 | 2.1 |
| 19195 | 1.15038 | 2.1 |

| | | |
|---|---|---|
| 19196 | 1.24687 | 2.1 |
| 19197 | 1.83613 | 2.1 |
| 19198 | 1.99157 | 2.1 |
| 19199 | 1.45789 | 0.05 |
| 19200 | 1.1706 | 0.05 |
| 19201 | 1.74746 | 0.05 |
| 19202 | 1.1706 | 0.05 |
| 19203 | 1.96383 | 0.05 |
| 19204 | 1.67727 | 0.05 |
| 19205 | 1.67727 | 0.05 |
| 19206 | 1.60329 | 0.05 |
| 19207 | 1.18803 | 2.1 |
| 19208 | 1.3227 | 2.1 |
| 19209 | 1.3227 | 2.1 |
| 19210 | 1.40844 | 2.1 |
| 19211 | 1.07889 | 2.1 |
| 19212 | 1.07721 | 2.1 |
| 19213 | 1.07721 | 2.1 |
| 19214 | 1.00832 | 2.1 |
| 19215 | 0.953523 | 0.16146 |
| 19216 | 1.62875 | 0.118043 |
| 19217 | 1.07427 | 0.737004 |
| 19218 | 1.19722 | 0.62975 |
| 19219 | 1.1972 | 0.237124 |
| 19220 | 1.1599 | 0.263505 |
| 19221 | 2.97463 | 0.05 |
| 19222 | 1.9477 | 0.05 |
| 19223 | 1.95222 | 0.05 |
| 19224 | 1.75083 | 0.05 |
| 19225 | 3.46787 | 2.1 |
| 19226 | 3.02896 | 2.1 |
| 19227 | 3.02896 | 2.1 |
| 19228 | 2.46145 | 2.1 |
| 19229 | 0.970962 | 0.05 |
| 19230 | 1.0227 | 0.05 |
| 19231 | 1.12328 | 0.05 |
| 19232 | 1.02269 | 0.05 |
| 19233 | 0.904978 | 2.1 |
| 19234 | 0.725887 | 2.1 |
| 19235 | 0.725887 | 2.1 |
| 19236 | 0.601592 | 2.1 |
| 19237 | 0.956294 | 2.1 |
| 19238 | 1.13179 | 2.1 |
| 19239 | 0.956294 | 2.1 |
| 19240 | 1.13179 | 2.1 |
| 19241 | 1.05556 | 2.1 |
| 19242 | 1.13179 | 2.1 |
| 19243 | 1.38859 | 2.1 |
| 19244 | 1.13179 | 2.1 |
| 19245 | 1.06659 | 2.1 |
| 19246 | 1.05776 | 2.1 |
| 19247 | 1.06659 | 2.1 |
| 19248 | 1.04291 | 0.703646 |
| 19249 | 1.54699 | 1.12608 |
| 19250 | 1.42307 | 1.20698 |
| 19251 | 1.5654 | 2.08205 |
| 19252 | 1.97906 | 2.1 |
| 19253 | 1.15354 | 0.05 |

| | | |
|---|---|---|
| 19254 | 1.15505 | 0.05 |
| 19255 | 1.26455 | 0.05 |
| 19256 | 2.50042 | 0.05 |
| 19257 | 2.50042 | 0.05 |
| 19258 | 3.22585 | 0.05 |
| 19259 | 1.19258 | 0.05 |
| 19260 | 1.34562 | 0.05 |
| 19261 | 1.49515 | 0.05 |
| 19262 | 1.66295 | 0.05 |
| 19263 | 1.02395 | 2.1 |
| 19264 | 1.51067 | 2.1 |
| 19265 | 1.35821 | 2.1 |
| 19266 | 2.10671 | 2.1 |
| 19267 | 1.0305 | 2.0039 |
| 19268 | 1.07855 | 1.63796 |
| 19269 | 1.20761 | 2.1 |
| 19270 | 0.98488 | 2.1 |
| 19271 | 1.4162 | 2.1 |
| 19272 | 1.14825 | 2.1 |
| 19273 | 1.29415 | 2.1 |
| 19274 | 1.6067 | 2.1 |
| 19275 | 1.23851 | 0.05 |
| 19276 | 1.09651 | 0.05 |
| 19277 | 1.60636 | 2.1 |
| 19278 | 1.53521 | 2.1 |
| 19279 | 1.53521 | 2.1 |
| 19280 | 1.46279 | 2.1 |
| 19281 | 1.28044 | 0.05 |
| 19282 | 1.64147 | 0.05 |
| 19283 | 1.18422 | 0.05 |
| 19284 | 1.68208 | 0.05 |
| 19285 | 1.09726 | 2.1 |
| 19286 | 1.234 | 2.1 |
| 19287 | 1.234 | 2.1 |
| 19288 | 1.09483 | 2.1 |
| 19289 | 1.89318 | 2.1 |
| 19290 | 1.98448 | 2.1 |
| 19291 | 1.98448 | 2.1 |
| 19292 | 2.02612 | 2.1 |
| 19293 | 1.22026 | 0.975627 |
| 19294 | 1.23527 | 0.557603 |
| 19295 | 1.09044 | 0.281043 |
| 19296 | 0.993366 | 0.05 |
| 19297 | 1.22026 | 1.49568 |
| 19298 | 0.993368 | 1.27845 |
| 19299 | 2.61357 | 2.1 |
| 19300 | 2.48208 | 2.1 |
| 19301 | 2.48208 | 2.1 |
| 19302 | 2.62868 | 2.1 |
| 19303 | 1.85439 | 0.906517 |
| 19304 | 1.81776 | 0.232577 |
| 19305 | 1.81776 | 0.139593 |
| 19306 | 2.1587 | 0.317786 |
| 19307 | 1.8914 | 0.65518 |
| 19308 | 2.23961 | 0.335793 |
| 19309 | 1.49357 | 0.05 |
| 19310 | 2.23961 | 0.373803 |
| 19311 | 1.78014 | 0.534814 |

| | | |
|---|---|---|
| 19312 | 1.67635 | 0.575318 |
| 19313 | 1.66219 | 0.206444 |
| 19314 | 1.56036 | 0.189825 |
| 19315 | 1.14721 | 0.05 |
| 19316 | 2.22495 | 0.221717 |
| 19317 | 1.88509 | 0.0881609 |
| 19318 | 1.95249 | 0.05 |
| 19319 | 2.11959 | 0.05 |
| 19320 | 1.37427 | 0.05 |
| 19321 | 1.39379 | 0.05 |
| 19322 | 1.17614 | 0.05 |
| 19323 | 1.39379 | 2.1 |
| 19324 | 1.14397 | 0.05 |
| 19325 | 1.09651 | 2.1 |
| 19326 | 1.42843 | 2.1 |
| 19327 | 1.7069 | 2.1 |
| 19328 | 2.31293 | 2.1 |
| 19329 | 1.56839 | 2.1 |
| 19330 | 1.78252 | 0.17737 |
| 19331 | 1.78254 | 0.0630977 |
| 19332 | 1.09142 | 0.146197 |
| 19333 | 0.99829 | 0.05 |
| 19334 | 1.09372 | 0.05 |
| 19335 | 1.88175 | 0.05 |
| 19336 | 0.898452 | 0.05 |
| 19337 | 1.06936 | 2.1 |
| 19338 | 1.4162 | 2.1 |
| 19339 | 1.17989 | 2.1 |
| 19340 | 1.47411 | 2.1 |
| 19341 | 1.25376 | 2.1 |
| 19342 | 1.00757 | 0.797926 |
| 19343 | 1.4185 | 0.05 |
| 19344 | 1.02011 | 0.0586444 |
| 19345 | 2.22495 | 0.0566588 |
| 19346 | 1.79542 | 0.05 |
| 19347 | 1.79542 | 0.05 |
| 19348 | 1.19352 | 0.05 |
| 19349 | 2.38566 | 2.1 |
| 19350 | 1.83255 | 1.44751 |
| 19351 | 1.62667 | 2.1 |
| 19352 | 1.17879 | 1.63018 |
| 19353 | 2.79168 | 2.1 |
| 19354 | 2.28162 | 2.1 |
| 19355 | 2.28162 | 2.1 |
| 19356 | 1.90905 | 2.1 |
| 19357 | 1.40117 | 2.1 |
| 19358 | 1.28268 | 2.1 |
| 19359 | 1.41096 | 2.1 |
| 19360 | 1.22978 | 2.1 |
| 19361 | 3.20034 | 2.1 |
| 19362 | 3.42052 | 2.1 |
| 19363 | 3.42052 | 2.1 |
| 19364 | 3.50562 | 2.1 |
| 19365 | 1.46279 | 2.1 |
| 19366 | 1.3864 | 2.1 |
| 19367 | 1.3864 | 2.1 |
| 19368 | 1.3201 | 2.1 |
| 19369 | 1.35561 | 0.05 |

| | | |
|---|---|---|
| 19370 | 0.974077 | 0.05 |
| 19371 | 0.967673 | 0.240811 |
| 19372 | 1.22482 | 0.0803871 |
| 19373 | 1.02305 | 0.05 |
| 19374 | 1.22482 | 0.0882453 |
| 19375 | 0.999492 | 2.1 |
| 19376 | 1.14657 | 2.1 |
| 19377 | 1.14657 | 2.1 |
| 19378 | 1.31346 | 2.1 |
| 19379 | 1.84417 | 0.05 |
| 19380 | 1.31777 | 0.05 |
| 19381 | 1.19796 | 0.05 |
| 19382 | 0.691742 | 0.05 |
| 19383 | 1.16725 | 2.1 |
| 19384 | 1.9679 | 2.1 |
| 19385 | 1.08848 | 0.05 |
| 19386 | 1.15356 | 0.05 |
| 19387 | 1.58713 | 2.1 |
| 19388 | 1.58742 | 2.1 |
| 19389 | 1.58742 | 2.1 |
| 19390 | 1.49662 | 2.1 |
| 19391 | 1.32201 | 0.05 |
| 19392 | 1.32097 | 1.31698 |
| 19393 | 1.32097 | 0.165053 |
| 19394 | 1.31825 | 1.63125 |
| 19395 | 1.12028 | 2.1 |
| 19396 | 1.15852 | 2.1 |
| 19397 | 1.15852 | 2.1 |
| 19398 | 1.16239 | 2.1 |
| 19399 | 1.07624 | 2.1 |
| 19400 | 1.30047 | 2.1 |
| 19401 | 1.44847 | 0.854808 |
| 19402 | 1.55292 | 0.831518 |
| 19403 | 1.21125 | 0.05 |
| 19404 | 1.15262 | 2.1 |
| 19405 | 1.05025 | 2.1 |
| 19406 | 1.55351 | 2.1 |
| 19407 | 1.5329 | 0.05 |
| 19408 | 2.26758 | 0.05 |
| 19409 | 1.94968 | 0.05 |
| 19410 | 1.65747 | 0.05 |
| 19411 | 1.42549 | 0.05 |
| 19412 | 1.57656 | 0.05 |
| 19413 | 2.11157 | 0.510893 |
| 19414 | 1.56531 | 0.534862 |
| 19415 | 1.69279 | 0.405298 |
| 19416 | 1.06204 | 0.573614 |
| 19417 | 0.947863 | 0.546963 |
| 19418 | 1.19854 | 1.15492 |
| 19419 | 0.947862 | 0.136089 |
| 19420 | 1.31126 | 0.0964839 |
| 19421 | 1.3425 | 1.525 |
| 19422 | 1.29976 | 1.17714 |
| 19423 | 1.05129 | 0.05 |
| 19424 | 1.51026 | 0.05 |
| 19425 | 1.05129 | 0.05 |
| 19426 | 1.2951 | 0.05 |
| 19427 | 1.7155 | 2.1 |

| | | |
|---|---|---|
| 19428 | 1.72196 | 2.1 |
| 19429 | 1.72196 | 2.1 |
| 19430 | 1.77867 | 2.1 |
| 19431 | 0.964755 | 2.1 |
| 19432 | 1.14142 | 2.1 |
| 19433 | 1.03766 | 2.1 |
| 19434 | 0.986723 | 2.1 |
| 19435 | 1.28072 | 0.306592 |
| 19436 | 1.10609 | 1.81716 |
| 19437 | 1.28072 | 0.05 |
| 19438 | 1.16776 | 2.1 |
| 19439 | 1.61687 | 2.1 |
| 19440 | 1.60965 | 2.1 |
| 19441 | 1.60965 | 2.1 |
| 19442 | 1.59654 | 2.1 |
| 19443 | 1.26512 | 2.1 |
| 19444 | 1.23964 | 2.1 |
| 19445 | 2.07997 | 2.1 |
| 19446 | 2.60048 | 2.1 |
| 19447 | 1.23732 | 2.1 |
| 19448 | 1.3372 | 2.1 |
| 19449 | 2.62253 | 2.1 |
| 19450 | 2.47056 | 2.1 |
| 19451 | 0.92782 | 0.05 |
| 19452 | 1.40335 | 0.05 |
| 19453 | 1.26301 | 0.05 |
| 19454 | 1.52668 | 0.05 |
| 19455 | 0.99818 | 0.05 |
| 19456 | 1.51165 | 0.113006 |
| 19457 | 1.66213 | 0.05 |
| 19458 | 1.55246 | 0.0881416 |
| 19459 | 2.31255 | 2.1 |
| 19460 | 1.34366 | 2.1 |
| 19461 | 1.90577 | 2.1 |
| 19462 | 0.969739 | 2.1 |
| 19463 | 3.4245 | 2.1 |
| 19464 | 2.8415 | 2.1 |
| 19465 | 1.97559 | 2.1 |
| 19466 | 1.82362 | 2.1 |
| 19467 | 1.48162 | 2.1 |
| 19468 | 1.23204 | 2.1 |
| 19469 | 1.23204 | 2.1 |
| 19470 | 0.95947 | 2.1 |
| 19471 | 1.05267 | 2.1 |
| 19472 | 1.44091 | 1.10838 |
| 19473 | 1.05267 | 1.54344 |
| 19474 | 1.5795 | 2.1 |
| 19475 | 0.738711 | 2.1 |
| 19476 | 1.28549 | 2.1 |
| 19477 | 1.15694 | 2.1 |
| 19478 | 1.72061 | 2.1 |
| 19479 | 1.69858 | 2.1 |
| 19480 | 0.954563 | 2.1 |
| 19481 | 0.859087 | 2.1 |
| 19482 | 0.788886 | 2.1 |
| 19483 | 1.08732 | 0.05 |
| 19484 | 1.3357 | 0.05 |
| 19485 | 1.3357 | 0.05 |

| | | |
|---|---|---|
| 19486 | 1.48676 | 0.05 |
| 19487 | 2.15079 | 2.1 |
| 19488 | 2.12414 | 2.1 |
| 19489 | 2.12414 | 2.1 |
| 19490 | 2.04664 | 2.1 |
| 19491 | 1.98859 | 2.1 |
| 19492 | 0.992159 | 2.1 |
| 19493 | 1.48064 | 2.1 |
| 19494 | 1.2163 | 2.1 |
| 19495 | 1.26337 | 2.1 |
| 19496 | 1.63159 | 2.1 |
| 19497 | 1.00366 | 0.05 |
| 19498 | 1.65452 | 0.05 |
| 19499 | 1.10408 | 2.1 |
| 19500 | 1.37704 | 2.1 |
| 19501 | 1.50224 | 2.1 |
| 19502 | 1.42866 | 2.1 |
| 19503 | 1.43939 | 2.1 |
| 19504 | 1.02784 | 2.1 |
| 19505 | 1.00763 | 0.05 |
| 19506 | 1.23746 | 0.05 |
| 19507 | 1.23747 | 0.05 |
| 19508 | 1.13642 | 0.05 |
| 19509 | 1.49906 | 0.05 |
| 19510 | 1.46506 | 0.05 |
| 19511 | 1.46506 | 0.05 |
| 19512 | 1.33061 | 0.05 |
| 19513 | 1.08166 | 0.05 |
| 19514 | 1.01052 | 0.05 |
| 19515 | 1.02836 | 0.05 |
| 19516 | 1.08166 | 0.05 |
| 19517 | 1.25334 | 0.05 |
| 19518 | 1.23749 | 0.05 |
| 19519 | 1.37501 | 0.05 |
| 19520 | 1.47354 | 0.05 |
| 19521 | 1.30167 | 2.1 |
| 19522 | 1.29897 | 2.1 |
| 19523 | 1.51042 | 2.1 |
| 19524 | 1.07684 | 2.1 |
| 19525 | 1.07684 | 2.1 |
| 19526 | 0.765121 | 2.1 |
| 19527 | 1.5535 | 1.26881 |
| 19528 | 1.40602 | 2.1 |
| 19529 | 1.40603 | 2.1 |
| 19530 | 1.25669 | 2.1 |
| 19531 | 1.08276 | 2.1 |
| 19532 | 1.60948 | 2.1 |
| 19533 | 1.26929 | 2.1 |
| 19534 | 1.09847 | 2.1 |
| 19535 | 0.827082 | 0.05 |
| 19536 | 1.15841 | 0.05 |
| 19537 | 1.25063 | 0.0547147 |
| 19538 | 1.14397 | 0.05 |
| 19539 | 1.25063 | 0.454519 |
| 19540 | 1.10476 | 0.05 |
| 19541 | 1.14872 | 0.05 |
| 19542 | 1.3659 | 0.05 |
| 19543 | 1.36589 | 0.05 |

| | | |
|---|---|---|
| 19544 | 1.4677 | 0.05 |
| 19545 | 0.590354 | 2.1 |
| 19546 | 0.655202 | 2.1 |
| 19547 | 3.48926 | 2.1 |
| 19548 | 3.32709 | 2.1 |
| 19549 | 3.32709 | 2.1 |
| 19550 | 3.13938 | 2.1 |
| 19551 | 1.24458 | 0.145011 |
| 19552 | 1.29681 | 0.109254 |
| 19553 | 1.40915 | 0.05 |
| 19554 | 1.17891 | 0.314549 |
| 19555 | 1.33171 | 0.05 |
| 19556 | 1.37681 | 0.212856 |
| 19557 | 1.37681 | 2.1 |
| 19558 | 1.4516 | 2.1 |
| 19559 | 2.68241 | 0.05 |
| 19560 | 1.61031 | 0.05 |
| 19561 | 1.6488 | 2.1 |
| 19562 | 1.6585 | 2.1 |
| 19563 | 1.65848 | 2.1 |
| 19564 | 1.67061 | 2.1 |
| 19565 | 1.15152 | 2.1 |
| 19566 | 1.32548 | 2.1 |
| 19567 | 1.43677 | 2.1 |
| 19568 | 1.10264 | 2.1 |
| 19569 | 1.01472 | 2.1 |
| 19570 | 0.966964 | 2.1 |
| 19571 | 2.867 | 2.1 |
| 19572 | 2.94897 | 2.1 |
| 19573 | 1.65815 | 2.1 |
| 19574 | 1.4502 | 2.1 |
| 19575 | 1.4502 | 2.1 |
| 19576 | 1.26755 | 2.1 |
| 19577 | 1.37138 | 2.1 |
| 19578 | 1.45026 | 2.1 |
| 19579 | 1.45026 | 2.1 |
| 19580 | 1.53446 | 2.1 |
| 19581 | 0.973502 | 2.1 |
| 19582 | 1.08542 | 2.1 |
| 19583 | 1.13387 | 0.05 |
| 19584 | 1.71031 | 0.05 |
| 19585 | 1.08911 | 2.1 |
| 19586 | 1.10748 | 2.1 |
| 19587 | 1.21823 | 2.1 |
| 19588 | 1.25005 | 2.1 |
| 19589 | 0.657014 | 2.1 |
| 19590 | 1.38139 | 2.1 |
| 19591 | 1.01485 | 2.1 |
| 19592 | 1.26045 | 2.1 |
| 19593 | 1.12468 | 2.1 |
| 19594 | 1.26045 | 2.1 |
| 19595 | 1.89278 | 0.1741 |
| 19596 | 1.06433 | 0.0585688 |
| 19597 | 1.87861 | 0.067665 |
| 19598 | 0.967544 | 0.05 |
| 19599 | 1.50319 | 2.1 |
| 19600 | 3.47245 | 2.1 |
| 19601 | 2.2354 | 0.595738 |

| | | |
|---|---|---|
| 19602 | 1.16701 | 0.498658 |
| 19603 | 1.3592 | 2.1 |
| 19604 | 1.3305 | 0.742023 |
| 19605 | 1.3305 | 1.91643 |
| 19606 | 1.15008 | 0.288165 |
| 19607 | 1.12457 | 0.05 |
| 19608 | 1.40707 | 0.05 |
| 19609 | 1.01778 | 2.1 |
| 19610 | 1.35733 | 2.1 |
| 19611 | 1.01779 | 2.1 |
| 19612 | 1.12064 | 2.1 |
| 19613 | 1.30694 | 0.899335 |
| 19614 | 1.42716 | 1.26574 |
| 19615 | 1.42717 | 1.78824 |
| 19616 | 1.04321 | 1.19994 |
| 19617 | 1.19352 | 0.05 |
| 19618 | 1.32634 | 0.05 |
| 19619 | 1.14605 | 2.1 |
| 19620 | 1.0417 | 2.1 |
| 19621 | 1.19676 | 0.0650712 |
| 19622 | 1.03804 | 0.0787728 |
| 19623 | 2.09455 | 0.174573 |
| 19624 | 1.15338 | 0.329777 |
| 19625 | 2.21119 | 2.1 |
| 19626 | 1.26097 | 2.1 |
| 19627 | 1.88394 | 0.05 |
| 19628 | 1.24106 | 0.05 |
| 19629 | 2.98143 | 2.1 |
| 19630 | 2.56248 | 1.3938 |
| 19631 | 1.88394 | 1.77087 |
| 19632 | 1.68703 | 0.05 |
| 19633 | 1.5846 | 2.1 |
| 19634 | 1.94799 | 2.1 |
| 19635 | 1.20091 | 2.1 |
| 19636 | 1.40393 | 2.1 |
| 19637 | 3.09031 | 2.1 |
| 19638 | 2.90243 | 2.1 |
| 19639 | 1.27227 | 2.1 |
| 19640 | 1.32592 | 2.1 |
| 19641 | 2.90247 | 2.1 |
| 19642 | 2.71057 | 2.1 |
| 19643 | 1.32594 | 2.1 |
| 19644 | 1.34817 | 2.1 |
| 19645 | 1.4092 | 2.1 |
| 19646 | 1.44859 | 2.1 |
| 19647 | 1.52008 | 2.1 |
| 19648 | 1.401 2.1 | |
| 19649 | 1.40099 | 2.1 |
| 19650 | 1.28936 | 2.1 |
| 19651 | 1.56002 | 0.05 |
| 19652 | 1.08811 | 0.05 |
| 19653 | 1.46041 | 0.05 |
| 19654 | 1.56002 | 0.05 |
| 19655 | 1.50524 | 2.1 |
| 19656 | 1.21304 | 2.1 |
| 19657 | 1.21304 | 2.1 |
| 19658 | 0.947115 | 2.1 |
| 19659 | 1.60665 | 2.1 |

| | | |
|---|---|---|
| 19660 | 1.85179 | 1.75321 |
| 19661 | 1.85178 | 1.89921 |
| 19662 | 2.06277 | 0.933874 |
| 19663 | 2.17534 | 2.1 |
| 19664 | 1.23007 | 2.1 |
| 19665 | 2.17533 | 2.1 |
| 19666 | 1.66635 | 2.1 |
| 19667 | 2.00869 | 2.1 |
| 19668 | 0.986724 | 2.1 |
| 19669 | 2.00475 | 2.1 |
| 19670 | 1.99394 | 2.1 |
| 19671 | 1.67244 | 0.05 |
| 19672 | 1.39604 | 0.05 |
| 19673 | 1.39604 | 0.05 |
| 19674 | 0.954046 | 0.05 |
| 19675 | 0.988863 | 2.1 |
| 19676 | 1.9864 | 2.1 |
| 19677 | 1.9864 | 2.1 |
| 19678 | 3.38645 | 2.1 |
| 19679 | 1.46932 | 0.05 |
| 19680 | 1.50847 | 0.05 |
| 19681 | 1.50848 | 0.05 |
| 19682 | 1.42705 | 0.05 |
| 19683 | 1.50108 | 2.1 |
| 19684 | 1.51621 | 0.813199 |
| 19685 | 1.94317 | 0.850512 |
| 19686 | 1.18382 | 0.05 |
| 19687 | 2.05091 | 0.05 |
| 19688 | 1.88593 | 0.05 |
| 19689 | 1.54299 | 0.05 |
| 19690 | 0.987265 | 0.05 |
| 19691 | 1.27766 | 2.1 |
| 19692 | 1.5178 | 2.1 |
| 19693 | 1.5178 | 2.1 |
| 19694 | 1.81518 | 2.1 |
| 19695 | 1.04626 | 0.900097 |
| 19696 | 1.31057 | 0.95625 |
| 19697 | 1.31057 | 0.942603 |
| 19698 | 1.22297 | 1.09214 |
| 19699 | 1.67844 | 2.07205 |
| 19700 | 1.62862 | 2.1 |
| 19701 | 1.62112 | 2.1 |
| 19702 | 1.87598 | 2.1 |
| 19703 | 1.17844 | 2.1 |
| 19704 | 1.37063 | 2.1 |
| 19705 | 1.37063 | 2.1 |
| 19706 | 1.68723 | 2.1 |
| 19707 | 1.99943 | 0.05 |
| 19708 | 1.17631 | 0.05 |
| 19709 | 1.4263 | 0.312071 |
| 19710 | 1.31023 | 2.1 |
| 19711 | 1.31023 | 0.345629 |
| 19712 | 1.20871 | 2.1 |
| 19713 | 1.50356 | 2.1 |
| 19714 | 1.69311 | 2.1 |
| 19715 | 1.69599 | 2.1 |
| 19716 | 1.6801 | 2.1 |
| 19717 | 1.5843 | 0.05 |

| | | |
|---|---|---|
| 19718 | 0.840233 | 0.05 |
| 19719 | 1.95358 | 0.05 |
| 19720 | 1.40216 | 0.05 |
| 19721 | 1.39078 | 2.1 |
| 19722 | 1.18948 | 2.07302 |
| 19723 | 1.01606 | 1.41481 |
| 19724 | 1.18948 | 1.91818 |
| 19725 | 1.63823 | 2.1 |
| 19726 | 1.67687 | 2.1 |
| 19727 | 1.67687 | 2.1 |
| 19728 | 1.52855 | 2.1 |
| 19729 | 1.11048 | 2.1 |
| 19730 | 1.25346 | 2.1 |
| 19731 | 2.42984 | 2.0916 |
| 19732 | 3.41731 | 1.03159 |
| 19733 | 1.2258 | 2.1 |
| 19734 | 1.2966 | 2.1 |
| 19735 | 1.2966 | 2.1 |
| 19736 | 1.37392 | 2.1 |
| 19737 | 1.41758 | 2.1 |
| 19738 | 1.35504 | 2.1 |
| 19739 | 1.35504 | 2.1 |
| 19740 | 1.30347 | 2.1 |
| 19741 | 1.50877 | 2.1 |
| 19742 | 1.11193 | 2.1 |
| 19743 | 1.40984 | 2.1 |
| 19744 | 1.54537 | 2.1 |
| 19745 | 1.71638 | 2.1 |
| 19746 | 1.77795 | 1.43604 |
| 19747 | 2.07593 | 2.1 |
| 19748 | 1.93387 | 2.1 |
| 19749 | 1.9339 | 2.1 |
| 19750 | 1.75669 | 2.1 |
| 19751 | 1.80299 | 1.05084 |
| 19752 | 1.31251 | 0.942357 |
| 19753 | 1.31249 | 0.817513 |
| 19754 | 1.13411 | 0.753547 |
| 19755 | 1.06313 | 2.1 |
| 19756 | 1.04921 | 2.1 |
| 19757 | 1.55766 | 2.1 |
| 19758 | 1.53728 | 2.1 |
| 19759 | 1.53729 | 2.1 |
| 19760 | 1.51789 | 2.1 |
| 19761 | 0.993021 | 0.05 |
| 19762 | 1.02478 | 0.0601647 |
| 19763 | 1.03408 | 0.05 |
| 19764 | 1.09056 | 0.05 |
| 19765 | 0.697755 | 2.1 |
| 19766 | 0.632901 | 2.1 |
| 19767 | 1.34072 | 0.05 |
| 19768 | 1.80841 | 0.05 |
| 19769 | 1.80841 | 0.0505394 |
| 19770 | 1.17793 | 0.0559094 |
| 19771 | 1.75032 | 2.1 |
| 19772 | 1.13076 | 2.1 |
| 19773 | 2.11851 | 2.1 |
| 19774 | 1.73255 | 2.1 |
| 19775 | 1.73255 | 2.1 |

| | | |
|---|---|---|
| 19776 | 1.11181 | 2.1 |
| 19777 | 1.43772 | 2.1 |
| 19778 | 1.67381 | 2.1 |
| 19779 | 1.52164 | 2.1 |
| 19780 | 1.60861 | 2.1 |
| 19781 | 1.30588 | 0.05 |
| 19782 | 1.75241 | 0.05 |
| 19783 | 1.33026 | 1.43214 |
| 19784 | 0.962705 | 0.790007 |
| 19785 | 0.99678 | 0.05 |
| 19786 | 1.30572 | 0.05 |
| 19787 | 1.41943 | 0.05 |
| 19788 | 1.2683 | 0.05 |
| 19789 | 1.14145 | 0.05 |
| 19790 | 1.01361 | 0.05 |
| 19791 | 1.29943 | 2.1 |
| 19792 | 1.3483 | 2.1 |
| 19793 | 1.34832 | 2.1 |
| 19794 | 1.83488 | 2.1 |
| 19795 | 1.71065 | 2.1 |
| 19796 | 1.66178 | 2.1 |
| 19797 | 1.39076 | 2.1 |
| 19798 | 1.37456 | 2.1 |
| 19799 | 1.66126 | 2.1 |
| 19800 | 1.562 | 2.1 |
| 19801 | 1.37456 | 2.1 |
| 19802 | 1.36065 | 2.1 |
| 19803 | 1.04026 | 0.351478 |
| 19804 | 1.51898 | 0.386023 |
| 19805 | 1.67088 | 0.0868418 |
| 19806 | 1.9726 | 0.211199 |
| 19807 | 1.74852 | 0.349537 |
| 19808 | 1.95512 | 0.672328 |
| 19809 | 1.16165 | 0.86475 |
| 19810 | 1.06584 | 0.279988 |
| 19811 | 1.39327 | 2.1 |
| 19812 | 1.41394 | 2.1 |
| 19813 | 1.41394 | 2.1 |
| 19814 | 1.45506 | 2.1 |
| 19815 | 1.39523 | 2.1 |
| 19816 | 1.21119 | 2.1 |
| 19817 | 1.09005 | 2.1 |
| 19818 | 0.921311 | 2.1 |
| 19819 | 1.58164 | 2.1 |
| 19820 | 1.77574 | 2.1 |
| 19821 | 1.73184 | 2.1 |
| 19822 | 1.59423 | 2.1 |
| 19823 | 1.8214 | 2.1 |
| 19824 | 3.10544 | 1.06668 |
| 19825 | 1.01243 | 2.1 |
| 19826 | 1.30896 | 0.935587 |
| 19827 | 2.16956 | 1.17031 |
| 19828 | 1.29608 | 0.860673 |
| 19829 | 1.38617 | 2.02601 |
| 19830 | 1.49536 | 1.68293 |
| 19831 | 2.35239 | 0.969654 |
| 19832 | 1.24613 | 1.81255 |
| 19833 | 1.94351 | 2.1 |

| | | |
|---|---|---|
| 19834 | 2.05361 | 2.1 |
| 19835 | 2.05358 | 2.1 |
| 19836 | 2.38994 | 2.1 |
| 19837 | 1.13533 | 2.1 |
| 19838 | 1.08276 | 2.1 |
| 19839 | 1.55611 | 0.05 |
| 19840 | 1.33957 | 0.05 |
| 19841 | 1.33957 | 0.05 |
| 19842 | 1.69011 | 0.111242 |
| 19843 | 1.5515 | 0.847431 |
| 19844 | 1.59106 | 0.819235 |
| 19845 | 1.02958 | 2.1 |
| 19846 | 1.12266 | 2.1 |
| 19847 | 1.12266 | 2.1 |
| 19848 | 1.24925 | 2.1 |
| 19849 | 1.80223 | 2.1 |
| 19850 | 1.20242 | 2.0813 |
| 19851 | 2.13665 | 0.57347 |
| 19852 | 1.3924 | 0.32592 |
| 19853 | 0.904894 | 1.15875 |
| 19854 | 1.82633 | 2.1 |
| 19855 | 1.94138 | 0.05 |
| 19856 | 1.34009 | 0.05 |
| 19857 | 1.15096 | 2.1 |
| 19858 | 0.994292 | 2.1 |
| 19859 | 1.10101 | 2.1 |
| 19860 | 1.15096 | 2.1 |
| 19861 | 1.50438 | 2.1 |
| 19862 | 1.67825 | 2.1 |
| 19863 | 1.67825 | 2.1 |
| 19864 | 1.91894 | 2.1 |
| 19865 | 2.04664 | 2.1 |
| 19866 | 1.92063 | 2.1 |
| 19867 | 1.72857 | 2.1 |
| 19868 | 1.89757 | 2.1 |
| 19869 | 1.61843 | 0.05 |
| 19870 | 1.49431 | 0.05 |
| 19871 | 2.33925 | 2.1 |
| 19872 | 1.80683 | 2.1 |
| 19873 | 1.64257 | 2.1 |
| 19874 | 1.50108 | 2.1 |
| 19875 | 2.82561 | 2.1 |
| 19876 | 2.97847 | 2.1 |
| 19877 | 2.97848 | 2.1 |
| 19878 | 3.20034 | 2.1 |
| 19879 | 0.66055 | 2.1 |
| 19880 | 1.95919 | 2.1 |
| 19881 | 1.07976 | 2.1 |
| 19882 | 1.30237 | 2.1 |
| 19883 | 1.99943 | 0.05 |
| 19884 | 1.31802 | 0.05 |
| 19885 | 1.53336 | 0.05 |
| 19886 | 1.31802 | 0.05 |
| 19887 | 1.27737 | 0.825602 |
| 19888 | 1.44848 | 0.808227 |
| 19889 | 2.35499 | 0.05 |
| 19890 | 1.30041 | 0.05 |
| 19891 | 2.35499 | 0.05 |

| | | |
|---|---|---|
| 19892 | 1.65381 | 0.05 |
| 19893 | 0.996083 | 2.1 |
| 19894 | 1.28754 | 2.1 |
| 19895 | 0.996081 | 2.1 |
| 19896 | 1.44859 | 2.1 |
| 19897 | 1.24763 | 1.89954 |
| 19898 | 1.03697 | 2.1 |
| 19899 | 0.951273 | 1.42767 |
| 19900 | 1.60018 | 1.04999 |
| 19901 | 2.0815 | 2.1 |
| 19902 | 1.20488 | 2.1 |
| 19903 | 2.95785 | 2.1 |
| 19904 | 2.52245 | 2.1 |
| 19905 | 1.68455 | 0.05 |
| 19906 | 1.12971 | 0.05 |
| 19907 | 1.1952 | 0.05 |
| 19908 | 2.0815 | 0.05 |
| 19909 | 1.3963 | 2.1 |
| 19910 | 1.4934 | 2.1 |
| 19911 | 1.47973 | 2.1 |
| 19912 | 1.25559 | 2.1 |
| 19913 | 1.1233 | 2.1 |
| 19914 | 1.15009 | 2.1 |
| 19915 | 1.15009 | 2.1 |
| 19916 | 0.977775 | 2.1 |
| 19917 | 1.16482 | 1.06202 |
| 19918 | 1.29313 | 0.645244 |
| 19919 | 1.29313 | 0.765532 |
| 19920 | 1.294 | 0.648816 |
| 19921 | 1.48693 | 0.05 |
| 19922 | 1.47608 | 0.05 |
| 19923 | 1.36005 | 2.1 |
| 19924 | 1.47109 | 2.1 |
| 19925 | 1.47109 | 2.1 |
| 19926 | 1.3515 | 2.1 |
| 19927 | 2.48828 | 2.1 |
| 19928 | 2.32378 | 2.1 |
| 19929 | 2.32378 | 2.1 |
| 19930 | 2.1692 | 2.1 |
| 19931 | 1.03217 | 0.235607 |
| 19932 | 0.953521 | 0.553701 |
| 19933 | 1.4515 | 0.05 |
| 19934 | 3.72406 | 0.05 |
| 19935 | 0.963395 | 1.91624 |
| 19936 | 1.01346 | 1.07223 |
| 19937 | 1.62241 | 2.1 |
| 19938 | 1.62027 | 2.1 |
| 19939 | 1.62027 | 2.1 |
| 19940 | 1.61687 | 2.1 |
| 19941 | 1.2593 | 0.05 |
| 19942 | 0.981586 | 0.05 |
| 19943 | 0.721961 | 2.1 |
| 19944 | 0.888955 | 2.1 |
| 19945 | 1.22031 | 0.05 |
| 19946 | 1.27566 | 2.1 |
| 19947 | 2.50066 | 2.1 |
| 19948 | 1.19814 | 2.1 |
| 19949 | 1.42278 | 0.05 |

| | | |
|---|---|---|
| 19950 | 2.02657 | 0.05 |
| 19951 | 1.0756 | 0.316881 |
| 19952 | 1.10049 | 0.276749 |
| 19953 | 2.26155 | 2.1 |
| 19954 | 2.15634 | 2.1 |
| 19955 | 2.15634 | 2.1 |
| 19956 | 2.01613 | 2.1 |
| 19957 | 1.94233 | 1.2532 |
| 19958 | 1.18127 | 1.79231 |
| 19959 | 1.36865 | 1.96342 |
| 19960 | 1.75755 | 1.61892 |
| 19961 | 2.01613 | 2.1 |
| 19962 | 1.78958 | 2.1 |
| 19963 | 1.97995 | 2.1 |
| 19964 | 1.79198 | 2.1 |
| 19965 | 1.20841 | 2.1 |
| 19966 | 1.6519 | 2.1 |
| 19967 | 1.80207 | 2.1 |
| 19968 | 1.66774 | 2.1 |
| 19969 | 1.17935 | 0.05 |
| 19970 | 1.88071 | 0.05 |
| 19971 | 1.58723 | 0.05 |
| 19972 | 1.7361 | 0.05 |
| 19973 | 0.794165 | 2.1 |
| 19974 | 1.21327 | 2.1 |
| 19975 | 1.21327 | 2.1 |
| 19976 | 1.34268 | 2.1 |
| 19977 | 1.34497 | 0.05 |
| 19978 | 0.793303 | 0.05 |
| 19979 | 1.12323 | 0.05 |
| 19980 | 1.34496 | 0.05 |
| 19981 | 0.928226 | 0.0816018 |
| 19982 | 1.89804 | 0.18859 |
| 19983 | 2.60141 | 0.05 |
| 19984 | 2.60142 | 2.1 |
| 19985 | 2.60772 | 0.0574886 |
| 19986 | 0.99747 | 2.1 |
| 19987 | 1.27096 | 0.161188 |
| 19988 | 1.27096 | 0.05 |
| 19989 | 1.03472 | 0.0829513 |
| 19990 | 1.09962 | 0.358624 |
| 19991 | 1.21148 | 2.1 |
| 19992 | 1.21148 | 0.772141 |
| 19993 | 1.35025 | 2.1 |
| 19994 | 1.09795 | 2.1 |
| 19995 | 1.00775 | 2.1 |
| 19996 | 1.07162 | 2.1 |
| 19997 | 1.60665 | 2.1 |
| 19998 | 1.52984 | 2.1 |
| 19999 | 1.61179 | 2.1 |
| 20000 | 1.70057 | 2.1 |
| 20001 | 1.76063 | 2.1 |
| 20002 | 1.14547 | 0.05 |
| 20003 | 0.964782 | 0.05 |
| 20004 | 1.17208 | 0.05 |
| 20005 | 0.964769 | 0.05 |
| 20006 | 1.04268 | 2.1 |
| 20007 | 1.57459 | 2.1 |

| | | |
|---|---|---|
| 20008 | 1.57459 | 2.1 |
| 20009 | 1.32449 | 2.1 |
| 20010 | 2.07527 | 0.881338 |
| 20011 | 1.00013 | 0.726088 |
| 20012 | 0.813711 | 0.05 |
| 20013 | 1.05677 | 2.1 |
| 20014 | 1.30664 | 1.3635 |
| 20015 | 1.96011 | 2.1 |
| 20016 | 1.7151 | 1.15242 |
| 20017 | 1.61309 | 1.19473 |
| 20018 | 1.18954 | 0.05 |
| 20019 | 1.05447 | 2.1 |
| 20020 | 1.05446 | 0.241253 |
| 20021 | 1.11787 | 0.187903 |
| 20022 | 1.44437 | 2.1 |
| 20023 | 1.84739 | 2.1 |
| 20024 | 1.84739 | 2.1 |
| 20025 | 1.06203 | 2.1 |
| 20026 | 1.78391 | 2.1 |
| 20027 | 1.89267 | 2.1 |
| 20028 | 1.9212 | 2.1 |
| 20029 | 2.02997 | 2.1 |
| 20030 | 1.32051 | 0.05 |
| 20031 | 1.47169 | 0.05 |
| 20032 | 1.20691 | 0.05 |
| 20033 | 1.72271 | 0.05 |
| 20034 | 1.2858 | 0.255126 |
| 20035 | 1.31011 | 0.772138 |
| 20036 | 1.31011 | 0.522353 |
| 20037 | 1.34124 | 0.742337 |
| 20038 | 1.294 | 0.840329 |
| 20039 | 1.03079 | 0.665037 |
| 20040 | 1.83192 | 2.1 |
| 20041 | 1.81979 | 2.1 |
| 20042 | 1.81979 | 2.1 |
| 20043 | 1.57829 | 2.1 |
| 20044 | 1.35435 | 1.57925 |
| 20045 | 1.19023 | 2.06613 |
| 20046 | 0.991115 | 2.1 |
| 20047 | 1.27725 | 2.1 |
| 20048 | 1.38204 | 2.1 |
| 20049 | 1.48013 | 2.1 |
| 20050 | 0.983493 | 2.1 |
| 20051 | 2.26155 | 2.1 |
| 20052 | 1.02311 | 0.620256 |
| 20053 | 1.55485 | 0.422959 |
| 20054 | 1.71033 | 0.346781 |
| 20055 | 2.07319 | 0.200671 |
| 20056 | 2.29074 | 0.0672974 |
| 20057 | 1.50822 | 0.117159 |
| 20058 | 2.08147 | 2.1 |
| 20059 | 1.1585 | 2.1 |
| 20060 | 3.74092 | 0.335936 |
| 20061 | 2.40799 | 2.1 |
| 20062 | 2.05349 | 2.1 |
| 20063 | 1.92656 | 2.1 |
| 20064 | 1.09634 | 0.05 |
| 20065 | 1.45502 | 0.05 |

| | | |
|---|---|---|
| 20066 | 1.17261 | 0.05 |
| 20067 | 1.29383 | 0.05 |
| 20068 | 1.29383 | 0.05 |
| 20069 | 1.47689 | 0.05 |
| 20070 | 1.85717 | 0.0780051 |
| 20071 | 1.53798 | 0.05 |
| 20072 | 1.9493 | 2.1 |
| 20073 | 2.0371 | 2.0553 |
| 20074 | 1.8519 | 2.1 |
| 20075 | 1.92119 | 1.26149 |
| 20076 | 1.00764 | 0.05 |
| 20077 | 1.0302 | 0.05 |
| 20078 | 1.0302 | 0.05 |
| 20079 | 0.90047 | 0.05 |
| 20080 | 1.49155 | 0.05 |
| 20081 | 2.26403 | 0.05 |
| 20082 | 2.06158 | 0.05 |
| 20083 | 1.09506 | 0.05 |
| 20084 | 1.9212 | 1.364 |
| 20085 | 1.99586 | 1.0121 |
| 20086 | 1.99586 | 1.05663 |
| 20087 | 2.07527 | 0.971546 |
| 20088 | 1.20478 | 2.1 |
| 20089 | 1.09662 | 2.1 |
| 20090 | 1.55961 | 2.1 |
| 20091 | 1.03472 | 2.1 |
| 20092 | 1.54983 | 2.1 |
| 20093 | 2.02953 | 2.1 |
| 20094 | 1.84502 | 2.1 |
| 20095 | 2.1221 | 2.1 |
| 20096 | 1.02969 | 2.08742 |
| 20097 | 2.23523 | 2.1 |
| 20098 | 1.2377 | 0.169219 |
| 20099 | 1.03818 | 0.11 |
| 20100 | 1.978 2.1 | |
| 20101 | 1.78159 | 2.1 |
| 20102 | 1.45762 | 2.1 |
| 20103 | 1.34234 | 2.1 |
| 20104 | 2.01624 | 2.1 |
| 20105 | 2.52072 | 2.1 |
| 20106 | 1.23013 | 2.1 |
| 20107 | 2.5593 | 2.1 |
| 20108 | 3.06464 | 1.30378 |
| 20109 | 2.63697 | 0.845316 |
| 20110 | 2.63697 | 1.06718 |
| 20111 | 1.87111 | 0.717502 |
| 20112 | 1.04574 | 0.05 |
| 20113 | 2.13428 | 0.05 |
| 20114 | 1.61715 | 2.1 |
| 20115 | 1.44137 | 2.1 |
| 20116 | 1.44867 | 2.1 |
| 20117 | 1.18283 | 2.1 |
| 20118 | 1.50584 | 0.05 |
| 20119 | 1.45466 | 0.05 |
| 20120 | 1.4959 | 0.05 |
| 20121 | 1.93489 | 0.05 |
| 20122 | 2.60772 | 2.1 |
| 20123 | 2.68868 | 2.1 |

| | | |
|---|---|---|
| 20124 | 2.68868 | 2.1 |
| 20125 | 2.82561 | 2.1 |
| 20126 | 1.49654 | 2.1 |
| 20127 | 1.38672 | 2.1 |
| 20128 | 1.36813 | 2.1 |
| 20129 | 1.98396 | 2.1 |
| 20130 | 1.93852 | 1.77862 |
| 20131 | 2.14237 | 1.07458 |
| 20132 | 1.44421 | 0.05 |
| 20133 | 1.21749 | 0.05 |
| 20134 | 1.09572 | 0.05 |
| 20135 | 1.11081 | 0.05 |
| 20136 | 2.32363 | 2.1 |
| 20137 | 1.24375 | 2.1 |
| 20138 | 3.79242 | 2.1 |
| 20139 | 3.46787 | 2.1 |
| 20140 | 1.19854 | 1.55529 |
| 20141 | 1.35487 | 1.53738 |
| 20142 | 1.35486 | 1.7577 |
| 20143 | 1.38616 | 1.70798 |
| 20144 | 1.67265 | 0.05 |
| 20145 | 0.909487 | 0.05 |
| 20146 | 2.53562 | 2.1 |
| 20147 | 1.09564 | 2.1 |
| 20148 | 2.9068 | 2.1 |
| 20149 | 3.57307 | 0.128357 |
| 20150 | 2.50369 | 1.46923 |
| 20151 | 1.47348 | 2.1 |
| 20152 | 1.24925 | 2.1 |
| 20153 | 1.3991 | 2.1 |
| 20154 | 1.3991 | 2.1 |
| 20155 | 1.52984 | 2.1 |
| 20156 | 1.60428 | 2.1 |
| 20157 | 1.50097 | 2.1 |
| 20158 | 1.50097 | 2.1 |
| 20159 | 1.40239 | 2.1 |
| 20160 | 2.19621 | 2.1 |
| 20161 | 2.35003 | 2.1 |
| 20162 | 1.57932 | 0.05 |
| 20163 | 1.61194 | 0.05 |
| 20164 | 2.0815 | 0.05 |
| 20165 | 1.18539 | 0.05 |
| 20166 | 1.5795 | 0.975228 |
| 20167 | 1.53064 | 1.16095 |
| 20168 | 1.89227 | 0.05 |
| 20169 | 3.01037 | 0.05 |
| 20170 | 2.08149 | 2.1 |
| 20171 | 2.06678 | 2.1 |
| 20172 | 1.89226 | 0.05 |
| 20173 | 3.47888 | 0.05 |
| 20174 | 1.89226 | 0.05 |
| 20175 | 3.65842 | 0.05 |
| 20176 | 3.88546 | 0.05 |
| 20177 | 1.89226 | 0.05 |
| 20178 | 1.11239 | 2.1 |
| 20179 | 1.29133 | 0.05 |
| 20180 | 0.889309 | 0.05 |
| 20181 | 0.881934 | 0.05 |

| | | |
|---|---|---|
| 20182 | 1.88406 | 2.1 |
| 20183 | 0.636946 | 2.1 |
| 20184 | 0.636945 | 2.08326 |
| 20185 | 1.28073 | 2.1 |
| 20186 | 1.28075 | 2.1 |
| 20187 | 0.942007 | 2.1 |
| 20188 | 1.68436 | 2.1 |
| 20189 | 1.4677 | 0.05 |
| 20190 | 0.851542 | 0.05 |
| 20191 | 1.39063 | 0.05 |
| 20192 | 0.851539 | 0.05 |
| 20193 | 1.11597 | 0.05 |
| 20194 | 1.35403 | 0.05 |
| 20195 | 0.959473 | 2.1 |
| 20196 | 1.07976 | 2.1 |
| 20197 | 0.946129 | 1.62923 |
| 20198 | 0.946129 | 0.05 |
| 20199 | 1.38464 | 0.585665 |
| 20200 | 1.36991 | 0.052018 |
| 20201 | 0.946131 | 0.198008 |
| 20202 | 0.94613 | 1.01599 |
| 20203 | 1.34493 | 0.399529 |
| 20204 | 1.36991 | 0.37023 |
| 20205 | 1.36991 | 0.0726453 |
| 20206 | 0.946131 | 0.36214 |
| 20207 | 0.78392 | 0.898447 |
| 20208 | 0.80282 | 0.869395 |
| 20209 | 1.10351 | 0.891537 |
| 20210 | 0.80846 | 0.966223 |
| 20211 | 0.838026 | 0.880142 |
| 20212 | 1.08558 | 0.888022 |
| 20213 | 0.718203 | 0.950112 |
| 20214 | 0.501866 | 0.759864 |
| 20215 | 0.85426 | 0.972446 |
| 20216 | 1.24249 | 0.05 |
| 20217 | 1.37052 | 0.05 |
| 20218 | 0.851541 | 0.05 |
| 20219 | 1.0092 | 0.05 |
| 20220 | 0.851554 | 0.05 |
| 20221 | 1.49826 | 0.05 |
| 20222 | 1.13727 | 0.05 |
| 20223 | 0.778149 | 0.0547416 |
| 20224 | 1.20257 | 0.094367 |
| 20225 | 0.946133 | 0.05 |
| 20226 | 0.778149 | 0.05 |
| 20227 | 0.77815 | 0.214581 |
| 20228 | 0.946132 | 2.1 |
| 20229 | 1.17602 | 2.1 |
| 20230 | 0.946132 | 2.1 |
| 20231 | 1.31257 | 2.1 |
| 20232 | 1.17602 | 2.1 |
| 20233 | 1.35821 | 0.05 |
| 20234 | 1.19993 | 0.05 |
| 20235 | 0.946131 | 0.05 |
| 20236 | 0.946131 | 0.05 |
| 20237 | 1.08848 | 0.05 |
| 20238 | 1.00509 | 2.1 |
| 20239 | 1.19629 | 2.1 |

| | | |
|---|---|---|
| 20240 | 0.867593 | 2.1 |
| 20241 | 0.757921 | 2.1 |
| 20242 | 0.729116 | 0.05 |
| 20243 | 0.89162 | 0.05 |
| 20244 | 1.16811 | 0.05 |
| 20245 | 1.3676 | 2.1 |
| 20246 | 1.2638 | 0.185829 |
| 20247 | 0.946132 | 2.1 |
| 20248 | 0.946132 | 2.1 |
| 20249 | 1.2638 | 2.1 |
| 20250 | 1.00185 | 2.1 |
| 20251 | 1.60948 | 2.1 |
| 20252 | 0.602075 | 2.1 |
| 20253 | 0.942611 | 2.1 |
| 20254 | 0.946132 | 2.1 |
| 20255 | 0.942611 | 2.1 |
| 20256 | 1.11822 | 2.1 |
| 20257 | 1.21558 | 0.05 |
| 20258 | 0.607788 | 0.05 |
| 20259 | 0.827029 | 0.05 |
| 20260 | 1.00691 | 0.0581402 |
| 20261 | 0.607789 | 0.05 |
| 20262 | 0.607775 | 0.0689493 |
| 20263 | 1.23248 | 0.956951 |
| 20264 | 0.902653 | 1.15513 |
| 20265 | 0.639143 | 1.22326 |
| 20266 | 0.851545 | 0.984365 |
| 20267 | 0.605153 | 0.978769 |
| 20268 | 1.06826 | 2.1 |
| 20269 | 1.29365 | 2.1 |
| 20270 | 1.41232 | 2.1 |
| 20271 | 0.946131 | 2.1 |
| 20272 | 1.20854 | 2.1 |
| 20273 | 0.946131 | 2.1 |
| 20274 | 0.909005 | 0.05 |
| 20275 | 1.16672 | 0.05 |
| 20276 | 0.810959 | 0.05 |
| 20277 | 0.946131 | 0.05 |
| 20278 | 1.40104 | 0.05 |
| 20279 | 0.946131 | 0.05 |
| 20280 | 1.35403 | 0.05 |
| 20281 | 1.40104 | 0.05 |
| 20282 | 1.92535 | 0.05 |
| 20283 | 2.14534 | 0.05 |
| 20284 | 1.77532 | 0.05 |
| 20285 | 0.851541 | 0.05 |
| 20286 | 1.05585 | 0.05 |
| 20287 | 1.27098 | 0.05 |
| 20288 | 0.870925 | 0.05 |
| 20289 | 1.15547 | 0.05 |
| 20290 | 1.47583 | 0.05 |
| 20291 | 1.35007 | 0.05 |
| 20292 | 0.740095 | 0.05 |
| 20293 | 0.644912 | 0.05 |
| 20294 | 0.819489 | 0.05 |
| 20295 | 0.983028 | 0.05 |
| 20296 | 0.861849 | 0.05 |
| 20297 | 1.40511 | 0.05 |

| | | |
|---|---|---|
| 20298 | 0.86182 | 0.05 |
| 20299 | 0.9705 | 0.05 |
| 20300 | 1.40511 | 0.05 |
| 20301 | 1.15539 | 2.1 |
| 20302 | 0.946119 | 2.1 |
| 20303 | 0.736382 | 2.1 |
| 20304 | 0.736384 | 2.1 |
| 20305 | 1.41561 | 2.1 |
| 20306 | 0.785651 | 2.1 |
| 20307 | 1.34479 | 2.1 |
| 20308 | 1.38885 | 2.1 |
| 20309 | 0.94613 | 2.1 |
| 20310 | 1.29461 | 2.1 |
| 20311 | 0.94613 | 2.1 |
| 20312 | 0.776179 | 2.1 |
| 20313 | 0.858611 | 2.1 |
| 20314 | 0.946128 | 2.1 |
| 20315 | 1.04073 | 2.1 |
| 20316 | 1.16399 | 2.1 |
| 20317 | 0.990019 | 2.1 |
| 20318 | 1.15564 | 2.1 |
| 20319 | 1.28956 | 0.05 |
| 20320 | 1.35145 | 0.05 |
| 20321 | 1.09662 | 2.1 |
| 20322 | 1.8155 | 0.05 |
| 20323 | 0.811459 | 0.05 |
| 20324 | 0.731982 | 0.05 |
| 20325 | 0.900554 | 0.05 |
| 20326 | 0.946127 | 0.44499 |
| 20327 | 0.817066 | 0.805927 |
| 20328 | 0.817064 | 0.663971 |
| 20329 | 1.41798 | 0.601495 |
| 20330 | 0.871606 | 0.711613 |
| 20331 | 1.30265 | 0.05 |
| 20332 | 0.849402 | 1.77387 |
| 20333 | 0.868582 | 0.689629 |
| 20334 | 0.909137 | 1.18683 |
| 20335 | 0.807016 | 1.32832 |
| 20336 | 0.83393 | 0.05 |
| 20337 | 0.9034 | 0.229029 |
| 20338 | 1.53515 | 0.175778 |
| 20339 | 1.17665 | 0.05 |
| 20340 | 0.946132 | 0.05 |
| 20341 | 0.946129 | 0.05 |
| 20342 | 1.35977 | 0.05 |
| 20343 | 1.34254 | 0.05 |
| 20344 | 1.14241 | 0.05 |
| 20345 | 0.946132 | 0.05 |
| 20346 | 0.946132 | 0.05 |
| 20347 | 1.27376 | 0.05 |
| 20348 | 1.35978 | 0.05 |
| 20349 | 1.01976 | 0.05 |
| 20350 | 1.23498 | 0.152436 |
| 20351 | 1.35002 | 1.00583 |
| 20352 | 1.11683 | 1.17642 |
| 20353 | 0.88359 | 1.82345 |
| 20354 | 0.751374 | 1.30935 |
| 20355 | 0.791714 | 1.27352 |

| | | |
|---|---|---|
| 20356 | 1.6067 | 2.1 |
| 20357 | 0.923782 | 2.1 |
| 20358 | 1.44689 | 2.1 |
| 20359 | 0.94613 | 2.1 |
| 20360 | 0.946131 | 2.1 |
| 20361 | 1.5261 | 2.1 |
| 20362 | 1.26247 | 2.1 |
| 20363 | 1.80085 | 0.05 |
| 20364 | 1.7693 | 0.05 |
| 20365 | 0.900422 | 0.05 |
| 20366 | 1.31357 | 0.05 |
| 20367 | 0.94613 | 0.05 |
| 20368 | 0.900424 | 0.05 |
| 20369 | 0.900397 | 0.05 |
| 20370 | 1.39448 | 0.05 |
| 20371 | 1.36769 | 0.05 |
| 20372 | 0.713524 | 0.05 |
| 20373 | 0.713554 | 0.05 |
| 20374 | 0.946124 | 0.05 |
| 20375 | 1.24979 | 0.05 |
| 20376 | 1.04765 | 0.05 |
| 20377 | 0.70054 | 0.05 |
| 20378 | 0.776991 | 0.05 |
| 20379 | 0.738905 | 0.05 |
| 20380 | 1.32605 | 0.05 |
| 20381 | 0.784957 | 0.05 |
| 20382 | 0.803955 | 0.05 |
| 20383 | 0.819486 | 0.05 |
| 20384 | 1.79306 | 0.05 |
| 20385 | 1.18275 | 0.05 |
| 20386 | 1.25626 | 0.05 |
| 20387 | 0.936806 | 0.05 |
| 20388 | 0.653124 | 0.05 |
| 20389 | 0.946132 | 0.05 |
| 20390 | 0.653151 | 0.05 |
| 20391 | 1.10748 | 0.05 |
| 20392 | 1.95244 | 2.1 |
| 20393 | 1.40967 | 2.08885 |
| 20394 | 1.27916 | 1.81729 |
| 20395 | 0.946132 | 0.05 |
| 20396 | 0.946132 | 0.320112 |
| 20397 | 1.30555 | 0.460618 |
| 20398 | 1.38651 | 1.47182 |
| 20399 | 0.946132 | 0.142491 |
| 20400 | 1.23571 | 0.05 |
| 20401 | 1.21261 | 0.774645 |
| 20402 | 1.34225 | 0.285222 |
| 20403 | 0.946132 | 1.52856 |
| 20404 | 0.734059 | 0.05 |
| 20405 | 0.734084 | 0.372655 |
| 20406 | 0.78438 | 0.05 |
| 20407 | 1.13748 | 0.05 |
| 20408 | 0.946131 | 0.243097 |
| 20409 | 0.78438 | 0.05 |
| 20410 | 0.784392 | 0.0584492 |
| 20411 | 0.626495 | 2.1 |
| 20412 | 0.694635 | 2.1 |
| 20413 | 0.946125 | 2.1 |

| | | |
|---|---|---|
| 20414 | 0.694632 | 2.1 |
| 20415 | 1.38521 | 2.1 |
| 20416 | 0.795469 | 2.1 |
| 20417 | 0.850877 | 2.1 |
| 20418 | 1.37098 | 2.1 |
| 20419 | 0.946131 | 2.1 |
| 20420 | 0.79547 | 2.1 |
| 20421 | 0.946132 | 0.881522 |
| 20422 | 1.30448 | 1.7132 |
| 20423 | 0.628361 | 0.742261 |
| 20424 | 0.686964 | 0.785595 |
| 20425 | 0.493261 | 0.829568 |
| 20426 | 1.90912 | 2.1 |
| 20427 | 0.946127 | 1.48346 |
| 20428 | 1.36604 | 0.695847 |
| 20429 | 0.671687 | 0.839801 |
| 20430 | 0.946128 | 0.992332 |
| 20431 | 0.671699 | 0.777082 |
| 20432 | 1.04973 | 2.1 |
| 20433 | 0.982858 | 0.05 |
| 20434 | 0.974081 | 0.05 |
| 20435 | 1.69993 | 0.05 |
| 20436 | 1.00763 | 0.534293 |
| 20437 | 0.755566 | 2.1 |
| 20438 | 0.946129 | 2.1 |
| 20439 | 1.04184 | 2.1 |
| 20440 | 1.13509 | 2.1 |
| 20441 | 0.755566 | 2.1 |
| 20442 | 1.114 | 2.1 |
| 20443 | 0.810946 | 2.1 |
| 20444 | 0.786259 | 2.1 |
| 20445 | 1.43455 | 2.1 |
| 20446 | 1.05071 | 2.1 |
| 20447 | 0.746095 | 2.1 |
| 20448 | 0.82737 | 2.1 |
| 20449 | 0.946126 | 2.1 |
| 20450 | 1.04071 | 2.1 |
| 20451 | 1.0852 | 2.1 |
| 20452 | 1.59019 | 0.05 |
| 20453 | 0.621972 | 0.05 |
| 20454 | 1.14475 | 0.05 |
| 20455 | 0.691062 | 0.05 |
| 20456 | 1.20057 | 0.05 |
| 20457 | 0.846868 | 0.05 |
| 20458 | 1.26062 | 0.05 |
| 20459 | 0.795093 | 0.05 |
| 20460 | 1.20056 | 0.05 |
| 20461 | 0.795065 | 0.05 |
| 20462 | 1.18203 | 0.05 |
| 20463 | 1.20057 | 0.05 |
| 20464 | 0.991869 | 0.0699916 |
| 20465 | 0.841088 | 2.1 |
| 20466 | 0.946131 | 2.1 |
| 20467 | 0.869097 | 2.1 |
| 20468 | 0.946131 | 2.1 |
| 20469 | 0.841089 | 2.1 |
| 20470 | 1.77867 | 2.1 |
| 20471 | 1.99296 | 0.05 |

| | | |
|---|---|---|
| 20472 | 1.91703 | 2.1 |
| 20473 | 0.651334 | 0.05 |
| 20474 | 1.31467 | 0.05 |
| 20475 | 0.651332 | 0.05 |
| 20476 | 0.651332 | 0.05 |
| 20477 | 1.03079 | 0.05 |
| 20478 | 0.808519 | 0.05 |
| 20479 | 1.41417 | 0.05 |
| 20480 | 1.03165 | 0.05 |
| 20481 | 1.43577 | 2.1 |
| 20482 | 1.39053 | 2.1 |
| 20483 | 1.07006 | 2.1 |
| 20484 | 0.946132 | 2.1 |
| 20485 | 0.946133 | 2.1 |
| 20486 | 1.07006 | 2.1 |
| 20487 | 1.12717 | 2.1 |
| 20488 | 0.994293 | 2.1 |
| 20489 | 0.946132 | 2.1 |
| 20490 | 1.31912 | 2.1 |
| 20491 | 0.946131 | 2.1 |
| 20492 | 1.40571 | 2.1 |
| 20493 | 1.31912 | 2.1 |
| 20494 | 1.12292 | 0.05 |
| 20495 | 1.07755 | 0.05 |
| 20496 | 0.851539 | 0.05 |
| 20497 | 0.946131 | 0.05 |
| 20498 | 0.929834 | 0.05 |
| 20499 | 1.83919 | 0.05 |
| 20500 | 1.04713 | 0.05 |
| 20501 | 0.929726 | 0.05 |
| 20502 | 1.50985 | 0.05 |
| 20503 | 0.946127 | 0.05 |
| 20504 | 1.0227 | 0.05 |
| 20505 | 1.2582 | 0.05 |
| 20506 | 1.1391 | 0.669843 |
| 20507 | 0.780831 | 0.929734 |
| 20508 | 0.78086 | 0.710321 |
| 20509 | 0.946133 | 0.922439 |
| 20510 | 0.780831 | 0.680529 |
| 20511 | 1.37762 | 0.757506 |
| 20512 | 0.946116 | 2.1 |
| 20513 | 1.22882 | 2.1 |
| 20514 | 1.40015 | 2.1 |
| 20515 | 1.40017 | 2.1 |
| 20516 | 0.946105 | 2.1 |
| 20517 | 0.553567 | 0.05 |
| 20518 | 1.19263 | 0.05 |
| 20519 | 0.946132 | 0.05 |
| 20520 | 0.553594 | 0.05 |
| 20521 | 1.19263 | 0.05 |
| 20522 | 1.18237 | 0.05 |
| 20523 | 0.946133 | 0.05 |
| 20524 | 0.978467 | 0.05 |
| 20525 | 0.56262 | 0.05 |
| 20526 | 0.946132 | 0.05 |
| 20527 | 0.562633 | 0.05 |
| 20528 | 0.900175 | 0.05 |
| 20529 | 1.04627 | 0.623613 |

| | | |
|---|---|---|
| 20530 | 0.549928 | 0.721998 |
| 20531 | 1.17765 | 0.860795 |
| 20532 | 1.11479 | 0.975324 |
| 20533 | 0.398261 | 0.879332 |
| 20534 | 0.927608 | 1.02278 |
| 20535 | 1.38467 | 0.745794 |
| 20536 | 1.04824 | 0.532875 |
| 20537 | 1.33893 | 0.856495 |
| 20538 | 1.55775 | 0.849179 |
| 20539 | 0.816895 | 0.962469 |
| 20540 | 1.96057 | 1.0177 |
| 20541 | 1.35798 | 2.1 |
| 20542 | 1.29262 | 2.1 |
| 20543 | 0.94613 | 2.1 |
| 20544 | 0.94613 | 2.1 |
| 20545 | 1.29261 | 2.1 |
| 20546 | 1.18067 | 2.1 |
| 20547 | 0.946131 | 2.1 |
| 20548 | 0.946132 | 2.1 |
| 20549 | 1.27809 | 2.1 |
| 20550 | 1.35799 | 2.1 |
| 20551 | 1.03933 | 2.1 |
| 20552 | 1.49438 | 2.1 |
| 20553 | 0.946132 | 2.1 |
| 20554 | 1.13537 | 2.1 |
| 20555 | 1.41666 | 2.1 |
| 20556 | 1.74237 | 2.1 |
| 20557 | 0.946132 | 2.1 |
| 20558 | 1.04291 | 0.05 |
| 20559 | 1.63802 | 2.1 |
| 20560 | 1.24714 | 2.09099 |
| 20561 | 0.842013 | 2.1 |
| 20562 | 0.810959 | 2.1 |
| 20563 | 0.767923 | 0.084257 |
| 20564 | 1.19583 | 0.123796 |
| 20565 | 0.767921 | 0.05 |
| 20566 | 0.767921 | 0.0550663 |
| 20567 | 0.961348 | 0.177698 |
| 20568 | 2.08687 | 2.09173 |
| 20569 | 0.739729 | 0.626269 |
| 20570 | 1.04342 | 0.562578 |
| 20571 | 0.739757 | 0.593282 |
| 20572 | 0.946129 | 0.63489 |
| 20573 | 1.04341 | 0.838987 |
| 20574 | 1.38683 | 0.05 |
| 20575 | 1.34153 | 0.0697287 |
| 20576 | 0.946129 | 0.05 |
| 20577 | 0.946131 | 0.05 |
| 20578 | 1.02028 | 0.05 |
| 20579 | 1.47606 | 0.05 |
| 20580 | 1.20739 | 0.05 |
| 20581 | 0.957684 | 0.05 |
| 20582 | 0.946116 | 0.0675382 |
| 20583 | 0.851528 | 0.0564889 |
| 20584 | 0.957685 | 0.0655502 |
| 20585 | 1.04504 | 2.1 |
| 20586 | 0.902938 | 2.1 |
| 20587 | 0.946132 | 2.1 |

| | | |
|---|---|---|
| 20588 | 1.07566 | 2.1 |
| 20589 | 1.4558 | 2.1 |
| 20590 | 1.93286 | 2.1 |
| 20591 | 1.27241 | 2.1 |
| 20592 | 0.828671 | 2.1 |
| 20593 | 0.810933 | 2.1 |
| 20594 | 1.31335 | 0.05 |
| 20595 | 1.40305 | 0.05 |
| 20596 | 0.912698 | 0.05 |
| 20597 | 0.810946 | 0.05 |
| 20598 | 1.11608 | 0.05 |
| 20599 | 1.26409 | 0.156525 |
| 20600 | 0.946132 | 0.207007 |
| 20601 | 1.27376 | 0.306659 |
| 20602 | 0.946132 | 0.384866 |
| 20603 | 1.03333 | 0.464426 |
| 20604 | 1.27376 | 0.433936 |
| 20605 | 0.94613 | 0.05 |
| 20606 | 0.812384 | 0.05 |
| 20607 | 0.687744 | 0.05 |
| 20608 | 0.946132 | 0.05 |
| 20609 | 0.687758 | 0.05 |
| 20610 | 1.09667 | 0.05 |
| 20611 | 1.56977 | 2.1 |
| 20612 | 0.851544 | 2.1 |
| 20613 | 1.01136 | 2.1 |
| 20614 | 0.851481 | 2.1 |
| 20615 | 0.946131 | 2.1 |
| 20616 | 0.705409 | 2.1 |
| 20617 | 1.32449 | 0.05 |
| 20618 | 1.13508 | 0.05 |
| 20619 | 0.94613 | 0.05 |
| 20620 | 0.94613 | 0.05 |
| 20621 | 0.965822 | 0.05 |
| 20622 | 1.95301 | 0.05 |
| 20623 | 0.851538 | 0.0531719 |
| 20624 | 1.29262 | 0.05 |
| 20625 | 0.95417 | 0.05 |
| 20626 | 0.851541 | 0.0694254 |
| 20627 | 0.94613 | 0.0604877 |
| 20628 | 1.02727 | 0.240448 |
| 20629 | 1.37675 | 1.5394 |
| 20630 | 0.955646 | 0.374907 |
| 20631 | 0.95114 | 0.740744 |
| 20632 | 0.851528 | 0.836225 |
| 20633 | 1.76513 | 1.28244 |
| 20634 | 0.87155 | 2.1 |
| 20635 | 0.94613 | 2.1 |
| 20636 | 0.871519 | 2.1 |
| 20637 | 0.966631 | 2.1 |
| 20638 | 1.37791 | 2.1 |
| 20639 | 1.19122 | 0.05 |
| 20640 | 0.818971 | 0.349158 |
| 20641 | 0.628799 | 0.05 |
| 20642 | 0.861027 | 0.0890883 |
| 20643 | 0.800034 | 0.05 |
| 20644 | 0.91846 | 0.05 |
| 20645 | 0.765645 | 0.05 |

| | | |
|---|---|---|
| 20646 | 1.13709 | 0.381504 |
| 20647 | 1.29054 | 0.810681 |
| 20648 | 0.755336 | 0.978401 |
| 20649 | 0.755334 | 0.677408 |
| 20650 | 0.946133 | 1.03414 |
| 20651 | 0.755336 | 0.336615 |
| 20652 | 1.14444 | 0.845949 |
| 20653 | 0.872823 | 0.931075 |
| 20654 | 0.533645 | 0.645926 |
| 20655 | 0.946132 | 0.804259 |
| 20656 | 0.533645 | 0.108064 |
| 20657 | 1.02311 | 0.461323 |
| 20658 | 1.00278 | 2.1 |
| 20659 | 1.1016 | 0.05 |
| 20660 | 0.629615 | 0.05 |
| 20661 | 1.12628 | 0.05 |
| 20662 | 0.946131 | 0.05 |
| 20663 | 0.629615 | 0.05 |
| 20664 | 0.629641 | 0.05 |
| 20665 | 1.67046 | 1.87485 |
| 20666 | 1.81667 | 2.1 |
| 20667 | 1.7145 | 2.1 |
| 20668 | 0.94613 | 2.1 |
| 20669 | 1.29322 | 2.1 |
| 20670 | 1.00365 | 2.1 |
| 20671 | 0.946131 | 2.1 |
| 20672 | 1.29322 | 2.1 |
| 20673 | 1.27581 | 2.1 |
| 20674 | 1.33916 | 2.1 |
| 20675 | 1.32784 | 2.1 |
| 20676 | 1.74787 | 2.1 |
| 20677 | 2.28084 | 2.1 |
| 20678 | 2.98092 | 2.1 |
| 20679 | 0.946132 | 0.778047 |
| 20680 | 1.2435 | 2.1 |
| 20681 | 0.946132 | 2.1 |
| 20682 | 1.4099 | 1.39363 |
| 20683 | 1.2435 | 2.1 |
| 20684 | 1.26438 | 2.1 |
| 20685 | 0.745287 | 2.1 |
| 20686 | 0.696827 | 2.1 |
| 20687 | 0.696781 | 2.1 |
| 20688 | 1.41195 | 2.1 |
| 20689 | 0.946131 | 2.1 |
| 20690 | 1.8758 | 1.06159 |
| 20691 | 0.832979 | 0.348043 |
| 20692 | 0.75054 | 0.05 |
| 20693 | 0.810958 | 0.0789644 |
| 20694 | 0.922889 | 0.65434 |
| 20695 | 0.694844 | 0.0633828 |
| 20696 | 0.694814 | 0.250516 |
| 20697 | 0.946131 | 0.0884307 |
| 20698 | 0.694844 | 0.153921 |
| 20699 | 1.02369 | 2.1 |
| 20700 | 1.4468 | 0.05 |
| 20701 | 0.946125 | 0.05 |
| 20702 | 0.93487 | 0.05 |
| 20703 | 1.23608 | 0.063323 |

| | | |
|---|---|---|
| 20704 | 1.34257 | 0.05 |
| 20705 | 0.851525 | 0.05 |
| 20706 | 1.03298 | 0.05 |
| 20707 | 1.22418 | 1.0544 |
| 20708 | 0.946131 | 2.1 |
| 20709 | 1.41833 | 1.13254 |
| 20710 | 1.23452 | 0.918497 |
| 20711 | 0.946131 | 0.419122 |
| 20712 | 1.41833 | 0.840593 |
| 20713 | 1.13077 | 0.05 |
| 20714 | 0.942874 | 2.1 |
| 20715 | 0.946132 | 1.61112 |
| 20716 | 0.504366 | 2.05922 |
| 20717 | 0.593647 | 2.1 |
| 20718 | 0.898829 | 2.0902 |
| 20719 | 1.27921 | 2.1 |
| 20720 | 1.12503 | 2.1 |
| 20721 | 1.22539 | 2.1 |
| 20722 | 1.10332 | 1.00205 |
| 20723 | 0.851541 | 2.1 |
| 20724 | 0.94613 | 2.1 |
| 20725 | 0.945109 | 2.1 |
| 20726 | 0.607962 | 2.1 |
| 20727 | 0.946133 | 2.1 |
| 20728 | 0.607961 | 2.1 |
| 20729 | 0.661956 | 2.1 |
| 20730 | 1.35048 | 2.1 |
| 20731 | 1.66018 | 2.1 |
| 20732 | 0.956467 | 2.1 |
| 20733 | 0.851542 | 2.1 |
| 20734 | 0.890734 | 2.1 |
| 20735 | 0.802455 | 2.1 |
| 20736 | 1.35943 | 2.1 |
| 20737 | 1.04798 | 2.1 |
| 20738 | 1.26426 | 0.05 |
| 20739 | 1.3689 | 0.05 |
| 20740 | 0.946133 | 0.05 |
| 20741 | 0.946133 | 0.05 |
| 20742 | 1.09729 | 0.05 |
| 20743 | 0.765259 | 2.1 |
| 20744 | 0.810945 | 2.1 |
| 20745 | 0.810957 | 2.1 |
| 20746 | 1.10284 | 2.09608 |
| 20747 | 1.09368 | 2.1 |
| 20748 | 0.772601 | 2.1 |
| 20749 | 0.788011 | 2.1 |
| 20750 | 0.810934 | 2.1 |
| 20751 | 0.694357 | 0.94427 |
| 20752 | 1.22641 | 1.021 |
| 20753 | 0.946132 | 2.03558 |
| 20754 | 0.946132 | 0.980633 |
| 20755 | 0.946133 | 0.843045 |
| 20756 | 1.42754 | 1.78155 |
| 20757 | 0.776414 | 0.765636 |
| 20758 | 0.806525 | 1.59774 |
| 20759 | 0.575339 | 0.848768 |
| 20760 | 0.97154 | 0.729858 |
| 20761 | 0.8217 | 1.07987 |

| | | |
|---|---|---|
| 20762 | 0.838039 | 1.63977 |
| 20763 | 0.94613 | 0.450291 |
| 20764 | 0.946132 | 0.104162 |
| 20765 | 1.39575 | 0.05 |
| 20766 | 1.39575 | 0.0560438 |
| 20767 | 1.12818 | 0.05 |
| 20768 | 0.946132 | 1.16228 |
| 20769 | 0.946133 | 0.260985 |
| 20770 | 1.06879 | 0.05 |
| 20771 | 1.06879 | 0.141656 |
| 20772 | 0.970358 | 0.0763896 |
| 20773 | 1.2474 | 0.05 |
| 20774 | 1.01919 | 0.05 |
| 20775 | 0.94613 | 0.05 |
| 20776 | 1.28952 | 0.05 |
| 20777 | 1.00809 | 0.05 |
| 20778 | 0.849344 | 0.05 |
| 20779 | 0.640304 | 0.05 |
| 20780 | 1.35065 | 0.05 |
| 20781 | 0.946132 | 0.05 |
| 20782 | 0.640333 | 0.05 |
| 20783 | 0.946132 | 0.05 |
| 20784 | 0.685489 | 2.1 |
| 20785 | 0.94613 | 2.1 |
| 20786 | 0.68546 | 2.1 |
| 20787 | 0.874264 | 2.1 |
| 20788 | 1.27676 | 2.1 |
| 20789 | 0.946129 | 0.0873996 |
| 20790 | 1.09024 | 0.05 |
| 20791 | 0.714563 | 0.05 |
| 20792 | 1.12335 | 0.05 |
| 20793 | 1.07931 | 0.05 |
| 20794 | 1.17799 | 0.05 |
| 20795 | 1.8795 | 0.05 |
| 20796 | 2.15229 | 0.05 |
| 20797 | 1.13859 | 0.05 |
| 20798 | 0.946132 | 0.54434 |
| 20799 | 1.11374 | 0.05 |
| 20800 | 1.1386 | 0.05 |
| 20801 | 0.615713 | 0.0528295 |
| 20802 | 1.19322 | 0.05 |
| 20803 | 1.3377 | 0.05 |
| 20804 | 0.670801 | 0.05 |
| 20805 | 0.94613 | 0.172643 |
| 20806 | 0.66617 | 0.05 |
| 20807 | 1.36173 | 0.05 |
| 20808 | 0.980473 | 0.05 |
| 20809 | 1.39132 | 0.05 |
| 20810 | 0.864653 | 0.05 |
| 20811 | 1.23837 | 0.05 |
| 20812 | 0.810959 | 0.05 |
| 20813 | 1.1483 | 0.05 |
| 20814 | 1.00381 | 1.83072 |
| 20815 | 0.946128 | 1.86849 |
| 20816 | 1.39696 | 1.22989 |
| 20817 | 0.946131 | 1.08351 |
| 20818 | 0.946131 | 1.18383 |
| 20819 | 1.38613 | 1.24978 |

| | | |
|---|---|---|
| 20820 | 0.973501 | 1.89154 |
| 20821 | 0.946127 | 1.08055 |
| 20822 | 1.35968 | 1.11982 |
| 20823 | 0.946131 | 0.87894 |
| 20824 | 0.946129 | 1.30322 |
| 20825 | 1.39696 | 1.22124 |
| 20826 | 1.12088 | 2.1 |
| 20827 | 1.41122 | 2.1 |
| 20828 | 1.14464 | 0.155721 |
| 20829 | 0.946131 | 2.1 |
| 20830 | 0.946131 | 2.06586 |
| 20831 | 1.14464 | 2.1 |
| 20832 | 1.33266 | 1.93707 |
| 20833 | 1.07341 | 0.967645 |
| 20834 | 0.827287 | 1.91786 |
| 20835 | 0.946132 | 1.60538 |
| 20836 | 0.827287 | 2.1 |
| 20837 | 1.15644 | 1.37683 |
| 20838 | 1.27558 | 0.238679 |
| 20839 | 1.15067 | 0.05 |
| 20840 | 0.946131 | 2.1 |
| 20841 | 1.29143 | 0.05 |
| 20842 | 1.36281 | 2.1 |
| 20843 | 1.36281 | 0.156153 |
| 20844 | 0.946131 | 0.139774 |
| 20845 | 1.40863 | 2.1 |
| 20846 | 1.20143 | 0.05 |
| 20847 | 1.19755 | 0.05 |
| 20848 | 0.566153 | 0.05 |
| 20849 | 0.946131 | 0.05 |
| 20850 | 1.07979 | 0.05 |
| 20851 | 1.00463 | 0.05 |
| 20852 | 0.566153 | 0.05 |
| 20853 | 0.768212 | 2.1 |
| 20854 | 1.32461 | 2.1 |
| 20855 | 1.13268 | 2.1 |
| 20856 | 1.13268 | 2.1 |
| 20857 | 0.946133 | 2.1 |
| 20858 | 1.53642 | 2.1 |
| 20859 | 1.5791 | 2.1 |
| 20860 | 0.726635 | 2.1 |
| 20861 | 1.2334 | 2.1 |
| 20862 | 1.29198 | 2.1 |
| 20863 | 1.29198 | 1.15792 |
| 20864 | 0.946131 | 0.938196 |
| 20865 | 0.956585 | 2.1 |
| 20866 | 1.25294 | 2.1 |
| 20867 | 0.946133 | 2.1 |
| 20868 | 1.35287 | 2.1 |
| 20869 | 1.23877 | 2.1 |
| 20870 | 0.946131 | 2.1 |
| 20871 | 1.35287 | 2.1 |
| 20872 | 1.01843 | 2.1 |
| 20873 | 3.77569 | 2.1 |
| 20874 | 0.86707 | 2.1 |
| 20875 | 0.867066 | 2.1 |
| 20876 | 1.38639 | 2.1 |
| 20877 | 1.38639 | 2.1 |

| | | |
|---|---|---|
| 20878 | 0.896168 | 2.1 |
| 20879 | 0.974716 | 0.05 |
| 20880 | 1.09411 | 0.05 |
| 20881 | 0.663171 | 0.05 |
| 20882 | 0.663171 | 0.05 |
| 20883 | 0.946132 | 0.05 |
| 20884 | 0.66317 | 0.05 |
| 20885 | 1.47104 | 0.05 |
| 20886 | 1.13629 | 0.05 |
| 20887 | 0.772342 | 0.05 |
| 20888 | 0.772312 | 0.05 |
| 20889 | 0.94613 | 0.05 |
| 20890 | 0.77234 | 0.05 |
| 20891 | 1.43154 | 2.1 |
| 20892 | 1.37704 | 2.1 |
| 20893 | 1.40025 | 2.1 |
| 20894 | 1.3063 | 2.1 |
| 20895 | 0.946132 | 2.1 |
| 20896 | 1.04075 | 2.1 |
| 20897 | 0.946131 | 2.1 |
| 20898 | 0.946118 | 2.1 |
| 20899 | 0.750011 | 0.05 |
| 20900 | 1.0971 | 0.0902491 |
| 20901 | 0.889667 | 0.05 |
| 20902 | 0.709598 | 0.267419 |
| 20903 | 2.39737 | 0.102074 |
| 20904 | 1.19974 | 0.05 |
| 20905 | 0.94613 | 0.05 |
| 20906 | 1.25809 | 0.05 |
| 20907 | 1.19974 | 0.05 |
| 20908 | 0.73005 | 0.05 |
| 20909 | 1.50764 | 0.05 |
| 20910 | 1.25809 | 0.05 |
| 20911 | 0.946133 | 0.05 |
| 20912 | 1.10357 | 0.05 |
| 20913 | 1.25809 | 0.05 |
| 20914 | 0.628177 | 0.05 |
| 20915 | 1.3543 | 0.05 |
| 20916 | 0.793711 | 0.05 |
| 20917 | 1.18455 | 0.05 |
| 20918 | 1.03966 | 0.05 |
| 20919 | 0.946131 | 0.05 |
| 20920 | 0.767453 | 0.05 |
| 20921 | 0.612292 | 0.05 |
| 20922 | 0.946131 | 0.05 |
| 20923 | 1.24128 | 0.05 |
| 20924 | 1.08761 | 0.05 |
| 20925 | 0.762059 | 0.05 |
| 20926 | 1.005 2.1 | |
| 20927 | 1.17054 | 2.1 |
| 20928 | 1.12665 | 2.1 |
| 20929 | 0.750368 | 2.1 |
| 20930 | 0.851542 | 2.1 |
| 20931 | 0.975436 | 2.1 |
| 20932 | 0.975442 | 2.1 |
| 20933 | 0.675367 | 2.1 |
| 20934 | 1.51973 | 0.05 |
| 20935 | 0.94613 | 0.05 |

| | | |
|---|---|---|
| 20936 | 1.38954 | 0.05 |
| 20937 | 0.946131 | 0.05 |
| 20938 | 1.07912 | 0.05 |
| 20939 | 1.38954 | 0.05 |
| 20940 | 2.0219 | 2.1 |
| 20941 | 1.18347 | 0.05 |
| 20942 | 0.946132 | 0.195997 |
| 20943 | 0.94613 | 0.05 |
| 20944 | 1.00202 | 0.05 |
| 20945 | 1.00201 | 0.05 |
| 20946 | 0.946132 | 0.05 |
| 20947 | 0.946132 | 0.456281 |
| 20948 | 1.37981 | 0.0634322 |
| 20949 | 1.26874 | 0.05 |
| 20950 | 1.26874 | 0.05 |
| 20951 | 0.946132 | 0.05 |
| 20952 | 2.8687 | 0.05 |
| 20953 | 1.42884 | 2.1 |
| 20954 | 1.05188 | 2.1 |
| 20955 | 0.625307 | 2.1 |
| 20956 | 0.810956 | 2.1 |
| 20957 | 0.526656 | 2.1 |
| 20958 | 0.526684 | 2.1 |
| 20959 | 0.724122 | 2.1 |
| 20960 | 0.946132 | 2.1 |
| 20961 | 0.526684 | 2.1 |
| 20962 | 1.05337 | 2.1 |
| 20963 | 1.0593 | 2.1 |
| 20964 | 1.47981 | 2.1 |
| 20965 | 1.74431 | 2.08801 |
| 20966 | 0.885612 | 2.1 |
| 20967 | 0.810946 | 0.923001 |
| 20968 | 0.845185 | 1.65084 |
| 20969 | 1.38622 | 0.520073 |
| 20970 | 0.723194 | 0.261718 |
| 20971 | 0.554302 | 0.495964 |
| 20972 | 0.709597 | 0.252137 |
| 20973 | 0.909507 | 0.348979 |
| 20974 | 0.912754 | 0.207868 |
| 20975 | 1.15174 | 0.05 |
| 20976 | 0.946131 | 0.351047 |
| 20977 | 0.912753 | 0.177623 |
| 20978 | 1.15174 | 0.126362 |
| 20979 | 1.02011 | 0.05 |
| 20980 | 0.792754 | 2.04496 |
| 20981 | 0.946129 | 2.1 |
| 20982 | 0.792754 | 1.9282 |
| 20983 | 1.27575 | 1.7764 |
| 20984 | 0.94613 | 1.79495 |
| 20985 | 1.60132 | 2.1 |
| 20986 | 0.922455 | 2.1 |
| 20987 | 1.18713 | 1.45467 |
| 20988 | 0.926957 | 1.70896 |
| 20989 | 1.66525 | 1.85637 |
| 20990 | 0.946132 | 2.1 |
| 20991 | 0.851541 | 2.1 |
| 20992 | 0.961191 | 2.1 |
| 20993 | 1.27534 | 2.1 |

| | | |
|---|---|---|
| 20994 | 0.851554 | 2.1 |
| 20995 | 1.0499 | 2.1 |
| 20996 | 1.01776 | 0.05 |
| 20997 | 0.97622 | 0.05 |
| 20998 | 0.810934 | 0.05 |
| 20999 | 2.6802 | 0.05 |
| 21000 | 1.31485 | 0.05 |
| 21001 | 1.09699 | 0.05 |
| 21002 | 0.681649 | 0.05 |
| 21003 | 0.790369 | 0.05 |
| 21004 | 0.946131 | 0.05 |
| 21005 | 0.838026 | 1.14727 |
| 21006 | 0.567833 | 0.05 |
| 21007 | 0.891492 | 0.498339 |
| 21008 | 0.986984 | 0.05 |
| 21009 | 0.946132 | 0.592372 |
| 21010 | 1.19028 | 0.05 |
| 21011 | 0.986984 | 0.05 |
| 21012 | 0.595623 | 0.0561522 |
| 21013 | 0.946131 | 2.1 |
| 21014 | 1.36502 | 2.1 |
| 21015 | 1.08109 | 2.1 |
| 21016 | 0.946129 | 2.1 |
| 21017 | 1.36503 | 2.1 |
| 21018 | 0.773897 | 2.1 |
| 21019 | 0.946133 | 1.00532 |
| 21020 | 0.613447 | 2.1 |
| 21021 | 0.613449 | 2.1 |
| 21022 | 0.946131 | 2.1 |
| 21023 | 1.11163 | 2.1 |
| 21024 | 0.966114 | 2.1 |
| 21025 | 0.735699 | 2.1 |
| 21026 | 0.662147 | 2.1 |
| 21027 | 1.04072 | 2.1 |
| 21028 | 0.946131 | 2.1 |
| 21029 | 0.862978 | 2.1 |
| 21030 | 1.48839 | 0.905484 |
| 21031 | 0.936141 | 0.05 |
| 21032 | 0.946131 | 1.05939 |
| 21033 | 0.936142 | 2.1 |
| 21034 | 1.40595 | 2.1 |
| 21035 | 0.946131 | 2.04961 |
| 21036 | 1.05297 | 0.05 |
| 21037 | 3.09035 | 2.1 |
| 21038 | 1.19952 | 2.1 |
| 21039 | 0.907198 | 2.1 |
| 21040 | 0.849144 | 2.1 |
| 21041 | 0.612469 | 0.107826 |
| 21042 | 0.551237 | 0.50917 |
| 21043 | 0.985256 | 0.684897 |
| 21044 | 0.551209 | 0.629309 |
| 21045 | 0.85153 | 0.845331 |
| 21046 | 0.624712 | 2.1 |
| 21047 | 0.900018 | 2.1 |
| 21048 | 0.851542 | 2.1 |
| 21049 | 0.864787 | 2.1 |
| 21050 | 1.27669 | 2.1 |
| 21051 | 0.946711 | 0.803202 |

| | | |
|---|---|---|
| 21052 | 0.810947 | 0.868162 |
| 21053 | 0.841547 | 0.903937 |
| 21054 | 0.764114 | 0.845068 |
| 21055 | 1.26692 | 1.6103 |
| 21056 | 0.946132 | 0.911748 |
| 21057 | 1.37675 | 1.18365 |
| 21058 | 1.26692 | 0.9308 |
| 21059 | 1.37545 | 1.36089 |
| 21060 | 1.49637 | 0.05 |
| 21061 | 0.709598 | 0.05 |
| 21062 | 0.751252 | 0.05 |
| 21063 | 1.36811 | 0.05 |
| 21064 | 0.635625 | 0.05 |
| 21065 | 1.31713 | 0.05 |
| 21066 | 1.18977 | 2.1 |
| 21067 | 0.893377 | 1.63785 |
| 21068 | 0.707344 | 2.1 |
| 21069 | 0.946131 | 2.1 |
| 21070 | 1.20221 | 2.1 |
| 21071 | 1.20221 | 2.1 |
| 21072 | 0.707345 | 2.1 |
| 21073 | 1.26786 | 2.1 |
| 21074 | 0.988048 | 2.1 |
| 21075 | 0.751293 | 2.1 |
| 21076 | 0.632008 | 2.1 |
| 21077 | 1.51067 | 2.1 |
| 21078 | 1.13534 | 2.1 |
| 21079 | 0.946133 | 2.1 |
| 21080 | 0.946131 | 2.1 |
| 21081 | 1.25716 | 2.1 |
| 21082 | 1.3199 | 2.1 |
| 21083 | 1.09616 | 2.1 |
| 21084 | 1.36677 | 1.29637 |
| 21085 | 1.34752 | 1.97297 |
| 21086 | 0.838041 | 1.06787 |
| 21087 | 1.67023 | 2.1 |
| 21088 | 1.02247 | 0.133759 |
| 21089 | 1.03079 | 0.155032 |
| 21090 | 0.928982 | 0.05 |
| 21091 | 1.13635 | 0.05 |
| 21092 | 0.946132 | 0.05 |
| 21093 | 0.92898 | 0.05 |
| 21094 | 1.13635 | 0.05 |
| 21095 | 1.1643 | 0.05 |
| 21096 | 0.851541 | 0.05 |
| 21097 | 1.39939 | 0.05 |
| 21098 | 0.946129 | 0.05 |
| 21099 | 1.07775 | 0.05 |
| 21100 | 1.14503 | 0.05 |
| 21101 | 1.78573 | 0.05 |
| 21102 | 1.29827 | 0.05 |
| 21103 | 0.872373 | 0.05 |
| 21104 | 1.01162 | 0.05 |
| 21105 | 0.83804 | 0.05 |
| 21106 | 1.48851 | 0.05 |
| 21107 | 1.47385 | 0.05 |
| 21108 | 0.843858 | 0.05 |
| 21109 | 0.938626 | 0.05 |

| | | |
|---|---|---|
| 21110 | 0.89567 | 0.05 |
| 21111 | 1.59457 | 0.690633 |
| 21112 | 2.09666 | 0.05 |
| 21113 | 0.775132 | 0.05 |
| 21114 | 0.927005 | 0.05 |
| 21115 | 0.854224 | 0.05 |
| 21116 | 0.775142 | 0.05 |
| 21117 | 0.946106 | 0.05 |
| 21118 | 1.80784 | 2.1 |
| 21119 | 2.04593 | 2.1 |
| 21120 | 1.32282 | 2.1 |
| 21121 | 0.952138 | 2.1 |
| 21122 | 0.946132 | 2.1 |
| 21123 | 0.946132 | 2.1 |
| 21124 | 0.952138 | 2.1 |
| 21125 | 1.46988 | 2.1 |
| 21126 | 0.80465 | 0.05 |
| 21127 | 1.14155 | 0.05 |
| 21128 | 0.810931 | 0.05 |
| 21129 | 1.66203 | 0.275007 |
| 21130 | 0.674605 | 0.121752 |
| 21131 | 0.946132 | 0.35005 |
| 21132 | 1.26706 | 0.199513 |
| 21133 | 0.674633 | 0.0799043 |
| 21134 | 0.946132 | 0.600971 |
| 21135 | 0.980664 | 0.350326 |
| 21136 | 1.34927 | 0.182567 |
| 21137 | 0.758454 | 0.05 |
| 21138 | 0.946133 | 0.05 |
| 21139 | 0.985371 | 0.05 |
| 21140 | 1.28632 | 0.05 |
| 21141 | 0.946133 | 0.0792084 |
| 21142 | 1.46868 | 0.05 |
| 21143 | 1.7522 | 0.05 |
| 21144 | 0.737431 | 2.1 |
| 21145 | 0.663679 | 2.1 |
| 21146 | 1.16541 | 1.79777 |
| 21147 | 1.3179 | 2.05083 |
| 21148 | 1.04081 | 2.06269 |
| 21149 | 0.887866 | 1.74604 |
| 21150 | 0.806961 | 0.454006 |
| 21151 | 0.929061 | 1.52675 |
| 21152 | 1.28418 | 0.05 |
| 21153 | 1.38752 | 0.05 |
| 21154 | 1.33038 | 0.0754718 |
| 21155 | 0.946132 | 0.05 |
| 21156 | 0.946132 | 0.05 |
| 21157 | 1.14897 | 0.05 |
| 21158 | 1.1685 | 0.05 |
| 21159 | 1.18774 | 2.1 |
| 21160 | 0.961899 | 2.1 |
| 21161 | 1.48841 | 2.1 |
| 21162 | 1.79105 | 2.1 |
| 21163 | 3.51614 | 2.1 |
| 21164 | 0.682342 | 2.1 |
| 21165 | 0.783184 | 2.1 |
| 21166 | 0.946132 | 2.1 |
| 21167 | 0.868396 | 2.1 |

| | | |
|---|---|---|
| 21168 | 1.21813 | 2.1 |
| 21169 | 0.848305 | 0.482403 |
| 21170 | 0.94559 | 0.720322 |
| 21171 | 0.805691 | 0.824143 |
| 21172 | 0.752217 | 2.1 |
| 21173 | 0.946132 | 2.1 |
| 21174 | 0.839123 | 2.1 |
| 21175 | 0.946131 | 2.1 |
| 21176 | 0.752187 | 2.1 |
| 21177 | 1.96491 | 2.1 |
| 21178 | 0.520997 | 2.1 |
| 21179 | 0.946132 | 2.1 |
| 21180 | 0.73446 | 2.1 |
| 21181 | 0.946133 | 2.1 |
| 21182 | 0.520995 | 2.1 |
| 21183 | 1.88522 | 2.1 |
| 21184 | 1.8361 | 2.1 |
| 21185 | 1.11285 | 2.1 |
| 21186 | 1.25334 | 1.21452 |
| 21187 | 2.00756 | 2.1 |
| 21188 | 1.44252 | 0.05 |
| 21189 | 1.00255 | 0.988445 |
| 21190 | 1.64516 | 2.1 |
| 21191 | 1.12399 | 0.05 |
| 21192 | 1.13542 | 0.05 |
| 21193 | 1.09038 | 0.05 |
| 21194 | 0.746903 | 0.05 |
| 21195 | 0.672241 | 0.05 |
| 21196 | 0.871774 | 0.05 |
| 21197 | 1.73513 | 1.12237 |
| 21198 | 0.949995 | 2.1 |
| 21199 | 1.17244 | 2.1 |
| 21200 | 0.946127 | 2.1 |
| 21201 | 1.3028 | 2.1 |
| 21202 | 1.17244 | 2.1 |
| 21203 | 0.774101 | 2.1 |
| 21204 | 0.946127 | 2.1 |
| 21205 | 0.939289 | 2.1 |
| 21206 | 0.946129 | 2.1 |
| 21207 | 0.774101 | 2.1 |
| 21208 | 1.87858 | 2.1 |
| 21209 | 1.44293 | 0.05 |
| 21210 | 1.43213 | 0.05 |
| 21211 | 0.946131 | 2.1 |
| 21212 | 0.716614 | 2.1 |
| 21213 | 0.716644 | 2.1 |
| 21214 | 1.35828 | 2.1 |
| 21215 | 0.790935 | 2.1 |
| 21216 | 0.730157 | 2.1 |
| 21217 | 1.41149 | 2.1 |
| 21218 | 0.730186 | 2.1 |
| 21219 | 0.730186 | 2.1 |
| 21220 | 0.946131 | 2.1 |
| 21221 | 0.946132 | 1.19751 |
| 21222 | 0.946132 | 0.427926 |
| 21223 | 0.990713 | 0.05 |
| 21224 | 1.40779 | 0.121877 |
| 21225 | 0.946132 | 0.10431 |

| | | |
|---|---|---|
| 21226 | 1.06486 | 0.05 |
| 21227 | 0.946131 | 0.959293 |
| 21228 | 0.946133 | 0.183766 |
| 21229 | 1.40779 | 0.05 |
| 21230 | 1.26874 | 0.105628 |
| 21231 | 0.946131 | 0.0990873 |
| 21232 | 1.02588 | 0.560562 |
| 21233 | 0.826073 | 0.0850701 |
| 21234 | 1.41233 | 0.0785927 |
| 21235 | 0.861269 | 0.075858 |
| 21236 | 1.04073 | 0.203358 |
| 21237 | 1.27111 | 0.275817 |
| 21238 | 1.56016 | 1.78668 |
| 21239 | 1.60866 | 0.05 |
| 21240 | 0.94613 | 0.05 |
| 21241 | 0.946129 | 0.05 |
| 21242 | 1.36376 | 0.05 |
| 21243 | 1.22187 | 0.05 |
| 21244 | 0.94613 | 0.05 |
| 21245 | 1.82581 | 1.32756 |
| 21246 | 1.19705 | 1.14496 |
| 21247 | 1.7513 | 2.1 |
| 21248 | 1.85017 | 2.1 |
| 21249 | 1.72116 | 2.1 |
| 21250 | 1.12058 | 2.1 |
| 21251 | 0.899991 | 2.1 |
| 21252 | 0.616288 | 2.1 |
| 21253 | 1.07289 | 2.1 |
| 21254 | 1.7092 | 0.523258 |
| 21255 | 0.946132 | 0.463535 |
| 21256 | 0.946133 | 0.05 |
| 21257 | 1.18717 | 0.05 |
| 21258 | 1.40029 | 0.190402 |
| 21259 | 0.946132 | 0.05 |
| 21260 | 0.94613 | 0.271469 |
| 21261 | 1.18547 | 0.195499 |
| 21262 | 0.960326 | 0.205856 |
| 21263 | 0.960345 | 0.179381 |
| 21264 | 0.942557 | 0.229168 |
| 21265 | 1.18601 | 0.05 |
| 21266 | 0.946132 | 0.09808 |
| 21267 | 1.30307 | 0.078042 |
| 21268 | 1.08169 | 0.054837 |
| 21269 | 1.08169 | 0.069493 |
| 21270 | 1.31716 | 0.0807092 |
| 21271 | 1.51065 | 0.531686 |
| 21272 | 0.877642 | 0.05 |
| 21273 | 0.617384 | 0.05 |
| 21274 | 0.575741 | 0.05 |
| 21275 | 0.972448 | 0.05 |
| 21276 | 1.11334 | 0.05 |
| 21277 | 1.13092 | 2.1 |
| 21278 | 0.946127 | 0.611406 |
| 21279 | 1.31724 | 0.744576 |
| 21280 | 1.39751 | 0.348794 |
| 21281 | 1.39751 | 0.392355 |
| 21282 | 0.946128 | 0.525571 |
| 21283 | 0.946132 | 0.411375 |

| | | |
|---|---|---|
| 21284 | 0.713284 | 0.104229 |
| 21285 | 1.40668 | 0.451214 |
| 21286 | 1.03547 | 0.0681277 |
| 21287 | 0.713298 | 0.385438 |
| 21288 | 1.88774 | 0.05 |
| 21289 | 0.830164 | 2.1 |
| 21290 | 1.05211 | 2.1 |
| 21291 | 0.946119 | 1.55559 |
| 21292 | 0.946107 | 2.1 |
| 21293 | 1.283 2.1 | |
| 21294 | 2.63609 | 2.1 |
| 21295 | 1.28418 | 1.01108 |
| 21296 | 0.946128 | 1.02178 |
| 21297 | 1.10335 | 0.919252 |
| 21298 | 0.598377 | 1.43068 |
| 21299 | 1.18818 | 0.937041 |
| 21300 | 1.10335 | 0.894394 |
| 21301 | 1.58972 | 1.09254 |
| 21302 | 1.90194 | 2.1 |
| 21303 | 0.946118 | 2.1 |
| 21304 | 0.946115 | 2.1 |
| 21305 | 0.950969 | 2.1 |
| 21306 | 1.36918 | 2.1 |
| 21307 | 0.950955 | 2.1 |
| 21308 | 1.4263 | 2.1 |
| 21309 | 1.11978 | 0.904996 |
| 21310 | 0.946132 | 1.32686 |
| 21311 | 0.946133 | 1.69729 |
| 21312 | 1.37658 | 0.925539 |
| 21313 | 1.2364 | 1.03117 |
| 21314 | 1.05496 | 0.880606 |
| 21315 | 0.946132 | 1.86155 |
| 21316 | 0.946132 | 1.19031 |
| 21317 | 1.21858 | 0.844893 |
| 21318 | 1.37658 | 1.08015 |
| 21319 | 1.09194 | 1.90359 |
| 21320 | 1.14721 | 0.05 |
| 21321 | 1.53814 | 0.05 |
| 21322 | 0.546751 | 0.810653 |
| 21323 | 0.492063 | 1.24332 |
| 21324 | 1.16889 | 2.1 |
| 21325 | 1.00349 | 2.07921 |
| 21326 | 0.863559 | 0.978718 |
| 21327 | 1.38102 | 1.67132 |
| 21328 | 0.597449 | 1.02829 |
| 21329 | 0.537748 | 0.790817 |
| 21330 | 1.16297 | 1.46424 |
| 21331 | 0.999366 | 1.26178 |
| 21332 | 0.896554 | 0.927 |
| 21333 | 0.566962 | 0.05 |
| 21334 | 0.946129 | 0.05 |
| 21335 | 0.452729 | 0.05 |
| 21336 | 0.696552 | 0.05 |
| 21337 | 0.852524 | 0.05 |
| 21338 | 1.57992 | 0.05 |
| 21339 | 1.64985 | 0.05 |
| 21340 | 0.946128 | 2.1 |
| 21341 | 1.21847 | 2.1 |

| | | |
|---|---|---|
| 21342 | 0.946129 | 2.1 |
| 21343 | 1.41131 | 2.1 |
| 21344 | 1.21847 | 2.1 |
| 21345 | 1.21269 | 0.05 |
| 21346 | 1.0417 | 2.1 |
| 21347 | 1.15821 | 0.992627 |
| 21348 | 0.946132 | 0.518704 |
| 21349 | 0.946132 | 2.1 |
| 21350 | 1.37967 | 0.05 |
| 21351 | 0.851529 | 0.115114 |
| 21352 | 1.20455 | 2.1 |
| 21353 | 1.4282 | 0.05 |
| 21354 | 1.26593 | 2.1 |
| 21355 | 0.838026 | 0.05 |
| 21356 | 0.985096 | 0.05 |
| 21357 | 0.83804 | 0.05 |
| 21358 | 0.822395 | 1.06227 |
| 21359 | 0.420272 | 0.0725174 |
| 21360 | 0.946131 | 2.1 |
| 21361 | 1.17461 | 2.1 |
| 21362 | 0.94613 | 2.1 |
| 21363 | 1.40225 | 2.1 |
| 21364 | 1.17461 | 2.1 |
| 21365 | 1.09847 | 2.1 |
| 21366 | 1.22222 | 0.930207 |
| 21367 | 1.41798 | 0.0987985 |
| 21368 | 0.791658 | 0.713882 |
| 21369 | 0.791656 | 0.563803 |
| 21370 | 0.946129 | 0.431238 |
| 21371 | 0.817064 | 0.449417 |
| 21372 | 0.946114 | 0.156975 |
| 21373 | 0.75824 | 0.233506 |
| 21374 | 0.75821 | 0.266325 |
| 21375 | 1.41778 | 0.111395 |
| 21376 | 0.791635 | 0.220496 |
| 21377 | 1.04164 | 2.1 |
| 21378 | 0.946131 | 0.636728 |
| 21379 | 1.11172 | 0.270757 |
| 21380 | 1.30076 | 0.05 |
| 21381 | 1.35721 | 0.0835443 |
| 21382 | 1.11172 | 0.0811349 |
| 21383 | 1.87218 | 0.05 |
| 21384 | 0.987248 | 0.05 |
| 21385 | 0.729695 | 0.425634 |
| 21386 | 0.974389 | 0.05 |
| 21387 | 1.18002 | 0.05 |
| 21388 | 0.822612 | 0.05 |
| 21389 | 1.35721 | 0.05 |
| 21390 | 0.634295 | 0.732566 |
| 21391 | 0.634326 | 0.73737 |
| 21392 | 1.13245 | 1.15549 |
| 21393 | 1.13245 | 0.957602 |
| 21394 | 0.937961 | 0.668353 |
| 21395 | 1.18769 | 0.841215 |
| 21396 | 0.854949 | 0.521273 |
| 21397 | 0.967357 | 0.65887 |
| 21398 | 0.810934 | 0.518276 |
| 21399 | 3.91082 | 2.1 |

| | | |
|---|---|---|
| 21400 | 0.946132 | 2.1 |
| 21401 | 0.946132 | 2.1 |
| 21402 | 1.05383 | 2.1 |
| 21403 | 1.05383 | 2.1 |
| 21404 | 0.986498 | 2.1 |
| 21405 | 1.50732 | 0.05 |
| 21406 | 0.753659 | 0.05 |
| 21407 | 1.22144 | 0.05 |
| 21408 | 1.30463 | 0.05 |
| 21409 | 0.753659 | 0.05 |
| 21410 | 1.30463 | 0.05 |
| 21411 | 0.886189 | 0.05 |
| 21412 | 1.04321 | 0.05 |
| 21413 | 0.909928 | 0.05 |
| 21414 | 1.18659 | 0.05 |
| 21415 | 1.19785 | 0.05 |
| 21416 | 1.40969 | 0.05 |
| 21417 | 1.24094 | 0.05 |
| 21418 | 0.946126 | 0.05 |
| 21419 | 1.2929 | 0.05 |
| 21420 | 1.24093 | 0.05 |
| 21421 | 0.867944 | 0.05 |
| 21422 | 1.41903 | 2.1 |
| 21423 | 2.47881 | 0.05 |
| 21424 | 1.12943 | 0.05 |
| 21425 | 0.764229 | 2.1 |
| 21426 | 0.764256 | 2.1 |
| 21427 | 0.94613 | 2.1 |
| 21428 | 0.94613 | 2.1 |
| 21429 | 1.1995 | 2.1 |
| 21430 | 1.83423 | 2.1 |
| 21431 | 0.634816 | 2.1 |
| 21432 | 0.877837 | 2.1 |
| 21433 | 0.94613 | 2.1 |
| 21434 | 1.04073 | 2.1 |
| 21435 | 1.06485 | 2.1 |
| 21436 | 0.94613 | 0.05 |
| 21437 | 0.62725 | 0.05 |
| 21438 | 0.798212 | 0.05 |
| 21439 | 1.33241 | 0.05 |
| 21440 | 0.62725 | 0.05 |
| 21441 | 1.82301 | 0.05 |
| 21442 | 1.40615 | 2.1 |
| 21443 | 0.782504 | 2.1 |
| 21444 | 0.748635 | 2.1 |
| 21445 | 0.946131 | 2.1 |
| 21446 | 0.748607 | 2.1 |
| 21447 | 0.988456 | 2.1 |
| 21448 | 1.64833 | 0.05 |
| 21449 | 1.35657 | 0.05 |
| 21450 | 1.26155 | 0.05 |
| 21451 | 0.915755 | 0.05 |
| 21452 | 0.915755 | 0.05 |
| 21453 | 0.946132 | 0.05 |
| 21454 | 1.49386 | 0.05 |
| 21455 | 1.01346 | 2.1 |
| 21456 | 0.946131 | 0.750132 |
| 21457 | 0.946132 | 2.1 |

| | | |
|---|---|---|
| 21458 | 1.38651 | 2.1 |
| 21459 | 1.39523 | 2.1 |
| 21460 | 0.946132 | 2.1 |
| 21461 | 1.06312 | 2.1 |
| 21462 | 0.946131 | 2.1 |
| 21463 | 0.946129 | 2.1 |
| 21464 | 1.39523 | 2.1 |
| 21465 | 1.37372 | 2.1 |
| 21466 | 0.946131 | 2.1 |
| 21467 | 1.2929 | 0.05 |
| 21468 | 0.94613 | 0.05 |
| 21469 | 1.24448 | 0.05 |
| 21470 | 1.2929 | 0.05 |
| 21471 | 0.862011 | 0.05 |
| 21472 | 1.73583 | 0.05 |
| 21473 | 1.724 | 0.05 |
| 21474 | 1.03824 | 2.1 |
| 21475 | 1.534 | 2.1 |
| 21476 | 0.725492 | 1.91351 |
| 21477 | 0.771967 | 2.09023 |
| 21478 | 0.61731 | 1.15298 |
| 21479 | 0.475743 | 1.61191 |
| 21480 | 0.719694 | 1.75972 |
| 21481 | 1.14878 | 1.07835 |
| 21482 | 0.946117 | 1.89311 |
| 21483 | 0.94613 | 2.0235 |
| 21484 | 0.946105 | 1.98042 |
| 21485 | 0.482666 | 1.1388 |
| 21486 | 0.609172 | 1.15544 |
| 21487 | 0.946131 | 2.1 |
| 21488 | 1.39509 | 0.723367 |
| 21489 | 1.07439 | 2.1 |
| 21490 | 0.946131 | 1.30545 |
| 21491 | 1.39509 | 1.4175 |
| 21492 | 0.963571 | 1.75278 |
| 21493 | 0.591011 | 0.121626 |
| 21494 | 1.1357 | 0.05 |
| 21495 | 2.11193 | 0.240023 |
| 21496 | 1.60936 | 2.1 |
| 21497 | 1.34118 | 0.05 |
| 21498 | 1.27696 | 0.0628635 |
| 21499 | 0.94613 | 0.0533589 |
| 21500 | 0.94613 | 0.05 |
| 21501 | 1.04257 | 0.05 |
| 21502 | 1.53336 | 0.05 |
| 21503 | 1.0631 | 0.05 |
| 21504 | 1.04072 | 0.05 |
| 21505 | 0.670678 | 0.05 |
| 21506 | 0.709531 | 0.05 |
| 21507 | 0.891275 | 0.05 |
| 21508 | 0.683583 | 0.05 |
| 21509 | 0.902478 | 0.05 |
| 21510 | 0.807671 | 0.05 |
| 21511 | 1.11024 | 2.1 |
| 21512 | 1.08171 | 2.1 |
| 21513 | 1.16725 | 2.1 |
| 21514 | 0.946128 | 2.1 |
| 21515 | 1.13537 | 2.1 |

| | | |
|---|---|---|
| 21516 | 1.40525 | 2.1 |
| 21517 | 1.28365 | 2.1 |
| 21518 | 1.04523 | 2.1 |
| 21519 | 0.960106 | 2.1 |
| 21520 | 0.946128 | 2.1 |
| 21521 | 1.23452 | 2.1 |
| 21522 | 0.946129 | 2.1 |
| 21523 | 1.3704 | 2.1 |
| 21524 | 1.23452 | 2.1 |
| 21525 | 1.16906 | 0.895726 |
| 21526 | 0.946131 | 1.79919 |
| 21527 | 0.867246 | 0.999693 |
| 21528 | 0.867217 | 2.03969 |
| 21529 | 0.946131 | 1.23138 |
| 21530 | 1.13438 | 1.69588 |
| 21531 | 1.28684 | 1.77987 |
| 21532 | 0.946132 | 2.05785 |
| 21533 | 0.883935 | 1.46199 |
| 21534 | 0.883938 | 2.1 |
| 21535 | 0.946133 | 1.49003 |
| 21536 | 1.73444 | 1.15019 |
| 21537 | 1.21835 | 2.1 |
| 21538 | 1.28228 | 2.1 |
| 21539 | 1.8643 | 1.17097 |
| 21540 | 0.743379 | 0.117111 |
| 21541 | 1.34921 | 0.11116 |
| 21542 | 0.743381 | 0.118372 |
| 21543 | 0.743381 | 0.119652 |
| 21544 | 0.946131 | 0.146052 |
| 21545 | 0.915528 | 2.1 |
| 21546 | 0.982742 | 2.1 |
| 21547 | 0.975965 | 2.1 |
| 21548 | 0.891132 | 0.05 |
| 21549 | 1.27533 | 0.05 |
| 21550 | 0.946132 | 0.05 |
| 21551 | 1.25215 | 0.05 |
| 21552 | 0.590343 | 0.05 |
| 21553 | 0.946133 | 0.05 |
| 21554 | 0.590331 | 0.05 |
| 21555 | 1.62375 | 2.1 |
| 21556 | 0.687769 | 0.565214 |
| 21557 | 1.14374 | 0.43118 |
| 21558 | 1.20885 | 0.348502 |
| 21559 | 0.946132 | 0.605406 |
| 21560 | 1.14374 | 0.699635 |
| 21561 | 1.09308 | 0.130916 |
| 21562 | 1.02055 | 0.0785865 |
| 21563 | 0.810934 | 0.05 |
| 21564 | 0.917609 | 1.44317 |
| 21565 | 1.13094 | 1.70866 |
| 21566 | 0.70959 | 1.11977 |
| 21567 | 1.11223 | 2.1 |
| 21568 | 0.83051 | 1.25989 |
| 21569 | 0.810946 | 1.72852 |
| 21570 | 0.810959 | 1.1616 |
| 21571 | 1.5773 | 0.05 |
| 21572 | 1.96133 | 0.922092 |
| 21573 | 1.0214 | 1.07623 |

| | | |
|---|---|---|
| 21574 | 0.762783 | 1.62414 |
| 21575 | 0.838036 | 0.95647 |
| 21576 | 0.63591 | 1.93558 |
| 21577 | 0.967411 | 0.706331 |
| 21578 | 0.946132 | 2.1 |
| 21579 | 0.946132 | 2.1 |
| 21580 | 1.39699 | 1.72227 |
| 21581 | 1.61642 | 2.1 |
| 21582 | 0.860954 | 0.945708 |
| 21583 | 0.957411 | 2.06151 |
| 21584 | 0.660856 | 1.15542 |
| 21585 | 1.10003 | 2.1 |
| 21586 | 0.968249 | 2.1 |
| 21587 | 0.910184 | 1.23324 |
| 21588 | 1.30029 | 0.139011 |
| 21589 | 0.754119 | 0.05 |
| 21590 | 0.754121 | 0.603691 |
| 21591 | 0.94613 | 1.15915 |
| 21592 | 1.08039 | 2.1 |
| 21593 | 0.810942 | 0.05 |
| 21594 | 1.15153 | 0.05 |
| 21595 | 1.06968 | 0.05 |
| 21596 | 1.38456 | 2.1 |
| 21597 | 1.04118 | 2.1 |
| 21598 | 0.838027 | 2.1 |
| 21599 | 1.04073 | 2.1 |
| 21600 | 0.754209 | 2.1 |
| 21601 | 1.332 | 0.05 |
| 21602 | 1.26193 | 2.1 |
| 21603 | 1.15317 | 0.05 |
| 21604 | 3.86179 | 0.05 |
| 21605 | 1.56005 | 2.1 |
| 21606 | 0.683035 | 0.494812 |
| 21607 | 0.931463 | 0.462666 |
| 21608 | 0.556019 | 0.716647 |
| 21609 | 0.929172 | 0.589464 |
| 21610 | 0.756874 | 0.140193 |
| 21611 | 1.23527 | 0.647816 |
| 21612 | 2.05961 | 0.704197 |
| 21613 | 0.946132 | 2.1 |
| 21614 | 1.16684 | 2.1 |
| 21615 | 1.3937 | 2.1 |
| 21616 | 1.3937 | 2.1 |
| 21617 | 0.946132 | 2.1 |
| 21618 | 1.17885 | 2.1 |
| 21619 | 1.62507 | 0.05 |
| 21620 | 1.58874 | 2.1 |
| 21621 | 0.819953 | 2.1 |
| 21622 | 1.16023 | 2.1 |
| 21623 | 1.34782 | 2.1 |
| 21624 | 1.16023 | 2.1 |
| 21625 | 0.819955 | 2.1 |
| 21626 | 0.79437 | 2.1 |
| 21627 | 0.794373 | 2.1 |
| 21628 | 1.20056 | 2.1 |
| 21629 | 1.25 | 2.1 |
| 21630 | 1.20056 | 2.1 |
| 21631 | 1.35787 | 2.1 |

| | | |
|---|---|---|
| 21632 | 0.946131 | 2.1 |
| 21633 | 1.38585 | 2.1 |
| 21634 | 0.946128 | 0.849562 |
| 21635 | 0.946129 | 1.54968 |
| 21636 | 1.35714 | 1.7296 |
| 21637 | 1.04147 | 2.1 |
| 21638 | 0.946133 | 1.74713 |
| 21639 | 1.30382 | 1.30986 |
| 21640 | 0.946131 | 0.35801 |
| 21641 | 0.946131 | 1.13912 |
| 21642 | 1.38585 | 1.39535 |
| 21643 | 1.22305 | 1.43922 |
| 21644 | 1.00763 | 0.05 |
| 21645 | 0.813199 | 0.05 |
| 21646 | 0.810948 | 0.05 |
| 21647 | 1.15928 | 0.05 |
| 21648 | 0.944166 | 0.05 |
| 21649 | 0.94613 | 0.05 |
| 21650 | 0.946133 | 0.05 |
| 21651 | 1.4179 | 0.05 |
| 21652 | 0.946132 | 0.05 |
| 21653 | 1.36965 | 0.05 |
| 21654 | 1.05444 | 0.05 |
| 21655 | 0.690302 | 0.05 |
| 21656 | 0.838038 | 0.05 |
| 21657 | 0.802821 | 0.05 |
| 21658 | 1.40944 | 0.0613637 |
| 21659 | 0.946126 | 0.360062 |
| 21660 | 0.799998 | 0.05 |
| 21661 | 0.800035 | 0.05 |
| 21662 | 1.1766 | 2.1 |
| 21663 | 1.00319 | 2.1 |
| 21664 | 1.01606 | 1.60609 |
| 21665 | 0.872158 | 2.1 |
| 21666 | 1.04884 | 2.1 |
| 21667 | 0.679494 | 2.1 |
| 21668 | 0.946131 | 2.1 |
| 21669 | 1.37609 | 2.1 |
| 21670 | 0.94613 | 2.1 |
| 21671 | 0.946131 | 2.1 |
| 21672 | 1.18486 | 2.1 |
| 21673 | 0.726287 | 2.1 |
| 21674 | 0.946131 | 2.1 |
| 21675 | 0.726286 | 2.1 |
| 21676 | 1.30318 | 2.1 |
| 21677 | 1.01712 | 2.1 |
| 21678 | 0.946132 | 2.1 |
| 21679 | 1.04404 | 2.1 |
| 21680 | 1.37017 | 2.1 |
| 21681 | 1.37017 | 2.1 |
| 21682 | 0.946133 | 2.1 |
| 21683 | 0.992084 | 1.06912 |
| 21684 | 0.946131 | 0.890159 |
| 21685 | 0.529655 | 2.02308 |
| 21686 | 0.529686 | 0.989896 |
| 21687 | 0.946131 | 0.773738 |
| 21688 | 1.05937 | 1.14012 |
| 21689 | 1.14622 | 0.625045 |

| | | |
|---|---|---|
| 21690 | 0.946131 | 0.542274 |
| 21691 | 0.584575 | 1.43296 |
| 21692 | 0.584575 | 0.703428 |
| 21693 | 0.946129 | 0.47133 |
| 21694 | 0.709714 | 2.1 |
| 21695 | 0.946132 | 2.1 |
| 21696 | 0.709714 | 2.1 |
| 21697 | 1.37199 | 2.1 |
| 21698 | 0.82899 | 2.1 |
| 21699 | 1.00133 | 2.1 |
| 21700 | 0.778424 | 0.05 |
| 21701 | 0.946132 | 0.05 |
| 21702 | 1.24094 | 0.05 |
| 21703 | 1.00691 | 0.05 |
| 21704 | 0.64868 | 0.05 |
| 21705 | 1.29736 | 0.05 |
| 21706 | 1.24767 | 0.144143 |
| 21707 | 1.28953 | 0.790387 |
| 21708 | 1.34612 | 0.807762 |
| 21709 | 0.946133 | 0.622288 |
| 21710 | 1.14187 | 1.19184 |
| 21711 | 0.946132 | 0.707065 |
| 21712 | 0.946129 | 0.636411 |
| 21713 | 1.4099 | 2.1 |
| 21714 | 0.946131 | 0.294286 |
| 21715 | 1.34612 | 0.112209 |
| 21716 | 1.4099 | 0.0841496 |
| 21717 | 0.956003 | 0.05 |
| 21718 | 1.40572 | 0.05 |
| 21719 | 3.54443 | 2.1 |
| 21720 | 0.994001 | 0.05 |
| 21721 | 1.03679 | 2.1 |
| 21722 | 1.08467 | 2.1 |
| 21723 | 1.56875 | 0.05 |
| 21724 | 0.968463 | 2.1 |
| 21725 | 0.974889 | 2.1 |
| 21726 | 0.946131 | 2.1 |
| 21727 | 1.30203 | 2.1 |
| 21728 | 1.40199 | 2.1 |
| 21729 | 1.40199 | 2.1 |
| 21730 | 0.946132 | 2.1 |
| 21731 | 0.946132 | 2.1 |
| 21732 | 1.36994 | 2.1 |
| 21733 | 1.37372 | 2.1 |
| 21734 | 1.37372 | 2.1 |
| 21735 | 0.946132 | 2.1 |
| 21736 | 0.928979 | 1.03181 |
| 21737 | 0.565509 | 1.27772 |
| 21738 | 1.60196 | 0.688349 |
| 21739 | 1.16239 | 0.835993 |
| 21740 | 1.10746 | 0.141619 |
| 21741 | 1.15412 | 2.1 |
| 21742 | 1.15171 | 2.1 |
| 21743 | 0.946133 | 2.1 |
| 21744 | 0.940877 | 2.1 |
| 21745 | 0.940878 | 2.1 |
| 21746 | 1.41432 | 2.1 |
| 21747 | 1.04687 | 2.1 |

| | | |
|---|---|---|
| 21748 | 2.75524 | 0.05 |
| 21749 | 0.946132 | 0.05 |
| 21750 | 0.766452 | 0.05 |
| 21751 | 0.766427 | 0.05 |
| 21752 | 1.36004 | 0.05 |
| 21753 | 1.06648 | 0.05 |
| 21754 | 1.62483 | 2.1 |
| 21755 | 1.0577 | 2.1 |
| 21756 | 1.39158 | 2.1 |
| 21757 | 0.94255 | 2.1 |
| 21758 | 1.05029 | 2.1 |
| 21759 | 1.03735 | 2.06792 |
| 21760 | 0.931055 | 2.1 |
| 21761 | 1.03797 | 2.1 |
| 21762 | 1.43687 | 2.02923 |
| 21763 | 1.28765 | 1.91254 |
| 21764 | 1.1811 | 1.09923 |
| 21765 | 0.946132 | 1.41906 |
| 21766 | 0.946132 | 1.50659 |
| 21767 | 1.1811 | 1.33941 |
| 21768 | 0.67805 | 0.249762 |
| 21769 | 1.50998 | 2.00131 |
| 21770 | 1.16661 | 2.1 |
| 21771 | 1.36749 | 2.1 |
| 21772 | 0.810945 | 0.651688 |
| 21773 | 1.14745 | 1.20284 |
| 21774 | 0.865588 | 0.475064 |
| 21775 | 1.36665 | 0.124662 |
| 21776 | 0.506964 | 1.37459 |
| 21777 | 0.506966 | 0.91165 |
| 21778 | 0.552786 | 0.294033 |
| 21779 | 0.946133 | 0.062204 |
| 21780 | 1.21336 | 0.05 |
| 21781 | 0.552786 | 0.684437 |
| 21782 | 0.552757 | 1.78488 |
| 21783 | 0.653468 | 0.173918 |
| 21784 | 1.36665 | 1.4111 |
| 21785 | 1.27922 | 0.05 |
| 21786 | 1.08133 | 0.05 |
| 21787 | 1.05348 | 0.05 |
| 21788 | 0.946132 | 0.05 |
| 21789 | 1.19601 | 0.05 |
| 21790 | 1.13276 | 0.05 |
| 21791 | 0.7856 | 0.05 |
| 21792 | 1.57125 | 0.05 |
| 21793 | 1.19601 | 0.05 |
| 21794 | 0.946131 | 0.0989544 |
| 21795 | 1.34534 | 0.05 |
| 21796 | 1.19601 | 0.05 |
| 21797 | 0.870489 | 0.05 |
| 21798 | 1.49404 | 2.1 |
| 21799 | 1.35943 | 1.32074 |
| 21800 | 1.10427 | 2.09498 |
| 21801 | 0.94613 | 1.1563 |
| 21802 | 0.946131 | 1.51155 |
| 21803 | 1.10427 | 1.34379 |
| 21804 | 1.2064 | 0.05 |
| 21805 | 0.946132 | 0.0808537 |

| | | |
|---|---|---|
| 21806 | 1.11288 | 0.394032 |
| 21807 | 1.34268 | 0.05 |
| 21808 | 1.34268 | 0.05 |
| 21809 | 0.946132 | 0.05 |
| 21810 | 0.946128 | 2.1 |
| 21811 | 0.946128 | 0.05 |
| 21812 | 1.30835 | 2.1 |
| 21813 | 1.38463 | 0.208905 |
| 21814 | 0.946129 | 0.278188 |
| 21815 | 1.43778 | 0.896701 |
| 21816 | 1.23943 | 0.5025 |
| 21817 | 1.4748 | 2.1 |
| 21818 | 0.71295 | 2.1 |
| 21819 | 0.712951 | 2.1 |
| 21820 | 0.946132 | 2.1 |
| 21821 | 0.946133 | 2.1 |
| 21822 | 1.13976 | 2.1 |
| 21823 | 1.71109 | 2.1 |
| 21824 | 1.32426 | 2.1 |
| 21825 | 0.671255 | 2.1 |
| 21826 | 1.29123 | 2.1 |
| 21827 | 0.946132 | 2.1 |
| 21828 | 0.946132 | 2.1 |
| 21829 | 1.25603 | 2.1 |
| 21830 | 2.21126 | 0.05 |
| 21831 | 0.892889 | 2.1 |
| 21832 | 0.946129 | 2.1 |
| 21833 | 0.892888 | 2.1 |
| 21834 | 0.92872 | 2.1 |
| 21835 | 1.34352 | 2.1 |
| 21836 | 0.622784 | 0.0660547 |
| 21837 | 1.02947 | 0.05 |
| 21838 | 0.810957 | 0.05 |
| 21839 | 1.91756 | 1.09979 |
| 21840 | 1.06012 | 0.176709 |
| 21841 | 1.09569 | 0.05 |
| 21842 | 1.5456 | 0.05 |
| 21843 | 1.1211 | 0.05 |
| 21844 | 1.21031 | 0.05 |
| 21845 | 1.03939 | 0.05 |
| 21846 | 0.519697 | 0.05 |
| 21847 | 0.933889 | 0.05 |
| 21848 | 0.519726 | 0.05 |
| 21849 | 1.16699 | 0.05 |
| 21850 | 0.933889 | 0.05 |
| 21851 | 0.840599 | 0.05 |
| 21852 | 0.94613 | 0.05 |
| 21853 | 0.840568 | 0.05 |
| 21854 | 1.26868 | 0.05 |
| 21855 | 1.16699 | 0.05 |
| 21856 | 1.17822 | 2.1 |
| 21857 | 0.922044 | 0.083365 |
| 21858 | 1.29322 | 0.437484 |
| 21859 | 0.946132 | 0.164919 |
| 21860 | 0.995178 | 0.524026 |
| 21861 | 0.922056 | 0.477855 |
| 21862 | 1.76232 | 0.561503 |
| 21863 | 1.77065 | 1.12085 |

| | | |
|---|---|---|
| 21864 | 1.38149 | 0.850696 |
| 21865 | 0.959301 | 1.13001 |
| 21866 | 0.946133 | 0.935595 |
| 21867 | 0.946132 | 1.0526 |
| 21868 | 0.959301 | 0.983077 |
| 21869 | 1.3612 | 0.730747 |
| 21870 | 0.885314 | 1.09291 |
| 21871 | 0.885309 | 0.733876 |
| 21872 | 0.94613 | 1.04094 |
| 21873 | 0.8853 | 0.730992 |
| 21874 | 1.56164 | 0.939049 |
| 21875 | 1.45506 | 0.0547581 |
| 21876 | 1.45257 | 2.1 |
| 21877 | 0.851541 | 0.05 |
| 21878 | 1.29018 | 0.05 |
| 21879 | 0.851541 | 0.05 |
| 21880 | 1.21087 | 0.05 |
| 21881 | 1.24979 | 0.05 |
| 21882 | 1.56983 | 0.05 |
| 21883 | 1.60151 | 0.05 |
| 21884 | 1.78952 | 2.1 |
| 21885 | 0.638164 | 2.1 |
| 21886 | 0.638163 | 2.1 |
| 21887 | 1.39754 | 2.1 |
| 21888 | 1.39754 | 2.1 |
| 21889 | 0.681589 | 2.1 |
| 21890 | 1.34993 | 2.1 |
| 21891 | 1.04525 | 2.1 |
| 21892 | 0.946133 | 2.1 |
| 21893 | 1.04072 | 2.1 |
| 21894 | 0.857805 | 2.1 |
| 21895 | 1.06053 | 2.1 |
| 21896 | 1.49066 | 2.1 |
| 21897 | 1.4185 | 1.03262 |
| 21898 | 0.934059 | 2.1 |
| 21899 | 0.93406 | 1.04075 |
| 21900 | 0.94613 | 1.61973 |
| 21901 | 0.93406 | 1.18951 |
| 21902 | 1.25277 | 1.22646 |
| 21903 | 1.00792 | 2.1 |
| 21904 | 0.609466 | 1.22951 |
| 21905 | 0.946132 | 1.85331 |
| 21906 | 0.609463 | 2.1 |
| 21907 | 1.30664 | 2.1 |
| 21908 | 0.810943 | 1.67149 |
| 21909 | 1.05741 | 0.673276 |
| 21910 | 1.3868 | 0.946265 |
| 21911 | 1.15558 | 0.05 |
| 21912 | 1.13537 | 2.1 |
| 21913 | 0.946132 | 2.1 |
| 21914 | 0.946132 | 2.1 |
| 21915 | 1.08266 | 2.1 |
| 21916 | 1.54934 | 2.1 |
| 21917 | 1.26016 | 2.1 |
| 21918 | 2.01915 | 2.1 |
| 21919 | 0.60346 | 1.27176 |
| 21920 | 0.651529 | 1.36788 |
| 21921 | 1.15581 | 2.08712 |

| | | |
|---|---|---|
| 21922 | 1.13037 | 1.10954 |
| 21923 | 1.04071 | 1.01745 |
| 21924 | 1.77796 | 2.1 |
| 21925 | 0.532308 | 1.2708 |
| 21926 | 0.532308 | 1.119 |
| 21927 | 1.15369 | 2.1 |
| 21928 | 1.1537 | 1.95105 |
| 21929 | 0.903288 | 1.37417 |
| 21930 | 0.804766 | 2.1 |
| 21931 | 0.804739 | 2.1 |
| 21932 | 0.946128 | 2.1 |
| 21933 | 0.946131 | 2.1 |
| 21934 | 1.16745 | 2.1 |
| 21935 | 1.04193 | 2.1 |
| 21936 | 0.98505 | 2.1 |
| 21937 | 1.40724 | 0.608516 |
| 21938 | 1.14216 | 0.545741 |
| 21939 | 0.946131 | 0.443631 |
| 21940 | 0.946131 | 0.507376 |
| 21941 | 1.10941 | 0.523083 |
| 21942 | 0.81268 | 0.05 |
| 21943 | 0.946131 | 0.05 |
| 21944 | 1.28673 | 0.05 |
| 21945 | 1.28673 | 0.05 |
| 21946 | 0.812681 | 0.05 |
| 21947 | 1.84716 | 0.05 |
| 21948 | 1.84716 | 0.05 |
| 21949 | 0.92361 | 0.05 |
| 21950 | 0.94613 | 0.05 |
| 21951 | 0.923582 | 0.05 |
| 21952 | 1.26227 | 0.05 |
| 21953 | 0.923581 | 0.05 |
| 21954 | 0.747394 | 2.1 |
| 21955 | 1.32582 | 2.1 |
| 21956 | 0.747365 | 2.1 |
| 21957 | 0.97587 | 2.1 |
| 21958 | 1.32582 | 2.1 |
| 21959 | 1.04182 | 0.05 |
| 21960 | 0.961743 | 0.05 |
| 21961 | 0.946126 | 0.05 |
| 21962 | 1.47463 | 0.05 |
| 21963 | 1.12183 | 0.05 |
| 21964 | 0.851552 | 0.05 |
| 21965 | 1.33177 | 0.05 |
| 21966 | 0.946131 | 0.05 |
| 21967 | 1.41512 | 0.05 |
| 21968 | 1.33178 | 0.05 |
| 21969 | 0.946131 | 0.05 |
| 21970 | 0.927735 | 0.0758953 |
| 21971 | 0.97206 | 0.05 |
| 21972 | 0.515566 | 0.05 |
| 21973 | 0.946129 | 0.05 |
| 21974 | 0.515538 | 0.05 |
| 21975 | 1.04482 | 0.05 |
| 21976 | 0.975535 | 0.05 |
| 21977 | 0.522411 | 0.05 |
| 21978 | 0.522382 | 0.05 |
| 21979 | 0.925346 | 0.05 |

| | | |
|---|---|---|
| 21980 | 0.575817 | 0.05 |
| 21981 | 0.946129 | 2.1 |
| 21982 | 1.14256 | 2.1 |
| 21983 | 1.32054 | 2.1 |
| 21984 | 1.40525 | 2.1 |
| 21985 | 1.14256 | 2.1 |
| 21986 | 1.59475 | 2.1 |
| 21987 | 0.773813 | 2.1 |
| 21988 | 1.4073 | 2.1 |
| 21989 | 0.773813 | 2.1 |
| 21990 | 0.773813 | 2.1 |
| 21991 | 1.4073 | 2.1 |
| 21992 | 0.993021 | 2.1 |
| 21993 | 1.50466 | 0.05 |
| 21994 | 0.851539 | 0.05 |
| 21995 | 1.20299 | 0.05 |
| 21996 | 0.946131 | 0.05 |
| 21997 | 1.54464 | 0.05 |
| 21998 | 1.20309 | 0.05 |
| 21999 | 2.00701 | 0.05 |
| 22000 | 0.810946 | 0.05 |
| 22001 | 1.07678 | 0.05 |
| 22002 | 0.900128 | 0.05 |
| 22003 | 1.26842 | 2.1 |
| 22004 | 1.11206 | 2.1 |
| 22005 | 0.946132 | 2.1 |
| 22006 | 1.04074 | 2.1 |
| 22007 | 0.948809 | 2.1 |
| 22008 | 0.946131 | 0.05 |
| 22009 | 0.929617 | 0.05 |
| 22010 | 1.24057 | 0.05 |
| 22011 | 1.36485 | 0.05 |
| 22012 | 0.851554 | 0.05 |
| 22013 | 1.96823 | 0.05 |
| 22014 | 0.914836 | 0.05 |
| 22015 | 0.94613 | 0.05 |
| 22016 | 1.38911 | 0.05 |
| 22017 | 0.914805 | 0.05 |
| 22018 | 0.946131 | 0.05 |
| 22019 | 0.946131 | 0.05 |
| 22020 | 1.04072 | 0.05 |
| 22021 | 1.26837 | 0.05 |
| 22022 | 0.970054 | 0.05 |
| 22023 | 0.923958 | 0.05 |
| 22024 | 1.16653 | 2.1 |
| 22025 | 1.34412 | 2.1 |
| 22026 | 0.851541 | 0.05 |
| 22027 | 1.38645 | 0.05 |
| 22028 | 0.891637 | 0.05 |
| 22029 | 0.851487 | 0.05 |
| 22030 | 0.890871 | 0.05 |
| 22031 | 1.56178 | 0.05 |
| 22032 | 0.946128 | 0.05 |
| 22033 | 1.26868 | 0.05 |
| 22034 | 0.946131 | 0.05 |
| 22035 | 1.18789 | 0.05 |
| 22036 | 1.26868 | 0.05 |
| 22037 | 1.26868 | 0.05 |

| | | |
|---|---|---|
| 22038 | 0.946128 | 0.05 |
| 22039 | 1.18716 | 0.05 |
| 22040 | 0.946131 | 0.05 |
| 22041 | 0.59358 | 0.05 |
| 22042 | 1.23923 | 0.05 |
| 22043 | 1.00024 | 0.05 |
| 22044 | 0.593593 | 0.05 |
| 22045 | 0.946131 | 0.05 |
| 22046 | 0.713645 | 0.05 |
| 22047 | 1.23175 | 0.05 |
| 22048 | 1.23923 | 0.05 |
| 22049 | 0.713633 | 0.05 |
| 22050 | 1.24687 | 0.05 |
| 22051 | 1.80662 | 2.1 |
| 22052 | 2.15762 | 2.1 |
| 22053 | 0.780455 | 2.1 |
| 22054 | 0.946131 | 2.1 |
| 22055 | 0.780455 | 2.1 |
| 22056 | 1.40583 | 2.1 |
| 22057 | 1.39852 | 2.1 |
| 22058 | 0.565619 | 2.1 |
| 22059 | 1.1428 | 2.1 |
| 22060 | 0.663168 | 0.05 |
| 22061 | 1.02163 | 0.05 |
| 22062 | 1.04072 | 0.05 |
| 22063 | 0.663169 | 0.05 |
| 22064 | 1.13511 | 0.05 |
| 22065 | 1.85277 | 0.05 |
| 22066 | 1.17701 | 0.109817 |
| 22067 | 0.993831 | 0.05 |
| 22068 | 1.0823 | 0.05 |
| 22069 | 0.795761 | 0.05 |
| 22070 | 1.24596 | 0.05 |
| 22071 | 0.566075 | 0.05 |
| 22072 | 0.94613 | 0.05 |
| 22073 | 0.56605 | 0.05 |
| 22074 | 0.774911 | 0.05 |
| 22075 | 0.946131 | 0.05 |
| 22076 | 1.24417 | 0.05 |
| 22077 | 0.774911 | 0.05 |
| 22078 | 0.946132 | 0.05 |
| 22079 | 1.54982 | 0.05 |
| 22080 | 1.82961 | 0.05 |
| 22081 | 1.12035 | 0.05 |
| 22082 | 0.876835 | 2.1 |
| 22083 | 0.946131 | 2.1 |
| 22084 | 0.876836 | 2.1 |
| 22085 | 1.12968 | 2.1 |
| 22086 | 1.23536 | 2.1 |
| 22087 | 2.15082 | 2.1 |
| 22088 | 0.58365 | 2.1 |
| 22089 | 0.946133 | 2.1 |
| 22090 | 0.583653 | 2.1 |
| 22091 | 0.992097 | 2.1 |
| 22092 | 1.12968 | 2.1 |
| 22093 | 1.0305 | 2.1 |
| 22094 | 0.82613 | 0.05 |
| 22095 | 0.879644 | 0.05 |

| | | |
|---|---|---|
| 22096 | 1.00688 | 0.05 |
| 22097 | 1.52776 | 2.1 |
| 22098 | 0.851537 | 1.76796 |
| 22099 | 1.00851 | 1.5727 |
| 22100 | 0.946128 | 0.991847 |
| 22101 | 0.851552 | 1.30547 |
| 22102 | 1.4895 | 1.98555 |
| 22103 | 0.761574 | 2.1 |
| 22104 | 0.946132 | 2.1 |
| 22105 | 1.01095 | 2.1 |
| 22106 | 1.01095 | 2.1 |
| 22107 | 0.761572 | 2.1 |
| 22108 | 1.8575 | 2.1 |
| 22109 | 1.19984 | 0.05 |
| 22110 | 1.25797 | 0.05 |
| 22111 | 0.946132 | 0.05 |
| 22112 | 0.946132 | 0.05 |
| 22113 | 0.95332 | 0.05 |
| 22114 | 0.946131 | 2.1 |
| 22115 | 0.851542 | 2.1 |
| 22116 | 1.03172 | 2.1 |
| 22117 | 1.22889 | 2.1 |
| 22118 | 1.0313 | 2.1 |
| 22119 | 1.15977 | 1.39066 |
| 22120 | 0.918875 | 0.518449 |
| 22121 | 0.8654 | 2.1 |
| 22122 | 0.865374 | 0.671153 |
| 22123 | 0.946132 | 0.560228 |
| 22124 | 0.985051 | 2.1 |
| 22125 | 1.31993 | 0.05 |
| 22126 | 1.33622 | 0.496731 |
| 22127 | 0.959992 | 0.255163 |
| 22128 | 0.926499 | 2.1 |
| 22129 | 1.03544 | 2.1 |
| 22130 | 0.946132 | 2.1 |
| 22131 | 0.946132 | 2.1 |
| 22132 | 1.40335 | 2.1 |
| 22133 | 0.772299 | 2.1 |
| 22134 | 0.851529 | 2.1 |
| 22135 | 0.857243 | 2.1 |
| 22136 | 1.38155 | 2.1 |
| 22137 | 1.05462 | 2.1 |
| 22138 | 0.832138 | 2.1 |
| 22139 | 0.946132 | 2.1 |
| 22140 | 0.832137 | 2.1 |
| 22141 | 0.959414 | 2.1 |
| 22142 | 1.36541 | 2.1 |
| 22143 | 1.91894 | 2.1 |
| 22144 | 1.04661 | 1.20815 |
| 22145 | 1.28355 | 2.1 |
| 22146 | 0.877353 | 0.742988 |
| 22147 | 0.877353 | 1.30463 |
| 22148 | 0.94613 | 0.846061 |
| 22149 | 1.20537 | 0.05 |
| 22150 | 1.09914 | 0.05 |
| 22151 | 0.94613 | 0.05 |
| 22152 | 0.499593 | 0.05 |
| 22153 | 0.499606 | 0.05 |

| | | |
|---|---|---|
| 22154 | 1.0959 | 0.05 |
| 22155 | 0.838027 | 0.05 |
| 22156 | 0.883644 | 0.05 |
| 22157 | 0.95019 | 0.05 |
| 22158 | 1.10638 | 2.1 |
| 22159 | 0.946132 | 2.1 |
| 22160 | 1.41744 | 2.1 |
| 22161 | 1.05842 | 2.1 |
| 22162 | 0.94613 | 2.1 |
| 22163 | 1.41744 | 2.1 |
| 22164 | 0.991985 | 2.1 |
| 22165 | 3.31755 | 1.49606 |
| 22166 | 1.05556 | 2.1 |
| 22167 | 0.624075 | 1.01266 |
| 22168 | 0.814554 | 0.504787 |
| 22169 | 0.946131 | 1.3102 |
| 22170 | 0.814554 | 1.60173 |
| 22171 | 1.31857 | 2.1 |
| 22172 | 1.26282 | 2.1 |
| 22173 | 0.946132 | 2.1 |
| 22174 | 1.14302 | 2.1 |
| 22175 | 0.946132 | 2.1 |
| 22176 | 0.946132 | 2.1 |
| 22177 | 1.40372 | 2.1 |
| 22178 | 1.24688 | 0.05 |
| 22179 | 0.89889 | 2.1 |
| 22180 | 1.17286 | 2.1 |
| 22181 | 0.71741 | 2.1 |
| 22182 | 0.491096 | 0.447835 |
| 22183 | 1.05432 | 0.05 |
| 22184 | 0.719531 | 2.1 |
| 22185 | 1.25145 | 2.1 |
| 22186 | 0.864686 | 2.1 |
| 22187 | 0.867974 | 2.1 |
| 22188 | 1.21734 | 2.1 |
| 22189 | 0.405517 | 0.05 |
| 22190 | 0.818878 | 0.325346 |
| 22191 | 0.502457 | 0.05 |
| 22192 | 1.05868 | 0.05 |
| 22193 | 0.912018 | 0.05 |
| 22194 | 1.40591 | 0.05 |
| 22195 | 0.957584 | 0.05 |
| 22196 | 0.946131 | 0.05 |
| 22197 | 1.11612 | 0.05 |
| 22198 | 1.3365 | 0.05 |
| 22199 | 0.946128 | 0.05 |
| 22200 | 1.16534 | 0.05 |
| 22201 | 1.38969 | 0.0573876 |
| 22202 | 0.891597 | 1.18938 |
| 22203 | 0.640032 | 2.1 |
| 22204 | 0.956758 | 1.48786 |
| 22205 | 1.3428 | 0.12023 |
| 22206 | 0.953295 | 2.1 |
| 22207 | 0.946131 | 0.05 |
| 22208 | 1.16167 | 0.05 |
| 22209 | 0.94613 | 0.0525596 |
| 22210 | 0.951675 | 0.05 |
| 22211 | 1.16167 | 0.05 |

| | | |
|---|---|---|
| 22212 | 1.24538 | 0.05 |
| 22213 | 1.13482 | 2.1 |
| 22214 | 1.38775 | 2.1 |
| 22215 | 0.934061 | 2.1 |
| 22216 | 0.91408 | 2.1 |
| 22217 | 1.06099 | 2.1 |
| 22218 | 0.980549 | 0.05 |
| 22219 | 0.946132 | 0.05 |
| 22220 | 1.10321 | 0.05 |
| 22221 | 0.99126 | 0.05 |
| 22222 | 0.991261 | 0.05 |
| 22223 | 1.35732 | 0.05 |
| 22224 | 1.1952 | 2.1 |
| 22225 | 0.946132 | 1.27936 |
| 22226 | 0.946132 | 2.1 |
| 22227 | 1.32819 | 1.5963 |
| 22228 | 1.30171 | 1.05182 |
| 22229 | 1.29667 | 0.05 |
| 22230 | 1.00982 | 0.05 |
| 22231 | 0.407589 | 0.146096 |
| 22232 | 1.18266 | 1.56329 |
| 22233 | 1.04037 | 0.131761 |
| 22234 | 0.956694 | 0.768485 |
| 22235 | 0.552639 | 0.0827055 |
| 22236 | 0.810943 | 2.1 |
| 22237 | 1.43144 | 0.796009 |
| 22238 | 0.918074 | 0.763958 |
| 22239 | 1.60976 | 2.1 |
| 22240 | 4.00847 | 2.1 |
| 22241 | 2.3995 | 0.05 |
| 22242 | 1.36203 | 2.1 |
| 22243 | 0.919619 | 2.1 |
| 22244 | 1.30188 | 2.1 |
| 22245 | 0.681073 | 2.1 |
| 22246 | 0.681043 | 2.1 |
| 22247 | 1.04687 | 2.1 |
| 22248 | 3.32085 | 2.1 |
| 22249 | 1.80396 | 0.05 |
| 22250 | 0.773007 | 0.05 |
| 22251 | 0.773034 | 0.05 |
| 22252 | 1.22141 | 0.0740054 |
| 22253 | 1.22141 | 0.113358 |
| 22254 | 0.946131 | 0.253153 |
| 22255 | 2.02071 | 0.05 |
| 22256 | 0.951912 | 0.05 |
| 22257 | 1.39027 | 0.05 |
| 22258 | 0.94613 | 0.05 |
| 22259 | 0.829922 | 0.05 |
| 22260 | 0.829946 | 0.05 |
| 22261 | 1.24902 | 1.3117 |
| 22262 | 2.8333 | 1.02851 |
| 22263 | 0.946131 | 0.05 |
| 22264 | 0.929725 | 0.05 |
| 22265 | 1.1134 | 0.05 |
| 22266 | 1.17056 | 0.05 |
| 22267 | 0.929713 | 0.05 |
| 22268 | 0.749096 | 2.1 |
| 22269 | 1.31961 | 2.1 |

| | | |
|---|---|---|
| 22270 | 0.870191 | 2.1 |
| 22271 | 1.04071 | 2.1 |
| 22272 | 1.1877 | 2.1 |
| 22273 | 1.03882 | 2.1 |
| 22274 | 1.44801 | 2.1 |
| 22275 | 3.55198 | 2.1 |
| 22276 | 1.11193 | 2.1 |
| 22277 | 0.663922 | 2.1 |
| 22278 | 0.663922 | 2.1 |
| 22279 | 0.946129 | 2.1 |
| 22280 | 0.946131 | 2.1 |
| 22281 | 0.97079 | 2.1 |
| 22282 | 1.36049 | 2.1 |
| 22283 | 1.06105 | 2.1 |
| 22284 | 0.92251 | 2.1 |
| 22285 | 0.94613 | 2.1 |
| 22286 | 0.922511 | 2.1 |
| 22287 | 1.04101 | 2.1 |
| 22288 | 0.810944 | 0.05 |
| 22289 | 0.994109 | 0.05 |
| 22290 | 0.623143 | 0.05 |
| 22291 | 0.864244 | 0.05 |
| 22292 | 1.14627 | 0.05 |
| 22293 | 0.64586 | 0.05 |
| 22294 | 0.721422 | 0.05 |
| 22295 | 0.870885 | 0.05 |
| 22296 | 0.855175 | 0.05 |
| 22297 | 0.946131 | 0.932299 |
| 22298 | 1.38157 | 2.1 |
| 22299 | 1.33266 | 0.123717 |
| 22300 | 0.946131 | 2.1 |
| 22301 | 1.38158 | 2.05836 |
| 22302 | 0.946132 | 2.1 |
| 22303 | 1.3268 | 2.1 |
| 22304 | 0.946132 | 2.1 |
| 22305 | 0.946132 | 2.1 |
| 22306 | 1.38158 | 2.1 |
| 22307 | 1.42186 | 2.1 |
| 22308 | 1.47204 | 2.1 |
| 22309 | 1.14665 | 2.1 |
| 22310 | 0.946132 | 1.18232 |
| 22311 | 0.94613 | 2.1 |
| 22312 | 1.30116 | 2.1 |
| 22313 | 1.32819 | 1.98513 |
| 22314 | 1.25388 | 2.1 |
| 22315 | 0.887808 | 1.28569 |
| 22316 | 0.946132 | 2.1 |
| 22317 | 1.23536 | 2.1 |
| 22318 | 1.34309 | 2.1 |
| 22319 | 1.07612 | 2.1 |
| 22320 | 0.728982 | 0.05 |
| 22321 | 1.3157 | 0.05 |
| 22322 | 0.946131 | 0.05 |
| 22323 | 0.686176 | 0.05 |
| 22324 | 1.3035 | 0.05 |
| 22325 | 0.686139 | 0.05 |
| 22326 | 0.906806 | 0.05 |
| 22327 | 0.794122 | 0.05 |

| | | |
|---|---|---|
| 22328 | 1.17173 | 0.05 |
| 22329 | 1.8194 | 0.05 |
| 22330 | 0.851543 | 2.1 |
| 22331 | 1.19345 | 2.1 |
| 22332 | 0.946133 | 2.1 |
| 22333 | 0.946132 | 2.1 |
| 22334 | 1.56179 | 2.1 |
| 22335 | 0.620263 | 2.1 |
| 22336 | 1.30226 | 2.1 |
| 22337 | 1.2334 | 1.2349 |
| 22338 | 1.2334 | 0.927316 |
| 22339 | 0.946133 | 1.00181 |
| 22340 | 1.24053 | 2.1 |
| 22341 | 1.4828 | 2.1 |
| 22342 | 0.957622 | 2.1 |
| 22343 | 1.00521 | 1.60327 |
| 22344 | 1.49463 | 2.1 |
| 22345 | 1.39621 | 2.1 |
| 22346 | 1.09861 | 2.1 |
| 22347 | 0.946131 | 2.1 |
| 22348 | 0.946131 | 2.1 |
| 22349 | 1.09861 | 2.1 |
| 22350 | 2.16987 | 0.05 |
| 22351 | 0.572182 | 0.05 |
| 22352 | 1.15802 | 0.05 |
| 22353 | 0.63574 | 0.05 |
| 22354 | 1.18897 | 0.05 |
| 22355 | 0.996733 | 0.05 |
| 22356 | 1.82402 | 1.66718 |
| 22357 | 0.969602 | 1.12236 |
| 22358 | 0.794777 | 1.92781 |
| 22359 | 1.02626 | 1.50955 |
| 22360 | 1.51396 | 0.05 |
| 22361 | 1.40574 | 0.05 |
| 22362 | 1.38683 | 0.05 |
| 22363 | 0.946126 | 0.05 |
| 22364 | 0.946128 | 0.05 |
| 22365 | 1.07138 | 0.05 |
| 22366 | 0.946131 | 2.1 |
| 22367 | 1.41331 | 2.1 |
| 22368 | 0.94613 | 2.1 |
| 22369 | 0.94613 | 2.1 |
| 22370 | 1.27451 | 2.1 |
| 22371 | 0.974196 | 2.1 |
| 22372 | 1.2049 | 0.05 |
| 22373 | 1.42757 | 2.1 |
| 22374 | 1.02461 | 2.1 |
| 22375 | 2.07087 | 2.1 |
| 22376 | 1.92515 | 0.958767 |
| 22377 | 0.802138 | 0.82656 |
| 22378 | 0.946128 | 2.01501 |
| 22379 | 0.802107 | 1.37119 |
| 22380 | 1.23726 | 1.31556 |
| 22381 | 1.21997 | 1.00972 |
| 22382 | 0.767577 | 0.146083 |
| 22383 | 0.946131 | 1.67124 |
| 22384 | 0.767577 | 0.989764 |
| 22385 | 1.3279 | 0.866968 |

| | | |
|---|---|---|
| 22386 | 1.23726 | 0.541379 |
| 22387 | 1.29729 | 2.1 |
| 22388 | 0.946128 | 2.1 |
| 22389 | 1.14186 | 2.1 |
| 22390 | 1.39214 | 2.1 |
| 22391 | 1.39214 | 2.1 |
| 22392 | 0.946129 | 2.1 |
| 22393 | 1.23012 | 0.05 |
| 22394 | 0.653228 | 0.0660567 |
| 22395 | 0.946133 | 1.86544 |
| 22396 | 1.15918 | 1.86616 |
| 22397 | 1.26893 | 0.70266 |
| 22398 | 0.653228 | 1.5118 |
| 22399 | 1.06848 | 1.6654 |
| 22400 | 0.615121 | 2.1 |
| 22401 | 0.841488 | 1.37995 |
| 22402 | 0.946131 | 2.1 |
| 22403 | 1.04071 | 2.1 |
| 22404 | 0.965717 | 2.1 |
| 22405 | 1.77893 | 1.45347 |
| 22406 | 1.11314 | 0.05 |
| 22407 | 1.65411 | 0.210255 |
| 22408 | 0.85472 | 0.05 |
| 22409 | 1.1604 | 0.05 |
| 22410 | 1.19141 | 0.05 |
| 22411 | 1.19141 | 0.05 |
| 22412 | 0.94613 | 0.05 |
| 22413 | 1.70944 | 0.05 |
| 22414 | 1.89082 | 0.05 |
| 22415 | 1.05175 | 0.827602 |
| 22416 | 0.815276 | 2.1 |
| 22417 | 0.843283 | 2.1 |
| 22418 | 0.946131 | 2.1 |
| 22419 | 0.843284 | 2.1 |
| 22420 | 1.38897 | 2.1 |
| 22421 | 1.01019 | 0.190111 |
| 22422 | 1.05197 | 0.0809836 |
| 22423 | 0.810959 | 0.134506 |
| 22424 | 1.33037 | 0.05 |
| 22425 | 1.3415 | 0.05 |
| 22426 | 0.946118 | 0.05 |
| 22427 | 0.946131 | 0.05 |
| 22428 | 1.09803 | 0.05 |
| 22429 | 0.930281 | 2.1 |
| 22430 | 1.3335 | 2.1 |
| 22431 | 0.784151 | 2.1 |
| 22432 | 0.78415 | 2.1 |
| 22433 | 0.946132 | 2.1 |
| 22434 | 1.6299 | 0.05 |
| 22435 | 0.995795 | 2.1 |
| 22436 | 1.84846 | 1.54631 |
| 22437 | 0.929873 | 1.953 |
| 22438 | 0.889245 | 1.99267 |
| 22439 | 0.808072 | 2.05485 |
| 22440 | 0.851541 | 1.91252 |
| 22441 | 1.51956 | 1.46032 |
| 22442 | 0.94613 | 1.46595 |
| 22443 | 0.851553 | 1.6447 |

| | | |
|---|---|---|
| 22444 | 0.946787 | 1.51195 |
| 22445 | 1.15544 | 1.76331 |
| 22446 | 1.30289 | 0.05 |
| 22447 | 1.28323 | 0.05 |
| 22448 | 0.85024 | 0.05 |
| 22449 | 1.09662 | 0.05 |
| 22450 | 1.67687 | 0.05 |
| 22451 | 0.946127 | 2.1 |
| 22452 | 1.40496 | 2.1 |
| 22453 | 1.22442 | 2.1 |
| 22454 | 0.946128 | 2.1 |
| 22455 | 1.40496 | 2.1 |
| 22456 | 1.00573 | 2.1 |
| 22457 | 0.636837 | 2.1 |
| 22458 | 0.988642 | 2.1 |
| 22459 | 0.573141 | 2.1 |
| 22460 | 0.946128 | 2.1 |
| 22461 | 0.786538 | 2.1 |
| 22462 | 2.28748 | 1.55511 |
| 22463 | 0.682805 | 2.1 |
| 22464 | 0.946132 | 2.1 |
| 22465 | 0.682776 | 2.1 |
| 22466 | 0.946132 | 2.1 |
| 22467 | 0.79775 | 2.1 |
| 22468 | 1.05403 | 0.05 |
| 22469 | 0.888273 | 0.05 |
| 22470 | 0.876373 | 0.05 |
| 22471 | 0.576083 | 0.05 |
| 22472 | 0.946132 | 0.05 |
| 22473 | 0.576057 | 0.05 |
| 22474 | 0.576058 | 0.05 |
| 22475 | 0.946131 | 0.05 |
| 22476 | 1.41724 | 0.05 |
| 22477 | 1.31678 | 0.21031 |
| 22478 | 0.946132 | 0.186513 |
| 22479 | 0.946132 | 0.104088 |
| 22480 | 1.31678 | 0.0844198 |
| 22481 | 0.935738 | 2.1 |
| 22482 | 0.946132 | 2.1 |
| 22483 | 0.935765 | 2.1 |
| 22484 | 1.32949 | 2.1 |
| 22485 | 0.946131 | 2.1 |
| 22486 | 1.02525 | 2.1 |
| 22487 | 0.946128 | 2.1 |
| 22488 | 1.41207 | 2.1 |
| 22489 | 1.04675 | 2.1 |
| 22490 | 0.946131 | 2.1 |
| 22491 | 1.41207 | 2.1 |
| 22492 | 0.946131 | 2.1 |
| 22493 | 0.946132 | 2.1 |
| 22494 | 1.41125 | 2.1 |
| 22495 | 1.41125 | 2.1 |
| 22496 | 1.27087 | 2.1 |
| 22497 | 0.924071 | 0.05 |
| 22498 | 0.608893 | 0.05 |
| 22499 | 0.528907 | 0.05 |
| 22500 | 1.78249 | 0.05 |
| 22501 | 0.720094 | 0.05 |

| | | |
|---|---|---|
| 22502 | 0.781667 | 2.1 |
| 22503 | 0.781638 | 2.1 |
| 22504 | 1.40756 | 2.1 |
| 22505 | 1.40756 | 2.1 |
| 22506 | 0.797635 | 2.1 |
| 22507 | 1.11946 | 0.05 |
| 22508 | 1.32068 | 0.05 |
| 22509 | 0.88757 | 0.05 |
| 22510 | 1.27891 | 0.05 |
| 22511 | 0.810957 | 0.05 |
| 22512 | 0.914656 | 0.05 |
| 22513 | 0.640819 | 0.05 |
| 22514 | 0.745643 | 0.05 |
| 22515 | 0.865108 | 0.05 |
| 22516 | 1.06883 | 0.05 |
| 22517 | 0.806001 | 0.05 |
| 22518 | 1.19462 | 2.1 |
| 22519 | 1.42763 | 0.05 |
| 22520 | 1.24682 | 0.05 |
| 22521 | 0.78392 | 0.05 |
| 22522 | 1.02874 | 0.05 |
| 22523 | 0.884512 | 0.05 |
| 22524 | 1.1658 | 2.1 |
| 22525 | 0.663026 | 0.05 |
| 22526 | 1.21174 | 0.05 |
| 22527 | 0.663056 | 0.05 |
| 22528 | 1.15968 | 0.05 |
| 22529 | 1.21174 | 0.05 |
| 22530 | 0.993976 | 0.05 |
| 22531 | 0.945495 | 0.05 |
| 22532 | 0.671202 | 0.05 |
| 22533 | 1.37312 | 2.1 |
| 22534 | 1.28499 | 2.1 |
| 22535 | 0.946133 | 2.1 |
| 22536 | 0.946132 | 2.1 |
| 22537 | 1.28499 | 2.1 |
| 22538 | 1.25081 | 2.1 |
| 22539 | 0.946132 | 2.1 |
| 22540 | 0.946132 | 2.1 |
| 22541 | 1.27985 | 2.1 |
| 22542 | 1.37312 | 2.1 |
| 22543 | 0.830638 | 2.1 |
| 22544 | 1.05358 | 2.1 |
| 22545 | 1.22209 | 2.1 |
| 22546 | 1.09874 | 2.1 |
| 22547 | 0.74758 | 2.1 |
| 22548 | 2.50011 | 1.84449 |
| 22549 | 0.6157 | 0.05 |
| 22550 | 0.674634 | 0.05 |
| 22551 | 0.946128 | 1.05904 |
| 22552 | 0.674633 | 0.124886 |
| 22553 | 0.946131 | 0.303199 |
| 22554 | 1.96192 | 1.61953 |
| 22555 | 1.31612 | 0.05 |
| 22556 | 1.10735 | 0.05 |
| 22557 | 0.936676 | 0.05 |
| 22558 | 0.808779 | 0.05 |
| 22559 | 0.808808 | 0.05 |

| | | |
|---|---|---|
| 22560 | 1.00245 | 0.05 |
| 22561 | 1.26179 | 0.05 |
| 22562 | 1.05758 | 2.1 |
| 22563 | 1.03408 | 2.1 |
| 22564 | 0.946132 | 0.05 |
| 22565 | 0.914368 | 0.05 |
| 22566 | 1.20524 | 0.05 |
| 22567 | 1.12743 | 0.05 |
| 22568 | 0.851555 | 0.05 |
| 22569 | 1.17885 | 0.466723 |
| 22570 | 0.867881 | 0.334919 |
| 22571 | 1.00122 | 0.2914 |
| 22572 | 0.867883 | 0.121359 |
| 22573 | 0.867881 | 0.226694 |
| 22574 | 1.19583 | 0.377013 |
| 22575 | 1.17743 | 2.1 |
| 22576 | 0.878684 | 1.89288 |
| 22577 | 0.687087 | 2.1 |
| 22578 | 0.955079 | 0.204377 |
| 22579 | 0.672977 | 0.688992 |
| 22580 | 0.735937 | 0.711553 |
| 22581 | 0.851531 | 0.722254 |
| 22582 | 0.672957 | 0.70624 |
| 22583 | 0.923974 | 0.683971 |
| 22584 | 1.2243 | 2.1 |
| 22585 | 0.808463 | 0.996262 |
| 22586 | 0.971078 | 1.0821 |
| 22587 | 1.06161 | 1.10984 |
| 22588 | 0.810947 | 1.06537 |
| 22589 | 0.886074 | 1.23205 |
| 22590 | 0.786829 | 1.30964 |
| 22591 | 0.55062 | 0.90745 |
| 22592 | 0.574458 | 1.89699 |
| 22593 | 0.946129 | 1.01982 |
| 22594 | 1.42526 | 1.16789 |
| 22595 | 1.15962 | 1.05345 |
| 22596 | 1.24526 | 0.05 |
| 22597 | 0.94613 | 0.05 |
| 22598 | 0.946128 | 0.05 |
| 22599 | 1.23279 | 0.164547 |
| 22600 | 1.38645 | 0.0618083 |
| 22601 | 0.94613 | 0.115093 |
| 22602 | 1.05042 | 2.1 |
| 22603 | 0.946128 | 2.1 |
| 22604 | 0.946129 | 2.1 |
| 22605 | 1.24286 | 2.1 |
| 22606 | 1.39659 | 2.1 |
| 22607 | 0.94613 | 2.1 |
| 22608 | 1.10407 | 2.1 |
| 22609 | 1.32809 | 1.99565 |
| 22610 | 0.993999 | 2.1 |
| 22611 | 0.810959 | 1.72176 |
| 22612 | 0.983643 | 1.8096 |
| 22613 | 0.869552 | 2.1 |
| 22614 | 0.747801 | 1.57736 |
| 22615 | 1.53429 | 1.03233 |
| 22616 | 1.13127 | 2.1 |
| 22617 | 1.38397 | 2.1 |

| | | |
|---|---|---|
| 22618 | 1.08366 | 2.1 |
| 22619 | 0.946129 | 2.1 |
| 22620 | 0.946131 | 2.1 |
| 22621 | 1.08366 | 2.1 |
| 22622 | 1.06007 | 2.1 |
| 22623 | 1.79641 | 2.1 |
| 22624 | 0.758799 | 2.1 |
| 22625 | 1.28442 | 2.1 |
| 22626 | 1.06957 | 2.1 |
| 22627 | 0.946133 | 2.1 |
| 22628 | 1.28442 | 2.1 |
| 22629 | 1.76788 | 2.1 |
| 22630 | 1.38669 | 2.1 |
| 22631 | 0.986095 | 2.1 |
| 22632 | 1.38478 | 2.1 |
| 22633 | 0.946132 | 1.0092 |
| 22634 | 1.24344 | 0.697632 |
| 22635 | 0.622283 | 1.99811 |
| 22636 | 1.10335 | 0.888389 |
| 22637 | 1.24344 | 0.934033 |
| 22638 | 1.1967 | 1.38023 |
| 22639 | 0.946131 | 0.05 |
| 22640 | 0.901005 | 0.05 |
| 22641 | 1.05751 | 0.05 |
| 22642 | 1.37771 | 0.05 |
| 22643 | 0.851527 | 0.05 |
| 22644 | 1.26643 | 1.16469 |
| 22645 | 1.08992 | 1.65302 |
| 22646 | 0.946132 | 1.48473 |
| 22647 | 0.946132 | 1.10247 |
| 22648 | 1.08992 | 1.65456 |
| 22649 | 1.53534 | 2.1 |
| 22650 | 1.58825 | 1.44595 |
| 22651 | 1.0447 | 2.1 |
| 22652 | 0.593818 | 1.67909 |
| 22653 | 0.89883 | 1.95665 |
| 22654 | 0.734088 | 2.05991 |
| 22655 | 0.998451 | 0.836071 |
| 22656 | 1.64156 | 0.0908167 |
| 22657 | 1.05654 | 0.663049 |
| 22658 | 1.37641 | 0.0528935 |
| 22659 | 1.74553 | 0.104605 |
| 22660 | 1.31112 | 0.05 |
| 22661 | 0.74518 | 0.175472 |
| 22662 | 0.851539 | 0.148854 |
| 22663 | 0.851554 | 0.05 |
| 22664 | 0.851475 | 0.05 |
| 22665 | 0.946132 | 0.05 |
| 22666 | 1.28895 | 0.05 |
| 22667 | 1.11356 | 0.05 |
| 22668 | 0.946132 | 0.05 |
| 22669 | 0.946132 | 0.05 |
| 22670 | 1.84214 | 2.1 |
| 22671 | 1.23851 | 0.05 |
| 22672 | 1.25866 | 0.05 |
| 22673 | 1.28655 | 0.179803 |
| 22674 | 0.946131 | 0.0871058 |
| 22675 | 0.946132 | 0.0548839 |

| | | |
|---|---|---|
| 22676 | 1.28655 | 0.05 |
| 22677 | 1.11929 | 0.05 |
| 22678 | 0.946132 | 0.101038 |
| 22679 | 0.946132 | 0.05 |
| 22680 | 1.34254 | 0.05 |
| 22681 | 1.25866 | 0.05 |
| 22682 | 0.946132 | 2.1 |
| 22683 | 1.27451 | 2.1 |
| 22684 | 1.19803 | 2.1 |
| 22685 | 0.94613 | 2.1 |
| 22686 | 1.27451 | 2.1 |
| 22687 | 0.946131 | 2.1 |
| 22688 | 1.36523 | 2.1 |
| 22689 | 1.14599 | 2.1 |
| 22690 | 0.946129 | 2.1 |
| 22691 | 1.36523 | 2.1 |
| 22692 | 1.09518 | 2.1 |
| 22693 | 0.977777 | 2.1 |
| 22694 | 0.583237 | 2.1 |
| 22695 | 0.583267 | 2.1 |
| 22696 | 0.903872 | 2.1 |
| 22697 | 0.946129 | 2.1 |
| 22698 | 0.583258 | 2.1 |
| 22699 | 1.58179 | 0.05 |
| 22700 | 0.946131 | 0.05 |
| 22701 | 1.35123 | 0.05 |
| 22702 | 0.655837 | 0.05 |
| 22703 | 1.12018 | 0.05 |
| 22704 | 1.35123 | 0.05 |
| 22705 | 0.62967 | 0.05 |
| 22706 | 0.860497 | 0.05 |
| 22707 | 0.816639 | 0.05 |
| 22708 | 0.746716 | 2.1 |
| 22709 | 0.810947 | 2.1 |
| 22710 | 0.810959 | 2.1 |
| 22711 | 0.653422 | 2.1 |
| 22712 | 0.289127 | 1.31908 |
| 22713 | 0.475716 | 2.1 |
| 22714 | 0.980651 | 1.35588 |
| 22715 | 0.946119 | 2.1 |
| 22716 | 0.946132 | 2.1 |
| 22717 | 1.08299 | 2.1 |
| 22718 | 1.34539 | 2.1 |
| 22719 | 1.28753 | 0.05 |
| 22720 | 0.946132 | 0.05 |
| 22721 | 0.946132 | 0.05 |
| 22722 | 1.19719 | 0.05 |
| 22723 | 0.946133 | 0.05 |
| 22724 | 0.958947 | 0.05 |
| 22725 | 1.3492 | 0.05 |
| 22726 | 0.946132 | 0.05 |
| 22727 | 0.946128 | 0.05 |
| 22728 | 1.22588 | 0.05 |
| 22729 | 0.946129 | 0.05 |
| 22730 | 0.991113 | 2.1 |
| 22731 | 1.35966 | 2.1 |
| 22732 | 0.961 2.1 | |
| 22733 | 0.946129 | 2.1 |

| | | |
|---|---|---|
| 22734 | 0.946131 | 2.1 |
| 22735 | 0.961001 | 2.1 |
| 22736 | 0.727096 | 1.92242 |
| 22737 | 0.810946 | 0.217496 |
| 22738 | 0.810959 | 0.77872 |
| 22739 | 1.31658 | 0.05 |
| 22740 | 1.23167 | 2.1 |
| 22741 | 1.44836 | 0.05 |
| 22742 | 1.50103 | 0.05 |
| 22743 | 1.23351 | 0.05 |
| 22744 | 1.35657 | 0.05 |
| 22745 | 0.851541 | 0.05 |
| 22746 | 0.93668 | 0.05 |
| 22747 | 0.946131 | 0.05 |
| 22748 | 3.7335 | 2.1 |
| 22749 | 1.1822 | 2.1 |
| 22750 | 1.31127 | 0.05 |
| 22751 | 1.3689 | 0.05 |
| 22752 | 1.35822 | 0.05 |
| 22753 | 0.946133 | 0.05 |
| 22754 | 0.946132 | 0.05 |
| 22755 | 1.14926 | 0.05 |
| 22756 | 1.40822 | 0.05 |
| 22757 | 1.04935 | 0.05 |
| 22758 | 0.704112 | 0.05 |
| 22759 | 0.704112 | 0.05 |
| 22760 | 0.946131 | 0.05 |
| 22761 | 0.704112 | 0.05 |
| 22762 | 1.37721 | 0.05 |
| 22763 | 0.85154 | 2.1 |
| 22764 | 1.06949 | 2.1 |
| 22765 | 0.851541 | 2.1 |
| 22766 | 0.946128 | 2.1 |
| 22767 | 1.1175 | 2.1 |
| 22768 | 1.44091 | 2.1 |
| 22769 | 0.96029 | 0.68014 |
| 22770 | 3.46486 | 1.14492 |
| 22771 | 0.603286 | 0.05 |
| 22772 | 0.432283 | 0.05 |
| 22773 | 0.851541 | 0.05 |
| 22774 | 1.09355 | 0.05 |
| 22775 | 0.851534 | 0.05 |
| 22776 | 1.04263 | 2.1 |
| 22777 | 0.851541 | 2.1 |
| 22778 | 0.965604 | 2.1 |
| 22779 | 0.94613 | 2.1 |
| 22780 | 1.23953 | 2.1 |
| 22781 | 0.869615 | 2.1 |
| 22782 | 1.02415 | 2.1 |
| 22783 | 0.983032 | 2.1 |
| 22784 | 1.35385 | 1.63993 |
| 22785 | 1.59971 | 0.05 |
| 22786 | 0.915178 | 2.1 |
| 22787 | 0.962435 | 2.1 |
| 22788 | 1.3116 | 2.1 |
| 22789 | 1.38302 | 2.1 |
| 22790 | 1.13227 | 2.1 |
| 22791 | 1.25923 | 0.05 |

| | | |
|---|---|---|
| 22792 | 0.988805 | 2.1 |
| 22793 | 0.611486 | 0.956591 |
| 22794 | 1.1019 | 1.02499 |
| 22795 | 1.11124 | 1.72349 |
| 22796 | 0.716713 | 0.938109 |
| 22797 | 0.946131 | 1.37267 |
| 22798 | 1.22297 | 1.16638 |
| 22799 | 1.12873 | 1.82958 |
| 22800 | 0.946131 | 1.71239 |
| 22801 | 0.946132 | 1.84245 |
| 22802 | 1.3469 | 2.03449 |
| 22803 | 1.25366 | 2.1 |
| 22804 | 1.1611 | 1.04356 |
| 22805 | 0.946132 | 1.43967 |
| 22806 | 0.946132 | 1.09482 |
| 22807 | 1.2854 | 1.47883 |
| 22808 | 1.3469 | 1.65881 |
| 22809 | 1.07468 | 1.19149 |
| 22810 | 1.79317 | 2.1 |
| 22811 | 1.07444 | 2.1 |
| 22812 | 1.69436 | 2.1 |
| 22813 | 0.851539 | 2.1 |
| 22814 | 0.870925 | 2.1 |
| 22815 | 0.851541 | 2.1 |
| 22816 | 0.870951 | 2.1 |
| 22817 | 1.26097 | 2.1 |
| 22818 | 1.26247 | 2.1 |
| 22819 | 0.946132 | 2.1 |
| 22820 | 1.24041 | 2.1 |
| 22821 | 0.946132 | 2.1 |
| 22822 | 0.946132 | 2.1 |
| 22823 | 1.40274 | 2.1 |
| 22824 | 0.946131 | 0.05 |
| 22825 | 0.797945 | 0.05 |
| 22826 | 1.12988 | 0.05 |
| 22827 | 1.00681 | 0.05 |
| 22828 | 0.797932 | 0.05 |
| 22829 | 0.977483 | 0.05 |
| 22830 | 0.943362 | 0.0753955 |
| 22831 | 0.9891 | 0.05 |
| 22832 | 1.1703 | 0.05 |
| 22833 | 0.663171 | 0.05 |
| 22834 | 1.40882 | 0.05 |
| 22835 | 1.32639 | 0.05 |
| 22836 | 1.24448 | 0.05 |
| 22837 | 0.946132 | 0.05 |
| 22838 | 1.11015 | 0.05 |
| 22839 | 1.24448 | 0.05 |
| 22840 | 0.754768 | 0.05 |
| 22841 | 1.13854 | 2.1 |
| 22842 | 1.23654 | 2.1 |
| 22843 | 0.714101 | 0.0534728 |
| 22844 | 0.851538 | 0.05 |
| 22845 | 0.79261 | 1.39837 |
| 22846 | 1.30598 | 0.668448 |
| 22847 | 1.05338 | 0.05 |
| 22848 | 1.41758 | 2.1 |
| 22849 | 0.83317 | 0.05 |

| | | |
|---|---|---|
| 22850 | 1.00058 | 0.05 |
| 22851 | 0.810958 | 0.05 |
| 22852 | 1.63791 | 0.05 |
| 22853 | 1.55116 | 2.1 |
| 22854 | 1.29735 | 1.3078 |
| 22855 | 0.945147 | 2.1 |
| 22856 | 0.94515 | 1.33162 |
| 22857 | 0.94613 | 2.03954 |
| 22858 | 0.945149 | 2.1 |
| 22859 | 1.02368 | 2.1 |
| 22860 | 1.12787 | 2.1 |
| 22861 | 1.04007 | 2.1 |
| 22862 | 3.78805 | 2.1 |
| 22863 | 0.816891 | 0.05 |
| 22864 | 0.943996 | 0.05 |
| 22865 | 0.870002 | 0.05 |
| 22866 | 0.564533 | 0.05 |
| 22867 | 0.564536 | 0.05 |
| 22868 | 0.946128 | 0.299335 |
| 22869 | 0.564535 | 0.05 |
| 22870 | 0.946128 | 0.139739 |
| 22871 | 1.15177 | 2.1 |
| 22872 | 1.16806 | 0.985198 |
| 22873 | 0.999083 | 1.14739 |
| 22874 | 0.946131 | 1.10868 |
| 22875 | 0.946131 | 1.09037 |
| 22876 | 0.999084 | 1.27196 |
| 22877 | 1.28918 | 0.90938 |
| 22878 | 1.0383 | 1.1566 |
| 22879 | 0.946133 | 1.04993 |
| 22880 | 0.946132 | 1.09716 |
| 22881 | 1.0383 | 1.14806 |
| 22882 | 1.27737 | 1.6934 |
| 22883 | 1.39296 | 0.302016 |
| 22884 | 0.998682 | 2.1 |
| 22885 | 3.98933 | 2.1 |
| 22886 | 1.05328 | 0.05 |
| 22887 | 1.23475 | 2.1 |
| 22888 | 0.900856 | 1.9063 |
| 22889 | 0.94001 | 0.979871 |
| 22890 | 0.707748 | 0.82451 |
| 22891 | 0.707751 | 1.36691 |
| 22892 | 0.946128 | 0.882568 |
| 22893 | 0.70775 | 1.08535 |
| 22894 | 0.996109 | 1.02592 |
| 22895 | 1.68551 | 2.1 |
| 22896 | 0.851539 | 2.1 |
| 22897 | 0.962688 | 2.1 |
| 22898 | 0.94613 | 0.996492 |
| 22899 | 1.04073 | 2.1 |
| 22900 | 1.44824 | 2.1 |
| 22901 | 0.794313 | 0.05 |
| 22902 | 0.838031 | 0.05 |
| 22903 | 1.11076 | 0.05 |
| 22904 | 1.38813 | 2.1 |
| 22905 | 1.17204 | 2.1 |
| 22906 | 0.946132 | 2.1 |
| 22907 | 0.946131 | 2.1 |

| | | |
|---|---|---|
| 22908 | 1.17204 | 2.1 |
| 22909 | 0.961779 | 0.05 |
| 22910 | 1.0685 | 0.05 |
| 22911 | 0.810947 | 2.1 |
| 22912 | 1.2899 | 1.01412 |
| 22913 | 0.890404 | 1.00804 |
| 22914 | 0.909981 | 2.1 |
| 22915 | 0.810946 | 1.28945 |
| 22916 | 0.890402 | 1.05401 |
| 22917 | 0.522844 | 2.1 |
| 22918 | 0.593846 | 2.1 |
| 22919 | 1.30226 | 1.17832 |
| 22920 | 1.30226 | 1.07248 |
| 22921 | 0.946132 | 1.46465 |
| 22922 | 1.03537 | 0.05 |
| 22923 | 1.50035 | 0.05 |
| 22924 | 0.940356 | 0.05 |
| 22925 | 0.946131 | 0.05 |
| 22926 | 1.03441 | 0.05 |
| 22927 | 1.21171 | 0.05 |
| 22928 | 1.35169 | 0.05 |
| 22929 | 1.32449 | 0.05 |
| 22930 | 0.946131 | 0.05 |
| 22931 | 0.946131 | 0.05 |
| 22932 | 1.06627 | 0.05 |
| 22933 | 1.03102 | 0.117012 |
| 22934 | 0.488712 | 0.05 |
| 22935 | 0.946131 | 0.05 |
| 22936 | 1.23146 | 0.05 |
| 22937 | 1.13242 | 0.05 |
| 22938 | 0.946131 | 0.05 |
| 22939 | 0.409199 | 0.05 |
| 22940 | 0.429959 | 0.05 |
| 22941 | 1.08357 | 0.05 |
| 22942 | 0.80926 | 0.05 |
| 22943 | 0.879791 | 0.05 |
| 22944 | 0.946129 | 1.14721 |
| 22945 | 1.28828 | 2.1 |
| 22946 | 0.946131 | 0.686483 |
| 22947 | 0.946131 | 2.1 |
| 22948 | 1.35969 | 2.1 |
| 22949 | 1.10049 | 1.99971 |
| 22950 | 0.923724 | 1.91954 |
| 22951 | 0.923695 | 2.1 |
| 22952 | 0.94613 | 1.74895 |
| 22953 | 0.94613 | 2.1 |
| 22954 | 1.28828 | 2.1 |
| 22955 | 1.73525 | 0.05 |
| 22956 | 0.656239 | 0.05 |
| 22957 | 1.2996 | 0.05 |
| 22958 | 0.812939 | 0.05 |
| 22959 | 0.65624 | 0.05 |
| 22960 | 1.2996 | 0.05 |
| 22961 | 1.67372 | 2.1 |
| 22962 | 0.946131 | 2.1 |
| 22963 | 0.946132 | 2.1 |
| 22964 | 1.05978 | 2.1 |
| 22965 | 1.31049 | 2.1 |

| | | |
|---|---|---|
| 22966 | 0.946132 | 2.1 |
| 22967 | 1.11077 | 2.1 |
| 22968 | 0.579725 | 0.05 |
| 22969 | 0.373532 | 0.05 |
| 22970 | 1.13537 | 0.071545 |
| 22971 | 1.20726 | 0.05 |
| 22972 | 0.946131 | 0.05 |
| 22973 | 1.45694 | 0.245124 |
| 22974 | 0.750367 | 0.05 |
| 22975 | 0.946126 | 0.05 |
| 22976 | 0.750365 | 0.05 |
| 22977 | 1.25698 | 0.05 |
| 22978 | 1.35726 | 0.05 |
| 22979 | 1.6812 | 0.05 |
| 22980 | 1.78665 | 1.70936 |
| 22981 | 1.46568 | 2.1 |
| 22982 | 1.29606 | 2.1 |
| 22983 | 1.25298 | 2.1 |
| 22984 | 0.946115 | 2.1 |
| 22985 | 0.946129 | 2.1 |
| 22986 | 1.25296 | 2.1 |
| 22987 | 1.27778 | 0.05 |
| 22988 | 1.17042 | 2.1 |
| 22989 | 1.25858 | 0.05 |
| 22990 | 0.93106 | 0.05 |
| 22991 | 0.810959 | 0.05 |
| 22992 | 1.38905 | 2.1 |
| 22993 | 1.19216 | 2.1 |
| 22994 | 0.946131 | 2.1 |
| 22995 | 0.946131 | 2.1 |
| 22996 | 1.19216 | 2.1 |
| 22997 | 1.58339 | 2.1 |
| 22998 | 0.81094 | 0.05 |
| 22999 | 0.845129 | 0.05 |
| 23000 | 0.918535 | 0.05 |
| 23001 | 0.946132 | 2.1 |
| 23002 | 0.946132 | 2.1 |
| 23003 | 1.31904 | 2.1 |
| 23004 | 1.31904 | 2.1 |
| 23005 | 1.21275 | 2.1 |
| 23006 | 1.15471 | 2.1 |
| 23007 | 1.33125 | 2.1 |
| 23008 | 0.702954 | 0.05 |
| 23009 | 0.942973 | 0.05 |
| 23010 | 0.94613 | 0.05 |
| 23011 | 0.641772 | 0.05 |
| 23012 | 0.702942 | 0.05 |
| 23013 | 0.991865 | 0.05 |
| 23014 | 0.639952 | 0.05 |
| 23015 | 1.17333 | 0.05 |
| 23016 | 0.946132 | 0.05 |
| 23017 | 0.490975 | 0.05 |
| 23018 | 0.495934 | 0.05 |
| 23019 | 1.19904 | 0.05 |
| 23020 | 1.30488 | 0.05 |
| 23021 | 1.02305 | 0.05 |
| 23022 | 1.49842 | 0.05 |
| 23023 | 1.54196 | 2.1 |

| | | |
|---|---|---|
| 23024 | 1.37224 | 2.1 |
| 23025 | 1.0953 | 0.05 |
| 23026 | 1.18952 | 2.1 |
| 23027 | 1.68851 | 0.465478 |
| 23028 | 0.946131 | 0.107242 |
| 23029 | 1.12491 | 0.143241 |
| 23030 | 0.583591 | 0.0996828 |
| 23031 | 1.28727 | 0.157214 |
| 23032 | 1.12491 | 0.277021 |
| 23033 | 0.884641 | 0.426703 |
| 23034 | 0.810944 | 0.36688 |
| 23035 | 0.917973 | 0.510514 |
| 23036 | 0.846671 | 0.501278 |
| 23037 | 0.596968 | 0.0604177 |
| 23038 | 0.716347 | 0.485355 |
| 23039 | 1.36255 | 0.0818481 |
| 23040 | 1.11077 | 2.1 |
| 23041 | 0.946132 | 2.1 |
| 23042 | 0.946132 | 2.1 |
| 23043 | 1.26267 | 2.1 |
| 23044 | 1.26441 | 2.1 |
| 23045 | 1.85238 | 2.1 |
| 23046 | 1.36693 | 2.1 |
| 23047 | 0.616123 | 2.1 |
| 23048 | 0.481844 | 2.1 |
| 23049 | 0.709599 | 2.1 |
| 23050 | 0.815889 | 2.1 |
| 23051 | 1.1502 | 2.1 |
| 23052 | 1.63937 | 0.0802026 |
| 23053 | 1.25797 | 0.05 |
| 23054 | 1.22878 | 0.05 |
| 23055 | 0.946132 | 0.05 |
| 23056 | 0.946132 | 0.05 |
| 23057 | 0.965737 | 0.05 |
| 23058 | 1.1893 | 0.05 |
| 23059 | 1.22877 | 0.05 |
| 23060 | 0.735217 | 0.05 |
| 23061 | 0.85154 | 0.05 |
| 23062 | 0.851553 | 0.05 |
| 23063 | 0.851473 | 0.05 |
| 23064 | 1.58916 | 0.05 |
| 23065 | 1.25545 | 0.05 |
| 23066 | 0.810942 | 0.05 |
| 23067 | 0.919314 | 0.05 |
| 23068 | 0.928633 | 0.05 |
| 23069 | 2.14514 | 0.05 |
| 23070 | 1.23389 | 2.1 |
| 23071 | 0.946128 | 0.05 |
| 23072 | 0.969504 | 0.05 |
| 23073 | 0.851539 | 0.05 |
| 23074 | 1.41503 | 0.05 |
| 23075 | 0.958517 | 0.05 |
| 23076 | 0.990615 | 0.05 |
| 23077 | 1.7943 | 0.05 |
| 23078 | 0.946133 | 0.05 |
| 23079 | 1.27581 | 0.05 |
| 23080 | 0.946132 | 0.05 |
| 23081 | 1.36362 | 0.05 |

| | | |
|---|---|---|
| 23082 | 1.36936 | 0.05 |
| 23083 | 1.71903 | 2.1 |
| 23084 | 0.751639 | 2.1 |
| 23085 | 0.77464 | 2.1 |
| 23086 | 0.946133 | 2.1 |
| 23087 | 1.04073 | 2.1 |
| 23088 | 1.23551 | 2.1 |
| 23089 | 0.94613 | 2.1 |
| 23090 | 1.18682 | 2.1 |
| 23091 | 0.946131 | 2.1 |
| 23092 | 0.94613 | 2.1 |
| 23093 | 1.40638 | 2.1 |
| 23094 | 0.483346 | 0.05 |
| 23095 | 0.432527 | 0.05 |
| 23096 | 0.946132 | 0.05 |
| 23097 | 0.483312 | 0.05 |
| 23098 | 0.946132 | 0.05 |
| 23099 | 0.878798 | 0.05 |
| 23100 | 0.810944 | 0.05 |
| 23101 | 1.14604 | 0.05 |
| 23102 | 0.946129 | 0.05 |
| 23103 | 1.35594 | 0.05 |
| 23104 | 0.538378 | 0.05 |
| 23105 | 0.946131 | 0.05 |
| 23106 | 0.538378 | 0.05 |
| 23107 | 1.00065 | 0.380694 |
| 23108 | 0.962878 | 0.132215 |
| 23109 | 1.32634 | 0.05 |
| 23110 | 1.28066 | 0.05 |
| 23111 | 0.785651 | 2.1 |
| 23112 | 1.20327 | 2.1 |
| 23113 | 1.47058 | 2.09872 |
| 23114 | 0.551834 | 1.08793 |
| 23115 | 1.04072 | 2.1 |
| 23116 | 0.506719 | 1.48774 |
| 23117 | 1.12786 | 1.93204 |
| 23118 | 1.45789 | 0.05 |
| 23119 | 0.681532 | 0.05 |
| 23120 | 1.21087 | 0.05 |
| 23121 | 0.94613 | 0.05 |
| 23122 | 0.681534 | 0.05 |
| 23123 | 1.21087 | 0.05 |
| 23124 | 1.98618 | 0.16244 |
| 23125 | 1.71742 | 1.02145 |
| 23126 | 1.07427 | 0.766441 |
| 23127 | 1.1972 | 0.353108 |
| 23128 | 0.933474 | 0.588976 |
| 23129 | 1.1745 | 0.675772 |
| 23130 | 0.94612 | 0.605248 |
| 23131 | 0.933485 | 0.671049 |
| 23132 | 1.17449 | 0.655339 |
| 23133 | 0.851541 | 0.05 |
| 23134 | 1.12774 | 0.05 |
| 23135 | 0.946129 | 0.05 |
| 23136 | 0.851486 | 0.05 |
| 23137 | 1.12517 | 0.05 |
| 23138 | 1.12328 | 0.05 |
| 23139 | 1.13179 | 2.1 |

| | | |
|---|---|---|
| 23140 | 1.02438 | 2.1 |
| 23141 | 1.05776 | 2.1 |
| 23142 | 1.41273 | 1.37378 |
| 23143 | 1.1229 | 2.1 |
| 23144 | 0.875912 | 1.38371 |
| 23145 | 0.946132 | 2.1 |
| 23146 | 0.875912 | 2.1 |
| 23147 | 1.42307 | 2.1 |
| 23148 | 1.15354 | 0.05 |
| 23149 | 0.848382 | 0.05 |
| 23150 | 1.2685 | 2.1 |
| 23151 | 0.938104 | 2.1 |
| 23152 | 0.793166 | 2.1 |
| 23153 | 1.07855 | 2.1 |
| 23154 | 0.98488 | 2.1 |
| 23155 | 1.4162 | 2.1 |
| 23156 | 1.09651 | 0.05 |
| 23157 | 1.98502 | 0.05 |
| 23158 | 1.41168 | 0.05 |
| 23159 | 1.33879 | 0.05 |
| 23160 | 0.946127 | 0.05 |
| 23161 | 1.13528 | 0.05 |
| 23162 | 0.946128 | 0.05 |
| 23163 | 1.09726 | 2.1 |
| 23164 | 1.22026 | 0.859382 |
| 23165 | 1.09044 | 2.1 |
| 23166 | 2.23961 | 0.305496 |
| 23167 | 1.14721 | 0.05 |
| 23168 | 1.17614 | 1.19821 |
| 23169 | 0.615065 | 2.1 |
| 23170 | 0.946131 | 2.1 |
| 23171 | 0.778488 | 2.1 |
| 23172 | 0.94613 | 2.1 |
| 23173 | 0.615066 | 2.1 |
| 23174 | 1.78254 | 2.1 |
| 23175 | 0.898452 | 0.05 |
| 23176 | 1.47411 | 2.1 |
| 23177 | 0.59676 | 0.05 |
| 23178 | 0.59676 | 0.05 |
| 23179 | 0.94613 | 0.05 |
| 23180 | 1.13511 | 0.05 |
| 23181 | 0.946131 | 0.05 |
| 23182 | 0.916275 | 2.1 |
| 23183 | 0.946132 | 2.1 |
| 23184 | 0.916274 | 1.12313 |
| 23185 | 1.36182 | 2.1 |
| 23186 | 0.946131 | 2.1 |
| 23187 | 0.857202 | 0.05 |
| 23188 | 0.941918 | 0.05 |
| 23189 | 0.943349 | 0.05 |
| 23190 | 1.2493 | 0.05 |
| 23191 | 0.946133 | 0.05 |
| 23192 | 1.01814 | 0.05 |
| 23193 | 0.927881 | 0.05 |
| 23194 | 1.11845 | 0.05 |
| 23195 | 1.2493 | 0.05 |
| 23196 | 1.11845 | 0.05 |
| 23197 | 0.946129 | 0.05 |

| | | |
|---|---|---|
| 23198 | 0.967673 | 0.0522127 |
| 23199 | 1.01332 | 0.05 |
| 23200 | 0.946133 | 0.05 |
| 23201 | 0.81938 | 0.05 |
| 23202 | 0.819377 | 0.05 |
| 23203 | 0.946133 | 0.05 |
| 23204 | 1.22474 | 0.05 |
| 23205 | 1.15321 | 0.05 |
| 23206 | 0.797489 | 0.05 |
| 23207 | 0.946131 | 0.05 |
| 23208 | 0.797461 | 0.05 |
| 23209 | 1.35328 | 0.871216 |
| 23210 | 1.03318 | 1.24639 |
| 23211 | 0.946131 | 0.997009 |
| 23212 | 0.94613 | 1.06231 |
| 23213 | 1.03319 | 1.0917 |
| 23214 | 1.37938 | 0.870687 |
| 23215 | 1.00512 | 1.2476 |
| 23216 | 0.946133 | 0.964975 |
| 23217 | 0.946132 | 1.06291 |
| 23218 | 1.00512 | 1.03032 |
| 23219 | 1.5515 | 1.28694 |
| 23220 | 1.55351 | 2.1 |
| 23221 | 0.94261 | 0.05 |
| 23222 | 0.828156 | 0.05 |
| 23223 | 0.916495 | 0.05 |
| 23224 | 3.06309 | 0.05 |
| 23225 | 0.619832 | 0.05 |
| 23226 | 0.705903 | 0.05 |
| 23227 | 1.2968 | 0.05 |
| 23228 | 0.946133 | 0.05 |
| 23229 | 0.705904 | 0.05 |
| 23230 | 1.2968 | 0.05 |
| 23231 | 1.01971 | 0.05 |
| 23232 | 0.60302 | 0.05 |
| 23233 | 1.07242 | 0.05 |
| 23234 | 1.26426 | 0.05 |
| 23235 | 0.946131 | 0.069474 |
| 23236 | 0.946131 | 0.0714855 |
| 23237 | 0.963109 | 0.0660865 |
| 23238 | 0.947862 | 0.0746364 |
| 23239 | 1.40248 | 1.11706 |
| 23240 | 0.767143 | 1.22258 |
| 23241 | 0.767114 | 1.18445 |
| 23242 | 0.946129 | 1.24834 |
| 23243 | 0.767143 | 0.774758 |
| 23244 | 1.22637 | 0.990808 |
| 23245 | 1.2281 | 1.10493 |
| 23246 | 0.590909 | 1.27147 |
| 23247 | 0.490832 | 1.12615 |
| 23248 | 0.804174 | 1.27435 |
| 23249 | 0.747368 | 0.969311 |
| 23250 | 1.05129 | 0.05 |
| 23251 | 1.10609 | 0.066543 |
| 23252 | 1.3639 | 0.0575917 |
| 23253 | 0.776117 | 0.133492 |
| 23254 | 0.752329 | 0.0599394 |
| 23255 | 1.04073 | 0.0738434 |

| | | |
|---|---|---|
| 23256 | 0.67713 | 0.0669309 |
| 23257 | 3.4245 | 2.1 |
| 23258 | 1.18526 | 2.1 |
| 23259 | 1.2161 | 1.22853 |
| 23260 | 0.876834 | 2.1 |
| 23261 | 0.876831 | 1.11539 |
| 23262 | 0.946132 | 1.96884 |
| 23263 | 0.876834 | 1.31395 |
| 23264 | 0.954563 | 2.1 |
| 23265 | 0.924132 | 2.1 |
| 23266 | 1.25075 | 2.1 |
| 23267 | 1.01652 | 2.1 |
| 23268 | 0.92413 | 2.1 |
| 23269 | 1.37579 | 2.1 |
| 23270 | 0.992159 | 2.1 |
| 23271 | 1.26092 | 2.1 |
| 23272 | 0.929435 | 2.1 |
| 23273 | 0.844958 | 2.1 |
| 23274 | 0.79598 | 2.1 |
| 23275 | 0.852559 | 2.1 |
| 23276 | 0.946131 | 0.117073 |
| 23277 | 1.28435 | 0.05 |
| 23278 | 0.827261 | 0.0968733 |
| 23279 | 0.946128 | 0.104386 |
| 23280 | 0.827298 | 0.150929 |
| 23281 | 1.65452 | 0.05 |
| 23282 | 1.79929 | 2.1 |
| 23283 | 0.791947 | 2.06502 |
| 23284 | 0.902072 | 1.19294 |
| 23285 | 0.882672 | 1.85785 |
| 23286 | 1.21703 | 2.1 |
| 23287 | 0.900338 | 2.1 |
| 23288 | 1.37712 | 2.1 |
| 23289 | 0.782591 | 2.1 |
| 23290 | 0.782562 | 2.1 |
| 23291 | 0.94613 | 2.1 |
| 23292 | 1.34749 | 2.1 |
| 23293 | 0.61359 | 2.1 |
| 23294 | 1.14397 | 0.05 |
| 23295 | 1.02963 | 0.05 |
| 23296 | 1.36589 | 0.05 |
| 23297 | 0.946132 | 0.05 |
| 23298 | 1.28644 | 0.05 |
| 23299 | 0.946131 | 0.05 |
| 23300 | 1.40104 | 0.05 |
| 23301 | 1.28644 | 0.05 |
| 23302 | 0.810946 | 0.05 |
| 23303 | 1.09178 | 0.05 |
| 23304 | 1.26956 | 0.05 |
| 23305 | 0.810943 | 0.05 |
| 23306 | 0.885633 | 0.05 |
| 23307 | 1.00844 | 0.05 |
| 23308 | 0.825671 | 0.05 |
| 23309 | 0.792093 | 0.05 |
| 23310 | 0.925037 | 0.05 |
| 23311 | 0.946131 | 0.429701 |
| 23312 | 0.946131 | 0.05 |
| 23313 | 1.18052 | 0.05 |

| | | |
|---|---|---|
| 23314 | 1.40265 | 0.05 |
| 23315 | 0.946132 | 0.485747 |
| 23316 | 1.17891 | 0.0722265 |
| 23317 | 0.734248 | 0.05 |
| 23318 | 0.810943 | 0.05 |
| 23319 | 0.731621 | 0.05 |
| 23320 | 0.799857 | 0.0522245 |
| 23321 | 0.834507 | 0.542886 |
| 23322 | 0.810932 | 0.0908191 |
| 23323 | 1.01316 | 0.05 |
| 23324 | 1.23884 | 0.05 |
| 23325 | 1.88438 | 2.1 |
| 23326 | 0.946129 | 2.1 |
| 23327 | 1.38614 | 2.1 |
| 23328 | 1.34245 | 1.15795 |
| 23329 | 0.946131 | 1.28733 |
| 23330 | 1.38614 | 1.21218 |
| 23331 | 0.946132 | 0.05 |
| 23332 | 0.910733 | 0.05 |
| 23333 | 1.24726 | 0.05 |
| 23334 | 1.13742 | 0.05 |
| 23335 | 0.851555 | 0.05 |
| 23336 | 1.50368 | 0.05 |
| 23337 | 0.94613 | 0.05 |
| 23338 | 1.08998 | 2.1 |
| 23339 | 1.36899 | 0.05 |
| 23340 | 1.36898 | 0.0550375 |
| 23341 | 0.946131 | 0.05 |
| 23342 | 1.26045 | 2.1 |
| 23343 | 1.06432 | 0.426679 |
| 23344 | 1.89016 | 0.05 |
| 23345 | 0.83514 | 1.7983 |
| 23346 | 1.36653 | 2.1 |
| 23347 | 1.23426 | 2.1 |
| 23348 | 0.94613 | 1.85512 |
| 23349 | 0.835112 | 2.1 |
| 23350 | 1.14567 | 1.06145 |
| 23351 | 1.12096 | 0.954185 |
| 23352 | 0.881106 | 1.16352 |
| 23353 | 1.17787 | 0.076934 |
| 23354 | 1.29738 | 0.856016 |
| 23355 | 0.940472 | 0.05 |
| 23356 | 1.03452 | 0.790593 |
| 23357 | 0.946131 | 0.0829652 |
| 23358 | 2.7489 | 0.566691 |
| 23359 | 1.01778 | 2.1 |
| 23360 | 1.04321 | 0.393931 |
| 23361 | 1.19352 | 0.05 |
| 23362 | 1.16912 | 1.76098 |
| 23363 | 0.946132 | 1.30455 |
| 23364 | 0.946132 | 2.08277 |
| 23365 | 1.40924 | 2.05441 |
| 23366 | 1.22927 | 2.1 |
| 23367 | 2.09455 | 0.05 |
| 23368 | 3.00575 | 2.1 |
| 23369 | 1.08928 | 0.05 |
| 23370 | 1.94799 | 2.1 |
| 23371 | 1.4092 | 2.1 |

| | | |
|---|---|---|
| 23372 | 0.946132 | 2.1 |
| 23373 | 1.16 | 2.1 |
| 23374 | 1.01985 | 2.1 |
| 23375 | 0.946133 | 2.1 |
| 23376 | 1.16 | 2.1 |
| 23377 | 1.15371 | 0.05 |
| 23378 | 1.56002 | 0.05 |
| 23379 | 1.23007 | 2.1 |
| 23380 | 1.9992 | 2.1 |
| 23381 | 0.652605 | 2.1 |
| 23382 | 0.937903 | 2.1 |
| 23383 | 0.946132 | 2.1 |
| 23384 | 0.937902 | 2.1 |
| 23385 | 1.228 | 2.1 |
| 23386 | 1.33694 | 1.45333 |
| 23387 | 1.00191 | 2.1 |
| 23388 | 0.716814 | 1.60619 |
| 23389 | 0.94613 | 2.1 |
| 23390 | 0.716816 | 2.1 |
| 23391 | 1.70388 | 1.93173 |
| 23392 | 0.946129 | 0.128457 |
| 23393 | 1.09362 | 0.339678 |
| 23394 | 0.946131 | 0.650331 |
| 23395 | 1.4185 | 0.541848 |
| 23396 | 1.09362 | 0.220381 |
| 23397 | 1.31057 | 0.774518 |
| 23398 | 1.17631 | 0.05 |
| 23399 | 1.19404 | 0.05 |
| 23400 | 0.94613 | 0.05 |
| 23401 | 0.946129 | 0.05 |
| 23402 | 0.99972 | 0.05 |
| 23403 | 0.94613 | 0.05 |
| 23404 | 0.835199 | 0.05 |
| 23405 | 1.23351 | 0.05 |
| 23406 | 0.582785 | 0.05 |
| 23407 | 0.701079 | 0.05 |
| 23408 | 0.946132 | 0.05 |
| 23409 | 2.17385 | 0.05 |
| 23410 | 1.18948 | 2.1 |
| 23411 | 1.25346 | 2.1 |
| 23412 | 1.18586 | 0.798509 |
| 23413 | 0.880876 | 0.0916738 |
| 23414 | 0.68632 | 0.830442 |
| 23415 | 0.946118 | 2.1 |
| 23416 | 0.966936 | 2.1 |
| 23417 | 0.946117 | 2.1 |
| 23418 | 1.41636 | 2.1 |
| 23419 | 1.03798 | 2.1 |
| 23420 | 0.729178 | 0.686234 |
| 23421 | 0.721888 | 0.954602 |
| 23422 | 0.946132 | 0.726248 |
| 23423 | 0.656286 | 0.880607 |
| 23424 | 1.13898 | 0.800697 |
| 23425 | 1.02351 | 0.05 |
| 23426 | 1.48803 | 0.05 |
| 23427 | 0.946131 | 0.05 |
| 23428 | 1.04921 | 2.1 |
| 23429 | 1.02478 | 0.05 |

| | | |
|---|---|---|
| 23430 | 0.993023 | 2.1 |
| 23431 | 1.80841 | 0.05 |
| 23432 | 1.29215 | 0.05 |
| 23433 | 0.904206 | 0.05 |
| 23434 | 0.904207 | 0.05 |
| 23435 | 0.946132 | 0.05 |
| 23436 | 0.904207 | 0.05 |
| 23437 | 1.07347 | 2.1 |
| 23438 | 0.555875 | 1.26492 |
| 23439 | 0.555904 | 1.74715 |
| 23440 | 1.30841 | 2.1 |
| 23441 | 1.30841 | 2.1 |
| 23442 | 0.91951 | 2.1 |
| 23443 | 1.33026 | 0.05 |
| 23444 | 1.43289 | 0.05 |
| 23445 | 1.15703 | 1.67418 |
| 23446 | 0.886594 | 2.1 |
| 23447 | 1.05129 | 1.88267 |
| 23448 | 1.08421 | 0.0511192 |
| 23449 | 1.35946 | 0.0701724 |
| 23450 | 0.882955 | 0.217165 |
| 23451 | 0.946131 | 0.180239 |
| 23452 | 0.882985 | 0.122153 |
| 23453 | 0.583501 | 0.285286 |
| 23454 | 0.532921 | 0.281687 |
| 23455 | 0.94613 | 1.91856 |
| 23456 | 0.532892 | 1.1848 |
| 23457 | 1.23224 | 1.20597 |
| 23458 | 1.12786 | 1.07985 |
| 23459 | 0.946131 | 0.738205 |
| 23460 | 1.23732 | 0.05 |
| 23461 | 1.40609 | 0.143452 |
| 23462 | 1.40609 | 0.05 |
| 23463 | 0.946131 | 0.05 |
| 23464 | 0.910672 | 1.49056 |
| 23465 | 1.08478 | 1.33781 |
| 23466 | 0.94613 | 0.712122 |
| 23467 | 0.94613 | 1.20229 |
| 23468 | 1.30382 | 1.16761 |
| 23469 | 3.10544 | 2.1 |
| 23470 | 0.679509 | 2.1 |
| 23471 | 1.15966 | 1.58201 |
| 23472 | 0.75499 | 2.1 |
| 23473 | 1.37511 | 1.77428 |
| 23474 | 1.01776 | 1.5588 |
| 23475 | 2.35239 | 0.93047 |
| 23476 | 0.92406 | 2.1 |
| 23477 | 0.946118 | 2.1 |
| 23478 | 0.92406 | 2.1 |
| 23479 | 1.07979 | 2.1 |
| 23480 | 1.29604 | 2.1 |
| 23481 | 1.29897 | 2.1 |
| 23482 | 0.902645 | 0.304828 |
| 23483 | 0.729493 | 1.51826 |
| 23484 | 0.906844 | 0.994477 |
| 23485 | 0.739518 | 0.05 |
| 23486 | 1.10564 | 0.05 |
| 23487 | 0.946131 | 0.05 |

| | | |
|---|---|---|
| 23488 | 0.739519 | 0.05 |
| 23489 | 0.739506 | 0.05 |
| 23490 | 1.79907 | 0.05 |
| 23491 | 1.59106 | 1.04248 |
| 23492 | 1.11067 | 0.778734 |
| 23493 | 0.795516 | 0.973771 |
| 23494 | 0.795519 | 0.838901 |
| 23495 | 0.946132 | 0.948541 |
| 23496 | 0.795506 | 0.814149 |
| 23497 | 1.80223 | 2.1 |
| 23498 | 1.95572 | 2.1 |
| 23499 | 1.16237 | 0.93232 |
| 23500 | 1.07518 | 2.1 |
| 23501 | 0.696199 | 0.906508 |
| 23502 | 0.94613 | 1.74028 |
| 23503 | 0.696199 | 1.15663 |
| 23504 | 1.6379 | 0.05 |
| 23505 | 0.946118 | 0.05 |
| 23506 | 0.851529 | 0.05 |
| 23507 | 1.08716 | 0.05 |
| 23508 | 0.973118 | 0.05 |
| 23509 | 0.851528 | 0.05 |
| 23510 | 1.15096 | 2.1 |
| 23511 | 0.65023 | 0.05 |
| 23512 | 0.94613 | 0.05 |
| 23513 | 0.650205 | 0.05 |
| 23514 | 0.650205 | 0.05 |
| 23515 | 0.811233 | 0.05 |
| 23516 | 0.778721 | 2.1 |
| 23517 | 0.810945 | 1.00386 |
| 23518 | 0.997518 | 1.00007 |
| 23519 | 0.838028 | 0.05 |
| 23520 | 0.952893 | 0.05 |
| 23521 | 0.838037 | 0.05 |
| 23522 | 1.58166 | 0.05 |
| 23523 | 1.44848 | 1.5378 |
| 23524 | 1.30041 | 0.05 |
| 23525 | 1.03697 | 0.05 |
| 23526 | 0.946132 | 1.44662 |
| 23527 | 1.0277 | 1.41309 |
| 23528 | 0.946131 | 1.58019 |
| 23529 | 1.00501 | 1.26729 |
| 23530 | 1.0277 | 1.1134 |
| 23531 | 1.64988 | 2.1 |
| 23532 | 0.946131 | 2.1 |
| 23533 | 0.94613 | 0.05 |
| 23534 | 1.27702 | 2.1 |
| 23535 | 1.55163 | 0.05 |
| 23536 | 0.897905 | 0.05 |
| 23537 | 1.12971 | 0.05 |
| 23538 | 1.35715 | 0.05 |
| 23539 | 0.881884 | 0.145954 |
| 23540 | 0.664007 | 0.05 |
| 23541 | 0.663978 | 0.0691074 |
| 23542 | 0.94613 | 0.05 |
| 23543 | 0.664006 | 0.0600193 |
| 23544 | 1.48693 | 0.05 |
| 23545 | 1.29289 | 0.05 |

| | | |
|---|---|---|
| 23546 | 1.32081 | 0.05 |
| 23547 | 0.946119 | 0.05 |
| 23548 | 0.946132 | 0.05 |
| 23549 | 1.02972 | 0.05 |
| 23550 | 0.946131 | 2.1 |
| 23551 | 0.946127 | 2.1 |
| 23552 | 1.23513 | 2.1 |
| 23553 | 1.38154 | 2.1 |
| 23554 | 0.94613 | 2.1 |
| 23555 | 1.25933 | 0.05 |
| 23556 | 0.917954 | 0.05 |
| 23557 | 0.868589 | 0.05 |
| 23558 | 3.0957 | 2.1 |
| 23559 | 0.711391 | 0.05 |
| 23560 | 0.711391 | 0.05 |
| 23561 | 0.991303 | 0.05 |
| 23562 | 1.37322 | 0.05 |
| 23563 | 0.729145 | 0.05 |
| 23564 | 2.55648 | 0.05 |
| 23565 | 1.0756 | 2.04456 |
| 23566 | 0.760299 | 1.37724 |
| 23567 | 0.763592 | 0.310765 |
| 23568 | 0.946126 | 0.655132 |
| 23569 | 0.76359 | 0.230642 |
| 23570 | 1.35862 | 1.21301 |
| 23571 | 0.946133 | 2.1 |
| 23572 | 1.00807 | 2.1 |
| 23573 | 0.946132 | 2.1 |
| 23574 | 1.39823 | 2.1 |
| 23575 | 1.07817 | 2.1 |
| 23576 | 1.44379 | 1.31113 |
| 23577 | 1.17934 | 0.05 |
| 23578 | 1.13451 | 0.206445 |
| 23579 | 0.94613 | 0.271513 |
| 23580 | 1.03974 | 2.1 |
| 23581 | 1.36835 | 0.706544 |
| 23582 | 1.36835 | 2.1 |
| 23583 | 0.94613 | 2.1 |
| 23584 | 1.27096 | 2.1 |
| 23585 | 0.853042 | 2.1 |
| 23586 | 0.946131 | 2.1 |
| 23587 | 1.13658 | 2.1 |
| 23588 | 1.06997 | 2.1 |
| 23589 | 0.94613 | 2.1 |
| 23590 | 1.41556 | 0.05 |
| 23591 | 1.21708 | 0.120315 |
| 23592 | 0.94613 | 0.141859 |
| 23593 | 0.946131 | 0.0694739 |
| 23594 | 1.21708 | 0.0733375 |
| 23595 | 0.964782 | 0.05 |
| 23596 | 0.946119 | 0.100566 |
| 23597 | 1.23447 | 0.0654221 |
| 23598 | 0.946118 | 0.0941038 |
| 23599 | 1.41844 | 0.0578162 |
| 23600 | 1.23446 | 0.0816509 |
| 23601 | 1.1357 | 0.05 |
| 23602 | 0.619856 | 0.05 |
| 23603 | 1.1509 | 0.05 |

| | | |
|---|---|---|
| 23604 | 0.946126 | 0.05 |
| 23605 | 1.19262 | 0.05 |
| 23606 | 1.1509 | 0.05 |
| 23607 | 1.04268 | 2.1 |
| 23608 | 2.07527 | 0.637662 |
| 23609 | 1.38259 | 0.957278 |
| 23610 | 0.980054 | 1.60469 |
| 23611 | 0.946124 | 1.02039 |
| 23612 | 0.946127 | 1.23785 |
| 23613 | 0.980056 | 1.08795 |
| 23614 | 1.62916 | 2.1 |
| 23615 | 0.946131 | 2.1 |
| 23616 | 1.40571 | 2.1 |
| 23617 | 0.946131 | 2.1 |
| 23618 | 1.38885 | 2.1 |
| 23619 | 1.40571 | 2.1 |
| 23620 | 1.11787 | 0.426156 |
| 23621 | 0.946129 | 0.05 |
| 23622 | 1.28831 | 2.1 |
| 23623 | 1.41498 | 0.05 |
| 23624 | 1.41498 | 0.350365 |
| 23625 | 0.946131 | 0.0965024 |
| 23626 | 0.850964 | 0.05 |
| 23627 | 0.921299 | 0.05 |
| 23628 | 1.37358 | 0.05 |
| 23629 | 0.946132 | 0.05 |
| 23630 | 0.850964 | 0.05 |
| 23631 | 0.823988 | 0.05 |
| 23632 | 0.823984 | 0.05 |
| 23633 | 1.36568 | 0.05 |
| 23634 | 1.36569 | 0.05 |
| 23635 | 0.921275 | 0.05 |
| 23636 | 1.84739 | 2.1 |
| 23637 | 1.47169 | 0.05 |
| 23638 | 0.946131 | 0.05 |
| 23639 | 0.735844 | 0.05 |
| 23640 | 1.11819 | 0.05 |
| 23641 | 1.10904 | 0.05 |
| 23642 | 0.735831 | 0.05 |
| 23643 | 0.946131 | 2.1 |
| 23644 | 0.946132 | 2.1 |
| 23645 | 1.23487 | 2.1 |
| 23646 | 1.3281 | 2.1 |
| 23647 | 0.946131 | 2.1 |
| 23648 | 1.72271 | 0.05 |
| 23649 | 0.861359 | 0.05 |
| 23650 | 0.946131 | 0.05 |
| 23651 | 0.861357 | 0.05 |
| 23652 | 1.1369 | 0.05 |
| 23653 | 1.26868 | 0.05 |
| 23654 | 0.789173 | 2.1 |
| 23655 | 0.789143 | 2.1 |
| 23656 | 0.946133 | 1.6445 |
| 23657 | 1.34386 | 2.00284 |
| 23658 | 0.789144 | 2.07041 |
| 23659 | 0.983493 | 2.1 |
| 23660 | 1.18944 | 0.599872 |
| 23661 | 1.05121 | 0.535837 |

| | | |
|---|---|---|
| 23662 | 0.777398 | 0.544995 |
| 23663 | 0.946132 | 0.832818 |
| 23664 | 0.777423 | 0.908668 |
| 23665 | 1.09841 | 0.05 |
| 23666 | 0.963919 | 0.05 |
| 23667 | 0.474855 | 0.05 |
| 23668 | 0.946131 | 0.05 |
| 23669 | 0.474855 | 0.05 |
| 23670 | 3.40074 | 1.17998 |
| 23671 | 1.29453 | 2.1 |
| 23672 | 1.01139 | 0.05 |
| 23673 | 0.946133 | 0.230209 |
| 23674 | 1.35381 | 0.05 |
| 23675 | 1.18322 | 0.071304 |
| 23676 | 0.946133 | 0.194965 |
| 23677 | 1.45502 | 0.05 |
| 23678 | 0.970088 | 1.24431 |
| 23679 | 0.960333 | 1.42417 |
| 23680 | 0.838036 | 1.47199 |
| 23681 | 1.30927 | 0.05 |
| 23682 | 0.828617 | 0.0741009 |
| 23683 | 0.714795 | 0.05 |
| 23684 | 0.946132 | 0.05 |
| 23685 | 0.714824 | 0.05 |
| 23686 | 0.946131 | 2.1 |
| 23687 | 0.851542 | 2.1 |
| 23688 | 1.38204 | 2.1 |
| 23689 | 1.24512 | 2.1 |
| 23690 | 1.04086 | 2.1 |
| 23691 | 0.723573 | 2.1 |
| 23692 | 0.946131 | 2.1 |
| 23693 | 0.982454 | 2.1 |
| 23694 | 0.982454 | 2.1 |
| 23695 | 0.723544 | 2.1 |
| 23696 | 1.17261 | 0.05 |
| 23697 | 1.34153 | 0.05 |
| 23698 | 1.41383 | 0.05 |
| 23699 | 0.946132 | 0.05 |
| 23700 | 0.946133 | 0.05 |
| 23701 | 1.12818 | 0.05 |
| 23702 | 1.29383 | 0.05 |
| 23703 | 0.946133 | 0.0678982 |
| 23704 | 1.0027 | 0.0694606 |
| 23705 | 0.768989 | 0.05 |
| 23706 | 0.946133 | 0.0957402 |
| 23707 | 0.768977 | 0.102263 |
| 23708 | 0.946132 | 0.12308 |
| 23709 | 1.24333 | 0.05 |
| 23710 | 0.928583 | 0.112747 |
| 23711 | 0.946132 | 0.117134 |
| 23712 | 0.928583 | 0.153501 |
| 23713 | 1.53798 | 0.05 |
| 23714 | 2.95436 | 0.05 |
| 23715 | 1.30649 | 0.05 |
| 23716 | 0.809789 | 0.05 |
| 23717 | 0.745744 | 0.05 |
| 23718 | 0.745774 | 0.05 |
| 23719 | 0.946128 | 0.05 |

| | | |
|---|---|---|
| 23720 | 1.00777 | 0.05 |
| 23721 | 1.40271 | 0.05 |
| 23722 | 0.768387 | 0.05 |
| 23723 | 0.946132 | 0.05 |
| 23724 | 0.922045 | 0.05 |
| 23725 | 0.946132 | 2.1 |
| 23726 | 1.39021 | 2.1 |
| 23727 | 1.10046 | 2.1 |
| 23728 | 0.946132 | 2.1 |
| 23729 | 1.39021 | 2.1 |
| 23730 | 2.06158 | 0.05 |
| 23731 | 0.913967 | 0.601502 |
| 23732 | 1.30107 | 0.82726 |
| 23733 | 0.946132 | 0.608546 |
| 23734 | 0.913966 | 0.659599 |
| 23735 | 1.30107 | 0.637347 |
| 23736 | 1.64117 | 2.1 |
| 23737 | 0.514872 | 1.91655 |
| 23738 | 0.514846 | 1.03388 |
| 23739 | 1.30811 | 2.1 |
| 23740 | 1.30812 | 0.617104 |
| 23741 | 0.888989 | 1.12626 |
| 23742 | 1.99279 | 1.6778 |
| 23743 | 0.910271 | 0.894453 |
| 23744 | 0.838027 | 0.966371 |
| 23745 | 0.928444 | 0.976539 |
| 23746 | 1.2377 | 1.26097 |
| 23747 | 0.851537 | 2.1 |
| 23748 | 0.899142 | 2.1 |
| 23749 | 0.810075 | 2.1 |
| 23750 | 1.3771 | 2.1 |
| 23751 | 1.03281 | 2.1 |
| 23752 | 0.719877 | 2.1 |
| 23753 | 0.901838 | 2.1 |
| 23754 | 1.01748 | 2.1 |
| 23755 | 1.12315 | 2.1 |
| 23756 | 0.64787 | 2.1 |
| 23757 | 1.173 2.1 | |
| 23758 | 0.876903 | 2.1 |
| 23759 | 0.597337 | 2.1 |
| 23760 | 1.00291 | 2.1 |
| 23761 | 0.597309 | 2.1 |
| 23762 | 1.09897 | 0.05 |
| 23763 | 0.946132 | 0.205351 |
| 23764 | 1.1386 | 0.05 |
| 23765 | 1.09897 | 0.05 |
| 23766 | 0.596652 | 0.05 |
| 23767 | 2.5593 | 2.1 |
| 23768 | 0.946133 | 0.730685 |
| 23769 | 1.29172 | 0.677652 |
| 23770 | 1.11337 | 0.293991 |
| 23771 | 0.946131 | 0.571095 |
| 23772 | 0.946131 | 0.321068 |
| 23773 | 0.96926 | 0.836687 |
| 23774 | 0.946132 | 1.07588 |
| 23775 | 0.946132 | 1.13929 |
| 23776 | 1.07118 | 0.71431 |
| 23777 | 1.29698 | 0.746902 |

| | | |
|---|---|---|
| 23778 | 1.37867 | 0.945859 |
| 23779 | 0.946119 | 1.615 |
| 23780 | 0.94612 | 2.1 |
| 23781 | 1.33154 | 0.950121 |
| 23782 | 0.946107 | 1.21641 |
| 23783 | 2.17757 | 1.5037 |
| 23784 | 0.946131 | 0.05 |
| 23785 | 0.68393 | 0.05 |
| 23786 | 0.68396 | 0.05 |
| 23787 | 1.33671 | 0.05 |
| 23788 | 1.10733 | 0.05 |
| 23789 | 2.31367 | 2.1 |
| 23790 | 0.989326 | 1.59896 |
| 23791 | 0.689952 | 2.1 |
| 23792 | 0.854787 | 1.52911 |
| 23793 | 3.73635 | 2.1 |
| 23794 | 1.41489 | 2.1 |
| 23795 | 1.25544 | 0.262254 |
| 23796 | 0.946128 | 2.1 |
| 23797 | 0.946129 | 2.1 |
| 23798 | 1.13536 | 2.1 |
| 23799 | 2.10284 | 1.26426 |
| 23800 | 0.851542 | 2.1 |
| 23801 | 0.828523 | 2.1 |
| 23802 | 0.946132 | 2.1 |
| 23803 | 0.946132 | 2.1 |
| 23804 | 1.50873 | 2.1 |
| 23805 | 0.989048 | 2.1 |
| 23806 | 0.925055 | 2.1 |
| 23807 | 0.838041 | 2.1 |
| 23808 | 0.807714 | 0.117073 |
| 23809 | 0.656831 | 0.05 |
| 23810 | 0.997603 | 0.0572512 |
| 23811 | 0.946131 | 0.925392 |
| 23812 | 1.02602 | 0.272941 |
| 23813 | 0.946129 | 1.0687 |
| 23814 | 1.35469 | 0.41703 |
| 23815 | 1.25083 | 0.620257 |
| 23816 | 0.851541 | 0.05 |
| 23817 | 0.851483 | 0.05 |
| 23818 | 0.851539 | 0.05 |
| 23819 | 1.19785 | 0.05 |
| 23820 | 1.07615 | 0.05 |
| 23821 | 0.714507 | 0.147506 |
| 23822 | 1.41461 | 0.119517 |
| 23823 | 0.714507 | 0.136816 |
| 23824 | 0.714507 | 0.144679 |
| 23825 | 0.946133 | 0.172198 |
| 23826 | 0.946131 | 0.682649 |
| 23827 | 0.972232 | 0.371308 |
| 23828 | 0.94613 | 1.50251 |
| 23829 | 1.25083 | 0.233819 |
| 23830 | 1.30165 | 0.565018 |
| 23831 | 1.05332 | 2.1 |
| 23832 | 0.574768 | 2.1 |
| 23833 | 1.94541 | 0.05 |
| 23834 | 0.789633 | 0.05 |
| 23835 | 0.946127 | 0.05 |

| | | |
|---|---|---|
| 23836 | 1.33798 | 0.05 |
| 23837 | 1.22903 | 0.05 |
| 23838 | 0.946128 | 0.05 |
| 23839 | 0.946128 | 2.1 |
| 23840 | 1.11882 | 2.1 |
| 23841 | 0.996487 | 2.1 |
| 23842 | 0.94613 | 2.1 |
| 23843 | 1.11883 | 2.1 |
| 23844 | 0.946127 | 2.1 |
| 23845 | 1.30082 | 2.1 |
| 23846 | 1.02608 | 2.1 |
| 23847 | 0.946128 | 2.1 |
| 23848 | 1.30081 | 2.1 |
| 23849 | 1.3507 | 1.88994 |
| 23850 | 1.19583 | 0.140643 |
| 23851 | 0.946127 | 2.1 |
| 23852 | 0.94613 | 1.36013 |
| 23853 | 1.19583 | 1.67443 |
| 23854 | 0.477652 | 0.05 |
| 23855 | 1.20416 | 0.05 |
| 23856 | 0.946131 | 0.05 |
| 23857 | 0.477652 | 0.05 |
| 23858 | 0.477691 | 0.05 |
| 23859 | 1.34536 | 0.05 |
| 23860 | 0.758971 | 0.05 |
| 23861 | 1.25124 | 0.05 |
| 23862 | 1.31467 | 0.05 |
| 23863 | 0.758971 | 0.05 |
| 23864 | 1.31467 | 0.05 |
| 23865 | 0.946131 | 2.1 |
| 23866 | 1.14184 | 2.1 |
| 23867 | 1.35028 | 2.1 |
| 23868 | 1.35028 | 2.1 |
| 23869 | 0.946131 | 2.1 |
| 23870 | 1.13537 | 2.1 |
| 23871 | 0.946132 | 1.23419 |
| 23872 | 0.946132 | 2.1 |
| 23873 | 1.23031 | 2.1 |
| 23874 | 1.27555 | 2.1 |
| 23875 | 1.53064 | 2.1 |
| 23876 | 1.28421 | 1.15203 |
| 23877 | 1.07707 | 1.05913 |
| 23878 | 0.946125 | 1.06446 |
| 23879 | 0.946127 | 1.16281 |
| 23880 | 1.07707 | 1.13353 |
| 23881 | 0.946133 | 0.05 |
| 23882 | 1.3495 | 0.05 |
| 23883 | 0.946132 | 0.05 |
| 23884 | 1.05704 | 0.05 |
| 23885 | 1.3495 | 0.05 |
| 23886 | 0.946132 | 0.05 |
| 23887 | 1.37794 | 0.05 |
| 23888 | 0.946132 | 0.05 |
| 23889 | 1.01367 | 0.05 |
| 23890 | 1.37794 | 0.05 |
| 23891 | 0.928516 | 2.1 |
| 23892 | 0.946133 | 2.1 |
| 23893 | 0.928518 | 2.1 |

| | | |
|---|---|---|
| 23894 | 1.14105 | 2.1 |
| 23895 | 1.26406 | 2.1 |
| 23896 | 0.797664 | 2.1 |
| 23897 | 0.946132 | 2.1 |
| 23898 | 0.797663 | 2.1 |
| 23899 | 1.34285 | 2.1 |
| 23900 | 0.929875 | 2.1 |
| 23901 | 0.946131 | 1.74857 |
| 23902 | 1.21139 | 0.459933 |
| 23903 | 1.41492 | 0.05 |
| 23904 | 1.41492 | 0.39927 |
| 23905 | 0.946131 | 0.05 |
| 23906 | 0.946131 | 2.1 |
| 23907 | 0.689388 | 2.1 |
| 23908 | 0.94365 | 2.1 |
| 23909 | 0.777345 | 2.1 |
| 23910 | 0.709599 | 2.1 |
| 23911 | 0.774968 | 2.1 |
| 23912 | 0.946132 | 2.1 |
| 23913 | 0.77497 | 2.1 |
| 23914 | 0.868145 | 2.1 |
| 23915 | 1.28401 | 2.1 |
| 23916 | 0.94613 | 0.316438 |
| 23917 | 1.19655 | 2.1 |
| 23918 | 1.39428 | 0.0570038 |
| 23919 | 1.39428 | 0.206427 |
| 23920 | 0.946131 | 0.05 |
| 23921 | 1.1848 | 2.1 |
| 23922 | 1.07591 | 2.1 |
| 23923 | 0.946131 | 2.1 |
| 23924 | 0.946131 | 2.1 |
| 23925 | 0.874788 | 2.1 |
| 23926 | 1.35703 | 2.1 |
| 23927 | 1.35357 | 2.1 |
| 23928 | 0.946132 | 2.1 |
| 23929 | 1.26022 | 2.1 |
| 23930 | 0.946132 | 2.1 |
| 23931 | 0.946131 | 2.1 |
| 23932 | 0.966426 | 2.1 |
| 23933 | 0.946133 | 2.1 |
| 23934 | 0.946131 | 2.1 |
| 23935 | 1.3484 | 2.1 |
| 23936 | 1.26441 | 2.1 |
| 23937 | 1.2079 | 2.1 |
| 23938 | 0.946133 | 2.1 |
| 23939 | 0.946132 | 2.1 |
| 23940 | 1.2079 | 2.1 |
| 23941 | 1.2736 | 2.1 |
| 23942 | 1.25774 | 2.1 |
| 23943 | 0.946133 | 2.1 |
| 23944 | 0.851555 | 2.1 |
| 23945 | 0.96172 | 2.1 |
| 23946 | 0.946128 | 2.1 |
| 23947 | 1.06879 | 2.1 |
| 23948 | 0.94613 | 2.1 |
| 23949 | 1.32608 | 2.1 |
| 23950 | 1.06879 | 2.1 |
| 23951 | 0.946132 | 2.1 |

| | | |
|---|---|---|
| 23952 | 1.32608 | 2.1 |
| 23953 | 0.946131 | 2.1 |
| 23954 | 1.40488 | 2.1 |
| 23955 | 1.32608 | 2.1 |
| 23956 | 0.94613 | 1.02608 |
| 23957 | 1.19234 | 0.0690002 |
| 23958 | 0.946129 | 0.615559 |
| 23959 | 0.94613 | 1.22282 |
| 23960 | 1.31649 | 1.43675 |
| 23961 | 0.912059 | 0.876964 |
| 23962 | 1.04514 | 0.874606 |
| 23963 | 0.946132 | 0.511404 |
| 23964 | 0.946132 | 1.99134 |
| 23965 | 1.34646 | 2.1 |
| 23966 | 1.01187 | 0.505751 |
| 23967 | 1.33728 | 0.616062 |
| 23968 | 0.761974 | 0.05 |
| 23969 | 0.761974 | 0.613057 |
| 23970 | 0.94613 | 0.507877 |
| 23971 | 1.40641 | 2.1 |
| 23972 | 0.955752 | 0.893014 |
| 23973 | 0.736738 | 2.1 |
| 23974 | 0.94613 | 1.04744 |
| 23975 | 0.736739 | 1.17185 |
| 23976 | 0.861471 | 2.1 |
| 23977 | 1.34556 | 2.1 |
| 23978 | 0.810957 | 2.1 |
| 23979 | 0.946131 | 2.1 |
| 23980 | 1.37811 | 2.1 |
| 23981 | 1.17949 | 2.1 |
| 23982 | 0.946132 | 2.1 |
| 23983 | 1.37811 | 2.1 |
| 23984 | 0.946131 | 2.1 |
| 23985 | 1.40447 | 2.1 |
| 23986 | 0.946132 | 2.1 |
| 23987 | 0.946132 | 2.1 |
| 23988 | 1.39826 | 2.1 |
| 23989 | 0.946131 | 2.1 |
| 23990 | 1.283 2.1 | |
| 23991 | 0.94613 | 2.1 |
| 23992 | 0.946131 | 2.1 |
| 23993 | 1.40447 | 2.1 |
| 23994 | 1.20541 | 2.1 |
| 23995 | 0.946131 | 2.1 |
| 23996 | 0.946127 | 2.1 |
| 23997 | 1.33607 | 2.1 |
| 23998 | 1.41021 | 2.1 |
| 23999 | 1.01271 | 2.1 |
| 24000 | 0.946131 | 2.1 |
| 24001 | 0.920606 | 2.1 |
| 24002 | 1.13078 | 2.1 |
| 24003 | 1.60331 | 2.1 |
| 24004 | 0.946118 | 0.829842 |
| 24005 | 1.09718 | 2.1 |
| 24006 | 0.946116 | 1.45046 |
| 24007 | 1.32662 | 0.870393 |
| 24008 | 1.0972 | 0.962265 |
| 24009 | 0.94613 | 0.840224 |

| | | |
|---|---|---|
| 24010 | 1.32666 | 1.3126 |
| 24011 | 0.946128 | 0.761177 |
| 24012 | 1.31381 | 0.72371 |
| 24013 | 1.32666 | 0.610891 |
| 24014 | 0.94612 | 2.1 |
| 24015 | 0.940748 | 2.1 |
| 24016 | 1.03561 | 2.1 |
| 24017 | 1.35111 | 2.1 |
| 24018 | 0.851527 | 2.1 |
| 24019 | 0.607156 | 2.1 |
| 24020 | 0.789158 | 2.1 |
| 24021 | 0.946129 | 1.07143 |
| 24022 | 1.04071 | 2.1 |
| 24023 | 1.13144 | 2.1 |
| 24024 | 0.946133 | 2.1 |
| 24025 | 1.35663 | 2.1 |
| 24026 | 0.946132 | 2.1 |
| 24027 | 0.946133 | 2.1 |
| 24028 | 1.34237 | 2.1 |
| 24029 | 0.919455 | 2.1 |
| 24030 | 1.01143 | 2.1 |
| 24031 | 0.946128 | 2.1 |
| 24032 | 0.946131 | 2.1 |
| 24033 | 1.62796 | 2.1 |
| 24034 | 1.17796 | 1.63221 |
| 24035 | 1.23024 | 2.1 |
| 24036 | 0.946132 | 1.15083 |
| 24037 | 0.85153 | 2.03361 |
| 24038 | 0.986867 | 2.1 |
| 24039 | 0.889308 | 2.1 |
| 24040 | 0.94613 | 2.1 |
| 24041 | 0.889338 | 2.1 |
| 24042 | 0.946131 | 2.1 |
| 24043 | 0.889337 | 2.1 |
| 24044 | 1.18396 | 1.18573 |
| 24045 | 0.94613 | 0.979613 |
| 24046 | 0.946125 | 2.07755 |
| 24047 | 1.39514 | 1.20817 |
| 24048 | 1.30809 | 1.49847 |
| 24049 | 0.803322 | 2.1 |
| 24050 | 0.925893 | 2.09585 |
| 24051 | 1.36183 | 1.29638 |
| 24052 | 0.925892 | 1.4806 |
| 24053 | 0.946133 | 1.48406 |
| 24054 | 1.38354 | 0.0758329 |
| 24055 | 1.16009 | 0.327683 |
| 24056 | 0.946132 | 0.146624 |
| 24057 | 0.946132 | 0.0747078 |
| 24058 | 1.01462 | 0.05 |
| 24059 | 1.18854 | 0.994136 |
| 24060 | 0.888265 | 1.99001 |
| 24061 | 0.745095 | 1.12753 |
| 24062 | 0.709597 | 1.63907 |
| 24063 | 0.936541 | 1.43653 |
| 24064 | 1.24142 | 0.05 |
| 24065 | 1.35059 | 0.05 |
| 24066 | 0.946128 | 0.05 |
| 24067 | 0.94613 | 0.05 |

| | | |
|---|---|---|
| 24068 | 1.16623 | 0.0540887 |
| 24069 | 1.24945 | 0.05 |
| 24070 | 1.33035 | 0.05 |
| 24071 | 0.946127 | 0.05 |
| 24072 | 0.946128 | 0.05 |
| 24073 | 1.13343 | 0.05 |
| 24074 | 0.637995 | 0.922958 |
| 24075 | 1.10455 | 1.03494 |
| 24076 | 1.22641 | 1.45555 |
| 24077 | 1.22641 | 2.05063 |
| 24078 | 0.946132 | 1.34722 |
| 24079 | 1.3665 | 0.05 |
| 24080 | 1.28507 | 0.05 |
| 24081 | 0.946127 | 0.05 |
| 24082 | 0.946129 | 0.05 |
| 24083 | 1.2051 | 0.05 |
| 24084 | 0.797374 | 2.1 |
| 24085 | 0.797376 | 2.1 |
| 24086 | 1.13113 | 2.1 |
| 24087 | 1.20057 | 2.1 |
| 24088 | 1.12049 | 2.1 |
| 24089 | 0.59387 | 0.808868 |
| 24090 | 1.13245 | 0.831649 |
| 24091 | 0.966134 | 1.01047 |
| 24092 | 0.593842 | 0.547137 |
| 24093 | 0.946128 | 0.991222 |
| 24094 | 0.641455 | 0.05 |
| 24095 | 0.916507 | 0.05 |
| 24096 | 1.04072 | 1.24694 |
| 24097 | 0.415585 | 0.05 |
| 24098 | 0.507244 | 0.97185 |
| 24099 | 0.678938 | 2.1 |
| 24100 | 1.24154 | 2.1 |
| 24101 | 1.25 | 1.72163 |
| 24102 | 1.24154 | 2.04484 |
| 24103 | 1.25 | 2.0266 |
| 24104 | 0.851009 | 1.38098 |
| 24105 | 0.946131 | 2.1 |
| 24106 | 0.851539 | 1.58431 |
| 24107 | 1.21997 | 1.34952 |
| 24108 | 1.45109 | 1.2994 |
| 24109 | 0.804877 | 0.911677 |
| 24110 | 1.00062 | 0.05 |
| 24111 | 0.851536 | 0.702468 |
| 24112 | 0.909673 | 0.0551692 |
| 24113 | 0.946129 | 0.758834 |
| 24114 | 1.20494 | 0.05 |
| 24115 | 1.09088 | 0.05 |
| 24116 | 0.887634 | 0.05 |
| 24117 | 0.81027 | 0.05 |
| 24118 | 0.900294 | 0.05 |
| 24119 | 0.747713 | 0.05 |
| 24120 | 0.975467 | 0.05 |
| 24121 | 0.747712 | 0.05 |
| 24122 | 1.39168 | 0.05 |
| 24123 | 0.975467 | 0.05 |
| 24124 | 0.94613 | 0.112568 |
| 24125 | 1.32827 | 0.0740037 |

| | | |
|---|---|---|
| 24126 | 0.946128 | 0.0808356 |
| 24127 | 1.41201 | 0.135208 |
| 24128 | 1.32827 | 0.115404 |
| 24129 | 0.981121 | 0.05 |
| 24130 | 0.936311 | 0.05 |
| 24131 | 1.00715 | 0.05 |
| 24132 | 1.15914 | 0.05 |
| 24133 | 0.704409 | 0.05 |
| 24134 | 0.713444 | 0.274742 |
| 24135 | 0.954538 | 0.0570781 |
| 24136 | 0.944508 | 0.0810114 |
| 24137 | 0.851165 | 0.659652 |
| 24138 | 0.946131 | 1.41668 |
| 24139 | 0.851192 | 0.377199 |
| 24140 | 0.946131 | 0.279924 |
| 24141 | 1.3279 | 0.273972 |
| 24142 | 1.33587 | 0.05 |
| 24143 | 0.946126 | 0.05 |
| 24144 | 1.27512 | 0.05 |
| 24145 | 1.33587 | 0.05 |
| 24146 | 0.748059 | 0.05 |
| 24147 | 1.17451 | 0.05 |
| 24148 | 1.04299 | 0.05 |
| 24149 | 0.851542 | 0.05 |
| 24150 | 0.946131 | 0.05 |
| 24151 | 0.892109 | 0.05 |
| 24152 | 0.946131 | 0.05 |
| 24153 | 0.851541 | 0.05 |
| 24154 | 1.2178 | 0.05 |
| 24155 | 1.2179 | 0.05 |
| 24156 | 0.946793 | 0.05 |
| 24157 | 0.709599 | 0.05 |
| 24158 | 0.987426 | 0.05 |
| 24159 | 1.39502 | 0.05 |
| 24160 | 1.13624 | 0.05 |
| 24161 | 0.73176 | 0.05 |
| 24162 | 1.36697 | 0.05 |
| 24163 | 1.33393 | 0.05 |
| 24164 | 0.946131 | 0.05 |
| 24165 | 0.946132 | 0.05 |
| 24166 | 1.23845 | 0.05 |
| 24167 | 0.50702 | 0.909114 |
| 24168 | 1.06442 | 0.888579 |
| 24169 | 0.946131 | 1.01937 |
| 24170 | 1.02363 | 1.04733 |
| 24171 | 1.13537 | 0.05 |
| 24172 | 1.25049 | 0.05 |
| 24173 | 0.94613 | 0.05 |
| 24174 | 1.14993 | 0.05 |
| 24175 | 1.38056 | 0.05 |
| 24176 | 0.946126 | 0.05 |
| 24177 | 1.24678 | 0.05 |
| 24178 | 0.684521 | 0.05 |
| 24179 | 0.946124 | 0.05 |
| 24180 | 0.684518 | 0.05 |
| 24181 | 0.851542 | 0.606012 |
| 24182 | 0.925459 | 0.575203 |
| 24183 | 0.946131 | 0.623589 |

| | | |
|---|---|---|
| 24184 | 0.851474 | 0.664352 |
| 24185 | 0.923305 | 0.664771 |
| 24186 | 1.4055 | 0.05 |
| 24187 | 1.32077 | 0.05 |
| 24188 | 0.946127 | 0.05 |
| 24189 | 1.21757 | 0.05 |
| 24190 | 0.946129 | 0.05 |
| 24191 | 0.711214 | 0.05 |
| 24192 | 1.30463 | 0.05 |
| 24193 | 0.711185 | 0.05 |
| 24194 | 0.711185 | 0.05 |
| 24195 | 1.22716 | 0.05 |
| 24196 | 1.29958 | 0.05 |
| 24197 | 1.26201 | 0.05 |
| 24198 | 0.568927 | 0.05 |
| 24199 | 0.568927 | 0.05 |
| 24200 | 0.946132 | 0.05 |
| 24201 | 0.851542 | 0.05 |
| 24202 | 1.44968 | 0.05 |
| 24203 | 0.851543 | 0.05 |
| 24204 | 1.35123 | 0.05 |
| 24205 | 1.40427 | 0.05 |
| 24206 | 0.593295 | 0.05 |
| 24207 | 0.946129 | 0.05 |
| 24208 | 1.22144 | 0.05 |
| 24209 | 0.908278 | 0.05 |
| 24210 | 1.22144 | 0.05 |
| 24211 | 0.867593 | 0.0629123 |
| 24212 | 1.23351 | 0.05 |
| 24213 | 0.946131 | 0.0717555 |
| 24214 | 1.05516 | 0.052052 |
| 24215 | 1.23351 | 0.05 |
| 24216 | 1.15564 | 0.05 |
| 24217 | 0.946131 | 0.05 |
| 24218 | 0.766652 | 0.05 |
| 24219 | 0.766681 | 0.05 |
| 24220 | 0.946131 | 0.05 |
| 24221 | 0.946131 | 0.05 |
| 24222 | 0.897851 | 0.05 |
| 24223 | 1.1751 | 0.05 |
| 24224 | 1.00273 | 0.05 |
| 24225 | 0.851554 | 0.05 |
| 24226 | 0.883272 | 0.05 |
| 24227 | 1.17111 | 0.118827 |
| 24228 | 0.851541 | 0.05 |
| 24229 | 1.01665 | 0.05 |
| 24230 | 0.851555 | 0.05 |
| 24231 | 0.946131 | 0.0813132 |
| 24232 | 0.911508 | 0.05 |
| 24233 | 1.08054 | 0.097235 |
| 24234 | 1.33007 | 0.05 |
| 24235 | 0.911471 | 0.05 |
| 24236 | 1.26787 | 2.1 |
| 24237 | 1.16641 | 1.77712 |
| 24238 | 0.946132 | 1.61371 |
| 24239 | 0.946133 | 1.34171 |
| 24240 | 1.16641 | 1.90566 |
| 24241 | 0.946131 | 2.1 |

| | | |
|---|---|---|
| 24242 | 1.25037 | 0.05 |
| 24243 | 1.34733 | 0.653884 |
| 24244 | 1.34733 | 0.151952 |
| 24245 | 0.946131 | 0.0988222 |
| 24246 | 0.946131 | 0.05 |
| 24247 | 1.41377 | 0.05 |
| 24248 | 0.94613 | 0.05 |
| 24249 | 1.30541 | 0.05 |
| 24250 | 1.41377 | 0.05 |
| 24251 | 1.3473 | 2.1 |
| 24252 | 1.11804 | 2.1 |
| 24253 | 0.94613 | 2.1 |
| 24254 | 0.946131 | 2.1 |
| 24255 | 1.11805 | 2.1 |
| 24256 | 1.32068 | 2.1 |
| 24257 | 0.946131 | 2.1 |
| 24258 | 0.946128 | 2.1 |
| 24259 | 1.39384 | 2.1 |
| 24260 | 1.4181 | 2.1 |
| 24261 | 1.12913 | 2.1 |
| 24262 | 0.94613 | 2.1 |
| 24263 | 0.946129 | 2.1 |
| 24264 | 1.20328 | 2.1 |
| 24265 | 1.39385 | 2.1 |
| 24266 | 1.3335 | 2.1 |
| 24267 | 1.40987 | 2.1 |
| 24268 | 0.94613 | 2.1 |
| 24269 | 1.21474 | 2.1 |
| 24270 | 0.94613 | 2.1 |
| 24271 | 0.946132 | 2.1 |
| 24272 | 1.15203 | 2.1 |
| 24273 | 1.41192 | 0.920507 |
| 24274 | 1.41192 | 2.1 |
| 24275 | 0.946132 | 2.1 |
| 24276 | 0.946131 | 2.1 |
| 24277 | 1.40684 | 2.1 |
| 24278 | 1.2597 | 2.1 |
| 24279 | 0.946131 | 2.1 |
| 24280 | 1.40684 | 2.09055 |
| 24281 | 0.946131 | 2.1 |
| 24282 | 1.39113 | 2.1 |
| 24283 | 1.34049 | 1.05787 |
| 24284 | 0.946131 | 1.72899 |
| 24285 | 1.39113 | 1.06996 |
| 24286 | 0.946132 | 2.04298 |
| 24287 | 1.31649 | 0.985137 |
| 24288 | 0.946132 | 0.948653 |
| 24289 | 0.946132 | 1.15547 |
| 24290 | 1.34297 | 1.21122 |
| 24291 | 0.946131 | 0.86157 |
| 24292 | 1.41766 | 0.836998 |
| 24293 | 0.954559 | 2.03978 |
| 24294 | 0.946131 | 0.734885 |
| 24295 | 0.946131 | 0.619904 |
| 24296 | 1.25447 | 0.05 |
| 24297 | 1.35559 | 0.05 |
| 24298 | 0.946127 | 0.05 |
| 24299 | 0.946128 | 0.05 |

| | | |
|---|---|---|
| 24300 | 1.14874 | 0.05 |
| 24301 | 1.33035 | 0.05 |
| 24302 | 1.3665 | 0.05 |
| 24303 | 0.946129 | 0.05 |
| 24304 | 0.94613 | 0.05 |
| 24305 | 1.20732 | 0.05 |
| 24306 | 0.946131 | 2.1 |
| 24307 | 0.946131 | 2.1 |
| 24308 | 1.32949 | 2.1 |
| 24309 | 1.34921 | 2.1 |
| 24310 | 0.946131 | 2.1 |
| 24311 | 0.946132 | 0.721986 |
| 24312 | 1.25479 | 0.709158 |
| 24313 | 1.38357 | 0.53099 |
| 24314 | 1.38357 | 0.332358 |
| 24315 | 0.946132 | 0.434922 |
| 24316 | 0.946131 | 1.97782 |
| 24317 | 1.31034 | 0.05 |
| 24318 | 1.37127 | 0.719364 |
| 24319 | 1.37127 | 0.05 |
| 24320 | 0.946131 | 0.538925 |
| 24321 | 1.23923 | 0.05 |
| 24322 | 1.41169 | 0.05 |
| 24323 | 0.94613 | 0.05 |
| 24324 | 1.12703 | 0.05 |
| 24325 | 0.946131 | 0.05 |
| 24326 | 0.946119 | 2.1 |
| 24327 | 1.41915 | 2.1 |
| 24328 | 1.02679 | 2.1 |
| 24329 | 0.946107 | 2.1 |
| 24330 | 1.41917 | 2.1 |
| 24331 | 0.946133 | 2.1 |
| 24332 | 1.36581 | 2.1 |
| 24333 | 0.946131 | 2.1 |
| 24334 | 1.36581 | 2.1 |
| 24335 | 0.946131 | 2.1 |
| 24336 | 0.946132 | 2.1 |
| 24337 | 1.04384 | 2.1 |
| 24338 | 1.34973 | 2.1 |
| 24339 | 1.34967 | 2.1 |
| 24340 | 0.851555 | 2.1 |
| 24341 | 0.946132 | 0.05 |
| 24342 | 1.3855 | 0.05 |
| 24343 | 1.08603 | 0.05 |
| 24344 | 1.08602 | 0.05 |
| 24345 | 1.16234 | 0.05 |
| 24346 | 0.946133 | 0.0705991 |
| 24347 | 1.29348 | 0.161676 |
| 24348 | 1.20111 | 0.05 |
| 24349 | 1.20112 | 0.0940169 |
| 24350 | 1.3855 | 0.064423 |
| 24351 | 0.946128 | 1.82496 |
| 24352 | 1.30798 | 2.1 |
| 24353 | 0.946129 | 2.1 |
| 24354 | 1.34828 | 2.1 |
| 24355 | 1.30797 | 2.1 |
| 24356 | 1.24932 | 0.05 |
| 24357 | 1.40549 | 0.05 |

| | | |
|---|---|---|
| 24358 | 0.946117 | 0.05 |
| 24359 | 1.19615 | 0.05 |
| 24360 | 0.946106 | 0.05 |
| 24361 | 0.946131 | 2.1 |
| 24362 | 1.29926 | 2.1 |
| 24363 | 1.25144 | 2.1 |
| 24364 | 0.946131 | 2.1 |
| 24365 | 1.29925 | 2.1 |
| 24366 | 0.946132 | 2.1 |
| 24367 | 1.39826 | 2.1 |
| 24368 | 0.946133 | 2.1 |
| 24369 | 0.946133 | 2.1 |
| 24370 | 1.29926 | 2.1 |
| 24371 | 0.946119 | 0.05 |
| 24372 | 1.33614 | 0.05 |
| 24373 | 0.946118 | 0.05 |
| 24374 | 1.41377 | 0.05 |
| 24375 | 1.33612 | 0.05 |
| 24376 | 0.946133 | 1.49649 |
| 24377 | 1.3933 | 2.1 |
| 24378 | 1.34999 | 0.990764 |
| 24379 | 0.946133 | 1.77105 |
| 24380 | 1.3933 | 1.24034 |
| 24381 | 0.946131 | 1.95311 |
| 24382 | 1.36139 | 2.1 |
| 24383 | 0.946132 | 1.18113 |
| 24384 | 0.946131 | 2.1 |
| 24385 | 1.39329 | 1.22713 |
| 24386 | 1.37449 | 1.47172 |
| 24387 | 1.27925 | 0.343289 |
| 24388 | 0.919796 | 0.180688 |
| 24389 | 1.01176 | 0.895483 |
| 24390 | 0.94613 | 1.01536 |
| 24391 | 0.946133 | 2.1 |
| 24392 | 1.27679 | 2.1 |
| 24393 | 0.946133 | 1.84463 |
| 24394 | 0.946132 | 2.1 |
| 24395 | 1.39731 | 2.1 |
| 24396 | 0.946119 | 2.1 |
| 24397 | 1.40765 | 1.95992 |
| 24398 | 1.1981 | 2.1 |
| 24399 | 0.946131 | 1.82565 |
| 24400 | 1.40764 | 2.1 |
| 24401 | 0.946127 | 2.1 |
| 24402 | 1.3557 | 2.1 |
| 24403 | 0.946127 | 2.1 |
| 24404 | 0.946128 | 2.1 |
| 24405 | 1.13485 | 2.1 |
| 24406 | 1.4008 | 2.1 |
| 24407 | 1.1587 | 2.1 |
| 24408 | 0.946131 | 2.1 |
| 24409 | 0.946131 | 2.1 |
| 24410 | 1.1587 | 2.1 |
| 24411 | 1.32082 | 0.05 |
| 24412 | 1.29215 | 0.05 |
| 24413 | 0.94613 | 0.05 |
| 24414 | 0.946131 | 0.05 |
| 24415 | 1.02366 | 0.05 |

| | | |
|---|---|---|
| 24416 | 1.27697 | 0.05 |
| 24417 | 1.2929 | 0.05 |
| 24418 | 0.946132 | 0.05 |
| 24419 | 0.946132 | 0.05 |
| 24420 | 1.01451 | 0.05 |
| 24421 | 0.946132 | 2.1 |
| 24422 | 1.27252 | 2.1 |
| 24423 | 0.946132 | 2.1 |
| 24424 | 1.37869 | 2.1 |
| 24425 | 1.27252 | 2.1 |
| 24426 | 1.15627 | 2.1 |
| 24427 | 1.35703 | 2.1 |
| 24428 | 0.946132 | 2.1 |
| 24429 | 1.03783 | 2.1 |
| 24430 | 0.946132 | 2.1 |
| 24431 | 0.946128 | 2.1 |
| 24432 | 1.37869 | 2.1 |
| 24433 | 0.946131 | 2.1 |
| 24434 | 1.35357 | 2.1 |
| 24435 | 1.37869 | 2.1 |
| 24436 | 0.946132 | 2.1 |
| 24437 | 0.946131 | 2.1 |
| 24438 | 1.33471 | 2.1 |
| 24439 | 0.946131 | 2.1 |
| 24440 | 1.36373 | 2.1 |
| 24441 | 1.41342 | 0.919022 |
| 24442 | 1.06832 | 1.05457 |
| 24443 | 0.946128 | 1.58585 |
| 24444 | 0.94613 | 0.767648 |
| 24445 | 1.06832 | 1.40754 |
| 24446 | 1.26929 | 2.1 |
| 24447 | 0.946131 | 1.24598 |
| 24448 | 0.946128 | 2.1 |
| 24449 | 1.41022 | 2.1 |
| 24450 | 1.37034 | 2.1 |
| 24451 | 1.37759 | 1.00364 |
| 24452 | 1.07612 | 1.20191 |
| 24453 | 0.946126 | 0.99625 |
| 24454 | 0.946129 | 1.18713 |
| 24455 | 1.07612 | 1.06277 |
| 24456 | 0.946131 | 0.711274 |
| 24457 | 0.94613 | 0.111042 |
| 24458 | 1.23848 | 0.0520772 |
| 24459 | 1.38897 | 0.05 |
| 24460 | 0.946131 | 0.0690924 |
| 24461 | 0.946132 | 0.438696 |
| 24462 | 0.946133 | 0.05 |
| 24463 | 1.34369 | 0.088912 |
| 24464 | 1.40332 | 0.05 |
| 24465 | 0.946132 | 0.0754349 |
| 24466 | 0.946131 | 2.1 |
| 24467 | 1.24023 | 2.1 |
| 24468 | 1.38244 | 2.1 |
| 24469 | 1.38244 | 2.1 |
| 24470 | 0.946131 | 2.1 |
| 24471 | 0.946132 | 2.1 |
| 24472 | 1.33468 | 2.1 |
| 24473 | 1.41619 | 2.1 |

| | | |
|---|---|---|
| 24474 | 1.41619 | 2.1 |
| 24475 | 0.946131 | 2.1 |
| 24476 | 1.1908 | 1.24168 |
| 24477 | 0.946128 | 1.05615 |
| 24478 | 0.946126 | 1.77366 |
| 24479 | 1.26654 | 1.98712 |
| 24480 | 1.39514 | 2.1 |
| 24481 | 0.93608 | 2.1 |
| 24482 | 1.04105 | 2.1 |
| 24483 | 1.39551 | 2.1 |
| 24484 | 1.52471 | 2.1 |
| 24485 | 1.04072 | 2.1 |
| 24486 | 1.33174 | 0.05 |
| 24487 | 0.946128 | 0.05 |
| 24488 | 0.94613 | 0.05 |
| 24489 | 1.17801 | 0.05 |
| 24490 | 0.94613 | 0.05 |
| 24491 | 0.946132 | 2.1 |
| 24492 | 0.946132 | 2.1 |
| 24493 | 1.29065 | 2.1 |
| 24494 | 1.29065 | 2.1 |
| 24495 | 0.975266 | 2.1 |
| 24496 | 0.946131 | 2.1 |
| 24497 | 1.26025 | 2.1 |
| 24498 | 0.955601 | 2.1 |
| 24499 | 0.946131 | 2.1 |
| 24500 | 1.26025 | 2.1 |
| 24501 | 0.94613 | 0.107564 |
| 24502 | 1.23146 | 0.0563188 |
| 24503 | 0.946127 | 0.0771994 |
| 24504 | 1.32827 | 0.105982 |
| 24505 | 1.23146 | 0.0855335 |
| 24506 | 1.25008 | 0.663732 |
| 24507 | 1.05018 | 0.754907 |
| 24508 | 0.94613 | 0.373067 |
| 24509 | 0.932744 | 0.899566 |
| 24510 | 1.04207 | 0.820937 |
| 24511 | 0.946132 | 0.0564532 |
| 24512 | 1.10023 | 0.0839084 |
| 24513 | 1.36748 | 0.05 |
| 24514 | 1.36748 | 0.516817 |
| 24515 | 0.946133 | 0.0625816 |
| 24516 | 1.06023 | 0.05 |
| 24517 | 1.08019 | 0.05 |
| 24518 | 0.94613 | 0.116081 |
| 24519 | 1.06023 | 0.05 |
| 24520 | 0.94613 | 0.05 |
| 24521 | 1.16727 | 0.112516 |
| 24522 | 1.35856 | 0.319747 |
| 24523 | 0.946132 | 0.457808 |
| 24524 | 1.16727 | 0.308948 |
| 24525 | 0.946132 | 0.248584 |
| 24526 | 0.946133 | 2.1 |
| 24527 | 0.946131 | 2.1 |
| 24528 | 1.21691 | 2.1 |
| 24529 | 1.39558 | 2.1 |
| 24530 | 0.946131 | 2.1 |
| 24531 | 1.09905 | 0.05 |

| | | |
|---|---|---|
| 24532 | 0.946132 | 0.05 |
| 24533 | 1.3365 | 0.05 |
| 24534 | 1.35381 | 0.05 |
| 24535 | 0.946132 | 0.05 |
| 24536 | 0.94613 | 0.05 |
| 24537 | 0.946128 | 0.05 |
| 24538 | 1.40332 | 0.05 |
| 24539 | 1.39041 | 0.05 |
| 24540 | 0.94613 | 0.05 |
| 24541 | 0.946132 | 0.05 |
| 24542 | 1.3659 | 1.71421 |
| 24543 | 1.39041 | 0.05 |
| 24544 | 1.39041 | 0.0584946 |
| 24545 | 0.946132 | 0.133307 |
| 24546 | 0.840292 | 2.1 |
| 24547 | 0.946133 | 1.9604 |
| 24548 | 0.851541 | 2.1 |
| 24549 | 1.28464 | 2.1 |
| 24550 | 1.28806 | 2.1 |
| 24551 | 1.12591 | 0.813716 |
| 24552 | 1.05033 | 0.946668 |
| 24553 | 0.946132 | 0.868115 |
| 24554 | 0.946132 | 1.00652 |
| 24555 | 1.13537 | 0.967594 |
| 24556 | 1.21177 | 1.1907 |
| 24557 | 0.946132 | 1.32726 |
| 24558 | 0.946132 | 2.1 |
| 24559 | 1.41134 | 1.2901 |
| 24560 | 1.27079 | 1.45653 |
| 24561 | 0.946131 | 0.05 |
| 24562 | 1.35403 | 0.05 |
| 24563 | 0.94613 | 0.05 |
| 24564 | 0.94613 | 0.05 |
| 24565 | 0.946131 | 0.05 |
| 24566 | 0.946131 | 0.05 |
| 24567 | 0.946132 | 0.05 |
| 24568 | 0.94613 | 0.05 |
| 24569 | 1.37052 | 0.05 |
| 24570 | 0.946131 | 0.05 |
| 24571 | 0.946133 | 0.05 |
| 24572 | 0.946132 | 0.05 |
| 24573 | 0.946133 | 0.05 |
| 24574 | 1.24249 | 0.05 |
| 24575 | 0.946132 | 0.05 |
| 24576 | 1.20608 | 2.1 |
| 24577 | 0.946132 | 2.1 |
| 24578 | 0.946131 | 2.1 |
| 24579 | 0.946132 | 2.1 |
| 24580 | 0.946132 | 2.1 |
| 24581 | 0.946131 | 2.1 |
| 24582 | 0.946128 | 2.1 |
| 24583 | 0.946129 | 2.1 |
| 24584 | 1.41232 | 2.1 |
| 24585 | 0.94613 | 2.1 |
| 24586 | 0.946132 | 0.05 |
| 24587 | 1.32449 | 0.05 |
| 24588 | 0.946131 | 0.05 |
| 24589 | 0.946131 | 0.05 |

| | | |
|---|---|---|
| 24590 | 1.32449 | 0.05 |
| 24591 | 0.946116 | 0.938026 |
| 24592 | 1.41563 | 2.1 |
| 24593 | 0.946118 | 2.1 |
| 24594 | 0.946105 | 2.09851 |
| 24595 | 0.946131 | 1.09518 |
| 24596 | 1.26563 | 2.1 |
| 24597 | 1.32819 | 2.1 |
| 24598 | 0.946131 | 0.107772 |
| 24599 | 0.946131 | 2.1 |
| 24600 | 0.946131 | 1.08412 |
| 24601 | 0.946131 | 2.1 |
| 24602 | 0.946133 | 1.44551 |
| 24603 | 1.30382 | 2.1 |
| 24604 | 0.946131 | 1.33802 |
| 24605 | 0.946131 | 1.38568 |
| 24606 | 1.30382 | 2.09763 |
| 24607 | 0.946133 | 0.05 |
| 24608 | 1.15803 | 0.05 |
| 24609 | 1.28895 | 0.05 |
| 24610 | 0.954071 | 0.05 |
| 24611 | 0.946132 | 0.05 |
| 24612 | 1.0264 | 0.05 |
| 24613 | 0.946132 | 1.54566 |
| 24614 | 0.946132 | 0.788139 |
| 24615 | 0.946131 | 1.36225 |
| 24616 | 0.946132 | 0.923593 |
| 24617 | 1.35977 | 0.05 |
| 24618 | 0.946132 | 0.05 |
| 24619 | 0.946129 | 0.05 |
| 24620 | 0.94613 | 0.05 |
| 24621 | 0.946129 | 0.05 |
| 24622 | 0.876631 | 0.196363 |
| 24623 | 0.946133 | 0.718649 |
| 24624 | 0.946132 | 1.09568 |
| 24625 | 0.946132 | 1.30418 |
| 24626 | 0.876606 | 1.0292 |
| 24627 | 0.946131 | 0.05 |
| 24628 | 1.24979 | 0.05 |
| 24629 | 0.94613 | 0.05 |
| 24630 | 0.94613 | 0.05 |
| 24631 | 0.94613 | 0.05 |
| 24632 | 0.921791 | 0.05 |
| 24633 | 0.946131 | 0.05 |
| 24634 | 0.921762 | 0.05 |
| 24635 | 0.946131 | 0.05 |
| 24636 | 0.946131 | 0.05 |
| 24637 | 0.810944 | 0.05 |
| 24638 | 0.810945 | 0.05 |
| 24639 | 0.810959 | 0.05 |
| 24640 | 0.946133 | 0.05 |
| 24641 | 1.25797 | 0.05 |
| 24642 | 0.946132 | 0.05 |
| 24643 | 0.946132 | 0.05 |
| 24644 | 1.25797 | 0.05 |
| 24645 | 1.13748 | 0.05 |
| 24646 | 0.851512 | 0.0586644 |
| 24647 | 0.94613 | 0.617444 |

| | | |
|---|---|---|
| 24648 | 1.02373 | 0.05 |
| 24649 | 0.946131 | 0.264795 |
| 24650 | 0.946122 | 2.1 |
| 24651 | 1.3852 | 2.1 |
| 24652 | 0.946124 | 2.1 |
| 24653 | 0.946118 | 2.1 |
| 24654 | 1.38521 | 2.1 |
| 24655 | 0.946131 | 0.71922 |
| 24656 | 0.946131 | 1.44249 |
| 24657 | 1.30448 | 0.949927 |
| 24658 | 0.946129 | 0.712524 |
| 24659 | 0.94613 | 0.71261 |
| 24660 | 0.946133 | 2.1 |
| 24661 | 0.94613 | 2.1 |
| 24662 | 0.946131 | 2.1 |
| 24663 | 0.946131 | 2.1 |
| 24664 | 1.36503 | 2.1 |
| 24665 | 0.810947 | 0.05 |
| 24666 | 0.810947 | 0.05 |
| 24667 | 0.810934 | 0.05 |
| 24668 | 3.34066 | 0.05 |
| 24669 | 0.946133 | 0.814587 |
| 24670 | 1.4185 | 0.513243 |
| 24671 | 0.946132 | 0.532777 |
| 24672 | 0.946133 | 0.511483 |
| 24673 | 0.946132 | 0.373278 |
| 24674 | 0.94613 | 2.1 |
| 24675 | 0.946128 | 2.1 |
| 24676 | 1.03925 | 2.1 |
| 24677 | 0.944743 | 2.1 |
| 24678 | 0.947518 | 2.1 |
| 24679 | 0.946132 | 2.1 |
| 24680 | 1.20573 | 2.1 |
| 24681 | 0.94613 | 2.1 |
| 24682 | 0.94613 | 2.1 |
| 24683 | 1.20573 | 2.1 |
| 24684 | 1.20056 | 0.05 |
| 24685 | 0.946132 | 0.05 |
| 24686 | 0.946131 | 0.05 |
| 24687 | 0.946131 | 0.05 |
| 24688 | 0.946131 | 0.05 |
| 24689 | 0.94613 | 2.1 |
| 24690 | 0.946128 | 2.1 |
| 24691 | 1.38302 | 2.1 |
| 24692 | 0.94613 | 2.1 |
| 24693 | 0.94613 | 2.1 |
| 24694 | 0.827866 | 0.05 |
| 24695 | 0.913552 | 0.05 |
| 24696 | 0.716526 | 0.05 |
| 24697 | 0.82784 | 0.05 |
| 24698 | 1.20634 | 0.05 |
| 24699 | 0.946131 | 0.05 |
| 24700 | 0.94613 | 0.05 |
| 24701 | 1.39502 | 0.05 |
| 24702 | 0.88146 | 0.05 |
| 24703 | 0.946132 | 2.1 |
| 24704 | 0.946132 | 2.1 |
| 24705 | 0.946132 | 2.1 |

| | | |
|---|---|---|
| 24706 | 0.946132 | 2.1 |
| 24707 | 1.39053 | 2.1 |
| 24708 | 0.946131 | 2.1 |
| 24709 | 1.40571 | 2.1 |
| 24710 | 0.946131 | 2.1 |
| 24711 | 0.946131 | 2.1 |
| 24712 | 0.946132 | 2.1 |
| 24713 | 0.94613 | 0.0567144 |
| 24714 | 1.2582 | 0.075634 |
| 24715 | 0.946127 | 0.0898176 |
| 24716 | 0.946129 | 0.0625016 |
| 24717 | 0.946129 | 0.0572216 |
| 24718 | 0.946129 | 1.01521 |
| 24719 | 0.946132 | 1.05157 |
| 24720 | 0.946131 | 0.628269 |
| 24721 | 0.946131 | 1.14029 |
| 24722 | 1.29054 | 0.82843 |
| 24723 | 0.810944 | 0.05 |
| 24724 | 0.810941 | 0.05 |
| 24725 | 0.810931 | 0.05 |
| 24726 | 0.946132 | 0.622316 |
| 24727 | 0.946132 | 0.650086 |
| 24728 | 0.946132 | 1.33302 |
| 24729 | 0.946132 | 0.82128 |
| 24730 | 0.927608 | 0.603587 |
| 24731 | 0.946128 | 2.1 |
| 24732 | 0.946133 | 2.1 |
| 24733 | 0.946131 | 2.1 |
| 24734 | 0.94613 | 2.1 |
| 24735 | 1.38813 | 2.1 |
| 24736 | 0.94613 | 0.05 |
| 24737 | 0.946128 | 2.1 |
| 24738 | 0.946132 | 0.05 |
| 24739 | 1.41666 | 2.1 |
| 24740 | 0.94613 | 1.52047 |
| 24741 | 0.946118 | 0.05 |
| 24742 | 1.34151 | 0.05 |
| 24743 | 0.946116 | 0.05 |
| 24744 | 0.946131 | 0.05 |
| 24745 | 1.34149 | 0.05 |
| 24746 | 0.946132 | 2.1 |
| 24747 | 1.31889 | 2.1 |
| 24748 | 0.946132 | 2.1 |
| 24749 | 0.946132 | 2.1 |
| 24750 | 1.31889 | 2.1 |
| 24751 | 0.864997 | 0.05 |
| 24752 | 0.928981 | 0.05 |
| 24753 | 0.872219 | 0.05 |
| 24754 | 0.946127 | 0.142785 |
| 24755 | 0.946131 | 0.11345 |
| 24756 | 1.37709 | 0.05 |
| 24757 | 0.946131 | 0.15817 |
| 24758 | 0.946129 | 0.0986096 |
| 24759 | 1.21882 | 0.05 |
| 24760 | 0.938168 | 0.05 |
| 24761 | 0.938161 | 0.05 |
| 24762 | 1.06327 | 0.05 |
| 24763 | 1.33457 | 0.05 |

| | | |
|---|---|---|
| 24764 | 0.946133 | 0.976685 |
| 24765 | 0.946132 | 0.960178 |
| 24766 | 0.946133 | 0.509723 |
| 24767 | 0.946132 | 1.25865 |
| 24768 | 1.14444 | 0.633698 |
| 24769 | 1.12628 | 0.05 |
| 24770 | 0.946132 | 0.05 |
| 24771 | 0.946131 | 0.05 |
| 24772 | 1.12628 | 0.05 |
| 24773 | 0.946131 | 0.05 |
| 24774 | 0.946132 | 2.1 |
| 24775 | 0.946132 | 2.1 |
| 24776 | 0.946133 | 2.1 |
| 24777 | 0.946132 | 2.1 |
| 24778 | 1.29322 | 2.1 |
| 24779 | 0.946131 | 2.1 |
| 24780 | 0.946132 | 2.1 |
| 24781 | 0.94613 | 2.1 |
| 24782 | 0.946131 | 2.1 |
| 24783 | 1.28441 | 2.1 |
| 24784 | 0.946118 | 2.1 |
| 24785 | 0.946119 | 2.1 |
| 24786 | 0.946117 | 2.1 |
| 24787 | 1.28075 | 2.1 |
| 24788 | 0.946105 | 2.1 |
| 24789 | 0.810945 | 2.1 |
| 24790 | 0.810942 | 2.1 |
| 24791 | 0.81093 | 2.1 |
| 24792 | 0.946127 | 2.1 |
| 24793 | 0.946127 | 2.1 |
| 24794 | 1.28228 | 2.1 |
| 24795 | 0.946129 | 2.1 |
| 24796 | 0.946132 | 2.1 |
| 24797 | 1.14042 | 2.1 |
| 24798 | 0.946132 | 2.1 |
| 24799 | 0.946133 | 2.1 |
| 24800 | 1.14042 | 2.1 |
| 24801 | 0.946133 | 0.05 |
| 24802 | 0.946132 | 0.05 |
| 24803 | 1.28632 | 0.05 |
| 24804 | 1.28632 | 0.05 |
| 24805 | 0.946133 | 0.05 |
| 24806 | 0.946131 | 1.41779 |
| 24807 | 0.946131 | 1.09524 |
| 24808 | 0.946131 | 1.26702 |
| 24809 | 1.31817 | 1.50001 |
| 24810 | 0.946131 | 1.11632 |
| 24811 | 1.35048 | 2.1 |
| 24812 | 0.946132 | 2.1 |
| 24813 | 0.946132 | 2.1 |
| 24814 | 0.946132 | 2.1 |
| 24815 | 0.946131 | 2.1 |
| 24816 | 0.946131 | 2.1 |
| 24817 | 1.35943 | 2.1 |
| 24818 | 0.94613 | 2.1 |
| 24819 | 0.946132 | 2.1 |
| 24820 | 0.946131 | 2.1 |
| 24821 | 0.990713 | 2.1 |

| | | |
|---|---|---|
| 24822 | 0.946132 | 2.1 |
| 24823 | 0.946132 | 2.1 |
| 24824 | 0.946133 | 2.1 |
| 24825 | 0.946132 | 2.1 |
| 24826 | 0.946133 | 1.16992 |
| 24827 | 1.01433 | 1.16786 |
| 24828 | 0.877931 | 1.22691 |
| 24829 | 0.96573 | 0.882938 |
| 24830 | 0.946131 | 0.881617 |
| 24831 | 0.946131 | 0.714449 |
| 24832 | 0.946133 | 0.278609 |
| 24833 | 1.39575 | 0.05 |
| 24834 | 0.946131 | 0.0629757 |
| 24835 | 0.94613 | 0.057594 |
| 24836 | 0.810943 | 0.131635 |
| 24837 | 0.810942 | 0.194471 |
| 24838 | 0.709589 | 0.122168 |
| 24839 | 0.924071 | 0.0512017 |
| 24840 | 1.04293 | 0.05 |
| 24841 | 1.15747 | 0.05 |
| 24842 | 0.946131 | 0.0567065 |
| 24843 | 1.04071 | 0.05 |
| 24844 | 0.810946 | 0.05 |
| 24845 | 0.810943 | 0.05 |
| 24846 | 0.810959 | 0.05 |
| 24847 | 0.946128 | 1.31586 |
| 24848 | 1.39696 | 1.94628 |
| 24849 | 0.94613 | 0.926778 |
| 24850 | 0.94613 | 0.969059 |
| 24851 | 1.39696 | 1.48262 |
| 24852 | 0.946131 | 0.884721 |
| 24853 | 0.946131 | 2.1 |
| 24854 | 0.946131 | 1.58128 |
| 24855 | 0.946131 | 1.10112 |
| 24856 | 1.29735 | 1.94959 |
| 24857 | 0.946128 | 1.32467 |
| 24858 | 0.946133 | 0.0960579 |
| 24859 | 0.94613 | 2.1 |
| 24860 | 0.946131 | 1.31907 |
| 24861 | 1.33266 | 0.718509 |
| 24862 | 0.946131 | 2.1 |
| 24863 | 0.94613 | 0.05 |
| 24864 | 0.946131 | 2.1 |
| 24865 | 1.40609 | 0.05 |
| 24866 | 0.946131 | 0.514809 |
| 24867 | 0.946131 | 1.64555 |
| 24868 | 0.946133 | 1.78981 |
| 24869 | 0.946129 | 1.99712 |
| 24870 | 1.23339 | 2.1 |
| 24871 | 0.946131 | 1.37228 |
| 24872 | 0.946132 | 2.1 |
| 24873 | 0.946133 | 2.1 |
| 24874 | 0.946132 | 2.1 |
| 24875 | 1.29198 | 2.1 |
| 24876 | 0.946132 | 2.1 |
| 24877 | 0.946131 | 2.1 |
| 24878 | 1.07297 | 2.1 |
| 24879 | 0.946127 | 2.1 |

| | | |
|---|---|---|
| 24880 | 0.94613 | 2.1 |
| 24881 | 1.07297 | 2.1 |
| 24882 | 0.946125 | 2.1 |
| 24883 | 0.946126 | 2.1 |
| 24884 | 0.946126 | 2.1 |
| 24885 | 1.3864 | 2.1 |
| 24886 | 0.946119 | 2.1 |
| 24887 | 0.946128 | 0.05 |
| 24888 | 1.09411 | 0.05 |
| 24889 | 0.946131 | 0.05 |
| 24890 | 0.946131 | 0.05 |
| 24891 | 1.09411 | 0.05 |
| 24892 | 0.946131 | 0.05 |
| 24893 | 1.25837 | 0.05 |
| 24894 | 0.946131 | 0.05 |
| 24895 | 0.946131 | 0.05 |
| 24896 | 1.25837 | 0.05 |
| 24897 | 0.810945 | 2.1 |
| 24898 | 0.810943 | 2.1 |
| 24899 | 0.810933 | 2.1 |
| 24900 | 0.94613 | 0.05 |
| 24901 | 0.946131 | 0.155095 |
| 24902 | 0.946132 | 0.05 |
| 24903 | 0.946129 | 0.05 |
| 24904 | 0.946131 | 0.05 |
| 24905 | 0.946131 | 0.05 |
| 24906 | 1.28911 | 0.05 |
| 24907 | 0.946129 | 0.08145 |
| 24908 | 0.946133 | 0.05 |
| 24909 | 0.946131 | 0.05 |
| 24910 | 0.94613 | 0.0587735 |
| 24911 | 1.38954 | 0.144154 |
| 24912 | 0.946132 | 0.05 |
| 24913 | 0.94613 | 0.0991137 |
| 24914 | 0.946131 | 0.0571953 |
| 24915 | 0.946131 | 0.552754 |
| 24916 | 0.946131 | 0.0572321 |
| 24917 | 0.946131 | 0.05 |
| 24918 | 1.37981 | 0.05 |
| 24919 | 0.946131 | 0.0529809 |
| 24920 | 0.946131 | 2.1 |
| 24921 | 1.26958 | 2.1 |
| 24922 | 0.946127 | 2.1 |
| 24923 | 0.946129 | 2.1 |
| 24924 | 1.26957 | 2.1 |
| 24925 | 0.94613 | 2.1 |
| 24926 | 1.16889 | 2.1 |
| 24927 | 0.946126 | 2.1 |
| 24928 | 0.946128 | 2.1 |
| 24929 | 1.16889 | 2.1 |
| 24930 | 0.946133 | 0.0907205 |
| 24931 | 1.13511 | 0.05 |
| 24932 | 0.946132 | 0.222657 |
| 24933 | 0.946133 | 0.0532193 |
| 24934 | 0.946132 | 0.0891802 |
| 24935 | 1.27575 | 0.588726 |
| 24936 | 0.946129 | 2.1 |
| 24937 | 0.946131 | 1.70649 |

| | | |
|---|---|---|
| 24938 | 0.94613 | 2.1 |
| 24939 | 0.946131 | 2.05691 |
| 24940 | 0.946131 | 2.1 |
| 24941 | 0.946128 | 2.1 |
| 24942 | 1.41712 | 2.1 |
| 24943 | 1.41712 | 2.1 |
| 24944 | 0.94613 | 2.1 |
| 24945 | 0.946129 | 0.05 |
| 24946 | 0.946132 | 0.05 |
| 24947 | 0.946131 | 0.05 |
| 24948 | 0.946131 | 0.05 |
| 24949 | 0.946131 | 0.05 |
| 24950 | 0.979045 | 1.95871 |
| 24951 | 0.946131 | 1.00412 |
| 24952 | 0.617549 | 2.1 |
| 24953 | 0.555844 | 2.0968 |
| 24954 | 1.04071 | 0.96642 |
| 24955 | 0.946132 | 1.55423 |
| 24956 | 0.946132 | 1.19466 |
| 24957 | 0.946131 | 2.1 |
| 24958 | 1.22618 | 1.01103 |
| 24959 | 0.946132 | 0.183133 |
| 24960 | 0.946131 | 2.1 |
| 24961 | 1.13976 | 2.1 |
| 24962 | 0.946129 | 2.1 |
| 24963 | 0.946131 | 2.1 |
| 24964 | 1.13976 | 2.1 |
| 24965 | 0.810945 | 0.65238 |
| 24966 | 0.810943 | 0.088029 |
| 24967 | 0.810932 | 0.792359 |
| 24968 | 1.25358 | 1.06933 |
| 24969 | 0.94613 | 0.174686 |
| 24970 | 0.946128 | 0.0557651 |
| 24971 | 1.25358 | 2.09848 |
| 24972 | 1.07811 | 0.683364 |
| 24973 | 0.946129 | 2.1 |
| 24974 | 1.09197 | 2.1 |
| 24975 | 0.946131 | 2.1 |
| 24976 | 0.946131 | 2.1 |
| 24977 | 1.09197 | 2.1 |
| 24978 | 0.594912 | 2.1 |
| 24979 | 0.993703 | 2.1 |
| 24980 | 1.10173 | 2.1 |
| 24981 | 0.9916 | 2.1 |
| 24982 | 0.946131 | 2.1 |
| 24983 | 0.946128 | 2.1 |
| 24984 | 0.946131 | 2.1 |
| 24985 | 0.94613 | 2.1 |
| 24986 | 0.946129 | 2.1 |
| 24987 | 0.946132 | 2.1 |
| 24988 | 1.40335 | 2.1 |
| 24989 | 0.946133 | 2.1 |
| 24990 | 0.946133 | 2.1 |
| 24991 | 1.40335 | 2.1 |
| 24992 | 0.946132 | 0.997448 |
| 24993 | 0.946132 | 2.1 |
| 24994 | 0.946132 | 1.07259 |
| 24995 | 0.946132 | 1.22551 |

| | | |
|---|---|---|
| 24996 | 1.411 | 1.84757 |
| 24997 | 0.611675 | 0.05 |
| 24998 | 0.946129 | 0.05 |
| 24999 | 1.31678 | 0.05 |
| 25000 | 0.946124 | 0.05 |
| 25001 | 0.946127 | 0.05 |
| 25002 | 0.946128 | 0.05 |
| 25003 | 0.946132 | 0.05 |
| 25004 | 0.946131 | 0.05 |
| 25005 | 0.946132 | 0.053379 |
| 25006 | 0.946132 | 0.05 |
| 25007 | 0.946132 | 0.05 |
| 25008 | 0.810946 | 0.05 |
| 25009 | 0.810943 | 0.05 |
| 25010 | 0.81096 | 0.05 |
| 25011 | 1.39301 | 0.05 |
| 25012 | 0.946132 | 0.05 |
| 25013 | 0.625812 | 0.05 |
| 25014 | 0.946132 | 0.05 |
| 25015 | 0.625812 | 0.05 |
| 25016 | 0.810946 | 1.10968 |
| 25017 | 0.810945 | 1.85404 |
| 25018 | 0.810931 | 2.1 |
| 25019 | 0.810943 | 2.1 |
| 25020 | 0.810944 | 2.1 |
| 25021 | 0.810958 | 2.1 |
| 25022 | 0.946131 | 0.282854 |
| 25023 | 0.946131 | 0.216712 |
| 25024 | 0.946128 | 0.344144 |
| 25025 | 0.946129 | 0.301392 |
| 25026 | 0.94613 | 0.215843 |
| 25027 | 0.946133 | 0.05 |
| 25028 | 0.946133 | 0.126181 |
| 25029 | 0.98537 | 0.05 |
| 25030 | 0.985371 | 0.05 |
| 25031 | 0.946132 | 0.200975 |
| 25032 | 0.946129 | 0.05 |
| 25033 | 1.38752 | 0.05 |
| 25034 | 0.946131 | 0.0600145 |
| 25035 | 0.946131 | 0.0523775 |
| 25036 | 1.38752 | 0.05 |
| 25037 | 0.946119 | 0.05 |
| 25038 | 1.33036 | 0.05 |
| 25039 | 0.946119 | 0.05 |
| 25040 | 0.946132 | 0.05 |
| 25041 | 1.33035 | 0.05 |
| 25042 | 1.3868 | 2.1 |
| 25043 | 0.946132 | 2.1 |
| 25044 | 0.946132 | 2.1 |
| 25045 | 0.946133 | 2.1 |
| 25046 | 0.946132 | 2.1 |
| 25047 | 0.810947 | 2.1 |
| 25048 | 0.810946 | 2.1 |
| 25049 | 0.81096 | 2.1 |
| 25050 | 0.810946 | 2.1 |
| 25051 | 0.810946 | 2.1 |
| 25052 | 0.810933 | 2.1 |
| 25053 | 0.946133 | 2.1 |

| | | |
|---|---|---|
| 25054 | 1.35342 | 2.1 |
| 25055 | 0.946133 | 2.1 |
| 25056 | 0.946132 | 2.1 |
| 25057 | 1.35342 | 2.1 |
| 25058 | 0.946132 | 2.1 |
| 25059 | 0.946132 | 2.1 |
| 25060 | 1.28569 | 2.1 |
| 25061 | 0.946132 | 2.1 |
| 25062 | 0.946133 | 2.1 |
| 25063 | 0.946132 | 2.1 |
| 25064 | 1.32547 | 2.1 |
| 25065 | 0.946132 | 2.1 |
| 25066 | 0.946132 | 2.1 |
| 25067 | 1.32547 | 2.1 |
| 25068 | 0.94613 | 0.05 |
| 25069 | 0.946133 | 0.05 |
| 25070 | 1.39664 | 0.05 |
| 25071 | 1.39664 | 0.05 |
| 25072 | 0.946131 | 0.05 |
| 25073 | 0.946131 | 1.18575 |
| 25074 | 0.94613 | 2.1 |
| 25075 | 0.946132 | 0.870581 |
| 25076 | 0.946131 | 2.1 |
| 25077 | 1.41833 | 1.76394 |
| 25078 | 0.946131 | 0.05 |
| 25079 | 0.946131 | 0.05 |
| 25080 | 0.946131 | 0.05 |
| 25081 | 1.26155 | 0.05 |
| 25082 | 0.946132 | 0.05 |
| 25083 | 0.810946 | 2.1 |
| 25084 | 0.810946 | 2.1 |
| 25085 | 0.810959 | 2.1 |
| 25086 | 0.94613 | 0.816086 |
| 25087 | 0.946132 | 0.1245 |
| 25088 | 1.40779 | 0.05 |
| 25089 | 1.40779 | 0.0607042 |
| 25090 | 0.94613 | 0.0739515 |
| 25091 | 0.94613 | 0.05 |
| 25092 | 0.946129 | 0.05 |
| 25093 | 1.36376 | 0.05 |
| 25094 | 1.36376 | 0.05 |
| 25095 | 0.946131 | 0.05 |
| 25096 | 0.946128 | 2.1 |
| 25097 | 0.946127 | 2.1 |
| 25098 | 0.946131 | 2.1 |
| 25099 | 1.40756 | 2.1 |
| 25100 | 0.94613 | 2.1 |
| 25101 | 0.946131 | 1.78656 |
| 25102 | 0.946132 | 2.1 |
| 25103 | 0.94613 | 1.84774 |
| 25104 | 0.946131 | 1.85326 |
| 25105 | 1.31947 | 2.1 |
| 25106 | 0.946131 | 2.1 |
| 25107 | 0.946131 | 2.1 |
| 25108 | 0.946129 | 2.1 |
| 25109 | 0.946131 | 2.1 |
| 25110 | 1.25768 | 2.1 |
| 25111 | 0.946132 | 0.35417 |

| | | |
|---|---|---|
| 25112 | 0.94613 | 0.39126 |
| 25113 | 0.946131 | 0.329785 |
| 25114 | 0.946131 | 0.302224 |
| 25115 | 0.946131 | 0.358658 |
| 25116 | 0.810943 | 0.924492 |
| 25117 | 0.810942 | 1.08952 |
| 25118 | 0.81093 | 2.1 |
| 25119 | 0.946128 | 0.975772 |
| 25120 | 0.946133 | 0.785294 |
| 25121 | 0.946131 | 1.01888 |
| 25122 | 0.946131 | 0.940573 |
| 25123 | 0.94613 | 1.02492 |
| 25124 | 0.946132 | 1.00765 |
| 25125 | 0.946131 | 0.833708 |
| 25126 | 0.946132 | 1.04466 |
| 25127 | 0.946132 | 0.999112 |
| 25128 | 0.946132 | 1.07434 |
| 25129 | 0.946119 | 2.1 |
| 25130 | 0.946119 | 2.1 |
| 25131 | 1.31523 | 2.1 |
| 25132 | 0.946132 | 2.1 |
| 25133 | 0.946107 | 2.1 |
| 25134 | 0.946133 | 2.1 |
| 25135 | 0.946132 | 0.102767 |
| 25136 | 0.946132 | 0.111907 |
| 25137 | 1.28803 | 1.90638 |
| 25138 | 0.946132 | 2.00071 |
| 25139 | 1.37658 | 1.14704 |
| 25140 | 0.946132 | 1.0063 |
| 25141 | 0.946133 | 1.87478 |
| 25142 | 0.946132 | 1.23889 |
| 25143 | 0.946132 | 1.53105 |
| 25144 | 0.946132 | 1.01998 |
| 25145 | 0.94613 | 1.11694 |
| 25146 | 0.94613 | 1.55764 |
| 25147 | 1.15541 | 0.974867 |
| 25148 | 0.92943 | 1.27211 |
| 25149 | 0.946126 | 0.05 |
| 25150 | 0.946129 | 0.05 |
| 25151 | 1.33849 | 0.05 |
| 25152 | 1.33849 | 0.05 |
| 25153 | 0.946129 | 0.05 |
| 25154 | 0.810946 | 0.05 |
| 25155 | 0.810944 | 0.05 |
| 25156 | 0.810959 | 0.05 |
| 25157 | 1.39027 | 0.05 |
| 25158 | 0.946131 | 0.05 |
| 25159 | 0.946131 | 0.05 |
| 25160 | 1.39027 | 0.05 |
| 25161 | 0.946131 | 0.05 |
| 25162 | 1.40924 | 2.1 |
| 25163 | 0.946131 | 1.27726 |
| 25164 | 0.946132 | 2.1 |
| 25165 | 0.946132 | 1.10339 |
| 25166 | 0.946132 | 1.84456 |
| 25167 | 0.946131 | 2.1 |
| 25168 | 0.94613 | 2.1 |
| 25169 | 0.946128 | 2.1 |

| | | |
|---|---|---|
| 25170 | 0.946128 | 2.1 |
| 25171 | 1.41744 | 2.1 |
| 25172 | 0.946132 | 0.0974791 |
| 25173 | 0.946128 | 0.0585116 |
| 25174 | 0.94613 | 0.807066 |
| 25175 | 1.37675 | 0.136736 |
| 25176 | 0.94613 | 0.789487 |
| 25177 | 0.946132 | 0.775276 |
| 25178 | 0.946132 | 0.0586676 |
| 25179 | 0.946132 | 0.05 |
| 25180 | 0.946132 | 0.0662667 |
| 25181 | 0.946131 | 0.05 |
| 25182 | 0.946132 | 0.861022 |
| 25183 | 0.946132 | 0.656082 |
| 25184 | 0.946133 | 1.2677 |
| 25185 | 1.10003 | 0.239299 |
| 25186 | 0.946132 | 0.9623 |
| 25187 | 0.946132 | 0.05 |
| 25188 | 0.946131 | 0.05 |
| 25189 | 0.946132 | 0.05 |
| 25190 | 0.946131 | 0.05 |
| 25191 | 0.946132 | 0.05 |
| 25192 | 0.946132 | 0.05 |
| 25193 | 0.946131 | 0.05 |
| 25194 | 0.946132 | 0.05 |
| 25195 | 0.946132 | 0.05 |
| 25196 | 0.946131 | 0.05 |
| 25197 | 1.24094 | 0.05 |
| 25198 | 0.946126 | 0.05 |
| 25199 | 0.946128 | 0.05 |
| 25200 | 0.946129 | 0.05 |
| 25201 | 0.946129 | 0.05 |
| 25202 | 1.2929 | 0.05 |
| 25203 | 0.946131 | 0.05 |
| 25204 | 0.946131 | 0.05 |
| 25205 | 0.946131 | 0.05 |
| 25206 | 0.946131 | 0.05 |
| 25207 | 0.810946 | 0.05 |
| 25208 | 0.810945 | 0.05 |
| 25209 | 0.810959 | 0.05 |
| 25210 | 0.810946 | 1.99004 |
| 25211 | 0.810943 | 1.99239 |
| 25212 | 0.810933 | 2.1 |
| 25213 | 0.946128 | 2.1 |
| 25214 | 1.18315 | 2.1 |
| 25215 | 0.94613 | 2.1 |
| 25216 | 0.94613 | 2.1 |
| 25217 | 1.18316 | 2.1 |
| 25218 | 0.946126 | 0.0665602 |
| 25219 | 1.3324 | 0.05 |
| 25220 | 0.946127 | 0.0696049 |
| 25221 | 0.94613 | 0.05 |
| 25222 | 0.946128 | 0.05 |
| 25223 | 0.94613 | 2.1 |
| 25224 | 0.946128 | 2.1 |
| 25225 | 1.39523 | 2.1 |
| 25226 | 1.39523 | 2.1 |
| 25227 | 0.946131 | 2.1 |

| | | |
|---|---|---|
| 25228 | 1.36541 | 2.1 |
| 25229 | 0.946132 | 2.1 |
| 25230 | 0.946132 | 2.1 |
| 25231 | 0.946132 | 2.1 |
| 25232 | 0.946132 | 2.1 |
| 25233 | 0.946131 | 2.1 |
| 25234 | 0.94613 | 1.06505 |
| 25235 | 0.946132 | 1.72374 |
| 25236 | 0.946131 | 0.110424 |
| 25237 | 1.39509 | 1.36221 |
| 25238 | 0.946132 | 2.1 |
| 25239 | 0.946132 | 2.1 |
| 25240 | 1.29319 | 2.1 |
| 25241 | 0.946132 | 2.1 |
| 25242 | 0.946133 | 2.1 |
| 25243 | 0.946132 | 0.05 |
| 25244 | 0.946131 | 0.05 |
| 25245 | 0.946132 | 0.05 |
| 25246 | 1.01196 | 0.05 |
| 25247 | 0.946132 | 0.05 |
| 25248 | 0.946116 | 2.1 |
| 25249 | 0.946117 | 2.1 |
| 25250 | 1.38153 | 2.1 |
| 25251 | 1.38155 | 2.1 |
| 25252 | 0.946105 | 2.1 |
| 25253 | 0.946132 | 2.0864 |
| 25254 | 0.946131 | 2.07986 |
| 25255 | 0.946132 | 1.87905 |
| 25256 | 1.28684 | 1.86319 |
| 25257 | 0.946132 | 1.36064 |
| 25258 | 1.10185 | 1.58032 |
| 25259 | 0.94613 | 1.83191 |
| 25260 | 0.946128 | 2.1 |
| 25261 | 0.94613 | 2.1 |
| 25262 | 0.94613 | 1.78442 |
| 25263 | 0.810947 | 0.05 |
| 25264 | 0.810945 | 0.05 |
| 25265 | 0.810932 | 0.05 |
| 25266 | 0.946119 | 0.0931772 |
| 25267 | 1.16717 | 0.05 |
| 25268 | 0.946117 | 0.149816 |
| 25269 | 0.94613 | 0.05 |
| 25270 | 1.16715 | 0.05 |
| 25271 | 0.94612 | 0.40771 |
| 25272 | 0.946119 | 0.349894 |
| 25273 | 0.946119 | 0.200001 |
| 25274 | 0.946132 | 0.05 |
| 25275 | 1.20882 | 0.132384 |
| 25276 | 1.07043 | 0.108894 |
| 25277 | 0.823268 | 0.0855822 |
| 25278 | 0.810959 | 0.114253 |
| 25279 | 0.946132 | 1.8211 |
| 25280 | 1.14879 | 0.293297 |
| 25281 | 0.94613 | 1.47539 |
| 25282 | 0.946131 | 1.71485 |
| 25283 | 1.14879 | 0.62485 |
| 25284 | 1.23837 | 2.1 |
| 25285 | 0.94612 | 2.1 |

| | | |
|---|---|---|
| 25286 | 0.946118 | 2.1 |
| 25287 | 0.946131 | 2.1 |
| 25288 | 0.946105 | 2.1 |
| 25289 | 0.810946 | 0.05 |
| 25290 | 0.810946 | 0.05 |
| 25291 | 0.810959 | 0.05 |
| 25292 | 0.810947 | 1.23146 |
| 25293 | 0.810947 | 0.495479 |
| 25294 | 0.810934 | 0.475192 |
| 25295 | 0.946128 | 2.1 |
| 25296 | 0.946131 | 2.1 |
| 25297 | 0.946128 | 2.1 |
| 25298 | 0.946129 | 2.1 |
| 25299 | 0.94613 | 2.1 |
| 25300 | 0.94613 | 2.1 |
| 25301 | 0.946131 | 2.1 |
| 25302 | 0.946131 | 2.1 |
| 25303 | 0.946131 | 2.1 |
| 25304 | 0.946131 | 2.1 |
| 25305 | 0.946131 | 1.91346 |
| 25306 | 1.38585 | 2.1 |
| 25307 | 0.946128 | 1.19201 |
| 25308 | 0.94613 | 2.01487 |
| 25309 | 1.38585 | 2.1 |
| 25310 | 0.946133 | 0.05 |
| 25311 | 0.946133 | 0.05 |
| 25312 | 0.946132 | 0.05 |
| 25313 | 0.946132 | 0.05 |
| 25314 | 0.946132 | 0.05 |
| 25315 | 0.918871 | 0.05 |
| 25316 | 0.810248 | 0.05 |
| 25317 | 0.838037 | 0.05 |
| 25318 | 0.946126 | 0.05 |
| 25319 | 0.943647 | 0.05 |
| 25320 | 1.13836 | 0.05 |
| 25321 | 1.03802 | 0.05 |
| 25322 | 0.946129 | 0.05 |
| 25323 | 0.946133 | 1.04121 |
| 25324 | 0.946132 | 0.498248 |
| 25325 | 0.946132 | 2.1 |
| 25326 | 1.1699 | 0.211607 |
| 25327 | 0.946132 | 0.129476 |
| 25328 | 0.946131 | 2.1 |
| 25329 | 0.946132 | 2.1 |
| 25330 | 1.37199 | 2.1 |
| 25331 | 1.37199 | 2.1 |
| 25332 | 0.946132 | 2.1 |
| 25333 | 0.946132 | 0.960238 |
| 25334 | 0.946133 | 0.592894 |
| 25335 | 0.946133 | 1.15093 |
| 25336 | 0.946132 | 0.585218 |
| 25337 | 1.38623 | 0.707087 |
| 25338 | 0.946132 | 2.1 |
| 25339 | 0.946132 | 2.1 |
| 25340 | 0.946132 | 2.1 |
| 25341 | 0.946132 | 2.1 |
| 25342 | 1.40098 | 2.1 |
| 25343 | 1.20787 | 0.669437 |

| | | |
|---|---|---|
| 25344 | 1.41894 | 0.629904 |
| 25345 | 0.946133 | 0.134019 |
| 25346 | 1.01777 | 0.717886 |
| 25347 | 0.946132 | 2.1 |
| 25348 | 0.946132 | 2.1 |
| 25349 | 0.946133 | 2.1 |
| 25350 | 1.40199 | 2.1 |
| 25351 | 0.946132 | 2.1 |
| 25352 | 0.810946 | 1.03535 |
| 25353 | 0.810947 | 1.93323 |
| 25354 | 0.810934 | 1.18521 |
| 25355 | 0.946126 | 0.92746 |
| 25356 | 0.94613 | 1.4491 |
| 25357 | 0.946125 | 1.33133 |
| 25358 | 1.40247 | 1.02776 |
| 25359 | 0.946128 | 0.985167 |
| 25360 | 1.25703 | 1.41199 |
| 25361 | 0.87821 | 1.01135 |
| 25362 | 0.878238 | 0.962889 |
| 25363 | 0.946119 | 2.01615 |
| 25364 | 0.946132 | 2.1 |
| 25365 | 0.946132 | 0.644505 |
| 25366 | 0.946132 | 0.852868 |
| 25367 | 0.946131 | 1.39312 |
| 25368 | 0.946132 | 0.95759 |
| 25369 | 1.28543 | 0.824019 |
| 25370 | 0.946115 | 2.1 |
| 25371 | 1.41638 | 2.1 |
| 25372 | 0.946116 | 2.1 |
| 25373 | 0.946104 | 2.1 |
| 25374 | 0.946129 | 2.1 |
| 25375 | 0.946132 | 2.1 |
| 25376 | 1.41432 | 2.1 |
| 25377 | 0.946132 | 2.1 |
| 25378 | 0.946132 | 2.1 |
| 25379 | 0.946132 | 2.1 |
| 25380 | 0.810947 | 0.05 |
| 25381 | 0.810947 | 0.05 |
| 25382 | 0.709567 | 0.05 |
| 25383 | 0.851542 | 0.05 |
| 25384 | 1.01217 | 0.05 |
| 25385 | 0.697993 | 0.05 |
| 25386 | 0.946133 | 0.05 |
| 25387 | 0.851488 | 0.05 |
| 25388 | 0.946129 | 2.1 |
| 25389 | 0.94613 | 2.1 |
| 25390 | 1.2254 | 1.71088 |
| 25391 | 0.946131 | 2.1 |
| 25392 | 0.946131 | 2.07723 |
| 25393 | 0.700785 | 2.1 |
| 25394 | 0.915893 | 1.77731 |
| 25395 | 0.838013 | 1.54163 |
| 25396 | 0.760188 | 0.717738 |
| 25397 | 1.19601 | 0.05 |
| 25398 | 0.946133 | 0.05 |
| 25399 | 0.946132 | 0.05 |
| 25400 | 0.946132 | 0.05 |
| 25401 | 0.946132 | 0.05 |

| | | |
|---|---|---|
| 25402 | 0.946132 | 0.942107 |
| 25403 | 0.946133 | 2.1 |
| 25404 | 0.946132 | 1.28271 |
| 25405 | 0.946133 | 2.1 |
| 25406 | 1.36956 | 2.1 |
| 25407 | 0.946131 | 0.539576 |
| 25408 | 0.94613 | 0.05 |
| 25409 | 0.946133 | 0.347518 |
| 25410 | 1.34268 | 0.05 |
| 25411 | 0.946131 | 0.05 |
| 25412 | 0.946132 | 2.1 |
| 25413 | 1.25603 | 2.1 |
| 25414 | 0.946132 | 2.1 |
| 25415 | 0.946132 | 2.1 |
| 25416 | 1.25603 | 2.1 |
| 25417 | 0.810946 | 1.0065 |
| 25418 | 0.810943 | 1.02747 |
| 25419 | 0.810933 | 0.970418 |
| 25420 | 0.496545 | 0.05 |
| 25421 | 0.805767 | 0.224146 |
| 25422 | 0.936671 | 0.151827 |
| 25423 | 0.530063 | 0.0889547 |
| 25424 | 1.05954 | 0.106067 |
| 25425 | 0.810946 | 0.05 |
| 25426 | 0.810943 | 0.05 |
| 25427 | 0.810959 | 0.05 |
| 25428 | 1.16699 | 0.05 |
| 25429 | 0.946131 | 0.05 |
| 25430 | 0.946129 | 0.05 |
| 25431 | 0.946131 | 0.05 |
| 25432 | 0.946131 | 0.05 |
| 25433 | 0.810943 | 1.17078 |
| 25434 | 0.810942 | 1.33167 |
| 25435 | 0.810933 | 1.19482 |
| 25436 | 0.946132 | 1.12867 |
| 25437 | 1.04832 | 1.11663 |
| 25438 | 0.870714 | 1.18519 |
| 25439 | 0.957819 | 1.4419 |
| 25440 | 0.94613 | 0.990279 |
| 25441 | 1.82931 | 2.1 |
| 25442 | 0.946132 | 0.822014 |
| 25443 | 0.946129 | 0.755854 |
| 25444 | 0.94613 | 0.857788 |
| 25445 | 0.946131 | 0.818359 |
| 25446 | 0.94613 | 0.684962 |
| 25447 | 0.946119 | 0.983934 |
| 25448 | 0.94612 | 1.11093 |
| 25449 | 0.94612 | 0.859814 |
| 25450 | 0.946132 | 1.05912 |
| 25451 | 1.36118 | 0.918412 |
| 25452 | 0.810947 | 0.194881 |
| 25453 | 0.810947 | 0.219579 |
| 25454 | 0.810934 | 0.25384 |
| 25455 | 0.946132 | 2.1 |
| 25456 | 0.946132 | 2.1 |
| 25457 | 0.946132 | 2.1 |
| 25458 | 1.39754 | 2.1 |
| 25459 | 0.946131 | 2.1 |

| | | |
|---|---|---|
| 25460 | 0.946129 | 2.1 |
| 25461 | 0.946133 | 2.1 |
| 25462 | 0.946131 | 2.1 |
| 25463 | 0.946131 | 2.1 |
| 25464 | 1.41732 | 2.1 |
| 25465 | 0.946132 | 0.902004 |
| 25466 | 0.946128 | 2.07805 |
| 25467 | 0.94613 | 1.04932 |
| 25468 | 0.94613 | 1.17313 |
| 25469 | 1.25277 | 1.53602 |
| 25470 | 0.94613 | 1.34154 |
| 25471 | 0.946128 | 2.04012 |
| 25472 | 1.21261 | 0.259446 |
| 25473 | 0.94613 | 1.45187 |
| 25474 | 0.94613 | 0.402153 |
| 25475 | 1.39407 | 2.1 |
| 25476 | 0.946133 | 2.1 |
| 25477 | 0.94613 | 2.1 |
| 25478 | 0.946131 | 2.1 |
| 25479 | 0.946131 | 2.1 |
| 25480 | 0.946119 | 1.03544 |
| 25481 | 0.946119 | 1.32342 |
| 25482 | 0.946119 | 1.55797 |
| 25483 | 1.1537 | 1.31407 |
| 25484 | 0.946106 | 0.948522 |
| 25485 | 0.946131 | 2.1 |
| 25486 | 1.11822 | 2.1 |
| 25487 | 0.946129 | 2.1 |
| 25488 | 0.946131 | 2.1 |
| 25489 | 1.11822 | 2.1 |
| 25490 | 1.46806 | 0.05 |
| 25491 | 0.498881 | 0.05 |
| 25492 | 0.996658 | 0.05 |
| 25493 | 1.2179 | 0.05 |
| 25494 | 0.991519 | 0.05 |
| 25495 | 0.946131 | 0.05 |
| 25496 | 0.946131 | 0.05 |
| 25497 | 1.28673 | 0.05 |
| 25498 | 0.946131 | 0.05 |
| 25499 | 0.946131 | 0.05 |
| 25500 | 1.33178 | 0.05 |
| 25501 | 0.946132 | 0.05 |
| 25502 | 0.946132 | 0.05 |
| 25503 | 0.946132 | 0.05 |
| 25504 | 0.946132 | 0.05 |
| 25505 | 0.946128 | 0.05 |
| 25506 | 0.972061 | 0.05 |
| 25507 | 0.946129 | 0.05 |
| 25508 | 0.946129 | 0.05 |
| 25509 | 0.972059 | 0.05 |
| 25510 | 0.946129 | 2.1 |
| 25511 | 0.94613 | 2.1 |
| 25512 | 0.94613 | 2.1 |
| 25513 | 0.94613 | 2.1 |
| 25514 | 0.94613 | 2.1 |
| 25515 | 0.946131 | 2.1 |
| 25516 | 0.946127 | 2.1 |
| 25517 | 0.946131 | 2.1 |

| | | |
|---|---|---|
| 25518 | 0.946129 | 2.1 |
| 25519 | 1.4073 | 2.1 |
| 25520 | 0.946131 | 0.05 |
| 25521 | 1.05016 | 0.05 |
| 25522 | 0.946132 | 0.05 |
| 25523 | 0.946131 | 0.05 |
| 25524 | 0.946132 | 0.05 |
| 25525 | 0.946128 | 0.05 |
| 25526 | 1.13398 | 0.05 |
| 25527 | 0.946131 | 0.05 |
| 25528 | 0.946131 | 0.05 |
| 25529 | 0.94613 | 0.05 |
| 25530 | 0.946131 | 0.05 |
| 25531 | 0.946131 | 0.05 |
| 25532 | 1.38911 | 0.05 |
| 25533 | 1.38911 | 0.05 |
| 25534 | 0.946132 | 0.05 |
| 25535 | 0.946131 | 0.05 |
| 25536 | 0.94613 | 0.05 |
| 25537 | 0.946131 | 0.05 |
| 25538 | 0.946131 | 0.05 |
| 25539 | 0.946131 | 0.05 |
| 25540 | 0.946131 | 0.05 |
| 25541 | 1.23923 | 0.05 |
| 25542 | 0.94613 | 0.05 |
| 25543 | 0.946131 | 0.05 |
| 25544 | 0.946131 | 0.05 |
| 25545 | 1.40511 | 0.05 |
| 25546 | 0.946131 | 0.05 |
| 25547 | 0.946131 | 0.05 |
| 25548 | 0.946132 | 0.05 |
| 25549 | 0.946131 | 0.05 |
| 25550 | 1.12968 | 2.1 |
| 25551 | 0.946132 | 2.1 |
| 25552 | 0.946132 | 2.1 |
| 25553 | 0.946131 | 2.1 |
| 25554 | 0.946132 | 2.1 |
| 25555 | 0.946132 | 1.66002 |
| 25556 | 1.4172 | 2.1 |
| 25557 | 0.94613 | 1.1855 |
| 25558 | 0.94613 | 2.02759 |
| 25559 | 1.4172 | 2.1 |
| 25560 | 0.946132 | 2.1 |
| 25561 | 0.946132 | 2.1 |
| 25562 | 1.31323 | 2.1 |
| 25563 | 0.946133 | 2.1 |
| 25564 | 0.946132 | 2.1 |
| 25565 | 0.946131 | 2.1 |
| 25566 | 0.94613 | 0.05 |
| 25567 | 1.36546 | 2.1 |
| 25568 | 1.36546 | 1.5942 |
| 25569 | 0.946131 | 0.822453 |
| 25570 | 0.946132 | 0.522729 |
| 25571 | 0.946133 | 0.565486 |
| 25572 | 0.946132 | 0.0689191 |
| 25573 | 1.36899 | 0.05 |
| 25574 | 0.946133 | 0.169759 |
| 25575 | 0.946131 | 0.05 |

| | | |
|---|---|---|
| 25576 | 0.946132 | 0.05 |
| 25577 | 1.03543 | 0.05 |
| 25578 | 1.1412 | 0.05 |
| 25579 | 0.941275 | 0.05 |
| 25580 | 1.28952 | 0.05 |
| 25581 | 0.94613 | 0.05 |
| 25582 | 0.946129 | 0.05 |
| 25583 | 0.946132 | 0.05 |
| 25584 | 0.94613 | 0.05 |
| 25585 | 0.94613 | 2.1 |
| 25586 | 0.94613 | 2.1 |
| 25587 | 0.946129 | 2.1 |
| 25588 | 0.94613 | 2.1 |
| 25589 | 1.35966 | 2.1 |
| 25590 | 0.810946 | 2.1 |
| 25591 | 0.810942 | 2.1 |
| 25592 | 0.810932 | 2.1 |
| 25593 | 0.94613 | 2.1 |
| 25594 | 1.40372 | 2.1 |
| 25595 | 0.946131 | 2.1 |
| 25596 | 0.94613 | 2.1 |
| 25597 | 1.40372 | 2.1 |
| 25598 | 0.946133 | 2.1 |
| 25599 | 0.946131 | 2.1 |
| 25600 | 0.94613 | 2.1 |
| 25601 | 0.946131 | 2.1 |
| 25602 | 0.946131 | 2.1 |
| 25603 | 1.31453 | 2.1 |
| 25604 | 0.94613 | 2.1 |
| 25605 | 0.946128 | 2.1 |
| 25606 | 0.94613 | 2.1 |
| 25607 | 0.94613 | 2.1 |
| 25608 | 0.946131 | 0.05 |
| 25609 | 1.12018 | 0.05 |
| 25610 | 0.94613 | 0.05 |
| 25611 | 0.94613 | 0.05 |
| 25612 | 0.94613 | 0.05 |
| 25613 | 0.946131 | 0.05 |
| 25614 | 1.16373 | 0.05 |
| 25615 | 0.946131 | 0.05 |
| 25616 | 0.94613 | 0.05 |
| 25617 | 0.946131 | 0.05 |
| 25618 | 0.946126 | 0.0516013 |
| 25619 | 0.946132 | 0.05 |
| 25620 | 0.946128 | 0.05 |
| 25621 | 0.946129 | 0.156948 |
| 25622 | 0.946129 | 0.116051 |
| 25623 | 0.946132 | 0.05 |
| 25624 | 0.946133 | 0.05 |
| 25625 | 0.946132 | 0.05 |
| 25626 | 0.946132 | 0.05 |
| 25627 | 0.946133 | 0.05 |
| 25628 | 0.946132 | 0.972651 |
| 25629 | 0.946129 | 1.88112 |
| 25630 | 0.94613 | 0.868072 |
| 25631 | 1.36399 | 1.03413 |
| 25632 | 0.946131 | 0.906993 |
| 25633 | 0.946132 | 2.1 |

| | | |
|---|---|---|
| 25634 | 0.946132 | 2.1 |
| 25635 | 0.946133 | 2.1 |
| 25636 | 1.38302 | 2.1 |
| 25637 | 0.946133 | 2.1 |
| 25638 | 0.810943 | 2.1 |
| 25639 | 0.810944 | 2.1 |
| 25640 | 0.810945 | 2.1 |
| 25641 | 0.946132 | 2.1 |
| 25642 | 0.946131 | 2.1 |
| 25643 | 0.946132 | 2.1 |
| 25644 | 1.30188 | 2.1 |
| 25645 | 0.946132 | 2.1 |
| 25646 | 0.810946 | 0.820465 |
| 25647 | 0.72985 | 1.74943 |
| 25648 | 0.810934 | 0.997944 |
| 25649 | 0.856243 | 0.607424 |
| 25650 | 0.946129 | 0.69899 |
| 25651 | 0.946132 | 0.755486 |
| 25652 | 1.19686 | 0.297118 |
| 25653 | 0.851527 | 0.846649 |
| 25654 | 0.946131 | 2.1 |
| 25655 | 0.94613 | 2.1 |
| 25656 | 0.94613 | 2.1 |
| 25657 | 0.94613 | 2.1 |
| 25658 | 1.31961 | 2.1 |
| 25659 | 0.946131 | 2.1 |
| 25660 | 0.946131 | 2.1 |
| 25661 | 0.94613 | 2.1 |
| 25662 | 0.946131 | 2.1 |
| 25663 | 1.3605 | 2.1 |
| 25664 | 1.30116 | 2.1 |
| 25665 | 0.946133 | 0.180536 |
| 25666 | 0.946131 | 2.1 |
| 25667 | 0.946131 | 1.84398 |
| 25668 | 0.94613 | 2.1 |
| 25669 | 0.946132 | 0.05 |
| 25670 | 1.3035 | 0.05 |
| 25671 | 0.94613 | 0.05 |
| 25672 | 0.946131 | 0.05 |
| 25673 | 0.946131 | 0.05 |
| 25674 | 0.946132 | 2.1 |
| 25675 | 1.37869 | 2.1 |
| 25676 | 1.2051 | 2.1 |
| 25677 | 0.946132 | 2.1 |
| 25678 | 1.2051 | 2.1 |
| 25679 | 0.810946 | 2.1 |
| 25680 | 0.810945 | 2.1 |
| 25681 | 0.810946 | 2.1 |
| 25682 | 1.34623 | 2.1 |
| 25683 | 0.946131 | 2.1 |
| 25684 | 0.946131 | 2.1 |
| 25685 | 1.34623 | 2.1 |
| 25686 | 1.22993 | 2.1 |
| 25687 | 1.32299 | 0.05 |
| 25688 | 0.946132 | 0.05 |
| 25689 | 0.946132 | 0.05 |
| 25690 | 0.946132 | 0.05 |
| 25691 | 0.946131 | 0.05 |

| | | |
|---|---|---|
| 25692 | 0.94613 | 0.05 |
| 25693 | 1.40574 | 0.05 |
| 25694 | 0.946128 | 0.05 |
| 25695 | 0.946131 | 0.0715043 |
| 25696 | 1.40574 | 0.05 |
| 25697 | 0.946132 | 2.1 |
| 25698 | 1.41331 | 2.1 |
| 25699 | 0.94613 | 2.1 |
| 25700 | 0.946131 | 2.1 |
| 25701 | 1.41331 | 2.1 |
| 25702 | 0.94613 | 2.1 |
| 25703 | 1.37609 | 2.1 |
| 25704 | 0.946129 | 2.1 |
| 25705 | 0.946131 | 2.1 |
| 25706 | 1.37609 | 2.1 |
| 25707 | 1.039 | 0.0906149 |
| 25708 | 0.946131 | 2.07465 |
| 25709 | 0.944514 | 1.39585 |
| 25710 | 0.946132 | 1.8355 |
| 25711 | 0.946132 | 1.57925 |
| 25712 | 1.23726 | 0.181501 |
| 25713 | 0.946128 | 1.64266 |
| 25714 | 0.946128 | 1.32099 |
| 25715 | 0.946129 | 1.64124 |
| 25716 | 0.94613 | 1.41695 |
| 25717 | 0.946129 | 2.1 |
| 25718 | 0.946131 | 2.1 |
| 25719 | 0.946131 | 2.1 |
| 25720 | 1.39214 | 2.1 |
| 25721 | 0.94613 | 2.1 |
| 25722 | 0.946118 | 0.404005 |
| 25723 | 0.946118 | 0.05 |
| 25724 | 1.41078 | 0.05 |
| 25725 | 1.41076 | 0.05 |
| 25726 | 0.946131 | 0.05 |
| 25727 | 0.946131 | 0.825328 |
| 25728 | 0.946131 | 0.706811 |
| 25729 | 0.946132 | 0.760124 |
| 25730 | 0.94613 | 0.659683 |
| 25731 | 0.946131 | 0.6259 |
| 25732 | 0.946132 | 0.05 |
| 25733 | 0.946132 | 0.05 |
| 25734 | 0.946133 | 0.05 |
| 25735 | 1.28924 | 0.05 |
| 25736 | 0.946132 | 0.05 |
| 25737 | 0.946133 | 0.104643 |
| 25738 | 1.09812 | 0.05 |
| 25739 | 0.946132 | 0.05 |
| 25740 | 0.946132 | 0.113786 |
| 25741 | 1.09812 | 0.0986038 |
| 25742 | 0.946132 | 0.657324 |
| 25743 | 0.946132 | 0.122002 |
| 25744 | 0.946132 | 0.594024 |
| 25745 | 0.946132 | 0.652945 |
| 25746 | 1.15388 | 0.460508 |
| 25747 | 0.946132 | 0.134237 |
| 25748 | 0.974688 | 0.05 |
| 25749 | 0.946132 | 0.05 |

| | | |
|---|---|---|
| 25750 | 0.946132 | 0.133389 |
| 25751 | 0.974687 | 0.133424 |
| 25752 | 1.0644 | 1.26426 |
| 25753 | 0.946133 | 1.9018 |
| 25754 | 0.946132 | 1.23568 |
| 25755 | 0.946133 | 1.31122 |
| 25756 | 1.26547 | 1.44573 |
| 25757 | 1.02905 | 1.09546 |
| 25758 | 0.911537 | 1.15692 |
| 25759 | 0.838038 | 1.34559 |
| 25760 | 0.946131 | 0.05 |
| 25761 | 0.94613 | 0.05 |
| 25762 | 0.946132 | 0.05 |
| 25763 | 0.946131 | 0.05 |
| 25764 | 1.33922 | 0.05 |
| 25765 | 0.946132 | 0.05 |
| 25766 | 0.946132 | 0.05 |
| 25767 | 1.40926 | 0.05 |
| 25768 | 1.40927 | 0.05 |
| 25769 | 0.946132 | 0.05 |
| 25770 | 0.946129 | 2.1 |
| 25771 | 0.946131 | 2.1 |
| 25772 | 0.946131 | 1.22775 |
| 25773 | 0.946131 | 0.995336 |
| 25774 | 1.40496 | 1.92656 |
| 25775 | 0.94613 | 0.949216 |
| 25776 | 0.94613 | 0.827409 |
| 25777 | 0.946127 | 0.876009 |
| 25778 | 0.946128 | 0.744671 |
| 25779 | 1.38914 | 0.778896 |
| 25780 | 2.675 | 0.05 |
| 25781 | 1.22266 | 0.05 |
| 25782 | 0.946131 | 0.05 |
| 25783 | 0.946132 | 0.05 |
| 25784 | 0.946132 | 0.05 |
| 25785 | 1.22266 | 0.05 |
| 25786 | 0.946124 | 0.05 |
| 25787 | 0.946128 | 0.05 |
| 25788 | 0.946126 | 0.05 |
| 25789 | 1.21428 | 0.05 |
| 25790 | 0.946127 | 0.05 |
| 25791 | 0.946131 | 0.05 |
| 25792 | 0.946131 | 0.05 |
| 25793 | 0.946132 | 0.05 |
| 25794 | 0.946129 | 0.05 |
| 25795 | 0.940118 | 0.05 |
| 25796 | 0.810943 | 2.1 |
| 25797 | 0.810945 | 2.1 |
| 25798 | 0.810958 | 2.1 |
| 25799 | 0.946133 | 0.05 |
| 25800 | 1.11539 | 0.05 |
| 25801 | 0.946132 | 0.055551 |
| 25802 | 0.946132 | 0.05 |
| 25803 | 0.946133 | 0.05 |
| 25804 | 0.946133 | 2.1 |
| 25805 | 1.41195 | 2.1 |
| 25806 | 0.946133 | 2.1 |
| 25807 | 0.946132 | 2.1 |

| | | |
|---|---|---|
| 25808 | 0.946133 | 2.1 |
| 25809 | 1.15968 | 0.05 |
| 25810 | 0.946131 | 0.05 |
| 25811 | 0.946131 | 0.05 |
| 25812 | 0.946131 | 0.05 |
| 25813 | 0.946131 | 0.05 |
| 25814 | 0.94613 | 2.1 |
| 25815 | 0.946128 | 2.1 |
| 25816 | 0.946132 | 1.3482 |
| 25817 | 0.94613 | 2.1 |
| 25818 | 0.94613 | 1.72805 |
| 25819 | 0.946131 | 2.1 |
| 25820 | 1.04211 | 2.03456 |
| 25821 | 0.850155 | 0.650629 |
| 25822 | 0.946131 | 1.67676 |
| 25823 | 0.946131 | 1.35393 |
| 25824 | 0.946131 | 0.05 |
| 25825 | 0.94613 | 0.05 |
| 25826 | 0.946129 | 0.05 |
| 25827 | 0.946131 | 0.05 |
| 25828 | 0.946131 | 0.05 |
| 25829 | 1.40193 | 0.05 |
| 25830 | 1.19756 | 0.05 |
| 25831 | 0.561936 | 0.05 |
| 25832 | 0.808778 | 0.05 |
| 25833 | 1.19756 | 0.05 |
| 25834 | 0.946132 | 0.05 |
| 25835 | 0.946132 | 0.05 |
| 25836 | 1.33916 | 0.05 |
| 25837 | 1.33916 | 0.05 |
| 25838 | 0.946133 | 0.05 |
| 25839 | 0.946127 | 0.23478 |
| 25840 | 0.946127 | 0.0726402 |
| 25841 | 0.946131 | 0.144371 |
| 25842 | 0.946128 | 0.101701 |
| 25843 | 0.946129 | 0.110206 |
| 25844 | 0.589428 | 0.604083 |
| 25845 | 0.851501 | 0.0518868 |
| 25846 | 0.868924 | 0.419942 |
| 25847 | 0.877703 | 0.408208 |
| 25848 | 0.85153 | 0.520829 |
| 25849 | 0.946132 | 1.74947 |
| 25850 | 1.25372 | 2.1 |
| 25851 | 0.946132 | 2.1 |
| 25852 | 0.946132 | 2.1 |
| 25853 | 1.25372 | 2.1 |
| 25854 | 0.946132 | 0.7501 |
| 25855 | 0.946128 | 2.1 |
| 25856 | 0.94613 | 1.03893 |
| 25857 | 0.94613 | 1.40853 |
| 25858 | 1.2778 | 1.97267 |
| 25859 | 0.946118 | 0.757423 |
| 25860 | 1.35629 | 0.77473 |
| 25861 | 0.946117 | 0.783748 |
| 25862 | 0.946131 | 0.743575 |
| 25863 | 0.946105 | 0.672794 |
| 25864 | 0.946132 | 0.05 |
| 25865 | 0.946132 | 0.451166 |

| | | |
|---|---|---|
| 25866 | 1.38646 | 0.305809 |
| 25867 | 1.38646 | 0.399425 |
| 25868 | 0.946132 | 0.702579 |
| 25869 | 0.946128 | 2.1 |
| 25870 | 0.946128 | 0.05 |
| 25871 | 1.39659 | 2.1 |
| 25872 | 1.39659 | 1.56281 |
| 25873 | 0.946129 | 0.461631 |
| 25874 | 0.946132 | 1.91357 |
| 25875 | 0.946131 | 1.43852 |
| 25876 | 0.946132 | 1.55383 |
| 25877 | 0.946132 | 1.56529 |
| 25878 | 1.15139 | 1.34811 |
| 25879 | 0.946133 | 2.1 |
| 25880 | 0.946132 | 2.1 |
| 25881 | 0.946132 | 2.1 |
| 25882 | 0.946132 | 2.1 |
| 25883 | 1.36151 | 2.1 |
| 25884 | 0.946127 | 2.1 |
| 25885 | 0.946131 | 2.1 |
| 25886 | 0.946128 | 2.1 |
| 25887 | 0.94613 | 2.1 |
| 25888 | 1.38397 | 2.1 |
| 25889 | 0.946131 | 0.05 |
| 25890 | 1.31112 | 0.05 |
| 25891 | 0.946127 | 0.28729 |
| 25892 | 0.946128 | 0.381464 |
| 25893 | 1.31112 | 0.0595756 |
| 25894 | 1.29778 | 0.05 |
| 25895 | 0.883417 | 0.05 |
| 25896 | 1.32613 | 0.05 |
| 25897 | 0.94613 | 0.05 |
| 25898 | 0.94613 | 0.05 |
| 25899 | 0.946132 | 2.1 |
| 25900 | 0.94613 | 2.1 |
| 25901 | 0.946131 | 2.1 |
| 25902 | 0.946131 | 2.1 |
| 25903 | 1.36523 | 2.1 |
| 25904 | 0.946132 | 0.05 |
| 25905 | 1.01653 | 0.05 |
| 25906 | 0.946132 | 0.05 |
| 25907 | 0.946131 | 0.05 |
| 25908 | 0.946131 | 0.05 |
| 25909 | 0.94612 | 2.1 |
| 25910 | 1.08301 | 1.65499 |
| 25911 | 0.946119 | 2.1 |
| 25912 | 0.946132 | 2.1 |
| 25913 | 1.08299 | 2.1 |
| 25914 | 0.810942 | 2.1 |
| 25915 | 0.810944 | 0.916571 |
| 25916 | 0.810957 | 1.80879 |
| 25917 | 0.946126 | 0.05 |
| 25918 | 0.946132 | 0.05 |
| 25919 | 0.946129 | 0.05 |
| 25920 | 1.3492 | 0.05 |
| 25921 | 0.94613 | 0.05 |
| 25922 | 0.946131 | 0.05 |
| 25923 | 1.24766 | 0.05 |

| | | |
|---|---|---|
| 25924 | 0.946128 | 0.166176 |
| 25925 | 0.946131 | 0.220897 |
| 25926 | 1.24766 | 0.05 |
| 25927 | 0.946132 | 0.05 |
| 25928 | 0.946131 | 0.05 |
| 25929 | 0.946131 | 0.05 |
| 25930 | 1.23351 | 0.05 |
| 25931 | 0.946132 | 0.05 |
| 25932 | 0.946131 | 0.05 |
| 25933 | 0.946132 | 0.05 |
| 25934 | 0.946131 | 0.05 |
| 25935 | 1.35657 | 0.05 |
| 25936 | 0.946131 | 0.05 |
| 25937 | 0.946132 | 0.05 |
| 25938 | 1.3689 | 0.05 |
| 25939 | 0.946132 | 0.05 |
| 25940 | 0.946132 | 0.05 |
| 25941 | 1.3689 | 0.05 |
| 25942 | 0.946133 | 0.05 |
| 25943 | 1.35822 | 0.05 |
| 25944 | 0.946133 | 0.05 |
| 25945 | 0.946132 | 0.05 |
| 25946 | 1.35822 | 0.05 |
| 25947 | 0.946131 | 0.744624 |
| 25948 | 0.946131 | 1.31861 |
| 25949 | 0.946131 | 1.23914 |
| 25950 | 0.946131 | 0.890594 |
| 25951 | 1.4101 | 1.60032 |
| 25952 | 0.810944 | 1.81605 |
| 25953 | 0.810939 | 1.88329 |
| 25954 | 0.810955 | 1.24146 |
| 25955 | 0.946132 | 2.1 |
| 25956 | 1.37733 | 2.1 |
| 25957 | 0.946132 | 2.1 |
| 25958 | 0.946131 | 2.1 |
| 25959 | 0.946131 | 2.1 |
| 25960 | 1.32582 | 2.1 |
| 25961 | 0.946132 | 2.1 |
| 25962 | 0.946132 | 1.8 |
| 25963 | 0.946132 | 2.1 |
| 25964 | 0.946132 | 2.1 |
| 25965 | 0.946132 | 2.1 |
| 25966 | 0.946132 | 2.1 |
| 25967 | 1.31401 | 2.1 |
| 25968 | 1.31401 | 2.1 |
| 25969 | 0.946131 | 2.1 |
| 25970 | 1.3469 | 1.50828 |
| 25971 | 0.946132 | 1.91529 |
| 25972 | 0.946132 | 2.09194 |
| 25973 | 0.946133 | 1.0452 |
| 25974 | 0.946132 | 1.25558 |
| 25975 | 0.946126 | 2.1 |
| 25976 | 0.946127 | 2.1 |
| 25977 | 0.94613 | 2.1 |
| 25978 | 0.946128 | 2.1 |
| 25979 | 1.41206 | 2.1 |
| 25980 | 0.946127 | 2.0647 |
| 25981 | 1.40109 | 2.1 |

| | | |
|---|---|---|
| 25982 | 0.946128 | 2.08535 |
| 25983 | 0.946129 | 2.1 |
| 25984 | 1.40109 | 2.1 |
| 25985 | 1.24448 | 0.05 |
| 25986 | 0.94613 | 0.05 |
| 25987 | 0.946132 | 0.05 |
| 25988 | 0.946129 | 0.05 |
| 25989 | 0.946131 | 0.05 |
| 25990 | 1.11015 | 0.05 |
| 25991 | 0.946133 | 0.05 |
| 25992 | 0.946132 | 0.05 |
| 25993 | 0.946133 | 0.05 |
| 25994 | 0.946132 | 0.05 |
| 25995 | 0.946132 | 2.1 |
| 25996 | 0.946131 | 2.1 |
| 25997 | 0.946131 | 2.1 |
| 25998 | 1.38775 | 2.1 |
| 25999 | 0.946131 | 2.1 |
| 26000 | 0.946133 | 0.0785155 |
| 26001 | 0.946131 | 0.0655085 |
| 26002 | 1.30598 | 1.52323 |
| 26003 | 1.30598 | 0.68699 |
| 26004 | 0.946131 | 0.464906 |
| 26005 | 0.946132 | 2.1 |
| 26006 | 0.946132 | 2.1 |
| 26007 | 0.946132 | 2.1 |
| 26008 | 1.40774 | 2.1 |
| 26009 | 0.946132 | 2.1 |
| 26010 | 0.798396 | 0.05 |
| 26011 | 0.946133 | 0.877067 |
| 26012 | 0.946128 | 1.67334 |
| 26013 | 0.946131 | 1.18155 |
| 26014 | 0.946131 | 1.18349 |
| 26015 | 1.28918 | 1.22293 |
| 26016 | 0.946132 | 0.43376 |
| 26017 | 1.40668 | 0.363223 |
| 26018 | 0.94613 | 0.418915 |
| 26019 | 0.94613 | 0.392818 |
| 26020 | 0.94613 | 0.415511 |
| 26021 | 0.810946 | 2.1 |
| 26022 | 0.810945 | 2.1 |
| 26023 | 0.810933 | 2.1 |
| 26024 | 0.946131 | 2.1 |
| 26025 | 1.30338 | 2.1 |
| 26026 | 0.94613 | 2.1 |
| 26027 | 1.30339 | 2.1 |
| 26028 | 0.946131 | 2.1 |
| 26029 | 0.946127 | 0.909607 |
| 26030 | 0.87161 | 1.33908 |
| 26031 | 0.946128 | 0.981383 |
| 26032 | 0.946129 | 1.13704 |
| 26033 | 0.827876 | 0.897462 |
| 26034 | 0.946131 | 2.1 |
| 26035 | 1.40796 | 2.1 |
| 26036 | 0.94613 | 2.1 |
| 26037 | 0.94613 | 2.1 |
| 26038 | 1.40796 | 2.1 |
| 26039 | 0.946132 | 0.0975345 |

| | | |
|---|---|---|
| 26040 | 0.946132 | 0.190492 |
| 26041 | 0.946132 | 0.0756549 |
| 26042 | 0.946132 | 0.05 |
| 26043 | 1.41724 | 0.05 |
| 26044 | 1.34254 | 0.05 |
| 26045 | 0.946132 | 0.05 |
| 26046 | 0.946132 | 0.05 |
| 26047 | 0.946132 | 0.05 |
| 26048 | 0.946133 | 0.05 |
| 26049 | 0.946133 | 2.1 |
| 26050 | 0.946132 | 1.66388 |
| 26051 | 0.946132 | 1.64975 |
| 26052 | 1.26501 | 2.0895 |
| 26053 | 0.946132 | 1.35518 |
| 26054 | 0.946131 | 0.05 |
| 26055 | 1.35169 | 0.05 |
| 26056 | 0.946131 | 0.05 |
| 26057 | 0.946131 | 0.05 |
| 26058 | 1.35169 | 0.05 |
| 26059 | 0.676844 | 0.05 |
| 26060 | 0.946131 | 0.05 |
| 26061 | 0.94613 | 0.05 |
| 26062 | 1.13242 | 0.05 |
| 26063 | 1.13242 | 0.05 |
| 26064 | 0.946131 | 0.05 |
| 26065 | 0.946128 | 1.08314 |
| 26066 | 1.28828 | 2.1 |
| 26067 | 0.946131 | 1.19657 |
| 26068 | 0.946131 | 1.36009 |
| 26069 | 1.28828 | 1.71274 |
| 26070 | 0.946131 | 0.135345 |
| 26071 | 1.2996 | 0.0725675 |
| 26072 | 0.946133 | 0.146093 |
| 26073 | 0.946131 | 0.0885228 |
| 26074 | 0.946131 | 0.104019 |
| 26075 | 0.946132 | 2.1 |
| 26076 | 1.40638 | 2.1 |
| 26077 | 0.946131 | 0.84389 |
| 26078 | 0.946132 | 2.1 |
| 26079 | 1.40638 | 2.1 |
| 26080 | 2.84909 | 0.05 |
| 26081 | 1.35726 | 0.05 |
| 26082 | 0.946131 | 0.05 |
| 26083 | 0.946131 | 0.05 |
| 26084 | 0.946131 | 0.05 |
| 26085 | 0.946131 | 0.05 |
| 26086 | 1.39852 | 2.1 |
| 26087 | 0.946132 | 2.1 |
| 26088 | 0.94613 | 2.1 |
| 26089 | 0.94613 | 2.1 |
| 26090 | 0.94613 | 2.1 |
| 26091 | 0.946126 | 0.05 |
| 26092 | 0.946131 | 0.05 |
| 26093 | 0.946128 | 0.05 |
| 26094 | 1.31666 | 0.05 |
| 26095 | 0.946128 | 0.05 |
| 26096 | 0.946126 | 0.05 |
| 26097 | 0.946131 | 0.05 |

| | | |
|---|---|---|
| 26098 | 0.946129 | 0.05 |
| 26099 | 1.32275 | 0.05 |
| 26100 | 0.946129 | 0.05 |
| 26101 | 0.946131 | 2.1 |
| 26102 | 0.946132 | 2.1 |
| 26103 | 1.41125 | 2.1 |
| 26104 | 0.946131 | 2.1 |
| 26105 | 0.946131 | 2.1 |
| 26106 | 0.946131 | 2.1 |
| 26107 | 0.946132 | 2.1 |
| 26108 | 1.31904 | 2.1 |
| 26109 | 0.946132 | 2.1 |
| 26110 | 0.946132 | 2.1 |
| 26111 | 0.946129 | 0.05 |
| 26112 | 0.946132 | 0.05 |
| 26113 | 0.94613 | 0.05 |
| 26114 | 1.02426 | 0.05 |
| 26115 | 0.9311 | 0.05 |
| 26116 | 0.946128 | 2.07705 |
| 26117 | 0.946133 | 1.26371 |
| 26118 | 0.946131 | 1.81103 |
| 26119 | 1.40092 | 1.95939 |
| 26120 | 0.946131 | 1.42853 |
| 26121 | 0.946117 | 0.0549223 |
| 26122 | 0.946118 | 2.1 |
| 26123 | 0.946118 | 0.05 |
| 26124 | 1.40017 | 1.86719 |
| 26125 | 0.946105 | 0.102823 |
| 26126 | 0.946132 | 0.36941 |
| 26127 | 1.03547 | 0.11637 |
| 26128 | 0.946132 | 0.266588 |
| 26129 | 0.946133 | 0.469369 |
| 26130 | 0.946132 | 0.43264 |
| 26131 | 0.946133 | 2.1 |
| 26132 | 0.946132 | 2.1 |
| 26133 | 0.946133 | 2.1 |
| 26134 | 0.946133 | 2.1 |
| 26135 | 1.36694 | 2.1 |
| 26136 | 0.946132 | 0.05 |
| 26137 | 1.22878 | 0.05 |
| 26138 | 0.946132 | 0.05 |
| 26139 | 0.946132 | 0.05 |
| 26140 | 1.22878 | 0.05 |
| 26141 | 1.11014 | 0.05 |
| 26142 | 0.913078 | 0.05 |
| 26143 | 1.11015 | 0.05 |
| 26144 | 0.49154 | 0.05 |
| 26145 | 0.946127 | 2.1 |
| 26146 | 0.946131 | 2.1 |
| 26147 | 0.946128 | 2.1 |
| 26148 | 0.946129 | 2.1 |
| 26149 | 1.38752 | 2.1 |
| 26150 | 0.946132 | 2.1 |
| 26151 | 1.37277 | 2.1 |
| 26152 | 0.946132 | 2.1 |
| 26153 | 0.946131 | 2.1 |
| 26154 | 1.37277 | 2.1 |
| 26155 | 0.94613 | 0.110039 |

| | | |
|---|---|---|
| 26156 | 1.16834 | 0.05 |
| 26157 | 0.946129 | 0.124041 |
| 26158 | 0.94613 | 0.05 |
| 26159 | 0.94613 | 0.0728369 |
| 26160 | 0.946127 | 0.0693611 |
| 26161 | 0.946131 | 0.05 |
| 26162 | 1.35594 | 0.05 |
| 26163 | 0.946128 | 0.0925241 |
| 26164 | 0.94613 | 0.05 |
| 26165 | 0.946128 | 1.39152 |
| 26166 | 0.946128 | 0.0978579 |
| 26167 | 1.38464 | 0.407424 |
| 26168 | 1.38463 | 0.0916327 |
| 26169 | 0.94613 | 0.358762 |
| 26170 | 0.946129 | 1.32146 |
| 26171 | 0.946131 | 0.287127 |
| 26172 | 0.94613 | 0.411744 |
| 26173 | 1.39751 | 0.118974 |
| 26174 | 0.946131 | 0.487194 |
| 26175 | 1.25837 | 0.05 |
| 26176 | 1.21134 | 0.05 |
| 26177 | 0.700013 | 0.05 |
| 26178 | 0.700041 | 0.05 |
| 26179 | 1.40583 | 2.1 |
| 26180 | 0.946132 | 2.1 |
| 26181 | 0.946132 | 2.1 |
| 26182 | 0.946133 | 2.1 |
| 26183 | 0.946132 | 2.1 |
| 26184 | 0.827865 | 2.1 |
| 26185 | 0.946131 | 2.1 |
| 26186 | 0.946131 | 2.07536 |
| 26187 | 0.946131 | 2.1 |
| 26188 | 0.946131 | 1.92795 |
| 26189 | 0.946132 | 0.05 |
| 26190 | 1.21087 | 0.05 |
| 26191 | 0.94613 | 0.05 |
| 26192 | 0.94613 | 0.05 |
| 26193 | 0.946131 | 0.05 |
| 26194 | 0.94613 | 0.671933 |
| 26195 | 1.17452 | 0.830583 |
| 26196 | 0.946132 | 0.679073 |
| 26197 | 0.94613 | 0.761791 |
| 26198 | 0.94613 | 0.659309 |
| 26199 | 0.946119 | 0.579389 |
| 26200 | 1.27374 | 0.633844 |
| 26201 | 0.946119 | 0.581021 |
| 26202 | 0.946131 | 0.60612 |
| 26203 | 0.946106 | 0.568381 |
| 26204 | 0.946133 | 2.1 |
| 26205 | 0.946132 | 2.1 |
| 26206 | 0.946133 | 2.1 |
| 26207 | 0.946132 | 2.1 |
| 26208 | 1.39621 | 2.1 |
| 26209 | 1.34352 | 2.1 |
| 26210 | 0.946132 | 2.1 |
| 26211 | 0.946132 | 2.1 |
| 26212 | 0.946132 | 2.1 |
| 26213 | 0.946132 | 2.1 |

| | | |
|---|---|---|
| 26214 | 0.946132 | 0.763876 |
| 26215 | 0.946128 | 2.1 |
| 26216 | 0.94613 | 1.26663 |
| 26217 | 0.94613 | 2.07488 |
| 26218 | 1.41273 | 2.1 |
| 26219 | 1.23536 | 2.1 |
| 26220 | 0.946131 | 1.3836 |
| 26221 | 0.946132 | 2.1 |
| 26222 | 0.946132 | 2.1 |
| 26223 | 0.946132 | 2.1 |
| 26224 | 0.946132 | 2.1 |
| 26225 | 1.41131 | 2.1 |
| 26226 | 0.946131 | 2.1 |
| 26227 | 0.946131 | 2.1 |
| 26228 | 0.946131 | 2.1 |
| 26229 | 0.946133 | 0.633935 |
| 26230 | 0.946132 | 2.09975 |
| 26231 | 0.946133 | 0.601755 |
| 26232 | 1.41894 | 0.521044 |
| 26233 | 0.946132 | 0.0694781 |
| 26234 | 0.946128 | 0.204026 |
| 26235 | 1.4099 | 1.21326 |
| 26236 | 0.946131 | 0.745768 |
| 26237 | 0.946131 | 2.1 |
| 26238 | 0.946131 | 0.05 |
| 26239 | 0.810947 | 2.09636 |
| 26240 | 0.810945 | 2.1 |
| 26241 | 0.810933 | 2.1 |
| 26242 | 0.946131 | 0.702513 |
| 26243 | 1.25765 | 2.1 |
| 26244 | 0.946128 | 1.14683 |
| 26245 | 0.946129 | 0.743008 |
| 26246 | 1.25765 | 1.19932 |
| 26247 | 1.21347 | 2.1 |
| 26248 | 0.946129 | 2.1 |
| 26249 | 0.94613 | 2.1 |
| 26250 | 1.21347 | 2.1 |
| 26251 | 0.94613 | 2.1 |
| 26252 | 0.94613 | 0.05 |
| 26253 | 0.946132 | 0.05 |
| 26254 | 1.2493 | 0.05 |
| 26255 | 1.2493 | 0.05 |
| 26256 | 0.94613 | 0.05 |
| 26257 | 0.946132 | 0.927879 |
| 26258 | 0.946128 | 1.3843 |
| 26259 | 0.946131 | 0.930617 |
| 26260 | 0.94613 | 1.11665 |
| 26261 | 1.37938 | 1.03271 |
| 26262 | 0.946133 | 0.05 |
| 26263 | 0.946132 | 0.05 |
| 26264 | 0.946133 | 0.05 |
| 26265 | 0.946132 | 0.05 |
| 26266 | 0.946133 | 0.05 |
| 26267 | 0.946129 | 0.05 |
| 26268 | 1.26426 | 0.05 |
| 26269 | 0.946131 | 0.0603766 |
| 26270 | 0.946131 | 0.064801 |
| 26271 | 1.26426 | 0.0581576 |

| | | |
|---|---|---|
| 26272 | 0.872447 | 2.08732 |
| 26273 | 0.946132 | 1.36448 |
| 26274 | 0.946132 | 1.79231 |
| 26275 | 0.946132 | 2.00048 |
| 26276 | 0.946133 | 1.51872 |
| 26277 | 0.946117 | 1.35097 |
| 26278 | 0.946116 | 1.16185 |
| 26279 | 0.946113 | 1.10379 |
| 26280 | 0.946129 | 1.28588 |
| 26281 | 1.27211 | 0.887767 |
| 26282 | 0.94613 | 0.220819 |
| 26283 | 0.946129 | 0.05 |
| 26284 | 1.40029 | 0.05 |
| 26285 | 1.40028 | 0.05 |
| 26286 | 0.94613 | 0.226234 |
| 26287 | 0.810942 | 2.1 |
| 26288 | 0.810943 | 2.1 |
| 26289 | 0.810932 | 2.1 |
| 26290 | 1.29521 | 2.1 |
| 26291 | 0.946129 | 2.1 |
| 26292 | 0.946131 | 2.1 |
| 26293 | 1.29521 | 2.1 |
| 26294 | 1.29521 | 2.1 |
| 26295 | 0.946132 | 0.0584533 |
| 26296 | 0.946129 | 0.05 |
| 26297 | 1.24596 | 0.05 |
| 26298 | 0.946131 | 0.0603287 |
| 26299 | 0.946131 | 0.05 |
| 26300 | 0.946131 | 2.1 |
| 26301 | 1.35616 | 2.1 |
| 26302 | 0.946133 | 2.1 |
| 26303 | 0.94613 | 2.1 |
| 26304 | 1.35616 | 2.1 |
| 26305 | 0.879548 | 0.783558 |
| 26306 | 1.01272 | 0.851788 |
| 26307 | 0.946133 | 0.881109 |
| 26308 | 0.946133 | 0.685769 |
| 26309 | 0.946132 | 0.870471 |
| 26310 | 0.946132 | 0.994243 |
| 26311 | 0.918009 | 0.727938 |
| 26312 | 0.91801 | 1.35781 |
| 26313 | 0.946132 | 0.903309 |
| 26314 | 0.946132 | 1.04783 |
| 26315 | 0.946132 | 0.05 |
| 26316 | 1.28644 | 0.05 |
| 26317 | 0.946131 | 0.05 |
| 26318 | 0.946131 | 0.05 |
| 26319 | 0.946132 | 0.05 |
| 26320 | 0.946131 | 0.05 |
| 26321 | 1.40103 | 0.05 |
| 26322 | 0.946131 | 0.05 |
| 26323 | 0.946131 | 0.05 |
| 26324 | 0.946131 | 0.05 |
| 26325 | 0.946128 | 0.05 |
| 26326 | 1.03327 | 0.05 |
| 26327 | 0.946129 | 0.05 |
| 26328 | 0.946131 | 0.05 |
| 26329 | 0.94613 | 0.05 |

| | | |
|---|---|---|
| 26330 | 0.946132 | 0.315167 |
| 26331 | 0.946133 | 0.05 |
| 26332 | 1.40265 | 0.05 |
| 26333 | 1.40265 | 0.05 |
| 26334 | 0.946133 | 0.05 |
| 26335 | 0.946131 | 0.05 |
| 26336 | 0.946131 | 0.05 |
| 26337 | 0.946131 | 0.05 |
| 26338 | 0.856655 | 0.05 |
| 26339 | 0.856616 | 0.05 |
| 26340 | 0.946132 | 0.113888 |
| 26341 | 0.946132 | 0.169613 |
| 26342 | 0.946132 | 0.528151 |
| 26343 | 1.15844 | 0.0586367 |
| 26344 | 0.946131 | 0.555195 |
| 26345 | 0.946131 | 2.1 |
| 26346 | 1.4031 | 2.1 |
| 26347 | 1.40304 | 2.1 |
| 26348 | 0.851554 | 2.1 |
| 26349 | 0.946131 | 0.05 |
| 26350 | 0.94613 | 0.05 |
| 26351 | 1.24417 | 0.05 |
| 26352 | 1.24416 | 0.05 |
| 26353 | 0.946131 | 0.05 |
| 26354 | 0.946132 | 0.161663 |
| 26355 | 1.20992 | 0.05 |
| 26356 | 0.946132 | 0.05 |
| 26357 | 0.946132 | 0.543788 |
| 26358 | 1.20992 | 0.223666 |
| 26359 | 0.946128 | 0.919821 |
| 26360 | 0.94613 | 1.03864 |
| 26361 | 0.94613 | 0.944283 |
| 26362 | 0.94613 | 1.03958 |
| 26363 | 1.23426 | 1.06328 |
| 26364 | 0.810943 | 0.578397 |
| 26365 | 0.810946 | 0.254197 |
| 26366 | 0.810933 | 0.437129 |
| 26367 | 0.810945 | 2.1 |
| 26368 | 0.810945 | 0.05 |
| 26369 | 0.810932 | 0.876651 |
| 26370 | 1.25104 | 1.2961 |
| 26371 | 0.946131 | 2.1 |
| 26372 | 0.946131 | 0.127442 |
| 26373 | 1.25104 | 1.34308 |
| 26374 | 1.25104 | 0.0665704 |
| 26375 | 1.25488 | 2.1 |
| 26376 | 0.946132 | 2.1 |
| 26377 | 0.946132 | 2.1 |
| 26378 | 0.946132 | 2.1 |
| 26379 | 0.946131 | 2.1 |
| 26380 | 0.946131 | 0.05 |
| 26381 | 0.946126 | 0.05 |
| 26382 | 0.946128 | 0.05 |
| 26383 | 1.32131 | 0.05 |
| 26384 | 0.946128 | 0.05 |
| 26385 | 0.946133 | 2.1 |
| 26386 | 0.946132 | 0.0799145 |
| 26387 | 0.946132 | 2.1 |

| | | |
|---|---|---|
| 26388 | 0.946133 | 2.1 |
| 26389 | 1.41123 | 1.8635 |
| 26390 | 0.946132 | 0.05 |
| 26391 | 0.946133 | 0.05 |
| 26392 | 0.946132 | 0.05 |
| 26393 | 1.32163 | 0.05 |
| 26394 | 0.946133 | 0.05 |
| 26395 | 0.946132 | 0.777579 |
| 26396 | 0.946132 | 2.1 |
| 26397 | 1.38651 | 2.1 |
| 26398 | 1.38651 | 2.1 |
| 26399 | 0.946132 | 2.1 |
| 26400 | 0.94613 | 0.05 |
| 26401 | 1.3639 | 0.05 |
| 26402 | 0.946128 | 0.0644772 |
| 26403 | 0.946131 | 0.05 |
| 26404 | 1.3639 | 0.05 |
| 26405 | 0.946133 | 0.744736 |
| 26406 | 1.40724 | 0.503162 |
| 26407 | 0.946132 | 0.400072 |
| 26408 | 0.946133 | 0.555449 |
| 26409 | 1.40724 | 0.564892 |
| 26410 | 1.21099 | 0.922174 |
| 26411 | 0.946128 | 1.60156 |
| 26412 | 0.946127 | 2.1 |
| 26413 | 0.946128 | 2.1 |
| 26414 | 0.946129 | 1.43899 |
| 26415 | 0.946132 | 0.05 |
| 26416 | 1.35946 | 0.05 |
| 26417 | 0.946131 | 0.204462 |
| 26418 | 0.946131 | 0.10935 |
| 26419 | 1.35946 | 0.0866901 |
| 26420 | 1.34794 | 0.050747 |
| 26421 | 0.517994 | 1.3987 |
| 26422 | 0.946132 | 0.0780192 |
| 26423 | 0.946132 | 0.426637 |
| 26424 | 0.517965 | 0.508305 |
| 26425 | 1.23224 | 0.40281 |
| 26426 | 0.94613 | 2.1 |
| 26427 | 0.946131 | 1.22525 |
| 26428 | 0.946131 | 0.966429 |
| 26429 | 0.946131 | 0.833112 |
| 26430 | 1.37511 | 0.486214 |
| 26431 | 0.946132 | 1.17136 |
| 26432 | 0.946133 | 2.1 |
| 26433 | 0.946133 | 1.96528 |
| 26434 | 0.946132 | 1.18814 |
| 26435 | 0.851106 | 0.0580281 |
| 26436 | 0.946129 | 0.119196 |
| 26437 | 0.946131 | 0.340307 |
| 26438 | 0.946131 | 1.03435 |
| 26439 | 0.851066 | 0.742271 |
| 26440 | 0.946131 | 0.05 |
| 26441 | 0.946131 | 0.05 |
| 26442 | 0.94613 | 0.05 |
| 26443 | 1.31357 | 0.05 |
| 26444 | 0.946131 | 0.05 |
| 26445 | 0.946133 | 0.979529 |

| | | |
|---|---|---|
| 26446 | 0.946132 | 1.30396 |
| 26447 | 0.946133 | 0.90144 |
| 26448 | 0.946132 | 1.09375 |
| 26449 | 1.38149 | 0.971221 |
| 26450 | 0.810944 | 0.866181 |
| 26451 | 0.810945 | 1.28714 |
| 26452 | 0.810958 | 2.1 |
| 26453 | 0.946118 | 0.05 |
| 26454 | 0.946118 | 0.05 |
| 26455 | 1.37835 | 0.05 |
| 26456 | 1.37837 | 0.05 |
| 26457 | 0.946106 | 0.05 |
| 26458 | 0.946128 | 0.05 |
| 26459 | 0.946131 | 0.05 |
| 26460 | 1.34831 | 0.05 |
| 26461 | 1.34831 | 0.05 |
| 26462 | 0.94613 | 0.05 |
| 26463 | 0.946132 | 0.05 |
| 26464 | 0.946132 | 0.05 |
| 26465 | 0.909981 | 0.05 |
| 26466 | 1.00099 | 0.05 |
| 26467 | 0.946131 | 0.05 |
| 26468 | 0.946132 | 0.842467 |
| 26469 | 0.946133 | 1.54888 |
| 26470 | 0.946133 | 0.989218 |
| 26471 | 0.946132 | 1.10192 |
| 26472 | 1.35328 | 1.08589 |
| 26473 | 0.946132 | 0.05 |
| 26474 | 0.946132 | 0.05 |
| 26475 | 1.35386 | 0.05 |
| 26476 | 1.35385 | 0.05 |
| 26477 | 0.946133 | 0.05 |
| 26478 | 0.946121 | 0.05 |
| 26479 | 0.946132 | 0.05 |
| 26480 | 0.900167 | 0.05 |
| 26481 | 1.01189 | 0.05 |
| 26482 | 0.946132 | 0.05 |
| 26483 | 0.946132 | 2.1 |
| 26484 | 1.41585 | 1.24547 |
| 26485 | 0.946131 | 1.64071 |
| 26486 | 0.946131 | 1.22883 |
| 26487 | 0.94613 | 1.26722 |
| 26488 | 0.810946 | 2.1 |
| 26489 | 0.810946 | 2.1 |
| 26490 | 0.810933 | 2.1 |
| 26491 | 1.34447 | 2.1 |
| 26492 | 0.946131 | 2.1 |
| 26493 | 0.946131 | 2.1 |
| 26494 | 1.34447 | 2.1 |
| 26495 | 1.19886 | 2.1 |
| 26496 | 0.946132 | 0.05 |
| 26497 | 0.946132 | 0.05 |
| 26498 | 0.946132 | 0.05 |
| 26499 | 1.35065 | 0.05 |
| 26500 | 0.946132 | 0.05 |
| 26501 | 0.946131 | 0.05 |
| 26502 | 1.3022 | 0.05 |
| 26503 | 0.94613 | 0.205659 |

| | | |
|---|---|---|
| 26504 | 0.946131 | 0.0740746 |
| 26505 | 1.3022 | 0.0620766 |
| 26506 | 0.946127 | 0.05 |
| 26507 | 1.38683 | 0.05 |
| 26508 | 0.946127 | 0.05 |
| 26509 | 0.946128 | 0.05 |
| 26510 | 1.38683 | 0.05 |
| 26511 | 0.810946 | 0.05 |
| 26512 | 0.810947 | 0.05 |
| 26513 | 0.810959 | 0.05 |
| 26514 | 0.946132 | 0.05 |
| 26515 | 0.946131 | 0.05 |
| 26516 | 0.946132 | 0.05 |
| 26517 | 0.946132 | 0.05 |
| 26518 | 0.946132 | 0.05 |
| 26519 | 0.946127 | 1.14163 |
| 26520 | 1.35968 | 2.06473 |
| 26521 | 0.946131 | 1.06401 |
| 26522 | 0.946129 | 0.995298 |
| 26523 | 1.35969 | 1.73826 |
| 26524 | 0.946133 | 2.1 |
| 26525 | 1.35862 | 1.72417 |
| 26526 | 0.946132 | 2.1 |
| 26527 | 0.946132 | 1.11375 |
| 26528 | 1.35862 | 1.62327 |
| 26529 | 0.946132 | 0.987328 |
| 26530 | 0.946132 | 0.856281 |
| 26531 | 0.946132 | 1.32551 |
| 26532 | 0.946131 | 0.640785 |
| 26533 | 0.946132 | 0.761329 |
| 26534 | 0.946131 | 0.216674 |
| 26535 | 0.946131 | 0.156371 |
| 26536 | 0.946128 | 0.248623 |
| 26537 | 0.946129 | 0.0504374 |
| 26538 | 1.41232 | 0.154715 |
| 26539 | 0.946128 | 0.780443 |
| 26540 | 1.29331 | 2.1 |
| 26541 | 0.946131 | 0.852977 |
| 26542 | 0.946131 | 2.06021 |
| 26543 | 1.29331 | 2.09375 |
| 26544 | 0.946133 | 0.112117 |
| 26545 | 0.946132 | 0.146449 |
| 26546 | 0.946132 | 0.10333 |
| 26547 | 0.946132 | 0.0628334 |
| 26548 | 1.41556 | 0.05 |
| 26549 | 0.94612 | 0.0854884 |
| 26550 | 0.946119 | 0.05 |
| 26551 | 0.946119 | 0.0833973 |
| 26552 | 0.946132 | 0.05 |
| 26553 | 0.946107 | 0.05 |
| 26554 | 0.946129 | 0.05 |
| 26555 | 1.1509 | 0.05 |
| 26556 | 0.946126 | 0.05 |
| 26557 | 0.946129 | 0.05 |
| 26558 | 0.94613 | 0.05 |
| 26559 | 0.946131 | 0.05 |
| 26560 | 1.19263 | 0.05 |
| 26561 | 0.946132 | 0.05 |

| | | |
|---|---|---|
| 26562 | 0.946132 | 0.05 |
| 26563 | 0.946132 | 0.05 |
| 26564 | 0.94613 | 0.630551 |
| 26565 | 1.36939 | 0.741915 |
| 26566 | 0.946128 | 0.546195 |
| 26567 | 0.946131 | 0.737454 |
| 26568 | 1.36939 | 0.458816 |
| 26569 | 0.946133 | 0.990753 |
| 26570 | 0.827865 | 2.03408 |
| 26571 | 0.946132 | 1.02822 |
| 26572 | 0.946132 | 1.40418 |
| 26573 | 1.40398 | 1.35378 |
| 26574 | 0.946127 | 0.456993 |
| 26575 | 0.946127 | 0.301949 |
| 26576 | 0.94613 | 0.179439 |
| 26577 | 1.41498 | 0.279064 |
| 26578 | 0.946128 | 0.230357 |
| 26579 | 0.94613 | 0.05 |
| 26580 | 0.946131 | 0.05 |
| 26581 | 0.946129 | 0.05 |
| 26582 | 0.94613 | 0.05 |
| 26583 | 0.94613 | 0.05 |
| 26584 | 0.946118 | 0.05 |
| 26585 | 0.946119 | 0.05 |
| 26586 | 0.946116 | 0.05 |
| 26587 | 1.36569 | 0.05 |
| 26588 | 0.946105 | 0.05 |
| 26589 | 0.946132 | 0.05 |
| 26590 | 1.23175 | 0.05 |
| 26591 | 0.94613 | 0.05 |
| 26592 | 0.946133 | 0.05 |
| 26593 | 0.946131 | 0.05 |
| 26594 | 1.25698 | 0.05 |
| 26595 | 0.946126 | 0.05 |
| 26596 | 0.946129 | 0.05 |
| 26597 | 0.946128 | 0.05 |
| 26598 | 0.946129 | 0.05 |
| 26599 | 0.810943 | 2.1 |
| 26600 | 0.810944 | 2.1 |
| 26601 | 0.810932 | 2.1 |
| 26602 | 0.946132 | 0.05 |
| 26603 | 1.09841 | 0.05 |
| 26604 | 0.946131 | 0.05 |
| 26605 | 0.946132 | 0.05 |
| 26606 | 1.09841 | 0.05 |
| 26607 | 0.810936 | 0.173715 |
| 26608 | 0.810935 | 0.05 |
| 26609 | 0.810934 | 0.757952 |
| 26610 | 1.23851 | 2.1 |
| 26611 | 0.946132 | 0.0792418 |
| 26612 | 0.946131 | 0.255346 |
| 26613 | 1.23851 | 2.1 |
| 26614 | 1.23851 | 2.0912 |
| 26615 | 0.946132 | 0.05 |
| 26616 | 0.946131 | 0.05 |
| 26617 | 0.946132 | 0.05 |
| 26618 | 0.946132 | 0.05 |
| 26619 | 0.946132 | 0.05 |

| | | |
|---|---|---|
| 26620 | 0.939141 | 1.17231 |
| 26621 | 0.953118 | 1.73791 |
| 26622 | 0.946126 | 1.08225 |
| 26623 | 0.946129 | 1.19242 |
| 26624 | 1.03306 | 1.27617 |
| 26625 | 0.946132 | 0.05 |
| 26626 | 1.30927 | 0.05 |
| 26627 | 0.946132 | 0.0659906 |
| 26628 | 0.946132 | 0.05 |
| 26629 | 1.30928 | 0.05 |
| 26630 | 0.94613 | 2.1 |
| 26631 | 0.946131 | 2.1 |
| 26632 | 1.24511 | 2.1 |
| 26633 | 0.94613 | 2.1 |
| 26634 | 0.94613 | 2.1 |
| 26635 | 0.946133 | 0.05 |
| 26636 | 1.34152 | 0.05 |
| 26637 | 0.946131 | 0.05 |
| 26638 | 0.946131 | 0.05 |
| 26639 | 1.34152 | 0.05 |
| 26640 | 0.946132 | 0.05 |
| 26641 | 1.41383 | 0.05 |
| 26642 | 0.946132 | 0.05 |
| 26643 | 0.946132 | 0.05 |
| 26644 | 1.41383 | 0.05 |
| 26645 | 0.946132 | 0.406403 |
| 26646 | 0.946131 | 0.22354 |
| 26647 | 1.41392 | 0.0697486 |
| 26648 | 0.946132 | 0.505991 |
| 26649 | 0.946132 | 0.310088 |
| 26650 | 0.946132 | 0.781761 |
| 26651 | 0.946132 | 0.699991 |
| 26652 | 1.24333 | 0.295455 |
| 26653 | 0.946133 | 0.871196 |
| 26654 | 0.946133 | 0.735084 |
| 26655 | 0.946124 | 0.05 |
| 26656 | 0.94613 | 0.05 |
| 26657 | 0.946126 | 0.05 |
| 26658 | 1.3065 | 0.05 |
| 26659 | 0.946127 | 0.05 |
| 26660 | 0.810946 | 0.05 |
| 26661 | 0.810943 | 0.05 |
| 26662 | 0.81096 | 0.05 |
| 26663 | 0.946131 | 0.05 |
| 26664 | 1.40271 | 0.05 |
| 26665 | 0.946131 | 0.05 |
| 26666 | 0.946131 | 0.05 |
| 26667 | 1.40271 | 0.05 |
| 26668 | 0.946132 | 0.0520036 |
| 26669 | 0.94613 | 0.085904 |
| 26670 | 0.946131 | 0.05 |
| 26671 | 0.94613 | 0.0634522 |
| 26672 | 0.946131 | 0.0522159 |
| 26673 | 0.946133 | 0.620699 |
| 26674 | 1.30107 | 0.641574 |
| 26675 | 0.946132 | 0.615453 |
| 26676 | 0.946132 | 0.644609 |
| 26677 | 0.946133 | 0.657501 |

| | | |
|---|---|---|
| 26678 | 0.810946 | 2.1 |
| 26679 | 0.810944 | 2.1 |
| 26680 | 0.810958 | 2.1 |
| 26681 | 0.946133 | 1.30931 |
| 26682 | 0.946131 | 1.09258 |
| 26683 | 0.946131 | 2.1 |
| 26684 | 1.30812 | 1.92625 |
| 26685 | 0.946131 | 1.00874 |
| 26686 | 0.94613 | 0.526888 |
| 26687 | 0.975002 | 1.0855 |
| 26688 | 0.917259 | 2.1 |
| 26689 | 1.00902 | 1.39353 |
| 26690 | 0.94613 | 0.315161 |
| 26691 | 0.946131 | 2.1 |
| 26692 | 0.946127 | 2.1 |
| 26693 | 1.398 | 2.1 |
| 26694 | 0.946129 | 2.1 |
| 26695 | 0.946129 | 2.1 |
| 26696 | 0.946133 | 2.1 |
| 26697 | 0.946132 | 2.1 |
| 26698 | 0.946132 | 2.1 |
| 26699 | 0.946132 | 2.1 |
| 26700 | 1.3033 | 2.1 |
| 26701 | 1.09897 | 0.05 |
| 26702 | 0.946131 | 0.0939721 |
| 26703 | 0.946132 | 0.05 |
| 26704 | 0.946129 | 0.05 |
| 26705 | 0.946131 | 0.0557242 |
| 26706 | 0.946131 | 2.1 |
| 26707 | 0.946132 | 2.1 |
| 26708 | 1.39858 | 2.1 |
| 26709 | 0.946131 | 2.1 |
| 26710 | 0.946131 | 2.1 |
| 26711 | 0.946119 | 0.732465 |
| 26712 | 1.08878 | 0.788045 |
| 26713 | 0.946119 | 1.41259 |
| 26714 | 1.0888 | 0.789672 |
| 26715 | 0.946107 | 1.33149 |
| 26716 | 0.946132 | 0.05 |
| 26717 | 1.10733 | 0.05 |
| 26718 | 0.946132 | 0.05 |
| 26719 | 0.946131 | 0.05 |
| 26720 | 0.946132 | 0.05 |
| 26721 | 0.946128 | 0.05 |
| 26722 | 1.33671 | 0.05 |
| 26723 | 0.94613 | 0.05 |
| 26724 | 0.94613 | 0.05 |
| 26725 | 0.94613 | 0.05 |
| 26726 | 0.810943 | 2.1 |
| 26727 | 0.810944 | 2.1 |
| 26728 | 0.810945 | 2.1 |
| 26729 | 1.35117 | 2.1 |
| 26730 | 0.946129 | 2.1 |
| 26731 | 0.94613 | 2.1 |
| 26732 | 1.35117 | 2.1 |
| 26733 | 1.27174 | 2.1 |
| 26734 | 0.946132 | 2.1 |
| 26735 | 0.946133 | 2.1 |

| | | |
|---|---|---|
| 26736 | 0.997296 | 1.16115 |
| 26737 | 0.946133 | 1.96365 |
| 26738 | 0.946132 | 1.48062 |
| 26739 | 0.810943 | 2.1 |
| 26740 | 0.810944 | 2.1 |
| 26741 | 0.810945 | 2.1 |
| 26742 | 0.810946 | 0.989905 |
| 26743 | 0.810943 | 2.1 |
| 26744 | 0.810933 | 1.47683 |
| 26745 | 0.946127 | 1.16906 |
| 26746 | 0.946131 | 0.529506 |
| 26747 | 0.946128 | 1.15454 |
| 26748 | 0.94613 | 1.16337 |
| 26749 | 0.946129 | 1.27251 |
| 26750 | 0.946131 | 0.05 |
| 26751 | 0.946131 | 0.05 |
| 26752 | 0.946129 | 0.05 |
| 26753 | 0.946131 | 0.05 |
| 26754 | 0.94613 | 0.05 |
| 26755 | 0.946133 | 0.342973 |
| 26756 | 0.946132 | 0.402606 |
| 26757 | 0.946132 | 0.369757 |
| 26758 | 0.946132 | 0.340993 |
| 26759 | 0.946133 | 0.402928 |
| 26760 | 0.946131 | 0.831063 |
| 26761 | 0.94613 | 0.324252 |
| 26762 | 0.946129 | 0.968896 |
| 26763 | 0.946131 | 0.302746 |
| 26764 | 0.946131 | 0.464851 |
| 26765 | 0.946132 | 0.908724 |
| 26766 | 0.946129 | 2.1 |
| 26767 | 0.94613 | 1.8875 |
| 26768 | 0.946131 | 1.37009 |
| 26769 | 1.39356 | 2.00389 |
| 26770 | 0.810946 | 2.1 |
| 26771 | 0.810947 | 2.1 |
| 26772 | 0.810934 | 2.1 |
| 26773 | 0.946125 | 0.05 |
| 26774 | 0.946127 | 0.05 |
| 26775 | 1.33798 | 0.05 |
| 26776 | 1.33797 | 0.05 |
| 26777 | 0.946128 | 0.05 |
| 26778 | 0.946131 | 0.05 |
| 26779 | 0.946131 | 0.05 |
| 26780 | 1.23146 | 0.05 |
| 26781 | 1.23146 | 0.05 |
| 26782 | 0.946131 | 0.05 |
| 26783 | 0.946129 | 2.1 |
| 26784 | 0.94613 | 2.1 |
| 26785 | 0.946131 | 2.1 |
| 26786 | 0.94613 | 2.1 |
| 26787 | 1.30082 | 2.1 |
| 26788 | 0.946131 | 0.05 |
| 26789 | 0.946132 | 0.05 |
| 26790 | 0.946131 | 0.05 |
| 26791 | 1.20416 | 0.05 |
| 26792 | 0.946132 | 0.05 |
| 26793 | 0.946132 | 0.05 |

| | | |
|---|---|---|
| 26794 | 0.946131 | 0.05 |
| 26795 | 0.946131 | 0.05 |
| 26796 | 1.18711 | 0.05 |
| 26797 | 0.946132 | 0.05 |
| 26798 | 0.946132 | 0.05 |
| 26799 | 0.946132 | 0.05 |
| 26800 | 0.946133 | 0.05 |
| 26801 | 0.946132 | 0.05 |
| 26802 | 0.946133 | 0.05 |
| 26803 | 0.946131 | 0.05 |
| 26804 | 0.946132 | 0.05 |
| 26805 | 0.94613 | 0.05 |
| 26806 | 0.946131 | 0.05 |
| 26807 | 0.946131 | 0.05 |
| 26808 | 0.946133 | 0.05 |
| 26809 | 0.946132 | 0.05 |
| 26810 | 0.946133 | 0.05 |
| 26811 | 0.946132 | 0.05 |
| 26812 | 0.946132 | 0.05 |
| 26813 | 0.946132 | 2.1 |
| 26814 | 0.946132 | 2.1 |
| 26815 | 0.946132 | 2.1 |
| 26816 | 1.36422 | 2.1 |
| 26817 | 0.946132 | 2.1 |
| 26818 | 0.946131 | 0.863365 |
| 26819 | 0.946132 | 1.9551 |
| 26820 | 0.946132 | 1.09912 |
| 26821 | 0.946132 | 1.13286 |
| 26822 | 1.4185 | 1.62669 |
| 26823 | 0.946133 | 0.05 |
| 26824 | 1.37794 | 0.05 |
| 26825 | 0.946132 | 0.05 |
| 26826 | 0.946133 | 0.05 |
| 26827 | 0.946132 | 0.05 |
| 26828 | 1.4045 | 2.1 |
| 26829 | 0.946132 | 2.1 |
| 26830 | 0.946132 | 2.1 |
| 26831 | 0.946132 | 2.1 |
| 26832 | 0.946132 | 2.1 |
| 26833 | 0.946131 | 2.1 |
| 26834 | 0.946132 | 2.1 |
| 26835 | 1.34285 | 2.1 |
| 26836 | 1.34285 | 2.1 |
| 26837 | 0.946132 | 2.1 |
| 26838 | 0.946131 | 2.1 |
| 26839 | 0.946132 | 2.1 |
| 26840 | 1.40739 | 2.1 |
| 26841 | 1.40739 | 2.1 |
| 26842 | 0.946131 | 2.1 |
| 26843 | 0.946128 | 2.1 |
| 26844 | 0.946132 | 2.1 |
| 26845 | 0.94613 | 2.1 |
| 26846 | 0.94613 | 2.1 |
| 26847 | 1.3164 | 2.1 |
| 26848 | 0.946132 | 2.1 |
| 26849 | 1.3484 | 2.1 |
| 26850 | 0.946132 | 1.63155 |
| 26851 | 0.946132 | 2.1 |

| | | |
|---|---|---|
| 26852 | 1.3484 | 2.1 |
| 26853 | 0.946132 | 2.1 |
| 26854 | 0.946132 | 2.1 |
| 26855 | 0.946133 | 2.1 |
| 26856 | 0.946133 | 2.1 |
| 26857 | 1.41507 | 2.1 |
| 26858 | 0.946132 | 2.1 |
| 26859 | 1.32608 | 2.1 |
| 26860 | 0.946131 | 2.1 |
| 26861 | 0.946131 | 2.1 |
| 26862 | 0.946132 | 2.1 |
| 26863 | 0.94613 | 0.972777 |
| 26864 | 1.34646 | 2.1 |
| 26865 | 0.946129 | 1.04471 |
| 26866 | 0.94613 | 0.936983 |
| 26867 | 1.34646 | 1.48996 |
| 26868 | 0.946131 | 0.382388 |
| 26869 | 0.946131 | 2.1 |
| 26870 | 0.94613 | 0.613379 |
| 26871 | 1.33728 | 0.05 |
| 26872 | 0.946131 | 0.178462 |
| 26873 | 0.946127 | 2.1 |
| 26874 | 0.946131 | 1.12763 |
| 26875 | 0.946128 | 2.1 |
| 26876 | 0.946129 | 2.1 |
| 26877 | 1.4064 | 2.08285 |
| 26878 | 0.946133 | 2.1 |
| 26879 | 0.946131 | 2.1 |
| 26880 | 0.946131 | 2.1 |
| 26881 | 0.946131 | 2.1 |
| 26882 | 0.94613 | 2.1 |
| 26883 | 1.34912 | 2.1 |
| 26884 | 0.946131 | 2.1 |
| 26885 | 0.946129 | 2.1 |
| 26886 | 0.946131 | 2.1 |
| 26887 | 0.94613 | 2.1 |
| 26888 | 0.946132 | 2.1 |
| 26889 | 1.40447 | 2.1 |
| 26890 | 0.946132 | 2.1 |
| 26891 | 0.946133 | 2.1 |
| 26892 | 1.40447 | 2.1 |
| 26893 | 1.33607 | 2.1 |
| 26894 | 0.946131 | 2.1 |
| 26895 | 0.946127 | 2.1 |
| 26896 | 0.94613 | 2.1 |
| 26897 | 0.946129 | 2.1 |
| 26898 | 0.946117 | 0.5369 |
| 26899 | 1.32664 | 1.11068 |
| 26900 | 0.946115 | 0.812627 |
| 26901 | 0.946105 | 1.76743 |
| 26902 | 0.94613 | 0.0807924 |
| 26903 | 0.946118 | 2.1 |
| 26904 | 0.946114 | 2.1 |
| 26905 | 1.35886 | 2.1 |
| 26906 | 1.35884 | 2.1 |
| 26907 | 0.946129 | 2.1 |
| 26908 | 0.946132 | 2.1 |
| 26909 | 1.2571 | 2.1 |

| | | |
|---|---|---|
| 26910 | 0.946132 | 2.1 |
| 26911 | 0.946132 | 2.1 |
| 26912 | 1.2571 | 2.1 |
| 26913 | 0.946133 | 2.1 |
| 26914 | 1.35663 | 2.1 |
| 26915 | 0.946132 | 2.1 |
| 26916 | 0.946132 | 2.1 |
| 26917 | 1.35663 | 2.1 |
| 26918 | 0.946133 | 0.897706 |
| 26919 | 0.946129 | 2.01323 |
| 26920 | 0.946131 | 1.29627 |
| 26921 | 0.946131 | 1.15243 |
| 26922 | 1.30881 | 2.0604 |
| 26923 | 0.946132 | 2.1 |
| 26924 | 0.94613 | 2.1 |
| 26925 | 1.34652 | 2.1 |
| 26926 | 0.94613 | 2.1 |
| 26927 | 0.94613 | 2.1 |
| 26928 | 0.946132 | 2.1 |
| 26929 | 0.946133 | 1.7737 |
| 26930 | 1.36183 | 0.793342 |
| 26931 | 1.36183 | 1.34781 |
| 26932 | 0.946132 | 1.64399 |
| 26933 | 0.946132 | 0.0749247 |
| 26934 | 1.38353 | 0.05 |
| 26935 | 0.94613 | 0.283205 |
| 26936 | 0.94613 | 0.150757 |
| 26937 | 1.38354 | 0.05 |
| 26938 | 0.946128 | 0.05 |
| 26939 | 1.24887 | 0.05 |
| 26940 | 0.94613 | 0.05 |
| 26941 | 0.94613 | 0.0933191 |
| 26942 | 1.24887 | 0.05 |
| 26943 | 0.946129 | 0.928522 |
| 26944 | 0.946126 | 1.64626 |
| 26945 | 0.946124 | 0.975704 |
| 26946 | 0.946128 | 1.04596 |
| 26947 | 1.32056 | 1.2231 |
| 26948 | 0.946129 | 1.01041 |
| 26949 | 0.946128 | 1.7467 |
| 26950 | 0.946126 | 0.999621 |
| 26951 | 0.946129 | 1.15881 |
| 26952 | 1.20351 | 1.22312 |
| 26953 | 0.946131 | 1.14877 |
| 26954 | 0.946131 | 1.28783 |
| 26955 | 0.946127 | 1.96843 |
| 26956 | 1.25591 | 1.78907 |
| 26957 | 0.94613 | 0.398497 |
| 26958 | 0.946132 | 1.34376 |
| 26959 | 0.946133 | 1.20915 |
| 26960 | 0.946132 | 1.83982 |
| 26961 | 1.15436 | 1.10144 |
| 26962 | 0.930306 | 1.02129 |
| 26963 | 0.946132 | 2.1 |
| 26964 | 0.946132 | 2.1 |
| 26965 | 0.946132 | 2.1 |
| 26966 | 0.946133 | 2.1 |
| 26967 | 0.946132 | 2.1 |

| | | |
|---|---|---|
| 26968 | 0.946129 | 1.32598 |
| 26969 | 0.946132 | 0.966082 |
| 26970 | 0.946126 | 1.49395 |
| 26971 | 1.13245 | 0.806153 |
| 26972 | 0.946129 | 0.971019 |
| 26973 | 0.94613 | 2.1 |
| 26974 | 0.946132 | 2.1 |
| 26975 | 0.946129 | 1.87669 |
| 26976 | 0.946131 | 2.1 |
| 26977 | 0.946131 | 2.1 |
| 26978 | 1.41279 | 2.1 |
| 26979 | 1.76254 | 2.1 |
| 26980 | 0.600454 | 2.00991 |
| 26981 | 0.858991 | 1.9851 |
| 26982 | 0.940722 | 2.0653 |
| 26983 | 1.28439 | 0.956294 |
| 26984 | 0.946131 | 1.49436 |
| 26985 | 0.94613 | 1.61254 |
| 26986 | 0.946131 | 2.1 |
| 26987 | 0.946131 | 2.1 |
| 26988 | 0.946132 | 1.00113 |
| 26989 | 1.39335 | 0.132591 |
| 26990 | 0.946132 | 0.885594 |
| 26991 | 0.946132 | 0.419284 |
| 26992 | 0.946132 | 0.832222 |
| 26993 | 1.33879 | 0.05 |
| 26994 | 0.946131 | 0.05 |
| 26995 | 0.94613 | 0.0692614 |
| 26996 | 1.33879 | 0.05 |
| 26997 | 0.946131 | 0.05 |
| 26998 | 0.946126 | 0.058532 |
| 26999 | 0.946131 | 0.117751 |
| 27000 | 1.37342 | 0.05 |
| 27001 | 1.37343 | 0.05 |
| 27002 | 0.946128 | 0.612048 |
| 27003 | 1.39168 | 0.05 |
| 27004 | 0.946131 | 0.05 |
| 27005 | 0.946131 | 0.05 |
| 27006 | 0.946131 | 0.05 |
| 27007 | 0.946131 | 0.05 |
| 27008 | 0.946131 | 0.103031 |
| 27009 | 1.412 | 0.05 |
| 27010 | 0.946127 | 0.0521069 |
| 27011 | 0.946128 | 0.0520148 |
| 27012 | 0.946128 | 0.099532 |
| 27013 | 1.1387 | 0.05 |
| 27014 | 0.943364 | 0.05 |
| 27015 | 0.946132 | 0.05 |
| 27016 | 0.946132 | 0.05 |
| 27017 | 0.946133 | 0.05 |
| 27018 | 1.21085 | 0.05 |
| 27019 | 0.88324 | 0.321586 |
| 27020 | 0.946129 | 0.102557 |
| 27021 | 0.946131 | 0.14639 |
| 27022 | 0.946131 | 0.0696174 |
| 27023 | 1.33587 | 0.05 |
| 27024 | 0.946125 | 0.05 |
| 27025 | 0.946129 | 0.05 |

| | | |
|---|---|---|
| 27026 | 0.946126 | 0.05 |
| 27027 | 0.946127 | 0.05 |
| 27028 | 1.27512 | 0.05 |
| 27029 | 0.946132 | 0.05 |
| 27030 | 0.946132 | 0.05 |
| 27031 | 0.946133 | 0.05 |
| 27032 | 0.946132 | 0.05 |
| 27033 | 0.946128 | 0.05 |
| 27034 | 1.30498 | 0.05 |
| 27035 | 0.946131 | 0.05 |
| 27036 | 0.94613 | 0.05 |
| 27037 | 1.30497 | 0.05 |
| 27038 | 0.946131 | 0.05 |
| 27039 | 1.25626 | 0.05 |
| 27040 | 0.946131 | 0.05 |
| 27041 | 0.946131 | 0.05 |
| 27042 | 1.25626 | 0.05 |
| 27043 | 0.946131 | 0.05 |
| 27044 | 0.94613 | 0.05 |
| 27045 | 1.35316 | 0.05 |
| 27046 | 0.946131 | 0.05 |
| 27047 | 0.946131 | 0.05 |
| 27048 | 0.94613 | 0.145136 |
| 27049 | 1.28727 | 0.100164 |
| 27050 | 0.946128 | 0.05 |
| 27051 | 0.946131 | 0.0656947 |
| 27052 | 0.94613 | 0.104881 |
| 27053 | 0.946131 | 0.05 |
| 27054 | 1.39502 | 0.05 |
| 27055 | 0.946132 | 0.05 |
| 27056 | 0.946132 | 0.05 |
| 27057 | 0.946132 | 0.05 |
| 27058 | 1.36173 | 0.05 |
| 27059 | 0.946131 | 0.0912655 |
| 27060 | 0.946126 | 0.05 |
| 27061 | 0.946127 | 0.05 |
| 27062 | 0.946128 | 0.05 |
| 27063 | 0.946129 | 0.946033 |
| 27064 | 0.946133 | 0.816976 |
| 27065 | 0.946131 | 0.968442 |
| 27066 | 0.946131 | 1.08122 |
| 27067 | 0.946131 | 1.01669 |
| 27068 | 0.946128 | 0.05 |
| 27069 | 0.946132 | 0.05 |
| 27070 | 1.24678 | 0.05 |
| 27071 | 0.94613 | 0.05 |
| 27072 | 0.94613 | 0.05 |
| 27073 | 0.946129 | 0.05 |
| 27074 | 0.94613 | 0.05 |
| 27075 | 1.25215 | 0.05 |
| 27076 | 0.946126 | 0.05 |
| 27077 | 0.946129 | 0.05 |
| 27078 | 0.946129 | 0.675795 |
| 27079 | 1.29689 | 0.657473 |
| 27080 | 0.946131 | 0.688747 |
| 27081 | 0.946131 | 0.638342 |
| 27082 | 0.946131 | 0.587642 |
| 27083 | 0.946128 | 0.05 |

| | | |
|---|---|---|
| 27084 | 0.94613 | 0.05 |
| 27085 | 0.946131 | 0.05 |
| 27086 | 0.94613 | 0.05 |
| 27087 | 0.946131 | 0.05 |
| 27088 | 0.946127 | 0.05 |
| 27089 | 0.946128 | 0.05 |
| 27090 | 0.94613 | 0.05 |
| 27091 | 0.946129 | 0.05 |
| 27092 | 0.946129 | 0.05 |
| 27093 | 0.946133 | 0.05 |
| 27094 | 0.946132 | 0.05 |
| 27095 | 0.946132 | 0.05 |
| 27096 | 1.29957 | 0.05 |
| 27097 | 0.946133 | 0.05 |
| 27098 | 0.946132 | 0.05 |
| 27099 | 0.946133 | 0.05 |
| 27100 | 0.946132 | 0.05 |
| 27101 | 1.26201 | 0.05 |
| 27102 | 0.946132 | 0.05 |
| 27103 | 0.946133 | 0.05 |
| 27104 | 1.40427 | 0.05 |
| 27105 | 0.946132 | 0.05 |
| 27106 | 0.946132 | 0.05 |
| 27107 | 0.946133 | 0.05 |
| 27108 | 0.946132 | 0.05 |
| 27109 | 1.35123 | 0.05 |
| 27110 | 0.946132 | 0.05 |
| 27111 | 0.946132 | 0.05 |
| 27112 | 0.946131 | 0.05 |
| 27113 | 0.946128 | 0.05 |
| 27114 | 0.946129 | 0.05 |
| 27115 | 0.946131 | 0.05 |
| 27116 | 0.94613 | 0.05 |
| 27117 | 0.946131 | 0.05 |
| 27118 | 0.946127 | 0.374944 |
| 27119 | 1.23351 | 0.188197 |
| 27120 | 0.946131 | 0.473908 |
| 27121 | 0.946129 | 0.210152 |
| 27122 | 0.94613 | 0.240603 |
| 27123 | 0.946131 | 0.05 |
| 27124 | 0.946131 | 0.05 |
| 27125 | 0.94613 | 0.05 |
| 27126 | 1.18119 | 0.05 |
| 27127 | 0.946131 | 0.05 |
| 27128 | 0.946132 | 0.05 |
| 27129 | 1.30563 | 0.05 |
| 27130 | 0.946133 | 0.05 |
| 27131 | 0.946132 | 0.05 |
| 27132 | 0.946133 | 0.05 |
| 27133 | 0.946132 | 0.05 |
| 27134 | 0.946132 | 0.05 |
| 27135 | 0.946133 | 0.712674 |
| 27136 | 1.20256 | 0.05 |
| 27137 | 0.946133 | 0.065801 |
| 27138 | 0.94613 | 0.1011 |
| 27139 | 1.33007 | 0.0521284 |
| 27140 | 0.946128 | 0.413052 |
| 27141 | 0.946132 | 0.0633271 |

| | | |
|---|---|---|
| 27142 | 0.946131 | 0.119647 |
| 27143 | 0.946131 | 2.1 |
| 27144 | 0.946131 | 2.1 |
| 27145 | 0.946128 | 2.1 |
| 27146 | 0.946129 | 2.1 |
| 27147 | 0.94613 | 2.1 |
| 27148 | 0.810942 | 2.1 |
| 27149 | 0.810942 | 2.1 |
| 27150 | 0.81093 | 2.1 |
| 27151 | 0.946132 | 2.1 |
| 27152 | 1.39823 | 2.1 |
| 27153 | 0.946132 | 2.1 |
| 27154 | 0.946131 | 2.1 |
| 27155 | 0.946132 | 2.1 |
| 27156 | 0.946131 | 2.1 |
| 27157 | 0.946131 | 2.1 |
| 27158 | 0.946131 | 2.1 |
| 27159 | 1.37713 | 2.1 |
| 27160 | 0.946131 | 2.1 |
| 27161 | 0.946133 | 1.26828 |
| 27162 | 0.946131 | 2.1 |
| 27163 | 1.40594 | 2.1 |
| 27164 | 1.40594 | 2.1 |
| 27165 | 0.94613 | 2.1 |
| 27166 | 0.946133 | 2.1 |
| 27167 | 0.946131 | 2.1 |
| 27168 | 1.38155 | 2.1 |
| 27169 | 1.38155 | 2.1 |
| 27170 | 0.946131 | 2.1 |
| 27171 | 0.946132 | 0.873071 |
| 27172 | 0.946133 | 2.1 |
| 27173 | 0.946132 | 1.0654 |
| 27174 | 0.946132 | 1.03499 |
| 27175 | 1.33694 | 1.82286 |
| 27176 | 0.946131 | 2.1 |
| 27177 | 1.228 2.1 | |
| 27178 | 0.946129 | 2.1 |
| 27179 | 0.946131 | 2.1 |
| 27180 | 1.228 2.1 | |
| 27181 | 0.946132 | 1.13861 |
| 27182 | 0.946132 | 1.63935 |
| 27183 | 0.946131 | 2.1 |
| 27184 | 0.946132 | 1.24448 |
| 27185 | 1.35943 | 2.04769 |
| 27186 | 0.946133 | 0.05 |
| 27187 | 0.946133 | 0.05 |
| 27188 | 1.02377 | 0.05 |
| 27189 | 1.02377 | 0.05 |
| 27190 | 0.946132 | 0.05 |
| 27191 | 0.94613 | 0.238789 |
| 27192 | 0.946131 | 0.11201 |
| 27193 | 0.946132 | 0.394388 |
| 27194 | 1.29322 | 0.0628844 |
| 27195 | 0.946131 | 0.41102 |
| 27196 | 0.946131 | 1.68071 |
| 27197 | 0.946131 | 0.05 |
| 27198 | 0.946132 | 1.46383 |
| 27199 | 1.41492 | 0.05 |

| | | |
|---|---|---|
| 27200 | 0.946131 | 0.0906408 |
| 27201 | 0.946132 | 2.1 |
| 27202 | 0.946131 | 2.1 |
| 27203 | 0.946132 | 2.1 |
| 27204 | 1.35028 | 2.1 |
| 27205 | 0.946132 | 2.1 |
| 27206 | 0.946132 | 2.1 |
| 27207 | 0.946132 | 2.1 |
| 27208 | 0.946131 | 2.1 |
| 27209 | 1.32461 | 2.1 |
| 27210 | 0.946132 | 2.1 |
| 27211 | 0.946131 | 2.1 |
| 27212 | 0.946132 | 2.1 |
| 27213 | 1.40369 | 2.1 |
| 27214 | 0.94613 | 2.1 |
| 27215 | 0.946131 | 2.1 |
| 27216 | 0.946132 | 0.05 |
| 27217 | 0.979597 | 0.05 |
| 27218 | 0.946131 | 0.05 |
| 27219 | 0.946132 | 0.05 |
| 27220 | 0.946132 | 0.05 |
| 27221 | 0.946131 | 2.1 |
| 27222 | 0.946131 | 2.1 |
| 27223 | 0.94613 | 2.1 |
| 27224 | 0.946131 | 2.1 |
| 27225 | 1.41897 | 2.1 |
| 27226 | 1.37791 | 2.1 |
| 27227 | 0.94613 | 2.1 |
| 27228 | 0.946129 | 2.1 |
| 27229 | 0.946131 | 2.1 |
| 27230 | 0.946131 | 2.1 |
| 27231 | 1.39384 | 2.1 |
| 27232 | 0.946131 | 2.1 |
| 27233 | 0.946128 | 2.1 |
| 27234 | 0.946129 | 2.1 |
| 27235 | 0.946129 | 2.1 |
| 27236 | 0.810945 | 1.571 |
| 27237 | 0.810944 | 2.1 |
| 27238 | 0.810958 | 1.83337 |
| 27239 | 0.946128 | 2.1 |
| 27240 | 0.946132 | 2.1 |
| 27241 | 0.94613 | 2.1 |
| 27242 | 1.3335 | 2.1 |
| 27243 | 0.946131 | 2.1 |
| 27244 | 0.94613 | 0.05 |
| 27245 | 0.946129 | 2.05516 |
| 27246 | 0.946132 | 0.05 |
| 27247 | 1.41192 | 0.806863 |
| 27248 | 0.94613 | 0.05 |
| 27249 | 0.946132 | 0.694305 |
| 27250 | 0.946133 | 0.610875 |
| 27251 | 0.946132 | 0.490555 |
| 27252 | 1.39428 | 0.425518 |
| 27253 | 0.946133 | 0.476124 |
| 27254 | 0.94613 | 2.06036 |
| 27255 | 0.946128 | 2.1 |
| 27256 | 0.946132 | 1.05036 |
| 27257 | 0.94613 | 2.1 |

| | | |
|---|---|---|
| 27258 | 1.40684 | 2.1 |
| 27259 | 0.946131 | 2.1 |
| 27260 | 0.946131 | 2.1 |
| 27261 | 0.946131 | 2.1 |
| 27262 | 0.946131 | 2.1 |
| 27263 | 1.39021 | 2.1 |
| 27264 | 0.946131 | 1.13221 |
| 27265 | 1.31649 | 2.1 |
| 27266 | 0.946131 | 0.96451 |
| 27267 | 0.946131 | 0.996725 |
| 27268 | 1.31649 | 1.41612 |
| 27269 | 0.810946 | 2.1 |
| 27270 | 0.810946 | 2.1 |
| 27271 | 0.810958 | 2.1 |
| 27272 | 1.16687 | 2.1 |
| 27273 | 0.946131 | 2.1 |
| 27274 | 0.946131 | 2.1 |
| 27275 | 1.16687 | 2.1 |
| 27276 | 1.16687 | 2.1 |
| 27277 | 0.946128 | 0.943653 |
| 27278 | 1.3564 | 0.990911 |
| 27279 | 0.946131 | 0.985264 |
| 27280 | 0.946131 | 1.05556 |
| 27281 | 0.94613 | 0.807302 |
| 27282 | 0.946128 | 2.1 |
| 27283 | 1.35828 | 2.1 |
| 27284 | 0.94613 | 2.1 |
| 27285 | 0.946131 | 2.1 |
| 27286 | 0.94613 | 2.1 |
| 27287 | 0.946133 | 0.141518 |
| 27288 | 1.36477 | 0.0598108 |
| 27289 | 0.946132 | 0.148444 |
| 27290 | 0.946132 | 0.73949 |
| 27291 | 1.36477 | 0.300664 |
| 27292 | 0.946132 | 0.979714 |
| 27293 | 1.31857 | 2.1 |
| 27294 | 0.946132 | 1.23136 |
| 27295 | 0.946132 | 1.73896 |
| 27296 | 1.31857 | 1.43005 |
| 27297 | 0.94613 | 0.0530119 |
| 27298 | 1.35059 | 0.05 |
| 27299 | 0.946128 | 0.05 |
| 27300 | 0.94613 | 0.05 |
| 27301 | 1.35059 | 0.05 |
| 27302 | 0.946126 | 0.05 |
| 27303 | 1.35559 | 0.05 |
| 27304 | 0.946127 | 0.05 |
| 27305 | 0.946128 | 0.05 |
| 27306 | 1.35559 | 0.05 |
| 27307 | 0.946131 | 0.05 |
| 27308 | 1.33035 | 0.05 |
| 27309 | 0.946127 | 0.05 |
| 27310 | 0.946128 | 0.05 |
| 27311 | 1.33035 | 0.05 |
| 27312 | 0.94613 | 0.05 |
| 27313 | 1.3665 | 0.05 |
| 27314 | 0.946129 | 0.05 |
| 27315 | 0.946131 | 0.05 |

| | | |
|---|---|---|
| 27316 | 1.3665 | 0.05 |
| 27317 | 0.94613 | 0.0892472 |
| 27318 | 1.32828 | 0.05 |
| 27319 | 0.946129 | 0.0558324 |
| 27320 | 0.94613 | 0.0618994 |
| 27321 | 0.946131 | 0.0900783 |
| 27322 | 0.946131 | 2.1 |
| 27323 | 0.946131 | 2.1 |
| 27324 | 1.32949 | 2.1 |
| 27325 | 1.32949 | 2.1 |
| 27326 | 0.946131 | 2.1 |
| 27327 | 0.946131 | 1.68201 |
| 27328 | 0.94613 | 0.05 |
| 27329 | 0.946133 | 0.705874 |
| 27330 | 1.38357 | 0.05 |
| 27331 | 0.946131 | 0.542705 |
| 27332 | 0.946131 | 2.1 |
| 27333 | 0.946128 | 2.1 |
| 27334 | 1.36919 | 2.1 |
| 27335 | 0.946131 | 2.1 |
| 27336 | 0.94613 | 2.1 |
| 27337 | 0.946131 | 0.856661 |
| 27338 | 1.3771 | 2.1 |
| 27339 | 0.946129 | 2.1 |
| 27340 | 0.94613 | 2.1 |
| 27341 | 0.946131 | 1.52817 |
| 27342 | 0.946131 | 1.32636 |
| 27343 | 0.94613 | 0.844156 |
| 27344 | 0.946129 | 0.504346 |
| 27345 | 1.30029 | 0.925619 |
| 27346 | 0.94613 | 0.600481 |
| 27347 | 1.21174 | 0.05 |
| 27348 | 0.946132 | 0.05 |
| 27349 | 0.946132 | 0.05 |
| 27350 | 0.946132 | 0.05 |
| 27351 | 0.946133 | 0.05 |
| 27352 | 0.810946 | 2.1 |
| 27353 | 0.810945 | 2.1 |
| 27354 | 0.810946 | 2.1 |
| 27355 | 1.1796 | 2.1 |
| 27356 | 0.946131 | 2.1 |
| 27357 | 0.946132 | 2.1 |
| 27358 | 1.1796 | 2.1 |
| 27359 | 1.1796 | 2.1 |
| 27360 | 0.946131 | 2.1 |
| 27361 | 0.946131 | 2.1 |
| 27362 | 0.946133 | 2.1 |
| 27363 | 0.946131 | 2.1 |
| 27364 | 1.40615 | 2.1 |
| 27365 | 0.810945 | 2.1 |
| 27366 | 0.810944 | 2.1 |
| 27367 | 0.810945 | 2.1 |
| 27368 | 1.40239 | 2.1 |
| 27369 | 0.94613 | 2.1 |
| 27370 | 1.35561 | 2.1 |
| 27371 | 0.946133 | 2.1 |
| 27372 | 1.35562 | 2.1 |
| 27373 | 1.41169 | 0.05 |

| | | |
|---|---|---|
| 27374 | 0.946131 | 0.05 |
| 27375 | 0.946127 | 0.0511237 |
| 27376 | 1.41169 | 0.05 |
| 27377 | 0.946128 | 0.05 |
| 27378 | 0.946119 | 2.1 |
| 27379 | 0.946119 | 2.1 |
| 27380 | 0.946119 | 2.1 |
| 27381 | 0.946107 | 2.1 |
| 27382 | 1.41917 | 2.1 |
| 27383 | 0.810946 | 1.14123 |
| 27384 | 0.810943 | 1.2515 |
| 27385 | 0.810934 | 2.06804 |
| 27386 | 0.946127 | 2.1 |
| 27387 | 1.1995 | 2.1 |
| 27388 | 0.946126 | 2.1 |
| 27389 | 0.946128 | 2.1 |
| 27390 | 1.1995 | 2.1 |
| 27391 | 0.810944 | 2.1 |
| 27392 | 0.810947 | 2.1 |
| 27393 | 0.81096 | 2.1 |
| 27394 | 0.946133 | 0.05 |
| 27395 | 1.3495 | 0.05 |
| 27396 | 0.946132 | 0.05 |
| 27397 | 0.946132 | 0.05 |
| 27398 | 0.946133 | 0.05 |
| 27399 | 0.946132 | 2.1 |
| 27400 | 0.946132 | 2.1 |
| 27401 | 0.946133 | 2.1 |
| 27402 | 1.39529 | 2.1 |
| 27403 | 0.946133 | 2.1 |
| 27404 | 0.946133 | 2.1 |
| 27405 | 1.28525 | 2.1 |
| 27406 | 0.946132 | 2.1 |
| 27407 | 0.946133 | 2.1 |
| 27408 | 1.28525 | 2.1 |
| 27409 | 0.810946 | 2.1 |
| 27410 | 0.810945 | 2.1 |
| 27411 | 0.810946 | 2.1 |
| 27412 | 1.33931 | 2.1 |
| 27413 | 0.946132 | 2.1 |
| 27414 | 0.946132 | 2.1 |
| 27415 | 1.33931 | 2.1 |
| 27416 | 1.24616 | 2.1 |
| 27417 | 0.946133 | 0.106711 |
| 27418 | 0.946133 | 0.157746 |
| 27419 | 0.946132 | 0.05 |
| 27420 | 0.946132 | 0.0920217 |
| 27421 | 0.946133 | 0.0620949 |
| 27422 | 0.946128 | 2.1 |
| 27423 | 1.40225 | 2.1 |
| 27424 | 0.946128 | 2.1 |
| 27425 | 0.94613 | 2.1 |
| 27426 | 0.94613 | 2.1 |
| 27427 | 0.946113 | 0.05 |
| 27428 | 0.946118 | 0.05 |
| 27429 | 0.946114 | 0.05 |
| 27430 | 1.40551 | 0.05 |
| 27431 | 0.946103 | 0.05 |

| | | |
|---|---|---|
| 27432 | 0.94613 | 2.1 |
| 27433 | 0.946132 | 2.1 |
| 27434 | 0.946129 | 2.1 |
| 27435 | 0.946131 | 2.1 |
| 27436 | 1.37811 | 2.1 |
| 27437 | 0.946132 | 2.1 |
| 27438 | 1.39826 | 2.1 |
| 27439 | 0.946133 | 2.1 |
| 27440 | 0.946132 | 2.1 |
| 27441 | 1.39826 | 2.1 |
| 27442 | 0.946118 | 2.1 |
| 27443 | 1.28298 | 2.1 |
| 27444 | 0.946117 | 2.1 |
| 27445 | 0.946105 | 2.1 |
| 27446 | 1.283 2.1 | |
| 27447 | 0.946118 | 0.05 |
| 27448 | 1.41375 | 0.05 |
| 27449 | 0.946117 | 0.05 |
| 27450 | 0.946131 | 0.05 |
| 27451 | 0.946106 | 0.05 |
| 27452 | 0.946132 | 0.532918 |
| 27453 | 0.946129 | 0.236772 |
| 27454 | 1.28436 | 0.165175 |
| 27455 | 0.946131 | 0.655339 |
| 27456 | 0.946131 | 0.476645 |
| 27457 | 0.810947 | 0.0573482 |
| 27458 | 0.810946 | 0.119597 |
| 27459 | 0.81096 | 0.0968377 |
| 27460 | 0.946132 | 0.05 |
| 27461 | 1.34338 | 0.05 |
| 27462 | 0.961435 | 0.05 |
| 27463 | 0.961435 | 0.05 |
| 27464 | 0.946132 | 0.05 |
| 27465 | 1.3028 | 2.0152 |
| 27466 | 0.946131 | 2.07314 |
| 27467 | 0.946127 | 2.1 |
| 27468 | 0.946128 | 2.1 |
| 27469 | 0.946129 | 2.1 |
| 27470 | 0.946132 | 0.565005 |
| 27471 | 1.37973 | 0.139686 |
| 27472 | 0.946132 | 0.677715 |
| 27473 | 0.946132 | 0.236622 |
| 27474 | 0.946132 | 0.335274 |
| 27475 | 0.946131 | 0.86853 |
| 27476 | 0.94613 | 0.743466 |
| 27477 | 0.946129 | 0.869304 |
| 27478 | 0.946131 | 0.587229 |
| 27479 | 1.41798 | 0.46692 |
| 27480 | 0.946118 | 0.644274 |
| 27481 | 0.946118 | 0.244454 |
| 27482 | 0.946117 | 0.651639 |
| 27483 | 0.946131 | 0.340559 |
| 27484 | 0.946105 | 0.285846 |
| 27485 | 0.946131 | 0.912018 |
| 27486 | 0.946131 | 0.618596 |
| 27487 | 0.94613 | 0.911073 |
| 27488 | 0.946131 | 0.611528 |
| 27489 | 0.946131 | 0.385895 |

| | | |
|---|---|---|
| 27490 | 0.946133 | 1.12207 |
| 27491 | 1.40274 | 2.1 |
| 27492 | 0.946133 | 0.273505 |
| 27493 | 0.946132 | 2.1 |
| 27494 | 1.40274 | 2.1 |
| 27495 | 0.946131 | 1.27448 |
| 27496 | 1.39731 | 2.1 |
| 27497 | 0.946132 | 0.952847 |
| 27498 | 0.94613 | 1.15692 |
| 27499 | 1.39731 | 1.93525 |
| 27500 | 0.94612 | 2.1 |
| 27501 | 0.94612 | 1.67437 |
| 27502 | 0.946119 | 1.48324 |
| 27503 | 0.946132 | 1.62865 |
| 27504 | 1.40764 | 2.1 |
| 27505 | 0.946128 | 2.1 |
| 27506 | 0.946132 | 2.1 |
| 27507 | 0.94613 | 2.1 |
| 27508 | 0.94613 | 2.1 |
| 27509 | 1.31213 | 2.1 |
| 27510 | 0.946127 | 2.01815 |
| 27511 | 0.94613 | 2.1 |
| 27512 | 0.946127 | 2.04154 |
| 27513 | 0.946128 | 2.1 |
| 27514 | 1.36653 | 2.1 |
| 27515 | 0.946127 | 2.1 |
| 27516 | 1.3557 | 2.1 |
| 27517 | 0.946128 | 2.1 |
| 27518 | 0.946128 | 2.1 |
| 27519 | 1.3557 | 2.1 |
| 27520 | 0.946133 | 2.1 |
| 27521 | 0.946132 | 2.1 |
| 27522 | 0.946133 | 2.1 |
| 27523 | 0.946132 | 2.1 |
| 27524 | 1.40081 | 2.1 |
| 27525 | 0.946128 | 2.1 |
| 27526 | 0.946133 | 2.1 |
| 27527 | 0.946131 | 2.1 |
| 27528 | 0.946131 | 2.1 |
| 27529 | 1.32282 | 2.1 |
| 27530 | 0.810945 | 2.1 |
| 27531 | 0.810943 | 2.1 |
| 27532 | 0.810933 | 2.1 |
| 27533 | 1.27676 | 2.1 |
| 27534 | 0.94613 | 2.1 |
| 27535 | 0.946128 | 2.1 |
| 27536 | 0.946131 | 2.1 |
| 27537 | 0.946131 | 2.1 |
| 27538 | 0.946131 | 0.554173 |
| 27539 | 0.946127 | 0.05 |
| 27540 | 1.41328 | 0.666902 |
| 27541 | 1.41327 | 0.621523 |
| 27542 | 0.946128 | 0.598967 |
| 27543 | 0.946132 | 0.05 |
| 27544 | 1.32083 | 0.05 |
| 27545 | 0.946132 | 0.05 |
| 27546 | 0.946133 | 0.05 |
| 27547 | 1.32082 | 0.05 |

| | | |
|---|---|---|
| 27548 | 0.946128 | 0.05 |
| 27549 | 1.29215 | 0.05 |
| 27550 | 0.94613 | 0.05 |
| 27551 | 0.94613 | 0.05 |
| 27552 | 1.29215 | 0.05 |
| 27553 | 0.946132 | 0.05 |
| 27554 | 1.27696 | 0.05 |
| 27555 | 0.94613 | 0.05 |
| 27556 | 0.94613 | 0.05 |
| 27557 | 1.27696 | 0.05 |
| 27558 | 0.94612 | 0.05 |
| 27559 | 1.29289 | 0.05 |
| 27560 | 0.946119 | 0.05 |
| 27561 | 0.946132 | 0.05 |
| 27562 | 1.29287 | 0.05 |
| 27563 | 0.946128 | 2.1 |
| 27564 | 1.37869 | 2.1 |
| 27565 | 0.946131 | 2.1 |
| 27566 | 0.94613 | 2.1 |
| 27567 | 0.946131 | 2.1 |
| 27568 | 0.946133 | 2.1 |
| 27569 | 0.946132 | 2.1 |
| 27570 | 0.946132 | 2.1 |
| 27571 | 1.35703 | 2.1 |
| 27572 | 0.946133 | 2.1 |
| 27573 | 0.946132 | 2.1 |
| 27574 | 0.946132 | 2.1 |
| 27575 | 0.946133 | 2.1 |
| 27576 | 1.37017 | 2.1 |
| 27577 | 0.946133 | 2.1 |
| 27578 | 0.946119 | 0.05 |
| 27579 | 0.94612 | 0.05 |
| 27580 | 1.38583 | 0.05 |
| 27581 | 1.38585 | 0.05 |
| 27582 | 0.946107 | 0.05 |
| 27583 | 0.946126 | 0.05 |
| 27584 | 0.946127 | 0.05 |
| 27585 | 1.22904 | 0.05 |
| 27586 | 1.22904 | 0.05 |
| 27587 | 0.946128 | 0.05 |
| 27588 | 1.3279 | 0.0514766 |
| 27589 | 0.946131 | 1.38191 |
| 27590 | 0.94613 | 1.09006 |
| 27591 | 0.946131 | 1.32441 |
| 27592 | 0.946131 | 1.09808 |
| 27593 | 0.946132 | 2.1 |
| 27594 | 0.946132 | 2.1 |
| 27595 | 0.946132 | 2.1 |
| 27596 | 0.946131 | 2.1 |
| 27597 | 1.40932 | 2.1 |
| 27598 | 1.26868 | 0.05 |
| 27599 | 0.946131 | 0.05 |
| 27600 | 0.94613 | 0.05 |
| 27601 | 0.946131 | 0.05 |
| 27602 | 0.946131 | 0.05 |
| 27603 | 0.810943 | 2.1 |
| 27604 | 0.810945 | 2.1 |
| 27605 | 0.810932 | 2.1 |

| | | |
|---|---|---|
| 27606 | 0.810943 | 0.556687 |
| 27607 | 0.810944 | 0.0518617 |
| 27608 | 0.810957 | 0.05 |
| 27609 | 1.31539 | 0.550704 |
| 27610 | 0.946132 | 1.9152 |
| 27611 | 0.946128 | 0.05 |
| 27612 | 1.3154 | 0.124266 |
| 27613 | 1.17856 | 0.117812 |
| 27614 | 1.21997 | 0.411772 |
| 27615 | 0.94613 | 1.93303 |
| 27616 | 0.946129 | 1.46009 |
| 27617 | 0.94613 | 2.08977 |
| 27618 | 0.94613 | 1.84927 |
| 27619 | 0.946131 | 1.05298 |
| 27620 | 0.946131 | 0.215272 |
| 27621 | 0.946129 | 1.12955 |
| 27622 | 0.94613 | 0.781685 |
| 27623 | 0.946131 | 1.21572 |
| 27624 | 0.946127 | 0.438203 |
| 27625 | 0.946131 | 0.328097 |
| 27626 | 0.946127 | 2.01647 |
| 27627 | 0.946129 | 1.05073 |
| 27628 | 1.41342 | 0.80498 |
| 27629 | 0.946131 | 0.795162 |
| 27630 | 0.946131 | 1.5113 |
| 27631 | 0.946128 | 1.887 |
| 27632 | 0.946131 | 0.900653 |
| 27633 | 1.29149 | 1.36485 |
| 27634 | 1.41022 | 2.1 |
| 27635 | 0.94613 | 0.0821153 |
| 27636 | 0.946128 | 2.1 |
| 27637 | 0.94613 | 2.1 |
| 27638 | 0.94613 | 2.1 |
| 27639 | 0.946128 | 0.05 |
| 27640 | 0.94613 | 0.05 |
| 27641 | 1.27864 | 0.05 |
| 27642 | 1.27864 | 0.05 |
| 27643 | 0.946131 | 0.05 |
| 27644 | 0.946129 | 0.832841 |
| 27645 | 0.946125 | 1.72336 |
| 27646 | 0.946124 | 1.17474 |
| 27647 | 0.946127 | 0.971393 |
| 27648 | 1.37759 | 1.40908 |
| 27649 | 0.946129 | 0.881603 |
| 27650 | 0.94613 | 1.71817 |
| 27651 | 0.946126 | 1.03029 |
| 27652 | 0.946129 | 1.01449 |
| 27653 | 1.38259 | 1.26675 |
| 27654 | 0.946131 | 0.05 |
| 27655 | 1.34118 | 0.05 |
| 27656 | 0.946132 | 0.0701505 |
| 27657 | 0.946132 | 0.05 |
| 27658 | 1.34118 | 0.05 |
| 27659 | 0.946131 | 0.05 |
| 27660 | 1.25017 | 0.05 |
| 27661 | 0.946128 | 0.05 |
| 27662 | 0.946131 | 0.05 |
| 27663 | 0.946131 | 0.05 |

| | | |
|---|---|---|
| 27664 | 0.946131 | 2.1 |
| 27665 | 0.946128 | 2.1 |
| 27666 | 0.946133 | 2.1 |
| 27667 | 1.3937 | 2.1 |
| 27668 | 0.94613 | 2.1 |
| 27669 | 0.946131 | 0.732852 |
| 27670 | 0.94613 | 0.195112 |
| 27671 | 1.40404 | 0.05 |
| 27672 | 1.40404 | 0.05 |
| 27673 | 0.946131 | 0.356263 |
| 27674 | 0.946132 | 0.553065 |
| 27675 | 0.946132 | 0.0726675 |
| 27676 | 1.38897 | 0.0729386 |
| 27677 | 1.38897 | 0.05 |
| 27678 | 0.946132 | 0.251647 |
| 27679 | 0.94613 | 0.416765 |
| 27680 | 0.946128 | 0.05 |
| 27681 | 1.40332 | 0.05 |
| 27682 | 1.40331 | 0.05 |
| 27683 | 0.94613 | 0.452799 |
| 27684 | 0.946132 | 0.05 |
| 27685 | 0.946132 | 0.510566 |
| 27686 | 0.946133 | 0.102306 |
| 27687 | 1.41619 | 1.56463 |
| 27688 | 0.946132 | 0.05 |
| 27689 | 0.946132 | 1.2663 |
| 27690 | 0.946132 | 2.1 |
| 27691 | 0.946132 | 2.1 |
| 27692 | 1.38244 | 2.1 |
| 27693 | 0.946132 | 2.1 |
| 27694 | 0.810946 | 1.11218 |
| 27695 | 0.810943 | 0.648154 |
| 27696 | 0.810934 | 0.184545 |
| 27697 | 0.810947 | 0.983283 |
| 27698 | 0.810943 | 0.261465 |
| 27699 | 0.810934 | 0.339453 |
| 27700 | 1.12786 | 1.17025 |
| 27701 | 0.946132 | 2.1 |
| 27702 | 0.946132 | 1.995 |
| 27703 | 0.946132 | 1.38518 |
| 27704 | 0.946133 | 1.34062 |
| 27705 | 0.946132 | 2.01762 |
| 27706 | 0.946132 | 2.01416 |
| 27707 | 0.946132 | 1.352 |
| 27708 | 1.27806 | 1.98976 |
| 27709 | 0.946133 | 1.25707 |
| 27710 | 0.946132 | 2.1 |
| 27711 | 0.946132 | 2.1 |
| 27712 | 0.946133 | 2.1 |
| 27713 | 0.946132 | 2.1 |
| 27714 | 0.946133 | 2.1 |
| 27715 | 1.39514 | 1.94553 |
| 27716 | 0.946129 | 1.06273 |
| 27717 | 0.946124 | 1.57838 |
| 27718 | 0.946127 | 0.919426 |
| 27719 | 0.946127 | 1.78 |
| 27720 | 0.94613 | 2.1 |
| 27721 | 0.946129 | 2.1 |

| | | |
|---|---|---|
| 27722 | 0.946132 | 2.1 |
| 27723 | 1.3991 | 2.1 |
| 27724 | 0.94613 | 2.1 |
| 27725 | 0.946132 | 0.134737 |
| 27726 | 0.946133 | 2.1 |
| 27727 | 0.946132 | 2.1 |
| 27728 | 1.36835 | 0.438961 |
| 27729 | 0.946132 | 0.306141 |
| 27730 | 0.946132 | 2.1 |
| 27731 | 0.946132 | 2.1 |
| 27732 | 0.946133 | 2.1 |
| 27733 | 0.946132 | 2.1 |
| 27734 | 1.39047 | 2.1 |
| 27735 | 0.946132 | 0.05 |
| 27736 | 0.946128 | 0.05 |
| 27737 | 0.94613 | 0.05 |
| 27738 | 1.33174 | 0.05 |
| 27739 | 0.946131 | 0.05 |
| 27740 | 0.946128 | 0.05 |
| 27741 | 0.946131 | 0.05 |
| 27742 | 0.94613 | 0.05 |
| 27743 | 0.94613 | 0.05 |
| 27744 | 0.94613 | 0.05 |
| 27745 | 0.946119 | 2.1 |
| 27746 | 0.946119 | 2.1 |
| 27747 | 1.29064 | 2.1 |
| 27748 | 0.946132 | 2.1 |
| 27749 | 0.946107 | 2.1 |
| 27750 | 0.946133 | 2.1 |
| 27751 | 0.94613 | 2.1 |
| 27752 | 1.30584 | 2.1 |
| 27753 | 0.946131 | 2.1 |
| 27754 | 0.946131 | 2.1 |
| 27755 | 0.946131 | 2.1 |
| 27756 | 0.946132 | 2.1 |
| 27757 | 0.94613 | 2.1 |
| 27758 | 0.946131 | 2.1 |
| 27759 | 1.39549 | 2.1 |
| 27760 | 0.913386 | 1.27172 |
| 27761 | 0.978873 | 1.08827 |
| 27762 | 0.946126 | 1.07769 |
| 27763 | 0.94613 | 1.32613 |
| 27764 | 1.00473 | 0.697177 |
| 27765 | 0.946132 | 0.810439 |
| 27766 | 0.946133 | 0.810106 |
| 27767 | 0.946132 | 0.543622 |
| 27768 | 0.946132 | 0.919729 |
| 27769 | 1.38894 | 0.556344 |
| 27770 | 0.946128 | 0.702119 |
| 27771 | 0.946131 | 0.502881 |
| 27772 | 0.94613 | 0.337125 |
| 27773 | 0.94613 | 0.645007 |
| 27774 | 1.23865 | 0.323074 |
| 27775 | 0.946126 | 1.31952 |
| 27776 | 0.946129 | 1.35324 |
| 27777 | 0.946124 | 0.941622 |
| 27778 | 0.946127 | 1.26924 |
| 27779 | 1.40248 | 1.02753 |

| | | |
|---|---|---|
| 27780 | 0.946133 | 0.860584 |
| 27781 | 0.946132 | 0.943327 |
| 27782 | 0.946132 | 0.795915 |
| 27783 | 0.946133 | 0.947834 |
| 27784 | 1.1391 | 0.794265 |
| 27785 | 0.827862 | 0.719999 |
| 27786 | 0.946133 | 0.777744 |
| 27787 | 0.946131 | 0.974444 |
| 27788 | 0.946131 | 0.796794 |
| 27789 | 0.94613 | 0.759545 |
| 27790 | 0.946128 | 0.593535 |
| 27791 | 0.946132 | 0.727298 |
| 27792 | 0.94613 | 0.789613 |
| 27793 | 1.21388 | 0.360506 |
| 27794 | 0.94613 | 0.746966 |
| 27795 | 0.810943 | 0.05 |
| 27796 | 0.810945 | 0.055261 |
| 27797 | 0.810932 | 0.0622592 |
| 27798 | 0.946131 | 0.269204 |
| 27799 | 0.946131 | 0.696862 |
| 27800 | 0.946133 | 1.09395 |
| 27801 | 1.36748 | 0.41593 |
| 27802 | 0.946132 | 0.05 |
| 27803 | 0.946128 | 0.100589 |
| 27804 | 0.946131 | 0.0540504 |
| 27805 | 0.94613 | 0.162779 |
| 27806 | 1.35856 | 0.05 |
| 27807 | 0.94613 | 0.102313 |
| 27808 | 0.946132 | 2.1 |
| 27809 | 0.946131 | 2.1 |
| 27810 | 0.946133 | 2.1 |
| 27811 | 0.946132 | 2.1 |
| 27812 | 0.946131 | 2.1 |
| 27813 | 0.946133 | 2.1 |
| 27814 | 0.946131 | 2.1 |
| 27815 | 1.39558 | 2.1 |
| 27816 | 1.39558 | 2.1 |
| 27817 | 0.94613 | 2.1 |
| 27818 | 0.946131 | 0.05 |
| 27819 | 0.94613 | 0.05 |
| 27820 | 0.946129 | 0.05 |
| 27821 | 1.41388 | 0.05 |
| 27822 | 0.946131 | 0.05 |
| 27823 | 0.946133 | 0.05 |
| 27824 | 0.946132 | 0.05 |
| 27825 | 0.946132 | 0.05 |
| 27826 | 0.946133 | 0.05 |
| 27827 | 0.93452 | 0.05 |
| 27828 | 0.946129 | 2.1 |
| 27829 | 1.38897 | 2.1 |
| 27830 | 0.946131 | 2.1 |
| 27831 | 0.946131 | 2.1 |
| 27832 | 1.38897 | 2.1 |
| 27833 | 0.946133 | 2.1 |
| 27834 | 0.946132 | 1.45042 |
| 27835 | 0.946133 | 2.1 |
| 27836 | 0.946132 | 0.767126 |
| 27837 | 1.38773 | 1.04678 |

| | | |
|---|---|---|
| 27838 | 1.41512 | 0.05 |
| 27839 | 0.946131 | 0.05 |
| 27840 | 0.946129 | 0.05 |
| 27841 | 0.946133 | 0.05 |
| 27842 | 0.946131 | 0.05 |
| 27843 | 0.946132 | 0.651901 |
| 27844 | 0.946132 | 2.1 |
| 27845 | 0.946132 | 0.714282 |
| 27846 | 0.946133 | 1.01525 |
| 27847 | 0.946133 | 2.1 |
| 27848 | 0.946126 | 0.245705 |
| 27849 | 0.946132 | 1.04433 |
| 27850 | 0.946129 | 0.277761 |
| 27851 | 0.946129 | 0.829799 |
| 27852 | 0.946129 | 1.06844 |
| 27853 | 0.939895 | 2.1 |
| 27854 | 1.03386 | 2.1 |
| 27855 | 0.94613 | 2.1 |
| 27856 | 0.946131 | 2.00663 |
| 27857 | 1.03386 | 2.0617 |
| 27858 | 0.946131 | 2.1 |
| 27859 | 1.39309 | 2.1 |
| 27860 | 0.946133 | 1.85578 |
| 27861 | 0.946131 | 2.1 |
| 27862 | 1.39309 | 2.1 |
| 27863 | 0.946133 | 2.1 |
| 27864 | 1.24853 | 2.1 |
| 27865 | 0.946132 | 2.1 |
| 27866 | 0.946132 | 2.1 |
| 27867 | 1.24853 | 2.1 |
| 27868 | 0.946133 | 2.1 |
| 27869 | 1.39699 | 0.788941 |
| 27870 | 0.946132 | 1.9172 |
| 27871 | 0.946132 | 2.1 |
| 27872 | 1.39699 | 1.2536 |
| 27873 | 0.946132 | 2.1 |
| 27874 | 0.946131 | 2.0701 |
| 27875 | 0.946133 | 2.1 |
| 27876 | 0.946131 | 2.1 |
| 27877 | 1.38969 | 2.1 |
| 27878 | 1.28464 | 2.1 |
| 27879 | 0.946133 | 2.1 |
| 27880 | 0.94613 | 2.1 |
| 27881 | 0.946131 | 2.06625 |
| 27882 | 0.946131 | 2.09863 |
| 27883 | 0.946119 | 0.867159 |
| 27884 | 0.94612 | 0.974336 |
| 27885 | 0.946119 | 0.871937 |
| 27886 | 0.946132 | 0.933542 |
| 27887 | 1.3749 | 0.797407 |
| 27888 | 0.810942 | 2.1 |
| 27889 | 0.810943 | 2.1 |
| 27890 | 0.810957 | 2.1 |
| 27891 | 1.41134 | 2.1 |
| 27892 | 0.946132 | 0.936776 |
| 27893 | 0.946132 | 2.08864 |
| 27894 | 0.946132 | 1.06319 |
| 27895 | 0.946132 | 2.0406 |

| | | |
|---|---|---|
| 27896 | 1.31338 | 2.1 |
| 27897 | 0.946132 | 1.12074 |
| 27898 | 0.946132 | 2.1 |
| 27899 | 0.946133 | 1.51701 |
| 27900 | 0.946132 | 2.08096 |
| 27901 | 0.946103 | 0.05 |
| 27902 | 0.752448 | 0.05 |
| 27903 | 0.946132 | 0.05 |
| 27904 | 0.946131 | 0.05 |
| 27905 | 1.20617 | 0.05 |
| 27906 | 0.946132 | 0.992142 |
| 27907 | 1.08054 | 0.606853 |
| 27908 | 1.25736 | 0.976003 |
| 27909 | 0.946132 | 1.00207 |
| 27910 | 0.946131 | 0.966407 |
| 27911 | 0.946128 | 2.1 |
| 27912 | 1.33275 | 0.346677 |
| 27913 | 1.1896 | 2.1 |
| 27914 | 0.946131 | 2.1 |
| 27915 | 1.18962 | 2.1 |
| 27916 | 1.21955 | 0.05 |
| 27917 | 0.946131 | 0.05 |
| 27918 | 1.33587 | 0.05 |
| 27919 | 0.741946 | 0.05 |
| 27920 | 1.07387 | 0.05 |
| 27921 | 0.945844 | 0.889875 |
| 27922 | 1.3292 | 2.1 |
| 27923 | 1.59472 | 2.1 |
| 27924 | 0.946131 | 1.00107 |
| 27925 | 1.04043 | 1.20916 |
| 27926 | 1.13529 | 0.05 |
| 27927 | 0.946132 | 0.0701683 |
| 27928 | 1.24893 | 0.05 |
| 27929 | 1.12675 | 0.05 |
| 27930 | 0.936652 | 0.0704664 |
| 27931 | 1.36968 | 2.1 |
| 27932 | 0.946131 | 2.1 |
| 27933 | 0.946132 | 2.1 |
| 27934 | 1.36968 | 2.1 |
| 27935 | 1.258 | 2.1 |
| 27936 | 3.17458 | 0.05 |
| 27937 | 0.946132 | 1.70604 |
| 27938 | 1.35022 | 2.1 |
| 27939 | 1.35022 | 2.1 |
| 27940 | 0.94613 | 2.1 |
| 27941 | 1.12356 | 2.1 |
| 27942 | 0.946131 | 2.1 |
| 27943 | 1.22401 | 2.1 |
| 27944 | 1.22401 | 2.1 |
| 27945 | 0.94613 | 2.1 |
| 27946 | 0.952455 | 2.1 |
| 27947 | 1.2107 | 2.1 |
| 27948 | 0.946132 | 2.1 |
| 27949 | 1.33047 | 2.1 |
| 27950 | 0.946131 | 2.1 |
| 27951 | 0.946132 | 2.1 |
| 27952 | 0.61957 | 2.1 |
| 27953 | 0.8636 | 2.1 |

| | | |
|---|---|---|
| 27954 | 0.946132 | 2.1 |
| 27955 | 1.19294 | 2.1 |
| 27956 | 0.946128 | 2.1 |
| 27957 | 1.36607 | 2.1 |
| 27958 | 0.94613 | 2.1 |
| 27959 | 1.39416 | 2.1 |
| 27960 | 1.36607 | 2.1 |
| 27961 | 1.21474 | 2.1 |
| 27962 | 0.946131 | 2.1 |
| 27963 | 0.946131 | 2.1 |
| 27964 | 1.16288 | 2.1 |
| 27965 | 1.08542 | 2.1 |
| 27966 | 0.851526 | 1.02628 |
| 27967 | 1.30374 | 2.1 |
| 27968 | 1.01708 | 2.1 |
| 27969 | 0.946114 | 1.02923 |
| 27970 | 0.96976 | 1.97081 |
| 27971 | 0.946132 | 0.05 |
| 27972 | 1.49997 | 0.05 |
| 27973 | 0.771735 | 0.05 |
| 27974 | 0.771764 | 0.05 |
| 27975 | 0.946133 | 0.05 |
| 27976 | 0.700863 | 1.14376 |
| 27977 | 1.42222 | 2.1 |
| 27978 | 0.946131 | 2.1 |
| 27979 | 0.946128 | 0.756132 |
| 27980 | 1.42509 | 2.1 |
| 27981 | 1.14298 | 0.904805 |
| 27982 | 0.837014 | 2.1 |
| 27983 | 0.946131 | 2.1 |
| 27984 | 1.29426 | 2.1 |
| 27985 | 0.836985 | 2.1 |
| 27986 | 0.946129 | 2.1 |
| 27987 | 1.22089 | 0.0722331 |
| 27988 | 0.946132 | 0.998118 |
| 27989 | 0.712486 | 0.133096 |
| 27990 | 0.6234 | 0.05 |
| 27991 | 0.946131 | 2.1 |
| 27992 | 1.40271 | 2.1 |
| 27993 | 1.32241 | 2.1 |
| 27994 | 0.946133 | 2.1 |
| 27995 | 1.32241 | 2.1 |
| 27996 | 0.769373 | 1.28742 |
| 27997 | 0.946129 | 2.1 |
| 27998 | 1.41302 | 2.1 |
| 27999 | 1.28254 | 2.1 |
| 28000 | 0.946131 | 2.1 |
| 28001 | 1.28254 | 2.1 |
| 28002 | 0.851543 | 1.90591 |
| 28003 | 1.23056 | 2.1 |
| 28004 | 1.24356 | 2.1 |
| 28005 | 0.851543 | 2.08553 |
| 28006 | 0.909417 | 2.1 |
| 28007 | 1.13808 | 0.505771 |
| 28008 | 0.946133 | 0.556984 |
| 28009 | 0.946131 | 0.133008 |
| 28010 | 1.13808 | 0.984041 |
| 28011 | 1.13808 | 0.516114 |

| | | |
|---|---|---|
| 28012 | 0.930941 | 1.33262 |
| 28013 | 1.37808 | 1.07768 |
| 28014 | 1.17573 | 0.734436 |
| 28015 | 0.946124 | 1.30389 |
| 28016 | 1.02404 | 0.968983 |
| 28017 | 0.946116 | 2.1 |
| 28018 | 1.3144 | 0.10157 |
| 28019 | 1.3144 | 2.1 |
| 28020 | 0.946118 | 2.1 |
| 28021 | 1.13089 | 2.1 |
| 28022 | 0.946128 | 0.05 |
| 28023 | 0.946101 | 0.05 |
| 28024 | 1.2606 | 0.05 |
| 28025 | 1.26055 | 0.05 |
| 28026 | 0.580823 | 0.476986 |
| 28027 | 0.946132 | 1.10513 |
| 28028 | 0.580825 | 0.435512 |
| 28029 | 1.23224 | 0.638032 |
| 28030 | 0.946128 | 2.1 |
| 28031 | 1.3719 | 2.1 |
| 28032 | 0.94613 | 2.1 |
| 28033 | 1.3719 | 2.1 |
| 28034 | 0.94613 | 2.1 |
| 28035 | 0.946132 | 1.21819 |
| 28036 | 1.35025 | 0.766298 |
| 28037 | 1.35025 | 1.0274 |
| 28038 | 0.946132 | 1.905 |
| 28039 | 1.1794 | 1.16844 |
| 28040 | 0.946133 | 2.1 |
| 28041 | 1.25598 | 2.1 |
| 28042 | 1.29249 | 2.1 |
| 28043 | 1.38154 | 2.1 |
| 28044 | 0.851541 | 2.1 |
| 28045 | 1.31508 | 2.1 |
| 28046 | 1.37558 | 2.1 |
| 28047 | 0.946129 | 2.1 |
| 28048 | 0.867663 | 2.1 |
| 28049 | 0.94613 | 1.39759 |
| 28050 | 1.35426 | 1.18808 |
| 28051 | 0.84872 | 1.19792 |
| 28052 | 0.946129 | 1.07148 |
| 28053 | 1.17195 | 2.1 |
| 28054 | 0.946133 | 2.1 |
| 28055 | 1.39009 | 2.1 |
| 28056 | 1.14342 | 2.1 |
| 28057 | 0.946133 | 2.1 |
| 28058 | 1.14343 | 2.1 |
| 28059 | 1.38819 | 2.1 |
| 28060 | 0.946133 | 2.1 |
| 28061 | 1.4116 | 2.1 |
| 28062 | 0.946131 | 2.1 |
| 28063 | 0.946131 | 2.1 |
| 28064 | 0.946127 | 2.1 |
| 28065 | 1.32244 | 2.1 |
| 28066 | 1.32244 | 2.1 |
| 28067 | 0.946131 | 2.1 |
| 28068 | 1.2176 | 2.1 |
| 28069 | 1.75823 | 2.1 |

| | | |
|---|---|---|
| 28070 | 1.11239 | 2.1 |
| 28071 | 2.17979 | 2.1 |
| 28072 | 1.65533 | 2.1 |
| 28073 | 3.41356 | 2.1 |
| 28074 | 1.94841 | 2.1 |
| 28075 | 1.91767 | 2.1 |
| 28076 | 1.76726 | 2.1 |
| 28077 | 1.70302 | 2.1 |
| 28078 | 3.76137 | 2.1 |
| 28079 | 1.94841 | 2.1 |
| 28080 | 1.33962 | 2.1 |
| 28081 | 1.31365 | 2.1 |
| 28082 | 1.70302 | 2.1 |
| 28083 | 3.2123 | 2.1 |
| 28084 | 1.76726 | 2.1 |
| 28085 | 1.70302 | 2.1 |
| 28086 | 1.47581 | 2.1 |
| 28087 | 1.7457 | 2.1 |
| 28088 | 3.22764 | 2.1 |
| 28089 | 1.20478 | 2.1 |
| 28090 | 1.70302 | 2.1 |
| 28091 | 1.27956 | 2.1 |
| 28092 | 1.18569 | 2.1 |
| 28093 | 1.63979 | 2.1 |
| 28094 | 1.51949 | 2.1 |
| 28095 | 1.53082 | 2.1 |
| 28096 | 1.92911 | 2.1 |
| 28097 | 1.32068 | 2.1 |
| 28098 | 1.74454 | 2.1 |
| 28099 | 3.27536 | 2.1 |
| 28100 | 1.08172 | 2.1 |
| 28101 | 1.74454 | 2.1 |
| 28102 | 1.52603 | 2.1 |
| 28103 | 1.52603 | 2.1 |
| 28104 | 1.32068 | 2.1 |
| 28105 | 1.32068 | 2.1 |
| 28106 | 1.89226 | 2.1 |
| 28107 | 2.37364 | 2.1 |
| 28108 | 1.89226 | 2.1 |
| 28109 | 2.42769 | 2.1 |
| 28110 | 4.31995 | 2.1 |
| 28111 | 2.37365 | 2.1 |
| 28112 | 1.5362 | 2.1 |
| 28113 | 2.4277 | 2.1 |
| 28114 | 1.56328 | 2.1 |
| 28115 | 3.9639 | 2.1 |
| 28116 | 1.89225 | 2.1 |
| 28117 | 2.42768 | 2.1 |
| 28118 | 1.89213 | 2.1 |
| 28119 | 1.89225 | 2.1 |
| 28120 | 2.42756 | 2.1 |
| 28121 | 2.42768 | 2.1 |
| 28122 | 2.42768 | 2.1 |
| 28123 | 1.58163 | 2.1 |
| 28124 | 1.56315 | 2.1 |
| 28125 | 1.56327 | 2.1 |
| 28126 | 2.42757 | 2.1 |
| 28127 | 2.42768 | 2.1 |

| | | |
|---|---|---|
| 28128 | 1.01641 | 2.1 |
| 28129 | 2.15005 | 2.1 |
| 28130 | 1.00099 | 2.1 |
| 28131 | 2.15114 | 2.1 |
| 28132 | 3.15104 | 2.1 |
| 28133 | 0.994813 | 2.1 |
| 28134 | 2.15114 | 2.1 |
| 28135 | 2.17159 | 2.1 |
| 28136 | 2.17159 | 2.1 |
| 28137 | 1.00099 | 2.1 |
| 28138 | 1.00099 | 2.1 |
| 28139 | 1.29608 | 2.1 |
| 28140 | 1.29608 | 2.1 |
| 28141 | 0.942035 | 2.1 |
| 28142 | 0.942035 | 1.734 |
| 28143 | 1.29198 | 2.1 |
| 28144 | 0.942033 | 2.1 |
| 28145 | 0.946132 | 2.1 |
| 28146 | 0.946132 | 2.1 |
| 28147 | 0.946132 | 2.02268 |
| 28148 | 0.942033 | 2.1 |
| 28149 | 0.946133 | 1.11674 |
| 28150 | 1.29198 | 2.1 |
| 28151 | 1.99985 | 2.1 |
| 28152 | 1.14207 | 2.1 |
| 28153 | 1.83584 | 2.1 |
| 28154 | 1.00232 | 2.1 |
| 28155 | 3.00217 | 2.1 |
| 28156 | 1.99867 | 0.05 |
| 28157 | 2.0815 | 0.05 |
| 28158 | 1.56806 | 0.05 |
| 28159 | 1.28828 | 0.05 |
| 28160 | 3.58519 | 0.05 |
| 28161 | 1.89226 | 0.05 |
| 28162 | 1.89226 | 0.05 |
| 28163 | 1.89226 | 0.05 |
| 28164 | 1.89226 | 0.05 |
| 28165 | 1.89226 | 0.05 |
| 28166 | 1.89226 | 0.05 |
| 28167 | 1.89226 | 0.05 |
| 28168 | 1.89226 | 0.05 |
| 28169 | 2.23193 | 0.05 |
| 28170 | 2.48978 | 0.05 |
| 28171 | 4.38204 | 0.05 |
| 28172 | 1.85293 | 0.765397 |
| 28173 | 1.64279 | 0.104668 |
| 28174 | 1.76268 | 0.0644084 |
| 28175 | 1.54214 | 0.05 |
| 28176 | 3.39507 | 0.0617592 |
| 28177 | 1.6428 | 0.224021 |
| 28178 | 1.17302 | 0.05 |
| 28179 | 1.17302 | 0.798113 |
| 28180 | 1.28419 | 0.24337 |
| 28181 | 1.54214 | 0.05 |
| 28182 | 1.65331 | 0.136234 |
| 28183 | 1.76268 | 0.807683 |
| 28184 | 1.54214 | 0.459712 |
| 28185 | 1.65324 | 0.108446 |

| | | |
|---|---|---|
| 28186 | 1.42491 | 0.05 |
| 28187 | 3.19538 | 0.05 |
| 28188 | 0.651043 | 0.286042 |
| 28189 | 0.651043 | 0.620529 |
| 28190 | 0.647925 | 0.07432 |
| 28191 | 0.647925 | 0.546447 |
| 28192 | 0.521747 | 0.0568143 |
| 28193 | 0.58651 | 0.110836 |
| 28194 | 0.77107 | 0.469042 |
| 28195 | 0.712457 | 0.189429 |
| 28196 | 0.712457 | 0.05 |
| 28197 | 0.521747 | 0.0648758 |
| 28198 | 0.58651 | 0.05 |
| 28199 | 0.77107 | 0.465346 |
| 28200 | 1.2873 | 2.1 |
| 28201 | 1.48751 | 2.1 |
| 28202 | 1.59659 | 2.1 |
| 28203 | 1.77596 | 2.06735 |
| 28204 | 3.06326 | 2.1 |
| 28205 | 1.07976 | 2.1 |
| 28206 | 1.59659 | 2.1 |
| 28207 | 1.30237 | 2.1 |
| 28208 | 1.77723 | 2.1 |
| 28209 | 2.89897 | 2.1 |
| 28210 | 1.77723 | 2.1 |
| 28211 | 1.77596 | 2.1 |
| 28212 | 1.82614 | 2.1 |
| 28213 | 1.59659 | 2.1 |
| 28214 | 2.0055 | 2.1 |
| 28215 | 1.77596 | 2.1 |
| 28216 | 2.64523 | 0.72159 |
| 28217 | 2.73982 | 0.318171 |
| 28218 | 2.50057 | 0.595733 |
| 28219 | 2.61606 | 0.167973 |
| 28220 | 5.26129 | 0.284579 |
| 28221 | 2.73982 | 0.397463 |
| 28222 | 2.68987 | 0.438041 |
| 28223 | 2.61607 | 0.397739 |
| 28224 | 2.57611 | 0.242631 |
| 28225 | 5.30593 | 0.271916 |
| 28226 | 1.89226 | 0.105771 |
| 28227 | 1.89226 | 0.752764 |
| 28228 | 1.89226 | 0.550037 |
| 28229 | 2.02178 | 0.531447 |
| 28230 | 3.78452 | 0.567826 |
| 28231 | 1.89226 | 0.05 |
| 28232 | 1.89226 | 0.05 |
| 28233 | 1.89226 | 1.31173 |
| 28234 | 1.89226 | 0.05 |
| 28235 | 1.89226 | 1.20933 |
| 28236 | 1.89226 | 0.05 |
| 28237 | 2.35204 | 2.1 |
| 28238 | 1.50108 | 2.1 |
| 28239 | 1.3715 | 2.1 |
| 28240 | 2.18469 | 2.1 |
| 28241 | 3.68577 | 2.1 |
| 28242 | 2.62514 | 2.1 |
| 28243 | 2.1847 | 2.1 |

| | | |
|---|---|---|
| 28244 | 2.0487 | 2.1 |
| 28245 | 2.45213 | 2.1 |
| 28246 | 4.63682 | 2.1 |
| 28247 | 1.25075 | 2.1 |
| 28248 | 2.18469 | 2.1 |
| 28249 | 1.3715 | 2.1 |
| 28250 | 1.3715 | 2.1 |
| 28251 | 2.0487 | 2.1 |
| 28252 | 2.0487 | 2.1 |
| 28253 | 1.28667 | 2.1 |
| 28254 | 1.11285 | 2.1 |
| 28255 | 1.03529 | 2.1 |
| 28256 | 1.03529 | 2.1 |
| 28257 | 1.30711 | 2.1 |
| 28258 | 1.30711 | 2.1 |
| 28259 | 2.35053 | 2.1 |
| 28260 | 1.89225 | 2.1 |
| 28261 | 1.89225 | 2.1 |
| 28262 | 2.51216 | 2.1 |
| 28263 | 4.24278 | 2.1 |
| 28264 | 1.89225 | 2.1 |
| 28265 | 1.89225 | 2.1 |
| 28266 | 1.89225 | 2.1 |
| 28267 | 1.89225 | 2.1 |
| 28268 | 3.7845 | 2.1 |
| 28269 | 1.92025 | 2.1 |
| 28270 | 1.32119 | 2.1 |
| 28271 | 1.44777 | 2.1 |
| 28272 | 1.33834 | 2.1 |
| 28273 | 1.92025 | 2.1 |
| 28274 | 1.81082 | 2.1 |
| 28275 | 1.89226 | 2.1 |
| 28276 | 2.67272 | 2.1 |
| 28277 | 2.51218 | 2.1 |
| 28278 | 2.51218 | 2.1 |
| 28279 | 1.89226 | 2.1 |
| 28280 | 1.89226 | 2.1 |
| 28281 | 1.89226 | 2.1 |
| 28282 | 1.89226 | 2.1 |
| 28283 | 1.89226 | 2.1 |
| 28284 | 1.89226 | 2.1 |
| 28285 | 3.78452 | 2.1 |
| 28286 | 1.92027 | 2.1 |
| 28287 | 1.44778 | 2.1 |
| 28288 | 1.61121 | 2.1 |
| 28289 | 2.01555 | 2.1 |
| 28290 | 3.53147 | 2.1 |
| 28291 | 1.90727 | 0.05 |
| 28292 | 2.29851 | 0.05 |
| 28293 | 2.71643 | 0.05 |
| 28294 | 2.17695 | 0.05 |
| 28295 | 4.47547 | 0.05 |
| 28296 | 1.90728 | 0.05 |
| 28297 | 1.56339 | 0.05 |
| 28298 | 1.58143 | 0.05 |
| 28299 | 2.17696 | 0.05 |
| 28300 | 3.74035 | 0.05 |
| 28301 | 1.91368 | 0.05 |

| | | |
|---|---|---|
| 28302 | 2.17696 | 0.0520306 |
| 28303 | 2.39986 | 0.05 |
| 28304 | 1.75904 | 0.05 |
| 28305 | 3.936 | 0.05 |
| 28306 | 1.18793 | 0.05 |
| 28307 | 2.74744 | 0.05 |
| 28308 | 1.38716 | 0.05 |
| 28309 | 2.57465 | 0.05 |
| 28310 | 3.93537 | 0.05 |
| 28311 | 1.3148 | 0.05 |
| 28312 | 2.74744 | 0.05 |
| 28313 | 2.70805 | 0.05 |
| 28314 | 1.38716 | 0.05 |
| 28315 | 2.70805 | 0.05 |
| 28316 | 1.38716 | 0.05 |
| 28317 | 1.20608 | 2.1 |
| 28318 | 0.975871 | 2.1 |
| 28319 | 0.946132 | 2.1 |
| 28320 | 0.946132 | 2.1 |
| 28321 | 0.946132 | 2.1 |
| 28322 | 0.946132 | 2.1 |
| 28323 | 0.975871 | 2.1 |
| 28324 | 0.975871 | 2.1 |
| 28325 | 1.20608 | 2.1 |
| 28326 | 0.946132 | 2.1 |
| 28327 | 0.946133 | 2.1 |
| 28328 | 0.975871 | 2.1 |
| 28329 | 2.8198 | 0.861355 |
| 28330 | 2.29967 | 2.1 |
| 28331 | 2.09542 | 2.1 |
| 28332 | 2.63784 | 0.741972 |
| 28333 | 4.93751 | 2.1 |
| 28334 | 2.09542 | 2.1 |
| 28335 | 1.39523 | 2.1 |
| 28336 | 1.21119 | 2.1 |
| 28337 | 1.39523 | 2.1 |
| 28338 | 2.11563 | 2.1 |
| 28339 | 2.29967 | 2.1 |
| 28340 | 1.88584 | 2.1 |
| 28341 | 2.63784 | 0.630665 |
| 28342 | 2.11262 | 1.76257 |
| 28343 | 1.91132 | 0.98469 |
| 28344 | 4.02153 | 2.1 |
| 28345 | 1.88584 | 2.1 |
| 28346 | 1.21119 | 2.1 |
| 28347 | 1.12606 | 2.1 |
| 28348 | 1.91132 | 1.0202 |
| 28349 | 3.1225 | 2.1 |
| 28350 | 2.18077 | 2.1 |
| 28351 | 1.78167 | 2.1 |
| 28352 | 2.2947 | 0.917442 |
| 28353 | 1.93139 | 1.58014 |
| 28354 | 4.07637 | 1.03763 |
| 28355 | 1.5709 | 1.28461 |
| 28356 | 1.03235 | 2.1 |
| 28357 | 2.05933 | 2.05911 |
| 28358 | 1.78168 | 1.10707 |
| 28359 | 1.31 | 0.242477 |

| | | |
|---|---|---|
| 28360 | 1.03235 | 0.585639 |
| 28361 | 2.2947 | 1.18076 |
| 28362 | 2.31785 | 2.1 |
| 28363 | 2.40627 | 2.1 |
| 28364 | 2.08288 | 1.01221 |
| 28365 | 4.37758 | 2.1 |
| 28366 | 1.95737 | 1.88796 |
| 28367 | 1.31 | 2.1 |
| 28368 | 2.08289 | 0.760548 |
| 28369 | 1.50108 | 0.965411 |
| 28370 | 3.39289 | 0.907388 |
| 28371 | 2.49676 | 2.1 |
| 28372 | 2.24781 | 2.1 |
| 28373 | 2.5276 | 0.789106 |
| 28374 | 2.7352 | 2.1 |
| 28375 | 5.02436 | 2.1 |
| 28376 | 2.49676 | 2.1 |
| 28377 | 2.43549 | 2.1 |
| 28378 | 2.6398 | 2.1 |
| 28379 | 2.7352 | 2.1 |
| 28380 | 5.13656 | 2.1 |
| 28381 | 1.89226 | 2.1 |
| 28382 | 2.7352 | 2.1 |
| 28383 | 1.89226 | 2.1 |
| 28384 | 2.6398 | 2.1 |
| 28385 | 1.89226 | 2.1 |
| 28386 | 2.6398 | 2.1 |
| 28387 | 2.16843 | 0.05 |
| 28388 | 1.00185 | 0.05 |
| 28389 | 1.90039 | 2.1 |
| 28390 | 3.1547 | 0.05 |
| 28391 | 2.54064 | 0.05 |
| 28392 | 2.46752 | 2.1 |
| 28393 | 1.90039 | 2.1 |
| 28394 | 2.26044 | 2.1 |
| 28395 | 4.36791 | 0.05 |
| 28396 | 1.65815 | 2.1 |
| 28397 | 2.48652 | 2.1 |
| 28398 | 1.65815 | 2.1 |
| 28399 | 1.9004 | 2.1 |
| 28400 | 2.21711 | 2.1 |
| 28401 | 2.5034 | 2.1 |
| 28402 | 2.37167 | 2.1 |
| 28403 | 2.59965 | 2.1 |
| 28404 | 2.62812 | 2.1 |
| 28405 | 2.40308 | 2.1 |
| 28406 | 4.99979 | 2.1 |
| 28407 | 2.50137 | 2.1 |
| 28408 | 2.59965 | 2.1 |
| 28409 | 2.62812 | 2.1 |
| 28410 | 2.5214 | 2.1 |
| 28411 | 5.12948 | 2.1 |
| 28412 | 2.62814 | 2.1 |
| 28413 | 2.4031 | 2.1 |
| 28414 | 2.43509 | 2.1 |
| 28415 | 2.65066 | 2.1 |
| 28416 | 5.06323 | 2.1 |
| 28417 | 2.62814 | 2.1 |

| | | |
|---|---|---|
| 28418 | 2.52142 | 2.1 |
| 28419 | 2.53008 | 2.1 |
| 28420 | 2.65066 | 2.1 |
| 28421 | 5.15822 | 2.1 |
| 28422 | 2.0815 | 2.1 |
| 28423 | 1.92067 | 2.1 |
| 28424 | 1.59611 | 2.1 |
| 28425 | 1.89226 | 2.1 |
| 28426 | 1.62453 | 2.1 |
| 28427 | 1.92067 | 2.1 |
| 28428 | 1.89226 | 2.1 |
| 28429 | 1.89226 | 2.1 |
| 28430 | 1.89226 | 2.1 |
| 28431 | 1.89226 | 2.1 |
| 28432 | 3.78453 | 2.1 |
| 28433 | 0.946131 | 2.1 |
| 28434 | 0.946131 | 2.1 |
| 28435 | 0.929501 | 2.1 |
| 28436 | 0.910761 | 2.1 |
| 28437 | 0.9461 | 2.1 |
| 28438 | 0.9461 | 2.1 |
| 28439 | 0.910792 | 2.1 |
| 28440 | 0.910792 | 2.1 |
| 28441 | 0.946131 | 2.1 |
| 28442 | 0.929501 | 2.1 |
| 28443 | 0.910761 | 2.1 |
| 28444 | 0.946131 | 2.1 |
| 28445 | 2.0815 | 2.1 |
| 28446 | 1.81661 | 2.1 |
| 28447 | 2.1341 | 2.1 |
| 28448 | 2.0815 | 2.1 |
| 28449 | 1.86921 | 2.1 |
| 28450 | 1.81661 | 2.1 |
| 28451 | 1.89226 | 2.1 |
| 28452 | 1.89226 | 2.1 |
| 28453 | 1.89226 | 2.1 |
| 28454 | 1.89226 | 2.1 |
| 28455 | 1.89226 | 2.1 |
| 28456 | 1.89226 | 2.1 |
| 28457 | 0.946131 | 2.1 |
| 28458 | 0.946131 | 2.1 |
| 28459 | 0.949104 | 2.1 |
| 28460 | 0.9295 | 2.1 |
| 28461 | 0.946132 | 2.1 |
| 28462 | 0.946132 | 2.1 |
| 28463 | 0.929499 | 2.1 |
| 28464 | 0.929499 | 2.1 |
| 28465 | 0.946131 | 2.1 |
| 28466 | 0.949105 | 2.1 |
| 28467 | 0.946131 | 2.1 |
| 28468 | 0.929501 | 2.1 |
| 28469 | 1.66104 | 2.1 |
| 28470 | 2.34044 | 2.1 |
| 28471 | 2.08889 | 2.1 |
| 28472 | 1.33264 | 2.1 |
| 28473 | 3.67308 | 2.1 |
| 28474 | 2.0889 | 2.1 |
| 28475 | 1.08011 | 2.1 |

| | | |
|---|---|---|
| 28476 | 1.08011 | 2.1 |
| 28477 | 1.33264 | 2.1 |
| 28478 | 1.87598 | 2.1 |
| 28479 | 2.12851 | 2.1 |
| 28480 | 2.27472 | 2.1 |
| 28481 | 1.89226 | 1.25652 |
| 28482 | 2.27703 | 2.1 |
| 28483 | 1.89226 | 0.861152 |
| 28484 | 4.16699 | 0.920817 |
| 28485 | 1.89226 | 2.1 |
| 28486 | 1.89226 | 1.43062 |
| 28487 | 1.89226 | 1.72067 |
| 28488 | 1.89226 | 1.13494 |
| 28489 | 3.78452 | 1.99 |
| 28490 | 1.00007 | 2.06033 |
| 28491 | 1.32316 | 1.06738 |
| 28492 | 1.29417 | 0.835293 |
| 28493 | 1.29417 | 1.79393 |
| 28494 | 1.02051 | 0.895653 |
| 28495 | 1.02051 | 1.29269 |
| 28496 | 2.2846 | 2.1 |
| 28497 | 1.89226 | 1.01724 |
| 28498 | 2.27703 | 0.960268 |
| 28499 | 2.27703 | 2.1 |
| 28500 | 1.89226 | 0.163253 |
| 28501 | 1.89226 | 2.1 |
| 28502 | 1.89226 | 2.1 |
| 28503 | 1.89226 | 2.05529 |
| 28504 | 1.89226 | 1.07224 |
| 28505 | 1.89226 | 0.589918 |
| 28506 | 3.78452 | 0.978491 |
| 28507 | 1.02051 | 2.1 |
| 28508 | 1.32316 | 1.99984 |
| 28509 | 1.03373 | 1.05035 |
| 28510 | 1.34066 | 1.0744 |
| 28511 | 2.35689 | 2.1 |
| 28512 | 1.703 | 2.1 |
| 28513 | 1.89224 | 2.1 |
| 28514 | 1.52266 | 2.1 |
| 28515 | 1.89224 | 2.1 |
| 28516 | 1.23344 | 2.1 |
| 28517 | 1.53282 | 2.1 |
| 28518 | 1.89223 | 2.1 |
| 28519 | 1.89223 | 2.1 |
| 28520 | 1.89223 | 2.1 |
| 28521 | 1.89223 | 2.1 |
| 28522 | 3.78447 | 2.1 |
| 28523 | 0.796845 | 2.1 |
| 28524 | 0.796845 | 2.1 |
| 28525 | 0.812146 | 2.1 |
| 28526 | 0.812146 | 2.1 |
| 28527 | 1.08006 | 2.1 |
| 28528 | 1.02679 | 2.1 |
| 28529 | 0.551538 | 2.1 |
| 28530 | 0.528929 | 2.1 |
| 28531 | 0.551529 | 2.1 |
| 28532 | 1.08005 | 2.1 |
| 28533 | 0.528935 | 2.1 |

| | | |
|---|---|---|
| 28534 | 1.0268 | 2.1 |
| 28535 | 1.5001 | 2.1 |
| 28536 | 1.70302 | 2.1 |
| 28537 | 2.0726 | 2.1 |
| 28538 | 1.70302 | 2.1 |
| 28539 | 1.99601 | 2.1 |
| 28540 | 1.70303 | 2.1 |
| 28541 | 1.89226 | 2.1 |
| 28542 | 1.89226 | 2.1 |
| 28543 | 1.89226 | 2.1 |
| 28544 | 1.89226 | 2.1 |
| 28545 | 1.89226 | 2.1 |
| 28546 | 1.89226 | 2.1 |
| 28547 | 1.17285 | 2.1 |
| 28548 | 2.16015 | 2.1 |
| 28549 | 2.23563 | 2.1 |
| 28550 | 2.23563 | 2.1 |
| 28551 | 1.10309 | 2.1 |
| 28552 | 1.10309 | 2.1 |
| 28553 | 0.946128 | 2.1 |
| 28554 | 0.946128 | 2.1 |
| 28555 | 1.21847 | 0.610898 |
| 28556 | 1.2884 | 2.1 |
| 28557 | 0.94613 | 2.1 |
| 28558 | 0.94613 | 2.1 |
| 28559 | 1.21847 | 2.1 |
| 28560 | 1.21847 | 0.614886 |
| 28561 | 0.946131 | 2.1 |
| 28562 | 1.21847 | 2.1 |
| 28563 | 0.94613 | 2.1 |
| 28564 | 1.2884 | 2.1 |
| 28565 | 1.83922 | 0.697485 |
| 28566 | 2.5768 | 2.1 |
| 28567 | 1.83488 | 2.1 |
| 28568 | 1.82846 | 0.05 |
| 28569 | 2.43693 | 2.1 |
| 28570 | 2.43693 | 2.1 |
| 28571 | 2.36134 | 2.1 |
| 28572 | 1.81656 | 2.1 |
| 28573 | 1.86454 | 2.1 |
| 28574 | 1.6697 | 2.1 |
| 28575 | 2.55618 | 2.1 |
| 28576 | 2.36134 | 2.1 |
| 28577 | 2.55618 | 2.1 |
| 28578 | 1.9323 | 2.1 |
| 28579 | 1.9302 | 2.1 |
| 28580 | 1.81656 | 2.1 |
| 28581 | 2.66983 | 2.1 |
| 28582 | 2.55618 | 2.1 |
| 28583 | 1.89226 | 0.855695 |
| 28584 | 1.70302 | 0.290612 |
| 28585 | 1.94935 | 1.01406 |
| 28586 | 1.70302 | 0.794974 |
| 28587 | 1.95569 | 0.319852 |
| 28588 | 1.70302 | 0.0576816 |
| 28589 | 2.82465 | 2.1 |
| 28590 | 2.41707 | 2.1 |
| 28591 | 2.24481 | 2.1 |

| | | |
|---|---|---|
| 28592 | 2.68958 | 2.1 |
| 28593 | 5.10665 | 2.1 |
| 28594 | 1.41232 | 2.1 |
| 28595 | 1.34479 | 2.1 |
| 28596 | 0.946129 | 2.1 |
| 28597 | 0.946129 | 2.1 |
| 28598 | 0.94613 | 2.1 |
| 28599 | 0.94613 | 2.1 |
| 28600 | 1.41232 | 2.1 |
| 28601 | 1.41232 | 2.1 |
| 28602 | 1.34479 | 2.1 |
| 28603 | 0.94613 | 2.1 |
| 28604 | 0.946131 | 2.1 |
| 28605 | 1.41232 | 2.1 |
| 28606 | 1.86859 | 0.05 |
| 28607 | 1.89226 | 0.05 |
| 28608 | 1.89226 | 0.05 |
| 28609 | 1.89226 | 0.05 |
| 28610 | 1.45875 | 0.05 |
| 28611 | 1.45875 | 0.05 |
| 28612 | 2.1769 | 0.64367 |
| 28613 | 2.50259 | 0.572053 |
| 28614 | 1.94955 | 0.407228 |
| 28615 | 2.25959 | 0.694911 |
| 28616 | 4.45214 | 0.753716 |
| 28617 | 1.89226 | 0.633574 |
| 28618 | 1.89226 | 0.668012 |
| 28619 | 1.89226 | 0.533732 |
| 28620 | 1.89226 | 0.671611 |
| 28621 | 3.78452 | 0.523654 |
| 28622 | 1.89226 | 0.722915 |
| 28623 | 1.89226 | 0.926084 |
| 28624 | 1.89226 | 0.664352 |
| 28625 | 1.89226 | 0.831505 |
| 28626 | 3.78452 | 1.53307 |
| 28627 | 2.50259 | 0.62148 |
| 28628 | 2.49445 | 0.710047 |
| 28629 | 2.25959 | 0.589249 |
| 28630 | 2.20843 | 0.610143 |
| 28631 | 4.71102 | 0.350122 |
| 28632 | 1.94955 | 0.450623 |
| 28633 | 2.25959 | 0.400449 |
| 28634 | 1.24689 | 0.1203 |
| 28635 | 1.79906 | 0.443536 |
| 28636 | 3.74861 | 0.180819 |
| 28637 | 1.89226 | 0.55648 |
| 28638 | 1.89226 | 0.573254 |
| 28639 | 1.89226 | 0.432762 |
| 28640 | 1.89226 | 0.512646 |
| 28641 | 3.78452 | 0.659021 |
| 28642 | 1.89226 | 0.577457 |
| 28643 | 1.89226 | 0.623526 |
| 28644 | 1.89226 | 0.486041 |
| 28645 | 1.89226 | 0.430072 |
| 28646 | 3.78452 | 0.423529 |
| 28647 | 2.25959 | 0.161378 |
| 28648 | 2.20843 | 0.20214 |
| 28649 | 1.79906 | 0.331082 |

| | | |
|---|---|---|
| 28650 | 1.98391 | 0.385814 |
| 28651 | 4.00748 | 0.383459 |
| 28652 | 1.89226 | 2.1 |
| 28653 | 1.89226 | 2.1 |
| 28654 | 1.89226 | 2.1 |
| 28655 | 1.89226 | 2.1 |
| 28656 | 3.78452 | 2.1 |
| 28657 | 1.89226 | 2.1 |
| 28658 | 1.89226 | 2.1 |
| 28659 | 1.89226 | 2.1 |
| 28660 | 1.89226 | 2.1 |
| 28661 | 3.78452 | 2.1 |
| 28662 | 1.64718 | 2.1 |
| 28663 | 1.23085 | 2.1 |
| 28664 | 1.46028 | 2.1 |
| 28665 | 1.39564 | 2.1 |
| 28666 | 1.68372 | 2.1 |
| 28667 | 1.64718 | 2.1 |
| 28668 | 1.64718 | 2.1 |
| 28669 | 1.64981 | 2.1 |
| 28670 | 1.70303 | 2.1 |
| 28671 | 1.6991 | 2.1 |
| 28672 | 3.3199 | 2.1 |
| 28673 | 1.89226 | 2.1 |
| 28674 | 1.89226 | 2.1 |
| 28675 | 1.89226 | 2.1 |
| 28676 | 1.89226 | 2.1 |
| 28677 | 3.78452 | 2.1 |
| 28678 | 1.89226 | 2.1 |
| 28679 | 1.89226 | 2.1 |
| 28680 | 1.89226 | 2.1 |
| 28681 | 1.89226 | 2.1 |
| 28682 | 3.78452 | 2.1 |
| 28683 | 0.666701 | 2.1 |
| 28684 | 0.65896 | 2.1 |
| 28685 | 0.920797 | 2.1 |
| 28686 | 0.895441 | 2.1 |
| 28687 | 0.775871 | 2.1 |
| 28688 | 0.687493 | 2.1 |
| 28689 | 0.777393 | 2.1 |
| 28690 | 0.777393 | 2.1 |
| 28691 | 0.677856 | 2.1 |
| 28692 | 0.90095 | 2.1 |
| 28693 | 0.621435 | 2.1 |
| 28694 | 0.858124 | 2.1 |
| 28695 | 1.70303 | 2.1 |
| 28696 | 1.70731 | 2.1 |
| 28697 | 1.79663 | 2.1 |
| 28698 | 1.68783 | 2.1 |
| 28699 | 3.17337 | 2.1 |
| 28700 | 1.22724 | 2.1 |
| 28701 | 2.0021 | 2.1 |
| 28702 | 1.90427 | 2.1 |
| 28703 | 1.08698 | 2.1 |
| 28704 | 3.08907 | 2.1 |
| 28705 | 2.41689 | 2.1 |
| 28706 | 2.00209 | 2.1 |
| 28707 | 1.90427 | 2.1 |

| | | |
|---|---|---|
| 28708 | 2.35245 | 2.1 |
| 28709 | 4.35454 | 2.1 |
| 28710 | 1.90427 | 2.1 |
| 28711 | 0.956294 | 2.1 |
| 28712 | 0.956294 | 2.1 |
| 28713 | 1.08698 | 2.1 |
| 28714 | 1.81488 | 2.1 |
| 28715 | 1.94556 | 2.1 |
| 28716 | 1.90427 | 2.1 |
| 28717 | 2.35245 | 2.1 |
| 28718 | 2.28979 | 2.1 |
| 28719 | 1.81488 | 2.1 |
| 28720 | 4.16733 | 2.1 |
| 28721 | 1.6272 | 2.1 |
| 28722 | 1.70302 | 2.1 |
| 28723 | 1.70303 | 2.1 |
| 28724 | 1.70302 | 2.1 |
| 28725 | 1.91167 | 2.1 |
| 28726 | 1.91167 | 2.1 |
| 28727 | 1.49063 | 2.1 |
| 28728 | 1.89226 | 2.1 |
| 28729 | 1.89226 | 2.1 |
| 28730 | 1.89226 | 2.1 |
| 28731 | 1.6272 | 2.1 |
| 28732 | 1.6272 | 2.1 |
| 28733 | 2.25988 | 1.11369 |
| 28734 | 1.89226 | 0.166547 |
| 28735 | 1.89226 | 1.00175 |
| 28736 | 2.23152 | 0.809514 |
| 28737 | 4.12379 | 0.715013 |
| 28738 | 2.25988 | 0.841569 |
| 28739 | 1.16361 | 1.06573 |
| 28740 | 1.15853 | 2.1 |
| 28741 | 2.23153 | 0.373757 |
| 28742 | 3.39513 | 1.14707 |
| 28743 | 1.89226 | 0.962521 |
| 28744 | 2.23153 | 0.569509 |
| 28745 | 2.21022 | 1.17196 |
| 28746 | 2.23153 | 0.823428 |
| 28747 | 1.87095 | 0.128362 |
| 28748 | 1.89226 | 0.0679671 |
| 28749 | 1.16239 | 1.85643 |
| 28750 | 2.23153 | 0.0676075 |
| 28751 | 1.15853 | 1.18356 |
| 28752 | 1.15853 | 0.200957 |
| 28753 | 2.21022 | 1.29307 |
| 28754 | 2.21022 | 0.182782 |
| 28755 | 1.78381 | 0.05 |
| 28756 | 1.70302 | 0.05 |
| 28757 | 1.15453 | 0.05 |
| 28758 | 1.35007 | 0.05 |
| 28759 | 1.82021 | 0.05 |
| 28760 | 2.12851 | 0.05 |
| 28761 | 1.89226 | 0.05 |
| 28762 | 1.89226 | 0.05 |
| 28763 | 1.89226 | 0.05 |
| 28764 | 1.89226 | 0.05 |
| 28765 | 1.89226 | 0.05 |

| | | |
|---|---|---|
| 28766 | 1.89226 | 0.05 |
| 28767 | 1.89258 | 0.05 |
| 28768 | 1.089 | 0.05 |
| 28769 | 1.22586 | 0.05 |
| 28770 | 1.79877 | 0.05 |
| 28771 | 2.88777 | 0.05 |
| 28772 | 1.91564 | 0.05 |
| 28773 | 1.79877 | 0.05 |
| 28774 | 1.96356 | 0.05 |
| 28775 | 1.70302 | 0.05 |
| 28776 | 2.37828 | 0.05 |
| 28777 | 2.04859 | 0.05 |
| 28778 | 1.89226 | 0.05 |
| 28779 | 1.89226 | 0.05 |
| 28780 | 1.89226 | 0.05 |
| 28781 | 1.89226 | 0.05 |
| 28782 | 3.78453 | 0.05 |
| 28783 | 1.89226 | 0.05 |
| 28784 | 1.89226 | 0.05 |
| 28785 | 1.89226 | 0.05 |
| 28786 | 1.89226 | 0.05 |
| 28787 | 1.89226 | 0.05 |
| 28788 | 1.89226 | 0.05 |
| 28789 | 2.28593 | 2.1 |
| 28790 | 1.89226 | 2.1 |
| 28791 | 2.2731 | 2.1 |
| 28792 | 1.89226 | 0.704014 |
| 28793 | 4.17819 | 1.30833 |
| 28794 | 1.89226 | 2.1 |
| 28795 | 1.89226 | 2.1 |
| 28796 | 1.89226 | 1.38628 |
| 28797 | 1.89226 | 1.13025 |
| 28798 | 3.78452 | 1.79069 |
| 28799 | 1.03933 | 2.1 |
| 28800 | 1.34355 | 1.0542 |
| 28801 | 1.35048 | 0.98945 |
| 28802 | 1.35048 | 2.1 |
| 28803 | 1.02975 | 0.867606 |
| 28804 | 1.02975 | 1.77374 |
| 28805 | 2.25105 | 2.1 |
| 28806 | 1.89226 | 1.8323 |
| 28807 | 2.27311 | 2.1 |
| 28808 | 2.27311 | 2.1 |
| 28809 | 1.89226 | 0.897595 |
| 28810 | 1.89226 | 2.1 |
| 28811 | 1.89226 | 2.1 |
| 28812 | 1.89226 | 2.1 |
| 28813 | 1.89226 | 1.67257 |
| 28814 | 1.89226 | 0.262746 |
| 28815 | 3.78452 | 1.0622 |
| 28816 | 1.02975 | 2.1 |
| 28817 | 1.34355 | 2.08357 |
| 28818 | 1.01121 | 1.23446 |
| 28819 | 1.33079 | 0.996865 |
| 28820 | 2.35476 | 2.1 |
| 28821 | 1.24526 | 0.05 |
| 28822 | 2.36406 | 0.05 |
| 28823 | 2.81021 | 0.05 |

| | | |
|---|---|---|
| 28824 | 1.7237 | 0.05 |
| 28825 | 4.08776 | 0.05 |
| 28826 | 1.89226 | 2.1 |
| 28827 | 1.89226 | 2.1 |
| 28828 | 1.89226 | 2.1 |
| 28829 | 1.89226 | 2.1 |
| 28830 | 3.78453 | 2.1 |
| 28831 | 1.79028 | 2.1 |
| 28832 | 1.89238 | 2.1 |
| 28833 | 1.65533 | 2.1 |
| 28834 | 1.77821 | 2.1 |
| 28835 | 3.5477 | 2.1 |
| 28836 | 1.89238 | 2.1 |
| 28837 | 1.7465 | 0.05 |
| 28838 | 1.77821 | 2.1 |
| 28839 | 1.63557 | 0.05 |
| 28840 | 3.52795 | 0.598441 |
| 28841 | 1.89226 | 2.1 |
| 28842 | 1.89226 | 0.05 |
| 28843 | 1.89226 | 2.1 |
| 28844 | 1.89226 | 0.05 |
| 28845 | 3.78452 | 0.05 |
| 28846 | 1.89226 | 2.1 |
| 28847 | 1.89226 | 2.1 |
| 28848 | 1.89226 | 2.1 |
| 28849 | 1.89226 | 2.1 |
| 28850 | 3.78452 | 2.1 |
| 28851 | 1.65532 | 2.1 |
| 28852 | 1.77821 | 2.1 |
| 28853 | 1.53278 | 2.1 |
| 28854 | 1.67571 | 2.1 |
| 28855 | 3.33103 | 2.1 |
| 28856 | 1.77821 | 2.1 |
| 28857 | 1.63557 | 0.05 |
| 28858 | 1.67571 | 2.1 |
| 28859 | 1.52615 | 0.05 |
| 28860 | 3.31128 | 0.0777544 |
| 28861 | 1.89226 | 2.1 |
| 28862 | 1.89226 | 0.05 |
| 28863 | 1.89226 | 2.1 |
| 28864 | 1.89226 | 0.05 |
| 28865 | 3.78452 | 0.471887 |
| 28866 | 2.83129 | 2.1 |
| 28867 | 1.89226 | 0.05 |
| 28868 | 1.89226 | 0.875369 |
| 28869 | 2.6491 | 1.51938 |
| 28870 | 4.54136 | 0.782026 |
| 28871 | 2.83129 | 2.1 |
| 28872 | 1.47278 | 2.1 |
| 28873 | 1.3909 | 2.1 |
| 28874 | 2.6491 | 2.1 |
| 28875 | 4.12188 | 2.1 |
| 28876 | 1.89225 | 2.1 |
| 28877 | 2.65393 | 2.1 |
| 28878 | 1.89225 | 2.1 |
| 28879 | 2.69591 | 2.1 |
| 28880 | 4.58816 | 2.1 |
| 28881 | 1.91703 | 2.1 |

| | | |
|---|---|---|
| 28882 | 2.00209 | 2.1 |
| 28883 | 2.65393 | 2.1 |
| 28884 | 2.65393 | 2.1 |
| 28885 | 2.69591 | 2.1 |
| 28886 | 2.00209 | 2.1 |
| 28887 | 1.89226 | 2.1 |
| 28888 | 2.69593 | 2.1 |
| 28889 | 1.93967 | 2.1 |
| 28890 | 1.89226 | 2.1 |
| 28891 | 2.74334 | 2.1 |
| 28892 | 2.69593 | 2.1 |
| 28893 | 2.74335 | 2.1 |
| 28894 | 2.0021 | 2.1 |
| 28895 | 2.76367 | 2.1 |
| 28896 | 2.69593 | 2.1 |
| 28897 | 2.06984 | 2.1 |
| 28898 | 2.0021 | 2.1 |
| 28899 | 2.24481 | 2.1 |
| 28900 | 2.58921 | 2.1 |
| 28901 | 2.68958 | 2.1 |
| 28902 | 2.68958 | 2.1 |
| 28903 | 2.11984 | 2.1 |
| 28904 | 2.11984 | 2.1 |
| 28905 | 2.11984 | 2.1 |
| 28906 | 2.58921 | 2.1 |
| 28907 | 2.46488 | 2.1 |
| 28908 | 1.98339 | 2.1 |
| 28909 | 4.5726 | 2.1 |
| 28910 | 2.7777 | 2.1 |
| 28911 | 2.58921 | 2.1 |
| 28912 | 2.46488 | 2.1 |
| 28913 | 2.70401 | 2.1 |
| 28914 | 5.29322 | 2.1 |
| 28915 | 2.17741 | 2.1 |
| 28916 | 2.54197 | 2.1 |
| 28917 | 2.54018 | 2.1 |
| 28918 | 2.18608 | 2.1 |
| 28919 | 4.72805 | 2.1 |
| 28920 | 2.17742 | 2.1 |
| 28921 | 1.58054 | 2.1 |
| 28922 | 1.59169 | 2.1 |
| 28923 | 2.18608 | 2.1 |
| 28924 | 3.76662 | 2.1 |
| 28925 | 2.54018 | 2.1 |
| 28926 | 2.18608 | 2.1 |
| 28927 | 2.19104 | 2.1 |
| 28928 | 2.5392 | 2.1 |
| 28929 | 4.72528 | 2.1 |
| 28930 | 1.60497 | 2.1 |
| 28931 | 2.18608 | 2.1 |
| 28932 | 1.59169 | 2.1 |
| 28933 | 1.59169 | 2.1 |
| 28934 | 2.19105 | 2.1 |
| 28935 | 2.19105 | 2.1 |
| 28936 | 2.58661 | 1.82234 |
| 28937 | 1.06203 | 2.1 |
| 28938 | 2.7043 | 2.1 |
| 28939 | 2.21397 | 2.1 |

| | | |
|---|---|---|
| 28940 | 1.55236 | 2.1 |
| 28941 | 1.06203 | 2.1 |
| 28942 | 1.89226 | 1.39771 |
| 28943 | 1.89226 | 2.0692 |
| 28944 | 1.89226 | 0.832065 |
| 28945 | 1.89226 | 2.1 |
| 28946 | 3.78453 | 1.5033 |
| 28947 | 1.08195 | 1.63739 |
| 28948 | 0.990019 | 2.1 |
| 28949 | 1.31976 | 2.1 |
| 28950 | 1.4539 | 0.951948 |
| 28951 | 2.40171 | 2.1 |
| 28952 | 1.89226 | 1.10958 |
| 28953 | 1.89226 | 2.1 |
| 28954 | 1.89226 | 1.15902 |
| 28955 | 1.89226 | 0.607788 |
| 28956 | 3.78453 | 1.33074 |
| 28957 | 1.89226 | 1.84047 |
| 28958 | 1.89226 | 1.41627 |
| 28959 | 1.89226 | 0.959767 |
| 28960 | 1.89226 | 2.1 |
| 28961 | 3.78453 | 1.29591 |
| 28962 | 1.43895 | 2.1 |
| 28963 | 1.31976 | 2.1 |
| 28964 | 1.70066 | 0.752339 |
| 28965 | 1.33783 | 2.1 |
| 28966 | 1.43895 | 2.1 |
| 28967 | 1.07612 | 2.1 |
| 28968 | 0.65988 | 2.08379 |
| 28969 | 0.850329 | 1.49672 |
| 28970 | 0.919598 | 1.02007 |
| 28971 | 0.726952 | 1.79427 |
| 28972 | 0.788106 | 0.891428 |
| 28973 | 0.788106 | 2.1 |
| 28974 | 0.789175 | 2.1 |
| 28975 | 0.789175 | 0.389406 |
| 28976 | 0.850329 | 1.08678 |
| 28977 | 0.919598 | 1.0392 |
| 28978 | 0.659879 | 1.75922 |
| 28979 | 0.726952 | 1.46965 |
| 28980 | 1.34389 | 2.1 |
| 28981 | 1.41279 | 2.1 |
| 28982 | 1.15564 | 2.1 |
| 28983 | 1.15564 | 2.1 |
| 28984 | 1.55998 | 2.07872 |
| 28985 | 1.55998 | 2.1 |
| 28986 | 1.89226 | 1.76758 |
| 28987 | 1.89226 | 2.1 |
| 28988 | 1.89226 | 2.1 |
| 28989 | 1.89226 | 1.17895 |
| 28990 | 1.89226 | 2.1 |
| 28991 | 1.89226 | 1.26367 |
| 28992 | 1.55998 | 2.1 |
| 28993 | 1.41279 | 2.1 |
| 28994 | 1.72901 | 2.06755 |
| 28995 | 1.62414 | 2.1 |
| 28996 | 3.1418 | 2.1 |
| 28997 | 1.89226 | 1.78232 |

| | | |
|---|---|---|
| 28998 | 1.89226 | 2.1 |
| 28999 | 1.89226 | 2.1 |
| 29000 | 1.89226 | 1.00593 |
| 29001 | 3.78452 | 2.1 |
| 29002 | 1.89226 | 0.832184 |
| 29003 | 1.89226 | 2.1 |
| 29004 | 1.89226 | 1.75907 |
| 29005 | 1.89226 | 2.1 |
| 29006 | 1.89226 | 0.996452 |
| 29007 | 1.89226 | 1.28821 |
| 29008 | 1.62414 | 2.1 |
| 29009 | 1.01243 | 2.1 |
| 29010 | 1.70932 | 2.1 |
| 29011 | 1.41279 | 1.76392 |
| 29012 | 1.30896 | 2.1 |
| 29013 | 1.01243 | 1.40888 |
| 29014 | 1.9719 | 2.1 |
| 29015 | 1.10725 | 2.1 |
| 29016 | 1.12411 | 2.1 |
| 29017 | 1.98691 | 2.1 |
| 29018 | 3.09601 | 2.1 |
| 29019 | 1.97189 | 2.1 |
| 29020 | 2.47932 | 2.1 |
| 29021 | 2.49092 | 2.1 |
| 29022 | 1.98691 | 2.1 |
| 29023 | 4.46282 | 2.1 |
| 29024 | 1.1486 | 2.1 |
| 29025 | 1.98691 | 2.1 |
| 29026 | 1.12411 | 2.1 |
| 29027 | 1.12411 | 2.1 |
| 29028 | 2.01602 | 2.1 |
| 29029 | 2.01602 | 2.1 |
| 29030 | 2.49093 | 2.1 |
| 29031 | 1.98691 | 2.1 |
| 29032 | 2.01601 | 2.1 |
| 29033 | 2.51651 | 2.1 |
| 29034 | 4.50694 | 2.1 |
| 29035 | 1.93147 | 0.05 |
| 29036 | 2.51593 | 0.05 |
| 29037 | 1.35146 | 0.05 |
| 29038 | 1.35146 | 0.05 |
| 29039 | 2.48665 | 0.05 |
| 29040 | 1.90664 | 0.05 |
| 29041 | 1.63979 | 2.1 |
| 29042 | 1.7457 | 2.1 |
| 29043 | 1.66046 | 2.1 |
| 29044 | 1.56558 | 2.1 |
| 29045 | 3.31128 | 2.1 |
| 29046 | 0.819895 | 2.1 |
| 29047 | 0.782792 | 2.1 |
| 29048 | 0.60239 | 2.1 |
| 29049 | 0.54831 | 2.1 |
| 29050 | 0.688347 | 2.1 |
| 29051 | 0.688347 | 2.1 |
| 29052 | 0.679857 | 2.1 |
| 29053 | 0.679857 | 2.1 |
| 29054 | 0.782792 | 2.1 |
| 29055 | 0.54831 | 2.1 |

| | | |
|---|---|---|
| 29056 | 0.60239 | 2.1 |
| 29057 | 0.819895 | 2.1 |
| 29058 | 1.66046 | 2.1 |
| 29059 | 1.42485 | 2.1 |
| 29060 | 1.56558 | 2.1 |
| 29061 | 1.56558 | 2.1 |
| 29062 | 1.52995 | 2.1 |
| 29063 | 1.52995 | 2.1 |
| 29064 | 2.58101 | 0.05 |
| 29065 | 2.09803 | 2.1 |
| 29066 | 2.408 0.05 | |
| 29067 | 1.86893 | 2.1 |
| 29068 | 4.44994 | 0.0521795 |
| 29069 | 2.36062 | 2.1 |
| 29070 | 1.25104 | 2.1 |
| 29071 | 1.25104 | 2.1 |
| 29072 | 1.39703 | 2.1 |
| 29073 | 1.86893 | 2.1 |
| 29074 | 2.01492 | 2.1 |
| 29075 | 2.408 0.05 | |
| 29076 | 1.86893 | 2.1 |
| 29077 | 2.22575 | 0.05 |
| 29078 | 1.70677 | 2.1 |
| 29079 | 4.09468 | 0.05 |
| 29080 | 0.737605 | 2.1 |
| 29081 | 0.737605 | 2.1 |
| 29082 | 0.741302 | 2.1 |
| 29083 | 0.741302 | 2.1 |
| 29084 | 0.55189 | 2.1 |
| 29085 | 0.625517 | 2.1 |
| 29086 | 0.934468 | 2.1 |
| 29087 | 0.853389 | 2.1 |
| 29088 | 0.853389 | 2.1 |
| 29089 | 0.55189 | 2.1 |
| 29090 | 0.625517 | 2.1 |
| 29091 | 0.934467 | 2.1 |
| 29092 | 2.35053 | 2.1 |
| 29093 | 1.09615 | 2.1 |
| 29094 | 1.25998 | 2.1 |
| 29095 | 2.51216 | 2.1 |
| 29096 | 3.61051 | 2.1 |
| 29097 | 1.33636 | 2.1 |
| 29098 | 1.25609 | 2.1 |
| 29099 | 0.698455 | 2.1 |
| 29100 | 0.629995 | 2.1 |
| 29101 | 0.986092 | 2.1 |
| 29102 | 0.986092 | 2.1 |
| 29103 | 0.980263 | 2.1 |
| 29104 | 0.980263 | 2.1 |
| 29105 | 1.33636 | 2.1 |
| 29106 | 0.698455 | 2.1 |
| 29107 | 0.629995 | 2.1 |
| 29108 | 1.25609 | 2.1 |
| 29109 | 1.89226 | 0.948182 |
| 29110 | 1.89226 | 1.0082 |
| 29111 | 1.89226 | 1.0756 |
| 29112 | 1.89226 | 0.979028 |
| 29113 | 3.78452 | 0.94766 |

| | | |
|---|---|---|
| 29114 | 1.74321 | 0.881587 |
| 29115 | 1.89226 | 0.67328 |
| 29116 | 1.91755 | 0.975247 |
| 29117 | 1.91755 | 0.866053 |
| 29118 | 1.89226 | 0.872451 |
| 29119 | 1.89226 | 0.680604 |
| 29120 | 0.946132 | 0.937933 |
| 29121 | 0.946132 | 0.882378 |
| 29122 | 0.949798 | 0.453203 |
| 29123 | 1.08054 | 0.905148 |
| 29124 | 1.08054 | 0.643248 |
| 29125 | 1.08054 | 0.902635 |
| 29126 | 0.946132 | 0.888438 |
| 29127 | 0.946132 | 0.912802 |
| 29128 | 0.946132 | 0.887257 |
| 29129 | 0.949798 | 0.353398 |
| 29130 | 1.08054 | 0.795327 |
| 29131 | 0.946131 | 1.01227 |
| 29132 | 1.89226 | 1.03869 |
| 29133 | 1.89226 | 0.888195 |
| 29134 | 1.89226 | 0.855506 |
| 29135 | 1.89226 | 0.86993 |
| 29136 | 1.89226 | 0.954261 |
| 29137 | 1.89226 | 0.975329 |
| 29138 | 2.39378 | 0.05 |
| 29139 | 2.29234 | 0.05 |
| 29140 | 2.29234 | 0.05 |
| 29141 | 2.7933 | 0.05 |
| 29142 | 1.18422 | 0.05 |
| 29143 | 1.68518 | 0.05 |
| 29144 | 2.79329 | 0.05 |
| 29145 | 1.89226 | 0.05 |
| 29146 | 2.29233 | 0.05 |
| 29147 | 1.89226 | 0.05 |
| 29148 | 4.18459 | 0.05 |
| 29149 | 0.857046 | 0.05 |
| 29150 | 0.857037 | 0.05 |
| 29151 | 0.94613 | 0.05 |
| 29152 | 0.94613 | 0.05 |
| 29153 | 0.851539 | 0.05 |
| 29154 | 0.851539 | 0.05 |
| 29155 | 1.0315 | 0.05 |
| 29156 | 0.952867 | 0.05 |
| 29157 | 0.851486 | 0.05 |
| 29158 | 0.952869 | 0.05 |
| 29159 | 1.03156 | 0.05 |
| 29160 | 0.851528 | 0.05 |
| 29161 | 1.61981 | 2.1 |
| 29162 | 1.93096 | 2.1 |
| 29163 | 2.00776 | 2.1 |
| 29164 | 1.62218 | 2.1 |
| 29165 | 3.62757 | 2.1 |
| 29166 | 2.00776 | 2.1 |
| 29167 | 1.68541 | 2.1 |
| 29168 | 1.83215 | 2.1 |
| 29169 | 1.62218 | 2.1 |
| 29170 | 2.21773 | 2.1 |
| 29171 | 2.00776 | 2.1 |

| | | |
|---|---|---|
| 29172 | 1.89188 | 0.05 |
| 29173 | 1.70303 | 0.05 |
| 29174 | 1.89226 | 0.05 |
| 29175 | 1.72465 | 0.05 |
| 29176 | 1.89226 | 0.05 |
| 29177 | 1.70552 | 0.05 |
| 29178 | 2.48659 | 0.05 |
| 29179 | 2.0815 | 0.05 |
| 29180 | 1.89226 | 0.05 |
| 29181 | 2.08111 | 0.05 |
| 29182 | 1.89226 | 0.05 |
| 29183 | 2.08111 | 0.05 |
| 29184 | 2.35332 | 0.05 |
| 29185 | 1.83284 | 0.05 |
| 29186 | 2.28483 | 0.05 |
| 29187 | 2.71955 | 0.05 |
| 29188 | 4.63815 | 0.05 |
| 29189 | 1.89227 | 2.1 |
| 29190 | 1.89227 | 2.1 |
| 29191 | 1.89227 | 2.1 |
| 29192 | 1.89227 | 2.1 |
| 29193 | 3.78453 | 2.1 |
| 29194 | 1.33506 | 2.1 |
| 29195 | 1.17129 | 2.1 |
| 29196 | 1.33506 | 2.1 |
| 29197 | 1.38415 | 2.1 |
| 29198 | 1.13664 | 2.1 |
| 29199 | 1.18573 | 2.1 |
| 29200 | 1.89226 | 2.1 |
| 29201 | 2.68785 | 2.1 |
| 29202 | 1.89226 | 2.1 |
| 29203 | 2.6692 | 2.1 |
| 29204 | 4.58011 | 2.1 |
| 29205 | 1.89226 | 2.1 |
| 29206 | 1.89226 | 2.1 |
| 29207 | 1.89226 | 2.1 |
| 29208 | 1.89226 | 2.1 |
| 29209 | 3.78453 | 2.1 |
| 29210 | 1.33506 | 2.1 |
| 29211 | 1.13664 | 2.1 |
| 29212 | 1.27575 | 2.1 |
| 29213 | 1.09627 | 2.1 |
| 29214 | 2.43133 | 2.1 |
| 29215 | 1.89226 | 2.1 |
| 29216 | 2.65349 | 2.1 |
| 29217 | 2.66919 | 2.1 |
| 29218 | 2.66919 | 2.1 |
| 29219 | 1.89226 | 2.1 |
| 29220 | 1.89226 | 2.1 |
| 29221 | 1.89226 | 2.1 |
| 29222 | 1.89226 | 2.1 |
| 29223 | 1.89226 | 2.1 |
| 29224 | 1.89226 | 2.1 |
| 29225 | 3.78453 | 2.1 |
| 29226 | 1.16834 | 2.1 |
| 29227 | 1.16834 | 2.1 |
| 29228 | 0.946132 | 2.1 |
| 29229 | 0.946132 | 2.1 |

| | | |
|---|---|---|
| 29230 | 1.26978 | 2.1 |
| 29231 | 1.16834 | 2.1 |
| 29232 | 0.94613 | 2.1 |
| 29233 | 0.94613 | 2.1 |
| 29234 | 0.94613 | 2.1 |
| 29235 | 1.26978 | 2.1 |
| 29236 | 0.94613 | 2.1 |
| 29237 | 1.16834 | 2.1 |
| 29238 | 2.14698 | 2.1 |
| 29239 | 1.89226 | 2.1 |
| 29240 | 1.89226 | 2.1 |
| 29241 | 1.89226 | 2.1 |
| 29242 | 2.40719 | 2.1 |
| 29243 | 2.40719 | 2.1 |
| 29244 | 1.89226 | 2.1 |
| 29245 | 1.89226 | 2.1 |
| 29246 | 1.89226 | 2.1 |
| 29247 | 1.89226 | 2.1 |
| 29248 | 3.78453 | 2.1 |
| 29249 | 1.26977 | 2.1 |
| 29250 | 1.26977 | 2.1 |
| 29251 | 0.946133 | 2.1 |
| 29252 | 0.946133 | 2.1 |
| 29253 | 1.37467 | 2.1 |
| 29254 | 1.26978 | 2.1 |
| 29255 | 0.946131 | 2.1 |
| 29256 | 0.946131 | 2.1 |
| 29257 | 1.37467 | 2.1 |
| 29258 | 0.94613 | 2.1 |
| 29259 | 0.946131 | 2.1 |
| 29260 | 1.26978 | 2.1 |
| 29261 | 1.2036 | 2.1 |
| 29262 | 1.34912 | 2.1 |
| 29263 | 0.946131 | 2.1 |
| 29264 | 0.946131 | 2.1 |
| 29265 | 1.34912 | 2.1 |
| 29266 | 1.34912 | 2.1 |
| 29267 | 0.946129 | 2.1 |
| 29268 | 0.946129 | 2.1 |
| 29269 | 1.34912 | 2.1 |
| 29270 | 0.94613 | 2.1 |
| 29271 | 0.946131 | 2.1 |
| 29272 | 1.2036 | 2.1 |
| 29273 | 2.47164 | 0.0512843 |
| 29274 | 1.89019 | 0.05 |
| 29275 | 1.79109 | 0.05 |
| 29276 | 2.41632 | 0.05 |
| 29277 | 4.26273 | 0.05 |
| 29278 | 1.54197 | 0.05 |
| 29279 | 2.41632 | 0.05 |
| 29280 | 1.79109 | 0.05 |
| 29281 | 1.79109 | 0.05 |
| 29282 | 2.20929 | 0.05 |
| 29283 | 2.20929 | 0.05 |
| 29284 | 1.70302 | 0.497672 |
| 29285 | 1.48104 | 0.918108 |
| 29286 | 1.99365 | 1.16444 |
| 29287 | 1.32897 | 0.372273 |

| | | |
|---|---|---|
| 29288 | 1.70302 | 1.20704 |
| 29289 | 1.14034 | 0.389729 |
| 29290 | 1.70302 | 0.686285 |
| 29291 | 1.99365 | 0.585972 |
| 29292 | 1.99297 | 1.11642 |
| 29293 | 2.36337 | 0.147084 |
| 29294 | 4.04804 | 0.430725 |
| 29295 | 1.51569 | 0.252178 |
| 29296 | 2.49093 | 0.21368 |
| 29297 | 1.6026 | 0.33535 |
| 29298 | 1.43229 | 0.0752152 |
| 29299 | 2.0815 | 0.582579 |
| 29300 | 1.83402 | 0.124544 |
| 29301 | 2.54665 | 2.1 |
| 29302 | 2.34044 | 2.1 |
| 29303 | 2.08889 | 2.1 |
| 29304 | 2.37653 | 2.1 |
| 29305 | 4.71697 | 2.1 |
| 29306 | 2.54665 | 2.1 |
| 29307 | 2.45212 | 2.1 |
| 29308 | 2.31012 | 2.1 |
| 29309 | 2.37653 | 2.1 |
| 29310 | 4.82865 | 2.1 |
| 29311 | 2.08889 | 2.1 |
| 29312 | 2.37653 | 2.1 |
| 29313 | 2.23366 | 2.1 |
| 29314 | 1.87598 | 2.1 |
| 29315 | 4.25251 | 2.1 |
| 29316 | 2.31012 | 2.1 |
| 29317 | 2.37653 | 2.1 |
| 29318 | 2.23367 | 2.1 |
| 29319 | 2.17384 | 2.1 |
| 29320 | 4.55037 | 2.1 |
| 29321 | 0.946131 | 2.1 |
| 29322 | 0.946131 | 2.1 |
| 29323 | 1.30338 | 2.1 |
| 29324 | 1.23186 | 2.1 |
| 29325 | 0.94613 | 2.1 |
| 29326 | 0.94613 | 2.1 |
| 29327 | 1.23187 | 2.1 |
| 29328 | 1.23187 | 2.1 |
| 29329 | 0.946131 | 2.1 |
| 29330 | 1.23186 | 2.1 |
| 29331 | 1.30338 | 2.1 |
| 29332 | 0.946131 | 2.1 |
| 29333 | 1.89226 | 2.1 |
| 29334 | 2.46373 | 2.1 |
| 29335 | 2.34725 | 2.1 |
| 29336 | 2.46373 | 2.1 |
| 29337 | 1.77579 | 2.1 |
| 29338 | 1.89226 | 2.1 |
| 29339 | 2.61901 | 2.1 |
| 29340 | 2.45899 | 2.1 |
| 29341 | 2.64067 | 2.1 |
| 29342 | 2.52708 | 2.1 |
| 29343 | 5.14609 | 2.1 |
| 29344 | 2.41795 | 2.1 |
| 29345 | 2.0815 | 2.1 |

| | | |
|---|---|---|
| 29346 | 2.52708 | 2.1 |
| 29347 | 1.92277 | 2.1 |
| 29348 | 4.31368 | 2.1 |
| 29349 | 2.64067 | 2.1 |
| 29350 | 2.52708 | 2.1 |
| 29351 | 2.58638 | 2.1 |
| 29352 | 2.51039 | 2.1 |
| 29353 | 5.11346 | 2.1 |
| 29354 | 2.52708 | 2.1 |
| 29355 | 2.13642 | 2.1 |
| 29356 | 2.51039 | 2.1 |
| 29357 | 2.16633 | 2.1 |
| 29358 | 4.69341 | 2.1 |
| 29359 | 1.61028 | 1.43655 |
| 29360 | 1.65712 | 0.55174 |
| 29361 | 1.35002 | 0.0702681 |
| 29362 | 1.35002 | 1.48343 |
| 29363 | 1.88637 | 0.45729 |
| 29364 | 1.88637 | 2.1 |
| 29365 | 2.57663 | 2.1 |
| 29366 | 2.24227 | 2.1 |
| 29367 | 2.48354 | 2.1 |
| 29368 | 2.14317 | 2.1 |
| 29369 | 4.7198 | 2.1 |
| 29370 | 2.24227 | 2.1 |
| 29371 | 1.77353 | 2.1 |
| 29372 | 2.14317 | 2.1 |
| 29373 | 1.69956 | 2.1 |
| 29374 | 3.9167 | 2.1 |
| 29375 | 2.48353 | 2.1 |
| 29376 | 2.14317 | 2.1 |
| 29377 | 2.39282 | 2.1 |
| 29378 | 1.79824 | 2.1 |
| 29379 | 4.53599 | 2.1 |
| 29380 | 2.14317 | 2.1 |
| 29381 | 1.69956 | 2.1 |
| 29382 | 1.91427 | 2.1 |
| 29383 | 1.6347 | 2.1 |
| 29384 | 3.77787 | 2.1 |
| 29385 | 1.89226 | 0.944337 |
| 29386 | 1.89226 | 1.20153 |
| 29387 | 1.89226 | 1.22235 |
| 29388 | 1.89226 | 1.07644 |
| 29389 | 3.78453 | 2.00064 |
| 29390 | 1.89226 | 0.882899 |
| 29391 | 1.89226 | 0.839517 |
| 29392 | 1.89226 | 0.994915 |
| 29393 | 1.89226 | 0.987118 |
| 29394 | 3.78452 | 0.562206 |
| 29395 | 1.89226 | 0.819111 |
| 29396 | 1.89652 | 1.64071 |
| 29397 | 1.89226 | 0.157513 |
| 29398 | 1.89226 | 1.02321 |
| 29399 | 1.19352 | 1.0326 |
| 29400 | 1.19352 | 2.1 |
| 29401 | 1.89226 | 1.03719 |
| 29402 | 1.89653 | 0.586508 |
| 29403 | 1.89226 | 1.85857 |

| | | |
|---|---|---|
| 29404 | 1.89226 | 1.29224 |
| 29405 | 1.89226 | 0.891369 |
| 29406 | 1.89226 | 0.951989 |
| 29407 | 1.89226 | 1.62137 |
| 29408 | 1.75326 | 0.874309 |
| 29409 | 1.89226 | 2.1 |
| 29410 | 1.12948 | 1.80681 |
| 29411 | 1.89226 | 0.720149 |
| 29412 | 1.12948 | 0.601119 |
| 29413 | 2.61064 | 2.1 |
| 29414 | 1.89226 | 2.1 |
| 29415 | 1.89226 | 2.1 |
| 29416 | 1.89226 | 2.1 |
| 29417 | 2.6085 | 2.1 |
| 29418 | 2.6085 | 2.1 |
| 29419 | 2.6085 | 2.1 |
| 29420 | 1.89226 | 2.1 |
| 29421 | 1.89226 | 2.1 |
| 29422 | 1.89226 | 2.1 |
| 29423 | 2.62161 | 2.1 |
| 29424 | 2.62161 | 2.1 |
| 29425 | 2.16535 | 2.1 |
| 29426 | 1.44603 | 2.1 |
| 29427 | 2.79784 | 2.1 |
| 29428 | 1.84756 | 2.1 |
| 29429 | 4.01292 | 2.1 |
| 29430 | 2.65262 | 2.1 |
| 29431 | 1.03391 | 2.1 |
| 29432 | 2.20239 | 2.1 |
| 29433 | 1.74416 | 2.1 |
| 29434 | 1.49213 | 2.1 |
| 29435 | 1.03391 | 2.1 |
| 29436 | 2.29066 | 2.1 |
| 29437 | 2.49174 | 2.1 |
| 29438 | 2.56502 | 2.1 |
| 29439 | 2.33802 | 2.1 |
| 29440 | 4.85568 | 2.1 |
| 29441 | 2.39663 | 2.1 |
| 29442 | 2.49174 | 2.1 |
| 29443 | 2.56502 | 2.1 |
| 29444 | 2.45929 | 2.1 |
| 29445 | 4.96165 | 2.1 |
| 29446 | 2.56502 | 2.1 |
| 29447 | 2.33802 | 2.1 |
| 29448 | 2.37168 | 2.1 |
| 29449 | 2.59967 | 2.1 |
| 29450 | 4.9367 | 2.1 |
| 29451 | 2.56502 | 2.1 |
| 29452 | 2.45929 | 2.1 |
| 29453 | 2.50138 | 2.1 |
| 29454 | 2.59967 | 2.1 |
| 29455 | 5.0664 | 2.1 |
| 29456 | 2.27009 | 1.02872 |
| 29457 | 1.26247 | 2.1 |
| 29458 | 1.86812 | 2.1 |
| 29459 | 2.837 | 1.44186 |
| 29460 | 4.13822 | 2.1 |
| 29461 | 1.89226 | 0.190839 |

| | | |
|---|---|---|
| 29462 | 1.89226 | 2.1 |
| 29463 | 1.89226 | 2.1 |
| 29464 | 1.89226 | 2.1 |
| 29465 | 3.78453 | 2.1 |
| 29466 | 1.75835 | 2.1 |
| 29467 | 1.72866 | 2.1 |
| 29468 | 1.65313 | 2.1 |
| 29469 | 1.64822 | 2.1 |
| 29470 | 3.3818 | 2.1 |
| 29471 | 1.72866 | 2.1 |
| 29472 | 1.58401 | 2.1 |
| 29473 | 1.64822 | 2.1 |
| 29474 | 1.52441 | 2.1 |
| 29475 | 3.25307 | 2.1 |
| 29476 | 1.89226 | 2.1 |
| 29477 | 1.89226 | 2.1 |
| 29478 | 1.89226 | 2.1 |
| 29479 | 1.89226 | 2.1 |
| 29480 | 3.78452 | 2.1 |
| 29481 | 1.89226 | 2.1 |
| 29482 | 1.89226 | 2.1 |
| 29483 | 1.89226 | 0.114233 |
| 29484 | 1.89226 | 2.1 |
| 29485 | 3.78453 | 2.1 |
| 29486 | 1.65313 | 2.1 |
| 29487 | 1.64822 | 2.1 |
| 29488 | 1.55779 | 2.1 |
| 29489 | 1.57968 | 2.1 |
| 29490 | 3.23281 | 2.1 |
| 29491 | 1.64822 | 2.1 |
| 29492 | 1.52441 | 2.1 |
| 29493 | 1.57967 | 2.1 |
| 29494 | 1.474 2.1 | |
| 29495 | 3.10408 | 2.1 |
| 29496 | 1.89226 | 2.1 |
| 29497 | 1.89226 | 2.1 |
| 29498 | 1.89226 | 2.1 |
| 29499 | 1.89226 | 2.1 |
| 29500 | 3.78452 | 2.1 |
| 29501 | 1.86067 | 1.27964 |
| 29502 | 1.58407 | 2.1 |
| 29503 | 2.07965 | 0.899833 |
| 29504 | 1.83123 | 1.83054 |
| 29505 | 3.66372 | 1.11944 |
| 29506 | 1.89226 | 0.981241 |
| 29507 | 1.89226 | 2.1 |
| 29508 | 1.89226 | 1.11846 |
| 29509 | 1.89226 | 2.08891 |
| 29510 | 3.78452 | 1.38512 |
| 29511 | 1.89226 | 0.250458 |
| 29512 | 1.89226 | 2.1 |
| 29513 | 1.89226 | 0.12647 |
| 29514 | 1.89226 | 2.1 |
| 29515 | 1.89226 | 0.0620453 |
| 29516 | 1.89226 | 2.1 |
| 29517 | 1.83123 | 0.920664 |
| 29518 | 1.03472 | 2.1 |
| 29519 | 1.91068 | 2.09035 |

| | | |
|---|---|---|
| 29520 | 1.58406 | 1.11477 |
| 29521 | 1.36133 | 1.1846 |
| 29522 | 1.03472 | 0.353921 |
| 29523 | 2.07965 | 1.07641 |
| 29524 | 1.83123 | 1.95433 |
| 29525 | 2.28385 | 1.11719 |
| 29526 | 2.09114 | 1.44644 |
| 29527 | 4.1708 | 1.69016 |
| 29528 | 1.89226 | 1.40339 |
| 29529 | 1.89226 | 2.1 |
| 29530 | 1.89226 | 0.902946 |
| 29531 | 1.89226 | 2.0042 |
| 29532 | 3.78452 | 1.14414 |
| 29533 | 1.89226 | 0.717468 |
| 29534 | 1.89226 | 2.1 |
| 29535 | 1.89226 | 1.04771 |
| 29536 | 1.89226 | 2.1 |
| 29537 | 3.78452 | 1.26872 |
| 29538 | 1.83123 | 0.930048 |
| 29539 | 1.36133 | 2.1 |
| 29540 | 2.09114 | 0.501913 |
| 29541 | 1.69436 | 2.08895 |
| 29542 | 3.45248 | 1.17254 |
| 29543 | 1.89226 | 2.1 |
| 29544 | 1.89226 | 2.1 |
| 29545 | 1.89226 | 2.1 |
| 29546 | 1.89226 | 2.1 |
| 29547 | 3.78453 | 2.1 |
| 29548 | 1.53278 | 2.1 |
| 29549 | 1.67571 | 2.1 |
| 29550 | 1.40182 | 2.1 |
| 29551 | 1.55686 | 2.1 |
| 29552 | 3.07752 | 2.1 |
| 29553 | 1.67571 | 2.1 |
| 29554 | 1.52615 | 0.05 |
| 29555 | 1.55686 | 2.1 |
| 29556 | 1.38963 | 0.05 |
| 29557 | 3.06534 | 0.685619 |
| 29558 | 1.89226 | 2.1 |
| 29559 | 1.89226 | 0.05 |
| 29560 | 1.89226 | 2.1 |
| 29561 | 1.89226 | 0.05 |
| 29562 | 3.78452 | 0.106229 |
| 29563 | 1.89226 | 2.1 |
| 29564 | 1.89226 | 2.1 |
| 29565 | 1.89226 | 2.1 |
| 29566 | 1.89226 | 2.1 |
| 29567 | 3.78453 | 2.1 |
| 29568 | 1.40182 | 2.1 |
| 29569 | 1.55686 | 2.1 |
| 29570 | 1.24509 | 2.1 |
| 29571 | 1.38426 | 2.1 |
| 29572 | 2.78608 | 2.1 |
| 29573 | 1.55686 | 2.1 |
| 29574 | 1.38963 | 0.05 |
| 29575 | 1.2064 | 2.1 |
| 29576 | 1.2064 | 0.05 |
| 29577 | 1.38426 | 2.1 |

| | | |
|---|---|---|
| 29578 | 1.56749 | 0.05 |
| 29579 | 0.810946 | 2.1 |
| 29580 | 0.810946 | 0.05 |
| 29581 | 0.810945 | 2.1 |
| 29582 | 0.810945 | 2.1 |
| 29583 | 0.810945 | 1.59361 |
| 29584 | 0.810945 | 0.50436 |
| 29585 | 1.89226 | 0.05 |
| 29586 | 1.35169 | 0.061204 |
| 29587 | 1.08132 | 2.1 |
| 29588 | 1.08132 | 0.05 |
| 29589 | 1.08132 | 0.706615 |
| 29590 | 1.28418 | 0.1045 |
| 29591 | 1.59382 | 0.0503237 |
| 29592 | 1.78289 | 0.0657627 |
| 29593 | 1.51021 | 0.05 |
| 29594 | 3.10403 | 0.05 |
| 29595 | 1.76897 | 0.713472 |
| 29596 | 1.59382 | 0.05 |
| 29597 | 1.78289 | 0.0627571 |
| 29598 | 1.94302 | 0.078532 |
| 29599 | 3.53684 | 0.181382 |
| 29600 | 1.89226 | 0.05 |
| 29601 | 1.89226 | 0.05 |
| 29602 | 1.89226 | 0.05 |
| 29603 | 1.89226 | 0.05 |
| 29604 | 3.78453 | 0.05 |
| 29605 | 1.89227 | 0.126534 |
| 29606 | 1.89227 | 0.0543567 |
| 29607 | 1.89227 | 0.05 |
| 29608 | 1.89227 | 0.05 |
| 29609 | 1.89227 | 0.05 |
| 29610 | 1.89227 | 0.0946111 |
| 29611 | 1.78286 | 0.197195 |
| 29612 | 1.51019 | 0.05 |
| 29613 | 1.79979 | 0.05 |
| 29614 | 2.02511 | 0.05 |
| 29615 | 3.5353 | 0.0916329 |
| 29616 | 1.78286 | 0.105381 |
| 29617 | 1.94299 | 0.0599414 |
| 29618 | 2.17831 | 0.353985 |
| 29619 | 2.02511 | 0.05 |
| 29620 | 3.9681 | 0.05 |
| 29621 | 1.89224 | 0.05 |
| 29622 | 1.89224 | 0.05 |
| 29623 | 1.89224 | 0.05 |
| 29624 | 1.89224 | 0.140573 |
| 29625 | 3.78448 | 0.0648231 |
| 29626 | 1.89224 | 0.05 |
| 29627 | 1.89224 | 0.0502221 |
| 29628 | 1.89224 | 0.05 |
| 29629 | 1.89224 | 0.05 |
| 29630 | 1.89224 | 0.0847981 |
| 29631 | 1.89224 | 0.123609 |
| 29632 | 1.83198 | 0.168359 |
| 29633 | 1.0961 | 0.05 |
| 29634 | 1.39448 | 0.138237 |
| 29635 | 1.99568 | 0.0592144 |

| | | |
|---|---|---|
| 29636 | 3.09178 | 0.196486 |
| 29637 | 1.25503 | 0.05 |
| 29638 | 1.99568 | 0.05 |
| 29639 | 2.3661 | 0.0922907 |
| 29640 | 1.70302 | 0.05 |
| 29641 | 3.55833 | 0.05 |
| 29642 | 2.69899 | 0.05 |
| 29643 | 1.85796 | 0.05 |
| 29644 | 2.11407 | 0.05 |
| 29645 | 1.36769 | 0.05 |
| 29646 | 3.97203 | 0.05 |
| 29647 | 1.89226 | 0.05 |
| 29648 | 1.89226 | 0.05 |
| 29649 | 1.89226 | 0.05 |
| 29650 | 1.89226 | 0.05 |
| 29651 | 3.78452 | 0.05 |
| 29652 | 1.89225 | 0.05 |
| 29653 | 2.63355 | 0.05 |
| 29654 | 2.09529 | 0.05 |
| 29655 | 2.09529 | 0.05 |
| 29656 | 1.89225 | 0.05 |
| 29657 | 1.89225 | 0.05 |
| 29658 | 1.89226 | 0.05 |
| 29659 | 1.89226 | 0.05 |
| 29660 | 1.89226 | 0.05 |
| 29661 | 1.89226 | 0.05 |
| 29662 | 1.89226 | 0.05 |
| 29663 | 1.89226 | 0.05 |
| 29664 | 2.12204 | 0.05 |
| 29665 | 1.89226 | 0.05 |
| 29666 | 2.05798 | 0.05 |
| 29667 | 2.01954 | 0.05 |
| 29668 | 1.70302 | 0.05 |
| 29669 | 1.3865 | 0.05 |
| 29670 | 1.28326 | 0.0687266 |
| 29671 | 1.33326 | 0.36663 |
| 29672 | 1.19052 | 0.05 |
| 29673 | 1.25369 | 0.532556 |
| 29674 | 2.53694 | 0.131439 |
| 29675 | 1.33327 | 0.05 |
| 29676 | 1.27339 | 2.1 |
| 29677 | 1.25369 | 0.05 |
| 29678 | 1.19733 | 2.1 |
| 29679 | 2.52708 | 0.0662196 |
| 29680 | 1.19052 | 0.05 |
| 29681 | 1.25369 | 0.396263 |
| 29682 | 1.10938 | 0.05 |
| 29683 | 1.17614 | 0.0695662 |
| 29684 | 2.36307 | 0.05 |
| 29685 | 1.25369 | 0.05 |
| 29686 | 1.19733 | 2.1 |
| 29687 | 1.17614 | 0.05 |
| 29688 | 1.11868 | 2.1 |
| 29689 | 2.37237 | 1.55065 |
| 29690 | 1.10747 | 0.05 |
| 29691 | 1.47688 | 0.05 |
| 29692 | 1.97732 | 0.05 |
| 29693 | 1.62206 | 0.05 |

| | | |
|---|---|---|
| 29694 | 3.09894 | 0.05 |
| 29695 | 1.89226 | 0.05 |
| 29696 | 1.89226 | 0.05 |
| 29697 | 1.89226 | 0.05 |
| 29698 | 1.89226 | 0.05 |
| 29699 | 1.89226 | 0.05 |
| 29700 | 1.89226 | 0.05 |
| 29701 | 1.62206 | 0.05 |
| 29702 | 1.70302 | 0.05 |
| 29703 | 1.22962 | 0.05 |
| 29704 | 1.1893 | 0.05 |
| 29705 | 2.15177 | 0.05 |
| 29706 | 2.08121 | 0.05 |
| 29707 | 1.97732 | 0.05 |
| 29708 | 2.08121 | 0.05 |
| 29709 | 1.62206 | 0.05 |
| 29710 | 1.62206 | 0.05 |
| 29711 | 2.41464 | 0.05 |
| 29712 | 2.41464 | 0.05 |
| 29713 | 1.89226 | 0.05 |
| 29714 | 1.89226 | 0.05 |
| 29715 | 1.89226 | 0.05 |
| 29716 | 1.89226 | 0.05 |
| 29717 | 3.78452 | 0.05 |
| 29718 | 0.946133 | 0.05 |
| 29719 | 0.946133 | 0.05 |
| 29720 | 0.946132 | 0.05 |
| 29721 | 0.946132 | 0.05 |
| 29722 | 0.946132 | 0.05 |
| 29723 | 0.946132 | 0.05 |
| 29724 | 0.946133 | 0.05 |
| 29725 | 0.946133 | 0.05 |
| 29726 | 0.946132 | 0.05 |
| 29727 | 0.946132 | 0.05 |
| 29728 | 0.946132 | 0.05 |
| 29729 | 0.946132 | 0.05 |
| 29730 | 1.83157 | 0.05 |
| 29731 | 2.19989 | 0.0606531 |
| 29732 | 2.20664 | 0.05 |
| 29733 | 1.81487 | 0.05 |
| 29734 | 4.03146 | 0.05 |
| 29735 | 2.41055 | 2.1 |
| 29736 | 2.19989 | 2.1 |
| 29737 | 2.20665 | 1.9205 |
| 29738 | 2.38023 | 1.35754 |
| 29739 | 4.61044 | 1.49381 |
| 29740 | 2.19989 | 2.1 |
| 29741 | 2.22558 | 2.1 |
| 29742 | 2.38023 | 1.98322 |
| 29743 | 2.07065 | 0.305681 |
| 29744 | 4.42547 | 1.24844 |
| 29745 | 2.19988 | 0.222375 |
| 29746 | 1.70303 | 0.05 |
| 29747 | 1.89939 | 0.05 |
| 29748 | 1.86357 | 0.05 |
| 29749 | 3.78536 | 0.05 |
| 29750 | 1.04921 | 2.1 |
| 29751 | 1.30711 | 2.1 |

| | | |
|---|---|---|
| 29752 | 1.53076 | 2.1 |
| 29753 | 1.22927 | 2.1 |
| 29754 | 2.57997 | 2.1 |
| 29755 | 1.53076 | 2.1 |
| 29756 | 1.46568 | 2.1 |
| 29757 | 1.56056 | 2.1 |
| 29758 | 1.22927 | 2.1 |
| 29759 | 1.86206 | 2.1 |
| 29760 | 1.53076 | 2.1 |
| 29761 | 1.18353 | 2.1 |
| 29762 | 2.28593 | 2.1 |
| 29763 | 1.16979 | 2.1 |
| 29764 | 2.2731 | 2.1 |
| 29765 | 3.45571 | 2.1 |
| 29766 | 0.854776 | 2.1 |
| 29767 | 0.854776 | 2.1 |
| 29768 | 0.855641 | 2.1 |
| 29769 | 0.855641 | 2.1 |
| 29770 | 1.12552 | 2.1 |
| 29771 | 1.13655 | 2.1 |
| 29772 | 0.57216 | 2.1 |
| 29773 | 0.584893 | 2.1 |
| 29774 | 0.57216 | 2.1 |
| 29775 | 1.12552 | 2.1 |
| 29776 | 0.584893 | 2.1 |
| 29777 | 1.13655 | 2.1 |
| 29778 | 1.54462 | 2.1 |
| 29779 | 2.76309 | 2.1 |
| 29780 | 1.34869 | 2.1 |
| 29781 | 2.48573 | 2.1 |
| 29782 | 4.03035 | 2.1 |
| 29783 | 1.35328 | 1.07927 |
| 29784 | 1.0383 | 0.962608 |
| 29785 | 1.03318 | 1.79742 |
| 29786 | 0.704834 | 1.57723 |
| 29787 | 1.0257 | 1.54731 |
| 29788 | 1.0257 | 1.01604 |
| 29789 | 1.03241 | 0.85025 |
| 29790 | 1.03241 | 1.99785 |
| 29791 | 1.35328 | 1.24904 |
| 29792 | 1.03319 | 2.00737 |
| 29793 | 1.0383 | 1.6178 |
| 29794 | 0.704835 | 1.37035 |
| 29795 | 2.27126 | 0.05 |
| 29796 | 2.77291 | 2.1 |
| 29797 | 1.81771 | 0.340977 |
| 29798 | 2.6111 | 0.05 |
| 29799 | 4.88236 | 0.224967 |
| 29800 | 2.77291 | 1.80323 |
| 29801 | 1.89227 | 0.171114 |
| 29802 | 1.89227 | 0.225961 |
| 29803 | 1.89227 | 2.1 |
| 29804 | 2.6111 | 0.05 |
| 29805 | 2.6111 | 2.1 |
| 29806 | 2.22061 | 0.0822481 |
| 29807 | 2.41522 | 0.0606429 |
| 29808 | 2.41522 | 0.05 |
| 29809 | 2.6111 | 1.55151 |

| | | |
|---|---|---|
| 29810 | 1.75765 | 0.05 |
| 29811 | 1.95353 | 2.1 |
| 29812 | 2.27502 | 0.124749 |
| 29813 | 1.89227 | 1.47083 |
| 29814 | 1.89227 | 0.11129 |
| 29815 | 2.8189 | 0.05 |
| 29816 | 4.71116 | 0.0547719 |
| 29817 | 1.89226 | 0.05 |
| 29818 | 1.89226 | 1.67615 |
| 29819 | 1.89226 | 0.185645 |
| 29820 | 1.89226 | 0.05 |
| 29821 | 1.89226 | 1.03009 |
| 29822 | 1.89226 | 0.24092 |
| 29823 | 1.89226 | 2.04073 |
| 29824 | 1.89226 | 1.92124 |
| 29825 | 1.89226 | 1.44256 |
| 29826 | 1.89226 | 1.12249 |
| 29827 | 3.78453 | 1.99769 |
| 29828 | 1.89226 | 1.88451 |
| 29829 | 2.57079 | 0.827384 |
| 29830 | 2.5932 | 1.1909 |
| 29831 | 1.89226 | 1.10494 |
| 29832 | 4.48546 | 1.01433 |
| 29833 | 0.589396 | 1.20288 |
| 29834 | 0.641454 | 0.922945 |
| 29835 | 0.814062 | 1.52602 |
| 29836 | 0.726518 | 1.2389 |
| 29837 | 0.675126 | 1.44156 |
| 29838 | 0.675125 | 1.32525 |
| 29839 | 0.692847 | 1.53071 |
| 29840 | 0.692847 | 1.23795 |
| 29841 | 0.641454 | 1.0177 |
| 29842 | 0.814062 | 1.4004 |
| 29843 | 0.726518 | 1.61705 |
| 29844 | 0.589396 | 1.22955 |
| 29845 | 1.89226 | 1.60914 |
| 29846 | 1.89226 | 1.12752 |
| 29847 | 1.89226 | 1.58022 |
| 29848 | 1.89226 | 2.09572 |
| 29849 | 1.89226 | 1.26789 |
| 29850 | 1.89226 | 2.08139 |
| 29851 | 1.16906 | 0.335739 |
| 29852 | 1.2966 | 1.1124 |
| 29853 | 0.946132 | 1.02469 |
| 29854 | 0.946132 | 1.93663 |
| 29855 | 1.16906 | 1.07245 |
| 29856 | 1.16906 | 0.604275 |
| 29857 | 0.946132 | 1.17866 |
| 29858 | 0.946132 | 1.28989 |
| 29859 | 1.16906 | 1.64046 |
| 29860 | 0.946131 | 1.34328 |
| 29861 | 1.2966 | 1.11298 |
| 29862 | 0.946132 | 1.97418 |
| 29863 | 0.641455 | 1.22386 |
| 29864 | 0.567191 | 0.904611 |
| 29865 | 0.807594 | 1.61736 |
| 29866 | 0.814062 | 1.28232 |
| 29867 | 0.673735 | 1.66147 |

| | | |
|---|---|---|
| 29868 | 0.673734 | 1.03518 |
| 29869 | 0.707519 | 1.24404 |
| 29870 | 0.707519 | 1.81435 |
| 29871 | 0.807594 | 1.46364 |
| 29872 | 0.56719 | 1.99289 |
| 29873 | 0.641455 | 1.48654 |
| 29874 | 0.814063 | 2.02459 |
| 29875 | 1.89225 | 2.1 |
| 29876 | 1.89225 | 2.1 |
| 29877 | 1.89225 | 2.1 |
| 29878 | 1.89225 | 2.1 |
| 29879 | 3.7845 | 2.1 |
| 29880 | 1.89225 | 2.1 |
| 29881 | 1.89225 | 2.1 |
| 29882 | 1.89225 | 2.1 |
| 29883 | 1.89225 | 2.1 |
| 29884 | 1.89225 | 2.1 |
| 29885 | 1.89225 | 2.1 |
| 29886 | 1.89226 | 2.1 |
| 29887 | 1.89226 | 2.1 |
| 29888 | 1.89226 | 2.1 |
| 29889 | 1.89226 | 2.1 |
| 29890 | 3.78452 | 2.1 |
| 29891 | 1.89226 | 2.1 |
| 29892 | 1.89226 | 2.1 |
| 29893 | 1.89226 | 2.1 |
| 29894 | 1.89226 | 2.1 |
| 29895 | 1.89226 | 2.1 |
| 29896 | 1.89226 | 2.1 |
| 29897 | 1.89226 | 2.1 |
| 29898 | 1.89226 | 2.1 |
| 29899 | 1.89226 | 2.1 |
| 29900 | 1.89226 | 2.1 |
| 29901 | 3.78452 | 2.1 |
| 29902 | 1.89226 | 2.1 |
| 29903 | 1.38928 | 2.1 |
| 29904 | 1.89226 | 2.1 |
| 29905 | 1.51378 | 2.1 |
| 29906 | 1.89061 | 2.1 |
| 29907 | 1.89226 | 2.1 |
| 29908 | 1.89226 | 2.1 |
| 29909 | 1.89226 | 2.1 |
| 29910 | 1.89226 | 2.1 |
| 29911 | 1.89226 | 2.1 |
| 29912 | 3.78453 | 2.1 |
| 29913 | 1.04572 | 2.1 |
| 29914 | 0.994213 | 2.1 |
| 29915 | 0.912466 | 2.1 |
| 29916 | 0.912466 | 2.1 |
| 29917 | 0.947743 | 2.1 |
| 29918 | 1.00388 | 2.1 |
| 29919 | 0.900913 | 2.1 |
| 29920 | 0.900913 | 2.1 |
| 29921 | 0.900915 | 2.1 |
| 29922 | 0.946131 | 2.1 |
| 29923 | 0.946131 | 2.1 |
| 29924 | 0.999196 | 2.1 |
| 29925 | 1.89226 | 2.1 |

| | | |
|---|---|---|
| 29926 | 1.89226 | 2.1 |
| 29927 | 1.89226 | 2.1 |
| 29928 | 1.89226 | 2.1 |
| 29929 | 3.78453 | 2.1 |
| 29930 | 0.946161 | 2.1 |
| 29931 | 1.1354 | 2.1 |
| 29932 | 0.850847 | 2.1 |
| 29933 | 0.850847 | 2.1 |
| 29934 | 1.01445 | 2.1 |
| 29935 | 1.02106 | 2.1 |
| 29936 | 0.946131 | 2.1 |
| 29937 | 0.946131 | 2.1 |
| 29938 | 1.13537 | 2.1 |
| 29939 | 1.01444 | 2.1 |
| 29940 | 0.850877 | 2.1 |
| 29941 | 0.946131 | 2.1 |
| 29942 | 1.80183 | 2.1 |
| 29943 | 1.89226 | 2.1 |
| 29944 | 1.89226 | 2.1 |
| 29945 | 1.89226 | 2.1 |
| 29946 | 1.79606 | 2.1 |
| 29947 | 1.79606 | 2.1 |
| 29948 | 1.24001 | 2.1 |
| 29949 | 1.41822 | 1.85335 |
| 29950 | 1.95665 | 0.92192 |
| 29951 | 1.77342 | 1.63328 |
| 29952 | 3.19666 | 1.24549 |
| 29953 | 1.95665 | 1.24646 |
| 29954 | 1.44859 | 2.1 |
| 29955 | 1.41821 | 2.06563 |
| 29956 | 1.41821 | 2.09615 |
| 29957 | 1.95543 | 1.23637 |
| 29958 | 1.95543 | 1.69351 |
| 29959 | 1.95665 | 1.08746 |
| 29960 | 1.77342 | 1.69446 |
| 29961 | 2.33611 | 1.41925 |
| 29962 | 2.49694 | 0.902934 |
| 29963 | 4.29276 | 1.95153 |
| 29964 | 1.95665 | 1.72617 |
| 29965 | 1.95544 | 1.82586 |
| 29966 | 2.4728 | 0.793894 |
| 29967 | 2.49693 | 1.42479 |
| 29968 | 4.42945 | 1.18458 |
| 29969 | 2.24937 | 2.1 |
| 29970 | 2.49053 | 2.1 |
| 29971 | 2.16323 | 2.1 |
| 29972 | 2.36377 | 2.1 |
| 29973 | 4.61314 | 2.1 |
| 29974 | 2.49053 | 2.1 |
| 29975 | 2.58639 | 2.1 |
| 29976 | 2.36377 | 2.1 |
| 29977 | 2.47395 | 2.1 |
| 29978 | 4.95016 | 2.1 |
| 29979 | 2.45054 | 2.1 |
| 29980 | 2.36377 | 2.1 |
| 29981 | 1.92911 | 2.1 |
| 29982 | 2.181 2.1 | |
| 29983 | 4.29288 | 2.1 |

| | | |
|---|---|---|
| 29984 | 2.36377 | 2.1 |
| 29985 | 2.47395 | 2.1 |
| 29986 | 2.181 | 2.1 |
| 29987 | 2.29881 | 2.1 |
| 29988 | 4.66258 | 2.1 |
| 29989 | 1.57885 | 2.1 |
| 29990 | 1.13339 | 2.1 |
| 29991 | 1.48594 | 2.1 |
| 29992 | 1.13322 | 2.1 |
| 29993 | 2.71207 | 2.1 |
| 29994 | 1.48596 | 2.1 |
| 29995 | 1.20761 | 2.1 |
| 29996 | 1.20761 | 2.1 |
| 29997 | 1.13323 | 2.1 |
| 29998 | 1.46568 | 2.1 |
| 29999 | 1.3913 | 2.1 |
| 30000 | 1.89226 | 0.579471 |
| 30001 | 1.89226 | 1.44503 |
| 30002 | 1.89226 | 0.674057 |
| 30003 | 1.89226 | 1.18365 |
| 30004 | 1.89226 | 0.535232 |
| 30005 | 1.89226 | 0.735006 |
| 30006 | 1.11712 | 0.964414 |
| 30007 | 0.946129 | 1.18344 |
| 30008 | 0.686934 | 0.750036 |
| 30009 | 0.946133 | 0.755337 |
| 30010 | 0.9461 | 0.938993 |
| 30011 | 1.10729 | 2.1 |
| 30012 | 0.686963 | 1.32385 |
| 30013 | 0.686963 | 0.57687 |
| 30014 | 1.10945 | 1.04256 |
| 30015 | 0.80397 | 2.1 |
| 30016 | 0.946131 | 0.75727 |
| 30017 | 0.946131 | 1.35029 |
| 30018 | 1.89226 | 2.1 |
| 30019 | 1.89226 | 1.69712 |
| 30020 | 1.89226 | 1.10807 |
| 30021 | 1.89226 | 2.1 |
| 30022 | 3.78452 | 1.93144 |
| 30023 | 1.22615 | 2.1 |
| 30024 | 1.3439 | 2.1 |
| 30025 | 1.47868 | 2.1 |
| 30026 | 1.55998 | 1.99565 |
| 30027 | 2.78613 | 2.1 |
| 30028 | 1.04973 | 2.1 |
| 30029 | 1.47868 | 2.1 |
| 30030 | 1.20149 | 2.1 |
| 30031 | 1.61721 | 2.1 |
| 30032 | 2.68017 | 2.1 |
| 30033 | 1.89226 | 2.1 |
| 30034 | 1.89226 | 2.1 |
| 30035 | 1.89226 | 2.1 |
| 30036 | 1.89226 | 0.754258 |
| 30037 | 3.78452 | 2.1 |
| 30038 | 0.946133 | 0.757427 |
| 30039 | 0.946133 | 2.1 |
| 30040 | 0.946128 | 2.1 |
| 30041 | 0.946128 | 2.1 |

| | | |
|---|---|---|
| 30042 | 0.946131 | 0.692593 |
| 30043 | 0.946131 | 1.9131 |
| 30044 | 0.946131 | 2.1 |
| 30045 | 0.946131 | 0.846579 |
| 30046 | 0.94613 | 2.1 |
| 30047 | 0.94613 | 1.42524 |
| 30048 | 0.946131 | 2.1 |
| 30049 | 0.946131 | 1.20774 |
| 30050 | 0.798126 | 2.1 |
| 30051 | 0.798126 | 2.1 |
| 30052 | 0.805719 | 2.1 |
| 30053 | 0.805719 | 2.1 |
| 30054 | 0.779992 | 2.1 |
| 30055 | 0.864507 | 2.1 |
| 30056 | 0.739338 | 2.1 |
| 30057 | 0.808607 | 2.1 |
| 30058 | 0.808606 | 2.1 |
| 30059 | 0.864506 | 2.1 |
| 30060 | 0.739339 | 2.1 |
| 30061 | 0.779993 | 2.1 |
| 30062 | 0.946128 | 2.1 |
| 30063 | 0.946128 | 2.1 |
| 30064 | 1.18959 | 1.01336 |
| 30065 | 1.00304 | 2.1 |
| 30066 | 1.00304 | 2.1 |
| 30067 | 1.00304 | 2.1 |
| 30068 | 0.94613 | 2.1 |
| 30069 | 0.94613 | 1.13712 |
| 30070 | 0.94613 | 2.1 |
| 30071 | 1.18959 | 2.1 |
| 30072 | 1.00304 | 2.1 |
| 30073 | 0.94613 | 2.1 |
| 30074 | 2.19163 | 2.1 |
| 30075 | 1.89226 | 2.1 |
| 30076 | 2.16541 | 2.1 |
| 30077 | 1.89226 | 1.27031 |
| 30078 | 4.08389 | 2.1 |
| 30079 | 1.89226 | 2.1 |
| 30080 | 1.89226 | 1.56827 |
| 30081 | 1.89226 | 2.1 |
| 30082 | 1.89226 | 1.092 |
| 30083 | 3.78452 | 2.1 |
| 30084 | 0.964782 | 2.1 |
| 30085 | 1.30151 | 1.02507 |
| 30086 | 1.30318 | 1.02404 |
| 30087 | 1.30318 | 2.1 |
| 30088 | 0.949134 | 0.912082 |
| 30089 | 0.949134 | 2.1 |
| 30090 | 2.15005 | 2.1 |
| 30091 | 1.89226 | 2.04073 |
| 30092 | 2.16541 | 2.1 |
| 30093 | 2.16541 | 2.1 |
| 30094 | 1.89226 | 1.00811 |
| 30095 | 1.89226 | 2.1 |
| 30096 | 1.89226 | 2.1 |
| 30097 | 1.89226 | 2.1 |
| 30098 | 1.89226 | 2.1 |
| 30099 | 1.89226 | 0.692401 |

| | | |
|---|---|---|
| 30100 | 3.78452 | 1.29564 |
| 30101 | 0.949134 | 2.1 |
| 30102 | 1.30151 | 2.1 |
| 30103 | 0.949134 | 2.1 |
| 30104 | 1.32143 | 1.05972 |
| 30105 | 2.25064 | 2.1 |
| 30106 | 0.982859 | 0.05 |
| 30107 | 1.28326 | 0.126418 |
| 30108 | 1.19052 | 0.05 |
| 30109 | 1.19052 | 0.372339 |
| 30110 | 1.10476 | 0.05 |
| 30111 | 1.10476 | 0.372685 |
| 30112 | 1.37265 | 0.404516 |
| 30113 | 1.38247 | 0.565853 |
| 30114 | 1.36636 | 1.16025 |
| 30115 | 1.3294 | 0.0990248 |
| 30116 | 2.71187 | 0.160224 |
| 30117 | 1.89226 | 0.980199 |
| 30118 | 1.89226 | 0.05 |
| 30119 | 1.89226 | 0.148343 |
| 30120 | 1.89226 | 0.0849568 |
| 30121 | 1.89226 | 0.661998 |
| 30122 | 1.89226 | 0.852785 |
| 30123 | 1.36636 | 1.58396 |
| 30124 | 1.3294 | 0.05 |
| 30125 | 1.30001 | 0.34157 |
| 30126 | 1.36416 | 1.08002 |
| 30127 | 2.69356 | 0.677427 |
| 30128 | 1.89226 | 1.2468 |
| 30129 | 1.89226 | 0.0603271 |
| 30130 | 1.89226 | 0.243463 |
| 30131 | 1.89226 | 0.856285 |
| 30132 | 1.89226 | 0.09988 |
| 30133 | 1.89226 | 0.351179 |
| 30134 | 2.83701 | 0.677003 |
| 30135 | 1.89226 | 0.728279 |
| 30136 | 1.89226 | 1.08716 |
| 30137 | 2.79312 | 0.135828 |
| 30138 | 4.68539 | 0.173494 |
| 30139 | 1.44288 | 2.1 |
| 30140 | 0.948677 | 2.1 |
| 30141 | 1.84076 | 2.1 |
| 30142 | 1.42884 | 2.1 |
| 30143 | 2.78943 | 2.1 |
| 30144 | 1.89226 | 2.1 |
| 30145 | 1.89226 | 2.1 |
| 30146 | 1.89226 | 2.1 |
| 30147 | 1.89226 | 2.1 |
| 30148 | 3.78452 | 2.1 |
| 30149 | 0.909402 | 2.1 |
| 30150 | 0.909402 | 2.1 |
| 30151 | 1.3063 | 2.1 |
| 30152 | 1.56757 | 2.1 |
| 30153 | 1.00038 | 2.1 |
| 30154 | 1.5235 | 2.1 |
| 30155 | 0.946127 | 2.1 |
| 30156 | 0.946127 | 2.1 |
| 30157 | 0.946128 | 2.1 |

| | | |
|---|---|---|
| 30158 | 1.26957 | 2.1 |
| 30159 | 1.00038 | 2.1 |
| 30160 | 0.946129 | 2.1 |
| 30161 | 1.17256 | 2.1 |
| 30162 | 2.2846 | 2.1 |
| 30163 | 1.1844 | 2.1 |
| 30164 | 2.28904 | 2.1 |
| 30165 | 3.469 2.1 | |
| 30166 | 0.867886 | 2.1 |
| 30167 | 0.867886 | 2.1 |
| 30168 | 0.867277 | 2.1 |
| 30169 | 0.867277 | 2.1 |
| 30170 | 1.14296 | 2.1 |
| 30171 | 1.14452 | 2.1 |
| 30172 | 0.591766 | 2.1 |
| 30173 | 0.592199 | 2.1 |
| 30174 | 0.591766 | 2.1 |
| 30175 | 1.14296 | 2.1 |
| 30176 | 0.5922 | 2.1 |
| 30177 | 1.14452 | 2.1 |
| 30178 | 1.27408 | 0.05 |
| 30179 | 1.56339 | 0.05 |
| 30180 | 1.52522 | 0.05 |
| 30181 | 1.34447 | 0.05 |
| 30182 | 2.90786 | 0.05 |
| 30183 | 1.27408 | 0.05 |
| 30184 | 1.01121 | 0.05 |
| 30185 | 0.991868 | 0.05 |
| 30186 | 1.34447 | 0.05 |
| 30187 | 2.35569 | 0.05 |
| 30188 | 0.944818 | 0.0607864 |
| 30189 | 0.883008 | 0.0507612 |
| 30190 | 0.66957 | 0.05 |
| 30191 | 0.616883 | 0.05 |
| 30192 | 0.755679 | 0.0522769 |
| 30193 | 0.755679 | 0.0563762 |
| 30194 | 0.791912 | 0.109145 |
| 30195 | 0.701943 | 0.05 |
| 30196 | 0.901541 | 0.05 |
| 30197 | 0.634847 | 0.05 |
| 30198 | 0.82781 | 0.0625645 |
| 30199 | 0.655405 | 0.0503639 |
| 30200 | 1.73819 | 2.1 |
| 30201 | 1.70303 | 2.1 |
| 30202 | 1.70303 | 2.1 |
| 30203 | 1.70303 | 2.1 |
| 30204 | 1.94516 | 2.1 |
| 30205 | 1.94517 | 2.1 |
| 30206 | 0.94994 | 2.1 |
| 30207 | 2.07469 | 2.1 |
| 30208 | 1.08506 | 2.1 |
| 30209 | 2.0815 | 2.1 |
| 30210 | 3.12902 | 2.1 |
| 30211 | 0.946131 | 2.1 |
| 30212 | 0.946131 | 2.1 |
| 30213 | 0.946131 | 2.1 |
| 30214 | 0.946131 | 2.1 |
| 30215 | 0.946128 | 2.1 |

| | | |
|---|---|---|
| 30216 | 0.946128 | 2.1 |
| 30217 | 0.946133 | 2.1 |
| 30218 | 0.946133 | 2.1 |
| 30219 | 0.946131 | 2.1 |
| 30220 | 0.946131 | 2.1 |
| 30221 | 0.94613 | 2.1 |
| 30222 | 0.94613 | 2.1 |
| 30223 | 2.51796 | 2.1 |
| 30224 | 1.64193 | 2.1 |
| 30225 | 2.51172 | 2.1 |
| 30226 | 1.68893 | 2.1 |
| 30227 | 4.20689 | 2.1 |
| 30228 | 2.51172 | 2.1 |
| 30229 | 1.70002 | 2.1 |
| 30230 | 1.70002 | 2.1 |
| 30231 | 1.68893 | 2.1 |
| 30232 | 2.48689 | 2.1 |
| 30233 | 2.4758 | 2.1 |
| 30234 | 2.27963 | 0.05 |
| 30235 | 2.21582 | 0.05 |
| 30236 | 2.60486 | 0.05 |
| 30237 | 2.5105 | 0.05 |
| 30238 | 4.79013 | 0.05 |
| 30239 | 2.21582 | 0.05 |
| 30240 | 2.02069 | 0.05 |
| 30241 | 2.5105 | 0.05 |
| 30242 | 2.28615 | 0.05 |
| 30243 | 4.53119 | 0.05 |
| 30244 | 2.60486 | 0.05 |
| 30245 | 2.5105 | 0.05 |
| 30246 | 2.55791 | 0.05 |
| 30247 | 2.47141 | 0.05 |
| 30248 | 5.06842 | 0.05 |
| 30249 | 2.5105 | 0.05 |
| 30250 | 2.28615 | 0.05 |
| 30251 | 2.47141 | 0.05 |
| 30252 | 2.23337 | 0.05 |
| 30253 | 4.74387 | 0.05 |
| 30254 | 2.06984 | 2.1 |
| 30255 | 0.967499 | 2.1 |
| 30256 | 2.12435 | 2.1 |
| 30257 | 2.0021 | 2.1 |
| 30258 | 1.08975 | 2.1 |
| 30259 | 0.967499 | 2.1 |
| 30260 | 1.89226 | 0.05 |
| 30261 | 1.89226 | 0.05 |
| 30262 | 1.89226 | 0.05 |
| 30263 | 1.89226 | 0.05 |
| 30264 | 1.89226 | 0.05 |
| 30265 | 1.89226 | 0.05 |
| 30266 | 1.89226 | 2.1 |
| 30267 | 1.89226 | 2.1 |
| 30268 | 1.89226 | 2.1 |
| 30269 | 1.89226 | 2.1 |
| 30270 | 3.78452 | 2.1 |
| 30271 | 1.89227 | 2.1 |
| 30272 | 1.89227 | 2.1 |
| 30273 | 1.89227 | 2.1 |

| | | |
|---|---|---|
| 30274 | 1.89227 | 2.1 |
| 30275 | 3.78453 | 2.1 |
| 30276 | 0.55882 | 2.1 |
| 30277 | 0.696462 | 2.1 |
| 30278 | 1.07003 | 2.1 |
| 30279 | 0.937179 | 2.1 |
| 30280 | 0.818077 | 2.1 |
| 30281 | 0.818077 | 2.1 |
| 30282 | 0.815565 | 2.1 |
| 30283 | 0.815565 | 2.1 |
| 30284 | 0.696462 | 2.1 |
| 30285 | 1.07003 | 2.1 |
| 30286 | 0.93718 | 2.1 |
| 30287 | 0.55882 | 2.1 |
| 30288 | 2.53857 | 2.1 |
| 30289 | 1.87436 | 2.1 |
| 30290 | 2.27541 | 2.1 |
| 30291 | 2.27541 | 2.1 |
| 30292 | 2.14005 | 2.1 |
| 30293 | 2.14005 | 2.1 |
| 30294 | 1.89226 | 2.1 |
| 30295 | 1.89226 | 2.1 |
| 30296 | 1.89226 | 2.1 |
| 30297 | 1.89226 | 2.1 |
| 30298 | 3.78452 | 2.1 |
| 30299 | 1.89227 | 2.1 |
| 30300 | 1.89227 | 2.1 |
| 30301 | 1.89227 | 2.1 |
| 30302 | 1.89227 | 2.1 |
| 30303 | 3.78453 | 2.1 |
| 30304 | 1.39292 | 2.1 |
| 30305 | 2.14006 | 2.1 |
| 30306 | 2.41083 | 2.1 |
| 30307 | 1.68761 | 2.1 |
| 30308 | 3.80376 | 2.1 |
| 30309 | 2.53857 | 2.1 |
| 30310 | 2.14005 | 2.1 |
| 30311 | 2.41083 | 2.1 |
| 30312 | 2.82043 | 2.1 |
| 30313 | 4.9494 | 2.1 |
| 30314 | 1.90439 | 0.05 |
| 30315 | 1.51835 | 0.05 |
| 30316 | 1.20473 | 0.05 |
| 30317 | 0.947693 | 0.05 |
| 30318 | 2.85208 | 0.05 |
| 30319 | 2.586 | 2.1 |
| 30320 | 1.46262 | 2.1 |
| 30321 | 2.14009 | 2.1 |
| 30322 | 2.78101 | 2.1 |
| 30323 | 4.72609 | 2.1 |
| 30324 | 1.89224 | 2.1 |
| 30325 | 2.78101 | 2.1 |
| 30326 | 2.586 | 2.1 |
| 30327 | 2.586 | 2.1 |
| 30328 | 1.89224 | 2.1 |
| 30329 | 1.89224 | 2.1 |
| 30330 | 0.994293 | 2.1 |
| 30331 | 1.19 | 2.1 |

| | | |
|---|---|---|
| 30332 | 1.09483 | 2.1 |
| 30333 | 1.09483 | 2.1 |
| 30334 | 1.06827 | 2.1 |
| 30335 | 1.06827 | 2.1 |
| 30336 | 1.89226 | 2.1 |
| 30337 | 1.89226 | 2.1 |
| 30338 | 1.89226 | 2.1 |
| 30339 | 1.89226 | 2.1 |
| 30340 | 3.78452 | 2.1 |
| 30341 | 0.946132 | 2.1 |
| 30342 | 0.946132 | 2.1 |
| 30343 | 1.31912 | 2.1 |
| 30344 | 1.3572 | 2.1 |
| 30345 | 0.946131 | 2.1 |
| 30346 | 0.946131 | 2.1 |
| 30347 | 1.31912 | 2.1 |
| 30348 | 1.31912 | 2.1 |
| 30349 | 0.946131 | 2.1 |
| 30350 | 1.31912 | 2.1 |
| 30351 | 1.35721 | 2.1 |
| 30352 | 0.946131 | 2.1 |
| 30353 | 2.24577 | 0.273786 |
| 30354 | 1.09408 | 0.05 |
| 30355 | 1.60239 | 0.05 |
| 30356 | 1.09408 | 0.319857 |
| 30357 | 2.39404 | 0.05 |
| 30358 | 2.07382 | 0.05 |
| 30359 | 1.70303 | 0.05 |
| 30360 | 1.3216 | 0.05 |
| 30361 | 1.23104 | 0.05 |
| 30362 | 1.05475 | 0.05 |
| 30363 | 2.60408 | 0.05 |
| 30364 | 1.89226 | 0.284194 |
| 30365 | 1.89226 | 0.257925 |
| 30366 | 1.89226 | 0.170148 |
| 30367 | 1.89226 | 0.155518 |
| 30368 | 3.78452 | 0.374051 |
| 30369 | 1.89226 | 0.05 |
| 30370 | 2.3845 | 0.05 |
| 30371 | 2.6875 | 0.05 |
| 30372 | 1.89226 | 0.05 |
| 30373 | 2.6875 | 0.05 |
| 30374 | 1.89226 | 0.05 |
| 30375 | 1.46759 | 2.1 |
| 30376 | 1.17585 | 2.1 |
| 30377 | 1.85189 | 2.1 |
| 30378 | 1.39223 | 2.1 |
| 30379 | 2.85981 | 2.1 |
| 30380 | 1.85189 | 2.1 |
| 30381 | 1.71255 | 2.1 |
| 30382 | 1.71255 | 2.1 |
| 30383 | 1.39223 | 2.1 |
| 30384 | 2.28511 | 2.1 |
| 30385 | 1.96479 | 2.1 |
| 30386 | 1.89227 | 0.854343 |
| 30387 | 1.89227 | 1.00835 |
| 30388 | 1.89227 | 1.12556 |
| 30389 | 1.89227 | 0.735091 |

| | | |
|---|---|---|
| 30390 | 1.89227 | 1.78203 |
| 30391 | 1.89227 | 0.918371 |
| 30392 | 1.89226 | 0.819317 |
| 30393 | 1.89226 | 0.971922 |
| 30394 | 1.89226 | 1.1548 |
| 30395 | 1.89226 | 0.497589 |
| 30396 | 1.89226 | 1.88344 |
| 30397 | 1.89226 | 0.886208 |
| 30398 | 1.89226 | 0.834112 |
| 30399 | 1.89226 | 1.07854 |
| 30400 | 1.89226 | 1.10427 |
| 30401 | 1.89226 | 0.60879 |
| 30402 | 1.89226 | 1.99591 |
| 30403 | 1.89226 | 0.930373 |
| 30404 | 1.89226 | 0.66824 |
| 30405 | 2.77788 | 0.852941 |
| 30406 | 2.21062 | 0.93595 |
| 30407 | 2.21062 | 0.539789 |
| 30408 | 1.89226 | 1.10086 |
| 30409 | 1.89226 | 0.792425 |
| 30410 | 0.868548 | 0.580363 |
| 30411 | 0.610104 | 0.834742 |
| 30412 | 0.796678 | 0.783397 |
| 30413 | 0.865021 | 0.446343 |
| 30414 | 1.0883 | 0.507779 |
| 30415 | 0.904938 | 0.805539 |
| 30416 | 2.21062 | 0.595665 |
| 30417 | 1.49548 | 0.662822 |
| 30418 | 1.2068 | 0.957047 |
| 30419 | 1.54764 | 0.698921 |
| 30420 | 0.790005 | 0.422777 |
| 30421 | 1.89226 | 2.1 |
| 30422 | 2.67457 | 2.1 |
| 30423 | 1.89226 | 2.1 |
| 30424 | 2.56352 | 2.1 |
| 30425 | 4.56683 | 2.1 |
| 30426 | 2.67456 | 2.1 |
| 30427 | 2.20011 | 2.1 |
| 30428 | 2.56351 | 2.1 |
| 30429 | 2.11083 | 2.1 |
| 30430 | 4.78539 | 2.1 |
| 30431 | 2.56348 | 2.1 |
| 30432 | 2.1108 | 2.1 |
| 30433 | 2.45763 | 2.1 |
| 30434 | 2.00986 | 2.1 |
| 30435 | 4.56843 | 2.1 |
| 30436 | 0.946133 | 0.05 |
| 30437 | 0.946133 | 0.05 |
| 30438 | 0.946132 | 0.05 |
| 30439 | 0.946132 | 0.05 |
| 30440 | 0.946132 | 0.05 |
| 30441 | 0.946132 | 0.05 |
| 30442 | 0.946132 | 0.05 |
| 30443 | 0.946132 | 0.05 |
| 30444 | 0.946132 | 0.05 |
| 30445 | 0.946132 | 0.05 |
| 30446 | 0.946133 | 0.05 |
| 30447 | 0.946133 | 0.05 |

| | | |
|---|---|---|
| 30448 | 1.45927 | 0.158058 |
| 30449 | 1.25133 | 1.04683 |
| 30450 | 1.00082 | 0.52295 |
| 30451 | 1.41689 | 0.868302 |
| 30452 | 1.0432 | 0.20958 |
| 30453 | 1.45927 | 0.718624 |
| 30454 | 1.41689 | 0.263963 |
| 30455 | 1.29579 | 1.44167 |
| 30456 | 1.34874 | 0.761998 |
| 30457 | 1.25133 | 0.804892 |
| 30458 | 2.76563 | 1.63827 |
| 30459 | 0.675555 | 1.34773 |
| 30460 | 0.674371 | 1.00351 |
| 30461 | 0.625664 | 0.724767 |
| 30462 | 0.523133 | 0.811525 |
| 30463 | 0.676509 | 1.03597 |
| 30464 | 0.676509 | 1.51186 |
| 30465 | 0.62471 | 0.973375 |
| 30466 | 0.62471 | 0.432052 |
| 30467 | 0.675556 | 0.471985 |
| 30468 | 0.523132 | 0.234902 |
| 30469 | 0.674373 | 0.305008 |
| 30470 | 0.625663 | 0.5307 |
| 30471 | 1.89226 | 0.81629 |
| 30472 | 1.89226 | 0.811039 |
| 30473 | 1.89226 | 0.773404 |
| 30474 | 1.89226 | 0.826272 |
| 30475 | 3.78453 | 1.11849 |
| 30476 | 0.851517 | 1.25783 |
| 30477 | 0.946133 | 0.860993 |
| 30478 | 0.946132 | 0.961596 |
| 30479 | 0.946132 | 1.23998 |
| 30480 | 0.946133 | 0.671365 |
| 30481 | 0.851517 | 2.07175 |
| 30482 | 0.946132 | 0.894605 |
| 30483 | 0.946132 | 0.779172 |
| 30484 | 0.851516 | 0.861476 |
| 30485 | 0.851516 | 0.76446 |
| 30486 | 0.946133 | 1.34961 |
| 30487 | 0.946133 | 0.878772 |
| 30488 | 0.569995 | 0.809104 |
| 30489 | 0.816895 | 1.08162 |
| 30490 | 1.33893 | 1.16576 |
| 30491 | 1.18795 | 0.945976 |
| 30492 | 1.18795 | 0.888475 |
| 30493 | 0.569995 | 1.64351 |
| 30494 | 0.816895 | 0.971132 |
| 30495 | 1.33893 | 1.09585 |
| 30496 | 0.859572 | 1.34351 |
| 30497 | 0.859572 | 1.12326 |
| 30498 | 1.04936 | 0.930689 |
| 30499 | 1.04936 | 0.813107 |
| 30500 | 1.89226 | 2.1 |
| 30501 | 1.89226 | 2.1 |
| 30502 | 2.55618 | 2.1 |
| 30503 | 2.55618 | 2.1 |
| 30504 | 2.66983 | 2.1 |
| 30505 | 2.00591 | 2.1 |

| | | |
|---|---|---|
| 30506 | 1.89226 | 0.05 |
| 30507 | 2.64586 | 2.1 |
| 30508 | 2.83331 | 2.1 |
| 30509 | 2.83331 | 0.0963364 |
| 30510 | 1.89226 | 2.1 |
| 30511 | 1.89226 | 0.05 |
| 30512 | 2.64586 | 2.1 |
| 30513 | 2.0815 | 2.1 |
| 30514 | 1.99985 | 2.1 |
| 30515 | 1.94005 | 2.1 |
| 30516 | 2.83332 | 2.1 |
| 30517 | 2.74859 | 2.1 |
| 30518 | 1.98061 | 2.1 |
| 30519 | 1.68356 | 2.1 |
| 30520 | 1.83879 | 2.1 |
| 30521 | 1.55987 | 2.1 |
| 30522 | 3.54048 | 2.1 |
| 30523 | 1.68356 | 2.1 |
| 30524 | 1.20969 | 2.1 |
| 30525 | 1.20969 | 2.1 |
| 30526 | 1.30751 | 2.1 |
| 30527 | 1.55987 | 2.1 |
| 30528 | 1.65769 | 2.1 |
| 30529 | 1.83879 | 2.1 |
| 30530 | 1.55987 | 2.1 |
| 30531 | 1.68443 | 2.1 |
| 30532 | 1.4449 | 2.1 |
| 30533 | 3.2443 | 2.1 |
| 30534 | 0.660368 | 2.1 |
| 30535 | 0.660368 | 2.1 |
| 30536 | 0.666922 | 2.1 |
| 30537 | 0.666922 | 2.1 |
| 30538 | 0.560377 | 2.1 |
| 30539 | 0.604842 | 2.1 |
| 30540 | 0.779935 | 2.1 |
| 30541 | 0.722448 | 2.1 |
| 30542 | 0.722446 | 2.1 |
| 30543 | 0.560377 | 2.1 |
| 30544 | 0.604844 | 2.1 |
| 30545 | 0.779934 | 2.1 |
| 30546 | 1.89227 | 2.1 |
| 30547 | 1.89227 | 2.1 |
| 30548 | 1.89227 | 2.1 |
| 30549 | 1.89227 | 2.1 |
| 30550 | 3.78453 | 2.1 |
| 30551 | 1.42896 | 2.1 |
| 30552 | 1.23037 | 2.1 |
| 30553 | 1.42896 | 2.1 |
| 30554 | 1.474 2.1 | |
| 30555 | 1.2016 | 2.1 |
| 30556 | 1.24665 | 2.1 |
| 30557 | 1.89226 | 2.1 |
| 30558 | 2.7188 | 2.1 |
| 30559 | 1.89226 | 2.1 |
| 30560 | 2.70702 | 2.1 |
| 30561 | 4.61106 | 2.1 |
| 30562 | 1.89226 | 2.1 |
| 30563 | 1.89226 | 2.1 |

| | | |
|---|---|---|
| 30564 | 1.89226 | 2.1 |
| 30565 | 1.89226 | 2.1 |
| 30566 | 3.78453 | 2.1 |
| 30567 | 0.644603 | 2.1 |
| 30568 | 0.644603 | 2.1 |
| 30569 | 0.648272 | 2.1 |
| 30570 | 0.648272 | 2.1 |
| 30571 | 0.585644 | 2.1 |
| 30572 | 0.600802 | 2.1 |
| 30573 | 0.692073 | 2.1 |
| 30574 | 0.714478 | 2.1 |
| 30575 | 0.585644 | 2.1 |
| 30576 | 0.692073 | 2.1 |
| 30577 | 0.714478 | 2.1 |
| 30578 | 0.600802 | 2.1 |
| 30579 | 1.35351 | 2.1 |
| 30580 | 1.35351 | 2.1 |
| 30581 | 0.946131 | 2.1 |
| 30582 | 0.946131 | 2.1 |
| 30583 | 1.34392 | 2.1 |
| 30584 | 1.35351 | 2.1 |
| 30585 | 0.946132 | 2.1 |
| 30586 | 0.946132 | 2.1 |
| 30587 | 0.946132 | 2.1 |
| 30588 | 1.34392 | 2.1 |
| 30589 | 0.946132 | 2.1 |
| 30590 | 1.35351 | 2.1 |
| 30591 | 1.87609 | 2.1 |
| 30592 | 1.79155 | 2.1 |
| 30593 | 1.79155 | 2.1 |
| 30594 | 1.89226 | 2.1 |
| 30595 | 1.89226 | 2.1 |
| 30596 | 1.99211 | 2.1 |
| 30597 | 1.89226 | 2.1 |
| 30598 | 1.71706 | 2.1 |
| 30599 | 1.71706 | 2.1 |
| 30600 | 1.79155 | 2.1 |
| 30601 | 1.89226 | 2.1 |
| 30602 | 1.96675 | 2.1 |
| 30603 | 1.46869 | 2.1 |
| 30604 | 1.89226 | 2.1 |
| 30605 | 1.89226 | 2.1 |
| 30606 | 1.89226 | 2.1 |
| 30607 | 1.5001 | 2.1 |
| 30608 | 1.5001 | 2.1 |
| 30609 | 1.89226 | 2.1 |
| 30610 | 1.89226 | 2.1 |
| 30611 | 1.89226 | 2.1 |
| 30612 | 1.89226 | 2.1 |
| 30613 | 1.89226 | 2.1 |
| 30614 | 1.89226 | 2.1 |
| 30615 | 1.21408 | 2.1 |
| 30616 | 2.23563 | 2.1 |
| 30617 | 2.34021 | 2.1 |
| 30618 | 2.34021 | 2.1 |
| 30619 | 1.17285 | 2.1 |
| 30620 | 1.17285 | 2.1 |
| 30621 | 1.89226 | 2.1 |

| | | |
|---|---|---|
| 30622 | 1.89226 | 2.1 |
| 30623 | 1.47937 | 2.1 |
| 30624 | 1.89226 | 2.1 |
| 30625 | 1.14822 | 2.1 |
| 30626 | 1.46869 | 2.1 |
| 30627 | 1.89226 | 2.1 |
| 30628 | 1.89226 | 2.1 |
| 30629 | 1.89226 | 2.1 |
| 30630 | 1.89226 | 2.1 |
| 30631 | 1.89226 | 2.1 |
| 30632 | 1.89226 | 2.1 |
| 30633 | 1.25716 | 2.1 |
| 30634 | 2.34021 | 2.1 |
| 30635 | 2.4542 | 2.1 |
| 30636 | 2.4542 | 2.1 |
| 30637 | 1.21408 | 2.1 |
| 30638 | 1.21408 | 2.1 |
| 30639 | 1.66824 | 2.1 |
| 30640 | 2.44883 | 2.1 |
| 30641 | 1.93003 | 2.1 |
| 30642 | 2.24795 | 2.1 |
| 30643 | 3.88907 | 2.1 |
| 30644 | 1.89319 | 2.1 |
| 30645 | 2.24795 | 2.1 |
| 30646 | 2.82724 | 2.1 |
| 30647 | 2.31318 | 2.1 |
| 30648 | 2.30868 | 2.1 |
| 30649 | 1.93003 | 2.1 |
| 30650 | 1.89226 | 1.05811 |
| 30651 | 1.42589 | 2.1 |
| 30652 | 1.42589 | 2.1 |
| 30653 | 1.80501 | 2.1 |
| 30654 | 1.89226 | 2.1 |
| 30655 | 2.27137 | 2.1 |
| 30656 | 1.21067 | 0.05 |
| 30657 | 2.39166 | 0.294039 |
| 30658 | 1.73577 | 0.148359 |
| 30659 | 1.73577 | 0.109328 |
| 30660 | 1.53584 | 0.0758624 |
| 30661 | 1.53584 | 0.05 |
| 30662 | 1.35025 | 1.84914 |
| 30663 | 2.0599 | 0.871043 |
| 30664 | 1.51084 | 0.987395 |
| 30665 | 2.27218 | 0.0825078 |
| 30666 | 3.57074 | 0.986517 |
| 30667 | 2.0599 | 0.680418 |
| 30668 | 2.51818 | 1.38468 |
| 30669 | 2.27218 | 0.05 |
| 30670 | 2.66532 | 0.742634 |
| 30671 | 4.72522 | 0.414734 |
| 30672 | 1.67115 | 1.85779 |
| 30673 | 2.27218 | 0.787968 |
| 30674 | 2.39605 | 1.4851 |
| 30675 | 2.39605 | 0.750356 |
| 30676 | 1.51084 | 1.04269 |
| 30677 | 1.51084 | 0.961085 |
| 30678 | 2.27218 | 0.678759 |
| 30679 | 2.66532 | 1.68772 |

| | | |
|---|---|---|
| 30680 | 2.39605 | 0.086358 |
| 30681 | 2.76829 | 0.174716 |
| 30682 | 5.06137 | 0.743468 |
| 30683 | 2.08684 | 0.83062 |
| 30684 | 2.36033 | 1.53789 |
| 30685 | 1.89224 | 0.751591 |
| 30686 | 1.89224 | 1.13722 |
| 30687 | 4.25257 | 0.84626 |
| 30688 | 2.41473 | 0.05 |
| 30689 | 1.00307 | 0.05 |
| 30690 | 2.01365 | 0.05 |
| 30691 | 1.00307 | 0.0924918 |
| 30692 | 3.05113 | 0.05 |
| 30693 | 2.04056 | 0.072633 |
| 30694 | 1.11099 | 2.1 |
| 30695 | 2.25103 | 2.1 |
| 30696 | 2.22227 | 2.1 |
| 30697 | 2.22227 | 2.1 |
| 30698 | 1.14431 | 2.1 |
| 30699 | 1.14431 | 2.1 |
| 30700 | 1.07485 | 2.1 |
| 30701 | 2.22229 | 2.1 |
| 30702 | 2.19163 | 2.1 |
| 30703 | 2.19163 | 2.1 |
| 30704 | 1.111 2.1 | |
| 30705 | 1.111 2.1 | |
| 30706 | 1.0547 | 2.1 |
| 30707 | 1.19886 | 2.1 |
| 30708 | 0.946131 | 2.1 |
| 30709 | 0.946131 | 2.1 |
| 30710 | 0.946131 | 2.1 |
| 30711 | 0.946131 | 2.1 |
| 30712 | 1.19886 | 2.1 |
| 30713 | 1.19886 | 2.1 |
| 30714 | 1.0547 | 2.1 |
| 30715 | 0.946131 | 2.1 |
| 30716 | 0.946132 | 2.1 |
| 30717 | 1.19886 | 2.1 |
| 30718 | 1.89417 | 2.1 |
| 30719 | 2.64424 | 0.05 |
| 30720 | 1.76585 | 2.1 |
| 30721 | 2.47695 | 0.122135 |
| 30722 | 4.37112 | 0.770084 |
| 30723 | 1.89226 | 2.1 |
| 30724 | 1.89226 | 0.730617 |
| 30725 | 1.89226 | 2.1 |
| 30726 | 1.89226 | 0.126696 |
| 30727 | 3.78453 | 1.32184 |
| 30728 | 0.930281 | 2.1 |
| 30729 | 1.0547 | 2.1 |
| 30730 | 0.946131 | 2.1 |
| 30731 | 0.946131 | 2.1 |
| 30732 | 0.930309 | 2.1 |
| 30733 | 0.930309 | 2.1 |
| 30734 | 1.07052 | 2.1 |
| 30735 | 1.07052 | 2.1 |
| 30736 | 0.93028 | 2.1 |
| 30737 | 0.946131 | 2.1 |

| | | |
|---|---|---|
| 30738 | 1.0547 | 2.1 |
| 30739 | 0.946131 | 2.1 |
| 30740 | 1.00015 | 0.05 |
| 30741 | 1.00015 | 1.70913 |
| 30742 | 1.01918 | 2.1 |
| 30743 | 1.01918 | 0.0653739 |
| 30744 | 1.17437 | 0.114528 |
| 30745 | 1.23847 | 0.05 |
| 30746 | 0.882926 | 2.1 |
| 30747 | 0.780859 | 1.29434 |
| 30748 | 0.780859 | 0.0648375 |
| 30749 | 1.17438 | 0.227674 |
| 30750 | 0.882925 | 0.341826 |
| 30751 | 1.23848 | 0.708968 |
| 30752 | 1.89226 | 2.1 |
| 30753 | 1.89226 | 0.0914765 |
| 30754 | 1.89226 | 0.733531 |
| 30755 | 1.89226 | 2.1 |
| 30756 | 1.89226 | 0.60006 |
| 30757 | 1.89226 | 2.1 |
| 30758 | 1.22083 | 2.1 |
| 30759 | 1.29579 | 2.1 |
| 30760 | 1.26236 | 2.1 |
| 30761 | 1.06191 | 2.1 |
| 30762 | 1.42128 | 2.1 |
| 30763 | 1.22083 | 2.1 |
| 30764 | 1.42128 | 2.1 |
| 30765 | 1.45806 | 2.1 |
| 30766 | 1.43502 | 2.1 |
| 30767 | 1.29579 | 2.1 |
| 30768 | 1.5605 | 2.1 |
| 30769 | 1.42128 | 2.1 |
| 30770 | 1.02813 | 2.1 |
| 30771 | 1.12121 | 2.1 |
| 30772 | 1.40557 | 2.1 |
| 30773 | 1.35298 | 2.1 |
| 30774 | 1.1314 | 2.1 |
| 30775 | 1.28679 | 2.1 |
| 30776 | 1.1715 | 2.1 |
| 30777 | 1.1715 | 2.1 |
| 30778 | 0.975656 | 2.1 |
| 30779 | 1.24908 | 2.1 |
| 30780 | 0.98956 | 2.1 |
| 30781 | 1.2681 | 2.1 |
| 30782 | 0.946132 | 2.1 |
| 30783 | 0.946132 | 2.1 |
| 30784 | 1.31889 | 2.1 |
| 30785 | 1.31889 | 2.1 |
| 30786 | 1.16546 | 2.1 |
| 30787 | 1.31889 | 2.1 |
| 30788 | 0.946133 | 2.1 |
| 30789 | 0.946133 | 2.1 |
| 30790 | 0.946133 | 2.1 |
| 30791 | 1.31889 | 2.1 |
| 30792 | 0.946132 | 2.1 |
| 30793 | 1.16546 | 2.1 |
| 30794 | 2.51518 | 0.05 |
| 30795 | 2.37584 | 0.05 |

| | | |
|---|---|---|
| 30796 | 1.11712 | 0.05 |
| 30797 | 1.31242 | 0.05 |
| 30798 | 3.68826 | 0.05 |
| 30799 | 2.51518 | 0.05 |
| 30800 | 1.26311 | 0.05 |
| 30801 | 2.48977 | 0.05 |
| 30802 | 2.48977 | 0.05 |
| 30803 | 1.31242 | 0.05 |
| 30804 | 1.31242 | 0.05 |
| 30805 | 1.89226 | 0.490089 |
| 30806 | 1.89226 | 0.818237 |
| 30807 | 1.89226 | 0.0609997 |
| 30808 | 1.89226 | 0.81555 |
| 30809 | 3.78452 | 0.275578 |
| 30810 | 2.04737 | 0.206593 |
| 30811 | 1.77122 | 0.804147 |
| 30812 | 1.94498 | 0.05 |
| 30813 | 1.65174 | 0.944857 |
| 30814 | 3.71621 | 0.197497 |
| 30815 | 1.41077 | 2.1 |
| 30816 | 1.29729 | 2.1 |
| 30817 | 1.77123 | 0.05 |
| 30818 | 1.77123 | 2.1 |
| 30819 | 1.65175 | 0.100284 |
| 30820 | 1.29129 | 0.467144 |
| 30821 | 1.89226 | 0.05 |
| 30822 | 1.89226 | 2.1 |
| 30823 | 1.89226 | 2.1 |
| 30824 | 1.89226 | 0.05 |
| 30825 | 1.89226 | 2.1 |
| 30826 | 1.89226 | 0.340845 |
| 30827 | 1.89226 | 0.159641 |
| 30828 | 1.89226 | 0.757971 |
| 30829 | 1.89226 | 0.05 |
| 30830 | 1.89226 | 0.651142 |
| 30831 | 3.78451 | 0.0853614 |
| 30832 | 1.94498 | 0.0647715 |
| 30833 | 1.65174 | 0.764996 |
| 30834 | 1.7968 | 0.05 |
| 30835 | 1.49831 | 0.283245 |
| 30836 | 3.44329 | 0.112495 |
| 30837 | 1.49831 | 0.05 |
| 30838 | 1.29729 | 2.1 |
| 30839 | 1.49831 | 2.1 |
| 30840 | 1.65175 | 0.05 |
| 30841 | 1.16858 | 2.1 |
| 30842 | 1.32201 | 0.0764438 |
| 30843 | 1.89226 | 0.05 |
| 30844 | 1.89226 | 2.1 |
| 30845 | 1.89226 | 0.0610025 |
| 30846 | 1.89226 | 2.1 |
| 30847 | 3.78452 | 0.230264 |
| 30848 | 1.89226 | 0.05 |
| 30849 | 1.89226 | 0.05 |
| 30850 | 1.89226 | 0.05 |
| 30851 | 1.89226 | 0.05 |
| 30852 | 1.89226 | 0.05 |
| 30853 | 1.89226 | 0.05 |

| | | |
|---|---|---|
| 30854 | 1.65324 | 0.697257 |
| 30855 | 1.26409 | 0.05 |
| 30856 | 1.42491 | 0.05 |
| 30857 | 1.42491 | 0.738706 |
| 30858 | 1.52268 | 0.05 |
| 30859 | 1.52268 | 0.71106 |
| 30860 | 1.52268 | 1.4816 |
| 30861 | 1.26409 | 0.370364 |
| 30862 | 1.32697 | 0.117871 |
| 30863 | 1.05724 | 0.05 |
| 30864 | 2.57991 | 0.191284 |
| 30865 | 1.89226 | 0.0670857 |
| 30866 | 1.96548 | 0.0718025 |
| 30867 | 2.0178 | 0.123843 |
| 30868 | 1.96548 | 0.0630485 |
| 30869 | 1.94458 | 0.0671897 |
| 30870 | 1.89226 | 0.05 |
| 30871 | 1.99366 | 0.0877303 |
| 30872 | 2.0178 | 0.0829598 |
| 30873 | 1.89226 | 0.116186 |
| 30874 | 2.0178 | 0.0846438 |
| 30875 | 1.75999 | 0.07191 |
| 30876 | 1.89226 | 0.05 |
| 30877 | 0.946126 | 2.1 |
| 30878 | 0.946126 | 2.1 |
| 30879 | 1.28228 | 2.1 |
| 30880 | 1.19444 | 2.1 |
| 30881 | 0.946127 | 2.1 |
| 30882 | 0.946127 | 2.1 |
| 30883 | 1.19444 | 2.1 |
| 30884 | 1.19444 | 2.1 |
| 30885 | 0.946128 | 2.1 |
| 30886 | 1.19444 | 2.1 |
| 30887 | 1.28228 | 2.1 |
| 30888 | 0.946128 | 2.1 |
| 30889 | 1.89226 | 2.1 |
| 30890 | 1.89226 | 2.1 |
| 30891 | 1.89226 | 2.1 |
| 30892 | 1.89226 | 2.1 |
| 30893 | 3.78452 | 2.1 |
| 30894 | 2.14104 | 2.1 |
| 30895 | 1.89226 | 2.1 |
| 30896 | 2.19948 | 2.1 |
| 30897 | 1.89226 | 2.1 |
| 30898 | 1.89226 | 2.1 |
| 30899 | 2.19948 | 2.1 |
| 30900 | 1.89226 | 2.1 |
| 30901 | 2.38888 | 2.1 |
| 30902 | 2.23932 | 2.1 |
| 30903 | 2.38888 | 2.1 |
| 30904 | 1.74269 | 2.1 |
| 30905 | 1.89226 | 2.1 |
| 30906 | 1.89226 | 2.1 |
| 30907 | 1.89226 | 2.1 |
| 30908 | 1.89226 | 2.1 |
| 30909 | 1.89226 | 2.1 |
| 30910 | 3.78452 | 2.1 |
| 30911 | 0.946132 | 2.1 |

| | | |
|---|---|---|
| 30912 | 0.946132 | 2.1 |
| 30913 | 1.07052 | 2.1 |
| 30914 | 1.07052 | 2.1 |
| 30915 | 0.946132 | 2.1 |
| 30916 | 0.946132 | 2.1 |
| 30917 | 1.05132 | 2.1 |
| 30918 | 1.07052 | 2.1 |
| 30919 | 1.05132 | 2.1 |
| 30920 | 0.946132 | 2.1 |
| 30921 | 1.07052 | 2.1 |
| 30922 | 0.946133 | 2.1 |
| 30923 | 2.40171 | 2.1 |
| 30924 | 1.89226 | 2.1 |
| 30925 | 1.89226 | 2.1 |
| 30926 | 1.89226 | 2.1 |
| 30927 | 2.42723 | 2.1 |
| 30928 | 2.42723 | 2.1 |
| 30929 | 1.89226 | 2.1 |
| 30930 | 1.89226 | 1.91905 |
| 30931 | 1.89226 | 2.1 |
| 30932 | 1.89226 | 2.1 |
| 30933 | 3.78452 | 2.1 |
| 30934 | 1.63661 | 2.1 |
| 30935 | 2.3532 | 1.49088 |
| 30936 | 2.33287 | 1.5527 |
| 30937 | 2.33287 | 2.1 |
| 30938 | 1.64158 | 0.872828 |
| 30939 | 1.64158 | 2.1 |
| 30940 | 2.38918 | 2.1 |
| 30941 | 1.89226 | 2.1 |
| 30942 | 1.89226 | 2.1 |
| 30943 | 1.89226 | 2.1 |
| 30944 | 2.40171 | 2.1 |
| 30945 | 2.40171 | 2.1 |
| 30946 | 1.89226 | 2.1 |
| 30947 | 1.89226 | 2.1 |
| 30948 | 1.89226 | 2.1 |
| 30949 | 1.89226 | 2.1 |
| 30950 | 1.89226 | 2.1 |
| 30951 | 1.89226 | 2.1 |
| 30952 | 1.64158 | 2.1 |
| 30953 | 2.3532 | 2.1 |
| 30954 | 1.6488 | 2.1 |
| 30955 | 2.37364 | 2.1 |
| 30956 | 4.002 2.1 | |
| 30957 | 2.54752 | 0.329044 |
| 30958 | 1.42445 | 0.561115 |
| 30959 | 1.86697 | 0.551093 |
| 30960 | 1.86697 | 0.281993 |
| 30961 | 2.06666 | 0.596206 |
| 30962 | 2.06666 | 0.521659 |
| 30963 | 2.50872 | 0.0536148 |
| 30964 | 2.06666 | 0.170944 |
| 30965 | 1.90439 | 0.0861253 |
| 30966 | 1.51835 | 0.0958973 |
| 30967 | 3.97105 | 0.0872643 |
| 30968 | 0.755161 | 0.126606 |
| 30969 | 0.755161 | 0.102176 |

| | | |
|---|---|---|
| 30970 | 0.793994 | 0.0797034 |
| 30971 | 0.793994 | 0.140117 |
| 30972 | 0.712225 | 0.0892798 |
| 30973 | 0.515826 | 0.0844014 |
| 30974 | 1.03333 | 0.170123 |
| 30975 | 0.759175 | 0.14644 |
| 30976 | 0.759173 | 0.101051 |
| 30977 | 0.515827 | 0.05 |
| 30978 | 0.712226 | 0.0591635 |
| 30979 | 1.03333 | 0.113652 |
| 30980 | 1.69633 | 0.05 |
| 30981 | 1.89226 | 0.05 |
| 30982 | 1.62477 | 0.05 |
| 30983 | 1.89226 | 0.05 |
| 30984 | 1.62477 | 0.05 |
| 30985 | 1.89226 | 0.05 |
| 30986 | 1.89226 | 0.05 |
| 30987 | 1.89226 | 0.147787 |
| 30988 | 1.89226 | 0.153325 |
| 30989 | 1.89226 | 0.104741 |
| 30990 | 3.78452 | 0.0960754 |
| 30991 | 2.28622 | 0.05 |
| 30992 | 1.09667 | 0.05 |
| 30993 | 1.17506 | 0.05 |
| 30994 | 2.24411 | 0.05 |
| 30995 | 1.17506 | 0.05 |
| 30996 | 2.24411 | 0.05 |
| 30997 | 2.24741 | 2.1 |
| 30998 | 1.70303 | 2.1 |
| 30999 | 2.49168 | 2.1 |
| 31000 | 1.57936 | 2.1 |
| 31001 | 2.53729 | 2.1 |
| 31002 | 1.70303 | 2.1 |
| 31003 | 2.24741 | 2.1 |
| 31004 | 2.47557 | 2.1 |
| 31005 | 1.89227 | 2.1 |
| 31006 | 1.89227 | 2.1 |
| 31007 | 4.36784 | 2.1 |
| 31008 | 2.27016 | 0.05 |
| 31009 | 1.10476 | 0.05 |
| 31010 | 1.44103 | 0.05 |
| 31011 | 1.10476 | 0.05 |
| 31012 | 2.26791 | 0.05 |
| 31013 | 1.93165 | 0.05 |
| 31014 | 2.27016 | 0.05 |
| 31015 | 1.89226 | 0.05 |
| 31016 | 1.89226 | 0.05 |
| 31017 | 2.4065 | 0.05 |
| 31018 | 4.29876 | 0.05 |
| 31019 | 2.27016 | 0.05 |
| 31020 | 1.44103 | 0.05 |
| 31021 | 1.53336 | 0.05 |
| 31022 | 2.4065 | 0.05 |
| 31023 | 3.84753 | 0.05 |
| 31024 | 2.7542 | 0.05 |
| 31025 | 1.89226 | 0.07633 |
| 31026 | 2.35027 | 0.0501455 |
| 31027 | 1.89226 | 0.0572633 |

| | | |
|---|---|---|
| 31028 | 2.35027 | 0.0540129 |
| 31029 | 1.89226 | 0.0601303 |
| 31030 | 2.3301 | 2.1 |
| 31031 | 2.36071 | 2.1 |
| 31032 | 2.07629 | 2.1 |
| 31033 | 2.01105 | 2.1 |
| 31034 | 4.37176 | 2.1 |
| 31035 | 1.86512 | 2.1 |
| 31036 | 2.01105 | 2.1 |
| 31037 | 2.01105 | 2.1 |
| 31038 | 2.3301 | 2.1 |
| 31039 | 1.57459 | 2.1 |
| 31040 | 1.89364 | 2.1 |
| 31041 | 2.52671 | 2.1 |
| 31042 | 2.01105 | 2.1 |
| 31043 | 1.78251 | 2.1 |
| 31044 | 1.70302 | 2.1 |
| 31045 | 3.98362 | 2.1 |
| 31046 | 1.227 | 2.1 |
| 31047 | 2.01105 | 2.1 |
| 31048 | 1.45869 | 2.1 |
| 31049 | 1.41713 | 2.1 |
| 31050 | 1.70302 | 2.1 |
| 31051 | 1.64994 | 2.1 |
| 31052 | 2.00989 | 2.1 |
| 31053 | 1.28938 | 2.1 |
| 31054 | 1.8769 | 2.1 |
| 31055 | 1.20969 | 2.1 |
| 31056 | 3.21958 | 2.1 |
| 31057 | 1.8769 | 2.1 |
| 31058 | 1.15864 | 2.1 |
| 31059 | 1.15864 | 2.1 |
| 31060 | 1.20969 | 2.1 |
| 31061 | 1.77353 | 2.1 |
| 31062 | 1.82458 | 2.1 |
| 31063 | 1.7986 | 2.1 |
| 31064 | 2.48717 | 2.1 |
| 31065 | 2.50161 | 2.1 |
| 31066 | 1.75869 | 2.1 |
| 31067 | 4.30021 | 2.1 |
| 31068 | 2.50161 | 2.1 |
| 31069 | 1.75251 | 2.1 |
| 31070 | 1.81678 | 2.1 |
| 31071 | 1.75869 | 2.1 |
| 31072 | 2.5597 | 2.1 |
| 31073 | 2.50161 | 2.1 |
| 31074 | 1.91334 | 0.322589 |
| 31075 | 1.85439 | 0.856941 |
| 31076 | 1.91334 | 0.515108 |
| 31077 | 1.70303 | 0.833582 |
| 31078 | 1.70303 | 0.192106 |
| 31079 | 1.49692 | 0.656491 |
| 31080 | 2.39986 | 0.05 |
| 31081 | 1.89226 | 0.0508916 |
| 31082 | 1.89226 | 0.05 |
| 31083 | 2.50756 | 0.05 |
| 31084 | 4.39982 | 0.05 |
| 31085 | 2.39986 | 0.056712 |

| | | |
|---|---|---|
| 31086 | 1.70303 | 0.05 |
| 31087 | 1.75564 | 0.05 |
| 31088 | 1.75904 | 0.05 |
| 31089 | 2.50272 | 0.0561889 |
| 31090 | 2.50756 | 0.0580724 |
| 31091 | 1.89226 | 0.05 |
| 31092 | 2.50756 | 0.05 |
| 31093 | 2.47857 | 0.05 |
| 31094 | 2.50756 | 0.0781704 |
| 31095 | 1.86327 | 0.05 |
| 31096 | 1.89226 | 0.05 |
| 31097 | 1.22241 | 0.0681916 |
| 31098 | 1.20827 | 0.0734624 |
| 31099 | 0.772416 | 0.05 |
| 31100 | 0.555208 | 0.0584866 |
| 31101 | 1.004 | 0.0510662 |
| 31102 | 1.004 | 0.0578397 |
| 31103 | 0.93504 | 0.143103 |
| 31104 | 0.744364 | 0.05 |
| 31105 | 1.2089 | 0.05 |
| 31106 | 0.654935 | 0.05 |
| 31107 | 0.773927 | 0.0503127 |
| 31108 | 1.03618 | 0.0656847 |
| 31109 | 1.89227 | 0.05 |
| 31110 | 1.89227 | 0.05 |
| 31111 | 1.89227 | 0.05 |
| 31112 | 1.89227 | 0.05 |
| 31113 | 3.78453 | 0.05 |
| 31114 | 1.89226 | 0.05 |
| 31115 | 1.89226 | 0.05 |
| 31116 | 1.89226 | 0.05 |
| 31117 | 1.89226 | 0.05 |
| 31118 | 3.78452 | 0.05 |
| 31119 | 1.82643 | 0.05 |
| 31120 | 2.7084 | 0.05 |
| 31121 | 2.12654 | 0.05 |
| 31122 | 2.65614 | 0.05 |
| 31123 | 1.82643 | 0.05 |
| 31124 | 2.35603 | 0.05 |
| 31125 | 1.29385 | 0.05 |
| 31126 | 0.946133 | 0.05 |
| 31127 | 1.05492 | 0.05 |
| 31128 | 0.946132 | 0.05 |
| 31129 | 1.05492 | 0.05 |
| 31130 | 1.29385 | 0.05 |
| 31131 | 0.946133 | 0.05 |
| 31132 | 0.946133 | 0.05 |
| 31133 | 1.29385 | 0.05 |
| 31134 | 1.29385 | 0.05 |
| 31135 | 0.946132 | 0.05 |
| 31136 | 0.946132 | 0.05 |
| 31137 | 0.946132 | 0.05 |
| 31138 | 1.27329 | 0.05 |
| 31139 | 1.29385 | 0.05 |
| 31140 | 0.946129 | 0.05 |
| 31141 | 1.29385 | 0.05 |
| 31142 | 1.27328 | 0.05 |
| 31143 | 0.946131 | 0.05 |

| | | |
|---|---|---|
| 31144 | 0.946131 | 0.05 |
| 31145 | 1.27328 | 0.05 |
| 31146 | 1.27328 | 0.05 |
| 31147 | 0.94613 | 0.05 |
| 31148 | 0.94613 | 0.05 |
| 31149 | 1.89226 | 2.03025 |
| 31150 | 1.89226 | 2.1 |
| 31151 | 1.89226 | 0.9468 |
| 31152 | 1.89226 | 1.3514 |
| 31153 | 3.78453 | 1.53344 |
| 31154 | 1.89226 | 1.69566 |
| 31155 | 1.89226 | 2.1 |
| 31156 | 1.89226 | 0.884326 |
| 31157 | 1.89226 | 1.87302 |
| 31158 | 3.78453 | 1.16327 |
| 31159 | 1.21488 | 2.09779 |
| 31160 | 1.14605 | 2.1 |
| 31161 | 1.62974 | 1.56311 |
| 31162 | 1.52399 | 1.24707 |
| 31163 | 2.84463 | 2.1 |
| 31164 | 0.577078 | 1.35307 |
| 31165 | 0.81251 | 2.1 |
| 31166 | 0.821003 | 1.82576 |
| 31167 | 0.543413 | 2.1 |
| 31168 | 0.683929 | 1.45138 |
| 31169 | 0.722433 | 2.1 |
| 31170 | 0.730674 | 2.1 |
| 31171 | 0.730674 | 1.25008 |
| 31172 | 0.857766 | 1.9342 |
| 31173 | 0.862119 | 1.40601 |
| 31174 | 0.600938 | 2.1 |
| 31175 | 0.596094 | 1.91746 |
| 31176 | 1.89226 | 1.12665 |
| 31177 | 1.89226 | 2.01025 |
| 31178 | 1.89226 | 0.87987 |
| 31179 | 1.89226 | 1.57092 |
| 31180 | 3.78453 | 1.19118 |
| 31181 | 1.89226 | 1.02539 |
| 31182 | 1.89226 | 2.1 |
| 31183 | 1.89226 | 1.01755 |
| 31184 | 1.89226 | 1.29661 |
| 31185 | 3.78453 | 1.72849 |
| 31186 | 1.62974 | 2.1 |
| 31187 | 1.56958 | 2.1 |
| 31188 | 2.2362 | 1.10536 |
| 31189 | 2.11424 | 1.73281 |
| 31190 | 3.86595 | 1.72528 |
| 31191 | 2.2362 | 1.22955 |
| 31192 | 1.85414 | 2.1 |
| 31193 | 2.28211 | 0.900391 |
| 31194 | 1.78367 | 1.04706 |
| 31195 | 2.2362 | 2.08222 |
| 31196 | 1.73256 | 2.1 |
| 31197 | 0.851539 | 1.70816 |
| 31198 | 0.851539 | 0.780519 |
| 31199 | 1.14454 | 1.19189 |
| 31200 | 0.938988 | 2.1 |
| 31201 | 0.851485 | 1.50924 |

| | | |
|---|---|---|
| 31202 | 0.920593 | 0.825831 |
| 31203 | 0.851541 | 0.05 |
| 31204 | 0.851541 | 0.918972 |
| 31205 | 1.04073 | 0.734944 |
| 31206 | 1.16342 | 0.797517 |
| 31207 | 0.825315 | 1.02769 |
| 31208 | 0.825316 | 0.832107 |
| 31209 | 1.89226 | 2.1 |
| 31210 | 1.89226 | 2.1 |
| 31211 | 1.89226 | 2.1 |
| 31212 | 1.89226 | 2.1 |
| 31213 | 3.78453 | 2.1 |
| 31214 | 2.57016 | 2.1 |
| 31215 | 1.74789 | 2.1 |
| 31216 | 2.57016 | 2.1 |
| 31217 | 2.522 | 2.1 |
| 31218 | 1.61727 | 2.1 |
| 31219 | 1.56911 | 2.1 |
| 31220 | 1.28075 | 2.1 |
| 31221 | 1.406 | 2.1 |
| 31222 | 0.946131 | 2.1 |
| 31223 | 0.946131 | 2.1 |
| 31224 | 1.28075 | 2.1 |
| 31225 | 1.00333 | 2.1 |
| 31226 | 0.94613 | 2.1 |
| 31227 | 0.94613 | 2.1 |
| 31228 | 1.041 | 2.1 |
| 31229 | 1.041 | 2.1 |
| 31230 | 0.943484 | 2.1 |
| 31231 | 1.27717 | 2.1 |
| 31232 | 1.89226 | 2.1 |
| 31233 | 1.89226 | 2.1 |
| 31234 | 1.89226 | 2.1 |
| 31235 | 1.89226 | 2.1 |
| 31236 | 1.89226 | 2.1 |
| 31237 | 1.89226 | 2.1 |
| 31238 | 2.57016 | 2.1 |
| 31239 | 1.61727 | 2.1 |
| 31240 | 2.74196 | 2.1 |
| 31241 | 1.59094 | 2.1 |
| 31242 | 4.16109 | 2.1 |
| 31243 | 1.89226 | 2.1 |
| 31244 | 2.0815 | 2.1 |
| 31245 | 2.2034 | 2.1 |
| 31246 | 1.82493 | 2.1 |
| 31247 | 1.87847 | 2.1 |
| 31248 | 2.16142 | 2.1 |
| 31249 | 1.89226 | 2.1 |
| 31250 | 1.89226 | 2.1 |
| 31251 | 1.89226 | 2.1 |
| 31252 | 1.89226 | 2.1 |
| 31253 | 3.78453 | 2.1 |
| 31254 | 0.985252 | 2.1 |
| 31255 | 0.985252 | 2.1 |
| 31256 | 0.946132 | 2.1 |
| 31257 | 0.946132 | 2.1 |
| 31258 | 1.0655 | 2.1 |
| 31259 | 0.985255 | 2.1 |

| | | |
|---|---|---|
| 31260 | 0.94613 | 2.1 |
| 31261 | 0.94613 | 2.1 |
| 31262 | 0.94613 | 2.1 |
| 31263 | 1.0655 | 2.1 |
| 31264 | 0.94613 | 2.1 |
| 31265 | 0.985255 | 2.1 |
| 31266 | 1.89226 | 2.1 |
| 31267 | 1.89226 | 2.1 |
| 31268 | 1.89226 | 2.1 |
| 31269 | 1.89226 | 2.1 |
| 31270 | 3.78453 | 2.1 |
| 31271 | 1.06549 | 2.1 |
| 31272 | 1.06549 | 2.1 |
| 31273 | 0.946133 | 2.1 |
| 31274 | 0.946133 | 2.1 |
| 31275 | 1.16834 | 2.1 |
| 31276 | 1.0655 | 2.1 |
| 31277 | 0.946131 | 2.1 |
| 31278 | 0.946131 | 2.1 |
| 31279 | 0.946131 | 2.1 |
| 31280 | 1.16834 | 2.1 |
| 31281 | 0.94613 | 2.1 |
| 31282 | 1.0655 | 2.1 |
| 31283 | 0.966631 | 2.1 |
| 31284 | 1.07349 | 2.1 |
| 31285 | 0.946131 | 2.1 |
| 31286 | 0.946131 | 2.1 |
| 31287 | 1.07349 | 2.1 |
| 31288 | 1.07349 | 2.1 |
| 31289 | 0.946129 | 2.1 |
| 31290 | 0.946129 | 2.1 |
| 31291 | 1.07349 | 2.1 |
| 31292 | 0.946131 | 2.1 |
| 31293 | 0.94613 | 2.1 |
| 31294 | 0.966631 | 2.1 |
| 31295 | 2.38055 | 0.0745511 |
| 31296 | 1.89226 | 1.32073 |
| 31297 | 1.89226 | 1.00462 |
| 31298 | 1.46724 | 0.05 |
| 31299 | 3.35949 | 0.12004 |
| 31300 | 1.42249 | 2.1 |
| 31301 | 2.05499 | 2.1 |
| 31302 | 1.39708 | 2.1 |
| 31303 | 2.09033 | 2.1 |
| 31304 | 3.45207 | 2.1 |
| 31305 | 2.05499 | 2.1 |
| 31306 | 2.57425 | 2.1 |
| 31307 | 2.51287 | 2.1 |
| 31308 | 2.47753 | 2.1 |
| 31309 | 2.09033 | 2.1 |
| 31310 | 2.05499 | 2.1 |
| 31311 | 1.39327 | 2.1 |
| 31312 | 2.09033 | 2.1 |
| 31313 | 2.16217 | 2.1 |
| 31314 | 2.16217 | 2.1 |
| 31315 | 1.39708 | 2.1 |
| 31316 | 1.39708 | 2.1 |
| 31317 | 2.16217 | 2.1 |

| | | |
|---|---|---|
| 31318 | 2.57426 | 2.1 |
| 31319 | 2.73006 | 2.1 |
| 31320 | 2.73006 | 2.1 |
| 31321 | 2.09033 | 2.1 |
| 31322 | 2.09033 | 2.1 |
| 31323 | 1.89226 | 0.05 |
| 31324 | 1.89226 | 0.05 |
| 31325 | 1.89226 | 0.05 |
| 31326 | 1.89226 | 0.05 |
| 31327 | 3.78452 | 0.05 |
| 31328 | 0.838749 | 0.05 |
| 31329 | 0.838749 | 0.05 |
| 31330 | 0.85824 | 0.05 |
| 31331 | 0.85824 | 0.05 |
| 31332 | 0.544644 | 0.05 |
| 31333 | 0.854718 | 0.05 |
| 31334 | 0.842271 | 0.05 |
| 31335 | 1.19141 | 0.05 |
| 31336 | 0.842272 | 0.05 |
| 31337 | 0.544643 | 0.05 |
| 31338 | 0.854718 | 0.05 |
| 31339 | 1.19141 | 0.05 |
| 31340 | 1.34214 | 0.05 |
| 31341 | 1.34214 | 0.05 |
| 31342 | 0.893236 | 0.05 |
| 31343 | 0.893236 | 0.05 |
| 31344 | 0.893265 | 0.05 |
| 31345 | 1.28924 | 0.05 |
| 31346 | 0.946133 | 0.05 |
| 31347 | 0.946133 | 0.05 |
| 31348 | 0.946132 | 0.05 |
| 31349 | 0.893265 | 0.05 |
| 31350 | 0.946133 | 0.05 |
| 31351 | 1.28924 | 0.05 |
| 31352 | 2.7013 | 0.05 |
| 31353 | 1.89226 | 0.05 |
| 31354 | 1.9216 | 0.05 |
| 31355 | 1.9216 | 0.05 |
| 31356 | 1.89226 | 0.05 |
| 31357 | 1.89226 | 0.05 |
| 31358 | 1.26397 | 2.1 |
| 31359 | 1.52995 | 2.1 |
| 31360 | 1.30116 | 2.1 |
| 31361 | 1.04084 | 2.1 |
| 31362 | 2.57079 | 2.1 |
| 31363 | 1.47013 | 2.1 |
| 31364 | 2.17459 | 2.1 |
| 31365 | 2.10668 | 2.1 |
| 31366 | 1.38328 | 2.1 |
| 31367 | 3.55787 | 2.1 |
| 31368 | 2.55006 | 2.1 |
| 31369 | 2.17459 | 2.1 |
| 31370 | 2.10668 | 2.1 |
| 31371 | 2.49168 | 2.1 |
| 31372 | 4.66626 | 2.1 |
| 31373 | 0.613622 | 2.1 |
| 31374 | 0.691639 | 2.1 |
| 31375 | 1.00105 | 2.1 |

| | | |
|---|---|---|
| 31376 | 1.05334 | 2.1 |
| 31377 | 0.839874 | 2.1 |
| 31378 | 0.839874 | 2.1 |
| 31379 | 0.852814 | 2.1 |
| 31380 | 0.852814 | 2.1 |
| 31381 | 0.613622 | 2.1 |
| 31382 | 1.00105 | 2.1 |
| 31383 | 1.05334 | 2.1 |
| 31384 | 0.691639 | 2.1 |
| 31385 | 1.00105 | 2.1 |
| 31386 | 1.05334 | 2.1 |
| 31387 | 1.20844 | 2.1 |
| 31388 | 1.24584 | 2.1 |
| 31389 | 1.12717 | 2.1 |
| 31390 | 1.12717 | 2.1 |
| 31391 | 1.13462 | 2.1 |
| 31392 | 1.13462 | 2.1 |
| 31393 | 1.20845 | 2.1 |
| 31394 | 1.00105 | 2.1 |
| 31395 | 1.05334 | 2.1 |
| 31396 | 1.24584 | 2.1 |
| 31397 | 2.22558 | 2.1 |
| 31398 | 1.85981 | 1.94651 |
| 31399 | 2.18943 | 1.47982 |
| 31400 | 1.89142 | 0.05 |
| 31401 | 4.0854 | 1.11911 |
| 31402 | 2.02641 | 2.1 |
| 31403 | 1.85981 | 1.22396 |
| 31404 | 2.18943 | 1.28935 |
| 31405 | 1.67346 | 1.11715 |
| 31406 | 3.88622 | 0.965771 |
| 31407 | 1.85981 | 0.05 |
| 31408 | 1.10736 | 0.05 |
| 31409 | 1.27421 | 0.05 |
| 31410 | 1.67346 | 0.05 |
| 31411 | 1.27421 | 0.05 |
| 31412 | 1.67346 | 0.905543 |
| 31413 | 1.85981 | 0.05 |
| 31414 | 1.27362 | 0.100305 |
| 31415 | 1.89142 | 0.127281 |
| 31416 | 1.27421 | 0.05 |
| 31417 | 3.13343 | 0.05 |
| 31418 | 1.81893 | 0.05 |
| 31419 | 1.64985 | 0.05 |
| 31420 | 1.23477 | 0.05 |
| 31421 | 2.25405 | 0.05 |
| 31422 | 3.48882 | 0.05 |
| 31423 | 2.23933 | 0.05 |
| 31424 | 1.89226 | 0.05 |
| 31425 | 1.89226 | 0.05 |
| 31426 | 2.78053 | 0.05 |
| 31427 | 1.89226 | 0.05 |
| 31428 | 2.78053 | 0.05 |
| 31429 | 0.946131 | 0.05 |
| 31430 | 0.946131 | 0.05 |
| 31431 | 0.946132 | 0.05 |
| 31432 | 0.946132 | 0.05 |
| 31433 | 0.946131 | 0.05 |

| | | |
|---|---|---|
| 31434 | 0.946131 | 0.05 |
| 31435 | 0.946132 | 0.05 |
| 31436 | 0.946132 | 0.05 |
| 31437 | 0.946131 | 0.05 |
| 31438 | 0.946131 | 0.05 |
| 31439 | 0.946132 | 0.05 |
| 31440 | 0.946132 | 0.05 |
| 31441 | 1.89226 | 2.1 |
| 31442 | 2.48596 | 2.1 |
| 31443 | 1.89226 | 2.1 |
| 31444 | 2.51506 | 2.1 |
| 31445 | 4.40732 | 2.1 |
| 31446 | 2.48596 | 2.1 |
| 31447 | 1.63823 | 2.1 |
| 31448 | 2.51507 | 2.1 |
| 31449 | 1.67687 | 2.1 |
| 31450 | 4.1533 | 2.1 |
| 31451 | 1.89226 | 2.1 |
| 31452 | 2.51506 | 2.1 |
| 31453 | 1.92044 | 2.1 |
| 31454 | 1.89226 | 2.1 |
| 31455 | 2.54325 | 2.1 |
| 31456 | 2.51506 | 2.1 |
| 31457 | 2.51507 | 2.1 |
| 31458 | 1.5704 | 2.1 |
| 31459 | 1.70505 | 2.1 |
| 31460 | 1.67687 | 2.1 |
| 31461 | 2.54325 | 2.1 |
| 31462 | 2.51507 | 2.1 |
| 31463 | 2.0815 | 2.1 |
| 31464 | 1.70303 | 2.1 |
| 31465 | 1.70303 | 2.1 |
| 31466 | 1.80354 | 2.1 |
| 31467 | 1.98172 | 2.1 |
| 31468 | 2.09868 | 2.1 |
| 31469 | 1.27944 | 2.1 |
| 31470 | 1.79028 | 2.1 |
| 31471 | 1.13179 | 2.1 |
| 31472 | 1.65533 | 2.1 |
| 31473 | 2.93477 | 2.1 |
| 31474 | 1.89226 | 2.1 |
| 31475 | 1.89226 | 2.1 |
| 31476 | 1.89226 | 2.1 |
| 31477 | 1.89226 | 2.1 |
| 31478 | 1.89226 | 2.1 |
| 31479 | 1.89226 | 2.1 |
| 31480 | 1.98171 | 2.1 |
| 31481 | 1.89226 | 2.1 |
| 31482 | 1.89226 | 2.1 |
| 31483 | 1.89226 | 2.1 |
| 31484 | 1.65798 | 2.1 |
| 31485 | 1.65797 | 2.1 |
| 31486 | 1.00133 | 2.1 |
| 31487 | 1.65532 | 2.1 |
| 31488 | 1.53278 | 2.1 |
| 31489 | 1.53278 | 2.1 |
| 31490 | 1.13179 | 2.1 |
| 31491 | 1.13179 | 2.1 |

| | | |
|---|---|---|
| 31492 | 0.810946 | 2.1 |
| 31493 | 0.810946 | 2.1 |
| 31494 | 0.810944 | 2.1 |
| 31495 | 0.810944 | 2.1 |
| 31496 | 0.810946 | 2.1 |
| 31497 | 0.810946 | 2.1 |
| 31498 | 1.89226 | 2.1 |
| 31499 | 1.35169 | 2.1 |
| 31500 | 1.08132 | 2.1 |
| 31501 | 1.08132 | 2.1 |
| 31502 | 1.08132 | 2.1 |
| 31503 | 1.99528 | 0.05 |
| 31504 | 1.70303 | 0.05 |
| 31505 | 2.08152 | 0.05 |
| 31506 | 1.70303 | 0.05 |
| 31507 | 2.33877 | 0.05 |
| 31508 | 1.93967 | 0.05 |
| 31509 | 1.89226 | 0.05 |
| 31510 | 1.89226 | 0.05 |
| 31511 | 1.89226 | 0.05 |
| 31512 | 1.89226 | 0.05 |
| 31513 | 3.78452 | 0.05 |
| 31514 | 1.26259 | 0.05 |
| 31515 | 2.17205 | 0.05 |
| 31516 | 2.32468 | 0.05 |
| 31517 | 2.32468 | 0.05 |
| 31518 | 1.28055 | 0.05 |
| 31519 | 1.28055 | 0.05 |
| 31520 | 0.946131 | 0.05 |
| 31521 | 0.946131 | 0.05 |
| 31522 | 0.997642 | 0.05 |
| 31523 | 0.997642 | 0.05 |
| 31524 | 0.946131 | 0.05 |
| 31525 | 0.946131 | 0.05 |
| 31526 | 0.989846 | 0.05 |
| 31527 | 0.997642 | 0.05 |
| 31528 | 0.946132 | 0.05 |
| 31529 | 0.989846 | 0.05 |
| 31530 | 0.997641 | 0.05 |
| 31531 | 0.946131 | 0.05 |
| 31532 | 1.89226 | 0.05 |
| 31533 | 1.89226 | 0.05 |
| 31534 | 1.89226 | 0.05 |
| 31535 | 1.89226 | 0.05 |
| 31536 | 3.78452 | 0.05 |
| 31537 | 1.28055 | 0.05 |
| 31538 | 2.17205 | 0.05 |
| 31539 | 1.14305 | 0.05 |
| 31540 | 1.92737 | 0.05 |
| 31541 | 3.3151 | 0.05 |
| 31542 | 0.954908 | 0.05 |
| 31543 | 0.9705 | 0.05 |
| 31544 | 0.946131 | 0.05 |
| 31545 | 0.946131 | 0.05 |
| 31546 | 0.946131 | 0.05 |
| 31547 | 0.946131 | 0.05 |
| 31548 | 0.9705 | 0.05 |
| 31549 | 0.9705 | 0.05 |

| | | |
|---|---|---|
| 31550 | 0.954908 | 0.05 |
| 31551 | 0.946131 | 0.05 |
| 31552 | 0.946131 | 0.05 |
| 31553 | 0.9705 | 0.05 |
| 31554 | 0.835187 | 0.05 |
| 31555 | 0.954896 | 0.05 |
| 31556 | 0.946118 | 0.05 |
| 31557 | 0.946118 | 0.05 |
| 31558 | 0.835186 | 0.05 |
| 31559 | 0.835186 | 0.05 |
| 31560 | 1.06583 | 0.05 |
| 31561 | 1.06583 | 0.05 |
| 31562 | 0.835198 | 0.05 |
| 31563 | 0.946131 | 0.05 |
| 31564 | 0.954883 | 0.05 |
| 31565 | 0.946106 | 0.05 |
| 31566 | 1.89227 | 2.1 |
| 31567 | 1.89227 | 2.1 |
| 31568 | 1.89227 | 2.1 |
| 31569 | 1.89227 | 2.1 |
| 31570 | 1.89227 | 2.1 |
| 31571 | 1.89227 | 2.1 |
| 31572 | 0.885265 | 2.1 |
| 31573 | 0.885265 | 2.1 |
| 31574 | 0.871467 | 2.1 |
| 31575 | 0.871467 | 2.1 |
| 31576 | 1.11883 | 2.1 |
| 31577 | 0.787875 | 2.1 |
| 31578 | 0.996487 | 2.1 |
| 31579 | 0.637907 | 2.1 |
| 31580 | 0.996487 | 2.1 |
| 31581 | 1.11883 | 2.1 |
| 31582 | 0.787875 | 2.1 |
| 31583 | 0.637906 | 2.1 |
| 31584 | 1.89226 | 2.1 |
| 31585 | 2.39258 | 2.1 |
| 31586 | 2.39258 | 2.1 |
| 31587 | 2.54729 | 2.1 |
| 31588 | 1.89226 | 2.1 |
| 31589 | 2.04697 | 2.1 |
| 31590 | 1.89226 | 2.1 |
| 31591 | 2.0815 | 2.1 |
| 31592 | 2.0815 | 2.1 |
| 31593 | 2.48371 | 2.1 |
| 31594 | 2.0815 | 2.1 |
| 31595 | 2.51469 | 2.1 |
| 31596 | 1.00035 | 0.05 |
| 31597 | 1.55755 | 0.05 |
| 31598 | 1.03534 | 0.05 |
| 31599 | 1.61333 | 0.05 |
| 31600 | 2.59289 | 0.05 |
| 31601 | 1.55755 | 0.05 |
| 31602 | 1.85217 | 2.1 |
| 31603 | 1.61333 | 0.05 |
| 31604 | 1.92875 | 0.335943 |
| 31605 | 3.48629 | 0.05 |
| 31606 | 1.09962 | 0.437147 |
| 31607 | 1.61334 | 0.05 |

| | | |
|---|---|---|
| 31608 | 1.70868 | 0.693201 |
| 31609 | 1.70868 | 0.168967 |
| 31610 | 1.03535 | 1.30838 |
| 31611 | 1.03535 | 0.05 |
| 31612 | 1.61334 | 0.05 |
| 31613 | 1.92876 | 0.449926 |
| 31614 | 1.70868 | 0.05 |
| 31615 | 2.05274 | 1.91082 |
| 31616 | 3.63744 | 0.05 |
| 31617 | 1.82233 | 0.05 |
| 31618 | 2.05768 | 0.303204 |
| 31619 | 1.89226 | 0.315367 |
| 31620 | 1.65406 | 0.05 |
| 31621 | 1.89226 | 0.331378 |
| 31622 | 1.65406 | 0.05 |
| 31623 | 2.26047 | 0.05 |
| 31624 | 2.0815 | 0.722011 |
| 31625 | 1.89226 | 0.541866 |
| 31626 | 2.01156 | 0.0512551 |
| 31627 | 1.89226 | 0.374012 |
| 31628 | 2.01156 | 0.0534781 |
| 31629 | 1.89226 | 2.1 |
| 31630 | 1.89226 | 2.1 |
| 31631 | 1.89226 | 2.1 |
| 31632 | 1.89226 | 2.1 |
| 31633 | 3.78453 | 2.1 |
| 31634 | 2.70055 | 2.1 |
| 31635 | 2.28367 | 2.1 |
| 31636 | 2.56456 | 2.1 |
| 31637 | 2.33565 | 2.1 |
| 31638 | 4.84823 | 2.1 |
| 31639 | 2.28367 | 2.1 |
| 31640 | 1.5791 | 2.1 |
| 31641 | 1.96966 | 2.1 |
| 31642 | 1.09506 | 2.1 |
| 31643 | 3.37873 | 2.1 |
| 31644 | 1.89226 | 2.1 |
| 31645 | 1.89226 | 2.1 |
| 31646 | 1.89226 | 2.1 |
| 31647 | 1.89226 | 2.1 |
| 31648 | 3.78452 | 2.1 |
| 31649 | 0.94612 | 2.1 |
| 31650 | 0.94612 | 2.1 |
| 31651 | 0.946119 | 2.1 |
| 31652 | 0.946119 | 2.1 |
| 31653 | 0.946119 | 2.1 |
| 31654 | 0.946119 | 2.1 |
| 31655 | 0.946119 | 2.1 |
| 31656 | 0.946119 | 2.1 |
| 31657 | 0.946132 | 2.1 |
| 31658 | 0.946132 | 2.1 |
| 31659 | 0.946107 | 2.1 |
| 31660 | 0.946107 | 2.1 |
| 31661 | 2.56452 | 2.1 |
| 31662 | 1.57307 | 2.1 |
| 31663 | 2.10299 | 2.1 |
| 31664 | 2.1115 | 2.1 |
| 31665 | 2.52196 | 2.1 |

| | | |
|---|---|---|
| 31666 | 2.52196 | 2.1 |
| 31667 | 1.89226 | 2.1 |
| 31668 | 1.89226 | 1.57529 |
| 31669 | 1.89226 | 2.1 |
| 31670 | 1.89226 | 2.1 |
| 31671 | 3.78452 | 2.1 |
| 31672 | 2.37665 | 2.1 |
| 31673 | 1.89226 | 2.1 |
| 31674 | 2.28084 | 2.1 |
| 31675 | 1.89226 | 2.1 |
| 31676 | 4.26891 | 2.1 |
| 31677 | 0.946129 | 1.33663 |
| 31678 | 0.946129 | 2.1 |
| 31679 | 0.946133 | 2.1 |
| 31680 | 0.946133 | 2.1 |
| 31681 | 0.946131 | 2.1 |
| 31682 | 0.946131 | 1.05963 |
| 31683 | 0.946131 | 2.1 |
| 31684 | 0.946131 | 2.1 |
| 31685 | 0.946131 | 2.1 |
| 31686 | 0.946131 | 2.1 |
| 31687 | 0.946131 | 2.1 |
| 31688 | 0.946131 | 2.1 |
| 31689 | 1.7207 | 0.05 |
| 31690 | 1.65815 | 0.05 |
| 31691 | 1.33593 | 0.05 |
| 31692 | 1.31508 | 0.05 |
| 31693 | 2.99408 | 0.05 |
| 31694 | 0.676047 | 0.05 |
| 31695 | 0.676047 | 0.05 |
| 31696 | 0.684362 | 0.05 |
| 31697 | 0.684362 | 0.05 |
| 31698 | 0.547994 | 0.05 |
| 31699 | 0.702871 | 0.05 |
| 31700 | 0.829076 | 0.05 |
| 31701 | 0.657538 | 0.05 |
| 31702 | 0.657538 | 0.05 |
| 31703 | 0.547994 | 0.05 |
| 31704 | 0.829077 | 0.05 |
| 31705 | 0.702871 | 0.05 |
| 31706 | 1.9518 | 2.1 |
| 31707 | 1.30648 | 2.1 |
| 31708 | 1.28846 | 2.1 |
| 31709 | 1.92033 | 2.1 |
| 31710 | 3.22681 | 2.1 |
| 31711 | 1.89226 | 2.1 |
| 31712 | 1.89226 | 2.1 |
| 31713 | 1.89226 | 2.1 |
| 31714 | 1.89226 | 2.1 |
| 31715 | 3.78453 | 2.1 |
| 31716 | 0.946128 | 2.1 |
| 31717 | 0.946128 | 2.1 |
| 31718 | 1.10113 | 2.1 |
| 31719 | 1.09587 | 2.1 |
| 31720 | 0.946131 | 2.1 |
| 31721 | 0.946131 | 2.1 |
| 31722 | 1.09587 | 2.1 |
| 31723 | 1.09587 | 2.1 |

| | | |
|---|---|---|
| 31724 | 0.946131 | 2.1 |
| 31725 | 1.09587 | 2.1 |
| 31726 | 1.10112 | 2.1 |
| 31727 | 0.94613 | 2.1 |
| 31728 | 1.26923 | 2.1 |
| 31729 | 1.92033 | 2.1 |
| 31730 | 1.28846 | 2.1 |
| 31731 | 1.28846 | 2.1 |
| 31732 | 1.89452 | 2.1 |
| 31733 | 1.89452 | 2.1 |
| 31734 | 1.89226 | 2.1 |
| 31735 | 1.89226 | 2.1 |
| 31736 | 1.89226 | 2.1 |
| 31737 | 1.89226 | 2.1 |
| 31738 | 3.78453 | 2.1 |
| 31739 | 1.89226 | 2.1 |
| 31740 | 2.19174 | 2.1 |
| 31741 | 2.15616 | 2.1 |
| 31742 | 2.19174 | 2.1 |
| 31743 | 1.85669 | 2.1 |
| 31744 | 1.89226 | 2.1 |
| 31745 | 1.89226 | 2.1 |
| 31746 | 1.89226 | 2.1 |
| 31747 | 1.89226 | 2.1 |
| 31748 | 1.89226 | 2.1 |
| 31749 | 3.78452 | 2.1 |
| 31750 | 1.89226 | 2.1 |
| 31751 | 1.89226 | 2.1 |
| 31752 | 1.89226 | 2.1 |
| 31753 | 1.89226 | 2.1 |
| 31754 | 3.78452 | 2.1 |
| 31755 | 1.51223 | 2.1 |
| 31756 | 1.41562 | 2.1 |
| 31757 | 1.5139 | 2.1 |
| 31758 | 1.42284 | 2.1 |
| 31759 | 1.6033 | 2.1 |
| 31760 | 1.51223 | 2.1 |
| 31761 | 1.51223 | 2.1 |
| 31762 | 1.25953 | 2.1 |
| 31763 | 1.38085 | 2.1 |
| 31764 | 1.6033 | 2.1 |
| 31765 | 2.89309 | 2.1 |
| 31766 | 0.946131 | 2.1 |
| 31767 | 0.946131 | 2.1 |
| 31768 | 0.946131 | 2.1 |
| 31769 | 0.946131 | 2.1 |
| 31770 | 0.946132 | 2.1 |
| 31771 | 0.946132 | 2.1 |
| 31772 | 0.946129 | 2.1 |
| 31773 | 0.946129 | 2.1 |
| 31774 | 0.946131 | 2.1 |
| 31775 | 0.946131 | 2.1 |
| 31776 | 0.94613 | 2.1 |
| 31777 | 0.94613 | 2.1 |
| 31778 | 1.89226 | 2.1 |
| 31779 | 1.89226 | 2.1 |
| 31780 | 1.89226 | 2.1 |
| 31781 | 1.89226 | 2.1 |

| | | |
|---|---|---|
| 31782 | 3.78452 | 2.1 |
| 31783 | 0.707806 | 2.1 |
| 31784 | 0.697816 | 2.1 |
| 31785 | 0.823593 | 2.1 |
| 31786 | 0.801649 | 2.1 |
| 31787 | 0.74182 | 2.1 |
| 31788 | 0.74182 | 2.1 |
| 31789 | 0.757645 | 2.1 |
| 31790 | 0.757645 | 2.1 |
| 31791 | 0.823592 | 2.1 |
| 31792 | 0.697818 | 2.1 |
| 31793 | 0.707807 | 2.1 |
| 31794 | 0.801647 | 2.1 |
| 31795 | 1.52534 | 2.1 |
| 31796 | 1.6033 | 2.1 |
| 31797 | 1.64719 | 2.1 |
| 31798 | 1.38085 | 2.1 |
| 31799 | 1.64719 | 2.1 |
| 31800 | 1.38085 | 2.1 |
| 31801 | 2.67041 | 2.1 |
| 31802 | 1.89226 | 0.728636 |
| 31803 | 1.89226 | 2.1 |
| 31804 | 1.89226 | 2.1 |
| 31805 | 2.487 | 2.1 |
| 31806 | 2.487 | 2.1 |
| 31807 | 2.70097 | 2.1 |
| 31808 | 1.70303 | 2.1 |
| 31809 | 1.71163 | 2.1 |
| 31810 | 2.487 | 2.1 |
| 31811 | 4.46723 | 2.1 |
| 31812 | 1.14984 | 2.1 |
| 31813 | 1.2435 | 2.1 |
| 31814 | 0.778 | 2.1 |
| 31815 | 0.697616 | 2.1 |
| 31816 | 0.967211 | 2.1 |
| 31817 | 0.967211 | 2.1 |
| 31818 | 0.973907 | 2.1 |
| 31819 | 0.973907 | 2.1 |
| 31820 | 0.697616 | 2.1 |
| 31821 | 1.14984 | 2.1 |
| 31822 | 0.778 | 2.1 |
| 31823 | 1.2435 | 2.1 |
| 31824 | 2.4158 | 2.1 |
| 31825 | 1.26438 | 2.1 |
| 31826 | 1.676 | 2.1 |
| 31827 | 2.77505 | 2.1 |
| 31828 | 4.0918 | 2.1 |
| 31829 | 2.58258 | 2.1 |
| 31830 | 1.89226 | 2.1 |
| 31831 | 1.89226 | 2.1 |
| 31832 | 1.89226 | 2.1 |
| 31833 | 2.47032 | 2.1 |
| 31834 | 2.47032 | 2.1 |
| 31835 | 1.23516 | 2.1 |
| 31836 | 1.18734 | 2.1 |
| 31837 | 0.946131 | 2.1 |
| 31838 | 0.946131 | 2.1 |
| 31839 | 1.18734 | 2.1 |

| | | |
|---|---|---|
| 31840 | 1.18734 | 2.1 |
| 31841 | 0.94613 | 2.1 |
| 31842 | 0.94613 | 2.1 |
| 31843 | 1.18734 | 2.1 |
| 31844 | 0.946131 | 2.1 |
| 31845 | 0.946131 | 2.1 |
| 31846 | 1.23516 | 2.1 |
| 31847 | 2.8239 | 2.1 |
| 31848 | 1.39361 | 2.1 |
| 31849 | 1.12342 | 2.1 |
| 31850 | 2.57275 | 2.1 |
| 31851 | 3.96637 | 2.1 |
| 31852 | 0.937929 | 0.825808 |
| 31853 | 0.937929 | 0.718885 |
| 31854 | 1.39734 | 0.228811 |
| 31855 | 1.39734 | 0.339518 |
| 31856 | 1.38914 | 0.342792 |
| 31857 | 0.94613 | 0.732963 |
| 31858 | 0.937902 | 0.776159 |
| 31859 | 0.94613 | 0.05 |
| 31860 | 0.94613 | 0.616996 |
| 31861 | 0.94613 | 0.661836 |
| 31862 | 1.38914 | 0.468102 |
| 31863 | 0.937902 | 0.379824 |
| 31864 | 1.89226 | 0.05 |
| 31865 | 2.17765 | 0.739932 |
| 31866 | 1.84577 | 0.47746 |
| 31867 | 1.84577 | 0.0922713 |
| 31868 | 1.89226 | 0.249173 |
| 31869 | 1.89226 | 0.0564733 |
| 31870 | 2.11262 | 1.45158 |
| 31871 | 1.57766 | 1.18128 |
| 31872 | 2.22689 | 2.1 |
| 31873 | 1.91132 | 0.05 |
| 31874 | 2.19624 | 2.1 |
| 31875 | 1.79689 | 0.05 |
| 31876 | 1.99656 | 0.05 |
| 31877 | 1.57766 | 0.946439 |
| 31878 | 1.39067 | 2.1 |
| 31879 | 1.73802 | 0.754171 |
| 31880 | 3.31568 | 1.01545 |
| 31881 | 1.89226 | 0.20263 |
| 31882 | 1.89226 | 0.05 |
| 31883 | 1.89226 | 0.05 |
| 31884 | 1.89226 | 0.2562 |
| 31885 | 1.89226 | 0.05 |
| 31886 | 1.89226 | 0.05 |
| 31887 | 1.84936 | 0.293975 |
| 31888 | 2.0815 | 0.05 |
| 31889 | 2.0815 | 0.05 |
| 31890 | 2.47217 | 0.119265 |
| 31891 | 1.04528 | 0.05 |
| 31892 | 1.62774 | 0.0758051 |
| 31893 | 1.89226 | 0.05 |
| 31894 | 1.89226 | 0.05 |
| 31895 | 1.89226 | 0.05 |
| 31896 | 1.89226 | 0.103784 |
| 31897 | 1.89226 | 0.05 |

| | | |
|---|---|---|
| 31898 | 1.89226 | 0.05 |
| 31899 | 1.69055 | 0.05 |
| 31900 | 2.68518 | 0.05 |
| 31901 | 1.69055 | 0.05 |
| 31902 | 2.25055 | 0.05 |
| 31903 | 2.39587 | 0.05 |
| 31904 | 3.01801 | 0.05 |
| 31905 | 1.89226 | 0.05 |
| 31906 | 1.89226 | 0.05 |
| 31907 | 1.89226 | 0.05 |
| 31908 | 1.89226 | 0.05 |
| 31909 | 3.78452 | 0.05 |
| 31910 | 1.24499 | 1.65334 |
| 31911 | 1.70302 | 2.1 |
| 31912 | 1.41207 | 2.1 |
| 31913 | 1.10574 | 0.0840745 |
| 31914 | 2.66056 | 1.85326 |
| 31915 | 1.70303 | 2.1 |
| 31916 | 2.39605 | 1.09368 |
| 31917 | 2.72674 | 1.48062 |
| 31918 | 2.46904 | 0.785498 |
| 31919 | 1.84557 | 1.92143 |
| 31920 | 1.67115 | 1.06219 |
| 31921 | 2.46904 | 0.148056 |
| 31922 | 2.76829 | 1.95383 |
| 31923 | 2.83666 | 0.051652 |
| 31924 | 2.83666 | 0.868744 |
| 31925 | 2.39605 | 0.759675 |
| 31926 | 2.39605 | 1.92716 |
| 31927 | 1.07323 | 0.05 |
| 31928 | 1.07323 | 2.1 |
| 31929 | 1.06715 | 2.1 |
| 31930 | 1.18052 | 0.900018 |
| 31931 | 1.30067 | 0.663534 |
| 31932 | 1.26542 | 0.744428 |
| 31933 | 0.914516 | 2.1 |
| 31934 | 0.839633 | 2.1 |
| 31935 | 0.936958 | 0.957367 |
| 31936 | 1.26751 | 0.955259 |
| 31937 | 0.847764 | 0.878523 |
| 31938 | 1.35395 | 1.02366 |
| 31939 | 2.69069 | 0.05 |
| 31940 | 1.03074 | 0.05 |
| 31941 | 1.74098 | 0.05 |
| 31942 | 1.97397 | 0.05 |
| 31943 | 3.71495 | 0.05 |
| 31944 | 2.44554 | 2.1 |
| 31945 | 1.89226 | 2.1 |
| 31946 | 1.89226 | 2.1 |
| 31947 | 1.89226 | 2.1 |
| 31948 | 2.40645 | 2.1 |
| 31949 | 2.40645 | 2.1 |
| 31950 | 1.89226 | 2.1 |
| 31951 | 1.89226 | 2.1 |
| 31952 | 1.89226 | 2.1 |
| 31953 | 1.89226 | 2.1 |
| 31954 | 3.78452 | 2.1 |
| 31955 | 1.82117 | 2.1 |

| | | |
|---|---|---|
| 31956 | 2.55786 | 2.09337 |
| 31957 | 2.51431 | 2.1 |
| 31958 | 2.51431 | 2.1 |
| 31959 | 1.85054 | 2.1 |
| 31960 | 1.84375 | 2.1 |
| 31961 | 2.38467 | 2.1 |
| 31962 | 1.89226 | 2.1 |
| 31963 | 1.89226 | 2.1 |
| 31964 | 1.89226 | 2.1 |
| 31965 | 2.44554 | 2.1 |
| 31966 | 2.44554 | 2.1 |
| 31967 | 1.89226 | 2.1 |
| 31968 | 1.89226 | 2.1 |
| 31969 | 1.89226 | 2.1 |
| 31970 | 1.89226 | 2.1 |
| 31971 | 1.89226 | 2.1 |
| 31972 | 1.89226 | 2.1 |
| 31973 | 2.02397 | 2.1 |
| 31974 | 2.55786 | 2.1 |
| 31975 | 1.77775 | 2.1 |
| 31976 | 2.61404 | 2.1 |
| 31977 | 4.33561 | 2.1 |
| 31978 | 1.89226 | 1.58418 |
| 31979 | 1.89226 | 1.47736 |
| 31980 | 1.89226 | 0.985636 |
| 31981 | 1.89226 | 1.17909 |
| 31982 | 3.78453 | 1.35519 |
| 31983 | 1.89226 | 1.65942 |
| 31984 | 2.80184 | 2.02019 |
| 31985 | 1.89226 | 1.20159 |
| 31986 | 1.89226 | 1.90874 |
| 31987 | 2.03045 | 2.06819 |
| 31988 | 2.03045 | 2.1 |
| 31989 | 2.52263 | 1.75861 |
| 31990 | 1.27921 | 2.1 |
| 31991 | 1.58429 | 2.1 |
| 31992 | 1.73571 | 2.1 |
| 31993 | 2.37121 | 2.1 |
| 31994 | 2.52263 | 1.93668 |
| 31995 | 0.764974 | 2.1 |
| 31996 | 0.650577 | 2.1 |
| 31997 | 0.712428 | 2.1 |
| 31998 | 0.562516 | 2.1 |
| 31999 | 0.67264 | 2.1 |
| 32000 | 0.67264 | 2.1 |
| 32001 | 0.654851 | 2.1 |
| 32002 | 0.654851 | 2.1 |
| 32003 | 0.562515 | 2.1 |
| 32004 | 0.650579 | 2.1 |
| 32005 | 0.712427 | 2.1 |
| 32006 | 0.764976 | 2.1 |
| 32007 | 1.89227 | 2.1 |
| 32008 | 1.89227 | 2.1 |
| 32009 | 1.89227 | 2.1 |
| 32010 | 1.89227 | 2.1 |
| 32011 | 3.78453 | 2.1 |
| 32012 | 1.89226 | 2.1 |
| 32013 | 1.89226 | 2.1 |

| | | |
|---|---|---|
| 32014 | 1.89226 | 2.1 |
| 32015 | 1.89226 | 2.1 |
| 32016 | 3.78453 | 2.1 |
| 32017 | 1.49248 | 2.1 |
| 32018 | 1.24959 | 2.1 |
| 32019 | 1.28211 | 2.1 |
| 32020 | 1.26432 | 2.1 |
| 32021 | 1.51027 | 2.1 |
| 32022 | 1.49248 | 2.1 |
| 32023 | 1.49248 | 2.1 |
| 32024 | 1.19017 | 2.1 |
| 32025 | 1.24977 | 2.1 |
| 32026 | 1.51027 | 2.1 |
| 32027 | 2.74225 | 2.1 |
| 32028 | 1.89226 | 2.1 |
| 32029 | 1.89226 | 2.1 |
| 32030 | 1.89226 | 2.1 |
| 32031 | 1.89226 | 2.1 |
| 32032 | 3.78453 | 2.1 |
| 32033 | 1.89226 | 2.1 |
| 32034 | 1.89226 | 2.1 |
| 32035 | 1.89226 | 2.1 |
| 32036 | 1.89226 | 2.1 |
| 32037 | 3.78453 | 2.1 |
| 32038 | 0.624796 | 2.1 |
| 32039 | 0.618472 | 2.1 |
| 32040 | 0.770147 | 2.1 |
| 32041 | 0.755132 | 2.1 |
| 32042 | 0.681445 | 2.1 |
| 32043 | 0.681445 | 2.1 |
| 32044 | 0.69216 | 2.1 |
| 32045 | 0.69216 | 2.1 |
| 32046 | 0.618472 | 2.1 |
| 32047 | 0.770147 | 2.1 |
| 32048 | 0.624796 | 2.1 |
| 32049 | 0.755132 | 2.1 |
| 32050 | 1.54029 | 2.1 |
| 32051 | 1.51027 | 2.1 |
| 32052 | 1.54029 | 2.1 |
| 32053 | 1.30682 | 2.1 |
| 32054 | 1.24977 | 2.1 |
| 32055 | 1.24977 | 2.1 |
| 32056 | 0.994293 | 2.1 |
| 32057 | 2.17159 | 2.1 |
| 32058 | 2.21149 | 2.1 |
| 32059 | 2.21149 | 2.1 |
| 32060 | 0.994814 | 2.1 |
| 32061 | 0.994814 | 2.1 |
| 32062 | 0.990481 | 2.1 |
| 32063 | 2.21149 | 2.1 |
| 32064 | 2.2671 | 2.1 |
| 32065 | 2.2671 | 2.1 |
| 32066 | 0.994293 | 2.1 |
| 32067 | 0.994293 | 2.1 |
| 32068 | 2.41579 | 2.1 |
| 32069 | 1.09847 | 2.1 |
| 32070 | 1.70302 | 1.33979 |
| 32071 | 2.45069 | 2.1 |

| | | |
|---|---|---|
| 32072 | 3.61169 | 2.1 |
| 32073 | 2.48689 | 2.1 |
| 32074 | 1.67969 | 2.1 |
| 32075 | 1.67969 | 2.1 |
| 32076 | 1.70002 | 2.1 |
| 32077 | 2.43826 | 2.1 |
| 32078 | 2.45859 | 2.1 |
| 32079 | 2.43827 | 2.1 |
| 32080 | 1.62408 | 2.1 |
| 32081 | 1.62408 | 2.1 |
| 32082 | 1.67969 | 2.1 |
| 32083 | 2.36729 | 2.1 |
| 32084 | 2.4229 | 2.1 |
| 32085 | 2.47199 | 2.1 |
| 32086 | 1.0823 | 2.1 |
| 32087 | 1.0607 | 2.1 |
| 32088 | 2.44276 | 2.1 |
| 32089 | 3.52506 | 2.1 |
| 32090 | 2.47199 | 2.1 |
| 32091 | 1.89226 | 2.1 |
| 32092 | 1.89226 | 2.1 |
| 32093 | 2.44277 | 2.1 |
| 32094 | 4.33503 | 2.1 |
| 32095 | 1.89226 | 2.1 |
| 32096 | 1.89226 | 2.1 |
| 32097 | 1.89226 | 2.1 |
| 32098 | 1.89226 | 2.1 |
| 32099 | 3.78453 | 2.1 |
| 32100 | 1.89226 | 2.1 |
| 32101 | 1.89226 | 2.1 |
| 32102 | 1.89226 | 2.1 |
| 32103 | 1.89226 | 2.1 |
| 32104 | 1.89226 | 2.1 |
| 32105 | 1.89226 | 2.1 |
| 32106 | 1.02986 | 2.1 |
| 32107 | 2.44276 | 2.1 |
| 32108 | 1.0607 | 2.1 |
| 32109 | 1.0607 | 2.1 |
| 32110 | 2.40055 | 2.1 |
| 32111 | 2.40055 | 2.1 |
| 32112 | 1.89226 | 2.1 |
| 32113 | 2.44277 | 2.1 |
| 32114 | 2.40056 | 2.1 |
| 32115 | 2.44277 | 2.1 |
| 32116 | 1.85005 | 2.1 |
| 32117 | 1.89226 | 2.1 |
| 32118 | 1.89226 | 2.1 |
| 32119 | 1.89226 | 2.1 |
| 32120 | 1.89226 | 2.1 |
| 32121 | 1.89226 | 2.1 |
| 32122 | 3.78453 | 2.1 |
| 32123 | 1.89226 | 2.1 |
| 32124 | 1.89226 | 2.1 |
| 32125 | 1.89226 | 2.1 |
| 32126 | 1.89226 | 2.1 |
| 32127 | 3.78452 | 2.1 |
| 32128 | 2.79502 | 0.244619 |
| 32129 | 2.63448 | 0.644281 |

| | | |
|---|---|---|
| 32130 | 2.68986 | 0.0807236 |
| 32131 | 2.53868 | 0.379396 |
| 32132 | 5.32434 | 0.300594 |
| 32133 | 2.63449 | 0.05 |
| 32134 | 2.39282 | 2.1 |
| 32135 | 2.53869 | 0.05 |
| 32136 | 2.3085 | 2.1 |
| 32137 | 4.94299 | 0.0787256 |
| 32138 | 2.68986 | 0.242947 |
| 32139 | 2.53868 | 0.369658 |
| 32140 | 2.5761 | 0.0724519 |
| 32141 | 2.43404 | 0.2919 |
| 32142 | 5.1239 | 0.155891 |
| 32143 | 2.53869 | 0.05 |
| 32144 | 2.3085 | 2.1 |
| 32145 | 2.43405 | 0.05 |
| 32146 | 2.22575 | 2.1 |
| 32147 | 4.74255 | 0.0698754 |
| 32148 | 1.89226 | 2.1 |
| 32149 | 1.89226 | 2.1 |
| 32150 | 1.89226 | 2.1 |
| 32151 | 1.89226 | 2.1 |
| 32152 | 3.78453 | 2.1 |
| 32153 | 1.32391 | 2.1 |
| 32154 | 1.44951 | 2.1 |
| 32155 | 1.44951 | 2.1 |
| 32156 | 1.89226 | 2.1 |
| 32157 | 1.89226 | 2.1 |
| 32158 | 2.20203 | 2.1 |
| 32159 | 0.507656 | 2.1 |
| 32160 | 0.578945 | 2.1 |
| 32161 | 1.05568 | 2.1 |
| 32162 | 0.959007 | 2.1 |
| 32163 | 0.76264 | 2.1 |
| 32164 | 0.76264 | 2.1 |
| 32165 | 0.775313 | 2.1 |
| 32166 | 0.775313 | 2.1 |
| 32167 | 0.578945 | 2.1 |
| 32168 | 1.05568 | 2.1 |
| 32169 | 0.959007 | 2.1 |
| 32170 | 0.507656 | 2.1 |
| 32171 | 1.89226 | 0.05 |
| 32172 | 2.4065 | 0.05 |
| 32173 | 2.36561 | 0.05 |
| 32174 | 2.4065 | 0.05 |
| 32175 | 1.85138 | 0.05 |
| 32176 | 1.89226 | 0.05 |
| 32177 | 1.40274 | 0.05 |
| 32178 | 2.4065 | 0.05 |
| 32179 | 1.53336 | 0.05 |
| 32180 | 1.53336 | 0.05 |
| 32181 | 2.36562 | 0.05 |
| 32182 | 2.36562 | 0.05 |
| 32183 | 0.946115 | 0.05 |
| 32184 | 0.946115 | 0.05 |
| 32185 | 1.06496 | 0.05 |
| 32186 | 1.18279 | 0.05 |
| 32187 | 0.946117 | 0.05 |

| | | |
|---|---|---|
| 32188 | 0.946117 | 0.05 |
| 32189 | 1.06496 | 0.05 |
| 32190 | 1.06496 | 0.05 |
| 32191 | 0.94613 | 0.05 |
| 32192 | 1.06497 | 0.05 |
| 32193 | 0.946105 | 0.05 |
| 32194 | 1.18278 | 0.05 |
| 32195 | 1.00213 | 0.05 |
| 32196 | 2.36559 | 0.05 |
| 32197 | 1.40272 | 0.05 |
| 32198 | 1.40272 | 0.05 |
| 32199 | 2.12992 | 0.05 |
| 32200 | 2.12992 | 0.05 |
| 32201 | 0.95646 | 2.1 |
| 32202 | 2.07201 | 2.1 |
| 32203 | 1.01808 | 2.1 |
| 32204 | 2.14876 | 1.59986 |
| 32205 | 3.09009 | 2.1 |
| 32206 | 1.89226 | 2.1 |
| 32207 | 1.89226 | 0.916399 |
| 32208 | 1.89226 | 2.1 |
| 32209 | 1.89226 | 2.1 |
| 32210 | 3.78453 | 2.1 |
| 32211 | 1.89227 | 2.1 |
| 32212 | 1.89227 | 2.1 |
| 32213 | 1.89227 | 2.1 |
| 32214 | 1.89227 | 2.1 |
| 32215 | 1.89227 | 2.1 |
| 32216 | 1.89227 | 2.1 |
| 32217 | 2.71886 | 2.1 |
| 32218 | 1.60497 | 2.1 |
| 32219 | 1.43935 | 2.1 |
| 32220 | 2.55151 | 2.1 |
| 32221 | 4.15648 | 2.1 |
| 32222 | 2.71886 | 2.1 |
| 32223 | 1.89226 | 2.1 |
| 32224 | 1.89226 | 2.1 |
| 32225 | 2.55151 | 2.1 |
| 32226 | 4.44377 | 2.1 |
| 32227 | 1.89226 | 2.1 |
| 32228 | 1.89226 | 2.1 |
| 32229 | 1.89226 | 2.1 |
| 32230 | 1.89226 | 1.72374 |
| 32231 | 3.78453 | 2.1 |
| 32232 | 1.89226 | 2.1 |
| 32233 | 1.89226 | 2.1 |
| 32234 | 1.89226 | 2.1 |
| 32235 | 1.89226 | 2.1 |
| 32236 | 3.78453 | 2.1 |
| 32237 | 1.58942 | 0.05 |
| 32238 | 1.92622 | 0.0953045 |
| 32239 | 2.52852 | 0.0849014 |
| 32240 | 2.19457 | 0.05 |
| 32241 | 4.12079 | 0.05 |
| 32242 | 1.92649 | 0.05 |
| 32243 | 1.44426 | 0.05 |
| 32244 | 1.87529 | 0.05 |
| 32245 | 2.19457 | 0.05 |

| | | |
|---|---|---|
| 32246 | 3.63883 | 0.05 |
| 32247 | 1.87529 | 0.05 |
| 32248 | 2.19457 | 0.05 |
| 32249 | 2.7378 | 0.05 |
| 32250 | 2.29852 | 0.05 |
| 32251 | 4.49309 | 0.05 |
| 32252 | 1.35976 | 2.1 |
| 32253 | 1.89452 | 2.1 |
| 32254 | 1.39616 | 2.1 |
| 32255 | 1.1423 | 2.1 |
| 32256 | 1.8996 | 2.1 |
| 32257 | 1.52068 | 2.1 |
| 32258 | 1.89226 | 2.1 |
| 32259 | 1.89226 | 2.1 |
| 32260 | 1.89226 | 2.1 |
| 32261 | 1.89226 | 2.1 |
| 32262 | 1.89226 | 2.1 |
| 32263 | 1.89226 | 2.1 |
| 32264 | 1.89226 | 2.1 |
| 32265 | 2.15617 | 2.1 |
| 32266 | 2.0953 | 2.1 |
| 32267 | 2.15617 | 2.1 |
| 32268 | 1.83139 | 2.1 |
| 32269 | 1.89226 | 2.1 |
| 32270 | 1.16817 | 2.1 |
| 32271 | 1.8996 | 2.1 |
| 32272 | 1.23614 | 2.1 |
| 32273 | 1.48338 | 2.1 |
| 32274 | 1.80218 | 2.1 |
| 32275 | 2.16263 | 2.1 |
| 32276 | 1.89226 | 2.1 |
| 32277 | 1.89226 | 2.1 |
| 32278 | 1.89226 | 2.1 |
| 32279 | 1.89226 | 2.1 |
| 32280 | 1.89226 | 2.1 |
| 32281 | 1.89226 | 2.1 |
| 32282 | 1.89226 | 2.1 |
| 32283 | 2.0953 | 2.1 |
| 32284 | 2.03074 | 2.1 |
| 32285 | 2.0953 | 2.1 |
| 32286 | 1.8277 | 2.1 |
| 32287 | 1.89226 | 2.1 |
| 32288 | 1.74455 | 2.1 |
| 32289 | 2.181 | 2.1 |
| 32290 | 1.74455 | 2.1 |
| 32291 | 1.92911 | 2.1 |
| 32292 | 1.98195 | 2.1 |
| 32293 | 2.16651 | 2.1 |
| 32294 | 2.181 | 2.1 |
| 32295 | 2.29881 | 2.1 |
| 32296 | 1.98195 | 2.1 |
| 32297 | 1.80729 | 2.1 |
| 32298 | 4.28075 | 2.1 |
| 32299 | 1.74455 | 2.1 |
| 32300 | 1.98195 | 2.1 |
| 32301 | 1.52603 | 2.1 |
| 32302 | 1.76603 | 2.1 |
| 32303 | 3.51058 | 2.1 |

| | | |
|---|---|---|
| 32304 | 1.76603 | 2.1 |
| 32305 | 1.94506 | 2.1 |
| 32306 | 1.76603 | 2.1 |
| 32307 | 1.98195 | 2.1 |
| 32308 | 1.86795 | 2.1 |
| 32309 | 2.08387 | 2.1 |
| 32310 | 0.984305 | 2.1 |
| 32311 | 1.9 | 2.1 |
| 32312 | 2.86619 | 2.1 |
| 32313 | 1.86189 | 2.1 |
| 32314 | 3.76189 | 2.1 |
| 32315 | 1.4621 | 2.1 |
| 32316 | 1.89226 | 2.1 |
| 32317 | 2.81374 | 2.1 |
| 32318 | 1.89226 | 2.1 |
| 32319 | 3.35437 | 2.1 |
| 32320 | 0.946133 | 2.1 |
| 32321 | 0.946133 | 2.1 |
| 32322 | 0.946132 | 2.1 |
| 32323 | 0.946132 | 2.1 |
| 32324 | 0.946132 | 2.1 |
| 32325 | 0.946132 | 2.1 |
| 32326 | 0.946133 | 2.1 |
| 32327 | 0.946133 | 2.1 |
| 32328 | 0.946132 | 2.1 |
| 32329 | 0.946132 | 2.1 |
| 32330 | 0.946132 | 2.1 |
| 32331 | 0.946132 | 2.1 |
| 32332 | 1.64268 | 2.1 |
| 32333 | 1.93673 | 2.1 |
| 32334 | 2.25284 | 2.1 |
| 32335 | 1.97548 | 2.1 |
| 32336 | 3.89552 | 2.1 |
| 32337 | 1.86206 | 2.1 |
| 32338 | 1.93673 | 2.1 |
| 32339 | 2.25284 | 2.1 |
| 32340 | 2.20075 | 2.1 |
| 32341 | 4.1149 | 2.1 |
| 32342 | 2.25284 | 2.1 |
| 32343 | 2.24781 | 2.1 |
| 32344 | 1.97548 | 2.1 |
| 32345 | 1.97548 | 2.1 |
| 32346 | 2.49676 | 2.1 |
| 32347 | 2.49676 | 2.1 |
| 32348 | 2.25284 | 2.1 |
| 32349 | 2.43549 | 2.1 |
| 32350 | 2.44467 | 2.1 |
| 32351 | 2.20075 | 2.1 |
| 32352 | 2.49676 | 2.1 |
| 32353 | 2.25284 | 2.1 |
| 32354 | 2.57928 | 2.1 |
| 32355 | 2.32606 | 2.1 |
| 32356 | 2.59522 | 2.1 |
| 32357 | 2.39022 | 2.1 |
| 32358 | 4.9695 | 2.1 |
| 32359 | 2.32606 | 2.1 |
| 32360 | 1.77677 | 2.1 |
| 32361 | 2.39022 | 2.1 |

| | | |
|---|---|---|
| 32362 | 2.28669 | 2.1 |
| 32363 | 4.16699 | 2.1 |
| 32364 | 2.59522 | 2.1 |
| 32365 | 2.39022 | 2.1 |
| 32366 | 2.61901 | 2.1 |
| 32367 | 2.45899 | 2.1 |
| 32368 | 5.00923 | 2.1 |
| 32369 | 2.39022 | 2.1 |
| 32370 | 2.11575 | 2.1 |
| 32371 | 2.41795 | 2.1 |
| 32372 | 2.0815 | 2.1 |
| 32373 | 4.67085 | 2.1 |
| 32374 | 1.89226 | 2.1 |
| 32375 | 2.24723 | 2.1 |
| 32376 | 1.95405 | 2.1 |
| 32377 | 1.89226 | 2.1 |
| 32378 | 2.30902 | 2.1 |
| 32379 | 2.24723 | 2.1 |
| 32380 | 1.89226 | 2.1 |
| 32381 | 1.89226 | 2.1 |
| 32382 | 1.89226 | 2.1 |
| 32383 | 1.89226 | 2.1 |
| 32384 | 3.78453 | 2.1 |
| 32385 | 0.946131 | 2.1 |
| 32386 | 0.946131 | 2.1 |
| 32387 | 1.14643 | 2.1 |
| 32388 | 1.10673 | 2.1 |
| 32389 | 1.10673 | 2.1 |
| 32390 | 1.10673 | 2.1 |
| 32391 | 0.946133 | 2.1 |
| 32392 | 0.946133 | 2.1 |
| 32393 | 0.946131 | 2.1 |
| 32394 | 1.14643 | 2.1 |
| 32395 | 1.10673 | 2.1 |
| 32396 | 0.946131 | 2.1 |
| 32397 | 1.89226 | 2.1 |
| 32398 | 2.30902 | 2.1 |
| 32399 | 1.96635 | 2.1 |
| 32400 | 1.89226 | 2.1 |
| 32401 | 2.38311 | 2.1 |
| 32402 | 2.30902 | 2.1 |
| 32403 | 1.89226 | 2.1 |
| 32404 | 1.89226 | 2.1 |
| 32405 | 1.89226 | 2.1 |
| 32406 | 1.89226 | 2.1 |
| 32407 | 1.89226 | 2.1 |
| 32408 | 1.89226 | 2.1 |
| 32409 | 0.946131 | 2.1 |
| 32410 | 0.946131 | 2.1 |
| 32411 | 1.19156 | 2.1 |
| 32412 | 1.14643 | 2.1 |
| 32413 | 1.14643 | 2.1 |
| 32414 | 1.14643 | 2.1 |
| 32415 | 0.946133 | 2.1 |
| 32416 | 0.946133 | 2.1 |
| 32417 | 0.946131 | 2.1 |
| 32418 | 1.19156 | 2.1 |
| 32419 | 0.946131 | 2.1 |

| | | |
|---|---|---|
| 32420 | 1.14643 | 2.1 |
| 32421 | 1.89226 | 1.37232 |
| 32422 | 1.89226 | 0.808903 |
| 32423 | 1.89226 | 0.68858 |
| 32424 | 1.89226 | 1.16993 |
| 32425 | 1.89226 | 0.808257 |
| 32426 | 1.89226 | 1.87754 |
| 32427 | 1.89226 | 0.890951 |
| 32428 | 1.89226 | 0.839068 |
| 32429 | 1.89226 | 0.783815 |
| 32430 | 1.9645 | 0.918482 |
| 32431 | 3.78452 | 1.07737 |
| 32432 | 2.39489 | 2.1 |
| 32433 | 2.15715 | 2.1 |
| 32434 | 2.07821 | 2.1 |
| 32435 | 2.33541 | 2.1 |
| 32436 | 4.49256 | 2.1 |
| 32437 | 2.39489 | 2.1 |
| 32438 | 2.34898 | 2.1 |
| 32439 | 2.30729 | 2.1 |
| 32440 | 2.33541 | 2.1 |
| 32441 | 4.6844 | 2.1 |
| 32442 | 2.07821 | 2.1 |
| 32443 | 2.33541 | 2.1 |
| 32444 | 2.26415 | 2.1 |
| 32445 | 1.99632 | 2.1 |
| 32446 | 4.33174 | 2.1 |
| 32447 | 2.30729 | 2.1 |
| 32448 | 2.33542 | 2.1 |
| 32449 | 2.26416 | 2.1 |
| 32450 | 2.26618 | 2.1 |
| 32451 | 4.60159 | 2.1 |
| 32452 | 1.19837 | 1.737 |
| 32453 | 2.79151 | 0.05 |
| 32454 | 2.25636 | 0.454633 |
| 32455 | 2.25636 | 0.130193 |
| 32456 | 1.94072 | 0.180877 |
| 32457 | 1.94072 | 0.173985 |
| 32458 | 2.53725 | 0.05 |
| 32459 | 1.16699 | 0.05 |
| 32460 | 1.69864 | 0.05 |
| 32461 | 2.0815 | 0.05 |
| 32462 | 3.58273 | 0.05 |
| 32463 | 1.91646 | 2.1 |
| 32464 | 2.51686 | 2.1 |
| 32465 | 2.49872 | 2.1 |
| 32466 | 1.85819 | 2.1 |
| 32467 | 4.41518 | 2.1 |
| 32468 | 0.929098 | 2.1 |
| 32469 | 0.8993 | 2.1 |
| 32470 | 1.24359 | 2.1 |
| 32471 | 1.24936 | 2.1 |
| 32472 | 1.06832 | 2.1 |
| 32473 | 1.06832 | 2.1 |
| 32474 | 1.08034 | 2.1 |
| 32475 | 1.08034 | 2.1 |
| 32476 | 0.899298 | 2.1 |
| 32477 | 1.24359 | 2.1 |

| | | |
|---|---|---|
| 32478 | 1.24936 | 2.1 |
| 32479 | 0.929097 | 2.1 |
| 32480 | 2.0815 | 0.05 |
| 32481 | 1.89226 | 0.05 |
| 32482 | 2.0815 | 0.05 |
| 32483 | 1.82811 | 0.05 |
| 32484 | 1.94982 | 0.05 |
| 32485 | 1.71246 | 0.05 |
| 32486 | 2.3256 | 2.1 |
| 32487 | 1.89226 | 2.1 |
| 32488 | 1.89226 | 2.1 |
| 32489 | 1.89226 | 2.1 |
| 32490 | 2.38467 | 2.1 |
| 32491 | 2.38467 | 2.1 |
| 32492 | 1.89226 | 2.1 |
| 32493 | 1.89226 | 2.1 |
| 32494 | 1.89226 | 2.1 |
| 32495 | 1.89226 | 2.1 |
| 32496 | 1.89226 | 2.1 |
| 32497 | 1.89226 | 2.1 |
| 32498 | 1.89763 | 2.1 |
| 32499 | 2.61405 | 2.1 |
| 32500 | 2.15035 | 2.1 |
| 32501 | 2.68796 | 2.1 |
| 32502 | 1.42219 | 2.1 |
| 32503 | 1.77775 | 2.1 |
| 32504 | 2.26306 | 2.1 |
| 32505 | 1.89226 | 2.1 |
| 32506 | 1.89226 | 2.1 |
| 32507 | 1.89226 | 2.1 |
| 32508 | 2.3256 | 2.1 |
| 32509 | 2.3256 | 2.1 |
| 32510 | 1.89226 | 2.1 |
| 32511 | 1.89226 | 2.1 |
| 32512 | 1.89226 | 2.1 |
| 32513 | 1.89226 | 2.1 |
| 32514 | 1.89226 | 2.1 |
| 32515 | 1.89226 | 2.1 |
| 32516 | 1.92887 | 2.1 |
| 32517 | 2.41915 | 2.1 |
| 32518 | 2.50142 | 2.1 |
| 32519 | 2.50142 | 2.1 |
| 32520 | 1.90388 | 2.1 |
| 32521 | 1.89763 | 2.1 |
| 32522 | 1.89224 | 2.1 |
| 32523 | 1.89224 | 2.1 |
| 32524 | 1.89224 | 2.1 |
| 32525 | 1.89224 | 2.1 |
| 32526 | 3.78448 | 2.1 |
| 32527 | 2.25315 | 2.1 |
| 32528 | 1.05843 | 2.1 |
| 32529 | 2.38995 | 2.1 |
| 32530 | 1.31541 | 2.1 |
| 32531 | 3.56856 | 2.1 |
| 32532 | 0.517552 | 2.1 |
| 32533 | 0.68548 | 2.1 |
| 32534 | 1.27675 | 2.1 |
| 32535 | 1.13076 | 2.1 |

| | | |
|---|---|---|
| 32536 | 0.913611 | 2.1 |
| 32537 | 0.913612 | 2.1 |
| 32538 | 0.902633 | 2.1 |
| 32539 | 0.902633 | 2.1 |
| 32540 | 0.68547 | 2.1 |
| 32541 | 1.27673 | 2.1 |
| 32542 | 1.13078 | 2.1 |
| 32543 | 0.517559 | 2.1 |
| 32544 | 1.89226 | 2.1 |
| 32545 | 1.89226 | 2.1 |
| 32546 | 1.89226 | 2.1 |
| 32547 | 1.89226 | 2.1 |
| 32548 | 3.78453 | 2.1 |
| 32549 | 0.962936 | 2.1 |
| 32550 | 0.962936 | 2.1 |
| 32551 | 0.996141 | 2.1 |
| 32552 | 0.996141 | 2.1 |
| 32553 | 0.830609 | 2.1 |
| 32554 | 0.657714 | 2.1 |
| 32555 | 1.30136 | 2.1 |
| 32556 | 1.19499 | 2.1 |
| 32557 | 1.30136 | 2.1 |
| 32558 | 0.830609 | 2.1 |
| 32559 | 1.19499 | 2.1 |
| 32560 | 0.657713 | 2.1 |
| 32561 | 1.60057 | 0.05 |
| 32562 | 1.39615 | 0.05 |
| 32563 | 1.06474 | 0.05 |
| 32564 | 1.39615 | 0.111057 |
| 32565 | 1.64655 | 0.05 |
| 32566 | 2.04424 | 0.0530237 |
| 32567 | 1.89226 | 0.0726117 |
| 32568 | 1.89226 | 0.358127 |
| 32569 | 1.89226 | 0.199357 |
| 32570 | 1.89226 | 0.166587 |
| 32571 | 1.89226 | 0.162072 |
| 32572 | 1.89226 | 0.157641 |
| 32573 | 2.56322 | 0.05 |
| 32574 | 1.89226 | 0.201155 |
| 32575 | 1.89226 | 0.0736718 |
| 32576 | 1.89226 | 0.0610869 |
| 32577 | 2.18047 | 0.0560837 |
| 32578 | 2.18047 | 0.05 |
| 32579 | 1.89226 | 0.05 |
| 32580 | 1.89226 | 0.0629718 |
| 32581 | 1.89226 | 0.120217 |
| 32582 | 1.65573 | 0.05 |
| 32583 | 1.89226 | 0.10608 |
| 32584 | 1.65573 | 0.05 |
| 32585 | 1.05353 | 0.05 |
| 32586 | 1.83158 | 0.05 |
| 32587 | 1.50282 | 0.05 |
| 32588 | 1.20669 | 0.05 |
| 32589 | 2.88511 | 0.05 |
| 32590 | 1.44807 | 2.1 |
| 32591 | 1.93592 | 2.1 |
| 32592 | 1.80703 | 2.1 |
| 32593 | 1.27806 | 2.1 |

| | | |
|---|---|---|
| 32594 | 3.21398 | 2.1 |
| 32595 | 2.0815 | 2.1 |
| 32596 | 1.87465 | 2.1 |
| 32597 | 1.87465 | 2.1 |
| 32598 | 1.50566 | 2.1 |
| 32599 | 2.0815 | 2.1 |
| 32600 | 1.69263 | 2.1 |
| 32601 | 1.89226 | 2.1 |
| 32602 | 1.89226 | 2.1 |
| 32603 | 1.89226 | 2.1 |
| 32604 | 1.89226 | 2.1 |
| 32605 | 1.89226 | 2.1 |
| 32606 | 1.89226 | 2.1 |
| 32607 | 1.62632 | 2.1 |
| 32608 | 0.994294 | 2.1 |
| 32609 | 0.994294 | 2.1 |
| 32610 | 1.27806 | 2.1 |
| 32611 | 1.81921 | 2.1 |
| 32612 | 2.10776 | 2.1 |
| 32613 | 2.0815 | 2.1 |
| 32614 | 1.70303 | 2.1 |
| 32615 | 1.70303 | 2.1 |
| 32616 | 1.85749 | 2.1 |
| 32617 | 1.90451 | 2.1 |
| 32618 | 2.07724 | 2.1 |
| 32619 | 1.89226 | 2.1 |
| 32620 | 1.89226 | 2.1 |
| 32621 | 1.89226 | 2.1 |
| 32622 | 1.89226 | 2.1 |
| 32623 | 1.89226 | 2.1 |
| 32624 | 1.89226 | 2.1 |
| 32625 | 2.69773 | 2.1 |
| 32626 | 1.88129 | 2.1 |
| 32627 | 2.55196 | 2.1 |
| 32628 | 2.26469 | 2.1 |
| 32629 | 4.94367 | 2.1 |
| 32630 | 1.88129 | 2.1 |
| 32631 | 1.25889 | 2.1 |
| 32632 | 1.26614 | 2.1 |
| 32633 | 1.05914 | 2.1 |
| 32634 | 2.26469 | 2.1 |
| 32635 | 1.88129 | 2.1 |
| 32636 | 2.55196 | 2.1 |
| 32637 | 2.0588 | 2.1 |
| 32638 | 2.6536 | 2.1 |
| 32639 | 2.15287 | 2.1 |
| 32640 | 4.70484 | 2.1 |
| 32641 | 0.8516 | 2.1 |
| 32642 | 0.8516 | 2.1 |
| 32643 | 0.849002 | 2.1 |
| 32644 | 0.849002 | 2.1 |
| 32645 | 0.629445 | 2.1 |
| 32646 | 0.671197 | 2.1 |
| 32647 | 1.0294 | 2.1 |
| 32648 | 1.07644 | 2.1 |
| 32649 | 1.07644 | 2.1 |
| 32650 | 0.671197 | 2.1 |
| 32651 | 0.629446 | 2.1 |

| | | |
|---|---|---|
| 32652 | 1.0294 | 2.1 |
| 32653 | 2.02115 | 2.1 |
| 32654 | 1.18382 | 2.1 |
| 32655 | 2.29107 | 0.337669 |
| 32656 | 1.46903 | 1.50908 |
| 32657 | 3.47488 | 1.00503 |
| 32658 | 2.60763 | 1.2374 |
| 32659 | 2.29107 | 1.83061 |
| 32660 | 1.82141 | 1.41427 |
| 32661 | 1.82141 | 2.1 |
| 32662 | 1.46903 | 2.1 |
| 32663 | 2.29107 | 2.1 |
| 32664 | 1.64007 | 2.1 |
| 32665 | 2.2024 | 2.1 |
| 32666 | 1.44044 | 2.1 |
| 32667 | 2.02634 | 2.1 |
| 32668 | 3.6664 | 2.1 |
| 32669 | 1.25565 | 2.1 |
| 32670 | 2.02635 | 2.1 |
| 32671 | 1.87228 | 2.1 |
| 32672 | 1.87228 | 2.1 |
| 32673 | 1.44045 | 2.1 |
| 32674 | 1.44045 | 2.1 |
| 32675 | 1.89226 | 0.05 |
| 32676 | 1.78503 | 0.05 |
| 32677 | 1.78503 | 0.05 |
| 32678 | 1.40048 | 0.05 |
| 32679 | 2.0815 | 0.05 |
| 32680 | 1.62972 | 0.05 |
| 32681 | 1.27875 | 2.1 |
| 32682 | 1.1483 | 0.231696 |
| 32683 | 1.40014 | 0.228211 |
| 32684 | 1.40014 | 2.1 |
| 32685 | 1.04598 | 1.62965 |
| 32686 | 1.04598 | 2.1 |
| 32687 | 2.63443 | 1.24579 |
| 32688 | 2.26444 | 2.1 |
| 32689 | 2.79392 | 0.173296 |
| 32690 | 2.4863 | 2.1 |
| 32691 | 5.12073 | 1.92376 |
| 32692 | 1.83158 | 0.05 |
| 32693 | 1.18508 | 0.05 |
| 32694 | 1.20669 | 0.05 |
| 32695 | 1.81488 | 0.05 |
| 32696 | 1.20669 | 0.05 |
| 32697 | 1.81488 | 0.05 |
| 32698 | 1.08134 | 0.05 |
| 32699 | 1.70303 | 0.05 |
| 32700 | 1.24027 | 0.05 |
| 32701 | 1.30359 | 0.05 |
| 32702 | 1.89939 | 0.05 |
| 32703 | 1.70303 | 0.05 |
| 32704 | 1.89226 | 2.1 |
| 32705 | 2.69027 | 2.1 |
| 32706 | 1.89226 | 2.1 |
| 32707 | 2.2078 | 2.1 |
| 32708 | 1.89226 | 2.1 |
| 32709 | 2.2078 | 2.1 |

| | | |
|---|---|---|
| 32710 | 1.89226 | 2.1 |
| 32711 | 2.78977 | 2.1 |
| 32712 | 1.89226 | 2.1 |
| 32713 | 2.69027 | 2.1 |
| 32714 | 1.89226 | 2.1 |
| 32715 | 2.69027 | 2.1 |
| 32716 | 1.89226 | 2.1 |
| 32717 | 1.89226 | 2.1 |
| 32718 | 1.89226 | 1.70524 |
| 32719 | 1.89226 | 2.1 |
| 32720 | 3.78452 | 2.1 |
| 32721 | 1.43687 | 2.1 |
| 32722 | 1.71059 | 2.1 |
| 32723 | 2.53014 | 2.1 |
| 32724 | 2.6521 | 2.1 |
| 32725 | 4.24073 | 2.1 |
| 32726 | 2.6521 | 2.1 |
| 32727 | 1.66762 | 2.1 |
| 32728 | 2.50299 | 2.1 |
| 32729 | 2.50299 | 2.1 |
| 32730 | 1.71059 | 2.1 |
| 32731 | 1.71059 | 2.1 |
| 32732 | 1.89226 | 2.1 |
| 32733 | 1.89226 | 2.1 |
| 32734 | 1.89226 | 2.1 |
| 32735 | 1.89226 | 2.1 |
| 32736 | 1.89226 | 2.1 |
| 32737 | 1.89226 | 2.1 |
| 32738 | 2.10662 | 0.63429 |
| 32739 | 2.77417 | 0.559836 |
| 32740 | 2.78873 | 0.968641 |
| 32741 | 2.11661 | 0.591916 |
| 32742 | 4.89078 | 0.63761 |
| 32743 | 2.10662 | 0.761922 |
| 32744 | 1.22857 | 0.0958276 |
| 32745 | 1.25849 | 0.797186 |
| 32746 | 2.11661 | 0.665406 |
| 32747 | 3.34518 | 0.690476 |
| 32748 | 2.78873 | 0.80293 |
| 32749 | 2.11661 | 0.549619 |
| 32750 | 2.12475 | 0.636444 |
| 32751 | 2.79029 | 0.641596 |
| 32752 | 4.9069 | 0.633928 |
| 32753 | 1.2858 | 0.688282 |
| 32754 | 2.11661 | 0.601357 |
| 32755 | 1.25849 | 0.640501 |
| 32756 | 1.25849 | 0.679406 |
| 32757 | 2.12475 | 0.621936 |
| 32758 | 2.12475 | 0.657828 |
| 32759 | 1.13537 | 2.1 |
| 32760 | 1.20714 | 2.1 |
| 32761 | 0.946129 | 2.1 |
| 32762 | 0.946129 | 2.1 |
| 32763 | 0.946131 | 2.1 |
| 32764 | 0.946131 | 2.1 |
| 32765 | 1.29521 | 2.1 |
| 32766 | 1.20713 | 2.1 |
| 32767 | 1.13537 | 2.1 |

| | | |
|---|---|---|
| 32768 | 0.946131 | 2.1 |
| 32769 | 0.94613 | 2.1 |
| 32770 | 1.29521 | 2.1 |
| 32771 | 1.00533 | 2.1 |
| 32772 | 0.876055 | 2.1 |
| 32773 | 0.946128 | 2.1 |
| 32774 | 0.946128 | 2.1 |
| 32775 | 0.913908 | 2.1 |
| 32776 | 0.913908 | 2.1 |
| 32777 | 1.17081 | 2.1 |
| 32778 | 0.896413 | 2.1 |
| 32779 | 0.955905 | 2.1 |
| 32780 | 0.893715 | 2.1 |
| 32781 | 1.20327 | 2.1 |
| 32782 | 0.998544 | 2.1 |
| 32783 | 1.89226 | 1.99032 |
| 32784 | 1.89226 | 2.1 |
| 32785 | 1.89226 | 0.835643 |
| 32786 | 1.89226 | 1.40876 |
| 32787 | 3.78452 | 1.05607 |
| 32788 | 0.540975 | 1.35274 |
| 32789 | 0.726949 | 2.1 |
| 32790 | 0.712198 | 2.1 |
| 32791 | 0.506675 | 1.22209 |
| 32792 | 0.611916 | 1.29058 |
| 32793 | 0.611916 | 2.1 |
| 32794 | 0.621708 | 1.98814 |
| 32795 | 0.621708 | 1.20966 |
| 32796 | 0.726951 | 1.54675 |
| 32797 | 0.712196 | 1.28398 |
| 32798 | 0.506674 | 1.73311 |
| 32799 | 0.540976 | 1.51197 |
| 32800 | 1.89226 | 1.21949 |
| 32801 | 1.89226 | 2.1 |
| 32802 | 1.89226 | 1.25815 |
| 32803 | 1.89226 | 1.31633 |
| 32804 | 3.78453 | 1.98273 |
| 32805 | 1.89226 | 1.11235 |
| 32806 | 1.89226 | 1.30677 |
| 32807 | 1.89226 | 0.834352 |
| 32808 | 1.89226 | 2.1 |
| 32809 | 1.89226 | 0.899087 |
| 32810 | 1.89226 | 2.1 |
| 32811 | 0.919594 | 1.18065 |
| 32812 | 0.726949 | 2.01085 |
| 32813 | 0.898318 | 1.25439 |
| 32814 | 0.712198 | 1.98089 |
| 32815 | 0.817559 | 1.01769 |
| 32816 | 0.817559 | 2.1 |
| 32817 | 0.814234 | 1.00943 |
| 32818 | 0.814234 | 2.06148 |
| 32819 | 0.919597 | 1.06799 |
| 32820 | 0.898316 | 0.918673 |
| 32821 | 0.726951 | 2.0496 |
| 32822 | 0.712196 | 1.5034 |
| 32823 | 1.79663 | 0.976891 |
| 32824 | 1.12088 | 2.1 |
| 32825 | 1.54699 | 1.28402 |

| | | |
|---|---|---|
| 32826 | 1.17475 | 1.36161 |
| 32827 | 1.79663 | 2.1 |
| 32828 | 1.42439 | 2.1 |
| 32829 | 2.28928 | 1.16157 |
| 32830 | 1.23077 | 1.5813 |
| 32831 | 1.65458 | 1.04316 |
| 32832 | 2.66533 | 2.09668 |
| 32833 | 3.94385 | 1.329 |
| 32834 | 1.89226 | 2.1 |
| 32835 | 1.89226 | 2.1 |
| 32836 | 1.89226 | 2.1 |
| 32837 | 1.89226 | 2.1 |
| 32838 | 3.78452 | 2.1 |
| 32839 | 1.38426 | 2.1 |
| 32840 | 1.15067 | 2.1 |
| 32841 | 1.38426 | 2.1 |
| 32842 | 1.01595 | 2.1 |
| 32843 | 1.24509 | 2.1 |
| 32844 | 1.01595 | 2.1 |
| 32845 | 2.62097 | 2.1 |
| 32846 | 1.89226 | 2.1 |
| 32847 | 2.72561 | 2.1 |
| 32848 | 2.72561 | 2.1 |
| 32849 | 1.89226 | 2.1 |
| 32850 | 1.89226 | 2.1 |
| 32851 | 1.90103 | 0.05 |
| 32852 | 1.41902 | 2.1 |
| 32853 | 1.77249 | 0.05 |
| 32854 | 1.30272 | 2.1 |
| 32855 | 3.20375 | 0.05 |
| 32856 | 1.77249 | 0.05 |
| 32857 | 1.19548 | 2.1 |
| 32858 | 1.19548 | 0.524604 |
| 32859 | 1.30272 | 1.78811 |
| 32860 | 1.65815 | 0.05 |
| 32861 | 1.76539 | 0.782542 |
| 32862 | 2.62161 | 2.1 |
| 32863 | 1.89226 | 2.1 |
| 32864 | 1.89226 | 2.1 |
| 32865 | 1.89226 | 2.1 |
| 32866 | 2.6446 | 2.1 |
| 32867 | 2.6446 | 2.1 |
| 32868 | 2.64459 | 2.1 |
| 32869 | 1.89226 | 2.1 |
| 32870 | 1.89226 | 2.1 |
| 32871 | 1.89226 | 2.1 |
| 32872 | 2.58257 | 2.1 |
| 32873 | 2.58257 | 2.1 |
| 32874 | 1.89226 | 0.05 |
| 32875 | 1.89226 | 0.924471 |
| 32876 | 2.31301 | 0.822679 |
| 32877 | 2.76309 | 0.0723462 |
| 32878 | 1.89226 | 0.513411 |
| 32879 | 2.26047 | 0.0660282 |
| 32880 | 1.89226 | 0.05 |
| 32881 | 2.31301 | 1.03485 |
| 32882 | 2.17765 | 0.740398 |
| 32883 | 2.17765 | 0.090371 |

| | | |
|---|---|---|
| 32884 | 1.89226 | 0.498783 |
| 32885 | 1.89226 | 0.0681731 |
| 32886 | 2.18516 | 2.1 |
| 32887 | 1.85196 | 2.1 |
| 32888 | 1.78595 | 2.1 |
| 32889 | 2.14606 | 2.1 |
| 32890 | 3.99802 | 2.1 |
| 32891 | 2.18516 | 2.1 |
| 32892 | 2.21189 | 2.1 |
| 32893 | 2.18937 | 2.1 |
| 32894 | 2.14606 | 2.1 |
| 32895 | 4.35796 | 2.1 |
| 32896 | 1.60735 | 2.1 |
| 32897 | 2.14606 | 2.1 |
| 32898 | 2.15714 | 2.1 |
| 32899 | 1.70302 | 2.1 |
| 32900 | 3.70055 | 2.1 |
| 32901 | 2.18937 | 2.1 |
| 32902 | 2.14606 | 2.1 |
| 32903 | 2.0927 | 2.1 |
| 32904 | 2.162 2.1 | |
| 32905 | 4.30806 | 2.1 |
| 32906 | 2.17326 | 2.1 |
| 32907 | 2.58517 | 2.1 |
| 32908 | 2.55318 | 2.1 |
| 32909 | 2.1665 | 2.1 |
| 32910 | 4.75167 | 2.1 |
| 32911 | 2.17326 | 2.1 |
| 32912 | 1.53556 | 2.1 |
| 32913 | 1.56253 | 2.1 |
| 32914 | 2.1665 | 2.1 |
| 32915 | 3.70206 | 2.1 |
| 32916 | 2.55317 | 2.1 |
| 32917 | 2.1665 | 2.1 |
| 32918 | 2.17741 | 2.1 |
| 32919 | 2.54197 | 2.1 |
| 32920 | 4.70847 | 2.1 |
| 32921 | 2.17742 | 2.1 |
| 32922 | 2.1665 | 2.1 |
| 32923 | 2.18452 | 2.1 |
| 32924 | 1.58054 | 2.1 |
| 32925 | 1.56253 | 2.1 |
| 32926 | 1.56253 | 2.1 |
| 32927 | 1.71051 | 0.05 |
| 32928 | 1.89807 | 0.05 |
| 32929 | 1.19755 | 0.05 |
| 32930 | 1.19755 | 0.05 |
| 32931 | 2.25632 | 0.05 |
| 32932 | 2.25632 | 0.05 |
| 32933 | 0.876046 | 0.05 |
| 32934 | 1.27293 | 0.05 |
| 32935 | 0.946132 | 0.05 |
| 32936 | 0.946132 | 0.05 |
| 32937 | 0.946131 | 0.05 |
| 32938 | 0.869312 | 0.05 |
| 32939 | 1.12767 | 0.05 |
| 32940 | 0.946132 | 0.05 |
| 32941 | 1.22225 | 0.05 |

| | | |
|---|---|---|
| 32942 | 1.37968 | 0.05 |
| 32943 | 0.851555 | 0.05 |
| 32944 | 0.851555 | 0.05 |
| 32945 | 0.946133 | 1.63589 |
| 32946 | 0.946133 | 2.1 |
| 32947 | 0.946132 | 2.1 |
| 32948 | 0.946132 | 0.837123 |
| 32949 | 0.946133 | 1.86358 |
| 32950 | 0.946133 | 1.92025 |
| 32951 | 0.946132 | 2.1 |
| 32952 | 0.946132 | 2.073 |
| 32953 | 0.946132 | 2.1 |
| 32954 | 0.946132 | 2.1 |
| 32955 | 0.946133 | 2.08214 |
| 32956 | 0.946133 | 2.1 |
| 32957 | 1.35152 | 2.1 |
| 32958 | 0.948936 | 2.1 |
| 32959 | 1.90166 | 2.1 |
| 32960 | 1.70303 | 2.1 |
| 32961 | 2.90858 | 2.1 |
| 32962 | 1.89226 | 2.1 |
| 32963 | 1.89226 | 2.1 |
| 32964 | 1.89226 | 2.1 |
| 32965 | 1.89226 | 2.1 |
| 32966 | 3.78452 | 2.1 |
| 32967 | 1.90166 | 2.1 |
| 32968 | 1.70303 | 2.1 |
| 32969 | 2.52454 | 2.1 |
| 32970 | 2.13919 | 2.1 |
| 32971 | 3.99529 | 2.1 |
| 32972 | 1.89226 | 1.77588 |
| 32973 | 1.89226 | 2.1 |
| 32974 | 1.89226 | 2.1 |
| 32975 | 1.89226 | 2.1 |
| 32976 | 3.78452 | 2.1 |
| 32977 | 1.89226 | 2.1 |
| 32978 | 1.89226 | 2.1 |
| 32979 | 1.89226 | 2.1 |
| 32980 | 1.89226 | 2.1 |
| 32981 | 1.89226 | 1.02812 |
| 32982 | 1.89226 | 2.1 |
| 32983 | 2.13918 | 2.1 |
| 32984 | 0.992674 | 2.1 |
| 32985 | 2.3397 | 2.1 |
| 32986 | 1.8023 | 2.1 |
| 32987 | 1.47769 | 2.1 |
| 32988 | 0.992674 | 2.1 |
| 32989 | 1.89226 | 0.05 |
| 32990 | 1.89226 | 0.05 |
| 32991 | 1.89226 | 0.05 |
| 32992 | 1.89226 | 0.05 |
| 32993 | 3.78452 | 0.05 |
| 32994 | 1.89226 | 0.05 |
| 32995 | 2.54752 | 0.05 |
| 32996 | 2.63974 | 0.05 |
| 32997 | 1.89226 | 0.05 |
| 32998 | 4.53201 | 0.05 |
| 32999 | 0.498412 | 0.05 |

| | | |
|---|---|---|
| 33000 | 0.500955 | 0.05 |
| 33001 | 0.57955 | 0.05 |
| 33002 | 0.563873 | 0.05 |
| 33003 | 0.529107 | 0.05 |
| 33004 | 0.529107 | 0.05 |
| 33005 | 0.53572 | 0.05 |
| 33006 | 0.53572 | 0.05 |
| 33007 | 0.500956 | 0.05 |
| 33008 | 0.579548 | 0.05 |
| 33009 | 0.563871 | 0.05 |
| 33010 | 0.498414 | 0.05 |
| 33011 | 1.89223 | 0.05 |
| 33012 | 1.89223 | 0.05 |
| 33013 | 1.89223 | 0.05 |
| 33014 | 1.89223 | 0.05 |
| 33015 | 1.89223 | 0.05 |
| 33016 | 1.89223 | 0.05 |
| 33017 | 1.28752 | 0.05 |
| 33018 | 1.31985 | 0.05 |
| 33019 | 0.946119 | 0.05 |
| 33020 | 0.946119 | 0.05 |
| 33021 | 1.28752 | 0.05 |
| 33022 | 1.28752 | 0.05 |
| 33023 | 0.946119 | 0.05 |
| 33024 | 0.946119 | 0.05 |
| 33025 | 1.28753 | 0.05 |
| 33026 | 0.946132 | 0.05 |
| 33027 | 1.31984 | 0.05 |
| 33028 | 0.946107 | 0.05 |
| 33029 | 0.500948 | 0.05 |
| 33030 | 0.479465 | 0.05 |
| 33031 | 0.562335 | 0.05 |
| 33032 | 0.579542 | 0.05 |
| 33033 | 0.528436 | 0.05 |
| 33034 | 0.528436 | 0.05 |
| 33035 | 0.530571 | 0.05 |
| 33036 | 0.530571 | 0.05 |
| 33037 | 0.56234 | 0.05 |
| 33038 | 0.479473 | 0.05 |
| 33039 | 0.500943 | 0.05 |
| 33040 | 0.579533 | 0.05 |
| 33041 | 1.89226 | 2.1 |
| 33042 | 1.89226 | 2.1 |
| 33043 | 1.89226 | 2.1 |
| 33044 | 1.89226 | 2.1 |
| 33045 | 3.78452 | 2.1 |
| 33046 | 1.50767 | 2.1 |
| 33047 | 1.14496 | 2.1 |
| 33048 | 1.50767 | 2.1 |
| 33049 | 1.58539 | 2.1 |
| 33050 | 1.07653 | 2.1 |
| 33051 | 1.15425 | 2.1 |
| 33052 | 1.89226 | 2.1 |
| 33053 | 2.43826 | 2.1 |
| 33054 | 2.43826 | 2.1 |
| 33055 | 2.48688 | 2.1 |
| 33056 | 1.89226 | 2.1 |
| 33057 | 1.94088 | 2.1 |

| | | |
|---|---|---|
| 33058 | 1.89226 | 2.1 |
| 33059 | 1.89226 | 2.1 |
| 33060 | 1.89226 | 2.1 |
| 33061 | 1.89226 | 2.1 |
| 33062 | 3.78452 | 2.1 |
| 33063 | 1.50767 | 2.1 |
| 33064 | 1.07653 | 2.1 |
| 33065 | 1.41077 | 2.1 |
| 33066 | 0.991407 | 2.1 |
| 33067 | 2.49907 | 2.1 |
| 33068 | 1.21913 | 2.1 |
| 33069 | 1.21913 | 2.1 |
| 33070 | 0.946128 | 2.1 |
| 33071 | 0.946128 | 2.1 |
| 33072 | 1.21913 | 2.1 |
| 33073 | 1.18364 | 2.1 |
| 33074 | 0.946131 | 2.1 |
| 33075 | 0.946131 | 2.1 |
| 33076 | 1.18364 | 2.1 |
| 33077 | 0.946129 | 2.1 |
| 33078 | 1.21913 | 2.1 |
| 33079 | 0.94613 | 2.1 |
| 33080 | 1.89226 | 0.05 |
| 33081 | 1.89226 | 2.1 |
| 33082 | 1.89226 | 0.655703 |
| 33083 | 1.89226 | 2.1 |
| 33084 | 3.78452 | 2.1 |
| 33085 | 1.93823 | 0.636919 |
| 33086 | 1.60976 | 2.1 |
| 33087 | 1.93823 | 2.1 |
| 33088 | 2.20045 | 0.0525812 |
| 33089 | 1.36999 | 2.1 |
| 33090 | 1.63222 | 0.880593 |
| 33091 | 1.38302 | 2.1 |
| 33092 | 1.38302 | 2.1 |
| 33093 | 0.946132 | 2.1 |
| 33094 | 0.946132 | 2.1 |
| 33095 | 1.27258 | 2.1 |
| 33096 | 1.38302 | 2.1 |
| 33097 | 0.946133 | 2.1 |
| 33098 | 0.946133 | 2.1 |
| 33099 | 0.946133 | 2.1 |
| 33100 | 1.27258 | 2.1 |
| 33101 | 0.946132 | 2.1 |
| 33102 | 1.38302 | 2.1 |
| 33103 | 1.89226 | 0.05 |
| 33104 | 1.89226 | 2.1 |
| 33105 | 1.89226 | 0.0817179 |
| 33106 | 1.89226 | 2.1 |
| 33107 | 3.78452 | 2.1 |
| 33108 | 1.93823 | 0.05 |
| 33109 | 1.36999 | 2.1 |
| 33110 | 1.70423 | 0.0824186 |
| 33111 | 1.14195 | 2.1 |
| 33112 | 3.08018 | 1.05503 |
| 33113 | 1.27258 | 2.1 |
| 33114 | 1.27258 | 2.1 |
| 33115 | 0.946132 | 2.1 |

| | | |
|---|---|---|
| 33116 | 0.946132 | 2.1 |
| 33117 | 1.17233 | 2.1 |
| 33118 | 1.27258 | 2.1 |
| 33119 | 0.946132 | 2.1 |
| 33120 | 0.946132 | 2.1 |
| 33121 | 1.17233 | 2.1 |
| 33122 | 0.946132 | 2.1 |
| 33123 | 0.946132 | 2.1 |
| 33124 | 1.27258 | 2.1 |
| 33125 | 2.70575 | 2.1 |
| 33126 | 1.89226 | 0.93543 |
| 33127 | 2.71429 | 2.1 |
| 33128 | 2.71429 | 2.1 |
| 33129 | 1.89226 | 2.1 |
| 33130 | 1.89226 | 0.958399 |
| 33131 | 0.946133 | 0.822583 |
| 33132 | 0.946133 | 2.1 |
| 33133 | 1.28713 | 2.1 |
| 33134 | 1.28713 | 2.1 |
| 33135 | 0.946131 | 2.1 |
| 33136 | 0.946131 | 0.808204 |
| 33137 | 1.28713 | 2.1 |
| 33138 | 1.36503 | 2.1 |
| 33139 | 0.946131 | 2.1 |
| 33140 | 1.36503 | 2.1 |
| 33141 | 1.28713 | 2.1 |
| 33142 | 0.946131 | 2.1 |
| 33143 | 1.89226 | 2.1 |
| 33144 | 1.89226 | 2.05758 |
| 33145 | 1.89226 | 2.1 |
| 33146 | 1.89226 | 2.00088 |
| 33147 | 1.89226 | 2.1 |
| 33148 | 1.89226 | 2.04232 |
| 33149 | 1.22615 | 2.1 |
| 33150 | 1.04147 | 2.1 |
| 33151 | 1.3439 | 2.1 |
| 33152 | 1.3439 | 2.1 |
| 33153 | 0.969118 | 2.1 |
| 33154 | 0.969118 | 2.1 |
| 33155 | 0.980841 | 2.1 |
| 33156 | 0.992505 | 2.1 |
| 33157 | 1.34289 | 2.1 |
| 33158 | 1.70303 | 2.1 |
| 33159 | 2.56081 | 2.1 |
| 33160 | 1.89225 | 2.1 |
| 33161 | 2.66785 | 2.1 |
| 33162 | 2.79506 | 2.1 |
| 33163 | 2.79506 | 2.1 |
| 33164 | 1.89225 | 2.1 |
| 33165 | 1.89225 | 2.1 |
| 33166 | 0.978778 | 2.1 |
| 33167 | 0.978778 | 2.1 |
| 33168 | 0.993303 | 2.1 |
| 33169 | 0.993303 | 2.1 |
| 33170 | 0.603538 | 2.1 |
| 33171 | 0.638158 | 2.1 |
| 33172 | 1.33393 | 2.1 |
| 33173 | 1.39753 | 2.1 |

| | | |
|---|---|---|
| 33174 | 1.33391 | 2.1 |
| 33175 | 0.603535 | 2.1 |
| 33176 | 0.638164 | 2.1 |
| 33177 | 1.39754 | 2.1 |
| 33178 | 1.33393 | 2.1 |
| 33179 | 1.33393 | 2.1 |
| 33180 | 0.946132 | 2.1 |
| 33181 | 0.946132 | 2.1 |
| 33182 | 1.33393 | 2.1 |
| 33183 | 1.27365 | 2.1 |
| 33184 | 0.946132 | 2.1 |
| 33185 | 0.946132 | 2.1 |
| 33186 | 0.946132 | 2.1 |
| 33187 | 1.27365 | 2.1 |
| 33188 | 1.33393 | 2.1 |
| 33189 | 0.946131 | 2.1 |
| 33190 | 1.89226 | 2.1 |
| 33191 | 1.89226 | 2.1 |
| 33192 | 1.89226 | 2.1 |
| 33193 | 1.89226 | 2.1 |
| 33194 | 3.78452 | 2.1 |
| 33195 | 1.89226 | 2.1 |
| 33196 | 1.89226 | 2.1 |
| 33197 | 1.89226 | 2.1 |
| 33198 | 1.89226 | 2.1 |
| 33199 | 3.78453 | 2.1 |
| 33200 | 0.979364 | 2.1 |
| 33201 | 0.979364 | 2.1 |
| 33202 | 0.89618 | 2.1 |
| 33203 | 0.89618 | 2.1 |
| 33204 | 0.896178 | 2.1 |
| 33205 | 0.929411 | 2.1 |
| 33206 | 0.946133 | 2.1 |
| 33207 | 0.946133 | 2.1 |
| 33208 | 0.946131 | 2.1 |
| 33209 | 0.89618 | 2.1 |
| 33210 | 0.946131 | 2.1 |
| 33211 | 0.929413 | 2.1 |
| 33212 | 1.89226 | 2.1 |
| 33213 | 1.89226 | 2.1 |
| 33214 | 1.89226 | 2.1 |
| 33215 | 1.89226 | 2.1 |
| 33216 | 1.89226 | 2.1 |
| 33217 | 1.89226 | 2.1 |
| 33218 | 1.89225 | 2.1 |
| 33219 | 1.89225 | 2.1 |
| 33220 | 1.89225 | 2.1 |
| 33221 | 1.89225 | 2.1 |
| 33222 | 3.7845 | 2.1 |
| 33223 | 1.89225 | 2.1 |
| 33224 | 1.89225 | 2.1 |
| 33225 | 1.89225 | 2.1 |
| 33226 | 1.89225 | 2.1 |
| 33227 | 1.89225 | 2.1 |
| 33228 | 1.89225 | 2.1 |
| 33229 | 0.946128 | 0.05 |
| 33230 | 0.946128 | 0.05 |
| 33231 | 0.946132 | 0.05 |

| | | |
|---|---|---|
| 33232 | 0.946132 | 0.05 |
| 33233 | 0.94613 | 0.05 |
| 33234 | 0.94613 | 0.05 |
| 33235 | 0.946129 | 0.05 |
| 33236 | 0.946129 | 0.05 |
| 33237 | 0.94613 | 0.05 |
| 33238 | 0.94613 | 0.05 |
| 33239 | 0.94613 | 0.05 |
| 33240 | 0.94613 | 0.05 |
| 33241 | 1.89227 | 1.5973 |
| 33242 | 1.89227 | 1.48531 |
| 33243 | 1.89227 | 1.03893 |
| 33244 | 1.89227 | 0.949506 |
| 33245 | 3.78453 | 1.0695 |
| 33246 | 1.89226 | 1.43238 |
| 33247 | 2.43717 | 2.08725 |
| 33248 | 2.63259 | 1.1624 |
| 33249 | 1.89226 | 0.838696 |
| 33250 | 4.52485 | 1.51129 |
| 33251 | 0.616808 | 1.14664 |
| 33252 | 0.792277 | 1.63028 |
| 33253 | 1.04986 | 1.01018 |
| 33254 | 0.72303 | 1.53522 |
| 33255 | 0.702675 | 0.647884 |
| 33256 | 0.76862 | 2.1 |
| 33257 | 0.809154 | 2.05563 |
| 33258 | 0.809154 | 1.06436 |
| 33259 | 0.808757 | 1.59627 |
| 33260 | 1.07789 | 0.846726 |
| 33261 | 0.772568 | 1.0327 |
| 33262 | 0.598878 | 1.84203 |
| 33263 | 1.89226 | 1.52329 |
| 33264 | 1.89226 | 1.26872 |
| 33265 | 1.89226 | 1.06758 |
| 33266 | 1.89226 | 0.998236 |
| 33267 | 3.78453 | 1.41218 |
| 33268 | 1.36428 | 2.1 |
| 33269 | 1.31629 | 1.20515 |
| 33270 | 0.946132 | 1.28714 |
| 33271 | 0.946132 | 1.13234 |
| 33272 | 1.36428 | 0.892686 |
| 33273 | 1.36428 | 2.1 |
| 33274 | 0.946132 | 1.40477 |
| 33275 | 0.946132 | 2.07459 |
| 33276 | 1.36428 | 2.1 |
| 33277 | 0.946132 | 1.0076 |
| 33278 | 1.31629 | 1.89768 |
| 33279 | 0.946132 | 0.842322 |
| 33280 | 1.578 | 1.63456 |
| 33281 | 2.85336 | 0.863088 |
| 33282 | 2.11368 | 0.733898 |
| 33283 | 2.14375 | 1.1521 |
| 33284 | 1.93852 | 1.15035 |
| 33285 | 1.93852 | 1.86849 |
| 33286 | 2.34806 | 0.517464 |
| 33287 | 1.42913 | 0.05 |
| 33288 | 1.61539 | 0.591176 |
| 33289 | 2.45322 | 0.0757631 |

| | | |
|---|---|---|
| 33290 | 3.88235 | 0.505868 |
| 33291 | 2.34806 | 0.398187 |
| 33292 | 1.89226 | 0.123584 |
| 33293 | 1.89226 | 0.570826 |
| 33294 | 2.45322 | 0.393131 |
| 33295 | 4.34548 | 0.101706 |
| 33296 | 1.89226 | 0.386627 |
| 33297 | 1.89226 | 0.0959233 |
| 33298 | 1.89226 | 0.424623 |
| 33299 | 1.89226 | 0.39761 |
| 33300 | 3.78452 | 0.417685 |
| 33301 | 1.89226 | 0.106941 |
| 33302 | 1.89226 | 0.05 |
| 33303 | 1.89226 | 0.356865 |
| 33304 | 1.89226 | 0.398661 |
| 33305 | 3.78452 | 0.0671577 |
| 33306 | 1.25972 | 0.381902 |
| 33307 | 2.45322 | 0.05 |
| 33308 | 2.58823 | 0.541436 |
| 33309 | 1.79398 | 0.0563313 |
| 33310 | 4.2472 | 0.05 |
| 33311 | 1.89226 | 0.498324 |
| 33312 | 2.45322 | 0.0630184 |
| 33313 | 2.58823 | 0.179616 |
| 33314 | 2.45322 | 0.0528867 |
| 33315 | 2.02727 | 0.249237 |
| 33316 | 1.89226 | 0.0576129 |
| 33317 | 1.89226 | 0.199166 |
| 33318 | 1.89226 | 0.181791 |
| 33319 | 1.89226 | 0.43682 |
| 33320 | 1.89226 | 0.115286 |
| 33321 | 3.78452 | 0.0525017 |
| 33322 | 1.89226 | 0.05 |
| 33323 | 1.89226 | 0.080844 |
| 33324 | 1.89226 | 0.05 |
| 33325 | 1.89226 | 0.05 |
| 33326 | 1.89226 | 0.468514 |
| 33327 | 1.89226 | 0.349048 |
| 33328 | 1.70048 | 2.1 |
| 33329 | 2.1572 | 0.05 |
| 33330 | 1.45477 | 1.99024 |
| 33331 | 1.93973 | 0.05 |
| 33332 | 3.64021 | 0.263714 |
| 33333 | 1.17614 | 1.87537 |
| 33334 | 1.93973 | 0.05 |
| 33335 | 1.69661 | 2.1 |
| 33336 | 1.69661 | 0.05 |
| 33337 | 1.45477 | 2.1 |
| 33338 | 1.45477 | 0.05 |
| 33339 | 1.94343 | 2.1 |
| 33340 | 2.53782 | 1.49587 |
| 33341 | 2.59903 | 2.1 |
| 33342 | 2.59903 | 0.836727 |
| 33343 | 1.87598 | 2.1 |
| 33344 | 1.87598 | 0.910036 |
| 33345 | 0.946131 | 0.421223 |
| 33346 | 0.946131 | 1.25267 |
| 33347 | 1.26891 | 2.1 |

| | | |
|---|---|---|
| 33348 | 1.26891 | 0.05 |
| 33349 | 0.94613 | 1.53232 |
| 33350 | 0.94613 | 1.11075 |
| 33351 | 1.26891 | 1.45474 |
| 33352 | 1.29952 | 0.90661 |
| 33353 | 1.29952 | 0.221065 |
| 33354 | 0.946131 | 0.05 |
| 33355 | 1.26891 | 0.954414 |
| 33356 | 0.946131 | 0.964475 |
| 33357 | 2.00551 | 2.1 |
| 33358 | 2.59903 | 1.97009 |
| 33359 | 2.65811 | 2.1 |
| 33360 | 2.65811 | 1.02678 |
| 33361 | 1.94343 | 2.1 |
| 33362 | 1.94343 | 1.1031 |
| 33363 | 2.65811 | 0.89607 |
| 33364 | 1.89226 | 2.1 |
| 33365 | 1.89226 | 0.60153 |
| 33366 | 1.89226 | 1.32141 |
| 33367 | 2.59903 | 0.960089 |
| 33368 | 2.59903 | 2.1 |
| 33369 | 1.89227 | 2.1 |
| 33370 | 1.89227 | 2.1 |
| 33371 | 1.89227 | 2.1 |
| 33372 | 1.89227 | 2.1 |
| 33373 | 3.78453 | 2.1 |
| 33374 | 1.15471 | 2.1 |
| 33375 | 0.993889 | 2.1 |
| 33376 | 1.78919 | 2.1 |
| 33377 | 1.77325 | 2.1 |
| 33378 | 2.78308 | 2.1 |
| 33379 | 1.89226 | 2.1 |
| 33380 | 1.89226 | 2.1 |
| 33381 | 1.89226 | 2.1 |
| 33382 | 1.89226 | 2.1 |
| 33383 | 3.78453 | 2.1 |
| 33384 | 1.78919 | 2.1 |
| 33385 | 1.77325 | 2.1 |
| 33386 | 2.45969 | 2.1 |
| 33387 | 2.70835 | 2.1 |
| 33388 | 4.49753 | 2.1 |
| 33389 | 0.939314 | 2.1 |
| 33390 | 0.939315 | 2.1 |
| 33391 | 1.01133 | 2.1 |
| 33392 | 1.01133 | 2.1 |
| 33393 | 0.596471 | 2.1 |
| 33394 | 1.20813 | 2.1 |
| 33395 | 0.886619 | 2.1 |
| 33396 | 1.35417 | 2.1 |
| 33397 | 1.35417 | 2.1 |
| 33398 | 1.20813 | 2.1 |
| 33399 | 0.886621 | 2.1 |
| 33400 | 0.59647 | 2.1 |
| 33401 | 0.946131 | 2.1 |
| 33402 | 0.946131 | 2.1 |
| 33403 | 0.94613 | 2.1 |
| 33404 | 0.94613 | 2.1 |
| 33405 | 0.946133 | 2.1 |

| | | |
|---|---|---|
| 33406 | 0.946133 | 2.1 |
| 33407 | 0.946128 | 2.1 |
| 33408 | 0.946128 | 2.1 |
| 33409 | 0.946131 | 2.1 |
| 33410 | 0.946131 | 2.1 |
| 33411 | 0.946131 | 2.1 |
| 33412 | 0.946131 | 2.1 |
| 33413 | 1.03939 | 2.1 |
| 33414 | 1.03939 | 2.1 |
| 33415 | 0.946132 | 0.235003 |
| 33416 | 0.946132 | 1.20659 |
| 33417 | 0.946132 | 1.57288 |
| 33418 | 0.946132 | 2.1 |
| 33419 | 1.23851 | 0.403239 |
| 33420 | 1.03939 | 2.1 |
| 33421 | 1.03939 | 2.1 |
| 33422 | 0.946132 | 2.1 |
| 33423 | 0.946133 | 2.1 |
| 33424 | 1.23851 | 2.1 |
| 33425 | 0.902503 | 2.1 |
| 33426 | 0.946129 | 2.1 |
| 33427 | 0.946132 | 0.346312 |
| 33428 | 0.946132 | 2.1 |
| 33429 | 0.902472 | 2.02137 |
| 33430 | 0.902472 | 2.1 |
| 33431 | 1.08736 | 0.990904 |
| 33432 | 0.994093 | 2.1 |
| 33433 | 0.946133 | 2.1 |
| 33434 | 0.902504 | 2.1 |
| 33435 | 1.03939 | 2.1 |
| 33436 | 0.946132 | 2.1 |
| 33437 | 1.35306 | 1.16605 |
| 33438 | 1.29982 | 0.736882 |
| 33439 | 1.7216 | 0.728437 |
| 33440 | 1.89183 | 0.690872 |
| 33441 | 3.07466 | 0.742215 |
| 33442 | 0.485098 | 0.745178 |
| 33443 | 0.582401 | 0.774062 |
| 33444 | 0.649908 | 0.959519 |
| 33445 | 0.8608 | 0.794649 |
| 33446 | 0.587739 | 0.60713 |
| 33447 | 0.58774 | 0.867542 |
| 33448 | 0.644567 | 0.77724 |
| 33449 | 0.644567 | 1.76189 |
| 33450 | 0.58241 | 0.930655 |
| 33451 | 0.86081 | 0.642699 |
| 33452 | 0.649898 | 1.15898 |
| 33453 | 0.485093 | 0.774535 |
| 33454 | 2.25936 | 2.1 |
| 33455 | 2.71441 | 2.1 |
| 33456 | 2.63824 | 2.1 |
| 33457 | 2.20652 | 2.1 |
| 33458 | 4.92092 | 2.1 |
| 33459 | 2.25936 | 2.1 |
| 33460 | 1.57627 | 2.1 |
| 33461 | 1.5493 | 2.1 |
| 33462 | 2.20652 | 2.1 |
| 33463 | 3.78279 | 2.1 |

| | | |
|---|---|---|
| 33464 | 2.63824 | 2.1 |
| 33465 | 2.20652 | 2.1 |
| 33466 | 2.17326 | 2.1 |
| 33467 | 2.58517 | 2.1 |
| 33468 | 4.79169 | 2.1 |
| 33469 | 2.17326 | 2.1 |
| 33470 | 2.20652 | 2.1 |
| 33471 | 2.19278 | 2.1 |
| 33472 | 1.53556 | 2.1 |
| 33473 | 1.5493 | 2.1 |
| 33474 | 1.5493 | 2.1 |
| 33475 | 1.97465 | 2.1 |
| 33476 | 2.13925 | 2.1 |
| 33477 | 2.04483 | 2.1 |
| 33478 | 1.62784 | 2.1 |
| 33479 | 3.76709 | 2.1 |
| 33480 | 1.89226 | 2.1 |
| 33481 | 1.89226 | 2.1 |
| 33482 | 1.89226 | 2.1 |
| 33483 | 1.89226 | 2.1 |
| 33484 | 1.89226 | 2.1 |
| 33485 | 1.89226 | 2.1 |
| 33486 | 0.724037 | 2.1 |
| 33487 | 0.81392 | 2.1 |
| 33488 | 0.96796 | 2.1 |
| 33489 | 1.02242 | 2.1 |
| 33490 | 0.882091 | 2.1 |
| 33491 | 0.882091 | 2.1 |
| 33492 | 0.899789 | 2.1 |
| 33493 | 0.899789 | 2.1 |
| 33494 | 0.724036 | 2.1 |
| 33495 | 0.96796 | 2.1 |
| 33496 | 1.02242 | 2.1 |
| 33497 | 0.81392 | 2.1 |
| 33498 | 1.13537 | 2.1 |
| 33499 | 1.63054 | 2.1 |
| 33500 | 0.946131 | 2.1 |
| 33501 | 0.946131 | 2.1 |
| 33502 | 0.946131 | 2.1 |
| 33503 | 0.946131 | 2.1 |
| 33504 | 1.258 2.1 | |
| 33505 | 1.63054 | 2.1 |
| 33506 | 1.22632 | 2.1 |
| 33507 | 1.22632 | 2.1 |
| 33508 | 0.946131 | 2.1 |
| 33509 | 1.258 2.1 | |
| 33510 | 1.89226 | 2.1 |
| 33511 | 1.89226 | 2.1 |
| 33512 | 1.89226 | 2.1 |
| 33513 | 1.89226 | 2.1 |
| 33514 | 1.89226 | 2.1 |
| 33515 | 1.89226 | 2.1 |
| 33516 | 0.809739 | 2.1 |
| 33517 | 0.862674 | 2.1 |
| 33518 | 1.1995 | 2.1 |
| 33519 | 1.33899 | 2.1 |
| 33520 | 1.06324 | 2.1 |
| 33521 | 1.06324 | 2.1 |

| | | |
|---|---|---|
| 33522 | 0.981932 | 2.1 |
| 33523 | 1.19563 | 2.1 |
| 33524 | 0.809727 | 2.1 |
| 33525 | 1.14092 | 2.1 |
| 33526 | 1.33902 | 2.1 |
| 33527 | 0.863494 | 2.1 |
| 33528 | 2.23742 | 2.1 |
| 33529 | 1.84902 | 2.1 |
| 33530 | 1.88088 | 2.1 |
| 33531 | 2.20392 | 2.1 |
| 33532 | 4.08644 | 2.1 |
| 33533 | 1.3592 | 2.1 |
| 33534 | 1.84902 | 2.1 |
| 33535 | 1.88088 | 2.1 |
| 33536 | 1.3305 | 2.1 |
| 33537 | 1.88088 | 2.1 |
| 33538 | 1.3305 | 2.1 |
| 33539 | 1.84902 | 2.1 |
| 33540 | 1.15008 | 0.05 |
| 33541 | 1.3305 | 2.1 |
| 33542 | 1.70539 | 2.1 |
| 33543 | 2.9991 | 2.1 |
| 33544 | 1.84901 | 2.1 |
| 33545 | 2.0264 | 2.1 |
| 33546 | 2.20391 | 2.1 |
| 33547 | 1.70539 | 2.1 |
| 33548 | 3.87541 | 2.1 |
| 33549 | 1.03852 | 2.1 |
| 33550 | 1.38247 | 0.0515269 |
| 33551 | 1.36635 | 0.520379 |
| 33552 | 1.01537 | 1.98521 |
| 33553 | 2.39784 | 1.41172 |
| 33554 | 2.0815 | 2.1 |
| 33555 | 1.91744 | 2.06365 |
| 33556 | 1.91744 | 0.767975 |
| 33557 | 2.06496 | 2.1 |
| 33558 | 1.89227 | 1.07299 |
| 33559 | 2.03474 | 2.1 |
| 33560 | 1.89226 | 0.227444 |
| 33561 | 1.89226 | 1.19024 |
| 33562 | 1.89226 | 0.349029 |
| 33563 | 1.89226 | 2.1 |
| 33564 | 1.89226 | 0.0644964 |
| 33565 | 1.89226 | 2.06819 |
| 33566 | 1.36635 | 0.659891 |
| 33567 | 1.02923 | 2.1 |
| 33568 | 1.02923 | 0.732624 |
| 33569 | 1.01537 | 2.1 |
| 33570 | 1.36416 | 0.128239 |
| 33571 | 1.3503 | 2.1 |
| 33572 | 1.89226 | 2.1 |
| 33573 | 1.89359 | 2.1 |
| 33574 | 1.89359 | 1.80621 |
| 33575 | 1.91744 | 2.1 |
| 33576 | 1.89226 | 1.5907 |
| 33577 | 1.91611 | 2.1 |
| 33578 | 0.946128 | 2.1 |
| 33579 | 0.946128 | 1.88563 |

| | | |
|---|---|---|
| 33580 | 0.946133 | 0.487729 |
| 33581 | 0.946133 | 0.0867106 |
| 33582 | 0.94613 | 0.0637702 |
| 33583 | 0.94613 | 1.48395 |
| 33584 | 0.946131 | 2.1 |
| 33585 | 0.946131 | 0.104693 |
| 33586 | 0.946131 | 0.868894 |
| 33587 | 0.946131 | 0.994918 |
| 33588 | 0.946131 | 1.04359 |
| 33589 | 0.946131 | 1.14885 |
| 33590 | 2.689 | 2.1 |
| 33591 | 2.0815 | 2.1 |
| 33592 | 2.72786 | 2.1 |
| 33593 | 2.48295 | 2.1 |
| 33594 | 5.02397 | 2.1 |
| 33595 | 2.72786 | 2.1 |
| 33596 | 1.86424 | 2.1 |
| 33597 | 2.7188 | 2.1 |
| 33598 | 2.08981 | 2.1 |
| 33599 | 4.81768 | 2.1 |
| 33600 | 2.08982 | 2.1 |
| 33601 | 0.986987 | 2.1 |
| 33602 | 2.08982 | 2.1 |
| 33603 | 1.87249 | 2.1 |
| 33604 | 1.01964 | 2.1 |
| 33605 | 0.906115 | 2.1 |
| 33606 | 1.44247 | 2.1 |
| 33607 | 1.43872 | 2.1 |
| 33608 | 1.17054 | 2.1 |
| 33609 | 1.17054 | 2.1 |
| 33610 | 1.43872 | 2.1 |
| 33611 | 1.26045 | 2.1 |
| 33612 | 2.0815 | 0.05 |
| 33613 | 2.0815 | 0.05 |
| 33614 | 2.0815 | 0.05 |
| 33615 | 1.81468 | 0.05 |
| 33616 | 1.89065 | 0.05 |
| 33617 | 1.55625 | 0.05 |
| 33618 | 1.89062 | 0.05 |
| 33619 | 1.89224 | 0.05 |
| 33620 | 1.89224 | 0.05 |
| 33621 | 2.27073 | 0.05 |
| 33622 | 1.86856 | 0.05 |
| 33623 | 2.24232 | 0.05 |
| 33624 | 1.21419 | 2.1 |
| 33625 | 2.79716 | 2.1 |
| 33626 | 2.50005 | 2.1 |
| 33627 | 2.50005 | 2.1 |
| 33628 | 1.5008 | 2.1 |
| 33629 | 1.5008 | 2.1 |
| 33630 | 1.60139 | 0.05 |
| 33631 | 1.7103 | 0.05 |
| 33632 | 1.04159 | 0.05 |
| 33633 | 1.2489 | 0.05 |
| 33634 | 2.75189 | 0.05 |
| 33635 | 0.732812 | 0.05 |
| 33636 | 0.732812 | 0.05 |
| 33637 | 0.751986 | 0.05 |

| | | |
|---|---|---|
| 33638 | 0.751986 | 0.05 |
| 33639 | 0.860346 | 0.05 |
| 33640 | 0.667961 | 0.05 |
| 33641 | 0.85515 | 0.05 |
| 33642 | 0.624452 | 0.05 |
| 33643 | 0.62445 | 0.05 |
| 33644 | 0.667962 | 0.05 |
| 33645 | 0.855148 | 0.05 |
| 33646 | 0.860348 | 0.05 |
| 33647 | 1.89226 | 0.219911 |
| 33648 | 2.50872 | 0.192222 |
| 33649 | 2.77909 | 0.178012 |
| 33650 | 1.89226 | 0.247045 |
| 33651 | 2.77909 | 0.189952 |
| 33652 | 1.89226 | 0.268323 |
| 33653 | 2.15825 | 0.05 |
| 33654 | 2.50872 | 0.05 |
| 33655 | 1.90439 | 0.05 |
| 33656 | 1.90439 | 0.0605774 |
| 33657 | 2.77909 | 0.05 |
| 33658 | 2.77909 | 0.128955 |
| 33659 | 1.12468 | 2.1 |
| 33660 | 1.43872 | 2.1 |
| 33661 | 1.64926 | 2.1 |
| 33662 | 1.2724 | 2.1 |
| 33663 | 2.77395 | 2.1 |
| 33664 | 1.64926 | 2.1 |
| 33665 | 1.38438 | 2.1 |
| 33666 | 1.47244 | 2.1 |
| 33667 | 1.2724 | 2.1 |
| 33668 | 1.8493 | 2.1 |
| 33669 | 1.64926 | 2.1 |
| 33670 | 1.36416 | 0.270763 |
| 33671 | 1.0652 | 2.1 |
| 33672 | 1.0652 | 1.00454 |
| 33673 | 1.02923 | 2.1 |
| 33674 | 1.37496 | 0.336312 |
| 33675 | 1.33898 | 2.1 |
| 33676 | 1.89227 | 2.1 |
| 33677 | 1.90433 | 2.1 |
| 33678 | 1.90433 | 2.1 |
| 33679 | 1.89359 | 2.1 |
| 33680 | 1.89227 | 2.1 |
| 33681 | 1.88153 | 2.1 |
| 33682 | 0.946129 | 2.1 |
| 33683 | 0.946129 | 2.1 |
| 33684 | 0.946133 | 1.04471 |
| 33685 | 0.946133 | 0.218636 |
| 33686 | 0.946131 | 0.176329 |
| 33687 | 0.946131 | 2.1 |
| 33688 | 0.946131 | 2.1 |
| 33689 | 0.946131 | 0.23339 |
| 33690 | 0.946131 | 1.23113 |
| 33691 | 0.946131 | 1.50141 |
| 33692 | 0.946131 | 1.2996 |
| 33693 | 0.946131 | 1.38647 |
| 33694 | 1.37496 | 0.386616 |
| 33695 | 1.07519 | 2.1 |

| | | |
|---|---|---|
| 33696 | 1.07519 | 1.13121 |
| 33697 | 1.0652 | 2.1 |
| 33698 | 1.39725 | 0.392676 |
| 33699 | 1.38726 | 2.1 |
| 33700 | 1.89227 | 2.1 |
| 33701 | 1.9052 | 2.1 |
| 33702 | 1.9052 | 2.1 |
| 33703 | 1.90433 | 2.1 |
| 33704 | 1.89227 | 2.1 |
| 33705 | 1.8914 | 2.1 |
| 33706 | 0.946129 | 2.1 |
| 33707 | 0.946129 | 2.1 |
| 33708 | 0.946133 | 1.14141 |
| 33709 | 0.946133 | 0.26487 |
| 33710 | 0.946131 | 0.210746 |
| 33711 | 0.946131 | 2.1 |
| 33712 | 0.946131 | 2.1 |
| 33713 | 0.946131 | 0.239777 |
| 33714 | 0.946131 | 1.44599 |
| 33715 | 0.946131 | 1.89653 |
| 33716 | 0.946131 | 1.46474 |
| 33717 | 0.946131 | 1.76913 |
| 33718 | 1.91316 | 0.099131 |
| 33719 | 1.89226 | 0.108727 |
| 33720 | 1.89226 | 0.103521 |
| 33721 | 1.89226 | 0.144332 |
| 33722 | 1.99366 | 0.0669279 |
| 33723 | 1.99366 | 0.0897094 |
| 33724 | 1.93083 | 0.05 |
| 33725 | 1.89226 | 0.11748 |
| 33726 | 1.51379 | 0.054689 |
| 33727 | 1.89226 | 0.130381 |
| 33728 | 1.53051 | 0.05 |
| 33729 | 1.91316 | 0.113896 |
| 33730 | 2.16731 | 2.1 |
| 33731 | 1.21373 | 2.1 |
| 33732 | 1.47804 | 2.1 |
| 33733 | 2.37601 | 2.1 |
| 33734 | 3.64535 | 2.1 |
| 33735 | 1.7211 | 2.1 |
| 33736 | 2.37601 | 2.1 |
| 33737 | 1.47804 | 2.1 |
| 33738 | 1.47804 | 2.1 |
| 33739 | 2.56843 | 2.1 |
| 33740 | 2.56843 | 2.1 |
| 33741 | 2.58731 | 2.1 |
| 33742 | 1.59094 | 2.1 |
| 33743 | 1.59094 | 2.1 |
| 33744 | 1.76816 | 2.1 |
| 33745 | 2.41707 | 2.1 |
| 33746 | 2.5943 | 2.1 |
| 33747 | 0.718346 | 2.1 |
| 33748 | 0.795468 | 2.1 |
| 33749 | 1.20854 | 2.1 |
| 33750 | 1.1224 | 2.1 |
| 33751 | 0.961203 | 2.1 |
| 33752 | 0.961203 | 2.1 |
| 33753 | 0.95667 | 2.1 |

| | | |
|---|---|---|
| 33754 | 0.95667 | 2.1 |
| 33755 | 0.718346 | 2.1 |
| 33756 | 1.1224 | 2.1 |
| 33757 | 0.795469 | 2.1 |
| 33758 | 1.20854 | 2.1 |
| 33759 | 1.84076 | 2.1 |
| 33760 | 1.42884 | 2.1 |
| 33761 | 2.24274 | 2.1 |
| 33762 | 1.85415 | 2.1 |
| 33763 | 3.69491 | 2.1 |
| 33764 | 1.89226 | 2.1 |
| 33765 | 1.89226 | 2.1 |
| 33766 | 1.89226 | 2.1 |
| 33767 | 1.89226 | 2.1 |
| 33768 | 3.78452 | 2.1 |
| 33769 | 2.24273 | 2.1 |
| 33770 | 2.24412 | 2.1 |
| 33771 | 1.85415 | 2.1 |
| 33772 | 1.85415 | 2.1 |
| 33773 | 2.68473 | 2.1 |
| 33774 | 2.68473 | 2.1 |
| 33775 | 1.89226 | 2.07997 |
| 33776 | 1.89226 | 2.1 |
| 33777 | 1.89226 | 2.1 |
| 33778 | 1.89226 | 1.45566 |
| 33779 | 1.89226 | 2.1 |
| 33780 | 1.89226 | 1.65769 |
| 33781 | 1.89226 | 1.74577 |
| 33782 | 1.89226 | 2.1 |
| 33783 | 1.89226 | 2.1 |
| 33784 | 1.89226 | 2.1 |
| 33785 | 3.78451 | 2.1 |
| 33786 | 1.85415 | 2.1 |
| 33787 | 1.10961 | 2.1 |
| 33788 | 2.24411 | 2.1 |
| 33789 | 1.51067 | 2.1 |
| 33790 | 3.35373 | 2.1 |
| 33791 | 1.70302 | 2.1 |
| 33792 | 1.89226 | 2.1 |
| 33793 | 1.76399 | 2.1 |
| 33794 | 1.43577 | 2.1 |
| 33795 | 2.08149 | 2.1 |
| 33796 | 1.70302 | 2.1 |
| 33797 | 1.89226 | 2.1 |
| 33798 | 1.89226 | 2.1 |
| 33799 | 1.89226 | 2.1 |
| 33800 | 1.89226 | 2.1 |
| 33801 | 1.89226 | 2.1 |
| 33802 | 1.89226 | 2.1 |
| 33803 | 1.0593 | 2.1 |
| 33804 | 1.17289 | 2.1 |
| 33805 | 1.5379 | 2.1 |
| 33806 | 1.46757 | 2.1 |
| 33807 | 2.71079 | 2.1 |
| 33808 | 0.931235 | 2.1 |
| 33809 | 1.1151 | 2.1 |
| 33810 | 0.975236 | 2.1 |
| 33811 | 0.975236 | 2.1 |

| | | |
|---|---|---|
| 33812 | 1.31147 | 2.1 |
| 33813 | 0.762882 | 2.1 |
| 33814 | 1.13152 | 2.1 |
| 33815 | 0.505799 | 2.1 |
| 33816 | 1.31149 | 2.1 |
| 33817 | 1.07629 | 2.1 |
| 33818 | 0.884817 | 2.1 |
| 33819 | 0.505793 | 2.1 |
| 33820 | 2.69027 | 2.1 |
| 33821 | 1.60441 | 2.1 |
| 33822 | 2.69027 | 2.1 |
| 33823 | 1.64771 | 2.1 |
| 33824 | 2.78977 | 2.1 |
| 33825 | 1.72731 | 2.1 |
| 33826 | 0.77878 | 0.05 |
| 33827 | 0.843226 | 0.05 |
| 33828 | 0.845997 | 0.05 |
| 33829 | 0.926091 | 0.05 |
| 33830 | 0.77878 | 0.05 |
| 33831 | 0.926093 | 0.05 |
| 33832 | 1.3922 | 1.48479 |
| 33833 | 1.3922 | 1.15614 |
| 33834 | 0.872156 | 2.1 |
| 33835 | 0.872156 | 2.1 |
| 33836 | 0.872157 | 2.1 |
| 33837 | 0.946132 | 0.521058 |
| 33838 | 0.946129 | 0.753497 |
| 33839 | 1.31822 | 2.1 |
| 33840 | 0.94613 | 2.02792 |
| 33841 | 0.94613 | 2.05674 |
| 33842 | 1.31823 | 1.82784 |
| 33843 | 0.872157 | 2.05126 |
| 33844 | 1.81534 | 0.51688 |
| 33845 | 1.89226 | 0.436981 |
| 33846 | 1.59602 | 0.663362 |
| 33847 | 1.59602 | 0.860924 |
| 33848 | 1.89226 | 0.05 |
| 33849 | 1.89226 | 0.0840211 |
| 33850 | 1.89227 | 0.594289 |
| 33851 | 1.89227 | 0.615067 |
| 33852 | 1.89227 | 0.652092 |
| 33853 | 1.89227 | 0.556682 |
| 33854 | 3.78453 | 0.551601 |
| 33855 | 1.89226 | 0.615258 |
| 33856 | 1.89226 | 0.173853 |
| 33857 | 1.89226 | 0.506745 |
| 33858 | 1.89226 | 0.417016 |
| 33859 | 3.78453 | 0.629934 |
| 33860 | 2.30348 | 0.05 |
| 33861 | 1.4185 | 0.0679478 |
| 33862 | 1.82551 | 0.12552 |
| 33863 | 1.02011 | 0.05 |
| 33864 | 3.24401 | 0.05 |
| 33865 | 2.46702 | 0.05 |
| 33866 | 1.17129 | 0.05 |
| 33867 | 1.82551 | 0.05 |
| 33868 | 1.82551 | 0.05 |
| 33869 | 1.73525 | 0.05 |

| | | |
|---|---|---|
| 33870 | 1.73525 | 0.05 |
| 33871 | 1.89227 | 0.56982 |
| 33872 | 1.89227 | 0.146027 |
| 33873 | 1.89227 | 0.192861 |
| 33874 | 1.89227 | 0.261654 |
| 33875 | 1.89227 | 0.510702 |
| 33876 | 1.89227 | 0.558811 |
| 33877 | 2.80034 | 2.1 |
| 33878 | 2.45767 | 2.1 |
| 33879 | 2.67994 | 2.1 |
| 33880 | 2.35395 | 2.1 |
| 33881 | 5.1376 | 2.1 |
| 33882 | 2.45766 | 2.1 |
| 33883 | 1.8769 | 2.1 |
| 33884 | 2.00989 | 2.1 |
| 33885 | 2.00989 | 2.1 |
| 33886 | 2.35395 | 2.1 |
| 33887 | 2.35395 | 2.1 |
| 33888 | 2.57662 | 2.1 |
| 33889 | 2.35395 | 2.1 |
| 33890 | 2.57662 | 2.1 |
| 33891 | 2.67993 | 2.1 |
| 33892 | 2.24227 | 2.1 |
| 33893 | 2.34558 | 2.1 |
| 33894 | 1.02874 | 2.1 |
| 33895 | 1.02874 | 2.1 |
| 33896 | 1.03084 | 2.1 |
| 33897 | 1.03084 | 2.1 |
| 33898 | 0.938448 | 2.1 |
| 33899 | 0.886762 | 2.1 |
| 33900 | 1.17698 | 2.1 |
| 33901 | 1.12114 | 2.1 |
| 33902 | 1.12113 | 2.1 |
| 33903 | 0.886764 | 2.1 |
| 33904 | 0.93845 | 2.1 |
| 33905 | 1.17697 | 2.1 |
| 33906 | 1.91969 | 2.1 |
| 33907 | 2.5508 | 2.1 |
| 33908 | 1.88273 | 2.1 |
| 33909 | 2.76609 | 2.1 |
| 33910 | 4.68578 | 2.1 |
| 33911 | 2.83423 | 2.1 |
| 33912 | 1.89226 | 2.1 |
| 33913 | 2.76609 | 2.1 |
| 33914 | 1.89226 | 2.1 |
| 33915 | 4.65835 | 2.1 |
| 33916 | 1.56685 | 0.724201 |
| 33917 | 1.11712 | 0.483127 |
| 33918 | 1.07999 | 1.15441 |
| 33919 | 1.51448 | 0.793902 |
| 33920 | 2.6316 | 0.828186 |
| 33921 | 1.56685 | 0.125046 |
| 33922 | 1.67652 | 0.952452 |
| 33923 | 1.5955 | 1.13044 |
| 33924 | 1.51448 | 0.693518 |
| 33925 | 3.19099 | 0.844936 |
| 33926 | 1.89226 | 0.978144 |
| 33927 | 1.89226 | 0.098919 |

| | | |
|---|---|---|
| 33928 | 1.89226 | 0.720485 |
| 33929 | 1.89226 | 0.810063 |
| 33930 | 3.78453 | 0.806885 |
| 33931 | 1.89226 | 0.820111 |
| 33932 | 1.89226 | 0.606794 |
| 33933 | 1.89226 | 0.952343 |
| 33934 | 1.89226 | 0.940221 |
| 33935 | 1.89226 | 0.194263 |
| 33936 | 1.89226 | 0.204461 |
| 33937 | 1.0562 | 0.889437 |
| 33938 | 1.51448 | 0.562523 |
| 33939 | 1.07999 | 0.763389 |
| 33940 | 1.07999 | 0.408784 |
| 33941 | 1.46441 | 0.856279 |
| 33942 | 1.46441 | 0.674264 |
| 33943 | 1.5955 | 1.2195 |
| 33944 | 1.51448 | 0.649702 |
| 33945 | 1.46441 | 0.740202 |
| 33946 | 1.52909 | 0.903441 |
| 33947 | 3.04357 | 0.813796 |
| 33948 | 1.89226 | 0.686606 |
| 33949 | 1.89226 | 0.795514 |
| 33950 | 1.89226 | 0.922743 |
| 33951 | 1.89226 | 0.405661 |
| 33952 | 3.78453 | 0.45509 |
| 33953 | 1.89227 | 0.617828 |
| 33954 | 1.89227 | 0.05 |
| 33955 | 1.89227 | 0.899493 |
| 33956 | 1.89227 | 0.699619 |
| 33957 | 3.78453 | 0.742733 |
| 33958 | 1.89994 | 0.058824 |
| 33959 | 1.70303 | 0.05 |
| 33960 | 1.68576 | 0.05 |
| 33961 | 1.89608 | 0.05 |
| 33962 | 3.8237 | 0.05 |
| 33963 | 1.89994 | 0.260523 |
| 33964 | 2.31116 | 0.886307 |
| 33965 | 1.9386 | 0.05 |
| 33966 | 1.88653 | 1.03807 |
| 33967 | 3.74885 | 0.241613 |
| 33968 | 2.31117 | 0.972579 |
| 33969 | 2.20665 | 1.20575 |
| 33970 | 2.09617 | 0.952723 |
| 33971 | 2.41055 | 2.1 |
| 33972 | 2.09617 | 1.12597 |
| 33973 | 2.41055 | 2.1 |
| 33974 | 1.10777 | 0.05 |
| 33975 | 1.0523 | 0.05 |
| 33976 | 0.912098 | 0.05 |
| 33977 | 0.757533 | 0.05 |
| 33978 | 0.89287 | 0.05 |
| 33979 | 1.07571 | 0.05 |
| 33980 | 0.77485 | 0.05 |
| 33981 | 0.98645 | 0.05 |
| 33982 | 0.989171 | 0.05 |
| 33983 | 0.875089 | 0.05 |
| 33984 | 0.979686 | 0.05 |
| 33985 | 0.979686 | 0.05 |

| | | |
|---|---|---|
| 33986 | 2.52442 | 2.1 |
| 33987 | 1.49998 | 2.1 |
| 33988 | 2.51692 | 2.1 |
| 33989 | 1.56957 | 2.1 |
| 33990 | 4.09399 | 2.1 |
| 33991 | 1.01298 | 2.1 |
| 33992 | 1.01298 | 2.1 |
| 33993 | 1.03079 | 2.1 |
| 33994 | 1.03079 | 2.1 |
| 33995 | 0.820964 | 2.1 |
| 33996 | 0.784786 | 2.1 |
| 33997 | 1.25898 | 2.1 |
| 33998 | 1.25846 | 2.1 |
| 33999 | 1.25898 | 2.1 |
| 34000 | 0.820964 | 2.1 |
| 34001 | 1.25846 | 2.1 |
| 34002 | 0.784786 | 2.1 |
| 34003 | 1.89226 | 0.05 |
| 34004 | 1.89226 | 0.05 |
| 34005 | 2.6297 | 0.05 |
| 34006 | 1.89226 | 0.05 |
| 34007 | 3.78452 | 0.05 |
| 34008 | 1.89226 | 0.05 |
| 34009 | 1.89226 | 0.05 |
| 34010 | 1.89226 | 0.05 |
| 34011 | 1.89226 | 0.05 |
| 34012 | 1.89226 | 0.05 |
| 34013 | 1.89226 | 0.05 |
| 34014 | 2.19397 | 0.05 |
| 34015 | 1.89226 | 0.05 |
| 34016 | 2.34112 | 0.05 |
| 34017 | 2.34112 | 0.05 |
| 34018 | 1.89226 | 0.05 |
| 34019 | 1.89226 | 0.05 |
| 34020 | 2.23366 | 2.1 |
| 34021 | 1.87598 | 2.1 |
| 34022 | 1.64716 | 2.1 |
| 34023 | 2.06048 | 2.1 |
| 34024 | 3.93645 | 2.1 |
| 34025 | 2.23367 | 2.1 |
| 34026 | 2.17384 | 2.1 |
| 34027 | 2.04818 | 2.1 |
| 34028 | 2.06048 | 2.1 |
| 34029 | 4.23432 | 2.1 |
| 34030 | 1.9855 | 2.1 |
| 34031 | 2.06048 | 2.1 |
| 34032 | 1.93163 | 2.1 |
| 34033 | 1.50737 | 2.1 |
| 34034 | 3.56785 | 2.1 |
| 34035 | 2.04818 | 2.1 |
| 34036 | 2.06048 | 2.1 |
| 34037 | 2.27465 | 2.1 |
| 34038 | 1.92264 | 2.1 |
| 34039 | 3.98312 | 2.1 |
| 34040 | 2.08149 | 0.05 |
| 34041 | 1.89226 | 1.57896 |
| 34042 | 1.89226 | 0.826954 |
| 34043 | 1.33153 | 0.05 |

| | | |
|---|---|---|
| 34044 | 1.68038 | 1.94488 |
| 34045 | 1.18244 | 0.05 |
| 34046 | 1.89226 | 0.320116 |
| 34047 | 1.89226 | 1.82833 |
| 34048 | 1.89226 | 1.61959 |
| 34049 | 1.89226 | 0.0750305 |
| 34050 | 3.78452 | 0.451318 |
| 34051 | 2.69069 | 0.05 |
| 34052 | 1.89226 | 0.111912 |
| 34053 | 1.89226 | 0.151157 |
| 34054 | 1.97397 | 0.05 |
| 34055 | 1.89226 | 0.150616 |
| 34056 | 1.97397 | 0.05 |
| 34057 | 1.89226 | 1.86462 |
| 34058 | 2.38056 | 0.0780825 |
| 34059 | 1.89226 | 0.0844315 |
| 34060 | 1.89226 | 0.93718 |
| 34061 | 3.78452 | 0.540512 |
| 34062 | 0.904435 | 2.1 |
| 34063 | 0.904435 | 2.1 |
| 34064 | 0.925315 | 2.1 |
| 34065 | 0.925315 | 2.1 |
| 34066 | 1.13312 | 2.1 |
| 34067 | 1.08109 | 2.1 |
| 34068 | 0.70697 | 2.1 |
| 34069 | 0.696633 | 2.1 |
| 34070 | 0.70697 | 2.1 |
| 34071 | 1.13312 | 2.1 |
| 34072 | 0.696633 | 2.1 |
| 34073 | 1.08109 | 2.1 |
| 34074 | 1.45506 | 2.1 |
| 34075 | 2.26623 | 2.1 |
| 34076 | 2.38363 | 2.1 |
| 34077 | 2.38363 | 2.1 |
| 34078 | 1.41394 | 2.1 |
| 34079 | 1.41394 | 2.1 |
| 34080 | 0.946131 | 2.1 |
| 34081 | 0.946131 | 2.1 |
| 34082 | 1.13311 | 2.1 |
| 34083 | 1.13311 | 2.1 |
| 34084 | 0.946127 | 2.1 |
| 34085 | 0.946127 | 2.1 |
| 34086 | 1.13312 | 2.1 |
| 34087 | 1.19182 | 2.1 |
| 34088 | 1.19181 | 2.1 |
| 34089 | 0.946129 | 2.1 |
| 34090 | 1.13312 | 2.1 |
| 34091 | 0.94613 | 2.1 |
| 34092 | 1.89226 | 0.086111 |
| 34093 | 1.89226 | 2.1 |
| 34094 | 1.89226 | 0.645387 |
| 34095 | 1.89226 | 2.08408 |
| 34096 | 1.89226 | 0.750362 |
| 34097 | 1.89226 | 2.02082 |
| 34098 | 1.05168 | 2.1 |
| 34099 | 0.696527 | 2.1 |
| 34100 | 0.907442 | 0.418444 |
| 34101 | 0.907442 | 0.808872 |

| | | |
|---|---|---|
| 34102 | 0.953145 | 0.109341 |
| 34103 | 0.782285 | 2.1 |
| 34104 | 0.773928 | 2.1 |
| 34105 | 0.773928 | 0.343749 |
| 34106 | 0.696538 | 0.617239 |
| 34107 | 0.851554 | 0.99417 |
| 34108 | 1.04071 | 1.195 |
| 34109 | 1.12335 | 1.21666 |
| 34110 | 1.89226 | 0.830128 |
| 34111 | 1.89226 | 0.152977 |
| 34112 | 1.89226 | 0.307621 |
| 34113 | 1.89226 | 0.05 |
| 34114 | 3.78453 | 0.0647072 |
| 34115 | 1.89226 | 0.734065 |
| 34116 | 1.89226 | 0.124525 |
| 34117 | 1.89226 | 0.322858 |
| 34118 | 1.89226 | 0.0657165 |
| 34119 | 3.78453 | 0.628827 |
| 34120 | 2.50912 | 0.149169 |
| 34121 | 1.89226 | 0.05 |
| 34122 | 1.89226 | 0.0690991 |
| 34123 | 2.63195 | 0.05 |
| 34124 | 4.40139 | 0.05 |
| 34125 | 2.50912 | 0.189821 |
| 34126 | 1.3598 | 0.0595529 |
| 34127 | 1.70303 | 0.0599305 |
| 34128 | 2.36874 | 0.05 |
| 34129 | 3.82055 | 0.0985101 |
| 34130 | 1.89227 | 0.280019 |
| 34131 | 1.89227 | 0.0746409 |
| 34132 | 1.89227 | 0.079163 |
| 34133 | 1.89227 | 0.05 |
| 34134 | 3.78453 | 0.252716 |
| 34135 | 1.89227 | 0.303951 |
| 34136 | 1.89227 | 0.0595117 |
| 34137 | 1.89227 | 0.0660869 |
| 34138 | 1.89227 | 0.05 |
| 34139 | 3.78453 | 0.05 |
| 34140 | 1.89226 | 0.0598975 |
| 34141 | 2.67088 | 0.05 |
| 34142 | 2.63195 | 0.05 |
| 34143 | 2.63195 | 0.05 |
| 34144 | 1.89226 | 0.05 |
| 34145 | 1.89226 | 0.0844866 |
| 34146 | 1.30202 | 0.05 |
| 34147 | 1.28304 | 0.05 |
| 34148 | 0.809902 | 0.05 |
| 34149 | 0.888801 | 0.05 |
| 34150 | 1.13262 | 0.05 |
| 34151 | 0.968276 | 0.05 |
| 34152 | 1.0733 | 0.05 |
| 34153 | 1.0733 | 0.05 |
| 34154 | 1.35419 | 0.05 |
| 34155 | 0.801222 | 0.05 |
| 34156 | 0.899732 | 0.0636328 |
| 34157 | 1.23967 | 0.05 |
| 34158 | 1.31595 | 2.1 |
| 34159 | 0.869501 | 2.1 |

| | | |
|---|---|---|
| 34160 | 2.65473 | 2.1 |
| 34161 | 1.88782 | 2.1 |
| 34162 | 3.20377 | 2.1 |
| 34163 | 1.89227 | 2.1 |
| 34164 | 1.89227 | 2.1 |
| 34165 | 1.89227 | 2.1 |
| 34166 | 1.89227 | 2.1 |
| 34167 | 3.78453 | 2.1 |
| 34168 | 2.65473 | 2.1 |
| 34169 | 1.89226 | 2.1 |
| 34170 | 1.88781 | 2.1 |
| 34171 | 1.88782 | 2.1 |
| 34172 | 2.0815 | 2.1 |
| 34173 | 2.0815 | 2.1 |
| 34174 | 1.89226 | 2.1 |
| 34175 | 1.89226 | 2.1 |
| 34176 | 1.89226 | 2.1 |
| 34177 | 1.89226 | 2.1 |
| 34178 | 1.89226 | 2.1 |
| 34179 | 1.89226 | 2.1 |
| 34180 | 1.89226 | 2.1 |
| 34181 | 1.89226 | 2.1 |
| 34182 | 1.89226 | 2.1 |
| 34183 | 1.89226 | 2.1 |
| 34184 | 1.89226 | 2.1 |
| 34185 | 1.89226 | 2.1 |
| 34186 | 2.39057 | 2.1 |
| 34187 | 1.89226 | 0.452811 |
| 34188 | 1.89226 | 0.0763483 |
| 34189 | 1.89226 | 2.1 |
| 34190 | 2.14243 | 0.0504256 |
| 34191 | 2.14243 | 2.1 |
| 34192 | 1.89226 | 0.080624 |
| 34193 | 1.89226 | 2.1 |
| 34194 | 1.89226 | 0.151078 |
| 34195 | 1.89226 | 0.456761 |
| 34196 | 1.89226 | 0.853865 |
| 34197 | 1.89226 | 2.1 |
| 34198 | 1.89226 | 0.05 |
| 34199 | 1.89226 | 2.1 |
| 34200 | 1.89226 | 0.05 |
| 34201 | 1.89226 | 0.121086 |
| 34202 | 1.89226 | 0.141856 |
| 34203 | 1.89226 | 1.94063 |
| 34204 | 2.56323 | 0.05 |
| 34205 | 1.36324 | 0.05 |
| 34206 | 1.05296 | 0.05 |
| 34207 | 2.18048 | 0.05 |
| 34208 | 3.54372 | 0.05 |
| 34209 | 0.946128 | 2.1 |
| 34210 | 0.946128 | 2.1 |
| 34211 | 1.08733 | 2.1 |
| 34212 | 1.08733 | 2.1 |
| 34213 | 0.946131 | 2.1 |
| 34214 | 0.946131 | 2.1 |
| 34215 | 1.09197 | 2.1 |
| 34216 | 1.08732 | 2.1 |
| 34217 | 0.946131 | 2.1 |

| | | |
|---|---|---|
| 34218 | 1.09197 | 2.1 |
| 34219 | 1.08732 | 2.1 |
| 34220 | 0.946131 | 2.1 |
| 34221 | 1.35111 | 0.920972 |
| 34222 | 1.51327 | 0.636242 |
| 34223 | 1.64493 | 0.49326 |
| 34224 | 1.31058 | 0.951058 |
| 34225 | 2.99605 | 1.34701 |
| 34226 | 1.8124 | 0.496142 |
| 34227 | 1.51327 | 1.10438 |
| 34228 | 1.64493 | 1.27972 |
| 34229 | 1.31358 | 0.579832 |
| 34230 | 1.8124 | 1.21703 |
| 34231 | 1.48105 | 0.414028 |
| 34232 | 1.64493 | 0.671986 |
| 34233 | 1.31058 | 0.690217 |
| 34234 | 1.22297 | 0.586879 |
| 34235 | 1.70101 | 0.639246 |
| 34236 | 2.8679 | 0.360663 |
| 34237 | 1.70101 | 1.03819 |
| 34238 | 1.99297 | 0.62247 |
| 34239 | 1.64493 | 1.09357 |
| 34240 | 1.64493 | 0.456981 |
| 34241 | 1.99297 | 1.17919 |
| 34242 | 1.8124 | 0.205517 |
| 34243 | 2.0815 | 2.1 |
| 34244 | 1.80258 | 2.1 |
| 34245 | 1.80258 | 2.1 |
| 34246 | 1.83506 | 2.1 |
| 34247 | 2.0815 | 2.1 |
| 34248 | 2.1212 | 2.1 |
| 34249 | 2.0815 | 2.1 |
| 34250 | 1.82129 | 2.1 |
| 34251 | 1.82129 | 2.1 |
| 34252 | 1.80445 | 2.1 |
| 34253 | 2.0815 | 2.1 |
| 34254 | 2.06092 | 2.1 |
| 34255 | 1.89224 | 2.1 |
| 34256 | 1.703 2.1 | |
| 34257 | 1.97839 | 2.1 |
| 34258 | 1.703 2.1 | |
| 34259 | 1.97402 | 2.1 |
| 34260 | 1.703 2.1 | |
| 34261 | 1.89226 | 0.739318 |
| 34262 | 1.89226 | 0.924488 |
| 34263 | 1.89226 | 0.737388 |
| 34264 | 2.75351 | 0.830343 |
| 34265 | 3.78453 | 0.844925 |
| 34266 | 1.89226 | 0.753972 |
| 34267 | 3.02887 | 1.14933 |
| 34268 | 2.03149 | 0.701256 |
| 34269 | 2.03149 | 0.779564 |
| 34270 | 1.89226 | 0.889687 |
| 34271 | 1.89226 | 1.19721 |
| 34272 | 2.17979 | 2.1 |
| 34273 | 1.65533 | 2.1 |
| 34274 | 2.57212 | 2.1 |
| 34275 | 2.20346 | 2.1 |

| | | |
|---|---|---|
| 34276 | 4.22745 | 2.1 |
| 34277 | 1.89227 | 2.1 |
| 34278 | 2.20347 | 2.1 |
| 34279 | 1.89227 | 2.1 |
| 34280 | 2.57212 | 2.1 |
| 34281 | 1.89227 | 2.1 |
| 34282 | 2.57212 | 2.1 |
| 34283 | 2.05274 | 0.492495 |
| 34284 | 2.16783 | 2.1 |
| 34285 | 2.24429 | 0.494288 |
| 34286 | 2.38404 | 2.1 |
| 34287 | 4.41212 | 2.1 |
| 34288 | 2.16783 | 2.1 |
| 34289 | 2.05967 | 2.1 |
| 34290 | 2.38404 | 2.1 |
| 34291 | 2.24695 | 2.1 |
| 34292 | 4.41478 | 2.1 |
| 34293 | 2.24429 | 0.393245 |
| 34294 | 2.38404 | 2.1 |
| 34295 | 2.51819 | 0.759412 |
| 34296 | 2.60723 | 2.1 |
| 34297 | 4.85152 | 2.1 |
| 34298 | 2.38404 | 2.1 |
| 34299 | 2.24695 | 2.1 |
| 34300 | 2.60723 | 2.1 |
| 34301 | 2.45247 | 2.1 |
| 34302 | 4.85418 | 2.1 |
| 34303 | 1.89227 | 2.1 |
| 34304 | 1.89227 | 2.1 |
| 34305 | 1.89227 | 2.1 |
| 34306 | 1.89227 | 2.1 |
| 34307 | 3.78453 | 2.1 |
| 34308 | 1.89226 | 2.1 |
| 34309 | 1.89226 | 2.1 |
| 34310 | 1.89226 | 2.1 |
| 34311 | 1.89226 | 2.1 |
| 34312 | 3.78453 | 2.1 |
| 34313 | 0.618473 | 2.1 |
| 34314 | 0.619945 | 2.1 |
| 34315 | 0.797692 | 2.1 |
| 34316 | 0.770147 | 2.1 |
| 34317 | 0.68852 | 2.1 |
| 34318 | 0.68852 | 2.1 |
| 34319 | 0.701573 | 2.1 |
| 34320 | 0.701573 | 2.1 |
| 34321 | 0.619945 | 2.1 |
| 34322 | 0.797691 | 2.1 |
| 34323 | 0.618473 | 2.1 |
| 34324 | 0.770147 | 2.1 |
| 34325 | 0.797692 | 2.1 |
| 34326 | 0.770148 | 2.1 |
| 34327 | 0.694787 | 2.1 |
| 34328 | 0.653411 | 2.1 |
| 34329 | 0.722102 | 2.1 |
| 34330 | 0.722102 | 2.1 |
| 34331 | 0.729001 | 2.1 |
| 34332 | 0.729001 | 2.1 |
| 34333 | 0.797692 | 2.1 |

| | | |
|---|---|---|
| 34334 | 0.694787 | 2.1 |
| 34335 | 0.770148 | 2.1 |
| 34336 | 0.653412 | 2.1 |
| 34337 | 1.89227 | 2.1 |
| 34338 | 1.89227 | 2.1 |
| 34339 | 1.89227 | 2.1 |
| 34340 | 1.89227 | 2.1 |
| 34341 | 3.78453 | 2.1 |
| 34342 | 1.89226 | 2.1 |
| 34343 | 1.89226 | 2.1 |
| 34344 | 1.89226 | 2.1 |
| 34345 | 1.89226 | 2.1 |
| 34346 | 3.78453 | 2.1 |
| 34347 | 1.23989 | 2.1 |
| 34348 | 1.59538 | 2.1 |
| 34349 | 1.66647 | 2.1 |
| 34350 | 1.26599 | 2.1 |
| 34351 | 2.90636 | 2.1 |
| 34352 | 1.66647 | 2.1 |
| 34353 | 1.38957 | 2.1 |
| 34354 | 1.47446 | 2.1 |
| 34355 | 1.40337 | 2.1 |
| 34356 | 1.66647 | 2.1 |
| 34357 | 1.59538 | 2.1 |
| 34358 | 2.80619 | 2.1 |
| 34359 | 2.0815 | 2.1 |
| 34360 | 2.0815 | 2.1 |
| 34361 | 2.0815 | 2.1 |
| 34362 | 2.73721 | 2.1 |
| 34363 | 2.74192 | 2.1 |
| 34364 | 2.73721 | 2.1 |
| 34365 | 2.0815 | 2.1 |
| 34366 | 2.0815 | 2.1 |
| 34367 | 2.0815 | 2.1 |
| 34368 | 2.689 2.1 | |
| 34369 | 2.69166 | 2.1 |
| 34370 | 2.62369 | 2.1 |
| 34371 | 2.19717 | 2.1 |
| 34372 | 2.60405 | 2.1 |
| 34373 | 2.20566 | 2.1 |
| 34374 | 4.80122 | 2.1 |
| 34375 | 2.19717 | 2.1 |
| 34376 | 1.37392 | 2.1 |
| 34377 | 2.20566 | 2.1 |
| 34378 | 1.45408 | 2.1 |
| 34379 | 3.65125 | 2.1 |
| 34380 | 2.60405 | 2.1 |
| 34381 | 2.20566 | 2.1 |
| 34382 | 2.57466 | 2.1 |
| 34383 | 2.21715 | 2.1 |
| 34384 | 4.8212 | 2.1 |
| 34385 | 2.20566 | 2.1 |
| 34386 | 1.54249 | 2.1 |
| 34387 | 1.54249 | 2.1 |
| 34388 | 1.45408 | 2.1 |
| 34389 | 2.21715 | 2.1 |
| 34390 | 2.12874 | 2.1 |
| 34391 | 1.60647 | 2.1 |

| | | |
|---|---|---|
| 34392 | 1.31346 | 2.1 |
| 34393 | 1.82649 | 2.1 |
| 34394 | 1.51067 | 2.1 |
| 34395 | 3.13995 | 2.1 |
| 34396 | 1.89226 | 2.1 |
| 34397 | 1.89226 | 2.1 |
| 34398 | 1.89226 | 2.1 |
| 34399 | 1.89226 | 2.1 |
| 34400 | 3.78452 | 2.1 |
| 34401 | 1.89226 | 2.1 |
| 34402 | 2.6096 | 2.1 |
| 34403 | 2.8067 | 2.1 |
| 34404 | 2.8067 | 2.1 |
| 34405 | 1.89226 | 2.1 |
| 34406 | 1.89226 | 2.1 |
| 34407 | 1.82649 | 2.1 |
| 34408 | 1.3596 | 2.1 |
| 34409 | 2.17708 | 2.1 |
| 34410 | 1.70302 | 2.1 |
| 34411 | 3.40243 | 2.1 |
| 34412 | 1.89226 | 2.1 |
| 34413 | 1.89226 | 2.1 |
| 34414 | 1.89226 | 1.13248 |
| 34415 | 1.89226 | 2.1 |
| 34416 | 3.78452 | 2.1 |
| 34417 | 2.27067 | 2.1 |
| 34418 | 2.43549 | 2.1 |
| 34419 | 2.63979 | 2.1 |
| 34420 | 2.41828 | 2.1 |
| 34421 | 2.27067 | 2.1 |
| 34422 | 2.04916 | 2.1 |
| 34423 | 1.30047 | 2.1 |
| 34424 | 2.04916 | 2.1 |
| 34425 | 2.27068 | 2.1 |
| 34426 | 1.5586 | 2.1 |
| 34427 | 3.57115 | 2.1 |
| 34428 | 2.27068 | 2.1 |
| 34429 | 1.84214 | 2.1 |
| 34430 | 1.80224 | 2.1 |
| 34431 | 1.5586 | 2.1 |
| 34432 | 2.51432 | 2.1 |
| 34433 | 2.27068 | 2.1 |
| 34434 | 1.63719 | 2.1 |
| 34435 | 2.15714 | 2.1 |
| 34436 | 2.07821 | 2.1 |
| 34437 | 1.52204 | 2.1 |
| 34438 | 3.67919 | 2.1 |
| 34439 | 2.07821 | 2.1 |
| 34440 | 1.40118 | 2.1 |
| 34441 | 1.40118 | 2.1 |
| 34442 | 1.52204 | 2.1 |
| 34443 | 1.99632 | 2.1 |
| 34444 | 2.11718 | 2.1 |
| 34445 | 0.946131 | 2.1 |
| 34446 | 0.946131 | 2.1 |
| 34447 | 1.06957 | 2.1 |
| 34448 | 1.06957 | 2.1 |
| 34449 | 0.946132 | 2.1 |

| | | |
|---|---|---|
| 34450 | 0.946132 | 1.24726 |
| 34451 | 1.411 | 2.1 |
| 34452 | 1.06957 | 2.1 |
| 34453 | 0.946132 | 2.1 |
| 34454 | 1.411 | 2.1 |
| 34455 | 1.06957 | 2.1 |
| 34456 | 0.946132 | 2.1 |
| 34457 | 1.99875 | 0.759986 |
| 34458 | 2.57079 | 0.722468 |
| 34459 | 2.59319 | 1.16791 |
| 34460 | 1.9105 | 0.430484 |
| 34461 | 4.59194 | 0.808984 |
| 34462 | 1.99875 | 0.726626 |
| 34463 | 1.02247 | 0.05 |
| 34464 | 1.07017 | 0.151238 |
| 34465 | 1.15841 | 0.165395 |
| 34466 | 1.91051 | 0.114302 |
| 34467 | 1.99875 | 0.211326 |
| 34468 | 2.59319 | 1.06399 |
| 34469 | 1.9105 | 0.492695 |
| 34470 | 2.33812 | 0.698099 |
| 34471 | 2.59319 | 0.720786 |
| 34472 | 1.59457 | 0.836788 |
| 34473 | 1.59457 | 0.804433 |
| 34474 | 2.50906 | 0.704939 |
| 34475 | 2.0467 | 0.470764 |
| 34476 | 2.03357 | 0.619936 |
| 34477 | 2.53176 | 0.434842 |
| 34478 | 4.54263 | 0.581164 |
| 34479 | 1.294 | 0.742763 |
| 34480 | 1.66636 | 0.623103 |
| 34481 | 2.03357 | 0.575831 |
| 34482 | 1.03079 | 0.464792 |
| 34483 | 3.32757 | 0.595755 |
| 34484 | 1.68108 | 0.503218 |
| 34485 | 2.53176 | 0.339086 |
| 34486 | 2.36417 | 0.25683 |
| 34487 | 2.71666 | 0.171445 |
| 34488 | 1.68108 | 0.732729 |
| 34489 | 2.03357 | 0.647252 |
| 34490 | 1.89226 | 0.05 |
| 34491 | 2.59302 | 0.05 |
| 34492 | 2.2727 | 0.05 |
| 34493 | 2.2727 | 0.05 |
| 34494 | 1.89226 | 0.0621342 |
| 34495 | 1.89226 | 0.05 |
| 34496 | 1.89226 | 0.0676149 |
| 34497 | 1.89226 | 0.05 |
| 34498 | 1.89226 | 0.05 |
| 34499 | 1.89226 | 0.05 |
| 34500 | 3.78452 | 0.0601047 |
| 34501 | 1.89225 | 0.261406 |
| 34502 | 1.89225 | 0.05 |
| 34503 | 1.89225 | 0.05 |
| 34504 | 1.89225 | 0.05 |
| 34505 | 1.89225 | 0.05 |
| 34506 | 1.89225 | 0.05 |
| 34507 | 0.934114 | 0.05 |

| | | |
|---|---|---|
| 34508 | 0.934114 | 0.05 |
| 34509 | 0.944544 | 0.0582441 |
| 34510 | 0.944544 | 0.0581305 |
| 34511 | 0.721493 | 0.05 |
| 34512 | 0.58215 | 0.05 |
| 34513 | 1.2965 | 0.05 |
| 34514 | 1.13634 | 0.05 |
| 34515 | 1.13635 | 0.05 |
| 34516 | 0.582148 | 0.05 |
| 34517 | 1.29651 | 0.05 |
| 34518 | 0.721491 | 0.05 |
| 34519 | 1.89226 | 0.05 |
| 34520 | 1.89226 | 0.05 |
| 34521 | 1.89226 | 0.05 |
| 34522 | 1.89226 | 0.05 |
| 34523 | 3.78452 | 0.05 |
| 34524 | 0.946131 | 0.0510974 |
| 34525 | 0.946131 | 0.0521788 |
| 34526 | 0.946131 | 0.05 |
| 34527 | 0.946131 | 0.0665364 |
| 34528 | 0.946131 | 0.05 |
| 34529 | 0.946131 | 0.0557532 |
| 34530 | 0.946131 | 0.05 |
| 34531 | 0.946131 | 0.136844 |
| 34532 | 0.946131 | 0.05 |
| 34533 | 0.946131 | 0.05 |
| 34534 | 0.946131 | 0.06022 |
| 34535 | 0.946131 | 0.0570716 |
| 34536 | 1.89226 | 0.05 |
| 34537 | 1.89226 | 0.05 |
| 34538 | 2.78602 | 0.05 |
| 34539 | 1.89226 | 0.05 |
| 34540 | 3.78453 | 0.05 |
| 34541 | 1.52601 | 2.1 |
| 34542 | 1.76601 | 2.1 |
| 34543 | 1.27839 | 2.1 |
| 34544 | 1.56095 | 2.1 |
| 34545 | 3.0444 | 2.1 |
| 34546 | 1.76601 | 2.1 |
| 34547 | 1.86793 | 2.1 |
| 34548 | 1.56095 | 2.1 |
| 34549 | 1.68152 | 2.1 |
| 34550 | 3.44753 | 2.1 |
| 34551 | 1.27841 | 2.1 |
| 34552 | 1.56097 | 2.1 |
| 34553 | 1.0577 | 2.1 |
| 34554 | 1.36861 | 2.1 |
| 34555 | 2.64702 | 2.1 |
| 34556 | 1.56097 | 2.1 |
| 34557 | 1.68154 | 2.1 |
| 34558 | 1.36861 | 2.1 |
| 34559 | 1.53296 | 2.1 |
| 34560 | 3.05015 | 2.1 |
| 34561 | 1.89226 | 2.1 |
| 34562 | 1.89226 | 2.1 |
| 34563 | 1.89226 | 2.1 |
| 34564 | 1.89226 | 2.1 |
| 34565 | 3.78453 | 2.1 |

| | | |
|---|---|---|
| 34566 | 0.983075 | 2.1 |
| 34567 | 0.788361 | 2.1 |
| 34568 | 0.724726 | 2.1 |
| 34569 | 0.724726 | 2.1 |
| 34570 | 0.983027 | 2.1 |
| 34571 | 0.724734 | 2.1 |
| 34572 | 0.788364 | 2.1 |
| 34573 | 0.788364 | 2.1 |
| 34574 | 0.788335 | 2.1 |
| 34575 | 0.94613 | 2.1 |
| 34576 | 0.724757 | 2.1 |
| 34577 | 0.946131 | 2.1 |
| 34578 | 1.15789 | 2.1 |
| 34579 | 2.11135 | 2.1 |
| 34580 | 2.25826 | 2.1 |
| 34581 | 1.28845 | 2.1 |
| 34582 | 3.41615 | 2.1 |
| 34583 | 1.89226 | 2.1 |
| 34584 | 1.89226 | 2.1 |
| 34585 | 1.89226 | 2.1 |
| 34586 | 1.89226 | 2.1 |
| 34587 | 3.78453 | 2.1 |
| 34588 | 1.57667 | 2.1 |
| 34589 | 1.70002 | 2.1 |
| 34590 | 1.70002 | 2.1 |
| 34591 | 1.89226 | 2.1 |
| 34592 | 1.89226 | 2.1 |
| 34593 | 2.05244 | 2.1 |
| 34594 | 2.25826 | 2.1 |
| 34595 | 1.42214 | 2.1 |
| 34596 | 1.43675 | 2.1 |
| 34597 | 1.28845 | 2.1 |
| 34598 | 2.40656 | 2.1 |
| 34599 | 2.25826 | 2.1 |
| 34600 | 1.58066 | 2.1 |
| 34601 | 1.74247 | 2.1 |
| 34602 | 1.85097 | 2.1 |
| 34603 | 1.61155 | 2.1 |
| 34604 | 3.43163 | 2.1 |
| 34605 | 0.785612 | 2.1 |
| 34606 | 0.789638 | 2.1 |
| 34607 | 0.882561 | 2.1 |
| 34608 | 0.763529 | 2.1 |
| 34609 | 0.686921 | 2.1 |
| 34610 | 0.86048 | 2.1 |
| 34611 | 0.871812 | 2.1 |
| 34612 | 0.871812 | 2.1 |
| 34613 | 0.830183 | 2.1 |
| 34614 | 0.882572 | 2.1 |
| 34615 | 0.902315 | 2.1 |
| 34616 | 0.785602 | 2.1 |
| 34617 | 1.01972 | 0.05 |
| 34618 | 0.936657 | 0.05 |
| 34619 | 0.946131 | 0.05 |
| 34620 | 0.946131 | 0.05 |
| 34621 | 0.927018 | 0.05 |
| 34622 | 0.927018 | 0.05 |
| 34623 | 1.1376 | 0.05 |

| | | |
|---|---|---|
| 34624 | 0.953663 | 0.05 |
| 34625 | 1.1257 | 0.05 |
| 34626 | 1.04073 | 0.05 |
| 34627 | 1.00358 | 0.05 |
| 34628 | 0.851528 | 0.05 |
| 34629 | 1.73576 | 0.139012 |
| 34630 | 2.00244 | 0.68764 |
| 34631 | 1.17798 | 0.274915 |
| 34632 | 1.25796 | 0.0984015 |
| 34633 | 2.99373 | 0.127682 |
| 34634 | 2.00244 | 0.66831 |
| 34635 | 1.10719 | 0.348789 |
| 34636 | 2.03236 | 0.734724 |
| 34637 | 2.03236 | 0.628507 |
| 34638 | 1.25797 | 0.690974 |
| 34639 | 1.25797 | 0.366191 |
| 34640 | 1.89226 | 2.1 |
| 34641 | 1.89226 | 2.1 |
| 34642 | 1.89226 | 2.1 |
| 34643 | 1.89226 | 2.1 |
| 34644 | 3.78452 | 2.1 |
| 34645 | 2.3174 | 2.1 |
| 34646 | 1.50005 | 2.1 |
| 34647 | 1.90428 | 2.1 |
| 34648 | 2.64564 | 2.1 |
| 34649 | 4.22168 | 2.1 |
| 34650 | 1.89226 | 2.1 |
| 34651 | 1.89226 | 2.1 |
| 34652 | 1.89226 | 2.1 |
| 34653 | 1.89226 | 2.1 |
| 34654 | 1.89226 | 2.1 |
| 34655 | 1.89226 | 2.1 |
| 34656 | 1.32749 | 0.05 |
| 34657 | 1.70302 | 0.0698659 |
| 34658 | 2.08149 | 0.05 |
| 34659 | 1.70302 | 0.0771712 |
| 34660 | 2.19634 | 0.05 |
| 34661 | 1.93083 | 0.0594581 |
| 34662 | 1.89226 | 1.48972 |
| 34663 | 1.89226 | 2.1 |
| 34664 | 1.89226 | 0.831093 |
| 34665 | 1.89226 | 2.1 |
| 34666 | 3.78453 | 1.1321 |
| 34667 | 1.89226 | 1.38621 |
| 34668 | 1.89226 | 2.1 |
| 34669 | 1.89226 | 1.03615 |
| 34670 | 1.89226 | 1.58862 |
| 34671 | 3.78452 | 1.99817 |
| 34672 | 2.17499 | 1.12713 |
| 34673 | 1.79663 | 2.0559 |
| 34674 | 2.1538 | 0.655329 |
| 34675 | 1.818 | 1.15877 |
| 34676 | 2.17499 | 1.74708 |
| 34677 | 1.83919 | 2.1 |
| 34678 | 0.898316 | 2.1 |
| 34679 | 1.0769 | 1.26759 |
| 34680 | 0.968276 | 2.1 |
| 34681 | 0.773495 | 1.59216 |

| | | |
|---|---|---|
| 34682 | 0.921126 | 2.1 |
| 34683 | 0.921126 | 2.1 |
| 34684 | 0.92927 | 1.87705 |
| 34685 | 0.92927 | 2.01075 |
| 34686 | 1.0769 | 1.88703 |
| 34687 | 0.968276 | 1.32434 |
| 34688 | 0.898316 | 2.1 |
| 34689 | 0.773495 | 1.53501 |
| 34690 | 1.89226 | 1.01411 |
| 34691 | 1.89226 | 2.1 |
| 34692 | 1.89226 | 1.3396 |
| 34693 | 1.89226 | 1.22497 |
| 34694 | 3.78453 | 2.1 |
| 34695 | 1.89226 | 1.39948 |
| 34696 | 1.89226 | 2.1 |
| 34697 | 1.89226 | 0.906244 |
| 34698 | 1.89226 | 1.92796 |
| 34699 | 3.78452 | 1.20901 |
| 34700 | 2.17499 | 1.98902 |
| 34701 | 2.1538 | 2.1 |
| 34702 | 2.53574 | 1.19146 |
| 34703 | 2.54157 | 1.04225 |
| 34704 | 4.71073 | 2.01098 |
| 34705 | 1.0769 | 1.79183 |
| 34706 | 1.26787 | 1.16553 |
| 34707 | 1.16641 | 1.42459 |
| 34708 | 0.968276 | 2.1 |
| 34709 | 1.11625 | 1.49051 |
| 34710 | 1.11625 | 1.98772 |
| 34711 | 1.11989 | 2.1 |
| 34712 | 1.11989 | 0.928616 |
| 34713 | 1.26787 | 1.27147 |
| 34714 | 1.16641 | 2.1 |
| 34715 | 1.0769 | 2.1 |
| 34716 | 0.968277 | 2.00813 |
| 34717 | 1.89226 | 0.517825 |
| 34718 | 1.89226 | 0.410768 |
| 34719 | 1.89226 | 0.498681 |
| 34720 | 1.89226 | 0.469208 |
| 34721 | 3.78452 | 0.518229 |
| 34722 | 1.91739 | 0.336908 |
| 34723 | 2.20045 | 0.359988 |
| 34724 | 1.79005 | 0.443705 |
| 34725 | 2.33802 | 0.384156 |
| 34726 | 4.25539 | 0.325988 |
| 34727 | 2.52754 | 0.331542 |
| 34728 | 1.89226 | 0.435147 |
| 34729 | 2.69841 | 0.289207 |
| 34730 | 2.69841 | 0.374525 |
| 34731 | 1.89226 | 0.308397 |
| 34732 | 1.89226 | 0.427418 |
| 34733 | 1.89226 | 0.526915 |
| 34734 | 1.89226 | 0.556116 |
| 34735 | 1.89226 | 0.50149 |
| 34736 | 1.89226 | 0.565091 |
| 34737 | 3.78452 | 0.509218 |
| 34738 | 1.93544 | 0.514378 |
| 34739 | 2.33802 | 0.471995 |

| | | |
|---|---|---|
| 34740 | 1.55063 | 0.327817 |
| 34741 | 2.1769 | 0.427504 |
| 34742 | 3.88865 | 0.502899 |
| 34743 | 2.20929 | 0.05 |
| 34744 | 2.55162 | 0.05 |
| 34745 | 2.27807 | 0.05 |
| 34746 | 1.48831 | 0.05 |
| 34747 | 4.48736 | 0.05 |
| 34748 | 2.27807 | 0.05 |
| 34749 | 2.72723 | 0.05 |
| 34750 | 2.55162 | 0.05 |
| 34751 | 2.55162 | 0.05 |
| 34752 | 2.43157 | 0.05 |
| 34753 | 2.43157 | 0.05 |
| 34754 | 2.27807 | 0.05 |
| 34755 | 1.51612 | 0.05 |
| 34756 | 1.96048 | 0.05 |
| 34757 | 1.73022 | 0.05 |
| 34758 | 1.70302 | 0.05 |
| 34759 | 1.61016 | 0.05 |
| 34760 | 2.27807 | 0.05 |
| 34761 | 2.08048 | 0.05 |
| 34762 | 1.98522 | 0.05 |
| 34763 | 2.43157 | 0.05 |
| 34764 | 1.83798 | 0.05 |
| 34765 | 2.27807 | 0.05 |
| 34766 | 1.70302 | 0.05 |
| 34767 | 2.68507 | 0.05 |
| 34768 | 2.23856 | 0.05 |
| 34769 | 2.52174 | 0.05 |
| 34770 | 2.35331 | 0.05 |
| 34771 | 2.65099 | 0.05 |
| 34772 | 1.70303 | 0.05 |
| 34773 | 0.986848 | 0.05 |
| 34774 | 2.41566 | 0.05 |
| 34775 | 1.70303 | 0.05 |
| 34776 | 1.88139 | 0.05 |
| 34777 | 1.64955 | 0.05 |
| 34778 | 1.89226 | 2.1 |
| 34779 | 2.34978 | 1.79998 |
| 34780 | 1.89226 | 2.1 |
| 34781 | 2.68773 | 1.44312 |
| 34782 | 1.89226 | 2.1 |
| 34783 | 2.68773 | 1.70647 |
| 34784 | 1.89226 | 2.1 |
| 34785 | 1.89226 | 1.45748 |
| 34786 | 1.89226 | 1.56291 |
| 34787 | 1.89226 | 2.1 |
| 34788 | 3.78453 | 2.1 |
| 34789 | 1.00825 | 0.250358 |
| 34790 | 1.09847 | 0.468058 |
| 34791 | 0.939987 | 0.535365 |
| 34792 | 0.783516 | 1.90915 |
| 34793 | 0.838041 | 1.28426 |
| 34794 | 0.838041 | 1.91043 |
| 34795 | 1.49693 | 2.01116 |
| 34796 | 1.26602 | 1.40862 |
| 34797 | 1.10875 | 0.315292 |

| | | |
|---|---|---|
| 34798 | 1.05187 | 2.1 |
| 34799 | 1.28743 | 1.40132 |
| 34800 | 0.946132 | 0.940276 |
| 34801 | 0.946132 | 1.49829 |
| 34802 | 0.946132 | 2.1 |
| 34803 | 0.946132 | 2.1 |
| 34804 | 0.946132 | 0.29408 |
| 34805 | 1.31823 | 2.1 |
| 34806 | 0.946132 | 1.45053 |
| 34807 | 0.946132 | 2.1 |
| 34808 | 0.946131 | 2.1 |
| 34809 | 0.946131 | 2.1 |
| 34810 | 0.946132 | 1.88471 |
| 34811 | 1.31823 | 2.1 |
| 34812 | 1.51688 | 0.05 |
| 34813 | 1.79978 | 0.05 |
| 34814 | 1.1685 | 0.05 |
| 34815 | 1.1685 | 0.05 |
| 34816 | 1.85857 | 0.05 |
| 34817 | 1.51018 | 0.05 |
| 34818 | 0.946116 | 0.05 |
| 34819 | 0.946116 | 0.0930208 |
| 34820 | 0.94612 | 0.05 |
| 34821 | 0.94612 | 0.05 |
| 34822 | 0.946118 | 0.05 |
| 34823 | 0.946118 | 0.128877 |
| 34824 | 0.946118 | 0.05 |
| 34825 | 0.946118 | 0.0861195 |
| 34826 | 0.946131 | 0.05 |
| 34827 | 0.946131 | 0.0669161 |
| 34828 | 0.946105 | 0.05 |
| 34829 | 0.946105 | 0.0860268 |
| 34830 | 1.89224 | 2.1 |
| 34831 | 1.89224 | 2.1 |
| 34832 | 1.89224 | 2.1 |
| 34833 | 1.89224 | 2.1 |
| 34834 | 1.89224 | 2.1 |
| 34835 | 1.89224 | 2.1 |
| 34836 | 1.38678 | 2.1 |
| 34837 | 1.3349 | 2.1 |
| 34838 | 0.946119 | 2.1 |
| 34839 | 0.946119 | 2.1 |
| 34840 | 1.38678 | 2.1 |
| 34841 | 1.38678 | 2.1 |
| 34842 | 0.946119 | 2.1 |
| 34843 | 0.946119 | 2.1 |
| 34844 | 1.38676 | 2.1 |
| 34845 | 0.946107 | 2.1 |
| 34846 | 0.946132 | 2.1 |
| 34847 | 1.33491 | 2.1 |
| 34848 | 1.06455 | 2.1 |
| 34849 | 1.03932 | 2.1 |
| 34850 | 1.20649 | 2.1 |
| 34851 | 1.18773 | 2.1 |
| 34852 | 2.27105 | 2.1 |
| 34853 | 1.89226 | 2.1 |
| 34854 | 1.89226 | 2.1 |
| 34855 | 1.89226 | 2.1 |

| | | |
|---|---|---|
| 34856 | 1.89226 | 2.1 |
| 34857 | 1.89226 | 2.1 |
| 34858 | 1.89226 | 2.1 |
| 34859 | 1.36918 | 2.1 |
| 34860 | 1.18774 | 2.1 |
| 34861 | 1.31144 | 2.1 |
| 34862 | 1.20651 | 2.1 |
| 34863 | 1.31144 | 2.1 |
| 34864 | 1.20651 | 2.1 |
| 34865 | 2.29066 | 2.1 |
| 34866 | 1.76476 | 2.1 |
| 34867 | 1.79825 | 2.1 |
| 34868 | 2.33801 | 2.1 |
| 34869 | 4.08891 | 2.1 |
| 34870 | 1.79825 | 2.1 |
| 34871 | 2.33801 | 2.1 |
| 34872 | 2.37168 | 2.1 |
| 34873 | 1.49325 | 2.1 |
| 34874 | 4.16993 | 2.1 |
| 34875 | 1.13641 | 2.1 |
| 34876 | 1.43213 | 0.227537 |
| 34877 | 1.44085 | 1.94835 |
| 34878 | 1.18301 | 2.1 |
| 34879 | 2.61513 | 2.1 |
| 34880 | 1.89226 | 2.1 |
| 34881 | 2.23875 | 2.1 |
| 34882 | 2.23875 | 2.1 |
| 34883 | 2.10287 | 2.1 |
| 34884 | 1.89226 | 2.1 |
| 34885 | 1.75639 | 2.1 |
| 34886 | 1.89226 | 0.697127 |
| 34887 | 1.89226 | 2.1 |
| 34888 | 1.89226 | 0.988787 |
| 34889 | 1.89226 | 2.1 |
| 34890 | 1.89226 | 0.925407 |
| 34891 | 1.89226 | 2.1 |
| 34892 | 1.44085 | 2.1 |
| 34893 | 1.25173 | 2.1 |
| 34894 | 1.25173 | 1.42351 |
| 34895 | 1.18301 | 2.1 |
| 34896 | 1.4617 | 1.09302 |
| 34897 | 1.39298 | 2.1 |
| 34898 | 1.89227 | 2.1 |
| 34899 | 2.23875 | 2.1 |
| 34900 | 2.42504 | 2.1 |
| 34901 | 1.89227 | 2.1 |
| 34902 | 4.13101 | 2.1 |
| 34903 | 1.89226 | 0.685363 |
| 34904 | 1.89226 | 2.1 |
| 34905 | 1.89226 | 0.791956 |
| 34906 | 1.89226 | 2.1 |
| 34907 | 1.89226 | 0.307999 |
| 34908 | 1.89226 | 2.1 |
| 34909 | 1.89226 | 2.1 |
| 34910 | 2.41297 | 2.1 |
| 34911 | 2.70685 | 2.1 |
| 34912 | 2.70685 | 2.1 |
| 34913 | 1.89226 | 2.1 |

| | | |
|---|---|---|
| 34914 | 1.89226 | 2.1 |
| 34915 | 1.89226 | 2.1 |
| 34916 | 1.89226 | 2.1 |
| 34917 | 1.89226 | 2.1 |
| 34918 | 1.89226 | 2.1 |
| 34919 | 1.89226 | 2.1 |
| 34920 | 1.89226 | 2.1 |
| 34921 | 1.89226 | 2.1 |
| 34922 | 1.89226 | 2.1 |
| 34923 | 1.89226 | 2.1 |
| 34924 | 1.89226 | 2.1 |
| 34925 | 3.78453 | 2.1 |
| 34926 | 1.89226 | 2.1 |
| 34927 | 1.89226 | 2.1 |
| 34928 | 1.89226 | 2.1 |
| 34929 | 1.89226 | 2.1 |
| 34930 | 1.89226 | 2.1 |
| 34931 | 1.89226 | 2.1 |
| 34932 | 1.69044 | 0.05 |
| 34933 | 1.57979 | 0.05 |
| 34934 | 2.27963 | 0.05 |
| 34935 | 2.21582 | 0.05 |
| 34936 | 3.85942 | 0.05 |
| 34937 | 2.21582 | 0.05 |
| 34938 | 1.30722 | 0.05 |
| 34939 | 2.02069 | 0.05 |
| 34940 | 2.02069 | 0.05 |
| 34941 | 1.57979 | 0.05 |
| 34942 | 1.57979 | 0.05 |
| 34943 | 1.67825 | 2.1 |
| 34944 | 1.89226 | 2.1 |
| 34945 | 1.89226 | 2.1 |
| 34946 | 1.89226 | 2.1 |
| 34947 | 1.91894 | 2.1 |
| 34948 | 1.91894 | 2.1 |
| 34949 | 0.946133 | 2.1 |
| 34950 | 0.946133 | 2.1 |
| 34951 | 0.946132 | 2.1 |
| 34952 | 0.946132 | 2.1 |
| 34953 | 0.946132 | 2.1 |
| 34954 | 0.946132 | 2.1 |
| 34955 | 0.946133 | 2.1 |
| 34956 | 0.946133 | 2.1 |
| 34957 | 0.946133 | 2.1 |
| 34958 | 0.946133 | 2.1 |
| 34959 | 0.946132 | 2.1 |
| 34960 | 0.946132 | 2.1 |
| 34961 | 0.942609 | 2.1 |
| 34962 | 0.942609 | 2.1 |
| 34963 | 1.329 2.1 | |
| 34964 | 1.329 2.1 | |
| 34965 | 1.32547 | 2.1 |
| 34966 | 0.94261 | 2.1 |
| 34967 | 0.946132 | 2.1 |
| 34968 | 0.946132 | 2.1 |
| 34969 | 0.946132 | 2.1 |
| 34970 | 1.32547 | 2.1 |
| 34971 | 0.946132 | 2.1 |

| | | |
|---|---|---|
| 34972 | 0.94261 | 2.1 |
| 34973 | 1.55831 | 2.1 |
| 34974 | 1.07496 | 2.1 |
| 34975 | 1.80327 | 0.810337 |
| 34976 | 1.34851 | 0.969945 |
| 34977 | 2.87824 | 1.0814 |
| 34978 | 2.14346 | 0.588309 |
| 34979 | 2.77937 | 0.562014 |
| 34980 | 2.79173 | 0.766474 |
| 34981 | 2.17129 | 0.50653 |
| 34982 | 4.95066 | 0.555918 |
| 34983 | 2.14346 | 0.651974 |
| 34984 | 1.34124 | 0.05 |
| 34985 | 1.39818 | 0.56314 |
| 34986 | 2.1713 | 0.5477 |
| 34987 | 3.51253 | 0.54606 |
| 34988 | 2.79173 | 0.659434 |
| 34989 | 2.21882 | 0.328065 |
| 34990 | 2.17129 | 0.518293 |
| 34991 | 2.17129 | 0.535132 |
| 34992 | 2.81448 | 0.541483 |
| 34993 | 2.81448 | 0.556702 |
| 34994 | 1.10941 | 0.511508 |
| 34995 | 1.08565 | 0.565798 |
| 34996 | 0.699089 | 0.434669 |
| 34997 | 0.740176 | 0.506498 |
| 34998 | 0.908594 | 0.263751 |
| 34999 | 0.908594 | 0.411891 |
| 35000 | 0.91723 | 0.62695 |
| 35001 | 0.91723 | 0.456906 |
| 35002 | 1.10941 | 0.505245 |
| 35003 | 0.740176 | 0.489359 |
| 35004 | 0.699089 | 0.492452 |
| 35005 | 1.08565 | 0.482384 |
| 35006 | 1.89227 | 0.191051 |
| 35007 | 1.89227 | 0.05 |
| 35008 | 1.89227 | 0.05 |
| 35009 | 1.89227 | 0.05 |
| 35010 | 3.78453 | 0.05 |
| 35011 | 2.40032 | 0.05 |
| 35012 | 2.19526 | 0.05 |
| 35013 | 1.12907 | 0.05 |
| 35014 | 1.44253 | 0.05 |
| 35015 | 3.32433 | 0.05 |
| 35016 | 2.19526 | 0.05 |
| 35017 | 2.71093 | 0.05 |
| 35018 | 1.29826 | 0.05 |
| 35019 | 2.0815 | 0.05 |
| 35020 | 4.18911 | 0.05 |
| 35021 | 1.89226 | 0.05 |
| 35022 | 1.89226 | 0.05 |
| 35023 | 1.89226 | 0.05 |
| 35024 | 1.89226 | 0.05 |
| 35025 | 3.78452 | 0.05 |
| 35026 | 2.30822 | 0.05 |
| 35027 | 1.89226 | 0.05 |
| 35028 | 2.79329 | 0.05 |
| 35029 | 2.79329 | 0.05 |

| | | |
|---|---|---|
| 35030 | 1.89226 | 0.05 |
| 35031 | 1.89226 | 0.05 |
| 35032 | 0.946131 | 2.1 |
| 35033 | 0.946131 | 0.874549 |
| 35034 | 1.38414 | 2.1 |
| 35035 | 1.38414 | 0.05 |
| 35036 | 0.94613 | 2.1 |
| 35037 | 0.94613 | 2.1 |
| 35038 | 1.38415 | 0.807814 |
| 35039 | 1.41833 | 0.138794 |
| 35040 | 0.946132 | 1.58292 |
| 35041 | 1.41833 | 1.58717 |
| 35042 | 1.38414 | 1.06612 |
| 35043 | 0.946131 | 1.87956 |
| 35044 | 1.89226 | 2.1 |
| 35045 | 2.6536 | 2.1 |
| 35046 | 1.89226 | 2.1 |
| 35047 | 2.72238 | 2.1 |
| 35048 | 4.61465 | 2.1 |
| 35049 | 0.946132 | 2.1 |
| 35050 | 0.946132 | 2.1 |
| 35051 | 1.38657 | 2.1 |
| 35052 | 1.38657 | 2.1 |
| 35053 | 1.38657 | 2.1 |
| 35054 | 1.36119 | 2.1 |
| 35055 | 0.946131 | 1.87555 |
| 35056 | 0.946131 | 2.1 |
| 35057 | 0.946132 | 2.1 |
| 35058 | 1.38657 | 2.1 |
| 35059 | 0.946132 | 2.1 |
| 35060 | 1.36119 | 2.1 |
| 35061 | 1.89226 | 2.1 |
| 35062 | 2.4717 | 2.1 |
| 35063 | 2.5026 | 2.1 |
| 35064 | 2.4717 | 2.1 |
| 35065 | 1.92316 | 2.1 |
| 35066 | 1.89226 | 2.1 |
| 35067 | 1.89226 | 2.1 |
| 35068 | 1.89226 | 2.1 |
| 35069 | 1.89226 | 2.1 |
| 35070 | 1.89226 | 1.63409 |
| 35071 | 3.78453 | 2.1 |
| 35072 | 0.946128 | 2.1 |
| 35073 | 0.946128 | 1.28805 |
| 35074 | 1.3419 | 2.1 |
| 35075 | 1.3419 | 2.1 |
| 35076 | 0.946131 | 2.1 |
| 35077 | 0.946131 | 2.1 |
| 35078 | 1.36607 | 2.1 |
| 35079 | 1.3419 | 2.1 |
| 35080 | 0.946131 | 2.1 |
| 35081 | 1.36607 | 2.1 |
| 35082 | 1.3419 | 2.1 |
| 35083 | 0.94613 | 2.1 |
| 35084 | 1.89226 | 2.1 |
| 35085 | 2.50259 | 2.1 |
| 35086 | 2.53563 | 2.1 |
| 35087 | 2.50259 | 2.1 |

| | | |
|---|---|---|
| 35088 | 1.92529 | 2.1 |
| 35089 | 1.89226 | 2.1 |
| 35090 | 0.946132 | 2.1 |
| 35091 | 0.946132 | 2.1 |
| 35092 | 0.946131 | 2.1 |
| 35093 | 0.946131 | 2.1 |
| 35094 | 0.946132 | 2.1 |
| 35095 | 0.946132 | 2.1 |
| 35096 | 0.946131 | 2.1 |
| 35097 | 0.946131 | 2.1 |
| 35098 | 0.946131 | 2.1 |
| 35099 | 0.946131 | 2.1 |
| 35100 | 0.946131 | 2.1 |
| 35101 | 0.946131 | 2.1 |
| 35102 | 2.0815 | 2.1 |
| 35103 | 1.86506 | 2.1 |
| 35104 | 1.86506 | 2.1 |
| 35105 | 1.82567 | 2.1 |
| 35106 | 2.0815 | 2.1 |
| 35107 | 2.03335 | 2.1 |
| 35108 | 2.0815 | 2.1 |
| 35109 | 2.0815 | 2.1 |
| 35110 | 2.0815 | 2.1 |
| 35111 | 1.88668 | 2.1 |
| 35112 | 1.83221 | 2.1 |
| 35113 | 1.57275 | 2.1 |
| 35114 | 2.34061 | 0.05 |
| 35115 | 1.89226 | 0.05 |
| 35116 | 2.23254 | 0.05 |
| 35117 | 1.89226 | 0.05 |
| 35118 | 2.23254 | 0.05 |
| 35119 | 1.89226 | 0.05 |
| 35120 | 1.8619 | 0.05 |
| 35121 | 1.89226 | 0.05 |
| 35122 | 2.34061 | 0.05 |
| 35123 | 1.89226 | 0.05 |
| 35124 | 2.34061 | 0.05 |
| 35125 | 1.89226 | 0.05 |
| 35126 | 1.89227 | 0.05 |
| 35127 | 1.89227 | 0.05 |
| 35128 | 1.89227 | 0.05 |
| 35129 | 1.89227 | 0.05 |
| 35130 | 1.89227 | 0.05 |
| 35131 | 1.89227 | 0.05 |
| 35132 | 1.89227 | 0.05 |
| 35133 | 1.89227 | 0.05 |
| 35134 | 1.89227 | 0.05 |
| 35135 | 1.89227 | 0.05 |
| 35136 | 1.89227 | 0.05 |
| 35137 | 1.89227 | 0.05 |
| 35138 | 1.06365 | 0.05 |
| 35139 | 0.503305 | 0.05 |
| 35140 | 0.794912 | 0.05 |
| 35141 | 1.42752 | 0.05 |
| 35142 | 1.34826 | 0.05 |
| 35143 | 1.07163 | 0.05 |
| 35144 | 0.533113 | 0.05 |
| 35145 | 0.761277 | 0.05 |

| | | |
|---|---|---|
| 35146 | 0.848403 | 0.05 |
| 35147 | 0.876191 | 0.05 |
| 35148 | 0.963372 | 0.05 |
| 35149 | 0.963372 | 0.05 |
| 35150 | 1.55571 | 0.05 |
| 35151 | 2.76574 | 0.05 |
| 35152 | 2.92453 | 0.05 |
| 35153 | 1.70301 | 0.05 |
| 35154 | 2.33089 | 0.05 |
| 35155 | 1.06905 | 0.05 |
| 35156 | 2.28979 | 2.1 |
| 35157 | 1.81488 | 2.1 |
| 35158 | 1.63722 | 2.1 |
| 35159 | 2.22627 | 2.1 |
| 35160 | 4.04115 | 2.1 |
| 35161 | 1.98246 | 2.1 |
| 35162 | 2.15715 | 2.1 |
| 35163 | 2.22627 | 2.1 |
| 35164 | 2.22627 | 2.1 |
| 35165 | 1.63719 | 2.1 |
| 35166 | 1.63719 | 2.1 |
| 35167 | 2.1644 | 0.05 |
| 35168 | 1.903 0.05 | |
| 35169 | 1.23639 | 0.05 |
| 35170 | 1.42704 | 0.05 |
| 35171 | 3.61546 | 0.05 |
| 35172 | 1.89226 | 0.05 |
| 35173 | 1.89226 | 0.05 |
| 35174 | 1.89226 | 0.05 |
| 35175 | 1.89226 | 0.05 |
| 35176 | 3.78451 | 0.05 |
| 35177 | 2.61343 | 0.05 |
| 35178 | 1.89226 | 0.05 |
| 35179 | 2.08149 | 0.05 |
| 35180 | 1.74357 | 0.05 |
| 35181 | 2.08149 | 0.05 |
| 35182 | 1.74357 | 0.05 |
| 35183 | 1.63483 | 0.05 |
| 35184 | 1.8631 | 0.05 |
| 35185 | 1.8631 | 0.05 |
| 35186 | 1.70301 | 0.05 |
| 35187 | 2.0815 | 0.05 |
| 35188 | 1.94319 | 0.05 |
| 35189 | 2.0815 | 0.05 |
| 35190 | 1.89226 | 0.05 |
| 35191 | 1.89226 | 0.05 |
| 35192 | 2.05236 | 0.05 |
| 35193 | 2.23014 | 0.05 |
| 35194 | 2.41882 | 0.05 |
| 35195 | 2.14993 | 2.1 |
| 35196 | 1.87217 | 2.1 |
| 35197 | 2.34488 | 2.1 |
| 35198 | 2.5254 | 2.1 |
| 35199 | 4.39757 | 2.1 |
| 35200 | 0.949995 | 2.1 |
| 35201 | 0.630972 | 2.1 |
| 35202 | 0.936084 | 2.1 |
| 35203 | 1.17244 | 2.1 |

| | | |
|---|---|---|
| 35204 | 0.922394 | 2.1 |
| 35205 | 0.922394 | 2.1 |
| 35206 | 0.88102 | 2.1 |
| 35207 | 0.88102 | 2.1 |
| 35208 | 0.949998 | 2.1 |
| 35209 | 1.17244 | 2.1 |
| 35210 | 0.936081 | 2.1 |
| 35211 | 0.630973 | 2.1 |
| 35212 | 1.89226 | 2.1 |
| 35213 | 1.89226 | 2.1 |
| 35214 | 1.89226 | 2.1 |
| 35215 | 1.89226 | 2.1 |
| 35216 | 3.78452 | 2.1 |
| 35217 | 1.89226 | 2.0077 |
| 35218 | 1.89226 | 2.1 |
| 35219 | 1.89226 | 2.08344 |
| 35220 | 1.89226 | 2.1 |
| 35221 | 1.89226 | 1.98855 |
| 35222 | 1.89226 | 2.1 |
| 35223 | 2.34488 | 2.1 |
| 35224 | 2.5254 | 2.07643 |
| 35225 | 1.89226 | 1.99558 |
| 35226 | 1.89226 | 2.1 |
| 35227 | 4.41766 | 2.1 |
| 35228 | 1.89226 | 2.1 |
| 35229 | 1.89226 | 2.1 |
| 35230 | 1.89226 | 2.1 |
| 35231 | 1.89226 | 2.1 |
| 35232 | 3.78452 | 2.1 |
| 35233 | 1.89226 | 1.66053 |
| 35234 | 1.89226 | 2.1 |
| 35235 | 1.89226 | 1.80693 |
| 35236 | 1.89226 | 2.09237 |
| 35237 | 1.89226 | 1.92356 |
| 35238 | 1.89226 | 2.1 |
| 35239 | 1.5605 | 2.1 |
| 35240 | 1.15806 | 2.1 |
| 35241 | 1.31565 | 2.1 |
| 35242 | 1.65642 | 2.1 |
| 35243 | 2.87615 | 2.1 |
| 35244 | 1.46643 | 2.1 |
| 35245 | 1.65642 | 2.1 |
| 35246 | 1.31565 | 2.1 |
| 35247 | 1.31565 | 2.1 |
| 35248 | 1.74246 | 2.1 |
| 35249 | 1.74246 | 2.1 |
| 35250 | 1.89226 | 2.1 |
| 35251 | 1.89226 | 2.1 |
| 35252 | 1.89226 | 2.1 |
| 35253 | 1.89226 | 2.1 |
| 35254 | 1.89226 | 2.1 |
| 35255 | 1.89226 | 2.1 |
| 35256 | 1.15099 | 2.1 |
| 35257 | 1.15099 | 2.1 |
| 35258 | 0.939316 | 2.1 |
| 35259 | 0.939316 | 2.1 |
| 35260 | 0.946127 | 2.1 |
| 35261 | 0.946127 | 2.1 |

| | | |
|---|---|---|
| 35262 | 1.14418 | 2.1 |
| 35263 | 0.939289 | 2.1 |
| 35264 | 1.14417 | 2.1 |
| 35265 | 0.946129 | 2.1 |
| 35266 | 0.939287 | 2.1 |
| 35267 | 0.94613 | 2.1 |
| 35268 | 1.89226 | 2.1 |
| 35269 | 1.89226 | 2.1 |
| 35270 | 1.89226 | 2.1 |
| 35271 | 1.89226 | 2.1 |
| 35272 | 3.78452 | 2.1 |
| 35273 | 1.93164 | 0.05 |
| 35274 | 2.64898 | 0.05 |
| 35275 | 1.43213 | 0.05 |
| 35276 | 1.43213 | 0.05 |
| 35277 | 2.63206 | 0.05 |
| 35278 | 2.13254 | 0.05 |
| 35279 | 1.89226 | 2.1 |
| 35280 | 1.89226 | 2.1 |
| 35281 | 1.89226 | 2.1 |
| 35282 | 1.89226 | 2.1 |
| 35283 | 3.78452 | 2.1 |
| 35284 | 2.71655 | 2.1 |
| 35285 | 1.43323 | 2.1 |
| 35286 | 1.28413 | 2.1 |
| 35287 | 2.53614 | 2.1 |
| 35288 | 3.96937 | 2.1 |
| 35289 | 1.89226 | 2.1 |
| 35290 | 1.89226 | 2.1 |
| 35291 | 1.89226 | 2.1 |
| 35292 | 1.89226 | 2.1 |
| 35293 | 1.89226 | 2.1 |
| 35294 | 1.89226 | 2.1 |
| 35295 | 1.33356 | 2.1 |
| 35296 | 2.82297 | 2.1 |
| 35297 | 2.771 2.1 | |
| 35298 | 2.771 2.1 | |
| 35299 | 1.46037 | 2.1 |
| 35300 | 1.46037 | 2.1 |
| 35301 | 2.56034 | 2.1 |
| 35302 | 1.18474 | 2.1 |
| 35303 | 1.15113 | 2.1 |
| 35304 | 2.53724 | 2.1 |
| 35305 | 3.72198 | 2.1 |
| 35306 | 2.56034 | 2.1 |
| 35307 | 1.89226 | 2.1 |
| 35308 | 1.89226 | 2.1 |
| 35309 | 2.53725 | 2.1 |
| 35310 | 4.42951 | 2.1 |
| 35311 | 1.89226 | 2.1 |
| 35312 | 1.89226 | 2.1 |
| 35313 | 1.89226 | 2.1 |
| 35314 | 1.89226 | 2.1 |
| 35315 | 3.78453 | 2.1 |
| 35316 | 1.89226 | 2.1 |
| 35317 | 1.89226 | 2.1 |
| 35318 | 1.89226 | 2.1 |
| 35319 | 1.89226 | 2.1 |

| | | |
|---|---|---|
| 35320 | 3.78452 | 2.1 |
| 35321 | 1.1229 | 2.1 |
| 35322 | 2.53724 | 2.1 |
| 35323 | 1.15113 | 2.1 |
| 35324 | 1.15113 | 2.1 |
| 35325 | 2.51559 | 2.1 |
| 35326 | 2.51559 | 2.1 |
| 35327 | 1.89226 | 2.1 |
| 35328 | 2.53724 | 2.1 |
| 35329 | 2.51559 | 2.1 |
| 35330 | 2.53724 | 2.1 |
| 35331 | 1.87061 | 2.1 |
| 35332 | 1.89226 | 2.1 |
| 35333 | 1.89226 | 2.1 |
| 35334 | 1.89226 | 2.1 |
| 35335 | 1.89226 | 2.1 |
| 35336 | 1.89226 | 2.1 |
| 35337 | 3.78453 | 2.1 |
| 35338 | 1.89226 | 2.1 |
| 35339 | 1.89226 | 2.1 |
| 35340 | 1.89226 | 2.1 |
| 35341 | 1.89226 | 2.1 |
| 35342 | 3.78452 | 2.1 |
| 35343 | 1.98143 | 1.19359 |
| 35344 | 2.81559 | 0.05 |
| 35345 | 1.65411 | 0.0732353 |
| 35346 | 2.26675 | 0.485134 |
| 35347 | 4.24818 | 0.11553 |
| 35348 | 0.810656 | 0.58879 |
| 35349 | 0.810656 | 2.1 |
| 35350 | 0.821888 | 2.1 |
| 35351 | 0.821888 | 2.1 |
| 35352 | 1.1235 | 0.959146 |
| 35353 | 1.07439 | 2.1 |
| 35354 | 0.535693 | 2.1 |
| 35355 | 0.509042 | 2.1 |
| 35356 | 0.535693 | 2.1 |
| 35357 | 1.1235 | 2.1 |
| 35358 | 0.509043 | 2.1 |
| 35359 | 1.07439 | 2.1 |
| 35360 | 1.10378 | 2.1 |
| 35361 | 2.247 | 2.1 |
| 35362 | 2.27472 | 2.1 |
| 35363 | 2.27472 | 2.1 |
| 35364 | 1.07139 | 2.1 |
| 35365 | 1.07139 | 2.1 |
| 35366 | 2.08081 | 2.1 |
| 35367 | 1.40395 | 2.1 |
| 35368 | 1.34245 | 2.1 |
| 35369 | 2.00239 | 2.1 |
| 35370 | 3.40634 | 2.1 |
| 35371 | 1.89226 | 2.1 |
| 35372 | 1.89226 | 2.1 |
| 35373 | 1.89226 | 2.1 |
| 35374 | 1.89226 | 2.1 |
| 35375 | 3.78453 | 2.1 |
| 35376 | 0.946128 | 2.1 |
| 35377 | 0.946128 | 2.1 |

| | | |
|---|---|---|
| 35378 | 1.13785 | 2.1 |
| 35379 | 1.11496 | 2.1 |
| 35380 | 0.946131 | 2.1 |
| 35381 | 0.946131 | 2.1 |
| 35382 | 1.11495 | 2.1 |
| 35383 | 1.11495 | 2.1 |
| 35384 | 0.94613 | 2.1 |
| 35385 | 1.11495 | 2.1 |
| 35386 | 1.13785 | 2.1 |
| 35387 | 0.946131 | 2.1 |
| 35388 | 0.975888 | 2.1 |
| 35389 | 1.00118 | 2.1 |
| 35390 | 0.671218 | 2.1 |
| 35391 | 0.653229 | 2.1 |
| 35392 | 0.825372 | 2.1 |
| 35393 | 0.825372 | 2.1 |
| 35394 | 0.829038 | 2.1 |
| 35395 | 0.829038 | 2.1 |
| 35396 | 0.975901 | 2.1 |
| 35397 | 0.653238 | 2.1 |
| 35398 | 0.671209 | 2.1 |
| 35399 | 1.00117 | 2.1 |
| 35400 | 1.89224 | 2.1 |
| 35401 | 1.89224 | 2.1 |
| 35402 | 1.89224 | 2.1 |
| 35403 | 1.89224 | 2.1 |
| 35404 | 3.78448 | 2.1 |
| 35405 | 0.946115 | 2.1 |
| 35406 | 0.946115 | 2.1 |
| 35407 | 1.11494 | 2.1 |
| 35408 | 1.10111 | 2.1 |
| 35409 | 0.946118 | 2.1 |
| 35410 | 0.946118 | 2.1 |
| 35411 | 1.10111 | 2.1 |
| 35412 | 1.10111 | 2.1 |
| 35413 | 1.10112 | 2.1 |
| 35414 | 0.94613 | 2.1 |
| 35415 | 0.946105 | 2.1 |
| 35416 | 1.11492 | 2.1 |
| 35417 | 1.04028 | 0.456059 |
| 35418 | 2.27126 | 0.05 |
| 35419 | 2.0815 | 0.229665 |
| 35420 | 1.82249 | 0.624096 |
| 35421 | 1.33723 | 0.204756 |
| 35422 | 1.18684 | 0.405566 |
| 35423 | 0.812616 | 0.177097 |
| 35424 | 0.812616 | 0.132432 |
| 35425 | 0.997777 | 0.075509 |
| 35426 | 1.08122 | 0.214557 |
| 35427 | 0.94613 | 0.0959098 |
| 35428 | 0.94613 | 0.156345 |
| 35429 | 0.812648 | 0.133113 |
| 35430 | 0.932779 | 0.100772 |
| 35431 | 0.812648 | 0.0789696 |
| 35432 | 0.94613 | 0.142232 |
| 35433 | 0.849335 | 0.0717632 |
| 35434 | 0.94613 | 0.0843813 |
| 35435 | 1.80327 | 1.03534 |

| | | |
|---|---|---|
| 35436 | 1.56016 | 0.963627 |
| 35437 | 1.57731 | 2.0797 |
| 35438 | 1.34851 | 2.02102 |
| 35439 | 2.03207 | 1.09443 |
| 35440 | 1.80327 | 2.1 |
| 35441 | 2.03207 | 1.8794 |
| 35442 | 1.56016 | 1.74974 |
| 35443 | 2.18076 | 1.14994 |
| 35444 | 1.78167 | 1.14062 |
| 35445 | 3.81374 | 1.85137 |
| 35446 | 1.37597 | 2.07345 |
| 35447 | 1.60058 | 2.1 |
| 35448 | 1.70302 | 1.04344 |
| 35449 | 1.85448 | 1.2427 |
| 35450 | 3.15028 | 1.21422 |
| 35451 | 1.60058 | 1.02774 |
| 35452 | 1.4054 | 2.1 |
| 35453 | 1.66202 | 1.03853 |
| 35454 | 1.70302 | 2.1 |
| 35455 | 3.09979 | 1.41474 |
| 35456 | 1.70302 | 1.60065 |
| 35457 | 1.85448 | 1.65104 |
| 35458 | 2.21317 | 1.23416 |
| 35459 | 2.0961 | 1.18733 |
| 35460 | 4.01757 | 1.78638 |
| 35461 | 1.66202 | 1.22213 |
| 35462 | 1.70302 | 2.1 |
| 35463 | 2.09611 | 0.922312 |
| 35464 | 2.00892 | 2.02837 |
| 35465 | 3.6468 | 1.17953 |
| 35466 | 1.2966 | 0.05 |
| 35467 | 2.25495 | 0.05 |
| 35468 | 1.2966 | 0.05 |
| 35469 | 2.21117 | 0.05 |
| 35470 | 1.67687 | 0.05 |
| 35471 | 2.6167 | 0.05 |
| 35472 | 1.89227 | 0.05 |
| 35473 | 1.89227 | 0.05 |
| 35474 | 1.89227 | 0.05 |
| 35475 | 1.89227 | 0.05 |
| 35476 | 3.78453 | 0.05 |
| 35477 | 2.44375 | 0.05 |
| 35478 | 1.89227 | 0.05 |
| 35479 | 2.81853 | 0.05 |
| 35480 | 2.81853 | 0.05 |
| 35481 | 1.89227 | 0.05 |
| 35482 | 1.89227 | 0.05 |
| 35483 | 1.89226 | 0.992195 |
| 35484 | 1.75853 | 1.16441 |
| 35485 | 2.37636 | 0.963459 |
| 35486 | 2.37636 | 1.19984 |
| 35487 | 1.89226 | 0.868765 |
| 35488 | 1.89226 | 1.08144 |
| 35489 | 1.65814 | 0.05 |
| 35490 | 1.10187 | 1.22102 |
| 35491 | 1.10187 | 0.533977 |
| 35492 | 1.19547 | 1.54148 |
| 35493 | 1.56263 | 0.05 |

| | | |
|---|---|---|
| 35494 | 1.65624 | 0.98528 |
| 35495 | 1.56264 | 0.05 |
| 35496 | 1.01479 | 1.61044 |
| 35497 | 1.01479 | 0.62558 |
| 35498 | 1.10187 | 1.55339 |
| 35499 | 1.47561 | 0.05 |
| 35500 | 1.5627 | 1.29892 |
| 35501 | 1.89225 | 2.1 |
| 35502 | 1.89225 | 2.1 |
| 35503 | 1.89225 | 2.1 |
| 35504 | 1.89225 | 2.1 |
| 35505 | 3.7845 | 2.1 |
| 35506 | 2.54386 | 2.1 |
| 35507 | 1.91546 | 2.1 |
| 35508 | 2.46008 | 2.1 |
| 35509 | 1.82815 | 2.1 |
| 35510 | 4.37554 | 2.1 |
| 35511 | 1.91546 | 2.1 |
| 35512 | 1.04909 | 2.1 |
| 35513 | 1.82815 | 2.1 |
| 35514 | 0.983141 | 2.1 |
| 35515 | 2.8986 | 2.1 |
| 35516 | 1.89225 | 2.1 |
| 35517 | 1.89225 | 2.1 |
| 35518 | 1.89225 | 2.1 |
| 35519 | 1.89225 | 2.1 |
| 35520 | 3.78449 | 2.1 |
| 35521 | 1.89226 | 2.1 |
| 35522 | 1.89226 | 2.1 |
| 35523 | 1.89226 | 2.1 |
| 35524 | 1.89226 | 2.1 |
| 35525 | 1.89226 | 2.1 |
| 35526 | 1.89226 | 2.1 |
| 35527 | 2.46009 | 2.1 |
| 35528 | 1.7513 | 2.1 |
| 35529 | 1.82817 | 2.1 |
| 35530 | 1.82817 | 2.1 |
| 35531 | 2.38127 | 2.1 |
| 35532 | 2.38127 | 2.1 |
| 35533 | 2.55006 | 2.1 |
| 35534 | 2.64569 | 2.1 |
| 35535 | 2.60128 | 2.1 |
| 35536 | 2.49168 | 2.1 |
| 35537 | 5.13737 | 2.1 |
| 35538 | 2.51605 | 2.1 |
| 35539 | 2.64569 | 2.1 |
| 35540 | 2.60128 | 2.1 |
| 35541 | 2.48891 | 2.1 |
| 35542 | 5.1346 | 2.1 |
| 35543 | 2.60128 | 2.1 |
| 35544 | 2.49168 | 2.1 |
| 35545 | 2.41689 | 2.1 |
| 35546 | 2.55248 | 2.1 |
| 35547 | 5.04416 | 2.1 |
| 35548 | 2.60128 | 2.1 |
| 35549 | 2.48891 | 2.1 |
| 35550 | 2.45922 | 2.1 |
| 35551 | 2.55249 | 2.1 |

| | | |
|---|---|---|
| 35552 | 5.04139 | 2.1 |
| 35553 | 1.89227 | 2.1 |
| 35554 | 1.89227 | 2.1 |
| 35555 | 1.89227 | 2.1 |
| 35556 | 1.89227 | 2.1 |
| 35557 | 1.89227 | 2.1 |
| 35558 | 1.89227 | 2.1 |
| 35559 | 0.96063 | 2.1 |
| 35560 | 0.96063 | 2.1 |
| 35561 | 0.958806 | 2.1 |
| 35562 | 0.958806 | 2.1 |
| 35563 | 1.28892 | 2.1 |
| 35564 | 0.749042 | 2.1 |
| 35565 | 1.17409 | 2.1 |
| 35566 | 0.630516 | 2.1 |
| 35567 | 1.17409 | 2.1 |
| 35568 | 1.28892 | 2.1 |
| 35569 | 0.630515 | 2.1 |
| 35570 | 0.749042 | 2.1 |
| 35571 | 0.92511 | 2.1 |
| 35572 | 0.92511 | 2.1 |
| 35573 | 1.19511 | 2.1 |
| 35574 | 1.19511 | 2.1 |
| 35575 | 0.946132 | 2.1 |
| 35576 | 1.17409 | 2.1 |
| 35577 | 0.925081 | 2.1 |
| 35578 | 0.946129 | 2.1 |
| 35579 | 0.946131 | 1.83837 |
| 35580 | 0.946131 | 2.1 |
| 35581 | 1.17409 | 2.1 |
| 35582 | 0.925083 | 2.1 |
| 35583 | 1.89226 | 0.779783 |
| 35584 | 1.89226 | 0.949619 |
| 35585 | 1.89226 | 0.394065 |
| 35586 | 1.89226 | 0.893842 |
| 35587 | 1.89226 | 0.465573 |
| 35588 | 1.89226 | 0.857896 |
| 35589 | 1.89226 | 1.44119 |
| 35590 | 1.89226 | 1.64984 |
| 35591 | 1.89226 | 1.00629 |
| 35592 | 1.89226 | 1.81863 |
| 35593 | 3.78453 | 1.02151 |
| 35594 | 0.946132 | 2.1 |
| 35595 | 0.946132 | 2.1 |
| 35596 | 1.28892 | 2.1 |
| 35597 | 1.28892 | 2.1 |
| 35598 | 0.946132 | 2.1 |
| 35599 | 1.28892 | 2.1 |
| 35600 | 0.946133 | 2.1 |
| 35601 | 1.17409 | 2.1 |
| 35602 | 0.946132 | 2.1 |
| 35603 | 0.946132 | 2.1 |
| 35604 | 1.17409 | 2.1 |
| 35605 | 1.28892 | 2.1 |
| 35606 | 0.625229 | 2.1 |
| 35607 | 0.890321 | 2.1 |
| 35608 | 1.21636 | 2.1 |
| 35609 | 1.13022 | 2.1 |

| | | |
|---|---|---|
| 35610 | 1.07094 | 2.1 |
| 35611 | 1.03541 | 2.1 |
| 35612 | 0.942207 | 2.1 |
| 35613 | 0.942207 | 2.1 |
| 35614 | 0.898766 | 2.1 |
| 35615 | 1.26626 | 2.1 |
| 35616 | 1.0853 | 2.1 |
| 35617 | 0.600358 | 2.1 |
| 35618 | 0.719012 | 2.1 |
| 35619 | 0.719012 | 2.1 |
| 35620 | 1.17957 | 2.1 |
| 35621 | 1.17957 | 2.1 |
| 35622 | 0.719012 | 2.1 |
| 35623 | 0.952456 | 2.1 |
| 35624 | 0.946129 | 2.1 |
| 35625 | 0.946129 | 2.1 |
| 35626 | 0.946131 | 2.1 |
| 35627 | 0.952454 | 2.1 |
| 35628 | 0.946131 | 2.1 |
| 35629 | 0.719011 | 2.1 |
| 35630 | 0.94613 | 2.1 |
| 35631 | 0.94613 | 2.1 |
| 35632 | 0.94613 | 2.1 |
| 35633 | 0.94613 | 2.1 |
| 35634 | 0.946131 | 2.1 |
| 35635 | 0.946131 | 2.1 |
| 35636 | 0.946129 | 2.1 |
| 35637 | 0.946129 | 2.1 |
| 35638 | 0.94613 | 2.1 |
| 35639 | 0.94613 | 2.1 |
| 35640 | 0.94613 | 2.1 |
| 35641 | 0.94613 | 2.1 |
| 35642 | 1.1587 | 2.1 |
| 35643 | 0.973158 | 2.1 |
| 35644 | 0.750023 | 2.1 |
| 35645 | 0.536443 | 2.1 |
| 35646 | 0.84054 | 2.1 |
| 35647 | 0.84054 | 2.1 |
| 35648 | 0.854602 | 2.1 |
| 35649 | 0.854602 | 2.1 |
| 35650 | 1.1587 | 2.1 |
| 35651 | 0.750023 | 2.1 |
| 35652 | 0.973158 | 2.1 |
| 35653 | 0.536443 | 2.1 |
| 35654 | 1.89227 | 0.05 |
| 35655 | 1.89227 | 2.1 |
| 35656 | 1.89227 | 2.093 |
| 35657 | 1.89227 | 0.0966508 |
| 35658 | 3.78453 | 1.34639 |
| 35659 | 1.89226 | 2.1 |
| 35660 | 1.89226 | 2.1 |
| 35661 | 1.89226 | 1.23861 |
| 35662 | 1.89226 | 2.1 |
| 35663 | 3.78453 | 2.1 |
| 35664 | 1.7632 | 2.1 |
| 35665 | 1.61149 | 2.1 |
| 35666 | 1.98749 | 2.1 |
| 35667 | 2.11615 | 2.1 |

| | | |
|---|---|---|
| 35668 | 3.75068 | 2.1 |
| 35669 | 1.7632 | 2.1 |
| 35670 | 1.61149 | 2.1 |
| 35671 | 1.97733 | 0.094285 |
| 35672 | 2.11615 | 2.1 |
| 35673 | 3.74052 | 2.1 |
| 35674 | 1.89226 | 2.00116 |
| 35675 | 1.89226 | 0.593124 |
| 35676 | 1.89226 | 0.0812961 |
| 35677 | 1.89226 | 2.1 |
| 35678 | 3.78453 | 0.546555 |
| 35679 | 1.89226 | 0.415087 |
| 35680 | 1.89226 | 2.1 |
| 35681 | 1.89226 | 2.1 |
| 35682 | 1.89226 | 2.1 |
| 35683 | 3.78453 | 2.1 |
| 35684 | 1.98749 | 2.1 |
| 35685 | 2.11615 | 2.1 |
| 35686 | 2.43151 | 2.1 |
| 35687 | 2.27541 | 2.1 |
| 35688 | 4.41899 | 2.1 |
| 35689 | 1.97733 | 0.355256 |
| 35690 | 2.11615 | 2.1 |
| 35691 | 2.43151 | 2.1 |
| 35692 | 2.33986 | 0.0675487 |
| 35693 | 4.40883 | 2.1 |
| 35694 | 2.17892 | 0.0814739 |
| 35695 | 2.80057 | 0.648802 |
| 35696 | 1.88349 | 0.540597 |
| 35697 | 2.63062 | 0.0912466 |
| 35698 | 4.80954 | 0.472152 |
| 35699 | 1.89226 | 1.02783 |
| 35700 | 1.89226 | 0.301087 |
| 35701 | 1.89226 | 0.376814 |
| 35702 | 1.89226 | 0.132443 |
| 35703 | 3.78452 | 0.395875 |
| 35704 | 2.0155 | 0.901183 |
| 35705 | 1.97652 | 0.366528 |
| 35706 | 1.8247 | 0.317276 |
| 35707 | 1.80645 | 0.05 |
| 35708 | 3.80122 | 0.0915902 |
| 35709 | 1.97652 | 0.492244 |
| 35710 | 1.79681 | 0.504281 |
| 35711 | 1.80645 | 0.0540608 |
| 35712 | 1.59244 | 0.108859 |
| 35713 | 3.56896 | 0.129428 |
| 35714 | 1.89226 | 0.503597 |
| 35715 | 1.89226 | 0.797928 |
| 35716 | 1.89226 | 0.166198 |
| 35717 | 1.89226 | 0.224307 |
| 35718 | 3.78452 | 0.185144 |
| 35719 | 1.89223 | 0.55848 |
| 35720 | 1.89223 | 0.422646 |
| 35721 | 1.89223 | 0.808232 |
| 35722 | 1.89223 | 0.080452 |
| 35723 | 3.78447 | 0.394717 |
| 35724 | 1.82467 | 0.580522 |
| 35725 | 1.80643 | 0.0848874 |

| | | |
|---|---|---|
| 35726 | 1.55159 | 0.288546 |
| 35727 | 1.53969 | 0.0529212 |
| 35728 | 3.36437 | 0.0743193 |
| 35729 | 1.80643 | 0.151485 |
| 35730 | 1.59242 | 0.47823 |
| 35731 | 1.5397 | 0.102784 |
| 35732 | 1.40538 | 0.131 |
| 35733 | 3.13211 | 0.15892 |
| 35734 | 1.89224 | 0.481321 |
| 35735 | 1.89224 | 0.564006 |
| 35736 | 1.89224 | 0.14787 |
| 35737 | 1.89224 | 0.452597 |
| 35738 | 3.78447 | 0.369863 |
| 35739 | 1.37427 | 0.05 |
| 35740 | 2.37086 | 0.05 |
| 35741 | 2.37972 | 0.05 |
| 35742 | 1.94703 | 0.05 |
| 35743 | 2.19299 | 0.05 |
| 35744 | 1.91813 | 0.0818864 |
| 35745 | 1.07566 | 2.1 |
| 35746 | 1.12954 | 2.1 |
| 35747 | 0.946131 | 2.1 |
| 35748 | 0.946131 | 2.1 |
| 35749 | 1.12954 | 2.1 |
| 35750 | 1.12954 | 2.1 |
| 35751 | 0.946131 | 2.1 |
| 35752 | 0.946131 | 2.1 |
| 35753 | 1.12954 | 2.1 |
| 35754 | 0.946131 | 2.1 |
| 35755 | 0.946131 | 2.1 |
| 35756 | 1.07566 | 2.1 |
| 35757 | 2.25908 | 2.1 |
| 35758 | 1.89226 | 2.1 |
| 35759 | 1.89226 | 2.1 |
| 35760 | 1.89226 | 2.1 |
| 35761 | 2.37567 | 2.1 |
| 35762 | 2.37567 | 2.1 |
| 35763 | 1.37597 | 1.94595 |
| 35764 | 1.70302 | 0.886816 |
| 35765 | 1.24162 | 2.1 |
| 35766 | 1.12219 | 2.1 |
| 35767 | 1.37072 | 1.10383 |
| 35768 | 1.70302 | 2.1 |
| 35769 | 1.37072 | 2.1 |
| 35770 | 1.70302 | 1.27607 |
| 35771 | 1.54168 | 2.01662 |
| 35772 | 2.21318 | 1.17069 |
| 35773 | 3.39238 | 1.94418 |
| 35774 | 1.89226 | 0.05 |
| 35775 | 1.89226 | 0.05 |
| 35776 | 1.89226 | 0.05 |
| 35777 | 1.89226 | 0.05 |
| 35778 | 3.78452 | 0.05 |
| 35779 | 1.89227 | 0.05 |
| 35780 | 1.89227 | 0.05 |
| 35781 | 1.89227 | 0.05 |
| 35782 | 1.89227 | 0.05 |
| 35783 | 3.78453 | 0.05 |

| | | |
|---|---|---|
| 35784 | 1.89226 | 0.05 |
| 35785 | 1.89226 | 0.05 |
| 35786 | 1.89226 | 0.05 |
| 35787 | 1.89226 | 0.05 |
| 35788 | 3.78453 | 0.05 |
| 35789 | 1.89226 | 0.05 |
| 35790 | 2.21654 | 0.05 |
| 35791 | 2.72007 | 0.05 |
| 35792 | 2.72007 | 0.05 |
| 35793 | 1.89226 | 0.05 |
| 35794 | 1.89226 | 0.05 |
| 35795 | 0.946128 | 0.05 |
| 35796 | 0.946128 | 0.05 |
| 35797 | 0.946132 | 0.05 |
| 35798 | 0.946132 | 0.05 |
| 35799 | 0.94613 | 0.05 |
| 35800 | 0.94613 | 0.05 |
| 35801 | 0.946131 | 0.05 |
| 35802 | 0.946131 | 0.05 |
| 35803 | 0.94613 | 0.05 |
| 35804 | 0.94613 | 0.05 |
| 35805 | 0.94613 | 0.05 |
| 35806 | 0.94613 | 0.05 |
| 35807 | 1.89226 | 0.05 |
| 35808 | 1.89226 | 0.05 |
| 35809 | 1.89226 | 0.05 |
| 35810 | 1.89226 | 0.05 |
| 35811 | 3.78453 | 0.05 |
| 35812 | 1.33457 | 0.05 |
| 35813 | 0.946131 | 0.05 |
| 35814 | 1.08045 | 0.05 |
| 35815 | 0.946132 | 0.05 |
| 35816 | 1.08045 | 0.05 |
| 35817 | 1.08045 | 0.05 |
| 35818 | 0.946132 | 0.05 |
| 35819 | 0.946132 | 0.05 |
| 35820 | 1.33457 | 0.05 |
| 35821 | 1.08045 | 0.05 |
| 35822 | 0.946132 | 0.05 |
| 35823 | 0.946132 | 0.05 |
| 35824 | 1.53347 | 1.17935 |
| 35825 | 1.13092 | 2.1 |
| 35826 | 1.52938 | 2.1 |
| 35827 | 1.93568 | 1.7475 |
| 35828 | 3.06285 | 2.1 |
| 35829 | 1.89226 | 0.377833 |
| 35830 | 2.72959 | 0.328303 |
| 35831 | 2.82996 | 0.363567 |
| 35832 | 2.82996 | 0.324779 |
| 35833 | 1.89226 | 0.665205 |
| 35834 | 1.89226 | 0.393292 |
| 35835 | 2.7296 | 0.05 |
| 35836 | 2.57663 | 2.1 |
| 35837 | 2.48354 | 0.05 |
| 35838 | 2.48354 | 2.1 |
| 35839 | 2.82996 | 0.05 |
| 35840 | 2.82996 | 2.1 |
| 35841 | 2.63449 | 0.05 |

| | | |
|---|---|---|
| 35842 | 2.7296 | 0.05 |
| 35843 | 2.39282 | 0.05 |
| 35844 | 2.39282 | 2.1 |
| 35845 | 2.48354 | 2.1 |
| 35846 | 2.72521 | 2.1 |
| 35847 | 1.89224 | 2.1 |
| 35848 | 1.89224 | 2.1 |
| 35849 | 1.89224 | 0.893087 |
| 35850 | 1.89224 | 2.1 |
| 35851 | 3.78447 | 1.29461 |
| 35852 | 1.89226 | 2.1 |
| 35853 | 2.10425 | 2.1 |
| 35854 | 2.5142 | 2.1 |
| 35855 | 2.5142 | 2.1 |
| 35856 | 1.89226 | 2.1 |
| 35857 | 1.89226 | 2.1 |
| 35858 | 0.946133 | 0.989593 |
| 35859 | 0.946133 | 1.41753 |
| 35860 | 0.946128 | 2.1 |
| 35861 | 0.946128 | 2.1 |
| 35862 | 0.946131 | 2.1 |
| 35863 | 0.946131 | 0.948589 |
| 35864 | 0.94613 | 0.095202 |
| 35865 | 0.94613 | 2.1 |
| 35866 | 0.946131 | 2.03071 |
| 35867 | 0.946131 | 2.1 |
| 35868 | 0.946131 | 2.04896 |
| 35869 | 0.946131 | 2.1 |
| 35870 | 1.89226 | 0.598115 |
| 35871 | 1.89226 | 1.02117 |
| 35872 | 1.89226 | 0.829073 |
| 35873 | 1.89226 | 1.06298 |
| 35874 | 1.89226 | 0.791979 |
| 35875 | 1.89226 | 0.980296 |
| 35876 | 1.94446 | 0.714113 |
| 35877 | 2.50167 | 1.29358 |
| 35878 | 1.28418 | 0.952401 |
| 35879 | 1.94446 | 1.02394 |
| 35880 | 2.05205 | 1.04472 |
| 35881 | 2.05205 | 1.15249 |
| 35882 | 1.27835 | 2.1 |
| 35883 | 1.08341 | 2.1 |
| 35884 | 0.946132 | 2.1 |
| 35885 | 0.946132 | 2.1 |
| 35886 | 0.851542 | 2.1 |
| 35887 | 0.851542 | 2.1 |
| 35888 | 0.977356 | 2.1 |
| 35889 | 1.18368 | 2.1 |
| 35890 | 1.18378 | 2.1 |
| 35891 | 0.851555 | 2.1 |
| 35892 | 0.977346 | 2.1 |
| 35893 | 0.851476 | 2.1 |
| 35894 | 1.07369 | 2.1 |
| 35895 | 2.5813 | 2.1 |
| 35896 | 1.95053 | 2.1 |
| 35897 | 1.95053 | 2.1 |
| 35898 | 1.90196 | 2.1 |
| 35899 | 1.90196 | 2.1 |

| | | |
|---|---|---|
| 35900 | 1.89226 | 2.1 |
| 35901 | 1.89226 | 2.1 |
| 35902 | 1.89226 | 2.1 |
| 35903 | 1.89226 | 2.1 |
| 35904 | 3.78452 | 2.1 |
| 35905 | 1.89224 | 2.1 |
| 35906 | 1.89224 | 2.1 |
| 35907 | 1.89224 | 2.1 |
| 35908 | 1.89224 | 2.1 |
| 35909 | 1.89224 | 2.1 |
| 35910 | 1.89224 | 2.1 |
| 35911 | 1.97004 | 0.232963 |
| 35912 | 1.8072 | 1.57578 |
| 35913 | 1.5866 | 1.21973 |
| 35914 | 1.79478 | 0.391385 |
| 35915 | 3.60198 | 0.990493 |
| 35916 | 1.23267 | 2.1 |
| 35917 | 1.8072 | 0.166172 |
| 35918 | 1.58661 | 0.0965187 |
| 35919 | 0.947921 | 2.1 |
| 35920 | 2.75512 | 1.3717 |
| 35921 | 1.89226 | 0.218256 |
| 35922 | 1.89226 | 2.1 |
| 35923 | 1.89226 | 0.0828594 |
| 35924 | 1.89226 | 1.8344 |
| 35925 | 1.89226 | 0.05 |
| 35926 | 1.89226 | 2.1 |
| 35927 | 1.89226 | 1.59547 |
| 35928 | 1.89226 | 0.200447 |
| 35929 | 1.89226 | 0.616787 |
| 35930 | 1.89226 | 1.22831 |
| 35931 | 3.78452 | 0.884742 |
| 35932 | 1.4263 | 1.56168 |
| 35933 | 1.79478 | 0.425516 |
| 35934 | 1.67651 | 0.647751 |
| 35935 | 1.79478 | 1.83105 |
| 35936 | 1.46834 | 0.05 |
| 35937 | 1.5866 | 0.597004 |
| 35938 | 1.89226 | 0.739558 |
| 35939 | 1.89226 | 1.07919 |
| 35940 | 1.89226 | 0.262217 |
| 35941 | 1.89226 | 0.88626 |
| 35942 | 1.89226 | 0.414214 |
| 35943 | 1.89226 | 1.7879 |
| 35944 | 2.23956 | 1.9013 |
| 35945 | 1.81847 | 2.05816 |
| 35946 | 2.10991 | 1.0716 |
| 35947 | 2.75317 | 1.22292 |
| 35948 | 4.34947 | 1.65291 |
| 35949 | 1.60578 | 0.05 |
| 35950 | 1.94974 | 0.05 |
| 35951 | 1.68062 | 0.05 |
| 35952 | 1.14721 | 0.05 |
| 35953 | 3.2864 | 0.05 |
| 35954 | 1.3946 | 0.05 |
| 35955 | 1.692 | 0.05 |
| 35956 | 2.0154 | 0.05 |
| 35957 | 2.0154 | 0.05 |

| | | |
|---|---|---|
| 35958 | 1.11926 | 0.05 |
| 35959 | 1.11926 | 0.05 |
| 35960 | 1.90347 | 0.05 |
| 35961 | 1.83626 | 0.05 |
| 35962 | 1.84583 | 0.05 |
| 35963 | 1.89434 | 0.05 |
| 35964 | 3.79781 | 0.05 |
| 35965 | 1.89226 | 1.38349 |
| 35966 | 1.89226 | 1.07116 |
| 35967 | 1.89226 | 0.842553 |
| 35968 | 1.89226 | 1.31045 |
| 35969 | 3.78452 | 1.03888 |
| 35970 | 1.20986 | 1.51666 |
| 35971 | 1.20986 | 1.37687 |
| 35972 | 0.912635 | 1.23786 |
| 35973 | 0.912635 | 1.92154 |
| 35974 | 0.912667 | 0.973828 |
| 35975 | 1.17637 | 1.71827 |
| 35976 | 0.946125 | 1.93807 |
| 35977 | 0.946125 | 2.05717 |
| 35978 | 0.946128 | 2.06692 |
| 35979 | 0.912664 | 1.21558 |
| 35980 | 0.946127 | 2.1 |
| 35981 | 1.17637 | 1.33119 |
| 35982 | 1.89226 | 0.05 |
| 35983 | 1.65573 | 0.05 |
| 35984 | 1.65573 | 0.05 |
| 35985 | 1.89226 | 0.05 |
| 35986 | 1.89226 | 0.05 |
| 35987 | 2.12879 | 0.05 |
| 35988 | 1.89226 | 0.05 |
| 35989 | 1.89226 | 0.05 |
| 35990 | 1.89226 | 0.05 |
| 35991 | 1.89226 | 0.05 |
| 35992 | 1.89226 | 0.05 |
| 35993 | 1.89226 | 0.05 |
| 35994 | 2.40928 | 0.05 |
| 35995 | 1.89226 | 0.05 |
| 35996 | 1.95959 | 0.05 |
| 35997 | 1.70302 | 0.05 |
| 35998 | 3.47338 | 0.05 |
| 35999 | 1.89226 | 0.05 |
| 36000 | 1.89226 | 0.05 |
| 36001 | 1.89226 | 0.05 |
| 36002 | 1.89226 | 0.05 |
| 36003 | 3.78452 | 0.05 |
| 36004 | 1.64985 | 0.05 |
| 36005 | 2.47846 | 0.05 |
| 36006 | 2.25405 | 0.05 |
| 36007 | 1.80768 | 0.05 |
| 36008 | 4.06173 | 0.05 |
| 36009 | 1.98846 | 0.05 |
| 36010 | 0.912729 | 0.05 |
| 36011 | 1.05417 | 0.05 |
| 36012 | 1.70303 | 0.05 |
| 36013 | 2.82107 | 0.05 |
| 36014 | 2.47846 | 0.05 |
| 36015 | 1.70303 | 0.05 |

| | | |
|---|---|---|
| 36016 | 2.86918 | 0.05 |
| 36017 | 2.05973 | 0.05 |
| 36018 | 2.51808 | 0.05 |
| 36019 | 1.80768 | 0.05 |
| 36020 | 0.946131 | 0.05 |
| 36021 | 0.946131 | 0.05 |
| 36022 | 0.946132 | 0.05 |
| 36023 | 0.946132 | 0.05 |
| 36024 | 0.946131 | 0.05 |
| 36025 | 0.946131 | 0.05 |
| 36026 | 0.946133 | 0.05 |
| 36027 | 0.946133 | 0.05 |
| 36028 | 0.946131 | 0.05 |
| 36029 | 0.946131 | 0.05 |
| 36030 | 0.946132 | 0.05 |
| 36031 | 0.946132 | 0.05 |
| 36032 | 0.946133 | 0.05 |
| 36033 | 0.946133 | 0.05 |
| 36034 | 0.946132 | 0.05 |
| 36035 | 0.946132 | 0.05 |
| 36036 | 0.946133 | 0.05 |
| 36037 | 0.946133 | 0.05 |
| 36038 | 0.946132 | 0.05 |
| 36039 | 0.946132 | 0.05 |
| 36040 | 0.946132 | 0.05 |
| 36041 | 0.946132 | 0.05 |
| 36042 | 0.946133 | 0.05 |
| 36043 | 0.946133 | 0.05 |
| 36044 | 1.89227 | 0.05 |
| 36045 | 1.89227 | 0.05 |
| 36046 | 1.89227 | 0.05 |
| 36047 | 1.89227 | 0.05 |
| 36048 | 1.89227 | 0.05 |
| 36049 | 1.89227 | 0.05 |
| 36050 | 1.1656 | 2.1 |
| 36051 | 1.21847 | 2.1 |
| 36052 | 0.836006 | 2.1 |
| 36053 | 0.806007 | 2.1 |
| 36054 | 1.00653 | 2.1 |
| 36055 | 1.00653 | 2.1 |
| 36056 | 1.01794 | 2.1 |
| 36057 | 1.01794 | 2.1 |
| 36058 | 1.1656 | 2.1 |
| 36059 | 0.806007 | 2.1 |
| 36060 | 0.836006 | 2.1 |
| 36061 | 1.21847 | 2.1 |
| 36062 | 2.82263 | 2.1 |
| 36063 | 2.3312 | 2.1 |
| 36064 | 2.25936 | 2.1 |
| 36065 | 2.71441 | 2.1 |
| 36066 | 5.0456 | 2.1 |
| 36067 | 2.25936 | 2.1 |
| 36068 | 1.61201 | 2.1 |
| 36069 | 1.57627 | 2.1 |
| 36070 | 1.61201 | 2.1 |
| 36071 | 2.29545 | 2.1 |
| 36072 | 2.3312 | 2.1 |
| 36073 | 1.21269 | 0.05 |

| | | |
|---|---|---|
| 36074 | 1.48867 | 0.05 |
| 36075 | 1.68264 | 0.05 |
| 36076 | 1.9898 | 0.05 |
| 36077 | 3.17131 | 0.05 |
| 36078 | 2.0487 | 2.1 |
| 36079 | 2.45212 | 2.1 |
| 36080 | 2.31012 | 2.1 |
| 36081 | 1.94095 | 2.1 |
| 36082 | 4.39307 | 2.1 |
| 36083 | 1.02435 | 2.1 |
| 36084 | 0.970469 | 2.1 |
| 36085 | 0.625375 | 2.1 |
| 36086 | 0.567166 | 2.1 |
| 36087 | 0.796854 | 2.1 |
| 36088 | 0.796854 | 2.1 |
| 36089 | 0.79466 | 2.1 |
| 36090 | 0.79466 | 2.1 |
| 36091 | 0.970471 | 2.1 |
| 36092 | 0.567165 | 2.1 |
| 36093 | 0.625374 | 2.1 |
| 36094 | 1.02435 | 2.1 |
| 36095 | 2.31012 | 2.1 |
| 36096 | 1.94095 | 2.1 |
| 36097 | 1.4825 | 2.1 |
| 36098 | 2.17384 | 2.1 |
| 36099 | 4.11479 | 2.1 |
| 36100 | 0.946187 | 2.1 |
| 36101 | 0.803763 | 2.1 |
| 36102 | 0.581357 | 2.1 |
| 36103 | 0.510356 | 2.1 |
| 36104 | 0.745632 | 2.1 |
| 36105 | 0.745632 | 2.1 |
| 36106 | 0.734566 | 2.1 |
| 36107 | 0.734563 | 2.1 |
| 36108 | 0.896142 | 2.1 |
| 36109 | 0.510366 | 2.1 |
| 36110 | 0.946173 | 2.1 |
| 36111 | 0.552965 | 2.1 |
| 36112 | 1.89226 | 1.01843 |
| 36113 | 1.89226 | 1.81706 |
| 36114 | 1.89226 | 2.05293 |
| 36115 | 1.89226 | 2.09873 |
| 36116 | 1.89226 | 1.11302 |
| 36117 | 1.89226 | 1.85678 |
| 36118 | 1.50743 | 1.66194 |
| 36119 | 1.0417 | 2.1 |
| 36120 | 1.50743 | 2.1 |
| 36121 | 1.62974 | 1.15714 |
| 36122 | 1.14605 | 1.13936 |
| 36123 | 1.14605 | 2.1 |
| 36124 | 1.89226 | 0.928818 |
| 36125 | 1.89226 | 2.1 |
| 36126 | 1.89226 | 0.811616 |
| 36127 | 1.89226 | 1.61952 |
| 36128 | 1.89226 | 0.868276 |
| 36129 | 1.89226 | 2.00976 |
| 36130 | 1.89226 | 1.97284 |
| 36131 | 1.89226 | 1.50771 |

| | | |
|---|---|---|
| 36132 | 1.89226 | 0.732154 |
| 36133 | 1.89226 | 2.1 |
| 36134 | 3.78453 | 1.17667 |
| 36135 | 1.77186 | 1.94083 |
| 36136 | 1.35668 | 2.1 |
| 36137 | 1.70254 | 1.03835 |
| 36138 | 1.38756 | 2.1 |
| 36139 | 1.77186 | 2.1 |
| 36140 | 1.37814 | 2.1 |
| 36141 | 0.734857 | 2.1 |
| 36142 | 1.00549 | 1.98601 |
| 36143 | 0.835261 | 1.13456 |
| 36144 | 1.08357 | 2.1 |
| 36145 | 0.97665 | 1.67586 |
| 36146 | 0.795559 | 2.1 |
| 36147 | 0.891385 | 1.91635 |
| 36148 | 0.891385 | 0.825272 |
| 36149 | 0.874231 | 1.19069 |
| 36150 | 1.08356 | 1.53216 |
| 36151 | 0.734863 | 1.47963 |
| 36152 | 0.79449 | 2.05943 |
| 36153 | 1.89226 | 1.63211 |
| 36154 | 1.89226 | 0.0534778 |
| 36155 | 2.42671 | 0.444993 |
| 36156 | 1.98783 | 1.10441 |
| 36157 | 4.31897 | 0.28506 |
| 36158 | 0.579854 | 0.0750246 |
| 36159 | 0.965582 | 0.188015 |
| 36160 | 0.634403 | 0.978768 |
| 36161 | 1.17796 | 0.0892707 |
| 36162 | 1.00592 | 0.05364 |
| 36163 | 0.797259 | 1.77092 |
| 36164 | 0.905246 | 1.23668 |
| 36165 | 0.905246 | 0.0641178 |
| 36166 | 0.695715 | 0.0721932 |
| 36167 | 0.637106 | 0.546614 |
| 36168 | 1.24374 | 0.801918 |
| 36169 | 0.95617 | 0.28293 |
| 36170 | 2.0815 | 0.0755566 |
| 36171 | 1.60521 | 0.979505 |
| 36172 | 1.29556 | 0.794374 |
| 36173 | 2.32205 | 1.67763 |
| 36174 | 1.28445 | 0.05 |
| 36175 | 2.0815 | 0.877909 |
| 36176 | 2.3085 | 2.1 |
| 36177 | 2.21147 | 2.1 |
| 36178 | 2.3085 | 2.1 |
| 36179 | 2.39282 | 2.1 |
| 36180 | 1.98899 | 2.1 |
| 36181 | 2.0733 | 2.1 |
| 36182 | 2.3275 | 2.1 |
| 36183 | 1.63471 | 2.1 |
| 36184 | 1.98899 | 2.1 |
| 36185 | 1.56801 | 2.1 |
| 36186 | 3.62369 | 2.1 |
| 36187 | 2.3085 | 2.1 |
| 36188 | 1.98899 | 2.1 |
| 36189 | 2.22575 | 2.1 |

| | | |
|---|---|---|
| 36190 | 1.90502 | 2.1 |
| 36191 | 4.21353 | 2.1 |
| 36192 | 1.90502 | 2.1 |
| 36193 | 1.56801 | 2.1 |
| 36194 | 1.90502 | 2.1 |
| 36195 | 1.98899 | 2.1 |
| 36196 | 1.49733 | 2.1 |
| 36197 | 1.58129 | 2.1 |
| 36198 | 1.89226 | 1.02427 |
| 36199 | 2.6491 | 1.02317 |
| 36200 | 2.49266 | 2.1 |
| 36201 | 2.6491 | 0.952994 |
| 36202 | 1.73582 | 0.975711 |
| 36203 | 1.89226 | 0.145491 |
| 36204 | 1.31854 | 2.1 |
| 36205 | 2.6491 | 1.09523 |
| 36206 | 1.3909 | 2.1 |
| 36207 | 1.3909 | 2.1 |
| 36208 | 2.49266 | 2.1 |
| 36209 | 2.49266 | 2.1 |
| 36210 | 1.89226 | 1.12744 |
| 36211 | 2.49266 | 0.916565 |
| 36212 | 2.38634 | 2.1 |
| 36213 | 2.49266 | 0.883477 |
| 36214 | 1.78595 | 0.948487 |
| 36215 | 1.89226 | 0.171926 |
| 36216 | 1.23042 | 2.1 |
| 36217 | 2.49266 | 0.857748 |
| 36218 | 1.31854 | 2.1 |
| 36219 | 1.31854 | 1.9 |
| 36220 | 2.38635 | 2.1 |
| 36221 | 2.38635 | 2.1 |
| 36222 | 2.83487 | 2.1 |
| 36223 | 1.89226 | 2.1 |
| 36224 | 2.594 | 2.1 |
| 36225 | 2.594 | 2.1 |
| 36226 | 1.89226 | 2.1 |
| 36227 | 1.89226 | 2.1 |
| 36228 | 1.89226 | 2.1 |
| 36229 | 1.89226 | 2.1 |
| 36230 | 1.89226 | 2.1 |
| 36231 | 1.89226 | 2.1 |
| 36232 | 3.78452 | 2.1 |
| 36233 | 1.09518 | 2.1 |
| 36234 | 1.28158 | 2.1 |
| 36235 | 1.36844 | 2.1 |
| 36236 | 1.59972 | 2.1 |
| 36237 | 2.65003 | 2.1 |
| 36238 | 1.89226 | 2.1 |
| 36239 | 1.89226 | 2.1 |
| 36240 | 1.89226 | 2.1 |
| 36241 | 1.89226 | 2.1 |
| 36242 | 3.78452 | 2.1 |
| 36243 | 1.36844 | 2.1 |
| 36244 | 1.59972 | 2.1 |
| 36245 | 1.67352 | 2.1 |
| 36246 | 1.94291 | 2.1 |
| 36247 | 3.31135 | 2.1 |

| | | |
|---|---|---|
| 36248 | 1.52938 | 2.1 |
| 36249 | 1.93568 | 2.1 |
| 36250 | 2.36221 | 0.908337 |
| 36251 | 1.94723 | 2.1 |
| 36252 | 3.89158 | 1.92841 |
| 36253 | 1.94724 | 1.16317 |
| 36254 | 1.26593 | 2.1 |
| 36255 | 1.75367 | 2.1 |
| 36256 | 2.4322 | 1.53994 |
| 36257 | 3.7009 | 2.1 |
| 36258 | 2.36221 | 1.06537 |
| 36259 | 2.43219 | 2.1 |
| 36260 | 1.94723 | 1.44217 |
| 36261 | 1.94723 | 1.6821 |
| 36262 | 2.8202 | 1.8116 |
| 36263 | 2.8202 | 2.08683 |
| 36264 | 2.51604 | 2.1 |
| 36265 | 2.18533 | 2.1 |
| 36266 | 2.17228 | 2.1 |
| 36267 | 2.4889 | 2.1 |
| 36268 | 4.67423 | 2.1 |
| 36269 | 2.17228 | 2.1 |
| 36270 | 1.62183 | 2.1 |
| 36271 | 1.62316 | 2.1 |
| 36272 | 1.62183 | 2.1 |
| 36273 | 2.18666 | 2.1 |
| 36274 | 2.18533 | 2.1 |
| 36275 | 2.17227 | 2.1 |
| 36276 | 2.4889 | 2.1 |
| 36277 | 2.45922 | 2.1 |
| 36278 | 2.15686 | 2.1 |
| 36279 | 4.64576 | 2.1 |
| 36280 | 2.17228 | 2.1 |
| 36281 | 1.62316 | 2.1 |
| 36282 | 1.62241 | 2.1 |
| 36283 | 2.15686 | 2.1 |
| 36284 | 3.78002 | 2.1 |
| 36285 | 1.89226 | 0.657847 |
| 36286 | 1.89226 | 0.816978 |
| 36287 | 1.89226 | 0.420287 |
| 36288 | 1.89226 | 0.425473 |
| 36289 | 3.78453 | 1.44061 |
| 36290 | 1.89226 | 0.896466 |
| 36291 | 1.89226 | 0.845924 |
| 36292 | 1.89226 | 0.735713 |
| 36293 | 1.89226 | 0.0589221 |
| 36294 | 3.78452 | 0.284115 |
| 36295 | 1.89226 | 0.914969 |
| 36296 | 1.89226 | 0.648217 |
| 36297 | 1.89226 | 0.869382 |
| 36298 | 1.89226 | 0.825892 |
| 36299 | 1.89226 | 0.700228 |
| 36300 | 1.89226 | 0.733716 |
| 36301 | 1.89226 | 0.888725 |
| 36302 | 1.89226 | 0.975374 |
| 36303 | 2.53413 | 0.0543881 |
| 36304 | 1.89226 | 0.0664412 |
| 36305 | 3.78453 | 0.05 |

| | | |
|---|---|---|
| 36306 | 1.38048 | 0.0934883 |
| 36307 | 1.38048 | 0.255007 |
| 36308 | 0.490332 | 0.17851 |
| 36309 | 0.490332 | 0.0655333 |
| 36310 | 0.924679 | 0.05 |
| 36311 | 0.946131 | 1.7314 |
| 36312 | 0.946131 | 1.17241 |
| 36313 | 0.490332 | 0.05 |
| 36314 | 0.924678 | 0.05 |
| 36315 | 0.490332 | 0.05 |
| 36316 | 0.946131 | 0.653553 |
| 36317 | 0.946131 | 0.0627017 |
| 36318 | 1.89227 | 0.05 |
| 36319 | 1.89227 | 0.716622 |
| 36320 | 1.89227 | 0.534823 |
| 36321 | 1.89227 | 0.990135 |
| 36322 | 1.89227 | 0.05 |
| 36323 | 1.89227 | 0.161925 |
| 36324 | 1.30803 | 2.1 |
| 36325 | 2.55151 | 2.1 |
| 36326 | 1.43935 | 2.1 |
| 36327 | 1.43935 | 2.1 |
| 36328 | 2.34922 | 2.1 |
| 36329 | 2.34922 | 2.1 |
| 36330 | 0.946131 | 2.1 |
| 36331 | 0.946131 | 2.1 |
| 36332 | 1.27575 | 2.1 |
| 36333 | 1.17461 | 2.1 |
| 36334 | 0.94613 | 2.1 |
| 36335 | 0.94613 | 2.1 |
| 36336 | 1.17461 | 2.1 |
| 36337 | 1.17461 | 2.1 |
| 36338 | 0.946131 | 2.1 |
| 36339 | 1.17461 | 2.1 |
| 36340 | 0.946132 | 2.1 |
| 36341 | 1.27575 | 2.1 |
| 36342 | 1.89226 | 2.1 |
| 36343 | 1.89226 | 0.928758 |
| 36344 | 1.89226 | 2.1 |
| 36345 | 1.89226 | 2.1 |
| 36346 | 1.89226 | 2.1 |
| 36347 | 1.89226 | 2.1 |
| 36348 | 0.946133 | 2.1 |
| 36349 | 0.946133 | 2.1 |
| 36350 | 0.946132 | 2.1 |
| 36351 | 0.946132 | 2.1 |
| 36352 | 0.946133 | 2.1 |
| 36353 | 0.946133 | 2.1 |
| 36354 | 0.946132 | 2.1 |
| 36355 | 0.946132 | 2.1 |
| 36356 | 0.946132 | 2.1 |
| 36357 | 0.946132 | 2.1 |
| 36358 | 0.946133 | 2.1 |
| 36359 | 0.946133 | 2.1 |
| 36360 | 1.07043 | 2.1 |
| 36361 | 1.17461 | 2.1 |
| 36362 | 0.654016 | 2.1 |
| 36363 | 0.5829 | 2.1 |

| | | |
|---|---|---|
| 36364 | 0.870481 | 2.1 |
| 36365 | 0.870481 | 2.1 |
| 36366 | 0.887027 | 2.1 |
| 36367 | 0.887027 | 2.1 |
| 36368 | 1.07043 | 2.1 |
| 36369 | 0.5829 | 2.1 |
| 36370 | 0.654016 | 2.1 |
| 36371 | 1.17461 | 2.1 |
| 36372 | 1.89226 | 2.1 |
| 36373 | 1.89226 | 0.852254 |
| 36374 | 1.89226 | 2.1 |
| 36375 | 1.89226 | 2.1 |
| 36376 | 3.78453 | 2.1 |
| 36377 | 1.89226 | 2.1 |
| 36378 | 1.89226 | 2.1 |
| 36379 | 1.89226 | 2.1 |
| 36380 | 1.89226 | 2.1 |
| 36381 | 3.78453 | 2.1 |
| 36382 | 1.26929 | 2.1 |
| 36383 | 2.19313 | 2.1 |
| 36384 | 1.09847 | 2.1 |
| 36385 | 1.69916 | 2.1 |
| 36386 | 2.96844 | 2.1 |
| 36387 | 1.84124 | 2.1 |
| 36388 | 2.24937 | 2.1 |
| 36389 | 1.69915 | 2.1 |
| 36390 | 1.75691 | 2.1 |
| 36391 | 3.94852 | 2.1 |
| 36392 | 1.53082 | 2.1 |
| 36393 | 2.04422 | 2.1 |
| 36394 | 1.53082 | 2.1 |
| 36395 | 1.69915 | 2.1 |
| 36396 | 1.92911 | 2.1 |
| 36397 | 2.09744 | 2.1 |
| 36398 | 1.39263 | 0.758865 |
| 36399 | 1.0171 | 0.05 |
| 36400 | 1.1211 | 0.0756662 |
| 36401 | 1.46482 | 0.259484 |
| 36402 | 2.48192 | 0.44796 |
| 36403 | 1.39263 | 0.087946 |
| 36404 | 1.57962 | 0.403637 |
| 36405 | 1.65238 | 1.29959 |
| 36406 | 1.46482 | 0.0680031 |
| 36407 | 3.04443 | 0.0662164 |
| 36408 | 1.89226 | 0.19476 |
| 36409 | 1.89226 | 0.05 |
| 36410 | 1.89226 | 0.05 |
| 36411 | 1.89226 | 0.0768176 |
| 36412 | 3.78453 | 0.120332 |
| 36413 | 1.89227 | 0.05 |
| 36414 | 1.89227 | 0.05 |
| 36415 | 1.89227 | 0.0775494 |
| 36416 | 1.89227 | 0.05 |
| 36417 | 3.78453 | 0.05 |
| 36418 | 1.28418 | 0.138563 |
| 36419 | 1.46482 | 0.185071 |
| 36420 | 1.1211 | 0.05 |
| 36421 | 1.1211 | 0.55133 |

| | | |
|---|---|---|
| 36422 | 1.59382 | 0.05 |
| 36423 | 1.59382 | 0.575739 |
| 36424 | 1.76897 | 0.817419 |
| 36425 | 1.46482 | 0.0593784 |
| 36426 | 1.59382 | 0.120435 |
| 36427 | 1.46482 | 0.308449 |
| 36428 | 1.78138 | 0.05 |
| 36429 | 1.65238 | 0.0581874 |
| 36430 | 1.89226 | 0.05 |
| 36431 | 1.89226 | 0.0509116 |
| 36432 | 1.89226 | 0.05 |
| 36433 | 1.89226 | 0.434732 |
| 36434 | 1.89226 | 0.05 |
| 36435 | 1.89226 | 0.460734 |
| 36436 | 1.89226 | 0.060916 |
| 36437 | 1.89226 | 0.05 |
| 36438 | 1.89226 | 0.05 |
| 36439 | 1.89226 | 0.05 |
| 36440 | 1.89226 | 0.0900235 |
| 36441 | 1.89226 | 0.185451 |
| 36442 | 1.96832 | 2.1 |
| 36443 | 2.40858 | 1.87953 |
| 36444 | 1.75211 | 0.05 |
| 36445 | 2.20797 | 2.1 |
| 36446 | 4.1607 | 1.52506 |
| 36447 | 1.75211 | 0.05 |
| 36448 | 2.20797 | 2.1 |
| 36449 | 1.51592 | 2.1 |
| 36450 | 2.02064 | 2.09516 |
| 36451 | 3.77275 | 2.1 |
| 36452 | 2.71793 | 0.377249 |
| 36453 | 2.67099 | 0.0984089 |
| 36454 | 2.55105 | 0.176436 |
| 36455 | 2.71297 | 0.05 |
| 36456 | 5.26898 | 0.171462 |
| 36457 | 2.36417 | 0.428016 |
| 36458 | 2.67098 | 0.124771 |
| 36459 | 2.55104 | 0.209421 |
| 36460 | 1.83418 | 0.0554865 |
| 36461 | 4.91522 | 0.10778 |
| 36462 | 2.55105 | 0.110272 |
| 36463 | 2.71297 | 0.05 |
| 36464 | 2.58032 | 0.0837989 |
| 36465 | 2.28922 | 0.05 |
| 36466 | 5.13137 | 0.05 |
| 36467 | 2.55104 | 0.137319 |
| 36468 | 2.23787 | 0.05 |
| 36469 | 1.67265 | 0.0534625 |
| 36470 | 2.28922 | 0.05 |
| 36471 | 4.22369 | 0.0623918 |
| 36472 | 0.851542 | 0.839178 |
| 36473 | 0.851542 | 0.822182 |
| 36474 | 0.981419 | 0.561351 |
| 36475 | 0.854774 | 0.606776 |
| 36476 | 0.949431 | 0.377661 |
| 36477 | 0.753763 | 0.64487 |
| 36478 | 0.946131 | 0.826332 |
| 36479 | 0.946131 | 0.837494 |

| | | |
|---|---|---|
| 36480 | 1.04073 | 0.814818 |
| 36481 | 0.981433 | 0.177606 |
| 36482 | 0.851528 | 0.892812 |
| 36483 | 0.854828 | 0.750998 |
| 36484 | 1.89226 | 0.971124 |
| 36485 | 1.89226 | 0.805435 |
| 36486 | 1.89226 | 0.794776 |
| 36487 | 1.89226 | 0.805313 |
| 36488 | 1.89226 | 0.930466 |
| 36489 | 1.89226 | 0.94675 |
| 36490 | 0.814227 | 0.703952 |
| 36491 | 0.814227 | 0.725217 |
| 36492 | 0.946117 | 0.368609 |
| 36493 | 1.07285 | 0.367388 |
| 36494 | 0.879132 | 0.149961 |
| 36495 | 0.923293 | 0.182633 |
| 36496 | 0.946119 | 0.727248 |
| 36497 | 0.946119 | 0.736391 |
| 36498 | 0.946131 | 0.67274 |
| 36499 | 0.814236 | 0.05 |
| 36500 | 0.946106 | 0.764607 |
| 36501 | 0.870405 | 0.498151 |
| 36502 | 1.89224 | 0.858991 |
| 36503 | 1.89224 | 0.643848 |
| 36504 | 1.89224 | 0.65333 |
| 36505 | 1.89224 | 0.678809 |
| 36506 | 1.89224 | 0.830137 |
| 36507 | 1.89224 | 0.844633 |
| 36508 | 1.29388 | 1.3117 |
| 36509 | 1.04164 | 2.1 |
| 36510 | 1.64834 | 2.1 |
| 36511 | 1.82955 | 1.19755 |
| 36512 | 2.94222 | 2.1 |
| 36513 | 1.4934 | 0.179491 |
| 36514 | 2.41522 | 0.05 |
| 36515 | 2.20387 | 0.05 |
| 36516 | 2.20387 | 1.92638 |
| 36517 | 1.75765 | 0.05 |
| 36518 | 1.75765 | 1.52233 |
| 36519 | 2.41522 | 0.05 |
| 36520 | 2.77303 | 2.1 |
| 36521 | 2.20387 | 2.1 |
| 36522 | 2.58627 | 1.21077 |
| 36523 | 5.00149 | 2.03813 |
| 36524 | 1.22222 | 0.139652 |
| 36525 | 2.20387 | 0.0786079 |
| 36526 | 1.96831 | 0.05 |
| 36527 | 1.96831 | 2.07643 |
| 36528 | 1.4934 | 0.0513821 |
| 36529 | 1.4934 | 2.1 |
| 36530 | 1.96832 | 2.1 |
| 36531 | 2.58627 | 1.09733 |
| 36532 | 2.40858 | 0.0850187 |
| 36533 | 2.40858 | 2.1 |
| 36534 | 2.20387 | 0.334661 |
| 36535 | 2.20387 | 2.1 |
| 36536 | 1.5605 | 2.1 |
| 36537 | 1.53775 | 2.1 |

| | | |
|---|---|---|
| 36538 | 1.55398 | 2.1 |
| 36539 | 1.45806 | 2.1 |
| 36540 | 1.65642 | 2.1 |
| 36541 | 1.5605 | 2.1 |
| 36542 | 0.768876 | 2.1 |
| 36543 | 0.79033 | 2.1 |
| 36544 | 0.828211 | 2.1 |
| 36545 | 0.871233 | 2.1 |
| 36546 | 0.803843 | 2.1 |
| 36547 | 0.803843 | 2.1 |
| 36548 | 0.814698 | 2.1 |
| 36549 | 0.814698 | 2.1 |
| 36550 | 0.79033 | 2.1 |
| 36551 | 0.871233 | 2.1 |
| 36552 | 0.768876 | 2.1 |
| 36553 | 0.828212 | 2.1 |
| 36554 | 1.80252 | 2.1 |
| 36555 | 2.09715 | 0.710627 |
| 36556 | 1.9394 | 2.1 |
| 36557 | 1.50853 | 2.1 |
| 36558 | 3.60567 | 2.1 |
| 36559 | 1.96416 | 0.443223 |
| 36560 | 2.09715 | 0.772714 |
| 36561 | 1.88484 | 2.1 |
| 36562 | 1.57916 | 0.631164 |
| 36563 | 3.67631 | 0.910038 |
| 36564 | 1.89226 | 0.736089 |
| 36565 | 1.89226 | 0.05 |
| 36566 | 1.89226 | 0.645009 |
| 36567 | 1.89226 | 0.79534 |
| 36568 | 3.78453 | 0.581165 |
| 36569 | 1.89226 | 0.443735 |
| 36570 | 1.89226 | 0.698013 |
| 36571 | 1.89226 | 2.1 |
| 36572 | 1.89226 | 0.05 |
| 36573 | 1.89226 | 2.1 |
| 36574 | 1.89226 | 0.05 |
| 36575 | 1.5852 | 2.1 |
| 36576 | 1.50853 | 2.1 |
| 36577 | 1.2955 | 2.1 |
| 36578 | 1.44472 | 0.712264 |
| 36579 | 2.95325 | 2.1 |
| 36580 | 1.53065 | 1.73821 |
| 36581 | 1.57916 | 0.533949 |
| 36582 | 1.44472 | 0.732045 |
| 36583 | 1.78861 | 0.810608 |
| 36584 | 1.23527 | 0.864672 |
| 36585 | 1.23527 | 0.774463 |
| 36586 | 1.89227 | 0.53649 |
| 36587 | 1.89227 | 0.589469 |
| 36588 | 1.89227 | 0.925571 |
| 36589 | 1.89227 | 0.639302 |
| 36590 | 3.78453 | 0.616896 |
| 36591 | 1.89226 | 0.720274 |
| 36592 | 1.89226 | 0.439363 |
| 36593 | 1.89226 | 2.1 |
| 36594 | 1.89226 | 0.704398 |
| 36595 | 1.89226 | 0.843152 |

| | | |
|---|---|---|
| 36596 | 1.89226 | 0.05 |
| 36597 | 1.89227 | 1.10142 |
| 36598 | 1.89227 | 0.79641 |
| 36599 | 1.89227 | 0.805296 |
| 36600 | 1.89227 | 0.769604 |
| 36601 | 3.78453 | 0.767428 |
| 36602 | 0.943708 | 0.791384 |
| 36603 | 0.752219 | 0.716202 |
| 36604 | 0.946132 | 0.74971 |
| 36605 | 0.946132 | 0.796845 |
| 36606 | 0.946133 | 0.57565 |
| 36607 | 0.946133 | 1.18269 |
| 36608 | 0.752218 | 1.00487 |
| 36609 | 0.752218 | 0.828154 |
| 36610 | 0.752218 | 0.783511 |
| 36611 | 0.946132 | 0.676244 |
| 36612 | 0.946133 | 0.95339 |
| 36613 | 0.943708 | 0.813001 |
| 36614 | 0.943992 | 2.1 |
| 36615 | 0.943992 | 2.1 |
| 36616 | 1.23507 | 0.260724 |
| 36617 | 1.48209 | 2.1 |
| 36618 | 1.0384 | 2.1 |
| 36619 | 1.47953 | 2.1 |
| 36620 | 0.946128 | 0.427689 |
| 36621 | 0.946128 | 2.1 |
| 36622 | 0.946129 | 2.1 |
| 36623 | 1.23293 | 2.1 |
| 36624 | 1.0384 | 2.1 |
| 36625 | 0.946129 | 2.1 |
| 36626 | 1.89226 | 2.1 |
| 36627 | 2.46586 | 2.1 |
| 36628 | 2.80218 | 0.153156 |
| 36629 | 2.80218 | 2.1 |
| 36630 | 1.89226 | 0.396624 |
| 36631 | 1.89226 | 2.1 |
| 36632 | 1.32455 | 2.1 |
| 36633 | 1.89128 | 2.1 |
| 36634 | 1.32328 | 2.1 |
| 36635 | 1.97299 | 2.1 |
| 36636 | 3.29754 | 2.1 |
| 36637 | 1.89128 | 2.1 |
| 36638 | 2.4255 | 2.1 |
| 36639 | 1.973 | 2.1 |
| 36640 | 2.63807 | 2.1 |
| 36641 | 4.3985 | 2.1 |
| 36642 | 1.34008 | 2.1 |
| 36643 | 1.97299 | 2.1 |
| 36644 | 2.10766 | 2.1 |
| 36645 | 2.10766 | 2.1 |
| 36646 | 1.32328 | 2.1 |
| 36647 | 1.32328 | 2.1 |
| 36648 | 2.41055 | 2.1 |
| 36649 | 2.37491 | 2.1 |
| 36650 | 2.40754 | 2.1 |
| 36651 | 2.38023 | 2.1 |
| 36652 | 4.78546 | 2.1 |
| 36653 | 2.23743 | 2.1 |

| | | |
|---|---|---|
| 36654 | 2.37491 | 2.1 |
| 36655 | 2.40754 | 2.1 |
| 36656 | 2.20392 | 2.1 |
| 36657 | 4.61234 | 2.1 |
| 36658 | 2.37491 | 2.1 |
| 36659 | 2.02641 | 2.1 |
| 36660 | 2.20392 | 2.1 |
| 36661 | 2.18943 | 2.1 |
| 36662 | 4.40132 | 2.1 |
| 36663 | 2.37491 | 2.1 |
| 36664 | 2.22558 | 2.1 |
| 36665 | 2.38023 | 2.1 |
| 36666 | 2.18943 | 2.1 |
| 36667 | 4.60049 | 2.1 |
| 36668 | 1.89226 | 0.05 |
| 36669 | 1.89226 | 0.05 |
| 36670 | 1.89226 | 0.05 |
| 36671 | 1.89226 | 0.05 |
| 36672 | 1.89226 | 0.05 |
| 36673 | 1.89226 | 0.05 |
| 36674 | 1.03599 | 0.05 |
| 36675 | 0.706698 | 0.05 |
| 36676 | 1.41141 | 0.05 |
| 36677 | 1.17573 | 0.05 |
| 36678 | 1.11957 | 0.05 |
| 36679 | 0.765034 | 0.05 |
| 36680 | 1.05913 | 0.05 |
| 36681 | 1.45054 | 0.05 |
| 36682 | 1.04847 | 0.05 |
| 36683 | 1.04847 | 0.05 |
| 36684 | 1.15323 | 0.05 |
| 36685 | 1.05825 | 0.05 |
| 36686 | 1.89226 | 0.05 |
| 36687 | 1.89226 | 0.05 |
| 36688 | 1.89226 | 0.05 |
| 36689 | 1.89226 | 0.05 |
| 36690 | 1.89226 | 0.05 |
| 36691 | 1.89226 | 0.05 |
| 36692 | 2.22575 | 0.05 |
| 36693 | 1.70677 | 2.1 |
| 36694 | 1.90157 | 0.05 |
| 36695 | 1.5534 | 2.1 |
| 36696 | 3.77914 | 0.05 |
| 36697 | 1.70678 | 2.1 |
| 36698 | 0.968594 | 2.1 |
| 36699 | 0.968594 | 2.1 |
| 36700 | 1.10378 | 2.1 |
| 36701 | 1.5534 | 2.1 |
| 36702 | 1.68859 | 2.1 |
| 36703 | 2.31429 | 0.05 |
| 36704 | 1.5534 | 2.1 |
| 36705 | 1.90103 | 0.05 |
| 36706 | 1.41902 | 2.1 |
| 36707 | 3.45443 | 0.05 |
| 36708 | 2.53709 | 0.05 |
| 36709 | 1.209 | 0.05 |
| 36710 | 1.05417 | 0.05 |
| 36711 | 2.6513 | 0.05 |

| | | |
|---|---|---|
| 36712 | 3.70547 | 0.05 |
| 36713 | 1.02675 | 0.05 |
| 36714 | 1.16384 | 0.05 |
| 36715 | 2.22062 | 0.05 |
| 36716 | 2.48862 | 0.05 |
| 36717 | 3.38446 | 0.05 |
| 36718 | 1.89226 | 0.05 |
| 36719 | 1.89226 | 0.05 |
| 36720 | 1.89226 | 0.05 |
| 36721 | 1.89226 | 0.05 |
| 36722 | 1.89226 | 0.05 |
| 36723 | 1.89226 | 0.05 |
| 36724 | 1.89226 | 1.0329 |
| 36725 | 1.89226 | 2.1 |
| 36726 | 1.89226 | 2.1 |
| 36727 | 1.89226 | 1.45636 |
| 36728 | 1.89226 | 2.1 |
| 36729 | 1.89226 | 1.22081 |
| 36730 | 1.89226 | 0.931138 |
| 36731 | 1.89226 | 1.70174 |
| 36732 | 1.89226 | 2.01567 |
| 36733 | 1.89226 | 1.09479 |
| 36734 | 1.89226 | 1.71038 |
| 36735 | 1.89226 | 1.05221 |
| 36736 | 1.89227 | 1.15307 |
| 36737 | 1.89227 | 1.86016 |
| 36738 | 1.75032 | 2.08275 |
| 36739 | 1.75032 | 1.28634 |
| 36740 | 1.89227 | 1.8418 |
| 36741 | 1.89227 | 1.19986 |
| 36742 | 1.89226 | 0.05 |
| 36743 | 1.89226 | 0.086419 |
| 36744 | 1.89226 | 0.0604201 |
| 36745 | 1.89226 | 0.144851 |
| 36746 | 1.89226 | 0.0756571 |
| 36747 | 1.89226 | 0.05 |
| 36748 | 0.946132 | 0.05 |
| 36749 | 0.946132 | 0.05 |
| 36750 | 0.798215 | 0.05 |
| 36751 | 0.848163 | 0.05 |
| 36752 | 0.848163 | 0.05 |
| 36753 | 0.946131 | 0.05 |
| 36754 | 0.946132 | 0.05 |
| 36755 | 0.798216 | 0.05 |
| 36756 | 0.996105 | 0.05 |
| 36757 | 0.996105 | 0.05 |
| 36758 | 0.79819 | 0.05 |
| 36759 | 0.79819 | 0.05 |
| 36760 | 1.07763 | 0.05 |
| 36761 | 2.28622 | 0.05 |
| 36762 | 2.16109 | 0.05 |
| 36763 | 1.17506 | 0.05 |
| 36764 | 3.36385 | 0.05 |
| 36765 | 1.89226 | 2.1 |
| 36766 | 1.6663 | 2.1 |
| 36767 | 1.6663 | 2.1 |
| 36768 | 1.71706 | 2.1 |
| 36769 | 1.89226 | 2.1 |

| | | |
|---|---|---|
| 36770 | 1.94302 | 2.1 |
| 36771 | 1.89226 | 2.1 |
| 36772 | 1.62801 | 2.1 |
| 36773 | 1.62801 | 2.1 |
| 36774 | 1.6663 | 2.1 |
| 36775 | 1.89226 | 2.1 |
| 36776 | 1.93055 | 2.1 |
| 36777 | 1.48734 | 2.1 |
| 36778 | 1.99609 | 2.1 |
| 36779 | 1.52245 | 2.1 |
| 36780 | 2.04137 | 2.1 |
| 36781 | 3.51854 | 2.1 |
| 36782 | 1.99609 | 2.1 |
| 36783 | 2.28563 | 2.1 |
| 36784 | 2.04136 | 2.1 |
| 36785 | 2.35019 | 2.1 |
| 36786 | 4.34628 | 2.1 |
| 36787 | 1.50714 | 2.1 |
| 36788 | 2.04137 | 2.1 |
| 36789 | 2.04945 | 2.1 |
| 36790 | 2.04945 | 2.1 |
| 36791 | 1.52245 | 2.1 |
| 36792 | 1.52245 | 2.1 |
| 36793 | 2.04136 | 2.1 |
| 36794 | 2.35019 | 2.1 |
| 36795 | 2.04945 | 2.1 |
| 36796 | 2.38681 | 2.1 |
| 36797 | 4.39964 | 2.1 |
| 36798 | 2.50924 | 0.05 |
| 36799 | 1.38305 | 2.1 |
| 36800 | 2.56918 | 2.1 |
| 36801 | 1.44535 | 2.1 |
| 36802 | 3.95222 | 2.1 |
| 36803 | 1.89226 | 2.1 |
| 36804 | 1.89226 | 2.1 |
| 36805 | 1.89226 | 2.1 |
| 36806 | 1.89226 | 2.1 |
| 36807 | 3.78453 | 2.1 |
| 36808 | 1.40615 | 2.1 |
| 36809 | 1.38851 | 2.1 |
| 36810 | 0.748607 | 2.1 |
| 36811 | 0.724468 | 2.1 |
| 36812 | 1.06362 | 2.1 |
| 36813 | 1.06362 | 2.1 |
| 36814 | 1.067 | 2.1 |
| 36815 | 1.067 | 2.1 |
| 36816 | 1.40615 | 2.1 |
| 36817 | 0.748607 | 2.1 |
| 36818 | 0.724468 | 2.1 |
| 36819 | 1.38851 | 2.1 |
| 36820 | 2.56918 | 2.1 |
| 36821 | 1.53377 | 2.1 |
| 36822 | 1.53706 | 2.1 |
| 36823 | 1.44535 | 2.1 |
| 36824 | 2.66088 | 2.1 |
| 36825 | 2.56918 | 2.1 |
| 36826 | 1.89226 | 2.1 |
| 36827 | 1.89226 | 2.1 |

| | | |
|---|---|---|
| 36828 | 1.89226 | 2.1 |
| 36829 | 1.89226 | 2.1 |
| 36830 | 3.78453 | 2.1 |
| 36831 | 1.40239 | 2.1 |
| 36832 | 1.68466 | 2.1 |
| 36833 | 1.26091 | 2.1 |
| 36834 | 1.54641 | 2.1 |
| 36835 | 2.94881 | 2.1 |
| 36836 | 1.68466 | 2.1 |
| 36837 | 1.65324 | 0.156661 |
| 36838 | 1.54641 | 2.1 |
| 36839 | 1.52268 | 0.05 |
| 36840 | 3.19965 | 0.490388 |
| 36841 | 1.05776 | 2.1 |
| 36842 | 1.54641 | 2.1 |
| 36843 | 1.36595 | 2.1 |
| 36844 | 1.36595 | 2.1 |
| 36845 | 1.26091 | 2.1 |
| 36846 | 1.26091 | 2.1 |
| 36847 | 1.54641 | 2.1 |
| 36848 | 1.32697 | 0.05 |
| 36849 | 1.52268 | 0.05 |
| 36850 | 1.52268 | 2.1 |
| 36851 | 1.36595 | 0.05 |
| 36852 | 1.36595 | 2.1 |
| 36853 | 1.18647 | 1.62974 |
| 36854 | 0.988454 | 2.1 |
| 36855 | 1.4867 | 2.1 |
| 36856 | 1.63892 | 1.73594 |
| 36857 | 2.67317 | 2.1 |
| 36858 | 1.4867 | 2.1 |
| 36859 | 1.63892 | 2.05361 |
| 36860 | 2.09605 | 0.87644 |
| 36861 | 1.97645 | 2.1 |
| 36862 | 3.58275 | 1.48631 |
| 36863 | 1.29389 | 2.1 |
| 36864 | 1.97645 | 1.89854 |
| 36865 | 1.4867 | 1.90907 |
| 36866 | 1.4867 | 2.1 |
| 36867 | 1.82955 | 1.13655 |
| 36868 | 1.82955 | 2.1 |
| 36869 | 1.13454 | 1.09786 |
| 36870 | 0.983594 | 2.1 |
| 36871 | 1.16809 | 2.1 |
| 36872 | 1.06836 | 2.1 |
| 36873 | 1.08332 | 2.1 |
| 36874 | 0.983594 | 1.27194 |
| 36875 | 1.48751 | 2.1 |
| 36876 | 1.48047 | 1.8112 |
| 36877 | 1.77596 | 1.95951 |
| 36878 | 1.75506 | 2.03849 |
| 36879 | 3.25643 | 2.1 |
| 36880 | 1.48047 | 1.76466 |
| 36881 | 1.25334 | 2.1 |
| 36882 | 1.75505 | 1.53978 |
| 36883 | 1.55831 | 0.835289 |
| 36884 | 3.03878 | 2.04313 |
| 36885 | 1.77596 | 2.1 |

| | | |
|---|---|---|
| 36886 | 1.75506 | 2.1 |
| 36887 | 2.00551 | 2.1 |
| 36888 | 2.00083 | 1.00939 |
| 36889 | 3.77679 | 2.1 |
| 36890 | 1.75506 | 2.1 |
| 36891 | 1.80328 | 0.89213 |
| 36892 | 1.55831 | 2.0928 |
| 36893 | 1.55831 | 2.1 |
| 36894 | 2.00083 | 0.966175 |
| 36895 | 2.00083 | 1.31541 |
| 36896 | 2.58627 | 0.916902 |
| 36897 | 2.79047 | 2.1 |
| 36898 | 2.40858 | 2.1 |
| 36899 | 2.64748 | 2.1 |
| 36900 | 5.23375 | 2.1 |
| 36901 | 1.26755 | 2.1 |
| 36902 | 1.71717 | 2.1 |
| 36903 | 1.03824 | 2.1 |
| 36904 | 1.48901 | 2.1 |
| 36905 | 2.75656 | 2.1 |
| 36906 | 1.89226 | 2.1 |
| 36907 | 1.89226 | 2.1 |
| 36908 | 1.89226 | 2.1 |
| 36909 | 1.89226 | 2.1 |
| 36910 | 1.89226 | 2.1 |
| 36911 | 1.89226 | 2.1 |
| 36912 | 1.36541 | 2.1 |
| 36913 | 1.22156 | 2.1 |
| 36914 | 0.946132 | 2.1 |
| 36915 | 0.946132 | 2.1 |
| 36916 | 1.36541 | 2.1 |
| 36917 | 1.36541 | 2.1 |
| 36918 | 0.946132 | 2.1 |
| 36919 | 0.946132 | 2.1 |
| 36920 | 1.36541 | 2.1 |
| 36921 | 0.946133 | 2.1 |
| 36922 | 0.946132 | 2.1 |
| 36923 | 1.22156 | 2.1 |
| 36924 | 1.18901 | 2.1 |
| 36925 | 1.61149 | 2.1 |
| 36926 | 1.98748 | 2.1 |
| 36927 | 1.534 | 2.1 |
| 36928 | 3.1765 | 2.1 |
| 36929 | 1.89226 | 2.1 |
| 36930 | 1.89226 | 2.1 |
| 36931 | 1.89226 | 2.1 |
| 36932 | 1.89226 | 2.1 |
| 36933 | 3.78452 | 2.1 |
| 36934 | 1.98748 | 2.1 |
| 36935 | 1.534 | 2.1 |
| 36936 | 2.27541 | 2.1 |
| 36937 | 1.98748 | 2.1 |
| 36938 | 1.87436 | 2.1 |
| 36939 | 1.87436 | 2.1 |
| 36940 | 2.2988 | 2.1 |
| 36941 | 2.2604 | 2.1 |
| 36942 | 2.20831 | 2.1 |
| 36943 | 2.02802 | 2.1 |

| | | |
|---|---|---|
| 36944 | 4.32683 | 2.1 |
| 36945 | 2.26041 | 2.1 |
| 36946 | 1.98957 | 2.1 |
| 36947 | 2.02803 | 2.1 |
| 36948 | 1.75021 | 2.1 |
| 36949 | 4.0176 | 2.1 |
| 36950 | 2.34607 | 2.1 |
| 36951 | 2.02803 | 2.1 |
| 36952 | 1.86795 | 2.1 |
| 36953 | 1.81448 | 2.1 |
| 36954 | 3.89597 | 2.1 |
| 36955 | 2.02803 | 2.1 |
| 36956 | 1.75021 | 2.1 |
| 36957 | 1.81448 | 2.1 |
| 36958 | 1.57887 | 2.1 |
| 36959 | 3.6069 | 2.1 |
| 36960 | 0.946131 | 0.05 |
| 36961 | 0.946131 | 2.1 |
| 36962 | 1.1235 | 2.1 |
| 36963 | 1.1235 | 2.1 |
| 36964 | 0.946131 | 1.50432 |
| 36965 | 0.946131 | 0.837684 |
| 36966 | 1.1235 | 2.1 |
| 36967 | 1.13736 | 2.1 |
| 36968 | 1.13736 | 0.214179 |
| 36969 | 0.946131 | 1.65818 |
| 36970 | 1.1235 | 2.05833 |
| 36971 | 0.946131 | 1.4992 |
| 36972 | 1.89226 | 2.1 |
| 36973 | 1.89226 | 2.1 |
| 36974 | 1.89226 | 1.05329 |
| 36975 | 1.89226 | 0.883699 |
| 36976 | 3.78452 | 0.839515 |
| 36977 | 0.563266 | 2.1 |
| 36978 | 0.563266 | 1.16433 |
| 36979 | 0.565606 | 0.361232 |
| 36980 | 0.565606 | 2.1 |
| 36981 | 0.647086 | 2.08202 |
| 36982 | 0.624219 | 2.06779 |
| 36983 | 0.500035 | 1.1231 |
| 36984 | 0.481785 | 1.11872 |
| 36985 | 0.500034 | 0.451389 |
| 36986 | 0.647087 | 0.544722 |
| 36987 | 0.624219 | 1.61542 |
| 36988 | 0.481785 | 1.04734 |
| 36989 | 0.915788 | 0.05 |
| 36990 | 0.915788 | 0.05 |
| 36991 | 1.02732 | 0.402743 |
| 36992 | 1.02732 | 0.135217 |
| 36993 | 0.660831 | 0.197187 |
| 36994 | 1.11155 | 0.05 |
| 36995 | 0.713295 | 0.05 |
| 36996 | 1.26874 | 0.200161 |
| 36997 | 0.713295 | 0.109617 |
| 36998 | 0.660831 | 0.059808 |
| 36999 | 1.11155 | 0.0859231 |
| 37000 | 1.26874 | 0.05 |
| 37001 | 1.89226 | 0.749538 |

| | | |
|---|---|---|
| 37002 | 1.85091 | 0.05 |
| 37003 | 2.11193 | 0.453509 |
| 37004 | 1.85091 | 0.628737 |
| 37005 | 2.15328 | 0.0694822 |
| 37006 | 1.89226 | 0.0574327 |
| 37007 | 0.946131 | 0.05 |
| 37008 | 0.946131 | 0.122224 |
| 37009 | 1.18275 | 0.05 |
| 37010 | 1.05596 | 0.251067 |
| 37011 | 0.946127 | 0.0625298 |
| 37012 | 0.946127 | 0.05 |
| 37013 | 1.18275 | 0.362664 |
| 37014 | 1.18275 | 0.0518329 |
| 37015 | 0.946128 | 0.580617 |
| 37016 | 1.18275 | 0.05 |
| 37017 | 0.946129 | 0.167655 |
| 37018 | 1.05596 | 0.05 |
| 37019 | 1.60936 | 2.1 |
| 37020 | 2.35846 | 2.1 |
| 37021 | 2.06424 | 2.1 |
| 37022 | 2.57651 | 2.1 |
| 37023 | 4.42269 | 2.1 |
| 37024 | 1.89226 | 2.1 |
| 37025 | 1.89226 | 2.1 |
| 37026 | 1.89226 | 2.1 |
| 37027 | 1.89226 | 2.1 |
| 37028 | 3.78453 | 2.1 |
| 37029 | 1.89226 | 2.1 |
| 37030 | 1.89226 | 2.1 |
| 37031 | 1.89226 | 2.1 |
| 37032 | 1.89226 | 2.1 |
| 37033 | 1.89226 | 2.1 |
| 37034 | 1.89226 | 2.1 |
| 37035 | 1.95053 | 2.1 |
| 37036 | 2.06424 | 2.1 |
| 37037 | 1.95053 | 2.1 |
| 37038 | 1.30584 | 2.1 |
| 37039 | 2.5 | 2.1 |
| 37040 | 1.8553 | 2.1 |
| 37041 | 2.49999 | 2.1 |
| 37042 | 2.57651 | 2.1 |
| 37043 | 2.49999 | 2.1 |
| 37044 | 2.06423 | 2.1 |
| 37045 | 2.80738 | 2.1 |
| 37046 | 2.37162 | 2.1 |
| 37047 | 1.89226 | 2.1 |
| 37048 | 1.89226 | 2.1 |
| 37049 | 1.89226 | 2.1 |
| 37050 | 1.89226 | 2.1 |
| 37051 | 1.89226 | 2.1 |
| 37052 | 1.89226 | 2.1 |
| 37053 | 1.49295 | 0.05 |
| 37054 | 1.03824 | 0.05 |
| 37055 | 1.51396 | 0.0950201 |
| 37056 | 2.08514 | 0.05 |
| 37057 | 3.12338 | 0.05 |
| 37058 | 1.49798 | 0.05 |
| 37059 | 2.32017 | 0.05 |

| | | |
|---|---|---|
| 37060 | 2.68236 | 0.0716004 |
| 37061 | 2.08514 | 0.0514865 |
| 37062 | 4.4053 | 0.05 |
| 37063 | 1.89226 | 0.05 |
| 37064 | 1.89226 | 0.05 |
| 37065 | 1.89226 | 0.171944 |
| 37066 | 1.89226 | 0.05 |
| 37067 | 3.78452 | 0.0637015 |
| 37068 | 1.89226 | 0.0884081 |
| 37069 | 1.89226 | 0.05 |
| 37070 | 1.89226 | 0.0751332 |
| 37071 | 1.89226 | 0.05 |
| 37072 | 1.89226 | 0.05 |
| 37073 | 1.89226 | 0.05 |
| 37074 | 1.51396 | 0.05 |
| 37075 | 2.08514 | 0.05 |
| 37076 | 2.55393 | 0.05 |
| 37077 | 2.02901 | 0.05 |
| 37078 | 4.11415 | 0.05 |
| 37079 | 1.89226 | 0.05 |
| 37080 | 1.89226 | 0.05 |
| 37081 | 1.89226 | 0.0582844 |
| 37082 | 1.89226 | 0.05 |
| 37083 | 3.78451 | 0.05 |
| 37084 | 1.89226 | 0.05 |
| 37085 | 1.89226 | 0.05 |
| 37086 | 1.89226 | 0.05 |
| 37087 | 1.89226 | 0.05 |
| 37088 | 1.89226 | 0.0537201 |
| 37089 | 1.89226 | 0.05 |
| 37090 | 0.946131 | 0.05 |
| 37091 | 0.946131 | 0.05 |
| 37092 | 0.946132 | 0.05 |
| 37093 | 0.946132 | 0.05 |
| 37094 | 0.946132 | 0.05 |
| 37095 | 0.946132 | 0.05 |
| 37096 | 0.946132 | 0.05 |
| 37097 | 0.946132 | 0.05 |
| 37098 | 0.946132 | 0.05 |
| 37099 | 0.946132 | 0.05 |
| 37100 | 0.946132 | 0.05 |
| 37101 | 0.946132 | 0.05 |
| 37102 | 1.89226 | 2.1 |
| 37103 | 1.89226 | 2.1 |
| 37104 | 1.89226 | 2.1 |
| 37105 | 1.89226 | 2.1 |
| 37106 | 3.78452 | 2.1 |
| 37107 | 1.28367 | 2.1 |
| 37108 | 1.82707 | 2.1 |
| 37109 | 1.11025 | 2.1 |
| 37110 | 1.6365 | 2.1 |
| 37111 | 2.92016 | 2.1 |
| 37112 | 1.82707 | 2.1 |
| 37113 | 1.8247 | 0.05 |
| 37114 | 1.8247 | 2.1 |
| 37115 | 2.01549 | 0.05 |
| 37116 | 1.6365 | 2.09772 |
| 37117 | 1.82729 | 0.05 |

| | | |
|---|---|---|
| 37118 | 1.89226 | 2.1 |
| 37119 | 1.89226 | 0.05 |
| 37120 | 1.89226 | 0.05 |
| 37121 | 1.89226 | 2.1 |
| 37122 | 1.89226 | 0.05 |
| 37123 | 1.89226 | 2.1 |
| 37124 | 1.63648 | 2.1 |
| 37125 | 1.82467 | 0.05 |
| 37126 | 1.35877 | 2.1 |
| 37127 | 1.55159 | 0.05 |
| 37128 | 3.18345 | 0.0904246 |
| 37129 | 1.89223 | 2.1 |
| 37130 | 1.89223 | 0.05 |
| 37131 | 1.89223 | 2.1 |
| 37132 | 1.89223 | 0.0552076 |
| 37133 | 3.78447 | 0.832192 |
| 37134 | 1.59972 | 2.1 |
| 37135 | 1.28037 | 2.1 |
| 37136 | 1.63702 | 2.1 |
| 37137 | 1.28158 | 2.1 |
| 37138 | 1.63581 | 2.1 |
| 37139 | 1.28037 | 2.1 |
| 37140 | 1.89226 | 2.1 |
| 37141 | 1.89226 | 2.1 |
| 37142 | 1.89226 | 2.1 |
| 37143 | 1.89226 | 2.1 |
| 37144 | 1.89226 | 2.1 |
| 37145 | 1.89226 | 2.1 |
| 37146 | 1.89226 | 2.1 |
| 37147 | 1.89226 | 2.1 |
| 37148 | 1.89226 | 2.1 |
| 37149 | 1.89226 | 2.1 |
| 37150 | 3.78451 | 2.1 |
| 37151 | 1.28037 | 2.1 |
| 37152 | 1.05071 | 2.1 |
| 37153 | 1.63581 | 2.1 |
| 37154 | 1.44287 | 2.1 |
| 37155 | 2.72324 | 2.1 |
| 37156 | 1.59972 | 2.1 |
| 37157 | 1.63581 | 2.1 |
| 37158 | 1.94291 | 2.1 |
| 37159 | 2.00118 | 2.1 |
| 37160 | 3.57872 | 2.1 |
| 37161 | 1.89226 | 2.1 |
| 37162 | 1.89226 | 2.1 |
| 37163 | 1.89226 | 2.1 |
| 37164 | 1.89226 | 2.1 |
| 37165 | 3.78452 | 2.1 |
| 37166 | 0.946131 | 2.1 |
| 37167 | 0.946131 | 2.1 |
| 37168 | 0.946127 | 2.1 |
| 37169 | 0.946127 | 2.1 |
| 37170 | 0.94613 | 2.1 |
| 37171 | 0.94613 | 2.1 |
| 37172 | 0.946128 | 2.1 |
| 37173 | 0.946128 | 2.1 |
| 37174 | 0.946128 | 2.1 |
| 37175 | 0.946128 | 2.1 |

| | | |
|---|---|---|
| 37176 | 0.946129 | 2.1 |
| 37177 | 0.946129 | 2.1 |
| 37178 | 0.865056 | 2.1 |
| 37179 | 0.865056 | 2.1 |
| 37180 | 0.873224 | 2.1 |
| 37181 | 0.873224 | 2.1 |
| 37182 | 0.721437 | 2.1 |
| 37183 | 0.920378 | 2.1 |
| 37184 | 0.817903 | 2.1 |
| 37185 | 1.00059 | 2.1 |
| 37186 | 1.00059 | 2.1 |
| 37187 | 0.920376 | 2.1 |
| 37188 | 0.817904 | 2.1 |
| 37189 | 0.721437 | 2.1 |
| 37190 | 3.09157 | 2.1 |
| 37191 | 1.71255 | 2.1 |
| 37192 | 2.0815 | 2.1 |
| 37193 | 1.9918 | 2.1 |
| 37194 | 2.51364 | 2.1 |
| 37195 | 2.39396 | 2.1 |
| 37196 | 2.0815 | 2.1 |
| 37197 | 1.05051 | 2.1 |
| 37198 | 2.56724 | 2.1 |
| 37199 | 1.63991 | 2.1 |
| 37200 | 3.47929 | 2.1 |
| 37201 | 1.89226 | 0.05 |
| 37202 | 2.2973 | 0.0568726 |
| 37203 | 1.90705 | 0.05 |
| 37204 | 1.90705 | 0.05 |
| 37205 | 1.89226 | 0.05 |
| 37206 | 1.89226 | 0.05 |
| 37207 | 1.89226 | 0.05 |
| 37208 | 1.90705 | 0.05 |
| 37209 | 1.45321 | 0.05 |
| 37210 | 1.45321 | 0.05 |
| 37211 | 1.89226 | 0.05 |
| 37212 | 1.89226 | 0.05 |
| 37213 | 0.901146 | 2.1 |
| 37214 | 0.901146 | 2.1 |
| 37215 | 1.15227 | 2.1 |
| 37216 | 1.21279 | 2.1 |
| 37217 | 0.991265 | 2.1 |
| 37218 | 1.15003 | 2.1 |
| 37219 | 0.946132 | 2.1 |
| 37220 | 0.946132 | 2.1 |
| 37221 | 0.946132 | 2.1 |
| 37222 | 1.09997 | 2.1 |
| 37223 | 0.991266 | 2.1 |
| 37224 | 0.946132 | 2.1 |
| 37225 | 1.89226 | 2.1 |
| 37226 | 1.89226 | 2.1 |
| 37227 | 1.89226 | 2.1 |
| 37228 | 1.89226 | 2.1 |
| 37229 | 1.89226 | 2.1 |
| 37230 | 1.89226 | 2.1 |
| 37231 | 1.62229 | 2.1 |
| 37232 | 1.06053 | 2.1 |
| 37233 | 1.19698 | 2.1 |

| | | |
|---|---|---|
| 37234 | 1.70106 | 2.1 |
| 37235 | 2.81928 | 2.1 |
| 37236 | 0.946132 | 2.1 |
| 37237 | 0.946132 | 2.1 |
| 37238 | 1.17527 | 2.1 |
| 37239 | 1.09997 | 2.1 |
| 37240 | 1.09997 | 2.1 |
| 37241 | 1.09997 | 2.1 |
| 37242 | 0.946133 | 2.1 |
| 37243 | 0.946133 | 2.1 |
| 37244 | 1.17527 | 2.1 |
| 37245 | 0.946132 | 2.1 |
| 37246 | 0.946133 | 2.1 |
| 37247 | 1.09997 | 2.1 |
| 37248 | 1.89226 | 2.1 |
| 37249 | 1.89226 | 2.1 |
| 37250 | 1.89226 | 2.1 |
| 37251 | 1.89226 | 2.1 |
| 37252 | 1.89226 | 2.1 |
| 37253 | 1.89226 | 2.1 |
| 37254 | 0.905416 | 2.1 |
| 37255 | 0.850527 | 2.1 |
| 37256 | 0.660601 | 2.1 |
| 37257 | 0.598494 | 2.1 |
| 37258 | 0.750137 | 2.1 |
| 37259 | 0.750137 | 2.1 |
| 37260 | 0.753774 | 2.1 |
| 37261 | 0.753774 | 2.1 |
| 37262 | 0.660599 | 2.1 |
| 37263 | 0.905418 | 2.1 |
| 37264 | 0.598493 | 2.1 |
| 37265 | 0.850529 | 2.1 |
| 37266 | 2.43509 | 2.1 |
| 37267 | 2.65066 | 2.1 |
| 37268 | 2.66602 | 2.1 |
| 37269 | 2.46339 | 2.1 |
| 37270 | 5.10111 | 2.1 |
| 37271 | 2.53008 | 2.1 |
| 37272 | 2.65066 | 2.1 |
| 37273 | 2.66602 | 2.1 |
| 37274 | 2.52852 | 2.1 |
| 37275 | 5.1961 | 2.1 |
| 37276 | 2.66602 | 2.1 |
| 37277 | 2.46339 | 2.1 |
| 37278 | 2.47932 | 2.1 |
| 37279 | 2.67393 | 2.1 |
| 37280 | 5.14535 | 2.1 |
| 37281 | 2.66602 | 2.1 |
| 37282 | 2.52853 | 2.1 |
| 37283 | 2.51686 | 2.1 |
| 37284 | 2.67393 | 2.1 |
| 37285 | 5.18288 | 2.1 |
| 37286 | 1.89226 | 0.826584 |
| 37287 | 1.89226 | 0.324231 |
| 37288 | 1.89226 | 0.547288 |
| 37289 | 1.89226 | 0.129851 |
| 37290 | 3.78452 | 0.542032 |
| 37291 | 1.55161 | 0.465595 |

| | | |
|---|---|---|
| 37292 | 1.53972 | 0.233902 |
| 37293 | 1.32259 | 0.524012 |
| 37294 | 1.35082 | 0.05 |
| 37295 | 2.8623 | 0.253861 |
| 37296 | 1.53972 | 0.309476 |
| 37297 | 1.4054 | 0.340951 |
| 37298 | 1.35083 | 0.0658063 |
| 37299 | 1.24913 | 0.175547 |
| 37300 | 2.78885 | 0.151679 |
| 37301 | 1.89226 | 0.413213 |
| 37302 | 1.89226 | 0.790563 |
| 37303 | 1.89226 | 0.494884 |
| 37304 | 1.89226 | 0.196447 |
| 37305 | 3.78452 | 0.353012 |
| 37306 | 1.89226 | 0.124214 |
| 37307 | 1.89226 | 0.396232 |
| 37308 | 1.89226 | 0.801279 |
| 37309 | 1.89226 | 0.435307 |
| 37310 | 1.89226 | 0.191746 |
| 37311 | 1.89226 | 0.194157 |
| 37312 | 1.14599 | 0.0840439 |
| 37313 | 1.35082 | 0.241041 |
| 37314 | 1.14599 | 0.502155 |
| 37315 | 1.32259 | 0.72513 |
| 37316 | 1.19993 | 0.05 |
| 37317 | 1.37652 | 0.698018 |
| 37318 | 1.35083 | 0.202633 |
| 37319 | 1.24913 | 0.607721 |
| 37320 | 1.19993 | 0.239124 |
| 37321 | 1.11787 | 0.05 |
| 37322 | 2.44906 | 0.218086 |
| 37323 | 1.89226 | 0.56047 |
| 37324 | 1.89226 | 0.604314 |
| 37325 | 1.89226 | 0.396928 |
| 37326 | 1.89226 | 0.238488 |
| 37327 | 3.78452 | 0.442812 |
| 37328 | 1.51697 | 0.05 |
| 37329 | 2.57264 | 0.05 |
| 37330 | 1.30203 | 0.05 |
| 37331 | 2.41701 | 0.05 |
| 37332 | 3.93398 | 0.05 |
| 37333 | 1.17464 | 0.05 |
| 37334 | 2.41701 | 0.05 |
| 37335 | 2.32462 | 0.05 |
| 37336 | 2.32462 | 0.05 |
| 37337 | 1.30203 | 0.05 |
| 37338 | 1.30203 | 0.05 |
| 37339 | 2.08149 | 2.1 |
| 37340 | 1.9839 | 2.1 |
| 37341 | 1.71398 | 2.1 |
| 37342 | 1.99182 | 0.0743855 |
| 37343 | 3.71121 | 2.1 |
| 37344 | 2.16061 | 2.1 |
| 37345 | 2.01399 | 0.05 |
| 37346 | 1.99182 | 0.586782 |
| 37347 | 1.83895 | 0.536388 |
| 37348 | 4.00581 | 0.0770247 |
| 37349 | 1.90444 | 2.1 |

| | | |
|---|---|---|
| 37350 | 1.99182 | 0.290277 |
| 37351 | 1.71717 | 2.1 |
| 37352 | 1.805 | 0.925856 |
| 37353 | 3.70899 | 2.1 |
| 37354 | 1.99182 | 0.749698 |
| 37355 | 1.83896 | 0.424631 |
| 37356 | 1.805 | 2.1 |
| 37357 | 1.65243 | 0.0784533 |
| 37358 | 3.64425 | 0.416258 |
| 37359 | 1.18076 | 2.1 |
| 37360 | 1.27697 | 2.1 |
| 37361 | 1.05025 | 2.1 |
| 37362 | 1.27697 | 2.1 |
| 37363 | 1.25 | 2.1 |
| 37364 | 1.47671 | 2.1 |
| 37365 | 1.89226 | 2.1 |
| 37366 | 1.89226 | 2.1 |
| 37367 | 1.89226 | 2.1 |
| 37368 | 1.89226 | 2.1 |
| 37369 | 3.78452 | 2.1 |
| 37370 | 2.64124 | 2.1 |
| 37371 | 1.89226 | 2.1 |
| 37372 | 1.89226 | 2.1 |
| 37373 | 1.89226 | 2.1 |
| 37374 | 2.46904 | 2.1 |
| 37375 | 2.46904 | 2.1 |
| 37376 | 1.89226 | 2.1 |
| 37377 | 1.89226 | 2.1 |
| 37378 | 2.7408 | 2.1 |
| 37379 | 2.82858 | 2.1 |
| 37380 | 4.72084 | 2.1 |
| 37381 | 1.87875 | 2.1 |
| 37382 | 1.37704 | 2.1 |
| 37383 | 1.77042 | 2.1 |
| 37384 | 2.27801 | 2.1 |
| 37385 | 3.64916 | 2.1 |
| 37386 | 1.77042 | 2.1 |
| 37387 | 2.27801 | 2.1 |
| 37388 | 2.58523 | 2.1 |
| 37389 | 1.90178 | 2.1 |
| 37390 | 4.35565 | 2.1 |
| 37391 | 1.28667 | 2.1 |
| 37392 | 1.30711 | 2.1 |
| 37393 | 1.53077 | 2.1 |
| 37394 | 1.55635 | 2.1 |
| 37395 | 2.81743 | 2.1 |
| 37396 | 1.55635 | 2.1 |
| 37397 | 1.28228 | 2.1 |
| 37398 | 1.64268 | 2.1 |
| 37399 | 1.93673 | 2.1 |
| 37400 | 3.19903 | 2.1 |
| 37401 | 1.53077 | 2.1 |
| 37402 | 1.93673 | 2.1 |
| 37403 | 1.55635 | 2.1 |
| 37404 | 1.55635 | 2.1 |
| 37405 | 1.86206 | 2.1 |
| 37406 | 1.86206 | 2.1 |
| 37407 | 1.64892 | 2.1 |

| | | |
|---|---|---|
| 37408 | 1.60532 | 2.1 |
| 37409 | 1.85282 | 2.1 |
| 37410 | 1.96716 | 2.1 |
| 37411 | 3.61608 | 2.1 |
| 37412 | 1.60531 | 2.1 |
| 37413 | 1.32414 | 2.1 |
| 37414 | 1.96716 | 2.1 |
| 37415 | 1.75823 | 2.1 |
| 37416 | 3.2913 | 2.1 |
| 37417 | 1.91219 | 2.1 |
| 37418 | 1.96716 | 2.1 |
| 37419 | 2.0815 | 2.1 |
| 37420 | 2.51396 | 2.1 |
| 37421 | 4.30843 | 2.1 |
| 37422 | 1.96716 | 2.1 |
| 37423 | 2.17979 | 2.1 |
| 37424 | 1.75823 | 2.1 |
| 37425 | 1.75823 | 2.1 |
| 37426 | 2.28541 | 2.1 |
| 37427 | 2.28541 | 2.1 |
| 37428 | 1.89226 | 1.28795 |
| 37429 | 1.89226 | 2.1 |
| 37430 | 1.89226 | 1.33161 |
| 37431 | 1.89226 | 1.19122 |
| 37432 | 3.78453 | 1.4326 |
| 37433 | 1.89226 | 1.63106 |
| 37434 | 1.89226 | 1.70551 |
| 37435 | 1.89226 | 1.30747 |
| 37436 | 1.89226 | 1.84933 |
| 37437 | 1.89226 | 1.39304 |
| 37438 | 1.89226 | 2.00728 |
| 37439 | 1.61016 | 1.35869 |
| 37440 | 1.45303 | 2.1 |
| 37441 | 1.62812 | 1.51568 |
| 37442 | 1.86379 | 1.47771 |
| 37443 | 3.23828 | 1.55987 |
| 37444 | 1.61016 | 1.97988 |
| 37445 | 1.78716 | 2.1 |
| 37446 | 2.0815 | 1.10093 |
| 37447 | 1.6774 | 2.07357 |
| 37448 | 3.54454 | 2.01143 |
| 37449 | 1.89226 | 1.80143 |
| 37450 | 1.89226 | 1.6105 |
| 37451 | 1.89226 | 1.03806 |
| 37452 | 1.89226 | 1.96653 |
| 37453 | 3.78453 | 1.3946 |
| 37454 | 1.89226 | 1.89258 |
| 37455 | 1.89226 | 1.87957 |
| 37456 | 1.89226 | 1.45596 |
| 37457 | 1.89226 | 2.1 |
| 37458 | 1.89226 | 1.02997 |
| 37459 | 1.89226 | 1.34035 |
| 37460 | 0.807591 | 2.05123 |
| 37461 | 0.814059 | 1.50969 |
| 37462 | 1.00217 | 2.03139 |
| 37463 | 0.931896 | 1.58802 |
| 37464 | 0.850586 | 1.68374 |
| 37465 | 0.850586 | 2.1 |

| | | |
|---|---|---|
| 37466 | 0.8889 | 1.2901 |
| 37467 | 0.8889 | 1.99008 |
| 37468 | 0.807592 | 1.10401 |
| 37469 | 1.00217 | 2.01061 |
| 37470 | 0.814061 | 1.67899 |
| 37471 | 0.931894 | 2.1 |
| 37472 | 2.0815 | 1.31875 |
| 37473 | 1.6774 | 2.1 |
| 37474 | 2.00435 | 1.48194 |
| 37475 | 2.36104 | 1.41742 |
| 37476 | 3.86809 | 1.6425 |
| 37477 | 1.00532 | 0.168797 |
| 37478 | 1.00532 | 0.155721 |
| 37479 | 1.00183 | 0.206586 |
| 37480 | 1.00183 | 0.122589 |
| 37481 | 1.26377 | 0.167015 |
| 37482 | 1.34921 | 0.162928 |
| 37483 | 0.664872 | 0.16204 |
| 37484 | 0.743381 | 0.19164 |
| 37485 | 0.664872 | 0.167149 |
| 37486 | 1.26377 | 0.170771 |
| 37487 | 1.34921 | 0.168544 |
| 37488 | 0.743381 | 0.163951 |
| 37489 | 1.26826 | 0.05 |
| 37490 | 0.946131 | 2.1 |
| 37491 | 1.02404 | 2.1 |
| 37492 | 0.94613 | 2.1 |
| 37493 | 1.30281 | 2.1 |
| 37494 | 0.930941 | 2.1 |
| 37495 | 0.930941 | 2.1 |
| 37496 | 1.318 2.1 | |
| 37497 | 1.58161 | 2.1 |
| 37498 | 0.94613 | 2.1 |
| 37499 | 0.94613 | 2.09092 |
| 37500 | 1.56338 | 2.1 |
| 37501 | 1.02404 | 2.1 |
| 37502 | 2.81374 | 2.1 |
| 37503 | 1.89226 | 2.1 |
| 37504 | 1.89226 | 2.1 |
| 37505 | 1.89226 | 2.1 |
| 37506 | 3.78453 | 2.1 |
| 37507 | 0.946133 | 2.1 |
| 37508 | 0.946133 | 2.1 |
| 37509 | 0.946132 | 2.1 |
| 37510 | 0.946132 | 2.1 |
| 37511 | 0.946132 | 2.1 |
| 37512 | 0.946132 | 2.1 |
| 37513 | 0.946133 | 2.1 |
| 37514 | 0.946133 | 2.1 |
| 37515 | 0.946132 | 2.1 |
| 37516 | 0.946132 | 2.1 |
| 37517 | 0.946133 | 2.1 |
| 37518 | 0.946133 | 2.1 |
| 37519 | 1.89226 | 1.57913 |
| 37520 | 1.89226 | 2.1 |
| 37521 | 1.89226 | 2.02416 |
| 37522 | 1.89226 | 2.1 |
| 37523 | 3.78452 | 2.1 |

| | | |
|---|---|---|
| 37524 | 1.89226 | 2.1 |
| 37525 | 1.89226 | 2.1 |
| 37526 | 1.89226 | 2.1 |
| 37527 | 1.89226 | 2.07142 |
| 37528 | 1.89226 | 2.1 |
| 37529 | 1.89226 | 2.08757 |
| 37530 | 0.946133 | 2.1 |
| 37531 | 0.946133 | 2.1 |
| 37532 | 0.946132 | 2.1 |
| 37533 | 0.946132 | 2.1 |
| 37534 | 0.946132 | 2.1 |
| 37535 | 0.946132 | 2.1 |
| 37536 | 0.946133 | 2.1 |
| 37537 | 0.946133 | 2.1 |
| 37538 | 0.946132 | 2.1 |
| 37539 | 0.946132 | 2.1 |
| 37540 | 0.946132 | 2.1 |
| 37541 | 0.946132 | 2.1 |
| 37542 | 2.56953 | 2.1 |
| 37543 | 2.64148 | 2.1 |
| 37544 | 2.48631 | 2.1 |
| 37545 | 2.62213 | 2.1 |
| 37546 | 5.19166 | 2.1 |
| 37547 | 2.64148 | 2.1 |
| 37548 | 2.57929 | 2.1 |
| 37549 | 2.62213 | 2.1 |
| 37550 | 2.59522 | 2.1 |
| 37551 | 5.20142 | 2.1 |
| 37552 | 2.48631 | 2.1 |
| 37553 | 2.62213 | 2.1 |
| 37554 | 2.41176 | 2.1 |
| 37555 | 2.59262 | 2.1 |
| 37556 | 5.03389 | 2.1 |
| 37557 | 2.62213 | 2.1 |
| 37558 | 2.59522 | 2.1 |
| 37559 | 2.59262 | 2.1 |
| 37560 | 2.61901 | 2.1 |
| 37561 | 5.24114 | 2.1 |
| 37562 | 0.946132 | 0.05 |
| 37563 | 0.946132 | 0.05 |
| 37564 | 0.946133 | 0.05 |
| 37565 | 0.946133 | 0.05 |
| 37566 | 0.946132 | 0.05 |
| 37567 | 0.946132 | 0.05 |
| 37568 | 0.946133 | 0.05 |
| 37569 | 0.946133 | 0.05 |
| 37570 | 0.946132 | 0.05 |
| 37571 | 0.946132 | 0.05 |
| 37572 | 0.946133 | 0.05 |
| 37573 | 0.946133 | 0.05 |
| 37574 | 1.5681 | 0.05 |
| 37575 | 1.01248 | 0.05 |
| 37576 | 1.10749 | 0.05 |
| 37577 | 0.570913 | 0.05 |
| 37578 | 1.15589 | 0.05 |
| 37579 | 1.15588 | 0.05 |
| 37580 | 0.977549 | 0.05 |
| 37581 | 0.977549 | 0.05 |

| | | |
|---|---|---|
| 37582 | 1.01249 | 0.05 |
| 37583 | 0.654445 | 0.05 |
| 37584 | 1.36795 | 0.05 |
| 37585 | 1.10748 | 0.05 |
| 37586 | 1.73883 | 0.05 |
| 37587 | 1.87638 | 0.05 |
| 37588 | 1.85219 | 0.05 |
| 37589 | 1.93113 | 2.1 |
| 37590 | 3.7407 | 0.05 |
| 37591 | 1.87638 | 0.05 |
| 37592 | 1.73883 | 2.1 |
| 37593 | 1.93113 | 2.1 |
| 37594 | 1.80426 | 2.1 |
| 37595 | 3.66996 | 2.1 |
| 37596 | 2.02912 | 0.05 |
| 37597 | 1.44951 | 0.05 |
| 37598 | 1.50103 | 0.05 |
| 37599 | 2.02248 | 0.0723988 |
| 37600 | 3.47199 | 0.05 |
| 37601 | 1.89226 | 0.0602743 |
| 37602 | 1.89226 | 0.123307 |
| 37603 | 1.89226 | 0.125517 |
| 37604 | 1.89226 | 0.166311 |
| 37605 | 3.78453 | 0.0985855 |
| 37606 | 2.57881 | 0.05 |
| 37607 | 1.89226 | 0.128409 |
| 37608 | 1.89226 | 0.0962756 |
| 37609 | 1.89226 | 0.0759206 |
| 37610 | 2.6428 | 0.0688292 |
| 37611 | 2.6428 | 0.0557535 |
| 37612 | 1.01379 | 0.05 |
| 37613 | 0.985937 | 0.05 |
| 37614 | 0.769295 | 0.05 |
| 37615 | 0.715547 | 0.05 |
| 37616 | 0.853042 | 0.0519669 |
| 37617 | 0.853042 | 0.05 |
| 37618 | 0.864117 | 0.0561844 |
| 37619 | 0.864114 | 0.05 |
| 37620 | 0.715556 | 0.05 |
| 37621 | 1.03047 | 0.05 |
| 37622 | 0.731721 | 0.05 |
| 37623 | 0.985921 | 0.05 |
| 37624 | 1.89227 | 0.0832191 |
| 37625 | 1.89227 | 0.125375 |
| 37626 | 1.89227 | 0.100036 |
| 37627 | 1.89227 | 0.36792 |
| 37628 | 3.78453 | 0.115837 |
| 37629 | 0.946129 | 0.104362 |
| 37630 | 0.946129 | 0.0588243 |
| 37631 | 1.28161 | 0.05 |
| 37632 | 1.3214 | 0.0641834 |
| 37633 | 0.94613 | 0.0896856 |
| 37634 | 0.94613 | 0.0798362 |
| 37635 | 1.28161 | 0.05 |
| 37636 | 1.28161 | 0.05 |
| 37637 | 1.28161 | 0.05 |
| 37638 | 0.94613 | 0.0673737 |
| 37639 | 0.946131 | 0.0790365 |

| | | |
|---|---|---|
| 37640 | 1.3214 | 0.0565034 |
| 37641 | 0.946132 | 0.209152 |
| 37642 | 0.946132 | 0.222784 |
| 37643 | 0.706045 | 0.05 |
| 37644 | 1.16718 | 0.0784166 |
| 37645 | 0.706017 | 0.05 |
| 37646 | 0.706017 | 0.489712 |
| 37647 | 1.4073 | 0.0548838 |
| 37648 | 1.4073 | 0.05 |
| 37649 | 0.94613 | 0.0988766 |
| 37650 | 1.16719 | 0.0604595 |
| 37651 | 0.706046 | 0.122404 |
| 37652 | 0.946131 | 0.064435 |
| 37653 | 1.89226 | 0.540382 |
| 37654 | 1.89226 | 0.05 |
| 37655 | 1.89226 | 0.05 |
| 37656 | 1.89226 | 0.500561 |
| 37657 | 1.89226 | 0.05 |
| 37658 | 1.89226 | 0.59554 |
| 37659 | 0.946119 | 0.238069 |
| 37660 | 0.946119 | 0.301257 |
| 37661 | 0.946115 | 0.149946 |
| 37662 | 0.946115 | 0.05 |
| 37663 | 0.946117 | 0.131387 |
| 37664 | 0.946117 | 0.445554 |
| 37665 | 0.946117 | 0.184177 |
| 37666 | 0.946117 | 0.186067 |
| 37667 | 0.94613 | 0.071266 |
| 37668 | 0.94613 | 0.172563 |
| 37669 | 0.946105 | 0.143528 |
| 37670 | 0.946105 | 0.103685 |
| 37671 | 0.946132 | 0.758754 |
| 37672 | 0.946132 | 0.522852 |
| 37673 | 0.946131 | 0.466848 |
| 37674 | 0.946131 | 0.223532 |
| 37675 | 0.946132 | 0.749699 |
| 37676 | 0.946132 | 0.604608 |
| 37677 | 0.946132 | 0.494171 |
| 37678 | 0.827865 | 0.244612 |
| 37679 | 0.946132 | 0.280799 |
| 37680 | 0.946132 | 0.546003 |
| 37681 | 0.946132 | 0.553448 |
| 37682 | 0.827865 | 0.435689 |
| 37683 | 1.89226 | 1.42938 |
| 37684 | 1.89226 | 1.11423 |
| 37685 | 1.89226 | 1.52792 |
| 37686 | 1.89226 | 0.959485 |
| 37687 | 1.89226 | 2.0914 |
| 37688 | 1.89226 | 1.28238 |
| 37689 | 0.93094 | 0.99919 |
| 37690 | 0.93094 | 1.13357 |
| 37691 | 1.04649 | 1.01536 |
| 37692 | 0.614468 | 0.626772 |
| 37693 | 0.950358 | 0.663302 |
| 37694 | 0.799012 | 0.649782 |
| 37695 | 0.682742 | 1.03654 |
| 37696 | 0.682742 | 0.995359 |
| 37697 | 1.04073 | 1.6841 |

| | | |
|---|---|---|
| 37698 | 1.15596 | 1.29015 |
| 37699 | 0.614478 | 1.31642 |
| 37700 | 0.851524 | 1.12793 |
| 37701 | 2.22575 | 2.1 |
| 37702 | 1.90502 | 2.1 |
| 37703 | 2.10893 | 2.1 |
| 37704 | 1.80258 | 2.1 |
| 37705 | 4.01395 | 2.1 |
| 37706 | 1.90502 | 2.1 |
| 37707 | 1.40863 | 2.1 |
| 37708 | 1.49733 | 2.1 |
| 37709 | 1.49733 | 2.1 |
| 37710 | 1.80258 | 2.1 |
| 37711 | 1.80258 | 2.1 |
| 37712 | 2.10893 | 2.1 |
| 37713 | 1.68356 | 2.1 |
| 37714 | 1.80258 | 2.1 |
| 37715 | 1.80258 | 2.1 |
| 37716 | 1.98061 | 2.1 |
| 37717 | 1.98061 | 2.1 |
| 37718 | 1.80258 | 2.1 |
| 37719 | 1.40863 | 2.1 |
| 37720 | 1.68356 | 2.1 |
| 37721 | 1.30751 | 2.1 |
| 37722 | 3.11009 | 2.1 |
| 37723 | 1.35374 | 1.77473 |
| 37724 | 1.35374 | 2.07701 |
| 37725 | 0.746096 | 1.24367 |
| 37726 | 0.746096 | 1.21351 |
| 37727 | 1.1537 | 1.23683 |
| 37728 | 0.746095 | 1.67793 |
| 37729 | 0.946132 | 1.38852 |
| 37730 | 0.946132 | 1.02624 |
| 37731 | 0.946132 | 0.994177 |
| 37732 | 0.746095 | 1.14673 |
| 37733 | 0.946131 | 0.9523 |
| 37734 | 1.1537 | 1.04683 |
| 37735 | 2.15247 | 2.1 |
| 37736 | 1.70303 | 2.1 |
| 37737 | 1.41207 | 2.1 |
| 37738 | 1.43052 | 2.1 |
| 37739 | 3.22793 | 2.1 |
| 37740 | 0.940246 | 2.1 |
| 37741 | 0.728442 | 2.1 |
| 37742 | 0.650037 | 2.1 |
| 37743 | 0.539874 | 2.1 |
| 37744 | 0.694756 | 2.1 |
| 37745 | 0.694756 | 2.1 |
| 37746 | 0.776346 | 2.1 |
| 37747 | 0.656772 | 2.1 |
| 37748 | 0.733146 | 2.1 |
| 37749 | 0.563769 | 2.1 |
| 37750 | 0.66316 | 2.1 |
| 37751 | 0.899692 | 2.1 |
| 37752 | 1.21223 | 2.1 |
| 37753 | 1.22418 | 2.1 |
| 37754 | 1.43052 | 2.1 |
| 37755 | 1.43052 | 2.1 |

| | | |
|---|---|---|
| 37756 | 1.24353 | 2.1 |
| 37757 | 1.46181 | 2.1 |
| 37758 | 1.89226 | 0.05 |
| 37759 | 2.39894 | 0.05 |
| 37760 | 1.89226 | 0.05 |
| 37761 | 2.37417 | 0.05 |
| 37762 | 1.89226 | 0.05 |
| 37763 | 2.37417 | 0.05 |
| 37764 | 1.12767 | 0.05 |
| 37765 | 1.19947 | 0.05 |
| 37766 | 0.94613 | 0.05 |
| 37767 | 0.94613 | 0.05 |
| 37768 | 1.12766 | 0.05 |
| 37769 | 0.946128 | 0.05 |
| 37770 | 1.19947 | 0.05 |
| 37771 | 0.946132 | 0.05 |
| 37772 | 1.19947 | 0.05 |
| 37773 | 1.19947 | 0.05 |
| 37774 | 0.94613 | 0.05 |
| 37775 | 0.94613 | 0.05 |
| 37776 | 2.22621 | 0.05 |
| 37777 | 1.14533 | 2.1 |
| 37778 | 1.1131 | 2.1 |
| 37779 | 0.88238 | 2.1 |
| 37780 | 0.868521 | 2.1 |
| 37781 | 1.01153 | 2.1 |
| 37782 | 1.01153 | 2.1 |
| 37783 | 1.00232 | 2.1 |
| 37784 | 1.00232 | 2.1 |
| 37785 | 1.14533 | 2.1 |
| 37786 | 0.882378 | 2.1 |
| 37787 | 0.868519 | 2.1 |
| 37788 | 1.11311 | 2.1 |
| 37789 | 0.910183 | 1.99443 |
| 37790 | 1.07811 | 0.95098 |
| 37791 | 0.94613 | 0.0707335 |
| 37792 | 0.94613 | 0.113513 |
| 37793 | 0.910212 | 0.150514 |
| 37794 | 0.910212 | 2.1 |
| 37795 | 1.11403 | 0.075632 |
| 37796 | 1.11403 | 2.1 |
| 37797 | 0.910183 | 0.69055 |
| 37798 | 0.94613 | 0.989104 |
| 37799 | 0.94613 | 1.06709 |
| 37800 | 1.07811 | 1.06108 |
| 37801 | 1.87228 | 0.116505 |
| 37802 | 2.81189 | 2.1 |
| 37803 | 1.6197 | 2.1 |
| 37804 | 2.56716 | 2.1 |
| 37805 | 4.43944 | 2.1 |
| 37806 | 0.946132 | 0.537072 |
| 37807 | 0.946132 | 2.1 |
| 37808 | 0.946132 | 0.05 |
| 37809 | 0.946132 | 0.0891041 |
| 37810 | 0.946131 | 0.207558 |
| 37811 | 0.946131 | 2.1 |
| 37812 | 0.946132 | 0.0605762 |
| 37813 | 0.946132 | 1.15262 |

| | | |
|---|---|---|
| 37814 | 0.946132 | 0.221476 |
| 37815 | 0.946132 | 0.25195 |
| 37816 | 0.946132 | 0.373108 |
| 37817 | 0.946132 | 0.847152 |
| 37818 | 1.28724 | 2.1 |
| 37819 | 2.56716 | 2.1 |
| 37820 | 2.3014 | 1.17316 |
| 37821 | 2.3014 | 2.1 |
| 37822 | 1.6197 | 2.1 |
| 37823 | 1.6197 | 2.1 |
| 37824 | 0.946132 | 2.1 |
| 37825 | 0.946132 | 2.08302 |
| 37826 | 0.946132 | 0.05 |
| 37827 | 0.946132 | 0.217375 |
| 37828 | 0.946132 | 0.352735 |
| 37829 | 0.946132 | 2.1 |
| 37830 | 0.946133 | 2.1 |
| 37831 | 0.946133 | 0.05 |
| 37832 | 0.946133 | 0.887768 |
| 37833 | 0.946133 | 1.15784 |
| 37834 | 0.946132 | 0.947061 |
| 37835 | 0.946132 | 1.04285 |
| 37836 | 1.08039 | 2.1 |
| 37837 | 1.24445 | 2.1 |
| 37838 | 1.2873 | 2.1 |
| 37839 | 1.48751 | 2.1 |
| 37840 | 2.53175 | 2.1 |
| 37841 | 0.946125 | 0.504369 |
| 37842 | 0.946125 | 2.1 |
| 37843 | 1.12552 | 2.1 |
| 37844 | 1.12552 | 2.1 |
| 37845 | 0.946124 | 1.99168 |
| 37846 | 0.946124 | 2.1 |
| 37847 | 1.12552 | 2.1 |
| 37848 | 1.11114 | 2.1 |
| 37849 | 1.11113 | 2.1 |
| 37850 | 0.946118 | 2.1 |
| 37851 | 1.12552 | 2.1 |
| 37852 | 0.946131 | 2.1 |
| 37853 | 1.89225 | 1.0729 |
| 37854 | 1.89225 | 1.12175 |
| 37855 | 1.89225 | 2.1 |
| 37856 | 1.89225 | 2.1 |
| 37857 | 1.89225 | 0.752305 |
| 37858 | 1.89225 | 2.1 |
| 37859 | 0.987703 | 1.01371 |
| 37860 | 1.33078 | 1.93013 |
| 37861 | 1.31565 | 1.225 |
| 37862 | 1.31565 | 2.1 |
| 37863 | 1.01121 | 1.14395 |
| 37864 | 1.01121 | 2.1 |
| 37865 | 2.19163 | 2.1 |
| 37866 | 1.89226 | 2.1 |
| 37867 | 1.89226 | 2.1 |
| 37868 | 1.89226 | 2.0692 |
| 37869 | 2.22229 | 2.1 |
| 37870 | 2.22229 | 1.89985 |
| 37871 | 1.89226 | 2.1 |

| | | |
|---|---|---|
| 37872 | 1.89226 | 0.592308 |
| 37873 | 1.89226 | 0.241713 |
| 37874 | 1.89226 | 2.1 |
| 37875 | 1.89226 | 0.698581 |
| 37876 | 1.89226 | 2.1 |
| 37877 | 0.57141 | 2.1 |
| 37878 | 0.57141 | 0.892562 |
| 37879 | 0.574036 | 1.15429 |
| 37880 | 0.574036 | 2.1 |
| 37881 | 0.651591 | 2.1 |
| 37882 | 0.657827 | 1.17522 |
| 37883 | 0.482391 | 1.29185 |
| 37884 | 0.493855 | 2.1 |
| 37885 | 0.482391 | 0.827355 |
| 37886 | 0.651591 | 0.934145 |
| 37887 | 0.493855 | 1.67384 |
| 37888 | 0.657828 | 1.69896 |
| 37889 | 1.88428 | 2.1 |
| 37890 | 2.03975 | 2.1 |
| 37891 | 1.97316 | 2.1 |
| 37892 | 1.8266 | 2.1 |
| 37893 | 3.86635 | 2.1 |
| 37894 | 2.23053 | 2.1 |
| 37895 | 1.42255 | 2.1 |
| 37896 | 1.3868 | 2.1 |
| 37897 | 1.8266 | 2.1 |
| 37898 | 3.24915 | 2.1 |
| 37899 | 1.97316 | 2.1 |
| 37900 | 1.8266 | 2.1 |
| 37901 | 1.77128 | 2.1 |
| 37902 | 1.91767 | 2.1 |
| 37903 | 3.74427 | 2.1 |
| 37904 | 1.77128 | 2.1 |
| 37905 | 1.8266 | 2.1 |
| 37906 | 1.77942 | 2.1 |
| 37907 | 1.33962 | 2.1 |
| 37908 | 1.3868 | 2.1 |
| 37909 | 1.3868 | 2.1 |
| 37910 | 0.944111 | 2.1 |
| 37911 | 0.944111 | 2.1 |
| 37912 | 1.26036 | 2.1 |
| 37913 | 1.16968 | 2.1 |
| 37914 | 1.03853 | 2.1 |
| 37915 | 1.16646 | 2.1 |
| 37916 | 0.946129 | 2.1 |
| 37917 | 0.946129 | 2.1 |
| 37918 | 0.94613 | 2.1 |
| 37919 | 1.25768 | 2.1 |
| 37920 | 1.03853 | 2.1 |
| 37921 | 0.946131 | 2.1 |
| 37922 | 1.82441 | 0.05 |
| 37923 | 1.33061 | 0.05 |
| 37924 | 1.13272 | 0.05 |
| 37925 | 1.60249 | 0.05 |
| 37926 | 2.95712 | 0.05 |
| 37927 | 1.82441 | 0.05 |
| 37928 | 2.08757 | 0.05 |
| 37929 | 1.81292 | 0.05 |

| | | |
|---|---|---|
| 37930 | 1.60249 | 0.05 |
| 37931 | 3.63733 | 0.05 |
| 37932 | 1.81292 | 0.05 |
| 37933 | 1.332 | 0.05 |
| 37934 | 1.60249 | 0.05 |
| 37935 | 1.60249 | 0.05 |
| 37936 | 1.5344 | 0.05 |
| 37937 | 1.5344 | 0.05 |
| 37938 | 1.10107 | 2.1 |
| 37939 | 2.51559 | 2.1 |
| 37940 | 1.1229 | 2.1 |
| 37941 | 1.1229 | 2.1 |
| 37942 | 2.49456 | 2.1 |
| 37943 | 2.49456 | 2.1 |
| 37944 | 1.89226 | 2.1 |
| 37945 | 2.51559 | 2.1 |
| 37946 | 2.49457 | 2.1 |
| 37947 | 2.51559 | 2.1 |
| 37948 | 1.87124 | 2.1 |
| 37949 | 1.89226 | 2.1 |
| 37950 | 1.89226 | 2.1 |
| 37951 | 1.89226 | 2.1 |
| 37952 | 1.89226 | 2.1 |
| 37953 | 1.89226 | 2.1 |
| 37954 | 1.89226 | 2.1 |
| 37955 | 1.89226 | 2.1 |
| 37956 | 1.89226 | 2.1 |
| 37957 | 1.89226 | 2.1 |
| 37958 | 1.89226 | 2.1 |
| 37959 | 1.89226 | 2.1 |
| 37960 | 1.89226 | 2.1 |
| 37961 | 1.89226 | 2.1 |
| 37962 | 1.23599 | 2.1 |
| 37963 | 1.24728 | 2.1 |
| 37964 | 0.550534 | 2.1 |
| 37965 | 0.541151 | 2.1 |
| 37966 | 0.893754 | 2.1 |
| 37967 | 0.893754 | 2.1 |
| 37968 | 0.894678 | 2.1 |
| 37969 | 0.894678 | 2.1 |
| 37970 | 1.23599 | 2.1 |
| 37971 | 0.541149 | 2.1 |
| 37972 | 0.550533 | 2.1 |
| 37973 | 1.24728 | 2.1 |
| 37974 | 0.946132 | 2.1 |
| 37975 | 0.946132 | 2.1 |
| 37976 | 1.23599 | 2.1 |
| 37977 | 1.24728 | 2.1 |
| 37978 | 0.946132 | 2.1 |
| 37979 | 0.946132 | 2.1 |
| 37980 | 1.23599 | 2.1 |
| 37981 | 1.23599 | 2.1 |
| 37982 | 1.23599 | 2.1 |
| 37983 | 0.946132 | 2.1 |
| 37984 | 0.946132 | 2.1 |
| 37985 | 1.24728 | 2.1 |
| 37986 | 1.89226 | 2.1 |
| 37987 | 1.89226 | 2.1 |

| | | |
|---|---|---|
| 37988 | 1.89226 | 2.1 |
| 37989 | 1.89226 | 2.1 |
| 37990 | 1.89226 | 2.1 |
| 37991 | 1.89226 | 2.1 |
| 37992 | 0.946128 | 2.1 |
| 37993 | 0.946128 | 2.1 |
| 37994 | 0.946132 | 2.1 |
| 37995 | 0.946132 | 2.1 |
| 37996 | 0.94613 | 2.1 |
| 37997 | 0.94613 | 2.1 |
| 37998 | 0.946129 | 2.1 |
| 37999 | 0.946129 | 2.1 |
| 38000 | 0.94613 | 2.1 |
| 38001 | 0.94613 | 2.1 |
| 38002 | 0.94613 | 2.1 |
| 38003 | 0.94613 | 2.1 |
| 38004 | 2.58696 | 0.0518195 |
| 38005 | 2.57073 | 0.193965 |
| 38006 | 2.12585 | 0.0772131 |
| 38007 | 2.07607 | 0.281728 |
| 38008 | 4.69658 | 0.184405 |
| 38009 | 1.89226 | 0.278438 |
| 38010 | 1.89226 | 0.535655 |
| 38011 | 1.89226 | 0.193382 |
| 38012 | 1.89226 | 0.558494 |
| 38013 | 3.78452 | 0.243087 |
| 38014 | 1.89226 | 0.539189 |
| 38015 | 1.89226 | 0.619187 |
| 38016 | 1.89226 | 0.566145 |
| 38017 | 1.89226 | 0.599247 |
| 38018 | 3.78453 | 0.595295 |
| 38019 | 2.57074 | 0.273861 |
| 38020 | 2.32635 | 0.485272 |
| 38021 | 2.07608 | 0.331693 |
| 38022 | 1.73623 | 0.389106 |
| 38023 | 4.30697 | 0.358552 |
| 38024 | 2.12582 | 0.05 |
| 38025 | 1.51971 | 0.0619186 |
| 38026 | 1.57255 | 0.05 |
| 38027 | 2.07604 | 0.0750276 |
| 38028 | 1.62233 | 0.05 |
| 38029 | 2.12582 | 0.0708045 |
| 38030 | 1.89223 | 0.108425 |
| 38031 | 1.89223 | 0.377136 |
| 38032 | 1.89223 | 0.345521 |
| 38033 | 1.89223 | 0.168371 |
| 38034 | 1.89223 | 0.187747 |
| 38035 | 1.89223 | 0.0956561 |
| 38036 | 0.946119 | 0.5672 |
| 38037 | 0.946119 | 0.531496 |
| 38038 | 0.946119 | 0.175657 |
| 38039 | 0.946119 | 0.564671 |
| 38040 | 0.946119 | 0.572356 |
| 38041 | 0.946119 | 0.571058 |
| 38042 | 0.94612 | 0.54393 |
| 38043 | 0.94612 | 0.482503 |
| 38044 | 0.946132 | 0.545472 |
| 38045 | 0.946132 | 0.293843 |

| | | |
|---|---|---|
| 38046 | 0.946107 | 0.552273 |
| 38047 | 0.946107 | 0.545958 |
| 38048 | 0.811456 | 0.0941386 |
| 38049 | 0.811456 | 0.443212 |
| 38050 | 0.806521 | 0.0621634 |
| 38051 | 0.806521 | 0.4107 |
| 38052 | 0.868104 | 0.307286 |
| 38053 | 0.579949 | 0.133457 |
| 38054 | 0.759859 | 0.0720565 |
| 38055 | 1.03802 | 0.0772569 |
| 38056 | 0.579957 | 0.103326 |
| 38057 | 0.759868 | 0.129258 |
| 38058 | 1.03801 | 0.211151 |
| 38059 | 0.868093 | 0.150583 |
| 38060 | 2.7408 | 2.1 |
| 38061 | 2.82858 | 2.1 |
| 38062 | 2.57368 | 2.1 |
| 38063 | 2.52633 | 2.1 |
| 38064 | 5.3549 | 2.1 |
| 38065 | 2.7408 | 2.1 |
| 38066 | 2.31353 | 2.1 |
| 38067 | 2.14063 | 2.1 |
| 38068 | 2.52632 | 2.1 |
| 38069 | 4.83985 | 2.1 |
| 38070 | 2.57368 | 2.1 |
| 38071 | 2.52633 | 2.1 |
| 38072 | 2.3301 | 2.1 |
| 38073 | 2.36071 | 2.1 |
| 38074 | 4.88704 | 2.1 |
| 38075 | 1.86512 | 2.1 |
| 38076 | 2.52632 | 2.1 |
| 38077 | 2.14063 | 2.1 |
| 38078 | 2.14063 | 2.1 |
| 38079 | 2.3301 | 2.1 |
| 38080 | 2.3301 | 2.1 |
| 38081 | 1.86431 | 2.1 |
| 38082 | 1.89227 | 2.1 |
| 38083 | 1.68992 | 2.1 |
| 38084 | 1.68992 | 2.1 |
| 38085 | 1.89227 | 2.1 |
| 38086 | 1.89227 | 2.1 |
| 38087 | 2.0815 | 2.1 |
| 38088 | 1.89226 | 2.1 |
| 38089 | 2.04022 | 2.1 |
| 38090 | 1.86431 | 2.1 |
| 38091 | 2.07081 | 2.1 |
| 38092 | 1.89226 | 2.1 |
| 38093 | 1.91686 | 1.39329 |
| 38094 | 1.65683 | 2.07714 |
| 38095 | 1.86871 | 2.1 |
| 38096 | 1.65683 | 1.25888 |
| 38097 | 2.13075 | 2.1 |
| 38098 | 1.89226 | 0.546127 |
| 38099 | 1.89226 | 2.1 |
| 38100 | 1.89226 | 0.277758 |
| 38101 | 1.89226 | 0.831974 |
| 38102 | 1.89226 | 1.62944 |
| 38103 | 3.78452 | 0.896048 |

| | | |
|---|---|---|
| 38104 | 0.851541 | 2.1 |
| 38105 | 0.851541 | 2.1 |
| 38106 | 0.952803 | 2.1 |
| 38107 | 0.851573 | 2.1 |
| 38108 | 0.946129 | 2.1 |
| 38109 | 0.946129 | 1.25623 |
| 38110 | 0.929184 | 2.1 |
| 38111 | 0.757482 | 2.1 |
| 38112 | 0.836274 | 2.1 |
| 38113 | 0.851528 | 2.1 |
| 38114 | 0.952816 | 2.1 |
| 38115 | 1.04073 | 2.1 |
| 38116 | 1.70303 | 1.1132 |
| 38117 | 1.70302 | 0.953087 |
| 38118 | 1.5544 | 2.1 |
| 38119 | 1.70302 | 0.930683 |
| 38120 | 1.74957 | 1.09542 |
| 38121 | 1.91686 | 0.590691 |
| 38122 | 1.89226 | 0.769298 |
| 38123 | 1.89226 | 0.969229 |
| 38124 | 1.89226 | 1.71966 |
| 38125 | 1.89226 | 0.824923 |
| 38126 | 1.89226 | 0.628116 |
| 38127 | 1.89226 | 0.123889 |
| 38128 | 1.89226 | 2.1 |
| 38129 | 1.89226 | 2.1 |
| 38130 | 2.03472 | 2.1 |
| 38131 | 1.89226 | 2.1 |
| 38132 | 1.99827 | 2.1 |
| 38133 | 1.85836 | 1.6182 |
| 38134 | 1.00509 | 2.1 |
| 38135 | 1.55998 | 2.1 |
| 38136 | 1.83347 | 2.1 |
| 38137 | 1.45246 | 2.1 |
| 38138 | 3.01244 | 2.1 |
| 38139 | 1.23839 | 2.1 |
| 38140 | 0.884251 | 2.1 |
| 38141 | 0.946133 | 2.1 |
| 38142 | 0.946133 | 2.1 |
| 38143 | 0.884226 | 2.1 |
| 38144 | 0.884226 | 2.1 |
| 38145 | 0.946158 | 2.1 |
| 38146 | 0.946159 | 2.1 |
| 38147 | 1.23839 | 2.1 |
| 38148 | 0.946131 | 2.1 |
| 38149 | 0.946131 | 2.1 |
| 38150 | 0.884254 | 2.1 |
| 38151 | 1.89226 | 2.1 |
| 38152 | 1.89226 | 2.1 |
| 38153 | 1.89226 | 2.1 |
| 38154 | 1.89226 | 2.1 |
| 38155 | 1.89226 | 2.1 |
| 38156 | 1.89226 | 2.1 |
| 38157 | 2.1499 | 2.1 |
| 38158 | 1.45244 | 2.1 |
| 38159 | 1.83345 | 2.1 |
| 38160 | 1.83345 | 2.1 |
| 38161 | 1.87214 | 2.1 |

| | | |
|---|---|---|
| 38162 | 1.87214 | 2.1 |
| 38163 | 1.89224 | 2.1 |
| 38164 | 1.89224 | 2.1 |
| 38165 | 1.89224 | 2.1 |
| 38166 | 1.89224 | 2.1 |
| 38167 | 3.78448 | 2.1 |
| 38168 | 1.49155 | 0.566618 |
| 38169 | 2.0815 | 1.06396 |
| 38170 | 1.77515 | 1.12185 |
| 38171 | 1.72377 | 2.1 |
| 38172 | 3.50659 | 1.03941 |
| 38173 | 1.69876 | 0.05 |
| 38174 | 2.0815 | 2.1 |
| 38175 | 1.9133 | 0.187928 |
| 38176 | 1.68189 | 0.05 |
| 38177 | 2.17628 | 2.03985 |
| 38178 | 1.94335 | 2.1 |
| 38179 | 2.65129 | 0.05 |
| 38180 | 1.15317 | 0.05 |
| 38181 | 2.57085 | 0.05 |
| 38182 | 1.209 0.05 | |
| 38183 | 2.57085 | 0.05 |
| 38184 | 1.209 0.05 | |
| 38185 | 1.89226 | 0.05 |
| 38186 | 1.89226 | 0.05 |
| 38187 | 1.89226 | 0.05 |
| 38188 | 1.89226 | 0.05 |
| 38189 | 3.78452 | 0.05 |
| 38190 | 2.48861 | 0.05 |
| 38191 | 1.15252 | 0.05 |
| 38192 | 2.60515 | 0.05 |
| 38193 | 2.60515 | 0.05 |
| 38194 | 1.16384 | 0.05 |
| 38195 | 1.16384 | 0.05 |
| 38196 | 1.59903 | 0.812386 |
| 38197 | 1.26865 | 0.487533 |
| 38198 | 1.30486 | 1.36522 |
| 38199 | 1.70302 | 0.951831 |
| 38200 | 2.98573 | 0.918398 |
| 38201 | 1.59903 | 0.812928 |
| 38202 | 1.79478 | 0.908549 |
| 38203 | 1.79478 | 0.850843 |
| 38204 | 1.97005 | 0.942353 |
| 38205 | 1.50365 | 0.719647 |
| 38206 | 1.64192 | 1.30012 |
| 38207 | 1.89226 | 0.668527 |
| 38208 | 1.89226 | 0.777362 |
| 38209 | 1.89226 | 0.929524 |
| 38210 | 1.89226 | 0.636012 |
| 38211 | 3.78453 | 0.604482 |
| 38212 | 1.89226 | 0.843844 |
| 38213 | 1.89226 | 0.740189 |
| 38214 | 1.89226 | 0.847021 |
| 38215 | 1.89226 | 0.667789 |
| 38216 | 1.89226 | 0.877935 |
| 38217 | 1.89226 | 1.03398 |
| 38218 | 0.761672 | 0.696343 |
| 38219 | 0.896406 | 0.792529 |

| | | |
|---|---|---|
| 38220 | 0.603509 | 0.741837 |
| 38221 | 0.574071 | 0.968051 |
| 38222 | 0.695159 | 0.997424 |
| 38223 | 0.695159 | 0.219623 |
| 38224 | 0.762564 | 1.42194 |
| 38225 | 0.770159 | 0.543423 |
| 38226 | 0.803021 | 0.949383 |
| 38227 | 0.572529 | 1.17919 |
| 38228 | 0.696466 | 0.543072 |
| 38229 | 0.899733 | 0.705186 |
| 38230 | 1.66034 | 0.05 |
| 38231 | 1.79478 | 0.94264 |
| 38232 | 1.56685 | 0.687319 |
| 38233 | 1.68512 | 1.50482 |
| 38234 | 1.67652 | 0.765029 |
| 38235 | 1.67652 | 0.9575 |
| 38236 | 1.89226 | 0.961197 |
| 38237 | 1.89226 | 0.687266 |
| 38238 | 1.89226 | 0.829306 |
| 38239 | 1.89226 | 0.861222 |
| 38240 | 1.89226 | 0.856577 |
| 38241 | 1.89226 | 0.89255 |
| 38242 | 0.946133 | 0.505276 |
| 38243 | 0.946133 | 0.775912 |
| 38244 | 0.946132 | 0.948788 |
| 38245 | 0.946132 | 0.773007 |
| 38246 | 0.946132 | 0.834844 |
| 38247 | 0.946132 | 0.553343 |
| 38248 | 0.946133 | 1.56656 |
| 38249 | 0.946133 | 0.711955 |
| 38250 | 0.946133 | 0.816706 |
| 38251 | 0.946133 | 0.822129 |
| 38252 | 0.946132 | 0.640929 |
| 38253 | 0.946132 | 0.624672 |
| 38254 | 1.80424 | 2.1 |
| 38255 | 1.45158 | 2.1 |
| 38256 | 1.9098 | 2.1 |
| 38257 | 1.56003 | 2.1 |
| 38258 | 3.36138 | 2.1 |
| 38259 | 1.90982 | 2.1 |
| 38260 | 1.71606 | 2.1 |
| 38261 | 2.15314 | 2.1 |
| 38262 | 1.70302 | 2.1 |
| 38263 | 3.89051 | 2.1 |
| 38264 | 1.96416 | 0.81414 |
| 38265 | 1.23706 | 0.909787 |
| 38266 | 1.00053 | 0.645028 |
| 38267 | 1.38287 | 0.578832 |
| 38268 | 1.57916 | 0.585295 |
| 38269 | 1.9615 | 0.748425 |
| 38270 | 0.827865 | 0.593816 |
| 38271 | 0.946132 | 0.541558 |
| 38272 | 0.946132 | 0.478115 |
| 38273 | 0.946132 | 0.603935 |
| 38274 | 0.946132 | 0.694359 |
| 38275 | 0.946132 | 0.604786 |
| 38276 | 0.946131 | 0.787981 |
| 38277 | 0.946131 | 0.584713 |

| | | |
|---|---|---|
| 38278 | 0.827865 | 0.832478 |
| 38279 | 0.946132 | 0.498866 |
| 38280 | 0.946132 | 0.458635 |
| 38281 | 0.946132 | 0.523751 |
| 38282 | 1.89227 | 2.1 |
| 38283 | 1.89227 | 2.1 |
| 38284 | 1.89227 | 2.1 |
| 38285 | 1.89227 | 2.1 |
| 38286 | 3.78453 | 2.1 |
| 38287 | 2.3819 | 2.1 |
| 38288 | 1.58089 | 2.1 |
| 38289 | 1.58089 | 2.1 |
| 38290 | 1.6287 | 2.1 |
| 38291 | 2.33368 | 2.1 |
| 38292 | 2.3815 | 2.1 |
| 38293 | 0.946133 | 2.1 |
| 38294 | 0.946133 | 2.1 |
| 38295 | 0.946132 | 2.1 |
| 38296 | 0.946132 | 2.1 |
| 38297 | 0.946132 | 2.1 |
| 38298 | 0.946132 | 2.1 |
| 38299 | 0.946132 | 2.1 |
| 38300 | 0.946132 | 2.1 |
| 38301 | 0.946133 | 2.1 |
| 38302 | 0.946133 | 2.1 |
| 38303 | 0.946132 | 2.1 |
| 38304 | 0.946132 | 2.1 |
| 38305 | 1.89226 | 2.1 |
| 38306 | 1.89226 | 2.1 |
| 38307 | 1.89226 | 2.1 |
| 38308 | 1.89226 | 2.1 |
| 38309 | 3.78453 | 2.1 |
| 38310 | 2.7874 | 2.1 |
| 38311 | 2.33368 | 2.1 |
| 38312 | 2.71897 | 2.1 |
| 38313 | 2.27495 | 2.1 |
| 38314 | 5.05266 | 2.1 |
| 38315 | 0.970991 | 2.1 |
| 38316 | 0.970991 | 2.1 |
| 38317 | 0.956929 | 2.1 |
| 38318 | 0.956929 | 2.1 |
| 38319 | 0.732928 | 2.1 |
| 38320 | 0.790444 | 2.1 |
| 38321 | 1.16684 | 2.1 |
| 38322 | 1.13748 | 2.1 |
| 38323 | 1.13748 | 2.1 |
| 38324 | 0.732928 | 2.1 |
| 38325 | 0.790444 | 2.1 |
| 38326 | 1.16684 | 2.1 |
| 38327 | 0.946132 | 2.1 |
| 38328 | 0.946132 | 2.1 |
| 38329 | 0.946131 | 2.1 |
| 38330 | 0.946131 | 2.1 |
| 38331 | 0.946132 | 2.1 |
| 38332 | 0.946132 | 2.1 |
| 38333 | 0.946132 | 2.1 |
| 38334 | 0.946132 | 2.1 |
| 38335 | 0.946132 | 2.1 |

| | | |
|---|---|---|
| 38336 | 0.946132 | 2.1 |
| 38337 | 0.946132 | 2.1 |
| 38338 | 0.946132 | 2.1 |
| 38339 | 1.10747 | 0.189412 |
| 38340 | 2.30331 | 0.21419 |
| 38341 | 1.47954 | 0.079658 |
| 38342 | 2.63789 | 0.0681666 |
| 38343 | 3.78285 | 0.168042 |
| 38344 | 1.65215 | 0.109031 |
| 38345 | 2.63789 | 0.0794169 |
| 38346 | 2.82465 | 0.22587 |
| 38347 | 2.82465 | 0.136361 |
| 38348 | 1.47954 | 0.106059 |
| 38349 | 1.47954 | 0.0670979 |
| 38350 | 1.13456 | 2.1 |
| 38351 | 1.08334 | 2.1 |
| 38352 | 1.20674 | 0.932071 |
| 38353 | 1.17885 | 2.1 |
| 38354 | 2.29008 | 2.0142 |
| 38355 | 1.20674 | 1.55266 |
| 38356 | 1.17885 | 2.1 |
| 38357 | 1.28649 | 1.06188 |
| 38358 | 1.28516 | 1.99439 |
| 38359 | 2.4919 | 1.7321 |
| 38360 | 1.89226 | 2.1 |
| 38361 | 1.89226 | 2.1 |
| 38362 | 1.89226 | 2.1 |
| 38363 | 1.89226 | 2.1 |
| 38364 | 1.89226 | 2.1 |
| 38365 | 1.89226 | 2.1 |
| 38366 | 2.50288 | 1.72645 |
| 38367 | 2.02115 | 2.1 |
| 38368 | 2.7717 | 1.72832 |
| 38369 | 2.29107 | 0.911358 |
| 38370 | 4.79395 | 2.1 |
| 38371 | 1.27339 | 2.1 |
| 38372 | 1.10817 | 2.1 |
| 38373 | 1.19733 | 2.1 |
| 38374 | 1.032 | 2.1 |
| 38375 | 2.30539 | 2.1 |
| 38376 | 1.19733 | 2.1 |
| 38377 | 0.953637 | 2.1 |
| 38378 | 0.953637 | 2.1 |
| 38379 | 1.032 | 2.1 |
| 38380 | 1.11868 | 2.1 |
| 38381 | 1.19704 | 2.1 |
| 38382 | 2.01024 | 1.00188 |
| 38383 | 1.11938 | 1.13812 |
| 38384 | 1.9186 | 1.46853 |
| 38385 | 2.76298 | 0.978363 |
| 38386 | 3.92884 | 1.18484 |
| 38387 | 0.851541 | 0.05 |
| 38388 | 0.851541 | 0.05 |
| 38389 | 0.957577 | 0.05 |
| 38390 | 0.848952 | 0.05 |
| 38391 | 1.08043 | 0.05 |
| 38392 | 0.851541 | 0.05 |
| 38393 | 0.851484 | 0.05 |

| | | |
|---|---|---|
| 38394 | 0.957578 | 0.05 |
| 38395 | 1.20045 | 0.05 |
| 38396 | 0.726289 | 0.05 |
| 38397 | 0.946131 | 0.05 |
| 38398 | 0.946131 | 0.05 |
| 38399 | 1.50524 | 2.1 |
| 38400 | 2.38363 | 2.1 |
| 38401 | 2.49018 | 2.1 |
| 38402 | 2.49018 | 2.1 |
| 38403 | 1.45506 | 2.1 |
| 38404 | 1.45506 | 2.1 |
| 38405 | 2.49017 | 2.1 |
| 38406 | 1.89226 | 2.1 |
| 38407 | 1.89226 | 2.1 |
| 38408 | 1.89226 | 2.1 |
| 38409 | 2.38363 | 2.1 |
| 38410 | 2.38363 | 2.1 |
| 38411 | 1.54711 | 2.1 |
| 38412 | 2.49018 | 2.1 |
| 38413 | 2.56975 | 2.1 |
| 38414 | 2.56975 | 2.1 |
| 38415 | 1.50524 | 2.1 |
| 38416 | 1.50524 | 2.1 |
| 38417 | 2.56975 | 2.1 |
| 38418 | 1.89226 | 2.1 |
| 38419 | 1.89226 | 2.1 |
| 38420 | 1.89226 | 2.1 |
| 38421 | 2.49017 | 2.1 |
| 38422 | 2.49017 | 2.1 |
| 38423 | 1.89226 | 0.05 |
| 38424 | 1.89226 | 0.05 |
| 38425 | 1.89226 | 0.05 |
| 38426 | 1.89226 | 0.05 |
| 38427 | 1.89226 | 0.05 |
| 38428 | 1.89226 | 0.05 |
| 38429 | 1.89226 | 0.05 |
| 38430 | 1.89226 | 0.05 |
| 38431 | 1.89226 | 0.05 |
| 38432 | 1.89226 | 0.05 |
| 38433 | 1.89226 | 0.05 |
| 38434 | 1.89226 | 0.05 |
| 38435 | 1.89226 | 0.05 |
| 38436 | 1.89226 | 0.05 |
| 38437 | 2.8358 | 0.05 |
| 38438 | 1.89226 | 0.05 |
| 38439 | 3.78453 | 0.05 |
| 38440 | 1.60565 | 0.05 |
| 38441 | 1.89226 | 0.955891 |
| 38442 | 1.89226 | 0.292345 |
| 38443 | 1.40013 | 0.05 |
| 38444 | 1.89226 | 0.502321 |
| 38445 | 1.40013 | 0.0822925 |
| 38446 | 1.1766 | 2.1 |
| 38447 | 1.29435 | 2.1 |
| 38448 | 1.35735 | 2.1 |
| 38449 | 1.53509 | 2.1 |
| 38450 | 2.65169 | 2.1 |
| 38451 | 0.69701 | 2.1 |

| | | |
|---|---|---|
| 38452 | 0.69701 | 2.1 |
| 38453 | 0.711303 | 2.1 |
| 38454 | 0.711303 | 2.1 |
| 38455 | 0.761138 | 2.1 |
| 38456 | 0.612149 | 2.1 |
| 38457 | 0.647174 | 2.1 |
| 38458 | 0.767548 | 2.1 |
| 38459 | 0.767548 | 2.1 |
| 38460 | 0.761139 | 2.1 |
| 38461 | 0.612149 | 2.1 |
| 38462 | 0.647174 | 2.1 |
| 38463 | 1.29764 | 0.967116 |
| 38464 | 1.29579 | 1.61724 |
| 38465 | 1.34875 | 1.42906 |
| 38466 | 1.06717 | 1.64205 |
| 38467 | 1.29764 | 1.2892 |
| 38468 | 1.01606 | 1.02392 |
| 38469 | 0.756635 | 1.11448 |
| 38470 | 0.648821 | 1.17625 |
| 38471 | 0.675557 | 1.48498 |
| 38472 | 0.674374 | 1.35994 |
| 38473 | 0.74214 | 1.53914 |
| 38474 | 0.74214 | 1.36773 |
| 38475 | 0.688868 | 0.893985 |
| 38476 | 0.688868 | 1.30651 |
| 38477 | 0.675557 | 0.62252 |
| 38478 | 0.756634 | 0.970934 |
| 38479 | 0.648821 | 0.283143 |
| 38480 | 0.674374 | 0.802438 |
| 38481 | 1.51327 | 1.424 |
| 38482 | 1.48105 | 0.786252 |
| 38483 | 1.29764 | 1.45249 |
| 38484 | 1.29764 | 1.20902 |
| 38485 | 1.48105 | 1.41357 |
| 38486 | 1.05655 | 0.626847 |
| 38487 | 1.13537 | 2.1 |
| 38488 | 1.07326 | 2.1 |
| 38489 | 0.94613 | 2.1 |
| 38490 | 0.94613 | 2.1 |
| 38491 | 0.946132 | 2.1 |
| 38492 | 0.946132 | 2.1 |
| 38493 | 1.13537 | 2.1 |
| 38494 | 1.08927 | 2.1 |
| 38495 | 1.13537 | 2.1 |
| 38496 | 0.946131 | 2.1 |
| 38497 | 0.946131 | 2.1 |
| 38498 | 0.95947 | 2.1 |
| 38499 | 1.46892 | 2.1 |
| 38500 | 1.89226 | 2.1 |
| 38501 | 1.89226 | 2.1 |
| 38502 | 1.89226 | 2.1 |
| 38503 | 1.88031 | 2.1 |
| 38504 | 1.88031 | 2.1 |
| 38505 | 1.89226 | 2.1 |
| 38506 | 1.89226 | 2.1 |
| 38507 | 1.89226 | 2.1 |
| 38508 | 1.89226 | 2.1 |
| 38509 | 3.78453 | 2.1 |

| | | |
|---|---|---|
| 38510 | 2.18254 | 2.1 |
| 38511 | 1.89226 | 2.1 |
| 38512 | 2.50005 | 2.1 |
| 38513 | 1.89226 | 2.1 |
| 38514 | 4.39231 | 2.1 |
| 38515 | 1.88031 | 2.1 |
| 38516 | 2.0815 | 2.1 |
| 38517 | 2.0815 | 2.1 |
| 38518 | 1.92078 | 2.1 |
| 38519 | 2.0815 | 2.1 |
| 38520 | 1.9218 | 2.1 |
| 38521 | 1.89227 | 2.1 |
| 38522 | 1.89227 | 2.1 |
| 38523 | 1.89227 | 2.1 |
| 38524 | 1.89227 | 2.1 |
| 38525 | 1.89227 | 2.1 |
| 38526 | 1.89227 | 2.1 |
| 38527 | 1.98693 | 2.1 |
| 38528 | 1.82857 | 2.1 |
| 38529 | 2.32 | 2.1 |
| 38530 | 2.36972 | 2.1 |
| 38531 | 4.14857 | 2.1 |
| 38532 | 1.82857 | 2.1 |
| 38533 | 1.58688 | 2.1 |
| 38534 | 2.36971 | 2.1 |
| 38535 | 2.19093 | 2.1 |
| 38536 | 4.0195 | 2.1 |
| 38537 | 0.946132 | 2.1 |
| 38538 | 0.946132 | 2.1 |
| 38539 | 1.18486 | 2.1 |
| 38540 | 1.18486 | 2.1 |
| 38541 | 1.18486 | 2.1 |
| 38542 | 0.946132 | 2.1 |
| 38543 | 0.946132 | 2.1 |
| 38544 | 1.16 | 2.1 |
| 38545 | 0.946133 | 2.1 |
| 38546 | 0.946133 | 2.1 |
| 38547 | 1.16 | 2.1 |
| 38548 | 1.18486 | 2.1 |
| 38549 | 1.07798 | 1.59019 |
| 38550 | 1.08101 | 1.25979 |
| 38551 | 0.910786 | 2.1 |
| 38552 | 0.910786 | 2.1 |
| 38553 | 0.851537 | 2.1 |
| 38554 | 0.901377 | 1.27782 |
| 38555 | 1.14652 | 2.1 |
| 38556 | 1.04072 | 2.1 |
| 38557 | 0.946129 | 2.1 |
| 38558 | 0.946129 | 2.1 |
| 38559 | 1.0019 | 2.0676 |
| 38560 | 1.04263 | 2.1 |
| 38561 | 1.89226 | 2.1 |
| 38562 | 1.89226 | 2.1 |
| 38563 | 1.89226 | 2.1 |
| 38564 | 1.89226 | 2.1 |
| 38565 | 3.78452 | 2.1 |
| 38566 | 1.89226 | 2.1 |
| 38567 | 2.30146 | 2.1 |

| | | |
|---|---|---|
| 38568 | 1.89226 | 2.1 |
| 38569 | 2.18412 | 2.1 |
| 38570 | 4.19372 | 2.1 |
| 38571 | 2.30146 | 2.1 |
| 38572 | 1.39962 | 2.1 |
| 38573 | 2.18411 | 2.1 |
| 38574 | 1.28765 | 2.1 |
| 38575 | 3.5891 | 2.1 |
| 38576 | 1.89226 | 2.1 |
| 38577 | 1.89226 | 2.1 |
| 38578 | 1.89226 | 2.1 |
| 38579 | 1.89226 | 2.1 |
| 38580 | 1.89226 | 2.1 |
| 38581 | 1.89226 | 2.1 |
| 38582 | 1.89226 | 2.1 |
| 38583 | 1.89226 | 2.1 |
| 38584 | 1.89226 | 2.1 |
| 38585 | 1.89226 | 2.1 |
| 38586 | 3.78452 | 2.1 |
| 38587 | 2.18412 | 2.1 |
| 38588 | 1.20045 | 2.1 |
| 38589 | 1.20045 | 2.1 |
| 38590 | 1.28765 | 2.1 |
| 38591 | 2.08808 | 2.1 |
| 38592 | 2.17528 | 2.1 |
| 38593 | 0.946131 | 2.1 |
| 38594 | 0.946131 | 2.1 |
| 38595 | 0.946128 | 2.1 |
| 38596 | 0.946128 | 2.1 |
| 38597 | 0.946127 | 2.1 |
| 38598 | 0.946127 | 2.1 |
| 38599 | 0.946131 | 2.1 |
| 38600 | 0.946131 | 2.1 |
| 38601 | 0.946129 | 2.1 |
| 38602 | 0.946129 | 2.1 |
| 38603 | 0.94613 | 2.1 |
| 38604 | 0.94613 | 2.1 |
| 38605 | 1.89226 | 0.439944 |
| 38606 | 1.89226 | 1.38539 |
| 38607 | 1.89226 | 1.43848 |
| 38608 | 1.89226 | 0.56898 |
| 38609 | 3.78452 | 0.295416 |
| 38610 | 1.89227 | 0.125085 |
| 38611 | 1.89227 | 1.84815 |
| 38612 | 1.89227 | 0.05 |
| 38613 | 1.89227 | 1.26565 |
| 38614 | 1.89227 | 0.126122 |
| 38615 | 1.89227 | 1.60359 |
| 38616 | 1.89226 | 1.74088 |
| 38617 | 1.89226 | 0.754829 |
| 38618 | 1.89226 | 0.64558 |
| 38619 | 2.29245 | 0.763736 |
| 38620 | 3.78452 | 1.08307 |
| 38621 | 1.89226 | 1.65939 |
| 38622 | 1.89226 | 0.3559 |
| 38623 | 1.89226 | 0.233356 |
| 38624 | 1.89226 | 0.983432 |
| 38625 | 1.89226 | 0.05 |

| | | |
|---|---|---|
| 38626 | 1.89226 | 0.160387 |
| 38627 | 1.28095 | 2.1 |
| 38628 | 1.81846 | 2.1 |
| 38629 | 2.10991 | 1.05431 |
| 38630 | 1.34695 | 2.01648 |
| 38631 | 3.39086 | 2.08821 |
| 38632 | 2.10991 | 1.2932 |
| 38633 | 1.36457 | 2.06721 |
| 38634 | 1.67421 | 2.1 |
| 38635 | 1.34695 | 2.1 |
| 38636 | 2.43717 | 1.69167 |
| 38637 | 2.10991 | 1.91936 |
| 38638 | 2.69541 | 2.1 |
| 38639 | 1.48572 | 2.1 |
| 38640 | 2.63553 | 2.1 |
| 38641 | 1.45385 | 2.1 |
| 38642 | 4.14926 | 2.1 |
| 38643 | 2.63553 | 2.1 |
| 38644 | 1.44207 | 2.1 |
| 38645 | 1.44207 | 2.1 |
| 38646 | 1.45385 | 2.1 |
| 38647 | 2.588 2.1 | |
| 38648 | 2.59978 | 2.1 |
| 38649 | 1.89227 | 2.1 |
| 38650 | 1.89227 | 2.1 |
| 38651 | 1.89227 | 2.1 |
| 38652 | 1.89227 | 2.1 |
| 38653 | 3.78453 | 2.1 |
| 38654 | 1.89226 | 2.1 |
| 38655 | 1.89226 | 2.1 |
| 38656 | 1.89226 | 2.1 |
| 38657 | 1.89226 | 2.1 |
| 38658 | 3.78453 | 2.1 |
| 38659 | 2.15929 | 2.1 |
| 38660 | 1.38276 | 2.1 |
| 38661 | 1.43617 | 2.1 |
| 38662 | 1.35614 | 2.1 |
| 38663 | 2.23932 | 2.1 |
| 38664 | 2.15929 | 2.1 |
| 38665 | 2.15929 | 2.1 |
| 38666 | 2.25168 | 2.1 |
| 38667 | 2.3558 | 2.1 |
| 38668 | 2.23932 | 2.1 |
| 38669 | 4.51509 | 2.1 |
| 38670 | 1.89226 | 2.1 |
| 38671 | 1.89226 | 2.1 |
| 38672 | 1.89226 | 2.1 |
| 38673 | 1.89226 | 2.1 |
| 38674 | 3.78453 | 2.1 |
| 38675 | 1.89226 | 2.1 |
| 38676 | 1.89226 | 2.1 |
| 38677 | 1.89226 | 2.1 |
| 38678 | 1.89226 | 2.1 |
| 38679 | 1.89226 | 2.1 |
| 38680 | 1.89226 | 2.1 |
| 38681 | 2.23932 | 2.1 |
| 38682 | 1.43854 | 2.1 |
| 38683 | 1.49173 | 2.1 |

| | | |
|---|---|---|
| 38684 | 1.38276 | 2.1 |
| 38685 | 2.34829 | 2.1 |
| 38686 | 2.23932 | 2.1 |
| 38687 | 2.3558 | 2.1 |
| 38688 | 2.34829 | 2.1 |
| 38689 | 2.40685 | 2.1 |
| 38690 | 2.23932 | 2.1 |
| 38691 | 2.52332 | 2.1 |
| 38692 | 2.3558 | 2.1 |
| 38693 | 1.89226 | 0.05 |
| 38694 | 1.87361 | 0.05 |
| 38695 | 2.25082 | 0.05 |
| 38696 | 1.87361 | 0.05 |
| 38697 | 2.26947 | 0.05 |
| 38698 | 1.89226 | 0.05 |
| 38699 | 1.89226 | 0.05 |
| 38700 | 1.89226 | 0.05 |
| 38701 | 1.89226 | 0.05 |
| 38702 | 1.89226 | 0.05 |
| 38703 | 1.89226 | 0.05 |
| 38704 | 1.89226 | 0.05 |
| 38705 | 2.41314 | 0.05 |
| 38706 | 1.89226 | 0.05 |
| 38707 | 1.89226 | 0.05 |
| 38708 | 1.89226 | 0.05 |
| 38709 | 2.12204 | 0.05 |
| 38710 | 2.12204 | 0.05 |
| 38711 | 1.89226 | 0.05 |
| 38712 | 2.25082 | 0.05 |
| 38713 | 2.28189 | 0.05 |
| 38714 | 2.25082 | 0.05 |
| 38715 | 1.92333 | 0.05 |
| 38716 | 1.89226 | 0.05 |
| 38717 | 1.89226 | 0.05 |
| 38718 | 1.89226 | 0.05 |
| 38719 | 1.89226 | 0.05 |
| 38720 | 1.89226 | 0.05 |
| 38721 | 1.89226 | 0.05 |
| 38722 | 1.89226 | 0.05 |
| 38723 | 2.42521 | 0.05 |
| 38724 | 1.89226 | 0.05 |
| 38725 | 1.89226 | 0.05 |
| 38726 | 1.89226 | 0.05 |
| 38727 | 2.41314 | 0.05 |
| 38728 | 2.41314 | 0.05 |
| 38729 | 1.39725 | 1.06142 |
| 38730 | 1.07745 | 2.1 |
| 38731 | 1.07745 | 1.11756 |
| 38732 | 1.07519 | 2.1 |
| 38733 | 1.41336 | 0.274391 |
| 38734 | 1.41111 | 2.1 |
| 38735 | 1.89226 | 2.1 |
| 38736 | 1.92039 | 2.1 |
| 38737 | 1.92039 | 2.1 |
| 38738 | 1.9052 | 2.1 |
| 38739 | 1.89226 | 2.1 |
| 38740 | 1.87708 | 2.1 |
| 38741 | 0.946128 | 2.1 |

| | | |
|---|---|---|
| 38742 | 0.946128 | 2.1 |
| 38743 | 0.946133 | 0.98844 |
| 38744 | 0.946133 | 0.407155 |
| 38745 | 0.94613 | 0.29988 |
| 38746 | 0.94613 | 2.1 |
| 38747 | 0.946131 | 2.1 |
| 38748 | 0.946131 | 0.178321 |
| 38749 | 0.946131 | 1.48577 |
| 38750 | 0.946131 | 1.70337 |
| 38751 | 0.946131 | 1.38945 |
| 38752 | 0.946131 | 1.76045 |
| 38753 | 1.41336 | 0.67204 |
| 38754 | 1.08911 | 2.1 |
| 38755 | 1.08911 | 1.3877 |
| 38756 | 1.07745 | 2.1 |
| 38757 | 1.42381 | 0.899857 |
| 38758 | 1.41215 | 2.1 |
| 38759 | 1.89227 | 2.1 |
| 38760 | 1.95833 | 2.1 |
| 38761 | 1.95833 | 2.1 |
| 38762 | 1.92039 | 2.1 |
| 38763 | 1.89227 | 2.1 |
| 38764 | 1.85433 | 2.1 |
| 38765 | 0.946129 | 2.1 |
| 38766 | 0.946129 | 2.1 |
| 38767 | 0.946133 | 1.46535 |
| 38768 | 0.946133 | 0.371466 |
| 38769 | 0.946131 | 0.28145 |
| 38770 | 0.946131 | 2.1 |
| 38771 | 0.946131 | 2.1 |
| 38772 | 0.946131 | 0.454465 |
| 38773 | 0.946131 | 2.0107 |
| 38774 | 0.946131 | 2.07329 |
| 38775 | 0.946131 | 2.00624 |
| 38776 | 0.946131 | 2.09139 |
| 38777 | 1.41943 | 2.1 |
| 38778 | 2.74398 | 2.1 |
| 38779 | 1.18238 | 2.1 |
| 38780 | 2.53897 | 2.1 |
| 38781 | 3.9584 | 2.1 |
| 38782 | 2.74398 | 2.1 |
| 38783 | 1.89226 | 2.1 |
| 38784 | 1.89226 | 2.1 |
| 38785 | 1.89226 | 2.1 |
| 38786 | 2.53897 | 2.1 |
| 38787 | 2.53897 | 2.1 |
| 38788 | 1.89227 | 1.04624 |
| 38789 | 1.89227 | 0.540897 |
| 38790 | 1.89227 | 1.01791 |
| 38791 | 1.89227 | 0.391939 |
| 38792 | 3.78453 | 0.60237 |
| 38793 | 1.89226 | 0.598778 |
| 38794 | 2.08134 | 0.271115 |
| 38795 | 2.00632 | 0.157863 |
| 38796 | 2.00632 | 0.184583 |
| 38797 | 1.89226 | 0.512329 |
| 38798 | 1.89226 | 0.605496 |
| 38799 | 1.89227 | 0.835738 |

| | | |
|---|---|---|
| 38800 | 1.89227 | 0.352348 |
| 38801 | 1.89227 | 0.661606 |
| 38802 | 1.89227 | 0.150679 |
| 38803 | 3.78453 | 0.686055 |
| 38804 | 1.89227 | 0.972613 |
| 38805 | 1.89227 | 0.128388 |
| 38806 | 1.89227 | 0.119271 |
| 38807 | 1.89227 | 0.378043 |
| 38808 | 1.89227 | 0.600769 |
| 38809 | 1.89227 | 0.999627 |
| 38810 | 2.50912 | 0.167871 |
| 38811 | 1.89226 | 0.423305 |
| 38812 | 1.89226 | 0.05 |
| 38813 | 2.31261 | 0.330994 |
| 38814 | 2.31261 | 0.219334 |
| 38815 | 1.89226 | 0.694929 |
| 38816 | 2.50912 | 0.206943 |
| 38817 | 1.19999 | 0.0630912 |
| 38818 | 1.44056 | 0.05 |
| 38819 | 1.24405 | 0.0926487 |
| 38820 | 2.50912 | 0.150051 |
| 38821 | 2.31261 | 0.293203 |
| 38822 | 2.69224 | 0.797744 |
| 38823 | 2.28373 | 0.660518 |
| 38824 | 1.94788 | 1.63242 |
| 38825 | 2.4106 | 0.66823 |
| 38826 | 4.69434 | 0.964511 |
| 38827 | 2.69223 | 0.05 |
| 38828 | 2.63783 | 0.776209 |
| 38829 | 2.34737 | 1.6464 |
| 38830 | 2.4106 | 0.734554 |
| 38831 | 5.04843 | 0.838218 |
| 38832 | 1.89226 | 2.1 |
| 38833 | 1.99753 | 2.1 |
| 38834 | 1.87366 | 2.1 |
| 38835 | 1.99753 | 2.1 |
| 38836 | 1.76839 | 2.1 |
| 38837 | 1.89226 | 2.1 |
| 38838 | 1.89226 | 2.1 |
| 38839 | 1.89226 | 0.972815 |
| 38840 | 1.89226 | 2.1 |
| 38841 | 1.89226 | 2.1 |
| 38842 | 3.78452 | 2.1 |
| 38843 | 0.946132 | 2.1 |
| 38844 | 0.946132 | 2.1 |
| 38845 | 1.02744 | 2.1 |
| 38846 | 1.02744 | 2.1 |
| 38847 | 0.946132 | 2.1 |
| 38848 | 0.946132 | 2.1 |
| 38849 | 1.00989 | 2.1 |
| 38850 | 1.02744 | 2.1 |
| 38851 | 1.00989 | 2.1 |
| 38852 | 0.946133 | 2.1 |
| 38853 | 1.02744 | 2.1 |
| 38854 | 0.946132 | 2.1 |
| 38855 | 1.70302 | 2.1 |
| 38856 | 1.87366 | 2.1 |
| 38857 | 1.71618 | 2.1 |

| | | |
|---|---|---|
| 38858 | 1.87366 | 2.1 |
| 38859 | 1.73105 | 2.1 |
| 38860 | 1.89226 | 2.1 |
| 38861 | 1.89226 | 2.1 |
| 38862 | 1.89226 | 2.1 |
| 38863 | 1.89226 | 2.1 |
| 38864 | 1.89226 | 2.1 |
| 38865 | 1.89226 | 2.1 |
| 38866 | 1.89226 | 0.926019 |
| 38867 | 1.96624 | 2.1 |
| 38868 | 1.89226 | 2.1 |
| 38869 | 1.89226 | 2.1 |
| 38870 | 1.89226 | 2.1 |
| 38871 | 2.01977 | 2.1 |
| 38872 | 2.01977 | 2.1 |
| 38873 | 1.1032 | 1.59319 |
| 38874 | 1.15114 | 2.1 |
| 38875 | 1.14986 | 0.05 |
| 38876 | 1.19242 | 2.1 |
| 38877 | 2.301 | 2.1 |
| 38878 | 1.89226 | 1.39373 |
| 38879 | 1.89226 | 2.1 |
| 38880 | 1.89226 | 0.05 |
| 38881 | 1.89226 | 2.1 |
| 38882 | 3.78453 | 1.16091 |
| 38883 | 0.946132 | 2.1 |
| 38884 | 0.946132 | 2.1 |
| 38885 | 0.946132 | 2.07876 |
| 38886 | 0.946132 | 2.1 |
| 38887 | 0.946133 | 2.1 |
| 38888 | 0.946133 | 2.1 |
| 38889 | 0.946132 | 2.1 |
| 38890 | 0.946132 | 2.1 |
| 38891 | 0.946132 | 2.1 |
| 38892 | 0.946132 | 2.1 |
| 38893 | 0.946133 | 2.1 |
| 38894 | 0.946133 | 2.1 |
| 38895 | 1.19243 | 2.1 |
| 38896 | 0.953928 | 2.1 |
| 38897 | 1.18671 | 2.1 |
| 38898 | 1.15114 | 2.1 |
| 38899 | 0.9895 | 2.1 |
| 38900 | 0.953928 | 2.1 |
| 38901 | 1.19611 | 0.05 |
| 38902 | 1.19242 | 2.1 |
| 38903 | 1.24693 | 0.05 |
| 38904 | 1.24693 | 2.1 |
| 38905 | 1.14986 | 0.129348 |
| 38906 | 1.14986 | 2.1 |
| 38907 | 1.89225 | 0.05 |
| 38908 | 1.89225 | 2.1 |
| 38909 | 1.89225 | 2.1 |
| 38910 | 1.89225 | 0.955218 |
| 38911 | 1.89225 | 2.1 |
| 38912 | 1.89225 | 1.07281 |
| 38913 | 1.89225 | 2.1 |
| 38914 | 1.89225 | 2.1 |
| 38915 | 1.89225 | 2.1 |

| | | |
|---|---|---|
| 38916 | 1.89225 | 2.1 |
| 38917 | 1.89225 | 0.57317 |
| 38918 | 1.89225 | 2.1 |
| 38919 | 0.561532 | 2.1 |
| 38920 | 0.561532 | 2.1 |
| 38921 | 0.566382 | 2.1 |
| 38922 | 0.566382 | 2.1 |
| 38923 | 0.494747 | 2.1 |
| 38924 | 0.531705 | 2.1 |
| 38925 | 0.596208 | 2.1 |
| 38926 | 0.623464 | 1.38449 |
| 38927 | 0.623469 | 2.1 |
| 38928 | 0.531708 | 2.1 |
| 38929 | 0.596205 | 2.1 |
| 38930 | 0.494743 | 2.1 |
| 38931 | 1.25742 | 2.1 |
| 38932 | 1.18783 | 2.1 |
| 38933 | 0.946131 | 2.1 |
| 38934 | 0.946131 | 2.1 |
| 38935 | 1.25742 | 2.1 |
| 38936 | 1.25742 | 2.1 |
| 38937 | 0.946131 | 2.1 |
| 38938 | 0.946131 | 2.1 |
| 38939 | 1.25742 | 2.1 |
| 38940 | 0.946131 | 2.1 |
| 38941 | 0.946131 | 2.1 |
| 38942 | 1.18783 | 2.1 |
| 38943 | 1.35048 | 2.1 |
| 38944 | 1.25742 | 2.1 |
| 38945 | 0.946132 | 2.1 |
| 38946 | 0.946132 | 2.1 |
| 38947 | 1.35048 | 2.1 |
| 38948 | 1.35048 | 2.1 |
| 38949 | 0.946132 | 2.1 |
| 38950 | 0.946132 | 2.1 |
| 38951 | 1.35044 | 2.1 |
| 38952 | 0.946106 | 2.1 |
| 38953 | 1.25745 | 2.1 |
| 38954 | 0.946158 | 2.1 |
| 38955 | 1.13525 | 2.1 |
| 38956 | 2.70095 | 2.1 |
| 38957 | 2.51483 | 2.1 |
| 38958 | 2.51483 | 2.1 |
| 38959 | 1.21593 | 2.1 |
| 38960 | 1.21593 | 2.1 |
| 38961 | 1.93142 | 0.0903541 |
| 38962 | 2.8053 | 0.05 |
| 38963 | 1.559 | 0.50125 |
| 38964 | 2.60359 | 0.05 |
| 38965 | 4.53501 | 0.0584388 |
| 38966 | 2.80531 | 0.05 |
| 38967 | 1.89226 | 0.05 |
| 38968 | 2.60359 | 0.05 |
| 38969 | 1.89226 | 0.103122 |
| 38970 | 4.49586 | 0.05 |
| 38971 | 1.22938 | 0.109928 |
| 38972 | 2.60359 | 0.05 |
| 38973 | 2.37434 | 0.567796 |

| | | |
|---|---|---|
| 38974 | 2.37434 | 0.05 |
| 38975 | 1.55901 | 0.294753 |
| 38976 | 1.55901 | 0.05 |
| 38977 | 1.18717 | 0.433861 |
| 38978 | 1.18717 | 0.05 |
| 38979 | 0.946132 | 0.05 |
| 38980 | 0.946132 | 0.05 |
| 38981 | 0.946133 | 0.102343 |
| 38982 | 0.946133 | 0.379979 |
| 38983 | 1.3018 | 0.05 |
| 38984 | 1.18717 | 0.484437 |
| 38985 | 1.18714 | 0.05 |
| 38986 | 0.946107 | 0.132397 |
| 38987 | 1.30183 | 0.05 |
| 38988 | 0.946158 | 0.05 |
| 38989 | 1.89226 | 2.1 |
| 38990 | 2.80196 | 2.1 |
| 38991 | 2.67272 | 2.1 |
| 38992 | 2.67272 | 2.1 |
| 38993 | 1.89226 | 2.1 |
| 38994 | 1.89226 | 2.1 |
| 38995 | 1.89226 | 2.1 |
| 38996 | 1.89226 | 2.1 |
| 38997 | 1.89226 | 2.1 |
| 38998 | 1.89226 | 2.1 |
| 38999 | 1.89226 | 2.1 |
| 39000 | 1.89226 | 2.1 |
| 39001 | 1.74471 | 2.1 |
| 39002 | 2.01555 | 2.1 |
| 39003 | 1.61121 | 2.1 |
| 39004 | 1.61121 | 2.1 |
| 39005 | 2.13878 | 2.1 |
| 39006 | 2.13878 | 2.1 |
| 39007 | 1.89226 | 2.1 |
| 39008 | 1.89226 | 2.1 |
| 39009 | 1.89226 | 2.1 |
| 39010 | 1.89226 | 2.1 |
| 39011 | 1.89226 | 2.1 |
| 39012 | 1.89226 | 2.1 |
| 39013 | 1.09766 | 2.1 |
| 39014 | 1.04263 | 2.1 |
| 39015 | 0.881794 | 2.1 |
| 39016 | 0.894188 | 2.1 |
| 39017 | 0.795402 | 2.1 |
| 39018 | 0.99524 | 2.1 |
| 39019 | 1.00647 | 2.1 |
| 39020 | 1.00647 | 2.1 |
| 39021 | 1.15401 | 2.1 |
| 39022 | 0.881789 | 2.1 |
| 39023 | 0.850526 | 2.1 |
| 39024 | 1.04263 | 2.1 |
| 39025 | 1.4054 | 1.02573 |
| 39026 | 1.04788 | 2.1 |
| 39027 | 1.70302 | 0.660004 |
| 39028 | 1.41375 | 2.1 |
| 39029 | 2.82706 | 2.1 |
| 39030 | 1.70302 | 0.922391 |
| 39031 | 1.38776 | 2.1 |

| | | |
|---|---|---|
| 39032 | 1.59457 | 2.00133 |
| 39033 | 1.41375 | 2.1 |
| 39034 | 1.77261 | 0.335618 |
| 39035 | 1.70302 | 2.1 |
| 39036 | 1.89226 | 2.1 |
| 39037 | 1.89226 | 2.1 |
| 39038 | 1.89226 | 2.1 |
| 39039 | 1.89226 | 2.1 |
| 39040 | 3.78453 | 2.1 |
| 39041 | 2.18042 | 2.1 |
| 39042 | 1.74738 | 2.1 |
| 39043 | 2.08433 | 2.1 |
| 39044 | 1.66347 | 2.1 |
| 39045 | 3.83171 | 2.1 |
| 39046 | 1.74738 | 2.1 |
| 39047 | 1.1181 | 2.1 |
| 39048 | 1.66347 | 2.1 |
| 39049 | 1.06543 | 2.1 |
| 39050 | 2.81281 | 2.1 |
| 39051 | 1.89226 | 2.1 |
| 39052 | 1.89226 | 2.1 |
| 39053 | 1.89226 | 2.1 |
| 39054 | 1.89226 | 2.1 |
| 39055 | 3.78453 | 2.1 |
| 39056 | 1.89227 | 2.1 |
| 39057 | 1.89227 | 2.1 |
| 39058 | 1.89227 | 2.1 |
| 39059 | 1.89227 | 2.1 |
| 39060 | 3.78453 | 2.1 |
| 39061 | 2.08433 | 2.1 |
| 39062 | 1.66347 | 2.1 |
| 39063 | 1.97092 | 2.1 |
| 39064 | 1.57431 | 2.1 |
| 39065 | 3.65864 | 2.1 |
| 39066 | 1.66347 | 2.1 |
| 39067 | 1.01474 | 2.1 |
| 39068 | 1.01474 | 2.1 |
| 39069 | 1.06544 | 2.1 |
| 39070 | 1.57431 | 2.1 |
| 39071 | 1.625 2.1 | |
| 39072 | 1.89226 | 2.1 |
| 39073 | 1.89226 | 2.1 |
| 39074 | 1.89226 | 2.1 |
| 39075 | 1.89226 | 2.1 |
| 39076 | 1.89226 | 2.1 |
| 39077 | 1.89226 | 2.1 |
| 39078 | 1.08467 | 2.1 |
| 39079 | 1.87228 | 2.1 |
| 39080 | 1.73305 | 2.1 |
| 39081 | 1.73305 | 2.1 |
| 39082 | 1.25565 | 2.1 |
| 39083 | 1.25565 | 2.1 |
| 39084 | 0.695361 | 0.05 |
| 39085 | 0.799996 | 0.0678441 |
| 39086 | 1.13751 | 0.05 |
| 39087 | 1.40945 | 0.0640555 |
| 39088 | 1.40944 | 0.19247 |
| 39089 | 0.799999 | 0.05 |

| | | |
|---|---|---|
| 39090 | 1.13751 | 0.0500989 |
| 39091 | 0.695363 | 0.05 |
| 39092 | 1.01059 | 0.05 |
| 39093 | 1.01059 | 0.05 |
| 39094 | 0.926908 | 0.112361 |
| 39095 | 0.926909 | 0.05 |
| 39096 | 1.05481 | 2.1 |
| 39097 | 1.78069 | 2.1 |
| 39098 | 1.08074 | 2.1 |
| 39099 | 1.83284 | 2.1 |
| 39100 | 2.86143 | 2.1 |
| 39101 | 1.78069 | 2.1 |
| 39102 | 2.22044 | 2.1 |
| 39103 | 1.83284 | 2.1 |
| 39104 | 2.28696 | 1.07398 |
| 39105 | 4.06766 | 2.1 |
| 39106 | 1.07196 | 2.1 |
| 39107 | 1.64955 | 2.1 |
| 39108 | 2.02665 | 2.1 |
| 39109 | 2.0284 | 2.1 |
| 39110 | 1.08074 | 2.1 |
| 39111 | 1.08074 | 2.1 |
| 39112 | 1.64955 | 2.1 |
| 39113 | 2.28696 | 1.48959 |
| 39114 | 1.92033 | 2.1 |
| 39115 | 2.1004 | 2.1 |
| 39116 | 3.74988 | 2.1 |
| 39117 | 2.83787 | 0.641142 |
| 39118 | 1.70303 | 1.44237 |
| 39119 | 1.70303 | 0.605394 |
| 39120 | 1.83195 | 0.620747 |
| 39121 | 2.64075 | 0.613766 |
| 39122 | 2.70231 | 1.32045 |
| 39123 | 1.2087 | 2.1 |
| 39124 | 1.52909 | 0.219871 |
| 39125 | 1.43063 | 0.0913423 |
| 39126 | 1.10586 | 2.1 |
| 39127 | 2.63494 | 0.704908 |
| 39128 | 1.89227 | 0.722819 |
| 39129 | 2.10732 | 2.1 |
| 39130 | 2.10732 | 1.52437 |
| 39131 | 2.2365 | 2.1 |
| 39132 | 1.89227 | 0.85062 |
| 39133 | 2.02145 | 2.1 |
| 39134 | 1.89226 | 0.454159 |
| 39135 | 1.89226 | 1.95716 |
| 39136 | 1.89226 | 0.63057 |
| 39137 | 1.89226 | 1.72471 |
| 39138 | 1.89226 | 0.158162 |
| 39139 | 1.89226 | 2.1 |
| 39140 | 0.51926 | 2.1 |
| 39141 | 0.552927 | 2.1 |
| 39142 | 0.691235 | 0.5555 |
| 39143 | 0.715314 | 0.0719071 |
| 39144 | 0.619684 | 0.05 |
| 39145 | 0.619684 | 2.1 |
| 39146 | 0.624477 | 2.1 |
| 39147 | 0.624477 | 0.05 |

| | | |
|---|---|---|
| 39148 | 0.519262 | 0.421685 |
| 39149 | 0.691234 | 0.709393 |
| 39150 | 0.715313 | 1.11895 |
| 39151 | 0.552928 | 0.481823 |
| 39152 | 1.89226 | 1.97002 |
| 39153 | 1.87321 | 2.1 |
| 39154 | 1.87321 | 2.1 |
| 39155 | 1.89658 | 2.1 |
| 39156 | 2.0815 | 1.55644 |
| 39157 | 2.14789 | 2.1 |
| 39158 | 0.946128 | 2.1 |
| 39159 | 0.946128 | 2.00861 |
| 39160 | 0.946133 | 0.106686 |
| 39161 | 0.946133 | 0.0996099 |
| 39162 | 0.94613 | 0.05 |
| 39163 | 0.94613 | 1.90798 |
| 39164 | 0.946131 | 0.316665 |
| 39165 | 0.946131 | 2.1 |
| 39166 | 0.946131 | 0.248727 |
| 39167 | 0.946131 | 0.23418 |
| 39168 | 0.946131 | 0.702344 |
| 39169 | 0.946131 | 0.721662 |
| 39170 | 1.99297 | 1.07822 |
| 39171 | 2.19514 | 0.0871034 |
| 39172 | 2.46153 | 0.500204 |
| 39173 | 2.187 | 0.371531 |
| 39174 | 4.45451 | 0.385567 |
| 39175 | 2.0815 | 0.198823 |
| 39176 | 2.33402 | 0.109563 |
| 39177 | 2.46154 | 0.546272 |
| 39178 | 2.83046 | 0.05 |
| 39179 | 4.94496 | 0.143184 |
| 39180 | 2.46153 | 0.415245 |
| 39181 | 2.187 | 0.207416 |
| 39182 | 2.27351 | 0.757195 |
| 39183 | 2.63356 | 0.0608293 |
| 39184 | 4.73504 | 0.302389 |
| 39185 | 2.46154 | 0.457895 |
| 39186 | 2.83447 | 0.05 |
| 39187 | 2.74513 | 0.0947846 |
| 39188 | 2.5731 | 0.312873 |
| 39189 | 2.63357 | 0.0711125 |
| 39190 | 2.46154 | 0.213808 |
| 39191 | 2.80398 | 2.1 |
| 39192 | 2.60406 | 2.1 |
| 39193 | 2.73988 | 2.1 |
| 39194 | 2.57466 | 2.1 |
| 39195 | 5.34394 | 2.1 |
| 39196 | 1.89226 | 1.19202 |
| 39197 | 1.89226 | 0.840583 |
| 39198 | 1.89226 | 0.650191 |
| 39199 | 1.89226 | 1.34855 |
| 39200 | 1.89226 | 0.728986 |
| 39201 | 1.89226 | 2.1 |
| 39202 | 1.89224 | 1.38992 |
| 39203 | 1.89224 | 0.873359 |
| 39204 | 1.89224 | 0.757385 |
| 39205 | 1.89224 | 1.77706 |

| | | |
|---|---|---|
| 39206 | 1.89224 | 0.865507 |
| 39207 | 1.89224 | 2.06789 |
| 39208 | 1.40245 | 1.16233 |
| 39209 | 1.40246 | 1.93112 |
| 39210 | 0.946117 | 1.32203 |
| 39211 | 0.946117 | 2.1 |
| 39212 | 1.17635 | 0.981252 |
| 39213 | 1.40246 | 1.93279 |
| 39214 | 0.946112 | 0.92189 |
| 39215 | 0.946112 | 2.01812 |
| 39216 | 0.946127 | 1.37669 |
| 39217 | 1.17637 | 0.973942 |
| 39218 | 0.946102 | 2.1 |
| 39219 | 1.40244 | 1.18032 |
| 39220 | 2.53269 | 2.1 |
| 39221 | 1.70302 | 2.1 |
| 39222 | 1.56194 | 2.1 |
| 39223 | 2.17516 | 2.1 |
| 39224 | 4.19807 | 2.1 |
| 39225 | 1.89226 | 2.1 |
| 39226 | 1.89226 | 0.886017 |
| 39227 | 1.89226 | 2.1 |
| 39228 | 1.89226 | 2.1 |
| 39229 | 3.78453 | 2.1 |
| 39230 | 2.57276 | 2.1 |
| 39231 | 1.89226 | 2.1 |
| 39232 | 1.89226 | 2.1 |
| 39233 | 1.89226 | 2.1 |
| 39234 | 2.32525 | 2.1 |
| 39235 | 2.32525 | 2.1 |
| 39236 | 1.15171 | 2.1 |
| 39237 | 2.17517 | 2.1 |
| 39238 | 1.41995 | 2.1 |
| 39239 | 1.41995 | 2.1 |
| 39240 | 1.80252 | 2.1 |
| 39241 | 1.80252 | 2.1 |
| 39242 | 1.89226 | 2.1 |
| 39243 | 1.89226 | 2.1 |
| 39244 | 1.89226 | 2.1 |
| 39245 | 1.89226 | 0.735855 |
| 39246 | 3.78453 | 2.1 |
| 39247 | 0.946133 | 2.1 |
| 39248 | 0.946133 | 2.1 |
| 39249 | 1.16263 | 2.1 |
| 39250 | 1.04687 | 2.1 |
| 39251 | 0.946133 | 2.1 |
| 39252 | 0.946133 | 2.1 |
| 39253 | 1.04687 | 2.1 |
| 39254 | 1.04687 | 2.1 |
| 39255 | 0.946133 | 2.1 |
| 39256 | 1.04687 | 2.1 |
| 39257 | 0.946132 | 2.1 |
| 39258 | 1.16263 | 2.1 |
| 39259 | 0.682281 | 2.02361 |
| 39260 | 0.669233 | 2.1 |
| 39261 | 1.21859 | 2.1 |
| 39262 | 1.31629 | 1.62324 |
| 39263 | 0.916158 | 1.29706 |

| | | |
|---|---|---|
| 39264 | 0.916158 | 2.1 |
| 39265 | 0.971658 | 2.05429 |
| 39266 | 0.971658 | 2.1 |
| 39267 | 0.669234 | 2.1 |
| 39268 | 1.31629 | 1.71309 |
| 39269 | 0.682283 | 2.00867 |
| 39270 | 1.21858 | 2.1 |
| 39271 | 0.669232 | 1.6215 |
| 39272 | 0.623495 | 2.05981 |
| 39273 | 1.36428 | 1.91512 |
| 39274 | 1.31629 | 1.15823 |
| 39275 | 0.946475 | 1.04292 |
| 39276 | 0.946476 | 2.1 |
| 39277 | 0.99331 | 1.92638 |
| 39278 | 0.99331 | 1.91113 |
| 39279 | 1.36428 | 1.72547 |
| 39280 | 0.623497 | 1.25456 |
| 39281 | 0.669234 | 1.89479 |
| 39282 | 1.31629 | 1.36452 |
| 39283 | 2.02069 | 0.05 |
| 39284 | 1.46253 | 0.05 |
| 39285 | 2.51479 | 0.05 |
| 39286 | 1.87136 | 0.05 |
| 39287 | 3.89203 | 0.05 |
| 39288 | 1.46253 | 0.05 |
| 39289 | 1.38536 | 0.05 |
| 39290 | 1.38536 | 0.05 |
| 39291 | 1.05579 | 0.05 |
| 39292 | 1.73281 | 0.05 |
| 39293 | 1.46916 | 0.05 |
| 39294 | 2.51479 | 0.05 |
| 39295 | 1.87136 | 0.05 |
| 39296 | 2.23338 | 0.05 |
| 39297 | 2.15603 | 0.05 |
| 39298 | 4.55146 | 0.05 |
| 39299 | 0.822553 | 0.05 |
| 39300 | 0.822563 | 0.05 |
| 39301 | 0.842935 | 0.05 |
| 39302 | 0.842935 | 0.05 |
| 39303 | 0.744203 | 0.05 |
| 39304 | 0.675346 | 0.05 |
| 39305 | 0.874795 | 0.05 |
| 39306 | 1.00453 | 0.05 |
| 39307 | 1.00454 | 0.05 |
| 39308 | 0.782417 | 0.05 |
| 39309 | 0.675337 | 0.05 |
| 39310 | 0.858007 | 0.05 |
| 39311 | 0.946132 | 1.43774 |
| 39312 | 1.24847 | 0.920749 |
| 39313 | 0.946132 | 1.21369 |
| 39314 | 1.16806 | 1.47867 |
| 39315 | 1.24847 | 0.805023 |
| 39316 | 1.24847 | 2.08556 |
| 39317 | 0.946133 | 0.764557 |
| 39318 | 0.946133 | 1.96468 |
| 39319 | 0.946133 | 1.08036 |
| 39320 | 0.946133 | 0.833069 |
| 39321 | 1.16805 | 1.5853 |

| | | |
|---|---|---|
| 39322 | 1.24847 | 0.943179 |
| 39323 | 1.2364 | 1.63961 |
| 39324 | 0.946129 | 1.07665 |
| 39325 | 0.946133 | 1.48553 |
| 39326 | 1.24847 | 1.05115 |
| 39327 | 1.2364 | 0.980449 |
| 39328 | 1.2364 | 1.92231 |
| 39329 | 0.946131 | 0.818057 |
| 39330 | 0.946131 | 2.1 |
| 39331 | 0.946131 | 1.38955 |
| 39332 | 0.946131 | 0.9812 |
| 39333 | 1.2364 | 2.00697 |
| 39334 | 1.24847 | 1.06278 |
| 39335 | 1.89226 | 1.68659 |
| 39336 | 1.89226 | 1.42375 |
| 39337 | 1.89226 | 0.890655 |
| 39338 | 1.89226 | 0.993433 |
| 39339 | 1.89226 | 1.35221 |
| 39340 | 1.89226 | 1.88651 |
| 39341 | 1.89226 | 1.39029 |
| 39342 | 1.89226 | 1.00553 |
| 39343 | 1.89226 | 0.88107 |
| 39344 | 1.89226 | 1.4753 |
| 39345 | 1.89226 | 0.979794 |
| 39346 | 1.89226 | 1.70292 |
| 39347 | 1.69014 | 1.11251 |
| 39348 | 1.09194 | 1.62693 |
| 39349 | 1.13537 | 1.7903 |
| 39350 | 1.13537 | 1.67326 |
| 39351 | 1.58481 | 0.914323 |
| 39352 | 1.58481 | 1.07616 |
| 39353 | 1.58147 | 1.03428 |
| 39354 | 1.13537 | 1.14703 |
| 39355 | 1.69015 | 1.2537 |
| 39356 | 1.01179 | 0.889054 |
| 39357 | 1.69015 | 1.15046 |
| 39358 | 1.01179 | 1.55469 |
| 39359 | 0.946132 | 2.03884 |
| 39360 | 0.946132 | 1.22933 |
| 39361 | 1.28543 | 0.815934 |
| 39362 | 1.07341 | 1.95252 |
| 39363 | 1.07341 | 1.85099 |
| 39364 | 1.07341 | 0.05 |
| 39365 | 0.946132 | 1.04769 |
| 39366 | 0.946132 | 0.873711 |
| 39367 | 0.946133 | 0.910132 |
| 39368 | 1.28543 | 0.916123 |
| 39369 | 1.07341 | 0.917782 |
| 39370 | 0.946132 | 1.09022 |
| 39371 | 1.89226 | 0.737333 |
| 39372 | 1.89226 | 0.0764995 |
| 39373 | 1.89226 | 0.05 |
| 39374 | 1.89226 | 2.1 |
| 39375 | 1.89226 | 0.0555904 |
| 39376 | 1.89226 | 2.1 |
| 39377 | 0.805744 | 2.1 |
| 39378 | 0.988659 | 0.977029 |
| 39379 | 0.782044 | 0.05 |

| | | |
|---|---|---|
| 39380 | 0.578223 | 0.516176 |
| 39381 | 0.77823 | 0.05 |
| 39382 | 0.778231 | 2.1 |
| 39383 | 0.788653 | 0.05 |
| 39384 | 0.788653 | 1.75887 |
| 39385 | 0.988662 | 0.836593 |
| 39386 | 0.782042 | 0.481389 |
| 39387 | 0.805745 | 1.20968 |
| 39388 | 0.578222 | 0.737576 |
| 39389 | 1.89226 | 1.01299 |
| 39390 | 1.89226 | 0.488201 |
| 39391 | 1.89226 | 0.153731 |
| 39392 | 1.89226 | 2.1 |
| 39393 | 1.89226 | 0.109338 |
| 39394 | 1.89226 | 2.1 |
| 39395 | 1.97732 | 1.5111 |
| 39396 | 1.97819 | 0.471386 |
| 39397 | 1.56408 | 0.068233 |
| 39398 | 1.56408 | 2.1 |
| 39399 | 2.33986 | 0.449546 |
| 39400 | 2.33986 | 2.1 |
| 39401 | 1.84516 | 2.1 |
| 39402 | 2.21152 | 2.1 |
| 39403 | 1.90702 | 2.1 |
| 39404 | 1.54599 | 2.1 |
| 39405 | 3.75751 | 2.1 |
| 39406 | 1.89224 | 2.1 |
| 39407 | 1.89224 | 2.1 |
| 39408 | 1.89224 | 2.1 |
| 39409 | 1.89224 | 2.1 |
| 39410 | 1.89224 | 2.1 |
| 39411 | 1.89224 | 2.1 |
| 39412 | 1.47672 | 2.1 |
| 39413 | 1.54601 | 2.1 |
| 39414 | 1.27697 | 2.1 |
| 39415 | 1.54601 | 2.1 |
| 39416 | 1.638 2.1 | |
| 39417 | 1.90705 | 2.1 |
| 39418 | 0.946132 | 2.1 |
| 39419 | 0.946132 | 2.1 |
| 39420 | 0.946131 | 2.1 |
| 39421 | 0.946131 | 2.1 |
| 39422 | 0.946131 | 2.1 |
| 39423 | 0.946131 | 2.1 |
| 39424 | 0.946132 | 2.1 |
| 39425 | 0.946132 | 2.1 |
| 39426 | 0.946132 | 2.1 |
| 39427 | 0.946132 | 2.1 |
| 39428 | 0.946131 | 2.1 |
| 39429 | 0.946131 | 2.1 |
| 39430 | 0.946128 | 2.1 |
| 39431 | 0.946128 | 2.1 |
| 39432 | 1.32062 | 2.1 |
| 39433 | 1.4164 | 2.1 |
| 39434 | 0.946129 | 2.1 |
| 39435 | 0.946129 | 2.1 |
| 39436 | 1.32062 | 2.1 |
| 39437 | 1.32062 | 2.1 |

| | | |
|---|---|---|
| 39438 | 1.32062 | 2.1 |
| 39439 | 0.94613 | 2.1 |
| 39440 | 1.41639 | 2.1 |
| 39441 | 0.946129 | 2.1 |
| 39442 | 1.98979 | 0.05 |
| 39443 | 1.65977 | 0.05 |
| 39444 | 2.21772 | 0.05 |
| 39445 | 2.21772 | 0.05 |
| 39446 | 1.48866 | 0.05 |
| 39447 | 1.48866 | 0.05 |
| 39448 | 0.973129 | 0.05 |
| 39449 | 0.973129 | 0.05 |
| 39450 | 0.980954 | 0.05 |
| 39451 | 0.980954 | 0.05 |
| 39452 | 0.845218 | 0.05 |
| 39453 | 1.13981 | 0.05 |
| 39454 | 0.829886 | 0.05 |
| 39455 | 1.10886 | 0.05 |
| 39456 | 1.10886 | 0.05 |
| 39457 | 1.13982 | 0.05 |
| 39458 | 0.845219 | 0.05 |
| 39459 | 0.829886 | 0.05 |
| 39460 | 0.946132 | 0.889502 |
| 39461 | 0.946132 | 2.1 |
| 39462 | 1.3352 | 2.1 |
| 39463 | 1.41432 | 2.1 |
| 39464 | 0.946133 | 2.1 |
| 39465 | 0.946133 | 0.163026 |
| 39466 | 1.3352 | 2.1 |
| 39467 | 1.3352 | 2.1 |
| 39468 | 1.3352 | 2.1 |
| 39469 | 0.946132 | 2.1 |
| 39470 | 1.41432 | 2.1 |
| 39471 | 0.946133 | 2.1 |
| 39472 | 2.70097 | 2.1 |
| 39473 | 1.88175 | 2.1 |
| 39474 | 1.51482 | 2.1 |
| 39475 | 1.88175 | 2.1 |
| 39476 | 2.37784 | 2.1 |
| 39477 | 2.82864 | 2.1 |
| 39478 | 1.89227 | 0.05 |
| 39479 | 1.89227 | 0.05 |
| 39480 | 1.89227 | 0.05 |
| 39481 | 1.89227 | 0.05 |
| 39482 | 3.78453 | 0.05 |
| 39483 | 1.89226 | 0.05 |
| 39484 | 2.13295 | 0.05 |
| 39485 | 2.43203 | 0.05 |
| 39486 | 2.43203 | 0.05 |
| 39487 | 1.89226 | 0.05 |
| 39488 | 1.89226 | 0.05 |
| 39489 | 0.946133 | 0.05 |
| 39490 | 0.946133 | 0.05 |
| 39491 | 0.946132 | 0.05 |
| 39492 | 0.946132 | 0.05 |
| 39493 | 0.946132 | 0.05 |
| 39494 | 0.946132 | 0.05 |
| 39495 | 0.946133 | 0.05 |

| | | |
|---|---|---|
| 39496 | 0.946133 | 0.05 |
| 39497 | 0.946132 | 0.05 |
| 39498 | 0.946132 | 0.05 |
| 39499 | 0.946132 | 0.05 |
| 39500 | 0.946132 | 0.05 |
| 39501 | 1.54987 | 2.1 |
| 39502 | 1.24128 | 2.1 |
| 39503 | 1.24128 | 2.1 |
| 39504 | 1.50738 | 2.1 |
| 39505 | 1.75702 | 2.1 |
| 39506 | 2.02312 | 2.1 |
| 39507 | 1.89289 | 2.1 |
| 39508 | 1.92264 | 2.1 |
| 39509 | 1.72994 | 2.1 |
| 39510 | 1.75702 | 2.1 |
| 39511 | 3.67966 | 2.1 |
| 39512 | 1.75702 | 2.1 |
| 39513 | 0.994291 | 2.1 |
| 39514 | 0.994291 | 2.1 |
| 39515 | 1.24128 | 2.1 |
| 39516 | 1.4964 | 2.1 |
| 39517 | 1.74339 | 2.1 |
| 39518 | 1.72993 | 2.1 |
| 39519 | 1.49641 | 2.1 |
| 39520 | 1.49641 | 2.1 |
| 39521 | 1.75702 | 2.1 |
| 39522 | 1.3939 | 2.1 |
| 39523 | 1.65452 | 2.1 |
| 39524 | 1.19964 | 2.1 |
| 39525 | 1.53296 | 2.1 |
| 39526 | 1.19964 | 2.1 |
| 39527 | 1.36861 | 2.1 |
| 39528 | 1.42624 | 2.1 |
| 39529 | 1.59521 | 2.1 |
| 39530 | 1.19964 | 2.1 |
| 39531 | 1.42624 | 2.1 |
| 39532 | 1.07156 | 2.1 |
| 39533 | 1.36226 | 2.1 |
| 39534 | 2.5619 | 2.1 |
| 39535 | 2.14058 | 1.21706 |
| 39536 | 1.93569 | 2.1 |
| 39537 | 2.36221 | 1.53502 |
| 39538 | 2.5753 | 1.58189 |
| 39539 | 4.50279 | 2.1 |
| 39540 | 2.14057 | 1.99872 |
| 39541 | 2.20831 | 2.1 |
| 39542 | 2.64725 | 1.10611 |
| 39543 | 2.57529 | 1.9862 |
| 39544 | 4.78782 | 1.89944 |
| 39545 | 2.64725 | 1.3107 |
| 39546 | 2.57529 | 2.1 |
| 39547 | 1.89226 | 1.1307 |
| 39548 | 1.89226 | 0.941718 |
| 39549 | 4.53951 | 1.84013 |
| 39550 | 2.0815 | 2.1 |
| 39551 | 1.86137 | 2.1 |
| 39552 | 1.86137 | 2.1 |
| 39553 | 1.86159 | 2.1 |

| | | |
|---|---|---|
| 39554 | 2.0815 | 2.1 |
| 39555 | 2.10235 | 2.1 |
| 39556 | 1.33552 | 2.1 |
| 39557 | 2.32417 | 2.1 |
| 39558 | 1.18872 | 2.1 |
| 39559 | 1.93101 | 2.1 |
| 39560 | 3.26653 | 2.1 |
| 39561 | 1.89226 | 2.1 |
| 39562 | 1.89226 | 2.1 |
| 39563 | 1.89226 | 2.1 |
| 39564 | 1.89226 | 2.1 |
| 39565 | 1.89226 | 2.1 |
| 39566 | 1.89226 | 2.1 |
| 39567 | 0.955191 | 2.1 |
| 39568 | 1.03372 | 2.1 |
| 39569 | 0.946131 | 2.1 |
| 39570 | 0.946131 | 2.1 |
| 39571 | 0.930654 | 2.1 |
| 39572 | 0.930654 | 2.1 |
| 39573 | 1.04233 | 2.1 |
| 39574 | 1.05247 | 2.1 |
| 39575 | 0.955341 | 2.1 |
| 39576 | 0.946131 | 2.1 |
| 39577 | 1.02375 | 2.1 |
| 39578 | 1.13537 | 2.1 |
| 39579 | 1.1549 | 2.1 |
| 39580 | 1.93101 | 2.1 |
| 39581 | 1.91108 | 2.1 |
| 39582 | 1.52487 | 2.1 |
| 39583 | 1.3076 | 2.1 |
| 39584 | 1.06985 | 2.1 |
| 39585 | 1.89226 | 2.1 |
| 39586 | 1.89226 | 2.1 |
| 39587 | 1.89226 | 2.1 |
| 39588 | 1.89226 | 2.1 |
| 39589 | 1.89226 | 2.1 |
| 39590 | 1.89226 | 2.1 |
| 39591 | 1.58713 | 2.1 |
| 39592 | 2.6845 | 2.1 |
| 39593 | 1.58742 | 2.1 |
| 39594 | 2.74201 | 2.1 |
| 39595 | 4.27191 | 2.1 |
| 39596 | 2.68449 | 2.1 |
| 39597 | 1.89226 | 2.1 |
| 39598 | 2.74201 | 2.1 |
| 39599 | 1.89226 | 2.1 |
| 39600 | 4.57675 | 2.1 |
| 39601 | 1.57523 | 2.1 |
| 39602 | 2.74201 | 2.1 |
| 39603 | 2.8146 | 2.1 |
| 39604 | 2.8146 | 2.1 |
| 39605 | 1.58742 | 2.1 |
| 39606 | 1.58742 | 2.1 |
| 39607 | 0.946131 | 2.1 |
| 39608 | 0.946131 | 2.1 |
| 39609 | 1.371 | 2.1 |
| 39610 | 1.371 | 2.1 |
| 39611 | 0.946127 | 2.1 |

| | | |
|---|---|---|
| 39612 | 0.946127 | 2.1 |
| 39613 | 1.37101 | 2.1 |
| 39614 | 1.4073 | 2.1 |
| 39615 | 0.94613 | 2.1 |
| 39616 | 1.4073 | 2.1 |
| 39617 | 1.371 2.1 | |
| 39618 | 0.946129 | 2.1 |
| 39619 | 1.44801 | 2.04635 |
| 39620 | 1.06821 | 2.08369 |
| 39621 | 2.00542 | 1.92813 |
| 39622 | 2.27703 | 2.1 |
| 39623 | 3.34524 | 1.76945 |
| 39624 | 1.70301 | 0.0584565 |
| 39625 | 1.89226 | 0.66828 |
| 39626 | 1.65809 | 0.110129 |
| 39627 | 1.89226 | 0.616723 |
| 39628 | 1.62758 | 0.275095 |
| 39629 | 1.85744 | 0.601845 |
| 39630 | 1.89226 | 0.05 |
| 39631 | 1.89226 | 0.424516 |
| 39632 | 1.89226 | 0.285275 |
| 39633 | 1.89226 | 0.202155 |
| 39634 | 3.78452 | 0.195671 |
| 39635 | 1.89226 | 0.532782 |
| 39636 | 1.89226 | 0.0752012 |
| 39637 | 1.89226 | 0.218988 |
| 39638 | 1.89226 | 0.600887 |
| 39639 | 3.78452 | 0.442609 |
| 39640 | 0.851541 | 0.611223 |
| 39641 | 0.851541 | 0.493538 |
| 39642 | 0.91193 | 0.338466 |
| 39643 | 0.824837 | 0.533693 |
| 39644 | 0.854559 | 0.283797 |
| 39645 | 0.851534 | 0.850527 |
| 39646 | 0.81851 | 0.661653 |
| 39647 | 0.717219 | 0.0813535 |
| 39648 | 0.871743 | 0.56426 |
| 39649 | 0.837985 | 0.390654 |
| 39650 | 0.82902 | 0.425471 |
| 39651 | 0.82902 | 0.67866 |
| 39652 | 1.89226 | 0.602634 |
| 39653 | 1.92659 | 0.607401 |
| 39654 | 2.03235 | 0.620512 |
| 39655 | 1.89226 | 0.722749 |
| 39656 | 2.03235 | 0.518921 |
| 39657 | 1.89226 | 0.593887 |
| 39658 | 0.585557 | 0.805565 |
| 39659 | 0.585557 | 0.688014 |
| 39660 | 1.22612 | 0.675985 |
| 39661 | 1.09436 | 0.794847 |
| 39662 | 0.851537 | 0.906004 |
| 39663 | 0.526987 | 0.48144 |
| 39664 | 0.983879 | 0.61873 |
| 39665 | 1.04072 | 0.733605 |
| 39666 | 0.585526 | 0.703314 |
| 39667 | 0.792325 | 0.698129 |
| 39668 | 0.946129 | 0.724025 |
| 39669 | 0.946129 | 0.734755 |

| | | |
|---|---|---|
| 39670 | 1.68152 | 2.1 |
| 39671 | 1.81445 | 2.1 |
| 39672 | 1.68152 | 2.1 |
| 39673 | 1.86792 | 2.1 |
| 39674 | 1.65888 | 2.1 |
| 39675 | 1.84529 | 2.1 |
| 39676 | 1.81445 | 2.1 |
| 39677 | 1.57885 | 2.1 |
| 39678 | 1.65889 | 2.1 |
| 39679 | 1.48594 | 2.1 |
| 39680 | 3.23773 | 2.1 |
| 39681 | 1.68154 | 2.1 |
| 39682 | 1.65891 | 2.1 |
| 39683 | 1.53296 | 2.1 |
| 39684 | 1.56299 | 2.1 |
| 39685 | 3.24453 | 2.1 |
| 39686 | 1.56299 | 2.1 |
| 39687 | 1.48596 | 2.1 |
| 39688 | 1.56299 | 2.1 |
| 39689 | 1.65891 | 2.1 |
| 39690 | 1.46568 | 2.1 |
| 39691 | 1.5616 | 2.1 |
| 39692 | 2.68785 | 2.1 |
| 39693 | 2.08831 | 2.1 |
| 39694 | 2.66919 | 2.1 |
| 39695 | 2.1024 | 2.1 |
| 39696 | 4.79025 | 2.1 |
| 39697 | 2.08832 | 2.1 |
| 39698 | 1.15478 | 2.1 |
| 39699 | 1.15478 | 2.1 |
| 39700 | 1.09062 | 2.1 |
| 39701 | 2.10241 | 2.1 |
| 39702 | 2.03825 | 2.1 |
| 39703 | 2.65348 | 2.1 |
| 39704 | 2.1024 | 2.1 |
| 39705 | 2.12313 | 2.1 |
| 39706 | 2.12313 | 2.1 |
| 39707 | 2.66919 | 2.1 |
| 39708 | 2.66919 | 2.1 |
| 39709 | 0.813169 | 2.1 |
| 39710 | 0.813169 | 2.1 |
| 39711 | 0.825788 | 2.1 |
| 39712 | 0.825788 | 2.1 |
| 39713 | 0.612902 | 2.1 |
| 39714 | 0.577387 | 2.1 |
| 39715 | 1.0512 | 2.1 |
| 39716 | 1.06157 | 2.1 |
| 39717 | 1.06157 | 2.1 |
| 39718 | 0.612902 | 2.1 |
| 39719 | 0.577387 | 2.1 |
| 39720 | 1.0512 | 2.1 |
| 39721 | 1.27227 | 2.1 |
| 39722 | 1.91724 | 2.1 |
| 39723 | 1.32591 | 2.1 |
| 39724 | 1.99837 | 2.1 |
| 39725 | 3.24315 | 2.1 |
| 39726 | 1.89223 | 2.1 |
| 39727 | 1.89223 | 2.1 |

| | | |
|---|---|---|
| 39728 | 1.89223 | 2.1 |
| 39729 | 1.89223 | 2.1 |
| 39730 | 3.78446 | 2.1 |
| 39731 | 1.89224 | 2.1 |
| 39732 | 1.89224 | 1.04351 |
| 39733 | 1.89224 | 2.1 |
| 39734 | 1.89224 | 2.1 |
| 39735 | 3.78447 | 2.1 |
| 39736 | 1.91724 | 2.1 |
| 39737 | 2.321 | 2.1 |
| 39738 | 1.99838 | 2.1 |
| 39739 | 2.4149 | 2.1 |
| 39740 | 4.33214 | 2.1 |
| 39741 | 1.34817 | 2.1 |
| 39742 | 1.9984 | 2.1 |
| 39743 | 2.06238 | 2.1 |
| 39744 | 2.06238 | 2.1 |
| 39745 | 1.32593 | 2.1 |
| 39746 | 1.32593 | 2.1 |
| 39747 | 1.89226 | 2.1 |
| 39748 | 1.89226 | 2.1 |
| 39749 | 1.89226 | 2.1 |
| 39750 | 1.89226 | 2.1 |
| 39751 | 1.89226 | 2.1 |
| 39752 | 1.89226 | 2.1 |
| 39753 | 1.89226 | 2.1 |
| 39754 | 1.89226 | 2.1 |
| 39755 | 1.89226 | 2.1 |
| 39756 | 1.89226 | 1.09262 |
| 39757 | 3.78452 | 2.1 |
| 39758 | 1.9984 | 2.1 |
| 39759 | 2.41494 | 2.1 |
| 39760 | 2.06239 | 2.1 |
| 39761 | 2.5194 | 2.1 |
| 39762 | 4.47732 | 2.1 |
| 39763 | 1.19901 | 1.05026 |
| 39764 | 1.16661 | 2.1 |
| 39765 | 1.56859 | 2.1 |
| 39766 | 1.61029 | 1.29957 |
| 39767 | 2.7676 | 2.1 |
| 39768 | 1.56859 | 2.1 |
| 39769 | 1.88637 | 0.387242 |
| 39770 | 1.61029 | 0.0619839 |
| 39771 | 1.61029 | 2.1 |
| 39772 | 1.856 | 0.0711658 |
| 39773 | 1.856 | 2.1 |
| 39774 | 1.856 | 0.127101 |
| 39775 | 1.57916 | 2.1 |
| 39776 | 1.56859 | 0.471705 |
| 39777 | 1.56859 | 2.1 |
| 39778 | 1.57916 | 2.1 |
| 39779 | 1.23475 | 2.1 |
| 39780 | 0.890924 | 0.05 |
| 39781 | 1.07608 | 0.0690577 |
| 39782 | 0.72877 | 0.0860298 |
| 39783 | 0.910106 | 0.249456 |
| 39784 | 1.00196 | 0.05 |
| 39785 | 1.09519 | 0.414946 |

| | | |
|---|---|---|
| 39786 | 0.839967 | 0.05 |
| 39787 | 0.719043 | 0.05 |
| 39788 | 0.893859 | 0.05 |
| 39789 | 0.942408 | 0.108751 |
| 39790 | 0.873497 | 0.05 |
| 39791 | 0.873497 | 0.124508 |
| 39792 | 1.53046 | 0.05 |
| 39793 | 1.89994 | 0.05 |
| 39794 | 1.049 | 0.05 |
| 39795 | 1.049 | 0.05 |
| 39796 | 1.52512 | 0.05 |
| 39797 | 1.89994 | 0.05 |
| 39798 | 1.65573 | 0.972847 |
| 39799 | 1.89226 | 0.292078 |
| 39800 | 1.89226 | 0.154116 |
| 39801 | 2.73329 | 1.15893 |
| 39802 | 3.548 | 1.13571 |
| 39803 | 1.65573 | 0.385183 |
| 39804 | 2.42671 | 0.89285 |
| 39805 | 2.73329 | 0.197788 |
| 39806 | 1.89226 | 0.0519478 |
| 39807 | 2.73329 | 1.06033 |
| 39808 | 1.89226 | 0.577623 |
| 39809 | 0.941078 | 0.0660483 |
| 39810 | 1.01599 | 0.118716 |
| 39811 | 0.818936 | 0.05 |
| 39812 | 0.651554 | 0.119949 |
| 39813 | 0.904729 | 0.111609 |
| 39814 | 0.665756 | 0.129925 |
| 39815 | 0.843423 | 0.05 |
| 39816 | 0.918649 | 0.167957 |
| 39817 | 0.855806 | 0.39575 |
| 39818 | 0.814384 | 0.05 |
| 39819 | 0.844437 | 0.212976 |
| 39820 | 0.844437 | 0.198626 |
| 39821 | 2.22558 | 1.13011 |
| 39822 | 1.95785 | 0.249706 |
| 39823 | 2.07065 | 0.747201 |
| 39824 | 1.89142 | 0.664742 |
| 39825 | 2.07065 | 1.79742 |
| 39826 | 1.89142 | 1.46855 |
| 39827 | 0.640813 | 0.231635 |
| 39828 | 0.951654 | 0.800876 |
| 39829 | 0.797442 | 0.0945628 |
| 39830 | 0.535364 | 0.406771 |
| 39831 | 0.525269 | 0.725288 |
| 39832 | 0.620874 | 0.555596 |
| 39833 | 0.916953 | 0.777906 |
| 39834 | 0.969374 | 0.487015 |
| 39835 | 0.788925 | 1.23528 |
| 39836 | 0.69596 | 0.05 |
| 39837 | 0.743154 | 0.72975 |
| 39838 | 0.743154 | 0.818201 |
| 39839 | 1.89226 | 2.1 |
| 39840 | 1.89226 | 1.86804 |
| 39841 | 1.89226 | 0.897371 |
| 39842 | 1.89226 | 2.1 |
| 39843 | 3.78452 | 1.17386 |

| | | |
|---|---|---|
| 39844 | 1.32541 | 1.75166 |
| 39845 | 1.13722 | 1.21107 |
| 39846 | 0.91103 | 2.1 |
| 39847 | 0.881123 | 2.1 |
| 39848 | 1.03363 | 2.1 |
| 39849 | 1.03364 | 2.09097 |
| 39850 | 1.1505 | 1.99893 |
| 39851 | 1.1505 | 2.1 |
| 39852 | 1.32539 | 2.1 |
| 39853 | 1.07662 | 2.1 |
| 39854 | 0.881121 | 2.1 |
| 39855 | 0.962303 | 2.02581 |
| 39856 | 1.89226 | 1.04627 |
| 39857 | 1.89226 | 2.1 |
| 39858 | 1.89226 | 2.1 |
| 39859 | 1.89226 | 1.24771 |
| 39860 | 3.78453 | 2.1 |
| 39861 | 1.89226 | 0.858125 |
| 39862 | 1.89226 | 2.1 |
| 39863 | 1.89226 | 1.76754 |
| 39864 | 1.89226 | 1.32455 |
| 39865 | 1.89226 | 1.88683 |
| 39866 | 1.89226 | 1.87976 |
| 39867 | 1.27606 | 2.0532 |
| 39868 | 1.07664 | 1.3676 |
| 39869 | 0.905895 | 2.1 |
| 39870 | 0.696099 | 2.1 |
| 39871 | 0.984902 | 2.1 |
| 39872 | 0.984905 | 2.0265 |
| 39873 | 1.01878 | 2.1 |
| 39874 | 1.01878 | 0.909027 |
| 39875 | 1.27604 | 2.1 |
| 39876 | 1.06044 | 2.1 |
| 39877 | 1.13192 | 2.1 |
| 39878 | 0.696111 | 2.1 |
| 39879 | 1.89226 | 0.113679 |
| 39880 | 1.89226 | 0.05 |
| 39881 | 1.89226 | 0.05 |
| 39882 | 1.89226 | 0.481111 |
| 39883 | 1.89226 | 0.05 |
| 39884 | 1.89226 | 0.250308 |
| 39885 | 1.52615 | 0.0559057 |
| 39886 | 1.01294 | 0.05 |
| 39887 | 1.01294 | 0.693405 |
| 39888 | 1.18549 | 0.05 |
| 39889 | 1.38963 | 0.05 |
| 39890 | 1.56218 | 0.05 |
| 39891 | 1.89226 | 0.152418 |
| 39892 | 2.40801 | 0.05 |
| 39893 | 2.58101 | 0.429317 |
| 39894 | 2.58101 | 0.407596 |
| 39895 | 1.89226 | 0.05 |
| 39896 | 1.89226 | 0.05 |
| 39897 | 2.24071 | 2.1 |
| 39898 | 2.79317 | 2.1 |
| 39899 | 1.99136 | 2.1 |
| 39900 | 2.6167 | 2.1 |
| 39901 | 4.85741 | 2.1 |

| | | |
|---|---|---|
| 39902 | 2.79317 | 2.1 |
| 39903 | 1.89226 | 0.05 |
| 39904 | 1.89226 | 2.1 |
| 39905 | 1.89226 | 0.0647583 |
| 39906 | 2.6167 | 2.1 |
| 39907 | 2.6167 | 0.05 |
| 39908 | 1.08354 | 2.1 |
| 39909 | 1.08354 | 2.1 |
| 39910 | 1.10222 | 2.1 |
| 39911 | 1.10222 | 2.1 |
| 39912 | 1.2275 | 2.1 |
| 39913 | 1.30835 | 2.1 |
| 39914 | 0.995678 | 2.1 |
| 39915 | 0.877412 | 2.1 |
| 39916 | 0.877411 | 2.1 |
| 39917 | 1.2275 | 2.1 |
| 39918 | 0.995678 | 2.1 |
| 39919 | 1.30835 | 2.1 |
| 39920 | 1.05793 | 0.806889 |
| 39921 | 1.46441 | 0.228422 |
| 39922 | 1.0562 | 0.704744 |
| 39923 | 1.0562 | 0.45396 |
| 39924 | 1.41787 | 0.784174 |
| 39925 | 1.41787 | 0.653978 |
| 39926 | 1.41787 | 0.627518 |
| 39927 | 1.52909 | 0.957487 |
| 39928 | 1.46441 | 0.417006 |
| 39929 | 1.46441 | 0.897653 |
| 39930 | 1.43063 | 0.305446 |
| 39931 | 1.43063 | 0.643752 |
| 39932 | 1.89226 | 0.977413 |
| 39933 | 1.89226 | 0.437493 |
| 39934 | 1.89226 | 0.632063 |
| 39935 | 1.89226 | 0.958345 |
| 39936 | 3.78453 | 0.800434 |
| 39937 | 1.89227 | 0.799751 |
| 39938 | 1.89227 | 0.682888 |
| 39939 | 1.89227 | 0.613411 |
| 39940 | 1.89227 | 0.572677 |
| 39941 | 1.89227 | 0.663129 |
| 39942 | 1.89227 | 0.660412 |
| 39943 | 1.00763 | 0.664148 |
| 39944 | 1.41787 | 0.397923 |
| 39945 | 1.05793 | 0.586935 |
| 39946 | 1.05793 | 0.618324 |
| 39947 | 1.37265 | 0.705726 |
| 39948 | 1.37265 | 0.656414 |
| 39949 | 1.37265 | 0.510631 |
| 39950 | 1.43063 | 0.202293 |
| 39951 | 1.41787 | 0.402752 |
| 39952 | 1.41787 | 1.07707 |
| 39953 | 1.38247 | 0.211095 |
| 39954 | 1.38247 | 0.963704 |
| 39955 | 0.946132 | 0.511324 |
| 39956 | 0.946132 | 0.12469 |
| 39957 | 0.946132 | 0.678942 |
| 39958 | 0.946132 | 0.05 |
| 39959 | 0.946131 | 0.05 |

| | | |
|---|---|---|
| 39960 | 0.946131 | 0.130829 |
| 39961 | 0.946132 | 0.697817 |
| 39962 | 0.946132 | 0.590423 |
| 39963 | 0.946132 | 0.252845 |
| 39964 | 0.946132 | 0.154853 |
| 39965 | 0.946132 | 0.218286 |
| 39966 | 0.946132 | 0.167042 |
| 39967 | 1.89226 | 0.722991 |
| 39968 | 1.89226 | 0.13899 |
| 39969 | 1.89226 | 0.435922 |
| 39970 | 1.89226 | 0.826007 |
| 39971 | 1.89226 | 0.582543 |
| 39972 | 1.89226 | 0.777863 |
| 39973 | 1.19645 | 1.04195 |
| 39974 | 1.89183 | 0.636071 |
| 39975 | 1.91106 | 0.850646 |
| 39976 | 1.35306 | 0.684512 |
| 39977 | 1.75446 | 0.870572 |
| 39978 | 1.19645 | 0.689821 |
| 39979 | 2.37576 | 0.132687 |
| 39980 | 1.90139 | 1.70352 |
| 39981 | 2.1845 | 2.1 |
| 39982 | 1.86568 | 0.746341 |
| 39983 | 4.24145 | 1.97787 |
| 39984 | 2.29435 | 0.716552 |
| 39985 | 1.40602 | 2.1 |
| 39986 | 1.40602 | 0.335077 |
| 39987 | 1.55351 | 1.30936 |
| 39988 | 1.86568 | 2.1 |
| 39989 | 2.01317 | 2.1 |
| 39990 | 2.18452 | 2.1 |
| 39991 | 1.8657 | 0.05 |
| 39992 | 2.01399 | 0.551531 |
| 39993 | 1.70141 | 2.1 |
| 39994 | 3.87969 | 0.563119 |
| 39995 | 0.774999 | 2.1 |
| 39996 | 0.774999 | 0.561575 |
| 39997 | 0.778721 | 0.773345 |
| 39998 | 0.778721 | 2.1 |
| 39999 | 0.628348 | 2.1 |
| 40000 | 0.703016 | 2.1 |
| 40001 | 0.932848 | 0.601607 |
| 40002 | 0.850704 | 1.24009 |
| 40003 | 0.850704 | 1.64646 |
| 40004 | 0.628348 | 0.938763 |
| 40005 | 0.703015 | 0.611547 |
| 40006 | 0.932848 | 0.666074 |
| 40007 | 1.65243 | 0.25175 |
| 40008 | 1.34412 | 0.217015 |
| 40009 | 1.45569 | 0.557809 |
| 40010 | 1.1315 | 0.741456 |
| 40011 | 2.78393 | 0.445253 |
| 40012 | 1.48901 | 2.1 |
| 40013 | 1.805 | 1.2475 |
| 40014 | 1.48901 | 1.25109 |
| 40015 | 1.71717 | 2.1 |
| 40016 | 1.60635 | 0.05 |
| 40017 | 1.83451 | 2.1 |

| | | |
|---|---|---|
| 40018 | 1.805 | 2.09146 |
| 40019 | 1.65243 | 0.05 |
| 40020 | 1.60635 | 0.812082 |
| 40021 | 1.45569 | 0.196631 |
| 40022 | 3.25879 | 0.05 |
| 40023 | 1.27875 | 2.1 |
| 40024 | 1.60635 | 0.196706 |
| 40025 | 1.27875 | 1.90291 |
| 40026 | 1.48901 | 2.1 |
| 40027 | 1.40014 | 0.0809787 |
| 40028 | 1.6104 | 2.1 |
| 40029 | 0.715199 | 0.172009 |
| 40030 | 0.715199 | 1.93744 |
| 40031 | 0.712714 | 0.968666 |
| 40032 | 0.712714 | 0.05 |
| 40033 | 0.727843 | 0.05 |
| 40034 | 0.619713 | 0.05 |
| 40035 | 0.803177 | 1.24133 |
| 40036 | 0.70007 | 0.11845 |
| 40037 | 0.700069 | 0.0546918 |
| 40038 | 0.619713 | 0.05 |
| 40039 | 0.803177 | 0.05 |
| 40040 | 0.727844 | 0.0563748 |
| 40041 | 1.14357 | 2.1 |
| 40042 | 2.3506 | 2.1 |
| 40043 | 1.14357 | 2.1 |
| 40044 | 2.68773 | 2.1 |
| 40045 | 1.57829 | 2.1 |
| 40046 | 2.68773 | 2.1 |
| 40047 | 2.00551 | 2.1 |
| 40048 | 2.65811 | 1.68504 |
| 40049 | 2.07203 | 2.1 |
| 40050 | 2.72117 | 0.05 |
| 40051 | 4.72667 | 2.1 |
| 40052 | 0.946131 | 0.605992 |
| 40053 | 0.946131 | 2.1 |
| 40054 | 1.32905 | 2.1 |
| 40055 | 1.32905 | 0.106944 |
| 40056 | 0.94613 | 1.20508 |
| 40057 | 0.94613 | 1.62246 |
| 40058 | 1.32905 | 1.69369 |
| 40059 | 1.36058 | 1.2128 |
| 40060 | 1.36058 | 0.350889 |
| 40061 | 0.946131 | 0.076115 |
| 40062 | 1.32905 | 1.07893 |
| 40063 | 0.946131 | 1.97747 |
| 40064 | 1.21649 | 0.05 |
| 40065 | 1.21649 | 2.1 |
| 40066 | 1.21459 | 2.1 |
| 40067 | 1.21459 | 2.1 |
| 40068 | 1.39508 | 1.10772 |
| 40069 | 1.36057 | 0.833074 |
| 40070 | 1.03601 | 2.1 |
| 40071 | 1.07438 | 2.1 |
| 40072 | 1.07439 | 1.63369 |
| 40073 | 1.39509 | 1.90417 |
| 40074 | 1.036 | 1.73989 |
| 40075 | 1.36056 | 1.05079 |

| | | |
|---|---|---|
| 40076 | 2.79015 | 0.846823 |
| 40077 | 2.72114 | 2.1 |
| 40078 | 1.89225 | 0.909543 |
| 40079 | 1.89225 | 2.1 |
| 40080 | 1.89225 | 1.90341 |
| 40081 | 2.72114 | 0.66265 |
| 40082 | 0.876258 | 2.1 |
| 40083 | 0.892889 | 2.1 |
| 40084 | 1.34352 | 2.1 |
| 40085 | 1.27985 | 2.1 |
| 40086 | 1.07462 | 2.1 |
| 40087 | 1.07462 | 2.1 |
| 40088 | 1.09812 | 2.1 |
| 40089 | 1.09812 | 2.1 |
| 40090 | 0.892888 | 2.1 |
| 40091 | 1.34352 | 2.1 |
| 40092 | 0.876256 | 2.1 |
| 40093 | 1.27985 | 2.1 |
| 40094 | 1.39725 | 2.1 |
| 40095 | 1.30029 | 0.0842948 |
| 40096 | 1.31415 | 0.46956 |
| 40097 | 1.41336 | 1.47923 |
| 40098 | 2.71366 | 1.01078 |
| 40099 | 1.89226 | 0.380866 |
| 40100 | 1.89226 | 0.92835 |
| 40101 | 1.89226 | 1.98931 |
| 40102 | 1.89226 | 0.294402 |
| 40103 | 3.78453 | 0.440229 |
| 40104 | 0.946133 | 0.964417 |
| 40105 | 0.946133 | 0.761244 |
| 40106 | 1.38709 | 0.162432 |
| 40107 | 1.39437 | 0.803851 |
| 40108 | 0.946132 | 0.204279 |
| 40109 | 0.946132 | 0.712604 |
| 40110 | 1.39437 | 1.09004 |
| 40111 | 1.39437 | 0.336324 |
| 40112 | 0.946132 | 0.704176 |
| 40113 | 1.39437 | 0.237929 |
| 40114 | 1.38709 | 0.690352 |
| 40115 | 0.946132 | 0.68737 |
| 40116 | 0.706681 | 2.1 |
| 40117 | 0.711908 | 1.62627 |
| 40118 | 0.657077 | 0.11691 |
| 40119 | 0.661003 | 0.890089 |
| 40120 | 0.684188 | 0.112767 |
| 40121 | 0.684188 | 1.42103 |
| 40122 | 0.683496 | 2.1 |
| 40123 | 0.683496 | 0.150514 |
| 40124 | 0.711908 | 0.699786 |
| 40125 | 0.661003 | 0.762716 |
| 40126 | 0.657076 | 0.942926 |
| 40127 | 0.706681 | 0.879852 |
| 40128 | 0.946132 | 1.93421 |
| 40129 | 0.946132 | 0.595739 |
| 40130 | 0.946132 | 0.784549 |
| 40131 | 0.946132 | 0.144788 |
| 40132 | 0.946132 | 0.17031 |
| 40133 | 0.946132 | 0.947512 |

| | | |
|---|---|---|
| 40134 | 0.946133 | 0.427259 |
| 40135 | 0.946133 | 1.97546 |
| 40136 | 0.946133 | 0.378691 |
| 40137 | 0.946133 | 0.570279 |
| 40138 | 0.946132 | 0.825442 |
| 40139 | 0.946132 | 0.89663 |
| 40140 | 2.79029 | 0.398093 |
| 40141 | 1.89227 | 1.02574 |
| 40142 | 2.78873 | 0.664622 |
| 40143 | 2.78873 | 0.230156 |
| 40144 | 1.89383 | 0.762239 |
| 40145 | 1.89227 | 0.70956 |
| 40146 | 1.43669 | 2.1 |
| 40147 | 2.11984 | 2.1 |
| 40148 | 2.24481 | 2.1 |
| 40149 | 2.24481 | 2.1 |
| 40150 | 1.20311 | 2.1 |
| 40151 | 1.20311 | 2.1 |
| 40152 | 0.49703 | 2.1 |
| 40153 | 0.601553 | 2.1 |
| 40154 | 1.05992 | 2.1 |
| 40155 | 0.991694 | 2.1 |
| 40156 | 0.787558 | 2.1 |
| 40157 | 0.787558 | 2.1 |
| 40158 | 0.805689 | 2.1 |
| 40159 | 0.805689 | 2.1 |
| 40160 | 0.991694 | 2.1 |
| 40161 | 0.49703 | 2.1 |
| 40162 | 1.05992 | 2.1 |
| 40163 | 0.601553 | 2.1 |
| 40164 | 2.45905 | 2.1 |
| 40165 | 1.89226 | 2.1 |
| 40166 | 1.89226 | 2.1 |
| 40167 | 1.89226 | 2.1 |
| 40168 | 2.47395 | 2.1 |
| 40169 | 2.47395 | 2.1 |
| 40170 | 1.89226 | 2.1 |
| 40171 | 1.89226 | 2.05352 |
| 40172 | 1.89226 | 2.1 |
| 40173 | 1.89226 | 1.47923 |
| 40174 | 3.78452 | 2.1 |
| 40175 | 1.38427 | 2.1 |
| 40176 | 2.31203 | 1.06423 |
| 40177 | 2.28743 | 0.924049 |
| 40178 | 2.28743 | 2.1 |
| 40179 | 1.63384 | 0.854641 |
| 40180 | 1.63384 | 2.1 |
| 40181 | 2.42723 | 2.1 |
| 40182 | 1.89226 | 2.1 |
| 40183 | 1.89226 | 2.1 |
| 40184 | 1.89226 | 2.1 |
| 40185 | 2.45905 | 2.1 |
| 40186 | 2.45905 | 2.1 |
| 40187 | 0.946131 | 0.248548 |
| 40188 | 0.946131 | 2.1 |
| 40189 | 0.946131 | 2.1 |
| 40190 | 0.946131 | 2.1 |
| 40191 | 0.94613 | 1.32656 |

| | | |
|---|---|---|
| 40192 | 0.94613 | 2.1 |
| 40193 | 0.946132 | 2.1 |
| 40194 | 0.946132 | 2.1 |
| 40195 | 0.946131 | 2.1 |
| 40196 | 0.946131 | 2.1 |
| 40197 | 0.946131 | 2.1 |
| 40198 | 0.946131 | 2.1 |
| 40199 | 0.989441 | 2.1 |
| 40200 | 0.989441 | 2.1 |
| 40201 | 0.993918 | 2.1 |
| 40202 | 0.993918 | 2.1 |
| 40203 | 1.16644 | 2.1 |
| 40204 | 1.15601 | 1.05865 |
| 40205 | 0.818308 | 2.1 |
| 40206 | 0.816922 | 2.1 |
| 40207 | 0.818307 | 2.1 |
| 40208 | 1.16644 | 2.1 |
| 40209 | 1.15601 | 2.1 |
| 40210 | 0.816922 | 2.1 |
| 40211 | 0.996778 | 0.05 |
| 40212 | 2.44375 | 0.05 |
| 40213 | 0.996778 | 0.05 |
| 40214 | 1.92547 | 0.05 |
| 40215 | 1.56091 | 0.05 |
| 40216 | 2.4896 | 0.05 |
| 40217 | 1.56091 | 0.05 |
| 40218 | 2.44375 | 0.05 |
| 40219 | 1.90456 | 0.05 |
| 40220 | 1.90456 | 0.05 |
| 40221 | 2.81853 | 0.05 |
| 40222 | 2.47487 | 0.05 |
| 40223 | 1.32143 | 2.07015 |
| 40224 | 1.43294 | 2.1 |
| 40225 | 1.37444 | 0.955677 |
| 40226 | 1.51691 | 2.1 |
| 40227 | 2.80738 | 1.88144 |
| 40228 | 1.89226 | 1.4279 |
| 40229 | 1.89226 | 2.1 |
| 40230 | 1.89226 | 1.04964 |
| 40231 | 1.89226 | 1.63467 |
| 40232 | 3.78453 | 1.34708 |
| 40233 | 1.89226 | 1.46693 |
| 40234 | 1.89226 | 2.1 |
| 40235 | 1.89226 | 1.11344 |
| 40236 | 1.89226 | 2.1 |
| 40237 | 3.78453 | 2.1 |
| 40238 | 1.43294 | 1.41424 |
| 40239 | 1.3268 | 2.1 |
| 40240 | 1.51691 | 1.38687 |
| 40241 | 1.44091 | 2.1 |
| 40242 | 2.87385 | 2.1 |
| 40243 | 1.37444 | 2.1 |
| 40244 | 1.51691 | 2.1 |
| 40245 | 1.4714 | 0.931498 |
| 40246 | 1.65434 | 1.79895 |
| 40247 | 3.02878 | 1.65537 |
| 40248 | 1.89226 | 2.1 |
| 40249 | 1.89226 | 2.09981 |

| | | |
|---|---|---|
| 40250 | 1.89226 | 1.23706 |
| 40251 | 1.89226 | 1.9022 |
| 40252 | 3.78452 | 1.61455 |
| 40253 | 1.89226 | 1.28752 |
| 40254 | 1.89226 | 2.1 |
| 40255 | 1.89226 | 1.15256 |
| 40256 | 1.89226 | 2.1 |
| 40257 | 3.78453 | 2.1 |
| 40258 | 1.51691 | 1.98214 |
| 40259 | 1.44091 | 2.1 |
| 40260 | 1.65434 | 1.1776 |
| 40261 | 1.60647 | 2.1 |
| 40262 | 3.09526 | 2.1 |
| 40263 | 1.05013 | 2.1 |
| 40264 | 1.05013 | 2.1 |
| 40265 | 0.94613 | 2.1 |
| 40266 | 0.94613 | 2.1 |
| 40267 | 1.04959 | 2.1 |
| 40268 | 1.05014 | 2.1 |
| 40269 | 0.946129 | 2.1 |
| 40270 | 0.946129 | 2.1 |
| 40271 | 0.946131 | 2.1 |
| 40272 | 1.04958 | 2.1 |
| 40273 | 0.946131 | 2.1 |
| 40274 | 1.05013 | 2.1 |
| 40275 | 1.04959 | 2.1 |
| 40276 | 1.04959 | 2.1 |
| 40277 | 0.946131 | 2.1 |
| 40278 | 0.946131 | 2.1 |
| 40279 | 1.04765 | 2.1 |
| 40280 | 1.04959 | 2.1 |
| 40281 | 0.94613 | 2.1 |
| 40282 | 0.94613 | 2.1 |
| 40283 | 0.946131 | 2.1 |
| 40284 | 1.04765 | 2.1 |
| 40285 | 0.946131 | 2.1 |
| 40286 | 1.04959 | 2.1 |
| 40287 | 1.85744 | 2.1 |
| 40288 | 1.89226 | 2.1 |
| 40289 | 1.89226 | 2.1 |
| 40290 | 1.89226 | 2.1 |
| 40291 | 1.94839 | 2.1 |
| 40292 | 1.94839 | 2.1 |
| 40293 | 0.97419 | 2.1 |
| 40294 | 0.822054 | 2.1 |
| 40295 | 0.946123 | 2.1 |
| 40296 | 0.946123 | 2.1 |
| 40297 | 1.04202 | 2.1 |
| 40298 | 0.84898 | 2.1 |
| 40299 | 0.933307 | 2.1 |
| 40300 | 0.933307 | 2.1 |
| 40301 | 0.933285 | 2.1 |
| 40302 | 1.05018 | 2.1 |
| 40303 | 0.851528 | 2.1 |
| 40304 | 0.876787 | 2.1 |
| 40305 | 1.87528 | 0.05 |
| 40306 | 1.21137 | 0.05 |
| 40307 | 1.57893 | 0.05 |

| | | |
|---|---|---|
| 40308 | 1.21137 | 0.0513167 |
| 40309 | 1.81182 | 0.05 |
| 40310 | 1.44426 | 0.05 |
| 40311 | 0.993833 | 0.05 |
| 40312 | 1.21137 | 0.05 |
| 40313 | 1.57893 | 0.05 |
| 40314 | 1.31774 | 0.05 |
| 40315 | 2.5291 | 0.05 |
| 40316 | 1.87528 | 0.05 |
| 40317 | 1.57893 | 0.05 |
| 40318 | 1.90728 | 0.05 |
| 40319 | 2.29851 | 0.05 |
| 40320 | 3.87744 | 0.05 |
| 40321 | 1.90728 | 0.05 |
| 40322 | 1.31774 | 0.05 |
| 40323 | 1.56339 | 0.05 |
| 40324 | 1.31774 | 0.05 |
| 40325 | 1.82458 | 0.05 |
| 40326 | 1.57893 | 0.05 |
| 40327 | 1.89226 | 0.87844 |
| 40328 | 1.89226 | 1.05433 |
| 40329 | 1.89226 | 1.0002 |
| 40330 | 1.89226 | 1.05693 |
| 40331 | 1.89226 | 1.02562 |
| 40332 | 1.89226 | 1.08932 |
| 40333 | 1.89226 | 0.949089 |
| 40334 | 1.89226 | 1.13596 |
| 40335 | 1.89226 | 1.22063 |
| 40336 | 1.89226 | 1.03234 |
| 40337 | 3.78452 | 1.10691 |
| 40338 | 1.89226 | 1.03688 |
| 40339 | 2.17251 | 1.02196 |
| 40340 | 2.45575 | 0.6286 |
| 40341 | 2.45575 | 0.882916 |
| 40342 | 1.89226 | 0.873379 |
| 40343 | 1.89226 | 1.02818 |
| 40344 | 1.08625 | 1.13485 |
| 40345 | 1.22788 | 0.968053 |
| 40346 | 0.54447 | 0.91522 |
| 40347 | 0.794861 | 1.03707 |
| 40348 | 0.967729 | 1.12163 |
| 40349 | 0.967729 | 1.13857 |
| 40350 | 0.913386 | 0.995171 |
| 40351 | 0.913386 | 0.947009 |
| 40352 | 1.08625 | 1.09235 |
| 40353 | 0.544469 | 1.00697 |
| 40354 | 0.794862 | 1.08581 |
| 40355 | 1.22788 | 0.996666 |
| 40356 | 0.946128 | 1.07474 |
| 40357 | 0.946128 | 1.17009 |
| 40358 | 0.946132 | 1.02689 |
| 40359 | 0.946132 | 0.994888 |
| 40360 | 0.94613 | 1.0227 |
| 40361 | 0.94613 | 1.05637 |
| 40362 | 0.94613 | 1.18641 |
| 40363 | 0.94613 | 0.787835 |
| 40364 | 0.94613 | 1.05684 |
| 40365 | 0.94613 | 1.05114 |

| | | |
|---|---|---|
| 40366 | 0.94613 | 1.05403 |
| 40367 | 0.94613 | 1.0698 |
| 40368 | 0.946127 | 0.936546 |
| 40369 | 0.946127 | 0.869776 |
| 40370 | 1.08625 | 0.687156 |
| 40371 | 0.958779 | 1.05237 |
| 40372 | 1.08625 | 0.699879 |
| 40373 | 1.08625 | 0.89241 |
| 40374 | 0.946128 | 0.88488 |
| 40375 | 0.946128 | 0.908695 |
| 40376 | 0.946129 | 0.953086 |
| 40377 | 0.958777 | 1.00245 |
| 40378 | 1.08625 | 0.855487 |
| 40379 | 0.946129 | 0.990676 |
| 40380 | 2.16338 | 0.05 |
| 40381 | 1.94002 | 0.05 |
| 40382 | 1.98836 | 0.05 |
| 40383 | 2.60614 | 0.05 |
| 40384 | 1.5456 | 0.05 |
| 40385 | 2.16338 | 0.05 |
| 40386 | 1.19952 | 0.05 |
| 40387 | 2.34061 | 0.05 |
| 40388 | 1.21031 | 0.05 |
| 40389 | 2.1711 | 0.05 |
| 40390 | 3.54013 | 0.05 |
| 40391 | 1.13624 | 2.1 |
| 40392 | 1.70302 | 2.1 |
| 40393 | 1.8267 | 2.1 |
| 40394 | 1.11545 | 2.1 |
| 40395 | 2.85455 | 2.1 |
| 40396 | 1.82671 | 2.1 |
| 40397 | 1.91767 | 2.1 |
| 40398 | 1.81563 | 2.1 |
| 40399 | 1.91767 | 2.1 |
| 40400 | 1.71362 | 2.1 |
| 40401 | 1.83277 | 2.1 |
| 40402 | 1.66064 | 2.1 |
| 40403 | 0.996776 | 2.1 |
| 40404 | 0.996776 | 2.1 |
| 40405 | 1.11545 | 2.1 |
| 40406 | 1.54278 | 2.1 |
| 40407 | 1.66145 | 2.1 |
| 40408 | 1.66064 | 2.1 |
| 40409 | 1.7457 | 2.1 |
| 40410 | 1.81563 | 2.1 |
| 40411 | 1.81563 | 2.1 |
| 40412 | 1.54278 | 2.1 |
| 40413 | 1.54278 | 2.1 |
| 40414 | 2.71672 | 2.1 |
| 40415 | 2.70402 | 2.1 |
| 40416 | 2.64996 | 2.1 |
| 40417 | 2.69443 | 2.1 |
| 40418 | 5.39845 | 2.1 |
| 40419 | 2.71672 | 2.1 |
| 40420 | 2.54198 | 2.1 |
| 40421 | 2.54019 | 2.1 |
| 40422 | 2.69443 | 2.1 |
| 40423 | 5.23641 | 2.1 |

| | | |
|---|---|---|
| 40424 | 2.64996 | 2.1 |
| 40425 | 2.69443 | 2.1 |
| 40426 | 2.68213 | 2.1 |
| 40427 | 2.61243 | 2.1 |
| 40428 | 5.30686 | 2.1 |
| 40429 | 2.54019 | 2.1 |
| 40430 | 2.69443 | 2.1 |
| 40431 | 2.68213 | 2.1 |
| 40432 | 2.5392 | 2.1 |
| 40433 | 5.23363 | 2.1 |
| 40434 | 1.89226 | 2.1 |
| 40435 | 2.34725 | 2.1 |
| 40436 | 2.22414 | 2.1 |
| 40437 | 2.34725 | 2.1 |
| 40438 | 1.76914 | 2.1 |
| 40439 | 1.89226 | 2.1 |
| 40440 | 0.946131 | 2.1 |
| 40441 | 0.946131 | 2.1 |
| 40442 | 1.11207 | 2.1 |
| 40443 | 1.03798 | 2.1 |
| 40444 | 0.94613 | 2.1 |
| 40445 | 0.94613 | 2.1 |
| 40446 | 1.03798 | 2.1 |
| 40447 | 1.03798 | 2.1 |
| 40448 | 0.946131 | 2.1 |
| 40449 | 1.03798 | 2.1 |
| 40450 | 0.946131 | 2.1 |
| 40451 | 1.11207 | 2.1 |
| 40452 | 1.89226 | 0.05 |
| 40453 | 1.89226 | 0.05 |
| 40454 | 1.89226 | 0.05 |
| 40455 | 1.89226 | 0.05 |
| 40456 | 1.89226 | 0.05 |
| 40457 | 1.89226 | 0.05 |
| 40458 | 1.89226 | 0.05 |
| 40459 | 1.89226 | 0.05 |
| 40460 | 1.89226 | 0.05 |
| 40461 | 1.89226 | 0.05 |
| 40462 | 1.89226 | 0.05 |
| 40463 | 1.89226 | 0.05 |
| 40464 | 1.89226 | 0.05 |
| 40465 | 1.89226 | 0.05 |
| 40466 | 1.89226 | 0.05 |
| 40467 | 1.89226 | 0.05 |
| 40468 | 1.89226 | 0.05 |
| 40469 | 1.89226 | 0.05 |
| 40470 | 1.44085 | 2.1 |
| 40471 | 1.31825 | 0.0591737 |
| 40472 | 1.31923 | 0.633542 |
| 40473 | 1.31825 | 1.64872 |
| 40474 | 1.43311 | 0.203034 |
| 40475 | 1.43213 | 1.04126 |
| 40476 | 1.89226 | 0.115383 |
| 40477 | 1.89226 | 0.999562 |
| 40478 | 1.89226 | 0.523547 |
| 40479 | 1.89226 | 1.04687 |
| 40480 | 1.89226 | 0.114745 |
| 40481 | 1.89226 | 1.31571 |

| | | |
|---|---|---|
| 40482 | 0.946133 | 0.831967 |
| 40483 | 0.946133 | 0.694455 |
| 40484 | 1.38969 | 0.420463 |
| 40485 | 1.39587 | 0.688764 |
| 40486 | 0.946132 | 0.544166 |
| 40487 | 0.946132 | 0.66258 |
| 40488 | 1.39587 | 0.863594 |
| 40489 | 1.39587 | 0.138778 |
| 40490 | 0.946132 | 0.63339 |
| 40491 | 1.39587 | 0.615652 |
| 40492 | 1.38969 | 0.640987 |
| 40493 | 0.946132 | 0.643116 |
| 40494 | 1.44085 | 2.1 |
| 40495 | 1.31923 | 0.05 |
| 40496 | 1.33026 | 0.651257 |
| 40497 | 1.4617 | 1.48412 |
| 40498 | 2.78093 | 1.06414 |
| 40499 | 1.89226 | 0.0945345 |
| 40500 | 1.89226 | 0.88987 |
| 40501 | 1.89226 | 0.446027 |
| 40502 | 1.89226 | 1.03879 |
| 40503 | 1.89226 | 0.0766585 |
| 40504 | 1.89226 | 1.43428 |
| 40505 | 0.946133 | 0.762954 |
| 40506 | 0.946133 | 0.65286 |
| 40507 | 1.39587 | 0.606605 |
| 40508 | 1.40724 | 0.181499 |
| 40509 | 0.946132 | 0.358071 |
| 40510 | 0.946132 | 0.634613 |
| 40511 | 1.40724 | 0.780035 |
| 40512 | 1.40724 | 0.148785 |
| 40513 | 0.946132 | 0.601828 |
| 40514 | 1.40724 | 0.538664 |
| 40515 | 0.946133 | 0.694961 |
| 40516 | 1.39587 | 0.644134 |
| 40517 | 1.15818 | 2.1 |
| 40518 | 1.81846 | 2.05196 |
| 40519 | 1.52782 | 1.11816 |
| 40520 | 1.52782 | 2.1 |
| 40521 | 1.28095 | 0.9603 |
| 40522 | 1.28095 | 2.1 |
| 40523 | 2.56709 | 2.1 |
| 40524 | 1.4349 | 0.05 |
| 40525 | 1.4349 | 0.375206 |
| 40526 | 1.75471 | 0.748215 |
| 40527 | 2.29909 | 0.495165 |
| 40528 | 2.61889 | 1.82554 |
| 40529 | 2.5671 | 2.1 |
| 40530 | 2.82858 | 2.1 |
| 40531 | 2.57368 | 2.1 |
| 40532 | 2.29909 | 0.532353 |
| 40533 | 5.12767 | 2.1 |
| 40534 | 2.29909 | 2.1 |
| 40535 | 1.09148 | 0.05 |
| 40536 | 1.09148 | 0.371682 |
| 40537 | 1.4349 | 0.334155 |
| 40538 | 1.99776 | 0.0858919 |
| 40539 | 2.34119 | 0.794156 |

| | | |
|---|---|---|
| 40540 | 2.57368 | 2.1 |
| 40541 | 1.99777 | 0.237636 |
| 40542 | 1.99777 | 2.1 |
| 40543 | 2.29909 | 2.1 |
| 40544 | 2.36071 | 2.1 |
| 40545 | 2.66203 | 2.1 |
| 40546 | 1.89226 | 0.756526 |
| 40547 | 1.89226 | 0.979291 |
| 40548 | 1.89226 | 0.881091 |
| 40549 | 1.89226 | 0.817114 |
| 40550 | 3.78453 | 0.805557 |
| 40551 | 0.946128 | 0.962748 |
| 40552 | 0.946128 | 1.55766 |
| 40553 | 0.946133 | 0.873773 |
| 40554 | 0.946133 | 0.890623 |
| 40555 | 0.946131 | 1.03976 |
| 40556 | 0.946131 | 1.11532 |
| 40557 | 0.946131 | 1.32601 |
| 40558 | 0.946131 | 0.965843 |
| 40559 | 0.94613 | 1.37475 |
| 40560 | 0.94613 | 1.06609 |
| 40561 | 0.946131 | 1.00138 |
| 40562 | 0.946131 | 1.16023 |
| 40563 | 1.89226 | 1.017 |
| 40564 | 1.89226 | 1.15972 |
| 40565 | 1.89226 | 0.842649 |
| 40566 | 1.89226 | 1.1485 |
| 40567 | 3.78453 | 1.16896 |
| 40568 | 1.89226 | 1.08086 |
| 40569 | 1.89226 | 0.921532 |
| 40570 | 1.89226 | 0.805188 |
| 40571 | 1.89226 | 0.947268 |
| 40572 | 3.78453 | 0.997276 |
| 40573 | 1.89226 | 0.957831 |
| 40574 | 2.61682 | 1.69064 |
| 40575 | 1.89226 | 1.30384 |
| 40576 | 1.89226 | 1.45283 |
| 40577 | 1.89226 | 0.955134 |
| 40578 | 1.89226 | 1.31375 |
| 40579 | 1.89226 | 0.573133 |
| 40580 | 1.89226 | 0.571638 |
| 40581 | 1.89226 | 0.647072 |
| 40582 | 1.89226 | 0.673124 |
| 40583 | 3.78452 | 0.752116 |
| 40584 | 1.89226 | 0.56674 |
| 40585 | 1.89226 | 1.13807 |
| 40586 | 1.89226 | 0.590184 |
| 40587 | 1.89226 | 0.701416 |
| 40588 | 1.89226 | 0.744316 |
| 40589 | 1.89226 | 1.16335 |
| 40590 | 1.89227 | 0.584215 |
| 40591 | 1.89227 | 0.493743 |
| 40592 | 1.89227 | 0.781803 |
| 40593 | 1.89227 | 0.679069 |
| 40594 | 1.89227 | 0.592005 |
| 40595 | 1.89227 | 0.570257 |
| 40596 | 0.80447 | 0.942054 |
| 40597 | 0.534241 | 0.928206 |

| | | |
|---|---|---|
| 40598 | 0.995175 | 0.960883 |
| 40599 | 0.92204 | 0.7475 |
| 40600 | 0.922042 | 0.228012 |
| 40601 | 0.534241 | 0.489937 |
| 40602 | 0.804468 | 0.87775 |
| 40603 | 0.995176 | 0.403359 |
| 40604 | 0.71543 | 0.748013 |
| 40605 | 0.71543 | 0.05 |
| 40606 | 0.813988 | 0.586857 |
| 40607 | 0.813988 | 0.182355 |
| 40608 | 0.922045 | 0.141725 |
| 40609 | 0.942899 | 0.602163 |
| 40610 | 0.946132 | 0.459373 |
| 40611 | 0.946132 | 0.213516 |
| 40612 | 1.05169 | 0.05 |
| 40613 | 0.936661 | 0.573892 |
| 40614 | 0.922045 | 0.542031 |
| 40615 | 0.946133 | 0.360665 |
| 40616 | 1.01665 | 0.217739 |
| 40617 | 1.13008 | 0.05 |
| 40618 | 0.851529 | 0.121548 |
| 40619 | 0.851529 | 0.512443 |
| 40620 | 1.41908 | 2.1 |
| 40621 | 1.77723 | 2.1 |
| 40622 | 1.90485 | 2.1 |
| 40623 | 1.90485 | 2.1 |
| 40624 | 1.30237 | 2.1 |
| 40625 | 1.30237 | 2.1 |
| 40626 | 1.77723 | 2.1 |
| 40627 | 2.0055 | 2.1 |
| 40628 | 1.90485 | 2.1 |
| 40629 | 2.17158 | 2.1 |
| 40630 | 3.91035 | 2.1 |
| 40631 | 0.850906 | 2.1 |
| 40632 | 0.850906 | 2.1 |
| 40633 | 0.85668 | 2.1 |
| 40634 | 0.85668 | 2.1 |
| 40635 | 0.998045 | 2.1 |
| 40636 | 0.952425 | 2.1 |
| 40637 | 0.74367 | 2.1 |
| 40638 | 0.709541 | 2.1 |
| 40639 | 0.74367 | 2.1 |
| 40640 | 0.998045 | 2.1 |
| 40641 | 0.709541 | 2.1 |
| 40642 | 0.952425 | 2.1 |
| 40643 | 1.90485 | 2.1 |
| 40644 | 2.28563 | 2.1 |
| 40645 | 2.26282 | 2.1 |
| 40646 | 2.17158 | 2.1 |
| 40647 | 1.99609 | 2.1 |
| 40648 | 1.90485 | 1.7493 |
| 40649 | 2.00098 | 0.0610318 |
| 40650 | 1.90462 | 0.053668 |
| 40651 | 1.60057 | 0.0553996 |
| 40652 | 1.79224 | 0.287677 |
| 40653 | 3.69686 | 0.0610007 |
| 40654 | 2.65654 | 0.0827229 |
| 40655 | 1.79224 | 0.156136 |

| | | |
|---|---|---|
| 40656 | 2.22332 | 0.124389 |
| 40657 | 2.22332 | 0.118952 |
| 40658 | 2.24163 | 0.110298 |
| 40659 | 2.24163 | 0.124698 |
| 40660 | 1.89226 | 0.166028 |
| 40661 | 1.89226 | 0.195766 |
| 40662 | 1.89226 | 0.201892 |
| 40663 | 1.89226 | 0.408222 |
| 40664 | 3.78452 | 0.202777 |
| 40665 | 1.89226 | 0.0841706 |
| 40666 | 1.89226 | 0.356814 |
| 40667 | 1.89226 | 0.221684 |
| 40668 | 1.89226 | 0.316459 |
| 40669 | 1.89226 | 0.35535 |
| 40670 | 1.89226 | 0.300536 |
| 40671 | 2.24163 | 0.186477 |
| 40672 | 1.03645 | 0.236229 |
| 40673 | 1.79224 | 0.345362 |
| 40674 | 1.79224 | 0.194809 |
| 40675 | 1.54283 | 0.225704 |
| 40676 | 1.54283 | 0.190004 |
| 40677 | 0.946132 | 0.285048 |
| 40678 | 0.946132 | 0.193367 |
| 40679 | 0.946129 | 0.382733 |
| 40680 | 0.946129 | 0.29661 |
| 40681 | 0.94613 | 0.392944 |
| 40682 | 0.94613 | 0.247541 |
| 40683 | 0.946131 | 0.39361 |
| 40684 | 0.946131 | 0.249275 |
| 40685 | 0.94613 | 0.388417 |
| 40686 | 0.94613 | 0.388241 |
| 40687 | 0.946131 | 0.297515 |
| 40688 | 0.946131 | 0.202356 |
| 40689 | 1.89226 | 2.1 |
| 40690 | 1.89226 | 2.1 |
| 40691 | 1.89226 | 2.1 |
| 40692 | 1.89226 | 2.1 |
| 40693 | 3.78453 | 2.1 |
| 40694 | 1.52441 | 2.1 |
| 40695 | 1.30347 | 2.1 |
| 40696 | 1.52441 | 2.1 |
| 40697 | 1.58401 | 2.1 |
| 40698 | 1.2627 | 2.1 |
| 40699 | 1.3223 | 2.1 |
| 40700 | 1.89226 | 2.1 |
| 40701 | 2.72787 | 2.1 |
| 40702 | 2.72787 | 2.1 |
| 40703 | 2.74762 | 2.1 |
| 40704 | 1.89226 | 2.1 |
| 40705 | 1.91202 | 2.1 |
| 40706 | 1.89227 | 2.1 |
| 40707 | 1.89227 | 2.1 |
| 40708 | 1.89227 | 2.1 |
| 40709 | 1.89227 | 2.1 |
| 40710 | 3.78453 | 2.1 |
| 40711 | 0.56414 | 2.1 |
| 40712 | 0.561557 | 2.1 |
| 40713 | 0.527284 | 2.1 |

| | | |
|---|---|---|
| 40714 | 0.541143 | 2.1 |
| 40715 | 0.527276 | 2.1 |
| 40716 | 0.631685 | 2.1 |
| 40717 | 1.52441 | 2.1 |
| 40718 | 1.00232 | 2.1 |
| 40719 | 0.784495 | 2.1 |
| 40720 | 0.842304 | 2.1 |
| 40721 | 0.721569 | 2.1 |
| 40722 | 1.36393 | 2.1 |
| 40723 | 1.36393 | 2.1 |
| 40724 | 0.946132 | 2.1 |
| 40725 | 0.946132 | 2.1 |
| 40726 | 1.36393 | 2.1 |
| 40727 | 1.3594 | 2.1 |
| 40728 | 0.946132 | 2.1 |
| 40729 | 0.946132 | 2.1 |
| 40730 | 0.946107 | 2.1 |
| 40731 | 1.35936 | 2.1 |
| 40732 | 1.36397 | 2.1 |
| 40733 | 0.946158 | 2.1 |
| 40734 | 1.89226 | 2.1 |
| 40735 | 1.89226 | 2.1 |
| 40736 | 1.89226 | 2.1 |
| 40737 | 1.89226 | 2.1 |
| 40738 | 3.78453 | 2.1 |
| 40739 | 2.20802 | 2.1 |
| 40740 | 1.05677 | 2.1 |
| 40741 | 2.30479 | 2.1 |
| 40742 | 0.978699 | 2.1 |
| 40743 | 3.18672 | 2.1 |
| 40744 | 0.94613 | 2.1 |
| 40745 | 0.94613 | 2.1 |
| 40746 | 0.669866 | 2.1 |
| 40747 | 0.669866 | 2.1 |
| 40748 | 0.681588 | 2.1 |
| 40749 | 0.669865 | 2.1 |
| 40750 | 0.946133 | 2.1 |
| 40751 | 0.946133 | 2.1 |
| 40752 | 0.94613 | 2.1 |
| 40753 | 0.681589 | 2.1 |
| 40754 | 0.946131 | 2.1 |
| 40755 | 0.669867 | 2.1 |
| 40756 | 2.73514 | 2.1 |
| 40757 | 1.78301 | 2.1 |
| 40758 | 1.90618 | 2.1 |
| 40759 | 2.83464 | 2.1 |
| 40760 | 4.64132 | 2.1 |
| 40761 | 2.73514 | 2.1 |
| 40762 | 1.89226 | 2.1 |
| 40763 | 1.89226 | 2.1 |
| 40764 | 2.83464 | 2.1 |
| 40765 | 4.6274 | 2.1 |
| 40766 | 2.03915 | 2.1 |
| 40767 | 2.26616 | 2.1 |
| 40768 | 2.23549 | 2.1 |
| 40769 | 2.01704 | 2.1 |
| 40770 | 4.2832 | 2.1 |
| 40771 | 2.03916 | 2.1 |

| | | |
|---|---|---|
| 40772 | 1.55766 | 2.1 |
| 40773 | 1.53728 | 2.1 |
| 40774 | 2.01704 | 2.1 |
| 40775 | 3.5747 | 2.1 |
| 40776 | 2.23551 | 2.1 |
| 40777 | 2.01705 | 2.1 |
| 40778 | 1.99823 | 2.1 |
| 40779 | 2.21189 | 2.1 |
| 40780 | 4.22894 | 2.1 |
| 40781 | 1.51788 | 2.1 |
| 40782 | 2.01705 | 2.1 |
| 40783 | 1.53729 | 2.1 |
| 40784 | 1.53729 | 2.1 |
| 40785 | 1.99823 | 2.1 |
| 40786 | 1.99823 | 2.1 |
| 40787 | 2.588 2.1 | |
| 40788 | 1.45581 | 2.1 |
| 40789 | 1.45581 | 2.1 |
| 40790 | 1.44207 | 2.1 |
| 40791 | 2.54949 | 2.1 |
| 40792 | 2.53574 | 2.1 |
| 40793 | 0.98641 | 2.1 |
| 40794 | 0.98641 | 2.1 |
| 40795 | 1.00371 | 2.1 |
| 40796 | 1.00371 | 2.1 |
| 40797 | 0.749992 | 2.1 |
| 40798 | 0.727904 | 2.1 |
| 40799 | 1.26221 | 2.1 |
| 40800 | 1.27474 | 2.1 |
| 40801 | 1.26221 | 2.1 |
| 40802 | 0.749993 | 2.1 |
| 40803 | 0.727904 | 2.1 |
| 40804 | 1.27474 | 2.1 |
| 40805 | 1.23105 | 0.0673537 |
| 40806 | 1.35435 | 1.89694 |
| 40807 | 1.89226 | 0.966072 |
| 40808 | 1.89226 | 0.285453 |
| 40809 | 1.89226 | 1.66912 |
| 40810 | 1.7043 | 0.769309 |
| 40811 | 1.89226 | 2.1 |
| 40812 | 1.89226 | 0.932705 |
| 40813 | 1.89226 | 0.055658 |
| 40814 | 1.89226 | 2.08307 |
| 40815 | 3.78453 | 1.51679 |
| 40816 | 1.89226 | 0.05 |
| 40817 | 1.89226 | 2.09374 |
| 40818 | 1.89226 | 0.704972 |
| 40819 | 1.89226 | 0.819449 |
| 40820 | 1.89226 | 1.453 |
| 40821 | 1.89226 | 1.57489 |
| 40822 | 0.946131 | 0.152541 |
| 40823 | 1.40945 | 0.0924952 |
| 40824 | 0.94613 | 0.0838729 |
| 40825 | 0.94613 | 1.56444 |
| 40826 | 0.946129 | 1.65676 |
| 40827 | 0.946129 | 0.462943 |
| 40828 | 0.946132 | 1.75281 |
| 40829 | 1.40945 | 0.0521708 |

| | | |
|---|---|---|
| 40830 | 0.94613 | 0.475256 |
| 40831 | 0.94613 | 0.290707 |
| 40832 | 0.946131 | 0.255096 |
| 40833 | 0.94613 | 0.100832 |
| 40834 | 0.946133 | 0.269495 |
| 40835 | 0.946133 | 0.19616 |
| 40836 | 0.946132 | 1.36633 |
| 40837 | 0.946132 | 2.04738 |
| 40838 | 0.946132 | 2.00459 |
| 40839 | 0.946132 | 0.435732 |
| 40840 | 0.946133 | 1.39959 |
| 40841 | 0.946133 | 0.243402 |
| 40842 | 0.946132 | 1.18857 |
| 40843 | 0.946132 | 1.05756 |
| 40844 | 0.946133 | 0.543726 |
| 40845 | 0.946133 | 0.628069 |
| 40846 | 1.89226 | 0.6361 |
| 40847 | 1.89226 | 2.1 |
| 40848 | 1.89226 | 1.50865 |
| 40849 | 1.89226 | 1.33216 |
| 40850 | 1.89226 | 2.00558 |
| 40851 | 1.89226 | 1.62643 |
| 40852 | 1.89226 | 2.1 |
| 40853 | 1.89226 | 2.1 |
| 40854 | 1.89226 | 2.1 |
| 40855 | 1.89226 | 2.1 |
| 40856 | 1.89226 | 2.1 |
| 40857 | 1.89226 | 2.1 |
| 40858 | 1.89226 | 2.1 |
| 40859 | 1.89226 | 2.1 |
| 40860 | 1.89226 | 2.1 |
| 40861 | 1.89226 | 2.1 |
| 40862 | 3.78453 | 2.1 |
| 40863 | 1.66647 | 2.1 |
| 40864 | 1.15399 | 2.1 |
| 40865 | 1.25443 | 2.1 |
| 40866 | 1.26599 | 2.1 |
| 40867 | 1.58697 | 2.1 |
| 40868 | 1.66647 | 2.1 |
| 40869 | 1.66647 | 2.1 |
| 40870 | 1.47446 | 2.1 |
| 40871 | 1.55433 | 2.1 |
| 40872 | 1.74241 | 2.1 |
| 40873 | 3.2208 | 2.1 |
| 40874 | 1.89227 | 2.1 |
| 40875 | 1.89227 | 2.1 |
| 40876 | 1.89227 | 2.1 |
| 40877 | 1.89227 | 2.1 |
| 40878 | 3.78453 | 2.1 |
| 40879 | 0.946132 | 2.1 |
| 40880 | 0.946132 | 2.1 |
| 40881 | 0.946132 | 2.1 |
| 40882 | 0.946132 | 2.1 |
| 40883 | 0.946132 | 2.1 |
| 40884 | 0.946132 | 2.1 |
| 40885 | 0.946133 | 2.1 |
| 40886 | 0.946133 | 2.1 |
| 40887 | 0.946132 | 2.1 |

| | | |
|---|---|---|
| 40888 | 0.946132 | 2.1 |
| 40889 | 0.946133 | 2.1 |
| 40890 | 0.946133 | 2.1 |
| 40891 | 0.625067 | 2.1 |
| 40892 | 0.61907 | 2.1 |
| 40893 | 0.896913 | 2.1 |
| 40894 | 0.946199 | 2.1 |
| 40895 | 0.806287 | 2.1 |
| 40896 | 0.677685 | 2.1 |
| 40897 | 0.750657 | 2.1 |
| 40898 | 0.750657 | 2.1 |
| 40899 | 0.646004 | 2.1 |
| 40900 | 0.890236 | 2.1 |
| 40901 | 0.61049 | 2.1 |
| 40902 | 0.888979 | 2.1 |
| 40903 | 1.6223 | 2.1 |
| 40904 | 1.98034 | 2.1 |
| 40905 | 1.75003 | 2.1 |
| 40906 | 1.55433 | 2.1 |
| 40907 | 1.75003 | 2.1 |
| 40908 | 1.55433 | 2.1 |
| 40909 | 1.88602 | 2.1 |
| 40910 | 2.08149 | 2.1 |
| 40911 | 2.07991 | 2.1 |
| 40912 | 1.88473 | 2.1 |
| 40913 | 2.08149 | 2.1 |
| 40914 | 1.88602 | 2.1 |
| 40915 | 1.88458 | 2.1 |
| 40916 | 2.08149 | 2.1 |
| 40917 | 1.7069 | 2.1 |
| 40918 | 1.56284 | 2.1 |
| 40919 | 2.08149 | 2.1 |
| 40920 | 1.88458 | 2.1 |
| 40921 | 1.5955 | 2.1 |
| 40922 | 1.89226 | 2.1 |
| 40923 | 1.91975 | 2.1 |
| 40924 | 1.62299 | 2.1 |
| 40925 | 1.89226 | 2.1 |
| 40926 | 1.5955 | 2.1 |
| 40927 | 1.89226 | 2.1 |
| 40928 | 1.89226 | 2.1 |
| 40929 | 1.89226 | 2.1 |
| 40930 | 1.89226 | 2.1 |
| 40931 | 1.89226 | 1.2515 |
| 40932 | 1.89226 | 2.1 |
| 40933 | 1.89226 | 2.1 |
| 40934 | 1.89226 | 2.1 |
| 40935 | 1.89226 | 2.1 |
| 40936 | 1.89226 | 2.1 |
| 40937 | 1.89226 | 2.1 |
| 40938 | 1.89226 | 2.1 |
| 40939 | 1.62299 | 2.1 |
| 40940 | 1.89226 | 2.1 |
| 40941 | 1.71752 | 2.1 |
| 40942 | 1.44824 | 2.1 |
| 40943 | 1.89226 | 2.1 |
| 40944 | 1.62299 | 2.1 |
| 40945 | 1.89226 | 0.798277 |

| | | |
|---|---|---|
| 40946 | 2.8317 | 0.333928 |
| 40947 | 1.89226 | 0.801777 |
| 40948 | 2.64055 | 0.119003 |
| 40949 | 4.53281 | 0.781993 |
| 40950 | 2.8317 | 0.845308 |
| 40951 | 1.83151 | 0.0563903 |
| 40952 | 2.01001 | 0.211393 |
| 40953 | 2.01001 | 0.939165 |
| 40954 | 2.64055 | 0.05 |
| 40955 | 2.64055 | 0.322689 |
| 40956 | 0.946132 | 0.0656333 |
| 40957 | 0.946132 | 0.383227 |
| 40958 | 1.16167 | 0.165513 |
| 40959 | 1.16167 | 0.216234 |
| 40960 | 1.16167 | 0.119298 |
| 40961 | 1.32027 | 0.05 |
| 40962 | 0.946131 | 0.0747648 |
| 40963 | 0.946131 | 0.434168 |
| 40964 | 0.94613 | 0.055205 |
| 40965 | 1.16167 | 0.05 |
| 40966 | 0.946131 | 0.137736 |
| 40967 | 1.32027 | 0.117198 |
| 40968 | 2.32335 | 0.150176 |
| 40969 | 1.83151 | 0.0563207 |
| 40970 | 2.38525 | 0.104452 |
| 40971 | 2.64055 | 0.0874033 |
| 40972 | 1.57621 | 0.05 |
| 40973 | 1.83151 | 0.05 |
| 40974 | 2.25677 | 2.1 |
| 40975 | 1.88158 | 2.1 |
| 40976 | 1.86856 | 2.1 |
| 40977 | 2.0815 | 2.1 |
| 40978 | 1.70303 | 2.1 |
| 40979 | 1.88158 | 2.1 |
| 40980 | 2.50381 | 2.1 |
| 40981 | 1.89227 | 2.1 |
| 40982 | 2.25677 | 2.1 |
| 40983 | 2.25677 | 2.1 |
| 40984 | 1.89227 | 2.1 |
| 40985 | 1.89227 | 2.1 |
| 40986 | 1.89226 | 0.05 |
| 40987 | 1.50899 | 0.05 |
| 40988 | 2.13659 | 0.05 |
| 40989 | 1.50899 | 0.0506321 |
| 40990 | 2.51986 | 0.05 |
| 40991 | 1.89226 | 0.05 |
| 40992 | 0.946131 | 0.05 |
| 40993 | 0.946131 | 0.05 |
| 40994 | 1.06829 | 0.05 |
| 40995 | 0.895375 | 0.05 |
| 40996 | 0.94613 | 0.05 |
| 40997 | 0.94613 | 0.05 |
| 40998 | 1.33922 | 0.05 |
| 40999 | 0.895376 | 0.05 |
| 41000 | 0.946131 | 0.05 |
| 41001 | 1.33922 | 0.05 |
| 41002 | 0.946131 | 0.05 |
| 41003 | 1.06829 | 0.05 |

| | | |
|---|---|---|
| 41004 | 0.841467 | 0.182441 |
| 41005 | 0.578428 | 0.293947 |
| 41006 | 1.18079 | 0.159374 |
| 41007 | 0.920237 | 0.303927 |
| 41008 | 0.772317 | 0.0795366 |
| 41009 | 0.856555 | 0.45432 |
| 41010 | 0.910271 | 0.203148 |
| 41011 | 0.910271 | 0.489728 |
| 41012 | 0.975752 | 0.214098 |
| 41013 | 0.536929 | 0.109431 |
| 41014 | 0.81952 | 0.352563 |
| 41015 | 1.15252 | 0.138819 |
| 41016 | 1.89226 | 2.1 |
| 41017 | 2.51431 | 2.1 |
| 41018 | 1.89226 | 2.1 |
| 41019 | 1.89226 | 2.1 |
| 41020 | 2.78815 | 2.1 |
| 41021 | 2.78815 | 2.1 |
| 41022 | 2.0815 | 2.1 |
| 41023 | 2.0516 | 2.1 |
| 41024 | 2.0815 | 2.1 |
| 41025 | 3.06699 | 2.1 |
| 41026 | 2.76576 | 2.1 |
| 41027 | 2.8002 | 2.1 |
| 41028 | 1.94112 | 2.1 |
| 41029 | 1.89226 | 2.1 |
| 41030 | 2.20358 | 2.1 |
| 41031 | 1.89226 | 2.1 |
| 41032 | 4.09584 | 2.1 |
| 41033 | 1.09154 | 2.1 |
| 41034 | 2.62647 | 2.1 |
| 41035 | 2.20358 | 2.1 |
| 41036 | 2.20358 | 2.1 |
| 41037 | 1.52314 | 2.1 |
| 41038 | 1.52314 | 2.1 |
| 41039 | 1.31323 | 2.1 |
| 41040 | 1.31323 | 2.1 |
| 41041 | 0.946132 | 2.1 |
| 41042 | 0.946132 | 2.1 |
| 41043 | 0.946132 | 2.1 |
| 41044 | 0.946132 | 2.1 |
| 41045 | 1.31323 | 2.1 |
| 41046 | 1.10179 | 2.1 |
| 41047 | 1.31323 | 2.1 |
| 41048 | 0.946132 | 2.1 |
| 41049 | 1.10179 | 2.1 |
| 41050 | 0.946133 | 2.1 |
| 41051 | 1.89226 | 0.800002 |
| 41052 | 1.89226 | 1.15659 |
| 41053 | 1.89226 | 1.10825 |
| 41054 | 1.89226 | 1.29902 |
| 41055 | 1.89226 | 0.701665 |
| 41056 | 1.89226 | 1.03973 |
| 41057 | 1.35126 | 2.02589 |
| 41058 | 1.35126 | 2.1 |
| 41059 | 0.903285 | 1.25271 |
| 41060 | 0.903285 | 1.76393 |
| 41061 | 1.30841 | 1.60564 |

| | | |
|---|---|---|
| 41062 | 0.903313 | 2.1 |
| 41063 | 0.946132 | 1.47891 |
| 41064 | 0.946132 | 1.12314 |
| 41065 | 0.946132 | 1.55125 |
| 41066 | 0.903312 | 1.43064 |
| 41067 | 1.30841 | 1.31634 |
| 41068 | 0.946133 | 1.24373 |
| 41069 | 2.62097 | 2.1 |
| 41070 | 2.72561 | 2.1 |
| 41071 | 2.39016 | 2.1 |
| 41072 | 2.51062 | 2.1 |
| 41073 | 5.11577 | 2.1 |
| 41074 | 2.51062 | 2.1 |
| 41075 | 2.58286 | 0.0686292 |
| 41076 | 2.40107 | 2.1 |
| 41077 | 2.40107 | 0.064162 |
| 41078 | 2.72561 | 2.1 |
| 41079 | 2.72561 | 0.138755 |
| 41080 | 2.39016 | 2.1 |
| 41081 | 2.51062 | 2.1 |
| 41082 | 2.20242 | 2.1 |
| 41083 | 2.34107 | 2.1 |
| 41084 | 4.73124 | 2.1 |
| 41085 | 2.51062 | 2.1 |
| 41086 | 2.40107 | 0.05 |
| 41087 | 2.34107 | 2.1 |
| 41088 | 2.25197 | 0.05 |
| 41089 | 4.74215 | 0.118545 |
| 41090 | 2.75218 | 2.1 |
| 41091 | 1.89226 | 2.1 |
| 41092 | 2.3349 | 2.1 |
| 41093 | 2.3349 | 2.1 |
| 41094 | 1.89226 | 2.1 |
| 41095 | 1.89226 | 2.1 |
| 41096 | 1.46129 | 2.1 |
| 41097 | 0.996308 | 2.1 |
| 41098 | 1.88649 | 2.1 |
| 41099 | 1.70302 | 2.1 |
| 41100 | 2.95419 | 2.1 |
| 41101 | 1.89227 | 2.1 |
| 41102 | 1.89227 | 2.1 |
| 41103 | 1.89227 | 2.1 |
| 41104 | 1.89227 | 2.1 |
| 41105 | 3.78453 | 2.1 |
| 41106 | 0.646558 | 2.1 |
| 41107 | 0.646557 | 2.1 |
| 41108 | 0.60697 | 2.1 |
| 41109 | 0.60697 | 2.1 |
| 41110 | 0.587917 | 2.1 |
| 41111 | 0.519747 | 2.1 |
| 41112 | 0.586443 | 2.1 |
| 41113 | 0.733782 | 2.1 |
| 41114 | 0.733793 | 2.1 |
| 41115 | 0.587924 | 2.1 |
| 41116 | 0.519739 | 2.1 |
| 41117 | 0.586436 | 2.1 |
| 41118 | 2.1642 | 2.1 |
| 41119 | 1.42867 | 2.1 |

| | | |
|---|---|---|
| 41120 | 2.28847 | 2.1 |
| 41121 | 1.68299 | 2.1 |
| 41122 | 3.71714 | 2.1 |
| 41123 | 1.89226 | 2.1 |
| 41124 | 1.89226 | 2.1 |
| 41125 | 1.89226 | 2.1 |
| 41126 | 1.89226 | 2.1 |
| 41127 | 3.78453 | 2.1 |
| 41128 | 0.946131 | 2.1 |
| 41129 | 0.946131 | 2.1 |
| 41130 | 1.24994 | 2.1 |
| 41131 | 1.24994 | 2.1 |
| 41132 | 1.11582 | 2.1 |
| 41133 | 1.24994 | 2.1 |
| 41134 | 0.946133 | 2.1 |
| 41135 | 0.946133 | 2.1 |
| 41136 | 0.946131 | 2.1 |
| 41137 | 1.24994 | 2.1 |
| 41138 | 0.94613 | 2.1 |
| 41139 | 1.11582 | 2.1 |
| 41140 | 2.28847 | 2.1 |
| 41141 | 1.53629 | 2.1 |
| 41142 | 1.57498 | 2.1 |
| 41143 | 1.47417 | 2.1 |
| 41144 | 2.42221 | 2.1 |
| 41145 | 2.28847 | 2.1 |
| 41146 | 1.89226 | 2.1 |
| 41147 | 1.89226 | 2.1 |
| 41148 | 1.89226 | 2.1 |
| 41149 | 1.89226 | 2.1 |
| 41150 | 3.78453 | 2.1 |
| 41151 | 1.89226 | 2.1 |
| 41152 | 2.49988 | 2.1 |
| 41153 | 2.71233 | 2.1 |
| 41154 | 2.71233 | 2.1 |
| 41155 | 1.89226 | 2.1 |
| 41156 | 1.89226 | 2.1 |
| 41157 | 0.924534 | 2.1 |
| 41158 | 0.924534 | 2.1 |
| 41159 | 0.990395 | 2.1 |
| 41160 | 0.990395 | 2.1 |
| 41161 | 0.946131 | 2.1 |
| 41162 | 0.946131 | 2.1 |
| 41163 | 0.924505 | 2.1 |
| 41164 | 0.968797 | 2.1 |
| 41165 | 0.924505 | 2.1 |
| 41166 | 0.946131 | 2.1 |
| 41167 | 0.968797 | 2.1 |
| 41168 | 0.946131 | 2.1 |
| 41169 | 0.924533 | 2.1 |
| 41170 | 0.924533 | 2.1 |
| 41171 | 0.948264 | 2.1 |
| 41172 | 0.945345 | 2.1 |
| 41173 | 0.851541 | 2.1 |
| 41174 | 0.851541 | 2.1 |
| 41175 | 0.958934 | 2.1 |
| 41176 | 1.02617 | 2.1 |
| 41177 | 0.936668 | 2.1 |

| | | |
|---|---|---|
| 41178 | 1.04829 | 2.1 |
| 41179 | 0.924505 | 2.1 |
| 41180 | 0.946131 | 2.1 |
| 41181 | 2.28431 | 0.567489 |
| 41182 | 1.48035 | 0.100897 |
| 41183 | 1.59971 | 0.482312 |
| 41184 | 1.48035 | 0.0553136 |
| 41185 | 2.33818 | 0.460211 |
| 41186 | 2.21882 | 0.245286 |
| 41187 | 2.28431 | 0.458349 |
| 41188 | 1.89226 | 0.112253 |
| 41189 | 1.89226 | 0.587611 |
| 41190 | 2.3968 | 0.05 |
| 41191 | 4.28906 | 0.05 |
| 41192 | 2.28431 | 0.412037 |
| 41193 | 1.70303 | 0.05 |
| 41194 | 1.5428 | 0.05 |
| 41195 | 1.59971 | 0.05 |
| 41196 | 2.31152 | 0.313692 |
| 41197 | 2.3968 | 0.451143 |
| 41198 | 1.99823 | 2.1 |
| 41199 | 2.21189 | 2.1 |
| 41200 | 2.18937 | 2.1 |
| 41201 | 1.979 | 2.1 |
| 41202 | 4.19089 | 2.1 |
| 41203 | 0.999114 | 2.1 |
| 41204 | 0.9895 | 2.1 |
| 41205 | 0.758942 | 2.1 |
| 41206 | 0.749358 | 2.1 |
| 41207 | 0.874236 | 2.1 |
| 41208 | 0.874236 | 2.1 |
| 41209 | 0.874236 | 2.1 |
| 41210 | 0.874236 | 2.1 |
| 41211 | 0.9895 | 2.1 |
| 41212 | 0.749358 | 2.1 |
| 41213 | 0.758942 | 2.1 |
| 41214 | 0.999114 | 2.1 |
| 41215 | 2.18937 | 2.1 |
| 41216 | 1.979 | 2.1 |
| 41217 | 1.95416 | 2.1 |
| 41218 | 2.162 | 2.1 |
| 41219 | 4.14099 | 2.1 |
| 41220 | 1.95416 | 2.1 |
| 41221 | 1.49871 | 2.1 |
| 41222 | 1.47712 | 2.1 |
| 41223 | 1.49871 | 2.1 |
| 41224 | 1.9574 | 2.1 |
| 41225 | 1.979 | 2.1 |
| 41226 | 1.68508 | 0.05 |
| 41227 | 1.64984 | 0.05 |
| 41228 | 2.67711 | 0.05 |
| 41229 | 2.76407 | 0.05 |
| 41230 | 4.41391 | 0.05 |
| 41231 | 1.84227 | 0.05 |
| 41232 | 2.76407 | 0.05 |
| 41233 | 2.76407 | 0.05 |
| 41234 | 1.85007 | 0.05 |
| 41235 | 2.72769 | 0.05 |

| | | |
|---|---|---|
| 41236 | 1.80812 | 0.05 |
| 41237 | 1.94516 | 2.1 |
| 41238 | 1.89226 | 2.1 |
| 41239 | 1.89226 | 2.1 |
| 41240 | 1.89226 | 2.1 |
| 41241 | 1.98853 | 2.1 |
| 41242 | 1.98852 | 2.1 |
| 41243 | 1.28095 | 2.1 |
| 41244 | 2.0815 | 2.1 |
| 41245 | 2.29123 | 2.1 |
| 41246 | 2.06833 | 2.1 |
| 41247 | 1.08212 | 2.1 |
| 41248 | 0.976848 | 2.1 |
| 41249 | 1.89226 | 2.1 |
| 41250 | 1.89226 | 2.1 |
| 41251 | 1.89226 | 2.1 |
| 41252 | 1.89226 | 2.1 |
| 41253 | 1.89226 | 2.1 |
| 41254 | 1.89226 | 2.1 |
| 41255 | 1.13537 | 2.1 |
| 41256 | 1.18233 | 2.1 |
| 41257 | 0.946132 | 2.1 |
| 41258 | 0.946132 | 2.1 |
| 41259 | 0.946132 | 2.1 |
| 41260 | 0.946132 | 2.1 |
| 41261 | 0.994264 | 2.1 |
| 41262 | 1.18233 | 2.1 |
| 41263 | 1.13537 | 2.1 |
| 41264 | 0.946131 | 2.1 |
| 41265 | 0.946132 | 2.1 |
| 41266 | 0.994264 | 2.1 |
| 41267 | 0.953204 | 2.1 |
| 41268 | 0.953204 | 2.1 |
| 41269 | 0.966515 | 2.1 |
| 41270 | 0.966515 | 2.1 |
| 41271 | 1.30583 | 2.1 |
| 41272 | 1.14561 | 2.1 |
| 41273 | 0.640475 | 2.1 |
| 41274 | 0.774103 | 2.1 |
| 41275 | 1.30584 | 2.1 |
| 41276 | 0.774101 | 2.1 |
| 41277 | 0.640474 | 2.1 |
| 41278 | 1.14562 | 2.1 |
| 41279 | 0.946131 | 2.1 |
| 41280 | 0.946131 | 2.1 |
| 41281 | 0.946131 | 2.1 |
| 41282 | 0.946131 | 2.1 |
| 41283 | 0.946128 | 2.1 |
| 41284 | 0.946128 | 2.1 |
| 41285 | 0.946133 | 2.1 |
| 41286 | 0.946133 | 2.1 |
| 41287 | 0.94613 | 2.1 |
| 41288 | 0.94613 | 2.1 |
| 41289 | 0.946131 | 2.1 |
| 41290 | 0.946131 | 2.1 |
| 41291 | 0.946132 | 0.05 |
| 41292 | 0.946132 | 0.05 |
| 41293 | 0.946131 | 0.05 |

| | | |
|---|---|---|
| 41294 | 0.946131 | 0.05 |
| 41295 | 0.946131 | 0.05 |
| 41296 | 0.946131 | 0.05 |
| 41297 | 0.946132 | 0.05 |
| 41298 | 0.946132 | 0.05 |
| 41299 | 0.946131 | 0.05 |
| 41300 | 0.946131 | 0.05 |
| 41301 | 0.946132 | 0.05 |
| 41302 | 0.946132 | 0.05 |
| 41303 | 0.946131 | 0.05 |
| 41304 | 0.946131 | 0.05 |
| 41305 | 0.946131 | 0.05 |
| 41306 | 0.946131 | 0.05 |
| 41307 | 0.94613 | 0.05 |
| 41308 | 0.94613 | 0.05 |
| 41309 | 0.946132 | 0.05 |
| 41310 | 0.946132 | 0.05 |
| 41311 | 0.946131 | 0.05 |
| 41312 | 0.946131 | 0.05 |
| 41313 | 0.946131 | 0.05 |
| 41314 | 0.946131 | 0.05 |
| 41315 | 2.57882 | 0.05 |
| 41316 | 1.48676 | 0.05 |
| 41317 | 1.48797 | 0.05 |
| 41318 | 2.64281 | 0.0605866 |
| 41319 | 4.12957 | 0.05 |
| 41320 | 2.56323 | 0.05 |
| 41321 | 2.64281 | 0.05 |
| 41322 | 2.51807 | 0.05 |
| 41323 | 1.36324 | 0.05 |
| 41324 | 1.48797 | 0.0621751 |
| 41325 | 1.48797 | 0.059048 |
| 41326 | 1.04367 | 2.1 |
| 41327 | 2.42504 | 2.1 |
| 41328 | 2.65164 | 2.1 |
| 41329 | 1.49479 | 2.1 |
| 41330 | 3.91983 | 2.1 |
| 41331 | 2.15622 | 1.7503 |
| 41332 | 1.70539 | 2.1 |
| 41333 | 2.02369 | 2.1 |
| 41334 | 2.39039 | 2.1 |
| 41335 | 4.17992 | 2.1 |
| 41336 | 0.966979 | 2.1 |
| 41337 | 1.7054 | 2.1 |
| 41338 | 2.0237 | 2.1 |
| 41339 | 1.31502 | 2.1 |
| 41340 | 2.99067 | 2.1 |
| 41341 | 2.02369 | 2.1 |
| 41342 | 2.65637 | 2.1 |
| 41343 | 2.39039 | 1.49275 |
| 41344 | 2.39039 | 2.1 |
| 41345 | 2.29332 | 1.53297 |
| 41346 | 2.29332 | 2.1 |
| 41347 | 2.0237 | 2.1 |
| 41348 | 1.72116 | 2.1 |
| 41349 | 1.58465 | 2.1 |
| 41350 | 1.31502 | 2.1 |
| 41351 | 2.29332 | 2.1 |

| | | |
|---|---|---|
| 41352 | 2.0237 | 2.1 |
| 41353 | 1.0372 | 0.05 |
| 41354 | 2.32462 | 0.05 |
| 41355 | 2.2291 | 0.05 |
| 41356 | 2.2291 | 0.05 |
| 41357 | 1.17464 | 0.05 |
| 41358 | 1.17464 | 0.05 |
| 41359 | 2.68289 | 0.05 |
| 41360 | 1.07805 | 0.05 |
| 41361 | 1.70302 | 0.05 |
| 41362 | 2.06653 | 0.05 |
| 41363 | 3.5779 | 0.05 |
| 41364 | 1.89226 | 0.05 |
| 41365 | 1.89226 | 0.05 |
| 41366 | 1.89226 | 0.05 |
| 41367 | 1.89226 | 0.05 |
| 41368 | 3.78453 | 0.05 |
| 41369 | 1.89226 | 0.05 |
| 41370 | 1.89226 | 0.05 |
| 41371 | 1.89226 | 0.05 |
| 41372 | 1.89226 | 0.05 |
| 41373 | 1.89226 | 0.05 |
| 41374 | 1.89226 | 0.05 |
| 41375 | 1.89225 | 0.05 |
| 41376 | 1.89225 | 0.05 |
| 41377 | 1.89225 | 0.05 |
| 41378 | 1.89225 | 0.05 |
| 41379 | 1.89225 | 0.05 |
| 41380 | 1.89225 | 0.05 |
| 41381 | 0.94613 | 0.05 |
| 41382 | 0.94613 | 0.05 |
| 41383 | 0.94613 | 0.05 |
| 41384 | 0.94613 | 0.05 |
| 41385 | 0.946128 | 0.05 |
| 41386 | 0.946128 | 0.05 |
| 41387 | 0.946132 | 0.05 |
| 41388 | 0.946132 | 0.05 |
| 41389 | 0.94613 | 0.05 |
| 41390 | 0.94613 | 0.05 |
| 41391 | 0.94613 | 0.05 |
| 41392 | 0.94613 | 0.05 |
| 41393 | 1.29417 | 2.1 |
| 41394 | 1.32732 | 2.1 |
| 41395 | 1.32316 | 0.956163 |
| 41396 | 1.34973 | 2.1 |
| 41397 | 2.65048 | 2.1 |
| 41398 | 1.89226 | 1.23094 |
| 41399 | 1.89226 | 2.1 |
| 41400 | 1.89226 | 0.969979 |
| 41401 | 1.89226 | 2.1 |
| 41402 | 3.78453 | 1.72754 |
| 41403 | 1.89226 | 2.1 |
| 41404 | 1.89226 | 2.1 |
| 41405 | 1.89226 | 1.08073 |
| 41406 | 1.89226 | 2.1 |
| 41407 | 3.78453 | 2.1 |
| 41408 | 1.32732 | 2.1 |
| 41409 | 1.13006 | 2.1 |

| | | |
|---|---|---|
| 41410 | 1.13358 | 2.1 |
| 41411 | 1.11117 | 2.1 |
| 41412 | 1.34973 | 2.1 |
| 41413 | 1.32732 | 2.1 |
| 41414 | 1.32316 | 1.92893 |
| 41415 | 1.34973 | 2.1 |
| 41416 | 1.34066 | 1.0075 |
| 41417 | 1.36601 | 2.1 |
| 41418 | 2.68918 | 2.1 |
| 41419 | 1.89226 | 1.82667 |
| 41420 | 1.89226 | 2.1 |
| 41421 | 1.89226 | 0.554482 |
| 41422 | 1.89226 | 2.1 |
| 41423 | 3.78453 | 2.04478 |
| 41424 | 1.89226 | 1.93341 |
| 41425 | 1.89226 | 2.1 |
| 41426 | 1.89226 | 2.1 |
| 41427 | 1.89226 | 2.1 |
| 41428 | 1.89226 | 1.5334 |
| 41429 | 1.89226 | 2.1 |
| 41430 | 0.624134 | 2.1 |
| 41431 | 0.624134 | 2.1 |
| 41432 | 0.624334 | 2.07839 |
| 41433 | 0.624334 | 2.1 |
| 41434 | 0.56503 | 2.1 |
| 41435 | 0.573605 | 2.1 |
| 41436 | 0.674864 | 2.1 |
| 41437 | 0.683007 | 2.1 |
| 41438 | 0.683007 | 2.1 |
| 41439 | 0.573605 | 2.1 |
| 41440 | 0.565029 | 2.1 |
| 41441 | 0.674864 | 2.1 |
| 41442 | 2.16633 | 2.1 |
| 41443 | 1.40846 | 2.1 |
| 41444 | 2.13977 | 2.1 |
| 41445 | 1.40771 | 2.1 |
| 41446 | 3.57404 | 2.1 |
| 41447 | 2.13977 | 2.1 |
| 41448 | 1.29932 | 2.1 |
| 41449 | 1.29932 | 2.1 |
| 41450 | 1.40771 | 2.1 |
| 41451 | 1.98957 | 2.1 |
| 41452 | 2.09796 | 2.1 |
| 41453 | 2.6044 | 0.05 |
| 41454 | 1.85548 | 0.0614923 |
| 41455 | 1.89226 | 0.05 |
| 41456 | 1.89226 | 0.0964837 |
| 41457 | 3.74774 | 0.05 |
| 41458 | 1.89226 | 0.05 |
| 41459 | 1.89226 | 0.118748 |
| 41460 | 1.89226 | 0.05 |
| 41461 | 1.89226 | 0.05 |
| 41462 | 3.78453 | 0.05 |
| 41463 | 1.89226 | 2.1 |
| 41464 | 2.64107 | 2.1 |
| 41465 | 1.89226 | 2.1 |
| 41466 | 2.78977 | 2.1 |
| 41467 | 1.89226 | 2.1 |

| | | |
|---|---|---|
| 41468 | 2.78977 | 2.1 |
| 41469 | 1.89226 | 2.1 |
| 41470 | 1.89226 | 2.1 |
| 41471 | 1.89226 | 2.1 |
| 41472 | 1.89226 | 2.1 |
| 41473 | 1.89226 | 2.1 |
| 41474 | 1.89226 | 2.1 |
| 41475 | 2.43173 | 2.1 |
| 41476 | 1.41238 | 2.1 |
| 41477 | 2.0815 | 2.1 |
| 41478 | 1.98048 | 2.1 |
| 41479 | 1.62014 | 2.1 |
| 41480 | 1.50086 | 2.1 |
| 41481 | 0.946129 | 0.180711 |
| 41482 | 0.946129 | 0.181147 |
| 41483 | 1.27636 | 0.321061 |
| 41484 | 1.1984 | 0.05 |
| 41485 | 0.946131 | 0.05 |
| 41486 | 0.946131 | 0.0878991 |
| 41487 | 1.27636 | 0.240838 |
| 41488 | 1.27636 | 0.05 |
| 41489 | 0.946131 | 0.228837 |
| 41490 | 1.27636 | 0.05 |
| 41491 | 0.946131 | 0.0631748 |
| 41492 | 1.1984 | 0.05 |
| 41493 | 1.99569 | 0.078191 |
| 41494 | 2.55272 | 0.321534 |
| 41495 | 1.77403 | 0.05 |
| 41496 | 1.70303 | 0.05 |
| 41497 | 3.05772 | 0.188637 |
| 41498 | 2.55121 | 0.05 |
| 41499 | 0.946128 | 0.0635709 |
| 41500 | 0.946128 | 0.0967008 |
| 41501 | 1.38752 | 0.179234 |
| 41502 | 1.27636 | 0.05 |
| 41503 | 0.94613 | 0.05 |
| 41504 | 0.94613 | 0.05 |
| 41505 | 1.38752 | 0.179927 |
| 41506 | 1.38752 | 0.05 |
| 41507 | 0.946131 | 0.0610851 |
| 41508 | 1.38752 | 0.0655636 |
| 41509 | 0.946131 | 0.353701 |
| 41510 | 1.27636 | 0.05 |
| 41511 | 2.77504 | 0.0710903 |
| 41512 | 1.99569 | 0.05 |
| 41513 | 2.29794 | 0.05 |
| 41514 | 1.99569 | 0.05 |
| 41515 | 2.85497 | 0.05 |
| 41516 | 2.55272 | 0.05 |
| 41517 | 1.53284 | 2.1 |
| 41518 | 1.89226 | 2.1 |
| 41519 | 1.89226 | 2.1 |
| 41520 | 1.89226 | 2.1 |
| 41521 | 1.54763 | 2.1 |
| 41522 | 1.54763 | 2.1 |
| 41523 | 0.946131 | 2.1 |
| 41524 | 0.946131 | 2.1 |
| 41525 | 0.946127 | 2.1 |

| | | |
|---|---|---|
| 41526 | 0.946127 | 2.1 |
| 41527 | 0.946127 | 2.1 |
| 41528 | 0.946127 | 2.1 |
| 41529 | 0.946131 | 2.1 |
| 41530 | 0.946131 | 2.1 |
| 41531 | 0.94613 | 2.1 |
| 41532 | 0.94613 | 2.1 |
| 41533 | 0.946129 | 2.1 |
| 41534 | 0.946129 | 2.1 |
| 41535 | 0.751754 | 2.1 |
| 41536 | 0.751754 | 2.1 |
| 41537 | 0.771562 | 2.1 |
| 41538 | 0.771561 | 2.1 |
| 41539 | 1.0268 | 2.1 |
| 41540 | 0.95482 | 2.1 |
| 41541 | 0.528937 | 2.1 |
| 41542 | 0.496512 | 2.1 |
| 41543 | 0.528936 | 2.1 |
| 41544 | 1.0268 | 2.1 |
| 41545 | 0.954821 | 2.1 |
| 41546 | 0.49651 | 2.1 |
| 41547 | 2.64075 | 0.406807 |
| 41548 | 1.35181 | 1.12106 |
| 41549 | 1.35181 | 0.0663877 |
| 41550 | 1.83823 | 0.551685 |
| 41551 | 2.28373 | 0.68045 |
| 41552 | 2.88566 | 1.59613 |
| 41553 | 0.493018 | 1.10396 |
| 41554 | 0.675903 | 0.960439 |
| 41555 | 1.14187 | 0.623549 |
| 41556 | 0.973937 | 0.555143 |
| 41557 | 0.821166 | 0.627215 |
| 41558 | 0.821166 | 1.65016 |
| 41559 | 0.828674 | 2.1 |
| 41560 | 0.828674 | 0.369661 |
| 41561 | 0.973937 | 0.777279 |
| 41562 | 0.493018 | 0.795998 |
| 41563 | 0.675903 | 0.716691 |
| 41564 | 1.14187 | 0.814045 |
| 41565 | 1.89226 | 2.1 |
| 41566 | 2.11667 | 2.1 |
| 41567 | 1.91184 | 2.1 |
| 41568 | 1.89226 | 2.1 |
| 41569 | 2.13625 | 2.1 |
| 41570 | 2.11667 | 2.1 |
| 41571 | 1.89226 | 2.1 |
| 41572 | 1.89226 | 2.1 |
| 41573 | 1.89226 | 2.1 |
| 41574 | 1.89226 | 2.1 |
| 41575 | 3.78453 | 2.1 |
| 41576 | 0.907729 | 2.1 |
| 41577 | 0.907729 | 2.1 |
| 41578 | 1.04459 | 2.1 |
| 41579 | 0.890955 | 2.1 |
| 41580 | 0.988181 | 2.1 |
| 41581 | 0.841856 | 2.1 |
| 41582 | 0.946133 | 2.1 |
| 41583 | 0.946133 | 2.1 |

| | | |
|---|---|---|
| 41584 | 1.04072 | 2.1 |
| 41585 | 1.1064 | 2.1 |
| 41586 | 0.89099 | 2.1 |
| 41587 | 0.851541 | 2.1 |
| 41588 | 1.89225 | 2.1 |
| 41589 | 2.13623 | 2.1 |
| 41590 | 1.91044 | 2.1 |
| 41591 | 1.89225 | 2.1 |
| 41592 | 2.15442 | 2.1 |
| 41593 | 2.13623 | 2.1 |
| 41594 | 1.89225 | 2.1 |
| 41595 | 1.89225 | 2.1 |
| 41596 | 1.89225 | 2.1 |
| 41597 | 1.89225 | 2.1 |
| 41598 | 1.89225 | 2.1 |
| 41599 | 1.89225 | 2.1 |
| 41600 | 0.907723 | 2.1 |
| 41601 | 0.907723 | 2.1 |
| 41602 | 1.07536 | 2.1 |
| 41603 | 1.07814 | 2.1 |
| 41604 | 0.998499 | 2.1 |
| 41605 | 1.03575 | 2.1 |
| 41606 | 0.946126 | 2.1 |
| 41607 | 0.946126 | 2.1 |
| 41608 | 1.04072 | 2.1 |
| 41609 | 1.14025 | 2.1 |
| 41610 | 0.851528 | 2.1 |
| 41611 | 0.898284 | 2.1 |
| 41612 | 1.89226 | 0.05 |
| 41613 | 1.89226 | 0.05 |
| 41614 | 1.89226 | 0.05 |
| 41615 | 1.89226 | 0.05 |
| 41616 | 1.89226 | 0.05 |
| 41617 | 1.89226 | 0.05 |
| 41618 | 2.53008 | 2.1 |
| 41619 | 1.94534 | 2.1 |
| 41620 | 1.98702 | 2.1 |
| 41621 | 1.98858 | 2.1 |
| 41622 | 2.52852 | 2.1 |
| 41623 | 2.53008 | 2.1 |
| 41624 | 1.03267 | 2.1 |
| 41625 | 1.12591 | 2.1 |
| 41626 | 1.2959 | 2.1 |
| 41627 | 1.28992 | 2.1 |
| 41628 | 1.10851 | 2.1 |
| 41629 | 1.33078 | 2.1 |
| 41630 | 1.10661 | 2.1 |
| 41631 | 1.10661 | 2.1 |
| 41632 | 0.982208 | 2.1 |
| 41633 | 1.28992 | 2.1 |
| 41634 | 1.03267 | 2.1 |
| 41635 | 1.23262 | 2.1 |
| 41636 | 2.2712 | 2.1 |
| 41637 | 1.89226 | 2.1 |
| 41638 | 1.95954 | 2.1 |
| 41639 | 1.95954 | 2.1 |
| 41640 | 1.89226 | 2.1 |
| 41641 | 1.89226 | 2.1 |

| | | |
|---|---|---|
| 41642 | 1.89226 | 2.1 |
| 41643 | 1.89226 | 2.1 |
| 41644 | 1.89226 | 2.1 |
| 41645 | 1.89226 | 2.1 |
| 41646 | 3.78453 | 2.1 |
| 41647 | 1.33887 | 2.1 |
| 41648 | 1.25395 | 2.1 |
| 41649 | 0.88327 | 2.1 |
| 41650 | 0.676665 | 2.1 |
| 41651 | 0.84881 | 2.1 |
| 41652 | 0.987503 | 2.1 |
| 41653 | 1.13243 | 2.1 |
| 41654 | 1.13243 | 2.1 |
| 41655 | 1.15338 | 2.1 |
| 41656 | 0.849966 | 2.1 |
| 41657 | 0.793074 | 2.1 |
| 41658 | 1.11957 | 2.1 |
| 41659 | 0.946129 | 2.1 |
| 41660 | 0.946129 | 2.1 |
| 41661 | 1.28254 | 2.1 |
| 41662 | 1.1356 | 2.1 |
| 41663 | 1.1356 | 2.1 |
| 41664 | 1.1356 | 2.1 |
| 41665 | 0.946131 | 2.1 |
| 41666 | 0.946131 | 2.1 |
| 41667 | 0.946131 | 2.1 |
| 41668 | 1.28254 | 2.1 |
| 41669 | 1.1356 | 2.1 |
| 41670 | 0.946131 | 2.1 |
| 41671 | 1.89227 | 2.1 |
| 41672 | 1.89227 | 2.1 |
| 41673 | 1.89227 | 2.1 |
| 41674 | 1.89227 | 2.1 |
| 41675 | 3.78453 | 2.1 |
| 41676 | 0.936287 | 0.05 |
| 41677 | 1.01399 | 0.05 |
| 41678 | 0.959415 | 0.05 |
| 41679 | 0.959415 | 0.05 |
| 41680 | 0.910786 | 0.05 |
| 41681 | 1.38301 | 0.05 |
| 41682 | 1.0286 | 0.05 |
| 41683 | 0.535742 | 0.05 |
| 41684 | 0.505202 | 0.05 |
| 41685 | 0.937685 | 0.05 |
| 41686 | 1.11066 | 0.05 |
| 41687 | 1.46907 | 0.05 |
| 41688 | 1.00546 | 0.05 |
| 41689 | 1.01524 | 0.05 |
| 41690 | 0.94613 | 0.05 |
| 41691 | 0.94613 | 0.05 |
| 41692 | 1.05018 | 0.05 |
| 41693 | 0.914025 | 0.05 |
| 41694 | 0.913512 | 0.05 |
| 41695 | 0.851542 | 0.05 |
| 41696 | 1.05385 | 0.05 |
| 41697 | 1.05494 | 0.05 |
| 41698 | 0.91404 | 0.05 |
| 41699 | 0.91404 | 0.05 |

| | | |
|---|---|---|
| 41700 | 2.03253 | 0.749017 |
| 41701 | 1.82325 | 0.0914881 |
| 41702 | 1.93938 | 0.359216 |
| 41703 | 1.73756 | 0.05 |
| 41704 | 3.77009 | 0.071342 |
| 41705 | 1.82325 | 0.174104 |
| 41706 | 1.47562 | 0.655955 |
| 41707 | 1.73756 | 0.05 |
| 41708 | 1.3868 | 0.05 |
| 41709 | 3.21317 | 0.05 |
| 41710 | 1.93938 | 0.556479 |
| 41711 | 1.73756 | 0.179128 |
| 41712 | 1.85293 | 0.659499 |
| 41713 | 1.64279 | 0.05 |
| 41714 | 3.59049 | 0.123218 |
| 41715 | 1.73756 | 0.14602 |
| 41716 | 1.3868 | 0.577442 |
| 41717 | 1.64279 | 0.05 |
| 41718 | 1.28419 | 0.0815589 |
| 41719 | 3.02174 | 0.139081 |
| 41720 | 1.35048 | 2.1 |
| 41721 | 1.37999 | 2.1 |
| 41722 | 1.34355 | 1.03901 |
| 41723 | 1.37854 | 2.1 |
| 41724 | 2.72354 | 2.05053 |
| 41725 | 1.89226 | 1.19589 |
| 41726 | 1.89226 | 2.1 |
| 41727 | 1.89226 | 0.966052 |
| 41728 | 1.89226 | 2.04182 |
| 41729 | 3.78452 | 1.14111 |
| 41730 | 1.89226 | 2.1 |
| 41731 | 1.89226 | 2.1 |
| 41732 | 1.89226 | 0.393033 |
| 41733 | 1.89226 | 2.1 |
| 41734 | 3.78453 | 2.1 |
| 41735 | 1.37999 | 2.1 |
| 41736 | 1.18059 | 2.1 |
| 41737 | 1.17117 | 2.1 |
| 41738 | 1.17262 | 2.1 |
| 41739 | 1.37855 | 2.1 |
| 41740 | 1.37999 | 2.1 |
| 41741 | 1.34355 | 2.05071 |
| 41742 | 1.37854 | 2.1 |
| 41743 | 1.33079 | 1.04438 |
| 41744 | 1.3741 | 2.1 |
| 41745 | 2.71765 | 2.1 |
| 41746 | 1.89226 | 1.57823 |
| 41747 | 1.89226 | 2.1 |
| 41748 | 1.89226 | 0.816095 |
| 41749 | 1.89226 | 2.1 |
| 41750 | 3.78453 | 1.95292 |
| 41751 | 1.89226 | 1.08368 |
| 41752 | 1.89226 | 2.1 |
| 41753 | 1.89226 | 1.29092 |
| 41754 | 1.89226 | 2.1 |
| 41755 | 3.78453 | 2.1 |
| 41756 | 0.640305 | 2.1 |
| 41757 | 0.640305 | 2.1 |

| | | |
|---|---|---|
| 41758 | 0.643507 | 0.265975 |
| 41759 | 0.643507 | 2.1 |
| 41760 | 0.590294 | 2.1 |
| 41761 | 0.594539 | 2.1 |
| 41762 | 0.689273 | 1.18835 |
| 41763 | 0.687048 | 2.1 |
| 41764 | 0.687048 | 2.1 |
| 41765 | 0.594538 | 2.1 |
| 41766 | 0.590294 | 2.1 |
| 41767 | 0.689273 | 2.1 |
| 41768 | 2.01399 | 0.165498 |
| 41769 | 1.70141 | 0.469692 |
| 41770 | 1.83896 | 1.9618 |
| 41771 | 1.52482 | 1.32934 |
| 41772 | 3.53881 | 0.52765 |
| 41773 | 1.70141 | 0.547024 |
| 41774 | 1.0752 | 2.1 |
| 41775 | 1.0752 | 0.520211 |
| 41776 | 1.25669 | 0.675049 |
| 41777 | 1.52482 | 0.669996 |
| 41778 | 1.70632 | 2.1 |
| 41779 | 1.83896 | 1.48347 |
| 41780 | 1.52482 | 0.05 |
| 41781 | 1.65244 | 0.500977 |
| 41782 | 1.34412 | 1.54524 |
| 41783 | 3.17725 | 0.488594 |
| 41784 | 1.26782 | 0.05 |
| 41785 | 2.90353 | 0.05 |
| 41786 | 2.54203 | 0.05 |
| 41787 | 2.41979 | 0.05 |
| 41788 | 1.77007 | 0.05 |
| 41789 | 1.703 0.05 | |
| 41790 | 0.946118 | 0.05 |
| 41791 | 0.946118 | 0.05 |
| 41792 | 1.38643 | 0.05 |
| 41793 | 1.38644 | 0.05 |
| 41794 | 0.946117 | 0.05 |
| 41795 | 0.946117 | 0.05 |
| 41796 | 1.38644 | 0.05 |
| 41797 | 1.15545 | 0.05 |
| 41798 | 0.946131 | 0.05 |
| 41799 | 1.15546 | 0.05 |
| 41800 | 1.38642 | 0.05 |
| 41801 | 0.946105 | 0.05 |
| 41802 | 2.42256 | 0.0769013 |
| 41803 | 2.63056 | 0.05 |
| 41804 | 2.43174 | 0.05 |
| 41805 | 2.25035 | 0.05 |
| 41806 | 4.8543 | 0.05 |
| 41807 | 2.58031 | 0.05 |
| 41808 | 2.63056 | 0.05 |
| 41809 | 2.43174 | 0.05 |
| 41810 | 2.32127 | 0.05 |
| 41811 | 5.01205 | 0.05 |
| 41812 | 2.43174 | 0.05 |
| 41813 | 2.25035 | 0.05 |
| 41814 | 2.11465 | 0.05 |
| 41815 | 2.13042 | 0.05 |

| | | |
|---|---|---|
| 41816 | 4.54639 | 0.05 |
| 41817 | 2.43174 | 0.05 |
| 41818 | 2.32127 | 0.05 |
| 41819 | 1.92997 | 0.05 |
| 41820 | 2.13041 | 0.05 |
| 41821 | 4.36171 | 0.05 |
| 41822 | 1.89227 | 2.1 |
| 41823 | 1.89227 | 2.1 |
| 41824 | 1.89227 | 2.1 |
| 41825 | 1.89227 | 2.1 |
| 41826 | 3.78453 | 2.1 |
| 41827 | 0.568724 | 2.1 |
| 41828 | 0.568724 | 2.1 |
| 41829 | 0.57678 | 2.1 |
| 41830 | 0.57678 | 2.1 |
| 41831 | 0.523796 | 2.1 |
| 41832 | 0.548137 | 2.1 |
| 41833 | 0.597368 | 2.1 |
| 41834 | 0.637877 | 2.1 |
| 41835 | 0.597367 | 2.1 |
| 41836 | 0.523796 | 2.1 |
| 41837 | 0.637877 | 2.1 |
| 41838 | 0.548137 | 2.1 |
| 41839 | 1.32674 | 2.1 |
| 41840 | 1.32674 | 2.1 |
| 41841 | 0.946132 | 2.1 |
| 41842 | 0.946132 | 2.1 |
| 41843 | 1.3195 | 2.1 |
| 41844 | 1.32674 | 2.1 |
| 41845 | 0.946132 | 2.1 |
| 41846 | 0.946132 | 2.1 |
| 41847 | 0.946132 | 2.1 |
| 41848 | 1.3195 | 2.1 |
| 41849 | 1.32674 | 2.1 |
| 41850 | 0.946133 | 2.1 |
| 41851 | 1.89227 | 2.1 |
| 41852 | 1.89227 | 2.1 |
| 41853 | 1.89227 | 2.1 |
| 41854 | 1.89227 | 2.1 |
| 41855 | 3.78453 | 2.1 |
| 41856 | 1.19473 | 2.1 |
| 41857 | 0.987132 | 2.1 |
| 41858 | 0.987132 | 2.1 |
| 41859 | 1.04759 | 2.1 |
| 41860 | 1.0894 | 2.1 |
| 41861 | 1.14986 | 2.1 |
| 41862 | 1.3195 | 2.1 |
| 41863 | 1.3195 | 2.1 |
| 41864 | 0.946132 | 2.1 |
| 41865 | 0.946132 | 2.1 |
| 41866 | 1.31185 | 2.1 |
| 41867 | 1.3195 | 2.1 |
| 41868 | 0.946132 | 2.1 |
| 41869 | 0.946132 | 2.1 |
| 41870 | 1.31185 | 2.1 |
| 41871 | 0.946133 | 2.1 |
| 41872 | 0.946132 | 2.1 |
| 41873 | 1.3195 | 2.1 |

| | | |
|---|---|---|
| 41874 | 1.11256 | 0.566013 |
| 41875 | 1.70101 | 0.368885 |
| 41876 | 1.22297 | 0.566725 |
| 41877 | 1.22297 | 0.525571 |
| 41878 | 1.7252 | 0.633276 |
| 41879 | 1.7252 | 0.588303 |
| 41880 | 1.99298 | 0.619714 |
| 41881 | 1.70101 | 0.661342 |
| 41882 | 1.7252 | 0.875719 |
| 41883 | 2.18701 | 0.225464 |
| 41884 | 3.71818 | 0.421197 |
| 41885 | 1.03218 | 0.484629 |
| 41886 | 1.7252 | 0.24646 |
| 41887 | 1.11256 | 0.556402 |
| 41888 | 1.11256 | 0.429997 |
| 41889 | 1.7457 | 0.561799 |
| 41890 | 1.7457 | 0.445493 |
| 41891 | 1.7457 | 0.685334 |
| 41892 | 2.187 | 0.132268 |
| 41893 | 1.7252 | 0.449467 |
| 41894 | 1.7252 | 0.50789 |
| 41895 | 2.27351 | 0.352881 |
| 41896 | 2.27351 | 0.418616 |
| 41897 | 1.95544 | 1.05947 |
| 41898 | 1.35897 | 2.1 |
| 41899 | 1.44859 | 1.67211 |
| 41900 | 1.44859 | 2.06944 |
| 41901 | 1.80709 | 1.17529 |
| 41902 | 1.80709 | 1.9761 |
| 41903 | 1.80709 | 0.975531 |
| 41904 | 1.17822 | 2.1 |
| 41905 | 1.35897 | 1.67988 |
| 41906 | 1.35897 | 2.1 |
| 41907 | 1.52782 | 0.968907 |
| 41908 | 1.52782 | 1.89486 |
| 41909 | 1.11978 | 2.00402 |
| 41910 | 0.903546 | 1.13665 |
| 41911 | 0.977719 | 2.08003 |
| 41912 | 1.2364 | 1.14261 |
| 41913 | 1.03812 | 0.971878 |
| 41914 | 1.03812 | 2.1 |
| 41915 | 1.05938 | 0.918036 |
| 41916 | 1.05938 | 2.1 |
| 41917 | 1.11978 | 1.38409 |
| 41918 | 1.2364 | 0.933027 |
| 41919 | 0.97772 | 1.76359 |
| 41920 | 0.903546 | 1.30056 |
| 41921 | 1.80709 | 2.1 |
| 41922 | 1.81847 | 1.55309 |
| 41923 | 1.96029 | 2.0514 |
| 41924 | 1.52782 | 1.844 |
| 41925 | 2.23956 | 1.23776 |
| 41926 | 1.80709 | 1.71881 |
| 41927 | 1.89226 | 0.172937 |
| 41928 | 2.32962 | 0.05 |
| 41929 | 2.5992 | 0.05 |
| 41930 | 1.89226 | 0.05 |
| 41931 | 2.5992 | 0.151954 |

| | | |
|---|---|---|
| 41932 | 1.89226 | 0.174044 |
| 41933 | 2.55791 | 0.05 |
| 41934 | 2.47141 | 0.05 |
| 41935 | 2.13515 | 0.05 |
| 41936 | 2.10939 | 0.05 |
| 41937 | 4.66731 | 0.05 |
| 41938 | 2.47141 | 0.05 |
| 41939 | 2.23337 | 0.05 |
| 41940 | 2.10939 | 0.05 |
| 41941 | 2.03438 | 0.05 |
| 41942 | 4.34276 | 0.05 |
| 41943 | 2.13512 | 0.05 |
| 41944 | 1.71027 | 0.05 |
| 41945 | 1.71027 | 0.05 |
| 41946 | 2.10936 | 0.05 |
| 41947 | 1.60137 | 0.05 |
| 41948 | 2.00046 | 0.05 |
| 41949 | 1.71027 | 0.05 |
| 41950 | 2.03435 | 0.05 |
| 41951 | 1.71027 | 0.05 |
| 41952 | 2.10936 | 0.05 |
| 41953 | 1.72067 | 0.05 |
| 41954 | 2.11976 | 0.05 |
| 41955 | 1.177 0.05 | |
| 41956 | 1.36405 | 0.314975 |
| 41957 | 2.25635 | 0.353306 |
| 41958 | 2.60151 | 0.05 |
| 41959 | 3.6204 | 0.108799 |
| 41960 | 0.658869 | 0.05 |
| 41961 | 0.781697 | 0.05 |
| 41962 | 0.637037 | 0.05 |
| 41963 | 0.541148 | 0.05 |
| 41964 | 0.654682 | 0.05 |
| 41965 | 0.654682 | 0.05 |
| 41966 | 0.641224 | 0.05 |
| 41967 | 0.641224 | 0.05 |
| 41968 | 0.637039 | 0.05 |
| 41969 | 0.781696 | 0.05 |
| 41970 | 0.658867 | 0.05 |
| 41971 | 0.541149 | 0.05 |
| 41972 | 1.89226 | 0.05 |
| 41973 | 1.59152 | 0.05 |
| 41974 | 1.89226 | 0.05 |
| 41975 | 1.82125 | 0.05 |
| 41976 | 1.89226 | 0.05 |
| 41977 | 1.82125 | 0.05 |
| 41978 | 2.18967 | 0.05 |
| 41979 | 1.89227 | 0.05 |
| 41980 | 2.72752 | 0.05 |
| 41981 | 2.72752 | 0.05 |
| 41982 | 1.89227 | 0.05 |
| 41983 | 1.89227 | 0.05 |
| 41984 | 2.18967 | 0.05 |
| 41985 | 1.92547 | 0.05 |
| 41986 | 1.92547 | 0.05 |
| 41987 | 2.72752 | 0.05 |
| 41988 | 1.30572 | 0.05 |
| 41989 | 2.10777 | 0.05 |

| | | |
|---|---|---|
| 41990 | 2.39663 | 2.1 |
| 41991 | 1.92293 | 2.1 |
| 41992 | 1.96889 | 2.1 |
| 41993 | 2.45928 | 2.1 |
| 41994 | 4.36552 | 2.1 |
| 41995 | 1.02605 | 2.1 |
| 41996 | 1.92293 | 2.1 |
| 41997 | 1.9689 | 2.1 |
| 41998 | 1.04679 | 2.1 |
| 41999 | 2.99495 | 2.1 |
| 42000 | 1.96889 | 2.1 |
| 42001 | 2.45928 | 2.1 |
| 42002 | 2.50138 | 2.1 |
| 42003 | 1.99782 | 2.1 |
| 42004 | 4.47027 | 2.1 |
| 42005 | 1.9689 | 2.1 |
| 42006 | 1.03449 | 2.1 |
| 42007 | 1.07572 | 2.1 |
| 42008 | 1.04679 | 2.1 |
| 42009 | 1.99783 | 2.1 |
| 42010 | 1.9689 | 2.1 |
| 42011 | 1.72162 | 0.924563 |
| 42012 | 1.89186 | 0.702247 |
| 42013 | 2.29771 | 0.772621 |
| 42014 | 1.80149 | 0.55201 |
| 42015 | 4.01933 | 0.706355 |
| 42016 | 1.16482 | 0.961544 |
| 42017 | 1.97003 | 0.610415 |
| 42018 | 1.72162 | 0.558923 |
| 42019 | 1.72162 | 0.68304 |
| 42020 | 1.29313 | 0.704311 |
| 42021 | 1.29313 | 0.903805 |
| 42022 | 2.29771 | 0.82217 |
| 42023 | 2.84159 | 0.460672 |
| 42024 | 2.19622 | 0.595922 |
| 42025 | 1.97366 | 0.588605 |
| 42026 | 2.50906 | 0.657586 |
| 42027 | 2.29771 | 0.690399 |
| 42028 | 1.29313 | 0.886888 |
| 42029 | 2.4483 | 0.587661 |
| 42030 | 2.14223 | 0.579343 |
| 42031 | 2.14228 | 0.670785 |
| 42032 | 1.294 | 0.66838 |
| 42033 | 1.294 | 0.787738 |
| 42034 | 1.21495 | 0.05 |
| 42035 | 1.70303 | 0.05 |
| 42036 | 1.7924 | 0.05 |
| 42037 | 1.64002 | 0.05 |
| 42038 | 3.11963 | 0.05 |
| 42039 | 2.81021 | 0.05 |
| 42040 | 1.89226 | 0.05 |
| 42041 | 1.89226 | 0.05 |
| 42042 | 1.89226 | 0.05 |
| 42043 | 2.36406 | 0.05 |
| 42044 | 2.36406 | 0.05 |
| 42045 | 1.89226 | 0.05 |
| 42046 | 1.89226 | 0.05 |
| 42047 | 1.89226 | 0.05 |

| | | |
|---|---|---|
| 42048 | 1.89226 | 0.05 |
| 42049 | 1.89226 | 0.05 |
| 42050 | 1.89226 | 0.05 |
| 42051 | 2.29976 | 0.05 |
| 42052 | 1.64002 | 0.05 |
| 42053 | 1.8959 | 0.05 |
| 42054 | 1.90873 | 0.05 |
| 42055 | 1.85594 | 0.05 |
| 42056 | 1.85594 | 0.05 |
| 42057 | 1.89226 | 0.05 |
| 42058 | 1.89226 | 0.05 |
| 42059 | 1.89226 | 0.05 |
| 42060 | 1.89226 | 0.05 |
| 42061 | 3.78453 | 0.05 |
| 42062 | 1.89226 | 0.940189 |
| 42063 | 2.52823 | 2.1 |
| 42064 | 2.83441 | 2.0027 |
| 42065 | 2.83441 | 2.1 |
| 42066 | 1.89226 | 1.38538 |
| 42067 | 1.89226 | 2.1 |
| 42068 | 1.04522 | 1.91823 |
| 42069 | 1.04522 | 1.55834 |
| 42070 | 1.12831 | 2.08848 |
| 42071 | 1.18364 | 2.1 |
| 42072 | 0.640525 | 1.85785 |
| 42073 | 0.924882 | 1.85843 |
| 42074 | 1.27877 | 2.1 |
| 42075 | 1.43363 | 1.01462 |
| 42076 | 0.93474 | 1.86637 |
| 42077 | 1.58926 | 1.56099 |
| 42078 | 1.29573 | 2.1 |
| 42079 | 0.664247 | 2.07554 |
| 42080 | 2.02191 | 2.1 |
| 42081 | 1.89226 | 2.1 |
| 42082 | 1.89226 | 2.1 |
| 42083 | 1.89226 | 2.1 |
| 42084 | 2.58639 | 2.1 |
| 42085 | 2.58639 | 2.1 |
| 42086 | 1.16759 | 2.1 |
| 42087 | 2.11828 | 2.1 |
| 42088 | 1.60936 | 2.1 |
| 42089 | 2.35846 | 2.1 |
| 42090 | 3.52605 | 2.1 |
| 42091 | 1.89226 | 2.1 |
| 42092 | 1.89226 | 2.1 |
| 42093 | 1.89226 | 2.1 |
| 42094 | 1.89226 | 2.1 |
| 42095 | 3.78453 | 2.1 |
| 42096 | 2.82765 | 0.05 |
| 42097 | 1.89809 | 0.05 |
| 42098 | 2.15971 | 0.05 |
| 42099 | 1.89809 | 0.05 |
| 42100 | 2.29233 | 0.05 |
| 42101 | 2.03071 | 0.05 |
| 42102 | 1.90664 | 0.05 |
| 42103 | 2.15971 | 0.05 |
| 42104 | 1.28956 | 0.05 |
| 42105 | 1.28956 | 0.05 |

| | | |
|---|---|---|
| 42106 | 2.64414 | 0.05 |
| 42107 | 2.02706 | 0.05 |
| 42108 | 2.82765 | 0.05 |
| 42109 | 1.89226 | 0.05 |
| 42110 | 2.39968 | 0.05 |
| 42111 | 2.39968 | 0.05 |
| 42112 | 1.89226 | 0.05 |
| 42113 | 1.89226 | 0.05 |
| 42114 | 0.978643 | 2.1 |
| 42115 | 0.703968 | 2.1 |
| 42116 | 0.946129 | 2.1 |
| 42117 | 0.946129 | 2.1 |
| 42118 | 0.703939 | 2.1 |
| 42119 | 0.703939 | 2.1 |
| 42120 | 0.946159 | 2.1 |
| 42121 | 0.946159 | 2.1 |
| 42122 | 0.978642 | 2.1 |
| 42123 | 0.94613 | 2.1 |
| 42124 | 0.94613 | 2.1 |
| 42125 | 0.703968 | 2.1 |
| 42126 | 1.89226 | 2.1 |
| 42127 | 2.17309 | 0.669457 |
| 42128 | 2.17309 | 2.1 |
| 42129 | 1.47475 | 2.1 |
| 42130 | 1.89226 | 2.1 |
| 42131 | 1.19393 | 1.369 |
| 42132 | 1.89226 | 2.1 |
| 42133 | 1.89226 | 2.1 |
| 42134 | 1.89226 | 2.1 |
| 42135 | 1.89226 | 2.1 |
| 42136 | 3.78452 | 2.1 |
| 42137 | 1.17426 | 2.1 |
| 42138 | 0.978644 | 2.1 |
| 42139 | 0.946129 | 2.1 |
| 42140 | 0.946129 | 2.1 |
| 42141 | 0.946131 | 2.1 |
| 42142 | 0.946131 | 2.1 |
| 42143 | 0.978643 | 2.1 |
| 42144 | 0.978642 | 2.1 |
| 42145 | 1.17426 | 2.1 |
| 42146 | 0.946131 | 2.1 |
| 42147 | 0.978642 | 2.1 |
| 42148 | 0.94613 | 2.1 |
| 42149 | 1.89226 | 2.1 |
| 42150 | 1.89226 | 0.659117 |
| 42151 | 1.89226 | 2.1 |
| 42152 | 2.17309 | 1.04963 |
| 42153 | 2.56346 | 2.1 |
| 42154 | 2.94389 | 1.92281 |
| 42155 | 0.946131 | 2.1 |
| 42156 | 0.946131 | 2.1 |
| 42157 | 0.946128 | 2.1 |
| 42158 | 0.946128 | 2.1 |
| 42159 | 0.946131 | 2.1 |
| 42160 | 0.946131 | 2.1 |
| 42161 | 0.946128 | 2.1 |
| 42162 | 0.946128 | 2.1 |
| 42163 | 0.94613 | 2.1 |

| | | |
|---|---|---|
| 42164 | 0.94613 | 2.1 |
| 42165 | 0.946129 | 2.1 |
| 42166 | 0.946129 | 2.1 |
| 42167 | 1.89226 | 0.538484 |
| 42168 | 1.89226 | 0.598489 |
| 42169 | 1.89226 | 0.997467 |
| 42170 | 1.89226 | 0.501752 |
| 42171 | 3.78453 | 0.133849 |
| 42172 | 1.89226 | 0.915371 |
| 42173 | 1.89226 | 0.463589 |
| 42174 | 1.89226 | 1.09058 |
| 42175 | 1.89226 | 0.901024 |
| 42176 | 1.89226 | 0.739544 |
| 42177 | 1.89226 | 0.510369 |
| 42178 | 1.89226 | 0.455697 |
| 42179 | 1.30733 | 0.92677 |
| 42180 | 1.89226 | 1.11674 |
| 42181 | 1.92403 | 0.404753 |
| 42182 | 3.38635 | 0.406699 |
| 42183 | 1.89226 | 0.585771 |
| 42184 | 3.02887 | 0.740758 |
| 42185 | 2.0815 | 0.0983049 |
| 42186 | 1.88422 | 0.767148 |
| 42187 | 2.0815 | 0.374187 |
| 42188 | 1.88422 | 1.73833 |
| 42189 | 1.89226 | 1.27644 |
| 42190 | 1.70221 | 0.386461 |
| 42191 | 1.89226 | 1.76729 |
| 42192 | 1.20068 | 1.13902 |
| 42193 | 1.89226 | 0.970933 |
| 42194 | 1.20068 | 0.367284 |
| 42195 | 1.01393 | 1.13481 |
| 42196 | 1.20067 | 0.34135 |
| 42197 | 1.89226 | 1.30743 |
| 42198 | 2.44958 | 1.67464 |
| 42199 | 1.01393 | 0.595304 |
| 42200 | 1.89226 | 0.105066 |
| 42201 | 1.89226 | 2.1 |
| 42202 | 1.89226 | 2.1 |
| 42203 | 1.89226 | 2.1 |
| 42204 | 1.89226 | 2.1 |
| 42205 | 1.89226 | 2.1 |
| 42206 | 1.89226 | 2.1 |
| 42207 | 1.89226 | 2.1 |
| 42208 | 1.89226 | 0.05 |
| 42209 | 1.89226 | 0.0597619 |
| 42210 | 1.89226 | 2.1 |
| 42211 | 1.89226 | 0.136859 |
| 42212 | 1.89226 | 2.1 |
| 42213 | 1.89226 | 2.1 |
| 42214 | 1.89226 | 2.1 |
| 42215 | 1.89226 | 2.1 |
| 42216 | 1.89226 | 2.1 |
| 42217 | 1.89226 | 2.1 |
| 42218 | 1.89226 | 2.1 |
| 42219 | 1.91161 | 2.1 |
| 42220 | 2.45782 | 2.1 |
| 42221 | 1.39653 | 2.1 |

| | | |
|---|---|---|
| 42222 | 2.44652 | 2.1 |
| 42223 | 4.35812 | 2.1 |
| 42224 | 2.73092 | 2.07992 |
| 42225 | 1.89226 | 0.05 |
| 42226 | 1.89226 | 2.1 |
| 42227 | 1.89226 | 0.05 |
| 42228 | 2.44652 | 2.1 |
| 42229 | 2.44652 | 0.05 |
| 42230 | 1.89226 | 2.1 |
| 42231 | 1.89226 | 0.177946 |
| 42232 | 1.89226 | 0.70479 |
| 42233 | 1.89226 | 2.1 |
| 42234 | 1.89226 | 0.73415 |
| 42235 | 1.89226 | 2.1 |
| 42236 | 2.64924 | 2.1 |
| 42237 | 1.31109 | 2.1 |
| 42238 | 1.31109 | 2.1 |
| 42239 | 1.49958 | 2.1 |
| 42240 | 1.95039 | 2.1 |
| 42241 | 2.14451 | 2.1 |
| 42242 | 0.825157 | 2.1 |
| 42243 | 0.634856 | 2.1 |
| 42244 | 0.816836 | 2.1 |
| 42245 | 0.816836 | 2.1 |
| 42246 | 0.556743 | 2.1 |
| 42247 | 0.639142 | 2.1 |
| 42248 | 0.985106 | 2.1 |
| 42249 | 0.795045 | 2.1 |
| 42250 | 0.967461 | 2.1 |
| 42251 | 0.552494 | 2.1 |
| 42252 | 0.676829 | 2.1 |
| 42253 | 0.98803 | 2.1 |
| 42254 | 2.28564 | 2.1 |
| 42255 | 2.34084 | 1.42291 |
| 42256 | 2.3502 | 2.09536 |
| 42257 | 2.42908 | 2.1 |
| 42258 | 4.69104 | 2.1 |
| 42259 | 2.34084 | 2.1 |
| 42260 | 2.18077 | 2.1 |
| 42261 | 2.42908 | 2.1 |
| 42262 | 2.2947 | 1.04133 |
| 42263 | 4.63554 | 2.1 |
| 42264 | 2.3502 | 2.1 |
| 42265 | 2.42908 | 2.1 |
| 42266 | 2.38681 | 2.1 |
| 42267 | 2.50438 | 1.3481 |
| 42268 | 4.85458 | 2.1 |
| 42269 | 2.42908 | 2.1 |
| 42270 | 2.2947 | 1.81231 |
| 42271 | 2.50438 | 1.11724 |
| 42272 | 2.40627 | 2.1 |
| 42273 | 4.79908 | 1.86354 |
| 42274 | 1.89226 | 0.0704098 |
| 42275 | 2.20711 | 1.33976 |
| 42276 | 1.89226 | 0.0514658 |
| 42277 | 1.89226 | 1.9475 |
| 42278 | 2.02693 | 0.060316 |
| 42279 | 2.02693 | 1.7791 |

| | | |
|---|---|---|
| 42280 | 1.2269 | 2.1 |
| 42281 | 2.20711 | 0.456835 |
| 42282 | 2.0815 | 0.193818 |
| 42283 | 1.11506 | 2.1 |
| 42284 | 3.22109 | 2.1 |
| 42285 | 0.545802 | 1.66083 |
| 42286 | 0.547489 | 2.1 |
| 42287 | 0.85914 | 0.596771 |
| 42288 | 1.11641 | 0.841337 |
| 42289 | 0.899974 | 0.91703 |
| 42290 | 0.849893 | 2.1 |
| 42291 | 0.747885 | 1.83262 |
| 42292 | 0.747885 | 0.245468 |
| 42293 | 0.557184 | 2.1 |
| 42294 | 0.887039 | 2.1 |
| 42295 | 1.08948 | 2.05045 |
| 42296 | 0.59815 | 2.08552 |
| 42297 | 0.974139 | 2.1 |
| 42298 | 1.33385 | 2.1 |
| 42299 | 1.02368 | 2.1 |
| 42300 | 1.02368 | 2.1 |
| 42301 | 1.00833 | 2.1 |
| 42302 | 1.00833 | 2.1 |
| 42303 | 1.89226 | 0.539438 |
| 42304 | 1.89226 | 0.35809 |
| 42305 | 1.89226 | 0.203113 |
| 42306 | 1.89226 | 0.05 |
| 42307 | 3.78453 | 0.05 |
| 42308 | 1.74651 | 0.104197 |
| 42309 | 1.44605 | 0.05 |
| 42310 | 1.63557 | 0.05 |
| 42311 | 1.31993 | 0.05 |
| 42312 | 3.08162 | 0.05 |
| 42313 | 1.89226 | 0.0880656 |
| 42314 | 1.89226 | 0.170606 |
| 42315 | 1.89226 | 0.053634 |
| 42316 | 1.89226 | 0.05 |
| 42317 | 3.78452 | 0.05 |
| 42318 | 1.63557 | 0.05 |
| 42319 | 1.31992 | 0.05 |
| 42320 | 1.52615 | 0.05 |
| 42321 | 1.18549 | 0.05 |
| 42322 | 2.82106 | 0.05 |
| 42323 | 1.89226 | 0.05 |
| 42324 | 2.58101 | 0.05 |
| 42325 | 2.73797 | 0.351583 |
| 42326 | 2.73797 | 0.184938 |
| 42327 | 1.89226 | 0.05 |
| 42328 | 1.89226 | 0.05 |
| 42329 | 1.91762 | 0.237669 |
| 42330 | 1.44354 | 0.304142 |
| 42331 | 1.33622 | 0.244876 |
| 42332 | 1.33622 | 0.128093 |
| 42333 | 2.20248 | 0.649248 |
| 42334 | 2.20248 | 0.330867 |
| 42335 | 1.11712 | 2.1 |
| 42336 | 1.11712 | 2.1 |
| 42337 | 1.11421 | 2.1 |

| | | |
|---|---|---|
| 42338 | 1.11421 | 2.1 |
| 42339 | 0.833871 | 2.1 |
| 42340 | 0.926528 | 2.1 |
| 42341 | 1.3048 | 2.1 |
| 42342 | 1.40335 | 2.1 |
| 42343 | 0.926527 | 2.1 |
| 42344 | 1.40335 | 2.1 |
| 42345 | 1.3048 | 2.1 |
| 42346 | 0.833871 | 2.1 |
| 42347 | 0.879657 | 2.1 |
| 42348 | 0.937695 | 2.1 |
| 42349 | 0.946129 | 2.1 |
| 42350 | 0.946129 | 2.1 |
| 42351 | 0.851541 | 2.1 |
| 42352 | 0.851541 | 2.1 |
| 42353 | 1.1126 | 2.1 |
| 42354 | 1.04519 | 2.1 |
| 42355 | 0.851486 | 2.1 |
| 42356 | 0.980501 | 2.1 |
| 42357 | 0.898251 | 2.1 |
| 42358 | 0.851528 | 2.1 |
| 42359 | 1.37756 | 2.1 |
| 42360 | 2.73081 | 2.1 |
| 42361 | 2.44311 | 2.1 |
| 42362 | 2.44311 | 2.1 |
| 42363 | 1.66428 | 2.1 |
| 42364 | 1.66428 | 2.1 |
| 42365 | 1.89226 | 2.1 |
| 42366 | 1.89226 | 2.1 |
| 42367 | 1.89226 | 2.1 |
| 42368 | 1.89226 | 2.1 |
| 42369 | 3.78452 | 2.1 |
| 42370 | 1.65781 | 2.1 |
| 42371 | 1.89837 | 2.1 |
| 42372 | 1.46713 | 2.1 |
| 42373 | 1.98027 | 2.1 |
| 42374 | 3.63808 | 2.1 |
| 42375 | 2.29054 | 2.1 |
| 42376 | 2.26369 | 0.05 |
| 42377 | 1.98027 | 2.1 |
| 42378 | 2.15617 | 0.05 |
| 42379 | 4.24396 | 0.194104 |
| 42380 | 1.89226 | 2.1 |
| 42381 | 1.89226 | 0.05 |
| 42382 | 1.89226 | 2.1 |
| 42383 | 1.89226 | 0.0612848 |
| 42384 | 3.78452 | 1.02753 |
| 42385 | 0.946131 | 2.1 |
| 42386 | 0.946131 | 2.1 |
| 42387 | 0.946128 | 2.1 |
| 42388 | 0.946128 | 2.1 |
| 42389 | 0.946129 | 2.1 |
| 42390 | 0.946129 | 2.1 |
| 42391 | 0.94613 | 2.1 |
| 42392 | 0.94613 | 2.1 |
| 42393 | 0.94613 | 2.1 |
| 42394 | 0.94613 | 2.1 |
| 42395 | 0.94613 | 2.1 |

| | | |
|---|---|---|
| 42396 | 0.94613 | 2.1 |
| 42397 | 0.812186 | 2.1 |
| 42398 | 0.812186 | 2.1 |
| 42399 | 0.819781 | 2.1 |
| 42400 | 0.819781 | 2.1 |
| 42401 | 0.913532 | 2.1 |
| 42402 | 0.990134 | 2.1 |
| 42403 | 0.733564 | 2.1 |
| 42404 | 0.641833 | 2.1 |
| 42405 | 0.641832 | 2.1 |
| 42406 | 0.913533 | 2.1 |
| 42407 | 0.733564 | 2.1 |
| 42408 | 0.990134 | 2.1 |
| 42409 | 0.994262 | 0.05 |
| 42410 | 0.994262 | 2.1 |
| 42411 | 0.997354 | 2.1 |
| 42412 | 0.997354 | 0.05 |
| 42413 | 1.00775 | 0.05 |
| 42414 | 1.07808 | 0.05 |
| 42415 | 0.913533 | 2.1 |
| 42416 | 0.990135 | 2.1 |
| 42417 | 0.913532 | 0.211073 |
| 42418 | 1.00775 | 0.158849 |
| 42419 | 0.990134 | 0.272216 |
| 42420 | 1.07808 | 0.137998 |
| 42421 | 0.946131 | 2.1 |
| 42422 | 0.946131 | 0.254903 |
| 42423 | 0.946128 | 2.1 |
| 42424 | 0.946128 | 0.0786955 |
| 42425 | 0.946128 | 0.05 |
| 42426 | 0.946128 | 0.05 |
| 42427 | 0.94613 | 2.1 |
| 42428 | 0.94613 | 2.1 |
| 42429 | 0.946129 | 1.32823 |
| 42430 | 0.946129 | 1.5663 |
| 42431 | 0.946129 | 0.745915 |
| 42432 | 0.946129 | 0.780554 |
| 42433 | 0.816232 | 2.1 |
| 42434 | 0.760038 | 2.1 |
| 42435 | 0.845999 | 2.1 |
| 42436 | 0.845999 | 2.1 |
| 42437 | 1.0631 | 2.1 |
| 42438 | 1.28131 | 2.1 |
| 42439 | 0.474065 | 2.1 |
| 42440 | 0.591905 | 2.1 |
| 42441 | 0.601756 | 2.1 |
| 42442 | 1.23903 | 2.1 |
| 42443 | 0.988592 | 2.1 |
| 42444 | 0.49051 | 2.1 |
| 42445 | 2.57021 | 2.1 |
| 42446 | 1.89226 | 2.1 |
| 42447 | 2.68172 | 2.1 |
| 42448 | 1.89226 | 1.44866 |
| 42449 | 4.46247 | 2.1 |
| 42450 | 1.89226 | 2.1 |
| 42451 | 1.89226 | 1.8519 |
| 42452 | 1.89226 | 1.91663 |
| 42453 | 1.89226 | 1.86326 |

| | | |
|---|---|---|
| 42454 | 3.78452 | 1.99461 |
| 42455 | 1.12573 | 2.1 |
| 42456 | 1.65504 | 1.54532 |
| 42457 | 1.38848 | 2.1 |
| 42458 | 1.38848 | 2.1 |
| 42459 | 1.30567 | 1.07673 |
| 42460 | 1.30567 | 2.1 |
| 42461 | 2.82413 | 2.1 |
| 42462 | 1.89226 | 1.92983 |
| 42463 | 2.68172 | 2.1 |
| 42464 | 2.68172 | 2.1 |
| 42465 | 1.89226 | 2.1 |
| 42466 | 1.89226 | 2.1 |
| 42467 | 1.89226 | 2.1 |
| 42468 | 1.89226 | 2.1 |
| 42469 | 1.89226 | 2.1 |
| 42470 | 1.89226 | 1.04322 |
| 42471 | 3.78452 | 2.07893 |
| 42472 | 1.30567 | 2.1 |
| 42473 | 1.65504 | 2.08641 |
| 42474 | 1.52724 | 2.1 |
| 42475 | 1.89249 | 2.1 |
| 42476 | 3.18228 | 2.1 |
| 42477 | 2.57386 | 0.05 |
| 42478 | 1.89226 | 0.05 |
| 42479 | 1.89226 | 0.05 |
| 42480 | 2.66753 | 0.05 |
| 42481 | 1.89226 | 0.05 |
| 42482 | 2.66753 | 0.05 |
| 42483 | 1.89226 | 0.05 |
| 42484 | 1.89226 | 0.05 |
| 42485 | 2.2973 | 0.05 |
| 42486 | 3.12479 | 0.05 |
| 42487 | 1.89226 | 0.05 |
| 42488 | 2.57385 | 0.05 |
| 42489 | 0.489293 | 2.1 |
| 42490 | 0.672698 | 2.1 |
| 42491 | 0.59038 | 2.1 |
| 42492 | 0.738357 | 2.1 |
| 42493 | 0.62269 | 2.1 |
| 42494 | 0.62269 | 2.1 |
| 42495 | 0.640388 | 2.1 |
| 42496 | 0.640388 | 2.1 |
| 42497 | 0.59038 | 2.1 |
| 42498 | 0.489293 | 2.1 |
| 42499 | 0.738357 | 2.1 |
| 42500 | 0.672698 | 2.1 |
| 42501 | 1.89226 | 2.1 |
| 42502 | 1.89226 | 2.1 |
| 42503 | 1.89226 | 2.1 |
| 42504 | 1.89226 | 0.699449 |
| 42505 | 3.78452 | 2.1 |
| 42506 | 2.82858 | 2.1 |
| 42507 | 1.89226 | 0.749703 |
| 42508 | 2.5671 | 2.1 |
| 42509 | 1.89226 | 2.1 |
| 42510 | 2.5671 | 2.1 |
| 42511 | 1.89226 | 2.1 |

| | | |
|---|---|---|
| 42512 | 2.06581 | 0.05 |
| 42513 | 2.47308 | 0.05 |
| 42514 | 1.89433 | 0.05 |
| 42515 | 1.67571 | 0.05 |
| 42516 | 3.87274 | 0.05 |
| 42517 | 2.55791 | 0.05 |
| 42518 | 2.10276 | 0.05 |
| 42519 | 2.47308 | 0.05 |
| 42520 | 2.47308 | 0.05 |
| 42521 | 1.83415 | 0.05 |
| 42522 | 1.90922 | 0.05 |
| 42523 | 1.18421 | 0.05 |
| 42524 | 2.03277 | 0.05 |
| 42525 | 1.18421 | 0.05 |
| 42526 | 2.01275 | 0.05 |
| 42527 | 1.42818 | 0.05 |
| 42528 | 2.25673 | 0.05 |
| 42529 | 0.882904 | 0.05 |
| 42530 | 0.958492 | 0.05 |
| 42531 | 0.905756 | 0.05 |
| 42532 | 0.905756 | 0.05 |
| 42533 | 1.06352 | 0.05 |
| 42534 | 0.797654 | 0.05 |
| 42535 | 1.11319 | 0.05 |
| 42536 | 0.716795 | 0.05 |
| 42537 | 0.820721 | 0.05 |
| 42538 | 0.731963 | 0.05 |
| 42539 | 1.13143 | 0.05 |
| 42540 | 1.10528 | 0.05 |
| 42541 | 1.89226 | 0.05 |
| 42542 | 1.89226 | 0.05 |
| 42543 | 1.89226 | 0.05 |
| 42544 | 1.89226 | 0.05 |
| 42545 | 1.89226 | 0.05 |
| 42546 | 1.89226 | 0.05 |
| 42547 | 1.01241 | 0.05 |
| 42548 | 0.703929 | 0.05 |
| 42549 | 0.946133 | 0.05 |
| 42550 | 0.946133 | 0.05 |
| 42551 | 1.04072 | 0.05 |
| 42552 | 1.163 0.05 | |
| 42553 | 0.639952 | 0.05 |
| 42554 | 0.851543 | 0.05 |
| 42555 | 0.946132 | 0.05 |
| 42556 | 0.920761 | 0.05 |
| 42557 | 0.711038 | 0.05 |
| 42558 | 0.711038 | 0.05 |
| 42559 | 1.89226 | 0.05 |
| 42560 | 1.89226 | 0.05 |
| 42561 | 1.89226 | 0.05 |
| 42562 | 1.89226 | 0.05 |
| 42563 | 1.89226 | 0.05 |
| 42564 | 1.89226 | 0.05 |
| 42565 | 1.89226 | 0.05 |
| 42566 | 1.89226 | 0.05 |
| 42567 | 2.41075 | 0.05 |
| 42568 | 1.89226 | 0.05 |
| 42569 | 3.78451 | 0.05 |

| | | |
|---|---|---|
| 42570 | 0.968476 | 2.01878 |
| 42571 | 1.20674 | 1.00095 |
| 42572 | 1.13456 | 0.986351 |
| 42573 | 1.13456 | 2.1 |
| 42574 | 1.02074 | 0.860723 |
| 42575 | 1.02074 | 2.07635 |
| 42576 | 1.02074 | 2.1 |
| 42577 | 1.20674 | 1.98337 |
| 42578 | 1.07866 | 1.25181 |
| 42579 | 1.28649 | 1.08464 |
| 42580 | 2.2854 | 2.1 |
| 42581 | 1.94221 | 0.05 |
| 42582 | 1.36717 | 0.05 |
| 42583 | 1.45535 | 0.05 |
| 42584 | 1.93771 | 0.05 |
| 42585 | 3.39756 | 0.05 |
| 42586 | 1.94222 | 0.0513059 |
| 42587 | 2.25035 | 0.05 |
| 42588 | 2.41806 | 0.05 |
| 42589 | 2.42256 | 0.05 |
| 42590 | 1.93772 | 0.05 |
| 42591 | 1.94222 | 0.05 |
| 42592 | 1.49906 | 0.05 |
| 42593 | 1.93771 | 0.05 |
| 42594 | 1.45535 | 0.05 |
| 42595 | 1.45535 | 0.05 |
| 42596 | 1.91484 | 0.05 |
| 42597 | 1.91484 | 0.05 |
| 42598 | 1.91484 | 0.05 |
| 42599 | 2.25035 | 0.05 |
| 42600 | 1.93772 | 0.05 |
| 42601 | 1.93772 | 0.05 |
| 42602 | 2.11465 | 0.05 |
| 42603 | 2.11465 | 0.05 |
| 42604 | 2.61735 | 0.05 |
| 42605 | 1.5082 | 0.05 |
| 42606 | 2.36481 | 0.05 |
| 42607 | 1.58597 | 0.05 |
| 42608 | 2.36481 | 0.05 |
| 42609 | 1.46653 | 0.05 |
| 42610 | 2.36482 | 0.05 |
| 42611 | 1.08133 | 0.05 |
| 42612 | 1.5082 | 0.05 |
| 42613 | 2.01868 | 0.05 |
| 42614 | 3.52688 | 0.05 |
| 42615 | 0.94613 | 0.05 |
| 42616 | 0.94613 | 0.05 |
| 42617 | 0.946131 | 0.05 |
| 42618 | 0.946131 | 0.05 |
| 42619 | 0.946129 | 0.05 |
| 42620 | 0.946129 | 0.05 |
| 42621 | 0.946132 | 0.05 |
| 42622 | 0.946132 | 0.05 |
| 42623 | 0.94613 | 0.05 |
| 42624 | 0.94613 | 0.05 |
| 42625 | 0.946131 | 0.05 |
| 42626 | 0.946131 | 0.05 |
| 42627 | 2.53725 | 0.05 |

| | | |
|---|---|---|
| 42628 | 1.89227 | 0.05 |
| 42629 | 1.89227 | 0.05 |
| 42630 | 2.27073 | 0.05 |
| 42631 | 1.89227 | 0.05 |
| 42632 | 2.27073 | 0.05 |
| 42633 | 1.89227 | 0.05 |
| 42634 | 1.89227 | 0.05 |
| 42635 | 1.89227 | 0.05 |
| 42636 | 1.89227 | 0.05 |
| 42637 | 1.89227 | 0.05 |
| 42638 | 1.89227 | 0.05 |
| 42639 | 1.86766 | 2.1 |
| 42640 | 2.83487 | 2.1 |
| 42641 | 2.84318 | 2.1 |
| 42642 | 2.59401 | 2.1 |
| 42643 | 2.11684 | 2.1 |
| 42644 | 1.86766 | 2.1 |
| 42645 | 1.10638 | 2.1 |
| 42646 | 2.11684 | 2.1 |
| 42647 | 1.29111 | 2.1 |
| 42648 | 2.37439 | 2.1 |
| 42649 | 3.40796 | 2.1 |
| 42650 | 2.56843 | 2.1 |
| 42651 | 1.89226 | 2.1 |
| 42652 | 1.89226 | 2.1 |
| 42653 | 2.71932 | 2.1 |
| 42654 | 4.46069 | 2.1 |
| 42655 | 1.89226 | 2.1 |
| 42656 | 1.89226 | 2.1 |
| 42657 | 1.89226 | 2.1 |
| 42658 | 1.89226 | 2.1 |
| 42659 | 3.78452 | 2.1 |
| 42660 | 0.6416 | 2.1 |
| 42661 | 0.697585 | 2.1 |
| 42662 | 0.670706 | 2.1 |
| 42663 | 0.598927 | 2.1 |
| 42664 | 0.644313 | 2.1 |
| 42665 | 0.644313 | 2.1 |
| 42666 | 0.652199 | 2.1 |
| 42667 | 0.652199 | 2.1 |
| 42668 | 0.697587 | 2.1 |
| 42669 | 0.670705 | 2.1 |
| 42670 | 0.598926 | 2.1 |
| 42671 | 0.641601 | 2.1 |
| 42672 | 1.89226 | 2.1 |
| 42673 | 1.89226 | 2.1 |
| 42674 | 1.89226 | 2.1 |
| 42675 | 1.89226 | 2.1 |
| 42676 | 1.89226 | 2.1 |
| 42677 | 1.89226 | 2.1 |
| 42678 | 0.697585 | 2.1 |
| 42679 | 0.730617 | 2.1 |
| 42680 | 0.712082 | 2.1 |
| 42681 | 0.670706 | 2.1 |
| 42682 | 0.69854 | 2.1 |
| 42683 | 0.69854 | 2.1 |
| 42684 | 0.702783 | 2.1 |
| 42685 | 0.702783 | 2.1 |

| | | |
|---|---|---|
| 42686 | 0.730618 | 2.1 |
| 42687 | 0.712081 | 2.1 |
| 42688 | 0.697586 | 2.1 |
| 42689 | 0.670705 | 2.1 |
| 42690 | 1.89226 | 2.1 |
| 42691 | 1.89226 | 2.1 |
| 42692 | 1.89226 | 0.332633 |
| 42693 | 1.89226 | 2.1 |
| 42694 | 1.89226 | 0.237934 |
| 42695 | 1.89226 | 2.1 |
| 42696 | 2.0815 | 2.1 |
| 42697 | 1.12076 | 2.1 |
| 42698 | 1.38662 | 1.86669 |
| 42699 | 2.01244 | 2.1 |
| 42700 | 3.41508 | 2.1 |
| 42701 | 1.89226 | 2.1 |
| 42702 | 2.54324 | 2.1 |
| 42703 | 1.91981 | 2.1 |
| 42704 | 1.89226 | 2.1 |
| 42705 | 2.57079 | 2.1 |
| 42706 | 2.54324 | 2.1 |
| 42707 | 2.54325 | 2.1 |
| 42708 | 1.94779 | 2.1 |
| 42709 | 1.76879 | 2.1 |
| 42710 | 1.73811 | 2.1 |
| 42711 | 2.5708 | 2.1 |
| 42712 | 2.54325 | 2.1 |
| 42713 | 1.89226 | 2.1 |
| 42714 | 2.57079 | 2.1 |
| 42715 | 1.91894 | 2.1 |
| 42716 | 1.89226 | 2.1 |
| 42717 | 2.59747 | 2.1 |
| 42718 | 2.57079 | 2.1 |
| 42719 | 2.5708 | 2.1 |
| 42720 | 1.82268 | 2.1 |
| 42721 | 1.79738 | 2.1 |
| 42722 | 1.77071 | 2.1 |
| 42723 | 2.59747 | 2.1 |
| 42724 | 2.5708 | 2.1 |
| 42725 | 1.89226 | 0.0888256 |
| 42726 | 1.89226 | 0.713484 |
| 42727 | 1.89226 | 0.05 |
| 42728 | 1.89226 | 0.415562 |
| 42729 | 3.78451 | 0.0699493 |
| 42730 | 2.21582 | 0.05 |
| 42731 | 1.94412 | 0.688076 |
| 42732 | 2.13428 | 0.05 |
| 42733 | 1.86292 | 0.0923994 |
| 42734 | 4.07839 | 0.0506538 |
| 42735 | 1.94412 | 0.05 |
| 42736 | 1.5854 | 2.1 |
| 42737 | 1.86293 | 0.05 |
| 42738 | 1.50767 | 2.1 |
| 42739 | 3.45179 | 1.95415 |
| 42740 | 1.89226 | 0.05 |
| 42741 | 1.89226 | 2.1 |
| 42742 | 1.89226 | 0.052842 |
| 42743 | 1.89226 | 2.1 |

| | | |
|---|---|---|
| 42744 | 3.78452 | 2.1 |
| 42745 | 1.89226 | 0.239341 |
| 42746 | 1.89226 | 0.985606 |
| 42747 | 1.89226 | 0.05 |
| 42748 | 1.89226 | 0.582941 |
| 42749 | 3.78452 | 0.102753 |
| 42750 | 2.13428 | 0.0740708 |
| 42751 | 1.86293 | 0.169269 |
| 42752 | 2.04737 | 0.05 |
| 42753 | 1.77122 | 0.671236 |
| 42754 | 3.9055 | 0.088367 |
| 42755 | 1.86293 | 0.05 |
| 42756 | 1.50767 | 2.1 |
| 42757 | 1.41077 | 0.05 |
| 42758 | 1.41077 | 2.1 |
| 42759 | 1.77123 | 0.16136 |
| 42760 | 1.86813 | 2.1 |
| 42761 | 0.810943 | 0.05 |
| 42762 | 0.810943 | 2.1 |
| 42763 | 0.810945 | 0.05 |
| 42764 | 0.810945 | 0.0652306 |
| 42765 | 0.810958 | 2.1 |
| 42766 | 0.810958 | 1.82896 |
| 42767 | 1.89226 | 2.1 |
| 42768 | 1.35168 | 2.1 |
| 42769 | 1.08132 | 0.0801079 |
| 42770 | 1.08132 | 2.1 |
| 42771 | 1.0813 | 1.97596 |
| 42772 | 0.745979 | 2.1 |
| 42773 | 0.745979 | 2.1 |
| 42774 | 0.778781 | 2.1 |
| 42775 | 0.778781 | 2.1 |
| 42776 | 0.59549 | 2.1 |
| 42777 | 0.649658 | 2.1 |
| 42778 | 0.994784 | 2.1 |
| 42779 | 0.875102 | 2.1 |
| 42780 | 0.875102 | 2.1 |
| 42781 | 0.59549 | 2.1 |
| 42782 | 0.649658 | 2.1 |
| 42783 | 0.994784 | 2.1 |
| 42784 | 1.57887 | 2.1 |
| 42785 | 1.75021 | 2.1 |
| 42786 | 1.13341 | 2.1 |
| 42787 | 1.13341 | 2.1 |
| 42788 | 1.19098 | 2.1 |
| 42789 | 1.63644 | 2.1 |
| 42790 | 2.5761 | 0.0829292 |
| 42791 | 2.43404 | 0.378962 |
| 42792 | 2.44813 | 0.05 |
| 42793 | 2.31751 | 0.313233 |
| 42794 | 4.88218 | 0.111673 |
| 42795 | 2.43405 | 0.05 |
| 42796 | 2.22575 | 2.1 |
| 42797 | 2.31751 | 0.05 |
| 42798 | 2.10893 | 2.1 |
| 42799 | 4.54298 | 0.0609163 |
| 42800 | 2.44813 | 0.0616869 |
| 42801 | 2.31751 | 0.25523 |

| | | |
|---|---|---|
| 42802 | 2.30908 | 0.05 |
| 42803 | 2.19815 | 0.165662 |
| 42804 | 4.64628 | 0.05 |
| 42805 | 2.31751 | 0.05 |
| 42806 | 2.10893 | 2.1 |
| 42807 | 2.19815 | 0.05 |
| 42808 | 1.98061 | 2.1 |
| 42809 | 4.30708 | 0.05 |
| 42810 | 0.94613 | 2.1 |
| 42811 | 0.94613 | 2.1 |
| 42812 | 1.40372 | 2.1 |
| 42813 | 1.40372 | 2.1 |
| 42814 | 1.40372 | 2.1 |
| 42815 | 1.37167 | 2.1 |
| 42816 | 0.946132 | 2.1 |
| 42817 | 0.946132 | 2.1 |
| 42818 | 0.946131 | 2.1 |
| 42819 | 1.40372 | 2.1 |
| 42820 | 0.94613 | 2.1 |
| 42821 | 1.37167 | 2.1 |
| 42822 | 2.80744 | 2.1 |
| 42823 | 2.06984 | 2.1 |
| 42824 | 2.83418 | 2.1 |
| 42825 | 2.74335 | 2.1 |
| 42826 | 2.16067 | 2.1 |
| 42827 | 2.06984 | 2.1 |
| 42828 | 1.077 0.05 | |
| 42829 | 0.809116 | 0.05 |
| 42830 | 0.600419 | 0.05 |
| 42831 | 0.790277 | 0.05 |
| 42832 | 0.927647 | 0.05 |
| 42833 | 0.758598 | 0.05 |
| 42834 | 0.85749 | 0.05 |
| 42835 | 0.85749 | 0.05 |
| 42836 | 1.02417 | 0.05 |
| 42837 | 0.600411 | 0.05 |
| 42838 | 0.751701 | 0.05 |
| 42839 | 1.07702 | 0.05 |
| 42840 | 1.89226 | 2.1 |
| 42841 | 1.89226 | 2.1 |
| 42842 | 1.89226 | 2.1 |
| 42843 | 1.89226 | 2.1 |
| 42844 | 1.89226 | 2.1 |
| 42845 | 1.89226 | 2.1 |
| 42846 | 1.89226 | 2.1 |
| 42847 | 1.89226 | 2.1 |
| 42848 | 1.89226 | 2.1 |
| 42849 | 1.89226 | 2.1 |
| 42850 | 3.78453 | 2.1 |
| 42851 | 1.89226 | 2.1 |
| 42852 | 1.89226 | 2.1 |
| 42853 | 1.89226 | 2.1 |
| 42854 | 1.89226 | 2.1 |
| 42855 | 1.89226 | 2.1 |
| 42856 | 1.89226 | 2.1 |
| 42857 | 0.946131 | 2.1 |
| 42858 | 0.946131 | 2.1 |
| 42859 | 0.946131 | 2.1 |

| | | |
|---|---|---|
| 42860 | 0.946131 | 2.1 |
| 42861 | 0.946133 | 2.1 |
| 42862 | 0.946133 | 2.1 |
| 42863 | 0.946128 | 2.1 |
| 42864 | 0.946128 | 2.1 |
| 42865 | 0.946131 | 2.1 |
| 42866 | 0.946131 | 2.1 |
| 42867 | 0.94613 | 2.1 |
| 42868 | 0.94613 | 2.1 |
| 42869 | 2.17159 | 2.1 |
| 42870 | 2.00083 | 2.1 |
| 42871 | 2.21305 | 2.1 |
| 42872 | 2.00551 | 2.1 |
| 42873 | 2.20837 | 2.1 |
| 42874 | 2.00083 | 2.1 |
| 42875 | 2.20837 | 2.1 |
| 42876 | 1.80328 | 1.16958 |
| 42877 | 2.22962 | 2.1 |
| 42878 | 2.00083 | 1.60735 |
| 42879 | 2.03207 | 1.07205 |
| 42880 | 1.80328 | 1.02922 |
| 42881 | 2.28564 | 2.1 |
| 42882 | 2.20837 | 1.74294 |
| 42883 | 2.30406 | 2.1 |
| 42884 | 2.17159 | 2.1 |
| 42885 | 2.34084 | 2.1 |
| 42886 | 2.20837 | 2.1 |
| 42887 | 1.09523 | 0.933558 |
| 42888 | 1.09523 | 2.1 |
| 42889 | 1.09934 | 2.1 |
| 42890 | 1.09934 | 2.1 |
| 42891 | 1.01603 | 0.881352 |
| 42892 | 1.09038 | 2.1 |
| 42893 | 1.10419 | 1.03059 |
| 42894 | 1.17042 | 2.1 |
| 42895 | 1.17042 | 2.1 |
| 42896 | 1.09038 | 1.92931 |
| 42897 | 1.10419 | 1.23673 |
| 42898 | 1.01603 | 2.1 |
| 42899 | 2.60152 | 0.05 |
| 42900 | 0.976334 | 0.1371 |
| 42901 | 2.22344 | 0.281344 |
| 42902 | 2.22344 | 0.05 |
| 42903 | 1.36405 | 0.223161 |
| 42904 | 1.36405 | 0.05 |
| 42905 | 1.89226 | 2.1 |
| 42906 | 1.70302 | 2.1 |
| 42907 | 1.69019 | 2.1 |
| 42908 | 1.70302 | 2.1 |
| 42909 | 1.73933 | 2.1 |
| 42910 | 1.70303 | 1.0165 |
| 42911 | 1.89226 | 2.1 |
| 42912 | 1.89226 | 0.678522 |
| 42913 | 1.89226 | 2.03329 |
| 42914 | 1.89226 | 2.1 |
| 42915 | 3.78452 | 2.1 |
| 42916 | 1.89226 | 2.02863 |
| 42917 | 1.59036 | 2.1 |

| | | |
|---|---|---|
| 42918 | 1.65683 | 2.1 |
| 42919 | 1.59036 | 2.1 |
| 42920 | 1.95873 | 2.1 |
| 42921 | 1.89226 | 0.812719 |
| 42922 | 0.946131 | 0.694835 |
| 42923 | 0.946131 | 0.820264 |
| 42924 | 0.946127 | 2.1 |
| 42925 | 0.946127 | 1.9661 |
| 42926 | 0.946127 | 2.03256 |
| 42927 | 0.946127 | 0.131542 |
| 42928 | 0.946131 | 2.1 |
| 42929 | 0.946131 | 0.848961 |
| 42930 | 0.946129 | 0.944217 |
| 42931 | 0.946129 | 0.959313 |
| 42932 | 0.94613 | 1.01721 |
| 42933 | 0.94613 | 0.955831 |
| 42934 | 0.851541 | 2.1 |
| 42935 | 0.851541 | 2.1 |
| 42936 | 0.851483 | 2.1 |
| 42937 | 1.15713 | 2.1 |
| 42938 | 0.851539 | 2.1 |
| 42939 | 0.851539 | 2.04541 |
| 42940 | 0.942127 | 2.1 |
| 42941 | 1.14455 | 2.1 |
| 42942 | 0.838683 | 2.1 |
| 42943 | 0.946131 | 2.1 |
| 42944 | 0.957503 | 2.1 |
| 42945 | 0.957504 | 2.1 |
| 42946 | 1.89226 | 0.233984 |
| 42947 | 1.89226 | 0.05 |
| 42948 | 1.89226 | 0.427371 |
| 42949 | 1.89226 | 1.69813 |
| 42950 | 3.78452 | 0.05 |
| 42951 | 1.89226 | 0.05 |
| 42952 | 1.89226 | 0.05 |
| 42953 | 1.89226 | 0.05 |
| 42954 | 1.89226 | 0.05 |
| 42955 | 1.89226 | 0.05 |
| 42956 | 1.89226 | 0.05 |
| 42957 | 1.89226 | 0.05 |
| 42958 | 1.89226 | 0.05 |
| 42959 | 1.89226 | 0.05 |
| 42960 | 1.89226 | 0.05 |
| 42961 | 3.78453 | 0.05 |
| 42962 | 1.89226 | 0.05 |
| 42963 | 1.89226 | 2.1 |
| 42964 | 1.89226 | 2.1 |
| 42965 | 1.89226 | 0.934242 |
| 42966 | 3.78453 | 1.98666 |
| 42967 | 1.22575 | 0.202288 |
| 42968 | 1.05438 | 0.4606 |
| 42969 | 0.946131 | 0.606166 |
| 42970 | 0.946131 | 0.540887 |
| 42971 | 0.946129 | 1.57443 |
| 42972 | 1.22575 | 0.05 |
| 42973 | 1.05438 | 0.566083 |
| 42974 | 0.946132 | 0.05 |
| 42975 | 1.05438 | 0.550893 |

| | | |
|---|---|---|
| 42976 | 1.05438 | 0.813362 |
| 42977 | 0.946131 | 0.211185 |
| 42978 | 0.946131 | 0.389938 |
| 42979 | 2.4515 | 0.0788825 |
| 42980 | 1.13726 | 0.05 |
| 42981 | 2.10875 | 0.05 |
| 42982 | 1.49515 | 0.05 |
| 42983 | 2.10875 | 0.05 |
| 42984 | 1.49515 | 0.05 |
| 42985 | 0.831477 | 0.05 |
| 42986 | 0.747575 | 0.05 |
| 42987 | 1.22575 | 0.05 |
| 42988 | 1.28174 | 0.05 |
| 42989 | 1.28174 | 0.05 |
| 42990 | 0.831476 | 0.0600072 |
| 42991 | 1.22575 | 0.05 |
| 42992 | 0.747575 | 0.05 |
| 42993 | 1.03561 | 0.05 |
| 42994 | 1.03561 | 0.0834482 |
| 42995 | 1.02162 | 0.05 |
| 42996 | 1.02162 | 0.0837978 |
| 42997 | 1.89227 | 2.1 |
| 42998 | 2.56348 | 0.0791516 |
| 42999 | 1.89227 | 2.1 |
| 43000 | 2.45151 | 1.49883 |
| 43001 | 1.89227 | 2.1 |
| 43002 | 2.45151 | 0.0582014 |
| 43003 | 1.40013 | 0.05 |
| 43004 | 1.89226 | 1.58291 |
| 43005 | 1.89226 | 0.185993 |
| 43006 | 0.946643 | 0.05 |
| 43007 | 1.89226 | 0.936486 |
| 43008 | 0.946643 | 0.05 |
| 43009 | 1.89226 | 0.05 |
| 43010 | 1.89226 | 0.05 |
| 43011 | 1.89226 | 0.05 |
| 43012 | 1.89226 | 0.05 |
| 43013 | 3.78453 | 0.05 |
| 43014 | 1.89226 | 0.05 |
| 43015 | 1.89226 | 0.05 |
| 43016 | 1.89226 | 0.05 |
| 43017 | 1.89226 | 0.05 |
| 43018 | 3.78453 | 0.05 |
| 43019 | 0.946131 | 0.05 |
| 43020 | 0.946131 | 0.05 |
| 43021 | 0.946128 | 0.05 |
| 43022 | 0.946128 | 0.05 |
| 43023 | 0.94613 | 0.05 |
| 43024 | 0.94613 | 0.05 |
| 43025 | 0.946129 | 0.05 |
| 43026 | 0.946129 | 0.05 |
| 43027 | 0.94613 | 0.05 |
| 43028 | 0.94613 | 0.05 |
| 43029 | 0.94613 | 0.05 |
| 43030 | 0.94613 | 0.05 |
| 43031 | 1.89226 | 0.05 |
| 43032 | 1.89226 | 0.05 |
| 43033 | 1.17244 | 0.05 |

| | | |
|---|---|---|
| 43034 | 1.89226 | 0.05 |
| 43035 | 0.975291 | 0.05 |
| 43036 | 1.57408 | 0.05 |
| 43037 | 0.946132 | 0.05 |
| 43038 | 0.946132 | 0.05 |
| 43039 | 0.946131 | 0.05 |
| 43040 | 0.946131 | 0.05 |
| 43041 | 0.946132 | 0.05 |
| 43042 | 0.946132 | 0.05 |
| 43043 | 0.946131 | 0.05 |
| 43044 | 0.946131 | 0.05 |
| 43045 | 0.946131 | 0.05 |
| 43046 | 0.946131 | 0.05 |
| 43047 | 0.946132 | 0.05 |
| 43048 | 0.946132 | 0.05 |
| 43049 | 2.74744 | 0.05 |
| 43050 | 1.89226 | 0.05 |
| 43051 | 2.70805 | 0.05 |
| 43052 | 1.89226 | 0.05 |
| 43053 | 2.70805 | 0.05 |
| 43054 | 1.89226 | 0.05 |
| 43055 | 2.74744 | 0.05 |
| 43056 | 1.89226 | 0.05 |
| 43057 | 2.57465 | 0.05 |
| 43058 | 1.89226 | 0.05 |
| 43059 | 4.6397 | 0.05 |
| 43060 | 1.89226 | 0.05 |
| 43061 | 1.89226 | 0.05 |
| 43062 | 1.89226 | 0.05 |
| 43063 | 1.89226 | 0.154303 |
| 43064 | 3.78452 | 0.05 |
| 43065 | 1.89226 | 0.05 |
| 43066 | 1.89226 | 0.079678 |
| 43067 | 1.89226 | 0.05 |
| 43068 | 1.89226 | 0.05 |
| 43069 | 1.89226 | 0.295403 |
| 43070 | 1.89226 | 0.132907 |
| 43071 | 2.16206 | 0.05 |
| 43072 | 2.67861 | 0.05 |
| 43073 | 3.11996 | 0.05 |
| 43074 | 1.70303 | 0.05 |
| 43075 | 2.89015 | 0.05 |
| 43076 | 1.486 0.05 | |
| 43077 | 2.12839 | 0.05 |
| 43078 | 2.83631 | 0.05 |
| 43079 | 3.08096 | 0.05 |
| 43080 | 1.84157 | 0.05 |
| 43081 | 3.08096 | 0.05 |
| 43082 | 1.84157 | 0.05 |
| 43083 | 1.89225 | 0.950699 |
| 43084 | 1.89225 | 0.395372 |
| 43085 | 1.89225 | 0.566239 |
| 43086 | 1.89225 | 1.00601 |
| 43087 | 1.89225 | 0.398436 |
| 43088 | 1.89225 | 0.934084 |
| 43089 | 1.89226 | 0.141056 |
| 43090 | 1.89226 | 0.217424 |
| 43091 | 1.89226 | 0.923643 |

| | | |
|---|---|---|
| 43092 | 1.89226 | 0.105031 |
| 43093 | 3.78452 | 0.0733063 |
| 43094 | 1.89226 | 0.05 |
| 43095 | 2.49041 | 0.05 |
| 43096 | 1.89226 | 0.05 |
| 43097 | 1.89226 | 0.05 |
| 43098 | 2.27073 | 0.05 |
| 43099 | 2.27073 | 0.05 |
| 43100 | 1.09904 | 0.05 |
| 43101 | 0.677147 | 0.05 |
| 43102 | 1.3365 | 0.05 |
| 43103 | 0.95759 | 0.05 |
| 43104 | 0.957591 | 0.05 |
| 43105 | 0.677148 | 0.05 |
| 43106 | 1.09904 | 0.05 |
| 43107 | 1.3365 | 0.05 |
| 43108 | 0.996015 | 0.05 |
| 43109 | 0.996015 | 0.05 |
| 43110 | 1.01764 | 0.05 |
| 43111 | 1.01764 | 0.05 |
| 43112 | 2.77349 | 0.05 |
| 43113 | 2.72723 | 0.05 |
| 43114 | 2.43156 | 0.05 |
| 43115 | 2.4564 | 0.05 |
| 43116 | 5.20505 | 0.05 |
| 43117 | 2.77349 | 0.05 |
| 43118 | 2.68508 | 0.05 |
| 43119 | 2.33761 | 0.05 |
| 43120 | 2.4564 | 0.05 |
| 43121 | 5.1111 | 0.05 |
| 43122 | 2.43156 | 0.05 |
| 43123 | 2.70208 | 0.05 |
| 43124 | 1.85779 | 0.05 |
| 43125 | 2.10695 | 0.05 |
| 43126 | 4.77572 | 0.05 |
| 43127 | 2.33761 | 0.05 |
| 43128 | 2.70208 | 0.05 |
| 43129 | 1.85779 | 0.05 |
| 43130 | 1.61597 | 0.05 |
| 43131 | 4.19542 | 0.05 |
| 43132 | 0.946132 | 0.642321 |
| 43133 | 0.946132 | 1.43588 |
| 43134 | 0.746729 | 1.5754 |
| 43135 | 0.746729 | 2.0876 |
| 43136 | 0.946132 | 2.1 |
| 43137 | 0.915958 | 1.65747 |
| 43138 | 0.946133 | 0.460665 |
| 43139 | 0.74673 | 2.1 |
| 43140 | 0.946133 | 1.66742 |
| 43141 | 0.946133 | 1.66759 |
| 43142 | 0.915958 | 1.67696 |
| 43143 | 0.746729 | 1.55398 |
| 43144 | 1.50743 | 2.1 |
| 43145 | 2.45501 | 2.1 |
| 43146 | 2.29118 | 2.1 |
| 43147 | 2.29118 | 2.1 |
| 43148 | 1.75482 | 2.1 |
| 43149 | 1.75482 | 2.1 |

| | | |
|---|---|---|
| 43150 | 2.45501 | 2.1 |
| 43151 | 2.76927 | 0.05 |
| 43152 | 2.29118 | 2.1 |
| 43153 | 2.64523 | 0.05 |
| 43154 | 5.10024 | 0.745576 |
| 43155 | 1.25559 | 2.1 |
| 43156 | 2.29118 | 2.1 |
| 43157 | 2.0815 | 2.1 |
| 43158 | 2.26387 | 2.1 |
| 43159 | 1.35668 | 2.1 |
| 43160 | 1.47667 | 2.1 |
| 43161 | 2.28481 | 2.1 |
| 43162 | 2.64523 | 0.0944191 |
| 43163 | 2.50057 | 2.1 |
| 43164 | 2.50057 | 0.05 |
| 43165 | 2.29118 | 2.1 |
| 43166 | 2.29118 | 0.0854903 |
| 43167 | 0.946129 | 0.05 |
| 43168 | 0.946129 | 0.05 |
| 43169 | 1.23929 | 0.05 |
| 43170 | 1.15246 | 0.05 |
| 43171 | 0.946131 | 0.0579332 |
| 43172 | 0.946131 | 0.0836747 |
| 43173 | 1.15246 | 0.05 |
| 43174 | 1.15246 | 0.05 |
| 43175 | 0.946131 | 0.05 |
| 43176 | 1.15246 | 0.0966467 |
| 43177 | 0.946131 | 0.05 |
| 43178 | 1.23928 | 0.0503692 |
| 43179 | 0.946128 | 0.155625 |
| 43180 | 0.946128 | 0.0864494 |
| 43181 | 1.15246 | 0.05 |
| 43182 | 0.942814 | 0.152591 |
| 43183 | 0.946131 | 0.0986807 |
| 43184 | 0.946131 | 0.215754 |
| 43185 | 0.961347 | 0.0652992 |
| 43186 | 0.942811 | 0.05 |
| 43187 | 0.946131 | 0.0575168 |
| 43188 | 0.961347 | 0.05 |
| 43189 | 0.946131 | 0.05 |
| 43190 | 1.15246 | 0.05 |
| 43191 | 2.32334 | 0.05 |
| 43192 | 1.57621 | 0.05 |
| 43193 | 1.90335 | 0.05 |
| 43194 | 1.24538 | 0.05 |
| 43195 | 3.47956 | 0.05 |
| 43196 | 0.965762 | 0.05 |
| 43197 | 0.965762 | 0.05 |
| 43198 | 1.00245 | 0.05 |
| 43199 | 1.00245 | 0.05 |
| 43200 | 0.92786 | 0.05 |
| 43201 | 0.705297 | 0.05 |
| 43202 | 0.856414 | 0.05 |
| 43203 | 1.28968 | 0.05 |
| 43204 | 1.00306 | 0.05 |
| 43205 | 0.705303 | 0.05 |
| 43206 | 1.28967 | 0.05 |
| 43207 | 0.975478 | 0.05 |

| | | |
|---|---|---|
| 43208 | 3.05308 | 2.1 |
| 43209 | 1.76816 | 2.1 |
| 43210 | 1.76816 | 2.1 |
| 43211 | 1.69129 | 2.1 |
| 43212 | 2.58731 | 2.1 |
| 43213 | 2.51043 | 2.1 |
| 43214 | 1.89226 | 0.05 |
| 43215 | 2.71464 | 0.05 |
| 43216 | 2.34627 | 0.05 |
| 43217 | 1.89226 | 0.05 |
| 43218 | 2.34627 | 0.05 |
| 43219 | 1.89226 | 0.05 |
| 43220 | 2.34627 | 0.05 |
| 43221 | 2.71464 | 0.05 |
| 43222 | 1.98252 | 0.05 |
| 43223 | 1.98252 | 0.05 |
| 43224 | 1.51026 | 0.05 |
| 43225 | 2.34627 | 0.05 |
| 43226 | 2.38698 | 1.12921 |
| 43227 | 2.35142 | 2.1 |
| 43228 | 2.60343 | 1.32671 |
| 43229 | 2.6636 | 1.24685 |
| 43230 | 4.99041 | 1.46769 |
| 43231 | 1.07811 | 2.1 |
| 43232 | 1.1952 | 2.1 |
| 43233 | 1.17571 | 2.1 |
| 43234 | 1.30171 | 1.05335 |
| 43235 | 1.18322 | 0.988509 |
| 43236 | 1.18322 | 2.1 |
| 43237 | 1.18769 | 2.1 |
| 43238 | 1.18769 | 1.12361 |
| 43239 | 1.1952 | 2.1 |
| 43240 | 1.30171 | 2.1 |
| 43241 | 1.17571 | 2.1 |
| 43242 | 1.07811 | 2.1 |
| 43243 | 1.89226 | 1.60093 |
| 43244 | 2.6636 | 2.01826 |
| 43245 | 1.89226 | 0.522481 |
| 43246 | 2.60343 | 1.41363 |
| 43247 | 1.89226 | 1.3985 |
| 43248 | 2.60343 | 2.1 |
| 43249 | 1.5344 | 0.05 |
| 43250 | 1.4516 | 0.05 |
| 43251 | 1.17816 | 0.05 |
| 43252 | 1.21056 | 0.05 |
| 43253 | 2.71257 | 0.05 |
| 43254 | 1.89226 | 2.1 |
| 43255 | 1.89226 | 0.789969 |
| 43256 | 1.89226 | 1.07691 |
| 43257 | 1.89226 | 1.24095 |
| 43258 | 3.78452 | 0.960226 |
| 43259 | 1.89226 | 1.27971 |
| 43260 | 1.89226 | 0.129296 |
| 43261 | 1.89226 | 0.779754 |
| 43262 | 1.89226 | 0.695899 |
| 43263 | 1.65573 | 0.956852 |
| 43264 | 1.65573 | 0.727282 |
| 43265 | 1.89226 | 0.986749 |

| | | |
|---|---|---|
| 43266 | 1.89226 | 1.30322 |
| 43267 | 1.89226 | 2.1 |
| 43268 | 2.64212 | 0.489489 |
| 43269 | 3.78452 | 1.65568 |
| 43270 | 1.89226 | 1.38292 |
| 43271 | 1.89226 | 2.1 |
| 43272 | 1.89226 | 1.61266 |
| 43273 | 1.89226 | 2.1 |
| 43274 | 3.78452 | 2.1 |
| 43275 | 2.7367 | 0.05 |
| 43276 | 2.07947 | 2.1 |
| 43277 | 2.47319 | 1.37741 |
| 43278 | 1.85057 | 2.1 |
| 43279 | 4.55267 | 2.1 |
| 43280 | 2.07948 | 2.1 |
| 43281 | 1.3294 | 2.1 |
| 43282 | 1.85057 | 2.1 |
| 43283 | 1.09962 | 2.1 |
| 43284 | 3.1791 | 2.1 |
| 43285 | 1.89226 | 2.1 |
| 43286 | 1.89226 | 2.1 |
| 43287 | 1.89226 | 2.1 |
| 43288 | 1.89226 | 2.1 |
| 43289 | 3.78453 | 2.1 |
| 43290 | 0.94613 | 2.1 |
| 43291 | 0.94613 | 0.324007 |
| 43292 | 0.94613 | 0.787382 |
| 43293 | 0.94613 | 2.1 |
| 43294 | 0.946128 | 2.1 |
| 43295 | 0.946128 | 2.1 |
| 43296 | 0.946132 | 0.179488 |
| 43297 | 0.946132 | 0.154351 |
| 43298 | 0.94613 | 2.1 |
| 43299 | 0.94613 | 2.1 |
| 43300 | 0.946131 | 2.1 |
| 43301 | 0.946131 | 2.1 |
| 43302 | 1.00875 | 2.1 |
| 43303 | 1.00875 | 0.170842 |
| 43304 | 1.01676 | 2.1 |
| 43305 | 1.01676 | 0.05 |
| 43306 | 0.925282 | 2.1 |
| 43307 | 0.804877 | 2.1 |
| 43308 | 1.2366 | 0.739 |
| 43309 | 1.10023 | 0.729343 |
| 43310 | 0.804878 | 2.1 |
| 43311 | 1.10023 | 2.1 |
| 43312 | 1.2366 | 2.1 |
| 43313 | 0.925284 | 2.1 |
| 43314 | 1.89226 | 2.1 |
| 43315 | 1.89226 | 2.1 |
| 43316 | 1.89226 | 2.1 |
| 43317 | 1.89226 | 2.1 |
| 43318 | 3.78453 | 2.1 |
| 43319 | 1.89226 | 2.1 |
| 43320 | 1.89226 | 2.1 |
| 43321 | 1.89226 | 2.1 |
| 43322 | 1.89226 | 2.1 |
| 43323 | 1.89226 | 2.1 |

| | | |
|---|---|---|
| 43324 | 1.89226 | 2.1 |
| 43325 | 1.09766 | 2.1 |
| 43326 | 1.14842 | 2.1 |
| 43327 | 1.02909 | 2.1 |
| 43328 | 1.07659 | 2.1 |
| 43329 | 1.16718 | 2.1 |
| 43330 | 0.954189 | 2.1 |
| 43331 | 1.06653 | 2.1 |
| 43332 | 1.06653 | 2.1 |
| 43333 | 1.20738 | 2.1 |
| 43334 | 1.02909 | 2.1 |
| 43335 | 1.09767 | 2.1 |
| 43336 | 0.815951 | 2.1 |
| 43337 | 2.00793 | 2.1 |
| 43338 | 1.14048 | 2.1 |
| 43339 | 1.19086 | 2.1 |
| 43340 | 1.13723 | 2.1 |
| 43341 | 2.00793 | 2.1 |
| 43342 | 2.11772 | 2.1 |
| 43343 | 1.89226 | 2.1 |
| 43344 | 1.89226 | 2.1 |
| 43345 | 1.89226 | 2.1 |
| 43346 | 1.89226 | 2.1 |
| 43347 | 1.89226 | 2.1 |
| 43348 | 1.89226 | 2.1 |
| 43349 | 1.89226 | 2.1 |
| 43350 | 1.89226 | 2.1 |
| 43351 | 1.89226 | 2.1 |
| 43352 | 1.89226 | 2.1 |
| 43353 | 1.89226 | 2.1 |
| 43354 | 1.89226 | 2.1 |
| 43355 | 1.1779 | 2.1 |
| 43356 | 1.26166 | 2.1 |
| 43357 | 1.0875 | 2.1 |
| 43358 | 1.00397 | 2.1 |
| 43359 | 1.13286 | 2.1 |
| 43360 | 1.13286 | 2.1 |
| 43361 | 1.13277 | 2.1 |
| 43362 | 1.13277 | 2.1 |
| 43363 | 1.26166 | 2.1 |
| 43364 | 1.08749 | 2.1 |
| 43365 | 1.1779 | 2.1 |
| 43366 | 1.00396 | 2.1 |
| 43367 | 1.0875 | 2.1 |
| 43368 | 1.00397 | 2.1 |
| 43369 | 0.685749 | 2.1 |
| 43370 | 0.595432 | 2.1 |
| 43371 | 0.83976 | 2.1 |
| 43372 | 0.83976 | 2.1 |
| 43373 | 0.843169 | 2.1 |
| 43374 | 0.843169 | 2.1 |
| 43375 | 1.0875 | 2.1 |
| 43376 | 0.685749 | 2.1 |
| 43377 | 1.00397 | 2.1 |
| 43378 | 0.595432 | 2.1 |
| 43379 | 1.89226 | 2.1 |
| 43380 | 1.86443 | 2.1 |
| 43381 | 2.4988 | 2.1 |

| | | |
|---|---|---|
| 43382 | 2.0815 | 2.1 |
| 43383 | 2.28173 | 2.1 |
| 43384 | 1.86443 | 2.1 |
| 43385 | 1.89226 | 2.1 |
| 43386 | 1.89226 | 2.1 |
| 43387 | 1.89226 | 2.1 |
| 43388 | 1.89226 | 2.1 |
| 43389 | 1.89226 | 2.1 |
| 43390 | 1.89226 | 2.1 |
| 43391 | 0.946131 | 2.1 |
| 43392 | 0.946131 | 2.1 |
| 43393 | 0.969491 | 2.1 |
| 43394 | 0.949105 | 2.1 |
| 43395 | 0.949103 | 2.1 |
| 43396 | 0.949103 | 2.1 |
| 43397 | 0.946133 | 2.1 |
| 43398 | 0.946133 | 2.1 |
| 43399 | 0.946131 | 2.1 |
| 43400 | 0.969492 | 2.1 |
| 43401 | 0.946131 | 2.1 |
| 43402 | 0.949105 | 2.1 |
| 43403 | 1.89226 | 2.1 |
| 43404 | 2.07428 | 2.1 |
| 43405 | 1.93464 | 2.1 |
| 43406 | 1.89226 | 2.1 |
| 43407 | 2.11667 | 2.1 |
| 43408 | 2.07428 | 2.1 |
| 43409 | 0.946132 | 2.1 |
| 43410 | 0.946132 | 2.1 |
| 43411 | 0.946131 | 2.1 |
| 43412 | 0.946131 | 2.1 |
| 43413 | 0.946132 | 2.1 |
| 43414 | 0.946132 | 2.1 |
| 43415 | 0.946132 | 2.1 |
| 43416 | 0.946132 | 2.1 |
| 43417 | 0.946132 | 2.1 |
| 43418 | 0.946132 | 2.1 |
| 43419 | 0.946132 | 2.1 |
| 43420 | 0.946132 | 2.1 |
| 43421 | 1.97992 | 2.1 |
| 43422 | 1.89226 | 2.1 |
| 43423 | 1.89226 | 2.1 |
| 43424 | 1.93898 | 2.1 |
| 43425 | 1.93898 | 2.1 |
| 43426 | 1.89226 | 2.1 |
| 43427 | 2.12475 | 0.576505 |
| 43428 | 2.79029 | 0.628471 |
| 43429 | 2.7803 | 0.98945 |
| 43430 | 2.13052 | 0.546041 |
| 43431 | 4.92081 | 0.59747 |
| 43432 | 1.06238 | 0.772078 |
| 43433 | 1.06526 | 0.590606 |
| 43434 | 0.642901 | 0.53701 |
| 43435 | 0.655055 | 0.640788 |
| 43436 | 0.856391 | 0.558473 |
| 43437 | 0.856391 | 0.553889 |
| 43438 | 0.86104 | 1.38687 |
| 43439 | 0.86104 | 0.540674 |

| | | |
|---|---|---|
| 43440 | 1.06526 | 0.610723 |
| 43441 | 0.655055 | 0.600497 |
| 43442 | 0.642901 | 0.593969 |
| 43443 | 1.06238 | 0.569784 |
| 43444 | 2.7803 | 0.758992 |
| 43445 | 2.13053 | 0.233535 |
| 43446 | 2.14346 | 0.598648 |
| 43447 | 2.77937 | 0.601141 |
| 43448 | 4.9099 | 0.602222 |
| 43449 | 2.14346 | 0.67462 |
| 43450 | 1.31011 | 0.475418 |
| 43451 | 1.34124 | 0.590157 |
| 43452 | 1.31011 | 0.642309 |
| 43453 | 2.16166 | 0.529654 |
| 43454 | 2.13053 | 0.547491 |
| 43455 | 1.89226 | 2.1 |
| 43456 | 2.58176 | 2.1 |
| 43457 | 2.01422 | 2.1 |
| 43458 | 1.89226 | 2.1 |
| 43459 | 2.70373 | 2.1 |
| 43460 | 2.58176 | 2.1 |
| 43461 | 1.89226 | 2.1 |
| 43462 | 1.89226 | 2.1 |
| 43463 | 1.89226 | 2.1 |
| 43464 | 1.89226 | 2.1 |
| 43465 | 1.89226 | 2.1 |
| 43466 | 1.89226 | 2.1 |
| 43467 | 0.946128 | 2.1 |
| 43468 | 0.946128 | 2.1 |
| 43469 | 1.38521 | 2.1 |
| 43470 | 1.38521 | 2.1 |
| 43471 | 1.38521 | 2.1 |
| 43472 | 1.35186 | 2.1 |
| 43473 | 0.946131 | 2.1 |
| 43474 | 0.946131 | 2.1 |
| 43475 | 0.94613 | 2.1 |
| 43476 | 1.38521 | 2.1 |
| 43477 | 0.946131 | 2.1 |
| 43478 | 1.35186 | 2.1 |
| 43479 | 0.946133 | 2.1 |
| 43480 | 0.946133 | 2.1 |
| 43481 | 0.946132 | 2.1 |
| 43482 | 0.946132 | 2.1 |
| 43483 | 0.946132 | 2.1 |
| 43484 | 0.946132 | 2.1 |
| 43485 | 0.946132 | 2.1 |
| 43486 | 0.946132 | 2.1 |
| 43487 | 0.946132 | 2.1 |
| 43488 | 0.946132 | 2.1 |
| 43489 | 0.946133 | 2.1 |
| 43490 | 0.946133 | 2.1 |
| 43491 | 2.33374 | 2.1 |
| 43492 | 1.89226 | 2.1 |
| 43493 | 1.89226 | 2.1 |
| 43494 | 1.89226 | 2.1 |
| 43495 | 2.60377 | 2.1 |
| 43496 | 2.60377 | 2.1 |
| 43497 | 2.6771 | 0.05 |

| | | |
|---|---|---|
| 43498 | 1.36416 | 0.05 |
| 43499 | 1.64983 | 0.05 |
| 43500 | 1.64983 | 0.05 |
| 43501 | 2.44131 | 0.05 |
| 43502 | 2.44131 | 0.05 |
| 43503 | 2.58321 | 0.05 |
| 43504 | 1.89226 | 0.243584 |
| 43505 | 2.44283 | 0.05 |
| 43506 | 2.44283 | 0.05 |
| 43507 | 1.89226 | 0.564402 |
| 43508 | 1.89226 | 0.845973 |
| 43509 | 2.58321 | 0.143487 |
| 43510 | 2.44283 | 0.05 |
| 43511 | 1.49358 | 0.05 |
| 43512 | 1.57702 | 0.05 |
| 43513 | 3.93641 | 0.05 |
| 43514 | 0.951913 | 0.05 |
| 43515 | 0.81556 | 0.05 |
| 43516 | 0.946132 | 0.05 |
| 43517 | 0.946132 | 0.05 |
| 43518 | 1.04072 | 0.05 |
| 43519 | 1.12182 | 0.05 |
| 43520 | 0.856745 | 0.05 |
| 43521 | 0.851542 | 0.05 |
| 43522 | 0.923163 | 0.05 |
| 43523 | 0.923163 | 0.05 |
| 43524 | 1.04304 | 0.05 |
| 43525 | 0.85098 | 0.05 |
| 43526 | 2.70125 | 1.12849 |
| 43527 | 2.37734 | 1.07211 |
| 43528 | 2.63495 | 0.948348 |
| 43529 | 2.30539 | 2.06292 |
| 43530 | 5.01229 | 1.9134 |
| 43531 | 1.89226 | 1.89467 |
| 43532 | 1.89226 | 2.1 |
| 43533 | 1.89226 | 1.30628 |
| 43534 | 1.89226 | 2.1 |
| 43535 | 3.78452 | 0.955993 |
| 43536 | 1.89226 | 2.1 |
| 43537 | 1.89226 | 2.1 |
| 43538 | 1.89226 | 2.1 |
| 43539 | 1.89226 | 2.1 |
| 43540 | 3.78452 | 2.1 |
| 43541 | 2.37734 | 1.7429 |
| 43542 | 1.79346 | 2.1 |
| 43543 | 2.53594 | 1.75647 |
| 43544 | 1.70302 | 2.1 |
| 43545 | 4.27682 | 2.1 |
| 43546 | 2.63495 | 0.799081 |
| 43547 | 2.30539 | 1.30486 |
| 43548 | 2.49445 | 0.965692 |
| 43549 | 2.12089 | 0.819201 |
| 43550 | 4.75584 | 0.835238 |
| 43551 | 1.89226 | 0.985297 |
| 43552 | 1.89226 | 2.06961 |
| 43553 | 1.89226 | 1.004 |
| 43554 | 1.89226 | 2.1 |
| 43555 | 3.78452 | 2.1 |

| | | |
|---|---|---|
| 43556 | 1.89226 | 1.88247 |
| 43557 | 1.89226 | 2.1 |
| 43558 | 1.89226 | 2.1 |
| 43559 | 1.89226 | 2.1 |
| 43560 | 1.89226 | 1.10282 |
| 43561 | 1.89226 | 1.16348 |
| 43562 | 2.53594 | 1.07867 |
| 43563 | 1.60017 | 1.1196 |
| 43564 | 1.57079 | 1.30859 |
| 43565 | 1.70302 | 0.942228 |
| 43566 | 2.15772 | 1.12084 |
| 43567 | 2.33299 | 0.811459 |
| 43568 | 2.09114 | 2.1 |
| 43569 | 2.34898 | 2.1 |
| 43570 | 2.30729 | 2.1 |
| 43571 | 2.06556 | 2.1 |
| 43572 | 4.41454 | 2.1 |
| 43573 | 2.09114 | 2.1 |
| 43574 | 1.59654 | 2.1 |
| 43575 | 1.57823 | 2.1 |
| 43576 | 2.06556 | 2.1 |
| 43577 | 3.6621 | 2.1 |
| 43578 | 2.30729 | 2.1 |
| 43579 | 2.06556 | 2.1 |
| 43580 | 2.03917 | 2.1 |
| 43581 | 2.26617 | 2.1 |
| 43582 | 4.33173 | 2.1 |
| 43583 | 2.03917 | 2.1 |
| 43584 | 2.06556 | 2.1 |
| 43585 | 2.045 2.1 | |
| 43586 | 1.55767 | 2.1 |
| 43587 | 1.57823 | 2.1 |
| 43588 | 1.57823 | 2.1 |
| 43589 | 1.89225 | 2.1 |
| 43590 | 1.09726 | 2.1 |
| 43591 | 1.61196 | 2.1 |
| 43592 | 1.28597 | 2.1 |
| 43593 | 1.28597 | 0.05 |
| 43594 | 1.45633 | 2.1 |
| 43595 | 1.45633 | 2.1 |
| 43596 | 1.9544 | 0.05 |
| 43597 | 1.61196 | 2.1 |
| 43598 | 1.45633 | 2.1 |
| 43599 | 1.53735 | 2.1 |
| 43600 | 3.14931 | 2.1 |
| 43601 | 1.89226 | 2.1 |
| 43602 | 1.89226 | 2.1 |
| 43603 | 1.89226 | 2.1 |
| 43604 | 1.89226 | 2.1 |
| 43605 | 3.78453 | 2.1 |
| 43606 | 0.810946 | 2.1 |
| 43607 | 0.810946 | 2.1 |
| 43608 | 0.810947 | 2.1 |
| 43609 | 0.810947 | 2.1 |
| 43610 | 0.810933 | 2.1 |
| 43611 | 0.810933 | 2.1 |
| 43612 | 1.89226 | 2.1 |
| 43613 | 1.3517 | 2.1 |

| | | |
|---|---|---|
| 43614 | 1.08132 | 2.1 |
| 43615 | 1.08132 | 2.1 |
| 43616 | 1.08133 | 2.1 |
| 43617 | 0.851539 | 2.1 |
| 43618 | 0.851539 | 2.1 |
| 43619 | 0.972053 | 2.1 |
| 43620 | 1.05632 | 2.1 |
| 43621 | 0.94613 | 2.1 |
| 43622 | 0.94613 | 2.1 |
| 43623 | 1.0279 | 2.1 |
| 43624 | 0.94991 | 2.1 |
| 43625 | 1.0279 | 2.1 |
| 43626 | 0.946131 | 2.1 |
| 43627 | 0.865643 | 2.1 |
| 43628 | 0.94613 | 2.1 |
| 43629 | 1.89226 | 2.1 |
| 43630 | 1.89226 | 2.1 |
| 43631 | 1.89226 | 2.1 |
| 43632 | 1.89226 | 2.1 |
| 43633 | 3.78452 | 2.1 |
| 43634 | 1.03882 | 2.1 |
| 43635 | 1.91121 | 2.1 |
| 43636 | 1.41833 | 2.1 |
| 43637 | 2.34547 | 2.1 |
| 43638 | 3.32954 | 2.1 |
| 43639 | 2.3979 | 2.1 |
| 43640 | 1.89226 | 2.1 |
| 43641 | 1.89226 | 2.1 |
| 43642 | 1.89226 | 2.1 |
| 43643 | 2.0558 | 2.1 |
| 43644 | 2.0558 | 2.1 |
| 43645 | 1.89226 | 2.1 |
| 43646 | 1.89226 | 1.07225 |
| 43647 | 1.89226 | 2.1 |
| 43648 | 1.89226 | 2.1 |
| 43649 | 3.78452 | 2.1 |
| 43650 | 1.05816 | 2.1 |
| 43651 | 1.05816 | 2.1 |
| 43652 | 0.842045 | 2.1 |
| 43653 | 1.05252 | 2.1 |
| 43654 | 1.36057 | 2.1 |
| 43655 | 1.14339 | 2.1 |
| 43656 | 0.873397 | 2.1 |
| 43657 | 0.585073 | 2.1 |
| 43658 | 0.873388 | 2.1 |
| 43659 | 1.15129 | 2.1 |
| 43660 | 0.69143 | 2.1 |
| 43661 | 1.1434 | 2.1 |
| 43662 | 1.93338 | 2.1 |
| 43663 | 1.85883 | 2.1 |
| 43664 | 1.51714 | 2.1 |
| 43665 | 1.85883 | 2.1 |
| 43666 | 1.54795 | 2.1 |
| 43667 | 1.89226 | 2.1 |
| 43668 | 1.89226 | 2.1 |
| 43669 | 1.89226 | 2.1 |
| 43670 | 1.89226 | 2.1 |
| 43671 | 1.89226 | 2.1 |

| | | |
|---|---|---|
| 43672 | 3.78452 | 2.1 |
| 43673 | 0.844726 | 2.1 |
| 43674 | 0.844726 | 2.1 |
| 43675 | 0.960524 | 2.1 |
| 43676 | 1.16288 | 2.1 |
| 43677 | 0.851541 | 2.1 |
| 43678 | 0.851541 | 2.1 |
| 43679 | 0.929147 | 2.1 |
| 43680 | 1.15533 | 2.1 |
| 43681 | 0.844697 | 2.1 |
| 43682 | 0.946131 | 2.1 |
| 43683 | 0.851632 | 2.1 |
| 43684 | 0.946131 | 2.1 |
| 43685 | 1.89226 | 2.1 |
| 43686 | 1.70303 | 2.1 |
| 43687 | 1.74006 | 2.1 |
| 43688 | 1.70303 | 2.1 |
| 43689 | 1.99471 | 2.1 |
| 43690 | 1.93338 | 2.1 |
| 43691 | 0.946131 | 2.1 |
| 43692 | 0.946131 | 2.1 |
| 43693 | 0.946131 | 2.1 |
| 43694 | 0.946131 | 2.1 |
| 43695 | 0.94613 | 2.1 |
| 43696 | 0.94613 | 2.1 |
| 43697 | 0.946132 | 2.1 |
| 43698 | 0.946132 | 2.1 |
| 43699 | 0.946131 | 2.1 |
| 43700 | 0.946131 | 2.1 |
| 43701 | 0.946131 | 2.1 |
| 43702 | 0.946131 | 2.1 |
| 43703 | 1.58158 | 2.1 |
| 43704 | 1.89226 | 2.1 |
| 43705 | 1.89226 | 2.1 |
| 43706 | 1.89226 | 2.1 |
| 43707 | 1.68939 | 2.1 |
| 43708 | 1.68939 | 2.1 |
| 43709 | 0.946132 | 0.05 |
| 43710 | 0.946132 | 0.05 |
| 43711 | 1.12988 | 0.05 |
| 43712 | 1.30783 | 0.05 |
| 43713 | 1.30783 | 0.05 |
| 43714 | 0.946133 | 0.05 |
| 43715 | 0.946132 | 0.05 |
| 43716 | 1.12988 | 0.05 |
| 43717 | 1.30783 | 0.05 |
| 43718 | 1.30783 | 0.05 |
| 43719 | 0.946133 | 0.05 |
| 43720 | 0.946133 | 0.05 |
| 43721 | 1.89227 | 0.05 |
| 43722 | 1.89227 | 0.05 |
| 43723 | 1.89227 | 0.05 |
| 43724 | 1.89227 | 0.05 |
| 43725 | 1.89227 | 0.05 |
| 43726 | 1.89227 | 0.05 |
| 43727 | 1.89226 | 0.05 |
| 43728 | 1.89226 | 0.05 |
| 43729 | 1.89226 | 0.05 |

| | | |
|---|---|---|
| 43730 | 1.89226 | 0.05 |
| 43731 | 1.89226 | 0.05 |
| 43732 | 1.89226 | 0.05 |
| 43733 | 0.946131 | 0.05 |
| 43734 | 0.946131 | 0.05 |
| 43735 | 1.30783 | 0.05 |
| 43736 | 1.37715 | 0.05 |
| 43737 | 1.37715 | 0.05 |
| 43738 | 0.946133 | 0.05 |
| 43739 | 1.30783 | 0.05 |
| 43740 | 0.946129 | 0.05 |
| 43741 | 1.37715 | 0.05 |
| 43742 | 1.37715 | 0.05 |
| 43743 | 0.946131 | 0.05 |
| 43744 | 0.946131 | 0.05 |
| 43745 | 1.17056 | 0.05 |
| 43746 | 0.591757 | 0.05 |
| 43747 | 0.522025 | 0.05 |
| 43748 | 0.929727 | 0.05 |
| 43749 | 0.929728 | 0.05 |
| 43750 | 1.17056 | 0.05 |
| 43751 | 0.591756 | 0.05 |
| 43752 | 0.522025 | 0.05 |
| 43753 | 0.889054 | 0.05 |
| 43754 | 0.889054 | 0.05 |
| 43755 | 0.803535 | 0.05 |
| 43756 | 0.803535 | 0.05 |
| 43757 | 1.49814 | 2.1 |
| 43758 | 2.37439 | 2.1 |
| 43759 | 2.63923 | 2.1 |
| 43760 | 2.63923 | 2.1 |
| 43761 | 1.29111 | 2.1 |
| 43762 | 1.29111 | 2.1 |
| 43763 | 0.946132 | 2.1 |
| 43764 | 0.946132 | 2.1 |
| 43765 | 1.18719 | 2.1 |
| 43766 | 1.18719 | 2.1 |
| 43767 | 0.94613 | 2.1 |
| 43768 | 0.94613 | 2.1 |
| 43769 | 1.1872 | 2.1 |
| 43770 | 1.31961 | 2.1 |
| 43771 | 0.946131 | 2.1 |
| 43772 | 1.31961 | 2.1 |
| 43773 | 1.1872 | 2.1 |
| 43774 | 0.94613 | 2.1 |
| 43775 | 2.49445 | 0.507912 |
| 43776 | 2.12089 | 0.505471 |
| 43777 | 2.20843 | 0.560761 |
| 43778 | 1.82805 | 0.756322 |
| 43779 | 4.32932 | 0.613661 |
| 43780 | 1.89226 | 0.850572 |
| 43781 | 1.89226 | 1.21039 |
| 43782 | 1.89226 | 0.744268 |
| 43783 | 1.89226 | 0.73593 |
| 43784 | 3.78452 | 0.654487 |
| 43785 | 0.94613 | 0.781209 |
| 43786 | 0.94613 | 0.738512 |
| 43787 | 0.946128 | 0.674326 |

| | | |
|---|---|---|
| 43788 | 0.946128 | 0.899317 |
| 43789 | 0.946131 | 0.865852 |
| 43790 | 0.946131 | 0.898278 |
| 43791 | 0.946127 | 0.900331 |
| 43792 | 0.946127 | 0.675978 |
| 43793 | 0.946129 | 0.779073 |
| 43794 | 0.946129 | 0.704055 |
| 43795 | 0.946129 | 0.802532 |
| 43796 | 0.946129 | 0.803335 |
| 43797 | 1.82804 | 0.489739 |
| 43798 | 1.60017 | 0.817601 |
| 43799 | 1.87679 | 0.770003 |
| 43800 | 2.12088 | 0.222699 |
| 43801 | 1.35608 | 0.891056 |
| 43802 | 1.60017 | 0.709309 |
| 43803 | 2.20843 | 0.19454 |
| 43804 | 1.3216 | 0.0666222 |
| 43805 | 1.44426 | 0.0843064 |
| 43806 | 1.82805 | 0.125701 |
| 43807 | 1.80399 | 0.222894 |
| 43808 | 2.20843 | 0.290667 |
| 43809 | 1.89226 | 0.572348 |
| 43810 | 1.89226 | 0.545548 |
| 43811 | 1.89226 | 0.759069 |
| 43812 | 1.89226 | 0.597491 |
| 43813 | 1.89226 | 0.632346 |
| 43814 | 1.89226 | 0.501374 |
| 43815 | 1.89226 | 0.701675 |
| 43816 | 1.89226 | 0.296077 |
| 43817 | 1.89226 | 0.590457 |
| 43818 | 1.89226 | 0.581196 |
| 43819 | 1.89226 | 0.713891 |
| 43820 | 1.89226 | 0.396127 |
| 43821 | 0.627705 | 0.05 |
| 43822 | 0.634941 | 0.0561394 |
| 43823 | 0.629317 | 0.05 |
| 43824 | 0.783422 | 0.05 |
| 43825 | 0.629328 | 0.05 |
| 43826 | 0.448764 | 0.0669839 |
| 43827 | 1.35608 | 0.0866833 |
| 43828 | 1.00805 | 0.0888284 |
| 43829 | 0.814295 | 0.05 |
| 43830 | 0.598375 | 0.0610147 |
| 43831 | 1.04461 | 0.0538304 |
| 43832 | 1.09795 | 2.05609 |
| 43833 | 1.00775 | 2.1 |
| 43834 | 1.61202 | 2.1 |
| 43835 | 1.63419 | 2.01094 |
| 43836 | 2.64194 | 1.65245 |
| 43837 | 1.44801 | 1.75462 |
| 43838 | 1.61201 | 2.1 |
| 43839 | 2.35614 | 1.92119 |
| 43840 | 2.27703 | 2.1 |
| 43841 | 3.88904 | 2.06786 |
| 43842 | 2.35615 | 1.72202 |
| 43843 | 1.63419 | 1.58627 |
| 43844 | 2.29852 | 1.56054 |
| 43845 | 1.63419 | 1.59562 |

| | | |
|---|---|---|
| 43846 | 2.27634 | 1.6197 |
| 43847 | 1.61202 | 2.1 |
| 43848 | 0.973793 | 2.1 |
| 43849 | 2.2671 | 2.1 |
| 43850 | 2.32981 | 2.1 |
| 43851 | 2.32981 | 2.1 |
| 43852 | 0.990481 | 2.1 |
| 43853 | 0.990481 | 2.1 |
| 43854 | 1.50877 | 2.1 |
| 43855 | 1.11193 | 2.1 |
| 43856 | 2.29667 | 2.1 |
| 43857 | 1.94158 | 2.1 |
| 43858 | 3.45035 | 2.1 |
| 43859 | 2.29667 | 2.1 |
| 43860 | 1.89227 | 2.1 |
| 43861 | 1.94158 | 2.1 |
| 43862 | 1.94158 | 2.1 |
| 43863 | 1.89227 | 2.1 |
| 43864 | 1.89227 | 2.1 |
| 43865 | 2.51379 | 2.1 |
| 43866 | 1.30026 | 2.1 |
| 43867 | 1.84502 | 2.1 |
| 43868 | 2.72099 | 2.1 |
| 43869 | 4.35882 | 2.1 |
| 43870 | 1.89226 | 2.1 |
| 43871 | 2.721 | 2.1 |
| 43872 | 2.5138 | 2.1 |
| 43873 | 2.5138 | 2.1 |
| 43874 | 1.89226 | 2.1 |
| 43875 | 1.89226 | 2.1 |
| 43876 | 1.89226 | 0.05 |
| 43877 | 1.89226 | 0.05 |
| 43878 | 2.59101 | 0.05 |
| 43879 | 1.89226 | 0.05 |
| 43880 | 3.78453 | 0.05 |
| 43881 | 0.851541 | 0.05 |
| 43882 | 0.851541 | 0.05 |
| 43883 | 1.42502 | 0.05 |
| 43884 | 0.948679 | 0.05 |
| 43885 | 0.946132 | 0.05 |
| 43886 | 0.946132 | 0.05 |
| 43887 | 0.853617 | 0.05 |
| 43888 | 0.853618 | 0.05 |
| 43889 | 0.851475 | 0.05 |
| 43890 | 0.948666 | 0.05 |
| 43891 | 1.166 | 0.05 |
| 43892 | 0.851554 | 0.05 |
| 43893 | 2.51818 | 0.75229 |
| 43894 | 2.60723 | 2.1 |
| 43895 | 2.66532 | 0.112801 |
| 43896 | 2.76315 | 2.1 |
| 43897 | 5.27255 | 1.41924 |
| 43898 | 2.60723 | 2.1 |
| 43899 | 2.45247 | 2.1 |
| 43900 | 2.76315 | 2.1 |
| 43901 | 2.55197 | 2.1 |
| 43902 | 5.1592 | 2.1 |
| 43903 | 1.23768 | 0.696777 |

| | | |
|---|---|---|
| 43904 | 1.11433 | 0.799569 |
| 43905 | 1.32037 | 1.45255 |
| 43906 | 1.32037 | 0.855919 |
| 43907 | 1.0142 | 0.996233 |
| 43908 | 1.0142 | 0.549396 |
| 43909 | 0.886711 | 1.0311 |
| 43910 | 1.16806 | 0.979679 |
| 43911 | 0.999083 | 2.1 |
| 43912 | 0.710926 | 1.37143 |
| 43913 | 0.937757 | 1.64074 |
| 43914 | 0.937757 | 1.18262 |
| 43915 | 0.941224 | 0.787481 |
| 43916 | 0.941224 | 2.1 |
| 43917 | 1.16805 | 1.24176 |
| 43918 | 0.999084 | 2.1 |
| 43919 | 0.886709 | 1.9597 |
| 43920 | 0.710928 | 1.43911 |
| 43921 | 2.29332 | 2.1 |
| 43922 | 1.54904 | 2.1 |
| 43923 | 2.0815 | 2.1 |
| 43924 | 2.44199 | 2.1 |
| 43925 | 4.00418 | 2.1 |
| 43926 | 1.53873 | 0.05 |
| 43927 | 1.53422 | 0.0827991 |
| 43928 | 1.39661 | 0.05 |
| 43929 | 1.42387 | 0.438909 |
| 43930 | 2.9626 | 0.05 |
| 43931 | 1.53423 | 0.05 |
| 43932 | 1.44091 | 2.1 |
| 43933 | 1.42387 | 0.05 |
| 43934 | 1.35215 | 2.1 |
| 43935 | 2.86479 | 0.073312 |
| 43936 | 1.39661 | 0.05 |
| 43937 | 1.42387 | 0.310968 |
| 43938 | 1.28325 | 0.0527857 |
| 43939 | 1.33326 | 0.561473 |
| 43940 | 2.70713 | 0.0702679 |
| 43941 | 1.42387 | 0.05 |
| 43942 | 1.35215 | 2.1 |
| 43943 | 1.33327 | 0.05 |
| 43944 | 1.27339 | 2.1 |
| 43945 | 2.69726 | 0.506178 |
| 43946 | 2.58442 | 2.1 |
| 43947 | 1.1468 | 2.1 |
| 43948 | 1.18775 | 2.1 |
| 43949 | 2.60047 | 2.1 |
| 43950 | 3.74728 | 2.1 |
| 43951 | 2.58442 | 2.1 |
| 43952 | 1.89226 | 2.1 |
| 43953 | 1.89226 | 2.1 |
| 43954 | 2.60048 | 2.1 |
| 43955 | 4.49274 | 2.1 |
| 43956 | 1.89226 | 2.1 |
| 43957 | 1.89226 | 2.1 |
| 43958 | 1.89226 | 2.1 |
| 43959 | 1.89226 | 2.1 |
| 43960 | 3.78453 | 2.1 |
| 43961 | 1.89226 | 2.1 |

| | | |
|---|---|---|
| 43962 | 1.89226 | 2.1 |
| 43963 | 1.89226 | 2.1 |
| 43964 | 1.89226 | 0.81245 |
| 43965 | 3.78452 | 2.1 |
| 43966 | 1.22262 | 2.1 |
| 43967 | 2.60047 | 2.1 |
| 43968 | 1.18775 | 2.1 |
| 43969 | 1.18775 | 2.1 |
| 43970 | 2.61433 | 2.1 |
| 43971 | 2.61433 | 2.1 |
| 43972 | 1.89226 | 2.1 |
| 43973 | 2.60048 | 2.1 |
| 43974 | 2.61434 | 2.1 |
| 43975 | 2.60048 | 2.1 |
| 43976 | 1.90612 | 2.1 |
| 43977 | 1.89226 | 2.1 |
| 43978 | 1.89226 | 2.1 |
| 43979 | 1.89226 | 2.1 |
| 43980 | 1.89226 | 2.1 |
| 43981 | 1.89226 | 2.1 |
| 43982 | 3.78453 | 2.1 |
| 43983 | 1.89226 | 2.1 |
| 43984 | 1.89226 | 2.1 |
| 43985 | 1.89226 | 2.1 |
| 43986 | 1.89226 | 2.1 |
| 43987 | 3.78452 | 2.1 |
| 43988 | 2.12556 | 2.1 |
| 43989 | 0.991113 | 2.1 |
| 43990 | 1.27725 | 2.1 |
| 43991 | 2.31081 | 2.1 |
| 43992 | 3.40281 | 2.1 |
| 43993 | 1.89226 | 2.1 |
| 43994 | 2.31082 | 2.1 |
| 43995 | 2.12556 | 2.1 |
| 43996 | 2.12556 | 2.1 |
| 43997 | 1.89226 | 2.1 |
| 43998 | 1.89226 | 2.1 |
| 43999 | 1.22544 | 2.1 |
| 44000 | 1.12649 | 2.1 |
| 44001 | 0.775254 | 2.1 |
| 44002 | 0.643133 | 2.1 |
| 44003 | 0.917924 | 2.1 |
| 44004 | 0.934955 | 2.1 |
| 44005 | 0.975579 | 2.1 |
| 44006 | 0.975579 | 2.1 |
| 44007 | 1.23113 | 2.1 |
| 44008 | 0.685272 | 2.1 |
| 44009 | 0.651719 | 2.1 |
| 44010 | 1.1791 | 2.1 |
| 44011 | 1.89226 | 2.1 |
| 44012 | 2.5138 | 2.1 |
| 44013 | 2.31082 | 2.1 |
| 44014 | 2.31082 | 2.1 |
| 44015 | 1.89226 | 2.1 |
| 44016 | 1.89226 | 2.1 |
| 44017 | 1.89226 | 2.1 |
| 44018 | 1.89226 | 2.1 |
| 44019 | 1.89226 | 2.1 |

| | | |
|---|---|---|
| 44020 | 1.89226 | 2.1 |
| 44021 | 3.78453 | 2.1 |
| 44022 | 0.922224 | 2.1 |
| 44023 | 0.922224 | 2.1 |
| 44024 | 1.23555 | 2.1 |
| 44025 | 1.19715 | 2.1 |
| 44026 | 1.01445 | 2.1 |
| 44027 | 1.16061 | 2.1 |
| 44028 | 0.946132 | 2.1 |
| 44029 | 0.946132 | 2.1 |
| 44030 | 0.946131 | 2.1 |
| 44031 | 1.2051 | 2.1 |
| 44032 | 1.13537 | 2.1 |
| 44033 | 1.01445 | 2.1 |
| 44034 | 0.946133 | 2.1 |
| 44035 | 0.946133 | 2.1 |
| 44036 | 1.20648 | 2.1 |
| 44037 | 1.20648 | 2.1 |
| 44038 | 1.06454 | 2.1 |
| 44039 | 1.20648 | 2.1 |
| 44040 | 0.946133 | 2.1 |
| 44041 | 0.946133 | 2.1 |
| 44042 | 1.20649 | 2.1 |
| 44043 | 0.946133 | 2.1 |
| 44044 | 1.06454 | 2.1 |
| 44045 | 0.946132 | 2.1 |
| 44046 | 0.946132 | 2.1 |
| 44047 | 0.946132 | 2.1 |
| 44048 | 0.946132 | 2.1 |
| 44049 | 0.946132 | 2.1 |
| 44050 | 0.946132 | 2.1 |
| 44051 | 0.946132 | 2.1 |
| 44052 | 0.946133 | 2.1 |
| 44053 | 0.946133 | 2.1 |
| 44054 | 0.946133 | 2.1 |
| 44055 | 0.946133 | 2.1 |
| 44056 | 0.946132 | 2.1 |
| 44057 | 0.946132 | 2.1 |
| 44058 | 1.33078 | 2.06549 |
| 44059 | 1.37409 | 2.1 |
| 44060 | 1.31565 | 0.290906 |
| 44061 | 1.37114 | 2.1 |
| 44062 | 2.68973 | 2.1 |
| 44063 | 1.89225 | 1.93835 |
| 44064 | 1.89225 | 2.1 |
| 44065 | 1.89225 | 0.368686 |
| 44066 | 1.89225 | 2.1 |
| 44067 | 3.7845 | 1.22946 |
| 44068 | 1.89225 | 1.22695 |
| 44069 | 1.89225 | 2.1 |
| 44070 | 1.89225 | 1.05086 |
| 44071 | 1.89225 | 2.1 |
| 44072 | 3.7845 | 2.1 |
| 44073 | 1.37409 | 1.6969 |
| 44074 | 1.20408 | 2.1 |
| 44075 | 1.18612 | 2.1 |
| 44076 | 1.18907 | 2.1 |
| 44077 | 1.37114 | 1.15275 |

| | | |
|---|---|---|
| 44078 | 1.37409 | 2.1 |
| 44079 | 1.31566 | 2.1 |
| 44080 | 1.37115 | 2.1 |
| 44081 | 1.30318 | 0.258149 |
| 44082 | 1.37433 | 2.1 |
| 44083 | 2.68998 | 1.73839 |
| 44084 | 1.89226 | 2.1 |
| 44085 | 1.89226 | 2.1 |
| 44086 | 1.89226 | 0.84205 |
| 44087 | 1.89226 | 2.1 |
| 44088 | 3.78453 | 1.30883 |
| 44089 | 1.89226 | 1.15531 |
| 44090 | 1.89226 | 2.1 |
| 44091 | 1.89226 | 2.1 |
| 44092 | 1.89226 | 2.1 |
| 44093 | 1.89226 | 0.921429 |
| 44094 | 1.89226 | 2.1 |
| 44095 | 0.647001 | 2.1 |
| 44096 | 0.647001 | 2.1 |
| 44097 | 0.651851 | 0.874411 |
| 44098 | 0.651851 | 2.1 |
| 44099 | 0.602046 | 2.1 |
| 44100 | 0.613277 | 2.1 |
| 44101 | 0.685576 | 1.10221 |
| 44102 | 0.687164 | 1.63963 |
| 44103 | 0.687164 | 2.1 |
| 44104 | 0.613276 | 2.1 |
| 44105 | 0.602046 | 2.1 |
| 44106 | 0.685576 | 2.1 |
| 44107 | 0.946128 | 0.945454 |
| 44108 | 0.946128 | 0.287954 |
| 44109 | 1.15307 | 2.1 |
| 44110 | 1.19317 | 1.0087 |
| 44111 | 0.94613 | 1.33143 |
| 44112 | 0.94613 | 0.05 |
| 44113 | 1.15307 | 1.2697 |
| 44114 | 1.15307 | 2.08852 |
| 44115 | 0.946131 | 1.04457 |
| 44116 | 1.15307 | 0.940995 |
| 44117 | 0.946131 | 0.8735 |
| 44118 | 1.19317 | 0.596003 |
| 44119 | 1.18278 | 2.1 |
| 44120 | 2.38635 | 0.777719 |
| 44121 | 1.23042 | 2.1 |
| 44122 | 1.23042 | 1.43904 |
| 44123 | 2.30614 | 2.1 |
| 44124 | 2.30614 | 1.96694 |
| 44125 | 0.946129 | 0.812741 |
| 44126 | 0.946129 | 0.677375 |
| 44127 | 1.12994 | 1.10893 |
| 44128 | 1.15307 | 0.878166 |
| 44129 | 0.946131 | 1.06558 |
| 44130 | 0.946131 | 0.05 |
| 44131 | 1.12994 | 0.84985 |
| 44132 | 1.12994 | 0.956654 |
| 44133 | 1.12994 | 0.931415 |
| 44134 | 0.946131 | 0.975306 |
| 44135 | 0.946131 | 0.780254 |

| | | |
|---|---|---|
| 44136 | 1.15307 | 0.695847 |
| 44137 | 2.25988 | 1.18251 |
| 44138 | 2.30614 | 0.211558 |
| 44139 | 2.28696 | 2.1 |
| 44140 | 1.16361 | 2.1 |
| 44141 | 1.18278 | 2.1 |
| 44142 | 1.18278 | 1.53549 |
| 44143 | 2.3771 | 2.1 |
| 44144 | 1.90121 | 2.1 |
| 44145 | 1.81191 | 2.1 |
| 44146 | 2.46904 | 2.1 |
| 44147 | 4.37025 | 2.1 |
| 44148 | 1.15676 | 2.1 |
| 44149 | 1.23452 | 2.1 |
| 44150 | 0.823592 | 2.1 |
| 44151 | 0.737606 | 2.1 |
| 44152 | 0.988113 | 2.1 |
| 44153 | 0.988113 | 2.1 |
| 44154 | 0.984014 | 2.1 |
| 44155 | 0.984014 | 2.1 |
| 44156 | 1.15676 | 2.1 |
| 44157 | 0.737605 | 2.1 |
| 44158 | 0.823592 | 2.1 |
| 44159 | 1.23452 | 2.1 |
| 44160 | 1.30318 | 2.1 |
| 44161 | 1.37433 | 2.1 |
| 44162 | 1.30151 | 1.03517 |
| 44163 | 1.39067 | 2.1 |
| 44164 | 2.67584 | 1.80432 |
| 44165 | 1.89226 | 1.87176 |
| 44166 | 1.89226 | 2.1 |
| 44167 | 1.89226 | 1.00574 |
| 44168 | 1.89226 | 1.69168 |
| 44169 | 3.78453 | 1.20227 |
| 44170 | 1.89226 | 0.874244 |
| 44171 | 1.89226 | 2.1 |
| 44172 | 1.89226 | 0.349495 |
| 44173 | 1.89226 | 2.1 |
| 44174 | 1.89226 | 0.999148 |
| 44175 | 1.89226 | 2.1 |
| 44176 | 0.656819 | 2.1 |
| 44177 | 0.656819 | 0.934416 |
| 44178 | 0.661755 | 1.01739 |
| 44179 | 0.661755 | 2.1 |
| 44180 | 0.613277 | 2.1 |
| 44181 | 0.63141 | 2.1 |
| 44182 | 0.687164 | 0.973726 |
| 44183 | 0.695336 | 2.0552 |
| 44184 | 0.695337 | 2.1 |
| 44185 | 0.63141 | 2.1 |
| 44186 | 0.687164 | 2.1 |
| 44187 | 0.613276 | 2.1 |
| 44188 | 1.30151 | 2.07075 |
| 44189 | 1.39067 | 2.1 |
| 44190 | 1.32143 | 1.02323 |
| 44191 | 1.43294 | 2.1 |
| 44192 | 2.73445 | 2.1 |
| 44193 | 1.89226 | 1.88793 |

| | | |
|---|---|---|
| 44194 | 1.89226 | 2.1 |
| 44195 | 1.89226 | 0.880109 |
| 44196 | 1.89226 | 2.1 |
| 44197 | 3.78452 | 1.49102 |
| 44198 | 1.89226 | 1.42525 |
| 44199 | 1.89226 | 1.13736 |
| 44200 | 1.89226 | 0.983329 |
| 44201 | 1.89226 | 2.1 |
| 44202 | 1.89226 | 2.1 |
| 44203 | 1.89226 | 2.1 |
| 44204 | 1.43294 | 1.35045 |
| 44205 | 1.26282 | 2.1 |
| 44206 | 1.3268 | 0.936831 |
| 44207 | 1.3268 | 2.1 |
| 44208 | 1.39067 | 1.96412 |
| 44209 | 1.39067 | 2.1 |
| 44210 | 1.5511 | 0.05 |
| 44211 | 1.00665 | 0.05 |
| 44212 | 1.28239 | 0.05 |
| 44213 | 1.88631 | 0.05 |
| 44214 | 2.89296 | 0.05 |
| 44215 | 1.89227 | 0.05 |
| 44216 | 1.89227 | 0.05 |
| 44217 | 1.89227 | 0.0720709 |
| 44218 | 1.89227 | 0.0509189 |
| 44219 | 1.89227 | 0.05 |
| 44220 | 1.89227 | 0.05 |
| 44221 | 2.08848 | 0.05 |
| 44222 | 1.89226 | 0.05 |
| 44223 | 1.89226 | 0.05 |
| 44224 | 2.37947 | 0.0620792 |
| 44225 | 4.27173 | 0.05 |
| 44226 | 1.01456 | 0.05 |
| 44227 | 0.943157 | 0.05 |
| 44228 | 0.641198 | 0.05 |
| 44229 | 0.724757 | 0.05 |
| 44230 | 0.830923 | 0.05 |
| 44231 | 0.830923 | 0.05 |
| 44232 | 0.836991 | 0.05 |
| 44233 | 0.836991 | 0.05 |
| 44234 | 1.01456 | 0.05 |
| 44235 | 0.724757 | 0.0539798 |
| 44236 | 0.641197 | 0.05 |
| 44237 | 0.943157 | 0.05 |
| 44238 | 1.89227 | 0.05 |
| 44239 | 1.89227 | 0.0670239 |
| 44240 | 1.89227 | 0.0649053 |
| 44241 | 1.89227 | 0.085371 |
| 44242 | 3.78453 | 0.0642431 |
| 44243 | 0.946129 | 0.0517202 |
| 44244 | 0.946129 | 0.05 |
| 44245 | 1.28941 | 0.0572721 |
| 44246 | 1.18974 | 0.05 |
| 44247 | 0.94613 | 0.0569969 |
| 44248 | 0.94613 | 0.05 |
| 44249 | 1.28941 | 0.05 |
| 44250 | 1.28941 | 0.05 |
| 44251 | 1.28941 | 0.05 |

| | | |
|---|---|---|
| 44252 | 0.946131 | 0.0600167 |
| 44253 | 0.94613 | 0.05 |
| 44254 | 1.18974 | 0.05 |
| 44255 | 0.710636 | 2.1 |
| 44256 | 0.780249 | 2.1 |
| 44257 | 0.579033 | 2.1 |
| 44258 | 0.478812 | 2.1 |
| 44259 | 0.621822 | 2.1 |
| 44260 | 0.621822 | 2.1 |
| 44261 | 0.63724 | 2.1 |
| 44262 | 0.63724 | 2.1 |
| 44263 | 0.780251 | 2.1 |
| 44264 | 0.579032 | 2.1 |
| 44265 | 0.710637 | 2.1 |
| 44266 | 0.478811 | 2.1 |
| 44267 | 1.05499 | 2.1 |
| 44268 | 1.00521 | 2.1 |
| 44269 | 1.24445 | 2.03871 |
| 44270 | 1.23082 | 1.03513 |
| 44271 | 2.28581 | 2.1 |
| 44272 | 1.48751 | 2.1 |
| 44273 | 1.23083 | 1.61298 |
| 44274 | 1.4941 | 2.1 |
| 44275 | 1.24445 | 1.43275 |
| 44276 | 1.48047 | 1.37259 |
| 44277 | 1.23083 | 1.36625 |
| 44278 | 0.621185 | 2.0653 |
| 44279 | 0.621185 | 2.1 |
| 44280 | 0.620899 | 1.25842 |
| 44281 | 0.620899 | 2.1 |
| 44282 | 0.502515 | 1.77351 |
| 44283 | 0.626672 | 1.28053 |
| 44284 | 0.615412 | 1.06793 |
| 44285 | 0.740234 | 2.1 |
| 44286 | 0.740233 | 1.10182 |
| 44287 | 0.626672 | 0.995113 |
| 44288 | 0.615413 | 1.23521 |
| 44289 | 0.502516 | 1.50488 |
| 44290 | 1.95844 | 2.1 |
| 44291 | 2.6562 | 2.1 |
| 44292 | 1.95844 | 2.1 |
| 44293 | 2.20883 | 2.1 |
| 44294 | 2.38225 | 2.1 |
| 44295 | 2.63264 | 2.1 |
| 44296 | 1.89226 | 2.1 |
| 44297 | 1.89226 | 2.1 |
| 44298 | 1.89226 | 2.1 |
| 44299 | 1.89226 | 2.1 |
| 44300 | 3.78453 | 2.1 |
| 44301 | 1.95844 | 2.1 |
| 44302 | 2.38225 | 2.1 |
| 44303 | 1.71082 | 2.1 |
| 44304 | 2.14647 | 2.1 |
| 44305 | 4.1049 | 2.1 |
| 44306 | 0.946131 | 2.1 |
| 44307 | 0.946131 | 2.1 |
| 44308 | 0.946131 | 2.1 |
| 44309 | 0.946131 | 2.1 |

| | | |
|---|---|---|
| 44310 | 0.946131 | 2.1 |
| 44311 | 0.946131 | 2.1 |
| 44312 | 0.946131 | 2.1 |
| 44313 | 0.946131 | 2.1 |
| 44314 | 0.946131 | 2.1 |
| 44315 | 0.946131 | 2.1 |
| 44316 | 0.946131 | 2.1 |
| 44317 | 0.946131 | 2.1 |
| 44318 | 2.12597 | 2.1 |
| 44319 | 1.2295 | 2.1 |
| 44320 | 1.28014 | 2.1 |
| 44321 | 2.19723 | 2.1 |
| 44322 | 3.40611 | 2.1 |
| 44323 | 2.12597 | 2.1 |
| 44324 | 2.79242 | 2.1 |
| 44325 | 2.73375 | 2.1 |
| 44326 | 2.66249 | 2.1 |
| 44327 | 2.19723 | 2.1 |
| 44328 | 2.12597 | 2.1 |
| 44329 | 1.13716 | 2.1 |
| 44330 | 1.09861 | 2.1 |
| 44331 | 0.666692 | 2.1 |
| 44332 | 0.640071 | 2.1 |
| 44333 | 0.891617 | 2.1 |
| 44334 | 0.891617 | 2.1 |
| 44335 | 0.885614 | 2.1 |
| 44336 | 0.885614 | 2.1 |
| 44337 | 1.13716 | 2.1 |
| 44338 | 0.666692 | 2.1 |
| 44339 | 0.640071 | 2.1 |
| 44340 | 1.09861 | 2.1 |
| 44341 | 1.89226 | 0.05 |
| 44342 | 1.70303 | 0.05 |
| 44343 | 1.89226 | 0.05 |
| 44344 | 2.64598 | 0.05 |
| 44345 | 1.81091 | 0.05 |
| 44346 | 2.53222 | 0.05 |
| 44347 | 2.32422 | 0.05 |
| 44348 | 1.89226 | 0.05 |
| 44349 | 2.78336 | 0.05 |
| 44350 | 2.78336 | 0.05 |
| 44351 | 1.89226 | 0.05 |
| 44352 | 1.89226 | 0.05 |
| 44353 | 0.946132 | 0.05 |
| 44354 | 0.946132 | 0.05 |
| 44355 | 0.946131 | 0.05 |
| 44356 | 0.946131 | 0.05 |
| 44357 | 0.946132 | 0.05 |
| 44358 | 0.946132 | 0.05 |
| 44359 | 0.946131 | 0.05 |
| 44360 | 0.946131 | 0.05 |
| 44361 | 0.946131 | 0.05 |
| 44362 | 0.946131 | 0.05 |
| 44363 | 0.946131 | 0.05 |
| 44364 | 0.946131 | 0.05 |
| 44365 | 0.946131 | 0.05 |
| 44366 | 0.946131 | 0.05 |
| 44367 | 0.946132 | 0.05 |

| | | |
|---|---|---|
| 44368 | 0.946132 | 0.05 |
| 44369 | 0.946132 | 0.05 |
| 44370 | 0.946132 | 0.05 |
| 44371 | 0.946131 | 0.05 |
| 44372 | 0.946131 | 0.05 |
| 44373 | 0.946131 | 0.05 |
| 44374 | 0.946131 | 0.05 |
| 44375 | 0.946131 | 0.05 |
| 44376 | 0.946131 | 0.05 |
| 44377 | 2.24323 | 0.962713 |
| 44378 | 1.89226 | 2.1 |
| 44379 | 2.75021 | 1.39865 |
| 44380 | 2.75021 | 1.64788 |
| 44381 | 1.89226 | 0.212554 |
| 44382 | 1.89226 | 0.711382 |
| 44383 | 2.30908 | 0.05 |
| 44384 | 2.19815 | 0.131134 |
| 44385 | 2.1609 | 0.05 |
| 44386 | 1.83796 | 0.05 |
| 44387 | 4.35904 | 0.05 |
| 44388 | 2.19815 | 0.05 |
| 44389 | 2.02268 | 2.1 |
| 44390 | 2.17869 | 2.1 |
| 44391 | 1.9559 | 0.05 |
| 44392 | 2.0815 | 2.1 |
| 44393 | 1.83424 | 0.05 |
| 44394 | 2.1609 | 0.132613 |
| 44395 | 1.88877 | 0.509591 |
| 44396 | 1.98413 | 0.05 |
| 44397 | 1.87355 | 0.444265 |
| 44398 | 4.03445 | 0.0683704 |
| 44399 | 1.87355 | 0.05 |
| 44400 | 2.02268 | 2.1 |
| 44401 | 1.87355 | 2.1 |
| 44402 | 2.10488 | 0.05 |
| 44403 | 1.68443 | 2.1 |
| 44404 | 1.89448 | 0.05 |
| 44405 | 0.946131 | 0.05 |
| 44406 | 0.946131 | 0.05 |
| 44407 | 1.40574 | 0.05 |
| 44408 | 1.12373 | 0.05 |
| 44409 | 0.946129 | 0.05 |
| 44410 | 0.946129 | 0.05 |
| 44411 | 1.40574 | 0.0505845 |
| 44412 | 1.40574 | 0.05 |
| 44413 | 0.946131 | 0.05 |
| 44414 | 1.40574 | 0.05 |
| 44415 | 1.12373 | 0.05 |
| 44416 | 0.946131 | 0.05 |
| 44417 | 2.14277 | 0.05 |
| 44418 | 2.81148 | 0.084229 |
| 44419 | 1.42762 | 0.05 |
| 44420 | 1.42762 | 0.05 |
| 44421 | 2.96261 | 0.05 |
| 44422 | 2.24746 | 0.05 |
| 44423 | 1.89226 | 0.837712 |
| 44424 | 1.89226 | 0.893512 |
| 44425 | 1.89226 | 0.855885 |

| | | |
|---|---|---|
| 44426 | 1.89226 | 1.08528 |
| 44427 | 3.78453 | 1.66988 |
| 44428 | 1.89226 | 0.653882 |
| 44429 | 1.89226 | 0.87167 |
| 44430 | 1.89226 | 0.879395 |
| 44431 | 1.89226 | 1.01304 |
| 44432 | 3.78452 | 0.588513 |
| 44433 | 0.68433 | 0.682785 |
| 44434 | 0.946127 | 1.16056 |
| 44435 | 0.543921 | 1.65393 |
| 44436 | 0.946131 | 0.898173 |
| 44437 | 0.946131 | 0.679306 |
| 44438 | 0.946131 | 2.1 |
| 44439 | 0.543918 | 1.05831 |
| 44440 | 0.543918 | 1.12612 |
| 44441 | 0.684332 | 1.01642 |
| 44442 | 0.54392 | 2.1 |
| 44443 | 0.946129 | 0.202364 |
| 44444 | 0.946129 | 1.0211 |
| 44445 | 1.89226 | 0.923252 |
| 44446 | 1.08784 | 1.21382 |
| 44447 | 1.19352 | 0.0806192 |
| 44448 | 1.89226 | 1.00473 |
| 44449 | 1.19352 | 2.0967 |
| 44450 | 1.89226 | 1.72421 |
| 44451 | 0.635711 | 0.756632 |
| 44452 | 0.946132 | 1.29301 |
| 44453 | 0.946132 | 1.76687 |
| 44454 | 0.56474 | 0.937421 |
| 44455 | 0.750045 | 1.01603 |
| 44456 | 1.25657 | 2.1 |
| 44457 | 0.635682 | 0.96435 |
| 44458 | 0.635682 | 0.71868 |
| 44459 | 0.891954 | 0.879988 |
| 44460 | 1.32751 | 2.1 |
| 44461 | 0.564766 | 0.769448 |
| 44462 | 0.564766 | 1.60457 |
| 44463 | 0.858345 | 0.94856 |
| 44464 | 0.946128 | 1.46981 |
| 44465 | 0.635712 | 0.988837 |
| 44466 | 0.946133 | 0.959527 |
| 44467 | 0.977198 | 1.11201 |
| 44468 | 0.944521 | 2.1 |
| 44469 | 0.635682 | 1.28636 |
| 44470 | 0.635682 | 0.550742 |
| 44471 | 1.02924 | 1.06598 |
| 44472 | 0.705625 | 2.1 |
| 44473 | 0.851528 | 0.474012 |
| 44474 | 0.851528 | 2.1 |
| 44475 | 2.18903 | 2.1 |
| 44476 | 2.10182 | 2.1 |
| 44477 | 2.10182 | 2.1 |
| 44478 | 1.89226 | 2.1 |
| 44479 | 1.89226 | 2.1 |
| 44480 | 1.64983 | 2.1 |
| 44481 | 1.89226 | 2.1 |
| 44482 | 1.83867 | 2.1 |
| 44483 | 1.83867 | 2.1 |

| | | |
|---|---|---|
| 44484 | 1.89164 | 2.1 |
| 44485 | 2.0815 | 2.1 |
| 44486 | 2.17638 | 2.1 |
| 44487 | 2.57957 | 1.01924 |
| 44488 | 2.3506 | 2.1 |
| 44489 | 2.69056 | 0.671362 |
| 44490 | 2.49439 | 1.66341 |
| 44491 | 5.04116 | 0.915685 |
| 44492 | 2.3506 | 0.751686 |
| 44493 | 1.8911 | 2.1 |
| 44494 | 2.49439 | 1.07493 |
| 44495 | 2.06689 | 2.1 |
| 44496 | 4.41748 | 2.1 |
| 44497 | 2.69056 | 0.992115 |
| 44498 | 2.49439 | 1.83532 |
| 44499 | 2.77227 | 0.55953 |
| 44500 | 2.63443 | 1.60565 |
| 44501 | 5.32499 | 1.67724 |
| 44502 | 2.49439 | 1.09657 |
| 44503 | 2.06689 | 2.1 |
| 44504 | 2.63443 | 0.0926597 |
| 44505 | 2.26444 | 2.1 |
| 44506 | 4.70131 | 1.84589 |
| 44507 | 2.46327 | 2.1 |
| 44508 | 2.54347 | 2.1 |
| 44509 | 2.52869 | 2.1 |
| 44510 | 2.16534 | 2.1 |
| 44511 | 2.82661 | 2.1 |
| 44512 | 2.46327 | 2.1 |
| 44513 | 0.946131 | 2.1 |
| 44514 | 0.946131 | 2.1 |
| 44515 | 1.27174 | 2.1 |
| 44516 | 1.27174 | 2.1 |
| 44517 | 1.27174 | 2.1 |
| 44518 | 0.94613 | 2.1 |
| 44519 | 0.946128 | 2.1 |
| 44520 | 1.4133 | 2.1 |
| 44521 | 0.946129 | 2.1 |
| 44522 | 0.946129 | 2.1 |
| 44523 | 1.27174 | 2.1 |
| 44524 | 1.4133 | 2.1 |
| 44525 | 1.42757 | 2.1 |
| 44526 | 1.7457 | 2.1 |
| 44527 | 1.66047 | 2.1 |
| 44528 | 1.7457 | 2.1 |
| 44529 | 1.45754 | 2.1 |
| 44530 | 1.54278 | 2.1 |
| 44531 | 1.42757 | 2.1 |
| 44532 | 1.52996 | 2.1 |
| 44533 | 1.66047 | 2.1 |
| 44534 | 1.66047 | 2.1 |
| 44535 | 1.26397 | 2.1 |
| 44536 | 1.26397 | 2.1 |
| 44537 | 0.968536 | 2.1 |
| 44538 | 1.02461 | 2.1 |
| 44539 | 1.57575 | 2.1 |
| 44540 | 1.62391 | 2.1 |
| 44541 | 2.59245 | 2.1 |

| | | |
|---|---|---|
| 44542 | 0.946131 | 2.1 |
| 44543 | 0.946131 | 2.1 |
| 44544 | 0.946132 | 2.1 |
| 44545 | 0.946132 | 2.1 |
| 44546 | 0.946132 | 2.1 |
| 44547 | 0.946132 | 2.1 |
| 44548 | 0.946132 | 2.1 |
| 44549 | 0.946132 | 2.1 |
| 44550 | 0.946132 | 2.1 |
| 44551 | 0.946132 | 2.1 |
| 44552 | 0.946132 | 2.1 |
| 44553 | 0.946132 | 2.1 |
| 44554 | 2.23765 | 2.1 |
| 44555 | 1.62391 | 2.1 |
| 44556 | 2.22154 | 2.1 |
| 44557 | 1.57575 | 2.1 |
| 44558 | 2.2697 | 2.1 |
| 44559 | 1.62391 | 2.1 |
| 44560 | 1.89226 | 2.1 |
| 44561 | 1.89226 | 2.1 |
| 44562 | 1.89226 | 2.1 |
| 44563 | 1.89226 | 2.1 |
| 44564 | 1.89226 | 2.1 |
| 44565 | 1.89226 | 2.1 |
| 44566 | 1.89226 | 2.1 |
| 44567 | 1.89226 | 2.1 |
| 44568 | 1.89226 | 2.1 |
| 44569 | 1.89226 | 2.1 |
| 44570 | 1.89226 | 2.1 |
| 44571 | 1.89226 | 2.1 |
| 44572 | 2.2697 | 2.1 |
| 44573 | 1.46129 | 2.1 |
| 44574 | 2.25873 | 2.1 |
| 44575 | 1.62391 | 2.1 |
| 44576 | 2.09611 | 2.1 |
| 44577 | 1.46129 | 2.1 |
| 44578 | 1.89226 | 2.1 |
| 44579 | 2.07088 | 2.1 |
| 44580 | 1.89226 | 2.1 |
| 44581 | 2.31884 | 2.1 |
| 44582 | 4.21111 | 2.1 |
| 44583 | 1.89226 | 2.1 |
| 44584 | 2.31884 | 2.1 |
| 44585 | 2.11713 | 2.1 |
| 44586 | 1.89226 | 2.1 |
| 44587 | 2.54371 | 2.1 |
| 44588 | 2.31884 | 2.1 |
| 44589 | 2.54371 | 2.1 |
| 44590 | 1.24041 | 2.1 |
| 44591 | 2.55208 | 2.1 |
| 44592 | 2.31884 | 2.1 |
| 44593 | 1.23712 | 2.1 |
| 44594 | 1.00388 | 2.1 |
| 44595 | 2.72769 | 0.05 |
| 44596 | 2.57507 | 0.05 |
| 44597 | 1.89226 | 0.05 |
| 44598 | 1.89226 | 0.05 |
| 44599 | 4.46733 | 0.05 |

| | | |
|---|---|---|
| 44600 | 1.89226 | 0.05 |
| 44601 | 2.57506 | 0.05 |
| 44602 | 1.89226 | 0.05 |
| 44603 | 2.30076 | 0.05 |
| 44604 | 1.89226 | 0.05 |
| 44605 | 2.30076 | 0.05 |
| 44606 | 1.21171 | 0.05 |
| 44607 | 1.48589 | 0.05 |
| 44608 | 2.09166 | 0.05 |
| 44609 | 1.78381 | 0.05 |
| 44610 | 3.2697 | 0.05 |
| 44611 | 2.09166 | 0.05 |
| 44612 | 1.70302 | 0.05 |
| 44613 | 1.48589 | 0.05 |
| 44614 | 1.18027 | 0.05 |
| 44615 | 2.26571 | 0.05 |
| 44616 | 1.79969 | 0.05 |
| 44617 | 1.89226 | 0.05 |
| 44618 | 1.89226 | 0.05 |
| 44619 | 1.89226 | 0.05 |
| 44620 | 1.89226 | 0.05 |
| 44621 | 1.89226 | 0.05 |
| 44622 | 1.89226 | 0.05 |
| 44623 | 0.946131 | 0.05 |
| 44624 | 0.946131 | 0.05 |
| 44625 | 0.946128 | 0.05 |
| 44626 | 0.946128 | 0.05 |
| 44627 | 0.946129 | 0.05 |
| 44628 | 0.946129 | 0.05 |
| 44629 | 0.94613 | 0.05 |
| 44630 | 0.94613 | 0.05 |
| 44631 | 0.94613 | 0.05 |
| 44632 | 0.94613 | 0.05 |
| 44633 | 0.94613 | 0.05 |
| 44634 | 0.94613 | 0.05 |
| 44635 | 1.89226 | 1.65942 |
| 44636 | 1.89226 | 1.86942 |
| 44637 | 1.89226 | 2.1 |
| 44638 | 1.89226 | 1.35676 |
| 44639 | 1.89226 | 2.1 |
| 44640 | 1.89226 | 1.30017 |
| 44641 | 1.89226 | 1.78954 |
| 44642 | 1.89226 | 1.87007 |
| 44643 | 1.89226 | 2.1 |
| 44644 | 1.89226 | 1.34547 |
| 44645 | 1.89226 | 2.1 |
| 44646 | 1.89226 | 1.31094 |
| 44647 | 1.89226 | 1.39795 |
| 44648 | 1.89226 | 1.49086 |
| 44649 | 1.89226 | 2.03512 |
| 44650 | 1.89226 | 1.01668 |
| 44651 | 1.89226 | 2.03153 |
| 44652 | 1.89226 | 1.00539 |
| 44653 | 1.79103 | 0.907423 |
| 44654 | 0.968249 | 2.1 |
| 44655 | 1.44807 | 2.05787 |
| 44656 | 2.17984 | 0.170056 |
| 44657 | 3.2391 | 2.1 |

| | | |
|---|---|---|
| 44658 | 1.29735 | 1.59299 |
| 44659 | 1.08992 | 1.43762 |
| 44660 | 0.945147 | 2.1 |
| 44661 | 0.724033 | 2.1 |
| 44662 | 1.00723 | 2.1 |
| 44663 | 1.00723 | 1.9298 |
| 44664 | 1.01416 | 0.337947 |
| 44665 | 1.01416 | 2.1 |
| 44666 | 1.29735 | 2.02827 |
| 44667 | 0.94515 | 2.1 |
| 44668 | 0.724034 | 2.1 |
| 44669 | 1.08992 | 2.1 |
| 44670 | 1.39214 | 2.1 |
| 44671 | 1.39214 | 2.1 |
| 44672 | 0.946129 | 2.1 |
| 44673 | 0.946129 | 2.1 |
| 44674 | 1.33728 | 2.1 |
| 44675 | 1.39214 | 2.1 |
| 44676 | 0.94613 | 2.1 |
| 44677 | 0.94613 | 2.1 |
| 44678 | 0.94613 | 2.1 |
| 44679 | 1.33728 | 2.1 |
| 44680 | 0.94613 | 2.1 |
| 44681 | 1.39214 | 2.1 |
| 44682 | 2.78428 | 2.1 |
| 44683 | 2.28373 | 2.1 |
| 44684 | 2.67456 | 2.1 |
| 44685 | 2.20011 | 2.1 |
| 44686 | 4.95829 | 2.1 |
| 44687 | 1.90165 | 0.05 |
| 44688 | 2.24206 | 0.05 |
| 44689 | 1.66137 | 0.05 |
| 44690 | 2.07108 | 0.05 |
| 44691 | 3.90343 | 0.05 |
| 44692 | 1.89224 | 0.0677776 |
| 44693 | 1.89224 | 0.05 |
| 44694 | 1.89224 | 0.05 |
| 44695 | 1.89224 | 0.05 |
| 44696 | 3.78448 | 0.05 |
| 44697 | 2.82159 | 0.05 |
| 44698 | 1.89226 | 0.0569613 |
| 44699 | 2.50138 | 0.05 |
| 44700 | 1.89226 | 0.05 |
| 44701 | 4.39365 | 0.05 |
| 44702 | 1.66139 | 0.05 |
| 44703 | 1.86399 | 0.05 |
| 44704 | 1.56005 | 0.05 |
| 44705 | 1.90324 | 0.05 |
| 44706 | 3.56463 | 0.05 |
| 44707 | 0.946133 | 0.05 |
| 44708 | 0.946133 | 0.05 |
| 44709 | 0.946132 | 0.05 |
| 44710 | 0.946132 | 0.264831 |
| 44711 | 0.946133 | 0.05 |
| 44712 | 0.946133 | 0.05 |
| 44713 | 0.946132 | 0.05 |
| 44714 | 0.946132 | 0.05 |
| 44715 | 0.946133 | 0.05 |

| | | |
|---|---|---|
| 44716 | 0.946133 | 0.05 |
| 44717 | 0.946132 | 0.05 |
| 44718 | 0.946132 | 0.128236 |
| 44719 | 1.5176 | 0.76931 |
| 44720 | 1.97964 | 0.344797 |
| 44721 | 2.32889 | 0.482413 |
| 44722 | 1.91762 | 0.538894 |
| 44723 | 3.84649 | 0.494632 |
| 44724 | 2.3376 | 0.607404 |
| 44725 | 1.97963 | 0.450312 |
| 44726 | 2.32889 | 0.724469 |
| 44727 | 2.58303 | 0.337855 |
| 44728 | 4.66649 | 0.603876 |
| 44729 | 2.32889 | 0.461425 |
| 44730 | 2.20248 | 0.371044 |
| 44731 | 1.91762 | 0.252547 |
| 44732 | 1.91762 | 0.118553 |
| 44733 | 2.5615 | 0.676234 |
| 44734 | 2.5615 | 0.387386 |
| 44735 | 2.32889 | 0.713643 |
| 44736 | 2.58303 | 0.243895 |
| 44737 | 2.71793 | 0.537941 |
| 44738 | 2.56149 | 0.348675 |
| 44739 | 5.04681 | 0.451181 |
| 44740 | 1.89226 | 0.679269 |
| 44741 | 2.53785 | 2.1 |
| 44742 | 1.89226 | 0.739851 |
| 44743 | 2.60895 | 0.629949 |
| 44744 | 1.89226 | 1.51185 |
| 44745 | 2.60895 | 0.802502 |
| 44746 | 0.493248 | 0.688724 |
| 44747 | 1.30448 | 1.3048 |
| 44748 | 1.26893 | 2.1 |
| 44749 | 0.653226 | 2.00093 |
| 44750 | 0.653227 | 0.968525 |
| 44751 | 0.493247 | 2.1 |
| 44752 | 1.26893 | 2.1 |
| 44753 | 1.30448 | 0.739044 |
| 44754 | 0.832232 | 2.1 |
| 44755 | 0.832233 | 2.1 |
| 44756 | 0.929944 | 0.576197 |
| 44757 | 0.929944 | 0.954495 |
| 44758 | 1.89226 | 2.1 |
| 44759 | 1.47937 | 2.1 |
| 44760 | 1.50143 | 2.1 |
| 44761 | 1.47937 | 2.1 |
| 44762 | 1.91432 | 2.1 |
| 44763 | 1.89226 | 2.1 |
| 44764 | 1.89226 | 2.1 |
| 44765 | 1.89226 | 2.1 |
| 44766 | 1.89226 | 2.1 |
| 44767 | 1.89226 | 2.1 |
| 44768 | 1.89226 | 2.1 |
| 44769 | 1.89226 | 1.02177 |
| 44770 | 1.33419 | 2.1 |
| 44771 | 2.4542 | 2.1 |
| 44772 | 2.59834 | 2.1 |
| 44773 | 2.59834 | 2.1 |

| | | |
|---|---|---|
| 44774 | 1.25716 | 2.1 |
| 44775 | 1.25716 | 2.1 |
| 44776 | 1.70303 | 2.1 |
| 44777 | 1.50143 | 2.1 |
| 44778 | 1.60878 | 2.1 |
| 44779 | 1.50143 | 2.1 |
| 44780 | 1.93599 | 2.1 |
| 44781 | 1.89226 | 1.80555 |
| 44782 | 0.946131 | 1.08138 |
| 44783 | 0.946131 | 2.1 |
| 44784 | 0.946127 | 2.1 |
| 44785 | 0.946127 | 2.1 |
| 44786 | 0.946127 | 2.1 |
| 44787 | 0.946127 | 0.766656 |
| 44788 | 0.946131 | 2.1 |
| 44789 | 0.946131 | 2.1 |
| 44790 | 0.94613 | 2.1 |
| 44791 | 0.94613 | 2.1 |
| 44792 | 0.946129 | 2.1 |
| 44793 | 0.946129 | 2.1 |
| 44794 | 1.43912 | 2.1 |
| 44795 | 2.59834 | 2.1 |
| 44796 | 2.78093 | 2.1 |
| 44797 | 2.78093 | 2.1 |
| 44798 | 1.33419 | 2.1 |
| 44799 | 1.33419 | 2.1 |
| 44800 | 1.89226 | 2.1 |
| 44801 | 2.26322 | 2.1 |
| 44802 | 2.55682 | 2.1 |
| 44803 | 2.55682 | 2.1 |
| 44804 | 1.89226 | 2.1 |
| 44805 | 1.89226 | 2.1 |
| 44806 | 2.58032 | 0.0623157 |
| 44807 | 2.28922 | 0.05 |
| 44808 | 1.88424 | 0.05 |
| 44809 | 2.32127 | 0.05 |
| 44810 | 4.46456 | 0.05 |
| 44811 | 1.67265 | 0.0571908 |
| 44812 | 2.28922 | 0.05 |
| 44813 | 1.88424 | 0.05 |
| 44814 | 1.11314 | 0.05 |
| 44815 | 3.55689 | 0.05 |
| 44816 | 1.43751 | 0.05 |
| 44817 | 2.32127 | 0.05 |
| 44818 | 1.92998 | 0.05 |
| 44819 | 2.37671 | 0.05 |
| 44820 | 1.43751 | 0.05 |
| 44821 | 1.88424 | 0.05 |
| 44822 | 2.36729 | 2.1 |
| 44823 | 1.56714 | 2.1 |
| 44824 | 1.56714 | 2.1 |
| 44825 | 1.62408 | 2.1 |
| 44826 | 2.28373 | 2.1 |
| 44827 | 2.34067 | 2.1 |
| 44828 | 0.937268 | 2.1 |
| 44829 | 0.937268 | 2.1 |
| 44830 | 0.94636 | 2.1 |
| 44831 | 0.94636 | 2.1 |

| | | |
|---|---|---|
| 44832 | 0.76004 | 2.1 |
| 44833 | 0.783572 | 2.1 |
| 44834 | 1.14187 | 2.1 |
| 44835 | 1.10006 | 2.1 |
| 44836 | 1.10006 | 2.1 |
| 44837 | 0.76004 | 2.1 |
| 44838 | 0.783572 | 2.1 |
| 44839 | 1.14187 | 2.1 |
| 44840 | 1.28753 | 2.1 |
| 44841 | 1.36838 | 2.1 |
| 44842 | 1.62617 | 2.1 |
| 44843 | 1.82857 | 2.1 |
| 44844 | 3.1161 | 2.1 |
| 44845 | 1.82856 | 2.1 |
| 44846 | 1.17996 | 2.1 |
| 44847 | 1.53417 | 2.1 |
| 44848 | 1.56618 | 2.1 |
| 44849 | 1.36838 | 2.1 |
| 44850 | 1.36838 | 2.1 |
| 44851 | 1.35065 | 0.878154 |
| 44852 | 1.42659 | 0.05 |
| 44853 | 2.26675 | 0.128144 |
| 44854 | 2.26675 | 0.174394 |
| 44855 | 1.62137 | 0.0762422 |
| 44856 | 2.53747 | 0.122137 |
| 44857 | 1.89226 | 0.05 |
| 44858 | 1.89226 | 0.05 |
| 44859 | 1.89226 | 0.05 |
| 44860 | 1.89226 | 0.05 |
| 44861 | 3.78452 | 0.05 |
| 44862 | 1.36569 | 0.05 |
| 44863 | 1.36569 | 0.05 |
| 44864 | 0.946131 | 0.05 |
| 44865 | 0.946131 | 0.05 |
| 44866 | 1.16041 | 0.05 |
| 44867 | 1.36569 | 0.05 |
| 44868 | 0.946129 | 0.05 |
| 44869 | 0.946129 | 0.05 |
| 44870 | 1.1604 | 0.05 |
| 44871 | 0.94613 | 0.05 |
| 44872 | 0.946131 | 0.05 |
| 44873 | 1.36569 | 0.05 |
| 44874 | 2.73139 | 0.05 |
| 44875 | 1.25265 | 0.05 |
| 44876 | 1.25265 | 0.05 |
| 44877 | 1.64799 | 0.05 |
| 44878 | 2.32081 | 0.05 |
| 44879 | 2.71615 | 0.05 |
| 44880 | 0.946133 | 0.05 |
| 44881 | 0.946133 | 0.05 |
| 44882 | 0.946132 | 0.05 |
| 44883 | 0.946132 | 0.05 |
| 44884 | 0.946132 | 0.05 |
| 44885 | 0.946132 | 0.05 |
| 44886 | 0.946133 | 0.05 |
| 44887 | 0.946133 | 0.05 |
| 44888 | 0.946132 | 0.05 |
| 44889 | 0.946132 | 0.05 |

| | | |
|---|---|---|
| 44890 | 0.946133 | 0.05 |
| 44891 | 0.946133 | 0.05 |
| 44892 | 0.946131 | 0.05 |
| 44893 | 0.946131 | 0.05 |
| 44894 | 0.94613 | 0.05 |
| 44895 | 0.94613 | 0.05 |
| 44896 | 0.946132 | 0.05 |
| 44897 | 0.946132 | 0.05 |
| 44898 | 0.946129 | 0.05 |
| 44899 | 0.946129 | 0.05 |
| 44900 | 0.946131 | 0.05 |
| 44901 | 0.946131 | 0.05 |
| 44902 | 0.946131 | 0.05 |
| 44903 | 0.946131 | 0.05 |
| 44904 | 1.89226 | 0.05 |
| 44905 | 2.48503 | 2.1 |
| 44906 | 2.64586 | 2.1 |
| 44907 | 2.64586 | 0.05 |
| 44908 | 1.89226 | 2.1 |
| 44909 | 1.89226 | 0.05 |
| 44910 | 2.48504 | 2.1 |
| 44911 | 1.99985 | 2.1 |
| 44912 | 1.83584 | 2.1 |
| 44913 | 1.83584 | 2.1 |
| 44914 | 2.64586 | 2.1 |
| 44915 | 2.64586 | 2.1 |
| 44916 | 1.89225 | 0.05 |
| 44917 | 2.33095 | 2.1 |
| 44918 | 2.48501 | 2.1 |
| 44919 | 2.48501 | 0.0844201 |
| 44920 | 1.89225 | 2.1 |
| 44921 | 1.89225 | 0.05 |
| 44922 | 2.33096 | 2.1 |
| 44923 | 1.83583 | 2.1 |
| 44924 | 1.68436 | 2.1 |
| 44925 | 1.68436 | 2.1 |
| 44926 | 2.48502 | 2.1 |
| 44927 | 2.48502 | 2.1 |
| 44928 | 1.14807 | 2.1 |
| 44929 | 1.76545 | 2.1 |
| 44930 | 1.21292 | 2.1 |
| 44931 | 1.83936 | 2.1 |
| 44932 | 2.97837 | 2.1 |
| 44933 | 1.89226 | 2.1 |
| 44934 | 1.89226 | 2.1 |
| 44935 | 1.89226 | 2.1 |
| 44936 | 1.89226 | 2.1 |
| 44937 | 3.78451 | 2.1 |
| 44938 | 1.89226 | 2.1 |
| 44939 | 1.89226 | 1.17654 |
| 44940 | 1.89226 | 2.1 |
| 44941 | 1.89226 | 2.1 |
| 44942 | 3.78452 | 2.1 |
| 44943 | 1.76545 | 2.1 |
| 44944 | 2.12608 | 2.1 |
| 44945 | 1.83937 | 2.1 |
| 44946 | 2.22529 | 2.09305 |
| 44947 | 3.99074 | 2.1 |

| | | |
|---|---|---|
| 44948 | 0.780222 | 2.1 |
| 44949 | 0.780222 | 2.1 |
| 44950 | 0.784871 | 2.1 |
| 44951 | 0.784871 | 2.1 |
| 44952 | 0.95863 | 2.1 |
| 44953 | 0.919681 | 2.1 |
| 44954 | 0.636145 | 2.1 |
| 44955 | 0.606461 | 2.1 |
| 44956 | 0.636144 | 2.1 |
| 44957 | 0.958631 | 2.1 |
| 44958 | 0.60646 | 2.1 |
| 44959 | 0.919683 | 2.1 |
| 44960 | 1.89226 | 2.1 |
| 44961 | 1.89226 | 2.1 |
| 44962 | 1.89226 | 2.1 |
| 44963 | 1.89226 | 2.1 |
| 44964 | 3.78452 | 2.1 |
| 44965 | 1.89226 | 2.1 |
| 44966 | 1.89226 | 2.1 |
| 44967 | 1.89226 | 2.1 |
| 44968 | 1.89226 | 1.0084 |
| 44969 | 3.78452 | 2.1 |
| 44970 | 1.03789 | 2.1 |
| 44971 | 1.03789 | 2.1 |
| 44972 | 1.04231 | 2.1 |
| 44973 | 1.04231 | 2.1 |
| 44974 | 1.16052 | 2.1 |
| 44975 | 1.11265 | 0.943415 |
| 44976 | 0.958634 | 2.1 |
| 44977 | 0.919685 | 2.1 |
| 44978 | 0.958634 | 2.1 |
| 44979 | 1.16052 | 2.1 |
| 44980 | 0.919685 | 2.1 |
| 44981 | 1.11265 | 2.1 |
| 44982 | 1.43837 | 2.1 |
| 44983 | 2.57085 | 2.1 |
| 44984 | 1.39332 | 2.1 |
| 44985 | 2.58228 | 2.1 |
| 44986 | 3.96417 | 2.1 |
| 44987 | 1.89226 | 2.1 |
| 44988 | 1.89226 | 2.1 |
| 44989 | 1.89226 | 2.1 |
| 44990 | 1.89226 | 2.1 |
| 44991 | 1.89226 | 2.1 |
| 44992 | 1.89226 | 2.1 |
| 44993 | 1.89226 | 2.1 |
| 44994 | 1.89226 | 1.02846 |
| 44995 | 1.89226 | 2.1 |
| 44996 | 1.89226 | 2.1 |
| 44997 | 3.78452 | 2.1 |
| 44998 | 2.57085 | 2.1 |
| 44999 | 1.89226 | 2.1 |
| 45000 | 2.58229 | 2.1 |
| 45001 | 1.89226 | 2.1 |
| 45002 | 4.46312 | 2.1 |
| 45003 | 1.32368 | 2.1 |
| 45004 | 2.58228 | 2.1 |
| 45005 | 2.55352 | 2.1 |

| | | |
|---|---|---|
| 45006 | 2.55352 | 2.1 |
| 45007 | 1.39332 | 2.1 |
| 45008 | 1.39332 | 2.1 |
| 45009 | 1.89226 | 2.1 |
| 45010 | 1.89226 | 2.1 |
| 45011 | 1.89226 | 2.1 |
| 45012 | 1.89226 | 2.1 |
| 45013 | 1.89226 | 2.1 |
| 45014 | 1.89226 | 2.1 |
| 45015 | 1.89226 | 2.1 |
| 45016 | 1.89226 | 2.1 |
| 45017 | 1.89226 | 2.1 |
| 45018 | 1.89226 | 2.1 |
| 45019 | 1.89226 | 2.1 |
| 45020 | 1.89226 | 2.1 |
| 45021 | 2.55353 | 2.1 |
| 45022 | 1.89226 | 2.1 |
| 45023 | 2.58228 | 2.1 |
| 45024 | 1.89226 | 2.1 |
| 45025 | 1.89226 | 2.1 |
| 45026 | 2.58228 | 2.1 |
| 45027 | 1.40574 | 1.73163 |
| 45028 | 1.05175 | 1.25697 |
| 45029 | 1.71613 | 1.91173 |
| 45030 | 1.99961 | 1.22267 |
| 45031 | 3.12187 | 1.53978 |
| 45032 | 1.71613 | 1.90858 |
| 45033 | 2.50733 | 1.27917 |
| 45034 | 1.99961 | 2.06194 |
| 45035 | 1.99961 | 1.96636 |
| 45036 | 2.25746 | 1.20035 |
| 45037 | 2.25746 | 1.23409 |
| 45038 | 2.25746 | 1.12566 |
| 45039 | 1.89526 | 1.15545 |
| 45040 | 1.71613 | 1.71937 |
| 45041 | 1.71613 | 0.868001 |
| 45042 | 1.89526 | 1.20981 |
| 45043 | 1.27558 | 0.875895 |
| 45044 | 1.07536 | 2.1 |
| 45045 | 1.07536 | 2.1 |
| 45046 | 1.07034 | 2.1 |
| 45047 | 1.07034 | 2.1 |
| 45048 | 0.766879 | 2.1 |
| 45049 | 0.81527 | 2.1 |
| 45050 | 1.38896 | 2.1 |
| 45051 | 1.33043 | 2.1 |
| 45052 | 0.815264 | 2.1 |
| 45053 | 1.38895 | 2.1 |
| 45054 | 0.766884 | 2.1 |
| 45055 | 1.33044 | 2.1 |
| 45056 | 1.89225 | 2.1 |
| 45057 | 1.89225 | 2.1 |
| 45058 | 1.89225 | 2.1 |
| 45059 | 1.89225 | 2.1 |
| 45060 | 1.89225 | 2.1 |
| 45061 | 1.89225 | 2.1 |
| 45062 | 1.64059 | 2.1 |
| 45063 | 1.89225 | 2.1 |

| | | |
|---|---|---|
| 45064 | 1.565 | 2.1 |
| 45065 | 1.565 | 2.1 |
| 45066 | 1.89225 | 2.1 |
| 45067 | 1.89225 | 2.1 |
| 45068 | 1.89226 | 2.1 |
| 45069 | 1.89226 | 2.1 |
| 45070 | 1.89226 | 2.1 |
| 45071 | 1.89226 | 2.1 |
| 45072 | 1.89226 | 2.1 |
| 45073 | 1.89226 | 2.1 |
| 45074 | 1.67906 | 2.1 |
| 45075 | 1.89226 | 2.1 |
| 45076 | 1.6406 | 2.1 |
| 45077 | 1.6406 | 2.1 |
| 45078 | 1.89226 | 2.1 |
| 45079 | 1.89226 | 2.1 |
| 45080 | 2.18106 | 1.73862 |
| 45081 | 2.20831 | 2.1 |
| 45082 | 2.64725 | 1.11641 |
| 45083 | 2.63102 | 1.58229 |
| 45084 | 4.82831 | 1.84174 |
| 45085 | 2.18106 | 1.38768 |
| 45086 | 2.09605 | 2.1 |
| 45087 | 2.5601 | 1.65799 |
| 45088 | 2.63102 | 1.55468 |
| 45089 | 4.74116 | 2.1 |
| 45090 | 2.64725 | 1.31705 |
| 45091 | 1.89226 | 1.59943 |
| 45092 | 1.87603 | 2.1 |
| 45093 | 2.63102 | 1.33451 |
| 45094 | 1.89226 | 2.04176 |
| 45095 | 2.64725 | 1.08594 |
| 45096 | 0.946132 | 1.76307 |
| 45097 | 1.28005 | 1.289 |
| 45098 | 0.946132 | 2.1 |
| 45099 | 1.31551 | 1.11583 |
| 45100 | 1.28005 | 1.84359 |
| 45101 | 1.28005 | 2.1 |
| 45102 | 0.946132 | 1.14856 |
| 45103 | 0.946132 | 1.54091 |
| 45104 | 0.946132 | 1.20875 |
| 45105 | 0.946132 | 1.38663 |
| 45106 | 1.28005 | 1.76097 |
| 45107 | 1.31551 | 1.91603 |
| 45108 | 2.0815 | 2.1 |
| 45109 | 1.59318 | 2.1 |
| 45110 | 2.0815 | 2.1 |
| 45111 | 1.40161 | 2.1 |
| 45112 | 2.49782 | 2.1 |
| 45113 | 1.68553 | 2.1 |
| 45114 | 0.819885 | 2.1 |
| 45115 | 0.935082 | 2.1 |
| 45116 | 0.867363 | 2.1 |
| 45117 | 0.867363 | 2.1 |
| 45118 | 0.715431 | 2.1 |
| 45119 | 0.902777 | 2.1 |
| 45120 | 0.738933 | 2.1 |
| 45121 | 0.980053 | 2.1 |

| | | |
|---|---|---|
| 45122 | 0.936716 | 2.1 |
| 45123 | 0.917651 | 2.1 |
| 45124 | 0.775853 | 2.1 |
| 45125 | 0.74037 | 2.1 |
| 45126 | 2.78977 | 2.1 |
| 45127 | 2.38239 | 2.1 |
| 45128 | 2.78977 | 2.1 |
| 45129 | 1.96468 | 2.1 |
| 45130 | 2.64107 | 2.1 |
| 45131 | 1.80733 | 2.1 |
| 45132 | 1.80141 | 2.1 |
| 45133 | 2.28512 | 2.1 |
| 45134 | 2.28512 | 2.1 |
| 45135 | 1.8519 | 2.1 |
| 45136 | 2.64107 | 2.1 |
| 45137 | 2.20785 | 2.1 |
| 45138 | 0.946133 | 0.13135 |
| 45139 | 0.946133 | 0.121285 |
| 45140 | 0.946132 | 0.05 |
| 45141 | 0.946132 | 0.05 |
| 45142 | 0.946132 | 0.05 |
| 45143 | 0.946132 | 0.447487 |
| 45144 | 0.946132 | 0.115977 |
| 45145 | 0.946132 | 0.05 |
| 45146 | 0.946132 | 0.05 |
| 45147 | 0.946132 | 0.0602143 |
| 45148 | 0.946133 | 0.0770765 |
| 45149 | 0.946133 | 0.161315 |
| 45150 | 1.89226 | 0.0683711 |
| 45151 | 1.89226 | 0.05 |
| 45152 | 1.89226 | 0.05 |
| 45153 | 1.89226 | 0.05 |
| 45154 | 1.89226 | 0.05 |
| 45155 | 1.89226 | 0.110349 |
| 45156 | 1.89226 | 0.05 |
| 45157 | 1.89226 | 0.05 |
| 45158 | 1.89226 | 0.05 |
| 45159 | 1.89226 | 0.103141 |
| 45160 | 3.78453 | 0.05 |
| 45161 | 1.89226 | 0.0804798 |
| 45162 | 1.89226 | 0.12186 |
| 45163 | 1.89226 | 0.05 |
| 45164 | 1.89226 | 0.287625 |
| 45165 | 1.89226 | 0.109374 |
| 45166 | 1.89226 | 0.407425 |
| 45167 | 2.12925 | 0.05 |
| 45168 | 2.51397 | 0.05 |
| 45169 | 2.22488 | 0.05 |
| 45170 | 1.86113 | 0.05 |
| 45171 | 4.3751 | 0.05 |
| 45172 | 2.82754 | 0.05 |
| 45173 | 2.51397 | 0.05 |
| 45174 | 2.22489 | 0.05 |
| 45175 | 2.47291 | 0.05 |
| 45176 | 4.98688 | 0.05 |
| 45177 | 1.89226 | 0.05 |
| 45178 | 1.89226 | 0.05 |
| 45179 | 1.89226 | 0.05 |

| | | |
|---|---|---|
| 45180 | 1.89226 | 0.05 |
| 45181 | 3.78453 | 0.05 |
| 45182 | 1.89226 | 0.05 |
| 45183 | 1.89226 | 0.05 |
| 45184 | 1.89226 | 0.05 |
| 45185 | 1.89226 | 0.05 |
| 45186 | 3.78452 | 0.05 |
| 45187 | 0.90848 | 0.05 |
| 45188 | 1.11244 | 0.05 |
| 45189 | 0.930566 | 0.05 |
| 45190 | 0.73385 | 0.05 |
| 45191 | 0.921298 | 0.05 |
| 45192 | 0.921298 | 0.05 |
| 45193 | 0.924994 | 0.05 |
| 45194 | 0.924994 | 0.05 |
| 45195 | 0.908478 | 0.05 |
| 45196 | 0.733851 | 0.05 |
| 45197 | 1.11244 | 0.05 |
| 45198 | 0.930567 | 0.05 |
| 45199 | 2.22489 | 0.05 |
| 45200 | 2.0815 | 0.05 |
| 45201 | 2.0815 | 0.05 |
| 45202 | 2.30674 | 0.05 |
| 45203 | 1.99867 | 0.05 |
| 45204 | 2.33358 | 0.05 |
| 45205 | 1.89226 | 0.05 |
| 45206 | 1.89226 | 0.05 |
| 45207 | 1.89226 | 0.05 |
| 45208 | 1.89226 | 0.05 |
| 45209 | 1.89226 | 0.05 |
| 45210 | 1.89226 | 0.05 |
| 45211 | 0.946131 | 0.05 |
| 45212 | 0.946131 | 0.05 |
| 45213 | 0.946128 | 0.05 |
| 45214 | 0.946128 | 0.05 |
| 45215 | 0.946129 | 0.05 |
| 45216 | 0.946129 | 0.05 |
| 45217 | 0.94613 | 0.05 |
| 45218 | 0.94613 | 0.05 |
| 45219 | 0.94613 | 0.05 |
| 45220 | 0.94613 | 0.05 |
| 45221 | 0.94613 | 0.05 |
| 45222 | 0.94613 | 0.05 |
| 45223 | 2.28904 | 2.1 |
| 45224 | 1.89226 | 1.29636 |
| 45225 | 2.2846 | 1.95533 |
| 45226 | 2.2846 | 2.1 |
| 45227 | 1.89226 | 0.692484 |
| 45228 | 1.89226 | 2.1 |
| 45229 | 1.89226 | 1.27289 |
| 45230 | 1.89226 | 1.20343 |
| 45231 | 1.89226 | 2.1 |
| 45232 | 1.89226 | 2.1 |
| 45233 | 1.89226 | 0.871793 |
| 45234 | 1.89226 | 2.1 |
| 45235 | 1.04037 | 1.0252 |
| 45236 | 1.34066 | 1.90377 |
| 45237 | 1.34955 | 1.0389 |

| | | |
|---|---|---|
| 45238 | 1.34955 | 2.1 |
| 45239 | 1.03373 | 1.1482 |
| 45240 | 1.03373 | 2.1 |
| 45241 | 0.946131 | 0.05 |
| 45242 | 0.946131 | 2.1 |
| 45243 | 1.14452 | 2.1 |
| 45244 | 1.14452 | 2.1 |
| 45245 | 0.946131 | 1.22823 |
| 45246 | 0.946131 | 1.05869 |
| 45247 | 1.14296 | 2.1 |
| 45248 | 1.14452 | 2.1 |
| 45249 | 1.14296 | 0.856898 |
| 45250 | 0.946131 | 2.1 |
| 45251 | 1.14452 | 2.1 |
| 45252 | 0.946132 | 1.84546 |
| 45253 | 1.89226 | 1.15564 |
| 45254 | 1.89226 | 2.1 |
| 45255 | 1.89226 | 1.90007 |
| 45256 | 1.89226 | 2.1 |
| 45257 | 1.89226 | 0.541166 |
| 45258 | 1.89226 | 2.1 |
| 45259 | 0.597454 | 1.10533 |
| 45260 | 0.597454 | 2.1 |
| 45261 | 0.597972 | 1.02328 |
| 45262 | 0.597972 | 2.1 |
| 45263 | 0.675238 | 2.1 |
| 45264 | 0.674776 | 1.20424 |
| 45265 | 0.519667 | 1.16598 |
| 45266 | 0.520188 | 1.38089 |
| 45267 | 0.519667 | 0.846332 |
| 45268 | 0.675238 | 0.954425 |
| 45269 | 0.520188 | 1.38748 |
| 45270 | 0.674777 | 1.37948 |
| 45271 | 1.89226 | 0.05 |
| 45272 | 1.82125 | 0.05 |
| 45273 | 1.89226 | 0.05 |
| 45274 | 1.84314 | 0.05 |
| 45275 | 1.89226 | 0.0564986 |
| 45276 | 1.84314 | 0.05 |
| 45277 | 1.89226 | 0.05 |
| 45278 | 1.84314 | 0.05 |
| 45279 | 1.89226 | 0.05 |
| 45280 | 1.65794 | 0.05 |
| 45281 | 1.89226 | 0.05 |
| 45282 | 1.65794 | 0.05 |
| 45283 | 1.348 | 2.1 |
| 45284 | 1.43485 | 2.1 |
| 45285 | 1.79929 | 1.09582 |
| 45286 | 1.75309 | 2.01722 |
| 45287 | 3.14728 | 2.02685 |
| 45288 | 1.348 | 1.40991 |
| 45289 | 1.63892 | 2.1 |
| 45290 | 1.59157 | 2.1 |
| 45291 | 1.18647 | 2.1 |
| 45292 | 1.75309 | 1.5631 |
| 45293 | 1.348 | 2.01677 |
| 45294 | 1.79929 | 1.69917 |
| 45295 | 1.75309 | 2.1 |

| | | |
|---|---|---|
| 45296 | 2.18106 | 1.38986 |
| 45297 | 2.20831 | 1.42391 |
| 45298 | 3.98034 | 2.1 |
| 45299 | 2.18106 | 1.19478 |
| 45300 | 1.63892 | 2.1 |
| 45301 | 1.75309 | 1.27181 |
| 45302 | 1.75309 | 2.1 |
| 45303 | 2.09605 | 1.16871 |
| 45304 | 2.09605 | 2.1 |
| 45305 | 2.66076 | 0.0541353 |
| 45306 | 1.70302 | 0.05 |
| 45307 | 2.41575 | 0.05 |
| 45308 | 1.70302 | 0.05 |
| 45309 | 3.08598 | 0.05 |
| 45310 | 2.3661 | 0.05 |
| 45311 | 1.71053 | 0.05 |
| 45312 | 2.41575 | 0.0541006 |
| 45313 | 1.20143 | 0.05 |
| 45314 | 1.20143 | 0.05 |
| 45315 | 2.2123 | 0.05 |
| 45316 | 1.66739 | 0.05 |
| 45317 | 2.25633 | 0.05 |
| 45318 | 2.68302 | 0.05 |
| 45319 | 1.71051 | 0.05 |
| 45320 | 1.71051 | 0.05 |
| 45321 | 2.74192 | 0.05 |
| 45322 | 2.1961 | 0.05 |
| 45323 | 1.32553 | 0.479338 |
| 45324 | 2.34875 | 0.05 |
| 45325 | 2.19139 | 2.01234 |
| 45326 | 2.19139 | 0.05 |
| 45327 | 1.56172 | 1.92687 |
| 45328 | 1.56172 | 0.05 |
| 45329 | 1.89226 | 2.1 |
| 45330 | 1.89226 | 0.05 |
| 45331 | 1.89226 | 2.1 |
| 45332 | 1.89226 | 0.134442 |
| 45333 | 3.78453 | 0.879733 |
| 45334 | 2.667 2.1 | |
| 45335 | 1.26097 | 2.1 |
| 45336 | 1.26097 | 2.1 |
| 45337 | 1.5683 | 2.1 |
| 45338 | 2.42949 | 2.1 |
| 45339 | 2.73682 | 2.1 |
| 45340 | 1.13213 | 0.169803 |
| 45341 | 2.19139 | 0.05 |
| 45342 | 2.0815 | 1.03877 |
| 45343 | 1.77615 | 0.05 |
| 45344 | 1.37767 | 0.939454 |
| 45345 | 1.19297 | 0.05 |
| 45346 | 0.946132 | 0.0651745 |
| 45347 | 0.946132 | 2.1 |
| 45348 | 0.946131 | 2.1 |
| 45349 | 0.946131 | 0.236092 |
| 45350 | 0.946131 | 0.0913926 |
| 45351 | 0.946131 | 0.05 |
| 45352 | 0.946132 | 2.1 |
| 45353 | 0.946132 | 2.1 |

| | | |
|---|---|---|
| 45354 | 0.946132 | 0.912362 |
| 45355 | 0.946132 | 0.915072 |
| 45356 | 0.946131 | 0.815449 |
| 45357 | 0.946131 | 0.84901 |
| 45358 | 2.02064 | 2.1 |
| 45359 | 2.3606 | 2.1 |
| 45360 | 1.47478 | 2.1 |
| 45361 | 2.22997 | 2.1 |
| 45362 | 4.25061 | 2.1 |
| 45363 | 2.36059 | 2.1 |
| 45364 | 2.56952 | 2.1 |
| 45365 | 2.22997 | 2.1 |
| 45366 | 2.48631 | 2.1 |
| 45367 | 4.79949 | 2.1 |
| 45368 | 1.84261 | 2.1 |
| 45369 | 2.22997 | 2.1 |
| 45370 | 1.65706 | 2.1 |
| 45371 | 1.78837 | 2.1 |
| 45372 | 3.88703 | 2.1 |
| 45373 | 2.22997 | 2.1 |
| 45374 | 2.48631 | 2.1 |
| 45375 | 2.19528 | 2.1 |
| 45376 | 2.41175 | 2.1 |
| 45377 | 4.64172 | 2.1 |
| 45378 | 1.6466 | 2.1 |
| 45379 | 1.89226 | 2.1 |
| 45380 | 1.35972 | 2.1 |
| 45381 | 1.35972 | 2.1 |
| 45382 | 1.89226 | 2.1 |
| 45383 | 1.89226 | 2.1 |
| 45384 | 1.89226 | 2.1 |
| 45385 | 1.89226 | 2.1 |
| 45386 | 1.89226 | 2.1 |
| 45387 | 1.89226 | 2.1 |
| 45388 | 3.78453 | 2.1 |
| 45389 | 2.44797 | 2.1 |
| 45390 | 1.07214 | 2.1 |
| 45391 | 1.31589 | 2.1 |
| 45392 | 1.1211 | 2.1 |
| 45393 | 2.44797 | 2.1 |
| 45394 | 2.25318 | 2.1 |
| 45395 | 1.95954 | 2.1 |
| 45396 | 1.89226 | 2.1 |
| 45397 | 1.6466 | 2.1 |
| 45398 | 1.6466 | 2.1 |
| 45399 | 1.89226 | 2.1 |
| 45400 | 1.89226 | 2.1 |
| 45401 | 0.946133 | 2.1 |
| 45402 | 0.946133 | 2.1 |
| 45403 | 0.946132 | 2.1 |
| 45404 | 0.946132 | 2.1 |
| 45405 | 0.946132 | 2.1 |
| 45406 | 0.946132 | 2.1 |
| 45407 | 0.946133 | 2.1 |
| 45408 | 0.946133 | 2.1 |
| 45409 | 0.946132 | 2.1 |
| 45410 | 0.946132 | 2.1 |
| 45411 | 0.946133 | 2.1 |

| | | |
|---|---|---|
| 45412 | 0.946133 | 2.1 |
| 45413 | 2.44797 | 2.1 |
| 45414 | 1.36904 | 2.1 |
| 45415 | 1.70303 | 2.1 |
| 45416 | 2.37586 | 2.1 |
| 45417 | 4.00507 | 2.1 |
| 45418 | 0.631553 | 0.05 |
| 45419 | 1.24489 | 0.05 |
| 45420 | 1.11597 | 0.05 |
| 45421 | 0.496799 | 0.05 |
| 45422 | 0.872272 | 0.05 |
| 45423 | 0.872272 | 0.05 |
| 45424 | 0.869415 | 0.05 |
| 45425 | 0.869415 | 0.05 |
| 45426 | 0.4968 | 0.05 |
| 45427 | 0.631553 | 0.05 |
| 45428 | 1.11597 | 0.05 |
| 45429 | 1.24489 | 0.05 |
| 45430 | 1.84253 | 2.1 |
| 45431 | 2.17384 | 2.1 |
| 45432 | 2.04818 | 2.1 |
| 45433 | 1.65925 | 2.1 |
| 45434 | 3.83309 | 2.1 |
| 45435 | 2.04818 | 2.1 |
| 45436 | 1.65925 | 2.1 |
| 45437 | 1.48162 | 2.1 |
| 45438 | 1.92264 | 2.1 |
| 45439 | 3.58189 | 2.1 |
| 45440 | 2.3305 | 0.281132 |
| 45441 | 1.89226 | 0.438153 |
| 45442 | 2.52753 | 0.345615 |
| 45443 | 2.52753 | 0.438501 |
| 45444 | 1.89226 | 0.375479 |
| 45445 | 1.89226 | 0.435597 |
| 45446 | 1.89226 | 0.411614 |
| 45447 | 1.89226 | 0.586764 |
| 45448 | 1.89226 | 0.520716 |
| 45449 | 1.89226 | 0.541687 |
| 45450 | 1.89226 | 0.48329 |
| 45451 | 1.89226 | 0.538958 |
| 45452 | 1.34563 | 0.256898 |
| 45453 | 2.1769 | 0.443026 |
| 45454 | 1.94955 | 0.391414 |
| 45455 | 1.94955 | 0.559624 |
| 45456 | 1.55063 | 0.330474 |
| 45457 | 1.55063 | 0.548175 |
| 45458 | 2.09455 | 0.168682 |
| 45459 | 1.89226 | 0.428277 |
| 45460 | 2.3305 | 0.305206 |
| 45461 | 2.3305 | 0.383071 |
| 45462 | 1.89226 | 0.329853 |
| 45463 | 1.89226 | 0.401459 |
| 45464 | 1.89226 | 0.367103 |
| 45465 | 1.89226 | 0.43902 |
| 45466 | 1.89226 | 0.503077 |
| 45467 | 1.89226 | 0.415331 |
| 45468 | 1.89226 | 0.429844 |
| 45469 | 1.89226 | 0.496666 |

| | | |
|---|---|---|
| 45470 | 0.773392 | 0.0996098 |
| 45471 | 0.612765 | 0.791741 |
| 45472 | 0.79504 | 0.426978 |
| 45473 | 0.575323 | 0.588585 |
| 45474 | 1.0819 | 0.159706 |
| 45475 | 1.06106 | 0.443964 |
| 45476 | 1.04675 | 0.261179 |
| 45477 | 0.86858 | 0.465179 |
| 45478 | 0.796873 | 0.372396 |
| 45479 | 0.557469 | 0.291874 |
| 45480 | 0.807663 | 0.436623 |
| 45481 | 2.2024 | 2.1 |
| 45482 | 2.34106 | 2.1 |
| 45483 | 2.02634 | 2.1 |
| 45484 | 2.20125 | 2.1 |
| 45485 | 4.36739 | 2.1 |
| 45486 | 2.34106 | 2.1 |
| 45487 | 2.25195 | 0.05 |
| 45488 | 2.20125 | 2.1 |
| 45489 | 2.13132 | 0.05 |
| 45490 | 4.47237 | 1.05287 |
| 45491 | 2.02635 | 2.1 |
| 45492 | 2.22465 | 2.1 |
| 45493 | 1.68504 | 2.1 |
| 45494 | 2.0815 | 2.1 |
| 45495 | 3.92673 | 2.1 |
| 45496 | 2.22465 | 2.1 |
| 45497 | 2.13133 | 0.0805966 |
| 45498 | 2.0815 | 2.1 |
| 45499 | 2.23579 | 0.05 |
| 45500 | 4.44862 | 1.12928 |
| 45501 | 1.89226 | 1.05436 |
| 45502 | 1.89226 | 1.4519 |
| 45503 | 1.89226 | 2.00042 |
| 45504 | 1.89226 | 1.13552 |
| 45505 | 1.89226 | 1.82006 |
| 45506 | 1.89226 | 1.08785 |
| 45507 | 1.93967 | 0.05 |
| 45508 | 1.70302 | 0.05 |
| 45509 | 1.21883 | 0.05 |
| 45510 | 1.70302 | 0.05 |
| 45511 | 1.19889 | 0.05 |
| 45512 | 1.70302 | 0.05 |
| 45513 | 0.946131 | 0.05 |
| 45514 | 0.946131 | 0.05 |
| 45515 | 0.946131 | 0.05 |
| 45516 | 0.946131 | 0.05 |
| 45517 | 0.94613 | 0.05 |
| 45518 | 0.94613 | 0.05 |
| 45519 | 0.946131 | 0.05 |
| 45520 | 0.946131 | 0.05 |
| 45521 | 0.946131 | 0.05 |
| 45522 | 0.946131 | 0.05 |
| 45523 | 0.946131 | 0.05 |
| 45524 | 0.946131 | 0.05 |
| 45525 | 0.883128 | 0.05 |
| 45526 | 0.883128 | 0.05 |
| 45527 | 0.827519 | 0.05 |

| | | |
|---|---|---|
| 45528 | 0.82752 | 0.05 |
| 45529 | 1.16234 | 0.05 |
| 45530 | 1.19063 | 0.05 |
| 45531 | 0.631294 | 0.05 |
| 45532 | 0.548311 | 0.05 |
| 45533 | 0.631293 | 0.05 |
| 45534 | 1.16234 | 0.05 |
| 45535 | 1.19063 | 0.05 |
| 45536 | 0.54831 | 0.05 |
| 45537 | 1.89226 | 0.05 |
| 45538 | 1.89226 | 0.05 |
| 45539 | 1.89226 | 0.05 |
| 45540 | 1.89226 | 0.05 |
| 45541 | 3.78453 | 0.05 |
| 45542 | 2.33328 | 0.05 |
| 45543 | 1.87991 | 0.05 |
| 45544 | 1.87991 | 0.05 |
| 45545 | 2.82938 | 0.05 |
| 45546 | 1.84457 | 0.05 |
| 45547 | 2.79403 | 0.05 |
| 45548 | 2.82937 | 0.05 |
| 45549 | 2.70881 | 0.05 |
| 45550 | 1.8799 | 0.05 |
| 45551 | 1.82739 | 0.05 |
| 45552 | 4.58874 | 0.05 |
| 45553 | 1.89226 | 0.05 |
| 45554 | 1.89226 | 0.05 |
| 45555 | 1.89226 | 0.05 |
| 45556 | 1.89226 | 0.05 |
| 45557 | 3.78452 | 0.05 |
| 45558 | 1.17897 | 0.05 |
| 45559 | 1.82739 | 0.05 |
| 45560 | 1.17897 | 0.05 |
| 45561 | 1.76199 | 0.05 |
| 45562 | 1.23885 | 0.05 |
| 45563 | 1.80989 | 0.05 |
| 45564 | 0.946131 | 0.05 |
| 45565 | 0.946131 | 0.05 |
| 45566 | 0.946131 | 0.05 |
| 45567 | 0.946131 | 0.05 |
| 45568 | 0.946129 | 0.05 |
| 45569 | 0.946129 | 0.05 |
| 45570 | 0.946133 | 0.05 |
| 45571 | 0.946133 | 0.05 |
| 45572 | 0.946131 | 0.05 |
| 45573 | 0.946131 | 0.05 |
| 45574 | 0.946131 | 0.05 |
| 45575 | 0.946131 | 0.05 |
| 45576 | 0.946126 | 0.494209 |
| 45577 | 0.946126 | 2.1 |
| 45578 | 1.24887 | 2.1 |
| 45579 | 1.24887 | 2.1 |
| 45580 | 0.946127 | 2.1 |
| 45581 | 0.946127 | 0.767761 |
| 45582 | 1.24887 | 2.1 |
| 45583 | 1.28511 | 2.1 |
| 45584 | 1.2851 | 2.1 |
| 45585 | 0.946128 | 2.1 |

| | | |
|---|---|---|
| 45586 | 1.24887 | 2.1 |
| 45587 | 0.946128 | 2.1 |
| 45588 | 1.89226 | 2.1 |
| 45589 | 1.89226 | 2.09545 |
| 45590 | 1.89226 | 2.1 |
| 45591 | 1.89226 | 0.867108 |
| 45592 | 3.78452 | 1.50732 |
| 45593 | 0.592142 | 2.1 |
| 45594 | 0.592142 | 2.1 |
| 45595 | 0.60496 | 2.1 |
| 45596 | 0.60496 | 2.1 |
| 45597 | 0.694237 | 2.1 |
| 45598 | 0.608714 | 1.10194 |
| 45599 | 0.562863 | 2.1 |
| 45600 | 0.502864 | 2.1 |
| 45601 | 0.562863 | 2.1 |
| 45602 | 0.694237 | 2.1 |
| 45603 | 0.608714 | 2.1 |
| 45604 | 0.502864 | 2.1 |
| 45605 | 1.89226 | 0.177193 |
| 45606 | 1.89226 | 2.1 |
| 45607 | 1.89226 | 2.1 |
| 45608 | 1.89226 | 2.1 |
| 45609 | 3.78453 | 2.1 |
| 45610 | 1.55779 | 2.1 |
| 45611 | 1.57968 | 2.1 |
| 45612 | 1.47186 | 2.1 |
| 45613 | 1.51725 | 2.1 |
| 45614 | 3.05154 | 2.1 |
| 45615 | 1.57967 | 2.1 |
| 45616 | 1.474 2.1 | |
| 45617 | 1.51725 | 2.1 |
| 45618 | 1.42895 | 2.1 |
| 45619 | 3.00863 | 2.1 |
| 45620 | 1.89226 | 2.1 |
| 45621 | 1.89226 | 2.1 |
| 45622 | 1.89226 | 2.1 |
| 45623 | 1.89226 | 2.1 |
| 45624 | 3.78452 | 2.1 |
| 45625 | 1.89226 | 2.1 |
| 45626 | 1.89226 | 2.1 |
| 45627 | 1.89226 | 0.120011 |
| 45628 | 1.89226 | 2.1 |
| 45629 | 3.78453 | 2.1 |
| 45630 | 1.47186 | 2.1 |
| 45631 | 1.51725 | 2.1 |
| 45632 | 1.38859 | 2.1 |
| 45633 | 1.45332 | 2.1 |
| 45634 | 2.92519 | 2.1 |
| 45635 | 1.51725 | 2.1 |
| 45636 | 1.42895 | 2.1 |
| 45637 | 1.45332 | 2.1 |
| 45638 | 1.38414 | 2.1 |
| 45639 | 2.88228 | 2.1 |
| 45640 | 1.89226 | 2.1 |
| 45641 | 1.89226 | 2.1 |
| 45642 | 1.89226 | 2.1 |
| 45643 | 1.89226 | 2.1 |

| | | |
|---|---|---|
| 45644 | 3.78452 | 2.1 |
| 45645 | 1.34497 | 0.05 |
| 45646 | 1.36747 | 0.05 |
| 45647 | 1.18919 | 0.05 |
| 45648 | 2.08149 | 0.05 |
| 45649 | 1.23556 | 0.05 |
| 45650 | 2.13702 | 0.05 |
| 45651 | 1.10106 | 0.05 |
| 45652 | 2.08001 | 0.05 |
| 45653 | 1.34497 | 0.05 |
| 45654 | 2.31605 | 0.05 |
| 45655 | 1.34497 | 0.05 |
| 45656 | 2.31605 | 0.05 |
| 45657 | 2.31605 | 0.05 |
| 45658 | 2.0815 | 0.05 |
| 45659 | 1.9419 | 0.05 |
| 45660 | 2.46665 | 0.05 |
| 45661 | 4.52922 | 0.05 |
| 45662 | 1.20349 | 0.05 |
| 45663 | 2.08149 | 0.05 |
| 45664 | 1.36948 | 0.05 |
| 45665 | 1.34737 | 0.05 |
| 45666 | 2.08149 | 0.05 |
| 45667 | 2.08149 | 0.05 |
| 45668 | 0.946132 | 0.05 |
| 45669 | 0.946132 | 0.05 |
| 45670 | 1.37715 | 0.05 |
| 45671 | 0.823199 | 0.05 |
| 45672 | 1.21598 | 0.05 |
| 45673 | 0.946132 | 0.05 |
| 45674 | 1.37715 | 0.05 |
| 45675 | 0.946132 | 0.05 |
| 45676 | 1.21598 | 0.05 |
| 45677 | 0.823198 | 0.05 |
| 45678 | 0.946133 | 0.05 |
| 45679 | 0.946133 | 0.05 |
| 45680 | 1.89226 | 0.05 |
| 45681 | 1.89226 | 0.05 |
| 45682 | 1.89226 | 0.05 |
| 45683 | 1.89226 | 0.05 |
| 45684 | 3.78453 | 0.05 |
| 45685 | 1.89227 | 0.05 |
| 45686 | 1.89227 | 0.05 |
| 45687 | 1.89227 | 0.05 |
| 45688 | 1.89227 | 0.05 |
| 45689 | 3.78453 | 0.05 |
| 45690 | 0.946132 | 0.05 |
| 45691 | 0.946132 | 0.05 |
| 45692 | 1.21599 | 0.05 |
| 45693 | 1.21599 | 0.05 |
| 45694 | 0.966749 | 0.05 |
| 45695 | 0.946133 | 0.05 |
| 45696 | 1.21598 | 0.05 |
| 45697 | 0.946132 | 0.05 |
| 45698 | 0.966748 | 0.05 |
| 45699 | 1.21598 | 0.05 |
| 45700 | 0.946133 | 0.05 |
| 45701 | 0.946133 | 0.05 |

| | | |
|---|---|---|
| 45702 | 0.946133 | 0.05 |
| 45703 | 0.946133 | 0.05 |
| 45704 | 1.1134 | 0.05 |
| 45705 | 1.11555 | 0.05 |
| 45706 | 1.11555 | 0.05 |
| 45707 | 0.946132 | 0.05 |
| 45708 | 0.946132 | 0.05 |
| 45709 | 1.1134 | 0.05 |
| 45710 | 1.11555 | 0.05 |
| 45711 | 1.11555 | 0.05 |
| 45712 | 0.946132 | 0.05 |
| 45713 | 0.946132 | 0.05 |
| 45714 | 0.946133 | 0.05 |
| 45715 | 0.946133 | 0.05 |
| 45716 | 1.11555 | 0.05 |
| 45717 | 0.978467 | 0.05 |
| 45718 | 0.978468 | 0.05 |
| 45719 | 0.946133 | 0.05 |
| 45720 | 1.11555 | 0.05 |
| 45721 | 0.946132 | 0.05 |
| 45722 | 0.978468 | 0.05 |
| 45723 | 0.978468 | 0.05 |
| 45724 | 0.946132 | 0.05 |
| 45725 | 0.946132 | 0.05 |
| 45726 | 0.851542 | 2.1 |
| 45727 | 0.851542 | 2.1 |
| 45728 | 1.14168 | 2.1 |
| 45729 | 1.27897 | 2.1 |
| 45730 | 1.02817 | 2.1 |
| 45731 | 1.13536 | 2.1 |
| 45732 | 0.946131 | 2.1 |
| 45733 | 0.861207 | 2.1 |
| 45734 | 0.94613 | 2.1 |
| 45735 | 0.94613 | 2.1 |
| 45736 | 0.868751 | 2.1 |
| 45737 | 1.15181 | 2.1 |
| 45738 | 0.946132 | 2.1 |
| 45739 | 0.946132 | 2.1 |
| 45740 | 0.946128 | 2.1 |
| 45741 | 0.946128 | 0.960164 |
| 45742 | 0.94613 | 2.1 |
| 45743 | 0.94613 | 2.09711 |
| 45744 | 0.946131 | 1.99274 |
| 45745 | 0.946131 | 0.942963 |
| 45746 | 0.94613 | 0.837961 |
| 45747 | 0.94613 | 2.1 |
| 45748 | 0.94613 | 1.44253 |
| 45749 | 0.94613 | 2.1 |
| 45750 | 2.53273 | 0.05 |
| 45751 | 1.89226 | 0.05 |
| 45752 | 1.89226 | 0.05 |
| 45753 | 1.89226 | 0.05 |
| 45754 | 1.89226 | 0.05 |
| 45755 | 1.89226 | 0.05 |
| 45756 | 1.89226 | 2.1 |
| 45757 | 1.89226 | 2.1 |
| 45758 | 1.89226 | 2.1 |
| 45759 | 1.89226 | 2.1 |

| | | |
|---|---|---|
| 45760 | 3.78452 | 2.1 |
| 45761 | 1.89226 | 2.1 |
| 45762 | 2.54394 | 2.1 |
| 45763 | 1.89226 | 2.1 |
| 45764 | 2.42221 | 2.1 |
| 45765 | 4.43621 | 2.1 |
| 45766 | 2.54394 | 2.1 |
| 45767 | 1.62743 | 2.1 |
| 45768 | 2.42221 | 2.1 |
| 45769 | 1.5437 | 2.1 |
| 45770 | 4.08764 | 2.1 |
| 45771 | 1.89226 | 2.1 |
| 45772 | 1.89226 | 2.1 |
| 45773 | 1.89226 | 2.1 |
| 45774 | 1.89226 | 2.1 |
| 45775 | 3.78452 | 2.1 |
| 45776 | 1.89226 | 2.1 |
| 45777 | 1.89226 | 2.1 |
| 45778 | 1.89226 | 2.1 |
| 45779 | 1.89226 | 2.1 |
| 45780 | 3.78452 | 2.1 |
| 45781 | 1.89226 | 2.1 |
| 45782 | 2.30146 | 2.1 |
| 45783 | 2.42221 | 2.1 |
| 45784 | 1.89226 | 2.1 |
| 45785 | 1.89226 | 2.1 |
| 45786 | 2.42221 | 2.1 |
| 45787 | 2.30146 | 2.1 |
| 45788 | 2.42221 | 2.1 |
| 45789 | 1.39962 | 2.1 |
| 45790 | 1.39962 | 2.1 |
| 45791 | 1.5437 | 2.1 |
| 45792 | 2.44553 | 2.1 |
| 45793 | 1.89226 | 2.1 |
| 45794 | 1.89226 | 2.1 |
| 45795 | 1.89226 | 2.1 |
| 45796 | 1.89226 | 2.1 |
| 45797 | 3.78452 | 2.1 |
| 45798 | 1.62196 | 2.1 |
| 45799 | 2.00695 | 2.1 |
| 45800 | 1.95288 | 2.1 |
| 45801 | 1.70303 | 2.1 |
| 45802 | 3.53316 | 2.1 |
| 45803 | 1.80703 | 2.1 |
| 45804 | 2.00695 | 2.1 |
| 45805 | 1.92991 | 2.1 |
| 45806 | 2.00695 | 2.1 |
| 45807 | 1.85888 | 2.1 |
| 45808 | 1.93592 | 2.1 |
| 45809 | 1.89226 | 2.1 |
| 45810 | 1.89226 | 2.1 |
| 45811 | 1.89226 | 2.1 |
| 45812 | 1.89226 | 2.1 |
| 45813 | 1.89226 | 2.1 |
| 45814 | 1.89226 | 2.1 |
| 45815 | 1.89226 | 2.1 |
| 45816 | 1.89226 | 2.1 |
| 45817 | 1.89226 | 2.1 |

| | | |
|---|---|---|
| 45818 | 1.89226 | 2.1 |
| 45819 | 3.78453 | 2.1 |
| 45820 | 1.95288 | 2.1 |
| 45821 | 1.50524 | 2.1 |
| 45822 | 1.83564 | 2.1 |
| 45823 | 1.70303 | 2.1 |
| 45824 | 2.00749 | 2.1 |
| 45825 | 1.84671 | 2.1 |
| 45826 | 1.62632 | 2.1 |
| 45827 | 1.82498 | 2.1 |
| 45828 | 1.92991 | 2.1 |
| 45829 | 1.92991 | 2.1 |
| 45830 | 1.47928 | 2.1 |
| 45831 | 1.47751 | 2.1 |
| 45832 | 0.946128 | 2.1 |
| 45833 | 0.946128 | 2.1 |
| 45834 | 0.946132 | 2.1 |
| 45835 | 0.946132 | 2.1 |
| 45836 | 0.94613 | 2.1 |
| 45837 | 0.94613 | 2.1 |
| 45838 | 0.946129 | 2.1 |
| 45839 | 0.946129 | 2.1 |
| 45840 | 0.94613 | 2.1 |
| 45841 | 0.94613 | 2.1 |
| 45842 | 0.94613 | 2.1 |
| 45843 | 0.94613 | 2.1 |
| 45844 | 1.89226 | 2.1 |
| 45845 | 1.89226 | 2.1 |
| 45846 | 1.89226 | 2.1 |
| 45847 | 1.89226 | 2.1 |
| 45848 | 1.89226 | 2.1 |
| 45849 | 1.89226 | 2.1 |
| 45850 | 2.27351 | 0.420505 |
| 45851 | 2.74068 | 0.0517632 |
| 45852 | 2.72278 | 0.115712 |
| 45853 | 2.63356 | 0.272056 |
| 45854 | 2.36273 | 0.106102 |
| 45855 | 2.27351 | 0.257784 |
| 45856 | 2.36273 | 0.247137 |
| 45857 | 2.83112 | 0.05 |
| 45858 | 2.81212 | 0.0817207 |
| 45859 | 2.74068 | 0.175422 |
| 45860 | 2.43416 | 0.0809189 |
| 45861 | 2.36273 | 0.182545 |
| 45862 | 1.89227 | 0.124537 |
| 45863 | 2.83112 | 0.05 |
| 45864 | 2.74069 | 0.0855736 |
| 45865 | 2.74069 | 0.05 |
| 45866 | 1.89227 | 0.222394 |
| 45867 | 1.89227 | 0.0764742 |
| 45868 | 2.40887 | 2.1 |
| 45869 | 1.4494 | 2.1 |
| 45870 | 1.52164 | 2.1 |
| 45871 | 2.47534 | 2.1 |
| 45872 | 3.93051 | 2.1 |
| 45873 | 1.89226 | 2.1 |
| 45874 | 2.47534 | 2.1 |
| 45875 | 2.40887 | 2.1 |

| | | |
|---|---|---|
| 45876 | 2.40887 | 2.1 |
| 45877 | 1.89226 | 2.1 |
| 45878 | 1.89226 | 2.1 |
| 45879 | 1.60861 | 2.1 |
| 45880 | 2.47534 | 2.1 |
| 45881 | 1.52164 | 2.1 |
| 45882 | 1.52164 | 2.1 |
| 45883 | 2.55301 | 2.1 |
| 45884 | 2.55301 | 2.1 |
| 45885 | 0.946128 | 2.1 |
| 45886 | 0.946128 | 2.1 |
| 45887 | 1.23767 | 2.1 |
| 45888 | 1.2765 | 2.1 |
| 45889 | 1.23767 | 2.1 |
| 45890 | 1.23767 | 2.1 |
| 45891 | 0.946131 | 2.1 |
| 45892 | 0.946131 | 2.1 |
| 45893 | 0.946131 | 2.1 |
| 45894 | 1.2765 | 2.1 |
| 45895 | 1.23767 | 2.1 |
| 45896 | 0.946131 | 2.1 |
| 45897 | 1.89226 | 2.1 |
| 45898 | 1.87511 | 2.1 |
| 45899 | 1.87511 | 2.1 |
| 45900 | 1.95852 | 2.1 |
| 45901 | 2.0815 | 2.1 |
| 45902 | 2.22169 | 2.1 |
| 45903 | 1.89226 | 2.1 |
| 45904 | 1.89226 | 2.1 |
| 45905 | 1.89226 | 2.1 |
| 45906 | 1.89226 | 2.1 |
| 45907 | 1.89226 | 2.1 |
| 45908 | 1.89226 | 2.1 |
| 45909 | 2.0815 | 2.1 |
| 45910 | 1.80067 | 2.1 |
| 45911 | 1.80067 | 2.1 |
| 45912 | 2.05518 | 2.1 |
| 45913 | 1.89226 | 2.1 |
| 45914 | 2.16509 | 2.1 |
| 45915 | 1.87153 | 2.1 |
| 45916 | 2.65897 | 2.1 |
| 45917 | 1.58724 | 2.1 |
| 45918 | 2.38924 | 2.1 |
| 45919 | 4.26077 | 2.1 |
| 45920 | 1.89226 | 2.1 |
| 45921 | 1.89226 | 2.1 |
| 45922 | 1.89226 | 2.1 |
| 45923 | 1.89226 | 2.1 |
| 45924 | 1.89226 | 2.1 |
| 45925 | 1.89226 | 2.1 |
| 45926 | 1.89226 | 0.901275 |
| 45927 | 2.21022 | 0.184849 |
| 45928 | 2.19769 | 1.01486 |
| 45929 | 2.21022 | 0.692963 |
| 45930 | 1.87973 | 0.294243 |
| 45931 | 1.89226 | 0.0565094 |
| 45932 | 1.18376 | 1.27889 |
| 45933 | 2.21022 | 0.05 |

| | | |
|---|---|---|
| 45934 | 1.16239 | 1.10518 |
| 45935 | 1.16239 | 0.05 |
| 45936 | 2.19769 | 1.12745 |
| 45937 | 2.19769 | 0.05 |
| 45938 | 0.946128 | 0.695341 |
| 45939 | 0.946128 | 0.264424 |
| 45940 | 1.09362 | 0.904714 |
| 45941 | 1.09884 | 0.345915 |
| 45942 | 0.94613 | 0.719178 |
| 45943 | 0.94613 | 0.05 |
| 45944 | 1.09362 | 0.820428 |
| 45945 | 1.09362 | 0.241751 |
| 45946 | 0.946131 | 0.755518 |
| 45947 | 1.09362 | 0.75454 |
| 45948 | 0.946131 | 0.286948 |
| 45949 | 1.09884 | 0.078445 |
| 45950 | 1.19323 | 1.09829 |
| 45951 | 2.19769 | 0.05 |
| 45952 | 1.18376 | 0.957557 |
| 45953 | 1.18376 | 0.0780462 |
| 45954 | 2.18724 | 0.976668 |
| 45955 | 2.18724 | 0.168452 |
| 45956 | 2.0815 | 2.1 |
| 45957 | 0.922714 | 2.1 |
| 45958 | 2.0815 | 2.1 |
| 45959 | 1.84882 | 2.1 |
| 45960 | 1.20787 | 2.1 |
| 45961 | 1.08321 | 2.1 |
| 45962 | 2.6238 | 0.05 |
| 45963 | 1.89226 | 0.05 |
| 45964 | 1.89226 | 0.05 |
| 45965 | 2.4866 | 0.05 |
| 45966 | 1.89226 | 0.05 |
| 45967 | 2.4866 | 0.05 |
| 45968 | 2.66753 | 0.05 |
| 45969 | 1.89226 | 0.05 |
| 45970 | 1.89226 | 0.05 |
| 45971 | 2.6238 | 0.05 |
| 45972 | 1.89226 | 0.05 |
| 45973 | 2.6238 | 0.05 |
| 45974 | 0.946129 | 2.1 |
| 45975 | 0.946129 | 2.1 |
| 45976 | 1.04676 | 2.1 |
| 45977 | 1.04676 | 2.1 |
| 45978 | 0.946131 | 2.1 |
| 45979 | 0.946131 | 2.1 |
| 45980 | 1.08732 | 2.1 |
| 45981 | 1.04676 | 2.1 |
| 45982 | 1.08732 | 2.1 |
| 45983 | 0.946131 | 2.1 |
| 45984 | 1.04676 | 2.1 |
| 45985 | 0.946131 | 2.1 |
| 45986 | 1.20958 | 2.1 |
| 45987 | 1.73802 | 0.748506 |
| 45988 | 1.39067 | 0.893647 |
| 45989 | 1.39067 | 0.630003 |
| 45990 | 1.50969 | 0.983376 |
| 45991 | 1.50969 | 1.03319 |

| | | |
|---|---|---|
| 45992 | 1.00382 | 1.26527 |
| 45993 | 1.50969 | 0.05 |
| 45994 | 1.20958 | 1.0539 |
| 45995 | 1.20958 | 0.126705 |
| 45996 | 1.32039 | 1.36338 |
| 45997 | 1.32039 | 0.767217 |
| 45998 | 2.0815 | 2.1 |
| 45999 | 2.51396 | 2.1 |
| 46000 | 2.19826 | 2.1 |
| 46001 | 2.54175 | 2.1 |
| 46002 | 4.79599 | 2.1 |
| 46003 | 2.54174 | 2.1 |
| 46004 | 2.17978 | 2.1 |
| 46005 | 2.54174 | 2.1 |
| 46006 | 2.2854 | 2.1 |
| 46007 | 2.57212 | 2.1 |
| 46008 | 2.31578 | 2.1 |
| 46009 | 2.27103 | 2.1 |
| 46010 | 2.54175 | 2.1 |
| 46011 | 2.4255 | 2.1 |
| 46012 | 2.82251 | 2.1 |
| 46013 | 4.96725 | 2.1 |
| 46014 | 2.60614 | 0.05 |
| 46015 | 2.75374 | 0.05 |
| 46016 | 1.94002 | 0.05 |
| 46017 | 2.08392 | 0.05 |
| 46018 | 4.69006 | 0.05 |
| 46019 | 2.75373 | 0.05 |
| 46020 | 2.6875 | 0.05 |
| 46021 | 2.08392 | 0.05 |
| 46022 | 1.99944 | 0.05 |
| 46023 | 4.77142 | 0.05 |
| 46024 | 1.1211 | 0.05 |
| 46025 | 2.08392 | 0.05 |
| 46026 | 1.17787 | 0.05 |
| 46027 | 1.94002 | 0.05 |
| 46028 | 1.32178 | 0.05 |
| 46029 | 2.08392 | 0.05 |
| 46030 | 2.08392 | 0.05 |
| 46031 | 1.99944 | 0.05 |
| 46032 | 1.32178 | 0.05 |
| 46033 | 1.25929 | 0.05 |
| 46034 | 3.34321 | 0.05 |
| 46035 | 1.22266 | 0.05 |
| 46036 | 0.779473 | 0.05 |
| 46037 | 0.94613 | 0.05 |
| 46038 | 0.94613 | 0.05 |
| 46039 | 1.04072 | 0.05 |
| 46040 | 1.1407 | 0.05 |
| 46041 | 1.10042 | 0.05 |
| 46042 | 0.851542 | 0.05 |
| 46043 | 0.942046 | 0.05 |
| 46044 | 0.942046 | 0.05 |
| 46045 | 1.04114 | 0.05 |
| 46046 | 0.826602 | 0.05 |
| 46047 | 2.37468 | 2.1 |
| 46048 | 1.89226 | 2.1 |
| 46049 | 1.89226 | 2.1 |

| | | |
|---|---|---|
| 46050 | 1.89226 | 2.1 |
| 46051 | 2.27917 | 2.1 |
| 46052 | 2.27917 | 2.1 |
| 46053 | 1.13959 | 2.1 |
| 46054 | 1.09451 | 2.1 |
| 46055 | 0.946131 | 2.1 |
| 46056 | 0.946131 | 2.1 |
| 46057 | 1.09451 | 2.1 |
| 46058 | 1.09451 | 2.1 |
| 46059 | 0.94613 | 2.1 |
| 46060 | 0.94613 | 2.1 |
| 46061 | 1.09451 | 2.1 |
| 46062 | 0.946131 | 2.1 |
| 46063 | 0.946131 | 2.1 |
| 46064 | 1.13959 | 2.1 |
| 46065 | 2.52454 | 0.801152 |
| 46066 | 2.12221 | 2.1 |
| 46067 | 2.67624 | 1.0281 |
| 46068 | 2.33865 | 2.1 |
| 46069 | 4.86319 | 2.1 |
| 46070 | 1.89226 | 1.96726 |
| 46071 | 1.89226 | 2.1 |
| 46072 | 1.89226 | 2.02611 |
| 46073 | 1.89226 | 2.1 |
| 46074 | 3.78452 | 2.1 |
| 46075 | 1.89226 | 2.1 |
| 46076 | 1.89226 | 2.1 |
| 46077 | 1.89226 | 2.1 |
| 46078 | 1.89226 | 2.1 |
| 46079 | 3.78452 | 2.1 |
| 46080 | 2.12221 | 2.1 |
| 46081 | 1.47769 | 2.1 |
| 46082 | 2.33865 | 2.1 |
| 46083 | 1.62898 | 2.1 |
| 46084 | 3.81634 | 2.1 |
| 46085 | 2.67624 | 2.1 |
| 46086 | 2.37734 | 1.78011 |
| 46087 | 2.33865 | 2.1 |
| 46088 | 2.33865 | 2.1 |
| 46089 | 2.70125 | 2.1 |
| 46090 | 2.70125 | 2.1 |
| 46091 | 1.89226 | 2.1 |
| 46092 | 1.89226 | 2.1 |
| 46093 | 1.89226 | 2.1 |
| 46094 | 1.89226 | 2.1 |
| 46095 | 1.89226 | 1.03543 |
| 46096 | 1.89226 | 1.49584 |
| 46097 | 1.89226 | 2.1 |
| 46098 | 1.89226 | 2.1 |
| 46099 | 1.89226 | 2.1 |
| 46100 | 1.89226 | 2.1 |
| 46101 | 1.89226 | 2.1 |
| 46102 | 1.89226 | 2.1 |
| 46103 | 2.37733 | 2.1 |
| 46104 | 1.95927 | 2.1 |
| 46105 | 1.82516 | 1.19284 |
| 46106 | 1.82516 | 2.1 |
| 46107 | 2.33865 | 2.1 |

| | | |
|---|---|---|
| 46108 | 2.33865 | 2.1 |
| 46109 | 2.57466 | 2.1 |
| 46110 | 2.21715 | 2.1 |
| 46111 | 2.55653 | 2.1 |
| 46112 | 2.24775 | 2.1 |
| 46113 | 4.77367 | 2.1 |
| 46114 | 0.937095 | 2.1 |
| 46115 | 0.937095 | 2.1 |
| 46116 | 0.958028 | 2.1 |
| 46117 | 0.958028 | 2.1 |
| 46118 | 0.828386 | 2.1 |
| 46119 | 0.771244 | 2.1 |
| 46120 | 1.10858 | 2.1 |
| 46121 | 1.12388 | 2.1 |
| 46122 | 1.12388 | 2.1 |
| 46123 | 0.828386 | 2.1 |
| 46124 | 0.771244 | 2.1 |
| 46125 | 1.10858 | 2.1 |
| 46126 | 2.55653 | 2.1 |
| 46127 | 2.32606 | 2.1 |
| 46128 | 2.24775 | 2.1 |
| 46129 | 2.24775 | 2.1 |
| 46130 | 2.57928 | 2.1 |
| 46131 | 2.57928 | 2.1 |
| 46132 | 0.990481 | 2.1 |
| 46133 | 0.990481 | 2.1 |
| 46134 | 1.00094 | 2.1 |
| 46135 | 1.00094 | 2.1 |
| 46136 | 0.888385 | 2.1 |
| 46137 | 0.828386 | 2.1 |
| 46138 | 1.16303 | 2.1 |
| 46139 | 1.12388 | 2.1 |
| 46140 | 1.16303 | 2.1 |
| 46141 | 0.888385 | 2.1 |
| 46142 | 1.12388 | 2.1 |
| 46143 | 0.828387 | 2.1 |
| 46144 | 0.962524 | 2.1 |
| 46145 | 0.962524 | 2.1 |
| 46146 | 0.7976 | 2.1 |
| 46147 | 0.7976 | 2.1 |
| 46148 | 0.797628 | 2.1 |
| 46149 | 0.814 2.1 | |
| 46150 | 0.946125 | 2.1 |
| 46151 | 0.946125 | 2.1 |
| 46152 | 0.946118 | 2.1 |
| 46153 | 0.797623 | 2.1 |
| 46154 | 0.814006 | 2.1 |
| 46155 | 0.946131 | 2.1 |
| 46156 | 1.8978 | 0.05 |
| 46157 | 2.23892 | 0.05 |
| 46158 | 1.71469 | 0.05 |
| 46159 | 4.13672 | 0.05 |
| 46160 | 2.64136 | 0.05 |
| 46161 | 2.23892 | 0.05 |
| 46162 | 2.37578 | 0.423579 |
| 46163 | 1.77489 | 2.1 |
| 46164 | 4.6147 | 0.05 |
| 46165 | 1.02284 | 2.1 |

| | | |
|---|---|---|
| 46166 | 0.898796 | 0.545173 |
| 46167 | 0.948554 | 0.766804 |
| 46168 | 0.948554 | 2.1 |
| 46169 | 0.878797 | 2.1 |
| 46170 | 0.796197 | 2.1 |
| 46171 | 1.09145 | 0.609847 |
| 46172 | 0.963109 | 0.631398 |
| 46173 | 1.12114 | 2.1 |
| 46174 | 0.782739 | 2.1 |
| 46175 | 1.11084 | 2.1 |
| 46176 | 0.850742 | 2.1 |
| 46177 | 2.48024 | 2.1 |
| 46178 | 2.405 | 2.1 |
| 46179 | 2.24585 | 2.1 |
| 46180 | 2.34795 | 2.1 |
| 46181 | 4.75295 | 2.1 |
| 46182 | 1.93794 | 2.1 |
| 46183 | 2.405 | 2.1 |
| 46184 | 2.24585 | 2.1 |
| 46185 | 1.67594 | 2.1 |
| 46186 | 4.08094 | 2.1 |
| 46187 | 1.89226 | 2.1 |
| 46188 | 1.89226 | 0.05 |
| 46189 | 1.89226 | 2.1 |
| 46190 | 1.89226 | 0.973526 |
| 46191 | 3.78452 | 2.1 |
| 46192 | 1.89226 | 2.1 |
| 46193 | 1.89226 | 2.1 |
| 46194 | 1.89226 | 2.1 |
| 46195 | 1.89226 | 1.2437 |
| 46196 | 3.78452 | 2.1 |
| 46197 | 2.24585 | 2.1 |
| 46198 | 2.34795 | 2.1 |
| 46199 | 2.21155 | 2.1 |
| 46200 | 2.06626 | 2.1 |
| 46201 | 4.4142 | 2.1 |
| 46202 | 2.24585 | 2.1 |
| 46203 | 1.41498 | 2.1 |
| 46204 | 1.41498 | 2.1 |
| 46205 | 1.67594 | 2.1 |
| 46206 | 2.06625 | 2.1 |
| 46207 | 2.32721 | 2.1 |
| 46208 | 1.89226 | 2.1 |
| 46209 | 1.89226 | 0.902475 |
| 46210 | 1.89226 | 2.1 |
| 46211 | 1.89226 | 0.751596 |
| 46212 | 1.89226 | 2.1 |
| 46213 | 1.89226 | 0.365874 |
| 46214 | 1.89226 | 2.1 |
| 46215 | 1.89226 | 0.966944 |
| 46216 | 1.89226 | 2.1 |
| 46217 | 1.89226 | 2.1 |
| 46218 | 3.78452 | 2.1 |
| 46219 | 1.89226 | 0.05 |
| 46220 | 1.89226 | 0.05 |
| 46221 | 1.89226 | 0.05 |
| 46222 | 1.89226 | 0.05 |
| 46223 | 3.78452 | 0.05 |

| | | |
|---|---|---|
| 46224 | 1.89226 | 0.05 |
| 46225 | 2.53655 | 0.05 |
| 46226 | 1.89226 | 0.05 |
| 46227 | 1.89226 | 0.05 |
| 46228 | 1.89226 | 0.05 |
| 46229 | 1.89226 | 0.05 |
| 46230 | 1.89227 | 2.1 |
| 46231 | 1.89227 | 2.1 |
| 46232 | 1.89227 | 2.1 |
| 46233 | 1.89227 | 2.1 |
| 46234 | 1.89227 | 2.1 |
| 46235 | 1.89227 | 2.1 |
| 46236 | 0.91339 | 2.1 |
| 46237 | 0.91339 | 2.1 |
| 46238 | 1.2158 | 2.1 |
| 46239 | 1.20629 | 2.1 |
| 46240 | 1.00473 | 2.1 |
| 46241 | 1.15749 | 2.1 |
| 46242 | 0.946132 | 2.1 |
| 46243 | 0.946132 | 2.1 |
| 46244 | 0.946133 | 2.1 |
| 46245 | 1.17513 | 2.1 |
| 46246 | 1.00473 | 2.1 |
| 46247 | 0.946132 | 2.1 |
| 46248 | 1.89226 | 2.1 |
| 46249 | 1.89226 | 2.1 |
| 46250 | 1.89226 | 2.1 |
| 46251 | 1.89226 | 2.1 |
| 46252 | 1.89226 | 2.1 |
| 46253 | 1.89226 | 2.1 |
| 46254 | 1.89226 | 2.1 |
| 46255 | 2.6066 | 2.1 |
| 46256 | 2.35026 | 2.1 |
| 46257 | 2.35026 | 2.1 |
| 46258 | 1.89226 | 2.1 |
| 46259 | 1.89226 | 2.1 |
| 46260 | 2.04056 | 0.05 |
| 46261 | 2.77366 | 0.0702763 |
| 46262 | 1.24682 | 0.05 |
| 46263 | 1.24682 | 0.0538459 |
| 46264 | 2.93651 | 0.05 |
| 46265 | 2.14277 | 0.05 |
| 46266 | 2.68524 | 1.99527 |
| 46267 | 2.81847 | 1.93262 |
| 46268 | 1.89226 | 1.06836 |
| 46269 | 1.89226 | 1.96896 |
| 46270 | 4.57751 | 1.29529 |
| 46271 | 2.68524 | 1.45722 |
| 46272 | 1.89226 | 2.1 |
| 46273 | 1.89226 | 2.1 |
| 46274 | 2.50732 | 1.65205 |
| 46275 | 1.89226 | 1.92484 |
| 46276 | 2.50732 | 2.09412 |
| 46277 | 1.89226 | 1.18348 |
| 46278 | 1.89226 | 2.1 |
| 46279 | 1.89226 | 1.74924 |
| 46280 | 1.89226 | 2.1 |
| 46281 | 1.89226 | 1.26404 |

| | | |
|---|---|---|
| 46282 | 1.89226 | 2.0573 |
| 46283 | 1.89226 | 2.1 |
| 46284 | 1.89226 | 1.66988 |
| 46285 | 1.89226 | 1.69089 |
| 46286 | 1.89226 | 2.1 |
| 46287 | 1.89226 | 0.975257 |
| 46288 | 1.89226 | 1.31574 |
| 46289 | 1.61016 | 1.31894 |
| 46290 | 1.07467 | 1.91289 |
| 46291 | 1.24474 | 1.20459 |
| 46292 | 1.24474 | 2.1 |
| 46293 | 1.45303 | 1.34 |
| 46294 | 1.45303 | 2.1 |
| 46295 | 1.61016 | 1.9023 |
| 46296 | 1.24474 | 2.1 |
| 46297 | 1.61016 | 2.00459 |
| 46298 | 1.77186 | 1.45147 |
| 46299 | 1.37813 | 1.43434 |
| 46300 | 1.37813 | 2.07635 |
| 46301 | 1.73305 | 2.1 |
| 46302 | 2.0815 | 2.1 |
| 46303 | 1.97658 | 2.1 |
| 46304 | 1.75791 | 2.1 |
| 46305 | 1.87228 | 2.1 |
| 46306 | 1.66515 | 2.1 |
| 46307 | 2.0815 | 2.1 |
| 46308 | 1.93938 | 0.05 |
| 46309 | 2.03253 | 0.05 |
| 46310 | 2.2142 | 2.1 |
| 46311 | 1.97658 | 0.05 |
| 46312 | 2.15324 | 2.1 |
| 46313 | 1.73305 | 2.1 |
| 46314 | 1.97658 | 2.1 |
| 46315 | 1.60428 | 2.1 |
| 46316 | 1.8589 | 2.1 |
| 46317 | 3.58085 | 2.1 |
| 46318 | 1.97657 | 2.1 |
| 46319 | 1.93938 | 0.125061 |
| 46320 | 1.51316 | 2.1 |
| 46321 | 1.85293 | 0.070322 |
| 46322 | 3.82951 | 1.38669 |
| 46323 | 1.70303 | 0.05 |
| 46324 | 1.96087 | 0.0546356 |
| 46325 | 1.42308 | 0.05 |
| 46326 | 1.96087 | 0.106399 |
| 46327 | 1.25023 | 0.05 |
| 46328 | 1.70303 | 0.05 |
| 46329 | 0.946132 | 0.141086 |
| 46330 | 0.946132 | 0.0622139 |
| 46331 | 0.946131 | 0.05 |
| 46332 | 0.946131 | 0.0859353 |
| 46333 | 0.946132 | 0.129765 |
| 46334 | 0.946132 | 0.137969 |
| 46335 | 0.946132 | 0.05 |
| 46336 | 0.946132 | 0.05 |
| 46337 | 0.946132 | 0.107895 |
| 46338 | 0.946132 | 0.0565903 |
| 46339 | 0.946132 | 0.0696961 |

| | | |
|---|---|---|
| 46340 | 0.946132 | 0.106098 |
| 46341 | 1.89226 | 0.319811 |
| 46342 | 1.89226 | 0.386319 |
| 46343 | 1.89226 | 0.318451 |
| 46344 | 1.89226 | 0.37942 |
| 46345 | 3.78452 | 0.34454 |
| 46346 | 1.89226 | 0.0783104 |
| 46347 | 1.89226 | 0.255731 |
| 46348 | 1.89226 | 0.146169 |
| 46349 | 1.89226 | 0.350267 |
| 46350 | 1.89226 | 0.05 |
| 46351 | 1.89226 | 0.23265 |
| 46352 | 1.89226 | 0.174768 |
| 46353 | 1.89226 | 0.34743 |
| 46354 | 1.89226 | 0.364888 |
| 46355 | 1.89226 | 0.319091 |
| 46356 | 1.89226 | 0.214858 |
| 46357 | 1.89226 | 0.132527 |
| 46358 | 1.89226 | 0.05 |
| 46359 | 1.89226 | 0.135426 |
| 46360 | 2.48255 | 0.05 |
| 46361 | 1.89226 | 0.0783138 |
| 46362 | 3.78452 | 0.05 |
| 46363 | 1.89226 | 2.1 |
| 46364 | 2.42758 | 2.1 |
| 46365 | 1.9209 | 2.1 |
| 46366 | 1.89226 | 2.1 |
| 46367 | 2.45622 | 2.1 |
| 46368 | 2.42758 | 2.1 |
| 46369 | 2.42758 | 2.1 |
| 46370 | 1.49987 | 2.1 |
| 46371 | 1.61028 | 2.1 |
| 46372 | 1.58164 | 2.1 |
| 46373 | 2.45622 | 2.1 |
| 46374 | 2.42758 | 2.1 |
| 46375 | 0.94613 | 2.1 |
| 46376 | 0.94613 | 2.1 |
| 46377 | 1.24298 | 2.1 |
| 46378 | 1.24298 | 2.1 |
| 46379 | 1.24298 | 2.1 |
| 46380 | 1.22811 | 2.1 |
| 46381 | 0.946131 | 2.1 |
| 46382 | 0.946131 | 2.1 |
| 46383 | 0.94613 | 2.1 |
| 46384 | 1.24298 | 2.1 |
| 46385 | 0.94613 | 2.1 |
| 46386 | 1.22811 | 2.1 |
| 46387 | 2.48596 | 2.1 |
| 46388 | 2.45622 | 2.1 |
| 46389 | 1.63823 | 2.1 |
| 46390 | 1.4304 | 2.1 |
| 46391 | 1.45739 | 2.1 |
| 46392 | 2.45622 | 2.1 |
| 46393 | 2.4169 | 2.1 |
| 46394 | 2.55249 | 2.1 |
| 46395 | 2.51125 | 2.1 |
| 46396 | 2.35245 | 2.1 |
| 46397 | 4.90494 | 2.1 |

| | | |
|---|---|---|
| 46398 | 2.45922 | 2.1 |
| 46399 | 2.55249 | 2.1 |
| 46400 | 2.51126 | 2.1 |
| 46401 | 2.43226 | 2.1 |
| 46402 | 4.98474 | 2.1 |
| 46403 | 2.51125 | 2.1 |
| 46404 | 2.35245 | 2.1 |
| 46405 | 2.28979 | 2.1 |
| 46406 | 2.47228 | 2.1 |
| 46407 | 4.82473 | 2.1 |
| 46408 | 2.51126 | 2.1 |
| 46409 | 2.43226 | 2.1 |
| 46410 | 2.40668 | 2.1 |
| 46411 | 2.47228 | 2.1 |
| 46412 | 4.90453 | 2.1 |
| 46413 | 2.8045 | 2.1 |
| 46414 | 1.93707 | 2.1 |
| 46415 | 2.14087 | 2.1 |
| 46416 | 2.14087 | 2.1 |
| 46417 | 2.61595 | 2.1 |
| 46418 | 2.61595 | 2.1 |
| 46419 | 1.89226 | 2.1 |
| 46420 | 1.89226 | 2.1 |
| 46421 | 1.89226 | 2.1 |
| 46422 | 1.89226 | 1.2474 |
| 46423 | 1.89226 | 2.1 |
| 46424 | 1.89226 | 0.787851 |
| 46425 | 1.22368 | 2.1 |
| 46426 | 1.32339 | 2.1 |
| 46427 | 0.957125 | 2.1 |
| 46428 | 0.750074 | 2.1 |
| 46429 | 1.08438 | 2.1 |
| 46430 | 1.08438 | 2.1 |
| 46431 | 1.13094 | 2.1 |
| 46432 | 0.970797 | 2.1 |
| 46433 | 1.17568 | 2.1 |
| 46434 | 0.764392 | 2.1 |
| 46435 | 0.944286 | 2.1 |
| 46436 | 1.27523 | 2.1 |
| 46437 | 1.89226 | 2.1 |
| 46438 | 1.89226 | 2.1 |
| 46439 | 1.89226 | 2.1 |
| 46440 | 1.89226 | 1.00918 |
| 46441 | 3.78453 | 2.1 |
| 46442 | 2.8239 | 2.1 |
| 46443 | 1.89227 | 2.1 |
| 46444 | 2.57276 | 2.1 |
| 46445 | 2.57276 | 2.1 |
| 46446 | 1.89227 | 2.1 |
| 46447 | 1.89227 | 2.1 |
| 46448 | 1.18139 | 2.1 |
| 46449 | 1.85195 | 2.1 |
| 46450 | 1.78595 | 2.1 |
| 46451 | 1.08172 | 2.1 |
| 46452 | 2.93367 | 2.1 |
| 46453 | 1.60735 | 2.1 |
| 46454 | 1.20356 | 2.1 |
| 46455 | 0.967034 | 2.1 |

| | | |
|---|---|---|
| 46456 | 1.08172 | 2.1 |
| 46457 | 1.67324 | 2.1 |
| 46458 | 1.77293 | 2.1 |
| 46459 | 1.89226 | 2.1 |
| 46460 | 1.89226 | 2.1 |
| 46461 | 1.8213 | 2.1 |
| 46462 | 1.82129 | 2.1 |
| 46463 | 2.14171 | 2.1 |
| 46464 | 2.40192 | 2.1 |
| 46465 | 0.810943 | 2.1 |
| 46466 | 0.810943 | 2.1 |
| 46467 | 0.798469 | 2.1 |
| 46468 | 0.788336 | 2.1 |
| 46469 | 0.804015 | 2.1 |
| 46470 | 0.810945 | 2.1 |
| 46471 | 1.947 | 2.1 |
| 46472 | 1.36506 | 2.1 |
| 46473 | 1.08686 | 2.1 |
| 46474 | 1.07208 | 2.1 |
| 46475 | 1.08132 | 2.1 |
| 46476 | 1.13537 | 0.05 |
| 46477 | 1.14754 | 1.48793 |
| 46478 | 0.946131 | 0.05 |
| 46479 | 0.946131 | 2.1 |
| 46480 | 0.94613 | 0.05 |
| 46481 | 0.94613 | 0.05 |
| 46482 | 1.25104 | 2.1 |
| 46483 | 1.14754 | 0.560148 |
| 46484 | 1.13537 | 0.0671321 |
| 46485 | 0.946131 | 0.0645358 |
| 46486 | 0.946131 | 0.697336 |
| 46487 | 1.25104 | 0.145755 |
| 46488 | 0.96542 | 0.05 |
| 46489 | 0.922284 | 0.41225 |
| 46490 | 0.946131 | 2.1 |
| 46491 | 0.946131 | 0.0746834 |
| 46492 | 0.946131 | 0.0705197 |
| 46493 | 0.946131 | 0.05 |
| 46494 | 1.13537 | 2.1 |
| 46495 | 0.924824 | 0.452938 |
| 46496 | 0.859476 | 0.0546617 |
| 46497 | 0.842305 | 0.0781854 |
| 46498 | 1.25996 | 0.148374 |
| 46499 | 1.04996 | 0.11886 |
| 46500 | 1.71007 | 0.05 |
| 46501 | 2.73283 | 0.05 |
| 46502 | 2.73283 | 0.05 |
| 46503 | 1.89226 | 0.05 |
| 46504 | 1.89226 | 0.05 |
| 46505 | 1.13262 | 0.05 |
| 46506 | 0.946131 | 0.05 |
| 46507 | 0.946131 | 0.05 |
| 46508 | 0.946131 | 0.05 |
| 46509 | 0.946131 | 0.05 |
| 46510 | 0.946132 | 0.05 |
| 46511 | 0.946132 | 0.05 |
| 46512 | 0.946131 | 0.05 |
| 46513 | 0.946131 | 0.05 |

| | | |
|---|---|---|
| 46514 | 0.946131 | 0.05 |
| 46515 | 0.946131 | 0.05 |
| 46516 | 0.946132 | 0.05 |
| 46517 | 0.946132 | 0.05 |
| 46518 | 0.946131 | 0.05 |
| 46519 | 1.36642 | 0.05 |
| 46520 | 0.946125 | 0.05 |
| 46521 | 0.946125 | 0.05 |
| 46522 | 0.946128 | 0.05 |
| 46523 | 0.946128 | 0.05 |
| 46524 | 0.946126 | 0.05 |
| 46525 | 0.946128 | 0.05 |
| 46526 | 0.946128 | 0.05 |
| 46527 | 0.946128 | 0.05 |
| 46528 | 1.36641 | 0.05 |
| 46529 | 0.946128 | 0.05 |
| 46530 | 1.89226 | 0.05 |
| 46531 | 1.89226 | 0.05 |
| 46532 | 1.89226 | 0.05 |
| 46533 | 1.89226 | 0.05 |
| 46534 | 1.89226 | 0.05 |
| 46535 | 1.89226 | 0.05 |
| 46536 | 2.69784 | 2.1 |
| 46537 | 2.51651 | 2.1 |
| 46538 | 2.56999 | 2.1 |
| 46539 | 2.74623 | 2.1 |
| 46540 | 5.26783 | 2.1 |
| 46541 | 2.69784 | 2.1 |
| 46542 | 2.48718 | 2.1 |
| 46543 | 2.50161 | 2.1 |
| 46544 | 2.74623 | 2.1 |
| 46545 | 5.19945 | 2.1 |
| 46546 | 1.89226 | 2.1 |
| 46547 | 1.89226 | 2.1 |
| 46548 | 1.89226 | 2.1 |
| 46549 | 1.89226 | 2.1 |
| 46550 | 3.78453 | 2.1 |
| 46551 | 0.874264 | 2.1 |
| 46552 | 1.09879 | 2.1 |
| 46553 | 0.946131 | 2.1 |
| 46554 | 0.946131 | 2.1 |
| 46555 | 1.17062 | 2.1 |
| 46556 | 1.17062 | 2.1 |
| 46557 | 0.874293 | 2.1 |
| 46558 | 0.874293 | 2.1 |
| 46559 | 1.09879 | 2.1 |
| 46560 | 0.94613 | 2.1 |
| 46561 | 0.946131 | 2.1 |
| 46562 | 0.874265 | 2.1 |
| 46563 | 1.89226 | 2.1 |
| 46564 | 1.89226 | 2.1 |
| 46565 | 1.89226 | 2.1 |
| 46566 | 1.89226 | 2.1 |
| 46567 | 3.78453 | 2.1 |
| 46568 | 0.977382 | 2.1 |
| 46569 | 1.16424 | 2.1 |
| 46570 | 0.946133 | 2.1 |
| 46571 | 0.946133 | 2.1 |

| | | |
|---|---|---|
| 46572 | 1.33012 | 2.1 |
| 46573 | 1.25589 | 2.1 |
| 46574 | 0.888498 | 2.1 |
| 46575 | 0.888498 | 2.1 |
| 46576 | 1.25375 | 2.1 |
| 46577 | 0.946131 | 2.1 |
| 46578 | 0.946131 | 2.1 |
| 46579 | 0.977384 | 2.1 |
| 46580 | 1.89226 | 2.1 |
| 46581 | 2.19757 | 2.1 |
| 46582 | 2.62906 | 2.1 |
| 46583 | 1.89226 | 2.1 |
| 46584 | 1.89226 | 2.1 |
| 46585 | 2.62906 | 2.1 |
| 46586 | 1.27944 | 1.2563 |
| 46587 | 1.27731 | 2.1 |
| 46588 | 0.615701 | 2.1 |
| 46589 | 0.615701 | 1.374 |
| 46590 | 0.615673 | 0.05 |
| 46591 | 0.946133 | 2.04165 |
| 46592 | 0.948264 | 0.512997 |
| 46593 | 0.946132 | 1.2351 |
| 46594 | 0.94752 | 0.710381 |
| 46595 | 0.61706 | 0.05 |
| 46596 | 0.946132 | 0.91262 |
| 46597 | 0.946132 | 0.569252 |
| 46598 | 2.76604 | 2.1 |
| 46599 | 1.66728 | 2.1 |
| 46600 | 1.66728 | 2.1 |
| 46601 | 1.83036 | 2.1 |
| 46602 | 2.54515 | 2.1 |
| 46603 | 2.70823 | 2.1 |
| 46604 | 2.54515 | 2.1 |
| 46605 | 1.49958 | 2.1 |
| 46606 | 1.49958 | 2.1 |
| 46607 | 1.66728 | 2.1 |
| 46608 | 2.34465 | 2.1 |
| 46609 | 2.51235 | 2.1 |
| 46610 | 1.21128 | 2.1 |
| 46611 | 1.21128 | 2.1 |
| 46612 | 0.946131 | 2.1 |
| 46613 | 0.946131 | 2.1 |
| 46614 | 0.946131 | 2.1 |
| 46615 | 0.946131 | 2.1 |
| 46616 | 1.31401 | 2.1 |
| 46617 | 1.21128 | 2.1 |
| 46618 | 1.21128 | 2.1 |
| 46619 | 0.946131 | 2.1 |
| 46620 | 0.946131 | 2.1 |
| 46621 | 1.31401 | 2.1 |
| 46622 | 1.67374 | 2.1 |
| 46623 | 2.29586 | 2.1 |
| 46624 | 1.50033 | 2.1 |
| 46625 | 2.16598 | 2.1 |
| 46626 | 3.83972 | 2.1 |
| 46627 | 1.89226 | 2.1 |
| 46628 | 1.89226 | 2.1 |
| 46629 | 1.89226 | 2.1 |

| | | |
|---|---|---|
| 46630 | 1.89226 | 2.1 |
| 46631 | 3.78452 | 2.1 |
| 46632 | 1.13537 | 2.1 |
| 46633 | 1.02188 | 2.1 |
| 46634 | 0.946132 | 2.1 |
| 46635 | 0.946132 | 2.1 |
| 46636 | 0.946132 | 2.1 |
| 46637 | 0.946132 | 2.1 |
| 46638 | 1.21128 | 2.1 |
| 46639 | 1.02188 | 2.1 |
| 46640 | 1.04672 | 2.1 |
| 46641 | 1.04672 | 2.1 |
| 46642 | 1.21128 | 2.1 |
| 46643 | 0.946132 | 2.1 |
| 46644 | 0.815411 | 2.1 |
| 46645 | 0.870464 | 2.1 |
| 46646 | 0.885437 | 2.1 |
| 46647 | 0.885437 | 2.1 |
| 46648 | 1.06395 | 2.1 |
| 46649 | 1.00483 | 2.1 |
| 46650 | 0.757125 | 2.1 |
| 46651 | 0.673956 | 2.1 |
| 46652 | 0.684461 | 2.1 |
| 46653 | 1.06979 | 2.1 |
| 46654 | 0.731403 | 2.1 |
| 46655 | 1.0559 | 2.1 |
| 46656 | 1.89226 | 2.1 |
| 46657 | 1.89226 | 2.1 |
| 46658 | 1.89226 | 2.1 |
| 46659 | 1.89226 | 2.1 |
| 46660 | 1.89226 | 2.1 |
| 46661 | 1.89226 | 2.1 |
| 46662 | 1.76897 | 1.01596 |
| 46663 | 1.76932 | 1.11854 |
| 46664 | 1.97455 | 2.1 |
| 46665 | 1.94302 | 0.0628451 |
| 46666 | 3.71233 | 0.297402 |
| 46667 | 1.83347 | 2.1 |
| 46668 | 1.76932 | 1.36666 |
| 46669 | 1.97455 | 2.1 |
| 46670 | 1.76932 | 2.1 |
| 46671 | 1.76522 | 2.1 |
| 46672 | 1.55999 | 2.1 |
| 46673 | 1.89227 | 1.34139 |
| 46674 | 1.89227 | 2.09407 |
| 46675 | 1.89227 | 2.1 |
| 46676 | 1.89227 | 0.463727 |
| 46677 | 3.78453 | 1.12499 |
| 46678 | 1.89226 | 1.08562 |
| 46679 | 1.89226 | 0.05 |
| 46680 | 1.89226 | 0.05 |
| 46681 | 1.89226 | 0.352863 |
| 46682 | 3.78452 | 0.284107 |
| 46683 | 1.97452 | 2.1 |
| 46684 | 1.94299 | 0.05 |
| 46685 | 2.17831 | 0.169528 |
| 46686 | 2.23195 | 0.174952 |
| 46687 | 4.17495 | 0.344401 |

| | | |
|---|---|---|
| 46688 | 2.23196 | 0.36737 |
| 46689 | 2.1499 | 2.1 |
| 46690 | 1.97452 | 2.1 |
| 46691 | 1.97452 | 2.1 |
| 46692 | 2.09088 | 2.1 |
| 46693 | 1.83345 | 2.1 |
| 46694 | 0.94612 | 1.93299 |
| 46695 | 0.94612 | 2.1 |
| 46696 | 0.946119 | 0.0973642 |
| 46697 | 0.946119 | 0.170211 |
| 46698 | 0.946119 | 0.195444 |
| 46699 | 0.946119 | 1.80237 |
| 46700 | 0.94612 | 0.248046 |
| 46701 | 0.94612 | 2.1 |
| 46702 | 0.946132 | 0.520869 |
| 46703 | 0.946132 | 0.264055 |
| 46704 | 0.946107 | 1.06859 |
| 46705 | 0.946107 | 0.917064 |
| 46706 | 1.89224 | 0.05 |
| 46707 | 1.89224 | 0.168015 |
| 46708 | 1.89224 | 0.0909759 |
| 46709 | 1.89224 | 0.05 |
| 46710 | 3.78447 | 0.05 |
| 46711 | 1.70302 | 0.106739 |
| 46712 | 1.056 | 0.118726 |
| 46713 | 1.21829 | 0.316502 |
| 46714 | 1.89906 | 0.103322 |
| 46715 | 3.07892 | 0.0914744 |
| 46716 | 2.20248 | 0.0839624 |
| 46717 | 1.56498 | 0.125126 |
| 46718 | 1.46539 | 0.0923677 |
| 46719 | 1.48787 | 0.0526716 |
| 46720 | 2.37625 | 0.391427 |
| 46721 | 2.37625 | 0.149283 |
| 46722 | 1.14104 | 0.0560238 |
| 46723 | 1.00675 | 0.0708876 |
| 46724 | 0.700689 | 0.0524631 |
| 46725 | 0.624389 | 0.0617145 |
| 46726 | 0.782149 | 0.05 |
| 46727 | 0.940197 | 0.05 |
| 46728 | 0.803437 | 0.05 |
| 46729 | 0.803437 | 0.0655882 |
| 46730 | 0.995423 | 0.05 |
| 46731 | 0.700699 | 0.05 |
| 46732 | 0.593895 | 0.162519 |
| 46733 | 1.00673 | 0.0586436 |
| 46734 | 1.18097 | 0.0739956 |
| 46735 | 1.15839 | 0.0541816 |
| 46736 | 0.995407 | 0.0576194 |
| 46737 | 0.865932 | 0.05 |
| 46738 | 1.17299 | 0.05 |
| 46739 | 0.957054 | 0.05 |
| 46740 | 1.08016 | 0.0797536 |
| 46741 | 1.08016 | 0.133419 |
| 46742 | 0.995425 | 0.103298 |
| 46743 | 1.24161 | 0.05 |
| 46744 | 0.796771 | 0.05 |
| 46745 | 1.15837 | 0.0933287 |

| | | |
|---|---|---|
| 46746 | 2.57264 | 0.05 |
| 46747 | 1.89226 | 0.05 |
| 46748 | 2.41701 | 0.05 |
| 46749 | 1.89226 | 0.05 |
| 46750 | 4.30928 | 0.05 |
| 46751 | 1.69055 | 0.05 |
| 46752 | 2.39587 | 0.05 |
| 46753 | 1.53463 | 0.05 |
| 46754 | 2.2053 | 0.05 |
| 46755 | 3.89585 | 0.05 |
| 46756 | 0.946129 | 0.05 |
| 46757 | 0.946129 | 0.05 |
| 46758 | 0.94613 | 0.05 |
| 46759 | 0.94613 | 0.05 |
| 46760 | 0.946132 | 0.05 |
| 46761 | 0.946132 | 0.05 |
| 46762 | 0.946126 | 0.05 |
| 46763 | 0.946126 | 0.05 |
| 46764 | 0.94613 | 0.05 |
| 46765 | 0.94613 | 0.05 |
| 46766 | 0.946129 | 0.05 |
| 46767 | 0.946129 | 0.05 |
| 46768 | 2.41701 | 0.05 |
| 46769 | 1.89226 | 0.05 |
| 46770 | 1.89226 | 0.05 |
| 46771 | 1.89226 | 0.05 |
| 46772 | 2.32462 | 0.05 |
| 46773 | 2.32462 | 0.05 |
| 46774 | 1.41942 | 0.05 |
| 46775 | 2.2053 | 0.05 |
| 46776 | 2.02097 | 0.05 |
| 46777 | 2.02097 | 0.05 |
| 46778 | 1.53463 | 0.05 |
| 46779 | 1.53463 | 0.05 |
| 46780 | 1.89226 | 0.05 |
| 46781 | 1.89226 | 0.05 |
| 46782 | 1.89226 | 0.05 |
| 46783 | 1.89226 | 0.05 |
| 46784 | 1.89226 | 0.05 |
| 46785 | 1.89226 | 0.05 |
| 46786 | 1.89226 | 0.765752 |
| 46787 | 1.89226 | 2.1 |
| 46788 | 1.89226 | 2.1 |
| 46789 | 1.89226 | 1.41515 |
| 46790 | 1.89226 | 2.01475 |
| 46791 | 1.89226 | 1.01481 |
| 46792 | 1.02816 | 0.536015 |
| 46793 | 1.02816 | 1.32844 |
| 46794 | 0.595141 | 1.59591 |
| 46795 | 0.595141 | 1.86185 |
| 46796 | 0.946132 | 0.848735 |
| 46797 | 0.595113 | 1.98888 |
| 46798 | 0.677174 | 1.86876 |
| 46799 | 0.946132 | 0.411071 |
| 46800 | 0.946132 | 1.5716 |
| 46801 | 0.946132 | 1.57293 |
| 46802 | 0.595113 | 1.54928 |
| 46803 | 0.677174 | 1.62769 |

| | | |
|---|---|---|
| 46804 | 1.89226 | 1.30954 |
| 46805 | 1.89226 | 2.1 |
| 46806 | 1.89226 | 2.09996 |
| 46807 | 1.89226 | 1.41424 |
| 46808 | 1.89226 | 2.1 |
| 46809 | 1.89226 | 1.56792 |
| 46810 | 0.946133 | 0.590713 |
| 46811 | 0.946133 | 0.193931 |
| 46812 | 0.946132 | 2.08025 |
| 46813 | 0.946132 | 2.09212 |
| 46814 | 0.946132 | 2.06698 |
| 46815 | 0.946132 | 0.861268 |
| 46816 | 0.946132 | 1.62557 |
| 46817 | 0.946132 | 0.247922 |
| 46818 | 0.946132 | 1.48416 |
| 46819 | 0.946132 | 1.66026 |
| 46820 | 0.946133 | 1.08843 |
| 46821 | 0.946133 | 1.16173 |
| 46822 | 0.946131 | 1.56464 |
| 46823 | 0.946131 | 1.51026 |
| 46824 | 0.946131 | 2.1 |
| 46825 | 0.946131 | 2.1 |
| 46826 | 0.946128 | 2.1 |
| 46827 | 0.946128 | 1.56814 |
| 46828 | 0.946133 | 2.1 |
| 46829 | 0.946133 | 1.44832 |
| 46830 | 0.94613 | 2.07828 |
| 46831 | 0.94613 | 2.1 |
| 46832 | 0.946131 | 2.1 |
| 46833 | 0.946131 | 1.87657 |
| 46834 | 2.0264 | 2.02257 |
| 46835 | 1.67345 | 1.12866 |
| 46836 | 2.0264 | 1.06396 |
| 46837 | 1.70539 | 0.05 |
| 46838 | 1.31611 | 0.808738 |
| 46839 | 1.31611 | 0.825326 |
| 46840 | 1.89226 | 0.0857541 |
| 46841 | 1.89226 | 0.0843421 |
| 46842 | 1.89226 | 0.208347 |
| 46843 | 1.89226 | 0.0745587 |
| 46844 | 3.78452 | 0.071709 |
| 46845 | 1.89226 | 0.217763 |
| 46846 | 1.89226 | 0.130825 |
| 46847 | 1.89226 | 0.125039 |
| 46848 | 1.89226 | 0.287868 |
| 46849 | 1.89226 | 0.151048 |
| 46850 | 1.89226 | 0.391052 |
| 46851 | 1.02645 | 0.05 |
| 46852 | 0.810942 | 0.05 |
| 46853 | 0.882781 | 0.05 |
| 46854 | 0.882781 | 0.05 |
| 46855 | 2.80386 | 0.05 |
| 46856 | 1.56719 | 0.05 |
| 46857 | 1.22499 | 0.05 |
| 46858 | 1.08132 | 0.05 |
| 46859 | 1.36865 | 0.05 |
| 46860 | 0.810957 | 0.05 |
| 46861 | 0.810957 | 0.05 |

| | | |
|---|---|---|
| 46862 | 0.946132 | 0.05 |
| 46863 | 0.946132 | 0.0635809 |
| 46864 | 0.946129 | 0.05 |
| 46865 | 0.946129 | 0.05 |
| 46866 | 0.946131 | 0.05 |
| 46867 | 0.946131 | 0.05 |
| 46868 | 0.946131 | 0.0583874 |
| 46869 | 0.946131 | 0.05 |
| 46870 | 0.94613 | 0.05 |
| 46871 | 0.94613 | 0.05 |
| 46872 | 0.946131 | 0.0698952 |
| 46873 | 0.946131 | 0.05 |
| 46874 | 1.89226 | 0.0635433 |
| 46875 | 1.89226 | 0.05 |
| 46876 | 1.89226 | 0.0677424 |
| 46877 | 1.89226 | 0.05 |
| 46878 | 3.78453 | 0.05 |
| 46879 | 1.30462 | 0.05 |
| 46880 | 1.79906 | 0.152313 |
| 46881 | 1.01299 | 0.120198 |
| 46882 | 1.44185 | 0.0821977 |
| 46883 | 1.3499 | 0.0656145 |
| 46884 | 1.79906 | 0.0869411 |
| 46885 | 1.89226 | 0.249126 |
| 46886 | 1.89226 | 0.386099 |
| 46887 | 1.89226 | 0.0708384 |
| 46888 | 1.89226 | 0.297758 |
| 46889 | 1.89226 | 0.355153 |
| 46890 | 1.89226 | 0.506188 |
| 46891 | 1.89226 | 0.222596 |
| 46892 | 1.89226 | 0.256072 |
| 46893 | 1.89226 | 0.357587 |
| 46894 | 1.89226 | 0.334653 |
| 46895 | 1.89226 | 0.194964 |
| 46896 | 1.89226 | 0.185388 |
| 46897 | 1.3499 | 0.05 |
| 46898 | 1.79905 | 0.0757863 |
| 46899 | 1.29001 | 0.0599716 |
| 46900 | 1.36005 | 0.05 |
| 46901 | 1.88294 | 0.05 |
| 46902 | 1.79905 | 0.0648971 |
| 46903 | 1.89226 | 0.113381 |
| 46904 | 2.75374 | 0.05 |
| 46905 | 2.60614 | 0.05 |
| 46906 | 1.89226 | 0.0674026 |
| 46907 | 2.60614 | 0.05 |
| 46908 | 1.89226 | 0.05 |
| 46909 | 0.946131 | 0.05 |
| 46910 | 0.946131 | 0.0525453 |
| 46911 | 1.37687 | 0.05 |
| 46912 | 1.37687 | 0.05 |
| 46913 | 0.946131 | 0.0619952 |
| 46914 | 1.34375 | 0.0506969 |
| 46915 | 1.37686 | 0.05 |
| 46916 | 0.946128 | 0.05 |
| 46917 | 1.37687 | 0.05 |
| 46918 | 1.34375 | 0.05 |
| 46919 | 0.94613 | 0.0612039 |

| | | |
|---|---|---|
| 46920 | 0.94613 | 0.05 |
| 46921 | 1.44247 | 2.1 |
| 46922 | 1.12665 | 2.1 |
| 46923 | 1.50044 | 2.1 |
| 46924 | 1.73651 | 2.1 |
| 46925 | 2.94291 | 2.1 |
| 46926 | 1.73652 | 2.1 |
| 46927 | 1.64926 | 2.1 |
| 46928 | 1.44247 | 2.1 |
| 46929 | 1.44247 | 2.1 |
| 46930 | 1.64926 | 2.1 |
| 46931 | 1.43872 | 2.1 |
| 46932 | 1.50044 | 2.1 |
| 46933 | 1.73651 | 2.1 |
| 46934 | 2.00955 | 2.1 |
| 46935 | 1.87875 | 2.1 |
| 46936 | 3.50999 | 2.1 |
| 46937 | 1.64926 | 2.1 |
| 46938 | 1.73652 | 2.1 |
| 46939 | 2.00955 | 2.1 |
| 46940 | 1.8493 | 2.1 |
| 46941 | 3.65881 | 2.1 |
| 46942 | 1.89226 | 0.05 |
| 46943 | 2.28188 | 0.05 |
| 46944 | 1.99182 | 0.05 |
| 46945 | 2.28188 | 0.05 |
| 46946 | 1.6022 | 0.05 |
| 46947 | 1.89226 | 0.05 |
| 46948 | 1.89226 | 0.05 |
| 46949 | 1.89226 | 0.05 |
| 46950 | 1.89226 | 0.05 |
| 46951 | 1.89226 | 0.05 |
| 46952 | 1.89226 | 0.05 |
| 46953 | 1.89226 | 0.05 |
| 46954 | 1.89226 | 0.05 |
| 46955 | 1.89226 | 0.05 |
| 46956 | 2.19578 | 0.05 |
| 46957 | 1.89226 | 0.05 |
| 46958 | 2.81422 | 0.05 |
| 46959 | 2.42521 | 0.05 |
| 46960 | 1.89224 | 0.05 |
| 46961 | 1.99179 | 0.05 |
| 46962 | 1.454 | 0.05 |
| 46963 | 1.99179 | 0.05 |
| 46964 | 1.35444 | 0.05 |
| 46965 | 1.89224 | 0.05 |
| 46966 | 1.89224 | 0.05 |
| 46967 | 1.89224 | 0.05 |
| 46968 | 1.89224 | 0.05 |
| 46969 | 1.89224 | 0.05 |
| 46970 | 1.89224 | 0.05 |
| 46971 | 1.89224 | 0.05 |
| 46972 | 1.703 | 0.05 |
| 46973 | 2.19575 | 0.05 |
| 46974 | 1.9911 | 0.05 |
| 46975 | 2.19575 | 0.05 |
| 46976 | 1.67811 | 0.05 |
| 46977 | 1.89223 | 0.05 |

| | | |
|---|---|---|
| 46978 | 1.69586 | 2.1 |
| 46979 | 2.55301 | 2.1 |
| 46980 | 1.60861 | 2.1 |
| 46981 | 1.60861 | 2.1 |
| 46982 | 2.65233 | 2.1 |
| 46983 | 2.65233 | 2.1 |
| 46984 | 0.946129 | 2.1 |
| 46985 | 0.946129 | 2.1 |
| 46986 | 1.2765 | 2.1 |
| 46987 | 1.32617 | 2.1 |
| 46988 | 1.2765 | 2.1 |
| 46989 | 1.2765 | 2.1 |
| 46990 | 0.946131 | 2.1 |
| 46991 | 0.946131 | 2.1 |
| 46992 | 0.946131 | 2.1 |
| 46993 | 1.32616 | 2.1 |
| 46994 | 0.946131 | 2.1 |
| 46995 | 1.2765 | 2.1 |
| 46996 | 1.36757 | 2.1 |
| 46997 | 1.32617 | 2.1 |
| 46998 | 0.891503 | 2.1 |
| 46999 | 0.847932 | 2.1 |
| 47000 | 1.10722 | 2.1 |
| 47001 | 1.10722 | 2.1 |
| 47002 | 1.10829 | 2.1 |
| 47003 | 1.10829 | 2.1 |
| 47004 | 1.36757 | 2.1 |
| 47005 | 0.891504 | 2.1 |
| 47006 | 0.847932 | 2.1 |
| 47007 | 1.32617 | 2.1 |
| 47008 | 0.946129 | 2.1 |
| 47009 | 0.946129 | 2.1 |
| 47010 | 1.36757 | 2.1 |
| 47011 | 1.32617 | 2.1 |
| 47012 | 1.32616 | 2.1 |
| 47013 | 1.32616 | 2.1 |
| 47014 | 0.946131 | 2.1 |
| 47015 | 0.946131 | 2.1 |
| 47016 | 1.36757 | 2.1 |
| 47017 | 0.946131 | 2.1 |
| 47018 | 0.946131 | 2.1 |
| 47019 | 1.32616 | 2.1 |
| 47020 | 1.89226 | 0.05 |
| 47021 | 1.89226 | 0.05 |
| 47022 | 1.89226 | 0.05 |
| 47023 | 1.89226 | 0.05 |
| 47024 | 3.78452 | 0.05 |
| 47025 | 2.36336 | 0.05 |
| 47026 | 1.89226 | 0.05 |
| 47027 | 2.67831 | 0.05 |
| 47028 | 2.67831 | 0.05 |
| 47029 | 1.89226 | 0.05 |
| 47030 | 1.89226 | 0.05 |
| 47031 | 1.89227 | 0.05 |
| 47032 | 1.89227 | 0.05 |
| 47033 | 1.89227 | 0.05 |
| 47034 | 1.89227 | 0.05 |
| 47035 | 3.78453 | 0.05 |

| | | |
|---|---|---|
| 47036 | 1.89226 | 0.05 |
| 47037 | 1.89226 | 0.05 |
| 47038 | 1.89226 | 0.05 |
| 47039 | 1.89226 | 0.05 |
| 47040 | 3.78452 | 0.05 |
| 47041 | 2.36336 | 0.05 |
| 47042 | 2.0815 | 0.05 |
| 47043 | 2.0815 | 0.05 |
| 47044 | 2.41048 | 0.05 |
| 47045 | 1.66267 | 0.05 |
| 47046 | 2.13452 | 0.05 |
| 47047 | 1.89226 | 0.05 |
| 47048 | 1.89226 | 0.05 |
| 47049 | 1.89226 | 0.05 |
| 47050 | 1.89226 | 0.05 |
| 47051 | 1.89226 | 0.05 |
| 47052 | 1.89226 | 0.05 |
| 47053 | 1.89226 | 0.0875549 |
| 47054 | 1.89226 | 0.178528 |
| 47055 | 1.89226 | 0.05 |
| 47056 | 1.89226 | 0.204128 |
| 47057 | 1.89226 | 0.05 |
| 47058 | 1.89226 | 0.283101 |
| 47059 | 0.946128 | 0.583231 |
| 47060 | 0.946128 | 0.562211 |
| 47061 | 1.00122 | 0.220373 |
| 47062 | 1.00122 | 0.181193 |
| 47063 | 0.94613 | 0.59844 |
| 47064 | 1.01618 | 0.467206 |
| 47065 | 0.946131 | 0.37871 |
| 47066 | 1.00122 | 0.231654 |
| 47067 | 1.00122 | 0.142778 |
| 47068 | 1.01618 | 0.244988 |
| 47069 | 0.946131 | 0.581215 |
| 47070 | 0.946131 | 0.502568 |
| 47071 | 1.89226 | 1.99951 |
| 47072 | 1.89226 | 0.888222 |
| 47073 | 1.89226 | 0.838744 |
| 47074 | 1.89226 | 2.1 |
| 47075 | 3.78452 | 0.992705 |
| 47076 | 1.89226 | 1.08998 |
| 47077 | 1.89226 | 2.1 |
| 47078 | 1.89226 | 2.1 |
| 47079 | 1.89226 | 1.37975 |
| 47080 | 1.89226 | 2.1 |
| 47081 | 1.89226 | 1.29793 |
| 47082 | 1.89226 | 0.559152 |
| 47083 | 1.89226 | 2.1 |
| 47084 | 1.89226 | 1.99184 |
| 47085 | 1.89226 | 0.857048 |
| 47086 | 1.89226 | 1.93587 |
| 47087 | 1.89226 | 0.876137 |
| 47088 | 1.89226 | 0.865461 |
| 47089 | 1.89226 | 2.1 |
| 47090 | 1.89226 | 2.1 |
| 47091 | 1.89226 | 1.05132 |
| 47092 | 1.89226 | 2.1 |
| 47093 | 1.89226 | 1.13757 |

| | | |
|---|---|---|
| 47094 | 2.71279 | 0.945748 |
| 47095 | 1.4155 | 0.840185 |
| 47096 | 2.28488 | 0.765772 |
| 47097 | 1.06122 | 0.890642 |
| 47098 | 3.70038 | 0.767071 |
| 47099 | 1.89226 | 1.03679 |
| 47100 | 1.89226 | 0.92109 |
| 47101 | 1.89226 | 0.86897 |
| 47102 | 1.89226 | 0.855287 |
| 47103 | 3.78452 | 1.61501 |
| 47104 | 0.851542 | 0.794559 |
| 47105 | 0.851542 | 0.754543 |
| 47106 | 0.851484 | 0.802177 |
| 47107 | 0.865567 | 0.948036 |
| 47108 | 0.981616 | 0.79278 |
| 47109 | 0.864767 | 0.848887 |
| 47110 | 0.851542 | 0.864584 |
| 47111 | 0.851542 | 0.785266 |
| 47112 | 0.851477 | 0.825753 |
| 47113 | 0.851477 | 0.891293 |
| 47114 | 0.865635 | 0.814093 |
| 47115 | 0.816472 | 0.76202 |
| 47116 | 1.68443 | 2.1 |
| 47117 | 1.44489 | 2.1 |
| 47118 | 1.54924 | 2.1 |
| 47119 | 1.34389 | 2.1 |
| 47120 | 3.02832 | 2.1 |
| 47121 | 1.44489 | 2.1 |
| 47122 | 1.03899 | 2.1 |
| 47123 | 1.03899 | 2.1 |
| 47124 | 1.12075 | 2.1 |
| 47125 | 1.34389 | 2.1 |
| 47126 | 1.42566 | 2.1 |
| 47127 | 1.54924 | 2.1 |
| 47128 | 1.34389 | 2.1 |
| 47129 | 1.44091 | 2.1 |
| 47130 | 1.25866 | 2.1 |
| 47131 | 2.7848 | 2.1 |
| 47132 | 0.573141 | 2.1 |
| 47133 | 0.573141 | 2.1 |
| 47134 | 0.575681 | 2.1 |
| 47135 | 0.575681 | 2.1 |
| 47136 | 0.481985 | 2.1 |
| 47137 | 0.519492 | 2.1 |
| 47138 | 0.671948 | 2.1 |
| 47139 | 0.629331 | 2.1 |
| 47140 | 0.629329 | 2.1 |
| 47141 | 0.481986 | 2.1 |
| 47142 | 0.519494 | 2.1 |
| 47143 | 0.671947 | 2.1 |
| 47144 | 1.89226 | 0.706297 |
| 47145 | 1.89226 | 1.14898 |
| 47146 | 1.89226 | 0.874004 |
| 47147 | 2.31925 | 0.87495 |
| 47148 | 3.78452 | 1.06034 |
| 47149 | 1.30916 | 2.1 |
| 47150 | 1.17426 | 2.1 |
| 47151 | 0.946129 | 2.1 |

| | | |
|---|---|---|
| 47152 | 0.946129 | 2.1 |
| 47153 | 0.94613 | 2.1 |
| 47154 | 0.94613 | 2.1 |
| 47155 | 1.17426 | 2.1 |
| 47156 | 1.17426 | 2.1 |
| 47157 | 1.30916 | 2.1 |
| 47158 | 0.94613 | 2.1 |
| 47159 | 1.17426 | 2.1 |
| 47160 | 0.94613 | 2.1 |
| 47161 | 2.56346 | 2.1 |
| 47162 | 1.89226 | 0.828434 |
| 47163 | 1.89226 | 2.1 |
| 47164 | 1.89226 | 1.41748 |
| 47165 | 2.6074 | 2.1 |
| 47166 | 2.6074 | 1.4165 |
| 47167 | 1.89226 | 2.1 |
| 47168 | 1.89226 | 2.1 |
| 47169 | 1.89226 | 2.1 |
| 47170 | 1.89226 | 2.1 |
| 47171 | 3.78452 | 2.1 |
| 47172 | 1.33064 | 2.1 |
| 47173 | 1.84121 | 2.1 |
| 47174 | 0.946129 | 2.1 |
| 47175 | 0.946129 | 2.1 |
| 47176 | 0.946131 | 2.1 |
| 47177 | 0.946131 | 2.1 |
| 47178 | 1.30916 | 2.1 |
| 47179 | 1.8412 | 2.1 |
| 47180 | 1.33064 | 2.1 |
| 47181 | 0.94613 | 2.1 |
| 47182 | 1.30916 | 2.1 |
| 47183 | 0.946131 | 2.1 |
| 47184 | 1.19283 | 0.923869 |
| 47185 | 1.19283 | 0.768291 |
| 47186 | 0.946131 | 2.1 |
| 47187 | 0.946131 | 2.1 |
| 47188 | 0.946132 | 2.1 |
| 47189 | 0.946132 | 0.822264 |
| 47190 | 1.3037 | 2.1 |
| 47191 | 1.19283 | 0.790877 |
| 47192 | 1.19283 | 2.1 |
| 47193 | 0.946131 | 2.1 |
| 47194 | 0.946131 | 2.1 |
| 47195 | 1.3037 | 2.1 |
| 47196 | 1.89226 | 2.1 |
| 47197 | 1.89226 | 2.1 |
| 47198 | 1.89226 | 2.1 |
| 47199 | 1.89226 | 2.1 |
| 47200 | 1.89226 | 2.1 |
| 47201 | 1.89226 | 2.1 |
| 47202 | 2.17708 | 2.0413 |
| 47203 | 1.70302 | 2.1 |
| 47204 | 2.28188 | 1.98778 |
| 47205 | 2.13621 | 2.1 |
| 47206 | 4.28253 | 2.1 |
| 47207 | 1.89226 | 1.06108 |
| 47208 | 1.89226 | 2.1 |
| 47209 | 1.89226 | 2.05847 |

| | | |
|---|---|---|
| 47210 | 1.89226 | 2.1 |
| 47211 | 3.78452 | 2.1 |
| 47212 | 0.946132 | 2.1 |
| 47213 | 0.946132 | 2.1 |
| 47214 | 0.946132 | 2.1 |
| 47215 | 0.946132 | 2.1 |
| 47216 | 0.946131 | 2.1 |
| 47217 | 0.946131 | 2.1 |
| 47218 | 0.946132 | 2.1 |
| 47219 | 0.946132 | 2.1 |
| 47220 | 0.946132 | 2.1 |
| 47221 | 0.946132 | 2.1 |
| 47222 | 0.946132 | 2.1 |
| 47223 | 0.946132 | 2.1 |
| 47224 | 1.74933 | 2.1 |
| 47225 | 1.11833 | 2.1 |
| 47226 | 1.90074 | 2.1 |
| 47227 | 1.669 | 2.1 |
| 47228 | 1.335 | 2.1 |
| 47229 | 1.11833 | 2.1 |
| 47230 | 2.28188 | 1.18453 |
| 47231 | 2.31804 | 2.1 |
| 47232 | 2.42758 | 2.0034 |
| 47233 | 2.14895 | 2.1 |
| 47234 | 4.43083 | 2.1 |
| 47235 | 1.89226 | 1.20886 |
| 47236 | 1.89226 | 2.1 |
| 47237 | 1.89226 | 1.16439 |
| 47238 | 1.89226 | 2.1 |
| 47239 | 3.78452 | 2.1 |
| 47240 | 1.89226 | 2.1 |
| 47241 | 1.89226 | 2.1 |
| 47242 | 1.89226 | 2.1 |
| 47243 | 1.89226 | 2.1 |
| 47244 | 1.89226 | 2.1 |
| 47245 | 1.89226 | 2.1 |
| 47246 | 0.864133 | 2.1 |
| 47247 | 0.864133 | 2.1 |
| 47248 | 0.967939 | 2.1 |
| 47249 | 0.793739 | 2.1 |
| 47250 | 0.650797 | 2.1 |
| 47251 | 0.757546 | 2.1 |
| 47252 | 1.08941 | 2.1 |
| 47253 | 1.10136 | 2.1 |
| 47254 | 1.10138 | 2.1 |
| 47255 | 0.796445 | 2.1 |
| 47256 | 0.866907 | 2.1 |
| 47257 | 0.650788 | 2.1 |
| 47258 | 2.63062 | 0.0770766 |
| 47259 | 1.89226 | 0.0564287 |
| 47260 | 1.89226 | 0.178381 |
| 47261 | 1.89226 | 0.249958 |
| 47262 | 2.46557 | 0.05 |
| 47263 | 2.46557 | 0.418583 |
| 47264 | 1.16777 | 0.05 |
| 47265 | 1.38859 | 2.1 |
| 47266 | 1.05042 | 2.07924 |
| 47267 | 1.28909 | 2.1 |

| | | |
|---|---|---|
| 47268 | 2.45686 | 2.1 |
| 47269 | 1.89227 | 0.0569735 |
| 47270 | 1.89227 | 0.185794 |
| 47271 | 1.89227 | 0.05 |
| 47272 | 1.89227 | 2.1 |
| 47273 | 3.78453 | 0.05 |
| 47274 | 2.15114 | 2.1 |
| 47275 | 1.89226 | 2.1 |
| 47276 | 2.15005 | 2.1 |
| 47277 | 2.15005 | 2.1 |
| 47278 | 1.89226 | 1.3734 |
| 47279 | 1.89226 | 2.1 |
| 47280 | 1.89226 | 2.1 |
| 47281 | 1.89226 | 1.07521 |
| 47282 | 1.89226 | 2.1 |
| 47283 | 1.89226 | 2.1 |
| 47284 | 1.89226 | 0.891897 |
| 47285 | 1.89226 | 2.1 |
| 47286 | 0.973447 | 2.1 |
| 47287 | 1.32143 | 2.1 |
| 47288 | 1.37444 | 2.1 |
| 47289 | 1.37444 | 2.1 |
| 47290 | 0.949134 | 2.1 |
| 47291 | 0.949134 | 2.1 |
| 47292 | 0.946131 | 0.889413 |
| 47293 | 0.946131 | 2.1 |
| 47294 | 1.07557 | 2.1 |
| 47295 | 1.07557 | 2.1 |
| 47296 | 0.946131 | 2.1 |
| 47297 | 0.946131 | 2.1 |
| 47298 | 1.07557 | 2.1 |
| 47299 | 1.08579 | 2.1 |
| 47300 | 1.08579 | 2.1 |
| 47301 | 0.946131 | 2.1 |
| 47302 | 1.07557 | 2.1 |
| 47303 | 0.946131 | 2.1 |
| 47304 | 0.946131 | 0.268919 |
| 47305 | 0.946131 | 2.1 |
| 47306 | 0.946131 | 2.1 |
| 47307 | 0.946131 | 2.05581 |
| 47308 | 0.94613 | 1.06567 |
| 47309 | 0.94613 | 1.36678 |
| 47310 | 0.946131 | 2.1 |
| 47311 | 0.946131 | 2.1 |
| 47312 | 0.946131 | 1.78071 |
| 47313 | 0.946131 | 1.71894 |
| 47314 | 0.946131 | 1.61283 |
| 47315 | 0.946131 | 2.1 |
| 47316 | 0.606115 | 2.1 |
| 47317 | 0.606115 | 1.99795 |
| 47318 | 0.616308 | 2.1 |
| 47319 | 0.616308 | 2.1 |
| 47320 | 0.68722 | 1.0922 |
| 47321 | 0.735699 | 2.1 |
| 47322 | 0.514789 | 2.1 |
| 47323 | 0.486724 | 2.1 |
| 47324 | 0.514788 | 1.39456 |
| 47325 | 0.735699 | 1.9814 |

| | | |
|---|---|---|
| 47326 | 0.486723 | 2.1 |
| 47327 | 0.687221 | 2.04393 |
| 47328 | 2.33761 | 0.781005 |
| 47329 | 2.51749 | 0.420916 |
| 47330 | 2.68092 | 0.650313 |
| 47331 | 2.58304 | 0.415508 |
| 47332 | 5.01853 | 0.612771 |
| 47333 | 2.50906 | 0.703188 |
| 47334 | 2.51749 | 0.527629 |
| 47335 | 2.68091 | 0.672912 |
| 47336 | 2.53176 | 0.349903 |
| 47337 | 5.18997 | 0.625749 |
| 47338 | 2.68092 | 0.612907 |
| 47339 | 2.58304 | 0.376557 |
| 47340 | 2.71793 | 0.582605 |
| 47341 | 2.67098 | 0.171582 |
| 47342 | 5.39885 | 0.41257 |
| 47343 | 2.68091 | 0.674804 |
| 47344 | 2.53175 | 0.228629 |
| 47345 | 2.36417 | 0.503998 |
| 47346 | 2.67098 | 0.200547 |
| 47347 | 5.04508 | 0.454167 |
| 47348 | 2.76309 | 2.1 |
| 47349 | 1.89226 | 2.1 |
| 47350 | 1.89226 | 2.1 |
| 47351 | 1.89226 | 2.1 |
| 47352 | 2.48573 | 2.1 |
| 47353 | 2.48573 | 2.1 |
| 47354 | 1.35879 | 2.1 |
| 47355 | 1.55161 | 0.05 |
| 47356 | 1.10407 | 2.1 |
| 47357 | 1.32259 | 0.05 |
| 47358 | 2.68138 | 1.21239 |
| 47359 | 1.89226 | 2.1 |
| 47360 | 1.89226 | 0.184923 |
| 47361 | 1.89226 | 2.1 |
| 47362 | 1.89226 | 0.05 |
| 47363 | 3.78452 | 0.208198 |
| 47364 | 1.19612 | 2.1 |
| 47365 | 1.24694 | 2.1 |
| 47366 | 1.24844 | 0.180524 |
| 47367 | 1.29747 | 2.1 |
| 47368 | 2.49358 | 2.1 |
| 47369 | 1.89226 | 2.1 |
| 47370 | 1.89226 | 2.1 |
| 47371 | 1.89226 | 0.05 |
| 47372 | 1.89226 | 2.1 |
| 47373 | 3.78453 | 1.73883 |
| 47374 | 1.89226 | 2.1 |
| 47375 | 1.89226 | 2.1 |
| 47376 | 1.89226 | 2.1 |
| 47377 | 1.89226 | 2.1 |
| 47378 | 1.89226 | 2.1 |
| 47379 | 1.89226 | 2.1 |
| 47380 | 0.587463 | 2.1 |
| 47381 | 0.587463 | 2.1 |
| 47382 | 0.581977 | 2.1 |
| 47383 | 0.581977 | 2.1 |

| | | |
|---|---|---|
| 47384 | 0.531709 | 2.1 |
| 47385 | 0.545972 | 2.1 |
| 47386 | 0.623469 | 2.1 |
| 47387 | 0.648733 | 2.1 |
| 47388 | 0.648733 | 2.1 |
| 47389 | 0.545971 | 2.1 |
| 47390 | 0.623469 | 2.1 |
| 47391 | 0.531709 | 2.1 |
| 47392 | 1.29417 | 0.0781178 |
| 47393 | 1.29747 | 2.1 |
| 47394 | 1.27829 | 2.1 |
| 47395 | 1.24844 | 1.01281 |
| 47396 | 1.32732 | 2.1 |
| 47397 | 1.29747 | 0.953631 |
| 47398 | 1.89226 | 0.808109 |
| 47399 | 1.89226 | 2.1 |
| 47400 | 1.89226 | 0.05 |
| 47401 | 1.89226 | 1.00978 |
| 47402 | 1.89226 | 1.17317 |
| 47403 | 1.89226 | 2.1 |
| 47404 | 1.89226 | 2.1 |
| 47405 | 1.89226 | 2.1 |
| 47406 | 1.89226 | 1.78975 |
| 47407 | 1.89226 | 2.1 |
| 47408 | 1.89226 | 2.1 |
| 47409 | 1.89226 | 0.92397 |
| 47410 | 0.60346 | 2.1 |
| 47411 | 0.60346 | 2.1 |
| 47412 | 0.60086 | 0.464266 |
| 47413 | 0.60086 | 2.1 |
| 47414 | 0.545971 | 2.1 |
| 47415 | 0.555587 | 2.1 |
| 47416 | 0.648733 | 2.1 |
| 47417 | 0.663661 | 2.1 |
| 47418 | 0.663661 | 2.1 |
| 47419 | 0.555587 | 2.1 |
| 47420 | 0.648733 | 2.1 |
| 47421 | 0.545971 | 2.1 |
| 47422 | 1.34066 | 1.97022 |
| 47423 | 1.36601 | 2.1 |
| 47424 | 1.34955 | 0.106494 |
| 47425 | 1.37623 | 2.1 |
| 47426 | 2.71556 | 2.1 |
| 47427 | 1.89226 | 1.16149 |
| 47428 | 1.89226 | 2.1 |
| 47429 | 1.89226 | 0.110231 |
| 47430 | 1.89226 | 2.1 |
| 47431 | 3.78452 | 1.10146 |
| 47432 | 0.946132 | 2.1 |
| 47433 | 0.946132 | 2.1 |
| 47434 | 0.946132 | 0.110448 |
| 47435 | 0.946132 | 2.1 |
| 47436 | 0.946132 | 2.1 |
| 47437 | 0.946132 | 2.1 |
| 47438 | 0.946131 | 1.03552 |
| 47439 | 0.946131 | 1.04234 |
| 47440 | 0.946132 | 2.1 |
| 47441 | 0.946132 | 2.1 |

| | | |
|---|---|---|
| 47442 | 0.946132 | 2.1 |
| 47443 | 0.946132 | 2.1 |
| 47444 | 0.631351 | 0.837505 |
| 47445 | 0.631351 | 2.1 |
| 47446 | 0.632421 | 2.1 |
| 47447 | 0.632421 | 2.1 |
| 47448 | 0.573604 | 2.1 |
| 47449 | 0.580765 | 2.1 |
| 47450 | 0.683006 | 0.648317 |
| 47451 | 0.688118 | 2.1 |
| 47452 | 0.688118 | 2.1 |
| 47453 | 0.580765 | 2.1 |
| 47454 | 0.683007 | 2.1 |
| 47455 | 0.573604 | 2.1 |
| 47456 | 1.34955 | 1.85376 |
| 47457 | 1.37624 | 2.1 |
| 47458 | 1.35048 | 0.114841 |
| 47459 | 1.37999 | 2.1 |
| 47460 | 2.72954 | 1.6485 |
| 47461 | 0.946131 | 2.1 |
| 47462 | 0.946131 | 1.14464 |
| 47463 | 0.946131 | 2.1 |
| 47464 | 0.946131 | 2.1 |
| 47465 | 0.94613 | 2.1 |
| 47466 | 0.94613 | 1.94318 |
| 47467 | 0.946133 | 1.00768 |
| 47468 | 0.946133 | 1.36343 |
| 47469 | 0.946131 | 1.89456 |
| 47470 | 0.946131 | 0.952286 |
| 47471 | 0.946132 | 1.31729 |
| 47472 | 0.946132 | 1.21572 |
| 47473 | 1.89226 | 2.1 |
| 47474 | 1.89226 | 2.09725 |
| 47475 | 1.89226 | 2.1 |
| 47476 | 1.89226 | 2.1 |
| 47477 | 1.89226 | 2.1 |
| 47478 | 1.89226 | 2.1 |
| 47479 | 0.532189 | 0.903931 |
| 47480 | 0.531141 | 2.1 |
| 47481 | 0.497784 | 2.1 |
| 47482 | 0.502536 | 2.1 |
| 47483 | 1.37999 | 2.1 |
| 47484 | 0.92433 | 2.1 |
| 47485 | 0.670081 | 2.1 |
| 47486 | 0.727155 | 2.1 |
| 47487 | 0.786438 | 0.965807 |
| 47488 | 0.589798 | 2.1 |
| 47489 | 0.497784 | 2.1 |
| 47490 | 1.89226 | 2.1 |
| 47491 | 2.72301 | 2.1 |
| 47492 | 2.4158 | 2.1 |
| 47493 | 2.4158 | 2.1 |
| 47494 | 1.89226 | 2.1 |
| 47495 | 1.89226 | 2.1 |
| 47496 | 1.89226 | 2.1 |
| 47497 | 1.89226 | 2.1 |
| 47498 | 1.89226 | 2.1 |
| 47499 | 1.89226 | 2.1 |

| | | |
|---|---|---|
| 47500 | 1.89226 | 2.1 |
| 47501 | 1.89226 | 2.1 |
| 47502 | 1.36919 | 2.1 |
| 47503 | 1.12717 | 2.1 |
| 47504 | 1.38634 | 2.1 |
| 47505 | 1.56541 | 2.1 |
| 47506 | 2.75552 | 2.1 |
| 47507 | 1.89226 | 2.1 |
| 47508 | 1.89226 | 2.1 |
| 47509 | 1.89226 | 2.1 |
| 47510 | 1.89226 | 2.1 |
| 47511 | 1.89226 | 2.1 |
| 47512 | 1.89226 | 2.1 |
| 47513 | 0.907036 | 2.1 |
| 47514 | 0.782706 | 2.1 |
| 47515 | 0.838808 | 2.1 |
| 47516 | 0.693168 | 2.1 |
| 47517 | 0.794747 | 2.1 |
| 47518 | 0.794747 | 2.1 |
| 47519 | 0.805458 | 2.1 |
| 47520 | 0.805458 | 2.1 |
| 47521 | 0.907036 | 2.1 |
| 47522 | 0.838808 | 2.1 |
| 47523 | 0.693169 | 2.1 |
| 47524 | 0.782707 | 2.1 |
| 47525 | 0.94613 | 2.1 |
| 47526 | 0.94613 | 2.1 |
| 47527 | 1.188 2.1 | |
| 47528 | 1.28421 | 2.1 |
| 47529 | 1.18801 | 2.1 |
| 47530 | 1.18801 | 2.1 |
| 47531 | 0.946129 | 2.1 |
| 47532 | 0.946129 | 2.1 |
| 47533 | 1.28421 | 2.1 |
| 47534 | 0.94613 | 2.1 |
| 47535 | 1.188 2.1 | |
| 47536 | 0.94613 | 2.1 |
| 47537 | 1.89226 | 2.1 |
| 47538 | 1.89226 | 2.1 |
| 47539 | 1.89226 | 2.1 |
| 47540 | 1.89226 | 2.1 |
| 47541 | 1.89226 | 2.1 |
| 47542 | 1.89226 | 2.1 |
| 47543 | 1.16817 | 2.1 |
| 47544 | 1.06007 | 2.1 |
| 47545 | 1.19785 | 2.1 |
| 47546 | 1.2832 | 2.1 |
| 47547 | 2.36602 | 2.1 |
| 47548 | 0.960106 | 2.1 |
| 47549 | 2.0073 | 2.1 |
| 47550 | 1.51754 | 2.1 |
| 47551 | 2.56883 | 2.1 |
| 47552 | 3.52484 | 2.1 |
| 47553 | 1.06756 | 2.1 |
| 47554 | 1.57913 | 2.1 |
| 47555 | 1.87532 | 2.1 |
| 47556 | 1.38666 | 2.1 |
| 47557 | 2.94287 | 2.1 |

| | | |
|---|---|---|
| 47558 | 2.15622 | 1.70025 |
| 47559 | 1.38668 | 2.1 |
| 47560 | 1.87534 | 2.1 |
| 47561 | 1.87534 | 2.1 |
| 47562 | 1.70539 | 2.1 |
| 47563 | 1.70539 | 2.1 |
| 47564 | 0.986095 | 2.1 |
| 47565 | 1.36226 | 2.1 |
| 47566 | 1.3331 | 2.1 |
| 47567 | 1.3331 | 2.1 |
| 47568 | 1.07156 | 2.1 |
| 47569 | 1.07156 | 2.1 |
| 47570 | 0.682081 | 1.61367 |
| 47571 | 0.68748 | 1.1541 |
| 47572 | 0.650003 | 0.0831218 |
| 47573 | 0.645873 | 0.554398 |
| 47574 | 0.666344 | 0.0904377 |
| 47575 | 0.666344 | 1.12308 |
| 47576 | 0.66161 | 2.1 |
| 47577 | 0.66161 | 0.0696077 |
| 47578 | 0.68748 | 0.306726 |
| 47579 | 0.645873 | 0.478013 |
| 47580 | 0.650004 | 0.640565 |
| 47581 | 0.682082 | 0.60934 |
| 47582 | 0.946132 | 1.22262 |
| 47583 | 0.946132 | 0.436886 |
| 47584 | 0.946132 | 0.371491 |
| 47585 | 0.946132 | 0.05 |
| 47586 | 0.946132 | 0.05 |
| 47587 | 0.946132 | 0.490599 |
| 47588 | 0.946133 | 0.420816 |
| 47589 | 0.946133 | 1.29394 |
| 47590 | 0.946132 | 0.329691 |
| 47591 | 0.946132 | 0.168146 |
| 47592 | 0.946133 | 0.698787 |
| 47593 | 0.946133 | 0.419976 |
| 47594 | 1.89227 | 1.04789 |
| 47595 | 2.79312 | 0.154576 |
| 47596 | 2.77591 | 0.744419 |
| 47597 | 2.79312 | 0.647549 |
| 47598 | 1.87505 | 0.283432 |
| 47599 | 1.89227 | 0.250095 |
| 47600 | 0.68748 | 1.88573 |
| 47601 | 0.698626 | 1.17885 |
| 47602 | 0.645873 | 0.135133 |
| 47603 | 0.650148 | 0.533826 |
| 47604 | 0.670561 | 0.140627 |
| 47605 | 0.670561 | 1.23507 |
| 47606 | 0.667067 | 2.1 |
| 47607 | 0.667067 | 0.0607145 |
| 47608 | 0.698627 | 0.456786 |
| 47609 | 0.650148 | 0.755347 |
| 47610 | 0.68748 | 0.583775 |
| 47611 | 0.645873 | 0.834884 |
| 47612 | 0.946132 | 1.51575 |
| 47613 | 0.946132 | 0.536402 |
| 47614 | 0.946132 | 0.78604 |
| 47615 | 0.946132 | 0.0906248 |

| | | |
|---|---|---|
| 47616 | 0.946132 | 0.0986887 |
| 47617 | 0.946132 | 0.833601 |
| 47618 | 0.946133 | 0.621476 |
| 47619 | 0.946133 | 1.67974 |
| 47620 | 0.946133 | 0.50651 |
| 47621 | 0.946133 | 0.631211 |
| 47622 | 0.946132 | 0.889963 |
| 47623 | 0.946132 | 0.863304 |
| 47624 | 0.946133 | 0.867987 |
| 47625 | 0.946133 | 0.228106 |
| 47626 | 1.38709 | 0.790458 |
| 47627 | 1.38795 | 0.136716 |
| 47628 | 0.946132 | 0.159785 |
| 47629 | 0.946132 | 0.507242 |
| 47630 | 1.38709 | 0.957055 |
| 47631 | 1.38709 | 0.1681 |
| 47632 | 1.38709 | 0.593872 |
| 47633 | 0.946133 | 0.730828 |
| 47634 | 0.946132 | 0.723008 |
| 47635 | 1.38795 | 0.543104 |
| 47636 | 1.70301 | 0.05 |
| 47637 | 2.07659 | 0.05 |
| 47638 | 1.89226 | 0.05 |
| 47639 | 1.89226 | 0.05 |
| 47640 | 1.76332 | 0.05 |
| 47641 | 1.76331 | 0.05 |
| 47642 | 1.89226 | 0.05 |
| 47643 | 1.89226 | 0.05 |
| 47644 | 1.89226 | 0.05 |
| 47645 | 1.89226 | 0.05 |
| 47646 | 1.89226 | 0.05 |
| 47647 | 1.89226 | 0.05 |
| 47648 | 1.89226 | 0.05 |
| 47649 | 1.89226 | 0.05 |
| 47650 | 1.89226 | 0.05 |
| 47651 | 1.89226 | 0.05 |
| 47652 | 3.78452 | 0.05 |
| 47653 | 1.89226 | 0.05 |
| 47654 | 2.12492 | 0.05 |
| 47655 | 1.89226 | 0.05 |
| 47656 | 1.89226 | 0.05 |
| 47657 | 2.07659 | 0.05 |
| 47658 | 2.07659 | 0.05 |
| 47659 | 0.94613 | 0.05 |
| 47660 | 0.94613 | 0.05 |
| 47661 | 1.17498 | 0.05 |
| 47662 | 1.32446 | 0.05 |
| 47663 | 1.32446 | 0.05 |
| 47664 | 0.94613 | 0.05 |
| 47665 | 0.946127 | 0.05 |
| 47666 | 1.17498 | 0.05 |
| 47667 | 1.32446 | 0.05 |
| 47668 | 1.32446 | 0.05 |
| 47669 | 0.946128 | 0.05 |
| 47670 | 0.946128 | 0.05 |
| 47671 | 1.89226 | 2.1 |
| 47672 | 1.86472 | 2.1 |
| 47673 | 2.11049 | 2.1 |

| | | |
|---|---|---|
| 47674 | 1.89226 | 2.1 |
| 47675 | 2.08295 | 2.1 |
| 47676 | 1.86472 | 2.1 |
| 47677 | 1.89226 | 2.1 |
| 47678 | 2.08295 | 2.1 |
| 47679 | 2.14023 | 2.1 |
| 47680 | 1.89226 | 2.1 |
| 47681 | 2.33091 | 2.1 |
| 47682 | 2.08295 | 2.1 |
| 47683 | 0.809215 | 2.1 |
| 47684 | 0.809215 | 2.1 |
| 47685 | 0.810915 | 2.1 |
| 47686 | 0.810915 | 2.1 |
| 47687 | 1.09581 | 2.1 |
| 47688 | 1.08271 | 2.1 |
| 47689 | 0.520996 | 2.1 |
| 47690 | 0.537426 | 2.1 |
| 47691 | 0.520997 | 2.1 |
| 47692 | 1.08271 | 2.1 |
| 47693 | 0.537426 | 2.1 |
| 47694 | 1.09581 | 2.1 |
| 47695 | 0.79674 | 2.1 |
| 47696 | 0.79674 | 2.1 |
| 47697 | 0.799279 | 2.1 |
| 47698 | 0.799279 | 2.1 |
| 47699 | 1.07502 | 2.1 |
| 47700 | 1.0827 | 2.1 |
| 47701 | 0.508206 | 2.1 |
| 47702 | 0.520996 | 2.1 |
| 47703 | 0.508206 | 2.1 |
| 47704 | 1.07502 | 2.1 |
| 47705 | 0.520996 | 2.1 |
| 47706 | 1.0827 | 2.1 |
| 47707 | 2.15714 | 2.1 |
| 47708 | 1.70302 | 2.1 |
| 47709 | 1.78732 | 2.1 |
| 47710 | 2.02219 | 2.1 |
| 47711 | 3.98971 | 2.1 |
| 47712 | 2.0927 | 2.1 |
| 47713 | 2.162 | 2.1 |
| 47714 | 2.11563 | 2.1 |
| 47715 | 2.02219 | 2.1 |
| 47716 | 4.18419 | 2.1 |
| 47717 | 1.62483 | 2.1 |
| 47718 | 2.02219 | 2.1 |
| 47719 | 1.93617 | 2.1 |
| 47720 | 1.52008 | 2.1 |
| 47721 | 3.54227 | 2.1 |
| 47722 | 2.11563 | 2.1 |
| 47723 | 2.02219 | 2.1 |
| 47724 | 2.29218 | 2.1 |
| 47725 | 2.03975 | 2.1 |
| 47726 | 4.06194 | 2.1 |
| 47727 | 2.007 | 2.1 |
| 47728 | 1.89226 | 2.1 |
| 47729 | 1.89226 | 2.1 |
| 47730 | 1.89226 | 2.1 |
| 47731 | 2.06776 | 2.1 |

| | | |
|---|---|---|
| 47732 | 2.06776 | 2.1 |
| 47733 | 0.946129 | 2.1 |
| 47734 | 0.946129 | 2.1 |
| 47735 | 1.0035 | 2.1 |
| 47736 | 1.0035 | 2.1 |
| 47737 | 0.946131 | 2.1 |
| 47738 | 0.946131 | 2.1 |
| 47739 | 0.968797 | 2.1 |
| 47740 | 1.0035 | 2.1 |
| 47741 | 0.968771 | 2.1 |
| 47742 | 0.946105 | 2.1 |
| 47743 | 1.00353 | 2.1 |
| 47744 | 0.946157 | 2.1 |
| 47745 | 1.79103 | 2.1 |
| 47746 | 2.53286 | 0.70287 |
| 47747 | 2.60781 | 2.1 |
| 47748 | 2.219 | 0.939704 |
| 47749 | 2.17985 | 1.61807 |
| 47750 | 1.79103 | 0.633629 |
| 47751 | 2.219 | 1.71749 |
| 47752 | 2.66359 | 1.70938 |
| 47753 | 2.70084 | 2.1 |
| 47754 | 2.38698 | 1.32858 |
| 47755 | 2.53285 | 1.05724 |
| 47756 | 2.219 | 0.901277 |
| 47757 | 1.33179 | 1.49448 |
| 47758 | 0.946128 | 0.973227 |
| 47759 | 0.946133 | 1.22625 |
| 47760 | 1.26643 | 2.09996 |
| 47761 | 1.3318 | 1.11325 |
| 47762 | 1.3318 | 1.99845 |
| 47763 | 0.946131 | 0.921114 |
| 47764 | 0.946131 | 1.41725 |
| 47765 | 0.946131 | 0.9115 |
| 47766 | 0.946131 | 0.699149 |
| 47767 | 1.33179 | 1.15417 |
| 47768 | 1.26643 | 2.1 |
| 47769 | 2.28979 | 2.1 |
| 47770 | 2.47227 | 2.1 |
| 47771 | 2.43451 | 2.1 |
| 47772 | 2.22627 | 2.1 |
| 47773 | 4.69855 | 2.1 |
| 47774 | 2.40667 | 2.1 |
| 47775 | 2.47228 | 2.1 |
| 47776 | 2.43451 | 2.1 |
| 47777 | 2.38069 | 2.1 |
| 47778 | 4.85296 | 2.1 |
| 47779 | 2.43451 | 2.1 |
| 47780 | 2.15715 | 2.1 |
| 47781 | 2.15715 | 2.1 |
| 47782 | 2.22627 | 2.1 |
| 47783 | 2.39489 | 2.1 |
| 47784 | 2.46402 | 2.1 |
| 47785 | 2.43451 | 2.1 |
| 47786 | 2.34899 | 2.1 |
| 47787 | 2.38069 | 2.1 |
| 47788 | 2.38069 | 2.1 |
| 47789 | 2.3949 | 2.1 |

| | | |
|---|---|---|
| 47790 | 2.3949 | 2.1 |
| 47791 | 2.85425 | 1.76251 |
| 47792 | 1.70302 | 0.535106 |
| 47793 | 1.70302 | 0.342706 |
| 47794 | 1.52254 | 0.563333 |
| 47795 | 2.34181 | 0.53821 |
| 47796 | 2.08499 | 0.64656 |
| 47797 | 1.89226 | 0.57209 |
| 47798 | 1.89226 | 0.586268 |
| 47799 | 1.89226 | 0.294137 |
| 47800 | 1.89226 | 0.501326 |
| 47801 | 1.89226 | 0.456314 |
| 47802 | 1.89226 | 0.664623 |
| 47803 | 2.34181 | 0.692733 |
| 47804 | 1.38287 | 0.701808 |
| 47805 | 1.38287 | 0.643638 |
| 47806 | 1.84132 | 0.586275 |
| 47807 | 1.96416 | 0.567339 |
| 47808 | 2.4907 | 0.63075 |
| 47809 | 0.931432 | 0.831558 |
| 47810 | 0.946135 | 0.490177 |
| 47811 | 0.946133 | 0.452424 |
| 47812 | 0.946133 | 0.450669 |
| 47813 | 0.931464 | 0.552855 |
| 47814 | 0.931464 | 0.850615 |
| 47815 | 1.15573 | 0.46455 |
| 47816 | 0.96378 | 1.29127 |
| 47817 | 0.931434 | 0.611082 |
| 47818 | 0.946131 | 0.487073 |
| 47819 | 0.946131 | 0.483294 |
| 47820 | 1.13808 | 0.465648 |
| 47821 | 0.946132 | 0.713778 |
| 47822 | 0.946132 | 0.549009 |
| 47823 | 0.946132 | 0.633536 |
| 47824 | 0.946132 | 0.419459 |
| 47825 | 0.946133 | 0.661643 |
| 47826 | 0.946133 | 0.502716 |
| 47827 | 0.946132 | 0.790541 |
| 47828 | 0.946132 | 0.596531 |
| 47829 | 0.946133 | 0.555826 |
| 47830 | 0.946133 | 0.734538 |
| 47831 | 0.946132 | 0.485911 |
| 47832 | 0.946132 | 0.146169 |
| 47833 | 1.68074 | 2.1 |
| 47834 | 2.29459 | 2.1 |
| 47835 | 2.23361 | 2.1 |
| 47836 | 1.57234 | 2.1 |
| 47837 | 3.86693 | 2.1 |
| 47838 | 2.61243 | 2.1 |
| 47839 | 2.29458 | 2.1 |
| 47840 | 2.2336 | 2.1 |
| 47841 | 2.58234 | 2.1 |
| 47842 | 4.87692 | 2.1 |
| 47843 | 0.73506 | 2.1 |
| 47844 | 0.786166 | 2.1 |
| 47845 | 1.08729 | 2.1 |
| 47846 | 1.11679 | 2.1 |
| 47847 | 0.93134 | 2.1 |

| | | |
|---|---|---|
| 47848 | 0.93134 | 2.1 |
| 47849 | 0.942113 | 2.1 |
| 47850 | 0.942113 | 2.1 |
| 47851 | 0.735065 | 2.1 |
| 47852 | 1.08729 | 2.1 |
| 47853 | 1.11679 | 2.1 |
| 47854 | 0.78616 | 2.1 |
| 47855 | 1.08729 | 2.1 |
| 47856 | 1.11679 | 2.1 |
| 47857 | 1.27502 | 2.1 |
| 47858 | 1.29116 | 2.1 |
| 47859 | 1.19259 | 2.1 |
| 47860 | 1.19259 | 2.1 |
| 47861 | 1.19923 | 2.1 |
| 47862 | 1.19923 | 2.1 |
| 47863 | 1.27503 | 2.1 |
| 47864 | 1.08729 | 2.1 |
| 47865 | 1.11679 | 2.1 |
| 47866 | 1.29115 | 2.1 |
| 47867 | 0.974715 | 0.931195 |
| 47868 | 1.43779 | 0.693067 |
| 47869 | 1.93915 | 0.730019 |
| 47870 | 1.53908 | 0.758899 |
| 47871 | 2.91387 | 0.734937 |
| 47872 | 1.53908 | 0.547241 |
| 47873 | 0.99845 | 0.728716 |
| 47874 | 1.5176 | 0.898284 |
| 47875 | 1.97963 | 0.501148 |
| 47876 | 3.05668 | 0.699838 |
| 47877 | 1.93915 | 0.710415 |
| 47878 | 1.97963 | 0.562512 |
| 47879 | 1.53908 | 0.688612 |
| 47880 | 1.53908 | 0.342144 |
| 47881 | 2.3376 | 0.873994 |
| 47882 | 2.3376 | 0.666444 |
| 47883 | 1.89226 | 2.1 |
| 47884 | 2.59747 | 2.1 |
| 47885 | 1.91802 | 2.1 |
| 47886 | 1.89226 | 2.1 |
| 47887 | 2.62322 | 2.1 |
| 47888 | 2.59747 | 2.1 |
| 47889 | 2.59747 | 2.1 |
| 47890 | 1.87119 | 2.1 |
| 47891 | 1.84844 | 2.1 |
| 47892 | 1.82268 | 2.1 |
| 47893 | 2.62323 | 2.1 |
| 47894 | 2.59747 | 2.1 |
| 47895 | 0.94613 | 2.1 |
| 47896 | 0.94613 | 2.1 |
| 47897 | 1.32697 | 2.1 |
| 47898 | 1.32697 | 2.1 |
| 47899 | 1.32697 | 2.1 |
| 47900 | 1.31161 | 2.1 |
| 47901 | 0.946132 | 2.1 |
| 47902 | 0.946132 | 2.1 |
| 47903 | 0.94613 | 2.1 |
| 47904 | 1.32697 | 2.1 |
| 47905 | 0.946131 | 2.1 |

| | | |
|---|---|---|
| 47906 | 1.31161 | 2.1 |
| 47907 | 2.62323 | 2.1 |
| 47908 | 1.91704 | 2.1 |
| 47909 | 1.90191 | 2.1 |
| 47910 | 1.87119 | 2.1 |
| 47911 | 2.65395 | 2.1 |
| 47912 | 2.62323 | 2.1 |
| 47913 | 1.89226 | 2.1 |
| 47914 | 1.89226 | 2.1 |
| 47915 | 2.15958 | 2.1 |
| 47916 | 2.15958 | 2.1 |
| 47917 | 2.46558 | 2.1 |
| 47918 | 2.19826 | 2.1 |
| 47919 | 2.46558 | 2.1 |
| 47920 | 1.89226 | 2.1 |
| 47921 | 1.89226 | 2.1 |
| 47922 | 1.89226 | 2.1 |
| 47923 | 2.61064 | 2.1 |
| 47924 | 2.61064 | 2.1 |
| 47925 | 1.81759 | 0.664434 |
| 47926 | 1.89526 | 0.809238 |
| 47927 | 2.32219 | 1.09253 |
| 47928 | 1.881 | 0.526507 |
| 47929 | 1.81759 | 0.886111 |
| 47930 | 1.3764 | 0.262099 |
| 47931 | 1.81759 | 0.757022 |
| 47932 | 2.57079 | 0.565944 |
| 47933 | 2.32219 | 1.37279 |
| 47934 | 2.32219 | 0.704554 |
| 47935 | 1.99875 | 0.867953 |
| 47936 | 1.99875 | 0.304447 |
| 47937 | 0.536182 | 0.165774 |
| 47938 | 0.579206 | 0.05 |
| 47939 | 0.908796 | 0.711016 |
| 47940 | 0.999375 | 0.277412 |
| 47941 | 0.732091 | 0.435818 |
| 47942 | 0.732091 | 0.05 |
| 47943 | 0.755911 | 0.163885 |
| 47944 | 0.755911 | 0.637976 |
| 47945 | 0.999376 | 0.280259 |
| 47946 | 0.579206 | 0.42011 |
| 47947 | 0.908796 | 0.282318 |
| 47948 | 0.536182 | 0.235115 |
| 47949 | 0.94613 | 0.0836404 |
| 47950 | 0.94613 | 0.213735 |
| 47951 | 1.31112 | 0.34876 |
| 47952 | 1.18275 | 0.05 |
| 47953 | 0.946126 | 0.0562595 |
| 47954 | 0.946126 | 0.05 |
| 47955 | 1.31112 | 0.304207 |
| 47956 | 1.31112 | 0.05 |
| 47957 | 0.946128 | 0.161204 |
| 47958 | 1.31112 | 0.176821 |
| 47959 | 0.946128 | 0.0778304 |
| 47960 | 1.18275 | 0.05 |
| 47961 | 2.64617 | 0.589283 |
| 47962 | 1.27737 | 0.05 |
| 47963 | 1.83076 | 0.121239 |

| | | |
|---|---|---|
| 47964 | 1.27737 | 0.447622 |
| 47965 | 3.03764 | 0.05 |
| 47966 | 2.3655 | 0.05 |
| 47967 | 1.12991 | 0.05 |
| 47968 | 1.22375 | 0.05 |
| 47969 | 1.35931 | 0.05 |
| 47970 | 1.04971 | 0.05 |
| 47971 | 1.0419 | 0.05 |
| 47972 | 1.15237 | 0.05 |
| 47973 | 1.19593 | 0.05 |
| 47974 | 1.30306 | 0.05 |
| 47975 | 1.12937 | 0.05 |
| 47976 | 1.31934 | 0.05 |
| 47977 | 1.15203 | 0.05 |
| 47978 | 1.15203 | 0.05 |
| 47979 | 2.65227 | 0.05 |
| 47980 | 1.46041 | 0.05 |
| 47981 | 2.22712 | 0.05 |
| 47982 | 2.04816 | 0.05 |
| 47983 | 2.22712 | 0.05 |
| 47984 | 2.04816 | 0.05 |
| 47985 | 1.89226 | 0.05 |
| 47986 | 1.89226 | 0.0585277 |
| 47987 | 1.89226 | 0.05 |
| 47988 | 1.89226 | 0.118617 |
| 47989 | 3.78452 | 0.0663854 |
| 47990 | 1.24653 | 0.05 |
| 47991 | 1.66554 | 0.05 |
| 47992 | 1.53538 | 0.05 |
| 47993 | 1.53538 | 0.781772 |
| 47994 | 1.40915 | 0.05 |
| 47995 | 1.40915 | 0.702933 |
| 47996 | 1.66555 | 0.05 |
| 47997 | 1.75835 | 2.1 |
| 47998 | 1.53538 | 1.87234 |
| 47999 | 1.65313 | 2.1 |
| 48000 | 3.31868 | 2.1 |
| 48001 | 1.89226 | 0.0518029 |
| 48002 | 1.89226 | 0.146949 |
| 48003 | 1.89226 | 0.05 |
| 48004 | 1.89226 | 2.1 |
| 48005 | 3.78453 | 0.05 |
| 48006 | 1.89226 | 0.05 |
| 48007 | 1.89226 | 0.05 |
| 48008 | 1.89226 | 0.05 |
| 48009 | 1.89226 | 0.351706 |
| 48010 | 3.78452 | 0.05 |
| 48011 | 0.658778 | 2.1 |
| 48012 | 0.658778 | 0.05 |
| 48013 | 0.661956 | 0.05 |
| 48014 | 0.661956 | 0.12851 |
| 48015 | 0.703822 | 0.05 |
| 48016 | 0.767689 | 1.97678 |
| 48017 | 0.623266 | 0.05 |
| 48018 | 0.553046 | 0.05 |
| 48019 | 0.703804 | 0.05 |
| 48020 | 0.55303 | 0.05 |
| 48021 | 0.623282 | 0.05 |

| | | |
|---|---|---|
| 48022 | 0.767711 | 0.05 |
| 48023 | 1.53538 | 0.615626 |
| 48024 | 1.65313 | 2.1 |
| 48025 | 1.40765 | 0.05 |
| 48026 | 1.55779 | 2.1 |
| 48027 | 3.06078 | 2.1 |
| 48028 | 1.89227 | 0.05 |
| 48029 | 1.89227 | 2.1 |
| 48030 | 1.89227 | 0.223819 |
| 48031 | 1.89227 | 0.0779952 |
| 48032 | 3.78453 | 0.372676 |
| 48033 | 1.10517 | 2.1 |
| 48034 | 2.16532 | 2.1 |
| 48035 | 1.94374 | 2.1 |
| 48036 | 1.94001 | 2.1 |
| 48037 | 1.37756 | 2.1 |
| 48038 | 1.37756 | 2.1 |
| 48039 | 1.7453 | 0.05 |
| 48040 | 1.83284 | 0.05 |
| 48041 | 2.28483 | 0.05 |
| 48042 | 1.77804 | 0.05 |
| 48043 | 1.7453 | 0.05 |
| 48044 | 1.23851 | 0.05 |
| 48045 | 1.7453 | 0.05 |
| 48046 | 2.54752 | 0.05 |
| 48047 | 2.28483 | 0.05 |
| 48048 | 2.28483 | 0.05 |
| 48049 | 2.0189 | 0.05 |
| 48050 | 2.0189 | 0.05 |
| 48051 | 1.12457 | 0.05 |
| 48052 | 2.0189 | 0.05 |
| 48053 | 1.7453 | 0.05 |
| 48054 | 1.7453 | 0.05 |
| 48055 | 1.40707 | 0.05 |
| 48056 | 1.40707 | 0.05 |
| 48057 | 2.41176 | 2.1 |
| 48058 | 2.59262 | 2.1 |
| 48059 | 2.34085 | 2.1 |
| 48060 | 2.55827 | 2.1 |
| 48061 | 4.97003 | 2.1 |
| 48062 | 2.59262 | 2.1 |
| 48063 | 2.61901 | 2.1 |
| 48064 | 2.55827 | 2.1 |
| 48065 | 2.64067 | 2.1 |
| 48066 | 5.17728 | 2.1 |
| 48067 | 2.34084 | 2.1 |
| 48068 | 2.55827 | 2.1 |
| 48069 | 2.24937 | 2.1 |
| 48070 | 2.49053 | 2.1 |
| 48071 | 4.80764 | 2.1 |
| 48072 | 2.55826 | 2.1 |
| 48073 | 2.64067 | 2.1 |
| 48074 | 2.49053 | 2.1 |
| 48075 | 2.58639 | 2.1 |
| 48076 | 5.14465 | 2.1 |
| 48077 | 2.27918 | 0.05 |
| 48078 | 2.44132 | 0.05 |
| 48079 | 1.89226 | 0.05 |

| | | |
|---|---|---|
| 48080 | 1.89226 | 0.05 |
| 48081 | 4.33359 | 0.05 |
| 48082 | 1.89226 | 0.05 |
| 48083 | 2.44132 | 0.05 |
| 48084 | 2.6771 | 0.05 |
| 48085 | 2.6771 | 0.05 |
| 48086 | 1.89226 | 0.05 |
| 48087 | 1.89226 | 0.05 |
| 48088 | 1.60329 | 0.05 |
| 48089 | 1.38246 | 0.05 |
| 48090 | 1.98182 | 0.05 |
| 48091 | 2.2217 | 0.05 |
| 48092 | 3.60417 | 0.05 |
| 48093 | 2.05362 | 0.05 |
| 48094 | 1.02663 | 0.05 |
| 48095 | 1.43255 | 0.05 |
| 48096 | 1.52072 | 0.05 |
| 48097 | 1.70302 | 0.05 |
| 48098 | 1.7685 | 0.05 |
| 48099 | 0.946131 | 0.05 |
| 48100 | 0.946131 | 0.05 |
| 48101 | 0.946131 | 0.05 |
| 48102 | 0.946131 | 0.05 |
| 48103 | 0.946131 | 0.05 |
| 48104 | 0.946131 | 0.05 |
| 48105 | 0.946131 | 0.05 |
| 48106 | 0.946131 | 0.05 |
| 48107 | 0.946131 | 0.05 |
| 48108 | 0.946131 | 0.05 |
| 48109 | 0.946131 | 0.05 |
| 48110 | 0.946131 | 0.05 |
| 48111 | 1.89226 | 0.05 |
| 48112 | 1.89226 | 0.05 |
| 48113 | 1.89226 | 0.05 |
| 48114 | 1.89226 | 0.05 |
| 48115 | 1.89226 | 0.05 |
| 48116 | 1.89226 | 0.05 |
| 48117 | 1.92645 | 0.0769819 |
| 48118 | 2.83045 | 0.0511361 |
| 48119 | 1.92225 | 0.0738865 |
| 48120 | 2.01873 | 0.05 |
| 48121 | 2.51732 | 0.182739 |
| 48122 | 2.51732 | 0.05 |
| 48123 | 2.51732 | 0.05 |
| 48124 | 2.23857 | 0.05 |
| 48125 | 1.92645 | 0.05 |
| 48126 | 1.92645 | 0.05 |
| 48127 | 2.23857 | 0.0795777 |
| 48128 | 1.50865 | 0.05 |
| 48129 | 2.02687 | 2.1 |
| 48130 | 2.39604 | 2.1 |
| 48131 | 2.29198 | 2.1 |
| 48132 | 2.73046 | 2.1 |
| 48133 | 4.68803 | 2.1 |
| 48134 | 1.21148 | 0.05 |
| 48135 | 1.87957 | 0.63588 |
| 48136 | 2.03555 | 0.116999 |
| 48137 | 2.03555 | 0.507509 |

| | | |
|---|---|---|
| 48138 | 1.09962 | 0.269999 |
| 48139 | 1.09962 | 0.630352 |
| 48140 | 1.92558 | 0.05 |
| 48141 | 2.05274 | 1.99433 |
| 48142 | 2.2443 | 0.05 |
| 48143 | 2.24429 | 0.583242 |
| 48144 | 1.70869 | 0.05 |
| 48145 | 1.70868 | 1.51556 |
| 48146 | 0.818397 | 0.05 |
| 48147 | 0.818397 | 0.811076 |
| 48148 | 0.827911 | 0.05 |
| 48149 | 0.827908 | 0.716606 |
| 48150 | 1.00418 | 0.293709 |
| 48151 | 1.02054 | 0.5801 |
| 48152 | 0.692018 | 0.0998947 |
| 48153 | 0.620899 | 0.0897491 |
| 48154 | 0.65823 | 0.124906 |
| 48155 | 1.00418 | 0.05 |
| 48156 | 0.620899 | 0.202791 |
| 48157 | 1.0262 | 0.05 |
| 48158 | 1.92558 | 0.700879 |
| 48159 | 2.51818 | 1.30941 |
| 48160 | 1.94816 | 0.05 |
| 48161 | 2.01985 | 0.843084 |
| 48162 | 1.85389 | 0.05 |
| 48163 | 1.92558 | 0.792828 |
| 48164 | 0.903844 | 2.1 |
| 48165 | 0.903844 | 2.1 |
| 48166 | 0.946148 | 2.1 |
| 48167 | 0.946151 | 2.1 |
| 48168 | 0.946118 | 2.1 |
| 48169 | 0.903876 | 2.1 |
| 48170 | 0.946118 | 2.1 |
| 48171 | 1.10235 | 2.1 |
| 48172 | 0.946131 | 2.1 |
| 48173 | 0.946131 | 2.1 |
| 48174 | 1.10234 | 2.1 |
| 48175 | 0.903863 | 2.1 |
| 48176 | 1.63471 | 2.1 |
| 48177 | 1.08022 | 2.1 |
| 48178 | 1.56801 | 2.1 |
| 48179 | 1.03437 | 2.1 |
| 48180 | 2.66908 | 2.1 |
| 48181 | 1.56801 | 2.1 |
| 48182 | 0.97541 | 2.1 |
| 48183 | 0.97541 | 2.1 |
| 48184 | 1.03437 | 2.1 |
| 48185 | 1.49733 | 2.1 |
| 48186 | 1.55629 | 2.1 |
| 48187 | 1.89226 | 0.05 |
| 48188 | 1.89226 | 0.05 |
| 48189 | 1.89226 | 0.05 |
| 48190 | 1.89226 | 0.05 |
| 48191 | 3.78452 | 0.05 |
| 48192 | 0.946131 | 0.05 |
| 48193 | 1.16373 | 0.05 |
| 48194 | 0.946131 | 0.05 |
| 48195 | 0.734637 | 0.05 |

| | | |
|---|---|---|
| 48196 | 0.946132 | 0.05 |
| 48197 | 0.946132 | 0.05 |
| 48198 | 1.16373 | 0.05 |
| 48199 | 0.734636 | 0.05 |
| 48200 | 0.946093 | 0.05 |
| 48201 | 0.946093 | 0.05 |
| 48202 | 0.734675 | 0.05 |
| 48203 | 0.734675 | 0.05 |
| 48204 | 1.98413 | 0.05 |
| 48205 | 1.87355 | 0.198536 |
| 48206 | 1.72774 | 0.05 |
| 48207 | 1.67865 | 0.05 |
| 48208 | 3.60128 | 0.05 |
| 48209 | 1.87355 | 0.05 |
| 48210 | 1.54924 | 2.1 |
| 48211 | 1.68443 | 2.1 |
| 48212 | 1.68443 | 0.05 |
| 48213 | 1.67866 | 2.1 |
| 48214 | 1.67866 | 0.05 |
| 48215 | 1.72774 | 0.05 |
| 48216 | 1.53422 | 0.110315 |
| 48217 | 1.67865 | 0.05 |
| 48218 | 1.67865 | 0.05 |
| 48219 | 1.53873 | 0.0536615 |
| 48220 | 1.53873 | 0.05 |
| 48221 | 1.67866 | 0.05 |
| 48222 | 1.54924 | 2.1 |
| 48223 | 1.53423 | 0.05 |
| 48224 | 1.44091 | 2.1 |
| 48225 | 3.11957 | 0.12695 |
| 48226 | 1.89226 | 2.1 |
| 48227 | 1.89226 | 1.71493 |
| 48228 | 1.65573 | 2.08271 |
| 48229 | 1.65573 | 2.1 |
| 48230 | 1.89226 | 2.1 |
| 48231 | 1.89226 | 2.1 |
| 48232 | 1.89226 | 2.1 |
| 48233 | 1.89614 | 1.34552 |
| 48234 | 1.7508 | 1.71282 |
| 48235 | 1.72666 | 1.83702 |
| 48236 | 1.89226 | 1.96784 |
| 48237 | 1.89226 | 2.1 |
| 48238 | 1.89223 | 2.1 |
| 48239 | 1.89223 | 1.44039 |
| 48240 | 1.89223 | 1.48467 |
| 48241 | 1.89223 | 2.02999 |
| 48242 | 1.89223 | 1.71948 |
| 48243 | 1.89223 | 2.1 |
| 48244 | 1.89224 | 2.1 |
| 48245 | 1.89224 | 0.957871 |
| 48246 | 1.89224 | 1.41585 |
| 48247 | 1.89224 | 1.46709 |
| 48248 | 1.89224 | 1.72461 |
| 48249 | 1.89224 | 2.05338 |
| 48250 | 2.09773 | 2.1 |
| 48251 | 2.06623 | 2.1 |
| 48252 | 2.43268 | 2.1 |
| 48253 | 2.33713 | 2.1 |

| | | |
|---|---|---|
| 48254 | 1.703 2.1 | |
| 48255 | 1.61372 | 2.1 |
| 48256 | 0.646929 | 2.1 |
| 48257 | 0.689776 | 2.1 |
| 48258 | 1.05897 | 2.1 |
| 48259 | 0.956364 | 2.1 |
| 48260 | 0.905594 | 2.1 |
| 48261 | 0.904315 | 2.1 |
| 48262 | 0.727014 | 2.1 |
| 48263 | 0.850757 | 2.1 |
| 48264 | 0.798445 | 2.1 |
| 48265 | 0.654101 | 2.1 |
| 48266 | 0.717539 | 2.1 |
| 48267 | 1.0478 | 2.1 |
| 48268 | 1.89223 | 2.1 |
| 48269 | 1.89223 | 0.864093 |
| 48270 | 1.89223 | 2.1 |
| 48271 | 1.6557 | 0.05 |
| 48272 | 3.54794 | 0.922978 |
| 48273 | 0.946118 | 1.91519 |
| 48274 | 0.946118 | 2.1 |
| 48275 | 0.946118 | 2.1 |
| 48276 | 0.946118 | 2.1 |
| 48277 | 0.946117 | 2.1 |
| 48278 | 0.946117 | 0.768105 |
| 48279 | 0.946119 | 2.1 |
| 48280 | 0.946119 | 2.1 |
| 48281 | 0.946105 | 2.1 |
| 48282 | 0.946105 | 2.1 |
| 48283 | 0.946131 | 2.1 |
| 48284 | 0.946131 | 2.1 |
| 48285 | 1.47671 | 2.1 |
| 48286 | 1.70303 | 2.1 |
| 48287 | 1.90705 | 2.1 |
| 48288 | 1.98945 | 2.1 |
| 48289 | 1.3454 | 2.1 |
| 48290 | 1.40353 | 2.1 |
| 48291 | 1.89226 | 2.1 |
| 48292 | 1.89226 | 2.1 |
| 48293 | 1.89226 | 2.1 |
| 48294 | 1.89226 | 1.73704 |
| 48295 | 1.89226 | 2.1 |
| 48296 | 1.89226 | 0.543948 |
| 48297 | 2.39438 | 0.05 |
| 48298 | 2.69842 | 0.05 |
| 48299 | 2.45177 | 0.05 |
| 48300 | 2.28563 | 0.05 |
| 48301 | 4.84615 | 0.05 |
| 48302 | 1.28421 | 2.1 |
| 48303 | 1.35966 | 2.1 |
| 48304 | 0.961001 | 2.1 |
| 48305 | 0.860549 | 2.1 |
| 48306 | 1.1039 | 2.1 |
| 48307 | 1.1039 | 2.1 |
| 48308 | 1.11631 | 2.1 |
| 48309 | 1.11631 | 2.1 |
| 48310 | 1.35966 | 2.1 |
| 48311 | 0.961001 | 2.1 |

| | | |
|---|---|---|
| 48312 | 0.860549 | 2.1 |
| 48313 | 1.28421 | 2.1 |
| 48314 | 1.89226 | 1.46225 |
| 48315 | 1.89226 | 2.1 |
| 48316 | 1.89226 | 2.1 |
| 48317 | 1.89226 | 0.909362 |
| 48318 | 3.78452 | 1.50286 |
| 48319 | 1.48702 | 0.160308 |
| 48320 | 1.40656 | 0.211215 |
| 48321 | 0.626442 | 1.72415 |
| 48322 | 0.626442 | 1.52417 |
| 48323 | 0.626442 | 1.9023 |
| 48324 | 0.946132 | 0.21505 |
| 48325 | 1.05489 | 0.0576628 |
| 48326 | 1.05012 | 2.02629 |
| 48327 | 0.946131 | 1.29597 |
| 48328 | 0.946131 | 1.49484 |
| 48329 | 0.626442 | 1.52485 |
| 48330 | 1.05012 | 1.01394 |
| 48331 | 1.89226 | 2.1 |
| 48332 | 1.97212 | 0.552976 |
| 48333 | 1.89226 | 0.863465 |
| 48334 | 1.89226 | 1.59344 |
| 48335 | 3.78453 | 1.6804 |
| 48336 | 1.89226 | 2.1 |
| 48337 | 1.89226 | 1.17669 |
| 48338 | 1.89226 | 1.34399 |
| 48339 | 1.89226 | 2.01909 |
| 48340 | 3.78452 | 1.86743 |
| 48341 | 1.89226 | 0.914583 |
| 48342 | 1.89226 | 1.55836 |
| 48343 | 1.89226 | 1.95596 |
| 48344 | 1.89226 | 0.171105 |
| 48345 | 1.89226 | 1.94599 |
| 48346 | 1.89226 | 0.148853 |
| 48347 | 0.946161 | 0.0936679 |
| 48348 | 0.946161 | 0.988147 |
| 48349 | 0.932156 | 1.586 |
| 48350 | 0.932156 | 0.791587 |
| 48351 | 0.932184 | 1.45125 |
| 48352 | 0.946132 | 0.05 |
| 48353 | 0.946133 | 1.55878 |
| 48354 | 0.946133 | 0.195514 |
| 48355 | 0.946132 | 0.447702 |
| 48356 | 0.946132 | 0.620811 |
| 48357 | 0.932185 | 0.426753 |
| 48358 | 0.946133 | 0.146262 |
| 48359 | 0.946132 | 0.867409 |
| 48360 | 0.946132 | 0.124576 |
| 48361 | 0.946132 | 2.1 |
| 48362 | 0.946132 | 1.61881 |
| 48363 | 0.946132 | 1.534 |
| 48364 | 0.946132 | 1.00124 |
| 48365 | 0.946133 | 1.14895 |
| 48366 | 0.946133 | 1.94114 |
| 48367 | 0.946132 | 1.4357 |
| 48368 | 0.946132 | 1.65184 |
| 48369 | 0.946133 | 0.96027 |

| | | |
|---|---|---|
| 48370 | 0.946133 | 1.30803 |
| 48371 | 1.79397 | 0.0682917 |
| 48372 | 1.00636 | 0.05 |
| 48373 | 1.31658 | 0.476144 |
| 48374 | 2.02924 | 0.05 |
| 48375 | 3.0356 | 0.203821 |
| 48376 | 1.89226 | 0.197566 |
| 48377 | 1.89226 | 0.05 |
| 48378 | 1.89226 | 0.236091 |
| 48379 | 1.89226 | 0.05 |
| 48380 | 1.89226 | 0.0673235 |
| 48381 | 1.89226 | 0.05 |
| 48382 | 0.946131 | 0.05 |
| 48383 | 0.946131 | 0.0852122 |
| 48384 | 1.24766 | 0.207236 |
| 48385 | 1.03691 | 0.05 |
| 48386 | 0.946129 | 0.05 |
| 48387 | 0.946129 | 0.05 |
| 48388 | 1.24766 | 0.203403 |
| 48389 | 1.24766 | 0.05 |
| 48390 | 0.946131 | 0.0907175 |
| 48391 | 1.24766 | 0.126464 |
| 48392 | 1.03691 | 0.05 |
| 48393 | 0.946131 | 0.05 |
| 48394 | 1.44824 | 0.05 |
| 48395 | 2.02924 | 0.05 |
| 48396 | 1.7065 | 0.103997 |
| 48397 | 1.38604 | 0.217537 |
| 48398 | 2.32017 | 0.05 |
| 48399 | 2.32017 | 0.05 |
| 48400 | 1.89226 | 0.05 |
| 48401 | 1.89226 | 0.05 |
| 48402 | 1.89226 | 0.05 |
| 48403 | 1.89226 | 0.05 |
| 48404 | 1.89226 | 0.315083 |
| 48405 | 1.89226 | 0.113918 |
| 48406 | 0.478349 | 0.634897 |
| 48407 | 0.612092 | 2.1 |
| 48408 | 0.606115 | 2.1 |
| 48409 | 0.506213 | 2.1 |
| 48410 | 0.550677 | 2.1 |
| 48411 | 0.550677 | 0.24031 |
| 48412 | 0.567628 | 2.1 |
| 48413 | 0.567628 | 2.1 |
| 48414 | 0.506213 | 2.1 |
| 48415 | 0.478349 | 2.1 |
| 48416 | 0.606116 | 2.1 |
| 48417 | 0.612092 | 2.1 |
| 48418 | 1.52226 | 2.1 |
| 48419 | 1.54241 | 2.1 |
| 48420 | 1.23167 | 2.1 |
| 48421 | 1.23167 | 2.1 |
| 48422 | 1.75555 | 2.1 |
| 48423 | 1.75555 | 2.1 |
| 48424 | 1.75557 | 2.1 |
| 48425 | 1.54243 | 2.1 |
| 48426 | 1.9943 | 2.1 |
| 48427 | 1.85871 | 2.1 |

| | | |
|---|---|---|
| 48428 | 3.53673 | 2.1 |
| 48429 | 1.85871 | 2.1 |
| 48430 | 1.06936 | 2.1 |
| 48431 | 1.88926 | 2.1 |
| 48432 | 1.54243 | 2.1 |
| 48433 | 1.41619 | 2.1 |
| 48434 | 1.06936 | 2.1 |
| 48435 | 1.28649 | 1.87748 |
| 48436 | 1.28516 | 2.1 |
| 48437 | 1.38986 | 0.314342 |
| 48438 | 1.42399 | 1.87588 |
| 48439 | 2.67502 | 1.46905 |
| 48440 | 1.42399 | 0.884173 |
| 48441 | 1.07283 | 2.1 |
| 48442 | 1.46528 | 2.1 |
| 48443 | 1.28517 | 1.08274 |
| 48444 | 1.25294 | 2.1 |
| 48445 | 1.07283 | 0.967897 |
| 48446 | 1.38986 | 2.1 |
| 48447 | 1.42399 | 2.1 |
| 48448 | 1.52885 | 1.03245 |
| 48449 | 1.60058 | 1.31332 |
| 48450 | 2.99043 | 1.41994 |
| 48451 | 0.715316 | 2.1 |
| 48452 | 0.715316 | 1.50466 |
| 48453 | 0.719126 | 0.122002 |
| 48454 | 0.719126 | 2.1 |
| 48455 | 0.722447 | 2.1 |
| 48456 | 0.626469 | 2.1 |
| 48457 | 0.711995 | 0.92284 |
| 48458 | 0.800291 | 1.05793 |
| 48459 | 0.80029 | 1.94268 |
| 48460 | 0.722447 | 1.27953 |
| 48461 | 0.711995 | 1.96645 |
| 48462 | 0.62647 | 1.93923 |
| 48463 | 2.05632 | 0.05 |
| 48464 | 1.70303 | 0.05 |
| 48465 | 2.3923 | 0.05 |
| 48466 | 2.61277 | 0.05 |
| 48467 | 1.44836 | 0.05 |
| 48468 | 1.58184 | 0.05 |
| 48469 | 1.89226 | 0.05 |
| 48470 | 1.89226 | 0.05 |
| 48471 | 1.89226 | 0.05 |
| 48472 | 1.89226 | 0.05 |
| 48473 | 3.78453 | 0.05 |
| 48474 | 1.44091 | 2.1 |
| 48475 | 1.1822 | 2.1 |
| 48476 | 1.25866 | 2.1 |
| 48477 | 1.25866 | 2.1 |
| 48478 | 1.35215 | 2.1 |
| 48479 | 1.35215 | 2.1 |
| 48480 | 1.27338 | 2.1 |
| 48481 | 1.1822 | 2.1 |
| 48482 | 1.27338 | 2.1 |
| 48483 | 1.35215 | 2.1 |
| 48484 | 1.10817 | 2.1 |
| 48485 | 1.18694 | 2.1 |

| | | |
|---|---|---|
| 48486 | 0.946132 | 0.05 |
| 48487 | 0.946132 | 0.05 |
| 48488 | 0.946132 | 0.05 |
| 48489 | 0.946132 | 0.05 |
| 48490 | 0.946133 | 0.05 |
| 48491 | 0.946133 | 0.0508716 |
| 48492 | 0.946132 | 0.05 |
| 48493 | 0.946132 | 0.05 |
| 48494 | 0.946132 | 0.05 |
| 48495 | 0.946132 | 0.05 |
| 48496 | 0.946133 | 0.05 |
| 48497 | 0.946133 | 0.05 |
| 48498 | 2.6875 | 0.05 |
| 48499 | 2.40558 | 0.05 |
| 48500 | 2.1994 | 0.05 |
| 48501 | 1.70303 | 0.05 |
| 48502 | 4.68305 | 0.05 |
| 48503 | 0.915523 | 0.05 |
| 48504 | 0.915523 | 0.05 |
| 48505 | 0.861057 | 0.05 |
| 48506 | 0.946796 | 0.05 |
| 48507 | 0.838652 | 0.05 |
| 48508 | 0.654182 | 0.05 |
| 48509 | 1.14044 | 0.05 |
| 48510 | 0.80252 | 0.05 |
| 48511 | 0.836435 | 0.05 |
| 48512 | 0.608659 | 0.05 |
| 48513 | 1.22198 | 0.05 |
| 48514 | 0.863472 | 0.05 |
| 48515 | 2.1994 | 0.05 |
| 48516 | 0.981586 | 0.05 |
| 48517 | 1.59619 | 0.05 |
| 48518 | 1.70303 | 0.05 |
| 48519 | 1.24816 | 0.05 |
| 48520 | 1.38523 | 0.05 |
| 48521 | 2.71141 | 2.1 |
| 48522 | 1.70302 | 2.1 |
| 48523 | 2.56527 | 2.1 |
| 48524 | 1.88649 | 2.1 |
| 48525 | 2.41279 | 2.1 |
| 48526 | 1.70302 | 2.1 |
| 48527 | 1.89226 | 2.1 |
| 48528 | 1.89226 | 2.1 |
| 48529 | 1.89226 | 2.1 |
| 48530 | 1.89226 | 2.1 |
| 48531 | 3.78453 | 2.1 |
| 48532 | 1.89226 | 2.1 |
| 48533 | 1.89226 | 2.1 |
| 48534 | 1.89226 | 2.1 |
| 48535 | 1.89226 | 2.1 |
| 48536 | 1.89226 | 2.1 |
| 48537 | 1.89226 | 2.1 |
| 48538 | 2.18867 | 2.1 |
| 48539 | 0.983723 | 2.1 |
| 48540 | 2.42263 | 2.1 |
| 48541 | 1.8014 | 2.1 |
| 48542 | 1.62992 | 2.1 |
| 48543 | 0.983723 | 2.1 |

| | | |
|---|---|---|
| 48544 | 2.71141 | 2.1 |
| 48545 | 1.89226 | 2.1 |
| 48546 | 2.31157 | 2.1 |
| 48547 | 2.31157 | 2.1 |
| 48548 | 1.89226 | 2.1 |
| 48549 | 1.89226 | 2.1 |
| 48550 | 1.89227 | 2.1 |
| 48551 | 1.89227 | 1.47398 |
| 48552 | 1.89227 | 1.33693 |
| 48553 | 1.89227 | 2.1 |
| 48554 | 3.78453 | 2.1 |
| 48555 | 1.89226 | 2.0828 |
| 48556 | 1.89226 | 2.1 |
| 48557 | 1.89226 | 2.1 |
| 48558 | 1.89226 | 1.83315 |
| 48559 | 1.89226 | 2.1 |
| 48560 | 1.89226 | 1.67921 |
| 48561 | 1.89226 | 1.70438 |
| 48562 | 1.89226 | 1.36588 |
| 48563 | 1.89226 | 2.1 |
| 48564 | 1.89226 | 0.946356 |
| 48565 | 1.89226 | 1.46722 |
| 48566 | 1.89226 | 0.739167 |
| 48567 | 1.1427 | 2.1 |
| 48568 | 1.14956 | 2.1 |
| 48569 | 1.70302 | 1.30507 |
| 48570 | 1.81267 | 2.1 |
| 48571 | 2.88983 | 2.1 |
| 48572 | 1.89226 | 0.943144 |
| 48573 | 1.89226 | 2.1 |
| 48574 | 1.89226 | 0.976075 |
| 48575 | 1.89226 | 1.4551 |
| 48576 | 3.78453 | 1.55847 |
| 48577 | 1.89226 | 1.63454 |
| 48578 | 1.89226 | 2.05532 |
| 48579 | 1.89226 | 0.808112 |
| 48580 | 1.89226 | 1.52957 |
| 48581 | 3.78452 | 1.11836 |
| 48582 | 0.884197 | 2.1 |
| 48583 | 1.1125 | 1.46114 |
| 48584 | 1.13273 | 1.44405 |
| 48585 | 0.844557 | 1.94496 |
| 48586 | 0.981137 | 1.19357 |
| 48587 | 1.13971 | 2.07725 |
| 48588 | 0.980432 | 2.1 |
| 48589 | 0.980432 | 1.25351 |
| 48590 | 1.15859 | 1.31503 |
| 48591 | 1.12206 | 0.984175 |
| 48592 | 0.898619 | 2.1 |
| 48593 | 0.94426 | 1.54625 |
| 48594 | 2.21016 | 1.24485 |
| 48595 | 1.44091 | 2.1 |
| 48596 | 1.98408 | 1.29025 |
| 48597 | 1.4218 | 1.13669 |
| 48598 | 2.21016 | 2.1 |
| 48599 | 1.64788 | 2.1 |
| 48600 | 1.07526 | 0.414986 |
| 48601 | 1.91531 | 2.1 |

| | | |
|---|---|---|
| 48602 | 1.49155 | 2.1 |
| 48603 | 1.58551 | 0.0797355 |
| 48604 | 1.49155 | 0.302984 |
| 48605 | 1.58551 | 2.075 |
| 48606 | 1.98979 | 0.05 |
| 48607 | 2.21773 | 0.05 |
| 48608 | 2.30533 | 0.05 |
| 48609 | 2.55895 | 0.05 |
| 48610 | 4.52305 | 0.05 |
| 48611 | 2.55896 | 0.05 |
| 48612 | 2.27963 | 0.05 |
| 48613 | 2.60486 | 0.05 |
| 48614 | 2.60486 | 0.05 |
| 48615 | 2.21773 | 0.05 |
| 48616 | 2.21773 | 0.05 |
| 48617 | 2.30533 | 0.05 |
| 48618 | 2.55895 | 0.05 |
| 48619 | 2.29539 | 0.05 |
| 48620 | 2.47308 | 0.05 |
| 48621 | 4.7784 | 0.05 |
| 48622 | 2.55896 | 0.05 |
| 48623 | 2.55792 | 0.05 |
| 48624 | 2.55792 | 0.05 |
| 48625 | 2.60486 | 0.05 |
| 48626 | 2.47308 | 0.05 |
| 48627 | 2.52003 | 0.05 |
| 48628 | 0.946133 | 1.9517 |
| 48629 | 0.946133 | 2.1 |
| 48630 | 1.20851 | 1.36426 |
| 48631 | 1.20851 | 1.30653 |
| 48632 | 0.967411 | 1.2907 |
| 48633 | 1.20851 | 1.06333 |
| 48634 | 0.946132 | 2.1 |
| 48635 | 0.946132 | 1.96709 |
| 48636 | 0.946132 | 2.08728 |
| 48637 | 1.20851 | 1.2895 |
| 48638 | 0.967412 | 1.34856 |
| 48639 | 0.946133 | 1.89818 |
| 48640 | 1.89226 | 2.02718 |
| 48641 | 1.89226 | 1.18828 |
| 48642 | 1.89226 | 1.1838 |
| 48643 | 1.89226 | 1.21935 |
| 48644 | 1.89226 | 2.0899 |
| 48645 | 1.89226 | 2.0902 |
| 48646 | 1.89226 | 2.03534 |
| 48647 | 2.41701 | 1.76524 |
| 48648 | 2.71724 | 1.15076 |
| 48649 | 2.71724 | 1.56316 |
| 48650 | 1.89226 | 2.1 |
| 48651 | 1.89226 | 2.1 |
| 48652 | 0.946131 | 2.1 |
| 48653 | 0.946131 | 2.1 |
| 48654 | 0.946128 | 1.72128 |
| 48655 | 0.946128 | 2.02062 |
| 48656 | 0.94613 | 1.0535 |
| 48657 | 0.94613 | 1.24142 |
| 48658 | 0.94613 | 2.1 |
| 48659 | 0.94613 | 2.1 |

| | | |
|---|---|---|
| 48660 | 0.94613 | 1.95063 |
| 48661 | 0.94613 | 1.15951 |
| 48662 | 0.94613 | 1.43923 |
| 48663 | 0.94613 | 2.1 |
| 48664 | 0.851541 | 0.05 |
| 48665 | 0.851541 | 0.05 |
| 48666 | 0.851481 | 0.05 |
| 48667 | 0.997219 | 0.05 |
| 48668 | 0.997112 | 0.05 |
| 48669 | 0.992853 | 0.05 |
| 48670 | 0.85154 | 0.05 |
| 48671 | 0.85154 | 0.05 |
| 48672 | 0.894824 | 0.05 |
| 48673 | 0.894824 | 0.05 |
| 48674 | 0.94613 | 0.05 |
| 48675 | 0.906485 | 0.05 |
| 48676 | 0.802947 | 2.1 |
| 48677 | 0.845556 | 2.1 |
| 48678 | 0.582339 | 2.1 |
| 48679 | 0.519756 | 2.1 |
| 48680 | 0.714508 | 2.1 |
| 48681 | 0.714508 | 2.1 |
| 48682 | 0.628932 | 2.1 |
| 48683 | 0.708518 | 2.1 |
| 48684 | 0.801358 | 2.1 |
| 48685 | 0.476016 | 2.1 |
| 48686 | 0.569477 | 2.1 |
| 48687 | 0.878553 | 2.1 |
| 48688 | 1.89226 | 2.1 |
| 48689 | 1.89226 | 2.1 |
| 48690 | 1.89226 | 2.1 |
| 48691 | 1.89226 | 2.1 |
| 48692 | 1.89226 | 2.1 |
| 48693 | 1.89226 | 2.1 |
| 48694 | 0.946128 | 2.1 |
| 48695 | 0.946128 | 2.1 |
| 48696 | 1.01537 | 2.1 |
| 48697 | 0.9662 | 2.1 |
| 48698 | 0.946131 | 2.1 |
| 48699 | 0.946131 | 2.1 |
| 48700 | 0.966198 | 2.1 |
| 48701 | 0.966198 | 2.1 |
| 48702 | 0.94613 | 2.1 |
| 48703 | 0.966198 | 2.1 |
| 48704 | 0.946131 | 2.1 |
| 48705 | 1.01537 | 2.1 |
| 48706 | 1.89226 | 2.1 |
| 48707 | 1.89226 | 2.1 |
| 48708 | 1.91883 | 2.1 |
| 48709 | 1.91883 | 2.1 |
| 48710 | 2.15905 | 2.1 |
| 48711 | 2.13249 | 2.1 |
| 48712 | 1.07953 | 2.1 |
| 48713 | 1.2107 | 2.1 |
| 48714 | 0.946132 | 2.1 |
| 48715 | 0.946132 | 2.1 |
| 48716 | 1.2107 | 2.1 |
| 48717 | 1.2107 | 2.1 |

| | | |
|---|---|---|
| 48718 | 0.946132 | 2.1 |
| 48719 | 0.946132 | 2.1 |
| 48720 | 1.2107 | 2.1 |
| 48721 | 0.946133 | 2.1 |
| 48722 | 0.946132 | 2.1 |
| 48723 | 1.07953 | 2.1 |
| 48724 | 2.46752 | 2.1 |
| 48725 | 2.53733 | 2.1 |
| 48726 | 2.26044 | 2.1 |
| 48727 | 2.33222 | 1.73982 |
| 48728 | 4.79974 | 2.1 |
| 48729 | 2.53733 | 2.1 |
| 48730 | 2.37576 | 0.05 |
| 48731 | 2.33222 | 2.1 |
| 48732 | 2.1845 | 2.1 |
| 48733 | 4.70798 | 2.1 |
| 48734 | 2.48652 | 2.1 |
| 48735 | 2.33224 | 1.58008 |
| 48736 | 2.08149 | 2.1 |
| 48737 | 1.9839 | 2.1 |
| 48738 | 4.45623 | 2.1 |
| 48739 | 2.33223 | 2.06065 |
| 48740 | 2.18452 | 1.43676 |
| 48741 | 2.16061 | 2.1 |
| 48742 | 2.01399 | 0.143113 |
| 48743 | 4.34623 | 0.940086 |
| 48744 | 1.4617 | 1.16092 |
| 48745 | 1.34701 | 2.1 |
| 48746 | 1.34701 | 1.34145 |
| 48747 | 1.25173 | 2.1 |
| 48748 | 1.5117 | 1.04123 |
| 48749 | 1.41642 | 2.1 |
| 48750 | 2.65164 | 2.1 |
| 48751 | 1.89227 | 2.1 |
| 48752 | 1.89227 | 2.1 |
| 48753 | 1.89227 | 2.1 |
| 48754 | 2.42504 | 2.1 |
| 48755 | 2.42504 | 2.1 |
| 48756 | 1.89226 | 0.549171 |
| 48757 | 1.89226 | 2.04375 |
| 48758 | 1.89226 | 2.1 |
| 48759 | 1.89226 | 0.198466 |
| 48760 | 3.78452 | 0.723827 |
| 48761 | 1.5117 | 1.29617 |
| 48762 | 1.34701 | 2.1 |
| 48763 | 1.47001 | 2.1 |
| 48764 | 1.57961 | 0.338697 |
| 48765 | 2.92663 | 2.05833 |
| 48766 | 1.89226 | 2.1 |
| 48767 | 1.89226 | 0.287987 |
| 48768 | 1.89226 | 0.255619 |
| 48769 | 1.89226 | 1.87837 |
| 48770 | 3.78452 | 1.09514 |
| 48771 | 0.412773 | 0.05 |
| 48772 | 0.570839 | 0.05 |
| 48773 | 0.50721 | 0.05 |
| 48774 | 0.513638 | 0.05 |
| 48775 | 0.518805 | 0.05 |

| | | |
|---|---|---|
| 48776 | 0.41278 | 0.05 |
| 48777 | 1.5344 | 0.05 |
| 48778 | 0.939282 | 0.05 |
| 48779 | 0.720051 | 0.05 |
| 48780 | 0.691767 | 0.05 |
| 48781 | 0.761152 | 0.05 |
| 48782 | 1.89226 | 2.1 |
| 48783 | 1.89226 | 2.1 |
| 48784 | 1.89226 | 2.1 |
| 48785 | 1.89226 | 2.1 |
| 48786 | 1.89226 | 2.1 |
| 48787 | 1.89226 | 2.1 |
| 48788 | 1.24618 | 2.1 |
| 48789 | 2.10585 | 2.1 |
| 48790 | 1.08134 | 2.1 |
| 48791 | 1.93032 | 2.1 |
| 48792 | 3.1765 | 2.1 |
| 48793 | 1.98474 | 2.1 |
| 48794 | 2.57777 | 2.1 |
| 48795 | 1.93031 | 2.1 |
| 48796 | 2.19041 | 2.1 |
| 48797 | 3.99251 | 2.1 |
| 48798 | 1.89226 | 2.1 |
| 48799 | 1.89226 | 2.1 |
| 48800 | 1.89226 | 2.1 |
| 48801 | 1.89226 | 2.1 |
| 48802 | 3.78452 | 2.1 |
| 48803 | 1.67374 | 2.1 |
| 48804 | 2.19042 | 2.1 |
| 48805 | 1.67374 | 2.1 |
| 48806 | 2.09769 | 2.1 |
| 48807 | 2.29586 | 2.1 |
| 48808 | 2.7198 | 2.1 |
| 48809 | 1.89226 | 2.1 |
| 48810 | 1.89226 | 2.1 |
| 48811 | 1.89226 | 2.1 |
| 48812 | 1.89226 | 2.1 |
| 48813 | 1.89226 | 2.1 |
| 48814 | 1.89226 | 2.1 |
| 48815 | 0.946132 | 0.295392 |
| 48816 | 0.946132 | 0.146802 |
| 48817 | 0.947763 | 0.131881 |
| 48818 | 0.909304 | 0.166937 |
| 48819 | 0.946127 | 0.0924463 |
| 48820 | 0.947831 | 0.05 |
| 48821 | 0.907673 | 0.0603207 |
| 48822 | 0.907673 | 0.245064 |
| 48823 | 0.94613 | 0.110923 |
| 48824 | 0.947834 | 0.05 |
| 48825 | 0.907671 | 0.465425 |
| 48826 | 0.94613 | 0.05 |
| 48827 | 0.906519 | 0.0744786 |
| 48828 | 0.906519 | 0.05 |
| 48829 | 0.946097 | 0.05 |
| 48830 | 0.946097 | 0.0590528 |
| 48831 | 0.947834 | 0.0518562 |
| 48832 | 0.906485 | 0.05 |
| 48833 | 0.946131 | 0.05 |

| | | |
|---|---|---|
| 48834 | 0.946131 | 0.064711 |
| 48835 | 0.94613 | 0.0951153 |
| 48836 | 0.906485 | 0.05 |
| 48837 | 0.946131 | 0.0912151 |
| 48838 | 0.947835 | 0.05 |
| 48839 | 2.58638 | 2.1 |
| 48840 | 2.51039 | 2.1 |
| 48841 | 2.47395 | 2.1 |
| 48842 | 2.41539 | 2.1 |
| 48843 | 5.00178 | 2.1 |
| 48844 | 2.51039 | 2.1 |
| 48845 | 2.16633 | 2.1 |
| 48846 | 2.4154 | 2.1 |
| 48847 | 2.13977 | 2.1 |
| 48848 | 4.58173 | 2.1 |
| 48849 | 2.47395 | 2.1 |
| 48850 | 2.41539 | 2.1 |
| 48851 | 2.2988 | 2.1 |
| 48852 | 2.2604 | 2.1 |
| 48853 | 4.7142 | 2.1 |
| 48854 | 2.4154 | 2.1 |
| 48855 | 2.13977 | 2.1 |
| 48856 | 2.26041 | 2.1 |
| 48857 | 1.98957 | 2.1 |
| 48858 | 4.40497 | 2.1 |
| 48859 | 2.15721 | 0.05 |
| 48860 | 2.31843 | 0.0530412 |
| 48861 | 1.93973 | 0.104546 |
| 48862 | 2.13243 | 0.05 |
| 48863 | 4.25816 | 0.05 |
| 48864 | 2.31844 | 0.387809 |
| 48865 | 2.30908 | 0.05 |
| 48866 | 2.13243 | 0.05 |
| 48867 | 2.1609 | 0.0680439 |
| 48868 | 4.47934 | 0.05 |
| 48869 | 1.93973 | 0.572916 |
| 48870 | 2.13243 | 0.0554838 |
| 48871 | 1.69661 | 0.072704 |
| 48872 | 1.9339 | 0.05 |
| 48873 | 3.87363 | 0.05 |
| 48874 | 2.13243 | 0.11386 |
| 48875 | 2.1609 | 0.186519 |
| 48876 | 1.9339 | 0.05 |
| 48877 | 1.98414 | 0.322495 |
| 48878 | 4.0948 | 0.152866 |
| 48879 | 1.88857 | 0.05 |
| 48880 | 2.34107 | 0.05 |
| 48881 | 0.502431 | 0.05 |
| 48882 | 0.71416 | 0.05 |
| 48883 | 0.50243 | 0.05 |
| 48884 | 0.944284 | 0.05 |
| 48885 | 1.88856 | 2.1 |
| 48886 | 2.34107 | 2.1 |
| 48887 | 2.39662 | 2.1 |
| 48888 | 1.92293 | 2.1 |
| 48889 | 4.28519 | 2.1 |
| 48890 | 1.00486 | 2.1 |
| 48891 | 1.92293 | 2.1 |

| | | |
|---|---|---|
| 48892 | 1.88857 | 2.1 |
| 48893 | 1.88857 | 2.1 |
| 48894 | 1.02605 | 2.1 |
| 48895 | 1.02605 | 2.1 |
| 48896 | 2.53728 | 2.1 |
| 48897 | 1.70303 | 2.1 |
| 48898 | 2.03734 | 2.1 |
| 48899 | 1.3624 | 2.1 |
| 48900 | 2.54706 | 2.1 |
| 48901 | 1.70303 | 2.1 |
| 48902 | 2.54706 | 2.1 |
| 48903 | 1.50876 | 2.1 |
| 48904 | 2.74067 | 2.1 |
| 48905 | 1.85391 | 2.1 |
| 48906 | 2.29667 | 2.1 |
| 48907 | 1.50876 | 2.1 |
| 48908 | 0.946133 | 2.1 |
| 48909 | 0.946133 | 2.1 |
| 48910 | 1.24191 | 2.1 |
| 48911 | 1.24191 | 2.1 |
| 48912 | 0.946132 | 2.1 |
| 48913 | 1.24191 | 2.1 |
| 48914 | 1.23779 | 2.1 |
| 48915 | 0.946132 | 2.1 |
| 48916 | 0.946132 | 2.1 |
| 48917 | 0.946132 | 2.1 |
| 48918 | 1.23779 | 2.1 |
| 48919 | 1.24191 | 2.1 |
| 48920 | 0.946131 | 2.1 |
| 48921 | 0.946131 | 2.1 |
| 48922 | 1.14833 | 2.1 |
| 48923 | 1.14833 | 2.1 |
| 48924 | 1.14834 | 2.1 |
| 48925 | 0.946132 | 2.1 |
| 48926 | 0.94613 | 2.1 |
| 48927 | 1.24191 | 2.1 |
| 48928 | 0.946131 | 2.1 |
| 48929 | 0.946131 | 2.1 |
| 48930 | 1.24191 | 2.1 |
| 48931 | 1.14833 | 2.1 |
| 48932 | 2.08149 | 2.1 |
| 48933 | 2.31116 | 2.1 |
| 48934 | 1.9701 | 1.22141 |
| 48935 | 2.18328 | 2.1 |
| 48936 | 4.49563 | 2.1 |
| 48937 | 2.08149 | 2.1 |
| 48938 | 2.14059 | 2.1 |
| 48939 | 1.72377 | 2.1 |
| 48940 | 2.18328 | 2.1 |
| 48941 | 3.88931 | 2.1 |
| 48942 | 2.14059 | 2.1 |
| 48943 | 2.40755 | 2.1 |
| 48944 | 2.31145 | 2.1 |
| 48945 | 2.23743 | 2.1 |
| 48946 | 2.31145 | 2.1 |
| 48947 | 2.23743 | 2.1 |
| 48948 | 2.31117 | 2.1 |
| 48949 | 2.40754 | 2.1 |

| | | |
|---|---|---|
| 48950 | 2.41055 | 2.1 |
| 48951 | 2.31145 | 2.1 |
| 48952 | 2.41055 | 2.1 |
| 48953 | 2.31145 | 2.1 |
| 48954 | 2.5615 | 0.0791905 |
| 48955 | 2.37625 | 0.120512 |
| 48956 | 2.20248 | 0.0634762 |
| 48957 | 2.20248 | 0.05 |
| 48958 | 2.65967 | 0.317223 |
| 48959 | 2.65967 | 0.171684 |
| 48960 | 2.71793 | 0.297461 |
| 48961 | 2.65966 | 0.0978912 |
| 48962 | 2.56149 | 0.05 |
| 48963 | 2.56149 | 0.05 |
| 48964 | 2.71296 | 0.168172 |
| 48965 | 2.71296 | 0.160643 |
| 48966 | 1.31528 | 0.103467 |
| 48967 | 1.32983 | 0.0622756 |
| 48968 | 1.21128 | 0.0866405 |
| 48969 | 1.18812 | 0.0574251 |
| 48970 | 1.24232 | 0.0537701 |
| 48971 | 1.24232 | 0.051354 |
| 48972 | 1.26109 | 0.108151 |
| 48973 | 1.26109 | 0.156494 |
| 48974 | 1.21128 | 0.158122 |
| 48975 | 1.31528 | 0.05 |
| 48976 | 1.18812 | 0.05 |
| 48977 | 1.32983 | 0.0953348 |
| 48978 | 2.63056 | 0.05 |
| 48979 | 2.58032 | 0.0597748 |
| 48980 | 2.65966 | 0.05 |
| 48981 | 2.65966 | 0.0617663 |
| 48982 | 2.58032 | 0.104264 |
| 48983 | 2.71296 | 0.161693 |
| 48984 | 1.92039 | 2.1 |
| 48985 | 1.06226 | 2.1 |
| 48986 | 1.09252 | 2.1 |
| 48987 | 1.96052 | 2.1 |
| 48988 | 3.01291 | 2.1 |
| 48989 | 1.92039 | 2.1 |
| 48990 | 2.46338 | 2.1 |
| 48991 | 2.47522 | 2.1 |
| 48992 | 2.43508 | 2.1 |
| 48993 | 1.96052 | 2.1 |
| 48994 | 1.92039 | 2.1 |
| 48995 | 0.985949 | 2.1 |
| 48996 | 0.980261 | 2.1 |
| 48997 | 0.553625 | 2.1 |
| 48998 | 0.546262 | 2.1 |
| 48999 | 0.765671 | 2.1 |
| 49000 | 0.765671 | 2.1 |
| 49001 | 0.766539 | 2.1 |
| 49002 | 0.766539 | 2.1 |
| 49003 | 0.985949 | 2.1 |
| 49004 | 0.553625 | 2.1 |
| 49005 | 0.546261 | 2.1 |
| 49006 | 0.980261 | 2.1 |
| 49007 | 1.96052 | 2.1 |

| | | |
|---|---|---|
| 49008 | 2.46338 | 2.1 |
| 49009 | 1.97189 | 2.1 |
| 49010 | 1.96052 | 2.1 |
| 49011 | 2.47932 | 2.1 |
| 49012 | 2.47932 | 2.1 |
| 49013 | 0.964608 | 0.05 |
| 49014 | 0.691524 | 0.05 |
| 49015 | 1.25462 | 0.05 |
| 49016 | 0.691524 | 0.05 |
| 49017 | 1.89226 | 0.05 |
| 49018 | 1.89226 | 0.05 |
| 49019 | 1.44894 | 0.05 |
| 49020 | 2.77655 | 0.05 |
| 49021 | 1.4237 | 0.05 |
| 49022 | 2.77701 | 0.05 |
| 49023 | 2.77701 | 0.05 |
| 49024 | 1.38847 | 2.1 |
| 49025 | 1.46031 | 2.0014 |
| 49026 | 1.65504 | 1.38736 |
| 49027 | 1.75396 | 1.30921 |
| 49028 | 3.11535 | 1.67837 |
| 49029 | 1.89226 | 1.81987 |
| 49030 | 1.89226 | 2.1 |
| 49031 | 1.89226 | 1.54797 |
| 49032 | 1.89226 | 1.06436 |
| 49033 | 3.78452 | 1.8775 |
| 49034 | 1.89226 | 1.51233 |
| 49035 | 1.89226 | 2.1 |
| 49036 | 1.89226 | 0.862177 |
| 49037 | 1.89226 | 1.94189 |
| 49038 | 3.78452 | 1.07367 |
| 49039 | 1.46031 | 1.27878 |
| 49040 | 1.34851 | 2.1 |
| 49041 | 1.75395 | 1.17513 |
| 49042 | 1.62287 | 1.39627 |
| 49043 | 3.08318 | 1.89932 |
| 49044 | 1.65504 | 1.83425 |
| 49045 | 1.75396 | 1.97026 |
| 49046 | 1.89249 | 2.08306 |
| 49047 | 1.9857 | 1.20975 |
| 49048 | 3.64073 | 1.89292 |
| 49049 | 1.89226 | 2.06259 |
| 49050 | 1.89226 | 1.79599 |
| 49051 | 1.89226 | 1.14131 |
| 49052 | 1.89226 | 1.34119 |
| 49053 | 3.78452 | 1.24194 |
| 49054 | 1.89226 | 1.13388 |
| 49055 | 1.89226 | 2.1 |
| 49056 | 1.89226 | 1.25367 |
| 49057 | 1.89226 | 1.77285 |
| 49058 | 3.78452 | 2.07602 |
| 49059 | 1.75395 | 1.68912 |
| 49060 | 1.62287 | 1.97193 |
| 49061 | 1.98569 | 1.06417 |
| 49062 | 1.86067 | 1.29147 |
| 49063 | 3.60856 | 1.23544 |
| 49064 | 1.35697 | 2.1 |
| 49065 | 2.02064 | 2.1 |

| | | |
|---|---|---|
| 49066 | 1.89989 | 0.195498 |
| 49067 | 1.49571 | 2.1 |
| 49068 | 1.66753 | 1.01967 |
| 49069 | 1.36431 | 2.1 |
| 49070 | 0.744004 | 2.1 |
| 49071 | 0.743997 | 2.1 |
| 49072 | 0.717972 | 0.176416 |
| 49073 | 0.717972 | 2.1 |
| 49074 | 0.997982 | 2.1 |
| 49075 | 0.807796 | 2.1 |
| 49076 | 0.632235 | 0.748455 |
| 49077 | 0.488575 | 2.1 |
| 49078 | 0.849275 | 2.1 |
| 49079 | 0.488581 | 2.1 |
| 49080 | 0.724729 | 2.1 |
| 49081 | 0.997967 | 2.1 |
| 49082 | 2.5276 | 2.1 |
| 49083 | 1.70791 | 2.08792 |
| 49084 | 1.82872 | 0.178759 |
| 49085 | 1.56362 | 2.1 |
| 49086 | 2.5276 | 1.15106 |
| 49087 | 2.24781 | 2.1 |
| 49088 | 1.37591 | 2.1 |
| 49089 | 1.23217 | 1.89122 |
| 49090 | 1.00442 | 2.0106 |
| 49091 | 0.945282 | 0.792952 |
| 49092 | 1.15446 | 1.11943 |
| 49093 | 1.09123 | 2.1 |
| 49094 | 1.21506 | 2.1 |
| 49095 | 1.21506 | 1.15519 |
| 49096 | 1.39452 | 1.27663 |
| 49097 | 1.13108 | 2.0055 |
| 49098 | 1.27876 | 1.19239 |
| 49099 | 0.929006 | 1.01507 |
| 49100 | 1.46505 | 0.05 |
| 49101 | 1.91484 | 0.05 |
| 49102 | 1.49906 | 0.05 |
| 49103 | 1.49906 | 0.05 |
| 49104 | 1.90196 | 0.05 |
| 49105 | 1.90196 | 0.05 |
| 49106 | 1.90196 | 0.05 |
| 49107 | 2.11465 | 0.05 |
| 49108 | 1.91484 | 0.05 |
| 49109 | 1.91484 | 0.05 |
| 49110 | 2.04016 | 0.05 |
| 49111 | 2.04016 | 0.05 |
| 49112 | 0.912201 | 0.05 |
| 49113 | 0.950978 | 0.05 |
| 49114 | 0.665307 | 0.05 |
| 49115 | 0.732528 | 0.05 |
| 49116 | 0.829512 | 0.05 |
| 49117 | 0.829512 | 0.05 |
| 49118 | 0.815217 | 0.05 |
| 49119 | 0.815217 | 0.05 |
| 49120 | 0.912202 | 0.05 |
| 49121 | 0.665305 | 0.05 |
| 49122 | 0.732527 | 0.05 |
| 49123 | 0.95098 | 0.05 |

| | | |
|---|---|---|
| 49124 | 1.90196 | 0.05 |
| 49125 | 2.08757 | 0.05 |
| 49126 | 1.9626 | 0.05 |
| 49127 | 2.04016 | 0.05 |
| 49128 | 1.82441 | 0.05 |
| 49129 | 1.90196 | 0.05 |
| 49130 | 1.13048 | 2.1 |
| 49131 | 0.821162 | 2.1 |
| 49132 | 0.946131 | 2.1 |
| 49133 | 0.946131 | 2.1 |
| 49134 | 1.04567 | 2.1 |
| 49135 | 0.882347 | 2.1 |
| 49136 | 0.897276 | 2.1 |
| 49137 | 0.897276 | 2.1 |
| 49138 | 0.863109 | 2.1 |
| 49139 | 1.01055 | 2.1 |
| 49140 | 0.940305 | 2.1 |
| 49141 | 0.942166 | 2.1 |
| 49142 | 2.23933 | 0.05 |
| 49143 | 0.976217 | 0.05 |
| 49144 | 2.78053 | 0.05 |
| 49145 | 2.78053 | 0.05 |
| 49146 | 0.976217 | 0.05 |
| 49147 | 1.65984 | 0.05 |
| 49148 | 1.54168 | 2.1 |
| 49149 | 2.01197 | 1.85098 |
| 49150 | 1.69136 | 2.1 |
| 49151 | 2.13284 | 0.979122 |
| 49152 | 3.67452 | 2.1 |
| 49153 | 0.988805 | 2.1 |
| 49154 | 1.78069 | 2.1 |
| 49155 | 1.69136 | 2.1 |
| 49156 | 1.69136 | 2.1 |
| 49157 | 1.05481 | 2.1 |
| 49158 | 1.05481 | 2.1 |
| 49159 | 1.69136 | 2.1 |
| 49160 | 2.13284 | 1.28848 |
| 49161 | 1.78069 | 2.1 |
| 49162 | 2.22044 | 1.72076 |
| 49163 | 3.91353 | 2.1 |
| 49164 | 2.69379 | 0.990708 |
| 49165 | 2.32219 | 1.10551 |
| 49166 | 2.25746 | 2.06735 |
| 49167 | 2.25746 | 0.953384 |
| 49168 | 2.32219 | 1.1405 |
| 49169 | 1.89526 | 0.875868 |
| 49170 | 1.4556 | 2.1 |
| 49171 | 1.85028 | 2.1 |
| 49172 | 2.4909 | 0.0861706 |
| 49173 | 2.03799 | 2.1 |
| 49174 | 2.73495 | 1.70112 |
| 49175 | 1.85028 | 2.1 |
| 49176 | 2.71938 | 0.162246 |
| 49177 | 1.85028 | 2.1 |
| 49178 | 3.36051 | 2.1 |
| 49179 | 2.73495 | 2.03936 |
| 49180 | 2.47584 | 2.1 |
| 49181 | 1.85028 | 0.558904 |

| | | |
|---|---|---|
| 49182 | 2.47584 | 2.1 |
| 49183 | 1.04413 | 2.1 |
| 49184 | 2.1461 | 2.1 |
| 49185 | 1.74586 | 2.1 |
| 49186 | 1.44437 | 2.1 |
| 49187 | 1.04413 | 2.1 |
| 49188 | 1.89226 | 2.1 |
| 49189 | 1.89226 | 1.29367 |
| 49190 | 1.89226 | 2.1 |
| 49191 | 1.89226 | 2.02549 |
| 49192 | 3.78452 | 2.1 |
| 49193 | 1.52724 | 2.1 |
| 49194 | 1.89249 | 2.1 |
| 49195 | 1.68143 | 2.1 |
| 49196 | 2.02433 | 1.66686 |
| 49197 | 3.55157 | 2.1 |
| 49198 | 1.07444 | 2.1 |
| 49199 | 1.76545 | 2.1 |
| 49200 | 1.68142 | 2.1 |
| 49201 | 1.68142 | 2.1 |
| 49202 | 1.14807 | 2.1 |
| 49203 | 1.14807 | 2.1 |
| 49204 | 1.89226 | 2.1 |
| 49205 | 1.89226 | 2.1 |
| 49206 | 1.89226 | 2.1 |
| 49207 | 1.89226 | 2.1 |
| 49208 | 1.89226 | 2.1 |
| 49209 | 1.89226 | 0.844903 |
| 49210 | 1.89226 | 2.1 |
| 49211 | 1.89226 | 2.1 |
| 49212 | 1.89226 | 2.1 |
| 49213 | 1.89226 | 0.978888 |
| 49214 | 3.78452 | 2.1 |
| 49215 | 1.68143 | 2.1 |
| 49216 | 2.02433 | 2.05601 |
| 49217 | 1.76545 | 2.1 |
| 49218 | 2.12608 | 2.1 |
| 49219 | 3.78978 | 2.1 |
| 49220 | 2.62871 | 2.1 |
| 49221 | 1.49149 | 2.1 |
| 49222 | 1.36814 | 2.1 |
| 49223 | 2.44761 | 2.08769 |
| 49224 | 3.9391 | 2.1 |
| 49225 | 1.89226 | 2.1 |
| 49226 | 1.89226 | 0.577038 |
| 49227 | 1.89226 | 2.1 |
| 49228 | 1.89226 | 2.1 |
| 49229 | 3.78452 | 2.1 |
| 49230 | 0.851542 | 2.1 |
| 49231 | 0.851542 | 2.1 |
| 49232 | 1.00897 | 2.1 |
| 49233 | 0.931224 | 2.1 |
| 49234 | 0.838952 | 2.1 |
| 49235 | 0.838952 | 2.08367 |
| 49236 | 0.956848 | 2.1 |
| 49237 | 0.942474 | 2.1 |
| 49238 | 0.946132 | 2.1 |
| 49239 | 0.838952 | 2.1 |

| | | |
|---|---|---|
| 49240 | 0.878743 | 2.1 |
| 49241 | 0.946132 | 2.1 |
| 49242 | 1.15541 | 2.1 |
| 49243 | 2.44761 | 0.832966 |
| 49244 | 1.36814 | 2.1 |
| 49245 | 1.36814 | 2.1 |
| 49246 | 2.19439 | 2.1 |
| 49247 | 2.19439 | 2.1 |
| 49248 | 1.89226 | 0.828446 |
| 49249 | 1.89226 | 2.1 |
| 49250 | 1.89226 | 2.1 |
| 49251 | 1.89226 | 0.774009 |
| 49252 | 3.78452 | 2.1 |
| 49253 | 1.89226 | 2.1 |
| 49254 | 1.67791 | 2.1 |
| 49255 | 1.5713 | 2.1 |
| 49256 | 1.67791 | 2.1 |
| 49257 | 1.78566 | 2.1 |
| 49258 | 1.89226 | 2.1 |
| 49259 | 1.89226 | 0.102598 |
| 49260 | 1.62911 | 2.1 |
| 49261 | 2.24602 | 2.0907 |
| 49262 | 1.89226 | 2.1 |
| 49263 | 1.98287 | 0.914781 |
| 49264 | 1.62911 | 0.974971 |
| 49265 | 1.89226 | 2.1 |
| 49266 | 1.89226 | 2.1 |
| 49267 | 1.89226 | 0.721671 |
| 49268 | 1.89226 | 2.1 |
| 49269 | 3.78452 | 1.41217 |
| 49270 | 1.26688 | 2.1 |
| 49271 | 1.11804 | 2.1 |
| 49272 | 0.852467 | 1.19204 |
| 49273 | 0.693314 | 2.1 |
| 49274 | 0.977459 | 2.1 |
| 49275 | 0.977459 | 2.1 |
| 49276 | 0.982741 | 1.07126 |
| 49277 | 0.982741 | 2.1 |
| 49278 | 1.26689 | 2.1 |
| 49279 | 0.852464 | 2.1 |
| 49280 | 0.693312 | 2.1 |
| 49281 | 1.11804 | 2.1 |
| 49282 | 0.946131 | 2.1 |
| 49283 | 0.946131 | 0.88279 |
| 49284 | 1.1794 | 0.890294 |
| 49285 | 1.1794 | 2.1 |
| 49286 | 0.991434 | 2.1 |
| 49287 | 1.1794 | 1.06069 |
| 49288 | 0.946131 | 0.443964 |
| 49289 | 0.946131 | 0.87321 |
| 49290 | 0.946131 | 2.1 |
| 49291 | 1.1794 | 1.64819 |
| 49292 | 0.946131 | 2.1 |
| 49293 | 0.991434 | 1.25339 |
| 49294 | 1.89226 | 0.849878 |
| 49295 | 1.89226 | 2.1 |
| 49296 | 1.89226 | 0.718466 |
| 49297 | 1.89226 | 1.36651 |

| | | |
|---|---|---|
| 49298 | 3.78452 | 2.1 |
| 49299 | 2.53377 | 2.1 |
| 49300 | 1.53443 | 2.1 |
| 49301 | 2.0815 | 0.384632 |
| 49302 | 2.82979 | 2.1 |
| 49303 | 4.35462 | 2.1 |
| 49304 | 1.96192 | 0.440871 |
| 49305 | 1.23602 | 2.1 |
| 49306 | 2.16506 | 0.364806 |
| 49307 | 1.56841 | 2.03708 |
| 49308 | 3.40108 | 1.62285 |
| 49309 | 1.89226 | 1.66094 |
| 49310 | 1.89226 | 2.1 |
| 49311 | 1.89226 | 0.432525 |
| 49312 | 1.89226 | 2.1 |
| 49313 | 3.78452 | 1.71476 |
| 49314 | 0.946129 | 2.1 |
| 49315 | 0.946129 | 2.1 |
| 49316 | 1.13067 | 1.02202 |
| 49317 | 1.12952 | 2.1 |
| 49318 | 0.946129 | 2.1 |
| 49319 | 1.13089 | 2.1 |
| 49320 | 0.944978 | 1.0102 |
| 49321 | 0.944978 | 2.1 |
| 49322 | 0.946131 | 2.1 |
| 49323 | 1.13089 | 2.1 |
| 49324 | 0.944977 | 2.1 |
| 49325 | 0.946131 | 2.1 |
| 49326 | 0.710208 | 2.1 |
| 49327 | 0.710208 | 2.1 |
| 49328 | 0.718894 | 2.1 |
| 49329 | 0.718894 | 2.1 |
| 49330 | 0.559773 | 2.1 |
| 49331 | 0.579321 | 2.1 |
| 49332 | 0.886767 | 2.1 |
| 49333 | 0.849781 | 2.1 |
| 49334 | 0.849781 | 2.1 |
| 49335 | 0.559774 | 2.1 |
| 49336 | 0.579321 | 2.1 |
| 49337 | 0.886766 | 2.1 |
| 49338 | 1.63471 | 2.1 |
| 49339 | 1.69956 | 2.1 |
| 49340 | 1.08022 | 2.1 |
| 49341 | 1.08022 | 2.1 |
| 49342 | 1.11955 | 2.1 |
| 49343 | 1.67403 | 2.1 |
| 49344 | 1.03766 | 2.1 |
| 49345 | 2.02825 | 2.1 |
| 49346 | 2.02794 | 2.1 |
| 49347 | 2.0348 | 2.1 |
| 49348 | 1.07196 | 2.1 |
| 49349 | 1.07196 | 2.1 |
| 49350 | 1.9276 | 2.1 |
| 49351 | 2.1004 | 2.1 |
| 49352 | 2.1306 | 2.1 |
| 49353 | 2.1306 | 2.1 |
| 49354 | 1.92178 | 2.1 |
| 49355 | 1.92032 | 2.1 |

| | | |
|---|---|---|
| 49356 | 0.986724 | 2.1 |
| 49357 | 2.03064 | 2.1 |
| 49358 | 2.01385 | 2.1 |
| 49359 | 2.02404 | 2.1 |
| 49360 | 1.03766 | 2.1 |
| 49361 | 1.03766 | 2.1 |
| 49362 | 1.92027 | 2.1 |
| 49363 | 2.1306 | 2.1 |
| 49364 | 2.15388 | 2.1 |
| 49365 | 2.15388 | 2.1 |
| 49366 | 1.92614 | 2.1 |
| 49367 | 1.9276 | 2.1 |
| 49368 | 1.09578 | 2.1 |
| 49369 | 0.963018 | 2.1 |
| 49370 | 0.782956 | 1.24431 |
| 49371 | 0.529693 | 2.1 |
| 49372 | 0.6994 | 1.67461 |
| 49373 | 0.862532 | 2.1 |
| 49374 | 0.898605 | 2.1 |
| 49375 | 0.898605 | 1.63292 |
| 49376 | 0.776756 | 2.1 |
| 49377 | 1.14805 | 2.1 |
| 49378 | 0.966521 | 2.1 |
| 49379 | 0.617676 | 2.1 |
| 49380 | 2.67185 | 2.1 |
| 49381 | 2.099 | 2.1 |
| 49382 | 2.80548 | 2.1 |
| 49383 | 2.2727 | 2.1 |
| 49384 | 4.94455 | 2.1 |
| 49385 | 1.89226 | 2.1 |
| 49386 | 1.89226 | 2.1 |
| 49387 | 1.89226 | 2.1 |
| 49388 | 1.89226 | 2.1 |
| 49389 | 3.78453 | 2.1 |
| 49390 | 1.89226 | 2.1 |
| 49391 | 1.89226 | 2.1 |
| 49392 | 1.89226 | 2.1 |
| 49393 | 1.89226 | 2.1 |
| 49394 | 1.89226 | 2.1 |
| 49395 | 1.89226 | 2.1 |
| 49396 | 0.884801 | 2.1 |
| 49397 | 0.884801 | 2.1 |
| 49398 | 0.895199 | 2.1 |
| 49399 | 0.895199 | 2.1 |
| 49400 | 0.622774 | 2.1 |
| 49401 | 0.730501 | 2.1 |
| 49402 | 1.0495 | 2.1 |
| 49403 | 1.13635 | 2.1 |
| 49404 | 1.13635 | 2.1 |
| 49405 | 0.730503 | 2.1 |
| 49406 | 1.0495 | 2.1 |
| 49407 | 0.622775 | 2.1 |
| 49408 | 1.89226 | 2.1 |
| 49409 | 1.89226 | 2.1 |
| 49410 | 1.89226 | 2.1 |
| 49411 | 1.89226 | 2.1 |
| 49412 | 1.89226 | 2.1 |
| 49413 | 1.89226 | 2.1 |

| | | |
|---|---|---|
| 49414 | 1.89226 | 2.1 |
| 49415 | 1.89226 | 2.1 |
| 49416 | 1.89226 | 2.1 |
| 49417 | 1.89226 | 2.1 |
| 49418 | 1.89226 | 2.1 |
| 49419 | 1.89226 | 2.1 |
| 49420 | 2.2727 | 2.1 |
| 49421 | 1.89586 | 2.1 |
| 49422 | 1.66913 | 2.1 |
| 49423 | 1.46101 | 2.1 |
| 49424 | 2.48082 | 2.1 |
| 49425 | 2.2727 | 2.1 |
| 49426 | 1.89226 | 2.1 |
| 49427 | 1.89226 | 2.1 |
| 49428 | 1.84641 | 2.1 |
| 49429 | 1.89226 | 2.1 |
| 49430 | 1.30868 | 2.1 |
| 49431 | 1.34118 | 1.13499 |
| 49432 | 1.89226 | 0.85188 |
| 49433 | 1.89226 | 0.945679 |
| 49434 | 1.89226 | 1.09309 |
| 49435 | 1.89226 | 0.838491 |
| 49436 | 1.89226 | 0.822014 |
| 49437 | 1.89226 | 0.227128 |
| 49438 | 1.89226 | 0.983535 |
| 49439 | 1.89226 | 0.0551695 |
| 49440 | 1.89226 | 0.773827 |
| 49441 | 1.89226 | 0.95844 |
| 49442 | 3.78452 | 0.820569 |
| 49443 | 0.946132 | 2.1 |
| 49444 | 0.946132 | 1.59331 |
| 49445 | 0.946131 | 2.1 |
| 49446 | 0.946131 | 2.1 |
| 49447 | 0.946132 | 2.1 |
| 49448 | 0.946132 | 0.995881 |
| 49449 | 0.946132 | 2.1 |
| 49450 | 0.946132 | 2.1 |
| 49451 | 0.946132 | 2.1 |
| 49452 | 0.946132 | 2.1 |
| 49453 | 0.946132 | 2.1 |
| 49454 | 0.946132 | 2.1 |
| 49455 | 1.89227 | 1.48128 |
| 49456 | 1.84641 | 1.75431 |
| 49457 | 2.29517 | 2.1 |
| 49458 | 1.84641 | 1.13942 |
| 49459 | 2.34102 | 1.68825 |
| 49460 | 1.89227 | 0.9028 |
| 49461 | 1.89226 | 0.734059 |
| 49462 | 1.89226 | 0.190046 |
| 49463 | 1.89226 | 0.882595 |
| 49464 | 1.89226 | 0.698614 |
| 49465 | 1.89226 | 0.430941 |
| 49466 | 1.89226 | 0.0750889 |
| 49467 | 1.89226 | 0.644467 |
| 49468 | 1.89226 | 0.521689 |
| 49469 | 1.89226 | 0.81147 |
| 49470 | 1.89226 | 0.660316 |
| 49471 | 1.89226 | 0.199564 |

| | | |
|---|---|---|
| 49472 | 1.89226 | 0.05 |
| 49473 | 1.89226 | 1.71068 |
| 49474 | 1.89226 | 1.00017 |
| 49475 | 1.89226 | 2.1 |
| 49476 | 1.89226 | 0.891034 |
| 49477 | 1.89226 | 1.43143 |
| 49478 | 1.89226 | 0.888314 |
| 49479 | 0.851544 | 0.05 |
| 49480 | 0.757542 | 0.05 |
| 49481 | 1.13421 | 0.05 |
| 49482 | 1.04072 | 0.05 |
| 49483 | 0.946132 | 0.05 |
| 49484 | 0.946132 | 0.05 |
| 49485 | 0.841686 | 0.05 |
| 49486 | 0.834428 | 0.05 |
| 49487 | 1.13649 | 0.05 |
| 49488 | 0.937687 | 0.05 |
| 49489 | 0.841713 | 0.05 |
| 49490 | 0.841713 | 0.05 |
| 49491 | 1.89226 | 0.05 |
| 49492 | 1.89226 | 0.05 |
| 49493 | 1.89226 | 0.05 |
| 49494 | 1.89226 | 0.05 |
| 49495 | 1.89226 | 0.05 |
| 49496 | 1.89226 | 0.05 |
| 49497 | 2.31988 | 1.24149 |
| 49498 | 2.00082 | 2.1 |
| 49499 | 2.4594 | 0.640946 |
| 49500 | 2.17638 | 2.1 |
| 49501 | 4.46022 | 1.2141 |
| 49502 | 2.00082 | 0.598925 |
| 49503 | 1.41829 | 2.1 |
| 49504 | 1.61207 | 1.72852 |
| 49505 | 1.43652 | 2.1 |
| 49506 | 2.17637 | 0.343925 |
| 49507 | 2.00082 | 2.1 |
| 49508 | 2.4594 | 0.898172 |
| 49509 | 2.17638 | 2.1 |
| 49510 | 2.57957 | 0.923146 |
| 49511 | 2.3506 | 2.1 |
| 49512 | 4.81 | 1.34027 |
| 49513 | 2.3506 | 0.960392 |
| 49514 | 1.57368 | 2.1 |
| 49515 | 1.89111 | 0.0602191 |
| 49516 | 1.89111 | 2.1 |
| 49517 | 2.17638 | 0.0709556 |
| 49518 | 2.17638 | 2.1 |
| 49519 | 1.32813 | 2.1 |
| 49520 | 1.7237 | 2.1 |
| 49521 | 1.32461 | 2.1 |
| 49522 | 1.81656 | 2.1 |
| 49523 | 3.14468 | 2.1 |
| 49524 | 1.55132 | 2.1 |
| 49525 | 2.0815 | 2.1 |
| 49526 | 1.81656 | 2.1 |
| 49527 | 2.19826 | 2.1 |
| 49528 | 3.65818 | 2.1 |
| 49529 | 0.775951 | 2.1 |

| | | |
|---|---|---|
| 49530 | 0.775951 | 2.1 |
| 49531 | 0.794601 | 2.1 |
| 49532 | 0.794601 | 2.1 |
| 49533 | 0.945641 | 2.1 |
| 49534 | 0.90828 | 2.1 |
| 49535 | 0.662302 | 2.1 |
| 49536 | 0.662273 | 2.1 |
| 49537 | 0.662274 | 2.1 |
| 49538 | 0.945639 | 2.1 |
| 49539 | 0.662304 | 2.1 |
| 49540 | 0.908279 | 2.1 |
| 49541 | 1.03062 | 2.1 |
| 49542 | 1.03062 | 2.1 |
| 49543 | 1.05054 | 2.1 |
| 49544 | 1.05054 | 2.1 |
| 49545 | 1.13551 | 2.1 |
| 49546 | 1.21275 | 2.1 |
| 49547 | 0.945641 | 2.1 |
| 49548 | 0.90828 | 2.1 |
| 49549 | 0.945641 | 2.1 |
| 49550 | 1.21275 | 2.1 |
| 49551 | 0.90828 | 2.1 |
| 49552 | 1.13552 | 2.1 |
| 49553 | 1.89226 | 2.1 |
| 49554 | 1.79883 | 2.1 |
| 49555 | 1.95341 | 2.1 |
| 49556 | 1.89226 | 2.1 |
| 49557 | 1.85998 | 2.1 |
| 49558 | 1.79883 | 2.1 |
| 49559 | 1.89226 | 2.1 |
| 49560 | 1.89226 | 2.1 |
| 49561 | 1.89226 | 2.1 |
| 49562 | 1.89226 | 2.1 |
| 49563 | 3.78453 | 2.1 |
| 49564 | 0.851541 | 2.1 |
| 49565 | 0.851541 | 2.1 |
| 49566 | 0.970398 | 2.1 |
| 49567 | 0.954365 | 2.1 |
| 49568 | 0.855356 | 2.1 |
| 49569 | 0.855357 | 2.1 |
| 49570 | 0.946133 | 2.1 |
| 49571 | 0.946133 | 2.1 |
| 49572 | 1.04072 | 2.1 |
| 49573 | 0.970397 | 2.1 |
| 49574 | 0.950003 | 2.1 |
| 49575 | 0.851538 | 2.1 |
| 49576 | 1.89226 | 2.1 |
| 49577 | 1.85998 | 2.1 |
| 49578 | 1.95296 | 2.1 |
| 49579 | 1.89226 | 2.1 |
| 49580 | 1.92067 | 2.1 |
| 49581 | 1.85998 | 2.1 |
| 49582 | 0.946133 | 2.1 |
| 49583 | 0.946133 | 2.1 |
| 49584 | 0.946132 | 2.1 |
| 49585 | 0.946132 | 2.1 |
| 49586 | 0.946132 | 2.1 |
| 49587 | 0.946132 | 2.1 |

| | | |
|---|---|---|
| 49588 | 0.946132 | 2.1 |
| 49589 | 0.946132 | 2.1 |
| 49590 | 0.946132 | 2.1 |
| 49591 | 0.946132 | 2.1 |
| 49592 | 0.946133 | 2.1 |
| 49593 | 0.946133 | 2.1 |
| 49594 | 1.82152 | 2.1 |
| 49595 | 1.89226 | 2.1 |
| 49596 | 1.89226 | 2.1 |
| 49597 | 2.11152 | 2.1 |
| 49598 | 1.7472 | 2.1 |
| 49599 | 1.89226 | 2.1 |
| 49600 | 2.02687 | 2.1 |
| 49601 | 2.29199 | 2.1 |
| 49602 | 1.2734 | 2.1 |
| 49603 | 1.41077 | 2.1 |
| 49604 | 1.47381 | 2.1 |
| 49605 | 2.29199 | 2.1 |
| 49606 | 1.46889 | 2.1 |
| 49607 | 2.29199 | 2.1 |
| 49608 | 1.86766 | 2.1 |
| 49609 | 2.59401 | 2.1 |
| 49610 | 4.15965 | 2.1 |
| 49611 | 1.58995 | 2.1 |
| 49612 | 1.74246 | 2.1 |
| 49613 | 1.46643 | 2.1 |
| 49614 | 1.46643 | 2.1 |
| 49615 | 1.85097 | 2.1 |
| 49616 | 1.85097 | 2.1 |
| 49617 | 1.58995 | 2.1 |
| 49618 | 1.66584 | 2.1 |
| 49619 | 1.89214 | 2.1 |
| 49620 | 1.91787 | 2.1 |
| 49621 | 3.48208 | 2.1 |
| 49622 | 1.18474 | 2.1 |
| 49623 | 1.8144 | 2.1 |
| 49624 | 1.1486 | 2.1 |
| 49625 | 1.1486 | 2.1 |
| 49626 | 2.13071 | 2.1 |
| 49627 | 2.1257 | 2.1 |
| 49628 | 2.0815 | 2.1 |
| 49629 | 2.51651 | 2.1 |
| 49630 | 1.73681 | 2.1 |
| 49631 | 2.01601 | 2.1 |
| 49632 | 2.21406 | 2.1 |
| 49633 | 2.56998 | 2.1 |
| 49634 | 1.19995 | 2.1 |
| 49635 | 1.06256 | 2.1 |
| 49636 | 0.620677 | 2.1 |
| 49637 | 0.598084 | 2.1 |
| 49638 | 0.831426 | 2.1 |
| 49639 | 0.946056 | 2.1 |
| 49640 | 0.827692 | 2.1 |
| 49641 | 0.827692 | 2.1 |
| 49642 | 1.08958 | 2.1 |
| 49643 | 0.630132 | 2.1 |
| 49644 | 0.577547 | 2.1 |
| 49645 | 1.06104 | 2.1 |

| | | |
|---|---|---|
| 49646 | 2.0815 | 2.1 |
| 49647 | 2.66249 | 2.1 |
| 49648 | 2.78966 | 2.1 |
| 49649 | 2.47108 | 2.1 |
| 49650 | 2.33858 | 2.1 |
| 49651 | 2.0815 | 2.1 |
| 49652 | 1.53735 | 2.1 |
| 49653 | 1.42278 | 2.1 |
| 49654 | 1.23654 | 2.1 |
| 49655 | 1.34678 | 2.1 |
| 49656 | 2.76955 | 2.1 |
| 49657 | 1.89226 | 2.1 |
| 49658 | 1.89226 | 2.1 |
| 49659 | 1.89226 | 2.1 |
| 49660 | 1.89226 | 2.1 |
| 49661 | 3.78452 | 2.1 |
| 49662 | 1.23654 | 2.1 |
| 49663 | 1.34678 | 2.1 |
| 49664 | 1.19 2.1 | |
| 49665 | 1.08576 | 2.1 |
| 49666 | 2.43254 | 2.1 |
| 49667 | 1.38776 | 2.1 |
| 49668 | 1.29366 | 2.1 |
| 49669 | 0.946131 | 2.1 |
| 49670 | 0.946131 | 2.1 |
| 49671 | 0.946131 | 2.1 |
| 49672 | 0.946131 | 2.1 |
| 49673 | 1.38775 | 2.1 |
| 49674 | 1.38775 | 2.1 |
| 49675 | 1.29366 | 2.1 |
| 49676 | 0.946132 | 2.1 |
| 49677 | 0.946131 | 2.1 |
| 49678 | 1.38775 | 2.1 |
| 49679 | 0.946131 | 2.1 |
| 49680 | 0.946131 | 2.1 |
| 49681 | 0.946129 | 2.1 |
| 49682 | 0.946129 | 2.1 |
| 49683 | 0.94613 | 2.1 |
| 49684 | 0.94613 | 2.1 |
| 49685 | 0.946131 | 2.1 |
| 49686 | 0.946131 | 2.1 |
| 49687 | 0.946131 | 2.1 |
| 49688 | 0.946131 | 2.1 |
| 49689 | 0.94613 | 2.1 |
| 49690 | 0.94613 | 2.1 |
| 49691 | 1.89226 | 0.05 |
| 49692 | 1.89226 | 1.12319 |
| 49693 | 1.89226 | 0.268854 |
| 49694 | 1.89226 | 0.624703 |
| 49695 | 1.89226 | 0.706782 |
| 49696 | 1.89226 | 1.10942 |
| 49697 | 1.89226 | 0.147812 |
| 49698 | 1.89226 | 1.99634 |
| 49699 | 1.89226 | 0.05 |
| 49700 | 1.89226 | 0.086702 |
| 49701 | 1.89226 | 0.166351 |
| 49702 | 1.89226 | 1.07441 |
| 49703 | 1.89226 | 0.593591 |

| | | |
|---|---|---|
| 49704 | 1.89226 | 1.56323 |
| 49705 | 1.89226 | 0.601262 |
| 49706 | 1.89226 | 0.919274 |
| 49707 | 1.89226 | 0.593188 |
| 49708 | 1.89226 | 1.18111 |
| 49709 | 0.92338 | 2.1 |
| 49710 | 0.92338 | 0.335475 |
| 49711 | 0.94428 | 0.28598 |
| 49712 | 0.94428 | 0.0662664 |
| 49713 | 1.19528 | 0.90934 |
| 49714 | 1.30598 | 2.1 |
| 49715 | 0.714073 | 0.101813 |
| 49716 | 0.561679 | 1.79184 |
| 49717 | 1.19528 | 0.938512 |
| 49718 | 0.561678 | 0.94946 |
| 49719 | 0.714072 | 1.25524 |
| 49720 | 1.30598 | 1.17921 |
| 49721 | 2.61197 | 2.1 |
| 49722 | 1.89226 | 0.804521 |
| 49723 | 1.89226 | 0.550973 |
| 49724 | 1.89226 | 2.1 |
| 49725 | 2.39056 | 0.167972 |
| 49726 | 2.39056 | 2.1 |
| 49727 | 0.946132 | 2.05863 |
| 49728 | 0.946132 | 0.548723 |
| 49729 | 0.946132 | 0.0841345 |
| 49730 | 0.946132 | 0.181011 |
| 49731 | 0.946131 | 0.169664 |
| 49732 | 0.946131 | 2.1 |
| 49733 | 0.946132 | 0.97741 |
| 49734 | 0.946132 | 0.476613 |
| 49735 | 0.946132 | 0.766797 |
| 49736 | 0.946132 | 0.520156 |
| 49737 | 0.946132 | 0.232227 |
| 49738 | 0.946132 | 0.83464 |
| 49739 | 1.89224 | 2.1 |
| 49740 | 1.89224 | 2.1 |
| 49741 | 1.89224 | 2.1 |
| 49742 | 1.89224 | 2.1 |
| 49743 | 3.78448 | 2.1 |
| 49744 | 1.97089 | 2.1 |
| 49745 | 1.57428 | 2.1 |
| 49746 | 1.84991 | 2.1 |
| 49747 | 1.49078 | 2.1 |
| 49748 | 3.42419 | 2.1 |
| 49749 | 0.621927 | 2.1 |
| 49750 | 0.621927 | 2.1 |
| 49751 | 0.630824 | 2.1 |
| 49752 | 0.630824 | 2.1 |
| 49753 | 0.483483 | 2.1 |
| 49754 | 0.507361 | 2.1 |
| 49755 | 0.787142 | 2.1 |
| 49756 | 0.745391 | 2.1 |
| 49757 | 0.745381 | 2.1 |
| 49758 | 0.483476 | 2.1 |
| 49759 | 0.507368 | 2.1 |
| 49760 | 0.787152 | 2.1 |
| 49761 | 1.89224 | 2.1 |

| | | |
|---|---|---|
| 49762 | 1.89224 | 2.1 |
| 49763 | 1.89224 | 2.1 |
| 49764 | 1.89224 | 2.1 |
| 49765 | 1.89224 | 2.1 |
| 49766 | 1.89224 | 2.1 |
| 49767 | 1.89227 | 2.1 |
| 49768 | 1.89227 | 2.1 |
| 49769 | 1.89227 | 2.1 |
| 49770 | 1.89227 | 2.1 |
| 49771 | 1.89227 | 2.1 |
| 49772 | 1.89227 | 2.1 |
| 49773 | 1.84994 | 2.1 |
| 49774 | 1.41758 | 2.1 |
| 49775 | 1.4908 | 2.1 |
| 49776 | 1.4908 | 2.1 |
| 49777 | 1.74391 | 2.1 |
| 49778 | 1.74391 | 2.1 |
| 49779 | 1.89226 | 2.1 |
| 49780 | 1.89226 | 2.1 |
| 49781 | 1.89226 | 2.1 |
| 49782 | 1.89226 | 2.1 |
| 49783 | 3.78452 | 2.1 |
| 49784 | 1.01323 | 2.1 |
| 49785 | 1.01323 | 2.1 |
| 49786 | 1.01445 | 2.1 |
| 49787 | 1.01445 | 2.1 |
| 49788 | 0.839531 | 2.1 |
| 49789 | 0.875652 | 2.1 |
| 49790 | 1.19063 | 2.1 |
| 49791 | 1.15203 | 2.1 |
| 49792 | 1.15203 | 2.1 |
| 49793 | 0.839531 | 2.1 |
| 49794 | 0.875652 | 2.1 |
| 49795 | 1.19063 | 2.1 |
| 49796 | 1.89226 | 2.1 |
| 49797 | 2.75587 | 2.1 |
| 49798 | 2.81512 | 2.1 |
| 49799 | 2.81512 | 2.1 |
| 49800 | 1.89226 | 2.1 |
| 49801 | 1.89226 | 2.1 |
| 49802 | 1.89226 | 2.1 |
| 49803 | 1.89226 | 2.1 |
| 49804 | 1.89226 | 2.1 |
| 49805 | 1.89226 | 2.1 |
| 49806 | 3.78452 | 2.1 |
| 49807 | 0.974831 | 2.1 |
| 49808 | 0.974831 | 2.1 |
| 49809 | 0.976681 | 2.1 |
| 49810 | 0.976681 | 2.1 |
| 49811 | 0.803179 | 2.1 |
| 49812 | 0.83953 | 2.1 |
| 49813 | 1.11198 | 2.1 |
| 49814 | 1.15203 | 2.1 |
| 49815 | 1.11198 | 2.1 |
| 49816 | 0.803179 | 2.1 |
| 49817 | 1.15203 | 2.1 |
| 49818 | 0.83953 | 2.1 |
| 49819 | 1.37794 | 2.1 |

| | | |
|---|---|---|
| 49820 | 1.37794 | 2.1 |
| 49821 | 0.946128 | 2.1 |
| 49822 | 0.946128 | 2.1 |
| 49823 | 1.3477 | 2.1 |
| 49824 | 1.37793 | 2.1 |
| 49825 | 0.946131 | 2.1 |
| 49826 | 0.946131 | 2.1 |
| 49827 | 0.946129 | 2.1 |
| 49828 | 1.3477 | 2.1 |
| 49829 | 1.37793 | 2.1 |
| 49830 | 0.946129 | 2.1 |
| 49831 | 2.38577 | 1.60294 |
| 49832 | 1.20247 | 2.1 |
| 49833 | 1.75182 | 2.1 |
| 49834 | 2.82546 | 1.71705 |
| 49835 | 4.1376 | 2.1 |
| 49836 | 2.82546 | 2.1 |
| 49837 | 1.89226 | 0.809773 |
| 49838 | 2.38577 | 2.1 |
| 49839 | 2.38577 | 1.9598 |
| 49840 | 1.89226 | 2.1 |
| 49841 | 1.89226 | 1.69951 |
| 49842 | 1.035 2.1 | |
| 49843 | 2.15429 | 2.1 |
| 49844 | 0.970998 | 2.1 |
| 49845 | 2.0815 | 2.1 |
| 49846 | 2.89672 | 2.1 |
| 49847 | 0.946131 | 0.0994135 |
| 49848 | 0.946131 | 0.0716903 |
| 49849 | 0.853249 | 0.0649391 |
| 49850 | 1.02715 | 0.106853 |
| 49851 | 0.851537 | 0.0734429 |
| 49852 | 0.851537 | 0.078223 |
| 49853 | 1.02663 | 0.0571619 |
| 49854 | 1.13139 | 0.05 |
| 49855 | 0.946128 | 0.0534392 |
| 49856 | 0.932039 | 0.118123 |
| 49857 | 0.843985 | 0.05 |
| 49858 | 0.946129 | 0.0546001 |
| 49859 | 1.89226 | 0.0557114 |
| 49860 | 1.86408 | 0.0683513 |
| 49861 | 1.96548 | 0.129954 |
| 49862 | 1.86408 | 0.0778542 |
| 49863 | 1.99366 | 0.0554383 |
| 49864 | 1.89226 | 0.05 |
| 49865 | 2.70702 | 2.1 |
| 49866 | 2.08981 | 2.1 |
| 49867 | 2.08693 | 2.1 |
| 49868 | 2.08693 | 2.1 |
| 49869 | 2.7188 | 2.1 |
| 49870 | 2.7188 | 2.1 |
| 49871 | 2.08982 | 2.1 |
| 49872 | 1.01964 | 2.1 |
| 49873 | 2.08693 | 2.1 |
| 49874 | 1.04615 | 2.1 |
| 49875 | 3.13597 | 2.1 |
| 49876 | 2.70702 | 2.1 |
| 49877 | 2.08693 | 2.1 |

| | | |
|---|---|---|
| 49878 | 2.68784 | 2.1 |
| 49879 | 2.08831 | 2.1 |
| 49880 | 4.77477 | 2.1 |
| 49881 | 0.778233 | 2.1 |
| 49882 | 0.778233 | 2.1 |
| 49883 | 0.789001 | 2.1 |
| 49884 | 0.789001 | 2.1 |
| 49885 | 0.545307 | 2.1 |
| 49886 | 0.523075 | 2.1 |
| 49887 | 1.04347 | 2.1 |
| 49888 | 1.04416 | 2.1 |
| 49889 | 1.04416 | 2.1 |
| 49890 | 0.545308 | 2.1 |
| 49891 | 1.04347 | 2.1 |
| 49892 | 0.523075 | 2.1 |
| 49893 | 1.89226 | 0.547966 |
| 49894 | 1.89226 | 0.620884 |
| 49895 | 1.89226 | 0.403908 |
| 49896 | 1.89226 | 0.312075 |
| 49897 | 3.78453 | 0.290535 |
| 49898 | 1.39237 | 0.59621 |
| 49899 | 1.17933 | 1.6303 |
| 49900 | 0.755796 | 0.842921 |
| 49901 | 0.755796 | 1.22127 |
| 49902 | 0.755767 | 0.05 |
| 49903 | 0.94613 | 1.17487 |
| 49904 | 1.15918 | 0.499418 |
| 49905 | 0.94613 | 0.768242 |
| 49906 | 1.11632 | 0.557042 |
| 49907 | 0.92595 | 0.05 |
| 49908 | 0.94613 | 0.685389 |
| 49909 | 0.94613 | 0.65833 |
| 49910 | 1.89226 | 0.628084 |
| 49911 | 1.89226 | 0.327115 |
| 49912 | 1.89226 | 0.168052 |
| 49913 | 1.89226 | 0.05 |
| 49914 | 3.78452 | 0.400107 |
| 49915 | 0.950067 | 0.05 |
| 49916 | 0.75071 | 0.05 |
| 49917 | 0.906516 | 0.05 |
| 49918 | 0.906516 | 0.05 |
| 49919 | 0.81586 | 0.05 |
| 49920 | 0.851541 | 0.150383 |
| 49921 | 0.962101 | 0.05 |
| 49922 | 0.851476 | 0.05 |
| 49923 | 0.962105 | 0.05 |
| 49924 | 0.815808 | 0.05 |
| 49925 | 0.851529 | 0.05 |
| 49926 | 0.851529 | 0.05 |
| 49927 | 1.05032 | 0.05 |
| 49928 | 0.972326 | 0.05 |
| 49929 | 0.906515 | 0.05 |
| 49930 | 0.906515 | 0.05 |
| 49931 | 1.12245 | 0.05 |
| 49932 | 1.04072 | 0.05 |
| 49933 | 0.815857 | 0.05 |
| 49934 | 0.851539 | 0.0545075 |
| 49935 | 1.04451 | 0.05 |

| | | |
|---|---|---|
| 49936 | 0.815853 | 0.0590686 |
| 49937 | 0.919795 | 0.05 |
| 49938 | 0.919795 | 0.05 |
| 49939 | 1.5855 | 2.1 |
| 49940 | 2.14058 | 2.1 |
| 49941 | 1.9153 | 2.1 |
| 49942 | 1.79922 | 2.1 |
| 49943 | 3.72608 | 2.1 |
| 49944 | 1.58551 | 2.1 |
| 49945 | 1.24201 | 2.1 |
| 49946 | 0.960287 | 2.03537 |
| 49947 | 1.79922 | 2.1 |
| 49948 | 2.82752 | 2.1 |
| 49949 | 0.679602 | 2.1 |
| 49950 | 0.621009 | 2.1 |
| 49951 | 0.899614 | 2.1 |
| 49952 | 0.940441 | 2.1 |
| 49953 | 0.679602 | 2.1 |
| 49954 | 0.94044 | 2.1 |
| 49955 | 0.621009 | 2.1 |
| 49956 | 0.899615 | 2.1 |
| 49957 | 0.794027 | 2.1 |
| 49958 | 0.794027 | 2.1 |
| 49959 | 0.785189 | 2.1 |
| 49960 | 0.785189 | 1.97362 |
| 49961 | 2.14058 | 2.1 |
| 49962 | 1.79922 | 2.1 |
| 49963 | 2.23742 | 2.1 |
| 49964 | 2.23742 | 2.1 |
| 49965 | 1.78404 | 2.1 |
| 49966 | 1.88088 | 2.1 |
| 49967 | 1.15177 | 2.1 |
| 49968 | 2.0815 | 2.1 |
| 49969 | 1.93765 | 2.1 |
| 49970 | 1.78342 | 2.1 |
| 49971 | 1.39511 | 2.1 |
| 49972 | 1.28406 | 2.1 |
| 49973 | 1.97719 | 2.1 |
| 49974 | 2.50057 | 0.05 |
| 49975 | 2.35349 | 2.1 |
| 49976 | 2.35349 | 0.05 |
| 49977 | 2.31686 | 2.1 |
| 49978 | 2.32857 | 0.05 |
| 49979 | 1.93765 | 2.1 |
| 49980 | 2.35349 | 0.05 |
| 49981 | 1.70048 | 2.1 |
| 49982 | 2.15721 | 0.05 |
| 49983 | 4.05397 | 0.06607 |
| 49984 | 0.850388 | 2.1 |
| 49985 | 0.850388 | 2.1 |
| 49986 | 0.840019 | 2.1 |
| 49987 | 0.840019 | 2.1 |
| 49988 | 1.13736 | 2.1 |
| 49989 | 1.13852 | 2.1 |
| 49990 | 0.573661 | 2.1 |
| 49991 | 0.551891 | 2.1 |
| 49992 | 0.573661 | 2.1 |
| 49993 | 1.13852 | 2.1 |

| | | |
|---|---|---|
| 49994 | 0.551892 | 2.1 |
| 49995 | 1.13736 | 2.1 |
| 49996 | 0.860203 | 2.1 |
| 49997 | 0.860203 | 2.1 |
| 49998 | 0.855758 | 2.1 |
| 49999 | 0.855758 | 2.1 |
| 50000 | 1.1423 | 2.1 |
| 50001 | 1.13852 | 2.1 |
| 50002 | 0.58628 | 2.1 |
| 50003 | 0.573661 | 2.1 |
| 50004 | 0.586281 | 2.1 |
| 50005 | 1.1423 | 2.1 |
| 50006 | 0.573661 | 2.1 |
| 50007 | 1.13852 | 2.1 |
| 50008 | 1.28918 | 1.09925 |
| 50009 | 0.999086 | 0.978623 |
| 50010 | 1.0383 | 2.05447 |
| 50011 | 0.736019 | 1.50902 |
| 50012 | 1.00954 | 1.53676 |
| 50013 | 1.00954 | 1.04518 |
| 50014 | 1.01566 | 0.808183 |
| 50015 | 1.01566 | 2.09707 |
| 50016 | 1.28918 | 1.22115 |
| 50017 | 1.0383 | 2.1 |
| 50018 | 0.999086 | 1.79749 |
| 50019 | 0.736019 | 1.40074 |
| 50020 | 2.20797 | 2.1 |
| 50021 | 2.64748 | 2.1 |
| 50022 | 2.50375 | 1.94413 |
| 50023 | 2.50375 | 2.1 |
| 50024 | 2.40858 | 2.1 |
| 50025 | 2.40858 | 2.1 |
| 50026 | 2.64748 | 2.1 |
| 50027 | 2.74744 | 2.1 |
| 50028 | 2.50375 | 2.1 |
| 50029 | 2.6521 | 2.1 |
| 50030 | 5.29958 | 2.1 |
| 50031 | 2.20797 | 2.1 |
| 50032 | 2.50375 | 2.1 |
| 50033 | 2.02064 | 1.85863 |
| 50034 | 2.3606 | 2.1 |
| 50035 | 4.52439 | 2.1 |
| 50036 | 2.36059 | 2.1 |
| 50037 | 2.6521 | 2.1 |
| 50038 | 2.56952 | 2.1 |
| 50039 | 2.56952 | 2.1 |
| 50040 | 2.50375 | 2.1 |
| 50041 | 2.50375 | 2.1 |
| 50042 | 2.49439 | 0.0882807 |
| 50043 | 1.89226 | 0.328326 |
| 50044 | 2.81337 | 0.249435 |
| 50045 | 1.89226 | 0.407849 |
| 50046 | 4.38665 | 0.376285 |
| 50047 | 1.89226 | 0.384643 |
| 50048 | 1.89226 | 0.475929 |
| 50049 | 1.89226 | 0.549763 |
| 50050 | 1.89226 | 0.536044 |
| 50051 | 3.78452 | 0.471671 |

| | | |
|---|---|---|
| 50052 | 1.89226 | 0.349231 |
| 50053 | 1.89226 | 0.414161 |
| 50054 | 1.89226 | 0.416203 |
| 50055 | 1.89226 | 0.461643 |
| 50056 | 3.78452 | 0.4175 |
| 50057 | 1.93948 | 0.618466 |
| 50058 | 1.98874 | 0.414264 |
| 50059 | 1.62494 | 0.592766 |
| 50060 | 2.14218 | 0.700026 |
| 50061 | 1.62494 | 0.128817 |
| 50062 | 2.14218 | 0.431842 |
| 50063 | 1.47354 | 0.454264 |
| 50064 | 1.98874 | 0.104716 |
| 50065 | 2.01865 | 0.461981 |
| 50066 | 1.62495 | 0.122345 |
| 50067 | 2.01865 | 0.51266 |
| 50068 | 1.62495 | 0.0877699 |
| 50069 | 1.89226 | 0.14231 |
| 50070 | 1.89226 | 2.1 |
| 50071 | 1.89226 | 2.1 |
| 50072 | 1.89226 | 2.1 |
| 50073 | 3.78453 | 2.1 |
| 50074 | 2.05257 | 2.1 |
| 50075 | 1.94441 | 2.1 |
| 50076 | 1.88487 | 2.1 |
| 50077 | 1.82441 | 2.1 |
| 50078 | 3.82928 | 2.1 |
| 50079 | 1.94441 | 2.1 |
| 50080 | 1.74391 | 2.1 |
| 50081 | 1.82441 | 2.1 |
| 50082 | 1.65636 | 2.1 |
| 50083 | 3.60077 | 2.1 |
| 50084 | 1.89226 | 2.1 |
| 50085 | 1.89226 | 2.1 |
| 50086 | 1.89226 | 2.1 |
| 50087 | 1.89226 | 2.1 |
| 50088 | 3.78452 | 2.1 |
| 50089 | 1.89226 | 2.1 |
| 50090 | 1.89226 | 2.1 |
| 50091 | 1.89226 | 0.0976096 |
| 50092 | 1.89226 | 2.1 |
| 50093 | 3.78453 | 2.1 |
| 50094 | 1.88487 | 2.1 |
| 50095 | 1.82441 | 2.1 |
| 50096 | 1.75835 | 2.1 |
| 50097 | 1.72866 | 2.1 |
| 50098 | 3.61354 | 2.1 |
| 50099 | 1.82441 | 2.1 |
| 50100 | 1.65636 | 2.1 |
| 50101 | 1.72866 | 2.1 |
| 50102 | 1.58401 | 2.1 |
| 50103 | 3.38503 | 2.1 |
| 50104 | 1.89226 | 2.1 |
| 50105 | 1.89226 | 2.1 |
| 50106 | 1.89226 | 2.1 |
| 50107 | 1.89226 | 2.1 |
| 50108 | 3.78452 | 2.1 |
| 50109 | 2.27432 | 2.1 |

| | | |
|---|---|---|
| 50110 | 1.33339 | 2.1 |
| 50111 | 1.38905 | 2.1 |
| 50112 | 2.34471 | 2.1 |
| 50113 | 3.66337 | 2.1 |
| 50114 | 1.89226 | 2.1 |
| 50115 | 2.34471 | 2.1 |
| 50116 | 2.27432 | 2.1 |
| 50117 | 2.27432 | 2.1 |
| 50118 | 1.89226 | 2.1 |
| 50119 | 1.89226 | 2.1 |
| 50120 | 1.20443 | 2.1 |
| 50121 | 1.17235 | 2.1 |
| 50122 | 0.724695 | 2.1 |
| 50123 | 0.694522 | 2.1 |
| 50124 | 0.949994 | 2.1 |
| 50125 | 0.949993 | 2.1 |
| 50126 | 0.948955 | 2.1 |
| 50127 | 0.948955 | 2.1 |
| 50128 | 1.20443 | 2.1 |
| 50129 | 0.7247 | 2.1 |
| 50130 | 0.694517 | 2.1 |
| 50131 | 1.17234 | 2.1 |
| 50132 | 1.89225 | 2.1 |
| 50133 | 2.40885 | 2.1 |
| 50134 | 2.3447 | 2.1 |
| 50135 | 2.3447 | 2.1 |
| 50136 | 1.89225 | 2.1 |
| 50137 | 1.89225 | 2.1 |
| 50138 | 1.37138 | 2.1 |
| 50139 | 1.82597 | 2.1 |
| 50140 | 1.45027 | 2.1 |
| 50141 | 1.92772 | 2.1 |
| 50142 | 3.27624 | 2.1 |
| 50143 | 1.89226 | 2.1 |
| 50144 | 1.89226 | 2.1 |
| 50145 | 1.89226 | 2.1 |
| 50146 | 1.89226 | 2.1 |
| 50147 | 3.78452 | 2.1 |
| 50148 | 1.89226 | 2.1 |
| 50149 | 1.89226 | 0.971364 |
| 50150 | 1.89226 | 2.1 |
| 50151 | 1.89226 | 2.01209 |
| 50152 | 3.78452 | 2.1 |
| 50153 | 1.82597 | 2.1 |
| 50154 | 2.02312 | 0.367185 |
| 50155 | 1.92772 | 2.1 |
| 50156 | 2.15888 | 2.1 |
| 50157 | 3.98485 | 2.1 |
| 50158 | 1.53446 | 2.1 |
| 50159 | 1.92772 | 2.1 |
| 50160 | 2.03975 | 2.1 |
| 50161 | 2.03975 | 2.1 |
| 50162 | 1.45027 | 2.1 |
| 50163 | 1.45027 | 2.1 |
| 50164 | 1.89226 | 2.1 |
| 50165 | 1.89226 | 2.1 |
| 50166 | 1.89226 | 2.1 |
| 50167 | 1.89226 | 2.1 |

| | | |
|---|---|---|
| 50168 | 1.89226 | 2.1 |
| 50169 | 1.89226 | 2.1 |
| 50170 | 1.89226 | 2.1 |
| 50171 | 1.89226 | 2.1 |
| 50172 | 1.89226 | 2.1 |
| 50173 | 1.89226 | 1.92426 |
| 50174 | 3.78452 | 2.1 |
| 50175 | 1.92772 | 2.1 |
| 50176 | 2.15888 | 2.1 |
| 50177 | 2.03975 | 2.1 |
| 50178 | 2.3111 | 2.1 |
| 50179 | 4.19863 | 2.1 |
| 50180 | 1.89226 | 2.1 |
| 50181 | 1.89226 | 2.1 |
| 50182 | 1.89226 | 2.1 |
| 50183 | 1.89226 | 2.1 |
| 50184 | 3.78452 | 2.1 |
| 50185 | 1.78376 | 2.1 |
| 50186 | 1.3201 | 2.1 |
| 50187 | 1.78376 | 2.1 |
| 50188 | 1.86102 | 2.1 |
| 50189 | 1.2679 | 2.1 |
| 50190 | 1.34517 | 2.1 |
| 50191 | 1.89226 | 2.1 |
| 50192 | 2.52442 | 2.1 |
| 50193 | 1.89226 | 2.1 |
| 50194 | 2.51691 | 2.1 |
| 50195 | 4.41667 | 2.1 |
| 50196 | 1.89226 | 2.1 |
| 50197 | 1.89226 | 2.1 |
| 50198 | 1.89226 | 2.1 |
| 50199 | 1.89226 | 2.1 |
| 50200 | 3.78452 | 2.1 |
| 50201 | 1.78376 | 2.1 |
| 50202 | 1.2679 | 2.1 |
| 50203 | 1.71839 | 2.1 |
| 50204 | 1.23504 | 2.1 |
| 50205 | 3.0188 | 2.1 |
| 50206 | 1.89226 | 2.1 |
| 50207 | 2.51795 | 2.1 |
| 50208 | 2.51691 | 2.1 |
| 50209 | 2.51691 | 2.1 |
| 50210 | 1.89226 | 2.1 |
| 50211 | 1.89226 | 2.1 |
| 50212 | 1.89226 | 0.869214 |
| 50213 | 1.89226 | 1.56067 |
| 50214 | 1.89226 | 1.01671 |
| 50215 | 1.89226 | 0.963967 |
| 50216 | 3.78452 | 0.92802 |
| 50217 | 1.89227 | 0.88804 |
| 50218 | 1.89227 | 0.942126 |
| 50219 | 3.26088 | 1.1451 |
| 50220 | 2.79543 | 0.98055 |
| 50221 | 2.20733 | 0.937167 |
| 50222 | 1.89227 | 0.890697 |
| 50223 | 1.89226 | 1.12069 |
| 50224 | 1.89226 | 0.935248 |
| 50225 | 1.89226 | 0.848338 |

| | | |
|---|---|---|
| 50226 | 1.89226 | 0.948271 |
| 50227 | 1.89226 | 0.967975 |
| 50228 | 1.89226 | 1.29212 |
| 50229 | 1.89227 | 0.878811 |
| 50230 | 2.20733 | 0.898538 |
| 50231 | 1.46973 | 1.22483 |
| 50232 | 1.89227 | 0.876075 |
| 50233 | 1.7848 | 1.10033 |
| 50234 | 2.20733 | 0.83644 |
| 50235 | 1.89226 | 2.1 |
| 50236 | 2.55682 | 2.1 |
| 50237 | 2.81593 | 2.1 |
| 50238 | 2.81593 | 2.1 |
| 50239 | 1.89226 | 2.1 |
| 50240 | 1.89226 | 2.1 |
| 50241 | 1.0607 | 2.1 |
| 50242 | 1.0607 | 2.1 |
| 50243 | 1.12617 | 2.1 |
| 50244 | 0.986957 | 2.1 |
| 50245 | 0.699328 | 2.1 |
| 50246 | 0.785567 | 2.1 |
| 50247 | 1.28855 | 2.1 |
| 50248 | 1.41913 | 2.1 |
| 50249 | 0.766737 | 1.13869 |
| 50250 | 1.34711 | 2.1 |
| 50251 | 1.24643 | 2.1 |
| 50252 | 0.687289 | 2.1 |
| 50253 | 3.11063 | 0.05 |
| 50254 | 1.89226 | 0.102367 |
| 50255 | 1.96536 | 0.05 |
| 50256 | 1.89226 | 0.05 |
| 50257 | 1.96536 | 0.05 |
| 50258 | 1.89226 | 0.05 |
| 50259 | 1.89226 | 0.686792 |
| 50260 | 1.89226 | 0.160912 |
| 50261 | 1.89226 | 0.259721 |
| 50262 | 1.89226 | 0.146077 |
| 50263 | 3.78452 | 0.679994 |
| 50264 | 1.89226 | 0.0516472 |
| 50265 | 1.96256 | 0.05 |
| 50266 | 1.89226 | 0.05 |
| 50267 | 1.89226 | 0.05 |
| 50268 | 2.49192 | 0.05 |
| 50269 | 2.49192 | 0.05 |
| 50270 | 1.85716 | 0.05 |
| 50271 | 2.56871 | 0.05 |
| 50272 | 1.03043 | 0.05 |
| 50273 | 1.03043 | 0.05 |
| 50274 | 2.48125 | 0.05 |
| 50275 | 1.65452 | 0.05 |
| 50276 | 1.89226 | 2.1 |
| 50277 | 2.09622 | 2.1 |
| 50278 | 2.09622 | 2.1 |
| 50279 | 2.08508 | 2.1 |
| 50280 | 1.89226 | 2.1 |
| 50281 | 1.88112 | 2.1 |
| 50282 | 1.04811 | 2.1 |
| 50283 | 1.04811 | 2.1 |

| | | |
|---|---|---|
| 50284 | 0.946131 | 2.1 |
| 50285 | 0.946131 | 2.1 |
| 50286 | 1.05014 | 2.1 |
| 50287 | 1.04811 | 2.1 |
| 50288 | 0.94613 | 2.1 |
| 50289 | 0.94613 | 2.1 |
| 50290 | 0.946131 | 2.1 |
| 50291 | 1.05013 | 2.1 |
| 50292 | 1.04811 | 2.1 |
| 50293 | 0.946131 | 2.1 |
| 50294 | 1.398 | 2.1 |
| 50295 | 2.10766 | 2.1 |
| 50296 | 2.3018 | 2.1 |
| 50297 | 2.3018 | 2.1 |
| 50298 | 1.34008 | 2.1 |
| 50299 | 1.34008 | 2.1 |
| 50300 | 1.1509 | 2.1 |
| 50301 | 1.1509 | 2.1 |
| 50302 | 0.946132 | 2.1 |
| 50303 | 0.946132 | 2.1 |
| 50304 | 0.946132 | 2.1 |
| 50305 | 0.946132 | 2.1 |
| 50306 | 1.05383 | 2.1 |
| 50307 | 1.1509 | 2.1 |
| 50308 | 1.1509 | 2.1 |
| 50309 | 0.946133 | 2.1 |
| 50310 | 0.946132 | 2.1 |
| 50311 | 1.05383 | 2.1 |
| 50312 | 0.931141 | 2.1 |
| 50313 | 0.931141 | 2.1 |
| 50314 | 0.971946 | 2.1 |
| 50315 | 0.971946 | 2.1 |
| 50316 | 1.28569 | 2.1 |
| 50317 | 1.1509 | 2.1 |
| 50318 | 0.699 | 2.1 |
| 50319 | 0.752185 | 2.1 |
| 50320 | 1.28568 | 2.1 |
| 50321 | 0.752186 | 2.1 |
| 50322 | 0.699001 | 2.1 |
| 50323 | 1.1509 | 2.1 |
| 50324 | 2.30181 | 2.1 |
| 50325 | 1.89226 | 2.1 |
| 50326 | 1.89226 | 2.1 |
| 50327 | 1.89226 | 2.1 |
| 50328 | 2.57137 | 2.1 |
| 50329 | 2.57137 | 2.1 |
| 50330 | 2.0815 | 2.1 |
| 50331 | 1.21962 | 2.1 |
| 50332 | 1.55173 | 2.1 |
| 50333 | 2.57849 | 2.1 |
| 50334 | 3.79001 | 2.1 |
| 50335 | 1.38779 | 2.1 |
| 50336 | 1.2923 | 2.1 |
| 50337 | 1.03974 | 2.1 |
| 50338 | 1.28832 | 2.1 |
| 50339 | 1.33566 | 2.1 |
| 50340 | 1.21116 | 2.1 |
| 50341 | 1.22343 | 2.1 |

| | | |
|---|---|---|
| 50342 | 1.22343 | 2.1 |
| 50343 | 1.42288 | 2.1 |
| 50344 | 1.20138 | 2.1 |
| 50345 | 1.26025 | 2.1 |
| 50346 | 1.00644 | 2.1 |
| 50347 | 1.26066 | 2.1 |
| 50348 | 1.14271 | 2.1 |
| 50349 | 0.886635 | 2.1 |
| 50350 | 0.795277 | 2.1 |
| 50351 | 1.02966 | 2.1 |
| 50352 | 1.02967 | 2.1 |
| 50353 | 1.05216 | 2.1 |
| 50354 | 1.05216 | 2.1 |
| 50355 | 1.26068 | 2.1 |
| 50356 | 0.790042 | 2.1 |
| 50357 | 0.795269 | 2.1 |
| 50358 | 1.20135 | 2.1 |
| 50359 | 1.89226 | 0.053326 |
| 50360 | 1.89226 | 0.05 |
| 50361 | 1.89226 | 0.0511629 |
| 50362 | 1.89226 | 0.05 |
| 50363 | 1.89226 | 0.0947158 |
| 50364 | 1.89226 | 0.05 |
| 50365 | 1.89227 | 0.05 |
| 50366 | 1.89227 | 0.05 |
| 50367 | 1.89227 | 0.05 |
| 50368 | 1.89227 | 0.05 |
| 50369 | 1.89227 | 0.05 |
| 50370 | 1.89227 | 0.05 |
| 50371 | 0.563873 | 0.05 |
| 50372 | 0.50988 | 0.05 |
| 50373 | 0.598693 | 0.05 |
| 50374 | 0.558819 | 0.05 |
| 50375 | 0.564855 | 0.05 |
| 50376 | 0.564855 | 0.05 |
| 50377 | 0.557838 | 0.05 |
| 50378 | 0.557838 | 0.05 |
| 50379 | 0.563873 | 0.05 |
| 50380 | 0.598694 | 0.05 |
| 50381 | 0.558819 | 0.05 |
| 50382 | 0.50988 | 0.05 |
| 50383 | 0.598693 | 0.05 |
| 50384 | 0.558819 | 0.05 |
| 50385 | 0.572737 | 0.05 |
| 50386 | 0.534248 | 0.05 |
| 50387 | 0.566788 | 0.05 |
| 50388 | 0.566788 | 0.05 |
| 50389 | 0.566153 | 0.05 |
| 50390 | 0.566153 | 0.05 |
| 50391 | 0.598694 | 0.05 |
| 50392 | 0.572737 | 0.05 |
| 50393 | 0.534248 | 0.05 |
| 50394 | 0.558819 | 0.05 |
| 50395 | 1.85217 | 2.1 |
| 50396 | 1.93111 | 2.1 |
| 50397 | 1.92875 | 0.684343 |
| 50398 | 2.02391 | 2.1 |
| 50399 | 3.85986 | 2.1 |

| | | |
|---|---|---|
| 50400 | 1.93112 | 2.1 |
| 50401 | 1.80425 | 2.1 |
| 50402 | 2.02392 | 2.1 |
| 50403 | 1.90981 | 2.1 |
| 50404 | 3.84092 | 2.1 |
| 50405 | 1.92876 | 0.232421 |
| 50406 | 2.02393 | 2.1 |
| 50407 | 2.05274 | 1.71061 |
| 50408 | 2.16783 | 2.1 |
| 50409 | 4.09659 | 2.1 |
| 50410 | 2.02393 | 2.1 |
| 50411 | 1.90982 | 2.1 |
| 50412 | 2.16784 | 2.1 |
| 50413 | 2.05967 | 2.1 |
| 50414 | 4.07766 | 2.1 |
| 50415 | 1.89226 | 1.36595 |
| 50416 | 1.89226 | 1.17435 |
| 50417 | 1.89226 | 1.40871 |
| 50418 | 1.89226 | 1.2715 |
| 50419 | 3.78453 | 1.00069 |
| 50420 | 1.89226 | 0.99941 |
| 50421 | 1.89226 | 1.49271 |
| 50422 | 1.89226 | 2.1 |
| 50423 | 2.44829 | 0.814129 |
| 50424 | 3.78453 | 2.09361 |
| 50425 | 1.89226 | 1.33158 |
| 50426 | 1.89226 | 1.5698 |
| 50427 | 1.89226 | 1.09159 |
| 50428 | 2.60452 | 1.0033 |
| 50429 | 3.78453 | 2.1 |
| 50430 | 1.58723 | 0.05 |
| 50431 | 1.88071 | 0.05 |
| 50432 | 2.97376 | 0.05 |
| 50433 | 2.13255 | 0.05 |
| 50434 | 4.43982 | 0.05 |
| 50435 | 2.20641 | 0.05 |
| 50436 | 1.33575 | 0.05 |
| 50437 | 2.15824 | 0.05 |
| 50438 | 2.15824 | 0.05 |
| 50439 | 1.2951 | 0.05 |
| 50440 | 1.2951 | 0.05 |
| 50441 | 0.825383 | 0.05 |
| 50442 | 0.825383 | 0.05 |
| 50443 | 0.856104 | 0.05 |
| 50444 | 0.856104 | 0.05 |
| 50445 | 0.952196 | 0.05 |
| 50446 | 0.602363 | 0.05 |
| 50447 | 0.667877 | 0.05 |
| 50448 | 1.07913 | 0.05 |
| 50449 | 0.602363 | 0.05 |
| 50450 | 0.667876 | 0.05 |
| 50451 | 1.07913 | 0.05 |
| 50452 | 0.952197 | 0.05 |
| 50453 | 2.57657 | 0.844193 |
| 50454 | 1.44437 | 2.1 |
| 50455 | 2.65378 | 2.1 |
| 50456 | 2.25076 | 2.1 |
| 50457 | 1.84739 | 2.1 |

| | | |
|---|---|---|
| 50458 | 1.44437 | 2.1 |
| 50459 | 2.0189 | 0.05 |
| 50460 | 2.54752 | 0.05 |
| 50461 | 2.63975 | 0.05 |
| 50462 | 2.31049 | 0.05 |
| 50463 | 4.65865 | 0.05 |
| 50464 | 1.40707 | 0.05 |
| 50465 | 1.89605 | 0.05 |
| 50466 | 2.0189 | 0.05 |
| 50467 | 2.0189 | 0.05 |
| 50468 | 1.50438 | 0.05 |
| 50469 | 1.50438 | 0.05 |
| 50470 | 2.63971 | 0.05 |
| 50471 | 2.38246 | 0.05 |
| 50472 | 2.13402 | 0.05 |
| 50473 | 2.57503 | 0.05 |
| 50474 | 4.77373 | 0.05 |
| 50475 | 1.50436 | 0.05 |
| 50476 | 1.96803 | 0.05 |
| 50477 | 2.13402 | 0.05 |
| 50478 | 1.45446 | 0.05 |
| 50479 | 3.63838 | 0.05 |
| 50480 | 2.30793 | 0.05 |
| 50481 | 2.11032 | 0.05 |
| 50482 | 1.24082 | 0.05 |
| 50483 | 1.05689 | 0.05 |
| 50484 | 3.36482 | 0.05 |
| 50485 | 1.89226 | 0.0642658 |
| 50486 | 1.89226 | 0.119395 |
| 50487 | 1.89226 | 0.1041 |
| 50488 | 1.89226 | 0.0703686 |
| 50489 | 3.78453 | 0.0743063 |
| 50490 | 0.812967 | 0.05 |
| 50491 | 0.812967 | 0.05 |
| 50492 | 1.00181 | 0.05 |
| 50493 | 1.08141 | 0.05 |
| 50494 | 0.946132 | 0.05 |
| 50495 | 0.812938 | 0.05 |
| 50496 | 0.932815 | 0.05 |
| 50497 | 1.05577 | 0.05 |
| 50498 | 0.812938 | 0.05 |
| 50499 | 0.932812 | 0.05 |
| 50500 | 0.946133 | 0.05 |
| 50501 | 0.946133 | 0.05 |
| 50502 | 2.53897 | 2.1 |
| 50503 | 1.89226 | 2.1 |
| 50504 | 1.89226 | 2.1 |
| 50505 | 1.89226 | 2.1 |
| 50506 | 2.3245 | 2.1 |
| 50507 | 2.3245 | 2.1 |
| 50508 | 1.00255 | 1.06374 |
| 50509 | 1.06561 | 2.1 |
| 50510 | 1.05481 | 0.05 |
| 50511 | 1.11077 | 2.1 |
| 50512 | 2.12042 | 2.1 |
| 50513 | 1.89226 | 1.58925 |
| 50514 | 1.89226 | 2.1 |
| 50515 | 1.89226 | 0.05 |

| | | |
|---|---|---|
| 50516 | 1.89226 | 2.1 |
| 50517 | 3.78453 | 1.35465 |
| 50518 | 1.89226 | 2.1 |
| 50519 | 2.77314 | 2.1 |
| 50520 | 2.81276 | 2.1 |
| 50521 | 2.81276 | 2.1 |
| 50522 | 1.89226 | 2.1 |
| 50523 | 1.89226 | 2.1 |
| 50524 | 1.11077 | 2.1 |
| 50525 | 1.15114 | 2.1 |
| 50526 | 1.10274 | 0.873037 |
| 50527 | 1.1032 | 2.1 |
| 50528 | 1.05481 | 2.08165 |
| 50529 | 1.1032 | 0.974649 |
| 50530 | 1.89226 | 1.11023 |
| 50531 | 1.89226 | 2.1 |
| 50532 | 1.89226 | 0.05 |
| 50533 | 1.89226 | 2.1 |
| 50534 | 3.78452 | 1.12391 |
| 50535 | 0.721957 | 0.180675 |
| 50536 | 0.721957 | 0.196608 |
| 50537 | 0.946103 | 0.355179 |
| 50538 | 0.946103 | 0.05 |
| 50539 | 0.814237 | 0.05 |
| 50540 | 0.721929 | 0.05 |
| 50541 | 0.946132 | 0.296756 |
| 50542 | 0.946132 | 0.49432 |
| 50543 | 0.721928 | 0.286784 |
| 50544 | 0.946131 | 0.593268 |
| 50545 | 0.946132 | 0.66862 |
| 50546 | 0.814237 | 0.161789 |
| 50547 | 1.89226 | 0.767462 |
| 50548 | 1.89226 | 0.213082 |
| 50549 | 1.89226 | 0.220522 |
| 50550 | 1.89226 | 0.261958 |
| 50551 | 1.89226 | 0.726915 |
| 50552 | 1.89226 | 0.741808 |
| 50553 | 1.37196 | 0.0762279 |
| 50554 | 2.83556 | 0.05 |
| 50555 | 1.5165 | 0.0562829 |
| 50556 | 1.5165 | 0.0900969 |
| 50557 | 2.74923 | 0.05 |
| 50558 | 2.74923 | 0.0546967 |
| 50559 | 0.946128 | 0.162856 |
| 50560 | 0.946128 | 0.340883 |
| 50561 | 0.827866 | 0.051418 |
| 50562 | 0.946132 | 0.100266 |
| 50563 | 0.94613 | 0.05 |
| 50564 | 0.94613 | 0.0520455 |
| 50565 | 0.94613 | 0.377077 |
| 50566 | 0.94613 | 0.339793 |
| 50567 | 0.94613 | 0.283324 |
| 50568 | 0.827864 | 0.485353 |
| 50569 | 0.94613 | 0.224902 |
| 50570 | 0.94613 | 0.633422 |
| 50571 | 1.36208 | 0.05 |
| 50572 | 2.74923 | 0.05 |
| 50573 | 1.37196 | 0.05 |

| | | |
|---|---|---|
| 50574 | 1.37196 | 0.05 |
| 50575 | 2.53256 | 0.05 |
| 50576 | 2.53256 | 0.05 |
| 50577 | 1.07705 | 2.1 |
| 50578 | 1.07705 | 2.1 |
| 50579 | 1.08253 | 2.1 |
| 50580 | 1.08253 | 2.1 |
| 50581 | 0.762985 | 2.1 |
| 50582 | 0.781638 | 2.1 |
| 50583 | 1.37794 | 2.1 |
| 50584 | 1.40756 | 2.1 |
| 50585 | 1.37794 | 2.1 |
| 50586 | 0.762986 | 2.1 |
| 50587 | 0.781638 | 2.1 |
| 50588 | 1.40756 | 2.1 |
| 50589 | 2.69541 | 2.1 |
| 50590 | 2.75587 | 2.1 |
| 50591 | 1.48572 | 2.1 |
| 50592 | 1.48572 | 2.1 |
| 50593 | 1.52597 | 2.1 |
| 50594 | 2.73566 | 2.1 |
| 50595 | 2.0554 | 1.52724 |
| 50596 | 1.00387 | 0.903342 |
| 50597 | 2.01001 | 0.707528 |
| 50598 | 0.994002 | 0.669402 |
| 50599 | 3.01389 | 0.242974 |
| 50600 | 1.89226 | 2.1 |
| 50601 | 2.41579 | 2.1 |
| 50602 | 2.12209 | 2.1 |
| 50603 | 2.12209 | 2.1 |
| 50604 | 1.89226 | 2.1 |
| 50605 | 1.89226 | 2.1 |
| 50606 | 1.89226 | 2.1 |
| 50607 | 1.89226 | 2.1 |
| 50608 | 1.89226 | 2.1 |
| 50609 | 1.89226 | 2.1 |
| 50610 | 1.89226 | 2.1 |
| 50611 | 1.89226 | 2.1 |
| 50612 | 0.88209 | 2.1 |
| 50613 | 0.899471 | 2.1 |
| 50614 | 0.651501 | 2.1 |
| 50615 | 0.533531 | 2.1 |
| 50616 | 0.724105 | 2.1 |
| 50617 | 0.711243 | 2.1 |
| 50618 | 0.692622 | 2.1 |
| 50619 | 0.692622 | 2.1 |
| 50620 | 0.890668 | 2.1 |
| 50621 | 0.738066 | 2.1 |
| 50622 | 0.778223 | 2.1 |
| 50623 | 0.531121 | 2.1 |
| 50624 | 1.89226 | 2.1 |
| 50625 | 2.77504 | 2.1 |
| 50626 | 2.4158 | 2.1 |
| 50627 | 2.4158 | 2.1 |
| 50628 | 1.89226 | 2.1 |
| 50629 | 1.89226 | 2.1 |
| 50630 | 1.89226 | 2.1 |
| 50631 | 1.89226 | 2.1 |

| | | |
|---|---|---|
| 50632 | 1.89226 | 2.1 |
| 50633 | 1.89226 | 2.1 |
| 50634 | 1.89226 | 2.1 |
| 50635 | 1.89226 | 2.1 |
| 50636 | 1.70303 | 2.1 |
| 50637 | 1.39832 | 2.1 |
| 50638 | 1.51749 | 2.1 |
| 50639 | 1.68783 | 2.1 |
| 50640 | 3.24844 | 2.1 |
| 50641 | 1.89226 | 2.1 |
| 50642 | 2.38311 | 2.1 |
| 50643 | 1.98321 | 2.1 |
| 50644 | 1.89226 | 2.1 |
| 50645 | 2.47406 | 2.1 |
| 50646 | 2.38311 | 2.1 |
| 50647 | 1.89226 | 2.1 |
| 50648 | 1.89226 | 2.1 |
| 50649 | 1.89226 | 2.1 |
| 50650 | 1.89226 | 2.1 |
| 50651 | 1.89226 | 2.1 |
| 50652 | 1.89226 | 2.1 |
| 50653 | 0.946131 | 2.1 |
| 50654 | 0.946131 | 2.1 |
| 50655 | 1.25167 | 2.1 |
| 50656 | 1.19156 | 2.1 |
| 50657 | 1.19156 | 2.1 |
| 50658 | 1.19156 | 2.1 |
| 50659 | 0.946133 | 2.1 |
| 50660 | 0.946133 | 2.1 |
| 50661 | 0.946131 | 2.1 |
| 50662 | 1.25167 | 2.1 |
| 50663 | 0.946131 | 2.1 |
| 50664 | 1.19156 | 2.1 |
| 50665 | 1.89226 | 2.1 |
| 50666 | 2.47406 | 2.1 |
| 50667 | 1.99996 | 2.1 |
| 50668 | 1.89226 | 2.1 |
| 50669 | 2.58176 | 2.1 |
| 50670 | 2.47406 | 2.1 |
| 50671 | 1.89226 | 2.1 |
| 50672 | 1.89226 | 2.1 |
| 50673 | 1.89226 | 2.1 |
| 50674 | 1.89226 | 2.1 |
| 50675 | 1.89226 | 2.1 |
| 50676 | 1.89226 | 2.1 |
| 50677 | 0.946131 | 2.1 |
| 50678 | 0.946131 | 2.1 |
| 50679 | 1.32241 | 2.1 |
| 50680 | 1.25167 | 2.1 |
| 50681 | 1.25167 | 2.1 |
| 50682 | 1.25167 | 2.1 |
| 50683 | 0.946133 | 2.1 |
| 50684 | 0.946133 | 2.1 |
| 50685 | 0.946131 | 2.1 |
| 50686 | 1.32241 | 2.1 |
| 50687 | 0.946131 | 2.1 |
| 50688 | 1.25167 | 2.1 |
| 50689 | 1.65151 | 2.1 |

| | | |
|---|---|---|
| 50690 | 1.92471 | 2.1 |
| 50691 | 1.81834 | 2.1 |
| 50692 | 2.13272 | 2.1 |
| 50693 | 3.74306 | 2.1 |
| 50694 | 1.92472 | 2.1 |
| 50695 | 1.9943 | 2.1 |
| 50696 | 2.13273 | 2.1 |
| 50697 | 2.24475 | 2.1 |
| 50698 | 4.16947 | 2.1 |
| 50699 | 1.81834 | 2.1 |
| 50700 | 2.13272 | 2.1 |
| 50701 | 2.02687 | 2.1 |
| 50702 | 2.39605 | 2.1 |
| 50703 | 4.21439 | 2.1 |
| 50704 | 2.13273 | 2.1 |
| 50705 | 2.24475 | 2.1 |
| 50706 | 2.39605 | 2.1 |
| 50707 | 2.54903 | 2.1 |
| 50708 | 4.6408 | 2.1 |
| 50709 | 1.10534 | 2.1 |
| 50710 | 1.22993 | 2.1 |
| 50711 | 0.946132 | 2.1 |
| 50712 | 0.946132 | 2.1 |
| 50713 | 0.946132 | 2.1 |
| 50714 | 0.946132 | 2.1 |
| 50715 | 1.22993 | 2.1 |
| 50716 | 1.22993 | 2.1 |
| 50717 | 1.10534 | 2.1 |
| 50718 | 0.946131 | 2.1 |
| 50719 | 0.946132 | 2.1 |
| 50720 | 1.22993 | 2.1 |
| 50721 | 1.47221 | 2.1 |
| 50722 | 2.14647 | 2.1 |
| 50723 | 1.95567 | 2.1 |
| 50724 | 1.95567 | 2.1 |
| 50725 | 1.71082 | 2.1 |
| 50726 | 1.71082 | 2.1 |
| 50727 | 0.946131 | 2.1 |
| 50728 | 0.946131 | 2.1 |
| 50729 | 0.946131 | 2.1 |
| 50730 | 0.946131 | 2.1 |
| 50731 | 0.946132 | 2.1 |
| 50732 | 0.946132 | 2.1 |
| 50733 | 0.946131 | 2.1 |
| 50734 | 0.946131 | 2.1 |
| 50735 | 0.946131 | 2.1 |
| 50736 | 0.946131 | 2.1 |
| 50737 | 0.946132 | 2.1 |
| 50738 | 0.946132 | 2.1 |
| 50739 | 0.998086 | 2.1 |
| 50740 | 1.09429 | 2.1 |
| 50741 | 0.946125 | 2.1 |
| 50742 | 0.946125 | 2.1 |
| 50743 | 0.90732 | 2.1 |
| 50744 | 0.90732 | 2.1 |
| 50745 | 1.14414 | 2.1 |
| 50746 | 1.16553 | 2.1 |
| 50747 | 1.05018 | 2.1 |

| | | |
|---|---|---|
| 50748 | 0.907332 | 2.1 |
| 50749 | 0.99482 | 2.1 |
| 50750 | 0.851529 | 2.1 |
| 50751 | 0.80534 | 2.1 |
| 50752 | 0.80534 | 2.1 |
| 50753 | 0.812206 | 2.1 |
| 50754 | 0.812206 | 2.1 |
| 50755 | 0.895134 | 2.1 |
| 50756 | 0.977828 | 2.1 |
| 50757 | 0.736099 | 2.1 |
| 50758 | 0.639718 | 2.1 |
| 50759 | 0.639722 | 2.1 |
| 50760 | 0.89514 | 2.1 |
| 50761 | 0.736095 | 2.1 |
| 50762 | 0.977821 | 2.1 |
| 50763 | 0.946125 | 2.1 |
| 50764 | 0.946125 | 2.1 |
| 50765 | 0.946125 | 2.1 |
| 50766 | 0.946125 | 2.1 |
| 50767 | 0.946125 | 2.1 |
| 50768 | 0.946125 | 2.1 |
| 50769 | 0.946124 | 2.1 |
| 50770 | 0.946124 | 2.1 |
| 50771 | 0.946131 | 2.1 |
| 50772 | 0.946131 | 2.1 |
| 50773 | 0.946118 | 2.1 |
| 50774 | 0.946118 | 2.1 |
| 50775 | 2.22622 | 0.05 |
| 50776 | 2.43347 | 0.05 |
| 50777 | 2.34108 | 0.05 |
| 50778 | 1.24587 | 2.1 |
| 50779 | 1.21674 | 2.1 |
| 50780 | 1.14533 | 2.1 |
| 50781 | 1.11311 | 2.1 |
| 50782 | 1.17874 | 2.1 |
| 50783 | 1.17874 | 2.1 |
| 50784 | 1.18024 | 2.1 |
| 50785 | 1.18024 | 2.1 |
| 50786 | 1.14533 | 2.1 |
| 50787 | 1.24587 | 2.1 |
| 50788 | 1.21674 | 2.1 |
| 50789 | 1.11311 | 2.1 |
| 50790 | 2.43347 | 2.1 |
| 50791 | 2.34107 | 2.1 |
| 50792 | 2.39663 | 2.1 |
| 50793 | 2.49174 | 2.1 |
| 50794 | 4.8301 | 2.1 |
| 50795 | 1.86206 | 2.1 |
| 50796 | 1.41058 | 2.1 |
| 50797 | 1.70302 | 2.1 |
| 50798 | 2.42083 | 2.1 |
| 50799 | 3.68129 | 2.1 |
| 50800 | 1.70302 | 2.1 |
| 50801 | 2.43549 | 2.1 |
| 50802 | 2.20075 | 2.1 |
| 50803 | 2.50358 | 2.1 |
| 50804 | 2.04916 | 2.1 |
| 50805 | 2.33113 | 2.1 |

| | | |
|---|---|---|
| 50806 | 1.07624 | 2.1 |
| 50807 | 2.12621 | 2.1 |
| 50808 | 1.57297 | 2.1 |
| 50809 | 1.60383 | 2.1 |
| 50810 | 1.30047 | 2.1 |
| 50811 | 1.30047 | 2.1 |
| 50812 | 2.33379 | 2.1 |
| 50813 | 2.56149 | 1.2802 |
| 50814 | 2.36734 | 1.42133 |
| 50815 | 2.62796 | 1.41945 |
| 50816 | 4.92883 | 1.81538 |
| 50817 | 2.5615 | 1.36005 |
| 50818 | 2.57957 | 1.52567 |
| 50819 | 2.62796 | 1.22511 |
| 50820 | 2.69056 | 1.15942 |
| 50821 | 5.25206 | 1.84665 |
| 50822 | 2.39322 | 2.1 |
| 50823 | 2.62796 | 2.1 |
| 50824 | 2.6849 | 2.1 |
| 50825 | 2.6849 | 2.0771 |
| 50826 | 2.36734 | 2.1 |
| 50827 | 2.36734 | 2.09845 |
| 50828 | 2.62796 | 1.84179 |
| 50829 | 2.69056 | 1.88008 |
| 50830 | 2.6849 | 0.916512 |
| 50831 | 2.77227 | 0.848502 |
| 50832 | 5.37546 | 1.07123 |
| 50833 | 1.89226 | 0.05 |
| 50834 | 1.89226 | 0.05 |
| 50835 | 1.89226 | 0.05 |
| 50836 | 1.89226 | 0.05 |
| 50837 | 1.89226 | 0.05 |
| 50838 | 1.89226 | 0.05 |
| 50839 | 1.06974 | 0.05 |
| 50840 | 1.06974 | 0.05 |
| 50841 | 1.02299 | 0.05 |
| 50842 | 1.02299 | 0.05 |
| 50843 | 1.35299 | 0.05 |
| 50844 | 1.26227 | 0.05 |
| 50845 | 0.646252 | 0.05 |
| 50846 | 0.830464 | 0.05 |
| 50847 | 0.830463 | 0.05 |
| 50848 | 1.35299 | 0.05 |
| 50849 | 1.26227 | 0.05 |
| 50850 | 0.646252 | 0.05 |
| 50851 | 2.52454 | 0.05 |
| 50852 | 1.89226 | 0.05 |
| 50853 | 2.70598 | 0.05 |
| 50854 | 1.89226 | 0.05 |
| 50855 | 4.59824 | 0.05 |
| 50856 | 1.89226 | 0.05 |
| 50857 | 1.89226 | 0.05 |
| 50858 | 1.89226 | 0.05 |
| 50859 | 1.89226 | 0.05 |
| 50860 | 1.89226 | 0.05 |
| 50861 | 1.89226 | 0.05 |
| 50862 | 1.89226 | 0.05 |
| 50863 | 1.89226 | 0.05 |

| | | |
|---|---|---|
| 50864 | 1.89226 | 0.05 |
| 50865 | 1.89226 | 0.05 |
| 50866 | 1.89226 | 0.05 |
| 50867 | 1.89226 | 0.05 |
| 50868 | 1.7666 | 0.05 |
| 50869 | 2.70598 | 0.05 |
| 50870 | 2.65378 | 0.05 |
| 50871 | 2.65378 | 0.05 |
| 50872 | 1.66093 | 0.05 |
| 50873 | 1.66093 | 0.05 |
| 50874 | 2.70598 | 0.05 |
| 50875 | 1.89226 | 0.05 |
| 50876 | 1.89226 | 0.05 |
| 50877 | 1.89226 | 0.05 |
| 50878 | 2.65377 | 0.05 |
| 50879 | 2.65377 | 0.05 |
| 50880 | 0.946132 | 0.05 |
| 50881 | 0.946132 | 0.05 |
| 50882 | 0.946131 | 0.05 |
| 50883 | 0.946131 | 0.05 |
| 50884 | 0.946131 | 0.05 |
| 50885 | 0.946131 | 0.05 |
| 50886 | 0.946132 | 0.05 |
| 50887 | 0.946132 | 0.05 |
| 50888 | 0.946132 | 0.05 |
| 50889 | 0.946132 | 0.05 |
| 50890 | 0.946131 | 0.05 |
| 50891 | 0.946131 | 0.05 |
| 50892 | 1.69858 | 2.1 |
| 50893 | 2.18238 | 2.1 |
| 50894 | 2.506 | 2.1 |
| 50895 | 1.93586 | 2.1 |
| 50896 | 4.20458 | 2.1 |
| 50897 | 2.21772 | 2.1 |
| 50898 | 2.18238 | 2.1 |
| 50899 | 2.506 | 2.1 |
| 50900 | 2.59215 | 2.1 |
| 50901 | 4.72372 | 2.1 |
| 50902 | 2.50596 | 2.1 |
| 50903 | 1.93584 | 2.1 |
| 50904 | 2.16728 | 2.1 |
| 50905 | 2.7679 | 2.1 |
| 50906 | 4.67325 | 2.1 |
| 50907 | 1.53296 | 2.1 |
| 50908 | 1.56299 | 2.1 |
| 50909 | 1.42624 | 2.1 |
| 50910 | 1.52961 | 2.1 |
| 50911 | 2.98923 | 2.1 |
| 50912 | 1.56299 | 2.1 |
| 50913 | 1.46568 | 2.1 |
| 50914 | 1.52961 | 2.1 |
| 50915 | 1.51916 | 2.1 |
| 50916 | 3.08215 | 2.1 |
| 50917 | 1.36226 | 2.1 |
| 50918 | 1.52961 | 2.1 |
| 50919 | 1.36226 | 2.1 |
| 50920 | 1.42624 | 2.1 |
| 50921 | 1.55369 | 2.1 |

| | | |
|---|---|---|
| 50922 | 1.61768 | 2.1 |
| 50923 | 1.52961 | 2.1 |
| 50924 | 1.51916 | 2.1 |
| 50925 | 1.55369 | 2.1 |
| 50926 | 1.64892 | 2.1 |
| 50927 | 3.07285 | 2.1 |
| 50928 | 2.13405 | 0.05 |
| 50929 | 2.57507 | 0.05 |
| 50930 | 2.39438 | 0.05 |
| 50931 | 1.93408 | 0.05 |
| 50932 | 4.52843 | 0.05 |
| 50933 | 2.13405 | 0.05 |
| 50934 | 1.27778 | 0.05 |
| 50935 | 1.2545 | 0.05 |
| 50936 | 1.45448 | 0.05 |
| 50937 | 1.93408 | 0.05 |
| 50938 | 2.13405 | 0.05 |
| 50939 | 2.39438 | 0.05 |
| 50940 | 1.93407 | 0.05 |
| 50941 | 1.60578 | 0.05 |
| 50942 | 1.87985 | 0.05 |
| 50943 | 4.00016 | 0.05 |
| 50944 | 1.0525 | 2.1 |
| 50945 | 1.05758 | 2.1 |
| 50946 | 1.43484 | 1.03617 |
| 50947 | 1.38506 | 2.06999 |
| 50948 | 2.48734 | 2.1 |
| 50949 | 1.20455 | 2.1 |
| 50950 | 1.67352 | 2.1 |
| 50951 | 1.52852 | 2.1 |
| 50952 | 2.05164 | 2.1 |
| 50953 | 3.20203 | 2.1 |
| 50954 | 1.89226 | 2.1 |
| 50955 | 1.89226 | 2.1 |
| 50956 | 1.89226 | 2.1 |
| 50957 | 1.89226 | 2.1 |
| 50958 | 3.78452 | 2.1 |
| 50959 | 1.89226 | 2.1 |
| 50960 | 1.89226 | 2.1 |
| 50961 | 1.89226 | 2.1 |
| 50962 | 1.89226 | 2.1 |
| 50963 | 1.89226 | 2.1 |
| 50964 | 1.89226 | 2.1 |
| 50965 | 2.05165 | 2.1 |
| 50966 | 1.94291 | 2.1 |
| 50967 | 2.09398 | 2.1 |
| 50968 | 1.67352 | 2.1 |
| 50969 | 2.36337 | 2.1 |
| 50970 | 1.94291 | 2.1 |
| 50971 | 1.52851 | 2.1 |
| 50972 | 2.52049 | 2.1 |
| 50973 | 2.43433 | 2.1 |
| 50974 | 2.05164 | 2.1 |
| 50975 | 1.9112 | 2.1 |
| 50976 | 1.52851 | 2.1 |
| 50977 | 1.89226 | 2.1 |
| 50978 | 1.89226 | 2.1 |
| 50979 | 1.89226 | 2.1 |

| | | |
|---|---|---|
| 50980 | 1.89226 | 2.1 |
| 50981 | 3.78451 | 2.1 |
| 50982 | 1.89226 | 2.1 |
| 50983 | 1.89226 | 2.1 |
| 50984 | 1.89226 | 2.1 |
| 50985 | 1.89226 | 2.1 |
| 50986 | 3.78452 | 2.1 |
| 50987 | 2.5205 | 2.1 |
| 50988 | 2.36337 | 2.1 |
| 50989 | 2.4605 | 2.1 |
| 50990 | 2.05165 | 2.1 |
| 50991 | 2.77222 | 2.1 |
| 50992 | 2.36337 | 2.1 |
| 50993 | 1.89226 | 2.1 |
| 50994 | 1.89226 | 2.1 |
| 50995 | 1.89226 | 2.1 |
| 50996 | 1.89226 | 2.1 |
| 50997 | 1.89226 | 2.1 |
| 50998 | 1.89226 | 2.1 |
| 50999 | 1.89226 | 2.1 |
| 51000 | 1.89226 | 2.1 |
| 51001 | 1.89226 | 2.1 |
| 51002 | 1.89226 | 2.1 |
| 51003 | 3.78452 | 2.1 |
| 51004 | 1.38905 | 2.1 |
| 51005 | 1.26166 | 2.1 |
| 51006 | 1.19216 | 2.1 |
| 51007 | 1.0875 | 2.1 |
| 51008 | 1.24393 | 2.1 |
| 51009 | 1.24393 | 2.1 |
| 51010 | 1.23261 | 2.1 |
| 51011 | 1.23261 | 2.1 |
| 51012 | 1.38905 | 2.1 |
| 51013 | 1.19216 | 2.1 |
| 51014 | 1.26166 | 2.1 |
| 51015 | 1.0875 | 2.1 |
| 51016 | 1.16233 | 2.1 |
| 51017 | 1.06028 | 2.1 |
| 51018 | 0.777395 | 2.1 |
| 51019 | 0.705508 | 2.1 |
| 51020 | 0.940018 | 2.1 |
| 51021 | 0.936465 | 2.1 |
| 51022 | 0.954216 | 2.1 |
| 51023 | 0.954216 | 2.1 |
| 51024 | 1.16125 | 2.1 |
| 51025 | 0.685505 | 2.1 |
| 51026 | 1.1157 | 2.1 |
| 51027 | 0.703534 | 2.1 |
| 51028 | 1.89226 | 2.1 |
| 51029 | 1.89226 | 2.1 |
| 51030 | 1.89226 | 2.1 |
| 51031 | 1.89226 | 2.1 |
| 51032 | 1.89226 | 2.1 |
| 51033 | 1.89226 | 2.1 |
| 51034 | 1.89226 | 2.1 |
| 51035 | 1.89226 | 2.1 |
| 51036 | 1.89226 | 2.1 |
| 51037 | 1.89226 | 2.1 |

| | | |
|---|---|---|
| 51038 | 1.89226 | 2.1 |
| 51039 | 1.89226 | 2.1 |
| 51040 | 2.38433 | 2.1 |
| 51041 | 1.43456 | 2.1 |
| 51042 | 1.93881 | 2.1 |
| 51043 | 2.36952 | 2.1 |
| 51044 | 4.32313 | 2.1 |
| 51045 | 1.8493 | 2.1 |
| 51046 | 1.5927 | 2.1 |
| 51047 | 1.45535 | 2.1 |
| 51048 | 1.38438 | 2.1 |
| 51049 | 1.89657 | 2.1 |
| 51050 | 1.8493 | 2.1 |
| 51051 | 2.0815 | 2.1 |
| 51052 | 1.6697 | 2.1 |
| 51053 | 2.0657 | 2.1 |
| 51054 | 1.5927 | 2.1 |
| 51055 | 2.59749 | 2.1 |
| 51056 | 2.0815 | 2.1 |
| 51057 | 2.76597 | 2.1 |
| 51058 | 1.89226 | 2.1 |
| 51059 | 1.89226 | 2.1 |
| 51060 | 1.89226 | 2.1 |
| 51061 | 2.6126 | 2.1 |
| 51062 | 2.6126 | 2.1 |
| 51063 | 2.62513 | 2.1 |
| 51064 | 1.89226 | 2.1 |
| 51065 | 1.89226 | 2.1 |
| 51066 | 1.89226 | 2.1 |
| 51067 | 2.76598 | 2.1 |
| 51068 | 2.76598 | 2.1 |
| 51069 | 2.54665 | 2.1 |
| 51070 | 2.6126 | 2.1 |
| 51071 | 2.76598 | 2.1 |
| 51072 | 2.76598 | 2.1 |
| 51073 | 2.34044 | 2.1 |
| 51074 | 2.34044 | 2.1 |
| 51075 | 2.62514 | 2.1 |
| 51076 | 2.76598 | 2.1 |
| 51077 | 2.54666 | 2.1 |
| 51078 | 2.45212 | 2.1 |
| 51079 | 5.2181 | 2.1 |
| 51080 | 2.14063 | 2.1 |
| 51081 | 1.47521 | 2.1 |
| 51082 | 1.27679 | 2.1 |
| 51083 | 1.47521 | 2.1 |
| 51084 | 2.11511 | 2.1 |
| 51085 | 2.31353 | 2.1 |
| 51086 | 0.93256 | 2.1 |
| 51087 | 1.07032 | 2.1 |
| 51088 | 0.638396 | 2.1 |
| 51089 | 0.521342 | 2.1 |
| 51090 | 0.790674 | 2.1 |
| 51091 | 0.790674 | 2.1 |
| 51092 | 0.80098 | 2.1 |
| 51093 | 0.800981 | 2.1 |
| 51094 | 0.932535 | 2.1 |
| 51095 | 0.521328 | 2.1 |

| | | |
|---|---|---|
| 51096 | 1.07034 | 2.1 |
| 51097 | 0.638414 | 2.1 |
| 51098 | 1.89226 | 2.1 |
| 51099 | 1.89226 | 1.47794 |
| 51100 | 1.89226 | 1.81338 |
| 51101 | 1.89226 | 2.1 |
| 51102 | 3.78453 | 2.1 |
| 51103 | 1.89226 | 2.1 |
| 51104 | 1.9842 | 2.1 |
| 51105 | 1.89226 | 2.1 |
| 51106 | 1.89226 | 2.1 |
| 51107 | 2.79705 | 2.1 |
| 51108 | 2.79705 | 2.1 |
| 51109 | 0.469955 | 2.1 |
| 51110 | 0.791665 | 2.1 |
| 51111 | 0.989115 | 2.04776 |
| 51112 | 0.763235 | 2.1 |
| 51113 | 0.776356 | 2.1 |
| 51114 | 0.805461 | 2.1 |
| 51115 | 0.776759 | 2.1 |
| 51116 | 0.776759 | 2.1 |
| 51117 | 0.799151 | 2.1 |
| 51118 | 0.940869 | 2.1 |
| 51119 | 0.78449 | 2.1 |
| 51120 | 0.489635 | 2.1 |
| 51121 | 1.89226 | 1.36845 |
| 51122 | 1.89226 | 2.1 |
| 51123 | 1.89226 | 1.36543 |
| 51124 | 1.89226 | 2.1 |
| 51125 | 1.89226 | 1.27293 |
| 51126 | 1.89226 | 2.1 |
| 51127 | 2.46058 | 2.1 |
| 51128 | 2.28991 | 2.1 |
| 51129 | 1.8525 | 2.1 |
| 51130 | 1.87697 | 2.1 |
| 51131 | 2.28991 | 2.1 |
| 51132 | 1.62397 | 2.1 |
| 51133 | 0.946131 | 0.05 |
| 51134 | 0.974033 | 0.05 |
| 51135 | 1.31667 | 0.05 |
| 51136 | 0.946131 | 0.05 |
| 51137 | 0.885497 | 0.05 |
| 51138 | 0.885497 | 0.05 |
| 51139 | 1.0468 | 0.05 |
| 51140 | 1.04678 | 0.05 |
| 51141 | 1.31667 | 0.05 |
| 51142 | 0.974019 | 0.05 |
| 51143 | 0.946131 | 0.05 |
| 51144 | 0.946131 | 0.05 |
| 51145 | 1.89226 | 0.05 |
| 51146 | 1.89226 | 0.05 |
| 51147 | 1.89226 | 0.05 |
| 51148 | 1.89226 | 0.05 |
| 51149 | 3.78452 | 0.05 |
| 51150 | 0.946125 | 0.05 |
| 51151 | 0.946125 | 0.05 |
| 51152 | 0.946131 | 0.05 |
| 51153 | 0.946131 | 0.05 |

| | | |
|---|---|---|
| 51154 | 0.946128 | 0.05 |
| 51155 | 0.946128 | 0.05 |
| 51156 | 0.946128 | 0.05 |
| 51157 | 0.946128 | 0.05 |
| 51158 | 0.946128 | 0.05 |
| 51159 | 0.946128 | 0.05 |
| 51160 | 0.946128 | 0.05 |
| 51161 | 0.946128 | 0.05 |
| 51162 | 0.946131 | 0.05 |
| 51163 | 0.98453 | 0.05 |
| 51164 | 1.32276 | 0.05 |
| 51165 | 0.946131 | 0.05 |
| 51166 | 0.946132 | 0.05 |
| 51167 | 0.946132 | 0.05 |
| 51168 | 0.98453 | 0.05 |
| 51169 | 1.32276 | 0.05 |
| 51170 | 1.32276 | 0.05 |
| 51171 | 1.32276 | 0.05 |
| 51172 | 0.946132 | 0.05 |
| 51173 | 0.946132 | 0.05 |
| 51174 | 1.89227 | 2.1 |
| 51175 | 1.89227 | 2.1 |
| 51176 | 1.89227 | 2.1 |
| 51177 | 1.89227 | 2.1 |
| 51178 | 1.89227 | 2.1 |
| 51179 | 1.89227 | 2.1 |
| 51180 | 1.89226 | 2.1 |
| 51181 | 1.89226 | 2.1 |
| 51182 | 1.89226 | 2.1 |
| 51183 | 1.89226 | 2.1 |
| 51184 | 1.89226 | 2.1 |
| 51185 | 1.89226 | 2.1 |
| 51186 | 1.05556 | 2.1 |
| 51187 | 1.38859 | 2.1 |
| 51188 | 1.46996 | 2.1 |
| 51189 | 1.25364 | 2.1 |
| 51190 | 2.52552 | 2.1 |
| 51191 | 0.694296 | 2.1 |
| 51192 | 0.73498 | 2.1 |
| 51193 | 0.561881 | 2.1 |
| 51194 | 0.512189 | 2.1 |
| 51195 | 0.621304 | 2.1 |
| 51196 | 0.621304 | 2.1 |
| 51197 | 0.625865 | 2.1 |
| 51198 | 0.625865 | 2.1 |
| 51199 | 0.734979 | 2.1 |
| 51200 | 0.561881 | 2.1 |
| 51201 | 0.51219 | 2.1 |
| 51202 | 0.694296 | 2.1 |
| 51203 | 0.946126 | 2.1 |
| 51204 | 0.946126 | 2.1 |
| 51205 | 0.946126 | 2.1 |
| 51206 | 0.946126 | 2.1 |
| 51207 | 0.946126 | 2.1 |
| 51208 | 0.946126 | 2.1 |
| 51209 | 0.946126 | 2.1 |
| 51210 | 0.946126 | 2.1 |
| 51211 | 0.946132 | 2.1 |

| | | |
|---|---|---|
| 51212 | 0.946132 | 2.1 |
| 51213 | 0.94612 | 2.1 |
| 51214 | 0.94612 | 2.1 |
| 51215 | 1.89225 | 2.1 |
| 51216 | 1.89225 | 2.1 |
| 51217 | 1.89225 | 2.1 |
| 51218 | 1.89225 | 2.1 |
| 51219 | 1.89225 | 2.1 |
| 51220 | 1.89225 | 2.1 |
| 51221 | 0.626814 | 2.1 |
| 51222 | 0.632155 | 2.1 |
| 51223 | 0.746234 | 2.1 |
| 51224 | 0.734974 | 2.1 |
| 51225 | 0.68202 | 2.1 |
| 51226 | 0.68202 | 2.1 |
| 51227 | 0.685108 | 2.1 |
| 51228 | 0.685108 | 2.1 |
| 51229 | 0.746239 | 2.1 |
| 51230 | 0.632159 | 2.1 |
| 51231 | 0.734969 | 2.1 |
| 51232 | 0.62681 | 2.1 |
| 51233 | 0.746235 | 2.1 |
| 51234 | 0.734975 | 2.1 |
| 51235 | 0.595081 | 2.1 |
| 51236 | 0.561877 | 2.1 |
| 51237 | 0.648584 | 2.1 |
| 51238 | 0.648584 | 2.1 |
| 51239 | 0.659528 | 2.1 |
| 51240 | 0.659528 | 2.1 |
| 51241 | 0.746239 | 2.1 |
| 51242 | 0.595085 | 2.1 |
| 51243 | 0.734969 | 2.1 |
| 51244 | 0.561874 | 2.1 |
| 51245 | 1.89249 | 2.1 |
| 51246 | 2.2137 | 2.1 |
| 51247 | 1.33125 | 2.1 |
| 51248 | 1.06499 | 2.1 |
| 51249 | 2.12571 | 2.1 |
| 51250 | 1.78918 | 2.1 |
| 51251 | 0.946131 | 2.1 |
| 51252 | 0.946131 | 2.1 |
| 51253 | 0.94613 | 2.1 |
| 51254 | 0.94613 | 2.1 |
| 51255 | 0.946132 | 2.1 |
| 51256 | 0.946132 | 2.1 |
| 51257 | 0.946129 | 2.1 |
| 51258 | 0.946129 | 2.1 |
| 51259 | 0.946131 | 2.1 |
| 51260 | 0.946131 | 2.1 |
| 51261 | 0.946131 | 2.1 |
| 51262 | 0.946131 | 2.1 |
| 51263 | 1.89227 | 2.1 |
| 51264 | 1.89227 | 2.1 |
| 51265 | 1.89227 | 2.1 |
| 51266 | 1.89227 | 2.1 |
| 51267 | 3.78453 | 2.1 |
| 51268 | 1.89226 | 2.1 |
| 51269 | 1.89226 | 2.1 |

| | | |
|---|---|---|
| 51270 | 1.89226 | 2.1 |
| 51271 | 1.89226 | 2.1 |
| 51272 | 1.89226 | 2.1 |
| 51273 | 1.89226 | 2.1 |
| 51274 | 2.01388 | 2.1 |
| 51275 | 1.38757 | 2.1 |
| 51276 | 1.24229 | 2.1 |
| 51277 | 1.3309 | 2.1 |
| 51278 | 1.88011 | 2.1 |
| 51279 | 2.01388 | 2.1 |
| 51280 | 2.01388 | 2.1 |
| 51281 | 2.01555 | 2.1 |
| 51282 | 2.13879 | 2.1 |
| 51283 | 1.80766 | 2.1 |
| 51284 | 4.15267 | 2.1 |
| 51285 | 0.946133 | 2.1 |
| 51286 | 0.946133 | 2.1 |
| 51287 | 0.946132 | 2.1 |
| 51288 | 0.946132 | 2.1 |
| 51289 | 0.946132 | 2.1 |
| 51290 | 0.946132 | 2.1 |
| 51291 | 0.946133 | 2.1 |
| 51292 | 0.946133 | 2.1 |
| 51293 | 0.946132 | 2.1 |
| 51294 | 0.946132 | 2.1 |
| 51295 | 0.946132 | 2.1 |
| 51296 | 0.946132 | 2.1 |
| 51297 | 1.89226 | 2.1 |
| 51298 | 1.89226 | 2.1 |
| 51299 | 1.89226 | 2.1 |
| 51300 | 1.89226 | 2.1 |
| 51301 | 1.89226 | 2.1 |
| 51302 | 1.89226 | 2.1 |
| 51303 | 0.676745 | 2.1 |
| 51304 | 0.68382 | 2.1 |
| 51305 | 1.07048 | 2.1 |
| 51306 | 1.1327 | 2.1 |
| 51307 | 0.929324 | 2.1 |
| 51308 | 0.871438 | 2.1 |
| 51309 | 0.867362 | 2.1 |
| 51310 | 0.867362 | 2.1 |
| 51311 | 1.10666 | 2.1 |
| 51312 | 0.695036 | 2.1 |
| 51313 | 0.744098 | 2.1 |
| 51314 | 1.10144 | 2.1 |
| 51315 | 2.25168 | 2.1 |
| 51316 | 2.22499 | 2.1 |
| 51317 | 2.15929 | 2.1 |
| 51318 | 2.13879 | 2.1 |
| 51319 | 2.15929 | 2.1 |
| 51320 | 2.13879 | 2.1 |
| 51321 | 2.15314 | 2.1 |
| 51322 | 1.70302 | 2.1 |
| 51323 | 2.24694 | 2.1 |
| 51324 | 1.65245 | 2.1 |
| 51325 | 3.82722 | 2.1 |
| 51326 | 1.65245 | 2.1 |
| 51327 | 1.00371 | 2.1 |

| | | |
|---|---|---|
| 51328 | 1.7437 | 2.1 |
| 51329 | 1.65748 | 2.1 |
| 51330 | 1.10459 | 2.1 |
| 51331 | 1.00371 | 2.1 |
| 51332 | 2.24694 | 2.1 |
| 51333 | 1.65246 | 2.1 |
| 51334 | 2.69773 | 2.1 |
| 51335 | 1.88129 | 2.1 |
| 51336 | 4.12824 | 2.1 |
| 51337 | 1.65246 | 2.1 |
| 51338 | 1.10459 | 2.1 |
| 51339 | 1.88129 | 2.1 |
| 51340 | 1.05914 | 2.1 |
| 51341 | 2.76496 | 2.1 |
| 51342 | 1.89226 | 2.1 |
| 51343 | 1.89226 | 2.1 |
| 51344 | 1.89226 | 2.1 |
| 51345 | 1.89226 | 2.1 |
| 51346 | 3.78452 | 2.1 |
| 51347 | 0.718577 | 2.1 |
| 51348 | 0.718577 | 2.1 |
| 51349 | 0.725249 | 2.1 |
| 51350 | 0.725249 | 2.1 |
| 51351 | 0.597916 | 2.1 |
| 51352 | 0.61752 | 2.1 |
| 51353 | 0.826306 | 2.1 |
| 51354 | 0.859194 | 2.1 |
| 51355 | 0.826306 | 2.1 |
| 51356 | 0.597916 | 2.1 |
| 51357 | 0.859194 | 2.1 |
| 51358 | 0.61752 | 2.1 |
| 51359 | 1.25898 | 2.1 |
| 51360 | 1.25898 | 2.1 |
| 51361 | 0.946128 | 2.1 |
| 51362 | 0.946128 | 2.1 |
| 51363 | 1.25897 | 2.1 |
| 51364 | 1.25585 | 2.1 |
| 51365 | 0.946131 | 2.1 |
| 51366 | 0.946131 | 2.1 |
| 51367 | 0.946129 | 2.1 |
| 51368 | 1.25586 | 2.1 |
| 51369 | 1.25898 | 2.1 |
| 51370 | 0.946129 | 2.1 |
| 51371 | 1.89226 | 2.1 |
| 51372 | 1.89226 | 2.1 |
| 51373 | 1.89226 | 2.1 |
| 51374 | 1.89226 | 2.1 |
| 51375 | 3.78452 | 2.1 |
| 51376 | 0.693258 | 2.1 |
| 51377 | 0.693258 | 2.1 |
| 51378 | 0.697356 | 2.1 |
| 51379 | 0.697356 | 2.1 |
| 51380 | 0.572479 | 2.1 |
| 51381 | 0.597916 | 2.1 |
| 51382 | 0.792698 | 2.1 |
| 51383 | 0.826306 | 2.1 |
| 51384 | 0.572478 | 2.1 |
| 51385 | 0.792697 | 2.1 |

| | | |
|---|---|---|
| 51386 | 0.826307 | 2.1 |
| 51387 | 0.597917 | 2.1 |
| 51388 | 1.25586 | 2.1 |
| 51389 | 1.25586 | 2.1 |
| 51390 | 0.946128 | 2.1 |
| 51391 | 0.946128 | 2.1 |
| 51392 | 1.25585 | 2.1 |
| 51393 | 1.24344 | 2.1 |
| 51394 | 0.946131 | 2.1 |
| 51395 | 0.946131 | 2.1 |
| 51396 | 0.946129 | 2.1 |
| 51397 | 1.24344 | 2.1 |
| 51398 | 0.94613 | 2.1 |
| 51399 | 1.25586 | 2.1 |
| 51400 | 1.65151 | 2.1 |
| 51401 | 1.81835 | 2.1 |
| 51402 | 1.12064 | 2.1 |
| 51403 | 1.12064 | 2.1 |
| 51404 | 1.24965 | 2.1 |
| 51405 | 1.81835 | 2.1 |
| 51406 | 1.24965 | 2.1 |
| 51407 | 1.81835 | 2.1 |
| 51408 | 1.41077 | 2.1 |
| 51409 | 2.02687 | 2.1 |
| 51410 | 3.22911 | 2.1 |
| 51411 | 1.89122 | 1.16228 |
| 51412 | 1.38776 | 2.1 |
| 51413 | 2.37014 | 0.972582 |
| 51414 | 1.80818 | 2.1 |
| 51415 | 3.6994 | 2.1 |
| 51416 | 2.15466 | 0.976388 |
| 51417 | 2.00083 | 2.1 |
| 51418 | 1.80818 | 1.88333 |
| 51419 | 1.80818 | 1.0156 |
| 51420 | 2.31988 | 2.1 |
| 51421 | 2.31988 | 1.0236 |
| 51422 | 1.80818 | 0.0925004 |
| 51423 | 1.18041 | 2.1 |
| 51424 | 2.00082 | 1.98124 |
| 51425 | 1.43652 | 2.1 |
| 51426 | 3.18123 | 2.1 |
| 51427 | 1.89226 | 1.0745 |
| 51428 | 1.89226 | 2.0124 |
| 51429 | 1.89226 | 1.20662 |
| 51430 | 1.89226 | 1.06952 |
| 51431 | 3.78453 | 1.19856 |
| 51432 | 1.89226 | 1.89307 |
| 51433 | 1.89226 | 1.90182 |
| 51434 | 1.89226 | 1.16322 |
| 51435 | 1.89226 | 1.22844 |
| 51436 | 3.78452 | 2.0169 |
| 51437 | 0.80759 | 1.45856 |
| 51438 | 0.686119 | 1.96232 |
| 51439 | 0.972147 | 1.54142 |
| 51440 | 1.00217 | 1.88995 |
| 51441 | 0.821334 | 2.1 |
| 51442 | 0.821334 | 1.99465 |
| 51443 | 0.866957 | 1.4617 |

| | | |
|---|---|---|
| 51444 | 0.866957 | 1.46331 |
| 51445 | 0.972145 | 2.03575 |
| 51446 | 0.686121 | 2.1 |
| 51447 | 0.807592 | 2.1 |
| 51448 | 1.00217 | 2.1 |
| 51449 | 0.972146 | 1.69394 |
| 51450 | 1.00217 | 1.96976 |
| 51451 | 1.26654 | 2.1 |
| 51452 | 1.1908 | 1.57564 |
| 51453 | 1.05501 | 1.94966 |
| 51454 | 1.05501 | 2.1 |
| 51455 | 1.10794 | 1.54849 |
| 51456 | 1.10794 | 0.97546 |
| 51457 | 0.972146 | 1.01787 |
| 51458 | 1.26654 | 1.97911 |
| 51459 | 1.00217 | 1.57367 |
| 51460 | 1.1908 | 2.1 |
| 51461 | 1.89226 | 1.46394 |
| 51462 | 1.89226 | 0.980727 |
| 51463 | 1.89226 | 1.07465 |
| 51464 | 1.89226 | 2.1 |
| 51465 | 1.89226 | 0.875097 |
| 51466 | 1.89226 | 1.25905 |
| 51467 | 0.972147 | 1.74557 |
| 51468 | 0.867854 | 2.01511 |
| 51469 | 1.26132 | 1.2781 |
| 51470 | 1.26654 | 1.88468 |
| 51471 | 1.0424 | 2.0518 |
| 51472 | 1.0424 | 2.1 |
| 51473 | 1.092 | 0.9406 |
| 51474 | 1.092 | 1.85351 |
| 51475 | 1.26132 | 1.99449 |
| 51476 | 0.867854 | 2.1 |
| 51477 | 0.972147 | 2.1 |
| 51478 | 1.26654 | 2.1 |
| 51479 | 1.4375 | 0.05 |
| 51480 | 1.92998 | 0.05 |
| 51481 | 1.66953 | 0.05 |
| 51482 | 1.0953 | 0.05 |
| 51483 | 3.10703 | 0.05 |
| 51484 | 1.89226 | 2.1 |
| 51485 | 1.89226 | 2.1 |
| 51486 | 1.89226 | 2.1 |
| 51487 | 1.89226 | 2.1 |
| 51488 | 3.78452 | 2.1 |
| 51489 | 2.74034 | 2.1 |
| 51490 | 2.08808 | 2.1 |
| 51491 | 2.63859 | 2.1 |
| 51492 | 2.00343 | 2.1 |
| 51493 | 4.72667 | 2.1 |
| 51494 | 0.799825 | 2.1 |
| 51495 | 0.799825 | 2.1 |
| 51496 | 0.80211 | 2.1 |
| 51497 | 0.80211 | 2.1 |
| 51498 | 0.562428 | 2.1 |
| 51499 | 0.600223 | 2.1 |
| 51500 | 1.04404 | 2.1 |
| 51501 | 1.00171 | 2.1 |

| | | |
|---|---|---|
| 51502 | 1.00171 | 2.1 |
| 51503 | 0.56243 | 2.1 |
| 51504 | 0.600225 | 2.1 |
| 51505 | 1.04404 | 2.1 |
| 51506 | 0.94613 | 2.1 |
| 51507 | 0.94613 | 2.1 |
| 51508 | 0.946128 | 2.1 |
| 51509 | 0.946128 | 2.1 |
| 51510 | 0.946127 | 2.1 |
| 51511 | 0.946127 | 2.1 |
| 51512 | 0.946131 | 2.1 |
| 51513 | 0.946131 | 2.1 |
| 51514 | 0.946129 | 2.1 |
| 51515 | 0.946129 | 2.1 |
| 51516 | 0.946129 | 2.1 |
| 51517 | 0.946129 | 2.1 |
| 51518 | 1.89226 | 2.1 |
| 51519 | 1.89226 | 2.1 |
| 51520 | 1.89226 | 2.1 |
| 51521 | 1.89226 | 2.1 |
| 51522 | 3.78452 | 2.1 |
| 51523 | 2.63859 | 2.1 |
| 51524 | 2.00343 | 2.1 |
| 51525 | 2.54388 | 2.1 |
| 51526 | 1.91548 | 2.1 |
| 51527 | 4.55407 | 2.1 |
| 51528 | 1.91548 | 2.1 |
| 51529 | 2.00342 | 2.1 |
| 51530 | 1.04909 | 2.1 |
| 51531 | 1.04909 | 2.1 |
| 51532 | 1.12486 | 2.1 |
| 51533 | 1.99124 | 2.1 |
| 51534 | 0.810942 | 2.1 |
| 51535 | 0.810942 | 2.1 |
| 51536 | 0.810942 | 2.1 |
| 51537 | 0.810942 | 2.1 |
| 51538 | 1.89226 | 2.1 |
| 51539 | 1.35169 | 2.1 |
| 51540 | 1.08131 | 2.1 |
| 51541 | 1.08132 | 2.1 |
| 51542 | 1.08132 | 2.1 |
| 51543 | 0.810944 | 2.1 |
| 51544 | 0.810944 | 2.1 |
| 51545 | 1.89226 | 0.175741 |
| 51546 | 1.89226 | 0.690656 |
| 51547 | 1.89226 | 0.068084 |
| 51548 | 1.89226 | 0.567372 |
| 51549 | 3.78452 | 0.136361 |
| 51550 | 1.7968 | 0.0679746 |
| 51551 | 1.49831 | 0.615663 |
| 51552 | 1.59244 | 0.05 |
| 51553 | 1.3395 | 0.101119 |
| 51554 | 3.09074 | 0.05 |
| 51555 | 1.49831 | 0.05 |
| 51556 | 1.16858 | 2.1 |
| 51557 | 1.33951 | 0.05 |
| 51558 | 1.0361 | 2.1 |
| 51559 | 2.53442 | 0.52343 |

| | | |
|---|---|---|
| 51560 | 1.89226 | 0.05 |
| 51561 | 1.89226 | 0.05 |
| 51562 | 1.89226 | 0.103459 |
| 51563 | 1.89226 | 2.1 |
| 51564 | 1.89226 | 2.1 |
| 51565 | 1.89226 | 2.1 |
| 51566 | 1.89223 | 0.340126 |
| 51567 | 1.89223 | 0.299323 |
| 51568 | 1.89223 | 0.540716 |
| 51569 | 1.89223 | 0.682028 |
| 51570 | 1.89223 | 0.05 |
| 51571 | 1.89223 | 0.250681 |
| 51572 | 1.59241 | 0.0909632 |
| 51573 | 1.18952 | 0.240058 |
| 51574 | 1.33949 | 0.130854 |
| 51575 | 1.33949 | 0.174457 |
| 51576 | 1.40538 | 0.192097 |
| 51577 | 1.40538 | 0.181891 |
| 51578 | 1.89226 | 0.0539135 |
| 51579 | 1.89226 | 0.362005 |
| 51580 | 1.89226 | 0.12823 |
| 51581 | 1.89226 | 0.187476 |
| 51582 | 1.89226 | 0.365896 |
| 51583 | 1.89226 | 0.411184 |
| 51584 | 1.89226 | 2.1 |
| 51585 | 1.89226 | 2.1 |
| 51586 | 1.89226 | 2.1 |
| 51587 | 1.89226 | 2.1 |
| 51588 | 3.78452 | 2.1 |
| 51589 | 1.89226 | 2.1 |
| 51590 | 1.89226 | 2.1 |
| 51591 | 1.89226 | 2.1 |
| 51592 | 1.89226 | 2.1 |
| 51593 | 3.78452 | 2.1 |
| 51594 | 2.01867 | 2.1 |
| 51595 | 1.46955 | 2.1 |
| 51596 | 1.58476 | 2.1 |
| 51597 | 1.38189 | 2.1 |
| 51598 | 2.22153 | 2.1 |
| 51599 | 2.01867 | 2.1 |
| 51600 | 2.01867 | 2.1 |
| 51601 | 2.52881 | 2.1 |
| 51602 | 2.21732 | 2.1 |
| 51603 | 2.21732 | 2.1 |
| 51604 | 2.22154 | 2.1 |
| 51605 | 2.22154 | 2.1 |
| 51606 | 1.89226 | 2.1 |
| 51607 | 1.89226 | 2.1 |
| 51608 | 1.89226 | 2.1 |
| 51609 | 1.89226 | 2.1 |
| 51610 | 1.89226 | 2.1 |
| 51611 | 1.89226 | 2.1 |
| 51612 | 1.89226 | 2.1 |
| 51613 | 1.89226 | 2.1 |
| 51614 | 1.89226 | 2.1 |
| 51615 | 1.89226 | 2.1 |
| 51616 | 1.89226 | 2.1 |
| 51617 | 1.89226 | 2.1 |

| | | |
|---|---|---|
| 51618 | 2.22153 | 2.1 |
| 51619 | 1.63361 | 2.1 |
| 51620 | 1.77336 | 2.1 |
| 51621 | 1.46955 | 2.1 |
| 51622 | 2.52534 | 2.1 |
| 51623 | 2.22153 | 2.1 |
| 51624 | 2.34431 | 2.1 |
| 51625 | 1.62905 | 2.1 |
| 51626 | 1.49693 | 2.1 |
| 51627 | 2.19197 | 2.1 |
| 51628 | 3.82103 | 2.1 |
| 51629 | 1.89226 | 2.1 |
| 51630 | 1.89226 | 2.1 |
| 51631 | 1.89226 | 2.1 |
| 51632 | 1.89226 | 2.1 |
| 51633 | 3.78453 | 2.1 |
| 51634 | 0.946128 | 2.1 |
| 51635 | 0.946128 | 2.1 |
| 51636 | 1.22372 | 2.1 |
| 51637 | 1.17331 | 2.1 |
| 51638 | 0.946131 | 2.1 |
| 51639 | 0.946131 | 2.1 |
| 51640 | 1.17331 | 2.1 |
| 51641 | 1.17331 | 2.1 |
| 51642 | 0.946131 | 2.1 |
| 51643 | 1.17331 | 2.1 |
| 51644 | 1.22372 | 2.1 |
| 51645 | 0.94613 | 2.1 |
| 51646 | 1.04041 | 2.1 |
| 51647 | 1.09599 | 2.1 |
| 51648 | 0.748462 | 2.1 |
| 51649 | 0.701976 | 2.1 |
| 51650 | 0.896701 | 2.1 |
| 51651 | 0.896701 | 2.1 |
| 51652 | 0.901262 | 2.1 |
| 51653 | 0.901262 | 2.1 |
| 51654 | 1.04041 | 2.1 |
| 51655 | 0.701977 | 2.1 |
| 51656 | 0.748463 | 2.1 |
| 51657 | 1.09599 | 2.1 |
| 51658 | 1.89226 | 2.1 |
| 51659 | 1.89226 | 2.1 |
| 51660 | 1.89226 | 2.1 |
| 51661 | 1.89226 | 2.1 |
| 51662 | 3.78453 | 2.1 |
| 51663 | 0.946128 | 2.1 |
| 51664 | 0.946128 | 2.1 |
| 51665 | 1.17331 | 2.1 |
| 51666 | 1.13785 | 2.1 |
| 51667 | 0.94613 | 2.1 |
| 51668 | 0.94613 | 2.1 |
| 51669 | 1.13785 | 2.1 |
| 51670 | 1.13785 | 2.1 |
| 51671 | 1.13785 | 2.1 |
| 51672 | 0.94613 | 2.1 |
| 51673 | 0.94613 | 2.1 |
| 51674 | 1.17331 | 2.1 |
| 51675 | 2.50381 | 2.1 |

| | | |
|---|---|---|
| 51676 | 1.89226 | 2.1 |
| 51677 | 1.89226 | 2.1 |
| 51678 | 2.73387 | 2.1 |
| 51679 | 4.39607 | 2.1 |
| 51680 | 1.80045 | 2.1 |
| 51681 | 1.99777 | 0.187651 |
| 51682 | 2.45134 | 2.1 |
| 51683 | 2.12463 | 2.1 |
| 51684 | 1.70302 | 2.1 |
| 51685 | 1.42655 | 2.1 |
| 51686 | 2.20231 | 2.1 |
| 51687 | 1.70302 | 2.1 |
| 51688 | 1.93128 | 2.1 |
| 51689 | 1.66768 | 2.1 |
| 51690 | 1.24499 | 0.676729 |
| 51691 | 1.70302 | 0.174605 |
| 51692 | 2.55572 | 0.05 |
| 51693 | 1.22811 | 0.05 |
| 51694 | 1.82584 | 0.05 |
| 51695 | 1.22811 | 0.05 |
| 51696 | 2.62764 | 0.05 |
| 51697 | 1.93147 | 0.05 |
| 51698 | 2.14513 | 0.05 |
| 51699 | 1.89226 | 0.05 |
| 51700 | 2.66481 | 0.05 |
| 51701 | 1.89226 | 0.05 |
| 51702 | 2.66481 | 0.05 |
| 51703 | 1.89226 | 0.05 |
| 51704 | 2.11649 | 2.1 |
| 51705 | 1.89226 | 2.1 |
| 51706 | 1.89226 | 2.1 |
| 51707 | 1.89226 | 2.1 |
| 51708 | 2.15426 | 2.1 |
| 51709 | 2.15426 | 2.1 |
| 51710 | 2.06776 | 2.1 |
| 51711 | 1.89226 | 2.1 |
| 51712 | 1.89226 | 2.1 |
| 51713 | 1.89226 | 2.1 |
| 51714 | 2.11649 | 2.1 |
| 51715 | 2.11649 | 2.1 |
| 51716 | 1.89226 | 2.1 |
| 51717 | 1.89226 | 2.1 |
| 51718 | 1.89226 | 2.1 |
| 51719 | 1.89226 | 2.1 |
| 51720 | 1.89226 | 2.1 |
| 51721 | 1.89226 | 2.1 |
| 51722 | 0.998134 | 2.1 |
| 51723 | 0.918904 | 2.1 |
| 51724 | 0.894708 | 2.1 |
| 51725 | 0.758742 | 2.1 |
| 51726 | 0.864276 | 2.1 |
| 51727 | 0.864276 | 2.1 |
| 51728 | 0.8926 | 2.1 |
| 51729 | 0.8926 | 2.1 |
| 51730 | 0.998134 | 2.1 |
| 51731 | 0.894709 | 2.1 |
| 51732 | 0.758742 | 2.1 |
| 51733 | 0.918904 | 2.1 |

| | | |
|---|---|---|
| 51734 | 1.89226 | 2.1 |
| 51735 | 1.89226 | 2.1 |
| 51736 | 1.89226 | 2.1 |
| 51737 | 1.89226 | 2.1 |
| 51738 | 1.89226 | 2.1 |
| 51739 | 1.89226 | 2.1 |
| 51740 | 1.89226 | 2.1 |
| 51741 | 1.89226 | 2.1 |
| 51742 | 1.89226 | 2.1 |
| 51743 | 1.89226 | 2.1 |
| 51744 | 1.89226 | 2.1 |
| 51745 | 1.89226 | 2.1 |
| 51746 | 1.99627 | 2.1 |
| 51747 | 1.61047 | 2.1 |
| 51748 | 2.0815 | 2.1 |
| 51749 | 2.07569 | 2.1 |
| 51750 | 3.75063 | 2.1 |
| 51751 | 0.848077 | 2.1 |
| 51752 | 1.07748 | 2.1 |
| 51753 | 0.802498 | 2.1 |
| 51754 | 0.626808 | 2.1 |
| 51755 | 0.875552 | 2.1 |
| 51756 | 0.737527 | 2.1 |
| 51757 | 0.835602 | 2.1 |
| 51758 | 0.835602 | 2.1 |
| 51759 | 0.983245 | 2.1 |
| 51760 | 0.781108 | 2.1 |
| 51761 | 0.872306 | 2.1 |
| 51762 | 0.601497 | 2.1 |
| 51763 | 2.74745 | 2.1 |
| 51764 | 2.73988 | 2.1 |
| 51765 | 2.6521 | 2.1 |
| 51766 | 2.67792 | 2.1 |
| 51767 | 5.39198 | 2.1 |
| 51768 | 2.73988 | 2.1 |
| 51769 | 2.57466 | 2.1 |
| 51770 | 2.67791 | 2.1 |
| 51771 | 2.55653 | 2.1 |
| 51772 | 5.29641 | 2.1 |
| 51773 | 2.6521 | 2.1 |
| 51774 | 2.64148 | 2.1 |
| 51775 | 2.67792 | 2.1 |
| 51776 | 2.67792 | 2.1 |
| 51777 | 2.56953 | 2.1 |
| 51778 | 2.56953 | 2.1 |
| 51779 | 2.64148 | 2.1 |
| 51780 | 2.55653 | 2.1 |
| 51781 | 2.57929 | 2.1 |
| 51782 | 2.57929 | 2.1 |
| 51783 | 2.67792 | 2.1 |
| 51784 | 2.67792 | 2.1 |
| 51785 | 1.89226 | 0.532186 |
| 51786 | 1.89226 | 0.0609608 |
| 51787 | 1.89226 | 0.149419 |
| 51788 | 1.89226 | 0.05 |
| 51789 | 3.78453 | 0.05 |
| 51790 | 1.68703 | 0.531837 |
| 51791 | 2.50385 | 0.05 |

| | | |
|---|---|---|
| 51792 | 1.45958 | 0.05 |
| 51793 | 2.0815 | 0.05 |
| 51794 | 3.93806 | 0.05 |
| 51795 | 2.50385 | 0.05 |
| 51796 | 2.57414 | 0.05 |
| 51797 | 2.0815 | 0.05 |
| 51798 | 2.39921 | 0.05 |
| 51799 | 4.91181 | 0.05 |
| 51800 | 1.89226 | 0.458559 |
| 51801 | 1.89226 | 0.05 |
| 51802 | 1.89226 | 0.05 |
| 51803 | 1.89226 | 0.05 |
| 51804 | 3.78453 | 0.05 |
| 51805 | 1.89226 | 0.324266 |
| 51806 | 1.89226 | 0.05 |
| 51807 | 1.89226 | 0.05 |
| 51808 | 1.89226 | 0.126423 |
| 51809 | 1.89226 | 0.05 |
| 51810 | 1.89226 | 0.424486 |
| 51811 | 1.23013 | 0.05 |
| 51812 | 2.0815 | 0.05 |
| 51813 | 1.90167 | 0.05 |
| 51814 | 1.71243 | 0.05 |
| 51815 | 1.45887 | 0.05 |
| 51816 | 1.31369 | 0.05 |
| 51817 | 2.0815 | 0.0625184 |
| 51818 | 2.39921 | 0.05 |
| 51819 | 1.7115 | 0.05 |
| 51820 | 2.2421 | 0.05 |
| 51821 | 4.09829 | 0.05 |
| 51822 | 1.89227 | 0.05 |
| 51823 | 1.89227 | 0.05 |
| 51824 | 1.89227 | 0.05 |
| 51825 | 1.89227 | 0.05 |
| 51826 | 3.78453 | 0.05 |
| 51827 | 2.39697 | 0.05 |
| 51828 | 1.85594 | 0.05 |
| 51829 | 1.91161 | 0.05 |
| 51830 | 2.49867 | 0.05 |
| 51831 | 4.35461 | 0.05 |
| 51832 | 1.12035 | 0.05 |
| 51833 | 1.85594 | 0.05 |
| 51834 | 1.91161 | 0.05 |
| 51835 | 1.15483 | 0.05 |
| 51836 | 3.01077 | 0.05 |
| 51837 | 1.89226 | 0.05 |
| 51838 | 1.89226 | 0.05 |
| 51839 | 1.89226 | 0.05 |
| 51840 | 1.89226 | 0.05 |
| 51841 | 1.89226 | 0.05 |
| 51842 | 1.89226 | 0.05 |
| 51843 | 1.89226 | 0.05 |
| 51844 | 1.89226 | 0.05 |
| 51845 | 1.89226 | 0.05 |
| 51846 | 1.89226 | 0.05 |
| 51847 | 1.89226 | 0.05 |
| 51848 | 1.89226 | 0.05 |
| 51849 | 0.936516 | 0.05 |

| | | |
|---|---|---|
| 51850 | 1.03496 | 0.05 |
| 51851 | 1.22607 | 0.05 |
| 51852 | 1.28058 | 0.05 |
| 51853 | 1.06317 | 0.05 |
| 51854 | 1.06317 | 0.05 |
| 51855 | 1.19735 | 0.05 |
| 51856 | 1.0813 | 0.05 |
| 51857 | 0.858168 | 0.05 |
| 51858 | 1.32278 | 0.05 |
| 51859 | 0.957674 | 0.05 |
| 51860 | 1.25178 | 0.05 |
| 51861 | 1.92659 | 0.05 |
| 51862 | 0.998957 | 0.05 |
| 51863 | 0.998957 | 0.05 |
| 51864 | 1.15481 | 0.05 |
| 51865 | 1.6031 | 0.05 |
| 51866 | 1.74459 | 0.05 |
| 51867 | 0.946119 | 0.05 |
| 51868 | 0.946119 | 0.05 |
| 51869 | 0.946118 | 0.05 |
| 51870 | 0.946118 | 0.05 |
| 51871 | 0.946118 | 0.05 |
| 51872 | 0.946118 | 0.05 |
| 51873 | 0.946119 | 0.05 |
| 51874 | 0.946119 | 0.05 |
| 51875 | 0.946131 | 0.05 |
| 51876 | 0.946131 | 0.05 |
| 51877 | 0.946106 | 0.05 |
| 51878 | 0.946106 | 0.05 |
| 51879 | 0.946118 | 0.05 |
| 51880 | 0.946118 | 0.05 |
| 51881 | 0.946119 | 0.05 |
| 51882 | 0.946119 | 0.05 |
| 51883 | 0.946118 | 0.05 |
| 51884 | 0.946118 | 0.05 |
| 51885 | 0.946119 | 0.05 |
| 51886 | 0.946119 | 0.05 |
| 51887 | 0.946131 | 0.05 |
| 51888 | 0.946131 | 0.05 |
| 51889 | 0.946106 | 0.05 |
| 51890 | 0.946106 | 0.05 |
| 51891 | 2.47164 | 0.05 |
| 51892 | 2.83689 | 0.05 |
| 51893 | 2.73872 | 0.0707847 |
| 51894 | 2.41632 | 0.05 |
| 51895 | 5.21036 | 0.05 |
| 51896 | 0.946133 | 0.05 |
| 51897 | 0.946133 | 0.0587426 |
| 51898 | 1.41845 | 0.05 |
| 51899 | 1.36936 | 0.05 |
| 51900 | 1.41845 | 0.05 |
| 51901 | 1.41845 | 0.05 |
| 51902 | 0.946132 | 0.057166 |
| 51903 | 0.946132 | 0.0543779 |
| 51904 | 0.946132 | 0.05 |
| 51905 | 1.36936 | 0.05 |
| 51906 | 1.41845 | 0.05 |
| 51907 | 0.946133 | 0.05 |

| | | |
|---|---|---|
| 51908 | 1.36936 | 0.05 |
| 51909 | 1.27581 | 0.05 |
| 51910 | 1.20816 | 0.05 |
| 51911 | 1.10464 | 0.05 |
| 51912 | 1.24454 | 0.05 |
| 51913 | 1.24454 | 0.05 |
| 51914 | 1.23943 | 0.05 |
| 51915 | 1.23943 | 0.05 |
| 51916 | 1.10465 | 0.05 |
| 51917 | 1.27581 | 0.05 |
| 51918 | 1.36936 | 0.05 |
| 51919 | 1.20816 | 0.05 |
| 51920 | 0.946131 | 2.1 |
| 51921 | 0.946131 | 2.1 |
| 51922 | 1.37277 | 2.1 |
| 51923 | 1.37277 | 2.1 |
| 51924 | 1.37277 | 2.1 |
| 51925 | 1.27186 | 2.1 |
| 51926 | 0.946131 | 2.1 |
| 51927 | 0.946131 | 2.1 |
| 51928 | 0.946131 | 2.1 |
| 51929 | 1.37277 | 2.1 |
| 51930 | 0.946131 | 2.1 |
| 51931 | 1.27186 | 2.1 |
| 51932 | 2.74554 | 2.1 |
| 51933 | 1.23712 | 2.1 |
| 51934 | 2.80992 | 2.1 |
| 51935 | 2.54371 | 2.1 |
| 51936 | 1.50333 | 2.1 |
| 51937 | 1.23712 | 2.1 |
| 51938 | 2.77314 | 2.1 |
| 51939 | 2.33316 | 2.1 |
| 51940 | 2.28748 | 2.1 |
| 51941 | 2.28748 | 2.1 |
| 51942 | 2.81275 | 2.1 |
| 51943 | 2.81275 | 2.1 |
| 51944 | 2.28749 | 2.1 |
| 51945 | 1.45887 | 2.1 |
| 51946 | 2.33317 | 2.1 |
| 51947 | 1.50022 | 2.1 |
| 51948 | 3.79204 | 2.1 |
| 51949 | 2.37364 | 2.1 |
| 51950 | 2.33317 | 2.1 |
| 51951 | 1.5362 | 2.1 |
| 51952 | 1.5362 | 2.1 |
| 51953 | 1.50022 | 2.1 |
| 51954 | 2.33317 | 2.1 |
| 51955 | 1.89226 | 0.316424 |
| 51956 | 1.89226 | 0.27015 |
| 51957 | 1.89226 | 0.126179 |
| 51958 | 1.89226 | 0.27397 |
| 51959 | 1.89226 | 0.194284 |
| 51960 | 1.89226 | 0.494279 |
| 51961 | 0.946128 | 0.05 |
| 51962 | 0.946128 | 0.0504745 |
| 51963 | 1.0644 | 0.05 |
| 51964 | 0.946133 | 0.05 |
| 51965 | 1.0644 | 0.05 |

| | | |
|---|---|---|
| 51966 | 0.94613 | 0.05 |
| 51967 | 0.946131 | 0.0604351 |
| 51968 | 0.946131 | 0.05 |
| 51969 | 1.0644 | 0.05 |
| 51970 | 1.0644 | 0.05 |
| 51971 | 0.946131 | 0.05 |
| 51972 | 0.946131 | 0.05 |
| 51973 | 1.89226 | 1.12793 |
| 51974 | 1.89226 | 0.470354 |
| 51975 | 1.89226 | 0.382303 |
| 51976 | 1.89226 | 0.05 |
| 51977 | 3.78452 | 0.107583 |
| 51978 | 1.14599 | 1.00296 |
| 51979 | 1.19993 | 0.21201 |
| 51980 | 1.00065 | 0.121391 |
| 51981 | 1.07497 | 0.05 |
| 51982 | 2.22096 | 0.122718 |
| 51983 | 0.546551 | 0.276292 |
| 51984 | 0.546551 | 0.50771 |
| 51985 | 0.54987 | 0.269719 |
| 51986 | 0.54987 | 0.692815 |
| 51987 | 0.503067 | 0.181575 |
| 51988 | 0.558937 | 0.221134 |
| 51989 | 0.599966 | 0.176695 |
| 51990 | 0.537484 | 0.400539 |
| 51991 | 0.503067 | 0.0582688 |
| 51992 | 0.537485 | 0.0569704 |
| 51993 | 0.599966 | 0.421898 |
| 51994 | 0.558936 | 0.0903519 |
| 51995 | 0.946132 | 0.217403 |
| 51996 | 0.946132 | 0.575136 |
| 51997 | 0.94613 | 0.282173 |
| 51998 | 0.94613 | 0.675413 |
| 51999 | 0.946131 | 0.480619 |
| 52000 | 0.946131 | 0.291584 |
| 52001 | 0.946131 | 0.474305 |
| 52002 | 0.946131 | 0.478065 |
| 52003 | 0.946131 | 0.146367 |
| 52004 | 0.946131 | 0.237543 |
| 52005 | 0.946131 | 0.479766 |
| 52006 | 0.946131 | 0.517445 |
| 52007 | 2.19104 | 2.1 |
| 52008 | 2.5392 | 2.1 |
| 52009 | 2.53302 | 2.1 |
| 52010 | 2.19226 | 2.1 |
| 52011 | 4.73146 | 2.1 |
| 52012 | 1.09552 | 2.1 |
| 52013 | 1.09613 | 2.1 |
| 52014 | 0.802484 | 2.1 |
| 52015 | 0.807941 | 2.1 |
| 52016 | 0.95052 | 2.1 |
| 52017 | 0.95052 | 2.1 |
| 52018 | 0.952944 | 2.1 |
| 52019 | 0.952944 | 2.1 |
| 52020 | 1.09613 | 2.1 |
| 52021 | 0.807941 | 2.1 |
| 52022 | 0.802484 | 2.1 |
| 52023 | 1.09552 | 2.1 |

| | | |
|---|---|---|
| 52024 | 2.533 | 2.1 |
| 52025 | 2.18531 | 2.1 |
| 52026 | 2.19224 | 2.1 |
| 52027 | 2.19224 | 2.1 |
| 52028 | 2.51603 | 2.1 |
| 52029 | 2.51603 | 2.1 |
| 52030 | 1.09266 | 2.1 |
| 52031 | 1.09612 | 2.1 |
| 52032 | 0.807935 | 2.1 |
| 52033 | 0.81091 | 2.1 |
| 52034 | 0.951897 | 2.1 |
| 52035 | 0.951897 | 2.1 |
| 52036 | 0.955134 | 2.1 |
| 52037 | 0.955134 | 2.1 |
| 52038 | 0.810915 | 2.1 |
| 52039 | 1.09266 | 2.1 |
| 52040 | 1.09611 | 2.1 |
| 52041 | 0.80793 | 2.1 |
| 52042 | 2.12741 | 0.679811 |
| 52043 | 2.79312 | 0.655862 |
| 52044 | 2.77591 | 1.04555 |
| 52045 | 2.10714 | 0.631155 |
| 52046 | 4.90026 | 0.399598 |
| 52047 | 2.12741 | 0.687058 |
| 52048 | 1.20859 | 0.0560139 |
| 52049 | 1.21073 | 1.0118 |
| 52050 | 2.10714 | 0.689188 |
| 52051 | 3.31573 | 0.741405 |
| 52052 | 1.38708 | 0.840709 |
| 52053 | 1.38795 | 0.320892 |
| 52054 | 1.05331 | 0.678579 |
| 52055 | 1.05357 | 0.594831 |
| 52056 | 1.22049 | 0.617487 |
| 52057 | 1.22049 | 0.672347 |
| 52058 | 1.22078 | 1.33269 |
| 52059 | 1.22078 | 0.181181 |
| 52060 | 1.05331 | 0.708707 |
| 52061 | 1.38709 | 0.725435 |
| 52062 | 1.38795 | 0.61066 |
| 52063 | 1.05357 | 0.647721 |
| 52064 | 2.10662 | 0.867422 |
| 52065 | 2.10714 | 0.57662 |
| 52066 | 2.12499 | 0.616281 |
| 52067 | 1.22857 | 0.803508 |
| 52068 | 1.21073 | 0.630951 |
| 52069 | 1.21073 | 0.1075 |
| 52070 | 1.82014 | 0.05 |
| 52071 | 2.08757 | 0.05 |
| 52072 | 1.81292 | 0.05 |
| 52073 | 1.67485 | 0.05 |
| 52074 | 3.63306 | 0.05 |
| 52075 | 1.82013 | 0.05 |
| 52076 | 1.21748 | 0.05 |
| 52077 | 1.29891 | 0.05 |
| 52078 | 1.4442 | 0.05 |
| 52079 | 1.67484 | 0.05 |
| 52080 | 1.82013 | 0.05 |
| 52081 | 1.81292 | 0.05 |

| | | |
|---|---|---|
| 52082 | 1.4516 | 0.05 |
| 52083 | 1.39633 | 0.05 |
| 52084 | 1.67485 | 0.05 |
| 52085 | 1.5344 | 0.05 |
| 52086 | 1.81292 | 0.05 |
| 52087 | 1.67484 | 0.05 |
| 52088 | 1.00982 | 0.05 |
| 52089 | 0.994235 | 0.05 |
| 52090 | 1.21748 | 0.05 |
| 52091 | 1.45159 | 0.05 |
| 52092 | 1.67484 | 0.05 |
| 52093 | 1.55216 | 2.1 |
| 52094 | 1.34407 | 2.1 |
| 52095 | 1.34407 | 2.1 |
| 52096 | 1.52008 | 2.1 |
| 52097 | 1.81783 | 2.1 |
| 52098 | 1.99384 | 2.1 |
| 52099 | 1.90817 | 2.1 |
| 52100 | 2.03975 | 2.1 |
| 52101 | 1.97317 | 2.1 |
| 52102 | 1.81783 | 2.1 |
| 52103 | 3.85758 | 2.1 |
| 52104 | 0.599049 | 2.1 |
| 52105 | 0.660673 | 2.1 |
| 52106 | 0.785063 | 2.1 |
| 52107 | 0.924278 | 2.1 |
| 52108 | 0.760416 | 2.1 |
| 52109 | 0.760416 | 2.1 |
| 52110 | 0.719088 | 2.1 |
| 52111 | 0.694532 | 2.1 |
| 52112 | 0.522441 | 2.1 |
| 52113 | 0.796789 | 2.1 |
| 52114 | 0.655207 | 2.1 |
| 52115 | 0.886155 | 2.1 |
| 52116 | 1.97317 | 2.1 |
| 52117 | 1.81783 | 2.1 |
| 52118 | 1.65098 | 2.1 |
| 52119 | 1.91767 | 2.1 |
| 52120 | 3.7355 | 2.1 |
| 52121 | 1.15038 | 1.25505 |
| 52122 | 1.28649 | 2.04489 |
| 52123 | 1.38986 | 1.53092 |
| 52124 | 1.38986 | 2.1 |
| 52125 | 1.07866 | 1.79536 |
| 52126 | 1.07866 | 2.1 |
| 52127 | 0.664498 | 0.524549 |
| 52128 | 0.664498 | 2.1 |
| 52129 | 0.675122 | 2.1 |
| 52130 | 0.675122 | 2.1 |
| 52131 | 0.69493 | 0.978301 |
| 52132 | 0.764429 | 1.87477 |
| 52133 | 0.623438 | 1.46726 |
| 52134 | 0.575191 | 2.1 |
| 52135 | 0.764429 | 1.15602 |
| 52136 | 0.623438 | 1.05297 |
| 52137 | 0.575191 | 2.1 |
| 52138 | 0.69493 | 1.97227 |
| 52139 | 1.89226 | 0.619661 |

| | | |
|---|---|---|
| 52140 | 1.89226 | 0.05 |
| 52141 | 1.89226 | 0.859095 |
| 52142 | 1.89226 | 0.05 |
| 52143 | 3.78453 | 0.05 |
| 52144 | 2.50075 | 0.0808764 |
| 52145 | 2.15276 | 0.05 |
| 52146 | 2.24902 | 0.770187 |
| 52147 | 2.30422 | 0.05 |
| 52148 | 4.40178 | 0.05 |
| 52149 | 1.70424 | 0.105084 |
| 52150 | 1.34494 | 0.551786 |
| 52151 | 2.15276 | 0.05 |
| 52152 | 2.15276 | 0.0787416 |
| 52153 | 2.0793 | 0.05 |
| 52154 | 1.63078 | 0.248892 |
| 52155 | 1.89226 | 0.05 |
| 52156 | 1.89226 | 0.450591 |
| 52157 | 1.89226 | 0.863994 |
| 52158 | 1.89226 | 0.05 |
| 52159 | 1.89226 | 0.620292 |
| 52160 | 1.89226 | 0.48567 |
| 52161 | 1.89226 | 0.126651 |
| 52162 | 1.89226 | 0.0560312 |
| 52163 | 1.89226 | 0.05 |
| 52164 | 1.89226 | 0.05 |
| 52165 | 3.78452 | 0.05 |
| 52166 | 2.24902 | 0.0814858 |
| 52167 | 1.91497 | 0.05 |
| 52168 | 1.97135 | 0.0864389 |
| 52169 | 1.73957 | 0.05 |
| 52170 | 3.98859 | 0.05 |
| 52171 | 1.73957 | 0.05 |
| 52172 | 1.34494 | 0.344439 |
| 52173 | 1.73957 | 0.902998 |
| 52174 | 1.69006 | 0.05 |
| 52175 | 1.3126 | 0.0670825 |
| 52176 | 1.34848 | 0.341164 |
| 52177 | 0.946132 | 0.05 |
| 52178 | 0.946132 | 0.429701 |
| 52179 | 0.946132 | 0.05 |
| 52180 | 0.946132 | 0.995396 |
| 52181 | 0.946132 | 0.121065 |
| 52182 | 0.946132 | 0.05 |
| 52183 | 0.946131 | 0.05 |
| 52184 | 0.946131 | 0.05 |
| 52185 | 0.946132 | 0.05 |
| 52186 | 0.946132 | 0.05 |
| 52187 | 0.946132 | 0.0545605 |
| 52188 | 0.946132 | 0.356594 |
| 52189 | 1.89226 | 2.1 |
| 52190 | 1.89226 | 1.88222 |
| 52191 | 1.89226 | 2.1 |
| 52192 | 1.89226 | 2.06943 |
| 52193 | 3.78453 | 2.1 |
| 52194 | 1.89226 | 2.1 |
| 52195 | 2.82624 | 1.75094 |
| 52196 | 1.89226 | 2.1 |
| 52197 | 2.02536 | 1.99558 |

| | | |
|---|---|---|
| 52198 | 1.89226 | 2.1 |
| 52199 | 2.02536 | 1.66405 |
| 52200 | 2.25027 | 0.05 |
| 52201 | 1.1706 | 0.05 |
| 52202 | 2.02733 | 0.05 |
| 52203 | 2.02733 | 0.05 |
| 52204 | 1.61462 | 0.05 |
| 52205 | 1.69832 | 0.05 |
| 52206 | 1.89226 | 0.05 |
| 52207 | 1.89226 | 0.05 |
| 52208 | 1.89226 | 0.05 |
| 52209 | 1.89226 | 0.05 |
| 52210 | 3.78452 | 0.05 |
| 52211 | 0.946131 | 0.05 |
| 52212 | 0.946131 | 0.05 |
| 52213 | 1.18182 | 0.05 |
| 52214 | 1.18182 | 0.05 |
| 52215 | 0.973819 | 0.05 |
| 52216 | 0.94613 | 0.05 |
| 52217 | 1.18182 | 0.05 |
| 52218 | 0.94613 | 0.05 |
| 52219 | 0.973819 | 0.05 |
| 52220 | 1.18182 | 0.05 |
| 52221 | 0.94613 | 0.05 |
| 52222 | 0.94613 | 0.05 |
| 52223 | 2.23944 | 2.1 |
| 52224 | 1.52903 | 2.1 |
| 52225 | 1.35977 | 2.1 |
| 52226 | 2.04847 | 2.1 |
| 52227 | 3.5775 | 2.1 |
| 52228 | 2.23944 | 2.1 |
| 52229 | 2.34795 | 2.1 |
| 52230 | 2.34795 | 2.1 |
| 52231 | 2.48025 | 2.1 |
| 52232 | 2.04847 | 2.1 |
| 52233 | 2.18077 | 2.1 |
| 52234 | 1.89226 | 2.1 |
| 52235 | 1.89226 | 2.1 |
| 52236 | 1.89226 | 2.1 |
| 52237 | 1.89226 | 2.1 |
| 52238 | 3.78452 | 2.1 |
| 52239 | 0.946127 | 2.1 |
| 52240 | 0.946127 | 2.1 |
| 52241 | 0.946131 | 2.1 |
| 52242 | 0.946131 | 2.1 |
| 52243 | 0.946128 | 2.1 |
| 52244 | 0.946128 | 2.1 |
| 52245 | 0.94613 | 2.1 |
| 52246 | 0.94613 | 2.1 |
| 52247 | 0.946129 | 2.1 |
| 52248 | 0.946129 | 2.1 |
| 52249 | 0.946129 | 2.1 |
| 52250 | 0.946129 | 2.1 |
| 52251 | 1.26956 | 2.1 |
| 52252 | 2.04847 | 2.1 |
| 52253 | 1.49576 | 2.1 |
| 52254 | 1.22377 | 2.1 |
| 52255 | 1.91172 | 2.1 |

| | | |
|---|---|---|
| 52256 | 1.50198 | 2.1 |
| 52257 | 1.48568 | 2.1 |
| 52258 | 2.34795 | 2.1 |
| 52259 | 2.04847 | 2.1 |
| 52260 | 2.04847 | 2.1 |
| 52261 | 2.21155 | 2.1 |
| 52262 | 2.21155 | 2.1 |
| 52263 | 1.89226 | 2.1 |
| 52264 | 1.89226 | 2.1 |
| 52265 | 1.89226 | 2.1 |
| 52266 | 1.89226 | 2.1 |
| 52267 | 1.89226 | 2.1 |
| 52268 | 1.89226 | 2.1 |
| 52269 | 0.810943 | 2.1 |
| 52270 | 0.810943 | 2.1 |
| 52271 | 0.810944 | 2.1 |
| 52272 | 0.810944 | 2.1 |
| 52273 | 0.810957 | 2.1 |
| 52274 | 0.810957 | 2.1 |
| 52275 | 1.89226 | 2.1 |
| 52276 | 1.35168 | 2.1 |
| 52277 | 1.08132 | 2.1 |
| 52278 | 1.08132 | 2.1 |
| 52279 | 1.0813 | 2.1 |
| 52280 | 0.946132 | 0.05 |
| 52281 | 1.33855 | 0.05 |
| 52282 | 0.946131 | 0.05 |
| 52283 | 1.38204 | 0.05 |
| 52284 | 0.946131 | 0.05 |
| 52285 | 0.946131 | 0.05 |
| 52286 | 1.38204 | 0.05 |
| 52287 | 1.38204 | 0.05 |
| 52288 | 0.946131 | 0.05 |
| 52289 | 0.946131 | 0.05 |
| 52290 | 1.33855 | 0.05 |
| 52291 | 1.38204 | 0.05 |
| 52292 | 1.89226 | 0.05 |
| 52293 | 2.76407 | 0.05 |
| 52294 | 1.89226 | 0.05 |
| 52295 | 2.72769 | 0.05 |
| 52296 | 1.89226 | 0.05 |
| 52297 | 2.72769 | 0.05 |
| 52298 | 2.03914 | 0.05 |
| 52299 | 1.67727 | 0.05 |
| 52300 | 1.94831 | 0.05 |
| 52301 | 1.81591 | 0.05 |
| 52302 | 2.32098 | 0.05 |
| 52303 | 2.32098 | 0.05 |
| 52304 | 0.801647 | 0.05 |
| 52305 | 1.11086 | 0.05 |
| 52306 | 0.838634 | 0.05 |
| 52307 | 1.16049 | 0.05 |
| 52308 | 0.977891 | 0.05 |
| 52309 | 0.977891 | 0.05 |
| 52310 | 0.984246 | 0.05 |
| 52311 | 0.984246 | 0.05 |
| 52312 | 1.11086 | 0.05 |
| 52313 | 1.16049 | 0.05 |

| | | |
|---|---|---|
| 52314 | 0.801647 | 0.05 |
| 52315 | 0.838634 | 0.05 |
| 52316 | 0.946132 | 0.05 |
| 52317 | 0.946132 | 0.05 |
| 52318 | 0.946131 | 0.05 |
| 52319 | 0.946131 | 0.05 |
| 52320 | 0.946131 | 0.05 |
| 52321 | 0.946131 | 0.05 |
| 52322 | 0.946132 | 0.05 |
| 52323 | 0.946132 | 0.05 |
| 52324 | 0.946131 | 0.05 |
| 52325 | 0.946131 | 0.05 |
| 52326 | 0.946131 | 0.05 |
| 52327 | 0.946131 | 0.05 |
| 52328 | 0.946131 | 0.05 |
| 52329 | 0.946131 | 0.05 |
| 52330 | 0.946131 | 0.05 |
| 52331 | 0.946131 | 0.05 |
| 52332 | 0.946131 | 0.05 |
| 52333 | 0.946131 | 0.05 |
| 52334 | 0.946131 | 0.05 |
| 52335 | 0.946131 | 0.05 |
| 52336 | 0.946131 | 0.05 |
| 52337 | 0.946131 | 0.05 |
| 52338 | 0.946131 | 0.05 |
| 52339 | 0.946131 | 0.05 |
| 52340 | 2.0815 | 2.1 |
| 52341 | 1.20787 | 2.1 |
| 52342 | 1.92277 | 2.1 |
| 52343 | 1.3227 | 2.1 |
| 52344 | 3.29401 | 2.1 |
| 52345 | 2.16633 | 2.1 |
| 52346 | 2.13642 | 2.1 |
| 52347 | 1.40846 | 2.1 |
| 52348 | 1.40846 | 2.1 |
| 52349 | 1.3227 | 2.1 |
| 52350 | 2.08058 | 2.1 |
| 52351 | 0.938106 | 2.1 |
| 52352 | 0.790258 | 2.1 |
| 52353 | 0.742975 | 2.1 |
| 52354 | 0.742975 | 2.1 |
| 52355 | 0.874389 | 2.1 |
| 52356 | 1.17364 | 2.1 |
| 52357 | 0.525948 | 2.1 |
| 52358 | 0.52513 | 2.1 |
| 52359 | 0.553732 | 2.1 |
| 52360 | 0.876996 | 2.1 |
| 52361 | 0.530954 | 2.1 |
| 52362 | 1.095 2.1 | |
| 52363 | 1.00833 | 2.1 |
| 52364 | 1.70608 | 2.1 |
| 52365 | 1.33385 | 2.1 |
| 52366 | 1.33385 | 2.1 |
| 52367 | 1.07722 | 2.1 |
| 52368 | 1.07722 | 2.1 |
| 52369 | 0.953523 | 0.491786 |
| 52370 | 1.7457 | 0.203654 |
| 52371 | 1.03218 | 0.422336 |

| | | |
|---|---|---|
| 52372 | 1.03218 | 0.309088 |
| 52373 | 1.79675 | 0.390498 |
| 52374 | 1.79675 | 0.293648 |
| 52375 | 1.13676 | 0.448867 |
| 52376 | 1.18136 | 0.125273 |
| 52377 | 0.87285 | 0.267222 |
| 52378 | 0.898375 | 0.496517 |
| 52379 | 1.03191 | 0.218825 |
| 52380 | 1.03191 | 0.0919635 |
| 52381 | 1.0223 | 0.36171 |
| 52382 | 1.0223 | 0.36612 |
| 52383 | 1.18136 | 0.227401 |
| 52384 | 0.898374 | 0.148363 |
| 52385 | 0.87285 | 0.215318 |
| 52386 | 1.13676 | 0.206711 |
| 52387 | 2.36273 | 0.209882 |
| 52388 | 1.46965 | 0.180041 |
| 52389 | 1.79675 | 0.0843507 |
| 52390 | 1.79675 | 0.05 |
| 52391 | 2.43417 | 0.255376 |
| 52392 | 2.43417 | 0.125452 |
| 52393 | 1.4714 | 2.1 |
| 52394 | 1.65434 | 2.1 |
| 52395 | 1.62322 | 1.02824 |
| 52396 | 1.86068 | 2.08691 |
| 52397 | 3.27756 | 1.6292 |
| 52398 | 1.89226 | 2.1 |
| 52399 | 1.89226 | 2.1 |
| 52400 | 1.89226 | 1.07173 |
| 52401 | 1.89226 | 1.27634 |
| 52402 | 3.78453 | 1.31641 |
| 52403 | 1.89226 | 1.31755 |
| 52404 | 1.89226 | 2.1 |
| 52405 | 1.89226 | 1.0323 |
| 52406 | 1.89226 | 2.1 |
| 52407 | 3.78453 | 1.83989 |
| 52408 | 1.65434 | 1.36112 |
| 52409 | 1.60647 | 2.1 |
| 52410 | 1.86068 | 1.19856 |
| 52411 | 1.82649 | 2.1 |
| 52412 | 3.48083 | 2.1 |
| 52413 | 1.62322 | 2.1 |
| 52414 | 1.86068 | 2.1 |
| 52415 | 1.84368 | 1.0072 |
| 52416 | 2.18862 | 1.40496 |
| 52417 | 3.81184 | 1.59065 |
| 52418 | 1.89226 | 2.1 |
| 52419 | 1.89226 | 2.01795 |
| 52420 | 1.89226 | 0.9565 |
| 52421 | 1.89226 | 1.49277 |
| 52422 | 3.78453 | 1.52036 |
| 52423 | 1.89226 | 1.37367 |
| 52424 | 1.89226 | 2.1 |
| 52425 | 1.89226 | 1.12997 |
| 52426 | 1.89226 | 2.1 |
| 52427 | 3.78453 | 2.1 |
| 52428 | 1.86068 | 1.21075 |
| 52429 | 1.82649 | 2.1 |

| | | |
|---|---|---|
| 52430 | 2.18862 | 1.10744 |
| 52431 | 2.1128 | 2.1 |
| 52432 | 4.01511 | 1.79915 |
| 52433 | 1.74351 | 2.1 |
| 52434 | 1.13854 | 2.1 |
| 52435 | 1.40233 | 2.1 |
| 52436 | 1.922 | 2.1 |
| 52437 | 3.14584 | 2.1 |
| 52438 | 1.922 | 2.1 |
| 52439 | 2.00775 | 2.1 |
| 52440 | 1.7435 | 2.1 |
| 52441 | 2.09224 | 2.1 |
| 52442 | 2.00775 | 2.1 |
| 52443 | 2.09295 | 2.1 |
| 52444 | 1.69858 | 2.1 |
| 52445 | 1.922 | 2.1 |
| 52446 | 1.40233 | 2.1 |
| 52447 | 1.40233 | 2.1 |
| 52448 | 2.18238 | 2.1 |
| 52449 | 2.18238 | 2.1 |
| 52450 | 1.00387 | 2.1 |
| 52451 | 1.10886 | 2.1 |
| 52452 | 0.961001 | 2.1 |
| 52453 | 1.09119 | 2.1 |
| 52454 | 1.02868 | 2.1 |
| 52455 | 1.02868 | 2.1 |
| 52456 | 1.04118 | 2.1 |
| 52457 | 1.04118 | 2.1 |
| 52458 | 1.10886 | 2.1 |
| 52459 | 1.09119 | 2.1 |
| 52460 | 0.960999 | 2.1 |
| 52461 | 1.00388 | 2.1 |
| 52462 | 0.885958 | 0.635231 |
| 52463 | 0.885958 | 0.580648 |
| 52464 | 0.875826 | 0.293295 |
| 52465 | 0.875826 | 0.758499 |
| 52466 | 0.913965 | 0.764345 |
| 52467 | 0.598609 | 0.603262 |
| 52468 | 1.16317 | 0.60285 |
| 52469 | 0.868115 | 0.570659 |
| 52470 | 0.868115 | 0.643177 |
| 52471 | 0.598609 | 0.611254 |
| 52472 | 1.16317 | 0.638541 |
| 52473 | 0.913965 | 0.657061 |
| 52474 | 0.946131 | 0.651354 |
| 52475 | 0.946131 | 0.621143 |
| 52476 | 0.946131 | 0.694317 |
| 52477 | 0.946131 | 0.624685 |
| 52478 | 0.946131 | 0.676588 |
| 52479 | 0.946131 | 0.662773 |
| 52480 | 0.946131 | 0.625186 |
| 52481 | 0.946131 | 0.614638 |
| 52482 | 0.946131 | 0.655281 |
| 52483 | 0.946131 | 0.662921 |
| 52484 | 0.946131 | 0.643501 |
| 52485 | 0.946131 | 0.645333 |
| 52486 | 0.946133 | 0.05 |
| 52487 | 0.946133 | 0.05 |

| | | |
|---|---|---|
| 52488 | 1.06246 | 0.05 |
| 52489 | 1.06246 | 0.05 |
| 52490 | 0.973851 | 0.05 |
| 52491 | 0.946133 | 0.05 |
| 52492 | 1.06246 | 0.05 |
| 52493 | 0.946132 | 0.05 |
| 52494 | 0.97385 | 0.05 |
| 52495 | 1.06246 | 0.05 |
| 52496 | 0.946133 | 0.05 |
| 52497 | 0.946133 | 0.05 |
| 52498 | 1.89226 | 0.05 |
| 52499 | 1.89226 | 0.05 |
| 52500 | 1.89226 | 0.05 |
| 52501 | 1.89226 | 0.05 |
| 52502 | 1.89226 | 0.05 |
| 52503 | 1.89226 | 0.05 |
| 52504 | 0.946131 | 0.05 |
| 52505 | 0.946131 | 0.05 |
| 52506 | 0.946127 | 0.05 |
| 52507 | 0.946127 | 0.05 |
| 52508 | 0.946131 | 0.05 |
| 52509 | 0.946131 | 0.05 |
| 52510 | 0.946127 | 0.05 |
| 52511 | 0.946127 | 0.05 |
| 52512 | 0.946128 | 0.05 |
| 52513 | 0.946128 | 0.05 |
| 52514 | 0.946129 | 0.05 |
| 52515 | 0.946129 | 0.05 |
| 52516 | 0.946132 | 0.05 |
| 52517 | 0.946132 | 0.05 |
| 52518 | 1.32446 | 0.05 |
| 52519 | 0.794912 | 0.05 |
| 52520 | 1.32429 | 0.05 |
| 52521 | 0.946131 | 0.05 |
| 52522 | 1.32446 | 0.05 |
| 52523 | 0.94613 | 0.05 |
| 52524 | 1.32429 | 0.05 |
| 52525 | 0.794915 | 0.05 |
| 52526 | 0.94613 | 0.05 |
| 52527 | 0.94613 | 0.05 |
| 52528 | 1.89226 | 0.05 |
| 52529 | 2.36365 | 0.05 |
| 52530 | 2.64857 | 0.05 |
| 52531 | 1.89226 | 0.05 |
| 52532 | 2.64857 | 0.05 |
| 52533 | 1.89226 | 0.05 |
| 52534 | 1.18463 | 2.1 |
| 52535 | 1.27174 | 2.1 |
| 52536 | 0.946129 | 2.1 |
| 52537 | 0.946129 | 2.1 |
| 52538 | 0.946131 | 2.1 |
| 52539 | 0.946131 | 2.1 |
| 52540 | 1.27174 | 2.1 |
| 52541 | 1.27174 | 2.1 |
| 52542 | 1.18463 | 2.1 |
| 52543 | 0.94613 | 2.1 |
| 52544 | 0.946131 | 2.1 |
| 52545 | 1.27174 | 2.1 |

| | | |
|---|---|---|
| 52546 | 2.57997 | 2.1 |
| 52547 | 1.89226 | 2.1 |
| 52548 | 1.89226 | 2.1 |
| 52549 | 1.89226 | 2.1 |
| 52550 | 2.41915 | 2.1 |
| 52551 | 2.41915 | 2.1 |
| 52552 | 1.89226 | 2.1 |
| 52553 | 1.89226 | 2.1 |
| 52554 | 1.89226 | 2.1 |
| 52555 | 1.89226 | 2.1 |
| 52556 | 3.78452 | 2.1 |
| 52557 | 1.08807 | 2.1 |
| 52558 | 1.18463 | 2.1 |
| 52559 | 0.946129 | 2.1 |
| 52560 | 0.946129 | 2.1 |
| 52561 | 0.946131 | 2.1 |
| 52562 | 0.946131 | 2.1 |
| 52563 | 1.18463 | 2.1 |
| 52564 | 1.18463 | 2.1 |
| 52565 | 0.946131 | 2.1 |
| 52566 | 1.08807 | 2.1 |
| 52567 | 1.18463 | 2.1 |
| 52568 | 0.94613 | 2.1 |
| 52569 | 1.07017 | 2.1 |
| 52570 | 1.07017 | 2.1 |
| 52571 | 0.946131 | 2.1 |
| 52572 | 0.946131 | 2.1 |
| 52573 | 0.946132 | 2.1 |
| 52574 | 0.946132 | 2.1 |
| 52575 | 1.20957 | 2.1 |
| 52576 | 1.07017 | 2.1 |
| 52577 | 1.07017 | 2.1 |
| 52578 | 0.946132 | 2.1 |
| 52579 | 0.946131 | 2.1 |
| 52580 | 1.20957 | 2.1 |
| 52581 | 1.89226 | 2.1 |
| 52582 | 1.89226 | 2.1 |
| 52583 | 1.89226 | 2.1 |
| 52584 | 1.89226 | 2.1 |
| 52585 | 1.89226 | 2.1 |
| 52586 | 1.89226 | 2.1 |
| 52587 | 1.16181 | 0.05 |
| 52588 | 1.0227 | 0.05 |
| 52589 | 1.0227 | 0.05 |
| 52590 | 0.970959 | 0.05 |
| 52591 | 1.24295 | 0.05 |
| 52592 | 1.19121 | 0.05 |
| 52593 | 1.12076 | 2.1 |
| 52594 | 2.53614 | 2.1 |
| 52595 | 1.28413 | 2.1 |
| 52596 | 1.28413 | 2.1 |
| 52597 | 2.36423 | 2.1 |
| 52598 | 2.36423 | 2.1 |
| 52599 | 1.89226 | 2.1 |
| 52600 | 1.89226 | 2.1 |
| 52601 | 1.89226 | 2.1 |
| 52602 | 1.89226 | 2.1 |
| 52603 | 3.78452 | 2.1 |

| | | |
|---|---|---|
| 52604 | 1.22557 | 2.1 |
| 52605 | 2.771 2.1 | |
| 52606 | 2.73012 | 2.1 |
| 52607 | 2.73012 | 2.1 |
| 52608 | 1.33356 | 2.1 |
| 52609 | 1.33356 | 2.1 |
| 52610 | 0.998682 | 2.1 |
| 52611 | 2.36423 | 2.1 |
| 52612 | 1.12076 | 2.1 |
| 52613 | 1.12076 | 2.1 |
| 52614 | 2.13757 | 2.1 |
| 52615 | 2.13757 | 2.1 |
| 52616 | 1.89226 | 2.1 |
| 52617 | 1.89226 | 2.1 |
| 52618 | 1.89226 | 2.1 |
| 52619 | 1.89226 | 2.1 |
| 52620 | 1.89226 | 2.1 |
| 52621 | 1.89226 | 2.1 |
| 52622 | 1.13987 | 2.1 |
| 52623 | 2.73011 | 2.1 |
| 52624 | 2.70875 | 2.1 |
| 52625 | 2.70875 | 2.1 |
| 52626 | 1.22557 | 2.1 |
| 52627 | 1.22557 | 2.1 |
| 52628 | 1.89227 | 2.1 |
| 52629 | 2.79243 | 2.1 |
| 52630 | 2.6625 | 2.1 |
| 52631 | 2.6625 | 2.1 |
| 52632 | 1.89227 | 2.1 |
| 52633 | 1.89227 | 2.1 |
| 52634 | 1.89226 | 2.1 |
| 52635 | 2.55971 | 2.1 |
| 52636 | 1.89226 | 2.1 |
| 52637 | 1.89226 | 2.1 |
| 52638 | 2.68704 | 2.1 |
| 52639 | 2.68704 | 2.1 |
| 52640 | 2.22043 | 2.05492 |
| 52641 | 2.38975 | 1.83528 |
| 52642 | 2.28696 | 1.28287 |
| 52643 | 2.48682 | 1.00812 |
| 52644 | 4.67671 | 1.22978 |
| 52645 | 2.38976 | 1.14482 |
| 52646 | 2.31988 | 1.8144 |
| 52647 | 2.48683 | 1.08421 |
| 52648 | 2.4594 | 1.51958 |
| 52649 | 4.84915 | 1.43124 |
| 52650 | 2.28696 | 1.95185 |
| 52651 | 2.48682 | 1.58878 |
| 52652 | 2.33379 | 2.1 |
| 52653 | 2.56149 | 0.897026 |
| 52654 | 4.84845 | 1.47298 |
| 52655 | 2.48683 | 1.77407 |
| 52656 | 2.4594 | 1.63883 |
| 52657 | 2.5615 | 0.948661 |
| 52658 | 2.57957 | 1.22809 |
| 52659 | 5.0209 | 1.09481 |
| 52660 | 1.06659 | 2.1 |
| 52661 | 1.04291 | 0.0516718 |

| | | |
|---|---|---|
| 52662 | 1.36595 | 0.164392 |
| 52663 | 1.36595 | 2.1 |
| 52664 | 1.02761 | 2.1 |
| 52665 | 1.32697 | 0.187479 |
| 52666 | 0.946132 | 2.00749 |
| 52667 | 0.946132 | 1.82923 |
| 52668 | 1.36956 | 2.1 |
| 52669 | 1.1229 | 2.1 |
| 52670 | 1.1229 | 1.52182 |
| 52671 | 1.1229 | 2.1 |
| 52672 | 0.946132 | 0.930595 |
| 52673 | 0.946132 | 2.1 |
| 52674 | 0.946132 | 2.1 |
| 52675 | 1.36956 | 2.1 |
| 52676 | 1.1229 | 2.1 |
| 52677 | 0.946133 | 2.1 |
| 52678 | 0.946133 | 1.10755 |
| 52679 | 0.946133 | 1.01837 |
| 52680 | 0.946128 | 2.1 |
| 52681 | 0.946128 | 2.1 |
| 52682 | 0.946131 | 2.1 |
| 52683 | 0.946131 | 1.5894 |
| 52684 | 0.946131 | 0.455627 |
| 52685 | 0.946131 | 2.1 |
| 52686 | 0.94613 | 2.1 |
| 52687 | 0.94613 | 2.05111 |
| 52688 | 0.946131 | 1.95665 |
| 52689 | 0.946131 | 1.90543 |
| 52690 | 0.966427 | 1.31428 |
| 52691 | 0.802265 | 2.1 |
| 52692 | 0.803714 | 2.1 |
| 52693 | 0.808476 | 1.79217 |
| 52694 | 1.94371 | 2.1 |
| 52695 | 1.36851 | 2.1 |
| 52696 | 0.853864 | 2.1 |
| 52697 | 1.26635 | 1.80652 |
| 52698 | 1.09085 | 1.45304 |
| 52699 | 0.857653 | 2.1 |
| 52700 | 0.630243 | 2.00608 |
| 52701 | 1.20132 | 0.05 |
| 52702 | 1.24295 | 0.05 |
| 52703 | 1.24295 | 0.05 |
| 52704 | 1.16182 | 0.05 |
| 52705 | 1.28465 | 0.05 |
| 52706 | 1.20351 | 0.05 |
| 52707 | 0.600658 | 0.05 |
| 52708 | 0.642323 | 0.05 |
| 52709 | 0.54424 | 0.05 |
| 52710 | 0.576779 | 0.05 |
| 52711 | 0.590985 | 0.05 |
| 52712 | 0.590985 | 0.05 |
| 52713 | 0.586452 | 0.05 |
| 52714 | 0.586452 | 0.05 |
| 52715 | 0.642323 | 0.05 |
| 52716 | 0.576779 | 0.05 |
| 52717 | 0.600658 | 0.05 |
| 52718 | 0.54424 | 0.05 |
| 52719 | 1.28463 | 0.05 |

| | | |
|---|---|---|
| 52720 | 1.24294 | 0.05 |
| 52721 | 1.12328 | 0.05 |
| 52722 | 1.15505 | 0.05 |
| 52723 | 2.39798 | 0.05 |
| 52724 | 1.98431 | 0.05 |
| 52725 | 2.18735 | 0.05 |
| 52726 | 1.98431 | 0.05 |
| 52727 | 1.70303 | 0.05 |
| 52728 | 1.70302 | 0.05 |
| 52729 | 1.37788 | 0.05 |
| 52730 | 1.89226 | 0.05 |
| 52731 | 2.37417 | 0.05 |
| 52732 | 1.89226 | 0.05 |
| 52733 | 2.18735 | 0.05 |
| 52734 | 1.89226 | 0.05 |
| 52735 | 2.18735 | 0.05 |
| 52736 | 1.89226 | 0.05 |
| 52737 | 1.89226 | 0.0873158 |
| 52738 | 1.89226 | 0.05 |
| 52739 | 1.89226 | 0.058232 |
| 52740 | 3.78452 | 0.122758 |
| 52741 | 1.8613 | 0.05 |
| 52742 | 2.03576 | 0.05 |
| 52743 | 1.61652 | 0.05 |
| 52744 | 1.83082 | 1.6302 |
| 52745 | 3.65228 | 0.05 |
| 52746 | 2.03576 | 0.05 |
| 52747 | 2.05257 | 2.1 |
| 52748 | 1.83082 | 0.736367 |
| 52749 | 1.88487 | 2.1 |
| 52750 | 3.92064 | 1.8916 |
| 52751 | 1.89226 | 0.05 |
| 52752 | 1.89226 | 0.156958 |
| 52753 | 1.89226 | 0.05 |
| 52754 | 1.89226 | 2.1 |
| 52755 | 3.78453 | 0.05 |
| 52756 | 0.946131 | 0.05 |
| 52757 | 0.946131 | 0.05 |
| 52758 | 0.94613 | 0.05 |
| 52759 | 0.94613 | 0.0771003 |
| 52760 | 0.946129 | 0.05 |
| 52761 | 0.946129 | 0.05 |
| 52762 | 0.946132 | 0.05 |
| 52763 | 0.946132 | 0.05 |
| 52764 | 0.94613 | 0.05 |
| 52765 | 0.94613 | 0.05 |
| 52766 | 0.946131 | 0.05 |
| 52767 | 0.946131 | 0.05 |
| 52768 | 0.804708 | 2.1 |
| 52769 | 0.804708 | 0.05 |
| 52770 | 0.815274 | 0.05 |
| 52771 | 0.815274 | 0.05 |
| 52772 | 0.83277 | 0.05 |
| 52773 | 0.915407 | 1.71805 |
| 52774 | 0.80826 | 0.05 |
| 52775 | 0.704574 | 0.05 |
| 52776 | 0.832771 | 0.05 |
| 52777 | 0.704573 | 0.05 |

| | | |
|---|---|---|
| 52778 | 0.808259 | 0.055803 |
| 52779 | 0.915409 | 0.05 |
| 52780 | 1.83082 | 0.545843 |
| 52781 | 1.88487 | 2.1 |
| 52782 | 1.66555 | 0.05 |
| 52783 | 1.75835 | 2.1 |
| 52784 | 3.55042 | 2.04928 |
| 52785 | 0.810947 | 0.05 |
| 52786 | 0.810947 | 0.173591 |
| 52787 | 0.810947 | 0.05 |
| 52788 | 0.810947 | 0.05 |
| 52789 | 0.810934 | 0.0735308 |
| 52790 | 0.810934 | 0.0743865 |
| 52791 | 1.89227 | 2.1 |
| 52792 | 1.3517 | 0.117317 |
| 52793 | 1.08132 | 0.0624332 |
| 52794 | 1.08132 | 0.11248 |
| 52795 | 1.08133 | 0.05 |
| 52796 | 1.89226 | 2.06954 |
| 52797 | 1.70303 | 2.1 |
| 52798 | 1.89226 | 2.1 |
| 52799 | 2.24712 | 2.1 |
| 52800 | 2.07363 | 2.1 |
| 52801 | 1.74617 | 2.1 |
| 52802 | 1.89226 | 2.1 |
| 52803 | 1.89226 | 1.46869 |
| 52804 | 1.89226 | 1.12781 |
| 52805 | 1.89226 | 2.1 |
| 52806 | 3.78452 | 1.7468 |
| 52807 | 1.50108 | 2.1 |
| 52808 | 1.43894 | 2.1 |
| 52809 | 1.8322 | 0.984577 |
| 52810 | 1.40967 | 2.1 |
| 52811 | 1.50108 | 2.1 |
| 52812 | 1.07854 | 2.1 |
| 52813 | 1.89226 | 1.04191 |
| 52814 | 1.89226 | 2.1 |
| 52815 | 1.89226 | 2.1 |
| 52816 | 1.89226 | 0.538835 |
| 52817 | 3.78452 | 2.1 |
| 52818 | 1.89226 | 2.1 |
| 52819 | 1.89226 | 1.31587 |
| 52820 | 1.89226 | 1.29792 |
| 52821 | 1.89226 | 2.1 |
| 52822 | 3.78453 | 1.80707 |
| 52823 | 1.54457 | 2.1 |
| 52824 | 1.35097 | 2.08899 |
| 52825 | 1.70754 | 0.821136 |
| 52826 | 1.29629 | 2.1 |
| 52827 | 1.54457 | 2.1 |
| 52828 | 1.0305 | 2.1 |
| 52829 | 0.731763 | 2.1 |
| 52830 | 1.00043 | 2.1 |
| 52831 | 1.13883 | 1.26121 |
| 52832 | 0.939026 | 2.09863 |
| 52833 | 1.04003 | 1.26151 |
| 52834 | 0.853868 | 2.1 |
| 52835 | 0.927188 | 2.1 |

| | | |
|---|---|---|
| 52836 | 0.927188 | 0.224951 |
| 52837 | 0.861565 | 1.69044 |
| 52838 | 1.13882 | 2.1 |
| 52839 | 0.731769 | 2.1 |
| 52840 | 0.89319 | 2.1 |
| 52841 | 1.46568 | 2.1 |
| 52842 | 1.20761 | 2.1 |
| 52843 | 1.51916 | 2.1 |
| 52844 | 1.36261 | 2.1 |
| 52845 | 2.72677 | 2.1 |
| 52846 | 1.51916 | 2.1 |
| 52847 | 1.60532 | 2.1 |
| 52848 | 1.36261 | 2.1 |
| 52849 | 1.36261 | 2.1 |
| 52850 | 1.64892 | 2.1 |
| 52851 | 1.64892 | 2.1 |
| 52852 | 1.3626 | 2.1 |
| 52853 | 0.98488 | 2.1 |
| 52854 | 1.60532 | 2.1 |
| 52855 | 1.32414 | 2.1 |
| 52856 | 2.68675 | 2.1 |
| 52857 | 1.78711 | 2.1 |
| 52858 | 1.14825 | 2.1 |
| 52859 | 2.16535 | 2.1 |
| 52860 | 1.6067 | 2.1 |
| 52861 | 3.3136 | 2.1 |
| 52862 | 1.89226 | 2.1 |
| 52863 | 1.89226 | 2.1 |
| 52864 | 1.89226 | 2.1 |
| 52865 | 1.89226 | 2.1 |
| 52866 | 3.78452 | 2.1 |
| 52867 | 2.34552 | 2.1 |
| 52868 | 1.53521 | 2.1 |
| 52869 | 1.53521 | 2.1 |
| 52870 | 1.60636 | 2.1 |
| 52871 | 1.92797 | 2.1 |
| 52872 | 1.99589 | 2.1 |
| 52873 | 1.34771 | 2.1 |
| 52874 | 1.34771 | 2.1 |
| 52875 | 0.946128 | 2.1 |
| 52876 | 0.946128 | 2.1 |
| 52877 | 1.3477 | 2.1 |
| 52878 | 1.31776 | 2.1 |
| 52879 | 0.946131 | 2.1 |
| 52880 | 0.946131 | 2.1 |
| 52881 | 0.94613 | 2.1 |
| 52882 | 1.31776 | 2.1 |
| 52883 | 0.946129 | 2.1 |
| 52884 | 1.3477 | 2.1 |
| 52885 | 1.89226 | 2.1 |
| 52886 | 1.89226 | 2.1 |
| 52887 | 1.89226 | 2.1 |
| 52888 | 1.89226 | 2.1 |
| 52889 | 3.78452 | 2.1 |
| 52890 | 0.92843 | 2.1 |
| 52891 | 0.672114 | 2.1 |
| 52892 | 0.89514 | 2.1 |
| 52893 | 0.89514 | 2.1 |

| | | |
|---|---|---|
| 52894 | 0.720104 | 2.1 |
| 52895 | 0.755753 | 2.1 |
| 52896 | 0.789158 | 2.1 |
| 52897 | 0.967302 | 2.1 |
| 52898 | 1.06565 | 2.1 |
| 52899 | 0.7397 | 2.1 |
| 52900 | 0.758893 | 2.1 |
| 52901 | 0.94672 | 2.1 |
| 52902 | 1.89226 | 2.1 |
| 52903 | 2.588 2.1 | |
| 52904 | 2.588 2.1 | |
| 52905 | 2.63552 | 2.1 |
| 52906 | 1.89226 | 2.1 |
| 52907 | 1.93978 | 2.1 |
| 52908 | 1.49347 | 0.05 |
| 52909 | 2.67757 | 0.05 |
| 52910 | 1.80061 | 0.05 |
| 52911 | 2.27056 | 0.05 |
| 52912 | 4.07117 | 0.05 |
| 52913 | 1.1016 | 0.05 |
| 52914 | 2.27057 | 0.05 |
| 52915 | 1.49348 | 0.05 |
| 52916 | 1.81893 | 0.05 |
| 52917 | 1.49348 | 0.05 |
| 52918 | 1.81893 | 0.05 |
| 52919 | 1.81893 | 0.05 |
| 52920 | 2.82339 | 0.05 |
| 52921 | 2.27057 | 0.05 |
| 52922 | 2.25405 | 0.05 |
| 52923 | 4.52462 | 0.05 |
| 52924 | 1.89226 | 0.05 |
| 52925 | 1.89226 | 0.05 |
| 52926 | 1.89226 | 0.05 |
| 52927 | 1.89226 | 0.05 |
| 52928 | 1.89226 | 0.05 |
| 52929 | 1.89226 | 0.05 |
| 52930 | 1.89226 | 0.05 |
| 52931 | 1.89226 | 0.05 |
| 52932 | 1.89226 | 0.05 |
| 52933 | 1.89226 | 0.05 |
| 52934 | 3.78453 | 0.05 |
| 52935 | 1.89226 | 0.05 |
| 52936 | 1.89226 | 0.05 |
| 52937 | 1.89226 | 0.05 |
| 52938 | 1.89226 | 0.05 |
| 52939 | 3.78452 | 0.05 |
| 52940 | 1.89226 | 0.05 |
| 52941 | 1.89226 | 0.05 |
| 52942 | 1.89226 | 0.05 |
| 52943 | 1.89226 | 0.05 |
| 52944 | 3.78451 | 0.05 |
| 52945 | 1.45633 | 2.1 |
| 52946 | 1.34678 | 2.1 |
| 52947 | 1.34678 | 2.1 |
| 52948 | 1.53735 | 2.1 |
| 52949 | 1.234 2.1 | |
| 52950 | 1.42457 | 2.1 |
| 52951 | 1.89226 | 2.1 |

| | | |
|---|---|---|
| 52952 | 1.89226 | 2.1 |
| 52953 | 1.89226 | 2.1 |
| 52954 | 1.89226 | 2.1 |
| 52955 | 1.89226 | 2.1 |
| 52956 | 1.89226 | 2.1 |
| 52957 | 1.09483 | 2.1 |
| 52958 | 1.34678 | 2.1 |
| 52959 | 1.234 | 2.1 |
| 52960 | 1.234 | 2.1 |
| 52961 | 1.19 | 2.1 |
| 52962 | 1.19 | 2.1 |
| 52963 | 0.946131 | 2.1 |
| 52964 | 0.946131 | 2.1 |
| 52965 | 0.946131 | 2.1 |
| 52966 | 0.946131 | 2.1 |
| 52967 | 0.946131 | 2.1 |
| 52968 | 0.946131 | 2.1 |
| 52969 | 0.946132 | 2.1 |
| 52970 | 0.946132 | 2.1 |
| 52971 | 0.946132 | 2.1 |
| 52972 | 0.946132 | 2.1 |
| 52973 | 0.946131 | 2.1 |
| 52974 | 0.946131 | 2.1 |
| 52975 | 2.82263 | 2.1 |
| 52976 | 1.89226 | 2.1 |
| 52977 | 2.71441 | 2.1 |
| 52978 | 2.71441 | 2.1 |
| 52979 | 1.89226 | 2.1 |
| 52980 | 1.89226 | 2.1 |
| 52981 | 1.1412 | 2.1 |
| 52982 | 1.1412 | 2.1 |
| 52983 | 1.14626 | 2.1 |
| 52984 | 1.14626 | 2.1 |
| 52985 | 1.28511 | 2.1 |
| 52986 | 1.34086 | 2.1 |
| 52987 | 0.992242 | 2.1 |
| 52988 | 0.946593 | 2.1 |
| 52989 | 0.992242 | 2.1 |
| 52990 | 1.34086 | 2.1 |
| 52991 | 0.946594 | 2.1 |
| 52992 | 1.28511 | 2.1 |
| 52993 | 1.18954 | 2.1 |
| 52994 | 1.18954 | 2.1 |
| 52995 | 1.21477 | 2.1 |
| 52996 | 1.21477 | 2.1 |
| 52997 | 1.41207 | 2.1 |
| 52998 | 1.34086 | 2.1 |
| 52999 | 1.01306 | 2.1 |
| 53000 | 0.992242 | 2.1 |
| 53001 | 1.01306 | 2.1 |
| 53002 | 1.41207 | 2.1 |
| 53003 | 0.992242 | 2.1 |
| 53004 | 1.34086 | 2.1 |
| 53005 | 1.09044 | 2.1 |
| 53006 | 1.44472 | 0.312458 |
| 53007 | 1.2955 | 2.1 |
| 53008 | 1.2955 | 0.806645 |
| 53009 | 1.22025 | 2.1 |

| | | |
|---|---|---|
| 53010 | 1.22025 | 2.1 |
| 53011 | 0.810944 | 0.231146 |
| 53012 | 0.810944 | 2.1 |
| 53013 | 0.810946 | 0.828247 |
| 53014 | 0.810946 | 0.05 |
| 53015 | 0.810959 | 0.772131 |
| 53016 | 0.810959 | 0.237062 |
| 53017 | 1.89226 | 0.05 |
| 53018 | 1.35167 | 0.635263 |
| 53019 | 1.08132 | 2.1 |
| 53020 | 1.08132 | 0.05 |
| 53021 | 1.0813 | 0.34462 |
| 53022 | 1.41894 | 0.693311 |
| 53023 | 1.28953 | 0.528601 |
| 53024 | 0.946132 | 0.229028 |
| 53025 | 0.946132 | 1.58522 |
| 53026 | 0.946133 | 0.988621 |
| 53027 | 0.946133 | 0.590998 |
| 53028 | 1.41894 | 0.761636 |
| 53029 | 1.41894 | 1.39107 |
| 53030 | 1.28953 | 0.626367 |
| 53031 | 0.946132 | 0.867561 |
| 53032 | 0.946132 | 0.792394 |
| 53033 | 1.41894 | 0.589926 |
| 53034 | 1.89226 | 2.1 |
| 53035 | 2.15444 | 2.1 |
| 53036 | 1.93239 | 2.1 |
| 53037 | 1.89226 | 2.1 |
| 53038 | 2.19457 | 2.1 |
| 53039 | 2.15444 | 2.1 |
| 53040 | 1.89226 | 2.1 |
| 53041 | 1.89226 | 2.1 |
| 53042 | 1.89226 | 2.1 |
| 53043 | 1.89226 | 2.1 |
| 53044 | 1.89226 | 2.1 |
| 53045 | 1.89226 | 2.1 |
| 53046 | 0.938507 | 2.1 |
| 53047 | 0.938507 | 2.1 |
| 53048 | 1.07871 | 2.1 |
| 53049 | 1.15801 | 2.1 |
| 53050 | 1.03236 | 2.1 |
| 53051 | 1.14766 | 2.1 |
| 53052 | 0.946133 | 2.1 |
| 53053 | 0.946133 | 2.1 |
| 53054 | 0.946131 | 2.1 |
| 53055 | 1.07009 | 2.1 |
| 53056 | 0.946131 | 2.1 |
| 53057 | 1.03237 | 2.1 |
| 53058 | 1.89226 | 2.1 |
| 53059 | 2.19457 | 2.1 |
| 53060 | 1.94492 | 2.1 |
| 53061 | 1.89226 | 2.1 |
| 53062 | 2.24723 | 2.1 |
| 53063 | 2.19457 | 2.1 |
| 53064 | 0.946132 | 2.1 |
| 53065 | 0.946132 | 2.1 |
| 53066 | 0.946131 | 2.1 |
| 53067 | 0.946131 | 2.1 |

| | | |
|---|---|---|
| 53068 | 0.946132 | 2.1 |
| 53069 | 0.946132 | 2.1 |
| 53070 | 0.946132 | 2.1 |
| 53071 | 0.946132 | 2.1 |
| 53072 | 0.946132 | 2.1 |
| 53073 | 0.946132 | 2.1 |
| 53074 | 0.946132 | 2.1 |
| 53075 | 0.946132 | 2.1 |
| 53076 | 2.21345 | 2.1 |
| 53077 | 1.89226 | 2.1 |
| 53078 | 1.89226 | 2.1 |
| 53079 | 2.14017 | 2.1 |
| 53080 | 2.14017 | 2.1 |
| 53081 | 1.89226 | 2.1 |
| 53082 | 1.89226 | 0.0816055 |
| 53083 | 1.89226 | 0.873151 |
| 53084 | 1.89226 | 0.226965 |
| 53085 | 1.85439 | 0.804308 |
| 53086 | 2.23961 | 0.05 |
| 53087 | 2.19479 | 0.411468 |
| 53088 | 1.2916 | 0.160771 |
| 53089 | 1.2916 | 0.0647649 |
| 53090 | 0.946128 | 0.5045 |
| 53091 | 0.946128 | 0.115065 |
| 53092 | 1.2916 | 0.05 |
| 53093 | 0.946131 | 0.1823 |
| 53094 | 0.946126 | 0.787179 |
| 53095 | 1.1198 | 0.05 |
| 53096 | 1.2916 | 0.0528897 |
| 53097 | 1.1198 | 0.434465 |
| 53098 | 0.946128 | 0.687046 |
| 53099 | 0.946128 | 0.532243 |
| 53100 | 1.89226 | 0.702821 |
| 53101 | 2.71262 | 0.444233 |
| 53102 | 1.89226 | 0.633755 |
| 53103 | 2.49959 | 0.643984 |
| 53104 | 4.39185 | 0.765675 |
| 53105 | 2.71262 | 0.582304 |
| 53106 | 1.34592 | 0.404522 |
| 53107 | 2.49959 | 0.629931 |
| 53108 | 1.14721 | 0.0791525 |
| 53109 | 3.84551 | 0.167698 |
| 53110 | 0.946131 | 0.458709 |
| 53111 | 0.946131 | 0.503181 |
| 53112 | 1.11247 | 0.548017 |
| 53113 | 1.11247 | 0.406545 |
| 53114 | 1.24979 | 0.345945 |
| 53115 | 1.11247 | 0.308854 |
| 53116 | 0.946129 | 0.618283 |
| 53117 | 0.946129 | 0.591384 |
| 53118 | 0.946131 | 0.214298 |
| 53119 | 1.11247 | 0.05 |
| 53120 | 0.946131 | 0.148936 |
| 53121 | 1.24979 | 0.190044 |
| 53122 | 0.946131 | 2.1 |
| 53123 | 0.946131 | 2.1 |
| 53124 | 0.94613 | 2.1 |
| 53125 | 0.94613 | 2.1 |

| | | |
|---|---|---|
| 53126 | 0.946132 | 2.1 |
| 53127 | 0.946132 | 2.1 |
| 53128 | 0.946129 | 2.1 |
| 53129 | 0.946129 | 2.1 |
| 53130 | 0.946131 | 2.1 |
| 53131 | 0.946131 | 2.1 |
| 53132 | 0.946131 | 2.1 |
| 53133 | 0.946131 | 2.1 |
| 53134 | 2.77816 | 2.1 |
| 53135 | 1.89226 | 2.1 |
| 53136 | 1.89226 | 2.1 |
| 53137 | 1.89226 | 2.1 |
| 53138 | 3.78453 | 2.1 |
| 53139 | 1.89226 | 2.1 |
| 53140 | 1.89226 | 2.1 |
| 53141 | 1.89226 | 2.1 |
| 53142 | 1.89226 | 2.1 |
| 53143 | 1.89226 | 2.1 |
| 53144 | 1.89226 | 2.1 |
| 53145 | 1.89226 | 2.1 |
| 53146 | 1.89226 | 2.1 |
| 53147 | 1.89226 | 2.1 |
| 53148 | 1.89226 | 2.1 |
| 53149 | 3.78452 | 2.1 |
| 53150 | 1.55698 | 2.1 |
| 53151 | 1.89226 | 2.1 |
| 53152 | 1.89226 | 2.1 |
| 53153 | 1.89226 | 2.1 |
| 53154 | 1.8575 | 2.1 |
| 53155 | 1.8575 | 2.1 |
| 53156 | 1.89226 | 2.1 |
| 53157 | 1.89226 | 2.1 |
| 53158 | 1.89226 | 2.1 |
| 53159 | 1.89226 | 2.1 |
| 53160 | 1.89226 | 2.1 |
| 53161 | 1.89226 | 2.1 |
| 53162 | 1.89227 | 2.1 |
| 53163 | 1.89227 | 2.1 |
| 53164 | 1.89227 | 2.1 |
| 53165 | 1.89227 | 2.1 |
| 53166 | 1.89227 | 2.1 |
| 53167 | 1.89227 | 2.1 |
| 53168 | 1.35509 | 2.1 |
| 53169 | 2.06238 | 2.1 |
| 53170 | 2.12244 | 2.1 |
| 53171 | 2.12244 | 2.1 |
| 53172 | 1.34817 | 2.1 |
| 53173 | 1.34817 | 2.1 |
| 53174 | 0.946126 | 2.1 |
| 53175 | 0.946126 | 2.1 |
| 53176 | 0.946131 | 2.1 |
| 53177 | 0.946131 | 2.1 |
| 53178 | 0.946127 | 2.1 |
| 53179 | 0.946127 | 2.1 |
| 53180 | 0.94613 | 2.1 |
| 53181 | 0.94613 | 2.1 |
| 53182 | 0.946128 | 2.1 |
| 53183 | 0.946128 | 2.1 |

| | | |
|---|---|---|
| 53184 | 0.946128 | 2.1 |
| 53185 | 0.946128 | 2.1 |
| 53186 | 1.89226 | 2.1 |
| 53187 | 1.89226 | 1.17062 |
| 53188 | 1.89226 | 2.1 |
| 53189 | 1.89226 | 2.1 |
| 53190 | 1.89226 | 2.1 |
| 53191 | 1.89226 | 2.1 |
| 53192 | 1.16742 | 2.1 |
| 53193 | 1.16742 | 2.1 |
| 53194 | 1.18122 | 2.1 |
| 53195 | 1.18122 | 2.1 |
| 53196 | 1.31745 | 2.1 |
| 53197 | 1.2597 | 1.37019 |
| 53198 | 1.03119 | 2.1 |
| 53199 | 1.06122 | 2.1 |
| 53200 | 1.06122 | 2.1 |
| 53201 | 1.31745 | 2.1 |
| 53202 | 1.03119 | 2.1 |
| 53203 | 1.2597 | 2.1 |
| 53204 | 1.36849 | 2.1 |
| 53205 | 2.12244 | 2.1 |
| 53206 | 2.20092 | 2.1 |
| 53207 | 2.20092 | 2.1 |
| 53208 | 1.35509 | 2.1 |
| 53209 | 1.35509 | 2.1 |
| 53210 | 1.89226 | 2.1 |
| 53211 | 1.89226 | 2.1 |
| 53212 | 1.89226 | 2.1 |
| 53213 | 1.89226 | 2.1 |
| 53214 | 1.89226 | 2.1 |
| 53215 | 1.89226 | 2.1 |
| 53216 | 1.89226 | 2.1 |
| 53217 | 1.89226 | 0.979293 |
| 53218 | 1.89226 | 2.1 |
| 53219 | 1.89226 | 2.1 |
| 53220 | 3.78452 | 2.1 |
| 53221 | 1.21731 | 2.1 |
| 53222 | 1.21731 | 2.1 |
| 53223 | 1.23412 | 2.1 |
| 53224 | 1.23412 | 2.1 |
| 53225 | 1.39021 | 2.1 |
| 53226 | 1.31745 | 2.1 |
| 53227 | 1.06122 | 2.1 |
| 53228 | 1.10046 | 2.1 |
| 53229 | 1.10046 | 2.1 |
| 53230 | 1.39021 | 2.1 |
| 53231 | 1.06122 | 2.1 |
| 53232 | 1.31745 | 2.1 |
| 53233 | 2.16503 | 0.543867 |
| 53234 | 1.78252 | 1.26887 |
| 53235 | 1.77293 | 1.00162 |
| 53236 | 1.56839 | 2.1 |
| 53237 | 2.36957 | 0.0509901 |
| 53238 | 2.16503 | 2.1 |
| 53239 | 1.89223 | 0.62703 |
| 53240 | 1.89223 | 2.1 |
| 53241 | 1.89223 | 0.0946518 |

| | | |
|---|---|---|
| 53242 | 1.89223 | 1.24276 |
| 53243 | 3.78447 | 2.1 |
| 53244 | 2.3696 | 1.2179 |
| 53245 | 1.78254 | 1.69074 |
| 53246 | 2.63298 | 0.208779 |
| 53247 | 2.07312 | 2.1 |
| 53248 | 4.44272 | 1.46713 |
| 53249 | 1.89226 | 1.3582 |
| 53250 | 1.89226 | 2.1 |
| 53251 | 1.89226 | 1.2016 |
| 53252 | 1.89226 | 2.1 |
| 53253 | 1.89226 | 0.05 |
| 53254 | 1.89226 | 2.1 |
| 53255 | 1.89226 | 0.699865 |
| 53256 | 1.89226 | 0.481557 |
| 53257 | 1.89226 | 0.71549 |
| 53258 | 1.89226 | 0.05 |
| 53259 | 3.78452 | 0.170969 |
| 53260 | 2.26369 | 0.698806 |
| 53261 | 2.2168 | 0.136664 |
| 53262 | 2.15617 | 0.0690423 |
| 53263 | 2.10864 | 0.05 |
| 53264 | 4.37234 | 0.05 |
| 53265 | 2.2168 | 0.389254 |
| 53266 | 1.94499 | 0.125868 |
| 53267 | 1.94499 | 0.572987 |
| 53268 | 2.04737 | 0.504142 |
| 53269 | 2.10864 | 0.0905966 |
| 53270 | 2.21103 | 0.159334 |
| 53271 | 1.89226 | 0.672571 |
| 53272 | 1.89226 | 0.203767 |
| 53273 | 1.89226 | 0.516382 |
| 53274 | 1.89226 | 0.5117 |
| 53275 | 1.89226 | 0.624815 |
| 53276 | 1.89226 | 0.539315 |
| 53277 | 1.89226 | 0.509968 |
| 53278 | 1.89226 | 0.458827 |
| 53279 | 1.89226 | 0.526921 |
| 53280 | 1.89226 | 0.740167 |
| 53281 | 1.89226 | 0.05 |
| 53282 | 1.89226 | 0.752516 |
| 53283 | 1.02995 | 0.05 |
| 53284 | 1.02995 | 0.711163 |
| 53285 | 1.03212 | 0.406669 |
| 53286 | 1.03212 | 0.379684 |
| 53287 | 0.988256 | 0.150679 |
| 53288 | 1.05432 | 0.05 |
| 53289 | 1.00775 | 0.214884 |
| 53290 | 1.07809 | 0.512982 |
| 53291 | 1.00775 | 0.05 |
| 53292 | 0.988257 | 0.0818485 |
| 53293 | 1.07808 | 0.491361 |
| 53294 | 1.05432 | 0.384541 |
| 53295 | 2.10864 | 0.505062 |
| 53296 | 1.94499 | 0.3252 |
| 53297 | 1.97652 | 0.0841354 |
| 53298 | 1.79681 | 0.366154 |
| 53299 | 3.9215 | 0.272434 |

| | | |
|---|---|---|
| 53300 | 0.810943 | 0.17141 |
| 53301 | 0.810943 | 0.74211 |
| 53302 | 0.810943 | 0.268815 |
| 53303 | 0.810943 | 0.357613 |
| 53304 | 0.810958 | 0.0579523 |
| 53305 | 0.810958 | 0.103547 |
| 53306 | 1.89226 | 0.416885 |
| 53307 | 1.35168 | 0.128323 |
| 53308 | 1.08132 | 0.235059 |
| 53309 | 1.08132 | 0.445221 |
| 53310 | 1.0813 | 0.378436 |
| 53311 | 0.657403 | 0.05 |
| 53312 | 1.35403 | 0.05 |
| 53313 | 1.24038 | 0.05 |
| 53314 | 0.499144 | 0.05 |
| 53315 | 1.24039 | 0.05 |
| 53316 | 1.35403 | 0.05 |
| 53317 | 0.657402 | 0.05 |
| 53318 | 0.499144 | 0.05 |
| 53319 | 0.915415 | 0.05 |
| 53320 | 0.915415 | 0.05 |
| 53321 | 0.937755 | 0.05 |
| 53322 | 0.937755 | 0.05 |
| 53323 | 1.72526 | 2.1 |
| 53324 | 1.17989 | 2.1 |
| 53325 | 1.96035 | 2.1 |
| 53326 | 1.47412 | 2.1 |
| 53327 | 3.14024 | 2.1 |
| 53328 | 1.89226 | 1.3499 |
| 53329 | 1.89226 | 2.1 |
| 53330 | 1.89226 | 2.1 |
| 53331 | 1.89226 | 0.957237 |
| 53332 | 3.78452 | 2.1 |
| 53333 | 0.946131 | 1.87013 |
| 53334 | 0.946131 | 1.763 |
| 53335 | 1.25764 | 2.1 |
| 53336 | 1.25764 | 2.1 |
| 53337 | 1.07087 | 1.25213 |
| 53338 | 1.25765 | 1.30645 |
| 53339 | 0.946128 | 1.21288 |
| 53340 | 0.946128 | 0.567539 |
| 53341 | 0.94613 | 1.03054 |
| 53342 | 1.25765 | 1.22423 |
| 53343 | 1.07086 | 1.47995 |
| 53344 | 0.946129 | 1.40914 |
| 53345 | 1.96035 | 2.1 |
| 53346 | 1.32668 | 2.1 |
| 53347 | 2.29301 | 2.1 |
| 53348 | 1.70303 | 2.1 |
| 53349 | 3.56428 | 2.1 |
| 53350 | 1.89226 | 2.1 |
| 53351 | 1.89226 | 1.89934 |
| 53352 | 1.89226 | 0.675617 |
| 53353 | 1.89226 | 2.1 |
| 53354 | 3.78452 | 0.641538 |
| 53355 | 0.897738 | 0.159002 |
| 53356 | 0.897738 | 0.20421 |
| 53357 | 0.946102 | 0.213499 |

| | | |
|---|---|---|
| 53358 | 0.946102 | 0.05 |
| 53359 | 1.11247 | 0.05 |
| 53360 | 0.89771 | 0.05 |
| 53361 | 0.94613 | 0.281501 |
| 53362 | 0.94613 | 0.267547 |
| 53363 | 0.897709 | 0.203776 |
| 53364 | 0.946131 | 0.369005 |
| 53365 | 0.946131 | 0.521983 |
| 53366 | 1.11247 | 0.0686248 |
| 53367 | 1.89226 | 2.1 |
| 53368 | 1.89226 | 2.1 |
| 53369 | 1.89226 | 2.1 |
| 53370 | 1.89226 | 2.1 |
| 53371 | 1.89226 | 2.1 |
| 53372 | 1.89226 | 2.1 |
| 53373 | 2.15429 | 2.1 |
| 53374 | 1.89226 | 2.1 |
| 53375 | 1.89226 | 2.1 |
| 53376 | 1.89226 | 2.1 |
| 53377 | 2.27074 | 2.1 |
| 53378 | 2.27074 | 2.1 |
| 53379 | 0.993311 | 1.0847 |
| 53380 | 0.993311 | 2.02824 |
| 53381 | 0.904322 | 2.1 |
| 53382 | 0.904322 | 2.1 |
| 53383 | 0.803324 | 2.1 |
| 53384 | 1.36183 | 1.12385 |
| 53385 | 0.916274 | 2.1 |
| 53386 | 0.535808 | 1.5466 |
| 53387 | 0.535808 | 1.95525 |
| 53388 | 0.803322 | 2.1 |
| 53389 | 0.916276 | 2.04854 |
| 53390 | 1.36183 | 2.1 |
| 53391 | 1.89226 | 2.1 |
| 53392 | 1.89226 | 2.1 |
| 53393 | 1.89226 | 2.1 |
| 53394 | 1.89226 | 2.1 |
| 53395 | 1.89226 | 2.1 |
| 53396 | 1.89226 | 2.1 |
| 53397 | 2.11955 | 2.1 |
| 53398 | 2.62097 | 2.1 |
| 53399 | 1.85542 | 2.1 |
| 53400 | 2.39016 | 2.1 |
| 53401 | 4.50971 | 2.1 |
| 53402 | 2.39016 | 2.1 |
| 53403 | 2.20242 | 2.1 |
| 53404 | 2.39016 | 2.1 |
| 53405 | 1.64008 | 2.1 |
| 53406 | 1.85542 | 2.1 |
| 53407 | 1.64008 | 2.1 |
| 53408 | 1.79667 | 2.1 |
| 53409 | 1.28267 | 2.1 |
| 53410 | 1.28267 | 2.1 |
| 53411 | 1.40117 | 2.1 |
| 53412 | 1.74514 | 2.1 |
| 53413 | 1.83994 | 2.1 |
| 53414 | 0.575914 | 2.1 |
| 53415 | 0.625292 | 2.1 |

| | | |
|---|---|---|
| 53416 | 0.949144 | 2.1 |
| 53417 | 0.882782 | 2.1 |
| 53418 | 0.773697 | 2.1 |
| 53419 | 0.773697 | 2.1 |
| 53420 | 0.786497 | 2.1 |
| 53421 | 0.786494 | 2.1 |
| 53422 | 0.605477 | 2.1 |
| 53423 | 0.949148 | 2.1 |
| 53424 | 0.625291 | 2.1 |
| 53425 | 0.862374 | 2.1 |
| 53426 | 1.89226 | 2.1 |
| 53427 | 2.44918 | 2.1 |
| 53428 | 2.4616 | 2.1 |
| 53429 | 2.44918 | 2.1 |
| 53430 | 1.90468 | 2.1 |
| 53431 | 1.89226 | 2.1 |
| 53432 | 1.89226 | 2.1 |
| 53433 | 1.89226 | 2.1 |
| 53434 | 1.89226 | 2.1 |
| 53435 | 1.89226 | 0.770789 |
| 53436 | 3.78453 | 2.1 |
| 53437 | 0.946128 | 2.1 |
| 53438 | 0.946128 | 0.534922 |
| 53439 | 1.23631 | 2.1 |
| 53440 | 1.23631 | 2.1 |
| 53441 | 0.946131 | 2.1 |
| 53442 | 0.946131 | 2.1 |
| 53443 | 1.3147 | 2.1 |
| 53444 | 1.23631 | 2.1 |
| 53445 | 1.3147 | 2.1 |
| 53446 | 0.946131 | 2.1 |
| 53447 | 1.23631 | 2.1 |
| 53448 | 0.94613 | 2.1 |
| 53449 | 1.89226 | 2.1 |
| 53450 | 2.4616 | 2.1 |
| 53451 | 2.4717 | 2.1 |
| 53452 | 2.4616 | 2.1 |
| 53453 | 1.90237 | 2.1 |
| 53454 | 1.89226 | 2.1 |
| 53455 | 0.946132 | 2.06678 |
| 53456 | 0.946132 | 2.1 |
| 53457 | 0.946131 | 2.1 |
| 53458 | 0.946131 | 2.1 |
| 53459 | 0.946132 | 2.1 |
| 53460 | 0.946132 | 2.1 |
| 53461 | 0.946131 | 2.1 |
| 53462 | 0.946131 | 2.1 |
| 53463 | 0.946131 | 2.1 |
| 53464 | 0.946131 | 2.1 |
| 53465 | 0.946131 | 2.1 |
| 53466 | 0.946131 | 2.1 |
| 53467 | 0.946128 | 2.1 |
| 53468 | 0.946128 | 2.1 |
| 53469 | 1.3147 | 1.58258 |
| 53470 | 1.3147 | 2.1 |
| 53471 | 0.94613 | 1.09596 |
| 53472 | 0.94613 | 2.1 |
| 53473 | 1.3419 | 2.1 |

| | | |
|---|---|---|
| 53474 | 1.3147 | 2.1 |
| 53475 | 1.3419 | 2.1 |
| 53476 | 0.94613 | 2.1 |
| 53477 | 1.3147 | 2.1 |
| 53478 | 0.94613 | 2.1 |
| 53479 | 1.89226 | 2.1 |
| 53480 | 1.89226 | 2.1 |
| 53481 | 1.89226 | 2.1 |
| 53482 | 1.89226 | 2.1 |
| 53483 | 3.78452 | 2.1 |
| 53484 | 2.3333 | 2.1 |
| 53485 | 1.3864 | 2.1 |
| 53486 | 1.3864 | 2.1 |
| 53487 | 1.4628 | 2.1 |
| 53488 | 1.95763 | 2.1 |
| 53489 | 2.03403 | 2.1 |
| 53490 | 1.294 | 2.1 |
| 53491 | 1.294 | 2.1 |
| 53492 | 0.946128 | 2.1 |
| 53493 | 0.946128 | 2.1 |
| 53494 | 1.294 | 2.1 |
| 53495 | 1.27474 | 2.1 |
| 53496 | 0.946131 | 2.1 |
| 53497 | 0.946131 | 2.1 |
| 53498 | 0.946129 | 2.1 |
| 53499 | 1.27474 | 2.1 |
| 53500 | 1.294 | 2.1 |
| 53501 | 0.94613 | 2.1 |
| 53502 | 1.89226 | 2.1 |
| 53503 | 1.89226 | 2.1 |
| 53504 | 1.89226 | 2.1 |
| 53505 | 1.89226 | 2.1 |
| 53506 | 1.89226 | 2.1 |
| 53507 | 1.89226 | 2.1 |
| 53508 | 0.808088 | 2.1 |
| 53509 | 0.808088 | 2.1 |
| 53510 | 0.815623 | 2.1 |
| 53511 | 0.815623 | 2.1 |
| 53512 | 0.660053 | 2.1 |
| 53513 | 0.693199 | 2.1 |
| 53514 | 0.930512 | 2.1 |
| 53515 | 0.978817 | 2.1 |
| 53516 | 0.660053 | 2.1 |
| 53517 | 0.930513 | 2.1 |
| 53518 | 0.693198 | 2.1 |
| 53519 | 0.978817 | 2.1 |
| 53520 | 1.27474 | 2.1 |
| 53521 | 1.27474 | 2.1 |
| 53522 | 0.946128 | 2.1 |
| 53523 | 0.946128 | 2.1 |
| 53524 | 1.26221 | 2.1 |
| 53525 | 1.27474 | 2.1 |
| 53526 | 0.946131 | 2.1 |
| 53527 | 0.946131 | 2.1 |
| 53528 | 0.94613 | 2.1 |
| 53529 | 1.26221 | 2.1 |
| 53530 | 0.946129 | 2.1 |
| 53531 | 1.27474 | 2.1 |

| | | |
|---|---|---|
| 53532 | 0.90207 | 0.05 |
| 53533 | 0.90207 | 0.05 |
| 53534 | 0.895719 | 0.05 |
| 53535 | 0.895719 | 0.05 |
| 53536 | 0.927855 | 0.05 |
| 53537 | 1.2493 | 0.05 |
| 53538 | 0.85723 | 0.05 |
| 53539 | 0.548484 | 0.05 |
| 53540 | 0.548485 | 0.05 |
| 53541 | 0.927855 | 0.05 |
| 53542 | 0.85723 | 0.05 |
| 53543 | 1.2493 | 0.05 |
| 53544 | 1.41319 | 0.0938095 |
| 53545 | 1.22482 | 0.05 |
| 53546 | 1.46629 | 0.05 |
| 53547 | 1.92622 | 0.111274 |
| 53548 | 3.15104 | 0.077526 |
| 53549 | 1.02305 | 0.05 |
| 53550 | 1.22482 | 0.05 |
| 53551 | 1.80336 | 0.05 |
| 53552 | 1.44426 | 0.05 |
| 53553 | 2.66908 | 0.05 |
| 53554 | 1.3268 | 2.1 |
| 53555 | 0.999489 | 2.1 |
| 53556 | 1.44091 | 2.1 |
| 53557 | 1.14657 | 2.1 |
| 53558 | 2.4404 | 2.1 |
| 53559 | 1.89226 | 2.1 |
| 53560 | 1.89226 | 2.1 |
| 53561 | 1.89226 | 2.1 |
| 53562 | 1.89226 | 2.1 |
| 53563 | 3.78452 | 2.1 |
| 53564 | 1.89226 | 2.1 |
| 53565 | 2.28605 | 2.1 |
| 53566 | 2.43157 | 2.1 |
| 53567 | 2.43157 | 2.1 |
| 53568 | 1.89226 | 2.1 |
| 53569 | 1.89226 | 2.1 |
| 53570 | 1.60647 | 2.1 |
| 53571 | 1.44091 | 2.1 |
| 53572 | 1.31346 | 2.1 |
| 53573 | 1.31346 | 2.1 |
| 53574 | 1.14657 | 2.1 |
| 53575 | 1.44091 | 2.1 |
| 53576 | 1.89226 | 2.1 |
| 53577 | 1.89226 | 2.1 |
| 53578 | 1.89226 | 2.1 |
| 53579 | 1.89226 | 2.1 |
| 53580 | 3.78452 | 2.1 |
| 53581 | 0.946132 | 2.1 |
| 53582 | 0.946132 | 2.1 |
| 53583 | 1.3048 | 2.1 |
| 53584 | 1.3048 | 2.1 |
| 53585 | 1.3048 | 2.1 |
| 53586 | 1.21578 | 2.1 |
| 53587 | 0.946132 | 2.1 |
| 53588 | 0.946132 | 2.1 |
| 53589 | 1.3048 | 2.1 |

| | | |
|---|---|---|
| 53590 | 0.946132 | 2.1 |
| 53591 | 0.946132 | 2.1 |
| 53592 | 1.21578 | 2.1 |
| 53593 | 1.89227 | 0.05 |
| 53594 | 1.89227 | 0.05 |
| 53595 | 1.89227 | 0.05 |
| 53596 | 1.89227 | 0.05 |
| 53597 | 3.78453 | 0.05 |
| 53598 | 1.6671 | 0.05 |
| 53599 | 1.87338 | 0.05 |
| 53600 | 1.6671 | 0.05 |
| 53601 | 2.02098 | 0.05 |
| 53602 | 1.38328 | 0.05 |
| 53603 | 1.73715 | 0.05 |
| 53604 | 1.89226 | 0.05 |
| 53605 | 1.89226 | 0.05 |
| 53606 | 1.89226 | 0.05 |
| 53607 | 1.89226 | 0.05 |
| 53608 | 3.78453 | 0.05 |
| 53609 | 1.6671 | 0.05 |
| 53610 | 1.38328 | 0.05 |
| 53611 | 1.35562 | 0.05 |
| 53612 | 1.01012 | 0.05 |
| 53613 | 2.67722 | 0.05 |
| 53614 | 0.819377 | 0.05 |
| 53615 | 0.647925 | 0.05 |
| 53616 | 1.17802 | 0.05 |
| 53617 | 1.33174 | 0.05 |
| 53618 | 0.98543 | 0.05 |
| 53619 | 0.98543 | 0.05 |
| 53620 | 0.994237 | 0.05 |
| 53621 | 0.994237 | 0.05 |
| 53622 | 1.17802 | 0.05 |
| 53623 | 0.647926 | 0.05 |
| 53624 | 1.33174 | 0.05 |
| 53625 | 0.819377 | 0.05 |
| 53626 | 1.27806 | 0.05 |
| 53627 | 1.68264 | 0.05 |
| 53628 | 1.44593 | 0.05 |
| 53629 | 1.878 0.05 | |
| 53630 | 3.15606 | 0.05 |
| 53631 | 1.878 0.05 | |
| 53632 | 1.9898 | 0.05 |
| 53633 | 1.878 0.05 | |
| 53634 | 1.68264 | 0.05 |
| 53635 | 2.30533 | 0.05 |
| 53636 | 2.10997 | 0.05 |
| 53637 | 0.814587 | 0.05 |
| 53638 | 0.814596 | 0.05 |
| 53639 | 0.82775 | 0.05 |
| 53640 | 0.82775 | 0.05 |
| 53641 | 1.0107 | 0.05 |
| 53642 | 0.91549 | 0.05 |
| 53643 | 0.741058 | 0.05 |
| 53644 | 0.714748 | 0.05 |
| 53645 | 0.714758 | 0.05 |
| 53646 | 1.03225 | 0.05 |
| 53647 | 0.704864 | 0.05 |

| | | |
|---|---|---|
| 53648 | 0.915488 | 0.05 |
| 53649 | 1.90347 | 0.05 |
| 53650 | 2.06584 | 0.05 |
| 53651 | 1.878 | 0.05 |
| 53652 | 1.50236 | 0.05 |
| 53653 | 2.05749 | 0.05 |
| 53654 | 2.30533 | 0.05 |
| 53655 | 2.24475 | 2.1 |
| 53656 | 1.85871 | 2.1 |
| 53657 | 2.13711 | 2.1 |
| 53658 | 2.13711 | 2.1 |
| 53659 | 1.9943 | 2.1 |
| 53660 | 1.9943 | 2.1 |
| 53661 | 1.85871 | 2.1 |
| 53662 | 1.7871 | 2.1 |
| 53663 | 1.41619 | 2.1 |
| 53664 | 1.41619 | 2.1 |
| 53665 | 2.13711 | 2.1 |
| 53666 | 2.13711 | 2.1 |
| 53667 | 2.24475 | 2.1 |
| 53668 | 2.13711 | 2.1 |
| 53669 | 2.54902 | 2.1 |
| 53670 | 2.46327 | 2.1 |
| 53671 | 4.70802 | 2.1 |
| 53672 | 1.06913 | 2.1 |
| 53673 | 1.06913 | 2.1 |
| 53674 | 1.0821 | 2.1 |
| 53675 | 1.0821 | 2.1 |
| 53676 | 0.893552 | 2.1 |
| 53677 | 1.08267 | 2.1 |
| 53678 | 1.06855 | 2.1 |
| 53679 | 1.23163 | 2.1 |
| 53680 | 1.23163 | 2.1 |
| 53681 | 1.08267 | 2.1 |
| 53682 | 0.893551 | 2.1 |
| 53683 | 1.06855 | 2.1 |
| 53684 | 1.67046 | 1.07109 |
| 53685 | 2.4106 | 0.536545 |
| 53686 | 2.05835 | 1.10622 |
| 53687 | 2.4106 | 1.23465 |
| 53688 | 1.59562 | 0.49649 |
| 53689 | 1.94788 | 0.495752 |
| 53690 | 2.34737 | 1.07923 |
| 53691 | 2.4106 | 0.580938 |
| 53692 | 2.05834 | 1.25403 |
| 53693 | 1.99655 | 1.08505 |
| 53694 | 4.40715 | 0.836476 |
| 53695 | 1.67046 | 0.769258 |
| 53696 | 2.05835 | 0.180529 |
| 53697 | 1.588 | 0.976013 |
| 53698 | 1.34857 | 1.5378 |
| 53699 | 3.40692 | 0.753463 |
| 53700 | 1.73802 | 0.688681 |
| 53701 | 1.89668 | 0.949331 |
| 53702 | 1.99655 | 2.06265 |
| 53703 | 1.99655 | 0.613278 |
| 53704 | 1.79981 | 0.817919 |
| 53705 | 2.05834 | 0.346351 |

| | | |
|---|---|---|
| 53706 | 1.89226 | 1.27559 |
| 53707 | 1.89226 | 0.9999 |
| 53708 | 1.89226 | 0.281123 |
| 53709 | 1.89226 | 2.08779 |
| 53710 | 1.89226 | 0.671037 |
| 53711 | 1.89226 | 2.1 |
| 53712 | 1.89226 | 1.01162 |
| 53713 | 1.89226 | 2.1 |
| 53714 | 1.89226 | 1.99418 |
| 53715 | 1.89226 | 0.264941 |
| 53716 | 3.78453 | 1.93146 |
| 53717 | 0.719473 | 2.1 |
| 53718 | 0.916103 | 1.90579 |
| 53719 | 1.02744 | 1.12351 |
| 53720 | 0.850328 | 2.1 |
| 53721 | 0.888094 | 1.06239 |
| 53722 | 0.888094 | 2.1 |
| 53723 | 0.878337 | 2.1 |
| 53724 | 0.878337 | 0.131519 |
| 53725 | 0.916103 | 1.28956 |
| 53726 | 1.02744 | 1.45192 |
| 53727 | 0.719473 | 1.89724 |
| 53728 | 0.850328 | 2.06838 |
| 53729 | 1.02744 | 1.33386 |
| 53730 | 0.850329 | 2.1 |
| 53731 | 1.0875 | 1.16554 |
| 53732 | 0.919597 | 1.44765 |
| 53733 | 0.975841 | 0.893386 |
| 53734 | 0.975841 | 2.1 |
| 53735 | 0.971196 | 0.868657 |
| 53736 | 0.971196 | 2.1 |
| 53737 | 1.02744 | 1.85279 |
| 53738 | 1.0875 | 1.13068 |
| 53739 | 0.850328 | 2.07875 |
| 53740 | 0.919598 | 1.78821 |
| 53741 | 1.89226 | 2.1 |
| 53742 | 1.89226 | 1.63831 |
| 53743 | 1.89226 | 0.853015 |
| 53744 | 1.89226 | 1.97451 |
| 53745 | 3.78453 | 1.39154 |
| 53746 | 1.89226 | 1.52055 |
| 53747 | 1.89226 | 0.992148 |
| 53748 | 1.89226 | 0.143061 |
| 53749 | 1.89226 | 2.1 |
| 53750 | 1.89226 | 0.26635 |
| 53751 | 1.89226 | 2.1 |
| 53752 | 1.11103 | 1.66175 |
| 53753 | 0.916103 | 2.1 |
| 53754 | 1.21177 | 1.26469 |
| 53755 | 1.02744 | 1.35791 |
| 53756 | 1.07188 | 0.950753 |
| 53757 | 1.07188 | 2.1 |
| 53758 | 1.06659 | 0.91515 |
| 53759 | 1.06659 | 2.1 |
| 53760 | 1.11103 | 2.1 |
| 53761 | 1.21177 | 1.61051 |
| 53762 | 0.916104 | 2.0969 |
| 53763 | 1.02744 | 2.1 |

| | | |
|---|---|---|
| 53764 | 2.54158 | 1.08919 |
| 53765 | 2.42354 | 1.46264 |
| 53766 | 2.17499 | 0.853612 |
| 53767 | 2.17499 | 2.1 |
| 53768 | 2.42354 | 1.99229 |
| 53769 | 2.05488 | 2.1 |
| 53770 | 1.89226 | 0.05 |
| 53771 | 1.89226 | 0.05 |
| 53772 | 1.89226 | 0.05 |
| 53773 | 1.89226 | 0.05 |
| 53774 | 3.78453 | 0.05 |
| 53775 | 1.89226 | 0.05 |
| 53776 | 1.89226 | 0.05 |
| 53777 | 1.89226 | 0.05 |
| 53778 | 1.89226 | 0.05 |
| 53779 | 1.89226 | 0.05 |
| 53780 | 1.89226 | 0.05 |
| 53781 | 1.89226 | 0.05 |
| 53782 | 1.89226 | 0.05 |
| 53783 | 1.89226 | 0.05 |
| 53784 | 1.89226 | 0.05 |
| 53785 | 1.89226 | 0.05 |
| 53786 | 1.89226 | 0.05 |
| 53787 | 1.89226 | 0.05 |
| 53788 | 1.89226 | 0.05 |
| 53789 | 1.89226 | 0.05 |
| 53790 | 1.89226 | 0.05 |
| 53791 | 3.78452 | 0.05 |
| 53792 | 0.802544 | 0.05 |
| 53793 | 0.946132 | 0.05 |
| 53794 | 0.946132 | 0.05 |
| 53795 | 0.787038 | 0.05 |
| 53796 | 0.787039 | 0.05 |
| 53797 | 0.787039 | 0.05 |
| 53798 | 0.961637 | 0.05 |
| 53799 | 0.961637 | 0.05 |
| 53800 | 0.787038 | 0.05 |
| 53801 | 0.802544 | 0.05 |
| 53802 | 0.946132 | 0.05 |
| 53803 | 0.946132 | 0.05 |
| 53804 | 1.89226 | 0.05 |
| 53805 | 1.60509 | 0.05 |
| 53806 | 1.89226 | 0.05 |
| 53807 | 1.89226 | 0.05 |
| 53808 | 1.3905 | 0.05 |
| 53809 | 1.3905 | 0.05 |
| 53810 | 0.946132 | 0.05 |
| 53811 | 0.946132 | 0.05 |
| 53812 | 0.946131 | 0.05 |
| 53813 | 0.946131 | 0.05 |
| 53814 | 0.946132 | 0.05 |
| 53815 | 0.946132 | 0.05 |
| 53816 | 0.946131 | 0.05 |
| 53817 | 0.946131 | 0.05 |
| 53818 | 0.946131 | 0.05 |
| 53819 | 0.946131 | 0.05 |
| 53820 | 0.946132 | 0.05 |
| 53821 | 0.946132 | 0.05 |

| | | |
|---|---|---|
| 53822 | 0.946131 | 0.05 |
| 53823 | 0.946131 | 0.05 |
| 53824 | 0.946131 | 0.05 |
| 53825 | 0.946131 | 0.05 |
| 53826 | 0.946132 | 0.05 |
| 53827 | 0.946132 | 0.05 |
| 53828 | 0.946131 | 0.05 |
| 53829 | 0.946131 | 0.05 |
| 53830 | 0.946131 | 0.05 |
| 53831 | 0.946131 | 0.05 |
| 53832 | 0.946132 | 0.05 |
| 53833 | 0.946132 | 0.05 |
| 53834 | 1.42382 | 2.1 |
| 53835 | 1.32201 | 0.05 |
| 53836 | 1.32096 | 0.697597 |
| 53837 | 1.42866 | 1.89204 |
| 53838 | 2.75067 | 1.08838 |
| 53839 | 1.89226 | 2.1 |
| 53840 | 1.89226 | 0.05 |
| 53841 | 1.89226 | 0.630952 |
| 53842 | 1.89226 | 0.952806 |
| 53843 | 1.89226 | 0.109913 |
| 53844 | 1.89226 | 0.289616 |
| 53845 | 1.89226 | 1.44235 |
| 53846 | 2.79029 | 0.569657 |
| 53847 | 2.7803 | 0.686937 |
| 53848 | 2.79029 | 0.72604 |
| 53849 | 1.88227 | 0.2105 |
| 53850 | 1.89226 | 0.475106 |
| 53851 | 0.716065 | 1.40405 |
| 53852 | 0.714333 | 2.1 |
| 53853 | 0.660483 | 0.240366 |
| 53854 | 0.659126 | 1.0042 |
| 53855 | 0.687538 | 0.267109 |
| 53856 | 0.687538 | 1.25235 |
| 53857 | 0.685921 | 2.1 |
| 53858 | 0.685921 | 0.163952 |
| 53859 | 0.659126 | 0.510082 |
| 53860 | 0.716065 | 0.757445 |
| 53861 | 0.660483 | 1.0608 |
| 53862 | 0.714333 | 1.04006 |
| 53863 | 1.89226 | 1.24418 |
| 53864 | 1.89226 | 0.05 |
| 53865 | 1.89226 | 0.667625 |
| 53866 | 1.89226 | 0.915602 |
| 53867 | 3.78453 | 0.895802 |
| 53868 | 0.946133 | 0.179238 |
| 53869 | 0.946133 | 0.797265 |
| 53870 | 1.39015 | 0.565764 |
| 53871 | 1.38969 | 0.0971019 |
| 53872 | 0.946132 | 0.103368 |
| 53873 | 0.946132 | 0.530057 |
| 53874 | 1.38969 | 0.836263 |
| 53875 | 1.38969 | 0.651185 |
| 53876 | 1.38969 | 0.234172 |
| 53877 | 0.946132 | 0.637978 |
| 53878 | 0.946133 | 0.746105 |
| 53879 | 1.39015 | 0.628771 |

| | | |
|---|---|---|
| 53880 | 2.72157 | 0.05 |
| 53881 | 1.22176 | 0.05 |
| 53882 | 1.59492 | 0.05 |
| 53883 | 1.22176 | 0.05 |
| 53884 | 2.40743 | 0.05 |
| 53885 | 2.03426 | 0.05 |
| 53886 | 0.946131 | 0.05 |
| 53887 | 0.946131 | 0.05 |
| 53888 | 1.36079 | 0.05 |
| 53889 | 1.01713 | 0.05 |
| 53890 | 0.94613 | 0.05 |
| 53891 | 0.94613 | 0.05 |
| 53892 | 1.36079 | 0.05 |
| 53893 | 1.36079 | 0.05 |
| 53894 | 0.946131 | 0.05 |
| 53895 | 1.36079 | 0.05 |
| 53896 | 1.01713 | 0.05 |
| 53897 | 0.946131 | 0.05 |
| 53898 | 1.89226 | 0.05 |
| 53899 | 1.89226 | 0.05 |
| 53900 | 1.89226 | 0.05 |
| 53901 | 1.89226 | 0.0604359 |
| 53902 | 3.78453 | 0.05 |
| 53903 | 1.89226 | 0.05 |
| 53904 | 1.89226 | 0.05 |
| 53905 | 1.89226 | 0.05 |
| 53906 | 1.89226 | 0.0785475 |
| 53907 | 1.89226 | 0.05 |
| 53908 | 1.89226 | 0.05 |
| 53909 | 0.946131 | 0.05 |
| 53910 | 0.946131 | 0.05 |
| 53911 | 0.946131 | 0.05 |
| 53912 | 1.36079 | 0.05 |
| 53913 | 0.94613 | 0.05 |
| 53914 | 0.94613 | 0.05 |
| 53915 | 0.946133 | 0.05 |
| 53916 | 0.946133 | 0.05 |
| 53917 | 0.946131 | 0.05 |
| 53918 | 0.946131 | 0.05 |
| 53919 | 0.946132 | 0.05 |
| 53920 | 1.36079 | 0.05 |
| 53921 | 1.89226 | 0.05 |
| 53922 | 1.89226 | 0.0564855 |
| 53923 | 1.89226 | 0.05 |
| 53924 | 1.89226 | 0.05 |
| 53925 | 1.89226 | 0.05 |
| 53926 | 1.89226 | 0.05 |
| 53927 | 1.89227 | 0.05 |
| 53928 | 1.89227 | 0.05 |
| 53929 | 1.89227 | 0.05 |
| 53930 | 1.89227 | 0.0676645 |
| 53931 | 1.89227 | 0.05 |
| 53932 | 1.89227 | 0.05 |
| 53933 | 1.60428 | 2.1 |
| 53934 | 2.2367 | 2.1 |
| 53935 | 1.50097 | 2.1 |
| 53936 | 1.80125 | 2.1 |
| 53937 | 3.40553 | 2.1 |

| | | |
|---|---|---|
| 53938 | 1.89095 | 2.1 |
| 53939 | 1.85293 | 0.242584 |
| 53940 | 1.80125 | 2.1 |
| 53941 | 1.76268 | 0.05 |
| 53942 | 3.65418 | 1.3998 |
| 53943 | 0.803177 | 2.1 |
| 53944 | 0.803177 | 2.1 |
| 53945 | 0.798644 | 2.1 |
| 53946 | 0.798644 | 2.1 |
| 53947 | 0.84233 | 2.1 |
| 53948 | 0.900624 | 2.1 |
| 53949 | 0.750483 | 2.1 |
| 53950 | 0.701197 | 2.1 |
| 53951 | 0.701197 | 2.1 |
| 53952 | 0.84233 | 2.1 |
| 53953 | 0.750483 | 2.1 |
| 53954 | 0.900625 | 2.1 |
| 53955 | 0.860925 | 0.05 |
| 53956 | 0.860925 | 2.1 |
| 53957 | 0.862743 | 2.1 |
| 53958 | 0.862743 | 0.229567 |
| 53959 | 0.881338 | 0.0747491 |
| 53960 | 0.826621 | 0.05 |
| 53961 | 0.84233 | 2.1 |
| 53962 | 0.900625 | 2.1 |
| 53963 | 0.84233 | 0.196585 |
| 53964 | 0.826621 | 0.378851 |
| 53965 | 0.900624 | 0.840972 |
| 53966 | 0.881339 | 0.446732 |
| 53967 | 1.37938 | 1.04941 |
| 53968 | 1.03319 | 1.03315 |
| 53969 | 1.00512 | 1.39819 |
| 53970 | 0.639582 | 1.5019 |
| 53971 | 1.00463 | 1.39579 |
| 53972 | 1.00463 | 0.9905 |
| 53973 | 1.01434 | 0.938762 |
| 53974 | 1.01434 | 1.6154 |
| 53975 | 1.37938 | 1.19847 |
| 53976 | 1.00512 | 1.53176 |
| 53977 | 1.03319 | 1.31537 |
| 53978 | 0.639582 | 1.28063 |
| 53979 | 0.728713 | 0.05 |
| 53980 | 0.728713 | 0.05 |
| 53981 | 0.73425 | 0.494194 |
| 53982 | 0.73425 | 0.674249 |
| 53983 | 0.525122 | 2.1 |
| 53984 | 0.605622 | 0.05 |
| 53985 | 0.948901 | 0.05 |
| 53986 | 0.857342 | 0.05 |
| 53987 | 0.85734 | 0.05 |
| 53988 | 0.525123 | 0.594102 |
| 53989 | 0.948899 | 0.05 |
| 53990 | 0.605624 | 0.05 |
| 53991 | 1.75449 | 0.737408 |
| 53992 | 1.4378 | 0.787891 |
| 53993 | 1.93916 | 0.891373 |
| 53994 | 2.22512 | 0.601539 |
| 53995 | 3.69364 | 0.807329 |

| | | |
|---|---|---|
| 53996 | 1.75448 | 0.942777 |
| 53997 | 1.89186 | 0.643767 |
| 53998 | 2.29771 | 0.723113 |
| 53999 | 2.22512 | 0.65008 |
| 54000 | 4.05219 | 0.703222 |
| 54001 | 1.93916 | 0.874891 |
| 54002 | 2.51749 | 0.487523 |
| 54003 | 2.62357 | 0.680293 |
| 54004 | 2.22512 | 0.600352 |
| 54005 | 2.33761 | 0.813933 |
| 54006 | 1.93916 | 0.711066 |
| 54007 | 2.29771 | 0.765014 |
| 54008 | 2.51749 | 0.562348 |
| 54009 | 2.22512 | 0.597613 |
| 54010 | 2.22512 | 0.489822 |
| 54011 | 2.50905 | 0.805577 |
| 54012 | 2.50905 | 0.711517 |
| 54013 | 0.87382 | 0.05 |
| 54014 | 1.33662 | 0.05 |
| 54015 | 1.20756 | 0.05 |
| 54016 | 0.890467 | 0.05 |
| 54017 | 1.08859 | 0.05 |
| 54018 | 1.08859 | 0.05 |
| 54019 | 1.13084 | 0.05 |
| 54020 | 1.08793 | 0.05 |
| 54021 | 1.34722 | 0.05 |
| 54022 | 1.27184 | 0.05 |
| 54023 | 0.917678 | 0.05 |
| 54024 | 0.97821 | 0.05 |
| 54025 | 1.28396 | 0.05 |
| 54026 | 1.70302 | 0.05 |
| 54027 | 2.30076 | 0.05 |
| 54028 | 1.20417 | 0.05 |
| 54029 | 2.57506 | 0.05 |
| 54030 | 2.2656 | 0.05 |
| 54031 | 1.03642 | 0.967532 |
| 54032 | 1.03642 | 0.05 |
| 54033 | 1.07029 | 0.0662234 |
| 54034 | 1.07029 | 0.161063 |
| 54035 | 1.31531 | 0.790304 |
| 54036 | 1.23279 | 0.0864056 |
| 54037 | 0.941743 | 0.120082 |
| 54038 | 0.791399 | 0.678795 |
| 54039 | 0.791399 | 0.0524101 |
| 54040 | 1.23279 | 0.0652208 |
| 54041 | 0.941744 | 0.395933 |
| 54042 | 1.31531 | 0.460471 |
| 54043 | 0.937354 | 1.07702 |
| 54044 | 0.937354 | 0.05 |
| 54045 | 0.954794 | 0.100301 |
| 54046 | 0.954794 | 0.0839089 |
| 54047 | 1.13563 | 0.0717096 |
| 54048 | 1.23279 | 0.792514 |
| 54049 | 0.791398 | 0.301348 |
| 54050 | 0.659359 | 0.646128 |
| 54051 | 1.13563 | 0.0607267 |
| 54052 | 0.659359 | 0.0640913 |
| 54053 | 0.791399 | 0.390757 |

| | | |
|---|---|---|
| 54054 | 1.23279 | 0.289938 |
| 54055 | 2.29852 | 0.189271 |
| 54056 | 1.89227 | 0.260051 |
| 54057 | 2.14485 | 0.1004 |
| 54058 | 2.14485 | 1.108 |
| 54059 | 1.89227 | 0.0621739 |
| 54060 | 1.89227 | 0.813461 |
| 54061 | 0.947863 | 0.221186 |
| 54062 | 1.19854 | 0.88754 |
| 54063 | 2.16841 | 0.309362 |
| 54064 | 1.76597 | 0.377667 |
| 54065 | 2.96451 | 0.246615 |
| 54066 | 1.89227 | 0.107825 |
| 54067 | 1.89227 | 0.170712 |
| 54068 | 1.89227 | 0.0847522 |
| 54069 | 1.89227 | 0.680272 |
| 54070 | 1.89227 | 0.0648182 |
| 54071 | 1.89227 | 0.548942 |
| 54072 | 2.46489 | 2.1 |
| 54073 | 1.98339 | 2.1 |
| 54074 | 1.81667 | 2.1 |
| 54075 | 2.36914 | 2.1 |
| 54076 | 4.35253 | 2.1 |
| 54077 | 2.46488 | 2.1 |
| 54078 | 2.70401 | 2.1 |
| 54079 | 2.64996 | 2.1 |
| 54080 | 2.36914 | 2.1 |
| 54081 | 5.07315 | 2.1 |
| 54082 | 1.81667 | 2.1 |
| 54083 | 2.29459 | 2.1 |
| 54084 | 2.36914 | 2.1 |
| 54085 | 2.36914 | 2.1 |
| 54086 | 1.68073 | 2.1 |
| 54087 | 1.68073 | 2.1 |
| 54088 | 2.64996 | 2.1 |
| 54089 | 2.29458 | 2.1 |
| 54090 | 2.29458 | 2.1 |
| 54091 | 2.36914 | 2.1 |
| 54092 | 2.61243 | 2.1 |
| 54093 | 2.68698 | 2.1 |
| 54094 | 1.89226 | 1.38876 |
| 54095 | 1.89226 | 0.798581 |
| 54096 | 1.89226 | 1.54162 |
| 54097 | 1.89226 | 1.13874 |
| 54098 | 1.89226 | 1.75436 |
| 54099 | 1.89226 | 1.18232 |
| 54100 | 1.89224 | 1.74287 |
| 54101 | 1.89224 | 0.736305 |
| 54102 | 1.65571 | 1.26332 |
| 54103 | 1.65571 | 0.915164 |
| 54104 | 1.89224 | 1.49507 |
| 54105 | 1.89224 | 1.23281 |
| 54106 | 1.89224 | 1.36217 |
| 54107 | 1.89224 | 1.14444 |
| 54108 | 1.89224 | 1.49077 |
| 54109 | 1.89224 | 0.774086 |
| 54110 | 1.89224 | 1.93859 |
| 54111 | 1.89224 | 1.13995 |

| | | |
|---|---|---|
| 54112 | 1.98252 | 0.05 |
| 54113 | 2.20641 | 0.05 |
| 54114 | 1.05129 | 0.05 |
| 54115 | 1.2951 | 0.05 |
| 54116 | 3.27762 | 0.05 |
| 54117 | 1.85598 | 2.1 |
| 54118 | 1.88635 | 1.1743 |
| 54119 | 2.12346 | 1.03733 |
| 54120 | 2.10838 | 2.1 |
| 54121 | 3.97943 | 1.38021 |
| 54122 | 2.10838 | 1.93475 |
| 54123 | 1.87532 | 2.1 |
| 54124 | 1.85597 | 0.0901286 |
| 54125 | 1.85597 | 2.1 |
| 54126 | 1.87532 | 2.08478 |
| 54127 | 1.57914 | 2.1 |
| 54128 | 2.12348 | 1.18856 |
| 54129 | 2.35141 | 2.1 |
| 54130 | 2.37191 | 1.35527 |
| 54131 | 2.10841 | 1.67216 |
| 54132 | 2.38698 | 1.08822 |
| 54133 | 2.12348 | 1.92243 |
| 54134 | 1.07811 | 2.1 |
| 54135 | 0.937672 | 2.1 |
| 54136 | 1.17571 | 2.1 |
| 54137 | 1.05421 | 0.271847 |
| 54138 | 1.07087 | 1.29486 |
| 54139 | 1.07087 | 2.1 |
| 54140 | 1.06145 | 2.1 |
| 54141 | 1.06145 | 1.08842 |
| 54142 | 1.17571 | 2.1 |
| 54143 | 1.07811 | 2.1 |
| 54144 | 1.05421 | 2.1 |
| 54145 | 0.937672 | 2.1 |
| 54146 | 1.67334 | 2.1 |
| 54147 | 2.76609 | 2.1 |
| 54148 | 2.74144 | 2.1 |
| 54149 | 2.74144 | 2.1 |
| 54150 | 1.71157 | 2.1 |
| 54151 | 1.71157 | 2.1 |
| 54152 | 2.76609 | 2.1 |
| 54153 | 1.89226 | 2.1 |
| 54154 | 1.89226 | 2.1 |
| 54155 | 1.89226 | 2.1 |
| 54156 | 2.74143 | 2.1 |
| 54157 | 2.74143 | 2.1 |
| 54158 | 1.10393 | 2.1 |
| 54159 | 1.10393 | 2.1 |
| 54160 | 1.10788 | 2.1 |
| 54161 | 1.10788 | 2.1 |
| 54162 | 1.38302 | 2.1 |
| 54163 | 1.37072 | 2.1 |
| 54164 | 0.836672 | 2.1 |
| 54165 | 0.841089 | 2.1 |
| 54166 | 1.38302 | 2.1 |
| 54167 | 0.84109 | 2.1 |
| 54168 | 0.836671 | 2.1 |
| 54169 | 1.37072 | 2.1 |

| | | |
|---|---|---|
| 54170 | 1.38302 | 2.1 |
| 54171 | 1.38302 | 2.1 |
| 54172 | 0.946131 | 2.1 |
| 54173 | 0.946131 | 2.1 |
| 54174 | 0.946128 | 2.1 |
| 54175 | 0.946128 | 2.1 |
| 54176 | 1.38302 | 2.1 |
| 54177 | 1.37072 | 2.1 |
| 54178 | 1.38302 | 2.1 |
| 54179 | 0.946131 | 2.1 |
| 54180 | 0.94613 | 2.1 |
| 54181 | 1.37072 | 2.1 |
| 54182 | 1.28072 | 0.115301 |
| 54183 | 1.55779 | 2.1 |
| 54184 | 1.28072 | 2.1 |
| 54185 | 1.40765 | 0.592693 |
| 54186 | 1.47186 | 2.1 |
| 54187 | 1.59879 | 2.1 |
| 54188 | 1.89227 | 0.440396 |
| 54189 | 1.89227 | 0.0984207 |
| 54190 | 1.89227 | 0.05 |
| 54191 | 1.89227 | 2.1 |
| 54192 | 1.89227 | 0.05 |
| 54193 | 1.89227 | 2.1 |
| 54194 | 2.80057 | 0.0570155 |
| 54195 | 1.89226 | 0.0513865 |
| 54196 | 1.89226 | 0.497244 |
| 54197 | 1.89226 | 0.540435 |
| 54198 | 2.63061 | 0.05 |
| 54199 | 2.63061 | 0.593696 |
| 54200 | 1.28072 | 0.05 |
| 54201 | 1.38859 | 2.1 |
| 54202 | 1.47186 | 2.1 |
| 54203 | 1.47186 | 1.04093 |
| 54204 | 1.16777 | 2.1 |
| 54205 | 1.16777 | 2.1 |
| 54206 | 1.89226 | 0.0791977 |
| 54207 | 1.89226 | 0.108418 |
| 54208 | 1.89226 | 0.0673405 |
| 54209 | 1.89226 | 2.1 |
| 54210 | 1.89226 | 0.05 |
| 54211 | 1.89226 | 2.1 |
| 54212 | 1.83221 | 2.1 |
| 54213 | 2.0815 | 2.1 |
| 54214 | 2.0815 | 2.1 |
| 54215 | 2.08153 | 2.1 |
| 54216 | 1.86703 | 2.1 |
| 54217 | 1.87402 | 2.1 |
| 54218 | 1.86703 | 2.1 |
| 54219 | 2.08508 | 2.1 |
| 54220 | 2.08508 | 2.1 |
| 54221 | 2.29001 | 2.1 |
| 54222 | 1.89226 | 2.1 |
| 54223 | 2.08048 | 2.1 |
| 54224 | 2.7278 | 0.05 |
| 54225 | 1.10909 | 0.05 |
| 54226 | 1.50472 | 0.100068 |
| 54227 | 1.10909 | 0.05 |

| | | |
|---|---|---|
| 54228 | 2.69881 | 0.05 |
| 54229 | 2.30319 | 0.0644658 |
| 54230 | 1.23204 | 2.1 |
| 54231 | 1.92264 | 2.1 |
| 54232 | 1.48162 | 2.1 |
| 54233 | 1.48162 | 2.1 |
| 54234 | 1.72994 | 2.1 |
| 54235 | 1.72994 | 2.1 |
| 54236 | 0.864968 | 2.1 |
| 54237 | 0.696952 | 2.1 |
| 54238 | 0.61602 | 2.1 |
| 54239 | 0.479736 | 2.1 |
| 54240 | 0.66444 | 2.1 |
| 54241 | 0.66444 | 2.1 |
| 54242 | 0.680264 | 2.1 |
| 54243 | 0.680264 | 2.1 |
| 54244 | 0.696952 | 2.1 |
| 54245 | 0.479736 | 2.1 |
| 54246 | 0.61602 | 2.1 |
| 54247 | 0.864968 | 2.1 |
| 54248 | 1.89226 | 0.692612 |
| 54249 | 2.43219 | 2.1 |
| 54250 | 1.89226 | 1.31679 |
| 54251 | 2.82019 | 1.44178 |
| 54252 | 1.89226 | 2.07071 |
| 54253 | 2.82019 | 1.68991 |
| 54254 | 1.89226 | 0.80693 |
| 54255 | 2.83701 | 2.1 |
| 54256 | 2.2701 | 1.67354 |
| 54257 | 2.2701 | 1.12852 |
| 54258 | 1.89226 | 1.88814 |
| 54259 | 1.89226 | 1.43625 |
| 54260 | 1.89226 | 1.91081 |
| 54261 | 1.89226 | 0.806867 |
| 54262 | 1.89226 | 0.953225 |
| 54263 | 1.89226 | 1.33783 |
| 54264 | 1.89226 | 1.32839 |
| 54265 | 1.89226 | 1.28043 |
| 54266 | 1.5795 | 1.94493 |
| 54267 | 1.44091 | 1.8668 |
| 54268 | 1.05267 | 2.1 |
| 54269 | 1.05267 | 1.4008 |
| 54270 | 1.98408 | 1.99269 |
| 54271 | 1.98408 | 1.26628 |
| 54272 | 2.20624 | 2.1 |
| 54273 | 1.08831 | 2.1 |
| 54274 | 2.49053 | 2.1 |
| 54275 | 1.42486 | 2.1 |
| 54276 | 3.57883 | 2.1 |
| 54277 | 1.89226 | 2.1 |
| 54278 | 1.89226 | 2.1 |
| 54279 | 1.89226 | 2.1 |
| 54280 | 1.89226 | 2.1 |
| 54281 | 3.78453 | 2.1 |
| 54282 | 0.946132 | 2.1 |
| 54283 | 0.946132 | 2.1 |
| 54284 | 0.853591 | 2.1 |
| 54285 | 0.642207 | 2.1 |

| | | |
|---|---|---|
| 54286 | 0.94613 | 2.1 |
| 54287 | 0.94613 | 2.1 |
| 54288 | 0.642209 | 2.1 |
| 54289 | 0.642209 | 2.1 |
| 54290 | 0.946131 | 2.1 |
| 54291 | 0.853591 | 2.1 |
| 54292 | 0.642207 | 2.1 |
| 54293 | 0.946132 | 2.1 |
| 54294 | 1.0506 | 2.1 |
| 54295 | 1.0506 | 2.1 |
| 54296 | 1.0879 | 2.1 |
| 54297 | 0.960424 | 2.1 |
| 54298 | 0.708687 | 2.1 |
| 54299 | 0.782927 | 2.1 |
| 54300 | 1.40041 | 2.1 |
| 54301 | 1.2518 | 2.1 |
| 54302 | 0.767748 | 2.1 |
| 54303 | 1.31801 | 2.1 |
| 54304 | 1.21412 | 2.1 |
| 54305 | 0.69461 | 2.1 |
| 54306 | 1.89226 | 2.1 |
| 54307 | 1.89226 | 2.1 |
| 54308 | 1.89226 | 2.1 |
| 54309 | 1.89226 | 2.1 |
| 54310 | 1.89226 | 2.1 |
| 54311 | 1.89226 | 2.1 |
| 54312 | 0.922223 | 2.1 |
| 54313 | 0.922223 | 2.1 |
| 54314 | 1.04548 | 2.1 |
| 54315 | 0.768229 | 2.1 |
| 54316 | 0.948441 | 2.1 |
| 54317 | 0.806483 | 2.1 |
| 54318 | 0.85362 | 2.1 |
| 54319 | 0.85362 | 2.1 |
| 54320 | 1.05942 | 2.1 |
| 54321 | 0.922187 | 2.1 |
| 54322 | 0.851554 | 2.1 |
| 54323 | 0.768264 | 2.1 |
| 54324 | 2.16728 | 2.1 |
| 54325 | 0.95455 | 2.1 |
| 54326 | 1.21372 | 2.1 |
| 54327 | 0.98227 | 2.1 |
| 54328 | 2.16728 | 2.1 |
| 54329 | 1.93584 | 2.1 |
| 54330 | 2.08849 | 0.05 |
| 54331 | 1.08732 | 0.05 |
| 54332 | 1.3357 | 0.05 |
| 54333 | 2.37948 | 0.05 |
| 54334 | 3.4668 | 0.05 |
| 54335 | 2.57882 | 0.05 |
| 54336 | 2.37948 | 0.05 |
| 54337 | 2.53055 | 0.05 |
| 54338 | 1.48676 | 0.053074 |
| 54339 | 1.3357 | 0.0611922 |
| 54340 | 1.3357 | 0.0508076 |
| 54341 | 1.89226 | 2.1 |
| 54342 | 1.89226 | 2.1 |
| 54343 | 2.17471 | 2.1 |

| | | |
|---|---|---|
| 54344 | 1.89226 | 2.1 |
| 54345 | 2.60085 | 2.1 |
| 54346 | 2.26306 | 2.1 |
| 54347 | 0.946131 | 2.1 |
| 54348 | 0.946131 | 2.1 |
| 54349 | 0.946131 | 2.1 |
| 54350 | 0.946131 | 2.1 |
| 54351 | 0.94613 | 2.1 |
| 54352 | 0.94613 | 2.1 |
| 54353 | 0.946132 | 2.1 |
| 54354 | 0.946132 | 2.1 |
| 54355 | 0.946131 | 2.1 |
| 54356 | 0.946131 | 2.1 |
| 54357 | 0.946131 | 2.1 |
| 54358 | 0.946131 | 2.1 |
| 54359 | 1.89226 | 2.1 |
| 54360 | 2.17471 | 2.1 |
| 54361 | 2.08196 | 2.1 |
| 54362 | 2.17471 | 2.1 |
| 54363 | 1.79952 | 2.1 |
| 54364 | 1.89226 | 2.1 |
| 54365 | 1.89226 | 2.1 |
| 54366 | 1.89226 | 2.1 |
| 54367 | 1.89226 | 2.1 |
| 54368 | 1.89226 | 2.1 |
| 54369 | 1.89226 | 2.1 |
| 54370 | 1.89226 | 2.1 |
| 54371 | 1.86016 | 2.1 |
| 54372 | 1.89226 | 2.1 |
| 54373 | 1.89226 | 2.1 |
| 54374 | 1.89226 | 2.1 |
| 54375 | 1.84826 | 2.1 |
| 54376 | 1.84826 | 2.1 |
| 54377 | 2.50136 | 2.1 |
| 54378 | 1.99781 | 2.1 |
| 54379 | 1.99729 | 2.1 |
| 54380 | 2.5214 | 2.1 |
| 54381 | 4.49865 | 2.1 |
| 54382 | 0.51724 | 2.1 |
| 54383 | 0.496076 | 2.1 |
| 54384 | 0.998906 | 2.1 |
| 54385 | 0.998645 | 2.1 |
| 54386 | 0.742249 | 2.1 |
| 54387 | 0.742249 | 2.1 |
| 54388 | 0.752734 | 2.1 |
| 54389 | 0.752734 | 2.1 |
| 54390 | 0.998639 | 2.1 |
| 54391 | 0.496073 | 2.1 |
| 54392 | 0.517243 | 2.1 |
| 54393 | 0.998913 | 2.1 |
| 54394 | 0.998653 | 2.1 |
| 54395 | 0.994293 | 2.1 |
| 54396 | 1.26071 | 2.1 |
| 54397 | 1.26504 | 2.1 |
| 54398 | 1.12529 | 2.1 |
| 54399 | 1.12529 | 2.1 |
| 54400 | 1.12971 | 2.1 |
| 54401 | 1.12971 | 2.1 |

| | | |
|---|---|---|
| 54402 | 1.26504 | 2.1 |
| 54403 | 0.994292 | 2.1 |
| 54404 | 0.998651 | 2.1 |
| 54405 | 1.26071 | 2.1 |
| 54406 | 1.89226 | 2.1 |
| 54407 | 1.89226 | 2.1 |
| 54408 | 1.89226 | 2.1 |
| 54409 | 1.89226 | 2.1 |
| 54410 | 3.78452 | 2.1 |
| 54411 | 1.89226 | 2.1 |
| 54412 | 1.89226 | 2.1 |
| 54413 | 1.89226 | 2.1 |
| 54414 | 1.89226 | 2.1 |
| 54415 | 3.78452 | 2.1 |
| 54416 | 0.675872 | 2.1 |
| 54417 | 0.675641 | 2.1 |
| 54418 | 0.943072 | 2.1 |
| 54419 | 0.898317 | 2.1 |
| 54420 | 0.775717 | 2.1 |
| 54421 | 0.775717 | 2.1 |
| 54422 | 0.79824 | 2.1 |
| 54423 | 0.79824 | 2.1 |
| 54424 | 0.675642 | 2.1 |
| 54425 | 0.94307 | 2.1 |
| 54426 | 0.675874 | 2.1 |
| 54427 | 0.898315 | 2.1 |
| 54428 | 0.943071 | 2.1 |
| 54429 | 0.898316 | 2.1 |
| 54430 | 0.998133 | 2.1 |
| 54431 | 0.918903 | 2.1 |
| 54432 | 0.922369 | 2.1 |
| 54433 | 0.922369 | 2.1 |
| 54434 | 0.939606 | 2.1 |
| 54435 | 0.939606 | 2.1 |
| 54436 | 0.943071 | 2.1 |
| 54437 | 0.998133 | 2.1 |
| 54438 | 0.898316 | 2.1 |
| 54439 | 0.918903 | 2.1 |
| 54440 | 0.946131 | 2.1 |
| 54441 | 0.946131 | 2.1 |
| 54442 | 0.946131 | 2.1 |
| 54443 | 0.946131 | 2.1 |
| 54444 | 0.946132 | 2.1 |
| 54445 | 0.946132 | 2.1 |
| 54446 | 0.946129 | 2.1 |
| 54447 | 0.946129 | 2.1 |
| 54448 | 0.94613 | 2.1 |
| 54449 | 0.94613 | 2.1 |
| 54450 | 0.946131 | 2.1 |
| 54451 | 0.946131 | 2.1 |
| 54452 | 1.89226 | 2.1 |
| 54453 | 1.89226 | 2.1 |
| 54454 | 1.89226 | 2.1 |
| 54455 | 1.89226 | 2.1 |
| 54456 | 1.89226 | 2.1 |
| 54457 | 1.89226 | 2.1 |
| 54458 | 1.35129 | 2.1 |
| 54459 | 1.88614 | 2.1 |

| | | |
|---|---|---|
| 54460 | 2.01867 | 2.1 |
| 54461 | 1.38189 | 2.1 |
| 54462 | 3.36995 | 2.1 |
| 54463 | 0.943071 | 2.1 |
| 54464 | 1.00934 | 2.1 |
| 54465 | 0.998134 | 2.1 |
| 54466 | 1.10866 | 2.1 |
| 54467 | 0.992617 | 2.1 |
| 54468 | 0.992617 | 2.1 |
| 54469 | 1.01485 | 2.1 |
| 54470 | 1.01485 | 2.1 |
| 54471 | 1.00934 | 2.1 |
| 54472 | 1.10866 | 2.1 |
| 54473 | 0.943072 | 2.1 |
| 54474 | 0.998134 | 2.1 |
| 54475 | 2.67272 | 2.1 |
| 54476 | 1.61859 | 2.1 |
| 54477 | 1.70303 | 2.1 |
| 54478 | 2.52175 | 2.1 |
| 54479 | 4.5326 | 2.1 |
| 54480 | 2.00954 | 2.1 |
| 54481 | 2.27801 | 2.1 |
| 54482 | 1.87875 | 2.1 |
| 54483 | 1.87875 | 2.1 |
| 54484 | 2.37837 | 2.1 |
| 54485 | 2.37837 | 2.1 |
| 54486 | 2.00955 | 2.1 |
| 54487 | 1.78 | 2.1 |
| 54488 | 2.21813 | 2.1 |
| 54489 | 1.8493 | 2.1 |
| 54490 | 2.37838 | 2.1 |
| 54491 | 2.00955 | 2.1 |
| 54492 | 2.37838 | 2.1 |
| 54493 | 2.27801 | 2.1 |
| 54494 | 2.58523 | 2.1 |
| 54495 | 2.71597 | 2.1 |
| 54496 | 4.9636 | 2.1 |
| 54497 | 2.37838 | 2.1 |
| 54498 | 2.71597 | 2.1 |
| 54499 | 1.72709 | 2.1 |
| 54500 | 2.0815 | 2.1 |
| 54501 | 2.59749 | 2.1 |
| 54502 | 2.78034 | 2.1 |
| 54503 | 0.94613 | 0.72704 |
| 54504 | 0.94613 | 0.828894 |
| 54505 | 0.946128 | 0.517888 |
| 54506 | 0.946128 | 0.329851 |
| 54507 | 0.94613 | 0.309843 |
| 54508 | 0.94613 | 0.525424 |
| 54509 | 0.946128 | 0.828847 |
| 54510 | 0.946128 | 0.154299 |
| 54511 | 0.946129 | 0.616263 |
| 54512 | 0.946129 | 0.566801 |
| 54513 | 0.946129 | 0.644204 |
| 54514 | 0.946129 | 0.670095 |
| 54515 | 1.89226 | 0.97524 |
| 54516 | 1.89226 | 0.872736 |
| 54517 | 1.89226 | 0.957385 |

| | | |
|---|---|---|
| 54518 | 1.89226 | 0.763395 |
| 54519 | 3.78452 | 0.784582 |
| 54520 | 0.946133 | 0.140564 |
| 54521 | 0.946133 | 0.182787 |
| 54522 | 0.946132 | 0.0615507 |
| 54523 | 0.946132 | 0.0885916 |
| 54524 | 0.946132 | 0.0922184 |
| 54525 | 0.946132 | 0.0893994 |
| 54526 | 0.946133 | 0.166308 |
| 54527 | 0.946133 | 0.143216 |
| 54528 | 0.946132 | 0.115748 |
| 54529 | 0.946132 | 0.05 |
| 54530 | 0.946132 | 0.0984681 |
| 54531 | 0.946132 | 0.109841 |
| 54532 | 1.89226 | 0.464153 |
| 54533 | 2.10495 | 0.110906 |
| 54534 | 1.89226 | 0.0835293 |
| 54535 | 2.30932 | 0.0857053 |
| 54536 | 1.89226 | 0.482053 |
| 54537 | 2.30932 | 0.594422 |
| 54538 | 2.30932 | 0.0989946 |
| 54539 | 1.00366 | 0.05 |
| 54540 | 2.10496 | 0.05 |
| 54541 | 1.24728 | 0.05 |
| 54542 | 3.31298 | 0.05 |
| 54543 | 1.87557 | 2.1 |
| 54544 | 1.07167 | 2.1 |
| 54545 | 2.03045 | 2.1 |
| 54546 | 1.25185 | 2.1 |
| 54547 | 3.10212 | 2.1 |
| 54548 | 1.89226 | 2.1 |
| 54549 | 1.89226 | 2.1 |
| 54550 | 1.89226 | 2.1 |
| 54551 | 1.89226 | 2.1 |
| 54552 | 3.78453 | 2.1 |
| 54553 | 0.851541 | 2.1 |
| 54554 | 0.851541 | 2.1 |
| 54555 | 1.12236 | 2.1 |
| 54556 | 1.05783 | 2.1 |
| 54557 | 0.870663 | 2.1 |
| 54558 | 0.951856 | 2.1 |
| 54559 | 0.946132 | 2.1 |
| 54560 | 0.946132 | 2.1 |
| 54561 | 1.04073 | 2.1 |
| 54562 | 1.1296 | 2.1 |
| 54563 | 0.776062 | 2.1 |
| 54564 | 0.851527 | 2.1 |
| 54565 | 0.859395 | 2.1 |
| 54566 | 0.859395 | 2.1 |
| 54567 | 0.870165 | 2.1 |
| 54568 | 0.870165 | 2.1 |
| 54569 | 0.625924 | 2.1 |
| 54570 | 0.714333 | 2.1 |
| 54571 | 1.0821 | 2.1 |
| 54572 | 1.01523 | 2.1 |
| 54573 | 1.0821 | 2.1 |
| 54574 | 0.714333 | 2.1 |
| 54575 | 1.01523 | 2.1 |

| | | |
|---|---|---|
| 54576 | 0.625924 | 2.1 |
| 54577 | 1.89226 | 2.1 |
| 54578 | 1.89226 | 2.1 |
| 54579 | 1.89226 | 2.1 |
| 54580 | 1.89226 | 2.1 |
| 54581 | 3.78453 | 2.1 |
| 54582 | 1.89226 | 2.1 |
| 54583 | 1.89226 | 2.1 |
| 54584 | 2.23164 | 2.1 |
| 54585 | 2.67802 | 2.1 |
| 54586 | 2.03663 | 2.1 |
| 54587 | 1.89226 | 2.1 |
| 54588 | 2.42221 | 2.1 |
| 54589 | 1.61933 | 2.1 |
| 54590 | 2.80896 | 2.1 |
| 54591 | 1.87043 | 2.1 |
| 54592 | 4.29262 | 2.1 |
| 54593 | 1.89226 | 2.1 |
| 54594 | 1.89226 | 2.1 |
| 54595 | 1.89226 | 2.1 |
| 54596 | 1.89226 | 2.1 |
| 54597 | 1.89226 | 2.1 |
| 54598 | 1.89226 | 2.1 |
| 54599 | 2.80892 | 2.1 |
| 54600 | 1.87041 | 2.1 |
| 54601 | 2.67182 | 2.1 |
| 54602 | 2.30891 | 2.1 |
| 54603 | 5.07665 | 2.1 |
| 54604 | 1.89224 | 2.1 |
| 54605 | 1.89224 | 2.1 |
| 54606 | 1.89224 | 2.1 |
| 54607 | 1.89224 | 2.1 |
| 54608 | 3.78448 | 2.1 |
| 54609 | 0.946118 | 2.1 |
| 54610 | 0.946118 | 2.1 |
| 54611 | 0.946118 | 2.1 |
| 54612 | 0.946118 | 2.1 |
| 54613 | 0.946115 | 2.1 |
| 54614 | 0.946115 | 2.1 |
| 54615 | 0.94612 | 2.1 |
| 54616 | 0.94612 | 2.1 |
| 54617 | 0.94613 | 2.1 |
| 54618 | 0.94613 | 2.1 |
| 54619 | 0.946105 | 2.1 |
| 54620 | 0.946105 | 2.1 |
| 54621 | 2.3089 | 2.1 |
| 54622 | 1.02783 | 2.1 |
| 54623 | 1.98533 | 2.1 |
| 54624 | 1.76762 | 2.1 |
| 54625 | 1.24553 | 2.1 |
| 54626 | 1.02783 | 2.1 |
| 54627 | 2.2049 | 0.05 |
| 54628 | 2.34673 | 0.05 |
| 54629 | 1.00763 | 0.05 |
| 54630 | 1.23746 | 0.05 |
| 54631 | 3.44236 | 0.05 |
| 54632 | 1.89226 | 0.05 |
| 54633 | 1.89226 | 0.0817106 |

| | | |
|---|---|---|
| 54634 | 1.89226 | 0.05 |
| 54635 | 1.89226 | 0.0650246 |
| 54636 | 3.78452 | 0.066974 |
| 54637 | 1.89226 | 0.0842666 |
| 54638 | 1.89226 | 0.05 |
| 54639 | 1.89226 | 0.067821 |
| 54640 | 1.89226 | 0.0724985 |
| 54641 | 3.78453 | 0.0756324 |
| 54642 | 0.891618 | 0.05 |
| 54643 | 0.891618 | 0.05 |
| 54644 | 0.902158 | 0.05 |
| 54645 | 0.902158 | 0.05 |
| 54646 | 1.15396 | 0.05 |
| 54647 | 0.620408 | 0.05 |
| 54648 | 1.17337 | 0.05 |
| 54649 | 0.618733 | 0.05 |
| 54650 | 0.620408 | 0.05 |
| 54651 | 0.618733 | 0.05 |
| 54652 | 1.17337 | 0.05 |
| 54653 | 1.15396 | 0.05 |
| 54654 | 0.69978 | 0.05 |
| 54655 | 0.69978 | 0.05 |
| 54656 | 0.946102 | 0.05 |
| 54657 | 0.946102 | 0.05 |
| 54658 | 0.831702 | 0.05 |
| 54659 | 0.946129 | 0.05 |
| 54660 | 0.699752 | 0.05 |
| 54661 | 0.946132 | 0.05 |
| 54662 | 0.831703 | 0.05 |
| 54663 | 0.699751 | 0.05 |
| 54664 | 0.946131 | 0.05 |
| 54665 | 0.946131 | 0.05 |
| 54666 | 0.831675 | 0.05 |
| 54667 | 0.831675 | 0.05 |
| 54668 | 0.946163 | 0.05 |
| 54669 | 0.864397 | 0.05 |
| 54670 | 0.853471 | 0.05 |
| 54671 | 0.962623 | 0.05 |
| 54672 | 0.748555 | 0.05 |
| 54673 | 0.851543 | 0.05 |
| 54674 | 0.864714 | 0.05 |
| 54675 | 0.760411 | 0.05 |
| 54676 | 0.946133 | 0.05 |
| 54677 | 0.946133 | 0.05 |
| 54678 | 1.89226 | 0.423142 |
| 54679 | 1.89226 | 0.817223 |
| 54680 | 1.89226 | 0.710031 |
| 54681 | 1.89226 | 0.655134 |
| 54682 | 3.78452 | 0.71948 |
| 54683 | 1.89226 | 0.758315 |
| 54684 | 1.89226 | 0.646997 |
| 54685 | 1.89226 | 0.635095 |
| 54686 | 1.89226 | 0.903829 |
| 54687 | 3.78452 | 0.685931 |
| 54688 | 0.946132 | 0.857957 |
| 54689 | 0.946132 | 0.951995 |
| 54690 | 0.946132 | 0.725807 |
| 54691 | 0.946132 | 0.781462 |

| | | |
|---|---|---|
| 54692 | 0.946132 | 0.717244 |
| 54693 | 0.946132 | 1.14109 |
| 54694 | 0.946133 | 0.632977 |
| 54695 | 0.946133 | 0.91726 |
| 54696 | 0.946133 | 0.763945 |
| 54697 | 0.946133 | 0.724373 |
| 54698 | 0.946132 | 0.79731 |
| 54699 | 0.946132 | 0.940047 |
| 54700 | 1.89226 | 0.819853 |
| 54701 | 2.39715 | 0.822608 |
| 54702 | 1.89226 | 0.97622 |
| 54703 | 1.89226 | 1.0887 |
| 54704 | 1.89226 | 0.665782 |
| 54705 | 1.89226 | 0.734516 |
| 54706 | 0.94613 | 0.88393 |
| 54707 | 0.94613 | 0.825928 |
| 54708 | 0.94613 | 0.742006 |
| 54709 | 0.94613 | 0.700062 |
| 54710 | 0.946131 | 0.742024 |
| 54711 | 0.946131 | 1.01004 |
| 54712 | 0.946129 | 0.826782 |
| 54713 | 0.946129 | 0.69749 |
| 54714 | 0.94613 | 0.700452 |
| 54715 | 0.94613 | 0.76933 |
| 54716 | 0.94613 | 0.812048 |
| 54717 | 0.94613 | 0.817855 |
| 54718 | 2.54204 | 2.1 |
| 54719 | 2.3148 | 2.1 |
| 54720 | 2.55328 | 2.1 |
| 54721 | 2.39646 | 2.1 |
| 54722 | 4.71126 | 2.1 |
| 54723 | 1.62611 | 2.1 |
| 54724 | 2.3148 | 2.1 |
| 54725 | 2.16986 | 2.1 |
| 54726 | 1.43745 | 2.1 |
| 54727 | 3.75225 | 2.1 |
| 54728 | 1.89226 | 2.1 |
| 54729 | 1.89226 | 2.1 |
| 54730 | 1.89226 | 2.1 |
| 54731 | 1.89226 | 2.1 |
| 54732 | 3.78453 | 2.1 |
| 54733 | 1.89226 | 2.1 |
| 54734 | 1.89226 | 2.1 |
| 54735 | 1.89226 | 2.1 |
| 54736 | 1.89226 | 2.1 |
| 54737 | 3.78453 | 2.1 |
| 54738 | 2.12125 | 2.1 |
| 54739 | 2.39642 | 2.1 |
| 54740 | 2.2941 | 2.1 |
| 54741 | 1.9933 | 2.1 |
| 54742 | 4.38972 | 2.1 |
| 54743 | 1.73784 | 2.1 |
| 54744 | 1.26892 | 2.1 |
| 54745 | 1.26892 | 2.1 |
| 54746 | 1.43743 | 2.1 |
| 54747 | 1.9933 | 2.1 |
| 54748 | 2.1618 | 2.1 |
| 54749 | 0.946118 | 2.1 |

| | | |
|---|---|---|
| 54750 | 0.946118 | 2.1 |
| 54751 | 0.946118 | 2.1 |
| 54752 | 0.946118 | 2.1 |
| 54753 | 0.946119 | 2.1 |
| 54754 | 0.946119 | 2.1 |
| 54755 | 0.946118 | 2.1 |
| 54756 | 0.946118 | 2.1 |
| 54757 | 0.946131 | 2.1 |
| 54758 | 0.946131 | 2.1 |
| 54759 | 0.946106 | 2.1 |
| 54760 | 0.946106 | 2.1 |
| 54761 | 1.89224 | 2.1 |
| 54762 | 1.89224 | 2.1 |
| 54763 | 1.89224 | 2.1 |
| 54764 | 1.89224 | 2.1 |
| 54765 | 3.78448 | 2.1 |
| 54766 | 2.45922 | 2.1 |
| 54767 | 2.15686 | 2.1 |
| 54768 | 2.14259 | 2.1 |
| 54769 | 2.43225 | 2.1 |
| 54770 | 4.58911 | 2.1 |
| 54771 | 1.62027 | 2.1 |
| 54772 | 2.15686 | 2.1 |
| 54773 | 1.62241 | 2.1 |
| 54774 | 1.62241 | 2.1 |
| 54775 | 2.1426 | 2.1 |
| 54776 | 2.1426 | 2.1 |
| 54777 | 2.14259 | 2.1 |
| 54778 | 2.43225 | 2.1 |
| 54779 | 2.40667 | 2.1 |
| 54780 | 2.1281 | 2.1 |
| 54781 | 4.56035 | 2.1 |
| 54782 | 1.0713 | 2.1 |
| 54783 | 1.06405 | 2.1 |
| 54784 | 0.810136 | 2.1 |
| 54785 | 0.808433 | 2.1 |
| 54786 | 0.938508 | 2.1 |
| 54787 | 0.938508 | 2.1 |
| 54788 | 0.941223 | 2.1 |
| 54789 | 0.941223 | 2.1 |
| 54790 | 1.06405 | 2.1 |
| 54791 | 0.808433 | 2.1 |
| 54792 | 0.810135 | 2.1 |
| 54793 | 1.0713 | 2.1 |
| 54794 | 1.89226 | 0.05 |
| 54795 | 1.89226 | 0.05 |
| 54796 | 1.89226 | 0.05 |
| 54797 | 1.89226 | 0.05 |
| 54798 | 3.78453 | 0.05 |
| 54799 | 1.89227 | 0.05 |
| 54800 | 1.89227 | 0.05 |
| 54801 | 1.89227 | 0.05 |
| 54802 | 1.89227 | 0.05 |
| 54803 | 3.78453 | 0.05 |
| 54804 | 1.19 0.05 | |
| 54805 | 1.12469 | 0.05 |
| 54806 | 1.01052 | 0.05 |
| 54807 | 1.08166 | 0.05 |

| | | |
|---|---|---|
| 54808 | 2.20051 | 0.05 |
| 54809 | 0.594999 | 0.05 |
| 54810 | 0.540832 | 0.05 |
| 54811 | 0.514182 | 0.05 |
| 54812 | 0.567857 | 0.05 |
| 54813 | 0.554259 | 0.05 |
| 54814 | 0.554259 | 0.05 |
| 54815 | 0.55443 | 0.05 |
| 54816 | 0.55443 | 0.05 |
| 54817 | 0.540832 | 0.05 |
| 54818 | 0.514182 | 0.05 |
| 54819 | 0.567857 | 0.05 |
| 54820 | 0.594999 | 0.05 |
| 54821 | 1.38674 | 0.05 |
| 54822 | 0.946132 | 0.05 |
| 54823 | 1.36361 | 0.05 |
| 54824 | 0.946132 | 0.05 |
| 54825 | 1.36362 | 0.05 |
| 54826 | 1.36362 | 0.05 |
| 54827 | 0.946131 | 0.05 |
| 54828 | 0.946131 | 0.05 |
| 54829 | 1.38674 | 0.05 |
| 54830 | 1.36361 | 0.05 |
| 54831 | 0.946132 | 0.05 |
| 54832 | 0.946132 | 0.05 |
| 54833 | 1.89226 | 0.05 |
| 54834 | 2.68508 | 0.05 |
| 54835 | 2.77349 | 0.05 |
| 54836 | 1.89226 | 0.05 |
| 54837 | 2.77349 | 0.05 |
| 54838 | 1.89226 | 0.05 |
| 54839 | 2.15315 | 0.05 |
| 54840 | 1.96254 | 0.0858924 |
| 54841 | 1.51377 | 0.0510931 |
| 54842 | 1.89226 | 0.052246 |
| 54843 | 1.47799 | 0.05 |
| 54844 | 1.89226 | 0.05 |
| 54845 | 2.49439 | 0.0509569 |
| 54846 | 1.89226 | 0.355845 |
| 54847 | 2.15315 | 0.211465 |
| 54848 | 1.89226 | 0.162597 |
| 54849 | 2.15315 | 0.130943 |
| 54850 | 1.89226 | 0.0608268 |
| 54851 | 1.89226 | 0.376245 |
| 54852 | 1.89226 | 0.393355 |
| 54853 | 1.89226 | 0.373205 |
| 54854 | 1.89226 | 0.346509 |
| 54855 | 1.89226 | 0.430335 |
| 54856 | 1.89226 | 0.405791 |
| 54857 | 1.89226 | 0.23115 |
| 54858 | 1.89226 | 0.25618 |
| 54859 | 1.89226 | 0.146261 |
| 54860 | 1.89226 | 0.220106 |
| 54861 | 1.89226 | 0.0834189 |
| 54862 | 1.89226 | 0.224768 |
| 54863 | 1.375 | 0.05 |
| 54864 | 2.01865 | 0.0931893 |
| 54865 | 2.12047 | 0.05 |

| | | |
|---|---|---|
| 54866 | 1.47354 | 0.05 |
| 54867 | 2.12047 | 0.162714 |
| 54868 | 1.47354 | 0.0649283 |
| 54869 | 0.687499 | 0.05 |
| 54870 | 1.06023 | 0.05 |
| 54871 | 1.08019 | 0.05 |
| 54872 | 0.604229 | 0.05 |
| 54873 | 1.08018 | 0.05 |
| 54874 | 1.06023 | 0.05 |
| 54875 | 0.687502 | 0.05 |
| 54876 | 0.60423 | 0.05 |
| 54877 | 0.80646 | 0.05 |
| 54878 | 0.806461 | 0.05 |
| 54879 | 0.858001 | 0.05 |
| 54880 | 0.858001 | 0.05 |
| 54881 | 2.26414 | 2.1 |
| 54882 | 1.87342 | 2.1 |
| 54883 | 1.87342 | 2.1 |
| 54884 | 1.79667 | 2.1 |
| 54885 | 2.44045 | 2.1 |
| 54886 | 2.31924 | 2.1 |
| 54887 | 2.26414 | 2.1 |
| 54888 | 2.26616 | 2.1 |
| 54889 | 2.2355 | 2.1 |
| 54890 | 2.21858 | 2.1 |
| 54891 | 4.48474 | 2.1 |
| 54892 | 2.18516 | 2.1 |
| 54893 | 1.87344 | 2.1 |
| 54894 | 2.21859 | 2.1 |
| 54895 | 2.66233 | 2.1 |
| 54896 | 1.85196 | 2.1 |
| 54897 | 2.22236 | 2.1 |
| 54898 | 2.23551 | 2.1 |
| 54899 | 2.21859 | 2.1 |
| 54900 | 2.18516 | 2.1 |
| 54901 | 2.21189 | 2.1 |
| 54902 | 4.43049 | 2.1 |
| 54903 | 1.17171 | 2.1 |
| 54904 | 2.38924 | 2.1 |
| 54905 | 2.0815 | 2.1 |
| 54906 | 2.33348 | 2.1 |
| 54907 | 1.42851 | 2.1 |
| 54908 | 1.5959 | 2.1 |
| 54909 | 1.89226 | 2.1 |
| 54910 | 1.89226 | 2.1 |
| 54911 | 1.89226 | 2.1 |
| 54912 | 1.89226 | 2.1 |
| 54913 | 3.78452 | 2.1 |
| 54914 | 2.75425 | 2.1 |
| 54915 | 1.34366 | 2.1 |
| 54916 | 1.34366 | 2.1 |
| 54917 | 1.56512 | 2.1 |
| 54918 | 2.54186 | 2.1 |
| 54919 | 2.76332 | 2.1 |
| 54920 | 1.07751 | 2.1 |
| 54921 | 2.0815 | 2.1 |
| 54922 | 1.97259 | 2.1 |
| 54923 | 1.76455 | 2.1 |

| | | |
|---|---|---|
| 54924 | 1.30191 | 2.1 |
| 54925 | 1.16461 | 2.1 |
| 54926 | 0.946131 | 2.1 |
| 54927 | 0.946131 | 2.1 |
| 54928 | 0.946129 | 2.1 |
| 54929 | 0.946129 | 2.1 |
| 54930 | 0.946131 | 2.1 |
| 54931 | 0.946131 | 2.1 |
| 54932 | 0.94613 | 2.1 |
| 54933 | 0.94613 | 2.1 |
| 54934 | 0.94613 | 2.1 |
| 54935 | 0.94613 | 2.1 |
| 54936 | 0.946131 | 2.1 |
| 54937 | 0.946131 | 2.1 |
| 54938 | 1.89226 | 0.0840516 |
| 54939 | 1.89226 | 0.05 |
| 54940 | 1.89226 | 0.05 |
| 54941 | 1.89226 | 0.05 |
| 54942 | 3.78453 | 0.05 |
| 54943 | 1.89226 | 0.0631917 |
| 54944 | 1.89226 | 0.05 |
| 54945 | 1.89226 | 0.05 |
| 54946 | 1.89226 | 0.05 |
| 54947 | 3.78453 | 0.05 |
| 54948 | 0.946132 | 0.05 |
| 54949 | 0.946132 | 0.05 |
| 54950 | 1.30327 | 0.05 |
| 54951 | 1.33544 | 0.05 |
| 54952 | 1.33544 | 0.05 |
| 54953 | 1.33544 | 0.05 |
| 54954 | 0.946132 | 0.05 |
| 54955 | 0.946132 | 0.05 |
| 54956 | 0.946132 | 0.05 |
| 54957 | 1.30327 | 0.05 |
| 54958 | 1.33544 | 0.05 |
| 54959 | 0.946132 | 0.05 |
| 54960 | 1.30327 | 0.05 |
| 54961 | 1.33544 | 0.05 |
| 54962 | 0.709426 | 0.05 |
| 54963 | 0.790129 | 0.05 |
| 54964 | 1.05886 | 0.05 |
| 54965 | 1.05886 | 0.05 |
| 54966 | 1.03454 | 0.05 |
| 54967 | 1.03454 | 0.05 |
| 54968 | 1.30327 | 0.05 |
| 54969 | 0.709426 | 0.05 |
| 54970 | 1.33544 | 0.05 |
| 54971 | 0.790128 | 0.05 |
| 54972 | 0.946132 | 0.05 |
| 54973 | 0.946132 | 0.05 |
| 54974 | 0.827866 | 0.05 |
| 54975 | 0.946133 | 0.05 |
| 54976 | 0.946132 | 0.05 |
| 54977 | 0.946132 | 0.05 |
| 54978 | 0.946133 | 0.05 |
| 54979 | 0.946133 | 0.05 |
| 54980 | 0.946132 | 0.05 |
| 54981 | 0.827866 | 0.05 |

| | | |
|---|---|---|
| 54982 | 0.946133 | 0.05 |
| 54983 | 0.946133 | 0.05 |
| 54984 | 1.89227 | 0.05 |
| 54985 | 1.89227 | 0.05 |
| 54986 | 1.89227 | 0.05 |
| 54987 | 1.89227 | 0.05 |
| 54988 | 1.89227 | 0.05 |
| 54989 | 1.89227 | 0.05 |
| 54990 | 0.946133 | 0.05 |
| 54991 | 0.946133 | 0.05 |
| 54992 | 1.21602 | 0.05 |
| 54993 | 1.30327 | 0.05 |
| 54994 | 1.30327 | 0.05 |
| 54995 | 1.30327 | 0.05 |
| 54996 | 0.946132 | 0.05 |
| 54997 | 0.946132 | 0.05 |
| 54998 | 0.946132 | 0.05 |
| 54999 | 1.21602 | 0.05 |
| 55000 | 0.946133 | 0.05 |
| 55001 | 1.30327 | 0.05 |
| 55002 | 1.04227 | 0.05 |
| 55003 | 1.11706 | 0.05 |
| 55004 | 0.968413 | 0.05 |
| 55005 | 0.945482 | 0.05 |
| 55006 | 1.3204 | 0.05 |
| 55007 | 1.23215 | 0.05 |
| 55008 | 0.810791 | 0.05 |
| 55009 | 1.38978 | 0.05 |
| 55010 | 1.07728 | 0.05 |
| 55011 | 1.11704 | 0.05 |
| 55012 | 0.608052 | 0.05 |
| 55013 | 1.89226 | 0.05 |
| 55014 | 1.89226 | 0.05 |
| 55015 | 1.89226 | 0.05 |
| 55016 | 1.89226 | 0.05 |
| 55017 | 3.78453 | 0.05 |
| 55018 | 1.89226 | 0.05 |
| 55019 | 1.89226 | 0.05 |
| 55020 | 1.89226 | 0.05 |
| 55021 | 1.89226 | 0.05 |
| 55022 | 3.78451 | 0.05 |
| 55023 | 2.58476 | 0.05 |
| 55024 | 1.56598 | 0.05 |
| 55025 | 1.55045 | 0.05 |
| 55026 | 1.5124 | 0.05 |
| 55027 | 2.62281 | 0.05 |
| 55028 | 2.58476 | 0.05 |
| 55029 | 2.58477 | 0.05 |
| 55030 | 1.89226 | 0.05 |
| 55031 | 1.89226 | 0.05 |
| 55032 | 1.89226 | 0.05 |
| 55033 | 2.62282 | 0.05 |
| 55034 | 2.62282 | 0.05 |
| 55035 | 1.89224 | 0.05 |
| 55036 | 1.89224 | 0.05 |
| 55037 | 1.89224 | 0.05 |
| 55038 | 1.89224 | 0.05 |
| 55039 | 3.78448 | 0.05 |

| | | |
|---|---|---|
| 55040 | 1.89223 | 0.05 |
| 55041 | 1.89223 | 0.05 |
| 55042 | 1.89223 | 0.05 |
| 55043 | 1.89223 | 0.05 |
| 55044 | 1.89223 | 0.05 |
| 55045 | 1.89223 | 0.05 |
| 55046 | 2.62278 | 0.05 |
| 55047 | 1.45053 | 0.05 |
| 55048 | 1.49678 | 0.05 |
| 55049 | 1.56596 | 0.05 |
| 55050 | 2.5536 | 0.05 |
| 55051 | 2.62278 | 0.05 |
| 55052 | 1.31139 | 0.05 |
| 55053 | 1.2768 | 0.05 |
| 55054 | 0.946118 | 0.05 |
| 55055 | 0.946118 | 0.05 |
| 55056 | 1.2768 | 0.05 |
| 55057 | 1.2768 | 0.05 |
| 55058 | 0.946116 | 0.05 |
| 55059 | 0.946116 | 0.05 |
| 55060 | 1.27682 | 0.05 |
| 55061 | 0.94613 | 0.05 |
| 55062 | 0.946105 | 0.05 |
| 55063 | 1.31138 | 0.05 |
| 55064 | 1.38506 | 1.76046 |
| 55065 | 1.53348 | 2.1 |
| 55066 | 1.17042 | 1.83997 |
| 55067 | 1.17042 | 2.1 |
| 55068 | 1.73669 | 1.91017 |
| 55069 | 1.73669 | 2.1 |
| 55070 | 1.73669 | 1.55171 |
| 55071 | 1.79928 | 0.998959 |
| 55072 | 1.38506 | 1.35187 |
| 55073 | 1.38506 | 2.1 |
| 55074 | 1.79928 | 2.09521 |
| 55075 | 1.43484 | 2.1 |
| 55076 | 1.73669 | 1.30215 |
| 55077 | 1.93568 | 2.1 |
| 55078 | 1.53347 | 1.21762 |
| 55079 | 1.53347 | 2.0946 |
| 55080 | 2.14058 | 1.29876 |
| 55081 | 2.14058 | 2.1 |
| 55082 | 0.89964 | 0.984847 |
| 55083 | 1.10415 | 2.1 |
| 55084 | 0.868346 | 1.45272 |
| 55085 | 1.07029 | 2.1 |
| 55086 | 0.98687 | 1.7084 |
| 55087 | 0.98687 | 2.1 |
| 55088 | 0.985627 | 0.723638 |
| 55089 | 0.985627 | 2.1 |
| 55090 | 1.07029 | 2.07264 |
| 55091 | 1.10415 | 1.65082 |
| 55092 | 0.868344 | 2.1 |
| 55093 | 0.899642 | 2.1 |
| 55094 | 2.40667 | 2.1 |
| 55095 | 2.1281 | 2.1 |
| 55096 | 2.11176 | 2.1 |
| 55097 | 2.38069 | 2.1 |

| | | |
|---|---|---|
| 55098 | 4.50878 | 2.1 |
| 55099 | 2.11176 | 2.1 |
| 55100 | 1.61687 | 2.1 |
| 55101 | 1.60965 | 2.1 |
| 55102 | 1.61687 | 2.1 |
| 55103 | 2.12088 | 2.1 |
| 55104 | 2.1281 | 2.1 |
| 55105 | 2.11176 | 2.1 |
| 55106 | 2.34898 | 2.1 |
| 55107 | 2.34898 | 2.1 |
| 55108 | 2.38069 | 2.1 |
| 55109 | 2.09114 | 2.1 |
| 55110 | 2.12285 | 2.1 |
| 55111 | 1.05588 | 2.1 |
| 55112 | 1.04557 | 2.1 |
| 55113 | 0.804824 | 2.1 |
| 55114 | 0.798269 | 2.1 |
| 55115 | 0.92615 | 2.1 |
| 55116 | 0.92615 | 2.1 |
| 55117 | 0.927999 | 2.1 |
| 55118 | 0.927999 | 2.1 |
| 55119 | 1.04557 | 2.1 |
| 55120 | 0.798269 | 2.1 |
| 55121 | 1.05588 | 2.1 |
| 55122 | 0.804824 | 2.1 |
| 55123 | 1.43548 | 0.140868 |
| 55124 | 1.53873 | 0.338221 |
| 55125 | 1.25063 | 0.196427 |
| 55126 | 1.39662 | 0.200313 |
| 55127 | 2.78937 | 0.0655286 |
| 55128 | 1.25063 | 0.38137 |
| 55129 | 1.39662 | 0.177917 |
| 55130 | 1.10476 | 0.05 |
| 55131 | 1.28326 | 0.144171 |
| 55132 | 2.53389 | 0.140088 |
| 55133 | 1.51147 | 0.05 |
| 55134 | 1.86114 | 0.05 |
| 55135 | 1.86114 | 0.05 |
| 55136 | 2.12926 | 0.05 |
| 55137 | 1.3659 | 0.05 |
| 55138 | 1.63401 | 0.05 |
| 55139 | 1.89226 | 0.05 |
| 55140 | 1.89226 | 0.05 |
| 55141 | 1.89226 | 0.05 |
| 55142 | 1.89226 | 0.05 |
| 55143 | 1.89226 | 0.05 |
| 55144 | 1.89226 | 0.05 |
| 55145 | 1.89226 | 0.05 |
| 55146 | 1.89226 | 0.05 |
| 55147 | 2.46286 | 0.05 |
| 55148 | 1.89226 | 0.05 |
| 55149 | 3.78452 | 0.05 |
| 55150 | 1.89226 | 0.05 |
| 55151 | 1.89226 | 0.05 |
| 55152 | 1.89226 | 0.05 |
| 55153 | 1.89226 | 0.05 |
| 55154 | 1.89226 | 0.05 |
| 55155 | 1.89226 | 0.05 |

| | | |
|---|---|---|
| 55156 | 1.89226 | 0.05 |
| 55157 | 1.89226 | 0.05 |
| 55158 | 2.28992 | 0.05 |
| 55159 | 1.89226 | 0.05 |
| 55160 | 3.78452 | 0.05 |
| 55161 | 1.13537 | 0.05 |
| 55162 | 0.886595 | 0.0532012 |
| 55163 | 0.946131 | 0.05 |
| 55164 | 0.946131 | 0.05 |
| 55165 | 0.94613 | 0.0911775 |
| 55166 | 0.94613 | 0.05 |
| 55167 | 1.25069 | 0.05 |
| 55168 | 0.841855 | 0.112437 |
| 55169 | 1.27436 | 0.05 |
| 55170 | 1.06196 | 0.05 |
| 55171 | 1.09758 | 0.05 |
| 55172 | 0.830306 | 0.05 |
| 55173 | 0.826931 | 0.471975 |
| 55174 | 0.826933 | 0.05 |
| 55175 | 0.764631 | 0.05 |
| 55176 | 0.764631 | 0.05 |
| 55177 | 1.00427 | 0.05 |
| 55178 | 0.788034 | 0.05 |
| 55179 | 0.726852 | 0.05 |
| 55180 | 0.604206 | 0.359844 |
| 55181 | 0.828475 | 0.05 |
| 55182 | 0.604198 | 0.05 |
| 55183 | 0.8332 | 0.05 |
| 55184 | 1.00428 | 0.05 |
| 55185 | 1.89226 | 0.0510683 |
| 55186 | 1.89226 | 0.05 |
| 55187 | 1.89226 | 0.05 |
| 55188 | 1.89226 | 0.05 |
| 55189 | 1.89226 | 0.05 |
| 55190 | 1.89226 | 0.0531484 |
| 55191 | 0.768789 | 2.07562 |
| 55192 | 0.849132 | 0.05 |
| 55193 | 0.666423 | 1.99172 |
| 55194 | 0.688993 | 0.424137 |
| 55195 | 0.868671 | 0.05 |
| 55196 | 0.89117 | 1.64641 |
| 55197 | 0.705632 | 2.1 |
| 55198 | 1.80425 | 2.1 |
| 55199 | 1.73883 | 2.1 |
| 55200 | 1.45159 | 2.1 |
| 55201 | 1.45159 | 2.1 |
| 55202 | 1.37681 | 2.1 |
| 55203 | 1.73883 | 2.1 |
| 55204 | 1.39679 | 0.05 |
| 55205 | 2.08149 | 0.05 |
| 55206 | 1.89226 | 0.05 |
| 55207 | 2.32763 | 0.05 |
| 55208 | 1.89226 | 0.05 |
| 55209 | 2.31274 | 0.05 |
| 55210 | 1.89226 | 0.05 |
| 55211 | 1.89226 | 0.05 |
| 55212 | 1.89226 | 0.05 |
| 55213 | 1.89226 | 0.05 |

| | | |
|---|---|---|
| 55214 | 1.89226 | 0.05 |
| 55215 | 1.89226 | 0.05 |
| 55216 | 1.89226 | 0.05 |
| 55217 | 1.89226 | 0.05 |
| 55218 | 1.89226 | 0.05 |
| 55219 | 1.89226 | 0.05 |
| 55220 | 1.89226 | 0.05 |
| 55221 | 1.89226 | 0.05 |
| 55222 | 1.95181 | 0.05 |
| 55223 | 1.89226 | 0.05 |
| 55224 | 1.89226 | 0.05 |
| 55225 | 1.89226 | 0.05 |
| 55226 | 3.78451 | 0.05 |
| 55227 | 0.946131 | 0.05 |
| 55228 | 0.946131 | 0.05 |
| 55229 | 0.946125 | 0.05 |
| 55230 | 0.946125 | 0.05 |
| 55231 | 0.946127 | 0.05 |
| 55232 | 0.946127 | 0.05 |
| 55233 | 0.946128 | 0.05 |
| 55234 | 0.946128 | 0.05 |
| 55235 | 0.946128 | 0.05 |
| 55236 | 0.946128 | 0.05 |
| 55237 | 0.946128 | 0.05 |
| 55238 | 0.946128 | 0.05 |
| 55239 | 1.89226 | 0.05 |
| 55240 | 1.89226 | 0.05 |
| 55241 | 1.89226 | 0.05 |
| 55242 | 1.89226 | 0.05 |
| 55243 | 1.89226 | 0.05 |
| 55244 | 1.89226 | 0.05 |
| 55245 | 1.89226 | 0.752228 |
| 55246 | 1.89226 | 0.716028 |
| 55247 | 1.89226 | 0.607595 |
| 55248 | 1.89226 | 0.564395 |
| 55249 | 3.78453 | 0.651112 |
| 55250 | 1.89226 | 0.767247 |
| 55251 | 1.89226 | 0.674854 |
| 55252 | 1.89226 | 0.669634 |
| 55253 | 1.89226 | 0.555817 |
| 55254 | 3.78452 | 0.665587 |
| 55255 | 1.21387 | 0.58564 |
| 55256 | 0.8164 | 0.139297 |
| 55257 | 0.946133 | 0.544372 |
| 55258 | 0.946133 | 0.581098 |
| 55259 | 0.946102 | 0.130395 |
| 55260 | 0.946102 | 0.623886 |
| 55261 | 0.816431 | 0.80002 |
| 55262 | 0.816431 | 0.652583 |
| 55263 | 0.816401 | 0.469755 |
| 55264 | 0.94613 | 0.316792 |
| 55265 | 0.946131 | 0.936784 |
| 55266 | 1.21388 | 0.560963 |
| 55267 | 0.946132 | 0.398291 |
| 55268 | 0.946132 | 0.399022 |
| 55269 | 0.946132 | 0.57946 |
| 55270 | 0.946132 | 0.522203 |
| 55271 | 0.946132 | 0.145048 |

| | | |
|---|---|---|
| 55272 | 0.946132 | 0.41772 |
| 55273 | 0.946132 | 0.68727 |
| 55274 | 0.946132 | 0.515385 |
| 55275 | 0.946131 | 0.131206 |
| 55276 | 0.946131 | 0.523485 |
| 55277 | 0.946132 | 0.692442 |
| 55278 | 0.946132 | 0.536092 |
| 55279 | 1.89226 | 0.664195 |
| 55280 | 1.89226 | 0.152287 |
| 55281 | 1.89226 | 0.130277 |
| 55282 | 1.89226 | 0.656389 |
| 55283 | 1.89226 | 0.241694 |
| 55284 | 1.89226 | 0.848501 |
| 55285 | 1.89226 | 0.73703 |
| 55286 | 1.89226 | 0.64839 |
| 55287 | 2.15871 | 0.066575 |
| 55288 | 2.15871 | 0.712227 |
| 55289 | 1.81454 | 0.05 |
| 55290 | 1.54809 | 0.761923 |
| 55291 | 1.89226 | 0.990816 |
| 55292 | 1.89226 | 1.77876 |
| 55293 | 1.89226 | 1.03448 |
| 55294 | 1.89226 | 0.937866 |
| 55295 | 3.78453 | 1.27691 |
| 55296 | 1.89226 | 1.26976 |
| 55297 | 1.89226 | 0.854944 |
| 55298 | 1.89226 | 0.799487 |
| 55299 | 1.89226 | 1.18205 |
| 55300 | 1.89226 | 1.88449 |
| 55301 | 1.89226 | 1.33639 |
| 55302 | 2.29205 | 1.18785 |
| 55303 | 1.42633 | 1.14169 |
| 55304 | 1.69015 | 1.44681 |
| 55305 | 1.69015 | 1.54569 |
| 55306 | 2.10364 | 0.773745 |
| 55307 | 2.05344 | 0.818879 |
| 55308 | 2.29205 | 0.918066 |
| 55309 | 1.58147 | 1.08029 |
| 55310 | 2.16529 | 0.793933 |
| 55311 | 1.56339 | 0.7191 |
| 55312 | 2.29205 | 1.62622 |
| 55313 | 1.69015 | 1.38318 |
| 55314 | 1.89226 | 1.36149 |
| 55315 | 1.89226 | 1.11004 |
| 55316 | 1.89226 | 0.867333 |
| 55317 | 1.89226 | 1.06905 |
| 55318 | 3.78453 | 0.93915 |
| 55319 | 1.89226 | 1.02858 |
| 55320 | 1.89226 | 1.67562 |
| 55321 | 1.89226 | 1.17058 |
| 55322 | 1.89226 | 0.88867 |
| 55323 | 3.78452 | 1.72945 |
| 55324 | 2.29205 | 1.59188 |
| 55325 | 2.85335 | 0.693916 |
| 55326 | 2.55409 | 1.19663 |
| 55327 | 2.04345 | 0.838894 |
| 55328 | 2.76338 | 1.06434 |
| 55329 | 2.29205 | 0.929756 |

| | | |
|---|---|---|
| 55330 | 2.29205 | 1.08616 |
| 55331 | 2.16529 | 1.37482 |
| 55332 | 2.72418 | 1.04332 |
| 55333 | 2.76338 | 0.632025 |
| 55334 | 5.01623 | 1.105 |
| 55335 | 1.89227 | 0.05 |
| 55336 | 1.89227 | 0.05 |
| 55337 | 1.89227 | 0.05 |
| 55338 | 1.89227 | 0.05 |
| 55339 | 3.78453 | 0.05 |
| 55340 | 2.39588 | 0.05 |
| 55341 | 2.14918 | 0.05 |
| 55342 | 2.14918 | 0.05 |
| 55343 | 2.37907 | 0.05 |
| 55344 | 2.20531 | 0.05 |
| 55345 | 2.4352 | 0.05 |
| 55346 | 1.89226 | 0.05 |
| 55347 | 2.64327 | 0.05 |
| 55348 | 2.36175 | 0.05 |
| 55349 | 1.89226 | 0.05 |
| 55350 | 4.25402 | 0.05 |
| 55351 | 1.89226 | 0.05 |
| 55352 | 1.89226 | 0.05 |
| 55353 | 1.89226 | 0.05 |
| 55354 | 1.89226 | 0.05 |
| 55355 | 1.89226 | 0.05 |
| 55356 | 1.89226 | 0.05 |
| 55357 | 1.05395 | 0.05 |
| 55358 | 1.05395 | 0.05 |
| 55359 | 1.03114 | 0.05 |
| 55360 | 1.03114 | 0.05 |
| 55361 | 0.936689 | 0.05 |
| 55362 | 1.07459 | 0.05 |
| 55363 | 1.10265 | 0.05 |
| 55364 | 1.01049 | 0.05 |
| 55365 | 0.93669 | 0.05 |
| 55366 | 1.01049 | 0.05 |
| 55367 | 1.07459 | 0.05 |
| 55368 | 1.10265 | 0.05 |
| 55369 | 1.18088 | 0.05 |
| 55370 | 0.776509 | 0.05 |
| 55371 | 0.946132 | 0.05 |
| 55372 | 0.946132 | 0.05 |
| 55373 | 1.04225 | 0.05 |
| 55374 | 0.830392 | 0.05 |
| 55375 | 0.931352 | 0.05 |
| 55376 | 0.931352 | 0.05 |
| 55377 | 1.13006 | 0.05 |
| 55378 | 1.04074 | 0.05 |
| 55379 | 1.0628 | 0.05 |
| 55380 | 0.851529 | 0.05 |
| 55381 | 2.40032 | 2.1 |
| 55382 | 1.89226 | 2.1 |
| 55383 | 1.89226 | 2.1 |
| 55384 | 1.89226 | 2.1 |
| 55385 | 2.38918 | 2.1 |
| 55386 | 2.38918 | 2.1 |
| 55387 | 1.89226 | 2.1 |

| | | |
|---|---|---|
| 55388 | 1.89226 | 2.1 |
| 55389 | 1.89226 | 2.1 |
| 55390 | 1.89226 | 2.1 |
| 55391 | 1.89226 | 2.1 |
| 55392 | 1.89226 | 2.1 |
| 55393 | 1.6585 | 2.1 |
| 55394 | 2.37364 | 2.1 |
| 55395 | 2.39622 | 2.1 |
| 55396 | 2.39622 | 2.1 |
| 55397 | 1.6488 | 2.1 |
| 55398 | 1.6488 | 2.1 |
| 55399 | 2.44603 | 2.1 |
| 55400 | 1.89224 | 2.1 |
| 55401 | 1.89224 | 2.1 |
| 55402 | 1.89224 | 2.1 |
| 55403 | 2.40029 | 2.1 |
| 55404 | 2.40029 | 2.1 |
| 55405 | 1.89224 | 2.1 |
| 55406 | 1.89224 | 2.1 |
| 55407 | 1.89224 | 2.1 |
| 55408 | 1.89224 | 2.1 |
| 55409 | 1.89224 | 2.1 |
| 55410 | 1.89224 | 2.1 |
| 55411 | 1.01946 | 2.1 |
| 55412 | 1.01946 | 2.1 |
| 55413 | 1.02497 | 2.1 |
| 55414 | 1.02497 | 2.1 |
| 55415 | 1.21519 | 2.1 |
| 55416 | 1.19809 | 2.1 |
| 55417 | 0.835303 | 2.1 |
| 55418 | 0.82924 | 2.1 |
| 55419 | 0.835314 | 2.1 |
| 55420 | 1.2152 | 2.1 |
| 55421 | 0.829228 | 2.1 |
| 55422 | 1.19808 | 2.1 |
| 55423 | 1.91027 | 2.1 |
| 55424 | 1.05809 | 2.1 |
| 55425 | 1.00659 | 2.1 |
| 55426 | 2.06727 | 2.1 |
| 55427 | 3.11819 | 2.1 |
| 55428 | 2.37166 | 2.1 |
| 55429 | 2.06726 | 2.1 |
| 55430 | 1.66994 | 2.1 |
| 55431 | 1.67623 | 2.1 |
| 55432 | 2.40308 | 2.1 |
| 55433 | 2.40308 | 2.1 |
| 55434 | 0.960194 | 2.1 |
| 55435 | 0.939664 | 2.1 |
| 55436 | 0.531131 | 2.1 |
| 55437 | 0.503298 | 2.1 |
| 55438 | 0.729927 | 2.1 |
| 55439 | 0.729927 | 2.1 |
| 55440 | 0.733565 | 2.1 |
| 55441 | 0.733565 | 2.1 |
| 55442 | 0.960195 | 2.1 |
| 55443 | 0.531132 | 2.1 |
| 55444 | 0.503297 | 2.1 |
| 55445 | 0.939663 | 2.1 |

| | | |
|---|---|---|
| 55446 | 1.20154 | 2.1 |
| 55447 | 1.21754 | 2.1 |
| 55448 | 0.960195 | 2.1 |
| 55449 | 0.939665 | 2.1 |
| 55450 | 1.07745 | 2.1 |
| 55451 | 1.07745 | 2.1 |
| 55452 | 1.07976 | 2.1 |
| 55453 | 1.07976 | 2.1 |
| 55454 | 0.960194 | 2.1 |
| 55455 | 1.21754 | 2.1 |
| 55456 | 0.939662 | 2.1 |
| 55457 | 1.20155 | 2.1 |
| 55458 | 1.89224 | 2.1 |
| 55459 | 1.89224 | 2.1 |
| 55460 | 2.08609 | 2.1 |
| 55461 | 2.08609 | 2.1 |
| 55462 | 2.09527 | 2.1 |
| 55463 | 1.90142 | 2.1 |
| 55464 | 1.65815 | 2.1 |
| 55465 | 1.71398 | 2.1 |
| 55466 | 2.2001 | 2.1 |
| 55467 | 2.23791 | 2.1 |
| 55468 | 1.4502 | 2.1 |
| 55469 | 1.4502 | 2.1 |
| 55470 | 0.793587 | 2.1 |
| 55471 | 0.793587 | 2.1 |
| 55472 | 0.792408 | 2.1 |
| 55473 | 0.792408 | 2.1 |
| 55474 | 0.952221 | 2.1 |
| 55475 | 0.858585 | 2.1 |
| 55476 | 0.725101 | 2.1 |
| 55477 | 0.633774 | 2.1 |
| 55478 | 0.633775 | 2.1 |
| 55479 | 0.858585 | 2.1 |
| 55480 | 0.952221 | 2.1 |
| 55481 | 0.725101 | 2.1 |
| 55482 | 1.5735 | 2.1 |
| 55483 | 2.56975 | 2.1 |
| 55484 | 2.63166 | 2.1 |
| 55485 | 2.63166 | 2.1 |
| 55486 | 1.54711 | 2.1 |
| 55487 | 1.54711 | 2.1 |
| 55488 | 0.946131 | 2.1 |
| 55489 | 0.946131 | 2.1 |
| 55490 | 1.28487 | 2.1 |
| 55491 | 1.28487 | 2.1 |
| 55492 | 0.946127 | 2.1 |
| 55493 | 0.946127 | 2.1 |
| 55494 | 1.28488 | 2.1 |
| 55495 | 1.31583 | 2.1 |
| 55496 | 1.31583 | 2.1 |
| 55497 | 0.94613 | 2.1 |
| 55498 | 1.28487 | 2.1 |
| 55499 | 0.946129 | 2.1 |
| 55500 | 1.0596 | 2.1 |
| 55501 | 1.0596 | 2.1 |
| 55502 | 1.06939 | 2.1 |
| 55503 | 1.06939 | 2.1 |

| | | |
|---|---|---|
| 55504 | 1.34225 | 2.1 |
| 55505 | 1.31583 | 2.1 |
| 55506 | 0.793563 | 2.1 |
| 55507 | 0.786748 | 2.1 |
| 55508 | 0.793563 | 2.1 |
| 55509 | 1.34225 | 2.1 |
| 55510 | 0.786748 | 2.1 |
| 55511 | 1.31583 | 2.1 |
| 55512 | 0.946131 | 2.1 |
| 55513 | 0.946131 | 2.1 |
| 55514 | 1.31582 | 2.1 |
| 55515 | 1.31582 | 2.1 |
| 55516 | 0.946127 | 2.1 |
| 55517 | 0.946127 | 2.1 |
| 55518 | 1.34225 | 2.1 |
| 55519 | 1.31583 | 2.1 |
| 55520 | 1.34225 | 2.1 |
| 55521 | 0.946129 | 2.1 |
| 55522 | 1.31583 | 2.1 |
| 55523 | 0.946129 | 2.1 |
| 55524 | 1.89226 | 2.1 |
| 55525 | 1.89226 | 2.1 |
| 55526 | 1.89226 | 2.1 |
| 55527 | 1.89226 | 2.1 |
| 55528 | 1.89226 | 2.1 |
| 55529 | 1.89226 | 2.1 |
| 55530 | 1.89226 | 2.1 |
| 55531 | 1.89226 | 2.1 |
| 55532 | 1.89226 | 2.1 |
| 55533 | 1.89226 | 2.1 |
| 55534 | 1.89226 | 2.1 |
| 55535 | 1.89226 | 2.1 |
| 55536 | 1.26992 | 2.1 |
| 55537 | 1.75003 | 2.1 |
| 55538 | 1.79883 | 2.1 |
| 55539 | 1.26732 | 2.1 |
| 55540 | 3.06875 | 2.1 |
| 55541 | 0.875017 | 2.1 |
| 55542 | 0.899414 | 2.1 |
| 55543 | 0.811147 | 2.1 |
| 55544 | 0.85053 | 2.1 |
| 55545 | 0.851513 | 2.1 |
| 55546 | 0.851513 | 2.1 |
| 55547 | 0.859048 | 2.1 |
| 55548 | 0.859048 | 2.1 |
| 55549 | 0.899413 | 2.1 |
| 55550 | 0.85053 | 2.1 |
| 55551 | 0.811147 | 2.1 |
| 55552 | 0.875016 | 2.1 |
| 55553 | 1.89227 | 2.1 |
| 55554 | 1.89227 | 2.1 |
| 55555 | 1.89227 | 2.1 |
| 55556 | 1.89227 | 2.1 |
| 55557 | 1.89227 | 2.1 |
| 55558 | 1.89227 | 2.1 |
| 55559 | 1.89226 | 2.1 |
| 55560 | 1.89226 | 2.1 |
| 55561 | 1.89226 | 2.1 |

| | | |
|---|---|---|
| 55562 | 1.89226 | 2.1 |
| 55563 | 1.89226 | 2.1 |
| 55564 | 1.89226 | 2.1 |
| 55565 | 0.633661 | 2.1 |
| 55566 | 0.642005 | 2.1 |
| 55567 | 0.93285 | 2.1 |
| 55568 | 0.899413 | 2.1 |
| 55569 | 0.764487 | 2.1 |
| 55570 | 0.764487 | 2.1 |
| 55571 | 0.776931 | 2.1 |
| 55572 | 0.776931 | 2.1 |
| 55573 | 0.642004 | 2.1 |
| 55574 | 0.93285 | 2.1 |
| 55575 | 0.899414 | 2.1 |
| 55576 | 0.633661 | 2.1 |
| 55577 | 0.932851 | 2.1 |
| 55578 | 0.899414 | 2.1 |
| 55579 | 0.905419 | 2.1 |
| 55580 | 0.85053 | 2.1 |
| 55581 | 0.886336 | 2.1 |
| 55582 | 0.886336 | 2.1 |
| 55583 | 0.897045 | 2.1 |
| 55584 | 0.897045 | 2.1 |
| 55585 | 0.93285 | 2.1 |
| 55586 | 0.905419 | 2.1 |
| 55587 | 0.899414 | 2.1 |
| 55588 | 0.850531 | 2.1 |
| 55589 | 2.39322 | 2.1 |
| 55590 | 2.74149 | 0.865019 |
| 55591 | 2.70944 | 2.1 |
| 55592 | 2.6849 | 0.938636 |
| 55593 | 2.41776 | 2.1 |
| 55594 | 2.39322 | 0.715841 |
| 55595 | 2.41776 | 2.1 |
| 55596 | 2.80992 | 2.1 |
| 55597 | 2.77256 | 2.1 |
| 55598 | 2.74149 | 2.1 |
| 55599 | 2.44882 | 2.1 |
| 55600 | 2.41776 | 0.831279 |
| 55601 | 2.80992 | 2.1 |
| 55602 | 1.89226 | 1.81537 |
| 55603 | 2.74149 | 1.89458 |
| 55604 | 2.74149 | 2.1 |
| 55605 | 1.89226 | 1.06994 |
| 55606 | 1.89226 | 1.6432 |
| 55607 | 1.89226 | 0.05 |
| 55608 | 1.89226 | 0.05 |
| 55609 | 1.89226 | 0.05 |
| 55610 | 1.89226 | 0.05 |
| 55611 | 1.89226 | 0.05 |
| 55612 | 1.89226 | 0.05 |
| 55613 | 2.38796 | 0.05 |
| 55614 | 2.0815 | 0.05 |
| 55615 | 2.0815 | 0.05 |
| 55616 | 2.49452 | 0.05 |
| 55617 | 1.55994 | 0.05 |
| 55618 | 2.14721 | 0.05 |
| 55619 | 1.89227 | 0.05 |

| | | |
|---|---|---|
| 55620 | 1.89227 | 0.05 |
| 55621 | 1.89227 | 0.05 |
| 55622 | 1.89227 | 0.05 |
| 55623 | 1.89227 | 0.05 |
| 55624 | 1.89227 | 0.05 |
| 55625 | 1.13387 | 0.05 |
| 55626 | 2.27494 | 0.05 |
| 55627 | 1.13387 | 0.05 |
| 55628 | 2.19489 | 0.05 |
| 55629 | 1.60867 | 0.05 |
| 55630 | 2.6994 | 0.05 |
| 55631 | 1.89226 | 0.05 |
| 55632 | 1.89226 | 0.05 |
| 55633 | 1.89226 | 0.05 |
| 55634 | 1.89226 | 0.05 |
| 55635 | 3.78453 | 0.05 |
| 55636 | 1.42381 | 1.56094 |
| 55637 | 1.10747 | 2.1 |
| 55638 | 1.10747 | 1.12322 |
| 55639 | 1.08911 | 2.1 |
| 55640 | 1.42866 | 0.420279 |
| 55641 | 1.4103 | 2.1 |
| 55642 | 1.89226 | 2.1 |
| 55643 | 1.88181 | 2.1 |
| 55644 | 1.88181 | 2.1 |
| 55645 | 1.76249 | 2.1 |
| 55646 | 2.0815 | 2.1 |
| 55647 | 1.97539 | 2.1 |
| 55648 | 0.946128 | 2.1 |
| 55649 | 0.946128 | 2.1 |
| 55650 | 0.946133 | 1.15531 |
| 55651 | 0.946133 | 0.10731 |
| 55652 | 0.94613 | 0.104869 |
| 55653 | 0.94613 | 2.1 |
| 55654 | 0.946131 | 2.1 |
| 55655 | 0.946131 | 0.18365 |
| 55656 | 0.946131 | 1.8668 |
| 55657 | 0.946131 | 2.03123 |
| 55658 | 0.946131 | 1.97257 |
| 55659 | 0.946131 | 2.03513 |
| 55660 | 1.42866 | 1.22627 |
| 55661 | 1.43213 | 1.0845 |
| 55662 | 1.10748 | 2.1 |
| 55663 | 1.10748 | 2.1 |
| 55664 | 1.13641 | 2.1 |
| 55665 | 1.43213 | 2.1 |
| 55666 | 2.0815 | 2.1 |
| 55667 | 2.10287 | 2.1 |
| 55668 | 2.10287 | 2.1 |
| 55669 | 2.0921 | 2.1 |
| 55670 | 1.89227 | 2.1 |
| 55671 | 1.83938 | 2.1 |
| 55672 | 0.810944 | 0.78244 |
| 55673 | 0.810944 | 0.228545 |
| 55674 | 0.810945 | 0.192656 |
| 55675 | 0.810945 | 2.1 |
| 55676 | 0.810933 | 1.58181 |
| 55677 | 0.810933 | 1.59673 |

| | | |
|---|---|---|
| 55678 | 1.89226 | 2.1 |
| 55679 | 1.3517 | 2.1 |
| 55680 | 1.08132 | 1.50025 |
| 55681 | 1.08132 | 2.1 |
| 55682 | 1.08133 | 1.24556 |
| 55683 | 2.33097 | 2.1 |
| 55684 | 1.53822 | 2.1 |
| 55685 | 1.53822 | 2.1 |
| 55686 | 1.68437 | 2.1 |
| 55687 | 2.17996 | 2.1 |
| 55688 | 2.32612 | 2.1 |
| 55689 | 0.99189 | 2.1 |
| 55690 | 1.18948 | 0.0593055 |
| 55691 | 1.19756 | 0.05 |
| 55692 | 1.19756 | 2.1 |
| 55693 | 0.978851 | 2.1 |
| 55694 | 0.926995 | 2.1 |
| 55695 | 1.26924 | 0.05 |
| 55696 | 1.34642 | 0.05 |
| 55697 | 1.3228 | 0.05 |
| 55698 | 0.990899 | 0.0555228 |
| 55699 | 1.0627 | 0.50825 |
| 55700 | 1.33473 | 0.655344 |
| 55701 | 1.96196 | 2.1 |
| 55702 | 1.39702 | 2.1 |
| 55703 | 1.39702 | 2.1 |
| 55704 | 1.53822 | 2.1 |
| 55705 | 2.17372 | 2.1 |
| 55706 | 2.31011 | 2.1 |
| 55707 | 1.89226 | 0.633909 |
| 55708 | 1.89226 | 0.135502 |
| 55709 | 1.89226 | 0.510583 |
| 55710 | 1.89226 | 0.05 |
| 55711 | 3.78453 | 0.064239 |
| 55712 | 2.38475 | 0.517947 |
| 55713 | 1.73957 | 0.346132 |
| 55714 | 1.88452 | 0.288637 |
| 55715 | 1.58695 | 0.05 |
| 55716 | 3.6241 | 0.05 |
| 55717 | 1.73958 | 0.0853562 |
| 55718 | 1.3126 | 0.582841 |
| 55719 | 1.58695 | 0.05 |
| 55720 | 1.14946 | 0.05 |
| 55721 | 2.88903 | 0.05 |
| 55722 | 1.89226 | 0.100166 |
| 55723 | 1.89226 | 0.658775 |
| 55724 | 1.89226 | 0.05 |
| 55725 | 1.89226 | 0.130685 |
| 55726 | 3.78453 | 0.060279 |
| 55727 | 1.89225 | 0.122939 |
| 55728 | 1.89225 | 0.130284 |
| 55729 | 1.89225 | 0.103354 |
| 55730 | 1.89225 | 0.05 |
| 55731 | 3.7845 | 0.05 |
| 55732 | 1.88451 | 0.0738296 |
| 55733 | 1.58694 | 0.05 |
| 55734 | 1.74649 | 0.118344 |
| 55735 | 1.44604 | 0.05 |

| | | |
|---|---|---|
| 55736 | 3.33055 | 0.05 |
| 55737 | 1.58694 | 0.05 |
| 55738 | 0.995727 | 0.05 |
| 55739 | 0.995727 | 0.480071 |
| 55740 | 1.14945 | 0.416802 |
| 55741 | 1.44604 | 0.05 |
| 55742 | 1.59976 | 0.242693 |
| 55743 | 1.89225 | 0.05 |
| 55744 | 1.89225 | 0.05 |
| 55745 | 1.89225 | 0.561687 |
| 55746 | 1.89225 | 0.280614 |
| 55747 | 1.89225 | 0.352119 |
| 55748 | 1.89225 | 0.169028 |
| 55749 | 2.01197 | 1.90687 |
| 55750 | 2.0961 | 1.70319 |
| 55751 | 2.13283 | 1.18279 |
| 55752 | 2.25399 | 0.932797 |
| 55753 | 4.22894 | 1.38529 |
| 55754 | 2.09611 | 1.09184 |
| 55755 | 2.37014 | 1.19659 |
| 55756 | 2.29031 | 1.59463 |
| 55757 | 2.10084 | 2.1 |
| 55758 | 2.25399 | 0.914173 |
| 55759 | 2.09611 | 2.1 |
| 55760 | 2.13283 | 1.64732 |
| 55761 | 2.25399 | 2.1 |
| 55762 | 2.22044 | 1.54821 |
| 55763 | 2.38975 | 0.96475 |
| 55764 | 4.52258 | 1.44388 |
| 55765 | 2.38976 | 0.77276 |
| 55766 | 2.15466 | 1.31718 |
| 55767 | 2.31988 | 0.114488 |
| 55768 | 2.31988 | 2.08482 |
| 55769 | 2.25399 | 0.576226 |
| 55770 | 2.25399 | 2.1 |
| 55771 | 2.58286 | 0.638655 |
| 55772 | 2.29193 | 0.0773195 |
| 55773 | 2.40107 | 0.610577 |
| 55774 | 2.13815 | 0.05 |
| 55775 | 4.72101 | 0.05 |
| 55776 | 2.29193 | 0.169295 |
| 55777 | 1.90104 | 0.0952951 |
| 55778 | 2.13815 | 0.05 |
| 55779 | 1.77249 | 0.263959 |
| 55780 | 4.03919 | 0.05 |
| 55781 | 2.25197 | 0.119355 |
| 55782 | 2.13815 | 0.05 |
| 55783 | 2.00625 | 0.0680802 |
| 55784 | 2.00625 | 0.05 |
| 55785 | 2.40107 | 0.192938 |
| 55786 | 2.40107 | 0.130098 |
| 55787 | 2.13815 | 0.05 |
| 55788 | 1.77249 | 0.320768 |
| 55789 | 2.00625 | 0.05 |
| 55790 | 1.65815 | 0.05 |
| 55791 | 3.7963 | 0.05 |
| 55792 | 2.43417 | 0.154074 |
| 55793 | 2.06653 | 0.101993 |

| | | |
|---|---|---|
| 55794 | 1.64587 | 0.123225 |
| 55795 | 1.65284 | 0.0718032 |
| 55796 | 2.46898 | 0.203841 |
| 55797 | 2.46898 | 0.10557 |
| 55798 | 2.46895 | 0.0897552 |
| 55799 | 1.89016 | 0.05 |
| 55800 | 1.87861 | 0.05 |
| 55801 | 1.87861 | 0.05 |
| 55802 | 2.47161 | 0.106812 |
| 55803 | 2.47161 | 0.0571778 |
| 55804 | 0.946132 | 0.566708 |
| 55805 | 0.946132 | 0.313001 |
| 55806 | 0.863311 | 0.336595 |
| 55807 | 1.34768 | 0.295626 |
| 55808 | 0.851542 | 0.410077 |
| 55809 | 0.851542 | 0.361006 |
| 55810 | 1.30451 | 0.50073 |
| 55811 | 1.45304 | 0.251341 |
| 55812 | 0.946132 | 0.315064 |
| 55813 | 1.20992 | 0.347691 |
| 55814 | 0.855162 | 0.459397 |
| 55815 | 0.946132 | 0.220821 |
| 55816 | 1.89226 | 0.184485 |
| 55817 | 1.89226 | 0.58425 |
| 55818 | 1.89226 | 0.150628 |
| 55819 | 1.89226 | 0.2299 |
| 55820 | 1.89226 | 0.33237 |
| 55821 | 1.89226 | 0.634782 |
| 55822 | 0.946132 | 0.511819 |
| 55823 | 0.946132 | 0.05 |
| 55824 | 0.946133 | 0.0621365 |
| 55825 | 0.946133 | 0.05 |
| 55826 | 0.946132 | 0.05 |
| 55827 | 0.946132 | 0.05 |
| 55828 | 0.946132 | 0.067455 |
| 55829 | 0.946132 | 0.05 |
| 55830 | 0.946132 | 0.107553 |
| 55831 | 0.946132 | 0.05 |
| 55832 | 0.946133 | 0.05 |
| 55833 | 0.946133 | 0.0528398 |
| 55834 | 2.35574 | 0.804311 |
| 55835 | 1.89226 | 0.576584 |
| 55836 | 1.89226 | 2.1 |
| 55837 | 1.89226 | 0.254305 |
| 55838 | 2.67456 | 0.845309 |
| 55839 | 2.67456 | 0.05 |
| 55840 | 0.583505 | 0.442471 |
| 55841 | 0.761972 | 0.50007 |
| 55842 | 1.17787 | 0.726906 |
| 55843 | 1.33728 | 0.123456 |
| 55844 | 0.965128 | 0.687878 |
| 55845 | 0.965128 | 0.46953 |
| 55846 | 0.974714 | 0.574247 |
| 55847 | 0.974715 | 0.701085 |
| 55848 | 1.17787 | 0.505888 |
| 55849 | 0.583506 | 0.706974 |
| 55850 | 1.33728 | 0.05 |
| 55851 | 0.761974 | 0.527738 |

| | | |
|---|---|---|
| 55852 | 1.89226 | 0.760974 |
| 55853 | 1.89226 | 0.422161 |
| 55854 | 1.89226 | 0.342348 |
| 55855 | 1.89226 | 0.119439 |
| 55856 | 1.89226 | 0.427651 |
| 55857 | 1.89226 | 0.604867 |
| 55858 | 1.89226 | 0.077716 |
| 55859 | 1.89226 | 0.05 |
| 55860 | 1.89226 | 0.1051 |
| 55861 | 1.89226 | 0.480005 |
| 55862 | 3.78453 | 0.0734891 |
| 55863 | 0.946132 | 0.0956457 |
| 55864 | 0.946132 | 0.465972 |
| 55865 | 0.946132 | 0.170146 |
| 55866 | 0.946132 | 0.05 |
| 55867 | 0.946133 | 0.538477 |
| 55868 | 0.946133 | 0.16465 |
| 55869 | 0.946132 | 0.634433 |
| 55870 | 0.946132 | 0.0713715 |
| 55871 | 0.946133 | 0.205073 |
| 55872 | 0.946133 | 0.653352 |
| 55873 | 0.946132 | 0.05 |
| 55874 | 0.946132 | 0.542696 |
| 55875 | 1.01779 | 2.1 |
| 55876 | 1.50685 | 2.1 |
| 55877 | 1.12064 | 2.1 |
| 55878 | 1.65151 | 2.1 |
| 55879 | 2.6275 | 2.1 |
| 55880 | 1.35733 | 2.1 |
| 55881 | 1.53507 | 2.1 |
| 55882 | 1.50683 | 2.1 |
| 55883 | 1.73626 | 2.1 |
| 55884 | 3.09359 | 2.1 |
| 55885 | 1.73626 | 2.1 |
| 55886 | 1.52226 | 2.1 |
| 55887 | 1.75555 | 2.1 |
| 55888 | 1.75555 | 2.1 |
| 55889 | 1.53507 | 2.1 |
| 55890 | 1.53507 | 2.1 |
| 55891 | 1.65151 | 2.1 |
| 55892 | 1.73628 | 2.1 |
| 55893 | 1.69528 | 2.1 |
| 55894 | 1.50685 | 2.1 |
| 55895 | 1.92471 | 2.1 |
| 55896 | 1.73628 | 2.1 |
| 55897 | 1.73629 | 2.1 |
| 55898 | 1.75558 | 2.1 |
| 55899 | 1.9943 | 2.1 |
| 55900 | 1.9943 | 2.1 |
| 55901 | 1.92471 | 2.1 |
| 55902 | 1.73629 | 2.1 |
| 55903 | 2.5601 | 1.16766 |
| 55904 | 1.97645 | 2.1 |
| 55905 | 2.09605 | 1.07121 |
| 55906 | 2.09605 | 2.09347 |
| 55907 | 2.45853 | 1.22406 |
| 55908 | 2.45853 | 2.1 |
| 55909 | 2.33824 | 2.08164 |

| | | |
|---|---|---|
| 55910 | 1.97646 | 1.92738 |
| 55911 | 2.45853 | 1.04031 |
| 55912 | 1.94984 | 2.1 |
| 55913 | 2.33824 | 2.09994 |
| 55914 | 1.82955 | 2.1 |
| 55915 | 1.22926 | 2.1 |
| 55916 | 0.946128 | 2.1 |
| 55917 | 0.946133 | 2.1 |
| 55918 | 1.28005 | 1.5652 |
| 55919 | 1.22927 | 1.63334 |
| 55920 | 1.22926 | 2.1 |
| 55921 | 0.946131 | 2.03929 |
| 55922 | 0.946131 | 1.62626 |
| 55923 | 0.946131 | 1.77987 |
| 55924 | 0.946131 | 1.43288 |
| 55925 | 1.28005 | 1.85486 |
| 55926 | 1.22926 | 1.62306 |
| 55927 | 2.35349 | 0.291908 |
| 55928 | 2.61606 | 0.388531 |
| 55929 | 2.48209 | 0.462293 |
| 55930 | 2.48209 | 0.133612 |
| 55931 | 2.50057 | 0.469197 |
| 55932 | 2.50057 | 0.262662 |
| 55933 | 2.61607 | 0.452109 |
| 55934 | 2.57611 | 0.21922 |
| 55935 | 2.4821 | 0.107467 |
| 55936 | 2.44814 | 0.116557 |
| 55937 | 5.06421 | 0.138844 |
| 55938 | 2.15721 | 0.05 |
| 55939 | 2.48209 | 0.282876 |
| 55940 | 2.31844 | 0.348039 |
| 55941 | 2.31844 | 0.05 |
| 55942 | 2.35349 | 0.353587 |
| 55943 | 2.35349 | 0.05 |
| 55944 | 2.44814 | 0.126334 |
| 55945 | 2.30908 | 0.0670235 |
| 55946 | 2.44814 | 0.0555602 |
| 55947 | 2.31844 | 0.0724752 |
| 55948 | 2.4821 | 0.349169 |
| 55949 | 2.31844 | 0.207034 |
| 55950 | 0.91766 | 0.179323 |
| 55951 | 0.91766 | 0.200116 |
| 55952 | 0.912463 | 0.10151 |
| 55953 | 0.912463 | 0.233219 |
| 55954 | 1.16525 | 0.18415 |
| 55955 | 1.26377 | 0.187943 |
| 55956 | 0.576692 | 0.186994 |
| 55957 | 0.664871 | 0.220655 |
| 55958 | 1.16525 | 0.139874 |
| 55959 | 0.576692 | 0.183035 |
| 55960 | 0.664871 | 0.254409 |
| 55961 | 1.26377 | 0.215979 |
| 55962 | 1.72901 | 2.1 |
| 55963 | 1.62414 | 2.1 |
| 55964 | 1.88971 | 2.1 |
| 55965 | 1.8411 | 1.79947 |
| 55966 | 3.57011 | 2.1 |
| 55967 | 1.89226 | 2.1 |

| | | |
|---|---|---|
| 55968 | 1.89226 | 1.99908 |
| 55969 | 1.89226 | 0.908264 |
| 55970 | 1.89226 | 2.1 |
| 55971 | 3.78452 | 1.01327 |
| 55972 | 1.89226 | 1.07394 |
| 55973 | 1.89226 | 2.1 |
| 55974 | 1.89226 | 2.1 |
| 55975 | 1.89226 | 1.16919 |
| 55976 | 3.78452 | 2.1 |
| 55977 | 1.62414 | 2.1 |
| 55978 | 1.30896 | 2.1 |
| 55979 | 1.8411 | 1.21578 |
| 55980 | 1.52776 | 2.1 |
| 55981 | 3.15006 | 2.07503 |
| 55982 | 1.51448 | 2.1 |
| 55983 | 1.8411 | 2.1 |
| 55984 | 2.02311 | 2.1 |
| 55985 | 1.99309 | 0.886038 |
| 55986 | 3.86421 | 1.98075 |
| 55987 | 1.89226 | 0.925004 |
| 55988 | 1.89226 | 2.1 |
| 55989 | 1.89226 | 2.1 |
| 55990 | 1.89226 | 1.11291 |
| 55991 | 3.78452 | 2.1 |
| 55992 | 1.89226 | 2.1 |
| 55993 | 1.89226 | 1.90911 |
| 55994 | 1.89226 | 1.38492 |
| 55995 | 1.89226 | 2.1 |
| 55996 | 3.78452 | 2.1 |
| 55997 | 1.8411 | 2.1 |
| 55998 | 1.52776 | 2.1 |
| 55999 | 1.99309 | 2.1 |
| 56000 | 1.72526 | 2.1 |
| 56001 | 3.56636 | 2.1 |
| 56002 | 1.89226 | 2.1 |
| 56003 | 1.89226 | 2.01773 |
| 56004 | 1.89226 | 2.1 |
| 56005 | 1.89226 | 2.1 |
| 56006 | 3.78453 | 2.1 |
| 56007 | 1.0424 | 2.1 |
| 56008 | 1.12935 | 2.1 |
| 56009 | 0.929383 | 2.1 |
| 56010 | 0.929383 | 2.1 |
| 56011 | 1.24941 | 2.1 |
| 56012 | 1.12686 | 2.1 |
| 56013 | 0.946132 | 2.1 |
| 56014 | 0.946132 | 2.1 |
| 56015 | 1.12944 | 2.1 |
| 56016 | 0.851554 | 2.1 |
| 56017 | 1.05017 | 2.1 |
| 56018 | 0.929315 | 2.1 |
| 56019 | 0.898029 | 2.1 |
| 56020 | 0.973994 | 2.1 |
| 56021 | 0.946131 | 2.06851 |
| 56022 | 0.946131 | 2.1 |
| 56023 | 1.0221 | 2.1 |
| 56024 | 1.0221 | 2.1 |
| 56025 | 0.898028 | 1.90579 |

| | | |
|---|---|---|
| 56026 | 0.898028 | 2.1 |
| 56027 | 0.973994 | 2.1 |
| 56028 | 0.946131 | 2.1 |
| 56029 | 0.946131 | 2.1 |
| 56030 | 0.898029 | 2.1 |
| 56031 | 1.89226 | 2.1 |
| 56032 | 1.89226 | 2.1 |
| 56033 | 1.89226 | 1.69103 |
| 56034 | 1.89226 | 2.1 |
| 56035 | 1.89226 | 1.88549 |
| 56036 | 1.89226 | 2.1 |
| 56037 | 1.13537 | 2.1 |
| 56038 | 0.879308 | 2.1 |
| 56039 | 0.946131 | 2.04965 |
| 56040 | 0.946131 | 1.95774 |
| 56041 | 1.13537 | 2.0018 |
| 56042 | 0.85187 | 2.1 |
| 56043 | 0.94613 | 2.1 |
| 56044 | 0.94613 | 1.51285 |
| 56045 | 1.05777 | 2.1 |
| 56046 | 1.05777 | 2.1 |
| 56047 | 0.799318 | 2.1 |
| 56048 | 0.776452 | 2.1 |
| 56049 | 0.946116 | 2.1 |
| 56050 | 0.946116 | 2.1 |
| 56051 | 1.09531 | 2.1 |
| 56052 | 1.09531 | 2.1 |
| 56053 | 0.946118 | 2.1 |
| 56054 | 0.946118 | 2.1 |
| 56055 | 1.09135 | 2.1 |
| 56056 | 1.09531 | 2.1 |
| 56057 | 1.09134 | 2.1 |
| 56058 | 0.946105 | 2.1 |
| 56059 | 1.09532 | 2.1 |
| 56060 | 0.946131 | 2.1 |
| 56061 | 0.946129 | 2.1 |
| 56062 | 0.946129 | 2.1 |
| 56063 | 1.09137 | 2.1 |
| 56064 | 1.09137 | 2.1 |
| 56065 | 0.946131 | 2.1 |
| 56066 | 0.946131 | 2.1 |
| 56067 | 1.07713 | 2.1 |
| 56068 | 1.09137 | 2.1 |
| 56069 | 0.946131 | 2.1 |
| 56070 | 1.07713 | 2.1 |
| 56071 | 1.09137 | 2.1 |
| 56072 | 0.946131 | 2.1 |
| 56073 | 0.938916 | 2.1 |
| 56074 | 0.938916 | 2.1 |
| 56075 | 0.936995 | 2.1 |
| 56076 | 1.00447 | 2.1 |
| 56077 | 0.979819 | 2.1 |
| 56078 | 1.16821 | 2.1 |
| 56079 | 1.01263 | 2.1 |
| 56080 | 0.719167 | 2.1 |
| 56081 | 1.04536 | 2.1 |
| 56082 | 1.18901 | 2.1 |
| 56083 | 0.758057 | 2.1 |

| | | |
|---|---|---|
| 56084 | 0.924111 | 2.1 |
| 56085 | 1.00532 | 2.1 |
| 56086 | 1.00532 | 2.1 |
| 56087 | 0.9925 | 2.1 |
| 56088 | 0.9925 | 2.1 |
| 56089 | 1.29322 | 2.1 |
| 56090 | 1.01985 | 2.1 |
| 56091 | 1.00365 | 2.1 |
| 56092 | 0.704601 | 2.1 |
| 56093 | 1.00365 | 2.1 |
| 56094 | 1.29322 | 2.1 |
| 56095 | 1.01985 | 2.1 |
| 56096 | 0.704602 | 2.1 |
| 56097 | 2.20011 | 2.1 |
| 56098 | 1.401 2.1 | |
| 56099 | 1.401 2.1 | |
| 56100 | 1.52008 | 2.1 |
| 56101 | 2.11084 | 2.1 |
| 56102 | 2.22991 | 2.1 |
| 56103 | 0.854068 | 2.1 |
| 56104 | 0.854068 | 2.1 |
| 56105 | 0.851357 | 2.1 |
| 56106 | 0.851357 | 2.1 |
| 56107 | 0.644681 | 2.1 |
| 56108 | 0.700493 | 2.1 |
| 56109 | 1.0554 | 2.1 |
| 56110 | 1.00493 | 2.1 |
| 56111 | 1.00495 | 2.1 |
| 56112 | 0.64469 | 2.1 |
| 56113 | 0.700483 | 2.1 |
| 56114 | 1.05539 | 2.1 |
| 56115 | 2.46665 | 0.05 |
| 56116 | 2.22713 | 0.05 |
| 56117 | 1.85877 | 0.05 |
| 56118 | 1.8967 | 0.05 |
| 56119 | 1.56002 | 0.05 |
| 56120 | 2.22713 | 0.05 |
| 56121 | 1.89225 | 2.1 |
| 56122 | 1.89225 | 2.1 |
| 56123 | 1.89225 | 2.1 |
| 56124 | 1.89225 | 2.1 |
| 56125 | 1.89225 | 2.1 |
| 56126 | 1.89225 | 2.1 |
| 56127 | 1.89225 | 2.1 |
| 56128 | 1.89225 | 2.1 |
| 56129 | 1.89225 | 2.1 |
| 56130 | 1.89225 | 2.1 |
| 56131 | 1.89225 | 2.1 |
| 56132 | 1.89225 | 2.1 |
| 56133 | 1.284 2.1 | |
| 56134 | 1.86569 | 2.1 |
| 56135 | 1.95398 | 2.1 |
| 56136 | 1.31091 | 2.1 |
| 56137 | 3.23798 | 2.1 |
| 56138 | 0.932844 | 2.1 |
| 56139 | 0.976992 | 2.1 |
| 56140 | 0.960128 | 2.1 |
| 56141 | 0.905413 | 2.1 |

| | | |
|---|---|---|
| 56142 | 0.938531 | 2.1 |
| 56143 | 0.938531 | 2.1 |
| 56144 | 0.943874 | 2.1 |
| 56145 | 0.943874 | 2.1 |
| 56146 | 0.976985 | 2.1 |
| 56147 | 0.960122 | 2.1 |
| 56148 | 0.93285 | 2.1 |
| 56149 | 0.905419 | 2.1 |
| 56150 | 1.89227 | 2.1 |
| 56151 | 1.89227 | 2.1 |
| 56152 | 1.89227 | 2.1 |
| 56153 | 1.89227 | 2.1 |
| 56154 | 3.78453 | 2.1 |
| 56155 | 1.89226 | 2.1 |
| 56156 | 1.89226 | 2.1 |
| 56157 | 1.89226 | 2.1 |
| 56158 | 1.89226 | 2.1 |
| 56159 | 1.89226 | 2.1 |
| 56160 | 1.89226 | 2.1 |
| 56161 | 0.65546 | 2.1 |
| 56162 | 0.665451 | 2.1 |
| 56163 | 1.00694 | 2.1 |
| 56164 | 0.976998 | 2.1 |
| 56165 | 0.816261 | 2.1 |
| 56166 | 0.816261 | 2.1 |
| 56167 | 0.826189 | 2.1 |
| 56168 | 0.826189 | 2.1 |
| 56169 | 1.00694 | 2.1 |
| 56170 | 0.665451 | 2.1 |
| 56171 | 0.976998 | 2.1 |
| 56172 | 0.65546 | 2.1 |
| 56173 | 2.01555 | 2.1 |
| 56174 | 2.01388 | 2.1 |
| 56175 | 1.92027 | 2.1 |
| 56176 | 1.92027 | 2.1 |
| 56177 | 2.01388 | 2.1 |
| 56178 | 1.954 | 2.1 |
| 56179 | 2.75466 | 2.1 |
| 56180 | 1.89226 | 2.1 |
| 56181 | 1.89226 | 2.1 |
| 56182 | 2.54931 | 2.1 |
| 56183 | 4.44158 | 2.1 |
| 56184 | 2.47917 | 2.1 |
| 56185 | 1.92772 | 2.1 |
| 56186 | 1.80682 | 2.1 |
| 56187 | 2.54931 | 2.1 |
| 56188 | 4.47702 | 2.1 |
| 56189 | 0.946132 | 2.1 |
| 56190 | 0.946132 | 2.1 |
| 56191 | 1.17602 | 2.1 |
| 56192 | 1.27466 | 2.1 |
| 56193 | 0.946132 | 2.1 |
| 56194 | 0.946132 | 2.1 |
| 56195 | 1.17602 | 2.1 |
| 56196 | 1.17602 | 2.1 |
| 56197 | 0.946132 | 2.1 |
| 56198 | 1.17602 | 2.1 |
| 56199 | 0.946131 | 2.1 |

| | | |
|---|---|---|
| 56200 | 1.27466 | 2.1 |
| 56201 | 2.35204 | 2.1 |
| 56202 | 2.54931 | 2.1 |
| 56203 | 2.40783 | 2.1 |
| 56204 | 1.50108 | 2.1 |
| 56205 | 1.64256 | 2.1 |
| 56206 | 1.64256 | 2.1 |
| 56207 | 1.89226 | 2.1 |
| 56208 | 1.89226 | 0.658563 |
| 56209 | 2.31012 | 2.1 |
| 56210 | 2.17534 | 2.1 |
| 56211 | 4.0676 | 2.1 |
| 56212 | 2.17533 | 2.1 |
| 56213 | 1.89226 | 0.66246 |
| 56214 | 2.17533 | 1.18432 |
| 56215 | 1.89226 | 0.929437 |
| 56216 | 1.66635 | 2.1 |
| 56217 | 1.38328 | 2.05887 |
| 56218 | 1.92027 | 2.1 |
| 56219 | 2.17597 | 2.1 |
| 56220 | 2.14619 | 2.1 |
| 56221 | 2.15388 | 2.1 |
| 56222 | 1.91087 | 2.1 |
| 56223 | 1.92027 | 1.33317 |
| 56224 | 1.08622 | 1.04781 |
| 56225 | 1.08622 | 2.1 |
| 56226 | 0.971184 | 2.1 |
| 56227 | 0.972761 | 2.1 |
| 56228 | 1.14845 | 2.1 |
| 56229 | 1.16321 | 2.1 |
| 56230 | 0.894769 | 2.1 |
| 56231 | 0.894967 | 2.1 |
| 56232 | 0.894775 | 2.1 |
| 56233 | 1.28563 | 2.1 |
| 56234 | 1.14844 | 2.1 |
| 56235 | 1.0167 | 2.1 |
| 56236 | 1.89226 | 1.01423 |
| 56237 | 1.89226 | 2.1 |
| 56238 | 1.89226 | 2.1 |
| 56239 | 1.89226 | 1.14285 |
| 56240 | 1.89226 | 2.1 |
| 56241 | 1.89226 | 1.28218 |
| 56242 | 0.937933 | 2.1 |
| 56243 | 0.937933 | 2.1 |
| 56244 | 1.26192 | 2.1 |
| 56245 | 1.26192 | 2.1 |
| 56246 | 0.937903 | 2.1 |
| 56247 | 1.25372 | 2.1 |
| 56248 | 0.946132 | 2.1 |
| 56249 | 0.946132 | 2.1 |
| 56250 | 0.946133 | 2.1 |
| 56251 | 1.25372 | 2.1 |
| 56252 | 0.946132 | 2.1 |
| 56253 | 0.937902 | 2.1 |
| 56254 | 0.946132 | 1.06795 |
| 56255 | 0.946132 | 0.869219 |
| 56256 | 0.946128 | 2.1 |
| 56257 | 0.946128 | 2.1 |

| | | |
|---|---|---|
| 56258 | 0.94613 | 2.1 |
| 56259 | 0.94613 | 1.04367 |
| 56260 | 0.94613 | 0.457979 |
| 56261 | 0.94613 | 2.1 |
| 56262 | 0.946131 | 1.52694 |
| 56263 | 0.946131 | 1.8708 |
| 56264 | 0.94613 | 1.66448 |
| 56265 | 0.94613 | 1.61781 |
| 56266 | 0.946132 | 1.27614 |
| 56267 | 0.946132 | 1.04035 |
| 56268 | 1.2778 | 2.1 |
| 56269 | 1.00191 | 2.1 |
| 56270 | 1.00191 | 1.20157 |
| 56271 | 1.00191 | 2.1 |
| 56272 | 0.94613 | 2.1 |
| 56273 | 0.94613 | 1.19692 |
| 56274 | 0.946131 | 2.1 |
| 56275 | 1.2778 | 2.1 |
| 56276 | 1.00191 | 2.1 |
| 56277 | 0.94613 | 2.05479 |
| 56278 | 1.50847 | 0.05 |
| 56279 | 2.0815 | 0.05 |
| 56280 | 1.56806 | 0.05 |
| 56281 | 2.0815 | 0.05 |
| 56282 | 1.52043 | 0.05 |
| 56283 | 1.9224 | 0.05 |
| 56284 | 1.06882 | 0.05 |
| 56285 | 0.69567 | 0.05 |
| 56286 | 0.773112 | 0.05 |
| 56287 | 1.09473 | 0.05 |
| 56288 | 0.8941 | 0.05 |
| 56289 | 0.8941 | 0.05 |
| 56290 | 0.937064 | 0.05 |
| 56291 | 0.981131 | 0.05 |
| 56292 | 0.763762 | 0.05 |
| 56293 | 0.722535 | 0.05 |
| 56294 | 1.2153 | 0.05 |
| 56295 | 1.07279 | 0.05 |
| 56296 | 1.89226 | 0.05 |
| 56297 | 1.89226 | 0.05 |
| 56298 | 1.89226 | 0.05 |
| 56299 | 1.89226 | 0.05 |
| 56300 | 1.89226 | 0.05 |
| 56301 | 1.89226 | 0.05 |
| 56302 | 1.89226 | 0.05 |
| 56303 | 1.89226 | 0.05 |
| 56304 | 1.89226 | 0.05 |
| 56305 | 1.89226 | 0.05 |
| 56306 | 3.78453 | 0.05 |
| 56307 | 2.51518 | 0.05 |
| 56308 | 1.89226 | 0.05 |
| 56309 | 1.89226 | 0.05 |
| 56310 | 1.89226 | 0.05 |
| 56311 | 2.37584 | 0.05 |
| 56312 | 2.37584 | 0.05 |
| 56313 | 1.25759 | 0.05 |
| 56314 | 0.946131 | 0.05 |
| 56315 | 0.946131 | 0.05 |

| | | |
|---|---|---|
| 56316 | 1.24489 | 0.05 |
| 56317 | 0.946131 | 0.05 |
| 56318 | 0.946131 | 0.05 |
| 56319 | 1.25759 | 0.05 |
| 56320 | 1.25759 | 0.05 |
| 56321 | 1.25759 | 0.05 |
| 56322 | 1.24489 | 0.05 |
| 56323 | 0.946131 | 0.05 |
| 56324 | 0.946131 | 0.05 |
| 56325 | 1.08028 | 0.46364 |
| 56326 | 1.09362 | 0.651906 |
| 56327 | 0.596616 | 0.292377 |
| 56328 | 0.606432 | 0.807038 |
| 56329 | 0.844265 | 0.823999 |
| 56330 | 0.844265 | 0.05 |
| 56331 | 0.855786 | 1.03883 |
| 56332 | 0.855786 | 0.161868 |
| 56333 | 1.08028 | 0.837599 |
| 56334 | 0.606433 | 0.245902 |
| 56335 | 0.596616 | 0.218792 |
| 56336 | 1.09362 | 0.368033 |
| 56337 | 2.837 | 0.854428 |
| 56338 | 2.16055 | 0.0698929 |
| 56339 | 2.12741 | 0.745211 |
| 56340 | 2.79312 | 0.69711 |
| 56341 | 4.95367 | 0.726419 |
| 56342 | 2.12741 | 0.797732 |
| 56343 | 1.21286 | 0.0786158 |
| 56344 | 1.20859 | 0.893376 |
| 56345 | 1.21286 | 0.95293 |
| 56346 | 2.15628 | 0.651473 |
| 56347 | 2.16056 | 0.0880016 |
| 56348 | 1.89226 | 2.1 |
| 56349 | 1.89226 | 2.1 |
| 56350 | 1.89226 | 2.1 |
| 56351 | 1.89226 | 2.1 |
| 56352 | 3.78453 | 2.1 |
| 56353 | 0.946102 | 2.1 |
| 56354 | 0.946102 | 2.1 |
| 56355 | 0.850039 | 2.1 |
| 56356 | 0.850039 | 2.1 |
| 56357 | 0.914891 | 2.1 |
| 56358 | 0.85001 | 2.1 |
| 56359 | 0.946131 | 2.1 |
| 56360 | 0.946131 | 2.1 |
| 56361 | 0.94613 | 2.1 |
| 56362 | 0.91489 | 2.1 |
| 56363 | 0.850011 | 2.1 |
| 56364 | 0.946131 | 2.1 |
| 56365 | 0.71107 | 2.1 |
| 56366 | 0.784813 | 2.1 |
| 56367 | 1.28453 | 2.1 |
| 56368 | 1.20328 | 2.1 |
| 56369 | 0.992128 | 2.1 |
| 56370 | 0.992128 | 2.1 |
| 56371 | 0.995965 | 2.1 |
| 56372 | 0.995965 | 2.1 |
| 56373 | 0.784813 | 2.1 |

| | | |
|---|---|---|
| 56374 | 1.28453 | 2.1 |
| 56375 | 0.711071 | 2.1 |
| 56376 | 1.20328 | 2.1 |
| 56377 | 1.89226 | 2.1 |
| 56378 | 1.89226 | 2.1 |
| 56379 | 1.89226 | 2.1 |
| 56380 | 1.89226 | 2.1 |
| 56381 | 3.78453 | 2.1 |
| 56382 | 0.946132 | 2.1 |
| 56383 | 0.946132 | 2.1 |
| 56384 | 0.914889 | 2.1 |
| 56385 | 0.914889 | 2.1 |
| 56386 | 0.985255 | 2.1 |
| 56387 | 0.914891 | 2.1 |
| 56388 | 0.946131 | 2.1 |
| 56389 | 0.946131 | 2.1 |
| 56390 | 0.946131 | 2.1 |
| 56391 | 0.985255 | 2.1 |
| 56392 | 0.94613 | 2.1 |
| 56393 | 0.91489 | 2.1 |
| 56394 | 0.784813 | 2.1 |
| 56395 | 0.871551 | 2.1 |
| 56396 | 1.37791 | 2.1 |
| 56397 | 1.28453 | 2.1 |
| 56398 | 1.07644 | 2.1 |
| 56399 | 1.07644 | 2.1 |
| 56400 | 1.07964 | 2.1 |
| 56401 | 1.07964 | 2.1 |
| 56402 | 0.871551 | 2.1 |
| 56403 | 1.37791 | 2.1 |
| 56404 | 0.784813 | 2.1 |
| 56405 | 1.28453 | 2.1 |
| 56406 | 1.73075 | 1.54394 |
| 56407 | 2.10582 | 0.184528 |
| 56408 | 1.89319 | 0.127792 |
| 56409 | 1.38766 | 1.96779 |
| 56410 | 3.27777 | 0.219147 |
| 56411 | 1.08715 | 1.59237 |
| 56412 | 1.54185 | 1.33113 |
| 56413 | 1.73074 | 0.778797 |
| 56414 | 1.73074 | 2.07253 |
| 56415 | 1.18948 | 0.20849 |
| 56416 | 1.18948 | 1.36272 |
| 56417 | 1.89319 | 0.102063 |
| 56418 | 1.41689 | 1.72461 |
| 56419 | 1.11619 | 0.234602 |
| 56420 | 1.38766 | 1.15873 |
| 56421 | 1.6052 | 0.146108 |
| 56422 | 1.89319 | 1.17992 |
| 56423 | 0.647894 | 1.41095 |
| 56424 | 0.594738 | 1.64818 |
| 56425 | 0.708445 | 1.28358 |
| 56426 | 0.770926 | 0.669649 |
| 56427 | 0.738326 | 1.26451 |
| 56428 | 0.738326 | 2.03333 |
| 56429 | 0.680494 | 1.24217 |
| 56430 | 0.680494 | 0.136081 |
| 56431 | 0.708443 | 0.448383 |

| | | |
|---|---|---|
| 56432 | 0.647895 | 0.517774 |
| 56433 | 0.770925 | 0.532873 |
| 56434 | 0.59474 | 0.0765506 |
| 56435 | 1.89111 | 0.533026 |
| 56436 | 1.06994 | 2.1 |
| 56437 | 2.06689 | 2.1 |
| 56438 | 1.37063 | 2.1 |
| 56439 | 3.13683 | 2.1 |
| 56440 | 2.06689 | 2.1 |
| 56441 | 1.39295 | 2.1 |
| 56442 | 1.56818 | 2.1 |
| 56443 | 1.37063 | 2.1 |
| 56444 | 2.26444 | 1.84312 |
| 56445 | 2.06689 | 2.1 |
| 56446 | 1.49808 | 2.1 |
| 56447 | 1.50201 | 2.1 |
| 56448 | 2.57784 | 2.1 |
| 56449 | 2.58615 | 2.1 |
| 56450 | 4.08424 | 2.1 |
| 56451 | 0.946133 | 2.1 |
| 56452 | 0.946133 | 2.1 |
| 56453 | 0.946132 | 2.1 |
| 56454 | 0.946132 | 2.1 |
| 56455 | 0.946132 | 2.1 |
| 56456 | 0.946132 | 2.1 |
| 56457 | 0.946132 | 2.1 |
| 56458 | 0.946132 | 2.1 |
| 56459 | 0.946133 | 2.1 |
| 56460 | 0.946133 | 2.1 |
| 56461 | 0.946132 | 2.1 |
| 56462 | 0.946132 | 2.1 |
| 56463 | 1.89226 | 2.1 |
| 56464 | 1.89226 | 2.1 |
| 56465 | 1.89226 | 2.1 |
| 56466 | 1.89226 | 2.1 |
| 56467 | 3.78452 | 2.1 |
| 56468 | 0.977201 | 2.1 |
| 56469 | 0.977201 | 2.1 |
| 56470 | 0.980489 | 2.1 |
| 56471 | 0.980489 | 2.1 |
| 56472 | 0.657973 | 2.1 |
| 56473 | 1.20669 | 2.1 |
| 56474 | 0.751002 | 2.1 |
| 56475 | 1.29307 | 2.1 |
| 56476 | 1.29308 | 2.1 |
| 56477 | 1.20669 | 2.1 |
| 56478 | 0.751003 | 2.1 |
| 56479 | 0.657972 | 2.1 |
| 56480 | 0.946131 | 2.1 |
| 56481 | 0.946131 | 2.1 |
| 56482 | 1.29308 | 2.1 |
| 56483 | 1.29308 | 2.1 |
| 56484 | 0.946132 | 2.1 |
| 56485 | 1.29308 | 2.1 |
| 56486 | 0.946132 | 2.1 |
| 56487 | 1.28892 | 2.1 |
| 56488 | 0.946132 | 2.1 |
| 56489 | 0.946132 | 2.1 |

| | | |
|---|---|---|
| 56490 | 1.28892 | 2.1 |
| 56491 | 1.29308 | 2.1 |
| 56492 | 0.946132 | 2.1 |
| 56493 | 0.946132 | 1.40987 |
| 56494 | 0.946131 | 2.1 |
| 56495 | 0.946131 | 2.1 |
| 56496 | 0.946132 | 2.1 |
| 56497 | 0.946132 | 2.1 |
| 56498 | 0.946132 | 1.94662 |
| 56499 | 0.946132 | 1.3965 |
| 56500 | 0.946132 | 1.53884 |
| 56501 | 0.946132 | 2.1 |
| 56502 | 0.946132 | 2.1 |
| 56503 | 0.946132 | 2.1 |
| 56504 | 1.89226 | 2.1 |
| 56505 | 1.89226 | 2.1 |
| 56506 | 1.89226 | 2.1 |
| 56507 | 1.89226 | 2.05775 |
| 56508 | 1.89226 | 2.1 |
| 56509 | 1.89226 | 2.01168 |
| 56510 | 0.94613 | 2.1 |
| 56511 | 0.94613 | 2.1 |
| 56512 | 1.20669 | 2.1 |
| 56513 | 1.20669 | 2.1 |
| 56514 | 1.20669 | 2.1 |
| 56515 | 0.946132 | 2.1 |
| 56516 | 0.946129 | 2.1 |
| 56517 | 1.29307 | 2.1 |
| 56518 | 0.946131 | 2.1 |
| 56519 | 0.946131 | 2.1 |
| 56520 | 1.29308 | 2.1 |
| 56521 | 1.20669 | 2.1 |
| 56522 | 1.89226 | 0.05 |
| 56523 | 1.89226 | 0.05 |
| 56524 | 1.89226 | 0.05 |
| 56525 | 1.89226 | 0.05 |
| 56526 | 3.78453 | 0.05 |
| 56527 | 0.946132 | 0.05 |
| 56528 | 0.946132 | 0.05 |
| 56529 | 0.946126 | 0.05 |
| 56530 | 0.946126 | 0.05 |
| 56531 | 0.946129 | 0.05 |
| 56532 | 0.946129 | 0.05 |
| 56533 | 0.946129 | 0.05 |
| 56534 | 0.946129 | 0.05 |
| 56535 | 0.946128 | 0.05 |
| 56536 | 0.946128 | 0.05 |
| 56537 | 0.946129 | 0.05 |
| 56538 | 0.946129 | 0.05 |
| 56539 | 0.725277 | 0.05 |
| 56540 | 0.588155 | 0.05 |
| 56541 | 1.19403 | 0.05 |
| 56542 | 1.27681 | 0.05 |
| 56543 | 0.918903 | 0.05 |
| 56544 | 0.918903 | 0.05 |
| 56545 | 0.946068 | 0.05 |
| 56546 | 0.946068 | 0.05 |
| 56547 | 1.19404 | 0.05 |

| | | |
|---|---|---|
| 56548 | 0.588153 | 0.05 |
| 56549 | 0.725275 | 0.05 |
| 56550 | 1.27682 | 0.05 |
| 56551 | 1.19404 | 0.05 |
| 56552 | 1.27682 | 0.05 |
| 56553 | 0.946131 | 0.05 |
| 56554 | 0.946131 | 0.05 |
| 56555 | 1.19404 | 0.05 |
| 56556 | 1.19404 | 0.05 |
| 56557 | 0.946129 | 0.05 |
| 56558 | 0.946129 | 0.05 |
| 56559 | 1.19404 | 0.05 |
| 56560 | 0.94613 | 0.05 |
| 56561 | 1.27682 | 0.05 |
| 56562 | 0.946131 | 0.05 |
| 56563 | 1.89226 | 0.05 |
| 56564 | 1.89226 | 0.05 |
| 56565 | 1.89226 | 0.05 |
| 56566 | 1.89226 | 0.05 |
| 56567 | 1.89226 | 0.05 |
| 56568 | 1.89226 | 0.05 |
| 56569 | 1.4263 | 1.97974 |
| 56570 | 1.67651 | 0.05 |
| 56571 | 1.5955 | 0.532705 |
| 56572 | 1.31022 | 2.08826 |
| 56573 | 2.98674 | 1.01035 |
| 56574 | 1.18824 | 2.1 |
| 56575 | 1.28802 | 2.1 |
| 56576 | 0.946132 | 0.300118 |
| 56577 | 0.946132 | 1.18761 |
| 56578 | 0.946132 | 0.757505 |
| 56579 | 0.946132 | 2.1 |
| 56580 | 1.28802 | 0.0611981 |
| 56581 | 1.28802 | 2.1 |
| 56582 | 1.18824 | 2.1 |
| 56583 | 0.946132 | 2.1 |
| 56584 | 0.946132 | 2.1 |
| 56585 | 1.28802 | 2.1 |
| 56586 | 1.89226 | 0.176791 |
| 56587 | 1.89226 | 1.41057 |
| 56588 | 1.89226 | 2.1 |
| 56589 | 1.89226 | 0.253683 |
| 56590 | 3.78452 | 0.281622 |
| 56591 | 1.5955 | 0.777295 |
| 56592 | 1.52909 | 0.106777 |
| 56593 | 1.31022 | 0.145798 |
| 56594 | 1.31022 | 2.1 |
| 56595 | 1.2087 | 2.1 |
| 56596 | 1.52909 | 2.1 |
| 56597 | 1.11825 | 2.1 |
| 56598 | 1.18824 | 2.1 |
| 56599 | 0.946132 | 1.32315 |
| 56600 | 0.946132 | 0.82513 |
| 56601 | 0.946132 | 0.591916 |
| 56602 | 0.946132 | 2.1 |
| 56603 | 1.18824 | 0.187088 |
| 56604 | 1.18824 | 2.1 |
| 56605 | 0.946132 | 2.0263 |

| | | |
|---|---|---|
| 56606 | 1.11825 | 2.1 |
| 56607 | 1.18824 | 2.1 |
| 56608 | 0.946133 | 2.1 |
| 56609 | 0.810943 | 1.9442 |
| 56610 | 0.810943 | 0.771518 |
| 56611 | 0.810945 | 0.496858 |
| 56612 | 0.810945 | 1.17586 |
| 56613 | 1.89226 | 0.200637 |
| 56614 | 1.35168 | 0.823367 |
| 56615 | 1.08132 | 0.51741 |
| 56616 | 1.0813 | 0.946042 |
| 56617 | 1.08132 | 1.80844 |
| 56618 | 0.810959 | 0.19287 |
| 56619 | 0.810959 | 0.92182 |
| 56620 | 1.89226 | 0.05 |
| 56621 | 1.89226 | 0.05 |
| 56622 | 1.89226 | 0.05 |
| 56623 | 1.89226 | 0.05 |
| 56624 | 3.78453 | 0.05 |
| 56625 | 1.89226 | 0.05 |
| 56626 | 1.89226 | 0.05 |
| 56627 | 1.89226 | 0.05 |
| 56628 | 1.89226 | 0.05 |
| 56629 | 1.89226 | 0.05 |
| 56630 | 1.89226 | 0.05 |
| 56631 | 0.946131 | 0.05 |
| 56632 | 0.946131 | 0.05 |
| 56633 | 0.946126 | 0.05 |
| 56634 | 0.946126 | 0.05 |
| 56635 | 0.946128 | 0.05 |
| 56636 | 0.946128 | 0.05 |
| 56637 | 0.946128 | 0.05 |
| 56638 | 0.946128 | 0.05 |
| 56639 | 0.946128 | 0.05 |
| 56640 | 0.946128 | 0.05 |
| 56641 | 0.946128 | 0.05 |
| 56642 | 0.946128 | 0.05 |
| 56643 | 1.89226 | 0.05 |
| 56644 | 1.89226 | 0.05 |
| 56645 | 1.89226 | 0.05 |
| 56646 | 1.89226 | 0.05 |
| 56647 | 1.89226 | 0.05 |
| 56648 | 1.89226 | 0.05 |
| 56649 | 1.89226 | 0.05 |
| 56650 | 1.89226 | 0.05 |
| 56651 | 1.89226 | 0.05 |
| 56652 | 1.89226 | 0.05 |
| 56653 | 3.78453 | 0.05 |
| 56654 | 1.07176 | 0.05 |
| 56655 | 1.07176 | 0.05 |
| 56656 | 0.946126 | 0.05 |
| 56657 | 0.946126 | 0.05 |
| 56658 | 1.07176 | 0.05 |
| 56659 | 0.946129 | 0.05 |
| 56660 | 1.27474 | 0.05 |
| 56661 | 0.94613 | 0.05 |
| 56662 | 1.27474 | 0.05 |
| 56663 | 1.07176 | 0.05 |

| | | |
|---|---|---|
| 56664 | 0.94613 | 0.05 |
| 56665 | 0.94613 | 0.05 |
| 56666 | 0.723486 | 0.05 |
| 56667 | 0.94613 | 0.05 |
| 56668 | 0.946126 | 0.05 |
| 56669 | 0.946126 | 0.05 |
| 56670 | 0.946129 | 0.05 |
| 56671 | 0.723484 | 0.05 |
| 56672 | 1.07176 | 0.05 |
| 56673 | 0.946129 | 0.05 |
| 56674 | 1.32398 | 0.05 |
| 56675 | 1.19835 | 0.05 |
| 56676 | 0.723471 | 0.05 |
| 56677 | 0.723471 | 0.05 |
| 56678 | 0.810946 | 0.05 |
| 56679 | 0.810946 | 0.05 |
| 56680 | 0.810946 | 0.05 |
| 56681 | 0.810946 | 0.05 |
| 56682 | 0.810933 | 0.05 |
| 56683 | 0.810933 | 0.05 |
| 56684 | 1.89226 | 0.05 |
| 56685 | 1.3517 | 0.05 |
| 56686 | 1.08132 | 0.05 |
| 56687 | 1.08132 | 0.05 |
| 56688 | 1.08133 | 0.05 |
| 56689 | 2.39923 | 2.1 |
| 56690 | 1.89226 | 2.1 |
| 56691 | 1.89226 | 2.1 |
| 56692 | 1.89226 | 2.1 |
| 56693 | 2.44606 | 2.1 |
| 56694 | 2.44606 | 2.1 |
| 56695 | 1.89226 | 2.1 |
| 56696 | 1.89226 | 2.1 |
| 56697 | 1.89226 | 2.1 |
| 56698 | 1.89226 | 2.1 |
| 56699 | 1.89226 | 2.1 |
| 56700 | 1.89226 | 2.1 |
| 56701 | 1.70302 | 2.1 |
| 56702 | 2.43041 | 2.1 |
| 56703 | 2.25275 | 2.1 |
| 56704 | 2.4747 | 2.1 |
| 56705 | 1.52079 | 2.1 |
| 56706 | 1.67063 | 2.1 |
| 56707 | 2.40644 | 2.1 |
| 56708 | 1.89226 | 2.1 |
| 56709 | 1.89226 | 2.1 |
| 56710 | 1.89226 | 2.1 |
| 56711 | 2.39923 | 2.1 |
| 56712 | 2.39923 | 2.1 |
| 56713 | 0.946131 | 0.905909 |
| 56714 | 0.946131 | 2.1 |
| 56715 | 0.946131 | 2.1 |
| 56716 | 0.946131 | 2.1 |
| 56717 | 0.94613 | 2.1 |
| 56718 | 0.94613 | 2.1 |
| 56719 | 0.946131 | 2.1 |
| 56720 | 0.946131 | 2.1 |
| 56721 | 0.946131 | 2.1 |

| | | |
|---|---|---|
| 56722 | 0.946131 | 2.1 |
| 56723 | 0.946131 | 2.1 |
| 56724 | 0.946131 | 2.1 |
| 56725 | 1.04938 | 2.1 |
| 56726 | 1.04938 | 2.1 |
| 56727 | 1.111 2.1 | |
| 56728 | 1.06978 | 2.1 |
| 56729 | 1.2748 | 2.1 |
| 56730 | 1.25472 | 2.1 |
| 56731 | 0.88782 | 2.1 |
| 56732 | 0.882987 | 2.1 |
| 56733 | 0.973779 | 2.1 |
| 56734 | 1.31997 | 2.1 |
| 56735 | 0.913905 | 2.1 |
| 56736 | 1.31312 | 2.1 |
| 56737 | 2.6536 | 2.1 |
| 56738 | 2.15287 | 2.1 |
| 56739 | 2.72238 | 2.1 |
| 56740 | 2.22887 | 2.1 |
| 56741 | 4.87525 | 2.1 |
| 56742 | 2.15288 | 2.1 |
| 56743 | 1.40719 | 2.1 |
| 56744 | 1.41839 | 2.1 |
| 56745 | 1.34239 | 2.1 |
| 56746 | 2.22887 | 2.1 |
| 56747 | 2.15288 | 2.1 |
| 56748 | 2.72237 | 2.1 |
| 56749 | 2.22887 | 2.1 |
| 56750 | 2.77314 | 2.1 |
| 56751 | 2.28748 | 2.1 |
| 56752 | 5.00985 | 2.1 |
| 56753 | 0.922802 | 2.1 |
| 56754 | 0.922802 | 2.1 |
| 56755 | 0.92107 | 2.1 |
| 56756 | 0.92107 | 2.1 |
| 56757 | 0.703593 | 2.1 |
| 56758 | 0.729436 | 2.1 |
| 56759 | 1.11444 | 2.1 |
| 56760 | 1.14374 | 2.1 |
| 56761 | 1.14374 | 2.1 |
| 56762 | 0.729436 | 2.1 |
| 56763 | 0.703593 | 2.1 |
| 56764 | 1.11444 | 2.1 |
| 56765 | 1.89226 | 2.1 |
| 56766 | 1.89226 | 2.1 |
| 56767 | 1.89226 | 2.1 |
| 56768 | 1.89226 | 2.1 |
| 56769 | 1.89226 | 2.1 |
| 56770 | 1.89226 | 2.1 |
| 56771 | 0.489927 | 2.1 |
| 56772 | 0.63008 | 2.1 |
| 56773 | 0.697385 | 2.1 |
| 56774 | 0.55524 | 2.1 |
| 56775 | 0.592141 | 2.1 |
| 56776 | 0.592141 | 2.1 |
| 56777 | 0.593179 | 2.1 |
| 56778 | 0.593179 | 2.1 |
| 56779 | 0.63008 | 2.1 |

| | | |
|---|---|---|
| 56780 | 0.697385 | 2.1 |
| 56781 | 0.55524 | 2.1 |
| 56782 | 0.489927 | 2.1 |
| 56783 | 0.946133 | 2.1 |
| 56784 | 0.946133 | 2.1 |
| 56785 | 0.946132 | 1.83294 |
| 56786 | 0.946132 | 0.11264 |
| 56787 | 0.946133 | 0.944021 |
| 56788 | 0.946133 | 2.1 |
| 56789 | 0.946132 | 2.1 |
| 56790 | 0.946132 | 0.575448 |
| 56791 | 0.946132 | 1.67148 |
| 56792 | 0.946132 | 1.18795 |
| 56793 | 0.946132 | 0.638353 |
| 56794 | 0.946132 | 1.2244 |
| 56795 | 1.89226 | 2.1 |
| 56796 | 1.89226 | 2.1 |
| 56797 | 1.89226 | 2.1 |
| 56798 | 1.89226 | 2.1 |
| 56799 | 1.89226 | 2.1 |
| 56800 | 1.89226 | 2.1 |
| 56801 | 0.805746 | 2.1 |
| 56802 | 0.63008 | 2.1 |
| 56803 | 0.881598 | 2.1 |
| 56804 | 0.697385 | 2.1 |
| 56805 | 0.749417 | 2.1 |
| 56806 | 0.749417 | 2.1 |
| 56807 | 0.753714 | 2.1 |
| 56808 | 0.753714 | 2.1 |
| 56809 | 0.881598 | 2.1 |
| 56810 | 0.805746 | 2.1 |
| 56811 | 0.63008 | 2.1 |
| 56812 | 0.697385 | 2.1 |
| 56813 | 0.881599 | 2.1 |
| 56814 | 0.697385 | 2.1 |
| 56815 | 0.805746 | 2.1 |
| 56816 | 0.626731 | 0.973342 |
| 56817 | 0.755479 | 2.0466 |
| 56818 | 0.755479 | 2.1 |
| 56819 | 0.752851 | 0.947072 |
| 56820 | 0.752851 | 2.1 |
| 56821 | 0.881598 | 2.1 |
| 56822 | 0.805747 | 2.1 |
| 56823 | 0.697385 | 2.1 |
| 56824 | 0.626731 | 2.1 |
| 56825 | 2.65348 | 2.1 |
| 56826 | 2.12314 | 2.1 |
| 56827 | 2.63899 | 2.1 |
| 56828 | 2.13898 | 2.1 |
| 56829 | 4.76213 | 2.1 |
| 56830 | 0.763518 | 2.1 |
| 56831 | 0.765889 | 2.1 |
| 56832 | 0.84086 | 2.1 |
| 56833 | 0.84086 | 2.1 |
| 56834 | 0.632078 | 2.1 |
| 56835 | 0.597565 | 2.1 |
| 56836 | 1.08813 | 2.1 |
| 56837 | 0.985643 | 2.1 |

| | | |
|---|---|---|
| 56838 | 0.982464 | 2.1 |
| 56839 | 0.66404 | 2.1 |
| 56840 | 1.0358 | 2.1 |
| 56841 | 0.598023 | 2.1 |
| 56842 | 1.29293 | 2.1 |
| 56843 | 1.29628 | 2.1 |
| 56844 | 1.19266 | 2.1 |
| 56845 | 1.19266 | 2.1 |
| 56846 | 1.07109 | 2.1 |
| 56847 | 1.13093 | 2.1 |
| 56848 | 1.34467 | 2.1 |
| 56849 | 1.35251 | 2.1 |
| 56850 | 1.34369 | 2.1 |
| 56851 | 1.12525 | 2.1 |
| 56852 | 1.28746 | 2.1 |
| 56853 | 1.13511 | 2.1 |
| 56854 | 2.19717 | 2.1 |
| 56855 | 2.16547 | 2.1 |
| 56856 | 1.37392 | 2.1 |
| 56857 | 1.37392 | 2.1 |
| 56858 | 1.2966 | 2.1 |
| 56859 | 2.11985 | 2.1 |
| 56860 | 1.89226 | 2.1 |
| 56861 | 1.89226 | 2.1 |
| 56862 | 1.89226 | 2.1 |
| 56863 | 1.89226 | 2.1 |
| 56864 | 1.89226 | 2.1 |
| 56865 | 1.89226 | 2.1 |
| 56866 | 1.65637 | 2.1 |
| 56867 | 1.41758 | 2.1 |
| 56868 | 1.65637 | 2.1 |
| 56869 | 1.74391 | 2.1 |
| 56870 | 1.35504 | 2.1 |
| 56871 | 1.44258 | 2.1 |
| 56872 | 1.40774 | 2.1 |
| 56873 | 1.40774 | 2.1 |
| 56874 | 0.946131 | 2.1 |
| 56875 | 0.946131 | 2.1 |
| 56876 | 1.40774 | 2.1 |
| 56877 | 1.38882 | 2.1 |
| 56878 | 0.946132 | 2.1 |
| 56879 | 0.946132 | 2.1 |
| 56880 | 0.946132 | 2.1 |
| 56881 | 1.38882 | 2.1 |
| 56882 | 0.946132 | 2.1 |
| 56883 | 1.40774 | 2.1 |
| 56884 | 1.89227 | 2.1 |
| 56885 | 1.89227 | 2.1 |
| 56886 | 1.89227 | 2.1 |
| 56887 | 1.89227 | 2.1 |
| 56888 | 3.78453 | 2.1 |
| 56889 | 0.603148 | 2.1 |
| 56890 | 0.600177 | 2.1 |
| 56891 | 0.558615 | 2.1 |
| 56892 | 0.580714 | 2.1 |
| 56893 | 0.558606 | 2.1 |
| 56894 | 0.67883 | 2.1 |
| 56895 | 1.65637 | 2.1 |

| | | |
|---|---|---|
| 56896 | 1.07933 | 2.1 |
| 56897 | 0.842649 | 2.1 |
| 56898 | 0.905168 | 2.1 |
| 56899 | 0.774334 | 2.1 |
| 56900 | 1.38882 | 2.1 |
| 56901 | 1.38882 | 2.1 |
| 56902 | 0.946132 | 2.1 |
| 56903 | 0.946132 | 2.1 |
| 56904 | 1.38882 | 2.1 |
| 56905 | 1.37381 | 2.1 |
| 56906 | 0.946132 | 2.1 |
| 56907 | 0.946132 | 2.1 |
| 56908 | 0.946132 | 2.1 |
| 56909 | 1.37381 | 2.1 |
| 56910 | 0.946133 | 2.1 |
| 56911 | 1.38882 | 2.1 |
| 56912 | 2.3771 | 2.1 |
| 56913 | 1.9339 | 2.1 |
| 56914 | 1.70782 | 2.1 |
| 56915 | 1.9339 | 2.1 |
| 56916 | 2.42693 | 2.1 |
| 56917 | 2.83279 | 2.1 |
| 56918 | 2.34901 | 0.685104 |
| 56919 | 1.45833 | 0.708959 |
| 56920 | 1.86695 | 0.632539 |
| 56921 | 1.03101 | 0.737495 |
| 56922 | 3.32528 | 0.662125 |
| 56923 | 1.89226 | 0.05 |
| 56924 | 1.89226 | 0.05 |
| 56925 | 1.89226 | 0.05 |
| 56926 | 1.89226 | 0.05 |
| 56927 | 3.78453 | 0.05 |
| 56928 | 2.71753 | 0.05 |
| 56929 | 1.89226 | 0.05 |
| 56930 | 2.76228 | 0.05 |
| 56931 | 2.76228 | 0.05 |
| 56932 | 1.89226 | 0.05 |
| 56933 | 1.89226 | 0.145293 |
| 56934 | 0.946131 | 0.05 |
| 56935 | 0.946131 | 0.05 |
| 56936 | 0.946132 | 0.05 |
| 56937 | 0.946132 | 0.05 |
| 56938 | 0.946132 | 0.05 |
| 56939 | 0.946132 | 0.05 |
| 56940 | 0.946132 | 0.05 |
| 56941 | 0.946132 | 0.05 |
| 56942 | 0.946132 | 0.05 |
| 56943 | 0.946132 | 0.05 |
| 56944 | 0.946132 | 0.05 |
| 56945 | 0.946132 | 0.05 |
| 56946 | 1.89227 | 0.05 |
| 56947 | 1.89227 | 0.05 |
| 56948 | 1.89227 | 0.05 |
| 56949 | 1.89227 | 0.05 |
| 56950 | 1.89227 | 0.05 |
| 56951 | 1.89227 | 0.05 |
| 56952 | 2.71753 | 0.05 |
| 56953 | 2.37907 | 0.05 |

| | | |
|---|---|---|
| 56954 | 2.37907 | 0.05 |
| 56955 | 2.76228 | 0.05 |
| 56956 | 2.39588 | 0.05 |
| 56957 | 2.77909 | 0.05 |
| 56958 | 1.6571 | 0.945907 |
| 56959 | 1.11682 | 1.77465 |
| 56960 | 1.47132 | 1.07462 |
| 56961 | 1.93763 | 0.0719331 |
| 56962 | 3.12842 | 1.40929 |
| 56963 | 1.93762 | 0.743566 |
| 56964 | 2.12345 | 1.17308 |
| 56965 | 1.65709 | 0.272373 |
| 56966 | 1.65709 | 1.89899 |
| 56967 | 2.12345 | 1.05875 |
| 56968 | 1.88635 | 2.1 |
| 56969 | 1.47134 | 2.1 |
| 56970 | 2.219 | 0.765727 |
| 56971 | 2.25734 | 2.1 |
| 56972 | 1.93765 | 0.860049 |
| 56973 | 1.79103 | 1.36316 |
| 56974 | 1.47134 | 0.439404 |
| 56975 | 1.06174 | 1.01363 |
| 56976 | 1.19349 | 2.1 |
| 56977 | 1.1095 | 2.02761 |
| 56978 | 0.968827 | 0.901599 |
| 56979 | 1.07892 | 0.857379 |
| 56980 | 1.07892 | 2.1 |
| 56981 | 1.0834 | 0.645309 |
| 56982 | 1.0834 | 1.12919 |
| 56983 | 1.1095 | 1.25168 |
| 56984 | 1.19349 | 1.08973 |
| 56985 | 0.968825 | 1.16718 |
| 56986 | 1.06174 | 1.16533 |
| 56987 | 2.40627 | 2.1 |
| 56988 | 2.08288 | 1.45568 |
| 56989 | 2.5444 | 0.914009 |
| 56990 | 2.29285 | 1.83925 |
| 56991 | 4.62729 | 1.07635 |
| 56992 | 2.08289 | 0.92803 |
| 56993 | 1.65703 | 1.10321 |
| 56994 | 1.71105 | 2.1 |
| 56995 | 1.50108 | 2.07773 |
| 56996 | 2.29286 | 2.1 |
| 56997 | 2.08289 | 2.1 |
| 56998 | 2.5444 | 1.11227 |
| 56999 | 2.29285 | 2.1 |
| 57000 | 2.71429 | 2.1 |
| 57001 | 2.50288 | 0.849346 |
| 57002 | 5.04728 | 2.1 |
| 57003 | 2.50288 | 0.880815 |
| 57004 | 2.00927 | 2.1 |
| 57005 | 2.02116 | 1.20185 |
| 57006 | 2.02116 | 2.1 |
| 57007 | 2.29286 | 1.05417 |
| 57008 | 2.29286 | 2.02416 |
| 57009 | 1.89226 | 2.1 |
| 57010 | 1.89226 | 2.1 |
| 57011 | 1.89226 | 0.116271 |

| | | |
|---|---|---|
| 57012 | 1.89226 | 2.1 |
| 57013 | 3.78453 | 2.1 |
| 57014 | 1.17418 | 2.1 |
| 57015 | 1.29741 | 2.1 |
| 57016 | 1.04921 | 2.1 |
| 57017 | 1.19352 | 2.1 |
| 57018 | 2.3677 | 2.1 |
| 57019 | 1.29741 | 2.1 |
| 57020 | 1.27575 | 2.1 |
| 57021 | 1.19352 | 2.1 |
| 57022 | 1.19473 | 2.1 |
| 57023 | 2.46927 | 2.1 |
| 57024 | 1.89226 | 2.1 |
| 57025 | 1.89226 | 2.1 |
| 57026 | 1.89226 | 2.1 |
| 57027 | 1.89226 | 2.1 |
| 57028 | 1.89226 | 2.1 |
| 57029 | 1.89226 | 2.1 |
| 57030 | 0.561332 | 2.1 |
| 57031 | 0.561332 | 2.1 |
| 57032 | 0.567597 | 2.1 |
| 57033 | 0.567597 | 2.1 |
| 57034 | 0.597365 | 2.1 |
| 57035 | 0.544699 | 2.1 |
| 57036 | 0.596761 | 2.1 |
| 57037 | 0.531564 | 2.1 |
| 57038 | 0.5447 | 2.1 |
| 57039 | 0.531563 | 2.1 |
| 57040 | 0.596759 | 2.1 |
| 57041 | 0.597366 | 2.1 |
| 57042 | 0.946131 | 2.1 |
| 57043 | 0.946131 | 2.1 |
| 57044 | 0.946131 | 2.1 |
| 57045 | 0.946131 | 2.1 |
| 57046 | 0.946129 | 2.1 |
| 57047 | 0.946129 | 2.1 |
| 57048 | 0.946133 | 2.1 |
| 57049 | 0.946133 | 2.1 |
| 57050 | 0.946131 | 2.1 |
| 57051 | 0.946131 | 2.1 |
| 57052 | 0.946131 | 2.1 |
| 57053 | 0.946131 | 2.1 |
| 57054 | 1.10938 | 0.05 |
| 57055 | 1.17614 | 0.110607 |
| 57056 | 1.02478 | 0.05 |
| 57057 | 1.09056 | 0.160537 |
| 57058 | 2.19994 | 0.144074 |
| 57059 | 1.17614 | 0.05 |
| 57060 | 1.11868 | 2.1 |
| 57061 | 1.03408 | 0.05 |
| 57062 | 1.03408 | 2.1 |
| 57063 | 1.09056 | 0.05 |
| 57064 | 1.17516 | 2.1 |
| 57065 | 1.89226 | 0.05 |
| 57066 | 1.89226 | 0.05 |
| 57067 | 1.89226 | 0.05 |
| 57068 | 1.89226 | 0.05 |
| 57069 | 1.89226 | 0.0756886 |

| | | |
|---|---|---|
| 57070 | 1.89226 | 0.05 |
| 57071 | 1.89226 | 0.05 |
| 57072 | 1.89226 | 0.05 |
| 57073 | 1.89226 | 0.05 |
| 57074 | 1.89226 | 0.05 |
| 57075 | 1.89226 | 0.05 |
| 57076 | 1.89226 | 0.05 |
| 57077 | 0.579551 | 0.05 |
| 57078 | 0.563873 | 0.05 |
| 57079 | 0.611341 | 0.05 |
| 57080 | 0.598693 | 0.05 |
| 57081 | 0.589856 | 0.05 |
| 57082 | 0.589856 | 0.05 |
| 57083 | 0.588387 | 0.05 |
| 57084 | 0.588387 | 0.05 |
| 57085 | 0.57955 | 0.05 |
| 57086 | 0.611341 | 0.05 |
| 57087 | 0.563873 | 0.05 |
| 57088 | 0.598693 | 0.05 |
| 57089 | 0.611341 | 0.05 |
| 57090 | 0.598693 | 0.05 |
| 57091 | 0.586048 | 0.05 |
| 57092 | 0.572737 | 0.05 |
| 57093 | 0.591824 | 0.05 |
| 57094 | 0.591824 | 0.05 |
| 57095 | 0.592254 | 0.05 |
| 57096 | 0.592254 | 0.05 |
| 57097 | 0.611341 | 0.05 |
| 57098 | 0.586048 | 0.05 |
| 57099 | 0.598693 | 0.05 |
| 57100 | 0.572737 | 0.05 |
| 57101 | 0.946119 | 0.05 |
| 57102 | 0.946119 | 0.05 |
| 57103 | 0.946119 | 0.05 |
| 57104 | 0.946119 | 0.05 |
| 57105 | 0.94612 | 0.05 |
| 57106 | 0.94612 | 0.05 |
| 57107 | 0.946119 | 0.05 |
| 57108 | 0.946119 | 0.0590974 |
| 57109 | 0.946132 | 0.05 |
| 57110 | 0.946132 | 0.05 |
| 57111 | 0.946107 | 0.05 |
| 57112 | 0.946107 | 0.05 |
| 57113 | 1.89224 | 0.05 |
| 57114 | 1.89224 | 0.05 |
| 57115 | 1.89224 | 0.05 |
| 57116 | 1.89224 | 0.05 |
| 57117 | 1.89224 | 0.05 |
| 57118 | 1.89224 | 0.05 |
| 57119 | 0.562335 | 0.05 |
| 57120 | 0.579543 | 0.05 |
| 57121 | 0.59499 | 0.05 |
| 57122 | 0.611333 | 0.05 |
| 57123 | 0.586619 | 0.05 |
| 57124 | 0.586619 | 0.05 |
| 57125 | 0.587049 | 0.05 |
| 57126 | 0.587049 | 0.05 |
| 57127 | 0.594999 | 0.05 |

| | | |
|---|---|---|
| 57128 | 0.562342 | 0.05 |
| 57129 | 0.579535 | 0.05 |
| 57130 | 0.611325 | 0.05 |
| 57131 | 0.59499 | 0.05 |
| 57132 | 0.611333 | 0.05 |
| 57133 | 0.567849 | 0.05 |
| 57134 | 0.58604 | 0.05 |
| 57135 | 0.590978 | 0.05 |
| 57136 | 0.590978 | 0.05 |
| 57137 | 0.590052 | 0.05 |
| 57138 | 0.590052 | 0.05 |
| 57139 | 0.567857 | 0.05 |
| 57140 | 0.594998 | 0.05 |
| 57141 | 0.611325 | 0.05 |
| 57142 | 0.586032 | 0.05 |
| 57143 | 1.69661 | 0.515114 |
| 57144 | 1.9339 | 0.0603591 |
| 57145 | 1.39379 | 0.0547596 |
| 57146 | 1.69107 | 0.05 |
| 57147 | 3.32768 | 0.05 |
| 57148 | 1.9339 | 0.210406 |
| 57149 | 1.98414 | 0.194978 |
| 57150 | 1.69107 | 0.05 |
| 57151 | 1.72774 | 0.0656506 |
| 57152 | 3.66164 | 0.05 |
| 57153 | 1.39379 | 0.634473 |
| 57154 | 1.69107 | 0.05 |
| 57155 | 1.14397 | 0.05 |
| 57156 | 1.43548 | 0.05 |
| 57157 | 2.82927 | 0.05 |
| 57158 | 1.69107 | 0.05 |
| 57159 | 1.72774 | 0.241946 |
| 57160 | 1.53873 | 0.05 |
| 57161 | 1.53873 | 0.205104 |
| 57162 | 1.43548 | 0.05 |
| 57163 | 1.62449 | 0.05 |
| 57164 | 2.58431 | 0.05 |
| 57165 | 1.34072 | 0.05 |
| 57166 | 1.80841 | 0.0583737 |
| 57167 | 1.34072 | 0.05 |
| 57168 | 2.51501 | 0.05 |
| 57169 | 2.04732 | 0.05 |
| 57170 | 1.89226 | 0.05 |
| 57171 | 1.89226 | 0.05 |
| 57172 | 1.89226 | 0.0619896 |
| 57173 | 1.89226 | 0.05 |
| 57174 | 1.89226 | 0.05 |
| 57175 | 1.89226 | 0.05 |
| 57176 | 0.946131 | 0.05 |
| 57177 | 0.946131 | 0.05 |
| 57178 | 1.3639 | 0.0625948 |
| 57179 | 1.1516 | 0.05 |
| 57180 | 0.946129 | 0.05 |
| 57181 | 0.946129 | 0.0673728 |
| 57182 | 1.36391 | 0.05 |
| 57183 | 1.36391 | 0.0676551 |
| 57184 | 0.946131 | 0.0582599 |
| 57185 | 1.3639 | 0.05 |

| | | |
|---|---|---|
| 57186 | 1.1516 | 0.05 |
| 57187 | 0.946131 | 0.05 |
| 57188 | 0.946131 | 0.0581652 |
| 57189 | 0.946131 | 0.05 |
| 57190 | 0.94613 | 0.0642941 |
| 57191 | 0.94613 | 0.05 |
| 57192 | 0.946128 | 0.0684075 |
| 57193 | 0.946128 | 0.0524325 |
| 57194 | 0.946133 | 0.05 |
| 57195 | 0.946133 | 0.05 |
| 57196 | 0.946131 | 0.05 |
| 57197 | 0.946131 | 0.0979214 |
| 57198 | 0.946131 | 0.05 |
| 57199 | 0.946131 | 0.05 |
| 57200 | 1.89226 | 1.22139 |
| 57201 | 2.63634 | 2.1 |
| 57202 | 1.89226 | 1.31506 |
| 57203 | 1.83902 | 1.67021 |
| 57204 | 3.73128 | 1.23717 |
| 57205 | 1.47059 | 2.1 |
| 57206 | 2.02928 | 2.1 |
| 57207 | 1.47059 | 2.1 |
| 57208 | 1.65706 | 2.1 |
| 57209 | 1.98628 | 2.1 |
| 57210 | 2.17274 | 2.1 |
| 57211 | 2.43618 | 2.1 |
| 57212 | 2.41176 | 2.1 |
| 57213 | 1.98627 | 2.1 |
| 57214 | 2.34084 | 2.1 |
| 57215 | 4.39803 | 2.1 |
| 57216 | 1.47059 | 2.1 |
| 57217 | 1.82453 | 2.1 |
| 57218 | 1.98627 | 2.1 |
| 57219 | 1.98627 | 2.1 |
| 57220 | 1.26929 | 2.1 |
| 57221 | 1.26929 | 2.1 |
| 57222 | 1.98627 | 2.1 |
| 57223 | 2.34084 | 2.1 |
| 57224 | 1.47264 | 2.1 |
| 57225 | 2.24937 | 2.1 |
| 57226 | 4.23564 | 2.1 |
| 57227 | 1.33026 | 0.114622 |
| 57228 | 1.51171 | 1.38833 |
| 57229 | 1.51171 | 0.141042 |
| 57230 | 1.4617 | 1.71725 |
| 57231 | 1.35382 | 0.0811441 |
| 57232 | 1.30382 | 2.1 |
| 57233 | 1.89226 | 0.0512895 |
| 57234 | 1.89226 | 0.760645 |
| 57235 | 1.89226 | 0.44517 |
| 57236 | 1.89226 | 0.982204 |
| 57237 | 1.89226 | 0.06324 |
| 57238 | 1.89226 | 1.38623 |
| 57239 | 1.39263 | 0.80007 |
| 57240 | 0.962704 | 0.05 |
| 57241 | 1.0171 | 0.536081 |
| 57242 | 0.962704 | 0.664557 |
| 57243 | 1.40822 | 0.05 |

| | | |
|---|---|---|
| 57244 | 1.35382 | 0.05 |
| 57245 | 0.755853 | 2.1 |
| 57246 | 0.789808 | 0.565268 |
| 57247 | 0.696315 | 0.801258 |
| 57248 | 0.676912 | 0.05 |
| 57249 | 0.729751 | 0.0583016 |
| 57250 | 0.729751 | 0.825185 |
| 57251 | 0.722417 | 2.1 |
| 57252 | 0.722417 | 0.05 |
| 57253 | 0.696315 | 0.384514 |
| 57254 | 0.789808 | 0.247684 |
| 57255 | 0.755853 | 0.714353 |
| 57256 | 0.676912 | 0.474193 |
| 57257 | 0.946132 | 0.756396 |
| 57258 | 0.946132 | 0.301451 |
| 57259 | 0.946132 | 0.482302 |
| 57260 | 0.946132 | 0.05 |
| 57261 | 0.946131 | 0.0526057 |
| 57262 | 0.946131 | 0.716395 |
| 57263 | 0.946132 | 0.0549655 |
| 57264 | 0.946132 | 0.321953 |
| 57265 | 0.946132 | 0.05 |
| 57266 | 0.946132 | 0.544608 |
| 57267 | 0.946132 | 0.345287 |
| 57268 | 0.946132 | 0.295288 |
| 57269 | 0.946133 | 0.488739 |
| 57270 | 0.946133 | 0.05 |
| 57271 | 0.946132 | 0.05 |
| 57272 | 0.946132 | 0.120164 |
| 57273 | 0.946132 | 0.05 |
| 57274 | 0.946132 | 0.220875 |
| 57275 | 0.946133 | 0.05 |
| 57276 | 0.946133 | 0.576608 |
| 57277 | 0.946132 | 0.0985404 |
| 57278 | 0.946132 | 0.159968 |
| 57279 | 0.946132 | 0.193865 |
| 57280 | 0.946132 | 0.571629 |
| 57281 | 1.89227 | 2.0348 |
| 57282 | 2.53014 | 1.92382 |
| 57283 | 1.89227 | 2.1 |
| 57284 | 1.98241 | 1.59224 |
| 57285 | 1.89227 | 2.1 |
| 57286 | 1.98241 | 1.67399 |
| 57287 | 1.89226 | 1.40798 |
| 57288 | 1.89226 | 2.1 |
| 57289 | 1.89226 | 2.1 |
| 57290 | 1.89226 | 2.03901 |
| 57291 | 1.89226 | 2.1 |
| 57292 | 1.89226 | 2.1 |
| 57293 | 1.89226 | 1.3215 |
| 57294 | 1.89226 | 2.1 |
| 57295 | 1.89226 | 1.9298 |
| 57296 | 1.89226 | 1.85256 |
| 57297 | 3.78452 | 1.94211 |
| 57298 | 2.45304 | 0.803482 |
| 57299 | 1.89226 | 2.1 |
| 57300 | 1.89226 | 1.65421 |
| 57301 | 1.89226 | 1.58974 |

| | | |
|---|---|---|
| 57302 | 1.89226 | 1.37787 |
| 57303 | 1.89226 | 1.24647 |
| 57304 | 1.89227 | 2.1 |
| 57305 | 1.89227 | 1.90533 |
| 57306 | 1.89227 | 2.1 |
| 57307 | 1.89227 | 1.41523 |
| 57308 | 1.89227 | 2.1 |
| 57309 | 1.89227 | 1.46789 |
| 57310 | 1.89226 | 1.69441 |
| 57311 | 1.89226 | 2.1 |
| 57312 | 1.89226 | 2.1 |
| 57313 | 1.89226 | 2.1 |
| 57314 | 1.89226 | 2.1 |
| 57315 | 1.89226 | 2.1 |
| 57316 | 2.09871 | 0.05 |
| 57317 | 2.71891 | 0.0796444 |
| 57318 | 1.31126 | 0.05 |
| 57319 | 1.31126 | 0.0883223 |
| 57320 | 2.55341 | 0.05 |
| 57321 | 1.76597 | 0.250787 |
| 57322 | 1.89226 | 0.118524 |
| 57323 | 1.89226 | 0.173673 |
| 57324 | 1.89226 | 0.05 |
| 57325 | 1.89226 | 0.529779 |
| 57326 | 1.89226 | 0.05 |
| 57327 | 1.89226 | 0.676032 |
| 57328 | 0.946128 | 0.151995 |
| 57329 | 0.946128 | 0.05 |
| 57330 | 0.946133 | 0.05 |
| 57331 | 0.946133 | 0.05 |
| 57332 | 0.946131 | 0.05 |
| 57333 | 0.946131 | 0.109846 |
| 57334 | 0.94613 | 0.05 |
| 57335 | 0.94613 | 0.05 |
| 57336 | 0.946131 | 0.05 |
| 57337 | 0.946131 | 0.05 |
| 57338 | 0.946131 | 0.05 |
| 57339 | 0.946131 | 0.05 |
| 57340 | 2.71891 | 0.05 |
| 57341 | 1.89226 | 0.05 |
| 57342 | 2.09871 | 0.05 |
| 57343 | 2.09871 | 0.150768 |
| 57344 | 1.89226 | 0.05 |
| 57345 | 1.89226 | 0.0850468 |
| 57346 | 1.89227 | 0.141181 |
| 57347 | 1.89227 | 0.104186 |
| 57348 | 1.89227 | 0.0533392 |
| 57349 | 1.89227 | 0.436083 |
| 57350 | 1.89227 | 0.0599277 |
| 57351 | 1.89227 | 0.555287 |
| 57352 | 0.946129 | 0.105659 |
| 57353 | 0.946129 | 0.05 |
| 57354 | 0.946133 | 0.05 |
| 57355 | 0.946133 | 0.05 |
| 57356 | 0.946131 | 0.05 |
| 57357 | 0.946131 | 0.100728 |
| 57358 | 0.946131 | 0.05 |
| 57359 | 0.946131 | 0.0524998 |

| | | |
|---|---|---|
| 57360 | 0.946131 | 0.05 |
| 57361 | 0.946131 | 0.05 |
| 57362 | 0.946131 | 0.05 |
| 57363 | 0.946131 | 0.05 |
| 57364 | 2.32462 | 0.05 |
| 57365 | 1.89226 | 0.05 |
| 57366 | 1.89226 | 0.05 |
| 57367 | 1.89226 | 0.05 |
| 57368 | 2.2291 | 0.05 |
| 57369 | 2.2291 | 0.05 |
| 57370 | 0.847669 | 0.05 |
| 57371 | 0.847669 | 0.05 |
| 57372 | 0.796966 | 0.05 |
| 57373 | 0.796966 | 0.05 |
| 57374 | 0.833552 | 0.05 |
| 57375 | 1.01049 | 0.05 |
| 57376 | 0.709709 | 0.05 |
| 57377 | 0.634147 | 0.05 |
| 57378 | 0.634148 | 0.05 |
| 57379 | 0.833549 | 0.05 |
| 57380 | 0.709711 | 0.05 |
| 57381 | 1.01049 | 0.05 |
| 57382 | 1.89226 | 0.05 |
| 57383 | 1.89226 | 0.05 |
| 57384 | 1.89226 | 0.05 |
| 57385 | 1.89226 | 0.05 |
| 57386 | 1.89226 | 0.05 |
| 57387 | 1.89226 | 0.05 |
| 57388 | 1.03611 | 0.05 |
| 57389 | 0.936655 | 0.05 |
| 57390 | 0.946132 | 0.05 |
| 57391 | 0.946132 | 0.05 |
| 57392 | 0.941918 | 0.05 |
| 57393 | 0.941918 | 0.05 |
| 57394 | 1.00308 | 0.05 |
| 57395 | 1.04117 | 0.05 |
| 57396 | 1.1406 | 0.05 |
| 57397 | 1.04074 | 0.05 |
| 57398 | 0.851529 | 0.05 |
| 57399 | 1.00311 | 0.05 |
| 57400 | 1.01814 | 0.05 |
| 57401 | 1.6671 | 0.05 |
| 57402 | 1.35561 | 0.05 |
| 57403 | 1.35561 | 0.05 |
| 57404 | 1.2683 | 0.05 |
| 57405 | 1.2683 | 0.05 |
| 57406 | 0.946128 | 0.05 |
| 57407 | 0.946128 | 0.05 |
| 57408 | 0.946129 | 0.05 |
| 57409 | 0.946129 | 0.05 |
| 57410 | 0.946132 | 0.05 |
| 57411 | 0.946132 | 0.05 |
| 57412 | 0.946126 | 0.05 |
| 57413 | 0.946126 | 0.05 |
| 57414 | 0.946129 | 0.05 |
| 57415 | 0.946129 | 0.05 |
| 57416 | 0.946129 | 0.05 |
| 57417 | 0.946129 | 0.05 |

| | | |
|---|---|---|
| 57418 | 2.23338 | 0.05 |
| 57419 | 1.96 | 0.05 |
| 57420 | 2.03438 | 0.05 |
| 57421 | 1.87835 | 0.05 |
| 57422 | 4.11172 | 0.05 |
| 57423 | 1.96 | 0.05 |
| 57424 | 1.52661 | 0.05 |
| 57425 | 1.87834 | 0.05 |
| 57426 | 1.53175 | 0.05 |
| 57427 | 3.40495 | 0.05 |
| 57428 | 2.03435 | 0.05 |
| 57429 | 1.65813 | 0.05 |
| 57430 | 1.65813 | 0.05 |
| 57431 | 1.87832 | 0.05 |
| 57432 | 1.72067 | 0.05 |
| 57433 | 1.94086 | 0.05 |
| 57434 | 1.65813 | 0.05 |
| 57435 | 1.40572 | 0.05 |
| 57436 | 1.87832 | 0.05 |
| 57437 | 1.87832 | 0.05 |
| 57438 | 1.31154 | 0.05 |
| 57439 | 1.53173 | 0.05 |
| 57440 | 1.86662 | 2.1 |
| 57441 | 2.0815 | 2.1 |
| 57442 | 2.0815 | 2.1 |
| 57443 | 2.0815 | 2.1 |
| 57444 | 1.87246 | 2.1 |
| 57445 | 1.87362 | 2.1 |
| 57446 | 1.0273 | 2.1 |
| 57447 | 0.996347 | 2.1 |
| 57448 | 0.946129 | 2.1 |
| 57449 | 0.946129 | 2.1 |
| 57450 | 1.04427 | 2.1 |
| 57451 | 1.04196 | 2.1 |
| 57452 | 0.910674 | 2.1 |
| 57453 | 0.910674 | 2.1 |
| 57454 | 1.04072 | 2.1 |
| 57455 | 1.10931 | 2.1 |
| 57456 | 1.03132 | 2.1 |
| 57457 | 0.933698 | 2.1 |
| 57458 | 2.63789 | 0.213014 |
| 57459 | 1.89226 | 0.569804 |
| 57460 | 2.3033 | 0.154379 |
| 57461 | 2.3033 | 0.568225 |
| 57462 | 1.89226 | 0.129693 |
| 57463 | 1.89226 | 0.574396 |
| 57464 | 1.89226 | 0.617839 |
| 57465 | 1.89226 | 0.682088 |
| 57466 | 1.89226 | 0.458634 |
| 57467 | 1.89226 | 0.643958 |
| 57468 | 3.78452 | 0.673758 |
| 57469 | 1.51898 | 0.344874 |
| 57470 | 1.36006 | 0.647749 |
| 57471 | 2.03143 | 0.685195 |
| 57472 | 2.03143 | 0.428494 |
| 57473 | 1.04026 | 0.611724 |
| 57474 | 1.04026 | 0.593792 |
| 57475 | 0.94613 | 0.149563 |

| | | |
|---|---|---|
| 57476 | 0.94613 | 0.488936 |
| 57477 | 1.31894 | 0.13295 |
| 57478 | 1.31894 | 0.37433 |
| 57479 | 0.94613 | 0.129181 |
| 57480 | 0.94613 | 0.514297 |
| 57481 | 1.31894 | 0.305908 |
| 57482 | 1.41232 | 0.488792 |
| 57483 | 0.946129 | 0.275744 |
| 57484 | 1.41232 | 0.298626 |
| 57485 | 1.31895 | 0.322728 |
| 57486 | 0.946129 | 0.344455 |
| 57487 | 1.89226 | 0.348697 |
| 57488 | 1.89226 | 0.594118 |
| 57489 | 1.89226 | 0.135088 |
| 57490 | 1.89226 | 0.581535 |
| 57491 | 1.89226 | 0.129647 |
| 57492 | 1.89226 | 0.586794 |
| 57493 | 1.89804 | 0.217874 |
| 57494 | 2.03143 | 0.213314 |
| 57495 | 2.46286 | 0.441398 |
| 57496 | 2.46287 | 0.114417 |
| 57497 | 1.51898 | 0.418564 |
| 57498 | 1.51898 | 0.114735 |
| 57499 | 1.89226 | 2.1 |
| 57500 | 1.89226 | 2.1 |
| 57501 | 1.89226 | 2.1 |
| 57502 | 1.89226 | 2.1 |
| 57503 | 3.78452 | 2.1 |
| 57504 | 1.3864 | 2.1 |
| 57505 | 1.3864 | 2.1 |
| 57506 | 0.946132 | 2.1 |
| 57507 | 0.946132 | 2.1 |
| 57508 | 1.32905 | 2.1 |
| 57509 | 1.3864 | 2.1 |
| 57510 | 0.946132 | 2.1 |
| 57511 | 0.946132 | 2.1 |
| 57512 | 0.946132 | 2.1 |
| 57513 | 1.32905 | 2.1 |
| 57514 | 0.946133 | 2.1 |
| 57515 | 1.3864 | 2.1 |
| 57516 | 2.77279 | 2.1 |
| 57517 | 1.67883 | 2.1 |
| 57518 | 1.67883 | 2.1 |
| 57519 | 1.73415 | 2.1 |
| 57520 | 2.65811 | 2.1 |
| 57521 | 2.71343 | 2.1 |
| 57522 | 0.946131 | 2.1 |
| 57523 | 0.946131 | 2.1 |
| 57524 | 0.946128 | 2.1 |
| 57525 | 0.946128 | 2.1 |
| 57526 | 0.946127 | 2.1 |
| 57527 | 0.946127 | 2.1 |
| 57528 | 0.946131 | 2.1 |
| 57529 | 0.946131 | 2.1 |
| 57530 | 0.946129 | 2.1 |
| 57531 | 0.946129 | 2.1 |
| 57532 | 0.94613 | 2.1 |
| 57533 | 0.94613 | 2.1 |

| | | |
|---|---|---|
| 57534 | 1.89226 | 2.1 |
| 57535 | 1.89226 | 2.1 |
| 57536 | 1.89226 | 2.1 |
| 57537 | 1.89226 | 2.1 |
| 57538 | 3.78452 | 2.1 |
| 57539 | 1.89226 | 2.1 |
| 57540 | 2.54394 | 2.1 |
| 57541 | 2.54394 | 2.1 |
| 57542 | 2.65811 | 2.1 |
| 57543 | 1.89226 | 2.1 |
| 57544 | 2.00643 | 2.1 |
| 57545 | 2.54394 | 2.1 |
| 57546 | 2.6581 | 2.1 |
| 57547 | 1.62743 | 2.1 |
| 57548 | 1.62743 | 2.1 |
| 57549 | 1.67883 | 2.1 |
| 57550 | 2.59533 | 2.1 |
| 57551 | 1.89226 | 2.1 |
| 57552 | 1.89226 | 2.1 |
| 57553 | 1.89226 | 2.1 |
| 57554 | 1.89226 | 2.1 |
| 57555 | 1.89226 | 2.1 |
| 57556 | 1.89226 | 2.1 |
| 57557 | 1.89226 | 1.48675 |
| 57558 | 1.89226 | 1.24786 |
| 57559 | 1.89226 | 2.1 |
| 57560 | 1.89226 | 0.786462 |
| 57561 | 1.89226 | 2.1 |
| 57562 | 1.89226 | 0.355504 |
| 57563 | 1.89226 | 1.77748 |
| 57564 | 1.89226 | 1.25829 |
| 57565 | 1.89226 | 2.1 |
| 57566 | 1.89226 | 1.12732 |
| 57567 | 1.89226 | 2.1 |
| 57568 | 1.89226 | 0.87339 |
| 57569 | 1.89226 | 0.723418 |
| 57570 | 1.89226 | 0.238978 |
| 57571 | 1.89226 | 1.6509 |
| 57572 | 1.89226 | 0.100194 |
| 57573 | 1.89226 | 1.29118 |
| 57574 | 1.89226 | 0.0684674 |
| 57575 | 2.68537 | 0.362881 |
| 57576 | 2.22575 | 0.284485 |
| 57577 | 2.47464 | 0.05 |
| 57578 | 1.79093 | 0.0623759 |
| 57579 | 4.70039 | 0.05 |
| 57580 | 2.81217 | 0.150978 |
| 57581 | 2.47464 | 0.05 |
| 57582 | 2.58286 | 0.0629743 |
| 57583 | 2.29193 | 0.05 |
| 57584 | 5.0575 | 0.05 |
| 57585 | 2.47464 | 0.05 |
| 57586 | 2.20365 | 0.171925 |
| 57587 | 2.29193 | 0.05 |
| 57588 | 1.90104 | 0.05 |
| 57589 | 4.37568 | 0.05 |
| 57590 | 1.89226 | 0.717209 |
| 57591 | 2.16957 | 2.1 |

| | | |
|---|---|---|
| 57592 | 2.52823 | 1.19256 |
| 57593 | 2.52823 | 2.1 |
| 57594 | 1.89226 | 0.961717 |
| 57595 | 1.89226 | 1.95253 |
| 57596 | 1.36226 | 2.1 |
| 57597 | 1.55369 | 2.1 |
| 57598 | 1.33309 | 2.1 |
| 57599 | 1.62593 | 2.1 |
| 57600 | 2.98819 | 2.1 |
| 57601 | 0.802281 | 2.1 |
| 57602 | 0.802281 | 2.1 |
| 57603 | 0.835143 | 2.1 |
| 57604 | 0.835143 | 2.1 |
| 57605 | 0.824458 | 2.1 |
| 57606 | 0.92641 | 2.1 |
| 57607 | 0.776847 | 2.1 |
| 57608 | 0.812967 | 2.1 |
| 57609 | 0.812967 | 2.1 |
| 57610 | 0.926411 | 2.1 |
| 57611 | 0.776847 | 2.1 |
| 57612 | 0.824458 | 2.1 |
| 57613 | 1.33309 | 2.1 |
| 57614 | 1.62593 | 2.1 |
| 57615 | 1.32813 | 2.1 |
| 57616 | 1.7237 | 2.1 |
| 57617 | 2.95406 | 2.1 |
| 57618 | 0.814544 | 2.1 |
| 57619 | 0.875312 | 2.1 |
| 57620 | 0.910502 | 2.1 |
| 57621 | 0.910502 | 2.1 |
| 57622 | 0.886633 | 2.1 |
| 57623 | 0.975739 | 2.1 |
| 57624 | 0.792623 | 2.1 |
| 57625 | 0.840283 | 2.1 |
| 57626 | 0.796372 | 2.1 |
| 57627 | 1.06159 | 2.1 |
| 57628 | 0.81948 | 2.1 |
| 57629 | 0.933833 | 2.1 |
| 57630 | 2.63997 | 2.1 |
| 57631 | 1.29694 | 2.1 |
| 57632 | 1.28141 | 2.1 |
| 57633 | 2.62623 | 2.1 |
| 57634 | 3.92317 | 2.1 |
| 57635 | 2.63998 | 2.1 |
| 57636 | 1.89226 | 2.1 |
| 57637 | 1.89226 | 2.1 |
| 57638 | 2.62623 | 2.1 |
| 57639 | 4.5185 | 2.1 |
| 57640 | 1.89226 | 2.1 |
| 57641 | 1.89226 | 2.1 |
| 57642 | 1.89226 | 2.1 |
| 57643 | 1.89226 | 2.1 |
| 57644 | 3.78453 | 2.1 |
| 57645 | 1.89226 | 2.1 |
| 57646 | 1.89226 | 2.1 |
| 57647 | 1.89226 | 2.1 |
| 57648 | 1.89226 | 2.1 |
| 57649 | 1.89226 | 2.1 |

| | | |
|---|---|---|
| 57650 | 1.89226 | 2.1 |
| 57651 | 1.25468 | 2.1 |
| 57652 | 2.62623 | 2.1 |
| 57653 | 1.28141 | 2.1 |
| 57654 | 1.28141 | 2.1 |
| 57655 | 2.60665 | 2.1 |
| 57656 | 2.60665 | 2.1 |
| 57657 | 1.89226 | 2.1 |
| 57658 | 2.62623 | 2.1 |
| 57659 | 2.60666 | 2.1 |
| 57660 | 2.62623 | 2.1 |
| 57661 | 1.87269 | 2.1 |
| 57662 | 1.89226 | 2.1 |
| 57663 | 1.89226 | 2.1 |
| 57664 | 1.89226 | 2.1 |
| 57665 | 1.89226 | 2.1 |
| 57666 | 1.89226 | 2.1 |
| 57667 | 3.78453 | 2.1 |
| 57668 | 1.89226 | 2.1 |
| 57669 | 1.89226 | 2.1 |
| 57670 | 1.89226 | 2.1 |
| 57671 | 1.89226 | 2.1 |
| 57672 | 1.89226 | 2.1 |
| 57673 | 1.89226 | 2.1 |
| 57674 | 2.30147 | 1.37245 |
| 57675 | 2.27703 | 2.1 |
| 57676 | 1.89227 | 0.896677 |
| 57677 | 1.89227 | 2.03887 |
| 57678 | 4.1693 | 1.66863 |
| 57679 | 1.38617 | 1.17083 |
| 57680 | 1.24613 | 1.51115 |
| 57681 | 2.59886 | 0.767783 |
| 57682 | 2.6238 | 1.66363 |
| 57683 | 3.86993 | 1.20325 |
| 57684 | 0.946133 | 1.69564 |
| 57685 | 0.946133 | 0.475762 |
| 57686 | 0.946132 | 0.804268 |
| 57687 | 0.946132 | 2.1 |
| 57688 | 0.946133 | 2.1 |
| 57689 | 0.946133 | 2.01014 |
| 57690 | 0.946132 | 0.534526 |
| 57691 | 0.946132 | 1.18099 |
| 57692 | 0.946133 | 1.42465 |
| 57693 | 0.946133 | 1.35544 |
| 57694 | 0.946132 | 1.19837 |
| 57695 | 0.946132 | 1.2165 |
| 57696 | 2.21773 | 2.1 |
| 57697 | 2.59216 | 2.1 |
| 57698 | 1.68541 | 2.1 |
| 57699 | 1.68541 | 2.1 |
| 57700 | 1.84814 | 2.1 |
| 57701 | 2.38046 | 2.1 |
| 57702 | 1.89966 | 2.1 |
| 57703 | 1.70303 | 2.1 |
| 57704 | 1.2028 | 2.1 |
| 57705 | 1.29897 | 2.1 |
| 57706 | 2.02873 | 2.1 |
| 57707 | 2.19093 | 2.1 |

| | | |
|---|---|---|
| 57708 | 2.75218 | 2.1 |
| 57709 | 1.70303 | 2.1 |
| 57710 | 3.10254 | 2.1 |
| 57711 | 2.24783 | 2.1 |
| 57712 | 2.56839 | 2.1 |
| 57713 | 1.70303 | 2.1 |
| 57714 | 2.56839 | 2.1 |
| 57715 | 1.08276 | 2.1 |
| 57716 | 2.24061 | 2.1 |
| 57717 | 1.67491 | 2.1 |
| 57718 | 1.60948 | 2.1 |
| 57719 | 1.08276 | 2.1 |
| 57720 | 1.89226 | 0.889785 |
| 57721 | 1.89226 | 1.15967 |
| 57722 | 1.89226 | 0.67777 |
| 57723 | 1.89226 | 0.68474 |
| 57724 | 3.78453 | 0.337717 |
| 57725 | 1.89226 | 0.690189 |
| 57726 | 1.89226 | 1.08626 |
| 57727 | 1.89226 | 0.461041 |
| 57728 | 1.89226 | 0.639418 |
| 57729 | 3.78452 | 1.80256 |
| 57730 | 1.89226 | 1.04511 |
| 57731 | 2.7535 | 0.103966 |
| 57732 | 1.89226 | 1.3233 |
| 57733 | 1.89226 | 1.11155 |
| 57734 | 1.89226 | 0.229528 |
| 57735 | 1.89226 | 0.578124 |
| 57736 | 1.89226 | 1.10297 |
| 57737 | 1.89226 | 0.627522 |
| 57738 | 1.89226 | 0.11595 |
| 57739 | 1.89226 | 1.07854 |
| 57740 | 1.89226 | 0.296456 |
| 57741 | 1.89226 | 1.71059 |
| 57742 | 1.89227 | 1.02636 |
| 57743 | 1.89227 | 0.890958 |
| 57744 | 1.89227 | 0.637316 |
| 57745 | 1.89227 | 0.288497 |
| 57746 | 3.78453 | 1.87502 |
| 57747 | 1.89226 | 0.982717 |
| 57748 | 2.53412 | 0.0512141 |
| 57749 | 1.89226 | 1.906 |
| 57750 | 1.89226 | 0.153654 |
| 57751 | 1.89226 | 0.516342 |
| 57752 | 1.89226 | 0.0958219 |
| 57753 | 2.21126 | 0.05 |
| 57754 | 1.89226 | 0.05 |
| 57755 | 2.57464 | 0.05 |
| 57756 | 1.89226 | 0.05 |
| 57757 | 2.57464 | 0.05 |
| 57758 | 1.89226 | 0.05 |
| 57759 | 1.89226 | 0.05 |
| 57760 | 1.89226 | 0.05 |
| 57761 | 1.89226 | 0.399527 |
| 57762 | 1.89226 | 0.05 |
| 57763 | 1.89226 | 0.0764282 |
| 57764 | 1.89226 | 0.05 |
| 57765 | 1.1418 | 0.05 |

| | | |
|---|---|---|
| 57766 | 0.653021 | 0.05 |
| 57767 | 1.37282 | 0.05 |
| 57768 | 0.931386 | 0.05 |
| 57769 | 1.34568 | 0.0665208 |
| 57770 | 1.17669 | 0.05 |
| 57771 | 0.666595 | 0.05 |
| 57772 | 0.786895 | 0.05 |
| 57773 | 0.933982 | 0.05 |
| 57774 | 1.05351 | 0.05 |
| 57775 | 1.00792 | 0.05 |
| 57776 | 1.00792 | 0.05 |
| 57777 | 1.89226 | 2.1 |
| 57778 | 1.89226 | 2.1 |
| 57779 | 1.89226 | 2.1 |
| 57780 | 1.89226 | 2.1 |
| 57781 | 3.78452 | 2.1 |
| 57782 | 1.89226 | 2.1 |
| 57783 | 1.89226 | 2.1 |
| 57784 | 1.89226 | 2.1 |
| 57785 | 1.89226 | 2.1 |
| 57786 | 1.89226 | 2.1 |
| 57787 | 1.89226 | 2.1 |
| 57788 | 1.65642 | 2.1 |
| 57789 | 1.30248 | 2.1 |
| 57790 | 1.38888 | 2.1 |
| 57791 | 1.25987 | 2.1 |
| 57792 | 1.70611 | 2.1 |
| 57793 | 1.65642 | 2.1 |
| 57794 | 1.65642 | 2.1 |
| 57795 | 1.81407 | 2.1 |
| 57796 | 2.19428 | 2.1 |
| 57797 | 1.92555 | 2.1 |
| 57798 | 3.8507 | 2.1 |
| 57799 | 1.89226 | 2.1 |
| 57800 | 1.89226 | 2.1 |
| 57801 | 1.89226 | 2.1 |
| 57802 | 1.89226 | 2.1 |
| 57803 | 3.78452 | 2.1 |
| 57804 | 1.89226 | 2.1 |
| 57805 | 1.89226 | 2.1 |
| 57806 | 1.89226 | 2.1 |
| 57807 | 1.89226 | 2.1 |
| 57808 | 1.89226 | 2.1 |
| 57809 | 1.89226 | 2.1 |
| 57810 | 0.705495 | 2.1 |
| 57811 | 0.809317 | 2.1 |
| 57812 | 1.1655 | 2.1 |
| 57813 | 1.09605 | 2.1 |
| 57814 | 0.983375 | 2.1 |
| 57815 | 0.797301 | 2.1 |
| 57816 | 0.906633 | 2.1 |
| 57817 | 0.906633 | 2.1 |
| 57818 | 0.85085 | 2.1 |
| 57819 | 1.16548 | 2.1 |
| 57820 | 0.705501 | 2.1 |
| 57821 | 1.00314 | 2.1 |
| 57822 | 2.19428 | 2.1 |
| 57823 | 2.31862 | 2.1 |

| | | |
|---|---|---|
| 57824 | 2.27408 | 2.1 |
| 57825 | 2.45893 | 2.1 |
| 57826 | 4.46836 | 2.1 |
| 57827 | 1.89226 | 0.859349 |
| 57828 | 1.89226 | 1.14296 |
| 57829 | 1.89226 | 1.05695 |
| 57830 | 1.89226 | 0.843803 |
| 57831 | 1.89226 | 1.71109 |
| 57832 | 1.89226 | 1.09847 |
| 57833 | 2.0815 | 0.855684 |
| 57834 | 2.22136 | 0.967273 |
| 57835 | 1.5515 | 0.891476 |
| 57836 | 1.86323 | 0.76334 |
| 57837 | 2.74986 | 1.18318 |
| 57838 | 3.30238 | 0.888756 |
| 57839 | 1.89224 | 0.862808 |
| 57840 | 1.89224 | 1.05884 |
| 57841 | 1.89224 | 1.09032 |
| 57842 | 1.89224 | 0.815565 |
| 57843 | 1.89224 | 1.80178 |
| 57844 | 1.89224 | 0.98588 |
| 57845 | 1.89224 | 0.85936 |
| 57846 | 1.89224 | 1.25056 |
| 57847 | 1.89224 | 0.975969 |
| 57848 | 1.89224 | 0.799902 |
| 57849 | 1.89224 | 1.71549 |
| 57850 | 1.89224 | 1.04279 |
| 57851 | 1.17846 | 0.780789 |
| 57852 | 0.810935 | 0.789325 |
| 57853 | 0.93347 | 1.30176 |
| 57854 | 0.93347 | 0.806459 |
| 57855 | 0.810932 | 0.976585 |
| 57856 | 0.810932 | 0.846085 |
| 57857 | 2.22133 | 0.876425 |
| 57858 | 1.71919 | 0.864785 |
| 57859 | 1.32629 | 0.75981 |
| 57860 | 1.0813 | 0.978599 |
| 57861 | 1.57137 | 0.853619 |
| 57862 | 2.21149 | 2.1 |
| 57863 | 1.89226 | 2.1 |
| 57864 | 1.89226 | 2.1 |
| 57865 | 1.89226 | 2.1 |
| 57866 | 2.17159 | 2.1 |
| 57867 | 2.17159 | 2.1 |
| 57868 | 0.946131 | 0.0770233 |
| 57869 | 0.946131 | 2.1 |
| 57870 | 0.946131 | 2.1 |
| 57871 | 0.946131 | 2.1 |
| 57872 | 0.94613 | 1.11778 |
| 57873 | 0.94613 | 2.02426 |
| 57874 | 0.946132 | 2.1 |
| 57875 | 0.946132 | 2.1 |
| 57876 | 0.946131 | 1.58096 |
| 57877 | 0.946131 | 1.50341 |
| 57878 | 0.946131 | 2.08651 |
| 57879 | 0.946131 | 1.87045 |
| 57880 | 1.12266 | 2.1 |
| 57881 | 1.4714 | 2.06784 |

| | | |
|---|---|---|
| 57882 | 1.62322 | 2.1 |
| 57883 | 1.62322 | 1.92818 |
| 57884 | 1.02958 | 2.1 |
| 57885 | 1.02958 | 2.1 |
| 57886 | 2.2671 | 2.1 |
| 57887 | 1.89226 | 2.1 |
| 57888 | 1.89226 | 2.1 |
| 57889 | 1.89226 | 2.1 |
| 57890 | 2.21149 | 2.1 |
| 57891 | 2.21149 | 2.1 |
| 57892 | 1.89226 | 2.1 |
| 57893 | 1.89226 | 1.71233 |
| 57894 | 1.89226 | 2.1 |
| 57895 | 1.89226 | 1.31637 |
| 57896 | 1.89226 | 2.1 |
| 57897 | 1.89226 | 2.1 |
| 57898 | 0.736914 | 2.1 |
| 57899 | 0.736914 | 2.1 |
| 57900 | 0.822839 | 2.1 |
| 57901 | 0.822836 | 2.1 |
| 57902 | 0.791298 | 1.01016 |
| 57903 | 1.00003 | 2.1 |
| 57904 | 0.640254 | 2.1 |
| 57905 | 0.575379 | 2.1 |
| 57906 | 0.609001 | 1.66344 |
| 57907 | 1.00005 | 2.01521 |
| 57908 | 0.575371 | 2.1 |
| 57909 | 0.831908 | 1.66539 |
| 57910 | 1.89226 | 2.1 |
| 57911 | 1.89226 | 2.1 |
| 57912 | 1.89226 | 2.1 |
| 57913 | 1.89226 | 2.1 |
| 57914 | 1.89226 | 2.1 |
| 57915 | 1.89226 | 2.1 |
| 57916 | 1.89226 | 2.1 |
| 57917 | 1.89226 | 2.1 |
| 57918 | 1.89226 | 2.1 |
| 57919 | 1.89226 | 2.1 |
| 57920 | 1.89226 | 2.1 |
| 57921 | 1.89226 | 2.1 |
| 57922 | 0.730617 | 2.1 |
| 57923 | 0.71052 | 2.1 |
| 57924 | 0.7329 | 2.1 |
| 57925 | 0.712083 | 2.1 |
| 57926 | 0.701051 | 2.1 |
| 57927 | 0.701051 | 2.1 |
| 57928 | 0.721552 | 2.1 |
| 57929 | 0.721552 | 2.1 |
| 57930 | 0.710521 | 2.1 |
| 57931 | 0.732898 | 2.1 |
| 57932 | 0.730619 | 2.1 |
| 57933 | 0.712081 | 2.1 |
| 57934 | 0.7329 | 2.1 |
| 57935 | 0.712082 | 2.1 |
| 57936 | 0.559601 | 2.1 |
| 57937 | 0.495993 | 2.1 |
| 57938 | 0.603718 | 2.1 |
| 57939 | 0.603718 | 2.1 |

| | | |
|---|---|---|
| 57940 | 0.625174 | 2.1 |
| 57941 | 0.625174 | 2.1 |
| 57942 | 0.732899 | 2.1 |
| 57943 | 0.559601 | 2.1 |
| 57944 | 0.712082 | 2.1 |
| 57945 | 0.495993 | 2.1 |
| 57946 | 0.94613 | 2.1 |
| 57947 | 0.94613 | 2.1 |
| 57948 | 0.94613 | 2.1 |
| 57949 | 0.94613 | 2.1 |
| 57950 | 0.946131 | 2.1 |
| 57951 | 0.946131 | 2.1 |
| 57952 | 0.946129 | 2.1 |
| 57953 | 0.946129 | 2.1 |
| 57954 | 0.94613 | 2.1 |
| 57955 | 0.94613 | 2.1 |
| 57956 | 0.94613 | 2.1 |
| 57957 | 0.94613 | 2.1 |
| 57958 | 1.89226 | 2.1 |
| 57959 | 1.89226 | 2.1 |
| 57960 | 1.89226 | 2.1 |
| 57961 | 1.89226 | 2.1 |
| 57962 | 1.89226 | 2.1 |
| 57963 | 1.89226 | 2.1 |
| 57964 | 1.42104 | 2.1 |
| 57965 | 1.4658 | 2.1 |
| 57966 | 1.51223 | 2.1 |
| 57967 | 1.42283 | 2.1 |
| 57968 | 2.93327 | 2.1 |
| 57969 | 0.732899 | 2.1 |
| 57970 | 0.756115 | 2.1 |
| 57971 | 0.629763 | 2.1 |
| 57972 | 0.559601 | 2.1 |
| 57973 | 0.646134 | 2.1 |
| 57974 | 0.646134 | 2.1 |
| 57975 | 0.669582 | 2.1 |
| 57976 | 0.669582 | 2.1 |
| 57977 | 0.756115 | 2.1 |
| 57978 | 0.629763 | 2.1 |
| 57979 | 0.732899 | 2.1 |
| 57980 | 0.559601 | 2.1 |
| 57981 | 2.566 | 2.1 |
| 57982 | 1.20242 | 2.1 |
| 57983 | 2.64084 | 2.1 |
| 57984 | 2.18297 | 2.1 |
| 57985 | 1.66029 | 2.1 |
| 57986 | 1.20242 | 2.1 |
| 57987 | 0.946133 | 1.2636 |
| 57988 | 0.946133 | 1.72559 |
| 57989 | 0.946129 | 2.1 |
| 57990 | 0.946129 | 2.1 |
| 57991 | 0.946131 | 2.1 |
| 57992 | 0.946131 | 1.18238 |
| 57993 | 0.946131 | 0.076931 |
| 57994 | 0.946131 | 2.1 |
| 57995 | 0.946131 | 0.955335 |
| 57996 | 0.946131 | 2.1 |
| 57997 | 0.946131 | 1.69228 |

| | | |
|---|---|---|
| 57998 | 0.946131 | 2.03194 |
| 57999 | 1.89226 | 0.05 |
| 58000 | 1.89226 | 0.05 |
| 58001 | 1.89226 | 0.05 |
| 58002 | 1.89226 | 0.05 |
| 58003 | 3.78453 | 0.05 |
| 58004 | 1.03073 | 0.05 |
| 58005 | 1.03073 | 0.05 |
| 58006 | 1.04066 | 0.05 |
| 58007 | 1.04066 | 0.05 |
| 58008 | 1.20802 | 0.05 |
| 58009 | 1.32689 | 0.05 |
| 58010 | 0.883301 | 0.05 |
| 58011 | 0.744506 | 0.05 |
| 58012 | 1.20802 | 0.05 |
| 58013 | 0.744506 | 0.05 |
| 58014 | 0.883301 | 0.05 |
| 58015 | 1.32689 | 0.05 |
| 58016 | 1.20802 | 0.05 |
| 58017 | 1.20802 | 0.05 |
| 58018 | 0.946131 | 0.05 |
| 58019 | 0.946131 | 0.05 |
| 58020 | 0.94613 | 0.05 |
| 58021 | 0.94613 | 0.05 |
| 58022 | 1.20802 | 0.05 |
| 58023 | 1.32689 | 0.05 |
| 58024 | 1.20802 | 0.05 |
| 58025 | 0.946131 | 0.05 |
| 58026 | 0.946131 | 0.05 |
| 58027 | 1.32689 | 0.05 |
| 58028 | 1.89226 | 0.05 |
| 58029 | 1.89226 | 0.05 |
| 58030 | 1.89226 | 0.05 |
| 58031 | 1.89226 | 0.05 |
| 58032 | 1.89226 | 0.05 |
| 58033 | 1.89226 | 0.05 |
| 58034 | 0.946119 | 0.05 |
| 58035 | 0.946119 | 0.05 |
| 58036 | 0.946118 | 0.05 |
| 58037 | 0.946118 | 0.05 |
| 58038 | 0.946118 | 0.05 |
| 58039 | 0.946118 | 0.05 |
| 58040 | 0.946119 | 0.05 |
| 58041 | 0.946119 | 0.05 |
| 58042 | 0.946131 | 0.05 |
| 58043 | 0.946131 | 0.05 |
| 58044 | 0.946106 | 0.05 |
| 58045 | 0.946106 | 0.05 |
| 58046 | 1.3939 | 2.1 |
| 58047 | 1.49641 | 2.1 |
| 58048 | 1.15096 | 2.1 |
| 58049 | 1.10101 | 2.1 |
| 58050 | 2.59742 | 2.1 |
| 58051 | 1.22106 | 2.1 |
| 58052 | 2.60663 | 2.1 |
| 58053 | 1.25467 | 2.1 |
| 58054 | 1.25467 | 2.1 |
| 58055 | 2.58377 | 2.1 |

| | | |
|---|---|---|
| 58056 | 2.58377 | 2.1 |
| 58057 | 1.89225 | 2.1 |
| 58058 | 2.60664 | 2.1 |
| 58059 | 2.58377 | 2.1 |
| 58060 | 2.60664 | 2.1 |
| 58061 | 1.86938 | 2.1 |
| 58062 | 1.89225 | 2.1 |
| 58063 | 1.89225 | 2.1 |
| 58064 | 1.89225 | 2.1 |
| 58065 | 1.89225 | 2.1 |
| 58066 | 1.89225 | 2.1 |
| 58067 | 1.89225 | 2.1 |
| 58068 | 1.89225 | 2.1 |
| 58069 | 0.946121 | 2.1 |
| 58070 | 0.946121 | 2.1 |
| 58071 | 0.946126 | 2.1 |
| 58072 | 0.946126 | 2.1 |
| 58073 | 0.946124 | 2.1 |
| 58074 | 0.946124 | 2.1 |
| 58075 | 0.946123 | 2.1 |
| 58076 | 0.946123 | 2.1 |
| 58077 | 0.946117 | 2.1 |
| 58078 | 0.946117 | 2.1 |
| 58079 | 0.94613 | 2.1 |
| 58080 | 0.94613 | 2.1 |
| 58081 | 1.28017 | 2.1 |
| 58082 | 1.29189 | 2.1 |
| 58083 | 0.610534 | 2.1 |
| 58084 | 0.592373 | 2.1 |
| 58085 | 0.943764 | 2.1 |
| 58086 | 0.943764 | 2.1 |
| 58087 | 0.940501 | 2.1 |
| 58088 | 0.940501 | 2.1 |
| 58089 | 1.28017 | 2.1 |
| 58090 | 0.592371 | 2.1 |
| 58091 | 0.610533 | 2.1 |
| 58092 | 1.29189 | 2.1 |
| 58093 | 0.946133 | 2.1 |
| 58094 | 0.946133 | 2.1 |
| 58095 | 1.28017 | 2.1 |
| 58096 | 1.29189 | 2.1 |
| 58097 | 0.946132 | 2.1 |
| 58098 | 0.946132 | 2.1 |
| 58099 | 1.28017 | 2.1 |
| 58100 | 1.28017 | 2.1 |
| 58101 | 1.28017 | 2.1 |
| 58102 | 0.946132 | 2.1 |
| 58103 | 0.946133 | 2.1 |
| 58104 | 1.2919 | 2.1 |
| 58105 | 1.89226 | 2.1 |
| 58106 | 1.89226 | 2.1 |
| 58107 | 1.89226 | 2.1 |
| 58108 | 1.89226 | 2.1 |
| 58109 | 1.89226 | 2.1 |
| 58110 | 1.89226 | 2.1 |
| 58111 | 1.89226 | 2.1 |
| 58112 | 1.89226 | 2.1 |
| 58113 | 1.89226 | 2.1 |

| | | |
|---|---|---|
| 58114 | 1.89226 | 2.1 |
| 58115 | 3.78452 | 2.1 |
| 58116 | 1.89226 | 2.1 |
| 58117 | 2.08196 | 2.1 |
| 58118 | 1.97854 | 2.1 |
| 58119 | 2.08196 | 2.1 |
| 58120 | 1.78884 | 2.1 |
| 58121 | 1.89226 | 2.1 |
| 58122 | 1.89226 | 2.1 |
| 58123 | 1.89226 | 2.1 |
| 58124 | 1.89226 | 2.1 |
| 58125 | 1.89226 | 2.1 |
| 58126 | 1.89226 | 2.1 |
| 58127 | 1.89226 | 2.1 |
| 58128 | 1.83688 | 2.1 |
| 58129 | 1.89226 | 2.1 |
| 58130 | 1.89226 | 2.1 |
| 58131 | 1.89226 | 2.1 |
| 58132 | 1.86015 | 2.1 |
| 58133 | 1.86015 | 2.1 |
| 58134 | 1.89226 | 2.1 |
| 58135 | 1.97854 | 2.1 |
| 58136 | 1.85883 | 2.1 |
| 58137 | 1.97854 | 2.1 |
| 58138 | 1.77255 | 2.1 |
| 58139 | 1.89226 | 2.1 |
| 58140 | 0.946131 | 2.1 |
| 58141 | 0.946131 | 2.1 |
| 58142 | 0.946131 | 2.1 |
| 58143 | 0.946131 | 2.1 |
| 58144 | 0.94613 | 2.1 |
| 58145 | 0.94613 | 2.1 |
| 58146 | 0.946132 | 2.1 |
| 58147 | 0.946132 | 2.1 |
| 58148 | 0.946131 | 2.1 |
| 58149 | 0.946131 | 2.1 |
| 58150 | 0.946131 | 2.1 |
| 58151 | 0.946131 | 2.1 |
| 58152 | 0.918412 | 2.1 |
| 58153 | 0.918412 | 2.1 |
| 58154 | 0.94893 | 2.1 |
| 58155 | 0.750211 | 2.1 |
| 58156 | 0.851541 | 2.1 |
| 58157 | 0.851541 | 2.1 |
| 58158 | 0.970566 | 2.1 |
| 58159 | 0.826567 | 2.1 |
| 58160 | 0.970554 | 2.1 |
| 58161 | 1.04071 | 2.1 |
| 58162 | 0.826632 | 2.1 |
| 58163 | 0.851554 | 2.1 |
| 58164 | 1.89226 | 0.05 |
| 58165 | 1.89226 | 0.05 |
| 58166 | 1.89226 | 0.05 |
| 58167 | 1.89226 | 0.05 |
| 58168 | 3.78453 | 0.05 |
| 58169 | 0.946131 | 0.05 |
| 58170 | 0.946131 | 0.05 |
| 58171 | 0.811232 | 0.05 |

| | | |
|---|---|---|
| 58172 | 0.811232 | 0.05 |
| 58173 | 0.94613 | 0.05 |
| 58174 | 0.94613 | 0.05 |
| 58175 | 0.959701 | 0.05 |
| 58176 | 0.811233 | 0.05 |
| 58177 | 0.94613 | 0.05 |
| 58178 | 0.811233 | 0.05 |
| 58179 | 0.959702 | 0.05 |
| 58180 | 0.946131 | 0.05 |
| 58181 | 1.89226 | 0.05 |
| 58182 | 1.89226 | 0.05 |
| 58183 | 1.89226 | 0.05 |
| 58184 | 1.89226 | 0.05 |
| 58185 | 1.89226 | 0.05 |
| 58186 | 1.89226 | 0.05 |
| 58187 | 1.89227 | 0.05 |
| 58188 | 1.89227 | 0.05 |
| 58189 | 1.89227 | 0.05 |
| 58190 | 1.89227 | 0.05 |
| 58191 | 1.89227 | 0.05 |
| 58192 | 1.89227 | 0.05 |
| 58193 | 1.89226 | 0.05 |
| 58194 | 1.89226 | 0.05 |
| 58195 | 1.89226 | 0.05 |
| 58196 | 1.89226 | 0.05 |
| 58197 | 3.78453 | 0.05 |
| 58198 | 1.89226 | 0.05 |
| 58199 | 1.89226 | 0.05 |
| 58200 | 1.89226 | 0.05 |
| 58201 | 1.89226 | 0.05 |
| 58202 | 1.89226 | 0.05 |
| 58203 | 1.89226 | 0.05 |
| 58204 | 1.89226 | 2.1 |
| 58205 | 2.36204 | 2.1 |
| 58206 | 2.39853 | 2.1 |
| 58207 | 2.36204 | 2.1 |
| 58208 | 1.92876 | 2.1 |
| 58209 | 1.89226 | 2.1 |
| 58210 | 1.89226 | 2.1 |
| 58211 | 1.89226 | 2.1 |
| 58212 | 1.89226 | 2.1 |
| 58213 | 1.89226 | 0.0982788 |
| 58214 | 3.78453 | 2.1 |
| 58215 | 0.946128 | 2.1 |
| 58216 | 0.946128 | 0.0809403 |
| 58217 | 1.13182 | 1.19974 |
| 58218 | 1.13182 | 2.1 |
| 58219 | 0.94613 | 0.910705 |
| 58220 | 0.94613 | 2.08359 |
| 58221 | 1.1749 | 2.1 |
| 58222 | 1.13182 | 2.1 |
| 58223 | 1.1749 | 2.1 |
| 58224 | 0.94613 | 2.1 |
| 58225 | 1.13182 | 2.1 |
| 58226 | 0.94613 | 2.1 |
| 58227 | 1.89226 | 2.1 |
| 58228 | 2.39853 | 2.1 |
| 58229 | 2.44918 | 2.1 |

| | | |
|---|---|---|
| 58230 | 2.39853 | 2.1 |
| 58231 | 1.94291 | 2.1 |
| 58232 | 1.89226 | 2.1 |
| 58233 | 0.946132 | 0.743114 |
| 58234 | 0.946132 | 2.1 |
| 58235 | 0.946131 | 2.1 |
| 58236 | 0.946131 | 2.1 |
| 58237 | 0.946132 | 2.1 |
| 58238 | 0.946132 | 2.1 |
| 58239 | 0.946131 | 2.1 |
| 58240 | 0.946131 | 2.1 |
| 58241 | 0.946131 | 2.1 |
| 58242 | 0.946131 | 2.1 |
| 58243 | 0.946132 | 2.1 |
| 58244 | 0.946132 | 2.1 |
| 58245 | 0.946128 | 2.1 |
| 58246 | 0.946128 | 2.1 |
| 58247 | 1.1749 | 2.1 |
| 58248 | 1.1749 | 2.1 |
| 58249 | 0.946131 | 1.95396 |
| 58250 | 0.946131 | 2.1 |
| 58251 | 1.23631 | 2.1 |
| 58252 | 1.1749 | 2.1 |
| 58253 | 1.23631 | 2.1 |
| 58254 | 0.94613 | 2.1 |
| 58255 | 1.1749 | 2.1 |
| 58256 | 0.946131 | 2.1 |
| 58257 | 1.89226 | 0.1696 |
| 58258 | 1.89226 | 2.1 |
| 58259 | 1.89226 | 2.1 |
| 58260 | 1.89226 | 2.1 |
| 58261 | 3.78453 | 2.1 |
| 58262 | 1.38859 | 2.1 |
| 58263 | 1.45333 | 2.1 |
| 58264 | 1.28909 | 2.1 |
| 58265 | 1.38091 | 2.1 |
| 58266 | 2.74242 | 2.1 |
| 58267 | 1.45332 | 2.1 |
| 58268 | 1.38414 | 2.1 |
| 58269 | 1.38091 | 2.1 |
| 58270 | 1.33506 | 2.1 |
| 58271 | 2.78838 | 2.1 |
| 58272 | 1.89226 | 2.1 |
| 58273 | 1.89226 | 2.1 |
| 58274 | 1.89226 | 2.1 |
| 58275 | 1.89226 | 2.1 |
| 58276 | 3.78452 | 2.1 |
| 58277 | 1.89226 | 2.1 |
| 58278 | 1.89226 | 2.1 |
| 58279 | 1.89226 | 0.973287 |
| 58280 | 1.89226 | 2.1 |
| 58281 | 1.89226 | 1.46393 |
| 58282 | 1.89226 | 2.1 |
| 58283 | 1.17418 | 2.1 |
| 58284 | 1.38091 | 2.1 |
| 58285 | 1.17418 | 2.1 |
| 58286 | 1.28909 | 2.1 |
| 58287 | 1.29741 | 2.1 |

| | | |
|---|---|---|
| 58288 | 1.41232 | 2.1 |
| 58289 | 1.38091 | 2.1 |
| 58290 | 1.33506 | 2.1 |
| 58291 | 1.27575 | 2.1 |
| 58292 | 1.27575 | 2.1 |
| 58293 | 1.29741 | 2.1 |
| 58294 | 1.35671 | 2.1 |
| 58295 | 1.89226 | 2.1 |
| 58296 | 1.89226 | 2.1 |
| 58297 | 1.89226 | 2.1 |
| 58298 | 1.89226 | 2.1 |
| 58299 | 3.78452 | 2.1 |
| 58300 | 1.92737 | 0.05 |
| 58301 | 2.40223 | 0.05 |
| 58302 | 1.56178 | 0.05 |
| 58303 | 1.97605 | 0.05 |
| 58304 | 3.90343 | 0.05 |
| 58305 | 1.89226 | 0.05 |
| 58306 | 1.89226 | 0.05 |
| 58307 | 1.89226 | 0.05 |
| 58308 | 1.89226 | 0.05 |
| 58309 | 3.78452 | 0.05 |
| 58310 | 1.89226 | 0.05 |
| 58311 | 1.89226 | 0.151656 |
| 58312 | 1.89226 | 0.107487 |
| 58313 | 1.89226 | 0.230361 |
| 58314 | 1.89226 | 0.05 |
| 58315 | 1.89226 | 0.0837 |
| 58316 | 1.13641 | 0.05 |
| 58317 | 1.13641 | 0.05 |
| 58318 | 1.12763 | 0.05 |
| 58319 | 1.12763 | 0.0530253 |
| 58320 | 1.29348 | 0.05 |
| 58321 | 1.06293 | 0.0779306 |
| 58322 | 1.20111 | 0.05 |
| 58323 | 0.988028 | 0.05 |
| 58324 | 0.988028 | 0.05 |
| 58325 | 1.06293 | 0.05 |
| 58326 | 1.29348 | 0.05 |
| 58327 | 1.20111 | 0.05 |
| 58328 | 1.56176 | 0.05 |
| 58329 | 1.46387 | 0.05 |
| 58330 | 1.97603 | 0.05 |
| 58331 | 1.97603 | 0.05 |
| 58332 | 1.08211 | 0.05 |
| 58333 | 1.08211 | 0.05 |
| 58334 | 1.89224 | 0.05 |
| 58335 | 1.89224 | 0.05 |
| 58336 | 1.89224 | 0.05 |
| 58337 | 1.89224 | 0.05 |
| 58338 | 1.89224 | 0.05 |
| 58339 | 1.89224 | 0.05 |
| 58340 | 1.89224 | 0.05 |
| 58341 | 1.89224 | 0.0868463 |
| 58342 | 1.89224 | 0.05 |
| 58343 | 1.89224 | 0.0627153 |
| 58344 | 3.78448 | 0.05 |
| 58345 | 0.820185 | 0.05 |

| | | |
|---|---|---|
| 58346 | 0.818947 | 0.05 |
| 58347 | 0.911041 | 0.05 |
| 58348 | 0.695265 | 0.05 |
| 58349 | 1.46388 | 0.05 |
| 58350 | 1.23607 | 0.05 |
| 58351 | 0.927068 | 0.05 |
| 58352 | 1.02691 | 0.05 |
| 58353 | 1.12918 | 0.05 |
| 58354 | 0.846843 | 0.05 |
| 58355 | 0.911039 | 0.05 |
| 58356 | 1.29129 | 2.1 |
| 58357 | 1.45547 | 2.1 |
| 58358 | 1.67327 | 2.1 |
| 58359 | 1.66698 | 2.1 |
| 58360 | 1.20149 | 2.1 |
| 58361 | 1.20149 | 2.1 |
| 58362 | 0.946133 | 0.843977 |
| 58363 | 0.946133 | 2.1 |
| 58364 | 0.946129 | 2.1 |
| 58365 | 0.946129 | 2.1 |
| 58366 | 0.946131 | 2.1 |
| 58367 | 0.946131 | 0.835022 |
| 58368 | 0.946131 | 2.1 |
| 58369 | 0.946131 | 2.1 |
| 58370 | 0.946131 | 2.1 |
| 58371 | 0.946131 | 2.1 |
| 58372 | 0.946131 | 2.1 |
| 58373 | 0.946131 | 2.1 |
| 58374 | 1.89226 | 2.1 |
| 58375 | 1.89226 | 2.1 |
| 58376 | 1.89226 | 2.1 |
| 58377 | 1.89226 | 0.927833 |
| 58378 | 3.78452 | 2.08426 |
| 58379 | 0.86875 | 2.1 |
| 58380 | 0.86875 | 2.1 |
| 58381 | 0.899002 | 2.1 |
| 58382 | 0.966245 | 2.1 |
| 58383 | 0.849364 | 1.00099 |
| 58384 | 1.01918 | 2.1 |
| 58385 | 0.764737 | 2.1 |
| 58386 | 0.89249 | 2.1 |
| 58387 | 0.764624 | 2.1 |
| 58388 | 0.967869 | 2.1 |
| 58389 | 0.808606 | 2.1 |
| 58390 | 0.864506 | 2.1 |
| 58391 | 0.773929 | 2.1 |
| 58392 | 0.773929 | 2.1 |
| 58393 | 0.781991 | 2.1 |
| 58394 | 0.836559 | 2.1 |
| 58395 | 0.983761 | 2.1 |
| 58396 | 0.834687 | 2.1 |
| 58397 | 0.680545 | 2.1 |
| 58398 | 0.640799 | 2.1 |
| 58399 | 0.70286 | 2.1 |
| 58400 | 0.935844 | 2.1 |
| 58401 | 0.629479 | 2.1 |
| 58402 | 0.823655 | 2.1 |
| 58403 | 0.946133 | 1.56602 |

| | | |
|---|---|---|
| 58404 | 0.946133 | 2.1 |
| 58405 | 0.946128 | 2.1 |
| 58406 | 0.946128 | 2.1 |
| 58407 | 0.94613 | 2.1 |
| 58408 | 0.94613 | 1.0038 |
| 58409 | 0.946131 | 2.1 |
| 58410 | 0.946131 | 2.1 |
| 58411 | 0.946131 | 2.1 |
| 58412 | 0.946131 | 2.1 |
| 58413 | 0.946131 | 2.1 |
| 58414 | 0.946131 | 2.1 |
| 58415 | 1.89226 | 2.1 |
| 58416 | 1.89226 | 0.946286 |
| 58417 | 1.89226 | 2.1 |
| 58418 | 1.89226 | 2.1 |
| 58419 | 1.89226 | 1.24277 |
| 58420 | 1.89226 | 2.1 |
| 58421 | 0.931638 | 2.1 |
| 58422 | 0.931638 | 1.6532 |
| 58423 | 1.03559 | 2.1 |
| 58424 | 0.977442 | 2.1 |
| 58425 | 0.826487 | 1.45807 |
| 58426 | 0.993902 | 1.68859 |
| 58427 | 1.04922 | 2.1 |
| 58428 | 0.894324 | 2.1 |
| 58429 | 0.912984 | 2.1 |
| 58430 | 1.01156 | 2.1 |
| 58431 | 0.944692 | 2.1 |
| 58432 | 1.15456 | 2.1 |
| 58433 | 1.89226 | 0.05 |
| 58434 | 1.89226 | 0.05 |
| 58435 | 1.89226 | 0.05 |
| 58436 | 1.89226 | 0.05 |
| 58437 | 3.78453 | 0.05 |
| 58438 | 1.89226 | 0.05 |
| 58439 | 1.89226 | 0.05 |
| 58440 | 2.64262 | 0.05 |
| 58441 | 2.64262 | 0.05 |
| 58442 | 1.78052 | 0.05 |
| 58443 | 1.03015 | 0.05 |
| 58444 | 0.999721 | 0.05 |
| 58445 | 0.659011 | 0.05 |
| 58446 | 1.15564 | 0.05 |
| 58447 | 1.41388 | 0.05 |
| 58448 | 1.01583 | 0.05 |
| 58449 | 1.01583 | 0.05 |
| 58450 | 1.05706 | 0.05 |
| 58451 | 1.05706 | 0.05 |
| 58452 | 1.15564 | 0.05 |
| 58453 | 0.65901 | 0.05 |
| 58454 | 1.41388 | 0.05 |
| 58455 | 0.999721 | 0.05 |
| 58456 | 1.89226 | 2.1 |
| 58457 | 1.89226 | 2.1 |
| 58458 | 1.89226 | 2.1 |
| 58459 | 1.89226 | 2.1 |
| 58460 | 3.78453 | 2.1 |
| 58461 | 2.39022 | 2.1 |

| | | |
|---|---|---|
| 58462 | 1.90734 | 2.1 |
| 58463 | 2.27634 | 2.1 |
| 58464 | 1.82666 | 2.1 |
| 58465 | 4.18368 | 2.1 |
| 58466 | 1.90734 | 2.1 |
| 58467 | 1.21564 | 2.1 |
| 58468 | 1.82666 | 2.1 |
| 58469 | 1.16938 | 2.1 |
| 58470 | 3.07672 | 2.1 |
| 58471 | 1.89226 | 2.1 |
| 58472 | 1.89226 | 2.1 |
| 58473 | 1.89226 | 2.1 |
| 58474 | 1.89226 | 2.1 |
| 58475 | 1.89226 | 2.1 |
| 58476 | 1.89226 | 2.1 |
| 58477 | 1.89227 | 2.1 |
| 58478 | 1.89227 | 2.1 |
| 58479 | 1.89227 | 2.1 |
| 58480 | 1.89227 | 2.1 |
| 58481 | 3.78453 | 2.1 |
| 58482 | 2.27634 | 2.1 |
| 58483 | 1.82666 | 2.1 |
| 58484 | 2.18042 | 2.1 |
| 58485 | 1.74738 | 2.1 |
| 58486 | 4.02372 | 2.1 |
| 58487 | 1.74738 | 2.1 |
| 58488 | 1.82666 | 2.1 |
| 58489 | 1.1181 | 2.1 |
| 58490 | 1.1181 | 2.1 |
| 58491 | 1.16938 | 2.1 |
| 58492 | 1.79865 | 2.1 |
| 58493 | 1.89226 | 2.1 |
| 58494 | 1.89226 | 2.1 |
| 58495 | 1.89226 | 2.1 |
| 58496 | 1.89226 | 2.1 |
| 58497 | 1.89226 | 2.1 |
| 58498 | 1.89226 | 2.1 |
| 58499 | 1.89226 | 1.60943 |
| 58500 | 1.89226 | 0.83041 |
| 58501 | 1.89226 | 0.965345 |
| 58502 | 1.89226 | 1.02255 |
| 58503 | 1.89226 | 1.70988 |
| 58504 | 1.89226 | 1.04248 |
| 58505 | 0.790736 | 1.04012 |
| 58506 | 1.08265 | 0.916843 |
| 58507 | 0.938909 | 1.40437 |
| 58508 | 0.638683 | 1.02886 |
| 58509 | 0.858588 | 1.1082 |
| 58510 | 0.858588 | 0.888604 |
| 58511 | 0.862743 | 1.62426 |
| 58512 | 0.862743 | 1.14733 |
| 58513 | 1.08265 | 1.42828 |
| 58514 | 0.938911 | 0.86103 |
| 58515 | 0.790735 | 1.37734 |
| 58516 | 0.638685 | 0.8798 |
| 58517 | 1.89226 | 0.864262 |
| 58518 | 1.89226 | 1.35965 |
| 58519 | 1.89226 | 1.00481 |

| | | |
|---|---|---|
| 58520 | 1.89226 | 0.872711 |
| 58521 | 1.89226 | 1.68285 |
| 58522 | 1.89226 | 1.32962 |
| 58523 | 1.89226 | 0.922099 |
| 58524 | 1.89226 | 1.54563 |
| 58525 | 1.89226 | 0.903957 |
| 58526 | 1.89226 | 0.941938 |
| 58527 | 1.89226 | 1.24345 |
| 58528 | 1.89226 | 1.59663 |
| 58529 | 1.08265 | 0.868636 |
| 58530 | 1.36209 | 0.815328 |
| 58531 | 1.25233 | 0.881393 |
| 58532 | 0.938909 | 1.01745 |
| 58533 | 1.14195 | 0.817443 |
| 58534 | 1.14195 | 0.759758 |
| 58535 | 1.15904 | 1.48771 |
| 58536 | 1.15904 | 0.956024 |
| 58537 | 1.36209 | 1.1126 |
| 58538 | 1.25233 | 0.696274 |
| 58539 | 1.08265 | 1.09619 |
| 58540 | 0.938911 | 0.777974 |
| 58541 | 1.22077 | 0.791988 |
| 58542 | 0.915244 | 0.855211 |
| 58543 | 0.967835 | 1.11479 |
| 58544 | 0.742495 | 1.06599 |
| 58545 | 0.78648 | 1.05065 |
| 58546 | 0.985041 | 0.842559 |
| 58547 | 0.989098 | 1.73394 |
| 58548 | 0.989098 | 0.947826 |
| 58549 | 1.03206 | 0.911155 |
| 58550 | 0.967897 | 1.25031 |
| 58551 | 0.915404 | 1.18872 |
| 58552 | 0.706105 | 0.9033 |
| 58553 | 2.69662 | 0.05 |
| 58554 | 1.89226 | 0.05 |
| 58555 | 2.35499 | 0.05 |
| 58556 | 1.89226 | 0.05 |
| 58557 | 4.24725 | 0.05 |
| 58558 | 1.89227 | 0.05 |
| 58559 | 1.89227 | 0.05 |
| 58560 | 1.89227 | 0.05 |
| 58561 | 1.89227 | 0.05 |
| 58562 | 3.78453 | 0.05 |
| 58563 | 2.35499 | 0.05 |
| 58564 | 1.70302 | 0.05 |
| 58565 | 1.70302 | 0.05 |
| 58566 | 1.703 | 0.05 |
| 58567 | 1.97374 | 0.05 |
| 58568 | 1.97372 | 0.05 |
| 58569 | 0.946133 | 0.05 |
| 58570 | 0.946133 | 0.05 |
| 58571 | 0.946132 | 0.05 |
| 58572 | 0.946132 | 0.05 |
| 58573 | 0.946133 | 0.05 |
| 58574 | 0.946133 | 0.05 |
| 58575 | 0.946132 | 0.05 |
| 58576 | 0.946132 | 0.05 |
| 58577 | 0.946133 | 0.05 |

| | | |
|---|---|---|
| 58578 | 0.946133 | 0.05 |
| 58579 | 0.946132 | 0.05 |
| 58580 | 0.946132 | 0.05 |
| 58581 | 0.6794 | 2.1 |
| 58582 | 0.6794 | 2.1 |
| 58583 | 0.670812 | 2.1 |
| 58584 | 0.62182 | 2.1 |
| 58585 | 0.648326 | 2.1 |
| 58586 | 0.792154 | 2.1 |
| 58587 | 0.719167 | 2.1 |
| 58588 | 0.494515 | 2.1 |
| 58589 | 0.649421 | 2.1 |
| 58590 | 0.804869 | 2.1 |
| 58591 | 0.485585 | 2.1 |
| 58592 | 0.627666 | 2.1 |
| 58593 | 1.79347 | 2.1 |
| 58594 | 1.03968 | 2.03612 |
| 58595 | 1.70303 | 2.1 |
| 58596 | 0.933265 | 1.3705 |
| 58597 | 2.62068 | 1.21078 |
| 58598 | 1.89226 | 2.1 |
| 58599 | 1.89226 | 2.1 |
| 58600 | 1.89226 | 2.1 |
| 58601 | 1.89226 | 1.16418 |
| 58602 | 3.78452 | 2.1 |
| 58603 | 1.89226 | 2.08969 |
| 58604 | 2.24604 | 2.1 |
| 58605 | 2.0554 | 2.1 |
| 58606 | 2.0554 | 2.1 |
| 58607 | 1.89226 | 2.1 |
| 58608 | 1.89226 | 2.1 |
| 58609 | 1.89226 | 2.1 |
| 58610 | 1.89226 | 0.05 |
| 58611 | 1.89226 | 0.186524 |
| 58612 | 1.89226 | 2.1 |
| 58613 | 1.89226 | 0.40177 |
| 58614 | 1.89226 | 2.1 |
| 58615 | 2.0815 | 2.1 |
| 58616 | 2.64424 | 0.05 |
| 58617 | 2.80807 | 0.05 |
| 58618 | 3.232 | 2.03217 |
| 58619 | 1.89417 | 0.05 |
| 58620 | 2.18012 | 2.1 |
| 58621 | 0.946131 | 2.1 |
| 58622 | 0.946131 | 0.05 |
| 58623 | 0.946131 | 2.1 |
| 58624 | 0.946131 | 0.796877 |
| 58625 | 0.94613 | 0.748016 |
| 58626 | 0.94613 | 0.05 |
| 58627 | 0.946132 | 2.1 |
| 58628 | 0.946132 | 2.1 |
| 58629 | 0.946131 | 1.01646 |
| 58630 | 0.946131 | 1.80451 |
| 58631 | 0.946131 | 0.984134 |
| 58632 | 0.946131 | 0.968195 |
| 58633 | 1.89227 | 0.05 |
| 58634 | 1.89227 | 0.05 |
| 58635 | 1.89227 | 0.05 |

| | | |
|---|---|---|
| 58636 | 1.89227 | 0.05 |
| 58637 | 3.78453 | 0.05 |
| 58638 | 2.70881 | 0.05 |
| 58639 | 2.68687 | 0.05 |
| 58640 | 2.03046 | 0.05 |
| 58641 | 1.9682 | 0.05 |
| 58642 | 4.67701 | 0.05 |
| 58643 | 1.08518 | 0.05 |
| 58644 | 1.08518 | 0.05 |
| 58645 | 1.09033 | 0.05 |
| 58646 | 1.09033 | 0.05 |
| 58647 | 0.842273 | 0.05 |
| 58648 | 1.19141 | 0.05 |
| 58649 | 1.34343 | 0.05 |
| 58650 | 0.9841 | 0.05 |
| 58651 | 0.9841 | 0.05 |
| 58652 | 0.842273 | 0.05 |
| 58653 | 1.34344 | 0.05 |
| 58654 | 1.19141 | 0.05 |
| 58655 | 0.946133 | 0.05 |
| 58656 | 0.946133 | 0.05 |
| 58657 | 0.946132 | 0.05 |
| 58658 | 0.946132 | 0.05 |
| 58659 | 0.946132 | 0.05 |
| 58660 | 0.946132 | 0.05 |
| 58661 | 0.946132 | 0.05 |
| 58662 | 0.946132 | 0.05 |
| 58663 | 0.946132 | 0.05 |
| 58664 | 0.946132 | 0.05 |
| 58665 | 0.946133 | 0.05 |
| 58666 | 0.946133 | 0.05 |
| 58667 | 1.89227 | 0.05 |
| 58668 | 1.89227 | 0.05 |
| 58669 | 1.89227 | 0.05 |
| 58670 | 1.89227 | 0.05 |
| 58671 | 1.89227 | 0.05 |
| 58672 | 1.89227 | 0.05 |
| 58673 | 2.03046 | 0.05 |
| 58674 | 1.9682 | 0.05 |
| 58675 | 1.23886 | 0.05 |
| 58676 | 1.12971 | 0.05 |
| 58677 | 3.20706 | 0.05 |
| 58678 | 1.89226 | 0.05 |
| 58679 | 1.89226 | 0.676308 |
| 58680 | 1.89226 | 0.05 |
| 58681 | 1.89226 | 0.228271 |
| 58682 | 3.78452 | 0.109905 |
| 58683 | 2.45569 | 0.05 |
| 58684 | 1.89226 | 0.05 |
| 58685 | 2.22922 | 0.05 |
| 58686 | 2.0815 | 0.112027 |
| 58687 | 1.85074 | 0.05 |
| 58688 | 1.70303 | 0.05 |
| 58689 | 2.72856 | 0.05 |
| 58690 | 1.89226 | 0.224999 |
| 58691 | 1.89226 | 0.05 |
| 58692 | 1.89226 | 0.0597892 |
| 58693 | 3.78452 | 0.05 |

| | | |
|---|---|---|
| 58694 | 1.89226 | 0.0612811 |
| 58695 | 1.89226 | 0.322241 |
| 58696 | 1.89226 | 0.05 |
| 58697 | 1.89226 | 0.358922 |
| 58698 | 3.78452 | 0.0919297 |
| 58699 | 0.946131 | 0.256306 |
| 58700 | 0.946131 | 0.05 |
| 58701 | 0.946131 | 0.158458 |
| 58702 | 0.946131 | 0.05 |
| 58703 | 0.94613 | 0.117475 |
| 58704 | 0.94613 | 0.201736 |
| 58705 | 0.946132 | 0.05 |
| 58706 | 0.946132 | 0.05 |
| 58707 | 0.946131 | 0.05 |
| 58708 | 0.946131 | 0.0530479 |
| 58709 | 0.946131 | 0.0612299 |
| 58710 | 0.946131 | 0.05 |
| 58711 | 2.0595 | 0.05 |
| 58712 | 2.58581 | 0.05 |
| 58713 | 1.48694 | 0.05 |
| 58714 | 1.48694 | 0.05 |
| 58715 | 2.60157 | 0.05 |
| 58716 | 2.02901 | 0.05 |
| 58717 | 1.89226 | 0.05 |
| 58718 | 1.89226 | 0.05 |
| 58719 | 1.89226 | 0.05 |
| 58720 | 1.89226 | 0.05 |
| 58721 | 1.89226 | 0.05 |
| 58722 | 1.89226 | 0.05 |
| 58723 | 2.04728 | 0.05 |
| 58724 | 2.64161 | 0.05 |
| 58725 | 1.47606 | 0.05 |
| 58726 | 1.47606 | 0.05 |
| 58727 | 2.63069 | 0.05 |
| 58728 | 2.05947 | 0.05 |
| 58729 | 1.89224 | 0.05 |
| 58730 | 1.89224 | 0.05 |
| 58731 | 1.89224 | 0.05 |
| 58732 | 1.89224 | 0.05 |
| 58733 | 1.89224 | 0.05 |
| 58734 | 1.89224 | 0.05 |
| 58735 | 0.946114 | 0.05 |
| 58736 | 0.946114 | 0.05 |
| 58737 | 0.946118 | 0.05 |
| 58738 | 0.946118 | 0.05 |
| 58739 | 0.946114 | 0.05 |
| 58740 | 0.946114 | 0.05 |
| 58741 | 0.946118 | 0.05 |
| 58742 | 0.946118 | 0.05 |
| 58743 | 0.946128 | 0.05 |
| 58744 | 0.946128 | 0.05 |
| 58745 | 0.946103 | 0.05 |
| 58746 | 0.946103 | 0.05 |
| 58747 | 1.1233 | 2.1 |
| 58748 | 1.15009 | 2.1 |
| 58749 | 2.36036 | 2.1 |
| 58750 | 2.38225 | 2.1 |
| 58751 | 3.50554 | 2.1 |

| | | |
|---|---|---|
| 58752 | 2.38224 | 2.1 |
| 58753 | 0.977775 | 2.1 |
| 58754 | 2.20473 | 2.1 |
| 58755 | 2.20473 | 2.1 |
| 58756 | 1.15009 | 2.1 |
| 58757 | 1.15009 | 2.1 |
| 58758 | 0.946119 | 2.1 |
| 58759 | 0.946119 | 2.1 |
| 58760 | 1.19111 | 2.1 |
| 58761 | 1.19111 | 2.1 |
| 58762 | 0.946118 | 2.1 |
| 58763 | 1.19111 | 2.1 |
| 58764 | 1.18016 | 2.1 |
| 58765 | 0.946118 | 1.09653 |
| 58766 | 0.94613 | 0.658782 |
| 58767 | 0.94613 | 2.1 |
| 58768 | 1.18015 | 2.1 |
| 58769 | 1.19109 | 2.1 |
| 58770 | 0.946118 | 2.1 |
| 58771 | 0.946118 | 2.1 |
| 58772 | 1.10235 | 2.1 |
| 58773 | 1.10235 | 2.1 |
| 58774 | 0.946118 | 2.1 |
| 58775 | 1.10235 | 2.1 |
| 58776 | 0.946115 | 2.1 |
| 58777 | 1.1911 | 2.1 |
| 58778 | 0.946129 | 2.1 |
| 58779 | 0.946129 | 2.1 |
| 58780 | 1.19109 | 2.1 |
| 58781 | 1.10234 | 2.1 |
| 58782 | 1.89226 | 2.1 |
| 58783 | 2.23932 | 2.1 |
| 58784 | 2.11568 | 2.1 |
| 58785 | 2.23932 | 2.1 |
| 58786 | 1.76862 | 2.1 |
| 58787 | 1.89226 | 2.1 |
| 58788 | 1.89226 | 2.1 |
| 58789 | 1.89226 | 2.1 |
| 58790 | 1.89226 | 2.1 |
| 58791 | 1.89226 | 2.1 |
| 58792 | 1.89226 | 2.1 |
| 58793 | 1.89226 | 2.1 |
| 58794 | 2.07838 | 2.1 |
| 58795 | 1.89226 | 2.1 |
| 58796 | 1.89226 | 2.1 |
| 58797 | 1.89226 | 2.1 |
| 58798 | 2.10264 | 2.1 |
| 58799 | 2.10264 | 2.1 |
| 58800 | 1.89226 | 2.1 |
| 58801 | 2.11568 | 2.1 |
| 58802 | 1.99753 | 2.1 |
| 58803 | 2.11568 | 2.1 |
| 58804 | 1.77411 | 2.1 |
| 58805 | 1.89226 | 2.1 |
| 58806 | 0.946128 | 1.7861 |
| 58807 | 0.946128 | 2.1 |
| 58808 | 0.946133 | 2.1 |
| 58809 | 0.946133 | 2.1 |

| | | |
|---|---|---|
| 58810 | 0.94613 | 2.1 |
| 58811 | 0.94613 | 1.31007 |
| 58812 | 0.946131 | 2.1 |
| 58813 | 0.946131 | 2.1 |
| 58814 | 0.946131 | 2.1 |
| 58815 | 0.946131 | 2.1 |
| 58816 | 0.946131 | 2.1 |
| 58817 | 0.946131 | 2.1 |
| 58818 | 0.946132 | 2.1 |
| 58819 | 0.946132 | 2.1 |
| 58820 | 1.03919 | 2.1 |
| 58821 | 1.03919 | 2.1 |
| 58822 | 0.946131 | 2.1 |
| 58823 | 0.946131 | 2.1 |
| 58824 | 1.02744 | 2.1 |
| 58825 | 1.03919 | 2.1 |
| 58826 | 1.02744 | 2.1 |
| 58827 | 0.946132 | 2.1 |
| 58828 | 1.03919 | 2.1 |
| 58829 | 0.946132 | 2.1 |
| 58830 | 1.64834 | 2.1 |
| 58831 | 1.82955 | 2.02385 |
| 58832 | 2.33824 | 1.22617 |
| 58833 | 2.19301 | 2.1 |
| 58834 | 3.98658 | 1.88606 |
| 58835 | 1.40574 | 2.1 |
| 58836 | 2.19301 | 1.74176 |
| 58837 | 1.64834 | 2.02205 |
| 58838 | 1.64834 | 2.1 |
| 58839 | 1.99961 | 1.25777 |
| 58840 | 1.99961 | 1.69916 |
| 58841 | 2.33824 | 1.33967 |
| 58842 | 2.19301 | 2.1 |
| 58843 | 2.68524 | 1.92904 |
| 58844 | 2.81847 | 1.66282 |
| 58845 | 5.02349 | 2.03971 |
| 58846 | 1.99961 | 2.1 |
| 58847 | 2.19301 | 1.50509 |
| 58848 | 2.68524 | 1.45205 |
| 58849 | 2.50732 | 2.01276 |
| 58850 | 4.68485 | 1.74906 |
| 58851 | 1.89226 | 2.1 |
| 58852 | 1.89226 | 2.08042 |
| 58853 | 1.89226 | 1.58583 |
| 58854 | 1.89226 | 2.1 |
| 58855 | 3.78453 | 2.1 |
| 58856 | 1.75142 | 2.1 |
| 58857 | 2.47026 | 2.1 |
| 58858 | 1.46811 | 2.1 |
| 58859 | 2.26652 | 2.1 |
| 58860 | 4.01794 | 2.1 |
| 58861 | 1.89226 | 2.1 |
| 58862 | 1.89226 | 2.1 |
| 58863 | 1.89226 | 2.1 |
| 58864 | 1.89226 | 2.1 |
| 58865 | 1.89226 | 2.1 |
| 58866 | 1.89226 | 2.1 |
| 58867 | 1.89226 | 2.1 |

| | | |
|---|---|---|
| 58868 | 1.89226 | 2.1 |
| 58869 | 1.89226 | 2.1 |
| 58870 | 1.89226 | 2.1 |
| 58871 | 1.89226 | 2.1 |
| 58872 | 1.89226 | 2.1 |
| 58873 | 0.812082 | 2.1 |
| 58874 | 0.878759 | 2.1 |
| 58875 | 0.882782 | 2.1 |
| 58876 | 0.882782 | 2.1 |
| 58877 | 1.05572 | 2.1 |
| 58878 | 1.04037 | 2.1 |
| 58879 | 0.73849 | 2.1 |
| 58880 | 0.626857 | 2.1 |
| 58881 | 0.638677 | 2.1 |
| 58882 | 1.05581 | 2.1 |
| 58883 | 0.715695 | 2.1 |
| 58884 | 1.10492 | 2.1 |
| 58885 | 1.17058 | 2.1 |
| 58886 | 1.07863 | 2.1 |
| 58887 | 1.21113 | 2.1 |
| 58888 | 1.21113 | 2.1 |
| 58889 | 1.2811 | 2.1 |
| 58890 | 1.37319 | 2.1 |
| 58891 | 1.01788 | 2.1 |
| 58892 | 0.999492 | 2.1 |
| 58893 | 1.04352 | 2.1 |
| 58894 | 1.32112 | 2.1 |
| 58895 | 1.10492 | 2.1 |
| 58896 | 1.34699 | 2.1 |
| 58897 | 1.89226 | 2.1 |
| 58898 | 1.89226 | 2.1 |
| 58899 | 1.89226 | 2.1 |
| 58900 | 1.89226 | 2.1 |
| 58901 | 1.89226 | 2.1 |
| 58902 | 1.89226 | 2.1 |
| 58903 | 1.19813 | 0.05 |
| 58904 | 1.19813 | 0.05 |
| 58905 | 2.43432 | 0.05 |
| 58906 | 1.21716 | 0.05 |
| 58907 | 0.946124 | 0.05 |
| 58908 | 1.21715 | 0.05 |
| 58909 | 0.946118 | 0.05 |
| 58910 | 2.56627 | 2.1 |
| 58911 | 1.89226 | 2.1 |
| 58912 | 1.89226 | 2.1 |
| 58913 | 1.89226 | 2.1 |
| 58914 | 2.43434 | 2.1 |
| 58915 | 2.43434 | 2.1 |
| 58916 | 1.89227 | 0.05 |
| 58917 | 1.89227 | 0.05 |
| 58918 | 1.89227 | 0.05 |
| 58919 | 1.89227 | 0.05 |
| 58920 | 1.89227 | 0.05 |
| 58921 | 1.89227 | 0.05 |
| 58922 | 1.89226 | 0.05 |
| 58923 | 1.89226 | 0.05 |
| 58924 | 1.89226 | 0.05 |
| 58925 | 1.89226 | 0.05 |

| | | |
|---|---|---|
| 58926 | 3.78453 | 0.05 |
| 58927 | 1.89226 | 0.05 |
| 58928 | 2.0764 | 0.05 |
| 58929 | 1.89226 | 0.05 |
| 58930 | 2.81674 | 0.05 |
| 58931 | 1.89226 | 0.05 |
| 58932 | 2.81674 | 0.05 |
| 58933 | 0.946132 | 0.05 |
| 58934 | 0.946132 | 0.05 |
| 58935 | 0.946131 | 0.05 |
| 58936 | 0.946131 | 0.05 |
| 58937 | 0.946132 | 0.05 |
| 58938 | 0.946132 | 0.05 |
| 58939 | 0.946132 | 0.05 |
| 58940 | 0.946132 | 0.05 |
| 58941 | 0.946132 | 0.05 |
| 58942 | 0.946132 | 0.05 |
| 58943 | 0.946132 | 0.05 |
| 58944 | 0.946132 | 0.05 |
| 58945 | 0.578684 | 1.44738 |
| 58946 | 0.578684 | 2.1 |
| 58947 | 0.589487 | 1.90829 |
| 58948 | 0.589487 | 2.1 |
| 58949 | 0.625461 | 2.1 |
| 58950 | 0.5378 | 2.1 |
| 58951 | 0.608714 | 2.1 |
| 58952 | 0.54271 | 1.59189 |
| 58953 | 0.608714 | 1.57094 |
| 58954 | 0.625461 | 1.15944 |
| 58955 | 0.54271 | 2.03976 |
| 58956 | 0.537801 | 1.88907 |
| 58957 | 0.946128 | 1.18035 |
| 58958 | 0.946128 | 2.1 |
| 58959 | 0.946133 | 1.08376 |
| 58960 | 0.946133 | 2.1 |
| 58961 | 0.946131 | 1.34833 |
| 58962 | 0.946131 | 1.14731 |
| 58963 | 0.946131 | 1.0931 |
| 58964 | 0.946131 | 1.44735 |
| 58965 | 0.94613 | 1.29863 |
| 58966 | 0.94613 | 1.01761 |
| 58967 | 0.946131 | 1.29391 |
| 58968 | 0.946131 | 1.47369 |
| 58969 | 1.21743 | 2.1 |
| 58970 | 1.25092 | 2.1 |
| 58971 | 1.38847 | 1.17602 |
| 58972 | 1.46031 | 1.14164 |
| 58973 | 2.67774 | 1.73467 |
| 58974 | 0.946128 | 1.1219 |
| 58975 | 0.946128 | 2.1 |
| 58976 | 0.946132 | 2.1 |
| 58977 | 0.946132 | 1.19389 |
| 58978 | 0.94613 | 1.69113 |
| 58979 | 0.94613 | 1.12392 |
| 58980 | 0.94613 | 1.49923 |
| 58981 | 0.94613 | 1.19392 |
| 58982 | 0.94613 | 1.11235 |
| 58983 | 0.94613 | 1.03578 |

| | | |
|---|---|---|
| 58984 | 0.94613 | 1.56768 |
| 58985 | 0.94613 | 1.16475 |
| 58986 | 1.89226 | 1.04573 |
| 58987 | 1.89226 | 1.17994 |
| 58988 | 1.89226 | 0.476264 |
| 58989 | 1.89226 | 2.1 |
| 58990 | 1.89226 | 2.1 |
| 58991 | 1.89226 | 2.1 |
| 58992 | 0.539566 | 1.10049 |
| 58993 | 0.534046 | 2.1 |
| 58994 | 0.577914 | 1.99993 |
| 58995 | 0.471623 | 1.93725 |
| 58996 | 1.46031 | 0.900511 |
| 58997 | 0.945865 | 1.15554 |
| 58998 | 0.770603 | 0.954802 |
| 58999 | 0.737201 | 2.1 |
| 59000 | 0.714829 | 1.20272 |
| 59001 | 0.536085 | 1.54394 |
| 59002 | 0.471617 | 1.35474 |
| 59003 | 1.89226 | 2.1 |
| 59004 | 1.89226 | 0.813159 |
| 59005 | 1.89226 | 0.769852 |
| 59006 | 1.89226 | 1.35711 |
| 59007 | 1.89226 | 1.4001 |
| 59008 | 1.89226 | 2.1 |
| 59009 | 1.89225 | 1.76331 |
| 59010 | 1.89225 | 0.442584 |
| 59011 | 1.65572 | 0.428185 |
| 59012 | 1.65572 | 1.04256 |
| 59013 | 1.89225 | 1.05949 |
| 59014 | 1.89225 | 1.96257 |
| 59015 | 1.89226 | 1.44239 |
| 59016 | 1.89226 | 1.09784 |
| 59017 | 1.89226 | 1.1133 |
| 59018 | 1.89226 | 0.100175 |
| 59019 | 3.78453 | 1.56584 |
| 59020 | 1.89226 | 2.1 |
| 59021 | 1.89226 | 0.620048 |
| 59022 | 1.89226 | 0.506067 |
| 59023 | 1.89226 | 1.18203 |
| 59024 | 1.89226 | 1.12529 |
| 59025 | 1.89226 | 2.1 |
| 59026 | 1.89225 | 1.39238 |
| 59027 | 1.89225 | 0.195083 |
| 59028 | 1.89225 | 0.342988 |
| 59029 | 1.89225 | 0.904836 |
| 59030 | 1.89225 | 0.808474 |
| 59031 | 1.89225 | 1.64231 |
| 59032 | 0.763592 | 0.675828 |
| 59033 | 0.763592 | 1.29975 |
| 59034 | 0.987417 | 0.946636 |
| 59035 | 0.987417 | 0.984976 |
| 59036 | 0.763592 | 0.412938 |
| 59037 | 0.804881 | 0.157063 |
| 59038 | 0.946126 | 0.978242 |
| 59039 | 0.946126 | 0.656656 |
| 59040 | 0.804879 | 0.125661 |
| 59041 | 0.94613 | 0.792857 |

| | | |
|---|---|---|
| 59042 | 0.946129 | 1.05835 |
| 59043 | 0.76359 | 0.510626 |
| 59044 | 1.46055 | 2.1 |
| 59045 | 2.04945 | 2.1 |
| 59046 | 2.04587 | 2.1 |
| 59047 | 2.04587 | 2.1 |
| 59048 | 1.50714 | 2.1 |
| 59049 | 1.50714 | 2.1 |
| 59050 | 2.04587 | 2.1 |
| 59051 | 2.38681 | 2.1 |
| 59052 | 2.42082 | 2.1 |
| 59053 | 2.42082 | 2.1 |
| 59054 | 2.04945 | 2.1 |
| 59055 | 2.04945 | 2.1 |
| 59056 | 0.872964 | 2.1 |
| 59057 | 0.872964 | 2.1 |
| 59058 | 0.884806 | 2.1 |
| 59059 | 0.884806 | 2.1 |
| 59060 | 1.0275 | 2.1 |
| 59061 | 1.02293 | 2.1 |
| 59062 | 0.711243 | 2.1 |
| 59063 | 0.730273 | 2.1 |
| 59064 | 0.711244 | 2.1 |
| 59065 | 1.0275 | 2.1 |
| 59066 | 0.730273 | 2.1 |
| 59067 | 1.02293 | 2.1 |
| 59068 | 2.04587 | 2.1 |
| 59069 | 2.47753 | 2.1 |
| 59070 | 2.42994 | 2.1 |
| 59071 | 2.42082 | 2.1 |
| 59072 | 2.05499 | 2.1 |
| 59073 | 2.04587 | 2.1 |
| 59074 | 0.946132 | 2.1 |
| 59075 | 0.946132 | 2.1 |
| 59076 | 1.13078 | 2.1 |
| 59077 | 1.07817 | 2.1 |
| 59078 | 0.946132 | 2.1 |
| 59079 | 0.946132 | 2.1 |
| 59080 | 1.07817 | 2.1 |
| 59081 | 1.07817 | 2.1 |
| 59082 | 0.946132 | 2.1 |
| 59083 | 1.07817 | 2.1 |
| 59084 | 1.13078 | 2.1 |
| 59085 | 0.946132 | 2.1 |
| 59086 | 1.89226 | 2.1 |
| 59087 | 1.89226 | 2.1 |
| 59088 | 1.89226 | 2.1 |
| 59089 | 1.89226 | 2.1 |
| 59090 | 1.89226 | 2.1 |
| 59091 | 1.89226 | 2.1 |
| 59092 | 1.89226 | 2.1 |
| 59093 | 1.89226 | 2.1 |
| 59094 | 1.89226 | 2.1 |
| 59095 | 1.89226 | 2.1 |
| 59096 | 1.89226 | 2.1 |
| 59097 | 1.89226 | 2.1 |
| 59098 | 1.89226 | 2.1 |
| 59099 | 1.89226 | 2.1 |

| | | |
|---|---|---|
| 59100 | 1.8173 | 2.1 |
| 59101 | 2.66885 | 2.1 |
| 59102 | 2.7438 | 2.1 |
| 59103 | 2.7438 | 2.1 |
| 59104 | 1.89226 | 0.85866 |
| 59105 | 1.89226 | 0.561416 |
| 59106 | 1.89226 | 0.751901 |
| 59107 | 1.89226 | 1.07971 |
| 59108 | 3.78453 | 0.817018 |
| 59109 | 1.89226 | 0.290443 |
| 59110 | 1.89226 | 0.441961 |
| 59111 | 1.89226 | 0.146954 |
| 59112 | 1.89226 | 1.67657 |
| 59113 | 1.89226 | 0.05 |
| 59114 | 1.89226 | 0.479486 |
| 59115 | 1.89226 | 0.0663009 |
| 59116 | 1.97385 | 0.758934 |
| 59117 | 1.7531 | 0.170481 |
| 59118 | 1.89226 | 1.85129 |
| 59119 | 1.7531 | 0.05 |
| 59120 | 1.89226 | 0.207873 |
| 59121 | 0.946131 | 1.18221 |
| 59122 | 0.840904 | 0.222413 |
| 59123 | 0.684333 | 0.284308 |
| 59124 | 0.94613 | 0.879389 |
| 59125 | 0.972337 | 0.50205 |
| 59126 | 0.946278 | 1.82448 |
| 59127 | 0.684304 | 0.404045 |
| 59128 | 0.684304 | 0.94403 |
| 59129 | 0.999838 | 0.238424 |
| 59130 | 0.759605 | 1.84042 |
| 59131 | 0.851528 | 0.389753 |
| 59132 | 0.851528 | 1.39542 |
| 59133 | 1.89226 | 0.72092 |
| 59134 | 2.23818 | 1.31621 |
| 59135 | 1.79771 | 0.854673 |
| 59136 | 1.89226 | 1.38546 |
| 59137 | 1.79771 | 0.533552 |
| 59138 | 1.89226 | 0.863945 |
| 59139 | 2.79647 | 2.1 |
| 59140 | 2.01613 | 2.1 |
| 59141 | 1.46826 | 2.1 |
| 59142 | 2.54504 | 2.1 |
| 59143 | 4.56117 | 2.1 |
| 59144 | 1.89226 | 2.1 |
| 59145 | 1.89226 | 2.1 |
| 59146 | 1.89226 | 2.1 |
| 59147 | 1.89226 | 2.1 |
| 59148 | 1.89226 | 2.1 |
| 59149 | 1.89226 | 2.1 |
| 59150 | 1.89226 | 2.1 |
| 59151 | 2.66884 | 2.1 |
| 59152 | 2.56022 | 2.1 |
| 59153 | 2.66884 | 2.1 |
| 59154 | 1.78364 | 2.1 |
| 59155 | 1.89226 | 2.1 |
| 59156 | 1.14282 | 2.1 |
| 59157 | 1.24068 | 2.1 |

| | | |
|---|---|---|
| 59158 | 0.880748 | 2.1 |
| 59159 | 0.834916 | 2.1 |
| 59160 | 1.05539 | 2.1 |
| 59161 | 1.05539 | 2.1 |
| 59162 | 1.04312 | 2.1 |
| 59163 | 1.04313 | 2.1 |
| 59164 | 1.2015 | 2.1 |
| 59165 | 0.83492 | 2.1 |
| 59166 | 0.789149 | 2.1 |
| 59167 | 1.24066 | 2.1 |
| 59168 | 1.89226 | 2.1 |
| 59169 | 1.89226 | 2.1 |
| 59170 | 1.89226 | 2.1 |
| 59171 | 1.89226 | 2.1 |
| 59172 | 3.78453 | 2.1 |
| 59173 | 0.946128 | 2.1 |
| 59174 | 0.946128 | 2.1 |
| 59175 | 1.28011 | 2.1 |
| 59176 | 1.22372 | 2.1 |
| 59177 | 0.946131 | 2.1 |
| 59178 | 0.946131 | 2.1 |
| 59179 | 1.22372 | 2.1 |
| 59180 | 1.22372 | 2.1 |
| 59181 | 1.22372 | 2.1 |
| 59182 | 0.94613 | 2.1 |
| 59183 | 0.946131 | 2.1 |
| 59184 | 1.28011 | 2.1 |
| 59185 | 2.28604 | 2.1 |
| 59186 | 1.34268 | 2.1 |
| 59187 | 2.43157 | 2.1 |
| 59188 | 1.50172 | 2.1 |
| 59189 | 3.77425 | 2.1 |
| 59190 | 2.6096 | 2.1 |
| 59191 | 2.43157 | 2.1 |
| 59192 | 1.66774 | 2.1 |
| 59193 | 1.66774 | 2.1 |
| 59194 | 1.50172 | 2.1 |
| 59195 | 2.43157 | 2.1 |
| 59196 | 2.16067 | 2.1 |
| 59197 | 1.08975 | 2.1 |
| 59198 | 2.19336 | 2.1 |
| 59199 | 2.06984 | 2.1 |
| 59200 | 1.21327 | 2.1 |
| 59201 | 1.08975 | 2.1 |
| 59202 | 2.28604 | 2.1 |
| 59203 | 1.21327 | 2.1 |
| 59204 | 2.29008 | 2.1 |
| 59205 | 2.16067 | 2.1 |
| 59206 | 1.34268 | 2.1 |
| 59207 | 1.21327 | 2.1 |
| 59208 | 1.89226 | 0.45992 |
| 59209 | 1.89226 | 0.0946327 |
| 59210 | 1.89226 | 0.375345 |
| 59211 | 1.89226 | 0.264971 |
| 59212 | 3.78452 | 0.224113 |
| 59213 | 1.89804 | 0.173432 |
| 59214 | 2.21656 | 0.05 |
| 59215 | 1.70537 | 0.472482 |

| | | |
|---|---|---|
| 59216 | 2.50023 | 0.081685 |
| 59217 | 3.8982 | 0.136248 |
| 59218 | 1.03136 | 0.232901 |
| 59219 | 1.89486 | 0.062306 |
| 59220 | 0.949364 | 0.26738 |
| 59221 | 1.82283 | 0.183925 |
| 59222 | 2.84422 | 0.221749 |
| 59223 | 1.89226 | 0.0902301 |
| 59224 | 1.89226 | 0.0939892 |
| 59225 | 1.89226 | 0.423889 |
| 59226 | 1.89226 | 0.22912 |
| 59227 | 3.78452 | 0.246956 |
| 59228 | 0.946132 | 0.396048 |
| 59229 | 0.946132 | 0.37897 |
| 59230 | 0.946129 | 0.531707 |
| 59231 | 0.946129 | 0.165175 |
| 59232 | 0.94613 | 0.187435 |
| 59233 | 0.94613 | 0.543438 |
| 59234 | 0.946131 | 0.311066 |
| 59235 | 0.946131 | 0.493961 |
| 59236 | 0.946131 | 0.475226 |
| 59237 | 0.946131 | 0.464522 |
| 59238 | 0.946131 | 0.05 |
| 59239 | 0.946131 | 0.567405 |
| 59240 | 1.09466 | 0.403297 |
| 59241 | 1.09466 | 0.0592292 |
| 59242 | 0.866512 | 0.449764 |
| 59243 | 1.08352 | 0.213576 |
| 59244 | 1.19189 | 0.193316 |
| 59245 | 1.21883 | 0.327912 |
| 59246 | 0.971139 | 0.368021 |
| 59247 | 0.873213 | 0.201134 |
| 59248 | 0.873202 | 0.151568 |
| 59249 | 1.00855 | 0.345601 |
| 59250 | 0.923735 | 0.267028 |
| 59251 | 1.21885 | 0.131053 |
| 59252 | 1.89225 | 0.05 |
| 59253 | 2.40019 | 0.05 |
| 59254 | 1.89225 | 0.05 |
| 59255 | 1.89225 | 0.05 |
| 59256 | 1.89225 | 0.05 |
| 59257 | 1.89225 | 0.05 |
| 59258 | 1.89225 | 0.05 |
| 59259 | 1.89225 | 0.05 |
| 59260 | 2.05088 | 0.05 |
| 59261 | 1.89225 | 0.05 |
| 59262 | 1.89225 | 0.05 |
| 59263 | 1.89225 | 0.05 |
| 59264 | 2.03956 | 0.05 |
| 59265 | 2.03956 | 0.05 |
| 59266 | 1.89226 | 2.1 |
| 59267 | 2.40021 | 2.1 |
| 59268 | 2.38473 | 0.05 |
| 59269 | 2.40021 | 2.1 |
| 59270 | 1.87679 | 0.62075 |
| 59271 | 1.89226 | 2.1 |
| 59272 | 1.89226 | 2.1 |
| 59273 | 1.89226 | 0.208022 |

| | | |
|---|---|---|
| 59274 | 1.89226 | 2.1 |
| 59275 | 1.89226 | 0.05 |
| 59276 | 1.89226 | 2.1 |
| 59277 | 1.89226 | 0.05 |
| 59278 | 2.0692 | 2.1 |
| 59279 | 1.89226 | 0.54955 |
| 59280 | 1.89226 | 2.1 |
| 59281 | 1.89226 | 0.05 |
| 59282 | 2.05089 | 2.1 |
| 59283 | 2.05089 | 0.05 |
| 59284 | 1.89226 | 2.1 |
| 59285 | 1.89226 | 2.1 |
| 59286 | 1.89226 | 2.1 |
| 59287 | 1.89226 | 2.1 |
| 59288 | 3.78452 | 2.1 |
| 59289 | 1.89226 | 0.590285 |
| 59290 | 2.50051 | 2.1 |
| 59291 | 1.89226 | 2.1 |
| 59292 | 2.30076 | 2.1 |
| 59293 | 4.39277 | 2.1 |
| 59294 | 2.50052 | 2.1 |
| 59295 | 1.70377 | 2.1 |
| 59296 | 2.30076 | 2.1 |
| 59297 | 1.54231 | 2.1 |
| 59298 | 4.04283 | 2.1 |
| 59299 | 1.89226 | 2.1 |
| 59300 | 1.89226 | 2.1 |
| 59301 | 1.89226 | 2.1 |
| 59302 | 1.89226 | 2.1 |
| 59303 | 1.89226 | 2.1 |
| 59304 | 1.89226 | 2.1 |
| 59305 | 1.89226 | 0.560268 |
| 59306 | 1.89226 | 2.1 |
| 59307 | 1.89226 | 2.1 |
| 59308 | 1.89226 | 2.1 |
| 59309 | 3.78452 | 2.1 |
| 59310 | 2.07948 | 2.1 |
| 59311 | 2.30076 | 2.1 |
| 59312 | 1.3294 | 2.1 |
| 59313 | 1.3294 | 2.1 |
| 59314 | 1.54232 | 2.1 |
| 59315 | 2.29239 | 2.1 |
| 59316 | 0.810947 | 2.1 |
| 59317 | 0.810947 | 2.1 |
| 59318 | 0.810946 | 2.1 |
| 59319 | 0.810946 | 2.1 |
| 59320 | 1.89226 | 2.1 |
| 59321 | 1.35169 | 2.1 |
| 59322 | 1.08132 | 2.1 |
| 59323 | 1.08132 | 2.1 |
| 59324 | 1.08132 | 2.1 |
| 59325 | 0.810946 | 2.1 |
| 59326 | 0.810946 | 2.1 |
| 59327 | 1.34851 | 1.16607 |
| 59328 | 0.997471 | 2.1 |
| 59329 | 1.62287 | 0.862364 |
| 59330 | 1.27096 | 2.03946 |
| 59331 | 2.62034 | 1.21443 |

| | | |
|---|---|---|
| 59332 | 1.89226 | 1.05694 |
| 59333 | 1.89226 | 2.1 |
| 59334 | 1.89226 | 1.14747 |
| 59335 | 1.89226 | 2.1 |
| 59336 | 3.78452 | 1.4017 |
| 59337 | 0.946128 | 2.1 |
| 59338 | 0.946128 | 2.1 |
| 59339 | 1.29331 | 0.103857 |
| 59340 | 1.29331 | 2.1 |
| 59341 | 1.29331 | 2.1 |
| 59342 | 1.10699 | 2.1 |
| 59343 | 0.946131 | 0.103034 |
| 59344 | 0.946131 | 2.0068 |
| 59345 | 0.94613 | 2.1 |
| 59346 | 1.29331 | 2.1 |
| 59347 | 1.10699 | 2.1 |
| 59348 | 0.946131 | 2.1 |
| 59349 | 1.62287 | 1.05237 |
| 59350 | 1.27096 | 2.1 |
| 59351 | 1.86067 | 1.00687 |
| 59352 | 1.58407 | 2.1 |
| 59353 | 3.20694 | 1.6557 |
| 59354 | 1.89226 | 1.33066 |
| 59355 | 1.89226 | 2.1 |
| 59356 | 1.89226 | 0.625014 |
| 59357 | 1.89226 | 2.1 |
| 59358 | 3.78452 | 1.35 |
| 59359 | 2.62761 | 0.750413 |
| 59360 | 2.55849 | 1.04138 |
| 59361 | 2.12261 | 1.41454 |
| 59362 | 2.22939 | 0.813472 |
| 59363 | 4.78788 | 0.899984 |
| 59364 | 0.772522 | 2.06611 |
| 59365 | 0.839588 | 1.36457 |
| 59366 | 1.31126 | 0.0807603 |
| 59367 | 1.08787 | 0.159299 |
| 59368 | 1.01318 | 0.197089 |
| 59369 | 1.01318 | 2.1 |
| 59370 | 0.900815 | 2.1 |
| 59371 | 1.10799 | 0.05 |
| 59372 | 0.772511 | 0.779266 |
| 59373 | 1.03473 | 1.00941 |
| 59374 | 1.31126 | 0.781683 |
| 59375 | 0.841464 | 1.17621 |
| 59376 | 1.89227 | 0.169973 |
| 59377 | 1.89227 | 2.08371 |
| 59378 | 1.89227 | 0.216841 |
| 59379 | 1.89227 | 1.70756 |
| 59380 | 1.89227 | 0.107826 |
| 59381 | 1.89227 | 1.78387 |
| 59382 | 1.89226 | 0.799796 |
| 59383 | 1.89226 | 0.928039 |
| 59384 | 1.89226 | 0.733577 |
| 59385 | 1.89226 | 1.06467 |
| 59386 | 1.89226 | 0.59654 |
| 59387 | 1.89226 | 0.911077 |
| 59388 | 2.12261 | 1.19554 |
| 59389 | 2.22939 | 0.47808 |

| | | |
|---|---|---|
| 59390 | 1.97004 | 0.719298 |
| 59391 | 1.8072 | 1.20034 |
| 59392 | 4.03658 | 0.816996 |
| 59393 | 1.5847 | 2.09152 |
| 59394 | 1.8072 | 0.109691 |
| 59395 | 2.12262 | 0.755917 |
| 59396 | 2.12262 | 1.98903 |
| 59397 | 1.23267 | 0.338234 |
| 59398 | 1.23267 | 1.96554 |
| 59399 | 1.89227 | 0.152674 |
| 59400 | 1.89227 | 2.1 |
| 59401 | 1.89227 | 0.199516 |
| 59402 | 1.89227 | 1.83141 |
| 59403 | 1.89227 | 0.0808032 |
| 59404 | 1.89227 | 1.97415 |
| 59405 | 1.89226 | 0.515806 |
| 59406 | 1.89226 | 0.931966 |
| 59407 | 1.89226 | 1.20202 |
| 59408 | 1.89226 | 0.506736 |
| 59409 | 3.78452 | 0.697526 |
| 59410 | 2.49093 | 2.1 |
| 59411 | 2.67393 | 2.1 |
| 59412 | 2.47932 | 2.1 |
| 59413 | 2.47932 | 2.1 |
| 59414 | 2.67994 | 2.1 |
| 59415 | 2.67994 | 2.1 |
| 59416 | 2.49873 | 2.1 |
| 59417 | 2.67393 | 2.1 |
| 59418 | 2.67993 | 2.1 |
| 59419 | 2.69807 | 2.1 |
| 59420 | 2.49873 | 2.1 |
| 59421 | 2.51686 | 2.1 |
| 59422 | 2.49093 | 2.1 |
| 59423 | 2.67994 | 2.1 |
| 59424 | 2.69784 | 2.1 |
| 59425 | 2.51651 | 2.1 |
| 59426 | 5.18877 | 2.1 |
| 59427 | 1.24359 | 2.1 |
| 59428 | 1.24936 | 2.1 |
| 59429 | 1.33997 | 2.1 |
| 59430 | 1.34892 | 2.1 |
| 59431 | 1.28805 | 2.1 |
| 59432 | 1.28805 | 2.1 |
| 59433 | 1.2955 | 2.1 |
| 59434 | 1.2955 | 2.1 |
| 59435 | 1.34892 | 2.1 |
| 59436 | 1.24359 | 2.1 |
| 59437 | 1.24936 | 2.1 |
| 59438 | 1.33997 | 2.1 |
| 59439 | 1.15506 | 2.06714 |
| 59440 | 1.15506 | 2.1 |
| 59441 | 0.946128 | 2.1 |
| 59442 | 0.946128 | 2.1 |
| 59443 | 0.946131 | 2.1 |
| 59444 | 1.15506 | 2.0895 |
| 59445 | 1.06997 | 2.1 |
| 59446 | 0.946128 | 2.1 |
| 59447 | 1.06997 | 2.1 |

| | | |
|---|---|---|
| 59448 | 1.15506 | 2.1 |
| 59449 | 0.946129 | 2.1 |
| 59450 | 0.946129 | 2.1 |
| 59451 | 1.70608 | 2.1 |
| 59452 | 2.13994 | 2.1 |
| 59453 | 1.33384 | 2.1 |
| 59454 | 1.43635 | 2.1 |
| 59455 | 3.14243 | 2.1 |
| 59456 | 2.13994 | 2.1 |
| 59457 | 2.31012 | 2.1 |
| 59458 | 1.43635 | 2.1 |
| 59459 | 1.23007 | 2.1 |
| 59460 | 3.74646 | 2.1 |
| 59461 | 2.46361 | 2.1 |
| 59462 | 1.89226 | 2.1 |
| 59463 | 1.89226 | 2.1 |
| 59464 | 1.89226 | 2.1 |
| 59465 | 2.39045 | 2.1 |
| 59466 | 2.39045 | 2.1 |
| 59467 | 1.89226 | 2.1 |
| 59468 | 1.89226 | 1.85149 |
| 59469 | 1.89226 | 2.1 |
| 59470 | 1.89226 | 1.10151 |
| 59471 | 3.78452 | 2.1 |
| 59472 | 1.52984 | 2.1 |
| 59473 | 2.25682 | 1.01346 |
| 59474 | 2.20831 | 0.870569 |
| 59475 | 2.20831 | 2.1 |
| 59476 | 1.61178 | 0.760136 |
| 59477 | 1.61178 | 2.1 |
| 59478 | 2.47395 | 2.1 |
| 59479 | 1.89226 | 2.1 |
| 59480 | 1.89226 | 2.1 |
| 59481 | 1.89226 | 2.1 |
| 59482 | 2.46362 | 2.1 |
| 59483 | 2.46362 | 2.1 |
| 59484 | 0.946131 | 0.259504 |
| 59485 | 0.946131 | 2.1 |
| 59486 | 0.946131 | 2.1 |
| 59487 | 0.946131 | 1.8974 |
| 59488 | 0.94613 | 1.2886 |
| 59489 | 0.94613 | 2.1 |
| 59490 | 0.946132 | 2.1 |
| 59491 | 0.946132 | 2.1 |
| 59492 | 0.946131 | 2.05621 |
| 59493 | 0.946131 | 2.1 |
| 59494 | 0.946131 | 2.1 |
| 59495 | 0.946131 | 2.1 |
| 59496 | 0.982398 | 2.1 |
| 59497 | 0.982398 | 2.1 |
| 59498 | 0.931703 | 2.1 |
| 59499 | 0.97549 | 2.1 |
| 59500 | 1.11509 | 2.09105 |
| 59501 | 1.10017 | 1.06333 |
| 59502 | 0.774576 | 2.1 |
| 59503 | 0.789859 | 2.1 |
| 59504 | 0.716502 | 2.1 |
| 59505 | 1.12793 | 2.1 |

| | | |
|---|---|---|
| 59506 | 1.14398 | 2.1 |
| 59507 | 0.817011 | 1.928 |
| 59508 | 1.82499 | 2.1 |
| 59509 | 1.50524 | 2.1 |
| 59510 | 1.21304 | 2.1 |
| 59511 | 1.54191 | 2.1 |
| 59512 | 3.04715 | 2.1 |
| 59513 | 1.54191 | 2.1 |
| 59514 | 1.49823 | 2.1 |
| 59515 | 1.82499 | 2.1 |
| 59516 | 1.82499 | 2.1 |
| 59517 | 1.21147 | 2.1 |
| 59518 | 1.48977 | 2.1 |
| 59519 | 1.89226 | 2.1 |
| 59520 | 1.89226 | 2.1 |
| 59521 | 1.89226 | 2.1 |
| 59522 | 1.89226 | 2.1 |
| 59523 | 3.78452 | 2.1 |
| 59524 | 1.89226 | 2.1 |
| 59525 | 1.89226 | 2.1 |
| 59526 | 1.89226 | 2.1 |
| 59527 | 1.89226 | 2.1 |
| 59528 | 1.89226 | 2.1 |
| 59529 | 1.89226 | 2.1 |
| 59530 | 0.566674 | 2.1 |
| 59531 | 0.770957 | 2.1 |
| 59532 | 0.60652 | 2.1 |
| 59533 | 0.473557 | 2.1 |
| 59534 | 0.604441 | 2.1 |
| 59535 | 0.604441 | 2.1 |
| 59536 | 0.640073 | 2.1 |
| 59537 | 0.640073 | 2.1 |
| 59538 | 0.473557 | 2.1 |
| 59539 | 0.566674 | 2.1 |
| 59540 | 0.60652 | 2.1 |
| 59541 | 0.770956 | 2.1 |
| 59542 | 1.36203 | 2.1 |
| 59543 | 1.13335 | 2.1 |
| 59544 | 1.54191 | 2.1 |
| 59545 | 1.54191 | 2.1 |
| 59546 | 1.00965 | 2.1 |
| 59547 | 1.00965 | 2.1 |
| 59548 | 1.89226 | 2.1 |
| 59549 | 1.89226 | 2.1 |
| 59550 | 1.89226 | 2.1 |
| 59551 | 1.89226 | 2.1 |
| 59552 | 1.89226 | 2.1 |
| 59553 | 1.89226 | 2.1 |
| 59554 | 0.946131 | 2.1 |
| 59555 | 0.946131 | 2.1 |
| 59556 | 0.946131 | 2.1 |
| 59557 | 0.946131 | 2.1 |
| 59558 | 0.946132 | 2.1 |
| 59559 | 0.946132 | 2.1 |
| 59560 | 0.946131 | 2.1 |
| 59561 | 0.946131 | 2.1 |
| 59562 | 0.946132 | 2.1 |
| 59563 | 0.946132 | 2.1 |

| | | |
|---|---|---|
| 59564 | 0.946131 | 2.1 |
| 59565 | 0.946131 | 2.1 |
| 59566 | 0.946132 | 0.05 |
| 59567 | 0.946132 | 0.05 |
| 59568 | 0.946133 | 0.05 |
| 59569 | 0.946133 | 0.05 |
| 59570 | 0.946132 | 0.05 |
| 59571 | 0.946132 | 0.05 |
| 59572 | 0.946132 | 0.05 |
| 59573 | 0.946132 | 0.05 |
| 59574 | 0.946132 | 0.05 |
| 59575 | 0.946132 | 0.05 |
| 59576 | 0.946133 | 0.05 |
| 59577 | 0.946133 | 0.05 |
| 59578 | 1.89226 | 0.05 |
| 59579 | 1.89226 | 0.05 |
| 59580 | 1.89226 | 0.05 |
| 59581 | 1.89226 | 0.05 |
| 59582 | 3.78453 | 0.05 |
| 59583 | 1.89226 | 0.05 |
| 59584 | 1.89226 | 0.05 |
| 59585 | 1.89226 | 0.05 |
| 59586 | 1.89226 | 0.05 |
| 59587 | 1.89226 | 0.05 |
| 59588 | 1.89226 | 0.05 |
| 59589 | 0.946132 | 0.05 |
| 59590 | 0.946132 | 0.05 |
| 59591 | 0.946131 | 0.05 |
| 59592 | 0.946131 | 0.05 |
| 59593 | 0.946132 | 0.05 |
| 59594 | 0.946132 | 0.05 |
| 59595 | 0.946132 | 0.05 |
| 59596 | 0.946132 | 0.05 |
| 59597 | 0.946132 | 0.05 |
| 59598 | 0.946132 | 0.05 |
| 59599 | 0.946132 | 0.05 |
| 59600 | 0.946132 | 0.05 |
| 59601 | 0.946129 | 0.05 |
| 59602 | 0.946129 | 0.05 |
| 59603 | 0.946128 | 0.05 |
| 59604 | 0.946128 | 0.05 |
| 59605 | 0.946132 | 0.05 |
| 59606 | 0.946132 | 0.05 |
| 59607 | 0.946126 | 0.05 |
| 59608 | 0.946126 | 0.05 |
| 59609 | 0.946129 | 0.05 |
| 59610 | 0.946129 | 0.05 |
| 59611 | 0.946129 | 0.05 |
| 59612 | 0.946129 | 0.05 |
| 59613 | 1.20674 | 0.05 |
| 59614 | 1.79877 | 0.05 |
| 59615 | 1.22586 | 0.05 |
| 59616 | 1.22586 | 0.05 |
| 59617 | 1.7919 | 0.05 |
| 59618 | 1.7919 | 0.05 |
| 59619 | 2.27622 | 0.05 |
| 59620 | 1.79877 | 0.05 |
| 59621 | 1.7919 | 0.05 |

| | | |
|---|---|---|
| 59622 | 2.27102 | 0.05 |
| 59623 | 4.06979 | 0.05 |
| 59624 | 1.89226 | 0.05 |
| 59625 | 1.89226 | 0.05 |
| 59626 | 1.89226 | 0.05 |
| 59627 | 1.89226 | 0.05 |
| 59628 | 3.78452 | 0.05 |
| 59629 | 1.89226 | 0.05 |
| 59630 | 1.89226 | 0.05 |
| 59631 | 1.89226 | 0.05 |
| 59632 | 1.89226 | 0.05 |
| 59633 | 3.78453 | 0.05 |
| 59634 | 1.13259 | 0.05 |
| 59635 | 1.79187 | 0.05 |
| 59636 | 1.32741 | 0.05 |
| 59637 | 1.18784 | 0.05 |
| 59638 | 1.703 0.05 | |
| 59639 | 1.49575 | 0.05 |
| 59640 | 1.46201 | 0.05 |
| 59641 | 2.27099 | 0.05 |
| 59642 | 1.79187 | 0.05 |
| 59643 | 1.79187 | 0.05 |
| 59644 | 2.12923 | 0.05 |
| 59645 | 2.12923 | 0.05 |
| 59646 | 1.89224 | 0.05 |
| 59647 | 1.89224 | 0.05 |
| 59648 | 1.89224 | 0.05 |
| 59649 | 1.89224 | 0.05 |
| 59650 | 3.78447 | 0.05 |
| 59651 | 0.946119 | 0.05 |
| 59652 | 0.946119 | 0.05 |
| 59653 | 0.946118 | 0.05 |
| 59654 | 0.946118 | 0.05 |
| 59655 | 0.946118 | 0.05 |
| 59656 | 0.946118 | 0.05 |
| 59657 | 0.946119 | 0.05 |
| 59658 | 0.946119 | 0.05 |
| 59659 | 0.946131 | 0.05 |
| 59660 | 0.946131 | 0.05 |
| 59661 | 0.946106 | 0.05 |
| 59662 | 0.946106 | 0.05 |
| 59663 | 2.07526 | 0.287323 |
| 59664 | 1.89226 | 0.17537 |
| 59665 | 1.89226 | 0.742549 |
| 59666 | 2.13445 | 0.644606 |
| 59667 | 4.02671 | 0.142921 |
| 59668 | 1.89226 | 0.27583 |
| 59669 | 1.89226 | 0.545165 |
| 59670 | 1.89226 | 0.596894 |
| 59671 | 1.89226 | 0.05 |
| 59672 | 1.89226 | 0.715383 |
| 59673 | 1.89226 | 0.472138 |
| 59674 | 2.13446 | 0.261464 |
| 59675 | 1.00013 | 0.05 |
| 59676 | 1.1475 | 0.96545 |
| 59677 | 2.20046 | 0.270241 |
| 59678 | 3.20059 | 0.744434 |
| 59679 | 0.946133 | 0.535575 |

| | | |
|---|---|---|
| 59680 | 0.946133 | 0.622892 |
| 59681 | 1.06722 | 0.605214 |
| 59682 | 1.10023 | 0.490316 |
| 59683 | 0.946131 | 0.552547 |
| 59684 | 0.946131 | 0.05 |
| 59685 | 1.10023 | 0.671754 |
| 59686 | 1.10023 | 0.257376 |
| 59687 | 0.946131 | 0.645943 |
| 59688 | 1.10023 | 0.213182 |
| 59689 | 0.94613 | 0.58032 |
| 59690 | 1.06723 | 0.147024 |
| 59691 | 1.89226 | 0.155673 |
| 59692 | 1.89226 | 0.106258 |
| 59693 | 1.89226 | 0.592834 |
| 59694 | 1.89226 | 0.480299 |
| 59695 | 3.78452 | 0.51176 |
| 59696 | 1.89226 | 0.583593 |
| 59697 | 1.89226 | 0.05 |
| 59698 | 1.89226 | 0.664264 |
| 59699 | 1.89226 | 0.578436 |
| 59700 | 1.89226 | 0.135156 |
| 59701 | 1.89226 | 0.0984839 |
| 59702 | 1.89226 | 2.1 |
| 59703 | 1.89226 | 0.05 |
| 59704 | 1.89226 | 0.05 |
| 59705 | 1.89226 | 0.05 |
| 59706 | 1.89226 | 2.1 |
| 59707 | 0.828588 | 0.05 |
| 59708 | 0.828588 | 0.05 |
| 59709 | 0.841316 | 0.05 |
| 59710 | 0.841316 | 2.1 |
| 59711 | 0.528387 | 2.1 |
| 59712 | 0.565893 | 0.05 |
| 59713 | 1.10401 | 0.05 |
| 59714 | 1.10401 | 2.1 |
| 59715 | 0.528387 | 2.1 |
| 59716 | 1.89226 | 2.1 |
| 59717 | 1.36047 | 0.05 |
| 59718 | 1.33973 | 2.1 |
| 59719 | 1.33973 | 2.1 |
| 59720 | 1.89226 | 0.05 |
| 59721 | 2.28951 | 0.05 |
| 59722 | 1.89226 | 1.04052 |
| 59723 | 2.80796 | 2.07637 |
| 59724 | 2.01584 | 2.05631 |
| 59725 | 2.01584 | 1.24535 |
| 59726 | 1.89226 | 1.83724 |
| 59727 | 1.89226 | 1.19769 |
| 59728 | 1.89226 | 0.86278 |
| 59729 | 1.89226 | 1.98984 |
| 59730 | 1.89226 | 1.58246 |
| 59731 | 1.89226 | 1.00052 |
| 59732 | 1.89226 | 1.70511 |
| 59733 | 1.89226 | 1.05442 |
| 59734 | 2.15223 | 1.00461 |
| 59735 | 1.30664 | 2.1 |
| 59736 | 1.96011 | 2.1 |
| 59737 | 2.76516 | 1.06025 |

| | | |
|---|---|---|
| 59738 | 4.11234 | 2.1 |
| 59739 | 1.89226 | 0.813146 |
| 59740 | 1.89226 | 1.98134 |
| 59741 | 1.89226 | 1.3562 |
| 59742 | 1.89226 | 0.951384 |
| 59743 | 1.89226 | 1.53929 |
| 59744 | 1.89226 | 1.00871 |
| 59745 | 2.81143 | 2.1 |
| 59746 | 2.58517 | 2.1 |
| 59747 | 2.55318 | 2.1 |
| 59748 | 2.7542 | 2.1 |
| 59749 | 5.33937 | 2.1 |
| 59750 | 2.7777 | 2.1 |
| 59751 | 2.7542 | 2.1 |
| 59752 | 2.71672 | 2.1 |
| 59753 | 2.70401 | 2.1 |
| 59754 | 5.45821 | 2.1 |
| 59755 | 2.55318 | 2.1 |
| 59756 | 2.7542 | 2.1 |
| 59757 | 2.71672 | 2.1 |
| 59758 | 2.54198 | 2.1 |
| 59759 | 5.29618 | 2.1 |
| 59760 | 1.95416 | 2.1 |
| 59761 | 2.162 2.1 | |
| 59762 | 2.11563 | 2.1 |
| 59763 | 1.91813 | 2.1 |
| 59764 | 4.08013 | 2.1 |
| 59765 | 1.95417 | 2.1 |
| 59766 | 1.47712 | 2.1 |
| 59767 | 1.45182 | 2.1 |
| 59768 | 1.91813 | 2.1 |
| 59769 | 3.39525 | 2.1 |
| 59770 | 2.11562 | 2.1 |
| 59771 | 1.91813 | 2.1 |
| 59772 | 1.50979 | 2.1 |
| 59773 | 2.03974 | 2.1 |
| 59774 | 3.95787 | 2.1 |
| 59775 | 1.85605 | 2.1 |
| 59776 | 1.91813 | 2.1 |
| 59777 | 1.88885 | 2.1 |
| 59778 | 1.42255 | 2.1 |
| 59779 | 1.45182 | 2.1 |
| 59780 | 1.45182 | 2.1 |
| 59781 | 0.94613 | 0.517554 |
| 59782 | 0.94613 | 0.462011 |
| 59783 | 0.946128 | 0.05 |
| 59784 | 0.946128 | 0.85832 |
| 59785 | 0.946127 | 0.555384 |
| 59786 | 0.946127 | 0.306821 |
| 59787 | 0.946131 | 0.109206 |
| 59788 | 0.946131 | 0.113361 |
| 59789 | 0.946129 | 0.390123 |
| 59790 | 0.946129 | 0.402328 |
| 59791 | 0.946128 | 0.352981 |
| 59792 | 0.946128 | 0.548755 |
| 59793 | 0.543052 | 0.457619 |
| 59794 | 0.54608 | 0.186077 |
| 59795 | 0.602293 | 0.0594626 |

| | | |
|---|---|---|
| 59796 | 0.535325 | 0.331388 |
| 59797 | 1.05446 | 0.294846 |
| 59798 | 0.850496 | 0.2369 |
| 59799 | 0.699838 | 0.466139 |
| 59800 | 0.679751 | 0.539091 |
| 59801 | 0.713797 | 0.067105 |
| 59802 | 0.509794 | 0.348686 |
| 59803 | 0.602304 | 0.14436 |
| 59804 | 1.40017 | 0.05 |
| 59805 | 1.40017 | 2.1 |
| 59806 | 0.94613 | 0.05 |
| 59807 | 0.94613 | 2.1 |
| 59808 | 1.40017 | 2.1 |
| 59809 | 1.33997 | 2.1 |
| 59810 | 0.946131 | 0.05 |
| 59811 | 0.946131 | 0.05 |
| 59812 | 1.33997 | 0.181757 |
| 59813 | 0.946131 | 0.418245 |
| 59814 | 0.946131 | 0.227524 |
| 59815 | 1.40017 | 0.291516 |
| 59816 | 1.89226 | 0.05 |
| 59817 | 1.89226 | 0.05 |
| 59818 | 1.89226 | 0.05 |
| 59819 | 1.89226 | 0.05 |
| 59820 | 3.78452 | 0.05 |
| 59821 | 0.946133 | 0.05 |
| 59822 | 0.946133 | 0.05 |
| 59823 | 0.946132 | 0.05 |
| 59824 | 0.946132 | 0.05 |
| 59825 | 0.946132 | 0.05 |
| 59826 | 0.946132 | 0.05 |
| 59827 | 0.946133 | 0.05 |
| 59828 | 0.946133 | 0.05 |
| 59829 | 0.946132 | 0.05 |
| 59830 | 0.946132 | 0.05 |
| 59831 | 0.946132 | 0.05 |
| 59832 | 0.946132 | 0.05 |
| 59833 | 1.89224 | 0.05 |
| 59834 | 1.89224 | 0.05 |
| 59835 | 1.89224 | 0.05 |
| 59836 | 1.89224 | 0.05 |
| 59837 | 1.89224 | 0.05 |
| 59838 | 1.89224 | 0.05 |
| 59839 | 0.94612 | 0.05 |
| 59840 | 0.94612 | 0.05 |
| 59841 | 0.946119 | 0.05 |
| 59842 | 0.946119 | 0.05 |
| 59843 | 0.946119 | 0.05 |
| 59844 | 0.946119 | 0.05 |
| 59845 | 0.94612 | 0.05 |
| 59846 | 0.94612 | 0.05 |
| 59847 | 0.946132 | 0.05 |
| 59848 | 0.946132 | 0.05 |
| 59849 | 0.946107 | 0.05 |
| 59850 | 0.946107 | 0.05 |
| 59851 | 2.63939 | 2.1 |
| 59852 | 1.2862 | 2.1 |
| 59853 | 1.29891 | 2.1 |

| | | |
|---|---|---|
| 59854 | 2.64419 | 2.1 |
| 59855 | 3.93039 | 2.1 |
| 59856 | 2.6394 | 2.1 |
| 59857 | 1.89226 | 2.1 |
| 59858 | 1.89226 | 2.1 |
| 59859 | 2.64419 | 2.1 |
| 59860 | 4.53646 | 2.1 |
| 59861 | 1.89226 | 2.1 |
| 59862 | 1.89226 | 2.1 |
| 59863 | 1.89226 | 2.1 |
| 59864 | 1.89226 | 2.1 |
| 59865 | 3.78453 | 2.1 |
| 59866 | 1.89226 | 2.1 |
| 59867 | 1.89226 | 2.1 |
| 59868 | 1.89226 | 2.1 |
| 59869 | 1.89226 | 2.1 |
| 59870 | 1.89226 | 2.1 |
| 59871 | 1.89226 | 2.1 |
| 59872 | 1.31998 | 2.1 |
| 59873 | 1.32209 | 2.1 |
| 59874 | 0.649456 | 2.1 |
| 59875 | 0.648474 | 2.1 |
| 59876 | 0.984995 | 2.1 |
| 59877 | 0.984995 | 2.1 |
| 59878 | 0.985573 | 2.1 |
| 59879 | 0.985573 | 2.1 |
| 59880 | 1.31999 | 2.1 |
| 59881 | 0.648472 | 2.1 |
| 59882 | 0.649455 | 2.1 |
| 59883 | 1.32209 | 2.1 |
| 59884 | 0.946133 | 2.1 |
| 59885 | 0.946133 | 2.1 |
| 59886 | 1.31999 | 2.1 |
| 59887 | 1.3221 | 2.1 |
| 59888 | 0.946132 | 2.1 |
| 59889 | 0.946132 | 2.1 |
| 59890 | 1.31999 | 2.1 |
| 59891 | 1.31999 | 2.1 |
| 59892 | 1.31999 | 2.1 |
| 59893 | 0.946132 | 2.1 |
| 59894 | 0.946133 | 2.1 |
| 59895 | 1.3221 | 2.1 |
| 59896 | 0.946132 | 2.1 |
| 59897 | 0.946132 | 2.1 |
| 59898 | 0.946131 | 2.1 |
| 59899 | 0.946131 | 2.1 |
| 59900 | 0.946132 | 2.1 |
| 59901 | 0.946132 | 2.1 |
| 59902 | 0.946131 | 2.1 |
| 59903 | 0.946131 | 2.1 |
| 59904 | 0.946131 | 2.1 |
| 59905 | 0.946131 | 2.1 |
| 59906 | 0.946132 | 2.1 |
| 59907 | 0.946132 | 2.1 |
| 59908 | 0.946128 | 2.1 |
| 59909 | 0.946128 | 2.1 |
| 59910 | 0.946132 | 2.1 |
| 59911 | 0.946132 | 2.1 |

| | | |
|---|---|---|
| 59912 | 0.946131 | 2.1 |
| 59913 | 0.946131 | 2.1 |
| 59914 | 0.94613 | 2.1 |
| 59915 | 0.94613 | 2.1 |
| 59916 | 0.94613 | 2.1 |
| 59917 | 0.94613 | 2.1 |
| 59918 | 0.946131 | 2.1 |
| 59919 | 0.946131 | 2.1 |
| 59920 | 1.89226 | 0.05 |
| 59921 | 1.89226 | 0.141696 |
| 59922 | 1.89226 | 0.130759 |
| 59923 | 1.89226 | 0.0638629 |
| 59924 | 1.89226 | 0.05 |
| 59925 | 1.89226 | 0.05 |
| 59926 | 1.89226 | 0.0575456 |
| 59927 | 1.89226 | 0.83049 |
| 59928 | 1.89226 | 0.253178 |
| 59929 | 1.89226 | 0.573851 |
| 59930 | 3.78453 | 0.87014 |
| 59931 | 0.946133 | 0.140504 |
| 59932 | 0.946133 | 0.122619 |
| 59933 | 0.946132 | 0.05 |
| 59934 | 0.946132 | 0.05 |
| 59935 | 0.946133 | 0.152376 |
| 59936 | 0.946133 | 0.19979 |
| 59937 | 0.946132 | 0.158767 |
| 59938 | 0.946132 | 0.05 |
| 59939 | 0.946132 | 0.106786 |
| 59940 | 0.946132 | 0.241238 |
| 59941 | 0.946132 | 0.05 |
| 59942 | 0.946132 | 0.389845 |
| 59943 | 2.23638 | 0.05 |
| 59944 | 1.89226 | 0.05 |
| 59945 | 2.66014 | 0.0579127 |
| 59946 | 2.66014 | 0.0853769 |
| 59947 | 1.89226 | 0.0793809 |
| 59948 | 1.89226 | 0.05 |
| 59949 | 1.11819 | 0.05 |
| 59950 | 1.33007 | 0.05 |
| 59951 | 0.660255 | 0.05 |
| 59952 | 0.91151 | 0.05 |
| 59953 | 0.911511 | 0.05 |
| 59954 | 1.33007 | 0.05 |
| 59955 | 1.11819 | 0.05 |
| 59956 | 0.660255 | 0.05 |
| 59957 | 0.985327 | 0.05 |
| 59958 | 0.985327 | 0.05 |
| 59959 | 1.005 | 0.05 |
| 59960 | 1.005 | 0.05 |
| 59961 | 1.89226 | 2.1 |
| 59962 | 1.89226 | 2.1 |
| 59963 | 1.89226 | 2.1 |
| 59964 | 1.89226 | 2.1 |
| 59965 | 3.78453 | 2.1 |
| 59966 | 1.59533 | 2.1 |
| 59967 | 2.68571 | 2.1 |
| 59968 | 1.28291 | 2.1 |
| 59969 | 2.46974 | 2.1 |

| | | |
|---|---|---|
| 59970 | 4.06506 | 2.1 |
| 59971 | 2.68571 | 2.1 |
| 59972 | 1.89226 | 2.1 |
| 59973 | 1.89226 | 2.1 |
| 59974 | 1.89226 | 2.1 |
| 59975 | 2.46973 | 2.1 |
| 59976 | 2.46973 | 2.1 |
| 59977 | 1.89226 | 2.1 |
| 59978 | 1.89226 | 2.1 |
| 59979 | 1.89226 | 2.1 |
| 59980 | 1.89226 | 2.1 |
| 59981 | 1.89226 | 2.1 |
| 59982 | 1.89226 | 2.1 |
| 59983 | 1.89226 | 2.1 |
| 59984 | 1.89226 | 2.1 |
| 59985 | 1.89226 | 2.1 |
| 59986 | 1.89226 | 2.1 |
| 59987 | 1.89226 | 2.1 |
| 59988 | 1.89226 | 2.1 |
| 59989 | 0.883594 | 2.1 |
| 59990 | 0.883594 | 2.1 |
| 59991 | 0.876484 | 2.1 |
| 59992 | 0.876484 | 2.1 |
| 59993 | 1.10442 | 2.1 |
| 59994 | 1.23487 | 2.1 |
| 59995 | 0.641457 | 2.1 |
| 59996 | 0.52521 | 2.1 |
| 59997 | 1.10442 | 2.1 |
| 59998 | 0.52521 | 2.1 |
| 59999 | 0.641457 | 2.1 |
| 60000 | 1.23487 | 2.1 |
| 60001 | 0.946131 | 2.1 |
| 60002 | 0.946131 | 2.1 |
| 60003 | 0.946131 | 2.1 |
| 60004 | 0.946131 | 2.1 |
| 60005 | 0.946131 | 2.1 |
| 60006 | 0.946131 | 2.1 |
| 60007 | 0.946131 | 2.1 |
| 60008 | 0.946131 | 2.1 |
| 60009 | 0.946131 | 2.1 |
| 60010 | 0.946131 | 2.1 |
| 60011 | 0.946131 | 2.1 |
| 60012 | 0.946131 | 2.1 |
| 60013 | 2.04016 | 0.05 |
| 60014 | 2.13042 | 0.05 |
| 60015 | 2.11465 | 0.05 |
| 60016 | 2.11465 | 0.05 |
| 60017 | 1.95908 | 0.05 |
| 60018 | 1.95908 | 0.05 |
| 60019 | 1.66953 | 0.05 |
| 60020 | 2.13041 | 0.05 |
| 60021 | 1.95908 | 0.05 |
| 60022 | 2.21952 | 0.05 |
| 60023 | 1.66953 | 0.05 |
| 60024 | 1.92997 | 0.05 |
| 60025 | 2.04016 | 0.05 |
| 60026 | 1.95908 | 0.05 |
| 60027 | 1.82014 | 0.05 |

| | | |
|---|---|---|
| 60028 | 2.08757 | 0.05 |
| 60029 | 3.86029 | 0.05 |
| 60030 | 0.834764 | 0.05 |
| 60031 | 0.722102 | 0.05 |
| 60032 | 0.979537 | 0.05 |
| 60033 | 0.910065 | 0.05 |
| 60034 | 0.840053 | 0.05 |
| 60035 | 0.840052 | 0.05 |
| 60036 | 0.861587 | 0.05 |
| 60037 | 0.861587 | 0.05 |
| 60038 | 0.910067 | 0.05 |
| 60039 | 0.722101 | 0.05 |
| 60040 | 0.834763 | 0.05 |
| 60041 | 0.979539 | 0.05 |
| 60042 | 1.89226 | 0.05 |
| 60043 | 1.89226 | 0.05 |
| 60044 | 1.89226 | 0.05 |
| 60045 | 1.89226 | 0.05 |
| 60046 | 1.89226 | 0.05 |
| 60047 | 1.89226 | 0.05 |
| 60048 | 1.89226 | 0.05 |
| 60049 | 1.89226 | 0.05 |
| 60050 | 1.89226 | 0.05 |
| 60051 | 1.89226 | 0.05 |
| 60052 | 3.78453 | 0.05 |
| 60053 | 1.89226 | 0.05 |
| 60054 | 1.89226 | 0.05 |
| 60055 | 1.89226 | 0.05 |
| 60056 | 1.89226 | 0.05 |
| 60057 | 1.89226 | 0.05 |
| 60058 | 1.89226 | 0.05 |
| 60059 | 1.20691 | 0.05 |
| 60060 | 2.27379 | 0.05 |
| 60061 | 2.58476 | 0.05 |
| 60062 | 1.5124 | 0.05 |
| 60063 | 3.79167 | 0.05 |
| 60064 | 1.89226 | 0.05 |
| 60065 | 2.2738 | 0.05 |
| 60066 | 2.58477 | 0.05 |
| 60067 | 1.89226 | 0.05 |
| 60068 | 4.47703 | 0.05 |
| 60069 | 0.952225 | 1.36931 |
| 60070 | 0.952225 | 1.91614 |
| 60071 | 0.909865 | 2.08063 |
| 60072 | 0.909865 | 2.1 |
| 60073 | 0.909895 | 2.1 |
| 60074 | 0.946132 | 1.41887 |
| 60075 | 0.946132 | 2.01694 |
| 60076 | 0.915958 | 1.46376 |
| 60077 | 0.946132 | 1.91166 |
| 60078 | 0.946132 | 1.9537 |
| 60079 | 0.909894 | 1.83329 |
| 60080 | 0.915958 | 1.96024 |
| 60081 | 0.983493 | 2.1 |
| 60082 | 2.40055 | 2.1 |
| 60083 | 1.02986 | 2.1 |
| 60084 | 1.02986 | 2.1 |
| 60085 | 2.34217 | 2.1 |

| | | |
|---|---|---|
| 60086 | 2.34217 | 2.1 |
| 60087 | 1.89226 | 2.1 |
| 60088 | 2.40056 | 2.1 |
| 60089 | 2.34217 | 2.1 |
| 60090 | 2.40056 | 2.1 |
| 60091 | 1.83388 | 2.1 |
| 60092 | 1.89226 | 2.1 |
| 60093 | 1.89226 | 2.1 |
| 60094 | 1.89226 | 2.1 |
| 60095 | 1.89226 | 2.1 |
| 60096 | 1.89226 | 2.1 |
| 60097 | 1.89226 | 2.1 |
| 60098 | 1.89226 | 2.1 |
| 60099 | 1.89226 | 2.1 |
| 60100 | 1.89226 | 2.1 |
| 60101 | 1.89226 | 2.1 |
| 60102 | 1.89226 | 2.1 |
| 60103 | 1.89226 | 2.1 |
| 60104 | 1.89226 | 2.1 |
| 60105 | 0.946133 | 2.1 |
| 60106 | 0.946133 | 2.1 |
| 60107 | 1.17109 | 2.1 |
| 60108 | 1.13078 | 2.1 |
| 60109 | 0.946132 | 2.1 |
| 60110 | 0.946132 | 2.1 |
| 60111 | 1.13078 | 2.1 |
| 60112 | 1.13078 | 2.1 |
| 60113 | 1.13078 | 2.1 |
| 60114 | 0.946133 | 2.1 |
| 60115 | 0.946132 | 2.1 |
| 60116 | 1.17109 | 2.1 |
| 60117 | 1.89226 | 2.1 |
| 60118 | 1.89226 | 2.1 |
| 60119 | 1.89226 | 2.1 |
| 60120 | 1.89226 | 2.1 |
| 60121 | 1.89226 | 2.1 |
| 60122 | 1.89226 | 2.1 |
| 60123 | 2.33963 | 1.23085 |
| 60124 | 1.89227 | 0.735936 |
| 60125 | 1.74565 | 0.886329 |
| 60126 | 1.74565 | 0.948622 |
| 60127 | 1.89227 | 0.10928 |
| 60128 | 1.89227 | 0.200152 |
| 60129 | 1.89227 | 0.974104 |
| 60130 | 1.89227 | 0.762156 |
| 60131 | 1.89227 | 1.69479 |
| 60132 | 1.89227 | 0.846685 |
| 60133 | 3.78453 | 0.827749 |
| 60134 | 1.31398 | 0.670866 |
| 60135 | 1.10392 | 0.791032 |
| 60136 | 0.796878 | 0.719627 |
| 60137 | 0.524356 | 0.402418 |
| 60138 | 0.870614 | 0.722393 |
| 60139 | 0.945739 | 0.352929 |
| 60140 | 0.89208 | 0.814744 |
| 60141 | 0.89208 | 0.944937 |
| 60142 | 1.23425 | 0.47483 |
| 60143 | 0.774658 | 0.917738 |

| | | |
|---|---|---|
| 60144 | 1.03182 | 0.443015 |
| 60145 | 0.557289 | 0.224535 |
| 60146 | 2.33963 | 1.10855 |
| 60147 | 1.89226 | 0.0819544 |
| 60148 | 1.89226 | 1.07648 |
| 60149 | 2.77245 | 0.656715 |
| 60150 | 4.23189 | 0.942499 |
| 60151 | 1.89226 | 1.25787 |
| 60152 | 1.89226 | 0.468324 |
| 60153 | 1.89226 | 1.32655 |
| 60154 | 1.89226 | 1.13022 |
| 60155 | 1.89226 | 0.720452 |
| 60156 | 1.89226 | 0.405979 |
| 60157 | 1.89226 | 0.05 |
| 60158 | 1.89226 | 0.05 |
| 60159 | 1.89226 | 0.05 |
| 60160 | 1.89226 | 0.0936706 |
| 60161 | 1.89226 | 0.05 |
| 60162 | 1.89226 | 0.0627027 |
| 60163 | 1.89226 | 0.05 |
| 60164 | 1.89226 | 0.05 |
| 60165 | 1.89226 | 0.05 |
| 60166 | 1.89226 | 0.0622857 |
| 60167 | 1.89226 | 0.05 |
| 60168 | 1.89226 | 0.05 |
| 60169 | 0.946128 | 0.05 |
| 60170 | 0.946128 | 0.05 |
| 60171 | 0.963918 | 0.05 |
| 60172 | 1.37543 | 0.05 |
| 60173 | 0.946129 | 0.05 |
| 60174 | 0.946129 | 0.05 |
| 60175 | 1.37542 | 0.05 |
| 60176 | 1.37542 | 0.05 |
| 60177 | 0.94613 | 0.05 |
| 60178 | 1.37542 | 0.05 |
| 60179 | 0.963916 | 0.05 |
| 60180 | 0.94613 | 0.05 |
| 60181 | 0.946132 | 0.05 |
| 60182 | 0.946132 | 0.05 |
| 60183 | 0.946131 | 0.05 |
| 60184 | 0.946131 | 0.05 |
| 60185 | 0.946131 | 0.05 |
| 60186 | 0.946131 | 0.05 |
| 60187 | 0.946132 | 0.05 |
| 60188 | 0.946132 | 0.05 |
| 60189 | 0.946131 | 0.05 |
| 60190 | 0.946131 | 0.05 |
| 60191 | 0.946131 | 0.05 |
| 60192 | 0.946131 | 0.05 |
| 60193 | 1.89226 | 0.05 |
| 60194 | 1.89226 | 0.05 |
| 60195 | 1.89226 | 0.05 |
| 60196 | 1.89226 | 0.0597735 |
| 60197 | 3.78452 | 0.05 |
| 60198 | 0.946127 | 0.05 |
| 60199 | 0.946127 | 0.05 |
| 60200 | 0.946131 | 0.05 |
| 60201 | 0.946131 | 0.05 |

| | | |
|---|---|---|
| 60202 | 0.946128 | 0.05 |
| 60203 | 0.946128 | 0.05 |
| 60204 | 0.94613 | 0.05 |
| 60205 | 0.94613 | 0.05 |
| 60206 | 0.946129 | 0.05 |
| 60207 | 0.946129 | 0.05 |
| 60208 | 0.946129 | 0.05 |
| 60209 | 0.946129 | 0.05 |
| 60210 | 1.89226 | 0.111393 |
| 60211 | 1.89226 | 0.798062 |
| 60212 | 1.89226 | 2.1 |
| 60213 | 1.89226 | 0.05 |
| 60214 | 3.78453 | 0.05 |
| 60215 | 1.89226 | 0.0639766 |
| 60216 | 1.89226 | 0.05 |
| 60217 | 1.89226 | 0.05 |
| 60218 | 1.89226 | 0.05 |
| 60219 | 3.78453 | 0.106085 |
| 60220 | 0.946131 | 0.05 |
| 60221 | 0.946131 | 0.05 |
| 60222 | 0.946132 | 0.988227 |
| 60223 | 0.946132 | 0.112049 |
| 60224 | 0.946132 | 0.889536 |
| 60225 | 0.946132 | 0.05 |
| 60226 | 0.946132 | 0.895421 |
| 60227 | 0.946132 | 0.0737682 |
| 60228 | 0.946132 | 0.0731694 |
| 60229 | 0.946132 | 0.35966 |
| 60230 | 0.946132 | 0.55843 |
| 60231 | 0.946132 | 0.060251 |
| 60232 | 0.548169 | 0.05 |
| 60233 | 0.736603 | 0.05 |
| 60234 | 1.18322 | 0.05 |
| 60235 | 1.01139 | 0.05 |
| 60236 | 1.01139 | 0.05 |
| 60237 | 0.548168 | 0.05 |
| 60238 | 0.736603 | 0.05 |
| 60239 | 1.18322 | 0.05 |
| 60240 | 0.861589 | 0.05 |
| 60241 | 0.861589 | 0.05 |
| 60242 | 0.869798 | 0.05 |
| 60243 | 0.869798 | 0.05 |
| 60244 | 1.16037 | 0.05 |
| 60245 | 0.942012 | 0.05 |
| 60246 | 0.942012 | 0.05 |
| 60247 | 0.62356 | 0.05 |
| 60248 | 0.891932 | 0.05 |
| 60249 | 0.900255 | 0.05 |
| 60250 | 2.02279 | 0.05 |
| 60251 | 1.48677 | 0.05 |
| 60252 | 1.54724 | 0.05 |
| 60253 | 0.831456 | 0.05 |
| 60254 | 1.19775 | 0.05 |
| 60255 | 1.89226 | 1.34816 |
| 60256 | 2.76291 | 1.39049 |
| 60257 | 1.89226 | 1.67603 |
| 60258 | 1.89226 | 1.20268 |
| 60259 | 3.78452 | 1.47916 |

| | | |
|---|---|---|
| 60260 | 1.89226 | 0.960876 |
| 60261 | 1.89226 | 1.41964 |
| 60262 | 1.89226 | 1.94561 |
| 60263 | 1.89226 | 1.02758 |
| 60264 | 1.89226 | 1.90809 |
| 60265 | 1.89226 | 1.03003 |
| 60266 | 1.89226 | 1.65039 |
| 60267 | 1.89226 | 1.06825 |
| 60268 | 1.89226 | 1.3438 |
| 60269 | 1.89226 | 1.23955 |
| 60270 | 3.78452 | 1.17691 |
| 60271 | 1.89226 | 0.879299 |
| 60272 | 1.89226 | 1.61518 |
| 60273 | 1.89226 | 1.77753 |
| 60274 | 1.89226 | 1.00064 |
| 60275 | 3.78453 | 1.31643 |
| 60276 | 0.946128 | 1.01475 |
| 60277 | 0.946128 | 1.20294 |
| 60278 | 0.946129 | 1.69185 |
| 60279 | 0.946129 | 1.31106 |
| 60280 | 0.946132 | 0.929184 |
| 60281 | 0.946132 | 1.34525 |
| 60282 | 0.946126 | 1.41877 |
| 60283 | 0.946126 | 1.0027 |
| 60284 | 0.946129 | 1.28245 |
| 60285 | 0.946129 | 1.21872 |
| 60286 | 0.946129 | 1.1557 |
| 60287 | 0.946129 | 1.1002 |
| 60288 | 2.80495 | 1.26447 |
| 60289 | 1.89225 | 1.02579 |
| 60290 | 1.89225 | 1.48878 |
| 60291 | 1.87794 | 1.46089 |
| 60292 | 1.89225 | 1.18239 |
| 60293 | 1.87794 | 1.17449 |
| 60294 | 2.25195 | 0.182028 |
| 60295 | 2.00624 | 0.347819 |
| 60296 | 2.13132 | 0.204868 |
| 60297 | 1.90239 | 0.05 |
| 60298 | 4.13756 | 0.05 |
| 60299 | 0.804661 | 0.22755 |
| 60300 | 0.804663 | 0.05 |
| 60301 | 0.889591 | 0.05 |
| 60302 | 0.889591 | 0.59829 |
| 60303 | 0.808324 | 0.05 |
| 60304 | 0.761765 | 0.0926374 |
| 60305 | 1.02822 | 0.159782 |
| 60306 | 0.879624 | 0.05 |
| 60307 | 0.879617 | 0.0553369 |
| 60308 | 0.800864 | 0.05 |
| 60309 | 0.978023 | 0.156471 |
| 60310 | 0.808329 | 0.05 |
| 60311 | 2.13133 | 0.521388 |
| 60312 | 2.00558 | 0.05 |
| 60313 | 2.09316 | 0.05 |
| 60314 | 2.09316 | 0.720751 |
| 60315 | 2.03253 | 0.05 |
| 60316 | 2.03253 | 0.949121 |
| 60317 | 0.84577 | 0.05 |

| | | |
|---|---|---|
| 60318 | 0.845767 | 0.315012 |
| 60319 | 0.843917 | 0.05 |
| 60320 | 0.843917 | 0.683849 |
| 60321 | 0.76177 | 0.05 |
| 60322 | 0.719345 | 0.0736939 |
| 60323 | 0.934435 | 0.05 |
| 60324 | 0.884197 | 0.05 |
| 60325 | 0.934439 | 0.05 |
| 60326 | 0.75627 | 0.05 |
| 60327 | 0.849424 | 0.0703478 |
| 60328 | 0.76177 | 0.05 |
| 60329 | 2.61855 | 0.05 |
| 60330 | 1.17793 | 0.05 |
| 60331 | 1.42965 | 0.0776973 |
| 60332 | 1.17793 | 0.05 |
| 60333 | 2.50057 | 0.05 |
| 60334 | 2.24885 | 0.05 |
| 60335 | 0.946133 | 0.0547248 |
| 60336 | 0.946133 | 0.05 |
| 60337 | 1.30928 | 0.0558881 |
| 60338 | 1.12443 | 0.05 |
| 60339 | 0.946132 | 0.05 |
| 60340 | 0.946132 | 0.05 |
| 60341 | 1.30928 | 0.05 |
| 60342 | 1.30928 | 0.05 |
| 60343 | 0.946132 | 0.050573 |
| 60344 | 1.30928 | 0.05 |
| 60345 | 1.12443 | 0.05 |
| 60346 | 0.946133 | 0.05 |
| 60347 | 1.89226 | 0.05 |
| 60348 | 1.89226 | 0.0795013 |
| 60349 | 1.89226 | 0.119202 |
| 60350 | 1.89226 | 0.0949696 |
| 60351 | 3.78452 | 0.0808786 |
| 60352 | 1.89226 | 0.128624 |
| 60353 | 1.89226 | 0.0647815 |
| 60354 | 1.89226 | 0.05 |
| 60355 | 1.89226 | 0.0567519 |
| 60356 | 1.89226 | 0.0682808 |
| 60357 | 1.89226 | 0.05 |
| 60358 | 1.89226 | 0.0662878 |
| 60359 | 1.89226 | 0.0518983 |
| 60360 | 1.89226 | 0.0777702 |
| 60361 | 1.89226 | 0.223838 |
| 60362 | 3.78452 | 0.072131 |
| 60363 | 0.94613 | 0.071593 |
| 60364 | 0.94613 | 0.108243 |
| 60365 | 0.946131 | 0.131576 |
| 60366 | 0.946131 | 0.074446 |
| 60367 | 0.946128 | 0.0865675 |
| 60368 | 0.946128 | 0.0700186 |
| 60369 | 0.946133 | 0.0664885 |
| 60370 | 0.946133 | 0.0633092 |
| 60371 | 0.946131 | 0.0808231 |
| 60372 | 0.946131 | 0.128911 |
| 60373 | 0.946131 | 0.0653366 |
| 60374 | 0.946131 | 0.05 |
| 60375 | 1.89226 | 2.1 |

| | | |
|---|---|---|
| 60376 | 1.89226 | 2.1 |
| 60377 | 1.89226 | 2.1 |
| 60378 | 1.89226 | 2.1 |
| 60379 | 3.78453 | 2.1 |
| 60380 | 1.0465 | 2.1 |
| 60381 | 2.73926 | 2.1 |
| 60382 | 2.03721 | 2.1 |
| 60383 | 2.03721 | 2.1 |
| 60384 | 1.44709 | 2.1 |
| 60385 | 1.44709 | 2.1 |
| 60386 | 0.946131 | 2.1 |
| 60387 | 0.946131 | 2.1 |
| 60388 | 0.946131 | 2.1 |
| 60389 | 0.946131 | 2.1 |
| 60390 | 0.946132 | 2.1 |
| 60391 | 0.946132 | 2.1 |
| 60392 | 0.94613 | 2.1 |
| 60393 | 0.94613 | 2.1 |
| 60394 | 0.946131 | 2.1 |
| 60395 | 0.946131 | 2.1 |
| 60396 | 0.946131 | 2.1 |
| 60397 | 0.946131 | 2.1 |
| 60398 | 0.946132 | 2.1 |
| 60399 | 0.946132 | 2.1 |
| 60400 | 0.946131 | 2.1 |
| 60401 | 0.946131 | 2.1 |
| 60402 | 0.946132 | 2.1 |
| 60403 | 0.946132 | 2.1 |
| 60404 | 0.946132 | 2.1 |
| 60405 | 0.946132 | 2.1 |
| 60406 | 0.946132 | 2.1 |
| 60407 | 0.946132 | 2.1 |
| 60408 | 0.946132 | 2.1 |
| 60409 | 0.946132 | 2.1 |
| 60410 | 1.89226 | 2.1 |
| 60411 | 1.89226 | 2.1 |
| 60412 | 1.89226 | 2.1 |
| 60413 | 1.89226 | 2.1 |
| 60414 | 3.78452 | 2.1 |
| 60415 | 1.61969 | 0.05 |
| 60416 | 1.79374 | 0.05 |
| 60417 | 1.29382 | 0.05 |
| 60418 | 1.29382 | 0.05 |
| 60419 | 2.05759 | 0.05 |
| 60420 | 2.05759 | 0.05 |
| 60421 | 1.89226 | 0.05 |
| 60422 | 1.89226 | 0.05 |
| 60423 | 1.89226 | 0.05 |
| 60424 | 1.89226 | 0.05 |
| 60425 | 1.89226 | 0.05 |
| 60426 | 1.89226 | 0.05 |
| 60427 | 1.89226 | 0.474764 |
| 60428 | 1.89226 | 0.713952 |
| 60429 | 1.89226 | 0.525764 |
| 60430 | 1.89226 | 0.617503 |
| 60431 | 1.89226 | 0.414378 |
| 60432 | 1.89226 | 0.507609 |
| 60433 | 1.89226 | 1.20238 |

| | | |
|---|---|---|
| 60434 | 1.89226 | 0.814962 |
| 60435 | 1.89226 | 0.936547 |
| 60436 | 1.89226 | 1.04769 |
| 60437 | 3.78453 | 1.20494 |
| 60438 | 0.946131 | 0.758352 |
| 60439 | 0.946131 | 0.791003 |
| 60440 | 0.946131 | 0.553908 |
| 60441 | 0.946131 | 0.770834 |
| 60442 | 0.946131 | 0.689755 |
| 60443 | 0.946131 | 0.574975 |
| 60444 | 0.946131 | 0.790688 |
| 60445 | 0.946131 | 0.870963 |
| 60446 | 0.946131 | 0.726324 |
| 60447 | 0.946131 | 0.70225 |
| 60448 | 0.946131 | 0.775946 |
| 60449 | 0.946131 | 0.717461 |
| 60450 | 2.45177 | 0.05 |
| 60451 | 2.68507 | 0.05 |
| 60452 | 2.3376 | 0.05 |
| 60453 | 2.22086 | 0.05 |
| 60454 | 4.78937 | 0.05 |
| 60455 | 2.45178 | 0.05 |
| 60456 | 2.35552 | 0.05 |
| 60457 | 1.68062 | 0.05 |
| 60458 | 1.81902 | 0.05 |
| 60459 | 4.13239 | 0.05 |
| 60460 | 2.3376 | 0.05 |
| 60461 | 1.55612 | 0.05 |
| 60462 | 1.69811 | 0.05 |
| 60463 | 2.31527 | 0.05 |
| 60464 | 1.79553 | 0.05 |
| 60465 | 2.3376 | 0.05 |
| 60466 | 1.55613 | 0.05 |
| 60467 | 1.06336 | 0.05 |
| 60468 | 1.97516 | 0.05 |
| 60469 | 1.88966 | 0.05 |
| 60470 | 1.06336 | 0.05 |
| 60471 | 1.68062 | 0.05 |
| 60472 | 1.89226 | 0.993465 |
| 60473 | 1.91634 | 0.488566 |
| 60474 | 2.03709 | 1.57341 |
| 60475 | 1.91634 | 0.874222 |
| 60476 | 1.83185 | 0.882055 |
| 60477 | 1.70303 | 0.198229 |
| 60478 | 1.89226 | 0.736425 |
| 60479 | 1.89226 | 0.345171 |
| 60480 | 1.89226 | 1.18296 |
| 60481 | 1.89226 | 0.736425 |
| 60482 | 1.89226 | 0.332197 |
| 60483 | 1.89226 | 0.0738564 |
| 60484 | 1.89226 | 2.05264 |
| 60485 | 2.03472 | 2.1 |
| 60486 | 2.22183 | 2.1 |
| 60487 | 2.03472 | 1.23213 |
| 60488 | 2.07936 | 2.1 |
| 60489 | 1.89226 | 0.806382 |
| 60490 | 1.89226 | 0.945394 |
| 60491 | 1.8519 | 0.143521 |

| | | |
|---|---|---|
| 60492 | 1.92119 | 1.12325 |
| 60493 | 1.8519 | 0.790122 |
| 60494 | 1.96156 | 0.479982 |
| 60495 | 1.89226 | 0.119276 |
| 60496 | 1.89226 | 0.689002 |
| 60497 | 1.89226 | 0.515493 |
| 60498 | 1.89226 | 1.03001 |
| 60499 | 1.89226 | 0.71537 |
| 60500 | 1.89226 | 0.282708 |
| 60501 | 1.89226 | 0.0589619 |
| 60502 | 1.89226 | 1.26188 |
| 60503 | 2.22183 | 1.13907 |
| 60504 | 2.39541 | 2.1 |
| 60505 | 2.22183 | 0.924325 |
| 60506 | 2.06585 | 1.46817 |
| 60507 | 1.89226 | 0.765357 |
| 60508 | 1.47001 | 2.1 |
| 60509 | 1.57962 | 0.106747 |
| 60510 | 1.65238 | 1.20301 |
| 60511 | 1.60502 | 2.1 |
| 60512 | 3.18464 | 2.1 |
| 60513 | 1.02415 | 2.1 |
| 60514 | 1.60503 | 2.1 |
| 60515 | 1.47001 | 2.1 |
| 60516 | 1.47001 | 2.1 |
| 60517 | 1.28228 | 2.1 |
| 60518 | 1.28228 | 2.1 |
| 60519 | 0.946133 | 2.1 |
| 60520 | 0.946133 | 2.1 |
| 60521 | 0.946132 | 2.1 |
| 60522 | 0.946132 | 1.18637 |
| 60523 | 0.946132 | 1.19498 |
| 60524 | 0.946132 | 2.1 |
| 60525 | 0.946133 | 2.1 |
| 60526 | 0.946133 | 2.1 |
| 60527 | 0.946132 | 2.1 |
| 60528 | 0.946132 | 2.1 |
| 60529 | 0.946133 | 2.1 |
| 60530 | 0.946133 | 2.1 |
| 60531 | 1.89226 | 0.0795275 |
| 60532 | 1.89226 | 0.719192 |
| 60533 | 1.89226 | 2.08193 |
| 60534 | 1.89226 | 0.05 |
| 60535 | 3.78452 | 0.0965564 |
| 60536 | 1.76897 | 1.13714 |
| 60537 | 1.60502 | 1.39214 |
| 60538 | 1.65238 | 0.257836 |
| 60539 | 1.65238 | 2.1 |
| 60540 | 1.76932 | 0.153833 |
| 60541 | 1.76932 | 2.1 |
| 60542 | 1.55998 | 2.1 |
| 60543 | 1.60503 | 2.01364 |
| 60544 | 1.76932 | 2.1 |
| 60545 | 1.60503 | 2.1 |
| 60546 | 1.44657 | 2.1 |
| 60547 | 1.28228 | 2.1 |
| 60548 | 0.946133 | 2.1 |
| 60549 | 0.946133 | 2.1 |

| | | |
|---|---|---|
| 60550 | 0.946132 | 2.1 |
| 60551 | 0.946132 | 0.251111 |
| 60552 | 0.946132 | 0.185828 |
| 60553 | 0.946132 | 2.1 |
| 60554 | 0.946133 | 1.98482 |
| 60555 | 0.946133 | 2.1 |
| 60556 | 0.946132 | 2.1 |
| 60557 | 0.946132 | 2.1 |
| 60558 | 0.946132 | 2.1 |
| 60559 | 0.946132 | 2.1 |
| 60560 | 0.946128 | 1.48464 |
| 60561 | 0.946128 | 0.05 |
| 60562 | 0.946133 | 0.05 |
| 60563 | 0.946133 | 0.0806917 |
| 60564 | 0.94613 | 0.05 |
| 60565 | 0.94613 | 0.108688 |
| 60566 | 0.946131 | 1.48164 |
| 60567 | 0.946131 | 0.105224 |
| 60568 | 0.946131 | 0.0626545 |
| 60569 | 0.946131 | 0.131332 |
| 60570 | 0.946131 | 0.0677403 |
| 60571 | 0.946131 | 0.206534 |
| 60572 | 1.85779 | 0.05 |
| 60573 | 1.00763 | 0.05 |
| 60574 | 1.74874 | 0.05 |
| 60575 | 1.74873 | 0.05 |
| 60576 | 1.14467 | 0.05 |
| 60577 | 1.14466 | 0.05 |
| 60578 | 1.85779 | 0.05 |
| 60579 | 1.61597 | 0.05 |
| 60580 | 1.00053 | 0.05 |
| 60581 | 1.03018 | 0.05 |
| 60582 | 2.65819 | 0.05 |
| 60583 | 1.89226 | 0.05 |
| 60584 | 1.89226 | 0.05 |
| 60585 | 1.89226 | 0.0727798 |
| 60586 | 1.89226 | 0.05 |
| 60587 | 3.78451 | 0.05 |
| 60588 | 1.89226 | 0.05 |
| 60589 | 1.89226 | 0.05 |
| 60590 | 1.89226 | 0.05 |
| 60591 | 1.89226 | 0.05 |
| 60592 | 1.89226 | 0.05 |
| 60593 | 1.89226 | 0.05 |
| 60594 | 1.11983 | 0.05 |
| 60595 | 0.810945 | 0.05 |
| 60596 | 0.913906 | 0.05 |
| 60597 | 0.913906 | 0.05 |
| 60598 | 2.23303 | 0.05 |
| 60599 | 1.66057 | 0.05 |
| 60600 | 1.28724 | 0.05 |
| 60601 | 1.08132 | 0.05 |
| 60602 | 1.49316 | 0.05 |
| 60603 | 0.810958 | 0.05 |
| 60604 | 0.810958 | 0.05 |
| 60605 | 0.827861 | 0.05 |
| 60606 | 0.946126 | 0.05 |
| 60607 | 0.946132 | 0.05 |

| | | |
|---|---|---|
| 60608 | 0.946132 | 0.05 |
| 60609 | 0.827863 | 0.05 |
| 60610 | 0.827863 | 0.05 |
| 60611 | 0.946129 | 0.05 |
| 60612 | 0.946129 | 0.05 |
| 60613 | 0.827863 | 0.05 |
| 60614 | 0.946128 | 0.05 |
| 60615 | 0.946129 | 0.05 |
| 60616 | 0.946129 | 0.05 |
| 60617 | 0.974718 | 0.05 |
| 60618 | 1.53671 | 0.05 |
| 60619 | 2.80542 | 0.05 |
| 60620 | 2.27259 | 0.05 |
| 60621 | 3.8093 | 0.05 |
| 60622 | 1.89226 | 0.05 |
| 60623 | 1.89226 | 0.05 |
| 60624 | 1.89226 | 0.05 |
| 60625 | 1.89226 | 0.05 |
| 60626 | 3.78453 | 0.05 |
| 60627 | 0.946132 | 0.05 |
| 60628 | 0.946132 | 0.05 |
| 60629 | 0.946131 | 0.05 |
| 60630 | 0.946131 | 0.05 |
| 60631 | 0.946132 | 0.05 |
| 60632 | 0.946132 | 0.05 |
| 60633 | 0.946131 | 0.05 |
| 60634 | 0.946131 | 0.05 |
| 60635 | 0.946132 | 0.05 |
| 60636 | 0.946132 | 0.05 |
| 60637 | 0.946131 | 0.05 |
| 60638 | 0.946131 | 0.05 |
| 60639 | 2.80542 | 0.05 |
| 60640 | 1.89226 | 0.05 |
| 60641 | 2.27259 | 0.05 |
| 60642 | 2.27259 | 0.05 |
| 60643 | 1.89226 | 0.05 |
| 60644 | 1.89226 | 0.05 |
| 60645 | 1.89226 | 0.05 |
| 60646 | 1.89226 | 0.05 |
| 60647 | 1.89226 | 0.05 |
| 60648 | 1.89226 | 0.05 |
| 60649 | 1.89226 | 0.05 |
| 60650 | 1.89226 | 0.05 |
| 60651 | 0.946132 | 0.05 |
| 60652 | 0.946132 | 0.05 |
| 60653 | 0.946131 | 0.05 |
| 60654 | 0.946131 | 0.05 |
| 60655 | 0.946132 | 0.05 |
| 60656 | 0.946132 | 0.05 |
| 60657 | 0.946131 | 0.05 |
| 60658 | 0.946131 | 0.05 |
| 60659 | 0.946131 | 0.05 |
| 60660 | 0.946131 | 0.05 |
| 60661 | 0.946131 | 0.05 |
| 60662 | 0.946131 | 0.05 |
| 60663 | 2.68213 | 2.1 |
| 60664 | 2.58234 | 2.1 |
| 60665 | 2.58234 | 2.1 |

| | | |
|---|---|---|
| 60666 | 2.61243 | 2.1 |
| 60667 | 2.67035 | 2.1 |
| 60668 | 2.70044 | 2.1 |
| 60669 | 2.68213 | 2.1 |
| 60670 | 2.53921 | 2.1 |
| 60671 | 2.53303 | 2.1 |
| 60672 | 2.67035 | 2.1 |
| 60673 | 5.20956 | 2.1 |
| 60674 | 2.67033 | 2.1 |
| 60675 | 2.58232 | 2.1 |
| 60676 | 2.55004 | 2.1 |
| 60677 | 2.64567 | 2.1 |
| 60678 | 5.228 | 2.1 |
| 60679 | 2.53301 | 2.1 |
| 60680 | 2.64567 | 2.1 |
| 60681 | 2.64567 | 2.1 |
| 60682 | 2.67033 | 2.1 |
| 60683 | 2.51603 | 2.1 |
| 60684 | 2.54069 | 2.1 |
| 60685 | 0.907497 | 2.1 |
| 60686 | 0.907497 | 2.1 |
| 60687 | 0.926641 | 2.1 |
| 60688 | 0.926641 | 2.1 |
| 60689 | 1.14989 | 2.1 |
| 60690 | 1.10046 | 2.1 |
| 60691 | 0.695334 | 2.1 |
| 60692 | 0.684247 | 2.1 |
| 60693 | 0.695333 | 2.1 |
| 60694 | 1.14989 | 2.1 |
| 60695 | 0.684246 | 2.1 |
| 60696 | 1.10046 | 2.1 |
| 60697 | 0.946126 | 1.11517 |
| 60698 | 0.946126 | 2.1 |
| 60699 | 0.946131 | 2.1 |
| 60700 | 0.946131 | 2.1 |
| 60701 | 0.946127 | 2.1 |
| 60702 | 0.946127 | 2.1 |
| 60703 | 0.94613 | 2.1 |
| 60704 | 0.94613 | 2.1 |
| 60705 | 0.946128 | 2.1 |
| 60706 | 0.946128 | 2.1 |
| 60707 | 0.946128 | 2.1 |
| 60708 | 0.946128 | 2.1 |
| 60709 | 1.89226 | 2.1 |
| 60710 | 1.89226 | 2.1 |
| 60711 | 1.89226 | 2.1 |
| 60712 | 1.89226 | 0.958501 |
| 60713 | 1.89226 | 2.1 |
| 60714 | 1.89226 | 0.784735 |
| 60715 | 1.42174 | 2.1 |
| 60716 | 2.29978 | 2.1 |
| 60717 | 2.41233 | 2.1 |
| 60718 | 2.41233 | 2.1 |
| 60719 | 1.39067 | 2.1 |
| 60720 | 1.39067 | 2.1 |
| 60721 | 1.89226 | 2.1 |
| 60722 | 1.89226 | 2.1 |
| 60723 | 1.89226 | 2.1 |

| | | |
|---|---|---|
| 60724 | 1.89226 | 2.1 |
| 60725 | 3.78452 | 2.1 |
| 60726 | 1.89226 | 2.1 |
| 60727 | 1.89226 | 2.1 |
| 60728 | 1.89226 | 2.1 |
| 60729 | 1.89226 | 2.1 |
| 60730 | 1.89226 | 2.1 |
| 60731 | 1.89226 | 2.1 |
| 60732 | 2.41234 | 2.1 |
| 60733 | 1.89226 | 2.1 |
| 60734 | 2.29979 | 2.1 |
| 60735 | 1.89226 | 2.1 |
| 60736 | 1.89226 | 2.1 |
| 60737 | 2.29979 | 2.1 |
| 60738 | 1.09506 | 0.05 |
| 60739 | 2.82921 | 0.110174 |
| 60740 | 2.53251 | 0.05 |
| 60741 | 2.53251 | 0.156148 |
| 60742 | 1.42901 | 0.05 |
| 60743 | 1.42901 | 0.174808 |
| 60744 | 0.946132 | 0.312023 |
| 60745 | 0.946132 | 0.218295 |
| 60746 | 0.946128 | 0.067089 |
| 60747 | 0.946128 | 0.229187 |
| 60748 | 0.94613 | 0.262609 |
| 60749 | 0.94613 | 0.330653 |
| 60750 | 0.946131 | 0.169697 |
| 60751 | 0.946131 | 0.144899 |
| 60752 | 0.94613 | 0.269368 |
| 60753 | 0.94613 | 0.162391 |
| 60754 | 0.94613 | 0.243227 |
| 60755 | 0.94613 | 0.33934 |
| 60756 | 1.89226 | 0.372981 |
| 60757 | 1.89226 | 0.389203 |
| 60758 | 1.89226 | 0.370281 |
| 60759 | 1.89226 | 0.386031 |
| 60760 | 3.78452 | 0.371118 |
| 60761 | 2.53251 | 0.100044 |
| 60762 | 1.89226 | 0.37071 |
| 60763 | 2.82921 | 0.06512 |
| 60764 | 1.89226 | 0.14272 |
| 60765 | 1.89226 | 0.377699 |
| 60766 | 2.82921 | 0.38947 |
| 60767 | 1.89226 | 0.240689 |
| 60768 | 1.89226 | 0.177868 |
| 60769 | 1.89226 | 0.320783 |
| 60770 | 1.89226 | 0.305618 |
| 60771 | 1.89226 | 0.163772 |
| 60772 | 1.89226 | 0.15843 |
| 60773 | 2.53251 | 0.05 |
| 60774 | 1.89226 | 0.128457 |
| 60775 | 2.06158 | 0.05 |
| 60776 | 1.89226 | 0.0624511 |
| 60777 | 4.42477 | 0.05 |
| 60778 | 1.23296 | 2.1 |
| 60779 | 2.55352 | 2.1 |
| 60780 | 2.47476 | 2.1 |
| 60781 | 2.47476 | 2.1 |

| | | |
|---|---|---|
| 60782 | 1.32368 | 2.1 |
| 60783 | 1.32368 | 2.1 |
| 60784 | 1.89226 | 2.1 |
| 60785 | 1.89226 | 2.1 |
| 60786 | 1.89226 | 2.1 |
| 60787 | 1.89226 | 2.1 |
| 60788 | 1.89226 | 2.1 |
| 60789 | 1.89226 | 2.1 |
| 60790 | 1.89226 | 2.1 |
| 60791 | 1.89226 | 2.1 |
| 60792 | 1.89226 | 2.1 |
| 60793 | 1.89226 | 2.1 |
| 60794 | 1.89226 | 2.1 |
| 60795 | 1.89226 | 2.1 |
| 60796 | 2.47476 | 2.1 |
| 60797 | 1.89226 | 2.1 |
| 60798 | 1.89226 | 2.1 |
| 60799 | 1.89226 | 2.1 |
| 60800 | 2.55353 | 2.1 |
| 60801 | 2.55353 | 2.1 |
| 60802 | 1.05983 | 2.1 |
| 60803 | 2.47476 | 2.1 |
| 60804 | 2.37664 | 2.1 |
| 60805 | 2.37664 | 2.1 |
| 60806 | 1.23296 | 2.1 |
| 60807 | 1.23296 | 2.1 |
| 60808 | 0.946127 | 2.1 |
| 60809 | 0.946127 | 2.1 |
| 60810 | 0.946131 | 2.1 |
| 60811 | 0.946131 | 2.1 |
| 60812 | 0.946127 | 2.1 |
| 60813 | 0.946127 | 2.1 |
| 60814 | 0.946131 | 2.1 |
| 60815 | 0.946131 | 2.1 |
| 60816 | 0.946129 | 2.1 |
| 60817 | 0.946129 | 2.1 |
| 60818 | 0.946128 | 2.1 |
| 60819 | 0.946128 | 2.1 |
| 60820 | 1.89226 | 2.1 |
| 60821 | 1.89226 | 2.1 |
| 60822 | 1.89226 | 2.1 |
| 60823 | 1.89226 | 1.2804 |
| 60824 | 3.78452 | 2.1 |
| 60825 | 2.37665 | 2.1 |
| 60826 | 1.89226 | 2.1 |
| 60827 | 1.89226 | 2.1 |
| 60828 | 1.89226 | 2.1 |
| 60829 | 2.47476 | 2.1 |
| 60830 | 2.47476 | 2.1 |
| 60831 | 1.89226 | 0.817036 |
| 60832 | 1.92119 | 0.0749811 |
| 60833 | 1.99586 | 0.979108 |
| 60834 | 1.92119 | 0.770045 |
| 60835 | 1.96693 | 0.236762 |
| 60836 | 1.89226 | 0.104887 |
| 60837 | 1.89226 | 0.672517 |
| 60838 | 1.89226 | 0.489336 |
| 60839 | 1.89226 | 0.891077 |

| | | |
|---|---|---|
| 60840 | 1.89226 | 0.650901 |
| 60841 | 1.89226 | 0.496095 |
| 60842 | 1.89226 | 0.05 |
| 60843 | 1.89226 | 0.982419 |
| 60844 | 2.39541 | 0.766734 |
| 60845 | 2.57183 | 1.72821 |
| 60846 | 2.39541 | 0.831909 |
| 60847 | 2.06868 | 0.786191 |
| 60848 | 1.89226 | 0.401654 |
| 60849 | 0.946133 | 0.62758 |
| 60850 | 0.946133 | 0.650826 |
| 60851 | 0.997928 | 0.407242 |
| 60852 | 1.03763 | 0.734883 |
| 60853 | 0.946131 | 0.740961 |
| 60854 | 0.946131 | 0.05 |
| 60855 | 1.03763 | 0.693555 |
| 60856 | 1.03763 | 0.166452 |
| 60857 | 1.03763 | 0.70041 |
| 60858 | 0.946131 | 0.671983 |
| 60859 | 0.94613 | 0.640324 |
| 60860 | 0.99793 | 0.102197 |
| 60861 | 1.89226 | 0.191508 |
| 60862 | 1.89226 | 0.651657 |
| 60863 | 1.89226 | 0.709191 |
| 60864 | 1.89226 | 0.05 |
| 60865 | 1.89226 | 0.859586 |
| 60866 | 1.89226 | 0.179322 |
| 60867 | 0.946131 | 0.603399 |
| 60868 | 0.946131 | 0.677253 |
| 60869 | 1.36939 | 0.451713 |
| 60870 | 1.28592 | 0.935558 |
| 60871 | 0.946129 | 0.848109 |
| 60872 | 0.946129 | 0.05 |
| 60873 | 1.36939 | 0.720528 |
| 60874 | 1.36939 | 0.738147 |
| 60875 | 0.946131 | 0.557709 |
| 60876 | 1.36939 | 0.468561 |
| 60877 | 0.946131 | 0.744121 |
| 60878 | 1.28592 | 0.09538 |
| 60879 | 1.89226 | 0.566682 |
| 60880 | 1.89226 | 0.717495 |
| 60881 | 1.89226 | 0.636538 |
| 60882 | 1.89226 | 0.622656 |
| 60883 | 3.78453 | 0.648969 |
| 60884 | 1.89226 | 0.722149 |
| 60885 | 1.89226 | 0.719477 |
| 60886 | 1.89226 | 0.630367 |
| 60887 | 1.89226 | 0.728643 |
| 60888 | 3.78452 | 0.67417 |
| 60889 | 1.91005 | 0.786978 |
| 60890 | 1.89226 | 0.835527 |
| 60891 | 2.60215 | 0.64934 |
| 60892 | 2.60215 | 0.764148 |
| 60893 | 1.89226 | 0.692553 |
| 60894 | 1.89226 | 0.894026 |
| 60895 | 0.946132 | 0.695996 |
| 60896 | 0.946132 | 0.65813 |
| 60897 | 0.946132 | 0.705837 |

| | | |
|---|---|---|
| 60898 | 0.946132 | 0.629777 |
| 60899 | 0.946132 | 0.691811 |
| 60900 | 0.946132 | 0.722474 |
| 60901 | 0.946132 | 0.654553 |
| 60902 | 0.946132 | 0.604593 |
| 60903 | 0.946132 | 0.659202 |
| 60904 | 0.946132 | 0.661275 |
| 60905 | 0.946131 | 0.631855 |
| 60906 | 0.946131 | 0.657948 |
| 60907 | 1.89226 | 0.696128 |
| 60908 | 1.89226 | 0.753305 |
| 60909 | 1.89226 | 0.945596 |
| 60910 | 1.89226 | 0.729463 |
| 60911 | 1.89226 | 0.68212 |
| 60912 | 1.89226 | 0.896675 |
| 60913 | 0.780483 | 0.789388 |
| 60914 | 0.78928 | 1.05918 |
| 60915 | 0.818566 | 0.798517 |
| 60916 | 0.460387 | 1.00987 |
| 60917 | 1.82793 | 0.650572 |
| 60918 | 1.32039 | 0.841982 |
| 60919 | 0.613889 | 0.700914 |
| 60920 | 1.0592 | 0.848375 |
| 60921 | 1.48698 | 0.888416 |
| 60922 | 0.818555 | 0.812313 |
| 60923 | 1.11515 | 0.879148 |
| 60924 | 1.2802 | 0.825901 |
| 60925 | 2.20046 | 0.136491 |
| 60926 | 1.1475 | 0.652994 |
| 60927 | 1.1475 | 0.112538 |
| 60928 | 2.27062 | 0.674602 |
| 60929 | 2.27062 | 0.14223 |
| 60930 | 1.89226 | 0.653077 |
| 60931 | 2.20046 | 0.412654 |
| 60932 | 2.27062 | 0.617608 |
| 60933 | 2.20046 | 0.543156 |
| 60934 | 1.96242 | 0.523672 |
| 60935 | 1.89226 | 0.494833 |
| 60936 | 1.89226 | 0.485026 |
| 60937 | 1.89226 | 0.208073 |
| 60938 | 1.89226 | 0.539365 |
| 60939 | 1.89226 | 0.476216 |
| 60940 | 3.78452 | 0.416214 |
| 60941 | 1.89226 | 0.323198 |
| 60942 | 1.89226 | 0.05 |
| 60943 | 1.89226 | 0.64983 |
| 60944 | 1.89226 | 0.494402 |
| 60945 | 3.78452 | 0.51743 |
| 60946 | 1.17403 | 0.163643 |
| 60947 | 1.13531 | 0.613555 |
| 60948 | 0.6401 | 0.150902 |
| 60949 | 0.714566 | 0.596003 |
| 60950 | 0.915987 | 0.408063 |
| 60951 | 0.915987 | 0.05 |
| 60952 | 0.933891 | 0.707563 |
| 60953 | 0.933891 | 0.318806 |
| 60954 | 1.17403 | 0.651723 |
| 60955 | 0.714566 | 0.23227 |

| | | |
|---|---|---|
| 60956 | 0.6401 | 0.610812 |
| 60957 | 1.13531 | 0.439975 |
| 60958 | 0.946133 | 0.485757 |
| 60959 | 0.946133 | 0.548613 |
| 60960 | 1.17403 | 0.433905 |
| 60961 | 1.13531 | 0.473948 |
| 60962 | 0.946131 | 0.427872 |
| 60963 | 0.946131 | 0.143219 |
| 60964 | 1.17403 | 0.588155 |
| 60965 | 1.17403 | 0.0533931 |
| 60966 | 1.17403 | 0.409121 |
| 60967 | 0.94613 | 0.492466 |
| 60968 | 0.946131 | 0.517548 |
| 60969 | 1.13531 | 0.151586 |
| 60970 | 1.89226 | 0.388036 |
| 60971 | 1.89226 | 0.425966 |
| 60972 | 1.89226 | 0.121214 |
| 60973 | 1.89226 | 0.05 |
| 60974 | 1.89226 | 0.514016 |
| 60975 | 1.89226 | 0.418991 |
| 60976 | 0.946131 | 0.367173 |
| 60977 | 0.946131 | 0.081766 |
| 60978 | 0.946131 | 0.421952 |
| 60979 | 0.946131 | 0.349705 |
| 60980 | 0.946129 | 0.440313 |
| 60981 | 0.946129 | 0.0644827 |
| 60982 | 0.946133 | 0.635599 |
| 60983 | 0.946133 | 0.05 |
| 60984 | 0.946131 | 0.32776 |
| 60985 | 0.946131 | 0.402836 |
| 60986 | 0.946131 | 0.05 |
| 60987 | 0.946131 | 0.139985 |
| 60988 | 2.29413 | 2.1 |
| 60989 | 1.99332 | 2.1 |
| 60990 | 1.84774 | 2.1 |
| 60991 | 2.21582 | 2.1 |
| 60992 | 4.20915 | 2.1 |
| 60993 | 0.517359 | 2.1 |
| 60994 | 0.634469 | 2.1 |
| 60995 | 0.996661 | 2.1 |
| 60996 | 0.92387 | 2.1 |
| 60997 | 0.768098 | 2.1 |
| 60998 | 0.768098 | 2.1 |
| 60999 | 0.790242 | 2.1 |
| 61000 | 0.790242 | 2.1 |
| 61001 | 0.923871 | 2.1 |
| 61002 | 0.517359 | 2.1 |
| 61003 | 0.634469 | 2.1 |
| 61004 | 0.996661 | 2.1 |
| 61005 | 1.89226 | 2.1 |
| 61006 | 1.89226 | 2.1 |
| 61007 | 1.89226 | 2.1 |
| 61008 | 1.89226 | 2.1 |
| 61009 | 3.78453 | 2.1 |
| 61010 | 0.946132 | 2.1 |
| 61011 | 0.946132 | 2.1 |
| 61012 | 0.946131 | 2.1 |
| 61013 | 0.946131 | 2.1 |

| | | |
|---|---|---|
| 61014 | 0.946132 | 2.1 |
| 61015 | 0.946132 | 2.1 |
| 61016 | 0.946131 | 2.1 |
| 61017 | 0.946131 | 2.1 |
| 61018 | 0.946132 | 2.1 |
| 61019 | 0.946132 | 2.1 |
| 61020 | 0.946131 | 2.1 |
| 61021 | 0.946131 | 2.1 |
| 61022 | 1.62708 | 2.1 |
| 61023 | 2.21582 | 2.1 |
| 61024 | 2.13925 | 2.1 |
| 61025 | 2.21582 | 2.1 |
| 61026 | 1.77117 | 2.1 |
| 61027 | 1.84774 | 2.1 |
| 61028 | 0.946132 | 2.1 |
| 61029 | 0.946132 | 2.1 |
| 61030 | 0.946131 | 2.1 |
| 61031 | 0.946131 | 2.1 |
| 61032 | 0.946132 | 2.1 |
| 61033 | 0.946132 | 2.1 |
| 61034 | 0.946131 | 2.1 |
| 61035 | 0.946131 | 2.1 |
| 61036 | 0.946131 | 2.1 |
| 61037 | 0.946131 | 2.1 |
| 61038 | 0.946131 | 2.1 |
| 61039 | 0.946131 | 2.1 |
| 61040 | 1.89226 | 1.93962 |
| 61041 | 1.89226 | 0.772477 |
| 61042 | 1.89226 | 0.22797 |
| 61043 | 1.89226 | 1.00264 |
| 61044 | 3.78453 | 1.19503 |
| 61045 | 1.89226 | 0.482409 |
| 61046 | 1.89226 | 1.27141 |
| 61047 | 1.89226 | 1.25452 |
| 61048 | 1.89226 | 2.1 |
| 61049 | 1.89226 | 0.151845 |
| 61050 | 1.89226 | 1.0186 |
| 61051 | 1.89226 | 0.182653 |
| 61052 | 1.89226 | 2.1 |
| 61053 | 1.89226 | 0.05 |
| 61054 | 1.89226 | 1.67304 |
| 61055 | 1.89226 | 0.186814 |
| 61056 | 1.89226 | 2.1 |
| 61057 | 0.914369 | 0.362616 |
| 61058 | 0.914369 | 0.82339 |
| 61059 | 0.810945 | 2.1 |
| 61060 | 0.810945 | 0.612347 |
| 61061 | 2.26581 | 2.1 |
| 61062 | 1.66199 | 2.1 |
| 61063 | 1.49503 | 1.12748 |
| 61064 | 1.28816 | 2.1 |
| 61065 | 1.08133 | 1.41568 |
| 61066 | 0.810933 | 1.74491 |
| 61067 | 1.1212 | 1.44917 |
| 61068 | 1.89227 | 0.665704 |
| 61069 | 1.89227 | 0.64763 |
| 61070 | 1.89227 | 0.56474 |
| 61071 | 1.89227 | 0.752527 |

| | | |
|---|---|---|
| 61072 | 3.78453 | 0.603725 |
| 61073 | 1.89226 | 0.572904 |
| 61074 | 1.89226 | 0.76805 |
| 61075 | 1.89226 | 0.70873 |
| 61076 | 1.89226 | 0.642161 |
| 61077 | 3.78453 | 0.697881 |
| 61078 | 1.89226 | 0.77797 |
| 61079 | 1.89226 | 0.686208 |
| 61080 | 1.89226 | 0.646853 |
| 61081 | 1.89226 | 0.895575 |
| 61082 | 3.78453 | 0.692862 |
| 61083 | 1.89226 | 0.658809 |
| 61084 | 1.89226 | 0.944403 |
| 61085 | 1.89226 | 0.759617 |
| 61086 | 2.25318 | 0.748816 |
| 61087 | 3.78453 | 0.877405 |
| 61088 | 1.89226 | 0.726275 |
| 61089 | 1.89226 | 0.904908 |
| 61090 | 1.89226 | 0.719257 |
| 61091 | 1.9637 | 0.728824 |
| 61092 | 3.78453 | 0.82423 |
| 61093 | 1.89226 | 0.755353 |
| 61094 | 1.89226 | 0.742868 |
| 61095 | 1.89226 | 0.637775 |
| 61096 | 1.89226 | 0.8288 |
| 61097 | 3.78453 | 0.690709 |
| 61098 | 1.89226 | 0.752438 |
| 61099 | 1.89226 | 1.09162 |
| 61100 | 1.89226 | 0.833943 |
| 61101 | 2.53384 | 0.830565 |
| 61102 | 3.78453 | 0.950083 |
| 61103 | 1.55405 | 0.744708 |
| 61104 | 1.03818 | 1.42421 |
| 61105 | 1.03818 | 0.241631 |
| 61106 | 1.34857 | 0.664472 |
| 61107 | 1.46338 | 0.641758 |
| 61108 | 1.77377 | 1.22978 |
| 61109 | 1.86273 | 0.64895 |
| 61110 | 1.73802 | 0.736181 |
| 61111 | 1.50969 | 1.94337 |
| 61112 | 1.46337 | 1.04643 |
| 61113 | 3.20139 | 0.787625 |
| 61114 | 1.2377 | 1.11271 |
| 61115 | 1.46337 | 0.69814 |
| 61116 | 1.27408 | 0.800612 |
| 61117 | 1.32039 | 1.92928 |
| 61118 | 1.50969 | 0.949823 |
| 61119 | 1.50969 | 0.513708 |
| 61120 | 1.89226 | 2.1 |
| 61121 | 1.89226 | 2.1 |
| 61122 | 1.89226 | 2.1 |
| 61123 | 1.89226 | 2.1 |
| 61124 | 1.89226 | 2.1 |
| 61125 | 1.89226 | 0.97454 |
| 61126 | 1.89169 | 2.1 |
| 61127 | 1.89226 | 2.1 |
| 61128 | 1.89226 | 2.1 |
| 61129 | 1.89226 | 2.1 |

| | | |
|---|---|---|
| 61130 | 1.96624 | 2.1 |
| 61131 | 1.96624 | 2.1 |
| 61132 | 2.62871 | 2.1 |
| 61133 | 1.6201 | 2.1 |
| 61134 | 1.49149 | 2.1 |
| 61135 | 1.6201 | 2.1 |
| 61136 | 2.62559 | 2.1 |
| 61137 | 2.75419 | 2.1 |
| 61138 | 0.946129 | 0.715318 |
| 61139 | 0.946129 | 2.1 |
| 61140 | 0.946133 | 2.1 |
| 61141 | 0.946133 | 2.1 |
| 61142 | 0.946131 | 2.1 |
| 61143 | 0.946131 | 0.664682 |
| 61144 | 0.946131 | 2.1 |
| 61145 | 0.946131 | 2.1 |
| 61146 | 0.946131 | 2.1 |
| 61147 | 0.946131 | 2.1 |
| 61148 | 0.946131 | 2.1 |
| 61149 | 0.946131 | 2.1 |
| 61150 | 0.851543 | 2.1 |
| 61151 | 0.851543 | 2.1 |
| 61152 | 1.04043 | 2.1 |
| 61153 | 0.997939 | 2.1 |
| 61154 | 0.946132 | 2.1 |
| 61155 | 0.946132 | 2.1 |
| 61156 | 0.894185 | 2.1 |
| 61157 | 0.893901 | 2.1 |
| 61158 | 0.998267 | 2.1 |
| 61159 | 1.04074 | 2.1 |
| 61160 | 0.85127 | 2.1 |
| 61161 | 0.85153 | 2.1 |
| 61162 | 1.89226 | 2.1 |
| 61163 | 1.89226 | 2.1 |
| 61164 | 1.89226 | 2.1 |
| 61165 | 1.89226 | 2.1 |
| 61166 | 1.89226 | 2.1 |
| 61167 | 1.89226 | 2.1 |
| 61168 | 0.928691 | 2.1 |
| 61169 | 0.928691 | 2.1 |
| 61170 | 0.935332 | 2.1 |
| 61171 | 0.935332 | 2.1 |
| 61172 | 1.398 | 2.1 |
| 61173 | 1.14418 | 2.1 |
| 61174 | 0.479331 | 2.1 |
| 61175 | 0.719848 | 2.1 |
| 61176 | 1.398 | 2.1 |
| 61177 | 0.719848 | 2.1 |
| 61178 | 1.14417 | 2.1 |
| 61179 | 0.47933 | 2.1 |
| 61180 | 2.28835 | 2.1 |
| 61181 | 1.89226 | 2.1 |
| 61182 | 1.89226 | 2.1 |
| 61183 | 1.89226 | 2.1 |
| 61184 | 2.796 | 2.1 |
| 61185 | 2.796 | 2.1 |
| 61186 | 0.946131 | 2.1 |
| 61187 | 0.946131 | 2.1 |

| | | |
|---|---|---|
| 61188 | 0.946131 | 2.1 |
| 61189 | 0.946131 | 2.1 |
| 61190 | 0.946128 | 2.1 |
| 61191 | 0.946128 | 2.1 |
| 61192 | 0.946133 | 2.1 |
| 61193 | 0.946133 | 2.1 |
| 61194 | 0.946131 | 2.1 |
| 61195 | 0.946131 | 2.1 |
| 61196 | 0.94613 | 2.1 |
| 61197 | 0.94613 | 2.1 |
| 61198 | 0.946131 | 2.1 |
| 61199 | 0.946131 | 2.1 |
| 61200 | 0.946131 | 2.1 |
| 61201 | 0.946131 | 2.1 |
| 61202 | 0.946131 | 2.1 |
| 61203 | 0.946131 | 2.1 |
| 61204 | 0.946131 | 2.1 |
| 61205 | 0.946131 | 2.1 |
| 61206 | 0.946131 | 2.1 |
| 61207 | 0.946131 | 2.1 |
| 61208 | 0.946131 | 2.1 |
| 61209 | 0.946131 | 2.1 |
| 61210 | 1.89226 | 2.1 |
| 61211 | 1.89226 | 2.1 |
| 61212 | 1.89226 | 2.1 |
| 61213 | 1.89226 | 2.1 |
| 61214 | 3.78452 | 2.1 |
| 61215 | 2.05207 | 0.377749 |
| 61216 | 1.70302 | 0.623878 |
| 61217 | 1.33794 | 2.09793 |
| 61218 | 1.98194 | 0.829727 |
| 61219 | 3.36873 | 0.883796 |
| 61220 | 2.62761 | 0.745916 |
| 61221 | 1.98194 | 0.845547 |
| 61222 | 2.28009 | 1.36953 |
| 61223 | 2.28009 | 0.661532 |
| 61224 | 2.22939 | 1.13709 |
| 61225 | 2.22939 | 0.723235 |
| 61226 | 1.89226 | 0.856109 |
| 61227 | 1.89226 | 0.351412 |
| 61228 | 1.89226 | 0.726181 |
| 61229 | 1.89226 | 0.649937 |
| 61230 | 1.89226 | 0.814773 |
| 61231 | 1.89226 | 0.770046 |
| 61232 | 1.89227 | 0.837712 |
| 61233 | 1.89227 | 0.758513 |
| 61234 | 1.89227 | 2.00929 |
| 61235 | 1.89227 | 1.04799 |
| 61236 | 1.89227 | 0.174911 |
| 61237 | 1.89227 | 0.05 |
| 61238 | 0.888355 | 0.309399 |
| 61239 | 0.990973 | 0.998667 |
| 61240 | 0.71133 | 0.836811 |
| 61241 | 0.634324 | 1.14639 |
| 61242 | 0.806209 | 1.03922 |
| 61243 | 0.806209 | 0.05 |
| 61244 | 0.819087 | 1.66653 |
| 61245 | 0.819087 | 1.08283 |

| | | |
|---|---|---|
| 61246 | 0.888355 | 1.13772 |
| 61247 | 0.634324 | 1.19027 |
| 61248 | 0.71133 | 0.875581 |
| 61249 | 0.990972 | 0.900629 |
| 61250 | 1.11469 | 1.05116 |
| 61251 | 0.985023 | 0.896855 |
| 61252 | 0.888354 | 0.638061 |
| 61253 | 0.990972 | 1.11766 |
| 61254 | 0.994754 | 0.907597 |
| 61255 | 0.994754 | 0.725825 |
| 61256 | 1.0083 | 1.22833 |
| 61257 | 1.0083 | 0.521971 |
| 61258 | 0.888354 | 0.702704 |
| 61259 | 0.985024 | 0.875812 |
| 61260 | 0.990971 | 0.710356 |
| 61261 | 1.11469 | 0.63001 |
| 61262 | 0.946132 | 1.02652 |
| 61263 | 0.946132 | 0.627122 |
| 61264 | 0.946132 | 0.746426 |
| 61265 | 0.946132 | 0.717892 |
| 61266 | 0.946132 | 0.716791 |
| 61267 | 0.946132 | 0.661551 |
| 61268 | 0.946133 | 1.39214 |
| 61269 | 0.946133 | 0.80262 |
| 61270 | 0.946133 | 0.825136 |
| 61271 | 0.946133 | 0.770237 |
| 61272 | 0.946132 | 0.691955 |
| 61273 | 0.946132 | 0.733443 |
| 61274 | 0.810944 | 0.780958 |
| 61275 | 0.810944 | 0.630992 |
| 61276 | 0.810947 | 0.925126 |
| 61277 | 0.810947 | 0.05 |
| 61278 | 1.89227 | 1.76882 |
| 61279 | 1.35168 | 0.825488 |
| 61280 | 1.08132 | 0.789811 |
| 61281 | 1.08131 | 0.8979 |
| 61282 | 1.08132 | 0.338953 |
| 61283 | 0.810959 | 0.573118 |
| 61284 | 0.810959 | 0.495443 |
| 61285 | 1.70303 | 2.1 |
| 61286 | 2.03975 | 2.1 |
| 61287 | 1.99324 | 2.1 |
| 61288 | 2.17615 | 2.1 |
| 61289 | 1.40549 | 2.1 |
| 61290 | 1.53446 | 2.1 |
| 61291 | 0.946133 | 2.1 |
| 61292 | 0.946133 | 2.1 |
| 61293 | 0.946129 | 2.1 |
| 61294 | 0.946129 | 2.1 |
| 61295 | 0.946131 | 2.1 |
| 61296 | 0.946131 | 2.03604 |
| 61297 | 0.946131 | 2.1 |
| 61298 | 0.946131 | 2.1 |
| 61299 | 0.946131 | 2.1 |
| 61300 | 0.946131 | 2.1 |
| 61301 | 0.946131 | 2.1 |
| 61302 | 0.946131 | 2.1 |
| 61303 | 1.89226 | 2.1 |

| | | |
|---|---|---|
| 61304 | 1.89226 | 2.1 |
| 61305 | 1.89226 | 2.1 |
| 61306 | 1.89226 | 2.1 |
| 61307 | 1.89226 | 2.1 |
| 61308 | 1.89226 | 2.1 |
| 61309 | 2.17615 | 2.1 |
| 61310 | 2.3111 | 2.1 |
| 61311 | 2.50288 | 2.1 |
| 61312 | 2.50288 | 2.1 |
| 61313 | 2.03975 | 2.1 |
| 61314 | 2.03975 | 2.1 |
| 61315 | 1.76401 | 2.1 |
| 61316 | 1.95852 | 2.1 |
| 61317 | 2.32134 | 2.1 |
| 61318 | 2.12306 | 2.1 |
| 61319 | 1.8513 | 2.1 |
| 61320 | 1.70303 | 2.1 |
| 61321 | 1.89226 | 2.1 |
| 61322 | 1.89226 | 2.1 |
| 61323 | 1.89226 | 2.1 |
| 61324 | 1.89226 | 2.1 |
| 61325 | 3.78452 | 2.1 |
| 61326 | 1.89226 | 2.1 |
| 61327 | 1.89226 | 0.773781 |
| 61328 | 1.89226 | 2.1 |
| 61329 | 1.89226 | 2.1 |
| 61330 | 3.78452 | 2.1 |
| 61331 | 1.2243 | 2.1 |
| 61332 | 1.2243 | 2.1 |
| 61333 | 1.24189 | 2.1 |
| 61334 | 1.24189 | 2.1 |
| 61335 | 1.37811 | 2.1 |
| 61336 | 1.25144 | 2.1 |
| 61337 | 1.08807 | 2.1 |
| 61338 | 1.17949 | 2.1 |
| 61339 | 1.17949 | 2.1 |
| 61340 | 1.37811 | 2.1 |
| 61341 | 1.08807 | 2.1 |
| 61342 | 1.25144 | 2.1 |
| 61343 | 1.89226 | 2.1 |
| 61344 | 1.89226 | 2.1 |
| 61345 | 1.89226 | 2.1 |
| 61346 | 1.89226 | 2.1 |
| 61347 | 1.89226 | 2.1 |
| 61348 | 1.89226 | 2.1 |
| 61349 | 1.89227 | 2.1 |
| 61350 | 1.89227 | 2.1 |
| 61351 | 1.89227 | 2.1 |
| 61352 | 1.89227 | 2.1 |
| 61353 | 1.89227 | 2.1 |
| 61354 | 1.89227 | 2.1 |
| 61355 | 1.89224 | 2.1 |
| 61356 | 1.89224 | 2.1 |
| 61357 | 1.89224 | 2.1 |
| 61358 | 1.89224 | 2.1 |
| 61359 | 3.78447 | 2.1 |
| 61360 | 1.89224 | 2.1 |
| 61361 | 1.89224 | 2.1 |

| | | |
|---|---|---|
| 61362 | 1.89224 | 2.1 |
| 61363 | 1.89224 | 2.1 |
| 61364 | 1.89224 | 2.1 |
| 61365 | 1.89224 | 2.1 |
| 61366 | 1.89224 | 2.1 |
| 61367 | 1.89224 | 2.1 |
| 61368 | 1.89224 | 2.1 |
| 61369 | 1.89224 | 2.1 |
| 61370 | 3.78448 | 2.1 |
| 61371 | 1.89224 | 2.1 |
| 61372 | 1.703 2.1 | |
| 61373 | 1.89224 | 2.1 |
| 61374 | 1.72991 | 2.1 |
| 61375 | 1.07643 | 2.1 |
| 61376 | 1.36657 | 2.1 |
| 61377 | 1.44472 | 2.1 |
| 61378 | 2.41233 | 2.1 |
| 61379 | 2.51628 | 2.1 |
| 61380 | 2.51628 | 2.1 |
| 61381 | 1.42174 | 2.1 |
| 61382 | 1.42174 | 2.1 |
| 61383 | 1.89226 | 2.1 |
| 61384 | 1.89226 | 2.1 |
| 61385 | 1.89226 | 2.1 |
| 61386 | 1.89226 | 2.1 |
| 61387 | 1.89226 | 2.1 |
| 61388 | 1.89226 | 2.1 |
| 61389 | 1.89226 | 2.1 |
| 61390 | 1.89226 | 2.1 |
| 61391 | 1.89226 | 2.1 |
| 61392 | 1.89226 | 0.90178 |
| 61393 | 3.78452 | 2.1 |
| 61394 | 2.51628 | 2.1 |
| 61395 | 1.89226 | 2.1 |
| 61396 | 1.89226 | 2.1 |
| 61397 | 1.89226 | 2.1 |
| 61398 | 2.41234 | 2.1 |
| 61399 | 2.41234 | 2.1 |
| 61400 | 0.996254 | 2.1 |
| 61401 | 0.996254 | 2.1 |
| 61402 | 1.01153 | 2.1 |
| 61403 | 1.01153 | 2.1 |
| 61404 | 1.28542 | 2.1 |
| 61405 | 1.25814 | 2.1 |
| 61406 | 0.719184 | 2.1 |
| 61407 | 0.72236 | 2.1 |
| 61408 | 0.719183 | 2.1 |
| 61409 | 1.28542 | 2.1 |
| 61410 | 0.722359 | 2.1 |
| 61411 | 1.25814 | 2.1 |
| 61412 | 0.946126 | 2.1 |
| 61413 | 0.946126 | 2.1 |
| 61414 | 0.946131 | 2.1 |
| 61415 | 0.946131 | 2.1 |
| 61416 | 0.946127 | 2.1 |
| 61417 | 0.946127 | 2.1 |
| 61418 | 0.94613 | 2.1 |
| 61419 | 0.94613 | 2.1 |

| | | |
|---|---|---|
| 61420 | 0.946128 | 2.1 |
| 61421 | 0.946128 | 2.1 |
| 61422 | 0.946128 | 2.1 |
| 61423 | 0.946128 | 2.1 |
| 61424 | 1.89226 | 2.1 |
| 61425 | 1.89226 | 2.00485 |
| 61426 | 1.89226 | 2.1 |
| 61427 | 1.89226 | 2.1 |
| 61428 | 1.89226 | 2.1 |
| 61429 | 1.89226 | 2.1 |
| 61430 | 0.946132 | 2.1 |
| 61431 | 0.946132 | 2.1 |
| 61432 | 1.25814 | 2.1 |
| 61433 | 1.25814 | 2.1 |
| 61434 | 0.946131 | 2.1 |
| 61435 | 0.946131 | 2.1 |
| 61436 | 1.28543 | 2.1 |
| 61437 | 1.25814 | 2.1 |
| 61438 | 1.28543 | 2.1 |
| 61439 | 0.946132 | 2.1 |
| 61440 | 1.25814 | 2.1 |
| 61441 | 0.946132 | 2.1 |
| 61442 | 1.89226 | 1.2396 |
| 61443 | 1.89226 | 0.05 |
| 61444 | 1.89226 | 0.874021 |
| 61445 | 1.89226 | 0.442574 |
| 61446 | 1.89226 | 0.155722 |
| 61447 | 1.89226 | 0.0771025 |
| 61448 | 1.89226 | 1.02914 |
| 61449 | 2.27719 | 0.05 |
| 61450 | 1.89226 | 0.0502768 |
| 61451 | 1.89226 | 0.193709 |
| 61452 | 3.78452 | 0.141201 |
| 61453 | 1.89225 | 1.4223 |
| 61454 | 1.89225 | 0.0838138 |
| 61455 | 1.89225 | 0.589273 |
| 61456 | 1.89225 | 0.749503 |
| 61457 | 3.78451 | 0.658459 |
| 61458 | 1.89226 | 0.05 |
| 61459 | 1.89226 | 0.071066 |
| 61460 | 1.89226 | 0.352804 |
| 61461 | 1.89226 | 0.05 |
| 61462 | 3.78452 | 0.05 |
| 61463 | 0.946132 | 0.05 |
| 61464 | 0.946132 | 0.05 |
| 61465 | 0.946132 | 0.125678 |
| 61466 | 0.946132 | 0.05 |
| 61467 | 0.946131 | 0.05 |
| 61468 | 0.946131 | 0.05 |
| 61469 | 0.946132 | 0.0729204 |
| 61470 | 0.946132 | 0.05 |
| 61471 | 0.946132 | 0.05 |
| 61472 | 0.946132 | 0.05 |
| 61473 | 0.946132 | 0.05 |
| 61474 | 0.946132 | 0.05 |
| 61475 | 0.946126 | 0.0585293 |
| 61476 | 0.946126 | 0.05 |
| 61477 | 0.946132 | 1.3671 |

| | | |
|---|---|---|
| 61478 | 0.946132 | 0.294185 |
| 61479 | 0.946128 | 0.0616805 |
| 61480 | 0.946128 | 0.183944 |
| 61481 | 0.946129 | 0.0898311 |
| 61482 | 0.946129 | 0.670741 |
| 61483 | 0.946129 | 0.180636 |
| 61484 | 0.946129 | 0.385294 |
| 61485 | 0.946129 | 0.101677 |
| 61486 | 0.946129 | 0.154274 |
| 61487 | 1.39858 | 2.1 |
| 61488 | 1.39858 | 2.1 |
| 61489 | 0.94613 | 2.1 |
| 61490 | 0.94613 | 2.1 |
| 61491 | 0.946132 | 2.1 |
| 61492 | 0.946132 | 2.1 |
| 61493 | 1.39858 | 2.1 |
| 61494 | 1.25002 | 2.1 |
| 61495 | 1.39858 | 2.1 |
| 61496 | 0.946131 | 2.1 |
| 61497 | 1.25002 | 2.1 |
| 61498 | 0.946131 | 2.1 |
| 61499 | 2.0815 | 2.1 |
| 61500 | 1.89226 | 2.1 |
| 61501 | 1.89226 | 2.1 |
| 61502 | 2.05298 | 2.1 |
| 61503 | 2.5593 | 2.1 |
| 61504 | 2.77667 | 2.1 |
| 61505 | 0.946133 | 2.1 |
| 61506 | 0.946133 | 2.1 |
| 61507 | 0.946132 | 2.1 |
| 61508 | 0.946132 | 2.1 |
| 61509 | 0.946132 | 2.1 |
| 61510 | 0.946132 | 2.1 |
| 61511 | 0.946133 | 2.1 |
| 61512 | 0.946133 | 2.1 |
| 61513 | 0.946132 | 2.1 |
| 61514 | 0.946132 | 2.1 |
| 61515 | 0.946132 | 2.1 |
| 61516 | 0.946132 | 2.1 |
| 61517 | 1.38908 | 2.1 |
| 61518 | 1.38908 | 2.1 |
| 61519 | 0.946132 | 2.1 |
| 61520 | 0.946132 | 2.1 |
| 61521 | 0.946132 | 2.1 |
| 61522 | 0.946132 | 2.1 |
| 61523 | 1.38908 | 2.1 |
| 61524 | 1.27965 | 2.1 |
| 61525 | 1.38908 | 2.1 |
| 61526 | 0.946132 | 2.1 |
| 61527 | 0.946133 | 2.1 |
| 61528 | 1.27965 | 2.1 |
| 61529 | 0.946133 | 2.1 |
| 61530 | 0.946133 | 2.1 |
| 61531 | 0.946132 | 2.1 |
| 61532 | 0.946132 | 2.1 |
| 61533 | 0.946132 | 2.1 |
| 61534 | 0.946132 | 2.1 |
| 61535 | 0.946133 | 2.1 |

| | | |
|---|---|---|
| 61536 | 0.946133 | 2.1 |
| 61537 | 0.946132 | 2.1 |
| 61538 | 0.946132 | 2.1 |
| 61539 | 0.946133 | 2.1 |
| 61540 | 0.946133 | 2.1 |
| 61541 | 1.89227 | 1.65242 |
| 61542 | 1.89227 | 1.66037 |
| 61543 | 1.89227 | 1.0318 |
| 61544 | 1.89227 | 0.725738 |
| 61545 | 3.78453 | 1.08488 |
| 61546 | 1.33154 | 1.05869 |
| 61547 | 1.33154 | 1.21959 |
| 61548 | 0.946132 | 1.23311 |
| 61549 | 0.946132 | 0.974009 |
| 61550 | 1.19095 | 0.836523 |
| 61551 | 1.33154 | 1.29271 |
| 61552 | 0.946133 | 1.2257 |
| 61553 | 0.946133 | 1.41767 |
| 61554 | 0.946133 | 2.1 |
| 61555 | 1.19095 | 0.922594 |
| 61556 | 0.946132 | 1.98896 |
| 61557 | 1.33154 | 1.05718 |
| 61558 | 1.0888 | 0.936046 |
| 61559 | 1.04745 | 0.930429 |
| 61560 | 0.946132 | 0.909629 |
| 61561 | 0.946132 | 0.942554 |
| 61562 | 1.04745 | 0.709134 |
| 61563 | 1.04745 | 1.292 |
| 61564 | 0.946132 | 1.74357 |
| 61565 | 0.946132 | 0.734759 |
| 61566 | 1.04745 | 1.6049 |
| 61567 | 0.946133 | 0.773291 |
| 61568 | 0.946132 | 1.47267 |
| 61569 | 1.0888 | 0.941766 |
| 61570 | 1.89226 | 2.1 |
| 61571 | 1.89226 | 0.707057 |
| 61572 | 1.89226 | 0.678669 |
| 61573 | 1.89226 | 0.888078 |
| 61574 | 1.89226 | 0.824932 |
| 61575 | 1.89226 | 1.14259 |
| 61576 | 1.89226 | 1.66642 |
| 61577 | 1.87592 | 0.649797 |
| 61578 | 1.87592 | 0.748919 |
| 61579 | 2.3819 | 0.923053 |
| 61580 | 1.89226 | 1.11976 |
| 61581 | 2.39825 | 1.33057 |
| 61582 | 1.89226 | 0.75373 |
| 61583 | 2.0949 | 1.1524 |
| 61584 | 1.88741 | 0.628571 |
| 61585 | 1.89226 | 0.655914 |
| 61586 | 1.89226 | 0.725111 |
| 61587 | 1.88741 | 1.19207 |
| 61588 | 1.07508 | 1.09949 |
| 61589 | 1.63419 | 1.64295 |
| 61590 | 1.63419 | 1.28325 |
| 61591 | 1.09795 | 1.7231 |
| 61592 | 1.56137 | 1.02065 |
| 61593 | 1.02513 | 2.00498 |

| | | |
|---|---|---|
| 61594 | 0.967672 | 0.213376 |
| 61595 | 1.56137 | 0.558088 |
| 61596 | 1.56137 | 0.217186 |
| 61597 | 1.07508 | 0.685684 |
| 61598 | 1.41319 | 0.129501 |
| 61599 | 0.926902 | 1.10208 |
| 61600 | 1.56137 | 0.690288 |
| 61601 | 1.63419 | 1.30348 |
| 61602 | 2.29852 | 1.45983 |
| 61603 | 2.14485 | 0.663177 |
| 61604 | 3.77904 | 0.4615 |
| 61605 | 1.41319 | 0.0867304 |
| 61606 | 1.92622 | 0.0849422 |
| 61607 | 1.56137 | 0.05 |
| 61608 | 1.56137 | 0.524985 |
| 61609 | 2.14485 | 0.545103 |
| 61610 | 1.92622 | 0.210058 |
| 61611 | 1.89227 | 2.1 |
| 61612 | 1.89227 | 2.1 |
| 61613 | 1.89227 | 2.1 |
| 61614 | 1.89227 | 2.1 |
| 61615 | 3.78453 | 2.1 |
| 61616 | 2.56618 | 2.1 |
| 61617 | 2.08612 | 2.1 |
| 61618 | 2.48048 | 2.1 |
| 61619 | 1.99644 | 2.1 |
| 61620 | 4.5666 | 2.1 |
| 61621 | 2.08612 | 2.1 |
| 61622 | 1.30659 | 2.1 |
| 61623 | 1.99644 | 2.1 |
| 61624 | 1.25404 | 2.1 |
| 61625 | 3.34016 | 2.1 |
| 61626 | 1.89226 | 2.1 |
| 61627 | 1.89226 | 2.1 |
| 61628 | 1.89226 | 2.1 |
| 61629 | 1.89226 | 2.1 |
| 61630 | 1.89226 | 2.1 |
| 61631 | 1.89226 | 2.1 |
| 61632 | 1.89227 | 2.1 |
| 61633 | 1.89227 | 2.1 |
| 61634 | 1.89227 | 2.1 |
| 61635 | 1.89227 | 2.1 |
| 61636 | 3.78453 | 2.1 |
| 61637 | 2.48048 | 2.1 |
| 61638 | 1.99644 | 2.1 |
| 61639 | 2.39022 | 2.1 |
| 61640 | 1.90734 | 2.1 |
| 61641 | 4.38782 | 2.1 |
| 61642 | 1.90734 | 2.1 |
| 61643 | 1.99644 | 2.1 |
| 61644 | 1.21564 | 2.1 |
| 61645 | 1.21564 | 2.1 |
| 61646 | 1.25404 | 2.1 |
| 61647 | 1.94574 | 2.1 |
| 61648 | 0.810946 | 2.1 |
| 61649 | 0.810946 | 2.1 |
| 61650 | 0.810947 | 2.1 |
| 61651 | 0.810947 | 2.1 |

| | | |
|---|---|---|
| 61652 | 1.89226 | 2.1 |
| 61653 | 1.3517 | 2.1 |
| 61654 | 1.08133 | 2.1 |
| 61655 | 1.08132 | 2.1 |
| 61656 | 1.08132 | 2.1 |
| 61657 | 0.810934 | 2.1 |
| 61658 | 0.810934 | 2.1 |
| 61659 | 1.89226 | 2.1 |
| 61660 | 1.89226 | 0.286761 |
| 61661 | 1.89226 | 0.857926 |
| 61662 | 1.89226 | 2.1 |
| 61663 | 3.78453 | 2.1 |
| 61664 | 1.89226 | 0.05 |
| 61665 | 1.89226 | 0.05 |
| 61666 | 1.89226 | 1.08932 |
| 61667 | 1.89226 | 0.05 |
| 61668 | 1.89226 | 0.103956 |
| 61669 | 1.89226 | 0.05 |
| 61670 | 0.946131 | 0.0522025 |
| 61671 | 0.946131 | 0.05 |
| 61672 | 0.94613 | 0.0528136 |
| 61673 | 0.94613 | 0.05 |
| 61674 | 0.946128 | 0.05 |
| 61675 | 0.946128 | 0.05 |
| 61676 | 0.946133 | 0.05 |
| 61677 | 0.946133 | 0.054069 |
| 61678 | 0.946131 | 0.05 |
| 61679 | 0.946131 | 0.05 |
| 61680 | 0.946131 | 0.05 |
| 61681 | 0.946131 | 0.05 |
| 61682 | 1.89226 | 2.1 |
| 61683 | 1.89226 | 0.05 |
| 61684 | 1.89226 | 1.40683 |
| 61685 | 1.89226 | 0.923275 |
| 61686 | 1.89226 | 0.330936 |
| 61687 | 1.89226 | 0.0813484 |
| 61688 | 2.56348 | 0.077267 |
| 61689 | 1.89226 | 2.1 |
| 61690 | 1.89226 | 2.1 |
| 61691 | 2.53234 | 0.997871 |
| 61692 | 4.45574 | 0.521097 |
| 61693 | 2.53234 | 0.05 |
| 61694 | 1.65583 | 0.05 |
| 61695 | 2.56348 | 0.05 |
| 61696 | 2.56348 | 0.05 |
| 61697 | 1.66295 | 0.05 |
| 61698 | 1.65583 | 0.05 |
| 61699 | 0.736602 | 0.05 |
| 61700 | 0.827916 | 0.05 |
| 61701 | 1.26617 | 0.05 |
| 61702 | 1.18322 | 0.05 |
| 61703 | 0.7366 | 0.05 |
| 61704 | 1.18321 | 0.05 |
| 61705 | 1.26617 | 0.05 |
| 61706 | 0.827918 | 0.05 |
| 61707 | 0.99929 | 0.05 |
| 61708 | 0.99929 | 0.05 |
| 61709 | 1.00348 | 0.05 |

| | | |
|---|---|---|
| 61710 | 1.00348 | 0.05 |
| 61711 | 1.26617 | 0.05 |
| 61712 | 1.18322 | 0.05 |
| 61713 | 0.946131 | 2.1 |
| 61714 | 0.946131 | 0.119577 |
| 61715 | 0.946129 | 0.05 |
| 61716 | 1.18321 | 0.05 |
| 61717 | 0.946133 | 2.1 |
| 61718 | 1.26617 | 1.12394 |
| 61719 | 1.26617 | 1.11298 |
| 61720 | 1.26617 | 0.05 |
| 61721 | 0.946131 | 1.92549 |
| 61722 | 0.946131 | 0.0999202 |
| 61723 | 2.23267 | 0.05 |
| 61724 | 1.89226 | 0.05 |
| 61725 | 2.07099 | 0.05 |
| 61726 | 1.89226 | 0.05 |
| 61727 | 2.07099 | 0.05 |
| 61728 | 1.89226 | 0.05 |
| 61729 | 0.946132 | 0.05 |
| 61730 | 1.35403 | 0.05 |
| 61731 | 1.24038 | 0.05 |
| 61732 | 0.946131 | 0.05 |
| 61733 | 0.946131 | 0.05 |
| 61734 | 0.946131 | 0.05 |
| 61735 | 1.24039 | 0.05 |
| 61736 | 1.35403 | 0.05 |
| 61737 | 1.24038 | 0.05 |
| 61738 | 1.24038 | 0.05 |
| 61739 | 0.946131 | 0.05 |
| 61740 | 0.946131 | 0.05 |
| 61741 | 1.89226 | 0.05 |
| 61742 | 1.89226 | 0.0629897 |
| 61743 | 1.89226 | 0.05 |
| 61744 | 1.89226 | 0.05 |
| 61745 | 1.89226 | 0.182451 |
| 61746 | 1.89226 | 0.118138 |
| 61747 | 1.89226 | 0.05 |
| 61748 | 1.89226 | 0.0896242 |
| 61749 | 1.89226 | 0.05 |
| 61750 | 1.89226 | 0.05 |
| 61751 | 3.78452 | 0.0681001 |
| 61752 | 1.56971 | 0.05 |
| 61753 | 2.80086 | 0.05 |
| 61754 | 2.59806 | 0.05 |
| 61755 | 1.93489 | 0.05 |
| 61756 | 2.59806 | 0.05 |
| 61757 | 1.93489 | 0.05 |
| 61758 | 0.827467 | 0.05 |
| 61759 | 1.26652 | 0.05 |
| 61760 | 1.13679 | 0.05 |
| 61761 | 0.639035 | 0.05 |
| 61762 | 1.11547 | 0.05 |
| 61763 | 1.30656 | 0.05 |
| 61764 | 0.869062 | 0.05 |
| 61765 | 0.696608 | 0.05 |
| 61766 | 1.07205 | 0.05 |
| 61767 | 0.953656 | 0.05 |

| | | |
|---|---|---|
| 61768 | 0.970744 | 0.05 |
| 61769 | 0.970744 | 0.05 |
| 61770 | 2.38681 | 2.1 |
| 61771 | 2.50438 | 1.79645 |
| 61772 | 2.42082 | 2.1 |
| 61773 | 2.59106 | 2.1 |
| 61774 | 4.92521 | 2.1 |
| 61775 | 2.50438 | 1.29267 |
| 61776 | 2.40627 | 2.1 |
| 61777 | 2.59106 | 2.1 |
| 61778 | 2.5444 | 1.09601 |
| 61779 | 5.04879 | 2.1 |
| 61780 | 2.42082 | 2.1 |
| 61781 | 2.70575 | 2.1 |
| 61782 | 2.64778 | 2.1 |
| 61783 | 2.59106 | 2.1 |
| 61784 | 2.47753 | 2.1 |
| 61785 | 2.42082 | 1.41025 |
| 61786 | 2.70575 | 2.1 |
| 61787 | 2.5444 | 1.78496 |
| 61788 | 2.76095 | 2.1 |
| 61789 | 2.59106 | 2.1 |
| 61790 | 2.71429 | 2.1 |
| 61791 | 2.5444 | 2.1 |
| 61792 | 1.89226 | 2.1 |
| 61793 | 2.38473 | 2.1 |
| 61794 | 2.36689 | 2.1 |
| 61795 | 2.38473 | 2.1 |
| 61796 | 1.87442 | 2.1 |
| 61797 | 1.89226 | 2.1 |
| 61798 | 1.89226 | 2.1 |
| 61799 | 1.89226 | 0.697467 |
| 61800 | 1.89226 | 2.1 |
| 61801 | 1.89226 | 0.689832 |
| 61802 | 1.89226 | 2.1 |
| 61803 | 1.89226 | 1.38453 |
| 61804 | 2.14952 | 2.1 |
| 61805 | 1.89226 | 0.601381 |
| 61806 | 1.89226 | 2.1 |
| 61807 | 1.89226 | 0.532048 |
| 61808 | 2.0692 | 2.1 |
| 61809 | 2.0692 | 0.781846 |
| 61810 | 1.89226 | 2.1 |
| 61811 | 2.36689 | 2.1 |
| 61812 | 2.36204 | 2.1 |
| 61813 | 2.36689 | 2.1 |
| 61814 | 1.88742 | 2.1 |
| 61815 | 1.89226 | 2.1 |
| 61816 | 0.946132 | 0.459905 |
| 61817 | 0.946132 | 2.1 |
| 61818 | 0.946131 | 2.1 |
| 61819 | 0.946131 | 2.1 |
| 61820 | 0.946132 | 1.90051 |
| 61821 | 0.946132 | 2.1 |
| 61822 | 0.946131 | 2.1 |
| 61823 | 0.946131 | 2.1 |
| 61824 | 0.946132 | 2.1 |
| 61825 | 0.946132 | 2.1 |

| | | |
|---|---|---|
| 61826 | 0.946131 | 2.1 |
| 61827 | 0.946131 | 2.1 |
| 61828 | 0.946128 | 2.1 |
| 61829 | 0.946128 | 2.1 |
| 61830 | 1.07476 | 1.70912 |
| 61831 | 1.07476 | 2.1 |
| 61832 | 0.946131 | 1.17916 |
| 61833 | 0.946131 | 2.1 |
| 61834 | 1.13182 | 2.1 |
| 61835 | 1.07476 | 2.1 |
| 61836 | 1.13182 | 2.1 |
| 61837 | 0.946131 | 2.1 |
| 61838 | 1.07476 | 2.1 |
| 61839 | 0.94613 | 0.837472 |
| 61840 | 1.89227 | 0.473516 |
| 61841 | 1.89227 | 0.572885 |
| 61842 | 1.89227 | 0.713598 |
| 61843 | 1.89227 | 0.664027 |
| 61844 | 3.78453 | 0.556822 |
| 61845 | 1.89226 | 0.501184 |
| 61846 | 1.89226 | 0.571639 |
| 61847 | 1.89226 | 0.510486 |
| 61848 | 1.89226 | 0.836861 |
| 61849 | 3.78452 | 0.534268 |
| 61850 | 1.89226 | 0.564981 |
| 61851 | 1.89226 | 0.667245 |
| 61852 | 1.89226 | 0.996866 |
| 61853 | 1.89226 | 1.12951 |
| 61854 | 3.78452 | 0.608434 |
| 61855 | 1.89226 | 0.566954 |
| 61856 | 1.89226 | 0.683541 |
| 61857 | 1.89226 | 0.619275 |
| 61858 | 1.89226 | 0.633416 |
| 61859 | 1.89226 | 0.736211 |
| 61860 | 1.89226 | 0.713386 |
| 61861 | 2.58344 | 0.942311 |
| 61862 | 2.00267 | 0.196667 |
| 61863 | 2.14218 | 0.85591 |
| 61864 | 2.22674 | 1.28423 |
| 61865 | 2.14218 | 0.101856 |
| 61866 | 2.22674 | 0.412835 |
| 61867 | 2.83531 | 1.36085 |
| 61868 | 1.56043 | 0.641222 |
| 61869 | 2.22674 | 1.20274 |
| 61870 | 1.90912 | 1.90953 |
| 61871 | 2.22674 | 0.124334 |
| 61872 | 1.90912 | 0.57666 |
| 61873 | 0.780217 | 0.680859 |
| 61874 | 1.11337 | 0.730838 |
| 61875 | 1.00133 | 1.25132 |
| 61876 | 0.804472 | 0.806196 |
| 61877 | 0.804472 | 0.965731 |
| 61878 | 0.780217 | 0.58722 |
| 61879 | 1.00133 | 0.172853 |
| 61880 | 1.11337 | 0.945598 |
| 61881 | 0.856668 | 0.896624 |
| 61882 | 0.856668 | 1.65405 |
| 61883 | 0.924883 | 0.579656 |

| | | |
|---|---|---|
| 61884 | 0.924883 | 0.52472 |
| 61885 | 1.93107 | 2.1 |
| 61886 | 1.68657 | 2.1 |
| 61887 | 1.7237 | 2.1 |
| 61888 | 1.89226 | 2.1 |
| 61889 | 1.53436 | 2.1 |
| 61890 | 1.68657 | 2.1 |
| 61891 | 1.89226 | 2.1 |
| 61892 | 1.89226 | 2.1 |
| 61893 | 1.89226 | 2.1 |
| 61894 | 1.89226 | 2.1 |
| 61895 | 3.78453 | 2.1 |
| 61896 | 0.946131 | 2.1 |
| 61897 | 0.946131 | 2.1 |
| 61898 | 0.855092 | 2.1 |
| 61899 | 0.83953 | 2.1 |
| 61900 | 0.946131 | 2.1 |
| 61901 | 0.946131 | 2.1 |
| 61902 | 0.839531 | 2.1 |
| 61903 | 0.839531 | 2.1 |
| 61904 | 0.946131 | 2.1 |
| 61905 | 0.855093 | 2.1 |
| 61906 | 0.839531 | 2.1 |
| 61907 | 0.946131 | 2.1 |
| 61908 | 1.89226 | 2.1 |
| 61909 | 1.70302 | 2.1 |
| 61910 | 2.21419 | 2.1 |
| 61911 | 1.93107 | 2.1 |
| 61912 | 1.97872 | 2.1 |
| 61913 | 1.70302 | 2.1 |
| 61914 | 0.946133 | 2.1 |
| 61915 | 0.946133 | 2.1 |
| 61916 | 0.946132 | 2.1 |
| 61917 | 0.946132 | 2.1 |
| 61918 | 0.946132 | 2.1 |
| 61919 | 0.946132 | 2.1 |
| 61920 | 0.946132 | 2.1 |
| 61921 | 0.946132 | 2.1 |
| 61922 | 0.946133 | 2.1 |
| 61923 | 0.946133 | 2.1 |
| 61924 | 0.946132 | 2.1 |
| 61925 | 0.946132 | 2.1 |
| 61926 | 1.90081 | 2.1 |
| 61927 | 1.89226 | 2.1 |
| 61928 | 1.51379 | 2.1 |
| 61929 | 1.36813 | 2.1 |
| 61930 | 1.71019 | 2.1 |
| 61931 | 1.89226 | 2.1 |
| 61932 | 1.89227 | 1.64309 |
| 61933 | 1.89227 | 0.827663 |
| 61934 | 1.89227 | 0.820912 |
| 61935 | 1.89227 | 1.12935 |
| 61936 | 1.89227 | 1.71307 |
| 61937 | 1.89227 | 2.08192 |
| 61938 | 1.36428 | 1.27657 |
| 61939 | 1.37869 | 1.09637 |
| 61940 | 0.946132 | 0.89618 |
| 61941 | 0.946132 | 1.1917 |

| | | |
|---|---|---|
| 61942 | 1.37869 | 0.823318 |
| 61943 | 1.37869 | 2.1 |
| 61944 | 0.946133 | 1.4795 |
| 61945 | 0.946133 | 1.82905 |
| 61946 | 1.37869 | 0.826358 |
| 61947 | 0.946132 | 1.05519 |
| 61948 | 1.36428 | 1.37019 |
| 61949 | 0.946133 | 0.697984 |
| 61950 | 0.94612 | 1.09392 |
| 61951 | 0.94612 | 1.02158 |
| 61952 | 0.946119 | 1.05866 |
| 61953 | 0.946119 | 1.17566 |
| 61954 | 0.946119 | 0.733952 |
| 61955 | 0.946119 | 2.01662 |
| 61956 | 0.94612 | 1.6307 |
| 61957 | 0.94612 | 2.06233 |
| 61958 | 0.946132 | 1.0423 |
| 61959 | 0.946132 | 0.764235 |
| 61960 | 0.946107 | 0.797121 |
| 61961 | 0.946107 | 1.36984 |
| 61962 | 1.89225 | 0.05 |
| 61963 | 1.89225 | 2.1 |
| 61964 | 1.63564 | 0.05 |
| 61965 | 1.42278 | 2.1 |
| 61966 | 1.42278 | 2.1 |
| 61967 | 1.57534 | 0.05 |
| 61968 | 1.53735 | 2.1 |
| 61969 | 1.68991 | 0.05 |
| 61970 | 1.57535 | 0.05 |
| 61971 | 1.04442 | 2.1 |
| 61972 | 1.04442 | 2.1 |
| 61973 | 1.22031 | 0.05 |
| 61974 | 1.42278 | 2.1 |
| 61975 | 1.59868 | 0.05 |
| 61976 | 0.946132 | 2.1 |
| 61977 | 0.946132 | 2.1 |
| 61978 | 0.946131 | 2.1 |
| 61979 | 0.946131 | 2.1 |
| 61980 | 0.946131 | 2.1 |
| 61981 | 0.946131 | 2.1 |
| 61982 | 0.946132 | 2.1 |
| 61983 | 0.946132 | 2.1 |
| 61984 | 0.946132 | 2.1 |
| 61985 | 0.946132 | 2.1 |
| 61986 | 0.946131 | 2.1 |
| 61987 | 0.946131 | 2.1 |
| 61988 | 1.89226 | 2.1 |
| 61989 | 1.89226 | 2.1 |
| 61990 | 1.89226 | 2.1 |
| 61991 | 1.89226 | 2.1 |
| 61992 | 1.89226 | 2.1 |
| 61993 | 1.89226 | 2.1 |
| 61994 | 2.03143 | 0.761133 |
| 61995 | 1.47111 | 2.02868 |
| 61996 | 2.28685 | 1.93258 |
| 61997 | 2.28685 | 1.78159 |
| 61998 | 1.36006 | 0.905033 |
| 61999 | 1.36006 | 0.866783 |

| | | |
|---|---|---|
| 62000 | 1.89226 | 0.721273 |
| 62001 | 1.89226 | 1.27209 |
| 62002 | 1.89226 | 1.0484 |
| 62003 | 1.89226 | 1.16951 |
| 62004 | 1.89226 | 0.719191 |
| 62005 | 1.89226 | 0.848815 |
| 62006 | 1.89226 | 1.14334 |
| 62007 | 1.89226 | 2.1 |
| 62008 | 1.89226 | 2.1 |
| 62009 | 1.89226 | 2.1 |
| 62010 | 1.89226 | 1.24546 |
| 62011 | 1.89226 | 2.1 |
| 62012 | 0.902821 | 2.1 |
| 62013 | 0.902821 | 2.1 |
| 62014 | 0.889218 | 2.1 |
| 62015 | 0.889218 | 2.1 |
| 62016 | 0.675759 | 2.1 |
| 62017 | 1.05649 | 2.1 |
| 62018 | 0.735554 | 2.1 |
| 62019 | 1.14342 | 2.1 |
| 62020 | 1.14343 | 1.91567 |
| 62021 | 1.05649 | 2.1 |
| 62022 | 0.735554 | 2.1 |
| 62023 | 0.675758 | 2.1 |
| 62024 | 2.46286 | 0.574891 |
| 62025 | 2.28685 | 0.771296 |
| 62026 | 2.67843 | 0.813993 |
| 62027 | 2.67843 | 0.53848 |
| 62028 | 2.03143 | 0.682417 |
| 62029 | 2.03143 | 0.601641 |
| 62030 | 1.89226 | 0.648214 |
| 62031 | 1.89226 | 0.728787 |
| 62032 | 1.89226 | 0.647909 |
| 62033 | 1.89226 | 0.561448 |
| 62034 | 3.78452 | 0.743702 |
| 62035 | 0.94613 | 1.02553 |
| 62036 | 0.94613 | 1.00744 |
| 62037 | 0.946128 | 2.07417 |
| 62038 | 0.946128 | 0.851951 |
| 62039 | 0.94613 | 2.1 |
| 62040 | 0.94613 | 0.890833 |
| 62041 | 0.946128 | 0.485025 |
| 62042 | 0.946128 | 0.928623 |
| 62043 | 0.946129 | 0.077994 |
| 62044 | 0.946129 | 1.15584 |
| 62045 | 0.946129 | 0.134819 |
| 62046 | 0.946129 | 2.1 |
| 62047 | 2.67843 | 0.73177 |
| 62048 | 2.11297 | 2.00399 |
| 62049 | 2.52453 | 1.51691 |
| 62050 | 2.52453 | 2.1 |
| 62051 | 2.28685 | 0.947685 |
| 62052 | 2.28685 | 0.883963 |
| 62053 | 1.89226 | 0.104288 |
| 62054 | 1.89226 | 0.969258 |
| 62055 | 1.89226 | 0.0738901 |
| 62056 | 1.89226 | 2.05304 |
| 62057 | 3.78451 | 0.175875 |

| | | |
|---|---|---|
| 62058 | 2.39524 | 0.0786006 |
| 62059 | 2.10188 | 0.895581 |
| 62060 | 2.29839 | 0.05 |
| 62061 | 2.02242 | 1.84309 |
| 62062 | 4.40028 | 0.168388 |
| 62063 | 1.71839 | 2.1 |
| 62064 | 1.65261 | 2.1 |
| 62065 | 2.10189 | 0.05 |
| 62066 | 2.10189 | 2.1 |
| 62067 | 2.02243 | 0.464763 |
| 62068 | 1.63893 | 0.725119 |
| 62069 | 0.946132 | 2.1 |
| 62070 | 0.946132 | 0.193724 |
| 62071 | 0.946129 | 0.149793 |
| 62072 | 0.946129 | 2.1 |
| 62073 | 0.94613 | 2.1 |
| 62074 | 0.94613 | 2.1 |
| 62075 | 0.946131 | 0.05 |
| 62076 | 0.946131 | 0.0898058 |
| 62077 | 0.946131 | 2.1 |
| 62078 | 0.946131 | 2.1 |
| 62079 | 0.946131 | 2.1 |
| 62080 | 0.946131 | 2.1 |
| 62081 | 1.89226 | 0.152918 |
| 62082 | 1.89226 | 1.96361 |
| 62083 | 1.89226 | 0.05 |
| 62084 | 1.89226 | 0.75689 |
| 62085 | 3.78452 | 0.139769 |
| 62086 | 2.2984 | 0.05 |
| 62087 | 2.02242 | 1.60913 |
| 62088 | 2.21582 | 0.05 |
| 62089 | 1.94412 | 0.67254 |
| 62090 | 4.24251 | 0.0970389 |
| 62091 | 1.94412 | 0.05 |
| 62092 | 1.5854 | 2.1 |
| 62093 | 2.02243 | 2.1 |
| 62094 | 2.02243 | 0.136874 |
| 62095 | 1.57431 | 0.152214 |
| 62096 | 1.65261 | 2.1 |
| 62097 | 0.810943 | 0.102293 |
| 62098 | 0.810943 | 2.1 |
| 62099 | 0.810945 | 2.1 |
| 62100 | 0.810945 | 2.1 |
| 62101 | 1.89226 | 0.057791 |
| 62102 | 1.3517 | 1.99866 |
| 62103 | 1.08133 | 2.1 |
| 62104 | 1.08132 | 2.1 |
| 62105 | 1.08132 | 0.05 |
| 62106 | 0.810933 | 2.1 |
| 62107 | 0.810933 | 2.1 |
| 62108 | 1.89226 | 0.05 |
| 62109 | 1.89226 | 0.05 |
| 62110 | 1.89226 | 0.05 |
| 62111 | 1.89226 | 0.05 |
| 62112 | 1.89226 | 0.05 |
| 62113 | 1.89226 | 0.05 |
| 62114 | 1.89226 | 0.05 |
| 62115 | 1.89226 | 0.05 |

| | | |
|---|---|---|
| 62116 | 1.89226 | 0.05 |
| 62117 | 1.89226 | 0.05 |
| 62118 | 3.78452 | 0.05 |
| 62119 | 1.89226 | 0.05 |
| 62120 | 1.89226 | 0.05 |
| 62121 | 1.89226 | 0.05 |
| 62122 | 1.89226 | 0.05 |
| 62123 | 1.89226 | 0.05 |
| 62124 | 1.89226 | 0.05 |
| 62125 | 0.946128 | 0.05 |
| 62126 | 0.946128 | 0.05 |
| 62127 | 0.946132 | 0.05 |
| 62128 | 0.946132 | 0.05 |
| 62129 | 0.94613 | 0.05 |
| 62130 | 0.94613 | 0.05 |
| 62131 | 0.946129 | 0.05 |
| 62132 | 0.946129 | 0.05 |
| 62133 | 0.94613 | 0.05 |
| 62134 | 0.94613 | 0.05 |
| 62135 | 0.94613 | 0.05 |
| 62136 | 0.94613 | 0.05 |
| 62137 | 1.89226 | 0.05 |
| 62138 | 1.89226 | 0.05 |
| 62139 | 1.89226 | 0.05 |
| 62140 | 1.89226 | 0.05 |
| 62141 | 1.89226 | 0.05 |
| 62142 | 1.89226 | 0.05 |
| 62143 | 2.81478 | 2.1 |
| 62144 | 1.89226 | 2.1 |
| 62145 | 2.65557 | 2.1 |
| 62146 | 1.89226 | 2.1 |
| 62147 | 4.54783 | 2.1 |
| 62148 | 1.89226 | 2.1 |
| 62149 | 1.89226 | 2.1 |
| 62150 | 1.89226 | 2.1 |
| 62151 | 1.89226 | 2.1 |
| 62152 | 3.78452 | 2.1 |
| 62153 | 2.65557 | 2.1 |
| 62154 | 1.89226 | 2.1 |
| 62155 | 1.89226 | 2.1 |
| 62156 | 1.89226 | 2.1 |
| 62157 | 2.57997 | 2.1 |
| 62158 | 2.57997 | 2.1 |
| 62159 | 0.946131 | 2.1 |
| 62160 | 0.946131 | 2.1 |
| 62161 | 0.946128 | 2.1 |
| 62162 | 0.946128 | 2.1 |
| 62163 | 0.94613 | 2.1 |
| 62164 | 0.94613 | 2.1 |
| 62165 | 0.946129 | 2.1 |
| 62166 | 0.946129 | 2.1 |
| 62167 | 0.94613 | 2.1 |
| 62168 | 0.94613 | 2.1 |
| 62169 | 0.94613 | 2.1 |
| 62170 | 0.94613 | 2.1 |
| 62171 | 1.89226 | 0.426529 |
| 62172 | 2.27703 | 2.05125 |
| 62173 | 2.35615 | 2.1 |

| | | |
|---|---|---|
| 62174 | 2.35615 | 0.968701 |
| 62175 | 1.89226 | 2.1 |
| 62176 | 1.89226 | 1.06425 |
| 62177 | 0.946132 | 0.414907 |
| 62178 | 1.17807 | 2.1 |
| 62179 | 1.14926 | 0.390644 |
| 62180 | 0.946132 | 1.42693 |
| 62181 | 0.946133 | 1.29558 |
| 62182 | 0.946133 | 1.40608 |
| 62183 | 1.14926 | 0.551009 |
| 62184 | 1.14926 | 0.453462 |
| 62185 | 0.946132 | 1.28269 |
| 62186 | 0.946132 | 0.66864 |
| 62187 | 1.17807 | 1.39573 |
| 62188 | 1.14926 | 0.831481 |
| 62189 | 2.16841 | 0.515164 |
| 62190 | 1.35487 | 1.28534 |
| 62191 | 1.19854 | 0.866443 |
| 62192 | 1.19854 | 0.528939 |
| 62193 | 2.46078 | 1.02438 |
| 62194 | 2.46078 | 0.908357 |
| 62195 | 0.69308 | 0.299186 |
| 62196 | 1.3119 | 1.39335 |
| 62197 | 0.677433 | 1.41542 |
| 62198 | 1.23039 | 0.939281 |
| 62199 | 0.978178 | 1.4173 |
| 62200 | 0.978178 | 1.4306 |
| 62201 | 0.945294 | 0.710307 |
| 62202 | 0.945294 | 0.947499 |
| 62203 | 1.3119 | 1.11697 |
| 62204 | 1.23039 | 1.03966 |
| 62205 | 0.693081 | 1.15824 |
| 62206 | 0.677432 | 1.17889 |
| 62207 | 1.89226 | 0.624907 |
| 62208 | 1.89226 | 2.1 |
| 62209 | 1.89226 | 2.1 |
| 62210 | 1.89226 | 1.03469 |
| 62211 | 1.89226 | 2.06846 |
| 62212 | 1.89226 | 1.01366 |
| 62213 | 0.946132 | 0.376309 |
| 62214 | 0.946132 | 2.1 |
| 62215 | 0.946132 | 0.193574 |
| 62216 | 0.946132 | 1.14513 |
| 62217 | 0.946133 | 1.10352 |
| 62218 | 0.946133 | 1.38065 |
| 62219 | 0.946132 | 0.404226 |
| 62220 | 0.946132 | 0.263827 |
| 62221 | 0.946133 | 0.835689 |
| 62222 | 0.946133 | 0.361956 |
| 62223 | 0.946132 | 1.13032 |
| 62224 | 0.946132 | 0.666738 |
| 62225 | 2.60637 | 2.1 |
| 62226 | 1.89226 | 2.1 |
| 62227 | 1.89226 | 2.1 |
| 62228 | 1.89226 | 1.40581 |
| 62229 | 3.78452 | 2.1 |
| 62230 | 1.89226 | 0.199289 |
| 62231 | 1.8396 | 1.58061 |

| | | |
|---|---|---|
| 62232 | 1.89226 | 2.1 |
| 62233 | 1.89226 | 0.22138 |
| 62234 | 1.60993 | 2.1 |
| 62235 | 1.60993 | 0.183464 |
| 62236 | 0.946131 | 1.06565 |
| 62237 | 0.946131 | 2.03127 |
| 62238 | 0.946128 | 2.1 |
| 62239 | 0.946128 | 2.1 |
| 62240 | 0.946131 | 0.977477 |
| 62241 | 0.946131 | 1.97653 |
| 62242 | 0.946127 | 2.1 |
| 62243 | 0.946127 | 1.81738 |
| 62244 | 0.946129 | 2.1 |
| 62245 | 0.946129 | 2.1 |
| 62246 | 0.94613 | 2.1 |
| 62247 | 0.94613 | 2.1 |
| 62248 | 1.89226 | 2.1 |
| 62249 | 1.89226 | 1.76858 |
| 62250 | 1.89226 | 2.1 |
| 62251 | 1.89226 | 2.1 |
| 62252 | 1.89226 | 2.1 |
| 62253 | 1.89226 | 2.1 |
| 62254 | 1.89226 | 0.208738 |
| 62255 | 1.89226 | 1.28412 |
| 62256 | 1.89226 | 2.08707 |
| 62257 | 1.89226 | 0.228345 |
| 62258 | 1.89226 | 2.1 |
| 62259 | 1.89226 | 0.120521 |
| 62260 | 0.946131 | 0.946081 |
| 62261 | 0.946131 | 2.02975 |
| 62262 | 0.946128 | 2.1 |
| 62263 | 0.946128 | 2.00942 |
| 62264 | 0.946131 | 0.90763 |
| 62265 | 0.946131 | 1.74814 |
| 62266 | 0.946127 | 1.99072 |
| 62267 | 0.946127 | 2.1 |
| 62268 | 0.94613 | 2.1 |
| 62269 | 0.94613 | 2.1 |
| 62270 | 0.946129 | 2.1 |
| 62271 | 0.946129 | 2.1 |
| 62272 | 0.946133 | 2.1 |
| 62273 | 0.946133 | 2.1 |
| 62274 | 1.28153 | 2.1 |
| 62275 | 1.26781 | 2.1 |
| 62276 | 0.946132 | 2.1 |
| 62277 | 0.946132 | 2.1 |
| 62278 | 1.28153 | 2.1 |
| 62279 | 1.28153 | 2.1 |
| 62280 | 0.946132 | 2.1 |
| 62281 | 1.28153 | 2.1 |
| 62282 | 0.946133 | 2.1 |
| 62283 | 1.26781 | 2.1 |
| 62284 | 1.89226 | 2.1 |
| 62285 | 1.89226 | 2.1 |
| 62286 | 1.89226 | 2.1 |
| 62287 | 1.89226 | 2.1 |
| 62288 | 1.89226 | 2.1 |
| 62289 | 1.89226 | 2.1 |

| | | |
|---|---|---|
| 62290 | 2.5844 | 2.1 |
| 62291 | 1.09563 | 2.1 |
| 62292 | 1.1468 | 2.1 |
| 62293 | 1.09563 | 2.1 |
| 62294 | 2.6142 | 2.1 |
| 62295 | 2.56304 | 2.1 |
| 62296 | 0.946126 | 2.1 |
| 62297 | 0.946126 | 2.1 |
| 62298 | 1.2922 | 2.1 |
| 62299 | 1.28152 | 2.1 |
| 62300 | 0.946126 | 2.1 |
| 62301 | 0.946126 | 2.1 |
| 62302 | 1.2922 | 2.1 |
| 62303 | 1.2922 | 2.1 |
| 62304 | 1.29221 | 2.1 |
| 62305 | 0.946132 | 2.1 |
| 62306 | 0.946119 | 2.1 |
| 62307 | 1.28151 | 2.1 |
| 62308 | 1.89225 | 2.1 |
| 62309 | 1.89225 | 2.1 |
| 62310 | 1.89225 | 2.1 |
| 62311 | 1.89225 | 2.1 |
| 62312 | 1.89225 | 2.1 |
| 62313 | 1.89225 | 2.1 |
| 62314 | 0.946121 | 2.1 |
| 62315 | 0.946121 | 2.1 |
| 62316 | 0.946126 | 2.1 |
| 62317 | 0.946126 | 2.1 |
| 62318 | 0.946124 | 1.99258 |
| 62319 | 0.946124 | 2.1 |
| 62320 | 0.946123 | 2.1 |
| 62321 | 0.946123 | 2.1 |
| 62322 | 0.94613 | 2.1 |
| 62323 | 0.94613 | 2.1 |
| 62324 | 0.946117 | 2.1 |
| 62325 | 0.946117 | 2.1 |
| 62326 | 1.89226 | 0.990968 |
| 62327 | 1.89226 | 1.10505 |
| 62328 | 1.89226 | 0.906054 |
| 62329 | 1.89226 | 2.1 |
| 62330 | 1.89226 | 0.05 |
| 62331 | 1.89226 | 2.1 |
| 62332 | 1.89226 | 2.1 |
| 62333 | 1.89226 | 2.1 |
| 62334 | 1.89226 | 0.254057 |
| 62335 | 1.89226 | 2.1 |
| 62336 | 1.89226 | 0.05 |
| 62337 | 1.89226 | 2.1 |
| 62338 | 1.89226 | 2.1 |
| 62339 | 2.81281 | 2.1 |
| 62340 | 2.50369 | 0.74593 |
| 62341 | 2.50369 | 2.1 |
| 62342 | 1.89226 | 1.09063 |
| 62343 | 1.89226 | 2.1 |
| 62344 | 1.89226 | 2.1 |
| 62345 | 1.89226 | 2.1 |
| 62346 | 1.89226 | 2.1 |
| 62347 | 1.89226 | 2.1 |

| | | |
|---|---|---|
| 62348 | 1.89226 | 2.1 |
| 62349 | 1.89226 | 2.1 |
| 62350 | 1.89226 | 2.1 |
| 62351 | 1.89226 | 2.1 |
| 62352 | 1.89226 | 2.1 |
| 62353 | 1.89226 | 2.1 |
| 62354 | 1.89226 | 2.1 |
| 62355 | 1.89226 | 2.1 |
| 62356 | 0.907036 | 2.1 |
| 62357 | 1.09714 | 2.1 |
| 62358 | 0.994609 | 2.1 |
| 62359 | 0.838808 | 2.1 |
| 62360 | 0.976536 | 2.1 |
| 62361 | 0.976536 | 2.1 |
| 62362 | 0.959412 | 2.1 |
| 62363 | 0.959412 | 2.1 |
| 62364 | 1.09714 | 2.1 |
| 62365 | 0.994609 | 2.1 |
| 62366 | 0.907036 | 2.1 |
| 62367 | 0.838808 | 2.1 |
| 62368 | 0.99461 | 2.1 |
| 62369 | 0.838808 | 2.1 |
| 62370 | 0.738212 | 2.1 |
| 62371 | 0.565634 | 2.1 |
| 62372 | 0.775921 | 2.1 |
| 62373 | 0.775921 | 2.1 |
| 62374 | 0.784322 | 2.1 |
| 62375 | 0.784322 | 2.1 |
| 62376 | 0.994609 | 2.1 |
| 62377 | 0.738212 | 2.1 |
| 62378 | 0.838808 | 2.1 |
| 62379 | 0.565635 | 2.1 |
| 62380 | 1.89226 | 2.1 |
| 62381 | 1.89226 | 2.1 |
| 62382 | 1.89226 | 2.1 |
| 62383 | 1.89226 | 2.1 |
| 62384 | 1.89226 | 2.1 |
| 62385 | 1.89226 | 2.1 |
| 62386 | 1.89226 | 2.1 |
| 62387 | 1.89226 | 2.1 |
| 62388 | 1.89226 | 2.1 |
| 62389 | 1.89226 | 2.1 |
| 62390 | 1.89226 | 2.1 |
| 62391 | 1.89226 | 2.1 |
| 62392 | 2.19428 | 2.1 |
| 62393 | 1.98922 | 2.1 |
| 62394 | 2.27322 | 2.1 |
| 62395 | 2.45893 | 2.1 |
| 62396 | 4.4675 | 2.1 |
| 62397 | 0.99461 | 2.1 |
| 62398 | 1.13661 | 2.1 |
| 62399 | 0.902272 | 2.1 |
| 62400 | 0.738212 | 2.1 |
| 62401 | 0.931874 | 2.1 |
| 62402 | 0.931874 | 2.1 |
| 62403 | 0.942953 | 2.1 |
| 62404 | 0.942953 | 2.1 |
| 62405 | 1.13658 | 2.1 |

| | | |
|---|---|---|
| 62406 | 0.902248 | 2.1 |
| 62407 | 0.994637 | 2.1 |
| 62408 | 0.738233 | 2.1 |
| 62409 | 1.89226 | 2.1 |
| 62410 | 1.89226 | 1.9648 |
| 62411 | 1.89226 | 2.1 |
| 62412 | 1.89226 | 2.1 |
| 62413 | 1.89226 | 2.1 |
| 62414 | 1.89226 | 1.97263 |
| 62415 | 1.89227 | 2.1 |
| 62416 | 2.58246 | 1.17733 |
| 62417 | 1.89227 | 1.92807 |
| 62418 | 1.89227 | 2.02477 |
| 62419 | 3.78453 | 1.42036 |
| 62420 | 1.89226 | 2.1 |
| 62421 | 1.89226 | 1.881 |
| 62422 | 1.65573 | 2.1 |
| 62423 | 1.65573 | 2.1 |
| 62424 | 1.89226 | 2.1 |
| 62425 | 1.89226 | 2.1 |
| 62426 | 1.89226 | 1.71933 |
| 62427 | 1.89226 | 1.38596 |
| 62428 | 1.89226 | 1.61854 |
| 62429 | 1.89226 | 1.61297 |
| 62430 | 1.89226 | 1.45712 |
| 62431 | 1.89226 | 1.53754 |
| 62432 | 1.89226 | 1.3783 |
| 62433 | 1.89226 | 1.44591 |
| 62434 | 1.89226 | 1.34579 |
| 62435 | 1.89226 | 1.57037 |
| 62436 | 1.89226 | 1.34227 |
| 62437 | 1.89226 | 1.63985 |
| 62438 | 0.946132 | 2.1 |
| 62439 | 0.946132 | 2.1 |
| 62440 | 1.01023 | 1.26929 |
| 62441 | 1.13651 | 2.04527 |
| 62442 | 1.01023 | 1.23127 |
| 62443 | 0.946133 | 1.6832 |
| 62444 | 1.0644 | 1.94698 |
| 62445 | 1.0644 | 2.1 |
| 62446 | 0.946132 | 2.1 |
| 62447 | 0.946132 | 1.94701 |
| 62448 | 1.13651 | 1.6683 |
| 62449 | 0.946132 | 1.90503 |
| 62450 | 1.89226 | 0.092131 |
| 62451 | 1.89226 | 1.76221 |
| 62452 | 1.89226 | 0.880605 |
| 62453 | 1.89226 | 0.05 |
| 62454 | 3.78451 | 0.173945 |
| 62455 | 1.00934 | 0.05 |
| 62456 | 0.741561 | 0.05 |
| 62457 | 0.946131 | 0.05 |
| 62458 | 0.946131 | 0.085787 |
| 62459 | 0.946131 | 0.384186 |
| 62460 | 0.741561 | 0.05 |
| 62461 | 1.00934 | 0.05 |
| 62462 | 0.946131 | 0.176509 |
| 62463 | 0.741586 | 0.05 |

| | | |
|---|---|---|
| 62464 | 0.741586 | 0.05 |
| 62465 | 0.946106 | 0.05 |
| 62466 | 0.946106 | 0.05 |
| 62467 | 1.14895 | 0.05 |
| 62468 | 0.957449 | 0.113553 |
| 62469 | 0.934806 | 1.16443 |
| 62470 | 0.934806 | 0.630818 |
| 62471 | 1.13536 | 0.05 |
| 62472 | 1.02829 | 1.69516 |
| 62473 | 0.946129 | 1.96348 |
| 62474 | 0.946129 | 0.05 |
| 62475 | 0.946127 | 0.361773 |
| 62476 | 0.946127 | 0.487035 |
| 62477 | 0.946127 | 0.239305 |
| 62478 | 1.0283 | 0.411951 |
| 62479 | 1.89226 | 1.83038 |
| 62480 | 1.89226 | 0.138861 |
| 62481 | 1.89226 | 0.105951 |
| 62482 | 1.89226 | 1.33276 |
| 62483 | 3.78452 | 0.725678 |
| 62484 | 1.89226 | 0.05 |
| 62485 | 1.89226 | 0.323733 |
| 62486 | 1.89226 | 0.156728 |
| 62487 | 1.89226 | 0.05 |
| 62488 | 3.78453 | 0.05 |
| 62489 | 1.89226 | 0.328708 |
| 62490 | 2.69068 | 0.05 |
| 62491 | 1.89226 | 0.05 |
| 62492 | 1.89226 | 0.159377 |
| 62493 | 3.78452 | 0.092799 |
| 62494 | 1.89226 | 0.145163 |
| 62495 | 1.89226 | 1.63289 |
| 62496 | 1.89226 | 1.59835 |
| 62497 | 1.89226 | 0.45763 |
| 62498 | 1.89226 | 1.2081 |
| 62499 | 1.89226 | 0.181959 |
| 62500 | 1.89226 | 2.1 |
| 62501 | 1.89226 | 2.1 |
| 62502 | 1.89226 | 2.1 |
| 62503 | 1.89226 | 2.1 |
| 62504 | 1.89226 | 2.1 |
| 62505 | 1.89226 | 2.1 |
| 62506 | 1.89226 | 2.1 |
| 62507 | 1.89226 | 2.1 |
| 62508 | 1.89226 | 2.1 |
| 62509 | 1.89226 | 2.1 |
| 62510 | 1.89226 | 2.1 |
| 62511 | 1.89226 | 2.1 |
| 62512 | 0.985109 | 2.1 |
| 62513 | 1.31144 | 2.1 |
| 62514 | 1.45864 | 2.1 |
| 62515 | 1.10667 | 2.1 |
| 62516 | 2.44375 | 2.1 |
| 62517 | 0.655718 | 2.1 |
| 62518 | 0.729316 | 2.1 |
| 62519 | 0.684594 | 2.1 |
| 62520 | 0.782707 | 2.1 |
| 62521 | 0.700849 | 2.1 |

| | | |
|---|---|---|
| 62522 | 0.700849 | 2.1 |
| 62523 | 0.71306 | 2.1 |
| 62524 | 0.71306 | 2.1 |
| 62525 | 0.729318 | 2.1 |
| 62526 | 0.782705 | 2.1 |
| 62527 | 0.684592 | 2.1 |
| 62528 | 0.655719 | 2.1 |
| 62529 | 0.946128 | 2.1 |
| 62530 | 0.946128 | 2.1 |
| 62531 | 0.946133 | 2.1 |
| 62532 | 0.946133 | 2.1 |
| 62533 | 0.946131 | 2.1 |
| 62534 | 0.946131 | 2.1 |
| 62535 | 0.946131 | 2.1 |
| 62536 | 0.946131 | 2.1 |
| 62537 | 0.94613 | 2.1 |
| 62538 | 0.94613 | 2.1 |
| 62539 | 0.946131 | 2.1 |
| 62540 | 0.946131 | 2.1 |
| 62541 | 1.89226 | 2.1 |
| 62542 | 1.89226 | 2.1 |
| 62543 | 1.89226 | 2.1 |
| 62544 | 1.89226 | 2.1 |
| 62545 | 1.89226 | 2.1 |
| 62546 | 1.89226 | 2.1 |
| 62547 | 0.553334 | 2.1 |
| 62548 | 0.629936 | 2.1 |
| 62549 | 0.828211 | 2.1 |
| 62550 | 0.729319 | 2.1 |
| 62551 | 0.674025 | 2.1 |
| 62552 | 0.674025 | 2.1 |
| 62553 | 0.68523 | 2.1 |
| 62554 | 0.68523 | 2.1 |
| 62555 | 0.828211 | 2.1 |
| 62556 | 0.629935 | 2.1 |
| 62557 | 0.72932 | 2.1 |
| 62558 | 0.553334 | 2.1 |
| 62559 | 0.828208 | 2.1 |
| 62560 | 0.729316 | 2.1 |
| 62561 | 0.907037 | 2.1 |
| 62562 | 0.782707 | 2.1 |
| 62563 | 0.799048 | 2.1 |
| 62564 | 0.799048 | 2.1 |
| 62565 | 0.811867 | 2.1 |
| 62566 | 0.811867 | 2.1 |
| 62567 | 0.828209 | 2.1 |
| 62568 | 0.907035 | 2.1 |
| 62569 | 0.729318 | 2.1 |
| 62570 | 0.782706 | 2.1 |
| 62571 | 2.32981 | 2.1 |
| 62572 | 1.89226 | 2.1 |
| 62573 | 1.89226 | 2.1 |
| 62574 | 1.89226 | 2.1 |
| 62575 | 2.2671 | 2.1 |
| 62576 | 2.2671 | 2.1 |
| 62577 | 1.89226 | 2.1 |
| 62578 | 1.89226 | 2.1 |
| 62579 | 1.89226 | 2.1 |

| | | |
|---|---|---|
| 62580 | 1.89226 | 1.50305 |
| 62581 | 1.89226 | 2.1 |
| 62582 | 1.89226 | 2.1 |
| 62583 | 1.3991 | 2.1 |
| 62584 | 1.81793 | 2.02676 |
| 62585 | 2.07226 | 2.1 |
| 62586 | 2.07226 | 1.48926 |
| 62587 | 1.24924 | 2.1 |
| 62588 | 1.24924 | 2.1 |
| 62589 | 0.946131 | 0.858811 |
| 62590 | 0.946131 | 2.1 |
| 62591 | 1.16491 | 2.1 |
| 62592 | 1.16491 | 2.1 |
| 62593 | 0.946131 | 2.1 |
| 62594 | 0.946131 | 2.1 |
| 62595 | 1.16491 | 2.1 |
| 62596 | 1.19523 | 2.1 |
| 62597 | 0.946132 | 2.1 |
| 62598 | 1.19523 | 2.1 |
| 62599 | 1.16491 | 2.1 |
| 62600 | 0.946131 | 2.1 |
| 62601 | 1.89226 | 2.1 |
| 62602 | 1.89226 | 1.81422 |
| 62603 | 1.89226 | 2.1 |
| 62604 | 1.89226 | 0.669688 |
| 62605 | 3.78452 | 1.26692 |
| 62606 | 0.901204 | 2.1 |
| 62607 | 0.901204 | 2.1 |
| 62608 | 0.902504 | 2.1 |
| 62609 | 0.902504 | 2.1 |
| 62610 | 1.10416 | 2.06436 |
| 62611 | 1.03613 | 1.06154 |
| 62612 | 0.764922 | 2.1 |
| 62613 | 0.699552 | 2.1 |
| 62614 | 0.764921 | 2.1 |
| 62615 | 1.10416 | 2.1 |
| 62616 | 0.699551 | 2.1 |
| 62617 | 1.03613 | 1.56895 |
| 62618 | 1.89226 | 0.420293 |
| 62619 | 1.89226 | 1.07758 |
| 62620 | 1.89226 | 1.09886 |
| 62621 | 1.89226 | 0.771626 |
| 62622 | 3.78453 | 0.980281 |
| 62623 | 1.89226 | 0.98728 |
| 62624 | 1.89226 | 1.19649 |
| 62625 | 1.89226 | 0.886077 |
| 62626 | 1.89226 | 1.44944 |
| 62627 | 1.89226 | 0.880273 |
| 62628 | 1.89226 | 1.4732 |
| 62629 | 0.946131 | 1.09388 |
| 62630 | 0.946131 | 1.11011 |
| 62631 | 0.946131 | 0.961079 |
| 62632 | 0.946131 | 0.924666 |
| 62633 | 0.946131 | 0.953604 |
| 62634 | 0.946131 | 1.10409 |
| 62635 | 0.946131 | 1.13527 |
| 62636 | 0.946131 | 0.554737 |
| 62637 | 0.946131 | 0.957353 |

| | | |
|---|---|---|
| 62638 | 0.946131 | 0.954075 |
| 62639 | 0.946131 | 0.949963 |
| 62640 | 0.946131 | 0.981966 |
| 62641 | 1.89226 | 1.00416 |
| 62642 | 1.89226 | 1.20024 |
| 62643 | 1.89226 | 1.05535 |
| 62644 | 1.89226 | 1.28021 |
| 62645 | 1.89226 | 1.05634 |
| 62646 | 1.89226 | 1.28371 |
| 62647 | 0.946127 | 1.04328 |
| 62648 | 0.946127 | 1.01528 |
| 62649 | 1.22788 | 0.666725 |
| 62650 | 1.35469 | 0.739011 |
| 62651 | 1.35469 | 0.912173 |
| 62652 | 1.35469 | 0.777927 |
| 62653 | 0.946128 | 1.05191 |
| 62654 | 0.946128 | 0.512394 |
| 62655 | 0.946129 | 0.84327 |
| 62656 | 1.22788 | 0.765404 |
| 62657 | 1.35469 | 0.836795 |
| 62658 | 0.946129 | 0.885618 |
| 62659 | 1.22788 | 1.09798 |
| 62660 | 1.35469 | 1.14735 |
| 62661 | 1.02602 | 0.862647 |
| 62662 | 0.794861 | 1.06996 |
| 62663 | 1.15301 | 1.13087 |
| 62664 | 1.15301 | 1.16214 |
| 62665 | 1.10089 | 0.920511 |
| 62666 | 1.10089 | 1.06863 |
| 62667 | 0.794861 | 1.02918 |
| 62668 | 1.22788 | 1.12273 |
| 62669 | 1.02602 | 1.10766 |
| 62670 | 1.35469 | 1.10769 |
| 62671 | 1.89226 | 0.137139 |
| 62672 | 1.89226 | 0.47309 |
| 62673 | 1.89226 | 1.38522 |
| 62674 | 1.89226 | 0.481218 |
| 62675 | 3.78452 | 0.626714 |
| 62676 | 1.13536 | 2.1 |
| 62677 | 0.942524 | 0.05 |
| 62678 | 0.946131 | 0.0986892 |
| 62679 | 0.946131 | 0.053678 |
| 62680 | 0.946127 | 0.15666 |
| 62681 | 0.946127 | 2.1 |
| 62682 | 1.26282 | 0.05 |
| 62683 | 0.917471 | 1.50251 |
| 62684 | 1.21023 | 0.384182 |
| 62685 | 1.00851 | 0.401463 |
| 62686 | 1.17955 | 0.243238 |
| 62687 | 0.883746 | 0.0623472 |
| 62688 | 1.89226 | 0.084462 |
| 62689 | 1.89226 | 2.1 |
| 62690 | 1.89226 | 0.159485 |
| 62691 | 1.89226 | 0.822105 |
| 62692 | 1.89226 | 0.108004 |
| 62693 | 1.89226 | 1.45664 |
| 62694 | 1.89226 | 0.339996 |
| 62695 | 1.89226 | 0.766572 |

| | | |
|---|---|---|
| 62696 | 1.89226 | 0.10137 |
| 62697 | 1.89226 | 0.274559 |
| 62698 | 1.89226 | 0.150165 |
| 62699 | 1.89226 | 0.790828 |
| 62700 | 0.94613 | 0.909533 |
| 62701 | 0.94613 | 0.599948 |
| 62702 | 0.94613 | 0.510811 |
| 62703 | 0.94613 | 0.576554 |
| 62704 | 0.946128 | 0.598475 |
| 62705 | 0.946128 | 0.963293 |
| 62706 | 0.946131 | 1.19413 |
| 62707 | 0.946131 | 0.442437 |
| 62708 | 0.94613 | 0.678904 |
| 62709 | 0.94613 | 0.7276 |
| 62710 | 0.94613 | 0.683101 |
| 62711 | 0.94613 | 0.653575 |
| 62712 | 0.883758 | 2.01991 |
| 62713 | 1.03041 | 0.4323 |
| 62714 | 0.946131 | 0.694619 |
| 62715 | 0.946131 | 0.148825 |
| 62716 | 0.851537 | 0.193917 |
| 62717 | 0.851537 | 1.9944 |
| 62718 | 1.23942 | 0.05 |
| 62719 | 1.14906 | 1.86706 |
| 62720 | 0.851484 | 0.491452 |
| 62721 | 0.985518 | 0.209203 |
| 62722 | 1.0156 | 0.460616 |
| 62723 | 0.851526 | 0.487179 |
| 62724 | 1.89226 | 0.0686455 |
| 62725 | 1.89226 | 2.1 |
| 62726 | 1.89226 | 0.0936667 |
| 62727 | 1.89226 | 0.892517 |
| 62728 | 1.89226 | 0.0687014 |
| 62729 | 1.89226 | 1.22713 |
| 62730 | 1.89226 | 0.579973 |
| 62731 | 1.89226 | 0.430775 |
| 62732 | 1.89226 | 0.482719 |
| 62733 | 1.89226 | 0.765092 |
| 62734 | 1.89226 | 0.120335 |
| 62735 | 1.89226 | 0.770918 |
| 62736 | 1.89227 | 0.418542 |
| 62737 | 1.89227 | 0.47635 |
| 62738 | 1.89227 | 0.464364 |
| 62739 | 1.89227 | 0.572532 |
| 62740 | 3.78453 | 0.459392 |
| 62741 | 1.89226 | 0.476718 |
| 62742 | 1.89226 | 0.47972 |
| 62743 | 1.89226 | 0.542218 |
| 62744 | 1.89226 | 0.694155 |
| 62745 | 3.78452 | 0.550838 |
| 62746 | 0.946132 | 0.589325 |
| 62747 | 0.946132 | 0.620726 |
| 62748 | 0.946128 | 0.783781 |
| 62749 | 0.946128 | 0.619106 |
| 62750 | 0.94613 | 0.556487 |
| 62751 | 0.94613 | 0.631148 |
| 62752 | 0.94613 | 0.473246 |
| 62753 | 0.94613 | 0.660516 |

| | | |
|---|---|---|
| 62754 | 0.946131 | 0.596838 |
| 62755 | 0.946131 | 0.826363 |
| 62756 | 0.94613 | 0.513693 |
| 62757 | 0.94613 | 0.556234 |
| 62758 | 1.89226 | 0.589765 |
| 62759 | 1.89226 | 0.539616 |
| 62760 | 1.89226 | 0.587013 |
| 62761 | 1.89226 | 0.493209 |
| 62762 | 1.89226 | 0.634404 |
| 62763 | 1.89226 | 0.514524 |
| 62764 | 1.98874 | 0.112474 |
| 62765 | 2.33455 | 0.398292 |
| 62766 | 2.01865 | 0.520227 |
| 62767 | 2.07024 | 0.523288 |
| 62768 | 4.32329 | 0.539383 |
| 62769 | 1.98874 | 0.445057 |
| 62770 | 2.00266 | 0.287085 |
| 62771 | 2.14218 | 0.690143 |
| 62772 | 2.07024 | 0.82719 |
| 62773 | 3.9914 | 0.371181 |
| 62774 | 1.00133 | 0.60551 |
| 62775 | 0.804471 | 0.410085 |
| 62776 | 1.03512 | 0.686844 |
| 62777 | 0.995176 | 0.63834 |
| 62778 | 0.80447 | 1.09238 |
| 62779 | 0.995178 | 0.516031 |
| 62780 | 1.00133 | 0.550142 |
| 62781 | 1.03512 | 0.950672 |
| 62782 | 0.880587 | 0.918908 |
| 62783 | 0.880587 | 0.77381 |
| 62784 | 0.959002 | 0.446627 |
| 62785 | 0.959002 | 0.502512 |
| 62786 | 2.33455 | 0.431892 |
| 62787 | 1.99036 | 0.453641 |
| 62788 | 2.07024 | 0.44082 |
| 62789 | 2.58645 | 0.55069 |
| 62790 | 4.32491 | 0.151863 |
| 62791 | 1.89226 | 0.333947 |
| 62792 | 1.89226 | 0.206204 |
| 62793 | 1.89226 | 0.286609 |
| 62794 | 1.89226 | 0.0957986 |
| 62795 | 3.78453 | 0.0967027 |
| 62796 | 1.89226 | 0.699776 |
| 62797 | 1.89226 | 0.200979 |
| 62798 | 1.89226 | 0.406449 |
| 62799 | 1.89226 | 0.288324 |
| 62800 | 3.78453 | 0.750052 |
| 62801 | 1.89226 | 0.0747181 |
| 62802 | 1.89226 | 0.05 |
| 62803 | 1.89226 | 0.05 |
| 62804 | 1.89226 | 0.0609851 |
| 62805 | 1.89226 | 0.05 |
| 62806 | 1.89226 | 0.0553919 |
| 62807 | 1.89226 | 0.072373 |
| 62808 | 2.1523 | 0.05 |
| 62809 | 2.53257 | 0.05 |
| 62810 | 2.53257 | 0.05 |
| 62811 | 1.89226 | 0.0879491 |

| | | |
|---|---|---|
| 62812 | 1.89226 | 0.0727957 |
| 62813 | 0.946133 | 0.0529591 |
| 62814 | 0.946133 | 0.115821 |
| 62815 | 0.946132 | 0.05 |
| 62816 | 0.946132 | 0.05 |
| 62817 | 0.946132 | 0.05 |
| 62818 | 0.946132 | 0.05 |
| 62819 | 0.946132 | 0.133322 |
| 62820 | 0.946132 | 0.05 |
| 62821 | 0.946133 | 0.0620406 |
| 62822 | 0.946133 | 0.0736431 |
| 62823 | 0.946132 | 0.135907 |
| 62824 | 0.946132 | 0.0717167 |
| 62825 | 0.946132 | 0.112588 |
| 62826 | 0.946132 | 0.289446 |
| 62827 | 0.946132 | 0.0844213 |
| 62828 | 0.946132 | 0.18984 |
| 62829 | 0.946132 | 0.0796655 |
| 62830 | 0.946132 | 0.0896607 |
| 62831 | 0.946132 | 0.332614 |
| 62832 | 0.946132 | 0.162099 |
| 62833 | 0.946132 | 0.131329 |
| 62834 | 0.946132 | 0.124769 |
| 62835 | 0.946131 | 0.214067 |
| 62836 | 0.946131 | 0.184526 |
| 62837 | 1.62772 | 0.126869 |
| 62838 | 1.70303 | 0.129783 |
| 62839 | 1.70303 | 0.167903 |
| 62840 | 1.70303 | 0.170982 |
| 62841 | 1.90116 | 0.112222 |
| 62842 | 1.90116 | 0.10276 |
| 62843 | 1.89226 | 0.263664 |
| 62844 | 1.89226 | 0.3123 |
| 62845 | 1.89226 | 0.384495 |
| 62846 | 1.89226 | 0.397189 |
| 62847 | 1.89226 | 0.186603 |
| 62848 | 1.89226 | 0.328094 |
| 62849 | 1.89226 | 0.424715 |
| 62850 | 1.89226 | 0.448714 |
| 62851 | 1.89226 | 0.387671 |
| 62852 | 1.89226 | 0.447786 |
| 62853 | 3.78452 | 0.44052 |
| 62854 | 1.89226 | 0.383732 |
| 62855 | 1.89226 | 0.410287 |
| 62856 | 1.89226 | 0.40349 |
| 62857 | 1.89226 | 0.417148 |
| 62858 | 1.89226 | 0.385064 |
| 62859 | 1.89226 | 0.407244 |
| 62860 | 0.946132 | 0.36988 |
| 62861 | 0.946132 | 0.363161 |
| 62862 | 0.946129 | 0.361483 |
| 62863 | 0.946129 | 0.363658 |
| 62864 | 0.94613 | 0.338558 |
| 62865 | 0.94613 | 0.368784 |
| 62866 | 0.946131 | 0.335667 |
| 62867 | 0.946131 | 0.29595 |
| 62868 | 0.94613 | 0.312721 |
| 62869 | 0.94613 | 0.368528 |

| | | |
|---|---|---|
| 62870 | 0.946131 | 0.164046 |
| 62871 | 0.946131 | 0.389776 |
| 62872 | 1.89226 | 0.390247 |
| 62873 | 1.89226 | 0.401038 |
| 62874 | 1.89226 | 0.44059 |
| 62875 | 1.89226 | 0.438431 |
| 62876 | 1.89226 | 0.349817 |
| 62877 | 1.89226 | 0.347562 |
| 62878 | 1.89226 | 0.0628077 |
| 62879 | 1.89226 | 0.931575 |
| 62880 | 1.89226 | 0.895286 |
| 62881 | 1.89226 | 0.0514898 |
| 62882 | 3.78452 | 0.188543 |
| 62883 | 1.89226 | 0.331902 |
| 62884 | 1.89226 | 0.916422 |
| 62885 | 1.89226 | 0.121697 |
| 62886 | 1.89226 | 0.97839 |
| 62887 | 1.89226 | 0.168795 |
| 62888 | 1.89226 | 1.18447 |
| 62889 | 1.30165 | 0.173922 |
| 62890 | 0.946127 | 0.602996 |
| 62891 | 1.09659 | 0.0768443 |
| 62892 | 0.946131 | 0.143064 |
| 62893 | 1.09659 | 0.0639111 |
| 62894 | 1.09659 | 1.29379 |
| 62895 | 0.946128 | 1.67505 |
| 62896 | 0.946128 | 0.05 |
| 62897 | 1.30165 | 0.148129 |
| 62898 | 1.09659 | 0.840763 |
| 62899 | 0.946129 | 0.157618 |
| 62900 | 0.946129 | 0.656068 |
| 62901 | 0.946128 | 0.871994 |
| 62902 | 0.946128 | 0.935991 |
| 62903 | 0.946133 | 0.372627 |
| 62904 | 0.946133 | 0.792545 |
| 62905 | 0.946131 | 0.514588 |
| 62906 | 0.946131 | 1.15889 |
| 62907 | 0.946131 | 0.933072 |
| 62908 | 0.946131 | 0.29489 |
| 62909 | 0.94613 | 0.513566 |
| 62910 | 0.94613 | 0.327035 |
| 62911 | 0.946131 | 0.363359 |
| 62912 | 0.946131 | 0.811212 |
| 62913 | 0.946133 | 0.931626 |
| 62914 | 0.946133 | 0.987696 |
| 62915 | 0.946132 | 0.479373 |
| 62916 | 0.946132 | 0.837281 |
| 62917 | 0.946132 | 0.407745 |
| 62918 | 0.946132 | 1.0556 |
| 62919 | 0.946132 | 0.997619 |
| 62920 | 0.946132 | 0.450455 |
| 62921 | 0.946132 | 0.659581 |
| 62922 | 0.946132 | 0.707412 |
| 62923 | 0.946133 | 0.866506 |
| 62924 | 0.946133 | 0.919501 |
| 62925 | 1.30165 | 1.2138 |
| 62926 | 0.972228 | 1.12354 |
| 62927 | 1.09659 | 0.905406 |

| | | |
|---|---|---|
| 62928 | 0.656268 | 1.30753 |
| 62929 | 1.06217 | 0.895031 |
| 62930 | 1.06217 | 1.69978 |
| 62931 | 1.00665 | 0.395195 |
| 62932 | 1.00665 | 1.36657 |
| 62933 | 0.97223 | 0.48646 |
| 62934 | 0.656267 | 0.553677 |
| 62935 | 1.30165 | 0.643919 |
| 62936 | 1.09659 | 1.01695 |
| 62937 | 0.946132 | 1.43192 |
| 62938 | 0.946132 | 1.84333 |
| 62939 | 1.19895 | 2.1 |
| 62940 | 1.19895 | 2.1 |
| 62941 | 0.94613 | 2.1 |
| 62942 | 0.94613 | 0.819566 |
| 62943 | 1.39356 | 2.1 |
| 62944 | 1.19895 | 2.1 |
| 62945 | 0.94613 | 2.09779 |
| 62946 | 1.39356 | 2.1 |
| 62947 | 1.19895 | 2.1 |
| 62948 | 0.946131 | 2.1 |
| 62949 | 1.89226 | 0.896906 |
| 62950 | 1.89226 | 2.1 |
| 62951 | 1.89226 | 2.1 |
| 62952 | 1.89226 | 0.845432 |
| 62953 | 1.89226 | 2.1 |
| 62954 | 1.89226 | 0.884833 |
| 62955 | 0.946132 | 0.770203 |
| 62956 | 0.946132 | 0.724115 |
| 62957 | 0.946128 | 1.09329 |
| 62958 | 0.946128 | 1.71229 |
| 62959 | 0.94613 | 1.10172 |
| 62960 | 0.94613 | 0.0548787 |
| 62961 | 0.94613 | 0.810949 |
| 62962 | 0.94613 | 1.13266 |
| 62963 | 0.946131 | 0.549942 |
| 62964 | 0.946131 | 0.789948 |
| 62965 | 0.94613 | 0.848012 |
| 62966 | 0.94613 | 0.902736 |
| 62967 | 1.89226 | 0.05 |
| 62968 | 1.89226 | 0.05 |
| 62969 | 1.89226 | 0.05 |
| 62970 | 1.89226 | 0.05 |
| 62971 | 1.89226 | 0.05 |
| 62972 | 1.89226 | 0.05 |
| 62973 | 1.29881 | 0.05 |
| 62974 | 1.09985 | 0.05 |
| 62975 | 0.946126 | 0.05 |
| 62976 | 0.946126 | 0.05 |
| 62977 | 0.851539 | 0.05 |
| 62978 | 1.20421 | 0.05 |
| 62979 | 0.851483 | 0.05 |
| 62980 | 0.999426 | 0.05 |
| 62981 | 1.47172 | 0.05 |
| 62982 | 0.999413 | 0.05 |
| 62983 | 0.851552 | 0.05 |
| 62984 | 0.851552 | 0.05 |
| 62985 | 1.89226 | 0.05 |

| | | |
|---|---|---|
| 62986 | 1.89226 | 0.05 |
| 62987 | 1.89226 | 0.05 |
| 62988 | 1.89226 | 0.05 |
| 62989 | 1.89226 | 0.05 |
| 62990 | 1.89226 | 0.05 |
| 62991 | 1.89226 | 0.05 |
| 62992 | 1.89226 | 0.05 |
| 62993 | 1.89226 | 0.05 |
| 62994 | 1.89226 | 0.05 |
| 62995 | 1.89226 | 0.05 |
| 62996 | 1.89226 | 0.05 |
| 62997 | 1.33915 | 2.1 |
| 62998 | 1.99297 | 2.1 |
| 62999 | 2.05216 | 2.1 |
| 63000 | 2.60163 | 2.1 |
| 63001 | 4.04513 | 2.1 |
| 63002 | 1.89226 | 1.87786 |
| 63003 | 1.89226 | 2.1 |
| 63004 | 1.89226 | 2.1 |
| 63005 | 1.89226 | 2.1 |
| 63006 | 3.78452 | 2.1 |
| 63007 | 0.946132 | 2.1 |
| 63008 | 0.946132 | 2.1 |
| 63009 | 0.946133 | 2.1 |
| 63010 | 0.946133 | 2.1 |
| 63011 | 0.946132 | 2.1 |
| 63012 | 0.946132 | 1.14828 |
| 63013 | 0.946133 | 2.1 |
| 63014 | 0.946133 | 2.1 |
| 63015 | 0.946132 | 2.1 |
| 63016 | 0.946132 | 2.1 |
| 63017 | 0.946132 | 2.1 |
| 63018 | 0.946132 | 2.1 |
| 63019 | 1.89226 | 1.90398 |
| 63020 | 1.89226 | 0.887071 |
| 63021 | 1.89226 | 1.0003 |
| 63022 | 1.89226 | 2.05764 |
| 63023 | 3.78452 | 1.30951 |
| 63024 | 1.89227 | 0.977322 |
| 63025 | 1.89227 | 2.1 |
| 63026 | 1.89227 | 2.1 |
| 63027 | 1.89227 | 0.874045 |
| 63028 | 1.89227 | 2.1 |
| 63029 | 1.89227 | 0.928201 |
| 63030 | 2.29301 | 2.1 |
| 63031 | 1.70303 | 2.1 |
| 63032 | 2.46749 | 2.1 |
| 63033 | 2.30728 | 2.1 |
| 63034 | 4.53937 | 2.1 |
| 63035 | 1.89226 | 0.894817 |
| 63036 | 1.89226 | 2.1 |
| 63037 | 1.89226 | 2.1 |
| 63038 | 1.89226 | 1.06709 |
| 63039 | 3.78452 | 2.1 |
| 63040 | 1.89226 | 0.746527 |
| 63041 | 1.89226 | 2.1 |
| 63042 | 1.89226 | 2.1 |
| 63043 | 1.89226 | 2.1 |

| | | |
|---|---|---|
| 63044 | 1.89226 | 0.865503 |
| 63045 | 1.89226 | 1.3165 |
| 63046 | 2.30728 | 2.1 |
| 63047 | 1.00757 | 2.0956 |
| 63048 | 2.0833 | 2.1 |
| 63049 | 1.65318 | 2.1 |
| 63050 | 1.417 2.1 | |
| 63051 | 1.00757 | 2.1 |
| 63052 | 2.46749 | 2.1 |
| 63053 | 2.0975 | 2.1 |
| 63054 | 2.79653 | 2.1 |
| 63055 | 2.44785 | 2.1 |
| 63056 | 4.91534 | 2.1 |
| 63057 | 1.89226 | 2.1 |
| 63058 | 1.89226 | 2.1 |
| 63059 | 1.89226 | 0.769873 |
| 63060 | 1.89226 | 2.1 |
| 63061 | 3.78452 | 1.02111 |
| 63062 | 1.89226 | 1.12458 |
| 63063 | 1.89226 | 2.1 |
| 63064 | 1.89226 | 2.1 |
| 63065 | 1.89226 | 2.06566 |
| 63066 | 1.89226 | 2.1 |
| 63067 | 1.89226 | 2.04461 |
| 63068 | 0.970559 | 2.1 |
| 63069 | 0.970559 | 2.1 |
| 63070 | 0.979305 | 2.1 |
| 63071 | 0.979305 | 2.1 |
| 63072 | 0.708502 | 2.1 |
| 63073 | 0.901117 | 2.1 |
| 63074 | 1.04875 | 2.1 |
| 63075 | 1.22392 | 2.1 |
| 63076 | 1.22392 | 2.1 |
| 63077 | 0.901117 | 2.1 |
| 63078 | 1.04875 | 2.1 |
| 63079 | 0.7085 | 2.1 |
| 63080 | 1.89226 | 1.41761 |
| 63081 | 1.89226 | 0.795061 |
| 63082 | 1.89226 | 0.964402 |
| 63083 | 1.89226 | 1.02997 |
| 63084 | 3.78452 | 1.51895 |
| 63085 | 1.89226 | 1.14343 |
| 63086 | 1.89226 | 0.230264 |
| 63087 | 1.89226 | 1.00267 |
| 63088 | 1.89226 | 0.872342 |
| 63089 | 3.78452 | 0.610277 |
| 63090 | 1.89226 | 0.05 |
| 63091 | 1.89226 | 0.05 |
| 63092 | 1.89226 | 0.05 |
| 63093 | 1.89226 | 0.05 |
| 63094 | 3.78453 | 0.05 |
| 63095 | 1.89226 | 0.411413 |
| 63096 | 1.89226 | 0.14032 |
| 63097 | 1.89226 | 0.0751183 |
| 63098 | 1.89226 | 0.0628982 |
| 63099 | 1.89226 | 0.084906 |
| 63100 | 1.89226 | 0.05 |
| 63101 | 1.89226 | 0.697578 |

| | | |
|---|---|---|
| 63102 | 2.35441 | 0.684766 |
| 63103 | 1.89226 | 0.976078 |
| 63104 | 2.30931 | 0.05 |
| 63105 | 4.24667 | 0.1584 |
| 63106 | 1.11612 | 0.05 |
| 63107 | 0.946131 | 0.05 |
| 63108 | 1.1772 | 0.0578019 |
| 63109 | 0.946131 | 0.105012 |
| 63110 | 1.17721 | 0.153571 |
| 63111 | 1.11612 | 0.05 |
| 63112 | 0.94613 | 0.05 |
| 63113 | 0.94613 | 0.183483 |
| 63114 | 1.11612 | 0.05 |
| 63115 | 1.11612 | 0.05 |
| 63116 | 0.946131 | 0.05 |
| 63117 | 0.946131 | 0.05 |
| 63118 | 1.11612 | 0.05 |
| 63119 | 0.582675 | 0.05 |
| 63120 | 1.17721 | 0.05 |
| 63121 | 0.651969 | 0.05 |
| 63122 | 0.651969 | 0.05 |
| 63123 | 0.582674 | 0.05 |
| 63124 | 1.17721 | 0.05 |
| 63125 | 1.11612 | 0.05 |
| 63126 | 0.877955 | 0.05 |
| 63127 | 0.877955 | 0.05 |
| 63128 | 0.881925 | 0.05 |
| 63129 | 0.881925 | 0.05 |
| 63130 | 1.17721 | 0.05 |
| 63131 | 0.651969 | 0.05 |
| 63132 | 1.15466 | 0.05 |
| 63133 | 0.623642 | 0.05 |
| 63134 | 0.623642 | 0.05 |
| 63135 | 0.651969 | 0.05 |
| 63136 | 1.17721 | 0.05 |
| 63137 | 1.15466 | 0.05 |
| 63138 | 0.904714 | 0.05 |
| 63139 | 0.904714 | 0.05 |
| 63140 | 0.901914 | 0.05 |
| 63141 | 0.901914 | 0.05 |
| 63142 | 1.89226 | 1.1715 |
| 63143 | 1.89226 | 1.00641 |
| 63144 | 1.89226 | 0.828909 |
| 63145 | 1.89226 | 1.38788 |
| 63146 | 1.89226 | 0.280308 |
| 63147 | 1.89226 | 2.1 |
| 63148 | 1.89226 | 1.19835 |
| 63149 | 1.89226 | 0.214256 |
| 63150 | 1.89226 | 0.106962 |
| 63151 | 1.89226 | 2.1 |
| 63152 | 1.89226 | 0.0968018 |
| 63153 | 1.89226 | 2.1 |
| 63154 | 2.33986 | 2.1 |
| 63155 | 1.97819 | 0.971569 |
| 63156 | 2.39166 | 0.05 |
| 63157 | 2.70141 | 2.1 |
| 63158 | 4.73151 | 0.88514 |
| 63159 | 0.989095 | 1.14532 |

| | | |
|---|---|---|
| 63160 | 1.19583 | 0.547568 |
| 63161 | 0.851194 | 0.763198 |
| 63162 | 0.581197 | 0.832895 |
| 63163 | 0.872704 | 0.108034 |
| 63164 | 0.872704 | 1.30312 |
| 63165 | 0.904322 | 0.829638 |
| 63166 | 0.904322 | 0.963638 |
| 63167 | 1.19583 | 0.328665 |
| 63168 | 0.851193 | 0.507774 |
| 63169 | 0.989095 | 0.890755 |
| 63170 | 0.581197 | 0.758539 |
| 63171 | 1.89226 | 0.90527 |
| 63172 | 1.89226 | 1.07021 |
| 63173 | 1.89226 | 0.966954 |
| 63174 | 1.89226 | 1.52308 |
| 63175 | 1.89226 | 0.75505 |
| 63176 | 1.89226 | 2.1 |
| 63177 | 1.89226 | 1.18737 |
| 63178 | 1.89226 | 0.306146 |
| 63179 | 1.89226 | 0.195768 |
| 63180 | 1.89226 | 2.1 |
| 63181 | 1.89226 | 0.0846054 |
| 63182 | 1.89226 | 2.1 |
| 63183 | 2.39166 | 0.895086 |
| 63184 | 1.70239 | 1.11896 |
| 63185 | 2.13665 | 0.847733 |
| 63186 | 2.82684 | 0.855174 |
| 63187 | 4.52831 | 0.892658 |
| 63188 | 1.89226 | 0.05 |
| 63189 | 1.89226 | 0.05 |
| 63190 | 1.89226 | 0.05 |
| 63191 | 1.89226 | 0.05 |
| 63192 | 3.78453 | 0.05 |
| 63193 | 1.89226 | 0.05 |
| 63194 | 1.89226 | 0.05 |
| 63195 | 1.89226 | 0.05 |
| 63196 | 1.89226 | 0.05 |
| 63197 | 1.89226 | 0.05 |
| 63198 | 1.89226 | 0.05 |
| 63199 | 1.89226 | 0.05 |
| 63200 | 1.89226 | 0.05 |
| 63201 | 1.89226 | 0.05 |
| 63202 | 1.89226 | 0.05 |
| 63203 | 1.89226 | 0.05 |
| 63204 | 1.89226 | 0.05 |
| 63205 | 1.89226 | 0.05 |
| 63206 | 1.89226 | 0.05 |
| 63207 | 1.89226 | 0.05 |
| 63208 | 1.89226 | 0.05 |
| 63209 | 1.89226 | 0.0531431 |
| 63210 | 1.89226 | 0.05 |
| 63211 | 1.89226 | 0.05 |
| 63212 | 1.89226 | 0.05 |
| 63213 | 1.89226 | 0.05 |
| 63214 | 1.89226 | 0.05 |
| 63215 | 1.89226 | 0.0509631 |
| 63216 | 1.89226 | 0.05 |
| 63217 | 1.5169 | 0.0553324 |

| | | |
|---|---|---|
| 63218 | 1.61969 | 0.05 |
| 63219 | 1.17262 | 0.05 |
| 63220 | 1.17262 | 0.05 |
| 63221 | 1.88868 | 0.05 |
| 63222 | 1.88868 | 0.0532909 |
| 63223 | 1.79981 | 0.05 |
| 63224 | 1.88868 | 0.05 |
| 63225 | 1.5169 | 0.05 |
| 63226 | 1.5169 | 0.05 |
| 63227 | 2.12891 | 0.05 |
| 63228 | 2.12891 | 0.102303 |
| 63229 | 1.89226 | 0.105174 |
| 63230 | 1.89226 | 0.05 |
| 63231 | 1.89226 | 0.05 |
| 63232 | 1.89226 | 0.05 |
| 63233 | 3.78452 | 0.05 |
| 63234 | 0.946132 | 0.05 |
| 63235 | 0.946132 | 0.05 |
| 63236 | 0.946132 | 0.05 |
| 63237 | 0.946132 | 0.05 |
| 63238 | 0.946132 | 0.05 |
| 63239 | 0.946132 | 0.0582145 |
| 63240 | 0.946133 | 0.05 |
| 63241 | 0.946133 | 0.05 |
| 63242 | 0.946133 | 0.05 |
| 63243 | 0.946133 | 0.05 |
| 63244 | 0.946132 | 0.05 |
| 63245 | 0.946132 | 0.05 |
| 63246 | 1.88868 | 0.05 |
| 63247 | 2.05759 | 0.05 |
| 63248 | 1.61969 | 0.05 |
| 63249 | 1.61969 | 0.05 |
| 63250 | 2.29747 | 0.05 |
| 63251 | 2.29747 | 0.05 |
| 63252 | 2.12891 | 0.0844562 |
| 63253 | 2.29747 | 0.05 |
| 63254 | 1.88868 | 0.05 |
| 63255 | 1.88868 | 0.05 |
| 63256 | 2.50894 | 0.05 |
| 63257 | 2.50894 | 0.05 |
| 63258 | 1.89226 | 0.05 |
| 63259 | 1.89226 | 0.05 |
| 63260 | 1.89226 | 0.05 |
| 63261 | 1.89226 | 0.0755012 |
| 63262 | 1.89226 | 0.05 |
| 63263 | 1.89226 | 0.104679 |
| 63264 | 0.946132 | 0.05 |
| 63265 | 0.946132 | 0.05 |
| 63266 | 0.946132 | 0.05 |
| 63267 | 0.946132 | 0.05 |
| 63268 | 0.946131 | 0.05 |
| 63269 | 0.946131 | 0.0548503 |
| 63270 | 0.946132 | 0.05 |
| 63271 | 0.946132 | 0.05 |
| 63272 | 0.946132 | 0.05 |
| 63273 | 0.946132 | 0.05 |
| 63274 | 0.946132 | 0.05 |
| 63275 | 0.946132 | 0.05 |

| | | |
|---|---|---|
| 63276 | 2.42758 | 1.25489 |
| 63277 | 2.14895 | 2.1 |
| 63278 | 2.56975 | 1.22624 |
| 63279 | 2.28142 | 2.1 |
| 63280 | 4.71869 | 2.1 |
| 63281 | 1.89226 | 1.12666 |
| 63282 | 1.89226 | 2.1 |
| 63283 | 1.89226 | 1.25778 |
| 63284 | 1.89226 | 2.1 |
| 63285 | 3.78452 | 2.1 |
| 63286 | 1.89226 | 2.1 |
| 63287 | 1.89226 | 2.1 |
| 63288 | 1.89226 | 2.1 |
| 63289 | 1.89226 | 2.1 |
| 63290 | 1.89226 | 2.1 |
| 63291 | 1.89226 | 2.1 |
| 63292 | 0.960916 | 2.1 |
| 63293 | 0.960916 | 2.1 |
| 63294 | 0.978118 | 2.1 |
| 63295 | 0.875674 | 2.1 |
| 63296 | 0.775661 | 2.1 |
| 63297 | 0.773613 | 2.1 |
| 63298 | 1.07631 | 2.1 |
| 63299 | 1.14266 | 2.1 |
| 63300 | 1.16927 | 2.1 |
| 63301 | 0.898612 | 2.1 |
| 63302 | 1.04757 | 2.1 |
| 63303 | 0.757536 | 2.1 |
| 63304 | 2.56975 | 1.10008 |
| 63305 | 2.28142 | 2.1 |
| 63306 | 2.64384 | 1.32077 |
| 63307 | 2.40621 | 2.1 |
| 63308 | 4.97596 | 2.1 |
| 63309 | 1.89226 | 1.11719 |
| 63310 | 1.89226 | 2.1 |
| 63311 | 1.89226 | 0.110219 |
| 63312 | 1.89226 | 2.1 |
| 63313 | 3.78452 | 1.99348 |
| 63314 | 1.89226 | 2.1 |
| 63315 | 1.89226 | 2.1 |
| 63316 | 1.89226 | 2.1 |
| 63317 | 1.89226 | 2.1 |
| 63318 | 1.89226 | 2.1 |
| 63319 | 1.89226 | 2.1 |
| 63320 | 1.03328 | 2.1 |
| 63321 | 1.03328 | 2.1 |
| 63322 | 1.04321 | 2.1 |
| 63323 | 1.15424 | 2.1 |
| 63324 | 0.936183 | 2.1 |
| 63325 | 0.944491 | 2.1 |
| 63326 | 1.14266 | 2.1 |
| 63327 | 1.20517 | 2.1 |
| 63328 | 1.23323 | 2.1 |
| 63329 | 0.961268 | 2.1 |
| 63330 | 1.11215 | 2.1 |
| 63331 | 0.827247 | 2.1 |
| 63332 | 1.89226 | 2.1 |
| 63333 | 1.89226 | 1.47532 |

| | | |
|---|---|---|
| 63334 | 1.89226 | 2.1 |
| 63335 | 1.89226 | 1.88778 |
| 63336 | 3.78453 | 2.1 |
| 63337 | 2.14647 | 2.1 |
| 63338 | 2.20589 | 2.1 |
| 63339 | 1.95567 | 2.1 |
| 63340 | 2.03224 | 1.94923 |
| 63341 | 4.17871 | 2.1 |
| 63342 | 2.06035 | 2.1 |
| 63343 | 1.88452 | 0.05 |
| 63344 | 1.88452 | 2.1 |
| 63345 | 2.26997 | 0.05 |
| 63346 | 2.03224 | 1.91879 |
| 63347 | 2.42272 | 0.05 |
| 63348 | 1.89226 | 2.1 |
| 63349 | 1.89226 | 0.05 |
| 63350 | 1.89226 | 0.05 |
| 63351 | 1.89226 | 2.1 |
| 63352 | 1.89226 | 0.05 |
| 63353 | 1.89226 | 2.1 |
| 63354 | 1.89225 | 2.1 |
| 63355 | 1.89225 | 2.1 |
| 63356 | 1.89225 | 2.1 |
| 63357 | 1.89225 | 2.1 |
| 63358 | 1.89225 | 2.1 |
| 63359 | 1.89225 | 2.1 |
| 63360 | 0.952418 | 2.1 |
| 63361 | 0.952418 | 2.1 |
| 63362 | 0.95883 | 2.1 |
| 63363 | 0.95883 | 2.1 |
| 63364 | 0.946182 | 2.1 |
| 63365 | 1.01611 | 2.1 |
| 63366 | 0.895134 | 2.1 |
| 63367 | 0.977828 | 2.1 |
| 63368 | 0.89514 | 2.1 |
| 63369 | 0.946189 | 2.1 |
| 63370 | 0.977821 | 2.1 |
| 63371 | 1.01611 | 2.1 |
| 63372 | 2.03223 | 2.1 |
| 63373 | 1.88451 | 0.0555549 |
| 63374 | 1.89236 | 2.1 |
| 63375 | 1.74649 | 0.05 |
| 63376 | 3.77687 | 0.05 |
| 63377 | 0.81094 | 2.1 |
| 63378 | 0.81094 | 0.05 |
| 63379 | 0.810939 | 2.1 |
| 63380 | 0.810939 | 2.1 |
| 63381 | 0.810945 | 0.05 |
| 63382 | 0.810945 | 0.211567 |
| 63383 | 1.89225 | 0.05 |
| 63384 | 1.35167 | 0.05 |
| 63385 | 1.08131 | 2.1 |
| 63386 | 1.08131 | 0.05 |
| 63387 | 1.0813 | 0.0629451 |
| 63388 | 1.89226 | 0.05 |
| 63389 | 1.89226 | 0.05 |
| 63390 | 1.89226 | 0.05 |
| 63391 | 1.89226 | 0.05 |

| | | |
|---|---|---|
| 63392 | 1.89226 | 0.05 |
| 63393 | 1.89226 | 0.05 |
| 63394 | 1.01842 | 0.05 |
| 63395 | 0.936656 | 0.05 |
| 63396 | 0.946131 | 0.05 |
| 63397 | 0.946131 | 0.05 |
| 63398 | 1.11157 | 0.05 |
| 63399 | 1.04281 | 0.05 |
| 63400 | 0.925803 | 0.05 |
| 63401 | 0.925803 | 0.05 |
| 63402 | 0.851553 | 0.05 |
| 63403 | 1.11158 | 0.05 |
| 63404 | 1.05017 | 0.05 |
| 63405 | 0.925798 | 0.05 |
| 63406 | 1.89226 | 0.05 |
| 63407 | 1.89226 | 0.05 |
| 63408 | 1.89226 | 0.05 |
| 63409 | 1.89226 | 0.05 |
| 63410 | 1.89226 | 0.05 |
| 63411 | 1.89226 | 0.05 |
| 63412 | 1.23504 | 0.05 |
| 63413 | 1.23504 | 0.05 |
| 63414 | 0.946131 | 0.05 |
| 63415 | 0.946131 | 0.05 |
| 63416 | 1.37358 | 0.05 |
| 63417 | 1.23504 | 0.05 |
| 63418 | 0.946129 | 0.05 |
| 63419 | 0.946129 | 0.05 |
| 63420 | 1.37358 | 0.05 |
| 63421 | 0.946131 | 0.05 |
| 63422 | 0.94613 | 0.05 |
| 63423 | 1.23504 | 0.05 |
| 63424 | 1.00261 | 0.05 |
| 63425 | 1.00261 | 0.05 |
| 63426 | 1.02952 | 0.05 |
| 63427 | 1.02952 | 0.05 |
| 63428 | 0.850964 | 0.05 |
| 63429 | 0.658553 | 0.05 |
| 63430 | 1.37358 | 0.05 |
| 63431 | 1.23504 | 0.05 |
| 63432 | 1.37358 | 0.05 |
| 63433 | 0.850965 | 0.05 |
| 63434 | 1.23504 | 0.05 |
| 63435 | 0.658553 | 0.05 |
| 63436 | 0.946133 | 0.05 |
| 63437 | 0.946133 | 0.05 |
| 63438 | 0.946132 | 0.05 |
| 63439 | 0.946132 | 0.05 |
| 63440 | 0.946132 | 0.05 |
| 63441 | 0.946132 | 0.05 |
| 63442 | 0.946133 | 0.05 |
| 63443 | 0.946133 | 0.05 |
| 63444 | 0.946133 | 0.05 |
| 63445 | 0.946133 | 0.05 |
| 63446 | 0.946132 | 0.05 |
| 63447 | 0.946132 | 0.05 |
| 63448 | 1.89226 | 0.0666334 |
| 63449 | 1.89226 | 0.090246 |

| | | |
|---|---|---|
| 63450 | 1.89226 | 0.05 |
| 63451 | 1.89226 | 0.05 |
| 63452 | 3.78452 | 0.0876134 |
| 63453 | 1.89226 | 0.0930225 |
| 63454 | 1.89226 | 0.0928587 |
| 63455 | 1.89226 | 0.05 |
| 63456 | 1.89226 | 0.05 |
| 63457 | 3.78452 | 0.05 |
| 63458 | 1.89227 | 0.05 |
| 63459 | 1.89227 | 0.05 |
| 63460 | 1.89227 | 0.05 |
| 63461 | 1.89227 | 0.05 |
| 63462 | 1.89227 | 0.05 |
| 63463 | 1.89227 | 0.05 |
| 63464 | 0.94613 | 0.05 |
| 63465 | 0.94613 | 0.05 |
| 63466 | 0.946127 | 0.05 |
| 63467 | 0.946127 | 0.05 |
| 63468 | 0.94613 | 0.05 |
| 63469 | 0.94613 | 0.05 |
| 63470 | 0.946127 | 0.05 |
| 63471 | 0.946127 | 0.05 |
| 63472 | 0.946128 | 0.05 |
| 63473 | 0.946128 | 0.05 |
| 63474 | 0.946128 | 0.05 |
| 63475 | 0.946128 | 0.05 |
| 63476 | 0.946132 | 0.05 |
| 63477 | 0.946132 | 0.05 |
| 63478 | 0.946128 | 0.05 |
| 63479 | 0.946128 | 0.05 |
| 63480 | 0.94613 | 0.05 |
| 63481 | 0.94613 | 0.05 |
| 63482 | 0.946131 | 0.05 |
| 63483 | 0.946131 | 0.05 |
| 63484 | 0.94613 | 0.05 |
| 63485 | 0.94613 | 0.05 |
| 63486 | 0.94613 | 0.05 |
| 63487 | 0.94613 | 0.05 |
| 63488 | 1.89226 | 2.1 |
| 63489 | 1.89226 | 2.1 |
| 63490 | 1.89226 | 2.1 |
| 63491 | 1.89226 | 2.1 |
| 63492 | 3.78453 | 2.1 |
| 63493 | 2.72844 | 2.1 |
| 63494 | 1.89226 | 2.1 |
| 63495 | 2.26536 | 2.1 |
| 63496 | 2.26536 | 2.1 |
| 63497 | 1.89226 | 2.1 |
| 63498 | 1.89226 | 2.1 |
| 63499 | 1.89226 | 2.1 |
| 63500 | 1.89226 | 2.1 |
| 63501 | 1.89226 | 2.1 |
| 63502 | 1.89226 | 2.1 |
| 63503 | 1.89226 | 2.1 |
| 63504 | 1.89226 | 2.1 |
| 63505 | 1.89226 | 2.1 |
| 63506 | 1.89226 | 2.1 |
| 63507 | 1.89226 | 2.1 |

| | | |
|---|---|---|
| 63508 | 1.89226 | 2.1 |
| 63509 | 1.89226 | 2.1 |
| 63510 | 1.89226 | 2.1 |
| 63511 | 2.72844 | 2.1 |
| 63512 | 2.26536 | 2.1 |
| 63513 | 2.28367 | 2.1 |
| 63514 | 1.5791 | 2.1 |
| 63515 | 4.54903 | 2.1 |
| 63516 | 0.810945 | 2.1 |
| 63517 | 0.810945 | 2.1 |
| 63518 | 0.810943 | 2.1 |
| 63519 | 0.810943 | 2.1 |
| 63520 | 1.89226 | 2.1 |
| 63521 | 1.3517 | 2.1 |
| 63522 | 1.08133 | 2.1 |
| 63523 | 1.08132 | 2.1 |
| 63524 | 1.08132 | 2.1 |
| 63525 | 0.810933 | 2.1 |
| 63526 | 0.810933 | 2.1 |
| 63527 | 1.89227 | 2.1 |
| 63528 | 1.89227 | 2.1 |
| 63529 | 1.89227 | 2.1 |
| 63530 | 1.89227 | 2.1 |
| 63531 | 3.78453 | 2.1 |
| 63532 | 2.71897 | 2.1 |
| 63533 | 2.27495 | 2.1 |
| 63534 | 2.648 | 2.1 |
| 63535 | 2.20832 | 2.1 |
| 63536 | 4.92729 | 2.1 |
| 63537 | 2.27495 | 2.1 |
| 63538 | 1.38455 | 2.1 |
| 63539 | 1.38455 | 2.1 |
| 63540 | 1.46586 | 2.1 |
| 63541 | 2.20831 | 2.1 |
| 63542 | 2.28962 | 2.1 |
| 63543 | 0.946133 | 2.1 |
| 63544 | 0.946133 | 2.1 |
| 63545 | 0.946132 | 2.1 |
| 63546 | 0.946132 | 2.1 |
| 63547 | 0.946132 | 2.1 |
| 63548 | 0.946132 | 2.1 |
| 63549 | 0.946132 | 2.1 |
| 63550 | 0.946132 | 2.1 |
| 63551 | 0.946132 | 2.1 |
| 63552 | 0.946132 | 2.1 |
| 63553 | 0.946133 | 2.1 |
| 63554 | 0.946133 | 2.1 |
| 63555 | 1.89226 | 2.1 |
| 63556 | 1.89226 | 2.1 |
| 63557 | 1.89226 | 2.1 |
| 63558 | 1.89226 | 2.1 |
| 63559 | 1.89226 | 2.1 |
| 63560 | 1.89226 | 2.1 |
| 63561 | 2.648 | 2.1 |
| 63562 | 2.08612 | 2.1 |
| 63563 | 2.08612 | 2.1 |
| 63564 | 2.20831 | 2.1 |
| 63565 | 2.56618 | 2.1 |

| | | |
|---|---|---|
| 63566 | 2.68837 | 2.1 |
| 63567 | 2.08612 | 2.1 |
| 63568 | 2.20831 | 2.1 |
| 63569 | 1.30659 | 2.1 |
| 63570 | 1.30659 | 2.1 |
| 63571 | 1.38455 | 2.1 |
| 63572 | 2.16408 | 2.1 |
| 63573 | 0.810946 | 2.1 |
| 63574 | 0.810946 | 2.1 |
| 63575 | 0.810946 | 2.1 |
| 63576 | 0.810946 | 2.1 |
| 63577 | 1.89226 | 2.1 |
| 63578 | 1.3517 | 2.1 |
| 63579 | 1.08133 | 2.1 |
| 63580 | 1.08132 | 2.1 |
| 63581 | 1.08132 | 2.1 |
| 63582 | 0.810934 | 2.1 |
| 63583 | 0.810934 | 2.1 |
| 63584 | 1.89226 | 1.88686 |
| 63585 | 1.89226 | 1.44747 |
| 63586 | 1.89226 | 0.955791 |
| 63587 | 1.89226 | 2.1 |
| 63588 | 3.78452 | 1.24907 |
| 63589 | 1.89226 | 0.860344 |
| 63590 | 1.89226 | 2.1 |
| 63591 | 1.89226 | 2.1 |
| 63592 | 1.89226 | 1.27567 |
| 63593 | 3.78453 | 2.1 |
| 63594 | 1.08733 | 2.1 |
| 63595 | 0.856615 | 2.1 |
| 63596 | 1.25964 | 1.49575 |
| 63597 | 1.12378 | 1.48986 |
| 63598 | 1.23551 | 1.17845 |
| 63599 | 0.969894 | 2.1 |
| 63600 | 0.985977 | 2.1 |
| 63601 | 0.985977 | 1.17263 |
| 63602 | 1.16169 | 1.88439 |
| 63603 | 1.21072 | 2.07568 |
| 63604 | 0.765175 | 2.09497 |
| 63605 | 0.863419 | 2.03269 |
| 63606 | 2.39934 | 2.1 |
| 63607 | 2.44427 | 1.59826 |
| 63608 | 2.64003 | 1.00979 |
| 63609 | 2.39077 | 2.1 |
| 63610 | 2.39934 | 2.1 |
| 63611 | 2.10194 | 2.1 |
| 63612 | 1.89226 | 0.996498 |
| 63613 | 1.89226 | 2.1 |
| 63614 | 1.89226 | 1.91628 |
| 63615 | 1.89226 | 0.635652 |
| 63616 | 3.78452 | 2.02284 |
| 63617 | 0.946132 | 2.1 |
| 63618 | 0.946132 | 2.1 |
| 63619 | 0.946131 | 1.08352 |
| 63620 | 0.946131 | 1.12004 |
| 63621 | 0.946132 | 1.07486 |
| 63622 | 0.946132 | 2.1 |
| 63623 | 0.946132 | 2.1 |

| | | |
|---|---|---|
| 63624 | 0.946132 | 1.16648 |
| 63625 | 0.946132 | 1.80225 |
| 63626 | 0.946132 | 2.09013 |
| 63627 | 0.946132 | 1.44917 |
| 63628 | 0.946132 | 2.08228 |
| 63629 | 1.19967 | 2.1 |
| 63630 | 0.946128 | 1.93918 |
| 63631 | 0.946133 | 1.12557 |
| 63632 | 1.32002 | 1.70333 |
| 63633 | 1.19967 | 2.1 |
| 63634 | 1.19967 | 2.1 |
| 63635 | 0.94613 | 2.1 |
| 63636 | 0.94613 | 0.411885 |
| 63637 | 0.946131 | 1.29175 |
| 63638 | 0.946131 | 1.48382 |
| 63639 | 1.19967 | 1.74802 |
| 63640 | 1.32001 | 1.68465 |
| 63641 | 1.89226 | 0.445239 |
| 63642 | 1.89226 | 0.805699 |
| 63643 | 1.89226 | 0.052149 |
| 63644 | 1.89226 | 2.1 |
| 63645 | 3.78451 | 0.331357 |
| 63646 | 2.6402 | 0.116249 |
| 63647 | 2.28552 | 0.802297 |
| 63648 | 2.50958 | 0.05 |
| 63649 | 2.18948 | 0.751025 |
| 63650 | 4.79509 | 0.206628 |
| 63651 | 2.28552 | 0.05 |
| 63652 | 1.86102 | 2.1 |
| 63653 | 2.18949 | 0.05 |
| 63654 | 1.78376 | 2.1 |
| 63655 | 4.06928 | 2.1 |
| 63656 | 1.89226 | 0.05 |
| 63657 | 1.89226 | 2.1 |
| 63658 | 1.89226 | 0.790053 |
| 63659 | 1.89226 | 2.1 |
| 63660 | 3.78452 | 2.1 |
| 63661 | 1.89226 | 0.05 |
| 63662 | 1.89226 | 2.1 |
| 63663 | 1.89226 | 0.05 |
| 63664 | 1.89226 | 1.87885 |
| 63665 | 3.78452 | 0.0732309 |
| 63666 | 2.50958 | 0.05 |
| 63667 | 2.18948 | 0.610676 |
| 63668 | 2.39524 | 0.05 |
| 63669 | 2.10188 | 2.00596 |
| 63670 | 4.61146 | 0.0533303 |
| 63671 | 2.18949 | 0.05 |
| 63672 | 1.78376 | 2.1 |
| 63673 | 1.71839 | 0.422769 |
| 63674 | 1.71839 | 2.1 |
| 63675 | 2.10189 | 0.70695 |
| 63676 | 2.16726 | 2.1 |
| 63677 | 0.810943 | 0.261452 |
| 63678 | 0.810943 | 2.1 |
| 63679 | 0.810945 | 0.0608577 |
| 63680 | 0.810945 | 0.313049 |
| 63681 | 0.810959 | 2.1 |

| | | |
|---|---|---|
| 63682 | 0.810959 | 2.1 |
| 63683 | 1.89226 | 2.1 |
| 63684 | 1.35168 | 2.1 |
| 63685 | 1.08132 | 0.57361 |
| 63686 | 1.08132 | 2.1 |
| 63687 | 1.0813 | 2.1 |
| 63688 | 1.89226 | 0.84203 |
| 63689 | 1.89226 | 2.00899 |
| 63690 | 1.89226 | 1.11293 |
| 63691 | 1.89226 | 1.15958 |
| 63692 | 1.89226 | 1.5242 |
| 63693 | 1.89226 | 2.0545 |
| 63694 | 1.28421 | 1.60724 |
| 63695 | 0.992041 | 0.901482 |
| 63696 | 1.07707 | 2.1 |
| 63697 | 0.789746 | 1.98194 |
| 63698 | 1.03815 | 2.1 |
| 63699 | 1.03815 | 1.91322 |
| 63700 | 1.03581 | 2.0675 |
| 63701 | 1.03581 | 1.3443 |
| 63702 | 1.28421 | 1.72714 |
| 63703 | 1.07707 | 2.1 |
| 63704 | 0.789748 | 1.92478 |
| 63705 | 0.992038 | 1.27801 |
| 63706 | 1.89226 | 0.854007 |
| 63707 | 1.89226 | 2.09533 |
| 63708 | 1.89226 | 1.26453 |
| 63709 | 1.89226 | 1.03812 |
| 63710 | 1.89226 | 1.57464 |
| 63711 | 1.89226 | 1.24122 |
| 63712 | 1.89226 | 0.841377 |
| 63713 | 1.89226 | 1.49234 |
| 63714 | 1.89226 | 0.962586 |
| 63715 | 1.89226 | 1.0791 |
| 63716 | 1.89226 | 1.13391 |
| 63717 | 1.89226 | 1.85913 |
| 63718 | 0.92236 | 2.1 |
| 63719 | 0.655971 | 2.08492 |
| 63720 | 0.916772 | 2.1 |
| 63721 | 0.913834 | 1.36869 |
| 63722 | 1.18074 | 2.07181 |
| 63723 | 0.922346 | 2.1 |
| 63724 | 2.15414 | 1.0088 |
| 63725 | 1.53528 | 2.01755 |
| 63726 | 1.22748 | 2.1 |
| 63727 | 1.57442 | 1.10216 |
| 63728 | 0.874683 | 2.03258 |
| 63729 | 1.25935 | 2.1 |
| 63730 | 2.61433 | 2.1 |
| 63731 | 1.22262 | 2.1 |
| 63732 | 1.22262 | 2.1 |
| 63733 | 2.62813 | 2.1 |
| 63734 | 2.62813 | 2.1 |
| 63735 | 1.89226 | 2.1 |
| 63736 | 2.61434 | 2.1 |
| 63737 | 2.62814 | 2.1 |
| 63738 | 2.61434 | 2.1 |
| 63739 | 1.90607 | 2.1 |

| | | |
|---|---|---|
| 63740 | 1.89226 | 2.1 |
| 63741 | 1.89226 | 2.1 |
| 63742 | 1.89226 | 2.1 |
| 63743 | 1.89226 | 2.1 |
| 63744 | 1.89226 | 2.1 |
| 63745 | 1.89226 | 2.1 |
| 63746 | 1.89226 | 2.1 |
| 63747 | 1.89226 | 2.1 |
| 63748 | 1.89226 | 2.1 |
| 63749 | 1.89226 | 2.1 |
| 63750 | 1.89226 | 2.1 |
| 63751 | 1.89226 | 2.1 |
| 63752 | 1.89226 | 2.1 |
| 63753 | 1.31969 | 2.1 |
| 63754 | 1.31406 | 2.1 |
| 63755 | 0.629677 | 2.1 |
| 63756 | 0.643104 | 2.1 |
| 63757 | 0.976621 | 2.1 |
| 63758 | 0.976621 | 2.1 |
| 63759 | 0.98055 | 2.1 |
| 63760 | 0.98055 | 2.1 |
| 63761 | 1.3197 | 2.1 |
| 63762 | 0.643102 | 2.1 |
| 63763 | 0.629675 | 2.1 |
| 63764 | 1.31407 | 2.1 |
| 63765 | 0.946133 | 2.1 |
| 63766 | 0.946133 | 2.1 |
| 63767 | 1.3197 | 2.1 |
| 63768 | 1.31407 | 2.1 |
| 63769 | 0.946132 | 2.1 |
| 63770 | 0.946132 | 2.1 |
| 63771 | 1.3197 | 2.1 |
| 63772 | 1.3197 | 2.1 |
| 63773 | 1.3197 | 2.1 |
| 63774 | 0.946133 | 2.1 |
| 63775 | 0.946132 | 2.1 |
| 63776 | 1.31407 | 2.1 |
| 63777 | 1.89226 | 2.1 |
| 63778 | 1.89226 | 2.1 |
| 63779 | 1.89226 | 2.1 |
| 63780 | 1.89226 | 2.1 |
| 63781 | 1.89226 | 2.1 |
| 63782 | 1.89226 | 2.1 |
| 63783 | 1.89226 | 2.1 |
| 63784 | 1.89226 | 2.1 |
| 63785 | 1.89226 | 2.1 |
| 63786 | 1.89226 | 2.1 |
| 63787 | 1.89226 | 2.1 |
| 63788 | 1.89226 | 2.1 |
| 63789 | 1.89226 | 0.05 |
| 63790 | 1.89226 | 0.05 |
| 63791 | 1.89226 | 0.05 |
| 63792 | 1.89226 | 0.05 |
| 63793 | 3.78452 | 0.05 |
| 63794 | 1.89226 | 0.05 |
| 63795 | 1.89226 | 0.05 |
| 63796 | 1.89226 | 0.05 |
| 63797 | 1.89226 | 0.05 |

| | | |
|---|---|---|
| 63798 | 1.89226 | 0.05 |
| 63799 | 1.89226 | 0.05 |
| 63800 | 0.946131 | 0.05 |
| 63801 | 0.946131 | 0.05 |
| 63802 | 0.946128 | 0.05 |
| 63803 | 0.946128 | 0.05 |
| 63804 | 0.94613 | 0.05 |
| 63805 | 0.94613 | 0.05 |
| 63806 | 0.94613 | 0.05 |
| 63807 | 0.94613 | 0.05 |
| 63808 | 0.94613 | 0.05 |
| 63809 | 0.94613 | 0.05 |
| 63810 | 0.94613 | 0.05 |
| 63811 | 0.94613 | 0.05 |
| 63812 | 1.89226 | 0.05 |
| 63813 | 1.89226 | 0.05 |
| 63814 | 1.89226 | 0.05 |
| 63815 | 1.89226 | 0.05 |
| 63816 | 1.89226 | 0.05 |
| 63817 | 1.89226 | 0.05 |
| 63818 | 0.924614 | 0.05 |
| 63819 | 0.914275 | 0.05 |
| 63820 | 0.906003 | 0.05 |
| 63821 | 0.62479 | 0.05 |
| 63822 | 2.02733 | 0.05 |
| 63823 | 1.48332 | 0.05 |
| 63824 | 0.833106 | 0.05 |
| 63825 | 1.19364 | 0.05 |
| 63826 | 1.57482 | 0.05 |
| 63827 | 1.18103 | 0.05 |
| 63828 | 0.905988 | 0.05 |
| 63829 | 1.89226 | 2.1 |
| 63830 | 1.89226 | 2.1 |
| 63831 | 1.89226 | 2.1 |
| 63832 | 1.89226 | 2.1 |
| 63833 | 3.78452 | 2.1 |
| 63834 | 1.89226 | 2.1 |
| 63835 | 1.89226 | 2.1 |
| 63836 | 1.89226 | 2.1 |
| 63837 | 1.89226 | 2.1 |
| 63838 | 1.89226 | 2.1 |
| 63839 | 1.89226 | 2.1 |
| 63840 | 0.816805 | 2.1 |
| 63841 | 0.928516 | 2.1 |
| 63842 | 1.4045 | 2.1 |
| 63843 | 1.26268 | 2.1 |
| 63844 | 1.0881 | 2.1 |
| 63845 | 1.0881 | 2.1 |
| 63846 | 1.10309 | 2.1 |
| 63847 | 1.10309 | 2.1 |
| 63848 | 0.928518 | 2.1 |
| 63849 | 1.4045 | 2.1 |
| 63850 | 0.816807 | 2.1 |
| 63851 | 1.26267 | 2.1 |
| 63852 | 1.89226 | 2.1 |
| 63853 | 2.52535 | 2.1 |
| 63854 | 1.89226 | 2.1 |
| 63855 | 1.89226 | 2.1 |

| | | |
|---|---|---|
| 63856 | 2.809 | 2.1 |
| 63857 | 2.809 | 2.1 |
| 63858 | 1.89226 | 2.1 |
| 63859 | 1.89226 | 2.1 |
| 63860 | 1.89226 | 2.1 |
| 63861 | 1.89226 | 2.1 |
| 63862 | 3.78452 | 2.1 |
| 63863 | 0.946127 | 2.1 |
| 63864 | 0.946127 | 2.1 |
| 63865 | 0.946131 | 2.1 |
| 63866 | 0.946131 | 2.1 |
| 63867 | 0.94613 | 2.1 |
| 63868 | 0.94613 | 2.1 |
| 63869 | 0.946128 | 2.1 |
| 63870 | 0.946128 | 2.1 |
| 63871 | 0.946129 | 2.1 |
| 63872 | 0.946129 | 2.1 |
| 63873 | 0.946129 | 2.1 |
| 63874 | 0.946129 | 2.1 |
| 63875 | 1.89224 | 0.0908107 |
| 63876 | 1.89224 | 2.1 |
| 63877 | 1.89224 | 2.1 |
| 63878 | 1.89224 | 2.1 |
| 63879 | 3.78448 | 2.1 |
| 63880 | 2.39244 | 2.1 |
| 63881 | 2.27036 | 2.1 |
| 63882 | 2.25945 | 2.1 |
| 63883 | 2.27398 | 2.1 |
| 63884 | 4.52981 | 2.1 |
| 63885 | 2.27036 | 2.1 |
| 63886 | 1.97088 | 2.1 |
| 63887 | 1.86302 | 2.1 |
| 63888 | 1.84991 | 2.1 |
| 63889 | 4.12027 | 2.1 |
| 63890 | 1.89224 | 2.1 |
| 63891 | 1.89224 | 2.1 |
| 63892 | 1.89224 | 2.1 |
| 63893 | 1.89224 | 2.1 |
| 63894 | 3.78447 | 2.1 |
| 63895 | 1.89226 | 2.1 |
| 63896 | 1.89226 | 2.1 |
| 63897 | 1.89226 | 0.137872 |
| 63898 | 1.89226 | 2.1 |
| 63899 | 3.78453 | 2.1 |
| 63900 | 2.25948 | 2.1 |
| 63901 | 2.33611 | 2.1 |
| 63902 | 2.05257 | 2.1 |
| 63903 | 1.94441 | 2.1 |
| 63904 | 4.20389 | 2.1 |
| 63905 | 1.92514 | 2.1 |
| 63906 | 1.84993 | 2.1 |
| 63907 | 1.94441 | 2.1 |
| 63908 | 1.74391 | 2.1 |
| 63909 | 3.79434 | 2.1 |
| 63910 | 1.89226 | 2.1 |
| 63911 | 1.89226 | 2.1 |
| 63912 | 1.89226 | 2.1 |
| 63913 | 1.89226 | 2.1 |

| | | |
|---|---|---|
| 63914 | 3.78452 | 2.1 |
| 63915 | 15.1381 | 0.265576 |
| 63916 | 15.1381 | 0.503304 |
| 63917 | 15.1381 | 0.127782 |
| 63918 | 15.1381 | 0.930427 |
| 63919 | 30.2762 | 0.154265 |
| 63920 | 15.1381 | 0.43512 |
| 63921 | 15.1381 | 0.697235 |
| 63922 | 15.1381 | 0.504935 |
| 63923 | 15.1381 | 0.670639 |
| 63924 | 30.2762 | 0.636019 |
| 63925 | 15.1381 | 2.1 |
| 63926 | 15.1381 | 2.1 |
| 63927 | 15.1381 | 1.97507 |
| 63928 | 15.1381 | 2.1 |
| 63929 | 30.2762 | 2.1 |
| 63930 | 15.1381 | 0.337629 |
| 63931 | 15.1381 | 0.534347 |
| 63932 | 15.1381 | 0.493453 |
| 63933 | 15.1381 | 0.503989 |
| 63934 | 30.2762 | 0.28164 |
| 63935 | 1.89225 | 2.1 |
| 63936 | 1.89225 | 2.1 |
| 63937 | 1.89225 | 2.1 |
| 63938 | 1.89225 | 2.1 |
| 63939 | 3.78449 | 2.1 |
| 63940 | 1.39909 | 2.1 |
| 63941 | 1.39909 | 2.1 |
| 63942 | 0.946123 | 2.1 |
| 63943 | 0.946123 | 2.1 |
| 63944 | 1.3291 | 2.1 |
| 63945 | 1.39908 | 2.1 |
| 63946 | 0.946126 | 2.1 |
| 63947 | 0.946126 | 2.1 |
| 63948 | 0.946118 | 2.1 |
| 63949 | 1.32909 | 2.1 |
| 63950 | 0.94613 | 2.1 |
| 63951 | 1.3991 | 2.1 |
| 63952 | 3.07801 | 2.1 |
| 63953 | 1.84322 | 2.1 |
| 63954 | 1.84322 | 2.1 |
| 63955 | 1.68788 | 2.1 |
| 63956 | 2.6582 | 2.1 |
| 63957 | 2.50287 | 2.1 |
| 63958 | 1.89225 | 2.1 |
| 63959 | 1.89225 | 2.1 |
| 63960 | 1.89225 | 2.1 |
| 63961 | 1.89225 | 2.1 |
| 63962 | 3.7845 | 2.1 |
| 63963 | 1.89226 | 2.1 |
| 63964 | 1.89226 | 2.1 |
| 63965 | 1.89226 | 2.1 |
| 63966 | 1.89226 | 2.1 |
| 63967 | 3.78452 | 2.1 |
| 63968 | 1.89226 | 2.1 |
| 63969 | 2.50051 | 2.1 |
| 63970 | 2.50051 | 2.1 |
| 63971 | 2.65822 | 2.1 |

| | | |
|---|---|---|
| 63972 | 1.89226 | 2.1 |
| 63973 | 2.04997 | 2.1 |
| 63974 | 2.50052 | 2.1 |
| 63975 | 2.65822 | 2.1 |
| 63976 | 1.70377 | 2.1 |
| 63977 | 1.70377 | 2.1 |
| 63978 | 1.84324 | 2.1 |
| 63979 | 2.63998 | 2.1 |
| 63980 | 0.810947 | 2.1 |
| 63981 | 0.810947 | 2.1 |
| 63982 | 0.810946 | 2.1 |
| 63983 | 0.810946 | 2.1 |
| 63984 | 1.89226 | 2.1 |
| 63985 | 1.35169 | 2.1 |
| 63986 | 1.08132 | 2.1 |
| 63987 | 1.08132 | 2.1 |
| 63988 | 1.08132 | 2.1 |
| 63989 | 0.810946 | 2.1 |
| 63990 | 0.810946 | 2.1 |
| 63991 | 1.89226 | 2.1 |
| 63992 | 1.89226 | 2.1 |
| 63993 | 1.89226 | 2.1 |
| 63994 | 1.89226 | 2.1 |
| 63995 | 3.78453 | 2.1 |
| 63996 | 0.946164 | 2.1 |
| 63997 | 0.946164 | 2.1 |
| 63998 | 0.929842 | 2.1 |
| 63999 | 0.929842 | 2.1 |
| 64000 | 0.946132 | 2.1 |
| 64001 | 0.946132 | 2.1 |
| 64002 | 1.04101 | 2.1 |
| 64003 | 0.929874 | 2.1 |
| 64004 | 0.946132 | 2.1 |
| 64005 | 0.929874 | 2.1 |
| 64006 | 1.04101 | 2.1 |
| 64007 | 0.946132 | 2.1 |
| 64008 | 1.89226 | 2.1 |
| 64009 | 1.89226 | 2.1 |
| 64010 | 1.89226 | 2.1 |
| 64011 | 1.89226 | 2.1 |
| 64012 | 3.78453 | 2.1 |
| 64013 | 1.89226 | 2.1 |
| 64014 | 1.89226 | 2.1 |
| 64015 | 1.89226 | 2.1 |
| 64016 | 1.89226 | 2.1 |
| 64017 | 1.89226 | 2.1 |
| 64018 | 1.89226 | 2.1 |
| 64019 | 1.89226 | 2.1 |
| 64020 | 1.89226 | 2.1 |
| 64021 | 1.89226 | 2.1 |
| 64022 | 1.89226 | 2.1 |
| 64023 | 1.89226 | 2.1 |
| 64024 | 1.89226 | 2.1 |
| 64025 | 0.946131 | 2.1 |
| 64026 | 0.946131 | 2.1 |
| 64027 | 0.946131 | 2.1 |
| 64028 | 0.946131 | 2.1 |
| 64029 | 0.946132 | 2.1 |

| | | |
|---|---|---|
| 64030 | 0.946132 | 2.1 |
| 64031 | 0.946131 | 2.1 |
| 64032 | 0.946131 | 2.1 |
| 64033 | 0.946131 | 2.1 |
| 64034 | 0.946131 | 2.1 |
| 64035 | 0.946132 | 2.1 |
| 64036 | 0.946132 | 2.1 |
| 64037 | 1.89226 | 1.17977 |
| 64038 | 1.89226 | 0.258853 |
| 64039 | 1.89226 | 0.05 |
| 64040 | 1.89226 | 0.556749 |
| 64041 | 3.78453 | 0.647161 |
| 64042 | 2.73497 | 0.05 |
| 64043 | 1.93823 | 0.18258 |
| 64044 | 1.93823 | 0.05 |
| 64045 | 2.20046 | 0.527839 |
| 64046 | 2.42279 | 0.05 |
| 64047 | 2.68501 | 1.95285 |
| 64048 | 1.89226 | 0.05 |
| 64049 | 1.89226 | 0.324354 |
| 64050 | 1.89226 | 2.1 |
| 64051 | 1.89226 | 0.05 |
| 64052 | 1.89226 | 1.14226 |
| 64053 | 1.89226 | 0.05 |
| 64054 | 1.89226 | 0.05 |
| 64055 | 1.89226 | 0.0633764 |
| 64056 | 1.89226 | 0.05 |
| 64057 | 1.89226 | 1.44615 |
| 64058 | 1.89226 | 0.0736168 |
| 64059 | 1.89226 | 0.27971 |
| 64060 | 2.82984 | 0.478884 |
| 64061 | 2.15276 | 0.05 |
| 64062 | 2.15276 | 0.399639 |
| 64063 | 2.42278 | 0.0642521 |
| 64064 | 2.50074 | 0.05 |
| 64065 | 2.77077 | 0.185781 |
| 64066 | 2.42279 | 0.05 |
| 64067 | 1.93823 | 0.467365 |
| 64068 | 1.70423 | 0.05 |
| 64069 | 1.70423 | 0.230576 |
| 64070 | 2.15276 | 0.05 |
| 64071 | 2.38676 | 0.31809 |
| 64072 | 0.810946 | 0.05 |
| 64073 | 0.810946 | 0.620811 |
| 64074 | 0.810944 | 0.05 |
| 64075 | 0.810944 | 0.05 |
| 64076 | 0.810946 | 0.435374 |
| 64077 | 0.810946 | 0.277304 |
| 64078 | 1.89226 | 0.592775 |
| 64079 | 1.35169 | 0.05 |
| 64080 | 1.08132 | 0.0788416 |
| 64081 | 1.08132 | 0.05 |
| 64082 | 1.08132 | 0.05 |
| 64083 | 1.89226 | 2.1 |
| 64084 | 1.89226 | 2.1 |
| 64085 | 1.89226 | 2.1 |
| 64086 | 1.89226 | 2.1 |
| 64087 | 3.78452 | 2.1 |

| | | |
|---|---|---|
| 64088 | 1.89226 | 2.1 |
| 64089 | 1.89226 | 2.1 |
| 64090 | 1.89226 | 2.1 |
| 64091 | 1.89226 | 2.1 |
| 64092 | 3.78453 | 2.1 |
| 64093 | 1.04144 | 2.1 |
| 64094 | 1.04144 | 2.1 |
| 64095 | 0.938972 | 2.1 |
| 64096 | 0.938972 | 2.1 |
| 64097 | 0.969661 | 2.1 |
| 64098 | 1.03285 | 2.1 |
| 64099 | 0.946133 | 2.1 |
| 64100 | 0.946133 | 2.1 |
| 64101 | 0.946131 | 2.1 |
| 64102 | 0.969663 | 2.1 |
| 64103 | 0.946131 | 2.1 |
| 64104 | 1.03284 | 2.1 |
| 64105 | 0.946131 | 2.1 |
| 64106 | 0.946131 | 2.1 |
| 64107 | 0.946128 | 2.1 |
| 64108 | 0.946128 | 2.1 |
| 64109 | 0.946127 | 2.1 |
| 64110 | 0.946127 | 2.1 |
| 64111 | 0.946131 | 2.1 |
| 64112 | 0.946131 | 2.1 |
| 64113 | 0.94613 | 2.1 |
| 64114 | 0.94613 | 2.1 |
| 64115 | 0.946129 | 2.1 |
| 64116 | 0.946129 | 2.1 |
| 64117 | 1.89226 | 2.1 |
| 64118 | 1.89226 | 2.1 |
| 64119 | 1.89226 | 2.1 |
| 64120 | 1.89226 | 2.1 |
| 64121 | 3.78452 | 2.1 |
| 64122 | 1.89226 | 2.1 |
| 64123 | 1.89226 | 2.1 |
| 64124 | 1.89226 | 2.1 |
| 64125 | 1.89226 | 2.1 |
| 64126 | 3.78453 | 2.1 |
| 64127 | 1.89226 | 2.1 |
| 64128 | 1.89226 | 2.1 |
| 64129 | 1.85882 | 2.1 |
| 64130 | 1.85882 | 2.1 |
| 64131 | 1.93932 | 2.1 |
| 64132 | 1.97276 | 2.1 |
| 64133 | 0.94613 | 2.1 |
| 64134 | 0.94613 | 2.1 |
| 64135 | 0.946128 | 2.1 |
| 64136 | 0.946128 | 2.1 |
| 64137 | 0.946127 | 2.1 |
| 64138 | 0.946127 | 2.1 |
| 64139 | 0.946131 | 2.1 |
| 64140 | 0.946131 | 2.1 |
| 64141 | 0.946129 | 2.1 |
| 64142 | 0.946129 | 2.1 |
| 64143 | 0.946129 | 2.1 |
| 64144 | 0.946129 | 2.1 |
| 64145 | 1.89225 | 2.1 |

| | | |
|---|---|---|
| 64146 | 1.89225 | 2.1 |
| 64147 | 1.89225 | 2.1 |
| 64148 | 1.89225 | 2.1 |
| 64149 | 3.7845 | 2.1 |
| 64150 | 1.0086 | 2.1 |
| 64151 | 1.09028 | 2.1 |
| 64152 | 0.946125 | 2.1 |
| 64153 | 0.946125 | 2.1 |
| 64154 | 0.916906 | 2.1 |
| 64155 | 0.916906 | 2.1 |
| 64156 | 1.13051 | 2.1 |
| 64157 | 1.1505 | 2.1 |
| 64158 | 1.11557 | 2.1 |
| 64159 | 1.04071 | 2.1 |
| 64160 | 0.851542 | 2.1 |
| 64161 | 0.991196 | 2.1 |
| 64162 | 1.89225 | 2.1 |
| 64163 | 1.89225 | 2.1 |
| 64164 | 1.89225 | 2.1 |
| 64165 | 1.89225 | 2.1 |
| 64166 | 3.7845 | 2.1 |
| 64167 | 1.89225 | 2.1 |
| 64168 | 1.89225 | 2.1 |
| 64169 | 1.89225 | 2.1 |
| 64170 | 1.89225 | 2.1 |
| 64171 | 1.89225 | 2.1 |
| 64172 | 1.89225 | 2.1 |
| 64173 | 1.89226 | 2.1 |
| 64174 | 1.89226 | 2.1 |
| 64175 | 1.89226 | 2.1 |
| 64176 | 1.89226 | 2.1 |
| 64177 | 3.78452 | 2.1 |
| 64178 | 0.890962 | 2.1 |
| 64179 | 0.952547 | 2.1 |
| 64180 | 0.946132 | 2.1 |
| 64181 | 0.946132 | 2.1 |
| 64182 | 0.851543 | 2.1 |
| 64183 | 0.851543 | 2.1 |
| 64184 | 1.11558 | 2.1 |
| 64185 | 1.06304 | 2.1 |
| 64186 | 0.994315 | 2.1 |
| 64187 | 1.04072 | 2.1 |
| 64188 | 0.900943 | 2.1 |
| 64189 | 0.851542 | 2.1 |
| 64190 | 1.89226 | 2.1 |
| 64191 | 1.89226 | 2.1 |
| 64192 | 1.89226 | 2.1 |
| 64193 | 1.89226 | 2.1 |
| 64194 | 1.89226 | 2.1 |
| 64195 | 1.89226 | 2.1 |
| 64196 | 1.89226 | 2.1 |
| 64197 | 1.89226 | 2.1 |
| 64198 | 1.89226 | 2.1 |
| 64199 | 1.89226 | 2.1 |
| 64200 | 3.78452 | 2.1 |
| 64201 | 1.89226 | 2.1 |
| 64202 | 1.89226 | 2.1 |
| 64203 | 1.89226 | 2.1 |

| | | |
|---|---|---|
| 64204 | 1.89226 | 2.1 |
| 64205 | 3.78452 | 2.1 |
| 64206 | 0.782015 | 2.1 |
| 64207 | 0.929718 | 2.1 |
| 64208 | 0.946132 | 2.1 |
| 64209 | 0.946132 | 2.1 |
| 64210 | 0.782016 | 2.1 |
| 64211 | 0.782016 | 2.1 |
| 64212 | 1.06706 | 2.1 |
| 64213 | 1.11785 | 2.1 |
| 64214 | 0.782015 | 2.1 |
| 64215 | 0.946133 | 2.1 |
| 64216 | 0.878931 | 2.1 |
| 64217 | 0.946132 | 2.1 |
| 64218 | 1.89226 | 2.1 |
| 64219 | 1.89226 | 2.1 |
| 64220 | 1.89226 | 2.1 |
| 64221 | 1.89226 | 2.1 |
| 64222 | 1.89226 | 2.1 |
| 64223 | 1.89226 | 2.1 |
| 64224 | 0.946131 | 2.1 |
| 64225 | 0.946131 | 2.1 |
| 64226 | 0.946129 | 2.1 |
| 64227 | 0.946129 | 2.1 |
| 64228 | 0.946131 | 2.1 |
| 64229 | 0.946131 | 2.1 |
| 64230 | 0.94613 | 2.1 |
| 64231 | 0.94613 | 2.1 |
| 64232 | 0.946131 | 2.1 |
| 64233 | 0.946131 | 2.1 |
| 64234 | 0.94613 | 2.1 |
| 64235 | 0.94613 | 2.1 |
| 64236 | 0.827866 | 2.1 |
| 64237 | 0.946131 | 2.1 |
| 64238 | 0.946129 | 2.1 |
| 64239 | 0.946129 | 2.1 |
| 64240 | 0.94613 | 2.1 |
| 64241 | 0.94613 | 2.1 |
| 64242 | 0.94613 | 2.1 |
| 64243 | 0.94613 | 2.1 |
| 64244 | 0.827865 | 2.1 |
| 64245 | 0.94613 | 2.1 |
| 64246 | 0.94613 | 2.1 |
| 64247 | 0.94613 | 2.1 |
| 64248 | 1.89226 | 2.1 |
| 64249 | 1.89226 | 2.1 |
| 64250 | 1.89226 | 2.1 |
| 64251 | 1.89226 | 2.1 |
| 64252 | 3.78452 | 2.1 |
| 64253 | 1.89226 | 2.1 |
| 64254 | 1.89226 | 2.1 |
| 64255 | 1.89226 | 2.1 |
| 64256 | 1.89226 | 2.1 |
| 64257 | 1.89226 | 2.1 |
| 64258 | 1.89226 | 2.1 |
| 64259 | 1.89226 | 2.1 |
| 64260 | 1.89226 | 2.1 |
| 64261 | 1.89226 | 2.1 |

| | | |
|---|---|---|
| 64262 | 1.89226 | 2.1 |
| 64263 | 3.78453 | 2.1 |
| 64264 | 1.89226 | 2.1 |
| 64265 | 1.89226 | 2.1 |
| 64266 | 1.89226 | 2.1 |
| 64267 | 1.89226 | 2.1 |
| 64268 | 3.78452 | 2.1 |
| 64269 | 0.744377 | 2.1 |
| 64270 | 0.541919 | 2.1 |
| 64271 | 0.652022 | 2.1 |
| 64272 | 0.544198 | 2.1 |
| 64273 | 0.764633 | 2.1 |
| 64274 | 0.673254 | 2.1 |
| 64275 | 1.89226 | 2.1 |
| 64276 | 1.31149 | 2.1 |
| 64277 | 1.05423 | 2.1 |
| 64278 | 1.02189 | 2.1 |
| 64279 | 0.870602 | 2.1 |
| 64280 | 1.89226 | 2.1 |
| 64281 | 1.89226 | 2.1 |
| 64282 | 1.89226 | 2.1 |
| 64283 | 1.89226 | 2.1 |
| 64284 | 1.89226 | 2.1 |
| 64285 | 1.89226 | 2.1 |
| 64286 | 1.89226 | 2.1 |
| 64287 | 1.89226 | 2.1 |
| 64288 | 1.89226 | 2.1 |
| 64289 | 1.89226 | 2.1 |
| 64290 | 1.89226 | 2.1 |
| 64291 | 1.89226 | 2.1 |
| 64292 | 1.89226 | 2.1 |
| 64293 | 1.89226 | 2.1 |
| 64294 | 1.89226 | 2.1 |
| 64295 | 1.89226 | 2.1 |
| 64296 | 1.89226 | 2.1 |
| 64297 | 1.89226 | 2.1 |
| 64298 | 0.815314 | 2.1 |
| 64299 | 0.815313 | 2.1 |
| 64300 | 0.714029 | 2.1 |
| 64301 | 0.782951 | 2.1 |
| 64302 | 0.810944 | 2.1 |
| 64303 | 0.782954 | 2.1 |
| 64304 | 1.89226 | 2.1 |
| 64305 | 1.30999 | 2.1 |
| 64306 | 1.08132 | 2.1 |
| 64307 | 1.03962 | 2.1 |
| 64308 | 0.95209 | 2.1 |
| 64309 | 1.89226 | 2.1 |
| 64310 | 1.89226 | 2.1 |
| 64311 | 1.89226 | 2.1 |
| 64312 | 1.89226 | 2.1 |
| 64313 | 1.89226 | 2.1 |
| 64314 | 1.89226 | 2.1 |
| 64315 | 1.89226 | 2.1 |
| 64316 | 1.89226 | 2.1 |
| 64317 | 1.89226 | 2.1 |
| 64318 | 1.89226 | 2.1 |
| 64319 | 1.89226 | 2.1 |

| | | |
|---|---|---|
| 64320 | 1.89226 | 2.1 |
| 64321 | 1.89226 | 2.1 |
| 64322 | 1.89226 | 2.1 |
| 64323 | 1.89226 | 2.1 |
| 64324 | 1.89226 | 2.1 |
| 64325 | 1.89226 | 2.1 |
| 64326 | 1.89226 | 2.1 |
| 64327 | 0.871558 | 2.1 |
| 64328 | 0.713078 | 2.1 |
| 64329 | 0.756858 | 2.1 |
| 64330 | 0.703674 | 2.1 |
| 64331 | 0.838025 | 2.1 |
| 64332 | 0.862196 | 2.1 |
| 64333 | 1.89226 | 2.1 |
| 64334 | 1.33194 | 2.1 |
| 64335 | 1.10834 | 2.1 |
| 64336 | 1.06163 | 2.1 |
| 64337 | 1.01785 | 2.1 |
| 64338 | 1.89226 | 2.1 |
| 64339 | 1.89226 | 2.1 |
| 64340 | 1.89226 | 2.1 |
| 64341 | 1.89226 | 2.1 |
| 64342 | 3.78452 | 2.1 |
| 64343 | 1.89226 | 2.1 |
| 64344 | 1.89226 | 2.1 |
| 64345 | 1.89226 | 2.1 |
| 64346 | 1.89226 | 2.1 |
| 64347 | 1.89226 | 2.1 |
| 64348 | 1.89226 | 2.1 |
| 64349 | 1.89226 | 2.1 |
| 64350 | 1.89226 | 2.1 |
| 64351 | 1.89226 | 2.1 |
| 64352 | 1.89226 | 2.1 |
| 64353 | 1.89226 | 2.1 |
| 64354 | 1.89226 | 2.1 |
| 64355 | 0.958406 | 2.1 |
| 64356 | 1.1383 | 2.1 |
| 64357 | 0.865636 | 2.1 |
| 64358 | 1.10093 | 2.1 |
| 64359 | 0.838024 | 2.1 |
| 64360 | 0.975154 | 2.1 |
| 64361 | 1.89226 | 2.1 |
| 64362 | 1.38553 | 2.1 |
| 64363 | 1.10834 | 2.1 |
| 64364 | 1.11522 | 2.1 |
| 64365 | 1.15271 | 2.1 |
| 64366 | 1.89227 | 2.1 |
| 64367 | 1.89227 | 2.1 |
| 64368 | 1.89227 | 2.1 |
| 64369 | 1.89227 | 2.1 |
| 64370 | 3.78453 | 2.1 |
| 64371 | 0.946132 | 2.1 |
| 64372 | 0.946132 | 2.1 |
| 64373 | 0.946132 | 2.1 |
| 64374 | 0.946132 | 2.1 |
| 64375 | 0.946132 | 2.1 |
| 64376 | 0.946132 | 2.1 |
| 64377 | 0.946133 | 2.1 |

| | | |
|---|---|---|
| 64378 | 0.946133 | 2.1 |
| 64379 | 0.946132 | 2.1 |
| 64380 | 0.946132 | 2.1 |
| 64381 | 0.946133 | 2.1 |
| 64382 | 0.946133 | 2.1 |
| 64383 | 0.719271 | 2.1 |
| 64384 | 0.774968 | 2.1 |
| 64385 | 1.28401 | 2.1 |
| 64386 | 1.17415 | 2.1 |
| 64387 | 0.960999 | 2.1 |
| 64388 | 0.960999 | 2.1 |
| 64389 | 0.988115 | 2.1 |
| 64390 | 0.988115 | 2.1 |
| 64391 | 0.774968 | 2.1 |
| 64392 | 1.28401 | 2.1 |
| 64393 | 0.719271 | 2.1 |
| 64394 | 1.17415 | 2.1 |
| 64395 | 2.77811 | 2.1 |
| 64396 | 2.34829 | 2.1 |
| 64397 | 2.56802 | 2.1 |
| 64398 | 2.52333 | 2.1 |
| 64399 | 2.56802 | 2.1 |
| 64400 | 2.52333 | 2.1 |
| 64401 | 1.89227 | 2.1 |
| 64402 | 1.89227 | 2.1 |
| 64403 | 1.89227 | 2.1 |
| 64404 | 1.89227 | 2.1 |
| 64405 | 3.78453 | 2.1 |
| 64406 | 1.89226 | 2.1 |
| 64407 | 1.89226 | 2.1 |
| 64408 | 1.89226 | 2.1 |
| 64409 | 1.89226 | 2.1 |
| 64410 | 1.89226 | 2.1 |
| 64411 | 1.89226 | 2.1 |
| 64412 | 1.89226 | 2.1 |
| 64413 | 2.77811 | 2.1 |
| 64414 | 1.89226 | 2.1 |
| 64415 | 2.56802 | 2.1 |
| 64416 | 1.89226 | 2.1 |
| 64417 | 2.56802 | 2.1 |
| 64418 | 1.89226 | 0.119152 |
| 64419 | 1.89226 | 2.1 |
| 64420 | 1.89226 | 0.05 |
| 64421 | 1.89226 | 2.1 |
| 64422 | 3.78452 | 0.112028 |
| 64423 | 2.41802 | 0.252091 |
| 64424 | 1.95764 | 2.1 |
| 64425 | 1.95764 | 1.40849 |
| 64426 | 2.27727 | 2.1 |
| 64427 | 2.39311 | 0.195115 |
| 64428 | 2.73961 | 2.1 |
| 64429 | 0.946132 | 2.1 |
| 64430 | 0.946132 | 0.752536 |
| 64431 | 0.94613 | 0.843287 |
| 64432 | 0.94613 | 2.1 |
| 64433 | 0.946131 | 2.1 |
| 64434 | 0.946131 | 2.1 |
| 64435 | 0.946131 | 0.25955 |

| | | |
|---|---|---|
| 64436 | 0.946131 | 0.64123 |
| 64437 | 0.946131 | 2.1 |
| 64438 | 0.946131 | 2.1 |
| 64439 | 0.946131 | 2.1 |
| 64440 | 0.946131 | 2.1 |
| 64441 | 1.89226 | 0.108277 |
| 64442 | 1.89226 | 0.823139 |
| 64443 | 1.89226 | 2.1 |
| 64444 | 1.89226 | 0.0825871 |
| 64445 | 1.89226 | 2.1 |
| 64446 | 1.89226 | 0.257762 |
| 64447 | 2.78856 | 0.0688253 |
| 64448 | 2.28552 | 0.89459 |
| 64449 | 2.28552 | 0.0625386 |
| 64450 | 2.3931 | 0.595369 |
| 64451 | 2.6402 | 0.05 |
| 64452 | 2.74778 | 0.201962 |
| 64453 | 2.39311 | 0.05 |
| 64454 | 1.95764 | 2.1 |
| 64455 | 2.28552 | 0.0732583 |
| 64456 | 1.86103 | 2.1 |
| 64457 | 4.24316 | 2.1 |
| 64458 | 1.89226 | 0.0936731 |
| 64459 | 1.89226 | 2.1 |
| 64460 | 1.89226 | 0.735267 |
| 64461 | 1.89226 | 2.1 |
| 64462 | 1.89226 | 0.754111 |
| 64463 | 1.89226 | 2.1 |
| 64464 | 1.47412 | 2.1 |
| 64465 | 2.18862 | 1.78771 |
| 64466 | 2.07226 | 1.12238 |
| 64467 | 2.33645 | 1.39803 |
| 64468 | 4.26088 | 1.34156 |
| 64469 | 1.89226 | 1.02704 |
| 64470 | 1.89226 | 1.90225 |
| 64471 | 1.89226 | 1.18676 |
| 64472 | 1.89226 | 2.1 |
| 64473 | 1.89226 | 1.50775 |
| 64474 | 1.89226 | 2.1 |
| 64475 | 1.89226 | 1.23737 |
| 64476 | 1.89226 | 2.1 |
| 64477 | 1.89226 | 0.05 |
| 64478 | 1.89226 | 2.1 |
| 64479 | 3.78453 | 1.2908 |
| 64480 | 2.18862 | 1.22267 |
| 64481 | 2.1128 | 2.1 |
| 64482 | 2.33645 | 1.05544 |
| 64483 | 2.28188 | 2.1 |
| 64484 | 4.47051 | 2.1 |
| 64485 | 2.07226 | 2.1 |
| 64486 | 2.33645 | 2.01801 |
| 64487 | 2.20831 | 1.00299 |
| 64488 | 2.4646 | 1.09322 |
| 64489 | 4.53686 | 1.25456 |
| 64490 | 0.946131 | 0.9329 |
| 64491 | 0.946131 | 2.1 |
| 64492 | 0.946131 | 2.1 |
| 64493 | 0.946131 | 1.30572 |

| | | |
|---|---|---|
| 64494 | 0.94613 | 2.1 |
| 64495 | 0.94613 | 1.20499 |
| 64496 | 0.946133 | 2.1 |
| 64497 | 0.946133 | 2.1 |
| 64498 | 0.946132 | 0.945779 |
| 64499 | 0.946132 | 0.885394 |
| 64500 | 0.946131 | 2.1 |
| 64501 | 0.946131 | 1.66362 |
| 64502 | 1.89226 | 1.93322 |
| 64503 | 1.89226 | 2.1 |
| 64504 | 1.89226 | 0.969421 |
| 64505 | 1.89226 | 2.1 |
| 64506 | 3.78453 | 1.61479 |
| 64507 | 2.33646 | 1.21422 |
| 64508 | 2.28189 | 2.1 |
| 64509 | 2.4646 | 0.938884 |
| 64510 | 2.42758 | 2.1 |
| 64511 | 4.74648 | 1.3745 |
| 64512 | 1.89226 | 2.1 |
| 64513 | 1.89226 | 2.1 |
| 64514 | 1.89226 | 2.1 |
| 64515 | 1.89226 | 2.1 |
| 64516 | 1.89226 | 2.1 |
| 64517 | 1.89226 | 2.1 |
| 64518 | 1.89226 | 2.1 |
| 64519 | 1.89226 | 2.1 |
| 64520 | 1.89226 | 2.1 |
| 64521 | 1.89226 | 2.1 |
| 64522 | 1.89226 | 2.1 |
| 64523 | 1.89226 | 2.1 |
| 64524 | 1.89226 | 2.1 |
| 64525 | 1.89226 | 2.1 |
| 64526 | 1.89226 | 2.1 |
| 64527 | 1.89226 | 2.1 |
| 64528 | 3.78453 | 2.1 |
| 64529 | 1.89226 | 2.1 |
| 64530 | 1.89226 | 2.1 |
| 64531 | 1.89226 | 2.1 |
| 64532 | 1.89226 | 2.1 |
| 64533 | 1.89226 | 2.1 |
| 64534 | 1.89226 | 2.1 |
| 64535 | 1.89226 | 2.1 |
| 64536 | 1.89226 | 2.1 |
| 64537 | 1.89226 | 2.1 |
| 64538 | 1.89226 | 2.1 |
| 64539 | 3.78452 | 2.1 |
| 64540 | 0.877238 | 2.1 |
| 64541 | 0.877238 | 2.1 |
| 64542 | 1.24746 | 2.1 |
| 64543 | 1.04764 | 2.1 |
| 64544 | 0.964969 | 2.1 |
| 64545 | 1.1521 | 2.1 |
| 64546 | 0.946132 | 2.1 |
| 64547 | 0.946132 | 2.1 |
| 64548 | 0.946132 | 2.1 |
| 64549 | 1.12659 | 2.1 |
| 64550 | 0.964969 | 2.1 |
| 64551 | 0.946131 | 2.1 |

| | | |
|---|---|---|
| 64552 | 1.89226 | 2.1 |
| 64553 | 1.89226 | 2.1 |
| 64554 | 1.89226 | 2.1 |
| 64555 | 1.89226 | 2.1 |
| 64556 | 1.89226 | 2.1 |
| 64557 | 1.89226 | 2.1 |
| 64558 | 1.89226 | 2.1 |
| 64559 | 1.89226 | 2.1 |
| 64560 | 1.89226 | 2.1 |
| 64561 | 1.89226 | 2.1 |
| 64562 | 3.78453 | 2.1 |
| 64563 | 0.868144 | 2.1 |
| 64564 | 0.992762 | 2.1 |
| 64565 | 0.946131 | 2.1 |
| 64566 | 0.946131 | 2.1 |
| 64567 | 1.07072 | 2.1 |
| 64568 | 1.07072 | 2.1 |
| 64569 | 0.868171 | 2.1 |
| 64570 | 0.868171 | 2.1 |
| 64571 | 0.992762 | 2.1 |
| 64572 | 0.946131 | 2.1 |
| 64573 | 0.946131 | 2.1 |
| 64574 | 0.868144 | 2.1 |
| 64575 | 0.946132 | 2.1 |
| 64576 | 0.946132 | 2.1 |
| 64577 | 0.946131 | 2.1 |
| 64578 | 0.946131 | 2.1 |
| 64579 | 0.946132 | 2.1 |
| 64580 | 0.946132 | 2.1 |
| 64581 | 0.946132 | 2.1 |
| 64582 | 0.946132 | 2.1 |
| 64583 | 0.946132 | 2.1 |
| 64584 | 0.946132 | 2.1 |
| 64585 | 0.946132 | 2.1 |
| 64586 | 0.946132 | 2.1 |
| 64587 | 1.89227 | 2.1 |
| 64588 | 1.89227 | 2.1 |
| 64589 | 1.89227 | 2.1 |
| 64590 | 1.89227 | 2.1 |
| 64591 | 1.89227 | 2.1 |
| 64592 | 1.89227 | 2.1 |
| 64593 | 1.89226 | 2.1 |
| 64594 | 1.89226 | 2.1 |
| 64595 | 1.89226 | 2.1 |
| 64596 | 1.89226 | 2.1 |
| 64597 | 1.89226 | 2.1 |
| 64598 | 1.89226 | 2.1 |
| 64599 | 1.98552 | 2.1 |
| 64600 | 2.22298 | 2.1 |
| 64601 | 2.22298 | 2.1 |
| 64602 | 1.89226 | 2.1 |
| 64603 | 1.89226 | 2.1 |
| 64604 | 1.54525 | 2.1 |
| 64605 | 0.810946 | 2.1 |
| 64606 | 0.810946 | 2.1 |
| 64607 | 0.810947 | 2.1 |
| 64608 | 0.810947 | 2.1 |
| 64609 | 0.81096 | 2.1 |

| | | |
|---|---|---|
| 64610 | 0.81096 | 2.1 |
| 64611 | 1.89226 | 2.1 |
| 64612 | 1.35168 | 2.1 |
| 64613 | 1.08132 | 2.1 |
| 64614 | 1.08132 | 2.1 |
| 64615 | 1.0813 | 2.1 |
| 64616 | 1.89226 | 2.1 |
| 64617 | 1.89226 | 2.1 |
| 64618 | 1.89226 | 2.1 |
| 64619 | 1.89226 | 2.1 |
| 64620 | 3.78452 | 2.1 |
| 64621 | 1.7753 | 2.1 |
| 64622 | 2.39703 | 2.1 |
| 64623 | 1.90577 | 2.1 |
| 64624 | 2.03445 | 2.1 |
| 64625 | 3.82335 | 2.1 |
| 64626 | 2.39703 | 2.1 |
| 64627 | 2.47949 | 0.0704636 |
| 64628 | 2.26051 | 2.1 |
| 64629 | 2.36891 | 0.05 |
| 64630 | 4.76594 | 0.919752 |
| 64631 | 1.89226 | 2.1 |
| 64632 | 1.89226 | 0.580141 |
| 64633 | 1.89226 | 2.1 |
| 64634 | 1.89226 | 0.05 |
| 64635 | 3.78452 | 0.220199 |
| 64636 | 1.89226 | 2.1 |
| 64637 | 1.89226 | 2.1 |
| 64638 | 1.89226 | 2.1 |
| 64639 | 1.89226 | 2.1 |
| 64640 | 1.89226 | 2.1 |
| 64641 | 1.89226 | 2.1 |
| 64642 | 0.883745 | 2.1 |
| 64643 | 0.973143 | 2.1 |
| 64644 | 0.988173 | 2.1 |
| 64645 | 0.988173 | 2.1 |
| 64646 | 0.941927 | 2.1 |
| 64647 | 1.02971 | 2.1 |
| 64648 | 0.854065 | 2.1 |
| 64649 | 0.82702 | 2.1 |
| 64650 | 0.847835 | 2.1 |
| 64651 | 0.981126 | 2.1 |
| 64652 | 1.02996 | 2.1 |
| 64653 | 1.10704 | 2.1 |
| 64654 | 2.28031 | 2.1 |
| 64655 | 2.26369 | 0.055272 |
| 64656 | 2.26369 | 2.1 |
| 64657 | 2.36891 | 0.05 |
| 64658 | 2.30788 | 2.1 |
| 64659 | 2.42455 | 0.05 |
| 64660 | 1.89226 | 2.1 |
| 64661 | 1.89226 | 0.05 |
| 64662 | 1.89226 | 0.05 |
| 64663 | 1.89226 | 2.1 |
| 64664 | 1.89226 | 0.05 |
| 64665 | 1.89226 | 2.1 |
| 64666 | 2.71406 | 2.1 |
| 64667 | 1.83244 | 2.1 |

| | | |
|---|---|---|
| 64668 | 1.83244 | 2.1 |
| 64669 | 2.07567 | 2.1 |
| 64670 | 2.52044 | 2.1 |
| 64671 | 2.76367 | 2.1 |
| 64672 | 1.89226 | 2.1 |
| 64673 | 1.89226 | 2.1 |
| 64674 | 1.89226 | 2.1 |
| 64675 | 1.89226 | 2.1 |
| 64676 | 3.78453 | 2.1 |
| 64677 | 1.89226 | 2.1 |
| 64678 | 1.89226 | 2.1 |
| 64679 | 1.89226 | 2.1 |
| 64680 | 1.89226 | 2.1 |
| 64681 | 1.89226 | 2.1 |
| 64682 | 1.89226 | 2.1 |
| 64683 | 2.70714 | 2.1 |
| 64684 | 2.3148 | 2.1 |
| 64685 | 2.3148 | 2.1 |
| 64686 | 2.52044 | 2.1 |
| 64687 | 2.54204 | 2.1 |
| 64688 | 2.74768 | 2.1 |
| 64689 | 2.52044 | 2.1 |
| 64690 | 2.3148 | 2.1 |
| 64691 | 1.83244 | 2.1 |
| 64692 | 1.83244 | 2.1 |
| 64693 | 1.62611 | 2.1 |
| 64694 | 2.3148 | 2.1 |
| 64695 | 0.946131 | 2.1 |
| 64696 | 0.946131 | 2.1 |
| 64697 | 0.946131 | 2.1 |
| 64698 | 0.946131 | 2.1 |
| 64699 | 0.946131 | 2.1 |
| 64700 | 0.946131 | 2.1 |
| 64701 | 0.946131 | 2.1 |
| 64702 | 0.946131 | 2.1 |
| 64703 | 0.946131 | 2.1 |
| 64704 | 0.946131 | 2.1 |
| 64705 | 0.946131 | 2.1 |
| 64706 | 0.946131 | 2.1 |
| 64707 | 1.89226 | 2.1 |
| 64708 | 1.89226 | 2.1 |
| 64709 | 1.89226 | 2.1 |
| 64710 | 1.89226 | 2.1 |
| 64711 | 3.78453 | 2.1 |
| 64712 | 1.89226 | 2.1 |
| 64713 | 1.89226 | 2.1 |
| 64714 | 1.89226 | 2.1 |
| 64715 | 1.89226 | 2.1 |
| 64716 | 1.89226 | 2.1 |
| 64717 | 1.89226 | 2.1 |
| 64718 | 1.89227 | 2.1 |
| 64719 | 1.89227 | 2.1 |
| 64720 | 1.89227 | 2.1 |
| 64721 | 1.89227 | 2.1 |
| 64722 | 3.78453 | 2.1 |
| 64723 | 1.89226 | 2.1 |
| 64724 | 1.89226 | 2.1 |
| 64725 | 1.89226 | 2.1 |

| | | |
|---|---|---|
| 64726 | 1.89226 | 2.1 |
| 64727 | 3.78453 | 2.1 |
| 64728 | 0.915781 | 2.1 |
| 64729 | 0.863524 | 2.1 |
| 64730 | 0.717056 | 2.1 |
| 64731 | 0.830182 | 2.1 |
| 64732 | 0.865094 | 2.1 |
| 64733 | 0.918003 | 2.1 |
| 64734 | 1.89226 | 2.1 |
| 64735 | 1.29933 | 2.1 |
| 64736 | 1.11215 | 2.1 |
| 64737 | 1.05227 | 2.1 |
| 64738 | 0.966269 | 2.1 |
| 64739 | 1.89226 | 2.1 |
| 64740 | 1.89226 | 2.1 |
| 64741 | 1.89226 | 2.1 |
| 64742 | 1.89226 | 2.1 |
| 64743 | 1.89226 | 2.1 |
| 64744 | 1.89226 | 2.1 |
| 64745 | 1.89226 | 2.1 |
| 64746 | 1.89226 | 2.1 |
| 64747 | 1.89226 | 2.1 |
| 64748 | 1.89226 | 2.1 |
| 64749 | 1.89226 | 2.1 |
| 64750 | 1.89226 | 2.1 |
| 64751 | 1.89226 | 2.1 |
| 64752 | 1.89226 | 2.1 |
| 64753 | 1.89226 | 2.1 |
| 64754 | 1.89226 | 2.1 |
| 64755 | 1.89226 | 2.1 |
| 64756 | 1.89226 | 2.1 |
| 64757 | 1.01725 | 2.1 |
| 64758 | 0.997048 | 2.1 |
| 64759 | 0.818657 | 2.1 |
| 64760 | 0.960724 | 2.1 |
| 64761 | 0.838013 | 2.1 |
| 64762 | 1.04859 | 2.1 |
| 64763 | 1.89226 | 2.1 |
| 64764 | 1.41754 | 2.1 |
| 64765 | 1.17625 | 2.1 |
| 64766 | 1.0793 | 2.1 |
| 64767 | 1.18822 | 2.1 |
| 64768 | 2.16402 | 2.1 |
| 64769 | 1.89452 | 2.1 |
| 64770 | 1.50084 | 2.1 |
| 64771 | 2.09288 | 2.1 |
| 64772 | 3.9874 | 2.1 |
| 64773 | 2.16402 | 2.1 |
| 64774 | 2.13925 | 2.1 |
| 64775 | 2.04483 | 2.1 |
| 64776 | 2.09288 | 2.1 |
| 64777 | 4.23212 | 2.1 |
| 64778 | 1.89226 | 2.1 |
| 64779 | 1.89226 | 2.1 |
| 64780 | 1.89226 | 2.1 |
| 64781 | 1.89226 | 2.1 |
| 64782 | 3.78452 | 2.1 |
| 64783 | 1.89226 | 2.1 |

| | | |
|---|---|---|
| 64784 | 1.89226 | 2.1 |
| 64785 | 1.89226 | 2.1 |
| 64786 | 1.89226 | 2.1 |
| 64787 | 1.89226 | 2.1 |
| 64788 | 1.89226 | 2.1 |
| 64789 | 1.9824 | 2.1 |
| 64790 | 2.09288 | 2.1 |
| 64791 | 1.69889 | 2.1 |
| 64792 | 1.6817 | 2.1 |
| 64793 | 2.00695 | 2.1 |
| 64794 | 2.00695 | 2.1 |
| 64795 | 1.93592 | 2.1 |
| 64796 | 2.09288 | 2.1 |
| 64797 | 2.00695 | 2.1 |
| 64798 | 2.09288 | 2.1 |
| 64799 | 1.9589 | 2.1 |
| 64800 | 2.04483 | 2.1 |
| 64801 | 0.946128 | 2.1 |
| 64802 | 0.946128 | 2.1 |
| 64803 | 0.946132 | 2.1 |
| 64804 | 0.946132 | 2.1 |
| 64805 | 0.94613 | 2.1 |
| 64806 | 0.94613 | 2.1 |
| 64807 | 0.946129 | 2.1 |
| 64808 | 0.946129 | 2.1 |
| 64809 | 0.94613 | 2.1 |
| 64810 | 0.94613 | 2.1 |
| 64811 | 0.94613 | 2.1 |
| 64812 | 0.94613 | 2.1 |
| 64813 | 0.946131 | 2.1 |
| 64814 | 0.946131 | 2.1 |
| 64815 | 0.946131 | 2.1 |
| 64816 | 0.946131 | 2.1 |
| 64817 | 0.946131 | 2.1 |
| 64818 | 0.946131 | 2.1 |
| 64819 | 0.946131 | 2.1 |
| 64820 | 0.946131 | 2.1 |
| 64821 | 0.946131 | 2.1 |
| 64822 | 0.946131 | 2.1 |
| 64823 | 0.946131 | 2.1 |
| 64824 | 0.946131 | 2.1 |
| 64825 | 1.89226 | 2.1 |
| 64826 | 2.48083 | 2.1 |
| 64827 | 2.6968 | 2.1 |
| 64828 | 2.6968 | 2.1 |
| 64829 | 1.89226 | 2.1 |
| 64830 | 1.89226 | 2.1 |
| 64831 | 1.89226 | 2.1 |
| 64832 | 1.89226 | 2.1 |
| 64833 | 1.89226 | 2.1 |
| 64834 | 1.89226 | 2.1 |
| 64835 | 3.78453 | 2.1 |
| 64836 | 1.89226 | 2.1 |
| 64837 | 1.89226 | 2.1 |
| 64838 | 1.89226 | 2.1 |
| 64839 | 1.89226 | 2.1 |
| 64840 | 1.89226 | 2.1 |
| 64841 | 1.89226 | 2.1 |

| | | |
|---|---|---|
| 64842 | 1.1017 | 2.1 |
| 64843 | 1.1017 | 2.1 |
| 64844 | 1.10605 | 2.1 |
| 64845 | 1.01647 | 2.1 |
| 64846 | 0.868255 | 2.1 |
| 64847 | 0.883424 | 2.1 |
| 64848 | 1.24624 | 2.1 |
| 64849 | 1.35473 | 2.1 |
| 64850 | 0.942231 | 2.1 |
| 64851 | 1.31464 | 2.1 |
| 64852 | 0.910064 | 2.1 |
| 64853 | 1.27147 | 2.1 |
| 64854 | 1.89226 | 2.07399 |
| 64855 | 1.89226 | 2.1 |
| 64856 | 1.89226 | 0.116983 |
| 64857 | 1.89226 | 2.1 |
| 64858 | 3.78453 | 2.1 |
| 64859 | 1.89226 | 2.1 |
| 64860 | 1.89226 | 2.1 |
| 64861 | 1.89226 | 2.1 |
| 64862 | 1.89226 | 2.1 |
| 64863 | 1.89226 | 2.1 |
| 64864 | 1.89226 | 2.1 |
| 64865 | 1.89226 | 2.1 |
| 64866 | 1.89226 | 2.1 |
| 64867 | 1.89226 | 2.1 |
| 64868 | 1.89226 | 2.1 |
| 64869 | 3.78453 | 2.1 |
| 64870 | 1.89226 | 2.1 |
| 64871 | 2.70015 | 2.1 |
| 64872 | 1.89226 | 2.1 |
| 64873 | 2.61064 | 2.1 |
| 64874 | 4.59241 | 2.1 |
| 64875 | 2.70015 | 2.1 |
| 64876 | 2.3372 | 2.1 |
| 64877 | 2.61064 | 2.1 |
| 64878 | 1.85184 | 2.1 |
| 64879 | 4.55198 | 2.1 |
| 64880 | 1.89226 | 2.1 |
| 64881 | 1.89226 | 2.1 |
| 64882 | 1.89226 | 2.1 |
| 64883 | 1.89226 | 2.1 |
| 64884 | 1.89226 | 2.1 |
| 64885 | 1.89226 | 2.1 |
| 64886 | 1.89227 | 2.1 |
| 64887 | 1.89227 | 2.1 |
| 64888 | 1.89227 | 2.1 |
| 64889 | 1.89227 | 2.1 |
| 64890 | 3.78453 | 2.1 |
| 64891 | 1.89226 | 2.1 |
| 64892 | 2.52194 | 2.1 |
| 64893 | 2.61064 | 2.1 |
| 64894 | 2.61064 | 2.1 |
| 64895 | 1.89226 | 2.1 |
| 64896 | 1.89226 | 2.1 |
| 64897 | 1.095 | 2.1 |
| 64898 | 1.095 | 2.1 |
| 64899 | 1.09189 | 2.1 |

| | | |
|---|---|---|
| 64900 | 1.09189 | 2.1 |
| 64901 | 0.875334 | 2.1 |
| 64902 | 0.925919 | 2.1 |
| 64903 | 1.26097 | 2.1 |
| 64904 | 1.30532 | 2.1 |
| 64905 | 1.26097 | 2.1 |
| 64906 | 0.875334 | 2.1 |
| 64907 | 1.30532 | 2.1 |
| 64908 | 0.92592 | 2.1 |
| 64909 | 1.89226 | 2.1 |
| 64910 | 1.89226 | 2.1 |
| 64911 | 1.89226 | 2.1 |
| 64912 | 1.89226 | 2.1 |
| 64913 | 1.89226 | 2.1 |
| 64914 | 1.89226 | 2.1 |
| 64915 | 1.89226 | 2.1 |
| 64916 | 1.89226 | 2.1 |
| 64917 | 1.89226 | 2.1 |
| 64918 | 1.89226 | 2.1 |
| 64919 | 3.78453 | 2.1 |
| 64920 | 1.26097 | 2.1 |
| 64921 | 1.26097 | 2.1 |
| 64922 | 0.946132 | 2.1 |
| 64923 | 0.946132 | 2.1 |
| 64924 | 1.22176 | 2.1 |
| 64925 | 1.26097 | 2.1 |
| 64926 | 0.946132 | 2.1 |
| 64927 | 0.946132 | 2.1 |
| 64928 | 0.946132 | 2.1 |
| 64929 | 1.22176 | 2.1 |
| 64930 | 1.26097 | 2.1 |
| 64931 | 0.946132 | 2.1 |
| 64932 | 2.52194 | 2.1 |
| 64933 | 1.67381 | 2.1 |
| 64934 | 1.67381 | 2.1 |
| 64935 | 1.75067 | 2.1 |
| 64936 | 2.44352 | 2.1 |
| 64937 | 2.52038 | 2.1 |
| 64938 | 1.89226 | 2.1 |
| 64939 | 1.89226 | 2.1 |
| 64940 | 1.89226 | 2.1 |
| 64941 | 1.89226 | 2.1 |
| 64942 | 1.89226 | 2.1 |
| 64943 | 1.89226 | 2.1 |
| 64944 | 1.89227 | 2.1 |
| 64945 | 1.89227 | 2.1 |
| 64946 | 1.89227 | 2.1 |
| 64947 | 1.89227 | 2.1 |
| 64948 | 1.89227 | 2.1 |
| 64949 | 1.89227 | 2.1 |
| 64950 | 1.22176 | 2.1 |
| 64951 | 1.22176 | 2.1 |
| 64952 | 0.946132 | 2.1 |
| 64953 | 0.946132 | 2.1 |
| 64954 | 1.19095 | 2.1 |
| 64955 | 1.22176 | 2.1 |
| 64956 | 0.946133 | 2.1 |
| 64957 | 0.946133 | 2.1 |

| | | |
|---|---|---|
| 64958 | 1.19095 | 2.1 |
| 64959 | 0.946132 | 2.1 |
| 64960 | 0.946133 | 2.1 |
| 64961 | 1.22176 | 2.1 |
| 64962 | 2.44352 | 2.1 |
| 64963 | 1.6287 | 2.1 |
| 64964 | 1.6287 | 2.1 |
| 64965 | 1.67381 | 2.1 |
| 64966 | 2.3819 | 2.1 |
| 64967 | 2.427 | 2.1 |
| 64968 | 1.89226 | 2.1 |
| 64969 | 1.89226 | 2.1 |
| 64970 | 1.89226 | 2.1 |
| 64971 | 1.89226 | 2.1 |
| 64972 | 1.89226 | 2.1 |
| 64973 | 1.89226 | 2.1 |
| 64974 | 1.89226 | 2.1 |
| 64975 | 1.89226 | 2.1 |
| 64976 | 1.89226 | 2.1 |
| 64977 | 1.89226 | 2.1 |
| 64978 | 3.78453 | 2.1 |
| 64979 | 1.89226 | 2.1 |
| 64980 | 1.89226 | 2.1 |
| 64981 | 1.89226 | 2.1 |
| 64982 | 1.89226 | 2.1 |
| 64983 | 1.89226 | 2.1 |
| 64984 | 1.89226 | 2.1 |
| 64985 | 1.89226 | 2.1 |
| 64986 | 1.89226 | 2.1 |
| 64987 | 1.89226 | 2.1 |
| 64988 | 1.89226 | 2.1 |
| 64989 | 1.89226 | 2.1 |
| 64990 | 1.89226 | 2.1 |
| 64991 | 0.851542 | 2.1 |
| 64992 | 0.851542 | 2.1 |
| 64993 | 1.1736 | 2.1 |
| 64994 | 1.29261 | 2.1 |
| 64995 | 0.852708 | 2.1 |
| 64996 | 1.18843 | 2.1 |
| 64997 | 0.946132 | 2.1 |
| 64998 | 0.946132 | 2.1 |
| 64999 | 0.946132 | 2.1 |
| 65000 | 1.07901 | 2.1 |
| 65001 | 1.04208 | 2.1 |
| 65002 | 0.936669 | 2.1 |
| 65003 | 1.89227 | 2.1 |
| 65004 | 1.89227 | 2.1 |
| 65005 | 1.89227 | 2.1 |
| 65006 | 1.89227 | 2.1 |
| 65007 | 3.78453 | 2.1 |
| 65008 | 1.89226 | 2.1 |
| 65009 | 1.89226 | 2.1 |
| 65010 | 1.89226 | 2.1 |
| 65011 | 1.89226 | 2.1 |
| 65012 | 3.78453 | 2.1 |
| 65013 | 1.89226 | 2.1 |
| 65014 | 1.89226 | 2.1 |
| 65015 | 1.89226 | 2.1 |

| | | |
|---|---|---|
| 65016 | 1.89226 | 2.1 |
| 65017 | 1.89226 | 2.1 |
| 65018 | 1.89226 | 2.1 |
| 65019 | 0.946133 | 2.1 |
| 65020 | 0.946133 | 2.1 |
| 65021 | 1.28525 | 2.1 |
| 65022 | 1.28525 | 2.1 |
| 65023 | 1.07901 | 2.1 |
| 65024 | 1.28525 | 2.1 |
| 65025 | 0.946132 | 2.1 |
| 65026 | 0.946132 | 2.1 |
| 65027 | 0.946132 | 2.1 |
| 65028 | 1.28525 | 2.1 |
| 65029 | 0.946133 | 2.1 |
| 65030 | 1.07901 | 2.1 |
| 65031 | 1.89226 | 2.1 |
| 65032 | 1.89226 | 2.1 |
| 65033 | 1.89226 | 2.1 |
| 65034 | 1.89226 | 2.1 |
| 65035 | 1.89226 | 2.1 |
| 65036 | 1.89226 | 2.1 |
| 65037 | 1.89226 | 2.1 |
| 65038 | 1.89226 | 2.1 |
| 65039 | 1.89226 | 2.1 |
| 65040 | 1.89226 | 2.1 |
| 65041 | 1.89226 | 2.1 |
| 65042 | 1.89226 | 2.1 |
| 65043 | 1.24419 | 2.1 |
| 65044 | 1.09093 | 2.1 |
| 65045 | 1.22722 | 2.1 |
| 65046 | 1.18154 | 2.1 |
| 65047 | 1.27455 | 2.1 |
| 65048 | 1.08473 | 2.1 |
| 65049 | 1.15541 | 2.1 |
| 65050 | 1.15541 | 2.1 |
| 65051 | 1.29607 | 2.1 |
| 65052 | 1.23814 | 2.1 |
| 65053 | 1.13024 | 2.1 |
| 65054 | 1.12096 | 2.1 |
| 65055 | 1.18858 | 2.1 |
| 65056 | 1.13181 | 2.1 |
| 65057 | 0.988718 | 2.1 |
| 65058 | 0.802498 | 2.1 |
| 65059 | 0.994034 | 2.1 |
| 65060 | 1.15773 | 2.1 |
| 65061 | 1.0029 | 2.1 |
| 65062 | 1.0029 | 2.1 |
| 65063 | 1.23814 | 2.1 |
| 65064 | 0.997523 | 2.1 |
| 65065 | 1.11498 | 2.1 |
| 65066 | 0.899532 | 2.1 |
| 65067 | 1.89226 | 2.1 |
| 65068 | 1.89226 | 2.1 |
| 65069 | 1.89226 | 2.1 |
| 65070 | 1.89226 | 2.1 |
| 65071 | 1.89226 | 2.1 |
| 65072 | 1.89226 | 2.1 |
| 65073 | 1.89226 | 2.1 |

| | | |
|---|---|---|
| 65074 | 1.89226 | 2.1 |
| 65075 | 1.89226 | 2.1 |
| 65076 | 1.89226 | 2.1 |
| 65077 | 1.89226 | 2.1 |
| 65078 | 1.89226 | 2.1 |
| 65079 | 0.993139 | 2.1 |
| 65080 | 0.834108 | 2.1 |
| 65081 | 1.01147 | 2.1 |
| 65082 | 1.01358 | 2.1 |
| 65083 | 1.20067 | 2.1 |
| 65084 | 0.993156 | 2.1 |
| 65085 | 2.4158 | 2.1 |
| 65086 | 1.69956 | 2.1 |
| 65087 | 1.3544 | 2.1 |
| 65088 | 1.60095 | 2.1 |
| 65089 | 1.11219 | 2.1 |
| 65090 | 1.89226 | 2.1 |
| 65091 | 1.89226 | 2.1 |
| 65092 | 1.89226 | 2.1 |
| 65093 | 1.89226 | 2.1 |
| 65094 | 3.78452 | 2.1 |
| 65095 | 1.89226 | 2.1 |
| 65096 | 1.89226 | 2.1 |
| 65097 | 1.89226 | 2.1 |
| 65098 | 1.89226 | 2.1 |
| 65099 | 1.89226 | 2.1 |
| 65100 | 1.89226 | 2.1 |
| 65101 | 1.89226 | 2.1 |
| 65102 | 2.83014 | 2.1 |
| 65103 | 2.32404 | 2.1 |
| 65104 | 2.32404 | 2.1 |
| 65105 | 1.89226 | 2.1 |
| 65106 | 1.89226 | 2.1 |
| 65107 | 1.37966 | 2.1 |
| 65108 | 1.13294 | 2.1 |
| 65109 | 0.881723 | 2.1 |
| 65110 | 0.582304 | 2.1 |
| 65111 | 0.977944 | 2.1 |
| 65112 | 0.977948 | 2.1 |
| 65113 | 1.0264 | 2.1 |
| 65114 | 1.0264 | 2.1 |
| 65115 | 1.37967 | 2.1 |
| 65116 | 1.03896 | 2.1 |
| 65117 | 1.19108 | 2.1 |
| 65118 | 0.582298 | 2.1 |
| 65119 | 1.89226 | 2.1 |
| 65120 | 1.89226 | 2.1 |
| 65121 | 1.89226 | 2.1 |
| 65122 | 1.89226 | 2.1 |
| 65123 | 3.78452 | 2.1 |
| 65124 | 0.946131 | 2.1 |
| 65125 | 0.946131 | 2.1 |
| 65126 | 0.946131 | 2.1 |
| 65127 | 0.946131 | 2.1 |
| 65128 | 0.946132 | 2.1 |
| 65129 | 0.946132 | 2.1 |
| 65130 | 0.946129 | 2.1 |
| 65131 | 0.946129 | 2.1 |

| | | |
|---|---|---|
| 65132 | 0.946131 | 2.1 |
| 65133 | 0.946131 | 2.1 |
| 65134 | 0.94613 | 2.1 |
| 65135 | 0.94613 | 2.1 |
| 65136 | 1.89226 | 2.1 |
| 65137 | 1.89226 | 2.1 |
| 65138 | 1.89226 | 2.1 |
| 65139 | 1.89226 | 2.1 |
| 65140 | 3.78453 | 2.1 |
| 65141 | 1.89226 | 2.1 |
| 65142 | 1.89226 | 2.1 |
| 65143 | 1.89226 | 2.1 |
| 65144 | 1.89226 | 2.1 |
| 65145 | 1.89226 | 2.1 |
| 65146 | 1.89226 | 2.1 |
| 65147 | 2.65216 | 2.1 |
| 65148 | 1.93528 | 2.1 |
| 65149 | 1.78595 | 2.1 |
| 65150 | 2.48544 | 2.1 |
| 65151 | 4.42072 | 2.1 |
| 65152 | 1.89226 | 2.1 |
| 65153 | 1.89226 | 2.1 |
| 65154 | 1.89226 | 2.1 |
| 65155 | 1.89226 | 2.1 |
| 65156 | 1.89226 | 2.1 |
| 65157 | 1.89226 | 2.1 |
| 65158 | 1.89226 | 2.1 |
| 65159 | 1.89226 | 2.1 |
| 65160 | 1.89226 | 2.1 |
| 65161 | 1.89226 | 2.1 |
| 65162 | 3.78452 | 2.1 |
| 65163 | 1.66537 | 2.1 |
| 65164 | 2.48544 | 2.1 |
| 65165 | 1.78595 | 2.1 |
| 65166 | 1.78595 | 2.1 |
| 65167 | 2.36197 | 2.1 |
| 65168 | 2.36197 | 2.1 |
| 65169 | 2.80975 | 2.1 |
| 65170 | 2.48544 | 2.1 |
| 65171 | 2.36198 | 2.1 |
| 65172 | 2.65135 | 2.1 |
| 65173 | 5.13679 | 2.1 |
| 65174 | 1.89226 | 2.1 |
| 65175 | 1.89226 | 2.1 |
| 65176 | 1.89226 | 2.1 |
| 65177 | 1.89226 | 2.1 |
| 65178 | 1.89226 | 2.1 |
| 65179 | 1.89226 | 2.1 |
| 65180 | 0.946127 | 2.1 |
| 65181 | 0.946127 | 2.1 |
| 65182 | 0.946131 | 2.1 |
| 65183 | 0.946131 | 2.1 |
| 65184 | 0.946128 | 2.1 |
| 65185 | 0.946128 | 2.1 |
| 65186 | 0.94613 | 2.1 |
| 65187 | 0.94613 | 2.1 |
| 65188 | 0.946129 | 2.1 |
| 65189 | 0.946129 | 2.1 |

| | | |
|---|---|---|
| 65190 | 0.946129 | 2.1 |
| 65191 | 0.946129 | 2.1 |
| 65192 | 1.11972 | 2.1 |
| 65193 | 1.18099 | 2.1 |
| 65194 | 0.832687 | 2.1 |
| 65195 | 0.764517 | 2.1 |
| 65196 | 0.974426 | 2.1 |
| 65197 | 0.974426 | 2.1 |
| 65198 | 0.971078 | 2.1 |
| 65199 | 0.971078 | 2.1 |
| 65200 | 1.11972 | 2.1 |
| 65201 | 0.764515 | 2.1 |
| 65202 | 0.832686 | 2.1 |
| 65203 | 1.18099 | 2.1 |
| 65204 | 2.36198 | 2.1 |
| 65205 | 2.48025 | 2.1 |
| 65206 | 2.48025 | 2.1 |
| 65207 | 2.65135 | 2.1 |
| 65208 | 2.23944 | 2.1 |
| 65209 | 2.41055 | 2.1 |
| 65210 | 0.946132 | 2.1 |
| 65211 | 0.946132 | 2.1 |
| 65212 | 0.946131 | 2.1 |
| 65213 | 0.946131 | 2.1 |
| 65214 | 0.946131 | 2.1 |
| 65215 | 0.946131 | 2.1 |
| 65216 | 0.946132 | 2.1 |
| 65217 | 0.946132 | 2.1 |
| 65218 | 0.946131 | 2.1 |
| 65219 | 0.946131 | 2.1 |
| 65220 | 0.946132 | 2.1 |
| 65221 | 0.946132 | 2.1 |
| 65222 | 0.810942 | 2.1 |
| 65223 | 0.810942 | 2.1 |
| 65224 | 0.810944 | 2.1 |
| 65225 | 0.810944 | 2.1 |
| 65226 | 1.89226 | 2.1 |
| 65227 | 1.3517 | 2.1 |
| 65228 | 1.08132 | 2.1 |
| 65229 | 1.08133 | 2.1 |
| 65230 | 1.08132 | 2.1 |
| 65231 | 0.810932 | 2.1 |
| 65232 | 0.810932 | 2.1 |
| 65233 | 1.89249 | 2.1 |
| 65234 | 1.9857 | 1.77409 |
| 65235 | 2.02433 | 1.84264 |
| 65236 | 2.15807 | 1.67552 |
| 65237 | 4.01003 | 1.88402 |
| 65238 | 1.89226 | 1.42828 |
| 65239 | 1.89226 | 2.04885 |
| 65240 | 1.89226 | 2.1 |
| 65241 | 1.89226 | 1.07704 |
| 65242 | 3.78452 | 2.03321 |
| 65243 | 1.89226 | 1.87614 |
| 65244 | 1.89226 | 2.00125 |
| 65245 | 1.89226 | 0.893252 |
| 65246 | 1.89226 | 1.6078 |
| 65247 | 3.78452 | 1.09628 |

| | | |
|---|---|---|
| 65248 | 1.98569 | 1.45067 |
| 65249 | 1.86067 | 1.85564 |
| 65250 | 2.15807 | 1.31563 |
| 65251 | 2.07965 | 1.26177 |
| 65252 | 4.06534 | 2.05349 |
| 65253 | 2.02433 | 2.1 |
| 65254 | 2.15807 | 2.1 |
| 65255 | 2.12608 | 2.1 |
| 65256 | 2.3025 | 1.14038 |
| 65257 | 4.32683 | 2.1 |
| 65258 | 1.89226 | 2.1 |
| 65259 | 1.89226 | 1.60004 |
| 65260 | 1.89226 | 1.12935 |
| 65261 | 1.89226 | 1.23884 |
| 65262 | 3.78452 | 1.27475 |
| 65263 | 1.89226 | 1.15111 |
| 65264 | 1.89226 | 2.1 |
| 65265 | 1.89226 | 1.24082 |
| 65266 | 1.89226 | 1.26028 |
| 65267 | 3.78452 | 2.08928 |
| 65268 | 2.15807 | 2.09038 |
| 65269 | 2.07965 | 1.89465 |
| 65270 | 2.3025 | 1.08422 |
| 65271 | 2.28384 | 1.34604 |
| 65272 | 4.38215 | 1.17134 |
| 65273 | 1.89226 | 0.734022 |
| 65274 | 1.89226 | 0.18974 |
| 65275 | 1.89226 | 0.553096 |
| 65276 | 1.89226 | 0.05 |
| 65277 | 3.78453 | 0.05 |
| 65278 | 2.16905 | 0.267789 |
| 65279 | 2.56369 | 0.162205 |
| 65280 | 1.88394 | 0.215621 |
| 65281 | 2.41938 | 0.05 |
| 65282 | 4.44763 | 0.0613935 |
| 65283 | 2.56369 | 0.344203 |
| 65284 | 2.80663 | 0.188469 |
| 65285 | 2.41938 | 0.05 |
| 65286 | 2.69045 | 0.05 |
| 65287 | 5.25414 | 0.05 |
| 65288 | 1.89226 | 0.362987 |
| 65289 | 1.89226 | 0.356032 |
| 65290 | 1.89226 | 0.176266 |
| 65291 | 1.89226 | 0.05 |
| 65292 | 3.78453 | 0.231134 |
| 65293 | 1.89226 | 0.176482 |
| 65294 | 1.89226 | 0.0877321 |
| 65295 | 1.89226 | 0.705274 |
| 65296 | 1.89226 | 0.423014 |
| 65297 | 1.89226 | 0.313534 |
| 65298 | 1.89226 | 0.05 |
| 65299 | 1.01988 | 0.05 |
| 65300 | 1.01988 | 0.05 |
| 65301 | 1.03333 | 0.05 |
| 65302 | 1.03333 | 0.180503 |
| 65303 | 1.13811 | 0.05 |
| 65304 | 1.20969 | 0.05 |
| 65305 | 0.941974 | 0.05 |

| | | |
|---|---|---|
| 65306 | 0.843515 | 0.05 |
| 65307 | 0.843514 | 0.05 |
| 65308 | 1.13811 | 0.05 |
| 65309 | 0.941973 | 0.0551873 |
| 65310 | 1.20969 | 0.05 |
| 65311 | 2.41938 | 0.05 |
| 65312 | 2.57414 | 0.0856761 |
| 65313 | 2.57414 | 0.444307 |
| 65314 | 2.69045 | 0.329177 |
| 65315 | 2.27623 | 0.05 |
| 65316 | 2.39253 | 0.05 |
| 65317 | 1.89227 | 0.416083 |
| 65318 | 1.89227 | 0.05 |
| 65319 | 1.89227 | 0.292302 |
| 65320 | 1.89227 | 0.295527 |
| 65321 | 1.89227 | 0.05 |
| 65322 | 1.89227 | 0.0670272 |
| 65323 | 2.28385 | 1.49762 |
| 65324 | 2.09114 | 1.75911 |
| 65325 | 2.46968 | 0.89587 |
| 65326 | 2.32438 | 1.4467 |
| 65327 | 4.56082 | 0.984585 |
| 65328 | 1.89226 | 0.994027 |
| 65329 | 1.89226 | 2.1 |
| 65330 | 1.89226 | 1.12049 |
| 65331 | 1.89226 | 1.83948 |
| 65332 | 3.78452 | 1.62378 |
| 65333 | 1.89226 | 1.05234 |
| 65334 | 1.89226 | 2.1 |
| 65335 | 1.89226 | 0.507682 |
| 65336 | 1.89226 | 1.67321 |
| 65337 | 3.78452 | 1.25916 |
| 65338 | 2.09114 | 0.915978 |
| 65339 | 1.5249 | 2.1 |
| 65340 | 2.55684 | 0.926558 |
| 65341 | 1.86899 | 1.89165 |
| 65342 | 3.96011 | 1.34686 |
| 65343 | 2.46968 | 1.11527 |
| 65344 | 2.32439 | 1.97462 |
| 65345 | 2.62768 | 1.11854 |
| 65346 | 2.506 | 1.46325 |
| 65347 | 4.97568 | 2.1 |
| 65348 | 1.89226 | 1.48282 |
| 65349 | 1.89226 | 1.7594 |
| 65350 | 1.89226 | 0.758535 |
| 65351 | 1.89226 | 1.54477 |
| 65352 | 3.78452 | 1.02375 |
| 65353 | 1.89226 | 0.975585 |
| 65354 | 1.89226 | 1.81916 |
| 65355 | 1.89226 | 1.1505 |
| 65356 | 1.89226 | 2.1 |
| 65357 | 1.89226 | 0.202005 |
| 65358 | 1.89226 | 2.1 |
| 65359 | 2.55684 | 1.13983 |
| 65360 | 2.16506 | 1.65807 |
| 65361 | 1.86901 | 1.92828 |
| 65362 | 1.86899 | 1.13243 |
| 65363 | 2.75664 | 1.26986 |

| | | |
|---|---|---|
| 65364 | 2.75662 | 0.970971 |
| 65365 | 1.89224 | 0.05 |
| 65366 | 1.89224 | 0.149667 |
| 65367 | 1.89224 | 0.05 |
| 65368 | 1.89224 | 0.331611 |
| 65369 | 3.78447 | 0.05 |
| 65370 | 2.24206 | 0.05 |
| 65371 | 2.38862 | 0.169196 |
| 65372 | 2.07107 | 0.05 |
| 65373 | 2.23097 | 0.05 |
| 65374 | 4.47303 | 0.05 |
| 65375 | 2.38863 | 0.177762 |
| 65376 | 2.25945 | 2.1 |
| 65377 | 2.25945 | 0.05 |
| 65378 | 2.39244 | 2.1 |
| 65379 | 2.23098 | 0.05 |
| 65380 | 2.36397 | 2.1 |
| 65381 | 1.89224 | 0.05 |
| 65382 | 1.89224 | 2.1 |
| 65383 | 1.89224 | 0.156561 |
| 65384 | 1.89224 | 0.05 |
| 65385 | 1.89224 | 0.170689 |
| 65386 | 1.89224 | 0.457199 |
| 65387 | 1.89226 | 0.05 |
| 65388 | 1.89226 | 0.05 |
| 65389 | 1.89226 | 0.188068 |
| 65390 | 1.89226 | 0.05 |
| 65391 | 1.89226 | 0.05 |
| 65392 | 1.89226 | 0.05 |
| 65393 | 0.920975 | 1.12779 |
| 65394 | 0.920979 | 0.05 |
| 65395 | 1.03015 | 0.05 |
| 65396 | 1.03015 | 0.05 |
| 65397 | 0.992407 | 0.05 |
| 65398 | 1.08759 | 0.703159 |
| 65399 | 0.854346 | 0.05 |
| 65400 | 0.87241 | 0.05 |
| 65401 | 0.872397 | 0.05 |
| 65402 | 1.04334 | 0.05 |
| 65403 | 1.00965 | 0.05 |
| 65404 | 1.0876 | 0.05 |
| 65405 | 2.23101 | 0.56516 |
| 65406 | 2.25948 | 2.1 |
| 65407 | 2.03577 | 0.05 |
| 65408 | 2.05257 | 2.1 |
| 65409 | 4.29524 | 1.75173 |
| 65410 | 0.810947 | 0.05 |
| 65411 | 0.810947 | 0.121614 |
| 65412 | 0.810947 | 0.05 |
| 65413 | 0.810947 | 0.05 |
| 65414 | 0.810934 | 0.05 |
| 65415 | 0.810934 | 0.05 |
| 65416 | 1.89227 | 2.1 |
| 65417 | 1.3517 | 0.11934 |
| 65418 | 1.08132 | 0.067978 |
| 65419 | 1.08132 | 0.172105 |
| 65420 | 1.08133 | 0.05 |
| 65421 | 1.89226 | 1.63963 |

| | | |
|---|---|---|
| 65422 | 1.89226 | 2.1 |
| 65423 | 1.89226 | 1.00392 |
| 65424 | 1.89226 | 2.1 |
| 65425 | 1.89226 | 0.0667421 |
| 65426 | 1.89226 | 2.1 |
| 65427 | 1.89226 | 2.1 |
| 65428 | 1.89226 | 2.1 |
| 65429 | 1.89226 | 0.751054 |
| 65430 | 1.89226 | 2.1 |
| 65431 | 1.89226 | 0.437719 |
| 65432 | 1.89226 | 2.1 |
| 65433 | 0.879432 | 2.1 |
| 65434 | 0.879432 | 1.21541 |
| 65435 | 0.900223 | 0.0944976 |
| 65436 | 0.900223 | 2.1 |
| 65437 | 0.54571 | 2.1 |
| 65438 | 0.743092 | 2.0408 |
| 65439 | 1.03656 | 2.1 |
| 65440 | 1.19234 | 0.662002 |
| 65441 | 1.19234 | 1.86885 |
| 65442 | 0.743092 | 2.1 |
| 65443 | 1.03656 | 2.1 |
| 65444 | 0.545711 | 2.00237 |
| 65445 | 1.89226 | 0.818321 |
| 65446 | 2.38467 | 2.04711 |
| 65447 | 2.69293 | 0.851704 |
| 65448 | 2.69293 | 2.1 |
| 65449 | 1.89226 | 0.471241 |
| 65450 | 1.89226 | 2.00722 |
| 65451 | 1.89226 | 1.04783 |
| 65452 | 1.89226 | 2.0098 |
| 65453 | 1.89226 | 0.873217 |
| 65454 | 1.89226 | 2.1 |
| 65455 | 3.78452 | 1.31162 |
| 65456 | 0.946129 | 2.1 |
| 65457 | 0.946129 | 2.1 |
| 65458 | 0.946133 | 0.0863653 |
| 65459 | 0.946133 | 2.1 |
| 65460 | 0.946131 | 1.94843 |
| 65461 | 0.946131 | 2.1 |
| 65462 | 0.946131 | 0.0967792 |
| 65463 | 0.946131 | 1.87626 |
| 65464 | 0.946131 | 1.98158 |
| 65465 | 0.946131 | 1.98102 |
| 65466 | 0.946131 | 1.89243 |
| 65467 | 0.946131 | 1.67951 |
| 65468 | 1.04846 | 0.05 |
| 65469 | 1.04846 | 2.1 |
| 65470 | 1.05594 | 0.248208 |
| 65471 | 1.05594 | 2.1 |
| 65472 | 0.743092 | 2.1 |
| 65473 | 0.912061 | 1.53672 |
| 65474 | 1.19234 | 0.324153 |
| 65475 | 1.34647 | 0.546796 |
| 65476 | 0.912061 | 0.163082 |
| 65477 | 1.34646 | 1.14275 |
| 65478 | 1.19234 | 0.924048 |
| 65479 | 0.743093 | 0.990864 |

| | | |
|---|---|---|
| 65480 | 1.89226 | 0.982493 |
| 65481 | 1.89226 | 2.1 |
| 65482 | 1.89226 | 0.05 |
| 65483 | 1.89226 | 1.1277 |
| 65484 | 3.78452 | 1.12688 |
| 65485 | 0.946128 | 2.1 |
| 65486 | 0.946128 | 1.34291 |
| 65487 | 0.946133 | 2.09755 |
| 65488 | 0.946133 | 0.279098 |
| 65489 | 0.94613 | 2.1 |
| 65490 | 0.94613 | 2.1 |
| 65491 | 0.946131 | 1.85828 |
| 65492 | 0.946131 | 0.361543 |
| 65493 | 0.946131 | 2.1 |
| 65494 | 0.946131 | 2.1 |
| 65495 | 0.946131 | 1.88125 |
| 65496 | 0.946131 | 1.98969 |
| 65497 | 1.89226 | 1.32438 |
| 65498 | 1.89226 | 2.1 |
| 65499 | 1.89226 | 0.952413 |
| 65500 | 1.89226 | 1.82329 |
| 65501 | 3.78452 | 1.98992 |
| 65502 | 1.89226 | 1.23421 |
| 65503 | 1.89226 | 1.67705 |
| 65504 | 1.89226 | 1.06139 |
| 65505 | 1.89226 | 2.1 |
| 65506 | 1.89226 | 0.0851638 |
| 65507 | 1.89226 | 2.1 |
| 65508 | 1.89226 | 1.18905 |
| 65509 | 1.89226 | 2.1 |
| 65510 | 1.89226 | 0.890728 |
| 65511 | 1.89226 | 2.1 |
| 65512 | 1.89226 | 0.267696 |
| 65513 | 1.89226 | 2.1 |
| 65514 | 0.946132 | 2.1 |
| 65515 | 0.946132 | 1.26219 |
| 65516 | 1.17758 | 0.842659 |
| 65517 | 1.17759 | 2.1 |
| 65518 | 1.04514 | 2.1 |
| 65519 | 1.17758 | 1.15216 |
| 65520 | 0.946133 | 0.810079 |
| 65521 | 0.946133 | 0.998902 |
| 65522 | 0.946107 | 1.22462 |
| 65523 | 1.17755 | 1.23948 |
| 65524 | 1.04517 | 1.30785 |
| 65525 | 0.946158 | 1.18178 |
| 65526 | 1.89226 | 2.1 |
| 65527 | 2.10171 | 2.1 |
| 65528 | 1.95064 | 2.1 |
| 65529 | 2.10171 | 2.1 |
| 65530 | 1.7412 | 2.1 |
| 65531 | 1.89226 | 2.1 |
| 65532 | 1.89226 | 2.1 |
| 65533 | 1.89226 | 2.1 |
| 65534 | 1.89226 | 2.1 |
| 65535 | 1.89226 | 2.1 |
| 65536 | 1.89226 | 2.1 |
| 65537 | 1.89226 | 2.1 |

| | | |
|---|---|---|
| 65538 | 1.89226 | 2.1 |
| 65539 | 1.89226 | 0.870934 |
| 65540 | 1.89226 | 2.1 |
| 65541 | 1.89226 | 2.1 |
| 65542 | 3.78453 | 2.1 |
| 65543 | 0.946133 | 2.1 |
| 65544 | 0.946133 | 2.1 |
| 65545 | 0.946132 | 2.1 |
| 65546 | 0.946132 | 2.1 |
| 65547 | 0.946133 | 2.1 |
| 65548 | 0.946133 | 2.1 |
| 65549 | 0.946132 | 2.1 |
| 65550 | 0.946132 | 2.1 |
| 65551 | 0.946132 | 2.1 |
| 65552 | 0.946132 | 2.1 |
| 65553 | 0.946132 | 2.1 |
| 65554 | 0.946132 | 2.1 |
| 65555 | 1.70303 | 2.1 |
| 65556 | 1.95064 | 2.1 |
| 65557 | 1.74039 | 2.1 |
| 65558 | 1.95064 | 2.1 |
| 65559 | 1.69368 | 2.1 |
| 65560 | 1.89226 | 2.1 |
| 65561 | 1.89226 | 2.1 |
| 65562 | 1.89226 | 2.1 |
| 65563 | 1.89226 | 2.1 |
| 65564 | 1.89226 | 2.1 |
| 65565 | 3.78453 | 2.1 |
| 65566 | 0.946132 | 0.758694 |
| 65567 | 0.946132 | 2.1 |
| 65568 | 0.946132 | 2.1 |
| 65569 | 0.946132 | 2.1 |
| 65570 | 0.946133 | 2.1 |
| 65571 | 0.946133 | 0.888189 |
| 65572 | 0.946132 | 2.1 |
| 65573 | 0.946132 | 2.1 |
| 65574 | 0.946107 | 2.1 |
| 65575 | 0.946107 | 2.1 |
| 65576 | 0.946158 | 2.1 |
| 65577 | 0.946158 | 2.1 |
| 65578 | 0.946133 | 2.1 |
| 65579 | 0.946133 | 2.1 |
| 65580 | 0.946132 | 2.1 |
| 65581 | 0.946132 | 2.1 |
| 65582 | 0.946133 | 2.1 |
| 65583 | 0.946133 | 2.1 |
| 65584 | 0.946132 | 2.1 |
| 65585 | 0.946132 | 2.1 |
| 65586 | 0.946107 | 2.1 |
| 65587 | 0.946107 | 2.1 |
| 65588 | 0.946158 | 2.1 |
| 65589 | 0.946158 | 2.1 |
| 65590 | 1.89226 | 2.1 |
| 65591 | 1.89226 | 2.1 |
| 65592 | 1.89226 | 2.1 |
| 65593 | 1.89226 | 2.1 |
| 65594 | 1.89226 | 2.1 |
| 65595 | 1.89226 | 2.1 |

| | | |
|---|---|---|
| 65596 | 1.89226 | 2.1 |
| 65597 | 1.89226 | 2.1 |
| 65598 | 1.89226 | 2.1 |
| 65599 | 1.89226 | 2.1 |
| 65600 | 1.89226 | 2.1 |
| 65601 | 1.89226 | 2.1 |
| 65602 | 2.5198 | 2.1 |
| 65603 | 2.45893 | 2.1 |
| 65604 | 2.27322 | 2.1 |
| 65605 | 2.27322 | 2.1 |
| 65606 | 2.63576 | 2.1 |
| 65607 | 2.63576 | 2.1 |
| 65608 | 1.24045 | 2.1 |
| 65609 | 1.11906 | 2.1 |
| 65610 | 1.13578 | 2.1 |
| 65611 | 0.916205 | 2.1 |
| 65612 | 1.12539 | 2.1 |
| 65613 | 1.08404 | 2.1 |
| 65614 | 1.08484 | 2.1 |
| 65615 | 1.08484 | 2.1 |
| 65616 | 1.22836 | 2.1 |
| 65617 | 0.919363 | 2.1 |
| 65618 | 1.16507 | 2.1 |
| 65619 | 0.924864 | 2.1 |
| 65620 | 1.89226 | 2.1 |
| 65621 | 1.89226 | 2.1 |
| 65622 | 1.89226 | 1.82732 |
| 65623 | 1.89226 | 2.1 |
| 65624 | 1.89226 | 0.953411 |
| 65625 | 1.89226 | 2.1 |
| 65626 | 1.89226 | 2.1 |
| 65627 | 1.89226 | 2.1 |
| 65628 | 1.89226 | 2.1 |
| 65629 | 1.89226 | 2.1 |
| 65630 | 1.89226 | 2.1 |
| 65631 | 1.89226 | 2.1 |
| 65632 | 2.6946 | 2.1 |
| 65633 | 2.63576 | 2.1 |
| 65634 | 2.5198 | 2.1 |
| 65635 | 2.5198 | 2.1 |
| 65636 | 2.8362 | 2.1 |
| 65637 | 2.8362 | 2.1 |
| 65638 | 1.3265 | 2.1 |
| 65639 | 1.24045 | 2.1 |
| 65640 | 0.983223 | 2.1 |
| 65641 | 0.954429 | 2.1 |
| 65642 | 1.06046 | 2.1 |
| 65643 | 1.10594 | 2.1 |
| 65644 | 1.1915 | 2.1 |
| 65645 | 1.1915 | 2.1 |
| 65646 | 1.31357 | 2.1 |
| 65647 | 1.09005 | 2.1 |
| 65648 | 1.29145 | 2.1 |
| 65649 | 0.995825 | 2.1 |
| 65650 | 1.89226 | 2.1 |
| 65651 | 1.89226 | 2.1 |
| 65652 | 1.89226 | 2.1 |
| 65653 | 1.89226 | 2.1 |

| | | |
|---|---|---|
| 65654 | 3.78452 | 2.1 |
| 65655 | 1.89226 | 2.1 |
| 65656 | 1.89226 | 2.1 |
| 65657 | 1.89226 | 2.1 |
| 65658 | 1.89226 | 2.1 |
| 65659 | 3.78453 | 2.1 |
| 65660 | 1.91802 | 2.1 |
| 65661 | 2.27409 | 2.1 |
| 65662 | 2.48048 | 2.1 |
| 65663 | 2.22264 | 2.1 |
| 65664 | 1.91802 | 2.1 |
| 65665 | 1.66018 | 2.1 |
| 65666 | 1.13704 | 2.1 |
| 65667 | 1.24023 | 2.1 |
| 65668 | 1.22947 | 2.1 |
| 65669 | 1.31788 | 2.1 |
| 65670 | 1.23859 | 2.1 |
| 65671 | 1.23859 | 2.1 |
| 65672 | 1.23111 | 2.1 |
| 65673 | 1.23111 | 2.1 |
| 65674 | 1.24024 | 2.1 |
| 65675 | 1.31788 | 2.1 |
| 65676 | 1.13704 | 2.1 |
| 65677 | 1.22947 | 2.1 |
| 65678 | 1.89226 | 2.1 |
| 65679 | 1.89226 | 2.1 |
| 65680 | 1.89226 | 2.1 |
| 65681 | 1.89226 | 2.1 |
| 65682 | 1.89226 | 2.1 |
| 65683 | 1.89226 | 2.1 |
| 65684 | 1.89226 | 2.1 |
| 65685 | 1.89226 | 2.1 |
| 65686 | 1.89226 | 2.1 |
| 65687 | 1.89226 | 2.1 |
| 65688 | 3.78453 | 2.1 |
| 65689 | 1.05568 | 2.1 |
| 65690 | 0.959008 | 2.1 |
| 65691 | 1.32068 | 2.1 |
| 65692 | 1.24024 | 2.1 |
| 65693 | 1.15206 | 2.1 |
| 65694 | 1.15206 | 2.1 |
| 65695 | 1.14386 | 2.1 |
| 65696 | 1.14386 | 2.1 |
| 65697 | 1.05568 | 2.1 |
| 65698 | 1.32068 | 2.1 |
| 65699 | 0.959008 | 2.1 |
| 65700 | 1.24024 | 2.1 |
| 65701 | 1.32068 | 2.1 |
| 65702 | 1.24023 | 2.1 |
| 65703 | 1.31788 | 2.1 |
| 65704 | 1.4181 | 2.1 |
| 65705 | 1.31436 | 2.1 |
| 65706 | 1.31436 | 2.1 |
| 65707 | 1.3242 | 2.1 |
| 65708 | 1.3242 | 2.1 |
| 65709 | 1.32068 | 2.1 |
| 65710 | 1.4181 | 2.1 |
| 65711 | 1.24024 | 2.1 |

| | | |
|---|---|---|
| 65712 | 1.31788 | 2.1 |
| 65713 | 1.89226 | 1.36383 |
| 65714 | 1.89226 | 0.05 |
| 65715 | 1.89226 | 0.535689 |
| 65716 | 1.89226 | 0.05 |
| 65717 | 1.89226 | 0.355748 |
| 65718 | 1.89226 | 0.582291 |
| 65719 | 1.02925 | 0.05 |
| 65720 | 1.16678 | 0.47329 |
| 65721 | 0.946132 | 0.05 |
| 65722 | 0.946132 | 0.632572 |
| 65723 | 0.935677 | 0.527533 |
| 65724 | 0.935677 | 0.05 |
| 65725 | 1.18902 | 0.101123 |
| 65726 | 1.21562 | 0.606718 |
| 65727 | 1.13433 | 0.157899 |
| 65728 | 1.04071 | 0.355872 |
| 65729 | 0.851553 | 0.181534 |
| 65730 | 1.06075 | 0.05 |
| 65731 | 1.89226 | 0.523242 |
| 65732 | 1.89226 | 0.05 |
| 65733 | 1.89226 | 0.216329 |
| 65734 | 1.89226 | 0.05 |
| 65735 | 1.89226 | 0.05 |
| 65736 | 1.89226 | 0.391177 |
| 65737 | 1.89226 | 0.719458 |
| 65738 | 1.89226 | 0.05 |
| 65739 | 1.89226 | 0.05 |
| 65740 | 1.89226 | 0.106949 |
| 65741 | 3.78453 | 0.05 |
| 65742 | 0.946128 | 0.582049 |
| 65743 | 0.946128 | 0.168475 |
| 65744 | 0.946133 | 0.199133 |
| 65745 | 0.946133 | 0.05 |
| 65746 | 0.946131 | 0.078484 |
| 65747 | 0.946131 | 0.0947878 |
| 65748 | 0.94613 | 0.555762 |
| 65749 | 0.94613 | 0.114036 |
| 65750 | 0.946131 | 0.503646 |
| 65751 | 0.946131 | 0.445277 |
| 65752 | 0.946131 | 0.05 |
| 65753 | 0.946131 | 0.05 |
| 65754 | 0.946132 | 0.630654 |
| 65755 | 0.946132 | 0.0762343 |
| 65756 | 0.946132 | 0.672176 |
| 65757 | 0.946132 | 0.05 |
| 65758 | 0.946132 | 0.510693 |
| 65759 | 0.946132 | 0.05 |
| 65760 | 0.946131 | 0.547176 |
| 65761 | 0.946131 | 0.241459 |
| 65762 | 0.946132 | 0.089186 |
| 65763 | 0.946132 | 0.648686 |
| 65764 | 0.946132 | 0.05 |
| 65765 | 0.946132 | 0.376004 |
| 65766 | 0.946131 | 2.1 |
| 65767 | 0.946131 | 1.19893 |
| 65768 | 0.946131 | 2.1 |
| 65769 | 0.946131 | 0.854485 |

| | | |
|---|---|---|
| 65770 | 0.946131 | 0.990192 |
| 65771 | 0.946131 | 2.1 |
| 65772 | 0.946131 | 0.97319 |
| 65773 | 0.946131 | 0.808137 |
| 65774 | 0.946131 | 1.14089 |
| 65775 | 0.946131 | 1.14026 |
| 65776 | 0.946131 | 1.23895 |
| 65777 | 0.946131 | 1.21676 |
| 65778 | 1.89226 | 1.0597 |
| 65779 | 1.89226 | 1.35754 |
| 65780 | 1.89226 | 0.868908 |
| 65781 | 1.89226 | 2.1 |
| 65782 | 1.89226 | 0.641214 |
| 65783 | 1.89226 | 2.1 |
| 65784 | 1.89226 | 1.01709 |
| 65785 | 1.89226 | 1.01925 |
| 65786 | 1.89226 | 1.07734 |
| 65787 | 1.89226 | 2.1 |
| 65788 | 1.89226 | 0.852638 |
| 65789 | 1.89226 | 2.1 |
| 65790 | 1.89226 | 1.16334 |
| 65791 | 1.89226 | 1.03115 |
| 65792 | 1.89226 | 0.737938 |
| 65793 | 1.89226 | 2.1 |
| 65794 | 1.89226 | 0.0982603 |
| 65795 | 1.89226 | 2.1 |
| 65796 | 1.89226 | 2.1 |
| 65797 | 1.89226 | 2.1 |
| 65798 | 1.89226 | 0.415004 |
| 65799 | 1.89226 | 2.1 |
| 65800 | 3.78452 | 1.82787 |
| 65801 | 0.946131 | 2.1 |
| 65802 | 0.946131 | 0.609374 |
| 65803 | 1.17195 | 0.851644 |
| 65804 | 1.17195 | 2.1 |
| 65805 | 0.958489 | 2.1 |
| 65806 | 1.17195 | 1.4956 |
| 65807 | 0.946129 | 0.87458 |
| 65808 | 0.946129 | 1.37282 |
| 65809 | 0.946131 | 1.23778 |
| 65810 | 1.17195 | 1.25356 |
| 65811 | 0.958487 | 1.31874 |
| 65812 | 0.94613 | 1.29293 |
| 65813 | 2.69656 | 2.1 |
| 65814 | 2.17516 | 2.1 |
| 65815 | 2.41885 | 2.1 |
| 65816 | 2.41885 | 1.99715 |
| 65817 | 2.46476 | 2.1 |
| 65818 | 2.46476 | 0.930915 |
| 65819 | 2.69657 | 2.1 |
| 65820 | 2.30233 | 0.643451 |
| 65821 | 2.30233 | 1.8947 |
| 65822 | 2.59476 | 0.847082 |
| 65823 | 2.46477 | 1.95874 |
| 65824 | 2.7572 | 1.0038 |
| 65825 | 1.89226 | 2.1 |
| 65826 | 1.89226 | 0.483235 |
| 65827 | 1.89226 | 0.215941 |

| | | |
|---|---|---|
| 65828 | 1.89226 | 0.475614 |
| 65829 | 1.89226 | 0.199661 |
| 65830 | 1.89226 | 0.690275 |
| 65831 | 1.89226 | 1.95083 |
| 65832 | 1.89226 | 0.823476 |
| 65833 | 1.89226 | 2.1 |
| 65834 | 1.89226 | 0.134509 |
| 65835 | 3.78452 | 0.917486 |
| 65836 | 2.46477 | 2.1 |
| 65837 | 2.17516 | 2.1 |
| 65838 | 1.80252 | 2.1 |
| 65839 | 2.09715 | 0.688597 |
| 65840 | 4.27231 | 2.1 |
| 65841 | 2.46477 | 2.1 |
| 65842 | 2.09715 | 0.7316 |
| 65843 | 2.30233 | 0.858441 |
| 65844 | 2.30233 | 0.829436 |
| 65845 | 1.96416 | 0.069092 |
| 65846 | 2.09715 | 0.0835264 |
| 65847 | 1.89227 | 0.7138 |
| 65848 | 1.89227 | 0.05 |
| 65849 | 1.89227 | 0.252705 |
| 65850 | 1.89227 | 0.0713619 |
| 65851 | 1.89227 | 0.675511 |
| 65852 | 1.89227 | 0.814562 |
| 65853 | 1.89226 | 2.1 |
| 65854 | 1.89226 | 0.05 |
| 65855 | 1.89226 | 0.662984 |
| 65856 | 1.89226 | 0.229554 |
| 65857 | 3.78452 | 0.677836 |
| 65858 | 0.946132 | 2.1 |
| 65859 | 0.946132 | 2.1 |
| 65860 | 0.946132 | 2.1 |
| 65861 | 0.946132 | 2.1 |
| 65862 | 0.946132 | 2.1 |
| 65863 | 0.946132 | 2.1 |
| 65864 | 0.946132 | 2.1 |
| 65865 | 0.946132 | 2.1 |
| 65866 | 0.946132 | 2.1 |
| 65867 | 0.946132 | 2.1 |
| 65868 | 0.946131 | 2.1 |
| 65869 | 0.946131 | 2.1 |
| 65870 | 1.89226 | 2.1 |
| 65871 | 1.89226 | 2.1 |
| 65872 | 1.89226 | 2.1 |
| 65873 | 1.89226 | 2.1 |
| 65874 | 3.78453 | 2.1 |
| 65875 | 1.89226 | 2.1 |
| 65876 | 1.89226 | 2.1 |
| 65877 | 1.89226 | 2.1 |
| 65878 | 1.89226 | 2.1 |
| 65879 | 1.89226 | 2.1 |
| 65880 | 1.89226 | 2.1 |
| 65881 | 1.89226 | 2.1 |
| 65882 | 1.63707 | 2.1 |
| 65883 | 1.63707 | 2.1 |
| 65884 | 1.38859 | 2.1 |
| 65885 | 1.89226 | 2.1 |

| | | |
|---|---|---|
| 65886 | 1.64377 | 2.1 |
| 65887 | 0.94613 | 2.1 |
| 65888 | 0.94613 | 2.1 |
| 65889 | 0.94613 | 2.1 |
| 65890 | 0.94613 | 2.1 |
| 65891 | 0.946131 | 2.1 |
| 65892 | 0.946131 | 2.1 |
| 65893 | 0.946129 | 2.1 |
| 65894 | 0.946129 | 2.1 |
| 65895 | 0.94613 | 2.1 |
| 65896 | 0.94613 | 2.1 |
| 65897 | 0.94613 | 2.1 |
| 65898 | 0.94613 | 2.1 |
| 65899 | 2.15888 | 2.1 |
| 65900 | 1.99309 | 2.1 |
| 65901 | 2.16841 | 2.1 |
| 65902 | 2.16841 | 2.1 |
| 65903 | 2.02311 | 0.867349 |
| 65904 | 2.02311 | 0.798933 |
| 65905 | 1.89226 | 0.887164 |
| 65906 | 1.89226 | 2.05117 |
| 65907 | 1.89226 | 2.1 |
| 65908 | 1.89226 | 1.30458 |
| 65909 | 1.89226 | 2.1 |
| 65910 | 1.89226 | 0.767426 |
| 65911 | 1.89226 | 2.1 |
| 65912 | 1.89226 | 2.1 |
| 65913 | 1.89226 | 2.1 |
| 65914 | 1.89226 | 2.1 |
| 65915 | 1.89226 | 2.1 |
| 65916 | 1.89226 | 2.1 |
| 65917 | 1.99309 | 2.1 |
| 65918 | 1.96035 | 2.1 |
| 65919 | 1.72526 | 2.1 |
| 65920 | 1.72526 | 2.1 |
| 65921 | 2.16841 | 2.1 |
| 65922 | 2.16841 | 2.1 |
| 65923 | 2.15888 | 2.1 |
| 65924 | 2.16841 | 2.1 |
| 65925 | 2.3111 | 2.1 |
| 65926 | 2.35938 | 2.1 |
| 65927 | 4.51826 | 2.1 |
| 65928 | 1.89226 | 2.1 |
| 65929 | 1.89226 | 0.88575 |
| 65930 | 1.89226 | 0.796431 |
| 65931 | 1.89226 | 0.05 |
| 65932 | 3.78452 | 2.1 |
| 65933 | 1.89226 | 2.1 |
| 65934 | 1.89226 | 2.1 |
| 65935 | 1.89226 | 2.1 |
| 65936 | 1.89226 | 0.635476 |
| 65937 | 3.78452 | 2.1 |
| 65938 | 2.16841 | 2.1 |
| 65939 | 1.96035 | 2.1 |
| 65940 | 2.35938 | 2.1 |
| 65941 | 2.18493 | 2.1 |
| 65942 | 4.31973 | 2.1 |
| 65943 | 1.89226 | 2.1 |

| | | |
|---|---|---|
| 65944 | 1.89226 | 2.1 |
| 65945 | 1.89226 | 2.1 |
| 65946 | 1.89226 | 2.1 |
| 65947 | 3.78453 | 2.1 |
| 65948 | 1.89226 | 2.1 |
| 65949 | 1.89226 | 2.1 |
| 65950 | 1.89226 | 2.1 |
| 65951 | 1.89226 | 2.1 |
| 65952 | 3.78452 | 2.1 |
| 65953 | 0.946158 | 2.1 |
| 65954 | 0.946158 | 2.1 |
| 65955 | 0.818509 | 2.1 |
| 65956 | 0.818509 | 2.1 |
| 65957 | 0.933975 | 2.1 |
| 65958 | 0.818539 | 2.1 |
| 65959 | 0.946128 | 2.1 |
| 65960 | 0.946128 | 2.1 |
| 65961 | 0.94613 | 2.1 |
| 65962 | 0.933974 | 2.1 |
| 65963 | 0.818537 | 2.1 |
| 65964 | 0.946129 | 2.1 |
| 65965 | 0.946131 | 2.1 |
| 65966 | 0.946131 | 2.1 |
| 65967 | 0.946131 | 2.1 |
| 65968 | 0.946131 | 2.1 |
| 65969 | 0.946132 | 2.1 |
| 65970 | 0.946132 | 2.1 |
| 65971 | 0.946129 | 2.1 |
| 65972 | 0.946129 | 2.1 |
| 65973 | 0.946131 | 2.1 |
| 65974 | 0.946131 | 2.1 |
| 65975 | 0.94613 | 2.1 |
| 65976 | 0.94613 | 2.1 |
| 65977 | 1.89226 | 2.1 |
| 65978 | 1.89226 | 2.1 |
| 65979 | 1.89226 | 2.1 |
| 65980 | 1.89226 | 2.1 |
| 65981 | 3.78453 | 2.1 |
| 65982 | 1.89226 | 2.1 |
| 65983 | 1.89226 | 2.1 |
| 65984 | 1.89226 | 2.1 |
| 65985 | 1.89226 | 2.1 |
| 65986 | 3.78452 | 2.1 |
| 65987 | 1.89226 | 2.1 |
| 65988 | 1.8706 | 2.1 |
| 65989 | 1.8706 | 2.1 |
| 65990 | 1.68112 | 2.1 |
| 65991 | 2.0815 | 2.1 |
| 65992 | 1.88715 | 2.1 |
| 65993 | 0.94613 | 2.1 |
| 65994 | 0.94613 | 2.1 |
| 65995 | 0.94613 | 2.1 |
| 65996 | 0.94613 | 2.1 |
| 65997 | 0.946131 | 2.1 |
| 65998 | 0.946131 | 2.1 |
| 65999 | 0.946129 | 2.1 |
| 66000 | 0.946129 | 2.1 |
| 66001 | 0.94613 | 2.1 |

| | | |
|---|---|---|
| 66002 | 0.94613 | 2.1 |
| 66003 | 0.94613 | 2.1 |
| 66004 | 0.94613 | 2.1 |
| 66005 | 1.76401 | 2.1 |
| 66006 | 2.60983 | 2.1 |
| 66007 | 2.50461 | 2.1 |
| 66008 | 2.35897 | 2.1 |
| 66009 | 1.90965 | 2.1 |
| 66010 | 1.76401 | 2.1 |
| 66011 | 1.89226 | 2.1 |
| 66012 | 1.89226 | 2.1 |
| 66013 | 1.89226 | 2.1 |
| 66014 | 1.89226 | 2.1 |
| 66015 | 3.78452 | 2.1 |
| 66016 | 1.89226 | 2.1 |
| 66017 | 1.89226 | 2.1 |
| 66018 | 1.89226 | 2.1 |
| 66019 | 1.89226 | 0.806116 |
| 66020 | 1.89226 | 2.1 |
| 66021 | 1.89226 | 1.33005 |
| 66022 | 1.90965 | 2.1 |
| 66023 | 2.83834 | 2.1 |
| 66024 | 2.7538 | 2.1 |
| 66025 | 2.60983 | 2.1 |
| 66026 | 2.05361 | 2.1 |
| 66027 | 1.90965 | 2.1 |
| 66028 | 1.89226 | 2.1 |
| 66029 | 1.89226 | 2.1 |
| 66030 | 1.89226 | 2.1 |
| 66031 | 1.89226 | 2.1 |
| 66032 | 1.89226 | 2.1 |
| 66033 | 1.89226 | 2.1 |
| 66034 | 1.89226 | 2.1 |
| 66035 | 1.89226 | 2.1 |
| 66036 | 1.89226 | 2.1 |
| 66037 | 1.89226 | 0.801466 |
| 66038 | 3.78452 | 2.1 |
| 66039 | 2.83834 | 2.1 |
| 66040 | 1.89226 | 2.1 |
| 66041 | 2.60983 | 2.1 |
| 66042 | 2.60983 | 2.1 |
| 66043 | 1.89226 | 2.1 |
| 66044 | 1.89226 | 2.1 |
| 66045 | 1.89226 | 2.1 |
| 66046 | 1.89226 | 2.1 |
| 66047 | 1.89226 | 2.1 |
| 66048 | 1.89226 | 2.1 |
| 66049 | 1.89226 | 2.1 |
| 66050 | 1.89226 | 2.1 |
| 66051 | 1.89226 | 1.94419 |
| 66052 | 1.89226 | 2.1 |
| 66053 | 1.89226 | 2.1 |
| 66054 | 1.89226 | 1.70617 |
| 66055 | 1.89226 | 2.1 |
| 66056 | 1.89226 | 2.09337 |
| 66057 | 2.44785 | 2.1 |
| 66058 | 1.80223 | 2.1 |
| 66059 | 2.80894 | 2.1 |

| | | |
|---|---|---|
| 66060 | 2.18296 | 2.1 |
| 66061 | 4.63081 | 2.1 |
| 66062 | 1.89226 | 2.1 |
| 66063 | 1.89226 | 2.1 |
| 66064 | 1.89226 | 2.1 |
| 66065 | 1.89226 | 2.02834 |
| 66066 | 1.89226 | 2.1 |
| 66067 | 1.89226 | 2.01598 |
| 66068 | 0.946131 | 1.11023 |
| 66069 | 0.946131 | 2.1 |
| 66070 | 0.946127 | 2.1 |
| 66071 | 0.946127 | 2.1 |
| 66072 | 0.946131 | 2.06363 |
| 66073 | 0.946131 | 2.1 |
| 66074 | 0.946128 | 2.1 |
| 66075 | 0.946128 | 2.1 |
| 66076 | 0.94613 | 2.1 |
| 66077 | 0.94613 | 2.1 |
| 66078 | 0.946129 | 2.1 |
| 66079 | 0.946129 | 2.1 |
| 66080 | 1.89226 | 2.09838 |
| 66081 | 1.89226 | 2.1 |
| 66082 | 1.89226 | 2.1 |
| 66083 | 1.89226 | 2.1 |
| 66084 | 3.78452 | 2.1 |
| 66085 | 1.89226 | 2.1 |
| 66086 | 1.89226 | 2.1 |
| 66087 | 1.89226 | 2.1 |
| 66088 | 1.89226 | 2.1 |
| 66089 | 3.78453 | 2.1 |
| 66090 | 2.41083 | 2.1 |
| 66091 | 2.93937 | 2.1 |
| 66092 | 1.68761 | 2.1 |
| 66093 | 1.68761 | 2.1 |
| 66094 | 1.67248 | 2.1 |
| 66095 | 2.3957 | 2.1 |
| 66096 | 1.89226 | 2.1 |
| 66097 | 1.89226 | 2.1 |
| 66098 | 1.89226 | 1.67006 |
| 66099 | 1.89226 | 2.1 |
| 66100 | 1.89226 | 1.35191 |
| 66101 | 1.89226 | 2.1 |
| 66102 | 1.89227 | 2.1 |
| 66103 | 1.89227 | 2.1 |
| 66104 | 1.89227 | 2.1 |
| 66105 | 1.89227 | 2.1 |
| 66106 | 1.89227 | 2.1 |
| 66107 | 1.89227 | 2.1 |
| 66108 | 1.89224 | 0.719597 |
| 66109 | 1.89224 | 0.126317 |
| 66110 | 1.89224 | 0.964251 |
| 66111 | 1.89224 | 0.725876 |
| 66112 | 3.78448 | 0.625408 |
| 66113 | 1.89224 | 1.13344 |
| 66114 | 1.89224 | 0.669339 |
| 66115 | 1.89224 | 1.02 |
| 66116 | 1.89224 | 0.100091 |
| 66117 | 1.89224 | 1.75269 |

| | | |
|---|---|---|
| 66118 | 1.89224 | 0.83082 |
| 66119 | 1.11152 | 0.544813 |
| 66120 | 1.29346 | 0.927141 |
| 66121 | 0.978596 | 0.892522 |
| 66122 | 0.918324 | 1.24551 |
| 66123 | 1.06821 | 1.1714 |
| 66124 | 1.06821 | 0.19081 |
| 66125 | 0.985654 | 1.9781 |
| 66126 | 1.15278 | 1.14235 |
| 66127 | 1.07693 | 1.14079 |
| 66128 | 0.902432 | 1.29496 |
| 66129 | 0.968939 | 1.53679 |
| 66130 | 1.34644 | 0.864482 |
| 66131 | 0.946132 | 0.498217 |
| 66132 | 0.946132 | 0.768213 |
| 66133 | 0.946132 | 0.803802 |
| 66134 | 0.946132 | 0.618405 |
| 66135 | 0.946131 | 0.676689 |
| 66136 | 0.946131 | 0.0910107 |
| 66137 | 0.946132 | 0.998127 |
| 66138 | 0.946132 | 0.457975 |
| 66139 | 0.946132 | 0.738994 |
| 66140 | 0.946132 | 0.68568 |
| 66141 | 0.946132 | 0.201421 |
| 66142 | 0.946132 | 0.557406 |
| 66143 | 0.946133 | 0.813311 |
| 66144 | 0.946133 | 0.142544 |
| 66145 | 0.946132 | 0.755287 |
| 66146 | 0.946132 | 1.0172 |
| 66147 | 0.946132 | 0.839988 |
| 66148 | 0.946132 | 0.05 |
| 66149 | 0.946133 | 2.03248 |
| 66150 | 0.946133 | 0.433807 |
| 66151 | 0.946132 | 0.938435 |
| 66152 | 0.946132 | 0.789343 |
| 66153 | 0.946132 | 0.356471 |
| 66154 | 0.946132 | 0.285628 |
| 66155 | 1.89224 | 0.0535373 |
| 66156 | 1.89224 | 2.1 |
| 66157 | 1.89224 | 0.963344 |
| 66158 | 1.89224 | 2.1 |
| 66159 | 1.89224 | 0.594167 |
| 66160 | 1.89224 | 2.1 |
| 66161 | 1.89224 | 0.982556 |
| 66162 | 1.89224 | 2.1 |
| 66163 | 1.89224 | 2.1 |
| 66164 | 1.89224 | 2.1 |
| 66165 | 1.89224 | 2.1 |
| 66166 | 1.89224 | 2.1 |
| 66167 | 1.89226 | 0.501942 |
| 66168 | 1.89226 | 2.1 |
| 66169 | 1.89226 | 2.1 |
| 66170 | 1.89226 | 1.27234 |
| 66171 | 3.78453 | 2.1 |
| 66172 | 1.11056 | 2.1 |
| 66173 | 1.21595 | 2.1 |
| 66174 | 1.12763 | 2.1 |
| 66175 | 1.12763 | 2.1 |

| | | |
|---|---|---|
| 66176 | 1.23339 | 2.1 |
| 66177 | 1.35697 | 2.1 |
| 66178 | 1.03581 | 2.1 |
| 66179 | 0.95954 | 2.1 |
| 66180 | 0.89761 | 2.1 |
| 66181 | 1.26602 | 2.1 |
| 66182 | 1.12749 | 2.1 |
| 66183 | 1.45307 | 2.1 |
| 66184 | 2.71775 | 2.1 |
| 66185 | 1.89226 | 2.1 |
| 66186 | 1.89226 | 2.1 |
| 66187 | 1.89226 | 2.1 |
| 66188 | 2.47025 | 2.1 |
| 66189 | 2.47025 | 2.1 |
| 66190 | 0.94613 | 2.1 |
| 66191 | 0.94613 | 2.1 |
| 66192 | 0.94613 | 1.8425 |
| 66193 | 0.94613 | 2.1 |
| 66194 | 0.946128 | 2.1 |
| 66195 | 0.946128 | 2.1 |
| 66196 | 0.946132 | 2.1 |
| 66197 | 0.946132 | 2.1 |
| 66198 | 0.94613 | 2.1 |
| 66199 | 0.94613 | 2.1 |
| 66200 | 0.94613 | 2.1 |
| 66201 | 0.94613 | 2.1 |
| 66202 | 1.89226 | 0.907948 |
| 66203 | 1.89226 | 2.1 |
| 66204 | 1.89226 | 2.1 |
| 66205 | 1.89226 | 1.14855 |
| 66206 | 1.89226 | 2.1 |
| 66207 | 1.89226 | 1.5154 |
| 66208 | 1.89226 | 1.04845 |
| 66209 | 1.89226 | 2.1 |
| 66210 | 1.89226 | 2.1 |
| 66211 | 1.89226 | 1.05129 |
| 66212 | 1.89226 | 2.1 |
| 66213 | 1.89226 | 1.3105 |
| 66214 | 0.946132 | 2.1 |
| 66215 | 0.946132 | 2.1 |
| 66216 | 0.946133 | 2.1 |
| 66217 | 0.946133 | 2.1 |
| 66218 | 0.946133 | 2.1 |
| 66219 | 0.946133 | 1.64153 |
| 66220 | 0.946132 | 1.04283 |
| 66221 | 0.946132 | 2.1 |
| 66222 | 0.946133 | 2.1 |
| 66223 | 0.946133 | 2.1 |
| 66224 | 0.946132 | 2.1 |
| 66225 | 0.946132 | 2.1 |
| 66226 | 1.89226 | 1.00633 |
| 66227 | 1.89226 | 2.1 |
| 66228 | 1.89226 | 2.1 |
| 66229 | 1.89226 | 1.66134 |
| 66230 | 1.89226 | 2.1 |
| 66231 | 1.89226 | 1.73056 |
| 66232 | 0.946131 | 1.55649 |
| 66233 | 0.946131 | 1.06799 |

| | | |
|---|---|---|
| 66234 | 0.946127 | 2.1 |
| 66235 | 0.946127 | 2.1 |
| 66236 | 0.946131 | 1.46534 |
| 66237 | 0.946131 | 2.1 |
| 66238 | 0.946127 | 2.1 |
| 66239 | 0.946127 | 1.37356 |
| 66240 | 0.94613 | 2.1 |
| 66241 | 0.94613 | 2.1 |
| 66242 | 0.946129 | 2.1 |
| 66243 | 0.946129 | 2.1 |
| 66244 | 1.94291 | 2.1 |
| 66245 | 2.00118 | 2.1 |
| 66246 | 2.36337 | 2.1 |
| 66247 | 2.41217 | 2.1 |
| 66248 | 4.35508 | 2.1 |
| 66249 | 1.89226 | 2.1 |
| 66250 | 1.89226 | 2.1 |
| 66251 | 1.89226 | 2.1 |
| 66252 | 1.89226 | 2.1 |
| 66253 | 3.78452 | 2.1 |
| 66254 | 1.89226 | 2.1 |
| 66255 | 1.89226 | 2.1 |
| 66256 | 1.89226 | 2.1 |
| 66257 | 1.89226 | 2.1 |
| 66258 | 3.78452 | 2.1 |
| 66259 | 1.06209 | 2.1 |
| 66260 | 1.06209 | 2.1 |
| 66261 | 1.05987 | 2.1 |
| 66262 | 1.05987 | 2.1 |
| 66263 | 0.920378 | 2.1 |
| 66264 | 1.12137 | 2.1 |
| 66265 | 1.00059 | 2.1 |
| 66266 | 1.20608 | 2.1 |
| 66267 | 1.20608 | 2.1 |
| 66268 | 1.12137 | 2.1 |
| 66269 | 1.00059 | 2.1 |
| 66270 | 0.920376 | 2.1 |
| 66271 | 2.77222 | 2.1 |
| 66272 | 2.41216 | 2.1 |
| 66273 | 2.77002 | 2.1 |
| 66274 | 2.36337 | 2.1 |
| 66275 | 2.81882 | 2.1 |
| 66276 | 2.41216 | 2.1 |
| 66277 | 1.89226 | 2.1 |
| 66278 | 1.89226 | 2.1 |
| 66279 | 1.89226 | 2.1 |
| 66280 | 1.89226 | 2.1 |
| 66281 | 1.89226 | 2.1 |
| 66282 | 1.89226 | 2.0227 |
| 66283 | 1.89226 | 2.1 |
| 66284 | 1.89226 | 1.91219 |
| 66285 | 1.89226 | 2.1 |
| 66286 | 1.89226 | 2.1 |
| 66287 | 3.78451 | 2.1 |
| 66288 | 2.41216 | 2.1 |
| 66289 | 2.24273 | 2.1 |
| 66290 | 2.81882 | 2.1 |
| 66291 | 2.68473 | 2.1 |

| | | |
|---|---|---|
| 66292 | 5.09689 | 2.1 |
| 66293 | 1.89226 | 2.1 |
| 66294 | 1.89226 | 2.1 |
| 66295 | 1.89226 | 2.1 |
| 66296 | 1.89226 | 2.1 |
| 66297 | 3.78453 | 2.1 |
| 66298 | 2.17614 | 2.1 |
| 66299 | 2.65326 | 2.1 |
| 66300 | 1.93101 | 2.1 |
| 66301 | 2.58552 | 2.1 |
| 66302 | 4.58427 | 2.1 |
| 66303 | 2.65326 | 2.1 |
| 66304 | 1.89226 | 2.1 |
| 66305 | 1.89226 | 2.1 |
| 66306 | 1.89226 | 2.1 |
| 66307 | 2.58552 | 2.1 |
| 66308 | 2.58552 | 2.1 |
| 66309 | 1.89226 | 2.1 |
| 66310 | 1.89226 | 2.1 |
| 66311 | 1.89226 | 2.1 |
| 66312 | 1.89226 | 2.1 |
| 66313 | 3.78453 | 2.1 |
| 66314 | 1.89226 | 2.1 |
| 66315 | 1.89226 | 2.1 |
| 66316 | 1.89226 | 2.1 |
| 66317 | 1.89226 | 2.1 |
| 66318 | 3.78453 | 2.1 |
| 66319 | 1.93101 | 2.1 |
| 66320 | 2.58552 | 2.1 |
| 66321 | 1.46507 | 2.1 |
| 66322 | 2.52893 | 2.1 |
| 66323 | 4.45994 | 2.1 |
| 66324 | 1.26446 | 2.1 |
| 66325 | 1.26446 | 2.1 |
| 66326 | 0.946132 | 2.1 |
| 66327 | 0.946132 | 2.1 |
| 66328 | 0.94613 | 2.1 |
| 66329 | 0.94613 | 2.1 |
| 66330 | 1.29276 | 2.1 |
| 66331 | 1.26446 | 2.1 |
| 66332 | 1.26446 | 2.1 |
| 66333 | 0.94613 | 2.1 |
| 66334 | 0.94613 | 2.1 |
| 66335 | 1.29276 | 2.1 |
| 66336 | 1.89226 | 2.1 |
| 66337 | 1.89226 | 2.1 |
| 66338 | 1.89226 | 2.1 |
| 66339 | 1.89226 | 2.1 |
| 66340 | 3.78453 | 2.1 |
| 66341 | 1.89226 | 2.1 |
| 66342 | 1.89226 | 2.1 |
| 66343 | 1.89226 | 2.1 |
| 66344 | 1.89226 | 2.1 |
| 66345 | 3.78453 | 2.1 |
| 66346 | 1.83378 | 2.1 |
| 66347 | 2.52893 | 2.1 |
| 66348 | 1.75592 | 2.1 |
| 66349 | 2.49844 | 2.1 |

| | | |
|---|---|---|
| 66350 | 4.28485 | 2.1 |
| 66351 | 1.24922 | 2.1 |
| 66352 | 1.24922 | 2.1 |
| 66353 | 0.946133 | 2.1 |
| 66354 | 0.946133 | 2.1 |
| 66355 | 0.946131 | 2.1 |
| 66356 | 0.946131 | 2.1 |
| 66357 | 1.26446 | 2.1 |
| 66358 | 1.24922 | 2.1 |
| 66359 | 1.24922 | 2.1 |
| 66360 | 0.946131 | 2.1 |
| 66361 | 1.26446 | 2.1 |
| 66362 | 0.94613 | 2.1 |
| 66363 | 0.946132 | 2.1 |
| 66364 | 0.946132 | 2.1 |
| 66365 | 0.946132 | 2.1 |
| 66366 | 0.946132 | 2.1 |
| 66367 | 0.946132 | 2.1 |
| 66368 | 0.946132 | 2.1 |
| 66369 | 0.946132 | 2.1 |
| 66370 | 0.946132 | 2.1 |
| 66371 | 0.946132 | 2.1 |
| 66372 | 0.946132 | 2.1 |
| 66373 | 0.946131 | 2.1 |
| 66374 | 0.946131 | 2.1 |
| 66375 | 1.89226 | 2.1 |
| 66376 | 1.89226 | 2.1 |
| 66377 | 1.89226 | 2.1 |
| 66378 | 1.89226 | 2.1 |
| 66379 | 1.89226 | 2.1 |
| 66380 | 1.89226 | 2.1 |
| 66381 | 1.0465 | 2.1 |
| 66382 | 1.0465 | 2.1 |
| 66383 | 1.06047 | 2.1 |
| 66384 | 1.06047 | 2.1 |
| 66385 | 1.25687 | 2.1 |
| 66386 | 1.24922 | 2.1 |
| 66387 | 0.87796 | 2.1 |
| 66388 | 0.857749 | 2.1 |
| 66389 | 0.857749 | 2.1 |
| 66390 | 1.25687 | 2.1 |
| 66391 | 0.877959 | 2.1 |
| 66392 | 1.24922 | 2.1 |
| 66393 | 2.49843 | 2.1 |
| 66394 | 1.89226 | 2.1 |
| 66395 | 2.51374 | 2.1 |
| 66396 | 1.89226 | 2.1 |
| 66397 | 4.406 2.1 | |
| 66398 | 1.89226 | 2.1 |
| 66399 | 1.89226 | 2.1 |
| 66400 | 1.89226 | 2.1 |
| 66401 | 1.89226 | 2.1 |
| 66402 | 1.89226 | 2.1 |
| 66403 | 1.89226 | 2.1 |
| 66404 | 1.89226 | 1.0216 |
| 66405 | 1.89226 | 2.1 |
| 66406 | 1.89226 | 2.1 |
| 66407 | 1.89226 | 1.27892 |

| | | |
|---|---|---|
| 66408 | 1.89226 | 2.1 |
| 66409 | 1.89226 | 1.96346 |
| 66410 | 1.89226 | 2.1 |
| 66411 | 1.89226 | 1.89988 |
| 66412 | 1.89226 | 2.1 |
| 66413 | 1.89226 | 1.40713 |
| 66414 | 1.89226 | 2.1 |
| 66415 | 1.89226 | 2.00294 |
| 66416 | 1.03835 | 2.1 |
| 66417 | 1.03835 | 2.1 |
| 66418 | 1.15388 | 2.1 |
| 66419 | 0.94267 | 2.1 |
| 66420 | 0.736433 | 2.1 |
| 66421 | 0.849239 | 2.1 |
| 66422 | 1.15014 | 2.1 |
| 66423 | 1.39058 | 2.1 |
| 66424 | 0.838639 | 2.1 |
| 66425 | 1.39056 | 2.1 |
| 66426 | 1.09399 | 2.1 |
| 66427 | 0.736444 | 2.1 |
| 66428 | 1.89226 | 0.951331 |
| 66429 | 1.89226 | 2.1 |
| 66430 | 1.89226 | 2.1 |
| 66431 | 1.89226 | 0.98485 |
| 66432 | 1.89226 | 2.1 |
| 66433 | 1.89226 | 1.04912 |
| 66434 | 0.94613 | 1.0824 |
| 66435 | 0.94613 | 1.05196 |
| 66436 | 0.946127 | 2.1 |
| 66437 | 0.946127 | 2.1 |
| 66438 | 0.946131 | 2.1 |
| 66439 | 0.946131 | 1.02761 |
| 66440 | 0.946126 | 2.1 |
| 66441 | 0.946126 | 1.11327 |
| 66442 | 0.946128 | 2.1 |
| 66443 | 0.946128 | 2.1 |
| 66444 | 0.946128 | 2.1 |
| 66445 | 0.946128 | 2.1 |
| 66446 | 1.89226 | 2.1 |
| 66447 | 1.89226 | 1.19254 |
| 66448 | 1.89226 | 0.754245 |
| 66449 | 1.89226 | 2.1 |
| 66450 | 3.78453 | 1.20576 |
| 66451 | 0.946133 | 1.9519 |
| 66452 | 0.946133 | 0.781924 |
| 66453 | 0.946128 | 2.1 |
| 66454 | 0.946128 | 1.83106 |
| 66455 | 0.946131 | 1.99587 |
| 66456 | 0.946131 | 1.6695 |
| 66457 | 0.946131 | 0.34414 |
| 66458 | 0.946131 | 1.73122 |
| 66459 | 0.946131 | 1.2535 |
| 66460 | 0.946131 | 1.20907 |
| 66461 | 0.94613 | 1.34157 |
| 66462 | 0.94613 | 1.46465 |
| 66463 | 1.89226 | 0.902549 |
| 66464 | 1.89226 | 2.1 |
| 66465 | 1.89226 | 2.1 |

| | | |
|---|---|---|
| 66466 | 1.89226 | 1.11689 |
| 66467 | 1.89226 | 2.1 |
| 66468 | 1.89226 | 1.3439 |
| 66469 | 1.89226 | 0.882411 |
| 66470 | 1.89226 | 2.1 |
| 66471 | 1.89226 | 1.59861 |
| 66472 | 1.89226 | 1.10674 |
| 66473 | 1.89226 | 1.73747 |
| 66474 | 1.89226 | 1.71851 |
| 66475 | 1.89226 | 0.888673 |
| 66476 | 1.89226 | 2.1 |
| 66477 | 1.89226 | 1.27474 |
| 66478 | 1.89226 | 1.42097 |
| 66479 | 1.89226 | 1.35813 |
| 66480 | 1.89226 | 1.94829 |
| 66481 | 1.89226 | 0.974484 |
| 66482 | 2.67388 | 2.1 |
| 66483 | 2.23684 | 2.1 |
| 66484 | 2.23684 | 1.60978 |
| 66485 | 1.89226 | 2.1 |
| 66486 | 1.89226 | 1.99145 |
| 66487 | 1.89226 | 2.1 |
| 66488 | 1.89226 | 2.1 |
| 66489 | 1.89226 | 2.1 |
| 66490 | 1.89226 | 2.1 |
| 66491 | 1.89226 | 2.1 |
| 66492 | 1.89226 | 2.1 |
| 66493 | 1.72197 | 2.1 |
| 66494 | 2.51374 | 2.1 |
| 66495 | 2.58004 | 2.1 |
| 66496 | 2.58004 | 2.1 |
| 66497 | 1.7155 | 2.1 |
| 66498 | 1.7155 | 2.1 |
| 66499 | 2.51374 | 2.1 |
| 66500 | 1.89226 | 2.1 |
| 66501 | 1.89226 | 2.1 |
| 66502 | 1.89226 | 2.1 |
| 66503 | 2.58003 | 2.1 |
| 66504 | 2.58003 | 2.1 |
| 66505 | 0.946132 | 2.1 |
| 66506 | 0.946132 | 2.1 |
| 66507 | 0.946132 | 2.1 |
| 66508 | 0.946132 | 2.1 |
| 66509 | 0.946132 | 2.1 |
| 66510 | 0.946132 | 2.1 |
| 66511 | 0.946132 | 2.1 |
| 66512 | 0.946132 | 2.1 |
| 66513 | 0.946131 | 2.1 |
| 66514 | 0.946131 | 2.1 |
| 66515 | 0.946132 | 2.1 |
| 66516 | 0.946132 | 2.1 |
| 66517 | 1.89226 | 2.1 |
| 66518 | 1.89226 | 2.1 |
| 66519 | 1.89226 | 2.1 |
| 66520 | 1.89226 | 2.1 |
| 66521 | 3.78453 | 2.1 |
| 66522 | 1.08262 | 2.1 |
| 66523 | 1.08262 | 2.1 |

| | | |
|---|---|---|
| 66524 | 1.09674 | 2.1 |
| 66525 | 1.09674 | 2.1 |
| 66526 | 1.34652 | 2.1 |
| 66527 | 1.29002 | 2.1 |
| 66528 | 0.860984 | 2.1 |
| 66529 | 0.889338 | 2.1 |
| 66530 | 1.34652 | 2.1 |
| 66531 | 0.889338 | 2.1 |
| 66532 | 0.860983 | 2.1 |
| 66533 | 1.29002 | 2.1 |
| 66534 | 1.34652 | 2.1 |
| 66535 | 1.34652 | 2.1 |
| 66536 | 0.946133 | 2.1 |
| 66537 | 0.946133 | 2.1 |
| 66538 | 0.946131 | 2.1 |
| 66539 | 0.946131 | 2.1 |
| 66540 | 1.34652 | 2.1 |
| 66541 | 1.29002 | 2.1 |
| 66542 | 1.34652 | 2.1 |
| 66543 | 0.946131 | 2.1 |
| 66544 | 0.94613 | 2.1 |
| 66545 | 1.29002 | 2.1 |
| 66546 | 1.89226 | 2.1 |
| 66547 | 1.89226 | 2.1 |
| 66548 | 1.89226 | 2.1 |
| 66549 | 1.89226 | 2.1 |
| 66550 | 1.89226 | 2.1 |
| 66551 | 1.89226 | 2.1 |
| 66552 | 1.89226 | 2.1 |
| 66553 | 1.89226 | 2.1 |
| 66554 | 1.89226 | 2.1 |
| 66555 | 1.89226 | 2.1 |
| 66556 | 1.89226 | 2.1 |
| 66557 | 1.89226 | 2.1 |
| 66558 | 0.946131 | 2.1 |
| 66559 | 0.946131 | 2.1 |
| 66560 | 0.946131 | 2.1 |
| 66561 | 0.946131 | 2.1 |
| 66562 | 0.946131 | 2.1 |
| 66563 | 0.946131 | 2.1 |
| 66564 | 0.946131 | 2.1 |
| 66565 | 0.946131 | 2.1 |
| 66566 | 0.946131 | 2.1 |
| 66567 | 0.946131 | 2.1 |
| 66568 | 0.946131 | 2.1 |
| 66569 | 0.946131 | 2.1 |
| 66570 | 1.89226 | 2.1 |
| 66571 | 1.89226 | 2.1 |
| 66572 | 1.89226 | 2.1 |
| 66573 | 1.89226 | 2.1 |
| 66574 | 1.89226 | 2.1 |
| 66575 | 1.89226 | 2.1 |
| 66576 | 1.04103 | 2.1 |
| 66577 | 1.13849 | 2.1 |
| 66578 | 0.943014 | 2.1 |
| 66579 | 0.943014 | 2.1 |
| 66580 | 0.943591 | 2.1 |
| 66581 | 0.943591 | 2.1 |

| | | |
|---|---|---|
| 66582 | 1.03757 | 2.1 |
| 66583 | 1.04098 | 2.1 |
| 66584 | 1.03761 | 2.1 |
| 66585 | 0.946131 | 2.1 |
| 66586 | 0.943041 | 2.1 |
| 66587 | 0.946131 | 2.1 |
| 66588 | 0.946128 | 2.1 |
| 66589 | 0.946128 | 2.1 |
| 66590 | 0.946133 | 2.1 |
| 66591 | 0.946133 | 2.1 |
| 66592 | 0.946131 | 2.1 |
| 66593 | 0.946131 | 2.1 |
| 66594 | 0.946131 | 2.1 |
| 66595 | 0.946131 | 2.1 |
| 66596 | 0.946131 | 2.1 |
| 66597 | 0.946131 | 2.1 |
| 66598 | 0.94613 | 2.1 |
| 66599 | 0.94613 | 2.1 |
| 66600 | 0.946132 | 2.1 |
| 66601 | 0.946132 | 2.1 |
| 66602 | 0.946132 | 2.1 |
| 66603 | 0.946132 | 2.1 |
| 66604 | 0.946132 | 2.1 |
| 66605 | 0.946132 | 2.1 |
| 66606 | 0.946132 | 2.1 |
| 66607 | 0.946132 | 2.1 |
| 66608 | 0.946132 | 2.1 |
| 66609 | 0.946132 | 2.1 |
| 66610 | 0.946131 | 2.1 |
| 66611 | 0.946131 | 2.1 |
| 66612 | 1.89226 | 0.95619 |
| 66613 | 1.89226 | 2.1 |
| 66614 | 1.89226 | 1.08204 |
| 66615 | 1.89226 | 1.06237 |
| 66616 | 3.78452 | 1.6061 |
| 66617 | 0.946132 | 1.30349 |
| 66618 | 0.946132 | 1.76673 |
| 66619 | 0.946131 | 1.06939 |
| 66620 | 0.946131 | 1.00149 |
| 66621 | 0.946132 | 1.06629 |
| 66622 | 0.946132 | 2.1 |
| 66623 | 0.946132 | 1.34078 |
| 66624 | 0.946132 | 1.25776 |
| 66625 | 0.946132 | 1.22045 |
| 66626 | 0.946132 | 1.24002 |
| 66627 | 0.946132 | 1.33104 |
| 66628 | 0.946132 | 1.4996 |
| 66629 | 0.935482 | 2.1 |
| 66630 | 1.08999 | 1.99589 |
| 66631 | 1.24315 | 1.46096 |
| 66632 | 1.12559 | 2.05037 |
| 66633 | 1.21343 | 1.36124 |
| 66634 | 0.889397 | 2.1 |
| 66635 | 1.01265 | 0.912415 |
| 66636 | 1.01265 | 2.1 |
| 66637 | 1.07332 | 1.18882 |
| 66638 | 1.34292 | 1.95396 |
| 66639 | 0.865649 | 1.88968 |

| | | |
|---|---|---|
| 66640 | 0.840024 | 2.1 |
| 66641 | 1.15433 | 1.78403 |
| 66642 | 1.02858 | 2.1 |
| 66643 | 1.34082 | 1.65398 |
| 66644 | 1.08357 | 1.75565 |
| 66645 | 1.10938 | 1.06808 |
| 66646 | 1.33349 | 2.1 |
| 66647 | 1.12232 | 2.1 |
| 66648 | 1.12232 | 0.933713 |
| 66649 | 1.21357 | 1.73472 |
| 66650 | 1.3408 | 1.97523 |
| 66651 | 1.02859 | 1.98654 |
| 66652 | 1.24211 | 2.1 |
| 66653 | 1.89226 | 1.33848 |
| 66654 | 1.89226 | 0.794571 |
| 66655 | 1.89226 | 0.96303 |
| 66656 | 1.89226 | 2.1 |
| 66657 | 1.89226 | 0.770324 |
| 66658 | 1.89226 | 1.60275 |
| 66659 | 1.89226 | 0.930901 |
| 66660 | 1.89226 | 1.16813 |
| 66661 | 1.89226 | 0.940787 |
| 66662 | 1.89226 | 2.03056 |
| 66663 | 1.89226 | 1.23776 |
| 66664 | 1.89226 | 2.08612 |
| 66665 | 2.0815 | 1.75887 |
| 66666 | 2.79029 | 1.79943 |
| 66667 | 2.36793 | 1.27461 |
| 66668 | 2.58428 | 2.1 |
| 66669 | 2.38161 | 0.887071 |
| 66670 | 2.59922 | 2.08699 |
| 66671 | 1.89226 | 2.1 |
| 66672 | 1.89226 | 1.68268 |
| 66673 | 1.89226 | 2.1 |
| 66674 | 1.89226 | 2.1 |
| 66675 | 1.89226 | 2.1 |
| 66676 | 1.89226 | 2.1 |
| 66677 | 1.89226 | 1.89664 |
| 66678 | 1.89226 | 0.126127 |
| 66679 | 1.89226 | 0.882072 |
| 66680 | 1.89226 | 0.915577 |
| 66681 | 3.78452 | 0.758844 |
| 66682 | 1.89226 | 2.1 |
| 66683 | 1.89226 | 0.82608 |
| 66684 | 1.89226 | 1.99559 |
| 66685 | 1.89226 | 1.89018 |
| 66686 | 1.89226 | 1.77857 |
| 66687 | 1.89226 | 1.89069 |
| 66688 | 0.946132 | 0.967364 |
| 66689 | 0.946132 | 2.1 |
| 66690 | 0.94613 | 2.1 |
| 66691 | 0.94613 | 2.1 |
| 66692 | 0.946131 | 2.1 |
| 66693 | 0.946131 | 0.865734 |
| 66694 | 0.946131 | 2.1 |
| 66695 | 0.946131 | 2.1 |
| 66696 | 0.946131 | 2.1 |
| 66697 | 0.946131 | 2.1 |

| | | |
|---|---|---|
| 66698 | 0.946131 | 2.1 |
| 66699 | 0.946131 | 2.1 |
| 66700 | 1.85178 | 2.1 |
| 66701 | 1.89226 | 0.731579 |
| 66702 | 2.0815 | 0.338464 |
| 66703 | 2.27054 | 1.39706 |
| 66704 | 1.70303 | 0.293148 |
| 66705 | 1.5217 | 0.462022 |
| 66706 | 1.89226 | 2.1 |
| 66707 | 1.89226 | 0.700676 |
| 66708 | 1.89226 | 1.69449 |
| 66709 | 1.89226 | 1.14662 |
| 66710 | 1.89226 | 2.1 |
| 66711 | 1.89226 | 1.70824 |
| 66712 | 2.58823 | 0.227093 |
| 66713 | 2.02924 | 0.0961747 |
| 66714 | 1.79398 | 0.05 |
| 66715 | 1.79398 | 0.05 |
| 66716 | 2.76708 | 0.485951 |
| 66717 | 2.76708 | 0.250099 |
| 66718 | 0.946133 | 0.337286 |
| 66719 | 0.946133 | 0.231332 |
| 66720 | 1.29411 | 0.199657 |
| 66721 | 1.38353 | 0.247916 |
| 66722 | 0.946131 | 0.145668 |
| 66723 | 0.946131 | 0.306811 |
| 66724 | 1.38354 | 0.363189 |
| 66725 | 1.38354 | 0.094723 |
| 66726 | 0.946131 | 0.193595 |
| 66727 | 1.38354 | 0.171213 |
| 66728 | 0.94613 | 0.394313 |
| 66729 | 1.29411 | 0.0823263 |
| 66730 | 1.89226 | 0.124332 |
| 66731 | 1.89226 | 0.05 |
| 66732 | 1.89226 | 0.322072 |
| 66733 | 1.89226 | 0.160464 |
| 66734 | 3.78452 | 0.26399 |
| 66735 | 0.946131 | 0.05 |
| 66736 | 0.946131 | 0.117783 |
| 66737 | 0.946131 | 0.271255 |
| 66738 | 0.946131 | 0.05 |
| 66739 | 0.946129 | 0.05 |
| 66740 | 0.946129 | 0.05 |
| 66741 | 0.946133 | 0.263001 |
| 66742 | 0.946133 | 0.121356 |
| 66743 | 0.946131 | 0.327574 |
| 66744 | 0.946131 | 0.05 |
| 66745 | 0.946131 | 0.05 |
| 66746 | 0.946131 | 0.05 |
| 66747 | 1.89226 | 0.05 |
| 66748 | 1.89226 | 0.05 |
| 66749 | 1.89226 | 0.131026 |
| 66750 | 1.89226 | 0.198698 |
| 66751 | 3.78452 | 0.05 |
| 66752 | 0.94613 | 0.05 |
| 66753 | 0.94613 | 0.0862636 |
| 66754 | 0.946131 | 0.05 |
| 66755 | 0.946131 | 0.14861 |

| | | |
|---|---|---|
| 66756 | 0.946128 | 0.05 |
| 66757 | 0.946128 | 0.05 |
| 66758 | 0.946133 | 0.05 |
| 66759 | 0.946133 | 0.05 |
| 66760 | 0.946131 | 0.21474 |
| 66761 | 0.946131 | 0.05 |
| 66762 | 0.946131 | 0.131919 |
| 66763 | 0.946131 | 0.05 |
| 66764 | 0.946129 | 0.289003 |
| 66765 | 0.946129 | 0.103991 |
| 66766 | 1.13486 | 0.05 |
| 66767 | 0.859753 | 0.109197 |
| 66768 | 0.851541 | 0.0976476 |
| 66769 | 0.851541 | 0.154163 |
| 66770 | 1.34346 | 0.13136 |
| 66771 | 1.26212 | 0.05 |
| 66772 | 0.851553 | 0.053509 |
| 66773 | 1.12403 | 0.0729505 |
| 66774 | 0.851474 | 0.05 |
| 66775 | 0.956163 | 0.0500803 |
| 66776 | 0.946132 | 0.143784 |
| 66777 | 0.946132 | 0.05 |
| 66778 | 0.946133 | 0.05 |
| 66779 | 0.946133 | 0.05 |
| 66780 | 0.946132 | 0.05 |
| 66781 | 0.946132 | 0.05 |
| 66782 | 0.946133 | 0.05 |
| 66783 | 0.946133 | 0.05 |
| 66784 | 0.946133 | 0.07685 |
| 66785 | 0.946133 | 0.05 |
| 66786 | 0.946132 | 0.05 |
| 66787 | 0.946132 | 0.05 |
| 66788 | 1.89226 | 0.05 |
| 66789 | 1.89226 | 0.05 |
| 66790 | 1.89226 | 0.131616 |
| 66791 | 1.89226 | 0.0801567 |
| 66792 | 3.78453 | 0.05 |
| 66793 | 0.946128 | 0.672426 |
| 66794 | 0.946128 | 0.591552 |
| 66795 | 0.946132 | 0.0913498 |
| 66796 | 0.946132 | 0.107768 |
| 66797 | 0.94613 | 0.0987868 |
| 66798 | 0.94613 | 0.1646 |
| 66799 | 0.94613 | 0.269664 |
| 66800 | 0.94613 | 0.475284 |
| 66801 | 0.946131 | 0.729718 |
| 66802 | 0.946131 | 0.128295 |
| 66803 | 0.94613 | 0.0720236 |
| 66804 | 0.94613 | 0.448606 |
| 66805 | 0.946132 | 0.0574576 |
| 66806 | 0.946132 | 0.05 |
| 66807 | 0.946133 | 0.0568266 |
| 66808 | 0.946133 | 0.05 |
| 66809 | 0.946132 | 0.05 |
| 66810 | 0.946132 | 0.05 |
| 66811 | 0.946133 | 0.05 |
| 66812 | 0.946133 | 0.05 |
| 66813 | 0.946132 | 0.0692698 |

| | | |
|---|---|---|
| 66814 | 0.946132 | 0.05 |
| 66815 | 0.946132 | 0.05 |
| 66816 | 0.946132 | 0.05 |
| 66817 | 1.89226 | 0.05 |
| 66818 | 1.89226 | 0.05 |
| 66819 | 1.89226 | 0.128504 |
| 66820 | 1.89226 | 0.05 |
| 66821 | 1.89226 | 0.410792 |
| 66822 | 1.89226 | 0.05 |
| 66823 | 1.89226 | 0.05 |
| 66824 | 1.89226 | 0.102263 |
| 66825 | 1.89226 | 0.05 |
| 66826 | 1.89226 | 0.05 |
| 66827 | 1.89226 | 0.101679 |
| 66828 | 1.89226 | 0.124885 |
| 66829 | 1.89226 | 2.1 |
| 66830 | 1.89226 | 2.1 |
| 66831 | 1.89226 | 2.1 |
| 66832 | 1.89226 | 2.1 |
| 66833 | 3.78452 | 2.1 |
| 66834 | 1.07652 | 2.1 |
| 66835 | 1.07652 | 2.1 |
| 66836 | 0.928749 | 2.1 |
| 66837 | 0.928749 | 2.1 |
| 66838 | 0.946132 | 2.1 |
| 66839 | 0.946132 | 2.1 |
| 66840 | 1.05914 | 2.1 |
| 66841 | 0.928746 | 2.1 |
| 66842 | 1.05914 | 2.1 |
| 66843 | 0.946131 | 2.1 |
| 66844 | 0.94613 | 2.1 |
| 66845 | 0.928747 | 2.1 |
| 66846 | 1.89226 | 2.1 |
| 66847 | 1.89226 | 2.1 |
| 66848 | 1.89226 | 2.1 |
| 66849 | 1.89226 | 2.1 |
| 66850 | 1.89226 | 2.1 |
| 66851 | 1.89226 | 2.1 |
| 66852 | 1.89227 | 2.1 |
| 66853 | 1.89227 | 2.1 |
| 66854 | 1.89227 | 2.1 |
| 66855 | 1.89227 | 2.1 |
| 66856 | 1.89227 | 2.1 |
| 66857 | 1.89227 | 2.1 |
| 66858 | 1.89226 | 2.1 |
| 66859 | 1.89226 | 2.1 |
| 66860 | 1.89226 | 2.1 |
| 66861 | 1.89226 | 2.1 |
| 66862 | 1.89226 | 2.1 |
| 66863 | 1.89226 | 2.1 |
| 66864 | 2.11828 | 2.1 |
| 66865 | 1.89226 | 2.1 |
| 66866 | 2.35845 | 2.1 |
| 66867 | 1.89226 | 2.1 |
| 66868 | 4.25071 | 2.1 |
| 66869 | 1.89226 | 2.1 |
| 66870 | 1.89226 | 2.1 |
| 66871 | 1.89226 | 2.1 |

| | | |
|---|---|---|
| 66872 | 1.89226 | 2.1 |
| 66873 | 3.78453 | 2.1 |
| 66874 | 1.89226 | 2.1 |
| 66875 | 1.89226 | 2.1 |
| 66876 | 1.89226 | 2.1 |
| 66877 | 1.89226 | 2.1 |
| 66878 | 1.89226 | 2.1 |
| 66879 | 1.89226 | 2.1 |
| 66880 | 1.89226 | 0.900297 |
| 66881 | 1.89226 | 1.98323 |
| 66882 | 1.65573 | 1.86969 |
| 66883 | 1.65573 | 0.990599 |
| 66884 | 1.89226 | 1.81229 |
| 66885 | 1.89226 | 1.00112 |
| 66886 | 1.89226 | 0.79269 |
| 66887 | 1.89226 | 1.78689 |
| 66888 | 1.89226 | 1.27552 |
| 66889 | 1.89226 | 0.92858 |
| 66890 | 1.89226 | 1.47853 |
| 66891 | 1.89226 | 0.975008 |
| 66892 | 1.89226 | 0.858577 |
| 66893 | 1.89226 | 1.80938 |
| 66894 | 1.89226 | 1.63544 |
| 66895 | 1.89226 | 0.960886 |
| 66896 | 1.89226 | 1.7408 |
| 66897 | 1.89226 | 0.985848 |
| 66898 | 1.89226 | 0.776255 |
| 66899 | 1.89226 | 1.87166 |
| 66900 | 1.89226 | 1.24949 |
| 66901 | 1.89226 | 0.893216 |
| 66902 | 1.89226 | 1.40657 |
| 66903 | 1.89226 | 0.936535 |
| 66904 | 0.946128 | 0.981949 |
| 66905 | 0.946128 | 0.850348 |
| 66906 | 0.946125 | 1.37733 |
| 66907 | 0.946125 | 1.41134 |
| 66908 | 0.94613 | 0.940166 |
| 66909 | 0.94613 | 2.1 |
| 66910 | 0.946124 | 1.92521 |
| 66911 | 0.946124 | 0.929746 |
| 66912 | 0.946127 | 1.1103 |
| 66913 | 0.946127 | 1.11059 |
| 66914 | 0.946127 | 1.14327 |
| 66915 | 0.946127 | 1.20349 |
| 66916 | 1.89226 | 0.822261 |
| 66917 | 1.89226 | 1.43663 |
| 66918 | 1.89226 | 0.809049 |
| 66919 | 1.89226 | 0.721922 |
| 66920 | 3.78452 | 0.86091 |
| 66921 | 1.89226 | 1.10272 |
| 66922 | 1.89226 | 1.16024 |
| 66923 | 1.89226 | 0.849336 |
| 66924 | 1.89226 | 1.14919 |
| 66925 | 3.78453 | 1.06241 |
| 66926 | 1.05926 | 2.05557 |
| 66927 | 0.891358 | 2.1 |
| 66928 | 0.946129 | 1.39666 |
| 66929 | 0.946129 | 2.1 |

| | | |
|---|---|---|
| 66930 | 0.946101 | 1.28677 |
| 66931 | 0.946101 | 2.1 |
| 66932 | 0.891386 | 1.698 |
| 66933 | 0.891386 | 2.02032 |
| 66934 | 0.891357 | 2.0661 |
| 66935 | 0.946129 | 1.78667 |
| 66936 | 0.94613 | 1.9576 |
| 66937 | 1.05926 | 1.93832 |
| 66938 | 0.946126 | 0.958547 |
| 66939 | 0.946126 | 1.18915 |
| 66940 | 0.94613 | 0.943078 |
| 66941 | 0.94613 | 1.28622 |
| 66942 | 0.946131 | 1.06971 |
| 66943 | 0.946131 | 1.24768 |
| 66944 | 0.946125 | 1.41836 |
| 66945 | 0.946125 | 0.724386 |
| 66946 | 0.946127 | 0.991119 |
| 66947 | 0.946127 | 0.946215 |
| 66948 | 0.946128 | 1.29257 |
| 66949 | 0.946128 | 1.08879 |
| 66950 | 1.89226 | 0.904814 |
| 66951 | 1.89226 | 0.757909 |
| 66952 | 1.89226 | 0.673394 |
| 66953 | 1.89226 | 1.11808 |
| 66954 | 1.89226 | 0.729384 |
| 66955 | 1.89226 | 1.53608 |
| 66956 | 1.89226 | 0.946766 |
| 66957 | 1.89226 | 1.35679 |
| 66958 | 1.89226 | 1.13561 |
| 66959 | 1.89226 | 1.30272 |
| 66960 | 1.89226 | 0.935658 |
| 66961 | 1.89226 | 1.27556 |
| 66962 | 1.16109 | 2.1 |
| 66963 | 1.23243 | 2.1 |
| 66964 | 0.659819 | 1.65095 |
| 66965 | 0.659819 | 2.1 |
| 66966 | 0.697965 | 1.10967 |
| 66967 | 1.00087 | 2.1 |
| 66968 | 0.891386 | 2.1 |
| 66969 | 0.891386 | 1.6724 |
| 66970 | 0.946129 | 2.1 |
| 66971 | 0.65979 | 1.30927 |
| 66972 | 0.891357 | 2.06017 |
| 66973 | 0.946129 | 1.78465 |
| 66974 | 1.89225 | 0.803024 |
| 66975 | 1.89225 | 1.12638 |
| 66976 | 1.89225 | 1.60516 |
| 66977 | 1.89225 | 0.716983 |
| 66978 | 1.89225 | 2.1 |
| 66979 | 1.89225 | 0.948531 |
| 66980 | 1.79981 | 0.05 |
| 66981 | 2.02514 | 0.05 |
| 66982 | 2.33247 | 0.188191 |
| 66983 | 2.12891 | 0.05 |
| 66984 | 4.15405 | 0.05 |
| 66985 | 2.48284 | 0.260405 |
| 66986 | 2.02514 | 0.05 |
| 66987 | 2.33247 | 0.05 |

| | | |
|---|---|---|
| 66988 | 2.02514 | 0.114592 |
| 66989 | 2.48567 | 0.05 |
| 66990 | 2.17834 | 0.0561682 |
| 66991 | 1.89226 | 0.05 |
| 66992 | 1.89226 | 0.144145 |
| 66993 | 1.89226 | 0.224863 |
| 66994 | 1.89226 | 0.05 |
| 66995 | 3.78453 | 0.05 |
| 66996 | 1.89227 | 0.05 |
| 66997 | 1.89227 | 0.05 |
| 66998 | 1.89227 | 0.05 |
| 66999 | 1.89227 | 0.0675368 |
| 67000 | 1.89227 | 0.05 |
| 67001 | 1.89227 | 0.214581 |
| 67002 | 2.50894 | 0.05 |
| 67003 | 2.70118 | 0.0561461 |
| 67004 | 2.12891 | 0.05 |
| 67005 | 2.12891 | 0.05 |
| 67006 | 2.7125 | 0.05 |
| 67007 | 2.33247 | 0.05 |
| 67008 | 1.89226 | 0.133536 |
| 67009 | 1.89226 | 0.05 |
| 67010 | 1.89226 | 0.05 |
| 67011 | 1.89226 | 0.116079 |
| 67012 | 1.89226 | 0.05 |
| 67013 | 1.89226 | 0.0648473 |
| 67014 | 0.946133 | 0.05 |
| 67015 | 0.946133 | 0.0508095 |
| 67016 | 0.946132 | 0.05 |
| 67017 | 0.946132 | 0.05 |
| 67018 | 0.946132 | 0.05 |
| 67019 | 0.946132 | 0.099919 |
| 67020 | 0.946133 | 0.05 |
| 67021 | 0.946133 | 0.0630358 |
| 67022 | 0.946132 | 0.05 |
| 67023 | 0.946132 | 0.05 |
| 67024 | 0.946132 | 0.05 |
| 67025 | 0.946132 | 0.05 |
| 67026 | 1.90098 | 0.0756629 |
| 67027 | 1.36463 | 0.148751 |
| 67028 | 1.56656 | 0.152658 |
| 67029 | 1.36463 | 0.077475 |
| 67030 | 2.04386 | 0.069076 |
| 67031 | 1.89227 | 0.0715596 |
| 67032 | 1.89226 | 0.0760059 |
| 67033 | 1.89226 | 0.099424 |
| 67034 | 1.89226 | 0.163822 |
| 67035 | 1.89226 | 0.097179 |
| 67036 | 1.89226 | 0.0772598 |
| 67037 | 1.89226 | 0.0527861 |
| 67038 | 1.89226 | 0.128275 |
| 67039 | 1.89226 | 0.0795176 |
| 67040 | 1.89226 | 0.25221 |
| 67041 | 1.89226 | 0.123472 |
| 67042 | 1.89226 | 0.161561 |
| 67043 | 1.89226 | 0.097334 |
| 67044 | 1.89226 | 0.10967 |
| 67045 | 1.89226 | 0.146594 |

| | | |
|---|---|---|
| 67046 | 1.89226 | 0.264007 |
| 67047 | 1.89226 | 0.101926 |
| 67048 | 1.89226 | 0.220142 |
| 67049 | 1.89226 | 0.0877609 |
| 67050 | 1.89226 | 0.0646567 |
| 67051 | 1.70303 | 0.130781 |
| 67052 | 2.1292 | 0.156231 |
| 67053 | 1.70303 | 0.0764863 |
| 67054 | 2.32302 | 0.077996 |
| 67055 | 1.90098 | 0.0559199 |
| 67056 | 1.89226 | 0.056795 |
| 67057 | 1.89226 | 0.109583 |
| 67058 | 1.89226 | 0.200199 |
| 67059 | 1.89226 | 0.101377 |
| 67060 | 1.89226 | 0.0781495 |
| 67061 | 1.89226 | 0.05 |
| 67062 | 1.89226 | 0.142596 |
| 67063 | 1.89226 | 0.156613 |
| 67064 | 1.89226 | 0.250592 |
| 67065 | 1.89226 | 0.0993086 |
| 67066 | 1.89226 | 0.163968 |
| 67067 | 1.89226 | 0.0689825 |
| 67068 | 1.89226 | 0.106047 |
| 67069 | 1.89226 | 0.239004 |
| 67070 | 1.89226 | 0.281304 |
| 67071 | 1.89226 | 0.112966 |
| 67072 | 1.89226 | 0.182386 |
| 67073 | 1.89226 | 0.0737201 |
| 67074 | 1.89226 | 0.331604 |
| 67075 | 1.89226 | 1.23814 |
| 67076 | 1.89226 | 1.08151 |
| 67077 | 1.89226 | 0.798474 |
| 67078 | 3.78453 | 0.467031 |
| 67079 | 1.89226 | 0.647003 |
| 67080 | 1.89226 | 1.85311 |
| 67081 | 1.89226 | 0.109731 |
| 67082 | 1.89226 | 2.1 |
| 67083 | 1.89226 | 1.02182 |
| 67084 | 1.89226 | 2.1 |
| 67085 | 1.89226 | 1.22599 |
| 67086 | 1.89226 | 0.809515 |
| 67087 | 1.89226 | 0.71129 |
| 67088 | 1.89226 | 0.914079 |
| 67089 | 3.78452 | 1.03679 |
| 67090 | 1.89224 | 1.07134 |
| 67091 | 1.89224 | 0.75301 |
| 67092 | 1.89224 | 0.858823 |
| 67093 | 1.89224 | 1.1949 |
| 67094 | 1.89224 | 0.251947 |
| 67095 | 1.89224 | 0.77322 |
| 67096 | 1.89224 | 0.67332 |
| 67097 | 1.89224 | 2.1 |
| 67098 | 1.89224 | 1.24273 |
| 67099 | 1.89224 | 2.1 |
| 67100 | 1.89224 | 0.0833073 |
| 67101 | 1.89224 | 2.1 |
| 67102 | 1.31307 | 2.1 |
| 67103 | 1.31307 | 2.1 |

| | | |
|---|---|---|
| 67104 | 0.888948 | 1.29174 |
| 67105 | 0.888948 | 1.07756 |
| 67106 | 1.2559 | 1.3158 |
| 67107 | 0.888979 | 2.1 |
| 67108 | 0.946114 | 2.1 |
| 67109 | 0.946114 | 1.0193 |
| 67110 | 0.946129 | 0.899277 |
| 67111 | 0.888989 | 1.17859 |
| 67112 | 0.946104 | 0.248621 |
| 67113 | 1.25588 | 0.988627 |
| 67114 | 1.89224 | 0.398421 |
| 67115 | 1.89224 | 1.22957 |
| 67116 | 1.89224 | 0.786884 |
| 67117 | 1.89224 | 1.15444 |
| 67118 | 1.89224 | 0.929818 |
| 67119 | 1.89224 | 1.93022 |
| 67120 | 1.79375 | 0.05 |
| 67121 | 1.47689 | 0.05 |
| 67122 | 1.97732 | 0.05 |
| 67123 | 2.26687 | 0.05 |
| 67124 | 3.74375 | 0.05 |
| 67125 | 2.05759 | 0.05 |
| 67126 | 2.26686 | 0.05 |
| 67127 | 1.79374 | 0.05 |
| 67128 | 1.79374 | 0.05 |
| 67129 | 2.49889 | 0.05 |
| 67130 | 2.49889 | 0.05 |
| 67131 | 1.89226 | 0.05 |
| 67132 | 1.89226 | 0.05 |
| 67133 | 1.89226 | 0.05 |
| 67134 | 1.89226 | 0.05 |
| 67135 | 3.78452 | 0.05 |
| 67136 | 1.89227 | 0.05 |
| 67137 | 1.89227 | 0.05 |
| 67138 | 1.89227 | 0.05 |
| 67139 | 1.89227 | 0.05 |
| 67140 | 1.89227 | 0.05 |
| 67141 | 1.89227 | 0.05 |
| 67142 | 1.97732 | 0.05 |
| 67143 | 2.26687 | 0.05 |
| 67144 | 2.6607 | 0.05 |
| 67145 | 2.41464 | 0.05 |
| 67146 | 4.68151 | 0.05 |
| 67147 | 1.89226 | 0.05 |
| 67148 | 1.89226 | 0.05 |
| 67149 | 1.89226 | 0.05 |
| 67150 | 1.89226 | 0.05 |
| 67151 | 3.78452 | 0.05 |
| 67152 | 1.89226 | 0.05 |
| 67153 | 1.89226 | 0.05 |
| 67154 | 1.89226 | 0.05 |
| 67155 | 1.89226 | 0.05 |
| 67156 | 1.89226 | 0.05 |
| 67157 | 1.89226 | 0.05 |
| 67158 | 1.89226 | 0.770473 |
| 67159 | 1.89226 | 1.41175 |
| 67160 | 1.89226 | 0.910043 |
| 67161 | 1.89226 | 0.808221 |

| | | |
|---|---|---|
| 67162 | 3.78452 | 0.813258 |
| 67163 | 1.89226 | 0.847713 |
| 67164 | 1.89226 | 1.59097 |
| 67165 | 1.89226 | 0.859208 |
| 67166 | 1.89226 | 1.12151 |
| 67167 | 1.89226 | 0.909932 |
| 67168 | 1.89226 | 1.69867 |
| 67169 | 1.89226 | 1.38984 |
| 67170 | 1.89226 | 0.814878 |
| 67171 | 1.89226 | 0.750941 |
| 67172 | 1.89226 | 1.20069 |
| 67173 | 1.89226 | 0.795061 |
| 67174 | 1.89226 | 1.26932 |
| 67175 | 1.89226 | 0.911169 |
| 67176 | 1.89226 | 0.799255 |
| 67177 | 1.89226 | 0.717186 |
| 67178 | 1.89226 | 1.18504 |
| 67179 | 1.89226 | 0.766174 |
| 67180 | 1.89226 | 1.42413 |
| 67181 | 1.89226 | 0.853592 |
| 67182 | 2.45282 | 1.1536 |
| 67183 | 1.89226 | 1.26469 |
| 67184 | 1.89226 | 1.74764 |
| 67185 | 1.89226 | 0.819661 |
| 67186 | 1.89226 | 1.0774 |
| 67187 | 1.89226 | 0.816344 |
| 67188 | 1.89226 | 1.33492 |
| 67189 | 1.89226 | 0.803478 |
| 67190 | 1.89226 | 0.889409 |
| 67191 | 1.89226 | 1.36442 |
| 67192 | 1.89226 | 2.03901 |
| 67193 | 1.87308 | 0.05 |
| 67194 | 1.88631 | 0.05 |
| 67195 | 1.63908 | 0.05 |
| 67196 | 1.70302 | 0.05 |
| 67197 | 2.08745 | 0.05 |
| 67198 | 2.16916 | 0.05 |
| 67199 | 2.41019 | 0.05 |
| 67200 | 2.733 | 0.05 |
| 67201 | 2.08121 | 0.05 |
| 67202 | 2.08121 | 0.05 |
| 67203 | 2.74362 | 0.05 |
| 67204 | 2.41464 | 0.05 |
| 67205 | 1.89226 | 0.05 |
| 67206 | 1.89226 | 0.05 |
| 67207 | 1.89226 | 0.05 |
| 67208 | 1.89226 | 0.05 |
| 67209 | 1.89226 | 0.05 |
| 67210 | 1.89226 | 0.05 |
| 67211 | 0.946133 | 0.05 |
| 67212 | 0.946133 | 0.05 |
| 67213 | 0.946132 | 0.0575468 |
| 67214 | 0.946132 | 0.05 |
| 67215 | 0.946132 | 0.05 |
| 67216 | 0.946132 | 0.05 |
| 67217 | 0.946133 | 0.05 |
| 67218 | 0.946133 | 0.05 |
| 67219 | 0.946132 | 0.05 |

| | | |
|---|---|---|
| 67220 | 0.946132 | 0.0745986 |
| 67221 | 0.946133 | 0.05 |
| 67222 | 0.946133 | 0.05 |
| 67223 | 2.02912 | 0.05 |
| 67224 | 2.57016 | 0.05 |
| 67225 | 1.88631 | 0.05 |
| 67226 | 1.88631 | 0.05 |
| 67227 | 2.55301 | 0.05 |
| 67228 | 2.4102 | 0.05 |
| 67229 | 1.89226 | 0.05 |
| 67230 | 1.89226 | 0.0500181 |
| 67231 | 1.89226 | 0.0509298 |
| 67232 | 1.89226 | 0.0769206 |
| 67233 | 3.78452 | 0.0566567 |
| 67234 | 1.89227 | 0.05 |
| 67235 | 1.89227 | 0.0982947 |
| 67236 | 1.89227 | 0.0647756 |
| 67237 | 1.89227 | 0.073827 |
| 67238 | 1.89227 | 0.0682929 |
| 67239 | 1.89227 | 0.068236 |
| 67240 | 1.89226 | 2.1 |
| 67241 | 1.89226 | 2.1 |
| 67242 | 1.89226 | 2.1 |
| 67243 | 1.89226 | 2.1 |
| 67244 | 3.78452 | 2.1 |
| 67245 | 1.2515 | 2.1 |
| 67246 | 1.34788 | 2.1 |
| 67247 | 0.946132 | 2.1 |
| 67248 | 0.946132 | 2.1 |
| 67249 | 0.973043 | 2.1 |
| 67250 | 1.16766 | 2.1 |
| 67251 | 0.919222 | 2.06393 |
| 67252 | 1.2159 | 2.1 |
| 67253 | 0.946132 | 2.1 |
| 67254 | 0.946132 | 2.1 |
| 67255 | 1.33746 | 2.1 |
| 67256 | 1.13537 | 2.1 |
| 67257 | 1.89226 | 2.1 |
| 67258 | 1.89226 | 2.1 |
| 67259 | 1.89226 | 2.1 |
| 67260 | 1.89226 | 2.1 |
| 67261 | 1.89226 | 2.1 |
| 67262 | 1.89226 | 2.1 |
| 67263 | 1.89226 | 2.1 |
| 67264 | 1.89226 | 2.1 |
| 67265 | 1.89226 | 2.1 |
| 67266 | 1.89226 | 2.1 |
| 67267 | 3.78452 | 2.1 |
| 67268 | 1.89227 | 2.1 |
| 67269 | 1.89227 | 1.71732 |
| 67270 | 1.89227 | 2.1 |
| 67271 | 1.89227 | 2.1 |
| 67272 | 1.89227 | 2.1 |
| 67273 | 1.89227 | 2.1 |
| 67274 | 0.946132 | 2.1 |
| 67275 | 0.946132 | 2.1 |
| 67276 | 0.946129 | 2.1 |
| 67277 | 0.946129 | 2.1 |

| | | |
|---|---|---|
| 67278 | 0.946131 | 2.1 |
| 67279 | 0.946131 | 2.1 |
| 67280 | 0.946131 | 2.1 |
| 67281 | 0.946131 | 2.1 |
| 67282 | 0.946131 | 2.1 |
| 67283 | 0.946131 | 2.1 |
| 67284 | 0.94613 | 2.1 |
| 67285 | 0.94613 | 2.1 |
| 67286 | 1.89226 | 1.12648 |
| 67287 | 1.89226 | 1.02774 |
| 67288 | 1.89226 | 1.15529 |
| 67289 | 1.89226 | 0.897017 |
| 67290 | 3.78453 | 0.968109 |
| 67291 | 0.946132 | 1.89341 |
| 67292 | 0.946132 | 0.981513 |
| 67293 | 0.946132 | 1.17638 |
| 67294 | 0.946132 | 1.02625 |
| 67295 | 0.946132 | 0.778568 |
| 67296 | 0.946132 | 1.93529 |
| 67297 | 0.946132 | 1.05306 |
| 67298 | 0.946132 | 1.11043 |
| 67299 | 0.946131 | 2.1 |
| 67300 | 0.946131 | 0.90155 |
| 67301 | 0.946132 | 1.35842 |
| 67302 | 0.946132 | 0.968464 |
| 67303 | 1.89226 | 1.32179 |
| 67304 | 1.89226 | 0.773316 |
| 67305 | 1.89226 | 0.726376 |
| 67306 | 1.89226 | 0.8958 |
| 67307 | 1.89226 | 1.02979 |
| 67308 | 1.89226 | 1.65231 |
| 67309 | 1.89226 | 1.79133 |
| 67310 | 1.89226 | 0.85468 |
| 67311 | 1.89226 | 0.756364 |
| 67312 | 1.89226 | 0.855523 |
| 67313 | 3.78453 | 0.882361 |
| 67314 | 0.96614 | 1.03382 |
| 67315 | 0.96614 | 0.957395 |
| 67316 | 0.946131 | 0.675505 |
| 67317 | 0.946131 | 0.841621 |
| 67318 | 0.946131 | 0.899176 |
| 67319 | 0.96614 | 0.847572 |
| 67320 | 1.37736 | 1.04861 |
| 67321 | 0.946131 | 1.28055 |
| 67322 | 1.37736 | 1.67558 |
| 67323 | 0.96614 | 0.804052 |
| 67324 | 0.946131 | 1.44987 |
| 67325 | 0.946131 | 0.775276 |
| 67326 | 0.946129 | 1.43981 |
| 67327 | 0.946129 | 0.923369 |
| 67328 | 0.946128 | 1.00225 |
| 67329 | 0.946128 | 1.40673 |
| 67330 | 0.946132 | 0.728313 |
| 67331 | 0.946132 | 1.98586 |
| 67332 | 0.946126 | 1.07249 |
| 67333 | 0.946126 | 0.920012 |
| 67334 | 0.946129 | 0.767655 |
| 67335 | 0.946129 | 0.854584 |

| | | |
|---|---|---|
| 67336 | 0.946129 | 0.768223 |
| 67337 | 0.946129 | 1.01444 |
| 67338 | 1.89226 | 0.0501951 |
| 67339 | 1.65723 | 0.05 |
| 67340 | 1.83117 | 0.0812373 |
| 67341 | 1.65723 | 0.0692713 |
| 67342 | 2.0662 | 0.05 |
| 67343 | 1.89226 | 0.05 |
| 67344 | 1.89226 | 0.0662808 |
| 67345 | 1.89226 | 0.0721535 |
| 67346 | 1.89226 | 0.132571 |
| 67347 | 1.89226 | 0.107846 |
| 67348 | 1.89226 | 0.05 |
| 67349 | 1.89226 | 0.05 |
| 67350 | 1.89226 | 0.159282 |
| 67351 | 1.89226 | 0.179353 |
| 67352 | 1.89226 | 0.301162 |
| 67353 | 1.89226 | 0.219267 |
| 67354 | 1.89226 | 0.104287 |
| 67355 | 1.89226 | 0.0653222 |
| 67356 | 1.89226 | 0.106718 |
| 67357 | 1.89226 | 0.0758502 |
| 67358 | 1.89226 | 0.198504 |
| 67359 | 1.89226 | 0.154426 |
| 67360 | 1.89226 | 0.0742572 |
| 67361 | 1.89226 | 0.0528333 |
| 67362 | 1.89226 | 0.0784321 |
| 67363 | 1.83117 | 0.0621295 |
| 67364 | 1.92807 | 0.0878251 |
| 67365 | 1.83117 | 0.099391 |
| 67366 | 1.98916 | 0.05 |
| 67367 | 1.89226 | 0.05 |
| 67368 | 1.89226 | 0.05 |
| 67369 | 1.89226 | 0.105379 |
| 67370 | 1.89226 | 0.102922 |
| 67371 | 1.89226 | 0.140926 |
| 67372 | 3.78453 | 0.0889691 |
| 67373 | 1.89226 | 0.260971 |
| 67374 | 1.89226 | 0.234179 |
| 67375 | 1.89226 | 0.3883 |
| 67376 | 1.89226 | 0.34237 |
| 67377 | 1.89226 | 0.144162 |
| 67378 | 1.89226 | 0.129081 |
| 67379 | 1.89226 | 0.179404 |
| 67380 | 1.89226 | 0.139057 |
| 67381 | 1.89226 | 0.254234 |
| 67382 | 1.89226 | 0.204085 |
| 67383 | 1.89226 | 0.101029 |
| 67384 | 1.89226 | 0.0770558 |
| 67385 | 1.88037 | 0.0822137 |
| 67386 | 1.89227 | 0.0842046 |
| 67387 | 1.89227 | 0.151366 |
| 67388 | 1.89227 | 0.0667523 |
| 67389 | 1.98599 | 0.110625 |
| 67390 | 1.98599 | 0.05 |
| 67391 | 1.89226 | 0.142041 |
| 67392 | 1.89226 | 0.157752 |
| 67393 | 1.89226 | 0.306119 |

| | | |
|---|---|---|
| 67394 | 1.89226 | 0.189082 |
| 67395 | 1.89226 | 0.163911 |
| 67396 | 1.89226 | 0.0832285 |
| 67397 | 1.89226 | 0.232818 |
| 67398 | 1.89226 | 0.223789 |
| 67399 | 1.89226 | 0.377034 |
| 67400 | 1.89226 | 0.451027 |
| 67401 | 3.78452 | 0.262751 |
| 67402 | 1.89226 | 0.189913 |
| 67403 | 1.89226 | 0.335772 |
| 67404 | 1.89226 | 0.294197 |
| 67405 | 1.89226 | 0.241908 |
| 67406 | 1.89226 | 0.24807 |
| 67407 | 1.89226 | 0.203164 |
| 67408 | 1.90116 | 0.0956712 |
| 67409 | 1.89227 | 0.0873219 |
| 67410 | 1.89226 | 0.1476 |
| 67411 | 1.89227 | 0.0855911 |
| 67412 | 1.88036 | 0.110786 |
| 67413 | 1.88037 | 0.0557531 |
| 67414 | 1.89226 | 0.220674 |
| 67415 | 1.89226 | 0.19895 |
| 67416 | 1.89226 | 0.32408 |
| 67417 | 1.89226 | 0.208415 |
| 67418 | 1.89226 | 0.205207 |
| 67419 | 1.89226 | 0.10183 |
| 67420 | 0.946132 | 0.433319 |
| 67421 | 0.946132 | 0.395925 |
| 67422 | 0.946128 | 0.423033 |
| 67423 | 0.946128 | 0.368415 |
| 67424 | 0.94613 | 0.420299 |
| 67425 | 0.94613 | 0.437232 |
| 67426 | 0.94613 | 0.366354 |
| 67427 | 0.94613 | 0.383765 |
| 67428 | 0.946131 | 0.418432 |
| 67429 | 0.946131 | 0.438279 |
| 67430 | 0.94613 | 0.256343 |
| 67431 | 0.94613 | 0.452977 |
| 67432 | 1.89226 | 0.295539 |
| 67433 | 1.89226 | 0.330524 |
| 67434 | 1.89226 | 0.326711 |
| 67435 | 1.89226 | 0.304369 |
| 67436 | 1.89226 | 0.355124 |
| 67437 | 1.89226 | 0.39136 |
| 67438 | 1.89226 | 0.0616829 |
| 67439 | 1.89226 | 1.42884 |
| 67440 | 2.35239 | 1.3882 |
| 67441 | 2.27873 | 0.123158 |
| 67442 | 1.89226 | 1.32519 |
| 67443 | 1.83301 | 0.229021 |
| 67444 | 2.68449 | 0.400014 |
| 67445 | 1.89226 | 2.1 |
| 67446 | 1.89226 | 0.639641 |
| 67447 | 1.89226 | 1.08589 |
| 67448 | 3.78453 | 0.799172 |
| 67449 | 0.946132 | 2.1 |
| 67450 | 0.946132 | 0.907253 |
| 67451 | 0.946129 | 0.281784 |

| | | |
|---|---|---|
| 67452 | 0.946129 | 2.08714 |
| 67453 | 0.946131 | 2.09595 |
| 67454 | 0.946131 | 1.36066 |
| 67455 | 0.94613 | 0.777403 |
| 67456 | 0.94613 | 1.22069 |
| 67457 | 0.946131 | 1.41517 |
| 67458 | 0.946131 | 1.05773 |
| 67459 | 0.946131 | 1.18177 |
| 67460 | 0.946131 | 1.49259 |
| 67461 | 1.89226 | 0.116621 |
| 67462 | 1.89226 | 1.16206 |
| 67463 | 1.89226 | 1.4186 |
| 67464 | 1.89226 | 0.175489 |
| 67465 | 1.89226 | 1.36036 |
| 67466 | 1.89226 | 0.280173 |
| 67467 | 1.70303 | 0.171929 |
| 67468 | 1.89226 | 1.32922 |
| 67469 | 1.89226 | 0.12254 |
| 67470 | 1.89226 | 0.368764 |
| 67471 | 3.78453 | 0.245432 |
| 67472 | 1.89227 | 1.15807 |
| 67473 | 1.89227 | 1.29854 |
| 67474 | 1.89227 | 0.229025 |
| 67475 | 1.89227 | 2.01253 |
| 67476 | 3.78453 | 1.24661 |
| 67477 | 1.89226 | 0.939617 |
| 67478 | 1.89226 | 2.1 |
| 67479 | 1.89226 | 1.13229 |
| 67480 | 1.89226 | 1.70663 |
| 67481 | 3.78452 | 1.43458 |
| 67482 | 1.89226 | 2.1 |
| 67483 | 1.89226 | 2.1 |
| 67484 | 1.89226 | 2.1 |
| 67485 | 1.89226 | 2.1 |
| 67486 | 3.78452 | 2.1 |
| 67487 | 0.946132 | 1.73619 |
| 67488 | 0.946132 | 2.1 |
| 67489 | 0.946129 | 2.1 |
| 67490 | 0.946129 | 2.1 |
| 67491 | 0.94613 | 2.1 |
| 67492 | 0.94613 | 2.0851 |
| 67493 | 0.946131 | 2.1 |
| 67494 | 0.946131 | 2.1 |
| 67495 | 0.946131 | 2.1 |
| 67496 | 0.946131 | 2.1 |
| 67497 | 0.946131 | 2.1 |
| 67498 | 0.946131 | 2.06085 |
| 67499 | 1.89226 | 2.1 |
| 67500 | 1.89226 | 2.08574 |
| 67501 | 1.89226 | 2.1 |
| 67502 | 1.89226 | 2.1 |
| 67503 | 3.78452 | 2.1 |
| 67504 | 1.89226 | 1.54605 |
| 67505 | 1.89226 | 2.1 |
| 67506 | 1.89226 | 2.1 |
| 67507 | 1.89226 | 1.63216 |
| 67508 | 1.89226 | 2.1 |
| 67509 | 1.89226 | 1.57054 |

| | | |
|---|---|---|
| 67510 | 1.89227 | 2.1 |
| 67511 | 2.48308 | 1.39955 |
| 67512 | 1.89227 | 1.63971 |
| 67513 | 1.89227 | 2.1 |
| 67514 | 3.78453 | 2.1 |
| 67515 | 1.89226 | 2.04989 |
| 67516 | 1.89226 | 2.1 |
| 67517 | 1.89226 | 2.1 |
| 67518 | 1.89226 | 1.84513 |
| 67519 | 1.89226 | 2.1 |
| 67520 | 1.89226 | 1.62645 |
| 67521 | 1.89226 | 2.02467 |
| 67522 | 1.89226 | 1.88048 |
| 67523 | 1.89226 | 2.1 |
| 67524 | 1.89226 | 1.89582 |
| 67525 | 3.78453 | 2.1 |
| 67526 | 0.946131 | 1.82823 |
| 67527 | 0.946131 | 2.1 |
| 67528 | 0.946131 | 2.1 |
| 67529 | 0.946131 | 2.1 |
| 67530 | 0.946131 | 2.1 |
| 67531 | 0.946131 | 2.09954 |
| 67532 | 0.946131 | 2.1 |
| 67533 | 0.946131 | 2.1 |
| 67534 | 0.946131 | 2.1 |
| 67535 | 0.946131 | 2.1 |
| 67536 | 0.946131 | 2.1 |
| 67537 | 0.946131 | 1.98613 |
| 67538 | 1.23719 | 2.1 |
| 67539 | 1.28439 | 1.96134 |
| 67540 | 0.94613 | 1.744 |
| 67541 | 0.94613 | 1.92467 |
| 67542 | 1.28439 | 2.0669 |
| 67543 | 1.6431 | 2.1 |
| 67544 | 0.946129 | 1.84555 |
| 67545 | 0.946129 | 1.52947 |
| 67546 | 1.28439 | 1.16553 |
| 67547 | 0.946131 | 1.03921 |
| 67548 | 1.21038 | 0.343146 |
| 67549 | 1.21038 | 1.21445 |
| 67550 | 1.89226 | 2.1 |
| 67551 | 1.89226 | 1.70379 |
| 67552 | 1.89226 | 1.97416 |
| 67553 | 1.89226 | 1.89382 |
| 67554 | 1.89226 | 1.79589 |
| 67555 | 1.89226 | 1.7282 |
| 67556 | 1.89226 | 2.04882 |
| 67557 | 1.89226 | 2.1 |
| 67558 | 1.89226 | 2.1 |
| 67559 | 1.89226 | 1.6527 |
| 67560 | 1.89226 | 2.1 |
| 67561 | 1.89226 | 1.49986 |
| 67562 | 1.89226 | 1.13459 |
| 67563 | 1.89226 | 0.161967 |
| 67564 | 1.89226 | 0.236147 |
| 67565 | 1.89226 | 0.804051 |
| 67566 | 1.89226 | 0.678234 |
| 67567 | 1.89226 | 1.1823 |

| | | |
|---|---|---|
| 67568 | 0.946128 | 0.406434 |
| 67569 | 0.946128 | 0.830258 |
| 67570 | 0.946125 | 0.05 |
| 67571 | 0.946125 | 0.384363 |
| 67572 | 0.94613 | 0.0763786 |
| 67573 | 0.94613 | 0.232864 |
| 67574 | 0.946124 | 0.681444 |
| 67575 | 0.946124 | 0.843301 |
| 67576 | 0.946127 | 0.132484 |
| 67577 | 0.946127 | 0.05 |
| 67578 | 0.946127 | 0.659163 |
| 67579 | 0.946127 | 0.823915 |
| 67580 | 1.89226 | 0.0521503 |
| 67581 | 2.78671 | 0.0539241 |
| 67582 | 2.59302 | 0.05 |
| 67583 | 2.59302 | 0.0525251 |
| 67584 | 1.89226 | 0.268949 |
| 67585 | 1.89226 | 0.138418 |
| 67586 | 1.89226 | 0.120841 |
| 67587 | 1.89226 | 0.25084 |
| 67588 | 1.89226 | 0.0812915 |
| 67589 | 1.89226 | 0.0820501 |
| 67590 | 1.89226 | 0.575561 |
| 67591 | 1.89226 | 0.325947 |
| 67592 | 1.89225 | 0.0943808 |
| 67593 | 1.89225 | 0.05 |
| 67594 | 1.89225 | 0.315808 |
| 67595 | 1.89225 | 0.188171 |
| 67596 | 1.89225 | 0.470198 |
| 67597 | 1.89225 | 0.0580116 |
| 67598 | 0.979536 | 0.05 |
| 67599 | 0.816597 | 0.0622957 |
| 67600 | 1.15446 | 0.05 |
| 67601 | 1.07423 | 0.05 |
| 67602 | 1.08452 | 0.0674183 |
| 67603 | 0.603522 | 0.05 |
| 67604 | 1.70302 | 0.05 |
| 67605 | 1.29344 | 0.05 |
| 67606 | 1.21806 | 0.05 |
| 67607 | 0.723758 | 0.05 |
| 67608 | 1.37562 | 0.05 |
| 67609 | 1.89226 | 0.0593148 |
| 67610 | 1.92807 | 0.0806416 |
| 67611 | 1.90843 | 0.0682777 |
| 67612 | 1.92807 | 0.124123 |
| 67613 | 1.87263 | 0.05 |
| 67614 | 1.89226 | 0.0664955 |
| 67615 | 0.946132 | 0.147382 |
| 67616 | 0.946132 | 0.111336 |
| 67617 | 0.946131 | 0.0967623 |
| 67618 | 0.946131 | 0.123253 |
| 67619 | 0.946132 | 0.139633 |
| 67620 | 0.946132 | 0.128943 |
| 67621 | 0.946132 | 0.05 |
| 67622 | 0.946132 | 0.0872629 |
| 67623 | 0.946132 | 0.136168 |
| 67624 | 0.946132 | 0.17545 |
| 67625 | 0.946132 | 0.104209 |

| | | |
|---|---|---|
| 67626 | 0.946132 | 0.147188 |
| 67627 | 1.89226 | 0.225446 |
| 67628 | 1.89226 | 0.361095 |
| 67629 | 1.89226 | 0.343672 |
| 67630 | 1.89226 | 0.391908 |
| 67631 | 3.78452 | 0.349316 |
| 67632 | 1.89226 | 0.229218 |
| 67633 | 1.89226 | 0.23627 |
| 67634 | 1.89226 | 0.273088 |
| 67635 | 1.89226 | 0.280403 |
| 67636 | 1.89226 | 0.1306 |
| 67637 | 1.89226 | 0.163569 |
| 67638 | 1.70303 | 0.0503868 |
| 67639 | 1.90843 | 0.0584541 |
| 67640 | 1.77002 | 0.0547196 |
| 67641 | 1.90843 | 0.128718 |
| 67642 | 1.75709 | 0.05 |
| 67643 | 1.89226 | 0.0605315 |
| 67644 | 1.89226 | 0.0708368 |
| 67645 | 1.89226 | 0.137287 |
| 67646 | 1.89226 | 0.089421 |
| 67647 | 1.89226 | 0.273946 |
| 67648 | 3.78453 | 0.111176 |
| 67649 | 1.89226 | 0.30708 |
| 67650 | 1.89226 | 0.387529 |
| 67651 | 1.89226 | 0.350321 |
| 67652 | 1.89226 | 0.403557 |
| 67653 | 3.78452 | 0.380589 |
| 67654 | 0.946131 | 0.190949 |
| 67655 | 0.946131 | 0.320324 |
| 67656 | 0.946131 | 0.107217 |
| 67657 | 0.946131 | 0.242923 |
| 67658 | 0.946129 | 0.258238 |
| 67659 | 0.946129 | 0.26164 |
| 67660 | 0.946133 | 0.0990429 |
| 67661 | 0.946133 | 0.162874 |
| 67662 | 0.946131 | 0.238769 |
| 67663 | 0.946131 | 0.251936 |
| 67664 | 0.946131 | 0.170385 |
| 67665 | 0.946131 | 0.269812 |
| 67666 | 1.89226 | 0.05 |
| 67667 | 2.10275 | 0.239387 |
| 67668 | 1.76377 | 0.0934811 |
| 67669 | 1.76377 | 0.05 |
| 67670 | 1.89226 | 0.0600457 |
| 67671 | 1.89226 | 0.05 |
| 67672 | 1.89226 | 0.05 |
| 67673 | 1.89226 | 0.203639 |
| 67674 | 2.10275 | 0.13261 |
| 67675 | 2.55889 | 0.05 |
| 67676 | 1.89226 | 0.0820256 |
| 67677 | 2.30274 | 0.05 |
| 67678 | 1.89226 | 0.05 |
| 67679 | 1.89226 | 0.967612 |
| 67680 | 1.89226 | 0.780195 |
| 67681 | 1.89226 | 0.0625915 |
| 67682 | 1.89226 | 0.577825 |
| 67683 | 1.89226 | 0.0654264 |

| | | |
|---|---|---|
| 67684 | 1.89226 | 0.05 |
| 67685 | 1.89226 | 1.02517 |
| 67686 | 1.89226 | 0.726901 |
| 67687 | 1.89226 | 0.085148 |
| 67688 | 1.89226 | 0.474844 |
| 67689 | 1.89226 | 0.0747166 |
| 67690 | 1.89226 | 0.05 |
| 67691 | 1.89226 | 0.235379 |
| 67692 | 1.89226 | 0.101253 |
| 67693 | 1.89226 | 0.05 |
| 67694 | 1.89226 | 0.0882039 |
| 67695 | 1.89226 | 0.05 |
| 67696 | 1.89226 | 0.05 |
| 67697 | 1.89226 | 0.171867 |
| 67698 | 1.89226 | 0.106019 |
| 67699 | 1.89226 | 0.05 |
| 67700 | 1.89226 | 0.0693984 |
| 67701 | 1.89226 | 0.05 |
| 67702 | 1.89226 | 0.05 |
| 67703 | 1.89226 | 0.895059 |
| 67704 | 1.89226 | 0.630765 |
| 67705 | 1.89226 | 0.0812788 |
| 67706 | 1.89226 | 0.562591 |
| 67707 | 1.89226 | 0.0919581 |
| 67708 | 1.89226 | 0.513317 |
| 67709 | 1.89226 | 0.924646 |
| 67710 | 1.89226 | 0.062784 |
| 67711 | 1.89226 | 0.153747 |
| 67712 | 1.89226 | 0.0570478 |
| 67713 | 1.89226 | 0.466941 |
| 67714 | 0.946131 | 0.05 |
| 67715 | 0.976399 | 0.05 |
| 67716 | 1.10124 | 0.05 |
| 67717 | 0.946131 | 0.05 |
| 67718 | 0.887633 | 0.05 |
| 67719 | 0.887633 | 0.05 |
| 67720 | 1.16933 | 0.05 |
| 67721 | 1.23163 | 0.05 |
| 67722 | 0.946131 | 0.05 |
| 67723 | 0.946131 | 0.05 |
| 67724 | 0.976399 | 0.05 |
| 67725 | 1.14992 | 0.05 |
| 67726 | 1.89226 | 0.05 |
| 67727 | 1.89226 | 0.291121 |
| 67728 | 1.89226 | 0.05 |
| 67729 | 1.89226 | 0.05 |
| 67730 | 3.78452 | 0.05 |
| 67731 | 1.89226 | 0.05 |
| 67732 | 1.89226 | 0.0866605 |
| 67733 | 1.89226 | 0.05 |
| 67734 | 1.89226 | 0.05 |
| 67735 | 3.78453 | 0.05 |
| 67736 | 0.946132 | 0.05 |
| 67737 | 0.946132 | 0.05 |
| 67738 | 0.946131 | 0.05 |
| 67739 | 0.946131 | 0.05 |
| 67740 | 0.946131 | 0.05 |
| 67741 | 0.946131 | 0.05 |

| | | |
|---|---|---|
| 67742 | 0.946132 | 0.05 |
| 67743 | 0.946132 | 0.05 |
| 67744 | 0.946131 | 0.05 |
| 67745 | 0.946131 | 0.05 |
| 67746 | 0.946132 | 0.05 |
| 67747 | 0.946132 | 0.05 |
| 67748 | 0.946132 | 0.0525907 |
| 67749 | 0.946132 | 0.05 |
| 67750 | 0.946131 | 0.05 |
| 67751 | 0.946131 | 0.050502 |
| 67752 | 0.946131 | 0.0553988 |
| 67753 | 0.946131 | 0.05 |
| 67754 | 0.946132 | 0.05 |
| 67755 | 0.946132 | 0.05 |
| 67756 | 0.946131 | 0.05 |
| 67757 | 0.946131 | 0.05 |
| 67758 | 0.946132 | 0.05 |
| 67759 | 0.946132 | 0.05 |
| 67760 | 1.89226 | 0.0625637 |
| 67761 | 1.89226 | 0.134921 |
| 67762 | 1.89226 | 0.05 |
| 67763 | 1.89226 | 0.207675 |
| 67764 | 3.78452 | 0.0934069 |
| 67765 | 1.89226 | 0.824762 |
| 67766 | 1.89226 | 0.734062 |
| 67767 | 1.89226 | 0.66974 |
| 67768 | 1.89226 | 0.296742 |
| 67769 | 3.78453 | 0.291285 |
| 67770 | 1.89226 | 0.135745 |
| 67771 | 1.89226 | 0.790899 |
| 67772 | 1.89226 | 0.678507 |
| 67773 | 1.89226 | 0.831964 |
| 67774 | 1.89226 | 0.217029 |
| 67775 | 1.89226 | 0.532907 |
| 67776 | 1.89225 | 0.331954 |
| 67777 | 1.89225 | 0.403456 |
| 67778 | 1.89225 | 0.535674 |
| 67779 | 1.89225 | 0.780587 |
| 67780 | 1.89225 | 0.074315 |
| 67781 | 1.89225 | 0.487409 |
| 67782 | 1.89226 | 0.571535 |
| 67783 | 1.89226 | 0.571877 |
| 67784 | 1.89226 | 0.584905 |
| 67785 | 1.89226 | 0.748923 |
| 67786 | 1.89226 | 0.114674 |
| 67787 | 1.89226 | 0.299431 |
| 67788 | 1.89226 | 0.851442 |
| 67789 | 1.89226 | 0.18386 |
| 67790 | 1.89226 | 0.05 |
| 67791 | 1.89226 | 0.05 |
| 67792 | 3.78453 | 0.106869 |
| 67793 | 1.39491 | 0.05 |
| 67794 | 1.39491 | 0.05 |
| 67795 | 0.924649 | 0.05 |
| 67796 | 0.924649 | 0.05 |
| 67797 | 0.946131 | 0.172191 |
| 67798 | 1.37343 | 0.05 |
| 67799 | 0.924678 | 0.05 |

| | | |
|---|---|---|
| 67800 | 0.946131 | 0.593997 |
| 67801 | 0.924678 | 0.05 |
| 67802 | 1.37343 | 0.05 |
| 67803 | 0.946131 | 0.312554 |
| 67804 | 0.946131 | 0.370613 |
| 67805 | 1.89226 | 0.497418 |
| 67806 | 1.89226 | 0.05 |
| 67807 | 1.89226 | 0.0935933 |
| 67808 | 1.89226 | 0.229681 |
| 67809 | 1.89226 | 0.05 |
| 67810 | 1.89226 | 0.0528569 |
| 67811 | 0.946126 | 0.05 |
| 67812 | 0.975461 | 0.05 |
| 67813 | 0.946132 | 0.05 |
| 67814 | 1.12674 | 0.05 |
| 67815 | 0.946129 | 0.05 |
| 67816 | 0.946129 | 0.05 |
| 67817 | 1.12673 | 0.05 |
| 67818 | 1.12673 | 0.05 |
| 67819 | 0.946129 | 0.05 |
| 67820 | 0.946129 | 0.05 |
| 67821 | 0.975464 | 0.05 |
| 67822 | 1.12673 | 0.05 |
| 67823 | 1.89226 | 0.05 |
| 67824 | 1.89226 | 0.05 |
| 67825 | 1.89226 | 0.05 |
| 67826 | 1.89226 | 0.05 |
| 67827 | 1.89226 | 0.05 |
| 67828 | 1.89226 | 0.05 |
| 67829 | 0.946126 | 0.05 |
| 67830 | 0.946126 | 0.05 |
| 67831 | 0.946131 | 0.05 |
| 67832 | 0.946131 | 0.05 |
| 67833 | 0.946129 | 0.05 |
| 67834 | 0.946129 | 0.05 |
| 67835 | 0.946128 | 0.05 |
| 67836 | 0.946128 | 0.05 |
| 67837 | 0.946129 | 0.05 |
| 67838 | 0.946129 | 0.05 |
| 67839 | 0.946128 | 0.05 |
| 67840 | 0.946128 | 0.05 |
| 67841 | 1.89226 | 0.05 |
| 67842 | 1.89226 | 0.05 |
| 67843 | 1.89226 | 0.05 |
| 67844 | 1.89226 | 0.05 |
| 67845 | 3.78451 | 0.05 |
| 67846 | 1.89226 | 0.05 |
| 67847 | 1.89226 | 0.05 |
| 67848 | 1.89226 | 0.05 |
| 67849 | 1.89226 | 0.05 |
| 67850 | 3.78452 | 0.05 |
| 67851 | 0.946125 | 0.05 |
| 67852 | 0.946125 | 0.05 |
| 67853 | 0.946131 | 0.05 |
| 67854 | 0.946131 | 0.05 |
| 67855 | 0.946128 | 0.05 |
| 67856 | 0.946128 | 0.05 |
| 67857 | 0.946128 | 0.05 |

| | | |
|---|---|---|
| 67858 | 0.946128 | 0.05 |
| 67859 | 0.946128 | 0.05 |
| 67860 | 0.946128 | 0.05 |
| 67861 | 0.946128 | 0.05 |
| 67862 | 0.946128 | 0.05 |
| 67863 | 1.89226 | 0.0551437 |
| 67864 | 1.89226 | 0.182197 |
| 67865 | 1.89226 | 0.132231 |
| 67866 | 1.89226 | 0.111693 |
| 67867 | 3.78452 | 0.131822 |
| 67868 | 1.89226 | 0.11331 |
| 67869 | 1.89226 | 0.275931 |
| 67870 | 1.89226 | 0.213969 |
| 67871 | 1.89226 | 0.20783 |
| 67872 | 1.89226 | 0.167969 |
| 67873 | 1.89226 | 0.178507 |
| 67874 | 1.12492 | 0.190573 |
| 67875 | 1.41201 | 0.132975 |
| 67876 | 1.12082 | 0.151247 |
| 67877 | 0.771416 | 0.231273 |
| 67878 | 1.10725 | 0.232748 |
| 67879 | 1.10725 | 0.14153 |
| 67880 | 1.07618 | 0.388492 |
| 67881 | 1.07618 | 0.05 |
| 67882 | 1.12492 | 0.152722 |
| 67883 | 0.771415 | 0.219224 |
| 67884 | 1.12082 | 0.121589 |
| 67885 | 1.41201 | 0.210375 |
| 67886 | 2.82401 | 0.0660315 |
| 67887 | 1.89226 | 0.181832 |
| 67888 | 1.89226 | 0.0944272 |
| 67889 | 1.89226 | 0.084223 |
| 67890 | 2.24983 | 0.179074 |
| 67891 | 2.24983 | 0.131674 |
| 67892 | 0.946133 | 0.290876 |
| 67893 | 0.946133 | 0.15542 |
| 67894 | 0.946129 | 0.133371 |
| 67895 | 0.946129 | 0.207122 |
| 67896 | 0.946131 | 0.297092 |
| 67897 | 0.946131 | 0.312142 |
| 67898 | 0.946131 | 0.206902 |
| 67899 | 0.946131 | 0.0780865 |
| 67900 | 0.946131 | 0.106517 |
| 67901 | 0.946131 | 0.139941 |
| 67902 | 0.946131 | 0.131681 |
| 67903 | 0.946131 | 0.177688 |
| 67904 | 0.946132 | 0.295296 |
| 67905 | 0.946132 | 0.2891 |
| 67906 | 0.946133 | 0.275496 |
| 67907 | 0.946133 | 0.225599 |
| 67908 | 0.946132 | 0.381317 |
| 67909 | 0.946132 | 0.334048 |
| 67910 | 0.946133 | 0.327626 |
| 67911 | 0.946133 | 0.186927 |
| 67912 | 0.946132 | 0.325374 |
| 67913 | 0.946132 | 0.23431 |
| 67914 | 0.946133 | 0.374523 |
| 67915 | 0.946133 | 0.245416 |

| | | |
|---|---|---|
| 67916 | 1.89227 | 0.05 |
| 67917 | 1.89227 | 0.05 |
| 67918 | 1.89227 | 0.05 |
| 67919 | 1.89227 | 0.05 |
| 67920 | 1.89227 | 0.05 |
| 67921 | 1.89227 | 0.05 |
| 67922 | 1.42982 | 0.05 |
| 67923 | 1.19944 | 0.05 |
| 67924 | 0.936311 | 0.05 |
| 67925 | 0.936311 | 0.05 |
| 67926 | 0.946132 | 0.05 |
| 67927 | 1.02993 | 0.05 |
| 67928 | 1.41513 | 0.05 |
| 67929 | 0.946133 | 0.05 |
| 67930 | 0.946133 | 0.05 |
| 67931 | 0.946133 | 0.05 |
| 67932 | 1.02993 | 0.05 |
| 67933 | 1.18181 | 0.05 |
| 67934 | 1.89226 | 0.05 |
| 67935 | 1.89226 | 0.05 |
| 67936 | 1.89226 | 0.0937806 |
| 67937 | 1.89226 | 0.05 |
| 67938 | 3.78453 | 0.05 |
| 67939 | 1.89226 | 0.05 |
| 67940 | 1.89226 | 0.05 |
| 67941 | 1.89226 | 0.05 |
| 67942 | 1.89226 | 0.05 |
| 67943 | 1.89226 | 0.05 |
| 67944 | 1.89226 | 0.05 |
| 67945 | 1.89226 | 0.05 |
| 67946 | 1.89226 | 0.05 |
| 67947 | 1.89226 | 0.05 |
| 67948 | 1.89226 | 0.05 |
| 67949 | 1.89226 | 0.05 |
| 67950 | 1.89226 | 0.05 |
| 67951 | 0.946131 | 0.05 |
| 67952 | 0.946131 | 0.05 |
| 67953 | 0.946132 | 0.05 |
| 67954 | 0.946132 | 0.05 |
| 67955 | 0.946132 | 0.05 |
| 67956 | 0.946132 | 0.05 |
| 67957 | 0.946132 | 0.0552452 |
| 67958 | 0.946132 | 0.05 |
| 67959 | 0.946132 | 0.05 |
| 67960 | 0.946132 | 0.05 |
| 67961 | 0.946132 | 0.05 |
| 67962 | 0.946132 | 0.05 |
| 67963 | 1.89226 | 1.09286 |
| 67964 | 1.89226 | 1.17287 |
| 67965 | 1.89226 | 1.43951 |
| 67966 | 1.89226 | 0.408596 |
| 67967 | 1.89226 | 1.4449 |
| 67968 | 1.89226 | 0.480534 |
| 67969 | 1.89226 | 1.37042 |
| 67970 | 2.22749 | 0.0507376 |
| 67971 | 1.89226 | 0.111304 |
| 67972 | 1.89226 | 0.35914 |
| 67973 | 3.78453 | 0.395724 |

| | | |
|---|---|---|
| 67974 | 1.89226 | 1.15982 |
| 67975 | 1.89226 | 1.28606 |
| 67976 | 1.89226 | 1.51265 |
| 67977 | 1.89226 | 0.434736 |
| 67978 | 3.78453 | 0.990764 |
| 67979 | 1.89226 | 0.251869 |
| 67980 | 1.89226 | 0.448454 |
| 67981 | 1.89226 | 1.00631 |
| 67982 | 1.89226 | 0.102186 |
| 67983 | 1.89226 | 1.10789 |
| 67984 | 1.89226 | 0.12472 |
| 67985 | 0.94613 | 0.0917304 |
| 67986 | 0.94613 | 1.05005 |
| 67987 | 0.94613 | 1.37862 |
| 67988 | 0.94613 | 0.831477 |
| 67989 | 0.946131 | 0.469153 |
| 67990 | 0.946131 | 0.126015 |
| 67991 | 0.946129 | 1.26728 |
| 67992 | 0.946129 | 0.390006 |
| 67993 | 0.94613 | 0.435695 |
| 67994 | 0.94613 | 0.44238 |
| 67995 | 0.94613 | 0.522184 |
| 67996 | 0.94613 | 0.21227 |
| 67997 | 1.89226 | 0.05 |
| 67998 | 1.89226 | 0.05 |
| 67999 | 1.89226 | 0.05 |
| 68000 | 1.89226 | 0.05 |
| 68001 | 1.89226 | 0.05 |
| 68002 | 1.89226 | 0.05 |
| 68003 | 1.89226 | 0.05 |
| 68004 | 1.89226 | 0.05 |
| 68005 | 1.89226 | 0.05 |
| 68006 | 1.89226 | 0.05 |
| 68007 | 3.78452 | 0.05 |
| 68008 | 1.89225 | 0.05 |
| 68009 | 1.89225 | 0.05 |
| 68010 | 1.89225 | 0.05 |
| 68011 | 1.89225 | 0.05 |
| 68012 | 1.89225 | 0.05 |
| 68013 | 1.89225 | 0.05 |
| 68014 | 1.89226 | 0.05 |
| 68015 | 1.89226 | 0.05 |
| 68016 | 1.89226 | 0.05 |
| 68017 | 1.89226 | 0.05 |
| 68018 | 1.89226 | 0.05 |
| 68019 | 1.89226 | 0.05 |
| 68020 | 0.946132 | 0.05 |
| 68021 | 0.946132 | 0.05 |
| 68022 | 0.946132 | 0.05 |
| 68023 | 0.946132 | 0.05 |
| 68024 | 0.946131 | 0.05 |
| 68025 | 0.946131 | 0.05 |
| 68026 | 0.946132 | 0.05 |
| 68027 | 0.946132 | 0.05 |
| 68028 | 0.946132 | 0.05 |
| 68029 | 0.946132 | 0.05 |
| 68030 | 0.946132 | 0.05 |
| 68031 | 0.946132 | 0.05 |

| | | |
|---|---|---|
| 68032 | 1.89226 | 0.05 |
| 68033 | 1.89226 | 0.05 |
| 68034 | 1.89226 | 0.05 |
| 68035 | 1.89226 | 0.05 |
| 68036 | 1.89226 | 0.05 |
| 68037 | 1.89226 | 0.05 |
| 68038 | 1.89226 | 0.05 |
| 68039 | 1.89226 | 0.05 |
| 68040 | 1.89226 | 0.05 |
| 68041 | 1.89226 | 0.05 |
| 68042 | 1.89226 | 0.05 |
| 68043 | 1.89226 | 0.05 |
| 68044 | 1.89226 | 0.05 |
| 68045 | 1.89226 | 0.05 |
| 68046 | 1.89226 | 0.05 |
| 68047 | 1.89226 | 0.05 |
| 68048 | 1.89226 | 0.05 |
| 68049 | 1.89226 | 0.05 |
| 68050 | 1.89226 | 0.05 |
| 68051 | 1.89226 | 0.05 |
| 68052 | 1.89226 | 0.05 |
| 68053 | 1.89226 | 0.05 |
| 68054 | 3.78452 | 0.05 |
| 68055 | 1.89226 | 0.05 |
| 68056 | 1.89226 | 0.05 |
| 68057 | 1.89226 | 0.05 |
| 68058 | 1.89226 | 0.05 |
| 68059 | 1.89226 | 0.05 |
| 68060 | 1.89226 | 0.05 |
| 68061 | 1.89226 | 0.05 |
| 68062 | 1.89226 | 0.05 |
| 68063 | 1.89226 | 0.05 |
| 68064 | 1.89226 | 0.05 |
| 68065 | 3.78453 | 0.05 |
| 68066 | 1.89226 | 0.05 |
| 68067 | 2.48862 | 0.05 |
| 68068 | 1.89226 | 0.05 |
| 68069 | 2.22062 | 0.05 |
| 68070 | 1.89226 | 0.05 |
| 68071 | 2.22062 | 0.05 |
| 68072 | 1.89227 | 0.05 |
| 68073 | 1.89227 | 0.05 |
| 68074 | 1.89227 | 0.05 |
| 68075 | 1.89227 | 0.05 |
| 68076 | 1.89227 | 0.05 |
| 68077 | 1.89227 | 0.05 |
| 68078 | 0.946132 | 0.05 |
| 68079 | 0.946132 | 0.05 |
| 68080 | 0.946131 | 0.05 |
| 68081 | 0.946131 | 0.05 |
| 68082 | 0.94613 | 0.05 |
| 68083 | 0.94613 | 0.05 |
| 68084 | 0.946133 | 0.05 |
| 68085 | 0.946133 | 0.05 |
| 68086 | 0.946131 | 0.05 |
| 68087 | 0.946131 | 0.05 |
| 68088 | 0.946131 | 0.05 |
| 68089 | 0.946131 | 0.05 |

| | | |
|---|---|---|
| 68090 | 1.89226 | 0.05 |
| 68091 | 1.89226 | 0.05 |
| 68092 | 1.89226 | 0.05 |
| 68093 | 1.89226 | 0.05 |
| 68094 | 3.78453 | 0.05 |
| 68095 | 1.89226 | 0.05 |
| 68096 | 1.89226 | 0.05 |
| 68097 | 1.89226 | 0.05 |
| 68098 | 1.89226 | 0.05 |
| 68099 | 3.78453 | 0.05 |
| 68100 | 1.89226 | 0.05 |
| 68101 | 1.89226 | 0.0662598 |
| 68102 | 1.89226 | 0.0640762 |
| 68103 | 1.89226 | 0.166139 |
| 68104 | 1.89226 | 0.05 |
| 68105 | 1.89226 | 0.0607081 |
| 68106 | 1.89226 | 0.221443 |
| 68107 | 1.89226 | 0.171699 |
| 68108 | 1.89226 | 0.0616199 |
| 68109 | 1.89226 | 0.369349 |
| 68110 | 3.78452 | 0.12607 |
| 68111 | 1.89226 | 0.05 |
| 68112 | 1.89226 | 0.300557 |
| 68113 | 1.89226 | 0.112079 |
| 68114 | 1.89226 | 0.20024 |
| 68115 | 3.78453 | 0.154469 |
| 68116 | 2.57454 | 0.140083 |
| 68117 | 1.89226 | 0.222119 |
| 68118 | 1.3663 | 0.131653 |
| 68119 | 1.89226 | 0.216222 |
| 68120 | 3.25856 | 0.237618 |
| 68121 | 1.89226 | 0.136514 |
| 68122 | 1.89226 | 0.116744 |
| 68123 | 1.89226 | 0.101032 |
| 68124 | 1.89226 | 0.258688 |
| 68125 | 3.78451 | 0.0886854 |
| 68126 | 1.89226 | 0.108419 |
| 68127 | 1.89226 | 0.536948 |
| 68128 | 1.89226 | 0.0705353 |
| 68129 | 1.89226 | 0.151626 |
| 68130 | 1.89226 | 0.114057 |
| 68131 | 1.89226 | 0.273156 |
| 68132 | 0.810946 | 0.265664 |
| 68133 | 0.810946 | 0.20695 |
| 68134 | 0.810946 | 0.425775 |
| 68135 | 0.810946 | 0.507453 |
| 68136 | 0.810959 | 0.165594 |
| 68137 | 0.810959 | 0.0956823 |
| 68138 | 1.89226 | 0.229169 |
| 68139 | 1.35168 | 0.145275 |
| 68140 | 1.08132 | 0.144776 |
| 68141 | 1.08132 | 0.483119 |
| 68142 | 1.0813 | 0.229101 |
| 68143 | 1.89226 | 0.12432 |
| 68144 | 1.89226 | 0.467279 |
| 68145 | 1.89226 | 0.610161 |
| 68146 | 1.89226 | 0.483173 |
| 68147 | 3.78453 | 0.453996 |

| | | |
|---|---|---|
| 68148 | 1.89226 | 0.389357 |
| 68149 | 1.89226 | 0.411273 |
| 68150 | 1.89226 | 0.472795 |
| 68151 | 1.89226 | 0.604134 |
| 68152 | 1.89226 | 0.0669438 |
| 68153 | 1.89226 | 0.290343 |
| 68154 | 1.89226 | 0.427487 |
| 68155 | 1.89226 | 0.49704 |
| 68156 | 1.89226 | 0.604844 |
| 68157 | 1.89226 | 0.494776 |
| 68158 | 3.78452 | 0.475451 |
| 68159 | 1.89226 | 0.465829 |
| 68160 | 1.89226 | 0.471804 |
| 68161 | 1.89226 | 0.491082 |
| 68162 | 1.89226 | 0.692383 |
| 68163 | 3.78452 | 0.500844 |
| 68164 | 2.142 | 0.684618 |
| 68165 | 1.93949 | 0.146154 |
| 68166 | 1.39296 | 0.645986 |
| 68167 | 2.55711 | 0.783955 |
| 68168 | 1.39296 | 0.05 |
| 68169 | 2.55711 | 0.346176 |
| 68170 | 1.89226 | 0.364094 |
| 68171 | 2.5571 | 0.05 |
| 68172 | 2.142 | 0.5153 |
| 68173 | 1.89226 | 0.799216 |
| 68174 | 4.44936 | 0.523429 |
| 68175 | 0.946131 | 0.462651 |
| 68176 | 0.946131 | 0.489668 |
| 68177 | 1.27855 | 0.549794 |
| 68178 | 1.29171 | 0.512523 |
| 68179 | 1.29171 | 0.582586 |
| 68180 | 0.946127 | 0.438335 |
| 68181 | 1.27855 | 0.05 |
| 68182 | 0.946131 | 0.735131 |
| 68183 | 1.29172 | 0.584497 |
| 68184 | 1.29172 | 0.393226 |
| 68185 | 0.946129 | 0.25555 |
| 68186 | 0.946129 | 0.342083 |
| 68187 | 1.27855 | 0.579184 |
| 68188 | 1.29172 | 0.536483 |
| 68189 | 0.969743 | 0.613332 |
| 68190 | 1.07109 | 0.574223 |
| 68191 | 1.29172 | 0.950182 |
| 68192 | 1.07109 | 0.283038 |
| 68193 | 0.969745 | 0.120206 |
| 68194 | 1.27855 | 0.742623 |
| 68195 | 1.10873 | 0.711403 |
| 68196 | 1.10873 | 0.785775 |
| 68197 | 1.15273 | 0.462748 |
| 68198 | 1.15273 | 0.495188 |
| 68199 | 1.89227 | 0.05 |
| 68200 | 1.89227 | 0.05 |
| 68201 | 1.89227 | 0.0616848 |
| 68202 | 1.89227 | 0.05 |
| 68203 | 2.1288 | 0.05 |
| 68204 | 2.1288 | 0.05 |
| 68205 | 1.89227 | 0.110573 |

| | | |
|---|---|---|
| 68206 | 1.89227 | 0.246827 |
| 68207 | 1.89227 | 0.111766 |
| 68208 | 1.89227 | 0.161845 |
| 68209 | 3.78453 | 0.168809 |
| 68210 | 1.89226 | 0.366954 |
| 68211 | 1.89226 | 0.372133 |
| 68212 | 1.89226 | 0.212639 |
| 68213 | 1.89226 | 0.326784 |
| 68214 | 3.78453 | 0.297365 |
| 68215 | 1.89226 | 0.112673 |
| 68216 | 1.89226 | 0.27666 |
| 68217 | 1.89226 | 0.0961781 |
| 68218 | 1.89226 | 0.0680184 |
| 68219 | 1.89226 | 0.115932 |
| 68220 | 1.89226 | 0.221591 |
| 68221 | 1.94432 | 0.05 |
| 68222 | 1.89226 | 0.05 |
| 68223 | 2.07911 | 0.05 |
| 68224 | 1.89226 | 0.05 |
| 68225 | 2.07911 | 0.05 |
| 68226 | 1.89226 | 0.05 |
| 68227 | 1.89226 | 0.05 |
| 68228 | 1.89226 | 0.05 |
| 68229 | 1.89226 | 0.05 |
| 68230 | 1.89226 | 0.05 |
| 68231 | 1.89226 | 0.05 |
| 68232 | 1.89226 | 0.05 |
| 68233 | 1.89226 | 0.0647379 |
| 68234 | 1.89226 | 0.103931 |
| 68235 | 1.89226 | 0.217609 |
| 68236 | 1.89226 | 0.05 |
| 68237 | 1.89226 | 0.285468 |
| 68238 | 1.89226 | 0.05 |
| 68239 | 1.89226 | 0.05 |
| 68240 | 1.89226 | 0.05 |
| 68241 | 1.89226 | 0.05 |
| 68242 | 1.89226 | 0.05 |
| 68243 | 1.89226 | 0.05 |
| 68244 | 1.89226 | 0.05 |
| 68245 | 1.86778 | 0.05 |
| 68246 | 2.72348 | 0.05 |
| 68247 | 1.89226 | 0.05 |
| 68248 | 1.89226 | 0.05 |
| 68249 | 3.76004 | 0.05 |
| 68250 | 1.89226 | 0.0753848 |
| 68251 | 1.89226 | 0.115465 |
| 68252 | 1.89226 | 0.549887 |
| 68253 | 1.89226 | 0.05 |
| 68254 | 3.78451 | 0.0688923 |
| 68255 | 1.89226 | 0.05 |
| 68256 | 1.89226 | 0.05 |
| 68257 | 1.89226 | 0.05 |
| 68258 | 1.89226 | 0.05 |
| 68259 | 1.89226 | 0.05 |
| 68260 | 1.89226 | 0.05 |
| 68261 | 0.94613 | 0.05 |
| 68262 | 0.94613 | 0.05 |
| 68263 | 0.94613 | 0.05 |

| | | |
|---|---|---|
| 68264 | 0.94613 | 0.05 |
| 68265 | 0.946131 | 0.05 |
| 68266 | 0.946131 | 0.05 |
| 68267 | 0.946129 | 0.05 |
| 68268 | 0.946129 | 0.05 |
| 68269 | 0.94613 | 0.05 |
| 68270 | 0.94613 | 0.05 |
| 68271 | 0.94613 | 0.05 |
| 68272 | 0.94613 | 0.05 |
| 68273 | 0.946124 | 0.05 |
| 68274 | 0.946124 | 0.05 |
| 68275 | 0.94613 | 0.164964 |
| 68276 | 0.94613 | 0.05 |
| 68277 | 0.946128 | 0.05 |
| 68278 | 0.946128 | 0.05 |
| 68279 | 0.946126 | 0.128844 |
| 68280 | 0.946126 | 0.05 |
| 68281 | 0.946127 | 0.05 |
| 68282 | 0.946127 | 0.05 |
| 68283 | 0.946127 | 0.05 |
| 68284 | 0.946127 | 0.05 |
| 68285 | 2.09166 | 0.05 |
| 68286 | 2.1285 | 0.05 |
| 68287 | 1.78381 | 0.05 |
| 68288 | 1.78381 | 0.05 |
| 68289 | 2.47689 | 0.05 |
| 68290 | 2.47689 | 0.05 |
| 68291 | 2.09167 | 0.05 |
| 68292 | 2.26572 | 0.05 |
| 68293 | 2.73393 | 0.05 |
| 68294 | 2.4769 | 0.05 |
| 68295 | 4.74261 | 0.05 |
| 68296 | 1.89226 | 0.05 |
| 68297 | 1.89226 | 0.05 |
| 68298 | 1.89226 | 0.05 |
| 68299 | 1.89226 | 0.05 |
| 68300 | 1.89226 | 0.05 |
| 68301 | 1.89226 | 0.05 |
| 68302 | 0.946131 | 0.05 |
| 68303 | 0.946131 | 0.05 |
| 68304 | 0.946129 | 0.05 |
| 68305 | 0.946129 | 0.05 |
| 68306 | 0.94613 | 0.05 |
| 68307 | 0.94613 | 0.05 |
| 68308 | 0.946131 | 0.05 |
| 68309 | 0.946131 | 0.05 |
| 68310 | 0.94613 | 0.05 |
| 68311 | 0.94613 | 0.05 |
| 68312 | 0.946131 | 0.05 |
| 68313 | 0.946131 | 0.05 |
| 68314 | 2.27622 | 0.05 |
| 68315 | 2.66786 | 0.05 |
| 68316 | 2.1285 | 0.05 |
| 68317 | 2.1285 | 0.05 |
| 68318 | 2.62461 | 0.05 |
| 68319 | 2.47689 | 0.05 |
| 68320 | 1.89226 | 0.05 |
| 68321 | 1.89226 | 0.05 |

| | | |
|---|---|---|
| 68322 | 1.89226 | 0.05 |
| 68323 | 1.89226 | 0.05 |
| 68324 | 3.78453 | 0.05 |
| 68325 | 1.89226 | 0.05 |
| 68326 | 1.89226 | 0.05 |
| 68327 | 1.89226 | 0.05 |
| 68328 | 1.89226 | 0.05 |
| 68329 | 1.89226 | 0.05 |
| 68330 | 1.89226 | 0.05 |
| 68331 | 1.89226 | 0.752951 |
| 68332 | 1.89226 | 0.963959 |
| 68333 | 1.89226 | 0.768164 |
| 68334 | 1.89226 | 0.910314 |
| 68335 | 1.89226 | 0.778669 |
| 68336 | 1.89226 | 0.924558 |
| 68337 | 1.89226 | 1.04779 |
| 68338 | 1.89226 | 0.80548 |
| 68339 | 1.89226 | 0.797288 |
| 68340 | 1.89226 | 1.0951 |
| 68341 | 3.78452 | 0.904683 |
| 68342 | 1.89227 | 0.796013 |
| 68343 | 1.89227 | 1.05032 |
| 68344 | 1.89227 | 1.02595 |
| 68345 | 1.89227 | 0.898747 |
| 68346 | 3.78453 | 0.950156 |
| 68347 | 0.946127 | 1.05522 |
| 68348 | 0.946127 | 0.950163 |
| 68349 | 1.06438 | 1.75749 |
| 68350 | 0.946131 | 0.869866 |
| 68351 | 1.06438 | 1.05775 |
| 68352 | 0.94613 | 1.10976 |
| 68353 | 0.946128 | 0.969338 |
| 68354 | 0.946128 | 0.826942 |
| 68355 | 1.06438 | 1.04633 |
| 68356 | 1.06438 | 1.65466 |
| 68357 | 0.946129 | 1.01649 |
| 68358 | 0.946129 | 0.977138 |
| 68359 | 1.89226 | 0.820255 |
| 68360 | 1.89226 | 1.0305 |
| 68361 | 1.89226 | 0.900327 |
| 68362 | 1.89226 | 1.00792 |
| 68363 | 1.89226 | 0.929855 |
| 68364 | 1.89226 | 1.05133 |
| 68365 | 0.946133 | 0.97681 |
| 68366 | 0.946133 | 0.915108 |
| 68367 | 1.39771 | 1.13714 |
| 68368 | 0.946132 | 0.860533 |
| 68369 | 1.39771 | 1.12547 |
| 68370 | 1.39772 | 0.899932 |
| 68371 | 0.946133 | 0.94166 |
| 68372 | 0.946133 | 0.83474 |
| 68373 | 0.946132 | 1.09889 |
| 68374 | 1.39772 | 1.14543 |
| 68375 | 0.946132 | 1.04096 |
| 68376 | 0.946132 | 0.944396 |
| 68377 | 1.98182 | 0.05 |
| 68378 | 2.2217 | 0.05 |
| 68379 | 2.79889 | 0.05 |

| | | |
|---|---|---|
| 68380 | 2.50097 | 0.05 |
| 68381 | 4.72267 | 0.05 |
| 68382 | 2.05362 | 0.05 |
| 68383 | 1.70302 | 0.05 |
| 68384 | 2.75109 | 0.05 |
| 68385 | 2.138 0.05 | |
| 68386 | 2.0815 | 0.05 |
| 68387 | 1.84751 | 0.05 |
| 68388 | 0.946131 | 0.05 |
| 68389 | 0.946131 | 0.05 |
| 68390 | 0.946131 | 0.05 |
| 68391 | 0.946131 | 0.05 |
| 68392 | 0.946131 | 0.05 |
| 68393 | 0.946131 | 0.05 |
| 68394 | 0.946132 | 0.05 |
| 68395 | 0.946132 | 0.05 |
| 68396 | 0.946131 | 0.05 |
| 68397 | 0.946131 | 0.05 |
| 68398 | 0.946132 | 0.05 |
| 68399 | 0.946132 | 0.05 |
| 68400 | 1.89226 | 0.05 |
| 68401 | 1.89226 | 0.05 |
| 68402 | 1.89226 | 0.05 |
| 68403 | 1.89226 | 0.05 |
| 68404 | 3.78451 | 0.05 |
| 68405 | 0.946126 | 0.05 |
| 68406 | 1.25048 | 0.05 |
| 68407 | 0.946131 | 0.05 |
| 68408 | 1.39945 | 0.05 |
| 68409 | 0.946127 | 0.05 |
| 68410 | 0.946127 | 0.05 |
| 68411 | 1.39944 | 0.05 |
| 68412 | 1.39944 | 0.05 |
| 68413 | 0.946129 | 0.05 |
| 68414 | 0.946129 | 0.05 |
| 68415 | 1.39944 | 0.05 |
| 68416 | 1.25048 | 0.05 |
| 68417 | 1.89226 | 0.05 |
| 68418 | 1.89226 | 0.05 |
| 68419 | 1.89226 | 0.05 |
| 68420 | 1.89226 | 0.05 |
| 68421 | 1.89226 | 0.05 |
| 68422 | 1.89226 | 0.05 |
| 68423 | 0.946126 | 0.05 |
| 68424 | 0.946126 | 0.05 |
| 68425 | 0.946131 | 0.05 |
| 68426 | 0.946131 | 0.05 |
| 68427 | 0.946128 | 0.05 |
| 68428 | 0.946128 | 0.05 |
| 68429 | 0.946129 | 0.05 |
| 68430 | 0.946129 | 0.05 |
| 68431 | 0.946129 | 0.05 |
| 68432 | 0.946129 | 0.05 |
| 68433 | 0.946128 | 0.05 |
| 68434 | 0.946128 | 0.05 |
| 68435 | 1.89226 | 0.05 |
| 68436 | 1.89226 | 0.05 |
| 68437 | 1.89226 | 0.05 |

| | | |
|---|---|---|
| 68438 | 1.89226 | 0.05 |
| 68439 | 1.89226 | 0.05 |
| 68440 | 1.89226 | 0.05 |
| 68441 | 1.01393 | 0.05 |
| 68442 | 0.775245 | 0.05 |
| 68443 | 0.946126 | 0.05 |
| 68444 | 0.946126 | 0.05 |
| 68445 | 0.78305 | 0.05 |
| 68446 | 0.78305 | 0.05 |
| 68447 | 0.946159 | 0.05 |
| 68448 | 0.929267 | 0.05 |
| 68449 | 1.14956 | 0.05 |
| 68450 | 1.04073 | 0.05 |
| 68451 | 0.851526 | 0.05 |
| 68452 | 0.704781 | 0.05 |
| 68453 | 1.89226 | 0.05 |
| 68454 | 1.89226 | 0.05 |
| 68455 | 1.89226 | 0.05 |
| 68456 | 1.89226 | 0.05 |
| 68457 | 3.78451 | 0.05 |
| 68458 | 1.89226 | 0.05 |
| 68459 | 1.89226 | 0.05 |
| 68460 | 1.89226 | 0.05 |
| 68461 | 1.89226 | 0.05 |
| 68462 | 1.89226 | 0.05 |
| 68463 | 1.89226 | 0.05 |
| 68464 | 1.89226 | 0.05 |
| 68465 | 1.89226 | 0.05 |
| 68466 | 1.89226 | 0.05 |
| 68467 | 1.89226 | 0.05 |
| 68468 | 1.89226 | 0.05 |
| 68469 | 1.89226 | 0.05 |
| 68470 | 1.16317 | 0.05 |
| 68471 | 1.0292 | 0.05 |
| 68472 | 0.946126 | 0.05 |
| 68473 | 0.946126 | 0.05 |
| 68474 | 0.93562 | 0.05 |
| 68475 | 0.93562 | 0.05 |
| 68476 | 1.0418 | 0.05 |
| 68477 | 1.04181 | 0.05 |
| 68478 | 1.16317 | 0.05 |
| 68479 | 0.946129 | 0.05 |
| 68480 | 1.02919 | 0.05 |
| 68481 | 0.946128 | 0.05 |
| 68482 | 0.946131 | 0.05 |
| 68483 | 0.946131 | 0.05 |
| 68484 | 0.946126 | 0.05 |
| 68485 | 0.946126 | 0.05 |
| 68486 | 0.946129 | 0.05 |
| 68487 | 0.946129 | 0.05 |
| 68488 | 0.946128 | 0.05 |
| 68489 | 0.946128 | 0.05 |
| 68490 | 0.946129 | 0.05 |
| 68491 | 0.946129 | 0.05 |
| 68492 | 0.946128 | 0.05 |
| 68493 | 0.946128 | 0.05 |
| 68494 | 1.89226 | 0.05 |
| 68495 | 1.89226 | 0.05 |

| | | |
|---|---|---|
| 68496 | 1.89226 | 0.05 |
| 68497 | 1.89226 | 0.05 |
| 68498 | 1.89226 | 0.05 |
| 68499 | 1.89226 | 0.05 |
| 68500 | 1.89226 | 0.05 |
| 68501 | 1.89226 | 0.05 |
| 68502 | 1.89226 | 0.05 |
| 68503 | 1.89226 | 0.05 |
| 68504 | 1.89226 | 0.05 |
| 68505 | 1.89226 | 0.05 |
| 68506 | 1.89226 | 0.05 |
| 68507 | 1.89226 | 0.05 |
| 68508 | 1.89226 | 0.05 |
| 68509 | 1.89226 | 0.05 |
| 68510 | 3.78453 | 0.05 |
| 68511 | 1.89226 | 0.05 |
| 68512 | 1.89226 | 0.05 |
| 68513 | 1.89226 | 0.05 |
| 68514 | 1.89226 | 0.05 |
| 68515 | 3.78452 | 0.05 |
| 68516 | 1.89225 | 0.05 |
| 68517 | 1.89225 | 0.05 |
| 68518 | 1.89225 | 0.05 |
| 68519 | 1.89225 | 0.05 |
| 68520 | 1.89225 | 0.05 |
| 68521 | 1.89225 | 0.05 |
| 68522 | 0.946132 | 0.05 |
| 68523 | 0.946132 | 0.05 |
| 68524 | 0.946133 | 0.05 |
| 68525 | 0.946133 | 0.05 |
| 68526 | 0.946132 | 0.05 |
| 68527 | 0.946132 | 0.05 |
| 68528 | 0.946132 | 0.05 |
| 68529 | 0.946132 | 0.05 |
| 68530 | 0.946133 | 0.05 |
| 68531 | 0.946133 | 0.05 |
| 68532 | 0.946132 | 0.05 |
| 68533 | 0.946132 | 0.05 |
| 68534 | 1.89226 | 0.05 |
| 68535 | 1.89226 | 0.05 |
| 68536 | 1.89226 | 0.05 |
| 68537 | 1.89226 | 0.05 |
| 68538 | 3.78453 | 0.05 |
| 68539 | 1.13537 | 0.05 |
| 68540 | 1.09222 | 0.05 |
| 68541 | 0.946126 | 0.05 |
| 68542 | 0.946126 | 0.05 |
| 68543 | 0.946128 | 0.05 |
| 68544 | 0.946128 | 0.05 |
| 68545 | 1.16317 | 0.05 |
| 68546 | 1.13034 | 0.05 |
| 68547 | 1.30155 | 0.05 |
| 68548 | 1.08462 | 0.05 |
| 68549 | 0.992915 | 0.05 |
| 68550 | 0.807642 | 0.05 |
| 68551 | 0.946131 | 0.05 |
| 68552 | 0.946131 | 0.05 |
| 68553 | 0.946125 | 0.05 |

| | | |
|---|---|---|
| 68554 | 0.946125 | 0.05 |
| 68555 | 0.946128 | 0.05 |
| 68556 | 0.946128 | 0.05 |
| 68557 | 0.946128 | 0.05 |
| 68558 | 0.946128 | 0.05 |
| 68559 | 0.946128 | 0.05 |
| 68560 | 0.946128 | 0.05 |
| 68561 | 0.946128 | 0.05 |
| 68562 | 0.946128 | 0.05 |
| 68563 | 1.89226 | 0.05 |
| 68564 | 1.89226 | 0.05 |
| 68565 | 1.89226 | 0.05 |
| 68566 | 1.89226 | 0.05 |
| 68567 | 1.89226 | 0.05 |
| 68568 | 1.89226 | 0.05 |
| 68569 | 1.89224 | 0.05 |
| 68570 | 1.89224 | 0.05 |
| 68571 | 1.89224 | 0.05 |
| 68572 | 1.89224 | 0.05 |
| 68573 | 1.89224 | 0.05 |
| 68574 | 1.89224 | 0.05 |
| 68575 | 0.946086 | 0.05 |
| 68576 | 1.17919 | 0.05 |
| 68577 | 0.89738 | 0.05 |
| 68578 | 0.89738 | 0.05 |
| 68579 | 0.946116 | 0.05 |
| 68580 | 0.946116 | 0.05 |
| 68581 | 1.13048 | 0.05 |
| 68582 | 1.11844 | 0.05 |
| 68583 | 0.946128 | 0.05 |
| 68584 | 0.897362 | 0.05 |
| 68585 | 1.1282 | 0.05 |
| 68586 | 0.946103 | 0.05 |
| 68587 | 1.89223 | 0.05 |
| 68588 | 1.89223 | 0.05 |
| 68589 | 1.89223 | 0.05 |
| 68590 | 1.89223 | 0.05 |
| 68591 | 3.78446 | 0.05 |
| 68592 | 0.946118 | 0.05 |
| 68593 | 0.946118 | 0.05 |
| 68594 | 0.946118 | 0.05 |
| 68595 | 0.946118 | 0.05 |
| 68596 | 0.946119 | 0.05 |
| 68597 | 0.946119 | 0.05 |
| 68598 | 0.946118 | 0.05 |
| 68599 | 0.946118 | 0.05 |
| 68600 | 0.946131 | 0.05 |
| 68601 | 0.946131 | 0.05 |
| 68602 | 0.946106 | 0.05 |
| 68603 | 0.946106 | 0.05 |
| 68604 | 1.89227 | 0.795885 |
| 68605 | 1.89227 | 0.875803 |
| 68606 | 1.89227 | 0.837115 |
| 68607 | 1.89227 | 0.747795 |
| 68608 | 3.78453 | 0.737241 |
| 68609 | 1.89226 | 0.882558 |
| 68610 | 1.89226 | 0.651979 |
| 68611 | 1.89226 | 0.642766 |

| | | |
|---|---|---|
| 68612 | 1.89226 | 0.677383 |
| 68613 | 1.89226 | 0.811492 |
| 68614 | 1.89226 | 0.864215 |
| 68615 | 1.89226 | 0.574516 |
| 68616 | 2.59378 | 0.205225 |
| 68617 | 2.30348 | 0.167454 |
| 68618 | 2.30348 | 0.476141 |
| 68619 | 1.89226 | 0.569277 |
| 68620 | 1.89226 | 0.621307 |
| 68621 | 1.89226 | 0.766244 |
| 68622 | 1.89226 | 0.659905 |
| 68623 | 1.89226 | 0.675499 |
| 68624 | 1.89226 | 0.666834 |
| 68625 | 3.78453 | 0.770079 |
| 68626 | 1.89226 | 0.667287 |
| 68627 | 1.89226 | 0.592271 |
| 68628 | 1.89226 | 0.575607 |
| 68629 | 1.89226 | 0.623633 |
| 68630 | 1.89226 | 0.691788 |
| 68631 | 1.89226 | 0.759089 |
| 68632 | 0.904406 | 0.430126 |
| 68633 | 1.01667 | 0.457894 |
| 68634 | 1.11974 | 0.261533 |
| 68635 | 0.994417 | 0.503172 |
| 68636 | 1.02013 | 0.327193 |
| 68637 | 0.628203 | 0.527881 |
| 68638 | 1.70302 | 0.441812 |
| 68639 | 1.49898 | 0.235676 |
| 68640 | 1.14559 | 0.463927 |
| 68641 | 0.900571 | 0.277222 |
| 68642 | 1.58886 | 0.440142 |
| 68643 | 2.81102 | 0.05 |
| 68644 | 2.3923 | 0.05 |
| 68645 | 2.05632 | 0.05 |
| 68646 | 2.43514 | 0.05 |
| 68647 | 4.82744 | 0.05 |
| 68648 | 1.89226 | 0.05 |
| 68649 | 1.89226 | 0.05 |
| 68650 | 1.89226 | 0.05 |
| 68651 | 1.89226 | 0.05 |
| 68652 | 1.89226 | 0.05 |
| 68653 | 1.89226 | 0.05 |
| 68654 | 1.89226 | 0.05 |
| 68655 | 1.89226 | 0.05 |
| 68656 | 1.89226 | 0.05 |
| 68657 | 1.89226 | 0.05 |
| 68658 | 3.78451 | 0.05 |
| 68659 | 2.50803 | 0.05 |
| 68660 | 1.903 | 0.05 |
| 68661 | 1.903 | 0.05 |
| 68662 | 2.19162 | 0.05 |
| 68663 | 2.09934 | 0.05 |
| 68664 | 2.39063 | 0.05 |
| 68665 | 1.00243 | 0.05 |
| 68666 | 0.731731 | 0.05 |
| 68667 | 0.876616 | 0.05 |
| 68668 | 1.00451 | 0.05 |
| 68669 | 0.986901 | 0.05 |

| | | |
|---|---|---|
| 68670 | 0.986901 | 0.05 |
| 68671 | 0.887729 | 0.05 |
| 68672 | 0.887732 | 0.05 |
| 68673 | 0.876605 | 0.05 |
| 68674 | 0.769283 | 0.05 |
| 68675 | 1.00245 | 0.05 |
| 68676 | 1.18712 | 0.05 |
| 68677 | 0.946131 | 0.05 |
| 68678 | 0.946131 | 0.05 |
| 68679 | 0.946131 | 0.05 |
| 68680 | 0.946131 | 0.05 |
| 68681 | 0.946131 | 0.05 |
| 68682 | 0.946131 | 0.05 |
| 68683 | 0.946131 | 0.05 |
| 68684 | 0.946131 | 0.05 |
| 68685 | 0.946131 | 0.05 |
| 68686 | 0.946131 | 0.05 |
| 68687 | 0.946131 | 0.05 |
| 68688 | 0.946131 | 0.05 |
| 68689 | 1.89226 | 0.05 |
| 68690 | 1.89226 | 0.05 |
| 68691 | 1.89226 | 0.05 |
| 68692 | 1.89226 | 0.05 |
| 68693 | 1.89226 | 0.05 |
| 68694 | 1.89226 | 0.05 |
| 68695 | 1.89226 | 0.05 |
| 68696 | 1.89226 | 0.05 |
| 68697 | 1.89226 | 0.05 |
| 68698 | 1.89226 | 0.05 |
| 68699 | 3.78453 | 0.05 |
| 68700 | 1.89226 | 0.05 |
| 68701 | 1.89226 | 0.05 |
| 68702 | 1.89226 | 0.05 |
| 68703 | 1.89226 | 0.05 |
| 68704 | 3.78453 | 0.05 |
| 68705 | 1.89226 | 0.05 |
| 68706 | 1.89226 | 0.05 |
| 68707 | 1.89226 | 0.05 |
| 68708 | 1.89226 | 0.05 |
| 68709 | 1.89226 | 0.05 |
| 68710 | 1.89226 | 0.05 |
| 68711 | 0.946132 | 0.05 |
| 68712 | 0.946132 | 0.05 |
| 68713 | 0.946131 | 0.05 |
| 68714 | 0.946131 | 0.05 |
| 68715 | 0.946131 | 0.05 |
| 68716 | 0.946131 | 0.05 |
| 68717 | 0.946132 | 0.05 |
| 68718 | 0.946132 | 0.05 |
| 68719 | 0.946131 | 0.05 |
| 68720 | 0.946131 | 0.05 |
| 68721 | 0.946131 | 0.05 |
| 68722 | 0.946131 | 0.05 |
| 68723 | 0.946131 | 0.05 |
| 68724 | 0.946131 | 0.05 |
| 68725 | 0.946131 | 0.05 |
| 68726 | 0.946131 | 0.05 |
| 68727 | 0.94613 | 0.05 |

| | | |
|---|---|---|
| 68728 | 0.94613 | 0.05 |
| 68729 | 0.946132 | 0.05 |
| 68730 | 0.946132 | 0.05 |
| 68731 | 0.946131 | 0.05 |
| 68732 | 0.946131 | 0.05 |
| 68733 | 0.946131 | 0.05 |
| 68734 | 0.946131 | 0.05 |
| 68735 | 1.89226 | 0.05 |
| 68736 | 1.89226 | 0.05 |
| 68737 | 1.89226 | 0.05 |
| 68738 | 1.89226 | 0.05 |
| 68739 | 1.89226 | 0.05 |
| 68740 | 1.89226 | 0.05 |
| 68741 | 1.89226 | 0.05 |
| 68742 | 1.89226 | 0.05 |
| 68743 | 1.89226 | 0.05 |
| 68744 | 1.89226 | 0.05 |
| 68745 | 3.78453 | 0.05 |
| 68746 | 1.89226 | 0.05 |
| 68747 | 1.89226 | 0.05 |
| 68748 | 1.89226 | 0.05 |
| 68749 | 1.89226 | 0.05 |
| 68750 | 1.89226 | 0.05 |
| 68751 | 1.89226 | 0.05 |
| 68752 | 0.946132 | 0.05 |
| 68753 | 0.946132 | 0.05 |
| 68754 | 0.946131 | 0.05 |
| 68755 | 0.946131 | 0.05 |
| 68756 | 0.946132 | 0.05 |
| 68757 | 0.946132 | 0.05 |
| 68758 | 0.946131 | 0.05 |
| 68759 | 0.946131 | 0.05 |
| 68760 | 0.946132 | 0.05 |
| 68761 | 0.946132 | 0.05 |
| 68762 | 0.946131 | 0.05 |
| 68763 | 0.946131 | 0.05 |
| 68764 | 0.946131 | 0.05 |
| 68765 | 0.946131 | 0.05 |
| 68766 | 0.946132 | 0.05 |
| 68767 | 0.946132 | 0.05 |
| 68768 | 0.946132 | 0.05 |
| 68769 | 0.946132 | 0.05 |
| 68770 | 0.946131 | 0.05 |
| 68771 | 0.946131 | 0.05 |
| 68772 | 0.946132 | 0.05 |
| 68773 | 0.946132 | 0.05 |
| 68774 | 0.946131 | 0.05 |
| 68775 | 0.946131 | 0.05 |
| 68776 | 1.89226 | 0.596507 |
| 68777 | 1.89226 | 0.528798 |
| 68778 | 1.89226 | 0.651286 |
| 68779 | 1.89226 | 0.211002 |
| 68780 | 3.78452 | 0.203991 |
| 68781 | 1.89226 | 0.867692 |
| 68782 | 1.89226 | 0.421503 |
| 68783 | 1.89226 | 0.724969 |
| 68784 | 1.89226 | 0.744889 |
| 68785 | 3.78452 | 0.873344 |

| | | |
|---|---|---|
| 68786 | 1.89226 | 0.444595 |
| 68787 | 1.89226 | 0.625718 |
| 68788 | 1.89226 | 0.720047 |
| 68789 | 1.89226 | 0.251726 |
| 68790 | 3.78452 | 0.231469 |
| 68791 | 1.89226 | 0.546581 |
| 68792 | 2.75945 | 0.052451 |
| 68793 | 1.89226 | 0.136701 |
| 68794 | 2.35026 | 0.0967413 |
| 68795 | 4.24252 | 0.614268 |
| 68796 | 0.946131 | 0.187613 |
| 68797 | 0.946131 | 0.33924 |
| 68798 | 0.946131 | 0.0560843 |
| 68799 | 0.946131 | 0.103904 |
| 68800 | 0.946132 | 0.056779 |
| 68801 | 0.946132 | 0.078982 |
| 68802 | 0.946129 | 0.436613 |
| 68803 | 0.946129 | 0.116348 |
| 68804 | 0.946131 | 0.240374 |
| 68805 | 0.946131 | 0.247785 |
| 68806 | 0.94613 | 0.483477 |
| 68807 | 0.94613 | 0.218693 |
| 68808 | 1.89226 | 0.632421 |
| 68809 | 1.89226 | 0.625849 |
| 68810 | 1.89226 | 0.391789 |
| 68811 | 1.89226 | 0.660191 |
| 68812 | 1.89226 | 0.60624 |
| 68813 | 1.89226 | 0.707692 |
| 68814 | 0.946129 | 0.121052 |
| 68815 | 0.946129 | 0.225496 |
| 68816 | 0.946133 | 0.166196 |
| 68817 | 0.946133 | 0.0847867 |
| 68818 | 0.946131 | 0.0693418 |
| 68819 | 0.946131 | 0.075412 |
| 68820 | 0.946131 | 0.229598 |
| 68821 | 0.946131 | 0.10143 |
| 68822 | 0.946131 | 0.211488 |
| 68823 | 0.946131 | 0.199237 |
| 68824 | 0.946131 | 0.308243 |
| 68825 | 0.946131 | 0.17118 |
| 68826 | 1.89226 | 0.05 |
| 68827 | 2.35026 | 0.05 |
| 68828 | 1.32126 | 0.05 |
| 68829 | 1.89226 | 0.05 |
| 68830 | 1.77925 | 0.05 |
| 68831 | 2.35026 | 0.05 |
| 68832 | 1.89226 | 0.05 |
| 68833 | 1.89226 | 0.05 |
| 68834 | 1.89226 | 0.05 |
| 68835 | 1.89226 | 0.05 |
| 68836 | 1.89226 | 0.05 |
| 68837 | 1.89226 | 0.05 |
| 68838 | 1.89226 | 0.05 |
| 68839 | 1.89226 | 0.05 |
| 68840 | 1.89226 | 0.05 |
| 68841 | 1.89226 | 0.05 |
| 68842 | 3.78453 | 0.05 |
| 68843 | 1.89226 | 0.05 |

| | | |
|---|---|---|
| 68844 | 1.89226 | 0.05 |
| 68845 | 1.89226 | 0.05 |
| 68846 | 1.89226 | 0.05 |
| 68847 | 1.89226 | 0.05 |
| 68848 | 1.89226 | 0.05 |
| 68849 | 0.946131 | 0.05 |
| 68850 | 0.946131 | 0.05 |
| 68851 | 0.946131 | 0.05 |
| 68852 | 0.946131 | 0.05 |
| 68853 | 0.946132 | 0.05 |
| 68854 | 0.946132 | 0.05 |
| 68855 | 0.946131 | 0.05 |
| 68856 | 0.946131 | 0.05 |
| 68857 | 0.946132 | 0.05 |
| 68858 | 0.946132 | 0.05 |
| 68859 | 0.946131 | 0.05 |
| 68860 | 0.946131 | 0.05 |
| 68861 | 1.89226 | 0.05 |
| 68862 | 1.89226 | 0.05 |
| 68863 | 1.89226 | 0.05 |
| 68864 | 1.89226 | 0.05 |
| 68865 | 1.89226 | 0.05 |
| 68866 | 1.89226 | 0.05 |
| 68867 | 1.89226 | 0.05 |
| 68868 | 1.89226 | 0.0518857 |
| 68869 | 1.89226 | 0.05 |
| 68870 | 1.89226 | 0.05 |
| 68871 | 3.78453 | 0.05 |
| 68872 | 1.89227 | 0.0535365 |
| 68873 | 1.89227 | 0.0760785 |
| 68874 | 1.89227 | 0.05 |
| 68875 | 1.89227 | 0.146901 |
| 68876 | 3.78453 | 0.05 |
| 68877 | 1.89226 | 0.05 |
| 68878 | 1.89226 | 0.05 |
| 68879 | 1.89226 | 0.05 |
| 68880 | 1.89226 | 0.05 |
| 68881 | 3.78452 | 0.05 |
| 68882 | 0.946132 | 0.05 |
| 68883 | 0.946132 | 0.05 |
| 68884 | 0.946131 | 0.05 |
| 68885 | 0.946131 | 0.05 |
| 68886 | 0.946131 | 0.05 |
| 68887 | 0.946131 | 0.05 |
| 68888 | 0.946133 | 0.05 |
| 68889 | 0.946133 | 0.05 |
| 68890 | 0.946132 | 0.05 |
| 68891 | 0.946132 | 0.05 |
| 68892 | 0.946131 | 0.05 |
| 68893 | 0.946131 | 0.05 |
| 68894 | 1.89227 | 0.05 |
| 68895 | 1.89227 | 0.0834001 |
| 68896 | 1.89227 | 0.05 |
| 68897 | 1.89227 | 0.05 |
| 68898 | 1.89227 | 0.0675451 |
| 68899 | 1.89227 | 0.0683451 |
| 68900 | 2.20491 | 0.05 |
| 68901 | 1.81656 | 0.05 |

| | | |
|---|---|---|
| 68902 | 1.81656 | 0.05 |
| 68903 | 1.89226 | 0.05 |
| 68904 | 1.89226 | 0.05 |
| 68905 | 1.89226 | 0.05 |
| 68906 | 1.89226 | 0.239148 |
| 68907 | 1.89226 | 0.447363 |
| 68908 | 1.89226 | 0.323215 |
| 68909 | 1.89226 | 0.167195 |
| 68910 | 3.78452 | 0.184585 |
| 68911 | 1.89226 | 0.66186 |
| 68912 | 1.89226 | 0.622376 |
| 68913 | 1.89226 | 0.611704 |
| 68914 | 1.89226 | 0.674218 |
| 68915 | 3.78453 | 0.663835 |
| 68916 | 1.89226 | 0.628755 |
| 68917 | 1.89226 | 0.633118 |
| 68918 | 1.89226 | 0.606663 |
| 68919 | 1.89226 | 0.636211 |
| 68920 | 1.89226 | 0.636472 |
| 68921 | 1.89226 | 0.671815 |
| 68922 | 1.89226 | 0.0886669 |
| 68923 | 2.11032 | 0.05 |
| 68924 | 2.30793 | 0.05 |
| 68925 | 1.89226 | 0.05 |
| 68926 | 2.30793 | 0.0626975 |
| 68927 | 1.89226 | 0.0831906 |
| 68928 | 1.89226 | 0.536551 |
| 68929 | 1.89226 | 0.518712 |
| 68930 | 1.89226 | 0.292319 |
| 68931 | 1.89226 | 0.444102 |
| 68932 | 1.89226 | 0.512205 |
| 68933 | 1.89226 | 0.586628 |
| 68934 | 0.810943 | 0.441156 |
| 68935 | 0.810943 | 0.319865 |
| 68936 | 0.810943 | 0.430821 |
| 68937 | 0.810943 | 0.309007 |
| 68938 | 0.810933 | 0.352938 |
| 68939 | 0.810933 | 0.390691 |
| 68940 | 1.89226 | 0.319285 |
| 68941 | 1.3517 | 0.41151 |
| 68942 | 1.08132 | 0.335869 |
| 68943 | 1.08132 | 0.565557 |
| 68944 | 1.08133 | 0.379474 |
| 68945 | 1.89226 | 0.05 |
| 68946 | 1.89226 | 0.05 |
| 68947 | 1.89226 | 0.05 |
| 68948 | 1.89226 | 0.05 |
| 68949 | 3.78453 | 0.05 |
| 68950 | 1.89226 | 0.05 |
| 68951 | 1.89226 | 0.05 |
| 68952 | 1.89226 | 0.05 |
| 68953 | 1.89226 | 0.05 |
| 68954 | 1.89226 | 0.05 |
| 68955 | 1.89226 | 0.05 |
| 68956 | 0.810945 | 0.05 |
| 68957 | 0.810945 | 0.05 |
| 68958 | 0.870829 | 0.05 |
| 68959 | 0.870829 | 0.05 |

| | | |
|---|---|---|
| 68960 | 0.990502 | 0.05 |
| 68961 | 0.810933 | 0.05 |
| 68962 | 2.82777 | 0.05 |
| 68963 | 1.53126 | 0.05 |
| 68964 | 1.32076 | 0.05 |
| 68965 | 1.201 | 0.05 |
| 68966 | 1.08133 | 0.05 |
| 68967 | 1.89226 | 0.05 |
| 68968 | 1.89226 | 0.05 |
| 68969 | 1.89226 | 0.05 |
| 68970 | 1.89226 | 0.05 |
| 68971 | 1.89226 | 0.05 |
| 68972 | 1.89226 | 0.05 |
| 68973 | 0.946132 | 0.05 |
| 68974 | 0.946132 | 0.05 |
| 68975 | 0.946133 | 0.05 |
| 68976 | 0.946133 | 0.05 |
| 68977 | 0.946132 | 0.05 |
| 68978 | 0.946132 | 0.05 |
| 68979 | 0.946132 | 0.05 |
| 68980 | 0.946132 | 0.05 |
| 68981 | 0.946133 | 0.05 |
| 68982 | 0.946133 | 0.05 |
| 68983 | 0.946132 | 0.05 |
| 68984 | 0.946132 | 0.05 |
| 68985 | 0.946133 | 0.05 |
| 68986 | 0.946133 | 0.05 |
| 68987 | 0.946132 | 0.05 |
| 68988 | 0.946132 | 0.05 |
| 68989 | 0.946132 | 0.05 |
| 68990 | 0.946132 | 0.05 |
| 68991 | 0.946132 | 0.05 |
| 68992 | 0.946132 | 0.05 |
| 68993 | 0.946133 | 0.05 |
| 68994 | 0.946133 | 0.05 |
| 68995 | 0.946132 | 0.05 |
| 68996 | 0.946132 | 0.05 |
| 68997 | 1.89226 | 0.05 |
| 68998 | 1.89226 | 0.05 |
| 68999 | 1.89226 | 0.05 |
| 69000 | 1.89226 | 0.05 |
| 69001 | 1.89226 | 0.05 |
| 69002 | 1.89226 | 0.05 |
| 69003 | 1.89226 | 0.05 |
| 69004 | 1.89226 | 0.113202 |
| 69005 | 1.89226 | 0.05 |
| 69006 | 1.89226 | 0.0959457 |
| 69007 | 3.78452 | 0.127503 |
| 69008 | 1.89226 | 0.101231 |
| 69009 | 1.89226 | 0.160849 |
| 69010 | 1.89226 | 0.0965239 |
| 69011 | 1.89226 | 0.56629 |
| 69012 | 3.78452 | 0.0843283 |
| 69013 | 1.89226 | 0.05 |
| 69014 | 1.89226 | 0.179824 |
| 69015 | 1.89226 | 0.05 |
| 69016 | 1.89226 | 0.05 |
| 69017 | 1.89226 | 0.203845 |

| | | |
|---|---|---|
| 69018 | 1.89226 | 0.193138 |
| 69019 | 0.946128 | 0.05 |
| 69020 | 0.946128 | 0.05 |
| 69021 | 0.946132 | 0.05 |
| 69022 | 0.946132 | 0.05 |
| 69023 | 0.946129 | 0.05 |
| 69024 | 0.946129 | 0.05 |
| 69025 | 0.946131 | 0.05 |
| 69026 | 0.946131 | 0.05 |
| 69027 | 0.94613 | 0.05 |
| 69028 | 0.94613 | 0.05 |
| 69029 | 0.946131 | 0.05 |
| 69030 | 0.946131 | 0.05 |
| 69031 | 0.946129 | 0.0510368 |
| 69032 | 0.946129 | 0.05 |
| 69033 | 0.946133 | 0.0529884 |
| 69034 | 0.946133 | 0.05 |
| 69035 | 0.946131 | 0.0550363 |
| 69036 | 0.946131 | 0.0604519 |
| 69037 | 0.946131 | 0.05 |
| 69038 | 0.946131 | 0.05 |
| 69039 | 0.946131 | 0.0520162 |
| 69040 | 0.946131 | 0.05 |
| 69041 | 0.946131 | 0.05 |
| 69042 | 0.946131 | 0.05 |
| 69043 | 1.89226 | 0.05 |
| 69044 | 1.89226 | 0.05 |
| 69045 | 1.89226 | 0.05 |
| 69046 | 1.89226 | 0.05 |
| 69047 | 3.78452 | 0.05 |
| 69048 | 1.89225 | 0.05 |
| 69049 | 1.89225 | 0.05 |
| 69050 | 1.89225 | 0.05 |
| 69051 | 1.89225 | 0.05 |
| 69052 | 1.89225 | 0.05 |
| 69053 | 1.89225 | 0.05 |
| 69054 | 1.89226 | 0.05 |
| 69055 | 1.89226 | 0.05 |
| 69056 | 1.89226 | 0.05 |
| 69057 | 1.89226 | 0.05 |
| 69058 | 3.78452 | 0.05 |
| 69059 | 1.89226 | 0.05 |
| 69060 | 1.89226 | 0.05 |
| 69061 | 1.89226 | 0.05 |
| 69062 | 1.89226 | 0.05 |
| 69063 | 3.78453 | 0.05 |
| 69064 | 1.89226 | 0.05 |
| 69065 | 1.89226 | 0.05 |
| 69066 | 1.89226 | 0.05 |
| 69067 | 1.89226 | 0.05 |
| 69068 | 3.78452 | 0.05 |
| 69069 | 1.22164 | 0.05 |
| 69070 | 1.22164 | 0.05 |
| 69071 | 0.806032 | 0.05 |
| 69072 | 0.806032 | 0.05 |
| 69073 | 1.08154 | 0.05 |
| 69074 | 0.946126 | 0.05 |
| 69075 | 0.946129 | 0.05 |

| | | |
|---|---|---|
| 69076 | 0.806065 | 0.05 |
| 69077 | 0.806063 | 0.05 |
| 69078 | 1.08155 | 0.05 |
| 69079 | 0.946128 | 0.05 |
| 69080 | 0.946128 | 0.05 |
| 69081 | 1.28554 | 0.05 |
| 69082 | 1.28554 | 0.05 |
| 69083 | 0.946126 | 0.05 |
| 69084 | 0.946126 | 0.05 |
| 69085 | 1.28554 | 0.05 |
| 69086 | 0.946129 | 0.05 |
| 69087 | 1.08155 | 0.05 |
| 69088 | 0.94613 | 0.05 |
| 69089 | 1.08155 | 0.05 |
| 69090 | 1.28554 | 0.05 |
| 69091 | 0.94613 | 0.05 |
| 69092 | 0.94613 | 0.05 |
| 69093 | 0.946132 | 0.05 |
| 69094 | 0.946132 | 0.05 |
| 69095 | 0.946132 | 0.05 |
| 69096 | 0.946132 | 0.05 |
| 69097 | 0.946132 | 0.05 |
| 69098 | 0.946132 | 0.05 |
| 69099 | 0.946133 | 0.05 |
| 69100 | 0.946133 | 0.05 |
| 69101 | 0.946132 | 0.05 |
| 69102 | 0.946132 | 0.05 |
| 69103 | 0.946133 | 0.05 |
| 69104 | 0.946133 | 0.05 |
| 69105 | 1.89226 | 0.323744 |
| 69106 | 1.89226 | 0.108061 |
| 69107 | 1.89226 | 0.493804 |
| 69108 | 1.89226 | 0.0902609 |
| 69109 | 3.78453 | 0.386185 |
| 69110 | 1.89226 | 0.215514 |
| 69111 | 1.89226 | 0.05 |
| 69112 | 1.89226 | 0.05 |
| 69113 | 1.89226 | 0.0635736 |
| 69114 | 1.89226 | 0.05 |
| 69115 | 1.89226 | 0.0627016 |
| 69116 | 1.89226 | 0.05 |
| 69117 | 1.89226 | 0.05 |
| 69118 | 1.89226 | 0.05 |
| 69119 | 1.89226 | 0.05 |
| 69120 | 1.89226 | 0.05 |
| 69121 | 1.89226 | 0.05 |
| 69122 | 1.89226 | 0.05 |
| 69123 | 1.89226 | 0.05 |
| 69124 | 1.89226 | 0.05 |
| 69125 | 1.89226 | 0.05 |
| 69126 | 1.89226 | 0.05 |
| 69127 | 1.89226 | 0.05 |
| 69128 | 0.946132 | 0.0820347 |
| 69129 | 0.946132 | 0.109323 |
| 69130 | 0.946132 | 0.05 |
| 69131 | 0.946132 | 0.0716513 |
| 69132 | 0.946132 | 0.05 |
| 69133 | 0.946132 | 0.05 |

| | | |
|---|---|---|
| 69134 | 0.946132 | 0.0837108 |
| 69135 | 0.946132 | 0.05 |
| 69136 | 0.946131 | 0.05 |
| 69137 | 0.946131 | 0.0581161 |
| 69138 | 0.946132 | 0.05 |
| 69139 | 0.946132 | 0.05 |
| 69140 | 1.89226 | 0.05 |
| 69141 | 1.89226 | 0.05 |
| 69142 | 1.89226 | 0.05 |
| 69143 | 1.89226 | 0.05 |
| 69144 | 3.78453 | 0.05 |
| 69145 | 0.851541 | 0.05 |
| 69146 | 0.72883 | 0.05 |
| 69147 | 0.991276 | 0.05 |
| 69148 | 0.851485 | 0.05 |
| 69149 | 1.00003 | 0.05 |
| 69150 | 1.00003 | 0.05 |
| 69151 | 0.809788 | 0.05 |
| 69152 | 0.809788 | 0.05 |
| 69153 | 0.890738 | 0.05 |
| 69154 | 0.991264 | 0.05 |
| 69155 | 0.851554 | 0.05 |
| 69156 | 0.851554 | 0.05 |
| 69157 | 0.8628 | 0.05 |
| 69158 | 0.78387 | 0.05 |
| 69159 | 0.830233 | 0.05 |
| 69160 | 0.830233 | 0.05 |
| 69161 | 0.808312 | 0.05 |
| 69162 | 0.817238 | 0.05 |
| 69163 | 1.70303 | 0.05 |
| 69164 | 1.24116 | 0.05 |
| 69165 | 0.995299 | 0.05 |
| 69166 | 1.0989 | 0.05 |
| 69167 | 1.02714 | 0.05 |
| 69168 | 1.89226 | 0.078296 |
| 69169 | 1.89226 | 0.0830949 |
| 69170 | 1.89226 | 0.0501356 |
| 69171 | 1.89226 | 0.950297 |
| 69172 | 3.78451 | 0.05 |
| 69173 | 0.946131 | 0.186374 |
| 69174 | 0.946131 | 0.05 |
| 69175 | 0.946131 | 0.0958138 |
| 69176 | 0.946131 | 0.0771479 |
| 69177 | 0.94613 | 0.0974924 |
| 69178 | 0.94613 | 0.05 |
| 69179 | 0.946132 | 0.05 |
| 69180 | 0.946132 | 0.05 |
| 69181 | 0.946131 | 0.05 |
| 69182 | 0.946131 | 0.139808 |
| 69183 | 0.946131 | 0.05 |
| 69184 | 0.946131 | 0.05 |
| 69185 | 1.89226 | 0.05 |
| 69186 | 1.89226 | 1.56434 |
| 69187 | 1.89226 | 1.02822 |
| 69188 | 1.89226 | 1.29636 |
| 69189 | 3.78453 | 1.0498 |
| 69190 | 1.89226 | 2.1 |
| 69191 | 1.89226 | 1.96803 |

| | | |
|---|---|---|
| 69192 | 1.89226 | 2.1 |
| 69193 | 1.89226 | 0.839713 |
| 69194 | 1.89226 | 2.1 |
| 69195 | 1.89226 | 0.752433 |
| 69196 | 0.946126 | 0.05 |
| 69197 | 1.17111 | 0.05 |
| 69198 | 1.3393 | 0.05 |
| 69199 | 0.946131 | 0.05 |
| 69200 | 0.946127 | 0.05 |
| 69201 | 0.946127 | 0.05 |
| 69202 | 1.3393 | 0.05 |
| 69203 | 1.17111 | 0.0581117 |
| 69204 | 1.3393 | 0.05 |
| 69205 | 1.3393 | 0.05 |
| 69206 | 0.946129 | 0.05 |
| 69207 | 0.946129 | 0.05 |
| 69208 | 0.946126 | 2.1 |
| 69209 | 0.946126 | 0.593629 |
| 69210 | 0.946132 | 2.1 |
| 69211 | 0.946132 | 2.1 |
| 69212 | 0.94613 | 2.1 |
| 69213 | 0.94613 | 0.05 |
| 69214 | 0.946129 | 1.59079 |
| 69215 | 0.946129 | 0.935128 |
| 69216 | 0.946129 | 0.05 |
| 69217 | 0.946129 | 2.1 |
| 69218 | 0.94613 | 1.0354 |
| 69219 | 0.94613 | 0.232779 |
| 69220 | 0.946131 | 0.186452 |
| 69221 | 0.946131 | 0.063323 |
| 69222 | 0.94613 | 0.0968488 |
| 69223 | 0.94613 | 0.109431 |
| 69224 | 0.946128 | 0.202889 |
| 69225 | 0.946128 | 0.287907 |
| 69226 | 0.946132 | 0.0734601 |
| 69227 | 0.946132 | 0.0981471 |
| 69228 | 0.94613 | 0.17786 |
| 69229 | 0.94613 | 0.0544388 |
| 69230 | 0.94613 | 0.117654 |
| 69231 | 0.94613 | 0.124904 |
| 69232 | 0.94613 | 1.04631 |
| 69233 | 0.94613 | 0.487934 |
| 69234 | 0.94613 | 0.806641 |
| 69235 | 0.94613 | 0.486827 |
| 69236 | 0.946128 | 1.1016 |
| 69237 | 0.946128 | 1.13183 |
| 69238 | 0.946131 | 0.472606 |
| 69239 | 0.946131 | 0.268222 |
| 69240 | 0.94613 | 1.03708 |
| 69241 | 0.94613 | 0.361085 |
| 69242 | 0.94613 | 0.749598 |
| 69243 | 0.94613 | 1.03104 |
| 69244 | 1.89226 | 1.06109 |
| 69245 | 1.89226 | 0.804464 |
| 69246 | 1.89226 | 0.990535 |
| 69247 | 1.89226 | 1.82598 |
| 69248 | 3.78453 | 0.986682 |
| 69249 | 1.89226 | 0.776996 |

| | | |
|---|---|---|
| 69250 | 1.89226 | 0.772607 |
| 69251 | 1.89226 | 0.169154 |
| 69252 | 1.89226 | 0.772988 |
| 69253 | 1.89226 | 0.251958 |
| 69254 | 1.89226 | 0.754484 |
| 69255 | 1.89226 | 0.241757 |
| 69256 | 1.89226 | 1.01628 |
| 69257 | 1.89226 | 0.05 |
| 69258 | 1.89226 | 0.221868 |
| 69259 | 1.89226 | 0.140005 |
| 69260 | 1.89226 | 0.570153 |
| 69261 | 1.89227 | 0.0669455 |
| 69262 | 2.28622 | 0.135495 |
| 69263 | 2.16109 | 0.0844385 |
| 69264 | 1.89227 | 0.174781 |
| 69265 | 4.17849 | 0.0800153 |
| 69266 | 15.1381 | 0.39043 |
| 69267 | 15.1381 | 0.538569 |
| 69268 | 15.1381 | 0.417972 |
| 69269 | 15.1381 | 0.478867 |
| 69270 | 30.2762 | 0.386719 |
| 69271 | 15.1381 | 0.385179 |
| 69272 | 15.1381 | 0.523557 |
| 69273 | 15.1381 | 0.278425 |
| 69274 | 15.1381 | 0.469921 |
| 69275 | 30.2762 | 0.378444 |
| 69276 | 15.1381 | 0.593758 |
| 69277 | 15.1381 | 0.585856 |
| 69278 | 15.1381 | 0.05 |
| 69279 | 15.1381 | 0.594754 |
| 69280 | 30.2762 | 0.546167 |
| 69281 | 15.1381 | 0.400175 |
| 69282 | 15.1381 | 0.60006 |
| 69283 | 15.1381 | 0.275546 |
| 69284 | 15.1381 | 0.438625 |
| 69285 | 30.2762 | 0.439263 |
| 69286 | 15.138 | 0.601187 |
| 69287 | 15.138 | 2.08251 |
| 69288 | 15.138 | 0.594166 |
| 69289 | 15.138 | 2.1 |
| 69290 | 30.276 | 1.6362 |
| 69291 | 15.138 | 2.1 |
| 69292 | 15.138 | 2.1 |
| 69293 | 15.138 | 2.1 |
| 69294 | 15.138 | 2.1 |
| 69295 | 30.276 | 2.1 |
| 69296 | 15.138 | 1.74332 |
| 69297 | 15.138 | 1.26398 |
| 69298 | 15.138 | 0.499936 |
| 69299 | 15.138 | 2.1 |
| 69300 | 30.2759 | 1.94866 |
| 69301 | 15.138 | 1.61229 |
| 69302 | 15.138 | 2.1 |
| 69303 | 15.138 | 2.1 |
| 69304 | 15.138 | 1.29209 |
| 69305 | 30.2759 | 2.04743 |
| 69306 | 15.138 | 2.1 |
| 69307 | 15.138 | 2.1 |

| | | |
|---|---|---|
| 69308 | 15.138 | 2.1 |
| 69309 | 15.138 | 2.1 |
| 69310 | 30.276 | 2.1 |
| 69311 | 15.1381 | 2.1 |
| 69312 | 15.1381 | 2.1 |
| 69313 | 15.1381 | 2.1 |
| 69314 | 15.1381 | 2.1 |
| 69315 | 30.2762 | 2.1 |
| 69316 | 15.1381 | 2.1 |
| 69317 | 15.1381 | 2.1 |
| 69318 | 15.1381 | 2.1 |
| 69319 | 15.1381 | 2.1 |
| 69320 | 30.2762 | 2.1 |
| 69321 | 15.138 | 1.97888 |
| 69322 | 15.138 | 2.1 |
| 69323 | 15.138 | 2.1 |
| 69324 | 15.138 | 2.1 |
| 69325 | 30.276 | 2.1 |
| 69326 | 15.138 | 2.1 |
| 69327 | 15.138 | 2.1 |
| 69328 | 15.138 | 2.1 |
| 69329 | 15.138 | 2.1 |
| 69330 | 30.276 | 2.1 |
| 69331 | 15.1381 | 1.19945 |
| 69332 | 15.1381 | 2.1 |
| 69333 | 15.1381 | 2.1 |
| 69334 | 15.1381 | 1.62967 |
| 69335 | 30.2762 | 2.0733 |
| 69336 | 15.1381 | 2.1 |
| 69337 | 15.1381 | 2.1 |
| 69338 | 15.1381 | 2.08945 |
| 69339 | 15.1381 | 2.1 |
| 69340 | 30.2762 | 2.1 |
| 69341 | 15.1381 | 2.1 |
| 69342 | 15.1381 | 2.1 |
| 69343 | 15.1381 | 2.1 |
| 69344 | 15.1381 | 2.1 |
| 69345 | 30.2762 | 2.1 |
| 69346 | 15.138 | 0.417467 |
| 69347 | 15.138 | 0.460628 |
| 69348 | 15.138 | 0.1715 |
| 69349 | 15.138 | 0.636463 |
| 69350 | 30.276 | 0.401952 |
| 69351 | 1.89226 | 2.1 |
| 69352 | 1.89226 | 2.1 |
| 69353 | 1.89226 | 2.1 |
| 69354 | 1.89226 | 2.1 |
| 69355 | 3.78453 | 2.1 |
| 69356 | 1.89226 | 2.1 |
| 69357 | 1.89226 | 2.1 |
| 69358 | 1.89226 | 2.1 |
| 69359 | 1.89226 | 2.1 |
| 69360 | 1.89226 | 2.1 |
| 69361 | 1.89226 | 2.1 |
| 69362 | 1.89226 | 2.1 |
| 69363 | 1.89226 | 2.1 |
| 69364 | 1.89226 | 2.1 |
| 69365 | 1.89226 | 2.1 |

| | | |
|---|---|---|
| 69366 | 1.89226 | 2.1 |
| 69367 | 1.89226 | 2.1 |
| 69368 | 1.89226 | 2.1 |
| 69369 | 1.89226 | 2.1 |
| 69370 | 1.89226 | 2.1 |
| 69371 | 1.89226 | 2.1 |
| 69372 | 3.78453 | 2.1 |
| 69373 | 1.89226 | 2.1 |
| 69374 | 1.89226 | 2.1 |
| 69375 | 1.89226 | 2.1 |
| 69376 | 1.89226 | 2.1 |
| 69377 | 1.89226 | 2.1 |
| 69378 | 1.89226 | 2.1 |
| 69379 | 0.946127 | 2.1 |
| 69380 | 0.946127 | 2.1 |
| 69381 | 0.946131 | 2.1 |
| 69382 | 0.946131 | 2.1 |
| 69383 | 0.946131 | 2.1 |
| 69384 | 0.946131 | 2.1 |
| 69385 | 0.946128 | 2.1 |
| 69386 | 0.946128 | 2.1 |
| 69387 | 0.94613 | 2.1 |
| 69388 | 0.94613 | 2.1 |
| 69389 | 0.946129 | 2.1 |
| 69390 | 0.946129 | 2.1 |
| 69391 | 1.89226 | 2.1 |
| 69392 | 1.89226 | 2.1 |
| 69393 | 1.89226 | 2.1 |
| 69394 | 1.89226 | 2.1 |
| 69395 | 1.89226 | 2.1 |
| 69396 | 1.89226 | 2.1 |
| 69397 | 1.89226 | 2.1 |
| 69398 | 1.89226 | 2.1 |
| 69399 | 1.89226 | 2.1 |
| 69400 | 1.89226 | 2.1 |
| 69401 | 3.78452 | 2.1 |
| 69402 | 1.89226 | 2.1 |
| 69403 | 1.89226 | 2.1 |
| 69404 | 1.89226 | 2.1 |
| 69405 | 1.89226 | 2.1 |
| 69406 | 1.89226 | 2.1 |
| 69407 | 1.89226 | 2.1 |
| 69408 | 0.946129 | 2.1 |
| 69409 | 0.946129 | 2.1 |
| 69410 | 0.946133 | 2.1 |
| 69411 | 0.946133 | 2.1 |
| 69412 | 0.946131 | 2.1 |
| 69413 | 0.946131 | 2.1 |
| 69414 | 0.946131 | 2.1 |
| 69415 | 0.946131 | 2.1 |
| 69416 | 0.946131 | 2.1 |
| 69417 | 0.946131 | 2.1 |
| 69418 | 0.946131 | 2.1 |
| 69419 | 0.946131 | 2.1 |
| 69420 | 0.946132 | 2.1 |
| 69421 | 0.946132 | 2.1 |
| 69422 | 0.946132 | 2.1 |
| 69423 | 0.946132 | 2.1 |

| | | |
|---|---|---|
| 69424 | 0.946132 | 2.1 |
| 69425 | 0.946132 | 2.1 |
| 69426 | 0.946133 | 2.1 |
| 69427 | 0.946133 | 2.1 |
| 69428 | 0.946132 | 2.1 |
| 69429 | 0.946132 | 2.1 |
| 69430 | 0.946133 | 2.1 |
| 69431 | 0.946133 | 2.1 |
| 69432 | 1.89226 | 2.1 |
| 69433 | 1.89226 | 2.1 |
| 69434 | 1.89226 | 2.1 |
| 69435 | 1.89226 | 2.1 |
| 69436 | 3.78452 | 2.1 |
| 69437 | 1.89226 | 2.1 |
| 69438 | 1.89226 | 2.1 |
| 69439 | 1.89226 | 2.1 |
| 69440 | 1.89226 | 2.1 |
| 69441 | 1.89226 | 2.1 |
| 69442 | 1.89226 | 2.1 |
| 69443 | 0.946129 | 2.1 |
| 69444 | 0.946129 | 2.1 |
| 69445 | 0.946133 | 2.1 |
| 69446 | 0.946133 | 2.1 |
| 69447 | 0.946131 | 2.1 |
| 69448 | 0.946131 | 2.1 |
| 69449 | 0.946131 | 2.1 |
| 69450 | 0.946131 | 2.1 |
| 69451 | 0.946131 | 2.1 |
| 69452 | 0.946131 | 2.1 |
| 69453 | 0.946131 | 2.1 |
| 69454 | 0.946131 | 2.1 |
| 69455 | 0.946132 | 2.1 |
| 69456 | 0.946132 | 2.1 |
| 69457 | 0.946132 | 2.1 |
| 69458 | 0.946132 | 2.1 |
| 69459 | 0.946132 | 2.1 |
| 69460 | 0.946132 | 2.1 |
| 69461 | 0.946133 | 2.1 |
| 69462 | 0.946133 | 2.1 |
| 69463 | 0.946133 | 2.1 |
| 69464 | 0.946133 | 2.1 |
| 69465 | 0.946132 | 2.1 |
| 69466 | 0.946132 | 2.1 |
| 69467 | 1.89226 | 2.1 |
| 69468 | 1.89226 | 2.1 |
| 69469 | 1.89226 | 2.1 |
| 69470 | 1.89226 | 2.1 |
| 69471 | 3.78453 | 2.1 |
| 69472 | 0.946128 | 2.1 |
| 69473 | 0.946128 | 2.1 |
| 69474 | 0.946132 | 2.1 |
| 69475 | 0.946132 | 2.1 |
| 69476 | 0.94613 | 2.1 |
| 69477 | 0.94613 | 2.1 |
| 69478 | 0.94613 | 2.1 |
| 69479 | 0.94613 | 2.1 |
| 69480 | 0.94613 | 2.1 |
| 69481 | 0.94613 | 2.1 |

| | | |
|---|---|---|
| 69482 | 0.94613 | 2.1 |
| 69483 | 0.94613 | 2.1 |
| 69484 | 0.946127 | 2.1 |
| 69485 | 0.946127 | 2.1 |
| 69486 | 0.946131 | 2.1 |
| 69487 | 0.946131 | 2.1 |
| 69488 | 0.94613 | 2.1 |
| 69489 | 0.94613 | 2.1 |
| 69490 | 0.946128 | 2.1 |
| 69491 | 0.946128 | 2.1 |
| 69492 | 0.946129 | 2.1 |
| 69493 | 0.946129 | 2.1 |
| 69494 | 0.946129 | 2.1 |
| 69495 | 0.946129 | 2.1 |
| 69496 | 0.94613 | 2.1 |
| 69497 | 0.94613 | 2.1 |
| 69498 | 0.94613 | 2.1 |
| 69499 | 0.94613 | 2.1 |
| 69500 | 0.946131 | 2.1 |
| 69501 | 0.946131 | 2.1 |
| 69502 | 0.946129 | 2.1 |
| 69503 | 0.946129 | 2.1 |
| 69504 | 0.94613 | 2.1 |
| 69505 | 0.94613 | 2.1 |
| 69506 | 0.94613 | 2.1 |
| 69507 | 0.94613 | 2.1 |
| 69508 | 1.89226 | 2.1 |
| 69509 | 1.89226 | 2.1 |
| 69510 | 1.89226 | 2.1 |
| 69511 | 1.89226 | 2.1 |
| 69512 | 3.78453 | 2.1 |
| 69513 | 1.89226 | 2.1 |
| 69514 | 1.89226 | 2.1 |
| 69515 | 1.89226 | 2.1 |
| 69516 | 1.89226 | 2.1 |
| 69517 | 1.89226 | 2.1 |
| 69518 | 1.89226 | 2.1 |
| 69519 | 1.89226 | 2.1 |
| 69520 | 1.89226 | 2.1 |
| 69521 | 1.89226 | 2.1 |
| 69522 | 1.89226 | 2.1 |
| 69523 | 1.89226 | 2.1 |
| 69524 | 1.89226 | 2.1 |
| 69525 | 0.946131 | 2.1 |
| 69526 | 0.946131 | 2.1 |
| 69527 | 0.946131 | 2.1 |
| 69528 | 0.946131 | 2.1 |
| 69529 | 0.946132 | 2.1 |
| 69530 | 0.946132 | 2.1 |
| 69531 | 0.946129 | 2.1 |
| 69532 | 0.946129 | 2.1 |
| 69533 | 0.946131 | 2.1 |
| 69534 | 0.946131 | 2.1 |
| 69535 | 0.94613 | 2.1 |
| 69536 | 0.94613 | 2.1 |
| 69537 | 1.89224 | 2.1 |
| 69538 | 1.89224 | 2.1 |
| 69539 | 1.89224 | 2.1 |

| | | |
|---|---|---|
| 69540 | 1.89224 | 2.1 |
| 69541 | 3.78448 | 2.1 |
| 69542 | 1.89224 | 2.1 |
| 69543 | 1.89224 | 2.1 |
| 69544 | 1.89224 | 2.1 |
| 69545 | 1.89224 | 2.1 |
| 69546 | 3.78447 | 2.1 |
| 69547 | 1.89223 | 2.1 |
| 69548 | 2.38995 | 2.1 |
| 69549 | 2.25315 | 2.1 |
| 69550 | 2.25315 | 2.1 |
| 69551 | 1.89223 | 2.1 |
| 69552 | 1.89223 | 2.1 |
| 69553 | 1.89224 | 2.1 |
| 69554 | 1.89224 | 2.1 |
| 69555 | 1.89224 | 2.1 |
| 69556 | 1.89224 | 2.1 |
| 69557 | 1.89224 | 2.1 |
| 69558 | 1.89224 | 2.1 |
| 69559 | 1.89226 | 2.1 |
| 69560 | 1.89226 | 2.1 |
| 69561 | 1.89226 | 2.1 |
| 69562 | 1.89226 | 2.1 |
| 69563 | 3.78453 | 2.1 |
| 69564 | 1.89226 | 2.1 |
| 69565 | 1.89226 | 2.1 |
| 69566 | 1.89226 | 2.1 |
| 69567 | 1.89226 | 2.1 |
| 69568 | 1.89226 | 2.1 |
| 69569 | 1.89226 | 2.1 |
| 69570 | 1.89226 | 2.1 |
| 69571 | 2.60272 | 2.1 |
| 69572 | 2.38998 | 2.1 |
| 69573 | 2.38998 | 2.1 |
| 69574 | 1.89226 | 2.1 |
| 69575 | 1.89226 | 2.1 |
| 69576 | 0.94613 | 2.1 |
| 69577 | 0.94613 | 2.1 |
| 69578 | 0.94613 | 2.1 |
| 69579 | 0.94613 | 2.1 |
| 69580 | 0.946131 | 2.1 |
| 69581 | 0.946131 | 2.1 |
| 69582 | 0.946129 | 2.1 |
| 69583 | 0.946129 | 2.1 |
| 69584 | 0.94613 | 2.1 |
| 69585 | 0.94613 | 2.1 |
| 69586 | 0.94613 | 2.1 |
| 69587 | 0.94613 | 2.1 |
| 69588 | 1.89226 | 2.1 |
| 69589 | 1.89226 | 2.1 |
| 69590 | 1.89226 | 2.1 |
| 69591 | 1.89226 | 2.1 |
| 69592 | 3.78452 | 2.1 |
| 69593 | 1.89226 | 2.1 |
| 69594 | 1.89226 | 2.1 |
| 69595 | 1.89226 | 2.1 |
| 69596 | 1.89226 | 2.1 |
| 69597 | 3.78452 | 2.1 |

| | | |
|---|---|---|
| 69598 | 1.89226 | 2.1 |
| 69599 | 1.89226 | 2.1 |
| 69600 | 1.89226 | 2.1 |
| 69601 | 1.89226 | 2.1 |
| 69602 | 1.89226 | 2.1 |
| 69603 | 1.89226 | 2.1 |
| 69604 | 0.946132 | 2.1 |
| 69605 | 0.946132 | 2.1 |
| 69606 | 0.946132 | 2.1 |
| 69607 | 0.946132 | 2.1 |
| 69608 | 0.946131 | 2.1 |
| 69609 | 0.946131 | 2.1 |
| 69610 | 0.946132 | 2.1 |
| 69611 | 0.946132 | 2.1 |
| 69612 | 0.946132 | 2.1 |
| 69613 | 0.946132 | 2.1 |
| 69614 | 0.946132 | 2.1 |
| 69615 | 0.946132 | 2.1 |
| 69616 | 1.89226 | 2.1 |
| 69617 | 1.89226 | 2.1 |
| 69618 | 1.89226 | 2.1 |
| 69619 | 1.89226 | 2.1 |
| 69620 | 3.78452 | 2.1 |
| 69621 | 1.89226 | 2.1 |
| 69622 | 1.89226 | 2.1 |
| 69623 | 1.89226 | 2.1 |
| 69624 | 1.89226 | 2.1 |
| 69625 | 3.78452 | 2.1 |
| 69626 | 0.946129 | 2.1 |
| 69627 | 0.946129 | 2.1 |
| 69628 | 0.946133 | 2.1 |
| 69629 | 0.946133 | 2.1 |
| 69630 | 0.946131 | 2.1 |
| 69631 | 0.946131 | 2.1 |
| 69632 | 0.946131 | 2.1 |
| 69633 | 0.946131 | 2.1 |
| 69634 | 0.946131 | 2.1 |
| 69635 | 0.946131 | 2.1 |
| 69636 | 0.946131 | 2.1 |
| 69637 | 0.946131 | 2.1 |
| 69638 | 0.946132 | 2.1 |
| 69639 | 0.946132 | 2.1 |
| 69640 | 0.946132 | 2.1 |
| 69641 | 0.946132 | 2.1 |
| 69642 | 0.946132 | 2.1 |
| 69643 | 0.946132 | 2.1 |
| 69644 | 0.946133 | 2.1 |
| 69645 | 0.946133 | 2.1 |
| 69646 | 0.946133 | 2.1 |
| 69647 | 0.946133 | 2.1 |
| 69648 | 0.946132 | 2.1 |
| 69649 | 0.946132 | 2.1 |
| 69650 | 1.89224 | 2.1 |
| 69651 | 1.89224 | 2.1 |
| 69652 | 1.89224 | 2.1 |
| 69653 | 1.89224 | 2.1 |
| 69654 | 3.78447 | 2.1 |
| 69655 | 1.89223 | 2.1 |

| | | |
|---|---|---|
| 69656 | 1.89223 | 2.1 |
| 69657 | 1.89223 | 2.1 |
| 69658 | 1.89223 | 2.1 |
| 69659 | 1.89223 | 2.1 |
| 69660 | 1.89223 | 2.1 |
| 69661 | 1.89224 | 2.1 |
| 69662 | 1.89224 | 2.1 |
| 69663 | 1.89224 | 2.1 |
| 69664 | 1.89224 | 2.1 |
| 69665 | 3.78447 | 2.1 |
| 69666 | 1.89224 | 2.1 |
| 69667 | 1.89224 | 2.1 |
| 69668 | 1.89224 | 2.1 |
| 69669 | 1.89224 | 2.1 |
| 69670 | 3.78448 | 2.1 |
| 69671 | 1.89226 | 2.1 |
| 69672 | 1.89226 | 2.1 |
| 69673 | 1.89226 | 2.1 |
| 69674 | 1.89226 | 2.1 |
| 69675 | 3.78452 | 2.1 |
| 69676 | 1.89226 | 2.1 |
| 69677 | 1.89226 | 2.1 |
| 69678 | 1.89226 | 2.1 |
| 69679 | 1.89226 | 2.1 |
| 69680 | 1.89226 | 2.1 |
| 69681 | 1.89226 | 2.1 |
| 69682 | 1.89226 | 2.1 |
| 69683 | 1.89226 | 2.1 |
| 69684 | 1.89226 | 2.1 |
| 69685 | 1.89226 | 2.1 |
| 69686 | 1.89226 | 2.1 |
| 69687 | 1.89226 | 2.1 |
| 69688 | 0.810946 | 2.1 |
| 69689 | 0.810946 | 2.1 |
| 69690 | 0.810946 | 2.1 |
| 69691 | 0.810946 | 2.1 |
| 69692 | 0.810933 | 2.1 |
| 69693 | 0.810933 | 2.1 |
| 69694 | 1.89226 | 2.1 |
| 69695 | 1.3517 | 2.1 |
| 69696 | 1.08132 | 2.1 |
| 69697 | 1.08132 | 2.1 |
| 69698 | 1.08133 | 2.1 |
| 69699 | 1.89226 | 2.1 |
| 69700 | 1.89226 | 2.1 |
| 69701 | 1.89226 | 2.1 |
| 69702 | 1.89226 | 2.1 |
| 69703 | 3.78453 | 2.1 |
| 69704 | 1.89226 | 2.1 |
| 69705 | 1.89226 | 2.1 |
| 69706 | 1.89226 | 2.1 |
| 69707 | 1.89226 | 2.1 |
| 69708 | 3.78452 | 2.1 |
| 69709 | 1.89226 | 2.1 |
| 69710 | 1.89226 | 2.1 |
| 69711 | 1.89226 | 2.1 |
| 69712 | 1.89226 | 2.1 |
| 69713 | 1.89226 | 2.1 |

| | | |
|---|---|---|
| 69714 | 1.89226 | 2.1 |
| 69715 | 0.94613 | 2.1 |
| 69716 | 0.94613 | 2.1 |
| 69717 | 0.94613 | 2.1 |
| 69718 | 0.94613 | 2.1 |
| 69719 | 0.946131 | 2.1 |
| 69720 | 0.946131 | 2.1 |
| 69721 | 0.946129 | 2.1 |
| 69722 | 0.946129 | 2.1 |
| 69723 | 0.94613 | 2.1 |
| 69724 | 0.94613 | 2.1 |
| 69725 | 0.94613 | 2.1 |
| 69726 | 0.94613 | 2.1 |
| 69727 | 1.89226 | 2.1 |
| 69728 | 1.89226 | 2.1 |
| 69729 | 1.89226 | 2.1 |
| 69730 | 1.89226 | 2.1 |
| 69731 | 3.78453 | 2.1 |
| 69732 | 1.89226 | 2.1 |
| 69733 | 1.89226 | 2.1 |
| 69734 | 1.89226 | 2.1 |
| 69735 | 1.89226 | 2.1 |
| 69736 | 3.78452 | 2.1 |
| 69737 | 1.89226 | 2.1 |
| 69738 | 1.89226 | 2.1 |
| 69739 | 1.89226 | 2.1 |
| 69740 | 1.89226 | 2.1 |
| 69741 | 3.78452 | 2.1 |
| 69742 | 1.89226 | 2.1 |
| 69743 | 1.89226 | 2.1 |
| 69744 | 1.89226 | 2.1 |
| 69745 | 1.89226 | 2.1 |
| 69746 | 1.89226 | 2.1 |
| 69747 | 1.89226 | 2.1 |
| 69748 | 1.89226 | 2.1 |
| 69749 | 1.89226 | 2.1 |
| 69750 | 1.89226 | 2.1 |
| 69751 | 1.89226 | 2.1 |
| 69752 | 3.78452 | 2.1 |
| 69753 | 1.89226 | 2.1 |
| 69754 | 1.89226 | 2.1 |
| 69755 | 1.89226 | 2.1 |
| 69756 | 1.89226 | 2.1 |
| 69757 | 3.78452 | 2.1 |
| 69758 | 0.946128 | 2.1 |
| 69759 | 0.946128 | 2.1 |
| 69760 | 0.946133 | 2.1 |
| 69761 | 0.946133 | 2.1 |
| 69762 | 0.94613 | 2.1 |
| 69763 | 0.94613 | 2.1 |
| 69764 | 0.946131 | 2.1 |
| 69765 | 0.946131 | 2.1 |
| 69766 | 0.946131 | 2.1 |
| 69767 | 0.946131 | 2.1 |
| 69768 | 0.946131 | 2.1 |
| 69769 | 0.946131 | 2.1 |
| 69770 | 0.946132 | 2.1 |
| 69771 | 0.946132 | 2.1 |

| | | |
|---|---|---|
| 69772 | 0.946132 | 2.1 |
| 69773 | 0.946132 | 2.1 |
| 69774 | 0.946131 | 2.1 |
| 69775 | 0.946131 | 2.1 |
| 69776 | 0.946132 | 2.1 |
| 69777 | 0.946132 | 2.1 |
| 69778 | 0.946132 | 2.1 |
| 69779 | 0.946132 | 2.1 |
| 69780 | 0.946132 | 2.1 |
| 69781 | 0.946132 | 2.1 |
| 69782 | 1.89226 | 2.1 |
| 69783 | 1.89226 | 2.1 |
| 69784 | 1.89226 | 2.1 |
| 69785 | 1.89226 | 2.1 |
| 69786 | 3.78452 | 2.1 |
| 69787 | 1.89226 | 2.1 |
| 69788 | 1.89226 | 2.1 |
| 69789 | 1.89226 | 2.1 |
| 69790 | 1.89226 | 2.1 |
| 69791 | 3.78452 | 2.1 |
| 69792 | 0.946129 | 2.1 |
| 69793 | 0.946129 | 2.1 |
| 69794 | 0.946133 | 2.1 |
| 69795 | 0.946133 | 2.1 |
| 69796 | 0.946131 | 2.1 |
| 69797 | 0.946131 | 2.1 |
| 69798 | 0.946131 | 2.1 |
| 69799 | 0.946131 | 2.1 |
| 69800 | 0.946131 | 2.1 |
| 69801 | 0.946131 | 2.1 |
| 69802 | 0.946131 | 2.1 |
| 69803 | 0.946131 | 2.1 |
| 69804 | 0.946132 | 2.1 |
| 69805 | 0.946132 | 2.1 |
| 69806 | 0.946132 | 2.1 |
| 69807 | 0.946132 | 2.1 |
| 69808 | 0.946132 | 2.1 |
| 69809 | 0.946132 | 2.1 |
| 69810 | 0.946133 | 2.1 |
| 69811 | 0.946133 | 2.1 |
| 69812 | 0.946132 | 2.1 |
| 69813 | 0.946132 | 2.1 |
| 69814 | 0.946133 | 2.1 |
| 69815 | 0.946133 | 2.1 |
| 69816 | 1.89226 | 2.1 |
| 69817 | 1.89226 | 2.1 |
| 69818 | 1.89226 | 2.1 |
| 69819 | 1.89226 | 2.1 |
| 69820 | 1.89226 | 2.1 |
| 69821 | 1.89226 | 2.1 |
| 69822 | 0.946128 | 2.1 |
| 69823 | 0.946128 | 2.1 |
| 69824 | 0.946133 | 2.1 |
| 69825 | 0.946133 | 2.1 |
| 69826 | 0.946131 | 2.1 |
| 69827 | 0.946131 | 2.1 |
| 69828 | 0.94613 | 2.1 |
| 69829 | 0.94613 | 2.1 |

| | | |
|---|---|---|
| 69830 | 0.946131 | 2.1 |
| 69831 | 0.946131 | 2.1 |
| 69832 | 0.946131 | 2.1 |
| 69833 | 0.946131 | 2.1 |
| 69834 | 1.89226 | 2.1 |
| 69835 | 1.89226 | 2.1 |
| 69836 | 1.89226 | 2.1 |
| 69837 | 1.89226 | 2.1 |
| 69838 | 1.89226 | 2.1 |
| 69839 | 1.89226 | 2.1 |
| 69840 | 1.89226 | 2.1 |
| 69841 | 1.89226 | 2.1 |
| 69842 | 1.89226 | 2.1 |
| 69843 | 1.89226 | 2.07599 |
| 69844 | 3.78453 | 2.1 |
| 69845 | 1.89226 | 2.1 |
| 69846 | 1.89226 | 2.1 |
| 69847 | 1.89226 | 2.1 |
| 69848 | 1.89226 | 2.1 |
| 69849 | 1.89226 | 2.1 |
| 69850 | 1.89226 | 2.1 |
| 69851 | 0.946127 | 2.1 |
| 69852 | 0.946127 | 2.1 |
| 69853 | 0.946131 | 2.1 |
| 69854 | 0.946131 | 2.1 |
| 69855 | 0.946131 | 2.1 |
| 69856 | 0.946131 | 2.1 |
| 69857 | 0.946127 | 2.1 |
| 69858 | 0.946127 | 2.1 |
| 69859 | 0.946129 | 2.1 |
| 69860 | 0.946129 | 2.1 |
| 69861 | 0.94613 | 2.1 |
| 69862 | 0.94613 | 2.1 |
| 69863 | 0.946131 | 2.1 |
| 69864 | 0.946131 | 2.1 |
| 69865 | 0.946131 | 2.1 |
| 69866 | 0.946131 | 2.1 |
| 69867 | 0.946133 | 2.1 |
| 69868 | 0.946133 | 2.1 |
| 69869 | 0.946128 | 2.1 |
| 69870 | 0.946128 | 2.0401 |
| 69871 | 0.94613 | 2.1 |
| 69872 | 0.94613 | 2.1 |
| 69873 | 0.946131 | 2.1 |
| 69874 | 0.946131 | 2.1 |
| 69875 | 1.89226 | 2.1 |
| 69876 | 1.89226 | 2.1 |
| 69877 | 1.89226 | 2.1 |
| 69878 | 1.89226 | 2.1 |
| 69879 | 1.89226 | 2.1 |
| 69880 | 1.89226 | 2.1 |
| 69881 | 1.89226 | 2.1 |
| 69882 | 1.89226 | 2.1 |
| 69883 | 1.89226 | 2.1 |
| 69884 | 1.89226 | 2.1 |
| 69885 | 3.78452 | 2.1 |
| 69886 | 1.89226 | 2.1 |
| 69887 | 1.89226 | 2.1 |

| | | |
|---|---|---|
| 69888 | 1.89226 | 2.1 |
| 69889 | 1.89226 | 2.1 |
| 69890 | 3.78452 | 2.1 |
| 69891 | 1.89226 | 2.1 |
| 69892 | 1.89226 | 2.1 |
| 69893 | 1.89226 | 2.1 |
| 69894 | 1.89226 | 2.1 |
| 69895 | 1.89226 | 2.1 |
| 69896 | 1.89226 | 2.1 |
| 69897 | 1.89226 | 2.1 |
| 69898 | 1.89226 | 2.1 |
| 69899 | 1.89226 | 2.1 |
| 69900 | 1.89226 | 2.1 |
| 69901 | 3.78453 | 2.1 |
| 69902 | 1.89226 | 2.1 |
| 69903 | 1.89226 | 2.1 |
| 69904 | 1.89226 | 2.1 |
| 69905 | 1.89226 | 2.1 |
| 69906 | 3.78452 | 2.1 |
| 69907 | 1.89226 | 2.1 |
| 69908 | 1.89226 | 2.1 |
| 69909 | 1.89226 | 2.1 |
| 69910 | 1.89226 | 2.1 |
| 69911 | 3.78452 | 2.1 |
| 69912 | 1.89226 | 2.1 |
| 69913 | 1.89226 | 2.1 |
| 69914 | 1.89226 | 2.1 |
| 69915 | 1.89226 | 2.1 |
| 69916 | 1.89226 | 2.1 |
| 69917 | 1.89226 | 2.1 |
| 69918 | 0.946131 | 2.1 |
| 69919 | 0.946131 | 2.1 |
| 69920 | 0.946131 | 2.1 |
| 69921 | 0.946131 | 2.1 |
| 69922 | 0.946131 | 2.1 |
| 69923 | 0.946131 | 2.1 |
| 69924 | 0.946131 | 2.1 |
| 69925 | 0.946131 | 2.1 |
| 69926 | 0.946131 | 2.1 |
| 69927 | 0.946131 | 2.1 |
| 69928 | 0.946131 | 2.1 |
| 69929 | 0.946131 | 2.1 |
| 69930 | 1.89226 | 2.1 |
| 69931 | 1.89226 | 2.1 |
| 69932 | 1.89226 | 2.1 |
| 69933 | 1.89226 | 2.1 |
| 69934 | 1.89226 | 2.1 |
| 69935 | 1.89226 | 2.1 |
| 69936 | 1.89226 | 2.1 |
| 69937 | 1.89226 | 2.1 |
| 69938 | 1.89226 | 2.1 |
| 69939 | 1.89226 | 2.1 |
| 69940 | 1.89226 | 2.1 |
| 69941 | 1.89226 | 2.1 |
| 69942 | 0.946131 | 2.1 |
| 69943 | 0.946131 | 2.1 |
| 69944 | 0.946131 | 2.1 |
| 69945 | 0.946131 | 2.1 |

| | | |
|---|---|---|
| 69946 | 0.946131 | 2.1 |
| 69947 | 0.946131 | 2.1 |
| 69948 | 0.946131 | 2.1 |
| 69949 | 0.946131 | 2.1 |
| 69950 | 0.946131 | 2.1 |
| 69951 | 0.946131 | 2.1 |
| 69952 | 0.946131 | 2.1 |
| 69953 | 0.946131 | 2.1 |
| 69954 | 1.89226 | 2.1 |
| 69955 | 1.89226 | 2.1 |
| 69956 | 1.89226 | 2.1 |
| 69957 | 1.89226 | 2.1 |
| 69958 | 1.89226 | 2.1 |
| 69959 | 1.89226 | 2.1 |
| 69960 | 0.946128 | 2.1 |
| 69961 | 0.946128 | 2.1 |
| 69962 | 0.946133 | 2.1 |
| 69963 | 0.946133 | 2.1 |
| 69964 | 0.946131 | 2.1 |
| 69965 | 0.946131 | 2.1 |
| 69966 | 0.946131 | 2.1 |
| 69967 | 0.946131 | 2.1 |
| 69968 | 0.946131 | 2.1 |
| 69969 | 0.946131 | 2.1 |
| 69970 | 0.94613 | 2.1 |
| 69971 | 0.94613 | 2.1 |
| 69972 | 1.89226 | 2.1 |
| 69973 | 1.89226 | 2.1 |
| 69974 | 1.89226 | 2.1 |
| 69975 | 1.89226 | 2.1 |
| 69976 | 1.89226 | 2.1 |
| 69977 | 1.89226 | 2.1 |
| 69978 | 0.946131 | 2.1 |
| 69979 | 0.946131 | 2.1 |
| 69980 | 0.946131 | 2.1 |
| 69981 | 0.946131 | 2.1 |
| 69982 | 0.946132 | 2.1 |
| 69983 | 0.946132 | 2.1 |
| 69984 | 0.946131 | 2.1 |
| 69985 | 0.946131 | 2.1 |
| 69986 | 0.946132 | 2.1 |
| 69987 | 0.946132 | 2.1 |
| 69988 | 0.946131 | 2.1 |
| 69989 | 0.946131 | 2.1 |
| 69990 | 1.89226 | 2.1 |
| 69991 | 1.89226 | 2.1 |
| 69992 | 1.89226 | 2.1 |
| 69993 | 1.89226 | 2.1 |
| 69994 | 1.89226 | 2.1 |
| 69995 | 1.89226 | 2.1 |
| 69996 | 1.89226 | 2.1 |
| 69997 | 1.89226 | 2.1 |
| 69998 | 1.89226 | 2.1 |
| 69999 | 1.89226 | 2.1 |
| 70000 | 3.78452 | 2.1 |
| 70001 | 1.89226 | 2.1 |
| 70002 | 1.89226 | 2.1 |
| 70003 | 1.89226 | 2.1 |

| | | |
|---|---|---|
| 70004 | 1.89226 | 2.1 |
| 70005 | 3.78452 | 2.1 |
| 70006 | 0.946131 | 2.1 |
| 70007 | 0.946131 | 2.1 |
| 70008 | 0.946131 | 2.1 |
| 70009 | 0.946131 | 2.1 |
| 70010 | 0.946132 | 2.1 |
| 70011 | 0.946132 | 2.1 |
| 70012 | 0.946131 | 2.1 |
| 70013 | 0.946131 | 2.1 |
| 70014 | 0.946132 | 2.1 |
| 70015 | 0.946132 | 2.1 |
| 70016 | 0.946131 | 2.1 |
| 70017 | 0.946131 | 2.1 |
| 70018 | 1.89226 | 2.1 |
| 70019 | 1.89226 | 2.1 |
| 70020 | 1.89226 | 2.1 |
| 70021 | 1.89226 | 2.1 |
| 70022 | 1.89226 | 2.1 |
| 70023 | 1.89226 | 2.1 |
| 70024 | 1.89226 | 2.1 |
| 70025 | 1.89226 | 2.1 |
| 70026 | 1.89226 | 2.1 |
| 70027 | 1.89226 | 2.1 |
| 70028 | 3.78452 | 2.1 |
| 70029 | 1.89226 | 2.1 |
| 70030 | 1.89226 | 2.1 |
| 70031 | 1.89226 | 2.1 |
| 70032 | 1.89226 | 2.1 |
| 70033 | 3.78452 | 2.1 |
| 70034 | 0.946131 | 2.1 |
| 70035 | 0.946131 | 2.1 |
| 70036 | 0.946131 | 2.1 |
| 70037 | 0.946131 | 2.1 |
| 70038 | 0.946131 | 2.1 |
| 70039 | 0.946131 | 2.1 |
| 70040 | 0.946131 | 2.1 |
| 70041 | 0.946131 | 2.1 |
| 70042 | 0.946131 | 2.1 |
| 70043 | 0.946131 | 2.1 |
| 70044 | 0.946131 | 2.1 |
| 70045 | 0.946131 | 2.1 |
| 70046 | 1.89226 | 2.1 |
| 70047 | 1.89226 | 2.1 |
| 70048 | 1.89226 | 2.1 |
| 70049 | 1.89226 | 2.1 |
| 70050 | 3.78453 | 2.1 |
| 70051 | 1.89226 | 2.1 |
| 70052 | 1.89226 | 2.1 |
| 70053 | 1.89226 | 2.1 |
| 70054 | 1.89226 | 2.1 |
| 70055 | 1.89226 | 2.1 |
| 70056 | 1.89226 | 2.1 |
| 70057 | 1.89226 | 2.1 |
| 70058 | 1.89226 | 2.1 |
| 70059 | 1.89226 | 2.1 |
| 70060 | 1.89226 | 2.1 |
| 70061 | 3.78453 | 2.1 |

| | | |
|---|---|---|
| 70062 | 1.89226 | 2.1 |
| 70063 | 1.89226 | 2.1 |
| 70064 | 1.89226 | 2.1 |
| 70065 | 1.89226 | 2.1 |
| 70066 | 3.78453 | 2.1 |
| 70067 | 1.89226 | 2.1 |
| 70068 | 1.89226 | 2.1 |
| 70069 | 1.89226 | 2.1 |
| 70070 | 1.89226 | 2.1 |
| 70071 | 3.78453 | 2.1 |
| 70072 | 0.946132 | 2.1 |
| 70073 | 0.946132 | 2.1 |
| 70074 | 0.946132 | 2.1 |
| 70075 | 0.946132 | 2.1 |
| 70076 | 0.946132 | 2.1 |
| 70077 | 0.946132 | 2.1 |
| 70078 | 0.946132 | 2.1 |
| 70079 | 0.946132 | 2.1 |
| 70080 | 0.946132 | 2.1 |
| 70081 | 0.946132 | 2.1 |
| 70082 | 0.946131 | 2.1 |
| 70083 | 0.946131 | 2.1 |
| 70084 | 0.946131 | 2.1 |
| 70085 | 0.946131 | 2.1 |
| 70086 | 0.946131 | 2.1 |
| 70087 | 0.946131 | 2.1 |
| 70088 | 0.946132 | 2.1 |
| 70089 | 0.946132 | 2.1 |
| 70090 | 0.946131 | 2.1 |
| 70091 | 0.946131 | 2.1 |
| 70092 | 0.946132 | 2.1 |
| 70093 | 0.946132 | 2.1 |
| 70094 | 0.946131 | 2.1 |
| 70095 | 0.946131 | 2.1 |
| 70096 | 1.89226 | 2.1 |
| 70097 | 1.89226 | 2.1 |
| 70098 | 1.89226 | 2.1 |
| 70099 | 1.89226 | 2.1 |
| 70100 | 3.78453 | 2.1 |
| 70101 | 1.89226 | 2.1 |
| 70102 | 1.89226 | 2.1 |
| 70103 | 1.89226 | 2.1 |
| 70104 | 1.89226 | 2.1 |
| 70105 | 3.78453 | 2.1 |
| 70106 | 0.946131 | 2.1 |
| 70107 | 0.946131 | 2.1 |
| 70108 | 0.946131 | 2.1 |
| 70109 | 0.946131 | 2.1 |
| 70110 | 0.946131 | 2.1 |
| 70111 | 0.946131 | 2.1 |
| 70112 | 0.946131 | 2.1 |
| 70113 | 0.946131 | 2.1 |
| 70114 | 0.946131 | 2.1 |
| 70115 | 0.946131 | 2.1 |
| 70116 | 0.946131 | 2.1 |
| 70117 | 0.946131 | 2.1 |
| 70118 | 1.89226 | 2.1 |
| 70119 | 1.89226 | 2.1 |

| | | |
|---|---|---|
| 70120 | 1.89226 | 2.1 |
| 70121 | 1.89226 | 2.1 |
| 70122 | 3.78452 | 2.1 |
| 70123 | 0.946132 | 2.1 |
| 70124 | 0.946132 | 2.1 |
| 70125 | 0.946131 | 2.1 |
| 70126 | 0.946131 | 2.1 |
| 70127 | 0.946132 | 2.1 |
| 70128 | 0.946132 | 2.1 |
| 70129 | 0.946131 | 2.1 |
| 70130 | 0.946131 | 2.1 |
| 70131 | 0.946131 | 2.1 |
| 70132 | 0.946131 | 2.1 |
| 70133 | 0.946131 | 2.1 |
| 70134 | 0.946131 | 2.1 |
| 70135 | 1.89226 | 2.1 |
| 70136 | 1.89226 | 2.1 |
| 70137 | 1.89226 | 2.1 |
| 70138 | 1.89226 | 2.1 |
| 70139 | 1.89226 | 2.1 |
| 70140 | 1.89226 | 2.1 |
| 70141 | 0.946132 | 2.1 |
| 70142 | 0.946132 | 2.1 |
| 70143 | 0.946132 | 2.1 |
| 70144 | 0.946132 | 2.1 |
| 70145 | 0.946132 | 2.1 |
| 70146 | 0.946132 | 2.1 |
| 70147 | 0.946132 | 2.1 |
| 70148 | 0.946132 | 2.1 |
| 70149 | 0.946132 | 2.1 |
| 70150 | 0.946132 | 2.1 |
| 70151 | 0.946131 | 2.1 |
| 70152 | 0.946131 | 2.1 |
| 70153 | 0.946131 | 2.1 |
| 70154 | 0.946131 | 2.1 |
| 70155 | 0.946131 | 2.1 |
| 70156 | 0.946131 | 2.1 |
| 70157 | 0.946132 | 2.1 |
| 70158 | 0.946132 | 2.1 |
| 70159 | 0.946131 | 2.1 |
| 70160 | 0.946131 | 2.1 |
| 70161 | 0.946132 | 2.1 |
| 70162 | 0.946132 | 2.1 |
| 70163 | 0.946131 | 2.1 |
| 70164 | 0.946131 | 2.1 |
| 70165 | 0.946132 | 2.1 |
| 70166 | 0.946132 | 2.1 |
| 70167 | 0.946131 | 2.1 |
| 70168 | 0.946131 | 2.1 |
| 70169 | 0.946132 | 2.1 |
| 70170 | 0.946132 | 2.1 |
| 70171 | 0.946131 | 2.1 |
| 70172 | 0.946131 | 2.1 |
| 70173 | 0.946132 | 2.1 |
| 70174 | 0.946132 | 2.1 |
| 70175 | 0.946131 | 2.1 |
| 70176 | 0.946131 | 2.1 |
| 70177 | 1.89226 | 2.1 |

| | | |
|---|---|---|
| 70178 | 1.89226 | 2.1 |
| 70179 | 1.89226 | 2.1 |
| 70180 | 1.89226 | 2.1 |
| 70181 | 3.78453 | 2.1 |
| 70182 | 1.89226 | 2.1 |
| 70183 | 1.89226 | 2.1 |
| 70184 | 1.89226 | 2.1 |
| 70185 | 1.89226 | 2.1 |
| 70186 | 3.78453 | 2.1 |
| 70187 | 1.89226 | 2.1 |
| 70188 | 1.89226 | 2.1 |
| 70189 | 1.89226 | 2.1 |
| 70190 | 1.89226 | 2.1 |
| 70191 | 1.89226 | 2.1 |
| 70192 | 1.89226 | 2.1 |
| 70193 | 1.89226 | 2.1 |
| 70194 | 1.89226 | 2.1 |
| 70195 | 1.89226 | 2.1 |
| 70196 | 1.89226 | 2.1 |
| 70197 | 3.78453 | 2.1 |
| 70198 | 1.89226 | 2.1 |
| 70199 | 1.89226 | 2.1 |
| 70200 | 1.89226 | 2.1 |
| 70201 | 1.89226 | 2.1 |
| 70202 | 3.78453 | 2.1 |
| 70203 | 1.89226 | 2.1 |
| 70204 | 1.89226 | 2.1 |
| 70205 | 1.89226 | 2.1 |
| 70206 | 1.89226 | 2.1 |
| 70207 | 1.89226 | 2.1 |
| 70208 | 1.89226 | 2.1 |
| 70209 | 1.89226 | 2.1 |
| 70210 | 1.89226 | 2.1 |
| 70211 | 1.89226 | 2.1 |
| 70212 | 1.89226 | 2.1 |
| 70213 | 1.89226 | 2.1 |
| 70214 | 1.89226 | 2.1 |
| 70215 | 1.89226 | 2.1 |
| 70216 | 1.89226 | 2.1 |
| 70217 | 1.89226 | 2.1 |
| 70218 | 1.89226 | 2.1 |
| 70219 | 3.78453 | 2.1 |
| 70220 | 1.89226 | 2.1 |
| 70221 | 1.89226 | 2.1 |
| 70222 | 1.89226 | 2.1 |
| 70223 | 1.89226 | 2.1 |
| 70224 | 1.89226 | 2.1 |
| 70225 | 1.89226 | 2.1 |
| 70226 | 1.89226 | 2.1 |
| 70227 | 1.89226 | 2.1 |
| 70228 | 1.89226 | 2.1 |
| 70229 | 1.89226 | 2.1 |
| 70230 | 1.89226 | 2.1 |
| 70231 | 1.89226 | 2.1 |
| 70232 | 1.89226 | 2.1 |
| 70233 | 1.89226 | 1.20979 |
| 70234 | 1.89226 | 2.1 |
| 70235 | 1.89226 | 0.945242 |

| | | |
|---|---|---|
| 70236 | 3.78453 | 1.93959 |
| 70237 | 1.89226 | 2.1 |
| 70238 | 1.89226 | 0.853962 |
| 70239 | 1.89226 | 0.138136 |
| 70240 | 1.89226 | 2.1 |
| 70241 | 1.89226 | 0.129755 |
| 70242 | 1.89226 | 2.1 |
| 70243 | 1.89226 | 2.1 |
| 70244 | 1.89226 | 2.1 |
| 70245 | 1.89226 | 2.1 |
| 70246 | 1.89226 | 2.1 |
| 70247 | 1.89226 | 2.1 |
| 70248 | 1.89226 | 2.1 |
| 70249 | 1.89226 | 2.1 |
| 70250 | 1.89226 | 2.1 |
| 70251 | 1.89226 | 2.1 |
| 70252 | 1.89226 | 2.1 |
| 70253 | 3.78453 | 2.1 |
| 70254 | 1.89226 | 2.1 |
| 70255 | 1.89226 | 1.51417 |
| 70256 | 1.89226 | 2.1 |
| 70257 | 1.89226 | 1.202 |
| 70258 | 1.89226 | 2.1 |
| 70259 | 1.89226 | 1.43658 |
| 70260 | 1.89226 | 2.1 |
| 70261 | 1.89226 | 1.23609 |
| 70262 | 1.89226 | 2.1 |
| 70263 | 1.89226 | 2.1 |
| 70264 | 1.89226 | 1.97049 |
| 70265 | 1.89226 | 1.24415 |
| 70266 | 1.89226 | 2.1 |
| 70267 | 1.89226 | 2.1 |
| 70268 | 1.89226 | 2.1 |
| 70269 | 1.89226 | 2.1 |
| 70270 | 1.89226 | 2.1 |
| 70271 | 1.89226 | 2.1 |
| 70272 | 1.89226 | 2.1 |
| 70273 | 1.89226 | 2.06824 |
| 70274 | 1.89226 | 2.1 |
| 70275 | 1.89226 | 1.92704 |
| 70276 | 3.78452 | 2.1 |
| 70277 | 1.89226 | 2.1 |
| 70278 | 1.89226 | 1.10356 |
| 70279 | 1.89226 | 2.1 |
| 70280 | 1.89226 | 0.423848 |
| 70281 | 3.78452 | 2.1 |
| 70282 | 0.946131 | 2.1 |
| 70283 | 0.946131 | 0.215341 |
| 70284 | 0.946131 | 1.61432 |
| 70285 | 0.946131 | 2.1 |
| 70286 | 0.94613 | 0.892678 |
| 70287 | 0.94613 | 1.99746 |
| 70288 | 0.946132 | 2.1 |
| 70289 | 0.946132 | 2.1 |
| 70290 | 0.946131 | 1.88753 |
| 70291 | 0.946131 | 1.04476 |
| 70292 | 0.946131 | 2.01907 |
| 70293 | 0.946131 | 1.22493 |

| | | |
|---|---|---|
| 70294 | 1.89226 | 2.1 |
| 70295 | 1.89226 | 2.1 |
| 70296 | 1.89226 | 2.1 |
| 70297 | 1.89226 | 2.1 |
| 70298 | 1.89226 | 2.1 |
| 70299 | 1.89226 | 2.1 |
| 70300 | 1.89226 | 2.1 |
| 70301 | 1.89226 | 2.1 |
| 70302 | 1.89226 | 2.1 |
| 70303 | 1.89226 | 2.07511 |
| 70304 | 3.78452 | 2.1 |
| 70305 | 1.89226 | 2.1 |
| 70306 | 1.89226 | 0.934775 |
| 70307 | 1.89226 | 2.1 |
| 70308 | 1.89226 | 0.05 |
| 70309 | 3.78452 | 0.913822 |
| 70310 | 0.946131 | 1.06465 |
| 70311 | 0.946131 | 2.1 |
| 70312 | 0.946131 | 2.1 |
| 70313 | 0.946131 | 1.19368 |
| 70314 | 0.94613 | 0.929096 |
| 70315 | 0.94613 | 0.221741 |
| 70316 | 0.946132 | 2.1 |
| 70317 | 0.946132 | 2.1 |
| 70318 | 0.946131 | 2.1 |
| 70319 | 0.946131 | 1.96728 |
| 70320 | 0.946131 | 1.94179 |
| 70321 | 0.946131 | 1.11925 |
| 70322 | 1.89226 | 2.1 |
| 70323 | 1.89226 | 2.1 |
| 70324 | 1.89226 | 2.1 |
| 70325 | 1.89226 | 2.1 |
| 70326 | 3.78452 | 2.1 |
| 70327 | 1.89226 | 2.1 |
| 70328 | 1.89226 | 2.1 |
| 70329 | 1.89226 | 2.1 |
| 70330 | 1.89226 | 2.1 |
| 70331 | 3.78453 | 2.1 |
| 70332 | 1.89226 | 2.1 |
| 70333 | 1.89226 | 2.1 |
| 70334 | 1.89226 | 2.1 |
| 70335 | 1.89226 | 0.803711 |
| 70336 | 3.78452 | 1.72901 |
| 70337 | 1.89226 | 2.1 |
| 70338 | 1.89226 | 2.1 |
| 70339 | 1.89226 | 2.1 |
| 70340 | 1.89226 | 1.23631 |
| 70341 | 1.89226 | 2.1 |
| 70342 | 1.89226 | 2.1 |
| 70343 | 1.89226 | 2.1 |
| 70344 | 1.89226 | 2.1 |
| 70345 | 1.89226 | 2.1 |
| 70346 | 1.89226 | 0.873384 |
| 70347 | 3.78452 | 2.1 |
| 70348 | 1.89226 | 2.1 |
| 70349 | 1.89226 | 2.1 |
| 70350 | 1.89226 | 2.1 |
| 70351 | 1.89226 | 2.1 |

| | | |
|---|---|---|
| 70352 | 3.78453 | 2.1 |
| 70353 | 1.89226 | 2.1 |
| 70354 | 1.89226 | 0.963371 |
| 70355 | 1.89226 | 2.1 |
| 70356 | 1.89226 | 0.254039 |
| 70357 | 3.78453 | 2.09544 |
| 70358 | 1.89226 | 2.1 |
| 70359 | 1.89226 | 0.837116 |
| 70360 | 1.89226 | 2.1 |
| 70361 | 1.89226 | 1.07604 |
| 70362 | 1.89226 | 2.1 |
| 70363 | 1.89226 | 0.096823 |
| 70364 | 1.89226 | 2.1 |
| 70365 | 1.89226 | 2.1 |
| 70366 | 1.89226 | 2.1 |
| 70367 | 1.89226 | 0.834862 |
| 70368 | 3.78452 | 2.1 |
| 70369 | 1.89226 | 2.1 |
| 70370 | 1.89226 | 2.1 |
| 70371 | 1.89226 | 2.1 |
| 70372 | 1.89226 | 0.863317 |
| 70373 | 3.78453 | 2.1 |
| 70374 | 1.89226 | 2.1 |
| 70375 | 1.89226 | 2.1 |
| 70376 | 1.89226 | 2.1 |
| 70377 | 1.89226 | 0.760712 |
| 70378 | 3.78453 | 1.26671 |
| 70379 | 1.89226 | 2.1 |
| 70380 | 1.89226 | 1.1848 |
| 70381 | 1.89226 | 1.86871 |
| 70382 | 1.89226 | 1.10898 |
| 70383 | 1.89226 | 2.1 |
| 70384 | 1.89226 | 2.1 |
| 70385 | 0.946128 | 1.06735 |
| 70386 | 0.946128 | 2.1 |
| 70387 | 0.946132 | 1.74229 |
| 70388 | 0.946132 | 2.1 |
| 70389 | 0.946129 | 2.1 |
| 70390 | 0.946129 | 2.1 |
| 70391 | 0.94613 | 2.1 |
| 70392 | 0.94613 | 2.1 |
| 70393 | 0.94613 | 2.1 |
| 70394 | 0.94613 | 2.1 |
| 70395 | 0.94613 | 2.1 |
| 70396 | 0.94613 | 2.1 |
| 70397 | 1.89226 | 2.1 |
| 70398 | 1.89226 | 2.1 |
| 70399 | 1.89226 | 2.1 |
| 70400 | 1.89226 | 2.1 |
| 70401 | 1.89226 | 2.1 |
| 70402 | 1.89226 | 0.857272 |
| 70403 | 1.89226 | 2.1 |
| 70404 | 1.89226 | 1.11117 |
| 70405 | 1.89226 | 1.27541 |
| 70406 | 1.89226 | 2.1 |
| 70407 | 1.89226 | 0.0759474 |
| 70408 | 1.89226 | 2.1 |
| 70409 | 0.946127 | 2.1 |

| | | |
|---|---|---|
| 70410 | 0.946127 | 1.28798 |
| 70411 | 0.946131 | 0.94047 |
| 70412 | 0.946131 | 2.1 |
| 70413 | 0.946128 | 2.04657 |
| 70414 | 0.946128 | 0.880274 |
| 70415 | 0.94613 | 2.1 |
| 70416 | 0.94613 | 2.1 |
| 70417 | 0.946129 | 2.01761 |
| 70418 | 0.946129 | 1.99419 |
| 70419 | 0.946129 | 2.1 |
| 70420 | 0.946129 | 2.1 |
| 70421 | 1.89226 | 2.1 |
| 70422 | 1.89226 | 2.1 |
| 70423 | 1.89226 | 2.1 |
| 70424 | 1.89226 | 0.933581 |
| 70425 | 3.78452 | 2.1 |
| 70426 | 1.89226 | 2.1 |
| 70427 | 1.89226 | 1.04491 |
| 70428 | 1.89226 | 2.1 |
| 70429 | 1.89226 | 2.1 |
| 70430 | 1.89226 | 2.1 |
| 70431 | 1.89226 | 2.1 |
| 70432 | 1.89226 | 2.1 |
| 70433 | 1.89226 | 0.916042 |
| 70434 | 1.89226 | 1.58835 |
| 70435 | 1.89226 | 2.1 |
| 70436 | 1.89226 | 0.169871 |
| 70437 | 1.89226 | 2.1 |
| 70438 | 1.89226 | 0.853779 |
| 70439 | 1.89226 | 2.1 |
| 70440 | 1.89226 | 2.1 |
| 70441 | 1.89226 | 1.06383 |
| 70442 | 1.89226 | 2.1 |
| 70443 | 1.89226 | 2.04591 |
| 70444 | 0.946131 | 1.59679 |
| 70445 | 0.946131 | 2.1 |
| 70446 | 0.946131 | 2.1 |
| 70447 | 0.946131 | 2.1 |
| 70448 | 0.946131 | 2.1 |
| 70449 | 0.946131 | 2.1 |
| 70450 | 0.946132 | 2.1 |
| 70451 | 0.946132 | 2.1 |
| 70452 | 0.946132 | 2.1 |
| 70453 | 0.946132 | 2.1 |
| 70454 | 0.946131 | 2.1 |
| 70455 | 0.946131 | 2.1 |
| 70456 | 1.89226 | 2.1 |
| 70457 | 1.89226 | 2.01255 |
| 70458 | 1.89226 | 2.1 |
| 70459 | 1.89226 | 2.1 |
| 70460 | 1.89226 | 2.1 |
| 70461 | 1.89226 | 2.1 |
| 70462 | 1.89226 | 2.1 |
| 70463 | 1.89226 | 0.725823 |
| 70464 | 1.89226 | 1.01836 |
| 70465 | 1.89226 | 2.1 |
| 70466 | 1.89226 | 0.05 |
| 70467 | 1.89226 | 2.1 |

| | | |
|---|---|---|
| 70468 | 0.946131 | 2.1 |
| 70469 | 0.946131 | 0.909531 |
| 70470 | 0.946131 | 0.95917 |
| 70471 | 0.946131 | 2.1 |
| 70472 | 0.94613 | 0.640966 |
| 70473 | 0.94613 | 0.638224 |
| 70474 | 0.946132 | 2.1 |
| 70475 | 0.946132 | 2.1 |
| 70476 | 0.946131 | 2.1 |
| 70477 | 0.946131 | 2.1 |
| 70478 | 0.946131 | 2.1 |
| 70479 | 0.946131 | 1.11945 |
| 70480 | 1.89226 | 2.1 |
| 70481 | 1.89226 | 2.1 |
| 70482 | 1.89226 | 2.1 |
| 70483 | 1.89226 | 0.914994 |
| 70484 | 3.78453 | 2.1 |
| 70485 | 1.89226 | 2.1 |
| 70486 | 1.89226 | 1.1194 |
| 70487 | 1.89226 | 2.1 |
| 70488 | 1.89226 | 2.1 |
| 70489 | 1.89226 | 2.1 |
| 70490 | 1.89226 | 0.587547 |
| 70491 | 1.89226 | 2.1 |
| 70492 | 1.89226 | 1.51879 |
| 70493 | 1.89226 | 2.1 |
| 70494 | 1.89226 | 0.320575 |
| 70495 | 3.78452 | 1.04903 |
| 70496 | 1.89226 | 2.1 |
| 70497 | 1.89226 | 1.36525 |
| 70498 | 1.89226 | 2.1 |
| 70499 | 1.89226 | 0.863068 |
| 70500 | 3.78452 | 2.1 |
| 70501 | 0.946131 | 0.250723 |
| 70502 | 0.946131 | 2.1 |
| 70503 | 0.946131 | 2.1 |
| 70504 | 0.946131 | 1.35862 |
| 70505 | 0.946131 | 1.48217 |
| 70506 | 0.946131 | 1.79993 |
| 70507 | 0.946131 | 2.1 |
| 70508 | 0.946131 | 2.1 |
| 70509 | 0.946131 | 2.1 |
| 70510 | 0.946131 | 2.1 |
| 70511 | 0.946131 | 2.1 |
| 70512 | 0.946131 | 2.1 |
| 70513 | 0.946128 | 0.389193 |
| 70514 | 0.946128 | 2.1 |
| 70515 | 0.946132 | 1.67125 |
| 70516 | 0.946132 | 2.1 |
| 70517 | 0.946129 | 1.72827 |
| 70518 | 0.946129 | 1.16167 |
| 70519 | 0.94613 | 2.1 |
| 70520 | 0.94613 | 2.1 |
| 70521 | 0.94613 | 2.1 |
| 70522 | 0.94613 | 2.1 |
| 70523 | 0.94613 | 2.1 |
| 70524 | 0.94613 | 2.1 |
| 70525 | 1.89226 | 2.1 |

| | | |
|---|---|---|
| 70526 | 1.89226 | 0.820697 |
| 70527 | 1.89226 | 0.868134 |
| 70528 | 1.89226 | 2.1 |
| 70529 | 1.89226 | 0.05 |
| 70530 | 1.89226 | 2.1 |
| 70531 | 0.810945 | 2.1 |
| 70532 | 0.810945 | 0.966789 |
| 70533 | 0.810944 | 1.0165 |
| 70534 | 0.810944 | 1.03939 |
| 70535 | 1.89226 | 2.1 |
| 70536 | 1.35169 | 2.1 |
| 70537 | 1.08132 | 1.17498 |
| 70538 | 1.08132 | 1.81647 |
| 70539 | 1.08132 | 2.1 |
| 70540 | 0.810945 | 1.16942 |
| 70541 | 0.810945 | 2.1 |
| 70542 | 0.946131 | 2.1 |
| 70543 | 0.946131 | 2.1 |
| 70544 | 0.946131 | 2.1 |
| 70545 | 0.946131 | 2.1 |
| 70546 | 0.946131 | 2.1 |
| 70547 | 0.946131 | 2.1 |
| 70548 | 0.946131 | 2.1 |
| 70549 | 0.946131 | 2.1 |
| 70550 | 0.946131 | 2.1 |
| 70551 | 0.946131 | 2.1 |
| 70552 | 0.946131 | 2.1 |
| 70553 | 0.946131 | 2.1 |
| 70554 | 1.89226 | 2.1 |
| 70555 | 1.89226 | 2.1 |
| 70556 | 1.89226 | 2.1 |
| 70557 | 1.89226 | 2.1 |
| 70558 | 1.89226 | 2.1 |
| 70559 | 1.89226 | 2.1 |
| 70560 | 1.89226 | 2.1 |
| 70561 | 1.89226 | 2.1 |
| 70562 | 1.89226 | 2.1 |
| 70563 | 1.89226 | 2.1 |
| 70564 | 1.89226 | 2.1 |
| 70565 | 1.89226 | 2.1 |
| 70566 | 0.946131 | 2.1 |
| 70567 | 0.946131 | 2.1 |
| 70568 | 0.946131 | 2.1 |
| 70569 | 0.946131 | 2.1 |
| 70570 | 0.946131 | 2.1 |
| 70571 | 0.946131 | 2.1 |
| 70572 | 0.946131 | 2.1 |
| 70573 | 0.946131 | 2.1 |
| 70574 | 0.946131 | 2.1 |
| 70575 | 0.946131 | 2.1 |
| 70576 | 0.946131 | 2.1 |
| 70577 | 0.946131 | 2.1 |
| 70578 | 1.89226 | 2.1 |
| 70579 | 1.89226 | 2.1 |
| 70580 | 1.89226 | 2.1 |
| 70581 | 1.89226 | 2.1 |
| 70582 | 1.89226 | 2.1 |
| 70583 | 1.89226 | 2.1 |

| | | |
|---|---|---|
| 70584 | 1.89226 | 2.1 |
| 70585 | 1.89226 | 2.1 |
| 70586 | 1.89226 | 2.1 |
| 70587 | 1.89226 | 2.1 |
| 70588 | 1.89226 | 2.1 |
| 70589 | 1.89226 | 2.1 |
| 70590 | 1.89226 | 2.1 |
| 70591 | 1.89226 | 2.1 |
| 70592 | 1.89226 | 2.1 |
| 70593 | 1.89226 | 2.1 |
| 70594 | 1.89226 | 2.1 |
| 70595 | 1.89226 | 2.1 |
| 70596 | 0.946132 | 2.1 |
| 70597 | 0.946132 | 2.1 |
| 70598 | 0.946131 | 2.1 |
| 70599 | 0.946131 | 2.1 |
| 70600 | 0.946132 | 2.1 |
| 70601 | 0.946132 | 2.1 |
| 70602 | 0.946132 | 2.1 |
| 70603 | 0.946132 | 2.1 |
| 70604 | 0.946132 | 2.1 |
| 70605 | 0.946132 | 2.1 |
| 70606 | 0.946132 | 2.1 |
| 70607 | 0.946132 | 2.1 |
| 70608 | 0.946131 | 2.1 |
| 70609 | 0.946131 | 2.1 |
| 70610 | 0.946131 | 2.1 |
| 70611 | 0.946131 | 2.1 |
| 70612 | 0.946131 | 2.1 |
| 70613 | 0.946131 | 2.1 |
| 70614 | 0.946131 | 2.1 |
| 70615 | 0.946131 | 2.1 |
| 70616 | 0.946131 | 2.1 |
| 70617 | 0.946131 | 2.1 |
| 70618 | 0.946131 | 2.1 |
| 70619 | 0.946131 | 2.1 |
| 70620 | 0.946132 | 2.1 |
| 70621 | 0.946132 | 2.1 |
| 70622 | 0.946131 | 2.1 |
| 70623 | 0.946131 | 2.1 |
| 70624 | 0.946132 | 2.1 |
| 70625 | 0.946132 | 2.1 |
| 70626 | 0.946131 | 2.1 |
| 70627 | 0.946131 | 2.1 |
| 70628 | 0.946131 | 2.1 |
| 70629 | 0.946131 | 2.1 |
| 70630 | 0.946131 | 2.1 |
| 70631 | 0.946131 | 2.1 |
| 70632 | 1.89226 | 2.1 |
| 70633 | 1.89226 | 2.1 |
| 70634 | 1.89226 | 2.1 |
| 70635 | 1.89226 | 2.1 |
| 70636 | 1.89226 | 2.1 |
| 70637 | 1.89226 | 2.1 |
| 70638 | 1.37713 | 2.1 |
| 70639 | 1.27093 | 2.1 |
| 70640 | 0.946132 | 2.1 |
| 70641 | 0.946132 | 2.1 |

| | | |
|---|---|---|
| 70642 | 0.946132 | 2.1 |
| 70643 | 0.946132 | 2.1 |
| 70644 | 1.37713 | 2.1 |
| 70645 | 1.37713 | 2.1 |
| 70646 | 1.27093 | 2.1 |
| 70647 | 0.946131 | 2.1 |
| 70648 | 0.946132 | 2.1 |
| 70649 | 1.37713 | 2.1 |
| 70650 | 0.946131 | 2.1 |
| 70651 | 0.946131 | 2.1 |
| 70652 | 0.946131 | 2.1 |
| 70653 | 0.946131 | 2.1 |
| 70654 | 0.946132 | 2.1 |
| 70655 | 0.946132 | 2.1 |
| 70656 | 0.946131 | 2.1 |
| 70657 | 0.946131 | 2.1 |
| 70658 | 0.946131 | 2.1 |
| 70659 | 0.946131 | 2.1 |
| 70660 | 0.946132 | 2.1 |
| 70661 | 0.946132 | 2.1 |
| 70662 | 0.946132 | 2.1 |
| 70663 | 0.946132 | 2.1 |
| 70664 | 0.946131 | 2.1 |
| 70665 | 0.946131 | 2.1 |
| 70666 | 0.946132 | 2.1 |
| 70667 | 0.946132 | 2.1 |
| 70668 | 0.946131 | 2.1 |
| 70669 | 0.946131 | 2.1 |
| 70670 | 0.946131 | 2.1 |
| 70671 | 0.946131 | 2.1 |
| 70672 | 0.946132 | 2.1 |
| 70673 | 0.946132 | 2.1 |
| 70674 | 1.89226 | 2.1 |
| 70675 | 1.89226 | 2.1 |
| 70676 | 1.89226 | 2.1 |
| 70677 | 1.89226 | 2.1 |
| 70678 | 1.89226 | 2.1 |
| 70679 | 1.89226 | 2.1 |
| 70680 | 1.89226 | 2.1 |
| 70681 | 1.89226 | 2.1 |
| 70682 | 1.89226 | 2.1 |
| 70683 | 1.89226 | 2.1 |
| 70684 | 1.89226 | 2.1 |
| 70685 | 1.89226 | 2.1 |
| 70686 | 1.89226 | 2.1 |
| 70687 | 1.89226 | 2.1 |
| 70688 | 1.89226 | 2.1 |
| 70689 | 1.89226 | 0.932208 |
| 70690 | 3.78452 | 2.1 |
| 70691 | 1.89226 | 2.1 |
| 70692 | 1.89226 | 2.1 |
| 70693 | 1.89226 | 2.1 |
| 70694 | 1.89226 | 1.33747 |
| 70695 | 1.89226 | 2.1 |
| 70696 | 1.89226 | 2.1 |
| 70697 | 1.89226 | 2.1 |
| 70698 | 1.89226 | 2.1 |
| 70699 | 1.89226 | 2.1 |

| | | |
|---|---|---|
| 70700 | 1.89226 | 2.1 |
| 70701 | 3.78452 | 2.1 |
| 70702 | 1.89226 | 2.1 |
| 70703 | 1.89226 | 2.1 |
| 70704 | 1.89226 | 2.1 |
| 70705 | 1.89226 | 2.1 |
| 70706 | 3.78453 | 2.1 |
| 70707 | 1.89226 | 2.1 |
| 70708 | 1.89226 | 1.02199 |
| 70709 | 1.89226 | 1.4247 |
| 70710 | 1.89226 | 2.1 |
| 70711 | 1.89226 | 0.39024 |
| 70712 | 1.89226 | 2.1 |
| 70713 | 1.89226 | 0.521731 |
| 70714 | 1.89226 | 2.1 |
| 70715 | 1.89226 | 2.1 |
| 70716 | 1.89226 | 1.23986 |
| 70717 | 1.89226 | 2.1 |
| 70718 | 1.89226 | 2.03085 |
| 70719 | 1.89226 | 2.1 |
| 70720 | 1.89226 | 2.1 |
| 70721 | 1.89226 | 2.1 |
| 70722 | 1.89226 | 2.1 |
| 70723 | 3.78452 | 2.1 |
| 70724 | 1.89226 | 2.1 |
| 70725 | 1.89226 | 1.97191 |
| 70726 | 1.89226 | 2.1 |
| 70727 | 1.89226 | 2.1 |
| 70728 | 1.89226 | 2.1 |
| 70729 | 1.89226 | 2.1 |
| 70730 | 1.89226 | 2.1 |
| 70731 | 1.89226 | 1.48413 |
| 70732 | 1.89226 | 1.61902 |
| 70733 | 1.89226 | 2.1 |
| 70734 | 1.89226 | 0.420208 |
| 70735 | 1.89226 | 2.1 |
| 70736 | 1.89226 | 1.12604 |
| 70737 | 1.89226 | 2.1 |
| 70738 | 1.89226 | 2.1 |
| 70739 | 1.89226 | 1.81226 |
| 70740 | 1.89226 | 2.1 |
| 70741 | 1.89226 | 2.1 |
| 70742 | 0.946131 | 1.96954 |
| 70743 | 0.946131 | 2.1 |
| 70744 | 0.946131 | 2.1 |
| 70745 | 0.946131 | 2.1 |
| 70746 | 0.946131 | 2.1 |
| 70747 | 0.946131 | 2.1 |
| 70748 | 0.946132 | 2.1 |
| 70749 | 0.946132 | 2.1 |
| 70750 | 0.946131 | 2.1 |
| 70751 | 0.946131 | 2.1 |
| 70752 | 0.946132 | 2.1 |
| 70753 | 0.946132 | 2.1 |
| 70754 | 1.89226 | 2.1 |
| 70755 | 1.89226 | 2.1 |
| 70756 | 1.89226 | 2.1 |
| 70757 | 1.89226 | 2.1 |

| | | |
|---|---|---|
| 70758 | 1.89226 | 2.1 |
| 70759 | 1.89226 | 2.1 |
| 70760 | 1.89226 | 2.1 |
| 70761 | 1.89226 | 0.856034 |
| 70762 | 1.89226 | 0.988726 |
| 70763 | 1.89226 | 2.1 |
| 70764 | 1.89226 | 0.05 |
| 70765 | 1.89226 | 2.1 |
| 70766 | 0.946131 | 2.1 |
| 70767 | 0.946131 | 1.02601 |
| 70768 | 0.946131 | 1.14967 |
| 70769 | 0.946131 | 2.1 |
| 70770 | 0.94613 | 1.02414 |
| 70771 | 0.94613 | 0.891216 |
| 70772 | 0.946132 | 2.1 |
| 70773 | 0.946132 | 2.1 |
| 70774 | 0.946131 | 1.09172 |
| 70775 | 0.946131 | 2.1 |
| 70776 | 0.946131 | 2.1 |
| 70777 | 0.946131 | 1.62799 |
| 70778 | 1.89226 | 2.1 |
| 70779 | 1.89226 | 0.422652 |
| 70780 | 1.89226 | 0.149888 |
| 70781 | 1.89226 | 2.1 |
| 70782 | 3.78453 | 1.57174 |
| 70783 | 2.81189 | 2.1 |
| 70784 | 1.89226 | 2.1 |
| 70785 | 1.89226 | 2.1 |
| 70786 | 1.89226 | 2.1 |
| 70787 | 2.56715 | 2.1 |
| 70788 | 2.56715 | 2.1 |
| 70789 | 1.89226 | 2.1 |
| 70790 | 1.89226 | 2.1 |
| 70791 | 1.89226 | 2.1 |
| 70792 | 1.89226 | 2.1 |
| 70793 | 3.78452 | 2.1 |
| 70794 | 1.89226 | 0.737564 |
| 70795 | 1.89226 | 2.1 |
| 70796 | 1.89226 | 2.1 |
| 70797 | 1.89226 | 2.1 |
| 70798 | 3.78452 | 2.1 |
| 70799 | 1.89226 | 0.243047 |
| 70800 | 1.89226 | 2.1 |
| 70801 | 1.89226 | 1.05439 |
| 70802 | 1.89226 | 2.1 |
| 70803 | 1.89226 | 0.563714 |
| 70804 | 1.89226 | 2.1 |
| 70805 | 1.1507 | 2.1 |
| 70806 | 1.1507 | 2.1 |
| 70807 | 0.946133 | 2.1 |
| 70808 | 0.946133 | 2.1 |
| 70809 | 0.946131 | 2.1 |
| 70810 | 0.946131 | 2.1 |
| 70811 | 1.28358 | 2.1 |
| 70812 | 1.1507 | 2.1 |
| 70813 | 1.1507 | 2.1 |
| 70814 | 0.946131 | 2.1 |
| 70815 | 0.94613 | 2.1 |

| | | |
|---|---|---|
| 70816 | 1.28358 | 2.1 |
| 70817 | 1.89226 | 2.1 |
| 70818 | 1.89226 | 2.1 |
| 70819 | 1.89226 | 2.1 |
| 70820 | 1.89226 | 2.1 |
| 70821 | 1.89226 | 2.1 |
| 70822 | 1.89226 | 2.1 |
| 70823 | 0.946131 | 2.1 |
| 70824 | 0.946131 | 2.1 |
| 70825 | 0.946131 | 0.79029 |
| 70826 | 0.946131 | 2.1 |
| 70827 | 0.946128 | 2.1 |
| 70828 | 0.946128 | 2.1 |
| 70829 | 0.946133 | 2.1 |
| 70830 | 0.946133 | 1.26226 |
| 70831 | 0.946131 | 2.1 |
| 70832 | 0.946131 | 2.1 |
| 70833 | 0.94613 | 2.1 |
| 70834 | 0.94613 | 2.1 |
| 70835 | 1.89226 | 0.0936163 |
| 70836 | 1.89226 | 2.1 |
| 70837 | 1.89226 | 1.94035 |
| 70838 | 1.89226 | 0.386481 |
| 70839 | 3.78453 | 0.878913 |
| 70840 | 1.89226 | 2.1 |
| 70841 | 1.89226 | 2.1 |
| 70842 | 1.89226 | 0.225096 |
| 70843 | 1.89226 | 2.1 |
| 70844 | 3.78452 | 2.1 |
| 70845 | 1.89226 | 2.1 |
| 70846 | 1.89226 | 2.1 |
| 70847 | 1.89226 | 2.1 |
| 70848 | 1.89226 | 2.1 |
| 70849 | 3.78452 | 2.1 |
| 70850 | 1.89226 | 2.1 |
| 70851 | 1.89226 | 1.47084 |
| 70852 | 1.89226 | 0.42952 |
| 70853 | 1.89226 | 2.1 |
| 70854 | 3.78452 | 2.1 |
| 70855 | 0.946132 | 2.1 |
| 70856 | 0.946132 | 1.54745 |
| 70857 | 0.946132 | 0.05 |
| 70858 | 0.946132 | 0.503149 |
| 70859 | 0.946132 | 0.375394 |
| 70860 | 0.946132 | 2.1 |
| 70861 | 0.946132 | 2.1 |
| 70862 | 0.946132 | 0.05 |
| 70863 | 0.946132 | 0.951145 |
| 70864 | 0.946132 | 0.928037 |
| 70865 | 0.946131 | 0.912379 |
| 70866 | 0.946131 | 1.02734 |
| 70867 | 1.89226 | 2.1 |
| 70868 | 1.89226 | 2.1 |
| 70869 | 1.89226 | 2.1 |
| 70870 | 1.89226 | 2.1 |
| 70871 | 1.89226 | 2.1 |
| 70872 | 1.89226 | 2.1 |
| 70873 | 1.89226 | 0.531544 |

| | | |
|---|---|---|
| 70874 | 1.89226 | 2.1 |
| 70875 | 1.89226 | 2.1 |
| 70876 | 1.89226 | 2.1 |
| 70877 | 1.89226 | 2.1 |
| 70878 | 1.89226 | 2.1 |
| 70879 | 1.89226 | 2.1 |
| 70880 | 1.89226 | 2.1 |
| 70881 | 1.89226 | 2.1 |
| 70882 | 1.89226 | 2.1 |
| 70883 | 3.78453 | 2.1 |
| 70884 | 1.89226 | 2.1 |
| 70885 | 1.89226 | 2.1 |
| 70886 | 1.89226 | 2.1 |
| 70887 | 1.89226 | 2.1 |
| 70888 | 3.78452 | 2.1 |
| 70889 | 1.89226 | 2.1 |
| 70890 | 1.89226 | 2.1 |
| 70891 | 1.89226 | 2.1 |
| 70892 | 1.89226 | 2.1 |
| 70893 | 1.89226 | 2.1 |
| 70894 | 1.89226 | 2.1 |
| 70895 | 1.89226 | 2.1 |
| 70896 | 1.89226 | 2.1 |
| 70897 | 1.89226 | 2.1 |
| 70898 | 1.89226 | 2.1 |
| 70899 | 3.78453 | 2.1 |
| 70900 | 1.89226 | 2.1 |
| 70901 | 1.89226 | 2.1 |
| 70902 | 1.89226 | 2.1 |
| 70903 | 1.89226 | 2.1 |
| 70904 | 1.89226 | 2.1 |
| 70905 | 1.89226 | 2.1 |
| 70906 | 1.89226 | 2.1 |
| 70907 | 1.89226 | 2.1 |
| 70908 | 1.89226 | 2.1 |
| 70909 | 1.89226 | 2.1 |
| 70910 | 3.78452 | 2.1 |
| 70911 | 1.89226 | 2.1 |
| 70912 | 1.89226 | 2.1 |
| 70913 | 1.89226 | 2.1 |
| 70914 | 1.89226 | 2.1 |
| 70915 | 3.78452 | 2.1 |
| 70916 | 1.89226 | 2.1 |
| 70917 | 1.89226 | 2.1 |
| 70918 | 1.89226 | 2.1 |
| 70919 | 1.89226 | 2.1 |
| 70920 | 3.78453 | 2.1 |
| 70921 | 1.89224 | 2.1 |
| 70922 | 1.89224 | 2.1 |
| 70923 | 1.89224 | 2.1 |
| 70924 | 1.89224 | 2.1 |
| 70925 | 3.78448 | 2.1 |
| 70926 | 1.89224 | 2.1 |
| 70927 | 1.89224 | 2.1 |
| 70928 | 1.89224 | 2.1 |
| 70929 | 1.89224 | 2.1 |
| 70930 | 1.89224 | 2.1 |
| 70931 | 1.89224 | 2.1 |

| | | |
|---|---|---|
| 70932 | 1.89224 | 2.1 |
| 70933 | 1.89224 | 2.1 |
| 70934 | 1.89224 | 2.1 |
| 70935 | 1.89224 | 2.1 |
| 70936 | 3.78447 | 2.1 |
| 70937 | 0.946119 | 2.1 |
| 70938 | 0.946119 | 2.1 |
| 70939 | 0.946119 | 2.1 |
| 70940 | 0.946119 | 2.1 |
| 70941 | 0.946119 | 2.1 |
| 70942 | 0.946119 | 2.1 |
| 70943 | 0.946119 | 2.1 |
| 70944 | 0.946119 | 2.1 |
| 70945 | 0.946106 | 2.1 |
| 70946 | 0.946106 | 2.1 |
| 70947 | 0.946131 | 2.1 |
| 70948 | 0.946131 | 2.1 |
| 70949 | 1.89226 | 2.1 |
| 70950 | 1.89226 | 2.1 |
| 70951 | 1.89226 | 2.1 |
| 70952 | 1.89226 | 2.1 |
| 70953 | 3.78453 | 2.1 |
| 70954 | 0.810943 | 2.1 |
| 70955 | 0.810943 | 2.1 |
| 70956 | 0.810945 | 2.1 |
| 70957 | 0.810945 | 2.1 |
| 70958 | 0.810932 | 2.1 |
| 70959 | 0.810932 | 2.1 |
| 70960 | 1.89226 | 2.1 |
| 70961 | 1.3517 | 2.1 |
| 70962 | 1.08132 | 2.1 |
| 70963 | 1.08132 | 2.1 |
| 70964 | 1.08133 | 2.1 |
| 70965 | 1.89226 | 2.1 |
| 70966 | 1.89226 | 2.1 |
| 70967 | 1.89226 | 2.1 |
| 70968 | 1.89226 | 2.1 |
| 70969 | 1.89226 | 2.1 |
| 70970 | 1.89226 | 2.1 |
| 70971 | 0.946131 | 2.1 |
| 70972 | 0.946131 | 2.1 |
| 70973 | 0.946131 | 2.1 |
| 70974 | 0.946131 | 2.1 |
| 70975 | 0.946131 | 2.1 |
| 70976 | 0.946131 | 2.1 |
| 70977 | 0.946131 | 2.1 |
| 70978 | 0.946131 | 2.1 |
| 70979 | 0.946131 | 2.1 |
| 70980 | 0.946131 | 2.1 |
| 70981 | 0.946131 | 2.1 |
| 70982 | 0.946131 | 2.1 |
| 70983 | 1.89226 | 1.86658 |
| 70984 | 1.89226 | 2.1 |
| 70985 | 1.89226 | 2.1 |
| 70986 | 1.89226 | 2.1 |
| 70987 | 3.78453 | 2.1 |
| 70988 | 1.89226 | 2.1 |
| 70989 | 1.89226 | 2.1 |

| | | |
|---|---|---|
| 70990 | 1.89226 | 2.1 |
| 70991 | 1.89226 | 2.1 |
| 70992 | 3.78452 | 2.1 |
| 70993 | 1.89226 | 2.1 |
| 70994 | 1.89226 | 2.1 |
| 70995 | 1.89226 | 2.1 |
| 70996 | 1.89226 | 2.1 |
| 70997 | 3.78452 | 2.1 |
| 70998 | 1.89226 | 2.1 |
| 70999 | 1.89226 | 2.1 |
| 71000 | 1.89226 | 2.1 |
| 71001 | 1.89226 | 2.1 |
| 71002 | 3.78453 | 2.1 |
| 71003 | 1.89226 | 2.1 |
| 71004 | 1.89226 | 2.1 |
| 71005 | 1.89226 | 2.1 |
| 71006 | 1.89226 | 2.1 |
| 71007 | 1.89226 | 2.1 |
| 71008 | 1.89226 | 2.1 |
| 71009 | 1.89226 | 2.1 |
| 71010 | 1.89226 | 2.1 |
| 71011 | 1.89226 | 2.1 |
| 71012 | 1.89226 | 2.1 |
| 71013 | 1.89226 | 2.1 |
| 71014 | 1.89226 | 2.1 |
| 71015 | 1.89226 | 2.1 |
| 71016 | 1.89226 | 2.1 |
| 71017 | 1.89226 | 2.1 |
| 71018 | 1.89226 | 2.1 |
| 71019 | 3.78452 | 2.1 |
| 71020 | 1.89226 | 2.1 |
| 71021 | 1.89226 | 2.1 |
| 71022 | 1.89226 | 2.1 |
| 71023 | 1.89226 | 2.1 |
| 71024 | 1.89226 | 2.1 |
| 71025 | 1.89226 | 2.1 |
| 71026 | 1.89226 | 2.1 |
| 71027 | 1.89226 | 2.1 |
| 71028 | 1.89226 | 2.1 |
| 71029 | 1.89226 | 2.1 |
| 71030 | 1.89226 | 2.1 |
| 71031 | 1.89226 | 2.1 |
| 71032 | 0.946128 | 2.1 |
| 71033 | 0.946128 | 2.1 |
| 71034 | 0.946133 | 2.1 |
| 71035 | 0.946133 | 2.1 |
| 71036 | 0.946131 | 2.1 |
| 71037 | 0.946131 | 2.1 |
| 71038 | 0.946131 | 2.1 |
| 71039 | 0.946131 | 2.1 |
| 71040 | 0.94613 | 2.1 |
| 71041 | 0.94613 | 2.1 |
| 71042 | 0.946131 | 2.1 |
| 71043 | 0.946131 | 2.1 |
| 71044 | 1.89226 | 2.1 |
| 71045 | 1.89226 | 2.1 |
| 71046 | 1.89226 | 2.1 |
| 71047 | 1.89226 | 2.1 |

| | | |
|---|---|---|
| 71048 | 1.89226 | 2.1 |
| 71049 | 1.89226 | 2.1 |
| 71050 | 1.89226 | 2.1 |
| 71051 | 1.89226 | 2.1 |
| 71052 | 1.89226 | 2.1 |
| 71053 | 1.89226 | 2.1 |
| 71054 | 1.89226 | 2.1 |
| 71055 | 1.89226 | 2.1 |
| 71056 | 2.76309 | 2.1 |
| 71057 | 1.89226 | 2.1 |
| 71058 | 2.57778 | 2.1 |
| 71059 | 1.89226 | 2.1 |
| 71060 | 4.47004 | 2.1 |
| 71061 | 0.946128 | 2.1 |
| 71062 | 0.946128 | 2.1 |
| 71063 | 0.946133 | 2.1 |
| 71064 | 0.946133 | 2.1 |
| 71065 | 0.946131 | 2.1 |
| 71066 | 0.946131 | 2.1 |
| 71067 | 0.946131 | 2.1 |
| 71068 | 0.946131 | 2.1 |
| 71069 | 0.946131 | 2.1 |
| 71070 | 0.946131 | 2.1 |
| 71071 | 0.94613 | 2.1 |
| 71072 | 0.94613 | 2.1 |
| 71073 | 0.946132 | 2.1 |
| 71074 | 0.946132 | 2.1 |
| 71075 | 0.946132 | 2.1 |
| 71076 | 0.946132 | 2.1 |
| 71077 | 0.946132 | 2.1 |
| 71078 | 0.946132 | 2.1 |
| 71079 | 0.946132 | 2.1 |
| 71080 | 0.946132 | 2.1 |
| 71081 | 0.946132 | 2.1 |
| 71082 | 0.946132 | 2.1 |
| 71083 | 0.946131 | 2.1 |
| 71084 | 0.946131 | 2.1 |
| 71085 | 1.89227 | 2.09349 |
| 71086 | 1.89227 | 0.783488 |
| 71087 | 1.89227 | 0.634214 |
| 71088 | 1.89227 | 2.1 |
| 71089 | 3.78453 | 0.898962 |
| 71090 | 1.89226 | 0.569229 |
| 71091 | 1.89226 | 1.99592 |
| 71092 | 1.89226 | 0.885062 |
| 71093 | 1.89226 | 0.843929 |
| 71094 | 3.78452 | 0.86226 |
| 71095 | 1.89226 | 2.1 |
| 71096 | 1.89226 | 0.724006 |
| 71097 | 1.89226 | 1.61659 |
| 71098 | 1.89226 | 1.89856 |
| 71099 | 1.89226 | 0.756442 |
| 71100 | 1.89226 | 0.791849 |
| 71101 | 0.946131 | 0.830137 |
| 71102 | 0.946131 | 0.609902 |
| 71103 | 0.946131 | 2.1 |
| 71104 | 0.946131 | 2.1 |
| 71105 | 0.946133 | 2.1 |

| | | |
|---|---|---|
| 71106 | 0.946133 | 0.897984 |
| 71107 | 0.946129 | 0.892625 |
| 71108 | 0.946129 | 2.1 |
| 71109 | 0.946131 | 1.08475 |
| 71110 | 0.946131 | 1.11445 |
| 71111 | 0.946131 | 1.04759 |
| 71112 | 0.946131 | 0.90997 |
| 71113 | 1.89226 | 0.515947 |
| 71114 | 1.89226 | 1.98811 |
| 71115 | 1.89226 | 2.06554 |
| 71116 | 1.89226 | 0.738562 |
| 71117 | 1.89226 | 1.64271 |
| 71118 | 1.89226 | 0.779847 |
| 71119 | 1.89226 | 0.873948 |
| 71120 | 1.89226 | 0.730711 |
| 71121 | 1.89226 | 0.566692 |
| 71122 | 1.89226 | 1.44031 |
| 71123 | 3.78452 | 0.707667 |
| 71124 | 1.89226 | 1.44858 |
| 71125 | 1.89226 | 0.89392 |
| 71126 | 1.89226 | 1.63702 |
| 71127 | 1.89226 | 0.684476 |
| 71128 | 1.89226 | 2.0995 |
| 71129 | 1.89226 | 0.822457 |
| 71130 | 0.94613 | 0.774673 |
| 71131 | 0.94613 | 0.864701 |
| 71132 | 0.946131 | 2.1 |
| 71133 | 0.946131 | 1.99005 |
| 71134 | 0.946133 | 0.884885 |
| 71135 | 0.946133 | 2.07248 |
| 71136 | 0.946128 | 2.08502 |
| 71137 | 0.946128 | 0.798588 |
| 71138 | 0.946131 | 1.2887 |
| 71139 | 0.946131 | 1.36624 |
| 71140 | 0.946131 | 0.913997 |
| 71141 | 0.946131 | 0.928595 |
| 71142 | 1.89226 | 0.803845 |
| 71143 | 1.89226 | 2.1 |
| 71144 | 1.89226 | 2.0967 |
| 71145 | 1.89226 | 0.990801 |
| 71146 | 1.89226 | 2.08018 |
| 71147 | 1.89226 | 1.05914 |
| 71148 | 1.89226 | 0.787895 |
| 71149 | 1.89226 | 2.0952 |
| 71150 | 1.89226 | 1.21621 |
| 71151 | 1.89226 | 1.07533 |
| 71152 | 1.89226 | 1.21939 |
| 71153 | 1.89226 | 1.32392 |
| 71154 | 0.946133 | 1.62633 |
| 71155 | 0.946133 | 0.756042 |
| 71156 | 0.946129 | 2.1 |
| 71157 | 0.946129 | 1.31577 |
| 71158 | 0.946131 | 2.1 |
| 71159 | 0.946131 | 1.08625 |
| 71160 | 0.946131 | 1.6248 |
| 71161 | 0.946131 | 1.02012 |
| 71162 | 0.946131 | 1.33349 |
| 71163 | 0.946131 | 1.24117 |

| | | |
|---|---|---|
| 71164 | 0.946131 | 1.25814 |
| 71165 | 0.946131 | 1.37147 |
| 71166 | 1.89226 | 0.731184 |
| 71167 | 1.89226 | 2.1 |
| 71168 | 1.89226 | 1.57545 |
| 71169 | 1.89226 | 0.929122 |
| 71170 | 1.89226 | 1.49769 |
| 71171 | 1.89226 | 1.02822 |
| 71172 | 1.89226 | 0.739223 |
| 71173 | 1.89226 | 2.03648 |
| 71174 | 1.89226 | 0.99347 |
| 71175 | 1.89226 | 0.937784 |
| 71176 | 1.89226 | 0.995996 |
| 71177 | 1.89226 | 1.14622 |
| 71178 | 1.89226 | 0.82849 |
| 71179 | 1.89226 | 2.1 |
| 71180 | 1.89226 | 1.151 |
| 71181 | 1.89226 | 1.20087 |
| 71182 | 1.89226 | 1.23126 |
| 71183 | 1.89226 | 1.8447 |
| 71184 | 1.89227 | 0.846168 |
| 71185 | 1.89227 | 2.1 |
| 71186 | 1.89227 | 1.86977 |
| 71187 | 1.89227 | 1.25286 |
| 71188 | 1.89227 | 1.95894 |
| 71189 | 1.89227 | 1.43587 |
| 71190 | 1.89227 | 2.1 |
| 71191 | 1.89227 | 2.1 |
| 71192 | 1.89227 | 2.1 |
| 71193 | 1.89227 | 2.1 |
| 71194 | 1.89227 | 2.1 |
| 71195 | 1.89227 | 2.1 |
| 71196 | 1.89226 | 0.833507 |
| 71197 | 1.89226 | 2.1 |
| 71198 | 1.89226 | 2.1 |
| 71199 | 1.89226 | 0.877872 |
| 71200 | 1.89226 | 2.1 |
| 71201 | 1.89226 | 0.909641 |
| 71202 | 1.89226 | 0.893703 |
| 71203 | 1.89226 | 2.1 |
| 71204 | 1.89226 | 2.1 |
| 71205 | 1.89226 | 0.961324 |
| 71206 | 1.89226 | 2.1 |
| 71207 | 1.89226 | 1.02014 |
| 71208 | 1.89227 | 2.08839 |
| 71209 | 1.89227 | 2.1 |
| 71210 | 1.89227 | 2.1 |
| 71211 | 1.89227 | 2.1 |
| 71212 | 3.78453 | 2.1 |
| 71213 | 1.89226 | 0.439218 |
| 71214 | 1.89226 | 1.89783 |
| 71215 | 1.89226 | 2.07438 |
| 71216 | 1.89226 | 0.759543 |
| 71217 | 1.89226 | 1.7595 |
| 71218 | 1.89226 | 0.750828 |
| 71219 | 1.89226 | 0.543376 |
| 71220 | 1.89226 | 2.1 |
| 71221 | 1.89226 | 2.05728 |

| | | |
|---|---|---|
| 71222 | 1.89226 | 0.592072 |
| 71223 | 3.78452 | 1.37592 |
| 71224 | 1.89226 | 2.1 |
| 71225 | 1.89226 | 2.1 |
| 71226 | 1.89226 | 2.1 |
| 71227 | 1.89226 | 2.1 |
| 71228 | 1.89226 | 2.1 |
| 71229 | 1.89226 | 2.1 |
| 71230 | 1.89227 | 0.606362 |
| 71231 | 1.89227 | 1.47761 |
| 71232 | 1.89227 | 1.08062 |
| 71233 | 1.89227 | 0.686682 |
| 71234 | 1.89227 | 1.05958 |
| 71235 | 1.89227 | 0.695555 |
| 71236 | 1.89226 | 0.493751 |
| 71237 | 1.89226 | 0.986829 |
| 71238 | 1.89226 | 0.839698 |
| 71239 | 1.89226 | 0.655306 |
| 71240 | 1.89226 | 0.788389 |
| 71241 | 1.89226 | 0.65296 |
| 71242 | 1.89226 | 1.1738 |
| 71243 | 1.89226 | 0.837096 |
| 71244 | 1.89226 | 1.20722 |
| 71245 | 1.89226 | 0.653976 |
| 71246 | 1.89226 | 1.93185 |
| 71247 | 1.89226 | 0.772828 |
| 71248 | 0.946131 | 0.711839 |
| 71249 | 0.946131 | 0.773105 |
| 71250 | 0.946131 | 1.31616 |
| 71251 | 0.946131 | 2.1 |
| 71252 | 0.946133 | 0.804948 |
| 71253 | 0.946133 | 2.03486 |
| 71254 | 0.946129 | 1.77126 |
| 71255 | 0.946129 | 0.741964 |
| 71256 | 0.946131 | 1.06528 |
| 71257 | 0.946131 | 1.08899 |
| 71258 | 0.946131 | 0.841725 |
| 71259 | 0.946131 | 0.862887 |
| 71260 | 1.89227 | 0.481754 |
| 71261 | 1.89227 | 0.985285 |
| 71262 | 1.89227 | 0.89918 |
| 71263 | 1.89227 | 0.639993 |
| 71264 | 1.89227 | 0.906984 |
| 71265 | 1.89227 | 0.644493 |
| 71266 | 1.89226 | 0.5763 |
| 71267 | 1.89226 | 0.858038 |
| 71268 | 1.89226 | 0.733457 |
| 71269 | 1.89226 | 0.602721 |
| 71270 | 1.89226 | 0.706364 |
| 71271 | 1.89226 | 0.608291 |
| 71272 | 1.89226 | 0.855645 |
| 71273 | 1.89226 | 0.786141 |
| 71274 | 1.89226 | 0.908864 |
| 71275 | 1.89226 | 0.606946 |
| 71276 | 1.89226 | 1.49312 |
| 71277 | 1.89226 | 0.744368 |
| 71278 | 0.946131 | 0.67144 |
| 71279 | 0.946131 | 0.713806 |

| | | |
|---|---|---|
| 71280 | 0.946131 | 0.976869 |
| 71281 | 0.946131 | 2.0108 |
| 71282 | 0.946133 | 0.731608 |
| 71283 | 0.946133 | 1.49252 |
| 71284 | 0.946129 | 1.03405 |
| 71285 | 0.946129 | 0.679294 |
| 71286 | 0.946131 | 0.917215 |
| 71287 | 0.946131 | 0.932824 |
| 71288 | 0.946131 | 0.737036 |
| 71289 | 0.946131 | 0.753446 |
| 71290 | 1.89227 | 0.856764 |
| 71291 | 1.89227 | 2.1 |
| 71292 | 1.89227 | 2.1 |
| 71293 | 1.89227 | 0.857883 |
| 71294 | 3.78453 | 2.09896 |
| 71295 | 1.89226 | 2.09204 |
| 71296 | 1.89226 | 0.978563 |
| 71297 | 1.89226 | 0.715787 |
| 71298 | 1.89226 | 2.1 |
| 71299 | 3.78452 | 0.898495 |
| 71300 | 1.89226 | 0.85243 |
| 71301 | 1.89226 | 2.1 |
| 71302 | 1.89226 | 2.0744 |
| 71303 | 1.89226 | 1.22954 |
| 71304 | 3.78452 | 2.1 |
| 71305 | 1.89226 | 2.1 |
| 71306 | 1.89226 | 1.13912 |
| 71307 | 1.89226 | 0.849937 |
| 71308 | 1.89226 | 2.1 |
| 71309 | 3.78452 | 1.2273 |
| 71310 | 1.89226 | 2.1 |
| 71311 | 1.89226 | 0.894608 |
| 71312 | 1.89226 | 2.1 |
| 71313 | 1.89226 | 0.921333 |
| 71314 | 1.89226 | 2.1 |
| 71315 | 1.89226 | 0.169976 |
| 71316 | 1.89226 | 0.720925 |
| 71317 | 1.89226 | 2.1 |
| 71318 | 1.89226 | 1.65769 |
| 71319 | 1.89226 | 0.772502 |
| 71320 | 1.89226 | 1.48718 |
| 71321 | 1.89226 | 0.824139 |
| 71322 | 0.946131 | 1.10523 |
| 71323 | 0.946131 | 0.751902 |
| 71324 | 0.946127 | 2.04362 |
| 71325 | 0.946127 | 2.1 |
| 71326 | 0.946131 | 1.0159 |
| 71327 | 0.946131 | 2.08184 |
| 71328 | 0.946127 | 2.06234 |
| 71329 | 0.946127 | 0.821197 |
| 71330 | 0.946129 | 1.61731 |
| 71331 | 0.946129 | 1.58079 |
| 71332 | 0.946129 | 1.00606 |
| 71333 | 0.946129 | 1.19539 |
| 71334 | 0.94613 | 0.896928 |
| 71335 | 0.94613 | 0.928901 |
| 71336 | 0.946131 | 2.1 |
| 71337 | 0.946131 | 2.1 |

| | | |
|---|---|---|
| 71338 | 0.946133 | 0.991915 |
| 71339 | 0.946133 | 2.1 |
| 71340 | 0.946128 | 0.614311 |
| 71341 | 0.946128 | 2.1 |
| 71342 | 0.946131 | 2.1 |
| 71343 | 0.946131 | 1.69635 |
| 71344 | 0.946131 | 1.80346 |
| 71345 | 0.946131 | 1.90919 |
| 71346 | 1.89226 | 2.02404 |
| 71347 | 1.89226 | 2.00425 |
| 71348 | 1.89226 | 0.746258 |
| 71349 | 1.89226 | 2.1 |
| 71350 | 3.78453 | 1.00377 |
| 71351 | 1.89226 | 1.56528 |
| 71352 | 1.89226 | 1.85984 |
| 71353 | 1.89226 | 0.879903 |
| 71354 | 1.89226 | 1.41136 |
| 71355 | 3.78452 | 1.27802 |
| 71356 | 1.89226 | 2.1 |
| 71357 | 1.89226 | 1.0511 |
| 71358 | 1.89226 | 1.4406 |
| 71359 | 1.89226 | 2.1 |
| 71360 | 1.89226 | 1.06946 |
| 71361 | 1.89226 | 0.88761 |
| 71362 | 0.946132 | 2.0972 |
| 71363 | 0.946132 | 1.21558 |
| 71364 | 0.946132 | 2.1 |
| 71365 | 0.946132 | 1.22139 |
| 71366 | 0.946133 | 2.1 |
| 71367 | 0.946133 | 1.90744 |
| 71368 | 0.946132 | 1.37139 |
| 71369 | 0.946132 | 1.28642 |
| 71370 | 0.946133 | 1.5005 |
| 71371 | 0.946133 | 1.2538 |
| 71372 | 0.946132 | 1.38365 |
[truncated: 386,108 more chars]
