# Supplementary material for: On the Use of Bone Remodelling Models to Estimate the Density Distribution of Bones. Uniqueness of the Solution
Source: PLoS One. 2016 Feb 9;11(2):e0148603. doi: 10.1371/journal.pone.0148603 (PMC4747586; doi:10.1371/journal.pone.0148603)
Supplement: S10 File — (PDF) [file pone.0148603.s010.pdf]

N° Elem	Volume (mm3)	Density (g/cm3)
1	1.32415	2.1
2	1.75823	2.1
3	0.984099	2.1
4	1.46442	2.1
5	1.53082	2.1
6	1.32068	2.1
7	1.91404	0.05
8	1.17036	0.05
9	0.929209	0.05
10	1.27254	0.05
11	2.0926	0.05
12	2.20407	0.05
13	1.21488	0.05
14	1.26024	0.05
15	0.794454	0.05
16	1.95087	0.05
17	0.953925	2.1
18	0.98949	2.1
19	1.27875	2.1
20	1.04598	2.1
21	0.594005	2.1
22	0.578861	2.1
23	1.09506	2.1
24	1.45327	2.1
25	2.58396	2.1
26	1.58581	2.1
27	1.5858	2.1
28	0.636946	2.1
29	0.94202	2.1
30	0.946119	2.1
31	0.636953	2.1
32	0.946107	2.1
33	1.09505	2.1
34	1.27389	2.1
35	1.00232	2.1
36	1.00231	2.1
37	1.83583	2.1
38	1.00231	2.1
39	1.28828	0.05
40	1.81696	0.05
41	1.28828	0.05
42	1.28828	0.05
43	1.0044	0.05
44	0.85148	0.05
45	0.851539	0.05
46	1.57767	0.05
47	1.52227	0.05
48	0.730657	0.05
49	0.94613	0.05
50	1.28228	2.1
51	1.64268	2.1
52	1.10101	2.1
53	1.3939	2.1
54	0.959471	2.1
55	1.10101	2.1

56	1.2873	2.1
57	1.59659	2.1
58	1.80841	0.284984
59	1.80841	0.31852
60	0.946129	0.05
61	1.36991	0.05
62	1.32262	0.977492
63	1.32261	0.05
64	1.38464	0.837745
65	0.962877	0.749841
66	0.962878	0.686499
67	1.82129	0.70913
68	1.82129	0.05
69	0.94613	0.52728
70	1.39751	0.242383
71	0.946131	0.74425
72	1.34493	0.167275
73	1.39751	0.49493
74	0.837577	0.781416
75	0.823478	0.690006
76	1.10889	1.25071
77	1.01686	0.774058
78	1.1942	1.32562
79	0.836129	0.770062
80	0.937165	0.68478
81	0.969438	0.851269
82	0.895386	0.747562
83	1.30554	1.41641
84	1.15798	0.786015
85	1.22578	1.86362
86	1.15255	0.828634
87	0.9765	0.478638
88	2.02178	1.82347
89	1.63755	1.63385
90	0.727713	0.881476
91	0.810945	0.81783
92	1.00559	1.47259
93	0.879098	0.945569
94	1.07852	1.52335
95	0.657764	0.838147
96	0.829406	0.768479
97	0.720201	0.05
98	0.946131	0.05
99	1.11827	0.05
100	1.50754	0.05
101	1.64835	0.05
102	1.71903	0.05
103	0.851554	0.05
104	1.75626	0.05
105	2.1646	0.05
106	1.5563	0.05
107	1.5563	0.05
108	2.10876	0.05
109	1.05438	0.0799279
110	0.946133	0.05
111	1.05438	0.665286
112	1.20256	0.05
113	1.47879	0.144092

114	1.47879	1.37323
115	0.77815	1.26879
116	0.947114	2.1
117	1.31257	2.1
118	1.09235	2.1
119	0.946132	2.1
120	1.54246	2.1
121	1.54246	2.1
122	1.09235	2.1
123	0.946131	2.1
124	1.29507	2.06544
125	1.0353	2.1
126	1.03529	2.1
127	1.28667	2.1
128	1.44777	2.1
129	1.61121	2.1
130	1.22176	0.0513487
131	0.946129	0.05
132	1.08848	0.05
133	0.946131	0.0789372
134	1.61201	0.05
135	1.61201	0.05
136	1.19993	0.05
137	1.35822	0.05
138	1.1539	0.05
139	1.00765	0.05
140	1.28228	2.1
141	2.65339	2.1
142	1.28228	2.1
143	1.55998	2.1
144	1.00509	2.1
145	0.93845	2.1
146	1.27674	2.1
147	0.810958	2.1
148	1.91381	2.1
149	1.1861	2.1
150	1.24886	2.1
151	2.02842	2.1
152	0.991436	0.05
153	1.34588	0.05
154	2.02729	0.05
155	0.72904	0.05
156	1.06139	0.05
157	0.835368	0.05
158	1.63819	0.05
159	1.10745	0.05
160	1.79925	0.05
161	1.00085	0.05
162	0.946133	2.1
163	1.41123	2.1
164	1.8327	2.1
165	1.8327	2.1
166	0.946132	0.652874
167	1.41123	2.1
168	1.3676	2.1
169	1.11048	2.1
170	1.11048	2.1
171	1.65815	2.1

172	1.9004	2.1
173	1.88691	2.1
174	1.82232	2.1
175	1.46591	2.1
176	1.09115	2.1
177	1.02051	1.20092
178	1.03373	1.32203
179	1.10307	2.1
180	1.05556	2.1
181	1.82533	1.12067
182	1.19496	1.01539
183	1.28597	2.1
184	1.20421	2.1
185	2.23644	2.1
186	1.08378	2.1
187	0.983724	2.1
188	0.602075	2.1
189	1.45876	2.1
190	1.45875	2.1
191	0.602103	2.1
192	1.11822	2.1
193	0.946133	2.1
194	0.602103	2.1
195	1.20421	2.1
196	1.26371	0.127266
197	1.55179	0.0904837
198	1.14042	0.05
199	1.56876	0.05
200	2.27496	0.083241
201	1.13748	0.0849763
202	1.02884	0.144716
203	1.25124	0.122859
204	0.82703	0.0963802
205	0.607775	0.059549
206	1.49242	0.106285
207	1.35674	0.19758
208	1.6697	2.1
209	1.81655	2.1
210	0.900108	1.25894
211	0.851537	1.03617
212	0.703076	1.34287
213	0.639143	1.1442
214	1.42358	1.28432
215	1.4976	1.60296
216	0.86284	1.17335
217	1.2965	1.26164
218	1.27835	2.1
219	1.00012	1.10251
220	1.74332	1.55835
221	1.08576	2.1
222	1.19 2.1	
223	1.08576	2.1
224	1.20854	2.1
225	0.946131	2.1
226	0.946131	2.1
227	1.75985	2.1
228	1.75985	2.1
229	1.41232	2.1

230	1.29365	2.1
231	0.973559	2.1
232	1.23698	0.05
233	1.45055	0.05
234	1.26947	0.05
235	0.856218	0.05
236	1.75303	0.05
237	1.14938	0.05
238	0.984711	0.05
239	2.05771	0.05
240	0.625171	0.05
241	0.965675	0.05
242	1.17631	0.05
243	1.4677	0.05
244	1.35403	0.05
245	0.975149	0.05
246	0.946131	0.05
247	1.80893	0.05
248	1.80893	0.05
249	0.975149	0.05
250	0.946132	0.05
251	1.9503	0.05
252	2.80207	0.05
253	1.76411	0.05
254	2.77469	0.05
255	1.9503	0.05
256	1.26784	0.05
257	2.31093	0.05
258	0.851541	0.05
259	1.39521	0.05
260	1.45613	0.05
261	0.851541	0.05
262	0.996013	0.05
263	1.07989	0.05
264	0.946131	0.05
265	1.08213	0.05
266	1.74289	0.05
267	1.75838	0.05
268	2.11163	0.05
269	1.23773	2.1
270	1.25398	2.1
271	1.0304	2.1
272	1.32368	2.1
273	1.11712	0.973959
274	1.07999	0.921284
275	1.1766	2.1
276	1.35735	2.1
277	1.089 0.05	
278	1.81193	0.05
279	1.11081	0.05
280	1.78069	0.05
281	0.717512	0.05
282	0.810946	0.05
283	0.703901	0.05
284	1.09028	0.05
285	0.958031	0.05
286	0.892585	0.05
287	0.873431	0.05

288	0.997755	0.05
289	1.85479	0.05
290	0.983029	0.05
291	1.27922	0.05
292	1.64346	0.272946
293	1.27922	0.0590638
294	1.09795	2.1
295	1.07508	0.711901
296	1.02975	1.50574
297	1.01121	2.1
298	1.72369	0.05
299	1.12035	0.05
300	2.13511	0.05
301	0.9705	0.05
302	0.946131	0.05
303	0.86182	0.05
304	1.51379	0.05
305	1.51379	0.05
306	0.946132	0.05
307	0.861849	0.05
308	1.34591	0.21938
309	1.14721	0.05
310	1.15539	2.1
311	1.90938	2.1
312	2.19436	2.1
313	1.41563	2.1
314	0.785641	2.1
315	0.736384	2.1
316	1.46489	2.1
317	1.46488	2.1
318	0.736372	2.1
319	0.946132	2.1
320	1.90941	1.68795
321	1.18907	2.1
322	1.20409	2.1
323	0.973559	2.1
324	1.29461	2.1
325	0.946129	2.1
326	0.94613	2.1
327	1.78751	2.1
328	1.78751	2.1
329	1.38885	2.1
330	1.34479	2.1
331	1.07283	2.1
332	1.6869	2.1
333	1.52544	2.1
334	1.07797	2.1
335	1.13819	0.978193
336	0.776179	2.1
337	1.61092	2.04233
338	1.36155	2.1
339	0.77618	2.1
340	1.2933	1.35955
341	1.15946	2.1
342	0.69857	2.1
343	1.87607	2.1
344	1.07612	2.1
345	1.31976	1.20763

346	1.01243	2.1
347	1.41279	1.3525
348	1.35146	0.05
349	1.90664	0.05
350	1.35145	0.05
351	1.22811	0.051669
352	1.93147	0.05
353	1.42486	2.1
354	1.56558	2.1
355	1.0476	0.05
356	2.31605	0.05
357	1.89367	0.05
358	0.804706	0.05
359	0.783919	0.05
360	1.3116	0.05
361	1.03891	0.05
362	0.960473	0.05
363	1.08462	0.05
364	0.785994	0.05
365	1.1318	0.05
366	1.29576	0.05
367	1.3946	0.05
368	1.11924	0.05
369	0.834322	2.1
370	1.0256	2.1
371	1.74321	0.831204
372	1.41798	0.838996
373	0.871608	0.915202
374	1.47253	0.726934
375	1.47252	0.715014
376	0.817064	0.795102
377	0.817064	0.834996
378	0.946129	0.593587
379	1.63413	0.598613
380	1.9194	0.05
381	1.93562	0.05
382	2.06307	0.05
383	1.46021	0.05
384	0.955002	1.26818
385	0.833409	1.76982
386	0.838039	1.09815
387	1.48702	1.24891
388	1.1697	1.24406
389	1.23549	1.31068
390	1.18373	1.23235
391	2.02254	0.671478
392	1.68867	1.35285
393	0.875481	0.34393
394	0.821635	1.58722
395	0.858878	0.2288
396	1.30245	0.599949
397	1.07476	0.665436
398	1.11907	0.617079
399	1.10453	0.337839
400	1.70233	0.672551
401	1.70233	0.593199
402	2.6558	1.38816
403	1.7818	2.1

404	1.87059	2.1
405	1.34412	0.05
406	1.98068	0.05
407	2.56369	0.05
408	1.49512	0.05
409	1.35977	0.05
410	1.34254	0.05
411	1.75618	0.05
412	1.75618	0.05
413	0.946129	0.0546359
414	0.946129	0.05
415	1.17665	0.05
416	1.01976	0.05
417	1.27376	0.05
418	1.35978	0.05
419	1.68741	0.05
420	1.68741	0.05
421	0.946132	0.05
422	0.946133	0.05
423	1.14241	0.05
424	0.996831	0.05
425	0.996828	0.05
426	1.12774	0.05
427	1.13664	2.1
428	1.09627	2.1
429	2.8814	2.1
430	3.49751	2.1
431	1.79109	0.066516
432	1.54197	0.05
433	2.75153	0.05
434	1.93019	0.05
435	1.51569	0.599794
436	1.55145	1.12863
437	1.78595	2.1
438	3.74452	2.1
439	1.66537	2.1
440	3.55161	2.1
441	1.35002	0.298347
442	1.19901	0.810867
443	1.61028	2.1
444	1.35002	0.952132
445	1.65712	0.895935
446	1.10585	0.664097
447	0.961347	0.987604
448	0.810944	0.919747
449	1.16148	1.81053
450	1.08066	1.77556
451	0.934577	0.660401
452	1.05164	1.39706
453	0.79216	0.783983
454	2.99194	2.1
455	1.2832	2.1
456	2.97842	2.1
457	1.39517	2.1
458	1.03391	2.1
459	1.74416	2.1
460	1.05071	2.1
461	0.923782	2.1

462	1.55369	2.1
463	1.86444	2.1
464	1.01617	2.1
465	1.27174	2.1
466	1.20573	2.1
467	1.01616	2.1
468	1.13621	2.1
469	1.75366	2.1
470	2.27009	1.96634
471	1.6813	2.1
472	1.47904	0.05
473	2.62714	0.05
474	1.80085	0.05
475	1.80085	0.05
476	2.74104	0.05
477	1.37052	0.05
478	0.946131	0.05
479	1.37052	0.05
480	1.31357	0.05
481	1.7837	0.05
482	1.7837	0.05
483	0.900397	0.05
484	1.74487	0.05
485	2.88672	2.1
486	2.88249	2.1
487	1.0961	0.05
488	1.20143	0.05
489	1.66739	0.05
490	1.85795	0.05
491	1.45789	0.05
492	2.11407	0.05
493	0.713524	0.05
494	1.58391	0.05
495	1.58391	0.05
496	1.04765	0.05
497	1.24979	0.05
498	0.713551	0.05
499	0.946127	0.05
500	1.36768	0.05
501	1.14433	0.05
502	1.66923	0.05
503	0.699479	0.05
504	0.70351	0.05
505	0.758416	0.05
506	1.07678	0.05
507	0.909874	0.05
508	0.940218	0.05
509	0.876604	0.05
510	1.27148	0.05
511	2.42348	0.05
512	1.19837	0.05
513	0.764764	0.05
514	0.829259	0.05
515	0.740584	0.05
516	1.2772	0.05
517	0.964465	0.05
518	1.06231	0.05
519	1.06924	0.05

520	1.17566	0.05
521	1.3063	0.05
522	2.51253	0.05
523	2.42267	0.05
524	1.89226	0.05
525	1.46492	0.05
526	0.946132	0.05
527	0.653151	0.05
528	0.653124	0.05
529	1.53995	0.05
530	1.53995	0.05
531	0.936806	0.05
532	1.25626	0.05
533	1.1893	0.05
534	1.62206	0.05
535	1.04921	2.1
536	1.22927	2.1
537	0.801765	2.1
538	0.774917	2.1
539	0.844623	2.1
540	0.737481	2.1
541	3.24379	2.1
542	2.48573	2.1
543	1.40967	1.27963
544	1.47203	1.51946
545	2.07659	1.11856
546	1.40967	1.52392
547	2.06638	1.1806
548	1.17418	2.1
549	1.04921	2.1
550	1.74593	2.1
551	1.74593	2.1
552	0.946132	2.1
553	1.38651	2.1
554	1.20761	0.05
555	1.20761	1.71395
556	1.30555	1.45751
557	1.46812	0.195391
558	2.41602	0.578822
559	1.46812	0.73317
560	1.46812	0.05
561	0.734059	0.132799
562	0.946132	1.65915
563	1.21261	1.00467
564	1.34225	0.796579
565	0.734084	0.658903
566	1.82077	0.715149
567	1.82077	0.382461
568	1.13751	0.0765189
569	0.946131	0.602045
570	1.13751	0.284544
571	1.13748	0.155392
572	0.784392	0.204958
573	1.4906	0.161799
574	1.4906	0.05
575	1.17879	1.25465
576	1.28291	1.22289
577	0.626495	2.1

578	1.45334	2.1
579	1.45334	2.1
580	0.626494	2.1
581	1.3852	2.1
582	0.946116	2.1
583	0.626499	2.1
584	1.47203	1.11622
585	1.42639	2.1
586	1.42639	2.1
587	0.795469	2.1
588	0.795469	2.1
589	0.946132	2.1
590	0.850877	2.1
591	1.37098	2.1
592	1.87194	2.1
593	1.76988	2.1
594	3.16295	0.05
595	3.26725	0.05
596	1.13322	2.1
597	1.20761	2.1
598	0.686933	0.92747
599	0.493248	0.772774
600	1.2053	2.09281
601	0.947149	0.823311
602	1.27591	0.900641
603	1.75732	2.07114
604	0.493261	0.918575
605	1.34337	2.1
606	2.73207	0.771805
607	1.19857	0.855577
608	0.671688	0.72651
609	0.946134	1.94754
610	1.36603	0.762364
611	2.07817	0.865175
612	1.82573	1.82658
613	0.671699	0.992568
614	1.47772	0.737495
615	0.975349	2.1
616	1.22615	2.1
617	1.47868	2.1
618	2.29351	2.1
619	1.47642	2.1
620	2.94443	1.97531
621	1.80454	2.1
622	0.949135	2.1
623	0.949134	2.1
624	1.10476	0.05
625	0.982857	0.05
626	1.10938	0.05
627	1.19052	0.105575
628	1.01012	0.05
629	1.35562	0.05
630	0.691742	0.05
631	2.39596	0.05
632	2.35604	0.05
633	0.974081	0.05
634	3.22444	0.05
635	1.3294	0.510923

636	1.37265	0.913942
637	1.3294	0.862228
638	1.51113	2.1
639	1.43643	2.1
640	1.72726	2.1
641	1.634	2.1
642	0.755566	2.1
643	1.29319	2.1
644	0.755567	2.1
645	0.946131	2.1
646	1.29319	2.1
647	0.973563	2.1
648	0.904398	2.1
649	0.904406	2.1
650	1.1538	2.1
651	1.10437	2.1
652	0.935418	2.1
653	1.16649	2.1
654	0.975667	2.1
655	1.17952	2.1
656	1.95053	2.1
657	0.948676	2.1
658	1.44287	2.1
659	0.746095	2.1
660	1.51224	2.1
661	1.27354	2.1
662	0.746066	2.1
663	1.20573	2.1
664	1.12442	2.1
665	0.671487	2.1
666	1.04355	2.1
667	0.948675	2.1
668	0.967673	0.537067
669	1.12931	0.05
670	1.38212	0.05
671	2.40113	0.05
672	1.08054	0.05
673	1.04072	0.05
674	0.691062	0.05
675	1.58052	0.05
676	1.33502	0.05
677	0.946131	0.05
678	0.691062	0.05
679	1.02663	0.05
680	1.80569	0.05
681	1.26062	0.05
682	1.18203	0.05
683	0.946131	0.05
684	0.795065	0.05
685	1.58753	0.05
686	1.58753	0.05
687	0.946131	0.05
688	0.795093	0.05
689	0.805057	2.1
690	0.812415	2.1
691	1.23868	0.136549
692	1.34448	0.05
693	1.67201	2.1

694	1.61202	2.1
695	1.41103	2.1
696	1.41103	2.1
697	0.841088	2.1
698	1.38302	2.1
699	0.841089	2.1
700	0.869097	2.1
701	1.38302	2.1
702	0.949941	2.1
703	0.94994	2.1
704	1.88608	2.1
705	0.854942	2.1
706	1.68893	2.1
707	1.70002	2.1
708	2.02107	0.05
709	2.04982	0.05
710	3.11063	0.05
711	2.03805	0.05
712	1.67638	2.1
713	0.967492	2.1
714	2.00209	2.1
715	0.967491	2.1
716	0.870742	2.1
717	1.30266	0.05
718	0.651334	0.05
719	1.69413	0.05
720	1.69413	0.05
721	0.946132	0.05
722	1.03079	0.05
723	1.31468	0.05
724	0.946132	0.05
725	0.919055	0.05
726	1.16815	0.05
727	2.79004	0.05
728	1.2136	0.05
729	2.62935	0.05
730	0.947692	0.05
731	1.51835	0.0634981
732	0.947693	0.05
733	0.947692	0.05
734	0.980841	2.1
735	1.43577	2.1
736	0.980841	2.1
737	1.67858	2.1
738	1.34124	0.141177
739	1.39818	0.0548554
740	1.2065	2.1
741	0.946133	2.1
742	1.2079	2.1
743	1.65229	2.1
744	1.65229	2.1
745	0.946132	2.1
746	1.2079	2.1
747	1.39053	2.1
748	1.20651	2.1
749	1.36919	2.1
750	1.12717	2.1
751	1.09483	2.1

752	0.994294	2.1
753	0.97356	2.1
754	1.06827	2.1
755	1.40571	2.1
756	1.29259	2.1
757	0.946131	2.1
758	1.7787	2.1
759	1.7787	2.1
760	1.29259	2.1
761	0.946132	2.1
762	1.09408	0.432948
763	1.03824	0.150356
764	0.851541	0.05
765	1.23608	0.05
766	0.851539	0.05
767	1.5021	0.0509539
768	1.54839	0.05
769	1.12373	0.05
770	1.22617	0.05
771	1.42762	0.05
772	2.24746	0.05
773	1.05475	0.05
774	1.46845	0.05
775	1.05475	0.05
776	0.929726	0.05
777	1.52072	0.05
778	1.82488	0.05
779	1.02271	0.05
780	1.19225	0.05
781	1.43071	0.05
782	0.94613	0.05
783	1.05475	0.05
784	1.39223	2.1
785	1.71255	2.1
786	0.946132	0.957257
787	1.10531	1.00798
788	1.46355	0.993753
789	1.46355	0.811234
790	0.78086	1.10128
791	1.10531	1.00217
792	1.1391	0.972589
793	1.56166	1.07623
794	2.21062	0.839632
795	1.0361	2.1
796	1.73577	2.1
797	1.73577	2.1
798	0.946116	2.1
799	1.28174	2.1
800	0.946118	2.1
801	1.22883	2.1
802	1.28172	2.1
803	1.03609	2.1
804	0.553567	0.05
805	1.60581	0.05
806	1.60581	0.05
807	0.553594	0.05
808	0.966748	0.05
809	0.966748	0.05

810	0.946133	0.05
811	1.05602	0.05
812	2.38525	0.05
813	1.14503	0.05
814	1.24678	0.05
815	0.56262	0.05
816	1.24678	0.05
817	0.978467	0.05
818	1.66262	0.05
819	1.66262	0.05
820	0.562633	0.05
821	1.09522	0.05
822	2.49356	0.05
823	0.592547	0.05
824	1.04321	0.799093
825	1.25133	1.30743
826	1.70508	1.21824
827	1.81043	1.01738
828	0.494934	1.1056
829	0.601047	1.06421
830	0.993428	1.06509
831	0.946573	1.16804
832	1.35039	1.01281
833	0.816895	1.41384
834	1.5063	0.966405
835	0.816895	0.90579
836	0.946132	0.911793
837	1.9067	1.18752
838	2.14504	1.31928
839	0.816895	0.889
840	2.29624	0.900634
841	2.76933	0.913406
842	1.254 1.34162	
843	0.946128	2.1
844	1.38813	2.1
845	1.79998	2.1
846	1.79998	2.1
847	0.94613	2.1
848	1.38813	2.1
849	1.35798	2.1
850	1.27809	2.1
851	1.35798	2.1
852	1.68994	2.1
853	1.68994	2.1
854	0.946132	2.1
855	0.946132	2.1
856	1.18067	2.1
857	0.958256	2.1
858	0.958255	2.1
859	0.958257	2.1
860	0.958255	2.1
861	1.06457	2.1
862	1.14195	2.1
863	1.70423	0.0652894
864	1.14195	2.1
865	1.64285	2.1
866	2.02057	2.1
867	0.946132	2.1

868	1.44186	2.1
869	0.946133	2.1
870	1.16366	2.1
871	1.17233	2.1
872	1.31259	0.786512
873	1.2016	2.1
874	1.17129	2.1
875	1.05724	0.05
876	1.32697	1.06651
877	1.05724	0.774611
878	1.04292	2.04716
879	3.39037	2.1
880	2.57275	2.1
881	0.629212	2.1
882	2.32525	2.1
883	1.76949	0.747787
884	1.65809	0.915957
885	1.27665	2.1
886	1.19861	2.1
887	1.20045	2.1
888	1.1635	2.1
889	0.953633	0.05
890	0.953634	0.05
891	1.21408	2.1
892	1.25716	2.1
893	2.01791	2.1
894	1.89318	2.1
895	0.947921	2.06631
896	0.939049	2.1
897	0.947921	2.1
898	0.740753	2.1
899	0.916104	2.1
900	1.16941	2.1
901	1.72038	2.1
902	2.04441	2.1
903	0.973182	2.1
904	1.11751	2.1
905	0.611089	2.1
906	1.53584	0.0597478
907	0.767923	0.237464
908	1.38925	0.179505
909	1.38926	0.271034
910	0.946131	0.382988
911	0.961347	0.126462
912	1.19583	0.505011
913	0.946131	0.517107
914	1.3663	0.605049
915	1.21067	0.153619
916	1.00166	0.05
917	1.40707	0.05
918	1.50435	0.05
919	1.47954	0.886372
920	1.1233	2.1
921	2.36036	1.66343
922	1.04024	0.869174
923	0.739729	0.904879
924	1.45533	0.839433
925	1.45532	0.824003

926	0.946118	0.885391
927	1.15163	0.523436
928	1.15165	0.917918
929	0.739748	0.860681
930	1.04025	1.08116
931	1.47608	0.0803878
932	0.946131	0.105181
933	1.02028	0.05
934	0.946129	0.05
935	1.78223	0.05
936	1.78223	0.05
937	1.34153	0.05
938	1.38683	0.05
939	1.3407	0.0838143
940	1.3407	0.0826704
941	2.04729	0.05
942	0.851529	0.114697
943	1.26671	0.0509208
944	0.946116	0.117425
945	1.4869	0.05
946	1.29553	0.05
947	1.03645	0.05
948	1.20731	0.05
949	0.73665	2.1
950	0.693596	2.1
951	2.11152	2.1
952	1.73627	2.1
953	1.06191	2.1
954	1.52226	2.1
955	1.76869	2.1
956	1.1881	2.1
957	1.32349	2.1
958	1.51041	2.1
959	1.61591	2.1
960	0.946132	2.1
961	0.946132	2.1
962	1.13537	2.1
963	3.0048	2.1
964	0.985109	2.1
965	1.68899	2.1
966	3.23261	2.1
967	1.08831	2.1
968	2.20623	2.1
969	0.91749	2.1
970	1.15515	2.1
971	0.550438	2.1
972	1.66294	2.1
973	1.08257	2.1
974	0.87851	2.1
975	2.09046	2.1
976	0.738711	2.1
977	1.31242	0.05
978	1.26311	0.05
979	1.67652	0.05
980	0.968594	2.1
981	0.968594	2.1
982	0.981099	0.05
983	1.32418	0.05

984	2.67175	0.05
985	2.09678	0.05
986	1.2511	0.05
987	0.912698	0.05
988	0.810946	0.05
989	0.810959	0.05
990	1.67479	0.05
991	1.65677	0.05
992	1.28469	0.05
993	1.48817	0.05
994	1.08131	0.05
995	1.04349	0.661254
996	1.42492	0.749464
997	1.05724	0.736451
998	1.65723	0.05
999	1.95313	0.05
1000	1.9293	0.0583698
1001	2.02729	0.05
1002	0.963393	1.11797
1003	0.963392	0.888105
1004	2.90171	2.1
1005	2.65447	2.1
1006	1.64158	2.1
1007	1.6488	2.1
1008	1.15991	0.0930281
1009	0.946132	0.43655
1010	1.58199	0.206866
1011	1.58199	0.254497
1012	1.03333	0.439646
1013	1.25436	0.330921
1014	1.25436	0.491741
1015	0.946132	0.725449
1016	1.3771	0.05
1017	0.687745	0.05
1018	1.37709	0.05
1019	0.812386	0.05
1020	1.50172	0.05
1021	1.50172	0.05
1022	0.687758	0.05
1023	1.17506	0.05
1024	2.00539	0.05
1025	2.2441	0.05
1026	1.09667	0.05
1027	1.43577	2.1
1028	2.24741	2.1
1029	1.26103	2.1
1030	0.851544	2.1
1031	1.5543	2.1
1032	1.59756	2.1
1033	0.851543	2.1
1034	1.44673	2.1
1035	1.31947	2.1
1036	1.1237	2.1
1037	1.26103	2.1
1038	1.089 0.05	
1039	0.946128	0.05
1040	0.965824	0.05
1041	0.94613	0.05

1042	1.51344	0.05
1043	1.51344	0.05
1044	1.13508	0.05
1045	1.32449	0.05
1046	1.089	0.05
1047	3.33706	0.05
1048	2.7542	0.05
1049	1.27256	0.0767075
1050	0.954172	0.0527292
1051	1.55527	0.05
1052	1.05765	0.144524
1053	1.57326	0.082201
1054	1.38251	0.05
1055	0.94613	0.0963012
1056	2.38829	0.05
1057	2.00539	0.05
1058	1.53798	0.05
1059	1.20969	2.1
1060	1.15864	2.1
1061	1.7986	2.1
1062	1.74533	2.1
1063	0.944827	1.9036
1064	0.946131	1.0758
1065	0.851543	1.02822
1066	1.52816	0.554496
1067	1.5314	1.42978
1068	1.39266	0.303468
1069	0.851528	0.455261
1070	1.39589	0.876947
1071	1.7348	0.767108
1072	1.85439	1.4574
1073	1.86367	1.1596
1074	1.90905	0.806787
1075	1.22176	0.0670596
1076	1.59492	0.072362
1077	2.97929	2.1
1078	2.1905	2.1
1079	1.24128	2.1
1080	0.994293	2.1
1081	2.49767	0.05
1082	2.37105	0.05
1083	1.10834	1.04361
1084	1.53214	0.935283
1085	1.417	2.1
1086	2.13599	0.834872
1087	1.61727	2.1
1088	1.75004	2.1
1089	1.96301	2.1
1090	0.966631	2.1
1091	1.37791	2.1
1092	1.47302	2.1
1093	1.47302	2.1
1094	0.871519	2.1
1095	0.94613	2.1
1096	0.87155	2.1
1097	2.38731	2.1
1098	2.12399	1.0469
1099	1.74865	0.872051

1100	1.46724	0.151767
1101	2.38056	0.0937088
1102	1.46724	0.180153
1103	0.818887	0.112154
1104	0.810944	1.60534
1105	0.805227	0.135846
1106	1.06558	0.478764
1107	0.841487	0.236612
1108	1.08456	0.560649
1109	0.903083	0.448419
1110	1.08929	0.05
1111	1.68454	0.05
1112	1.08929	0.05
1113	1.07658	0.05
1114	0.904439	0.05
1115	1.58501	0.05
1116	1.19729	0.05
1117	1.05427	0.05
1118	1.47764	0.05
1119	0.810959	0.05
1120	1.60946	1.11718
1121	2.28887	1.08628
1122	0.946133	1.10975
1123	1.14444	0.968138
1124	1.67964	0.593418
1125	1.67964	0.686288
1126	0.755334	1.1629
1127	1.14444	1.106
1128	1.29054	1.04773
1129	1.37762	0.887843
1130	0.946132	1.10413
1131	0.533645	0.882361
1132	1.48361	0.570882
1133	1.48361	0.893702
1134	0.533645	1.07655
1135	0.872823	1.11374
1136	1.14444	0.920169
1137	1.37762	0.948017
1138	2.10101	1.00787
1139	1.02311	1.03471
1140	1.04083	2.1
1141	1.04084	2.1
1142	1.04084	2.1
1143	1.30116	2.1
1144	1.23477	0.05
1145	1.81893	0.05
1146	1.23477	0.05
1147	1.23477	0.05
1148	1.11967	0.05
1149	0.946131	0.05
1150	1.11966	0.05
1151	1.12628	0.05
1152	0.629641	0.05
1153	1.6163	0.05
1154	1.6163	0.05
1155	1.11117	2.1
1156	1.13006	2.1
1157	1.15149	2.1

1158	1.13179	2.1
1159	0.585368	0.0551898
1160	0.986036	1.01741
1161	1.94788	0.429919
1162	1.34857	0.182632
1163	2.31806	2.1
1164	2.06363	2.1
1165	1.00423	2.1
1166	0.829065	2.1
1167	0.551816	2.1
1168	0.99406	2.1
1169	1.98339	2.1
1170	1.68073	2.1
1171	2.558 2.1	
1172	0.711814	2.09302
1173	2.97316	2.1
1174	2.41579	2.1
1175	1.15248	0.05
1176	1.14305	0.05
1177	1.15483	0.05
1178	1.90981	0.05
1179	0.998957	0.05
1180	1.83742	0.05
1181	1.27581	2.1
1182	0.94613	2.1
1183	1.6315	2.1
1184	1.6315	2.1
1185	0.946132	2.1
1186	1.28441	2.1
1187	1.28441	2.1
1188	1.00365	2.1
1189	0.968536	2.1
1190	1.57575	2.1
1191	1.27581	2.1
1192	1.99297	2.1
1193	1.32784	2.1
1194	1.4259	2.1
1195	2.27952	2.1
1196	1.5685	2.1
1197	1.54231	2.1
1198	3.54638	2.1
1199	1.3294	2.1
1200	2.98237	2.1
1201	2.01633	0.581901
1202	2.0985	0.807804
1203	2.26047	0.260703
1204	1.7913	0.141185
1205	1.09504	2.1
1206	1.74126	2.1
1207	1.05983	2.1
1208	2.37664	2.1
1209	2.28084	2.1
1210	2.19948	2.1
1211	1.33593	0.05
1212	1.31508	0.05
1213	1.30647	2.1
1214	1.28846	2.1
1215	1.2955	2.1

1216	1.09044	2.1
1217	1.4099	2.1
1218	1.14984	2.1
1219	0.946132	1.20967
1220	1.70727	2.1
1221	1.70727	2.1
1222	1.14984	2.1
1223	0.946132	2.1
1224	0.911818	2.1
1225	2.12209	2.1
1226	2.4158	2.1
1227	1.20867	2.1
1228	2.84085	2.1
1229	1.42284	2.1
1230	2.45689	2.1
1231	1.41562	2.1
1232	0.918358	2.1
1233	1.11222	2.1
1234	0.999083	2.1
1235	0.696781	2.1
1236	1.30869	2.1
1237	1.66132	2.1
1238	0.696807	2.1
1239	0.745315	2.1
1240	1.23296	2.1
1241	0.780548	2.1
1242	1.93242	1.17396
1243	1.27362	1.42824
1244	2.77828	1.58952
1245	1.02653	0.90655
1246	0.808576	0.886138
1247	0.744012	1.06346
1248	0.605174	0.678368
1249	1.16501	0.303622
1250	0.803432	0.272357
1251	0.961016	0.317539
1252	1.10425	0.816142
1253	1.3756	0.973492
1254	2.17765	0.821468
1255	1.84578	0.578518
1256	1.32801	0.324714
1257	1.76377	0.27745
1258	0.946131	0.155306
1259	1.3022	0.589299
1260	0.694814	0.627249
1261	1.53028	0.142288
1262	1.53028	0.168656
1263	1.3022	0.303493
1264	0.922889	0.461679
1265	1.57766	2.1
1266	2.06911	2.1
1267	1.57766	2.1
1268	1.37721	0.05
1269	1.72174	0.05
1270	1.4468	0.05
1271	1.37721	0.05
1272	1.73972	0.113618
1273	1.62324	0.118149

1274	0.946125	0.0905636
1275	1.34259	0.05
1276	1.04187	0.05
1277	1.05019	0.05
1278	1.2361	0.05
1279	1.69055	0.05
1280	2.25056	0.0635245
1281	1.10574	0.0629423
1282	1.21632	1.0405
1283	1.10574	1.08722
1284	1.46181	2.1
1285	0.946131	0.197381
1286	1.74111	1.44326
1287	1.74111	1.07442
1288	0.94613	1.0736
1289	1.26891	1.0021
1290	1.26891	0.932325
1291	1.23452	1.31981
1292	1.00255	0.639992
1293	1.72286	0.05
1294	1.12233	0.05
1295	1.30682	0.05
1296	1.97397	0.05
1297	1.67425	2.1
1298	1.77775	2.1
1299	1.73571	2.1
1300	0.426265	2.1
1301	1.31817	1.92127
1302	1.81042	2.1
1303	1.85456	2.1
1304	0.466532	1.94656
1305	1.28025	1.71058
1306	0.964467	1.55478
1307	1.27921	2.1
1308	2.11851	1.96345
1309	2.37121	2.1
1310	1.49357	0.22124
1311	1.42486	2.1
1312	1.00278	2.1
1313	1.00278	2.1
1314	1.12503	2.1
1315	0.568697	2.1
1316	0.555211	2.1
1317	0.982272	2.1
1318	1.57558	2.1
1319	0.851539	2.1
1320	1.07307	2.1
1321	1.58292	2.1
1322	1.50731	2.1
1323	0.851541	1.65941
1324	1.00304	2.1
1325	1.36003	2.1
1326	1.13127	2.1
1327	0.672234	2.1
1328	0.763044	2.1
1329	0.966979	2.1
1330	1.31502	2.1
1331	1.67969	2.1

1332	1.62408	2.1
1333	0.661955	2.1
1334	1.35048	2.1
1335	1.40447	2.1
1336	1.40447	2.1
1337	0.607961	2.1
1338	0.946131	2.1
1339	0.607963	2.1
1340	1.01531	2.1
1341	1.01531	2.1
1342	1.91802	2.1
1343	0.777749	2.1
1344	1.22585	0.05
1345	1.20672	0.05
1346	1.03166	0.05
1347	0.553805	2.1
1348	2.00551	2.1
1349	2.07203	2.1
1350	0.589479	2.1
1351	1.22352	2.1
1352	0.984227	2.1
1353	0.802455	2.1
1354	1.40942	2.1
1355	1.6662	2.1
1356	0.802485	2.1
1357	0.936669	2.1
1358	1.31127	0.195179
1359	0.946132	0.167488
1360	1.09729	0.0601839
1361	0.946133	0.225615
1362	1.68703	0.06037
1363	1.68703	0.05
1364	1.3689	0.110068
1365	1.26426	0.111775
1366	1.26923	2.1
1367	1.23614	2.1
1368	0.828371	2.1
1369	2.04556	2.1
1370	0.772648	2.1
1371	0.791059	2.1
1372	0.809958	2.1
1373	1.70012	2.1
1374	0.267032	2.1
1375	1.67604	2.1
1376	0.803823	2.1
1377	0.267305	2.1
1378	0.80246	2.1
1379	0.874582	2.1
1380	1.00188	2.1
1381	0.709588	2.1
1382	1.50679	2.1
1383	1.22456	2.1
1384	0.870622	2.1
1385	1.05754	2.1
1386	1.1689	2.1
1387	1.54556	2.1
1388	1.89226	2.1
1389	0.995311	2.1

1390	1.26195	2.1
1391	1.9	2.1
1392	0.845055	2.1
1393	0.737836	2.1
1394	0.823707	2.1
1395	0.626587	2.1
1396	1.16109	2.1
1397	0.85517	2.1
1398	0.932036	2.1
1399	1.08321	2.1
1400	1.43863	2.1
1401	2.72297	2.1
1402	2.90798	2.1
1403	1.47818	1.24974
1404	1.47819	1.27808
1405	0.694357	1.17736
1406	0.694382	1.2055
1407	0.946132	0.927154
1408	0.694382	1.09678
1409	1.22641	1.01665
1410	1.31155	1.52067
1411	2.45282	1.27872
1412	0.787843	1.02519
1413	0.808984	0.874293
1414	1.14232	1.1569
1415	0.795037	1.19964
1416	0.887382	1.07077
1417	1.14493	1.02348
1418	0.78393	0.950112
1419	1.09129	1.18238
1420	0.791482	1.10119
1421	1.1137	1.41997
1422	1.11819	1.2186
1423	0.960173	1.11806
1424	1.09447	1.53358
1425	0.874365	1.09308
1426	1.2474	0.05
1427	1.75038	0.05
1428	1.75038	0.104287
1429	0.94613	0.197307
1430	1.30076	0.05
1431	1.12817	0.692212
1432	0.94613	0.144341
1433	1.30076	0.114212
1434	1.51841	0.05
1435	1.51841	0.0751878
1436	0.946132	0.516252
1437	1.39575	0.05
1438	0.970358	1.32406
1439	0.946133	0.247003
1440	1.39575	0.155691
1441	1.06486	0.05
1442	1.06486	0.05
1443	1.38622	0.05
1444	1.04521	0.05
1445	1.00809	0.05
1446	1.28952	0.05
1447	0.849316	0.05

1448	0.946132	0.05
1449	0.849344	0.05
1450	1.53631	0.05
1451	1.53631	0.05
1452	1.77843	0.05
1453	1.69863	0.05
1454	1.91646	2.1
1455	1.8582	2.1
1456	2.8349	0.05
1457	1.36307	0.05
1458	1.23886	0.05
1459	1.12971	0.05
1460	1.59662	0.05
1461	1.59662	0.05
1462	0.640304	0.05
1463	0.640333	0.05
1464	0.886276	0.05
1465	0.886276	0.05
1466	1.35065	0.05
1467	2.09002	0.05
1468	1.35543	0.05
1469	1.94688	2.1
1470	1.97221	2.1
1471	0.719563	2.1
1472	1.20358	2.1
1473	0.874264	2.1
1474	1.27676	2.1
1475	1.46557	2.1
1476	1.46557	2.1
1477	0.68546	2.1
1478	0.94613	2.1
1479	0.685489	2.1
1480	1.39615	0.05
1481	1.05404	0.05
1482	1.06474	0.05
1483	1.00713	0.05
1484	0.714535	0.0556907
1485	0.714563	0.0966493
1486	1.41646	0.05
1487	1.52582	0.05
1488	0.721694	0.05
1489	0.93665	0.05
1490	1.21067	0.172104
1491	1.21067	0.111596
1492	1.73577	0.368701
1493	1.17799	0.107545
1494	3.21432	0.0639249
1495	2.41452	0.0678863
1496	1.36208	0.05
1497	2.53256	0.05
1498	0.834593	0.05
1499	2.5853	0.05
1500	1.77259	0.05
1501	0.615724	0.05
1502	1.11374	0.149291
1503	0.615726	0.05
1504	0.946129	0.412522
1505	0.615714	0.313614

1506	1.75489	0.138451
1507	1.75489	0.0767127
1508	1.23146	0.05
1509	2.2772	0.05
1510	0.74463	0.05
1511	1.3546	0.05
1512	1.80252	0.05
1513	2.38304	0.05
1514	1.19151	0.0953978
1515	1.1231	0.0621371
1516	1.82345	0.05
1517	1.79805	0.05
1518	0.761337	0.207962
1519	0.946127	0.0679901
1520	0.557726	0.0987016
1521	1.05353	0.05
1522	1.05353	0.05
1523	1.68599	0.05
1524	1.05353	0.05
1525	1.40588	2.1
1526	1.09373	2.1
1527	1.18382	1.12393
1528	1.46903	1.05673
1529	1.25103	2.1
1530	1.10378	2.1
1531	1.58941	2.1
1532	1.29639	2.1
1533	1.14874	0.05
1534	0.899817	0.05
1535	0.666898	0.05
1536	1.76519	0.05
1537	1.17289	0.05
1538	1.03526	0.05
1539	1.97708	0.05
1540	1.31711	0.05
1541	0.992517	0.05
1542	1.70193	0.05
1543	1.13852	0.05
1544	1.23943	0.05
1545	1.40014	2.1
1546	1.04598	2.1
1547	1.1483	2.1
1548	1.11433	1.01345
1549	1.32037	0.386481
1550	1.00381	0.92447
1551	1.11435	0.279941
1552	0.973502	1.06061
1553	0.946128	1.6409
1554	1.83697	1.24786
1555	1.83697	2.1
1556	1.31721	1.66027
1557	1.38614	1.29905
1558	1.39696	1.21979
1559	1.31721	1.66851
1560	1.0756	1.84783
1561	1.08542	1.09086
1562	0.946127	2.1
1563	1.81052	0.988443

1564	1.81052	2.1
1565	1.24315	2.1
1566	1.39696	0.95333
1567	1.35969	1.33831
1568	1.24315	1.33284
1569	1.0756	0.125424
1570	1.20669	0.05
1571	1.18508	0.05
1572	1.71059	2.1
1573	1.66762	2.1
1574	2.50853	2.1
1575	1.8236	2.1
1576	1.78872	2.1
1577	1.41721	2.1
1578	1.01335	1.55206
1579	1.42439	1.11523
1580	0.946129	0.89619
1581	1.33266	1.24145
1582	1.79776	1.43762
1583	1.79776	2.1
1584	0.946131	0.365419
1585	1.33266	1.49187
1586	1.41123	1.39668
1587	1.25346	2.1
1588	0.946132	0.982932
1589	0.827287	1.88593
1590	1.57878	1.21984
1591	1.57878	1.20125
1592	0.827287	1.10921
1593	1.07341	1.1415
1594	1.33266	1.21725
1595	1.25346	1.25344
1596	1.61149	2.1
1597	1.15644	1.9858
1598	1.27558	1.03874
1599	1.3764	0.305016
1600	1.89526	1.16889
1601	1.37641	0.318477
1602	1.2064	0.1456
1603	1.38426	2.1
1604	1.01595	2.1
1605	1.15067	2.1
1606	1.82276	2.1
1607	1.82276	0.05
1608	0.946131	0.882028
1609	1.40609	0.05
1610	0.946132	2.1
1611	1.29143	2.1
1612	1.40609	2.00072
1613	1.30272	2.1
1614	1.19548	2.1
1615	2.95451	2.1
1616	1.46124	2.1
1617	2.93215	2.1
1618	1.42105	2.1
1619	1.75477	0.850055
1620	2.31301	1.15891
1621	2.10275	0.357969

1622	1.80099	0.440537
1623	0.97541	2.1
1624	1.49733	2.1
1625	0.97541	2.1
1626	1.30751	2.1
1627	1.28614	0.05
1628	1.74759	0.05
1629	1.19756	0.05
1630	1.71053	0.05
1631	1.19757	0.0519887
1632	1.28954	0.05
1633	2.02703	0.05
1634	2.29453	0.05
1635	1.28265	0.05
1636	0.793267	0.05
1637	1.19974	0.05
1638	0.509551	0.05
1639	1.04072	0.05
1640	1.35302	0.05
1641	1.53133	0.05
1642	0.566153	0.05
1643	1.09634	0.05
1644	1.12792	0.05
1645	1.68908	2.1
1646	1.68908	2.1
1647	0.768212	2.1
1648	0.768213	2.1
1649	0.946132	2.1
1650	0.768212	2.1
1651	1.32461	2.1
1652	1.87291	2.1
1653	2.64921	2.1
1654	1.53642	2.1
1655	2.26537	2.1
1656	1.53642	2.1
1657	1.79874	2.1
1658	1.79874	2.1
1659	0.726635	2.01142
1660	0.726636	2.1
1661	0.946128	2.1
1662	0.726634	2.09619
1663	1.23339	1.77339
1664	0.992674	2.1
1665	1.8023	2.1
1666	1.12324	0.05
1667	1.29163	0.05
1668	0.996831	0.05
1669	1.0019	0.05
1670	0.993831	0.05
1671	1.07283	2.1
1672	1.04788	2.1
1673	1.44489	0.947213
1674	1.04788	2.1
1675	1.07652	2.1
1676	0.991406	2.1
1677	1.37	2.1
1678	1.14195	2.1
1679	1.47904	0.05

1680	1.26593	2.08015
1681	1.75366	1.58218
1682	1.57621	0.0848076
1683	1.24538	0.05
1684	0.969118	2.1
1685	0.946133	1.19682
1686	1.69387	2.1
1687	1.69387	2.1
1688	0.94613	2.1
1689	1.28713	2.1
1690	1.28713	2.1
1691	1.23877	2.1
1692	0.969118	2.1
1693	1.22615	2.1
1694	0.98084	2.1
1695	1.01843	2.1
1696	1.50876	2.1
1697	1.85391	2.1
1698	1.84322	2.1
1699	1.70377	2.1
1700	1.78919	2.1
1701	2.66787	2.1
1702	1.41549	2.1
1703	1.41549	2.1
1704	0.86707	2.1
1705	0.896172	2.1
1706	0.946126	2.1
1707	0.867074	2.1
1708	0.946118	2.1
1709	1.54468	0.05
1710	1.54468	0.05
1711	2.27259	0.05
1712	0.946131	0.05
1713	1.25837	0.05
1714	0.663171	0.0665615
1715	1.68931	0.05
1716	1.68931	0.05
1717	1.25837	0.05
1718	1.09411	0.05
1719	1.73571	0.05
1720	2.34894	0.05
1721	0.946128	0.05
1722	1.30497	0.05
1723	0.772312	0.05
1724	1.66895	0.05
1725	1.66895	0.05
1726	1.30497	0.05
1727	1.13629	0.05
1728	1.22852	0.933043
1729	1.578	0.919742
1730	2.07781	2.1
1731	1.5478	2.1
1732	1.70048	2.1
1733	1.42329	2.1
1734	1.05481	2.08693
1735	1.1032	1.20571
1736	1.19267	2.1
1737	1.77324	2.1

1738	1.01121	0.0553229
1739	0.991867	0.05
1740	2.0744	1.38998
1741	1.75142	2.1
1742	1.35418	1.7629
1743	1.40041	2.1
1744	1.50045	2.1
1745	1.37704	2.1
1746	1.50045	2.1
1747	1.87875	2.1
1748	1.35308	0.830161
1749	1.29983	0.822571
1750	1.62784	2.1
1751	1.44807	2.1
1752	1.00965	2.1
1753	2.69264	2.1
1754	1.04075	2.1
1755	0.946131	2.1
1756	0.946132	2.1
1757	1.52704	2.1
1758	1.83246	2.1
1759	1.56757	2.1
1760	1.16687	2.1
1761	2.04857	2.1
1762	1.32707	2.1
1763	1.25519	0.05
1764	1.32101	0.05
1765	1.25055	0.88918
1766	0.827853	0.05
1767	1.11835	0.05
1768	1.07281	2.1
1769	1.54302	0.05
1770	0.724064	0.837263
1771	1.32709	2.1
1772	3.44123	1.49346
1773	2.46889	0.05
1774	0.730076	0.05
1775	1.25809	0.05
1776	0.730077	0.05
1777	0.946129	0.05
1778	1.72777	0.05
1779	1.72777	0.05
1780	0.73005	0.05
1781	1.7522	0.05
1782	2.51618	0.05
1783	0.628177	0.05
1784	1.1068	0.05
1785	0.747435	0.05
1786	0.946133	0.05
1787	1.84668	0.05
1788	1.8563	0.05
1789	0.628177	0.05
1790	1.45501	0.05
1791	2.19809	0.05
1792	1.45501	0.05
1793	1.48974	0.05
1794	1.7709	2.1
1795	2.63288	2.1

1796	1.5362	2.1
1797	1.56327	2.1
1798	3.03945	0.05
1799	0.899979	0.05
1800	0.869386	0.05
1801	1.85864	0.05
1802	1.78028	0.05
1803	1.28719	0.05
1804	1.09532	0.05
1805	0.881898	0.05
1806	1.28911	0.05
1807	1.39644	0.05
1808	0.688829	0.05
1809	2.14611	0.05
1810	1.80313	0.05
1811	0.68883	0.05
1812	0.946131	0.05
1813	1.25334	0.05
1814	1.88987	0.05
1815	1.94432	0.05
1816	1.37592	0.05
1817	1.11691	2.1
1818	1.02923	2.1
1819	0.45824	0.05
1820	0.646512	0.05
1821	2.46292	0.05
1822	0.493451	0.05
1823	2.00826	2.1
1824	2.49784	2.1
1825	1.17054	2.1
1826	1.01485	2.1
1827	1.26045	2.1
1828	1.17054	2.1
1829	1.44247	2.1
1830	2.02687	0.05
1831	1.66365	0.05
1832	1.64656	0.05
1833	1.56597	0.05
1834	1.21419	2.1
1835	1.5008	2.1
1836	1.10711	2.1
1837	1.44287	2.1
1838	1.44287	2.1
1839	0.750368	2.1
1840	1.25876	2.1
1841	0.82542	2.1
1842	1.04074	2.1
1843	1.25874	2.1
1844	1.04159	0.05
1845	1.2489	0.05
1846	0.980547	0.05
1847	1.62235	0.05
1848	1.15991	0.05
1849	0.980547	0.05
1850	0.94613	0.0606911
1851	1.54662	0.05
1852	1.54661	0.0548678
1853	1.07912	0.0810471

1854	1.1032	0.05
1855	1.1032	0.05
1856	0.94613	0.0540884
1857	1.12468	2.1
1858	1.2724	2.1
1859	1.06521	2.1
1860	1.0752	2.1
1861	1.94936	0.0617866
1862	1.62772	0.05
1863	1.94733	0.05
1864	1.42901	0.05
1865	1.33022	2.1
1866	1.70839	2.1
1867	1.75048	2.1
1868	1.41201	2.1
1869	1.52314	2.1
1870	1.44039	2.1
1871	1.51113	2.1
1872	2.58638	2.1
1873	0.987247	0.05
1874	1.38622	0.05
1875	0.858359	0.05
1876	1.19967	0.05
1877	1.06414	0.05
1878	0.946132	0.05
1879	0.94613	0.05
1880	0.687079	0.0856161
1881	0.946132	0.05
1882	0.822611	0.05
1883	1.70242	0.05
1884	1.70242	0.05
1885	0.946132	0.179433
1886	1.11155	0.0626947
1887	0.946132	0.259215
1888	1.11155	0.107881
1889	1.37981	0.0751741
1890	1.18348	0.05
1891	2.44179	0.05
1892	2.00403	0.101339
1893	1.10961	2.1
1894	1.85415	2.1
1895	1.44825	2.1
1896	0.932272	2.1
1897	0.810942	2.1
1898	1.00189	2.1
1899	1.11779	2.1
1900	1.3359	2.1
1901	0.965279	2.1
1902	0.833779	2.1
1903	1.05337	2.1
1904	0.526656	2.1
1905	1.44599	2.1
1906	1.44599	2.1
1907	1.24853	2.1
1908	0.946132	2.1
1909	1.24853	2.1
1910	0.724122	2.1
1911	1.91547	2.1

1912	2.49705	2.1
1913	1.05931	2.1
1914	1.1729	2.1
1915	1.34526	2.1
1916	1.5379	2.1
1917	1.90855	2.1
1918	2.08597	2.1
1919	1.68165	2.1
1920	1.45887	2.1
1921	1.50022	2.1
1922	1.4748	2.1
1923	2.63645	2.07106
1924	1.74431	2.1
1925	1.25288	2.08018
1926	1.25289	1.77908
1927	0.970025	1.87102
1928	0.747542	2.1
1929	0.810959	1.60516
1930	1.15369	1.89449
1931	0.977472	1.86093
1932	1.12695	1.90289
1933	0.996762	1.86344
1934	1.28249	0.873462
1935	0.946132	0.944532
1936	0.528621	1.02515
1937	1.59186	0.685405
1938	1.44072	0.751709
1939	0.633488	0.751557
1940	0.598506	0.928912
1941	1.51618	1.00484
1942	1.19999	0.520831
1943	1.46511	0.659783
1944	1.44056	0.492622
1945	0.912754	0.144542
1946	1.4725	0.0556288
1947	1.4725	0.05
1948	0.912753	0.121925
1949	1.23351	0.17885
1950	1.23351	0.227948
1951	0.946131	0.385291
1952	1.17129	0.05
1953	1.82551	0.05
1954	1.27575	2.06373
1955	0.946129	2.1
1956	0.946129	2.1
1957	0.946128	2.1
1958	0.792754	2.1
1959	0.94613	1.88674
1960	0.792752	1.86145
1961	2.08054	2.1
1962	1.20091	2.1
1963	2.1669	2.1
1964	1.24383	1.91341
1965	0.904669	2.05532
1966	0.83804	1.62445
1967	1.90486	1.97333
1968	1.29162	1.82255
1969	1.37383	2.04211

1970	1.86533	1.83582
1971	1.76472	2.06524
1972	1.60428	1.82577
1973	2.43993	2.06853
1974	1.37833	2.1
1975	1.18632	2.1
1976	0.946132	2.1
1977	1.27544	2.1
1978	1.05085	2.1
1979	0.961178	2.1
1980	0.859844	2.1
1981	1.0499	2.1
1982	1.75592	2.1
1983	2.08668	2.1
1984	1.75592	2.1
1985	1.4402	2.1
1986	1.72285	2.1
1987	0.83312	0.05
1988	1.07295	0.05
1989	1.37607	0.05
1990	1.32097	0.05
1991	1.11332	0.05
1992	1.18568	0.05
1993	0.877482	0.05
1994	1.4458	0.05
1995	2.34112	0.05
1996	2.68021	0.05
1997	0.681677	0.05
1998	0.946131	0.05
1999	0.681649	0.05
2000	1.83115	0.05
2001	1.68029	0.05
2002	1.05482	0.05
2003	1.31485	0.05
2004	2.08247	0.674943
2005	1.33153	0.441649
2006	0.935062	0.597705
2007	0.806958	1.80662
2008	0.827683	0.497419
2009	1.01041	0.877438
2010	0.741832	0.449592
2011	1.15668	1.05485
2012	0.908649	0.645531
2013	0.59561	0.05
2014	1.19028	0.203295
2015	0.59561	0.257166
2016	0.946132	0.252652
2017	0.595623	0.524677
2018	1.58164	0.233834
2019	1.58164	0.329872
2020	0.946131	2.1
2021	1.55201	2.1
2022	1.55201	2.1
2023	0.946127	2.1
2024	1.13312	2.1
2025	1.13312	2.1
2026	1.08108	2.1
2027	1.04973	2.1

2028	1.20149	2.1
2029	1.86902	0.51225
2030	0.613447	2.1
2031	1.22618	1.21567
2032	1.38663	1.85437
2033	1.38662	2.1
2034	0.613447	1.46076
2035	1.22617	2.1
2036	0.773899	2.1
2037	1.23515	1.95843
2038	1.87823	1.35755
2039	1.70303	0.679518
2040	1.4714	2.1
2041	1.74067	2.1
2042	0.735699	2.1
2043	1.73461	2.1
2044	1.47224	2.1
2045	1.22827	2.1
2046	1.1634	2.1
2047	1.36471	2.1
2048	0.735701	2.1
2049	2.8386	0.199079
2050	2.39056	2.1
2051	2.44128	0.949784
2052	1.54102	2.1
2053	1.54102	2.1
2054	0.936141	2.1
2055	1.40594	2.1
2056	1.07121	0.229101
2057	0.936142	2.1
2058	1.07121	2.1
2059	1.22691	0.05
2060	1.42907	0.05
2061	2.18048	0.05
2062	0.947662	0.05
2063	2.18394	2.1
2064	1.21292	2.1
2065	2.18394	2.1
2066	2.19064	2.1
2067	1.27229	2.1
2068	1.20859	0.852121
2069	1.21073	0.869006
2070	1.73899	2.1
2071	1.3164	2.1
2072	1.61245	2.1
2073	1.41444	2.1
2074	2.55116	2.1
2075	1.26193	2.1
2076	1.89553	2.1
2077	0.916438	2.1
2078	1.26113	2.1
2079	0.810934	2.1
2080	2.00419	2.1
2081	1.27872	2.1
2082	1.35306	2.1
2083	1.96749	2.1
2084	1.07955	2.1
2085	1.31854	2.1

2086	1.29505	0.849093
2087	1.29504	0.8966
2088	0.612469	0.968799
2089	0.990053	0.967045
2090	1.04072	0.916375
2091	0.985266	0.907139
2092	0.990039	0.877759
2093	1.06892	1.058
2094	1.57158	0.737362
2095	1.895	0.723932
2096	1.05698	0.569863
2097	0.925053	2.1
2098	0.624712	2.1
2099	1.42465	2.1
2100	1.49014	2.1
2101	0.687198	2.1
2102	1.22058	2.1
2103	0.851488	2.1
2104	0.588128	2.1
2105	1.12452	2.1
2106	1.02783	2.1
2107	1.7083	2.1
2108	1.06977	0.921286
2109	0.818569	0.830953
2110	1.14386	1.23702
2111	0.983791	0.899381
2112	1.12214	1.34723
2113	1.0915	0.892366
2114	0.810934	0.851814
2115	0.764114	0.978255
2116	1.87956	0.945549
2117	1.87956	1.27903
2118	0.764141	0.992847
2119	1.37675	0.883599
2120	0.764141	0.960387
2121	0.946132	0.920589
2122	1.9637	1.71887
2123	2.10107	0.879362
2124	1.67814	1.03725
2125	3.02886	1.00542
2126	0.908982	0.05
2127	0.621727	0.05
2128	0.525963	0.05
2129	0.946128	0.05
2130	1.60979	0.05
2131	1.45596	0.05
2132	0.556172	0.05
2133	2.06779	0.05
2134	2.17911	0.05
2135	1.00205	0.05
2136	1.30685	0.05
2137	1.16534	0.05
2138	1.65532	2.1
2139	2.20346	2.1
2140	0.993889	2.1
2141	1.09328	2.1
2142	2.09372	2.1
2143	2.10036	2.1

2144	1.33727	1.41385
2145	2.53383	1.57046
2146	0.893379	1.86541
2147	1.52828	1.2933
2148	1.09599	0.05
2149	1.09599	0.05
2150	1.50524	2.1
2151	1.54711	2.1
2152	1.41469	2.1
2153	1.99459	1.9945
2154	1.79805	2.1
2155	1.79805	2.1
2156	0.707344	2.1
2157	1.30318	2.1
2158	0.707344	2.1
2159	0.946129	2.1
2160	1.30318	2.1
2161	2.40442	2.1
2162	1.89226	2.1
2163	1.40794	2.1
2164	0.898721	2.1
2165	0.791921	1.2972
2166	0.810933	2.1
2167	1.10904	2.00784
2168	0.991102	2.1
2169	0.808502	0.973875
2170	1.1398	2.1
2171	1.31346	2.1
2172	1.11833	2.1
2173	1.669 2.1	
2174	1.25715	2.1
2175	1.3199	2.1
2176	1.63092	2.1
2177	1.63092	2.1
2178	0.946131	2.1
2179	0.946131	2.1
2180	1.13534	2.1
2181	0.979853	2.1
2182	2.19994	2.1
2183	0.725844	2.1
2184	2.19994	2.1
2185	2.35055	2.1
2186	1.52204	2.1
2187	1.40118	2.1
2188	3.42285	2.1
2189	2.05217	2.1
2190	1.50319	2.1
2191	0.947516	1.4898
2192	0.903572	1.42799
2193	0.76996	1.56171
2194	1.90959	2.1
2195	1.18897	2.1
2196	1.24166	2.1
2197	2.26785	1.96264
2198	2.05217	2.1
2199	2.73306	2.1
2200	1.42729	0.05
2201	1.387 0.05	

2202	1.59458	0.361354
2203	1.91051	0.303619
2204	1.68108	0.753762
2205	2.03357	0.800157
2206	0.928982	0.05
2207	1.55687	0.05
2208	1.55687	0.05
2209	0.92898	0.05
2210	1.3495	0.05
2211	1.3495	0.05
2212	0.946133	0.05
2213	1.85795	0.05
2214	2.2727	0.0575218
2215	1.20324	1.04071
2216	1.07784	0.05
2217	0.957792	0.05
2218	1.2745	0.05
2219	1.4659	0.05
2220	0.946129	0.05
2221	0.95778	0.05
2222	0.851553	0.05
2223	1.74103	0.05
2224	2.28994	0.05
2225	2.08213	0.05
2226	2.1556	0.05
2227	2.20372	0.05
2228	0.838027	0.05
2229	1.11373	0.05
2230	1.2909	0.05
2231	0.998424	0.05
2232	1.40621	0.05
2233	1.19478	0.05
2234	1.11783	0.05
2235	1.6695	0.05
2236	1.7498	0.05
2237	0.477652	0.05
2238	1.77291	0.05
2239	0.590448	0.05
2240	1.07668	0.05
2241	0.810946	0.05
2242	1.1946	0.05
2243	2.03914	0.05
2244	1.25585	0.05
2245	2.02622	0.05
2246	1.39091	0.05
2247	1.31871	0.05
2248	1.15789	2.1
2249	1.15755	2.1
2250	1.82268	2.1
2251	1.87119	2.1
2252	3.10636	2.1
2253	2.96285	2.1
2254	1.73443	0.907212
2255	2.33812	0.918624
2256	1.59457	0.596835
2257	1.73443	1.13511
2258	2.00665	2.1
2259	1.99841	2.1

2260	1.19901	2.1
2261	1.16661	2.1
2262	2.15883	0.05
2263	1.20951	0.05
2264	2.49192	0.05
2265	0.65112	0.05
2266	2.45317	0.05
2267	1.73091	0.05
2268	2.0795	0.05
2269	1.45891	0.05
2270	1.6531	0.05
2271	1.78304	0.05
2272	0.775132	0.05
2273	1.35051	0.05
2274	1.0461	0.05
2275	1.37837	0.05
2276	1.00259	0.05
2277	1.25796	0.354655
2278	1.10719	0.468608
2279	1.51067	2.1
2280	2.65632	2.1
2281	1.30521	2.1
2282	2.456	2.1
2283	3.19218	2.1
2284	2.67387	2.1
2285	0.988862	2.1
2286	1.917	2.1
2287	0.946132	2.1
2288	1.16202	2.1
2289	1.53871	2.1
2290	1.53871	2.1
2291	0.946132	2.1
2292	1.16202	2.1
2293	1.32282	2.1
2294	2.46356	2.1
2295	2.32404	2.1
2296	1.37562	0.05
2297	0.985546	0.05
2298	1.28983	0.05
2299	1.05566	0.05
2300	0.895312	0.05
2301	1.53325	0.05
2302	1.04342	0.05
2303	1.80881	0.05
2304	0.876865	0.05
2305	0.5689	0.05
2306	1.09506	0.05
2307	1.51789	2.1
2308	1.49872	2.1
2309	0.949364	0.492879
2310	2.01233	0.563444
2311	1.55063	0.399273
2312	1.57783	0.314186
2313	1.57783	0.569546
2314	0.674605	0.511331
2315	0.98537	0.0565709
2316	0.674633	0.05
2317	0.98537	0.0595294

2318	1.26706	0.317432
2319	0.980664	0.670913
2320	1.03298	0.198006
2321	1.84936	0.577716
2322	1.51696	0.343293
2323	1.97074	0.246659
2324	1.03298	0.108832
2325	1.51324	0.05
2326	1.51324	0.102749
2327	0.758454	0.05
2328	1.28632	0.05
2329	0.758482	0.05
2330	0.758482	0.05
2331	0.985372	0.05
2332	1.14105	0.05
2333	1.59787	0.05
2334	2.46244	0.0683221
2335	1.70367	0.05
2336	2.13757	1.20641
2337	1.98142	1.21222
2338	1.35878	0.05
2339	2.39947	0.05
2340	1.24554	0.05
2341	1.7522	0.05
2342	1.49693	1.97347
2343	1.49652	2.04048
2344	1.6211	1.97533
2345	1.45938	2.06305
2346	0.737431	2.1
2347	1.18643	2.06827
2348	1.04072	2.1
2349	0.9459	2.00341
2350	0.66364	2.1
2351	0.985109	1.65802
2352	0.638368	2.04406
2353	0.83804	1.50921
2354	0.998024	1.65717
2355	0.914471	1.2476
2356	1.14281	1.72274
2357	0.833972	1.72812
2358	0.991865	0.4011
2359	1.42208	0.0726285
2360	1.70718	2.1
2361	1.79888	2.1
2362	1.16851	0.0732493
2363	1.16851	0.05
2364	1.51021	0.05
2365	0.946132	0.05
2366	1.14897	0.05
2367	0.946132	0.05
2368	1.77177	0.05
2369	1.77177	0.05
2370	1.33038	0.05
2371	1.38752	0.05
2372	1.1726	0.05
2373	1.51688	0.05
2374	1.06455	2.1
2375	0.98511	2.1

2376	1.31144	2.1
2377	1.79825	2.1
2378	1.79825	2.1
2379	1.73044	2.1
2380	1.59589	0.05
2381	1.59589	0.05
2382	2.78009	2.1
2383	1.68761	2.1
2384	3.05511	2.1
2385	2.25318	2.1
2386	0.578998	2.1
2387	1.03512	2.1
2388	2.26156	2.1
2389	1.44859	1.07331
2390	1.35897	1.40755
2391	1.18301	2.1
2392	1.25173	2.1
2393	1.98851	2.1
2394	2.70685	2.1
2395	0.682342	2.1
2396	1.52491	2.1
2397	1.37119	2.1
2398	0.682313	2.1
2399	1.35342	2.1
2400	0.947555	2.1
2401	0.614107	2.1
2402	1.23007	2.1
2403	1.5735	2.1
2404	1.58713	2.1
2405	1.57979	0.05
2406	1.30723	0.05
2407	0.935658	0.947816
2408	0.8952	0.906723
2409	1.27352	1.082
2410	1.0985	1.04058
2411	1.07163	0.918435
2412	1.15823	0.980859
2413	0.836451	0.904457
2414	2.08051	0.794186
2415	1.92601	1.20442
2416	2.0644	0.854166
2417	1.37259	2.1
2418	1.37259	2.1
2419	0.752217	2.1
2420	1.28569	2.1
2421	0.752186	2.1
2422	0.839124	2.1
2423	1.28568	2.1
2424	1.04199	2.1
2425	1.04199	2.1
2426	2.59268	2.1
2427	1.04199	2.1
2428	1.5098	2.1
2429	1.5098	2.1
2430	0.520997	2.1
2431	1.29634	2.1
2432	0.520996	2.1
2433	0.73446	2.1

2434	1.29634	2.1
2435	0.983724	2.1
2436	1.23914	2.1
2437	2.65095	2.1
2438	1.52851	2.1
2439	2.399 2.1	
2440	1.68137	2.1
2441	1.11285	2.1
2442	1.04921	2.1
2443	1.30711	2.1
2444	1.04921	2.1
2445	1.07496	1.13944
2446	1.55831	0.868172
2447	1.07497	0.0951496
2448	1.07497	1.01796
2449	1.27391	2.1
2450	2.5615	2.1
2451	1.2739	2.1
2452	1.75253	2.1
2453	1.12907	0.05
2454	1.9194	0.0593678
2455	1.96617	0.05
2456	1.01225	2.1
2457	0.947979	2.1
2458	1.01225	1.57157
2459	1.06561	2.1
2460	1.01225	2.1
2461	3.56876	2.1
2462	3.68767	2.1
2463	1.52301	2.1
2464	1.61013	2.1
2465	1.43726	2.1
2466	2.04275	2.1
2467	0.995734	2.1
2468	1.16199	2.1
2469	1.73704	0.05
2470	1.04019	0.05
2471	0.956294	2.1
2472	1.81488	2.1
2473	1.63719	2.1
2474	1.50103	0.05
2475	1.75289	0.05
2476	1.31491	0.05
2477	1.03567	0.05
2478	0.672257	0.05
2479	1.04072	0.05
2480	0.746903	0.05
2481	1.43317	0.05
2482	1.39986	0.05
2483	1.06385	0.05
2484	1.1624	0.05
2485	1.61667	0.05
2486	1.27258	0.05
2487	2.559 0.05	
2488	1.66093	0.05
2489	1.10719	0.768965
2490	2.03235	0.773861
2491	1.17105	1.00132

2492	1.10719	0.786595
2493	1.3028	2.1
2494	0.946131	2.1
2495	0.946127	2.1
2496	1.52912	2.1
2497	1.52912	2.1
2498	0.946128	2.1
2499	0.949997	2.1
2500	1.31565	2.1
2501	1.46643	2.1
2502	1.5482	2.1
2503	1.47102	2.1
2504	1.47102	2.1
2505	0.774101	2.1
2506	1.30583	2.1
2507	0.774103	2.1
2508	0.939287	2.1
2509	1.30584	2.1
2510	0.958661	2.1
2511	2.28835	2.1
2512	1.43213	0.05
2513	2.13255	0.05
2514	1.43213	0.05
2515	1.10476	0.05
2516	1.93165	0.05
2517	1.03235	1.07183
2518	1.31	1.0912
2519	1.22082	2.1
2520	1.35827	2.1
2521	0.790936	2.1
2522	0.716644	2.1
2523	1.43257	2.1
2524	1.43257	2.1
2525	0.716615	2.1
2526	0.946131	2.1
2527	0.730157	2.1
2528	1.47212	2.1
2529	1.47212	2.1
2530	0.946131	2.1
2531	0.790792	2.1
2532	1.41149	2.1
2533	0.790791	2.1
2534	1.01006	2.1
2535	1.53045	0.05
2536	1.53045	0.0755858
2537	0.946132	0.534201
2538	1.40779	0.0563509
2539	1.06879	1.11222
2540	0.990713	0.18766
2541	1.06879	0.24277
2542	1.06486	0.05
2543	1.18347	0.05
2544	1.38622	0.05
2545	1.18347	0.05
2546	1.7304	0.05
2547	1.7304	0.05
2548	0.946131	0.265216
2549	1.26874	0.0590989

2550	1.13337	0.952119
2551	1.13337	0.144981
2552	1.40779	0.146846
2553	1.695	2.1
2554	1.77071	2.1
2555	0.649313	2.1
2556	0.692829	2.1
2557	1.40395	2.1
2558	1.34243	2.1
2559	0.813292	0.0515163
2560	0.632691	0.05
2561	1.75766	0.75398
2562	2.25727	0.895933
2563	1.74206	0.05
2564	0.826073	0.159444
2565	1.30022	0.554758
2566	1.45641	0.124386
2567	0.946131	0.0835756
2568	0.752245	0.376629
2569	0.960952	0.414283
2570	0.743477	0.05
2571	1.04355	0.0579683
2572	1.6253	0.05
2573	0.960577	0.05
2574	1.34852	1.01302
2575	1.03235	1.06996
2576	1.78168	0.875043
2577	1.2966	0.05
2578	2.21116	0.05
2579	1.6395	0.05
2580	1.6395	0.05
2581	0.94613	0.05
2582	1.22187	0.05
2583	0.962737	0.05
2584	0.962735	0.05
2585	1.36376	0.05
2586	1.2966	0.05
2587	1.2966	0.05
2588	1.19669	1.0331
2589	2.37636	1.10322
2590	1.08894	1.18452
2591	1.62551	1.30871
2592	2.51472	1.22775
2593	1.89226	1.06962
2594	0.980038	1.26626
2595	1.10187	2.1
2596	1.01479	2.1
2597	1.81663	2.1
2598	1.83247	2.1
2599	0.921851	0.427892
2600	0.983148	2.1
2601	1.82816	2.1
2602	1.26103	2.1
2603	2.34818	2.1
2604	1.23152	2.1
2605	2.15738	2.1
2606	1.23152	2.1
2607	1.51042	2.1

2608	0.842995	2.1
2609	1.22257	2.1
2610	0.810959	2.1
2611	1.68242	2.1
2612	0.95701	2.1
2613	1.12389	2.1
2614	1.95255	2.1
2615	1.57974	2.1
2616	1.94632	2.1
2617	0.81534	2.1
2618	1.13996	2.1
2619	1.18958	0.05
2620	1.25929	0.05
2621	3.02933	0.873162
2622	2.37434	0.05
2623	1.10609	0.0788255
2624	1.64132	0.080341
2625	1.64132	0.801523
2626	0.946132	0.634457
2627	1.40029	0.05
2628	1.08946	0.124831
2629	1.08946	0.56805
2630	1.18717	0.05
2631	2.71421	0.05
2632	1.08189	0.126988
2633	0.94613	0.205706
2634	1.37109	0.172288
2635	1.17925	0.05
2636	0.851541	0.204305
2637	0.942512	0.167016
2638	1.18541	0.0560762
2639	0.851526	0.215953
2640	1.0819	0.05
2641	1.86009	0.28721
2642	0.946132	0.0650898
2643	1.67409	0.05
2644	1.67409	0.0592961
2645	0.946131	0.0834972
2646	1.31716	0.0573038
2647	1.30307	0.05
2648	0.946131	0.117475
2649	1.18601	0.05
2650	3.15646	2.1
2651	0.985109	2.1
2652	3.37048	2.1
2653	1.10667	2.1
2654	1.77065	1.33886
2655	1.45837	1.17652
2656	1.77063	1.19338
2657	2.58104	1.12683
2658	1.37652	2.1
2659	1.54168	2.1
2660	1.00224	0.05
2661	1.05851	0.05
2662	1.39093	0.05
2663	1.43358	0.05
2664	0.575741	0.05
2665	0.518161	0.05

2666	1.04071	0.05
2667	1.13092	1.31688
2668	1.17042	2.1
2669	1.53347	1.74601
2670	1.52938	1.37153
2671	1.00613	0.286874
2672	1.81618	0.393405
2673	1.81618	0.446248
2674	0.946127	0.844136
2675	1.36479	0.475778
2676	0.94613	0.705066
2677	1.31724	0.314625
2678	1.3648	0.688265
2679	1.00613	0.661015
2680	0.781375	0.05
2681	1.13431	0.05
2682	1.03547	0.104589
2683	1.40668	0.335022
2684	0.94613	0.455192
2685	0.713285	0.524862
2686	1.72885	0.556661
2687	1.72885	0.244497
2688	0.713298	0.561209
2689	3.30075	0.0826718
2690	2.81337	0.201959
2691	1.39296	0.421204
2692	0.830164	2.1
2693	1.50493	2.1
2694	1.50494	2.1
2695	0.830136	2.1
2696	1.28298	2.1
2697	1.0521	2.1
2698	0.830147	2.1
2699	2.0799	2.1
2700	1.52115	1.59153
2701	2.5142	2.1
2702	1.31253	0.90334
2703	1.94446	1.11986
2704	1.18817	1.05034
2705	0.598348	1.62691
2706	1.69315	1.12498
2707	1.69315	1.60374
2708	0.598377	0.937917
2709	0.598347	0.945298
2710	0.94613	0.91866
2711	1.28418	1.06718
2712	2.05205	1.17196
2713	1.28418	1.51452
2714	1.0737	2.1
2715	1.81024	2.1
2716	2.73834	2.1
2717	1.71369	2.1
2718	1.71368	2.1
2719	0.946118	2.1
2720	1.29063	2.1
2721	1.36917	2.1
2722	0.94613	2.1
2723	1.29062	2.1

2724	0.947921	2.1
2725	1.58661	0.0724095
2726	1.37658	1.74792
2727	1.2364	1.71857
2728	1.66685	0.957523
2729	1.66685	2.0566
2730	0.946133	0.906247
2731	0.946133	1.18431
2732	1.11978	1.59176
2733	1.09194	1.01122
2734	1.21858	2.0563
2735	1.37658	1.46086
2736	1.64903	0.908779
2737	1.64903	2.1
2738	0.946132	0.920812
2739	0.946132	1.13768
2740	1.05496	1.0887
2741	1.22851	0.909442
2742	1.22852	2.1
2743	1.58482	0.826388
2744	1.60578	0.05
2745	1.06336	0.05
2746	1.68062	0.05
2747	1.14721	0.05
2748	1.11924	0.05
2749	1.18421	0.05
2750	2.01276	0.05
2751	1.23502	2.09917
2752	1.34926	2.06344
2753	0.546751	1.51231
2754	0.790446	2.05026
2755	1.04072	1.51161
2756	0.555868	1.44282
2757	0.930305	1.42982
2758	1.09344	1.61198
2759	1.18351	1.90249
2760	1.97215	1.53262
2761	1.07416	2.03318
2762	1.33204	1.44236
2763	1.22038	1.37492
2764	0.597449	1.14357
2765	0.821422	1.57933
2766	1.04071	1.31154
2767	0.608026	1.06636
2768	0.929427	1.24499
2769	1.64929	0.05
2770	1.68541	0.05
2771	0.496093	0.05
2772	1.3385	0.05
2773	0.790798	0.05
2774	1.22971	0.05
2775	0.898853	0.05
2776	2.14361	0.05
2777	2.10813	0.05
2778	1.05417	0.05
2779	1.80768	0.05
2780	1.09725	2.1
2781	1.41131	2.1

2782	1.1656	2.1
2783	0.946129	2.1
2784	1.68365	2.1
2785	1.68365	2.1
2786	1.1656	2.1
2787	0.94613	2.1
2788	1.21269	0.05
2789	1.48867	0.05
2790	1.27806	0.05
2791	1.68264	0.05
2792	1.37813	1.83391
2793	1.50743	1.22788
2794	1.04121	0.700124
2795	1.12434	0.135184
2796	1.39553	0.0844961
2797	1.44721	1.48671
2798	0.946132	0.124301
2799	1.23943	0.229773
2800	1.05292	0.289209
2801	1.28607	0.10687
2802	1.16915	0.329868
2803	1.76306	1.12962
2804	1.42266	1.12038
2805	1.36844	2.1
2806	1.36844	2.1
2807	1.67352	2.1
2808	1.11256	1.03022
2809	1.03217	0.743687
2810	1.18422	0.05
2811	1.04159	0.05
2812	1.60139	0.05
2813	1.04159	0.05
2814	0.988805	2.1
2815	1.05481	2.1
2816	1.52938	2.1
2817	1.94724	1.73462
2818	0.896336	0.05
2819	0.911484	0.05
2820	1.18315	0.05
2821	1.10515	0.05
2822	1.04067	0.05
2823	1.12758	0.05
2824	0.904106	0.05
2825	1.6901	0.05
2826	1.68671	0.05
2827	1.99908	0.05
2828	0.772426	0.511661
2829	0.810945	2.09717
2830	0.667562	1.17973
2831	0.496617	0.154585
2832	0.847689	0.865085
2833	0.914378	1.60549
2834	0.934003	0.328715
2835	1.07459	0.944339
2836	1.40225	2.1
2837	1.07043	2.1
2838	0.94613	2.1
2839	1.63072	2.1

2840	1.63072	2.1
2841	1.07043	2.1
2842	0.946131	2.1
2843	1.53082	2.1
2844	1.69916	2.1
2845	1.75211	0.0854848
2846	1.96831	2.1
2847	1.75211	0.470456
2848	0.946127	0.460423
2849	0.817065	0.577744
2850	1.41798	0.461158
2851	1.41798	0.200561
2852	0.791656	0.588583
2853	0.791656	0.669636
2854	1.41798	0.402113
2855	1.58331	0.378788
2856	1.41776	0.195877
2857	0.791647	0.249657
2858	1.4512	0.15012
2859	1.45119	0.154246
2860	0.75821	0.30954
2861	0.758248	0.27352
2862	0.946104	0.216143
2863	1.36482	0.138285
2864	1.29389	2.1
2865	1.04164	1.71266
2866	1.40574	1.66801
2867	1.64833	1.52267
2868	1.71183	0.05
2869	1.71183	0.05
2870	0.946131	0.158058
2871	1.35721	0.0771276
2872	0.946131	0.863681
2873	0.946131	0.27741
2874	1.30076	0.135532
2875	1.2474	0.05
2876	2.85225	0.157651
2877	2.71441	0.05
2878	1.2474	0.05
2879	1.38622	0.05
2880	0.858359	0.05
2881	1.3682	0.05
2882	1.57364	0.05
2883	0.729695	0.0592033
2884	0.729723	0.05
2885	0.822611	0.358289
2886	0.946133	0.0796298
2887	0.729723	0.05
2888	0.974704	0.912309
2889	0.974702	0.896676
2890	1.81603	2.1
2891	1.96348	2.1
2892	1.43612	1.10263
2893	1.43612	1.20317
2894	0.634295	1.07095
2895	0.93796	1.37858
2896	0.946133	1.16228
2897	0.634326	0.969582

2898	0.946133	1.07094
2899	1.2654	0.893287
2900	1.26865	1.12305
2901	2.2649	1.0521
2902	1.00864	1.06819
2903	1.09689	0.871314
2904	0.832021	0.88236
2905	1.53229	0.85108
2906	1.1145	0.869374
2907	0.929583	0.890435
2908	1.613	0.838718
2909	0.644729	0.872109
2910	1.03471	1.34886
2911	2.7739	2.1
2912	1.36133	1.50318
2913	2.50215	2.1
2914	2.52195	2.1
2915	1.0465	2.1
2916	1.42673	2.1
2917	1.42673	2.1
2918	0.946132	2.1
2919	1.31904	2.1
2920	0.986499	2.1
2921	0.946132	2.1
2922	1.31904	2.1
2923	0.966978	2.1
2924	1.42237	0.05
2925	2.60925	0.05
2926	0.753659	0.05
2927	1.77241	0.05
2928	1.77241	0.05
2929	0.946129	0.05
2930	0.75366	0.05
2931	1.22144	0.05
2932	0.94613	0.05
2933	1.50732	0.05
2934	1.18514	0.05
2935	0.902187	0.05
2936	1.89858	0.05
2937	1.24294	0.05
2938	1.67211	0.05
2939	1.41786	0.05
2940	0.810959	0.05
2941	1.50732	0.05
2942	2.44288	0.05
2943	1.40969	0.05
2944	2.06654	0.05
2945	1.40969	0.05
2946	1.40882	0.05
2947	1.94063	0.05
2948	0.867921	0.05
2949	1.29289	0.05
2950	0.867918	0.05
2951	0.946128	0.05
2952	0.867944	0.05
2953	1.66589	0.05
2954	1.66589	0.05
2955	0.968595	2.1

2956	1.5534	2.1
2957	1.27793	2.1
2958	1.13433	2.1
2959	1.3439	0.05
2960	2.53709	0.05
2961	0.571998	0.05
2962	0.64837	0.05
2963	1.99755	0.05
2964	1.99854	0.05
2965	0.764229	2.1
2966	1.61842	2.1
2967	1.61842	2.1
2968	1.18315	2.1
2969	1.1995	2.1
2970	1.18315	2.1
2971	0.764256	2.1
2972	1.52364	2.1
2973	2.36631	2.1
2974	0.641912	2.1
2975	1.7181	2.1
2976	1.459 2.1	
2977	0.634845	1.98731
2978	1.18315	2.1
2979	1.17615	2.1
2980	0.571367	2.1
2981	1.3324	0.05
2982	0.798214	0.05
2983	0.946128	0.05
2984	0.627252	0.05
2985	1.50337	0.05
2986	1.50337	0.05
2987	0.62725	0.05
2988	1.07763	0.05
2989	1.07763	0.05
2990	2.16109	0.05
2991	1.07763	0.05
2992	1.12486	2.1
2993	1.11827	2.1
2994	1.0491	2.1
2995	1.09034	2.1
2996	1.09431	2.1
2997	0.946131	2.1
2998	0.748607	2.1
2999	1.44002	2.1
3000	1.44002	2.1
3001	0.748635	2.1
3002	0.782505	2.1
3003	1.40615	2.1
3004	1.19539	2.1
3005	1.29389	2.1
3006	1.4867	1.2549
3007	1.50103	0.05
3008	1.63025	0.05
3009	2.72109	0.05
3010	0.915755	0.05
3011	0.946132	0.05
3012	0.915755	0.05
3013	1.70236	0.05

3014	1.70236	0.05
3015	1.26155	0.05
3016	1.35657	0.05
3017	1.83151	0.05
3018	1.83151	0.05
3019	2.52309	0.05
3020	0.983607	2.1
3021	1.06838	0.217656
3022	0.983609	1.22069
3023	0.983596	0.694361
3024	2.74384	0.05
3025	1.6261	0.05
3026	1.15477	2.1
3027	1.02136	2.1
3028	1.83561	2.1
3029	1.83561	2.1
3030	0.946131	2.1
3031	1.39523	2.1
3032	1.29313	2.08259
3033	1.29313	2.1
3034	1.38651	2.1
3035	1.06313	2.1
3036	0.974888	2.1
3037	1.0894	2.1
3038	0.974888	2.1
3039	1.82282	2.1
3040	1.82282	2.1
3041	0.946131	2.1
3042	1.37372	2.1
3043	1.32374	2.1
3044	1.32374	2.1
3045	1.39523	2.1
3046	1.40882	0.05
3047	0.861998	0.05
3048	1.24448	0.05
3049	0.861999	0.05
3050	0.946129	0.05
3051	0.862011	0.05
3052	1.67537	0.05
3053	1.67537	0.05
3054	1.72399	0.05
3055	2.58579	0.05
3056	1.72399	0.05
3057	1.50951	0.05
3058	2.48897	0.05
3059	1.26755	2.1
3060	1.03824	2.1
3061	1.27875	2.1
3062	1.48901	2.1
3063	1.18902	2.1
3064	1.11764	2.1
3065	1.87436	2.1
3066	0.95345	2.1
3067	0.724191	1.68745
3068	0.86668	1.65957
3069	0.811523	1.85394
3070	1.04074	1.29602
3071	0.843097	1.51035

3072	0.833434	1.76029
3073	0.965727	1.54292
3074	0.85346	1.74655
3075	1.00185	1.04172
3076	1.00184	1.51969
3077	0.980651	1.794
3078	0.980664	1.49387
3079	0.475737	1.23775
3080	0.719696	1.29552
3081	0.946109	1.21156
3082	0.946116	1.34672
3083	0.719693	1.24132
3084	0.946117	1.34796
3085	1.14879	1.70439
3086	0.719683	1.60714
3087	0.900977	1.27532
3088	0.972364	1.40196
3089	1.22961	1.38312
3090	0.405384	1.28039
3091	1.34406	2.1
3092	1.2187	2.1
3093	0.963571	0.0887991
3094	0.946131	0.0522053
3095	1.57246	0.957382
3096	1.57246	2.1
3097	0.946131	0.975721
3098	1.1235	2.1
3099	1.1235	1.52211
3100	1.07439	1.73107
3101	1.19612	1.25899
3102	1.24844	1.20736
3103	1.00007	1.73845
3104	1.02429	2.1
3105	1.10459	2.1
3106	1.06724	0.0603439
3107	0.67519	0.293258
3108	0.49762	0.0522754
3109	1.43435	0.05
3110	0.833315	0.0549261
3111	0.58135	0.0540309
3112	1.14749	0.681414
3113	1.85091	1.28178
3114	1.2802	0.545896
3115	1.27737	0.562166
3116	2.3655	0.393965
3117	1.30584	2.1
3118	2.06424	2.1
3119	0.946131	0.05
3120	1.04257	0.05
3121	0.94613	0.05
3122	1.67201	0.05
3123	1.67201	0.05
3124	1.27696	0.05
3125	1.34118	0.05
3126	1.3413	0.05
3127	2.36238	0.05
3128	0.6036	0.05
3129	1.11745	0.05

3130	1.46126	0.05
3131	1.39612	0.05
3132	0.670678	0.05
3133	1.03402	0.05
3134	1.11271	0.05
3135	0.838028	0.05
3136	0.807019	0.05
3137	1.27787	0.05
3138	0.994705	0.05
3139	1.20014	0.05
3140	0.964793	0.05
3141	0.863868	0.05
3142	1.3413	0.05
3143	1.79549	0.05
3144	1.35877	2.1
3145	1.63648	2.1
3146	1.08171	2.1
3147	1.27839	2.1
3148	1.52601	2.1
3149	1.27841	2.1
3150	1.71255	2.1
3151	2.16307	2.1
3152	1.91592	2.1
3153	1.78508	2.1
3154	0.946128	2.1
3155	1.41199	2.1
3156	0.946128	2.1
3157	0.901088	2.1
3158	1.55244	2.1
3159	1.59475	2.1
3160	1.40921	2.1
3161	0.960106	2.1
3162	1.40921	2.1
3163	2.0073	2.1
3164	2.2973	0.0772759
3165	1.90705	0.0706442
3166	1.53671	0.05
3167	1.86035	0.05
3168	1.33747	2.1
3169	1.14007	2.1
3170	1.3212	2.1
3171	1.66867	0.05
3172	1.30203	0.05
3173	1.27696	2.1
3174	1.05025	2.1
3175	1.05025	2.1
3176	1.3704	2.1
3177	1.15677	2.1
3178	0.946129	2.1
3179	1.65879	2.1
3180	1.65879	2.1
3181	1.15676	2.1
3182	0.94613	2.1
3183	0.867217	1.22161
3184	1.28684	1.13534
3185	1.58866	1.20383
3186	1.58866	0.975267
3187	0.867246	1.9815

3188	1.28684	1.06325
3189	1.16906	1.2449
3190	1.13438	1.19506
3191	1.37224	1.24338
3192	1.61519	1.3305
3193	0.883939	2.02504
3194	1.40092	1.2545
3195	1.80383	2.00445
3196	1.80383	1.61157
3197	0.883935	1.92595
3198	1.40092	1.19199
3199	1.28684	1.84057
3200	1.76788	1.0566
3201	2.57368	1.20097
3202	1.37224	2.07108
3203	1.65411	0.192654
3204	1.77042	2.1
3205	1.77042	2.1
3206	1.90446	2.1
3207	1.28667	2.1
3208	1.55635	2.1
3209	1.30646	0.809185
3210	2.31836	1.4241
3211	1.30645	0.857026
3212	1.86429	1.58033
3213	1.48676	0.05
3214	0.743379	0.333684
3215	1.41848	0.39438
3216	1.41848	0.132512
3217	0.946131	0.394877
3218	0.812649	0.295012
3219	1.34921	0.21821
3220	0.812649	0.0986359
3221	0.949366	0.05
3222	1.32974	0.05
3223	0.794204	0.05
3224	0.634352	2.1
3225	0.838025	2.1
3226	1.08802	2.1
3227	0.838038	2.1
3228	1.18314	2.1
3229	1.12495	2.1
3230	1.22774	2.1
3231	1.04343	2.1
3232	0.921111	0.05
3233	2.1609	0.05
3234	1.09987	0.05
3235	2.5043	0.05
3236	1.05492	0.05
3237	0.590344	0.05
3238	1.05492	0.05
3239	1.25215	0.05
3240	1.71674	0.05
3241	1.71674	0.05
3242	0.590331	0.05
3243	1.17078	0.05
3244	0.625542	0.05
3245	0.724673	2.1

3246	1.08011	2.1
3247	1.87598	2.1
3248	1.50738	2.1
3249	1.44951	0.05
3250	1.50102	0.05
3251	1.8042	0.408049
3252	0.687769	0.911074
3253	1.66483	0.712337
3254	1.66483	0.838736
3255	0.946132	0.900381
3256	0.687798	0.817309
3257	1.20885	0.904348
3258	0.687799	0.877049
3259	0.886063	0.302402
3260	0.798751	0.286862
3261	0.584813	0.48477
3262	1.49483	0.0721204
3263	0.888005	0.129911
3264	1.03589	0.0948413
3265	1.70435	0.32584
3266	0.710414	2.1
3267	0.917609	1.46159
3268	0.810947	1.59364
3269	0.81096	1.52476
3270	1.48658	1.56042
3271	1.53653	1.57217
3272	1.19329	1.88111
3273	0.946153	1.54072
3274	1.50798	1.6753
3275	1.85017	2.1
3276	1.19768	2.1
3277	0.864304	2.1
3278	0.792906	1.7319
3279	0.989025	1.50523
3280	1.19768	1.80422
3281	1.54548	1.64878
3282	1.18798	2.02338
3283	1.31875	1.40945
3284	1.05725	1.55591
3285	2.17365	0.05
3286	2.48106	0.05
3287	1.87451	0.05
3288	1.27189	1.31115
3289	2.79399	1.90901
3290	0.859565	1.37565
3291	0.926174	1.27653
3292	0.604744	1.22899
3293	1.41832	0.709539
3294	1.05494	1.12491
3295	0.874749	1.16225
3296	1.50067	1.05858
3297	0.917817	1.48749
3298	0.63591	1.48836
3299	1.72849	1.40247
3300	1.72849	0.885208
3301	0.635943	1.20396
3302	1.39699	1.46204
3303	0.967411	1.99539

3304	0.635943	1.36348
3305	1.39908	1.26707
3306	1.26971	1.42168
3307	1.02969	1.23404
3308	1.15326	2.1
3309	2.30741	1.78662
3310	0.93545	1.28026
3311	0.802747	1.05089
3312	1.15466	1.28291
3313	0.859164	1.68308
3314	1.02616	1.57121
3315	1.23149	1.21679
3316	0.83804	1.11058
3317	1.13266	2.1
3318	1.64142	2.1
3319	1.21223	2.1
3320	1.43051	2.1
3321	1.32418	1.00644
3322	0.621319	1.07356
3323	1.47134	2.1
3324	1.79103	0.729738
3325	3.25951	0.05
3326	3.16764	0.05
3327	1.65583	0.05
3328	1.4732	0.05
3329	1.76476	2.1
3330	1.80114	1.45011
3331	0.754121	2.1
3332	0.94613	0.368619
3333	0.754119	2.1
3334	1.45636	2.1
3335	1.45636	0.448436
3336	1.30029	1.15457
3337	0.910183	0.534112
3338	1.29099	2.1
3339	1.08039	2.1
3340	1.05498	2.1
3341	1.24445	2.1
3342	1.2873	2.1
3343	0.987703	2.1
3344	0.964782	2.1
3345	1.73645	0.05
3346	0.871279	0.05
3347	0.884744	0.05
3348	1.85193	0.05
3349	1.68681	0.05
3350	1.33689	0.05
3351	1.23493	0.05
3352	0.892482	0.05
3353	2.95185	0.05
3354	2.10496	0.05
3355	1.87334	2.1
3356	0.94411	2.1
3357	0.754196	2.1
3358	1.43122	2.1
3359	1.58684	2.1
3360	0.838027	2.1
3361	1.1536	2.1

3362	1.24878	2.1
3363	1.23125	2.1
3364	2.86862	2.1
3365	1.57636	2.1
3366	0.998451	0.775134
3367	1.5176	0.592183
3368	1.13271	0.05
3369	1.60248	0.05
3370	1.22083	2.1
3371	1.79996	2.1
3372	1.19087	2.1
3373	2.00342	2.1
3374	1.3715	2.1
3375	2.79227	2.1
3376	2.7873	2.1
3377	0.987265	0.05
3378	1.62715	2.1
3379	1.70117	2.1
3380	1.27806	0.05
3381	1.45246	2.1
3382	1.45244	2.1
3383	1.87214	2.1
3384	0.995796	2.1
3385	1.68189	0.05
3386	1.49155	2.1
3387	1.58054	2.1
3388	1.59169	2.1
3389	0.736364	0.05
3390	0.70236	0.05
3391	2.24367	0.05
3392	2.57085	0.05
3393	1.70952	0.05
3394	2.60516	0.05
3395	1.45158	2.1
3396	1.00371	2.1
3397	1.65748	2.1
3398	1.23706	0.674307
3399	1.55834	0.850933
3400	1.72671	0.833312
3401	0.597656	0.747638
3402	1.39137	0.829033
3403	0.855578	0.269138
3404	1.48398	0.784937
3405	0.745117	0.712276
3406	1.23706	0.777156
3407	1.57916	0.354831
3408	0.893025	0.258254
3409	3.22433	0.769632
3410	2.47324	1.15347
3411	1.63852	2.1
3412	1.63852	2.1
3413	0.946132	2.1
3414	1.19095	2.1
3415	0.946133	2.1
3416	1.16684	2.1
3417	1.19095	2.1
3418	1.06698	0.05
3419	1.48692	0.0510102

3420	1.08334	1.13536
3421	1.07283	2.1
3422	1.28517	0.420113
3423	1.2746	0.05
3424	1.30723	0.05
3425	2.02069	0.05
3426	1.05579	0.05
3427	1.59475	2.1
3428	2.40113	2.1
3429	1.58875	2.1
3430	1.6881	2.1
3431	1.6881	2.1
3432	0.819953	2.1
3433	0.819954	2.1
3434	0.946131	2.1
3435	0.946131	2.1
3436	1.34782	2.1
3437	1.65619	2.1
3438	1.65619	2.1
3439	0.79437	2.1
3440	1.25	2.1
3441	0.94613	2.1
3442	0.946131	2.1
3443	0.794371	2.1
3444	1.58875	2.1
3445	2.5	2.1
3446	1.04147	2.1
3447	0.946131	1.00321
3448	1.79686	2.1
3449	1.79686	2.1
3450	1.25144	1.01006
3451	1.35714	1.57425
3452	1.38585	1.71228
3453	1.25144	2.1
3454	1.15564	2.1
3455	1.34389	2.1
3456	0.946133	1.21153
3457	1.74353	2.1
3458	1.74353	2.1
3459	1.14553	1.15265
3460	1.38585	1.13836
3461	1.30382	1.25342
3462	1.14553	2.1
3463	1.15564	2.1
3464	1.90618	2.1
3465	2.11056	2.1
3466	1.032	2.1
3467	0.953636	2.1
3468	1.23131	1.46724
3469	1.39763	1.47053
3470	2.01024	1.21737
3471	1.9186	1.24351
3472	1.83658	0.05
3473	1.81656	0.05
3474	2.2049	0.05
3475	2.16081	0.05
3476	1.12219	0.05
3477	0.599771	0.05

3478	1.61401	0.05
3479	1.05912	0.05
3480	0.951648	0.05
3481	1.89328	0.05
3482	0.913682	0.05
3483	1.00763	0.05
3484	1.29129	2.1
3485	1.37138	2.1
3486	1.41986	0.05
3487	1.41986	0.05
3488	0.944166	0.05
3489	1.41789	0.05
3490	0.944167	0.05
3491	0.944166	0.05
3492	0.946132	0.05
3493	1.98713	0.05
3494	1.23626	0.05
3495	0.932791	0.05
3496	0.810251	0.05
3497	0.986748	0.05
3498	0.898155	0.05
3499	0.92624	0.05
3500	1.1349	0.05
3501	0.934572	0.05
3502	1.23104	0.420796
3503	1.23104	0.613721
3504	0.799994	0.0589592
3505	0.94613	0.229803
3506	0.802822	0.05
3507	1.40944	0.851095
3508	0.800035	0.143509
3509	1.41223	0.199553
3510	1.41223	0.244213
3511	1.6	0.108875
3512	1.60565	0.05
3513	1.23042	2.1
3514	1.0645	2.1
3515	1.00319	2.1
3516	1.29435	2.1
3517	1.2243	2.1
3518	1.2243	2.1
3519	1.05655	1.64003
3520	1.29764	1.82092
3521	2.55252	2.1
3522	1.15676	2.1
3523	0.855295	2.1
3524	0.810959	2.1
3525	1.65857	2.1
3526	1.00849	2.1
3527	1.16021	2.1
3528	1.75869	2.1
3529	1.13775	2.1
3530	1.20091	2.1
3531	1.61582	2.1
3532	1.88031	2.1
3533	0.968536	2.1
3534	0.946131	2.1
3535	1.61481	2.1

3536	1.61481	2.1
3537	1.09547	2.1
3538	1.18486	2.1
3539	1.37609	2.1
3540	1.09546	2.1
3541	1.02461	2.1
3542	1.83223	2.1
3543	1.74002	2.1
3544	1.70939	2.1
3545	1.7094	2.1
3546	0.726287	2.1
3547	1.01712	2.09065
3548	1.30318	2.1
3549	0.946131	2.1
3550	0.87155	2.1
3551	1.77093	1.92362
3552	2.02356	1.93316
3553	1.32449	2.1
3554	1.10003	2.1
3555	1.5161	2.1
3556	1.5161	2.1
3557	0.946132	2.1
3558	1.09206	2.1
3559	0.946132	2.1
3560	1.04404	2.1
3561	1.09206	2.1
3562	0.635628	1.04846
3563	1.14623	0.518418
3564	1.73197	0.879013
3565	1.73197	1.07316
3566	0.529655	0.289017
3567	1.14623	0.773555
3568	0.992085	0.958225
3569	1.16915	1.24236
3570	2.29245	0.0631301
3571	0.584573	0.98404
3572	0.956756	0.636716
3573	1.52471	0.819183
3574	1.51409	1.15917
3575	0.584575	0.395186
3576	0.94613	0.646251
3577	1.14622	0.370863
3578	1.28096	1.19999
3579	1.34695	1.62274
3580	1.04193	2.1
3581	1.05378	2.1
3582	0.798209	2.1
3583	1.32007	2.1
3584	1.64327	0.05
3585	2.64674	2.09581
3586	2.38936	0.05
3587	2.88475	0.05
3588	1.07745	2.1
3589	1.08911	2.1
3590	1.49127	2.1
3591	1.49127	2.1
3592	0.709714	2.1
3593	1.37199	2.1

3594	0.82899	2.1
3595	0.709714	2.1
3596	0.946132	2.1
3597	1.00133	2.1
3598	1.40182	2.1
3599	1.53278	2.1
3600	1.40182	2.1
3601	1.31764	0.05
3602	1.00691	0.05
3603	1.24094	0.05
3604	0.64868	0.05
3605	0.946133	0.05
3606	0.64868	0.05
3607	1.71762	0.05
3608	1.71762	0.05
3609	1.41662	0.05
3610	0.975477	0.05
3611	1.73584	0.05
3612	2.48188	0.05
3613	2.2045	0.479053
3614	2.08134	0.442149
3615	0.993366	0.05
3616	0.993368	0.892685
3617	1.14187	0.77972
3618	0.946132	0.784297
3619	0.946133	0.944449
3620	1.68951	0.853828
3621	1.68951	1.03788
3622	1.34612	0.83589
3623	1.28953	0.775281
3624	1.34611	0.978949
3625	1.31892	0.277915
3626	0.946131	0.872218
3627	1.80988	0.350276
3628	1.80988	2.1
3629	1.31892	0.899431
3630	0.946131	0.901707
3631	2.13214	2.1
3632	1.94691	2.1
3633	1.52661	0.05
3634	1.53175	0.05
3635	0.956005	0.05
3636	0.955991	0.05
3637	1.40572	0.05
3638	0.955992	0.05
3639	1.51084	1.76908
3640	1.67115	2.1
3641	1.73143	2.1
3642	1.55234	2.1
3643	1.69377	2.1
3644	2.51483	2.1
3645	1.25987	2.1
3646	1.4472	2.1
3647	1.83151	0.659035
3648	2.01001	1.28366
3649	0.993998	0.598529
3650	1.83151	0.471334
3651	1.40915	0.05

3652	1.24653	0.258402
3653	1.74472	2.1
3654	1.74472	2.1
3655	1.74244	2.1
3656	1.04788	2.1
3657	1.28522	2.1
3658	1.03785	2.1
3659	0.896519	2.1
3660	1.60428	2.1
3661	1.73305	2.1
3662	1.39072	0.05
3663	2.27501	0.0553246
3664	1.39073	0.0825421
3665	1.39073	0.05
3666	1.06838	2.1
3667	0.968462	1.07429
3668	1.06837	1.52074
3669	1.13455	1.39236
3670	1.23527	0.837845
3671	2.71686	0.571399
3672	1.86881	0.183382
3673	1.10586	1.26718
3674	1.03852	2.1
3675	1.03899	2.1
3676	0.963973	2.1
3677	1.00319	2.1
3678	1.00319	2.1
3679	0.987132	2.1
3680	0.974889	2.1
3681	0.987131	2.1
3682	1.7677	2.1
3683	1.7677	2.1
3684	0.946131	2.1
3685	1.31185	2.1
3686	0.946132	2.1
3687	1.30203	2.1
3688	1.31185	2.1
3689	1.82958	2.1
3690	1.82958	2.1
3691	0.946132	2.1
3692	1.40199	2.1
3693	0.946133	2.1
3694	1.36994	2.1
3695	1.40199	2.1
3696	0.97489	2.1
3697	1.05939	2.1
3698	1.01922	2.1
3699	0.810941	1.92223
3700	1.05372	2.1
3701	0.754065	2.1
3702	0.926131	2.1
3703	1.35905	2.1
3704	0.810931	2.1
3705	1.75647	1.23384
3706	1.68265	2.1
3707	1.41995	2.1
3708	1.36457	1.5969
3709	0.933392	1.34225

3710	1.42255	2.1
3711	1.3868	2.1
3712	1.08074	2.1
3713	1.07196	2.1
3714	1.13537	1.02237
3715	1.01179	0.860821
3716	1.60196	0.744823
3717	1.72798	0.742394
3718	1.3715	0.749812
3719	1.56409	1.08355
3720	1.56409	0.809431
3721	1.97819	1.13259
3722	1.47954	0.803985
3723	0.798655	0.20013
3724	1.47952	0.813778
3725	2.30327	0.239452
3726	1.49111	2.1
3727	1.96013	2.1
3728	1.54599	2.1
3729	2.06632	2.1
3730	1.54601	2.1
3731	1.10557	0.605633
3732	1.17624	0.114292
3733	1.60497	2.1
3734	1.61587	2.1
3735	1.65977	0.05
3736	1.69044	0.05
3737	1.00659	2.1
3738	1.06226	2.1
3739	1.15171	2.1
3740	1.50853	2.1
3741	1.80252	2.1
3742	1.41432	2.1
3743	1.04687	2.1
3744	0.940878	2.1
3745	1.52031	2.1
3746	1.52031	2.1
3747	0.940877	2.1
3748	0.946132	2.1
3749	1.50853	2.1
3750	0.963973	2.1
3751	1.70562	0.05
3752	2.43203	0.05
3753	1.36004	0.05
3754	1.06648	0.05
3755	1.66009	0.05
3756	1.66009	0.05
3757	0.766427	0.05
3758	0.766452	0.05
3759	0.946132	0.05
3760	1.98012	0.05
3761	1.20357	2.1
3762	1.20356	2.1
3763	1.67324	2.1
3764	1.52008	2.1
3765	1.19964	2.1
3766	1.36861	2.1
3767	1.19964	2.1

3768	2.85415	2.1
3769	1.43687	2.1
3770	0.935852	2.1
3771	1.09972	2.1
3772	0.810933	2.1
3773	2.25974	2.1
3774	1.74149	2.1
3775	1.36984	2.1
3776	1.87602	2.1
3777	0.890748	2.1
3778	1.20587	2.05969
3779	0.810956	1.88025
3780	2.03174	2.08516
3781	1.34403	2.1
3782	1.58825	2.07523
3783	1.63096	2.06085
3784	2.53735	2.1
3785	1.98241	2.1
3786	2.53014	2.1
3787	1.1775	1.28093
3788	1.06538	1.10437
3789	0.946132	1.20826
3790	1.4101	2.1
3791	1.75162	2.1
3792	1.75162	1.11677
3793	0.946132	1.20899
3794	1.4101	1.1165
3795	1.28765	1.13727
3796	1.1427	1.05078
3797	1.1427	0.913445
3798	1.3109	2.1
3799	1.02721	1.53783
3800	0.846447	1.68474
3801	0.862076	2.1
3802	0.644098	1.73578
3803	0.581308	1.76852
3804	1.02306	1.63857
3805	0.322641	0.929405
3806	0.589346	1.03814
3807	2.25572	1.58441
3808	1.31372	2.1
3809	1.18873	2.1
3810	1.78225	2.1
3811	1.764 2.1	
3812	1.17503	2.1
3813	1.06821	2.1
3814	1.06821	1.97133
3815	1.7777	2.06011
3816	1.9096	0.951008
3817	1.70239	1.0895
3818	1.16182	0.05
3819	1.20132	0.05
3820	1.23475	2.1
3821	1.56859	2.08697
3822	2.46263	2.1
3823	0.528744	2.1
3824	2.93337	2.1
3825	2.42504	2.1

3826	1.38646	0.05
3827	1.61963	0.05
3828	1.88016	0.171078
3829	0.622283	0.652485
3830	1.03243	0.769777
3831	0.710307	1.03144
3832	0.798124	0.764785
3833	0.931073	0.964038
3834	0.892862	1.36107
3835	1.06791	0.826415
3836	0.552787	1.40632
3837	0.946132	0.813028
3838	1.36665	0.571099
3839	1.36665	1.37715
3840	0.506966	0.790256
3841	0.506964	1.55256
3842	1.36665	0.349841
3843	0.653469	0.68624
3844	1.36665	0.108476
3845	1.21339	0.0838193
3846	1.21339	0.988354
3847	0.552757	0.649359
3848	0.552786	1.70906
3849	1.21336	0.0531219
3850	1.70744	1.35612
3851	1.0775	0.05
3852	1.59527	0.286193
3853	1.48312	0.0794744
3854	2.01868	0.05
3855	1.18947	0.05
3856	1.48312	0.082107
3857	0.785626	0.05
3858	1.19601	0.05
3859	0.785626	0.05
3860	0.946132	0.05
3861	0.7856	0.05
3862	1.59087	0.05
3863	1.51158	0.05
3864	1.74098	0.05
3865	2.39202	0.05
3866	0.870489	0.05
3867	1.34534	0.05
3868	0.870488	0.05
3869	0.946133	0.05
3870	0.870489	0.107354
3871	1.67086	0.0537198
3872	1.67086	0.05
3873	2.11311	2.1
3874	1.98765	1.40997
3875	0.946133	1.03878
3876	1.30881	2.1
3877	1.72211	2.1
3878	1.72211	1.34438
3879	0.94613	2.08692
3880	1.30881	1.47295
3881	1.35943	1.12689
3882	1.01294	0.296276
3883	1.01294	0.05

3884	1.38963	0.888159
3885	1.01294	0.320317
3886	1.60056	0.64602
3887	1.60056	0.05
3888	0.946132	0.218322
3889	1.204	0.05
3890	0.946132	0.59529
3891	1.11288	0.05
3892	1.204	0.100666
3893	1.14599	0.0800662
3894	1.00065	0.46771
3895	1.74685	0.968515
3896	1.74685	2.1
3897	0.946128	0.957848
3898	1.38463	0.0613741
3899	1.2275	2.1
3900	1.2275	1.85999
3901	1.30835	1.87131
3902	2.04407	2.1
3903	2.50425	2.1
3904	1.19647	0.837646
3905	1.19647	0.858684
3906	1.19645	0.911843
3907	1.75446	0.84071
3908	1.1315	0.05
3909	1.1315	0.777673
3910	1.45569	0.355386
3911	1.1315	0.05
3912	1.14357	2.1
3913	1.25793	2.1
3914	1.14357	2.1
3915	2.08181	2.1
3916	1.4714	2.1
3917	0.71295	2.1
3918	1.68285	2.1
3919	1.68285	2.1
3920	1.25603	2.1
3921	1.13976	2.1
3922	1.25603	2.1
3923	0.71295	2.1
3924	1.61095	2.1
3925	2.51207	2.1
3926	1.63762	2.1
3927	2.74451	2.1
3928	0.671255	2.1
3929	1.87601	2.1
3930	1.87601	2.1
3931	0.671226	2.1
3932	1.25603	2.1
3933	1.29123	2.1
3934	0.671226	2.1
3935	1.63723	2.1
3936	0.872928	0.05
3937	1.47904	0.05
3938	1.18793	0.05
3939	2.57464	0.05
3940	1.14986	2.1
3941	1.19611	2.1

3942	0.96029	0.734636
3943	1.7961	2.1
3944	0.928721	2.1
3945	1.34352	2.1
3946	1.37935	2.1
3947	1.37935	2.1
3948	0.892888	2.1
3949	0.946131	2.1
3950	0.892888	2.1
3951	1.75422	2.1
3952	1.05556	2.1
3953	1.27362	0.101061
3954	1.27421	0.05
3955	1.3003	1.09123
3956	1.31415	0.781312
3957	1.01431	2.1
3958	0.673951	2.1
3959	1.63384	2.1
3960	1.63661	2.1
3961	1.52245	2.1
3962	1.50714	2.1
3963	0.996778	0.05
3964	1.56091	0.05
3965	1.06543	2.1
3966	2.66689	2.1
3967	1.01474	2.1
3968	2.76422	2.1
3969	1.45321	0.0698033
3970	0.974716	0.05
3971	0.810943	0.05
3972	0.909982	0.109901
3973	0.937793	0.05
3974	1.05138	0.05
3975	1.25044	0.05
3976	0.92095	0.05
3977	0.830414	0.0630904
3978	2.1402	0.05
3979	1.75938	2.1
3980	1.05556	2.1
3981	1.78558	2.1
3982	1.379 2.1	
3983	0.970214	0.846133
3984	0.9702	0.828328
3985	1.08894	1.11823
3986	2.17251	0.923873
3987	1.51569	0.407127
3988	1.06012	0.366932
3989	0.98966	0.206745
3990	1.55926	0.0677503
3991	1.92645	0.175316
3992	1.37427	0.05
3993	2.16338	0.05
3994	1.5456	0.05
3995	1.5456	0.05
3996	1.94002	0.05
3997	1.33135	0.05
3998	1.31948	0.05
3999	2.23255	0.05

4000	2.34061	0.05
4001	1.52903	2.1
4002	3.38327	2.1
4003	1.35977	2.1
4004	2.22414	2.1
4005	1.6812	0.05
4006	2.33397	0.05
4007	1.16699	0.05
4008	0.946131	0.05
4009	1.58115	0.05
4010	1.58115	0.05
4011	0.519726	0.05
4012	0.946131	0.05
4013	0.519697	0.05
4014	1.26868	0.05
4015	1.16699	0.05
4016	1.5951	0.05
4017	1.5951	0.05
4018	0.840568	0.05
4019	0.946131	0.05
4020	0.840598	0.05
4021	1.30047	2.1
4022	1.5586	2.1
4023	1.31825	1.06949
4024	1.31924	1.0367
4025	1.15818	2.1
4026	1.52782	1.05573
4027	1.15818	1.24815
4028	1.15818	1.25222
4029	0.99518	0.577484
4030	0.94613	0.410033
4031	0.922045	0.658703
4032	1.29322	0.475642
4033	1.29321	0.0642648
4034	1.29321	0.534219
4035	0.922056	0.601793
4036	1.06848	0.70054
4037	1.84409	0.578849
4038	1.9186	1.36247
4039	2.72244	1.14799
4040	0.946132	1.04624
4041	1.36122	1.16707
4042	1.79658	1.17241
4043	1.79658	1.04885
4044	0.946133	1.29091
4045	1.36122	1.12043
4046	1.38149	1.06249
4047	1.59106	1.04329
4048	0.946116	1.02296
4049	1.29052	1.0322
4050	1.76641	1.02398
4051	1.76641	0.836921
4052	0.885309	1.25431
4053	1.29054	1.10778
4054	1.36118	1.0666
4055	1.59103	1.11237
4056	2.27816	0.96487
4057	1.56164	0.844392

4058	1.41114	0.05
4059	2.04128	0.05
4060	0.986723	2.1
4061	1.03645	0.177281
4062	1.79224	0.05
4063	1.2627	2.1
4064	1.23036	2.1
4065	1.45257	2.1
4066	1.41469	2.1
4067	1.41469	2.1
4068	2.60636	2.1
4069	1.45789	0.05
4070	0.851541	0.05
4071	1.57877	0.05
4072	1.53134	0.05
4073	0.851479	0.05
4074	1.08982	0.05
4075	0.716284	0.05
4076	0.946131	0.05
4077	1.73193	0.05
4078	2.57309	0.05
4079	1.36307	0.05
4080	2.42175	0.05
4081	1.72948	0.05
4082	0.978698	2.1
4083	0.978699	2.1
4084	2.08398	2.1
4085	0.978698	2.1
4086	1.44096	2.1
4087	1.44096	2.1
4088	0.638164	2.1
4089	0.681588	2.1
4090	0.946133	2.1
4091	0.638165	2.1
4092	0.94613	2.1
4093	1.55432	2.1
4094	0.901147	2.1
4095	0.85777	2.1
4096	1.36073	2.1
4097	1.44004	2.1
4098	0.946133	2.1
4099	1.09982	2.1
4100	1.27562	2.1
4101	1.55432	2.1
4102	1.62229	2.1
4103	1.0101	2.1
4104	2.28459	2.1
4105	2.50554	2.1
4106	0.946131	1.14783
4107	1.25276	2.1
4108	1.73721	2.1
4109	1.73721	1.38115
4110	0.93406	2.1
4111	1.25277	1.77276
4112	1.4185	1.30346
4113	1.53064	1.32681
4114	0.946133	1.37307
4115	0.609462	2.1

4116	1.65123	2.1
4117	1.65123	1.88842
4118	0.609466	2.1
4119	1.00792	1.96575
4120	1.25277	1.70493
4121	1.53064	2.1
4122	2.15223	1.32736
4123	1.30664	1.96867
4124	1.10674	2.1
4125	1.22862	2.1
4126	1.49358	0.141267
4127	2.0207	0.05
4128	2.09374	2.1
4129	1.88175	2.1
4130	1.10938	0.05
4131	1.02478	0.102786
4132	1.13272	0.05
4133	1.13272	0.05
4134	1.03935	1.31785
4135	0.810945	1.7161
4136	0.527573	0.255457
4137	0.925011	1.24494
4138	1.03193	0.200044
4139	1.38587	0.413535
4140	0.703469	0.417323
4141	0.912282	1.25343
4142	1.75505	2.1
4143	1.78529	2.1
4144	1.60017	1.03207
4145	1.35608	0.274734
4146	1.01479	2.1
4147	1.47561	0.762153
4148	1.01479	2.1
4149	1.28418	0.179109
4150	2.62605	2.1
4151	1.71233	2.1
4152	3.22222	2.1
4153	1.97526	2.1
4154	0.949712	0.100366
4155	1.78986	0.052558
4156	2.72879	0.05
4157	2.66428	0.05
4158	1.68108	0.412037
4159	1.67265	0.209098
4160	2.24877	0.373598
4161	1.15558	0.383665
4162	1.39708	2.1
4163	1.39327	2.1
4164	1.69788	0.05
4165	1.1273	0.05
4166	1.17302	0.472635
4167	1.04349	0.05
4168	0.979853	2.1
4169	1.19094	2.1
4170	1.39408	2.1
4171	1.61857	2.1
4172	1.66523	2.1
4173	0.946132	2.1

4174	0.838554	2.1
4175	1.05151	2.1
4176	1.18901	2.1
4177	1.18901	2.1
4178	1.61149	2.1
4179	1.22857	0.878055
4180	1.25848	0.873028
4181	1.09154	2.1
4182	2.20358	2.1
4183	1.23013	2.1
4184	1.55698	2.1
4185	1.55828	2.1
4186	1.52391	2.1
4187	0.60346	1.9189
4188	1.06336	2.1
4189	0.870714	1.31086
4190	0.543109	1.91365
4191	1.10367	1.29841
4192	1.20686	1.87955
4193	1.20684	2.1
4194	2.5118	1.45928
4195	1.20684	2.1
4196	1.52468	2.1
4197	1.52469	2.1
4198	0.532308	1.78666
4199	0.903301	2.1
4200	0.946119	1.46111
4201	0.532315	1.53625
4202	0.946107	1.55709
4203	1.46129	2.1
4204	0.804766	2.1
4205	1.4809	2.1
4206	1.4809	2.1
4207	1.11822	2.1
4208	1.16744	2.1
4209	1.11822	2.1
4210	0.804737	2.1
4211	1.04193	2.1
4212	0.983723	2.1
4213	1.8014	2.1
4214	1.1729	2.1
4215	1.03951	2.1
4216	1.03951	2.1
4217	1.17583	2.1
4218	1.42866	2.1
4219	1.78503	2.1
4220	1.17822	1.19075
4221	1.89121	2.1
4222	1.44472	2.1
4223	1.62143	2.1
4224	1.43837	2.1
4225	1.01121	0.05
4226	0.962703	0.942733
4227	0.946129	0.184245
4228	1.10941	0.0922771
4229	0.946131	0.516235
4230	1.60326	0.827812
4231	1.60326	0.0522611

4232	1.14215	0.212191
4233	1.40724	0.588927
4234	0.962703	0.0861958
4235	1.53336	0.05
4236	1.40272	0.05
4237	1.64984	0.05
4238	1.67345	0.05
4239	1.08212	2.1
4240	1.40905	2.1
4241	0.946132	0.05
4242	0.946132	0.05
4243	0.81268	0.05
4244	0.946131	0.05
4245	0.812681	0.05
4246	0.946131	0.05
4247	0.946132	0.05
4248	0.997755	0.05
4249	1.94585	0.05
4250	1.2925	0.05
4251	2.52454	0.05
4252	1.69182	0.05
4253	1.69182	0.05
4254	0.92361	0.05
4255	1.35316	0.05
4256	1.26227	0.05
4257	0.946131	0.05
4258	1.35316	0.05
4259	1.48676	0.0596501
4260	1.33917	0.0647169
4261	0.757292	2.1
4262	1.02415	2.1
4263	1.48495	2.1
4264	0.97587	2.1
4265	0.946132	2.1
4266	0.747365	2.1
4267	1.55432	2.1
4268	1.55432	2.1
4269	0.946132	2.1
4270	0.747394	2.1
4271	1.17464	0.05
4272	1.0372	0.05
4273	2.16553	0.05
4274	2.00616	0.05
4275	1.12184	0.05
4276	1.33177	0.05
4277	0.936674	0.05
4278	0.946129	0.05
4279	0.851552	0.05
4280	1.60205	0.05
4281	1.77391	0.05
4282	1.03327	0.05
4283	1.41512	0.05
4284	1.03327	0.05
4285	0.946133	0.05
4286	0.946131	0.05
4287	1.80077	0.05
4288	1.80077	0.05
4289	1.54848	2.1

4290	1.29931	2.1
4291	1.04482	0.0940925
4292	2.08069	0.0989194
4293	0.946127	0.05
4294	0.515539	0.109434
4295	0.515566	0.126802
4296	1.38423	0.05
4297	1.38423	0.0616742
4298	0.972058	0.0626448
4299	0.927737	0.0901577
4300	0.94971	0.05
4301	2.19682	0.0596312
4302	0.946132	0.0861315
4303	1.09841	0.0644123
4304	0.522382	0.150075
4305	1.5962	0.05
4306	1.72791	0.05
4307	1.07428	0.054682
4308	1.05215	0.0661204
4309	1.54632	1.4483
4310	1.15693	1.61428
4311	1.41238	2.1
4312	1.41238	2.1
4313	1.77966	2.1
4314	1.77966	2.1
4315	0.946129	2.1
4316	1.40525	2.1
4317	0.946131	2.1
4318	0.94613	2.1
4319	1.32054	2.1
4320	1.41238	2.1
4321	1.94026	2.1
4322	1.0171	0.118576
4323	1.1211	0.05
4324	0.993022	2.1
4325	0.773813	2.1
4326	1.4073	2.1
4327	1.4073	2.1
4328	0.94613	2.1
4329	0.787615	2.1
4330	0.94613	2.1
4331	0.787615	2.1
4332	1.764	2.1
4333	1.90964	2.1
4334	1.05787	2.1
4335	1.403	0.0686999
4336	0.744189	0.467439
4337	2.32834	2.1
4338	2.34986	2.1
4339	1.17106	1.37661
4340	1.94826	0.05
4341	2.40606	0.05
4342	1.26387	0.05
4343	0.954013	0.05
4344	0.946131	0.05
4345	1.71	0.05
4346	1.51926	0.05
4347	0.953997	0.05

4348	0.851554	0.05
4349	1.96502	0.05
4350	1.72954	0.05
4351	2.89117	0.05
4352	0.971958	0.05
4353	0.985575	0.05
4354	1.41943	0.05
4355	1.20021	0.05
4356	1.29599	0.05
4357	1.15687	0.05
4358	0.810958	0.05
4359	1.12919	0.05
4360	1.98859	2.1
4361	1.82044	2.1
4362	1.90488	2.1
4363	0.851542	2.1
4364	1.02973	2.1
4365	1.39286	2.1
4366	1.44873	2.1
4367	0.946132	2.1
4368	1.11826	2.1
4369	1.26842	2.1
4370	2.09461	2.1
4371	1.76952	0.05
4372	1.6473	0.05
4373	0.946131	0.05
4374	1.36486	0.05
4375	1.04243	0.05
4376	1.05017	0.05
4377	1.24059	0.05
4378	2.11646	0.05
4379	1.50368	0.05
4380	2.38796	0.05
4381	1.50368	0.05
4382	1.71844	0.05
4383	1.71844	0.05
4384	0.914836	0.05
4385	1.24416	0.05
4386	0.914805	0.05
4387	1.24416	0.05
4388	1.38911	0.05
4389	1.46859	0.05
4390	1.35348	0.05
4391	0.946131	0.05
4392	1.1226	0.05
4393	0.857078	0.05
4394	0.9746	0.05
4395	1.37624	0.05
4396	1.15131	0.05
4397	1.139	0.05
4398	1.90456	0.05
4399	2.09692	0.05
4400	1.16655	2.1
4401	1.23017	2.1
4402	2.14592	2.1
4403	1.23017	2.1
4404	1.0752	2.1
4405	1.52482	0.05

4406	1.09252	2.1
4407	1.10725	2.1
4408	1.14305	0.05
4409	0.851541	0.05
4410	1.48073	0.05
4411	1.45607	0.05
4412	0.946131	0.05
4413	0.932823	0.05
4414	1.45828	0.05
4415	0.851528	0.05
4416	1.14305	0.05
4417	1.92737	0.05
4418	1.08211	0.05
4419	1.69831	0.05
4420	1.69831	0.05
4421	0.946131	0.05
4422	1.26868	0.05
4423	1.23377	0.05
4424	1.23377	0.05
4425	0.94723	0.05
4426	1.64322	0.05
4427	1.64322	0.05
4428	0.946131	0.05
4429	1.32068	0.05
4430	0.946129	0.05
4431	1.18789	0.0630672
4432	1.32068	0.05
4433	1.32813	2.1
4434	1.32461	2.1
4435	1.42729	0.05
4436	2.47846	0.05
4437	1.00024	0.05
4438	1.23923	0.05
4439	0.946131	0.05
4440	0.712304	0.05
4441	1.77501	0.05
4442	1.77501	0.05
4443	0.593593	0.05
4444	1.23923	0.05
4445	1.23175	0.05
4446	0.94613	0.05
4447	0.713646	0.05
4448	1.75735	0.05
4449	1.75735	0.05
4450	0.713633	0.065874
4451	1.37157	0.05
4452	1.47169	0.05
4453	2.21808	0.05
4454	1.47169	0.05
4455	1.04759	2.1
4456	0.987132	2.1
4457	1.11181	2.1
4458	2.61682	2.1
4459	1.11181	2.1
4460	1.06461	1.40009
4461	1.69733	2.1
4462	2.79705	2.1
4463	1.08245	2.1

4464	1.40583	2.1
4465	1.39852	2.1
4466	2.0239	2.1
4467	2.0239	2.1
4468	0.780455	2.1
4469	0.946132	2.1
4470	0.780455	2.1
4471	1.01885	2.1
4472	0.564217	2.1
4473	0.733461	2.1
4474	0.537126	2.1
4475	0.683838	2.1
4476	0.779662	2.1
4477	0.634141	2.1
4478	0.685337	2.1
4479	1.58848	2.1
4480	1.17129	0.05
4481	0.663168	0.05
4482	1.55587	0.05
4483	1.31342	0.05
4484	0.596868	0.05
4485	1.1666	0.0603819
4486	0.867261	0.0554784
4487	0.946132	0.0924943
4488	1.28843	0.05
4489	1.31248	0.05
4490	2.59921	0.05
4491	1.32634	0.05
4492	1.35564	0.05
4493	1.52661	0.05
4494	1.12323	0.05
4495	1.17701	0.754885
4496	1.36405	0.77642
4497	1.19837	0.345297
4498	2.25636	0.308445
4499	1.0823	0.05
4500	1.31774	0.05
4501	1.0823	0.05
4502	1.01121	0.05
4503	1.27408	0.05
4504	0.946131	0.05
4505	0.566074	0.05
4506	0.79576	0.05
4507	1.24596	0.0504697
4508	1.47567	0.0528378
4509	1.47567	0.05
4510	0.56605	0.0503323
4511	1.6567	0.05
4512	0.967792	0.05
4513	1.00366	0.05
4514	1.00366	0.05
4515	1.56409	0.05
4516	1.56409	0.05
4517	0.774911	0.05
4518	1.09483	0.05
4519	0.774912	0.05
4520	1.09483	0.05
4521	1.24417	0.05

4522	1.13387	0.05
4523	1.13387	0.05
4524	1.30572	0.05
4525	2.18966	0.05
4526	0.98575	0.05
4527	1.54982	0.05
4528	2.48833	0.05
4529	1.54982	0.05
4530	1.28954	0.05
4531	1.64002	0.05
4532	1.64002	0.05
4533	1.85594	0.05
4534	1.12968	2.1
4535	1.23536	2.1
4536	1.4882	2.1
4537	1.4882	2.1
4538	0.876836	2.1
4539	0.946131	2.1
4540	0.876835	2.1
4541	1.43166	2.1
4542	2.25936	2.1
4543	1.0305	2.1
4544	0.992095	2.1
4545	1.12968	2.1
4546	1.53813	2.1
4547	1.53812	2.1
4548	0.583653	2.1
4549	0.94613	2.1
4550	0.583651	2.1
4551	0.964033	2.1
4552	0.964034	2.1
4553	1.54457	2.1
4554	3.54109	0.05
4555	0.99974	0.05
4556	0.78814	0.05
4557	1.65729	0.05
4558	1.1283	0.05
4559	1.09407	0.05
4560	1.55091	0.05
4561	0.870773	0.05
4562	1.19967	0.05
4563	1.56521	0.05
4564	1.30896	1.40273
4565	1.17989	2.1
4566	1.72526	2.1
4567	0.851537	1.74154
4568	1.58944	2.1
4569	1.56915	2.1
4570	0.891039	1.56818
4571	1.4172	1.53773
4572	0.963818	1.42467
4573	0.851474	2.05104
4574	1.17989	2.1
4575	1.56261	2.1
4576	1.56261	2.1
4577	0.761574	2.1
4578	1.31323	2.1
4579	0.761573	2.1

4580	0.946133	2.1
4581	1.31323	2.1
4582	1.16759	2.1
4583	1.16759	2.1
4584	2.11829	2.1
4585	1.16759	2.1
4586	1.10748	0.05
4587	1.47688	0.05
4588	0.946133	0.05
4589	0.953319	0.05
4590	0.946132	0.05
4591	1.51168	0.05
4592	1.51168	0.05
4593	1.25797	0.05
4594	1.19984	0.05
4595	1.95729	2.1
4596	2.34853	2.1
4597	1.94343	2.1
4598	2.00551	2.1
4599	1.20067	0.713097
4600	1.87244	0.806204
4601	1.4178	2.1
4602	1.33538	2.1
4603	0.946131	2.1
4604	1.22895	2.1
4605	1.12433	2.1
4606	0.956757	2.1
4607	0.91826	2.1
4608	1.44221	2.1
4609	1.14206	2.1
4610	1.80514	0.207234
4611	0.890304	1.37087
4612	1.1362	0.205612
4613	1.32819	0.257771
4614	1.46311	2.1
4615	0.8654	0.184127
4616	1.1699	1.21212
4617	1.00267	1.24453
4618	1.02882	0.444622
4619	1.00832	2.1
4620	0.974137	2.1
4621	1.02368	2.1
4622	0.974138	2.1
4623	0.995734	0.05
4624	1.44605	0.384457
4625	1.18549	0.05
4626	1.33621	0.50535
4627	1.5176	0.826743
4628	1.91762	0.643173
4629	1.33621	0.462967
4630	1.6638	0.587029
4631	1.66774	2.1
4632	1.11833	2.1
4633	0.926499	2.1
4634	1.51229	2.1
4635	1.51229	2.1
4636	0.926528	2.1
4637	1.40335	2.1

4638	1.03544	2.1
4639	0.926527	2.1
4640	1.85305	2.1
4641	1.5812	2.1
4642	1.15529	2.1
4643	1.31086	2.1
4644	0.999206	2.1
4645	1.46714	2.1
4646	1.73087	2.1
4647	0.772299	2.1
4648	1.24341	2.1
4649	0.997867	2.1
4650	0.772311	2.1
4651	0.936657	2.1
4652	1.35986	2.1
4653	0.959414	2.1
4654	1.36541	2.1
4655	1.49268	2.1
4656	1.49268	2.1
4657	0.832137	2.1
4658	0.946132	2.1
4659	0.832138	2.1
4660	1.64523	2.1
4661	1.11764	2.1
4662	1.05776	2.1
4663	0.972465	2.1
4664	0.972464	2.1
4665	1.98425	2.1
4666	0.969822	2.1
4667	1.30566	2.1
4668	1.52724	2.1
4669	2.32237	0.05
4670	2.30274	0.0690271
4671	2.27761	0.05
4672	3.33012	0.05
4673	1.24001	0.929408
4674	1.41821	1.10623
4675	0.978584	2.1
4676	1.18076	2.1
4677	1.07645	2.1
4678	0.877351	0.05
4679	0.946132	1.13879
4680	0.877353	0.341277
4681	1.58444	2.1
4682	1.49614	2.1
4683	1.28355	1.96012
4684	1.13491	1.4526
4685	1.56875	0.05
4686	0.499592	0.05
4687	0.946131	0.05
4688	1.20538	0.05
4689	1.09913	0.05
4690	1.8049	0.05
4691	1.8049	0.05
4692	0.499606	0.05
4693	2.41076	0.05
4694	0.860202	0.05
4695	0.906798	0.05

4696	1.8626	0.05
4697	1.33986	0.05
4698	1.2992	0.05
4699	1.58702	0.05
4700	1.05939	0.05
4701	1.02074	2.1
4702	1.07866	2.1
4703	1.5082	0.05
4704	1.5522	0.05
4705	1.65903	0.05
4706	1.86766	2.1
4707	2.11684	2.1
4708	0.946132	2.1
4709	1.6585	2.1
4710	1.6585	2.1
4711	0.94613	2.1
4712	1.1872	2.1
4713	1.1872	2.1
4714	1.05842	2.1
4715	1.20455	2.1
4716	1.34141	2.1
4717	1.34141	2.1
4718	1.42416	2.1
4719	1.75818	2.1
4720	2.63715	1.1126
4721	1.80454	2.1
4722	2.26198	2.1
4723	0.696255	2.1
4724	0.624075	2.1
4725	1.50905	0.978795
4726	1.50905	2.1
4727	0.624075	2.1
4728	1.31857	0.706798
4729	0.946131	2.1
4730	0.624075	1.21432
4731	0.914628	2.1
4732	1.14721	2.1
4733	1.16153	2.1
4734	1.47664	2.1
4735	1.54791	2.1
4736	1.19098	2.1
4737	1.13341	2.1
4738	1.00503	0.45812
4739	1.00503	1.1334
4740	1.22655	2.1
4741	0.999489	2.1
4742	1.3268	2.1
4743	0.946132	2.1
4744	1.60061	2.1
4745	1.60061	2.1
4746	1.08034	2.1
4747	1.40372	2.1
4748	1.14302	2.1
4749	1.08034	2.1
4750	0.999491	2.1
4751	1.17152	0.05
4752	1.17152	0.05
4753	1.17152	0.05

4754	2.05423	0.05
4755	1.40984	2.1
4756	1.2489	2.1
4757	0.885089	2.1
4758	0.810931	2.1
4759	1.84128	2.1
4760	1.11206	2.1
4761	1.21926	2.1
4762	1.99084	2.1
4763	0.976334	0.664748
4764	0.599363	0.450636
4765	1.02621	0.17973
4766	0.462959	0.433987
4767	1.42612	0.142896
4768	0.299682	0.196943
4769	1.51379	2.1
4770	0.719531	2.1
4771	1.3702	2.1
4772	1.33293	2.1
4773	0.851539	2.1
4774	0.62998	2.1
4775	0.851543	2.1
4776	0.629966	2.1
4777	1.43132	2.1
4778	1.13328	1.23241
4779	1.93212	1.0333
4780	1.62345	1.01919
4781	1.38511	1.32974
4782	1.40013	0.313529
4783	1.04132	0.119279
4784	0.83473	1.06498
4785	0.868732	0.749735
4786	0.810595	1.75933
4787	0.729919	0.643684
4788	0.992428	0.774807
4789	0.577107	0.219334
4790	1.18464	0.810556
4791	0.791199	0.885549
4792	0.810943	0.05
4793	0.860723	0.05
4794	1.33348	0.05
4795	1.57467	0.05
4796	0.981032	0.05
4797	0.731569	0.05
4798	0.770312	0.05
4799	1.46055	2.1
4800	1.42249	2.1
4801	1.75999	0.05
4802	1.6868	0.05
4803	1.17042	1.00443
4804	1.01485	2.1
4805	1.19952	0.05
4806	1.40592	0.150229
4807	1.19952	0.05
4808	2.20145	0.05
4809	1.33649	0.05
4810	1.11612	0.073826
4811	0.957588	0.05

4812	0.946126	0.0658254
4813	1.50648	0.05
4814	1.50648	0.05
4815	0.946128	0.05
4816	2.10671	0.05
4817	1.91518	0.05
4818	2.23223	0.05
4819	1.53556	2.1
4820	1.56253	2.1
4821	1.27806	0.05
4822	1.44593	0.05
4823	1.03818	0.256607
4824	1.03818	0.0919957
4825	1.38969	0.476264
4826	1.85548	0.160427
4827	2.6044	0.437187
4828	1.85548	0.13573
4829	1.49346	2.1
4830	1.83192	2.00108
4831	0.834043	1.99904
4832	0.810943	0.589819
4833	0.887113	0.988272
4834	0.893827	1.16785
4835	0.991061	1.28196
4836	0.91939	1.53744
4837	0.793307	1.62115
4838	1.75291	1.6718
4839	1.35435	1.4421
4840	1.16989	0.0916045
4841	1.36717	0.087182
4842	2.12276	0.0719265
4843	2.30493	0.319861
4844	2.47857	0.169763
4845	1.92269	0.122344
4846	1.66426	0.429162
4847	0.956698	1.07624
4848	0.956698	2.1
4849	0.857957	2.1
4850	1.4706	2.1
4851	1.65706	2.1
4852	1.47059	2.1
4853	1.04713	0.05
4854	0.946131	0.100006
4855	1.47374	0.0969901
4856	1.47374	0.05
4857	0.951675	0.05
4858	1.2582	0.0958303
4859	1.2582	0.05
4860	0.94613	0.156433
4861	1.4468	0.05
4862	1.90335	0.05
4863	1.14886	2.1
4864	2.59689	2.1
4865	1.02747	2.1
4866	0.946131	2.1
4867	0.934061	2.1
4868	1.63979	2.1
4869	1.65206	2.1

4870	1.48	2.1
4871	1.27467	2.1
4872	2.20449	2.1
4873	1.1333	0.05
4874	1.95334	2.1
4875	2.20984	2.1
4876	1.46389	0.05
4877	1.4639	0.05
4878	1.4639	0.05
4879	0.946132	0.05
4880	1.5144	0.05
4881	1.5144	0.05
4882	0.946132	0.05
4883	1.35732	0.05
4884	1.10321	0.05
4885	0.946132	0.05
4886	0.980549	0.05
4887	1.32819	2.1
4888	1.30171	1.28452
4889	1.68377	1.13058
4890	1.68377	2.1
4891	0.946132	1.00205
4892	0.946132	2.1
4893	1.1952	1.35652
4894	0.990019	1.37278
4895	1.08195	1.81583
4896	1.2269	2.1
4897	1.19219	2.1
4898	1.01485	2.1
4899	1.57125	0.05
4900	2.10696	0.05
4901	1.29666	0.05
4902	1.57125	0.05
4903	1.47712	2.1
4904	1.45182	2.1
4905	1.17816	0.05
4906	1.4516	0.05
4907	1.17816	0.05
4908	1.17816	0.05
4909	0.596954	1.11705
4910	1.65133	0.401232
4911	0.583859	0.448556
4912	1.04072	1.16049
4913	1.28795	0.448571
4914	1.59423	0.279743
4915	0.483559	0.624008
4916	1.74088	0.338605
4917	1.09545	1.088
4918	1.02519	1.36076
4919	2.47579	1.02763
4920	1.54732	1.12622
4921	2.17663	1.00041
4922	1.75006	1.03775
4923	1.32354	1.10013
4924	1.09962	2.1
4925	1.85057	2.1
4926	1.91046	2.1
4927	2.17037	2.1

4928	1.99528	0.05
4929	1.97969	0.05
4930	3.82765	2.1
4931	1.85767	2.1
4932	2.77042	2.1
4933	1.37236	0.05
4934	2.23254	0.05
4935	1.10089	0.05
4936	0.994294	2.1
4937	1.81921	2.1
4938	1.00073	2.1
4939	0.681043	2.1
4940	0.946131	2.1
4941	0.681073	2.1
4942	1.51804	2.1
4943	1.60653	2.1
4944	1.30188	2.1
4945	0.919622	2.1
4946	1.00965	2.1
4947	1.93821	2.1
4948	2.60377	2.1
4949	1.36416	0.05
4950	1.36416	0.05
4951	1.36416	0.05
4952	2.44132	0.05
4953	2.15871	0.427845
4954	2.22171	0.164463
4955	1.70747	0.142847
4956	1.49515	0.0836964
4957	0.773007	0.186719
4958	0.946125	0.211759
4959	0.946131	0.405424
4960	0.773034	0.101954
4961	1.15844	0.215989
4962	1.98104	0.124363
4963	2.44283	0.120149
4964	1.05417	0.05
4965	1.1596	0.05
4966	0.829921	0.05
4967	0.946131	0.05
4968	0.951911	0.05
4969	1.39027	0.05
4970	0.829946	0.05
4971	1.51223	0.05
4972	1.51223	0.05
4973	1.65984	0.05
4974	1.90382	0.05
4975	1.93228	1.18536
4976	2.47924	1.14386
4977	1.73904	1.28531
4978	1.40547	0.921699
4979	2.37589	1.01616
4980	0.662605	1.24682
4981	2.33293	1.0812
4982	1.72249	2.1
4983	1.68478	2.1
4984	1.00514	0.390379
4985	1.4537	2.1

4986	1.92698	2.1
4987	1.46041	0.05
4988	2.04817	0.05
4989	1.65954	2.1
4990	1.42342	2.1
4991	1.04405	0.05
4992	1.17056	0.05
4993	1.11339	0.05
4994	0.946133	0.05
4995	0.929724	0.05
4996	1.35424	0.05
4997	1.35424	0.05
4998	0.929713	0.05
4999	1.85945	0.05
5000	1.38905	2.1
5001	1.39056	2.1
5002	1.52852	2.1
5003	1.06472	2.1
5004	0.749096	2.1
5005	1.46076	2.1
5006	1.29263	2.1
5007	0.94613	2.1
5008	0.69097	2.1
5009	0.982284	2.1
5010	0.67419	2.1
5011	1.52852	2.1
5012	1.91121	2.1
5013	1.00775	2.1
5014	1.61201	2.1
5015	0.529987	2.1
5016	0.506125	2.1
5017	1.44696	2.1
5018	2.32981	2.1
5019	1.32784	2.1
5020	1.94158	2.1
5021	1.31595	2.1
5022	0.663922	2.1
5023	1.44663	2.1
5024	1.44663	2.1
5025	1.13976	2.1
5026	0.970788	2.1
5027	1.13976	2.1
5028	0.663921	2.1
5029	0.966114	2.1
5030	1.05498	1.12233
5031	1.38086	2.1
5032	0.946127	2.1
5033	0.922513	2.1
5034	1.49903	2.1
5035	1.49903	2.1
5036	0.92251	2.1
5037	1.06105	2.1
5038	1.36049	2.1
5039	1.38086	2.1
5040	1.52534	2.1
5041	1.04101	2.1
5042	1.30861	0.05
5043	0.949112	0.05

5044	0.894275	0.05
5045	1.10232	0.05
5046	0.830889	0.05
5047	1.26554	0.05
5048	0.948347	0.05
5049	0.81096	0.05
5050	1.18338	0.05
5051	0.883764	0.05
5052	1.54793	0.05
5053	1.02867	0.05
5054	1.8573	0.05
5055	1.06065	0.05
5056	0.64842	0.05
5057	1.99319	0.05
5058	1.9476	0.05
5059	2.51436	0.05
5060	1.68337	0.05
5061	1.92321	0.05
5062	0.810942	0.05
5063	0.921001	0.05
5064	0.99758	0.05
5065	0.960785	0.05
5066	1.01565	0.05
5067	0.908624	0.05
5068	0.912835	0.05
5069	1.18351	0.05
5070	1.40239	2.1
5071	1.26091	2.1
5072	0.947979	2.1
5073	0.946131	2.1
5074	1.81959	2.1
5075	1.81959	1.25444
5076	0.94613	2.1
5077	1.38415	1.00262
5078	1.38414	2.1
5079	1.33266	2.1
5080	0.946132	2.1
5081	1.76225	2.1
5082	1.76225	2.1
5083	1.27599	2.1
5084	1.38158	2.1
5085	1.3268	2.1
5086	1.27599	2.1
5087	0.947979	2.1
5088	1.0142	0.389344
5089	1.01422	0.278662
5090	1.11435	0.859968
5091	1.42186	1.00023
5092	1.24001	1.15679
5093	1.77342	1.18957
5094	1.42186	1.37129
5095	1.99817	1.04851
5096	1.27361	2.1
5097	1.68914	2.1
5098	1.30116	2.1
5099	1.32819	1.55372
5100	1.68322	1.46232
5101	1.68322	2.1

5102	0.94613	0.275654
5103	0.94613	2.1
5104	1.14666	2.055
5105	1.07612	1.33012
5106	1.31151	2.1
5107	1.22095	2.1
5108	1.57256	2.1
5109	1.6679	2.1
5110	0.946132	1.31174
5111	0.946133	2.1
5112	1.13537	2.1
5113	1.07855	2.1
5114	1.07855	2.1
5115	1.43895	1.26404
5116	1.38481	0.05
5117	1.72385	0.05
5118	0.550441	0.05
5119	0.79807	0.05
5120	1.11627	0.05
5121	1.30349	0.05
5122	0.94613	0.05
5123	0.686176	0.05
5124	1.11627	0.05
5125	1.73363	0.05
5126	1.73363	0.05
5127	0.686139	0.05
5128	1.24932	0.05
5129	0.899582	0.05
5130	2.00472	0.05
5131	1.17121	0.05
5132	1.2874	0.05
5133	2.0194	0.05
5134	0.88601	0.05
5135	1.77409	0.05
5136	1.73293	0.05
5137	2.74045	0.05
5138	1.83787	0.05
5139	1.45448	0.05
5140	1.27778	0.05
5141	1.1192	2.1
5142	1.25953	2.1
5143	1.93611	2.1
5144	1.92104	2.1
5145	1.15506	0.05
5146	1.15506	0.05
5147	1.23943	0.729461
5148	0.851543	2.1
5149	1.6061	2.1
5150	1.80058	2.1
5151	0.869471	2.1
5152	1.3931	2.1
5153	1.06454	2.1
5154	0.93667	2.1
5155	2.16916	2.1
5156	3.10246	2.1
5157	1.91539	2.1
5158	1.91539	2.1
5159	0.620263	1.63401

5160	0.620265	2.1
5161	0.946132	1.88672
5162	0.620265	1.48435
5163	1.30226	1.35818
5164	1.87292	2.1
5165	1.18769	2.1
5166	2.60452	2.1
5167	1.26574	2.1
5168	2.46679	2.1
5169	1.36459	2.1
5170	1.26865	1.10799
5171	1.1744	0.958189
5172	1.15841	0.11548
5173	1.02247	0.05
5174	1.93649	2.1
5175	1.64718	2.1
5176	1.59654	2.1
5177	1.57823	2.1
5178	1.00665	0.05
5179	1.28239	0.05
5180	0.887895	2.1
5181	1.22083	2.1
5182	1.42127	2.1
5183	1.07374	2.1
5184	1.00503	1.38082
5185	1.23082	1.05072
5186	2.78663	2.1
5187	2.20883	2.1
5188	2.54588	2.1
5189	0.946128	2.1
5190	1.13716	2.1
5191	1.58724	2.1
5192	1.58724	2.1
5193	0.946131	2.1
5194	1.13716	2.1
5195	1.39621	2.1
5196	1.02438	2.1
5197	1.49543	0.05
5198	2.78336	0.05
5199	1.27148	0.05
5200	1.0906	0.05
5201	1.04072	0.05
5202	0.63574	0.05
5203	1.45506	0.05
5204	1.5863	0.05
5205	0.928352	0.05
5206	0.647687	0.05
5207	2.2375	2.025
5208	1.50998	1.78315
5209	2.75021	1.72319
5210	1.91912	1.57195
5211	1.99872	1.36477
5212	0.918127	1.91778
5213	1.03031	1.46588
5214	0.951169	0.745985
5215	1.27975	1.11594
5216	1.12735	1.43874
5217	1.15741	1.41078

5218	1.24998	1.39371
5219	1.65953	0.05
5220	0.967555	0.288469
5221	0.967542	0.05
5222	1.03824	0.05
5223	1.48693	0.0795401
5224	1.48693	0.05
5225	2.02901	0.05
5226	0.946127	0.05
5227	1.07139	0.05
5228	0.946126	0.05
5229	1.84644	0.05
5230	1.84644	0.05
5231	1.38683	0.05
5232	1.40574	0.05
5233	1.36866	0.907229
5234	0.649325	0.87107
5235	1.93631	2.1
5236	1.50145	2.1
5237	1.6287	2.1
5238	1.31302	2.1
5239	1.18041	2.1
5240	1.18041	2.1
5241	0.974196	2.1
5242	0.946131	2.1
5243	1.74169	2.1
5244	1.74169	2.1
5245	1.23163	2.1
5246	1.27451	2.1
5247	1.41331	2.1
5248	1.23163	2.1
5249	1.28037	2.1
5250	1.28158	2.1
5251	1.28037	2.1
5252	2.28015	0.05
5253	2.06561	0.05
5254	2.61343	0.05
5255	1.11712	0.05
5256	1.63689	1.04069
5257	1.01393	0.805334
5258	0.996775	2.1
5259	1.54277	2.1
5260	1.26397	2.1
5261	1.46129	2.1
5262	1.62391	2.1
5263	1.08848	0.05
5264	1.11833	2.1
5265	1.24041	2.1
5266	2.31884	2.1
5267	1.335	2.1
5268	1.48589	0.05
5269	1.77131	0.05
5270	1.8422	1.40619
5271	2.47453	1.86153
5272	1.23726	1.63911
5273	1.21996	1.65952
5274	1.65512	1.78176
5275	1.65512	1.74965

5276	0.802107	1.82395
5277	0.946129	1.23857
5278	0.802137	1.15468
5279	1.3279	1.3021
5280	1.23726	1.31477
5281	1.79759	1.24432
5282	1.79759	1.0693
5283	0.767577	1.6565
5284	0.946131	0.861069
5285	0.767577	0.775453
5286	1.03534	0.05
5287	1.09962	0.536283
5288	0.929217	1.36889
5289	1.70126	1.45516
5290	0.991407	2.1
5291	1.41077	2.1
5292	0.991406	2.1
5293	1.62966	2.1
5294	1.62966	2.1
5295	0.946128	2.1
5296	1.18364	2.1
5297	0.946131	2.1
5298	1.14186	2.1
5299	1.18364	2.1
5300	1.16858	2.1
5301	1.66137	0.19836
5302	1.90165	0.05
5303	1.66139	0.05
5304	1.26893	0.0859862
5305	1.15918	0.70412
5306	0.653228	0.737921
5307	0.946133	0.7
5308	1.77488	2.01076
5309	1.77489	0.979129
5310	0.653228	0.724578
5311	1.56043	0.713404
5312	1.60894	0.882025
5313	1.56043	0.665956
5314	1.06848	1.73645
5315	1.04307	2.1
5316	1.14188	2.1
5317	1.09195	2.1
5318	0.615121	2.1
5319	1.50867	2.1
5320	1.27152	2.1
5321	0.615094	2.1
5322	1.07297	2.1
5323	1.1372	2.1
5324	0.553608	2.1
5325	1.23019	2.1
5326	0.992675	2.1
5327	1.40984	1.58584
5328	2.55682	2.1
5329	3.66069	2.1
5330	3.58726	2.1
5331	1.4375	0.05
5332	1.88424	0.056582
5333	1.56714	2.1

5334	1.6721	2.1
5335	1.18474	2.1
5336	1.2295	2.1
5337	1.28754	2.1
5338	1.36838	2.1
5339	2.78661	2.1
5340	2.64055	2.1
5341	1.35065	0.92103
5342	2.26675	0.0987491
5343	1.35065	0.302144
5344	1.21558	0.220019
5345	1.49709	0.05
5346	1.4971	0.05
5347	0.85472	0.05
5348	0.854718	0.05
5349	0.946129	0.05
5350	0.854717	0.05
5351	1.1604	0.05
5352	1.25265	0.05
5353	2.32081	0.05
5354	1.15371	0.05
5355	1.14945	0.780333
5356	0.995725	0.819812
5357	1.00307	0.05
5358	1.10907	0.330207
5359	2.30315	0.0863003
5360	1.10908	0.249365
5361	1.27558	1.03789
5362	1.71613	1.43437
5363	1.32977	2.1
5364	0.815276	2.1
5365	1.41697	2.1
5366	1.41697	2.1
5367	0.815276	2.1
5368	1.38897	2.1
5369	0.946131	2.1
5370	0.815275	2.1
5371	1.44684	2.1
5372	1.21489	1.83286
5373	1.22476	1.80101
5374	0.870366	1.07435
5375	0.862572	0.891947
5376	0.835435	0.759619
5377	1.6993	0.565504
5378	1.23618	0.408237
5379	1.18624	0.724237
5380	1.53058	0.492731
5381	2.16108	1.09529
5382	2.21589	0.991248
5383	1.04037	2.1
5384	1.03933	2.1
5385	1.57753	0.05
5386	1.87644	0.05
5387	1.24728	0.05
5388	1.30394	0.05
5389	1.3538	2.1
5390	1.69858	2.1
5391	1.1726	0.05

5392	0.94612	0.05
5393	1.09805	0.05
5394	0.946118	0.05
5395	1.72576	0.05
5396	1.72575	0.05
5397	1.34152	0.05
5398	1.33034	0.05
5399	0.784151	2.1
5400	0.946131	2.1
5401	0.784151	2.1
5402	1.47963	2.1
5403	1.47963	2.1
5404	1.3335	2.1
5405	0.930281	2.1
5406	1.29435	2.1
5407	0.883308	1.97609
5408	1.31589	2.1
5409	1.67846	2.1
5410	0.993598	0.05
5411	2.23193	0.05
5412	0.9936	0.05
5413	0.615629	0.05
5414	1.65925	2.1
5415	2.05992	2.1
5416	1.65925	2.1
5417	4.08735	1.72218
5418	2.72856	1.81655
5419	0.869602	2.1
5420	3.42833	1.13471
5421	1.14397	0.05
5422	1.34563	0.513996
5423	1.08189	0.249658
5424	0.782128	2.1
5425	1.0022	2.1
5426	0.900427	2.09435
5427	1.51499	2.1
5428	1.05588	2.1
5429	1.13739	2.1
5430	1.37004	2.1
5431	1.14603	2.1
5432	0.851541	1.47431
5433	0.946799	2.04981
5434	1.14854	1.92379
5435	1.33793	1.5838
5436	0.94613	1.57009
5437	1.24336	1.8591
5438	0.851473	1.92286
5439	2.08792	1.84865
5440	1.01869	1.91972
5441	0.986953	0.05
5442	0.896238	0.05
5443	0.906574	0.05
5444	0.852145	0.05
5445	2.14191	0.05
5446	1.15979	0.05
5447	1.18327	0.05
5448	2.32742	0.05
5449	2.35961	0.05

5450	2.38126	0.05
5451	1.13632	0.05
5452	1.17897	0.05
5453	1.76199	0.05
5454	1.00573	2.1
5455	0.946127	0.936212
5456	1.7077	2.1
5457	1.7077	2.1
5458	0.946127	1.70785
5459	1.24887	2.1
5460	1.24887	2.1
5461	1.22441	2.1
5462	1.08542	2.1
5463	1.21743	2.02469
5464	1.12572	2.1
5465	1.05499	1.05762
5466	1.00521	1.03313
5467	2.86191	0.05
5468	2.27972	0.05
5469	1.01169	2.1
5470	1.03373	2.1
5471	1.39319	2.1
5472	0.636837	2.1
5473	1.63908	2.1
5474	1.45713	2.06072
5475	1.04072	2.1
5476	1.25025	2.08764
5477	1.38914	2.1
5478	0.636807	2.1
5479	1.50211	2.1
5480	1.36555	2.1
5481	2.77545	1.51416
5482	0.682805	2.1
5483	1.50267	2.1
5484	1.50267	2.1
5485	0.797749	2.1
5486	1.38773	2.1
5487	1.38773	2.1
5488	0.682776	2.1
5489	2.01245	0.05
5490	0.925918	0.05
5491	0.75689	0.05
5492	1.25823	0.05
5493	1.16505	0.05
5494	0.838779	0.05
5495	1.14722	0.05
5496	0.756849	0.05
5497	1.52298	0.05
5498	2.11034	0.05
5499	1.92174	0.05
5500	1.92174	0.05
5501	0.576083	0.05
5502	1.23146	0.05
5503	1.26637	0.05
5504	1.23146	0.05
5505	1.26637	0.05
5506	0.946133	0.394793
5507	1.37034	0.106268

5508	1.84145	0.218735
5509	1.84145	0.093972
5510	0.946132	0.730306
5511	1.37034	0.319773
5512	1.41724	0.301687
5513	1.0685	0.0918529
5514	1.14547	0.0971411
5515	1.24976	2.1
5516	1.30682	2.1
5517	2.12095	2.1
5518	1.46392	2.1
5519	1.46392	2.1
5520	0.935738	2.1
5521	1.32949	2.1
5522	1.07017	2.1
5523	0.935766	2.1
5524	1.07017	2.1
5525	1.78095	2.1
5526	1.05619	0.794871
5527	1.05793	0.813163
5528	1.77677	2.1
5529	2.04766	2.1
5530	1.02524	2.1
5531	1.18803	2.1
5532	1.70047	2.1
5533	1.556 2.1	
5534	1.24201	2.1
5535	1.3592	2.1
5536	2.11824	0.05
5537	2.20248	0.05
5538	3.43878	0.05
5539	3.12466	0.05
5540	2.09351	2.1
5541	0.946128	2.1
5542	1.51269	2.1
5543	1.51269	2.1
5544	0.946131	2.1
5545	1.01306	2.1
5546	1.04676	2.1
5547	1.01306	2.1
5548	1.07444	2.1
5549	2.17465	2.1
5550	1.14808	2.1
5551	1.39067	1.28647
5552	1.20958	1.19711
5553	0.662832	0.154949
5554	1.93512	0.835162
5555	1.15471	2.1
5556	1.75119	2.1
5557	1.75118	2.1
5558	0.946131	2.1
5559	1.28606	2.1
5560	1.27087	2.1
5561	0.946131	2.1
5562	1.28606	2.1
5563	1.58916	0.05
5564	0.927822	0.05
5565	2.24367	0.05

5566	1.8734	0.05
5567	3.51668	0.05
5568	2.68986	0.05
5569	2.32395	2.1
5570	1.39564	2.1
5571	2.10862	2.1
5572	1.36763	2.1
5573	0.970963	0.05
5574	0.97096	0.05
5575	1.11897	0.05
5576	1.26885	0.05
5577	1.11605	0.05
5578	2.06612	0.05
5579	1.717 0.05	
5580	0.59502	0.05
5581	0.825755	0.05
5582	0.709624	0.0544267
5583	1.65741	0.05
5584	0.307486	0.05
5585	1.4785	0.05
5586	0.983143	2.1
5587	1.08247	2.1
5588	1.42353	2.1
5589	1.42353	2.1
5590	0.781667	2.1
5591	0.797634	2.1
5592	0.946131	2.1
5593	0.781638	2.1
5594	0.946131	2.1
5595	0.98315	2.1
5596	1.083 2.1	
5597	1.61975	0.05
5598	1.11946	0.05
5599	1.18977	0.05
5600	0.996484	0.05
5601	2.24343	0.05
5602	1.4787	0.05
5603	1.66127	0.05
5604	1.86028	0.05
5605	1.09128	0.05
5606	1.03115	0.05
5607	0.838025	0.05
5608	1.3736	0.05
5609	1.44009	0.05
5610	0.92917	0.05
5611	1.03889	0.05
5612	0.956974	0.05
5613	3.23468	0.05
5614	0.953922	0.05
5615	0.957104	0.05
5616	1.66231	0.05
5617	1.26778	0.05
5618	1.47615	0.05
5619	1.28621	0.05
5620	1.08043	0.05
5621	2.35026	2.1
5622	2.35025	2.1
5623	2.60659	2.1

5624	1.57627	2.1
5625	1.5493	2.1
5626	1.24682	0.0677476
5627	2.14277	0.0897622
5628	1.24682	0.795806
5629	1.00307	0.112562
5630	2.04056	0.142421
5631	1.45408	2.1
5632	1.54249	2.1
5633	1.24474	1.5316
5634	1.37813	1.91348
5635	0.924158	0.05
5636	0.916623	0.05
5637	1.35344	0.05
5638	1.00859	0.05
5639	1.34786	0.05
5640	0.952457	0.05
5641	0.85404	0.05
5642	1.06341	2.1
5643	1.09194	2.1
5644	1.93707	2.1
5645	2.14087	2.1
5646	1.93707	2.1
5647	1.07519	2.1
5648	1.07519	1.04
5649	1.62518	2.1
5650	1.3592	2.1
5651	1.13183	2.1
5652	1.20357	2.1
5653	1.4858	2.1
5654	1.23694	2.1
5655	1.44011	2.1
5656	1.23989	2.1
5657	2.21604	2.1
5658	1.85574	0.05
5659	1.55649	2.1
5660	1.48861	0.05
5661	1.15968	0.05
5662	0.946132	0.05
5663	0.663056	0.05
5664	1.70837	0.05
5665	1.70837	0.05
5666	0.946131	0.05
5667	0.663026	0.05
5668	2.09604	0.05
5669	0.81094	0.05
5670	1.06559	0.05
5671	0.810956	0.05
5672	1.15474	0.05
5673	0.990051	0.05
5674	0.894971	0.05
5675	1.32536	0.05
5676	0.946133	2.1
5677	1.33125	2.1
5678	1.75823	2.1
5679	1.75823	2.1
5680	0.946133	2.1
5681	1.33125	2.1

5682	1.37312	2.1
5683	1.27985	2.1
5684	1.37312	2.1
5685	1.70684	2.1
5686	1.70684	2.1
5687	0.946132	2.1
5688	0.946132	2.1
5689	1.25081	2.1
5690	0.956293	2.1
5691	0.956294	2.1
5692	1.36035	2.1
5693	1.41944	2.1
5694	0.830638	2.1
5695	0.747568	2.1
5696	0.888498	2.1
5697	1.04071	2.1
5698	1.17128	2.1
5699	1.92339	2.1
5700	2.91208	2.1
5701	1.82208	0.981219
5702	1.89653	2.1
5703	1.82588	1.52466
5704	1.26703	0.287182
5705	1.26703	1.06432
5706	0.6157	0.272851
5707	1.26706	1.56406
5708	0.61567	0.05
5709	0.615671	0.063375
5710	1.26706	0.224746
5711	1.89158	0.895836
5712	1.23134	0.162004
5713	2.49497	0.05
5714	1.68103	0.05
5715	2.0554	2.1
5716	1.72647	2.1
5717	1.67375	2.1
5718	1.65037	2.1
5719	1.04349	0.556854
5720	1.69055	0.05
5721	1.53463	0.05
5722	1.19022	2.1
5723	1.89226	1.2548
5724	1.15009	0.982909
5725	1.7054	0.0755906
5726	1.31612	0.05
5727	1.31611	0.193972
5728	1.67345	0.05
5729	1.06577	0.05
5730	1.40193	0.05
5731	1.72411	0.05
5732	1.46123	0.05
5733	0.808808	0.05
5734	0.897714	0.05
5735	0.851554	0.05
5736	1.3499	0.05
5737	1.29001	0.05
5738	1.22083	2.1
5739	1.06191	2.1

5740	1.05758	1.36264
5741	1.348	1.97413
5742	1.43485	1.33064
5743	1.34799	1.78724
5744	1.36307	0.05
5745	2.28188	0.05
5746	1.7926	0.05
5747	1.38957	2.1
5748	1.47446	2.1
5749	0.953638	2.1
5750	1.11868	2.1
5751	0.953636	2.1
5752	1.03408	2.1
5753	1.52443	0.05
5754	1.38539	0.05
5755	0.946132	0.05
5756	1.12747	0.05
5757	1.04393	0.05
5758	1.05017	0.05
5759	1.20529	0.05
5760	1.3663	0.617772
5761	1.89226	0.722269
5762	1.17885	0.772139
5763	0.867881	0.688242
5764	1.32917	0.698228
5765	1.32917	0.390111
5766	0.946127	0.722874
5767	1.19583	0.477152
5768	1.00122	0.669473
5769	0.946128	0.708615
5770	1.71405	0.800973
5771	0.871815	2.1
5772	1.16435	2.1
5773	0.686619	2.1
5774	1.63102	2.1
5775	1.06589	2.1
5776	1.05375	2.1
5777	1.9023	2.1
5778	1.27394	0.944429
5779	1.88345	0.919873
5780	2.05636	0.970486
5781	0.672977	0.476804
5782	1.41274	0.699838
5783	1.65606	0.520725
5784	0.869329	0.484218
5785	1.2207	0.908535
5786	1.35631	0.782373
5787	0.908776	0.540995
5788	0.955081	0.111247
5789	1.2243	2.1
5790	1.23169	2.1
5791	1.52228	2.1
5792	1.23169	2.1
5793	0.990892	0.975193
5794	1.08376	0.810548
5795	1.79348	1.86494
5796	1.50043	0.897217
5797	1.32599	2.1

5798	1.37924	0.907486
5799	0.838014	0.797541
5800	0.87737	1.25792
5801	0.947086	0.902708
5802	1.27199	1.59223
5803	1.04918	0.898478
5804	1.04851	1.97702
5805	1.1699	1.09869
5806	0.810934	1.10354
5807	1.83602	2.1
5808	2.21909	2.00452
5809	0.619441	1.02965
5810	2.03755	0.937162
5811	1.83472	1.92597
5812	1.20416	1.38446
5813	1.26691	0.872641
5814	0.655795	1.04971
5815	0.94613	0.976097
5816	2.35169	1.33181
5817	3.54705	2.1
5818	2.60741	2.1
5819	1.59019	0.05
5820	1.24526	0.05
5821	1.59019	0.05
5822	2.36406	0.05
5823	1.16776	0.488627
5824	1.67311	1.23114
5825	1.67311	0.729077
5826	0.94613	0.847926
5827	1.38645	0.937989
5828	1.13563	0.0985308
5829	1.13563	0.848009
5830	1.23279	0.192145
5831	1.05042	2.1
5832	1.17418	2.1
5833	1.28909	2.1
5834	0.973448	2.1
5835	1.02958	2.1
5836	3.3005	2.1
5837	2.86325	2.1
5838	1.35879	2.1
5839	1.69332	2.1
5840	1.69332	2.1
5841	0.946128	2.1
5842	1.39659	2.1
5843	1.12035	2.1
5844	1.12035	2.1
5845	1.24286	2.1
5846	1.10407	2.1
5847	1.14599	2.1
5848	1.32259	0.915276
5849	0.788592	0.05
5850	2.4588	2.1
5851	0.909581	2.1
5852	1.35656	1.61463
5853	0.986895	1.96116
5854	2.30602	1.56435
5855	1.46873	1.88767

5856	1.55584	1.52904
5857	2.1547	1.66627
5858	1.87794	2.1
5859	0.808286	2.1
5860	0.928464	1.95508
5861	0.810959	1.83512
5862	1.50962	1.39299
5863	1.1053	1.66735
5864	1.0712	1.63389
5865	1.40504	1.61725
5866	1.87794	1.43854
5867	2.80496	1.59619
5868	1.41479	1.98949
5869	1.38634	2.1
5870	1.38634	2.1
5871	1.67762	2.1
5872	0.946131	2.1
5873	1.188 2.1	
5874	1.62585	2.1
5875	1.62585	2.1
5876	0.946129	2.1
5877	1.18801	2.1
5878	1.38397	2.1
5879	1.04546	2.1
5880	1.16817	2.1
5881	1.06007	2.1
5882	1.19785	2.1
5883	1.43652	2.1
5884	1.06994	2.1
5885	1.89111	2.1
5886	1.07144	2.1
5887	1.51754	2.1
5888	2.0873	2.1
5889	0.758799	2.1
5890	1.59518	2.1
5891	1.59518	2.1
5892	0.946132	2.1
5893	0.758771	2.1
5894	1.06957	2.1
5895	0.758771	2.1
5896	1.33916	2.1
5897	2.03045	1.28935
5898	2.80184	1.57632
5899	1.76788	2.06163
5900	1.27442	2.1
5901	1.06756	2.1
5902	0.966979	2.1
5903	1.7054	2.1
5904	1.07156	2.1
5905	0.986096	2.1
5906	1.32813	2.1
5907	1.3331	2.1
5908	2.08369	0.809282
5909	2.23818	1.98486
5910	1.10335	0.915505
5911	0.622314	1.5883
5912	1.72451	0.91384
5913	1.72451	1.87882

5914	0.622283	0.827235
5915	0.622314	0.82614
5916	0.946131	0.816817
5917	1.31254	2.1
5918	1.15704	2.1
5919	1.24463	0.88473
5920	2.2067	1.24086
5921	1.30001	0.681093
5922	1.29175	1.04043
5923	1.1233	0.05
5924	1.8689	0.05
5925	1.29494	0.05
5926	1.11895	0.05
5927	0.851539	0.05
5928	0.851474	0.05
5929	1.58352	0.05
5930	1.58228	0.05
5931	0.851527	0.05
5932	2.6389	0.05
5933	1.77548	0.05
5934	1.24555	2.1
5935	2.0512	2.1
5936	1.46101	2.1
5937	2.60635	2.1
5938	1.10476	0.05
5939	1.44103	0.05
5940	0.652391	2.1
5941	0.618322	2.1
5942	2.22915	2.1
5943	1.39067	2.1
5944	1.99737	2.1
5945	1.42174	2.1
5946	0.946132	0.945685
5947	1.29735	1.46206
5948	1.61765	2.1
5949	1.61765	1.07967
5950	0.946132	1.8258
5951	1.29735	1.06977
5952	1.26643	1.06035
5953	1.01335	1.2128
5954	1.01335	0.548782
5955	0.947669	2.1
5956	2.17167	2.1
5957	2.59733	2.1
5958	1.50954	2.1
5959	1.02638	1.78482
5960	0.555785	2.1
5961	1.91101	2.1
5962	2.09809	2.0566
5963	0.641974	2.1
5964	1.2022	2.0715
5965	1.36359	2.03702
5966	1.8128	2.1
5967	2.34348	0.0709193
5968	1.66343	0.292653
5969	0.974717	0.969088
5970	1.53908	0.866795
5971	1.27737	1.2303

5972	1.09408	0.32737
5973	2.07382	0.263373
5974	1.10466	1.46573
5975	1.05654	1.8088
5976	1.23498	1.25191
5977	1.48104	1.6012
5978	1.23498	1.16559
5979	1.17261	2.1
5980	1.18059	2.1
5981	2.43005	2.1
5982	1.04546	2.1
5983	2.81222	2.1
5984	1.16817	2.1
5985	1.75766	0.379964
5986	1.47021	0.81655
5987	1.07236	0.05
5988	1.81759	0.717523
5989	1.42913	0.215155
5990	1.00636	0.760825
5991	1.00636	0.157296
5992	1.79397	0.153508
5993	0.946131	0.0512691
5994	1.03691	0.05
5995	0.851539	0.158393
5996	1.46001	0.167301
5997	1.37878	0.05
5998	0.933258	0.05
5999	1.32423	0.05
6000	1.32614	0.05
6001	1.11356	0.05
6002	1.32614	0.05
6003	1.28895	0.05
6004	1.66895	0.05
6005	1.66895	0.05
6006	0.946132	0.05
6007	1.29576	0.05
6008	1.1318	0.05
6009	1.37621	1.60937
6010	1.94071	1.42066
6011	1.10517	2.1
6012	1.97098	2.1
6013	0.811 2.1	
6014	0.956127	2.1
6015	1.18647	2.1
6016	0.988455	1.4472
6017	1.12457	0.05
6018	1.7453	0.05
6019	0.92492	2.1
6020	1.24353	2.1
6021	1.84536	0.508528
6022	1.67662	0.201098
6023	1.38247	0.05
6024	1.02663	0.05
6025	0.946132	0.171749
6026	1.41723	0.0664973
6027	1.72976	0.17289
6028	1.72976	0.05
6029	0.946131	0.515605

6030	1.41723	0.178149
6031	1.25866	0.138683
6032	1.34254	0.05
6033	1.25866	0.0719407
6034	1.65507	0.05
6035	1.65507	0.05
6036	0.946132	0.190535
6037	0.946132	0.0646873
6038	1.11929	0.05
6039	0.961779	0.05
6040	0.961779	0.0516741
6041	0.946132	2.1
6042	1.69361	2.1
6043	1.69361	2.1
6044	0.94613	2.1
6045	1.36523	2.1
6046	1.36523	2.1
6047	1.19802	2.1
6048	0.974196	2.1
6049	1.09518	2.1
6050	0.946131	2.1
6051	1.7161	2.1
6052	1.7161	2.1
6053	0.94613	2.1
6054	1.297	2.1
6055	1.297	2.1
6056	1.14599	2.1
6057	0.974197	2.1
6058	0.974197	2.1
6059	1.80778	2.1
6060	2.20473	2.1
6061	1.80777	2.1
6062	1.3515	2.1
6063	0.583237	2.1
6064	1.39359	2.1
6065	1.39359	2.1
6066	1.07296	2.1
6067	0.946114	2.1
6068	1.07297	2.1
6069	0.903862	2.1
6070	0.956572	2.1
6071	1.03437	2.1
6072	0.975409	2.1
6073	1.31167	0.05
6074	2.70246	0.05
6075	1.12018	0.05
6076	0.655836	0.05
6077	0.655837	0.05
6078	1.81557	0.05
6079	1.81557	0.05
6080	0.655836	0.05
6081	0.946131	0.05
6082	1.01847	0.05
6083	1.61334	0.05
6084	0.810946	0.05
6085	0.8311	0.05
6086	1.2466	0.05
6087	1.20286	0.05

6088	0.97508	0.05
6089	0.870805	0.05
6090	0.760087	0.05
6091	0.853389	2.1
6092	0.820894	2.1
6093	0.928291	2.1
6094	1.06922	2.1
6095	1.32829	2.1
6096	1.12173	2.1
6097	1.16615	2.1
6098	0.957748	2.1
6099	0.612491	2.0801
6100	0.548141	2.1
6101	0.979614	2.1
6102	0.658184	2.1
6103	0.678456	1.85635
6104	0.529544	1.9893
6105	0.739013	2.1
6106	0.694037	2.1
6107	0.475716	2.04947
6108	1.58794	1.81005
6109	1.58794	1.4831
6110	0.475743	1.62747
6111	1.08301	2.1
6112	0.980664	2.1
6113	0.475737	2.1
6114	1.32709	2.1
6115	1.59396	2.1
6116	1.19719	0.05
6117	1.34921	0.05
6118	1.69061	0.05
6119	1.69061	0.05
6120	0.946132	0.05
6121	1.34921	0.05
6122	1.28753	0.05
6123	0.95895	0.05
6124	1.01051	0.05
6125	1.12468	0.05
6126	1.22588	0.05
6127	1.34254	0.05
6128	1.74561	0.05
6129	1.74561	0.05
6130	0.946128	0.05
6131	1.34253	0.05
6132	1.3492	0.05
6133	1.01051	0.05
6134	1.922	2.1
6135	1.922	2.1
6136	2.12556	2.1
6137	0.946131	2.1
6138	1.06278	2.1
6139	1.47631	2.1
6140	1.47631	2.1
6141	0.946129	2.1
6142	1.06278	2.1
6143	1.35966	2.1
6144	0.991985	2.1
6145	0.738507	1.42166

6146	0.536935	1.95107
6147	0.799004	1.53311
6148	1.04281	0.703456
6149	0.879197	0.671677
6150	1.06538	0.642876
6151	0.715942	1.37147
6152	1.00636	0.092633
6153	1.03824	0.05
6154	1.70147	0.0531047
6155	1.21223	2.1
6156	1.01243	2.1
6157	1.01242	2.1
6158	1.06935	2.1
6159	1.54241	2.1
6160	0.998971	0.05
6161	1.60312	0.05
6162	1.44836	0.05
6163	2.05632	0.05
6164	1.44836	0.05
6165	0.973765	0.05
6166	0.946131	0.05
6167	0.851541	0.05
6168	1.73854	0.05
6169	1.91752	0.05
6170	1.49622	0.05
6171	1.23351	0.05
6172	3.33289	2.1
6173	1.86606	2.1
6174	2.71233	2.1
6175	0.788816	2.1
6176	0.963972	2.1
6177	1.25866	2.1
6178	1.10817	2.1
6179	1.41908	2.1
6180	1.48734	2.1
6181	1.40822	0.0762338
6182	1.40822	0.0530774
6183	2.09871	0.141367
6184	0.946133	0.082011
6185	1.14926	0.05
6186	0.946133	0.140871
6187	1.78098	0.05
6188	1.78098	0.05
6189	1.35822	0.0570745
6190	1.3689	0.05
6191	1.22176	0.05
6192	2.03426	0.057693
6193	0.946131	0.0846659
6194	1.01713	0.05
6195	0.704112	0.104882
6196	1.36237	0.05
6197	1.36237	0.05
6198	1.01713	0.0681697
6199	1.04936	0.0784653
6200	1.87377	2.1
6201	1.60501	2.1
6202	0.981584	0.05
6203	1.69435	0.05

6204	0.85154	2.1
6205	1.47841	2.1
6206	1.4952	2.1
6207	1.31941	2.1
6208	1.04023	2.1
6209	1.1995	2.1
6210	0.914791	2.1
6211	1.1427	1.85031
6212	1.64787	1.19651
6213	1.07526	1.16154
6214	1.07527	0.591315
6215	1.07527	1.12326
6216	1.58551	0.865704
6217	2.77362	1.3905
6218	2.17974	1.05883
6219	2.71723	1.19739
6220	1.29868	0.05
6221	1.3594	0.05
6222	1.04786	0.05
6223	0.527876	0.05
6224	1.4085	0.05
6225	1.48538	0.05
6226	0.76829	0.05
6227	1.18514	0.05
6228	0.617954	0.05
6229	0.864161	0.05
6230	1.18835	0.05
6231	1.16817	2.1
6232	1.04263	2.1
6233	1.50524	2.1
6234	1.85355	2.1
6235	1.23963	2.1
6236	1.06279	2.1
6237	0.94613	2.1
6238	1.35229	2.1
6239	1.15947	2.1
6240	0.965593	2.1
6241	0.851553	2.1
6242	2.21296	2.1
6243	1.11764	2.1
6244	2.46399	2.1
6245	1.39292	2.1
6246	1.34701	2.1
6247	1.34701	2.1
6248	1.47001	2.1
6249	0.963166	0.05
6250	0.893445	0.05
6251	1.21056	0.05
6252	1.67375	2.1
6253	2.0977	2.1
6254	1.09154	2.1
6255	1.09154	2.1
6256	1.57122	1.02743
6257	1.60976	0.374975
6258	1.69533	0.136272
6259	1.6951	0.083741
6260	1.5831	0.0554084
6261	1.20969	2.1

6262	1.12076	2.1
6263	1.49808	2.1
6264	1.50201	2.1
6265	1.00278	2.1
6266	1.22811	0.05
6267	1.11545	2.1
6268	1.09646	2.1
6269	1.03107	0.05
6270	1.44293	0.05
6271	1.66752	1.98822
6272	1.11023	2.09712
6273	2.57831	2.1
6274	2.20191	1.28964
6275	2.18051	2.1
6276	1.48035	0.294461
6277	1.0961	0.0864504
6278	1.63241	0.053947
6279	1.0961	0.127191
6280	1.20959	2.1
6281	2.86136	2.1
6282	1.64933	2.1
6283	1.47217	2.1
6284	0.915178	2.1
6285	1.19074	2.1
6286	0.897276	2.1
6287	0.915177	2.1
6288	0.987037	2.1
6289	1.04288	2.1
6290	2.39323	2.1
6291	0.976218	0.05
6292	2.23933	0.05
6293	0.976215	0.05
6294	1.07384	0.05
6295	1.07652	2.1
6296	1.55678	2.1
6297	1.54168	2.1
6298	1.69136	2.1
6299	1.64391	1.53058
6300	1.76477	1.40205
6301	0.611486	1.66409
6302	0.683421	1.2043
6303	0.946131	1.32209
6304	1.1019	1.34757
6305	1.12946	1.20233
6306	1.54226	2.02234
6307	1.19496	1.4123
6308	1.96199	1.28937
6309	2.0775	1.82244
6310	1.22297	1.34302
6311	1.3469	1.07486
6312	1.25366	1.23054
6313	1.65443	1.25053
6314	1.65443	1.25255
6315	0.946132	1.52557
6316	0.946132	1.86497
6317	1.12873	1.38613
6318	1.07468	1.17294
6319	1.2854	0.831644

6320	1.3469	1.10435
6321	1.68616	1.07252
6322	1.68616	0.961124
6323	0.946132	1.47482
6324	0.946132	1.28719
6325	1.16109	1.15261
6326	1.17879	1.04373
6327	1.17879	1.04829
6328	1.45304	1.29787
6329	2.21058	2.1
6330	2.81552	2.1
6331	1.45535	0.05
6332	1.49906	0.05
6333	1.04413	2.1
6334	1.74586	2.1
6335	1.34526	2.1
6336	1.17402	1.16869
6337	1.74564	1.07203
6338	1.52724	2.1
6339	1.68142	2.1
6340	1.09651	2.1
6341	1.49149	2.1
6342	1.36814	2.1
6343	1.46783	2.1
6344	2.18116	2.1
6345	1.23602	1.13844
6346	1.74538	0.404264
6347	0.851539	2.1
6348	1.45775	2.1
6349	1.28919	2.1
6350	0.851483	2.1
6351	1.26102	2.1
6352	0.869889	2.1
6353	0.85151	2.1
6354	1.23451	0.813072
6355	1.23602	2.1
6356	1.11955	2.1
6357	1.08022	2.1
6358	0.977776	2.1
6359	1.27896	2.1
6360	1.64268	2.1
6361	1.97548	2.1
6362	1.72527	2.1
6363	0.946132	2.1
6364	1.69702	2.1
6365	1.69702	2.1
6366	1.13635	2.1
6367	1.40274	2.1
6368	1.24041	2.1
6369	1.13635	2.1
6370	1.78427	0.05
6371	1.68338	0.05
6372	1.00681	0.05
6373	1.12988	0.05
6374	0.946132	0.05
6375	0.957543	0.05
6376	1.40515	0.05
6377	1.40515	0.05

6378	0.797932	0.05
6379	1.30187	0.05
6380	1.14846	0.05
6381	0.665137	0.05
6382	1.09575	0.05
6383	0.596894	0.05
6384	1.04072	0.05
6385	0.663171	0.0909739
6386	1.47218	0.05
6387	1.32889	0.05
6388	1.32639	0.05
6389	1.94887	0.05
6390	1.32639	0.05
6391	1.19323	0.05
6392	2.19794	0.05
6393	0.754756	0.05
6394	1.11015	0.05
6395	0.754755	0.05
6396	0.946133	0.05
6397	0.754768	0.05
6398	1.59986	0.05
6399	1.59986	0.05
6400	1.84082	2.1
6401	1.79044	2.1
6402	1.75922	2.1
6403	1.86766	2.1
6404	1.58995	2.1
6405	1.58995	2.1
6406	1.74351	2.1
6407	1.04442	2.1
6408	1.42278	2.1
6409	1.08576	2.1
6410	0.994294	2.1
6411	1.44884	2.1
6412	1.71036	1.26878
6413	0.714101	0.43296
6414	1.17541	2.07057
6415	1.00872	0.05
6416	0.714072	0.631672
6417	0.936677	0.107354
6418	0.966979	2.1
6419	1.4908	2.1
6420	1.03108	0.132027
6421	0.949712	0.05
6422	0.810946	0.0607059
6423	0.88209	0.0773777
6424	0.646702	0.05
6425	0.972663	0.05
6426	0.862301	0.05
6427	0.868143	0.05
6428	1.11094	0.05
6429	1.35809	0.05
6430	1.32455	2.1
6431	1.32328	2.1
6432	1.69586	2.1
6433	1.78301	2.1
6434	1.67906	2.1
6435	1.60635	2.1

6436	1.57234	2.1
6437	1.47012	2.1
6438	1.22811	0.05
6439	1.08732	0.0844606
6440	2.08849	0.05
6441	1.08732	0.0828556
6442	1.30635	0.05
6443	0.77315	0.05
6444	1.25732	0.05
6445	2.43845	2.1
6446	2.38577	1.93167
6447	0.946132	1.03588
6448	1.19288	2.1
6449	1.54508	2.1
6450	1.54508	1.61332
6451	0.94515	2.1
6452	1.19288	2.06765
6453	1.29735	1.43049
6454	1.12087	0.987492
6455	1.54699	1.21196
6456	1.43635	2.1
6457	1.33385	2.1
6458	1.43635	2.1
6459	1.02368	2.1
6460	1.035 2.1	
6461	1.00128	2.1
6462	1.04009	2.1
6463	1.14052	2.1
6464	0.443552	2.1
6465	0.984351	2.1
6466	0.591443	2.1
6467	0.929561	2.1
6468	1.52079	2.1
6469	1.035 2.1	
6470	3.5282	2.1
6471	1.88288	2.1
6472	2.73877	1.38161
6473	0.906059	2.1
6474	1.23107	0.05
6475	0.906712	0.05
6476	0.841322	0.05
6477	1.53005	0.05
6478	1.10007	0.05
6479	1.38033	0.05
6480	1.03442	0.05
6481	0.787223	0.05
6482	1.42589	0.05
6483	2.35665	0.05
6484	1.17793	0.0560631
6485	1.81356	0.05
6486	1.42964	0.05
6487	1.95641	0.05
6488	1.39139	0.206835
6489	1.39139	0.595434
6490	0.564533	0.345294
6491	1.20016	0.672037
6492	0.755765	0.05
6493	0.755766	0.0535756

6494	1.20016	0.307442
6495	1.91011	0.546758
6496	1.4695	0.05
6497	1.70048	2.1
6498	1.93765	2.1
6499	0.741307	2.1
6500	0.780371	2.1
6501	0.946133	0.883274
6502	1.28918	1.24696
6503	1.5111	2.1
6504	1.5111	0.928581
6505	0.946131	1.55075
6506	1.28918	0.954764
6507	1.16805	0.87729
6508	1.01179	0.840906
6509	0.946132	0.914567
6510	1.35328	1.3177
6511	1.69633	2.00648
6512	1.69633	0.971543
6513	0.946133	1.66608
6514	1.35328	0.993988
6515	1.28918	0.918889
6516	1.01179	1.07273
6517	1.58147	1.16395
6518	1.27737	0.910427
6519	2.17223	0.05
6520	0.697168	0.05
6521	1.62495	0.124375
6522	1.62495	0.214754
6523	1.93948	0.671494
6524	0.601592	2.1
6525	1.93528	2.1
6526	2.13757	2.1
6527	1.67561	2.1
6528	2.70875	2.1
6529	1.93528	2.1
6530	3.98475	2.1
6531	1.12376	2.1
6532	1.19016	2.1
6533	1.67323	0.05
6534	1.74019	0.05
6535	1.84719	0.05
6536	1.00764	0.05
6537	1.23475	2.1
6538	1.06757	2.1
6539	1.57916	2.1
6540	1.06758	2.1
6541	1.28014	2.1
6542	1.33339	2.1
6543	1.2679	2.1
6544	1.23504	2.1
6545	1.50108	2.1
6546	1.3715	2.1
6547	0.58831	1.05791
6548	0.865971	1.05558
6549	0.813704	1.22281
6550	0.658856	1.11238
6551	0.495869	1.22031

6552	1.13845	1.11961
6553	0.780804	1.15108
6554	0.642023	1.17175
6555	0.707748	1.03266
6556	1.64476	0.992951
6557	1.64476	1.09054
6558	0.99611	1.04382
6559	1.35639	0.980503
6560	1.3564	1.06522
6561	0.946129	1.05359
6562	1.99222	1.0248
6563	1.4777	2.1
6564	1.03968	2.1
6565	1.82516	2.1
6566	0.851539	2.1
6567	1.63693	2.1
6568	1.59416	2.1
6569	0.878566	2.1
6570	1.40796	2.1
6571	1.13172	2.1
6572	0.851527	2.03009
6573	1.14366	2.1
6574	0.823822	0.0540632
6575	0.882001	0.062275
6576	1.09756	0.05
6577	1.11857	0.05
6578	1.0934	0.05
6579	0.960104	0.05
6580	0.982001	0.111554
6581	1.74319	0.05
6582	1.03043	0.05
6583	0.967194	0.05
6584	1.16938	2.1
6585	2.48377	2.1
6586	1.1181	2.1
6587	2.57349	2.1
6588	1.0465	2.1
6589	1.44709	2.1
6590	0.946132	2.1
6591	1.29302	2.1
6592	1.73502	2.1
6593	1.73502	2.1
6594	0.946132	2.1
6595	1.29302	2.1
6596	1.38813	2.1
6597	1.03933	2.1
6598	1.01976	0.05
6599	1.11764	0.0521776
6600	0.961779	0.130398
6601	1.0685	0.075397
6602	1.08426	1.30563
6603	0.99695	1.79755
6604	2.25593	1.66557
6605	1.47992	1.95979
6606	1.88989	1.2588
6607	1.65813	1.24113
6608	1.20278	1.43628
6609	0.981842	1.38836

6610	0.802655	2.1
6611	0.953489	2.1
6612	0.961719	2.1
6613	0.975614	2.06503
6614	0.991617	1.41213
6615	0.940748	1.60006
6616	2.25019	2.1
6617	2.30712	1.47987
6618	1.98823	2.1
6619	0.522844	1.45683
6620	0.946122	2.1
6621	0.946132	1.93668
6622	0.593846	1.39213
6623	1.26501	1.41383
6624	1.21171	0.05
6625	0.851542	0.05
6626	0.940327	0.05
6627	0.940356	0.05
6628	1.52117	0.05
6629	1.58072	0.05
6630	1.35169	0.05
6631	1.13889	0.05
6632	1.35007	0.05
6633	1.35007	0.05
6634	1.78381	0.05
6635	0.946132	0.05
6636	1.06627	0.05
6637	0.946131	0.05
6638	1.73005	0.05
6639	1.73005	0.05
6640	1.32449	0.05
6641	1.35169	0.05
6642	1.42446	0.574903
6643	1.86697	0.430412
6644	1.13413	0.0888275
6645	1.42446	0.0966119
6646	1.33575	0.05
6647	1.20473	0.05
6648	1.87517	0.05
6649	1.87517	0.05
6650	0.488712	0.05
6651	1.13242	0.05
6652	0.488713	0.05
6653	0.488713	0.05
6654	1.23146	0.05
6655	1.57932	0.05
6656	1.01698	0.05
6657	1.42085	0.05
6658	0.246725	0.05
6659	0.996283	0.05
6660	1.36965	0.05
6661	0.86361	0.05
6662	0.810945	0.05
6663	1.72938	0.05
6664	1.21649	0.05
6665	1.1865	0.05
6666	1.62387	0.05
6667	1.05302	0.05

6668	1.10049	0.0740197
6669	0.946129	2.1
6670	1.70184	0.586712
6671	1.70184	2.1
6672	1.12538	2.1
6673	1.35969	0.300934
6674	1.28828	2.1
6675	1.12538	1.62719
6676	0.99747	1.53929
6677	1.34851	0.764157
6678	0.923724	2.1
6679	1.47154	0.714279
6680	1.47154	2.1
6681	1.10698	2.1
6682	1.28828	0.263462
6683	1.10698	2.1
6684	0.923695	2.1
6685	0.99747	0.0514881
6686	1.65677	2.1
6687	1.77677	2.1
6688	1.05689	0.05
6689	2.11031	0.05
6690	1.07367	0.05
6691	0.656239	0.05
6692	1.4563	0.05
6693	1.4563	0.05
6694	0.656238	0.05
6695	0.946133	0.05
6696	0.812937	0.05
6697	0.946131	0.0555721
6698	1.05689	0.05
6699	1.34009	2.1
6700	1.398 2.1	
6701	3.29047	2.1
6702	2.53897	2.1
6703	1.24509	2.1
6704	3.06162	2.1
6705	1.01595	2.1
6706	1.52661	2.1
6707	1.5266	2.1
6708	0.946131	2.1
6709	1.31049	2.1
6710	1.16225	2.1
6711	1.05978	2.1
6712	1.16225	2.1
6713	1.06561	2.1
6714	0.953927	2.1
6715	1.15114	2.1
6716	1.1807	0.0545619
6717	0.507259	0.05
6718	1.47709	0.0544079
6719	1.36192	0.05
6720	0.487293	0.05
6721	1.65569	0.0873335
6722	0.679725	0.05
6723	0.721928	0.0584953
6724	1.03931	0.05
6725	1.0775	0.0797029

6726	1.0775	0.0833172
6727	2.30207	1.04079
6728	1.01814	0.981061
6729	2.33963	0.861903
6730	2.42589	1.01578
6731	1.33962	2.1
6732	1.27956	2.1
6733	2.14624	0.05
6734	1.6812	0.05
6735	1.25698	0.05
6736	1.35725	0.05
6737	1.86387	0.05
6738	1.86387	0.05
6739	0.750365	0.05
6740	0.946128	0.05
6741	0.750365	0.05
6742	1.72271	0.05
6743	1.72272	0.05
6744	2.53736	0.05
6745	1.07327	2.1
6746	1.05376	2.1
6747	1.00387	1.18014
6748	2.0554	1.15914
6749	1.00387	1.11969
6750	1.00387	1.07591
6751	1.27119	2.1
6752	1.51748	2.1
6753	3.15271	2.1
6754	3.45801	2.1
6755	1.99871	2.1
6756	1.41186	2.1
6757	1.07624	2.1
6758	1.5954	2.1
6759	1.19323	1.01792
6760	1.21286	0.9595
6761	0.946114	2.1
6762	1.38395	2.1
6763	1.73389	2.1
6764	1.7339	2.1
6765	0.946115	2.1
6766	1.38397	2.1
6767	1.29604	2.1
6768	1.04544	2.1
6769	1.60578	0.05
6770	1.93407	0.05
6771	1.0525	1.04273
6772	1.0525	1.30527
6773	1.0525	1.9839
6774	1.38506	1.77922
6775	2.34737	0.05
6776	1.3531	0.05
6777	1.2925	0.05
6778	0.871466	0.05
6779	1.26623	0.05
6780	0.700631	0.05
6781	1.88774	0.05
6782	1.14167	0.05
6783	1.18606	0.05

6784	2.1238	0.05
6785	1.42762	0.851191
6786	1.67791	0.05
6787	1.47118	0.05
6788	0.946128	2.1
6789	1.31641	2.1
6790	1.75933	2.1
6791	1.75933	2.1
6792	0.946131	2.1
6793	1.31641	2.1
6794	1.38905	2.1
6795	1.38437	2.1
6796	1.51829	2.1
6797	0.947114	2.1
6798	1.13335	2.1
6799	1.47521	2.1
6800	1.14911	2.1
6801	1.53822	2.1
6802	1.39702	2.1
6803	0.739166	2.1
6804	0.706056	2.1
6805	0.964033	2.1
6806	1.58993	2.1
6807	2.51892	2.1
6808	0.595905	0.05
6809	1.18155	0.05
6810	1.73364	0.05
6811	0.952735	0.05
6812	2.10381	0.05
6813	1.12115	0.05
6814	1.27826	0.05
6815	2.74021	0.05
6816	1.78416	2.1
6817	1.78416	2.1
6818	0.946132	2.1
6819	1.41126	2.1
6820	1.21275	2.1
6821	0.946133	2.1
6822	1.41126	2.1
6823	1.33125	2.1
6824	1.33125	2.1
6825	1.78918	2.1
6826	1.0465	2.1
6827	1.99715	2.1
6828	1.17683	2.1
6829	1.25889	2.1
6830	0.860834	0.05
6831	0.946131	0.05
6832	1.05008	0.05
6833	0.970444	0.05
6834	1.19401	0.05
6835	1.25989	0.05
6836	0.702942	0.0716822
6837	1.40591	0.234851
6838	1.72168	0.142651
6839	0.446352	0.0651626
6840	1.04072	0.05
6841	0.711038	0.320175

6842	0.980737	0.0682654
6843	1.05099	0.05
6844	1.26003	0.05
6845	0.495934	0.442402
6846	1.03114	0.05
6847	1.09911	0.05
6848	1.24825	0.05
6849	2.19827	0.05
6850	2.0953	0.05
6851	2.30437	0.05
6852	0.775923	0.05
6853	2.2283	0.05
6854	1.19583	2.1
6855	1.14496	2.1
6856	1.62183	2.1
6857	1.62316	2.1
6858	1.02305	0.0538808
6859	1.21136	0.05
6860	1.44426	0.05
6861	1.21137	0.05
6862	1.20143	0.05
6863	1.15009	0.05
6864	1.24965	2.1
6865	1.41077	2.1
6866	1.00052	0.05
6867	1.79553	0.05
6868	1.06336	0.05
6869	1.00053	0.05
6870	1.18041	2.1
6871	1.80818	2.1
6872	1.73571	1.34419
6873	1.94429	1.58258
6874	1.4375	0.05
6875	1.44421	0.05
6876	1.66953	0.05
6877	1.09529	0.05
6878	0.952395	2.1
6879	0.717337	2.1
6880	1.03609	2.1
6881	1.33949	0.430547
6882	1.2774	0.71086
6883	2.24983	0.133121
6884	1.28727	0.259241
6885	0.583592	0.666461
6886	0.583591	0.700449
6887	1.82859	0.175625
6888	1.82859	0.558437
6889	0.583593	0.72842
6890	0.946128	0.450421
6891	0.692233	0.420351
6892	1.12226	0.741492
6893	2.57454	0.710459
6894	0.810944	0.716576
6895	0.810945	0.678632
6896	1.12226	0.722214
6897	1.45867	0.716441
6898	1.22403	0.690013
6899	1.15262	0.717623

6900	1.08132	0.701947
6901	0.846671	0.680067
6902	0.480954	0.517262
6903	0.917975	0.6915
6904	0.917975	0.727748
6905	0.720957	0.155212
6906	0.795998	0.614918
6907	0.69316	0.737817
6908	0.641306	0.432575
6909	0.690817	0.634168
6910	0.961899	2.1
6911	2.49579	2.1
6912	2.31106	2.1
6913	1.70482	0.249153
6914	1.93156	0.05
6915	1.23868	0.05
6916	1.73878	0.199192
6917	1.26267	2.1
6918	1.26441	2.1
6919	1.58095	2.1
6920	1.58095	2.1
6921	0.946132	2.1
6922	0.946132	2.1
6923	1.11077	2.1
6924	1.62905	2.1
6925	1.49692	2.1
6926	3.49966	2.1
6927	2.39213	2.1
6928	2.12603	2.1
6929	0.946129	2.1
6930	0.461661	2.1
6931	1.52841	2.1
6932	1.39091	2.1
6933	0.550679	2.1
6934	0.509894	2.1
6935	1.49508	2.1
6936	2.05862	2.1
6937	2.47851	2.1
6938	1.07213	2.1
6939	1.09148	0.620933
6940	1.99776	0.676445
6941	1.38333	0.263293
6942	1.04412	2.1
6943	1.04412	2.1
6944	1.1893	0.05
6945	0.946132	0.05
6946	0.965737	0.05
6947	0.946132	0.05
6948	1.54061	0.05
6949	1.54061	0.05
6950	1.22878	0.05
6951	1.25797	0.05
6952	1.00665	0.05
6953	1.00665	0.05
6954	1.80369	0.05
6955	0.946128	0.05
6956	1.04424	0.0522903
6957	0.85154	0.068141

6958	1.39322	0.05
6959	1.35464	0.05
6960	0.939857	0.05
6961	1.28272	0.05
6962	1.19323	0.05
6963	1.03498	0.05
6964	2.22029	0.05
6965	0.540005	0.05
6966	0.970382	0.05
6967	2.14514	0.05
6968	1.31542	0.05
6969	1.05575	0.05
6970	0.810943	0.05
6971	1.31542	0.05
6972	1.70493	0.05
6973	1.22582	0.05
6974	1.31681	0.05
6975	1.40775	0.05
6976	0.928633	0.05
6977	1.2545	0.05
6978	2.66481	0.05
6979	1.32051	0.05
6980	1.32051	0.05
6981	2.66305	2.1
6982	1.3551	2.1
6983	2.45325	2.1
6984	1.3685	2.1
6985	1.51748	2.1
6986	1.78941	2.1
6987	1.02963	0.05
6988	1.28643	0.05
6989	0.86131	0.05
6990	0.851539	0.05
6991	1.46585	0.05
6992	1.38822	0.05
6993	0.996402	0.05
6994	0.851553	0.05
6995	1.73156	0.05
6996	1.91695	0.05
6997	1.92535	0.05
6998	2.57287	0.05
6999	1.77743	0.05
7000	1.02836	0.05
7001	1.36362	0.0529434
7002	1.36936	0.05
7003	1.78684	0.05
7004	1.78685	0.05
7005	0.946132	0.0776591
7006	1.27581	0.062476
7007	0.946132	0.116218
7008	1.02836	0.05
7009	1.55398	2.1
7010	1.07167	2.1
7011	1.87557	2.1
7012	0.751639	2.1
7013	1.50884	2.1
7014	1.33793	2.1
7015	0.751664	2.1

7016	1.37277	2.1
7017	0.979515	2.1
7018	0.676508	2.1
7019	1.09578	2.1
7020	0.953925	2.1
7021	0.94613	2.1
7022	1.64707	2.1
7023	1.64707	2.1
7024	1.16658	2.1
7025	1.40638	2.1
7026	1.18682	2.1
7027	1.16658	2.1
7028	0.543765	0.05
7029	1.57462	0.05
7030	1.57462	0.05
7031	1.00083	0.05
7032	1.16834	0.05
7033	1.16834	0.05
7034	0.889626	0.05
7035	1.25944	0.05
7036	0.824061	0.05
7037	1.9397	0.05
7038	1.21893	0.05
7039	1.91364	0.05
7040	1.33672	0.05
7041	0.928069	0.0640977
7042	1.41484	0.05
7043	0.538379	0.05
7044	1.41484	0.05
7045	1.35594	0.05
7046	2.11414	0.05
7047	2.11414	0.05
7048	0.538378	0.05
7049	1.38532	0.05
7050	0.962877	0.282595
7051	1.07497	0.749711
7052	1.00613	0.05
7053	0.962878	0.585864
7054	0.711447	2.1
7055	1.40008	0.05
7056	2.51674	0.05
7057	1.40008	0.05
7058	1.7263	0.05
7059	1.40008	0.0532603
7060	1.9216	0.05
7061	2.7013	0.05
7062	2.58584	0.05
7063	0.979296	0.05
7064	1.46459	0.05
7065	0.899254	0.05
7066	1.15038	2.1
7067	1.24687	2.1
7068	1.16738	2.1
7069	0.942323	2.1
7070	1.65573	1.66841
7071	0.844791	2.1
7072	0.54674	2.1
7073	0.795811	2.1

7074	1.08131	2.1
7075	1.03553	2.1
7076	0.758334	2.1
7077	0.708665	2.00487
7078	1.22626	1.93137
7079	0.835439	1.9357
7080	0.861131	1.69003
7081	0.68296	2.08347
7082	1.65224	1.96942
7083	2.82624	2.1
7084	1.0151	1.40897
7085	1.15905	1.83116
7086	1.71076	1.54538
7087	1.4529	1.28687
7088	0.494895	2.1
7089	0.946132	1.74064
7090	0.613132	1.51144
7091	0.863631	1.62645
7092	1.78391	2.1
7093	1.1706	0.05
7094	2.02734	0.05
7095	1.1706	0.05
7096	0.681532	0.05
7097	1.50316	0.05
7098	1.50316	0.05
7099	0.681534	0.05
7100	0.973819	0.05
7101	0.97382	0.05
7102	0.94613	0.05
7103	1.1706	0.05
7104	1.67727	0.05
7105	1.60329	0.05
7106	1.3227	2.1
7107	1.40846	2.1
7108	1.07889	2.1
7109	1.07721	2.1
7110	0.953523	0.356095
7111	1.79675	0.345146
7112	2.09859	1.02641
7113	1.21309	2.1
7114	1.54567	0.929429
7115	1.19722	0.867765
7116	1.82793	0.838164
7117	1.1599	0.404804
7118	1.73621	0.382072
7119	0.933474	0.801219
7120	1.51477	0.5025
7121	1.51477	0.637023
7122	0.93347	0.756111
7123	1.27374	0.768465
7124	1.27376	0.846067
7125	0.946105	0.856597
7126	1.1972	0.210445
7127	0.851541	0.05
7128	1.36971	0.05
7129	1.27832	0.05
7130	0.853489	0.05
7131	0.973847	0.05

7132	0.976308	0.05
7133	0.851528	0.05
7134	2.72654	0.05
7135	1.77867	0.05
7136	3.23032	2.1
7137	2.78208	2.1
7138	1.0227	0.05
7139	1.02269	0.05
7140	1.02269	0.05
7141	1.24293	0.05
7142	0.831695	2.1
7143	0.67513	2.1
7144	0.956294	2.1
7145	1.08623	2.1
7146	1.05556	2.1
7147	1.38859	2.1
7148	1.13179	2.1
7149	1.02438	2.1
7150	1.06659	2.1
7151	1.36595	2.1
7152	1.06659	2.1
7153	1.06659	1.46154
7154	0.946131	1.29008
7155	0.875912	2.1
7156	1.65971	2.1
7157	1.65971	2.1
7158	0.875912	2.1
7159	1.1229	2.1
7160	1.41273	2.1
7161	1.54699	1.95052
7162	1.93655	1.63021
7163	1.42307	1.22756
7164	1.97907	1.58818
7165	0.996774	2.1
7166	1.15505	0.05
7167	1.28463	0.05
7168	1.86353	0.05
7169	2.71398	0.05
7170	1.56787	0.05
7171	1.66295	0.05
7172	1.73432	1.59533
7173	1.05019	0.05
7174	1.42117	0.05
7175	0.888299	0.05
7176	1.38127	0.05
7177	1.72993	0.05
7178	0.664129	0.05
7179	0.99302	1.82972
7180	0.993023	1.70074
7181	1.13491	2.1
7182	0.876316	2.1
7183	1.2588	2.1
7184	0.895613	2.1
7185	2.07526	2.1
7186	1.23369	2.1
7187	1.25146	2.1
7188	2.15282	2.1
7189	1.22239	2.1

7190	1.10962	2.1
7191	1.77402	2.1
7192	1.0305	2.1
7193	1.50108	1.65837
7194	1.20761	2.1
7195	1.3626	2.1
7196	1.14825	2.1
7197	1.78711	2.1
7198	1.17933	2.1
7199	1.14824	2.1
7200	1.09651	0.05
7201	1.23851	0.05
7202	1.83284	0.05
7203	1.23851	0.05
7204	1.53521	2.1
7205	1.46279	2.1
7206	1.9233	0.05
7207	1.2966	0.05
7208	2.11443	0.05
7209	1.30265	0.05
7210	1.13528	0.05
7211	0.946131	0.0751286
7212	1.41169	0.05
7213	1.33879	0.05
7214	0.946128	0.05
7215	1.80435	0.05
7216	1.80435	0.05
7217	1.234 2.1	
7218	1.45633	2.1
7219	1.234 2.1	
7220	1.98448	2.1
7221	2.02612	2.1
7222	1.23527	0.905915
7223	1.44472	0.926826
7224	0.993366	1.11801
7225	1.22025	1.05152
7226	0.993368	0.908451
7227	2.43669	2.1
7228	2.56707	2.1
7229	1.80348	1.36916
7230	1.74406	0.745747
7231	1.90937	1.16999
7232	1.49357	0.05
7233	2.5832	0.394837
7234	1.82274	0.234323
7235	1.6957	0.211332
7236	2.22495	0.103641
7237	2.49959	0.191852
7238	2.01183	0.0547955
7239	1.37427	0.05
7240	1.15069	2.1
7241	1.39379	1.97676
7242	1.69661	0.05
7243	1.39379	0.0674242
7244	1.39654	2.1
7245	1.93505	2.1
7246	1.5525	2.1
7247	1.5525	2.1

7248	0.615065	2.1
7249	1.38908	2.1
7250	0.615065	2.1
7251	0.77849	2.1
7252	1.38908	2.1
7253	1.56839	1.35305
7254	1.09142	2.09528
7255	2.07313	1.16088
7256	0.998288	0.05
7257	1.31481	0.05
7258	1.88175	0.05
7259	2.23267	0.05
7260	1.06935	2.1
7261	1.06936	2.1
7262	1.17989	2.06435
7263	1.00757	1.09124
7264	1.65318	2.03077
7265	1.4185	0.0882046
7266	2.22495	0.0625832
7267	1.02011	0.05
7268	0.59676	0.05
7269	1.43606	0.05
7270	1.43606	0.05
7271	0.89771	0.05
7272	1.13511	0.0583371
7273	0.59676	0.05
7274	0.897709	0.05
7275	1.79542	0.05
7276	2.38566	2.1
7277	1.63838	0.490913
7278	1.63837	1.468
7279	0.916275	2.1
7280	1.36183	0.951412
7281	1.19282	2.1
7282	0.916275	2.05541
7283	1.19283	2.1
7284	2.01582	2.1
7285	1.00215	0.05
7286	2.60643	2.1
7287	2.15971	2.1
7288	1.28267	2.1
7289	1.29954	2.1
7290	3.40327	2.1
7291	3.49128	2.1
7292	1.3864	2.1
7293	1.3201	2.1
7294	1.45666	0.05
7295	1.55798	0.05
7296	0.857202	0.05
7297	1.24374	0.05
7298	1.04157	0.05
7299	0.85723	0.05
7300	1.02722	0.05
7301	1.01361	0.05
7302	0.974077	0.05
7303	1.01814	0.05
7304	0.974077	0.05
7305	1.43987	0.05

7306	1.43986	0.05
7307	0.927881	0.05
7308	0.946132	0.05
7309	0.927849	0.05
7310	0.927852	0.05
7311	1.2493	0.05
7312	1.22482	0.0944348
7313	1.41319	0.337426
7314	1.22482	0.0686736
7315	0.999492	2.1
7316	1.14657	2.1
7317	1.3617	0.05
7318	0.983269	0.05
7319	1.33174	0.05
7320	1.62816	0.05
7321	1.62817	0.05
7322	0.81938	0.05
7323	1.33174	0.05
7324	1.01332	0.05
7325	0.874788	0.05
7326	1.16725	2.1
7327	1.63991	2.1
7328	1.08848	0.05
7329	1.15354	0.05
7330	1.12665	2.1
7331	1.58742	2.1
7332	1.57523	2.1
7333	1.32201	1.09644
7334	1.32097	0.896914
7335	0.851542	0.05
7336	0.877188	0.05
7337	0.797489	0.0922966
7338	1.44721	0.05
7339	1.61761	0.05
7340	0.943799	0.05
7341	1.36079	0.05
7342	1.16361	2.1
7343	1.15852	2.05125
7344	1.07624	2.1
7345	1.07624	2.1
7346	0.946132	0.953628
7347	1.37938	1.31855
7348	1.78653	1.81595
7349	1.78653	1.02203
7350	0.946131	1.72571
7351	1.37938	1.05485
7352	1.35328	0.972793
7353	1.44847	0.96711
7354	0.946133	1.00558
7355	1.38149	1.25488
7356	1.81474	1.35403
7357	1.81474	1.05467
7358	0.946133	1.67168
7359	1.38149	1.10074
7360	1.37938	1.02589
7361	1.44847	1.11161
7362	1.93665	0.974283
7363	1.5515	1.00781

7364	1.05025	2.1
7365	1.05025	2.1
7366	1.71468	2.1
7367	1.04759	2.1
7368	1.04646	0.05
7369	1.00739	0.05
7370	1.37086	0.05
7371	1.12547	0.05
7372	1.03853	0.05
7373	1.34824	0.05
7374	0.799786	0.05
7375	2.10105	0.05
7376	2.72007	0.05
7377	0.589918	0.05
7378	0.589918	0.05
7379	1.1509	0.05
7380	0.619832	0.05
7381	1.1509	0.05
7382	0.705903	0.05
7383	1.53703	0.05
7384	1.53703	0.05
7385	0.705904	0.05
7386	0.946133	0.05
7387	0.946132	0.05
7388	0.946132	0.05
7389	0.588467	0.05
7390	1.1078	0.05
7391	0.408061	0.05
7392	1.38703	0.05
7393	1.68328	0.05
7394	1.87031	0.05
7395	1.86268	0.05
7396	1.27472	0.05
7397	0.947862	0.872473
7398	1.19854	1.3138
7399	0.946129	0.417004
7400	0.963111	0.196026
7401	0.946131	0.577215
7402	1.39055	0.162047
7403	1.39055	0.169005
7404	1.26426	0.272345
7405	1.07242	0.265318
7406	1.31126	0.158293
7407	1.76597	0.635841
7408	0.94613	1.3856
7409	1.27214	1.19372
7410	1.90751	1.16268
7411	1.90751	0.958361
7412	0.767114	1.30349
7413	1.27214	1.30566
7414	1.40248	1.24549
7415	1.41479	1.36712
7416	2.22625	1.38735
7417	0.913373	1.40497
7418	1.0158	1.1271
7419	1.61259	1.04532
7420	1.69051	0.675124
7421	0.560949	1.30721

7422	0.898559	1.34475
7423	1.28011	1.24119
7424	1.51026	0.05
7425	1.98252	0.05
7426	1.05129	0.05
7427	1.2951	0.05
7428	1.46041	0.05
7429	1.7155	2.1
7430	1.72196	2.1
7431	0.933885	2.1
7432	0.986723	2.1
7433	1.28072	0.05
7434	1.40765	2.1
7435	1.28072	0.706351
7436	1.61687	2.1
7437	1.60965	2.1
7438	1.21424	2.1
7439	2.07297	2.1
7440	1.19943	2.1
7441	2.38784	2.1
7442	1.10278	0.05
7443	1.43678	0.05
7444	1.1142	0.05
7445	1.18034	0.0836075
7446	0.94613	0.223543
7447	0.752356	0.05
7448	0.752329	0.0531866
7449	1.46634	0.05
7450	1.31789	0.05
7451	0.958159	0.0709413
7452	1.22752	0.05
7453	1.50472	0.05
7454	1.98974	2.1
7455	2.54186	2.1
7456	0.76512	2.1
7457	2.08777	2.1
7458	1.7753	2.1
7459	3.07372	2.1
7460	1.9756	2.1
7461	1.48162	2.1
7462	1.23204	2.1
7463	0.946131	1.09469
7464	1.13505	2.1
7465	1.47432	2.1
7466	1.47432	1.28322
7467	0.876831	2.1
7468	1.13505	1.88374
7469	1.2161	1.25742
7470	1.05267	1.74783
7471	1.05267	1.1765
7472	1.5795	1.20996
7473	0.98186	2.1
7474	1.56735	2.1
7475	0.859099	2.1
7476	1.69858	2.1
7477	1.93586	2.1
7478	1.09233	2.1
7479	1.08732	0.05

7480	1.3357	0.0953235
7481	1.84826	2.1
7482	0.924132	2.1
7483	1.39188	2.1
7484	1.43685	2.1
7485	0.851541	2.1
7486	1.16251	2.1
7487	0.946131	2.1
7488	1.05685	2.1
7489	2.13781	2.1
7490	1.98859	2.1
7491	1.99731	2.1
7492	1.51379	2.1
7493	1.48796	2.1
7494	0.851542	2.1
7495	0.802558	2.1
7496	1.09244	2.1
7497	1.33891	2.1
7498	0.844958	2.1
7499	0.710864	2.1
7500	1.26087	2.1
7501	1.03679	2.1
7502	1.51026	0.05
7503	1.05248	0.317756
7504	0.827258	0.305844
7505	1.05248	0.344519
7506	1.28435	0.333195
7507	1.50953	0.360376
7508	1.50953	0.05
7509	0.827298	0.402733
7510	1.00366	0.05
7511	2.10495	0.0626998
7512	1.17885	2.1
7513	1.25185	2.1
7514	1.02784	2.1
7515	1.76764	2.1
7516	1.23746	0.05
7517	1.24082	0.05
7518	1.46506	0.05
7519	1.33061	0.05
7520	0.768407	1.01104
7521	0.972058	0.814032
7522	1.16536	1.79843
7523	1.13325	0.905102
7524	0.978906	2.1
7525	1.02291	0.894745
7526	0.838015	0.809943
7527	1.01052	0.05
7528	1.08166	0.05
7529	1.42607	0.05
7530	1.47354	0.05
7531	1.21703	2.1
7532	1.29897	2.1
7533	1.83293	2.1
7534	1.29897	2.1
7535	1.24688	0.05
7536	0.782563	2.1
7537	0.946129	2.1

7538	0.782591	2.1
7539	1.49487	2.1
7540	1.49487	2.1
7541	1.37713	2.1
7542	0.900337	2.1
7543	1.31284	2.1
7544	0.924468	2.1
7545	1.40602	2.1
7546	1.25669	2.1
7547	0.986901	2.1
7548	0.958565	2.1
7549	1.37266	2.1
7550	1.64146	2.1
7551	0.49345	2.1
7552	1.08276	2.1
7553	1.08276	2.1
7554	1.26929	2.1
7555	1.09847	2.1
7556	0.827081	0.05
7557	1.07236	0.261985
7558	1.25063	0.05
7559	1.43548	0.05
7560	1.25063	0.05
7561	1.3659	0.05
7562	1.72441	0.05
7563	1.3659	0.05
7564	1.4677	0.05
7565	1.86114	0.05
7566	1.40104	0.05
7567	0.962675	0.05
7568	0.946131	0.05
7569	1.74134	0.05
7570	1.74134	0.05
7571	0.962676	0.05
7572	0.946131	0.05
7573	1.05548	0.05
7574	0.902187	0.05
7575	2.14178	0.05
7576	1.74998	0.05
7577	1.38541	0.05
7578	1.64468	0.05
7579	0.949317	0.05
7580	2.06654	0.05
7581	0.839626	0.05
7582	0.810945	0.05
7583	1.29203	0.05
7584	1.18091	0.05
7585	1.06872	0.05
7586	1.11956	0.05
7587	0.926911	0.05
7588	0.901716	0.05
7589	0.909146	0.05
7590	1.33776	0.05
7591	1.02048	0.05
7592	1.08365	0.05
7593	1.21042	0.05
7594	0.865634	0.05
7595	0.590354	2.1

7596	0.61173	2.1
7597	3.46825	2.1
7598	3.28404	2.1
7599	1.56004	0.05
7600	1.69344	0.05
7601	1.7151	0.267149
7602	0.946131	0.05
7603	1.40265	0.05
7604	1.13537	0.05
7605	0.965708	0.05
7606	1.13537	0.05
7607	1.29681	0.05
7608	1.40915	0.127911
7609	1.61652	0.107956
7610	1.33171	1.0042
7611	1.37681	2.1
7612	0.81094	0.05
7613	0.598606	0.05
7614	0.723111	0.05
7615	1.04562	0.05
7616	0.979052	0.05
7617	0.798185	0.05
7618	0.880117	0.05
7619	0.979341	0.384978
7620	0.800229	0.100739
7621	1.39126	0.301295
7622	1.13005	0.385608
7623	0.913877	0.497625
7624	1.26959	0.274258
7625	0.699741	0.454046
7626	1.19496	1.51179
7627	1.13392	0.05
7628	1.11913	0.05
7629	1.06866	0.05
7630	0.615546	0.05
7631	0.831006	0.05
7632	1.53286	0.05
7633	0.690977	0.05
7634	1.01956	0.05
7635	2.33818	0.05
7636	1.6585	2.1
7637	1.67061	2.1
7638	1.41414	2.1
7639	1.16756	2.1
7640	1.00898	0.05
7641	0.966964	2.1
7642	2.85443	2.1
7643	0.966977	2.1
7644	2.08427	2.1
7645	2.94901	2.1
7646	1.10735	0.05
7647	1.65815	2.1
7648	1.4502	2.1
7649	1.45026	2.1
7650	1.53446	2.1
7651	0.946129	1.08278
7652	1.81075	2.1
7653	1.81075	1.10568

7654	0.946131	1.54716
7655	1.37075	2.1
7656	1.37075	1.10341
7657	1.34245	1.88768
7658	0.973502	1.13183
7659	1.08542	2.1
7660	1.5814	0.05
7661	1.43836	0.05
7662	0.946132	0.05
7663	1.13747	0.05
7664	1.04429	0.05
7665	1.05017	0.05
7666	1.24732	0.05
7667	1.13387	0.05
7668	2.19489	0.05
7669	1.10748	2.1
7670	1.25006	2.1
7671	1.58834	2.1
7672	1.58834	0.0786482
7673	0.94613	2.1
7674	1.16548	2.1
7675	0.946132	0.05
7676	1.08998	2.02481
7677	1.16548	1.19995
7678	0.995732	0.139144
7679	0.657014	2.1
7680	0.957173	2.1
7681	1.01485	2.1
7682	1.12468	2.1
7683	1.43871	2.1
7684	1.23716	2.1
7685	1.89277	0.601411
7686	2.06653	0.173691
7687	0.967544	0.050121
7688	1.89016	0.109422
7689	2.22165	2.1
7690	0.83514	2.1
7691	1.76565	2.1
7692	1.76565	2.1
7693	0.94613	2.1
7694	0.835112	2.1
7695	1.23426	2.1
7696	1.36653	2.1
7697	0.895892	1.30986
7698	0.859278	1.2431
7699	0.791461	1.30718
7700	1.86249	1.54265
7701	1.14607	1.32638
7702	1.19904	1.47004
7703	1.84898	1.37415
7704	1.03452	1.01331
7705	0.946131	0.208699
7706	0.940472	0.625414
7707	1.53478	0.844236
7708	1.84175	0.739104
7709	1.55687	0.467321
7710	1.17787	0.05
7711	1.60589	0.713451

7712	2.35574	0.05
7713	1.3305	0.05
7714	1.15008	0.136704
7715	1.12457	0.05
7716	1.12457	0.05
7717	1.01778	2.1
7718	1.35733	2.1
7719	1.50683	2.1
7720	1.12064	2.1
7721	1.42716	0.278987
7722	1.42717	0.579505
7723	1.45927	1.1722
7724	1.04321	1.58296
7725	1.32634	0.05
7726	2.27022	0.05
7727	1.19352	0.05
7728	1.32634	0.05
7729	1.40924	2.06921
7730	1.22927	1.26204
7731	1.69237	1.75263
7732	1.69237	2.1
7733	0.946132	1.13002
7734	0.946133	1.7379
7735	1.16912	1.43944
7736	1.0417	1.29786
7737	1.14605	1.95531
7738	1.1233	2.1
7739	1.96882	1.94573
7740	1.03804	0.05
7741	1.15338	0.05
7742	1.15338	0.05
7743	2.33051	0.155622
7744	1.81507	2.1
7745	2.42949	2.1
7746	0.725879	0.874179
7747	1.88394	1.05148
7748	2.2699	0.05
7749	2.78952	2.1
7750	1.88394	0.05
7751	1.79606	2.1
7752	1.20091	2.1
7753	1.9614	2.1
7754	1.19901	2.01734
7755	3.07915	2.1
7756	1.32592	2.1
7757	2.86461	2.1
7758	1.34817	2.1
7759	1.44859	2.1
7760	2.03969	2.1
7761	0.946132	2.1
7762	1.50709	2.1
7763	1.50709	2.1
7764	0.946132	2.1
7765	1.29322	2.1
7766	1.29322	2.1
7767	1.01985	2.1
7768	0.968537	2.1
7769	1.401	2.1

7770	1.28936	2.1
7771	1.56002	0.05
7772	1.92548	0.05
7773	1.46041	0.05
7774	2.22712	0.05
7775	1.56001	0.05
7776	1.50524	2.1
7777	1.21304	2.1
7778	1.60665	1.73177
7779	1.85178	1.14185
7780	2.17534	2.1
7781	2.31012	2.1
7782	2.17533	1.80302
7783	0.986724	2.1
7784	2.01895	2.1
7785	2.00433	2.1
7786	1.45752	0.05
7787	1.12695	0.05
7788	0.652605	2.1
7789	1.5133	2.1
7790	1.5133	2.1
7791	0.652603	2.1
7792	1.228 2.1	
7793	0.946132	2.1
7794	0.652604	2.1
7795	0.946132	1.39487
7796	0.716815	2.1
7797	1.62203	2.1
7798	1.62203	2.1
7799	0.716814	2.1
7800	1.00191	2.1
7801	1.33694	2.1
7802	1.38216	2.1
7803	2.53338	2.1
7804	1.46932	0.05
7805	1.50848	0.05
7806	1.50108	1.22382
7807	1.18382	0.957987
7808	2.02115	0.860899
7809	0.921823	0.102749
7810	2.08356	0.05
7811	1.543 0.05	
7812	1.00763	0.129931
7813	1.4185	0.4042
7814	1.08028	0.398428
7815	0.946131	0.886597
7816	1.56599	0.320078
7817	1.56599	0.905634
7818	1.08028	0.706262
7819	0.946131	0.311081
7820	1.37518	2.1
7821	1.63453	2.1
7822	1.04627	1.23474
7823	1.04626	0.959341
7824	1.35111	1.31617
7825	1.22297	1.25547
7826	1.64499	2.1
7827	1.87598	2.1

7828	1.06994	2.1
7829	1.37063	2.1
7830	1.99943	0.05
7831	2.38807	0.05
7832	0.99972	0.05
7833	1.41388	0.05
7834	1.66179	0.05
7835	1.66179	0.05
7836	0.946129	0.05
7837	1.41388	0.05
7838	1.19404	0.05
7839	1.31023	0.363127
7840	1.20871	0.841539
7841	1.69731	2.1
7842	1.67971	2.1
7843	0.998971	0.05
7844	1.32988	0.05
7845	0.701079	0.05
7846	0.946131	0.05
7847	0.582785	0.05
7848	1.48592	0.05
7849	1.48592	0.05
7850	1.23351	0.05
7851	0.835199	0.05
7852	0.92427	0.05
7853	1.68262	0.05
7854	2.46702	0.05
7855	1.08715	2.1
7856	1.01606	1.88986
7857	1.29579	2.02206
7858	1.01606	2.1
7859	1.63823	2.1
7860	1.67687	2.1
7861	1.11048	2.1
7862	1.39477	2.1
7863	0.991114	2.1
7864	2.90978	0.941053
7865	0.906811	1.08491
7866	0.872849	0.521723
7867	1.17091	0.897429
7868	0.685763	0.862973
7869	1.6386	1.22311
7870	1.06907	0.888577
7871	1.05516	0.987694
7872	1.9189	0.983349
7873	1.04615	0.814431
7874	1.2966	2.1
7875	1.37392	2.1
7876	1.35504	2.1
7877	1.30347	2.1
7878	1.50877	2.1
7879	1.11193	2.1
7880	1.40984	2.1
7881	1.67682	2.1
7882	1.00371	2.1
7883	1.15412	2.1
7884	1.41638	2.1
7885	1.03796	2.1

7886	0.946117	2.1
7887	1.50823	2.1
7888	1.50822	2.1
7889	0.966923	2.1
7890	0.946131	2.1
7891	2.07593	2.1
7892	1.9339	2.1
7893	1.1817	0.854549
7894	0.729178	0.910492
7895	1.5079	0.880993
7896	1.26991	0.959919
7897	0.994049	0.916869
7898	1.17452	0.875376
7899	1.05711	0.930618
7900	1.04071	0.915749
7901	1.80299	0.911262
7902	1.60417	0.73011
7903	0.943405	0.05
7904	1.01129	0.05
7905	1.42972	0.05
7906	1.15904	0.05
7907	0.680409	0.05
7908	1.2816	0.05
7909	0.806176	0.05
7910	1.18954	0.05
7911	1.26055	0.05
7912	1.06313	2.1
7913	1.19352	2.1
7914	1.55766	2.1
7915	1.53729	2.1
7916	0.993021	0.05
7917	1.09056	0.512913
7918	1.03408	2.1
7919	0.993023	0.594965
7920	0.659901	2.1
7921	0.632905	2.1
7922	1.34072	0.05
7923	1.17793	0.135119
7924	2.24885	0.0672839
7925	0.946132	0.0643888
7926	1.12443	0.05
7927	0.904207	0.07375
7928	1.51237	0.05
7929	1.51237	0.05
7930	1.12443	0.05
7931	1.29215	0.05
7932	1.6943	2.1
7933	2.43858	2.1
7934	2.0589	1.95577
7935	1.67204	2.1
7936	1.67204	2.1
7937	0.555875	2.1
7938	0.91951	2.1
7939	0.946131	1.51989
7940	0.555904	2.1
7941	0.946131	2.09187
7942	1.6943	2.1
7943	1.04744	1.95585

7944	1.50938	2.1
7945	1.60861	2.1
7946	1.92406	0.05
7947	1.18716	0.05
7948	0.962705	0.741686
7949	1.35382	1.05591
7950	1.43632	0.05
7951	1.09386	0.05
7952	1.92547	0.05
7953	0.994164	0.05
7954	0.903974	2.1
7955	0.928829	1.93749
7956	0.810958	1.68905
7957	1.92853	1.91028
7958	1.598	1.86848
7959	1.28967	1.9866
7960	1.44993	1.86074
7961	0.946132	0.797578
7962	0.882984	0.615511
7963	0.882955	1.06089
7964	1.56071	0.153422
7965	1.56071	0.303765
7966	1.35946	0.553742
7967	1.08421	0.663826
7968	1.41943	0.05
7969	1.26265	0.05
7970	1.03235	0.142052
7971	1.50778	2.1
7972	1.70387	2.1
7973	1.65608	2.1
7974	1.39076	2.1
7975	1.6104	2.1
7976	1.37456	2.1
7977	1.51898	0.494226
7978	1.9726	0.13367
7979	2.13757	0.946835
7980	1.74856	1.11187
7981	0.635949	0.484192
7982	2.46448	1.15016
7983	1.23224	1.09658
7984	1.12786	1.25796
7985	1.82721	0.895102
7986	1.82721	0.412441
7987	0.532892	2.00455
7988	0.946131	1.30241
7989	0.53292	1.22855
7990	1.41394	2.1
7991	1.45506	2.1
7992	1.39523	2.1
7993	1.09006	2.1
7994	1.80264	0.05
7995	1.80264	0.595924
7996	0.946131	0.0527373
7997	1.34268	0.05
7998	0.946132	0.332079
7999	1.23732	0.05
8000	1.34268	0.0672857
8001	1.2064	0.967503

8002	1.58164	2.1
8003	1.73853	2.1
8004	1.01243	1.16999
8005	0.910672	1.62553
8006	1.47793	1.848
8007	1.47793	2.1
8008	0.910702	1.55722
8009	1.30382	1.1486
8010	1.08478	1.17559
8011	0.910702	2.0616
8012	2.60709	1.28025
8013	1.01243	1.22402
8014	1.85516	1.39261
8015	2.52823	0.973148
8016	1.24001	0.884689
8017	1.89793	0.587191
8018	1.73584	0.05
8019	1.24613	2.1
8020	1.37074	1.87316
8021	1.23763	1.65426
8022	1.04072	2.1
8023	0.75499	1.34818
8024	1.72329	1.77173
8025	1.46195	1.64861
8026	0.946132	1.46754
8027	0.747438	2.00463
8028	1.24613	1.61118
8029	2.59886	1.34349
8030	1.87279	2.1
8031	1.07977	2.1
8032	1.29606	2.1
8033	1.45178	2.1
8034	1.45177	2.1
8035	0.92406	2.1
8036	0.946131	2.1
8037	0.924047	2.1
8038	2.04529	2.1
8039	1.04544	2.1
8040	1.08276	2.1
8041	1.67491	2.1
8042	0.961826	0.941036
8043	0.810942	0.747842
8044	0.826622	1.794
8045	0.914378	1.93299
8046	0.89022	0.332416
8047	0.922383	0.981152
8048	0.988165	0.464165
8049	1.31357	0.05
8050	0.946131	0.05
8051	1.31357	0.05
8052	1.10564	0.05
8053	1.6797	0.05
8054	1.6797	0.05
8055	0.739506	0.05
8056	1.33957	0.05
8057	1.33957	0.05
8058	1.33957	0.05
8059	2.34223	0.05

8060	1.5515	1.0864
8061	2.22137	0.994463
8062	0.94612	0.933695
8063	1.13908	1.03052
8064	1.45423	0.940728
8065	1.45422	1.04556
8066	0.795519	1.13625
8067	1.1391	0.997618
8068	1.11065	0.972854
8069	1.12266	2.1
8070	1.24925	2.1
8071	1.20242	2.1
8072	2.18297	2.1
8073	3.00107	1.74217
8074	2.58297	0.565608
8075	0.884783	2.1
8076	0.851538	0.926174
8077	0.713587	2.08624
8078	1.47949	0.971327
8079	1.66673	2.1
8080	0.696199	2.1
8081	0.9383	1.22175
8082	1.29149	1.06839
8083	1.32266	2.028
8084	2.11428	0.05
8085	2.17439	0.05
8086	1.40489	0.05
8087	1.21071	0.05
8088	0.946118	0.05
8089	1.0746	0.05
8090	0.973119	0.05
8091	1.31333	0.05
8092	1.08723	0.05
8093	0.994292	2.1
8094	1.4964	2.1
8095	1.15096	2.1
8096	1.10101	2.1
8097	1.50438	2.1
8098	1.67825	2.1
8099	2.02103	2.1
8100	2.01978	2.1
8101	1.50931	0.05
8102	1.50931	0.05
8103	0.65023	0.05
8104	1.34831	0.05
8105	0.811233	0.05
8106	1.34831	0.05
8107	0.94613	0.05
8108	1.00754	1.2639
8109	0.714132	2.1
8110	1.28877	1.52791
8111	1.39905	1.46638
8112	1.03282	1.40525
8113	0.997769	1.26124
8114	1.11743	1.3238
8115	1.86863	1.04879
8116	3.30723	1.56
8117	1.66635	2.1

8118	1.35609	0.05
8119	1.38212	0.05
8120	1.95597	2.1
8121	1.64257	2.1
8122	2.93316	2.1
8123	3.13417	2.1
8124	0.733947	2.1
8125	1.15259	2.1
8126	1.07976	2.1
8127	1.30237	2.1
8128	1.99943	0.05
8129	0.907431	0.05
8130	0.878161	0.05
8131	1.07544	0.05
8132	1.03401	0.05
8133	0.991975	0.05
8134	1.03635	0.05
8135	0.788549	0.05
8136	1.44984	0.05
8137	1.84007	0.05
8138	2.31127	0.05
8139	1.27362	2.1
8140	1.27737	1.12185
8141	1.87783	0.958615
8142	2.35499	0.05
8143	2.69662	0.05
8144	2.24065	0.05
8145	0.996082	2.1
8146	0.996083	2.1
8147	1.44859	2.1
8148	1.03968	2.1
8149	1.60018	0.577695
8150	1.73082	1.73886
8151	0.946132	1.39153
8152	1.49742	1.89286
8153	1.49742	0.129941
8154	1.00501	1.20685
8155	1.41585	2.1
8156	1.41585	1.28481
8157	0.946131	1.68349
8158	1.03697	1.47577
8159	2.95786	2.1
8160	2.32185	2.1
8161	1.65783	0.166161
8162	1.772	2.1
8163	0.946131	0.14662
8164	1.40404	0.05
8165	1.13537	2.1
8166	1.04559	1.10691
8167	1.16091	1.15508
8168	2.63751	2.1
8169	1.68455	0.05
8170	1.9682	0.05
8171	2.16834	0.0941517
8172	2.45569	0.208937
8173	0.946128	0.0747695
8174	1.36428	0.250201
8175	0.663978	0.267076

8176	1.58218	0.0787039
8177	1.58219	0.0654378
8178	1.36428	0.140226
8179	0.881886	0.203336
8180	1.73849	2.1
8181	1.35668	2.1
8182	1.1233	2.1
8183	1.15009	2.1
8184	1.16482	0.812383
8185	1.29313	0.826701
8186	1.47608	0.0530843
8187	2.05949	0.05
8188	0.946119	0.05
8189	1.02974	0.05
8190	0.946119	0.05
8191	1.66758	0.05
8192	1.66757	0.05
8193	1.32082	0.05
8194	1.29287	0.05
8195	1.36005	2.1
8196	1.47109	2.1
8197	2.45958	2.1
8198	2.2975	2.1
8199	0.953521	0.558669
8200	0.953521	0.121227
8201	1.67054	2.1
8202	1.67054	2.1
8203	0.946131	2.1
8204	1.38154	2.1
8205	1.13326	2.1
8206	1.13326	2.1
8207	1.23513	2.1
8208	1.50998	2.1
8209	2.47982	0.05
8210	1.45091	0.05
8211	0.958953	0.05
8212	0.79239	0.05
8213	2.11929	0.05
8214	1.26511	0.05
8215	1.27733	0.05
8216	2.18127	0.05
8217	1.30169	0.05
8218	1.19259	0.05
8219	0.963395	1.20785
8220	0.999947	2.0806
8221	1.62241	2.1
8222	1.62027	2.1
8223	0.981586	0.05
8224	0.981585	0.05
8225	0.721961	2.1
8226	0.891321	2.1
8227	1.04442	2.1
8228	1.66554	2.1
8229	2.43434	2.1
8230	1.73944	0.05
8231	1.88032	0.05
8232	0.711391	0.05
8233	1.40837	0.05

8234	0.946133	0.05
8235	0.92252	0.05
8236	1.13964	0.05
8237	1.83394	0.05
8238	2.81674	0.05
8239	1.10049	1.56855
8240	1.25092	0.920071
8241	0.760299	1.16276
8242	1.48017	1.27935
8243	1.48017	1.38897
8244	0.760271	1.07877
8245	1.35861	1.21285
8246	0.946129	1.11514
8247	0.760269	1.09531
8248	2.26155	2.1
8249	2.15634	2.1
8250	1.39824	2.1
8251	1.07817	2.1
8252	0.946132	2.1
8253	1.53028	2.1
8254	1.53028	2.1
8255	1.00807	2.1
8256	0.946133	2.1
8257	2.06126	0.684648
8258	1.97385	1.79734
8259	1.75754	0.635229
8260	2.01613	2.1
8261	1.97995	2.1
8262	1.47696	2.1
8263	1.50172	2.1
8264	1.5177	0.05
8265	1.44146	0.05
8266	1.8807	0.05
8267	2.07068	0.05
8268	1.84267	0.05
8269	1.08975	2.1
8270	1.21327	2.1
8271	1.10106	0.05
8272	1.34496	0.05
8273	2.08452	0.718983
8274	1.70537	0.0510285
8275	2.62464	2.1
8276	1.5726	2.1
8277	1.5726	2.1
8278	0.94613	2.1
8279	1.15038	2.1
8280	0.946132	2.1
8281	1.03974	2.1
8282	1.15038	2.1
8283	0.99747	0.432732
8284	1.03472	1.89861
8285	1.58407	0.25576
8286	1.21148	0.857618
8287	1.35025	1.05212
8288	1.3428	0.246695
8289	1.00775	2.1
8290	1.00775	2.1
8291	1.3879	2.1

8292	1.39958	2.1
8293	0.853042	2.1
8294	1.06997	2.1
8295	0.853042	2.1
8296	0.853042	2.1
8297	1.12491	2.1
8298	1.17879	2.1
8299	1.07162	2.1
8300	1.61179	2.1
8301	1.76016	2.1
8302	0.946132	0.281235
8303	1.23449	0.09803
8304	1.70392	0.129927
8305	1.70392	0.0928602
8306	0.94613	0.384092
8307	1.23449	0.172142
8308	1.41556	0.177609
8309	1.14547	0.127129
8310	1.1721	0.05
8311	0.96478	0.0899314
8312	1.41843	0.113782
8313	1.2358	0.0579297
8314	1.70678	0.0547112
8315	1.70678	0.0569627
8316	0.946118	0.233832
8317	1.23582	0.14901
8318	0.946105	0.221831
8319	0.964769	0.05
8320	1.1357	0.05
8321	0.964767	0.05
8322	0.619856	0.05
8323	1.72366	0.05
8324	1.72367	0.05
8325	0.619831	0.05
8326	1.19262	0.05
8327	0.619829	0.05
8328	0.946129	0.05
8329	1.57459	2.1
8330	1.86512	2.1
8331	0.93841	2.1
8332	1.57459	2.1
8333	1.10476	0.05
8334	1.00013	0.809799
8335	2.13446	0.986001
8336	0.759781	2.1
8337	1.96011	1.43995
8338	0.946129	1.04174
8339	1.32056	1.95978
8340	1.75702	1.18325
8341	1.75702	2.1
8342	0.946124	1.81136
8343	1.32056	1.28773
8344	1.38258	1.09508
8345	1.96011	1.46668
8346	2.31099	1.39318
8347	0.97356	2.1
8348	1.38885	2.1
8349	1.3771	2.1

8350	0.946131	2.1
8351	1.84843	2.1
8352	1.84843	2.1
8353	1.3771	2.1
8354	0.946131	2.1
8355	1.00278	2.1
8356	1.05447	0.210142
8357	1.05446	0.446431
8358	1.24913	0.50817
8359	1.05447	0.208805
8360	1.80882	0.207441
8361	1.80882	2.1
8362	0.946129	2.1
8363	1.33997	2.1
8364	0.946131	0.05
8365	1.28831	1.91481
8366	1.33997	1.87832
8367	1.44391	0.05
8368	1.44391	0.05
8369	0.850964	0.05
8370	0.850963	0.05
8371	0.946132	0.05
8372	0.921299	0.05
8373	1.37358	0.05
8374	1.46297	0.05
8375	1.46298	0.05
8376	0.823988	0.05
8377	0.921287	0.05
8378	0.94612	0.05
8379	0.823995	0.05
8380	0.946107	0.05
8381	1.44437	2.1
8382	1.06203	2.1
8383	2.21397	2.1
8384	0.637677	2.1
8385	1.8892	2.1
8386	2.075 2.1	
8387	1.32051	0.05
8388	2.23637	0.05
8389	1.10904	0.0547445
8390	1.11819	0.05
8391	0.94613	0.0659224
8392	0.735845	0.05
8393	1.4914	0.0584139
8394	1.4914	0.05
8395	0.735831	0.112815
8396	1.61683	2.1
8397	1.61683	2.1
8398	0.946131	2.1
8399	1.3281	2.1
8400	1.10441	2.1
8401	1.10442	2.1
8402	1.23487	2.1
8403	1.09914	2.1
8404	1.20691	0.05
8405	2.27379	0.05
8406	1.1369	0.05
8407	1.26868	0.05

8408	1.54422	0.05
8409	1.54422	0.05
8410	0.861357	0.05
8411	0.94613	0.05
8412	0.861359	0.05
8413	1.2858	0.279649
8414	1.31011	0.826784
8415	1.294 0.793602	
8416	1.03079	0.876466
8417	1.81979	2.1
8418	1.57829	2.1
8419	1.19023	1.94332
8420	1.19022	2.0975
8421	1.46458	1.96375
8422	1.46458	2.1
8423	0.789173	2.1
8424	1.34386	2.1
8425	0.909896	2.1
8426	0.946131	2.03337
8427	0.909893	2.06722
8428	1.27725	2.1
8429	1.68451	2.1
8430	2.26155	2.1
8431	2.34217	2.1
8432	1.55485	0.953706
8433	0.90854	0.707561
8434	0.858256	0.704239
8435	1.47042	0.390515
8436	1.62771	0.395567
8437	0.777398	0.867168
8438	0.982525	0.720908
8439	1.23865	0.554284
8440	2.09928	0.736402
8441	0.946128	0.0511713
8442	0.474856	0.0630168
8443	0.474855	0.079196
8444	1.58747	0.05
8445	1.58747	0.05
8446	0.963917	0.05
8447	1.09841	0.05
8448	2.23904	2.1
8449	2.60055	0.458622
8450	1.02599	2.1
8451	2.17837	2.1
8452	2.07122	2.1
8453	2.7309	0.329171
8454	2.08482	2.1
8455	1.18322	0.05
8456	1.35381	0.731684
8457	1.01139	0.1734
8458	0.946133	1.17859
8459	1.59089	0.227734
8460	1.59089	0.05
8461	0.946133	0.258677
8462	1.09634	0.05
8463	2.02279	0.05
8464	1.03108	0.109763
8465	0.808978	1.52466

8466	1.07317	1.55814
8467	0.831695	1.50929
8468	1.22583	1.51677
8469	1.10215	1.42237
8470	1.07508	1.59198
8471	1.22204	1.46355
8472	1.04679	2.1
8473	1.92535	0.05
8474	1.11081	0.05
8475	0.946131	0.084295
8476	0.714824	0.056349
8477	0.714795	0.0853048
8478	1.4231	0.0620727
8479	1.4231	0.05
8480	0.828617	0.136195
8481	1.30928	0.05
8482	1.49894	2.1
8483	1.55702	2.1
8484	0.946131	2.1
8485	1.10689	2.1
8486	0.946227	2.1
8487	0.946131	2.1
8488	1.21754	2.1
8489	1.504 2.1	
8490	1.504 2.1	
8491	0.723573	2.1
8492	1.24512	2.1
8493	0.723544	2.1
8494	0.946131	2.1
8495	1.24512	2.1
8496	1.29383	0.05
8497	1.29383	0.05
8498	1.61969	0.05
8499	0.946132	0.05
8500	1.12818	0.05
8501	0.946132	0.05
8502	1.80922	0.05
8503	1.80922	0.05
8504	1.41383	0.05
8505	1.34153	0.05
8506	1.47689	0.05
8507	1.79375	0.05
8508	1.24333	0.139625
8509	0.768989	0.0848132
8510	1.24333	0.0643463
8511	1.0027	0.239718
8512	1.47705	0.187162
8513	1.47705	0.05
8514	0.768977	0.155373
8515	1.28436	0.323003
8516	0.928583	0.465046
8517	1.28436	0.0786562
8518	1.24333	0.420453
8519	1.5991	0.363209
8520	1.5991	0.05
8521	0.928583	0.479628
8522	1.85717	0.05
8523	2.48665	0.0674213

8524	1.18423	0.05
8525	1.9493	1.89548
8526	1.8519	1.23081
8527	1.68802	2.1
8528	1.0302	0.05
8529	0.900473	0.05
8530	1.99663	0.05
8531	2.61301	0.05
8532	0.745772	0.05
8533	0.946131	0.05
8534	1.48881	0.05
8535	1.48881	0.05
8536	0.745745	0.05
8537	0.809786	0.05
8538	1.3065	0.05
8539	0.946132	0.05
8540	0.768357	0.05
8541	0.768387	0.05
8542	1.76897	0.05
8543	1.76896	0.05
8544	1.40271	0.05
8545	1.00777	0.05
8546	1.37427	0.05
8547	1.1967	2.1
8548	0.946132	2.1
8549	1.59397	2.1
8550	1.59397	2.1
8551	0.946131	2.1
8552	1.14989	2.1
8553	1.14989	2.1
8554	1.10046	2.1
8555	1.09506	0.05
8556	2.53251	0.05
8557	1.9212	1.10277
8558	1.99586	0.988672
8559	0.913967	0.805075
8560	1.55028	0.780412
8561	1.55028	0.83784
8562	0.913966	0.812921
8563	1.16318	0.760522
8564	1.16318	0.783122
8565	0.946132	0.767929
8566	1.20478	2.1
8567	1.09662	2.1
8568	1.03472	2.1
8569	1.84774	2.1
8570	1.7984	2.1
8571	2.1221	2.1
8572	1.68223	2.1
8573	1.68224	2.1
8574	0.514872	1.99126
8575	0.888991	2.1
8576	0.946127	1.21025
8577	0.514845	1.98049
8578	0.946129	1.4721
8579	1.10127	1.8695
8580	2.61623	1.32703
8581	0.983581	0.918526

8582	0.869663	0.812359
8583	1.1818	1.56238
8584	1.10406	0.886044
8585	1.07133	1.87055
8586	1.09405	0.880057
8587	0.838014	0.826315
8588	0.842998	1.34684
8589	1.03818	1.05669
8590	1.46338	0.69934
8591	1.903 2.1	
8592	1.23942	2.1
8593	0.965933	2.1
8594	0.810075	2.1
8595	1.40571	2.1
8596	1.66219	2.1
8597	0.810047	2.1
8598	0.936678	2.1
8599	1.45808	2.1
8600	1.6384	2.1
8601	1.54699	2.1
8602	0.719877	2.1
8603	1.33256	2.1
8604	0.753547	2.1
8605	1.04073	2.1
8606	1.25822	2.1
8607	0.851542	2.1
8608	0.714102	2.1
8609	1.45886	2.1
8610	1.80171	2.1
8611	0.597337	2.1
8612	0.957713	2.1
8613	1.3033	2.1
8614	0.596613	0.05
8615	1.1386	0.0768999
8616	0.596612	0.05
8617	0.946133	0.108148
8618	0.596652	0.192774
8619	1.64092	0.079072
8620	1.64092	0.0684016
8621	1.991 2.1	
8622	1.23013	2.1
8623	2.77816	2.1
8624	2.74107	0.955981
8625	2.07681	0.900226
8626	1.47764	0.05
8627	1.72677	0.05
8628	1.52161	2.1
8629	1.28526	2.1
8630	1.90502	2.1
8631	1.87359	0.05
8632	1.93489	0.05
8633	2.70895	2.1
8634	2.83141	2.1
8635	1.41766	0.672462
8636	1.11337	0.66748
8637	1.41765	1.18562
8638	1.29172	0.680933
8639	1.76324	0.681008

8640	1.76324	0.880452
8641	0.946131	0.668671
8642	1.52428	2.1
8643	1.71459	2.1
8644	1.93852	0.862195
8645	1.07119	1.48973
8646	1.29698	1.14846
8647	1.42203	0.850618
8648	1.42203	1.45653
8649	0.946132	0.885527
8650	0.946132	0.969444
8651	0.96926	1.05431
8652	0.769913	1.90291
8653	0.946119	1.74015
8654	1.37867	1.11308
8655	1.94028	1.07941
8656	0.94612	0.915567
8657	0.946132	1.38888
8658	1.37865	1.03081
8659	2.14234	1.14535
8660	3.06459	1.32386
8661	1.44421	0.05
8662	1.33924	0.05
8663	1.33671	0.05
8664	1.10733	0.05
8665	0.68396	0.05
8666	1.76008	0.05
8667	1.76008	0.05
8668	0.68393	0.05
8669	0.946131	0.05
8670	3.79242	2.1
8671	2.46293	2.1
8672	3.56947	2.1
8673	1.35487	1.7466
8674	1.38616	1.99625
8675	0.954048	0.05
8676	1.36663	0.05
8677	0.909487	0.115356
8678	0.887288	2.1
8679	0.810947	0.496317
8680	0.810934	1.6422
8681	1.07046	1.81843
8682	1.13979	1.99553
8683	0.927838	0.733196
8684	0.919995	1.55682
8685	1.61447	2.1
8686	2.56305	2.1
8687	0.943414	2.1
8688	1.17016	2.1
8689	1.7238	2.1
8690	1.89334	2.1
8691	0.946128	0.394953
8692	1.25184	2.1
8693	1.55639	2.1
8694	3.12098	1.48402
8695	3.57307	1.05194
8696	2.81281	2.1
8697	0.851542	2.1

8698	1.27689	2.1
8699	1.52568	2.1
8700	0.839689	2.1
8701	1.36471	2.1
8702	0.827865	2.1
8703	0.93667	2.1
8704	0.900861	2.1
8705	0.943152	2.1
8706	1.00667	2.1
8707	1.18141	2.1
8708	1.15298	2.1
8709	1.20335	2.1
8710	1.00713	2.1
8711	0.597537	2.1
8712	1.0577	2.1
8713	0.967342	0.151237
8714	0.838027	1.09507
8715	0.893587	0.0878044
8716	1.06436	0.304916
8717	0.850048	0.0922294
8718	1.22026	0.517691
8719	0.988077	0.16921
8720	1.18769	0.05
8721	1.3991	2.1
8722	1.52984	2.1
8723	1.35469	1.30555
8724	1.25083	0.854081
8725	1.6594	1.45243
8726	1.6594	1.20549
8727	0.946129	0.565169
8728	1.02602	0.545171
8729	0.94613	0.318796
8730	1.29724	0.05
8731	1.14501	0.05
8732	1.13078	0.05
8733	1.48381	0.05
8734	1.45921	0.05
8735	0.851539	0.05
8736	0.890836	0.05
8737	0.946131	0.0575509
8738	1.60428	2.1
8739	1.50097	2.1
8740	0.714507	0.133038
8741	1.51396	0.0929314
8742	1.51396	0.0789455
8743	0.946132	0.123684
8744	0.813862	0.113188
8745	1.41461	0.0873717
8746	0.813862	0.073706
8747	0.921311	2.1
8748	1.25084	1.11741
8749	1.30165	0.842705
8750	1.60636	1.31048
8751	1.60636	1.06321
8752	0.94613	0.746979
8753	0.972232	0.486244
8754	0.946131	0.280676
8755	2.35003	2.1

8756	0.92546	2.1
8757	1.00713	2.1
8758	0.810947	2.1
8759	0.810934	2.1
8760	1.056	2.1
8761	0.926811	2.1
8762	0.766408	2.1
8763	1.34292	2.1
8764	1.57932	0.05
8765	2.67595	0.05
8766	1.77737	0.05
8767	1.77738	0.05
8768	0.789633	0.05
8769	1.22903	0.05
8770	0.789663	0.05
8771	0.789662	0.05
8772	1.33797	0.05
8773	0.946128	2.1
8774	1.47351	2.1
8775	1.47351	2.1
8776	0.94613	2.1
8777	1.30082	2.1
8778	1.30082	2.1
8779	0.996486	2.1
8780	0.946127	2.1
8781	1.72122	2.1
8782	1.72122	2.1
8783	0.94613	2.1
8784	1.36653	2.1
8785	1.36653	2.1
8786	1.02608	2.1
8787	0.709123	0.869105
8788	0.946127	0.908762
8789	1.41342	1.04163
8790	1.818	1.20105
8791	1.818	1.1262
8792	0.946127	0.413102
8793	1.41342	1.08156
8794	1.35071	1.02921
8795	1.8993	0.996142
8796	1.4748	2.1
8797	1.18711	0.05
8798	0.946131	0.05
8799	1.18711	0.05
8800	1.20416	0.05
8801	1.91358	0.05
8802	1.91358	0.05
8803	0.477691	0.05
8804	2.16798	0.05
8805	2.22307	0.05
8806	0.758971	0.05
8807	1.80694	0.05
8808	1.80694	0.05
8809	0.946132	0.05
8810	0.75897	0.05
8811	1.25124	0.05
8812	0.946131	0.05
8813	1.76837	2.1

8814	1.76837	2.1
8815	0.946131	2.1
8816	1.36422	2.1
8817	0.946131	2.1
8818	1.14184	2.1
8819	1.36422	2.1
8820	0.793303	0.05
8821	1.79584	0.05
8822	1.35333	2.1
8823	1.19967	1.39952
8824	1.55088	1.62353
8825	1.64246	2.1
8826	0.946132	0.94381
8827	0.886293	2.1
8828	1.25018	2.1
8829	1.5795	1.7927
8830	2.15414	1.30051
8831	0.94613	0.957457
8832	1.37758	1.67611
8833	1.71567	1.01965
8834	1.71567	2.1
8835	0.946125	1.20779
8836	1.37759	1.02386
8837	1.28421	0.942729
8838	0.933959	0.31937
8839	0.946133	0.05
8840	1.7813	0.05
8841	1.7813	0.05
8842	1.05704	0.05
8843	1.37794	0.05
8844	1.37794	0.05
8845	0.946132	0.05
8846	0.946132	0.05
8847	1.68197	0.05
8848	1.68197	0.05
8849	1.01367	0.05
8850	1.25017	0.05
8851	1.25017	0.05
8852	0.946132	0.05
8853	0.838953	2.1
8854	1.4045	2.1
8855	1.56388	2.1
8856	1.3182	2.1
8857	0.928518	2.1
8858	1.04073	2.1
8859	0.835676	2.1
8860	1.47507	2.1
8861	1.47506	2.1
8862	0.797664	2.1
8863	1.34285	2.1
8864	0.929875	2.1
8865	0.797663	2.1
8866	0.946133	2.1
8867	1.83628	0.131972
8868	1.83627	0.783955
8869	0.946131	0.698815
8870	1.36748	0.651494
8871	0.946133	0.05

8872	1.21139	0.754635
8873	1.36748	0.05
8874	1.06795	2.1
8875	1.08192	2.1
8876	0.827866	2.1
8877	1.02548	2.1
8878	0.95006	2.1
8879	1.53933	2.1
8880	0.586511	2.1
8881	0.868145	2.1
8882	1.28401	2.1
8883	1.37719	2.1
8884	1.37719	2.1
8885	0.77497	2.1
8886	0.946132	2.1
8887	0.774968	2.1
8888	1.7053	0.235364
8889	1.7053	2.03793
8890	0.94613	2.1
8891	1.25715	1.27524
8892	0.946132	0.255443
8893	1.19655	0.0738573
8894	1.25716	0.241807
8895	0.851542	2.1
8896	0.80043	2.1
8897	1.27239	2.1
8898	1.02719	2.1
8899	0.946131	2.1
8900	0.981318	2.1
8901	1.06628	2.1
8902	1.26022	2.1
8903	0.946132	2.1
8904	0.946132	2.1
8905	1.76447	2.1
8906	1.76447	2.1
8907	1.35357	2.1
8908	1.35703	2.1
8909	0.946131	2.1
8910	1.50538	2.1
8911	1.50538	2.1
8912	1.10311	2.1
8913	1.3484	2.1
8914	1.10312	2.1
8915	0.966428	2.1
8916	0.946133	2.1
8917	1.4008	2.1
8918	1.71908	2.1
8919	1.71908	2.1
8920	0.946133	2.1
8921	1.40081	2.1
8922	1.26441	2.1
8923	0.851543	2.1
8924	0.961732	2.1
8925	1.54576	2.1
8926	1.35364	2.1
8927	0.946133	2.1
8928	1.02913	2.1
8929	1.2735	2.1

8930	1.32608	2.1
8931	0.967643	2.1
8932	0.94613	2.1
8933	1.44873	2.1
8934	1.44873	2.1
8935	0.967641	2.1
8936	0.946131	2.1
8937	1.40488	2.1
8938	1.24272	2.1
8939	0.946131	2.1
8940	1.78483	2.1
8941	1.78483	2.1
8942	1.24272	2.1
8943	0.946131	2.1
8944	0.94613	2.1
8945	1.5627	0.946525
8946	1.5627	2.1
8947	1.03656	2.1
8948	1.31649	0.533498
8949	1.19233	1.26092
8950	1.03656	1.19
8951	0.912059	2.1
8952	1.47954	0.972543
8953	1.47954	2.1
8954	0.912061	2.1
8955	1.34647	0.692305
8956	1.04514	1.84291
8957	0.912061	1.18433
8958	0.761972	0.0546197
8959	0.946132	0.05
8960	0.761974	0.274435
8961	1.70225	0.11496
8962	1.70225	0.813701
8963	1.33728	0.123641
8964	1.01188	0.05
8965	0.94613	1.18149
8966	0.746361	1.37848
8967	1.6369	1.99393
8968	1.62454	1.48118
8969	0.736738	1.08635
8970	0.958487	1.13861
8971	1.40641	1.29671
8972	1.2284	2.1
8973	0.92609	2.1
8974	0.595102	2.1
8975	1.80871	2.1
8976	1.1516	2.1
8977	0.962474	2.1
8978	2.24024	2.1
8979	0.946131	2.1
8980	1.73689	2.1
8981	1.73689	2.1
8982	0.946132	2.1
8983	1.30492	2.1
8984	1.30492	2.1
8985	1.17949	2.1
8986	0.946131	2.1
8987	1.8566	2.1

8988	1.8566	2.1
8989	1.22392	2.1
8990	1.39826	2.1
8991	1.40447	2.1
8992	1.22392	2.1
8993	0.946131	2.1
8994	1.74134	2.1
8995	1.74134	2.1
8996	1.09148	2.1
8997	1.40447	2.1
8998	1.283	2.1
8999	1.09148	2.1
9000	1.33607	2.1
9001	1.41022	2.1
9002	1.80016	2.1
9003	1.80015	2.1
9004	0.946127	2.1
9005	0.946129	2.1
9006	1.20541	2.1
9007	1.13078	2.1
9008	1.33607	2.1
9009	1.54624	2.1
9010	1.85553	2.1
9011	0.920606	2.1
9012	0.946131	2.1
9013	1.01271	2.1
9014	1.32664	1.2888
9015	0.954692	1.26939
9016	0.946116	1.02233
9017	1.47771	1.03266
9018	1.4777	2.1
9019	0.95468	1.10143
9020	0.94613	1.10133
9021	1.31381	1.04892
9022	1.14005	0.869118
9023	0.946128	0.996
9024	1.69433	0.812295
9025	1.69433	1.23665
9026	1.14005	0.954026
9027	0.94613	0.851199
9028	1.521	2.1
9029	1.411	2.1
9030	0.94612	2.1
9031	1.35115	2.1
9032	1.04127	2.1
9033	1.05017	2.1
9034	1.03564	2.1
9035	0.607156	2.1
9036	1.54549	2.1
9037	1.36715	2.1
9038	0.607127	2.08715
9039	1.2571	2.1
9040	0.998701	2.1
9041	0.546436	2.1
9042	0.946133	2.1
9043	1.75286	2.1
9044	1.75286	2.1
9045	1.12206	2.1

9046	1.34237	2.1
9047	1.35663	2.1
9048	1.12206	2.1
9049	0.919455	2.1
9050	1.66517	2.1
9051	1.99822	2.1
9052	1.228 2.1	
9053	1.35662	2.1
9054	1.228 2.1	
9055	1.01143	2.1
9056	0.851543	1.13561
9057	0.986856	2.1
9058	1.42762	1.76556
9059	1.23095	2.1
9060	0.946132	2.1
9061	1.00659	2.0415
9062	1.1779	1.59626
9063	1.40025	2.1
9064	1.40025	2.1
9065	0.889308	2.1
9066	1.34652	2.1
9067	0.943042	2.1
9068	0.943041	2.1
9069	1.34652	2.1
9070	1.39514	1.51193
9071	1.30809	1.14071
9072	1.75711	1.10615
9073	1.7571	2.1
9074	0.946125	0.880594
9075	0.946127	1.2094
9076	1.18396	1.22765
9077	1.4844	0.564663
9078	1.4844	1.13578
9079	0.803322	1.91402
9080	0.946133	0.216889
9081	0.803324	2.1
9082	0.803324	1.5337
9083	1.36183	1.57828
9084	0.946131	0.0603008
9085	1.01462	0.05
9086	0.946132	0.05
9087	1.59749	0.0529795
9088	1.59749	0.05
9089	1.16009	0.05
9090	1.38354	0.05
9091	0.851539	1.13889
9092	1.36005	1.84357
9093	1.5532	1.3177
9094	1.80402	2.09954
9095	0.851537	1.93519
9096	0.902632	1.52823
9097	1.44437	1.30067
9098	0.633098	2.1
9099	1.00307	0.358188
9100	0.94613	0.12789
9101	1.16623	0.05
9102	0.946128	0.158266
9103	1.64588	0.0874272

9104	1.64588	0.0538307
9105	1.35059	0.0952923
9106	1.24142	0.101388
9107	0.946131	0.05
9108	1.13343	0.05
9109	0.946127	0.05
9110	1.63367	0.05
9111	1.63367	0.05
9112	1.33035	0.05
9113	1.24945	0.05
9114	1.78733	1.53728
9115	1.70856	1.9355
9116	0.637995	1.26455
9117	0.712454	1.28031
9118	0.946132	1.26017
9119	0.637964	1.19647
9120	1.13021	1.2612
9121	0.94613	0.05
9122	1.20509	0.05
9123	0.946127	0.0531378
9124	1.70545	0.05
9125	1.70545	0.05
9126	1.28508	0.05
9127	1.3665	0.05
9128	1.58612	2.1
9129	1.51845	2.1
9130	0.797374	2.1
9131	1.20057	2.1
9132	0.946132	2.1
9133	0.946132	2.1
9134	0.899819	2.1
9135	1.50471	1.10047
9136	1.50472	1.18633
9137	0.59387	1.18497
9138	0.593844	1.03116
9139	0.946126	1.09732
9140	0.966137	1.09601
9141	1.13245	1.04017
9142	1.20801	0.516992
9143	0.946131	1.1806
9144	1.47644	0.80301
9145	0.733223	0.594281
9146	0.563609	0.415051
9147	1.69218	0.652546
9148	1.6173	1.20839
9149	1.8126	2.08007
9150	1.8126	2.1
9151	0.678938	2.1
9152	0.678935	2.1
9153	0.946133	2.1
9154	0.946132	2.05646
9155	0.678936	2.1
9156	1.21997	1.84186
9157	1.28439	1.87849
9158	1.65282	2.07302
9159	1.86735	2.05406
9160	0.851539	1.99583
9161	0.94613	1.3371

9162	0.87688	1.12972
9163	0.804877	0.681726
9164	1.40747	0.936751
9165	1.41312	0.116964
9166	0.885349	0.328643
9167	1.25405	0.910238
9168	1.34463	0.403632
9169	0.804878	0.353565
9170	0.810299	0.05
9171	1.04072	0.05
9172	1.25601	0.05
9173	1.04943	0.05
9174	0.900294	0.05
9175	1.38777	0.05
9176	1.33131	0.05
9177	1.39168	0.05
9178	0.946132	0.05
9179	0.747712	0.05
9180	1.61944	0.05
9181	1.61944	0.05
9182	0.946132	0.05
9183	0.747712	0.05
9184	1.41201	0.05
9185	1.12082	0.260661
9186	0.946128	0.260826
9187	1.79415	0.05
9188	1.79415	0.121547
9189	1.12082	0.0625925
9190	0.94613	0.186772
9191	0.711721	0.05
9192	1.12316	0.05
9193	0.707038	0.05
9194	1.04072	0.05
9195	0.704409	0.05
9196	1.49036	0.05
9197	1.27689	0.05
9198	0.793044	0.111134
9199	0.810886	0.704244
9200	0.885957	0.178886
9201	1.38254	0.188684
9202	1.35991	0.149813
9203	1.01375	0.291745
9204	1.03169	0.35285
9205	0.946131	0.268345
9206	1.3279	0.822663
9207	1.42284	0.300959
9208	1.42284	0.199147
9209	0.851192	1.38688
9210	0.946131	0.996612
9211	0.851165	0.438828
9212	0.748062	0.05
9213	1.27511	0.05
9214	0.74806	0.05
9215	0.94613	0.05
9216	0.748059	0.05
9217	1.86293	0.05
9218	1.86293	0.05
9219	0.851542	0.05

9220	1.38186	0.05
9221	0.851542	0.05
9222	1.67869	0.05
9223	1.66579	0.05
9224	1.25626	0.05
9225	1.23046	0.05
9226	1.46023	0.05
9227	1.27083	0.05
9228	0.946131	0.05
9229	0.999304	0.05
9230	0.946803	0.05
9231	0.851554	0.05
9232	0.999227	0.05
9233	0.750097	0.05
9234	1.91815	0.05
9235	0.946132	0.05
9236	0.768117	0.05
9237	1.78003	0.05
9238	1.99572	0.05
9239	0.650453	0.05
9240	0.946132	0.05
9241	1.23845	0.05
9242	0.946131	0.05
9243	1.75477	0.05
9244	1.75477	0.05
9245	1.33393	0.05
9246	1.36697	0.05
9247	0.749018	1.09943
9248	1.02366	1.65077
9249	1.43924	1.18682
9250	1.00018	1.16039
9251	1.06438	0.992429
9252	0.946127	1.03463
9253	0.74906	1.04468
9254	0.946131	1.04194
9255	1.26488	0.05
9256	0.946131	0.05
9257	0.94613	0.05
9258	1.49051	0.05
9259	1.59837	0.05
9260	0.907797	0.05
9261	1.04455	0.05
9262	1.25214	0.05
9263	0.68452	0.05
9264	1.25215	0.05
9265	1.24677	0.05
9266	1.8144	0.05
9267	1.81441	0.05
9268	0.684518	0.05
9269	0.851542	0.543671
9270	1.52275	0.332796
9271	1.45223	0.271177
9272	1.0269	0.213684
9273	1.29689	0.78154
9274	1.32822	0.837624
9275	0.851553	0.911204
9276	1.21756	0.05
9277	0.946131	0.05

9278	0.946127	0.05
9279	1.78014	0.05
9280	1.78014	0.05
9281	1.32076	0.05
9282	1.40551	0.05
9283	0.711214	0.05
9284	1.82057	0.05
9285	1.82057	0.05
9286	0.946129	0.05
9287	1.22716	0.05
9288	1.30463	0.05
9289	0.94613	0.05
9290	0.568927	0.05
9291	0.946132	0.05
9292	0.568927	0.05
9293	1.99266	0.05
9294	1.99266	0.05
9295	1.26201	0.05
9296	1.29957	0.05
9297	1.21614	0.05
9298	0.851484	0.05
9299	0.851543	0.05
9300	1.85815	0.05
9301	1.81937	0.05
9302	0.697705	0.05
9303	0.946132	0.05
9304	0.593295	0.05
9305	1.53642	0.05
9306	1.53642	0.05
9307	0.908276	0.05
9308	0.593296	0.05
9309	0.593295	0.05
9310	0.94613	0.05
9311	0.867593	0.0874148
9312	1.42107	0.05
9313	1.42107	0.05
9314	0.867621	0.0598206
9315	1.05516	0.0828317
9316	0.867624	0.05
9317	0.946129	0.124778
9318	0.766681	0.05
9319	1.18119	0.05
9320	1.57018	0.05
9321	1.57018	0.05
9322	0.766652	0.05
9323	1.18119	0.05
9324	1.15564	0.05
9325	0.946131	0.05
9326	1.239 0.05	
9327	0.851541	0.05
9328	0.851487	0.05
9329	1.40021	0.05
9330	1.38373	0.05
9331	0.851554	0.05
9332	1.12266	0.05
9333	0.946131	0.05
9334	1.08234	0.0855123
9335	1.2882	0.05

9336	1.49963	0.183826
9337	1.46188	0.05
9338	0.851555	0.05
9339	1.33007	0.0991601
9340	1.08054	0.0780562
9341	0.946129	0.203197
9342	0.91151	0.05
9343	1.49914	0.119475
9344	1.49914	0.05
9345	0.911471	0.267465
9346	0.946132	1.09926
9347	1.35943	2.1
9348	1.68117	1.46821
9349	1.68117	2.1
9350	0.946132	1.66468
9351	1.35943	1.23482
9352	1.26787	1.08626
9353	1.81612	1.90746
9354	1.81612	0.139769
9355	0.946131	0.05
9356	1.41492	0.05
9357	0.946132	2.1
9358	1.25037	0.295031
9359	1.41492	0.05
9360	1.30541	0.05
9361	1.23646	0.05
9362	0.94613	0.05
9363	1.77304	0.05
9364	1.77304	0.05
9365	1.23646	0.05
9366	0.946132	0.05
9367	0.946131	2.1
9368	1.26689	2.1
9369	1.66806	2.1
9370	1.66806	2.1
9371	0.94613	2.1
9372	1.26689	2.1
9373	1.3473	2.1
9374	1.39384	2.1
9375	1.4181	2.1
9376	1.86581	2.1
9377	1.86581	2.1
9378	0.946128	2.1
9379	0.946129	2.1
9380	1.32068	2.1
9381	1.20328	2.1
9382	1.39384	2.1
9383	1.651	2.1
9384	1.651	2.1
9385	0.946129	2.1
9386	0.946131	2.1
9387	1.12913	2.1
9388	1.21474	2.1
9389	0.946132	2.1
9390	0.94613	2.1
9391	1.79724	2.1
9392	1.79724	2.1
9393	1.40987	2.1

9394	1.3335	2.1
9395	1.65642	2.1
9396	1.65642	2.1
9397	0.946132	2.1
9398	1.19063	2.1
9399	0.946133	2.1
9400	1.15203	2.1
9401	1.19063	2.1
9402	0.946131	1.0097
9403	1.77815	2.1
9404	1.77815	2.1
9405	0.946131	1.85496
9406	1.31745	2.1
9407	1.31745	2.1
9408	1.2597	2.1
9409	0.946131	0.897461
9410	1.85184	1.18963
9411	1.85184	2.1
9412	0.946128	1.38532
9413	1.40684	2.1
9414	1.40684	1.15977
9415	1.34049	1.54837
9416	0.946132	1.55923
9417	1.71333	1.20087
9418	1.71333	2.1
9419	1.1848	1.52893
9420	1.34297	1.10022
9421	1.31649	1.1782
9422	1.1848	1.77288
9423	1.36604	0.706347
9424	0.954559	0.719757
9425	1.36604	0.730968
9426	1.41766	0.690813
9427	1.83756	0.753064
9428	1.83756	1.57803
9429	0.946131	0.752509
9430	0.946127	0.05
9431	1.14874	0.05
9432	0.946127	0.05
9433	1.66393	0.05
9434	1.66393	0.05
9435	1.35559	0.05
9436	1.25447	0.05
9437	0.94613	0.05
9438	1.20732	0.05
9439	0.946129	0.05
9440	1.75073	0.05
9441	1.75073	0.05
9442	1.3665	0.05
9443	1.33035	0.05
9444	1.73256	2.1
9445	1.73256	2.1
9446	0.946131	2.1
9447	1.34921	2.1
9448	1.19462	2.1
9449	1.19462	2.1
9450	1.32949	2.1
9451	1.75754	0.741265

9452	1.75754	0.163116
9453	0.946132	0.75329
9454	1.3201	0.730319
9455	0.946132	0.735829
9456	1.25479	0.231204
9457	1.3201	0.71713
9458	1.8087	0.941838
9459	1.8087	0.05
9460	0.946131	0.244077
9461	1.38357	0.7167
9462	0.946133	0.923216
9463	1.31034	0.172284
9464	1.38357	0.814592
9465	1.12702	0.05
9466	0.946131	0.0551332
9467	1.23923	0.05
9468	1.4117	0.05
9469	0.946131	0.05
9470	1.70479	0.05
9471	1.70479	0.05
9472	0.946119	2.1
9473	1.55309	2.1
9474	1.55309	2.1
9475	0.946119	2.1
9476	1.08006	2.1
9477	1.08005	2.1
9478	1.02681	2.1
9479	1.36581	2.1
9480	1.93075	2.1
9481	0.946133	2.1
9482	0.772538	2.1
9483	0.946131	2.1
9484	0.946131	2.1
9485	1.33748	2.1
9486	1.44991	2.1
9487	1.31183	2.1
9488	0.946132	2.1
9489	0.944104	2.1
9490	0.915236	2.1
9491	1.05017	2.1
9492	0.944148	2.1
9493	0.946132	0.0563386
9494	1.60171	0.0623432
9495	1.60171	0.05
9496	0.946131	0.10545
9497	1.16234	0.05
9498	1.3855	0.107861
9499	0.946132	0.104703
9500	0.946133	0.198828
9501	1.73285	0.174919
9502	1.73285	0.05
9503	0.946133	0.328554
9504	1.3855	0.05
9505	1.29348	0.253455
9506	0.946132	0.367068
9507	1.34828	2.1
9508	1.20943	2.1
9509	0.946129	2.1

9510	1.71013	2.1
9511	1.71013	2.1
9512	1.20943	2.1
9513	0.94613	2.1
9514	1.19613	0.05
9515	0.946118	0.05
9516	0.946117	0.05
9517	1.70868	0.05
9518	1.70869	0.05
9519	1.40551	0.05
9520	1.2493	0.05
9521	0.946131	2.1
9522	1.73123	2.1
9523	1.73123	2.1
9524	0.946132	2.1
9525	1.37811	2.1
9526	1.37811	2.1
9527	1.25144	2.1
9528	0.946132	2.1
9529	1.75139	2.1
9530	1.75139	2.1
9531	1.23374	2.1
9532	1.29926	2.1
9533	1.39827	2.1
9534	1.23374	2.1
9535	1.41375	0.05
9536	1.25697	0.05
9537	0.946118	0.05
9538	1.80377	0.05
9539	1.80377	0.05
9540	1.25699	0.05
9541	0.946106	0.05
9542	0.946133	1.70154
9543	1.81806	1.25218
9544	1.81806	2.1
9545	0.946133	2.1
9546	1.37089	2.1
9547	1.37089	1.30071
9548	1.34999	2.1
9549	0.946131	2.1
9550	1.80855	1.21995
9551	1.80855	2.1
9552	1.31184	2.1
9553	1.3933	1.37887
9554	1.36139	2.01217
9555	1.31184	2.09839
9556	1.01175	1.31689
9557	0.94613	0.917567
9558	0.919796	1.80376
9559	1.50484	0.665419
9560	1.80584	1.94643
9561	1.53512	0.982809
9562	1.14539	0.877163
9563	0.946133	2.1
9564	1.72797	2.07086
9565	1.72797	2.1
9566	1.2111	2.1
9567	1.39731	1.35429

9568	1.27679	2.1
9569	1.21111	2.1
9570	0.946119	0.472227
9571	1.67673	1.23857
9572	1.67673	2.1
9573	0.946118	1.2575
9574	1.21519	2.1
9575	1.2152	2.1
9576	1.19808	2.1
9577	0.946127	2.1
9578	1.54442	2.1
9579	1.54442	2.1
9580	1.04806	2.1
9581	1.13485	2.1
9582	1.3557	2.1
9583	1.04805	2.1
9584	0.946129	2.1
9585	1.32282	2.1
9586	1.77749	2.1
9587	1.77749	2.1
9588	0.946131	2.1
9589	1.32282	2.1
9590	1.4008	2.1
9591	0.946128	0.0510029
9592	1.02366	0.05
9593	0.94613	0.05
9594	1.66684	0.05
9595	1.66684	0.05
9596	1.29215	0.05
9597	1.32082	0.05
9598	0.946133	0.05
9599	1.01451	0.05
9600	0.946132	0.05
9601	1.62374	0.05
9602	1.62374	0.05
9603	1.2929	0.05
9604	1.27697	0.05
9605	1.37869	2.1
9606	1.17215	2.1
9607	0.946132	2.1
9608	1.70508	2.1
9609	1.70508	2.1
9610	1.17215	2.1
9611	0.946132	2.1
9612	1.03784	2.1
9613	0.946132	2.1
9614	0.946132	2.1
9615	1.56718	2.1
9616	1.56717	2.1
9617	1.35703	2.1
9618	1.15628	2.1
9619	1.35356	2.1
9620	1.28976	2.1
9621	0.946131	2.1
9622	1.78612	2.1
9623	1.78612	2.1
9624	1.28976	2.1
9625	0.94613	2.1

9626	0.946132	2.1
9627	1.36373	2.1
9628	1.92382	2.1
9629	0.946131	2.1
9630	1.92382	2.1
9631	1.33471	2.1
9632	0.946132	2.1
9633	0.94613	0.852378
9634	1.29149	1.08132
9635	1.75878	0.917528
9636	1.75878	1.97014
9637	0.946128	1.04577
9638	1.29149	0.873802
9639	1.41342	0.721382
9640	1.41022	2.1
9641	1.37034	2.1
9642	1.83443	2.1
9643	1.83443	2.1
9644	0.946128	1.497
9645	0.946131	2.1
9646	1.26928	2.1
9647	0.946128	0.966806
9648	1.38259	1.95443
9649	1.81405	1.09078
9650	1.81405	2.1
9651	0.946126	1.41943
9652	1.38259	1.11992
9653	1.37759	0.992987
9654	1.68131	0.05
9655	1.68131	0.05
9656	0.946131	0.05
9657	1.38896	0.05
9658	1.17438	0.965323
9659	1.17438	0.05
9660	1.23848	0.658208
9661	1.80088	0.0544066
9662	1.80088	0.05
9663	0.946132	0.05
9664	1.40332	0.0547229
9665	1.28185	0.211406
9666	1.28185	0.05
9667	1.34369	0.05
9668	1.71939	2.1
9669	1.71939	2.1
9670	0.946131	2.1
9671	1.28308	2.1
9672	0.946133	2.1
9673	1.24024	2.1
9674	1.28309	2.1
9675	1.8525	2.1
9676	1.8525	2.1
9677	0.946132	2.1
9678	1.38244	2.1
9679	0.946132	2.1
9680	1.33469	2.1
9681	1.38244	2.1
9682	1.26654	1.26152
9683	1.39514	1.16403

9684	1.71556	1.14225
9685	1.71556	2.1
9686	0.946126	0.940766
9687	0.946129	1.60918
9688	1.1908	1.24093
9689	1.49995	2.1
9690	1.63875	2.1
9691	0.93608	2.1
9692	0.937208	2.1
9693	0.943708	2.1
9694	0.936134	2.1
9695	1.14341	2.1
9696	1.17802	0.05
9697	1.3542	0.05
9698	1.73981	0.05
9699	1.73981	0.05
9700	0.94613	0.05
9701	1.3542	0.05
9702	1.33174	0.05
9703	1.59452	2.1
9704	1.59452	2.1
9705	0.946132	2.1
9706	1.25 2.1	
9707	0.975266	2.1
9708	0.946132	2.1
9709	1.25 2.1	
9710	0.946131	2.1
9711	1.48685	2.1
9712	1.48685	2.1
9713	0.946132	2.1
9714	1.17273	2.1
9715	1.17273	2.1
9716	0.955602	2.1
9717	1.32827	0.05
9718	1.11166	0.151927
9719	0.946127	0.165787
9720	1.6136	0.05
9721	1.6136	0.0591735
9722	1.11166	0.0874436
9723	0.946129	0.132568
9724	0.851541	0.928603
9725	1.22107	0.84647
9726	1.64594	0.631566
9727	1.5271	0.773959
9728	0.94613	1.00602
9729	1.22112	0.97097
9730	1.25002	0.891117
9731	1.65795	0.05
9732	1.65795	2.1
9733	0.946132	2.1
9734	1.2366	0.473076
9735	0.946132	0.0818587
9736	1.10023	0.871835
9737	1.2366	0.0934244
9738	1.35856	0.05
9739	0.946132	0.05
9740	1.35856	0.05
9741	1.08019	0.05

9742	1.49261	0.05
9743	1.49261	0.05
9744	0.94613	0.05
9745	1.29322	0.238927
9746	0.946132	0.0910235
9747	1.29322	0.0523362
9748	1.35856	0.149275
9749	1.70565	0.05
9750	1.70565	0.11692
9751	0.946132	0.197344
9752	1.66635	2.1
9753	1.66635	2.1
9754	0.946133	2.1
9755	1.39558	2.1
9756	1.14793	2.1
9757	1.14793	2.1
9758	1.21691	2.1
9759	1.35381	0.05
9760	1.3365	0.394342
9761	1.09905	0.0664575
9762	0.946132	0.607999
9763	1.74418	0.08949
9764	1.74418	0.05
9765	0.946132	0.0704228
9766	1.84759	0.100897
9767	1.84759	0.05
9768	0.94613	0.05
9769	1.3904	0.110847
9770	1.34522	0.05
9771	1.34522	0.05
9772	1.40331	0.05
9773	1.86047	0.647381
9774	1.86047	2.1
9775	0.946132	2.1
9776	1.41619	2.1
9777	0.946132	0.05
9778	1.3659	1.13853
9779	1.41619	1.4109
9780	1.28464	2.1
9781	1.14496	1.54421
9782	1.57806	1.66991
9783	1.7753	2.02937
9784	0.851541	1.81012
9785	0.946131	2.06618
9786	0.872786	1.96568
9787	0.85062	0.864163
9788	1.52635	1.013
9789	1.63785	0.876677
9790	1.69972	1.14828
9791	0.946132	1.3568
9792	1.37493	0.896629
9793	1.23854	0.906193
9794	1.41134	2.1
9795	1.27079	1.96628
9796	1.736	1.37186
9797	1.736	2.1
9798	0.946132	1.02414
9799	0.946133	1.77781

9800	1.21177	2.05155
9801	0.946131	0.05
9802	0.946129	0.05
9803	0.94613	0.05
9804	0.946131	0.05
9805	0.946131	0.05
9806	1.35403	0.05
9807	0.946131	0.05
9808	1.37052	0.05
9809	0.946132	0.05
9810	0.94613	0.05
9811	0.946133	0.05
9812	0.946131	0.05
9813	0.946131	0.05
9814	0.946131	0.05
9815	1.24249	0.05
9816	0.946132	0.05
9817	0.946133	0.05
9818	0.946132	0.05
9819	0.946132	0.05
9820	0.946132	0.05
9821	0.946132	0.05
9822	0.946132	2.1
9823	0.946132	2.1
9824	0.946131	2.1
9825	0.946132	2.1
9826	0.946132	2.1
9827	0.946132	2.1
9828	1.20608	2.1
9829	1.41232	2.1
9830	0.946128	2.1
9831	0.946129	2.1
9832	0.94613	2.1
9833	0.94613	2.1
9834	0.94613	2.1
9835	0.94613	2.1
9836	0.946132	0.05
9837	0.946131	0.05
9838	0.946131	0.05
9839	0.946132	0.05
9840	0.946132	0.05
9841	0.946131	0.05
9842	0.946132	0.05
9843	0.946116	0.945693
9844	0.94612	2.1
9845	0.946118	1.23124
9846	0.946124	2.1
9847	0.946118	1.36617
9848	1.41561	2.1
9849	0.946131	1.99753
9850	0.945846	2.1
9851	1.03508	2.1
9852	0.77579	2.1
9853	0.658908	2.1
9854	1.17474	2.1
9855	0.94303	2.1
9856	0.849189	2.1
9857	0.94298	2.1

9858	1.03963	2.1
9859	0.946131	1.94648
9860	0.946131	1.39671
9861	0.946131	2.1
9862	0.946131	1.15578
9863	0.946131	0.0903737
9864	0.946131	1.29866
9865	1.32819	1.52903
9866	0.946133	1.1964
9867	0.946129	2.1
9868	0.946128	2.1
9869	0.94613	1.99643
9870	0.946131	2.1
9871	0.946131	1.1768
9872	0.946131	1.89216
9873	0.954072	0.05
9874	1.28895	0.05
9875	0.946133	0.05
9876	1.15803	0.05
9877	1.29689	0.05
9878	1.29689	0.05
9879	0.946132	0.05
9880	0.946132	0.964484
9881	0.946132	0.961732
9882	0.946132	0.574726
9883	0.946132	1.21994
9884	0.946132	1.63411
9885	0.946131	1.66477
9886	1.0264	1.64579
9887	0.946126	0.05
9888	0.946132	0.05
9889	0.946131	0.05
9890	0.94613	0.05
9891	0.946129	0.05
9892	0.94613	0.05
9893	1.35977	0.05
9894	0.946132	0.726491
9895	0.946133	0.728536
9896	0.876632	0.781827
9897	0.946132	0.746174
9898	1.01566	0.858752
9899	1.01566	0.775281
9900	0.876606	0.760696
9901	0.946131	0.05
9902	0.946128	0.05
9903	0.946128	0.05
9904	0.94613	0.05
9905	0.94613	0.05
9906	1.24979	0.05
9907	0.94613	0.05
9908	0.946131	0.05
9909	0.946131	0.05
9910	0.921762	0.05
9911	0.9705	0.05
9912	0.9705	0.05
9913	0.946131	0.05
9914	0.921791	0.05
9915	0.810944	0.05

9916	0.810945	0.05
9917	0.810959	0.05
9918	2.42267	0.05
9919	1.35168	0.05
9920	1.08132	0.05
9921	1.08132	0.05
9922	1.08131	0.05
9923	0.946133	0.05
9924	0.946132	0.05
9925	0.946132	0.05
9926	0.946133	0.05
9927	0.946133	0.05
9928	0.946132	0.05
9929	0.946133	0.05
9930	0.946131	0.324747
9931	0.946131	0.78997
9932	0.94613	0.0859421
9933	0.851514	0.167135
9934	0.946131	0.431855
9935	0.965268	0.73578
9936	0.851513	0.128076
9937	0.946122	2.1
9938	0.946123	2.1
9939	0.946126	2.1
9940	0.946124	2.1
9941	0.946124	2.1
9942	0.946118	2.1
9943	0.94613	2.1
9944	0.946131	0.680153
9945	1.30448	0.693285
9946	0.946132	0.724922
9947	0.946129	0.681759
9948	0.946131	0.926318
9949	0.946131	0.682147
9950	0.94613	0.74969
9951	0.946133	1.13068
9952	0.946128	2.1
9953	0.946128	2.1
9954	0.94613	2.1
9955	1.36503	2.1
9956	0.946131	2.1
9957	0.946131	2.1
9958	0.810947	0.05
9959	0.810947	0.05
9960	2.48233	0.05
9961	1.3517	0.05
9962	1.08132	0.05
9963	1.08132	0.05
9964	1.08133	0.05
9965	0.810934	0.05
9966	1.48874	0.05
9967	2.48233	0.05
9968	2.2369	0.05
9969	0.946133	0.258371
9970	0.946132	0.211992
9971	0.946132	0.89632
9972	0.946133	0.92524
9973	0.946133	0.619762

9974	1.41851	0.461727
9975	0.946132	0.305259
9976	1.03925	2.1
9977	0.946131	2.1
9978	0.94613	2.1
9979	0.946128	2.1
9980	0.946133	2.1
9981	0.944743	2.1
9982	1.13703	2.1
9983	0.946132	2.1
9984	0.946128	2.1
9985	0.946128	2.1
9986	0.946131	2.1
9987	0.94613	2.1
9988	0.94613	2.1
9989	0.94613	2.1
9990	0.946131	0.05
9991	0.946132	0.05
9992	0.946131	0.05
9993	0.946132	0.05
9994	0.946132	0.05
9995	0.946131	0.05
9996	1.20057	0.05
9997	0.946129	2.1
9998	0.94613	2.1
9999	0.94613	2.1
10000	0.946128	2.1
10001	0.946132	2.1
10002	0.94613	2.1
10003	1.38302	2.1
10004	0.856567	0.05
10005	1.14319	0.05
10006	0.716526	0.05
10007	1.20631	0.05
10008	0.626936	0.05
10009	0.946131	0.05
10010	0.94613	0.05
10011	1.39502	0.05
10012	0.881445	0.05
10013	0.946128	0.05
10014	0.94613	0.05
10015	0.881447	0.05
10016	0.946117	0.05
10017	0.946116	0.05
10018	0.88146	0.05
10019	0.946132	2.1
10020	1.39053	2.1
10021	0.946132	2.1
10022	0.946132	2.1
10023	0.946132	2.1
10024	0.946132	2.1
10025	0.946131	2.1
10026	0.946131	2.1
10027	0.946131	2.1
10028	0.946131	2.1
10029	0.946132	2.1
10030	0.946132	2.1
10031	1.40571	2.1

10032	0.946132	2.1
10033	0.94613	0.458787
10034	0.946127	0.37177
10035	0.946128	0.465715
10036	0.946131	0.651336
10037	0.946127	0.291438
10038	1.25819	0.269599
10039	0.946129	0.539074
10040	0.946129	0.966714
10041	1.29054	1.04944
10042	0.94613	0.936625
10043	0.94613	0.905855
10044	0.946131	1.29079
10045	0.946131	1.08301
10046	0.94613	1.04474
10047	0.810944	0.05
10048	0.810941	0.05
10049	1.61558	0.05
10050	1.3517	0.05
10051	1.08131	0.05
10052	1.08132	0.05
10053	1.08133	0.05
10054	0.810931	0.05
10055	1.02036	1.42414
10056	0.946132	1.06192
10057	1.02036	1.05021
10058	0.946132	0.856675
10059	1.1576	1.00384
10060	0.964656	1.07924
10061	0.927608	0.903893
10062	0.946128	2.1
10063	1.38813	2.1
10064	0.946129	2.1
10065	0.946131	2.1
10066	0.946131	2.1
10067	0.94613	2.1
10068	0.946131	2.1
10069	0.946131	2.1
10070	0.94613	0.824489
10071	0.94613	2.1
10072	1.41665	2.1
10073	0.946132	0.434779
10074	0.946131	2.1
10075	0.94613	2.1
10076	0.946118	0.0689972
10077	0.946118	0.05
10078	0.946116	0.0545204
10079	0.946126	0.05
10080	0.94612	0.05
10081	0.946131	0.0575734
10082	0.946105	0.05
10083	0.946132	2.1
10084	0.946132	2.1
10085	0.946132	2.1
10086	0.946131	2.1
10087	0.946132	2.1
10088	0.946132	2.1
10089	0.946132	2.1

10090	0.864997	0.05
10091	0.786573	0.05
10092	0.978561	0.05
10093	1.47385	0.05
10094	1.68652	0.05
10095	1.27803	0.05
10096	1.19771	0.05
10097	1.27595	0.05
10098	0.946127	0.21603
10099	1.3771	0.131217
10100	0.946127	0.129274
10101	0.946131	0.483056
10102	0.946129	0.0846086
10103	0.946129	0.259765
10104	0.946129	0.211204
10105	0.95697	0.05
10106	1.48133	0.05
10107	1.24499	0.05
10108	1.5295	0.05
10109	0.938161	0.05
10110	0.808717	0.05
10111	1.3542	0.05
10112	0.946133	1.0348
10113	1.14444	0.972058
10114	0.946132	0.727549
10115	0.946132	0.949513
10116	0.946133	1.24565
10117	0.946132	1.1002
10118	0.946132	1.02968
10119	0.946131	0.05
10120	0.946132	0.05
10121	0.946131	0.05
10122	0.946132	0.05
10123	0.946131	0.05
10124	0.946132	0.05
10125	0.946132	0.05
10126	0.946132	2.1
10127	0.946133	2.1
10128	0.946133	2.1
10129	0.946132	2.1
10130	1.29322	2.1
10131	0.946132	2.1
10132	0.946132	2.1
10133	0.946131	2.1
10134	0.946132	2.1
10135	0.946131	2.1
10136	0.946132	2.1
10137	1.28441	2.1
10138	0.946131	2.1
10139	0.946131	2.1
10140	0.946113	2.1
10141	0.946118	2.1
10142	0.946118	2.1
10143	1.28073	2.1
10144	0.946117	2.1
10145	0.946131	2.1
10146	0.946105	2.1
10147	0.810945	2.1

10148	0.810942	2.1
10149	2.75904	2.1
10150	1.3517	2.1
10151	1.08131	2.1
10152	1.08133	2.1
10153	1.08132	2.1
10154	0.81093	2.1
10155	0.946127	2.1
10156	1.37952	2.1
10157	1.86965	2.1
10158	0.946127	2.1
10159	1.86965	2.1
10160	1.28228	2.1
10161	1.37952	2.1
10162	0.946128	2.1
10163	0.946132	2.1
10164	1.14042	2.1
10165	0.946132	2.1
10166	1.14042	2.1
10167	1.09974	2.1
10168	1.09974	2.1
10169	0.946132	2.1
10170	0.946132	0.0562557
10171	0.946132	0.05
10172	0.946133	0.05
10173	0.946132	0.05
10174	0.946132	0.05
10175	0.946132	0.05
10176	0.946133	0.05
10177	1.31817	1.78611
10178	0.946131	1.27071
10179	0.946131	1.6232
10180	0.946131	1.4646
10181	0.946131	1.121
10182	0.946131	1.3155
10183	0.946131	1.32113
10184	0.946132	2.1
10185	0.946132	2.1
10186	0.946132	2.1
10187	0.946132	2.1
10188	0.946132	2.1
10189	0.946132	2.1
10190	1.35048	2.1
10191	0.946131	2.1
10192	0.946131	2.1
10193	0.94613	2.1
10194	0.946133	2.1
10195	0.946133	2.1
10196	1.35943	2.1
10197	0.946131	2.1
10198	0.946133	2.1
10199	0.946132	2.1
10200	0.946132	2.1
10201	0.946133	2.1
10202	0.946133	2.1
10203	0.946133	2.1
10204	0.990712	2.1
10205	0.965725	2.1

10206	0.946129	1.08677
10207	0.946133	1.60784
10208	1.21721	1.20608
10209	0.877931	0.962886
10210	0.946131	1.56348
10211	0.946131	1.05699
10212	0.946132	0.0612866
10213	0.946131	0.05
10214	0.946131	0.245358
10215	0.946133	0.0716486
10216	0.946128	0.883995
10217	0.946131	0.158593
10218	1.39575	0.123957
10219	0.810943	0.166121
10220	0.810942	0.204525
10221	2.58794	0.05
10222	1.21652	0.0711777
10223	0.946154	0.132535
10224	0.979952	0.133503
10225	1.0813	0.143933
10226	0.810959	0.192637
10227	0.924071	0.0740546
10228	1.16453	0.05
10229	1.28586	0.05
10230	0.946131	0.0890316
10231	1.1227	0.05
10232	1.16462	0.05
10233	0.851553	0.143075
10234	0.709578	0.05
10235	0.810943	0.0802723
10236	0.810959	0.0793894
10237	1.82615	0.05
10238	1.21651	0.05
10239	1.08132	0.05
10240	0.946155	0.05
10241	0.979937	0.0783217
10242	0.946128	1.36966
10243	0.946132	2.1
10244	0.946132	1.07775
10245	0.94613	2.1
10246	0.94613	2.02185
10247	0.94613	1.06384
10248	0.94613	1.61345
10249	0.946131	2.05737
10250	1.29735	2.1
10251	0.946131	2.1
10252	0.946131	1.101
10253	0.946131	1.6116
10254	0.946131	1.04503
10255	0.946131	2.1
10256	0.946128	2.1
10257	1.33266	1.20566
10258	0.94613	1.27559
10259	0.946131	2.1
10260	0.94613	0.180478
10261	0.946131	1.16863
10262	0.946131	1.15237
10263	0.946131	0.074017

10264	0.946131	2.1
10265	0.946131	0.135851
10266	1.40608	0.05
10267	0.946131	2.1
10268	0.946131	1.38224
10269	0.946131	1.68606
10270	0.946131	1.23613
10271	0.946131	2.1
10272	0.946131	1.37641
10273	1.2334	1.39436
10274	0.946129	1.91352
10275	0.946131	1.42908
10276	0.946131	2.1
10277	0.946133	2.1
10278	0.946133	2.1
10279	0.946132	1.92257
10280	1.29198	2.1
10281	0.946132	2.1
10282	0.946132	2.1
10283	0.946132	2.1
10284	0.946131	2.1
10285	0.946128	2.1
10286	0.946128	2.1
10287	0.946131	2.1
10288	0.946127	2.1
10289	0.94613	2.1
10290	0.946129	2.1
10291	0.946123	2.1
10292	0.946126	2.1
10293	0.946125	2.1
10294	1.38639	2.1
10295	0.946126	2.1
10296	0.946132	2.1
10297	0.946119	2.1
10298	0.946128	0.05
10299	0.946132	0.061235
10300	0.946131	0.0771247
10301	0.94613	0.05
10302	0.94613	0.05
10303	0.946131	0.0555865
10304	0.94613	0.05
10305	0.946131	0.05
10306	0.946131	0.05
10307	0.946131	0.0689853
10308	0.946131	0.05
10309	0.946131	0.05
10310	0.946131	0.05
10311	0.946131	0.05
10312	0.810945	2.1
10313	0.810943	2.1
10314	0.810933	2.1
10315	2.24688	2.1
10316	1.3517	2.1
10317	1.08132	2.1
10318	1.08132	2.1
10319	1.08133	2.1
10320	1.25809	0.05
10321	0.946131	0.261431

10322	1.25809	0.05
10323	0.946129	0.146216
10324	0.94613	0.05
10325	0.946131	0.117598
10326	0.946131	0.0720426
10327	0.946131	0.054318
10328	0.946131	0.05
10329	0.946129	0.0829049
10330	1.28911	0.05
10331	0.946131	0.05
10332	0.946131	0.05
10333	0.946131	0.05
10334	0.94613	0.1738
10335	0.946131	0.217079
10336	0.94613	0.0941235
10337	0.946128	0.204787
10338	0.946132	0.0812465
10339	1.38954	0.157571
10340	0.946131	0.140551
10341	0.946131	0.05
10342	0.946131	0.0994509
10343	0.946131	0.10462
10344	1.37981	0.0550099
10345	0.946131	0.552841
10346	0.946131	0.0818087
10347	0.946131	0.101875
10348	0.946131	2.1
10349	0.946127	2.1
10350	0.946127	2.1
10351	0.946131	2.1
10352	0.946127	2.1
10353	0.946129	2.1
10354	0.946128	2.1
10355	0.94613	2.1
10356	0.946126	2.1
10357	0.946127	2.1
10358	0.946131	2.1
10359	0.946126	2.1
10360	0.946128	2.1
10361	0.946128	2.1
10362	0.946133	0.101843
10363	0.946132	0.0680116
10364	0.946132	0.103788
10365	0.946132	0.066962
10366	0.946132	0.170019
10367	1.13511	0.05
10368	0.946132	0.0629748
10369	0.946132	1.87223
10370	0.946129	1.89714
10371	0.94613	1.64574
10372	0.946131	1.98418
10373	0.946131	2.1
10374	0.94613	2.1
10375	1.27575	2.1
10376	0.946129	2.1
10377	0.946131	2.1
10378	0.946131	2.1
10379	0.946128	2.1

10380	0.946133	2.1
10381	0.946131	2.1
10382	0.94613	2.1
10383	1.15581	0.05
10384	0.946132	0.05
10385	0.946131	0.05
10386	0.94613	0.05
10387	1.15581	0.05
10388	0.94613	0.05
10389	0.946131	0.05
10390	0.611407	1.16137
10391	0.946131	0.46438
10392	1.37335	1.08773
10393	1.15086	1.21549
10394	0.617549	0.902234
10395	0.851553	1.0969
10396	1.14592	1.21786
10397	1.22618	1.66175
10398	0.946132	0.36256
10399	0.946132	1.8325
10400	0.946132	0.680414
10401	0.946131	0.400435
10402	0.946132	1.20876
10403	0.946132	0.808923
10404	0.946131	2.1
10405	0.94613	2.1
10406	0.946131	2.1
10407	0.946133	2.1
10408	0.946129	2.1
10409	0.946131	2.1
10410	0.946131	2.1
10411	0.810945	0.880327
10412	0.810943	1.70022
10413	2.50715	1.12376
10414	1.3517	1.00904
10415	1.08133	0.971337
10416	1.08132	1.32937
10417	1.08132	0.388384
10418	0.810932	0.9074
10419	1.07811	1.30493
10420	0.94613	0.949177
10421	0.946128	1.43265
10422	1.25358	0.218134
10423	0.946131	0.88957
10424	1.25358	0.658426
10425	0.94613	0.753156
10426	0.946129	2.1
10427	1.09197	2.1
10428	0.946131	2.1
10429	1.09197	2.1
10430	1.09532	2.1
10431	1.09532	2.1
10432	0.946131	2.1
10433	1.58788	2.1
10434	1.34255	2.1
10435	0.594912	2.1
10436	0.58894	2.1
10437	0.946129	2.1

10438	1.16679	2.1
10439	1.04071	2.1
10440	0.535418	2.1
10441	0.946131	2.1
10442	0.946128	2.1
10443	0.946131	2.1
10444	1.20221	2.1
10445	0.946128	2.1
10446	0.94613	2.1
10447	1.20221	2.1
10448	0.946132	2.1
10449	0.946133	2.1
10450	0.946132	2.1
10451	0.946132	2.1
10452	0.946133	2.1
10453	0.946133	2.1
10454	0.946132	2.1
10455	0.946132	1.2572
10456	0.946132	1.89196
10457	0.946133	2.1
10458	0.946132	1.33475
10459	1.411	1.97693
10460	0.946132	1.51359
10461	0.946133	1.85777
10462	1.05575	0.05
10463	0.563734	0.05
10464	1.05575	0.05
10465	0.773833	0.05
10466	0.751686	0.05
10467	0.75884	0.05
10468	0.815612	0.05
10469	0.793465	0.05
10470	0.768563	0.05
10471	0.946129	0.05
10472	0.946125	0.05
10473	0.946126	0.05
10474	0.94613	0.05
10475	0.946124	0.0579626
10476	1.31678	0.05
10477	0.946128	0.05
10478	0.946132	0.0862762
10479	1.29914	0.05
10480	0.946132	0.05
10481	0.946132	0.0551538
10482	0.946132	0.0983495
10483	1.29914	0.05
10484	0.946132	0.0704316
10485	0.810946	0.074422
10486	0.810943	0.0699116
10487	2.21082	0.05
10488	1.35168	0.05
10489	1.08132	0.05
10490	1.08132	0.05
10491	1.0813	0.0750823
10492	0.81096	0.0800748
10493	0.625813	0.05
10494	0.946132	0.05
10495	0.946131	0.05

10496	0.946132	0.05
10497	0.946131	0.05
10498	1.39301	0.05
10499	0.625812	0.05
10500	0.810946	1.91518
10501	0.810945	2.1
10502	0.810931	2.1
10503	2.70043	2.1
10504	1.3517	2.1
10505	1.08131	2.1
10506	1.08131	2.1
10507	1.08133	2.1
10508	0.810943	2.1
10509	0.810944	2.1
10510	0.810958	2.1
10511	2.44802	2.1
10512	1.35168	2.1
10513	1.08132	2.1
10514	1.08132	2.1
10515	1.0813	2.1
10516	0.946131	0.715077
10517	0.946129	0.710206
10518	0.946128	0.655147
10519	0.946131	0.671918
10520	1.3492	0.714479
10521	1.3492	0.716132
10522	0.94613	0.704191
10523	0.946132	0.52093
10524	0.946132	0.0851098
10525	0.946133	0.348051
10526	0.946133	0.0762065
10527	0.946132	0.421801
10528	0.946132	0.0701956
10529	0.946132	0.386525
10530	0.946129	0.05
10531	0.946133	0.05
10532	0.946131	0.05
10533	0.94613	0.05
10534	0.946131	0.05
10535	0.946131	0.05
10536	0.946131	0.05
10537	0.946119	0.05
10538	0.946119	0.05
10539	0.946119	0.05
10540	0.946125	0.05
10541	0.94612	0.05
10542	0.946132	0.05
10543	0.946107	0.05
10544	0.946133	2.1
10545	0.946132	2.1
10546	0.946132	2.1
10547	0.946132	2.1
10548	0.946132	2.1
10549	0.946133	2.1
10550	1.3868	2.1
10551	0.810947	2.1
10552	0.810946	2.1
10553	0.81096	2.1

10554	2.66094	2.1
10555	1.35168	2.1
10556	1.08132	2.1
10557	1.08132	2.1
10558	1.0813	2.1
10559	0.810946	2.1
10560	0.810946	2.1
10561	0.810933	2.1
10562	2.75737	2.1
10563	1.3517	2.1
10564	1.08132	2.1
10565	1.08132	2.1
10566	1.08133	2.1
10567	0.946133	2.1
10568	0.946132	2.1
10569	0.946132	2.1
10570	0.946132	2.1
10571	0.946133	2.1
10572	0.946132	2.1
10573	0.946133	2.1
10574	0.946133	2.1
10575	0.946133	2.1
10576	0.946132	2.1
10577	0.946132	2.1
10578	0.946133	2.1
10579	0.946132	2.1
10580	1.28569	2.1
10581	0.946132	2.1
10582	0.946133	2.1
10583	0.946133	2.1
10584	0.946132	2.1
10585	0.946132	2.1
10586	0.946132	2.1
10587	0.946133	2.1
10588	0.946132	0.05
10589	0.946131	0.05
10590	0.94613	0.05
10591	0.946133	0.05
10592	0.946128	0.05
10593	0.946131	0.05
10594	0.946131	0.05
10595	0.946131	2.1
10596	0.94613	2.1
10597	0.946131	2.1
10598	0.94613	2.1
10599	1.41833	1.38723
10600	0.946131	2.07609
10601	0.946131	2.1
10602	1.26155	0.05
10603	0.946131	0.05
10604	0.946131	0.05
10605	0.946132	0.05
10606	0.946132	0.05
10607	0.946131	0.05
10608	0.946132	0.05
10609	0.810946	2.1
10610	0.810946	2.1
10611	0.810959	2.1

10612	2.47938	2.1
10613	1.35168	2.1
10614	1.08132	2.1
10615	1.08132	2.1
10616	1.08131	2.1
10617	0.946131	0.0516518
10618	0.94613	0.05
10619	0.94613	0.212076
10620	0.946132	0.05
10621	0.946128	0.650951
10622	0.94613	0.147099
10623	0.94613	0.128437
10624	0.94613	0.05
10625	0.946131	0.05
10626	0.94613	0.05
10627	0.946129	0.05
10628	0.946132	0.05
10629	0.94613	0.05
10630	0.946131	0.05
10631	0.946132	2.1
10632	0.946131	2.1
10633	0.946128	2.1
10634	1.40756	2.1
10635	0.946131	2.1
10636	0.946129	2.1
10637	0.94613	2.1
10638	0.946131	1.78006
10639	0.946131	1.86222
10640	0.946131	2.1
10641	0.946132	2.1
10642	1.31947	2.07952
10643	0.946131	2.1
10644	0.946131	2.1
10645	0.946131	2.1
10646	1.25768	2.1
10647	0.946132	2.1
10648	0.946132	2.1
10649	0.946129	2.1
10650	0.946131	2.1
10651	0.94613	2.1
10652	0.946132	0.623212
10653	0.946129	0.163162
10654	0.946129	0.659591
10655	0.94613	0.677402
10656	1.31716	0.344319
10657	1.31716	0.238694
10658	0.946131	0.702178
10659	0.810943	1.09831
10660	0.810942	2.1
10661	0.81093	2.08449
10662	2.37052	2.1
10663	1.3517	2.0787
10664	1.08132	1.03482
10665	1.08132	2.1
10666	1.08133	1.16274
10667	0.946128	0.999177
10668	1.10335	0.859132
10669	0.94613	0.855389

10670	0.946131	1.06817
10671	0.946131	0.857364
10672	1.10335	1.03859
10673	0.94613	1.08389
10674	0.946132	1.12863
10675	1.18818	1.01007
10676	0.946132	0.998981
10677	0.946132	1.14688
10678	0.946132	0.975309
10679	1.18818	1.15188
10680	0.946132	1.15908
10681	0.946125	2.1
10682	0.94612	2.1
10683	0.946119	2.1
10684	0.946119	2.1
10685	0.946119	2.1
10686	0.946132	2.1
10687	1.31522	2.1
10688	1.28803	2.1
10689	0.946132	0.27962
10690	0.946132	2.1
10691	0.946133	2.1
10692	0.946133	0.0563778
10693	0.946133	2.1
10694	0.946132	2.1
10695	0.946133	1.02254
10696	0.946132	1.46786
10697	0.946132	1.07798
10698	0.946132	0.898859
10699	0.946133	0.880402
10700	0.946132	0.871775
10701	1.37658	0.962564
10702	1.02239	1.83259
10703	0.946128	1.15165
10704	0.946132	1.54244
10705	1.02239	1.24705
10706	0.94613	1.06298
10707	0.96283	1.57505
10708	0.92943	1.12058
10709	0.946132	0.05
10710	0.946131	0.05
10711	0.946126	0.05
10712	0.946129	0.05
10713	0.946128	0.05
10714	0.946128	0.05
10715	0.946129	0.05
10716	0.810946	0.05
10717	0.810944	0.05
10718	2.22493	0.05
10719	1.21651	0.05
10720	1.0813	0.05
10721	0.946154	0.05
10722	0.979951	0.05
10723	0.70959	0.05
10724	0.946132	0.05
10725	0.946131	0.05
10726	0.946131	0.05
10727	0.946133	0.05

10728	0.946131	0.05
10729	0.946132	0.05
10730	0.946132	0.05
10731	0.946132	2.1
10732	0.946131	1.95481
10733	0.946132	2.1
10734	0.946132	1.20126
10735	0.946132	1.03022
10736	0.946132	1.25197
10737	1.40924	1.87585
10738	0.946131	2.1
10739	0.946128	2.1
10740	0.946127	2.1
10741	0.94613	2.1
10742	1.41743	2.1
10743	0.946128	2.1
10744	0.946129	2.1
10745	1.37675	0.244815
10746	0.946128	0.450986
10747	0.94613	0.259448
10748	0.94613	0.957901
10749	0.94613	0.255345
10750	0.946129	0.248937
10751	0.94613	0.897131
10752	0.946132	0.05
10753	0.946132	0.0650521
10754	0.946132	0.119194
10755	1.35721	0.05
10756	0.946132	0.628617
10757	0.946132	0.172337
10758	1.35721	0.0751028
10759	0.946133	0.98317
10760	0.946133	0.901381
10761	0.946132	0.952697
10762	1.10003	0.831713
10763	0.946133	0.840372
10764	0.946132	0.920258
10765	0.946132	0.898098
10766	0.946132	0.05
10767	0.946131	0.05
10768	0.946131	0.05
10769	0.946131	0.05
10770	1.0616	0.05
10771	1.0616	0.05
10772	0.946132	0.05
10773	0.946132	0.05
10774	0.946131	0.05
10775	0.946131	0.05
10776	0.946131	0.05
10777	1.09402	0.05
10778	1.09402	0.05
10779	0.946131	0.05
10780	0.946132	0.05
10781	0.946126	0.05
10782	1.24093	0.05
10783	0.946129	0.05
10784	0.946129	0.05
10785	0.946128	0.05

10786	0.946129	0.05
10787	0.946131	0.05
10788	0.946131	0.05
10789	1.2929	0.05
10790	0.946131	0.05
10791	0.946131	0.05
10792	0.946131	0.05
10793	0.946131	0.05
10794	0.810946	0.05
10795	0.810945	0.05
10796	0.810959	0.05
10797	2.41233	0.05
10798	1.35168	0.05
10799	1.08132	0.05
10800	1.08132	0.05
10801	1.0813	0.05
10802	0.810946	1.67172
10803	0.810943	2.1
10804	0.750103	2.1
10805	2.22778	2.1
10806	1.27059	2.1
10807	1.00021	2.1
10808	1.02049	2.1
10809	1.08133	2.1
10810	0.946128	2.1
10811	0.946132	2.1
10812	0.946132	2.1
10813	0.94613	2.1
10814	0.94613	2.1
10815	0.94613	2.1
10816	0.946131	2.1
10817	0.946126	0.181097
10818	0.946131	0.0734008
10819	0.946127	0.24296
10820	1.3324	0.051947
10821	0.946127	0.0832395
10822	0.946128	0.17098
10823	0.946128	0.381991
10824	0.94613	2.1
10825	0.946131	2.1
10826	0.94613	2.1
10827	0.946128	2.1
10828	0.946133	2.1
10829	0.946131	2.1
10830	0.946131	2.1
10831	0.946133	2.1
10832	0.946132	2.1
10833	0.946133	2.1
10834	0.946133	2.1
10835	0.946132	2.1
10836	0.946132	2.1
10837	1.36541	2.1
10838	0.946131	0.311652
10839	0.94613	2.1
10840	0.946131	1.12834
10841	0.94613	1.07376
10842	1.39509	1.86826
10843	0.946131	0.711684

10844	0.946131	2.03814
10845	0.946133	2.1
10846	0.946133	2.1
10847	0.946132	2.1
10848	0.946132	2.1
10849	0.946133	2.1
10850	0.946132	2.1
10851	1.29319	2.1
10852	1.01196	0.05
10853	0.946131	0.05
10854	0.946132	0.05
10855	0.946132	0.05
10856	0.946132	0.05
10857	0.946132	0.05
10858	0.946132	0.05
10859	0.946112	2.1
10860	0.946118	2.1
10861	0.946116	2.1
10862	0.946117	2.1
10863	0.946118	2.1
10864	0.94613	2.1
10865	0.946105	2.1
10866	1.28684	1.16339
10867	0.946131	1.21752
10868	0.946132	1.20591
10869	0.946132	1.47945
10870	0.946132	1.65634
10871	0.946132	1.25849
10872	0.946132	1.69825
10873	0.94613	2.1
10874	0.94613	2.1
10875	0.946128	2.01421
10876	0.946131	1.76445
10877	0.946131	2.00399
10878	0.94613	2.00867
10879	1.10185	2.1
10880	0.810947	0.05
10881	0.810945	0.05
10882	1.57348	0.05
10883	1.3517	0.05
10884	1.08131	0.05
10885	1.08132	0.05
10886	1.08133	0.05
10887	0.810932	0.05
10888	0.946119	0.325289
10889	0.946116	0.13834
10890	0.946117	0.245372
10891	0.946121	0.05
10892	0.946118	0.0606038
10893	0.94613	0.247635
10894	0.946105	0.167047
10895	0.94612	0.691102
10896	0.946122	0.275128
10897	0.946119	0.575933
10898	0.946119	0.796794
10899	1.20884	0.37634
10900	0.946132	0.762829
10901	0.946107	0.762575

10902	2.67849	1.32058
10903	1.17709	1.27278
10904	0.605418	1.51355
10905	0.286984	1.32942
10906	0.172654	1.36383
10907	0.810946	0.949023
10908	0.940878	0.813588
10909	0.941235	0.55773
10910	1.26696	0.538266
10911	1.56837	0.767922
10912	1.13737	0.576049
10913	1.34115	0.832295
10914	1.24889	0.322119
10915	0.946132	1.47677
10916	0.946129	1.18616
10917	0.946128	1.33506
10918	0.94613	1.26118
10919	0.94613	1.45852
10920	0.946131	1.38951
10921	0.94613	1.3409
10922	0.946116	2.1
10923	0.94612	2.1
10924	0.946118	2.1
10925	0.946123	2.1
10926	0.946118	2.1
10927	0.946131	2.1
10928	1.23836	2.1
10929	0.810946	0.05
10930	0.810946	0.05
10931	0.810959	0.05
10932	2.80677	0.05
10933	1.35168	0.05
10934	1.08132	0.05
10935	1.08132	0.05
10936	1.08131	0.05
10937	0.810947	0.719322
10938	0.810947	0.795941
10939	2.41574	0.799057
10940	1.21653	0.790647
10941	1.08133	0.756822
10942	0.946153	0.792761
10943	0.979949	0.783201
10944	0.709567	0.734579
10945	0.94613	2.1
10946	0.946131	2.1
10947	0.946128	2.1
10948	1.34782	2.1
10949	0.946128	2.1
10950	0.946129	2.1
10951	1.34782	2.1
10952	0.946132	2.1
10953	0.946132	2.1
10954	0.94613	2.1
10955	1.16023	2.1
10956	0.946131	2.1
10957	0.946131	2.1
10958	1.16023	2.1
10959	0.946131	1.14783

10960	0.946127	2.1
10961	0.946127	2.1
10962	0.946131	1.59525
10963	0.946128	2.1
10964	0.94613	1.2669
10965	0.946129	1.86646
10966	0.946133	0.05
10967	0.946132	0.05
10968	0.946132	0.05
10969	0.946133	0.0676416
10970	1.20045	0.05
10971	1.20045	0.05
10972	0.946132	0.0657891
10973	0.918871	0.05
10974	0.971961	0.05
10975	1.37324	0.05
10976	1.78255	0.05
10977	1.25155	0.05
10978	1.42172	0.05
10979	1.40554	0.05
10980	1.08862	0.05
10981	1.03803	0.05
10982	0.946132	0.05
10983	0.946126	0.05
10984	0.943647	0.05
10985	0.948611	0.05
10986	0.946129	0.05
10987	0.946129	0.05
10988	1.1699	0.275938
10989	0.946132	0.148552
10990	0.946133	0.82847
10991	0.946133	0.176242
10992	0.946132	0.0879092
10993	0.946133	0.297629
10994	0.946132	0.514459
10995	0.946132	2.1
10996	0.946131	2.1
10997	0.946131	2.1
10998	0.946132	2.1
10999	0.946131	2.1
11000	0.946131	2.1
11001	0.946132	2.1
11002	0.946132	1.0276
11003	1.38623	0.972371
11004	0.946132	0.870998
11005	0.946132	0.916193
11006	0.946133	0.979859
11007	0.946132	0.748648
11008	0.946133	1.09497
11009	0.946132	2.1
11010	1.40098	2.1
11011	0.946131	2.1
11012	0.946131	2.1
11013	0.946132	2.1
11014	0.946132	2.1
11015	0.946132	2.1
11016	1.01777	0.799782
11017	0.946132	0.692065

11018	0.946133	0.913438
11019	1.68068	0.767976
11020	1.68068	0.994785
11021	1.41894	0.761839
11022	1.20787	0.705839
11023	0.946132	0.740594
11024	0.946133	2.1
11025	0.946133	2.1
11026	0.946132	2.1
11027	1.40199	2.1
11028	0.946133	2.1
11029	0.946132	2.1
11030	0.946132	2.1
11031	0.810946	1.29564
11032	0.810947	1.03573
11033	2.37827	1.75737
11034	1.3517	1.33098
11035	1.08132	1.34735
11036	1.08132	1.38366
11037	1.08133	1.26474
11038	0.810934	1.23999
11039	0.946124	2.1
11040	0.94613	1.87397
11041	1.40248	1.27782
11042	0.946131	1.24057
11043	0.946125	0.977054
11044	0.946127	1.80004
11045	0.946128	1.06273
11046	1.25703	2.00295
11047	0.87821	1.30868
11048	1.18912	1.95902
11049	0.946119	1.18977
11050	0.87825	1.35943
11051	1.1891	1.49605
11052	0.946107	1.98352
11053	0.946132	2.1
11054	1.28543	1.99143
11055	0.946132	1.69594
11056	0.946132	1.34083
11057	0.946131	0.919054
11058	0.946132	0.927127
11059	0.946132	0.991806
11060	0.946115	2.1
11061	0.946119	2.1
11062	0.946116	2.1
11063	0.946123	2.1
11064	0.946118	2.1
11065	1.41636	2.1
11066	0.946129	2.1
11067	0.946132	1.03285
11068	0.946132	2.1
11069	0.946132	1.98674
11070	0.946131	2.1
11071	0.946131	2.1
11072	1.41432	2.1
11073	0.946132	2.1
11074	0.810947	0.05
11075	0.810947	0.05

11076	2.44433	0.05
11077	1.21654	0.05
11078	0.946154	0.05
11079	0.97995	0.05
11080	1.08133	0.05
11081	0.810934	0.05
11082	0.851542	0.05
11083	0.835698	0.05
11084	1.22216	0.05
11085	0.946133	0.05
11086	1.01217	0.05
11087	1.09996	0.05
11088	0.851529	0.05
11089	0.946132	1.7903
11090	0.946132	2.1
11091	0.946129	2.1
11092	0.94613	1.89778
11093	0.946131	2.1
11094	0.946131	2.1
11095	1.2254	2.1
11096	0.800897	1.41123
11097	0.838029	2.1
11098	0.838013	1.70484
11099	0.93679	1.18862
11100	1.20217	1.0586
11101	1.16923	1.43554
11102	0.94948	1.48403
11103	0.958118	1.65148
11104	0.36751	1.52302
11105	0.800535	1.82152
11106	0.272087	0.467632
11107	0.428084	0.943449
11108	0.986335	1.81347
11109	0.946132	0.05
11110	0.946133	0.0624002
11111	1.19601	0.05
11112	0.946132	0.05
11113	0.946132	0.05
11114	0.946133	0.0716441
11115	0.946133	0.05
11116	0.946132	1.86695
11117	1.36956	2.1
11118	0.946132	2.1
11119	0.946132	1.67742
11120	0.946132	2.1
11121	0.946133	2.1
11122	0.946132	2.1
11123	0.946132	0.05
11124	0.946131	0.879401
11125	0.946131	0.05
11126	1.34268	0.05
11127	0.946133	0.05
11128	0.946132	0.05
11129	0.946131	0.05
11130	0.946132	2.1
11131	0.946131	2.1
11132	0.946131	2.1
11133	0.946132	2.1

11134	0.946132	2.1
11135	0.946132	2.1
11136	0.946132	2.1
11137	0.810946	1.18954
11138	0.810943	1.08695
11139	2.51472	1.13634
11140	1.3517	1.04576
11141	1.08132	1.04359
11142	1.08132	1.18608
11143	1.08133	1.15
11144	0.810933	1.15526
11145	0.902316	0.217677
11146	1.04517	0.182637
11147	0.974924	0.05
11148	0.530063	0.699089
11149	1.1272	0.296555
11150	0.68207	0.243476
11151	0.477061	0.55561
11152	0.810946	0.05
11153	0.810943	0.05
11154	0.810959	0.05
11155	1.99211	0.05
11156	1.35168	0.05
11157	1.08132	0.05
11158	1.08132	0.05
11159	1.08131	0.05
11160	0.94613	0.05
11161	0.946131	0.05
11162	0.946132	0.05
11163	0.946132	0.05
11164	0.946129	0.05
11165	0.946131	0.05
11166	1.16699	0.05
11167	0.810943	1.36627
11168	0.810942	1.27385
11169	1.6943	2.07011
11170	1.27059	2.1
11171	1.00021	2.09675
11172	1.02049	1.89705
11173	1.08133	1.36979
11174	0.750103	1.34187
11175	0.957813	1.40117
11176	0.946128	1.70568
11177	0.946132	1.20155
11178	1.04832	1.8962
11179	0.870714	1.20806
11180	0.94613	1.27135
11181	0.94613	1.36837
11182	1.86374	2.1
11183	2.0815	1.3405
11184	1.15918	0.711447
11185	0.946129	0.704512
11186	1.15918	0.613147
11187	0.946131	1.02747
11188	0.946131	0.68293
11189	0.946131	0.781416
11190	0.94613	0.700718
11191	0.946119	1.01441

11192	1.3612	1.09154
11193	0.946125	1.05992
11194	0.946119	1.00782
11195	0.94612	1.27887
11196	0.946132	1.0644
11197	0.946107	1.05523
11198	0.810947	0.498026
11199	0.810947	0.562745
11200	2.50398	0.141682
11201	1.3517	0.164633
11202	1.08132	0.359498
11203	1.08133	0.479072
11204	1.08132	0.110607
11205	0.810934	0.464503
11206	0.946132	2.1
11207	0.946132	2.1
11208	0.946132	2.1
11209	1.39754	2.1
11210	0.946132	2.1
11211	0.946132	2.1
11212	0.946131	2.1
11213	0.946129	2.1
11214	1.41732	2.1
11215	0.946129	2.1
11216	0.946131	2.1
11217	0.946131	2.1
11218	0.946131	2.1
11219	0.946131	2.1
11220	0.946132	1.19225
11221	1.25276	2.07395
11222	0.946131	2.1
11223	0.94613	1.21266
11224	0.94613	1.97565
11225	0.94613	1.27999
11226	0.946131	1.42143
11227	1.21261	0.465969
11228	0.94613	0.999356
11229	0.94613	1.74977
11230	0.946128	0.115414
11231	0.946131	1.47788
11232	0.94613	1.17618
11233	0.946129	1.55155
11234	0.946128	2.1
11235	0.946133	2.1
11236	0.946132	2.1
11237	0.946131	2.1
11238	0.94613	2.1
11239	0.946131	2.1
11240	1.39407	2.1
11241	0.946116	1.44454
11242	0.946119	1.60741
11243	0.946119	1.17706
11244	1.15369	1.46965
11245	0.946119	1.06944
11246	0.946131	1.28777
11247	0.946106	1.27373
11248	0.946131	2.1
11249	0.946131	2.1

11250	0.946131	2.1
11251	0.946133	2.1
11252	0.946129	2.1
11253	0.946131	2.1
11254	0.946131	2.1
11255	1.19564	0.05
11256	0.498881	0.05
11257	1.33964	0.05
11258	0.498878	0.05
11259	0.923583	0.05
11260	0.831259	0.05
11261	0.553735	0.05
11262	0.946132	0.05
11263	0.946132	0.05
11264	0.946131	0.05
11265	0.946131	0.05
11266	0.946132	0.05
11267	0.946131	0.05
11268	1.28673	0.05
11269	0.946132	0.05
11270	0.946132	0.05
11271	1.33178	0.05
11272	0.946132	0.05
11273	0.946132	0.05
11274	0.946131	0.05
11275	0.946131	0.05
11276	0.946128	0.081431
11277	0.946132	0.0682326
11278	0.946129	0.182057
11279	0.946131	0.05
11280	0.946131	0.05
11281	0.946129	0.071266
11282	0.94613	0.05
11283	0.946131	2.1
11284	0.946131	2.1
11285	0.946129	2.1
11286	1.40525	2.1
11287	0.94613	2.1
11288	0.94613	2.1
11289	1.40525	2.1
11290	0.946131	2.1
11291	0.946126	2.1
11292	0.946127	2.1
11293	0.946127	2.1
11294	1.4073	2.1
11295	0.946129	2.1
11296	0.946129	2.1
11297	0.946131	0.05
11298	0.946131	0.05
11299	0.946132	0.05
11300	0.946131	0.05
11301	0.946131	0.05
11302	1.05016	0.05
11303	0.946132	0.05
11304	0.946128	0.05
11305	0.946132	0.05
11306	0.946131	0.05
11307	0.946129	0.05

11308	0.94613	0.05
11309	1.13398	0.05
11310	0.94613	0.05
11311	0.946132	0.05
11312	0.946132	0.05
11313	0.946131	0.05
11314	0.946131	0.05
11315	0.946132	0.05
11316	0.946131	0.05
11317	0.946132	0.05
11318	0.946131	0.05
11319	0.94613	0.05
11320	0.946131	0.05
11321	0.94613	0.0582559
11322	1.38645	0.05
11323	1.38645	0.05
11324	0.946131	0.05
11325	0.946131	0.0650784
11326	0.946131	0.05
11327	0.94613	0.0821099
11328	1.23923	0.05
11329	0.94613	0.05
11330	0.946131	0.0636317
11331	0.946131	0.102506
11332	0.946132	0.05
11333	0.946131	0.05
11334	0.946131	0.05
11335	0.946132	0.05
11336	0.946132	0.05
11337	0.946132	0.05
11338	1.40511	0.05
11339	0.946132	2.1
11340	0.946132	2.1
11341	0.946132	2.1
11342	0.946132	2.1
11343	0.946132	2.1
11344	0.946131	2.1
11345	1.12968	2.1
11346	0.946132	1.54683
11347	0.946128	2.1
11348	0.946128	2.1
11349	0.94613	1.96633
11350	0.94613	2.1
11351	0.94613	1.60831
11352	0.94613	2.1
11353	0.946133	2.1
11354	0.946133	2.1
11355	0.946132	2.1
11356	0.946132	2.1
11357	0.946132	2.1
11358	0.946133	2.1
11359	1.31323	2.1
11360	0.94613	2.1
11361	0.946131	0.986228
11362	0.946131	0.987498
11363	0.94613	0.071954
11364	0.946131	2.1
11365	0.946131	2.09036

11366	0.946131	1.71631
11367	0.946132	0.155157
11368	0.946132	0.4683
11369	0.946132	0.05
11370	1.36899	0.05
11371	0.946132	0.115317
11372	0.946132	0.05
11373	0.946133	0.151441
11374	1.03543	0.05
11375	0.946131	0.05
11376	0.946131	0.05
11377	0.946132	0.05
11378	0.94613	0.05
11379	0.950987	0.05
11380	0.941275	0.05
11381	0.946131	0.05
11382	0.94613	0.05
11383	1.28952	0.05
11384	0.946132	0.05
11385	0.94613	0.05
11386	0.946131	0.05
11387	0.946131	0.05
11388	0.94613	2.1
11389	1.35966	2.1
11390	0.946131	2.1
11391	0.946131	2.1
11392	0.946129	2.1
11393	0.94613	2.1
11394	0.94613	2.1
11395	0.810946	2.1
11396	0.810942	0.283684
11397	0.810932	2.1
11398	2.6655	2.1
11399	1.3517	2.1
11400	1.08132	2.1
11401	1.08131	0.236173
11402	1.08133	2.1
11403	0.94613	2.1
11404	0.946131	2.1
11405	0.94613	2.1
11406	0.946128	2.1
11407	0.946131	2.1
11408	0.94613	2.1
11409	0.94613	2.1
11410	0.946128	2.1
11411	0.946128	2.1
11412	0.946133	2.1
11413	1.25375	2.1
11414	0.94613	2.1
11415	0.946131	2.1
11416	1.25375	2.1
11417	0.94613	2.1
11418	0.94613	2.1
11419	0.946132	2.1
11420	0.946132	2.1
11421	0.946128	2.1
11422	0.94613	2.1
11423	1.31453	2.1

11424	0.946131	0.05
11425	0.946129	0.05
11426	0.94613	0.05
11427	0.94613	0.05
11428	0.94613	0.05
11429	1.12018	0.05
11430	0.94613	0.05
11431	0.946131	0.05
11432	0.946129	0.05
11433	0.946131	0.05
11434	1.16373	0.05
11435	0.94613	0.05
11436	0.94613	0.05
11437	0.946131	0.05
11438	1.36811	0.118132
11439	0.946132	0.150711
11440	1.36811	0.0793616
11441	0.946129	0.160164
11442	0.94613	0.170782
11443	0.946129	0.156719
11444	0.946129	0.12867
11445	0.946132	0.0679607
11446	0.946133	0.05
11447	0.946132	0.0536631
11448	0.946133	0.0843025
11449	1.35732	0.05
11450	1.35732	0.05
11451	0.946133	0.0600545
11452	1.36399	0.632564
11453	0.946129	1.02523
11454	0.94613	0.894823
11455	0.946131	1.04687
11456	0.94613	1.62249
11457	0.946131	0.985599
11458	0.946131	1.1004
11459	0.946133	2.1
11460	0.946132	2.1
11461	0.946132	2.1
11462	1.38302	2.1
11463	0.946133	2.1
11464	0.946132	2.1
11465	0.946133	2.1
11466	0.810943	2.1
11467	0.810944	2.1
11468	0.810945	2.1
11469	2.80542	2.1
11470	1.35169	2.1
11471	1.08132	2.1
11472	1.08132	2.1
11473	1.08132	2.1
11474	1.30189	2.1
11475	0.946131	2.1
11476	0.946132	2.1
11477	0.946131	2.1
11478	0.946131	2.1
11479	0.946131	2.1
11480	0.946132	2.1
11481	0.810946	1.01089

11482	0.810947	0.91295
11483	2.33293	0.961579
11484	1.24357	0.941999
11485	1.08132	0.95023
11486	0.973183	0.977044
11487	1.00023	0.979133
11488	0.810934	0.964338
11489	1.06584	0.90846
11490	0.946129	0.951772
11491	0.84731	0.769207
11492	1.16646	0.95248
11493	0.946126	0.979551
11494	1.2831	0.874446
11495	0.851527	0.938984
11496	0.946131	2.1
11497	0.946128	2.1
11498	0.946128	2.1
11499	0.94613	2.1
11500	1.31961	2.1
11501	0.94613	2.1
11502	0.94613	2.1
11503	0.946131	2.1
11504	1.3605	2.1
11505	0.946133	2.1
11506	0.946132	2.1
11507	0.94613	2.1
11508	0.946131	2.1
11509	0.946131	2.1
11510	0.946128	2.1
11511	0.946133	1.74859
11512	0.946132	2.1
11513	0.946131	1.3246
11514	0.946131	0.10463
11515	0.946131	2.08807
11516	1.30116	2.1
11517	0.946132	0.05
11518	0.946129	0.05
11519	0.94613	0.05
11520	1.3035	0.05
11521	0.946131	0.05
11522	0.94613	0.05
11523	0.946131	0.05
11524	0.946132	2.1
11525	1.2051	2.1
11526	1.2051	2.1
11527	0.946132	2.1
11528	1.37869	2.1
11529	0.946132	2.1
11530	0.946131	2.1
11531	0.810946	2.1
11532	0.810945	2.1
11533	2.69247	2.1
11534	1.35169	2.1
11535	1.08132	2.1
11536	1.08132	2.1
11537	1.08132	2.1
11538	0.810946	2.1
11539	1.22993	2.1

11540	0.946131	2.1
11541	0.946131	2.1
11542	1.34623	2.1
11543	0.946131	2.1
11544	1.34623	2.1
11545	0.946131	2.1
11546	0.946131	0.05
11547	0.946132	0.05
11548	0.946132	0.05
11549	0.946132	0.05
11550	0.946131	0.05
11551	0.946132	0.05
11552	1.32299	0.05
11553	0.94613	0.05
11554	0.946131	0.05
11555	0.946128	0.05
11556	0.946132	0.05
11557	0.946133	0.05
11558	0.946131	0.05
11559	0.946131	0.05
11560	0.946132	2.1
11561	0.946129	2.1
11562	0.946128	2.1
11563	0.94613	2.1
11564	0.94613	2.1
11565	0.946131	2.1
11566	0.94613	2.1
11567	0.94613	2.1
11568	0.94613	2.1
11569	0.946131	2.1
11570	0.946132	2.1
11571	0.946129	2.1
11572	0.946131	2.1
11573	0.946131	2.1
11574	0.946132	1.60768
11575	0.946131	1.64328
11576	1.13731	1.38011
11577	0.94775	1.74605
11578	0.944514	1.9081
11579	0.946132	2.04731
11580	1.039	2.00828
11581	0.946131	1.53505
11582	0.946128	1.46258
11583	0.94613	1.14533
11584	0.946131	1.54496
11585	0.946128	1.67921
11586	0.946129	1.89469
11587	1.23726	1.8322
11588	0.946132	2.1
11589	0.946132	2.1
11590	0.946129	2.1
11591	1.39214	2.1
11592	0.946131	2.1
11593	0.946131	2.1
11594	0.94613	2.1
11595	0.946125	0.211501
11596	0.946119	0.05
11597	0.946118	0.05

11598	0.946118	0.05
11599	0.94612	0.05
11600	0.946106	0.05
11601	0.946131	0.05
11602	1.26893	0.695752
11603	0.946131	0.687641
11604	1.26893	0.732414
11605	0.94613	0.678453
11606	0.946131	0.751304
11607	0.946131	0.687723
11608	0.946131	0.694257
11609	0.946133	0.05
11610	0.946133	0.05
11611	0.946132	0.05
11612	1.28924	0.05
11613	0.946133	0.05
11614	0.946132	0.05
11615	0.946132	0.05
11616	0.946133	0.091998
11617	0.946132	0.0902851
11618	0.946132	0.106361
11619	0.946133	0.0623383
11620	0.946133	0.145284
11621	0.946132	0.160716
11622	0.946133	0.094953
11623	0.946132	1.04724
11624	0.946133	0.227622
11625	0.946133	0.757634
11626	0.946132	1.0545
11627	1.15388	0.822337
11628	0.946132	1.03883
11629	0.946133	0.928254
11630	0.946132	0.208293
11631	0.946133	0.109078
11632	0.946132	0.2037
11633	0.946133	0.0807999
11634	0.946133	0.198533
11635	0.946132	0.2948
11636	0.946132	0.13463
11637	1.0644	1.29002
11638	1.12486	1.80989
11639	1.08674	1.91805
11640	0.946132	1.41078
11641	0.946132	1.75866
11642	0.946133	1.53903
11643	0.946132	1.51315
11644	0.808978	1.28493
11645	0.911537	1.56051
11646	0.932832	1.46144
11647	1.14604	1.80474
11648	1.56953	1.58489
11649	1.25866	1.41516
11650	1.18488	1.68222
11651	1.341	1.32196
11652	0.946131	0.05
11653	0.946131	0.05
11654	0.946131	0.05
11655	0.94613	0.05858

11656	1.33922	0.05
11657	0.946131	0.0585139
11658	0.946131	0.05
11659	0.946132	0.05
11660	0.946133	0.05
11661	0.946132	0.05
11662	0.946132	0.05
11663	0.946132	0.05
11664	0.946133	0.05
11665	0.946132	0.05
11666	0.946129	1.0189
11667	0.946133	2.1
11668	0.946133	1.14634
11669	0.946131	1.27086
11670	1.40496	2.1
11671	0.946131	1.04373
11672	0.946131	2.1
11673	0.94613	1.17654
11674	0.946127	0.941822
11675	0.946127	1.1033
11676	0.94613	1.20841
11677	1.38913	1.01557
11678	0.946128	1.12681
11679	0.946128	1.05845
11680	1.98863	0.05
11681	2.59915	0.05
11682	1.40338	0.05
11683	0.946131	0.05
11684	1.22266	0.05
11685	1.40338	0.05
11686	0.946132	0.05
11687	0.946132	0.05
11688	1.22266	0.05
11689	0.94613	0.05
11690	0.94613	0.05
11691	0.946124	0.05
11692	1.21428	0.05
11693	0.946126	0.05
11694	0.946127	0.05
11695	0.946127	0.05
11696	1.03411	0.05
11697	0.946131	0.05
11698	1.03411	0.05
11699	0.946129	0.05
11700	1.14258	0.05
11701	0.952143	0.05
11702	0.940118	0.05
11703	0.810943	2.1
11704	0.810945	2.1
11705	0.810958	2.1
11706	2.82603	2.1
11707	1.35168	2.1
11708	1.08132	2.1
11709	1.08132	2.1
11710	1.0813	2.1
11711	0.946133	0.0738922
11712	0.946132	0.0518642
11713	0.946132	0.0617755

11714	0.946132	0.05
11715	0.946132	0.05
11716	1.11539	0.05
11717	0.946133	0.0539321
11718	0.946133	2.1
11719	0.946132	2.1
11720	0.946133	2.1
11721	0.946133	2.1
11722	0.946132	2.1
11723	1.41195	2.1
11724	0.946133	2.1
11725	0.946131	0.05
11726	0.946131	0.05
11727	0.946131	0.05
11728	0.946131	0.05
11729	0.946131	0.05
11730	0.946131	0.05
11731	1.15968	0.05
11732	0.946131	1.05796
11733	0.94613	1.56871
11734	0.94613	2.1
11735	1.34386	1.68196
11736	0.946132	2.09819
11737	0.94613	2.1
11738	1.34386	1.93778
11739	0.946131	0.991622
11740	0.935202	0.51803
11741	0.946131	1.96589
11742	1.25054	1.24782
11743	0.850155	1.8188
11744	0.946131	1.83908
11745	0.935205	1.6968
11746	0.946131	0.0534033
11747	1.40193	0.05
11748	0.946132	0.05
11749	0.946132	0.05
11750	0.946129	0.0601047
11751	1.40193	0.05
11752	0.946131	0.0506902
11753	0.808779	0.05
11754	0.561937	0.05
11755	1.40193	0.05
11756	1.40193	0.05
11757	0.561936	0.05
11758	0.561937	0.05
11759	1.40193	0.05
11760	0.946132	0.05
11761	0.946132	0.05
11762	0.946132	0.05
11763	0.946132	0.05
11764	0.946133	0.05
11765	0.946132	0.05
11766	0.946133	0.05
11767	0.946127	0.673752
11768	0.946131	0.709176
11769	0.946131	0.401253
11770	0.946127	0.514627
11771	1.19583	0.330682

11772	1.19583	0.244301
11773	0.946129	0.485401
11774	0.589428	0.722624
11775	1.08737	0.69752
11776	1.10961	0.717758
11777	0.872901	0.705223
11778	0.541327	0.690405
11779	0.541312	0.708944
11780	0.85153	0.708482
11781	0.946132	2.09876
11782	0.946131	2.1
11783	0.946131	2.1
11784	0.946132	2.1
11785	0.946132	2.1
11786	0.946132	2.1
11787	0.946132	2.1
11788	0.946132	1.31321
11789	1.2778	2.1
11790	0.946131	2.1
11791	0.946131	1.3695
11792	0.94613	2.1
11793	0.94613	1.61024
11794	0.94613	1.73621
11795	0.946118	0.979288
11796	0.946112	0.99959
11797	0.946118	0.937386
11798	0.946119	0.956853
11799	0.946117	0.960305
11800	1.35631	0.737429
11801	0.946105	0.723204
11802	0.946133	1.71884
11803	0.946133	2.1
11804	0.946132	2.074
11805	0.946132	2.1
11806	0.946133	0.05
11807	0.946132	1.07344
11808	0.946132	1.0351
11809	0.94613	2.1
11810	0.946131	0.940069
11811	0.946128	0.852714
11812	0.946128	0.0634597
11813	0.94613	2.1
11814	0.946129	2.1
11815	0.946129	2.06342
11816	0.946132	1.41508
11817	1.15139	1.39397
11818	0.946132	1.1572
11819	0.946132	1.50486
11820	0.946132	1.50273
11821	0.946132	1.59481
11822	0.946132	1.43095
11823	0.946133	2.1
11824	1.36151	2.1
11825	0.946132	2.1
11826	0.946132	2.1
11827	0.946132	2.1
11828	0.946132	2.1
11829	0.946133	2.1

11830	0.946127	2.1
11831	1.38397	2.1
11832	0.94613	2.1
11833	0.946131	2.1
11834	0.946128	2.1
11835	0.94613	2.1
11836	0.946129	2.1
11837	0.946131	0.05
11838	0.946127	0.0580982
11839	0.946127	0.0639603
11840	0.946132	0.05
11841	0.946131	0.0647281
11842	0.946128	0.05
11843	0.946129	0.05
11844	1.0268	0.05
11845	1.23823	0.05
11846	1.2171	0.05
11847	0.883446	0.05
11848	1.23827	0.05
11849	1.32614	0.05
11850	0.94613	0.05
11851	0.946132	2.1
11852	0.946129	2.1
11853	0.946129	2.1
11854	0.94613	2.1
11855	1.36523	2.1
11856	0.946131	2.1
11857	0.94613	2.1
11858	0.946132	0.05
11859	0.946131	0.05
11860	0.946132	0.05
11861	0.946131	0.05
11862	0.946131	0.05
11863	1.01653	0.05
11864	0.946131	0.05
11865	0.94612	2.1
11866	0.946121	2.1
11867	0.946119	2.1
11868	0.946119	2.1
11869	0.946119	2.1
11870	0.946132	2.1
11871	0.946107	2.1
11872	0.810942	1.37054
11873	0.810944	0.861621
11874	2.60324	2.1
11875	1.21651	2.0756
11876	1.0813	1.76753
11877	0.946153	0.79043
11878	0.979948	2.1
11879	0.709588	2.06508
11880	1.3492	0.05
11881	0.946132	0.05
11882	0.946131	0.05
11883	0.94613	0.05
11884	0.946129	0.05
11885	0.946129	0.05
11886	0.94613	0.05
11887	0.946131	0.05

11888	0.94613	0.05
11889	0.946128	0.05
11890	0.946133	0.05
11891	0.946133	0.05
11892	0.946131	0.05
11893	0.946131	0.05
11894	1.23351	0.05
11895	0.946131	0.05
11896	0.946131	0.05
11897	0.946132	0.05
11898	0.946132	0.05
11899	0.946131	0.05
11900	0.946132	0.05
11901	1.35657	0.05
11902	0.946132	0.05
11903	0.946131	0.05
11904	0.946132	0.05
11905	0.946132	0.05
11906	0.946131	0.05
11907	0.946131	0.05
11908	0.946132	0.100377
11909	0.946132	0.0536605
11910	0.946132	0.123134
11911	0.946131	0.05
11912	0.946131	0.05
11913	0.946132	0.0586494
11914	0.946132	0.05
11915	0.946133	0.0603738
11916	0.946132	0.05
11917	0.946133	0.105341
11918	0.946132	0.05
11919	0.946132	0.05
11920	0.946132	0.05
11921	0.946133	0.05
11922	0.946131	1.07632
11923	1.4101	2.04391
11924	0.946131	2.1
11925	0.946131	0.912892
11926	0.946131	1.21598
11927	0.946131	0.906739
11928	0.946131	2.0625
11929	0.810944	1.48057
11930	0.810939	1.37113
11931	0.709586	1.15457
11932	2.71261	1.21576
11933	1.21651	1.28743
11934	0.946151	1.05048
11935	0.979943	1.29921
11936	1.0813	1.57758
11937	0.946132	2.1
11938	0.946131	2.1
11939	0.946132	2.1
11940	0.946131	2.1
11941	0.946131	2.1
11942	1.37733	2.1
11943	0.946131	2.1
11944	0.946133	2.1
11945	0.946132	2.1

11946	0.946132	2.1
11947	0.946133	2.1
11948	0.946133	2.1
11949	0.946132	2.1
11950	1.32582	2.1
11951	0.946132	2.1
11952	0.946132	2.1
11953	0.946132	2.1
11954	0.946132	2.1
11955	0.946132	2.1
11956	0.946132	2.1
11957	0.946131	2.1
11958	0.946133	2.02016
11959	0.946132	1.30883
11960	0.946132	1.58133
11961	0.946132	1.24384
11962	0.946132	1.29882
11963	0.946133	1.26447
11964	1.3469	1.26491
11965	0.946126	1.10035
11966	0.94613	2.1
11967	0.946131	2.1
11968	0.946127	2.1
11969	1.41206	2.1
11970	0.946128	2.1
11971	0.946128	2.1
11972	0.946127	2.1
11973	0.94613	2.1
11974	0.946131	0.40456
11975	0.94613	2.1
11976	0.946128	0.677185
11977	0.946129	2.1
11978	0.946129	2.1
11979	0.946131	0.05
11980	0.94613	0.05
11981	1.24448	0.05
11982	0.946129	0.05
11983	0.946131	0.05
11984	0.94613	0.05
11985	0.946131	0.05
11986	0.946132	0.05
11987	0.946133	0.05
11988	1.11015	0.05
11989	0.946133	0.0504013
11990	0.946132	0.0576431
11991	0.946132	0.0611729
11992	0.946132	0.05
11993	1.38775	2.1
11994	0.946131	2.1
11995	0.946131	2.1
11996	0.946132	2.1
11997	0.946132	2.1
11998	0.946131	2.1
11999	0.946131	2.1
12000	0.946129	2.1
12001	0.946129	2.1
12002	0.946133	1.33154
12003	0.946131	2.1

12004	0.946131	0.0527388
12005	0.946131	1.73178
12006	0.946131	1.37442
12007	0.946132	2.1
12008	0.946133	2.1
12009	0.946132	2.1
12010	1.40774	2.1
12011	0.946132	2.1
12012	0.946133	2.1
12013	0.946132	2.1
12014	0.610968	0.05
12015	0.688233	0.05
12016	0.715226	0.05
12017	0.792409	0.05
12018	1.13418	0.05
12019	0.956667	0.05
12020	0.757321	0.05
12021	0.968845	0.05
12022	0.595679	0.05
12023	0.946133	1.36655
12024	1.28917	1.55125
12025	0.946131	2.05409
12026	0.94613	0.968142
12027	0.946131	1.32039
12028	0.946131	1.17363
12029	0.946131	1.82103
12030	0.946132	0.598752
12031	0.946128	0.588262
12032	0.94613	0.641164
12033	1.40668	0.602009
12034	0.946131	0.603164
12035	0.946131	0.584671
12036	0.94613	0.616111
12037	0.810946	2.1
12038	0.810945	2.1
12039	2.78163	2.1
12040	1.3517	2.1
12041	1.08132	2.1
12042	1.08133	2.1
12043	1.08132	2.1
12044	0.810933	2.1
12045	0.946131	2.1
12046	1.39081	2.1
12047	0.94613	2.1
12048	1.30339	2.1
12049	1.30339	2.1
12050	1.39081	2.1
12051	0.946131	2.1
12052	0.946127	1.04049
12053	0.821631	1.09867
12054	0.946131	0.961901
12055	0.946131	1.04165
12056	0.946128	0.962296
12057	0.996109	1.00274
12058	0.827876	0.993443
12059	0.946131	2.1
12060	0.946129	2.1
12061	0.946129	2.1

12062	0.94613	2.1
12063	0.94613	2.1
12064	0.94613	2.1
12065	0.94613	2.1
12066	0.946132	0.110095
12067	1.41723	0.134333
12068	0.946132	0.0965995
12069	0.946133	0.05
12070	0.946132	0.412649
12071	0.946132	0.147255
12072	0.946133	0.144592
12073	0.946133	0.0743435
12074	0.946132	0.05
12075	0.946133	0.05
12076	0.946133	0.054659
12077	0.946132	0.16037
12078	0.946132	0.0889807
12079	1.34254	0.0887077
12080	1.26501	1.17691
12081	0.946132	1.27421
12082	0.946133	1.27738
12083	0.946133	1.64133
12084	0.946132	1.61878
12085	0.946133	1.28058
12086	0.946132	1.95168
12087	0.946131	0.05
12088	0.946131	0.05
12089	0.946131	0.05
12090	0.946131	0.05
12091	0.946131	0.05
12092	0.946131	0.05
12093	0.946131	0.05
12094	0.878843	0.05
12095	0.82561	0.05
12096	0.586974	0.05
12097	1.05343	0.05
12098	0.80631	0.05
12099	0.782663	0.05
12100	0.789405	0.05
12101	0.902491	0.05
12102	1.05344	0.05
12103	0.94613	0.05
12104	0.946131	0.05
12105	0.946131	0.05
12106	0.94613	0.05
12107	0.946131	0.05
12108	0.946131	0.05
12109	0.946131	0.05
12110	0.946128	2.1
12111	0.946133	2.1
12112	0.946133	0.631687
12113	0.94613	2.1
12114	0.946131	1.21468
12115	0.946131	1.3309
12116	0.946131	1.49565
12117	0.946131	0.150799
12118	0.946131	0.131411
12119	0.94613	0.134659

12120	0.946128	0.122689
12121	0.946133	0.165875
12122	1.2996	0.080964
12123	0.946131	0.120221
12124	0.946132	2.1
12125	0.946132	2.1
12126	0.946132	2.1
12127	0.946132	2.1
12128	0.946131	2.1
12129	0.946132	2.1
12130	0.946132	2.1
12131	1.99211	0.05
12132	2.55024	0.05
12133	0.946131	0.05
12134	0.946131	0.05
12135	0.946131	0.05
12136	0.946131	0.05
12137	0.946131	0.05
12138	0.946131	0.05
12139	1.35726	0.05
12140	0.946128	2.1
12141	0.946132	2.0136
12142	0.946131	2.1
12143	0.94613	2.1
12144	0.94613	2.1
12145	0.94613	2.1
12146	1.39852	2.1
12147	1.31666	0.05
12148	0.946131	0.05
12149	0.946128	0.05
12150	0.94613	0.05
12151	0.946128	0.05
12152	0.946128	0.05
12153	0.946128	0.05
12154	1.32275	0.05
12155	0.946131	0.05
12156	0.946129	0.05
12157	0.946128	0.05
12158	0.946129	0.05
12159	0.946128	0.05
12160	0.946129	0.05
12161	0.946132	2.1
12162	0.946131	2.1
12163	0.946131	2.1
12164	0.946132	2.1
12165	0.94613	2.1
12166	0.946131	2.1
12167	1.41125	2.1
12168	0.946132	2.1
12169	0.946132	2.1
12170	0.946131	2.1
12171	0.946132	2.1
12172	0.946132	2.1
12173	0.946132	2.1
12174	1.31904	2.1
12175	1.02425	0.05
12176	0.946132	0.05
12177	0.94613	0.05

12178	0.946131	0.05
12179	1.1534	0.05
12180	0.961161	0.05
12181	0.9311	0.0520972
12182	1.40092	1.23445
12183	0.946133	1.27961
12184	0.946132	1.2963
12185	0.946131	1.58817
12186	0.946131	1.23803
12187	0.94613	1.30338
12188	0.946131	1.76239
12189	0.946113	2.1
12190	0.946119	0.402836
12191	0.946117	2.1
12192	1.40015	2.1
12193	0.946118	0.0711976
12194	0.946131	2.1
12195	0.946105	2.06073
12196	0.946132	0.658903
12197	0.946133	0.637218
12198	0.946132	0.684505
12199	1.03547	0.674373
12200	0.946133	0.552368
12201	0.946133	0.662626
12202	0.946132	0.638316
12203	0.946133	2.1
12204	1.36694	2.1
12205	0.946133	2.1
12206	0.946132	2.1
12207	0.946133	2.1
12208	0.946133	2.1
12209	0.946132	2.1
12210	0.946132	0.05
12211	0.946133	0.05
12212	0.946132	0.05
12213	0.946133	0.05
12214	0.946133	0.05
12215	0.946132	0.0822018
12216	0.946132	0.05
12217	0.491552	0.05
12218	0.913078	0.05
12219	0.946132	0.0569677
12220	0.491552	0.05
12221	0.946132	0.05
12222	0.49154	0.05
12223	1.53168	0.05
12224	1.53168	0.05
12225	0.946127	2.1
12226	1.38752	2.1
12227	0.946129	2.1
12228	0.94613	2.1
12229	0.946128	2.1
12230	0.946129	2.1
12231	0.946129	2.1
12232	0.946132	2.1
12233	0.946132	2.1
12234	0.946132	2.1
12235	0.946132	2.1

12236	0.946132	2.1
12237	0.946131	2.1
12238	0.946132	2.1
12239	0.94613	0.108013
12240	0.94613	0.0746006
12241	0.94613	0.0851525
12242	0.946131	0.0734701
12243	0.946129	0.126347
12244	1.16834	0.05
12245	0.94613	0.10343
12246	0.946127	0.0954226
12247	1.35594	0.0503549
12248	0.946131	0.0576002
12249	0.946128	0.134145
12250	0.946127	0.05
12251	0.946129	0.0982996
12252	0.94613	0.084878
12253	0.94613	0.503825
12254	0.946131	0.720464
12255	0.946128	0.05
12256	0.946128	0.05
12257	0.946131	0.933974
12258	0.94613	0.05
12259	0.94613	0.817599
12260	0.946132	0.05
12261	0.946132	0.733872
12262	0.946129	0.645906
12263	1.39751	0.263831
12264	0.94613	0.755306
12265	0.946131	0.430629
12266	0.946131	0.724998
12267	0.946132	0.05
12268	0.700041	0.05
12269	0.700013	0.0535769
12270	1.7697	0.05
12271	1.7697	0.05
12272	1.21134	0.05
12273	0.946131	0.05
12274	1.25837	0.05
12275	0.946133	2.1
12276	0.946132	1.98512
12277	0.946132	1.6435
12278	0.946132	2.1
12279	0.946132	2.1
12280	0.946133	2.1
12281	1.40583	2.1
12282	0.946131	1.26483
12283	0.946131	2.03943
12284	0.946131	1.35466
12285	0.946131	2.1
12286	0.946131	2.08056
12287	0.827865	1.95569
12288	0.946131	2.02452
12289	0.946132	0.05
12290	0.946129	0.05
12291	0.946129	0.05
12292	0.946131	0.05
12293	0.94613	0.05

12294	1.21087	0.05
12295	0.946131	0.05
12296	0.94613	0.868516
12297	0.946131	0.891231
12298	0.94613	0.821808
12299	0.946128	0.86527
12300	0.946132	0.824653
12301	1.17452	0.82433
12302	0.94613	0.831533
12303	0.946119	0.808531
12304	0.946114	0.823905
12305	0.946119	0.633115
12306	0.946119	0.738524
12307	0.946119	0.752241
12308	1.27376	0.428089
12309	0.946106	0.511372
12310	0.946133	2.1
12311	1.39621	2.1
12312	0.946132	2.1
12313	0.946132	2.1
12314	0.946133	2.1
12315	0.946132	2.1
12316	0.946132	2.1
12317	0.946132	2.1
12318	0.946132	2.1
12319	0.946131	2.1
12320	0.946131	2.1
12321	0.946132	2.1
12322	0.946132	2.1
12323	1.34352	2.1
12324	0.946132	2.1
12325	1.41272	2.1
12326	0.946131	2.1
12327	0.94613	1.69554
12328	0.94613	2.1
12329	0.94613	1.91487
12330	0.94613	2.1
12331	0.946132	2.1
12332	0.946131	2.0558
12333	0.946132	2.1
12334	0.946132	1.9637
12335	0.946132	1.31637
12336	0.946132	2.1
12337	1.23536	2.1
12338	0.946132	2.1
12339	0.946131	2.1
12340	0.946131	2.1
12341	0.946132	2.1
12342	0.946132	2.1
12343	1.41131	2.1
12344	0.946131	2.1
12345	1.41894	0.906957
12346	0.946132	0.521745
12347	0.946133	0.792914
12348	0.946132	0.864246
12349	0.946132	0.811418
12350	0.946133	0.818745
12351	0.946132	0.801384

12352	0.946128	0.05
12353	0.946133	1.0027
12354	0.946131	0.883414
12355	0.946129	2.1
12356	0.94613	0.221179
12357	1.4099	1.07377
12358	0.946131	0.300458
12359	0.810947	2.1
12360	0.810945	1.12185
12361	0.810933	2.1
12362	2.62883	2.1
12363	1.3517	2.1
12364	1.08132	2.1
12365	1.08132	0.802937
12366	1.08133	2.1
12367	0.946131	1.07911
12368	0.946126	2.1
12369	0.946127	1.06524
12370	0.94613	1.09434
12371	0.946128	2.1
12372	0.946129	0.904067
12373	0.946129	1.0212
12374	1.21347	2.1
12375	0.946129	2.1
12376	0.94613	2.1
12377	1.21347	2.1
12378	1.33064	2.1
12379	0.94613	2.1
12380	1.33064	2.1
12381	0.94613	0.05
12382	0.946129	0.05
12383	0.94613	0.05
12384	0.946132	0.05
12385	0.946126	0.05
12386	0.946129	0.05
12387	0.94613	0.05
12388	0.946132	1.01921
12389	1.37938	1.20055
12390	0.946131	1.36373
12391	0.94613	0.969771
12392	0.946131	1.73004
12393	0.94613	1.03456
12394	0.94613	1.12636
12395	0.946133	0.05
12396	0.946133	0.05
12397	1.2968	0.05
12398	0.946132	0.05
12399	0.946133	0.05
12400	1.2968	0.05
12401	0.946133	0.05
12402	1.43643	0.05
12403	0.946129	0.284058
12404	0.946133	0.160205
12405	0.946131	0.398046
12406	0.94613	0.05
12407	0.946131	0.116038
12408	0.946131	0.194252
12409	0.946131	0.129992

12410	0.872447	1.40593
12411	0.946132	1.28181
12412	0.872447	1.10634
12413	0.827867	1.35796
12414	0.946132	1.43749
12415	0.827867	1.59722
12416	0.872447	1.57438
12417	0.946117	1.14071
12418	1.27213	1.09055
12419	0.946124	0.609938
12420	0.946119	1.06911
12421	0.946113	1.35678
12422	0.946129	1.21986
12423	0.946104	1.16258
12424	0.94613	0.327302
12425	0.946131	0.995968
12426	0.94613	0.472987
12427	0.946129	0.889262
12428	0.946132	0.05
12429	0.946131	0.05
12430	0.94613	0.05
12431	0.810942	2.1
12432	0.810943	2.1
12433	2.80212	2.1
12434	1.3517	2.1
12435	1.08133	2.1
12436	1.08132	2.1
12437	1.08132	2.1
12438	0.810932	2.1
12439	1.29521	2.1
12440	0.946129	2.1
12441	0.946131	2.1
12442	1.40106	2.1
12443	0.94613	2.1
12444	1.40106	2.1
12445	0.946131	2.1
12446	0.946132	0.173753
12447	1.24596	0.0890495
12448	0.94613	0.127826
12449	0.946131	0.157331
12450	0.946131	0.0556143
12451	0.946131	0.157627
12452	0.946131	0.168283
12453	0.946131	2.1
12454	0.946131	2.1
12455	0.946131	2.1
12456	0.946128	2.1
12457	0.946133	2.1
12458	0.94613	2.1
12459	0.946131	2.1
12460	0.879548	0.791456
12461	1.21527	0.748932
12462	0.967506	0.765765
12463	0.946132	0.92933
12464	0.946133	0.702259
12465	0.967511	1.02685
12466	0.946132	0.956217
12467	0.946132	0.978465

12468	0.946132	0.809885
12469	0.974253	0.83789
12470	0.974253	1.08369
12471	0.91801	0.828763
12472	0.918009	0.847886
12473	0.946132	0.962225
12474	0.946132	0.05
12475	0.946131	0.05
12476	0.946131	0.05
12477	0.946133	0.05
12478	0.946132	0.05
12479	1.28644	0.05
12480	0.946132	0.05
12481	0.946131	0.05
12482	0.946131	0.05
12483	0.946131	0.05
12484	0.946131	0.05
12485	0.946131	0.05
12486	1.40103	0.05
12487	0.946131	0.05
12488	0.946128	0.05
12489	0.946132	0.05
12490	0.946129	0.05
12491	1.03327	0.05
12492	0.946129	0.05
12493	0.946129	0.05
12494	0.94613	0.05
12495	0.946134	0.599307
12496	0.946133	0.0670466
12497	0.946132	0.05
12498	0.946133	0.0958982
12499	0.946132	0.05
12500	0.946132	0.05
12501	0.946133	0.05
12502	0.856654	0.05
12503	0.946131	0.05
12504	0.946131	0.05
12505	0.946132	0.05
12506	0.856616	0.05
12507	1.03565	0.05
12508	1.03565	0.05
12509	1.15844	0.510895
12510	0.946132	0.667371
12511	0.946132	0.221582
12512	0.946132	0.388072
12513	0.946132	0.287375
12514	0.946132	0.601687
12515	0.946131	0.530916
12516	1.4582	2.1
12517	1.35067	2.1
12518	0.946131	2.1
12519	1.04106	2.1
12520	0.938748	2.1
12521	0.943015	2.1
12522	1.05017	2.1
12523	0.938793	2.1
12524	0.946131	0.05
12525	0.946132	0.05

12526	0.946131	0.05
12527	0.94613	0.05
12528	0.946133	0.05
12529	0.946131	0.05
12530	0.946131	0.05
12531	0.946132	0.0869989
12532	0.946133	0.12014
12533	0.946132	0.0706468
12534	0.946133	0.275793
12535	0.946133	0.381497
12536	0.946132	0.12523
12537	0.946133	0.325969
12538	0.946128	1.19573
12539	0.946132	1.51443
12540	0.946132	1.20494
12541	0.94613	1.23573
12542	1.23426	1.31096
12543	0.94613	1.29182
12544	0.946131	1.31688
12545	0.810943	0.729133
12546	0.810946	0.770572
12547	2.60388	0.774568
12548	1.3517	0.752582
12549	1.08133	0.736379
12550	1.08132	0.773164
12551	1.08132	0.736239
12552	0.810933	0.329847
12553	0.810945	1.02673
12554	0.810945	0.0949909
12555	2.72377	2.1
12556	1.3517	1.22955
12557	1.08133	0.582533
12558	1.08132	0.914431
12559	1.08132	2.1
12560	0.810932	1.32088
12561	1.25104	0.05
12562	0.946131	0.998022
12563	0.946131	0.0758791
12564	1.36188	2.1
12565	0.946131	1.06635
12566	1.36188	1.36495
12567	0.946132	1.32975
12568	0.946132	2.1
12569	0.946132	2.1
12570	0.946132	2.1
12571	0.946132	2.1
12572	0.946132	2.1
12573	0.946132	2.1
12574	1.25488	2.1
12575	1.32132	0.05
12576	0.946126	0.05
12577	0.946127	0.05
12578	0.946128	0.05
12579	0.946128	0.05
12580	0.946128	0.05
12581	0.946128	0.05
12582	0.946133	2.1
12583	1.41123	2.03957

12584	0.946134	2.1
12585	0.946133	2.1
12586	0.946132	0.164733
12587	0.946133	2.1
12588	0.946132	2.1
12589	1.32163	0.05
12590	0.946133	0.05
12591	0.946133	0.05
12592	0.946132	0.05
12593	0.946132	0.05
12594	0.946132	0.05
12595	0.946133	0.05
12596	0.946133	2.1
12597	0.946133	2.1
12598	0.946132	2.1
12599	0.946132	2.1
12600	0.946132	1.84187
12601	0.946133	2.1
12602	0.946132	2.1
12603	0.94613	0.106483
12604	0.946131	0.0653976
12605	0.946128	0.0906026
12606	0.946133	0.05
12607	0.946133	0.05
12608	0.946131	0.116767
12609	0.946131	0.0716179
12610	0.946133	0.446365
12611	0.946132	0.0959542
12612	0.946132	0.617616
12613	0.946134	0.0788013
12614	0.946133	1.0589
12615	0.946133	0.05
12616	0.946132	0.555104
12617	0.94613	1.88712
12618	0.946128	2.1
12619	0.946127	1.58248
12620	0.94613	1.48616
12621	0.946131	1.79466
12622	0.946128	1.74964
12623	1.21099	2.05725
12624	0.946132	0.442336
12625	0.946131	0.514049
12626	0.946131	0.834272
12627	0.946133	0.05
12628	0.946133	0.103899
12629	0.946131	0.435227
12630	0.946132	0.234186
12631	1.34794	0.464695
12632	0.517994	0.300308
12633	0.517965	0.128561
12634	0.946132	0.73649
12635	0.9198	1.71742
12636	0.946132	1.20158
12637	0.9198	1.04596
12638	0.946132	1.1546
12639	0.94613	1.18721
12640	0.946131	0.392033
12641	0.946131	1.18172

12642	0.946131	1.604
12643	0.946131	1.19492
12644	1.23224	0.954738
12645	0.946132	1.89633
12646	0.946132	2.08154
12647	0.946133	1.54093
12648	0.946132	0.884955
12649	0.946132	1.38153
12650	0.946133	1.53837
12651	1.37511	1.62106
12652	0.946133	0.556267
12653	0.946129	0.613627
12654	0.851104	0.983507
12655	0.946131	0.660703
12656	1.0412	0.821425
12657	1.0412	0.815717
12658	0.851066	0.787134
12659	1.31357	0.05
12660	0.946131	0.05
12661	0.94613	0.05
12662	0.946131	0.05
12663	0.94613	0.05
12664	0.946131	0.05
12665	0.946131	0.05
12666	0.946133	1.00095
12667	1.38149	1.1412
12668	0.946132	1.14246
12669	0.946132	0.985376
12670	0.946133	1.69576
12671	0.946132	1.05058
12672	0.946133	1.07391
12673	0.810944	1.15721
12674	0.810945	2.1
12675	0.810958	2.1
12676	2.65782	2.1
12677	1.35168	2.1
12678	1.08132	1.17711
12679	1.08132	2.1
12680	1.0813	1.17608
12681	0.946113	0.05
12682	0.946119	0.05
12683	0.946118	0.05
12684	0.946118	0.05
12685	0.946119	0.05
12686	0.946131	0.05
12687	0.946106	0.05
12688	0.946132	0.05
12689	0.946132	0.05
12690	0.946128	0.05
12691	0.946131	0.05
12692	0.94613	0.05
12693	0.94613	0.05
12694	0.94613	0.05
12695	1.00099	0.05
12696	0.946132	0.05
12697	1.17876	0.05
12698	0.982282	0.05
12699	0.909981	0.05

12700	0.946132	0.05
12701	0.946131	0.05
12702	0.946132	1.04437
12703	1.35328	1.2903
12704	0.946132	1.89727
12705	0.946132	0.935858
12706	0.946133	1.71995
12707	0.946132	0.99521
12708	0.946133	1.482
12709	0.946133	0.05
12710	0.946133	0.05
12711	0.946132	0.05
12712	0.946132	0.05
12713	0.946132	0.05
12714	0.946132	0.05
12715	0.946133	0.05
12716	0.900195	0.05
12717	0.946132	0.05
12718	0.900167	0.05
12719	1.41963	0.05
12720	0.900195	0.05
12721	0.946133	0.05
12722	1.35385	0.05
12723	0.946132	1.29241
12724	0.946129	1.90551
12725	0.946129	1.84447
12726	0.946131	1.2682
12727	0.946131	1.9501
12728	1.41585	1.38708
12729	0.94613	2.03847
12730	0.810946	2.1
12731	0.810946	2.1
12732	2.68895	2.1
12733	1.3517	2.1
12734	1.08133	2.1
12735	1.08132	2.1
12736	1.08132	2.1
12737	0.810933	2.1
12738	1.19886	2.1
12739	0.946131	2.1
12740	0.946131	2.1
12741	1.34447	2.1
12742	0.946131	2.1
12743	1.34447	2.1
12744	0.946131	2.1
12745	0.946133	0.05
12746	0.946133	0.05
12747	0.946132	0.05
12748	1.35065	0.05
12749	0.946132	0.05
12750	0.946133	0.05
12751	0.946132	0.05
12752	0.946131	0.387489
12753	0.946131	0.56802
12754	0.94613	0.720865
12755	0.946131	0.05
12756	0.946131	0.0866781
12757	0.946131	0.379179

12758	0.946131	0.209861
12759	0.946127	0.05
12760	0.946131	0.05
12761	0.946127	0.05
12762	0.94613	0.05
12763	0.946131	0.05
12764	0.946128	0.05
12765	0.946129	0.05
12766	0.810946	0.05
12767	0.810947	0.05
12768	0.810959	0.05
12769	2.4391	0.05
12770	1.35168	0.05
12771	1.08132	0.05
12772	1.08132	0.05
12773	1.08131	0.05
12774	0.946132	0.05
12775	0.946132	0.05
12776	0.946132	0.05
12777	1.40837	0.05
12778	0.946132	0.05
12779	0.946132	0.05
12780	1.40837	0.05
12781	0.946127	2.09747
12782	0.946132	2.1
12783	0.946131	1.01083
12784	0.946127	2.1
12785	0.946131	1.51855
12786	0.946129	1.12125
12787	0.94613	1.5735
12788	0.946133	2.09024
12789	0.946132	1.69245
12790	0.946132	1.64342
12791	0.946132	1.38896
12792	0.946132	1.8516
12793	0.946132	1.19529
12794	0.946133	2.1
12795	0.946132	0.851837
12796	1.24344	0.694427
12797	0.946132	0.717119
12798	0.946132	0.993269
12799	0.946132	0.754855
12800	1.24344	0.746282
12801	0.946132	0.977731
12802	0.946131	0.517383
12803	0.946128	0.710771
12804	0.946128	0.338829
12805	0.946131	0.42957
12806	1.41232	0.369564
12807	0.946129	0.689262
12808	0.94613	0.543699
12809	0.946128	2.1
12810	0.946133	2.1
12811	0.946133	0.208317
12812	0.946131	2.1
12813	0.946131	0.61773
12814	0.946131	2.1
12815	0.94613	2.1

12816	0.946133	0.166801
12817	1.41556	0.112439
12818	0.946133	0.0710855
12819	0.946133	0.0563703
12820	0.946132	0.404686
12821	0.946132	0.107599
12822	0.946132	0.234448
12823	0.94612	0.114865
12824	1.41843	0.0617666
12825	0.946114	0.062027
12826	0.94612	0.05505
12827	0.946119	0.26355
12828	1.41845	0.0628345
12829	0.946107	0.201762
12830	0.946129	0.05
12831	0.946129	0.05
12832	0.94613	0.05
12833	0.946132	0.05
12834	0.946126	0.05
12835	1.1509	0.05
12836	0.94613	0.05
12837	0.946131	0.05
12838	0.946132	0.05
12839	0.946132	0.05
12840	0.946132	0.05
12841	0.946132	0.05
12842	1.19263	0.05
12843	0.946132	0.05
12844	0.94613	0.255139
12845	0.946131	0.311407
12846	0.946128	0.320296
12847	0.946133	0.556024
12848	0.946133	0.833496
12849	0.946131	0.201798
12850	0.946131	0.657592
12851	0.946133	1.22378
12852	1.22848	1.48565
12853	1.00336	1.47967
12854	0.827866	1.27265
12855	0.946132	1.85695
12856	0.827865	1.42391
12857	0.946133	1.33809
12858	0.946131	0.598841
12859	0.94613	0.70155
12860	0.946127	0.679992
12861	1.41498	0.761455
12862	0.94613	0.725713
12863	0.946128	0.40833
12864	0.946128	0.74906
12865	0.94613	0.05
12866	0.94613	0.05
12867	0.94613	0.05
12868	1.37358	0.05
12869	0.946129	0.05
12870	0.94613	0.05
12871	1.37357	0.05
12872	0.946111	0.05
12873	0.946118	0.05

12874	0.946118	0.05
12875	1.36568	0.05
12876	0.946116	0.05
12877	0.94613	0.05
12878	0.946105	0.05
12879	0.946132	0.0850052
12880	0.946131	0.05
12881	0.94613	0.214926
12882	1.23175	0.05
12883	0.946131	0.05
12884	0.946131	0.0819946
12885	0.946131	0.0963215
12886	0.946132	0.05
12887	0.946126	0.05
12888	0.946128	0.05
12889	0.946129	0.05
12890	0.946129	0.05
12891	0.946128	0.05
12892	1.25698	0.05
12893	0.810943	2.1
12894	0.810944	2.1
12895	2.81171	2.1
12896	1.3517	2.1
12897	1.08132	2.1
12898	1.08133	2.1
12899	1.08132	2.1
12900	0.810932	2.1
12901	0.946132	0.0552858
12902	0.946131	0.0683308
12903	0.946131	0.101636
12904	0.946132	0.05
12905	0.946132	0.05
12906	0.946132	0.0732871
12907	0.946131	0.05
12908	0.810936	0.189252
12909	0.810935	2.1
12910	2.80914	2.1
12911	1.35167	1.92837
12912	1.0813	1.20407
12913	1.0813	2.1
12914	1.0813	0.154124
12915	0.810934	1.40981
12916	1.23851	2.1
12917	0.946132	0.607506
12918	0.946131	2.1
12919	1.40459	0.21569
12920	0.946132	2.1
12921	1.40459	2.1
12922	0.946132	2.1
12923	1.19974	0.05
12924	0.946131	0.05
12925	1.19974	0.05
12926	0.946132	0.05
12927	0.946132	0.05
12928	0.946132	0.05
12929	0.946132	0.05
12930	0.939141	1.17081
12931	1.14375	1.43323

12932	1.03306	1.43326
12933	0.946132	1.24899
12934	0.946126	1.60181
12935	0.946129	1.34171
12936	0.94613	1.27207
12937	0.946132	0.0627847
12938	0.946131	0.0931075
12939	0.946132	0.0874538
12940	0.946132	0.05
12941	0.946132	0.0507788
12942	0.946132	0.215068
12943	0.946132	0.0884944
12944	0.946131	2.1
12945	0.94613	2.1
12946	0.94613	2.1
12947	0.946131	2.1
12948	0.946129	2.1
12949	0.94613	2.1
12950	1.24512	2.1
12951	0.946133	0.05
12952	0.946129	0.05
12953	0.946131	0.05
12954	0.946132	0.05
12955	0.946131	0.05
12956	0.946131	0.05
12957	0.946131	0.05
12958	0.946132	0.05
12959	0.946133	0.05
12960	0.946132	0.05
12961	0.946132	0.05
12962	0.946133	0.05
12963	0.946132	0.05
12964	0.946132	0.05
12965	0.946132	0.475442
12966	1.41392	0.21544
12967	0.946132	0.241493
12968	0.946132	0.714993
12969	0.946132	0.0931931
12970	0.946132	0.520698
12971	0.946132	0.454729
12972	0.946132	1.04385
12973	1.24333	0.749556
12974	0.946132	0.689908
12975	0.946133	1.14605
12976	0.946132	0.11934
12977	0.946132	1.11786
12978	0.946133	0.940695
12979	1.30649	0.05
12980	0.94613	0.05
12981	0.94613	0.05
12982	0.946128	0.05
12983	0.946126	0.05
12984	0.946127	0.05
12985	0.946127	0.05
12986	0.709578	0.05
12987	0.810943	0.05
12988	2.53303	0.05
12989	1.21651	0.05

12990	0.979951	0.05
12991	1.08132	0.05
12992	0.946141	0.05
12993	0.81096	0.05
12994	0.946131	0.05
12995	0.946131	0.05
12996	0.946131	0.05
12997	0.946131	0.05
12998	0.946131	0.05
12999	0.946131	0.05
13000	0.946131	0.05
13001	0.946132	0.0564444
13002	0.946129	0.0531536
13003	0.946129	0.05
13004	0.94613	0.05
13005	1.20634	0.05
13006	1.20634	0.05
13007	0.946131	0.0568547
13008	0.946133	0.792196
13009	0.946132	0.748622
13010	0.946132	0.73258
13011	0.946132	0.800274
13012	0.946132	0.726616
13013	1.30107	0.787661
13014	0.946133	0.792361
13015	0.810946	2.1
13016	0.810944	2.1
13017	2.73936	2.1
13018	1.35168	2.1
13019	1.0813	2.1
13020	1.08132	2.1
13021	1.08132	2.1
13022	0.810958	2.1
13023	0.946129	2.1
13024	0.946129	2.1
13025	0.946133	0.978998
13026	1.30812	2.1
13027	0.946131	1.32444
13028	0.946131	1.97073
13029	0.946131	2.02309
13030	1.00901	1.29804
13031	0.94613	1.90205
13032	0.94613	0.948923
13033	1.17001	1.41659
13034	0.917259	0.242292
13035	0.94613	1.14023
13036	0.94613	1.1648
13037	0.946126	2.1
13038	0.946127	2.1
13039	0.946131	2.1
13040	0.946127	2.1
13041	0.946131	2.1
13042	0.946129	2.1
13043	1.398	2.1
13044	0.946133	2.1
13045	1.3033	2.1
13046	0.946132	2.1
13047	0.946132	2.1

13048	0.946132	2.1
13049	0.946132	2.1
13050	0.946133	2.1
13051	0.946131	0.0911634
13052	0.946131	0.0990438
13053	1.09897	0.0527693
13054	0.946129	0.312314
13055	0.946131	0.184711
13056	0.94613	0.269553
13057	0.94613	0.0860983
13058	0.946132	2.1
13059	0.946131	2.1
13060	0.946131	2.1
13061	0.946132	2.1
13062	0.94613	2.1
13063	0.946131	2.1
13064	1.39858	2.1
13065	1.07117	0.908321
13066	1.08878	1.04754
13067	0.946119	1.11499
13068	1.08878	1.33784
13069	0.946119	0.826539
13070	0.946132	1.02771
13071	1.07116	0.920077
13072	0.946132	0.05
13073	0.946132	0.05
13074	0.946132	0.05
13075	0.946132	0.05
13076	0.946132	0.05
13077	1.10733	0.05
13078	0.946132	0.05
13079	0.946128	0.05
13080	0.946132	0.05
13081	0.94613	0.05
13082	0.946129	0.05
13083	0.94613	0.05
13084	1.3367	0.05
13085	0.94613	0.05
13086	0.810943	2.1
13087	0.810944	2.1
13088	2.70234	2.1
13089	1.21653	2.1
13090	1.08132	2.1
13091	0.946155	2.1
13092	0.979951	2.1
13093	0.709578	2.1
13094	1.27174	2.1
13095	0.946129	2.1
13096	0.94613	2.1
13097	1.35117	2.1
13098	0.94613	2.1
13099	1.35117	2.1
13100	0.94613	2.1
13101	0.946133	0.766306
13102	0.946133	0.639993
13103	0.946132	2.04133
13104	0.946133	0.423493
13105	0.946132	2.1

13106	0.946133	1.94053
13107	0.997296	2.01841
13108	0.810943	2.1
13109	0.810944	2.1
13110	0.810945	2.1
13111	2.78833	2.1
13112	1.35169	2.1
13113	1.08132	2.1
13114	1.08132	2.1
13115	1.08132	2.1
13116	0.810946	2.1
13117	0.810943	1.11226
13118	0.810933	1.58225
13119	2.70049	2.1
13120	1.3517	1.27703
13121	1.08132	2.1
13122	1.08132	0.90758
13123	1.08133	1.14367
13124	0.946127	1.34204
13125	1.35469	1.16383
13126	0.94613	1.10898
13127	0.946131	1.37172
13128	0.946128	0.853166
13129	1.35469	1.4329
13130	0.946129	1.49488
13131	0.946131	0.05
13132	1.2722	0.05
13133	0.946132	0.05
13134	0.946132	0.05
13135	0.946129	0.05
13136	1.2722	0.05
13137	0.94613	0.05
13138	0.946133	0.464464
13139	0.946132	0.0826383
13140	0.946132	0.420168
13141	0.946132	0.496157
13142	1.41461	0.305627
13143	1.41461	0.303483
13144	0.946133	0.659318
13145	0.946131	0.659891
13146	1.30165	0.167187
13147	0.946132	0.105102
13148	0.946132	1.06259
13149	0.946129	0.196906
13150	1.30165	1.00361
13151	0.946131	1.13172
13152	0.946132	1.18613
13153	0.946129	2.1
13154	0.94613	1.17358
13155	1.39356	2.1
13156	0.946131	1.2763
13157	0.946131	1.30716
13158	0.94613	2.05787
13159	0.810946	2.1
13160	0.810947	2.1
13161	0.810934	2.1
13162	2.35003	2.1
13163	1.3517	2.1

13164	1.08132	2.1
13165	1.08132	2.1
13166	1.08133	2.1
13167	0.946131	0.05
13168	0.946131	0.05
13169	0.946125	0.05
13170	0.946127	0.05
13171	0.946129	0.05
13172	0.946128	0.05
13173	0.946128	0.05
13174	0.946131	0.05
13175	0.946131	0.05
13176	0.946131	0.05
13177	0.946131	0.05
13178	0.946132	0.05
13179	0.946132	0.05
13180	0.946131	0.05
13181	0.946129	2.1
13182	0.946132	2.1
13183	0.946132	2.1
13184	0.94613	2.1
13185	1.30082	2.1
13186	0.94613	2.1
13187	0.946131	2.1
13188	1.20416	0.05
13189	0.946132	0.05
13190	0.946131	0.05
13191	0.946132	0.05
13192	0.946131	0.05
13193	0.946131	0.05
13194	0.946132	0.05
13195	1.18711	0.05
13196	0.946131	0.05
13197	0.946131	0.05
13198	0.946132	0.05
13199	0.946131	0.05
13200	0.946131	0.05
13201	0.946132	0.05
13202	0.946133	0.05
13203	0.946133	0.05
13204	0.946132	0.05
13205	1.30212	0.05
13206	0.946133	0.05
13207	0.946132	0.05
13208	1.30212	0.05
13209	0.946131	0.05
13210	0.946131	0.05
13211	0.946131	0.05
13212	0.946132	0.05
13213	1.31467	0.05
13214	1.31467	0.05
13215	0.946131	0.05
13216	0.946133	0.05
13217	0.946132	0.05
13218	0.946132	0.05
13219	0.946132	0.05
13220	1.25124	0.05
13221	1.25124	0.05

13222	0.946132	0.05
13223	0.946132	2.1
13224	0.946132	2.1
13225	0.946132	2.1
13226	1.36422	2.1
13227	0.946132	2.1
13228	0.946131	2.1
13229	0.946132	2.1
13230	0.946131	1.16384
13231	1.41851	2.02881
13232	0.946132	2.1
13233	0.946132	1.09594
13234	0.946132	1.76539
13235	0.946132	1.14088
13236	0.946132	1.66488
13237	0.946133	0.05
13238	0.946132	0.05
13239	0.946132	0.05
13240	0.946132	0.05
13241	0.946132	0.05
13242	1.37794	0.05
13243	0.946132	0.05
13244	0.946132	2.1
13245	0.946132	2.1
13246	0.946131	2.1
13247	0.946131	2.1
13248	0.946132	2.1
13249	0.946132	2.1
13250	1.4045	2.1
13251	0.946133	2.1
13252	0.946132	2.1
13253	0.946131	2.1
13254	0.946132	2.1
13255	0.946131	2.1
13256	0.946131	2.1
13257	0.946132	2.1
13258	0.946132	2.1
13259	0.946132	2.1
13260	0.946131	2.1
13261	0.946132	2.1
13262	0.946131	2.1
13263	0.946131	2.1
13264	0.946131	2.1
13265	0.946128	2.1
13266	1.31641	2.1
13267	0.946129	2.1
13268	0.94613	2.1
13269	0.94613	2.1
13270	0.94613	2.1
13271	0.94613	2.1
13272	0.946132	2.1
13273	0.946131	2.1
13274	0.946131	1.69184
13275	0.946132	2.1
13276	0.946132	2.1
13277	0.946132	2.1
13278	0.946132	2.1
13279	0.946132	2.1

13280	1.41507	2.1
13281	0.946132	2.1
13282	0.946132	2.1
13283	0.946133	2.1
13284	0.946133	2.1
13285	0.946132	2.1
13286	0.946132	2.1
13287	0.946131	2.1
13288	0.946131	2.1
13289	0.946133	2.1
13290	0.946132	2.1
13291	1.32608	2.1
13292	0.946132	2.1
13293	0.94613	2.03232
13294	0.94613	2.1
13295	0.94613	0.98729
13296	0.946131	2.1
13297	0.946129	1.38276
13298	0.94613	1.03042
13299	0.94613	1.30648
13300	1.33728	1.02858
13301	0.946131	0.931079
13302	0.94613	0.560979
13303	0.946132	1.02589
13304	0.946132	1.64237
13305	0.946131	0.908407
13306	0.946131	0.599884
13307	0.946127	2.1
13308	1.40641	2.1
13309	0.946129	2.1
13310	0.94613	2.1
13311	0.946128	1.26185
13312	0.946129	2.1
13313	0.946129	2.1
13314	0.946129	2.1
13315	0.946128	2.1
13316	0.946133	2.1
13317	1.37467	2.1
13318	0.946131	2.1
13319	0.946131	2.1
13320	1.37467	2.1
13321	0.946131	2.1
13322	0.946131	2.1
13323	0.946132	2.1
13324	0.946132	2.1
13325	0.946129	2.1
13326	0.946131	2.1
13327	1.34912	2.1
13328	0.946132	2.1
13329	0.946133	2.1
13330	0.946133	2.1
13331	0.946132	2.1
13332	0.946132	2.1
13333	0.946133	2.1
13334	0.946132	2.1
13335	0.946127	2.1
13336	0.946131	2.1
13337	0.946132	2.1

13338	0.946131	2.1
13339	0.946127	2.1
13340	0.94613	2.1
13341	1.33607	2.1
13342	0.946117	0.231532
13343	0.946117	0.896798
13344	0.946115	1.07109
13345	0.946124	2.1
13346	0.946119	0.238636
13347	1.32662	0.944199
13348	0.94613	0.653263
13349	0.946119	2.1
13350	0.946114	2.1
13351	0.946118	2.1
13352	0.946114	2.1
13353	0.946118	2.1
13354	0.946104	2.1
13355	0.946129	2.1
13356	0.946132	2.1
13357	0.946133	2.1
13358	0.946133	2.1
13359	0.946133	2.1
13360	0.946132	2.1
13361	0.946132	2.1
13362	0.946133	2.1
13363	0.946133	2.1
13364	0.946132	2.1
13365	0.946132	2.1
13366	0.946133	2.1
13367	0.946132	2.1
13368	0.946132	2.1
13369	0.946132	2.1
13370	0.946133	1.96854
13371	1.30881	2.1
13372	0.946132	2.1
13373	0.946131	1.28813
13374	0.946131	1.63666
13375	0.946131	1.20399
13376	0.946131	2.00887
13377	0.946128	2.1
13378	0.946128	2.1
13379	0.946132	2.1
13380	0.94613	2.1
13381	0.94613	2.1
13382	0.94613	2.1
13383	1.34652	2.1
13384	0.946133	0.660886
13385	0.946133	0.568164
13386	0.946132	2.04171
13387	0.946133	0.472236
13388	0.946132	2.1
13389	0.946132	1.29924
13390	0.946132	1.69477
13391	0.946132	0.05
13392	0.946128	0.0543463
13393	0.94613	0.05
13394	0.946131	0.05
13395	0.94613	0.05

13396	0.94613	0.05
13397	0.94613	0.05
13398	0.946128	0.0517116
13399	0.946132	0.05
13400	0.94613	0.05
13401	0.94613	0.05
13402	0.946131	0.070695
13403	0.94613	0.0547782
13404	0.94613	0.05
13405	0.946129	1.06023
13406	1.32056	1.47351
13407	0.946131	2.034
13408	0.94613	1.06093
13409	0.946124	1.68652
13410	0.946128	1.1174
13411	0.946127	1.21531
13412	0.946129	1.11687
13413	1.20351	1.43931
13414	0.946132	1.72549
13415	0.946132	1.15979
13416	0.946126	1.75845
13417	0.946129	1.24582
13418	0.946129	1.23287
13419	0.946127	1.65794
13420	0.946127	2.1
13421	0.946131	1.15312
13422	1.25592	1.84137
13423	0.946127	1.2061
13424	0.946129	1.34129
13425	0.94613	1.4603
13426	1.02332	2.06838
13427	0.946133	1.31479
13428	0.946132	1.34845
13429	1.02332	1.46465
13430	0.946132	1.05752
13431	0.96196	1.59127
13432	0.930306	1.19902
13433	0.946133	2.1
13434	0.946133	2.1
13435	0.946132	2.1
13436	1.20057	2.1
13437	0.946132	2.1
13438	0.946133	2.1
13439	1.20057	2.1
13440	0.946128	1.68742
13441	0.946129	1.03792
13442	0.946129	1.29409
13443	1.13245	1.04273
13444	0.946126	0.919026
13445	0.946128	1.15086
13446	0.946129	1.02744
13447	0.946131	1.6778
13448	0.946131	1.99488
13449	0.94613	2.1
13450	1.25	1.95236
13451	0.946129	2.1
13452	0.946131	2.1
13453	1.25	2.1

13454	0.540425	2.08264
13455	0.748493	2.09913
13456	1.46266	2.1
13457	1.20046	2.1
13458	0.600454	2.1
13459	1.41283	2.1
13460	0.582567	2.0999
13461	0.946131	1.81513
13462	0.946131	1.71287
13463	0.946132	1.66402
13464	0.946132	1.82332
13465	0.94613	1.58635
13466	0.946131	2.1
13467	1.28439	2.1
13468	0.946132	0.856977
13469	0.946131	0.676257
13470	0.946131	1.02603
13471	0.946132	0.706085
13472	0.946132	1.05885
13473	1.39335	0.521764
13474	0.946132	0.756456
13475	0.946131	0.0587726
13476	0.946131	0.166043
13477	0.94613	0.05
13478	0.946132	0.05
13479	0.946131	0.0959122
13480	0.946131	0.110724
13481	0.946131	0.05
13482	0.946131	0.0967143
13483	0.94613	0.127986
13484	0.946126	0.279931
13485	0.946131	0.0664017
13486	0.946125	0.761611
13487	0.946127	0.0751904
13488	0.946128	0.40222
13489	0.946131	0.05
13490	0.946131	0.05
13491	0.946131	0.05
13492	0.946131	0.05
13493	0.946131	0.05
13494	0.946131	0.05
13495	1.39168	0.05
13496	0.946131	0.106624
13497	0.946126	0.102597
13498	0.946127	0.27652
13499	0.946131	0.155205
13500	0.94613	0.0539729
13501	1.412	0.0754322
13502	0.946128	0.111789
13503	0.948901	0.05
13504	0.943364	0.05
13505	1.03768	0.05
13506	0.946132	0.0723073
13507	0.946133	0.0974831
13508	1.03767	0.0919004
13509	0.946132	0.05
13510	1.00902	0.183194
13511	0.88324	0.100414

13512	0.971584	0.079945
13513	0.946132	0.168764
13514	0.946129	0.834624
13515	0.946131	0.471657
13516	0.971572	0.616686
13517	0.946131	0.05
13518	0.946125	0.05
13519	1.33587	0.05
13520	0.946126	0.05
13521	0.946127	0.05
13522	0.946126	0.05
13523	0.946128	0.05
13524	0.946132	0.05
13525	0.946132	0.05
13526	1.27512	0.05
13527	0.946133	0.05
13528	0.946132	0.05
13529	0.946133	0.05
13530	0.946133	0.05
13531	0.946128	0.05
13532	0.946133	0.05
13533	0.946131	0.05
13534	0.94613	0.05
13535	0.946131	0.05
13536	0.94613	0.05
13537	0.946131	0.05
13538	0.946131	0.05
13539	0.946131	0.05
13540	0.946131	0.05
13541	0.946131	0.05
13542	0.946131	0.05
13543	0.946131	0.05
13544	0.946131	0.05
13545	0.94613	0.05
13546	0.946131	0.05
13547	0.946131	0.05
13548	0.94613	0.05
13549	0.946132	0.05
13550	0.946131	0.05
13551	1.35316	0.05
13552	0.94613	0.183488
13553	0.94613	0.17509
13554	0.946128	0.294885
13555	0.946132	0.149442
13556	0.946132	0.161059
13557	1.28727	0.140318
13558	0.94613	0.181311
13559	0.946131	0.05
13560	0.946132	0.05
13561	0.946132	0.05
13562	1.39502	0.05
13563	0.946132	0.05
13564	0.946132	0.05
13565	0.946132	0.05
13566	0.946125	0.081842
13567	0.946131	0.0581184
13568	0.94613	0.05
13569	0.946129	0.0849015

13570	0.946126	0.289908
13571	0.946127	0.160959
13572	1.36173	0.18478
13573	0.946129	1.05008
13574	1.02363	0.975652
13575	0.94613	0.971675
13576	0.946131	1.07009
13577	0.946131	0.912281
13578	1.02363	1.04363
13579	0.946131	1.03826
13580	0.946128	0.05
13581	1.24678	0.05
13582	0.94613	0.05
13583	0.94613	0.05
13584	0.94613	0.05
13585	0.946131	0.05
13586	0.94613	0.05
13587	0.946129	0.05
13588	1.25215	0.05
13589	0.946132	0.05
13590	0.946126	0.05
13591	0.946129	0.05
13592	0.94613	0.05
13593	0.946129	0.05
13594	0.946129	0.87895
13595	0.946133	0.893353
13596	0.946133	0.774582
13597	0.946131	0.776236
13598	0.946131	0.860004
13599	1.29689	0.349726
13600	0.946131	0.432189
13601	0.946128	0.05
13602	0.946132	0.05
13603	0.946132	0.05
13604	0.94613	0.05
13605	1.22716	0.05
13606	1.22716	0.05
13607	0.946131	0.05
13608	0.946127	0.05
13609	0.946131	0.05
13610	0.946131	0.05
13611	0.946128	0.05
13612	1.30463	0.05
13613	1.30463	0.05
13614	0.946129	0.05
13615	1.29958	0.05
13616	0.946132	0.05
13617	0.946132	0.05
13618	0.946132	0.05
13619	0.946132	0.05
13620	0.946132	0.05
13621	0.946133	0.05
13622	1.26201	0.05
13623	0.946133	0.05
13624	0.946132	0.05
13625	0.946132	0.05
13626	0.946132	0.05
13627	0.946133	0.05

13628	0.946132	0.05
13629	0.946133	0.05
13630	0.946132	0.05
13631	0.946132	0.05
13632	0.946133	0.05
13633	0.946132	0.05
13634	1.40427	0.05
13635	0.946133	0.05
13636	0.946132	0.05
13637	0.946132	0.05
13638	0.946132	0.05
13639	0.946132	0.05
13640	0.946132	0.05
13641	1.35123	0.05
13642	0.946131	0.05
13643	0.946128	0.05
13644	0.946132	0.05
13645	0.946132	0.05
13646	0.946129	0.05
13647	1.22144	0.05
13648	1.22144	0.05
13649	0.946131	0.05
13650	0.946127	0.237961
13651	0.946132	0.21672
13652	0.946131	0.203612
13653	0.946127	0.172875
13654	0.946131	0.287668
13655	1.23351	0.0831147
13656	0.94613	0.154814
13657	1.18119	0.05
13658	0.946131	0.05
13659	0.946132	0.05
13660	0.946132	0.05
13661	0.94613	0.05
13662	0.946131	0.05
13663	0.946131	0.05
13664	0.946132	0.076558
13665	0.946132	0.05
13666	0.946133	0.104688
13667	1.30563	0.05
13668	0.946132	0.05
13669	0.946132	0.0719365
13670	0.946133	0.150722
13671	1.20256	0.0954782
13672	0.946132	1.50925
13673	0.946133	1.11232
13674	0.946132	0.0697619
13675	0.946132	0.977981
13676	0.946132	1.20682
13677	0.946133	0.43887
13678	0.94613	0.875079
13679	0.94613	0.46548
13680	0.946128	1.17029
13681	1.33007	0.276514
13682	0.946129	0.312574
13683	0.94613	0.880067
13684	0.946131	1.18437
13685	0.946127	2.1

13686	0.946127	2.1
13687	0.946131	2.1
13688	1.30136	2.1
13689	0.946128	2.1
13690	0.946129	2.1
13691	1.30136	2.1
13692	0.810942	2.1
13693	0.810942	2.1
13694	0.81093	2.1
13695	2.3208	2.1
13696	1.3517	2.1
13697	1.08132	2.1
13698	1.08132	2.1
13699	1.08133	2.1
13700	0.946132	2.1
13701	0.946132	2.1
13702	0.946132	2.1
13703	0.946132	2.1
13704	0.946132	2.1
13705	1.39823	2.1
13706	0.946132	2.1
13707	1.37713	2.1
13708	0.946131	2.1
13709	0.946131	2.1
13710	0.946131	2.1
13711	0.946131	2.1
13712	0.946131	2.1
13713	0.946131	2.1
13714	0.946128	2.1
13715	0.946128	2.1
13716	0.946133	2.1
13717	0.946131	2.1
13718	0.946131	2.1
13719	0.946131	2.1
13720	0.94613	2.1
13721	0.946129	2.1
13722	0.946129	2.1
13723	0.946133	2.1
13724	0.946131	2.1
13725	0.946131	2.1
13726	0.946131	2.1
13727	0.946131	2.1
13728	0.946132	1.19545
13729	1.33694	2.1
13730	0.946133	2.1
13731	0.946133	1.15793
13732	0.946132	2.1
13733	0.946132	1.22669
13734	0.946133	1.63938
13735	0.946131	2.1
13736	0.94613	2.1
13737	0.946131	2.1
13738	0.946133	2.1
13739	0.946129	2.1
13740	0.946131	2.1
13741	0.946131	2.1
13742	0.946132	2.08539
13743	1.35943	2.1

13744	0.946132	2.1
13745	0.946132	1.22991
13746	0.946131	1.45647
13747	0.946132	1.14643
13748	0.946132	2.09502
13749	0.946132	0.05
13750	0.946132	0.05
13751	0.946133	0.05
13752	0.946133	0.05
13753	0.946132	0.05
13754	0.946132	0.05
13755	0.946132	0.05
13756	1.29322	0.269084
13757	0.946131	0.181789
13758	0.946132	0.05
13759	0.946129	0.515371
13760	0.946131	0.269797
13761	0.946131	0.0774506
13762	0.946131	0.500257
13763	0.946132	0.285675
13764	0.946132	2.1
13765	0.946131	0.05
13766	1.41492	0.05
13767	0.946132	1.40139
13768	0.946132	0.05
13769	0.946131	0.0736798
13770	0.946132	2.1
13771	0.946132	2.1
13772	0.946132	2.1
13773	1.35028	2.1
13774	0.946132	2.1
13775	0.946132	2.1
13776	0.946132	2.1
13777	0.946132	1.56813
13778	0.946132	2.1
13779	0.946132	2.1
13780	1.32461	2.1
13781	0.946131	2.1
13782	0.946132	2.1
13783	0.946132	2.1
13784	0.946131	2.1
13785	0.946131	2.1
13786	0.946131	2.1
13787	0.946132	2.1
13788	0.946129	2.1
13789	0.94613	2.1
13790	1.40369	2.1
13791	0.946132	0.05
13792	0.946131	0.05
13793	0.946131	0.05
13794	0.979598	0.05
13795	0.946131	0.05
13796	0.946131	0.05
13797	0.946132	0.05
13798	0.946131	2.1
13799	1.41897	2.1
13800	0.946132	2.1
13801	0.946132	2.1

13802	0.94613	2.1
13803	0.946131	2.1
13804	0.946131	2.1
13805	0.946131	2.1
13806	0.94613	2.1
13807	0.946132	2.1
13808	0.946132	2.1
13809	0.946129	2.1
13810	0.946131	2.1
13811	1.37791	2.1
13812	0.946127	2.1
13813	0.946131	2.1
13814	0.946131	2.1
13815	0.94613	2.1
13816	0.946128	2.1
13817	0.946129	2.1
13818	1.39384	2.1
13819	0.810945	2.1
13820	0.810944	1.42309
13821	0.810958	1.88055
13822	2.70852	2.1
13823	1.35168	1.61891
13824	1.08132	2.07184
13825	1.08132	1.20854
13826	1.0813	1.53796
13827	1.3335	2.1
13828	0.946132	2.1
13829	0.94613	2.1
13830	0.946132	2.1
13831	0.946131	2.1
13832	0.94613	2.1
13833	0.946131	2.1
13834	0.946132	2.1
13835	0.946131	0.0983142
13836	0.94613	2.1
13837	1.41192	2.1
13838	0.946132	0.0548943
13839	0.946131	1.75497
13840	0.94613	0.615909
13841	0.946133	0.777572
13842	0.946133	0.758021
13843	0.946132	0.703022
13844	1.39428	0.694442
13845	0.946132	0.725644
13846	0.946132	0.343287
13847	0.946133	0.732217
13848	0.94613	0.991547
13849	0.946129	2.1
13850	0.94613	1.15644
13851	0.946128	1.14251
13852	1.40684	2.1
13853	0.94613	1.11987
13854	0.94613	2.1
13855	0.946131	1.10155
13856	0.946131	2.1
13857	0.946131	2.1
13858	0.946131	2.07855
13859	1.39021	2.1

13860	0.946131	2.1
13861	0.946131	2.1
13862	0.946131	1.99894
13863	0.946131	2.1
13864	0.946131	1.19643
13865	0.946131	2.1
13866	0.946131	2.1
13867	0.946131	1.1542
13868	0.946131	1.41232
13869	0.810946	2.1
13870	0.810946	2.1
13871	2.69264	2.1
13872	1.35168	2.1
13873	1.0813	2.1
13874	1.08132	2.1
13875	1.08132	2.1
13876	0.810958	2.1
13877	1.16687	2.1
13878	0.946131	2.1
13879	0.946131	2.1
13880	1.34632	2.1
13881	0.946131	2.1
13882	1.34632	2.1
13883	0.946131	2.1
13884	0.946128	0.998493
13885	0.946133	1.01545
13886	0.946133	0.914765
13887	0.946131	0.981468
13888	0.946131	0.92881
13889	1.3564	0.932131
13890	0.94613	0.93207
13891	0.946128	2.1
13892	0.946132	2.1
13893	0.94613	2.1
13894	0.94613	2.1
13895	0.946131	2.1
13896	1.35827	2.1
13897	0.94613	2.1
13898	0.946133	0.0726794
13899	0.946132	0.563734
13900	0.946132	0.176333
13901	0.946133	0.411269
13902	0.946133	0.374765
13903	0.946132	0.278048
13904	0.946133	0.458417
13905	0.946132	2.1
13906	0.946132	2.1
13907	0.946132	0.989598
13908	0.946131	2.1
13909	0.946132	1.29309
13910	0.946132	1.25187
13911	0.946132	1.27384
13912	0.94613	0.107623
13913	0.94613	0.05
13914	0.946128	0.115098
13915	0.946132	0.05
13916	0.946132	0.0927571
13917	0.94613	0.0539668

13918	0.946131	0.05
13919	0.946126	0.05
13920	0.946131	0.05
13921	0.946127	0.05
13922	0.946129	0.05
13923	0.94613	0.05
13924	0.946128	0.05
13925	0.946128	0.05
13926	0.946131	0.05
13927	0.946127	0.05
13928	0.946127	0.05
13929	0.946131	0.05
13930	0.946131	0.05
13931	0.946128	0.05
13932	0.946129	0.05
13933	0.94613	0.05
13934	0.946131	0.05
13935	0.946129	0.05
13936	0.946132	0.05
13937	0.946132	0.05
13938	0.946131	0.05
13939	0.94613	0.05
13940	0.94613	0.0923821
13941	0.946131	0.101853
13942	0.946129	0.304338
13943	0.946132	0.144247
13944	0.946132	0.0758956
13945	1.32827	0.0963094
13946	0.946131	0.0922214
13947	0.946132	2.1
13948	0.946132	2.1
13949	0.946131	2.1
13950	0.946131	2.1
13951	0.946132	2.1
13952	0.946131	2.1
13953	0.946131	2.1
13954	0.946132	0.05
13955	0.946132	0.991494
13956	0.946131	0.0870068
13957	1.38356	0.05
13958	0.946133	0.869543
13959	0.946131	0.0734225
13960	0.946131	0.847574
13961	0.94613	2.1
13962	0.946131	2.1
13963	0.946131	2.1
13964	0.946128	2.1
13965	0.946133	2.1
13966	0.946131	2.1
13967	1.36918	2.1
13968	0.946131	0.947655
13969	0.94613	2.1
13970	0.946129	1.31969
13971	0.946133	2.1
13972	0.946132	1.59567
13973	1.3771	2.1
13974	0.946131	2.1
13975	1.30029	1.12996

13976	0.94613	0.878094
13977	0.946129	1.44192
13978	0.946132	1.42259
13979	0.946132	0.579652
13980	0.946131	1.05465
13981	0.94613	0.92326
13982	0.946133	0.05
13983	0.946132	0.05
13984	0.946132	0.05
13985	0.946132	0.05
13986	0.946132	0.05
13987	0.946132	0.05
13988	1.21174	0.05
13989	0.810946	2.1
13990	0.810945	2.1
13991	2.63045	2.1
13992	1.35169	2.1
13993	1.08132	2.1
13994	1.08132	2.1
13995	1.08132	2.1
13996	0.810946	2.1
13997	1.1796	2.1
13998	0.946131	2.1
13999	0.946132	2.1
14000	1.31522	2.1
14001	0.946132	2.1
14002	1.31522	2.1
14003	0.946132	2.1
14004	0.946131	2.1
14005	1.40615	2.1
14006	0.946129	2.1
14007	0.946128	2.1
14008	0.946133	2.1
14009	0.946131	2.1
14010	0.94613	2.1
14011	0.810945	2.1
14012	0.810944	2.1
14013	2.80479	2.1
14014	1.35169	2.1
14015	1.08132	2.1
14016	1.08132	2.1
14017	1.08132	2.1
14018	0.810945	2.1
14019	1.35562	2.1
14020	0.94613	2.1
14021	1.35561	2.1
14022	0.946133	2.1
14023	0.946131	2.1
14024	1.40239	2.1
14025	0.946131	2.1
14026	0.946125	0.05
14027	0.946131	0.0722996
14028	0.946127	0.05
14029	0.946129	0.05
14030	0.946128	0.0852494
14031	0.946129	0.135622
14032	0.946128	0.05
14033	0.946119	2.1

14034	0.946113	2.1
14035	0.94612	2.1
14036	0.946119	2.1
14037	1.41915	2.1
14038	0.946107	2.1
14039	0.946132	2.1
14040	0.810946	1.51644
14041	0.810943	2.1
14042	0.810934	2.1
14043	2.31411	2.1
14044	1.35171	2.1
14045	1.08132	1.95475
14046	1.08132	2.1
14047	1.08133	2.07847
14048	0.946127	2.1
14049	0.94613	2.1
14050	0.94613	2.1
14051	0.946131	2.1
14052	0.946126	2.1
14053	0.946128	2.1
14054	0.946128	2.1
14055	0.810944	2.1
14056	0.810947	2.1
14057	0.81096	2.1
14058	2.78019	2.1
14059	1.35168	2.1
14060	1.08132	2.1
14061	1.08132	2.1
14062	1.08131	2.1
14063	0.946133	0.05
14064	0.946132	0.05
14065	0.946132	0.05
14066	0.946133	0.05
14067	0.946132	0.05
14068	1.3495	0.05
14069	0.946133	0.05
14070	0.946133	2.1
14071	0.946132	2.1
14072	0.946132	2.1
14073	1.39529	2.1
14074	0.946133	2.1
14075	0.946132	2.1
14076	0.946133	2.1
14077	0.946133	2.1
14078	0.946132	2.1
14079	0.946132	2.1
14080	0.946132	2.1
14081	0.946132	2.1
14082	0.946133	2.1
14083	0.946132	2.1
14084	0.810946	2.1
14085	0.810945	2.1
14086	2.67861	2.1
14087	1.35169	2.1
14088	1.08132	2.1
14089	1.08132	2.1
14090	1.08132	2.1
14091	0.810946	2.1

14092	1.24616	2.1
14093	0.946132	2.1
14094	0.946132	2.1
14095	1.33931	2.1
14096	0.946132	2.1
14097	1.33931	2.1
14098	0.946133	2.1
14099	0.946133	0.411251
14100	0.946133	0.160179
14101	0.946132	0.208417
14102	0.946133	0.425726
14103	1.3855	0.109248
14104	1.3855	0.10427
14105	0.946133	0.331849
14106	0.946128	1.96529
14107	0.946132	2.1
14108	0.946128	2.1
14109	0.94613	2.1
14110	0.946131	2.1
14111	1.40225	2.1
14112	0.94613	2.1
14113	1.40548	0.05
14114	0.946118	0.05
14115	0.946114	0.05
14116	0.946112	0.05
14117	0.946117	0.05
14118	0.946128	0.05
14119	0.946103	0.05
14120	0.94613	2.1
14121	0.946132	2.1
14122	0.946131	2.1
14123	0.946132	2.1
14124	1.37811	2.1
14125	0.946131	2.1
14126	0.946131	2.1
14127	0.946132	2.1
14128	0.946133	2.1
14129	0.946133	2.1
14130	0.946132	2.1
14131	0.946133	2.1
14132	0.946132	2.1
14133	0.946132	2.1
14134	0.946118	2.1
14135	0.946113	2.1
14136	0.946118	2.1
14137	0.946119	2.1
14138	0.946117	2.1
14139	0.946105	2.1
14140	0.946131	2.1
14141	0.946118	0.05
14142	0.946118	0.05
14143	0.946117	0.05
14144	0.946113	0.05
14145	0.94612	0.05
14146	1.41377	0.05
14147	0.946106	0.05
14148	0.946132	1.09528
14149	1.28436	0.683267

14150	0.946131	0.726655
14151	0.946131	1.10979
14152	0.946131	0.113271
14153	0.94613	1.14752
14154	0.946131	0.925969
14155	0.810947	0.0996881
14156	0.810946	0.180062
14157	2.68675	0.05
14158	1.35168	0.0539924
14159	1.08132	0.0949596
14160	1.08132	0.0722368
14161	1.0813	0.147662
14162	0.81096	0.0907173
14163	0.946132	0.05
14164	1.34338	0.05
14165	0.946132	0.05
14166	1.34338	0.05
14167	0.946132	0.0599025
14168	1.34337	0.05
14169	0.946132	0.05
14170	0.946127	2.1
14171	0.946131	2.1
14172	0.946127	2.1
14173	0.946131	1.96084
14174	0.946131	2.1
14175	0.946128	2.1
14176	1.30281	2.1
14177	0.946132	0.406899
14178	0.946132	0.271876
14179	0.946132	0.371079
14180	0.946131	0.249985
14181	0.946132	0.57517
14182	1.37973	0.0561958
14183	0.946132	0.218933
14184	0.946131	0.898891
14185	1.41798	0.407075
14186	0.946132	0.166348
14187	0.946132	0.519068
14188	0.946129	1.0485
14189	0.946131	0.420826
14190	0.946131	1.00806
14191	0.946118	0.621396
14192	1.41776	0.243449
14193	0.946113	0.252012
14194	0.946119	0.238816
14195	0.946117	0.844837
14196	1.41778	0.245829
14197	0.946105	0.911577
14198	0.946131	1.00015
14199	1.41798	1.04915
14200	0.946132	1.14057
14201	0.946132	0.690635
14202	0.94613	1.0772
14203	1.41799	0.489097
14204	0.946131	0.602517
14205	0.946133	2.1
14206	0.946132	2.1
14207	0.946132	0.942536

14208	0.946132	2.1
14209	0.946133	1.56373
14210	0.946132	2.1
14211	0.946132	2.1
14212	0.946131	2.1
14213	0.946131	2.1
14214	0.94613	1.05234
14215	0.946129	2.1
14216	0.946132	1.34393
14217	0.94613	1.24483
14218	0.946131	2.1
14219	0.94612	1.36578
14220	0.946126	2.1
14221	0.946119	1.26187
14222	0.94612	1.68079
14223	1.40766	2.1
14224	0.946132	1.17558
14225	0.946107	2.1
14226	0.946128	2.1
14227	1.31213	2.1
14228	0.946129	2.1
14229	0.94613	2.1
14230	0.94613	2.1
14231	0.94613	2.1
14232	0.94613	2.1
14233	0.946127	2.1
14234	0.946131	2.1
14235	0.94613	2.1
14236	0.94613	2.1
14237	1.36653	2.1
14238	0.946128	2.1
14239	0.946128	2.1
14240	0.946127	2.1
14241	0.94613	2.1
14242	0.946131	2.1
14243	0.94613	2.1
14244	0.946128	2.1
14245	0.946128	2.1
14246	0.946129	2.1
14247	0.946133	2.1
14248	1.40081	2.1
14249	0.946132	2.1
14250	0.946132	2.1
14251	0.946133	2.1
14252	0.946132	2.1
14253	0.946133	2.1
14254	0.946128	2.1
14255	1.32282	2.1
14256	0.946129	2.1
14257	0.94613	2.1
14258	0.946131	2.1
14259	0.946131	2.1
14260	0.946131	2.1
14261	0.810945	2.1
14262	0.810943	2.1
14263	0.810933	2.1
14264	2.44097	2.1
14265	1.3517	2.1

14266	1.08132	2.1
14267	1.08132	2.1
14268	1.08133	2.1
14269	0.946131	2.1
14270	0.94613	2.1
14271	0.946133	2.1
14272	0.946133	2.1
14273	0.946128	2.1
14274	0.946131	2.1
14275	1.27676	2.1
14276	0.946126	0.914442
14277	0.946126	1.00149
14278	0.946131	0.839369
14279	0.946127	0.995212
14280	0.94613	0.367062
14281	0.946128	0.721428
14282	0.946128	0.140756
14283	0.946132	0.05
14284	0.946133	0.05
14285	0.946132	0.05
14286	0.946132	0.05
14287	0.946132	0.05
14288	0.946133	0.05
14289	0.946132	0.05
14290	0.946128	0.05
14291	0.946131	0.05
14292	0.94613	0.05
14293	0.946129	0.05
14294	0.94613	0.05
14295	0.94613	0.0908783
14296	0.94613	0.05
14297	0.946132	0.05
14298	0.946128	0.05
14299	0.94613	0.05
14300	0.946131	0.05
14301	0.94613	0.05
14302	0.94613	0.05
14303	0.946131	0.05
14304	0.94612	0.05
14305	0.946119	0.05
14306	0.946119	0.05
14307	0.946124	0.05
14308	0.946119	0.05
14309	0.946132	0.05
14310	0.946107	0.05
14311	0.946128	2.1
14312	0.946133	2.1
14313	0.946131	2.1
14314	0.94613	2.1
14315	0.946131	2.1
14316	1.37869	2.1
14317	0.946131	2.1
14318	1.35703	2.1
14319	0.946132	2.1
14320	0.946132	2.1
14321	0.946132	2.1
14322	0.946132	2.1
14323	0.946132	2.1

14324	0.946133	2.1
14325	0.946133	2.1
14326	0.946133	2.1
14327	0.946132	2.1
14328	1.37017	2.1
14329	0.946133	2.1
14330	0.946132	2.1
14331	0.946133	2.1
14332	0.946114	0.05
14333	0.946119	0.05
14334	0.946119	0.05
14335	0.94612	0.05
14336	0.946119	0.05
14337	0.946132	0.05
14338	0.946107	0.05
14339	0.946131	0.05
14340	0.946131	0.05
14341	0.946126	0.05
14342	0.946127	0.05
14343	0.94613	0.05
14344	0.946129	0.05
14345	0.946128	0.05
14346	0.946131	1.30145
14347	0.946131	1.03245
14348	0.946132	0.594977
14349	0.946132	1.27624
14350	0.94613	1.5237
14351	0.946131	1.5982
14352	1.3279	1.52889
14353	0.946132	2.1
14354	1.40932	2.1
14355	0.946132	2.1
14356	0.946132	2.1
14357	0.946132	2.1
14358	0.946131	2.1
14359	0.946132	2.1
14360	0.946131	0.05
14361	0.946131	0.05
14362	0.946132	0.05
14363	0.946132	0.05
14364	0.94613	0.05
14365	0.946131	0.05
14366	1.26868	0.05
14367	0.810943	2.1
14368	0.810945	2.1
14369	0.810932	2.1
14370	2.82321	2.1
14371	1.3517	2.1
14372	1.08132	2.1
14373	1.08132	2.1
14374	1.08133	2.1
14375	0.810943	0.05
14376	0.810944	0.15522
14377	2.63079	0.159549
14378	1.35168	0.264034
14379	1.0813	0.337667
14380	1.08132	0.067762
14381	1.08132	0.508304

14382	0.810957	0.234636
14383	1.17856	0.05
14384	0.946132	0.05
14385	0.946128	0.118795
14386	1.3154	0.266599
14387	0.94613	0.177
14388	1.31539	0.0830167
14389	0.94613	1.10578
14390	0.94613	1.69135
14391	0.94613	1.62501
14392	0.946131	1.4733
14393	0.946131	1.72038
14394	0.946129	1.78211
14395	0.94613	2.1
14396	1.21997	2.1
14397	0.946131	1.21549
14398	1.25083	0.501505
14399	0.946132	0.440579
14400	0.946132	1.30244
14401	0.946129	0.32319
14402	1.25083	1.33678
14403	0.946131	1.38489
14404	0.946127	1.34737
14405	1.41342	0.948408
14406	0.94613	1.2699
14407	0.946131	1.09484
14408	0.946127	0.672277
14409	0.946129	0.78263
14410	0.946129	1.0139
14411	0.946131	1.82965
14412	1.29149	1.84122
14413	0.946133	2.1
14414	0.946133	1.14151
14415	0.946128	1.62462
14416	0.946131	0.951601
14417	0.94613	1.37
14418	0.94613	2.1
14419	0.94613	1.94701
14420	0.946132	2.1
14421	0.946132	1.55249
14422	0.946128	0.162372
14423	0.94613	2.1
14424	1.41022	2.1
14425	0.946132	0.207753
14426	0.946132	0.05
14427	0.946128	0.131027
14428	0.94613	0.0901558
14429	0.94613	0.240053
14430	0.94613	0.0942949
14431	0.946131	0.122903
14432	0.946129	1.03631
14433	1.37758	1.84517
14434	0.946131	2.1
14435	0.94613	0.918793
14436	0.946124	1.58818
14437	0.946127	0.955562
14438	0.946127	1.94915
14439	0.946129	1.03713

14440	1.38259	1.67156
14441	0.946132	2.1
14442	0.946132	0.979117
14443	0.946126	1.74167
14444	0.946129	1.02777
14445	0.94613	1.52374
14446	0.946131	0.05
14447	0.946132	0.05
14448	0.946132	0.05
14449	0.946132	0.05
14450	0.946132	0.05
14451	0.946132	0.05
14452	0.946132	0.05
14453	0.946131	0.05
14454	0.94613	0.05
14455	0.94613	0.05
14456	0.946133	0.05
14457	0.946128	0.05
14458	1.25017	0.05
14459	0.946131	0.05
14460	0.946131	2.1
14461	0.946131	2.1
14462	0.946131	2.1
14463	1.3937	2.1
14464	0.946133	2.1
14465	0.946131	2.1
14466	0.94613	2.1
14467	0.946131	0.06496
14468	0.946131	0.320006
14469	0.946131	0.0579591
14470	0.94613	0.121377
14471	0.946133	0.902165
14472	0.946132	0.05
14473	0.946131	0.377384
14474	0.946132	0.0764824
14475	0.946132	0.48882
14476	0.946132	0.05
14477	0.946132	0.05
14478	0.946131	0.0555814
14479	0.946132	0.05
14480	0.946132	0.05
14481	0.946129	0.05
14482	0.94613	0.897437
14483	0.94613	0.0831081
14484	0.946128	0.560386
14485	0.946132	0.05
14486	0.946131	0.404625
14487	0.94613	0.05
14488	0.946132	2.1
14489	0.946132	0.371268
14490	0.946132	2.1
14491	1.41619	2.1
14492	0.946133	0.05
14493	0.946133	2.07743
14494	0.946132	1.10887
14495	0.946132	2.1
14496	0.946132	2.1
14497	0.946132	2.1

14498	1.38244	2.1
14499	0.946132	2.1
14500	0.946131	2.1
14501	0.946132	2.1
14502	0.810946	0.624913
14503	0.810943	1.11694
14504	0.810934	0.849427
14505	2.42741	0.145674
14506	1.35171	0.2689
14507	1.08132	0.559299
14508	1.08132	0.669589
14509	1.08133	0.817431
14510	0.810947	0.477979
14511	0.810943	1.05177
14512	2.44179	0.132348
14513	1.35171	0.328361
14514	1.08132	0.404248
14515	1.08132	0.537578
14516	1.08133	0.368438
14517	0.810934	0.369297
14518	0.946132	1.66052
14519	0.946132	1.58728
14520	0.946133	0.98032
14521	0.946133	1.55654
14522	0.946132	2.00544
14523	0.946132	1.59286
14524	1.12786	1.42945
14525	1.27806	0.995922
14526	0.946132	1.16233
14527	0.946133	1.05127
14528	0.946133	1.30051
14529	0.946132	1.9095
14530	0.946132	1.14789
14531	0.946133	1.74495
14532	0.946132	2.1
14533	0.946132	2.1
14534	0.946132	2.1
14535	1.37098	2.1
14536	0.946133	2.1
14537	0.946132	2.1
14538	1.37098	2.1
14539	0.946125	1.20181
14540	0.946129	1.08925
14541	0.946131	2.1
14542	0.94613	0.977192
14543	0.946124	0.900667
14544	0.946127	0.98056
14545	1.39514	1.22303
14546	0.946132	2.1
14547	0.946131	2.1
14548	0.94613	2.1
14549	1.39909	2.1
14550	0.946132	2.1
14551	0.946131	2.1
14552	0.94613	2.1
14553	0.946133	2.1
14554	0.946133	0.133072
14555	0.946132	2.1

14556	1.36835	2.1
14557	0.946132	0.87559
14558	0.946133	2.1
14559	0.946132	0.316852
14560	0.946132	2.1
14561	0.946133	2.1
14562	0.946133	2.1
14563	0.946132	2.1
14564	1.39047	2.1
14565	0.946132	2.1
14566	0.946132	2.1
14567	1.33174	0.05
14568	0.946128	0.05
14569	0.94613	0.05
14570	0.94613	0.05
14571	0.94613	0.05
14572	0.94613	0.05
14573	0.946131	0.05
14574	0.946128	0.05
14575	1.36004	0.05
14576	0.946129	0.05
14577	0.94613	0.05
14578	0.94613	0.05
14579	1.36003	0.05
14580	0.94613	0.05
14581	0.946124	2.1
14582	0.946119	2.1
14583	0.946119	2.1
14584	0.946119	2.1
14585	0.94612	2.1
14586	0.946132	2.1
14587	1.29062	2.1
14588	0.946128	2.1
14589	0.946128	2.1
14590	0.946133	2.1
14591	0.94613	2.1
14592	0.946131	2.1
14593	0.946131	2.1
14594	1.30584	2.1
14595	0.946131	2.1
14596	0.946131	2.1
14597	0.946131	2.1
14598	0.946132	2.1
14599	1.39549	2.1
14600	0.946131	2.1
14601	0.946131	2.1
14602	0.913386	1.14442
14603	1.17466	1.0715
14604	1.00473	0.735059
14605	0.946132	1.08264
14606	0.946126	1.38176
14607	0.94613	1.21282
14608	0.946129	1.15503
14609	0.946132	0.916578
14610	1.38894	0.93712
14611	0.946132	0.774525
14612	0.946132	0.797562
14613	0.946132	1.021

14614	0.946132	0.925374
14615	0.946133	0.925589
14616	0.946128	0.884991
14617	1.23865	0.915572
14618	0.946129	0.439535
14619	0.94613	0.675754
14620	0.94613	0.877226
14621	0.94613	0.728594
14622	0.94613	0.732697
14623	0.946126	1.094
14624	1.40248	1.2172
14625	0.946129	1.17925
14626	0.94613	1.10726
14627	0.946124	1.61227
14628	0.946127	1.22212
14629	0.946128	1.18532
14630	0.946133	0.931016
14631	1.1391	1.00029
14632	0.946132	0.986131
14633	0.946132	0.907604
14634	0.946132	1.11744
14635	0.946133	0.950695
14636	0.946132	0.964252
14637	1.17156	1.17676
14638	0.946133	1.23267
14639	1.33893	0.943263
14640	0.827864	0.949361
14641	0.995232	0.805749
14642	0.827864	1.11519
14643	0.94613	0.864288
14644	1.21387	0.83176
14645	0.946132	0.841349
14646	0.946131	0.761151
14647	0.94613	0.807997
14648	0.94613	0.790779
14649	0.94613	0.814599
14650	0.94613	0.809551
14651	0.810943	0.11476
14652	0.810945	0.05
14653	2.42463	0.05
14654	1.3517	0.05
14655	1.08132	0.0776132
14656	1.08132	0.0804851
14657	1.08133	0.0578309
14658	0.810932	0.116809
14659	0.946133	0.927099
14660	0.946132	0.74901
14661	0.946131	0.79131
14662	1.36748	0.881586
14663	0.946133	0.716197
14664	0.946131	0.772066
14665	0.946132	0.529368
14666	1.35856	0.0667383
14667	0.946131	0.0654607
14668	0.94613	0.05
14669	0.94613	0.0650881
14670	0.946131	0.0505626
14671	0.94613	0.0706573

14672	0.94613	0.122535
14673	0.946132	2.1
14674	0.946131	2.1
14675	0.946131	2.1
14676	0.946131	2.1
14677	1.41149	2.1
14678	1.41149	2.1
14679	0.946131	2.1
14680	0.946128	2.1
14681	0.946128	2.1
14682	0.946133	2.1
14683	0.946131	2.1
14684	0.946131	2.1
14685	0.946131	2.1
14686	0.94613	2.1
14687	1.41388	0.05
14688	0.94613	0.05
14689	0.946132	0.05
14690	0.946132	0.05
14691	0.946129	0.05
14692	0.946131	0.05
14693	0.946131	0.05
14694	1.02798	0.05
14695	0.946132	0.05
14696	1.02798	0.05
14697	0.946133	0.05
14698	1.14931	0.05
14699	0.957746	0.05
14700	0.93452	0.05
14701	0.946129	2.1
14702	0.946134	2.1
14703	0.946133	2.1
14704	0.946131	2.1
14705	0.946131	2.1
14706	0.946131	2.1
14707	0.946131	2.1
14708	0.946133	1.92653
14709	0.946132	1.12927
14710	0.946132	1.5025
14711	0.946132	2.1
14712	1.38773	1.15505
14713	0.946132	1.6918
14714	0.946133	1.25485
14715	0.946131	0.05
14716	0.946131	0.05
14717	1.41512	0.05
14718	0.946133	0.05
14719	0.946131	0.05
14720	0.946131	0.05
14721	0.946131	0.05
14722	1.35381	1.08917
14723	0.946132	2.1
14724	1.35381	0.560143
14725	0.946133	2.1
14726	0.946132	1.25825
14727	0.946132	2.1
14728	0.946133	1.62723
14729	1.3365	0.790341

14730	0.946132	1.4562
14731	1.3365	0.4347
14732	0.946129	1.2529
14733	0.94613	0.736594
14734	0.946128	1.3637
14735	0.946129	1.09698
14736	0.939895	1.90457
14737	1.14285	2.1
14738	0.952366	2.1
14739	0.946131	2.1
14740	0.94613	2.1
14741	0.946131	2.1
14742	0.94613	2.1
14743	0.946131	2.1
14744	0.946131	2.1
14745	0.946131	2.1
14746	0.946129	2.1
14747	0.946133	2.1
14748	0.946131	2.1
14749	0.946131	2.1
14750	0.946133	2.1
14751	0.946132	2.1
14752	0.946132	2.1
14753	0.946133	2.1
14754	0.946132	2.1
14755	0.946132	2.1
14756	0.946133	2.1
14757	0.946133	2.1
14758	0.946132	1.4859
14759	0.946132	2.1
14760	0.946133	2.00291
14761	0.946132	2.1
14762	0.946132	2.1
14763	0.946132	1.7042
14764	0.946132	1.11442
14765	0.946131	2.1
14766	0.946131	2.01563
14767	0.946131	1.93453
14768	1.38969	2.1
14769	0.946131	2.1
14770	0.946132	2.1
14771	0.946128	2.1
14772	0.946133	1.75851
14773	0.946132	2.1
14774	0.946131	1.66512
14775	0.94613	1.88142
14776	0.946131	2.06184
14777	1.28464	1.99128
14778	0.946119	0.916149
14779	1.37492	0.987481
14780	0.946125	1.03106
14781	0.946119	0.846503
14782	0.946119	1.21774
14783	0.946132	0.870387
14784	0.946107	0.980496
14785	0.810942	2.1
14786	0.810943	2.1
14787	0.709587	2.1

14788	2.64488	2.1
14789	1.21651	2.1
14790	0.946151	2.1
14791	0.979948	2.1
14792	1.0813	2.1
14793	0.946133	1.61332
14794	0.946132	2.1
14795	0.946132	2.1
14796	0.946133	1.16547
14797	0.946132	0.965994
14798	0.946132	1.09757
14799	1.41134	1.46384
14800	0.946133	2.1
14801	0.946132	1.85881
14802	0.946133	2.1
14803	0.946133	1.29548
14804	0.946132	0.596154
14805	0.946133	1.56917
14806	1.31338	1.85128
14807	2.07612	1.95201
14808	0.946102	0.05
14809	0.752448	0.05
14810	1.20617	0.05
14811	0.94613	0.05
14812	0.75242	0.05
14813	0.946131	0.05
14814	0.752419	0.05
14815	0.946132	1.17037
14816	1.25736	1.04738
14817	1.25736	0.870498
14818	0.946132	1.10332
14819	1.08054	0.563212
14820	1.25736	0.965825
14821	0.946132	1.1596
14822	0.946128	2.1
14823	1.1896	2.1
14824	1.18959	2.1
14825	0.946131	0.706636
14826	1.33271	2.1
14827	0.946105	2.1
14828	0.946156	2.1
14829	0.741946	0.05
14830	1.33587	0.05
14831	0.741947	0.05
14832	1.21954	0.05
14833	0.94613	0.05
14834	0.741946	0.05
14835	1.81347	0.05
14836	1.81347	0.05
14837	0.653322	0.05
14838	1.36935	0.05
14839	0.661882	0.05
14840	1.04753	0.05
14841	0.507464	0.05
14842	0.945844	1.11983
14843	1.59507	2.1
14844	1.04043	1.08038
14845	0.946131	2.1

14846	1.32891	2.02715
14847	0.946131	1.30629
14848	0.946131	1.06914
14849	1.28866	1.6724
14850	1.12682	0.05
14851	0.851543	0.274225
14852	1.04072	0.0746457
14853	0.946132	0.431108
14854	1.13437	0.05
14855	1.04167	0.05
14856	0.936652	0.115931
14857	1.258 2.1	
14858	0.946131	2.1
14859	0.946132	2.1
14860	1.36968	2.1
14861	0.946132	2.1
14862	1.36968	2.1
14863	0.946131	2.1
14864	2.30046	0.05
14865	2.82969	0.05
14866	0.946132	2.1
14867	1.35022	2.1
14868	1.12356	2.07682
14869	0.94613	2.1
14870	1.35022	2.1
14871	0.946131	2.1
14872	0.94613	2.1
14873	0.946131	2.1
14874	1.22401	2.1
14875	0.952456	2.1
14876	0.94613	2.1
14877	1.22401	2.1
14878	0.946131	2.1
14879	0.946131	2.1
14880	1.33047	2.1
14881	0.946132	2.1
14882	1.33047	2.1
14883	0.946131	2.1
14884	1.33047	2.1
14885	0.946132	2.1
14886	1.2107	2.1
14887	0.61957	2.1
14888	1.80666	2.1
14889	1.53743	2.1
14890	0.619572	2.05032
14891	1.32547	2.1
14892	1.16166	2.1
14893	1.04074	2.1
14894	0.557621	2.1
14895	0.946128	2.1
14896	1.36607	2.1
14897	0.94613	2.1
14898	1.36607	2.1
14899	1.39416	2.1
14900	0.94613	2.1
14901	0.94613	2.1
14902	1.23969	2.1
14903	0.946131	2.1

14904	0.946131	2.1
14905	1.13537	2.1
14906	1.05719	2.1
14907	0.806848	2.1
14908	1.30251	2.1
14909	0.851526	1.75385
14910	1.14176	2.1
14911	0.93666	1.25614
14912	0.946114	2.1
14913	1.15832	2.00124
14914	0.924624	1.95651
14915	0.967609	1.24763
14916	0.771764	0.05
14917	1.26652	0.05
14918	0.946133	0.05
14919	1.17957	0.05
14920	0.771735	0.05
14921	0.946122	0.05
14922	0.946133	0.05
14923	0.924325	1.28964
14924	0.656739	1.37443
14925	0.637876	1.3745
14926	0.530092	1.32767
14927	0.914415	1.25348
14928	0.821311	1.8632
14929	0.630589	1.59309
14930	0.738823	1.55222
14931	0.839109	1.40969
14932	1.55029	0.05
14933	1.13537	0.05
14934	0.946131	1.08578
14935	0.946128	0.94594
14936	1.30162	2.1
14937	0.952476	1.96034
14938	1.29289	2.07798
14939	0.939783	2.1
14940	0.94612	2.1
14941	0.946131	2.1
14942	1.07998	2.1
14943	0.836985	2.1
14944	1.1604	2.1
14945	0.94613	2.1
14946	0.837016	2.1
14947	0.712461	0.565744
14948	1.36399	0.532854
14949	1.8724	0.353636
14950	1.8724	0.286353
14951	0.623426	1.11405
14952	1.36399	0.3216
14953	1.22089	0.374288
14954	0.946132	0.317512
14955	0.946131	2.1
14956	1.32241	2.1
14957	1.32241	2.1
14958	0.946133	2.1
14959	1.40271	2.1
14960	0.946131	2.1
14961	0.946131	2.1

14962	1.1989	1.46474
14963	0.954106	1.69349
14964	0.820068	1.32962
14965	0.563139	1.5869
14966	1.06448	1.63203
14967	0.892472	2.0123
14968	0.862598	1.45574
14969	0.745851	2.04453
14970	1.13336	1.61577
14971	0.946129	2.1
14972	1.28254	2.1
14973	1.28254	2.1
14974	0.946131	2.1
14975	1.41302	2.1
14976	0.946131	2.1
14977	0.946131	2.1
14978	0.851543	2.09858
14979	1.21961	2.1
14980	0.851484	1.83281
14981	0.851543	2.1
14982	1.15705	2.1
14983	0.946133	2.1
14984	0.909417	2.1
14985	1.13808	0.782584
14986	0.946133	0.607649
14987	0.946131	0.789962
14988	1.30194	0.440129
14989	0.946131	0.736883
14990	1.30194	0.261571
14991	0.946131	0.723863
14992	0.930941	1.33877
14993	1.20185	1.10875
14994	1.02404	1.15815
14995	0.946124	1.42915
14996	1.3563	1.23225
14997	0.946127	1.51977
14998	0.946128	1.30426
14999	0.946116	2.1
15000	1.3144	2.1
15001	1.13088	2.1
15002	0.946118	0.338497
15003	1.31438	2.1
15004	0.946105	2.1
15005	0.946131	2.1
15006	1.76972	0.05
15007	1.42091	0.05
15008	0.946128	0.05
15009	0.946101	0.0655748
15010	0.863552	0.07188
15011	0.94613	0.05
15012	1.21232	0.05
15013	0.94613	0.103435
15014	1.2137	0.162071
15015	1.23224	0.861996
15016	1.86512	0.280591
15017	1.86512	0.118694
15018	0.580825	1.27957
15019	1.2137	1.00587

15020	0.946133	0.99466
15021	0.580822	0.745069
15022	0.946128	2.1
15023	1.40586	2.1
15024	0.94613	2.1
15025	1.3719	2.1
15026	1.3719	2.1
15027	1.40586	2.1
15028	0.94613	2.1
15029	0.946132	2.1
15030	1.35025	2.1
15031	1.1794	2.1
15032	0.946132	1.00968
15033	1.35025	1.21794
15034	0.946131	1.24085
15035	0.946132	1.1971
15036	0.946133	2.1
15037	1.60149	2.1
15038	1.67058	2.1
15039	0.851542	2.1
15040	1.20296	2.1
15041	1.17501	2.1
15042	0.946132	2.1
15043	1.04993	2.1
15044	0.851541	2.01205
15045	1.48219	2.1
15046	0.874565	1.86756
15047	0.946129	2.1
15048	1.22049	2.1
15049	0.946131	2.1
15050	0.946131	2.1
15051	0.94613	2.1
15052	0.848719	2.1
15053	1.17195	2.1
15054	0.946129	1.34258
15055	1.35426	1.41831
15056	0.94613	1.7357
15057	0.94613	1.96316
15058	0.946133	2.1
15059	1.14342	2.1
15060	1.14342	2.1
15061	0.946133	2.1
15062	1.39009	2.1
15063	0.946132	2.1
15064	0.946132	2.1
15065	1.38819	2.1
15066	0.946133	2.1
15067	1.38819	2.1
15068	0.946131	2.1
15069	1.4116	2.1
15070	0.946131	2.1
15071	1.38819	2.1
15072	0.946127	2.1
15073	1.32244	2.1
15074	1.2176	2.1
15075	0.946131	2.1
15076	1.32244	2.1
15077	0.94613	2.1

15078	0.946129	2.1
15079	1.32415	2.1
15080	1.11239	2.1
15081	0.984099	2.1
15082	1.65532	2.1
15083	1.53082	2.1
15084	1.32068	2.1
15085	1.32068	2.1
15086	1.08172	2.1
15087	3.76293	0.05
15088	1.36335	0.05
15089	0.953925	2.1
15090	0.989497	2.1
15091	0.98949	2.1
15092	1.06341	2.1
15093	1.27875	2.1
15094	1.04598	2.1
15095	0.603154	2.1
15096	0.582457	2.1
15097	0.582457	2.1
15098	0.568994	2.1
15099	1.5791	2.1
15100	1.09506	2.1
15101	1.45327	2.1
15102	1.88406	2.1
15103	1.88404	2.1
15104	1.27389	2.1
15105	1.14206	2.1
15106	1.00232	2.1
15107	1.00231	2.1
15108	1.68436	2.1
15109	1.4677	0.05
15110	1.28828	0.05
15111	1.28828	0.05
15112	1.46932	0.05
15113	1.28228	2.1
15114	1.64268	1.33883
15115	0.959473	2.1
15116	1.10101	2.1
15117	1.07976	2.1
15118	1.2873	2.1
15119	1.70303	0.05
15120	1.40066	0.05
15121	1.5563	0.05
15122	1.13727	0.05
15123	1.03529	2.1
15124	1.11285	2.1
15125	1.03529	2.1
15126	1.28667	2.1
15127	1.32119	2.1
15128	1.44777	2.1
15129	1.44778	2.1
15130	1.61121	2.1
15131	1.09372	0.05
15132	1.17664	0.05
15133	1.17664	0.05
15134	0.872931	0.05
15135	1.02415	2.1

15136	1.28228	2.1
15137	2.14692	2.1
15138	3.90469	2.1
15139	1.00509	2.1
15140	1.28228	2.1
15141	3.32936	0.05
15142	1.1333	0.05
15143	1.11048	2.1
15144	0.979854	2.1
15145	1.65815	2.1
15146	1.00185	2.1
15147	1.84887	2.1
15148	1.59597	2.1
15149	1.78468	2.1
15150	1.87508	2.1
15151	1.82716	2.1
15152	1.33429	2.1
15153	1.34626	2.1
15154	0.797142	2.1
15155	1.00007	1.34933
15156	1.02051	2.1
15157	1.02051	1.68014
15158	1.03373	2.1
15159	1.05786	2.1
15160	0.887631	2.1
15161	0.992773	2.1
15162	1.28462	2.1
15163	2.35274	1.98391
15164	1.82533	1.24114
15165	1.82533	1.76565
15166	1.19496	1.04821
15167	1.28597	0.05
15168	1.09725	2.1
15169	1.60948	2.1
15170	1.20421	2.1
15171	1.16566	2.1
15172	1.88522	2.1
15173	1.81948	0.10271
15174	1.14042	0.05
15175	1.21558	0.105906
15176	1.56876	0.05
15177	1.6697	2.1
15178	1.81655	2.1
15179	1.81655	2.1
15180	1.5956	2.1
15181	0.906059	2.1
15182	1.63045	2.1
15183	0.9533	1.23682
15184	1.1475	1.1281
15185	1.72231	2.1
15186	1.94936	1.6195
15187	1.06826	2.1
15188	1.08576	2.1
15189	1.06826	2.1
15190	0.973559	2.1
15191	1.62737	0.05
15192	0.965676	0.05
15193	1.45055	0.05

15194	1.17631	0.05
15195	1.92535	0.05
15196	1.9503	0.05
15197	1.99828	0.05
15198	1.70302	0.05
15199	1.65139	0.05
15200	1.26784	0.05
15201	1.35923	0.05
15202	1.70302	0.05
15203	1.33033	2.1
15204	1.12481	2.1
15205	1.29452	2.1
15206	1.25399	2.1
15207	1.12481	2.1
15208	0.918358	2.1
15209	1.39332	2.1
15210	1.32368	2.1
15211	1.11712	1.00368
15212	1.07999	1.01743
15213	1.07999	0.697339
15214	1.05619	0.9748
15215	1.1766	2.1
15216	1.35735	2.1
15217	1.42456	0.05
15218	1.089	0.05
15219	1.11081	0.05
15220	2.88521	0.05
15221	1.09754	0.05
15222	1.85479	0.05
15223	1.27922	0.689313
15224	1.03556	0.286049
15225	1.07508	1.9404
15226	1.09795	2.1
15227	0.967672	0.49445
15228	1.07508	1.62467
15229	1.03933	2.05912
15230	1.02975	2.1
15231	1.02975	2.1
15232	1.01121	2.1
15233	1.24526	0.05
15234	1.72368	0.05
15235	1.89607	0.05
15236	2.13512	0.05
15237	1.34591	0.0645982
15238	1.14721	0.208574
15239	1.15539	2.1
15240	1.90938	1.08464
15241	1.90941	2.1
15242	1.89242	2.1
15243	1.18907	2.1
15244	1.20408	2.1
15245	1.20409	2.1
15246	1.22655	2.1
15247	0.701447	2.1
15248	1.57567	2.1
15249	1.38806	2.1
15250	2.09407	2.1
15251	1.07612	1.19647

15252	0.990019	1.22934
15253	1.15564	2.1
15254	1.01243	1.74687
15255	1.28956	0.05
15256	1.35146	0.05
15257	1.35145	0.0543945
15258	1.22811	0.0569576
15259	1.09662	2.1
15260	1.42486	2.1
15261	1.10106	0.05
15262	1.90814	0.05
15263	1.3946	0.05
15264	1.11926	0.05
15265	0.725839	2.1
15266	0.925068	2.1
15267	0.925074	2.1
15268	1.43282	2.1
15269	1.91756	0.946527
15270	1.74321	1.16702
15271	1.74321	0.631514
15272	1.63413	0.97124
15273	1.9194	0.05
15274	1.62246	0.05
15275	1.90456	0.05
15276	1.06578	0.05
15277	1.46019	0.05
15278	1.17036	0.05
15279	1.70233	1.25757
15280	1.53515	0.777272
15281	2.03494	2.1
15282	1.78441	2.1
15283	1.75992	2.1
15284	1.94351	2.1
15285	1.51057	0.05
15286	0.991435	0.05
15287	1.98068	0.05
15288	1.64283	0.05
15289	2.96126	0.05
15290	2.20692	0.05
15291	1.95054	0.05
15292	1.21325	0.05
15293	0.996828	0.05
15294	1.01976	0.05
15295	1.17129	2.1
15296	1.13664	2.1
15297	1.13664	2.1
15298	1.09627	2.1
15299	2.65123	2.1
15300	3.23062	2.1
15301	3.23062	2.1
15302	3.92038	2.1
15303	1.89019	0.05
15304	1.79109	0.0594473
15305	1.79109	0.05
15306	1.54197	0.05
15307	3.41824	1.76405
15308	1.93553	0.05
15309	2.26215	1.80706

15310	2.0103	0.05
15311	1.51569	1.16941
15312	1.01683	0.770412
15313	1.78595	2.1
15314	1.66537	2.1
15315	3.73426	2.1
15316	3.53905	2.1
15317	1.66537	2.1
15318	1.52903	2.1
15319	3.53905	2.1
15320	3.3837	2.1
15321	1.19901	0.739922
15322	1.35002	1.89896
15323	1.35002	0.850148
15324	1.11683	0.720949
15325	1.75326	1.62386
15326	0.712404	1.44186
15327	2.97768	2.1
15328	2.98949	2.1
15329	1.2832	2.1
15330	1.39518	2.1
15331	2.98949	2.1
15332	2.99339	2.1
15333	1.39517	2.1
15334	1.46124	2.1
15335	1.28534	2.1
15336	1.03391	2.1
15337	1.75366	1.27758
15338	1.26247	1.96297
15339	1.13621	2.1
15340	2.43994	2.1
15341	1.47904	0.05
15342	1.80085	0.05
15343	1.80085	0.05
15344	1.64656	0.05
15345	2.87331	2.1
15346	2.82863	2.1
15347	2.82859	2.1
15348	2.84673	2.1
15349	1.0961	0.05
15350	1.39448	0.056991
15351	1.21366	0.0555265
15352	1.20143	0.05
15353	1.85795	0.05
15354	1.36768	0.05
15355	1.36769	0.05
15356	1.45789	0.05
15357	1.32605	0.05
15358	1.27148	0.05
15359	1.70218	0.05
15360	1.3063	0.05
15361	1.45603	0.05
15362	1.89226	0.05
15363	1.2742	0.05
15364	2.10112	0.05
15365	1.10748	0.05
15366	1.1893	0.05
15367	1.04921	2.1

15368	1.22927	2.1
15369	1.22927	2.1
15370	1.46568	2.1
15371	0.811186	2.1
15372	0.792162	2.1
15373	0.792162	2.1
15374	0.758749	2.1
15375	1.03581	2.1
15376	0.737481	2.1
15377	2.24071	2.1
15378	1.34868	2.1
15379	1.47203	1.03067
15380	1.40967	1.08801
15381	1.40967	1.14604
15382	1.27916	1.16486
15383	1.17418	1.96351
15384	1.04921	2.1
15385	1.28291	0.938114
15386	1.3213	0.05
15387	1.46812	0.456169
15388	2.42522	0.716235
15389	1.17879	0.972539
15390	1.28291	1.00212
15391	1.28291	0.974694
15392	1.13438	1.00929
15393	1.75004	2.1
15394	1.78247	2.1
15395	1.78247	2.1
15396	1.76421	2.1
15397	2.25975	0.05
15398	3.66111	0.05
15399	3.6611	0.05
15400	3.44043	0.05
15401	1.13339	2.1
15402	1.13322	2.1
15403	1.13323	2.1
15404	1.20761	2.1
15405	1.34337	0.737041
15406	1.90912	2.1
15407	1.04399	1.18224
15408	2.00578	2.1
15409	1.04973	2.1
15410	1.22615	2.1
15411	1.93384	2.1
15412	2.62816	0.190873
15413	1.13127	2.1
15414	1.47642	2.1
15415	2.62816	2.1
15416	3.31755	0.505618
15417	1.47642	2.1
15418	1.80454	2.1
15419	0.964782	2.1
15420	0.949135	2.1
15421	0.949134	2.1
15422	0.949134	2.1
15423	1.10476	0.519002
15424	0.982858	0.05
15425	1.10938	0.361958

15426	0.982858	0.0892928
15427	1.01012	0.05
15428	0.974081	0.05
15429	1.82643	0.05
15430	1.29585	0.05
15431	1.00763	0.91913
15432	1.3294	0.5955
15433	1.3294	0.404484
15434	1.30001	1.08673
15435	1.59151	2.1
15436	1.99443	2.1
15437	1.07369	2.1
15438	1.30584	2.1
15439	1.05071	2.1
15440	0.948676	2.1
15441	1.13842	2.1
15442	1.42884	2.1
15443	1.59019	0.05
15444	1.38212	0.05
15445	1.34102	0.05
15446	1.02663	0.05
15447	0.797011	2.1
15448	0.81301	2.1
15449	0.81301	2.1
15450	0.811187	2.1
15451	0.991869	0.0528734
15452	1.23868	0.0509641
15453	1.67201	2.1
15454	1.61201	2.1
15455	1.61202	2.1
15456	1.57627	2.1
15457	0.94994	2.1
15458	1.77867	2.1
15459	0.854945	2.1
15460	0.973905	2.1
15461	1.64193	2.1
15462	1.68893	2.1
15463	1.68893	2.1
15464	1.70002	2.1
15465	2.08149	0.0809774
15466	1.69456	0.05
15467	1.67399	0.05
15468	1.83922	2.1
15469	1.91703	2.1
15470	0.967492	2.1
15471	1.13317	2.1
15472	0.980766	2.1
15473	2.06158	0.05
15474	0.986498	0.05
15475	1.76289	0.05
15476	1.17248	0.05
15477	1.51794	0.05
15478	1.30266	0.05
15479	1.03165	0.05
15480	0.947692	0.0573151
15481	1.20473	0.05
15482	0.947693	0.05
15483	1.54357	2.1

15484	0.980841	2.1
15485	1.34124	0.31682
15486	1.39818	0.691953
15487	1.39818	0.05
15488	1.48035	0.53002
15489	1.03932	2.1
15490	1.2065	2.1
15491	1.20651	2.1
15492	1.12717	2.1
15493	1.09483	2.1
15494	0.994293	2.1
15495	0.994293	2.1
15496	0.97356	2.1
15497	0.995252	0.260203
15498	1.66294	0.515967
15499	1.82775	0.152608
15500	1.42762	0.05
15501	1.04713	0.05
15502	1.05475	0.05
15503	1.29002	0.05
15504	1.05475	0.05
15505	1.17585	2.1
15506	1.39223	2.1
15507	1.39223	2.1
15508	1.71255	2.1
15509	1.56166	1.02985
15510	1.37762	0.747179
15511	1.16858	2.1
15512	1.0361	2.1
15513	1.23966	0.05
15514	1.10718	0.05
15515	1.99957	0.05
15516	0.658392	0.05
15517	1.36904	0.05
15518	1.12524	0.05
15519	1.04321	1.41379
15520	1.04627	1.40954
15521	1.91351	0.922582
15522	1.63379	1.00053
15523	1.79718	1.11346
15524	0.59634	1.19224
15525	0.958257	2.1
15526	1.06457	2.1
15527	0.958255	2.1
15528	1.03933	2.1
15529	1.14195	0.0628079
15530	1.49438	0.082381
15531	1.49438	0.0577058
15532	1.31259	2.1
15533	1.23036	2.1
15534	1.2016	2.1
15535	1.2016	2.1
15536	1.17129	2.1
15537	1.05724	1.08904
15538	1.04291	2.09636
15539	1.12341	2.1
15540	2.32525	2.1
15541	3.39038	2.1

15542	2.09374	2.1
15543	1.76185	0.734385
15544	1.7934	0.780544
15545	1.65809	0.745635
15546	1.54852	1.16207
15547	1.38766	2.1
15548	1.28765	2.1
15549	1.26599	2.1
15550	1.21397	2.1
15551	1.28765	2.1
15552	1.20045	2.1
15553	1.21398	2.1
15554	1.15234	2.1
15555	0.953634	0.05
15556	1.39132	0.05
15557	1.17285	2.1
15558	1.21408	2.1
15559	1.21408	2.1
15560	0.982848	2.1
15561	2.00101	2.1
15562	2.00973	2.1
15563	2.20314	2.1
15564	1.89318	2.1
15565	1.23267	2.1
15566	0.947921	2.1
15567	1.18872	2.1
15568	0.740752	2.1
15569	1.92269	0.119204
15570	1.53584	0.086376
15571	1.53584	0.190764
15572	1.21067	0.0690387
15573	1.40707	0.05
15574	1.50437	0.05
15575	1.50435	0.05
15576	1.45446	0.05
15577	1.47954	0.929697
15578	2.08687	1.22658
15579	2.08687	2.1
15580	1.1233	2.1
15581	1.04025	0.978462
15582	1.36005	1.90284
15583	1.47606	0.0785943
15584	1.3407	0.0804173
15585	0.758743	2.1
15586	0.716917	2.1
15587	0.716922	2.1
15588	0.672964	2.1
15589	2.40014	2.1
15590	1.96848	2.1
15591	1.96848	2.1
15592	1.53555	2.1
15593	1.06191	2.1
15594	1.29579	2.1
15595	1.29579	2.1
15596	1.71289	2.1
15597	1.78584	2.1
15598	2.15211	2.1
15599	2.00427	2.1

15600	3.41615	2.1
15601	1.66408	2.1
15602	1.73954	2.1
15603	2.91663	2.1
15604	3.65818	2.1
15605	1.93286	2.1
15606	1.08831	2.1
15607	1.31242	0.05
15608	1.11712	0.05
15609	1.26311	0.05
15610	1.31242	0.05
15611	1.10378	2.1
15612	0.968594	2.1
15613	1.48389	0.05
15614	1.49613	0.05
15615	1.04349	0.767623
15616	1.26409	0.747646
15617	1.26409	0.74387
15618	1.05724	0.549712
15619	1.65723	0.05
15620	1.83116	0.0809138
15621	2.06284	0.05
15622	2.00512	0.0585328
15623	1.83116	0.0546151
15624	2.03139	0.0864124
15625	2.20564	0.05
15626	1.90048	0.05
15627	0.963392	2.1
15628	1.01346	0.798522
15629	2.98092	2.1
15630	2.70295	2.1
15631	2.70295	2.1
15632	2.48828	2.1
15633	1.63661	2.1
15634	1.64158	2.1
15635	1.64158	2.1
15636	1.6488	2.1
15637	1.17506	0.05
15638	1.09667	0.05
15639	2.00539	0.103969
15640	1.09667	0.05
15641	1.54946	2.1
15642	1.43577	2.1
15643	1.26103	2.1
15644	1.49808	2.1
15645	1.089 0.05	
15646	1.22586	0.05
15647	2.35027	0.0777109
15648	1.37549	0.05
15649	3.33706	0.05
15650	1.69894	0.05
15651	2.00539	0.05
15652	1.53798	0.05
15653	1.28938	2.1
15654	1.20969	2.1
15655	1.20969	2.1
15656	1.15864	2.1
15657	1.7986	2.1

15658	1.75869	2.1
15659	1.58281	2.1
15660	1.7392	2.1
15661	1.30733	1.00551
15662	1.5419	1.41137
15663	1.50883	1.17908
15664	1.85439	1.06185
15665	1.73531	0.868567
15666	1.70225	0.792354
15667	1.22176	0.05
15668	1.59492	0.0668851
15669	1.59492	0.05
15670	2.15542	0.0701716
15671	3.24379	2.1
15672	2.64775	2.1
15673	3.16707	2.1
15674	1.93032	2.1
15675	1.50738	2.1
15676	1.24128	2.1
15677	1.24128	2.1
15678	0.994293	2.1
15679	2.90808	0.05
15680	2.45797	0.05
15681	1.85501	0.05
15682	2.90809	0.05
15683	0.892835	1.29774
15684	1.55871	2.1
15685	1.04615	1.16522
15686	1.66643	0.556027
15687	1.70044	2.1
15688	1.80224	2.1
15689	1.70186	0.94833
15690	3.30123	0.914749
15691	1.74789	2.1
15692	1.77446	2.1
15693	1.77901	2.1
15694	1.57995	2.1
15695	1.81518	2.1
15696	2.17716	2.1
15697	2.17716	2.1
15698	2.65123	2.1
15699	2.01899	1.01527
15700	1.77479	0.758294
15701	1.19122	0.129172
15702	1.46724	0.057456
15703	1.70944	0.05
15704	1.08929	0.05
15705	1.68454	0.05
15706	1.08929	0.05
15707	1.51067	1.17379
15708	1.06729	0.989995
15709	1.37762	1.00228
15710	1.13992	0.812881
15711	1.26397	2.1
15712	1.04083	2.1
15713	1.00278	2.1
15714	1.04084	2.1
15715	1.1016	0.05

15716	1.23477	0.05
15717	1.23477	0.05
15718	1.64985	0.05
15719	1.11117	2.1
15720	1.13006	2.1
15721	1.13006	2.1
15722	1.14721	2.1
15723	1.21946	2.1
15724	1.0186	2.1
15725	1.13178	2.1
15726	1.00133	2.1
15727	1.67046	0.540846
15728	0.986036	0.264106
15729	1.67046	0.517522
15730	1.34857	1.76598
15731	2.46016	2.1
15732	2.07161	2.1
15733	2.10732	2.1
15734	2.08371	2.1
15735	1.35602	2.1
15736	0.829065	2.1
15737	1.81667	2.1
15738	0.99406	2.1
15739	1.81667	2.1
15740	1.68073	2.1
15741	2.05736	2.1
15742	2.97311	2.1
15743	2.14011	2.1
15744	1.23411	2.1
15745	1.13632	0.05
15746	1.40862	0.05
15747	1.28055	0.05
15748	1.14305	0.05
15749	1.12035	0.05
15750	1.15483	0.05
15751	1.94099	0.05
15752	1.82126	0.05
15753	1.15481	0.05
15754	0.998957	0.05
15755	2.10078	0.05
15756	1.81617	0.05
15757	1.27581	2.1
15758	0.968536	2.1
15759	1.33916	2.1
15760	1.27581	2.1
15761	1.32784	2.1
15762	1.4259	2.1
15763	1.70377	2.1
15764	1.54231	2.1
15765	3.77569	2.1
15766	3.42809	2.1
15767	1.54231	2.1
15768	1.51298	2.1
15769	3.55866	2.1
15770	2.86136	2.1
15771	1.83301	0.585698
15772	1.26371	0.375167
15773	1.98301	0.738945

15774	2.0609	0.442277
15775	2.26047	0.262496
15776	2.00537	0.172439
15777	1.7913	0.145552
15778	1.48864	0.0809048
15779	1.09504	2.1
15780	1.39827	2.1
15781	2.28084	2.1
15782	1.05983	2.1
15783	1.33593	0.05
15784	1.31508	0.05
15785	1.31508	0.05
15786	1.09599	0.05
15787	1.30647	2.1
15788	1.28846	2.1
15789	1.28846	2.1
15790	1.26923	2.1
15791	1.2955	2.1
15792	1.09044	2.1
15793	2.12209	2.1
15794	1.26438	2.1
15795	0.911819	2.1
15796	1.38752	2.1
15797	2.57704	2.1
15798	2.71736	2.1
15799	1.42284	2.1
15800	1.41562	2.1
15801	2.69131	2.1
15802	2.36178	2.1
15803	1.41562	2.1
15804	1.39564	2.1
15805	1.32368	2.1
15806	0.908053	2.1
15807	0.908053	2.1
15808	0.629213	2.1
15809	1.8758	1.76826
15810	1.27362	1.52263
15811	1.3756	0.935428
15812	2.28749	1.23719
15813	1.84577	0.864163
15814	2.17765	0.813106
15815	1.38969	0.580305
15816	1.84578	0.808518
15817	1.32801	0.2998
15818	1.76377	0.33925
15819	1.22845	2.1
15820	1.57766	1.07382
15821	1.57766	2.1
15822	1.39067	2.1
15823	1.15743	0.05
15824	1.37721	0.05
15825	1.69055	0.05
15826	1.14858	0.05
15827	1.38333	1.97652
15828	1.10575	0.280362
15829	1.22418	2.1
15830	1.10574	2.1
15831	1.91509	0.05

15832	1.02001	0.05
15833	1.03074	0.0636032
15834	1.19122	0.05
15835	1.67887	2.1
15836	1.67597	2.1
15837	1.84984	2.1
15838	1.77775	2.1
15839	1.73571	2.1
15840	1.27921	2.1
15841	2.11851	1.9852
15842	1.27921	2.1
15843	1.42486	2.1
15844	1.12503	2.1
15845	1.12503	2.1
15846	1.30116	2.1
15847	0.568995	2.1
15848	0.557348	2.1
15849	0.557348	2.1
15850	0.53907	2.1
15851	0.633575	2.1
15852	1.33756	1.11632
15853	1.51074	2.1
15854	1.93384	0.499989
15855	0.655202	2.1
15856	0.731934	2.1
15857	0.731934	2.1
15858	1.09061	2.1
15859	0.966979	2.1
15860	1.31502	2.1
15861	1.31502	2.1
15862	1.72116	2.1
15863	1.70002	2.1
15864	1.67969	2.1
15865	1.67969	2.1
15866	1.62408	2.1
15867	1.01531	2.1
15868	1.66018	2.1
15869	1.01531	2.1
15870	0.886968	2.1
15871	1.22585	0.05
15872	1.20674	0.05
15873	1.20672	0.05
15874	1.02962	0.05
15875	0.956467	2.1
15876	2.00551	2.1
15877	0.553801	2.1
15878	0.617006	2.1
15879	1.26923	2.1
15880	1.23614	2.1
15881	1.23614	2.1
15882	1.16817	2.1
15883	2.14482	2.1
15884	1.72637	2.1
15885	1.09368	2.1
15886	1.26195	2.1
15887	0.760546	2.1
15888	1.84094	2.1
15889	2.62868	2.1

15890	2.79784	2.1
15891	2.79784	2.1
15892	3.00129	2.1
15893	1.27593	1.0567
15894	1.38877	1.91488
15895	1.06486	0.05
15896	1.2474	0.05
15897	1.76234	0.05
15898	1.8685	0.05
15899	1.69863	0.05
15900	1.29666	0.05
15901	1.91646	2.1
15902	1.8582	2.1
15903	1.8582	2.1
15904	1.7986	2.1
15905	3.44043	0.05
15906	1.94764	0.05
15907	1.94764	0.05
15908	1.36307	0.05
15909	1.32075	0.05
15910	1.12971	0.05
15911	2.06319	0.05
15912	1.94982	0.05
15913	1.77255	0.05
15914	0.979297	0.05
15915	1.59997	2.1
15916	1.93506	2.1
15917	1.94409	2.1
15918	1.9597	2.1
15919	0.57899	2.1
15920	0.953036	2.1
15921	0.953049	2.1
15922	1.53503	2.1
15923	1.39615	0.05
15924	1.06474	0.05
15925	0.947564	0.05
15926	1.45506	0.05
15927	1.17799	0.476398
15928	1.21067	0.143698
15929	1.35945	0.05
15930	2.05648	0.0731077
15931	1.36208	0.05
15932	2.15229	0.05
15933	2.89288	0.05
15934	1.95719	0.05
15935	2.25169	0.05
15936	1.52756	0.05
15937	1.19322	0.05
15938	1.23146	0.05
15939	2.45024	0.108385
15940	0.794122	0.05
15941	1.6343	0.05
15942	1.34161	0.05
15943	1.05353	0.05
15944	1.50795	0.05
15945	1.05353	0.05
15946	1.20669	0.05
15947	1.59289	2.1

15948	0.989543	2.1
15949	1.40588	2.1
15950	0.991427	2.1
15951	0.921822	1.70011
15952	1.46903	0.948693
15953	1.46903	2.1
15954	1.8214	0.670769
15955	1.39702	2.1
15956	1.25103	2.1
15957	1.25103	2.1
15958	1.10378	2.1
15959	1.71813	2.1
15960	1.39595	2.1
15961	1.386 2.1	
15962	1.13008	2.1
15963	1.31711	0.05
15964	1.70191	0.05
15965	0.893249	0.05
15966	1.42669	0.05
15967	1.23943	2.1
15968	1.1483	2.1
15969	1.04598	2.1
15970	1.1483	2.1
15971	1.00381	0.141337
15972	1.11433	0.124335
15973	0.973501	1.10934
15974	1.0756	1.08782
15975	1.20669	0.05
15976	1.18508	0.05
15977	1.18508	0.05
15978	0.983027	0.05
15979	1.43687	2.1
15980	1.71059	2.1
15981	1.71059	2.1
15982	1.66762	2.1
15983	2.8415	2.1
15984	2.40555	2.1
15985	1.91546	2.1
15986	1.61385	2.1
15987	2.01569	2.1
15988	1.33183	2.1
15989	1.23218	2.1
15990	1.23999	2.1
15991	1.01335	0.929389
15992	1.12088	1.01734
15993	1.25346	2.1
15994	1.15644	2.1
15995	1.3764	1.1323
15996	1.27558	1.00394
15997	1.2064	2.1
15998	1.15067	2.1
15999	1.01595	2.1
16000	1.15067	2.1
16001	1.41902	2.1
16002	1.30272	2.1
16003	1.30272	2.1
16004	1.19548	2.1
16005	2.99339	2.1

16006	2.98041	2.1
16007	1.46124	2.1
16008	1.42105	2.1
16009	2.98041	2.1
16010	2.86339	2.1
16011	1.42105	2.1
16012	1.42284	2.1
16013	2.31301	0.689584
16014	1.80273	0.621078
16015	2.17765	0.960939
16016	2.31301	0.660962
16017	1.76378	0.360172
16018	2.10275	0.281766
16019	2.10275	0.342003
16020	2.26047	0.245887
16021	0.97541	2.1
16022	1.40863	2.1
16023	1.40863	2.1
16024	1.30751	2.1
16025	1.20143	0.05
16026	1.19756	0.05
16027	1.43707	0.05
16028	1.28954	0.05
16029	3.34254	0.05
16030	1.92916	0.05
16031	1.80089	0.05
16032	1.1323	0.05
16033	1.47321	0.05
16034	1.23756	0.05
16035	1.87291	2.1
16036	1.53642	2.1
16037	1.53642	2.1
16038	1.5791	2.1
16039	1.0446	2.1
16040	0.992674	2.1
16041	1.12324	0.05
16042	1.29163	0.05
16043	1.29163	0.05
16044	1.15371	0.05
16045	0.996831	0.05
16046	1.00192	0.05
16047	1.0019	0.05
16048	0.958937	0.05
16049	1.07283	2.1
16050	1.25294	1.18541
16051	1.25294	1.41822
16052	1.04788	1.37304
16053	1.14496	2.1
16054	1.07652	2.1
16055	1.07652	2.1
16056	0.991406	2.1
16057	1.60976	2.1
16058	1.37	2.1
16059	1.37	2.1
16060	1.14195	2.1
16061	1.26593	1.93691
16062	1.75366	2.1
16063	1.57621	0.0669903

16064	1.24538	0.05
16065	0.969118	2.1
16066	1.04147	2.1
16067	0.969118	2.1
16068	1.22615	2.1
16069	0.98084	2.1
16070	1.01843	2.1
16071	1.09435	2.1
16072	1.50876	2.1
16073	1.5976	2.1
16074	1.84322	2.1
16075	1.84324	2.1
16076	1.70377	2.1
16077	1.20709	2.1
16078	2.54729	2.1
16079	0.974716	0.05
16080	1.54468	0.05
16081	1.39021	0.05
16082	1.70303	0.05
16083	1.22852	1.89897
16084	1.578	1.12544
16085	1.578	1.90835
16086	1.93852	0.954739
16087	2.2506	2.1
16088	1.39302	2.1
16089	1.5478	2.1
16090	1.2269	2.1
16091	1.70048	2.1
16092	1.45477	2.1
16093	1.30929	2.1
16094	1.15069	2.1
16095	1.00255	2.1
16096	1.05481	0.0758141
16097	1.05481	2.1
16098	1.1032	0.074313
16099	0.993888	2.1
16100	1.19294	2.1
16101	1.01121	0.05
16102	0.991866	0.05
16103	2.20052	0.138778
16104	1.7409	2.1
16105	1.75142	2.1
16106	1.46811	2.1
16107	1.7409	1.45258
16108	1.23267	2.1
16109	1.46811	2.1
16110	1.18872	2.1
16111	1.12665	2.1
16112	1.50045	2.1
16113	1.50045	2.1
16114	1.37704	2.1
16115	1.35308	0.990223
16116	1.29983	0.774127
16117	1.29983	0.985127
16118	0.970212	0.790884
16119	1.82272	2.1
16120	1.62784	2.1
16121	1.62784	2.1

16122	1.87691	2.1
16123	3.65294	2.1
16124	2.33374	2.1
16125	2.12888	2.1
16126	1.62229	2.1
16127	1.41496	2.1
16128	1.32707	0.685744
16129	2.18871	0.143747
16130	1.4235	0.05
16131	1.46015	0.05
16132	1.25635	0.05
16133	1.45501	0.05
16134	1.3543	0.05
16135	1.38139	2.1
16136	1.98159	2.1
16137	2.15868	2.1
16138	4.04308	2.1
16139	1.5362	2.1
16140	1.56328	2.1
16141	1.56327	2.1
16142	1.58163	2.1
16143	1.88987	0.05
16144	1.08761	0.05
16145	0.862154	0.05
16146	1.77688	0.05
16147	1.14238	2.1
16148	1.11691	2.1
16149	1.01537	2.1
16150	1.02923	2.1
16151	0.977425	0.05
16152	1.15211	0.05
16153	2.0815	2.1
16154	1.18621	2.1
16155	1.01485	2.1
16156	1.17054	2.1
16157	1.17054	2.1
16158	1.12665	2.1
16159	1.87213	0.05
16160	1.82244	0.05
16161	1.45039	0.05
16162	1.7226	0.05
16163	1.841	0.05
16164	1.79011	0.05
16165	1.8792	0.05
16166	1.45053	0.05
16167	1.08947	2.1
16168	1.5008	2.1
16169	1.5008	2.1
16170	1.83186	2.1
16171	1.04159	0.05
16172	1.2489	0.05
16173	1.2489	0.05
16174	1.33592	0.05
16175	1.51973	0.05
16176	0.980547	0.05
16177	1.51974	0.05
16178	1.15991	0.0635895
16179	1.34965	2.1

16180	1.2724	2.1
16181	1.2724	2.1
16182	1.38438	2.1
16183	1.02923	2.1
16184	1.06521	2.1
16185	1.06521	2.1
16186	1.0752	2.1
16187	2.03139	0.0659124
16188	1.92951	0.0649092
16189	1.74852	0.05
16190	1.62772	0.05
16191	1.73655	0.0549654
16192	1.76809	0.05
16193	1.62772	0.05
16194	1.1432	0.05
16195	1.09235	2.1
16196	1.48081	2.1
16197	1.46712	2.1
16198	1.89321	2.1
16199	1.94548	2.1
16200	1.5636	2.1
16201	1.5636	2.1
16202	1.21124	2.1
16203	1.52314	2.1
16204	2.0219	2.1
16205	1.51113	2.1
16206	2.0219	2.1
16207	0.987247	0.0839939
16208	1.18347	0.05
16209	1.89226	0.271083
16210	1.61441	0.05
16211	1.42884	2.1
16212	1.10961	2.1
16213	1.56991	2.1
16214	1.05337	2.1
16215	1.05337	2.1
16216	1.91547	2.1
16217	1.05931	2.1
16218	1.1729	2.1
16219	1.34526	2.1
16220	1.0593	2.1
16221	1.0856	2.1
16222	1.90854	2.1
16223	1.91674	2.1
16224	1.11345	2.1
16225	1.75067	2.1
16226	1.50022	2.1
16227	1.50022	2.1
16228	1.53619	2.1
16229	1.4748	2.1
16230	1.74431	2.0572
16231	2.02581	1.76205
16232	1.25288	1.82218
16233	1.01814	1.00487
16234	1.2881	0.55769
16235	1.2881	0.719287
16236	1.54433	0.442886
16237	1.19999	0.585768

16238	1.44056	0.139091
16239	1.02011	0.05
16240	1.17129	0.05
16241	1.13775	2.1
16242	3.34815	2.1
16243	1.60428	2.00523
16244	1.85928	2.09478
16245	1.75592	2.1
16246	1.25989	2.1
16247	1.75592	2.1
16248	1.71549	2.1
16249	1.37634	2.1
16250	1.64693	2.1
16251	1.64693	2.1
16252	1.64193	2.1
16253	1.18351	0.05
16254	2.19398	0.05
16255	2.16272	0.49462
16256	1.19837	0.352476
16257	1.04973	2.1
16258	1.20149	2.1
16259	1.60574	2.1
16260	1.11164	0.782799
16261	1.87823	0.517844
16262	1.11164	1.14439
16263	0.772882	2.1
16264	1.4714	2.1
16265	2.14242	0.45381
16266	1.12336	2.1
16267	2.8386	0.167395
16268	2.13346	2.1
16269	1.06788	0.05
16270	0.947662	0.050136
16271	1.05297	0.05
16272	1.42907	0.05
16273	2.17465	2.1
16274	2.18394	2.1
16275	1.14808	2.1
16276	1.21292	2.1
16277	1.21292	2.1
16278	1.27229	2.1
16279	1.20859	0.399157
16280	1.21073	0.951174
16281	1.21073	0.232037
16282	1.22857	0.942036
16283	1.79158	2.1
16284	1.73899	2.1
16285	1.34012	2.1
16286	1.39991	2.1
16287	1.73898	2.1
16288	1.61245	2.1
16289	1.40136	2.1
16290	1.57234	2.1
16291	1.82162	2.1
16292	3.09875	2.1
16293	1.10264	2.1
16294	1.02339	2.1
16295	1.02339	2.1

16296	1.23042	2.1
16297	2.82266	0.908689
16298	1.05698	0.384582
16299	1.96384	0.851931
16300	1.10244	0.626358
16301	0.929061	2.1
16302	1.47007	2.1
16303	0.985303	2.1
16304	1.24554	2.1
16305	1.05993	2.1
16306	2.02074	2.1
16307	2.10106	0.968562
16308	1.18171	0.919204
16309	1.68111	0.989593
16310	1.84226	0.913315
16311	1.82131	0.05
16312	1.32371	0.05
16313	1.41902	0.05
16314	1.00205	0.05
16315	1.65532	2.1
16316	1.18977	2.1
16317	1.15471	2.1
16318	0.993889	2.1
16319	2.08521	2.1
16320	2.09885	2.1
16321	2.09497	2.1
16322	2.10604	2.1
16323	0.881028	1.23472
16324	1.52828	0.999087
16325	1.40574	0.05
16326	1.09599	0.05
16327	1.45506	2.1
16328	1.50524	2.1
16329	1.50524	2.1
16330	1.54711	2.1
16331	1.41469	2.1
16332	2.40442	2.1
16333	1.40794	2.1
16334	1.89226	2.1
16335	1.31346	2.1
16336	1.51067	2.1
16337	1.31363	2.1
16338	1.11833	2.1
16339	2.3373	2.1
16340	2.19994	2.1
16341	2.19994	2.1
16342	1.09616	2.1
16343	1.63719	2.1
16344	1.52204	2.1
16345	1.52205	2.1
16346	1.40118	2.1
16347	2.5946	2.1
16348	4.07065	2.1
16349	1.33916	2.1
16350	2.05217	2.1
16351	1.67023	2.1
16352	2.05217	2.1
16353	1.59458	0.437393

16354	1.02247	0.415656
16355	1.68108	0.792065
16356	1.03079	0.707531
16357	1.1643	0.0673538
16358	1.85795	0.05
16359	1.70302	0.05
16360	1.42076	0.05
16361	1.91351	0.05
16362	1.01148	0.05
16363	2.52402	0.05
16364	0.752625	0.05
16365	0.886968	2.1
16366	1.28845	2.1
16367	1.28845	2.1
16368	1.27766	2.1
16369	1.82268	2.1
16370	1.87118	2.1
16371	1.87119	2.1
16372	1.91704	2.1
16373	3.13938	2.1
16374	2.97839	2.1
16375	2.97839	2.1
16376	2.87331	2.1
16377	1.73443	1.05976
16378	1.59457	0.936561
16379	2.01827	2.1
16380	2.04586	2.1
16381	2.04586	2.1
16382	2.00865	2.1
16383	1.19901	2.1
16384	1.16661	2.1
16385	1.13215	0.05
16386	1.96256	0.05
16387	2.34043	0.05
16388	2.04154	0.05
16389	1.58991	0.05
16390	1.45893	0.05
16391	2.56818	0.05
16392	1.70532	0.05
16393	1.17798	0.119686
16394	1.25796	0.310107
16395	1.25797	0.346065
16396	1.10719	0.637277
16397	1.51067	2.1
16398	1.60707	2.1
16399	2.06946	2.1
16400	3.19218	2.1
16401	2.19965	2.1
16402	1.30521	2.1
16403	2.23684	1.47664
16404	1.43363	2.1
16405	1.51007	2.1
16406	2.46356	2.1
16407	1.90428	2.1
16408	1.13618	2.1
16409	2.13276	0.05
16410	0.998345	0.05
16411	1.2861	0.05

16412	1.20457	0.05
16413	1.51789	2.1
16414	1.49872	2.1
16415	1.49872	2.1
16416	1.47712	2.1
16417	1.87129	0.49767
16418	0.949364	0.177499
16419	1.83142	0.504791
16420	1.55063	0.370351
16421	1.03298	0.0644142
16422	0.980664	0.196359
16423	1.51696	0.05
16424	1.34927	0.647993
16425	1.70302	0.0594116
16426	1.65952	0.05
16427	1.76658	0.05
16428	0.986848	0.05
16429	2.13757	1.51979
16430	1.98142	0.0675777
16431	1.98142	1.02626
16432	1.65411	0.142869
16433	1.1323	0.05
16434	1.46015	0.05
16435	2.15376	1.94514
16436	1.64622	2.04166
16437	0.991867	0.0753307
16438	1.58074	0.152563
16439	1.69368	2.1
16440	1.76403	2.1
16441	1.76403	2.1
16442	1.83318	2.1
16443	1.28418	0.0926296
16444	1.16851	0.05
16445	1.1685	0.05
16446	1.1726	0.05
16447	1.06455	2.1
16448	1.18773	2.1
16449	0.98511	2.1
16450	1.18774	2.1
16451	1.76476	2.1
16452	1.79825	2.1
16453	1.79825	2.1
16454	0.961895	2.1
16455	1.59589	0.05
16456	2.25975	0.05
16457	2.76339	2.1
16458	3.0551	2.1
16459	1.68761	2.1
16460	1.59561	2.1
16461	2.17257	2.1
16462	1.05845	2.1
16463	1.03512	2.1
16464	2.19011	2.1
16465	1.44859	2.1
16466	1.35897	0.913951
16467	1.35897	2.1
16468	1.17822	1.17739
16469	1.13641	2.1

16470	1.18301	2.1
16471	1.18301	2.1
16472	1.25173	2.1
16473	2.41297	2.1
16474	1.36463	2.1
16475	1.54711	2.1
16476	1.5735	2.1
16477	1.5735	2.1
16478	1.58713	2.1
16479	1.69044	0.05
16480	1.57979	0.05
16481	1.57979	0.05
16482	1.14387	0.05
16483	1.04199	2.1
16484	1.96491	2.1
16485	1.04199	2.1
16486	1.46892	2.1
16487	0.983724	2.1
16488	1.70579	2.1
16489	1.88522	2.1
16490	1.23914	2.1
16491	1.79424	2.1
16492	1.52851	2.1
16493	1.04921	2.1
16494	1.11285	2.1
16495	1.25334	0.979179
16496	1.07496	0.896582
16497	1.07497	2.1
16498	1.34852	0.948377
16499	1.27391	2.1
16500	2.20307	2.1
16501	1.81311	2.1
16502	2.10305	2.1
16503	1.21059	0.0556438
16504	1.44253	0.0657493
16505	1.9194	0.05
16506	1.21159	0.05
16507	0.947979	2.1
16508	1.01225	0.0712201
16509	0.947979	2.1
16510	1.01225	2.1
16511	1.00255	2.1
16512	1.01225	2.1
16513	1.01225	2.1
16514	1.06561	2.1
16515	3.50562	2.1
16516	3.61336	2.1
16517	3.61336	2.1
16518	3.73635	2.1
16519	1.61245	2.1
16520	1.52301	2.1
16521	1.57612	2.1
16522	1.83035	2.1
16523	1.52301	2.1
16524	1.24789	2.1
16525	1.85156	2.1
16526	1.89072	2.1
16527	0.995735	2.1

16528	1.57738	2.1
16529	1.87141	0.05
16530	1.73388	0.05
16531	1.61344	0.05
16532	0.922676	0.05
16533	1.56614	2.1
16534	0.956294	2.1
16535	1.82589	2.1
16536	1.63719	2.1
16537	1.12399	0.05
16538	1.50103	0.05
16539	1.56976	0.05
16540	1.03567	0.05
16541	1.21776	0.05
16542	1.89708	0.05
16543	1.16323	0.05
16544	1.63533	0.05
16545	2.29397	0.05
16546	3.01596	0.05
16547	1.80145	0.05
16548	1.7666	0.05
16549	1.91621	0.786694
16550	1.10719	0.739557
16551	1.57828	0.991637
16552	1.17105	1.06241
16553	1.15806	2.1
16554	1.31565	2.1
16555	1.31565	2.1
16556	1.46643	2.1
16557	1.47507	2.1
16558	1.87858	2.1
16559	0.958661	2.1
16560	1.87858	2.1
16561	1.44293	0.05
16562	1.43213	0.05
16563	1.43213	0.05
16564	1.10476	0.05
16565	1.03235	2.1
16566	1.31	0.0826673
16567	1.31	2.1
16568	1.50108	0.977645
16569	1.32215	2.1
16570	1.10611	2.1
16571	1.10611	2.1
16572	0.904978	2.1
16573	1.06486	0.05
16574	1.18347	0.05
16575	1.70535	2.1
16576	1.94779	2.1
16577	1.77071	2.1
16578	1.82268	2.1
16579	0.61701	2.1
16580	0.672495	2.1
16581	0.672494	2.1
16582	0.713174	2.1
16583	1.40395	2.1
16584	1.34245	2.1
16585	1.34243	2.1

16586	1.30646	2.1
16587	1.06204	0.05
16588	0.632691	0.575842
16589	1.75766	0.123487
16590	1.14287	0.881368
16591	1.52101	0.0538187
16592	1.71414	0.057207
16593	1.86411	0.05
16594	1.6253	0.0913742
16595	1.04355	0.05
16596	1.0443	0.115084
16597	1.34852	2.1
16598	1.56016	0.692328
16599	1.56016	0.768078
16600	1.03235	0.863959
16601	1.2966	0.05
16602	1.82354	0.05
16603	1.2966	0.05
16604	1.67687	0.05
16605	1.19669	1.13651
16606	1.82979	1.42927
16607	1.58972	1.26836
16608	1.09219	1.21617
16609	1.42077	1.1884
16610	1.89226	1.07736
16611	1.19547	2.1
16612	1.10187	2.1
16613	1.10188	2.1
16614	1.01479	2.1
16615	1.81663	2.1
16616	1.81533	2.1
16617	1.83247	2.1
16618	1.51511	2.1
16619	0.983148	2.1
16620	1.7513	2.1
16621	1.85017	2.1
16622	1.26103	2.1
16623	1.23152	2.1
16624	1.65508	2.1
16625	1.23152	2.1
16626	2.15082	2.1
16627	1.57974	2.1
16628	1.07289	2.1
16629	0.81534	2.1
16630	1.51007	2.1
16631	1.00898	0.05
16632	1.18959	0.05
16633	1.32178	0.05
16634	1.25929	0.05
16635	2.17892	0.1075
16636	1.22938	0.05
16637	3.02933	0.603969
16638	2.34619	0.611351
16639	1.47679	0.259772
16640	1.0819	0.561873
16641	3.10558	2.1
16642	3.31457	2.1
16643	0.985109	2.1

16644	1.10667	2.1
16645	3.31457	2.1
16646	3.54443	2.1
16647	1.10667	2.1
16648	1.25987	2.1
16649	1.77063	1.22833
16650	1.51065	1.02488
16651	1.24688	2.1
16652	1.37652	2.1
16653	1.37652	2.1
16654	1.54168	2.1
16655	1.17042	1.09355
16656	1.13092	1.32501
16657	1.13092	1.598
16658	1.52938	2.1
16659	1.11787	0.535224
16660	1.00613	0.358903
16661	0.781373	0.138023
16662	1.6885	0.121835
16663	2.49439	0.073305
16664	1.42657	0.125436
16665	1.77903	2.1
16666	2.63606	2.1
16667	2.10425	2.1
16668	1.21425	2.1
16669	1.31253	1.22427
16670	1.28418	1.5915
16671	1.28418	1.19483
16672	1.58972	1.24284
16673	0.858948	2.1
16674	1.90196	2.1
16675	1.81024	2.1
16676	1.90194	2.1
16677	1.4263	0.0814346
16678	0.947921	0.0604538
16679	1.22852	0.963418
16680	1.09194	0.86965
16681	1.60578	0.05
16682	1.14721	0.05
16683	1.06336	0.05
16684	1.14721	0.05
16685	1.11924	0.05
16686	1.53814	0.05
16687	1.18421	0.05
16688	1.8458	0.05
16689	1.7565	2.1
16690	1.09344	1.53432
16691	1.09344	1.23192
16692	1.16971	2.1
16693	1.71465	0.05
16694	1.19412	0.05
16695	1.64985	0.05
16696	1.05417	0.05
16697	1.2127	0.05
16698	1.48867	0.05
16699	1.27806	0.05
16700	1.21269	0.05
16701	1.37813	1.14661

16702	1.0417	1.17718
16703	1.88626	0.107405
16704	1.14977	0.842004
16705	1.78803	2.1
16706	1.33793	1.2478
16707	1.42266	2.1
16708	1.26865	1.3562
16709	1.09518	2.1
16710	1.36844	2.1
16711	1.20455	2.1
16712	1.36844	2.1
16713	1.22297	0.985275
16714	1.11256	1.34569
16715	1.11256	0.746468
16716	1.03217	1.06447
16717	1.18422	0.05
16718	1.4282	0.05
16719	1.04159	0.05
16720	1.4282	0.05
16721	0.988805	2.1
16722	1.05481	2.1
16723	1.52938	1.06596
16724	1.26593	1.5062
16725	1.53082	2.1
16726	1.09847	2.1
16727	1.75211	0.115695
16728	1.22222	2.1
16729	1.75211	0.05
16730	1.51592	2.1
16731	1.63413	0.361447
16732	1.26662	0.543582
16733	1.42495	0.113253
16734	1.4356	0.160763
16735	1.29389	1.50781
16736	1.04164	2.1
16737	1.40574	1.53591
16738	1.04164	1.29524
16739	2.22344	0.760386
16740	1.45945	0.05
16741	0.987248	0.066851
16742	1.2474	0.05
16743	0.974702	0.985547
16744	1.43777	0.859887
16745	1.71289	2.1
16746	1.91529	2.1
16747	1.91529	2.1
16748	2.01827	2.1
16749	1.87111	1.27945
16750	1.00864	0.866718
16751	1.26865	0.947518
16752	1.18769	1.2346
16753	1.24167	2.1
16754	1.36133	0.126664
16755	2.28168	2.1
16756	3.98524	2.1
16757	1.36133	2.1
16758	1.69436	0.543258
16759	2.21926	2.1

16760	1.70302	2.1
16761	1.50732	0.05
16762	1.42237	0.05
16763	1.18659	0.05
16764	1.50732	0.05
16765	1.19785	0.05
16766	1.40969	0.05
16767	1.28339	0.05
16768	1.40882	0.05
16769	0.968595	2.1
16770	1.41903	2.1
16771	1.12567	2.1
16772	1.15898	2.1
16773	1.27241	2.1
16774	0.995796	2.1
16775	1.89983	0.05
16776	1.05417	0.05
16777	0.571999	0.05
16778	0.743597	0.05
16779	1.89353	0.05
16780	0.924068	0.05
16781	1.52851	2.1
16782	1.26969	2.1
16783	1.07763	0.05
16784	1.82301	0.05
16785	1.07763	0.05
16786	1.17506	0.05
16787	1.20045	2.1
16788	1.12486	2.1
16789	1.15234	2.1
16790	1.11598	2.1
16791	1.12486	2.1
16792	1.0491	2.1
16793	1.11598	2.1
16794	1.1047	2.1
16795	1.05902	2.1
16796	1.14361	2.1
16797	1.14361	2.1
16798	1.26851	2.1
16799	1.29389	1.24677
16800	0.988456	1.21163
16801	1.83688	0.05
16802	1.70302	0.05
16803	1.49386	0.05
16804	1.83151	0.05
16805	1.01346	0.224845
16806	0.983607	0.652429
16807	0.983596	2.1
16808	1.08332	0.400321
16809	2.36848	0.0591867
16810	2.97066	0.05
16811	1.12352	0.05
16812	2.5311	0.05
16813	1.09062	2.1
16814	0.962182	2.1
16815	0.962181	2.1
16816	1.2258	2.1
16817	0.974888	2.1

16818	1.06312	2.1
16819	1.73583	0.05
16820	1.72399	0.05
16821	1.724 0.05	
16822	1.50951	0.05
16823	1.26755	2.1
16824	1.03824	2.1
16825	1.27875	2.1
16826	1.03824	2.1
16827	1.18902	2.1
16828	1.534 2.1	
16829	1.11764	2.1
16830	1.534 2.1	
16831	1.52008	2.1
16832	1.34406	2.1
16833	1.34406	2.1
16834	1.2187	2.1
16835	0.963571	1.10293
16836	1.19612	1.0115
16837	0.963571	2.1
16838	1.00007	2.1
16839	0.903333	2.1
16840	1.12724	2.1
16841	1.23771	2.1
16842	1.17683	2.1
16843	1.20059	0.548872
16844	0.772023	0.0528233
16845	1.14749	0.490937
16846	1.2802	0.967599
16847	1.85091	1.5846
16848	2.11193	1.24986
16849	1.2802	0.139317
16850	1.42913	0.971962
16851	2.11193	1.16413
16852	1.27737	0.40282
16853	1.30584	2.1
16854	1.60936	2.1
16855	1.3413	0.05
16856	1.53336	0.05
16857	2.60679	0.05
16858	1.3413	0.05
16859	1.35877	2.1
16860	1.11024	2.1
16861	1.27839	2.1
16862	1.08171	2.1
16863	1.27841	2.1
16864	1.0577	2.1
16865	1.71255	2.1
16866	1.10611	2.1
16867	0.960106	2.1
16868	1.40921	2.1
16869	1.90705	0.0779791
16870	2.79564	0.0627381
16871	1.45321	0.0759393
16872	1.90705	0.0722993
16873	0.974718	0.05
16874	1.53671	0.05
16875	1.69039	0.05

16876	1.78229	0.05
16877	1.0101	2.1
16878	1.3167	2.1
16879	1.19699	2.1
16880	1.3212	2.1
16881	1.60811	0.0645643
16882	1.43224	0.05
16883	1.35401	0.0549725
16884	1.17464	0.05
16885	1.27696	2.1
16886	1.05025	2.1
16887	1.05025	2.1
16888	1.18076	2.1
16889	1.37224	1.64505
16890	1.13438	1.49071
16891	1.76788	1.37189
16892	1.73444	1.32513
16893	1.37704	2.1
16894	1.77042	2.1
16895	1.77042	2.1
16896	1.09778	2.1
16897	1.28667	2.1
16898	1.28228	2.1
16899	1.30646	0.777259
16900	1.68867	0.932855
16901	1.30645	1.09318
16902	0.986494	2.1
16903	1.6253	0.05
16904	1.48676	0.0646379
16905	1.48676	0.05
16906	1.32974	0.0564862
16907	0.634347	0.05
16908	1.15142	0.05
16909	1.11395	0.05
16910	1.18068	0.05
16911	1.36904	0.05
16912	0.764563	0.05
16913	2.09213	0.05
16914	1.56631	2.1
16915	1.08011	2.1
16916	1.80283	2.1
16917	1.50738	2.1
16918	1.44951	0.05
16919	1.50102	0.05
16920	1.50102	0.05
16921	1.39615	0.05
16922	1.02653	0.335776
16923	2.98258	0.479598
16924	2.0815	0.05
16925	1.39525	0.05
16926	1.82176	0.05
16927	0.977607	0.05
16928	1.96133	1.65068
16929	1.27189	1.35297
16930	0.826032	1.19804
16931	2.12831	1.16286
16932	1.77798	2.1
16933	1.02969	1.61807

16934	1.06463	1.43962
16935	1.49219	2.1
16936	1.10003	2.1
16937	1.21223	2.1
16938	1.00513	1.13066
16939	1.43788	2.05645
16940	1.47134	0.339801
16941	0.968249	0.823311
16942	3.22585	0.05
16943	3.36933	0.05
16944	3.36932	0.05
16945	3.03865	0.05
16946	1.66295	0.05
16947	1.65583	0.05
16948	1.65583	0.05
16949	1.4732	0.05
16950	1.73704	0.05
16951	1.76476	1.87449
16952	2.13345	0.284656
16953	1.55816	2.1
16954	1.55816	0.624701
16955	1.02601	2.1
16956	1.08039	2.1
16957	1.05498	1.13838
16958	1.08039	2.1
16959	1.2873	2.1
16960	1.01121	2.1
16961	0.987703	2.1
16962	0.98771	2.1
16963	0.964782	2.1
16964	1.35719	2.1
16965	2.61092	2.1
16966	2.6607	2.1
16967	3.41693	2.1
16968	1.23125	2.1
16969	1.73772	2.1
16970	0.99845	0.567503
16971	1.5176	0.869424
16972	1.13271	0.05
16973	1.332	0.05
16974	1.63159	2.1
16975	1.61342	2.1
16976	1.03679	2.1
16977	1.19087	2.1
16978	1.97172	2.1
16979	2.68634	2.1
16980	1.19086	2.1
16981	1.50091	2.1
16982	2.84677	2.1
16983	2.77894	2.1
16984	2.77894	2.1
16985	2.74206	2.1
16986	1.3284	2.1
16987	1.83524	2.1
16988	1.65853	2.1
16989	1.56741	2.1
16990	1.00509	2.1
16991	1.45246	2.1

16992	1.26193	2.1
16993	1.45244	2.1
16994	1.61963	0.05
16995	1.68189	0.05
16996	1.68189	1.0535
16997	1.49155	0.194455
16998	1.58054	2.1
16999	1.59169	2.1
17000	1.59169	2.1
17001	1.60497	2.1
17002	0.743595	0.05
17003	0.702359	0.05
17004	1.15317	0.05
17005	2.24367	0.05
17006	1.15252	0.05
17007	2.60354	0.05
17008	1.45158	2.1
17009	1.56004	2.1
17010	1.64527	2.1
17011	1.00371	2.1
17012	1.38287	0.435054
17013	0.893024	0.792359
17014	1.23527	0.863836
17015	1.23706	0.788257
17016	2.20993	0.753901
17017	1.54858	1.12033
17018	0.798666	0.101052
17019	1.22487	0.239152
17020	1.33158	0.05
17021	1.85902	0.303782
17022	1.08334	2.1
17023	1.17885	1.23524
17024	1.17885	0.201186
17025	1.07283	0.755611
17026	1.30723	0.05
17027	1.62507	0.05
17028	1.62507	0.05
17029	1.08854	0.05
17030	1.59475	2.1
17031	1.58874	2.1
17032	1.58875	2.1
17033	1.35787	2.1
17034	1.04147	2.1
17035	1.15564	2.1
17036	1.78301	2.1
17037	1.90618	2.1
17038	1.71555	2.1
17039	2.13143	2.1
17040	1.10817	2.1
17041	1.032	2.1
17042	1.032	2.1
17043	0.953636	2.1
17044	1.27916	1.25976
17045	1.11938	1.2087
17046	1.11937	1.21062
17047	1.9186	1.36406
17048	1.19967	0.05
17049	1.63489	0.05

17050	1.00763	0.05
17051	1.81656	0.05
17052	1.31814	0.05
17053	2.98183	0.05
17054	1.20149	2.1
17055	1.29129	2.1
17056	1.29129	2.1
17057	1.37138	2.1
17058	1.23104	0.270717
17059	1.75236	0.929647
17060	1.6	0.0806864
17061	1.60565	0.197474
17062	1.60565	0.14598
17063	1.40013	0.167184
17064	1.23042	2.1
17065	1.18278	2.1
17066	1.10853	2.1
17067	1.04724	2.1
17068	1.1766	2.1
17069	1.00319	2.1
17070	1.29435	2.1
17071	1.00319	2.1
17072	1.05655	1.75977
17073	1.01606	1.84752
17074	1.80391	2.1
17075	2.7699	2.1
17076	1.52073	2.1
17077	2.06835	2.1
17078	2.00202	2.1
17079	2.04906	2.1
17080	0.968536	2.1
17081	1.02461	2.1
17082	1.59784	2.1
17083	1.78249	2.1
17084	1.81409	2.1
17085	1.79844	2.1
17086	1.77093	2.01118
17087	2.02356	1.52587
17088	1.32449	2.1
17089	1.10003	2.1
17090	1.16915	0.31439
17091	1.05937	0.0992069
17092	1.28096	2.1
17093	1.34696	2.1
17094	1.34695	2.1
17095	1.36457	2.1
17096	1.06478	2.1
17097	1.06239	2.1
17098	1.06239	2.1
17099	1.09629	2.1
17100	0.798209	2.1
17101	1.8532	2.1
17102	2.10112	0.05
17103	2.8704	0.05
17104	2.8704	0.05
17105	2.92457	0.05
17106	1.0752	2.1
17107	1.07745	2.1

17108	1.07745	2.1
17109	1.08911	2.1
17110	1.40182	2.1
17111	1.00133	2.1
17112	1.40182	2.1
17113	1.24509	2.1
17114	2.22243	0.05
17115	1.07303	0.05
17116	1.29736	0.05
17117	1.73584	0.05
17118	2.0815	0.682679
17119	1.07999	0.16257
17120	2.1692	2.1
17121	1.99992	2.1
17122	1.99992	2.1
17123	1.78327	2.1
17124	1.52661	0.05
17125	0.956003	0.05
17126	1.53172	0.05
17127	0.955991	0.05
17128	1.35025	1.13383
17129	1.51084	2.1
17130	1.51084	2.1
17131	1.67115	2.1
17132	2.00977	2.1
17133	1.63632	2.1
17134	1.63632	2.1
17135	2.66128	2.1
17136	1.13525	2.1
17137	2.37566	2.1
17138	1.25987	2.1
17139	1.4472	2.1
17140	1.4472	2.1
17141	1.66018	2.1
17142	0.994001	1.37821
17143	1.83151	1.01593
17144	1.83151	0.34587
17145	1.57621	0.316934
17146	1.40915	0.05
17147	1.24653	0.46745
17148	1.24653	0.05
17149	1.10609	0.968243
17150	1.61121	2.1
17151	1.74472	2.1
17152	1.74472	2.1
17153	1.0368	2.1
17154	1.04788	2.1
17155	1.28522	2.1
17156	1.28522	2.1
17157	1.54196	2.1
17158	1.23387	2.1
17159	0.896519	2.1
17160	1.60428	2.1
17161	1.08467	2.1
17162	1.56875	0.061159
17163	1.39072	0.05
17164	1.39073	0.05
17165	1.6	0.15982

17166	1.01346	2.1
17167	1.06838	1.18139
17168	0.968463	1.25413
17169	1.06837	1.20815
17170	3.22433	0.610797
17171	2.05993	1.60289
17172	2.01836	0.0695673
17173	1.66154	1.71798
17174	1.20871	2.09607
17175	1.10586	2.1
17176	1.10586	2.07486
17177	1.03853	2.1
17178	1.12076	2.1
17179	1.03899	2.1
17180	1.03899	2.1
17181	0.963973	2.1
17182	0.987132	2.1
17183	1.0894	2.1
17184	2.37823	2.1
17185	1.75647	1.70888
17186	1.64625	2.1
17187	1.56195	2.1
17188	1.41995	2.1
17189	1.15171	2.1
17190	1.36457	2.1
17191	1.33847	2.1
17192	1.33847	2.1
17193	0.869601	1.80817
17194	1.42255	2.1
17195	1.3868	2.1
17196	1.3868	2.1
17197	1.33962	2.1
17198	1.05481	2.1
17199	1.08074	2.1
17200	1.08074	2.1
17201	0.857557	2.1
17202	1.13537	1.97373
17203	1.09194	0.861295
17204	1.01179	2.05291
17205	1.13537	0.819943
17206	1.15645	2.09832
17207	1.56409	0.05
17208	1.56409	0.729688
17209	1.16239	1.06641
17210	1.10746	0.44691
17211	1.47952	0.823305
17212	1.14733	2.1
17213	3.77068	2.1
17214	1.38497	2.1
17215	1.54599	2.1
17216	1.54601	2.1
17217	1.27697	2.1
17218	1.01393	1.7391
17219	1.10558	1.87
17220	0.995012	0.541267
17221	1.17624	1.37461
17222	1.60497	2.1
17223	1.61588	2.1

17224	1.61587	2.1
17225	1.62182	2.1
17226	1.48866	0.05
17227	1.65977	0.05
17228	1.65977	0.05
17229	1.69044	0.05
17230	0.961892	2.1
17231	1.00659	2.1
17232	1.0066	2.1
17233	1.06226	2.1
17234	1.15171	2.1
17235	1.50853	2.1
17236	1.50853	2.1
17237	1.2955	2.1
17238	1.3204	0.05
17239	2.13295	0.05
17240	2.75524	0.05
17241	1.18156	0.05
17242	1.73729	2.1
17243	1.20356	2.1
17244	1.62483	2.1
17245	1.52008	2.1
17246	1.19964	2.1
17247	1.0577	2.1
17248	1.19964	2.1
17249	1.07156	2.1
17250	1.98241	2.1
17251	1.43687	2.1
17252	1.27863	2.1
17253	1.19915	1.40396
17254	0.97269	1.49546
17255	1.0739	1.15064
17256	1.1427	2.1
17257	1.22476	1.0503
17258	1.06584	1.61061
17259	1.22626	1.37622
17260	1.31388	2.1
17261	1.16932	2.1
17262	1.28819	2.1
17263	1.0499	2.1
17264	1.55894	2.1
17265	1.78709	2.1
17266	1.75986	2.1
17267	1.764 2.1	
17268	1.44801	2.1
17269	1.28186	2.1
17270	1.20797	2.08649
17271	1.06821	2.1
17272	1.87287	0.831483
17273	2.08014	0.904507
17274	1.63225	0.935615
17275	1.39501	1.09581
17276	1.20132	0.05
17277	1.16182	0.05
17278	1.08848	0.05
17279	1.20132	0.05
17280	1.23475	2.03809
17281	1.16661	2.1

17282	2.31317	2.1
17283	2.50145	2.1
17284	1.04367	2.1
17285	2.23875	2.1
17286	0.953632	0.05
17287	1.38646	0.05
17288	1.38646	0.05
17289	1.61963	0.05
17290	1.62934	1.06741
17291	1.88626	0.644912
17292	1.0775	0.918742
17293	1.119	0.369766
17294	1.08133	0.0580744
17295	1.48312	0.05
17296	1.57125	0.05
17297	1.74098	0.05
17298	1.94372	1.2949
17299	1.22237	1.36389
17300	1.18549	0.699356
17301	1.01294	0.05
17302	1.01294	0.880597
17303	1.2064	0.954902
17304	1.14599	2.1
17305	1.00065	0.05
17306	1.76357	2.1
17307	2.23468	2.1
17308	2.21735	2.1
17309	3.32769	2.1
17310	1.19647	0.972629
17311	1.35307	0.801657
17312	1.19645	0.834229
17313	1.43778	0.809219
17314	1.34412	0.05
17315	1.1315	2.1
17316	1.1315	0.760246
17317	1.23943	0.495761
17318	1.57829	2.1
17319	1.22537	2.1
17320	1.14357	2.1
17321	1.32772	2.1
17322	1.4259	2.1
17323	1.34245	2.1
17324	1.47953	2.1
17325	1.70303	2.1
17326	2.21127	0.05
17327	1.47904	0.05
17328	1.18793	0.05
17329	2.21126	0.05
17330	1.1032	2.01252
17331	1.14986	0.359547
17332	1.14986	2.1
17333	1.19611	0.768795
17334	1.91883	2.1
17335	1.83019	2.1
17336	1.83019	2.1
17337	1.82462	2.1
17338	1.27362	0.05
17339	1.27421	0.05

17340	1.27421	0.05
17341	1.10736	0.05
17342	1.3003	0.318024
17343	1.31415	1.18709
17344	1.31415	0.220978
17345	1.32201	1.34694
17346	1.21124	2.1
17347	0.81568	2.1
17348	0.81568	2.1
17349	0.551816	2.1
17350	1.67119	2.1
17351	1.63384	2.1
17352	1.63384	2.1
17353	1.63661	2.1
17354	1.48734	2.1
17355	1.52245	2.1
17356	1.52245	2.1
17357	1.50714	2.1
17358	0.996778	0.05
17359	1.56091	0.05
17360	1.56091	0.05
17361	1.90457	0.05
17362	1.1181	2.1
17363	1.06543	2.1
17364	2.66828	2.1
17365	2.76932	2.1
17366	1.06543	2.1
17367	1.01474	2.1
17368	2.76932	2.1
17369	2.86704	2.1
17370	1.29724	0.05
17371	1.96938	0.05
17372	1.82462	2.1
17373	1.84553	2.1
17374	1.05556	2.1
17375	1.25363	2.1
17376	1.84552	2.1
17377	1.53208	2.1
17378	1.12826	2.1
17379	1.39075	2.1
17380	0.9702	1.06899
17381	1.16481	0.856047
17382	1.08894	0.925638
17383	1.91756	1.24073
17384	1.50865	0.137057
17385	1.06012	0.0558426
17386	1.5456	0.05
17387	1.37427	0.05
17388	1.1211	0.05
17389	1.5456	0.05
17390	1.33135	0.05
17391	1.07956	0.05
17392	2.23255	0.05
17393	1.21031	0.05
17394	1.52903	2.1
17395	1.35977	2.1
17396	3.3837	2.1
17397	3.20581	2.1

17398	1.35977	2.1
17399	1.15414	2.1
17400	3.20582	2.1
17401	2.07596	2.1
17402	1.6812	0.05
17403	1.03939	0.05
17404	1.30047	2.1
17405	1.5586	2.1
17406	1.5586	2.1
17407	1.84214	2.1
17408	1.31825	0.165856
17409	1.31923	1.64641
17410	1.31924	0.168254
17411	1.33026	1.1507
17412	1.15818	0.980948
17413	1.17822	1.12172
17414	1.15818	2.1
17415	1.28096	1.39277
17416	1.06848	0.7
17417	1.89639	0.700208
17418	1.9186	1.18846
17419	1.77065	1.10801
17420	1.59103	1.02548
17421	1.56164	0.942216
17422	1.09697	0.05
17423	2.38719	0.05
17424	2.22123	0.05
17425	2.46059	0.05
17426	1.45506	0.0675343
17427	1.03645	0.0577217
17428	1.30347	2.1
17429	1.2627	2.1
17430	1.2627	2.1
17431	1.23036	2.1
17432	1.41469	2.1
17433	1.45257	2.1
17434	1.46907	0.05
17435	1.70302	0.05
17436	1.49196	0.05
17437	1.36307	0.05
17438	1.05677	2.1
17439	0.978698	2.1
17440	0.978699	2.1
17441	2.01822	2.1
17442	1.47446	2.1
17443	1.55432	2.1
17444	1.55432	2.1
17445	1.06053	2.1
17446	1.86813	1.68085
17447	1.21892	2.1
17448	1.53064	1.1811
17449	1.30664	1.29231
17450	1.09629	2.1
17451	1.19244	2.1
17452	1.19244	2.1
17453	1.37635	2.1
17454	1.49358	0.168079
17455	1.57702	0.05

17456	2.09374	2.1
17457	1.88175	2.1
17458	1.88175	2.1
17459	1.51085	2.1
17460	1.10938	0.0530454
17461	1.02478	0.05
17462	1.33061	0.05
17463	1.13272	0.05
17464	1.75505	2.1
17465	1.78529	2.1
17466	1.6353	2.1
17467	1.59307	2.1
17468	1.60017	0.266611
17469	1.35608	1.09382
17470	1.35608	0.236338
17471	1.04713	0.132865
17472	1.01479	0.101969
17473	1.3868	0.486478
17474	1.3868	0.154679
17475	1.28418	2.1
17476	2.15949	2.1
17477	2.90429	2.1
17478	1.51902	2.1
17479	1.54755	2.1
17480	2.90429	2.1
17481	3.49966	2.1
17482	1.70842	2.1
17483	1.97526	2.1
17484	0.949712	0.05
17485	1.92784	0.05
17486	1.34093	0.05
17487	2.53426	0.0546241
17488	1.92783	0.05
17489	3.89373	0.05
17490	2.53426	0.05
17491	2.35961	0.0552725
17492	1.68108	0.584737
17493	1.15558	0.467014
17494	1.67265	0.36855
17495	1.27608	0.265997
17496	1.42249	2.1
17497	1.39708	2.1
17498	1.39708	2.1
17499	1.39327	2.1
17500	1.97046	0.05
17501	1.11688	0.05
17502	1.28418	0.162683
17503	1.17302	2.02761
17504	1.17302	0.05
17505	1.04349	2.02921
17506	0.979853	2.1
17507	1.26016	2.1
17508	1.18901	2.1
17509	1.26016	2.1
17510	1.22857	0.476309
17511	1.25848	0.926856
17512	1.25848	0.543002
17513	1.2858	0.901061

17514	1.09154	2.1
17515	2.01915	2.1
17516	1.23013	2.1
17517	1.55698	2.1
17518	1.55698	2.1
17519	1.8575	2.1
17520	2.01854	2.1
17521	1.20686	2.1
17522	1.20684	2.1
17523	1.77796	2.1
17524	1.46129	2.1
17525	1.04193	2.1
17526	1.10051	2.1
17527	0.983723	2.1
17528	1.1729	2.1
17529	0.98505	2.1
17530	0.985052	2.1
17531	1.03951	2.1
17532	1.03949	2.1
17533	1.17583	2.1
17534	1.42866	2.1
17535	1.97261	2.1
17536	1.73274	2.1
17537	1.53629	2.1
17538	1.98182	2.1
17539	1.72511	2.1
17540	1.42174	2.1
17541	1.44472	2.1
17542	1.72511	2.1
17543	1.45411	2.1
17544	1.44472	2.1
17545	1.43836	2.1
17546	0.962703	0.344943
17547	1.0171	0.756106
17548	1.53336	0.05
17549	1.40274	0.05
17550	1.40272	0.05
17551	1.00213	0.05
17552	1.50892	0.05
17553	1.64984	0.05
17554	1.67464	0.05
17555	1.67481	0.05
17556	1.08212	2.1
17557	1.28095	2.1
17558	1.46956	2.1
17559	1.70303	2.1
17560	1.2925	0.05
17561	1.84716	0.05
17562	1.48676	0.0534981
17563	1.48797	0.0866302
17564	1.33917	0.057114
17565	1.22691	0.0565244
17566	0.528744	2.1
17567	1.13728	2.1
17568	1.13728	2.1
17569	1.99843	2.1
17570	1.17464	0.05
17571	1.0372	0.05

17572	2.0815	0.05
17573	0.933462	0.05
17574	1.54931	2.1
17575	1.5485	2.1
17576	1.4077	2.1
17577	1.29931	2.1
17578	1.14931	0.10252
17579	2.02223	0.0859112
17580	1.04482	0.05
17581	0.94971	0.0522733
17582	1.54632	1.42738
17583	1.15693	1.48975
17584	1.66763	2.1
17585	1.41238	2.1
17586	1.41238	2.1
17587	1.48068	2.1
17588	1.0171	0.05
17589	1.1211	0.178983
17590	1.1211	0.05
17591	1.28418	0.0752248
17592	0.993021	2.1
17593	1.764 2.1	
17594	0.993023	2.1
17595	1.05787	2.1
17596	1.90914	0.05
17597	1.12229	1.59651
17598	1.02025	0.0605707
17599	0.585368	1.98964
17600	1.99323	2.1
17601	2.34988	2.1
17602	2.34988	2.1
17603	2.36987	2.1
17604	1.23107	0.05
17605	1.90664	0.05
17606	1.53496	0.05
17607	1.70302	0.05
17608	1.98859	2.1
17609	1.61057	2.1
17610	1.79844	2.1
17611	2.10811	2.1
17612	1.4618	2.1
17613	1.96722	2.1
17614	1.95191	2.1
17615	2.13418	2.1
17616	1.50368	0.05
17617	1.61417	0.05
17618	1.15008	0.05
17619	1.19685	0.05
17620	1.90456	0.05
17621	1.90899	0.05
17622	2.08864	0.05
17623	2.10447	0.05
17624	1.16653	2.1
17625	1.23017	2.1
17626	1.0752	0.0933902
17627	1.34412	0.05
17628	1.06226	2.1
17629	1.09252	2.1

17630	1.09252	2.1
17631	0.88579	2.1
17632	1.56178	0.05
17633	1.14305	0.05
17634	1.56175	0.05
17635	1.08211	0.05
17636	1.32813	2.1
17637	1.32461	2.1
17638	1.32461	2.1
17639	1.32455	2.1
17640	1.42729	0.05
17641	1.18716	0.05
17642	1.47169	0.05
17643	1.24687	0.05
17644	1.09627	2.1
17645	1.04759	2.1
17646	1.04759	2.1
17647	0.987132	2.1
17648	1.11181	1.96536
17649	1.80662	2.1
17650	1.8066	2.1
17651	1.06461	1.98462
17652	1.56091	2.1
17653	1.9842	2.1
17654	1.73525	0.05
17655	1.40557	0.05
17656	2.26906	0.05
17657	1.31248	0.05
17658	1.66832	0.05
17659	1.32634	0.05
17660	1.03314	0.05
17661	1.24682	0.05
17662	1.38536	0.05
17663	1.52661	0.05
17664	1.177	0.915721
17665	1.36405	0.332628
17666	1.19837	0.333864
17667	1.17701	0.05
17668	0.993831	0.05
17669	1.0823	0.05
17670	1.0823	0.05
17671	1.01121	0.05
17672	1.49653	0.05
17673	1.89583	0.05
17674	0.67242	0.05
17675	1.36047	0.05
17676	1.00366	0.05
17677	1.24728	0.05
17678	1.13387	0.05
17679	1.60867	0.05
17680	1.30572	0.05
17681	1.54982	0.05
17682	0.788592	0.05
17683	1.82961	0.05
17684	1.54982	0.05
17685	1.82961	0.05
17686	1.26062	0.05
17687	1.64002	0.05

17688	1.12035	0.05
17689	1.64002	0.05
17690	1.1673	2.1
17691	1.75367	2.1
17692	0.964034	2.1
17693	1.0305	2.1
17694	1.30896	2.1
17695	1.52776	1.99358
17696	1.52776	2.1
17697	1.17989	2.1
17698	1.16759	2.1
17699	1.8575	2.1
17700	1.16759	2.1
17701	1.60936	2.1
17702	1.10747	0.05
17703	1.47688	0.05
17704	1.40794	2.1
17705	1.95729	1.77695
17706	1.95729	2.1
17707	3.18156	2.0508
17708	1.87598	2.1
17709	1.94343	2.1
17710	1.94343	2.1
17711	2.00551	2.1
17712	1.20067	1.06693
17713	1.70744	1.92319
17714	1.87244	1.78959
17715	1.32075	1.36287
17716	1.64955	2.1
17717	1.44221	2.1
17718	1.31109	2.1
17719	1.14206	2.1
17720	1.60452	2.09194
17721	1.12111	0.535942
17722	1.00832	2.1
17723	0.974137	2.1
17724	0.974137	2.1
17725	0.98505	2.1
17726	1.02368	2.1
17727	0.985051	2.1
17728	0.995734	0.100691
17729	1.31993	0.0960969
17730	1.31993	0.0949582
17731	1.18549	0.05
17732	1.5176	0.664348
17733	1.33622	0.587573
17734	1.33621	0.486121
17735	1.02782	0.525017
17736	1.66774	2.1
17737	1.85305	2.1
17738	1.85305	2.1
17739	2.07088	2.1
17740	1.78873	2.1
17741	1.47533	2.1
17742	1.23152	2.1
17743	0.99922	2.1
17744	1.48493	2.1
17745	1.1394	2.1

17746	1.19179	2.1
17747	1.64289	2.1
17748	1.47858	2.1
17749	1.89687	2.1
17750	0.972464	2.1
17751	1.79473	2.1
17752	1.06971	2.1
17753	1.21089	2.1
17754	1.12572	2.1
17755	1.30566	2.1
17756	1.30566	2.1
17757	1.52724	2.1
17758	2.32237	0.05
17759	2.20248	0.05
17760	2.79564	0.0671315
17761	2.32237	0.0521975
17762	1.59354	0.05
17763	3.098 0.05	
17764	3.09801	0.05
17765	3.45132	0.05
17766	1.24001	2.09209
17767	1.41822	0.856329
17768	1.41821	2.1
17769	1.44859	0.915224
17770	1.34539	2.1
17771	1.06991	2.1
17772	1.18076	2.1
17773	0.978586	2.1
17774	0.999189	0.05
17775	2.41076	0.05
17776	0.968475	2.1
17777	1.02074	2.1
17778	1.02074	2.1
17779	1.07866	2.1
17780	0.817702	0.05
17781	1.5082	0.05
17782	1.48376	0.05
17783	1.18947	0.05
17784	1.10638	2.1
17785	1.86766	2.1
17786	1.19785	2.1
17787	1.34141	2.1
17788	1.34141	2.1
17789	0.991985	2.1
17790	2.35516	0.427368
17791	1.24815	0.160634
17792	1.80454	2.1
17793	1.22633	2.1
17794	0.696255	2.1
17795	1.19379	0.485728
17796	1.14721	2.1
17797	1.16153	2.1
17798	1.16153	2.1
17799	1.17261	2.1
17800	1.20814	2.1
17801	1.54791	2.1
17802	1.54791	2.1
17803	1.60477	2.1

17804	1.29931	2.1
17805	1.19098	2.1
17806	1.19098	2.1
17807	1.13341	2.1
17808	1.00503	2.1
17809	1.25334	0.621148
17810	1.22655	2.1
17811	1.26282	2.1
17812	1.26282	2.1
17813	0.999489	2.1
17814	1.54197	0.05
17815	1.17152	0.05
17816	1.17152	0.05
17817	1.49627	0.05
17818	1.36405	0.741898
17819	0.976334	0.250467
17820	1.12495	2.1
17821	1.54096	2.1
17822	1.75263	2.1
17823	1.49114	2.1
17824	0.842998	2.03227
17825	1.60244	1.01667
17826	1.48911	1.53518
17827	2.18685	1.02312
17828	1.75236	0.700716
17829	1.54143	1.49457
17830	1.40013	0.133084
17831	0.946646	0.123178
17832	1.50714	2.1
17833	1.46055	2.1
17834	1.46055	2.1
17835	1.42249	2.1
17836	2.12839	0.05
17837	1.75211	0.05
17838	1.76297	0.05
17839	1.70736	0.05
17840	1.19952	0.05
17841	1.56475	0.05
17842	1.74312	0.05
17843	1.48973	0.05
17844	1.91518	0.05
17845	1.16534	0.05
17846	1.53556	2.1
17847	1.56253	2.1
17848	1.56253	2.1
17849	1.58054	2.1
17850	1.27806	0.05
17851	1.44593	0.05
17852	1.44593	0.05
17853	1.3946	0.05
17854	1.34857	0.0750107
17855	1.03818	1.95114
17856	1.38969	0.13302
17857	1.85548	0.242952
17858	1.03108	0.115784
17859	1.85548	0.146248
17860	1.49346	1.98824
17861	1.83192	2.1

17862	1.75291	1.17903
17863	1.35435	2.01778
17864	0.921851	0.239726
17865	1.09646	0.129146
17866	1.2183	0.10888
17867	1.36717	0.0866558
17868	2.14179	0.05
17869	1.79805	0.0976834
17870	1.3428	0.16245
17871	2.30493	0.237029
17872	1.81545	0.0636065
17873	1.92269	0.122283
17874	2.30492	0.304443
17875	1.3663	0.344594
17876	1.22418	2.1
17877	0.956698	1.01746
17878	1.4706	2.1
17879	0.953295	2.1
17880	1.47059	2.1
17881	1.26929	2.1
17882	1.24538	0.05
17883	1.4468	0.05
17884	2.86762	2.1
17885	2.32911	2.1
17886	2.10864	2.1
17887	1.94548	2.1
17888	1.83134	2.1
17889	2.19519	2.1
17890	2.19853	2.1
17891	2.63855	2.1
17892	1.08213	0.05
17893	1.46389	0.05
17894	1.62235	0.05
17895	0.980549	0.05
17896	1.08195	2.08064
17897	0.99002	2.1
17898	1.2269	2.1
17899	1.0838	2.1
17900	0.889491	2.1
17901	1.19587	2.1
17902	1.18801	0.05
17903	1.57125	0.05
17904	1.47712	2.1
17905	1.45182	2.1
17906	1.45182	2.1
17907	1.42255	2.1
17908	1.17816	0.05
17909	1.00982	0.05
17910	1.21056	0.05
17911	1.17816	0.05
17912	3.80902	0.712874
17913	0.976991	0.48003
17914	1.09962	2.1
17915	1.60976	2.1
17916	1.87029	2.1
17917	2.33806	2.1
17918	2.12549	2.1
17919	2.21472	2.1

17920	2.16221	0.05
17921	1.99528	0.05
17922	1.99528	0.05
17923	1.97969	0.05
17924	3.60854	2.1
17925	4.00847	2.1
17926	2.70372	2.1
17927	1.253	2.1
17928	1.65739	2.1
17929	0.994294	2.1
17930	1.36203	2.1
17931	1.00965	2.1
17932	2.33374	2.1
17933	1.36209	2.1
17934	1.64983	0.05
17935	1.36416	0.05
17936	2.19766	0.05
17937	1.36416	0.05
17938	2.15871	0.487875
17939	1.81454	0.182292
17940	1.54607	0.05
17941	1.57702	0.244847
17942	0.948748	0.05
17943	1.04522	0.05
17944	1.60994	0.05
17945	1.90382	0.05
17946	1.90382	0.05
17947	1.57999	0.05
17948	1.73905	0.931569
17949	1.26535	1.24332
17950	2.12552	1.17936
17951	1.06891	1.08681
17952	1.13999	1.04985
17953	2.29812	1.19513
17954	2.83329	1.07608
17955	2.16272	0.918009
17956	1.71277	2.1
17957	1.68401	2.1
17958	1.68401	2.1
17959	1.69368	2.1
17960	1.06472	2.1
17961	1.56017	2.1
17962	1.2765	2.1
17963	1.99541	2.1
17964	1.46041	0.05
17965	2.04817	0.05
17966	2.04817	0.05
17967	2.33181	0.05
17968	2.06353	2.1
17969	1.35449	2.1
17970	1.52045	2.1
17971	1.45437	2.1
17972	1.04405	0.05
17973	1.85945	0.05
17974	1.85945	0.05
17975	2.22679	0.05
17976	1.33339	2.1
17977	1.19376	2.1

17978	1.33266	2.1
17979	1.44244	2.1
17980	1.20455	2.1
17981	1.52852	2.1
17982	1.03882	2.1
17983	1.52852	2.1
17984	1.44801	2.1
17985	1.00775	2.1
17986	0.53907	2.1
17987	0.506124	2.1
17988	2.39045	2.1
17989	0.973794	2.1
17990	1.11193	2.1
17991	1.32784	2.1
17992	1.31595	2.1
17993	0.966115	2.1
17994	1.25953	2.1
17995	1.38086	2.1
17996	1.38086	2.1
17997	1.04101	2.1
17998	1.99319	0.05
17999	1.23107	0.05
18000	3.3606	0.05
18001	1.23112	0.05
18002	1.40239	2.1
18003	1.26091	2.1
18004	1.26091	2.1
18005	1.05776	2.1
18006	1.23768	0.550794
18007	1.0142	1.3168
18008	1.11435	0.949941
18009	1.01422	0.858891
18010	1.24001	0.857676
18011	1.42186	1.05341
18012	1.42186	0.935063
18013	1.47204	1.0077
18014	1.27361	2.1
18015	1.56642	2.1
18016	1.71676	2.1
18017	2.16815	2.1
18018	1.07855	1.34492
18019	1.07612	1.31111
18020	1.703 0.05	
18021	1.46853	0.05
18022	1.45448	0.05
18023	1.27778	0.05
18024	0.991986	2.1
18025	1.1192	2.1
18026	1.1192	2.1
18027	1.25953	2.1
18028	2.08506	2.1
18029	1.75162	2.1
18030	1.94523	2.1
18031	1.88405	1.99984
18032	1.15506	0.05
18033	1.1233	0.05
18034	2.09736	2.1
18035	2.98303	2.1

18036	2.71184	2.1
18037	3.51614	2.1
18038	1.18769	2.1
18039	1.24053	2.1
18040	1.24053	2.1
18041	1.45327	2.1
18042	1.26865	1.56166
18043	1.1744	1.17647
18044	1.1744	1.04624
18045	1.11712	1.05612
18046	1.15841	0.05
18047	1.02247	0.05
18048	1.93649	2.1
18049	1.81191	2.1
18050	1.64718	2.1
18051	1.47521	2.1
18052	1.59654	2.1
18053	1.57823	2.1
18054	1.57823	2.1
18055	1.55768	2.1
18056	1.00665	0.05
18057	1.28239	0.05
18058	1.28239	0.05
18059	1.44951	0.05
18060	1.22083	2.1
18061	0.957622	2.1
18062	0.887896	2.1
18063	1.27387	2.1
18064	1.00521	1.08342
18065	1.00503	1.10938
18066	1.95844	2.1
18067	1.05042	2.1
18068	2.78662	2.1
18069	2.22399	2.1
18070	2.3356	0.05
18071	1.49543	0.05
18072	1.96854	0.05
18073	1.21681	0.05
18074	2.17803	1.85464
18075	1.50998	2.09097
18076	2.35239	1.58097
18077	2.26987	1.08504
18078	1.03824	0.05
18079	1.51396	0.0648303
18080	1.51396	0.05382
18081	1.48693	0.05
18082	1.08784	2.1
18083	1.36865	2.06693
18084	0.712405	2.1
18085	1.08784	2.1
18086	2.02522	2.1
18087	1.6738	2.1
18088	1.35175	2.1
18089	1.50094	2.1
18090	1.6738	2.1
18091	1.62871	2.1
18092	1.35084	2.1
18093	1.34276	2.1

18094	1.18041	2.1
18095	1.43652	2.1
18096	0.974196	2.1
18097	1.28037	2.1
18098	1.28037	2.1
18099	1.05071	2.1
18100	2.44737	0.05
18101	1.4672	0.05
18102	1.22883	0.05
18103	2.08149	0.05
18104	1.82208	1.9244
18105	1.59826	1.89543
18106	1.59826	1.22888
18107	1.01393	2.1
18108	1.42757	2.1
18109	0.996775	2.1
18110	1.42757	2.1
18111	1.26397	2.1
18112	1.02461	2.1
18113	1.46129	2.1
18114	1.11833	2.1
18115	1.335 2.1	
18116	2.07087	2.1
18117	1.24041	2.1
18118	1.335 2.1	
18119	1.55398	2.1
18120	1.21171	0.05
18121	1.48589	0.05
18122	1.32886	0.05
18123	1.8027	0.05
18124	1.53515	1.80626
18125	1.60428	1.72243
18126	1.00035	0.05
18127	1.03534	0.05
18128	1.03535	1.4823
18129	1.09962	2.1
18130	0.62132	1.30558
18131	1.21033	2.1
18132	1.30326	2.1
18133	2.6823	2.1
18134	0.991407	2.1
18135	1.29729	2.1
18136	1.29729	2.1
18137	1.16858	2.1
18138	1.66137	0.05
18139	1.23012	0.05
18140	1.66139	0.05
18141	1.41821	0.282357
18142	1.56043	0.691403
18143	1.06848	0.949157
18144	1.56043	1.8119
18145	1.90911	1.37164
18146	0.982847	2.1
18147	1.05863	2.1
18148	1.05863	2.1
18149	1.22352	2.1
18150	1.23019	1.69685
18151	1.60509	2.1

18152	1.19122	2.1
18153	1.47769	2.1
18154	2.18868	2.1
18155	1.40984	2.1
18156	3.55197	2.1
18157	3.57227	2.1
18158	3.57227	2.1
18159	3.48926	2.1
18160	1.4375	0.0655627
18161	1.11314	0.05
18162	1.62408	2.1
18163	1.74438	2.1
18164	1.72387	2.1
18165	1.50018	2.1
18166	1.1486	2.1
18167	1.18474	2.1
18168	1.18474	2.1
18169	1.2295	2.1
18170	1.28754	2.1
18171	1.36838	2.1
18172	1.36838	2.1
18173	1.21703	2.1
18174	2.74206	2.1
18175	2.70795	2.1
18176	2.70795	2.1
18177	2.55918	2.1
18178	1.35065	0.41708
18179	1.65411	0.0954586
18180	1.48572	0.370527
18181	1.28393	0.524099
18182	1.25265	0.05
18183	1.70944	0.05
18184	1.31259	0.549684
18185	1.14945	2.1
18186	1.14945	0.192628
18187	0.995725	2.1
18188	1.00307	0.05
18189	2.05786	0.209466
18190	1.56357	0.201299
18191	1.10907	0.168334
18192	1.27558	1.515
18193	1.05175	1.13703
18194	1.2685	2.1
18195	1.41369	2.1
18196	1.4137	2.1
18197	1.49654	2.1
18198	1.21489	2.1
18199	1.14605	1.59334
18200	1.22476	2.1
18201	1.21489	1.18181
18202	2.51472	1.22556
18203	2.52091	1.19424
18204	2.52091	1.09711
18205	1.79171	1.09898
18206	1.03373	2.1
18207	1.04037	2.1
18208	1.04038	2.1
18209	1.03933	2.1

18210	1.74123	0.05
18211	1.41902	0.05
18212	1.89777	0.05
18213	1.89899	0.05
18214	1.24728	0.05
18215	1.30394	0.05
18216	1.30394	0.05
18217	1.16535	0.05
18218	1.09914	2.1
18219	1.54259	2.1
18220	1.40233	2.1
18221	1.69858	2.1
18222	1.53555	2.1
18223	1.09859	1.7641
18224	1.09859	2.1
18225	0.725879	0.540625
18226	1.07213	2.1
18227	1.31589	2.1
18228	1.36904	2.1
18229	1.69711	2.1
18230	1.51028	0.05
18231	0.993598	0.05
18232	0.61563	0.05
18233	1.26311	0.05
18234	1.19496	2.1
18235	1.65925	2.1
18236	1.65925	2.1
18237	1.48162	2.1
18238	2.75738	1.48755
18239	1.24699	1.44985
18240	4.08729	1.98172
18241	2.35271	1.69576
18242	1.55063	0.493249
18243	1.34563	0.108803
18244	1.34563	0.244091
18245	1.08189	0.0502668
18246	1.09662	0.05
18247	2.35961	0.05
18248	0.986952	0.05
18249	0.813251	0.05
18250	1.17897	0.05
18251	1.67685	0.05
18252	1.00573	1.57124
18253	1.08542	1.11975
18254	1.00573	2.1
18255	1.12572	2.1
18256	1.05499	2.1
18257	1.00521	1.1511
18258	2.22679	0.05
18259	2.86746	0.05
18260	2.86746	0.05
18261	1.99958	0.05
18262	0.996517	2.1
18263	1.01169	2.1
18264	1.01169	2.1
18265	1.26346	2.1
18266	1.68274	2.1
18267	1.75505	2.1

18268	2.28748	2.02155
18269	1.36555	2.1
18270	3.47888	0.05
18271	0.692788	0.05
18272	1.0685	0.0724116
18273	1.14547	0.122621
18274	1.19017	2.1
18275	1.24976	2.1
18276	1.24976	2.1
18277	1.30682	2.1
18278	2.2153	2.1
18279	1.85457	2.1
18280	2.03267	2.1
18281	1.51042	2.1
18282	1.05619	0.427332
18283	1.05793	0.913036
18284	1.05793	0.197523
18285	1.00763	0.904042
18286	1.77677	2.1
18287	1.02524	2.1
18288	1.02524	2.1
18289	1.18803	2.1
18290	1.68669	2.1
18291	1.71162	2.1
18292	1.556	2.1
18293	1.39523	2.1
18294	0.960288	0.888473
18295	1.24201	1.99728
18296	1.24202	2.1
18297	1.3592	2.1
18298	2.14045	0.05
18299	1.62054	0.05
18300	2.20248	0.05
18301	2.34699	0.05
18302	3.45132	0.05
18303	3.38241	0.05
18304	3.38241	0.05
18305	2.96127	0.05
18306	2.02612	2.1
18307	2.09351	2.1
18308	2.09351	2.1
18309	2.17465	2.1
18310	1.07445	2.1
18311	1.14808	2.1
18312	1.39067	2.1
18313	1.20958	2.1
18314	1.20958	2.1
18315	1.00382	1.31893
18316	0.986496	2.1
18317	1.27142	2.1
18318	1.39857	2.06814
18319	2.26482	2.02023
18320	2.24367	0.05
18321	1.9234	0.05
18322	1.59076	0.05
18323	1.185	0.05
18324	3.86179	0.05
18325	3.0638	0.05

18326	2.7574	0.05
18327	1.83266	0.05
18328	2.59418	2.1
18329	2.23048	2.1
18330	1.39564	2.1
18331	1.36763	2.1
18332	2.23048	2.1
18333	2.02522	2.1
18334	1.36763	2.1
18335	1.35175	2.1
18336	1.16181	0.05
18337	0.97096	0.05
18338	2.05665	0.05
18339	0.483583	0.05
18340	0.601928	0.05
18341	3.88545	0.05
18342	1.04909	2.1
18343	0.983143	2.1
18344	1.10469	2.1
18345	1.10516	2.1
18346	1.10517	2.1
18347	1.09951	2.1
18348	2.33985	2.1
18349	2.35026	2.1
18350	2.35025	2.1
18351	1.19462	2.1
18352	1.57627	2.1
18353	1.5493	2.1
18354	1.5493	2.1
18355	1.53556	2.1
18356	1.42763	0.203912
18357	1.24682	0.127133
18358	1.24682	0.290092
18359	1.00307	0.268302
18360	1.37392	2.1
18361	1.45408	2.1
18362	1.45408	2.1
18363	1.54249	2.1
18364	1.24474	1.37701
18365	1.07467	1.43804
18366	1.37813	1.74182
18367	1.24474	1.74012
18368	1.06341	2.1
18369	1.09194	2.1
18370	1.09194	2.1
18371	1.11117	2.1
18372	1.1658	2.1
18373	1.93707	2.1
18374	1.93707	2.1
18375	1.47729	2.1
18376	1.25669	1.90651
18377	1.07519	2.1
18378	1.65417	2.1
18379	1.50956	2.1
18380	1.37231	2.1
18381	1.27285	2.1
18382	1.23612	2.1
18383	1.1899	2.1

18384	1.08172	2.1
18385	1.20357	2.1
18386	1.5664	2.1
18387	1.7472	2.1
18388	1.48434	2.1
18389	1.23989	2.1
18390	1.58836	2.1
18391	1.38766	2.1
18392	1.23989	2.1
18393	1.26599	2.1
18394	2.56248	2.1
18395	2.08673	1.14847
18396	1.87216	1.82854
18397	1.60476	0.05
18398	1.84355	2.1
18399	1.25971	1.16772
18400	1.44141	1.09971
18401	1.25521	0.05
18402	1.19837	0.05
18403	2.31936	0.05
18404	1.38153	2.1
18405	2.41734	2.1
18406	2.70856	2.1
18407	3.48385	2.1
18408	1.89226	0.972426
18409	1.64617	1.75674
18410	1.89112	1.28603
18411	1.23135	0.213563
18412	1.23134	0.62201
18413	1.34927	0.60865
18414	3.60096	0.05
18415	1.68103	0.05
18416	2.25644	2.1
18417	1.91955	2.1
18418	1.91955	2.1
18419	1.47688	2.1
18420	1.67375	2.1
18421	1.50033	2.1
18422	1.5098	2.1
18423	1.46908	2.1
18424	1.69055	0.05
18425	1.53463	0.05
18426	1.53463	0.05
18427	1.41943	0.05
18428	1.19022	1.96419
18429	1.95981	1.31141
18430	1.15009	1.31353
18431	1.31612	1.32661
18432	1.31611	0.05
18433	1.10735	0.05
18434	1.18601	0.05
18435	1.3499	0.05
18436	1.3499	0.05
18437	1.29001	0.05
18438	1.22083	2.1
18439	1.06191	2.1
18440	1.348	1.18042
18441	1.05758	1.54275

18442	1.34799	2.07867
18443	1.18647	1.63877
18444	2.92457	0.05
18445	1.99182	0.05
18446	2.25313	0.05
18447	1.30859	0.05
18448	1.30682	2.1
18449	1.38957	2.1
18450	1.38957	2.1
18451	1.47446	2.1
18452	0.953638	2.1
18453	1.03408	2.1
18454	1.17885	0.726904
18455	1.3663	0.66778
18456	1.08265	0.759847
18457	1.71948	0.821751
18458	1.29501	0.967671
18459	0.955093	1.0132
18460	0.955081	0.341004
18461	1.88345	0.88525
18462	1.2243	2.1
18463	1.23169	2.1
18464	2.04726	1.50261
18465	0.842869	1.80048
18466	3.18156	2.1
18467	3.60786	1.77041
18468	3.60786	2.1
18469	2.38566	1.26169
18470	1.24526	0.05
18471	1.59019	0.05
18472	1.16776	0.05
18473	1.05042	2.1
18474	1.17418	2.1
18475	1.05042	2.1
18476	0.949135	2.1
18477	0.973448	2.1
18478	0.973447	2.1
18479	1.02958	2.1
18480	3.57768	2.1
18481	3.04246	2.1
18482	3.04246	2.1
18483	2.47824	2.1
18484	1.35879	2.1
18485	1.10407	2.1
18486	1.14599	0.10778
18487	1.10407	0.948903
18488	1.87794	1.82432
18489	1.53429	1.54429
18490	1.12717	2.1
18491	1.38634	2.1
18492	1.38634	2.1
18493	1.13127	2.1
18494	1.04546	2.1
18495	1.06007	2.1
18496	1.06007	2.1
18497	1.19785	2.1
18498	1.43652	2.1
18499	1.71741	2.1

18500	1.70921	2.1
18501	1.06994	2.1
18502	0.960106	2.1
18503	1.48077	2.1
18504	1.48077	2.1
18505	2.5946	2.1
18506	2.03045	1.81745
18507	1.76788	2.06444
18508	2.03045	2.1
18509	0.633098	2.1
18510	1.06756	2.1
18511	1.38667	2.1
18512	0.966979	2.1
18513	1.38669	2.1
18514	1.07156	2.1
18515	0.986095	2.1
18516	1.32813	2.1
18517	0.986095	2.1
18518	1.98697	1.07154
18519	1.12016	2.09531
18520	1.24463	1.37149
18521	1.1967	2.06066
18522	1.30001	0.0621529
18523	1.29175	1.18612
18524	1.29175	0.157933
18525	1.3003	1.21933
18526	1.5606	0.05
18527	2.32098	0.05
18528	2.57888	0.05
18529	2.97463	0.05
18530	1.24555	2.1
18531	1.46101	2.1
18532	1.90723	2.1
18533	2.29126	2.1
18534	1.46101	2.1
18535	1.84089	2.1
18536	2.5519	2.1
18537	2.91663	2.1
18538	1.10476	0.05
18539	1.44103	0.05
18540	1.44103	0.05
18541	1.53336	0.05
18542	0.672964	2.1
18543	0.633884	2.1
18544	0.633884	2.1
18545	0.603154	2.1
18546	2.29663	2.1
18547	2.06892	2.1
18548	1.3685	2.1
18549	1.39067	2.1
18550	2.06892	2.1
18551	1.78208	2.1
18552	1.39067	2.1
18553	1.42174	2.1
18554	1.01335	1.91031
18555	1.08195	1.12234
18556	1.86463	2.1
18557	1.39827	2.1

18558	0.861515	2.1
18559	2.10074	2.1
18560	2.09511	0.782201
18561	2.04853	0.77136
18562	2.21488	0.799831
18563	1.38287	0.829428
18564	0.974717	0.894673
18565	0.998451	0.84591
18566	1.42281	1.29578
18567	1.82595	1.23314
18568	1.50458	0.698683
18569	1.09408	0.336698
18570	1.23498	1.59312
18571	1.05654	1.34904
18572	1.17261	2.1
18573	1.18058	2.1
18574	1.18059	2.1
18575	1.18907	2.1
18576	2.38998	2.1
18577	2.77437	2.1
18578	1.04546	2.1
18579	1.16817	2.1
18580	2.77437	2.1
18581	2.97768	2.1
18582	1.16817	2.1
18583	1.2832	2.1
18584	1.75766	0.05
18585	1.4934	0.962758
18586	1.4934	0.05
18587	1.20324	1.1648
18588	1.07236	0.688845
18589	1.37641	0.323616
18590	1.42913	0.05
18591	1.93373	0.618093
18592	1.42629	0.615132
18593	1.00636	0.156682
18594	0.933959	2.1
18595	2.22873	0.268256
18596	2.22873	1.03711
18597	2.13757	0.0586944
18598	1.10517	2.1
18599	1.86737	2.1
18600	0.811 2.1	
18601	1.19179	2.1
18602	1.18647	2.1
18603	0.988453	2.1
18604	1.12457	0.05
18605	1.23851	0.05
18606	0.92492	2.1
18607	1.45656	2.1
18608	1.61251	0.538797
18609	1.56696	0.853283
18610	1.90245	0.229203
18611	1.46046	0.221892
18612	1.60329	0.05
18613	1.38247	0.05
18614	1.38247	0.05
18615	1.23198	0.05

18616	1.09518	2.1
18617	1.28159	2.1
18618	0.977777	2.1
18619	1.80778	2.1
18620	1.80777	2.1
18621	1.16655	2.1
18622	1.3515	2.1
18623	0.956573	2.1
18624	1.08022	2.1
18625	1.03437	2.1
18626	1.03437	2.1
18627	0.975409	2.1
18628	1.47369	0.05
18629	1.31167	0.05
18630	1.01847	0.05
18631	2.75562	0.05
18632	1.13443	2.1
18633	1.32709	2.1
18634	1.01051	0.05
18635	0.958947	0.05
18636	1.7211	2.1
18637	1.922	2.1
18638	1.922	2.1
18639	0.991113	2.1
18640	1.00636	0.05
18641	1.31658	0.253417
18642	1.44627	0.0827194
18643	1.03824	0.0530901
18644	1.21223	2.1
18645	1.01242	2.1
18646	1.01243	2.1
18647	0.956698	2.1
18648	1.23167	2.1
18649	1.06935	2.1
18650	1.29215	0.05
18651	0.998971	0.05
18652	1.50103	0.05
18653	1.44836	0.05
18654	3.02513	2.1
18655	3.7335	2.1
18656	2.49988	2.1
18657	1.24948	2.1
18658	0.963972	2.1
18659	1.1822	2.1
18660	1.1822	2.1
18661	1.10817	2.1
18662	1.30237	2.1
18663	1.41908	2.1
18664	1.41908	2.1
18665	1.48734	2.1
18666	1.31127	0.115453
18667	1.40822	0.0838168
18668	1.40822	0.05
18669	1.22176	0.05
18670	1.82525	2.1
18671	1.59394	2.1
18672	1.22731	0.05
18673	0.981584	0.05

18674	1.1427	0.851962
18675	1.44091	1.04528
18676	1.49154	0.196313
18677	1.07526	0.714411
18678	1.07527	0.765793
18679	0.96029	0.666705
18680	2.40568	1.28836
18681	3.46486	1.1567
18682	2.417	1.25192
18683	1.52054	1.01922
18684	1.51638	0.0506123
18685	1.04786	0.05
18686	1.51404	0.0582074
18687	1.3461	0.05
18688	1.27696	0.05
18689	0.736457	0.05
18690	1.16817	2.1
18691	1.04263	2.1
18692	1.13449	2.1
18693	1.50524	2.1
18694	2.1182	2.1
18695	2.49001	2.1
18696	1.11764	2.1
18697	1.39292	2.1
18698	2.49001	2.1
18699	2.76339	2.1
18700	1.39292	2.1
18701	1.68761	2.1
18702	1.25173	2.1
18703	1.34701	2.1
18704	1.02415	2.1
18705	1.34701	2.1
18706	1.332	0.05
18707	0.989764	0.05
18708	0.963167	0.05
18709	1.21056	0.05
18710	1.67375	2.1
18711	1.17965	2.1
18712	1.09154	2.1
18713	1.52314	2.1
18714	1.54433	0.435842
18715	1.81969	0.251356
18716	1.3598	0.185877
18717	1.71041	0.0641858
18718	1.70934	0.102133
18719	1.33871	0.05
18720	1.79485	0.0772291
18721	1.51404	0.05
18722	1.30751	2.1
18723	1.20969	2.1
18724	1.20969	2.1
18725	1.12076	2.1
18726	1.49808	2.1
18727	1.50201	2.1
18728	1.50201	2.1
18729	1.31594	2.1
18730	1.2187	2.1
18731	1.11545	2.1

18732	1.227	2.1
18733	1.09645	2.1
18734	1.05902	0.05
18735	1.41595	0.05
18736	1.35696	2.1
18737	1.29446	0.144015
18738	0.857957	2.1
18739	1.21125	0.05
18740	2.43115	2.1
18741	2.1151	0.05
18742	2.0815	1.26901
18743	1.1723	2.1
18744	1.48035	0.0637245
18745	1.59971	0.610813
18746	1.33668	0.105195
18747	1.0961	0.0586121
18748	1.46235	2.1
18749	0.882195	2.1
18750	2.46624	2.1
18751	2.72161	2.1
18752	2.96094	2.1
18753	2.25644	2.1
18754	1.25923	0.05
18755	0.976218	0.05
18756	1.10319	0.05
18757	1.82583	0.05
18758	0.888955	2.1
18759	1.30817	2.1
18760	1.30817	2.1
18761	1.60787	2.1
18762	0.988805	2.1
18763	1.54168	1.09338
18764	1.19496	1.02849
18765	1.12224	1.79707
18766	1.93597	1.39497
18767	1.31113	1.32613
18768	1.17879	1.16481
18769	1.07468	1.15827
18770	2.0523	2.1
18771	2.41385	2.1
18772	2.41385	2.1
18773	3.0355	2.1
18774	1.36717	0.0799045
18775	1.45535	0.05
18776	1.45535	0.05
18777	1.49906	0.05
18778	1.37785	2.1
18779	1.04413	2.1
18780	1.06729	1.01773
18781	1.54305	0.904991
18782	1.07444	2.1
18783	1.52724	2.1
18784	1.49151	2.1
18785	1.36814	2.1
18786	1.36814	2.1
18787	1.15541	2.1
18788	1.19379	2.1
18789	1.78657	0.186074

18790	1.6079	2.1
18791	2.59469	0.336985
18792	1.35545	0.0639738
18793	1.23602	0.153071
18794	1.23452	2.1
18795	1.56842	0.883998
18796	1.15864	2.1
18797	1.11955	2.1
18798	1.11955	2.1
18799	1.08022	2.1
18800	1.64268	2.1
18801	1.26247	2.1
18802	1.16912	2.1
18803	2.1095	1.08872
18804	1.75549	0.05
18805	1.7491	0.05
18806	1.59833	0.05
18807	1.68338	0.05
18808	1.14846	0.05
18809	1.06515	0.05
18810	1.2721	0.05
18811	1.32639	0.05
18812	1.32639	0.05
18813	1.19323	0.05
18814	1.81034	2.1
18815	1.9034	2.1
18816	2.08245	2.1
18817	1.84887	2.1
18818	1.41448	2.1
18819	1.77326	2.1
18820	1.76872	2.1
18821	1.86766	2.1
18822	1.46643	2.1
18823	1.58995	2.1
18824	1.58995	2.1
18825	1.13854	2.1
18826	1.23654	2.1
18827	1.04442	2.1
18828	1.23654	2.1
18829	1.08576	2.1
18830	0.966979	2.1
18831	1.41758	2.1
18832	1.03108	0.05
18833	1.94412	0.0588771
18834	1.32455	2.1
18835	1.32328	2.1
18836	1.32328	2.1
18837	1.34008	2.1
18838	1.60861	2.1
18839	1.69586	2.1
18840	1.69586	2.1
18841	1.78301	2.1
18842	1.7513	2.1
18843	1.67906	2.1
18844	1.67906	2.1
18845	1.60635	2.1
18846	1.68073	2.1
18847	1.57234	2.1

18848	1.57233	2.1
18849	1.47012	2.1
18850	1.22811	0.05
18851	1.79683	0.05
18852	1.53417	0.057424
18853	1.08732	0.12572
18854	1.8903	1.55801
18855	1.20247	1.90258
18856	1.12087	1.59564
18857	1.54699	2.1
18858	1.43635	2.1
18859	1.02368	2.1
18860	1.43635	2.1
18861	1.23007	2.1
18862	1.035	2.1
18863	1.07889	2.1
18864	3.0355	2.1
18865	3.78805	2.1
18866	2.57183	2.01025
18867	1.27835	1.38111
18868	1.29813	0.05
18869	1.42965	0.1274
18870	1.69832	0.05
18871	2.18359	0.127399
18872	1.42964	0.05
18873	1.65723	0.095005
18874	1.98508	0.05
18875	2.06284	0.0735088
18876	1.50705	0.663648
18877	1.51153	0.409757
18878	1.36037	0.142703
18879	1.09767	0.308617
18880	1.70048	2.1
18881	1.15177	2.1
18882	0.713174	2.1
18883	0.763575	2.1
18884	0.763575	2.1
18885	0.797011	2.1
18886	1.01179	0.883587
18887	1.27737	0.893785
18888	3.16531	0.05
18889	1.0141	0.05
18890	1.47354	0.36231
18891	1.62495	0.112877
18892	1.62495	0.393167
18893	1.39296	0.584898
18894	0.998682	2.1
18895	1.93528	2.1
18896	2.71383	2.1
18897	1.13987	2.1
18898	1.93528	2.1
18899	1.78595	2.1
18900	3.98933	2.1
18901	3.73426	2.1
18902	1.02438	2.1
18903	1.12376	2.1
18904	1.12375	2.1
18905	1.19016	2.1

18906	1.37157	0.05
18907	1.60814	0.05
18908	1.70303	0.05
18909	1.01219	0.05
18910	1.06757	2.1
18911	1.23475	2.1
18912	1.2295	2.1
18913	1.28014	2.1
18914	1.28014	2.1
18915	1.33339	2.1
18916	1.3201	2.1
18917	1.2679	2.1
18918	1.2679	2.1
18919	1.23504	2.1
18920	1.50108	2.1
18921	1.3715	2.1
18922	1.3715	2.1
18923	1.25075	2.1
18924	1.99222	1.12326
18925	1.4155	1.09252
18926	1.4777	2.1
18927	1.52613	2.1
18928	1.91031	2.1
18929	1.03968	2.1
18930	2.04289	0.05
18931	0.967192	0.05
18932	1.03043	0.05
18933	1.65452	0.05
18934	1.21564	2.1
18935	1.16938	2.1
18936	2.47056	2.1
18937	2.56853	2.1
18938	1.16938	2.1
18939	1.1181	2.1
18940	2.56853	2.1
18941	2.66828	2.1
18942	1.0465	2.1
18943	1.44709	2.1
18944	1.44709	2.1
18945	1.96491	2.1
18946	1.01976	0.05
18947	0.961779	0.05
18948	1.11764	0.05
18949	0.961779	0.05
18950	1.21171	0.05
18951	1.35007	0.05
18952	1.03102	0.122109
18953	1.42446	0.477686
18954	1.42446	0.163721
18955	1.03166	0.0868878
18956	1.2951	0.05
18957	1.33575	0.05
18958	1.33575	0.05
18959	1.20473	0.05
18960	1.10049	0.802709
18961	0.99747	0.173407
18962	1.54249	2.1
18963	1.65677	2.1

18964	1.65677	2.1
18965	1.77677	2.1
18966	1.73525	0.05
18967	1.05689	0.05
18968	1.26051	0.05
18969	1.16259	0.05
18970	1.34009	2.1
18971	1.398	2.1
18972	1.398	2.1
18973	1.50437	2.1
18974	2.3245	2.1
18975	1.18237	2.1
18976	1.24509	2.1
18977	1.01595	2.1
18978	3.29047	2.1
18979	2.79168	2.1
18980	1.06561	2.1
18981	1.11077	2.1
18982	1.11077	2.1
18983	0.953927	2.1
18984	1.30509	0.05
18985	1.04685	0.05
18986	1.04685	0.05
18987	0.834593	0.05
18988	1.0775	0.05
18989	1.27362	0.05
18990	1.71762	0.972583
18991	2.5736	0.655626
18992	2.04717	1.0177
18993	1.45521	0.717387
18994	1.33962	2.1
18995	1.27956	2.1
18996	1.27956	2.1
18997	1.20478	2.1
18998	3.65841	0.05
18999	1.18656	0.05
19000	1.72272	0.05
19001	1.6812	0.05
19002	1.09951	2.1
19003	1.08231	2.1
19004	1.08231	2.1
19005	1.06478	2.1
19006	2.16196	1.62198
19007	1.00387	1.16578
19008	1.00387	1.1226
19009	0.993999	0.902041
19010	1.04101	2.1
19011	1.27119	2.1
19012	1.27119	2.1
19013	1.51748	2.1
19014	3.00129	2.1
19015	3.27303	2.1
19016	3.27303	2.1
19017	3.60855	2.1
19018	2.22399	2.1
19019	1.71995	2.1
19020	1.54793	2.1
19021	1.227	2.1

19022	1.07624	2.1
19023	1.264	2.1
19024	1.19323	1.18634
19025	1.21286	1.08386
19026	1.21286	0.701089
19027	1.20859	0.986241
19028	1.60578	0.05
19029	1.27778	0.05
19030	1.0525	2.1
19031	1.05758	1.25374
19032	1.0525	1.06506
19033	1.17042	1.41892
19034	1.62536	0.05
19035	3.71662	0.05
19036	1.78169	0.05
19037	1.91434	0.05
19038	2.12331	0.05
19039	1.33406	0.05
19040	1.38437	2.1
19041	1.51829	2.1
19042	1.51829	2.1
19043	1.6697	2.1
19044	0.947114	2.1
19045	1.13335	2.1
19046	1.13335	2.1
19047	1.00965	2.1
19048	1.47521	2.1
19049	1.27679	2.1
19050	1.14911	2.1
19051	0.93841	2.1
19052	1.68437	2.1
19053	1.53822	2.1
19054	1.53822	2.1
19055	1.39702	2.1
19056	0.726453	2.1
19057	0.754101	2.1
19058	0.754101	2.1
19059	0.69776	2.1
19060	0.964033	2.1
19061	1.62397	2.1
19062	1.70302	2.1
19063	1.78637	1.92609
19064	3.68158	0.05
19065	2.11685	0.05
19066	1.33125	2.1
19067	1.15471	2.1
19068	1.0465	2.1
19069	1.33125	2.1
19070	1.17683	2.1
19071	1.25889	2.1
19072	1.25889	2.1
19073	1.34239	2.1
19074	1.40591	0.05
19075	0.79348	0.05
19076	1.04019	0.05
19077	0.999187	0.05
19078	1.30218	0.05
19079	1.83339	0.05

19080	1.88574	0.05
19081	1.5177	0.05
19082	1.23504	2.1
19083	1.19583	2.1
19084	1.19583	2.1
19085	1.14496	2.1
19086	1.62183	2.1
19087	1.62316	2.1
19088	1.62316	2.1
19089	1.62241	2.1
19090	1.02305	0.05
19091	1.21136	0.05
19092	0.993832	0.05
19093	1.21137	0.05
19094	1.12064	2.1
19095	1.24965	2.1
19096	1.24965	2.1
19097	1.41077	2.1
19098	1.00052	0.05
19099	1.49842	0.05
19100	1.49843	0.05
19101	1.10801	0.05
19102	1.54196	1.09586
19103	1.18041	2.1
19104	1.73571	1.83413
19105	1.37224	2.07501
19106	1.4375	0.05
19107	1.09529	0.05
19108	1.44421	0.05
19109	1.0953	0.05
19110	1.0753	2.1
19111	0.815365	2.1
19112	0.815365	2.1
19113	0.597537	2.1
19114	1.03609	0.358319
19115	1.18952	0.122336
19116	1.54283	0.482423
19117	1.16719	0.658127
19118	1.16719	0.733668
19119	1.89226	0.719548
19120	2.55919	2.1
19121	2.37492	2.1
19122	2.37492	2.1
19123	2.07435	2.1
19124	1.36016	0.0618519
19125	1.70302	0.05
19126	1.85252	0.0649696
19127	1.2111	0.24512
19128	1.62905	2.1
19129	1.49692	2.1
19130	1.49692	2.1
19131	1.40395	2.1
19132	2.19083	2.1
19133	1.30542	2.1
19134	1.58736	2.1
19135	2.16579	2.1
19136	2.0815	2.1
19137	1.17935	2.1

19138	1.53803	2.1
19139	1.09148	0.784176
19140	1.81301	2.1
19141	1.38333	0.18871
19142	1.04412	2.1
19143	1.44437	2.1
19144	1.4675	0.05
19145	1.00665	0.0539017
19146	1.50951	0.05
19147	0.983105	0.05
19148	1.80252	0.05
19149	0.540005	0.05
19150	1.2545	0.05
19151	2.14514	0.05
19152	1.32051	0.05
19153	1.82302	0.05
19154	2.71057	2.1
19155	2.51107	2.1
19156	1.34817	2.1
19157	1.3551	2.1
19158	2.51107	2.1
19159	2.29663	2.1
19160	1.3551	2.1
19161	1.3685	2.1
19162	1.51748	2.1
19163	1.23389	2.1
19164	0.901928	0.05
19165	1.70302	0.05
19166	1.66787	0.05
19167	1.92535	0.05
19168	1.13571	0.05
19169	1.02836	0.05
19170	1.55398	2.1
19171	1.54179	2.1
19172	1.89608	2.1
19173	1.07167	2.1
19174	3.3488	0.05
19175	0.550044	0.05
19176	0.962877	0.319365
19177	1.00065	0.347317
19178	1.00613	0.744467
19179	1.07497	0.750775
19180	1.00486	0.05
19181	1.00486	0.05
19182	0.726453	2.1
19183	1.32634	0.05
19184	1.40008	0.05
19185	1.40008	0.05
19186	2.42267	0.05
19187	1.9216	0.05
19188	1.28066	0.05
19189	1.79102	1.0912
19190	1.18219	0.0559105
19191	1.18219	1.04095
19192	0.709123	0.132023
19193	1.07866	2.1
19194	1.15038	2.1
19195	1.15038	2.1

19196	1.24687	2.1
19197	1.83613	2.1
19198	1.99157	2.07824
19199	1.45789	0.05
19200	1.1706	0.05
19201	1.74746	0.05
19202	1.1706	0.05
19203	1.96383	0.05
19204	1.67727	0.05
19205	1.67727	0.05
19206	1.60329	0.05
19207	1.18803	2.1
19208	1.3227	2.1
19209	1.3227	2.1
19210	1.40844	2.1
19211	1.07889	2.1
19212	1.07721	2.1
19213	1.07721	2.1
19214	1.00832	2.1
19215	0.953523	0.332016
19216	1.62875	0.263238
19217	1.07427	0.512422
19218	1.19722	0.763006
19219	1.1972	0.175947
19220	1.1599	0.260808
19221	2.97463	0.05
19222	1.9477	0.05
19223	1.95222	0.05
19224	1.75083	0.05
19225	3.46787	2.1
19226	3.02896	2.1
19227	3.02896	2.1
19228	2.46145	2.1
19229	0.970962	0.05
19230	1.0227	0.05
19231	1.12328	0.05
19232	1.02269	0.05
19233	0.904978	2.1
19234	0.725887	2.1
19235	0.725887	2.1
19236	0.601592	2.1
19237	0.956294	2.1
19238	1.13179	2.1
19239	0.956294	2.1
19240	1.13179	2.1
19241	1.05556	2.1
19242	1.13179	2.1
19243	1.38859	2.1
19244	1.13179	2.1
19245	1.06659	2.1
19246	1.05776	2.1
19247	1.06659	2.1
19248	1.04291	1.04812
19249	1.54699	1.15119
19250	1.42307	1.25003
19251	1.5654	1.95151
19252	1.97906	2.1
19253	1.15354	0.05

19254	1.15505	0.05
19255	1.26455	0.05
19256	2.50042	0.05
19257	2.50042	0.05
19258	3.22585	0.05
19259	1.19258	0.05
19260	1.34562	0.05
19261	1.49515	0.05
19262	1.66295	0.05
19263	1.02395	2.1
19264	1.51067	2.1
19265	1.35821	2.1
19266	2.10671	2.1
19267	1.0305	1.96677
19268	1.07855	1.48386
19269	1.20761	2.1
19270	0.98488	2.1
19271	1.4162	2.1
19272	1.14825	2.1
19273	1.29415	2.1
19274	1.6067	2.1
19275	1.23851	0.05
19276	1.09651	0.05
19277	1.60636	2.1
19278	1.53521	2.1
19279	1.53521	2.1
19280	1.46279	2.1
19281	1.28044	0.05
19282	1.64147	0.0762741
19283	1.18422	0.05
19284	1.68208	0.05
19285	1.09726	2.1
19286	1.234 2.1	
19287	1.234 2.1	
19288	1.09483	2.1
19289	1.89318	2.1
19290	1.98448	2.1
19291	1.98448	2.1
19292	2.02612	2.1
19293	1.22026	1.12633
19294	1.23527	0.914876
19295	1.09044	1.03887
19296	0.993366	0.0541745
19297	1.22026	1.17064
19298	0.993368	0.929302
19299	2.61357	2.1
19300	2.48208	2.1
19301	2.48208	2.1
19302	2.62868	2.1
19303	1.85439	1.07207
19304	1.81776	0.618283
19305	1.81776	0.479591
19306	2.1587	0.599519
19307	1.8914	0.872298
19308	2.23961	0.569841
19309	1.49357	0.0708207
19310	2.23961	0.521765
19311	1.78014	0.390733

19312	1.67635	0.230371
19313	1.66219	0.178675
19314	1.56036	0.201947
19315	1.14721	0.05
19316	2.22495	0.175051
19317	1.88509	0.05
19318	1.95249	0.05
19319	2.11959	0.05
19320	1.37427	0.05
19321	1.39379	0.109961
19322	1.17614	0.138493
19323	1.39379	2.1
19324	1.14397	0.05
19325	1.09651	2.1
19326	1.42843	2.1
19327	1.7069	2.1
19328	2.31293	2.1
19329	1.56839	2.1
19330	1.78252	0.308304
19331	1.78254	0.131462
19332	1.09142	0.290077
19333	0.99829	0.05
19334	1.09372	0.05
19335	1.88175	0.05
19336	0.898452	0.05
19337	1.06936	2.1
19338	1.4162	2.1
19339	1.17989	2.1
19340	1.47411	2.1
19341	1.25376	2.1
19342	1.00757	0.995129
19343	1.4185	0.05
19344	1.02011	0.0755548
19345	2.22495	0.0626691
19346	1.79542	0.05
19347	1.79542	0.05
19348	1.19352	0.05
19349	2.38566	2.1
19350	1.83255	1.51651
19351	1.62667	2.1
19352	1.17879	1.7127
19353	2.79168	2.1
19354	2.28162	2.1
19355	2.28162	2.1
19356	1.90905	2.1
19357	1.40117	2.1
19358	1.28268	2.1
19359	1.41096	2.1
19360	1.22978	2.1
19361	3.20034	2.1
19362	3.42052	2.1
19363	3.42052	2.1
19364	3.50562	2.1
19365	1.46279	2.1
19366	1.3864	2.1
19367	1.3864	2.1
19368	1.3201	2.1
19369	1.35561	0.05

19370	0.974077	0.05
19371	0.967673	0.513218
19372	1.22482	0.180316
19373	1.02305	0.05
19374	1.22482	0.129507
19375	0.999492	2.1
19376	1.14657	2.1
19377	1.14657	2.1
19378	1.31346	2.1
19379	1.84417	0.05
19380	1.31777	0.05
19381	1.19796	0.05
19382	0.691742	0.05
19383	1.16725	2.1
19384	1.9679	2.1
19385	1.08848	0.05
19386	1.15356	0.05
19387	1.58713	2.1
19388	1.58742	2.1
19389	1.58742	2.1
19390	1.49662	2.1
19391	1.32201	0.226119
19392	1.32097	1.18111
19393	1.32097	0.144979
19394	1.31825	1.29856
19395	1.12028	2.1
19396	1.15852	2.1
19397	1.15852	2.1
19398	1.16239	2.1
19399	1.07624	2.1
19400	1.30047	2.1
19401	1.44847	0.946456
19402	1.55292	0.947333
19403	1.21125	0.05
19404	1.15262	2.1
19405	1.05025	2.1
19406	1.55351	2.1
19407	1.5329	0.05
19408	2.26758	0.05
19409	1.94968	0.05
19410	1.65747	0.05
19411	1.42549	0.05
19412	1.57656	0.05
19413	2.11157	0.241441
19414	1.56531	0.764134
19415	1.69279	0.05
19416	1.06204	0.778013
19417	0.947863	1.07667
19418	1.19854	1.57375
19419	0.947862	0.458196
19420	1.31126	0.273582
19421	1.3425	1.61858
19422	1.29976	1.34449
19423	1.05129	0.05
19424	1.51026	0.05
19425	1.05129	0.05
19426	1.2951	0.05
19427	1.7155	2.1

19428	1.72196	2.1
19429	1.72196	2.1
19430	1.77867	2.1
19431	0.964755	2.1
19432	1.14142	2.1
19433	1.03766	2.1
19434	0.986723	2.1
19435	1.28072	0.590659
19436	1.10609	2.1
19437	1.28072	0.05
19438	1.16776	2.1
19439	1.61687	2.1
19440	1.60965	2.1
19441	1.60965	2.1
19442	1.59654	2.1
19443	1.26512	2.1
19444	1.23964	2.1
19445	2.07997	2.1
19446	2.60048	2.1
19447	1.23732	2.1
19448	1.3372	2.1
19449	2.62253	2.1
19450	2.47056	2.1
19451	0.92782	0.05
19452	1.40335	0.05
19453	1.26301	0.05
19454	1.52668	0.05
19455	0.99818	0.05
19456	1.51165	0.326249
19457	1.66213	0.05
19458	1.55246	0.177767
19459	2.31255	2.1
19460	1.34366	2.1
19461	1.90577	2.1
19462	0.969739	2.1
19463	3.4245	2.1
19464	2.8415	2.1
19465	1.97559	2.1
19466	1.82362	2.1
19467	1.48162	2.1
19468	1.23204	2.1
19469	1.23204	2.1
19470	0.95947	2.1
19471	1.05267	2.1
19472	1.44091	1.10083
19473	1.05267	1.38802
19474	1.5795	2.1
19475	0.738711	2.1
19476	1.28549	2.1
19477	1.15694	2.1
19478	1.72061	2.1
19479	1.69858	2.1
19480	0.954563	2.1
19481	0.859087	2.1
19482	0.788886	2.1
19483	1.08732	0.05
19484	1.3357	0.0912108
19485	1.3357	0.0524243

19486	1.48676	0.0923686
19487	2.15079	2.1
19488	2.12414	2.1
19489	2.12414	2.1
19490	2.04664	2.1
19491	1.98859	2.1
19492	0.992159	2.1
19493	1.48064	2.1
19494	1.2163	2.1
19495	1.26337	2.1
19496	1.63159	2.1
19497	1.00366	0.103135
19498	1.65452	0.0697338
19499	1.10408	2.1
19500	1.37704	2.1
19501	1.50224	2.1
19502	1.42866	2.1
19503	1.43939	2.1
19504	1.02784	2.1
19505	1.00763	0.05
19506	1.23746	0.05
19507	1.23747	0.05
19508	1.13642	0.05
19509	1.49906	0.05
19510	1.46506	0.05
19511	1.46506	0.05
19512	1.33061	0.05
19513	1.08166	0.05
19514	1.01052	0.05
19515	1.02836	0.05
19516	1.08166	0.05
19517	1.25334	0.05
19518	1.23749	0.05
19519	1.37501	0.05
19520	1.47354	0.05
19521	1.30167	2.1
19522	1.29897	2.1
19523	1.51042	2.1
19524	1.07684	2.1
19525	1.07684	2.1
19526	0.765121	2.1
19527	1.5535	2.1
19528	1.40602	2.1
19529	1.40603	2.1
19530	1.25669	2.1
19531	1.08276	2.1
19532	1.60948	2.1
19533	1.26929	2.1
19534	1.09847	2.1
19535	0.827082	0.0555354
19536	1.15841	0.05
19537	1.25063	0.118373
19538	1.14397	0.05
19539	1.25063	0.365095
19540	1.10476	0.05
19541	1.14872	0.05
19542	1.3659	0.05
19543	1.36589	0.05

19544	1.4677	0.05
19545	0.590354	2.1
19546	0.655202	2.1
19547	3.48926	2.1
19548	3.32709	2.1
19549	3.32709	2.1
19550	3.13938	2.1
19551	1.24458	0.05
19552	1.29681	0.126746
19553	1.40915	0.05
19554	1.17891	0.227103
19555	1.33171	0.05
19556	1.37681	0.77953
19557	1.37681	2.1
19558	1.4516	2.1
19559	2.68241	0.05
19560	1.61031	0.05
19561	1.6488	2.1
19562	1.6585	2.1
19563	1.65848	2.1
19564	1.67061	2.1
19565	1.15152	2.1
19566	1.32548	2.1
19567	1.43677	2.1
19568	1.10264	2.1
19569	1.01472	2.1
19570	0.966964	2.1
19571	2.867 2.1	
19572	2.94897	2.1
19573	1.65815	2.1
19574	1.4502	2.1
19575	1.4502	2.1
19576	1.26755	2.1
19577	1.37138	2.1
19578	1.45026	2.1
19579	1.45026	2.1
19580	1.53446	2.1
19581	0.973502	2.1
19582	1.08542	2.1
19583	1.13387	0.05
19584	1.71031	0.05
19585	1.08911	2.1
19586	1.10748	2.1
19587	1.21823	2.1
19588	1.25005	2.1
19589	0.657014	2.1
19590	1.38139	2.1
19591	1.01485	2.1
19592	1.26045	2.1
19593	1.12468	2.1
19594	1.26045	2.1
19595	1.89278	0.274837
19596	1.06433	0.121325
19597	1.87861	0.128521
19598	0.967544	0.0593426
19599	1.50319	2.1
19600	3.47245	2.1
19601	2.2354	0.166429

19602	1.16701	0.577884
19603	1.3592	2.1
19604	1.3305	1.01772
19605	1.3305	2.08752
19606	1.15008	0.167506
19607	1.12457	0.05
19608	1.40707	0.05
19609	1.01778	2.1
19610	1.35733	2.1
19611	1.01779	2.1
19612	1.12064	2.1
19613	1.30694	0.965133
19614	1.42716	1.25499
19615	1.42717	1.58826
19616	1.04321	1.30946
19617	1.19352	0.05
19618	1.32634	0.05
19619	1.14605	2.1
19620	1.0417	2.00379
19621	1.19676	0.05
19622	1.03804	0.063272
19623	2.09455	0.16795
19624	1.15338	0.296323
19625	2.21119	2.1
19626	1.26097	2.1
19627	1.88394	0.0623253
19628	1.24106	0.05
19629	2.98143	2.1
19630	2.56248	1.99244
19631	1.88394	2.03934
19632	1.68703	0.05
19633	1.5846	2.1
19634	1.94799	2.1
19635	1.20091	2.1
19636	1.40393	2.1
19637	3.09031	2.1
19638	2.90243	2.1
19639	1.27227	2.1
19640	1.32592	2.1
19641	2.90247	2.1
19642	2.71057	2.1
19643	1.32594	2.1
19644	1.34817	2.1
19645	1.4092	2.1
19646	1.44859	2.1
19647	1.52008	2.1
19648	1.401	2.1
19649	1.40099	2.1
19650	1.28936	2.1
19651	1.56002	0.05
19652	1.08811	0.05
19653	1.46041	0.05
19654	1.56002	0.05
19655	1.50524	2.1
19656	1.21304	2.1
19657	1.21304	2.1
19658	0.947115	2.1
19659	1.60665	2.1

19660	1.85179	1.80661
19661	1.85178	1.83608
19662	2.06277	1.10134
19663	2.17534	2.1
19664	1.23007	2.1
19665	2.17533	2.1
19666	1.66635	2.1
19667	2.00869	2.1
19668	0.986724	2.1
19669	2.00475	2.1
19670	1.99394	2.1
19671	1.67244	0.05
19672	1.39604	0.05
19673	1.39604	0.05
19674	0.954046	0.05
19675	0.988863	2.1
19676	1.9864	2.1
19677	1.9864	2.1
19678	3.38645	2.1
19679	1.46932	0.05
19680	1.50847	0.05
19681	1.50848	0.05
19682	1.42705	0.05
19683	1.50108	2.1
19684	1.51621	0.918185
19685	1.94317	0.945539
19686	1.18382	0.117165
19687	2.05091	0.05
19688	1.88593	0.05
19689	1.54299	0.05
19690	0.987265	0.05
19691	1.27766	2.1
19692	1.5178	2.1
19693	1.5178	2.1
19694	1.81518	2.1
19695	1.04626	1.19064
19696	1.31057	1.30749
19697	1.31057	1.22771
19698	1.22297	1.35612
19699	1.67844	2.1
19700	1.62862	2.1
19701	1.62112	2.1
19702	1.87598	2.1
19703	1.17844	2.1
19704	1.37063	2.1
19705	1.37063	2.1
19706	1.68723	2.1
19707	1.99943	0.05
19708	1.17631	0.05
19709	1.4263	0.600871
19710	1.31023	2.1
19711	1.31023	0.947988
19712	1.20871	2.1
19713	1.50356	2.1
19714	1.69311	2.1
19715	1.69599	2.1
19716	1.6801	2.1
19717	1.5843	0.05

19718	0.840233	0.05
19719	1.95358	0.05
19720	1.40216	0.05
19721	1.39078	2.1
19722	1.18948	2.1
19723	1.01606	1.78485
19724	1.18948	2.1
19725	1.63823	2.1
19726	1.67687	2.1
19727	1.67687	2.1
19728	1.52855	2.1
19729	1.11048	2.1
19730	1.25346	2.1
19731	2.42984	1.92609
19732	3.41731	0.991963
19733	1.2258	2.1
19734	1.2966	2.1
19735	1.2966	2.1
19736	1.37392	2.1
19737	1.41758	2.1
19738	1.35504	2.1
19739	1.35504	2.1
19740	1.30347	2.1
19741	1.50877	2.1
19742	1.11193	2.1
19743	1.40984	2.1
19744	1.54537	2.1
19745	1.71638	2.1
19746	1.77795	1.6339
19747	2.07593	2.1
19748	1.93387	2.1
19749	1.9339	2.1
19750	1.75669	2.1
19751	1.80299	0.995848
19752	1.31251	0.957194
19753	1.31249	0.93891
19754	1.13411	0.797541
19755	1.06313	2.1
19756	1.04921	2.1
19757	1.55766	2.1
19758	1.53728	2.1
19759	1.53729	2.1
19760	1.51789	2.1
19761	0.993021	0.249953
19762	1.02478	0.32451
19763	1.03408	0.184765
19764	1.09056	0.948971
19765	0.697755	2.1
19766	0.632901	2.1
19767	1.34072	0.05
19768	1.80841	0.074558
19769	1.80841	0.113969
19770	1.17793	0.153964
19771	1.75032	2.1
19772	1.13076	2.1
19773	2.11851	2.1
19774	1.73255	2.1
19775	1.73255	2.1

19776	1.11181	2.1
19777	1.43772	2.1
19778	1.67381	2.1
19779	1.52164	2.1
19780	1.60861	2.1
19781	1.30588	0.05
19782	1.75241	0.05
19783	1.33026	1.27802
19784	0.962705	1.06436
19785	0.99678	0.05
19786	1.30572	0.05
19787	1.41943	0.05
19788	1.2683	0.05
19789	1.14145	0.05
19790	1.01361	0.05
19791	1.29943	2.1
19792	1.3483	2.1
19793	1.34832	2.1
19794	1.83488	2.1
19795	1.71065	2.1
19796	1.66178	2.1
19797	1.39076	2.1
19798	1.37456	2.1
19799	1.66126	2.1
19800	1.562 2.1	
19801	1.37456	2.1
19802	1.36065	2.1
19803	1.04026	0.76416
19804	1.51898	0.733995
19805	1.67088	0.177779
19806	1.9726	0.42684
19807	1.74852	0.623348
19808	1.95512	1.04541
19809	1.16165	1.1084
19810	1.06584	0.793881
19811	1.39327	2.1
19812	1.41394	2.1
19813	1.41394	2.1
19814	1.45506	2.1
19815	1.39523	2.1
19816	1.21119	2.1
19817	1.09005	2.1
19818	0.921311	2.1
19819	1.58164	2.1
19820	1.77574	2.1
19821	1.73184	2.1
19822	1.59423	2.1
19823	1.8214	2.1
19824	3.10544	1.07208
19825	1.01243	2.0712
19826	1.30896	1.11697
19827	2.16956	1.15524
19828	1.29608	0.972506
19829	1.38617	2.06474
19830	1.49536	1.80193
19831	2.35239	1.26606
19832	1.24613	1.74508
19833	1.94351	2.1

19834	2.05361	2.1
19835	2.05358	2.1
19836	2.38994	2.1
19837	1.13533	2.1
19838	1.08276	2.1
19839	1.55611	0.05
19840	1.33957	0.05
19841	1.33957	0.05
19842	1.69011	0.0705589
19843	1.5515	0.973999
19844	1.59106	0.973729
19845	1.02958	2.1
19846	1.12266	2.1
19847	1.12266	2.1
19848	1.24925	2.1
19849	1.80223	2.1
19850	1.20242	2.1
19851	2.13665	0.821779
19852	1.3924	0.659563
19853	0.904894	1.19191
19854	1.82633	2.1
19855	1.94138	0.05
19856	1.34009	0.05
19857	1.15096	2.1
19858	0.994292	2.1
19859	1.10101	2.1
19860	1.15096	2.1
19861	1.50438	2.1
19862	1.67825	2.1
19863	1.67825	2.1
19864	1.91894	2.1
19865	2.04664	2.1
19866	1.92063	2.1
19867	1.72857	2.1
19868	1.89757	2.1
19869	1.61843	0.05
19870	1.49431	0.05
19871	2.33925	2.1
19872	1.80683	2.1
19873	1.64257	2.1
19874	1.50108	2.1
19875	2.82561	2.1
19876	2.97847	2.1
19877	2.97848	2.1
19878	3.20034	2.1
19879	0.66055	2.1
19880	1.95919	2.1
19881	1.07976	2.1
19882	1.30237	2.1
19883	1.99943	0.05
19884	1.31802	0.05
19885	1.53336	0.05
19886	1.31802	0.05
19887	1.27737	0.899507
19888	1.44848	0.913142
19889	2.35499	0.05
19890	1.30041	0.05
19891	2.35499	0.05

19892	1.65381	0.05
19893	0.996083	2.1
19894	1.28754	2.1
19895	0.996081	2.1
19896	1.44859	2.1
19897	1.24763	2.1
19898	1.03697	2.1
19899	0.951273	1.7268
19900	1.60018	1.34974
19901	2.0815	2.1
19902	1.20488	2.1
19903	2.95785	2.1
19904	2.52245	2.1
19905	1.68455	0.05
19906	1.12971	0.05
19907	1.1952	0.0610156
19908	2.0815	0.144496
19909	1.3963	2.1
19910	1.4934	2.1
19911	1.47973	2.1
19912	1.25559	2.1
19913	1.1233	2.1
19914	1.15009	2.1
19915	1.15009	2.1
19916	0.977775	2.1
19917	1.16482	1.10489
19918	1.29313	0.818369
19919	1.29313	0.893078
19920	1.294	0.817666
19921	1.48693	0.0592788
19922	1.47608	0.05
19923	1.36005	2.1
19924	1.47109	2.1
19925	1.47109	2.1
19926	1.3515	2.1
19927	2.48828	2.1
19928	2.32378	2.1
19929	2.32378	2.1
19930	2.1692	2.1
19931	1.03217	0.404027
19932	0.953521	0.751984
19933	1.4515	0.05
19934	3.72406	0.05
19935	0.963395	1.93796
19936	1.01346	1.19688
19937	1.62241	2.1
19938	1.62027	2.1
19939	1.62027	2.1
19940	1.61687	2.1
19941	1.2593	0.05
19942	0.981586	0.05
19943	0.721961	2.1
19944	0.888955	2.1
19945	1.22031	0.05
19946	1.27566	2.1
19947	2.50066	2.1
19948	1.19814	2.1
19949	1.42278	0.05

19950	2.02657	0.05
19951	1.0756	0.872484
19952	1.10049	0.825836
19953	2.26155	2.1
19954	2.15634	2.1
19955	2.15634	2.1
19956	2.01613	2.1
19957	1.94233	1.1674
19958	1.18127	1.87243
19959	1.36865	1.66015
19960	1.75755	1.59567
19961	2.01613	2.1
19962	1.78958	2.1
19963	1.97995	2.1
19964	1.79198	2.1
19965	1.20841	2.1
19966	1.6519	2.1
19967	1.80207	2.1
19968	1.66774	2.1
19969	1.17935	0.05
19970	1.88071	0.05
19971	1.58723	0.05
19972	1.7361	0.05
19973	0.794165	2.1
19974	1.21327	2.1
19975	1.21327	2.1
19976	1.34268	2.1
19977	1.34497	0.05
19978	0.793303	0.05
19979	1.12323	0.05
19980	1.34496	0.05
19981	0.928226	0.0906189
19982	1.89804	0.181536
19983	2.60141	0.05
19984	2.60142	2.1
19985	2.60772	0.841207
19986	0.99747	2.1
19987	1.27096	0.356776
19988	1.27096	0.0922533
19989	1.03472	0.12482
19990	1.09962	0.859187
19991	1.21148	2.1
19992	1.21148	1.12181
19993	1.35025	2.1
19994	1.09795	2.1
19995	1.00775	2.1
19996	1.07162	2.1
19997	1.60665	2.1
19998	1.52984	2.1
19999	1.61179	2.1
20000	1.70057	2.1
20001	1.76063	2.1
20002	1.14547	0.0505795
20003	0.964782	0.0514159
20004	1.17208	0.05
20005	0.964769	0.05
20006	1.04268	2.1
20007	1.57459	2.1

20008	1.57459	2.1
20009	1.32449	2.1
20010	2.07527	1.01327
20011	1.00013	0.952655
20012	0.813711	0.05
20013	1.05677	2.1
20014	1.30664	1.51202
20015	1.96011	2.09891
20016	1.7151	1.26173
20017	1.61309	1.36048
20018	1.18954	0.143077
20019	1.05447	2.1
20020	1.05446	0.728017
20021	1.11787	0.458634
20022	1.44437	2.1
20023	1.84739	2.1
20024	1.84739	2.1
20025	1.06203	2.1
20026	1.78391	2.1
20027	1.89267	2.1
20028	1.9212	2.1
20029	2.02997	2.1
20030	1.32051	0.05
20031	1.47169	0.05
20032	1.20691	0.05
20033	1.72271	0.05
20034	1.2858	0.196542
20035	1.31011	0.894275
20036	1.31011	0.23553
20037	1.34124	0.885006
20038	1.294	0.943915
20039	1.03079	0.81916
20040	1.83192	2.1
20041	1.81979	2.1
20042	1.81979	2.1
20043	1.57829	2.1
20044	1.35435	1.90836
20045	1.19023	2.1
20046	0.991115	2.1
20047	1.27725	2.1
20048	1.38204	2.1
20049	1.48013	2.1
20050	0.983493	2.1
20051	2.26155	2.1
20052	1.02311	0.844385
20053	1.55485	0.668336
20054	1.71033	0.653397
20055	2.07319	0.404654
20056	2.29074	0.134424
20057	1.50822	0.174645
20058	2.08147	2.1
20059	1.1585	2.1
20060	3.74092	0.625255
20061	2.40799	2.1
20062	2.05349	2.1
20063	1.92656	2.1
20064	1.09634	0.05
20065	1.45502	0.05

20066	1.17261	0.05
20067	1.29383	0.05
20068	1.29383	0.05
20069	1.47689	0.05
20070	1.85717	0.232263
20071	1.53798	0.0565086
20072	1.9493	2.1
20073	2.0371	2.04453
20074	1.8519	2.1
20075	1.92119	1.30773
20076	1.00764	0.05
20077	1.0302	0.05
20078	1.0302	0.05
20079	0.90047	0.05
20080	1.49155	0.05
20081	2.26403	0.05
20082	2.06158	0.05
20083	1.09506	0.05
20084	1.9212	1.36204
20085	1.99586	1.12022
20086	1.99586	1.15119
20087	2.07527	1.07219
20088	1.20478	2.1
20089	1.09662	2.1
20090	1.55961	2.1
20091	1.03472	2.1
20092	1.54983	2.1
20093	2.02953	2.1
20094	1.84502	2.1
20095	2.1221	2.1
20096	1.02969	2.1
20097	2.23523	2.1
20098	1.2377	0.598267
20099	1.03818	0.47194
20100	1.978 2.1	
20101	1.78159	2.1
20102	1.45762	2.1
20103	1.34234	2.1
20104	2.01624	2.1
20105	2.52072	2.1
20106	1.23013	2.1
20107	2.5593	2.1
20108	3.06464	1.36317
20109	2.63697	0.969491
20110	2.63697	1.17056
20111	1.87111	0.898049
20112	1.04574	0.05
20113	2.13428	0.05
20114	1.61715	2.1
20115	1.44137	2.1
20116	1.44867	2.1
20117	1.18283	2.1
20118	1.50584	0.05
20119	1.45466	0.05
20120	1.4959	0.05
20121	1.93489	0.05
20122	2.60772	2.1
20123	2.68868	2.1

20124	2.68868	2.1
20125	2.82561	2.1
20126	1.49654	2.1
20127	1.38672	2.1
20128	1.36813	2.1
20129	1.98396	2.1
20130	1.93852	1.54714
20131	2.14237	1.07068
20132	1.44421	0.05
20133	1.21749	0.05
20134	1.09572	0.05
20135	1.11081	0.05
20136	2.32363	2.1
20137	1.24375	2.1
20138	3.79242	2.1
20139	3.46787	2.1
20140	1.19854	1.74001
20141	1.35487	1.66961
20142	1.35486	1.87132
20143	1.38616	1.81616
20144	1.67265	0.105343
20145	0.909487	0.116783
20146	2.53562	2.1
20147	1.09564	2.1
20148	2.9068	2.1
20149	3.57307	0.219024
20150	2.50369	1.57936
20151	1.47348	2.1
20152	1.24925	2.1
20153	1.3991	2.1
20154	1.3991	2.1
20155	1.52984	2.1
20156	1.60428	2.1
20157	1.50097	2.1
20158	1.50097	2.1
20159	1.40239	2.1
20160	2.19621	2.1
20161	2.35003	2.1
20162	1.57932	0.05
20163	1.61194	0.05
20164	2.0815	0.05
20165	1.18539	0.05
20166	1.5795	1.02402
20167	1.53064	1.18775
20168	1.89227	0.05
20169	3.01037	0.05
20170	2.08149	2.1
20171	2.06678	2.1
20172	1.89226	0.05
20173	3.47888	0.05
20174	1.89226	0.05
20175	3.65842	0.05
20176	3.88546	0.05
20177	1.89226	0.0560016
20178	1.11239	2.1
20179	1.29133	0.05
20180	0.889309	0.05
20181	0.881934	0.05

20182	1.88406	2.1
20183	0.636946	2.1
20184	0.636945	2.1
20185	1.28073	2.1
20186	1.28075	2.1
20187	0.942007	2.1
20188	1.68436	2.1
20189	1.4677	0.05
20190	0.851542	0.05
20191	1.39063	0.05
20192	0.851539	0.05
20193	1.11597	0.05
20194	1.35403	0.05
20195	0.959473	2.1
20196	1.07976	2.1
20197	0.946129	1.88046
20198	0.946129	0.399897
20199	1.38464	0.910296
20200	1.36991	0.109559
20201	0.946131	0.411169
20202	0.94613	0.817106
20203	1.34493	0.712593
20204	1.36991	0.678547
20205	1.36991	0.05
20206	0.946131	0.709979
20207	0.78392	0.814202
20208	0.80282	0.813123
20209	1.10351	0.787647
20210	0.80846	0.901626
20211	0.838026	0.857719
20212	1.08558	0.824062
20213	0.718203	0.884814
20214	0.501866	0.841177
20215	0.85426	0.880254
20216	1.24249	0.05
20217	1.37052	0.05
20218	0.851541	0.05
20219	1.0092	0.05
20220	0.851554	0.05
20221	1.49826	0.05
20222	1.13727	0.05
20223	0.778149	0.100201
20224	1.20257	0.0972489
20225	0.946133	0.05
20226	0.778149	0.0606446
20227	0.77815	0.205872
20228	0.946132	2.1
20229	1.17602	2.1
20230	0.946132	2.1
20231	1.31257	2.1
20232	1.17602	2.1
20233	1.35821	0.05
20234	1.19993	0.05
20235	0.946131	0.05
20236	0.946131	0.05
20237	1.08848	0.05
20238	1.00509	2.1
20239	1.19629	2.1

20240	0.867593	2.1
20241	0.757921	2.1
20242	0.729116	0.05
20243	0.89162	0.05
20244	1.16811	0.05
20245	1.3676	2.1
20246	1.2638	0.570104
20247	0.946132	2.1
20248	0.946132	2.1
20249	1.2638	2.1
20250	1.00185	2.1
20251	1.60948	2.1
20252	0.602075	2.1
20253	0.942611	2.1
20254	0.946132	2.1
20255	0.942611	2.1
20256	1.11822	2.1
20257	1.21558	0.05
20258	0.607788	0.05
20259	0.827029	0.05
20260	1.00691	0.175301
20261	0.607789	0.05
20262	0.607775	0.192854
20263	1.23248	1.16709
20264	0.902653	1.44326
20265	0.639143	1.51053
20266	0.851545	1.21756
20267	0.605153	1.23931
20268	1.06826	2.1
20269	1.29365	2.1
20270	1.41232	2.1
20271	0.946131	2.1
20272	1.20854	2.1
20273	0.946131	2.1
20274	0.909005	0.05
20275	1.16672	0.05
20276	0.810959	0.05
20277	0.946131	0.05
20278	1.40104	0.05
20279	0.946131	0.05
20280	1.35403	0.05
20281	1.40104	0.05
20282	1.92535	0.05
20283	2.14534	0.05
20284	1.77532	0.05
20285	0.851541	0.05
20286	1.05585	0.05
20287	1.27098	0.05
20288	0.870925	0.05
20289	1.15547	0.05
20290	1.47583	0.05
20291	1.35007	0.05
20292	0.740095	0.05
20293	0.644912	0.05
20294	0.819489	0.05
20295	0.983028	0.05
20296	0.861849	0.05
20297	1.40511	0.05

20298	0.86182	0.05
20299	0.9705	0.05
20300	1.40511	0.05
20301	1.15539	2.1
20302	0.946119	2.1
20303	0.736382	2.1
20304	0.736384	2.1
20305	1.41561	2.1
20306	0.785651	2.1
20307	1.34479	2.1
20308	1.38885	2.1
20309	0.94613	2.1
20310	1.29461	2.1
20311	0.94613	2.1
20312	0.776179	2.1
20313	0.858611	2.1
20314	0.946128	2.1
20315	1.04073	2.1
20316	1.16399	2.1
20317	0.990019	2.1
20318	1.15564	2.1
20319	1.28956	0.05
20320	1.35145	0.05
20321	1.09662	2.1
20322	1.8155	0.05
20323	0.811459	0.05
20324	0.731982	0.05
20325	0.900554	0.05
20326	0.946127	0.607209
20327	0.817066	0.894057
20328	0.817064	0.699886
20329	1.41798	0.728886
20330	0.871606	0.746213
20331	1.30265	0.05
20332	0.849402	1.86403
20333	0.868582	1.24526
20334	0.909137	1.51031
20335	0.807016	1.48735
20336	0.83393	0.11151
20337	0.9034	0.66085
20338	1.53515	0.554446
20339	1.17665	0.0514054
20340	0.946132	0.11272
20341	0.946129	0.05
20342	1.35977	0.05
20343	1.34254	0.05
20344	1.14241	0.05
20345	0.946132	0.05
20346	0.946132	0.05
20347	1.27376	0.05
20348	1.35978	0.05
20349	1.01976	0.05
20350	1.23498	0.48668
20351	1.35002	1.10013
20352	1.11683	1.09578
20353	0.88359	1.25745
20354	0.751374	0.965222
20355	0.791714	0.812742

20356	1.6067	2.1
20357	0.923782	2.1
20358	1.44689	2.1
20359	0.94613	2.1
20360	0.946131	2.1
20361	1.5261	2.1
20362	1.26247	2.1
20363	1.80085	0.05
20364	1.7693	0.05
20365	0.900422	0.05
20366	1.31357	0.05
20367	0.94613	0.05
20368	0.900424	0.05
20369	0.900397	0.05
20370	1.39448	0.05
20371	1.36769	0.05
20372	0.713524	0.05
20373	0.713554	0.05
20374	0.946124	0.05
20375	1.24979	0.05
20376	1.04765	0.05
20377	0.70054	0.05
20378	0.776991	0.05
20379	0.738905	0.05
20380	1.32605	0.05
20381	0.784957	0.05
20382	0.803955	0.05
20383	0.819486	0.05
20384	1.79306	0.05
20385	1.18275	0.05
20386	1.25626	0.05
20387	0.936806	0.05
20388	0.653124	0.05
20389	0.946132	0.05
20390	0.653151	0.05
20391	1.10748	0.05
20392	1.95244	2.1
20393	1.40967	2.07619
20394	1.27916	1.8679
20395	0.946132	0.0558607
20396	0.946132	1.76562
20397	1.30555	1.39203
20398	1.38651	1.96995
20399	0.946132	1.38339
20400	1.23571	0.16351
20401	1.21261	1.18298
20402	1.34225	0.715604
20403	0.946132	1.59989
20404	0.734059	0.0637177
20405	0.734084	0.676898
20406	0.78438	0.05
20407	1.13748	0.163575
20408	0.946131	0.562415
20409	0.78438	0.05
20410	0.784392	0.183046
20411	0.626495	2.1
20412	0.694635	2.1
20413	0.946125	2.1

20414	0.694632	2.1
20415	1.38521	2.1
20416	0.795469	2.1
20417	0.850877	2.1
20418	1.37098	2.1
20419	0.946131	2.1
20420	0.79547	2.1
20421	0.946132	0.780236
20422	1.30448	0.869107
20423	0.628361	0.357592
20424	0.686964	0.728406
20425	0.493261	0.734936
20426	1.90912	1.69537
20427	0.946127	1.05003
20428	1.36604	0.718615
20429	0.671687	0.754593
20430	0.946128	0.904201
20431	0.671699	0.760886
20432	1.04973	2.1
20433	0.982858	0.05
20434	0.974081	0.05
20435	1.69993	0.05
20436	1.00763	0.760968
20437	0.755566	2.1
20438	0.946129	2.1
20439	1.04184	2.1
20440	1.13509	2.1
20441	0.755566	2.1
20442	1.114	2.1
20443	0.810946	2.1
20444	0.786259	2.1
20445	1.43455	2.1
20446	1.05071	2.1
20447	0.746095	2.1
20448	0.82737	2.1
20449	0.946126	2.1
20450	1.04071	2.1
20451	1.0852	2.1
20452	1.59019	0.05
20453	0.621972	0.05
20454	1.14475	0.05
20455	0.691062	0.05
20456	1.20057	0.05
20457	0.846868	0.05
20458	1.26062	0.05
20459	0.795093	0.05
20460	1.20056	0.05
20461	0.795065	0.05
20462	1.18203	0.05
20463	1.20057	0.05
20464	0.991869	0.0903901
20465	0.841088	2.1
20466	0.946131	2.1
20467	0.869097	2.1
20468	0.946131	2.1
20469	0.841089	2.1
20470	1.77867	2.1
20471	1.99296	0.05

20472	1.91703	2.1
20473	0.651334	0.05
20474	1.31467	0.05
20475	0.651332	0.05
20476	0.651332	0.05
20477	1.03079	0.05
20478	0.808519	0.05
20479	1.41417	0.05
20480	1.03165	0.05
20481	1.43577	2.1
20482	1.39053	2.1
20483	1.07006	2.1
20484	0.946132	2.1
20485	0.946133	2.1
20486	1.07006	2.1
20487	1.12717	2.1
20488	0.994293	2.1
20489	0.946132	2.1
20490	1.31912	2.1
20491	0.946131	2.1
20492	1.40571	2.1
20493	1.31912	2.1
20494	1.12292	0.05
20495	1.07755	0.05
20496	0.851539	0.05
20497	0.946131	0.05
20498	0.929834	0.05
20499	1.83919	0.05
20500	1.04713	0.05
20501	0.929726	0.05
20502	1.50985	0.05
20503	0.946127	0.05
20504	1.0227	0.05
20505	1.2582	0.05
20506	1.1391	0.838307
20507	0.780831	1.04905
20508	0.78086	0.939222
20509	0.946133	1.03274
20510	0.780831	0.891267
20511	1.37762	0.992578
20512	0.946116	2.1
20513	1.22882	2.1
20514	1.40015	2.1
20515	1.40017	2.1
20516	0.946105	2.1
20517	0.553567	0.05
20518	1.19263	0.05
20519	0.946132	0.05
20520	0.553594	0.05
20521	1.19263	0.05
20522	1.18237	0.05
20523	0.946133	0.05
20524	0.978467	0.05
20525	0.56262	0.05
20526	0.946132	0.05
20527	0.562633	0.05
20528	0.900175	0.05
20529	1.04627	1.14043

20530	0.549928	0.922007
20531	1.17765	1.04485
20532	1.11479	1.09153
20533	0.398261	1.01683
20534	0.927608	1.11709
20535	1.38467	0.906912
20536	1.04824	0.845131
20537	1.33893	1.03999
20538	1.55775	0.939877
20539	0.816895	0.982522
20540	1.96057	1.06312
20541	1.35798	2.1
20542	1.29262	2.1
20543	0.94613	2.1
20544	0.94613	2.1
20545	1.29261	2.1
20546	1.18067	2.1
20547	0.946131	2.1
20548	0.946132	2.1
20549	1.27809	2.1
20550	1.35799	2.1
20551	1.03933	2.1
20552	1.49438	2.1
20553	0.946132	2.1
20554	1.13537	2.1
20555	1.41666	2.1
20556	1.74237	2.1
20557	0.946132	2.1
20558	1.04291	0.05
20559	1.63802	2.1
20560	1.24714	2.1
20561	0.842013	2.1
20562	0.810959	2.1
20563	0.767923	0.227205
20564	1.19583	0.424656
20565	0.767921	0.0659275
20566	0.767921	0.118361
20567	0.961348	0.250091
20568	2.08687	2.06295
20569	0.739729	0.867193
20570	1.04342	0.840535
20571	0.739757	0.845868
20572	0.946129	0.876981
20573	1.04341	1.01673
20574	1.38683	0.0550016
20575	1.34153	0.0871577
20576	0.946129	0.05
20577	0.946131	0.05
20578	1.02028	0.05
20579	1.47606	0.05
20580	1.20739	0.05
20581	0.957684	0.05
20582	0.946116	0.0730692
20583	0.851528	0.0655289
20584	0.957685	0.0771966
20585	1.04504	2.1
20586	0.902938	2.1
20587	0.946132	2.1

20588	1.07566	2.1
20589	1.4558	2.1
20590	1.93286	2.1
20591	1.27241	2.1
20592	0.828671	2.1
20593	0.810933	2.1
20594	1.31335	0.05
20595	1.40305	0.05
20596	0.912698	0.05
20597	0.810946	0.05
20598	1.11608	0.05
20599	1.26409	0.0915996
20600	0.946132	0.25217
20601	1.27376	0.246231
20602	0.946132	0.33672
20603	1.03333	0.401553
20604	1.27376	0.275079
20605	0.94613	0.0552286
20606	0.812384	0.05
20607	0.687744	0.05
20608	0.946132	0.0622108
20609	0.687758	0.0506253
20610	1.09667	0.05
20611	1.56977	2.1
20612	0.851544	2.1
20613	1.01136	2.1
20614	0.851481	2.1
20615	0.946131	2.1
20616	0.705409	2.1
20617	1.32449	0.05
20618	1.13508	0.05
20619	0.94613	0.05
20620	0.94613	0.05
20621	0.965822	0.05
20622	1.95301	0.05
20623	0.851538	0.116008
20624	1.29262	0.0993876
20625	0.95417	0.05
20626	0.851541	0.152086
20627	0.94613	0.129561
20628	1.02727	0.398497
20629	1.37675	1.41018
20630	0.955646	0.622263
20631	0.95114	0.878806
20632	0.851528	0.823691
20633	1.76513	1.33096
20634	0.87155	2.1
20635	0.94613	2.1
20636	0.871519	2.1
20637	0.966631	2.1
20638	1.37791	2.1
20639	1.19122	0.05
20640	0.818971	0.503923
20641	0.628799	0.05
20642	0.861027	0.309301
20643	0.800034	0.05
20644	0.91846	0.05
20645	0.765645	0.05

20646	1.13709	0.79449
20647	1.29054	1.04412
20648	0.755336	1.12191
20649	0.755334	0.9518
20650	0.946133	1.14933
20651	0.755336	0.503855
20652	1.14444	1.06136
20653	0.872823	1.06999
20654	0.533645	1.0113
20655	0.946132	0.992071
20656	0.533645	0.237825
20657	1.02311	0.710868
20658	1.00278	2.1
20659	1.1016	0.05
20660	0.629615	0.05
20661	1.12628	0.05
20662	0.946131	0.05
20663	0.629615	0.05
20664	0.629641	0.05
20665	1.67046	1.47847
20666	1.81667	2.1
20667	1.7145	2.1
20668	0.94613	2.1
20669	1.29322	2.1
20670	1.00365	2.1
20671	0.946131	2.1
20672	1.29322	2.1
20673	1.27581	2.1
20674	1.33916	2.1
20675	1.32784	2.1
20676	1.74787	2.1
20677	2.28084	2.1
20678	2.98092	2.1
20679	0.946132	1.0978
20680	1.2435	2.1
20681	0.946132	2.1
20682	1.4099	2.1
20683	1.2435	2.1
20684	1.26438	2.1
20685	0.745287	2.1
20686	0.696827	2.1
20687	0.696781	2.1
20688	1.41195	2.1
20689	0.946131	2.1
20690	1.8758	1.26739
20691	0.832979	0.709165
20692	0.75054	0.171365
20693	0.810958	0.411572
20694	0.922889	1.03089
20695	0.694844	0.262416
20696	0.694814	0.645941
20697	0.946131	0.348608
20698	0.694844	0.454927
20699	1.02369	2.1
20700	1.4468	0.05
20701	0.946125	0.132863
20702	0.93487	0.05
20703	1.23608	0.190432

20704	1.34257	0.05
20705	0.851525	0.05
20706	1.03298	0.0602333
20707	1.22418	1.47384
20708	0.946131	2.1
20709	1.41833	1.24099
20710	1.23452	1.10023
20711	0.946131	0.937879
20712	1.41833	1.09183
20713	1.13077	0.05
20714	0.942874	1.99347
20715	0.946132	1.63157
20716	0.504366	1.90543
20717	0.593647	2.1
20718	0.898829	2.00035
20719	1.27921	2.1
20720	1.12503	2.1
20721	1.22539	2.1
20722	1.10332	2.04437
20723	0.851541	2.1
20724	0.94613	2.1
20725	0.945109	2.1
20726	0.607962	2.1
20727	0.946133	2.1
20728	0.607961	2.1
20729	0.661956	2.1
20730	1.35048	2.1
20731	1.66018	2.1
20732	0.956467	2.1
20733	0.851542	2.1
20734	0.890734	2.1
20735	0.802455	2.1
20736	1.35943	2.1
20737	1.04798	2.1
20738	1.26426	0.0787139
20739	1.3689	0.05
20740	0.946133	0.0614624
20741	0.946133	0.0912747
20742	1.09729	0.0715014
20743	0.765259	2.1
20744	0.810945	2.1
20745	0.810957	2.1
20746	1.10284	2.1
20747	1.09368	2.1
20748	0.772601	2.1
20749	0.788011	2.1
20750	0.810934	2.1
20751	0.694357	1.11012
20752	1.22641	1.18207
20753	0.946132	1.92924
20754	0.946132	0.939118
20755	0.946133	0.980907
20756	1.42754	1.73516
20757	0.776414	0.963922
20758	0.806525	1.16953
20759	0.575339	1.10486
20760	0.97154	0.952055
20761	0.8217	1.1633

20762	0.838039	1.63587
20763	0.94613	0.673222
20764	0.946132	0.195903
20765	1.39575	0.05
20766	1.39575	0.131632
20767	1.12818	0.0850527
20768	0.946132	1.21704
20769	0.946133	0.439855
20770	1.06879	0.0542896
20771	1.06879	0.254836
20772	0.970358	0.173242
20773	1.2474	0.05
20774	1.01919	0.05
20775	0.94613	0.05
20776	1.28952	0.05
20777	1.00809	0.05
20778	0.849344	0.05
20779	0.640304	0.05
20780	1.35065	0.05
20781	0.946132	0.05
20782	0.640333	0.05
20783	0.946132	0.05
20784	0.685489	2.1
20785	0.94613	2.1
20786	0.68546	2.1
20787	0.874264	2.1
20788	1.27676	2.1
20789	0.946129	0.114026
20790	1.09024	0.05
20791	0.714563	0.0505261
20792	1.12335	0.05
20793	1.07931	0.0516001
20794	1.17799	0.0752393
20795	1.8795	0.05
20796	2.15229	0.05
20797	1.13859	0.0768525
20798	0.946132	1.00131
20799	1.11374	0.10689
20800	1.1386	0.162475
20801	0.615713	0.183163
20802	1.19322	0.05
20803	1.3377	0.05
20804	0.670801	0.05
20805	0.94613	0.411009
20806	0.66617	0.117601
20807	1.36173	0.118472
20808	0.980473	0.0548811
20809	1.39132	0.05
20810	0.864653	0.05
20811	1.23837	0.05
20812	0.810959	0.05
20813	1.1483	0.05
20814	1.00381	1.37442
20815	0.946128	1.90473
20816	1.39696	1.34774
20817	0.946131	1.1463
20818	0.946131	1.28221
20819	1.38613	1.35775

20820	0.973501	1.97556
20821	0.946127	1.27905
20822	1.35968	1.23525
20823	0.946131	1.02046
20824	0.946129	1.35808
20825	1.39696	1.31679
20826	1.12088	2.1
20827	1.41122	2.1
20828	1.14464	0.30985
20829	0.946131	2.1
20830	0.946131	1.9471
20831	1.14464	2.1
20832	1.33266	1.46564
20833	1.07341	1.05595
20834	0.827287	1.70423
20835	0.946132	1.3426
20836	0.827287	2.1
20837	1.15644	1.37009
20838	1.27558	0.751463
20839	1.15067	0.333956
20840	0.946131	2.1
20841	1.29143	0.139702
20842	1.36281	2.1
20843	1.36281	1.73181
20844	0.946131	2.01177
20845	1.40863	2.1
20846	1.20143	0.05
20847	1.19755	0.05
20848	0.566153	0.05
20849	0.946131	0.05
20850	1.07979	0.05
20851	1.00463	0.05
20852	0.566153	0.05
20853	0.768212	2.1
20854	1.32461	2.1
20855	1.13268	2.1
20856	1.13268	2.1
20857	0.946133	2.1
20858	1.53642	2.1
20859	1.5791	2.1
20860	0.726635	2.1
20861	1.2334	2.1
20862	1.29198	2.1
20863	1.29198	1.50845
20864	0.946131	1.26665
20865	0.956585	2.1
20866	1.25294	2.1
20867	0.946133	2.1
20868	1.35287	2.1
20869	1.23877	2.1
20870	0.946131	2.1
20871	1.35287	2.1
20872	1.01843	2.1
20873	3.77569	2.1
20874	0.86707	2.1
20875	0.867066	2.1
20876	1.38639	2.1
20877	1.38639	2.1

20878	0.896168	2.1
20879	0.974716	0.05
20880	1.09411	0.05
20881	0.663171	0.05
20882	0.663171	0.05
20883	0.946132	0.05
20884	0.66317	0.05
20885	1.47104	0.05
20886	1.13629	0.05
20887	0.772342	0.05
20888	0.772312	0.05
20889	0.94613	0.05
20890	0.77234	0.05
20891	1.43154	2.1
20892	1.37704	2.1
20893	1.40025	2.1
20894	1.3063	2.1
20895	0.946132	2.1
20896	1.04075	2.1
20897	0.946131	2.1
20898	0.946118	2.1
20899	0.750011	0.05
20900	1.0971	0.691241
20901	0.889667	0.0845074
20902	0.709598	0.674348
20903	2.39737	0.699457
20904	1.19974	0.05
20905	0.94613	0.05
20906	1.25809	0.05
20907	1.19974	0.05
20908	0.73005	0.05
20909	1.50764	0.05
20910	1.25809	0.05
20911	0.946133	0.05
20912	1.10357	0.05
20913	1.25809	0.05
20914	0.628177	0.05
20915	1.3543	0.05
20916	0.793711	0.05
20917	1.18455	0.05
20918	1.03966	0.05
20919	0.946131	0.05
20920	0.767453	0.05
20921	0.612292	0.05
20922	0.946131	0.05
20923	1.24128	0.05
20924	1.08761	0.05
20925	0.762059	0.05
20926	1.005 2.1	
20927	1.17054	2.1
20928	1.12665	2.1
20929	0.750368	2.1
20930	0.851542	2.1
20931	0.975436	2.1
20932	0.975442	2.1
20933	0.675367	2.1
20934	1.51973	0.05
20935	0.94613	0.05

20936	1.38954	0.055996
20937	0.946131	0.05
20938	1.07912	0.0573855
20939	1.38954	0.0508535
20940	2.0219	2.1
20941	1.18347	0.05
20942	0.946132	0.493689
20943	0.94613	0.05
20944	1.00202	0.05
20945	1.00201	0.05
20946	0.946132	0.0773941
20947	0.946132	0.702887
20948	1.37981	0.124259
20949	1.26874	0.0622013
20950	1.26874	0.0554668
20951	0.946132	0.0763511
20952	2.8687	0.05
20953	1.42884	2.1
20954	1.05188	2.1
20955	0.625307	2.1
20956	0.810956	2.1
20957	0.526656	2.1
20958	0.526684	2.1
20959	0.724122	2.1
20960	0.946132	2.1
20961	0.526684	2.1
20962	1.05337	2.1
20963	1.0593	2.1
20964	1.47981	2.1
20965	1.74431	2.1
20966	0.885612	2.1
20967	0.810946	1.36312
20968	0.845185	1.8791
20969	1.38622	0.865431
20970	0.723194	0.731237
20971	0.554302	0.931254
20972	0.709597	0.665891
20973	0.909507	0.736227
20974	0.912754	0.170372
20975	1.15174	0.05
20976	0.946131	0.218324
20977	0.912753	0.128525
20978	1.15174	0.094077
20979	1.02011	0.05
20980	0.792754	2.09303
20981	0.946129	2.1
20982	0.792754	2.04339
20983	1.27575	1.98227
20984	0.94613	2.01084
20985	1.60132	2.1
20986	0.922455	2.1
20987	1.18713	1.71969
20988	0.926957	1.89274
20989	1.66525	2.02436
20990	0.946132	2.1
20991	0.851541	2.1
20992	0.961191	2.1
20993	1.27534	2.1

20994	0.851554	2.1
20995	1.0499	2.1
20996	1.01776	0.05
20997	0.97622	0.05
20998	0.810934	0.05
20999	2.6802	0.05
21000	1.31485	0.05
21001	1.09699	0.05
21002	0.681649	0.05
21003	0.790369	0.05
21004	0.946131	0.05
21005	0.838026	1.38672
21006	0.567833	0.118282
21007	0.891492	0.895723
21008	0.986984	0.0562537
21009	0.946132	1.07912
21010	1.19028	0.114001
21011	0.986984	0.0956516
21012	0.595623	0.17836
21013	0.946131	2.1
21014	1.36502	2.1
21015	1.08109	2.1
21016	0.946129	2.1
21017	1.36503	2.1
21018	0.773897	2.1
21019	0.946133	1.48537
21020	0.613447	2.1
21021	0.613449	2.1
21022	0.946131	2.1
21023	1.11163	2.1
21024	0.966114	2.1
21025	0.735699	2.1
21026	0.662147	2.1
21027	1.04072	2.1
21028	0.946131	2.1
21029	0.862978	2.1
21030	1.48839	1.11444
21031	0.936141	0.173145
21032	0.946131	2.1
21033	0.936142	2.1
21034	1.40595	2.1
21035	0.946131	2.1
21036	1.05297	0.05
21037	3.09035	2.1
21038	1.19952	2.1
21039	0.907198	2.1
21040	0.849144	2.1
21041	0.612469	0.540682
21042	0.551237	0.78818
21043	0.985256	0.935442
21044	0.551209	0.822186
21045	0.85153	0.971264
21046	0.624712	2.1
21047	0.900018	2.1
21048	0.851542	2.1
21049	0.864787	2.1
21050	1.27669	2.1
21051	0.946711	0.854614

21052	0.810947	0.865449
21053	0.841547	0.891682
21054	0.764114	0.919534
21055	1.26692	1.27475
21056	0.946132	0.923516
21057	1.37675	0.945657
21058	1.26692	0.946166
21059	1.37545	1.2576
21060	1.49637	0.05
21061	0.709598	0.05
21062	0.751252	0.05
21063	1.36811	0.05
21064	0.635625	0.05
21065	1.31713	0.05
21066	1.18977	2.1
21067	0.893377	1.73107
21068	0.707344	2.1
21069	0.946131	2.1
21070	1.20221	2.1
21071	1.20221	2.1
21072	0.707345	2.1
21073	1.26786	2.1
21074	0.988048	2.1
21075	0.751293	2.1
21076	0.632008	2.1
21077	1.51067	2.1
21078	1.13534	2.1
21079	0.946133	2.1
21080	0.946131	2.1
21081	1.25716	2.1
21082	1.3199	2.1
21083	1.09616	2.1
21084	1.36677	1.58209
21085	1.34752	2.06639
21086	0.838041	1.39196
21087	1.67023	2.1
21088	1.02247	0.354274
21089	1.03079	0.640286
21090	0.928982	0.05
21091	1.13635	0.05
21092	0.946132	0.05
21093	0.92898	0.05
21094	1.13635	0.05
21095	1.1643	0.05
21096	0.851541	0.05
21097	1.39939	0.05
21098	0.946129	0.05
21099	1.07775	0.05
21100	1.14503	0.05
21101	1.78573	0.05
21102	1.29827	0.05
21103	0.872373	0.05
21104	1.01162	0.05
21105	0.83804	0.05
21106	1.48851	0.05
21107	1.47385	0.05
21108	0.843858	0.05
21109	0.938626	0.05

21110	0.89567	0.05
21111	1.59457	0.885166
21112	2.09666	0.05
21113	0.775132	0.05
21114	0.927005	0.05
21115	0.854224	0.05
21116	0.775142	0.05
21117	0.946106	0.05
21118	1.80784	2.1
21119	2.04593	2.1
21120	1.32282	2.1
21121	0.952138	2.1
21122	0.946132	2.1
21123	0.946132	2.1
21124	0.952138	2.1
21125	1.46988	2.1
21126	0.80465	0.05
21127	1.14155	0.05
21128	0.810931	0.05
21129	1.66203	0.546489
21130	0.674605	0.563515
21131	0.946132	0.551692
21132	1.26706	0.382061
21133	0.674633	0.201662
21134	0.946132	0.78928
21135	0.980664	0.618899
21136	1.34927	0.567296
21137	0.758454	0.0997042
21138	0.946133	0.05
21139	0.985371	0.05
21140	1.28632	0.097565
21141	0.946133	0.294451
21142	1.46868	0.05
21143	1.7522	0.05
21144	0.737431	2.1
21145	0.663679	2.1
21146	1.16541	1.94627
21147	1.3179	2.07755
21148	1.04081	2.099
21149	0.887866	1.8445
21150	0.806961	0.883303
21151	0.929061	1.68492
21152	1.28418	0.05
21153	1.38752	0.05
21154	1.33038	0.05
21155	0.946132	0.05
21156	0.946132	0.05
21157	1.14897	0.05
21158	1.1685	0.05
21159	1.18774	2.1
21160	0.961899	2.1
21161	1.48841	2.1
21162	1.79105	2.1
21163	3.51614	2.1
21164	0.682342	2.1
21165	0.783184	2.1
21166	0.946132	2.1
21167	0.868396	2.1

21168	1.21813	2.1
21169	0.848305	0.871191
21170	0.94559	0.888432
21171	0.805691	0.927471
21172	0.752217	2.1
21173	0.946132	2.1
21174	0.839123	2.1
21175	0.946131	2.1
21176	0.752187	2.1
21177	1.96491	2.1
21178	0.520997	2.1
21179	0.946132	2.1
21180	0.73446	2.1
21181	0.946133	2.1
21182	0.520995	2.1
21183	1.88522	2.1
21184	1.8361	2.1
21185	1.11285	2.1
21186	1.25334	1.25869
21187	2.00756	2.1
21188	1.44252	0.05
21189	1.00255	1.60172
21190	1.64516	2.1
21191	1.12399	0.05
21192	1.13542	0.05
21193	1.09038	0.05
21194	0.746903	0.05
21195	0.672241	0.05
21196	0.871774	0.05
21197	1.73513	0.971817
21198	0.949995	2.1
21199	1.17244	2.1
21200	0.946127	2.1
21201	1.3028	2.1
21202	1.17244	2.1
21203	0.774101	2.1
21204	0.946127	2.1
21205	0.939289	2.1
21206	0.946129	2.1
21207	0.774101	2.1
21208	1.87858	2.1
21209	1.44293	0.05
21210	1.43213	0.05
21211	0.946131	2.1
21212	0.716614	2.1
21213	0.716644	2.1
21214	1.35828	2.1
21215	0.790935	2.1
21216	0.730157	2.1
21217	1.41149	2.1
21218	0.730186	2.1
21219	0.730186	2.1
21220	0.946131	2.1
21221	0.946132	1.2254
21222	0.946132	0.632767
21223	0.990713	0.0609046
21224	1.40779	0.225879
21225	0.946132	0.222769

21226	1.06486	0.05
21227	0.946131	1.08211
21228	0.946133	0.262503
21229	1.40779	0.0782718
21230	1.26874	0.181879
21231	0.946131	0.181677
21232	1.02588	0.73963
21233	0.826073	0.13813
21234	1.41233	0.0518873
21235	0.861269	0.106192
21236	1.04073	0.37856
21237	1.27111	0.507537
21238	1.56016	1.55516
21239	1.60866	0.05
21240	0.94613	0.05
21241	0.946129	0.05
21242	1.36376	0.05
21243	1.22187	0.05
21244	0.94613	0.05
21245	1.82581	1.15268
21246	1.19705	1.27091
21247	1.7513	2.1
21248	1.85017	2.1
21249	1.72116	2.1
21250	1.12058	2.1
21251	0.899991	2.1
21252	0.616288	2.1
21253	1.07289	2.1
21254	1.7092	0.676217
21255	0.946132	0.715086
21256	0.946133	0.05
21257	1.18717	0.05
21258	1.40029	0.590045
21259	0.946132	0.05
21260	0.94613	0.246897
21261	1.18547	0.205655
21262	0.960326	0.172632
21263	0.960345	0.166337
21264	0.942557	0.213164
21265	1.18601	0.05
21266	0.946132	0.106213
21267	1.30307	0.0743593
21268	1.08169	0.0674611
21269	1.08169	0.0749963
21270	1.31716	0.0808148
21271	1.51065	0.907694
21272	0.877642	0.05
21273	0.617384	0.05
21274	0.575741	0.05
21275	0.972448	0.05
21276	1.11334	0.05
21277	1.13092	2.1
21278	0.946127	0.783995
21279	1.31724	0.852424
21280	1.39751	0.296581
21281	1.39751	0.333927
21282	0.946128	0.764995
21283	0.946132	0.544909

21284	0.713284	0.110278
21285	1.40668	0.562518
21286	1.03547	0.0701786
21287	0.713298	0.591417
21288	1.88774	0.0541811
21289	0.830164	2.1
21290	1.05211	2.1
21291	0.946119	2.1
21292	0.946107	2.1
21293	1.283 2.1	
21294	2.63609	2.1
21295	1.28418	0.951529
21296	0.946128	1.0332
21297	1.10335	0.946537
21298	0.598377	1.08989
21299	1.18818	0.939156
21300	1.10335	0.934069
21301	1.58972	1.1766
21302	1.90194	2.1
21303	0.946118	2.1
21304	0.946115	2.1
21305	0.950969	2.1
21306	1.36918	2.1
21307	0.950955	2.1
21308	1.4263	2.1
21309	1.11978	0.903122
21310	0.946132	1.13718
21311	0.946133	1.42421
21312	1.37658	0.910656
21313	1.2364	0.991394
21314	1.05496	0.953626
21315	0.946132	1.52389
21316	0.946132	1.08698
21317	1.21858	0.912126
21318	1.37658	1.05243
21319	1.09194	1.49634
21320	1.14721	0.05
21321	1.53814	0.05
21322	0.546751	1.05667
21323	0.492063	1.36578
21324	1.16889	2.09804
21325	1.00349	2.03836
21326	0.863559	1.19928
21327	1.38102	1.74473
21328	0.597449	1.14728
21329	0.537748	1.00371
21330	1.16297	1.51756
21331	0.999366	1.37618
21332	0.896554	1.1085
21333	0.566962	0.05
21334	0.946129	0.05
21335	0.452729	0.05
21336	0.696552	0.05
21337	0.852524	0.05
21338	1.57992	0.05
21339	1.64985	0.05
21340	0.946128	2.1
21341	1.21847	2.1

21342	0.946129	2.1
21343	1.41131	2.1
21344	1.21847	2.1
21345	1.21269	0.05
21346	1.0417	2.1
21347	1.15821	1.05414
21348	0.946132	0.685159
21349	0.946132	2.1
21350	1.37967	0.11778
21351	0.851529	0.224213
21352	1.20455	2.1
21353	1.4282	0.05
21354	1.26593	2.1
21355	0.838026	0.05
21356	0.985096	0.05
21357	0.83804	0.05
21358	0.822395	1.01069
21359	0.420272	0.456292
21360	0.946131	2.1
21361	1.17461	2.1
21362	0.94613	2.1
21363	1.40225	2.1
21364	1.17461	2.1
21365	1.09847	2.1
21366	1.22222	0.93065
21367	1.41798	0.16016
21368	0.791658	0.618891
21369	0.791656	0.579123
21370	0.946129	0.51772
21371	0.817064	0.494767
21372	0.946114	0.284967
21373	0.75824	0.305697
21374	0.75821	0.361142
21375	1.41778	0.199617
21376	0.791635	0.321192
21377	1.04164	1.95244
21378	0.946131	0.937264
21379	1.11172	0.561461
21380	1.30076	0.107178
21381	1.35721	0.166244
21382	1.11172	0.182855
21383	1.87218	0.05
21384	0.987248	0.05
21385	0.729695	0.82713
21386	0.974389	0.0658839
21387	1.18002	0.083849
21388	0.822612	0.05
21389	1.35721	0.0698525
21390	0.634295	0.915366
21391	0.634326	0.919183
21392	1.13245	1.24743
21393	1.13245	1.09741
21394	0.937961	0.893938
21395	1.18769	1.04247
21396	0.854949	0.834828
21397	0.967357	0.886176
21398	0.810934	0.823911
21399	3.91082	2.1

21400	0.946132	2.1
21401	0.946132	2.1
21402	1.05383	2.1
21403	1.05383	2.1
21404	0.986498	2.1
21405	1.50732	0.05
21406	0.753659	0.05
21407	1.22144	0.05
21408	1.30463	0.05
21409	0.753659	0.05
21410	1.30463	0.05
21411	0.886189	0.05
21412	1.04321	0.05
21413	0.909928	0.05
21414	1.18659	0.05
21415	1.19785	0.05
21416	1.40969	0.05
21417	1.24094	0.05
21418	0.946126	0.05
21419	1.2929	0.05
21420	1.24093	0.05
21421	0.867944	0.05
21422	1.41903	2.1
21423	2.47881	0.05
21424	1.12943	0.05
21425	0.764229	2.1
21426	0.764256	2.1
21427	0.94613	2.1
21428	0.94613	2.1
21429	1.1995	2.1
21430	1.83423	2.1
21431	0.634816	2.1
21432	0.877837	2.1
21433	0.94613	2.1
21434	1.04073	2.1
21435	1.06485	2.1
21436	0.94613	0.05
21437	0.62725	0.05
21438	0.798212	0.05
21439	1.33241	0.05
21440	0.62725	0.05
21441	1.82301	0.05
21442	1.40615	2.1
21443	0.782504	2.1
21444	0.748635	2.1
21445	0.946131	2.1
21446	0.748607	2.1
21447	0.988456	2.1
21448	1.64833	0.05
21449	1.35657	0.05
21450	1.26155	0.05
21451	0.915755	0.05
21452	0.915755	0.05
21453	0.946132	0.05
21454	1.49386	0.05
21455	1.01346	2.1
21456	0.946131	2.02979
21457	0.946132	2.1

21458	1.38651	2.1
21459	1.39523	2.1
21460	0.946132	2.1
21461	1.06312	2.1
21462	0.946131	2.1
21463	0.946129	2.1
21464	1.39523	2.1
21465	1.37372	2.1
21466	0.946131	2.1
21467	1.2929	0.05
21468	0.94613	0.05
21469	1.24448	0.05
21470	1.2929	0.05
21471	0.862011	0.05
21472	1.73583	0.05
21473	1.724	0.05
21474	1.03824	2.1
21475	1.534	2.1
21476	0.725492	1.95154
21477	0.771967	2.1
21478	0.61731	1.36196
21479	0.475743	1.81552
21480	0.719694	1.75208
21481	1.14878	1.28962
21482	0.946117	1.94244
21483	0.94613	2.00309
21484	0.946105	1.99803
21485	0.482666	1.31676
21486	0.609172	1.32674
21487	0.946131	2.1
21488	1.39509	1.00065
21489	1.07439	2.1
21490	0.946131	1.69555
21491	1.39509	1.74101
21492	0.963571	1.72701
21493	0.591011	0.139502
21494	1.1357	0.05
21495	2.11193	0.951177
21496	1.60936	2.1
21497	1.34118	0.05
21498	1.27696	0.05
21499	0.94613	0.05
21500	0.94613	0.05
21501	1.04257	0.05
21502	1.53336	0.05
21503	1.0631	0.05
21504	1.04072	0.05
21505	0.670678	0.05
21506	0.709531	0.05
21507	0.891275	0.05
21508	0.683583	0.05
21509	0.902478	0.05
21510	0.807671	0.05
21511	1.11024	2.1
21512	1.08171	2.1
21513	1.16725	2.1
21514	0.946128	2.1
21515	1.13537	2.1

21516	1.40525	2.1
21517	1.28365	2.1
21518	1.04523	2.1
21519	0.960106	2.1
21520	0.946128	2.1
21521	1.23452	2.1
21522	0.946129	2.1
21523	1.3704	2.1
21524	1.23452	2.1
21525	1.16906	0.9946
21526	0.946131	1.56287
21527	0.867246	1.05781
21528	0.867217	1.89991
21529	0.946131	1.16157
21530	1.13438	1.4851
21531	1.28684	1.55664
21532	0.946132	1.96005
21533	0.883935	1.30241
21534	0.883938	2.1
21535	0.946133	1.42226
21536	1.73444	1.17289
21537	1.21835	2.1
21538	1.28228	2.1
21539	1.8643	1.45899
21540	0.743379	0.245815
21541	1.34921	0.171635
21542	0.743381	0.250094
21543	0.743381	0.186891
21544	0.946131	0.390902
21545	0.915528	2.1
21546	0.982742	2.1
21547	0.975965	2.1
21548	0.891132	0.05
21549	1.27533	0.05
21550	0.946132	0.05
21551	1.25215	0.05
21552	0.590343	0.05
21553	0.946133	0.05
21554	0.590331	0.05
21555	1.62375	2.1
21556	0.687769	0.883484
21557	1.14374	0.870449
21558	1.20885	0.832386
21559	0.946132	0.895216
21560	1.14374	0.954628
21561	1.09308	0.409912
21562	1.02055	0.149399
21563	0.810934	0.05
21564	0.917609	1.63387
21565	1.13094	1.90588
21566	0.70959	1.39302
21567	1.11223	2.1
21568	0.83051	1.50444
21569	0.810946	1.91625
21570	0.810959	1.4219
21571	1.5773	0.05
21572	1.96133	1.20155
21573	1.0214	1.23292

21574	0.762783	1.65275
21575	0.838036	1.13065
21576	0.63591	1.86108
21577	0.967411	1.1053
21578	0.946132	2.1
21579	0.946132	2.1
21580	1.39699	1.8258
21581	1.61642	2.1
21582	0.860954	1.14279
21583	0.957411	1.87436
21584	0.660856	1.38491
21585	1.10003	2.1
21586	0.968249	2.1
21587	0.910184	1.17173
21588	1.30029	0.324832
21589	0.754119	0.05
21590	0.754121	0.592865
21591	0.94613	1.19069
21592	1.08039	2.1
21593	0.810942	0.05
21594	1.15153	0.05
21595	1.06968	0.05
21596	1.38456	2.1
21597	1.04118	2.1
21598	0.838027	2.1
21599	1.04073	2.1
21600	0.754209	2.1
21601	1.332 0.05	
21602	1.26193	2.1
21603	1.15317	0.05
21604	3.86179	0.05
21605	1.56005	2.1
21606	0.683035	0.726631
21607	0.931463	0.503887
21608	0.556019	0.825582
21609	0.929172	0.788587
21610	0.756874	0.114864
21611	1.23527	0.792424
21612	2.05961	0.829693
21613	0.946132	2.1
21614	1.16684	2.1
21615	1.3937	2.1
21616	1.3937	2.1
21617	0.946132	2.1
21618	1.17885	2.1
21619	1.62507	0.05
21620	1.58874	2.1
21621	0.819953	2.1
21622	1.16023	2.1
21623	1.34782	2.1
21624	1.16023	2.1
21625	0.819955	2.1
21626	0.79437	2.1
21627	0.794373	2.1
21628	1.20056	2.1
21629	1.25 2.1	
21630	1.20056	2.1
21631	1.35787	2.1

21632	0.946131	2.1
21633	1.38585	2.04098
21634	0.946128	1.04349
21635	0.946129	1.59392
21636	1.35714	1.74108
21637	1.04147	2.1
21638	0.946133	1.62452
21639	1.30382	1.20409
21640	0.946131	0.796445
21641	0.946131	1.19948
21642	1.38585	1.44806
21643	1.22305	1.48034
21644	1.00763	0.05
21645	0.813199	0.05
21646	0.810948	0.05
21647	1.15928	0.05
21648	0.944166	0.05
21649	0.94613	0.05
21650	0.946133	0.05
21651	1.4179	0.05
21652	0.946132	0.05
21653	1.36965	0.05
21654	1.05444	0.05
21655	0.690302	0.05
21656	0.838038	0.05
21657	0.802821	0.0856972
21658	1.40944	0.173345
21659	0.946126	0.661188
21660	0.799998	0.05
21661	0.800035	0.113479
21662	1.1766	2.1
21663	1.00319	2.1
21664	1.01606	1.8095
21665	0.872158	2.1
21666	1.04884	2.1
21667	0.679494	2.1
21668	0.946131	2.1
21669	1.37609	2.1
21670	0.94613	2.1
21671	0.946131	2.1
21672	1.18486	2.1
21673	0.726287	2.1
21674	0.946131	2.1
21675	0.726286	2.1
21676	1.30318	2.1
21677	1.01712	2.1
21678	0.946132	2.1
21679	1.04404	2.1
21680	1.37017	2.1
21681	1.37017	2.1
21682	0.946133	2.1
21683	0.992084	1.18032
21684	0.946131	0.863274
21685	0.529655	2.0095
21686	0.529686	1.10778
21687	0.946131	0.787043
21688	1.05937	1.1515
21689	1.14622	0.661129

21690	0.946131	0.620124
21691	0.584575	1.30962
21692	0.584575	0.753896
21693	0.946129	0.446503
21694	0.709714	2.1
21695	0.946132	2.1
21696	0.709714	2.1
21697	1.37199	2.1
21698	0.82899	2.1
21699	1.00133	2.1
21700	0.778424	0.05
21701	0.946132	0.05
21702	1.24094	0.05
21703	1.00691	0.05
21704	0.64868	0.05
21705	1.29736	0.05
21706	1.24767	0.334003
21707	1.28953	0.809699
21708	1.34612	0.860996
21709	0.946133	0.795306
21710	1.14187	1.00639
21711	0.946132	0.80573
21712	0.946129	0.847061
21713	1.4099	2.1
21714	0.946131	0.746117
21715	1.34612	0.0567983
21716	1.4099	0.541328
21717	0.956003	0.05
21718	1.40572	0.05
21719	3.54443	2.1
21720	0.994001	0.175769
21721	1.03679	2.1
21722	1.08467	2.1
21723	1.56875	0.05
21724	0.968463	2.1
21725	0.974889	2.1
21726	0.946131	2.1
21727	1.30203	2.1
21728	1.40199	2.1
21729	1.40199	2.1
21730	0.946132	2.1
21731	0.946132	2.1
21732	1.36994	2.1
21733	1.37372	2.1
21734	1.37372	2.1
21735	0.946132	2.1
21736	0.928979	1.1976
21737	0.565509	1.4147
21738	1.60196	0.738579
21739	1.16239	0.933581
21740	1.10746	0.528879
21741	1.15412	2.1
21742	1.15171	2.1
21743	0.946133	2.1
21744	0.940877	2.1
21745	0.940878	2.1
21746	1.41432	2.1
21747	1.04687	2.1

21748	2.75524	0.05
21749	0.946132	0.05
21750	0.766452	0.05
21751	0.766427	0.05
21752	1.36004	0.05
21753	1.06648	0.05
21754	1.62483	2.1
21755	1.0577	2.1
21756	1.39158	2.1
21757	0.94255	2.1
21758	1.05029	2.1
21759	1.03735	2.1
21760	0.931055	2.1
21761	1.03797	2.1
21762	1.43687	2.1
21763	1.28765	1.75033
21764	1.1811	1.15507
21765	0.946132	1.36437
21766	0.946132	1.35205
21767	1.1811	1.2685
21768	0.67805	0.757942
21769	1.50998	2.06514
21770	1.16661	2.1
21771	1.36749	2.1
21772	0.810945	0.825091
21773	1.14745	1.19126
21774	0.865588	0.776295
21775	1.36665	0.158072
21776	0.506964	1.16979
21777	0.506966	0.832864
21778	0.552786	0.507391
21779	0.946133	0.13931
21780	1.21336	0.0845842
21781	0.552786	0.752398
21782	0.552757	1.84383
21783	0.653468	0.448467
21784	1.36665	1.75461
21785	1.27922	0.05
21786	1.08133	0.05
21787	1.05348	0.05
21788	0.946132	0.05
21789	1.19601	0.05
21790	1.13276	0.05
21791	0.7856	0.05
21792	1.57125	0.05
21793	1.19601	0.05
21794	0.946131	0.250471
21795	1.34534	0.0902098
21796	1.19601	0.05
21797	0.870489	0.05
21798	1.49404	2.1
21799	1.35943	1.23394
21800	1.10427	2.01859
21801	0.94613	1.21672
21802	0.946131	1.52492
21803	1.10427	1.32392
21804	1.2064	0.05
21805	0.946132	0.7366

21806	1.11288	0.779337
21807	1.34268	0.110504
21808	1.34268	0.05
21809	0.946132	0.05
21810	0.946128	2.1
21811	0.946128	0.306167
21812	1.30835	2.1
21813	1.38463	1.33058
21814	0.946129	1.29999
21815	1.43778	0.95636
21816	1.23943	0.790713
21817	1.4748	2.1
21818	0.71295	2.1
21819	0.712951	2.1
21820	0.946132	2.1
21821	0.946133	2.1
21822	1.13976	2.1
21823	1.71109	2.1
21824	1.32426	2.1
21825	0.671255	2.1
21826	1.29123	2.1
21827	0.946132	2.1
21828	0.946132	2.1
21829	1.25603	2.1
21830	2.21126	0.05
21831	0.892889	2.1
21832	0.946129	2.1
21833	0.892888	2.1
21834	0.92872	2.1
21835	1.34352	2.1
21836	0.622784	0.208265
21837	1.02947	0.0533039
21838	0.810957	0.05
21839	1.91756	1.26789
21840	1.06012	0.491904
21841	1.09569	0.05
21842	1.5456	0.05
21843	1.1211	0.05
21844	1.21031	0.05
21845	1.03939	0.05
21846	0.519697	0.05
21847	0.933889	0.05
21848	0.519726	0.05
21849	1.16699	0.05
21850	0.933889	0.05
21851	0.840599	0.05
21852	0.94613	0.05
21853	0.840568	0.05
21854	1.26868	0.05
21855	1.16699	0.05
21856	1.17822	2.1
21857	0.922044	0.128603
21858	1.29322	0.369925
21859	0.946132	0.162246
21860	0.995178	0.598031
21861	0.922056	0.493508
21862	1.76232	0.703122
21863	1.77065	1.24711

21864	1.38149	1.01217
21865	0.959301	1.21162
21866	0.946133	1.06997
21867	0.946132	1.12844
21868	0.959301	1.08386
21869	1.3612	0.951319
21870	0.885314	1.18771
21871	0.885309	0.996452
21872	0.94613	1.14952
21873	0.8853	0.897391
21874	1.56164	1.07292
21875	1.45506	0.0604108
21876	1.45257	2.1
21877	0.851541	0.05
21878	1.29018	0.05
21879	0.851541	0.05
21880	1.21087	0.05
21881	1.24979	0.05
21882	1.56983	0.05
21883	1.60151	0.05
21884	1.78952	2.1
21885	0.638164	2.1
21886	0.638163	2.1
21887	1.39754	2.1
21888	1.39754	2.1
21889	0.681589	2.1
21890	1.34993	2.1
21891	1.04525	2.1
21892	0.946133	2.1
21893	1.04072	2.1
21894	0.857805	2.1
21895	1.06053	2.1
21896	1.49066	2.1
21897	1.4185	1.17735
21898	0.934059	2.05679
21899	0.93406	1.20678
21900	0.94613	1.52328
21901	0.93406	1.3121
21902	1.25277	1.42267
21903	1.00792	2.1
21904	0.609466	1.46952
21905	0.946132	1.91093
21906	0.609463	2.1
21907	1.30664	2.1
21908	0.810943	1.80081
21909	1.05741	1.07866
21910	1.3868	2.1
21911	1.15558	0.156976
21912	1.13537	2.1
21913	0.946132	2.1
21914	0.946132	2.1
21915	1.08266	2.1
21916	1.54934	2.1
21917	1.26016	2.1
21918	2.01915	2.1
21919	0.60346	1.44835
21920	0.651529	1.534
21921	1.15581	2.07131

21922	1.13037	1.28649
21923	1.04071	1.24599
21924	1.77796	2.1
21925	0.532308	1.4277
21926	0.532308	1.31154
21927	1.15369	2.1
21928	1.1537	1.91788
21929	0.903288	1.57689
21930	0.804766	2.1
21931	0.804739	2.1
21932	0.946128	2.1
21933	0.946131	2.1
21934	1.16745	2.1
21935	1.04193	2.1
21936	0.98505	2.1
21937	1.40724	0.533166
21938	1.14216	0.600671
21939	0.946131	0.108758
21940	0.946131	0.229548
21941	1.10941	0.146569
21942	0.81268	0.05
21943	0.946131	0.05
21944	1.28673	0.05
21945	1.28673	0.05
21946	0.812681	0.05
21947	1.84716	0.05
21948	1.84716	0.05
21949	0.92361	0.05
21950	0.94613	0.05
21951	0.923582	0.05
21952	1.26227	0.05
21953	0.923581	0.05
21954	0.747394	2.1
21955	1.32582	2.1
21956	0.747365	2.1
21957	0.97587	2.1
21958	1.32582	2.1
21959	1.04182	0.05
21960	0.961743	0.05
21961	0.946126	0.05
21962	1.47463	0.05
21963	1.12183	0.05
21964	0.851552	0.05
21965	1.33177	0.05
21966	0.946131	0.05
21967	1.41512	0.05
21968	1.33178	0.05
21969	0.946131	0.05
21970	0.927735	0.255713
21971	0.97206	0.0521285
21972	0.515566	0.0883745
21973	0.946129	0.0694802
21974	0.515538	0.0692989
21975	1.04482	0.05
21976	0.975535	0.05
21977	0.522411	0.05
21978	0.522382	0.0689557
21979	0.925346	0.0692763

21980	0.575817	0.076695
21981	0.946129	2.1
21982	1.14256	2.1
21983	1.32054	2.1
21984	1.40525	2.1
21985	1.14256	2.1
21986	1.59475	2.1
21987	0.773813	2.1
21988	1.4073	2.1
21989	0.773813	2.1
21990	0.773813	2.1
21991	1.4073	2.1
21992	0.993021	2.1
21993	1.50466	0.05
21994	0.851539	0.05
21995	1.20299	0.05
21996	0.946131	0.05
21997	1.54464	0.05
21998	1.20309	0.05
21999	2.00701	0.05
22000	0.810946	0.05
22001	1.07678	0.05
22002	0.900128	0.05
22003	1.26842	2.1
22004	1.11206	2.1
22005	0.946132	2.1
22006	1.04074	2.1
22007	0.948809	2.1
22008	0.946131	0.05
22009	0.929617	0.05
22010	1.24057	0.05
22011	1.36485	0.05
22012	0.851554	0.05
22013	1.96823	0.05
22014	0.914836	0.05
22015	0.94613	0.05
22016	1.38911	0.05
22017	0.914805	0.05
22018	0.946131	0.05
22019	0.946131	0.05
22020	1.04072	0.05
22021	1.26837	0.05
22022	0.970054	0.05
22023	0.923958	0.05
22024	1.16653	2.1
22025	1.34412	2.1
22026	0.851541	0.05
22027	1.38645	0.05
22028	0.891637	0.05
22029	0.851487	0.05
22030	0.890871	0.05
22031	1.56178	0.05
22032	0.946128	0.05
22033	1.26868	0.05
22034	0.946131	0.05
22035	1.18789	0.05
22036	1.26868	0.05
22037	1.26868	0.05

22038	0.946128	0.05
22039	1.18716	0.05
22040	0.946131	0.05
22041	0.59358	0.05
22042	1.23923	0.05
22043	1.00024	0.05
22044	0.593593	0.05
22045	0.946131	0.05
22046	0.713645	0.05
22047	1.23175	0.05
22048	1.23923	0.05
22049	0.713633	0.05
22050	1.24687	0.05
22051	1.80662	2.1
22052	2.15762	2.1
22053	0.780455	2.1
22054	0.946131	2.1
22055	0.780455	2.1
22056	1.40583	2.1
22057	1.39852	2.1
22058	0.565619	2.1
22059	1.1428	2.1
22060	0.663168	0.0571402
22061	1.02163	0.05
22062	1.04072	0.0685444
22063	0.663169	0.05
22064	1.13511	0.05
22065	1.85277	0.05
22066	1.17701	0.280632
22067	0.993831	0.05
22068	1.0823	0.05
22069	0.795761	0.05
22070	1.24596	0.05
22071	0.566075	0.05
22072	0.94613	0.05
22073	0.56605	0.0545253
22074	0.774911	0.05
22075	0.946131	0.05
22076	1.24417	0.05
22077	0.774911	0.05
22078	0.946132	0.05
22079	1.54982	0.05
22080	1.82961	0.05
22081	1.12035	0.05
22082	0.876835	2.1
22083	0.946131	2.1
22084	0.876836	2.1
22085	1.12968	2.1
22086	1.23536	2.1
22087	2.15082	2.1
22088	0.58365	2.1
22089	0.946133	2.1
22090	0.583653	2.1
22091	0.992097	2.1
22092	1.12968	2.1
22093	1.0305	2.1
22094	0.82613	0.05
22095	0.879644	0.05

22096	1.00688	0.05
22097	1.52776	2.1
22098	0.851537	1.67612
22099	1.00851	1.36172
22100	0.946128	1.11534
22101	0.851552	1.40009
22102	1.4895	1.82582
22103	0.761574	2.1
22104	0.946132	2.1
22105	1.01095	2.1
22106	1.01095	2.1
22107	0.761572	2.1
22108	1.8575	2.1
22109	1.19984	0.05
22110	1.25797	0.05
22111	0.946132	0.05
22112	0.946132	0.05
22113	0.95332	0.05
22114	0.946131	2.1
22115	0.851542	2.1
22116	1.03172	2.1
22117	1.22889	2.1
22118	1.0313	2.1
22119	1.15977	1.76343
22120	0.918875	0.840203
22121	0.8654	2.1
22122	0.865374	0.81407
22123	0.946132	0.760665
22124	0.985051	2.1
22125	1.31993	0.05
22126	1.33622	0.789022
22127	0.959992	0.56526
22128	0.926499	2.1
22129	1.03544	2.1
22130	0.946132	2.1
22131	0.946132	2.1
22132	1.40335	2.1
22133	0.772299	2.1
22134	0.851529	2.1
22135	0.857243	2.1
22136	1.38155	2.1
22137	1.05462	2.1
22138	0.832138	2.1
22139	0.946132	2.1
22140	0.832137	2.1
22141	0.959414	2.1
22142	1.36541	2.1
22143	1.91894	2.1
22144	1.04661	1.93017
22145	1.28355	2.1
22146	0.877353	1.12136
22147	0.877353	1.78307
22148	0.94613	1.54844
22149	1.20537	0.05
22150	1.09914	0.05
22151	0.94613	0.05
22152	0.499593	0.05
22153	0.499606	0.05

22154	1.0959	0.05
22155	0.838027	0.05
22156	0.883644	0.05
22157	0.95019	0.05
22158	1.10638	2.1
22159	0.946132	2.1
22160	1.41744	2.1
22161	1.05842	2.1
22162	0.94613	2.1
22163	1.41744	2.1
22164	0.991985	2.1
22165	3.31755	1.48805
22166	1.05556	2.1
22167	0.624075	1.25917
22168	0.814554	0.525188
22169	0.946131	1.32823
22170	0.814554	1.58156
22171	1.31857	2.1
22172	1.26282	2.1
22173	0.946132	2.1
22174	1.14302	2.1
22175	0.946132	2.1
22176	0.946132	2.1
22177	1.40372	2.1
22178	1.24688	0.05
22179	0.89889	2.1
22180	1.17286	2.1
22181	0.71741	2.1
22182	0.491096	0.772958
22183	1.05432	0.0716518
22184	0.719531	2.1
22185	1.25145	2.1
22186	0.864686	2.1
22187	0.867974	2.1
22188	1.21734	2.1
22189	0.405517	0.0930122
22190	0.818878	0.776237
22191	0.502457	0.05
22192	1.05868	0.05
22193	0.912018	0.05
22194	1.40591	0.05
22195	0.957584	0.05
22196	0.946131	0.05
22197	1.11612	0.05
22198	1.3365	0.05
22199	0.946128	0.0604388
22200	1.16534	0.05
22201	1.38969	0.221296
22202	0.891597	1.63638
22203	0.640032	2.0555
22204	0.956758	1.72197
22205	1.3428	0.229205
22206	0.953295	2.1
22207	0.946131	0.132766
22208	1.16167	0.0565706
22209	0.94613	0.259324
22210	0.951675	0.0764444
22211	1.16167	0.0933086

22212	1.24538	0.05
22213	1.13482	2.1
22214	1.38775	2.1
22215	0.934061	2.1
22216	0.91408	2.1
22217	1.06099	2.1
22218	0.980549	0.05
22219	0.946132	0.05
22220	1.10321	0.05
22221	0.99126	0.05
22222	0.991261	0.05
22223	1.35732	0.05
22224	1.1952	2.1
22225	0.946132	1.31972
22226	0.946132	2.1
22227	1.32819	1.52591
22228	1.30171	1.19018
22229	1.29667	0.05
22230	1.00982	0.05
22231	0.407589	0.364704
22232	1.18266	1.32531
22233	1.04037	0.309967
22234	0.956694	0.936814
22235	0.552639	0.129364
22236	0.810943	2.08619
22237	1.43144	1.01507
22238	0.918074	1.00044
22239	1.60976	2.1
22240	4.00847	2.1
22241	2.3995	0.05
22242	1.36203	2.1
22243	0.919619	2.1
22244	1.30188	2.1
22245	0.681073	2.1
22246	0.681043	2.1
22247	1.04687	2.1
22248	3.32085	2.1
22249	1.80396	0.05
22250	0.773007	0.132891
22251	0.773034	0.0598015
22252	1.22141	0.243088
22253	1.22141	0.311276
22254	0.946131	0.506196
22255	2.02071	0.05
22256	0.951912	0.05
22257	1.39027	0.05
22258	0.94613	0.05
22259	0.829922	0.05
22260	0.829946	0.05
22261	1.24902	1.40928
22262	2.8333	1.19582
22263	0.946131	0.05
22264	0.929725	0.05
22265	1.1134	0.05
22266	1.17056	0.05
22267	0.929713	0.05
22268	0.749096	2.1
22269	1.31961	2.1

22270	0.870191	2.1
22271	1.04071	2.1
22272	1.1877	2.1
22273	1.03882	2.1
22274	1.44801	2.1
22275	3.55198	2.1
22276	1.11193	2.1
22277	0.663922	2.1
22278	0.663922	2.1
22279	0.946129	2.1
22280	0.946131	2.1
22281	0.97079	2.1
22282	1.36049	2.1
22283	1.06105	2.1
22284	0.92251	2.1
22285	0.94613	2.1
22286	0.922511	2.1
22287	1.04101	2.1
22288	0.810944	0.05
22289	0.994109	0.05
22290	0.623143	0.05
22291	0.864244	0.05
22292	1.14627	0.05
22293	0.64586	0.05
22294	0.721422	0.05
22295	0.870885	0.05
22296	0.855175	0.05
22297	0.946131	1.14407
22298	1.38157	2.1
22299	1.33266	0.164437
22300	0.946131	2.1
22301	1.38158	2.1
22302	0.946132	2.1
22303	1.3268	2.1
22304	0.946132	2.1
22305	0.946132	2.1
22306	1.38158	2.1
22307	1.42186	2.1
22308	1.47204	2.08479
22309	1.14665	2.1
22310	0.946132	1.32694
22311	0.94613	2.1
22312	1.30116	2.1
22313	1.32819	2.03726
22314	1.25388	2.1
22315	0.887808	1.91772
22316	0.946132	2.1
22317	1.23536	2.1
22318	1.34309	2.1
22319	1.07612	2.1
22320	0.728982	0.05
22321	1.3157	0.05
22322	0.946131	0.05
22323	0.686176	0.05
22324	1.3035	0.05
22325	0.686139	0.05
22326	0.906806	0.05
22327	0.794122	0.05

22328	1.17173	0.05
22329	1.8194	0.05
22330	0.851543	2.1
22331	1.19345	2.1
22332	0.946133	2.1
22333	0.946132	2.1
22334	1.56179	2.1
22335	0.620263	2.1
22336	1.30226	2.1
22337	1.2334	1.43379
22338	1.2334	1.22064
22339	0.946133	1.23379
22340	1.24053	2.1
22341	1.4828	2.1
22342	0.957622	2.1
22343	1.00521	1.55701
22344	1.49463	2.1
22345	1.39621	2.1
22346	1.09861	2.1
22347	0.946131	2.1
22348	0.946131	2.1
22349	1.09861	2.1
22350	2.16987	0.05
22351	0.572182	0.05
22352	1.15802	0.05
22353	0.63574	0.05
22354	1.18897	0.05
22355	0.996733	0.05
22356	1.82402	1.84069
22357	0.969602	1.51633
22358	0.794777	1.89123
22359	1.02626	1.67404
22360	1.51396	0.05
22361	1.40574	0.05
22362	1.38683	0.05
22363	0.946126	0.05
22364	0.946128	0.05
22365	1.07138	0.05
22366	0.946131	2.1
22367	1.41331	2.1
22368	0.94613	2.1
22369	0.94613	2.1
22370	1.27451	2.1
22371	0.974196	2.1
22372	1.2049	0.05
22373	1.42757	2.1
22374	1.02461	2.1
22375	2.07087	2.1
22376	1.92515	1.50338
22377	0.802138	1.29175
22378	0.946128	1.94908
22379	0.802107	1.58404
22380	1.23726	1.53873
22381	1.21997	1.39845
22382	0.767577	0.403491
22383	0.946131	1.76031
22384	0.767577	1.33768
22385	1.3279	1.24448

22386	1.23726	0.964947
22387	1.29729	2.1
22388	0.946128	2.1
22389	1.14186	2.1
22390	1.39214	2.1
22391	1.39214	2.1
22392	0.946129	2.1
22393	1.23012	0.05
22394	0.653228	0.0649102
22395	0.946133	1.22861
22396	1.15918	1.48723
22397	1.26893	0.725951
22398	0.653228	0.776184
22399	1.06848	1.80874
22400	0.615121	2.1
22401	0.841488	1.93643
22402	0.946131	2.1
22403	1.04071	2.1
22404	0.965717	2.1
22405	1.77893	1.5695
22406	1.11314	0.05
22407	1.65411	0.312985
22408	0.85472	0.05
22409	1.1604	0.05
22410	1.19141	0.05
22411	1.19141	0.05
22412	0.94613	0.05
22413	1.70944	0.05
22414	1.89082	0.05
22415	1.05175	1.03483
22416	0.815276	2.1
22417	0.843283	2.1
22418	0.946131	2.1
22419	0.843284	2.1
22420	1.38897	2.1
22421	1.01019	0.574742
22422	1.05197	0.387653
22423	0.810959	0.461079
22424	1.33037	0.05
22425	1.3415	0.05
22426	0.946118	0.05
22427	0.946131	0.05
22428	1.09803	0.05
22429	0.930281	2.1
22430	1.3335	2.1
22431	0.784151	2.1
22432	0.78415	2.1
22433	0.946132	2.1
22434	1.6299	0.05
22435	0.995795	2.1
22436	1.84846	1.56573
22437	0.929873	2.1
22438	0.889245	2.1
22439	0.808072	2.1
22440	0.851541	1.95861
22441	1.51956	1.64907
22442	0.94613	1.73906
22443	0.851553	1.78881

22444	0.946787	1.7503
22445	1.15544	1.94156
22446	1.30289	0.05
22447	1.28323	0.05
22448	0.85024	0.05
22449	1.09662	0.05
22450	1.67687	0.05
22451	0.946127	2.1
22452	1.40496	2.1
22453	1.22442	2.1
22454	0.946128	2.1
22455	1.40496	2.1
22456	1.00573	2.1
22457	0.636837	2.1
22458	0.988642	2.1
22459	0.573141	2.1
22460	0.946128	2.1
22461	0.786538	2.1
22462	2.28748	1.59405
22463	0.682805	2.1
22464	0.946132	2.1
22465	0.682776	2.1
22466	0.946132	2.1
22467	0.79775	2.1
22468	1.05403	0.05
22469	0.888273	0.05
22470	0.876373	0.05
22471	0.576083	0.05
22472	0.946132	0.05
22473	0.576057	0.05
22474	0.576058	0.05
22475	0.946131	0.05
22476	1.41724	0.0806774
22477	1.31678	0.524587
22478	0.946132	0.557616
22479	0.946132	0.366963
22480	1.31678	0.290991
22481	0.935738	2.1
22482	0.946132	2.1
22483	0.935765	2.1
22484	1.32949	2.1
22485	0.946131	2.1
22486	1.02525	2.1
22487	0.946128	2.1
22488	1.41207	2.1
22489	1.04675	2.1
22490	0.946131	2.1
22491	1.41207	2.1
22492	0.946131	2.1
22493	0.946132	2.1
22494	1.41125	2.1
22495	1.41125	2.1
22496	1.27087	2.1
22497	0.924071	0.05
22498	0.608893	0.05
22499	0.528907	0.05
22500	1.78249	0.05
22501	0.720094	0.05

22502	0.781667	2.1
22503	0.781638	2.1
22504	1.40756	2.1
22505	1.40756	2.1
22506	0.797635	2.1
22507	1.11946	0.05
22508	1.32068	0.05
22509	0.88757	0.05
22510	1.27891	0.05
22511	0.810957	0.05
22512	0.914656	0.05
22513	0.640819	0.05
22514	0.745643	0.05
22515	0.865108	0.05
22516	1.06883	0.05
22517	0.806001	0.05
22518	1.19462	2.1
22519	1.42763	0.0629897
22520	1.24682	0.059957
22521	0.78392	0.05
22522	1.02874	0.05
22523	0.884512	0.05
22524	1.1658	2.1
22525	0.663026	0.05
22526	1.21174	0.05
22527	0.663056	0.05
22528	1.15968	0.05
22529	1.21174	0.05
22530	0.993976	0.05
22531	0.945495	0.05
22532	0.671202	0.05
22533	1.37312	2.1
22534	1.28499	2.1
22535	0.946133	2.1
22536	0.946132	2.1
22537	1.28499	2.1
22538	1.25081	2.1
22539	0.946132	2.1
22540	0.946132	2.1
22541	1.27985	2.1
22542	1.37312	2.1
22543	0.830638	2.1
22544	1.05358	2.1
22545	1.22209	2.1
22546	1.09874	2.1
22547	0.74758	2.1
22548	2.50011	1.91576
22549	0.6157	0.05
22550	0.674634	0.417438
22551	0.946128	1.01284
22552	0.674633	0.601442
22553	0.946131	0.398088
22554	1.96192	1.9566
22555	1.31612	0.05
22556	1.10735	0.05
22557	0.936676	0.05
22558	0.808779	0.05
22559	0.808808	0.05

22560	1.00245	0.05
22561	1.26179	0.05
22562	1.05758	2.1
22563	1.03408	2.1
22564	0.946132	0.05
22565	0.914368	0.05
22566	1.20524	0.05
22567	1.12743	0.05
22568	0.851555	0.05
22569	1.17885	0.718108
22570	0.867881	0.663712
22571	1.00122	0.702008
22572	0.867883	0.356223
22573	0.867881	0.6537
22574	1.19583	0.699666
22575	1.17743	2.1
22576	0.878684	2.1
22577	0.687087	2.1
22578	0.955079	0.123414
22579	0.672977	0.459103
22580	0.735937	0.539184
22581	0.851531	0.899665
22582	0.672957	0.524304
22583	0.923974	0.72862
22584	1.2243	2.1
22585	0.808463	0.941178
22586	0.971078	0.968368
22587	1.06161	0.973769
22588	0.810947	1.00578
22589	0.886074	1.03508
22590	0.786829	1.09375
22591	0.55062	0.950738
22592	0.574458	1.70051
22593	0.946129	0.963764
22594	1.42526	0.974986
22595	1.15962	0.990798
22596	1.24526	0.05
22597	0.94613	0.240386
22598	0.946128	0.05
22599	1.23279	0.159855
22600	1.38645	0.173736
22601	0.94613	0.156909
22602	1.05042	2.1
22603	0.946128	2.1
22604	0.946129	2.1
22605	1.24286	2.1
22606	1.39659	2.1
22607	0.94613	2.1
22608	1.10407	2.1
22609	1.32809	2.04833
22610	0.993999	2.1
22611	0.810959	1.85422
22612	0.983643	1.96788
22613	0.869552	2.1
22614	0.747801	1.74369
22615	1.53429	1.38751
22616	1.13127	2.1
22617	1.38397	2.1

22618	1.08366	2.1
22619	0.946129	2.1
22620	0.946131	2.1
22621	1.08366	2.1
22622	1.06007	2.1
22623	1.79641	2.1
22624	0.758799	2.1
22625	1.28442	2.1
22626	1.06957	2.1
22627	0.946133	2.1
22628	1.28442	2.1
22629	1.76788	2.00813
22630	1.38669	2.1
22631	0.986095	2.1
22632	1.38478	1.733
22633	0.946132	0.922171
22634	1.24344	0.76366
22635	0.622283	1.6173
22636	1.10335	0.824127
22637	1.24344	0.867633
22638	1.1967	1.0163
22639	0.946131	0.05
22640	0.901005	0.05
22641	1.05751	0.05
22642	1.37771	0.05
22643	0.851527	0.05
22644	1.26643	1.15633
22645	1.08992	1.6481
22646	0.946132	1.49543
22647	0.946132	1.11815
22648	1.08992	1.58637
22649	1.53534	2.1
22650	1.58825	1.73925
22651	1.0447	2.1
22652	0.593818	1.92836
22653	0.89883	2.02822
22654	0.734088	2.08196
22655	0.998451	0.975608
22656	1.64156	0.423726
22657	1.05654	1.17772
22658	1.37641	0.148263
22659	1.74553	0.0549934
22660	1.31112	0.0514922
22661	0.74518	0.0798853
22662	0.851539	0.05
22663	0.851554	0.05
22664	0.851475	0.05
22665	0.946132	0.05
22666	1.28895	0.05
22667	1.11356	0.05
22668	0.946132	0.05
22669	0.946132	0.05
22670	1.84214	2.1
22671	1.23851	0.05
22672	1.25866	0.05
22673	1.28655	0.429759
22674	0.946131	0.298488
22675	0.946132	0.19124

22676	1.28655	0.155601
22677	1.11929	0.151401
22678	0.946132	0.342206
22679	0.946132	0.0948717
22680	1.34254	0.0794717
22681	1.25866	0.0568188
22682	0.946132	2.1
22683	1.27451	2.1
22684	1.19803	2.1
22685	0.94613	2.1
22686	1.27451	2.1
22687	0.946131	2.1
22688	1.36523	2.1
22689	1.14599	2.1
22690	0.946129	2.1
22691	1.36523	2.1
22692	1.09518	2.1
22693	0.977777	2.1
22694	0.583237	2.1
22695	0.583267	2.1
22696	0.903872	2.1
22697	0.946129	2.1
22698	0.583258	2.1
22699	1.58179	0.05
22700	0.946131	0.05
22701	1.35123	0.05
22702	0.655837	0.05
22703	1.12018	0.05
22704	1.35123	0.05
22705	0.62967	0.05
22706	0.860497	0.05
22707	0.816639	0.05
22708	0.746716	2.1
22709	0.810947	2.1
22710	0.810959	2.1
22711	0.653422	2.1
22712	0.289127	1.60958
22713	0.475716	2.1
22714	0.980651	1.59281
22715	0.946119	2.1
22716	0.946132	2.1
22717	1.08299	2.1
22718	1.34539	2.1
22719	1.28753	0.05
22720	0.946132	0.05
22721	0.946132	0.05
22722	1.19719	0.05
22723	0.946133	0.05
22724	0.958947	0.05
22725	1.3492	0.05
22726	0.946132	0.05
22727	0.946128	0.05
22728	1.22588	0.05
22729	0.946129	0.05
22730	0.991113	2.1
22731	1.35966	2.1
22732	0.961 2.1	
22733	0.946129	2.1

22734	0.946131	2.1
22735	0.961001	2.1
22736	0.727096	1.92333
22737	0.810946	0.506858
22738	0.810959	1.19526
22739	1.31658	0.05
22740	1.23167	2.1
22741	1.44836	0.05
22742	1.50103	0.05
22743	1.23351	0.05
22744	1.35657	0.05
22745	0.851541	0.05
22746	0.93668	0.05
22747	0.946131	0.05
22748	3.7335	2.1
22749	1.1822	2.1
22750	1.31127	0.0697448
22751	1.3689	0.05
22752	1.35822	0.05
22753	0.946133	0.05
22754	0.946132	0.05
22755	1.14926	0.05
22756	1.40822	0.05
22757	1.04935	0.05
22758	0.704112	0.05
22759	0.704112	0.051629
22760	0.946131	0.0664462
22761	0.704112	0.05
22762	1.37721	0.05
22763	0.85154	2.1
22764	1.06949	2.1
22765	0.851541	2.1
22766	0.946128	2.1
22767	1.1175	2.1
22768	1.44091	2.1
22769	0.96029	0.845596
22770	3.46486	1.30907
22771	0.603286	0.0565303
22772	0.432283	0.05
22773	0.851541	0.05
22774	1.09355	0.05
22775	0.851534	0.05
22776	1.04263	2.1
22777	0.851541	2.1
22778	0.965604	2.1
22779	0.94613	2.1
22780	1.23953	2.1
22781	0.869615	2.1
22782	1.02415	2.1
22783	0.983032	2.1
22784	1.35385	1.57927
22785	1.59971	0.05
22786	0.915178	2.1
22787	0.962435	2.1
22788	1.3116	2.1
22789	1.38302	2.1
22790	1.13227	2.1
22791	1.25923	0.05

22792	0.988805	2.1
22793	0.611486	1.10763
22794	1.1019	1.19545
22795	1.11124	1.67709
22796	0.716713	1.11191
22797	0.946131	1.38769
22798	1.22297	1.33494
22799	1.12873	1.50735
22800	0.946131	1.55966
22801	0.946132	1.52773
22802	1.3469	1.87314
22803	1.25366	2.07899
22804	1.1611	1.09581
22805	0.946132	1.36309
22806	0.946132	1.12645
22807	1.2854	1.31757
22808	1.3469	1.4561
22809	1.07468	1.13657
22810	1.79317	2.1
22811	1.07444	2.1
22812	1.69436	2.1
22813	0.851539	2.1
22814	0.870925	2.1
22815	0.851541	2.1
22816	0.870951	2.1
22817	1.26097	2.1
22818	1.26247	2.1
22819	0.946132	2.1
22820	1.24041	2.1
22821	0.946132	2.1
22822	0.946132	2.1
22823	1.40274	2.1
22824	0.946131	0.05
22825	0.797945	0.05
22826	1.12988	0.05
22827	1.00681	0.05
22828	0.797932	0.05
22829	0.977483	0.05
22830	0.943362	0.17141
22831	0.9891	0.05
22832	1.1703	0.05
22833	0.663171	0.05
22834	1.40882	0.05
22835	1.32639	0.05
22836	1.24448	0.05
22837	0.946132	0.05
22838	1.11015	0.05
22839	1.24448	0.05
22840	0.754768	0.05
22841	1.13854	2.1
22842	1.23654	2.1
22843	0.714101	0.120154
22844	0.851538	0.0506887
22845	0.79261	1.32189
22846	1.30598	0.742953
22847	1.05338	0.0721641
22848	1.41758	2.1
22849	0.83317	0.05

22850	1.00058	0.05
22851	0.810958	0.05
22852	1.63791	0.05
22853	1.55116	2.1
22854	1.29735	1.23167
22855	0.945147	2.1
22856	0.94515	1.26672
22857	0.94613	2.01672
22858	0.945149	1.85875
22859	1.02368	2.1
22860	1.12787	2.1
22861	1.04007	2.1
22862	3.78805	2.1
22863	0.816891	0.05
22864	0.943996	0.05
22865	0.870002	0.05
22866	0.564533	0.05
22867	0.564536	0.302665
22868	0.946128	0.735652
22869	0.564535	0.386388
22870	0.946128	0.575523
22871	1.15177	2.1
22872	1.16806	0.955189
22873	0.999083	1.14323
22874	0.946131	1.02865
22875	0.946131	1.04033
22876	0.999084	1.11008
22877	1.28918	0.933995
22878	1.0383	1.18018
22879	0.946133	1.02103
22880	0.946132	1.05739
22881	1.0383	1.04571
22882	1.27737	1.67553
22883	1.39296	0.158096
22884	0.998682	2.1
22885	3.98933	2.1
22886	1.05328	0.05
22887	1.23475	2.1
22888	0.900856	1.35407
22889	0.94001	1.03608
22890	0.707748	0.974189
22891	0.707751	1.12838
22892	0.946128	0.980497
22893	0.70775	1.04431
22894	0.996109	1.03432
22895	1.68551	2.1
22896	0.851539	2.1
22897	0.962688	2.1
22898	0.94613	2.1
22899	1.04073	2.1
22900	1.44824	2.1
22901	0.794313	0.0699821
22902	0.838031	0.0708258
22903	1.11076	0.05
22904	1.38813	2.1
22905	1.17204	2.1
22906	0.946132	2.1
22907	0.946131	2.1

22908	1.17204	2.1
22909	0.961779	0.05
22910	1.0685	0.100033
22911	0.810947	2.1
22912	1.2899	1.16461
22913	0.890404	1.14795
22914	0.909981	2.1
22915	0.810946	1.41416
22916	0.890402	1.26813
22917	0.522844	2.1
22918	0.593846	2.1
22919	1.30226	1.28244
22920	1.30226	1.27458
22921	0.946132	1.52688
22922	1.03537	0.05
22923	1.50035	0.05
22924	0.940356	0.05
22925	0.946131	0.05
22926	1.03441	0.05
22927	1.21171	0.05
22928	1.35169	0.05
22929	1.32449	0.05
22930	0.946131	0.05
22931	0.946131	0.05
22932	1.06627	0.05
22933	1.03102	0.0839862
22934	0.488712	0.05
22935	0.946131	0.05
22936	1.23146	0.05
22937	1.13242	0.05
22938	0.946131	0.05
22939	0.409199	0.05
22940	0.429959	0.05
22941	1.08357	0.05
22942	0.80926	0.05
22943	0.879791	0.05
22944	0.946129	1.32533
22945	1.28828	2.1
22946	0.946131	0.721101
22947	0.946131	2.1
22948	1.35969	2.1
22949	1.10049	2.00359
22950	0.923724	2.1
22951	0.923695	2.1
22952	0.94613	2.1
22953	0.94613	2.1
22954	1.28828	2.1
22955	1.73525	0.05
22956	0.656239	0.05
22957	1.2996	0.05
22958	0.812939	0.0602446
22959	0.65624	0.05
22960	1.2996	0.05
22961	1.67372	2.1
22962	0.946131	2.1
22963	0.946132	2.1
22964	1.05978	2.1
22965	1.31049	2.1

22966	0.946132	2.1
22967	1.11077	2.1
22968	0.579725	0.0755783
22969	0.373532	0.05
22970	1.13537	0.108015
22971	1.20726	0.05
22972	0.946131	0.0627672
22973	1.45694	0.757988
22974	0.750367	0.05
22975	0.946126	0.05
22976	0.750365	0.05
22977	1.25698	0.05
22978	1.35726	0.05
22979	1.6812	0.05
22980	1.78665	1.86785
22981	1.46568	2.1
22982	1.29606	2.1
22983	1.25298	2.1
22984	0.946115	2.1
22985	0.946129	2.1
22986	1.25296	2.1
22987	1.27778	0.05
22988	1.17042	2.1
22989	1.25858	0.05
22990	0.93106	0.05
22991	0.810959	0.05
22992	1.38905	2.1
22993	1.19216	2.1
22994	0.946131	2.1
22995	0.946131	2.1
22996	1.19216	2.1
22997	1.58339	2.1
22998	0.81094	0.05
22999	0.845129	0.05
23000	0.918535	0.05
23001	0.946132	2.1
23002	0.946132	2.1
23003	1.31904	2.1
23004	1.31904	2.1
23005	1.21275	2.1
23006	1.15471	2.1
23007	1.33125	2.1
23008	0.702954	0.05
23009	0.942973	0.05
23010	0.94613	0.05
23011	0.641772	0.05
23012	0.702942	0.05
23013	0.991865	0.0531842
23014	0.639952	0.05
23015	1.17333	0.05
23016	0.946132	0.05
23017	0.490975	0.05
23018	0.495934	0.0895448
23019	1.19904	0.05
23020	1.30488	0.05
23021	1.02305	0.05
23022	1.49842	0.05
23023	1.54196	2.1

23024	1.37224	2.0923
23025	1.0953	0.05
23026	1.18952	2.1
23027	1.68851	0.696697
23028	0.946131	0.561643
23029	1.12491	0.169401
23030	0.583591	0.128558
23031	1.28727	0.656046
23032	1.12491	0.65939
23033	0.884641	0.726681
23034	0.810944	0.704252
23035	0.917973	0.697298
23036	0.846671	0.689997
23037	0.596968	0.119283
23038	0.716347	0.681311
23039	1.36255	0.0831423
23040	1.11077	2.1
23041	0.946132	2.1
23042	0.946132	2.1
23043	1.26267	2.1
23044	1.26441	2.1
23045	1.85238	2.1
23046	1.36693	2.1
23047	0.616123	2.1
23048	0.481844	2.1
23049	0.709599	2.1
23050	0.815889	2.1
23051	1.1502	2.1
23052	1.63937	0.568469
23053	1.25797	0.05
23054	1.22878	0.05
23055	0.946132	0.05
23056	0.946132	0.05
23057	0.965737	0.05
23058	1.1893	0.05
23059	1.22877	0.05
23060	0.735217	0.05
23061	0.85154	0.0509113
23062	0.851553	0.05
23063	0.851473	0.05
23064	1.58916	0.05
23065	1.25545	0.05
23066	0.810942	0.05
23067	0.919314	0.05
23068	0.928633	0.05
23069	2.14514	0.05
23070	1.23389	2.1
23071	0.946128	0.05
23072	0.969504	0.05
23073	0.851539	0.05
23074	1.41503	0.05
23075	0.958517	0.05
23076	0.990615	0.05
23077	1.7943	0.05
23078	0.946133	0.0819266
23079	1.27581	0.0951137
23080	0.946132	0.0867964
23081	1.36362	0.061149

23082	1.36936	0.05
23083	1.71903	2.1
23084	0.751639	2.1
23085	0.77464	2.1
23086	0.946133	2.1
23087	1.04073	2.1
23088	1.23551	2.1
23089	0.94613	2.1
23090	1.18682	2.1
23091	0.946131	2.1
23092	0.94613	2.1
23093	1.40638	2.1
23094	0.483346	0.05
23095	0.432527	0.05
23096	0.946132	0.05
23097	0.483312	0.05
23098	0.946132	0.05
23099	0.878798	0.05
23100	0.810944	0.05
23101	1.14604	0.05
23102	0.946129	0.05
23103	1.35594	0.05
23104	0.538378	0.05
23105	0.946131	0.05
23106	0.538378	0.05
23107	1.00065	0.148653
23108	0.962878	0.05
23109	1.32634	0.05
23110	1.28066	0.05
23111	0.785651	2.1
23112	1.20327	2.1
23113	1.47058	1.92783
23114	0.551834	1.26166
23115	1.04072	2.1
23116	0.506719	1.50541
23117	1.12786	1.78122
23118	1.45789	0.05
23119	0.681532	0.05
23120	1.21087	0.05
23121	0.94613	0.05
23122	0.681534	0.05
23123	1.21087	0.05
23124	1.98618	0.338402
23125	1.71742	1.14852
23126	1.07427	0.633791
23127	1.1972	0.193256
23128	0.933474	0.462438
23129	1.1745	0.595037
23130	0.94612	0.756173
23131	0.933485	0.77329
23132	1.17449	0.728956
23133	0.851541	0.05
23134	1.12774	0.05
23135	0.946129	0.05
23136	0.851486	0.05
23137	1.12517	0.05
23138	1.12328	0.05
23139	1.13179	2.1

23140	1.02438	2.1
23141	1.05776	2.1
23142	1.41273	1.34703
23143	1.1229	2.1
23144	0.875912	1.61323
23145	0.946132	2.1
23146	0.875912	2.1
23147	1.42307	2.1
23148	1.15354	0.05
23149	0.848382	0.05
23150	1.2685	2.1
23151	0.938104	2.1
23152	0.793166	2.1
23153	1.07855	2.1
23154	0.98488	2.1
23155	1.4162	2.1
23156	1.09651	0.05
23157	1.98502	0.05
23158	1.41168	0.05
23159	1.33879	0.05
23160	0.946127	0.0602452
23161	1.13528	0.05
23162	0.946128	0.05
23163	1.09726	2.1
23164	1.22026	0.949863
23165	1.09044	2.1
23166	2.23961	0.555417
23167	1.14721	0.05
23168	1.17614	2.1
23169	0.615065	2.1
23170	0.946131	2.1
23171	0.778488	2.1
23172	0.94613	2.1
23173	0.615066	2.1
23174	1.78254	2.1
23175	0.898452	0.05
23176	1.47411	2.1
23177	0.59676	0.05
23178	0.59676	0.05
23179	0.94613	0.0668172
23180	1.13511	0.05
23181	0.946131	0.05
23182	0.916275	2.1
23183	0.946132	2.1
23184	0.916274	1.24441
23185	1.36182	2.1
23186	0.946131	2.1
23187	0.857202	0.05
23188	0.941918	0.05
23189	0.943349	0.05
23190	1.2493	0.05
23191	0.946133	0.05
23192	1.01814	0.05
23193	0.927881	0.05
23194	1.11845	0.05
23195	1.2493	0.05
23196	1.11845	0.05
23197	0.946129	0.05

23198	0.967673	0.116777
23199	1.01332	0.05
23200	0.946133	0.05
23201	0.81938	0.05
23202	0.819377	0.05
23203	0.946133	0.05
23204	1.22474	0.05
23205	1.15321	0.05
23206	0.797489	0.05
23207	0.946131	0.05
23208	0.797461	0.05
23209	1.35328	0.958519
23210	1.03318	1.27039
23211	0.946131	1.0307
23212	0.94613	1.07413
23213	1.03319	1.0538
23214	1.37938	0.992388
23215	1.00512	1.27571
23216	0.946133	1.05675
23217	0.946132	1.10396
23218	1.00512	1.07329
23219	1.5515	1.29538
23220	1.55351	2.1
23221	0.94261	0.05
23222	0.828156	0.05
23223	0.916495	0.05
23224	3.06309	0.05
23225	0.619832	0.05
23226	0.705903	0.05
23227	1.2968	0.05
23228	0.946133	0.05
23229	0.705904	0.05
23230	1.2968	0.05
23231	1.01971	0.05
23232	0.60302	0.05
23233	1.07242	0.169181
23234	1.26426	0.158782
23235	0.946131	0.177969
23236	0.946131	0.202386
23237	0.963109	0.203916
23238	0.947862	0.270871
23239	1.40248	1.25891
23240	0.767143	1.33109
23241	0.767114	1.30892
23242	0.946129	1.34419
23243	0.767143	1.06345
23244	1.22637	1.27316
23245	1.2281	1.25171
23246	0.590909	1.36307
23247	0.490832	1.27185
23248	0.804174	1.36896
23249	0.747368	1.18434
23250	1.05129	0.05
23251	1.10609	0.111792
23252	1.3639	0.122354
23253	0.776117	0.314643
23254	0.752329	0.0602697
23255	1.04073	0.0852328

23256	0.67713	0.0741864
23257	3.4245	2.1
23258	1.18526	2.1
23259	1.2161	1.17424
23260	0.876834	2.04092
23261	0.876831	1.16607
23262	0.946132	1.85733
23263	0.876834	1.27083
23264	0.954563	2.1
23265	0.924132	2.1
23266	1.25075	2.1
23267	1.01652	2.1
23268	0.92413	2.1
23269	1.37579	2.1
23270	0.992159	2.1
23271	1.26092	2.1
23272	0.929435	2.1
23273	0.844958	2.1
23274	0.79598	2.1
23275	0.852559	2.1
23276	0.946131	0.375413
23277	1.28435	0.0734298
23278	0.827261	0.271528
23279	0.946128	0.35396
23280	0.827298	0.423072
23281	1.65452	0.05
23282	1.79929	2.1
23283	0.791947	1.48753
23284	0.902072	1.06803
23285	0.882672	1.10455
23286	1.21703	2.1
23287	0.900338	2.1
23288	1.37712	2.1
23289	0.782591	2.1
23290	0.782562	2.1
23291	0.94613	2.1
23292	1.34749	2.1
23293	0.61359	2.1
23294	1.14397	0.05
23295	1.02963	0.05
23296	1.36589	0.05
23297	0.946132	0.05
23298	1.28644	0.05
23299	0.946131	0.05
23300	1.40104	0.05
23301	1.28644	0.05
23302	0.810946	0.05
23303	1.09178	0.05
23304	1.26956	0.05
23305	0.810943	0.05
23306	0.885633	0.05
23307	1.00844	0.05
23308	0.825671	0.05
23309	0.792093	0.05
23310	0.925037	0.05
23311	0.946131	0.911131
23312	0.946131	0.05
23313	1.18052	0.05

23314	1.40265	0.155724
23315	0.946132	0.6925
23316	1.17891	0.137925
23317	0.734248	0.05
23318	0.810943	0.05
23319	0.731621	0.05
23320	0.799857	0.358139
23321	0.834507	0.711862
23322	0.810932	0.302301
23323	1.01316	0.05
23324	1.23884	0.05
23325	1.88438	2.1
23326	0.946129	2.1
23327	1.38614	2.1
23328	1.34245	1.31023
23329	0.946131	1.2636
23330	1.38614	1.30719
23331	0.946132	0.05
23332	0.910733	0.05
23333	1.24726	0.05
23334	1.13742	0.05
23335	0.851555	0.05
23336	1.50368	0.05
23337	0.94613	0.688916
23338	1.08998	2.1
23339	1.36899	0.05
23340	1.36898	0.534841
23341	0.946131	0.553443
23342	1.26045	2.1
23343	1.06432	0.642704
23344	1.89016	0.105906
23345	0.83514	2.1
23346	1.36653	2.1
23347	1.23426	2.1
23348	0.94613	2.1
23349	0.835112	2.1
23350	1.14567	1.35769
23351	1.12096	1.28455
23352	0.881106	1.46899
23353	1.17787	0.145985
23354	1.29738	1.00366
23355	0.940472	0.0503717
23356	1.03452	0.949953
23357	0.946131	0.165603
23358	2.7489	0.787882
23359	1.01778	2.1
23360	1.04321	0.754922
23361	1.19352	0.05
23362	1.16912	1.61716
23363	0.946132	1.25912
23364	0.946132	1.98591
23365	1.40924	1.93218
23366	1.22927	2.1
23367	2.09455	0.05
23368	3.00575	2.1
23369	1.08928	0.29777
23370	1.94799	2.1
23371	1.4092	2.1

23372	0.946132	2.1
23373	1.16	2.1
23374	1.01985	2.1
23375	0.946133	2.1
23376	1.16	2.1
23377	1.15371	0.05
23378	1.56002	0.05
23379	1.23007	2.1
23380	1.9992	2.1
23381	0.652605	2.1
23382	0.937903	2.1
23383	0.946132	2.1
23384	0.937902	2.1
23385	1.228	2.1
23386	1.33694	1.56626
23387	1.00191	2.1
23388	0.716814	1.95133
23389	0.94613	2.1
23390	0.716816	2.1
23391	1.70388	1.69433
23392	0.946129	0.136508
23393	1.09362	0.211946
23394	0.946131	0.55448
23395	1.4185	0.581199
23396	1.09362	0.226294
23397	1.31057	1.30304
23398	1.17631	0.05
23399	1.19404	0.05
23400	0.94613	0.05
23401	0.946129	0.05
23402	0.99972	0.05
23403	0.94613	0.05
23404	0.835199	0.05
23405	1.23351	0.05
23406	0.582785	0.05
23407	0.701079	0.05
23408	0.946132	0.05
23409	2.17385	0.05
23410	1.18948	2.1
23411	1.25346	2.1
23412	1.18586	0.866993
23413	0.880876	0.460826
23414	0.68632	0.80035
23415	0.946118	2.1
23416	0.966936	2.1
23417	0.946117	2.1
23418	1.41636	2.1
23419	1.03798	2.1
23420	0.729178	0.861841
23421	0.721888	0.95699
23422	0.946132	0.868279
23423	0.656286	0.92042
23424	1.13898	0.903002
23425	1.02351	0.05
23426	1.48803	0.05
23427	0.946131	0.05
23428	1.04921	2.1
23429	1.02478	0.05

23430	0.993023	2.1
23431	1.80841	0.05
23432	1.29215	0.05
23433	0.904206	0.05
23434	0.904207	0.0514652
23435	0.946132	0.05
23436	0.904207	0.05
23437	1.07347	2.1
23438	0.555875	1.40847
23439	0.555904	1.8474
23440	1.30841	2.1
23441	1.30841	2.1
23442	0.91951	2.1
23443	1.33026	0.0801656
23444	1.43289	0.05
23445	1.15703	1.84276
23446	0.886594	2.1
23447	1.05129	1.96927
23448	1.08421	0.200891
23449	1.35946	0.217553
23450	0.882955	0.508685
23451	0.946131	0.494535
23452	0.882985	0.371663
23453	0.583501	0.662706
23454	0.532921	0.622452
23455	0.94613	1.77907
23456	0.532892	1.25238
23457	1.23224	1.30707
23458	1.12786	1.24213
23459	0.946131	0.888976
23460	1.23732	0.312296
23461	1.40609	0.217372
23462	1.40609	0.05
23463	0.946131	0.0647291
23464	0.910672	1.46614
23465	1.08478	1.22842
23466	0.94613	0.906417
23467	0.94613	1.2496
23468	1.30382	1.26295
23469	3.10544	1.96155
23470	0.679509	2.1
23471	1.15966	1.71578
23472	0.75499	2.1
23473	1.37511	1.81452
23474	1.01776	1.69524
23475	2.35239	1.25093
23476	0.92406	2.1
23477	0.946118	2.1
23478	0.92406	2.1
23479	1.07979	2.1
23480	1.29604	2.1
23481	1.29897	2.1
23482	0.902645	0.336001
23483	0.729493	1.56859
23484	0.906844	0.951443
23485	0.739518	0.05
23486	1.10564	0.05
23487	0.946131	0.05

23488	0.739519	0.05
23489	0.739506	0.05
23490	1.79907	0.05
23491	1.59106	1.1311
23492	1.11067	0.920456
23493	0.795516	1.06588
23494	0.795519	0.956159
23495	0.946132	1.02865
23496	0.795506	0.962013
23497	1.80223	2.1
23498	1.95572	2.07951
23499	1.16237	0.970651
23500	1.07518	2.1
23501	0.696199	1.01908
23502	0.94613	1.41252
23503	0.696199	1.11239
23504	1.6379	0.05
23505	0.946118	0.05
23506	0.851529	0.05
23507	1.08716	0.05
23508	0.973118	0.05
23509	0.851528	0.05
23510	1.15096	2.1
23511	0.65023	0.05
23512	0.94613	0.05
23513	0.650205	0.05
23514	0.650205	0.05
23515	0.811233	0.05
23516	0.778721	2.1
23517	0.810945	1.1683
23518	0.997518	1.18205
23519	0.838028	0.05
23520	0.952893	0.05
23521	0.838037	0.05
23522	1.58166	0.05
23523	1.44848	1.55572
23524	1.30041	0.05
23525	1.03697	0.0961328
23526	0.946132	1.56464
23527	1.0277	1.71601
23528	0.946131	1.75291
23529	1.00501	1.52697
23530	1.0277	1.42434
23531	1.64988	2.1
23532	0.946131	2.1
23533	0.94613	0.122056
23534	1.27702	2.1
23535	1.55163	1.04382
23536	0.897905	0.655075
23537	1.12971	0.05
23538	1.35715	0.111692
23539	0.881884	0.624016
23540	0.664007	0.124833
23541	0.663978	0.241935
23542	0.94613	0.141906
23543	0.664006	0.268253
23544	1.48693	0.05
23545	1.29289	0.05

23546	1.32081	0.05
23547	0.946119	0.05
23548	0.946132	0.05
23549	1.02972	0.05
23550	0.946131	2.1
23551	0.946127	2.1
23552	1.23513	2.1
23553	1.38154	2.1
23554	0.94613	2.1
23555	1.25933	0.05
23556	0.917954	0.05
23557	0.868589	0.05
23558	3.0957	2.1
23559	0.711391	0.05
23560	0.711391	0.05
23561	0.991303	0.05
23562	1.37322	0.05
23563	0.729145	0.05
23564	2.55648	0.05
23565	1.0756	2.04229
23566	0.760299	1.44138
23567	0.763592	0.850259
23568	0.946126	1.01022
23569	0.76359	0.808601
23570	1.35862	1.36664
23571	0.946133	2.1
23572	1.00807	2.1
23573	0.946132	2.1
23574	1.39823	2.1
23575	1.07817	2.1
23576	1.44379	1.00762
23577	1.17934	0.05
23578	1.13451	0.52788
23579	0.94613	1.81463
23580	1.03974	2.1
23581	1.36835	1.45036
23582	1.36835	2.1
23583	0.94613	2.1
23584	1.27096	2.1
23585	0.853042	2.1
23586	0.946131	2.1
23587	1.13658	2.1
23588	1.06997	2.1
23589	0.94613	2.1
23590	1.41556	0.0760525
23591	1.21708	0.274583
23592	0.94613	0.363446
23593	0.946131	0.20696
23594	1.21708	0.20386
23595	0.964782	0.111447
23596	0.946119	0.248549
23597	1.23447	0.162255
23598	0.946118	0.242027
23599	1.41844	0.135379
23600	1.23446	0.187072
23601	1.1357	0.05
23602	0.619856	0.05
23603	1.1509	0.05

23604	0.946126	0.05
23605	1.19262	0.05
23606	1.1509	0.05
23607	1.04268	2.1
23608	2.07527	0.774352
23609	1.38259	1.05504
23610	0.980054	1.59335
23611	0.946124	1.11148
23612	0.946127	1.23228
23613	0.980056	1.14775
23614	1.62916	2.08461
23615	0.946131	2.1
23616	1.40571	2.1
23617	0.946131	2.1
23618	1.38885	2.1
23619	1.40571	2.1
23620	1.11787	0.563439
23621	0.946129	0.868745
23622	1.28831	2.1
23623	1.41498	0.05
23624	1.41498	1.70142
23625	0.946131	1.4826
23626	0.850964	0.05
23627	0.921299	0.05
23628	1.37358	0.05
23629	0.946132	0.05
23630	0.850964	0.05
23631	0.823988	0.05
23632	0.823984	0.05
23633	1.36568	0.05
23634	1.36569	0.05
23635	0.921275	0.05
23636	1.84739	2.1
23637	1.47169	0.05
23638	0.946131	0.053058
23639	0.735844	0.05
23640	1.11819	0.0666602
23641	1.10904	0.05
23642	0.735831	0.05
23643	0.946131	2.1
23644	0.946132	2.1
23645	1.23487	2.1
23646	1.3281	2.1
23647	0.946131	2.1
23648	1.72271	0.05
23649	0.861359	0.05
23650	0.946131	0.05
23651	0.861357	0.05
23652	1.1369	0.05
23653	1.26868	0.05
23654	0.789173	2.1
23655	0.789143	2.1
23656	0.946133	1.8552
23657	1.34386	2.07066
23658	0.789144	2.1
23659	0.983493	2.1
23660	1.18944	0.793931
23661	1.05121	0.82841

23662	0.777398	0.619176
23663	0.946132	0.969839
23664	0.777423	1.00961
23665	1.09841	0.05
23666	0.963919	0.05
23667	0.474855	0.05
23668	0.946131	0.05
23669	0.474855	0.05
23670	3.40074	1.36044
23671	1.29453	2.1
23672	1.01139	0.112255
23673	0.946133	0.899575
23674	1.35381	0.05
23675	1.18322	0.261504
23676	0.946133	0.756931
23677	1.45502	0.05
23678	0.970088	1.40014
23679	0.960333	1.52325
23680	0.838036	1.56573
23681	1.30927	0.0769811
23682	0.828617	0.15459
23683	0.714795	0.0604446
23684	0.946132	0.0715505
23685	0.714824	0.05
23686	0.946131	2.1
23687	0.851542	2.1
23688	1.38204	2.1
23689	1.24512	2.1
23690	1.04086	2.1
23691	0.723573	2.1
23692	0.946131	2.1
23693	0.982454	2.1
23694	0.982454	2.1
23695	0.723544	2.1
23696	1.17261	0.05
23697	1.34153	0.05
23698	1.41383	0.05
23699	0.946132	0.05
23700	0.946133	0.05
23701	1.12818	0.05
23702	1.29383	0.05
23703	0.946133	0.174019
23704	1.0027	0.167072
23705	0.768989	0.0538615
23706	0.946133	0.235389
23707	0.768977	0.221634
23708	0.946132	0.411512
23709	1.24333	0.118902
23710	0.928583	0.400108
23711	0.946132	0.44092
23712	0.928583	0.50659
23713	1.53798	0.05
23714	2.95436	0.05
23715	1.30649	0.05
23716	0.809789	0.05
23717	0.745744	0.05
23718	0.745774	0.05
23719	0.946128	0.05

23720	1.00777	0.05
23721	1.40271	0.05
23722	0.768387	0.05
23723	0.946132	0.05
23724	0.922045	0.05
23725	0.946132	2.1
23726	1.39021	2.1
23727	1.10046	2.1
23728	0.946132	2.1
23729	1.39021	2.1
23730	2.06158	0.05
23731	0.913967	0.754524
23732	1.30107	0.840426
23733	0.946132	0.776502
23734	0.913966	0.788496
23735	1.30107	0.782114
23736	1.64117	2.1
23737	0.514872	1.90674
23738	0.514846	1.24834
23739	1.30811	2.1
23740	1.30812	1.15573
23741	0.888989	1.38007
23742	1.99279	1.9944
23743	0.910271	0.897859
23744	0.838027	0.914889
23745	0.928444	0.923268
23746	1.2377	1.08617
23747	0.851537	2.1
23748	0.899142	2.1
23749	0.810075	2.1
23750	1.3771	2.1
23751	1.03281	2.1
23752	0.719877	2.1
23753	0.901838	2.1
23754	1.01748	2.1
23755	1.12315	2.1
23756	0.64787	2.1
23757	1.173 2.1	
23758	0.876903	2.1
23759	0.597337	2.1
23760	1.00291	2.1
23761	0.597309	2.1
23762	1.09897	0.05
23763	0.946132	0.526276
23764	1.1386	0.05
23765	1.09897	0.0561084
23766	0.596652	0.0721364
23767	2.5593	2.1
23768	0.946133	0.678701
23769	1.29172	0.669209
23770	1.11337	0.660231
23771	0.946131	0.677184
23772	0.946131	0.657754
23773	0.96926	0.925367
23774	0.946132	0.995955
23775	0.946132	1.0841
23776	1.07118	0.845777
23777	1.29698	0.861016

23778	1.37867	1.05036
23779	0.946119	1.35823
23780	0.94612	1.93868
23781	1.33154	1.0375
23782	0.946107	1.18657
23783	2.17757	1.51158
23784	0.946131	0.05
23785	0.68393	0.05
23786	0.68396	0.05
23787	1.33671	0.05
23788	1.10733	0.05
23789	2.31367	2.1
23790	0.989326	1.74916
23791	0.689952	2.1
23792	0.854787	1.64768
23793	3.73635	2.1
23794	1.41489	2.1
23795	1.25544	0.417118
23796	0.946128	2.1
23797	0.946129	2.1
23798	1.13536	2.1
23799	2.10284	1.28386
23800	0.851542	2.1
23801	0.828523	2.1
23802	0.946132	2.1
23803	0.946132	2.1
23804	1.50873	2.1
23805	0.989048	2.1
23806	0.925055	2.1
23807	0.838041	2.1
23808	0.807714	0.323219
23809	0.656831	0.05
23810	0.997603	0.190427
23811	0.946131	1.03742
23812	1.02602	0.458687
23813	0.946129	1.19712
23814	1.35469	0.575038
23815	1.25083	0.780424
23816	0.851541	0.05
23817	0.851483	0.05
23818	0.851539	0.05
23819	1.19785	0.05
23820	1.07615	0.05
23821	0.714507	0.109318
23822	1.41461	0.0976521
23823	0.714507	0.101462
23824	0.714507	0.112953
23825	0.946133	0.128719
23826	0.946131	0.958435
23827	0.972232	0.742666
23828	0.94613	1.17051
23829	1.25083	0.509056
23830	1.30165	0.884252
23831	1.05332	2.1
23832	0.574768	2.1
23833	1.94541	0.05
23834	0.789633	0.05
23835	0.946127	0.05

23836	1.33798	0.05
23837	1.22903	0.05
23838	0.946128	0.05
23839	0.946128	2.1
23840	1.11882	2.1
23841	0.996487	2.1
23842	0.94613	2.1
23843	1.11883	2.1
23844	0.946127	2.1
23845	1.30082	2.1
23846	1.02608	2.1
23847	0.946128	2.1
23848	1.30081	2.1
23849	1.3507	1.62984
23850	1.19583	0.47342
23851	0.946127	1.98246
23852	0.94613	1.19291
23853	1.19583	1.36981
23854	0.477652	0.05
23855	1.20416	0.05
23856	0.946131	0.05
23857	0.477652	0.05
23858	0.477691	0.05
23859	1.34536	0.05
23860	0.758971	0.05
23861	1.25124	0.05
23862	1.31467	0.05
23863	0.758971	0.05
23864	1.31467	0.05
23865	0.946131	2.1
23866	1.14184	2.1
23867	1.35028	2.1
23868	1.35028	2.1
23869	0.946131	2.1
23870	1.13537	2.1
23871	0.946132	1.29607
23872	0.946132	2.1
23873	1.23031	2.09528
23874	1.27555	2.04712
23875	1.53064	2.1
23876	1.28421	1.05778
23877	1.07707	1.10996
23878	0.946125	1.0458
23879	0.946127	1.10291
23880	1.07707	1.0704
23881	0.946133	0.05
23882	1.3495	0.05
23883	0.946132	0.05
23884	1.05704	0.05
23885	1.3495	0.05
23886	0.946132	0.05
23887	1.37794	0.05
23888	0.946132	0.05
23889	1.01367	0.05
23890	1.37794	0.05
23891	0.928516	2.1
23892	0.946133	2.1
23893	0.928518	2.1

23894	1.14105	2.1
23895	1.26406	2.1
23896	0.797664	2.1
23897	0.946132	2.1
23898	0.797663	2.1
23899	1.34285	2.1
23900	0.929875	2.1
23901	0.946131	1.789
23902	1.21139	0.322428
23903	1.41492	0.05
23904	1.41492	0.741873
23905	0.946131	0.05
23906	0.946131	2.1
23907	0.689388	2.1
23908	0.94365	2.1
23909	0.777345	2.1
23910	0.709599	2.1
23911	0.774968	2.1
23912	0.946132	2.1
23913	0.77497	2.1
23914	0.868145	2.1
23915	1.28401	2.1
23916	0.94613	0.703255
23917	1.19655	2.1
23918	1.39428	0.0503715
23919	1.39428	0.156385
23920	0.946131	0.0794084
23921	1.1848	2.1
23922	1.07591	2.1
23923	0.946131	2.1
23924	0.946131	2.1
23925	0.874788	2.1
23926	1.35703	2.1
23927	1.35357	2.1
23928	0.946132	2.1
23929	1.26022	2.1
23930	0.946132	2.1
23931	0.946131	2.1
23932	0.966426	2.1
23933	0.946133	2.1
23934	0.946131	2.1
23935	1.3484	2.1
23936	1.26441	2.1
23937	1.2079	2.1
23938	0.946133	2.1
23939	0.946132	2.1
23940	1.2079	2.1
23941	1.2736	2.1
23942	1.25774	2.1
23943	0.946133	2.1
23944	0.851555	2.1
23945	0.96172	2.1
23946	0.946128	2.1
23947	1.06879	2.1
23948	0.94613	2.1
23949	1.32608	2.1
23950	1.06879	2.1
23951	0.946132	2.1

23952	1.32608	2.1
23953	0.946131	2.1
23954	1.40488	2.1
23955	1.32608	2.1
23956	0.94613	1.20229
23957	1.19234	0.131006
23958	0.946129	0.583811
23959	0.94613	1.23927
23960	1.31649	1.36338
23961	0.912059	1.09081
23962	1.04514	0.958372
23963	0.946132	0.54267
23964	0.946132	1.94
23965	1.34646	2.03503
23966	1.01187	0.327304
23967	1.33728	0.307936
23968	0.761974	0.05
23969	0.761974	0.80594
23970	0.94613	0.464781
23971	1.40641	2.1
23972	0.955752	1.03032
23973	0.736738	2.01563
23974	0.94613	1.12727
23975	0.736739	1.21314
23976	0.861471	2.1
23977	1.34556	2.1
23978	0.810957	2.1
23979	0.946131	2.1
23980	1.37811	2.1
23981	1.17949	2.1
23982	0.946132	2.1
23983	1.37811	2.1
23984	0.946131	2.1
23985	1.40447	2.1
23986	0.946132	2.1
23987	0.946132	2.1
23988	1.39826	2.1
23989	0.946131	2.1
23990	1.283 2.1	
23991	0.94613	2.1
23992	0.946131	2.1
23993	1.40447	2.1
23994	1.20541	2.1
23995	0.946131	2.1
23996	0.946127	2.1
23997	1.33607	2.1
23998	1.41021	2.1
23999	1.01271	2.1
24000	0.946131	2.1
24001	0.920606	2.1
24002	1.13078	2.1
24003	1.60331	2.1
24004	0.946118	0.96132
24005	1.09718	2.1
24006	0.946116	1.27274
24007	1.32662	1.01714
24008	1.0972	1.10757
24009	0.94613	0.915296

24010	1.32666	1.16858
24011	0.946128	0.854925
24012	1.31381	0.850083
24013	1.32666	0.820742
24014	0.94612	2.1
24015	0.940748	2.1
24016	1.03561	2.1
24017	1.35111	2.1
24018	0.851527	2.1
24019	0.607156	2.1
24020	0.789158	2.1
24021	0.946129	1.69266
24022	1.04071	2.1
24023	1.13144	2.1
24024	0.946133	2.1
24025	1.35663	2.1
24026	0.946132	2.1
24027	0.946133	2.1
24028	1.34237	2.1
24029	0.919455	2.1
24030	1.01143	2.1
24031	0.946128	2.1
24032	0.946131	2.1
24033	1.62796	2.1
24034	1.17796	1.32389
24035	1.23024	2.1
24036	0.946132	1.25093
24037	0.85153	2.0207
24038	0.986867	1.86204
24039	0.889308	2.1
24040	0.94613	2.1
24041	0.889338	2.1
24042	0.946131	2.1
24043	0.889337	2.1
24044	1.18396	1.14579
24045	0.94613	0.985725
24046	0.946125	1.83019
24047	1.39514	1.11478
24048	1.30809	1.3087
24049	0.803322	2.1
24050	0.925893	2.0543
24051	1.36183	1.37938
24052	0.925892	1.54973
24053	0.946133	1.54987
24054	1.38354	0.05
24055	1.16009	0.0815914
24056	0.946132	0.0688468
24057	0.946132	0.05
24058	1.01462	0.05
24059	1.18854	1.14342
24060	0.888265	1.95723
24061	0.745095	1.21726
24062	0.709597	1.57012
24063	0.936541	1.43477
24064	1.24142	0.115497
24065	1.35059	0.0719805
24066	0.946128	0.05
24067	0.94613	0.0911602

24068	1.16623	0.0856326
24069	1.24945	0.05
24070	1.33035	0.05
24071	0.946127	0.05
24072	0.946128	0.05
24073	1.13343	0.05
24074	0.637995	1.11166
24075	1.10455	1.22357
24076	1.22641	1.55486
24077	1.22641	1.99922
24078	0.946132	1.43264
24079	1.3665	0.05
24080	1.28507	0.05
24081	0.946127	0.05
24082	0.946129	0.05
24083	1.2051	0.05
24084	0.797374	2.1
24085	0.797376	2.1
24086	1.13113	2.1
24087	1.20057	2.1
24088	1.12049	2.1
24089	0.59387	0.983709
24090	1.13245	1.02595
24091	0.966134	1.14748
24092	0.593842	0.963836
24093	0.946128	1.11185
24094	0.641455	0.246923
24095	0.916507	0.0895703
24096	1.04072	1.3372
24097	0.415585	0.173666
24098	0.507244	1.24192
24099	0.678938	2.1
24100	1.24154	2.1
24101	1.25	1.94329
24102	1.24154	2.09296
24103	1.25	2.1
24104	0.851009	1.82266
24105	0.946131	2.1
24106	0.851539	1.76212
24107	1.21997	1.62243
24108	1.45109	1.65681
24109	0.804877	1.05762
24110	1.00062	0.220363
24111	0.851536	0.967832
24112	0.909673	0.28222
24113	0.946129	0.988505
24114	1.20494	0.05
24115	1.09088	0.05
24116	0.887634	0.05
24117	0.81027	0.05
24118	0.900294	0.05
24119	0.747713	0.05
24120	0.975467	0.05
24121	0.747712	0.05
24122	1.39168	0.05
24123	0.975467	0.05
24124	0.94613	0.149075
24125	1.32827	0.0795853

24126	0.946128	0.0997882
24127	1.41201	0.417151
24128	1.32827	0.286777
24129	0.981121	0.05
24130	0.936311	0.05
24131	1.00715	0.05
24132	1.15914	0.05
24133	0.704409	0.05
24134	0.713444	0.49169
24135	0.954538	0.277024
24136	0.944508	0.309546
24137	0.851165	1.07104
24138	0.946131	1.55474
24139	0.851192	0.780426
24140	0.946131	0.661822
24141	1.3279	0.611912
24142	1.33587	0.05
24143	0.946126	0.05
24144	1.27512	0.05
24145	1.33587	0.05
24146	0.748059	0.05
24147	1.17451	0.05
24148	1.04299	0.05
24149	0.851542	0.05
24150	0.946131	0.05
24151	0.892109	0.05
24152	0.946131	0.05
24153	0.851541	0.05
24154	1.2178	0.05
24155	1.2179	0.05
24156	0.946793	0.05
24157	0.709599	0.05
24158	0.987426	0.05
24159	1.39502	0.05
24160	1.13624	0.05
24161	0.73176	0.05
24162	1.36697	0.05
24163	1.33393	0.05
24164	0.946131	0.05
24165	0.946132	0.05
24166	1.23845	0.05
24167	0.50702	1.02021
24168	1.06442	0.971754
24169	0.946131	1.02929
24170	1.02363	1.02816
24171	1.13537	0.05
24172	1.25049	0.05
24173	0.94613	0.05
24174	1.14993	0.05
24175	1.38056	0.05
24176	0.946126	0.05
24177	1.24678	0.05
24178	0.684521	0.05
24179	0.946124	0.05
24180	0.684518	0.05
24181	0.851542	0.508976
24182	0.925459	0.223376
24183	0.946131	0.765956

24184	0.851474	0.5883
24185	0.923305	0.661558
24186	1.4055	0.05
24187	1.32077	0.05
24188	0.946127	0.05
24189	1.21757	0.05
24190	0.946129	0.05
24191	0.711214	0.05
24192	1.30463	0.05
24193	0.711185	0.05
24194	0.711185	0.05
24195	1.22716	0.05
24196	1.29958	0.05
24197	1.26201	0.05
24198	0.568927	0.05
24199	0.568927	0.05
24200	0.946132	0.05
24201	0.851542	0.05
24202	1.44968	0.05
24203	0.851543	0.05
24204	1.35123	0.05
24205	1.40427	0.05
24206	0.593295	0.05
24207	0.946129	0.05
24208	1.22144	0.05
24209	0.908278	0.05
24210	1.22144	0.05
24211	0.867593	0.0920745
24212	1.23351	0.05
24213	0.946131	0.100825
24214	1.05516	0.0773878
24215	1.23351	0.0561611
24216	1.15564	0.05
24217	0.946131	0.05
24218	0.766652	0.05
24219	0.766681	0.05
24220	0.946131	0.05
24221	0.946131	0.05
24222	0.897851	0.05
24223	1.1751	0.05
24224	1.00273	0.05
24225	0.851554	0.05
24226	0.883272	0.05
24227	1.17111	0.319563
24228	0.851541	0.05
24229	1.01665	0.05
24230	0.851555	0.05
24231	0.946131	0.188401
24232	0.911508	0.0505647
24233	1.08054	0.238647
24234	1.33007	0.05
24235	0.911471	0.0744378
24236	1.26787	2.07873
24237	1.16641	1.702
24238	0.946132	1.55039
24239	0.946133	1.33082
24240	1.16641	1.79554
24241	0.946131	2.1

24242	1.25037	0.05
24243	1.34733	2.1
24244	1.34733	0.426438
24245	0.946131	0.206529
24246	0.946131	0.05
24247	1.41377	0.05
24248	0.94613	0.05
24249	1.30541	0.05
24250	1.41377	0.05
24251	1.3473	2.1
24252	1.11804	2.1
24253	0.94613	2.1
24254	0.946131	2.1
24255	1.11805	2.1
24256	1.32068	2.1
24257	0.946131	2.1
24258	0.946128	2.1
24259	1.39384	2.1
24260	1.4181	2.1
24261	1.12913	2.1
24262	0.94613	2.1
24263	0.946129	2.1
24264	1.20328	2.1
24265	1.39385	2.1
24266	1.3335	2.1
24267	1.40987	2.1
24268	0.94613	2.1
24269	1.21474	2.1
24270	0.94613	2.1
24271	0.946132	2.1
24272	1.15203	2.1
24273	1.41192	2.1
24274	1.41192	2.1
24275	0.946132	2.1
24276	0.946131	2.1
24277	1.40684	2.1
24278	1.2597	2.1
24279	0.946131	2.1
24280	1.40684	2.1
24281	0.946131	2.1
24282	1.39113	2.1
24283	1.34049	1.52192
24284	0.946131	1.35754
24285	1.39113	1.14291
24286	0.946132	2.00678
24287	1.31649	1.08261
24288	0.946132	1.00222
24289	0.946132	1.18568
24290	1.34297	1.24499
24291	0.946131	0.761309
24292	1.41766	0.732458
24293	0.954559	1.18335
24294	0.946131	0.724865
24295	0.946131	0.664263
24296	1.25447	0.05
24297	1.35559	0.05
24298	0.946127	0.05
24299	0.946128	0.05

24300	1.14874	0.05
24301	1.33035	0.05
24302	1.3665	0.05
24303	0.946129	0.05
24304	0.94613	0.05
24305	1.20732	0.05
24306	0.946131	2.1
24307	0.946131	2.1
24308	1.32949	2.1
24309	1.34921	2.1
24310	0.946131	2.1
24311	0.946132	0.776113
24312	1.25479	0.74318
24313	1.38357	0.749739
24314	1.38357	0.668253
24315	0.946132	0.70585
24316	0.946131	2.1
24317	1.31034	0.646721
24318	1.37127	0.965149
24319	1.37127	0.05
24320	0.946131	0.820339
24321	1.23923	0.05
24322	1.41169	0.05
24323	0.94613	0.05
24324	1.12703	0.05
24325	0.946131	0.05
24326	0.946119	2.1
24327	1.41915	2.1
24328	1.02679	2.1
24329	0.946107	2.1
24330	1.41917	2.1
24331	0.946133	2.1
24332	1.36581	2.1
24333	0.946131	2.1
24334	1.36581	2.1
24335	0.946131	2.1
24336	0.946132	2.1
24337	1.04384	2.1
24338	1.34973	2.1
24339	1.34967	2.1
24340	0.851555	2.1
24341	0.946132	0.05
24342	1.3855	0.109079
24343	1.08603	0.05
24344	1.08602	0.0546271
24345	1.16234	0.0616063
24346	0.946133	0.150341
24347	1.29348	0.377916
24348	1.20111	0.092336
24349	1.20112	0.209548
24350	1.3855	0.139292
24351	0.946128	2.1
24352	1.30798	2.1
24353	0.946129	2.1
24354	1.34828	2.1
24355	1.30797	2.1
24356	1.24932	0.05
24357	1.40549	0.05

24358	0.946117	0.05
24359	1.19615	0.05
24360	0.946106	0.05
24361	0.946131	2.1
24362	1.29926	2.1
24363	1.25144	2.1
24364	0.946131	2.1
24365	1.29925	2.1
24366	0.946132	2.1
24367	1.39826	2.1
24368	0.946133	2.1
24369	0.946133	2.1
24370	1.29926	2.1
24371	0.946119	0.05
24372	1.33614	0.05
24373	0.946118	0.05
24374	1.41377	0.05
24375	1.33612	0.05
24376	0.946133	1.60519
24377	1.3933	2.1
24378	1.34999	1.17831
24379	0.946133	1.63304
24380	1.3933	1.41351
24381	0.946131	2.06529
24382	1.36139	2.1
24383	0.946132	1.33667
24384	0.946131	2.1
24385	1.39329	1.60231
24386	1.37449	1.35799
24387	1.27925	0.81104
24388	0.919796	0.487056
24389	1.01176	1.04056
24390	0.94613	1.00729
24391	0.946133	2.1
24392	1.27679	2.1
24393	0.946133	2.08604
24394	0.946132	2.1
24395	1.39731	2.1
24396	0.946119	2.1
24397	1.40765	1.98405
24398	1.1981	2.1
24399	0.946131	1.92742
24400	1.40764	2.1
24401	0.946127	2.1
24402	1.3557	2.1
24403	0.946127	2.1
24404	0.946128	2.1
24405	1.13485	2.1
24406	1.4008	2.1
24407	1.1587	2.1
24408	0.946131	2.1
24409	0.946131	2.1
24410	1.1587	2.1
24411	1.32082	0.05
24412	1.29215	0.0619355
24413	0.94613	0.05
24414	0.946131	0.05
24415	1.02366	0.05

24416	1.27697	0.05
24417	1.2929	0.05
24418	0.946132	0.05
24419	0.946132	0.05
24420	1.01451	0.05
24421	0.946132	2.1
24422	1.27252	2.1
24423	0.946132	2.1
24424	1.37869	2.1
24425	1.27252	2.1
24426	1.15627	2.1
24427	1.35703	2.1
24428	0.946132	2.1
24429	1.03783	2.1
24430	0.946132	2.1
24431	0.946128	2.1
24432	1.37869	2.1
24433	0.946131	2.1
24434	1.35357	2.1
24435	1.37869	2.1
24436	0.946132	2.1
24437	0.946131	2.1
24438	1.33471	2.1
24439	0.946131	2.1
24440	1.36373	2.1
24441	1.41342	0.961545
24442	1.06832	1.005
24443	0.946128	1.46303
24444	0.94613	0.875946
24445	1.06832	1.28291
24446	1.26929	2.1
24447	0.946131	1.91378
24448	0.946128	2.1
24449	1.41022	2.1
24450	1.37034	2.1
24451	1.37759	1.00374
24452	1.07612	1.25543
24453	0.946126	1.04896
24454	0.946129	1.13677
24455	1.07612	1.07622
24456	0.946131	1.02132
24457	0.94613	0.0965838
24458	1.23848	0.0764639
24459	1.38897	0.07091
24460	0.946131	0.168969
24461	0.946132	0.781622
24462	0.946133	0.05
24463	1.34369	0.05
24464	1.40332	0.05
24465	0.946132	0.0763176
24466	0.946131	2.1
24467	1.24023	2.1
24468	1.38244	2.1
24469	1.38244	2.1
24470	0.946131	2.1
24471	0.946132	2.1
24472	1.33468	2.1
24473	1.41619	2.1

24474	1.41619	2.1
24475	0.946131	2.1
24476	1.1908	1.20599
24477	0.946128	1.0732
24478	0.946126	1.43477
24479	1.26654	1.70677
24480	1.39514	2.09399
24481	0.93608	2.1
24482	1.04105	2.1
24483	1.39551	2.1
24484	1.52471	2.1
24485	1.04072	2.1
24486	1.33174	0.05
24487	0.946128	0.05
24488	0.94613	0.05
24489	1.17801	0.05
24490	0.94613	0.05
24491	0.946132	2.1
24492	0.946132	2.1
24493	1.29065	2.1
24494	1.29065	2.1
24495	0.975266	2.1
24496	0.946131	2.1
24497	1.26025	2.1
24498	0.955601	2.1
24499	0.946131	2.1
24500	1.26025	2.1
24501	0.94613	0.108098
24502	1.23146	0.0595444
24503	0.946127	0.0998363
24504	1.32827	0.223816
24505	1.23146	0.137382
24506	1.25008	0.727934
24507	1.05018	0.936943
24508	0.94613	0.540405
24509	0.932744	1.00774
24510	1.04207	0.970326
24511	0.946132	0.118243
24512	1.10023	0.613737
24513	1.36748	0.063326
24514	1.36748	0.649718
24515	0.946133	0.241468
24516	1.06023	0.05
24517	1.08019	0.05
24518	0.94613	0.0654989
24519	1.06023	0.05
24520	0.94613	0.05
24521	1.16727	0.0542267
24522	1.35856	0.0983702
24523	0.946132	0.483524
24524	1.16727	0.118877
24525	0.946132	0.0825499
24526	0.946133	2.1
24527	0.946131	2.1
24528	1.21691	2.1
24529	1.39558	2.1
24530	0.946131	2.1
24531	1.09905	0.05

24532	0.946132	0.227649
24533	1.3365	0.05
24534	1.35381	0.08778
24535	0.946132	0.453791
24536	0.94613	0.05
24537	0.946128	0.05
24538	1.40332	0.05
24539	1.39041	0.05
24540	0.94613	0.05
24541	0.946132	0.500496
24542	1.3659	2.1
24543	1.39041	0.197233
24544	1.39041	1.41482
24545	0.946132	1.15197
24546	0.840292	2.08187
24547	0.946133	1.90449
24548	0.851541	2.1
24549	1.28464	2.06208
24550	1.28806	1.98953
24551	1.12591	0.879014
24552	1.05033	0.99438
24553	0.946132	0.922496
24554	0.946132	0.981701
24555	1.13537	0.958623
24556	1.21177	1.26146
24557	0.946132	1.3039
24558	0.946132	2.1
24559	1.41134	1.28847
24560	1.27079	1.43086
24561	0.946131	0.05
24562	1.35403	0.05
24563	0.94613	0.05
24564	0.94613	0.05
24565	0.946131	0.05
24566	0.946131	0.05
24567	0.946132	0.05
24568	0.94613	0.05
24569	1.37052	0.05
24570	0.946131	0.05
24571	0.946133	0.05
24572	0.946132	0.05
24573	0.946133	0.05
24574	1.24249	0.05
24575	0.946132	0.05
24576	1.20608	2.1
24577	0.946132	2.1
24578	0.946131	2.1
24579	0.946132	2.1
24580	0.946132	2.1
24581	0.946131	2.1
24582	0.946128	2.1
24583	0.946129	2.1
24584	1.41232	2.1
24585	0.94613	2.1
24586	0.946132	0.05
24587	1.32449	0.05
24588	0.946131	0.05
24589	0.946131	0.05

24590	1.32449	0.05
24591	0.946116	1.7079
24592	1.41563	2.1
24593	0.946118	2.1
24594	0.946105	2.1
24595	0.946131	1.97688
24596	1.26563	2.1
24597	1.32819	2.1
24598	0.946131	0.288813
24599	0.946131	2.1
24600	0.946131	1.21482
24601	0.946131	2.06663
24602	0.946133	1.44916
24603	1.30382	2.1
24604	0.946131	1.38608
24605	0.946131	1.34818
24606	1.30382	1.9225
24607	0.946133	0.05
24608	1.15803	0.05
24609	1.28895	0.05
24610	0.954071	0.05
24611	0.946132	0.05
24612	1.0264	0.103671
24613	0.946132	1.65942
24614	0.946132	1.17938
24615	0.946131	1.54926
24616	0.946132	1.26463
24617	1.35977	0.05
24618	0.946132	0.05
24619	0.946129	0.05
24620	0.94613	0.05
24621	0.946129	0.05
24622	0.876631	0.66825
24623	0.946133	0.714252
24624	0.946132	0.80387
24625	0.946132	0.764033
24626	0.876606	0.739352
24627	0.946131	0.05
24628	1.24979	0.05
24629	0.94613	0.05
24630	0.94613	0.05
24631	0.94613	0.05
24632	0.921791	0.05
24633	0.946131	0.05
24634	0.921762	0.05
24635	0.946131	0.05
24636	0.946131	0.05
24637	0.810944	0.05
24638	0.810945	0.05
24639	0.810959	0.05
24640	0.946133	0.05
24641	1.25797	0.05
24642	0.946132	0.05
24643	0.946132	0.05
24644	1.25797	0.05
24645	1.13748	0.0749371
24646	0.851512	0.201032
24647	0.94613	1.0126

24648	1.02373	0.0839519
24649	0.946131	0.579848
24650	0.946122	2.1
24651	1.3852	2.1
24652	0.946124	2.1
24653	0.946118	2.1
24654	1.38521	2.1
24655	0.946131	0.706284
24656	0.946131	0.747624
24657	1.30448	0.761315
24658	0.946129	0.699635
24659	0.94613	0.67209
24660	0.946133	2.1
24661	0.94613	2.1
24662	0.946131	2.1
24663	0.946131	2.1
24664	1.36503	2.1
24665	0.810947	0.05
24666	0.810947	0.05
24667	0.810934	0.05
24668	3.34066	0.05
24669	0.946133	0.586971
24670	1.4185	0.69182
24671	0.946132	0.62873
24672	0.946133	0.693307
24673	0.946132	0.230371
24674	0.94613	2.1
24675	0.946128	2.1
24676	1.03925	2.1
24677	0.944743	2.1
24678	0.947518	2.1
24679	0.946132	2.1
24680	1.20573	2.1
24681	0.94613	2.1
24682	0.94613	2.1
24683	1.20573	2.1
24684	1.20056	0.05
24685	0.946132	0.05
24686	0.946131	0.05
24687	0.946131	0.05
24688	0.946131	0.05
24689	0.94613	2.1
24690	0.946128	2.1
24691	1.38302	2.1
24692	0.94613	2.1
24693	0.94613	2.1
24694	0.827866	0.05
24695	0.913552	0.05
24696	0.716526	0.05
24697	0.82784	0.05
24698	1.20634	0.05
24699	0.946131	0.05
24700	0.94613	0.05
24701	1.39502	0.05
24702	0.88146	0.05
24703	0.946132	2.1
24704	0.946132	2.1
24705	0.946132	2.1

24706	0.946132	2.1
24707	1.39053	2.1
24708	0.946131	2.1
24709	1.40571	2.1
24710	0.946131	2.1
24711	0.946131	2.1
24712	0.946132	2.1
24713	0.94613	0.545643
24714	1.2582	0.26458
24715	0.946127	0.670376
24716	0.946129	0.254452
24717	0.946129	0.371798
24718	0.946129	1.07682
24719	0.946132	1.12306
24720	0.946131	0.907192
24721	0.946131	1.16603
24722	1.29054	0.997963
24723	0.810944	0.05
24724	0.810941	0.05
24725	0.810931	0.05
24726	0.946132	0.924657
24727	0.946132	0.881182
24728	0.946132	1.17228
24729	0.946132	0.931376
24730	0.927608	0.903822
24731	0.946128	2.1
24732	0.946133	2.1
24733	0.946131	2.1
24734	0.94613	2.1
24735	1.38813	2.1
24736	0.94613	0.40336
24737	0.946128	2.1
24738	0.946132	0.710507
24739	1.41666	2.1
24740	0.94613	2.1
24741	0.946118	0.05
24742	1.34151	0.05
24743	0.946116	0.05
24744	0.946131	0.05
24745	1.34149	0.05
24746	0.946132	2.1
24747	1.31889	2.1
24748	0.946132	2.1
24749	0.946132	2.1
24750	1.31889	2.1
24751	0.864997	0.05
24752	0.928981	0.05
24753	0.872219	0.05
24754	0.946127	0.388113
24755	0.946131	0.301235
24756	1.37709	0.114688
24757	0.946131	0.435871
24758	0.946129	0.279954
24759	1.21882	0.05
24760	0.938168	0.05
24761	0.938161	0.05
24762	1.06327	0.05
24763	1.33457	0.05

24764	0.946133	1.07159
24765	0.946132	1.07944
24766	0.946133	0.882362
24767	0.946132	1.23154
24768	1.14444	0.828903
24769	1.12628	0.05
24770	0.946132	0.05
24771	0.946131	0.05
24772	1.12628	0.05
24773	0.946131	0.05
24774	0.946132	2.1
24775	0.946132	2.1
24776	0.946133	2.1
24777	0.946132	2.1
24778	1.29322	2.1
24779	0.946131	2.1
24780	0.946132	2.1
24781	0.94613	2.1
24782	0.946131	2.1
24783	1.28441	2.1
24784	0.946118	2.1
24785	0.946119	2.1
24786	0.946117	2.1
24787	1.28075	2.1
24788	0.946105	2.1
24789	0.810945	2.1
24790	0.810942	2.1
24791	0.81093	2.1
24792	0.946127	2.1
24793	0.946127	2.1
24794	1.28228	2.1
24795	0.946129	2.1
24796	0.946132	2.1
24797	1.14042	2.1
24798	0.946132	2.1
24799	0.946133	2.1
24800	1.14042	2.1
24801	0.946133	0.0772064
24802	0.946132	0.05
24803	1.28632	0.05
24804	1.28632	0.05
24805	0.946133	0.05
24806	0.946131	1.39598
24807	0.946131	1.19653
24808	0.946131	1.28664
24809	1.31817	1.43148
24810	0.946131	1.20641
24811	1.35048	2.1
24812	0.946132	2.1
24813	0.946132	2.1
24814	0.946132	2.1
24815	0.946131	2.1
24816	0.946131	2.1
24817	1.35943	2.1
24818	0.94613	2.1
24819	0.946132	2.1
24820	0.946131	2.1
24821	0.990713	2.1

24822	0.946132	2.1
24823	0.946132	2.1
24824	0.946133	2.1
24825	0.946132	2.1
24826	0.946133	1.20624
24827	1.01433	1.24682
24828	0.877931	1.24873
24829	0.96573	1.06222
24830	0.946131	1.05676
24831	0.946131	0.941844
24832	0.946133	0.52374
24833	1.39575	0.0991542
24834	0.946131	0.139387
24835	0.94613	0.138818
24836	0.810943	0.214895
24837	0.810942	0.263066
24838	0.709589	0.192706
24839	0.924071	0.0895815
24840	1.04293	0.05
24841	1.15747	0.05
24842	0.946131	0.0983769
24843	1.04071	0.0677666
24844	0.810946	0.0938464
24845	0.810943	0.0562065
24846	0.810959	0.05
24847	0.946128	1.42695
24848	1.39696	1.95142
24849	0.94613	1.11093
24850	0.94613	1.12263
24851	1.39696	1.53526
24852	0.946131	1.00784
24853	0.946131	1.98758
24854	0.946131	1.58207
24855	0.946131	1.11946
24856	1.29735	1.81949
24857	0.946128	1.39686
24858	0.946133	0.190582
24859	0.94613	2.1
24860	0.946131	1.28637
24861	1.33266	0.835917
24862	0.946131	2.1
24863	0.94613	0.05
24864	0.946131	2.1
24865	1.40609	0.603217
24866	0.946131	1.68745
24867	0.946131	2.01171
24868	0.946133	1.98322
24869	0.946129	2.1
24870	1.23339	2.1
24871	0.946131	1.71728
24872	0.946132	2.1
24873	0.946133	2.1
24874	0.946132	2.1
24875	1.29198	2.1
24876	0.946132	2.1
24877	0.946131	2.1
24878	1.07297	2.1
24879	0.946127	2.1

24880	0.94613	2.1
24881	1.07297	2.1
24882	0.946125	2.1
24883	0.946126	2.1
24884	0.946126	2.1
24885	1.3864	2.1
24886	0.946119	2.1
24887	0.946128	0.05
24888	1.09411	0.05
24889	0.946131	0.067912
24890	0.946131	0.05
24891	1.09411	0.05
24892	0.946131	0.05
24893	1.25837	0.05
24894	0.946131	0.05
24895	0.946131	0.05
24896	1.25837	0.05
24897	0.810945	2.1
24898	0.810943	2.1
24899	0.810933	2.1
24900	0.94613	0.05
24901	0.946131	0.357222
24902	0.946132	0.0625531
24903	0.946129	0.05
24904	0.946131	0.0892909
24905	0.946131	0.05
24906	1.28911	0.05
24907	0.946129	0.0809693
24908	0.946133	0.05
24909	0.946131	0.05
24910	0.94613	0.108259
24911	1.38954	0.189602
24912	0.946132	0.0949175
24913	0.94613	0.168282
24914	0.946131	0.109585
24915	0.946131	0.782934
24916	0.946131	0.116026
24917	0.946131	0.109921
24918	1.37981	0.0778576
24919	0.946131	0.112549
24920	0.946131	2.1
24921	1.26958	2.1
24922	0.946127	2.1
24923	0.946129	2.1
24924	1.26957	2.1
24925	0.94613	2.1
24926	1.16889	2.1
24927	0.946126	2.1
24928	0.946128	2.1
24929	1.16889	2.1
24930	0.946133	0.115258
24931	1.13511	0.05
24932	0.946132	0.197106
24933	0.946133	0.0852988
24934	0.946132	0.107002
24935	1.27575	1.16006
24936	0.946129	2.1
24937	0.946131	1.80724

24938	0.94613	2.1
24939	0.946131	2.01656
24940	0.946131	2.1
24941	0.946128	2.1
24942	1.41712	2.1
24943	1.41712	2.1
24944	0.94613	2.1
24945	0.946129	0.05
24946	0.946132	0.05
24947	0.946131	0.05
24948	0.946131	0.05
24949	0.946131	0.05
24950	0.979045	2.06781
24951	0.946131	1.11254
24952	0.617549	2.1
24953	0.555844	2.1
24954	1.04071	1.08574
24955	0.946132	1.80004
24956	0.946132	1.1487
24957	0.946131	2.1
24958	1.22618	1.19784
24959	0.946132	0.281751
24960	0.946131	2.1
24961	1.13976	2.1
24962	0.946129	2.1
24963	0.946131	2.1
24964	1.13976	2.1
24965	0.810945	0.821695
24966	0.810943	0.228019
24967	0.810932	0.89326
24968	1.25358	1.1115
24969	0.94613	0.51534
24970	0.946128	0.127042
24971	1.25358	1.80038
24972	1.07811	0.988333
24973	0.946129	2.1
24974	1.09197	2.1
24975	0.946131	2.1
24976	0.946131	2.1
24977	1.09197	2.1
24978	0.594912	2.1
24979	0.993703	2.1
24980	1.10173	2.1
24981	0.9916	2.1
24982	0.946131	2.1
24983	0.946128	2.1
24984	0.946131	2.1
24985	0.94613	2.1
24986	0.946129	2.1
24987	0.946132	2.1
24988	1.40335	2.1
24989	0.946133	2.1
24990	0.946133	2.1
24991	1.40335	2.1
24992	0.946132	1.3405
24993	0.946132	2.1
24994	0.946132	1.41052
24995	0.946132	1.5369

24996	1.411	2.01558
24997	0.611675	0.05
24998	0.946129	0.0964935
24999	1.31678	0.05
25000	0.946124	0.0888624
25001	0.946127	0.05
25002	0.946128	0.0723871
25003	0.946132	0.12326
25004	0.946131	0.0625483
25005	0.946132	0.133617
25006	0.946132	0.0654972
25007	0.946132	0.0806109
25008	0.810946	0.077096
25009	0.810943	0.0800446
25010	0.81096	0.0802078
25011	1.39301	0.05
25012	0.946132	0.05
25013	0.625812	0.05
25014	0.946132	0.05
25015	0.625812	0.05
25016	0.810946	1.39753
25017	0.810945	2.09531
25018	0.810931	2.1
25019	0.810943	2.1
25020	0.810944	2.1
25021	0.810958	2.1
25022	0.946131	0.719753
25023	0.946131	0.71096
25024	0.946128	0.721405
25025	0.946129	0.715364
25026	0.94613	0.662895
25027	0.946133	0.17363
25028	0.946133	0.358918
25029	0.98537	0.0868807
25030	0.985371	0.05
25031	0.946132	0.441843
25032	0.946129	0.05
25033	1.38752	0.05
25034	0.946131	0.05
25035	0.946131	0.05
25036	1.38752	0.05
25037	0.946119	0.05
25038	1.33036	0.05
25039	0.946119	0.05
25040	0.946132	0.05
25041	1.33035	0.05
25042	1.3868	2.1
25043	0.946132	2.1
25044	0.946132	2.1
25045	0.946133	2.1
25046	0.946132	2.1
25047	0.810947	2.1
25048	0.810946	2.1
25049	0.81096	2.1
25050	0.810946	2.1
25051	0.810946	2.1
25052	0.810933	2.1
25053	0.946133	2.1

25054	1.35342	2.1
25055	0.946133	2.1
25056	0.946132	2.1
25057	1.35342	2.1
25058	0.946132	2.1
25059	0.946132	2.1
25060	1.28569	2.1
25061	0.946132	2.1
25062	0.946133	2.1
25063	0.946132	2.1
25064	1.32547	2.1
25065	0.946132	2.1
25066	0.946132	2.1
25067	1.32547	2.1
25068	0.94613	0.05
25069	0.946133	0.05
25070	1.39664	0.05
25071	1.39664	0.05
25072	0.946131	0.05
25073	0.946131	1.40449
25074	0.94613	2.1
25075	0.946132	1.12446
25076	0.946131	2.1
25077	1.41833	1.98776
25078	0.946131	0.05
25079	0.946131	0.05
25080	0.946131	0.05
25081	1.26155	0.05
25082	0.946132	0.05
25083	0.810946	2.1
25084	0.810946	2.1
25085	0.810959	2.1
25086	0.94613	1.04337
25087	0.946132	0.22423
25088	1.40779	0.0547876
25089	1.40779	0.120097
25090	0.94613	0.159415
25091	0.94613	0.05
25092	0.946129	0.05
25093	1.36376	0.05
25094	1.36376	0.05
25095	0.946131	0.05
25096	0.946128	2.1
25097	0.946127	2.1
25098	0.946131	2.1
25099	1.40756	2.1
25100	0.94613	2.1
25101	0.946131	1.9362
25102	0.946132	2.1
25103	0.94613	1.94796
25104	0.946131	1.90632
25105	1.31947	2.1
25106	0.946131	2.1
25107	0.946131	2.1
25108	0.946129	2.1
25109	0.946131	2.1
25110	1.25768	2.1
25111	0.946132	0.701992

25112	0.94613	0.696615
25113	0.946131	0.568702
25114	0.946131	0.467283
25115	0.946131	0.678173
25116	0.810943	0.988536
25117	0.810942	1.1529
25118	0.81093	2.06053
25119	0.946128	0.985129
25120	0.946133	0.868001
25121	0.946131	1.00582
25122	0.946131	0.943985
25123	0.94613	1.02478
25124	0.946132	1.10583
25125	0.946131	0.995769
25126	0.946132	1.11431
25127	0.946132	1.0992
25128	0.946132	1.16599
25129	0.946119	2.1
25130	0.946119	2.1
25131	1.31523	2.1
25132	0.946132	2.1
25133	0.946107	2.1
25134	0.946133	2.1
25135	0.946132	0.302362
25136	0.946132	0.572663
25137	1.28803	2.1
25138	0.946132	2.1
25139	1.37658	1.05297
25140	0.946132	0.94622
25141	0.946133	1.53486
25142	0.946132	1.04026
25143	0.946132	1.29025
25144	0.946132	1.13463
25145	0.94613	1.23621
25146	0.94613	1.4921
25147	1.15541	1.13583
25148	0.92943	1.31596
25149	0.946126	0.05
25150	0.946129	0.05
25151	1.33849	0.05
25152	1.33849	0.05
25153	0.946129	0.05
25154	0.810946	0.05
25155	0.810944	0.05
25156	0.810959	0.05
25157	1.39027	0.05
25158	0.946131	0.05
25159	0.946131	0.05
25160	1.39027	0.05
25161	0.946131	0.05
25162	1.40924	2.07376
25163	0.946131	1.2778
25164	0.946132	2.08735
25165	0.946132	1.15381
25166	0.946132	1.60559
25167	0.946131	2.1
25168	0.94613	2.1
25169	0.946128	2.1

25170	0.946128	2.1
25171	1.41744	2.1
25172	0.946132	0.231622
25173	0.946128	0.185115
25174	0.94613	0.97059
25175	1.37675	0.224443
25176	0.94613	0.913787
25177	0.946132	1.03114
25178	0.946132	0.117991
25179	0.946132	0.0632733
25180	0.946132	0.137409
25181	0.946131	0.121452
25182	0.946132	0.946094
25183	0.946132	0.872187
25184	0.946133	1.24679
25185	1.10003	0.801494
25186	0.946132	1.03037
25187	0.946132	0.05
25188	0.946131	0.05
25189	0.946132	0.05
25190	0.946131	0.05
25191	0.946132	0.05
25192	0.946132	0.05
25193	0.946131	0.05
25194	0.946132	0.05
25195	0.946132	0.05
25196	0.946131	0.05
25197	1.24094	0.05
25198	0.946126	0.05
25199	0.946128	0.05
25200	0.946129	0.05
25201	0.946129	0.05
25202	1.2929	0.05
25203	0.946131	0.05
25204	0.946131	0.05
25205	0.946131	0.05
25206	0.946131	0.05
25207	0.810946	0.05
25208	0.810945	0.05
25209	0.810959	0.05
25210	0.810946	2.07535
25211	0.810943	2.1
25212	0.810933	2.1
25213	0.946128	2.1
25214	1.18315	2.1
25215	0.94613	2.1
25216	0.94613	2.1
25217	1.18316	2.1
25218	0.946126	0.195794
25219	1.3324	0.05
25220	0.946127	0.226263
25221	0.94613	0.0545718
25222	0.946128	0.0687848
25223	0.94613	2.1
25224	0.946128	2.1
25225	1.39523	2.1
25226	1.39523	2.1
25227	0.946131	2.1

25228	1.36541	2.1
25229	0.946132	2.1
25230	0.946132	2.1
25231	0.946132	2.1
25232	0.946132	2.1
25233	0.946131	2.1
25234	0.94613	1.20672
25235	0.946132	1.50651
25236	0.946131	0.33647
25237	1.39509	1.49427
25238	0.946132	2.1
25239	0.946132	2.1
25240	1.29319	2.1
25241	0.946132	2.1
25242	0.946133	2.1
25243	0.946132	0.05
25244	0.946131	0.05
25245	0.946132	0.05
25246	1.01196	0.05
25247	0.946132	0.05
25248	0.946116	2.1
25249	0.946117	2.1
25250	1.38153	2.1
25251	1.38155	2.1
25252	0.946105	2.1
25253	0.946132	1.95154
25254	0.946131	2.0022
25255	0.946132	1.65282
25256	1.28684	1.71817
25257	0.946132	1.29737
25258	1.10185	1.7706
25259	0.94613	1.88895
25260	0.946128	2.1
25261	0.94613	2.1
25262	0.94613	1.87367
25263	0.810947	0.05
25264	0.810945	0.05
25265	0.810932	0.05
25266	0.946119	0.208233
25267	1.16717	0.05
25268	0.946117	0.197948
25269	0.94613	0.05
25270	1.16715	0.0571705
25271	0.94612	0.716196
25272	0.946119	0.787587
25273	0.946119	0.368256
25274	0.946132	0.0696317
25275	1.20882	0.305112
25276	1.07043	0.39938
25277	0.823268	0.380187
25278	0.810959	0.373142
25279	0.946132	1.76336
25280	1.14879	0.778331
25281	0.94613	1.52945
25282	0.946131	1.71866
25283	1.14879	1.02553
25284	1.23837	2.1
25285	0.94612	2.1

25286	0.946118	2.1
25287	0.946131	2.1
25288	0.946105	2.1
25289	0.810946	0.05
25290	0.810946	0.05
25291	0.810959	0.05
25292	0.810947	0.922258
25293	0.810947	0.529912
25294	0.810934	0.569745
25295	0.946128	2.1
25296	0.946131	2.1
25297	0.946128	2.1
25298	0.946129	2.1
25299	0.94613	2.1
25300	0.94613	2.1
25301	0.946131	2.1
25302	0.946131	2.1
25303	0.946131	2.1
25304	0.946131	2.1
25305	0.946131	1.8049
25306	1.38585	2.1
25307	0.946128	1.34759
25308	0.94613	1.90379
25309	1.38585	1.90233
25310	0.946133	0.05
25311	0.946133	0.05
25312	0.946132	0.05
25313	0.946132	0.05
25314	0.946132	0.05
25315	0.918871	0.05
25316	0.810248	0.05
25317	0.838037	0.05
25318	0.946126	0.05
25319	0.943647	0.05
25320	1.13836	0.05
25321	1.03802	0.05
25322	0.946129	0.05
25323	0.946133	1.10445
25324	0.946132	0.694758
25325	0.946132	2.1
25326	1.1699	0.402843
25327	0.946132	0.235487
25328	0.946131	2.1
25329	0.946132	2.1
25330	1.37199	2.1
25331	1.37199	2.1
25332	0.946132	2.1
25333	0.946132	1.05732
25334	0.946133	0.934393
25335	0.946133	1.16728
25336	0.946132	0.942884
25337	1.38623	0.967754
25338	0.946132	2.1
25339	0.946132	2.1
25340	0.946132	2.1
25341	0.946132	2.1
25342	1.40098	2.1
25343	1.20787	0.770316

25344	1.41894	0.767923
25345	0.946133	0.325006
25346	1.01777	0.826485
25347	0.946132	2.1
25348	0.946132	2.1
25349	0.946133	2.1
25350	1.40199	2.1
25351	0.946132	2.1
25352	0.810946	1.16707
25353	0.810947	1.66145
25354	0.810934	1.26233
25355	0.946126	1.0941
25356	0.94613	1.30116
25357	0.946125	1.27651
25358	1.40247	1.14529
25359	0.946128	1.10235
25360	1.25703	1.51173
25361	0.87821	1.18075
25362	0.878238	1.16791
25363	0.946119	1.95556
25364	0.946132	2.1
25365	0.946132	0.894326
25366	0.946132	0.871718
25367	0.946131	1.20958
25368	0.946132	0.95033
25369	1.28543	0.932597
25370	0.946115	2.1
25371	1.41638	2.1
25372	0.946116	2.1
25373	0.946104	2.1
25374	0.946129	2.1
25375	0.946132	2.1
25376	1.41432	2.1
25377	0.946132	2.1
25378	0.946132	2.1
25379	0.946132	2.1
25380	0.810947	0.05
25381	0.810947	0.05
25382	0.709567	0.05
25383	0.851542	0.05
25384	1.01217	0.05
25385	0.697993	0.05
25386	0.946133	0.05
25387	0.851488	0.05
25388	0.946129	2.1
25389	0.94613	2.1
25390	1.2254	1.90481
25391	0.946131	2.1
25392	0.946131	2.1
25393	0.700785	2.1
25394	0.915893	1.78036
25395	0.838013	1.68134
25396	0.760188	1.17609
25397	1.19601	0.05
25398	0.946133	0.05
25399	0.946132	0.0551306
25400	0.946132	0.05
25401	0.946132	0.0584211

25402	0.946132	1.1825
25403	0.946133	2.1
25404	0.946132	1.4147
25405	0.946133	2.1
25406	1.36956	2.1
25407	0.946131	0.906645
25408	0.94613	0.05
25409	0.946133	0.634625
25410	1.34268	0.05
25411	0.946131	0.05
25412	0.946132	2.1
25413	1.25603	2.1
25414	0.946132	2.1
25415	0.946132	2.1
25416	1.25603	2.1
25417	0.810946	1.15886
25418	0.810943	1.15696
25419	0.810933	1.15552
25420	0.496545	0.05
25421	0.805767	0.541088
25422	0.936671	0.445319
25423	0.530063	0.323813
25424	1.05954	0.332676
25425	0.810946	0.05
25426	0.810943	0.05
25427	0.810959	0.05
25428	1.16699	0.05
25429	0.946131	0.05
25430	0.946129	0.05
25431	0.946131	0.05
25432	0.946131	0.05
25433	0.810943	1.33426
25434	0.810942	1.42087
25435	0.810933	1.32948
25436	0.946132	1.31016
25437	1.04832	1.30976
25438	0.870714	1.40112
25439	0.957819	1.6444
25440	0.94613	1.22668
25441	1.82931	2.1
25442	0.946132	0.744536
25443	0.946129	0.726082
25444	0.94613	0.710845
25445	0.946131	0.760837
25446	0.94613	0.699349
25447	0.946119	1.04373
25448	0.94612	1.14663
25449	0.94612	1.01692
25450	0.946132	1.10713
25451	1.36118	1.04184
25452	0.810947	0.392025
25453	0.810947	0.314628
25454	0.810934	0.480917
25455	0.946132	2.1
25456	0.946132	2.1
25457	0.946132	2.1
25458	1.39754	2.1
25459	0.946131	2.1

25460	0.946129	2.1
25461	0.946133	2.1
25462	0.946131	2.1
25463	0.946131	2.1
25464	1.41732	2.1
25465	0.946132	1.06208
25466	0.946128	1.95807
25467	0.94613	1.15463
25468	0.94613	1.23515
25469	1.25277	1.45398
25470	0.94613	1.46631
25471	0.946128	1.98559
25472	1.21261	0.688602
25473	0.94613	1.53374
25474	0.94613	0.819523
25475	1.39407	2.1
25476	0.946133	2.1
25477	0.94613	2.1
25478	0.946131	2.1
25479	0.946131	2.1
25480	0.946119	1.20063
25481	0.946119	1.43789
25482	0.946119	1.49805
25483	1.1537	1.46393
25484	0.946106	1.15125
25485	0.946131	2.1
25486	1.11822	2.1
25487	0.946129	2.1
25488	0.946131	2.1
25489	1.11822	2.1
25490	1.46806	0.05
25491	0.498881	0.05
25492	0.996658	0.05
25493	1.2179	0.05
25494	0.991519	0.05
25495	0.946131	0.05
25496	0.946131	0.05
25497	1.28673	0.05
25498	0.946131	0.05
25499	0.946131	0.05
25500	1.33178	0.05
25501	0.946132	0.05
25502	0.946132	0.05
25503	0.946132	0.05
25504	0.946132	0.05
25505	0.946128	0.05
25506	0.972061	0.05
25507	0.946129	0.083074
25508	0.946129	0.0507704
25509	0.972059	0.0516895
25510	0.946129	2.1
25511	0.94613	2.1
25512	0.94613	2.1
25513	0.94613	2.1
25514	0.94613	2.1
25515	0.946131	2.1
25516	0.946127	2.1
25517	0.946131	2.1

25518	0.946129	2.1
25519	1.4073	2.1
25520	0.946131	0.05
25521	1.05016	0.05
25522	0.946132	0.05
25523	0.946131	0.05
25524	0.946132	0.05
25525	0.946128	0.05
25526	1.13398	0.05
25527	0.946131	0.05
25528	0.946131	0.05
25529	0.94613	0.05
25530	0.946131	0.05
25531	0.946131	0.05
25532	1.38911	0.05
25533	1.38911	0.05
25534	0.946132	0.05
25535	0.946131	0.05
25536	0.94613	0.05
25537	0.946131	0.05
25538	0.946131	0.05
25539	0.946131	0.05
25540	0.946131	0.05
25541	1.23923	0.05
25542	0.94613	0.05
25543	0.946131	0.05
25544	0.946131	0.05
25545	1.40511	0.05
25546	0.946131	0.05
25547	0.946131	0.05
25548	0.946132	0.05
25549	0.946131	0.05
25550	1.12968	2.1
25551	0.946132	2.1
25552	0.946132	2.1
25553	0.946131	2.1
25554	0.946132	2.1
25555	0.946132	1.636
25556	1.4172	2.1
25557	0.94613	1.3827
25558	0.94613	1.95213
25559	1.4172	2.1
25560	0.946132	2.1
25561	0.946132	2.1
25562	1.31323	2.1
25563	0.946133	2.1
25564	0.946132	2.1
25565	0.946131	2.1
25566	0.94613	0.166613
25567	1.36546	2.1
25568	1.36546	2.08559
25569	0.946131	1.82182
25570	0.946132	0.725375
25571	0.946133	0.207884
25572	0.946132	0.05
25573	1.36899	0.05
25574	0.946133	0.0916548
25575	0.946131	0.05

25576	0.946132	0.05
25577	1.03543	0.05
25578	1.1412	0.05
25579	0.941275	0.05
25580	1.28952	0.05
25581	0.94613	0.05
25582	0.946129	0.05
25583	0.946132	0.05
25584	0.94613	0.05
25585	0.94613	2.1
25586	0.94613	2.1
25587	0.946129	2.1
25588	0.94613	2.1
25589	1.35966	2.1
25590	0.810946	2.1
25591	0.810942	2.1
25592	0.810932	2.1
25593	0.94613	2.1
25594	1.40372	2.1
25595	0.946131	2.1
25596	0.94613	2.1
25597	1.40372	2.1
25598	0.946133	2.1
25599	0.946131	2.1
25600	0.94613	2.1
25601	0.946131	2.1
25602	0.946131	2.1
25603	1.31453	2.1
25604	0.94613	2.1
25605	0.946128	2.1
25606	0.94613	2.1
25607	0.94613	2.1
25608	0.946131	0.05
25609	1.12018	0.05
25610	0.94613	0.05
25611	0.94613	0.05
25612	0.94613	0.05
25613	0.946131	0.05
25614	1.16373	0.05
25615	0.946131	0.05
25616	0.94613	0.05
25617	0.946131	0.05
25618	0.946126	0.133002
25619	0.946132	0.134798
25620	0.946128	0.102985
25621	0.946129	0.347384
25622	0.946129	0.335375
25623	0.946132	0.05
25624	0.946133	0.0723199
25625	0.946132	0.05
25626	0.946132	0.0589189
25627	0.946133	0.053343
25628	0.946132	1.05729
25629	0.946129	1.50816
25630	0.94613	1.00219
25631	1.36399	1.11175
25632	0.946131	1.03591
25633	0.946132	2.1

25634	0.946132	2.1
25635	0.946133	2.1
25636	1.38302	2.1
25637	0.946133	2.1
25638	0.810943	2.1
25639	0.810944	2.1
25640	0.810945	2.1
25641	0.946132	2.1
25642	0.946131	2.1
25643	0.946132	2.1
25644	1.30188	2.1
25645	0.946132	2.1
25646	0.810946	0.952929
25647	0.72985	1.46292
25648	0.810934	1.05354
25649	0.856243	0.924936
25650	0.946129	0.901862
25651	0.946132	0.935615
25652	1.19686	0.74214
25653	0.851527	0.990314
25654	0.946131	2.1
25655	0.94613	2.1
25656	0.94613	2.1
25657	0.94613	2.1
25658	1.31961	2.1
25659	0.946131	2.1
25660	0.946131	2.1
25661	0.94613	2.1
25662	0.946131	2.1
25663	1.3605	2.1
25664	1.30116	2.1
25665	0.946133	0.390821
25666	0.946131	2.1
25667	0.946131	1.8217
25668	0.94613	2.1
25669	0.946132	0.05
25670	1.3035	0.05
25671	0.94613	0.05
25672	0.946131	0.05
25673	0.946131	0.05
25674	0.946132	2.1
25675	1.37869	2.1
25676	1.2051	2.1
25677	0.946132	2.1
25678	1.2051	2.1
25679	0.810946	2.1
25680	0.810945	2.1
25681	0.810946	2.1
25682	1.34623	2.1
25683	0.946131	2.1
25684	0.946131	2.1
25685	1.34623	2.1
25686	1.22993	2.1
25687	1.32299	0.05
25688	0.946132	0.05
25689	0.946132	0.05
25690	0.946132	0.05
25691	0.946131	0.05

25692	0.94613	0.05
25693	1.40574	0.05
25694	0.946128	0.05
25695	0.946131	0.05
25696	1.40574	0.05
25697	0.946132	2.1
25698	1.41331	2.1
25699	0.94613	2.1
25700	0.946131	2.1
25701	1.41331	2.1
25702	0.94613	2.1
25703	1.37609	2.1
25704	0.946129	2.1
25705	0.946131	2.1
25706	1.37609	2.1
25707	1.039	0.335471
25708	0.946131	1.96145
25709	0.944514	1.58393
25710	0.946132	1.90172
25711	0.946132	1.72687
25712	1.23726	0.476637
25713	0.946128	1.7088
25714	0.946128	1.50251
25715	0.946129	1.76794
25716	0.94613	1.59066
25717	0.946129	2.1
25718	0.946131	2.1
25719	0.946131	2.1
25720	1.39214	2.1
25721	0.94613	2.1
25722	0.946118	0.627728
25723	0.946118	0.05
25724	1.41078	0.05
25725	1.41076	0.05
25726	0.946131	0.05
25727	0.946131	0.704453
25728	0.946131	0.679206
25729	0.946132	0.701392
25730	0.94613	0.686121
25731	0.946131	0.674515
25732	0.946132	0.05
25733	0.946132	0.05
25734	0.946133	0.05
25735	1.28924	0.05
25736	0.946132	0.05
25737	0.946133	0.143117
25738	1.09812	0.05
25739	0.946132	0.0809936
25740	0.946132	0.18681
25741	1.09812	0.194003
25742	0.946132	1.04357
25743	0.946132	0.76978
25744	0.946132	1.0287
25745	0.946132	1.01863
25746	1.15388	1.03154
25747	0.946132	0.244483
25748	0.974688	0.05
25749	0.946132	0.103786

25750	0.946132	0.244072
25751	0.974687	0.31989
25752	1.0644	1.37238
25753	0.946133	1.80844
25754	0.946132	1.38936
25755	0.946133	1.40922
25756	1.26547	1.56077
25757	1.02905	1.25741
25758	0.911537	1.29916
25759	0.838038	1.40871
25760	0.946131	0.05
25761	0.94613	0.05
25762	0.946132	0.05
25763	0.946131	0.05
25764	1.33922	0.05
25765	0.946132	0.05
25766	0.946132	0.05
25767	1.40926	0.05
25768	1.40927	0.05
25769	0.946132	0.05
25770	0.946129	2.1
25771	0.946131	2.1
25772	0.946131	1.33484
25773	0.946131	1.1316
25774	1.40496	2.06672
25775	0.94613	1.13576
25776	0.94613	1.11136
25777	0.946127	1.09489
25778	0.946128	1.02567
25779	1.38914	1.0551
25780	2.675 0.05	
25781	1.22266	0.05
25782	0.946131	0.05
25783	0.946132	0.05
25784	0.946132	0.05
25785	1.22266	0.05
25786	0.946124	0.05
25787	0.946128	0.05
25788	0.946126	0.05
25789	1.21428	0.05
25790	0.946127	0.05
25791	0.946131	0.05
25792	0.946131	0.05
25793	0.946132	0.05
25794	0.946129	0.05
25795	0.940118	0.05
25796	0.810943	2.1
25797	0.810945	2.1
25798	0.810958	2.1
25799	0.946133	0.05
25800	1.11539	0.05
25801	0.946132	0.0715785
25802	0.946132	0.05
25803	0.946133	0.05
25804	0.946133	2.1
25805	1.41195	2.1
25806	0.946133	2.1
25807	0.946132	2.1

25808	0.946133	2.1
25809	1.15968	0.05
25810	0.946131	0.05
25811	0.946131	0.05
25812	0.946131	0.05
25813	0.946131	0.05
25814	0.94613	2.1
25815	0.946128	2.1
25816	0.946132	1.53268
25817	0.94613	2.1
25818	0.94613	1.78978
25819	0.946131	2.01107
25820	1.04211	1.9421
25821	0.850155	1.11642
25822	0.946131	1.75431
25823	0.946131	1.56048
25824	0.946131	0.0778766
25825	0.94613	0.05
25826	0.946129	0.078714
25827	0.946131	0.05
25828	0.946131	0.051262
25829	1.40193	0.05
25830	1.19756	0.05
25831	0.561936	0.05
25832	0.808778	0.05
25833	1.19756	0.05
25834	0.946132	0.05
25835	0.946132	0.05
25836	1.33916	0.05
25837	1.33916	0.05
25838	0.946133	0.05
25839	0.946127	0.491433
25840	0.946127	0.565831
25841	0.946131	0.371364
25842	0.946128	0.590987
25843	0.946129	0.455099
25844	0.589428	0.716361
25845	0.851501	0.688013
25846	0.868924	0.716952
25847	0.877703	0.706757
25848	0.85153	0.70799
25849	0.946132	2.08625
25850	1.25372	2.1
25851	0.946132	2.1
25852	0.946132	2.1
25853	1.25372	2.1
25854	0.946132	1.12394
25855	0.946128	2.1
25856	0.94613	1.25046
25857	0.94613	1.42267
25858	1.2778	1.89348
25859	0.946118	0.952522
25860	1.35629	0.963332
25861	0.946117	0.957072
25862	0.946131	0.939222
25863	0.946105	0.903893
25864	0.946132	0.119397
25865	0.946132	1.76216

25866	1.38646	1.07971
25867	1.38646	1.05399
25868	0.946132	1.15482
25869	0.946128	2.1
25870	0.946128	0.294506
25871	1.39659	2.1
25872	1.39659	2.1
25873	0.946129	2.03625
25874	0.946132	1.86428
25875	0.946131	1.52743
25876	0.946132	1.64478
25877	0.946132	1.58572
25878	1.15139	1.49923
25879	0.946133	2.1
25880	0.946132	2.1
25881	0.946132	2.1
25882	0.946132	2.1
25883	1.36151	2.1
25884	0.946127	2.1
25885	0.946131	2.1
25886	0.946128	2.1
25887	0.94613	2.1
25888	1.38397	2.1
25889	0.946131	0.05
25890	1.31112	0.05
25891	0.946127	0.0717084
25892	0.946128	0.123511
25893	1.31112	0.05
25894	1.29778	0.05
25895	0.883417	0.05
25896	1.32613	0.05
25897	0.94613	0.05
25898	0.94613	0.05
25899	0.946132	2.1
25900	0.94613	2.1
25901	0.946131	2.1
25902	0.946131	2.1
25903	1.36523	2.1
25904	0.946132	0.05
25905	1.01653	0.05
25906	0.946132	0.05
25907	0.946131	0.05
25908	0.946131	0.05
25909	0.94612	2.1
25910	1.08301	2.01075
25911	0.946119	2.1
25912	0.946132	2.1
25913	1.08299	2.1
25914	0.810942	2.1
25915	0.810944	1.44633
25916	0.810957	2.1
25917	0.946126	0.05
25918	0.946132	0.05
25919	0.946129	0.05
25920	1.3492	0.05
25921	0.94613	0.05
25922	0.946131	0.05
25923	1.24766	0.05

25924	0.946128	0.05
25925	0.946131	0.05
25926	1.24766	0.05
25927	0.946132	0.05
25928	0.946131	0.05
25929	0.946131	0.05
25930	1.23351	0.05
25931	0.946132	0.05
25932	0.946131	0.05
25933	0.946132	0.05
25934	0.946131	0.05
25935	1.35657	0.05
25936	0.946131	0.05
25937	0.946132	0.0533283
25938	1.3689	0.05
25939	0.946132	0.0642685
25940	0.946132	0.060339
25941	1.3689	0.05
25942	0.946133	0.05
25943	1.35822	0.05
25944	0.946133	0.05
25945	0.946132	0.05
25946	1.35822	0.05
25947	0.946131	0.855493
25948	0.946131	1.24602
25949	0.946131	1.18103
25950	0.946131	0.952419
25951	1.4101	1.48919
25952	0.810944	1.68424
25953	0.810939	1.80136
25954	0.810955	1.34961
25955	0.946132	2.1
25956	1.37733	2.1
25957	0.946132	2.1
25958	0.946131	2.1
25959	0.946131	2.1
25960	1.32582	2.1
25961	0.946132	2.1
25962	0.946132	2.1
25963	0.946132	2.1
25964	0.946132	2.1
25965	0.946132	2.1
25966	0.946132	2.1
25967	1.31401	2.1
25968	1.31401	2.1
25969	0.946131	2.1
25970	1.3469	1.34162
25971	0.946132	1.80507
25972	0.946132	1.94051
25973	0.946133	1.15
25974	0.946132	1.24262
25975	0.946126	2.1
25976	0.946127	2.1
25977	0.94613	2.1
25978	0.946128	2.1
25979	1.41206	2.1
25980	0.946127	2.1
25981	1.40109	2.1

25982	0.946128	2.1
25983	0.946129	2.1
25984	1.40109	2.1
25985	1.24448	0.05
25986	0.94613	0.05
25987	0.946132	0.05
25988	0.946129	0.05
25989	0.946131	0.05
25990	1.11015	0.05
25991	0.946133	0.05
25992	0.946132	0.05
25993	0.946133	0.05
25994	0.946132	0.05
25995	0.946132	2.1
25996	0.946131	2.1
25997	0.946131	2.1
25998	1.38775	2.1
25999	0.946131	2.1
26000	0.946133	0.203474
26001	0.946131	0.539172
26002	1.30598	2.1
26003	1.30598	1.64534
26004	0.946131	1.44869
26005	0.946132	2.1
26006	0.946132	2.1
26007	0.946132	2.1
26008	1.40774	2.1
26009	0.946132	2.1
26010	0.798396	0.05
26011	0.946133	0.897477
26012	0.946128	1.56172
26013	0.946131	1.0919
26014	0.946131	1.11789
26015	1.28918	1.12979
26016	0.946132	0.574081
26017	1.40668	0.446911
26018	0.94613	0.572366
26019	0.94613	0.512573
26020	0.94613	0.580613
26021	0.810946	2.1
26022	0.810945	2.1
26023	0.810933	2.1
26024	0.946131	2.1
26025	1.30338	2.1
26026	0.94613	2.1
26027	1.30339	2.1
26028	0.946131	2.1
26029	0.946127	0.984043
26030	0.87161	1.07003
26031	0.946128	0.987781
26032	0.946129	1.02166
26033	0.827876	0.998598
26034	0.946131	2.1
26035	1.40796	2.1
26036	0.94613	2.1
26037	0.94613	2.1
26038	1.40796	2.1
26039	0.946132	0.345656

26040	0.946132	0.594754
26041	0.946132	0.261531
26042	0.946132	0.169257
26043	1.41724	0.131309
26044	1.34254	0.05
26045	0.946132	0.117609
26046	0.946132	0.102752
26047	0.946132	0.0826208
26048	0.946133	0.0719072
26049	0.946133	2.03698
26050	0.946132	1.71985
26051	0.946132	1.49826
26052	1.26501	2.02946
26053	0.946132	1.38515
26054	0.946131	0.05
26055	1.35169	0.05
26056	0.946131	0.05
26057	0.946131	0.05
26058	1.35169	0.05
26059	0.676844	0.05
26060	0.946131	0.05
26061	0.94613	0.05
26062	1.13242	0.05
26063	1.13242	0.05
26064	0.946131	0.05
26065	0.946128	1.28212
26066	1.28828	2.1
26067	0.946131	1.2799
26068	0.946131	1.33652
26069	1.28828	1.56842
26070	0.946131	0.17749
26071	1.2996	0.101825
26072	0.946133	0.188206
26073	0.946131	0.145915
26074	0.946131	0.14744
26075	0.946132	2.1
26076	1.40638	2.1
26077	0.946131	2.1
26078	0.946132	2.1
26079	1.40638	2.1
26080	2.84909	0.05
26081	1.35726	0.05
26082	0.946131	0.05
26083	0.946131	0.05
26084	0.946131	0.05
26085	0.946131	0.05
26086	1.39852	2.1
26087	0.946132	2.1
26088	0.94613	2.1
26089	0.94613	2.1
26090	0.94613	2.1
26091	0.946126	0.05
26092	0.946131	0.05
26093	0.946128	0.05
26094	1.31666	0.05
26095	0.946128	0.05
26096	0.946126	0.05
26097	0.946131	0.05

26098	0.946129	0.05
26099	1.32275	0.05
26100	0.946129	0.05
26101	0.946131	2.1
26102	0.946132	2.1
26103	1.41125	2.1
26104	0.946131	2.1
26105	0.946131	2.1
26106	0.946131	2.1
26107	0.946132	2.1
26108	1.31904	2.1
26109	0.946132	2.1
26110	0.946132	2.1
26111	0.946129	0.05
26112	0.946132	0.05
26113	0.94613	0.05
26114	1.02426	0.05
26115	0.9311	0.05
26116	0.946128	1.96483
26117	0.946133	1.31933
26118	0.946131	1.65164
26119	1.40092	1.82928
26120	0.946131	1.40305
26121	0.946117	0.362821
26122	0.946118	2.1
26123	0.946118	0.076344
26124	1.40017	2.1
26125	0.946105	1.87491
26126	0.946132	0.589583
26127	1.03547	0.196482
26128	0.946132	0.521849
26129	0.946133	0.661591
26130	0.946132	0.634027
26131	0.946133	2.1
26132	0.946132	2.1
26133	0.946133	2.1
26134	0.946133	2.1
26135	1.36694	2.1
26136	0.946132	0.05
26137	1.22878	0.05
26138	0.946132	0.05
26139	0.946132	0.05
26140	1.22878	0.05
26141	1.11014	0.05
26142	0.913078	0.05
26143	1.11015	0.05
26144	0.49154	0.05
26145	0.946127	2.1
26146	0.946131	2.1
26147	0.946128	2.1
26148	0.946129	2.1
26149	1.38752	2.1
26150	0.946132	2.1
26151	1.37277	2.1
26152	0.946132	2.1
26153	0.946131	2.1
26154	1.37277	2.1
26155	0.94613	0.166068

26156	1.16834	0.0540999
26157	0.946129	0.173998
26158	0.94613	0.0855332
26159	0.94613	0.111397
26160	0.946127	0.1546
26161	0.946131	0.0786033
26162	1.35594	0.05
26163	0.946128	0.186324
26164	0.94613	0.104256
26165	0.946128	1.63332
26166	0.946128	0.556583
26167	1.38464	0.241891
26168	1.38463	0.0615115
26169	0.94613	0.727594
26170	0.946129	0.802566
26171	0.946131	0.710013
26172	0.94613	0.737783
26173	1.39751	0.0594431
26174	0.946131	0.72578
26175	1.25837	0.05
26176	1.21134	0.05
26177	0.700013	0.05
26178	0.700041	0.05
26179	1.40583	2.1
26180	0.946132	2.1
26181	0.946132	2.1
26182	0.946133	2.1
26183	0.946132	2.1
26184	0.827865	2.1
26185	0.946131	2.1
26186	0.946131	2.06167
26187	0.946131	2.1
26188	0.946131	2.00249
26189	0.946132	0.05
26190	1.21087	0.05
26191	0.94613	0.05
26192	0.94613	0.05
26193	0.946131	0.05
26194	0.94613	0.834244
26195	1.17452	0.872646
26196	0.946132	0.834839
26197	0.94613	0.852261
26198	0.94613	0.831185
26199	0.946119	0.739851
26200	1.27374	0.752936
26201	0.946119	0.773506
26202	0.946131	0.796582
26203	0.946106	0.680792
26204	0.946133	2.1
26205	0.946132	2.1
26206	0.946133	2.1
26207	0.946132	2.1
26208	1.39621	2.1
26209	1.34352	2.1
26210	0.946132	2.1
26211	0.946132	2.1
26212	0.946132	2.1
26213	0.946132	2.1

26214	0.946132	0.996764
26215	0.946128	2.1
26216	0.94613	1.27055
26217	0.94613	1.98706
26218	1.41273	2.1
26219	1.23536	2.1
26220	0.946131	1.69978
26221	0.946132	2.1
26222	0.946132	2.1
26223	0.946132	2.1
26224	0.946132	2.1
26225	1.41131	2.1
26226	0.946131	2.1
26227	0.946131	2.1
26228	0.946131	2.1
26229	0.946133	0.786511
26230	0.946132	1.84148
26231	0.946133	0.803348
26232	1.41894	0.723024
26233	0.946132	0.323772
26234	0.946128	0.599765
26235	1.4099	1.60096
26236	0.946131	0.997961
26237	0.946131	2.09453
26238	0.946131	0.104271
26239	0.810947	2.1
26240	0.810945	2.1
26241	0.810933	2.1
26242	0.946131	0.900401
26243	1.25765	2.1
26244	0.946128	1.10832
26245	0.946129	0.884976
26246	1.25765	1.05091
26247	1.21347	2.1
26248	0.946129	2.1
26249	0.94613	2.1
26250	1.21347	2.1
26251	0.94613	2.1
26252	0.94613	0.05
26253	0.946132	0.05
26254	1.2493	0.05
26255	1.2493	0.05
26256	0.94613	0.05
26257	0.946132	0.965819
26258	0.946128	1.28848
26259	0.946131	0.994578
26260	0.94613	1.08779
26261	1.37938	1.0583
26262	0.946133	0.05
26263	0.946132	0.05
26264	0.946133	0.05
26265	0.946132	0.05
26266	0.946133	0.05
26267	0.946129	0.128722
26268	1.26426	0.0780355
26269	0.946131	0.176598
26270	0.946131	0.175877
26271	1.26426	0.169789

26272	0.872447	2.04799
26273	0.946132	1.48342
26274	0.946132	1.92932
26275	0.946132	1.98506
26276	0.946133	1.62072
26277	0.946117	1.30995
26278	0.946116	1.23603
26279	0.946113	1.20696
26280	0.946129	1.32081
26281	1.27211	1.07356
26282	0.94613	0.464395
26283	0.946129	0.05
26284	1.40029	0.0614029
26285	1.40028	0.05
26286	0.94613	0.0525347
26287	0.810942	2.1
26288	0.810943	2.1
26289	0.810932	2.1
26290	1.29521	2.1
26291	0.946129	2.1
26292	0.946131	2.1
26293	1.29521	2.1
26294	1.29521	2.1
26295	0.946132	0.236421
26296	0.946129	0.114333
26297	1.24596	0.085671
26298	0.946131	0.244493
26299	0.946131	0.161667
26300	0.946131	2.1
26301	1.35616	2.1
26302	0.946133	2.1
26303	0.94613	2.1
26304	1.35616	2.1
26305	0.879548	0.825
26306	1.01272	0.776772
26307	0.946133	0.861526
26308	0.946133	0.705949
26309	0.946132	0.804682
26310	0.946132	0.960267
26311	0.918009	0.81318
26312	0.91801	1.00383
26313	0.946132	0.862323
26314	0.946132	0.916473
26315	0.946132	0.05
26316	1.28644	0.05
26317	0.946131	0.05
26318	0.946131	0.05
26319	0.946132	0.05
26320	0.946131	0.05
26321	1.40103	0.05
26322	0.946131	0.05
26323	0.946131	0.05
26324	0.946131	0.05
26325	0.946128	0.05
26326	1.03327	0.05
26327	0.946129	0.05
26328	0.946131	0.05
26329	0.94613	0.05

26330	0.946132	0.619386
26331	0.946133	0.05
26332	1.40265	0.051165
26333	1.40265	0.053082
26334	0.946133	0.05
26335	0.946131	0.05
26336	0.946131	0.05
26337	0.946131	0.05
26338	0.856655	0.05
26339	0.856616	0.05
26340	0.946132	0.485965
26341	0.946132	0.562439
26342	0.946132	0.797706
26343	1.15844	0.221039
26344	0.946131	0.779945
26345	0.946131	2.1
26346	1.4031	2.1
26347	1.40304	2.1
26348	0.851554	2.1
26349	0.946131	0.05
26350	0.94613	0.05
26351	1.24417	0.05
26352	1.24416	0.05
26353	0.946131	0.05
26354	0.946132	0.257988
26355	1.20992	0.250524
26356	0.946132	0.0999181
26357	0.946132	0.672472
26358	1.20992	0.434809
26359	0.946128	1.24981
26360	0.94613	1.37797
26361	0.94613	1.27589
26362	0.94613	1.31562
26363	1.23426	1.4022
26364	0.810943	0.770294
26365	0.810946	0.108616
26366	0.810933	0.655137
26367	0.810945	2.1
26368	0.810945	0.2697
26369	0.810932	1.30902
26370	1.25104	1.77573
26371	0.946131	2.1
26372	0.946131	0.95683
26373	1.25104	1.9716
26374	1.25104	0.594526
26375	1.25488	2.1
26376	0.946132	2.1
26377	0.946132	2.1
26378	0.946132	2.1
26379	0.946131	2.1
26380	0.946131	0.05
26381	0.946126	0.05
26382	0.946128	0.05
26383	1.32131	0.05
26384	0.946128	0.05
26385	0.946133	2.1
26386	0.946132	0.162719
26387	0.946132	2.1

26388	0.946133	2.1
26389	1.41123	2.1
26390	0.946132	0.05
26391	0.946133	0.05
26392	0.946132	0.05
26393	1.32163	0.05
26394	0.946133	0.05
26395	0.946132	2.04567
26396	0.946132	2.1
26397	1.38651	2.1
26398	1.38651	2.1
26399	0.946132	2.1
26400	0.94613	0.0705452
26401	1.3639	0.05
26402	0.946128	0.0779082
26403	0.946131	0.05
26404	1.3639	0.05
26405	0.946133	1.00293
26406	1.40724	0.228751
26407	0.946132	0.1422
26408	0.946133	0.241894
26409	1.40724	0.299017
26410	1.21099	1.45101
26411	0.946128	1.70777
26412	0.946127	2.1
26413	0.946128	2.08555
26414	0.946129	1.61794
26415	0.946132	0.117039
26416	1.35946	0.136254
26417	0.946131	0.532267
26418	0.946131	0.298291
26419	1.35946	0.279861
26420	1.34794	0.17578
26421	0.517994	1.46861
26422	0.946132	0.153311
26423	0.946132	0.880558
26424	0.517965	0.946674
26425	1.23224	0.745586
26426	0.94613	2.00209
26427	0.946131	1.23272
26428	0.946131	1.1324
26429	0.946131	1.03225
26430	1.37511	0.911615
26431	0.946132	1.30358
26432	0.946133	2.07475
26433	0.946133	1.84723
26434	0.946132	1.31863
26435	0.851106	0.195446
26436	0.946129	0.258008
26437	0.946131	0.610539
26438	0.946131	0.945832
26439	0.851066	0.832369
26440	0.946131	0.05
26441	0.946131	0.05
26442	0.94613	0.05
26443	1.31357	0.05
26444	0.946131	0.05
26445	0.946133	1.00936

26446	0.946132	1.23577
26447	0.946133	1.00315
26448	0.946132	1.09401
26449	1.38149	1.05308
26450	0.810944	0.973769
26451	0.810945	1.22588
26452	0.810958	2.1
26453	0.946118	0.05
26454	0.946118	0.05
26455	1.37835	0.05
26456	1.37837	0.05
26457	0.946106	0.05
26458	0.946128	0.05
26459	0.946131	0.05
26460	1.34831	0.05
26461	1.34831	0.05
26462	0.94613	0.05
26463	0.946132	0.05
26464	0.946132	0.05
26465	0.909981	0.05
26466	1.00099	0.05
26467	0.946131	0.05
26468	0.946132	0.898886
26469	0.946133	1.4019
26470	0.946133	0.986413
26471	0.946132	1.05402
26472	1.35328	1.05171
26473	0.946132	0.05
26474	0.946132	0.05
26475	1.35386	0.05
26476	1.35385	0.05
26477	0.946133	0.05
26478	0.946121	0.05
26479	0.946132	0.05
26480	0.900167	0.05
26481	1.01189	0.05
26482	0.946132	0.05
26483	0.946132	2.1
26484	1.41585	1.55848
26485	0.946131	1.69727
26486	0.946131	1.52015
26487	0.94613	1.52128
26488	0.810946	2.1
26489	0.810946	2.1
26490	0.810933	2.1
26491	1.34447	2.1
26492	0.946131	2.1
26493	0.946131	2.1
26494	1.34447	2.1
26495	1.19886	2.1
26496	0.946132	0.05
26497	0.946132	0.05
26498	0.946132	0.05
26499	1.35065	0.05
26500	0.946132	0.05
26501	0.946131	0.101386
26502	1.3022	0.149871
26503	0.94613	0.621113

26504	0.946131	0.258655
26505	1.3022	0.219158
26506	0.946127	0.05
26507	1.38683	0.05
26508	0.946127	0.05
26509	0.946128	0.05
26510	1.38683	0.05
26511	0.810946	0.05
26512	0.810947	0.05
26513	0.810959	0.05
26514	0.946132	0.05
26515	0.946131	0.05
26516	0.946132	0.05
26517	0.946132	0.05
26518	0.946132	0.05
26519	0.946127	1.31433
26520	1.35968	2.08149
26521	0.946131	1.19756
26522	0.946129	1.12649
26523	1.35969	1.61629
26524	0.946133	2.1
26525	1.35862	1.88478
26526	0.946132	2.1
26527	0.946132	1.31476
26528	1.35862	1.76656
26529	0.946132	0.905444
26530	0.946132	0.79182
26531	0.946132	0.951217
26532	0.946131	0.773758
26533	0.946132	0.835258
26534	0.946131	0.654453
26535	0.946131	0.251448
26536	0.946128	0.683017
26537	0.946129	0.0827148
26538	1.41232	0.382324
26539	0.946128	1.26823
26540	1.29331	2.1
26541	0.946131	1.32759
26542	0.946131	2.1
26543	1.29331	2.1
26544	0.946133	0.346521
26545	0.946132	0.433441
26546	0.946132	0.324855
26547	0.946132	0.205192
26548	1.41556	0.133773
26549	0.94612	0.242206
26550	0.946119	0.120592
26551	0.946119	0.240985
26552	0.946132	0.125429
26553	0.946107	0.102966
26554	0.946129	0.05
26555	1.1509	0.05
26556	0.946126	0.05
26557	0.946129	0.05
26558	0.94613	0.05
26559	0.946131	0.05
26560	1.19263	0.05
26561	0.946132	0.05

26562	0.946132	0.05
26563	0.946132	0.05
26564	0.94613	0.555599
26565	1.36939	0.778964
26566	0.946128	0.534751
26567	0.946131	0.980319
26568	1.36939	0.566634
26569	0.946133	1.13519
26570	0.827865	1.77146
26571	0.946132	1.18315
26572	0.946132	1.36342
26573	1.40398	1.4026
26574	0.946127	0.74064
26575	0.946127	0.807495
26576	0.94613	0.624193
26577	1.41498	0.429108
26578	0.946128	0.651761
26579	0.94613	0.05
26580	0.946131	0.05
26581	0.946129	0.05
26582	0.94613	0.05
26583	0.94613	0.05
26584	0.946118	0.05
26585	0.946119	0.05
26586	0.946116	0.05
26587	1.36569	0.05
26588	0.946105	0.05
26589	0.946132	0.05
26590	1.23175	0.05
26591	0.94613	0.05
26592	0.946133	0.05
26593	0.946131	0.05
26594	1.25698	0.05
26595	0.946126	0.05
26596	0.946129	0.05
26597	0.946128	0.05
26598	0.946129	0.05
26599	0.810943	2.1
26600	0.810944	2.1
26601	0.810932	2.1
26602	0.946132	0.05
26603	1.09841	0.05
26604	0.946131	0.0748446
26605	0.946132	0.05
26606	1.09841	0.055209
26607	0.810936	0.320302
26608	0.810935	0.163627
26609	0.810934	1.53111
26610	1.23851	2.1
26611	0.946132	0.187855
26612	0.946131	1.207
26613	1.23851	2.1
26614	1.23851	2.1
26615	0.946132	0.05
26616	0.946131	0.05
26617	0.946132	0.05
26618	0.946132	0.05
26619	0.946132	0.05

26620	0.939141	1.22507
26621	0.953118	1.63367
26622	0.946126	1.20748
26623	0.946129	1.26633
26624	1.03306	1.34634
26625	0.946132	0.0578935
26626	1.30927	0.0596078
26627	0.946132	0.101248
26628	0.946132	0.0612537
26629	1.30928	0.0691519
26630	0.94613	2.1
26631	0.946131	2.1
26632	1.24511	2.1
26633	0.94613	2.1
26634	0.94613	2.1
26635	0.946133	0.05
26636	1.34152	0.05
26637	0.946131	0.05
26638	0.946131	0.05
26639	1.34152	0.05
26640	0.946132	0.05
26641	1.41383	0.05
26642	0.946132	0.05
26643	0.946132	0.05
26644	1.41383	0.05
26645	0.946132	0.743728
26646	0.946131	0.43782
26647	1.41392	0.192083
26648	0.946132	0.831075
26649	0.946132	0.583677
26650	0.946132	1.0681
26651	0.946132	0.984435
26652	1.24333	0.654322
26653	0.946133	1.13603
26654	0.946133	1.0106
26655	0.946124	0.05
26656	0.94613	0.05
26657	0.946126	0.05
26658	1.3065	0.05
26659	0.946127	0.05
26660	0.810946	0.05
26661	0.810943	0.05
26662	0.81096	0.05
26663	0.946131	0.05
26664	1.40271	0.05
26665	0.946131	0.05
26666	0.946131	0.05
26667	1.40271	0.05
26668	0.946132	0.05
26669	0.94613	0.0553159
26670	0.946131	0.0506614
26671	0.94613	0.0539373
26672	0.946131	0.05
26673	0.946133	0.771221
26674	1.30107	0.759041
26675	0.946132	0.768969
26676	0.946132	0.771646
26677	0.946133	0.784538

26678	0.810946	2.1
26679	0.810944	2.1
26680	0.810958	2.1
26681	0.946133	1.50636
26682	0.946131	1.34501
26683	0.946131	2.1
26684	1.30812	2.1
26685	0.946131	1.39781
26686	0.94613	0.850519
26687	0.975002	1.18459
26688	0.917259	2.08187
26689	1.00902	1.6518
26690	0.94613	0.60062
26691	0.946131	2.1
26692	0.946127	2.1
26693	1.398	2.1
26694	0.946129	2.1
26695	0.946129	2.1
26696	0.946133	2.1
26697	0.946132	2.1
26698	0.946132	2.1
26699	0.946132	2.1
26700	1.3033	2.1
26701	1.09897	0.05
26702	0.946131	0.227803
26703	0.946132	0.109536
26704	0.946129	0.133359
26705	0.946131	0.1785
26706	0.946131	2.1
26707	0.946132	2.1
26708	1.39858	2.1
26709	0.946131	2.1
26710	0.946131	2.1
26711	0.946119	0.859002
26712	1.08878	0.891992
26713	0.946119	1.28362
26714	1.0888	0.891908
26715	0.946107	1.21549
26716	0.946132	0.05
26717	1.10733	0.05
26718	0.946132	0.05
26719	0.946131	0.05
26720	0.946132	0.05
26721	0.946128	0.05
26722	1.33671	0.05
26723	0.94613	0.05
26724	0.94613	0.05
26725	0.94613	0.05
26726	0.810943	2.1
26727	0.810944	2.1
26728	0.810945	2.1
26729	1.35117	2.1
26730	0.946129	2.1
26731	0.94613	2.1
26732	1.35117	2.1
26733	1.27174	2.1
26734	0.946132	2.1
26735	0.946133	2.1

26736	0.997296	1.3908
26737	0.946133	2.01814
26738	0.946132	1.63323
26739	0.810943	2.1
26740	0.810944	2.1
26741	0.810945	2.1
26742	0.810946	1.13293
26743	0.810943	2.0949
26744	0.810933	1.39547
26745	0.946127	1.3215
26746	0.946131	0.895438
26747	0.946128	1.30277
26748	0.94613	1.38057
26749	0.946129	1.46935
26750	0.946131	0.05
26751	0.946131	0.05
26752	0.946129	0.05
26753	0.946131	0.05
26754	0.94613	0.05
26755	0.946133	0.363149
26756	0.946132	0.672666
26757	0.946132	0.546909
26758	0.946132	0.389229
26759	0.946133	0.572285
26760	0.946131	0.907634
26761	0.94613	0.424302
26762	0.946129	1.08964
26763	0.946131	0.353899
26764	0.946131	0.888763
26765	0.946132	1.21364
26766	0.946129	2.1
26767	0.94613	2.1
26768	0.946131	1.75983
26769	1.39356	2.1
26770	0.810946	2.1
26771	0.810947	2.1
26772	0.810934	2.1
26773	0.946125	0.05
26774	0.946127	0.05
26775	1.33798	0.05
26776	1.33797	0.05
26777	0.946128	0.05
26778	0.946131	0.05
26779	0.946131	0.05
26780	1.23146	0.05
26781	1.23146	0.05
26782	0.946131	0.05
26783	0.946129	2.1
26784	0.94613	2.1
26785	0.946131	2.1
26786	0.94613	2.1
26787	1.30082	2.1
26788	0.946131	0.05
26789	0.946132	0.05
26790	0.946131	0.05
26791	1.20416	0.05
26792	0.946132	0.05
26793	0.946132	0.05

26794	0.946131	0.05
26795	0.946131	0.05
26796	1.18711	0.05
26797	0.946132	0.05
26798	0.946132	0.05
26799	0.946132	0.05
26800	0.946133	0.05
26801	0.946132	0.05
26802	0.946133	0.05
26803	0.946131	0.05
26804	0.946132	0.05
26805	0.94613	0.05
26806	0.946131	0.05
26807	0.946131	0.05
26808	0.946133	0.05
26809	0.946132	0.05
26810	0.946133	0.05
26811	0.946132	0.05
26812	0.946132	0.05
26813	0.946132	2.1
26814	0.946132	2.1
26815	0.946132	2.1
26816	1.36422	2.1
26817	0.946132	2.1
26818	0.946131	0.950048
26819	0.946132	1.73033
26820	0.946132	1.08514
26821	0.946132	1.12549
26822	1.4185	1.46554
26823	0.946133	0.05
26824	1.37794	0.05
26825	0.946132	0.05
26826	0.946133	0.05
26827	0.946132	0.05
26828	1.4045	2.1
26829	0.946132	2.1
26830	0.946132	2.1
26831	0.946132	2.1
26832	0.946132	2.1
26833	0.946131	2.1
26834	0.946132	2.1
26835	1.34285	2.1
26836	1.34285	2.1
26837	0.946132	2.1
26838	0.946131	2.1
26839	0.946132	2.1
26840	1.40739	2.1
26841	1.40739	2.1
26842	0.946131	2.1
26843	0.946128	2.1
26844	0.946132	2.1
26845	0.94613	2.1
26846	0.94613	2.1
26847	1.3164	2.1
26848	0.946132	2.1
26849	1.3484	2.1
26850	0.946132	2.07147
26851	0.946132	2.1

26852	1.3484	2.1
26853	0.946132	2.1
26854	0.946132	2.1
26855	0.946133	2.1
26856	0.946133	2.1
26857	1.41507	2.1
26858	0.946132	2.1
26859	1.32608	2.1
26860	0.946131	2.1
26861	0.946131	2.1
26862	0.946132	2.1
26863	0.94613	1.10081
26864	1.34646	2.1
26865	0.946129	1.11551
26866	0.94613	1.04179
26867	1.34646	1.34457
26868	0.946131	0.232198
26869	0.946131	2.1
26870	0.94613	0.966573
26871	1.33728	0.05
26872	0.946131	0.611326
26873	0.946127	2.1
26874	0.946131	1.22023
26875	0.946128	2.1
26876	0.946129	2.1
26877	1.4064	2.08472
26878	0.946133	2.1
26879	0.946131	2.1
26880	0.946131	2.1
26881	0.946131	2.1
26882	0.94613	2.1
26883	1.34912	2.1
26884	0.946131	2.1
26885	0.946129	2.1
26886	0.946131	2.1
26887	0.94613	2.1
26888	0.946132	2.1
26889	1.40447	2.1
26890	0.946132	2.1
26891	0.946133	2.1
26892	1.40447	2.1
26893	1.33607	2.1
26894	0.946131	2.1
26895	0.946127	2.1
26896	0.94613	2.1
26897	0.946129	2.1
26898	0.946117	0.621483
26899	1.32664	1.01713
26900	0.946115	0.896217
26901	0.946105	1.16919
26902	0.94613	0.197693
26903	0.946118	2.1
26904	0.946114	2.1
26905	1.35886	2.1
26906	1.35884	2.1
26907	0.946129	2.1
26908	0.946132	2.1
26909	1.2571	2.1

26910	0.946132	2.1
26911	0.946132	2.1
26912	1.2571	2.1
26913	0.946133	2.1
26914	1.35663	2.1
26915	0.946132	2.1
26916	0.946132	2.1
26917	1.35663	2.1
26918	0.946133	1.01078
26919	0.946129	1.88493
26920	0.946131	1.3028
26921	0.946131	1.19369
26922	1.30881	1.96822
26923	0.946132	2.1
26924	0.94613	2.1
26925	1.34652	2.1
26926	0.94613	2.1
26927	0.94613	2.1
26928	0.946132	2.1
26929	0.946133	1.83487
26930	1.36183	0.988046
26931	1.36183	1.32743
26932	0.946132	1.66612
26933	0.946132	0.05
26934	1.38353	0.05
26935	0.94613	0.0847717
26936	0.94613	0.0538246
26937	1.38354	0.05
26938	0.946128	0.0606943
26939	1.24887	0.05
26940	0.94613	0.05
26941	0.94613	0.0722465
26942	1.24887	0.0527531
26943	0.946129	1.00295
26944	0.946126	1.52457
26945	0.946124	1.05664
26946	0.946128	1.1002
26947	1.32056	1.21078
26948	0.946129	1.09602
26949	0.946128	1.63479
26950	0.946126	1.12196
26951	0.946129	1.2005
26952	1.20351	1.27311
26953	0.946131	1.38477
26954	0.946131	1.43603
26955	0.946127	2.06735
26956	1.25591	1.96112
26957	0.94613	0.958876
26958	0.946132	1.38214
26959	0.946133	1.33301
26960	0.946132	1.73478
26961	1.15436	1.24524
26962	0.930306	1.16937
26963	0.946132	2.1
26964	0.946132	2.1
26965	0.946132	2.1
26966	0.946133	2.1
26967	0.946132	2.1

26968	0.946129	1.17834
26969	0.946132	1.07192
26970	0.946126	1.39279
26971	1.13245	0.985569
26972	0.946129	1.0827
26973	0.94613	2.1
26974	0.946132	2.1
26975	0.946129	1.93261
26976	0.946131	2.1
26977	0.946131	2.1
26978	1.41279	2.1
26979	1.76254	2.1
26980	0.600454	2.09466
26981	0.858991	2.1
26982	0.940722	2.1
26983	1.28439	1.4307
26984	0.946131	1.76089
26985	0.94613	1.73623
26986	0.946131	2.1
26987	0.946131	2.1
26988	0.946132	1.16662
26989	1.39335	0.488342
26990	0.946132	1.0875
26991	0.946132	0.777904
26992	0.946132	1.05004
26993	1.33879	0.05
26994	0.946131	0.0577995
26995	0.94613	0.237
26996	1.33879	0.05
26997	0.946131	0.0873822
26998	0.946126	0.17858
26999	0.946131	0.238813
27000	1.37342	0.125226
27001	1.37343	0.128145
27002	0.946128	0.773637
27003	1.39168	0.05
27004	0.946131	0.05
27005	0.946131	0.05
27006	0.946131	0.05
27007	0.946131	0.05
27008	0.946131	0.096329
27009	1.412	0.05
27010	0.946127	0.05599
27011	0.946128	0.066603
27012	0.946128	0.0928069
27013	1.1387	0.05
27014	0.943364	0.0969148
27015	0.946132	0.05
27016	0.946132	0.05
27017	0.946133	0.05
27018	1.21085	0.05
27019	0.88324	0.597828
27020	0.946129	0.31554
27021	0.946131	0.399791
27022	0.946131	0.217322
27023	1.33587	0.05
27024	0.946125	0.05
27025	0.946129	0.05

27026	0.946126	0.05
27027	0.946127	0.05
27028	1.27512	0.05
27029	0.946132	0.05
27030	0.946132	0.05
27031	0.946133	0.05
27032	0.946132	0.05
27033	0.946128	0.05
27034	1.30498	0.05
27035	0.946131	0.05
27036	0.94613	0.05
27037	1.30497	0.05
27038	0.946131	0.05
27039	1.25626	0.05
27040	0.946131	0.05
27041	0.946131	0.05
27042	1.25626	0.05
27043	0.946131	0.05
27044	0.94613	0.05
27045	1.35316	0.05
27046	0.946131	0.05
27047	0.946131	0.05
27048	0.94613	0.241033
27049	1.28727	0.0997064
27050	0.946128	0.0673648
27051	0.946131	0.105755
27052	0.94613	0.249966
27053	0.946131	0.05
27054	1.39502	0.05
27055	0.946132	0.05
27056	0.946132	0.05
27057	0.946132	0.05
27058	1.36173	0.05
27059	0.946131	0.215652
27060	0.946126	0.118904
27061	0.946127	0.135188
27062	0.946128	0.0817598
27063	0.946129	0.996217
27064	0.946133	0.924044
27065	0.946131	0.999573
27066	0.946131	1.01558
27067	0.946131	1.04005
27068	0.946128	0.05
27069	0.946132	0.05
27070	1.24678	0.05
27071	0.94613	0.05
27072	0.94613	0.05
27073	0.946129	0.05
27074	0.94613	0.05
27075	1.25215	0.05
27076	0.946126	0.05
27077	0.946129	0.05
27078	0.946129	0.873916
27079	1.29689	0.789859
27080	0.946131	0.876658
27081	0.946131	0.850881
27082	0.946131	0.736709
27083	0.946128	0.05

27084	0.94613	0.05
27085	0.946131	0.05
27086	0.94613	0.05
27087	0.946131	0.05
27088	0.946127	0.05
27089	0.946128	0.05
27090	0.94613	0.05
27091	0.946129	0.05
27092	0.946129	0.05
27093	0.946133	0.05
27094	0.946132	0.05
27095	0.946132	0.05
27096	1.29957	0.05
27097	0.946133	0.05
27098	0.946132	0.05
27099	0.946133	0.05
27100	0.946132	0.05
27101	1.26201	0.05
27102	0.946132	0.05
27103	0.946133	0.05
27104	1.40427	0.05
27105	0.946132	0.05
27106	0.946132	0.05
27107	0.946133	0.05
27108	0.946132	0.05
27109	1.35123	0.05
27110	0.946132	0.05
27111	0.946132	0.05
27112	0.946131	0.05
27113	0.946128	0.05
27114	0.946129	0.05
27115	0.946131	0.05
27116	0.94613	0.05
27117	0.946131	0.05
27118	0.946127	0.278347
27119	1.23351	0.141023
27120	0.946131	0.345529
27121	0.946129	0.207658
27122	0.94613	0.213673
27123	0.946131	0.05
27124	0.946131	0.05
27125	0.94613	0.05
27126	1.18119	0.05
27127	0.946131	0.05
27128	0.946132	0.0634905
27129	1.30563	0.05
27130	0.946133	0.0981572
27131	0.946132	0.05
27132	0.946133	0.05
27133	0.946132	0.0682174
27134	0.946132	0.05
27135	0.946133	1.1693
27136	1.20256	0.0536446
27137	0.946133	0.103557
27138	0.94613	0.572939
27139	1.33007	0.275066
27140	0.946128	1.04178
27141	0.946132	0.308327

27142	0.946131	0.543513
27143	0.946131	2.1
27144	0.946131	2.1
27145	0.946128	2.1
27146	0.946129	2.1
27147	0.94613	2.1
27148	0.810942	2.1
27149	0.810942	2.1
27150	0.81093	2.1
27151	0.946132	2.1
27152	1.39823	2.1
27153	0.946132	2.1
27154	0.946131	2.1
27155	0.946132	2.1
27156	0.946131	2.1
27157	0.946131	2.1
27158	0.946131	2.1
27159	1.37713	2.1
27160	0.946131	2.1
27161	0.946133	2.1
27162	0.946131	2.1
27163	1.40594	2.1
27164	1.40594	2.1
27165	0.94613	2.1
27166	0.946133	2.1
27167	0.946131	2.1
27168	1.38155	2.1
27169	1.38155	2.1
27170	0.946131	2.1
27171	0.946132	1.05054
27172	0.946133	2.1
27173	0.946132	1.17578
27174	0.946132	1.20855
27175	1.33694	1.66674
27176	0.946131	2.1
27177	1.228	2.1
27178	0.946129	2.1
27179	0.946131	2.1
27180	1.228	2.1
27181	0.946132	1.16884
27182	0.946132	1.59305
27183	0.946131	2.07169
27184	0.946132	1.2405
27185	1.35943	1.97175
27186	0.946133	0.05
27187	0.946133	0.05
27188	1.02377	0.05
27189	1.02377	0.05
27190	0.946132	0.05
27191	0.94613	0.15157
27192	0.946131	0.0839916
27193	0.946132	0.280239
27194	1.29322	0.0568713
27195	0.946131	0.200189
27196	0.946131	2.1
27197	0.946131	0.05
27198	0.946132	2.1
27199	1.41492	0.05

27200	0.946131	0.247084
27201	0.946132	2.1
27202	0.946131	2.1
27203	0.946132	2.1
27204	1.35028	2.1
27205	0.946132	2.1
27206	0.946132	2.1
27207	0.946132	2.1
27208	0.946131	2.1
27209	1.32461	2.1
27210	0.946132	2.1
27211	0.946131	2.1
27212	0.946132	2.1
27213	1.40369	2.1
27214	0.94613	2.1
27215	0.946131	2.1
27216	0.946132	0.05
27217	0.979597	0.05
27218	0.946131	0.05
27219	0.946132	0.05
27220	0.946132	0.05
27221	0.946131	2.1
27222	0.946131	2.1
27223	0.94613	2.1
27224	0.946131	2.1
27225	1.41897	2.1
27226	1.37791	2.1
27227	0.94613	2.1
27228	0.946129	2.1
27229	0.946131	2.1
27230	0.946131	2.1
27231	1.39384	2.1
27232	0.946131	2.1
27233	0.946128	2.1
27234	0.946129	2.1
27235	0.946129	2.1
27236	0.810945	1.54383
27237	0.810944	2.1
27238	0.810958	1.65161
27239	0.946128	2.1
27240	0.946132	2.1
27241	0.94613	2.1
27242	1.3335	2.1
27243	0.946131	2.1
27244	0.94613	0.112175
27245	0.946129	2.1
27246	0.946132	0.05
27247	1.41192	1.61831
27248	0.94613	0.05
27249	0.946132	0.776247
27250	0.946133	0.746742
27251	0.946132	0.746678
27252	1.39428	0.710224
27253	0.946133	0.72039
27254	0.94613	1.89281
27255	0.946128	2.1
27256	0.946132	1.21494
27257	0.94613	1.90137

27258	1.40684	2.1
27259	0.946131	2.1
27260	0.946131	2.1
27261	0.946131	2.1
27262	0.946131	2.1
27263	1.39021	2.1
27264	0.946131	1.25088
27265	1.31649	2.1
27266	0.946131	1.09936
27267	0.946131	1.11606
27268	1.31649	1.40561
27269	0.810946	2.1
27270	0.810946	2.1
27271	0.810958	2.1
27272	1.16687	2.1
27273	0.946131	2.1
27274	0.946131	2.1
27275	1.16687	2.1
27276	1.16687	2.1
27277	0.946128	0.964403
27278	1.3564	0.982425
27279	0.946131	0.965097
27280	0.946131	0.96473
27281	0.94613	0.933545
27282	0.946128	2.1
27283	1.35828	2.1
27284	0.94613	2.1
27285	0.946131	2.1
27286	0.94613	2.1
27287	0.946133	0.255246
27288	1.36477	0.347263
27289	0.946132	0.622697
27290	0.946132	0.560865
27291	1.36477	0.571765
27292	0.946132	1.15602
27293	1.31857	2.1
27294	0.946132	1.26735
27295	0.946132	1.45614
27296	1.31857	1.26844
27297	0.94613	0.104831
27298	1.35059	0.05
27299	0.946128	0.05
27300	0.94613	0.057171
27301	1.35059	0.05
27302	0.946126	0.05
27303	1.35559	0.05
27304	0.946127	0.05
27305	0.946128	0.05
27306	1.35559	0.05
27307	0.946131	0.05
27308	1.33035	0.05
27309	0.946127	0.05
27310	0.946128	0.05
27311	1.33035	0.05
27312	0.94613	0.05
27313	1.3665	0.05
27314	0.946129	0.05
27315	0.946131	0.05

27316	1.3665	0.05
27317	0.94613	0.117975
27318	1.32828	0.05
27319	0.946129	0.05
27320	0.94613	0.0816
27321	0.946131	0.099722
27322	0.946131	2.1
27323	0.946131	2.1
27324	1.32949	2.1
27325	1.32949	2.1
27326	0.946131	2.1
27327	0.946131	1.93428
27328	0.94613	0.134468
27329	0.946133	0.947204
27330	1.38357	0.05
27331	0.946131	0.843397
27332	0.946131	2.1
27333	0.946128	2.1
27334	1.36919	2.1
27335	0.946131	2.1
27336	0.94613	2.1
27337	0.946131	1.70989
27338	1.3771	2.1
27339	0.946129	2.1
27340	0.94613	2.1
27341	0.946131	2.1
27342	0.946131	1.23602
27343	0.94613	0.905469
27344	0.946129	0.806977
27345	1.30029	0.990268
27346	0.94613	0.843753
27347	1.21174	0.05
27348	0.946132	0.05
27349	0.946132	0.05
27350	0.946132	0.05
27351	0.946133	0.05
27352	0.810946	2.1
27353	0.810945	2.1
27354	0.810946	2.1
27355	1.1796	2.1
27356	0.946131	2.1
27357	0.946132	2.1
27358	1.1796	2.1
27359	1.1796	2.1
27360	0.946131	2.1
27361	0.946131	2.1
27362	0.946133	2.1
27363	0.946131	2.1
27364	1.40615	2.1
27365	0.810945	2.1
27366	0.810944	2.1
27367	0.810945	2.1
27368	1.40239	2.1
27369	0.94613	2.1
27370	1.35561	2.1
27371	0.946133	2.1
27372	1.35562	2.1
27373	1.41169	0.05

27374	0.946131	0.05
27375	0.946127	0.152441
27376	1.41169	0.05
27377	0.946128	0.0866396
27378	0.946119	2.1
27379	0.946119	2.1
27380	0.946119	2.1
27381	0.946107	2.1
27382	1.41917	2.1
27383	0.810946	1.38637
27384	0.810943	1.6862
27385	0.810934	2.09909
27386	0.946127	2.1
27387	1.1995	2.1
27388	0.946126	2.1
27389	0.946128	2.1
27390	1.1995	2.1
27391	0.810944	2.1
27392	0.810947	2.1
27393	0.81096	2.1
27394	0.946133	0.0580107
27395	1.3495	0.05
27396	0.946132	0.05
27397	0.946132	0.05
27398	0.946133	0.05
27399	0.946132	2.1
27400	0.946132	2.1
27401	0.946133	2.1
27402	1.39529	2.1
27403	0.946133	2.1
27404	0.946133	2.1
27405	1.28525	2.1
27406	0.946132	2.1
27407	0.946133	2.1
27408	1.28525	2.1
27409	0.810946	2.1
27410	0.810945	2.1
27411	0.810946	2.1
27412	1.33931	2.1
27413	0.946132	2.1
27414	0.946132	2.1
27415	1.33931	2.1
27416	1.24616	2.1
27417	0.946133	0.196997
27418	0.946133	0.454727
27419	0.946132	0.10327
27420	0.946132	0.26829
27421	0.946133	0.169621
27422	0.946128	2.1
27423	1.40225	2.1
27424	0.946128	2.1
27425	0.94613	2.1
27426	0.94613	2.1
27427	0.946113	0.05
27428	0.946118	0.05
27429	0.946114	0.05
27430	1.40551	0.05
27431	0.946103	0.05

27432	0.94613	2.1
27433	0.946132	2.1
27434	0.946129	2.1
27435	0.946131	2.1
27436	1.37811	2.1
27437	0.946132	2.1
27438	1.39826	2.1
27439	0.946133	2.1
27440	0.946132	2.1
27441	1.39826	2.1
27442	0.946118	2.1
27443	1.28298	2.1
27444	0.946117	2.1
27445	0.946105	2.1
27446	1.283	2.1
27447	0.946118	0.05
27448	1.41375	0.05
27449	0.946117	0.05
27450	0.946131	0.05
27451	0.946106	0.05
27452	0.946132	1.10253
27453	0.946129	0.835219
27454	1.28436	0.641306
27455	0.946131	1.1082
27456	0.946131	0.994742
27457	0.810947	0.111402
27458	0.810946	0.182829
27459	0.81096	0.153327
27460	0.946132	0.0545361
27461	1.34338	0.05
27462	0.961435	0.05
27463	0.961435	0.05
27464	0.946132	0.0518331
27465	1.3028	2.06235
27466	0.946131	2.09069
27467	0.946127	2.1
27468	0.946128	2.1
27469	0.946129	2.1
27470	0.946132	0.528679
27471	1.37973	0.163961
27472	0.946132	0.721141
27473	0.946132	0.271673
27474	0.946132	0.330419
27475	0.946131	1.07026
27476	0.94613	0.935832
27477	0.946129	1.07207
27478	0.946131	0.856279
27479	1.41798	0.572273
27480	0.946118	0.868964
27481	0.946118	0.398929
27482	0.946117	0.885503
27483	0.946131	0.581474
27484	0.946105	0.442248
27485	0.946131	1.12825
27486	0.946131	0.827924
27487	0.94613	1.12469
27488	0.946131	0.963669
27489	0.946131	0.709163

27490	0.946133	1.27204
27491	1.40274	2.1
27492	0.946133	0.375229
27493	0.946132	2.1
27494	1.40274	2.1
27495	0.946131	1.50245
27496	1.39731	2.1
27497	0.946132	1.17215
27498	0.94613	1.29433
27499	1.39731	1.98424
27500	0.94612	2.1
27501	0.94612	1.6536
27502	0.946119	1.52508
27503	0.946132	1.60026
27504	1.40764	2.1
27505	0.946128	2.1
27506	0.946132	2.1
27507	0.94613	2.1
27508	0.94613	2.1
27509	1.31213	2.1
27510	0.946127	2.1
27511	0.94613	2.1
27512	0.946127	2.1
27513	0.946128	2.1
27514	1.36653	2.1
27515	0.946127	2.1
27516	1.3557	2.1
27517	0.946128	2.1
27518	0.946128	2.1
27519	1.3557	2.1
27520	0.946133	2.1
27521	0.946132	2.1
27522	0.946133	2.1
27523	0.946132	2.1
27524	1.40081	2.1
27525	0.946128	2.1
27526	0.946133	2.1
27527	0.946131	2.1
27528	0.946131	2.1
27529	1.32282	2.1
27530	0.810945	2.1
27531	0.810943	2.1
27532	0.810933	2.1
27533	1.27676	2.1
27534	0.94613	2.1
27535	0.946128	2.1
27536	0.946131	2.1
27537	0.946131	2.1
27538	0.946131	0.780739
27539	0.946127	0.0721114
27540	1.41328	0.856737
27541	1.41327	0.742329
27542	0.946128	0.323806
27543	0.946132	0.05
27544	1.32083	0.05
27545	0.946132	0.05
27546	0.946133	0.05
27547	1.32082	0.05

27548	0.946128	0.05
27549	1.29215	0.05
27550	0.94613	0.05
27551	0.94613	0.05
27552	1.29215	0.05
27553	0.946132	0.05
27554	1.27696	0.05
27555	0.94613	0.05
27556	0.94613	0.05
27557	1.27696	0.05
27558	0.94612	0.05
27559	1.29289	0.05
27560	0.946119	0.05
27561	0.946132	0.05
27562	1.29287	0.05
27563	0.946128	2.1
27564	1.37869	2.1
27565	0.946131	2.1
27566	0.94613	2.1
27567	0.946131	2.1
27568	0.946133	2.1
27569	0.946132	2.1
27570	0.946132	2.1
27571	1.35703	2.1
27572	0.946133	2.1
27573	0.946132	2.1
27574	0.946132	2.1
27575	0.946133	2.1
27576	1.37017	2.1
27577	0.946133	2.1
27578	0.946119	0.05
27579	0.94612	0.05
27580	1.38583	0.05
27581	1.38585	0.05
27582	0.946107	0.05
27583	0.946126	0.05
27584	0.946127	0.05
27585	1.22904	0.05
27586	1.22904	0.05
27587	0.946128	0.05
27588	1.3279	0.182003
27589	0.946131	1.52825
27590	0.94613	1.33205
27591	0.946131	1.51364
27592	0.946131	1.3299
27593	0.946132	2.1
27594	0.946132	2.1
27595	0.946132	2.1
27596	0.946131	2.1
27597	1.40932	2.1
27598	1.26868	0.05
27599	0.946131	0.05
27600	0.94613	0.05
27601	0.946131	0.05
27602	0.946131	0.05
27603	0.810943	2.1
27604	0.810945	2.1
27605	0.810932	2.1

27606	0.810943	1.06044
27607	0.810944	0.05
27608	0.810957	0.0558557
27609	1.31539	0.43179
27610	0.946132	1.88161
27611	0.946128	0.05
27612	1.3154	0.64163
27613	1.17856	0.0660767
27614	1.21997	0.850137
27615	0.94613	1.90698
27616	0.946129	1.62476
27617	0.94613	2.07347
27618	0.94613	1.87754
27619	0.946131	1.09565
27620	0.946131	0.391491
27621	0.946129	1.27226
27622	0.94613	0.910875
27623	0.946131	1.38059
27624	0.946127	0.684173
27625	0.946131	0.748637
27626	0.946127	1.66914
27627	0.946129	1.00731
27628	1.41342	0.936635
27629	0.946131	0.91747
27630	0.946131	1.31351
27631	0.946128	1.86673
27632	0.946131	0.966416
27633	1.29149	1.24588
27634	1.41022	2.1
27635	0.94613	0.217225
27636	0.946128	2.1
27637	0.94613	2.1
27638	0.94613	2.1
27639	0.946128	0.05
27640	0.94613	0.127208
27641	1.27864	0.0565818
27642	1.27864	0.05
27643	0.946131	0.0807551
27644	0.946129	0.876083
27645	0.946125	1.564
27646	0.946124	1.08536
27647	0.946127	0.981852
27648	1.37759	1.27164
27649	0.946129	0.930904
27650	0.94613	1.59781
27651	0.946126	1.02616
27652	0.946129	1.03672
27653	1.38259	1.20204
27654	0.946131	0.05
27655	1.34118	0.05
27656	0.946132	0.05
27657	0.946132	0.05
27658	1.34118	0.05
27659	0.946131	0.05
27660	1.25017	0.05
27661	0.946128	0.05
27662	0.946131	0.05
27663	0.946131	0.05

27664	0.946131	2.1
27665	0.946128	2.1
27666	0.946133	2.1
27667	1.3937	2.1
27668	0.94613	2.1
27669	0.946131	1.01859
27670	0.94613	0.749374
27671	1.40404	0.05
27672	1.40404	0.05
27673	0.946131	0.598183
27674	0.946132	0.826105
27675	0.946132	0.05
27676	1.38897	0.05
27677	1.38897	0.05
27678	0.946132	0.326098
27679	0.94613	0.745667
27680	0.946128	0.05
27681	1.40332	0.05
27682	1.40331	0.0597498
27683	0.94613	0.808786
27684	0.946132	0.202211
27685	0.946132	2.1
27686	0.946133	0.907295
27687	1.41619	2.1
27688	0.946132	1.02697
27689	0.946132	2.1
27690	0.946132	2.1
27691	0.946132	2.1
27692	1.38244	2.1
27693	0.946132	2.1
27694	0.810946	1.16959
27695	0.810943	0.937047
27696	0.810934	0.379762
27697	0.810947	1.06347
27698	0.810943	0.572595
27699	0.810934	0.555265
27700	1.12786	1.25463
27701	0.946132	2.1
27702	0.946132	1.77868
27703	0.946132	1.45502
27704	0.946133	1.39374
27705	0.946132	1.76081
27706	0.946132	1.88567
27707	0.946132	1.27397
27708	1.27806	1.81789
27709	0.946133	1.26014
27710	0.946132	2.1
27711	0.946132	2.1
27712	0.946133	2.1
27713	0.946132	2.1
27714	0.946133	2.1
27715	1.39514	1.67483
27716	0.946129	1.03235
27717	0.946124	1.3363
27718	0.946127	0.942827
27719	0.946127	1.50566
27720	0.94613	2.1
27721	0.946129	2.1

27722	0.946132	2.1
27723	1.3991	2.1
27724	0.94613	2.1
27725	0.946132	0.533651
27726	0.946133	2.1
27727	0.946132	2.1
27728	1.36835	0.994474
27729	0.946132	0.884972
27730	0.946132	2.1
27731	0.946132	2.1
27732	0.946133	2.1
27733	0.946132	2.1
27734	1.39047	2.1
27735	0.946132	0.05
27736	0.946128	0.05
27737	0.94613	0.05
27738	1.33174	0.05
27739	0.946131	0.05
27740	0.946128	0.05
27741	0.946131	0.05
27742	0.94613	0.05
27743	0.94613	0.05
27744	0.94613	0.05
27745	0.946119	2.1
27746	0.946119	2.1
27747	1.29064	2.1
27748	0.946132	2.1
27749	0.946107	2.1
27750	0.946133	2.1
27751	0.94613	2.1
27752	1.30584	2.1
27753	0.946131	2.1
27754	0.946131	2.1
27755	0.946131	2.1
27756	0.946132	2.1
27757	0.94613	2.1
27758	0.946131	2.1
27759	1.39549	2.1
27760	0.913386	1.28924
27761	0.978873	1.20702
27762	0.946126	1.20762
27763	0.94613	1.37755
27764	1.00473	0.9987
27765	0.946132	0.931307
27766	0.946133	0.952368
27767	0.946132	0.728404
27768	0.946132	0.978804
27769	1.38894	0.746792
27770	0.946128	0.922724
27771	0.946131	0.831534
27772	0.94613	0.443402
27773	0.94613	0.91337
27774	1.23865	0.541081
27775	0.946126	1.27812
27776	0.946129	1.3236
27777	0.946124	1.15163
27778	0.946127	1.27517
27779	1.40248	1.1667

27780	0.946133	0.948813
27781	0.946132	1.02452
27782	0.946132	0.936237
27783	0.946133	1.00078
27784	1.1391	0.941829
27785	0.827862	0.884524
27786	0.946133	0.883658
27787	0.946131	1.00359
27788	0.946131	0.900965
27789	0.94613	0.889727
27790	0.946128	0.801632
27791	0.946132	0.843338
27792	0.94613	0.874651
27793	1.21388	0.762575
27794	0.94613	0.850907
27795	0.810943	0.0811949
27796	0.810945	0.21711
27797	0.810932	0.217609
27798	0.946131	0.801378
27799	0.946131	0.779969
27800	0.946133	0.79452
27801	1.36748	0.740673
27802	0.946132	0.05
27803	0.946128	0.0566925
27804	0.946131	0.05
27805	0.94613	0.0706331
27806	1.35856	0.05
27807	0.94613	0.05
27808	0.946132	2.1
27809	0.946131	2.1
27810	0.946133	2.1
27811	0.946132	2.1
27812	0.946131	2.1
27813	0.946133	2.1
27814	0.946131	2.1
27815	1.39558	2.1
27816	1.39558	2.1
27817	0.94613	2.1
27818	0.946131	0.05
27819	0.94613	0.05
27820	0.946129	0.05
27821	1.41388	0.05
27822	0.946131	0.05
27823	0.946133	0.05
27824	0.946132	0.05
27825	0.946132	0.05
27826	0.946133	0.05
27827	0.93452	0.05
27828	0.946129	2.1
27829	1.38897	2.1
27830	0.946131	2.1
27831	0.946131	2.1
27832	1.38897	2.1
27833	0.946133	2.06704
27834	0.946132	1.54144
27835	0.946133	1.91111
27836	0.946132	1.0052
27837	1.38773	1.18868

27838	1.41512	0.05
27839	0.946131	0.05
27840	0.946129	0.05
27841	0.946133	0.05
27842	0.946131	0.05
27843	0.946132	1.09659
27844	0.946132	2.1
27845	0.946132	1.24573
27846	0.946133	1.29025
27847	0.946133	2.1
27848	0.946126	0.735001
27849	0.946132	1.3433
27850	0.946129	0.981044
27851	0.946129	1.16031
27852	0.946129	1.39765
27853	0.939895	2.1
27854	1.03386	2.1
27855	0.94613	2.1
27856	0.946131	2.1
27857	1.03386	2.1
27858	0.946131	2.1
27859	1.39309	2.1
27860	0.946133	2.1
27861	0.946131	2.1
27862	1.39309	2.1
27863	0.946133	2.1
27864	1.24853	2.1
27865	0.946132	2.1
27866	0.946132	2.1
27867	1.24853	2.1
27868	0.946133	2.1
27869	1.39699	1.19999
27870	0.946132	1.89699
27871	0.946132	2.1
27872	1.39699	1.41316
27873	0.946132	2.1
27874	0.946131	2.1
27875	0.946133	2.1
27876	0.946131	2.1
27877	1.38969	2.1
27878	1.28464	2.1
27879	0.946133	2.1
27880	0.94613	2.1
27881	0.946131	1.96074
27882	0.946131	1.9863
27883	0.946119	0.898421
27884	0.94612	0.976781
27885	0.946119	0.920017
27886	0.946132	0.942854
27887	1.3749	0.893522
27888	0.810942	2.1
27889	0.810943	2.1
27890	0.810957	2.1
27891	1.41134	2.1
27892	0.946132	1.06632
27893	0.946132	2.00588
27894	0.946132	1.15668
27895	0.946132	1.95143

27896	1.31338	2.1
27897	0.946132	1.24159
27898	0.946132	2.1
27899	0.946133	1.48933
27900	0.946132	1.9476
27901	0.946103	0.05
27902	0.752448	0.05
27903	0.946132	0.05
27904	0.946131	0.05
27905	1.20617	0.05
27906	0.946132	1.1224
27907	1.08054	0.650741
27908	1.25736	1.06422
27909	0.946132	1.1231
27910	0.946131	1.12316
27911	0.946128	2.1
27912	1.33275	0.705836
27913	1.1896	2.1
27914	0.946131	2.1
27915	1.18962	2.1
27916	1.21955	0.05
27917	0.946131	0.05
27918	1.33587	0.05
27919	0.741946	0.05
27920	1.07387	0.05
27921	0.945844	0.949578
27922	1.3292	2.1
27923	1.59472	1.9276
27924	0.946131	1.06626
27925	1.04043	1.10464
27926	1.13529	0.05
27927	0.946132	0.0937496
27928	1.24893	0.05
27929	1.12675	0.0719653
27930	0.936652	0.123092
27931	1.36968	2.1
27932	0.946131	2.1
27933	0.946132	2.1
27934	1.36968	2.1
27935	1.258	2.1
27936	3.17458	0.05
27937	0.946132	1.99028
27938	1.35022	2.1
27939	1.35022	2.1
27940	0.94613	2.1
27941	1.12356	2.1
27942	0.946131	2.1
27943	1.22401	2.1
27944	1.22401	2.1
27945	0.94613	2.1
27946	0.952455	2.1
27947	1.2107	2.1
27948	0.946132	2.1
27949	1.33047	2.1
27950	0.946131	2.1
27951	0.946132	2.1
27952	0.61957	2.1
27953	0.8636	2.1

27954	0.946132	2.1
27955	1.19294	2.1
27956	0.946128	2.1
27957	1.36607	2.1
27958	0.94613	2.1
27959	1.39416	2.1
27960	1.36607	2.1
27961	1.21474	2.1
27962	0.946131	2.1
27963	0.946131	2.1
27964	1.16288	2.1
27965	1.08542	2.1
27966	0.851526	1.03454
27967	1.30374	2.1
27968	1.01708	2.1
27969	0.946114	1.11646
27970	0.96976	1.43542
27971	0.946132	0.05
27972	1.49997	0.05
27973	0.771735	0.05
27974	0.771764	0.05
27975	0.946133	0.05
27976	0.700863	1.41879
27977	1.42222	2.1
27978	0.946131	2.1
27979	0.946128	1.36169
27980	1.42509	2.1
27981	1.14298	1.89909
27982	0.837014	2.1
27983	0.946131	2.1
27984	1.29426	2.1
27985	0.836985	2.1
27986	0.946129	2.1
27987	1.22089	0.265099
27988	0.946132	1.07301
27989	0.712486	0.240905
27990	0.6234	0.0947165
27991	0.946131	2.1
27992	1.40271	2.1
27993	1.32241	2.1
27994	0.946133	2.1
27995	1.32241	2.1
27996	0.769373	1.39029
27997	0.946129	2.1
27998	1.41302	2.1
27999	1.28254	2.1
28000	0.946131	2.1
28001	1.28254	2.1
28002	0.851543	2.05868
28003	1.23056	2.1
28004	1.24356	2.1
28005	0.851543	2.1
28006	0.909417	2.1
28007	1.13808	0.717547
28008	0.946133	0.776605
28009	0.946131	0.05
28010	1.13808	0.854244
28011	1.13808	0.73155

28012	0.930941	1.38056
28013	1.37808	1.23333
28014	1.17573	1.05921
28015	0.946124	1.40272
28016	1.02404	1.14828
28017	0.946116	2.1
28018	1.3144	0.381949
28019	1.3144	2.1
28020	0.946118	2.1
28021	1.13089	2.1
28022	0.946128	0.05
28023	0.946101	0.05
28024	1.2606	0.05
28025	1.26055	0.05
28026	0.580823	0.844167
28027	0.946132	1.18945
28028	0.580825	0.826328
28029	1.23224	0.993297
28030	0.946128	2.1
28031	1.3719	2.1
28032	0.94613	2.1
28033	1.3719	2.1
28034	0.94613	2.1
28035	0.946132	1.3571
28036	1.35025	0.975503
28037	1.35025	1.12739
28038	0.946132	1.98116
28039	1.1794	1.20227
28040	0.946133	2.1
28041	1.25598	2.1
28042	1.29249	2.1
28043	1.38154	2.1
28044	0.851541	2.1
28045	1.31508	2.1
28046	1.37558	2.1
28047	0.946129	2.1
28048	0.867663	2.1
28049	0.94613	1.43746
28050	1.35426	1.24923
28051	0.84872	1.23055
28052	0.946129	1.19985
28053	1.17195	1.97272
28054	0.946133	2.1
28055	1.39009	2.1
28056	1.14342	2.1
28057	0.946133	2.1
28058	1.14343	2.1
28059	1.38819	2.1
28060	0.946133	2.1
28061	1.4116	2.1
28062	0.946131	2.1
28063	0.946131	2.1
28064	0.946127	2.1
28065	1.32244	2.1
28066	1.32244	2.1
28067	0.946131	2.1
28068	1.2176	2.1
28069	1.75823	2.1

28070	1.11239	2.1
28071	2.17979	2.1
28072	1.65533	2.1
28073	3.41356	2.1
28074	1.94841	2.1
28075	1.91767	2.1
28076	1.76726	2.1
28077	1.70302	2.1
28078	3.76137	2.1
28079	1.94841	2.1
28080	1.33962	2.1
28081	1.31365	2.1
28082	1.70302	2.1
28083	3.2123	2.1
28084	1.76726	2.1
28085	1.70302	2.1
28086	1.47581	2.1
28087	1.7457	2.1
28088	3.22764	2.1
28089	1.20478	2.1
28090	1.70302	2.1
28091	1.27956	2.1
28092	1.18569	2.1
28093	1.63979	2.1
28094	1.51949	2.1
28095	1.53082	2.1
28096	1.92911	2.1
28097	1.32068	2.1
28098	1.74454	2.1
28099	3.27536	2.1
28100	1.08172	2.1
28101	1.74454	2.1
28102	1.52603	2.1
28103	1.52603	2.1
28104	1.32068	2.1
28105	1.32068	2.1
28106	1.89226	2.1
28107	2.37364	2.1
28108	1.89226	2.1
28109	2.42769	2.1
28110	4.31995	2.1
28111	2.37365	2.1
28112	1.5362	2.1
28113	2.4277	2.1
28114	1.56328	2.1
28115	3.9639	2.1
28116	1.89225	2.1
28117	2.42768	2.1
28118	1.89213	2.1
28119	1.89225	2.1
28120	2.42756	2.1
28121	2.42768	2.1
28122	2.42768	2.1
28123	1.58163	2.1
28124	1.56315	2.1
28125	1.56327	2.1
28126	2.42757	2.1
28127	2.42768	2.1

28128	1.01641	2.1
28129	2.15005	2.1
28130	1.00099	2.1
28131	2.15114	2.1
28132	3.15104	2.1
28133	0.994813	2.1
28134	2.15114	2.1
28135	2.17159	2.1
28136	2.17159	2.1
28137	1.00099	2.1
28138	1.00099	2.1
28139	1.29608	2.1
28140	1.29608	2.1
28141	0.942035	2.1
28142	0.942035	2.08647
28143	1.29198	2.1
28144	0.942033	2.1
28145	0.946132	2.1
28146	0.946132	2.1
28147	0.946132	2.1
28148	0.942033	2.1
28149	0.946133	1.96969
28150	1.29198	2.1
28151	1.99985	2.1
28152	1.14207	2.1
28153	1.83584	2.1
28154	1.00232	2.1
28155	3.00217	2.1
28156	1.99867	0.05
28157	2.0815	0.05
28158	1.56806	0.05
28159	1.28828	0.05
28160	3.58519	0.05
28161	1.89226	0.05
28162	1.89226	0.05
28163	1.89226	0.05
28164	1.89226	0.05
28165	1.89226	0.05
28166	1.89226	0.05
28167	1.89226	0.05
28168	1.89226	0.05
28169	2.23193	0.05
28170	2.48978	0.05
28171	4.38204	0.05
28172	1.85293	0.92603
28173	1.64279	0.630884
28174	1.76268	0.203029
28175	1.54214	0.05
28176	3.39507	0.335016
28177	1.6428	0.716416
28178	1.17302	0.05
28179	1.17302	0.872828
28180	1.28419	0.733702
28181	1.54214	0.05
28182	1.65331	0.0993512
28183	1.76268	1.0001
28184	1.54214	0.746441
28185	1.65324	0.162966

28186	1.42491	0.05
28187	3.19538	0.164727
28188	0.651043	0.442225
28189	0.651043	0.790504
28190	0.647925	0.05
28191	0.647925	0.761425
28192	0.521747	0.0945373
28193	0.58651	0.167218
28194	0.77107	0.723281
28195	0.712457	0.724835
28196	0.712457	0.05
28197	0.521747	0.0657157
28198	0.58651	0.434682
28199	0.77107	0.712182
28200	1.2873	2.1
28201	1.48751	2.1
28202	1.59659	2.1
28203	1.77596	2.05194
28204	3.06326	2.1
28205	1.07976	2.1
28206	1.59659	2.1
28207	1.30237	2.1
28208	1.77723	2.1
28209	2.89897	2.1
28210	1.77723	2.1
28211	1.77596	2.1
28212	1.82614	2.1
28213	1.59659	2.1
28214	2.0055	2.1
28215	1.77596	2.1
28216	2.64523	0.972539
28217	2.73982	0.730191
28218	2.50057	0.948348
28219	2.61606	0.0513898
28220	5.26129	0.187803
28221	2.73982	0.631091
28222	2.68987	0.628694
28223	2.61607	0.42577
28224	2.57611	0.05
28225	5.30593	0.212079
28226	1.89226	0.696522
28227	1.89226	0.739478
28228	1.89226	0.76068
28229	2.02178	0.736427
28230	3.78452	0.70459
28231	1.89226	0.05
28232	1.89226	0.116563
28233	1.89226	1.33305
28234	1.89226	0.05
28235	1.89226	1.41569
28236	1.89226	0.05
28237	2.35204	2.1
28238	1.50108	2.1
28239	1.3715	2.1
28240	2.18469	2.1
28241	3.68577	2.1
28242	2.62514	2.1
28243	2.1847	2.1

28244	2.0487	2.1
28245	2.45213	2.1
28246	4.63682	2.1
28247	1.25075	2.1
28248	2.18469	2.1
28249	1.3715	2.1
28250	1.3715	2.1
28251	2.0487	2.1
28252	2.0487	2.1
28253	1.28667	2.1
28254	1.11285	2.1
28255	1.03529	2.1
28256	1.03529	2.1
28257	1.30711	2.1
28258	1.30711	2.1
28259	2.35053	2.1
28260	1.89225	2.1
28261	1.89225	2.1
28262	2.51216	2.1
28263	4.24278	2.1
28264	1.89225	2.1
28265	1.89225	2.1
28266	1.89225	2.1
28267	1.89225	2.1
28268	3.7845	2.1
28269	1.92025	2.1
28270	1.32119	2.1
28271	1.44777	2.1
28272	1.33834	2.1
28273	1.92025	2.1
28274	1.81082	2.1
28275	1.89226	2.1
28276	2.67272	2.1
28277	2.51218	2.1
28278	2.51218	2.1
28279	1.89226	2.1
28280	1.89226	2.1
28281	1.89226	2.1
28282	1.89226	2.1
28283	1.89226	2.1
28284	1.89226	2.1
28285	3.78452	2.1
28286	1.92027	2.1
28287	1.44778	2.1
28288	1.61121	2.1
28289	2.01555	2.1
28290	3.53147	2.1
28291	1.90727	0.05
28292	2.29851	0.05
28293	2.71643	0.05
28294	2.17695	0.05
28295	4.47547	0.05
28296	1.90728	0.05
28297	1.56339	0.05
28298	1.58143	0.05
28299	2.17696	0.05
28300	3.74035	0.05
28301	1.91368	0.05

28302	2.17696	0.05
28303	2.39986	0.05
28304	1.75904	0.05
28305	3.936	0.05
28306	1.18793	0.05
28307	2.74744	0.05
28308	1.38716	0.05
28309	2.57465	0.05
28310	3.93537	0.05
28311	1.3148	0.05
28312	2.74744	0.05
28313	2.70805	0.05
28314	1.38716	0.05
28315	2.70805	0.05
28316	1.38716	0.05
28317	1.20608	2.1
28318	0.975871	2.1
28319	0.946132	2.1
28320	0.946132	2.1
28321	0.946132	2.1
28322	0.946132	2.1
28323	0.975871	2.1
28324	0.975871	2.1
28325	1.20608	2.1
28326	0.946132	2.1
28327	0.946133	2.1
28328	0.975871	2.1
28329	2.8198	1.7116
28330	2.29967	2.1
28331	2.09542	2.1
28332	2.63784	1.03202
28333	4.93751	2.1
28334	2.09542	2.1
28335	1.39523	2.1
28336	1.21119	2.1
28337	1.39523	2.1
28338	2.11563	2.1
28339	2.29967	2.1
28340	1.88584	2.1
28341	2.63784	0.938914
28342	2.11262	2.0332
28343	1.91132	1.36189
28344	4.02153	2.1
28345	1.88584	2.1
28346	1.21119	2.1
28347	1.12606	2.1
28348	1.91132	2.1
28349	3.1225	2.1
28350	2.18077	2.1
28351	1.78167	2.1
28352	2.2947	1.02891
28353	1.93139	1.37444
28354	4.07637	1.11908
28355	1.5709	1.13956
28356	1.03235	2.1
28357	2.05933	1.88865
28358	1.78168	1.15992
28359	1.31	0.560462

28360	1.03235	0.808803
28361	2.2947	1.24759
28362	2.31785	2.1
28363	2.40627	2.1
28364	2.08288	1.0864
28365	4.37758	2.1
28366	1.95737	1.52898
28367	1.31	2.1
28368	2.08289	0.90939
28369	1.50108	1.02442
28370	3.39289	0.998492
28371	2.49676	2.1
28372	2.24781	2.1
28373	2.5276	1.43225
28374	2.7352	2.1
28375	5.02436	2.1
28376	2.49676	2.1
28377	2.43549	2.1
28378	2.6398	2.1
28379	2.7352	2.1
28380	5.13656	2.1
28381	1.89226	2.1
28382	2.7352	2.1
28383	1.89226	2.1
28384	2.6398	2.1
28385	1.89226	2.1
28386	2.6398	2.1
28387	2.16843	0.05
28388	1.00185	0.05
28389	1.90039	2.1
28390	3.1547	0.05
28391	2.54064	0.05
28392	2.46752	2.1
28393	1.90039	2.1
28394	2.26044	2.1
28395	4.36791	0.246133
28396	1.65815	2.1
28397	2.48652	2.1
28398	1.65815	2.1
28399	1.9004	2.1
28400	2.21711	2.1
28401	2.5034	2.1
28402	2.37167	2.1
28403	2.59965	2.1
28404	2.62812	2.1
28405	2.40308	2.1
28406	4.99979	2.1
28407	2.50137	2.1
28408	2.59965	2.1
28409	2.62812	2.1
28410	2.5214	2.1
28411	5.12948	2.1
28412	2.62814	2.1
28413	2.4031	2.1
28414	2.43509	2.1
28415	2.65066	2.1
28416	5.06323	2.1
28417	2.62814	2.1

28418	2.52142	2.1
28419	2.53008	2.1
28420	2.65066	2.1
28421	5.15822	2.1
28422	2.0815	2.1
28423	1.92067	2.1
28424	1.59611	2.1
28425	1.89226	2.1
28426	1.62453	2.1
28427	1.92067	2.1
28428	1.89226	2.1
28429	1.89226	2.1
28430	1.89226	2.1
28431	1.89226	2.1
28432	3.78453	2.1
28433	0.946131	2.1
28434	0.946131	2.1
28435	0.929501	2.1
28436	0.910761	2.1
28437	0.9461	2.1
28438	0.9461	2.1
28439	0.910792	2.1
28440	0.910792	2.1
28441	0.946131	2.1
28442	0.929501	2.1
28443	0.910761	2.1
28444	0.946131	2.1
28445	2.0815	2.1
28446	1.81661	2.1
28447	2.1341	2.1
28448	2.0815	2.1
28449	1.86921	2.1
28450	1.81661	2.1
28451	1.89226	2.1
28452	1.89226	2.1
28453	1.89226	2.1
28454	1.89226	2.1
28455	1.89226	2.1
28456	1.89226	2.1
28457	0.946131	2.1
28458	0.946131	2.1
28459	0.949104	2.1
28460	0.9295	2.1
28461	0.946132	2.1
28462	0.946132	2.1
28463	0.929499	2.1
28464	0.929499	2.1
28465	0.946131	2.1
28466	0.949105	2.1
28467	0.946131	2.1
28468	0.929501	2.1
28469	1.66104	2.1
28470	2.34044	2.1
28471	2.08889	2.1
28472	1.33264	2.1
28473	3.67308	2.1
28474	2.0889	2.1
28475	1.08011	2.1

28476	1.08011	2.1
28477	1.33264	2.1
28478	1.87598	2.1
28479	2.12851	2.1
28480	2.27472	2.1
28481	1.89226	1.60427
28482	2.27703	2.1
28483	1.89226	1.04214
28484	4.16699	1.14513
28485	1.89226	2.1
28486	1.89226	1.53325
28487	1.89226	1.44122
28488	1.89226	1.22051
28489	3.78452	1.85701
28490	1.00007	2.0836
28491	1.32316	1.16285
28492	1.29417	0.949199
28493	1.29417	1.80435
28494	1.02051	1.04857
28495	1.02051	1.47117
28496	2.2846	2.1
28497	1.89226	1.25789
28498	2.27703	1.26405
28499	2.27703	2.1
28500	1.89226	0.722835
28501	1.89226	2.1
28502	1.89226	2.1
28503	1.89226	2.09189
28504	1.89226	1.13186
28505	1.89226	0.963598
28506	3.78452	1.17972
28507	1.02051	2.1
28508	1.32316	2.01381
28509	1.03373	1.10093
28510	1.34066	1.22559
28511	2.35689	2.1
28512	1.703 2.1	
28513	1.89224	2.1
28514	1.52266	2.1
28515	1.89224	2.1
28516	1.23344	2.1
28517	1.53282	2.1
28518	1.89223	2.1
28519	1.89223	2.1
28520	1.89223	2.1
28521	1.89223	2.1
28522	3.78447	2.1
28523	0.796845	2.1
28524	0.796845	2.1
28525	0.812146	2.1
28526	0.812146	2.1
28527	1.08006	2.1
28528	1.02679	2.1
28529	0.551538	2.1
28530	0.528929	2.1
28531	0.551529	2.1
28532	1.08005	2.1
28533	0.528935	2.1

28534	1.0268	2.1
28535	1.5001	2.1
28536	1.70302	2.1
28537	2.0726	2.1
28538	1.70302	2.1
28539	1.99601	2.1
28540	1.70303	2.1
28541	1.89226	2.1
28542	1.89226	2.1
28543	1.89226	2.1
28544	1.89226	2.1
28545	1.89226	2.1
28546	1.89226	2.1
28547	1.17285	2.1
28548	2.16015	2.1
28549	2.23563	2.1
28550	2.23563	2.1
28551	1.10309	2.1
28552	1.10309	2.1
28553	0.946128	2.1
28554	0.946128	2.1
28555	1.21847	0.870755
28556	1.2884	2.1
28557	0.94613	2.1
28558	0.94613	2.1
28559	1.21847	2.1
28560	1.21847	0.870217
28561	0.946131	2.1
28562	1.21847	2.1
28563	0.94613	2.1
28564	1.2884	2.1
28565	1.83922	1.79206
28566	2.5768	2.1
28567	1.83488	2.1
28568	1.82846	0.05
28569	2.43693	2.1
28570	2.43693	2.1
28571	2.36134	2.1
28572	1.81656	2.1
28573	1.86454	2.1
28574	1.6697	2.1
28575	2.55618	2.1
28576	2.36134	2.1
28577	2.55618	2.1
28578	1.9323	2.1
28579	1.9302	2.1
28580	1.81656	2.1
28581	2.66983	2.1
28582	2.55618	2.1
28583	1.89226	1.07135
28584	1.70302	0.767647
28585	1.94935	1.24739
28586	1.70302	1.06827
28587	1.95569	0.791741
28588	1.70302	0.180233
28589	2.82465	2.1
28590	2.41707	2.1
28591	2.24481	2.1

28592	2.68958	2.1
28593	5.10665	2.1
28594	1.41232	2.1
28595	1.34479	2.1
28596	0.946129	2.1
28597	0.946129	2.1
28598	0.94613	2.1
28599	0.94613	2.1
28600	1.41232	2.1
28601	1.41232	2.1
28602	1.34479	2.1
28603	0.94613	2.1
28604	0.946131	2.1
28605	1.41232	2.1
28606	1.86859	0.05
28607	1.89226	0.05
28608	1.89226	0.05
28609	1.89226	0.05
28610	1.45875	0.05
28611	1.45875	0.05
28612	2.1769	0.771983
28613	2.50259	0.756517
28614	1.94955	0.711418
28615	2.25959	0.800832
28616	4.45214	0.862726
28617	1.89226	0.780695
28618	1.89226	0.816005
28619	1.89226	0.724734
28620	1.89226	0.770298
28621	3.78452	0.737484
28622	1.89226	0.88009
28623	1.89226	0.972967
28624	1.89226	0.792847
28625	1.89226	0.969014
28626	3.78452	1.5423
28627	2.50259	0.810665
28628	2.49445	0.929216
28629	2.25959	0.730116
28630	2.20843	0.827879
28631	4.71102	0.776516
28632	1.94955	0.71463
28633	2.25959	0.705981
28634	1.24689	0.559508
28635	1.79906	0.707898
28636	3.74861	0.703825
28637	1.89226	0.727675
28638	1.89226	0.741409
28639	1.89226	0.706563
28640	1.89226	0.717439
28641	3.78452	0.743449
28642	1.89226	0.761396
28643	1.89226	0.822057
28644	1.89226	0.716324
28645	1.89226	0.747705
28646	3.78452	0.728367
28647	2.25959	0.564109
28648	2.20843	0.757829
28649	1.79906	0.707465

28650	1.98391	0.725479
28651	4.00748	0.719995
28652	1.89226	2.1
28653	1.89226	2.1
28654	1.89226	2.1
28655	1.89226	2.1
28656	3.78452	2.1
28657	1.89226	2.1
28658	1.89226	2.1
28659	1.89226	2.1
28660	1.89226	2.1
28661	3.78452	2.1
28662	1.64718	2.1
28663	1.23085	2.1
28664	1.46028	2.1
28665	1.39564	2.1
28666	1.68372	2.1
28667	1.64718	2.1
28668	1.64718	2.1
28669	1.64981	2.1
28670	1.70303	2.1
28671	1.6991	2.1
28672	3.3199	2.1
28673	1.89226	2.1
28674	1.89226	2.1
28675	1.89226	2.1
28676	1.89226	2.1
28677	3.78452	2.1
28678	1.89226	2.1
28679	1.89226	2.1
28680	1.89226	2.1
28681	1.89226	2.1
28682	3.78452	2.1
28683	0.666701	2.1
28684	0.65896	2.1
28685	0.920797	2.1
28686	0.895441	2.1
28687	0.775871	2.1
28688	0.687493	2.1
28689	0.777393	2.1
28690	0.777393	2.1
28691	0.677856	2.1
28692	0.90095	2.1
28693	0.621435	2.1
28694	0.858124	2.1
28695	1.70303	2.1
28696	1.70731	2.1
28697	1.79663	2.1
28698	1.68783	2.1
28699	3.17337	2.1
28700	1.22724	2.1
28701	2.0021	2.1
28702	1.90427	2.1
28703	1.08698	2.1
28704	3.08907	2.1
28705	2.41689	2.1
28706	2.00209	2.1
28707	1.90427	2.1

28708	2.35245	2.1
28709	4.35454	2.1
28710	1.90427	2.1
28711	0.956294	2.1
28712	0.956294	2.1
28713	1.08698	2.1
28714	1.81488	2.1
28715	1.94556	2.1
28716	1.90427	2.1
28717	2.35245	2.1
28718	2.28979	2.1
28719	1.81488	2.1
28720	4.16733	2.1
28721	1.6272	2.1
28722	1.70302	2.1
28723	1.70303	2.1
28724	1.70302	2.1
28725	1.91167	2.1
28726	1.91167	2.1
28727	1.49063	2.1
28728	1.89226	2.1
28729	1.89226	2.1
28730	1.89226	2.1
28731	1.6272	2.1
28732	1.6272	2.1
28733	2.25988	1.06622
28734	1.89226	0.217732
28735	1.89226	1.00484
28736	2.23152	0.948546
28737	4.12379	0.864196
28738	2.25988	1.03363
28739	1.16361	1.31788
28740	1.15853	2.1
28741	2.23153	0.90341
28742	3.39513	1.27748
28743	1.89226	0.968691
28744	2.23153	0.426225
28745	2.21022	1.07761
28746	2.23153	0.921053
28747	1.87095	0.20639
28748	1.89226	0.169675
28749	1.16239	1.73153
28750	2.23153	0.121214
28751	1.15853	1.29368
28752	1.15853	0.540365
28753	2.21022	1.33469
28754	2.21022	0.431073
28755	1.78381	0.05
28756	1.70302	0.05
28757	1.15453	0.05
28758	1.35007	0.05
28759	1.82021	0.05
28760	2.12851	0.05
28761	1.89226	0.05
28762	1.89226	0.05
28763	1.89226	0.05
28764	1.89226	0.05
28765	1.89226	0.05

28766	1.89226	0.05
28767	1.89258	0.05
28768	1.089 0.05	
28769	1.22586	0.05
28770	1.79877	0.05
28771	2.88777	0.05
28772	1.91564	0.05
28773	1.79877	0.05
28774	1.96356	0.05
28775	1.70302	0.05
28776	2.37828	0.05
28777	2.04859	0.05
28778	1.89226	0.05
28779	1.89226	0.05
28780	1.89226	0.05
28781	1.89226	0.05
28782	3.78453	0.05
28783	1.89226	0.05
28784	1.89226	0.05
28785	1.89226	0.05
28786	1.89226	0.05
28787	1.89226	0.05
28788	1.89226	0.05
28789	2.28593	2.1
28790	1.89226	2.1
28791	2.2731	2.1
28792	1.89226	1.01897
28793	4.17819	1.85385
28794	1.89226	2.1
28795	1.89226	2.1
28796	1.89226	1.30686
28797	1.89226	1.25853
28798	3.78452	1.73397
28799	1.03933	2.1
28800	1.34355	1.19803
28801	1.35048	1.1407
28802	1.35048	2.1
28803	1.02975	1.07133
28804	1.02975	1.95781
28805	2.25105	2.1
28806	1.89226	2.03274
28807	2.27311	2.1
28808	2.27311	2.1
28809	1.89226	1.17004
28810	1.89226	2.1
28811	1.89226	2.1
28812	1.89226	2.1
28813	1.89226	1.43639
28814	1.89226	0.821519
28815	3.78452	1.23526
28816	1.02975	2.1
28817	1.34355	2.1
28818	1.01121	1.24937
28819	1.33079	1.19934
28820	2.35476	2.1
28821	1.24526	0.05
28822	2.36406	0.05
28823	2.81021	0.05

28824	1.7237	0.05
28825	4.08776	0.05
28826	1.89226	2.1
28827	1.89226	2.1
28828	1.89226	2.1
28829	1.89226	2.1
28830	3.78453	2.1
28831	1.79028	2.1
28832	1.89238	2.1
28833	1.65533	2.1
28834	1.77821	2.1
28835	3.5477	2.1
28836	1.89238	2.1
28837	1.7465	0.05
28838	1.77821	2.1
28839	1.63557	0.0700144
28840	3.52795	1.82231
28841	1.89226	2.1
28842	1.89226	0.147593
28843	1.89226	2.1
28844	1.89226	0.05
28845	3.78452	0.175017
28846	1.89226	2.1
28847	1.89226	2.1
28848	1.89226	2.1
28849	1.89226	2.1
28850	3.78452	2.1
28851	1.65532	2.1
28852	1.77821	2.1
28853	1.53278	2.1
28854	1.67571	2.1
28855	3.33103	2.1
28856	1.77821	2.1
28857	1.63557	0.172577
28858	1.67571	2.1
28859	1.52615	0.05
28860	3.31128	1.66588
28861	1.89226	2.1
28862	1.89226	0.05
28863	1.89226	2.1
28864	1.89226	0.113584
28865	3.78452	1.93743
28866	2.83129	2.1
28867	1.89226	0.326955
28868	1.89226	1.12146
28869	2.6491	2.1
28870	4.54136	1.08561
28871	2.83129	2.1
28872	1.47278	2.1
28873	1.3909	2.1
28874	2.6491	2.1
28875	4.12188	2.1
28876	1.89225	2.1
28877	2.65393	2.1
28878	1.89225	2.1
28879	2.69591	2.1
28880	4.58816	2.1
28881	1.91703	2.1

28882	2.00209	2.1
28883	2.65393	2.1
28884	2.65393	2.1
28885	2.69591	2.1
28886	2.00209	2.1
28887	1.89226	2.1
28888	2.69593	2.1
28889	1.93967	2.1
28890	1.89226	2.1
28891	2.74334	2.1
28892	2.69593	2.1
28893	2.74335	2.1
28894	2.0021	2.1
28895	2.76367	2.1
28896	2.69593	2.1
28897	2.06984	2.1
28898	2.0021	2.1
28899	2.24481	2.1
28900	2.58921	2.1
28901	2.68958	2.1
28902	2.68958	2.1
28903	2.11984	2.1
28904	2.11984	2.1
28905	2.11984	2.1
28906	2.58921	2.1
28907	2.46488	2.1
28908	1.98339	2.1
28909	4.5726	2.1
28910	2.7777	2.1
28911	2.58921	2.1
28912	2.46488	2.1
28913	2.70401	2.1
28914	5.29322	2.1
28915	2.17741	2.1
28916	2.54197	2.1
28917	2.54018	2.1
28918	2.18608	2.1
28919	4.72805	2.1
28920	2.17742	2.1
28921	1.58054	2.1
28922	1.59169	2.1
28923	2.18608	2.1
28924	3.76662	2.1
28925	2.54018	2.1
28926	2.18608	2.1
28927	2.19104	2.1
28928	2.5392	2.1
28929	4.72528	2.1
28930	1.60497	2.1
28931	2.18608	2.1
28932	1.59169	2.1
28933	1.59169	2.1
28934	2.19105	2.1
28935	2.19105	2.1
28936	2.58661	2.1
28937	1.06203	2.1
28938	2.7043	2.1
28939	2.21397	2.1

28940	1.55236	2.1
28941	1.06203	2.1
28942	1.89226	1.43204
28943	1.89226	2.02629
28944	1.89226	0.96547
28945	1.89226	2.1
28946	3.78453	1.45938
28947	1.08195	1.66852
28948	0.990019	2.1
28949	1.31976	2.03212
28950	1.4539	1.06823
28951	2.40171	2.1
28952	1.89226	1.20222
28953	1.89226	2.1
28954	1.89226	1.19312
28955	1.89226	0.935575
28956	3.78453	1.32438
28957	1.89226	1.84612
28958	1.89226	1.42698
28959	1.89226	1.05466
28960	1.89226	2.1
28961	3.78453	1.34975
28962	1.43895	2.1
28963	1.31976	2.1
28964	1.70066	1.02174
28965	1.33783	2.1
28966	1.43895	2.1
28967	1.07612	2.1
28968	0.65988	2.00842
28969	0.850329	1.50084
28970	0.919598	1.12005
28971	0.726952	1.62314
28972	0.788106	1.01138
28973	0.788106	2.1
28974	0.789175	2.1
28975	0.789175	0.85856
28976	0.850329	1.14746
28977	0.919598	1.10394
28978	0.659879	1.70413
28979	0.726952	1.44862
28980	1.34389	2.1
28981	1.41279	1.96322
28982	1.15564	2.1
28983	1.15564	2.1
28984	1.55998	2.061
28985	1.55998	2.1
28986	1.89226	1.7428
28987	1.89226	2.1
28988	1.89226	2.1
28989	1.89226	1.23144
28990	1.89226	2.1
28991	1.89226	1.25366
28992	1.55998	2.1
28993	1.41279	2.1
28994	1.72901	2.1
28995	1.62414	2.1
28996	3.1418	2.1
28997	1.89226	1.81201

28998	1.89226	2.1
28999	1.89226	2.1
29000	1.89226	1.14048
29001	3.78452	2.06992
29002	1.89226	1.03229
29003	1.89226	2.1
29004	1.89226	1.488
29005	1.89226	2.1
29006	1.89226	1.12943
29007	1.89226	1.3571
29008	1.62414	2.1
29009	1.01243	2.1
29010	1.70932	2.1
29011	1.41279	1.59389
29012	1.30896	1.96536
29013	1.01243	1.40192
29014	1.9719	2.1
29015	1.10725	2.1
29016	1.12411	2.1
29017	1.98691	2.1
29018	3.09601	2.1
29019	1.97189	2.1
29020	2.47932	2.1
29021	2.49092	2.1
29022	1.98691	2.1
29023	4.46282	2.1
29024	1.1486	2.1
29025	1.98691	2.1
29026	1.12411	2.1
29027	1.12411	2.1
29028	2.01602	2.1
29029	2.01602	2.1
29030	2.49093	2.1
29031	1.98691	2.1
29032	2.01601	2.1
29033	2.51651	2.1
29034	4.50694	2.1
29035	1.93147	0.05
29036	2.51593	0.05
29037	1.35146	0.05
29038	1.35146	0.05
29039	2.48665	0.05
29040	1.90664	0.05
29041	1.63979	2.1
29042	1.7457	2.1
29043	1.66046	2.1
29044	1.56558	2.1
29045	3.31128	2.1
29046	0.819895	2.1
29047	0.782792	2.1
29048	0.60239	2.1
29049	0.54831	2.1
29050	0.688347	2.1
29051	0.688347	2.1
29052	0.679857	2.1
29053	0.679857	2.1
29054	0.782792	2.1
29055	0.54831	2.1

29056	0.60239	2.1
29057	0.819895	2.1
29058	1.66046	2.1
29059	1.42485	2.1
29060	1.56558	2.1
29061	1.56558	2.1
29062	1.52995	2.1
29063	1.52995	2.1
29064	2.58101	0.05
29065	2.09803	2.1
29066	2.408	0.236112
29067	1.86893	2.1
29068	4.44994	0.676086
29069	2.36062	2.1
29070	1.25104	2.1
29071	1.25104	2.1
29072	1.39703	2.1
29073	1.86893	2.1
29074	2.01492	2.1
29075	2.408	0.05
29076	1.86893	2.1
29077	2.22575	0.05
29078	1.70677	2.1
29079	4.09468	1.02663
29080	0.737605	2.1
29081	0.737605	2.1
29082	0.741302	2.1
29083	0.741302	2.1
29084	0.55189	2.1
29085	0.625517	2.1
29086	0.934468	2.1
29087	0.853389	2.1
29088	0.853389	2.1
29089	0.55189	2.1
29090	0.625517	2.1
29091	0.934467	2.1
29092	2.35053	2.1
29093	1.09615	2.1
29094	1.25998	2.1
29095	2.51216	2.1
29096	3.61051	2.1
29097	1.33636	2.1
29098	1.25609	2.1
29099	0.698455	2.1
29100	0.629995	2.1
29101	0.986092	2.1
29102	0.986092	2.1
29103	0.980263	2.1
29104	0.980263	2.1
29105	1.33636	2.1
29106	0.698455	2.1
29107	0.629995	2.1
29108	1.25609	2.1
29109	1.89226	1.12278
29110	1.89226	1.17311
29111	1.89226	1.2111
29112	1.89226	1.16536
29113	3.78452	1.14187

29114	1.74321	1.00369
29115	1.89226	0.849203
29116	1.91755	1.1556
29117	1.91755	0.932394
29118	1.89226	1.0309
29119	1.89226	0.824244
29120	0.946132	1.10877
29121	0.946132	1.07252
29122	0.949798	0.641939
29123	1.08054	0.926432
29124	1.08054	0.649241
29125	1.08054	0.916869
29126	0.946132	1.07495
29127	0.946132	1.07086
29128	0.946132	1.07653
29129	0.949798	0.490851
29130	1.08054	0.777869
29131	0.946131	1.11814
29132	1.89226	1.17255
29133	1.89226	1.09985
29134	1.89226	1.07381
29135	1.89226	1.08236
29136	1.89226	1.12228
29137	1.89226	1.13335
29138	2.39378	0.05
29139	2.29234	0.05
29140	2.29234	0.05
29141	2.7933	0.05
29142	1.18422	0.05
29143	1.68518	0.05
29144	2.79329	0.05
29145	1.89226	0.05
29146	2.29233	0.05
29147	1.89226	0.05
29148	4.18459	0.05
29149	0.857046	0.05
29150	0.857037	0.05
29151	0.94613	0.05
29152	0.94613	0.05
29153	0.851539	0.05
29154	0.851539	0.05
29155	1.0315	0.05
29156	0.952867	0.05
29157	0.851486	0.05
29158	0.952869	0.05
29159	1.03156	0.05
29160	0.851528	0.05
29161	1.61981	2.1
29162	1.93096	2.1
29163	2.00776	2.1
29164	1.62218	2.1
29165	3.62757	2.1
29166	2.00776	2.1
29167	1.68541	2.1
29168	1.83215	2.1
29169	1.62218	2.1
29170	2.21773	2.1
29171	2.00776	2.1

29172	1.89188	0.05
29173	1.70303	0.05
29174	1.89226	0.05
29175	1.72465	0.05
29176	1.89226	0.05
29177	1.70552	0.05
29178	2.48659	0.05
29179	2.0815	0.05
29180	1.89226	0.05
29181	2.08111	0.05
29182	1.89226	0.05
29183	2.08111	0.05
29184	2.35332	0.05
29185	1.83284	0.05
29186	2.28483	0.05
29187	2.71955	0.05
29188	4.63815	0.05
29189	1.89227	2.1
29190	1.89227	2.1
29191	1.89227	2.1
29192	1.89227	2.1
29193	3.78453	2.1
29194	1.33506	2.1
29195	1.17129	2.1
29196	1.33506	2.1
29197	1.38415	2.1
29198	1.13664	2.1
29199	1.18573	2.1
29200	1.89226	2.1
29201	2.68785	2.1
29202	1.89226	2.1
29203	2.6692	2.1
29204	4.58011	2.1
29205	1.89226	2.1
29206	1.89226	2.1
29207	1.89226	2.1
29208	1.89226	2.1
29209	3.78453	2.1
29210	1.33506	2.1
29211	1.13664	2.1
29212	1.27575	2.1
29213	1.09627	2.1
29214	2.43133	2.1
29215	1.89226	2.1
29216	2.65349	2.1
29217	2.66919	2.1
29218	2.66919	2.1
29219	1.89226	2.1
29220	1.89226	2.1
29221	1.89226	2.1
29222	1.89226	2.1
29223	1.89226	2.1
29224	1.89226	2.1
29225	3.78453	2.1
29226	1.16834	2.1
29227	1.16834	2.1
29228	0.946132	2.1
29229	0.946132	2.1

29230	1.26978	2.1
29231	1.16834	2.1
29232	0.94613	2.1
29233	0.94613	2.1
29234	0.94613	2.1
29235	1.26978	2.1
29236	0.94613	2.1
29237	1.16834	2.1
29238	2.14698	2.1
29239	1.89226	2.1
29240	1.89226	2.1
29241	1.89226	2.1
29242	2.40719	2.1
29243	2.40719	2.1
29244	1.89226	2.1
29245	1.89226	2.1
29246	1.89226	2.1
29247	1.89226	2.1
29248	3.78453	2.1
29249	1.26977	2.1
29250	1.26977	2.1
29251	0.946133	2.1
29252	0.946133	2.1
29253	1.37467	2.1
29254	1.26978	2.1
29255	0.946131	2.1
29256	0.946131	2.1
29257	1.37467	2.1
29258	0.94613	2.1
29259	0.946131	2.1
29260	1.26978	2.1
29261	1.2036	2.1
29262	1.34912	2.1
29263	0.946131	2.1
29264	0.946131	2.1
29265	1.34912	2.1
29266	1.34912	2.1
29267	0.946129	2.1
29268	0.946129	2.1
29269	1.34912	2.1
29270	0.94613	2.1
29271	0.946131	2.1
29272	1.2036	2.1
29273	2.47164	0.122724
29274	1.89019	0.05
29275	1.79109	0.101894
29276	2.41632	0.05
29277	4.26273	0.119099
29278	1.54197	0.0624675
29279	2.41632	0.05
29280	1.79109	0.05
29281	1.79109	0.0652265
29282	2.20929	0.05
29283	2.20929	0.05
29284	1.70302	0.916923
29285	1.48104	1.33711
29286	1.99365	1.45748
29287	1.32897	0.694881

29288	1.70302	1.47159
29289	1.14034	0.855003
29290	1.70302	1.11956
29291	1.99365	1.04631
29292	1.99297	1.34188
29293	2.36337	0.417377
29294	4.04804	0.921264
29295	1.51569	0.664363
29296	2.49093	0.507606
29297	1.6026	0.601868
29298	1.43229	0.242342
29299	2.0815	1.03673
29300	1.83402	0.455493
29301	2.54665	2.1
29302	2.34044	2.1
29303	2.08889	2.1
29304	2.37653	2.1
29305	4.71697	2.1
29306	2.54665	2.1
29307	2.45212	2.1
29308	2.31012	2.1
29309	2.37653	2.1
29310	4.82865	2.1
29311	2.08889	2.1
29312	2.37653	2.1
29313	2.23366	2.1
29314	1.87598	2.1
29315	4.25251	2.1
29316	2.31012	2.1
29317	2.37653	2.1
29318	2.23367	2.1
29319	2.17384	2.1
29320	4.55037	2.1
29321	0.946131	2.1
29322	0.946131	2.1
29323	1.30338	2.1
29324	1.23186	2.1
29325	0.94613	2.1
29326	0.94613	2.1
29327	1.23187	2.1
29328	1.23187	2.1
29329	0.946131	2.1
29330	1.23186	2.1
29331	1.30338	2.1
29332	0.946131	2.1
29333	1.89226	2.1
29334	2.46373	2.1
29335	2.34725	2.1
29336	2.46373	2.1
29337	1.77579	2.1
29338	1.89226	2.1
29339	2.61901	2.1
29340	2.45899	2.1
29341	2.64067	2.1
29342	2.52708	2.1
29343	5.14609	2.1
29344	2.41795	2.1
29345	2.0815	2.1

29346	2.52708	2.1
29347	1.92277	2.1
29348	4.31368	2.1
29349	2.64067	2.1
29350	2.52708	2.1
29351	2.58638	2.1
29352	2.51039	2.1
29353	5.11346	2.1
29354	2.52708	2.1
29355	2.13642	2.1
29356	2.51039	2.1
29357	2.16633	2.1
29358	4.69341	2.1
29359	1.61028	1.44725
29360	1.65712	0.789374
29361	1.35002	0.274654
29362	1.35002	1.4793
29363	1.88637	0.810667
29364	1.88637	2.1
29365	2.57663	2.1
29366	2.24227	2.1
29367	2.48354	2.1
29368	2.14317	2.1
29369	4.7198	2.1
29370	2.24227	2.1
29371	1.77353	2.1
29372	2.14317	2.1
29373	1.69956	2.1
29374	3.9167	2.1
29375	2.48353	2.1
29376	2.14317	2.1
29377	2.39282	2.1
29378	1.79824	2.1
29379	4.53599	2.1
29380	2.14317	2.1
29381	1.69956	2.1
29382	1.91427	2.1
29383	1.6347	2.1
29384	3.77787	2.1
29385	1.89226	0.726248
29386	1.89226	0.934308
29387	1.89226	0.789212
29388	1.89226	0.756922
29389	3.78453	1.47524
29390	1.89226	0.728014
29391	1.89226	0.768311
29392	1.89226	0.736457
29393	1.89226	0.82614
29394	3.78452	0.735118
29395	1.89226	0.740821
29396	1.89652	1.42811
29397	1.89226	0.1995
29398	1.89226	0.887503
29399	1.19352	0.830999
29400	1.19352	2.02052
29401	1.89226	0.789095
29402	1.89653	0.776462
29403	1.89226	1.66658

29404	1.89226	0.884114
29405	1.89226	0.780109
29406	1.89226	0.757084
29407	1.89226	1.34923
29408	1.75326	0.838545
29409	1.89226	2.07528
29410	1.12948	1.95174
29411	1.89226	0.71721
29412	1.12948	0.707288
29413	2.61064	2.1
29414	1.89226	2.1
29415	1.89226	2.1
29416	1.89226	2.1
29417	2.6085	2.1
29418	2.6085	2.1
29419	2.6085	2.1
29420	1.89226	2.1
29421	1.89226	2.1
29422	1.89226	2.1
29423	2.62161	2.1
29424	2.62161	2.1
29425	2.16535	2.1
29426	1.44603	2.1
29427	2.79784	2.1
29428	1.84756	2.1
29429	4.01292	2.1
29430	2.65262	2.1
29431	1.03391	2.1
29432	2.20239	2.1
29433	1.74416	2.1
29434	1.49213	2.1
29435	1.03391	2.1
29436	2.29066	2.1
29437	2.49174	2.1
29438	2.56502	2.1
29439	2.33802	2.1
29440	4.85568	2.1
29441	2.39663	2.1
29442	2.49174	2.1
29443	2.56502	2.1
29444	2.45929	2.1
29445	4.96165	2.1
29446	2.56502	2.1
29447	2.33802	2.1
29448	2.37168	2.1
29449	2.59967	2.1
29450	4.9367	2.1
29451	2.56502	2.1
29452	2.45929	2.1
29453	2.50138	2.1
29454	2.59967	2.1
29455	5.0664	2.1
29456	2.27009	1.21157
29457	1.26247	2.1
29458	1.86812	2.1
29459	2.837	1.51206
29460	4.13822	2.1
29461	1.89226	0.941727

29462	1.89226	2.1
29463	1.89226	2.1
29464	1.89226	2.1
29465	3.78453	2.1
29466	1.75835	2.1
29467	1.72866	2.1
29468	1.65313	2.1
29469	1.64822	2.1
29470	3.3818	2.1
29471	1.72866	2.1
29472	1.58401	2.1
29473	1.64822	2.1
29474	1.52441	2.1
29475	3.25307	2.1
29476	1.89226	2.1
29477	1.89226	2.1
29478	1.89226	2.1
29479	1.89226	2.1
29480	3.78452	2.1
29481	1.89226	2.1
29482	1.89226	2.1
29483	1.89226	0.88542
29484	1.89226	2.1
29485	3.78453	2.1
29486	1.65313	2.1
29487	1.64822	2.1
29488	1.55779	2.1
29489	1.57968	2.1
29490	3.23281	2.1
29491	1.64822	2.1
29492	1.52441	2.1
29493	1.57967	2.1
29494	1.474 2.1	
29495	3.10408	2.1
29496	1.89226	2.1
29497	1.89226	2.1
29498	1.89226	2.1
29499	1.89226	2.1
29500	3.78452	2.1
29501	1.86067	1.32568
29502	1.58407	2.1
29503	2.07965	1.01773
29504	1.83123	1.71372
29505	3.66372	1.16552
29506	1.89226	1.10544
29507	1.89226	2.1
29508	1.89226	1.10837
29509	1.89226	2.02717
29510	3.78452	1.40195
29511	1.89226	0.42159
29512	1.89226	2.1
29513	1.89226	0.199364
29514	1.89226	2.1
29515	1.89226	0.112507
29516	1.89226	2.1
29517	1.83123	1.02379
29518	1.03472	2.1
29519	1.91068	2.04185

29520	1.58406	1.26839
29521	1.36133	1.23325
29522	1.03472	0.554417
29523	2.07965	1.19127
29524	1.83123	1.89652
29525	2.28385	1.09859
29526	2.09114	1.37699
29527	4.1708	1.69416
29528	1.89226	1.36907
29529	1.89226	2.1
29530	1.89226	0.998573
29531	1.89226	1.80975
29532	3.78452	1.13661
29533	1.89226	0.879967
29534	1.89226	2.1
29535	1.89226	1.05335
29536	1.89226	2.1
29537	3.78452	1.3112
29538	1.83123	1.0804
29539	1.36133	2.1
29540	2.09114	0.81769
29541	1.69436	1.97969
29542	3.45248	1.1933
29543	1.89226	2.1
29544	1.89226	2.1
29545	1.89226	2.1
29546	1.89226	2.1
29547	3.78453	2.1
29548	1.53278	2.1
29549	1.67571	2.1
29550	1.40182	2.1
29551	1.55686	2.1
29552	3.07752	2.1
29553	1.67571	2.1
29554	1.52615	0.05
29555	1.55686	2.1
29556	1.38963	0.05
29557	3.06534	1.70888
29558	1.89226	2.1
29559	1.89226	0.588535
29560	1.89226	2.1
29561	1.89226	0.05
29562	3.78452	1.12425
29563	1.89226	2.1
29564	1.89226	2.1
29565	1.89226	2.1
29566	1.89226	2.1
29567	3.78453	2.1
29568	1.40182	2.1
29569	1.55686	2.1
29570	1.24509	2.1
29571	1.38426	2.1
29572	2.78608	2.1
29573	1.55686	2.1
29574	1.38963	0.0528675
29575	1.2064	2.1
29576	1.2064	0.0648147
29577	1.38426	2.1

29578	1.56749	0.552876
29579	0.810946	2.1
29580	0.810946	0.638683
29581	0.810945	2.1
29582	0.810945	2.1
29583	0.810945	2.1
29584	0.810945	1.53522
29585	1.89226	0.335562
29586	1.35169	0.981558
29587	1.08132	2.1
29588	1.08132	0.05
29589	1.08132	1.28684
29590	1.28418	0.0518569
29591	1.59382	0.05
29592	1.78289	0.0901185
29593	1.51021	0.05
29594	3.10403	0.05
29595	1.76897	0.971316
29596	1.59382	0.05
29597	1.78289	0.0791865
29598	1.94302	0.114434
29599	3.53684	0.193488
29600	1.89226	0.05
29601	1.89226	0.05
29602	1.89226	0.05
29603	1.89226	0.05
29604	3.78453	0.05
29605	1.89227	0.05
29606	1.89227	0.05
29607	1.89227	0.05
29608	1.89227	0.05
29609	1.89227	0.05
29610	1.89227	0.05
29611	1.78286	0.116027
29612	1.51019	0.05
29613	1.79979	0.05
29614	2.02511	0.050399
29615	3.5353	0.0622206
29616	1.78286	0.0965006
29617	1.94299	0.0867232
29618	2.17831	0.505063
29619	2.02511	0.05
29620	3.9681	0.05
29621	1.89224	0.05
29622	1.89224	0.05
29623	1.89224	0.05
29624	1.89224	0.113441
29625	3.78448	0.0871576
29626	1.89224	0.05
29627	1.89224	0.05
29628	1.89224	0.05
29629	1.89224	0.05
29630	1.89224	0.05
29631	1.89224	0.0542128
29632	1.83198	0.0596185
29633	1.0961	0.05
29634	1.39448	0.0707796
29635	1.99568	0.05

29636	3.09178	0.0667635
29637	1.25503	0.05
29638	1.99568	0.05
29639	2.3661	0.0560465
29640	1.70302	0.05
29641	3.55833	0.05
29642	2.69899	0.05
29643	1.85796	0.05
29644	2.11407	0.05
29645	1.36769	0.05
29646	3.97203	0.05
29647	1.89226	0.05
29648	1.89226	0.05
29649	1.89226	0.05
29650	1.89226	0.05
29651	3.78452	0.05
29652	1.89225	0.05
29653	2.63355	0.05
29654	2.09529	0.05
29655	2.09529	0.05
29656	1.89225	0.05
29657	1.89225	0.05
29658	1.89226	0.05
29659	1.89226	0.05
29660	1.89226	0.05
29661	1.89226	0.05
29662	1.89226	0.05
29663	1.89226	0.05
29664	2.12204	0.05
29665	1.89226	0.05
29666	2.05798	0.05
29667	2.01954	0.05
29668	1.70302	0.05
29669	1.3865	0.05
29670	1.28326	0.05
29671	1.33326	0.517757
29672	1.19052	0.05
29673	1.25369	0.391271
29674	2.53694	0.266736
29675	1.33327	0.178172
29676	1.27339	2.1
29677	1.25369	0.109022
29678	1.19733	2.1
29679	2.52708	2.1
29680	1.19052	0.05
29681	1.25369	0.42154
29682	1.10938	0.05
29683	1.17614	0.478776
29684	2.36307	0.05
29685	1.25369	0.05
29686	1.19733	2.1
29687	1.17614	0.555795
29688	1.11868	2.1
29689	2.37237	2.1
29690	1.10747	0.05
29691	1.47688	0.05
29692	1.97732	0.05
29693	1.62206	0.05

29694	3.09894	0.05
29695	1.89226	0.05
29696	1.89226	0.05
29697	1.89226	0.05
29698	1.89226	0.05
29699	1.89226	0.05
29700	1.89226	0.05
29701	1.62206	0.05
29702	1.70302	0.05
29703	1.22962	0.05
29704	1.1893	0.05
29705	2.15177	0.05
29706	2.08121	0.05
29707	1.97732	0.05
29708	2.08121	0.05
29709	1.62206	0.05
29710	1.62206	0.05
29711	2.41464	0.05
29712	2.41464	0.05
29713	1.89226	0.05
29714	1.89226	0.05
29715	1.89226	0.05
29716	1.89226	0.05
29717	3.78452	0.05
29718	0.946133	0.05
29719	0.946133	0.05
29720	0.946132	0.05
29721	0.946132	0.05
29722	0.946132	0.05
29723	0.946132	0.05
29724	0.946133	0.05
29725	0.946133	0.05
29726	0.946132	0.05
29727	0.946132	0.0535306
29728	0.946132	0.05
29729	0.946132	0.05
29730	1.83157	0.384722
29731	2.19989	0.371755
29732	2.20664	0.0934614
29733	1.81487	0.143916
29734	4.03146	0.158188
29735	2.41055	2.1
29736	2.19989	2.1
29737	2.20665	2.1
29738	2.38023	1.70032
29739	4.61044	1.98916
29740	2.19989	2.1
29741	2.22558	2.1
29742	2.38023	2.01335
29743	2.07065	1.12347
29744	4.42547	1.66324
29745	2.19988	1.17775
29746	1.70303	0.077602
29747	1.89939	0.440609
29748	1.86357	0.117724
29749	3.78536	0.514846
29750	1.04921	2.1
29751	1.30711	2.1

29752	1.53076	2.1
29753	1.22927	2.1
29754	2.57997	2.1
29755	1.53076	2.1
29756	1.46568	2.1
29757	1.56056	2.1
29758	1.22927	2.1
29759	1.86206	2.1
29760	1.53076	2.1
29761	1.18353	2.1
29762	2.28593	2.1
29763	1.16979	2.1
29764	2.2731	2.1
29765	3.45571	2.1
29766	0.854776	2.1
29767	0.854776	2.1
29768	0.855641	2.1
29769	0.855641	2.1
29770	1.12552	2.1
29771	1.13655	2.1
29772	0.57216	2.1
29773	0.584893	2.1
29774	0.57216	2.1
29775	1.12552	2.1
29776	0.584893	2.1
29777	1.13655	2.1
29778	1.54462	2.1
29779	2.76309	2.1
29780	1.34869	2.1
29781	2.48573	2.1
29782	4.03035	2.1
29783	1.35328	1.07314
29784	1.0383	1.00399
29785	1.03318	1.82427
29786	0.704834	1.54689
29787	1.0257	1.57502
29788	1.0257	1.07874
29789	1.03241	0.974465
29790	1.03241	1.82269
29791	1.35328	1.28719
29792	1.03319	1.88281
29793	1.0383	1.45288
29794	0.704835	1.32996
29795	2.27126	0.05
29796	2.77291	2.1
29797	1.81771	0.595258
29798	2.6111	0.267804
29799	4.88236	0.486134
29800	2.77291	2.1
29801	1.89227	1.21814
29802	1.89227	0.56124
29803	1.89227	2.1
29804	2.6111	0.102715
29805	2.6111	2.1
29806	2.22061	0.145788
29807	2.41522	0.132389
29808	2.41522	0.0758339
29809	2.6111	2.04852

29810	1.75765	0.060968
29811	1.95353	2.1
29812	2.27502	0.479792
29813	1.89227	1.68194
29814	1.89227	0.304995
29815	2.8189	0.0994744
29816	4.71116	0.162183
29817	1.89226	0.094913
29818	1.89226	1.59638
29819	1.89226	0.410339
29820	1.89226	0.112046
29821	1.89226	1.31292
29822	1.89226	0.677972
29823	1.89226	1.86314
29824	1.89226	1.73017
29825	1.89226	1.43959
29826	1.89226	1.12089
29827	3.78453	1.83963
29828	1.89226	1.71144
29829	2.57079	0.950602
29830	2.5932	1.16012
29831	1.89226	1.12903
29832	4.48546	1.07419
29833	0.589396	1.15182
29834	0.641454	0.998287
29835	0.814062	1.49108
29836	0.726518	1.1879
29837	0.675126	1.34406
29838	0.675125	1.13078
29839	0.692847	1.37129
29840	0.692847	1.16043
29841	0.641454	1.06053
29842	0.814062	1.26157
29843	0.726518	1.53911
29844	0.589396	1.19426
29845	1.89226	1.55108
29846	1.89226	1.12621
29847	1.89226	1.46284
29848	1.89226	1.9376
29849	1.89226	1.27083
29850	1.89226	1.86642
29851	1.16906	0.628745
29852	1.2966	1.10424
29853	0.946132	1.08592
29854	0.946132	1.8307
29855	1.16906	1.11008
29856	1.16906	0.905071
29857	0.946132	1.1241
29858	0.946132	1.26951
29859	1.16906	1.51204
29860	0.946131	1.22736
29861	1.2966	1.1536
29862	0.946132	1.89708
29863	0.641455	1.16603
29864	0.567191	1.01139
29865	0.807594	1.56568
29866	0.814062	1.24344
29867	0.673735	1.52865

29868	0.673734	1.07345
29869	0.707519	1.17743
29870	0.707519	1.62101
29871	0.807594	1.36109
29872	0.56719	1.86321
29873	0.641455	1.37628
29874	0.814063	1.91521
29875	1.89225	2.1
29876	1.89225	2.1
29877	1.89225	2.1
29878	1.89225	2.1
29879	3.7845	2.1
29880	1.89225	2.1
29881	1.89225	2.1
29882	1.89225	2.1
29883	1.89225	2.1
29884	1.89225	2.1
29885	1.89225	2.1
29886	1.89226	2.1
29887	1.89226	2.1
29888	1.89226	2.1
29889	1.89226	2.1
29890	3.78452	2.1
29891	1.89226	2.1
29892	1.89226	2.1
29893	1.89226	2.1
29894	1.89226	2.1
29895	1.89226	2.1
29896	1.89226	2.1
29897	1.89226	2.1
29898	1.89226	2.1
29899	1.89226	2.1
29900	1.89226	2.1
29901	3.78452	2.1
29902	1.89226	2.1
29903	1.38928	2.1
29904	1.89226	2.1
29905	1.51378	2.1
29906	1.89061	2.1
29907	1.89226	2.1
29908	1.89226	2.1
29909	1.89226	2.1
29910	1.89226	2.1
29911	1.89226	2.1
29912	3.78453	2.1
29913	1.04572	2.1
29914	0.994213	2.1
29915	0.912466	2.1
29916	0.912466	2.1
29917	0.947743	2.1
29918	1.00388	2.1
29919	0.900913	2.1
29920	0.900913	2.1
29921	0.900915	2.1
29922	0.946131	2.1
29923	0.946131	2.1
29924	0.999196	2.1
29925	1.89226	2.1

29926	1.89226	2.1
29927	1.89226	2.1
29928	1.89226	2.1
29929	3.78453	2.1
29930	0.946161	2.1
29931	1.1354	2.1
29932	0.850847	2.1
29933	0.850847	2.1
29934	1.01445	2.1
29935	1.02106	2.1
29936	0.946131	2.1
29937	0.946131	2.1
29938	1.13537	2.1
29939	1.01444	2.1
29940	0.850877	2.1
29941	0.946131	2.1
29942	1.80183	2.1
29943	1.89226	2.1
29944	1.89226	2.1
29945	1.89226	2.1
29946	1.79606	2.1
29947	1.79606	2.1
29948	1.24001	2.1
29949	1.41822	1.73184
29950	1.95665	0.887586
29951	1.77342	1.56032
29952	3.19666	1.13446
29953	1.95665	1.15945
29954	1.44859	2.09262
29955	1.41821	1.93535
29956	1.41821	1.97566
29957	1.95543	1.12578
29958	1.95543	1.52719
29959	1.95665	0.992749
29960	1.77342	1.55356
29961	2.33611	1.33212
29962	2.49694	0.881449
29963	4.29276	1.82588
29964	1.95665	1.53338
29965	1.95544	1.6558
29966	2.4728	0.827824
29967	2.49693	1.28137
29968	4.42945	1.07071
29969	2.24937	2.1
29970	2.49053	2.1
29971	2.16323	2.1
29972	2.36377	2.1
29973	4.61314	2.1
29974	2.49053	2.1
29975	2.58639	2.1
29976	2.36377	2.1
29977	2.47395	2.1
29978	4.95016	2.1
29979	2.45054	2.1
29980	2.36377	2.1
29981	1.92911	2.1
29982	2.181	2.1
29983	4.29288	2.1

29984	2.36377	2.1
29985	2.47395	2.1
29986	2.181 2.1	
29987	2.29881	2.1
29988	4.66258	2.1
29989	1.57885	2.1
29990	1.13339	2.1
29991	1.48594	2.1
29992	1.13322	2.1
29993	2.71207	2.1
29994	1.48596	2.1
29995	1.20761	2.1
29996	1.20761	2.1
29997	1.13323	2.1
29998	1.46568	2.1
29999	1.3913	2.1
30000	1.89226	0.680081
30001	1.89226	0.958669
30002	1.89226	0.711198
30003	1.89226	0.860486
30004	1.89226	0.688952
30005	1.89226	0.737481
30006	1.11712	0.812173
30007	0.946129	0.916247
30008	0.686934	0.728013
30009	0.946133	0.718828
30010	0.9461	0.804225
30011	1.10729	2.02406
30012	0.686963	0.967032
30013	0.686963	0.67207
30014	1.10945	0.863471
30015	0.80397	2.1
30016	0.946131	0.719173
30017	0.946131	0.982495
30018	1.89226	2.1
30019	1.89226	1.55123
30020	1.89226	1.33261
30021	1.89226	2.09204
30022	3.78452	2.03154
30023	1.22615	2.1
30024	1.3439	2.1
30025	1.47868	2.1
30026	1.55998	2.09367
30027	2.78613	2.1
30028	1.04973	2.1
30029	1.47868	2.1
30030	1.20149	2.1
30031	1.61721	2.1
30032	2.68017	2.1
30033	1.89226	2.1
30034	1.89226	2.1
30035	1.89226	2.1
30036	1.89226	1.1118
30037	3.78452	2.1
30038	0.946133	1.02708
30039	0.946133	2.1
30040	0.946128	2.1
30041	0.946128	2.1

30042	0.946131	0.990488
30043	0.946131	1.92871
30044	0.946131	2.1
30045	0.946131	1.1601
30046	0.94613	2.1
30047	0.94613	1.85012
30048	0.946131	2.1
30049	0.946131	1.72164
30050	0.798126	2.1
30051	0.798126	2.1
30052	0.805719	2.1
30053	0.805719	2.1
30054	0.779992	2.1
30055	0.864507	2.1
30056	0.739338	2.1
30057	0.808607	2.1
30058	0.808606	2.1
30059	0.864506	2.1
30060	0.739339	2.1
30061	0.779993	2.1
30062	0.946128	2.1
30063	0.946128	2.1
30064	1.18959	1.50771
30065	1.00304	2.1
30066	1.00304	2.1
30067	1.00304	2.1
30068	0.94613	2.1
30069	0.94613	1.81418
30070	0.94613	2.1
30071	1.18959	2.1
30072	1.00304	2.1
30073	0.94613	2.1
30074	2.19163	2.1
30075	1.89226	2.1
30076	2.16541	2.1
30077	1.89226	1.66491
30078	4.08389	2.1
30079	1.89226	2.1
30080	1.89226	1.77523
30081	1.89226	2.1
30082	1.89226	1.29443
30083	3.78452	2.1
30084	0.964782	2.1
30085	1.30151	1.24058
30086	1.30318	1.21108
30087	1.30318	2.1
30088	0.949134	1.14355
30089	0.949134	2.1
30090	2.15005	2.1
30091	1.89226	2.1
30092	2.16541	2.1
30093	2.16541	2.1
30094	1.89226	1.81151
30095	1.89226	2.1
30096	1.89226	2.1
30097	1.89226	2.1
30098	1.89226	2.1
30099	1.89226	0.991668

30100	3.78452	1.43742
30101	0.949134	2.1
30102	1.30151	2.1
30103	0.949134	2.1
30104	1.32143	1.29066
30105	2.25064	2.1
30106	0.982859	0.05
30107	1.28326	0.05
30108	1.19052	0.05
30109	1.19052	0.133456
30110	1.10476	0.05
30111	1.10476	0.12334
30112	1.37265	0.327401
30113	1.38247	0.350117
30114	1.36636	1.13399
30115	1.3294	0.198177
30116	2.71187	0.164678
30117	1.89226	0.866409
30118	1.89226	0.10066
30119	1.89226	0.278525
30120	1.89226	0.12562
30121	1.89226	0.798902
30122	1.89226	0.705922
30123	1.36636	1.4001
30124	1.3294	0.0523163
30125	1.30001	0.356018
30126	1.36416	1.10756
30127	2.69356	0.729298
30128	1.89226	1.27883
30129	1.89226	0.0960518
30130	1.89226	0.482514
30131	1.89226	1.09919
30132	1.89226	0.101781
30133	1.89226	0.305604
30134	2.83701	0.619112
30135	1.89226	0.467012
30136	1.89226	1.09516
30137	2.79312	0.247683
30138	4.68539	0.163297
30139	1.44288	2.1
30140	0.948677	2.1
30141	1.84076	2.1
30142	1.42884	2.1
30143	2.78943	2.1
30144	1.89226	2.1
30145	1.89226	2.1
30146	1.89226	2.1
30147	1.89226	2.1
30148	3.78452	2.1
30149	0.909402	2.1
30150	0.909402	2.1
30151	1.3063	2.1
30152	1.56757	2.1
30153	1.00038	2.1
30154	1.5235	2.1
30155	0.946127	2.1
30156	0.946127	2.1
30157	0.946128	2.1

30158	1.26957	2.1
30159	1.00038	2.1
30160	0.946129	2.1
30161	1.17256	2.1
30162	2.2846	2.1
30163	1.1844	2.1
30164	2.28904	2.1
30165	3.469	2.1
30166	0.867886	2.1
30167	0.867886	2.1
30168	0.867277	2.1
30169	0.867277	2.1
30170	1.14296	2.1
30171	1.14452	2.1
30172	0.591766	2.1
30173	0.592199	2.1
30174	0.591766	2.1
30175	1.14296	2.1
30176	0.5922	2.1
30177	1.14452	2.1
30178	1.27408	0.05
30179	1.56339	0.05
30180	1.52522	0.05
30181	1.34447	0.05
30182	2.90786	0.05
30183	1.27408	0.05
30184	1.01121	0.05
30185	0.991868	0.05
30186	1.34447	0.0507734
30187	2.35569	0.05
30188	0.944818	0.05
30189	0.883008	0.05
30190	0.66957	0.05
30191	0.616883	0.05
30192	0.755679	0.0627125
30193	0.755679	0.05
30194	0.791912	0.133983
30195	0.701943	0.05
30196	0.901541	0.05
30197	0.634847	0.05
30198	0.82781	0.05
30199	0.655405	0.05
30200	1.73819	2.1
30201	1.70303	2.1
30202	1.70303	2.1
30203	1.70303	2.1
30204	1.94516	2.1
30205	1.94517	2.1
30206	0.94994	2.1
30207	2.07469	2.1
30208	1.08506	2.1
30209	2.0815	2.1
30210	3.12902	2.1
30211	0.946131	2.1
30212	0.946131	2.1
30213	0.946131	2.1
30214	0.946131	2.1
30215	0.946128	2.1

30216	0.946128	2.1
30217	0.946133	2.1
30218	0.946133	2.1
30219	0.946131	2.1
30220	0.946131	2.1
30221	0.94613	2.1
30222	0.94613	2.1
30223	2.51796	2.1
30224	1.64193	2.1
30225	2.51172	2.1
30226	1.68893	2.1
30227	4.20689	2.1
30228	2.51172	2.1
30229	1.70002	2.1
30230	1.70002	2.1
30231	1.68893	2.1
30232	2.48689	2.1
30233	2.4758	2.1
30234	2.27963	0.05
30235	2.21582	0.05
30236	2.60486	0.05
30237	2.5105	0.05
30238	4.79013	0.05
30239	2.21582	0.05
30240	2.02069	0.05
30241	2.5105	0.05
30242	2.28615	0.05
30243	4.53119	0.05
30244	2.60486	0.05
30245	2.5105	0.05
30246	2.55791	0.05
30247	2.47141	0.05
30248	5.06842	0.05
30249	2.5105	0.05
30250	2.28615	0.05
30251	2.47141	0.05
30252	2.23337	0.05
30253	4.74387	0.05
30254	2.06984	2.1
30255	0.967499	2.1
30256	2.12435	2.1
30257	2.0021	2.1
30258	1.08975	2.1
30259	0.967499	2.1
30260	1.89226	0.05
30261	1.89226	0.05
30262	1.89226	0.05
30263	1.89226	0.05
30264	1.89226	0.05
30265	1.89226	0.05
30266	1.89226	2.1
30267	1.89226	2.1
30268	1.89226	2.1
30269	1.89226	2.1
30270	3.78452	2.1
30271	1.89227	2.1
30272	1.89227	2.1
30273	1.89227	2.1

30274	1.89227	2.1
30275	3.78453	2.1
30276	0.55882	2.1
30277	0.696462	2.1
30278	1.07003	2.1
30279	0.937179	2.1
30280	0.818077	2.1
30281	0.818077	2.1
30282	0.815565	2.1
30283	0.815565	2.1
30284	0.696462	2.1
30285	1.07003	2.1
30286	0.93718	2.1
30287	0.55882	2.1
30288	2.53857	2.1
30289	1.87436	2.1
30290	2.27541	2.1
30291	2.27541	2.1
30292	2.14005	2.1
30293	2.14005	2.1
30294	1.89226	2.1
30295	1.89226	2.1
30296	1.89226	2.1
30297	1.89226	2.1
30298	3.78452	2.1
30299	1.89227	2.1
30300	1.89227	2.1
30301	1.89227	2.1
30302	1.89227	2.1
30303	3.78453	2.1
30304	1.39292	2.1
30305	2.14006	2.1
30306	2.41083	2.1
30307	1.68761	2.1
30308	3.80376	2.1
30309	2.53857	2.1
30310	2.14005	2.1
30311	2.41083	2.1
30312	2.82043	2.1
30313	4.9494	2.1
30314	1.90439	0.05
30315	1.51835	0.05
30316	1.20473	0.05
30317	0.947693	0.05
30318	2.85208	0.05
30319	2.586	2.1
30320	1.46262	2.1
30321	2.14009	2.1
30322	2.78101	2.1
30323	4.72609	2.1
30324	1.89224	2.1
30325	2.78101	2.1
30326	2.586	2.1
30327	2.586	2.1
30328	1.89224	2.1
30329	1.89224	2.1
30330	0.994293	2.1
30331	1.19	2.1

30332	1.09483	2.1
30333	1.09483	2.1
30334	1.06827	2.1
30335	1.06827	2.1
30336	1.89226	2.1
30337	1.89226	2.1
30338	1.89226	2.1
30339	1.89226	2.1
30340	3.78452	2.1
30341	0.946132	2.1
30342	0.946132	2.1
30343	1.31912	2.1
30344	1.3572	2.1
30345	0.946131	2.1
30346	0.946131	2.1
30347	1.31912	2.1
30348	1.31912	2.1
30349	0.946131	2.1
30350	1.31912	2.1
30351	1.35721	2.1
30352	0.946131	2.1
30353	2.24577	0.182967
30354	1.09408	0.05
30355	1.60239	0.05
30356	1.09408	0.0812273
30357	2.39404	0.05
30358	2.07382	0.05
30359	1.70303	0.0855731
30360	1.3216	0.172523
30361	1.23104	0.05
30362	1.05475	0.146633
30363	2.60408	0.05
30364	1.89226	0.665365
30365	1.89226	0.732891
30366	1.89226	0.69942
30367	1.89226	0.648403
30368	3.78452	0.725479
30369	1.89226	0.105103
30370	2.3845	0.05
30371	2.6875	0.0539328
30372	1.89226	0.105184
30373	2.6875	0.05
30374	1.89226	0.05
30375	1.46759	2.1
30376	1.17585	2.1
30377	1.85189	2.1
30378	1.39223	2.1
30379	2.85981	2.1
30380	1.85189	2.1
30381	1.71255	2.1
30382	1.71255	2.1
30383	1.39223	2.1
30384	2.28511	2.1
30385	1.96479	2.1
30386	1.89227	0.987854
30387	1.89227	1.06814
30388	1.89227	1.12375
30389	1.89227	0.944161

30390	1.89227	1.33158
30391	1.89227	1.00126
30392	1.89226	0.991648
30393	1.89226	1.05528
30394	1.89226	1.15236
30395	1.89226	0.71968
30396	1.89226	1.41727
30397	1.89226	1.01106
30398	1.89226	0.978998
30399	1.89226	1.06466
30400	1.89226	1.0981
30401	1.89226	0.83798
30402	1.89226	1.54382
30403	1.89226	1.00494
30404	1.89226	0.889602
30405	2.77788	0.979941
30406	2.21062	1.01678
30407	2.21062	0.773989
30408	1.89226	1.07583
30409	1.89226	0.943848
30410	0.868548	0.810622
30411	0.610104	1.0131
30412	0.796678	0.959272
30413	0.865021	0.671755
30414	1.0883	0.673104
30415	0.904938	0.957292
30416	2.21062	0.804587
30417	1.49548	0.806446
30418	1.2068	1.05511
30419	1.54764	0.834316
30420	0.790005	0.594357
30421	1.89226	2.1
30422	2.67457	2.1
30423	1.89226	2.1
30424	2.56352	2.1
30425	4.56683	2.1
30426	2.67456	2.1
30427	2.20011	2.1
30428	2.56351	2.1
30429	2.11083	2.1
30430	4.78539	2.1
30431	2.56348	2.1
30432	2.1108	2.1
30433	2.45763	2.1
30434	2.00986	2.1
30435	4.56843	2.1
30436	0.946133	0.05
30437	0.946133	0.05
30438	0.946132	0.05
30439	0.946132	0.05
30440	0.946132	0.05
30441	0.946132	0.05
30442	0.946132	0.05
30443	0.946132	0.05
30444	0.946132	0.05
30445	0.946132	0.05
30446	0.946133	0.05
30447	0.946133	0.05

30448	1.45927	0.465568
30449	1.25133	1.37477
30450	1.00082	1.07707
30451	1.41689	1.14634
30452	1.0432	0.629169
30453	1.45927	1.16489
30454	1.41689	0.739541
30455	1.29579	1.68101
30456	1.34874	1.05564
30457	1.25133	1.41514
30458	2.76563	1.94966
30459	0.675555	1.58127
30460	0.674371	1.35883
30461	0.625664	1.23801
30462	0.523133	1.34244
30463	0.676509	1.49904
30464	0.676509	1.70337
30465	0.62471	1.3309
30466	0.62471	0.860416
30467	0.675556	0.899677
30468	0.523132	0.532379
30469	0.674373	0.546415
30470	0.625663	1.00093
30471	1.89226	0.926035
30472	1.89226	0.936879
30473	1.89226	0.90363
30474	1.89226	0.951432
30475	3.78453	1.04278
30476	0.851517	1.29677
30477	0.946133	0.992254
30478	0.946132	1.02749
30479	0.946132	1.27485
30480	0.946133	0.93039
30481	0.851517	1.94168
30482	0.946132	0.992996
30483	0.946132	0.951843
30484	0.851516	1.02346
30485	0.851516	0.940939
30486	0.946133	1.5598
30487	0.946133	0.985246
30488	0.569995	1.03623
30489	0.816895	1.1412
30490	1.33893	1.18632
30491	1.18795	1.11192
30492	1.18795	1.0557
30493	0.569995	1.14362
30494	0.816895	1.06839
30495	1.33893	1.21326
30496	0.859572	1.29838
30497	0.859572	1.2093
30498	1.04936	1.08243
30499	1.04936	0.97234
30500	1.89226	2.1
30501	1.89226	2.1
30502	2.55618	2.1
30503	2.55618	2.1
30504	2.66983	2.1
30505	2.00591	2.1

30506	1.89226	0.200543
30507	2.64586	2.1
30508	2.83331	2.1
30509	2.83331	0.660327
30510	1.89226	2.1
30511	1.89226	0.0520541
30512	2.64586	2.1
30513	2.0815	2.1
30514	1.99985	2.1
30515	1.94005	2.1
30516	2.83332	2.1
30517	2.74859	2.1
30518	1.98061	2.1
30519	1.68356	2.1
30520	1.83879	2.1
30521	1.55987	2.1
30522	3.54048	2.1
30523	1.68356	2.1
30524	1.20969	2.1
30525	1.20969	2.1
30526	1.30751	2.1
30527	1.55987	2.1
30528	1.65769	2.1
30529	1.83879	2.1
30530	1.55987	2.1
30531	1.68443	2.1
30532	1.4449	2.1
30533	3.2443	2.1
30534	0.660368	2.1
30535	0.660368	2.1
30536	0.666922	2.1
30537	0.666922	2.1
30538	0.560377	2.1
30539	0.604842	2.1
30540	0.779935	2.1
30541	0.722448	2.1
30542	0.722446	2.1
30543	0.560377	2.1
30544	0.604844	2.1
30545	0.779934	2.1
30546	1.89227	2.1
30547	1.89227	2.1
30548	1.89227	2.1
30549	1.89227	2.1
30550	3.78453	2.1
30551	1.42896	2.1
30552	1.23037	2.1
30553	1.42896	2.1
30554	1.474 2.1	
30555	1.2016	2.1
30556	1.24665	2.1
30557	1.89226	2.1
30558	2.7188	2.1
30559	1.89226	2.1
30560	2.70702	2.1
30561	4.61106	2.1
30562	1.89226	2.1
30563	1.89226	2.1

30564	1.89226	2.1
30565	1.89226	2.1
30566	3.78453	2.1
30567	0.644603	2.1
30568	0.644603	2.1
30569	0.648272	2.1
30570	0.648272	2.1
30571	0.585644	2.1
30572	0.600802	2.1
30573	0.692073	2.1
30574	0.714478	2.1
30575	0.585644	2.1
30576	0.692073	2.1
30577	0.714478	2.1
30578	0.600802	2.1
30579	1.35351	2.1
30580	1.35351	2.1
30581	0.946131	2.1
30582	0.946131	2.1
30583	1.34392	2.1
30584	1.35351	2.1
30585	0.946132	2.1
30586	0.946132	2.1
30587	0.946132	2.1
30588	1.34392	2.1
30589	0.946132	2.1
30590	1.35351	2.1
30591	1.87609	2.1
30592	1.79155	2.1
30593	1.79155	2.1
30594	1.89226	2.1
30595	1.89226	2.1
30596	1.99211	2.1
30597	1.89226	2.1
30598	1.71706	2.1
30599	1.71706	2.1
30600	1.79155	2.1
30601	1.89226	2.1
30602	1.96675	2.1
30603	1.46869	2.1
30604	1.89226	2.1
30605	1.89226	2.1
30606	1.89226	2.1
30607	1.5001	2.1
30608	1.5001	2.1
30609	1.89226	2.1
30610	1.89226	2.1
30611	1.89226	2.1
30612	1.89226	2.1
30613	1.89226	2.1
30614	1.89226	2.1
30615	1.21408	2.1
30616	2.23563	2.1
30617	2.34021	2.1
30618	2.34021	2.1
30619	1.17285	2.1
30620	1.17285	2.1
30621	1.89226	2.1

30622	1.89226	2.1
30623	1.47937	2.1
30624	1.89226	2.1
30625	1.14822	2.1
30626	1.46869	2.1
30627	1.89226	2.1
30628	1.89226	2.1
30629	1.89226	2.1
30630	1.89226	2.1
30631	1.89226	2.1
30632	1.89226	2.1
30633	1.25716	2.1
30634	2.34021	2.1
30635	2.4542	2.1
30636	2.4542	2.1
30637	1.21408	2.1
30638	1.21408	2.1
30639	1.66824	2.1
30640	2.44883	2.1
30641	1.93003	2.1
30642	2.24795	2.1
30643	3.88907	2.1
30644	1.89319	2.1
30645	2.24795	2.1
30646	2.82724	2.1
30647	2.31318	2.1
30648	2.30868	2.1
30649	1.93003	2.1
30650	1.89226	2.018
30651	1.42589	2.1
30652	1.42589	2.1
30653	1.80501	2.1
30654	1.89226	2.1
30655	2.27137	2.1
30656	1.21067	0.082621
30657	2.39166	0.456703
30658	1.73577	0.291641
30659	1.73577	0.297902
30660	1.53584	0.126881
30661	1.53584	0.110639
30662	1.35025	2.05481
30663	2.0599	1.16894
30664	1.51084	1.05429
30665	2.27218	0.465221
30666	3.57074	1.12027
30667	2.0599	1.07218
30668	2.51818	1.85923
30669	2.27218	0.24774
30670	2.66532	0.947299
30671	4.72522	0.644125
30672	1.67115	1.76087
30673	2.27218	1.09115
30674	2.39605	1.02661
30675	2.39605	1.01062
30676	1.51084	1.26521
30677	1.51084	1.30589
30678	2.27218	0.98191
30679	2.66532	1.8002

30680	2.39605	0.225107
30681	2.76829	0.440503
30682	5.06137	1.01899
30683	2.08684	1.03176
30684	2.36033	1.38164
30685	1.89224	0.95705
30686	1.89224	1.30188
30687	4.25257	1.01497
30688	2.41473	0.0941132
30689	1.00307	0.05
30690	2.01365	0.0614981
30691	1.00307	0.11378
30692	3.05113	0.05
30693	2.04056	0.0925803
30694	1.11099	2.1
30695	2.25103	2.1
30696	2.22227	2.1
30697	2.22227	2.1
30698	1.14431	2.1
30699	1.14431	2.1
30700	1.07485	2.1
30701	2.22229	2.1
30702	2.19163	2.1
30703	2.19163	2.1
30704	1.111 2.1	
30705	1.111 2.1	
30706	1.0547	2.1
30707	1.19886	2.1
30708	0.946131	2.1
30709	0.946131	2.1
30710	0.946131	2.1
30711	0.946131	2.1
30712	1.19886	2.1
30713	1.19886	2.1
30714	1.0547	2.1
30715	0.946131	2.1
30716	0.946132	2.1
30717	1.19886	2.1
30718	1.89417	2.1
30719	2.64424	0.05
30720	1.76585	2.1
30721	2.47695	0.620614
30722	4.37112	1.33546
30723	1.89226	2.1
30724	1.89226	1.08078
30725	1.89226	2.1
30726	1.89226	0.56607
30727	3.78453	2.1
30728	0.930281	2.1
30729	1.0547	2.1
30730	0.946131	2.1
30731	0.946131	2.1
30732	0.930309	2.1
30733	0.930309	2.1
30734	1.07052	2.1
30735	1.07052	2.1
30736	0.93028	2.1
30737	0.946131	2.1

30738	1.0547	2.1
30739	0.946131	2.1
30740	1.00015	0.05
30741	1.00015	2.1
30742	1.01918	2.1
30743	1.01918	0.291743
30744	1.17437	0.385094
30745	1.23847	0.05
30746	0.882926	2.1
30747	0.780859	2.1
30748	0.780859	0.117582
30749	1.17438	0.721148
30750	0.882925	1.01395
30751	1.23848	1.0565
30752	1.89226	2.1
30753	1.89226	0.495536
30754	1.89226	1.11591
30755	1.89226	2.1
30756	1.89226	1.08157
30757	1.89226	2.1
30758	1.22083	2.1
30759	1.29579	2.1
30760	1.26236	2.1
30761	1.06191	2.1
30762	1.42128	2.1
30763	1.22083	2.1
30764	1.42128	2.1
30765	1.45806	2.1
30766	1.43502	2.1
30767	1.29579	2.1
30768	1.5605	2.1
30769	1.42128	2.1
30770	1.02813	2.1
30771	1.12121	2.1
30772	1.40557	2.1
30773	1.35298	2.1
30774	1.1314	2.1
30775	1.28679	2.1
30776	1.1715	2.1
30777	1.1715	2.1
30778	0.975656	2.1
30779	1.24908	2.1
30780	0.98956	2.1
30781	1.2681	2.1
30782	0.946132	2.1
30783	0.946132	2.1
30784	1.31889	2.1
30785	1.31889	2.1
30786	1.16546	2.1
30787	1.31889	2.1
30788	0.946133	2.1
30789	0.946133	2.1
30790	0.946133	2.1
30791	1.31889	2.1
30792	0.946132	2.1
30793	1.16546	2.1
30794	2.51518	0.05
30795	2.37584	0.05

30796	1.11712	0.05
30797	1.31242	0.05
30798	3.68826	0.05
30799	2.51518	0.05
30800	1.26311	0.05
30801	2.48977	0.05
30802	2.48977	0.05
30803	1.31242	0.05
30804	1.31242	0.05
30805	1.89226	0.744049
30806	1.89226	1.37828
30807	1.89226	0.0631658
30808	1.89226	0.912085
30809	3.78452	0.542265
30810	2.04737	0.360791
30811	1.77122	1.37412
30812	1.94498	0.05
30813	1.65174	1.40011
30814	3.71621	0.240591
30815	1.41077	2.1
30816	1.29729	2.1
30817	1.77123	0.618399
30818	1.77123	2.1
30819	1.65175	1.34876
30820	1.29129	1.78824
30821	1.89226	0.300494
30822	1.89226	2.1
30823	1.89226	2.1
30824	1.89226	0.165886
30825	1.89226	2.1
30826	1.89226	1.83535
30827	1.89226	0.609597
30828	1.89226	0.912648
30829	1.89226	0.05
30830	1.89226	0.845805
30831	3.78451	0.0889548
30832	1.94498	0.0649122
30833	1.65174	1.77612
30834	1.7968	0.0605493
30835	1.49831	0.397728
30836	3.44329	0.154742
30837	1.49831	0.103435
30838	1.29729	2.1
30839	1.49831	2.1
30840	1.65175	0.425136
30841	1.16858	2.1
30842	1.32201	1.69352
30843	1.89226	0.0768601
30844	1.89226	2.1
30845	1.89226	0.199799
30846	1.89226	2.1
30847	3.78452	2.1
30848	1.89226	0.05
30849	1.89226	0.05
30850	1.89226	0.05
30851	1.89226	0.05
30852	1.89226	0.05
30853	1.89226	0.05

30854	1.65324	0.916219
30855	1.26409	0.05
30856	1.42491	0.05
30857	1.42491	0.936242
30858	1.52268	0.05
30859	1.52268	0.93519
30860	1.52268	1.81615
30861	1.26409	0.756715
30862	1.32697	0.274939
30863	1.05724	0.05
30864	2.57991	0.742272
30865	1.89226	0.0834483
30866	1.96548	0.0906807
30867	2.0178	0.277462
30868	1.96548	0.102937
30869	1.94458	0.0730097
30870	1.89226	0.05
30871	1.99366	0.112469
30872	2.0178	0.138597
30873	1.89226	0.280787
30874	2.0178	0.162658
30875	1.75999	0.0806309
30876	1.89226	0.0581786
30877	0.946126	2.1
30878	0.946126	2.1
30879	1.28228	2.1
30880	1.19444	2.1
30881	0.946127	2.1
30882	0.946127	2.1
30883	1.19444	2.1
30884	1.19444	2.1
30885	0.946128	2.1
30886	1.19444	2.1
30887	1.28228	2.1
30888	0.946128	2.1
30889	1.89226	2.1
30890	1.89226	2.1
30891	1.89226	2.1
30892	1.89226	2.1
30893	3.78452	2.1
30894	2.14104	2.1
30895	1.89226	2.1
30896	2.19948	2.1
30897	1.89226	2.1
30898	1.89226	2.1
30899	2.19948	2.1
30900	1.89226	2.1
30901	2.38888	2.1
30902	2.23932	2.1
30903	2.38888	2.1
30904	1.74269	2.1
30905	1.89226	2.1
30906	1.89226	2.1
30907	1.89226	2.1
30908	1.89226	2.1
30909	1.89226	2.1
30910	3.78452	2.1
30911	0.946132	2.1

30912	0.946132	2.1
30913	1.07052	2.1
30914	1.07052	2.1
30915	0.946132	2.1
30916	0.946132	2.1
30917	1.05132	2.1
30918	1.07052	2.1
30919	1.05132	2.1
30920	0.946132	2.1
30921	1.07052	2.1
30922	0.946133	2.1
30923	2.40171	2.1
30924	1.89226	2.1
30925	1.89226	2.1
30926	1.89226	2.1
30927	2.42723	2.1
30928	2.42723	2.1
30929	1.89226	2.1
30930	1.89226	2.06084
30931	1.89226	2.1
30932	1.89226	2.1
30933	3.78452	2.1
30934	1.63661	2.1
30935	2.3532	1.66766
30936	2.33287	1.94289
30937	2.33287	2.1
30938	1.64158	1.5118
30939	1.64158	2.1
30940	2.38918	2.1
30941	1.89226	2.1
30942	1.89226	2.1
30943	1.89226	2.1
30944	2.40171	2.1
30945	2.40171	2.1
30946	1.89226	2.1
30947	1.89226	2.1
30948	1.89226	2.1
30949	1.89226	2.1
30950	1.89226	2.1
30951	1.89226	2.1
30952	1.64158	2.1
30953	2.3532	2.1
30954	1.6488	2.1
30955	2.37364	2.1
30956	4.002 2.1	
30957	2.54752	0.245768
30958	1.42445	0.354382
30959	1.86697	0.343078
30960	1.86697	0.213288
30961	2.06666	0.582244
30962	2.06666	0.406389
30963	2.50872	0.070577
30964	2.06666	0.141997
30965	1.90439	0.103665
30966	1.51835	0.0966944
30967	3.97105	0.0971401
30968	0.755161	0.112115
30969	0.755161	0.0995632

30970	0.793994	0.0849344
30971	0.793994	0.119144
30972	0.712225	0.0817128
30973	0.515826	0.0718378
30974	1.03333	0.14073
30975	0.759175	0.124147
30976	0.759173	0.102768
30977	0.515827	0.05
30978	0.712226	0.0648898
30979	1.03333	0.108719
30980	1.69633	0.05
30981	1.89226	0.05
30982	1.62477	0.05
30983	1.89226	0.05
30984	1.62477	0.05
30985	1.89226	0.05
30986	1.89226	0.170541
30987	1.89226	0.429505
30988	1.89226	0.414063
30989	1.89226	0.281019
30990	3.78452	0.304601
30991	2.28622	0.05
30992	1.09667	0.05
30993	1.17506	0.05
30994	2.24411	0.05
30995	1.17506	0.05
30996	2.24411	0.0721254
30997	2.24741	2.1
30998	1.70303	2.1
30999	2.49168	2.1
31000	1.57936	2.1
31001	2.53729	2.1
31002	1.70303	2.1
31003	2.24741	2.1
31004	2.47557	2.1
31005	1.89227	2.1
31006	1.89227	2.1
31007	4.36784	2.1
31008	2.27016	0.05
31009	1.10476	0.05
31010	1.44103	0.05
31011	1.10476	0.05
31012	2.26791	0.05
31013	1.93165	0.05
31014	2.27016	0.05
31015	1.89226	0.05
31016	1.89226	0.05
31017	2.4065	0.05
31018	4.29876	0.05
31019	2.27016	0.05
31020	1.44103	0.05
31021	1.53336	0.05
31022	2.4065	0.05
31023	3.84753	0.05
31024	2.7542	0.0786353
31025	1.89226	0.197363
31026	2.35027	0.137394
31027	1.89226	0.160172

31028	2.35027	0.122496
31029	1.89226	0.145117
31030	2.3301	2.1
31031	2.36071	2.1
31032	2.07629	2.1
31033	2.01105	2.1
31034	4.37176	2.1
31035	1.86512	2.1
31036	2.01105	2.1
31037	2.01105	2.1
31038	2.3301	2.1
31039	1.57459	2.1
31040	1.89364	2.1
31041	2.52671	2.1
31042	2.01105	2.1
31043	1.78251	2.1
31044	1.70302	2.1
31045	3.98362	2.1
31046	1.227 2.1	
31047	2.01105	2.1
31048	1.45869	2.1
31049	1.41713	2.1
31050	1.70302	2.1
31051	1.64994	2.1
31052	2.00989	2.1
31053	1.28938	2.1
31054	1.8769	2.1
31055	1.20969	2.1
31056	3.21958	2.1
31057	1.8769	2.1
31058	1.15864	2.1
31059	1.15864	2.1
31060	1.20969	2.1
31061	1.77353	2.1
31062	1.82458	2.1
31063	1.7986	2.1
31064	2.48717	2.1
31065	2.50161	2.1
31066	1.75869	2.1
31067	4.30021	2.1
31068	2.50161	2.1
31069	1.75251	2.1
31070	1.81678	2.1
31071	1.75869	2.1
31072	2.5597	2.1
31073	2.50161	2.1
31074	1.91334	0.36063
31075	1.85439	0.968924
31076	1.91334	0.521198
31077	1.70303	0.947743
31078	1.70303	0.345631
31079	1.49692	0.81797
31080	2.39986	0.05
31081	1.89226	0.05
31082	1.89226	0.05
31083	2.50756	0.05
31084	4.39982	0.05
31085	2.39986	0.05

31086	1.70303	0.05
31087	1.75564	0.05
31088	1.75904	0.05
31089	2.50272	0.076638
31090	2.50756	0.05
31091	1.89226	0.05
31092	2.50756	0.05
31093	2.47857	0.05
31094	2.50756	0.0775146
31095	1.86327	0.05
31096	1.89226	0.05
31097	1.22241	0.0780039
31098	1.20827	0.071634
31099	0.772416	0.0683023
31100	0.555208	0.0749367
31101	1.004	0.0781496
31102	1.004	0.05
31103	0.93504	0.201334
31104	0.744364	0.05
31105	1.2089	0.0532898
31106	0.654935	0.05
31107	0.773927	0.05
31108	1.03618	0.0635974
31109	1.89227	0.05
31110	1.89227	0.05
31111	1.89227	0.05
31112	1.89227	0.05
31113	3.78453	0.05
31114	1.89226	0.05
31115	1.89226	0.05
31116	1.89226	0.05
31117	1.89226	0.05
31118	3.78452	0.05
31119	1.82643	0.05
31120	2.7084	0.05
31121	2.12654	0.05
31122	2.65614	0.05
31123	1.82643	0.05
31124	2.35603	0.05
31125	1.29385	0.05
31126	0.946133	0.05
31127	1.05492	0.05
31128	0.946132	0.05
31129	1.05492	0.05
31130	1.29385	0.05
31131	0.946133	0.05
31132	0.946133	0.05
31133	1.29385	0.05
31134	1.29385	0.05
31135	0.946132	0.05
31136	0.946132	0.05
31137	0.946132	0.05
31138	1.27329	0.05
31139	1.29385	0.05
31140	0.946129	0.05
31141	1.29385	0.05
31142	1.27328	0.05
31143	0.946131	0.05

31144	0.946131	0.05
31145	1.27328	0.05
31146	1.27328	0.05
31147	0.94613	0.05
31148	0.94613	0.05
31149	1.89226	1.87725
31150	1.89226	2.0647
31151	1.89226	1.00234
31152	1.89226	1.2511
31153	3.78453	1.40817
31154	1.89226	1.54366
31155	1.89226	2.01652
31156	1.89226	0.965729
31157	1.89226	1.58421
31158	3.78453	1.15293
31159	1.21488	1.961
31160	1.14605	2.1
31161	1.62974	1.38623
31162	1.52399	1.18365
31163	2.84463	2.01038
31164	0.577078	1.27228
31165	0.81251	2.1
31166	0.821003	1.58498
31167	0.543413	2.1
31168	0.683929	1.30628
31169	0.722433	2.1
31170	0.730674	2.1
31171	0.730674	1.19657
31172	0.857766	1.69461
31173	0.862119	1.27335
31174	0.600938	2.08115
31175	0.596094	1.65317
31176	1.89226	1.13397
31177	1.89226	1.79517
31178	1.89226	0.938006
31179	1.89226	1.35246
31180	3.78453	1.13021
31181	1.89226	1.06401
31182	1.89226	2.1
31183	1.89226	1.05045
31184	1.89226	1.18681
31185	3.78453	1.48995
31186	1.62974	2.09046
31187	1.56958	2.1
31188	2.2362	1.07301
31189	2.11424	1.48245
31190	3.86595	1.50133
31191	2.2362	1.16475
31192	1.85414	2.1
31193	2.28211	0.935492
31194	1.78367	1.02824
31195	2.2362	1.93445
31196	1.73256	2.1
31197	0.851539	1.13896
31198	0.851539	0.847474
31199	1.14454	1.0619
31200	0.938988	2.01864
31201	0.851485	1.14452

31202	0.920593	0.882219
31203	0.851541	0.0552166
31204	0.851541	0.950568
31205	1.04073	0.841707
31206	1.16342	0.876413
31207	0.825315	1.03621
31208	0.825316	0.877816
31209	1.89226	2.1
31210	1.89226	2.1
31211	1.89226	2.1
31212	1.89226	2.1
31213	3.78453	2.1
31214	2.57016	2.1
31215	1.74789	2.1
31216	2.57016	2.1
31217	2.522	2.1
31218	1.61727	2.1
31219	1.56911	2.1
31220	1.28075	2.1
31221	1.406	2.1
31222	0.946131	2.1
31223	0.946131	2.1
31224	1.28075	2.1
31225	1.00333	2.1
31226	0.94613	2.1
31227	0.94613	2.1
31228	1.041	2.1
31229	1.041	2.1
31230	0.943484	2.1
31231	1.27717	2.1
31232	1.89226	2.1
31233	1.89226	2.1
31234	1.89226	2.1
31235	1.89226	2.1
31236	1.89226	2.1
31237	1.89226	2.1
31238	2.57016	2.1
31239	1.61727	2.1
31240	2.74196	2.1
31241	1.59094	2.1
31242	4.16109	2.1
31243	1.89226	2.1
31244	2.0815	2.1
31245	2.2034	2.1
31246	1.82493	2.1
31247	1.87847	2.1
31248	2.16142	2.1
31249	1.89226	2.1
31250	1.89226	2.1
31251	1.89226	2.1
31252	1.89226	2.1
31253	3.78453	2.1
31254	0.985252	2.1
31255	0.985252	2.1
31256	0.946132	2.1
31257	0.946132	2.1
31258	1.0655	2.1
31259	0.985255	2.1

31260	0.94613	2.1
31261	0.94613	2.1
31262	0.94613	2.1
31263	1.0655	2.1
31264	0.94613	2.1
31265	0.985255	2.1
31266	1.89226	2.1
31267	1.89226	2.1
31268	1.89226	2.1
31269	1.89226	2.1
31270	3.78453	2.1
31271	1.06549	2.1
31272	1.06549	2.1
31273	0.946133	2.1
31274	0.946133	2.1
31275	1.16834	2.1
31276	1.0655	2.1
31277	0.946131	2.1
31278	0.946131	2.1
31279	0.946131	2.1
31280	1.16834	2.1
31281	0.94613	2.1
31282	1.0655	2.1
31283	0.966631	2.1
31284	1.07349	2.1
31285	0.946131	2.1
31286	0.946131	2.1
31287	1.07349	2.1
31288	1.07349	2.1
31289	0.946129	2.1
31290	0.946129	2.1
31291	1.07349	2.1
31292	0.946131	2.1
31293	0.94613	2.1
31294	0.966631	2.1
31295	2.38055	0.255965
31296	1.89226	1.52522
31297	1.89226	1.21227
31298	1.46724	0.05
31299	3.35949	0.367613
31300	1.42249	2.1
31301	2.05499	2.1
31302	1.39708	2.1
31303	2.09033	2.1
31304	3.45207	2.1
31305	2.05499	2.1
31306	2.57425	2.1
31307	2.51287	2.1
31308	2.47753	2.1
31309	2.09033	2.1
31310	2.05499	2.1
31311	1.39327	2.1
31312	2.09033	2.1
31313	2.16217	2.1
31314	2.16217	2.1
31315	1.39708	2.1
31316	1.39708	2.1
31317	2.16217	2.1

31318	2.57426	2.1
31319	2.73006	2.1
31320	2.73006	2.1
31321	2.09033	2.1
31322	2.09033	2.1
31323	1.89226	0.05
31324	1.89226	0.05
31325	1.89226	0.05
31326	1.89226	0.05
31327	3.78452	0.05
31328	0.838749	0.05
31329	0.838749	0.05
31330	0.85824	0.05
31331	0.85824	0.05
31332	0.544644	0.05
31333	0.854718	0.05
31334	0.842271	0.05
31335	1.19141	0.05
31336	0.842272	0.05
31337	0.544643	0.05
31338	0.854718	0.05
31339	1.19141	0.05
31340	1.34214	0.05
31341	1.34214	0.05
31342	0.893236	0.05
31343	0.893236	0.05
31344	0.893265	0.05
31345	1.28924	0.05
31346	0.946133	0.05
31347	0.946133	0.05
31348	0.946132	0.05
31349	0.893265	0.05
31350	0.946133	0.05
31351	1.28924	0.05
31352	2.7013	0.05
31353	1.89226	0.05
31354	1.9216	0.05
31355	1.9216	0.05
31356	1.89226	0.05
31357	1.89226	0.05
31358	1.26397	2.1
31359	1.52995	2.1
31360	1.30116	2.1
31361	1.04084	2.1
31362	2.57079	2.1
31363	1.47013	2.1
31364	2.17459	2.1
31365	2.10668	2.1
31366	1.38328	2.1
31367	3.55787	2.1
31368	2.55006	2.1
31369	2.17459	2.1
31370	2.10668	2.1
31371	2.49168	2.1
31372	4.66626	2.1
31373	0.613622	2.1
31374	0.691639	2.1
31375	1.00105	2.1

31376	1.05334	2.1
31377	0.839874	2.1
31378	0.839874	2.1
31379	0.852814	2.1
31380	0.852814	2.1
31381	0.613622	2.1
31382	1.00105	2.1
31383	1.05334	2.1
31384	0.691639	2.1
31385	1.00105	2.1
31386	1.05334	2.1
31387	1.20844	2.1
31388	1.24584	2.1
31389	1.12717	2.1
31390	1.12717	2.1
31391	1.13462	2.1
31392	1.13462	2.1
31393	1.20845	2.1
31394	1.00105	2.1
31395	1.05334	2.1
31396	1.24584	2.1
31397	2.22558	2.1
31398	1.85981	2.1
31399	2.18943	1.71152
31400	1.89142	0.0786644
31401	4.0854	1.46317
31402	2.02641	2.1
31403	1.85981	1.93513
31404	2.18943	1.66873
31405	1.67346	1.56248
31406	3.88622	1.45486
31407	1.85981	0.05
31408	1.10736	0.05
31409	1.27421	0.05
31410	1.67346	0.05
31411	1.27421	0.05
31412	1.67346	1.24721
31413	1.85981	0.0959889
31414	1.27362	0.368147
31415	1.89142	0.202055
31416	1.27421	0.05
31417	3.13343	0.05
31418	1.81893	0.05
31419	1.64985	0.05
31420	1.23477	0.05
31421	2.25405	0.05
31422	3.48882	0.05
31423	2.23933	0.05
31424	1.89226	0.05
31425	1.89226	0.05
31426	2.78053	0.05
31427	1.89226	0.05
31428	2.78053	0.05
31429	0.946131	0.05
31430	0.946131	0.05
31431	0.946132	0.05
31432	0.946132	0.05
31433	0.946131	0.05

31434	0.946131	0.05
31435	0.946132	0.05
31436	0.946132	0.05
31437	0.946131	0.05
31438	0.946131	0.05
31439	0.946132	0.05
31440	0.946132	0.05
31441	1.89226	2.1
31442	2.48596	2.1
31443	1.89226	2.1
31444	2.51506	2.1
31445	4.40732	2.1
31446	2.48596	2.1
31447	1.63823	2.1
31448	2.51507	2.1
31449	1.67687	2.1
31450	4.1533	2.1
31451	1.89226	2.1
31452	2.51506	2.1
31453	1.92044	2.1
31454	1.89226	2.1
31455	2.54325	2.1
31456	2.51506	2.1
31457	2.51507	2.1
31458	1.5704	2.1
31459	1.70505	2.1
31460	1.67687	2.1
31461	2.54325	2.1
31462	2.51507	2.1
31463	2.0815	2.1
31464	1.70303	2.1
31465	1.70303	2.1
31466	1.80354	2.1
31467	1.98172	2.1
31468	2.09868	2.1
31469	1.27944	2.1
31470	1.79028	2.1
31471	1.13179	2.1
31472	1.65533	2.1
31473	2.93477	2.1
31474	1.89226	2.1
31475	1.89226	2.1
31476	1.89226	2.1
31477	1.89226	2.1
31478	1.89226	2.1
31479	1.89226	2.1
31480	1.98171	2.1
31481	1.89226	2.1
31482	1.89226	2.1
31483	1.89226	2.1
31484	1.65798	2.1
31485	1.65797	2.1
31486	1.00133	2.1
31487	1.65532	2.1
31488	1.53278	2.1
31489	1.53278	2.1
31490	1.13179	2.1
31491	1.13179	2.1

31492	0.810946	2.1
31493	0.810946	2.1
31494	0.810944	2.1
31495	0.810944	2.1
31496	0.810946	2.1
31497	0.810946	2.1
31498	1.89226	2.1
31499	1.35169	2.1
31500	1.08132	2.1
31501	1.08132	2.1
31502	1.08132	2.1
31503	1.99528	0.05
31504	1.70303	0.05
31505	2.08152	0.05
31506	1.70303	0.05
31507	2.33877	0.05
31508	1.93967	0.05
31509	1.89226	0.05
31510	1.89226	0.05
31511	1.89226	0.05
31512	1.89226	0.055867
31513	3.78452	0.05
31514	1.26259	0.05
31515	2.17205	0.05
31516	2.32468	0.05
31517	2.32468	0.05
31518	1.28055	0.05
31519	1.28055	0.05
31520	0.946131	0.05
31521	0.946131	0.05
31522	0.997642	0.05
31523	0.997642	0.05
31524	0.946131	0.05
31525	0.946131	0.05
31526	0.989846	0.05
31527	0.997642	0.05
31528	0.946132	0.05
31529	0.989846	0.05
31530	0.997641	0.05
31531	0.946131	0.05
31532	1.89226	0.05
31533	1.89226	0.054133
31534	1.89226	0.05
31535	1.89226	0.0541974
31536	3.78452	0.05
31537	1.28055	0.05
31538	2.17205	0.05
31539	1.14305	0.05
31540	1.92737	0.05
31541	3.3151	0.05
31542	0.954908	0.05
31543	0.9705	0.05
31544	0.946131	0.05
31545	0.946131	0.05
31546	0.946131	0.05
31547	0.946131	0.05
31548	0.9705	0.05
31549	0.9705	0.05

31550	0.954908	0.05
31551	0.946131	0.05
31552	0.946131	0.05
31553	0.9705	0.05
31554	0.835187	0.05
31555	0.954896	0.05
31556	0.946118	0.05
31557	0.946118	0.05
31558	0.835186	0.05
31559	0.835186	0.05
31560	1.06583	0.05
31561	1.06583	0.05
31562	0.835198	0.05
31563	0.946131	0.05
31564	0.954883	0.05
31565	0.946106	0.05
31566	1.89227	2.1
31567	1.89227	2.1
31568	1.89227	2.1
31569	1.89227	2.1
31570	1.89227	2.1
31571	1.89227	2.1
31572	0.885265	2.1
31573	0.885265	2.1
31574	0.871467	2.1
31575	0.871467	2.1
31576	1.11883	2.1
31577	0.787875	2.1
31578	0.996487	2.1
31579	0.637907	2.1
31580	0.996487	2.1
31581	1.11883	2.1
31582	0.787875	2.1
31583	0.637906	2.1
31584	1.89226	2.1
31585	2.39258	2.1
31586	2.39258	2.1
31587	2.54729	2.1
31588	1.89226	2.1
31589	2.04697	2.1
31590	1.89226	2.1
31591	2.0815	2.1
31592	2.0815	2.1
31593	2.48371	2.1
31594	2.0815	2.1
31595	2.51469	2.1
31596	1.00035	0.05
31597	1.55755	0.05
31598	1.03534	0.05
31599	1.61333	0.05
31600	2.59289	0.05
31601	1.55755	0.05
31602	1.85217	2.1
31603	1.61333	0.05
31604	1.92875	0.931676
31605	3.48629	0.05
31606	1.09962	0.404191
31607	1.61334	0.05

31608	1.70868	0.808307
31609	1.70868	0.682645
31610	1.03535	1.68996
31611	1.03535	0.256302
31612	1.61334	0.05
31613	1.92876	1.40396
31614	1.70868	0.05
31615	2.05274	2.1
31616	3.63744	0.05
31617	1.82233	0.0771138
31618	2.05768	0.69369
31619	1.89226	0.599823
31620	1.65406	0.135429
31621	1.89226	0.589859
31622	1.65406	0.214595
31623	2.26047	0.123701
31624	2.0815	1.05324
31625	1.89226	0.845657
31626	2.01156	0.211717
31627	1.89226	0.736345
31628	2.01156	0.246515
31629	1.89226	2.1
31630	1.89226	2.1
31631	1.89226	2.1
31632	1.89226	2.1
31633	3.78453	2.1
31634	2.70055	2.1
31635	2.28367	2.1
31636	2.56456	2.1
31637	2.33565	2.1
31638	4.84823	2.1
31639	2.28367	2.1
31640	1.5791	2.1
31641	1.96966	2.1
31642	1.09506	2.1
31643	3.37873	2.1
31644	1.89226	2.1
31645	1.89226	2.1
31646	1.89226	2.1
31647	1.89226	2.1
31648	3.78452	2.1
31649	0.94612	2.1
31650	0.94612	2.1
31651	0.946119	2.1
31652	0.946119	2.1
31653	0.946119	2.1
31654	0.946119	2.1
31655	0.946119	2.1
31656	0.946119	2.1
31657	0.946132	2.1
31658	0.946132	2.1
31659	0.946107	2.1
31660	0.946107	2.1
31661	2.56452	2.1
31662	1.57307	2.1
31663	2.10299	2.1
31664	2.1115	2.1
31665	2.52196	2.1

31666	2.52196	2.1
31667	1.89226	2.1
31668	1.89226	2.1
31669	1.89226	2.1
31670	1.89226	2.1
31671	3.78452	2.1
31672	2.37665	2.1
31673	1.89226	2.1
31674	2.28084	2.1
31675	1.89226	2.1
31676	4.26891	2.1
31677	0.946129	2.1
31678	0.946129	2.1
31679	0.946133	2.1
31680	0.946133	2.1
31681	0.946131	2.1
31682	0.946131	2.1
31683	0.946131	2.1
31684	0.946131	2.1
31685	0.946131	2.1
31686	0.946131	2.1
31687	0.946131	2.1
31688	0.946131	2.1
31689	1.7207	0.05
31690	1.65815	0.05
31691	1.33593	0.05
31692	1.31508	0.05
31693	2.99408	0.05
31694	0.676047	0.05
31695	0.676047	0.05
31696	0.684362	0.05
31697	0.684362	0.05
31698	0.547994	0.05
31699	0.702871	0.05
31700	0.829076	0.05
31701	0.657538	0.05
31702	0.657538	0.05
31703	0.547994	0.05
31704	0.829077	0.05
31705	0.702871	0.05
31706	1.9518	2.1
31707	1.30648	2.1
31708	1.28846	2.1
31709	1.92033	2.1
31710	3.22681	2.1
31711	1.89226	2.1
31712	1.89226	2.1
31713	1.89226	2.1
31714	1.89226	2.1
31715	3.78453	2.1
31716	0.946128	2.1
31717	0.946128	2.1
31718	1.10113	2.1
31719	1.09587	2.1
31720	0.946131	2.1
31721	0.946131	2.1
31722	1.09587	2.1
31723	1.09587	2.1

31724	0.946131	2.1
31725	1.09587	2.1
31726	1.10112	2.1
31727	0.94613	2.1
31728	1.26923	2.1
31729	1.92033	2.1
31730	1.28846	2.1
31731	1.28846	2.1
31732	1.89452	2.1
31733	1.89452	2.1
31734	1.89226	2.1
31735	1.89226	2.1
31736	1.89226	2.1
31737	1.89226	2.1
31738	3.78453	2.1
31739	1.89226	2.1
31740	2.19174	2.1
31741	2.15616	2.1
31742	2.19174	2.1
31743	1.85669	2.1
31744	1.89226	2.1
31745	1.89226	2.1
31746	1.89226	2.1
31747	1.89226	2.1
31748	1.89226	2.1
31749	3.78452	2.1
31750	1.89226	2.1
31751	1.89226	2.1
31752	1.89226	2.1
31753	1.89226	2.1
31754	3.78452	2.1
31755	1.51223	2.1
31756	1.41562	2.1
31757	1.5139	2.1
31758	1.42284	2.1
31759	1.6033	2.1
31760	1.51223	2.1
31761	1.51223	2.1
31762	1.25953	2.1
31763	1.38085	2.1
31764	1.6033	2.1
31765	2.89309	2.1
31766	0.946131	2.1
31767	0.946131	2.1
31768	0.946131	2.1
31769	0.946131	2.1
31770	0.946132	2.1
31771	0.946132	2.1
31772	0.946129	2.1
31773	0.946129	2.1
31774	0.946131	2.1
31775	0.946131	2.1
31776	0.94613	2.1
31777	0.94613	2.1
31778	1.89226	2.1
31779	1.89226	2.1
31780	1.89226	2.1
31781	1.89226	2.1

31782	3.78452	2.1
31783	0.707806	2.1
31784	0.697816	2.1
31785	0.823593	2.1
31786	0.801649	2.1
31787	0.74182	2.1
31788	0.74182	2.1
31789	0.757645	2.1
31790	0.757645	2.1
31791	0.823592	2.1
31792	0.697818	2.1
31793	0.707807	2.1
31794	0.801647	2.1
31795	1.52534	2.1
31796	1.6033	2.1
31797	1.64719	2.1
31798	1.38085	2.1
31799	1.64719	2.1
31800	1.38085	2.1
31801	2.67041	2.1
31802	1.89226	1.06542
31803	1.89226	2.1
31804	1.89226	2.1
31805	2.487	2.1
31806	2.487	2.1
31807	2.70097	2.1
31808	1.70303	2.1
31809	1.71163	2.1
31810	2.487	2.1
31811	4.46723	2.1
31812	1.14984	2.1
31813	1.2435	2.1
31814	0.778	2.1
31815	0.697616	2.1
31816	0.967211	2.1
31817	0.967211	2.1
31818	0.973907	2.1
31819	0.973907	2.1
31820	0.697616	2.1
31821	1.14984	2.1
31822	0.778	2.1
31823	1.2435	2.1
31824	2.4158	2.1
31825	1.26438	2.1
31826	1.676	2.1
31827	2.77505	2.1
31828	4.0918	2.1
31829	2.58258	2.1
31830	1.89226	2.1
31831	1.89226	2.1
31832	1.89226	2.1
31833	2.47032	2.1
31834	2.47032	2.1
31835	1.23516	2.1
31836	1.18734	2.1
31837	0.946131	2.1
31838	0.946131	2.1
31839	1.18734	2.1

31840	1.18734	2.1
31841	0.94613	2.1
31842	0.94613	2.1
31843	1.18734	2.1
31844	0.946131	2.1
31845	0.946131	2.1
31846	1.23516	2.1
31847	2.8239	2.1
31848	1.39361	2.1
31849	1.12342	2.1
31850	2.57275	2.1
31851	3.96637	2.1
31852	0.937929	1.0693
31853	0.937929	0.997138
31854	1.39734	0.915308
31855	1.39734	0.886452
31856	1.38914	0.955701
31857	0.94613	1.01184
31858	0.937902	1.02462
31859	0.94613	0.0683013
31860	0.94613	0.908728
31861	0.94613	0.941264
31862	1.38914	0.964295
31863	0.937902	0.930952
31864	1.89226	0.113181
31865	2.17765	1.05897
31866	1.84577	0.85299
31867	1.84577	0.340047
31868	1.89226	0.624509
31869	1.89226	0.235183
31870	2.11262	1.4211
31871	1.57766	1.35723
31872	2.22689	2.1
31873	1.91132	0.167149
31874	2.19624	2.1
31875	1.79689	0.05
31876	1.99656	0.05
31877	1.57766	1.12901
31878	1.39067	2.1
31879	1.73802	0.910459
31880	3.31568	1.14167
31881	1.89226	0.522688
31882	1.89226	0.0613282
31883	1.89226	0.100543
31884	1.89226	0.734489
31885	1.89226	0.05
31886	1.89226	0.401759
31887	1.84936	0.49253
31888	2.0815	0.05
31889	2.0815	0.25052
31890	2.47217	0.326736
31891	1.04528	0.05
31892	1.62774	0.188769
31893	1.89226	0.181923
31894	1.89226	0.05
31895	1.89226	0.05
31896	1.89226	0.364563
31897	1.89226	0.05

31898	1.89226	0.160418
31899	1.69055	0.05
31900	2.68518	0.0737269
31901	1.69055	0.05
31902	2.25055	0.0547951
31903	2.39587	0.05
31904	3.01801	0.05
31905	1.89226	0.05
31906	1.89226	0.0722764
31907	1.89226	0.05
31908	1.89226	0.05
31909	3.78452	0.05
31910	1.24499	1.96472
31911	1.70302	2.1
31912	1.41207	2.1
31913	1.10574	0.382018
31914	2.66056	2.02589
31915	1.70303	2.1
31916	2.39605	1.35817
31917	2.72674	1.23852
31918	2.46904	1.05096
31919	1.84557	2.02625
31920	1.67115	1.36259
31921	2.46904	0.330632
31922	2.76829	2.04414
31923	2.83666	0.145337
31924	2.83666	1.10436
31925	2.39605	1.01123
31926	2.39605	1.98065
31927	1.07323	0.05
31928	1.07323	2.1
31929	1.06715	2.1
31930	1.18052	1.19057
31931	1.30067	0.968597
31932	1.26542	1.02097
31933	0.914516	2.1
31934	0.839633	2.1
31935	0.936958	1.08602
31936	1.26751	1.02579
31937	0.847764	1.15299
31938	1.35395	1.19504
31939	2.69069	0.0869347
31940	1.03074	0.0797412
31941	1.74098	0.05
31942	1.97397	0.05
31943	3.71495	0.05
31944	2.44554	2.1
31945	1.89226	2.1
31946	1.89226	2.1
31947	1.89226	2.1
31948	2.40645	2.1
31949	2.40645	2.1
31950	1.89226	2.1
31951	1.89226	2.1
31952	1.89226	2.1
31953	1.89226	2.1
31954	3.78452	2.1
31955	1.82117	2.1

31956	2.55786	2.1
31957	2.51431	2.1
31958	2.51431	2.1
31959	1.85054	2.1
31960	1.84375	2.1
31961	2.38467	2.1
31962	1.89226	2.1
31963	1.89226	2.1
31964	1.89226	2.1
31965	2.44554	2.1
31966	2.44554	2.1
31967	1.89226	2.1
31968	1.89226	2.1
31969	1.89226	2.1
31970	1.89226	2.1
31971	1.89226	2.1
31972	1.89226	2.1
31973	2.02397	2.1
31974	2.55786	2.1
31975	1.77775	2.1
31976	2.61404	2.1
31977	4.33561	2.1
31978	1.89226	1.475
31979	1.89226	1.37157
31980	1.89226	1.10954
31981	1.89226	1.22987
31982	3.78453	1.35713
31983	1.89226	1.67496
31984	2.80184	1.85033
31985	1.89226	1.28735
31986	1.89226	1.72185
31987	2.03045	1.92733
31988	2.03045	2.1
31989	2.52263	1.48008
31990	1.27921	2.1
31991	1.58429	2.1
31992	1.73571	2.1
31993	2.37121	1.92055
31994	2.52263	1.5779
31995	0.764974	2.1
31996	0.650577	2.1
31997	0.712428	2.1
31998	0.562516	2.1
31999	0.67264	2.1
32000	0.67264	2.1
32001	0.654851	2.1
32002	0.654851	2.1
32003	0.562515	2.1
32004	0.650579	2.1
32005	0.712427	2.1
32006	0.764976	2.1
32007	1.89227	2.1
32008	1.89227	2.1
32009	1.89227	2.1
32010	1.89227	2.1
32011	3.78453	2.1
32012	1.89226	2.1
32013	1.89226	2.1

32014	1.89226	2.1
32015	1.89226	2.1
32016	3.78453	2.1
32017	1.49248	2.1
32018	1.24959	2.1
32019	1.28211	2.1
32020	1.26432	2.1
32021	1.51027	2.1
32022	1.49248	2.1
32023	1.49248	2.1
32024	1.19017	2.1
32025	1.24977	2.1
32026	1.51027	2.1
32027	2.74225	2.1
32028	1.89226	2.1
32029	1.89226	2.1
32030	1.89226	2.1
32031	1.89226	2.1
32032	3.78453	2.1
32033	1.89226	2.1
32034	1.89226	2.1
32035	1.89226	2.1
32036	1.89226	2.1
32037	3.78453	2.1
32038	0.624796	2.1
32039	0.618472	2.1
32040	0.770147	2.1
32041	0.755132	2.1
32042	0.681445	2.1
32043	0.681445	2.1
32044	0.69216	2.1
32045	0.69216	2.1
32046	0.618472	2.1
32047	0.770147	2.1
32048	0.624796	2.1
32049	0.755132	2.1
32050	1.54029	2.1
32051	1.51027	2.1
32052	1.54029	2.1
32053	1.30682	2.1
32054	1.24977	2.1
32055	1.24977	2.1
32056	0.994293	2.1
32057	2.17159	2.1
32058	2.21149	2.1
32059	2.21149	2.1
32060	0.994814	2.1
32061	0.994814	2.1
32062	0.990481	2.1
32063	2.21149	2.1
32064	2.2671	2.1
32065	2.2671	2.1
32066	0.994293	2.1
32067	0.994293	2.1
32068	2.41579	2.1
32069	1.09847	2.1
32070	1.70302	2.1
32071	2.45069	2.1

32072	3.61169	2.1
32073	2.48689	2.1
32074	1.67969	2.1
32075	1.67969	2.1
32076	1.70002	2.1
32077	2.43826	2.1
32078	2.45859	2.1
32079	2.43827	2.1
32080	1.62408	2.1
32081	1.62408	2.1
32082	1.67969	2.1
32083	2.36729	2.1
32084	2.4229	2.1
32085	2.47199	2.1
32086	1.0823	2.1
32087	1.0607	2.1
32088	2.44276	2.1
32089	3.52506	2.1
32090	2.47199	2.1
32091	1.89226	2.1
32092	1.89226	2.1
32093	2.44277	2.1
32094	4.33503	2.1
32095	1.89226	2.1
32096	1.89226	2.1
32097	1.89226	2.1
32098	1.89226	2.1
32099	3.78453	2.1
32100	1.89226	2.1
32101	1.89226	2.1
32102	1.89226	2.1
32103	1.89226	2.1
32104	1.89226	2.1
32105	1.89226	2.1
32106	1.02986	2.1
32107	2.44276	2.1
32108	1.0607	2.1
32109	1.0607	2.1
32110	2.40055	2.1
32111	2.40055	2.1
32112	1.89226	2.1
32113	2.44277	2.1
32114	2.40056	2.1
32115	2.44277	2.1
32116	1.85005	2.1
32117	1.89226	2.1
32118	1.89226	2.1
32119	1.89226	2.1
32120	1.89226	2.1
32121	1.89226	2.1
32122	3.78453	2.1
32123	1.89226	2.1
32124	1.89226	2.1
32125	1.89226	2.1
32126	1.89226	2.1
32127	3.78452	2.1
32128	2.79502	0.456666
32129	2.63448	0.826755

32130	2.68986	0.05
32131	2.53868	0.501228
32132	5.32434	0.0812224
32133	2.63449	0.05
32134	2.39282	2.1
32135	2.53869	0.0741215
32136	2.3085	2.1
32137	4.94299	1.32765
32138	2.68986	0.347525
32139	2.53868	0.515017
32140	2.5761	0.05
32141	2.43404	0.2237
32142	5.1239	0.0561775
32143	2.53869	0.05
32144	2.3085	2.1
32145	2.43405	0.05
32146	2.22575	2.1
32147	4.74255	1.32716
32148	1.89226	2.1
32149	1.89226	2.1
32150	1.89226	2.1
32151	1.89226	2.1
32152	3.78453	2.1
32153	1.32391	2.1
32154	1.44951	2.1
32155	1.44951	2.1
32156	1.89226	2.1
32157	1.89226	2.1
32158	2.20203	2.1
32159	0.507656	2.1
32160	0.578945	2.1
32161	1.05568	2.1
32162	0.959007	2.1
32163	0.76264	2.1
32164	0.76264	2.1
32165	0.775313	2.1
32166	0.775313	2.1
32167	0.578945	2.1
32168	1.05568	2.1
32169	0.959007	2.1
32170	0.507656	2.1
32171	1.89226	0.05
32172	2.4065	0.05
32173	2.36561	0.05
32174	2.4065	0.05
32175	1.85138	0.05
32176	1.89226	0.05
32177	1.40274	0.05
32178	2.4065	0.05
32179	1.53336	0.05
32180	1.53336	0.05
32181	2.36562	0.05
32182	2.36562	0.05
32183	0.946115	0.05
32184	0.946115	0.05
32185	1.06496	0.05
32186	1.18279	0.05
32187	0.946117	0.05

32188	0.946117	0.05
32189	1.06496	0.05
32190	1.06496	0.05
32191	0.94613	0.05
32192	1.06497	0.05
32193	0.946105	0.05
32194	1.18278	0.05
32195	1.00213	0.05
32196	2.36559	0.05
32197	1.40272	0.05
32198	1.40272	0.05
32199	2.12992	0.05
32200	2.12992	0.05
32201	0.95646	2.1
32202	2.07201	2.1
32203	1.01808	2.1
32204	2.14876	2.1
32205	3.09009	2.1
32206	1.89226	2.1
32207	1.89226	2.07637
32208	1.89226	2.1
32209	1.89226	2.1
32210	3.78453	2.1
32211	1.89227	2.1
32212	1.89227	2.1
32213	1.89227	2.1
32214	1.89227	2.1
32215	1.89227	2.1
32216	1.89227	2.1
32217	2.71886	2.1
32218	1.60497	2.1
32219	1.43935	2.1
32220	2.55151	2.1
32221	4.15648	2.1
32222	2.71886	2.1
32223	1.89226	2.1
32224	1.89226	2.1
32225	2.55151	2.1
32226	4.44377	2.1
32227	1.89226	2.1
32228	1.89226	2.1
32229	1.89226	2.1
32230	1.89226	2.1
32231	3.78453	2.1
32232	1.89226	2.1
32233	1.89226	2.1
32234	1.89226	2.1
32235	1.89226	2.1
32236	3.78453	2.1
32237	1.58942	0.0670847
32238	1.92622	0.192883
32239	2.52852	0.189233
32240	2.19457	0.069119
32241	4.12079	0.0601723
32242	1.92649	0.0689928
32243	1.44426	0.05
32244	1.87529	0.05
32245	2.19457	0.0893699

32246	3.63883	0.0717456
32247	1.87529	0.05
32248	2.19457	0.0682179
32249	2.7378	0.0660566
32250	2.29852	0.05
32251	4.49309	0.05
32252	1.35976	2.1
32253	1.89452	2.1
32254	1.39616	2.1
32255	1.1423	2.1
32256	1.8996	2.1
32257	1.52068	2.1
32258	1.89226	2.1
32259	1.89226	2.1
32260	1.89226	2.1
32261	1.89226	2.1
32262	1.89226	2.1
32263	1.89226	2.1
32264	1.89226	2.1
32265	2.15617	2.1
32266	2.0953	2.1
32267	2.15617	2.1
32268	1.83139	2.1
32269	1.89226	2.1
32270	1.16817	2.1
32271	1.8996	2.1
32272	1.23614	2.1
32273	1.48338	2.1
32274	1.80218	2.1
32275	2.16263	2.1
32276	1.89226	2.1
32277	1.89226	2.1
32278	1.89226	2.1
32279	1.89226	2.1
32280	1.89226	2.1
32281	1.89226	2.1
32282	1.89226	2.1
32283	2.0953	2.1
32284	2.03074	2.1
32285	2.0953	2.1
32286	1.8277	2.1
32287	1.89226	2.1
32288	1.74455	2.1
32289	2.181	2.1
32290	1.74455	2.1
32291	1.92911	2.1
32292	1.98195	2.1
32293	2.16651	2.1
32294	2.181	2.1
32295	2.29881	2.1
32296	1.98195	2.1
32297	1.80729	2.1
32298	4.28075	2.1
32299	1.74455	2.1
32300	1.98195	2.1
32301	1.52603	2.1
32302	1.76603	2.1
32303	3.51058	2.1

32304	1.76603	2.1
32305	1.94506	2.1
32306	1.76603	2.1
32307	1.98195	2.1
32308	1.86795	2.1
32309	2.08387	2.1
32310	0.984305	2.1
32311	1.9	2.1
32312	2.86619	2.1
32313	1.86189	2.1
32314	3.76189	2.1
32315	1.4621	2.1
32316	1.89226	2.1
32317	2.81374	2.1
32318	1.89226	2.1
32319	3.35437	2.1
32320	0.946133	2.1
32321	0.946133	2.1
32322	0.946132	2.1
32323	0.946132	2.1
32324	0.946132	2.1
32325	0.946132	2.1
32326	0.946133	2.1
32327	0.946133	2.1
32328	0.946132	2.1
32329	0.946132	2.1
32330	0.946132	2.1
32331	0.946132	2.1
32332	1.64268	2.1
32333	1.93673	2.1
32334	2.25284	2.1
32335	1.97548	2.1
32336	3.89552	2.1
32337	1.86206	2.1
32338	1.93673	2.1
32339	2.25284	2.1
32340	2.20075	2.1
32341	4.1149	2.1
32342	2.25284	2.1
32343	2.24781	2.1
32344	1.97548	2.1
32345	1.97548	2.1
32346	2.49676	2.1
32347	2.49676	2.1
32348	2.25284	2.1
32349	2.43549	2.1
32350	2.44467	2.1
32351	2.20075	2.1
32352	2.49676	2.1
32353	2.25284	2.1
32354	2.57928	2.1
32355	2.32606	2.1
32356	2.59522	2.1
32357	2.39022	2.1
32358	4.9695	2.1
32359	2.32606	2.1
32360	1.77677	2.1
32361	2.39022	2.1

32362	2.28669	2.1
32363	4.16699	2.1
32364	2.59522	2.1
32365	2.39022	2.1
32366	2.61901	2.1
32367	2.45899	2.1
32368	5.00923	2.1
32369	2.39022	2.1
32370	2.11575	2.1
32371	2.41795	2.1
32372	2.0815	2.1
32373	4.67085	2.1
32374	1.89226	2.1
32375	2.24723	2.1
32376	1.95405	2.1
32377	1.89226	2.1
32378	2.30902	2.1
32379	2.24723	2.1
32380	1.89226	2.1
32381	1.89226	2.1
32382	1.89226	2.1
32383	1.89226	2.1
32384	3.78453	2.1
32385	0.946131	2.1
32386	0.946131	2.1
32387	1.14643	2.1
32388	1.10673	2.1
32389	1.10673	2.1
32390	1.10673	2.1
32391	0.946133	2.1
32392	0.946133	2.1
32393	0.946131	2.1
32394	1.14643	2.1
32395	1.10673	2.1
32396	0.946131	2.1
32397	1.89226	2.1
32398	2.30902	2.1
32399	1.96635	2.1
32400	1.89226	2.1
32401	2.38311	2.1
32402	2.30902	2.1
32403	1.89226	2.1
32404	1.89226	2.1
32405	1.89226	2.1
32406	1.89226	2.1
32407	1.89226	2.1
32408	1.89226	2.1
32409	0.946131	2.1
32410	0.946131	2.1
32411	1.19156	2.1
32412	1.14643	2.1
32413	1.14643	2.1
32414	1.14643	2.1
32415	0.946133	2.1
32416	0.946133	2.1
32417	0.946131	2.1
32418	1.19156	2.1
32419	0.946131	2.1

32420	1.14643	2.1
32421	1.89226	1.42087
32422	1.89226	0.96804
32423	1.89226	0.930672
32424	1.89226	1.21977
32425	1.89226	0.928073
32426	1.89226	1.83528
32427	1.89226	0.969176
32428	1.89226	0.997008
32429	1.89226	0.92278
32430	1.9645	1.0274
32431	3.78452	1.11049
32432	2.39489	2.1
32433	2.15715	2.1
32434	2.07821	2.1
32435	2.33541	2.1
32436	4.49256	2.1
32437	2.39489	2.1
32438	2.34898	2.1
32439	2.30729	2.1
32440	2.33541	2.1
32441	4.6844	2.1
32442	2.07821	2.1
32443	2.33541	2.1
32444	2.26415	2.1
32445	1.99632	2.1
32446	4.33174	2.1
32447	2.30729	2.1
32448	2.33542	2.1
32449	2.26416	2.1
32450	2.26618	2.1
32451	4.60159	2.1
32452	1.19837	1.68565
32453	2.79151	0.05
32454	2.25636	0.733359
32455	2.25636	0.26542
32456	1.94072	0.355054
32457	1.94072	0.278596
32458	2.53725	0.05
32459	1.16699	0.05
32460	1.69864	0.05
32461	2.0815	0.05
32462	3.58273	0.05
32463	1.91646	2.1
32464	2.51686	2.1
32465	2.49872	2.1
32466	1.85819	2.1
32467	4.41518	2.1
32468	0.929098	2.1
32469	0.8993	2.1
32470	1.24359	2.1
32471	1.24936	2.1
32472	1.06832	2.1
32473	1.06832	2.1
32474	1.08034	2.1
32475	1.08034	2.1
32476	0.899298	2.1
32477	1.24359	2.1

32478	1.24936	2.1
32479	0.929097	2.1
32480	2.0815	0.05
32481	1.89226	0.05
32482	2.0815	0.05
32483	1.82811	0.05
32484	1.94982	0.05
32485	1.71246	0.05
32486	2.3256	2.1
32487	1.89226	2.1
32488	1.89226	2.1
32489	1.89226	2.1
32490	2.38467	2.1
32491	2.38467	2.1
32492	1.89226	2.1
32493	1.89226	2.1
32494	1.89226	2.1
32495	1.89226	2.1
32496	1.89226	2.1
32497	1.89226	2.1
32498	1.89763	2.1
32499	2.61405	2.1
32500	2.15035	2.1
32501	2.68796	2.1
32502	1.42219	2.1
32503	1.77775	2.1
32504	2.26306	2.1
32505	1.89226	2.1
32506	1.89226	2.1
32507	1.89226	2.1
32508	2.3256	2.1
32509	2.3256	2.1
32510	1.89226	2.1
32511	1.89226	2.1
32512	1.89226	2.1
32513	1.89226	2.1
32514	1.89226	2.1
32515	1.89226	2.1
32516	1.92887	2.1
32517	2.41915	2.1
32518	2.50142	2.1
32519	2.50142	2.1
32520	1.90388	2.1
32521	1.89763	2.1
32522	1.89224	2.1
32523	1.89224	2.1
32524	1.89224	2.1
32525	1.89224	2.1
32526	3.78448	2.1
32527	2.25315	2.1
32528	1.05843	2.1
32529	2.38995	2.1
32530	1.31541	2.1
32531	3.56856	2.1
32532	0.517552	2.1
32533	0.68548	2.1
32534	1.27675	2.1
32535	1.13076	2.1

32536	0.913611	2.1
32537	0.913612	2.1
32538	0.902633	2.1
32539	0.902633	2.1
32540	0.68547	2.1
32541	1.27673	2.1
32542	1.13078	2.1
32543	0.517559	2.1
32544	1.89226	2.1
32545	1.89226	2.1
32546	1.89226	2.1
32547	1.89226	2.1
32548	3.78453	2.1
32549	0.962936	2.1
32550	0.962936	2.1
32551	0.996141	2.1
32552	0.996141	2.1
32553	0.830609	2.1
32554	0.657714	2.1
32555	1.30136	2.1
32556	1.19499	2.1
32557	1.30136	2.1
32558	0.830609	2.1
32559	1.19499	2.1
32560	0.657713	2.1
32561	1.60057	0.05
32562	1.39615	0.0539337
32563	1.06474	0.05
32564	1.39615	0.179606
32565	1.64655	0.05
32566	2.04424	0.0617704
32567	1.89226	0.165399
32568	1.89226	0.445677
32569	1.89226	0.312221
32570	1.89226	0.20423
32571	1.89226	0.364691
32572	1.89226	0.328829
32573	2.56322	0.0590462
32574	1.89226	0.233777
32575	1.89226	0.127012
32576	1.89226	0.0919404
32577	2.18047	0.120777
32578	2.18047	0.103437
32579	1.89226	0.15824
32580	1.89226	0.192411
32581	1.89226	0.269082
32582	1.65573	0.100176
32583	1.89226	0.30981
32584	1.65573	0.113504
32585	1.05353	0.05
32586	1.83158	0.05
32587	1.50282	0.05
32588	1.20669	0.05
32589	2.88511	0.05
32590	1.44807	2.1
32591	1.93592	2.1
32592	1.80703	2.1
32593	1.27806	2.1

32594	3.21398	2.1
32595	2.0815	2.1
32596	1.87465	2.1
32597	1.87465	2.1
32598	1.50566	2.1
32599	2.0815	2.1
32600	1.69263	2.1
32601	1.89226	2.1
32602	1.89226	2.1
32603	1.89226	2.1
32604	1.89226	2.1
32605	1.89226	2.1
32606	1.89226	2.1
32607	1.62632	2.1
32608	0.994294	2.1
32609	0.994294	2.1
32610	1.27806	2.1
32611	1.81921	2.1
32612	2.10776	2.1
32613	2.0815	2.1
32614	1.70303	2.1
32615	1.70303	2.1
32616	1.85749	2.1
32617	1.90451	2.1
32618	2.07724	2.1
32619	1.89226	2.1
32620	1.89226	2.1
32621	1.89226	2.1
32622	1.89226	2.1
32623	1.89226	2.1
32624	1.89226	2.1
32625	2.69773	2.1
32626	1.88129	2.1
32627	2.55196	2.1
32628	2.26469	2.1
32629	4.94367	2.1
32630	1.88129	2.1
32631	1.25889	2.1
32632	1.26614	2.1
32633	1.05914	2.1
32634	2.26469	2.1
32635	1.88129	2.1
32636	2.55196	2.1
32637	2.0588	2.1
32638	2.6536	2.1
32639	2.15287	2.1
32640	4.70484	2.1
32641	0.8516	2.1
32642	0.8516	2.1
32643	0.849002	2.1
32644	0.849002	2.1
32645	0.629445	2.1
32646	0.671197	2.1
32647	1.0294	2.1
32648	1.07644	2.1
32649	1.07644	2.1
32650	0.671197	2.1
32651	0.629446	2.1

32652	1.0294	2.1
32653	2.02115	2.06086
32654	1.18382	2.1
32655	2.29107	0.761769
32656	1.46903	1.28143
32657	3.47488	1.05526
32658	2.60763	1.19478
32659	2.29107	1.6305
32660	1.82141	1.24065
32661	1.82141	2.1
32662	1.46903	1.99889
32663	2.29107	2.02497
32664	1.64007	2.1
32665	2.2024	2.1
32666	1.44044	2.1
32667	2.02634	2.1
32668	3.6664	2.1
32669	1.25565	2.1
32670	2.02635	2.1
32671	1.87228	2.1
32672	1.87228	2.1
32673	1.44045	2.1
32674	1.44045	2.1
32675	1.89226	0.05
32676	1.78503	0.05
32677	1.78503	0.05
32678	1.40048	0.05
32679	2.0815	0.05
32680	1.62972	0.05
32681	1.27875	2.1
32682	1.1483	2.0635
32683	1.40014	2.1
32684	1.40014	2.1
32685	1.04598	2.1
32686	1.04598	2.1
32687	2.63443	1.39974
32688	2.26444	2.1
32689	2.79392	0.645098
32690	2.4863	2.1
32691	5.12073	1.89862
32692	1.83158	0.05
32693	1.18508	0.05
32694	1.20669	0.05
32695	1.81488	0.05
32696	1.20669	0.05
32697	1.81488	0.05
32698	1.08134	0.05
32699	1.70303	0.068091
32700	1.24027	0.05
32701	1.30359	0.05
32702	1.89939	0.0589499
32703	1.70303	0.05
32704	1.89226	2.1
32705	2.69027	2.1
32706	1.89226	2.1
32707	2.2078	2.1
32708	1.89226	2.1
32709	2.2078	2.1

32710	1.89226	2.1
32711	2.78977	2.1
32712	1.89226	2.1
32713	2.69027	2.1
32714	1.89226	2.1
32715	2.69027	2.1
32716	1.89226	2.1
32717	1.89226	2.1
32718	1.89226	1.9091
32719	1.89226	2.1
32720	3.78452	2.1
32721	1.43687	2.1
32722	1.71059	2.1
32723	2.53014	2.1
32724	2.6521	2.1
32725	4.24073	2.1
32726	2.6521	2.1
32727	1.66762	2.1
32728	2.50299	2.1
32729	2.50299	2.1
32730	1.71059	2.1
32731	1.71059	2.1
32732	1.89226	2.1
32733	1.89226	2.1
32734	1.89226	2.1
32735	1.89226	2.1
32736	1.89226	2.1
32737	1.89226	2.1
32738	2.10662	0.803578
32739	2.77417	0.227915
32740	2.78873	1.00771
32741	2.11661	0.809391
32742	4.89078	0.410726
32743	2.10662	0.86328
32744	1.22857	0.117893
32745	1.25849	0.904386
32746	2.11661	0.853098
32747	3.34518	0.848365
32748	2.78873	0.938113
32749	2.11661	0.615683
32750	2.12475	0.857021
32751	2.79029	0.637997
32752	4.9069	0.850481
32753	1.2858	0.8546
32754	2.11661	0.733475
32755	1.25849	0.824426
32756	1.25849	0.641936
32757	2.12475	0.828503
32758	2.12475	0.768479
32759	1.13537	2.1
32760	1.20714	2.1
32761	0.946129	2.1
32762	0.946129	2.1
32763	0.946131	2.1
32764	0.946131	2.1
32765	1.29521	2.1
32766	1.20713	2.1
32767	1.13537	2.1

32768	0.946131	2.1
32769	0.94613	2.1
32770	1.29521	2.1
32771	1.00533	2.1
32772	0.876055	2.1
32773	0.946128	2.1
32774	0.946128	2.1
32775	0.913908	2.1
32776	0.913908	2.1
32777	1.17081	2.1
32778	0.896413	2.1
32779	0.955905	2.1
32780	0.893715	2.1
32781	1.20327	2.1
32782	0.998544	2.1
32783	1.89226	1.80326
32784	1.89226	2.06464
32785	1.89226	0.951227
32786	1.89226	1.31928
32787	3.78452	1.13157
32788	0.540975	1.34782
32789	0.726949	2.1
32790	0.712198	2.1
32791	0.506675	1.22574
32792	0.611916	1.25218
32793	0.611916	2.1
32794	0.621708	1.90495
32795	0.621708	1.18701
32796	0.726951	1.45712
32797	0.712196	1.27374
32798	0.506674	1.67813
32799	0.540976	1.51592
32800	1.89226	1.2634
32801	1.89226	2.1
32802	1.89226	1.27983
32803	1.89226	1.25579
32804	3.78453	1.7358
32805	1.89226	1.17944
32806	1.89226	1.26726
32807	1.89226	0.957266
32808	1.89226	2.1
32809	1.89226	0.987231
32810	1.89226	2.1
32811	0.919594	1.23347
32812	0.726949	1.89696
32813	0.898318	1.24851
32814	0.712198	1.84433
32815	0.817559	1.06415
32816	0.817559	2.1
32817	0.814234	1.07856
32818	0.814234	2.00033
32819	0.919597	1.13651
32820	0.898316	1.00332
32821	0.726951	1.99669
32822	0.712196	1.4938
32823	1.79663	1.03636
32824	1.12088	2.1
32825	1.54699	1.26733

32826	1.17475	1.33515
32827	1.79663	2.1
32828	1.42439	2.1
32829	2.28928	1.25332
32830	1.23077	1.52327
32831	1.65458	1.10253
32832	2.66533	1.81577
32833	3.94385	1.34542
32834	1.89226	2.1
32835	1.89226	2.1
32836	1.89226	2.1
32837	1.89226	2.1
32838	3.78452	2.1
32839	1.38426	2.1
32840	1.15067	2.1
32841	1.38426	2.1
32842	1.01595	2.1
32843	1.24509	2.1
32844	1.01595	2.1
32845	2.62097	2.1
32846	1.89226	2.1
32847	2.72561	2.1
32848	2.72561	2.1
32849	1.89226	2.1
32850	1.89226	2.1
32851	1.90103	0.05
32852	1.41902	2.1
32853	1.77249	0.0562332
32854	1.30272	2.1
32855	3.20375	0.33231
32856	1.77249	0.05
32857	1.19548	2.1
32858	1.19548	0.808696
32859	1.30272	2.1
32860	1.65815	0.05
32861	1.76539	2.1
32862	2.62161	2.1
32863	1.89226	2.1
32864	1.89226	2.1
32865	1.89226	2.1
32866	2.6446	2.1
32867	2.6446	2.1
32868	2.64459	2.1
32869	1.89226	2.1
32870	1.89226	2.1
32871	1.89226	2.1
32872	2.58257	2.1
32873	2.58257	2.1
32874	1.89226	0.132912
32875	1.89226	1.17274
32876	2.31301	1.05393
32877	2.76309	0.281405
32878	1.89226	0.893112
32879	2.26047	0.291464
32880	1.89226	0.147103
32881	2.31301	1.1531
32882	2.17765	1.00395
32883	2.17765	0.335189

32884	1.89226	0.866293
32885	1.89226	0.290659
32886	2.18516	2.1
32887	1.85196	2.1
32888	1.78595	2.1
32889	2.14606	2.1
32890	3.99802	2.1
32891	2.18516	2.1
32892	2.21189	2.1
32893	2.18937	2.1
32894	2.14606	2.1
32895	4.35796	2.1
32896	1.60735	2.1
32897	2.14606	2.1
32898	2.15714	2.1
32899	1.70302	2.1
32900	3.70055	2.1
32901	2.18937	2.1
32902	2.14606	2.1
32903	2.0927	2.1
32904	2.162 2.1	
32905	4.30806	2.1
32906	2.17326	2.1
32907	2.58517	2.1
32908	2.55318	2.1
32909	2.1665	2.1
32910	4.75167	2.1
32911	2.17326	2.1
32912	1.53556	2.1
32913	1.56253	2.1
32914	2.1665	2.1
32915	3.70206	2.1
32916	2.55317	2.1
32917	2.1665	2.1
32918	2.17741	2.1
32919	2.54197	2.1
32920	4.70847	2.1
32921	2.17742	2.1
32922	2.1665	2.1
32923	2.18452	2.1
32924	1.58054	2.1
32925	1.56253	2.1
32926	1.56253	2.1
32927	1.71051	0.05
32928	1.89807	0.05
32929	1.19755	0.05
32930	1.19755	0.05
32931	2.25632	0.05
32932	2.25632	0.05
32933	0.876046	0.05
32934	1.27293	0.05
32935	0.946132	0.05
32936	0.946132	0.05
32937	0.946131	0.05
32938	0.869312	0.05
32939	1.12767	0.05
32940	0.946132	0.05
32941	1.22225	0.05

32942	1.37968	0.05
32943	0.851555	0.05
32944	0.851555	0.05
32945	0.946133	2.01783
32946	0.946133	2.1
32947	0.946132	2.1
32948	0.946132	1.33118
32949	0.946133	2.06411
32950	0.946133	2.1
32951	0.946132	2.1
32952	0.946132	2.1
32953	0.946132	2.1
32954	0.946132	2.1
32955	0.946133	2.1
32956	0.946133	2.1
32957	1.35152	2.1
32958	0.948936	2.1
32959	1.90166	2.1
32960	1.70303	2.1
32961	2.90858	2.1
32962	1.89226	2.1
32963	1.89226	2.1
32964	1.89226	2.1
32965	1.89226	2.1
32966	3.78452	2.1
32967	1.90166	2.1
32968	1.70303	2.1
32969	2.52454	2.1
32970	2.13919	2.1
32971	3.99529	2.1
32972	1.89226	1.32579
32973	1.89226	2.1
32974	1.89226	2.1
32975	1.89226	2.1
32976	3.78452	2.1
32977	1.89226	2.1
32978	1.89226	2.1
32979	1.89226	2.1
32980	1.89226	2.1
32981	1.89226	1.56254
32982	1.89226	2.1
32983	2.13918	2.1
32984	0.992674	2.1
32985	2.3397	2.1
32986	1.8023	2.1
32987	1.47769	2.1
32988	0.992674	2.1
32989	1.89226	0.05
32990	1.89226	0.05
32991	1.89226	0.0579534
32992	1.89226	0.05
32993	3.78452	0.05
32994	1.89226	0.05
32995	2.54752	0.05
32996	2.63974	0.05
32997	1.89226	0.05
32998	4.53201	0.05
32999	0.498412	0.05

33000	0.500955	0.05
33001	0.57955	0.05
33002	0.563873	0.05
33003	0.529107	0.05
33004	0.529107	0.05
33005	0.53572	0.05
33006	0.53572	0.05
33007	0.500956	0.05
33008	0.579548	0.05
33009	0.563871	0.05
33010	0.498414	0.05
33011	1.89223	0.05
33012	1.89223	0.05
33013	1.89223	0.05
33014	1.89223	0.05
33015	1.89223	0.05
33016	1.89223	0.05
33017	1.28752	0.05
33018	1.31985	0.05
33019	0.946119	0.05
33020	0.946119	0.05
33021	1.28752	0.05
33022	1.28752	0.05
33023	0.946119	0.05
33024	0.946119	0.0505421
33025	1.28753	0.05
33026	0.946132	0.05
33027	1.31984	0.05
33028	0.946107	0.05
33029	0.500948	0.05
33030	0.479465	0.05
33031	0.562335	0.05
33032	0.579542	0.05
33033	0.528436	0.05
33034	0.528436	0.05
33035	0.530571	0.05
33036	0.530571	0.05
33037	0.56234	0.05
33038	0.479473	0.05
33039	0.500943	0.05
33040	0.579533	0.05
33041	1.89226	2.1
33042	1.89226	2.1
33043	1.89226	2.1
33044	1.89226	2.1
33045	3.78452	2.1
33046	1.50767	2.1
33047	1.14496	2.1
33048	1.50767	2.1
33049	1.58539	2.1
33050	1.07653	2.1
33051	1.15425	2.1
33052	1.89226	2.1
33053	2.43826	2.1
33054	2.43826	2.1
33055	2.48688	2.1
33056	1.89226	2.1
33057	1.94088	2.1

33058	1.89226	2.1
33059	1.89226	2.1
33060	1.89226	2.1
33061	1.89226	2.1
33062	3.78452	2.1
33063	1.50767	2.1
33064	1.07653	2.1
33065	1.41077	2.1
33066	0.991407	2.1
33067	2.49907	2.1
33068	1.21913	2.1
33069	1.21913	2.1
33070	0.946128	2.1
33071	0.946128	2.1
33072	1.21913	2.1
33073	1.18364	2.1
33074	0.946131	2.1
33075	0.946131	2.1
33076	1.18364	2.1
33077	0.946129	2.1
33078	1.21913	2.1
33079	0.94613	2.1
33080	1.89226	0.582031
33081	1.89226	2.1
33082	1.89226	0.977239
33083	1.89226	2.1
33084	3.78452	2.1
33085	1.93823	0.964873
33086	1.60976	2.1
33087	1.93823	2.1
33088	2.20045	0.788465
33089	1.36999	2.1
33090	1.63222	2.1
33091	1.38302	2.1
33092	1.38302	2.1
33093	0.946132	2.1
33094	0.946132	2.1
33095	1.27258	2.1
33096	1.38302	2.1
33097	0.946133	2.1
33098	0.946133	2.1
33099	0.946133	2.1
33100	1.27258	2.1
33101	0.946132	2.1
33102	1.38302	2.1
33103	1.89226	0.158276
33104	1.89226	2.1
33105	1.89226	0.942594
33106	1.89226	2.1
33107	3.78452	2.1
33108	1.93823	0.0683044
33109	1.36999	2.1
33110	1.70423	0.887506
33111	1.14195	2.1
33112	3.08018	2.1
33113	1.27258	2.1
33114	1.27258	2.1
33115	0.946132	2.1

33116	0.946132	2.1
33117	1.17233	2.1
33118	1.27258	2.1
33119	0.946132	2.1
33120	0.946132	2.1
33121	1.17233	2.1
33122	0.946132	2.1
33123	0.946132	2.1
33124	1.27258	2.1
33125	2.70575	2.1
33126	1.89226	1.11413
33127	2.71429	2.1
33128	2.71429	2.1
33129	1.89226	2.1
33130	1.89226	1.16801
33131	0.946133	1.13606
33132	0.946133	2.1
33133	1.28713	2.1
33134	1.28713	2.1
33135	0.946131	2.1
33136	0.946131	1.11778
33137	1.28713	2.1
33138	1.36503	2.1
33139	0.946131	2.1
33140	1.36503	2.1
33141	1.28713	2.1
33142	0.946131	2.1
33143	1.89226	2.1
33144	1.89226	1.87798
33145	1.89226	2.1
33146	1.89226	2.1
33147	1.89226	2.1
33148	1.89226	2.06794
33149	1.22615	2.1
33150	1.04147	2.1
33151	1.3439	2.1
33152	1.3439	2.1
33153	0.969118	2.1
33154	0.969118	2.1
33155	0.980841	2.1
33156	0.992505	2.1
33157	1.34289	2.1
33158	1.70303	2.1
33159	2.56081	2.1
33160	1.89225	2.1
33161	2.66785	2.1
33162	2.79506	2.1
33163	2.79506	2.1
33164	1.89225	2.1
33165	1.89225	2.1
33166	0.978778	2.1
33167	0.978778	2.1
33168	0.993303	2.1
33169	0.993303	2.1
33170	0.603538	2.1
33171	0.638158	2.1
33172	1.33393	2.1
33173	1.39753	2.1

33174	1.33391	2.1
33175	0.603535	2.1
33176	0.638164	2.1
33177	1.39754	2.1
33178	1.33393	2.1
33179	1.33393	2.1
33180	0.946132	2.1
33181	0.946132	2.1
33182	1.33393	2.1
33183	1.27365	2.1
33184	0.946132	2.1
33185	0.946132	2.1
33186	0.946132	2.1
33187	1.27365	2.1
33188	1.33393	2.1
33189	0.946131	2.1
33190	1.89226	2.1
33191	1.89226	2.1
33192	1.89226	2.1
33193	1.89226	2.1
33194	3.78452	2.1
33195	1.89226	2.1
33196	1.89226	2.1
33197	1.89226	2.1
33198	1.89226	2.1
33199	3.78453	2.1
33200	0.979364	2.1
33201	0.979364	2.1
33202	0.89618	2.1
33203	0.89618	2.1
33204	0.896178	2.1
33205	0.929411	2.1
33206	0.946133	2.1
33207	0.946133	2.1
33208	0.946131	2.1
33209	0.89618	2.1
33210	0.946131	2.1
33211	0.929413	2.1
33212	1.89226	2.1
33213	1.89226	2.1
33214	1.89226	2.1
33215	1.89226	2.1
33216	1.89226	2.1
33217	1.89226	2.1
33218	1.89225	2.1
33219	1.89225	2.1
33220	1.89225	2.1
33221	1.89225	2.1
33222	3.7845	2.1
33223	1.89225	2.1
33224	1.89225	2.1
33225	1.89225	2.1
33226	1.89225	2.1
33227	1.89225	2.1
33228	1.89225	2.1
33229	0.946128	0.05
33230	0.946128	0.05
33231	0.946132	0.0613865

33232	0.946132	0.05
33233	0.94613	0.0594924
33234	0.94613	0.0701779
33235	0.946129	0.05
33236	0.946129	0.05
33237	0.94613	0.05
33238	0.94613	0.05
33239	0.94613	0.05
33240	0.94613	0.05
33241	1.89227	1.31218
33242	1.89227	1.23698
33243	1.89227	0.968179
33244	1.89227	0.932034
33245	3.78453	0.997036
33246	1.89226	1.13908
33247	2.43717	1.97676
33248	2.63259	1.09726
33249	1.89226	0.912341
33250	4.52485	1.2517
33251	0.616808	1.04751
33252	0.792277	1.47063
33253	1.04986	0.940598
33254	0.72303	1.37195
33255	0.702675	0.797598
33256	0.76862	2.1
33257	0.809154	1.8514
33258	0.809154	0.988207
33259	0.808757	1.34164
33260	1.07789	0.860193
33261	0.772568	0.987584
33262	0.598878	1.63889
33263	1.89226	1.24356
33264	1.89226	1.18934
33265	1.89226	1.00086
33266	1.89226	0.956993
33267	3.78453	1.11376
33268	1.36428	2.04812
33269	1.31629	1.15652
33270	0.946132	1.16451
33271	0.946132	1.06785
33272	1.36428	0.961041
33273	1.36428	2.05261
33274	0.946132	1.21812
33275	0.946132	1.80365
33276	1.36428	1.95463
33277	0.946132	1.02071
33278	1.31629	1.54622
33279	0.946132	0.947631
33280	1.578	1.54749
33281	2.85336	0.871587
33282	2.11368	0.81156
33283	2.14375	1.04783
33284	1.93852	1.03001
33285	1.93852	1.7999
33286	2.34806	0.328606
33287	1.42913	0.05
33288	1.61539	0.646983
33289	2.45322	0.0810015

33290	3.88235	0.357011
33291	2.34806	0.148509
33292	1.89226	0.0572759
33293	1.89226	0.695493
33294	2.45322	0.156745
33295	4.34548	0.0576481
33296	1.89226	0.0758897
33297	1.89226	0.05
33298	1.89226	0.264211
33299	1.89226	0.150322
33300	3.78452	0.346072
33301	1.89226	0.05
33302	1.89226	0.05
33303	1.89226	0.133673
33304	1.89226	0.160847
33305	3.78452	0.05
33306	1.25972	0.105542
33307	2.45322	0.05
33308	2.58823	0.410637
33309	1.79398	0.05
33310	4.2472	0.05
33311	1.89226	0.395579
33312	2.45322	0.05
33313	2.58823	0.0625352
33314	2.45322	0.05
33315	2.02727	0.05
33316	1.89226	0.05
33317	1.89226	0.05
33318	1.89226	0.05
33319	1.89226	0.393369
33320	1.89226	0.05
33321	3.78452	0.05
33322	1.89226	0.05
33323	1.89226	0.05
33324	1.89226	0.05
33325	1.89226	0.05
33326	1.89226	0.348842
33327	1.89226	0.072671
33328	1.70048	2.1
33329	2.1572	0.05
33330	1.45477	2.1
33331	1.93973	0.05
33332	3.64021	1.11695
33333	1.17614	2.1
33334	1.93973	0.0553091
33335	1.69661	2.1
33336	1.69661	0.0666977
33337	1.45477	2.1
33338	1.45477	0.381214
33339	1.94343	2.1
33340	2.53782	1.68397
33341	2.59903	2.1
33342	2.59903	1.05662
33343	1.87598	2.1
33344	1.87598	1.18127
33345	0.946131	0.367302
33346	0.946131	1.3318
33347	1.26891	2.1

33348	1.26891	0.11193
33349	0.94613	1.61971
33350	0.94613	1.22849
33351	1.26891	1.49549
33352	1.29952	1.06469
33353	1.29952	0.687185
33354	0.946131	0.153481
33355	1.26891	1.20337
33356	0.946131	1.21843
33357	2.00551	2.1
33358	2.59903	2.00431
33359	2.65811	2.1
33360	2.65811	1.20929
33361	1.94343	2.1
33362	1.94343	1.43103
33363	2.65811	0.890425
33364	1.89226	2.1
33365	1.89226	0.887593
33366	1.89226	1.39357
33367	2.59903	1.16366
33368	2.59903	2.1
33369	1.89227	2.1
33370	1.89227	2.1
33371	1.89227	2.1
33372	1.89227	2.1
33373	3.78453	2.1
33374	1.15471	2.1
33375	0.993889	2.1
33376	1.78919	2.1
33377	1.77325	2.1
33378	2.78308	2.1
33379	1.89226	2.1
33380	1.89226	2.1
33381	1.89226	2.1
33382	1.89226	2.1
33383	3.78453	2.1
33384	1.78919	2.1
33385	1.77325	2.1
33386	2.45969	2.1
33387	2.70835	2.1
33388	4.49753	2.1
33389	0.939314	2.1
33390	0.939315	2.1
33391	1.01133	2.1
33392	1.01133	2.1
33393	0.596471	2.1
33394	1.20813	2.1
33395	0.886619	2.1
33396	1.35417	2.1
33397	1.35417	2.1
33398	1.20813	2.1
33399	0.886621	2.1
33400	0.59647	2.1
33401	0.946131	2.1
33402	0.946131	2.1
33403	0.94613	2.1
33404	0.94613	2.1
33405	0.946133	2.1

33406	0.946133	2.1
33407	0.946128	2.1
33408	0.946128	2.1
33409	0.946131	2.1
33410	0.946131	2.1
33411	0.946131	2.1
33412	0.946131	2.1
33413	1.03939	2.1
33414	1.03939	2.1
33415	0.946132	0.692896
33416	0.946132	2.01929
33417	0.946132	2.1
33418	0.946132	2.1
33419	1.23851	1.33567
33420	1.03939	2.1
33421	1.03939	2.1
33422	0.946132	2.1
33423	0.946133	2.1
33424	1.23851	2.1
33425	0.902503	2.1
33426	0.946129	2.1
33427	0.946132	1.32804
33428	0.946132	2.1
33429	0.902472	2.1
33430	0.902472	2.1
33431	1.08736	1.73722
33432	0.994093	2.1
33433	0.946133	2.1
33434	0.902504	2.1
33435	1.03939	2.1
33436	0.946132	2.1
33437	1.35306	1.03208
33438	1.29982	0.848992
33439	1.7216	0.820884
33440	1.89183	0.818698
33441	3.07466	0.83207
33442	0.485098	0.842623
33443	0.582401	0.868102
33444	0.649908	0.937358
33445	0.8608	0.852734
33446	0.587739	0.784653
33447	0.58774	0.910187
33448	0.644567	0.849205
33449	0.644567	1.50394
33450	0.58241	0.912605
33451	0.86081	0.788321
33452	0.649898	1.07592
33453	0.485093	0.859023
33454	2.25936	2.1
33455	2.71441	2.1
33456	2.63824	2.1
33457	2.20652	2.1
33458	4.92092	2.1
33459	2.25936	2.1
33460	1.57627	2.1
33461	1.5493	2.1
33462	2.20652	2.1
33463	3.78279	2.1

33464	2.63824	2.1
33465	2.20652	2.1
33466	2.17326	2.1
33467	2.58517	2.1
33468	4.79169	2.1
33469	2.17326	2.1
33470	2.20652	2.1
33471	2.19278	2.1
33472	1.53556	2.1
33473	1.5493	2.1
33474	1.5493	2.1
33475	1.97465	2.1
33476	2.13925	2.1
33477	2.04483	2.1
33478	1.62784	2.1
33479	3.76709	2.1
33480	1.89226	2.1
33481	1.89226	2.1
33482	1.89226	2.1
33483	1.89226	2.1
33484	1.89226	2.1
33485	1.89226	2.1
33486	0.724037	2.1
33487	0.81392	2.1
33488	0.96796	2.1
33489	1.02242	2.1
33490	0.882091	2.1
33491	0.882091	2.1
33492	0.899789	2.1
33493	0.899789	2.1
33494	0.724036	2.1
33495	0.96796	2.1
33496	1.02242	2.1
33497	0.81392	2.1
33498	1.13537	2.1
33499	1.63054	2.1
33500	0.946131	2.1
33501	0.946131	2.1
33502	0.946131	2.1
33503	0.946131	2.1
33504	1.258	2.1
33505	1.63054	2.1
33506	1.22632	2.1
33507	1.22632	2.1
33508	0.946131	2.1
33509	1.258	2.1
33510	1.89226	2.1
33511	1.89226	2.1
33512	1.89226	2.1
33513	1.89226	2.1
33514	1.89226	2.1
33515	1.89226	2.1
33516	0.809739	2.1
33517	0.862674	2.1
33518	1.1995	2.1
33519	1.33899	2.1
33520	1.06324	2.1
33521	1.06324	2.1

33522	0.981932	2.1
33523	1.19563	2.1
33524	0.809727	2.1
33525	1.14092	2.1
33526	1.33902	2.1
33527	0.863494	2.1
33528	2.23742	2.1
33529	1.84902	2.1
33530	1.88088	2.1
33531	2.20392	2.1
33532	4.08644	2.1
33533	1.3592	2.1
33534	1.84902	2.1
33535	1.88088	2.1
33536	1.3305	2.1
33537	1.88088	2.1
33538	1.3305	2.1
33539	1.84902	2.1
33540	1.15008	0.05
33541	1.3305	2.1
33542	1.70539	2.1
33543	2.9991	2.1
33544	1.84901	2.1
33545	2.0264	2.1
33546	2.20391	2.1
33547	1.70539	2.1
33548	3.87541	2.1
33549	1.03852	2.1
33550	1.38247	0.104393
33551	1.36635	0.557175
33552	1.01537	2.1
33553	2.39784	1.54509
33554	2.0815	2.1
33555	1.91744	2.1
33556	1.91744	1.84367
33557	2.06496	2.1
33558	1.89227	2.01382
33559	2.03474	2.1
33560	1.89226	0.365241
33561	1.89226	1.95708
33562	1.89226	0.556954
33563	1.89226	2.1
33564	1.89226	0.147366
33565	1.89226	2.1
33566	1.36635	0.7799
33567	1.02923	2.1
33568	1.02923	1.23861
33569	1.01537	2.1
33570	1.36416	0.355987
33571	1.3503	2.1
33572	1.89226	2.1
33573	1.89359	2.1
33574	1.89359	2.1
33575	1.91744	2.1
33576	1.89226	2.1
33577	1.91611	2.1
33578	0.946128	2.1
33579	0.946128	2.1

33580	0.946133	0.804253
33581	0.946133	0.194096
33582	0.94613	0.151929
33583	0.94613	2.07049
33584	0.946131	2.1
33585	0.946131	0.238847
33586	0.946131	1.53268
33587	0.946131	1.72137
33588	0.946131	1.54742
33589	0.946131	1.70599
33590	2.689	2.1
33591	2.0815	2.1
33592	2.72786	2.1
33593	2.48295	2.1
33594	5.02397	2.1
33595	2.72786	2.1
33596	1.86424	2.1
33597	2.7188	2.1
33598	2.08981	2.1
33599	4.81768	2.1
33600	2.08982	2.1
33601	0.986987	2.1
33602	2.08982	2.1
33603	1.87249	2.1
33604	1.01964	2.1
33605	0.906115	2.1
33606	1.44247	2.1
33607	1.43872	2.1
33608	1.17054	2.1
33609	1.17054	2.1
33610	1.43872	2.1
33611	1.26045	2.1
33612	2.0815	0.05
33613	2.0815	0.05
33614	2.0815	0.05
33615	1.81468	0.05
33616	1.89065	0.05
33617	1.55625	0.05
33618	1.89062	0.05
33619	1.89224	0.05
33620	1.89224	0.05
33621	2.27073	0.05
33622	1.86856	0.05
33623	2.24232	0.05
33624	1.21419	2.1
33625	2.79716	2.1
33626	2.50005	2.1
33627	2.50005	2.1
33628	1.5008	2.1
33629	1.5008	2.1
33630	1.60139	0.05
33631	1.7103	0.05
33632	1.04159	0.05
33633	1.2489	0.05
33634	2.75189	0.05
33635	0.732812	0.05
33636	0.732812	0.05
33637	0.751986	0.05

33638	0.751986	0.05
33639	0.860346	0.05
33640	0.667961	0.05
33641	0.85515	0.05
33642	0.624452	0.05
33643	0.62445	0.05
33644	0.667962	0.05
33645	0.855148	0.05
33646	0.860348	0.05
33647	1.89226	0.279235
33648	2.50872	0.187215
33649	2.77909	0.178748
33650	1.89226	0.229791
33651	2.77909	0.230956
33652	1.89226	0.312971
33653	2.15825	0.0885916
33654	2.50872	0.0535473
33655	1.90439	0.05
33656	1.90439	0.0815145
33657	2.77909	0.0582648
33658	2.77909	0.153478
33659	1.12468	2.1
33660	1.43872	2.1
33661	1.64926	2.1
33662	1.2724	2.1
33663	2.77395	2.1
33664	1.64926	2.1
33665	1.38438	2.1
33666	1.47244	2.1
33667	1.2724	2.1
33668	1.8493	2.1
33669	1.64926	2.1
33670	1.36416	0.698785
33671	1.0652	2.1
33672	1.0652	1.35452
33673	1.02923	2.1
33674	1.37496	0.71421
33675	1.33898	2.1
33676	1.89227	2.1
33677	1.90433	2.1
33678	1.90433	2.1
33679	1.89359	2.1
33680	1.89227	2.1
33681	1.88153	2.1
33682	0.946129	2.1
33683	0.946129	2.1
33684	0.946133	1.26253
33685	0.946133	0.579055
33686	0.946131	0.519299
33687	0.946131	2.1
33688	0.946131	2.1
33689	0.946131	0.44385
33690	0.946131	1.80989
33691	0.946131	1.98982
33692	0.946131	1.75841
33693	0.946131	1.90186
33694	1.37496	0.912066
33695	1.07519	2.1

33696	1.07519	1.43587
33697	1.0652	2.1
33698	1.39725	0.843776
33699	1.38726	2.1
33700	1.89227	2.1
33701	1.9052	2.1
33702	1.9052	2.1
33703	1.90433	2.1
33704	1.89227	2.1
33705	1.8914	2.1
33706	0.946129	2.1
33707	0.946129	2.1
33708	0.946133	1.35591
33709	0.946133	0.73835
33710	0.946131	0.64988
33711	0.946131	2.1
33712	0.946131	2.1
33713	0.946131	0.54141
33714	0.946131	2.02499
33715	0.946131	2.1
33716	0.946131	1.98331
33717	0.946131	2.07529
33718	1.91316	0.120399
33719	1.89226	0.170759
33720	1.89226	0.200401
33721	1.89226	0.154617
33722	1.99366	0.0973916
33723	1.99366	0.076948
33724	1.93083	0.05
33725	1.89226	0.182712
33726	1.51379	0.0766967
33727	1.89226	0.0858854
33728	1.53051	0.0823014
33729	1.91316	0.0886567
33730	2.16731	2.1
33731	1.21373	2.1
33732	1.47804	2.1
33733	2.37601	2.1
33734	3.64535	2.1
33735	1.7211	2.1
33736	2.37601	2.1
33737	1.47804	2.1
33738	1.47804	2.1
33739	2.56843	2.1
33740	2.56843	2.1
33741	2.58731	2.1
33742	1.59094	2.1
33743	1.59094	2.1
33744	1.76816	2.1
33745	2.41707	2.1
33746	2.5943	2.1
33747	0.718346	2.1
33748	0.795468	2.1
33749	1.20854	2.1
33750	1.1224	2.1
33751	0.961203	2.1
33752	0.961203	2.1
33753	0.95667	2.1

33754	0.95667	2.1
33755	0.718346	2.1
33756	1.1224	2.1
33757	0.795469	2.1
33758	1.20854	2.1
33759	1.84076	2.1
33760	1.42884	2.1
33761	2.24274	2.1
33762	1.85415	2.1
33763	3.69491	2.1
33764	1.89226	2.1
33765	1.89226	2.1
33766	1.89226	2.1
33767	1.89226	2.1
33768	3.78452	2.1
33769	2.24273	2.1
33770	2.24412	2.1
33771	1.85415	2.1
33772	1.85415	2.1
33773	2.68473	2.1
33774	2.68473	2.1
33775	1.89226	2.1
33776	1.89226	2.1
33777	1.89226	2.1
33778	1.89226	2.09927
33779	1.89226	2.1
33780	1.89226	2.09242
33781	1.89226	2.09425
33782	1.89226	2.1
33783	1.89226	2.1
33784	1.89226	2.1
33785	3.78451	2.1
33786	1.85415	2.1
33787	1.10961	2.1
33788	2.24411	2.1
33789	1.51067	2.1
33790	3.35373	2.1
33791	1.70302	2.1
33792	1.89226	2.1
33793	1.76399	2.1
33794	1.43577	2.1
33795	2.08149	2.1
33796	1.70302	2.1
33797	1.89226	2.1
33798	1.89226	2.1
33799	1.89226	2.1
33800	1.89226	2.1
33801	1.89226	2.1
33802	1.89226	2.1
33803	1.0593	2.1
33804	1.17289	2.1
33805	1.5379	2.1
33806	1.46757	2.1
33807	2.71079	2.1
33808	0.931235	2.1
33809	1.1151	2.1
33810	0.975236	2.1
33811	0.975236	2.1

33812	1.31147	2.1
33813	0.762882	2.1
33814	1.13152	2.1
33815	0.505799	2.1
33816	1.31149	2.1
33817	1.07629	2.1
33818	0.884817	2.1
33819	0.505793	2.1
33820	2.69027	2.1
33821	1.60441	2.1
33822	2.69027	2.1
33823	1.64771	2.1
33824	2.78977	2.1
33825	1.72731	2.1
33826	0.77878	0.05
33827	0.843226	0.05
33828	0.845997	0.05
33829	0.926091	0.05
33830	0.77878	0.05
33831	0.926093	0.05
33832	1.3922	1.69852
33833	1.3922	1.58602
33834	0.872156	2.1
33835	0.872156	2.06653
33836	0.872157	2.1
33837	0.946132	1.05656
33838	0.946129	1.20486
33839	1.31822	2.1
33840	0.94613	1.99265
33841	0.94613	2.04124
33842	1.31823	1.93892
33843	0.872157	2.06254
33844	1.81534	0.744996
33845	1.89226	0.730643
33846	1.59602	0.948466
33847	1.59602	1.03133
33848	1.89226	0.182167
33849	1.89226	0.313922
33850	1.89227	0.77711
33851	1.89227	0.831004
33852	1.89227	0.880202
33853	1.89227	0.379169
33854	3.78453	0.456037
33855	1.89226	0.813009
33856	1.89226	0.0845359
33857	1.89226	0.323371
33858	1.89226	0.328606
33859	3.78453	0.766441
33860	2.30348	0.05
33861	1.4185	0.0682198
33862	1.82551	0.112595
33863	1.02011	0.05
33864	3.24401	0.05
33865	2.46702	0.05
33866	1.17129	0.05
33867	1.82551	0.05
33868	1.82551	0.05
33869	1.73525	0.05

33870	1.73525	0.0572742
33871	1.89227	0.635734
33872	1.89227	0.139677
33873	1.89227	0.167914
33874	1.89227	0.191706
33875	1.89227	0.488066
33876	1.89227	0.514788
33877	2.80034	2.1
33878	2.45767	2.1
33879	2.67994	2.1
33880	2.35395	2.1
33881	5.1376	2.1
33882	2.45766	2.1
33883	1.8769	2.1
33884	2.00989	2.1
33885	2.00989	2.1
33886	2.35395	2.1
33887	2.35395	2.1
33888	2.57662	2.1
33889	2.35395	2.1
33890	2.57662	2.1
33891	2.67993	2.1
33892	2.24227	2.1
33893	2.34558	2.1
33894	1.02874	2.1
33895	1.02874	2.1
33896	1.03084	2.1
33897	1.03084	2.1
33898	0.938448	2.1
33899	0.886762	2.1
33900	1.17698	2.1
33901	1.12114	2.1
33902	1.12113	2.1
33903	0.886764	2.1
33904	0.93845	2.1
33905	1.17697	2.1
33906	1.91969	2.1
33907	2.5508	2.1
33908	1.88273	2.1
33909	2.76609	2.1
33910	4.68578	2.1
33911	2.83423	2.1
33912	1.89226	2.1
33913	2.76609	2.1
33914	1.89226	2.1
33915	4.65835	2.1
33916	1.56685	0.832538
33917	1.11712	0.766637
33918	1.07999	1.01602
33919	1.51448	0.894569
33920	2.6316	0.880298
33921	1.56685	0.2937
33922	1.67652	0.937456
33923	1.5955	1.09957
33924	1.51448	0.849843
33925	3.19099	0.905407
33926	1.89226	0.954542
33927	1.89226	0.283427

33928	1.89226	0.870356
33929	1.89226	0.952044
33930	3.78453	0.893328
33931	1.89226	0.916969
33932	1.89226	0.721169
33933	1.89226	0.960041
33934	1.89226	0.982367
33935	1.89226	0.436331
33936	1.89226	0.565264
33937	1.0562	0.922332
33938	1.51448	0.467509
33939	1.07999	0.86906
33940	1.07999	0.427195
33941	1.46441	0.902137
33942	1.46441	0.610108
33943	1.5955	1.06281
33944	1.51448	0.651809
33945	1.46441	0.902805
33946	1.52909	1.01772
33947	3.04357	0.916425
33948	1.89226	0.781289
33949	1.89226	0.927998
33950	1.89226	0.951223
33951	1.89226	0.694544
33952	3.78453	0.476636
33953	1.89227	0.573714
33954	1.89227	0.109739
33955	1.89227	0.923978
33956	1.89227	0.778794
33957	3.78453	0.844767
33958	1.89994	0.13288
33959	1.70303	0.05
33960	1.68576	0.05
33961	1.89608	0.05
33962	3.8237	0.05
33963	1.89994	0.912764
33964	2.31116	1.23231
33965	1.9386	0.140094
33966	1.88653	1.40748
33967	3.74885	1.05115
33968	2.31117	2.06673
33969	2.20665	1.70028
33970	2.09617	1.40604
33971	2.41055	2.1
33972	2.09617	1.54141
33973	2.41055	2.1
33974	1.10777	0.05
33975	1.0523	0.05
33976	0.912098	0.05
33977	0.757533	0.05
33978	0.89287	0.103306
33979	1.07571	0.05
33980	0.77485	0.05
33981	0.98645	0.05
33982	0.989171	0.05
33983	0.875089	0.05
33984	0.979686	0.05
33985	0.979686	0.0695929

33986	2.52442	2.1
33987	1.49998	2.1
33988	2.51692	2.1
33989	1.56957	2.1
33990	4.09399	2.1
33991	1.01298	2.1
33992	1.01298	2.1
33993	1.03079	2.1
33994	1.03079	2.1
33995	0.820964	2.1
33996	0.784786	2.1
33997	1.25898	2.1
33998	1.25846	2.1
33999	1.25898	2.1
34000	0.820964	2.1
34001	1.25846	2.1
34002	0.784786	2.1
34003	1.89226	0.05
34004	1.89226	0.05
34005	2.6297	0.05
34006	1.89226	0.05
34007	3.78452	0.05
34008	1.89226	0.05
34009	1.89226	0.05
34010	1.89226	0.05
34011	1.89226	0.05
34012	1.89226	0.05
34013	1.89226	0.05
34014	2.19397	0.05
34015	1.89226	0.05
34016	2.34112	0.05
34017	2.34112	0.05
34018	1.89226	0.05
34019	1.89226	0.05
34020	2.23366	2.1
34021	1.87598	2.1
34022	1.64716	2.1
34023	2.06048	2.1
34024	3.93645	2.1
34025	2.23367	2.1
34026	2.17384	2.1
34027	2.04818	2.1
34028	2.06048	2.1
34029	4.23432	2.1
34030	1.9855	2.1
34031	2.06048	2.1
34032	1.93163	2.1
34033	1.50737	2.1
34034	3.56785	2.1
34035	2.04818	2.1
34036	2.06048	2.1
34037	2.27465	2.1
34038	1.92264	2.1
34039	3.98312	2.1
34040	2.08149	0.0632276
34041	1.89226	1.7154
34042	1.89226	1.25286
34043	1.33153	0.056169

34044	1.68038	1.92262
34045	1.18244	0.152678
34046	1.89226	0.651024
34047	1.89226	1.80575
34048	1.89226	1.63902
34049	1.89226	0.215019
34050	3.78452	0.828426
34051	2.69069	0.0942074
34052	1.89226	0.277791
34053	1.89226	0.303774
34054	1.97397	0.0915855
34055	1.89226	0.317081
34056	1.97397	0.0958246
34057	1.89226	1.80286
34058	2.38056	0.25467
34059	1.89226	0.262656
34060	1.89226	1.22574
34061	3.78452	0.940081
34062	0.904435	2.1
34063	0.904435	2.1
34064	0.925315	2.1
34065	0.925315	2.1
34066	1.13312	2.1
34067	1.08109	2.1
34068	0.70697	2.1
34069	0.696633	2.1
34070	0.70697	2.1
34071	1.13312	2.1
34072	0.696633	2.1
34073	1.08109	2.1
34074	1.45506	2.1
34075	2.26623	2.1
34076	2.38363	2.1
34077	2.38363	2.1
34078	1.41394	2.1
34079	1.41394	2.1
34080	0.946131	2.1
34081	0.946131	2.1
34082	1.13311	2.1
34083	1.13311	2.1
34084	0.946127	2.1
34085	0.946127	2.1
34086	1.13312	2.1
34087	1.19182	2.1
34088	1.19181	2.1
34089	0.946129	2.1
34090	1.13312	2.1
34091	0.94613	2.1
34092	1.89226	0.190523
34093	1.89226	2.09586
34094	1.89226	0.73035
34095	1.89226	2.01836
34096	1.89226	1.04343
34097	1.89226	2.08077
34098	1.05168	2.1
34099	0.696527	2.1
34100	0.907442	0.957845
34101	0.907442	1.37329

34102	0.953145	0.444301
34103	0.782285	2.1
34104	0.773928	2.1
34105	0.773928	0.876711
34106	0.696538	1.31936
34107	0.851554	1.47917
34108	1.04071	1.6018
34109	1.12335	1.5948
34110	1.89226	0.95002
34111	1.89226	0.36675
34112	1.89226	0.533322
34113	1.89226	0.112557
34114	3.78453	0.159048
34115	1.89226	0.870595
34116	1.89226	0.354501
34117	1.89226	0.56495
34118	1.89226	0.154917
34119	3.78453	0.840065
34120	2.50912	0.291059
34121	1.89226	0.112717
34122	1.89226	0.231234
34123	2.63195	0.087652
34124	4.40139	0.0949487
34125	2.50912	0.328583
34126	1.3598	0.118713
34127	1.70303	0.146615
34128	2.36874	0.05
34129	3.82055	0.231294
34130	1.89227	0.452874
34131	1.89227	0.186421
34132	1.89227	0.219915
34133	1.89227	0.05
34134	3.78453	0.522505
34135	1.89227	0.487061
34136	1.89227	0.160684
34137	1.89227	0.204819
34138	1.89227	0.0768039
34139	3.78453	0.0749591
34140	1.89226	0.218341
34141	2.67088	0.05
34142	2.63195	0.0509011
34143	2.63195	0.126059
34144	1.89226	0.0716424
34145	1.89226	0.265844
34146	1.30202	0.05
34147	1.28304	0.127206
34148	0.809902	0.0629879
34149	0.888801	0.0761119
34150	1.13262	0.05
34151	0.968276	0.05
34152	1.0733	0.103252
34153	1.0733	0.121019
34154	1.35419	0.05
34155	0.801222	0.05
34156	0.899732	0.176061
34157	1.23967	0.05
34158	1.31595	2.1
34159	0.869501	2.1

34160	2.65473	2.1
34161	1.88782	2.1
34162	3.20377	2.1
34163	1.89227	2.1
34164	1.89227	2.1
34165	1.89227	2.1
34166	1.89227	2.1
34167	3.78453	2.1
34168	2.65473	2.1
34169	1.89226	2.1
34170	1.88781	2.1
34171	1.88782	2.1
34172	2.0815	2.1
34173	2.0815	2.1
34174	1.89226	2.1
34175	1.89226	2.1
34176	1.89226	2.1
34177	1.89226	2.1
34178	1.89226	2.1
34179	1.89226	2.1
34180	1.89226	2.1
34181	1.89226	2.1
34182	1.89226	2.1
34183	1.89226	2.1
34184	1.89226	2.1
34185	1.89226	2.1
34186	2.39057	2.1
34187	1.89226	1.81622
34188	1.89226	0.327093
34189	1.89226	2.1
34190	2.14243	0.236229
34191	2.14243	2.1
34192	1.89226	0.266313
34193	1.89226	2.1
34194	1.89226	0.321424
34195	1.89226	0.679959
34196	1.89226	1.04694
34197	1.89226	2.1
34198	1.89226	0.093307
34199	1.89226	2.1
34200	1.89226	0.109907
34201	1.89226	0.333085
34202	1.89226	0.404277
34203	1.89226	2.0002
34204	2.56323	0.05
34205	1.36324	0.05
34206	1.05296	0.0752364
34207	2.18048	0.0577634
34208	3.54372	0.05
34209	0.946128	2.1
34210	0.946128	2.1
34211	1.08733	2.1
34212	1.08733	2.1
34213	0.946131	2.1
34214	0.946131	2.1
34215	1.09197	2.1
34216	1.08732	2.1
34217	0.946131	2.1

34218	1.09197	2.1
34219	1.08732	2.1
34220	0.946131	2.1
34221	1.35111	1.31874
34222	1.51327	1.15572
34223	1.64493	0.878073
34224	1.31058	1.38607
34225	2.99605	1.58261
34226	1.8124	0.899237
34227	1.51327	1.45376
34228	1.64493	1.5011
34229	1.31358	0.984717
34230	1.8124	1.4566
34231	1.48105	0.909958
34232	1.64493	1.07986
34233	1.31058	1.27335
34234	1.22297	1.11718
34235	1.70101	1.04942
34236	2.8679	0.776433
34237	1.70101	1.34488
34238	1.99297	1.04659
34239	1.64493	1.34145
34240	1.64493	0.933505
34241	1.99297	1.40188
34242	1.8124	0.533468
34243	2.0815	2.1
34244	1.80258	2.1
34245	1.80258	2.1
34246	1.83506	2.1
34247	2.0815	2.1
34248	2.1212	2.1
34249	2.0815	2.1
34250	1.82129	2.1
34251	1.82129	2.1
34252	1.80445	2.1
34253	2.0815	2.1
34254	2.06092	2.1
34255	1.89224	2.1
34256	1.703	2.1
34257	1.97839	2.1
34258	1.703	2.1
34259	1.97402	2.1
34260	1.703	2.1
34261	1.89226	0.82597
34262	1.89226	0.919986
34263	1.89226	0.878147
34264	2.75351	0.872461
34265	3.78453	0.88291
34266	1.89226	0.876261
34267	3.02887	1.00329
34268	2.03149	0.850474
34269	2.03149	0.90775
34270	1.89226	0.867181
34271	1.89226	0.930133
34272	2.17979	2.1
34273	1.65533	2.1
34274	2.57212	2.1
34275	2.20346	2.1

34276	4.22745	2.1
34277	1.89227	2.1
34278	2.20347	2.1
34279	1.89227	2.1
34280	2.57212	2.1
34281	1.89227	2.1
34282	2.57212	2.1
34283	2.05274	2.05146
34284	2.16783	2.1
34285	2.24429	0.96369
34286	2.38404	2.1
34287	4.41212	2.1
34288	2.16783	2.1
34289	2.05967	2.1
34290	2.38404	2.1
34291	2.24695	2.1
34292	4.41478	2.1
34293	2.24429	1.00215
34294	2.38404	2.1
34295	2.51819	1.19427
34296	2.60723	2.1
34297	4.85152	2.1
34298	2.38404	2.1
34299	2.24695	2.1
34300	2.60723	2.1
34301	2.45247	2.1
34302	4.85418	2.1
34303	1.89227	2.1
34304	1.89227	2.1
34305	1.89227	2.1
34306	1.89227	2.1
34307	3.78453	2.1
34308	1.89226	2.1
34309	1.89226	2.1
34310	1.89226	2.1
34311	1.89226	2.1
34312	3.78453	2.1
34313	0.618473	2.1
34314	0.619945	2.1
34315	0.797692	2.1
34316	0.770147	2.1
34317	0.68852	2.1
34318	0.68852	2.1
34319	0.701573	2.1
34320	0.701573	2.1
34321	0.619945	2.1
34322	0.797691	2.1
34323	0.618473	2.1
34324	0.770147	2.1
34325	0.797692	2.1
34326	0.770148	2.1
34327	0.694787	2.1
34328	0.653411	2.1
34329	0.722102	2.1
34330	0.722102	2.1
34331	0.729001	2.1
34332	0.729001	2.1
34333	0.797692	2.1

34334	0.694787	2.1
34335	0.770148	2.1
34336	0.653412	2.1
34337	1.89227	2.1
34338	1.89227	2.1
34339	1.89227	2.1
34340	1.89227	2.1
34341	3.78453	2.1
34342	1.89226	2.1
34343	1.89226	2.1
34344	1.89226	2.1
34345	1.89226	2.1
34346	3.78453	2.1
34347	1.23989	2.1
34348	1.59538	2.1
34349	1.66647	2.1
34350	1.26599	2.1
34351	2.90636	2.1
34352	1.66647	2.1
34353	1.38957	2.1
34354	1.47446	2.1
34355	1.40337	2.1
34356	1.66647	2.1
34357	1.59538	2.1
34358	2.80619	2.1
34359	2.0815	2.1
34360	2.0815	2.1
34361	2.0815	2.1
34362	2.73721	2.1
34363	2.74192	2.1
34364	2.73721	2.1
34365	2.0815	2.1
34366	2.0815	2.1
34367	2.0815	2.1
34368	2.689	2.1
34369	2.69166	2.1
34370	2.62369	2.1
34371	2.19717	2.1
34372	2.60405	2.1
34373	2.20566	2.1
34374	4.80122	2.1
34375	2.19717	2.1
34376	1.37392	2.1
34377	2.20566	2.1
34378	1.45408	2.1
34379	3.65125	2.1
34380	2.60405	2.1
34381	2.20566	2.1
34382	2.57466	2.1
34383	2.21715	2.1
34384	4.8212	2.1
34385	2.20566	2.1
34386	1.54249	2.1
34387	1.54249	2.1
34388	1.45408	2.1
34389	2.21715	2.1
34390	2.12874	2.1
34391	1.60647	2.1

34392	1.31346	2.1
34393	1.82649	2.1
34394	1.51067	2.1
34395	3.13995	2.1
34396	1.89226	2.1
34397	1.89226	2.1
34398	1.89226	2.1
34399	1.89226	2.1
34400	3.78452	2.1
34401	1.89226	2.1
34402	2.6096	2.1
34403	2.8067	2.1
34404	2.8067	2.1
34405	1.89226	2.1
34406	1.89226	2.1
34407	1.82649	2.1
34408	1.3596	2.1
34409	2.17708	2.1
34410	1.70302	2.1
34411	3.40243	2.1
34412	1.89226	2.1
34413	1.89226	2.1
34414	1.89226	1.65613
34415	1.89226	2.1
34416	3.78452	2.1
34417	2.27067	2.1
34418	2.43549	2.1
34419	2.63979	2.1
34420	2.41828	2.1
34421	2.27067	2.1
34422	2.04916	2.1
34423	1.30047	2.1
34424	2.04916	2.1
34425	2.27068	2.1
34426	1.5586	2.1
34427	3.57115	2.1
34428	2.27068	2.1
34429	1.84214	2.1
34430	1.80224	2.1
34431	1.5586	2.1
34432	2.51432	2.1
34433	2.27068	2.1
34434	1.63719	2.1
34435	2.15714	2.1
34436	2.07821	2.1
34437	1.52204	2.1
34438	3.67919	2.1
34439	2.07821	2.1
34440	1.40118	2.1
34441	1.40118	2.1
34442	1.52204	2.1
34443	1.99632	2.1
34444	2.11718	2.1
34445	0.946131	2.1
34446	0.946131	2.1
34447	1.06957	2.1
34448	1.06957	2.1
34449	0.946132	2.1

34450	0.946132	1.93768
34451	1.411	2.1
34452	1.06957	2.1
34453	0.946132	2.1
34454	1.411	2.1
34455	1.06957	2.1
34456	0.946132	2.1
34457	1.99875	0.932411
34458	2.57079	0.908441
34459	2.59319	1.15868
34460	1.9105	0.812482
34461	4.59194	0.945392
34462	1.99875	0.911487
34463	1.02247	0.11917
34464	1.07017	0.289188
34465	1.15841	0.385123
34466	1.91051	0.233499
34467	1.99875	0.464955
34468	2.59319	1.08926
34469	1.9105	0.849803
34470	2.33812	0.924384
34471	2.59319	0.914169
34472	1.59457	0.963002
34473	1.59457	0.954928
34474	2.50906	0.813222
34475	2.0467	0.75003
34476	2.03357	0.774158
34477	2.53176	0.747065
34478	4.54263	0.762528
34479	1.294	0.854366
34480	1.66636	0.791412
34481	2.03357	0.76992
34482	1.03079	0.758485
34483	3.32757	0.779359
34484	1.68108	0.758486
34485	2.53176	0.630815
34486	2.36417	0.711557
34487	2.71666	0.510395
34488	1.68108	0.827224
34489	2.03357	0.808265
34490	1.89226	0.0782831
34491	2.59302	0.0968447
34492	2.2727	0.05
34493	2.2727	0.05
34494	1.89226	0.146975
34495	1.89226	0.0896622
34496	1.89226	0.271228
34497	1.89226	0.10328
34498	1.89226	0.0931471
34499	1.89226	0.05
34500	3.78452	0.257434
34501	1.89225	0.514096
34502	1.89225	0.05
34503	1.89225	0.05
34504	1.89225	0.102125
34505	1.89225	0.0595139
34506	1.89225	0.140258
34507	0.934114	0.15689

34508	0.934114	0.05
34509	0.944544	0.110425
34510	0.944544	0.143827
34511	0.721493	0.05
34512	0.58215	0.05
34513	1.2965	0.0808447
34514	1.13634	0.05
34515	1.13635	0.05
34516	0.582148	0.05
34517	1.29651	0.0890115
34518	0.721491	0.05
34519	1.89226	0.0648835
34520	1.89226	0.05
34521	1.89226	0.05
34522	1.89226	0.05
34523	3.78452	0.05
34524	0.946131	0.139881
34525	0.946131	0.149269
34526	0.946131	0.117563
34527	0.946131	0.174498
34528	0.946131	0.114584
34529	0.946131	0.144803
34530	0.946131	0.140842
34531	0.946131	0.361779
34532	0.946131	0.112743
34533	0.946131	0.114185
34534	0.946131	0.172062
34535	0.946131	0.16158
34536	1.89226	0.05
34537	1.89226	0.05
34538	2.78602	0.05
34539	1.89226	0.05
34540	3.78453	0.05
34541	1.52601	2.1
34542	1.76601	2.1
34543	1.27839	2.1
34544	1.56095	2.1
34545	3.0444	2.1
34546	1.76601	2.1
34547	1.86793	2.1
34548	1.56095	2.1
34549	1.68152	2.1
34550	3.44753	2.1
34551	1.27841	2.1
34552	1.56097	2.1
34553	1.0577	2.1
34554	1.36861	2.1
34555	2.64702	2.1
34556	1.56097	2.1
34557	1.68154	2.1
34558	1.36861	2.1
34559	1.53296	2.1
34560	3.05015	2.1
34561	1.89226	2.1
34562	1.89226	2.1
34563	1.89226	2.1
34564	1.89226	2.1
34565	3.78453	2.1

34566	0.983075	2.1
34567	0.788361	2.1
34568	0.724726	2.1
34569	0.724726	2.1
34570	0.983027	2.1
34571	0.724734	2.1
34572	0.788364	2.1
34573	0.788364	2.1
34574	0.788335	2.1
34575	0.94613	2.1
34576	0.724757	2.1
34577	0.946131	2.1
34578	1.15789	2.1
34579	2.11135	2.1
34580	2.25826	2.1
34581	1.28845	2.1
34582	3.41615	2.1
34583	1.89226	2.1
34584	1.89226	2.1
34585	1.89226	2.1
34586	1.89226	2.1
34587	3.78453	2.1
34588	1.57667	2.1
34589	1.70002	2.1
34590	1.70002	2.1
34591	1.89226	2.1
34592	1.89226	2.1
34593	2.05244	2.1
34594	2.25826	2.1
34595	1.42214	2.1
34596	1.43675	2.1
34597	1.28845	2.1
34598	2.40656	2.1
34599	2.25826	2.1
34600	1.58066	2.1
34601	1.74247	2.1
34602	1.85097	2.1
34603	1.61155	2.1
34604	3.43163	2.1
34605	0.785612	2.1
34606	0.789638	2.1
34607	0.882561	2.1
34608	0.763529	2.1
34609	0.686921	2.1
34610	0.86048	2.1
34611	0.871812	2.1
34612	0.871812	2.1
34613	0.830183	2.1
34614	0.882572	2.1
34615	0.902315	2.1
34616	0.785602	2.1
34617	1.01972	0.05
34618	0.936657	0.05
34619	0.946131	0.05
34620	0.946131	0.05
34621	0.927018	0.05
34622	0.927018	0.05
34623	1.1376	0.05

34624	0.953663	0.05
34625	1.1257	0.05
34626	1.04073	0.05
34627	1.00358	0.05
34628	0.851528	0.05
34629	1.73576	0.2617
34630	2.00244	0.674137
34631	1.17798	0.288381
34632	1.25796	0.289843
34633	2.99373	0.275716
34634	2.00244	0.722715
34635	1.10719	0.696568
34636	2.03236	0.765882
34637	2.03236	0.736201
34638	1.25797	0.758373
34639	1.25797	0.699765
34640	1.89226	2.1
34641	1.89226	2.1
34642	1.89226	2.1
34643	1.89226	2.1
34644	3.78452	2.1
34645	2.3174	2.1
34646	1.50005	2.1
34647	1.90428	2.1
34648	2.64564	2.1
34649	4.22168	2.1
34650	1.89226	2.1
34651	1.89226	2.1
34652	1.89226	2.1
34653	1.89226	2.1
34654	1.89226	2.1
34655	1.89226	2.1
34656	1.32749	0.05
34657	1.70302	0.0572701
34658	2.08149	0.05
34659	1.70302	0.05
34660	2.19634	0.05
34661	1.93083	0.05
34662	1.89226	1.45818
34663	1.89226	2.09422
34664	1.89226	0.945205
34665	1.89226	2.1
34666	3.78453	1.18359
34667	1.89226	1.42872
34668	1.89226	2.08223
34669	1.89226	1.10566
34670	1.89226	1.44793
34671	3.78452	1.78045
34672	2.17499	1.16735
34673	1.79663	1.98674
34674	2.1538	0.956605
34675	1.818	1.21105
34676	2.17499	1.71489
34677	1.83919	2.1
34678	0.898316	2.1
34679	1.0769	1.28096
34680	0.968276	2.1
34681	0.773495	1.57623

34682	0.921126	2.0292
34683	0.921126	2.1
34684	0.92927	1.76346
34685	0.92927	1.81792
34686	1.0769	1.75609
34687	0.968276	1.32535
34688	0.898316	2.08772
34689	0.773495	1.53593
34690	1.89226	1.10386
34691	1.89226	2.1
34692	1.89226	1.32284
34693	1.89226	1.22643
34694	3.78453	2.1
34695	1.89226	1.39431
34696	1.89226	2.1
34697	1.89226	1.03217
34698	1.89226	1.69337
34699	3.78452	1.27121
34700	2.17499	1.95842
34701	2.1538	2.1
34702	2.53574	1.21816
34703	2.54157	1.11254
34704	4.71073	1.87722
34705	1.0769	1.64672
34706	1.26787	1.21781
34707	1.16641	1.42775
34708	0.968276	2.1
34709	1.11625	1.43443
34710	1.11625	1.94783
34711	1.11989	2.08582
34712	1.11989	1.01758
34713	1.26787	1.27376
34714	1.16641	2.1
34715	1.0769	2.1
34716	0.968277	1.87241
34717	1.89226	0.734834
34718	1.89226	0.745682
34719	1.89226	0.736245
34720	1.89226	0.72114
34721	3.78452	0.731025
34722	1.91739	0.720919
34723	2.20045	0.700668
34724	1.79005	0.703411
34725	2.33802	0.707048
34726	4.25539	0.710122
34727	2.52754	0.709527
34728	1.89226	0.714078
34729	2.69841	0.708365
34730	2.69841	0.71693
34731	1.89226	0.70614
34732	1.89226	0.713303
34733	1.89226	0.740265
34734	1.89226	0.737686
34735	1.89226	0.719736
34736	1.89226	0.73084
34737	3.78452	0.729591
34738	1.93544	0.710683
34739	2.33802	0.715597

34740	1.55063	0.713233
34741	2.1769	0.700683
34742	3.88865	0.705512
34743	2.20929	0.05
34744	2.55162	0.05
34745	2.27807	0.05
34746	1.48831	0.05
34747	4.48736	0.05
34748	2.27807	0.05
34749	2.72723	0.05
34750	2.55162	0.05
34751	2.55162	0.05
34752	2.43157	0.05
34753	2.43157	0.05
34754	2.27807	0.05
34755	1.51612	0.05
34756	1.96048	0.05
34757	1.73022	0.05
34758	1.70302	0.05
34759	1.61016	0.05
34760	2.27807	0.05
34761	2.08048	0.05
34762	1.98522	0.05
34763	2.43157	0.05
34764	1.83798	0.05
34765	2.27807	0.05
34766	1.70302	0.05
34767	2.68507	0.05
34768	2.23856	0.05
34769	2.52174	0.05
34770	2.35331	0.05
34771	2.65099	0.05
34772	1.70303	0.0542416
34773	0.986848	0.05
34774	2.41566	0.05
34775	1.70303	0.05
34776	1.88139	0.05
34777	1.64955	0.05
34778	1.89226	2.1
34779	2.34978	1.94833
34780	1.89226	2.1
34781	2.68773	1.65572
34782	1.89226	2.1
34783	2.68773	1.87014
34784	1.89226	2.1
34785	1.89226	1.61952
34786	1.89226	1.61462
34787	1.89226	2.1
34788	3.78453	2.06827
34789	1.00825	0.607189
34790	1.09847	0.925214
34791	0.939987	1.03892
34792	0.783516	1.87925
34793	0.838041	1.55565
34794	0.838041	1.9556
34795	1.49693	2.01021
34796	1.26602	1.55362
34797	1.10875	0.6269

34798	1.05187	2.1
34799	1.28743	1.62388
34800	0.946132	1.22417
34801	0.946132	1.65732
34802	0.946132	2.1
34803	0.946132	2.09629
34804	0.946132	0.638809
34805	1.31823	2.1
34806	0.946132	1.61088
34807	0.946132	2.1
34808	0.946131	2.1
34809	0.946131	2.1
34810	0.946132	1.9075
34811	1.31823	2.08041
34812	1.51688	0.05
34813	1.79978	0.05
34814	1.1685	0.05
34815	1.1685	0.05
34816	1.85857	0.05
34817	1.51018	0.05
34818	0.946116	0.05
34819	0.946116	0.05
34820	0.94612	0.05
34821	0.94612	0.05
34822	0.946118	0.05
34823	0.946118	0.05
34824	0.946118	0.05
34825	0.946118	0.05
34826	0.946131	0.05
34827	0.946131	0.05
34828	0.946105	0.05
34829	0.946105	0.05
34830	1.89224	2.1
34831	1.89224	2.1
34832	1.89224	2.1
34833	1.89224	2.1
34834	1.89224	2.1
34835	1.89224	2.1
34836	1.38678	2.1
34837	1.3349	2.1
34838	0.946119	2.1
34839	0.946119	2.1
34840	1.38678	2.1
34841	1.38678	2.1
34842	0.946119	2.1
34843	0.946119	2.1
34844	1.38676	2.1
34845	0.946107	2.1
34846	0.946132	2.1
34847	1.33491	2.1
34848	1.06455	2.1
34849	1.03932	2.1
34850	1.20649	2.1
34851	1.18773	2.1
34852	2.27105	2.1
34853	1.89226	2.1
34854	1.89226	2.1
34855	1.89226	2.1

34856	1.89226	2.1
34857	1.89226	2.1
34858	1.89226	2.1
34859	1.36918	2.1
34860	1.18774	2.1
34861	1.31144	2.1
34862	1.20651	2.1
34863	1.31144	2.1
34864	1.20651	2.1
34865	2.29066	2.1
34866	1.76476	2.1
34867	1.79825	2.1
34868	2.33801	2.1
34869	4.08891	2.1
34870	1.79825	2.1
34871	2.33801	2.1
34872	2.37168	2.1
34873	1.49325	2.1
34874	4.16993	2.1
34875	1.13641	2.1
34876	1.43213	0.804389
34877	1.44085	2.1
34878	1.18301	2.1
34879	2.61513	2.1
34880	1.89226	2.1
34881	2.23875	2.1
34882	2.23875	2.1
34883	2.10287	2.1
34884	1.89226	2.1
34885	1.75639	2.1
34886	1.89226	1.27117
34887	1.89226	2.1
34888	1.89226	1.29694
34889	1.89226	2.1
34890	1.89226	1.26787
34891	1.89226	2.1
34892	1.44085	2.1
34893	1.25173	2.1
34894	1.25173	1.90255
34895	1.18301	2.1
34896	1.4617	1.5056
34897	1.39298	2.1
34898	1.89227	2.1
34899	2.23875	2.1
34900	2.42504	2.1
34901	1.89227	2.1
34902	4.13101	2.1
34903	1.89226	1.27582
34904	1.89226	2.1
34905	1.89226	1.20751
34906	1.89226	2.1
34907	1.89226	1.19256
34908	1.89226	2.1
34909	1.89226	2.1
34910	2.41297	2.1
34911	2.70685	2.1
34912	2.70685	2.1
34913	1.89226	2.1

34914	1.89226	2.1
34915	1.89226	2.1
34916	1.89226	2.1
34917	1.89226	2.1
34918	1.89226	2.1
34919	1.89226	2.1
34920	1.89226	2.1
34921	1.89226	2.1
34922	1.89226	2.1
34923	1.89226	2.1
34924	1.89226	2.1
34925	3.78453	2.1
34926	1.89226	2.1
34927	1.89226	2.1
34928	1.89226	2.1
34929	1.89226	2.1
34930	1.89226	2.1
34931	1.89226	2.1
34932	1.69044	0.05
34933	1.57979	0.05
34934	2.27963	0.05
34935	2.21582	0.05
34936	3.85942	0.05
34937	2.21582	0.05
34938	1.30722	0.05
34939	2.02069	0.05
34940	2.02069	0.05
34941	1.57979	0.05
34942	1.57979	0.05
34943	1.67825	2.1
34944	1.89226	2.1
34945	1.89226	2.1
34946	1.89226	2.1
34947	1.91894	2.1
34948	1.91894	2.1
34949	0.946133	2.1
34950	0.946133	2.1
34951	0.946132	2.1
34952	0.946132	2.1
34953	0.946132	2.1
34954	0.946132	2.1
34955	0.946133	2.1
34956	0.946133	2.1
34957	0.946133	2.1
34958	0.946133	2.1
34959	0.946132	2.1
34960	0.946132	2.1
34961	0.942609	2.1
34962	0.942609	2.1
34963	1.329 2.1	
34964	1.329 2.1	
34965	1.32547	2.1
34966	0.94261	2.1
34967	0.946132	2.1
34968	0.946132	2.1
34969	0.946132	2.1
34970	1.32547	2.1
34971	0.946132	2.1

34972	0.94261	2.1
34973	1.55831	2.1
34974	1.07496	2.1
34975	1.80327	0.96029
34976	1.34851	1.05308
34977	2.87824	1.13609
34978	2.14346	0.827782
34979	2.77937	0.319416
34980	2.79173	0.972469
34981	2.17129	0.739999
34982	4.95066	0.255438
34983	2.14346	0.852233
34984	1.34124	0.05
34985	1.39818	0.80757
34986	2.1713	0.700244
34987	3.51253	0.798352
34988	2.79173	0.898526
34989	2.21882	0.202069
34990	2.17129	0.352926
34991	2.17129	0.321065
34992	2.81448	0.803291
34993	2.81448	0.731717
34994	1.10941	0.248025
34995	1.08565	0.288069
34996	0.699089	0.0864342
34997	0.740176	0.686039
34998	0.908594	0.0818971
34999	0.908594	0.0737569
35000	0.91723	0.867363
35001	0.91723	0.0976817
35002	1.10941	0.402057
35003	0.740176	0.188176
35004	0.699089	0.10566
35005	1.08565	0.10958
35006	1.89227	0.571089
35007	1.89227	0.428218
35008	1.89227	0.05
35009	1.89227	0.0637314
35010	3.78453	0.691963
35011	2.40032	0.411907
35012	2.19526	0.324096
35013	1.12907	0.05
35014	1.44253	0.596314
35015	3.32433	0.05
35016	2.19526	0.05
35017	2.71093	0.380602
35018	1.29826	0.111007
35019	2.0815	0.05
35020	4.18911	0.300978
35021	1.89226	0.05
35022	1.89226	0.144689
35023	1.89226	0.05
35024	1.89226	0.05
35025	3.78452	0.05
35026	2.30822	0.05
35027	1.89226	0.0502574
35028	2.79329	0.05
35029	2.79329	0.05

35030	1.89226	0.122575
35031	1.89226	0.05
35032	0.946131	2.1
35033	0.946131	1.14702
35034	1.38414	2.1
35035	1.38414	0.05
35036	0.94613	2.1
35037	0.94613	2.1
35038	1.38415	1.06626
35039	1.41833	0.224474
35040	0.946132	1.85915
35041	1.41833	1.89898
35042	1.38414	1.43574
35043	0.946131	2.09981
35044	1.89226	2.1
35045	2.6536	2.1
35046	1.89226	2.1
35047	2.72238	2.1
35048	4.61465	2.1
35049	0.946132	2.1
35050	0.946132	2.1
35051	1.38657	2.1
35052	1.38657	2.1
35053	1.38657	2.1
35054	1.36119	2.1
35055	0.946131	2.1
35056	0.946131	2.1
35057	0.946132	2.1
35058	1.38657	2.1
35059	0.946132	2.1
35060	1.36119	2.1
35061	1.89226	2.1
35062	2.4717	2.1
35063	2.5026	2.1
35064	2.4717	2.1
35065	1.92316	2.1
35066	1.89226	2.1
35067	1.89226	2.1
35068	1.89226	2.1
35069	1.89226	2.1
35070	1.89226	2.1
35071	3.78453	2.1
35072	0.946128	2.1
35073	0.946128	2.1
35074	1.3419	2.1
35075	1.3419	2.1
35076	0.946131	2.1
35077	0.946131	2.1
35078	1.36607	2.1
35079	1.3419	2.1
35080	0.946131	2.1
35081	1.36607	2.1
35082	1.3419	2.1
35083	0.94613	2.1
35084	1.89226	2.1
35085	2.50259	2.1
35086	2.53563	2.1
35087	2.50259	2.1

35088	1.92529	2.1
35089	1.89226	2.1
35090	0.946132	2.1
35091	0.946132	2.1
35092	0.946131	2.1
35093	0.946131	2.1
35094	0.946132	2.1
35095	0.946132	2.1
35096	0.946131	2.1
35097	0.946131	2.1
35098	0.946131	2.1
35099	0.946131	2.1
35100	0.946131	2.1
35101	0.946131	2.1
35102	2.0815	2.1
35103	1.86506	2.1
35104	1.86506	2.1
35105	1.82567	2.1
35106	2.0815	2.1
35107	2.03335	2.1
35108	2.0815	2.1
35109	2.0815	2.1
35110	2.0815	2.1
35111	1.88668	2.1
35112	1.83221	2.1
35113	1.57275	2.1
35114	2.34061	0.05
35115	1.89226	0.05
35116	2.23254	0.05
35117	1.89226	0.05
35118	2.23254	0.05
35119	1.89226	0.05
35120	1.8619	0.05
35121	1.89226	0.05
35122	2.34061	0.05
35123	1.89226	0.05
35124	2.34061	0.05
35125	1.89226	0.05
35126	1.89227	0.05
35127	1.89227	0.05
35128	1.89227	0.05
35129	1.89227	0.05
35130	1.89227	0.05
35131	1.89227	0.05
35132	1.89227	0.05
35133	1.89227	0.05
35134	1.89227	0.05
35135	1.89227	0.05
35136	1.89227	0.05
35137	1.89227	0.05
35138	1.06365	0.05
35139	0.503305	0.05
35140	0.794912	0.05
35141	1.42752	0.05
35142	1.34826	0.05
35143	1.07163	0.05
35144	0.533113	0.05
35145	0.761277	0.05

35146	0.848403	0.05
35147	0.876191	0.05
35148	0.963372	0.05
35149	0.963372	0.05
35150	1.55571	0.05
35151	2.76574	0.05
35152	2.92453	0.05
35153	1.70301	0.05
35154	2.33089	0.05
35155	1.06905	0.05
35156	2.28979	2.1
35157	1.81488	2.1
35158	1.63722	2.1
35159	2.22627	2.1
35160	4.04115	2.1
35161	1.98246	2.1
35162	2.15715	2.1
35163	2.22627	2.1
35164	2.22627	2.1
35165	1.63719	2.1
35166	1.63719	2.1
35167	2.1644	0.05
35168	1.903 0.05	
35169	1.23639	0.05
35170	1.42704	0.05
35171	3.61546	0.05
35172	1.89226	0.05
35173	1.89226	0.05
35174	1.89226	0.05
35175	1.89226	0.05
35176	3.78451	0.05
35177	2.61343	0.05
35178	1.89226	0.05
35179	2.08149	0.05
35180	1.74357	0.05
35181	2.08149	0.05
35182	1.74357	0.05
35183	1.63483	0.05
35184	1.8631	0.05
35185	1.8631	0.05
35186	1.70301	0.05
35187	2.0815	0.05
35188	1.94319	0.05
35189	2.0815	0.05
35190	1.89226	0.05
35191	1.89226	0.05
35192	2.05236	0.05
35193	2.23014	0.05
35194	2.41882	0.05
35195	2.14993	2.1
35196	1.87217	2.1
35197	2.34488	2.1
35198	2.5254	2.1
35199	4.39757	2.1
35200	0.949995	2.1
35201	0.630972	2.1
35202	0.936084	2.1
35203	1.17244	2.1

35204	0.922394	2.1
35205	0.922394	2.1
35206	0.88102	2.1
35207	0.88102	2.1
35208	0.949998	2.1
35209	1.17244	2.1
35210	0.936081	2.1
35211	0.630973	2.1
35212	1.89226	2.1
35213	1.89226	2.1
35214	1.89226	2.1
35215	1.89226	2.1
35216	3.78452	2.1
35217	1.89226	2.1
35218	1.89226	2.1
35219	1.89226	2.1
35220	1.89226	2.1
35221	1.89226	2.084
35222	1.89226	2.1
35223	2.34488	2.1
35224	2.5254	2.09224
35225	1.89226	2.1
35226	1.89226	2.1
35227	4.41766	2.1
35228	1.89226	2.1
35229	1.89226	2.1
35230	1.89226	2.1
35231	1.89226	2.1
35232	3.78452	2.1
35233	1.89226	1.84454
35234	1.89226	2.1
35235	1.89226	1.94168
35236	1.89226	2.08096
35237	1.89226	2.01633
35238	1.89226	2.1
35239	1.5605	2.1
35240	1.15806	2.1
35241	1.31565	2.1
35242	1.65642	2.1
35243	2.87615	2.1
35244	1.46643	2.1
35245	1.65642	2.1
35246	1.31565	2.1
35247	1.31565	2.1
35248	1.74246	2.1
35249	1.74246	2.1
35250	1.89226	2.1
35251	1.89226	2.1
35252	1.89226	2.1
35253	1.89226	2.1
35254	1.89226	2.1
35255	1.89226	2.1
35256	1.15099	2.1
35257	1.15099	2.1
35258	0.939316	2.1
35259	0.939316	2.1
35260	0.946127	2.1
35261	0.946127	2.1

35262	1.14418	2.1
35263	0.939289	2.1
35264	1.14417	2.1
35265	0.946129	2.1
35266	0.939287	2.1
35267	0.94613	2.1
35268	1.89226	2.1
35269	1.89226	2.1
35270	1.89226	2.1
35271	1.89226	2.1
35272	3.78452	2.1
35273	1.93164	0.05
35274	2.64898	0.05
35275	1.43213	0.05
35276	1.43213	0.05
35277	2.63206	0.05
35278	2.13254	0.05
35279	1.89226	2.1
35280	1.89226	2.1
35281	1.89226	2.1
35282	1.89226	2.1
35283	3.78452	2.1
35284	2.71655	2.1
35285	1.43323	2.1
35286	1.28413	2.1
35287	2.53614	2.1
35288	3.96937	2.1
35289	1.89226	2.1
35290	1.89226	2.1
35291	1.89226	2.1
35292	1.89226	2.1
35293	1.89226	2.1
35294	1.89226	2.1
35295	1.33356	2.1
35296	2.82297	2.1
35297	2.771 2.1	
35298	2.771 2.1	
35299	1.46037	2.1
35300	1.46037	2.1
35301	2.56034	2.1
35302	1.18474	2.1
35303	1.15113	2.1
35304	2.53724	2.1
35305	3.72198	2.1
35306	2.56034	2.1
35307	1.89226	2.1
35308	1.89226	2.1
35309	2.53725	2.1
35310	4.42951	2.1
35311	1.89226	2.1
35312	1.89226	2.1
35313	1.89226	2.1
35314	1.89226	2.1
35315	3.78453	2.1
35316	1.89226	2.1
35317	1.89226	2.1
35318	1.89226	2.1
35319	1.89226	2.1

35320	3.78452	2.1
35321	1.1229	2.1
35322	2.53724	2.1
35323	1.15113	2.1
35324	1.15113	2.1
35325	2.51559	2.1
35326	2.51559	2.1
35327	1.89226	2.1
35328	2.53724	2.1
35329	2.51559	2.1
35330	2.53724	2.1
35331	1.87061	2.1
35332	1.89226	2.1
35333	1.89226	2.1
35334	1.89226	2.1
35335	1.89226	2.1
35336	1.89226	2.1
35337	3.78453	2.1
35338	1.89226	2.1
35339	1.89226	2.1
35340	1.89226	2.1
35341	1.89226	2.1
35342	3.78452	2.1
35343	1.98143	1.24547
35344	2.81559	0.05
35345	1.65411	0.123722
35346	2.26675	0.717485
35347	4.24818	0.200152
35348	0.810656	2.1
35349	0.810656	2.1
35350	0.821888	2.1
35351	0.821888	2.1
35352	1.1235	2.1
35353	1.07439	2.1
35354	0.535693	2.1
35355	0.509042	2.1
35356	0.535693	2.1
35357	1.1235	2.1
35358	0.509043	2.1
35359	1.07439	2.1
35360	1.10378	2.1
35361	2.247 2.1	
35362	2.27472	2.1
35363	2.27472	2.1
35364	1.07139	2.1
35365	1.07139	2.1
35366	2.08081	2.1
35367	1.40395	2.1
35368	1.34245	2.1
35369	2.00239	2.1
35370	3.40634	2.1
35371	1.89226	2.1
35372	1.89226	2.1
35373	1.89226	2.1
35374	1.89226	2.1
35375	3.78453	2.1
35376	0.946128	2.1
35377	0.946128	2.1

35378	1.13785	2.1
35379	1.11496	2.1
35380	0.946131	2.1
35381	0.946131	2.1
35382	1.11495	2.1
35383	1.11495	2.1
35384	0.94613	2.1
35385	1.11495	2.1
35386	1.13785	2.1
35387	0.946131	2.1
35388	0.975888	2.1
35389	1.00118	2.1
35390	0.671218	2.1
35391	0.653229	2.1
35392	0.825372	2.1
35393	0.825372	2.1
35394	0.829038	2.1
35395	0.829038	2.1
35396	0.975901	2.1
35397	0.653238	2.1
35398	0.671209	2.1
35399	1.00117	2.1
35400	1.89224	2.1
35401	1.89224	2.1
35402	1.89224	2.1
35403	1.89224	2.1
35404	3.78448	2.1
35405	0.946115	2.1
35406	0.946115	2.1
35407	1.11494	2.1
35408	1.10111	2.1
35409	0.946118	2.1
35410	0.946118	2.1
35411	1.10111	2.1
35412	1.10111	2.1
35413	1.10112	2.1
35414	0.94613	2.1
35415	0.946105	2.1
35416	1.11492	2.1
35417	1.04028	0.769323
35418	2.27126	0.0531063
35419	2.0815	0.69963
35420	1.82249	0.3548
35421	1.33723	0.70429
35422	1.18684	0.239304
35423	0.812616	0.258725
35424	0.812616	0.332452
35425	0.997777	0.0790253
35426	1.08122	0.532399
35427	0.94613	0.0992969
35428	0.94613	0.280223
35429	0.812648	0.343838
35430	0.932779	0.139027
35431	0.812648	0.0767341
35432	0.94613	0.337833
35433	0.849335	0.101739
35434	0.94613	0.11819
35435	1.80327	1.13246

35436	1.56016	1.03111
35437	1.57731	1.98011
35438	1.34851	1.87219
35439	2.03207	1.1144
35440	1.80327	2.04788
35441	2.03207	1.85376
35442	1.56016	1.60767
35443	2.18076	1.17417
35444	1.78167	1.15184
35445	3.81374	1.66283
35446	1.37597	2.02411
35447	1.60058	2.1
35448	1.70302	1.11899
35449	1.85448	1.33597
35450	3.15028	1.31104
35451	1.60058	1.26915
35452	1.4054	2.1
35453	1.66202	1.15352
35454	1.70302	2.1
35455	3.09979	1.49418
35456	1.70302	1.5725
35457	1.85448	1.89554
35458	2.21317	1.24137
35459	2.0961	1.26339
35460	4.01757	1.72657
35461	1.66202	1.42137
35462	1.70302	2.1
35463	2.09611	1.06064
35464	2.00892	1.81622
35465	3.6468	1.29183
35466	1.2966	0.05
35467	2.25495	0.05
35468	1.2966	0.05
35469	2.21117	0.05
35470	1.67687	0.05
35471	2.6167	0.05
35472	1.89227	0.05
35473	1.89227	0.05
35474	1.89227	0.05
35475	1.89227	0.05
35476	3.78453	0.05
35477	2.44375	0.05
35478	1.89227	0.05
35479	2.81853	0.05
35480	2.81853	0.05
35481	1.89227	0.05
35482	1.89227	0.05
35483	1.89226	1.09125
35484	1.75853	1.1624
35485	2.37636	1.08476
35486	2.37636	1.22544
35487	1.89226	1.01043
35488	1.89226	1.13728
35489	1.65814	0.0818365
35490	1.10187	2.1
35491	1.10187	0.819741
35492	1.19547	2.1
35493	1.56263	0.05

35494	1.65624	2.1
35495	1.56264	0.352968
35496	1.01479	2.1
35497	1.01479	0.892301
35498	1.10187	2.1
35499	1.47561	0.05
35500	1.5627	2.1
35501	1.89225	2.1
35502	1.89225	2.1
35503	1.89225	2.1
35504	1.89225	2.1
35505	3.7845	2.1
35506	2.54386	2.1
35507	1.91546	2.1
35508	2.46008	2.1
35509	1.82815	2.1
35510	4.37554	2.1
35511	1.91546	2.1
35512	1.04909	2.1
35513	1.82815	2.1
35514	0.983141	2.1
35515	2.8986	2.1
35516	1.89225	2.1
35517	1.89225	2.1
35518	1.89225	2.1
35519	1.89225	2.1
35520	3.78449	2.1
35521	1.89226	2.1
35522	1.89226	2.1
35523	1.89226	2.1
35524	1.89226	2.1
35525	1.89226	2.1
35526	1.89226	2.1
35527	2.46009	2.1
35528	1.7513	2.1
35529	1.82817	2.1
35530	1.82817	2.1
35531	2.38127	2.1
35532	2.38127	2.1
35533	2.55006	2.1
35534	2.64569	2.1
35535	2.60128	2.1
35536	2.49168	2.1
35537	5.13737	2.1
35538	2.51605	2.1
35539	2.64569	2.1
35540	2.60128	2.1
35541	2.48891	2.1
35542	5.1346	2.1
35543	2.60128	2.1
35544	2.49168	2.1
35545	2.41689	2.1
35546	2.55248	2.1
35547	5.04416	2.1
35548	2.60128	2.1
35549	2.48891	2.1
35550	2.45922	2.1
35551	2.55249	2.1

35552	5.04139	2.1
35553	1.89227	2.1
35554	1.89227	2.1
35555	1.89227	2.1
35556	1.89227	2.1
35557	1.89227	2.1
35558	1.89227	2.1
35559	0.96063	2.1
35560	0.96063	2.1
35561	0.958806	2.1
35562	0.958806	2.1
35563	1.28892	2.1
35564	0.749042	2.1
35565	1.17409	2.1
35566	0.630516	2.1
35567	1.17409	2.1
35568	1.28892	2.1
35569	0.630515	2.1
35570	0.749042	2.1
35571	0.92511	2.1
35572	0.92511	2.1
35573	1.19511	2.1
35574	1.19511	2.1
35575	0.946132	2.1
35576	1.17409	2.1
35577	0.925081	2.1
35578	0.946129	2.1
35579	0.946131	2.00982
35580	0.946131	2.1
35581	1.17409	2.1
35582	0.925083	2.1
35583	1.89226	1.10239
35584	1.89226	1.27165
35585	1.89226	1.09531
35586	1.89226	1.17713
35587	1.89226	1.17075
35588	1.89226	1.17543
35589	1.89226	1.33474
35590	1.89226	1.81722
35591	1.89226	1.32033
35592	1.89226	1.63747
35593	3.78453	1.32958
35594	0.946132	2.1
35595	0.946132	2.1
35596	1.28892	2.1
35597	1.28892	2.1
35598	0.946132	2.1
35599	1.28892	2.1
35600	0.946133	2.1
35601	1.17409	2.1
35602	0.946132	2.1
35603	0.946132	2.1
35604	1.17409	2.1
35605	1.28892	2.1
35606	0.625229	2.1
35607	0.890321	2.1
35608	1.21636	2.1
35609	1.13022	2.1

35610	1.07094	2.1
35611	1.03541	2.1
35612	0.942207	2.1
35613	0.942207	2.1
35614	0.898766	2.1
35615	1.26626	2.1
35616	1.0853	2.1
35617	0.600358	2.1
35618	0.719012	2.1
35619	0.719012	2.1
35620	1.17957	2.1
35621	1.17957	2.1
35622	0.719012	2.1
35623	0.952456	2.1
35624	0.946129	2.1
35625	0.946129	2.1
35626	0.946131	2.1
35627	0.952454	2.1
35628	0.946131	2.1
35629	0.719011	2.1
35630	0.94613	2.1
35631	0.94613	2.1
35632	0.94613	2.1
35633	0.94613	2.1
35634	0.946131	2.1
35635	0.946131	2.1
35636	0.946129	2.1
35637	0.946129	2.1
35638	0.94613	2.1
35639	0.94613	2.1
35640	0.94613	2.1
35641	0.94613	2.1
35642	1.1587	2.1
35643	0.973158	2.1
35644	0.750023	2.1
35645	0.536443	2.1
35646	0.84054	2.1
35647	0.84054	2.1
35648	0.854602	2.1
35649	0.854602	2.1
35650	1.1587	2.1
35651	0.750023	2.1
35652	0.973158	2.1
35653	0.536443	2.1
35654	1.89227	0.0844643
35655	1.89227	2.1
35656	1.89227	2.1
35657	1.89227	0.144381
35658	3.78453	1.73662
35659	1.89226	2.1
35660	1.89226	2.1
35661	1.89226	2.09105
35662	1.89226	2.1
35663	3.78453	2.1
35664	1.7632	2.1
35665	1.61149	2.1
35666	1.98749	2.1
35667	2.11615	2.1

35668	3.75068	2.1
35669	1.7632	2.1
35670	1.61149	2.1
35671	1.97733	0.162172
35672	2.11615	2.1
35673	3.74052	2.1
35674	1.89226	2.07961
35675	1.89226	0.679681
35676	1.89226	0.121979
35677	1.89226	2.1
35678	3.78453	0.61564
35679	1.89226	1.54296
35680	1.89226	2.1
35681	1.89226	2.1
35682	1.89226	2.1
35683	3.78453	2.1
35684	1.98749	2.1
35685	2.11615	2.1
35686	2.43151	2.1
35687	2.27541	2.1
35688	4.41899	2.1
35689	1.97733	0.448276
35690	2.11615	2.1
35691	2.43151	2.1
35692	2.33986	0.122891
35693	4.40883	2.1
35694	2.17892	0.05
35695	2.80057	0.577219
35696	1.88349	0.794381
35697	2.63062	0.209608
35698	4.80954	0.604068
35699	1.89226	1.09739
35700	1.89226	0.746768
35701	1.89226	0.813658
35702	1.89226	0.165756
35703	3.78452	0.82773
35704	2.0155	1.39131
35705	1.97652	0.757793
35706	1.8247	0.529432
35707	1.80645	0.05
35708	3.80122	0.165453
35709	1.97652	0.740429
35710	1.79681	0.670867
35711	1.80645	0.0538093
35712	1.59244	0.115175
35713	3.56896	0.463791
35714	1.89226	0.745836
35715	1.89226	0.784063
35716	1.89226	0.566972
35717	1.89226	0.378351
35718	3.78452	0.669548
35719	1.89223	0.918627
35720	1.89223	0.754399
35721	1.89223	0.9263
35722	1.89223	0.0729499
35723	3.78447	0.710919
35724	1.82467	0.888034
35725	1.80643	0.375858

35726	1.55159	0.380086
35727	1.53969	0.05
35728	3.36437	0.0918036
35729	1.80643	0.363141
35730	1.59242	0.682896
35731	1.5397	0.181217
35732	1.40538	0.05
35733	3.13211	0.283057
35734	1.89224	0.744346
35735	1.89224	0.734021
35736	1.89224	0.39667
35737	1.89224	0.434054
35738	3.78447	0.714722
35739	1.37427	0.05
35740	2.37086	0.05
35741	2.37972	0.05
35742	1.94703	0.05
35743	2.19299	0.05
35744	1.91813	0.088769
35745	1.07566	2.1
35746	1.12954	2.1
35747	0.946131	2.1
35748	0.946131	2.1
35749	1.12954	2.1
35750	1.12954	2.1
35751	0.946131	2.1
35752	0.946131	2.1
35753	1.12954	2.1
35754	0.946131	2.1
35755	0.946131	2.1
35756	1.07566	2.1
35757	2.25908	2.1
35758	1.89226	2.1
35759	1.89226	2.1
35760	1.89226	2.1
35761	2.37567	2.1
35762	2.37567	2.1
35763	1.37597	2.02652
35764	1.70302	1.05259
35765	1.24162	2.1
35766	1.12219	2.1
35767	1.37072	1.25856
35768	1.70302	2.1
35769	1.37072	2.1
35770	1.70302	1.38073
35771	1.54168	2.0281
35772	2.21318	1.23828
35773	3.39238	2.017
35774	1.89226	0.05
35775	1.89226	0.05
35776	1.89226	0.05
35777	1.89226	0.05
35778	3.78452	0.05
35779	1.89227	0.05
35780	1.89227	0.05
35781	1.89227	0.05
35782	1.89227	0.05
35783	3.78453	0.05

35784	1.89226	0.05
35785	1.89226	0.05
35786	1.89226	0.05
35787	1.89226	0.05
35788	3.78453	0.05
35789	1.89226	0.05
35790	2.21654	0.05
35791	2.72007	0.05
35792	2.72007	0.05
35793	1.89226	0.05
35794	1.89226	0.05
35795	0.946128	0.05
35796	0.946128	0.05
35797	0.946132	0.05
35798	0.946132	0.05
35799	0.94613	0.05
35800	0.94613	0.05
35801	0.946131	0.05
35802	0.946131	0.05
35803	0.94613	0.05
35804	0.94613	0.05
35805	0.94613	0.05
35806	0.94613	0.05
35807	1.89226	0.05
35808	1.89226	0.05
35809	1.89226	0.05
35810	1.89226	0.05
35811	3.78453	0.05
35812	1.33457	0.05
35813	0.946131	0.05
35814	1.08045	0.05
35815	0.946132	0.05
35816	1.08045	0.05
35817	1.08045	0.05
35818	0.946132	0.05
35819	0.946132	0.05
35820	1.33457	0.05
35821	1.08045	0.05
35822	0.946132	0.05
35823	0.946132	0.05
35824	1.53347	1.19536
35825	1.13092	2.1
35826	1.52938	2.06398
35827	1.93568	1.67897
35828	3.06285	2.1
35829	1.89226	0.733688
35830	2.72959	0.439905
35831	2.82996	0.482773
35832	2.82996	0.713665
35833	1.89226	0.68348
35834	1.89226	0.715508
35835	2.7296	0.0866298
35836	2.57663	2.1
35837	2.48354	0.0643438
35838	2.48354	2.1
35839	2.82996	0.05
35840	2.82996	2.1
35841	2.63449	0.0579723

35842	2.7296	0.0895156
35843	2.39282	0.961407
35844	2.39282	2.1
35845	2.48354	2.1
35846	2.72521	2.1
35847	1.89224	2.1
35848	1.89224	2.00479
35849	1.89224	1.0491
35850	1.89224	2.1
35851	3.78447	1.40352
35852	1.89226	2.1
35853	2.10425	2.1
35854	2.5142	2.1
35855	2.5142	2.1
35856	1.89226	2.1
35857	1.89226	2.1
35858	0.946133	1.04398
35859	0.946133	1.31153
35860	0.946128	2.1
35861	0.946128	2.1
35862	0.946131	2.1
35863	0.946131	1.04935
35864	0.94613	0.313158
35865	0.94613	2.1
35866	0.946131	1.87405
35867	0.946131	2.05489
35868	0.946131	1.90092
35869	0.946131	1.89219
35870	1.89226	0.649939
35871	1.89226	1.12758
35872	1.89226	0.954284
35873	1.89226	1.10298
35874	1.89226	0.848487
35875	1.89226	1.09003
35876	1.94446	0.911928
35877	2.50167	1.25471
35878	1.28418	1.02
35879	1.94446	1.14481
35880	2.05205	1.10941
35881	2.05205	1.17368
35882	1.27835	2.1
35883	1.08341	2.1
35884	0.946132	2.1
35885	0.946132	2.1
35886	0.851542	2.1
35887	0.851542	2.1
35888	0.977356	2.1
35889	1.18368	2.1
35890	1.18378	2.1
35891	0.851555	2.1
35892	0.977346	2.1
35893	0.851476	2.1
35894	1.07369	2.1
35895	2.5813	2.1
35896	1.95053	2.1
35897	1.95053	2.1
35898	1.90196	2.1
35899	1.90196	2.1

35900	1.89226	2.1
35901	1.89226	2.1
35902	1.89226	2.1
35903	1.89226	2.1
35904	3.78452	2.1
35905	1.89224	2.1
35906	1.89224	2.1
35907	1.89224	2.1
35908	1.89224	2.1
35909	1.89224	2.1
35910	1.89224	2.1
35911	1.97004	0.596418
35912	1.8072	1.24713
35913	1.5866	1.21057
35914	1.79478	0.877134
35915	3.60198	1.04402
35916	1.23267	2.1
35917	1.8072	0.315988
35918	1.58661	0.219568
35919	0.947921	2.1
35920	2.75512	1.41233
35921	1.89226	0.465836
35922	1.89226	2.1
35923	1.89226	0.414766
35924	1.89226	2.03971
35925	1.89226	0.149474
35926	1.89226	2.1
35927	1.89226	1.25333
35928	1.89226	0.344871
35929	1.89226	0.897458
35930	1.89226	1.39226
35931	3.78452	0.989181
35932	1.4263	1.52203
35933	1.79478	0.578683
35934	1.67651	0.9679
35935	1.79478	1.73711
35936	1.46834	0.120785
35937	1.5866	0.745426
35938	1.89226	0.895617
35939	1.89226	1.23448
35940	1.89226	0.812511
35941	1.89226	1.10237
35942	1.89226	0.880881
35943	1.89226	1.62029
35944	2.23956	1.67664
35945	1.81847	1.91913
35946	2.10991	1.0113
35947	2.75317	1.10523
35948	4.34947	1.33473
35949	1.60578	0.05
35950	1.94974	0.05
35951	1.68062	0.05
35952	1.14721	0.05
35953	3.2864	0.05
35954	1.3946	0.05
35955	1.692 0.05	
35956	2.0154	0.05
35957	2.0154	0.05

35958	1.11926	0.05
35959	1.11926	0.05
35960	1.90347	0.05
35961	1.83626	0.05
35962	1.84583	0.05
35963	1.89434	0.05
35964	3.79781	0.05
35965	1.89226	1.39289
35966	1.89226	1.1617
35967	1.89226	1.01428
35968	1.89226	1.33432
35969	3.78452	1.13861
35970	1.20986	1.52711
35971	1.20986	1.49476
35972	0.912635	1.27197
35973	0.912635	1.78048
35974	0.912667	1.11803
35975	1.17637	1.82395
35976	0.946125	1.67531
35977	0.946125	1.86789
35978	0.946128	1.95403
35979	0.912664	1.28992
35980	0.946127	2.1
35981	1.17637	1.4115
35982	1.89226	0.05
35983	1.65573	0.05
35984	1.65573	0.05
35985	1.89226	0.05
35986	1.89226	0.05
35987	2.12879	0.05
35988	1.89226	0.05
35989	1.89226	0.05
35990	1.89226	0.05
35991	1.89226	0.05
35992	1.89226	0.05
35993	1.89226	0.05
35994	2.40928	0.05
35995	1.89226	0.05
35996	1.95959	0.05
35997	1.70302	0.05
35998	3.47338	0.05
35999	1.89226	0.05
36000	1.89226	0.05
36001	1.89226	0.05
36002	1.89226	0.05
36003	3.78452	0.05
36004	1.64985	0.05
36005	2.47846	0.05
36006	2.25405	0.05
36007	1.80768	0.05
36008	4.06173	0.05
36009	1.98846	0.05
36010	0.912729	0.05
36011	1.05417	0.05
36012	1.70303	0.05
36013	2.82107	0.05
36014	2.47846	0.05
36015	1.70303	0.05

36016	2.86918	0.05
36017	2.05973	0.05
36018	2.51808	0.05
36019	1.80768	0.05
36020	0.946131	0.05
36021	0.946131	0.05
36022	0.946132	0.05
36023	0.946132	0.05
36024	0.946131	0.05
36025	0.946131	0.05
36026	0.946133	0.05
36027	0.946133	0.05
36028	0.946131	0.05
36029	0.946131	0.05
36030	0.946132	0.05
36031	0.946132	0.05
36032	0.946133	0.05
36033	0.946133	0.05
36034	0.946132	0.05
36035	0.946132	0.05
36036	0.946133	0.05
36037	0.946133	0.05
36038	0.946132	0.05
36039	0.946132	0.05
36040	0.946132	0.05
36041	0.946132	0.05
36042	0.946133	0.05
36043	0.946133	0.05
36044	1.89227	0.05
36045	1.89227	0.05
36046	1.89227	0.05
36047	1.89227	0.05
36048	1.89227	0.05
36049	1.89227	0.05
36050	1.1656	2.1
36051	1.21847	2.1
36052	0.836006	2.1
36053	0.806007	2.1
36054	1.00653	2.1
36055	1.00653	2.1
36056	1.01794	2.1
36057	1.01794	2.1
36058	1.1656	2.1
36059	0.806007	2.1
36060	0.836006	2.1
36061	1.21847	2.1
36062	2.82263	2.1
36063	2.3312	2.1
36064	2.25936	2.1
36065	2.71441	2.1
36066	5.0456	2.1
36067	2.25936	2.1
36068	1.61201	2.1
36069	1.57627	2.1
36070	1.61201	2.1
36071	2.29545	2.1
36072	2.3312	2.1
36073	1.21269	0.05

36074	1.48867	0.05
36075	1.68264	0.05
36076	1.9898	0.05
36077	3.17131	0.05
36078	2.0487	2.1
36079	2.45212	2.1
36080	2.31012	2.1
36081	1.94095	2.1
36082	4.39307	2.1
36083	1.02435	2.1
36084	0.970469	2.1
36085	0.625375	2.1
36086	0.567166	2.1
36087	0.796854	2.1
36088	0.796854	2.1
36089	0.79466	2.1
36090	0.79466	2.1
36091	0.970471	2.1
36092	0.567165	2.1
36093	0.625374	2.1
36094	1.02435	2.1
36095	2.31012	2.1
36096	1.94095	2.1
36097	1.4825	2.1
36098	2.17384	2.1
36099	4.11479	2.1
36100	0.946187	2.1
36101	0.803763	2.1
36102	0.581357	2.1
36103	0.510356	2.1
36104	0.745632	2.1
36105	0.745632	2.1
36106	0.734566	2.1
36107	0.734563	2.1
36108	0.896142	2.1
36109	0.510366	2.1
36110	0.946173	2.1
36111	0.552965	2.1
36112	1.89226	1.09701
36113	1.89226	1.4837
36114	1.89226	1.83862
36115	1.89226	1.97487
36116	1.89226	1.12678
36117	1.89226	1.67933
36118	1.50743	1.41058
36119	1.0417	2.1
36120	1.50743	2.1
36121	1.62974	1.13229
36122	1.14605	1.12299
36123	1.14605	2.1
36124	1.89226	1.00013
36125	1.89226	1.96743
36126	1.89226	0.92408
36127	1.89226	1.5287
36128	1.89226	0.96469
36129	1.89226	1.89587
36130	1.89226	1.86243
36131	1.89226	1.33399

36132	1.89226	0.967022
36133	1.89226	1.96589
36134	3.78453	1.17694
36135	1.77186	1.66902
36136	1.35668	2.09533
36137	1.70254	1.09627
36138	1.38756	2.05345
36139	1.77186	1.88402
36140	1.37814	2.1
36141	0.734857	2.1
36142	1.00549	1.74024
36143	0.835261	1.13466
36144	1.08357	2.1
36145	0.97665	1.32791
36146	0.795559	2.1
36147	0.891385	1.63081
36148	0.891385	0.942226
36149	0.874231	1.14043
36150	1.08356	1.26809
36151	0.734863	1.35709
36152	0.79449	1.73782
36153	1.89226	1.53712
36154	1.89226	0.0989718
36155	2.42671	0.602368
36156	1.98783	1.10046
36157	4.31897	0.472119
36158	0.579854	0.616561
36159	0.965582	0.478816
36160	0.634403	1.05751
36161	1.17796	0.38501
36162	1.00592	0.174863
36163	0.797259	2.06007
36164	0.905246	1.16096
36165	0.905246	0.124831
36166	0.695715	0.237308
36167	0.637106	0.751618
36168	1.24374	0.835278
36169	0.95617	0.616491
36170	2.0815	0.257682
36171	1.60521	1.28148
36172	1.29556	1.01823
36173	2.32205	1.72082
36174	1.28445	0.276314
36175	2.0815	1.04842
36176	2.3085	2.1
36177	2.21147	2.1
36178	2.3085	2.1
36179	2.39282	2.1
36180	1.98899	2.1
36181	2.0733	2.1
36182	2.3275	2.1
36183	1.63471	2.1
36184	1.98899	2.1
36185	1.56801	2.1
36186	3.62369	2.1
36187	2.3085	2.1
36188	1.98899	2.1
36189	2.22575	2.1

36190	1.90502	2.1
36191	4.21353	2.1
36192	1.90502	2.1
36193	1.56801	2.1
36194	1.90502	2.1
36195	1.98899	2.1
36196	1.49733	2.1
36197	1.58129	2.1
36198	1.89226	1.15938
36199	2.6491	1.30881
36200	2.49266	2.1
36201	2.6491	1.1568
36202	1.73582	1.29151
36203	1.89226	0.895899
36204	1.31854	2.1
36205	2.6491	2.03589
36206	1.3909	2.1
36207	1.3909	2.1
36208	2.49266	2.1
36209	2.49266	2.1
36210	1.89226	1.1514
36211	2.49266	1.17854
36212	2.38634	2.1
36213	2.49266	1.07876
36214	1.78595	1.15866
36215	1.89226	0.885007
36216	1.23042	2.1
36217	2.49266	1.39433
36218	1.31854	2.1
36219	1.31854	2.1
36220	2.38635	2.1
36221	2.38635	2.1
36222	2.83487	2.1
36223	1.89226	2.1
36224	2.594	2.1
36225	2.594	2.1
36226	1.89226	2.1
36227	1.89226	2.1
36228	1.89226	2.1
36229	1.89226	2.1
36230	1.89226	2.1
36231	1.89226	2.1
36232	3.78452	2.1
36233	1.09518	2.1
36234	1.28158	2.1
36235	1.36844	2.1
36236	1.59972	2.1
36237	2.65003	2.1
36238	1.89226	2.1
36239	1.89226	2.1
36240	1.89226	2.1
36241	1.89226	2.1
36242	3.78452	2.1
36243	1.36844	2.1
36244	1.59972	2.1
36245	1.67352	2.1
36246	1.94291	2.1
36247	3.31135	2.1

36248	1.52938	2.1
36249	1.93568	2.07724
36250	2.36221	0.999182
36251	1.94723	2.1
36252	3.89158	1.84611
36253	1.94724	1.1811
36254	1.26593	2.1
36255	1.75367	2.1
36256	2.4322	1.40067
36257	3.7009	2.1
36258	2.36221	1.08259
36259	2.43219	2.1
36260	1.94723	1.46619
36261	1.94723	1.53433
36262	2.8202	1.7707
36263	2.8202	1.96709
36264	2.51604	2.1
36265	2.18533	2.1
36266	2.17228	2.1
36267	2.4889	2.1
36268	4.67423	2.1
36269	2.17228	2.1
36270	1.62183	2.1
36271	1.62316	2.1
36272	1.62183	2.1
36273	2.18666	2.1
36274	2.18533	2.1
36275	2.17227	2.1
36276	2.4889	2.1
36277	2.45922	2.1
36278	2.15686	2.1
36279	4.64576	2.1
36280	2.17228	2.1
36281	1.62316	2.1
36282	1.62241	2.1
36283	2.15686	2.1
36284	3.78002	2.1
36285	1.89226	0.762766
36286	1.89226	0.945378
36287	1.89226	0.501757
36288	1.89226	0.520916
36289	3.78453	1.34159
36290	1.89226	0.929199
36291	1.89226	0.913001
36292	1.89226	0.842749
36293	1.89226	0.101442
36294	3.78452	0.553643
36295	1.89226	0.964698
36296	1.89226	0.567392
36297	1.89226	0.889589
36298	1.89226	0.891001
36299	1.89226	0.685961
36300	1.89226	0.805698
36301	1.89226	0.898623
36302	1.89226	1.18065
36303	2.53413	0.0965549
36304	1.89226	0.193032
36305	3.78453	0.157992

36306	1.38048	0.36481
36307	1.38048	0.504022
36308	0.490332	0.476075
36309	0.490332	0.250812
36310	0.924679	0.05
36311	0.946131	1.70904
36312	0.946131	1.06163
36313	0.490332	0.05
36314	0.924678	0.0590218
36315	0.490332	0.05
36316	0.946131	0.6661
36317	0.946131	0.208225
36318	1.89227	0.0824407
36319	1.89227	0.84953
36320	1.89227	0.526715
36321	1.89227	1.03457
36322	1.89227	0.113439
36323	1.89227	0.412372
36324	1.30803	2.1
36325	2.55151	2.1
36326	1.43935	2.1
36327	1.43935	2.1
36328	2.34922	2.1
36329	2.34922	2.1
36330	0.946131	2.1
36331	0.946131	2.1
36332	1.27575	2.1
36333	1.17461	2.1
36334	0.94613	2.1
36335	0.94613	2.1
36336	1.17461	2.1
36337	1.17461	2.1
36338	0.946131	2.1
36339	1.17461	2.1
36340	0.946132	2.1
36341	1.27575	2.1
36342	1.89226	2.1
36343	1.89226	2.1
36344	1.89226	2.1
36345	1.89226	2.1
36346	1.89226	2.1
36347	1.89226	2.1
36348	0.946133	2.1
36349	0.946133	2.1
36350	0.946132	2.1
36351	0.946132	2.1
36352	0.946133	2.1
36353	0.946133	2.1
36354	0.946132	2.1
36355	0.946132	2.1
36356	0.946132	2.1
36357	0.946132	2.1
36358	0.946133	2.1
36359	0.946133	2.1
36360	1.07043	2.1
36361	1.17461	2.1
36362	0.654016	2.1
36363	0.5829	2.1

36364	0.870481	2.1
36365	0.870481	2.1
36366	0.887027	2.1
36367	0.887027	2.1
36368	1.07043	2.1
36369	0.5829	2.1
36370	0.654016	2.1
36371	1.17461	2.1
36372	1.89226	2.1
36373	1.89226	2.08069
36374	1.89226	2.1
36375	1.89226	2.1
36376	3.78453	2.1
36377	1.89226	2.1
36378	1.89226	2.1
36379	1.89226	2.1
36380	1.89226	2.1
36381	3.78453	2.1
36382	1.26929	2.1
36383	2.19313	2.1
36384	1.09847	2.1
36385	1.69916	2.1
36386	2.96844	2.1
36387	1.84124	2.1
36388	2.24937	2.1
36389	1.69915	2.1
36390	1.75691	2.1
36391	3.94852	2.1
36392	1.53082	2.1
36393	2.04422	2.1
36394	1.53082	2.1
36395	1.69915	2.1
36396	1.92911	2.1
36397	2.09744	2.1
36398	1.39263	0.474943
36399	1.0171	0.05
36400	1.1211	0.116607
36401	1.46482	0.180867
36402	2.48192	0.328376
36403	1.39263	0.204055
36404	1.57962	0.710042
36405	1.65238	1.63299
36406	1.46482	0.15121
36407	3.04443	0.16992
36408	1.89226	0.409294
36409	1.89226	0.05
36410	1.89226	0.0704745
36411	1.89226	0.122998
36412	3.78453	0.174407
36413	1.89227	0.05
36414	1.89227	0.05
36415	1.89227	0.079251
36416	1.89227	0.05
36417	3.78453	0.05
36418	1.28418	0.0827771
36419	1.46482	0.0981792
36420	1.1211	0.05
36421	1.1211	0.213625

36422	1.59382	0.05
36423	1.59382	0.205772
36424	1.76897	1.15707
36425	1.46482	0.0878282
36426	1.59382	0.198601
36427	1.46482	0.337919
36428	1.78138	0.077918
36429	1.65238	0.127967
36430	1.89226	0.0506722
36431	1.89226	0.0765172
36432	1.89226	0.05
36433	1.89226	0.211897
36434	1.89226	0.05
36435	1.89226	0.246957
36436	1.89226	0.0539021
36437	1.89226	0.05
36438	1.89226	0.05
36439	1.89226	0.05
36440	1.89226	0.05
36441	1.89226	0.0655408
36442	1.96832	2.1
36443	2.40858	2.1
36444	1.75211	0.0966109
36445	2.20797	2.1
36446	4.1607	2.1
36447	1.75211	0.18621
36448	2.20797	2.1
36449	1.51592	2.1
36450	2.02064	2.1
36451	3.77275	2.1
36452	2.71793	0.759692
36453	2.67099	0.277445
36454	2.55105	0.564591
36455	2.71297	0.124016
36456	5.26898	0.615076
36457	2.36417	0.727351
36458	2.67098	0.398598
36459	2.55104	0.612273
36460	1.83418	0.257976
36461	4.91522	0.319326
36462	2.55105	0.337531
36463	2.71297	0.143099
36464	2.58032	0.250319
36465	2.28922	0.0764348
36466	5.13137	0.0926947
36467	2.55104	0.402506
36468	2.23787	0.186797
36469	1.67265	0.199826
36470	2.28922	0.110263
36471	4.22369	0.298232
36472	0.851542	1.04368
36473	0.851542	1.02891
36474	0.981419	0.637667
36475	0.854774	0.740603
36476	0.949431	0.484192
36477	0.753763	0.710767
36478	0.946131	1.03055
36479	0.946131	1.03211

36480	1.04073	1.02633
36481	0.981433	0.266449
36482	0.851528	1.06404
36483	0.854828	0.765662
36484	1.89226	1.11608
36485	1.89226	1.01254
36486	1.89226	0.952328
36487	1.89226	0.989684
36488	1.89226	1.09194
36489	1.89226	1.10305
36490	0.814227	0.768722
36491	0.814227	0.833935
36492	0.946117	0.260089
36493	1.07285	0.297517
36494	0.879132	0.194377
36495	0.923293	0.224359
36496	0.946119	0.892586
36497	0.946119	0.753695
36498	0.946131	0.663968
36499	0.814236	0.0765009
36500	0.946106	0.983113
36501	0.870405	0.339697
36502	1.89224	1.04675
36503	1.89224	0.727204
36504	1.89224	0.650017
36505	1.89224	0.729099
36506	1.89224	1.02516
36507	1.89224	1.03581
36508	1.29388	1.34326
36509	1.04164	2.1
36510	1.64834	2.0461
36511	1.82955	1.24345
36512	2.94222	2.01775
36513	1.4934	0.299996
36514	2.41522	0.05
36515	2.20387	0.0714585
36516	2.20387	2.09703
36517	1.75765	0.100915
36518	1.75765	1.99067
36519	2.41522	0.05
36520	2.77303	2.1
36521	2.20387	2.1
36522	2.58627	2.1
36523	5.00149	2.1
36524	1.22222	0.394001
36525	2.20387	0.219846
36526	1.96831	0.102393
36527	1.96831	2.1
36528	1.4934	0.173207
36529	1.4934	2.1
36530	1.96832	2.1
36531	2.58627	2.1
36532	2.40858	0.42719
36533	2.40858	2.1
36534	2.20387	1.23035
36535	2.20387	2.1
36536	1.5605	2.1
36537	1.53775	2.1

36538	1.55398	2.1
36539	1.45806	2.1
36540	1.65642	2.1
36541	1.5605	2.1
36542	0.768876	2.1
36543	0.79033	2.1
36544	0.828211	2.1
36545	0.871233	2.1
36546	0.803843	2.1
36547	0.803843	2.1
36548	0.814698	2.1
36549	0.814698	2.1
36550	0.79033	2.1
36551	0.871233	2.1
36552	0.768876	2.1
36553	0.828212	2.1
36554	1.80252	2.1
36555	2.09715	1.03337
36556	1.9394	2.1
36557	1.50853	2.1
36558	3.60567	2.1
36559	1.96416	0.929266
36560	2.09715	1.02462
36561	1.88484	2.1
36562	1.57916	0.862409
36563	3.67631	1.05583
36564	1.89226	0.997458
36565	1.89226	0.05
36566	1.89226	0.867595
36567	1.89226	0.923481
36568	3.78453	0.842703
36569	1.89226	1.00309
36570	1.89226	1.13631
36571	1.89226	2.1
36572	1.89226	0.127229
36573	1.89226	2.1
36574	1.89226	0.05
36575	1.5852	2.1
36576	1.50853	2.1
36577	1.2955	2.1
36578	1.44472	1.02126
36579	2.95325	2.1
36580	1.53065	2.06038
36581	1.57916	0.507036
36582	1.44472	0.989043
36583	1.78861	0.989337
36584	1.23527	1.00624
36585	1.23527	0.978026
36586	1.89227	0.54728
36587	1.89227	0.837935
36588	1.89227	1.02588
36589	1.89227	0.789207
36590	3.78453	0.853025
36591	1.89226	1.02899
36592	1.89226	1.05358
36593	1.89226	2.1
36594	1.89226	1.00647
36595	1.89226	1.97248

36596	1.89226	0.05
36597	1.89227	1.08625
36598	1.89227	0.909849
36599	1.89227	0.900338
36600	1.89227	0.912019
36601	3.78453	0.893827
36602	0.943708	0.921773
36603	0.752219	0.877003
36604	0.946132	0.87322
36605	0.946132	0.89218
36606	0.946133	0.811483
36607	0.946133	1.23479
36608	0.752218	1.01727
36609	0.752218	0.937082
36610	0.752218	0.911103
36611	0.946132	0.844527
36612	0.946133	1.00435
36613	0.943708	0.928287
36614	0.943992	2.1
36615	0.943992	2.1
36616	1.23507	1.0339
36617	1.48209	2.1
36618	1.0384	2.1
36619	1.47953	2.1
36620	0.946128	0.952561
36621	0.946128	2.1
36622	0.946129	2.1
36623	1.23293	2.1
36624	1.0384	2.1
36625	0.946129	2.1
36626	1.89226	2.1
36627	2.46586	2.1
36628	2.80218	0.408773
36629	2.80218	2.1
36630	1.89226	1.08049
36631	1.89226	2.1
36632	1.32455	2.1
36633	1.89128	2.1
36634	1.32328	2.1
36635	1.97299	2.1
36636	3.29754	2.1
36637	1.89128	2.1
36638	2.4255	2.1
36639	1.973 2.1	
36640	2.63807	2.1
36641	4.3985	2.1
36642	1.34008	2.1
36643	1.97299	2.1
36644	2.10766	2.1
36645	2.10766	2.1
36646	1.32328	2.1
36647	1.32328	2.1
36648	2.41055	2.1
36649	2.37491	2.1
36650	2.40754	2.1
36651	2.38023	2.1
36652	4.78546	2.1
36653	2.23743	2.1

36654	2.37491	2.1
36655	2.40754	2.1
36656	2.20392	2.1
36657	4.61234	2.1
36658	2.37491	2.1
36659	2.02641	2.1
36660	2.20392	2.1
36661	2.18943	2.1
36662	4.40132	2.1
36663	2.37491	2.1
36664	2.22558	2.1
36665	2.38023	2.1
36666	2.18943	2.1
36667	4.60049	2.1
36668	1.89226	0.05
36669	1.89226	0.0557935
36670	1.89226	0.05
36671	1.89226	0.05
36672	1.89226	0.05
36673	1.89226	0.05
36674	1.03599	0.05
36675	0.706698	0.05
36676	1.41141	0.05
36677	1.17573	0.05
36678	1.11957	0.05
36679	0.765034	0.05
36680	1.05913	0.05
36681	1.45054	0.05
36682	1.04847	0.05
36683	1.04847	0.05
36684	1.15323	0.05
36685	1.05825	0.05
36686	1.89226	0.05
36687	1.89226	0.05
36688	1.89226	0.05
36689	1.89226	0.05
36690	1.89226	0.05
36691	1.89226	0.05
36692	2.22575	0.05
36693	1.70677	2.1
36694	1.90157	0.0570496
36695	1.5534	2.1
36696	3.77914	0.367244
36697	1.70678	2.1
36698	0.968594	2.1
36699	0.968594	2.1
36700	1.10378	2.1
36701	1.5534	2.1
36702	1.68859	2.1
36703	2.31429	0.05
36704	1.5534	2.1
36705	1.90103	0.05
36706	1.41902	2.1
36707	3.45443	0.776908
36708	2.53709	0.05
36709	1.209	0.05
36710	1.05417	0.05
36711	2.6513	0.05

36712	3.70547	0.05
36713	1.02675	0.05
36714	1.16384	0.05
36715	2.22062	0.05
36716	2.48862	0.05
36717	3.38446	0.05
36718	1.89226	0.05
36719	1.89226	0.05
36720	1.89226	0.05
36721	1.89226	0.05
36722	1.89226	0.05
36723	1.89226	0.05
36724	1.89226	1.31984
36725	1.89226	2.1
36726	1.89226	2.1
36727	1.89226	1.82279
36728	1.89226	2.1
36729	1.89226	1.51271
36730	1.89226	1.1961
36731	1.89226	1.8737
36732	1.89226	2.04804
36733	1.89226	1.32888
36734	1.89226	1.75369
36735	1.89226	1.2912
36736	1.89227	1.35421
36737	1.89227	2.02447
36738	1.75032	2.1
36739	1.75032	1.51547
36740	1.89227	1.90654
36741	1.89227	1.41845
36742	1.89226	0.1039
36743	1.89226	0.280095
36744	1.89226	0.198194
36745	1.89226	0.347674
36746	1.89226	0.218097
36747	1.89226	0.138314
36748	0.946132	0.05
36749	0.946132	0.05
36750	0.798215	0.05
36751	0.848163	0.05
36752	0.848163	0.05
36753	0.946131	0.05
36754	0.946132	0.05
36755	0.798216	0.05
36756	0.996105	0.05
36757	0.996105	0.05
36758	0.79819	0.05
36759	0.79819	0.05
36760	1.07763	0.05
36761	2.28622	0.05
36762	2.16109	0.05
36763	1.17506	0.05
36764	3.36385	0.05
36765	1.89226	2.1
36766	1.6663	2.1
36767	1.6663	2.1
36768	1.71706	2.1
36769	1.89226	2.1

36770	1.94302	2.1
36771	1.89226	2.1
36772	1.62801	2.1
36773	1.62801	2.1
36774	1.6663	2.1
36775	1.89226	2.1
36776	1.93055	2.1
36777	1.48734	2.1
36778	1.99609	2.1
36779	1.52245	2.1
36780	2.04137	2.1
36781	3.51854	2.1
36782	1.99609	2.1
36783	2.28563	2.1
36784	2.04136	2.1
36785	2.35019	2.1
36786	4.34628	2.1
36787	1.50714	2.1
36788	2.04137	2.1
36789	2.04945	2.1
36790	2.04945	2.1
36791	1.52245	2.1
36792	1.52245	2.1
36793	2.04136	2.1
36794	2.35019	2.1
36795	2.04945	2.1
36796	2.38681	2.1
36797	4.39964	2.1
36798	2.50924	0.05
36799	1.38305	2.1
36800	2.56918	2.1
36801	1.44535	2.1
36802	3.95222	2.1
36803	1.89226	2.1
36804	1.89226	2.1
36805	1.89226	2.1
36806	1.89226	2.1
36807	3.78453	2.1
36808	1.40615	2.1
36809	1.38851	2.1
36810	0.748607	2.1
36811	0.724468	2.1
36812	1.06362	2.1
36813	1.06362	2.1
36814	1.067 2.1	
36815	1.067 2.1	
36816	1.40615	2.1
36817	0.748607	2.1
36818	0.724468	2.1
36819	1.38851	2.1
36820	2.56918	2.1
36821	1.53377	2.1
36822	1.53706	2.1
36823	1.44535	2.1
36824	2.66088	2.1
36825	2.56918	2.1
36826	1.89226	2.1
36827	1.89226	2.1

36828	1.89226	2.1
36829	1.89226	2.1
36830	3.78453	2.1
36831	1.40239	2.1
36832	1.68466	2.1
36833	1.26091	2.1
36834	1.54641	2.1
36835	2.94881	2.1
36836	1.68466	2.1
36837	1.65324	0.422241
36838	1.54641	2.1
36839	1.52268	0.0787971
36840	3.19965	2.1
36841	1.05776	2.1
36842	1.54641	2.1
36843	1.36595	2.1
36844	1.36595	2.1
36845	1.26091	2.1
36846	1.26091	2.1
36847	1.54641	2.1
36848	1.32697	0.0797408
36849	1.52268	0.0962328
36850	1.52268	2.1
36851	1.36595	0.0679464
36852	1.36595	2.1
36853	1.18647	1.54511
36854	0.988454	2.1
36855	1.4867	2.08899
36856	1.63892	1.56827
36857	2.67317	2.1
36858	1.4867	2.1
36859	1.63892	1.84347
36860	2.09605	1.01255
36861	1.97645	2.1
36862	3.58275	1.40714
36863	1.29389	2.1
36864	1.97645	1.61829
36865	1.4867	1.55976
36866	1.4867	2.1
36867	1.82955	1.20342
36868	1.82955	2.1
36869	1.13454	1.27262
36870	0.983594	2.1
36871	1.16809	2.1
36872	1.06836	2.1
36873	1.08332	2.1
36874	0.983594	1.38903
36875	1.48751	2.1
36876	1.48047	1.75049
36877	1.77596	1.96275
36878	1.75506	1.94628
36879	3.25643	2.08517
36880	1.48047	1.6707
36881	1.25334	2.1
36882	1.75505	1.46025
36883	1.55831	0.987655
36884	3.03878	1.94619
36885	1.77596	2.08818

36886	1.75506	2.1
36887	2.00551	2.1
36888	2.00083	1.12903
36889	3.77679	2.1
36890	1.75506	2.0904
36891	1.80328	1.03041
36892	1.55831	1.96664
36893	1.55831	2.1
36894	2.00083	1.07736
36895	2.00083	1.30605
36896	2.58627	2.1
36897	2.79047	2.1
36898	2.40858	2.1
36899	2.64748	2.1
36900	5.23375	2.1
36901	1.26755	2.1
36902	1.71717	2.1
36903	1.03824	2.1
36904	1.48901	2.1
36905	2.75656	2.1
36906	1.89226	2.1
36907	1.89226	2.1
36908	1.89226	2.1
36909	1.89226	2.1
36910	1.89226	2.1
36911	1.89226	2.1
36912	1.36541	2.1
36913	1.22156	2.1
36914	0.946132	2.1
36915	0.946132	2.1
36916	1.36541	2.1
36917	1.36541	2.1
36918	0.946132	2.1
36919	0.946132	2.1
36920	1.36541	2.1
36921	0.946133	2.1
36922	0.946132	2.1
36923	1.22156	2.1
36924	1.18901	2.1
36925	1.61149	2.1
36926	1.98748	2.1
36927	1.534	2.1
36928	3.1765	2.1
36929	1.89226	2.1
36930	1.89226	2.1
36931	1.89226	2.1
36932	1.89226	2.1
36933	3.78452	2.1
36934	1.98748	2.1
36935	1.534	2.1
36936	2.27541	2.1
36937	1.98748	2.1
36938	1.87436	2.1
36939	1.87436	2.1
36940	2.2988	2.1
36941	2.2604	2.1
36942	2.20831	2.1
36943	2.02802	2.1

36944	4.32683	2.1
36945	2.26041	2.1
36946	1.98957	2.1
36947	2.02803	2.1
36948	1.75021	2.1
36949	4.0176	2.1
36950	2.34607	2.1
36951	2.02803	2.1
36952	1.86795	2.1
36953	1.81448	2.1
36954	3.89597	2.1
36955	2.02803	2.1
36956	1.75021	2.1
36957	1.81448	2.1
36958	1.57887	2.1
36959	3.6069	2.1
36960	0.946131	0.05
36961	0.946131	2.1
36962	1.1235	2.1
36963	1.1235	2.1
36964	0.946131	1.72389
36965	0.946131	1.07233
36966	1.1235	2.1
36967	1.13736	2.1
36968	1.13736	0.75294
36969	0.946131	1.52362
36970	1.1235	1.80012
36971	0.946131	1.64845
36972	1.89226	2.1
36973	1.89226	2.1
36974	1.89226	1.11918
36975	1.89226	1.05267
36976	3.78452	1.08278
36977	0.563266	2.1
36978	0.563266	1.1748
36979	0.565606	0.866394
36980	0.565606	2.1
36981	0.647086	2.09889
36982	0.624219	2.05291
36983	0.500035	1.2251
36984	0.481785	1.26305
36985	0.500034	0.995458
36986	0.647087	1.0312
36987	0.624219	1.83293
36988	0.481785	1.30694
36989	0.915788	0.05
36990	0.915788	0.0548857
36991	1.02732	0.506991
36992	1.02732	0.227663
36993	0.660831	0.387783
36994	1.11155	0.05
36995	0.713295	0.05
36996	1.26874	0.323949
36997	0.713295	0.180861
36998	0.660831	0.129691
36999	1.11155	0.157051
37000	1.26874	0.0882441
37001	1.89226	0.927609

37002	1.85091	0.113472
37003	2.11193	0.707085
37004	1.85091	0.659457
37005	2.15328	0.21033
37006	1.89226	0.116826
37007	0.946131	0.05865
37008	0.946131	0.166267
37009	1.18275	0.0532346
37010	1.05596	0.208776
37011	0.946127	0.0739221
37012	0.946127	0.05
37013	1.18275	0.30491
37014	1.18275	0.0892498
37015	0.946128	0.325982
37016	1.18275	0.05057
37017	0.946129	0.119719
37018	1.05596	0.05
37019	1.60936	2.1
37020	2.35846	2.1
37021	2.06424	2.1
37022	2.57651	2.1
37023	4.42269	2.1
37024	1.89226	2.1
37025	1.89226	2.1
37026	1.89226	2.1
37027	1.89226	2.1
37028	3.78453	2.1
37029	1.89226	2.1
37030	1.89226	2.1
37031	1.89226	2.1
37032	1.89226	2.1
37033	1.89226	2.1
37034	1.89226	2.1
37035	1.95053	2.1
37036	2.06424	2.1
37037	1.95053	2.1
37038	1.30584	2.1
37039	2.5	2.1
37040	1.8553	2.1
37041	2.49999	2.1
37042	2.57651	2.1
37043	2.49999	2.1
37044	2.06423	2.1
37045	2.80738	2.1
37046	2.37162	2.1
37047	1.89226	2.1
37048	1.89226	2.1
37049	1.89226	2.1
37050	1.89226	2.1
37051	1.89226	2.1
37052	1.89226	2.1
37053	1.49295	0.05
37054	1.03824	0.05
37055	1.51396	0.0500366
37056	2.08514	0.05
37057	3.12338	0.05
37058	1.49798	0.05
37059	2.32017	0.05

37060	2.68236	0.05
37061	2.08514	0.05
37062	4.4053	0.05
37063	1.89226	0.05
37064	1.89226	0.05
37065	1.89226	0.05
37066	1.89226	0.05
37067	3.78452	0.05
37068	1.89226	0.05
37069	1.89226	0.05
37070	1.89226	0.05
37071	1.89226	0.05
37072	1.89226	0.05
37073	1.89226	0.05
37074	1.51396	0.05
37075	2.08514	0.05
37076	2.55393	0.05
37077	2.02901	0.05
37078	4.11415	0.05
37079	1.89226	0.05
37080	1.89226	0.05
37081	1.89226	0.05
37082	1.89226	0.05
37083	3.78451	0.05
37084	1.89226	0.05
37085	1.89226	0.05
37086	1.89226	0.05
37087	1.89226	0.05
37088	1.89226	0.05
37089	1.89226	0.05
37090	0.946131	0.05
37091	0.946131	0.05
37092	0.946132	0.05
37093	0.946132	0.05
37094	0.946132	0.05
37095	0.946132	0.05
37096	0.946132	0.05
37097	0.946132	0.05
37098	0.946132	0.05
37099	0.946132	0.05
37100	0.946132	0.05
37101	0.946132	0.05
37102	1.89226	2.1
37103	1.89226	2.1
37104	1.89226	2.1
37105	1.89226	2.1
37106	3.78452	2.1
37107	1.28367	2.1
37108	1.82707	2.1
37109	1.11025	2.1
37110	1.6365	2.1
37111	2.92016	2.1
37112	1.82707	2.1
37113	1.8247	0.0910197
37114	1.8247	2.1
37115	2.01549	0.389466
37116	1.6365	2.1
37117	1.82729	0.117473

37118	1.89226	2.1
37119	1.89226	0.10399
37120	1.89226	0.05
37121	1.89226	2.1
37122	1.89226	0.0782931
37123	1.89226	2.1
37124	1.63648	2.1
37125	1.82467	0.142951
37126	1.35877	2.1
37127	1.55159	0.0615794
37128	3.18345	1.47168
37129	1.89223	2.1
37130	1.89223	0.143379
37131	1.89223	2.1
37132	1.89223	0.255342
37133	3.78447	2.1
37134	1.59972	2.1
37135	1.28037	2.1
37136	1.63702	2.1
37137	1.28158	2.1
37138	1.63581	2.1
37139	1.28037	2.1
37140	1.89226	2.1
37141	1.89226	2.1
37142	1.89226	2.1
37143	1.89226	2.1
37144	1.89226	2.1
37145	1.89226	2.1
37146	1.89226	2.1
37147	1.89226	2.1
37148	1.89226	2.1
37149	1.89226	2.1
37150	3.78451	2.1
37151	1.28037	2.1
37152	1.05071	2.1
37153	1.63581	2.1
37154	1.44287	2.1
37155	2.72324	2.1
37156	1.59972	2.1
37157	1.63581	2.1
37158	1.94291	2.1
37159	2.00118	2.1
37160	3.57872	2.1
37161	1.89226	2.1
37162	1.89226	2.1
37163	1.89226	2.1
37164	1.89226	2.1
37165	3.78452	2.1
37166	0.946131	2.1
37167	0.946131	2.1
37168	0.946127	2.1
37169	0.946127	2.1
37170	0.94613	2.1
37171	0.94613	2.1
37172	0.946128	2.1
37173	0.946128	2.1
37174	0.946128	2.1
37175	0.946128	2.1

37176	0.946129	2.1
37177	0.946129	2.1
37178	0.865056	2.1
37179	0.865056	2.1
37180	0.873224	2.1
37181	0.873224	2.1
37182	0.721437	2.1
37183	0.920378	2.1
37184	0.817903	2.1
37185	1.00059	2.1
37186	1.00059	2.1
37187	0.920376	2.1
37188	0.817904	2.1
37189	0.721437	2.1
37190	3.09157	2.1
37191	1.71255	2.1
37192	2.0815	2.1
37193	1.9918	2.1
37194	2.51364	2.1
37195	2.39396	2.1
37196	2.0815	2.1
37197	1.05051	2.1
37198	2.56724	2.1
37199	1.63991	2.1
37200	3.47929	2.1
37201	1.89226	0.05
37202	2.2973	0.174228
37203	1.90705	0.0818479
37204	1.90705	0.05
37205	1.89226	0.0579551
37206	1.89226	0.05
37207	1.89226	0.05
37208	1.90705	0.130228
37209	1.45321	0.100938
37210	1.45321	0.05
37211	1.89226	0.0564992
37212	1.89226	0.05
37213	0.901146	2.1
37214	0.901146	2.1
37215	1.15227	2.1
37216	1.21279	2.1
37217	0.991265	2.1
37218	1.15003	2.1
37219	0.946132	2.1
37220	0.946132	2.1
37221	0.946132	2.1
37222	1.09997	2.1
37223	0.991266	2.1
37224	0.946132	2.1
37225	1.89226	2.1
37226	1.89226	2.1
37227	1.89226	2.1
37228	1.89226	2.1
37229	1.89226	2.1
37230	1.89226	2.1
37231	1.62229	2.1
37232	1.06053	2.1
37233	1.19698	2.1

37234	1.70106	2.1
37235	2.81928	2.1
37236	0.946132	2.1
37237	0.946132	2.1
37238	1.17527	2.1
37239	1.09997	2.1
37240	1.09997	2.1
37241	1.09997	2.1
37242	0.946133	2.1
37243	0.946133	2.1
37244	1.17527	2.1
37245	0.946132	2.1
37246	0.946133	2.1
37247	1.09997	2.1
37248	1.89226	2.1
37249	1.89226	2.1
37250	1.89226	2.1
37251	1.89226	2.1
37252	1.89226	2.1
37253	1.89226	2.1
37254	0.905416	2.1
37255	0.850527	2.1
37256	0.660601	2.1
37257	0.598494	2.1
37258	0.750137	2.1
37259	0.750137	2.1
37260	0.753774	2.1
37261	0.753774	2.1
37262	0.660599	2.1
37263	0.905418	2.1
37264	0.598493	2.1
37265	0.850529	2.1
37266	2.43509	2.1
37267	2.65066	2.1
37268	2.66602	2.1
37269	2.46339	2.1
37270	5.10111	2.1
37271	2.53008	2.1
37272	2.65066	2.1
37273	2.66602	2.1
37274	2.52852	2.1
37275	5.1961	2.1
37276	2.66602	2.1
37277	2.46339	2.1
37278	2.47932	2.1
37279	2.67393	2.1
37280	5.14535	2.1
37281	2.66602	2.1
37282	2.52853	2.1
37283	2.51686	2.1
37284	2.67393	2.1
37285	5.18288	2.1
37286	1.89226	0.966387
37287	1.89226	0.754539
37288	1.89226	0.643426
37289	1.89226	0.391429
37290	3.78452	0.826248
37291	1.55161	0.681004

37292	1.53972	0.681235
37293	1.32259	0.851329
37294	1.35082	0.05
37295	2.8623	0.63759
37296	1.53972	0.689911
37297	1.4054	0.684055
37298	1.35083	0.074099
37299	1.24913	0.305233
37300	2.78885	0.472386
37301	1.89226	0.748882
37302	1.89226	0.765564
37303	1.89226	0.746701
37304	1.89226	0.350484
37305	3.78452	0.708297
37306	1.89226	0.305851
37307	1.89226	0.768402
37308	1.89226	0.964591
37309	1.89226	0.778934
37310	1.89226	0.537491
37311	1.89226	0.594037
37312	1.14599	0.123517
37313	1.35082	0.750934
37314	1.14599	0.814918
37315	1.32259	0.968169
37316	1.19993	0.05
37317	1.37652	1.02782
37318	1.35083	0.547095
37319	1.24913	0.734724
37320	1.19993	0.112117
37321	1.11787	0.05
37322	2.44906	0.617018
37323	1.89226	0.764488
37324	1.89226	0.735021
37325	1.89226	0.509122
37326	1.89226	0.427873
37327	3.78452	0.721463
37328	1.51697	0.05
37329	2.57264	0.05
37330	1.30203	0.05
37331	2.41701	0.05
37332	3.93398	0.05
37333	1.17464	0.05
37334	2.41701	0.05
37335	2.32462	0.05
37336	2.32462	0.05
37337	1.30203	0.05
37338	1.30203	0.05
37339	2.08149	2.1
37340	1.9839	2.1
37341	1.71398	2.1
37342	1.99182	0.776453
37343	3.71121	2.1
37344	2.16061	2.1
37345	2.01399	0.105771
37346	1.99182	1.00082
37347	1.83895	0.873595
37348	4.00581	0.874426
37349	1.90444	2.1

37350	1.99182	0.932297
37351	1.71717	2.1
37352	1.805	2.1
37353	3.70899	2.1
37354	1.99182	1.89527
37355	1.83896	0.910035
37356	1.805	2.1
37357	1.65243	0.683962
37358	3.64425	0.843471
37359	1.18076	2.1
37360	1.27697	2.1
37361	1.05025	2.1
37362	1.27697	2.1
37363	1.25	2.1
37364	1.47671	2.1
37365	1.89226	2.1
37366	1.89226	2.1
37367	1.89226	2.1
37368	1.89226	2.1
37369	3.78452	2.1
37370	2.64124	2.1
37371	1.89226	2.1
37372	1.89226	2.1
37373	1.89226	2.1
37374	2.46904	2.1
37375	2.46904	2.1
37376	1.89226	2.1
37377	1.89226	2.1
37378	2.7408	2.1
37379	2.82858	2.1
37380	4.72084	2.1
37381	1.87875	2.1
37382	1.37704	2.1
37383	1.77042	2.1
37384	2.27801	2.1
37385	3.64916	2.1
37386	1.77042	2.1
37387	2.27801	2.1
37388	2.58523	2.1
37389	1.90178	2.1
37390	4.35565	2.1
37391	1.28667	2.1
37392	1.30711	2.1
37393	1.53077	2.1
37394	1.55635	2.1
37395	2.81743	2.1
37396	1.55635	2.1
37397	1.28228	2.1
37398	1.64268	2.1
37399	1.93673	2.1
37400	3.19903	2.1
37401	1.53077	2.1
37402	1.93673	2.1
37403	1.55635	2.1
37404	1.55635	2.1
37405	1.86206	2.1
37406	1.86206	2.1
37407	1.64892	2.1

37408	1.60532	2.1
37409	1.85282	2.1
37410	1.96716	2.1
37411	3.61608	2.1
37412	1.60531	2.1
37413	1.32414	2.1
37414	1.96716	2.1
37415	1.75823	2.1
37416	3.2913	2.1
37417	1.91219	2.1
37418	1.96716	2.1
37419	2.0815	2.1
37420	2.51396	2.1
37421	4.30843	2.1
37422	1.96716	2.1
37423	2.17979	2.1
37424	1.75823	2.1
37425	1.75823	2.1
37426	2.28541	2.1
37427	2.28541	2.1
37428	1.89226	1.29829
37429	1.89226	2.02711
37430	1.89226	1.33343
37431	1.89226	1.17544
37432	3.78453	1.34432
37433	1.89226	1.57968
37434	1.89226	1.47126
37435	1.89226	1.35233
37436	1.89226	1.69639
37437	1.89226	1.36146
37438	1.89226	1.78088
37439	1.61016	1.32024
37440	1.45303	1.93903
37441	1.62812	1.41005
37442	1.86379	1.34587
37443	3.23828	1.38521
37444	1.61016	1.74788
37445	1.78716	2.01766
37446	2.0815	1.14023
37447	1.6774	1.84499
37448	3.54454	1.71825
37449	1.89226	1.62277
37450	1.89226	1.38345
37451	1.89226	1.12229
37452	1.89226	1.68318
37453	3.78453	1.3338
37454	1.89226	1.75873
37455	1.89226	1.67818
37456	1.89226	1.476
37457	1.89226	2.1
37458	1.89226	1.15398
37459	1.89226	1.25189
37460	0.807591	1.87471
37461	0.814059	1.33775
37462	1.00217	1.92016
37463	0.931896	1.43295
37464	0.850586	1.49238
37465	0.850586	2.03408

37466	0.8889	1.29479
37467	0.8889	1.79176
37468	0.807592	1.12949
37469	1.00217	1.82902
37470	0.814061	1.4736
37471	0.931894	2.08874
37472	2.0815	1.27594
37473	1.6774	2.1
37474	2.00435	1.35939
37475	2.36104	1.29995
37476	3.86809	1.35697
37477	1.00532	0.30659
37478	1.00532	0.273495
37479	1.00183	0.259777
37480	1.00183	0.188987
37481	1.26377	0.223316
37482	1.34921	0.313244
37483	0.664872	0.295646
37484	0.743381	0.338735
37485	0.664872	0.235333
37486	1.26377	0.232534
37487	1.34921	0.281945
37488	0.743381	0.332411
37489	1.26826	0.05
37490	0.946131	2.1
37491	1.02404	2.1
37492	0.94613	2.1
37493	1.30281	2.1
37494	0.930941	2.1
37495	0.930941	2.1
37496	1.318 2.1	
37497	1.58161	2.1
37498	0.94613	2.1
37499	0.94613	2.1
37500	1.56338	2.1
37501	1.02404	2.1
37502	2.81374	2.1
37503	1.89226	2.1
37504	1.89226	2.1
37505	1.89226	2.1
37506	3.78453	2.1
37507	0.946133	2.1
37508	0.946133	2.1
37509	0.946132	2.1
37510	0.946132	2.1
37511	0.946132	2.1
37512	0.946132	2.1
37513	0.946133	2.1
37514	0.946133	2.1
37515	0.946132	2.1
37516	0.946132	2.1
37517	0.946133	2.1
37518	0.946133	2.1
37519	1.89226	1.70338
37520	1.89226	2.1
37521	1.89226	1.97847
37522	1.89226	2.1
37523	3.78452	2.1

37524	1.89226	2.1
37525	1.89226	2.1
37526	1.89226	2.1
37527	1.89226	2.02151
37528	1.89226	2.1
37529	1.89226	2.03624
37530	0.946133	2.06863
37531	0.946133	2.1
37532	0.946132	2.04074
37533	0.946132	2.1
37534	0.946132	2.1
37535	0.946132	2.1
37536	0.946133	2.1
37537	0.946133	2.08052
37538	0.946132	2.1
37539	0.946132	2.1
37540	0.946132	2.1
37541	0.946132	2.1
37542	2.56953	2.1
37543	2.64148	2.1
37544	2.48631	2.1
37545	2.62213	2.1
37546	5.19166	2.1
37547	2.64148	2.1
37548	2.57929	2.1
37549	2.62213	2.1
37550	2.59522	2.1
37551	5.20142	2.1
37552	2.48631	2.1
37553	2.62213	2.1
37554	2.41176	2.1
37555	2.59262	2.1
37556	5.03389	2.1
37557	2.62213	2.1
37558	2.59522	2.1
37559	2.59262	2.1
37560	2.61901	2.1
37561	5.24114	2.1
37562	0.946132	0.05
37563	0.946132	0.05
37564	0.946133	0.05
37565	0.946133	0.05
37566	0.946132	0.05
37567	0.946132	0.05
37568	0.946133	0.05
37569	0.946133	0.05
37570	0.946132	0.05
37571	0.946132	0.05
37572	0.946133	0.05
37573	0.946133	0.05
37574	1.5681	0.05
37575	1.01248	0.05
37576	1.10749	0.05
37577	0.570913	0.05
37578	1.15589	0.05
37579	1.15588	0.05
37580	0.977549	0.05
37581	0.977549	0.05

37582	1.01249	0.05
37583	0.654445	0.05
37584	1.36795	0.05
37585	1.10748	0.05
37586	1.73883	0.05
37587	1.87638	0.05
37588	1.85219	0.05
37589	1.93113	2.1
37590	3.7407	0.05
37591	1.87638	0.05
37592	1.73883	2.1
37593	1.93113	2.1
37594	1.80426	2.1
37595	3.66996	2.1
37596	2.02912	0.05
37597	1.44951	0.0557833
37598	1.50103	0.0705615
37599	2.02248	0.112684
37600	3.47199	0.05
37601	1.89226	0.0865952
37602	1.89226	0.145571
37603	1.89226	0.226497
37604	1.89226	0.467202
37605	3.78453	0.157615
37606	2.57881	0.082499
37607	1.89226	0.213339
37608	1.89226	0.185568
37609	1.89226	0.114211
37610	2.6428	0.143976
37611	2.6428	0.0977171
37612	1.01379	0.0629021
37613	0.985937	0.05
37614	0.769295	0.0610731
37615	0.715547	0.05
37616	0.853042	0.077957
37617	0.853042	0.0624262
37618	0.864117	0.0882996
37619	0.864114	0.05
37620	0.715556	0.0785362
37621	1.03047	0.0717221
37622	0.731721	0.0827284
37623	0.985921	0.0764759
37624	1.89227	0.101887
37625	1.89227	0.204126
37626	1.89227	0.287613
37627	1.89227	0.515947
37628	3.78453	0.197182
37629	0.946129	0.112579
37630	0.946129	0.0886576
37631	1.28161	0.0649583
37632	1.3214	0.101361
37633	0.94613	0.124208
37634	0.94613	0.0967077
37635	1.28161	0.0877444
37636	1.28161	0.05
37637	1.28161	0.0960203
37638	0.94613	0.104289
37639	0.946131	0.0999162

37640	1.3214	0.0893582
37641	0.946132	0.399855
37642	0.946132	0.488941
37643	0.706045	0.133985
37644	1.16718	0.233595
37645	0.706017	0.0805797
37646	0.706017	0.778132
37647	1.4073	0.0855706
37648	1.4073	0.070254
37649	0.94613	0.231366
37650	1.16719	0.127015
37651	0.706046	0.260715
37652	0.946131	0.110004
37653	1.89226	0.843512
37654	1.89226	0.0924208
37655	1.89226	0.0518906
37656	1.89226	0.889328
37657	1.89226	0.0752187
37658	1.89226	0.883539
37659	0.946119	0.51031
37660	0.946119	0.665138
37661	0.946115	0.295165
37662	0.946115	0.0685618
37663	0.946117	0.241526
37664	0.946117	0.821194
37665	0.946117	0.280205
37666	0.946117	0.208353
37667	0.94613	0.0877941
37668	0.94613	0.383656
37669	0.946105	0.30059
37670	0.946105	0.127805
37671	0.946132	1.10026
37672	0.946132	0.976589
37673	0.946131	1.04162
37674	0.946131	0.676346
37675	0.946132	1.07653
37676	0.946132	1.0842
37677	0.946132	0.945388
37678	0.827865	0.715051
37679	0.946132	1.04497
37680	0.946132	1.04992
37681	0.946132	1.0483
37682	0.827865	0.944318
37683	1.89226	1.5058
37684	1.89226	1.2982
37685	1.89226	1.63631
37686	1.89226	1.20893
37687	1.89226	2.02069
37688	1.89226	1.40022
37689	0.93094	1.15396
37690	0.93094	1.23782
37691	1.04649	1.18311
37692	0.614468	0.991482
37693	0.950358	1.07118
37694	0.799012	0.996635
37695	0.682742	1.19052
37696	0.682742	1.1843
37697	1.04073	1.62793

37698	1.15596	1.39002
37699	0.614478	1.41644
37700	0.851524	1.24714
37701	2.22575	2.1
37702	1.90502	2.1
37703	2.10893	2.1
37704	1.80258	2.1
37705	4.01395	2.1
37706	1.90502	2.1
37707	1.40863	2.1
37708	1.49733	2.1
37709	1.49733	2.1
37710	1.80258	2.1
37711	1.80258	2.1
37712	2.10893	2.1
37713	1.68356	2.1
37714	1.80258	2.1
37715	1.80258	2.1
37716	1.98061	2.1
37717	1.98061	2.1
37718	1.80258	2.1
37719	1.40863	2.1
37720	1.68356	2.1
37721	1.30751	2.1
37722	3.11009	2.1
37723	1.35374	1.79883
37724	1.35374	2.01222
37725	0.746096	1.35896
37726	0.746096	1.30223
37727	1.1537	1.39971
37728	0.746095	1.86884
37729	0.946132	1.43212
37730	0.946132	1.18235
37731	0.946132	1.09435
37732	0.746095	1.27713
37733	0.946131	1.11311
37734	1.1537	1.20158
37735	2.15247	2.1
37736	1.70303	2.1
37737	1.41207	2.1
37738	1.43052	2.1
37739	3.22793	2.1
37740	0.940246	2.1
37741	0.728442	2.1
37742	0.650037	2.1
37743	0.539874	2.1
37744	0.694756	2.1
37745	0.694756	2.1
37746	0.776346	2.1
37747	0.656772	2.1
37748	0.733146	2.1
37749	0.563769	2.1
37750	0.66316	2.1
37751	0.899692	2.1
37752	1.21223	2.1
37753	1.22418	2.1
37754	1.43052	2.1
37755	1.43052	2.1

37756	1.24353	2.1
37757	1.46181	2.1
37758	1.89226	0.05
37759	2.39894	0.05
37760	1.89226	0.05
37761	2.37417	0.05
37762	1.89226	0.05
37763	2.37417	0.05
37764	1.12767	0.05
37765	1.19947	0.05
37766	0.94613	0.05
37767	0.94613	0.05
37768	1.12766	0.05
37769	0.946128	0.05
37770	1.19947	0.05
37771	0.946132	0.05
37772	1.19947	0.05
37773	1.19947	0.05
37774	0.94613	0.05
37775	0.94613	0.05
37776	2.22621	0.05
37777	1.14533	2.1
37778	1.1131	2.1
37779	0.88238	2.1
37780	0.868521	2.1
37781	1.01153	2.1
37782	1.01153	2.1
37783	1.00232	2.1
37784	1.00232	2.1
37785	1.14533	2.1
37786	0.882378	2.1
37787	0.868519	2.1
37788	1.11311	2.1
37789	0.910183	2.02544
37790	1.07811	0.870025
37791	0.94613	0.275707
37792	0.94613	0.327841
37793	0.910212	0.378798
37794	0.910212	2.1
37795	1.11403	0.291418
37796	1.11403	1.9962
37797	0.910183	0.915259
37798	0.94613	1.079
37799	0.94613	1.12764
37800	1.07811	1.11448
37801	1.87228	0.777672
37802	2.81189	2.1
37803	1.6197	2.1
37804	2.56716	2.1
37805	4.43944	2.1
37806	0.946132	1.06945
37807	0.946132	2.1
37808	0.946132	0.0802669
37809	0.946132	0.210961
37810	0.946131	0.617042
37811	0.946131	2.1
37812	0.946132	0.241594
37813	0.946132	1.30805

37814	0.946132	0.682567
37815	0.946132	0.746423
37816	0.946132	0.68168
37817	0.946132	1.12032
37818	1.28724	2.1
37819	2.56716	2.1
37820	2.3014	2.09373
37821	2.3014	2.1
37822	1.6197	2.1
37823	1.6197	2.1
37824	0.946132	2.1
37825	0.946132	2.1
37826	0.946132	0.0592778
37827	0.946132	0.662412
37828	0.946132	0.910773
37829	0.946132	2.1
37830	0.946133	2.1
37831	0.946133	0.0548009
37832	0.946133	1.28226
37833	0.946133	1.5062
37834	0.946132	1.41774
37835	0.946132	1.61741
37836	1.08039	2.1
37837	1.24445	2.1
37838	1.2873	2.1
37839	1.48751	2.1
37840	2.53175	2.1
37841	0.946125	1.00634
37842	0.946125	2.1
37843	1.12552	2.1
37844	1.12552	2.1
37845	0.946124	2.05946
37846	0.946124	2.1
37847	1.12552	2.1
37848	1.11114	2.1
37849	1.11113	2.1
37850	0.946118	2.1
37851	1.12552	2.1
37852	0.946131	2.1
37853	1.89225	1.21728
37854	1.89225	1.39024
37855	1.89225	2.1
37856	1.89225	2.1
37857	1.89225	1.10133
37858	1.89225	2.1
37859	0.987703	1.16339
37860	1.33078	2.06445
37861	1.31565	1.27022
37862	1.31565	2.1
37863	1.01121	1.2844
37864	1.01121	2.1
37865	2.19163	2.1
37866	1.89226	2.1
37867	1.89226	2.1
37868	1.89226	2.05874
37869	2.22229	2.1
37870	2.22229	2.09007
37871	1.89226	2.1

37872	1.89226	1.02809
37873	1.89226	0.665803
37874	1.89226	2.1
37875	1.89226	1.0636
37876	1.89226	2.1
37877	0.57141	2.1
37878	0.57141	1.07005
37879	0.574036	1.24232
37880	0.574036	2.1
37881	0.651591	2.1
37882	0.657827	1.32137
37883	0.482391	1.42714
37884	0.493855	2.09472
37885	0.482391	1.08345
37886	0.651591	1.14909
37887	0.493855	1.89769
37888	0.657828	1.81354
37889	1.88428	2.1
37890	2.03975	2.1
37891	1.97316	2.1
37892	1.8266	2.1
37893	3.86635	2.1
37894	2.23053	2.1
37895	1.42255	2.1
37896	1.3868	2.1
37897	1.8266	2.1
37898	3.24915	2.1
37899	1.97316	2.1
37900	1.8266	2.1
37901	1.77128	2.1
37902	1.91767	2.1
37903	3.74427	2.1
37904	1.77128	2.1
37905	1.8266	2.1
37906	1.77942	2.1
37907	1.33962	2.1
37908	1.3868	2.1
37909	1.3868	2.1
37910	0.944111	2.1
37911	0.944111	2.1
37912	1.26036	2.1
37913	1.16968	2.1
37914	1.03853	2.1
37915	1.16646	2.1
37916	0.946129	2.1
37917	0.946129	2.1
37918	0.94613	2.1
37919	1.25768	2.1
37920	1.03853	2.1
37921	0.946131	2.1
37922	1.82441	0.05
37923	1.33061	0.05
37924	1.13272	0.05
37925	1.60249	0.05
37926	2.95712	0.05
37927	1.82441	0.05
37928	2.08757	0.05
37929	1.81292	0.05

37930	1.60249	0.05
37931	3.63733	0.05
37932	1.81292	0.05
37933	1.332 0.05	
37934	1.60249	0.05
37935	1.60249	0.05
37936	1.5344	0.05
37937	1.5344	0.05
37938	1.10107	2.1
37939	2.51559	2.1
37940	1.1229	2.1
37941	1.1229	2.1
37942	2.49456	2.1
37943	2.49456	2.1
37944	1.89226	2.1
37945	2.51559	2.1
37946	2.49457	2.1
37947	2.51559	2.1
37948	1.87124	2.1
37949	1.89226	2.1
37950	1.89226	2.1
37951	1.89226	2.1
37952	1.89226	2.1
37953	1.89226	2.1
37954	1.89226	2.1
37955	1.89226	2.1
37956	1.89226	2.1
37957	1.89226	2.1
37958	1.89226	2.1
37959	1.89226	2.1
37960	1.89226	2.1
37961	1.89226	2.1
37962	1.23599	2.1
37963	1.24728	2.1
37964	0.550534	2.1
37965	0.541151	2.1
37966	0.893754	2.1
37967	0.893754	2.1
37968	0.894678	2.1
37969	0.894678	2.1
37970	1.23599	2.1
37971	0.541149	2.1
37972	0.550533	2.1
37973	1.24728	2.1
37974	0.946132	2.1
37975	0.946132	2.1
37976	1.23599	2.1
37977	1.24728	2.1
37978	0.946132	2.1
37979	0.946132	2.1
37980	1.23599	2.1
37981	1.23599	2.1
37982	1.23599	2.1
37983	0.946132	2.1
37984	0.946132	2.1
37985	1.24728	2.1
37986	1.89226	2.1
37987	1.89226	2.1

37988	1.89226	2.1
37989	1.89226	2.1
37990	1.89226	2.1
37991	1.89226	2.1
37992	0.946128	2.1
37993	0.946128	2.1
37994	0.946132	2.1
37995	0.946132	2.1
37996	0.94613	2.1
37997	0.94613	2.1
37998	0.946129	2.1
37999	0.946129	2.1
38000	0.94613	2.1
38001	0.94613	2.1
38002	0.94613	2.1
38003	0.94613	2.1
38004	2.58696	0.0868206
38005	2.57073	0.269885
38006	2.12585	0.137951
38007	2.07607	0.294452
38008	4.69658	0.242106
38009	1.89226	0.498131
38010	1.89226	0.700701
38011	1.89226	0.396226
38012	1.89226	0.730188
38013	3.78452	0.44983
38014	1.89226	0.72049
38015	1.89226	0.803015
38016	1.89226	0.762615
38017	1.89226	0.776813
38018	3.78453	0.778706
38019	2.57074	0.319686
38020	2.32635	0.356292
38021	2.07608	0.317642
38022	1.73623	0.415801
38023	4.30697	0.3647
38024	2.12582	0.0816562
38025	1.51971	0.097903
38026	1.57255	0.0845589
38027	2.07604	0.119501
38028	1.62233	0.0755221
38029	2.12582	0.103769
38030	1.89223	0.211626
38031	1.89223	0.515327
38032	1.89223	0.401319
38033	1.89223	0.272187
38034	1.89223	0.319595
38035	1.89223	0.218911
38036	0.946119	0.758089
38037	0.946119	0.572071
38038	0.946119	0.214633
38039	0.946119	0.585196
38040	0.946119	0.629096
38041	0.946119	0.674291
38042	0.94612	0.650623
38043	0.94612	0.538128
38044	0.946132	0.691328
38045	0.946132	0.286816

38046	0.946107	0.739034
38047	0.946107	0.527963
38048	0.811456	0.131776
38049	0.811456	0.371349
38050	0.806521	0.0854319
38051	0.806521	0.292229
38052	0.868104	0.25915
38053	0.579949	0.179307
38054	0.759859	0.109181
38055	1.03802	0.116545
38056	0.579957	0.133613
38057	0.759868	0.165957
38058	1.03801	0.216219
38059	0.868093	0.175114
38060	2.7408	2.1
38061	2.82858	2.1
38062	2.57368	2.1
38063	2.52633	2.1
38064	5.3549	2.1
38065	2.7408	2.1
38066	2.31353	2.1
38067	2.14063	2.1
38068	2.52632	2.1
38069	4.83985	2.1
38070	2.57368	2.1
38071	2.52633	2.1
38072	2.3301	2.1
38073	2.36071	2.1
38074	4.88704	2.1
38075	1.86512	2.1
38076	2.52632	2.1
38077	2.14063	2.1
38078	2.14063	2.1
38079	2.3301	2.1
38080	2.3301	2.1
38081	1.86431	2.1
38082	1.89227	2.1
38083	1.68992	2.1
38084	1.68992	2.1
38085	1.89227	2.1
38086	1.89227	2.1
38087	2.0815	2.1
38088	1.89226	2.1
38089	2.04022	2.1
38090	1.86431	2.1
38091	2.07081	2.1
38092	1.89226	2.1
38093	1.91686	1.39665
38094	1.65683	2.1
38095	1.86871	2.1
38096	1.65683	1.49167
38097	2.13075	2.1
38098	1.89226	0.998374
38099	1.89226	2.1
38100	1.89226	0.853239
38101	1.89226	1.11095
38102	1.89226	2.04475
38103	3.78452	1.22928

38104	0.851541	2.1
38105	0.851541	2.1
38106	0.952803	2.1
38107	0.851573	2.1
38108	0.946129	2.1
38109	0.946129	2.05788
38110	0.929184	2.1
38111	0.757482	2.1
38112	0.836274	2.1
38113	0.851528	2.1
38114	0.952816	2.1
38115	1.04073	2.1
38116	1.70303	1.16146
38117	1.70302	1.22598
38118	1.5544	2.1
38119	1.70302	1.13627
38120	1.74957	1.22847
38121	1.91686	0.942386
38122	1.89226	1.02516
38123	1.89226	1.3414
38124	1.89226	1.88015
38125	1.89226	1.09485
38126	1.89226	1.12491
38127	1.89226	0.442066
38128	1.89226	2.1
38129	1.89226	2.1
38130	2.03472	2.1
38131	1.89226	2.1
38132	1.99827	2.1
38133	1.85836	2.07692
38134	1.00509	2.1
38135	1.55998	2.1
38136	1.83347	2.1
38137	1.45246	2.1
38138	3.01244	2.1
38139	1.23839	2.1
38140	0.884251	2.1
38141	0.946133	2.1
38142	0.946133	2.1
38143	0.884226	2.1
38144	0.884226	2.1
38145	0.946158	2.1
38146	0.946159	2.1
38147	1.23839	2.1
38148	0.946131	2.1
38149	0.946131	2.1
38150	0.884254	2.1
38151	1.89226	2.1
38152	1.89226	2.1
38153	1.89226	2.1
38154	1.89226	2.1
38155	1.89226	2.1
38156	1.89226	2.1
38157	2.1499	2.1
38158	1.45244	2.1
38159	1.83345	2.1
38160	1.83345	2.1
38161	1.87214	2.1

38162	1.87214	2.1
38163	1.89224	2.1
38164	1.89224	2.1
38165	1.89224	2.1
38166	1.89224	2.1
38167	3.78448	2.1
38168	1.49155	1.13674
38169	2.0815	1.67389
38170	1.77515	1.69579
38171	1.72377	2.1
38172	3.50659	1.54036
38173	1.69876	0.05
38174	2.0815	2.1
38175	1.9133	0.235885
38176	1.68189	0.05
38177	2.17628	2.1
38178	1.94335	2.1
38179	2.65129	0.05
38180	1.15317	0.05
38181	2.57085	0.05
38182	1.209	0.05
38183	2.57085	0.05
38184	1.209	0.05
38185	1.89226	0.05
38186	1.89226	0.05
38187	1.89226	0.05
38188	1.89226	0.05
38189	3.78452	0.05
38190	2.48861	0.05
38191	1.15252	0.05
38192	2.60515	0.05
38193	2.60515	0.05
38194	1.16384	0.05
38195	1.16384	0.05
38196	1.59903	0.876614
38197	1.26865	0.76598
38198	1.30486	1.1668
38199	1.70302	0.949457
38200	2.98573	0.943057
38201	1.59903	0.843325
38202	1.79478	0.966775
38203	1.79478	0.878515
38204	1.97005	0.917478
38205	1.50365	0.85795
38206	1.64192	1.12058
38207	1.89226	0.793278
38208	1.89226	0.867593
38209	1.89226	0.987354
38210	1.89226	0.829262
38211	3.78453	0.820589
38212	1.89226	0.926602
38213	1.89226	0.858406
38214	1.89226	0.929738
38215	1.89226	0.818932
38216	1.89226	0.932433
38217	1.89226	0.980939
38218	0.761672	0.829745
38219	0.896406	0.873064

38220	0.603509	0.872711
38221	0.574071	0.942749
38222	0.695159	0.964913
38223	0.695159	0.421352
38224	0.762564	1.16623
38225	0.770159	0.722726
38226	0.803021	0.958616
38227	0.572529	1.03732
38228	0.696466	0.812961
38229	0.899733	0.817322
38230	1.66034	0.128109
38231	1.79478	0.943
38232	1.56685	0.853549
38233	1.68512	1.23557
38234	1.67652	0.887864
38235	1.67652	0.956144
38236	1.89226	0.960209
38237	1.89226	0.861795
38238	1.89226	0.924889
38239	1.89226	0.919559
38240	1.89226	0.940569
38241	1.89226	0.924135
38242	0.946133	0.811013
38243	0.946133	0.865998
38244	0.946132	0.91978
38245	0.946132	0.862888
38246	0.946132	0.88938
38247	0.946132	0.646418
38248	0.946133	1.24439
38249	0.946133	0.832145
38250	0.946133	0.89679
38251	0.946133	0.925854
38252	0.946132	0.791481
38253	0.946132	0.788729
38254	1.80424	2.1
38255	1.45158	2.1
38256	1.9098	2.1
38257	1.56003	2.1
38258	3.36138	2.1
38259	1.90982	2.1
38260	1.71606	2.1
38261	2.15314	2.1
38262	1.70302	2.1
38263	3.89051	2.1
38264	1.96416	0.943137
38265	1.23706	0.805659
38266	1.00053	0.651601
38267	1.38287	0.774092
38268	1.57916	0.812756
38269	1.9615	0.829644
38270	0.827865	0.745192
38271	0.946132	0.739108
38272	0.946132	0.353111
38273	0.946132	0.79066
38274	0.946132	0.798719
38275	0.946132	0.749296
38276	0.946131	0.800972
38277	0.946131	0.789412

38278	0.827865	0.853759
38279	0.946132	0.751333
38280	0.946132	0.33121
38281	0.946132	0.732131
38282	1.89227	2.1
38283	1.89227	2.1
38284	1.89227	2.1
38285	1.89227	2.1
38286	3.78453	2.1
38287	2.3819	2.1
38288	1.58089	2.1
38289	1.58089	2.1
38290	1.6287	2.1
38291	2.33368	2.1
38292	2.3815	2.1
38293	0.946133	2.1
38294	0.946133	2.1
38295	0.946132	2.1
38296	0.946132	2.1
38297	0.946132	2.1
38298	0.946132	2.1
38299	0.946132	2.1
38300	0.946132	2.1
38301	0.946133	2.1
38302	0.946133	2.1
38303	0.946132	2.1
38304	0.946132	2.1
38305	1.89226	2.1
38306	1.89226	2.1
38307	1.89226	2.1
38308	1.89226	2.1
38309	3.78453	2.1
38310	2.7874	2.1
38311	2.33368	2.1
38312	2.71897	2.1
38313	2.27495	2.1
38314	5.05266	2.1
38315	0.970991	2.1
38316	0.970991	2.1
38317	0.956929	2.1
38318	0.956929	2.1
38319	0.732928	2.1
38320	0.790444	2.1
38321	1.16684	2.1
38322	1.13748	2.1
38323	1.13748	2.1
38324	0.732928	2.1
38325	0.790444	2.1
38326	1.16684	2.1
38327	0.946132	2.1
38328	0.946132	2.1
38329	0.946131	2.1
38330	0.946131	2.1
38331	0.946132	2.1
38332	0.946132	2.1
38333	0.946132	2.1
38334	0.946132	2.1
38335	0.946132	2.1

38336	0.946132	2.1
38337	0.946132	2.1
38338	0.946132	2.1
38339	1.10747	0.510973
38340	2.30331	0.580256
38341	1.47954	0.100783
38342	2.63789	0.106604
38343	3.78285	0.379678
38344	1.65215	0.14868
38345	2.63789	0.102526
38346	2.82465	0.498487
38347	2.82465	0.168334
38348	1.47954	0.19936
38349	1.47954	0.0690854
38350	1.13456	2.1
38351	1.08334	2.1
38352	1.20674	1.14796
38353	1.17885	2.1
38354	2.29008	2.03474
38355	1.20674	1.70515
38356	1.17885	2.1
38357	1.28649	1.2236
38358	1.28516	1.81489
38359	2.4919	1.82114
38360	1.89226	2.1
38361	1.89226	2.1
38362	1.89226	2.1
38363	1.89226	2.1
38364	1.89226	2.1
38365	1.89226	2.1
38366	2.50288	1.72102
38367	2.02115	2.1
38368	2.7717	1.57872
38369	2.29107	1.02642
38370	4.79395	2.1
38371	1.27339	2.1
38372	1.10817	2.1
38373	1.19733	2.1
38374	1.032 2.1	
38375	2.30539	2.1
38376	1.19733	2.1
38377	0.953637	2.1
38378	0.953637	2.1
38379	1.032 2.1	
38380	1.11868	2.1
38381	1.19704	2.1
38382	2.01024	1.1223
38383	1.11938	1.24296
38384	1.9186	1.40999
38385	2.76298	1.13462
38386	3.92884	1.25041
38387	0.851541	0.05
38388	0.851541	0.05
38389	0.957577	0.05
38390	0.848952	0.05
38391	1.08043	0.05
38392	0.851541	0.05
38393	0.851484	0.05

38394	0.957578	0.05
38395	1.20045	0.05
38396	0.726289	0.05
38397	0.946131	0.05
38398	0.946131	0.05
38399	1.50524	2.1
38400	2.38363	2.1
38401	2.49018	2.1
38402	2.49018	2.1
38403	1.45506	2.1
38404	1.45506	2.1
38405	2.49017	2.1
38406	1.89226	2.1
38407	1.89226	2.1
38408	1.89226	2.1
38409	2.38363	2.1
38410	2.38363	2.1
38411	1.54711	2.1
38412	2.49018	2.1
38413	2.56975	2.1
38414	2.56975	2.1
38415	1.50524	2.1
38416	1.50524	2.1
38417	2.56975	2.1
38418	1.89226	2.1
38419	1.89226	2.1
38420	1.89226	2.1
38421	2.49017	2.1
38422	2.49017	2.1
38423	1.89226	0.05
38424	1.89226	0.05
38425	1.89226	0.05
38426	1.89226	0.05
38427	1.89226	0.05
38428	1.89226	0.05
38429	1.89226	0.05
38430	1.89226	0.05
38431	1.89226	0.05
38432	1.89226	0.05
38433	1.89226	0.05
38434	1.89226	0.05
38435	1.89226	0.05
38436	1.89226	0.05
38437	2.8358	0.05
38438	1.89226	0.071735
38439	3.78453	0.05
38440	1.60565	0.05
38441	1.89226	1.25864
38442	1.89226	0.69428
38443	1.40013	0.157579
38444	1.89226	0.821471
38445	1.40013	0.269063
38446	1.1766	2.1
38447	1.29435	2.1
38448	1.35735	2.1
38449	1.53509	2.1
38450	2.65169	2.1
38451	0.69701	2.1

38452	0.69701	2.1
38453	0.711303	2.1
38454	0.711303	2.1
38455	0.761138	2.1
38456	0.612149	2.1
38457	0.647174	2.1
38458	0.767548	2.1
38459	0.767548	2.1
38460	0.761139	2.1
38461	0.612149	2.1
38462	0.647174	2.1
38463	1.29764	1.35284
38464	1.29579	1.90641
38465	1.34875	1.70111
38466	1.06717	1.83921
38467	1.29764	1.5748
38468	1.01606	1.53059
38469	0.756635	1.4903
38470	0.648821	1.50851
38471	0.675557	1.77721
38472	0.674374	1.60953
38473	0.74214	1.85299
38474	0.74214	1.59662
38475	0.688868	1.24367
38476	0.688868	1.56232
38477	0.675557	1.05241
38478	0.756634	1.38581
38479	0.648821	0.714484
38480	0.674374	1.3722
38481	1.51327	1.65757
38482	1.48105	1.18285
38483	1.29764	1.66988
38484	1.29764	1.54337
38485	1.48105	1.64539
38486	1.05655	1.17536
38487	1.13537	2.1
38488	1.07326	2.1
38489	0.94613	2.1
38490	0.94613	2.1
38491	0.946132	2.1
38492	0.946132	2.1
38493	1.13537	2.1
38494	1.08927	2.1
38495	1.13537	2.1
38496	0.946131	2.1
38497	0.946131	2.1
38498	0.95947	2.1
38499	1.46892	2.1
38500	1.89226	2.1
38501	1.89226	2.1
38502	1.89226	2.1
38503	1.88031	2.1
38504	1.88031	2.1
38505	1.89226	2.1
38506	1.89226	2.1
38507	1.89226	2.1
38508	1.89226	2.1
38509	3.78453	2.1

38510	2.18254	2.1
38511	1.89226	2.1
38512	2.50005	2.1
38513	1.89226	2.1
38514	4.39231	2.1
38515	1.88031	2.1
38516	2.0815	2.1
38517	2.0815	2.1
38518	1.92078	2.1
38519	2.0815	2.1
38520	1.9218	2.1
38521	1.89227	2.1
38522	1.89227	2.1
38523	1.89227	2.1
38524	1.89227	2.1
38525	1.89227	2.1
38526	1.89227	2.1
38527	1.98693	2.1
38528	1.82857	2.1
38529	2.32	2.1
38530	2.36972	2.1
38531	4.14857	2.1
38532	1.82857	2.1
38533	1.58688	2.1
38534	2.36971	2.1
38535	2.19093	2.1
38536	4.0195	2.1
38537	0.946132	2.1
38538	0.946132	2.1
38539	1.18486	2.1
38540	1.18486	2.1
38541	1.18486	2.1
38542	0.946132	2.1
38543	0.946132	2.1
38544	1.16	2.1
38545	0.946133	2.1
38546	0.946133	2.1
38547	1.16	2.1
38548	1.18486	2.1
38549	1.07798	1.75477
38550	1.08101	1.495
38551	0.910786	2.1
38552	0.910786	2.1
38553	0.851537	2.1
38554	0.901377	1.49385
38555	1.14652	2.1
38556	1.04072	2.1
38557	0.946129	2.1
38558	0.946129	2.1
38559	1.0019	2.08255
38560	1.04263	2.1
38561	1.89226	2.1
38562	1.89226	2.1
38563	1.89226	2.1
38564	1.89226	2.1
38565	3.78452	2.1
38566	1.89226	2.1
38567	2.30146	2.1

38568	1.89226	2.1
38569	2.18412	2.1
38570	4.19372	2.1
38571	2.30146	2.1
38572	1.39962	2.1
38573	2.18411	2.1
38574	1.28765	2.1
38575	3.5891	2.1
38576	1.89226	2.1
38577	1.89226	2.1
38578	1.89226	2.1
38579	1.89226	2.1
38580	1.89226	2.1
38581	1.89226	2.1
38582	1.89226	2.1
38583	1.89226	2.1
38584	1.89226	2.1
38585	1.89226	2.1
38586	3.78452	2.1
38587	2.18412	2.1
38588	1.20045	2.1
38589	1.20045	2.1
38590	1.28765	2.1
38591	2.08808	2.1
38592	2.17528	2.1
38593	0.946131	2.1
38594	0.946131	2.1
38595	0.946128	2.1
38596	0.946128	2.1
38597	0.946127	2.1
38598	0.946127	2.1
38599	0.946131	2.1
38600	0.946131	2.1
38601	0.946129	2.1
38602	0.946129	2.1
38603	0.94613	2.1
38604	0.94613	2.1
38605	1.89226	0.751761
38606	1.89226	1.42065
38607	1.89226	1.25062
38608	1.89226	0.798882
38609	3.78452	0.788961
38610	1.89227	0.258546
38611	1.89227	1.90553
38612	1.89227	0.0740792
38613	1.89227	1.11825
38614	1.89227	0.285591
38615	1.89227	1.59429
38616	1.89226	1.76796
38617	1.89226	0.898455
38618	1.89226	0.80662
38619	2.29245	0.911479
38620	3.78452	1.07567
38621	1.89226	1.54724
38622	1.89226	0.460312
38623	1.89226	0.437916
38624	1.89226	0.976117
38625	1.89226	0.0966367

38626	1.89226	0.283839
38627	1.28095	1.84643
38628	1.81846	2.09721
38629	2.10991	1.04751
38630	1.34695	1.73183
38631	3.39086	1.86487
38632	2.10991	1.1771
38633	1.36457	1.89395
38634	1.67421	2.1
38635	1.34695	1.98012
38636	2.43717	1.34207
38637	2.10991	1.49896
38638	2.69541	2.1
38639	1.48572	2.1
38640	2.63553	2.1
38641	1.45385	2.1
38642	4.14926	2.1
38643	2.63553	2.1
38644	1.44207	2.1
38645	1.44207	2.1
38646	1.45385	2.1
38647	2.588 2.1	
38648	2.59978	2.1
38649	1.89227	2.1
38650	1.89227	2.1
38651	1.89227	2.1
38652	1.89227	2.1
38653	3.78453	2.1
38654	1.89226	2.1
38655	1.89226	2.1
38656	1.89226	2.1
38657	1.89226	2.1
38658	3.78453	2.1
38659	2.15929	2.1
38660	1.38276	2.1
38661	1.43617	2.1
38662	1.35614	2.1
38663	2.23932	2.1
38664	2.15929	2.1
38665	2.15929	2.1
38666	2.25168	2.1
38667	2.3558	2.1
38668	2.23932	2.1
38669	4.51509	2.1
38670	1.89226	2.1
38671	1.89226	2.1
38672	1.89226	2.1
38673	1.89226	2.1
38674	3.78453	2.1
38675	1.89226	2.1
38676	1.89226	2.1
38677	1.89226	2.1
38678	1.89226	2.1
38679	1.89226	2.1
38680	1.89226	2.1
38681	2.23932	2.1
38682	1.43854	2.1
38683	1.49173	2.1

38684	1.38276	2.1
38685	2.34829	2.1
38686	2.23932	2.1
38687	2.3558	2.1
38688	2.34829	2.1
38689	2.40685	2.1
38690	2.23932	2.1
38691	2.52332	2.1
38692	2.3558	2.1
38693	1.89226	0.05
38694	1.87361	0.05
38695	2.25082	0.05
38696	1.87361	0.05
38697	2.26947	0.05
38698	1.89226	0.05
38699	1.89226	0.05
38700	1.89226	0.05
38701	1.89226	0.05
38702	1.89226	0.05
38703	1.89226	0.05
38704	1.89226	0.05
38705	2.41314	0.05
38706	1.89226	0.05
38707	1.89226	0.05
38708	1.89226	0.05
38709	2.12204	0.05
38710	2.12204	0.05
38711	1.89226	0.05
38712	2.25082	0.05
38713	2.28189	0.05
38714	2.25082	0.05
38715	1.92333	0.05
38716	1.89226	0.05
38717	1.89226	0.05
38718	1.89226	0.05
38719	1.89226	0.05
38720	1.89226	0.05
38721	1.89226	0.05
38722	1.89226	0.05
38723	2.42521	0.05
38724	1.89226	0.05
38725	1.89226	0.05
38726	1.89226	0.05
38727	2.41314	0.05
38728	2.41314	0.05
38729	1.39725	1.47963
38730	1.07745	2.1
38731	1.07745	1.51463
38732	1.07519	2.1
38733	1.41336	0.826629
38734	1.41111	2.1
38735	1.89226	2.1
38736	1.92039	2.1
38737	1.92039	2.1
38738	1.9052	2.1
38739	1.89226	2.1
38740	1.87708	2.1
38741	0.946128	2.1

38742	0.946128	2.1
38743	0.946133	1.35102
38744	0.946133	0.9446
38745	0.94613	0.819148
38746	0.94613	2.1
38747	0.946131	2.1
38748	0.946131	0.501777
38749	0.946131	2.04488
38750	0.946131	2.1
38751	0.946131	2.00125
38752	0.946131	2.09189
38753	1.41336	1.40244
38754	1.08911	2.1
38755	1.08911	1.90209
38756	1.07745	2.1
38757	1.42381	1.39344
38758	1.41215	2.1
38759	1.89227	2.1
38760	1.95833	2.1
38761	1.95833	2.1
38762	1.92039	2.1
38763	1.89227	2.1
38764	1.85433	2.1
38765	0.946129	2.1
38766	0.946129	2.1
38767	0.946133	1.75069
38768	0.946133	0.995193
38769	0.946131	0.876051
38770	0.946131	2.1
38771	0.946131	2.1
38772	0.946131	0.919953
38773	0.946131	2.1
38774	0.946131	2.1
38775	0.946131	2.1
38776	0.946131	2.1
38777	1.41943	2.1
38778	2.74398	2.1
38779	1.18238	2.1
38780	2.53897	2.1
38781	3.9584	2.1
38782	2.74398	2.1
38783	1.89226	2.1
38784	1.89226	2.1
38785	1.89226	2.1
38786	2.53897	2.1
38787	2.53897	2.1
38788	1.89227	1.08197
38789	1.89227	0.911353
38790	1.89227	1.0435
38791	1.89227	0.82255
38792	3.78453	0.934486
38793	1.89226	0.847099
38794	2.08134	0.521959
38795	2.00632	0.350785
38796	2.00632	0.439945
38797	1.89226	0.784927
38798	1.89226	0.910974
38799	1.89227	0.976654

38800	1.89227	0.771038
38801	1.89227	0.902119
38802	1.89227	0.405933
38803	3.78453	0.931613
38804	1.89227	1.0162
38805	1.89227	0.391718
38806	1.89227	0.411191
38807	1.89227	0.824324
38808	1.89227	0.884903
38809	1.89227	1.04663
38810	2.50912	0.349692
38811	1.89226	0.757487
38812	1.89226	0.120479
38813	2.31261	0.574917
38814	2.31261	0.466223
38815	1.89226	0.871976
38816	2.50912	0.40431
38817	1.19999	0.161976
38818	1.44056	0.0963207
38819	1.24405	0.179754
38820	2.50912	0.351705
38821	2.31261	0.544694
38822	2.69224	0.859849
38823	2.28373	0.749883
38824	1.94788	0.987726
38825	2.4106	0.801348
38826	4.69434	0.868277
38827	2.69223	0.05
38828	2.63783	0.912872
38829	2.34737	1.29091
38830	2.4106	0.834499
38831	5.04843	0.935568
38832	1.89226	2.1
38833	1.99753	2.1
38834	1.87366	2.1
38835	1.99753	2.1
38836	1.76839	2.1
38837	1.89226	2.1
38838	1.89226	2.1
38839	1.89226	2.1
38840	1.89226	2.1
38841	1.89226	2.1
38842	3.78452	2.1
38843	0.946132	2.1
38844	0.946132	2.1
38845	1.02744	2.1
38846	1.02744	2.1
38847	0.946132	2.1
38848	0.946132	2.1
38849	1.00989	2.1
38850	1.02744	2.1
38851	1.00989	2.1
38852	0.946133	2.1
38853	1.02744	2.1
38854	0.946132	2.1
38855	1.70302	2.1
38856	1.87366	2.1
38857	1.71618	2.1

38858	1.87366	2.1
38859	1.73105	2.1
38860	1.89226	2.1
38861	1.89226	2.1
38862	1.89226	2.1
38863	1.89226	2.1
38864	1.89226	2.1
38865	1.89226	2.1
38866	1.89226	2.1
38867	1.96624	2.1
38868	1.89226	2.1
38869	1.89226	2.1
38870	1.89226	2.1
38871	2.01977	2.1
38872	2.01977	2.1
38873	1.1032	2.04288
38874	1.15114	2.1
38875	1.14986	0.0549685
38876	1.19242	2.1
38877	2.301	2.1
38878	1.89226	1.45468
38879	1.89226	2.1
38880	1.89226	0.0809786
38881	1.89226	2.1
38882	3.78453	1.5562
38883	0.946132	2.1
38884	0.946132	2.1
38885	0.946132	2.1
38886	0.946132	2.1
38887	0.946133	2.1
38888	0.946133	2.1
38889	0.946132	2.1
38890	0.946132	2.1
38891	0.946132	2.1
38892	0.946132	2.1
38893	0.946133	2.1
38894	0.946133	2.1
38895	1.19243	2.1
38896	0.953928	2.1
38897	1.18671	2.1
38898	1.15114	2.1
38899	0.9895	2.1
38900	0.953928	2.1
38901	1.19611	0.0836795
38902	1.19242	2.1
38903	1.24693	0.207269
38904	1.24693	2.1
38905	1.14986	0.909314
38906	1.14986	2.1
38907	1.89225	0.140894
38908	1.89225	2.1
38909	1.89225	2.1
38910	1.89225	1.28144
38911	1.89225	2.1
38912	1.89225	1.22909
38913	1.89225	2.1
38914	1.89225	2.1
38915	1.89225	2.1

38916	1.89225	2.1
38917	1.89225	2.07382
38918	1.89225	2.1
38919	0.561532	2.1
38920	0.561532	2.1
38921	0.566382	2.1
38922	0.566382	2.1
38923	0.494747	2.1
38924	0.531705	2.1
38925	0.596208	2.1
38926	0.623464	2.1
38927	0.623469	2.1
38928	0.531708	2.1
38929	0.596205	2.1
38930	0.494743	2.1
38931	1.25742	2.1
38932	1.18783	2.1
38933	0.946131	2.1
38934	0.946131	2.1
38935	1.25742	2.1
38936	1.25742	2.1
38937	0.946131	2.1
38938	0.946131	2.1
38939	1.25742	2.1
38940	0.946131	2.1
38941	0.946131	2.1
38942	1.18783	2.1
38943	1.35048	2.1
38944	1.25742	2.1
38945	0.946132	2.1
38946	0.946132	2.1
38947	1.35048	2.1
38948	1.35048	2.1
38949	0.946132	2.1
38950	0.946132	2.1
38951	1.35044	2.1
38952	0.946106	2.1
38953	1.25745	2.1
38954	0.946158	2.1
38955	1.13525	2.1
38956	2.70095	2.1
38957	2.51483	2.1
38958	2.51483	2.1
38959	1.21593	2.1
38960	1.21593	2.1
38961	1.93142	0.118046
38962	2.8053	0.05
38963	1.559 0.784612	
38964	2.60359	0.05
38965	4.53501	0.0962373
38966	2.80531	0.0509687
38967	1.89226	0.05
38968	2.60359	0.05
38969	1.89226	0.285165
38970	4.49586	0.05
38971	1.22938	0.342682
38972	2.60359	0.05
38973	2.37434	0.780321

38974	2.37434	0.05
38975	1.55901	0.349424
38976	1.55901	0.05
38977	1.18717	0.537568
38978	1.18717	0.05
38979	0.946132	0.05
38980	0.946132	0.05
38981	0.946133	0.316703
38982	0.946133	0.365852
38983	1.3018	0.05
38984	1.18717	0.702177
38985	1.18714	0.0793009
38986	0.946107	0.263921
38987	1.30183	0.05
38988	0.946158	0.05
38989	1.89226	2.1
38990	2.80196	2.1
38991	2.67272	2.1
38992	2.67272	2.1
38993	1.89226	2.1
38994	1.89226	2.1
38995	1.89226	2.1
38996	1.89226	2.1
38997	1.89226	2.1
38998	1.89226	2.1
38999	1.89226	2.1
39000	1.89226	2.1
39001	1.74471	2.1
39002	2.01555	2.1
39003	1.61121	2.1
39004	1.61121	2.1
39005	2.13878	2.1
39006	2.13878	2.1
39007	1.89226	2.1
39008	1.89226	2.1
39009	1.89226	2.1
39010	1.89226	2.1
39011	1.89226	2.1
39012	1.89226	2.1
39013	1.09766	2.1
39014	1.04263	2.1
39015	0.881794	2.1
39016	0.894188	2.1
39017	0.795402	2.1
39018	0.99524	2.1
39019	1.00647	2.1
39020	1.00647	2.1
39021	1.15401	2.1
39022	0.881789	2.1
39023	0.850526	2.1
39024	1.04263	2.1
39025	1.4054	1.24444
39026	1.04788	2.1
39027	1.70302	0.820451
39028	1.41375	2.1
39029	2.82706	2.1
39030	1.70302	1.09733
39031	1.38776	2.1

39032	1.59457	2.1
39033	1.41375	2.1
39034	1.77261	0.518799
39035	1.70302	2.1
39036	1.89226	2.1
39037	1.89226	2.1
39038	1.89226	2.1
39039	1.89226	2.1
39040	3.78453	2.1
39041	2.18042	2.1
39042	1.74738	2.1
39043	2.08433	2.1
39044	1.66347	2.1
39045	3.83171	2.1
39046	1.74738	2.1
39047	1.1181	2.1
39048	1.66347	2.1
39049	1.06543	2.1
39050	2.81281	2.1
39051	1.89226	2.1
39052	1.89226	2.1
39053	1.89226	2.1
39054	1.89226	2.1
39055	3.78453	2.1
39056	1.89227	2.1
39057	1.89227	2.1
39058	1.89227	2.1
39059	1.89227	2.1
39060	3.78453	2.1
39061	2.08433	2.1
39062	1.66347	2.1
39063	1.97092	2.1
39064	1.57431	2.1
39065	3.65864	2.1
39066	1.66347	2.1
39067	1.01474	2.1
39068	1.01474	2.1
39069	1.06544	2.1
39070	1.57431	2.1
39071	1.625 2.1	
39072	1.89226	2.1
39073	1.89226	2.1
39074	1.89226	2.1
39075	1.89226	2.1
39076	1.89226	2.1
39077	1.89226	2.1
39078	1.08467	2.1
39079	1.87228	2.1
39080	1.73305	2.1
39081	1.73305	2.1
39082	1.25565	2.1
39083	1.25565	2.1
39084	0.695361	0.0680275
39085	0.799996	0.19087
39086	1.13751	0.151622
39087	1.40945	0.176635
39088	1.40944	0.426849
39089	0.799999	0.05

39090	1.13751	0.126895
39091	0.695363	0.05
39092	1.01059	0.0762976
39093	1.01059	0.096394
39094	0.926908	0.426414
39095	0.926909	0.05
39096	1.05481	2.1
39097	1.78069	2.1
39098	1.08074	2.1
39099	1.83284	2.1
39100	2.86143	2.1
39101	1.78069	2.1
39102	2.22044	2.1
39103	1.83284	2.1
39104	2.28696	1.16485
39105	4.06766	2.1
39106	1.07196	2.1
39107	1.64955	2.1
39108	2.02665	2.1
39109	2.0284	2.1
39110	1.08074	2.1
39111	1.08074	2.1
39112	1.64955	2.1
39113	2.28696	1.72748
39114	1.92033	2.1
39115	2.1004	2.1
39116	3.74988	2.1
39117	2.83787	0.787306
39118	1.70303	1.01772
39119	1.70303	0.740768
39120	1.83195	0.745344
39121	2.64075	0.748306
39122	2.70231	0.867788
39123	1.2087	2.1
39124	1.52909	0.295364
39125	1.43063	0.163817
39126	1.10586	2.1
39127	2.63494	0.784804
39128	1.89227	1.4459
39129	2.10732	2.1
39130	2.10732	2.1
39131	2.2365	2.1
39132	1.89227	1.79935
39133	2.02145	2.1
39134	1.89226	0.495676
39135	1.89226	2.1
39136	1.89226	0.893013
39137	1.89226	1.93562
39138	1.89226	0.489473
39139	1.89226	2.1
39140	0.51926	2.1
39141	0.552927	2.1
39142	0.691235	0.528703
39143	0.715314	0.101767
39144	0.619684	0.0765306
39145	0.619684	2.1
39146	0.624477	2.1
39147	0.624477	0.0573141

39148	0.519262	0.67179
39149	0.691234	0.894569
39150	0.715313	1.12384
39151	0.552928	0.604218
39152	1.89226	2.1
39153	1.87321	2.1
39154	1.87321	2.1
39155	1.89658	2.1
39156	2.0815	2.1
39157	2.14789	2.1
39158	0.946128	2.1
39159	0.946128	2.1
39160	0.946133	0.169183
39161	0.946133	0.214523
39162	0.94613	0.0701924
39163	0.94613	2.1
39164	0.946131	0.668376
39165	0.946131	2.1
39166	0.946131	0.479725
39167	0.946131	0.482892
39168	0.946131	0.932569
39169	0.946131	0.878706
39170	1.99297	1.29508
39171	2.19514	0.27338
39172	2.46153	0.904677
39173	2.187	0.744237
39174	4.45451	0.756957
39175	2.0815	0.577145
39176	2.33402	0.308327
39177	2.46154	1.03015
39178	2.83046	0.108662
39179	4.94496	0.497731
39180	2.46153	0.787612
39181	2.187	0.481833
39182	2.27351	1.14389
39183	2.63356	0.168061
39184	4.73504	0.698645
39185	2.46154	0.908712
39186	2.83447	0.0985843
39187	2.74513	0.324642
39188	2.5731	0.68314
39189	2.63357	0.230413
39190	2.46154	0.514217
39191	2.80398	2.1
39192	2.60406	2.1
39193	2.73988	2.1
39194	2.57466	2.1
39195	5.34394	2.1
39196	1.89226	1.18871
39197	1.89226	0.989519
39198	1.89226	0.884536
39199	1.89226	1.29688
39200	1.89226	0.928872
39201	1.89226	1.98954
39202	1.89224	1.37354
39203	1.89224	1.02045
39204	1.89224	0.939638
39205	1.89224	1.69862

39206	1.89224	0.999701
39207	1.89224	1.98307
39208	1.40245	1.27886
39209	1.40246	1.90811
39210	0.946117	1.30613
39211	0.946117	1.96481
39212	1.17635	1.11939
39213	1.40246	1.98164
39214	0.946112	1.04623
39215	0.946112	1.88037
39216	0.946127	1.32621
39217	1.17637	1.12133
39218	0.946102	1.99103
39219	1.40244	1.28789
39220	2.53269	2.1
39221	1.70302	2.1
39222	1.56194	2.1
39223	2.17516	2.1
39224	4.19807	2.1
39225	1.89226	2.1
39226	1.89226	2.00194
39227	1.89226	2.1
39228	1.89226	2.1
39229	3.78453	2.1
39230	2.57276	2.1
39231	1.89226	2.1
39232	1.89226	2.1
39233	1.89226	2.1
39234	2.32525	2.1
39235	2.32525	2.1
39236	1.15171	2.1
39237	2.17517	2.1
39238	1.41995	2.1
39239	1.41995	2.1
39240	1.80252	2.1
39241	1.80252	2.1
39242	1.89226	2.1
39243	1.89226	2.1
39244	1.89226	2.1
39245	1.89226	1.09663
39246	3.78453	2.1
39247	0.946133	2.1
39248	0.946133	2.1
39249	1.16263	2.1
39250	1.04687	2.1
39251	0.946133	2.1
39252	0.946133	2.1
39253	1.04687	2.1
39254	1.04687	2.1
39255	0.946133	2.1
39256	1.04687	2.1
39257	0.946132	2.1
39258	1.16263	2.1
39259	0.682281	1.83299
39260	0.669233	2.1
39261	1.21859	2.1
39262	1.31629	1.41174
39263	0.916158	1.26145

39264	0.916158	2.1
39265	0.971658	1.79696
39266	0.971658	1.97916
39267	0.669234	1.93119
39268	1.31629	1.46706
39269	0.682283	1.8404
39270	1.21858	2.03168
39271	0.669232	1.51989
39272	0.623495	1.99737
39273	1.36428	1.75716
39274	1.31629	1.1727
39275	0.946475	1.14205
39276	0.946476	2.1
39277	0.99331	1.67389
39278	0.99331	1.62899
39279	1.36428	1.5839
39280	0.623497	1.29321
39281	0.669234	1.62686
39282	1.31629	1.35436
39283	2.02069	0.05
39284	1.46253	0.05
39285	2.51479	0.05
39286	1.87136	0.05
39287	3.89203	0.05
39288	1.46253	0.05
39289	1.38536	0.05
39290	1.38536	0.05
39291	1.05579	0.05
39292	1.73281	0.05
39293	1.46916	0.05
39294	2.51479	0.05
39295	1.87136	0.05
39296	2.23338	0.05
39297	2.15603	0.05
39298	4.55146	0.05
39299	0.822553	0.05
39300	0.822563	0.05
39301	0.842935	0.05
39302	0.842935	0.05
39303	0.744203	0.05
39304	0.675346	0.05
39305	0.874795	0.05
39306	1.00453	0.05
39307	1.00454	0.05
39308	0.782417	0.05
39309	0.675337	0.05
39310	0.858007	0.05
39311	0.946132	1.23245
39312	1.24847	0.887621
39313	0.946132	1.13485
39314	1.16806	1.33532
39315	1.24847	0.843607
39316	1.24847	1.97742
39317	0.946133	0.817427
39318	0.946133	1.82516
39319	0.946133	0.986461
39320	0.946133	0.84659
39321	1.16805	1.35596

39322	1.24847	0.917777
39323	1.2364	1.44234
39324	0.946129	0.986764
39325	0.946133	1.30426
39326	1.24847	0.986253
39327	1.2364	0.926686
39328	1.2364	1.77735
39329	0.946131	0.83605
39330	0.946131	2.08603
39331	0.946131	1.15275
39332	0.946131	0.919005
39333	1.2364	1.76506
39334	1.24847	1.02162
39335	1.89226	1.46735
39336	1.89226	1.36725
39337	1.89226	0.889604
39338	1.89226	0.946256
39339	1.89226	1.24474
39340	1.89226	1.70417
39341	1.89226	1.18141
39342	1.89226	0.953144
39343	1.89226	0.861609
39344	1.89226	1.34412
39345	1.89226	0.941615
39346	1.89226	1.51388
39347	1.69014	1.02788
39348	1.09194	1.38959
39349	1.13537	1.68299
39350	1.13537	1.49307
39351	1.58481	0.897117
39352	1.58481	0.971474
39353	1.58147	1.01754
39354	1.13537	1.03956
39355	1.69015	1.19706
39356	1.01179	0.914064
39357	1.69015	1.03889
39358	1.01179	1.42983
39359	0.946132	1.74831
39360	0.946132	1.18906
39361	1.28543	0.908877
39362	1.07341	1.58523
39363	1.07341	1.50924
39364	1.07341	0.112255
39365	0.946132	0.969425
39366	0.946132	0.937579
39367	0.946133	0.949213
39368	1.28543	0.937396
39369	1.07341	1.00719
39370	0.946132	1.11108
39371	1.89226	0.886238
39372	1.89226	0.145048
39373	1.89226	0.120295
39374	1.89226	2.02568
39375	1.89226	0.105016
39376	1.89226	2.1
39377	0.805744	2.1
39378	0.988659	1.14863
39379	0.782044	0.0796492

39380	0.578223	0.541139
39381	0.77823	0.0747157
39382	0.778231	2.1
39383	0.788653	0.0503592
39384	0.788653	1.54577
39385	0.988662	0.841946
39386	0.782042	0.523396
39387	0.805745	1.26698
39388	0.578222	0.82226
39389	1.89226	1.03953
39390	1.89226	0.476467
39391	1.89226	0.276276
39392	1.89226	2.05339
39393	1.89226	0.208337
39394	1.89226	2.1
39395	1.97732	1.43124
39396	1.97819	0.696442
39397	1.56408	0.10842
39398	1.56408	2.09316
39399	2.33986	0.507458
39400	2.33986	2.1
39401	1.84516	2.1
39402	2.21152	2.1
39403	1.90702	2.1
39404	1.54599	2.1
39405	3.75751	2.1
39406	1.89224	2.1
39407	1.89224	2.1
39408	1.89224	2.1
39409	1.89224	2.1
39410	1.89224	2.1
39411	1.89224	2.1
39412	1.47672	2.1
39413	1.54601	2.1
39414	1.27697	2.1
39415	1.54601	2.1
39416	1.638	2.1
39417	1.90705	2.1
39418	0.946132	2.1
39419	0.946132	2.1
39420	0.946131	2.1
39421	0.946131	2.1
39422	0.946131	2.1
39423	0.946131	2.1
39424	0.946132	2.1
39425	0.946132	2.1
39426	0.946132	2.1
39427	0.946132	2.1
39428	0.946131	2.1
39429	0.946131	2.1
39430	0.946128	2.1
39431	0.946128	2.1
39432	1.32062	2.1
39433	1.4164	2.1
39434	0.946129	2.1
39435	0.946129	2.1
39436	1.32062	2.1
39437	1.32062	2.1

39438	1.32062	2.1
39439	0.94613	2.1
39440	1.41639	2.1
39441	0.946129	2.1
39442	1.98979	0.05
39443	1.65977	0.05
39444	2.21772	0.05
39445	2.21772	0.05
39446	1.48866	0.05
39447	1.48866	0.05
39448	0.973129	0.05
39449	0.973129	0.05
39450	0.980954	0.05
39451	0.980954	0.05
39452	0.845218	0.05
39453	1.13981	0.05
39454	0.829886	0.05
39455	1.10886	0.05
39456	1.10886	0.05
39457	1.13982	0.05
39458	0.845219	0.05
39459	0.829886	0.05
39460	0.946132	1.71108
39461	0.946132	2.1
39462	1.3352	2.1
39463	1.41432	2.1
39464	0.946133	2.1
39465	0.946133	1.01083
39466	1.3352	2.1
39467	1.3352	2.1
39468	1.3352	2.1
39469	0.946132	2.1
39470	1.41432	2.1
39471	0.946133	2.1
39472	2.70097	2.1
39473	1.88175	2.1
39474	1.51482	2.1
39475	1.88175	2.1
39476	2.37784	2.1
39477	2.82864	2.1
39478	1.89227	0.05
39479	1.89227	0.05
39480	1.89227	0.05
39481	1.89227	0.05
39482	3.78453	0.05
39483	1.89226	0.05
39484	2.13295	0.05
39485	2.43203	0.05
39486	2.43203	0.05
39487	1.89226	0.05
39488	1.89226	0.05
39489	0.946133	0.05
39490	0.946133	0.05
39491	0.946132	0.05
39492	0.946132	0.05
39493	0.946132	0.05
39494	0.946132	0.05
39495	0.946133	0.05

39496	0.946133	0.05
39497	0.946132	0.05
39498	0.946132	0.05
39499	0.946132	0.05
39500	0.946132	0.05
39501	1.54987	2.1
39502	1.24128	2.1
39503	1.24128	2.1
39504	1.50738	2.1
39505	1.75702	2.1
39506	2.02312	2.1
39507	1.89289	2.1
39508	1.92264	2.1
39509	1.72994	2.1
39510	1.75702	2.1
39511	3.67966	2.1
39512	1.75702	2.1
39513	0.994291	2.1
39514	0.994291	2.1
39515	1.24128	2.1
39516	1.4964	2.1
39517	1.74339	2.1
39518	1.72993	2.1
39519	1.49641	2.1
39520	1.49641	2.1
39521	1.75702	2.1
39522	1.3939	2.1
39523	1.65452	2.1
39524	1.19964	2.1
39525	1.53296	2.1
39526	1.19964	2.1
39527	1.36861	2.1
39528	1.42624	2.1
39529	1.59521	2.1
39530	1.19964	2.1
39531	1.42624	2.1
39532	1.07156	2.1
39533	1.36226	2.1
39534	2.5619	2.1
39535	2.14058	1.2251
39536	1.93569	2.1
39537	2.36221	1.44556
39538	2.5753	1.38976
39539	4.50279	2.1
39540	2.14057	1.88135
39541	2.20831	2.1
39542	2.64725	1.12518
39543	2.57529	1.85398
39544	4.78782	1.79946
39545	2.64725	1.28755
39546	2.57529	2.04127
39547	1.89226	1.11872
39548	1.89226	0.999798
39549	4.53951	1.61078
39550	2.0815	2.1
39551	1.86137	2.1
39552	1.86137	2.1
39553	1.86159	2.1

39554	2.0815	2.1
39555	2.10235	2.1
39556	1.33552	2.1
39557	2.32417	2.1
39558	1.18872	2.1
39559	1.93101	2.1
39560	3.26653	2.1
39561	1.89226	2.1
39562	1.89226	2.1
39563	1.89226	2.1
39564	1.89226	2.1
39565	1.89226	2.1
39566	1.89226	2.1
39567	0.955191	2.1
39568	1.03372	2.1
39569	0.946131	2.1
39570	0.946131	2.1
39571	0.930654	2.1
39572	0.930654	2.1
39573	1.04233	2.1
39574	1.05247	2.1
39575	0.955341	2.1
39576	0.946131	2.1
39577	1.02375	2.1
39578	1.13537	2.1
39579	1.1549	2.1
39580	1.93101	2.1
39581	1.91108	2.1
39582	1.52487	2.1
39583	1.3076	2.1
39584	1.06985	2.1
39585	1.89226	2.1
39586	1.89226	2.1
39587	1.89226	2.1
39588	1.89226	2.1
39589	1.89226	2.1
39590	1.89226	2.1
39591	1.58713	2.1
39592	2.6845	2.1
39593	1.58742	2.1
39594	2.74201	2.1
39595	4.27191	2.1
39596	2.68449	2.1
39597	1.89226	2.1
39598	2.74201	2.1
39599	1.89226	2.1
39600	4.57675	2.1
39601	1.57523	2.1
39602	2.74201	2.1
39603	2.8146	2.1
39604	2.8146	2.1
39605	1.58742	2.1
39606	1.58742	2.1
39607	0.946131	2.1
39608	0.946131	2.1
39609	1.371 2.1	
39610	1.371 2.1	
39611	0.946127	2.1

39612	0.946127	2.1
39613	1.37101	2.1
39614	1.4073	2.1
39615	0.94613	2.1
39616	1.4073	2.1
39617	1.371 2.1	
39618	0.946129	2.1
39619	1.44801	2.09541
39620	1.06821	2.05749
39621	2.00542	1.99936
39622	2.27703	2.1
39623	3.34524	1.87297
39624	1.70301	0.681281
39625	1.89226	0.728314
39626	1.65809	0.696423
39627	1.89226	0.706148
39628	1.62758	0.702655
39629	1.85744	0.708732
39630	1.89226	0.670908
39631	1.89226	0.72016
39632	1.89226	0.715098
39633	1.89226	0.701646
39634	3.78452	0.704027
39635	1.89226	0.720922
39636	1.89226	0.691574
39637	1.89226	0.699711
39638	1.89226	0.756174
39639	3.78452	0.709788
39640	0.851541	0.721891
39641	0.851541	0.719642
39642	0.91193	0.681392
39643	0.824837	0.683702
39644	0.854559	0.683043
39645	0.851534	0.821563
39646	0.81851	0.723079
39647	0.717219	0.694144
39648	0.871743	0.709927
39649	0.837985	0.694262
39650	0.82902	0.690601
39651	0.82902	0.700319
39652	1.89226	0.736837
39653	1.92659	0.705372
39654	2.03235	0.708874
39655	1.89226	0.768576
39656	2.03235	0.687825
39657	1.89226	0.733416
39658	0.585557	0.820386
39659	0.585557	0.774604
39660	1.22612	0.718199
39661	1.09436	0.763525
39662	0.851537	0.810779
39663	0.526987	0.854934
39664	0.983879	0.722582
39665	1.04072	0.777573
39666	0.585526	0.78066
39667	0.792325	0.760644
39668	0.946129	0.770113
39669	0.946129	0.773746

39670	1.68152	2.1
39671	1.81445	2.1
39672	1.68152	2.1
39673	1.86792	2.1
39674	1.65888	2.1
39675	1.84529	2.1
39676	1.81445	2.1
39677	1.57885	2.1
39678	1.65889	2.1
39679	1.48594	2.1
39680	3.23773	2.1
39681	1.68154	2.1
39682	1.65891	2.1
39683	1.53296	2.1
39684	1.56299	2.1
39685	3.24453	2.1
39686	1.56299	2.1
39687	1.48596	2.1
39688	1.56299	2.1
39689	1.65891	2.1
39690	1.46568	2.1
39691	1.5616	2.1
39692	2.68785	2.1
39693	2.08831	2.1
39694	2.66919	2.1
39695	2.1024	2.1
39696	4.79025	2.1
39697	2.08832	2.1
39698	1.15478	2.1
39699	1.15478	2.1
39700	1.09062	2.1
39701	2.10241	2.1
39702	2.03825	2.1
39703	2.65348	2.1
39704	2.1024	2.1
39705	2.12313	2.1
39706	2.12313	2.1
39707	2.66919	2.1
39708	2.66919	2.1
39709	0.813169	2.1
39710	0.813169	2.1
39711	0.825788	2.1
39712	0.825788	2.1
39713	0.612902	2.1
39714	0.577387	2.1
39715	1.0512	2.1
39716	1.06157	2.1
39717	1.06157	2.1
39718	0.612902	2.1
39719	0.577387	2.1
39720	1.0512	2.1
39721	1.27227	2.1
39722	1.91724	2.1
39723	1.32591	2.1
39724	1.99837	2.1
39725	3.24315	2.1
39726	1.89223	2.1
39727	1.89223	2.1

39728	1.89223	2.1
39729	1.89223	2.1
39730	3.78446	2.1
39731	1.89224	2.1
39732	1.89224	1.21882
39733	1.89224	2.1
39734	1.89224	2.1
39735	3.78447	2.1
39736	1.91724	2.1
39737	2.321	2.1
39738	1.99838	2.1
39739	2.4149	2.1
39740	4.33214	2.1
39741	1.34817	2.1
39742	1.9984	2.1
39743	2.06238	2.1
39744	2.06238	2.1
39745	1.32593	2.1
39746	1.32593	2.1
39747	1.89226	2.1
39748	1.89226	2.1
39749	1.89226	2.1
39750	1.89226	2.1
39751	1.89226	2.1
39752	1.89226	2.1
39753	1.89226	2.1
39754	1.89226	2.1
39755	1.89226	2.1
39756	1.89226	1.41644
39757	3.78452	2.1
39758	1.9984	2.1
39759	2.41494	2.1
39760	2.06239	2.1
39761	2.5194	2.1
39762	4.47732	2.1
39763	1.19901	1.15542
39764	1.16661	2.1
39765	1.56859	2.1
39766	1.61029	1.29455
39767	2.7676	2.1
39768	1.56859	2.1
39769	1.88637	0.511418
39770	1.61029	0.131292
39771	1.61029	2.1
39772	1.856	0.141849
39773	1.856	2.1
39774	1.856	0.38189
39775	1.57916	2.1
39776	1.56859	1.12638
39777	1.56859	2.1
39778	1.57916	2.1
39779	1.23475	2.1
39780	0.890924	0.05
39781	1.07608	0.0681603
39782	0.72877	0.180623
39783	0.910106	0.675485
39784	1.00196	0.154187
39785	1.09519	0.990029

39786	0.839967	0.0621837
39787	0.719043	0.05
39788	0.893859	0.05
39789	0.942408	0.230004
39790	0.873497	0.199415
39791	0.873497	1.08744
39792	1.53046	0.05
39793	1.89994	0.117735
39794	1.049	0.05
39795	1.049	0.05
39796	1.52512	0.05
39797	1.89994	0.05
39798	1.65573	0.853647
39799	1.89226	0.547411
39800	1.89226	0.295141
39801	2.73329	1.23276
39802	3.548	0.946336
39803	1.65573	0.511554
39804	2.42671	0.877546
39805	2.73329	0.377303
39806	1.89226	0.148981
39807	2.73329	1.02453
39808	1.89226	0.692827
39809	0.941078	0.257687
39810	1.01599	0.511617
39811	0.818936	0.145442
39812	0.651554	0.56432
39813	0.904729	0.398648
39814	0.665756	0.406958
39815	0.843423	0.747936
39816	0.918649	0.491212
39817	0.855806	1.06649
39818	0.814384	0.05
39819	0.844437	0.785969
39820	0.844437	0.79278
39821	2.22558	1.41818
39822	1.95785	0.592708
39823	2.07065	1.14908
39824	1.89142	1.06803
39825	2.07065	1.94496
39826	1.89142	1.72674
39827	0.640813	0.675492
39828	0.951654	1.02558
39829	0.797442	0.251994
39830	0.535364	0.826364
39831	0.525269	1.06112
39832	0.620874	0.547153
39833	0.916953	1.08515
39834	0.969374	0.799783
39835	0.788925	1.44126
39836	0.69596	0.05
39837	0.743154	1.01078
39838	0.743154	1.0596
39839	1.89226	2.1
39840	1.89226	1.64454
39841	1.89226	1.00771
39842	1.89226	2.1
39843	3.78452	1.23822

39844	1.32541	1.52452
39845	1.13722	1.20932
39846	0.91103	2.1
39847	0.881123	2.1
39848	1.03363	2.1
39849	1.03364	2.02255
39850	1.1505	1.77728
39851	1.1505	2.1
39852	1.32539	2.1
39853	1.07662	2.1
39854	0.881121	2.1
39855	0.962303	2.0067
39856	1.89226	1.20098
39857	1.89226	2.1
39858	1.89226	2.1
39859	1.89226	1.29779
39860	3.78453	2.1
39861	1.89226	0.990855
39862	1.89226	2.1
39863	1.89226	1.69809
39864	1.89226	1.29078
39865	1.89226	1.76451
39866	1.89226	1.57181
39867	1.27606	1.7621
39868	1.07664	1.33772
39869	0.905895	2.1
39870	0.696099	2.1
39871	0.984902	2.1
39872	0.984905	1.89295
39873	1.01878	2.1
39874	1.01878	1.10874
39875	1.27604	2.1
39876	1.06044	2.1
39877	1.13192	2.1
39878	0.696111	2.1
39879	1.89226	0.424332
39880	1.89226	0.05
39881	1.89226	0.05
39882	1.89226	0.847047
39883	1.89226	0.05
39884	1.89226	0.672372
39885	1.52615	0.183678
39886	1.01294	0.05
39887	1.01294	0.923828
39888	1.18549	0.05
39889	1.38963	0.05
39890	1.56218	0.05
39891	1.89226	0.530863
39892	2.40801	0.05
39893	2.58101	0.181798
39894	2.58101	0.724274
39895	1.89226	0.05
39896	1.89226	0.05
39897	2.24071	2.1
39898	2.79317	2.1
39899	1.99136	2.1
39900	2.6167	2.1
39901	4.85741	2.1

39902	2.79317	2.1
39903	1.89226	0.0517353
39904	1.89226	2.1
39905	1.89226	0.981163
39906	2.6167	2.1
39907	2.6167	0.0977972
39908	1.08354	2.1
39909	1.08354	2.1
39910	1.10222	2.1
39911	1.10222	2.1
39912	1.2275	2.1
39913	1.30835	2.1
39914	0.995678	2.1
39915	0.877412	2.1
39916	0.877411	2.1
39917	1.2275	2.1
39918	0.995678	2.1
39919	1.30835	2.1
39920	1.05793	0.884598
39921	1.46441	0.147595
39922	1.0562	0.763614
39923	1.0562	0.281004
39924	1.41787	0.873858
39925	1.41787	0.411304
39926	1.41787	0.887715
39927	1.52909	1.01965
39928	1.46441	0.739371
39929	1.46441	0.614506
39930	1.43063	0.628682
39931	1.43063	0.454354
39932	1.89226	1.03108
39933	1.89226	0.438555
39934	1.89226	0.858941
39935	1.89226	1.10225
39936	3.78453	0.939402
39937	1.89227	0.886075
39938	1.89227	0.629561
39939	1.89227	0.707591
39940	1.89227	0.311696
39941	1.89227	0.774457
39942	1.89227	0.370723
39943	1.00763	0.791221
39944	1.41787	0.143522
39945	1.05793	0.748521
39946	1.05793	0.222648
39947	1.37265	0.831062
39948	1.37265	0.238554
39949	1.37265	0.62283
39950	1.43063	0.152631
39951	1.41787	0.578754
39952	1.41787	0.980183
39953	1.38247	0.399605
39954	1.38247	0.996383
39955	0.946132	0.43551
39956	0.946132	0.179401
39957	0.946132	0.776811
39958	0.946132	0.0870303
39959	0.946131	0.0948661

39960	0.946131	0.161879
39961	0.946132	0.455564
39962	0.946132	0.82299
39963	0.946132	0.440473
39964	0.946132	0.306845
39965	0.946132	0.249242
39966	0.946132	0.201167
39967	1.89226	0.854895
39968	1.89226	0.0893108
39969	1.89226	0.564276
39970	1.89226	0.355381
39971	1.89226	0.744016
39972	1.89226	0.519699
39973	1.19645	0.990184
39974	1.89183	0.791329
39975	1.91106	0.902092
39976	1.35306	0.822932
39977	1.75446	0.910672
39978	1.19645	0.820665
39979	2.37576	0.870954
39980	1.90139	2.1
39981	2.1845	2.1
39982	1.86568	1.70649
39983	4.24145	2.1
39984	2.29435	2.07235
39985	1.40602	2.1
39986	1.40602	0.936024
39987	1.55351	2.1
39988	1.86568	2.1
39989	2.01317	2.1
39990	2.18452	2.1
39991	1.8657	0.369662
39992	2.01399	0.899687
39993	1.70141	2.1
39994	3.87969	0.908569
39995	0.774999	2.1
39996	0.774999	0.921899
39997	0.778721	1.75319
39998	0.778721	2.1
39999	0.628348	2.1
40000	0.703016	2.1
40001	0.932848	0.980463
40002	0.850704	2.1
40003	0.850704	2.1
40004	0.628348	2.1
40005	0.703015	0.954447
40006	0.932848	2.1
40007	1.65243	0.720604
40008	1.34412	0.727489
40009	1.45569	0.83124
40010	1.1315	0.829544
40011	2.78393	0.759083
40012	1.48901	2.1
40013	1.805	2.1
40014	1.48901	2.1
40015	1.71717	2.1
40016	1.60635	0.624879
40017	1.83451	2.1

40018	1.805	2.1
40019	1.65243	0.0971387
40020	1.60635	2.07521
40021	1.45569	0.790905
40022	3.25879	0.05
40023	1.27875	2.1
40024	1.60635	2.1
40025	1.27875	2.1
40026	1.48901	2.1
40027	1.40014	1.95177
40028	1.6104	2.1
40029	0.715199	0.73148
40030	0.715199	2.1
40031	0.712714	2.1
40032	0.712714	0.05
40033	0.727843	0.05
40034	0.619713	0.13076
40035	0.803177	2.1
40036	0.70007	1.68433
40037	0.700069	0.297383
40038	0.619713	0.13641
40039	0.803177	0.0711485
40040	0.727844	0.126332
40041	1.14357	2.1
40042	2.3506	2.1
40043	1.14357	2.1
40044	2.68773	2.1
40045	1.57829	2.1
40046	2.68773	2.1
40047	2.00551	2.1
40048	2.65811	1.91844
40049	2.07203	2.1
40050	2.72117	0.05
40051	4.72667	2.1
40052	0.946131	0.605698
40053	0.946131	2.1
40054	1.32905	2.1
40055	1.32905	0.199365
40056	0.94613	1.22583
40057	0.94613	1.62267
40058	1.32905	1.53394
40059	1.36058	1.27801
40060	1.36058	0.973964
40061	0.946131	0.328058
40062	1.32905	1.31127
40063	0.946131	1.86088
40064	1.21649	0.05
40065	1.21649	2.1
40066	1.21459	2.1
40067	1.21459	2.1
40068	1.39508	1.29332
40069	1.36057	1.0751
40070	1.03601	2.1
40071	1.07438	2.1
40072	1.07439	1.59754
40073	1.39509	1.83678
40074	1.036	1.70036
40075	1.36056	1.24224

40076	2.79015	1.03552
40077	2.72114	2.1
40078	1.89225	1.14031
40079	1.89225	2.1
40080	1.89225	1.99001
40081	2.72114	0.941966
40082	0.876258	2.1
40083	0.892889	2.1
40084	1.34352	2.1
40085	1.27985	2.1
40086	1.07462	2.1
40087	1.07462	2.1
40088	1.09812	2.1
40089	1.09812	2.1
40090	0.892888	2.1
40091	1.34352	2.1
40092	0.876256	2.1
40093	1.27985	2.1
40094	1.39725	2.04983
40095	1.30029	0.218339
40096	1.31415	0.719197
40097	1.41336	1.61825
40098	2.71366	1.1617
40099	1.89226	0.498267
40100	1.89226	1.18683
40101	1.89226	1.88906
40102	1.89226	0.780948
40103	3.78453	0.789464
40104	0.946133	1.08403
40105	0.946133	0.987567
40106	1.38709	0.210571
40107	1.39437	0.942525
40108	0.946132	0.236552
40109	0.946132	0.522285
40110	1.39437	1.11751
40111	1.39437	0.335507
40112	0.946132	0.942517
40113	1.39437	0.350966
40114	1.38709	0.714287
40115	0.946132	0.892976
40116	0.706681	2.08889
40117	0.711908	1.73118
40118	0.657077	0.268015
40119	0.661003	1.13993
40120	0.684188	0.282684
40121	0.684188	1.54664
40122	0.683496	2.1
40123	0.683496	0.256503
40124	0.711908	1.07009
40125	0.661003	1.26051
40126	0.657076	1.17062
40127	0.706681	1.18237
40128	0.946132	1.9098
40129	0.946132	1.18374
40130	0.946132	1.03701
40131	0.946132	0.461865
40132	0.946132	0.556745
40133	0.946132	1.22043

40134	0.946133	0.852228
40135	0.946133	1.89756
40136	0.946133	0.868535
40137	0.946133	1.11177
40138	0.946132	0.923984
40139	0.946132	1.13089
40140	2.79029	0.300173
40141	1.89227	1.11033
40142	2.78873	0.530243
40143	2.78873	0.204921
40144	1.89383	0.906429
40145	1.89227	0.953482
40146	1.43669	2.1
40147	2.11984	2.1
40148	2.24481	2.1
40149	2.24481	2.1
40150	1.20311	2.1
40151	1.20311	2.1
40152	0.49703	2.1
40153	0.601553	2.1
40154	1.05992	2.1
40155	0.991694	2.1
40156	0.787558	2.1
40157	0.787558	2.1
40158	0.805689	2.1
40159	0.805689	2.1
40160	0.991694	2.1
40161	0.49703	2.1
40162	1.05992	2.1
40163	0.601553	2.1
40164	2.45905	2.1
40165	1.89226	2.1
40166	1.89226	2.1
40167	1.89226	2.1
40168	2.47395	2.1
40169	2.47395	2.1
40170	1.89226	2.1
40171	1.89226	2.08416
40172	1.89226	2.1
40173	1.89226	1.68374
40174	3.78452	2.1
40175	1.38427	2.1
40176	2.31203	1.20354
40177	2.28743	1.1953
40178	2.28743	2.1
40179	1.63384	1.11699
40180	1.63384	2.1
40181	2.42723	2.1
40182	1.89226	2.1
40183	1.89226	2.1
40184	1.89226	2.1
40185	2.45905	2.1
40186	2.45905	2.1
40187	0.946131	0.762823
40188	0.946131	2.1
40189	0.946131	2.1
40190	0.946131	2.1
40191	0.94613	1.35729

40192	0.94613	2.1
40193	0.946132	2.1
40194	0.946132	2.1
40195	0.946131	2.1
40196	0.946131	2.1
40197	0.946131	2.1
40198	0.946131	2.1
40199	0.989441	2.1
40200	0.989441	2.1
40201	0.993918	2.1
40202	0.993918	2.1
40203	1.16644	2.1
40204	1.15601	1.26467
40205	0.818308	2.1
40206	0.816922	2.1
40207	0.818307	2.1
40208	1.16644	2.1
40209	1.15601	2.1
40210	0.816922	2.1
40211	0.996778	0.05
40212	2.44375	0.05
40213	0.996778	0.05
40214	1.92547	0.05
40215	1.56091	0.05
40216	2.4896	0.05
40217	1.56091	0.05
40218	2.44375	0.05
40219	1.90456	0.05
40220	1.90456	0.05
40221	2.81853	0.05
40222	2.47487	0.05
40223	1.32143	2.1
40224	1.43294	2.1
40225	1.37444	1.12836
40226	1.51691	2.1
40227	2.80738	1.91417
40228	1.89226	1.55279
40229	1.89226	2.1
40230	1.89226	1.19501
40231	1.89226	1.42196
40232	3.78453	1.43267
40233	1.89226	1.79176
40234	1.89226	2.1
40235	1.89226	1.21
40236	1.89226	2.1
40237	3.78453	2.1
40238	1.43294	1.65701
40239	1.3268	2.1
40240	1.51691	1.52382
40241	1.44091	2.1
40242	2.87385	2.1
40243	1.37444	2.1
40244	1.51691	2.1
40245	1.4714	1.15336
40246	1.65434	1.60911
40247	3.02878	1.71853
40248	1.89226	2.1
40249	1.89226	2.0591

40250	1.89226	1.29162
40251	1.89226	1.79684
40252	3.78452	1.69602
40253	1.89226	1.49667
40254	1.89226	2.1
40255	1.89226	1.29594
40256	1.89226	2.1
40257	3.78453	2.1
40258	1.51691	2.09399
40259	1.44091	2.1
40260	1.65434	1.29363
40261	1.60647	2.1
40262	3.09526	2.1
40263	1.05013	2.1
40264	1.05013	2.1
40265	0.94613	2.1
40266	0.94613	2.1
40267	1.04959	2.1
40268	1.05014	2.1
40269	0.946129	2.1
40270	0.946129	2.1
40271	0.946131	2.1
40272	1.04958	2.1
40273	0.946131	2.1
40274	1.05013	2.1
40275	1.04959	2.1
40276	1.04959	2.1
40277	0.946131	2.1
40278	0.946131	2.1
40279	1.04765	2.1
40280	1.04959	2.1
40281	0.94613	2.1
40282	0.94613	2.1
40283	0.946131	2.1
40284	1.04765	2.1
40285	0.946131	2.1
40286	1.04959	2.1
40287	1.85744	2.1
40288	1.89226	2.1
40289	1.89226	2.1
40290	1.89226	2.1
40291	1.94839	2.1
40292	1.94839	2.1
40293	0.97419	2.1
40294	0.822054	2.1
40295	0.946123	2.1
40296	0.946123	2.1
40297	1.04202	2.1
40298	0.84898	2.1
40299	0.933307	2.1
40300	0.933307	2.1
40301	0.933285	2.1
40302	1.05018	2.1
40303	0.851528	2.1
40304	0.876787	2.1
40305	1.87528	0.05
40306	1.21137	0.05
40307	1.57893	0.05

40308	1.21137	0.0561979
40309	1.81182	0.05
40310	1.44426	0.05
40311	0.993833	0.05
40312	1.21137	0.05
40313	1.57893	0.05
40314	1.31774	0.05
40315	2.5291	0.05
40316	1.87528	0.05
40317	1.57893	0.05
40318	1.90728	0.05
40319	2.29851	0.05
40320	3.87744	0.05
40321	1.90728	0.05
40322	1.31774	0.05
40323	1.56339	0.05
40324	1.31774	0.05
40325	1.82458	0.05
40326	1.57893	0.05
40327	1.89226	0.850577
40328	1.89226	1.21246
40329	1.89226	1.14431
40330	1.89226	1.18139
40331	1.89226	1.14745
40332	1.89226	1.18566
40333	1.89226	1.13785
40334	1.89226	1.27038
40335	1.89226	1.31868
40336	1.89226	1.21177
40337	3.78452	1.26355
40338	1.89226	1.16867
40339	2.17251	1.20014
40340	2.45575	0.859663
40341	2.45575	1.10026
40342	1.89226	0.997545
40343	1.89226	1.21833
40344	1.08625	1.30452
40345	1.22788	1.02211
40346	0.54447	0.871608
40347	0.794861	1.14918
40348	0.967729	1.27824
40349	0.967729	1.29158
40350	0.913386	1.02204
40351	0.913386	0.951326
40352	1.08625	1.24325
40353	0.544469	1.10692
40354	0.794862	1.17522
40355	1.22788	1.11804
40356	0.946128	1.2295
40357	0.946128	1.26466
40358	0.946132	1.19695
40359	0.946132	1.16535
40360	0.94613	1.1973
40361	0.94613	1.22252
40362	0.94613	1.26845
40363	0.94613	1.00557
40364	0.94613	1.20428
40365	0.94613	1.20453

40366	0.94613	1.19494
40367	0.94613	1.20748
40368	0.946127	1.14091
40369	0.946127	1.05093
40370	1.08625	0.858961
40371	0.958779	1.18651
40372	1.08625	0.943684
40373	1.08625	1.08391
40374	0.946128	1.01189
40375	0.946128	1.06126
40376	0.946129	1.04389
40377	0.958777	1.11256
40378	1.08625	0.965121
40379	0.946129	1.15678
40380	2.16338	0.05
40381	1.94002	0.05
40382	1.98836	0.05
40383	2.60614	0.05
40384	1.5456	0.05
40385	2.16338	0.05
40386	1.19952	0.05
40387	2.34061	0.05
40388	1.21031	0.05
40389	2.1711	0.05
40390	3.54013	0.05
40391	1.13624	2.1
40392	1.70302	2.1
40393	1.8267	2.1
40394	1.11545	2.1
40395	2.85455	2.1
40396	1.82671	2.1
40397	1.91767	2.1
40398	1.81563	2.1
40399	1.91767	2.1
40400	1.71362	2.1
40401	1.83277	2.1
40402	1.66064	2.1
40403	0.996776	2.1
40404	0.996776	2.1
40405	1.11545	2.1
40406	1.54278	2.1
40407	1.66145	2.1
40408	1.66064	2.1
40409	1.7457	2.1
40410	1.81563	2.1
40411	1.81563	2.1
40412	1.54278	2.1
40413	1.54278	2.1
40414	2.71672	2.1
40415	2.70402	2.1
40416	2.64996	2.1
40417	2.69443	2.1
40418	5.39845	2.1
40419	2.71672	2.1
40420	2.54198	2.1
40421	2.54019	2.1
40422	2.69443	2.1
40423	5.23641	2.1

40424	2.64996	2.1
40425	2.69443	2.1
40426	2.68213	2.1
40427	2.61243	2.1
40428	5.30686	2.1
40429	2.54019	2.1
40430	2.69443	2.1
40431	2.68213	2.1
40432	2.5392	2.1
40433	5.23363	2.1
40434	1.89226	2.1
40435	2.34725	2.1
40436	2.22414	2.1
40437	2.34725	2.1
40438	1.76914	2.1
40439	1.89226	2.1
40440	0.946131	2.1
40441	0.946131	2.1
40442	1.11207	2.1
40443	1.03798	2.1
40444	0.94613	2.1
40445	0.94613	2.1
40446	1.03798	2.1
40447	1.03798	2.1
40448	0.946131	2.1
40449	1.03798	2.1
40450	0.946131	2.1
40451	1.11207	2.1
40452	1.89226	0.05
40453	1.89226	0.05
40454	1.89226	0.05
40455	1.89226	0.05
40456	1.89226	0.05
40457	1.89226	0.05
40458	1.89226	0.05
40459	1.89226	0.05
40460	1.89226	0.05
40461	1.89226	0.05
40462	1.89226	0.05
40463	1.89226	0.05
40464	1.89226	0.05
40465	1.89226	0.05
40466	1.89226	0.05
40467	1.89226	0.05
40468	1.89226	0.05
40469	1.89226	0.05
40470	1.44085	2.1
40471	1.31825	0.220299
40472	1.31923	1.16164
40473	1.31825	1.9768
40474	1.43311	0.470523
40475	1.43213	1.30109
40476	1.89226	0.350205
40477	1.89226	1.3211
40478	1.89226	0.995213
40479	1.89226	1.32329
40480	1.89226	0.515898
40481	1.89226	1.74049

40482	0.946133	1.0732
40483	0.946133	0.986493
40484	1.38969	0.363898
40485	1.39587	0.945353
40486	0.946132	0.434708
40487	0.946132	0.924606
40488	1.39587	1.0874
40489	1.39587	0.290307
40490	0.946132	0.935764
40491	1.39587	0.510374
40492	1.38969	0.890726
40493	0.946132	0.930087
40494	1.44085	2.1
40495	1.31923	0.0894923
40496	1.33026	1.07105
40497	1.4617	1.83442
40498	2.78093	1.27851
40499	1.89226	0.218621
40500	1.89226	1.23354
40501	1.89226	0.882272
40502	1.89226	1.35956
40503	1.89226	0.303901
40504	1.89226	1.82667
40505	0.946133	1.03344
40506	0.946133	0.970507
40507	1.39587	0.558605
40508	1.40724	0.141135
40509	0.946132	0.206369
40510	0.946132	0.57914
40511	1.40724	1.04587
40512	1.40724	0.272177
40513	0.946132	0.849944
40514	1.40724	0.696945
40515	0.946133	0.960138
40516	1.39587	0.870705
40517	1.15818	1.98463
40518	1.81846	1.81363
40519	1.52782	1.05525
40520	1.52782	2.1
40521	1.28095	0.978324
40522	1.28095	2.1
40523	2.56709	2.1
40524	1.4349	0.332671
40525	1.4349	1.08856
40526	1.75471	1.10033
40527	2.29909	1.11117
40528	2.61889	2.1
40529	2.5671	2.1
40530	2.82858	2.1
40531	2.57368	2.1
40532	2.29909	1.11392
40533	5.12767	2.1
40534	2.29909	2.1
40535	1.09148	0.0623806
40536	1.09148	1.04276
40537	1.4349	0.992295
40538	1.99776	0.71717
40539	2.34119	1.34767

40540	2.57368	2.1
40541	1.99777	1.04237
40542	1.99777	2.1
40543	2.29909	2.1
40544	2.36071	2.1
40545	2.66203	2.1
40546	1.89226	1.03896
40547	1.89226	1.16077
40548	1.89226	1.11694
40549	1.89226	1.0351
40550	3.78453	1.0283
40551	0.946128	1.21164
40552	0.946128	1.62402
40553	0.946133	1.12277
40554	0.946133	1.20877
40555	0.946131	1.23335
40556	0.946131	1.32779
40557	0.946131	1.43624
40558	0.946131	1.22816
40559	0.94613	1.49285
40560	0.94613	1.25957
40561	0.946131	1.19985
40562	0.946131	1.32595
40563	1.89226	1.17727
40564	1.89226	1.27751
40565	1.89226	1.0494
40566	1.89226	1.2859
40567	3.78453	1.30624
40568	1.89226	1.21621
40569	1.89226	1.11872
40570	1.89226	1.0185
40571	1.89226	1.14963
40572	3.78453	1.18912
40573	1.89226	1.14025
40574	2.61682	1.69871
40575	1.89226	1.40845
40576	1.89226	1.50277
40577	1.89226	1.14566
40578	1.89226	1.32735
40579	1.89226	0.64841
40580	1.89226	0.661651
40581	1.89226	0.645718
40582	1.89226	0.730362
40583	3.78452	0.708436
40584	1.89226	0.646542
40585	1.89226	1.01678
40586	1.89226	0.63126
40587	1.89226	0.700816
40588	1.89226	0.659377
40589	1.89226	0.688587
40590	1.89227	0.689706
40591	1.89227	0.60603
40592	1.89227	0.81109
40593	1.89227	0.782251
40594	1.89227	0.693885
40595	1.89227	0.615254
40596	0.80447	1.0297
40597	0.534241	0.969302

40598	0.995175	0.948736
40599	0.92204	0.7
40600	0.922042	0.645128
40601	0.534241	0.699832
40602	0.804468	0.694978
40603	0.995176	0.556829
40604	0.71543	0.692065
40605	0.71543	0.168199
40606	0.813988	0.663851
40607	0.813988	0.427793
40608	0.922045	0.291886
40609	0.942899	0.708724
40610	0.946132	0.495389
40611	0.946132	0.34309
40612	1.05169	0.0663124
40613	0.936661	0.7
40614	0.922045	0.653868
40615	0.946133	0.129462
40616	1.01665	0.071036
40617	1.13008	0.0656042
40618	0.851529	0.606687
40619	0.851529	0.628384
40620	1.41908	2.1
40621	1.77723	2.1
40622	1.90485	2.1
40623	1.90485	2.1
40624	1.30237	2.1
40625	1.30237	2.1
40626	1.77723	2.1
40627	2.0055	2.1
40628	1.90485	2.1
40629	2.17158	2.1
40630	3.91035	2.1
40631	0.850906	2.1
40632	0.850906	2.1
40633	0.85668	2.1
40634	0.85668	2.1
40635	0.998045	2.1
40636	0.952425	2.1
40637	0.74367	2.1
40638	0.709541	2.1
40639	0.74367	2.1
40640	0.998045	2.1
40641	0.709541	2.1
40642	0.952425	2.1
40643	1.90485	2.1
40644	2.28563	2.1
40645	2.26282	2.1
40646	2.17158	2.1
40647	1.99609	2.1
40648	1.90485	2.07215
40649	2.00098	0.070825
40650	1.90462	0.0719336
40651	1.60057	0.106721
40652	1.79224	0.482207
40653	3.69686	0.0838348
40654	2.65654	0.0579109
40655	1.79224	0.504972

40656	2.22332	0.149388
40657	2.22332	0.116654
40658	2.24163	0.165583
40659	2.24163	0.182053
40660	1.89226	0.223287
40661	1.89226	0.290133
40662	1.89226	0.534365
40663	1.89226	0.591381
40664	3.78452	0.451204
40665	1.89226	0.165225
40666	1.89226	0.486762
40667	1.89226	0.483215
40668	1.89226	0.493969
40669	1.89226	0.521333
40670	1.89226	0.405992
40671	2.24163	0.145995
40672	1.03645	0.519089
40673	1.79224	0.524986
40674	1.79224	0.249268
40675	1.54283	0.450504
40676	1.54283	0.465524
40677	0.946132	0.548763
40678	0.946132	0.337991
40679	0.946129	0.55466
40680	0.946129	0.465184
40681	0.94613	0.565578
40682	0.94613	0.415825
40683	0.946131	0.582368
40684	0.946131	0.45425
40685	0.94613	0.557638
40686	0.94613	0.556812
40687	0.946131	0.534111
40688	0.946131	0.46116
40689	1.89226	2.1
40690	1.89226	2.1
40691	1.89226	2.1
40692	1.89226	2.1
40693	3.78453	2.1
40694	1.52441	2.1
40695	1.30347	2.1
40696	1.52441	2.1
40697	1.58401	2.1
40698	1.2627	2.1
40699	1.3223	2.1
40700	1.89226	2.1
40701	2.72787	2.1
40702	2.72787	2.1
40703	2.74762	2.1
40704	1.89226	2.1
40705	1.91202	2.1
40706	1.89227	2.1
40707	1.89227	2.1
40708	1.89227	2.1
40709	1.89227	2.1
40710	3.78453	2.1
40711	0.56414	2.1
40712	0.561557	2.1
40713	0.527284	2.1

40714	0.541143	2.1
40715	0.527276	2.1
40716	0.631685	2.1
40717	1.52441	2.1
40718	1.00232	2.1
40719	0.784495	2.1
40720	0.842304	2.1
40721	0.721569	2.1
40722	1.36393	2.1
40723	1.36393	2.1
40724	0.946132	2.1
40725	0.946132	2.1
40726	1.36393	2.1
40727	1.3594	2.1
40728	0.946132	2.1
40729	0.946132	2.1
40730	0.946107	2.1
40731	1.35936	2.1
40732	1.36397	2.1
40733	0.946158	2.1
40734	1.89226	2.1
40735	1.89226	2.1
40736	1.89226	2.1
40737	1.89226	2.1
40738	3.78453	2.1
40739	2.20802	2.1
40740	1.05677	2.1
40741	2.30479	2.1
40742	0.978699	2.1
40743	3.18672	2.1
40744	0.94613	2.1
40745	0.94613	2.1
40746	0.669866	2.1
40747	0.669866	2.1
40748	0.681588	2.1
40749	0.669865	2.1
40750	0.946133	2.1
40751	0.946133	2.1
40752	0.94613	2.1
40753	0.681589	2.1
40754	0.946131	2.1
40755	0.669867	2.1
40756	2.73514	2.1
40757	1.78301	2.1
40758	1.90618	2.1
40759	2.83464	2.1
40760	4.64132	2.1
40761	2.73514	2.1
40762	1.89226	2.1
40763	1.89226	2.1
40764	2.83464	2.1
40765	4.6274	2.1
40766	2.03915	2.1
40767	2.26616	2.1
40768	2.23549	2.1
40769	2.01704	2.1
40770	4.2832	2.1
40771	2.03916	2.1

40772	1.55766	2.1
40773	1.53728	2.1
40774	2.01704	2.1
40775	3.5747	2.1
40776	2.23551	2.1
40777	2.01705	2.1
40778	1.99823	2.1
40779	2.21189	2.1
40780	4.22894	2.1
40781	1.51788	2.1
40782	2.01705	2.1
40783	1.53729	2.1
40784	1.53729	2.1
40785	1.99823	2.1
40786	1.99823	2.1
40787	2.588	2.1
40788	1.45581	2.1
40789	1.45581	2.1
40790	1.44207	2.1
40791	2.54949	2.1
40792	2.53574	2.1
40793	0.98641	2.1
40794	0.98641	2.1
40795	1.00371	2.1
40796	1.00371	2.1
40797	0.749992	2.1
40798	0.727904	2.1
40799	1.26221	2.1
40800	1.27474	2.1
40801	1.26221	2.1
40802	0.749993	2.1
40803	0.727904	2.1
40804	1.27474	2.1
40805	1.23105	0.242444
40806	1.35435	1.91864
40807	1.89226	1.36234
40808	1.89226	0.612516
40809	1.89226	1.78321
40810	1.7043	1.19331
40811	1.89226	2.08756
40812	1.89226	1.22848
40813	1.89226	0.12849
40814	1.89226	1.98375
40815	3.78453	1.62608
40816	1.89226	0.0981741
40817	1.89226	2.04428
40818	1.89226	0.892284
40819	1.89226	0.936995
40820	1.89226	1.47112
40821	1.89226	1.5591
40822	0.946131	0.464532
40823	1.40945	0.310052
40824	0.94613	0.238462
40825	0.94613	1.59436
40826	0.946129	1.62013
40827	0.946129	0.971088
40828	0.946132	1.86351
40829	1.40945	0.170176

40830	0.94613	0.831622
40831	0.94613	0.574413
40832	0.946131	0.761126
40833	0.94613	0.373802
40834	0.946133	0.669139
40835	0.946133	0.565396
40836	0.946132	1.43238
40837	0.946132	1.90451
40838	0.946132	1.85402
40839	0.946132	0.957164
40840	0.946133	1.45856
40841	0.946133	0.666737
40842	0.946132	1.41702
40843	0.946132	1.29012
40844	0.946133	0.979041
40845	0.946133	1.09425
40846	1.89226	0.809958
40847	1.89226	2.1
40848	1.89226	1.51436
40849	1.89226	1.40551
40850	1.89226	1.94005
40851	1.89226	1.62373
40852	1.89226	2.1
40853	1.89226	2.1
40854	1.89226	2.1
40855	1.89226	2.1
40856	1.89226	2.1
40857	1.89226	2.1
40858	1.89226	2.1
40859	1.89226	2.1
40860	1.89226	2.1
40861	1.89226	2.1
40862	3.78453	2.1
40863	1.66647	2.1
40864	1.15399	2.1
40865	1.25443	2.1
40866	1.26599	2.1
40867	1.58697	2.1
40868	1.66647	2.1
40869	1.66647	2.1
40870	1.47446	2.1
40871	1.55433	2.1
40872	1.74241	2.1
40873	3.2208	2.1
40874	1.89227	2.1
40875	1.89227	2.1
40876	1.89227	2.1
40877	1.89227	2.1
40878	3.78453	2.1
40879	0.946132	2.1
40880	0.946132	2.1
40881	0.946132	2.1
40882	0.946132	2.1
40883	0.946132	2.1
40884	0.946132	2.1
40885	0.946133	2.1
40886	0.946133	2.1
40887	0.946132	2.1

40888	0.946132	2.1
40889	0.946133	2.1
40890	0.946133	2.1
40891	0.625067	2.1
40892	0.61907	2.1
40893	0.896913	2.1
40894	0.946199	2.1
40895	0.806287	2.1
40896	0.677685	2.1
40897	0.750657	2.1
40898	0.750657	2.1
40899	0.646004	2.1
40900	0.890236	2.1
40901	0.61049	2.1
40902	0.888979	2.1
40903	1.6223	2.1
40904	1.98034	2.1
40905	1.75003	2.1
40906	1.55433	2.1
40907	1.75003	2.1
40908	1.55433	2.1
40909	1.88602	2.1
40910	2.08149	2.1
40911	2.07991	2.1
40912	1.88473	2.1
40913	2.08149	2.1
40914	1.88602	2.1
40915	1.88458	2.1
40916	2.08149	2.1
40917	1.7069	2.1
40918	1.56284	2.1
40919	2.08149	2.1
40920	1.88458	2.1
40921	1.5955	2.1
40922	1.89226	2.1
40923	1.91975	2.1
40924	1.62299	2.1
40925	1.89226	2.1
40926	1.5955	2.1
40927	1.89226	2.1
40928	1.89226	2.1
40929	1.89226	2.1
40930	1.89226	2.1
40931	1.89226	1.49472
40932	1.89226	2.1
40933	1.89226	2.1
40934	1.89226	2.1
40935	1.89226	2.1
40936	1.89226	2.1
40937	1.89226	2.1
40938	1.89226	2.1
40939	1.62299	2.1
40940	1.89226	2.1
40941	1.71752	2.1
40942	1.44824	2.1
40943	1.89226	2.1
40944	1.62299	2.1
40945	1.89226	1.06277

40946	2.8317	0.832243
40947	1.89226	1.1115
40948	2.64055	0.472576
40949	4.53281	1.07686
40950	2.8317	1.24097
40951	1.83151	0.3241
40952	2.01001	0.708613
40953	2.01001	1.18771
40954	2.64055	0.164516
40955	2.64055	0.845491
40956	0.946132	0.368554
40957	0.946132	0.826258
40958	1.16167	0.774741
40959	1.16167	0.507031
40960	1.16167	0.314169
40961	1.32027	0.232045
40962	0.946131	0.554001
40963	0.946131	0.894818
40964	0.94613	0.339099
40965	1.16167	0.213057
40966	0.946131	0.543565
40967	1.32027	0.553176
40968	2.32335	0.450144
40969	1.83151	0.255954
40970	2.38525	0.263696
40971	2.64055	0.378663
40972	1.57621	0.109523
40973	1.83151	0.198253
40974	2.25677	2.1
40975	1.88158	2.1
40976	1.86856	2.1
40977	2.0815	2.1
40978	1.70303	2.1
40979	1.88158	2.1
40980	2.50381	2.1
40981	1.89227	2.1
40982	2.25677	2.1
40983	2.25677	2.1
40984	1.89227	2.1
40985	1.89227	2.1
40986	1.89226	0.05
40987	1.50899	0.05
40988	2.13659	0.05
40989	1.50899	0.102388
40990	2.51986	0.05
40991	1.89226	0.05
40992	0.946131	0.05
40993	0.946131	0.05
40994	1.06829	0.05
40995	0.895375	0.05
40996	0.94613	0.05
40997	0.94613	0.05
40998	1.33922	0.05
40999	0.895376	0.05
41000	0.946131	0.05
41001	1.33922	0.05
41002	0.946131	0.05
41003	1.06829	0.05

41004	0.841467	0.539499
41005	0.578428	0.576358
41006	1.18079	0.398059
41007	0.920237	0.574486
41008	0.772317	0.293171
41009	0.856555	0.683841
41010	0.910271	0.580075
41011	0.910271	0.743215
41012	0.975752	0.507507
41013	0.536929	0.340587
41014	0.81952	0.635319
41015	1.15252	0.348058
41016	1.89226	2.1
41017	2.51431	2.1
41018	1.89226	2.1
41019	1.89226	2.1
41020	2.78815	2.1
41021	2.78815	2.1
41022	2.0815	2.1
41023	2.0516	2.1
41024	2.0815	2.1
41025	3.06699	2.1
41026	2.76576	2.1
41027	2.8002	2.1
41028	1.94112	2.1
41029	1.89226	2.1
41030	2.20358	2.1
41031	1.89226	2.1
41032	4.09584	2.1
41033	1.09154	2.1
41034	2.62647	2.1
41035	2.20358	2.1
41036	2.20358	2.1
41037	1.52314	2.1
41038	1.52314	2.1
41039	1.31323	2.1
41040	1.31323	2.1
41041	0.946132	2.1
41042	0.946132	2.1
41043	0.946132	2.1
41044	0.946132	2.1
41045	1.31323	2.1
41046	1.10179	2.1
41047	1.31323	2.1
41048	0.946132	2.1
41049	1.10179	2.1
41050	0.946133	2.1
41051	1.89226	1.04216
41052	1.89226	1.3236
41053	1.89226	1.25716
41054	1.89226	1.40502
41055	1.89226	1.02789
41056	1.89226	1.20573
41057	1.35126	2.01452
41058	1.35126	2.1
41059	0.903285	1.41131
41060	0.903285	1.76937
41061	1.30841	1.83074

41062	0.903313	2.1
41063	0.946132	1.55675
41064	0.946132	1.29711
41065	0.946132	1.49236
41066	0.903312	1.56728
41067	1.30841	1.45959
41068	0.946133	1.34376
41069	2.62097	2.1
41070	2.72561	2.1
41071	2.39016	2.1
41072	2.51062	2.1
41073	5.11577	2.1
41074	2.51062	2.1
41075	2.58286	0.884096
41076	2.40107	2.1
41077	2.40107	0.925034
41078	2.72561	2.1
41079	2.72561	1.22562
41080	2.39016	2.1
41081	2.51062	2.1
41082	2.20242	2.1
41083	2.34107	2.1
41084	4.73124	2.1
41085	2.51062	2.1
41086	2.40107	0.8599
41087	2.34107	2.1
41088	2.25197	0.137581
41089	4.74215	2.09131
41090	2.75218	2.1
41091	1.89226	2.1
41092	2.3349	2.1
41093	2.3349	2.1
41094	1.89226	2.1
41095	1.89226	2.1
41096	1.46129	2.1
41097	0.996308	2.1
41098	1.88649	2.1
41099	1.70302	2.1
41100	2.95419	2.1
41101	1.89227	2.1
41102	1.89227	2.1
41103	1.89227	2.1
41104	1.89227	2.1
41105	3.78453	2.1
41106	0.646558	2.1
41107	0.646557	2.1
41108	0.60697	2.1
41109	0.60697	2.1
41110	0.587917	2.1
41111	0.519747	2.1
41112	0.586443	2.1
41113	0.733782	2.1
41114	0.733793	2.1
41115	0.587924	2.1
41116	0.519739	2.1
41117	0.586436	2.1
41118	2.1642	2.1
41119	1.42867	2.1

41120	2.28847	2.1
41121	1.68299	2.1
41122	3.71714	2.1
41123	1.89226	2.1
41124	1.89226	2.1
41125	1.89226	2.1
41126	1.89226	2.1
41127	3.78453	2.1
41128	0.946131	2.1
41129	0.946131	2.1
41130	1.24994	2.1
41131	1.24994	2.1
41132	1.11582	2.1
41133	1.24994	2.1
41134	0.946133	2.1
41135	0.946133	2.1
41136	0.946131	2.1
41137	1.24994	2.1
41138	0.94613	2.1
41139	1.11582	2.1
41140	2.28847	2.1
41141	1.53629	2.1
41142	1.57498	2.1
41143	1.47417	2.1
41144	2.42221	2.1
41145	2.28847	2.1
41146	1.89226	2.1
41147	1.89226	2.1
41148	1.89226	2.1
41149	1.89226	2.1
41150	3.78453	2.1
41151	1.89226	2.1
41152	2.49988	2.1
41153	2.71233	2.1
41154	2.71233	2.1
41155	1.89226	2.1
41156	1.89226	2.1
41157	0.924534	2.1
41158	0.924534	2.1
41159	0.990395	2.1
41160	0.990395	2.1
41161	0.946131	2.1
41162	0.946131	2.1
41163	0.924505	2.1
41164	0.968797	2.1
41165	0.924505	2.1
41166	0.946131	2.1
41167	0.968797	2.1
41168	0.946131	2.1
41169	0.924533	2.1
41170	0.924533	2.1
41171	0.948264	2.1
41172	0.945345	2.1
41173	0.851541	2.1
41174	0.851541	2.1
41175	0.958934	2.1
41176	1.02617	2.1
41177	0.936668	2.1

41178	1.04829	2.1
41179	0.924505	2.1
41180	0.946131	2.1
41181	2.28431	0.550455
41182	1.48035	0.0507724
41183	1.59971	0.093757
41184	1.48035	0.0507998
41185	2.33818	0.161693
41186	2.21882	0.0745756
41187	2.28431	0.139821
41188	1.89226	0.05
41189	1.89226	0.279475
41190	2.3968	0.05
41191	4.28906	0.05
41192	2.28431	0.152108
41193	1.70303	0.05
41194	1.5428	0.05
41195	1.59971	0.05
41196	2.31152	0.260296
41197	2.3968	0.213661
41198	1.99823	2.1
41199	2.21189	2.1
41200	2.18937	2.1
41201	1.979 2.1	
41202	4.19089	2.1
41203	0.999114	2.1
41204	0.9895	2.1
41205	0.758942	2.1
41206	0.749358	2.1
41207	0.874236	2.1
41208	0.874236	2.1
41209	0.874236	2.1
41210	0.874236	2.1
41211	0.9895	2.1
41212	0.749358	2.1
41213	0.758942	2.1
41214	0.999114	2.1
41215	2.18937	2.1
41216	1.979 2.1	
41217	1.95416	2.1
41218	2.162 2.1	
41219	4.14099	2.1
41220	1.95416	2.1
41221	1.49871	2.1
41222	1.47712	2.1
41223	1.49871	2.1
41224	1.9574	2.1
41225	1.979 2.1	
41226	1.68508	0.05
41227	1.64984	0.05
41228	2.67711	0.05
41229	2.76407	0.05
41230	4.41391	0.05
41231	1.84227	0.05
41232	2.76407	0.05
41233	2.76407	0.05
41234	1.85007	0.05
41235	2.72769	0.05

41236	1.80812	0.05
41237	1.94516	2.1
41238	1.89226	2.1
41239	1.89226	2.1
41240	1.89226	2.1
41241	1.98853	2.1
41242	1.98852	2.1
41243	1.28095	2.1
41244	2.0815	2.1
41245	2.29123	2.1
41246	2.06833	2.1
41247	1.08212	2.1
41248	0.976848	2.1
41249	1.89226	2.1
41250	1.89226	2.1
41251	1.89226	2.1
41252	1.89226	2.1
41253	1.89226	2.1
41254	1.89226	2.1
41255	1.13537	2.1
41256	1.18233	2.1
41257	0.946132	2.1
41258	0.946132	2.1
41259	0.946132	2.1
41260	0.946132	2.1
41261	0.994264	2.1
41262	1.18233	2.1
41263	1.13537	2.1
41264	0.946131	2.1
41265	0.946132	2.1
41266	0.994264	2.1
41267	0.953204	2.1
41268	0.953204	2.1
41269	0.966515	2.1
41270	0.966515	2.1
41271	1.30583	2.1
41272	1.14561	2.1
41273	0.640475	2.1
41274	0.774103	2.1
41275	1.30584	2.1
41276	0.774101	2.1
41277	0.640474	2.1
41278	1.14562	2.1
41279	0.946131	2.1
41280	0.946131	2.1
41281	0.946131	2.1
41282	0.946131	2.1
41283	0.946128	2.1
41284	0.946128	2.1
41285	0.946133	2.1
41286	0.946133	2.1
41287	0.94613	2.1
41288	0.94613	2.1
41289	0.946131	2.1
41290	0.946131	2.1
41291	0.946132	0.05
41292	0.946132	0.05
41293	0.946131	0.05

41294	0.946131	0.05
41295	0.946131	0.05
41296	0.946131	0.05
41297	0.946132	0.05
41298	0.946132	0.05
41299	0.946131	0.05
41300	0.946131	0.05
41301	0.946132	0.05
41302	0.946132	0.05
41303	0.946131	0.05
41304	0.946131	0.05
41305	0.946131	0.05
41306	0.946131	0.05
41307	0.94613	0.05
41308	0.94613	0.05
41309	0.946132	0.05
41310	0.946132	0.05
41311	0.946131	0.05
41312	0.946131	0.05
41313	0.946131	0.05
41314	0.946131	0.05
41315	2.57882	0.05
41316	1.48676	0.0590221
41317	1.48797	0.0682098
41318	2.64281	0.111168
41319	4.12957	0.0608956
41320	2.56323	0.05
41321	2.64281	0.0631175
41322	2.51807	0.05
41323	1.36324	0.0994661
41324	1.48797	0.118453
41325	1.48797	0.156473
41326	1.04367	2.1
41327	2.42504	2.1
41328	2.65164	2.1
41329	1.49479	2.1
41330	3.91983	2.1
41331	2.15622	2.1
41332	1.70539	2.1
41333	2.02369	2.1
41334	2.39039	2.1
41335	4.17992	2.1
41336	0.966979	2.1
41337	1.7054	2.1
41338	2.0237	2.1
41339	1.31502	2.1
41340	2.99067	2.1
41341	2.02369	2.1
41342	2.65637	2.1
41343	2.39039	1.75009
41344	2.39039	2.1
41345	2.29332	1.84018
41346	2.29332	2.1
41347	2.0237	2.1
41348	1.72116	2.1
41349	1.58465	2.1
41350	1.31502	2.1
41351	2.29332	2.1

41352	2.0237	2.1
41353	1.0372	0.05
41354	2.32462	0.05
41355	2.2291	0.05
41356	2.2291	0.05
41357	1.17464	0.05
41358	1.17464	0.05
41359	2.68289	0.05
41360	1.07805	0.05
41361	1.70302	0.05
41362	2.06653	0.05
41363	3.5779	0.05
41364	1.89226	0.05
41365	1.89226	0.05
41366	1.89226	0.05
41367	1.89226	0.05
41368	3.78453	0.05
41369	1.89226	0.05
41370	1.89226	0.05
41371	1.89226	0.05
41372	1.89226	0.05
41373	1.89226	0.05
41374	1.89226	0.05
41375	1.89225	0.05
41376	1.89225	0.05
41377	1.89225	0.05
41378	1.89225	0.05
41379	1.89225	0.05
41380	1.89225	0.05
41381	0.94613	0.05
41382	0.94613	0.05
41383	0.94613	0.05
41384	0.94613	0.05
41385	0.946128	0.05
41386	0.946128	0.05
41387	0.946132	0.05
41388	0.946132	0.05
41389	0.94613	0.05
41390	0.94613	0.05
41391	0.94613	0.05
41392	0.94613	0.05
41393	1.29417	2.1
41394	1.32732	2.1
41395	1.32316	1.12153
41396	1.34973	2.1
41397	2.65048	2.1
41398	1.89226	1.41428
41399	1.89226	2.1
41400	1.89226	1.14906
41401	1.89226	2.1
41402	3.78453	1.78045
41403	1.89226	2.1
41404	1.89226	2.1
41405	1.89226	1.64889
41406	1.89226	2.1
41407	3.78453	2.1
41408	1.32732	2.1
41409	1.13006	2.1

41410	1.13358	2.1
41411	1.11117	2.1
41412	1.34973	2.1
41413	1.32732	2.1
41414	1.32316	2.00247
41415	1.34973	2.1
41416	1.34066	1.2203
41417	1.36601	2.1
41418	2.68918	2.1
41419	1.89226	2.00284
41420	1.89226	2.1
41421	1.89226	0.930868
41422	1.89226	2.1
41423	3.78453	2.00535
41424	1.89226	2.1
41425	1.89226	2.1
41426	1.89226	2.1
41427	1.89226	2.1
41428	1.89226	2.1
41429	1.89226	2.1
41430	0.624134	2.1
41431	0.624134	2.1
41432	0.624334	2.1
41433	0.624334	2.1
41434	0.56503	2.1
41435	0.573605	2.1
41436	0.674864	2.1
41437	0.683007	2.1
41438	0.683007	2.1
41439	0.573605	2.1
41440	0.565029	2.1
41441	0.674864	2.1
41442	2.16633	2.1
41443	1.40846	2.1
41444	2.13977	2.1
41445	1.40771	2.1
41446	3.57404	2.1
41447	2.13977	2.1
41448	1.29932	2.1
41449	1.29932	2.1
41450	1.40771	2.1
41451	1.98957	2.1
41452	2.09796	2.1
41453	2.6044	0.093319
41454	1.85548	0.241537
41455	1.89226	0.05
41456	1.89226	0.320207
41457	3.74774	0.11043
41458	1.89226	0.05
41459	1.89226	0.405995
41460	1.89226	0.05
41461	1.89226	0.122328
41462	3.78453	0.0900006
41463	1.89226	2.1
41464	2.64107	2.1
41465	1.89226	2.1
41466	2.78977	2.1
41467	1.89226	2.1

41468	2.78977	2.1
41469	1.89226	2.1
41470	1.89226	2.1
41471	1.89226	2.1
41472	1.89226	2.1
41473	1.89226	2.1
41474	1.89226	2.1
41475	2.43173	2.1
41476	1.41238	2.1
41477	2.0815	2.1
41478	1.98048	2.1
41479	1.62014	2.1
41480	1.50086	2.1
41481	0.946129	0.0542143
41482	0.946129	0.0548254
41483	1.27636	0.0797107
41484	1.1984	0.05
41485	0.946131	0.05
41486	0.946131	0.05
41487	1.27636	0.0722592
41488	1.27636	0.05
41489	0.946131	0.0860803
41490	1.27636	0.05
41491	0.946131	0.05
41492	1.1984	0.05
41493	1.99569	0.05
41494	2.55272	0.116754
41495	1.77403	0.05
41496	1.70303	0.05
41497	3.05772	0.0563776
41498	2.55121	0.05
41499	0.946128	0.05
41500	0.946128	0.05
41501	1.38752	0.0682084
41502	1.27636	0.05
41503	0.94613	0.05
41504	0.94613	0.05
41505	1.38752	0.0662379
41506	1.38752	0.05
41507	0.946131	0.05
41508	1.38752	0.05
41509	0.946131	0.055462
41510	1.27636	0.05
41511	2.77504	0.0505461
41512	1.99569	0.05
41513	2.29794	0.05
41514	1.99569	0.05
41515	2.85497	0.05
41516	2.55272	0.05
41517	1.53284	2.1
41518	1.89226	2.1
41519	1.89226	2.1
41520	1.89226	2.1
41521	1.54763	2.1
41522	1.54763	2.1
41523	0.946131	2.1
41524	0.946131	2.1
41525	0.946127	2.1

41526	0.946127	2.1
41527	0.946127	2.1
41528	0.946127	2.1
41529	0.946131	2.1
41530	0.946131	2.1
41531	0.94613	2.1
41532	0.94613	2.1
41533	0.946129	2.1
41534	0.946129	2.1
41535	0.751754	2.1
41536	0.751754	2.1
41537	0.771562	2.1
41538	0.771561	2.1
41539	1.0268	2.1
41540	0.95482	2.1
41541	0.528937	2.1
41542	0.496512	2.1
41543	0.528936	2.1
41544	1.0268	2.1
41545	0.954821	2.1
41546	0.49651	2.1
41547	2.64075	0.74175
41548	1.35181	1.00913
41549	1.35181	0.416518
41550	1.83823	0.800894
41551	2.28373	0.808214
41552	2.88566	1.04267
41553	0.493018	0.998654
41554	0.675903	0.956417
41555	1.14187	0.73549
41556	0.973937	0.726488
41557	0.821166	0.799584
41558	0.821166	1.09481
41559	0.828674	2.0054
41560	0.828674	0.72025
41561	0.973937	0.843682
41562	0.493018	0.855815
41563	0.675903	0.697618
41564	1.14187	0.792558
41565	1.89226	2.1
41566	2.11667	2.1
41567	1.91184	2.1
41568	1.89226	2.1
41569	2.13625	2.1
41570	2.11667	2.1
41571	1.89226	2.1
41572	1.89226	2.1
41573	1.89226	2.1
41574	1.89226	2.1
41575	3.78453	2.1
41576	0.907729	2.1
41577	0.907729	2.1
41578	1.04459	2.1
41579	0.890955	2.1
41580	0.988181	2.1
41581	0.841856	2.1
41582	0.946133	2.1
41583	0.946133	2.1

41584	1.04072	2.1
41585	1.1064	2.1
41586	0.89099	2.1
41587	0.851541	2.1
41588	1.89225	2.1
41589	2.13623	2.1
41590	1.91044	2.1
41591	1.89225	2.1
41592	2.15442	2.1
41593	2.13623	2.1
41594	1.89225	2.1
41595	1.89225	2.1
41596	1.89225	2.1
41597	1.89225	2.1
41598	1.89225	2.1
41599	1.89225	2.1
41600	0.907723	2.1
41601	0.907723	2.1
41602	1.07536	2.1
41603	1.07814	2.1
41604	0.998499	2.1
41605	1.03575	2.1
41606	0.946126	2.1
41607	0.946126	2.1
41608	1.04072	2.1
41609	1.14025	2.1
41610	0.851528	2.1
41611	0.898284	2.1
41612	1.89226	0.05
41613	1.89226	0.05
41614	1.89226	0.05
41615	1.89226	0.05
41616	1.89226	0.05
41617	1.89226	0.05
41618	2.53008	2.1
41619	1.94534	2.1
41620	1.98702	2.1
41621	1.98858	2.1
41622	2.52852	2.1
41623	2.53008	2.1
41624	1.03267	2.1
41625	1.12591	2.1
41626	1.2959	2.1
41627	1.28992	2.1
41628	1.10851	2.1
41629	1.33078	2.1
41630	1.10661	2.1
41631	1.10661	2.1
41632	0.982208	2.1
41633	1.28992	2.1
41634	1.03267	2.1
41635	1.23262	2.1
41636	2.2712	2.1
41637	1.89226	2.1
41638	1.95954	2.1
41639	1.95954	2.1
41640	1.89226	2.1
41641	1.89226	2.1

41642	1.89226	2.1
41643	1.89226	2.1
41644	1.89226	2.1
41645	1.89226	2.1
41646	3.78453	2.1
41647	1.33887	2.1
41648	1.25395	2.1
41649	0.88327	2.1
41650	0.676665	2.1
41651	0.84881	2.1
41652	0.987503	2.1
41653	1.13243	2.1
41654	1.13243	2.1
41655	1.15338	2.1
41656	0.849966	2.1
41657	0.793074	2.1
41658	1.11957	2.1
41659	0.946129	2.1
41660	0.946129	2.1
41661	1.28254	2.1
41662	1.1356	2.1
41663	1.1356	2.1
41664	1.1356	2.1
41665	0.946131	2.1
41666	0.946131	2.1
41667	0.946131	2.1
41668	1.28254	2.1
41669	1.1356	2.1
41670	0.946131	2.1
41671	1.89227	2.1
41672	1.89227	2.1
41673	1.89227	2.1
41674	1.89227	2.1
41675	3.78453	2.1
41676	0.936287	0.05
41677	1.01399	0.05
41678	0.959415	0.05
41679	0.959415	0.05
41680	0.910786	0.05
41681	1.38301	0.05
41682	1.0286	0.05
41683	0.535742	0.05
41684	0.505202	0.05
41685	0.937685	0.05
41686	1.11066	0.05
41687	1.46907	0.05
41688	1.00546	0.05
41689	1.01524	0.05
41690	0.94613	0.05
41691	0.94613	0.05
41692	1.05018	0.05
41693	0.914025	0.05
41694	0.913512	0.05
41695	0.851542	0.05
41696	1.05385	0.05
41697	1.05494	0.05
41698	0.91404	0.05
41699	0.91404	0.05

41700	2.03253	0.908943
41701	1.82325	0.510118
41702	1.93938	0.786958
41703	1.73756	0.05
41704	3.77009	0.187695
41705	1.82325	0.436724
41706	1.47562	0.76982
41707	1.73756	0.05
41708	1.3868	0.05
41709	3.21317	0.05
41710	1.93938	0.962346
41711	1.73756	0.535275
41712	1.85293	0.720083
41713	1.64279	0.05
41714	3.59049	0.146315
41715	1.73756	0.726744
41716	1.3868	0.741075
41717	1.64279	0.05
41718	1.28419	0.05
41719	3.02174	0.651536
41720	1.35048	2.1
41721	1.37999	2.1
41722	1.34355	1.18477
41723	1.37854	2.1
41724	2.72354	2.1
41725	1.89226	1.43361
41726	1.89226	2.1
41727	1.89226	1.15763
41728	1.89226	1.92309
41729	3.78452	1.27149
41730	1.89226	2.1
41731	1.89226	2.1
41732	1.89226	0.857351
41733	1.89226	2.1
41734	3.78453	2.1
41735	1.37999	2.1
41736	1.18059	2.1
41737	1.17117	2.1
41738	1.17262	2.1
41739	1.37855	2.1
41740	1.37999	2.1
41741	1.34355	2.08706
41742	1.37854	2.1
41743	1.33079	1.28202
41744	1.3741	2.1
41745	2.71765	2.1
41746	1.89226	1.85661
41747	1.89226	2.1
41748	1.89226	1.08569
41749	1.89226	2.1
41750	3.78453	1.90734
41751	1.89226	1.31622
41752	1.89226	2.1
41753	1.89226	1.65534
41754	1.89226	2.1
41755	3.78453	2.1
41756	0.640305	2.1
41757	0.640305	2.1

41758	0.643507	0.847367
41759	0.643507	2.1
41760	0.590294	2.1
41761	0.594539	2.1
41762	0.689273	2.05039
41763	0.687048	2.1
41764	0.687048	2.1
41765	0.594538	2.1
41766	0.590294	2.1
41767	0.689273	2.1
41768	2.01399	0.766509
41769	1.70141	0.80815
41770	1.83896	2.1
41771	1.52482	1.74504
41772	3.53881	0.842726
41773	1.70141	0.902183
41774	1.0752	2.1
41775	1.0752	0.846906
41776	1.25669	1.6729
41777	1.52482	1.02148
41778	1.70632	2.1
41779	1.83896	1.38244
41780	1.52482	0.134886
41781	1.65244	0.803121
41782	1.34412	1.45882
41783	3.17725	0.801354
41784	1.26782	0.05
41785	2.90353	0.05
41786	2.54203	0.05
41787	2.41979	0.05
41788	1.77007	0.05
41789	1.703 0.05	
41790	0.946118	0.05
41791	0.946118	0.05
41792	1.38643	0.05
41793	1.38644	0.05
41794	0.946117	0.05
41795	0.946117	0.05
41796	1.38644	0.05
41797	1.15545	0.05
41798	0.946131	0.05
41799	1.15546	0.05
41800	1.38642	0.05
41801	0.946105	0.05
41802	2.42256	0.216712
41803	2.63056	0.0682802
41804	2.43174	0.0629579
41805	2.25035	0.0518433
41806	4.8543	0.0773786
41807	2.58031	0.146479
41808	2.63056	0.0869831
41809	2.43174	0.060566
41810	2.32127	0.05
41811	5.01205	0.0756221
41812	2.43174	0.0775041
41813	2.25035	0.05
41814	2.11465	0.05
41815	2.13042	0.05

41816	4.54639	0.05
41817	2.43174	0.0776367
41818	2.32127	0.05
41819	1.92997	0.05
41820	2.13041	0.05
41821	4.36171	0.05
41822	1.89227	2.1
41823	1.89227	2.1
41824	1.89227	2.1
41825	1.89227	2.1
41826	3.78453	2.1
41827	0.568724	2.1
41828	0.568724	2.1
41829	0.57678	2.1
41830	0.57678	2.1
41831	0.523796	2.1
41832	0.548137	2.1
41833	0.597368	2.1
41834	0.637877	2.1
41835	0.597367	2.1
41836	0.523796	2.1
41837	0.637877	2.1
41838	0.548137	2.1
41839	1.32674	2.1
41840	1.32674	2.1
41841	0.946132	2.1
41842	0.946132	2.1
41843	1.3195	2.1
41844	1.32674	2.1
41845	0.946132	2.1
41846	0.946132	2.1
41847	0.946132	2.1
41848	1.3195	2.1
41849	1.32674	2.1
41850	0.946133	2.1
41851	1.89227	2.1
41852	1.89227	2.1
41853	1.89227	2.1
41854	1.89227	2.1
41855	3.78453	2.1
41856	1.19473	2.1
41857	0.987132	2.1
41858	0.987132	2.1
41859	1.04759	2.1
41860	1.0894	2.1
41861	1.14986	2.1
41862	1.3195	2.1
41863	1.3195	2.1
41864	0.946132	2.1
41865	0.946132	2.1
41866	1.31185	2.1
41867	1.3195	2.1
41868	0.946132	2.1
41869	0.946132	2.1
41870	1.31185	2.1
41871	0.946133	2.1
41872	0.946132	2.1
41873	1.3195	2.1

41874	1.11256	0.951134
41875	1.70101	0.71373
41876	1.22297	0.970068
41877	1.22297	0.896347
41878	1.7252	1.03781
41879	1.7252	0.948746
41880	1.99298	1.02684
41881	1.70101	1.04042
41882	1.7252	1.26396
41883	2.18701	0.545099
41884	3.71818	0.890739
41885	1.03218	0.8246
41886	1.7252	0.501105
41887	1.11256	0.880704
41888	1.11256	0.733438
41889	1.7457	0.892586
41890	1.7457	0.750409
41891	1.7457	1.08721
41892	2.187 0.338635	
41893	1.7252	0.879528
41894	1.7252	0.876213
41895	2.27351	0.68945
41896	2.27351	0.711145
41897	1.95544	1.00642
41898	1.35897	2.1
41899	1.44859	1.39651
41900	1.44859	1.9173
41901	1.80709	1.07221
41902	1.80709	1.76781
41903	1.80709	0.948603
41904	1.17822	2.03742
41905	1.35897	1.29512
41906	1.35897	2.05584
41907	1.52782	0.942908
41908	1.52782	1.58309
41909	1.11978	1.72829
41910	0.903546	1.03688
41911	0.977719	1.87269
41912	1.2364	1.05312
41913	1.03812	0.924382
41914	1.03812	2.1
41915	1.05938	0.902062
41916	1.05938	2.03394
41917	1.11978	1.15855
41918	1.2364	0.891285
41919	0.97772	1.50046
41920	0.903546	1.15868
41921	1.80709	2.00296
41922	1.81847	1.32518
41923	1.96029	1.95706
41924	1.52782	1.57815
41925	2.23956	1.12412
41926	1.80709	1.34038
41927	1.89226	0.204946
41928	2.32962	0.05
41929	2.5992	0.05
41930	1.89226	0.05
41931	2.5992	0.182469

41932	1.89226	0.217534
41933	2.55791	0.05
41934	2.47141	0.05
41935	2.13515	0.05
41936	2.10939	0.05
41937	4.66731	0.05
41938	2.47141	0.05
41939	2.23337	0.05
41940	2.10939	0.05
41941	2.03438	0.05
41942	4.34276	0.05
41943	2.13512	0.05
41944	1.71027	0.05
41945	1.71027	0.05
41946	2.10936	0.05
41947	1.60137	0.05
41948	2.00046	0.05
41949	1.71027	0.05
41950	2.03435	0.05
41951	1.71027	0.05
41952	2.10936	0.05
41953	1.72067	0.05
41954	2.11976	0.05
41955	1.177 0.05	
41956	1.36405	0.666418
41957	2.25635	0.616915
41958	2.60151	0.05
41959	3.6204	0.285874
41960	0.658869	0.05
41961	0.781697	0.05
41962	0.637037	0.05
41963	0.541148	0.05
41964	0.654682	0.05
41965	0.654682	0.05
41966	0.641224	0.05
41967	0.641224	0.05
41968	0.637039	0.05
41969	0.781696	0.05
41970	0.658867	0.05
41971	0.541149	0.05
41972	1.89226	0.0514992
41973	1.59152	0.05
41974	1.89226	0.05
41975	1.82125	0.05
41976	1.89226	0.05
41977	1.82125	0.05
41978	2.18967	0.05
41979	1.89227	0.05
41980	2.72752	0.05
41981	2.72752	0.05
41982	1.89227	0.05
41983	1.89227	0.05
41984	2.18967	0.05
41985	1.92547	0.05
41986	1.92547	0.05
41987	2.72752	0.05
41988	1.30572	0.05
41989	2.10777	0.05

41990	2.39663	2.1
41991	1.92293	2.1
41992	1.96889	2.1
41993	2.45928	2.1
41994	4.36552	2.1
41995	1.02605	2.1
41996	1.92293	2.1
41997	1.9689	2.1
41998	1.04679	2.1
41999	2.99495	2.1
42000	1.96889	2.1
42001	2.45928	2.1
42002	2.50138	2.1
42003	1.99782	2.1
42004	4.47027	2.1
42005	1.9689	2.1
42006	1.03449	2.1
42007	1.07572	2.1
42008	1.04679	2.1
42009	1.99783	2.1
42010	1.9689	2.1
42011	1.72162	0.917211
42012	1.89186	0.828064
42013	2.29771	0.838005
42014	1.80149	0.75382
42015	4.01933	0.820609
42016	1.16482	0.982012
42017	1.97003	0.779199
42018	1.72162	0.755428
42019	1.72162	0.818106
42020	1.29313	0.824157
42021	1.29313	0.975034
42022	2.29771	0.866926
42023	2.84159	0.73242
42024	2.19622	0.774404
42025	1.97366	0.768089
42026	2.50906	0.790294
42027	2.29771	0.798623
42028	1.29313	0.93086
42029	2.4483	0.769688
42030	2.14223	0.764119
42031	2.14228	0.813168
42032	1.294	0.803663
42033	1.294	0.88266
42034	1.21495	0.05
42035	1.70303	0.05
42036	1.7924	0.05
42037	1.64002	0.05
42038	3.11963	0.05
42039	2.81021	0.05
42040	1.89226	0.05
42041	1.89226	0.05
42042	1.89226	0.05
42043	2.36406	0.05
42044	2.36406	0.05
42045	1.89226	0.05
42046	1.89226	0.05
42047	1.89226	0.05

42048	1.89226	0.05
42049	1.89226	0.05
42050	1.89226	0.05
42051	2.29976	0.05
42052	1.64002	0.05
42053	1.8959	0.05
42054	1.90873	0.05
42055	1.85594	0.05
42056	1.85594	0.05
42057	1.89226	0.05
42058	1.89226	0.05
42059	1.89226	0.05
42060	1.89226	0.05
42061	3.78453	0.05
42062	1.89226	1.10949
42063	2.52823	2.1
42064	2.83441	1.65467
42065	2.83441	2.1
42066	1.89226	1.36025
42067	1.89226	2.07757
42068	1.04522	1.80625
42069	1.04522	1.51645
42070	1.12831	1.84745
42071	1.18364	2.1
42072	0.640525	1.74133
42073	0.924882	1.57448
42074	1.27877	1.89602
42075	1.43363	1.112
42076	0.93474	1.57016
42077	1.58926	1.447
42078	1.29573	1.94066
42079	0.664247	1.99679
42080	2.02191	2.1
42081	1.89226	2.1
42082	1.89226	2.1
42083	1.89226	2.1
42084	2.58639	2.1
42085	2.58639	2.1
42086	1.16759	2.1
42087	2.11828	2.1
42088	1.60936	2.1
42089	2.35846	2.1
42090	3.52605	2.1
42091	1.89226	2.1
42092	1.89226	2.1
42093	1.89226	2.1
42094	1.89226	2.1
42095	3.78453	2.1
42096	2.82765	0.05
42097	1.89809	0.05
42098	2.15971	0.05
42099	1.89809	0.05
42100	2.29233	0.05
42101	2.03071	0.05
42102	1.90664	0.05
42103	2.15971	0.05
42104	1.28956	0.05
42105	1.28956	0.05

42106	2.64414	0.05
42107	2.02706	0.05
42108	2.82765	0.05
42109	1.89226	0.05
42110	2.39968	0.05
42111	2.39968	0.05
42112	1.89226	0.05
42113	1.89226	0.05
42114	0.978643	2.1
42115	0.703968	2.1
42116	0.946129	2.1
42117	0.946129	2.1
42118	0.703939	2.1
42119	0.703939	2.1
42120	0.946159	2.1
42121	0.946159	2.1
42122	0.978642	2.1
42123	0.94613	2.1
42124	0.94613	2.1
42125	0.703968	2.1
42126	1.89226	2.1
42127	2.17309	0.974146
42128	2.17309	2.1
42129	1.47475	2.07284
42130	1.89226	2.1
42131	1.19393	1.4631
42132	1.89226	2.1
42133	1.89226	2.1
42134	1.89226	2.1
42135	1.89226	2.1
42136	3.78452	2.1
42137	1.17426	2.1
42138	0.978644	2.1
42139	0.946129	2.1
42140	0.946129	2.1
42141	0.946131	2.1
42142	0.946131	2.1
42143	0.978643	2.1
42144	0.978642	2.1
42145	1.17426	2.1
42146	0.946131	2.1
42147	0.978642	2.1
42148	0.94613	2.1
42149	1.89226	2.1
42150	1.89226	0.903667
42151	1.89226	2.1
42152	2.17309	1.19679
42153	2.56346	2.1
42154	2.94389	1.89037
42155	0.946131	2.1
42156	0.946131	2.1
42157	0.946128	2.1
42158	0.946128	2.1
42159	0.946131	2.1
42160	0.946131	2.1
42161	0.946128	2.1
42162	0.946128	2.1
42163	0.94613	2.1

42164	0.94613	2.1
42165	0.946129	2.1
42166	0.946129	2.1
42167	1.89226	0.727619
42168	1.89226	0.850921
42169	1.89226	0.894832
42170	1.89226	0.767993
42171	3.78453	0.543773
42172	1.89226	0.926512
42173	1.89226	0.822185
42174	1.89226	1.06973
42175	1.89226	1.04384
42176	1.89226	0.800946
42177	1.89226	0.719662
42178	1.89226	0.759676
42179	1.30733	0.960057
42180	1.89226	1.1005
42181	1.92403	0.784731
42182	3.38635	0.865972
42183	1.89226	0.839104
42184	3.02887	1.09322
42185	2.0815	0.318131
42186	1.88422	0.895914
42187	2.0815	0.561413
42188	1.88422	1.78394
42189	1.89226	1.11919
42190	1.70221	0.6412
42191	1.89226	1.80106
42192	1.20068	1.36534
42193	1.89226	0.853117
42194	1.20068	0.791579
42195	1.01393	1.00908
42196	1.20067	0.73812
42197	1.89226	1.54735
42198	2.44958	1.58907
42199	1.01393	0.794268
42200	1.89226	0.27262
42201	1.89226	2.1
42202	1.89226	2.1
42203	1.89226	2.1
42204	1.89226	2.1
42205	1.89226	2.1
42206	1.89226	2.1
42207	1.89226	2.1
42208	1.89226	0.723598
42209	1.89226	1.03662
42210	1.89226	2.1
42211	1.89226	1.05693
42212	1.89226	2.1
42213	1.89226	2.1
42214	1.89226	2.1
42215	1.89226	2.1
42216	1.89226	2.1
42217	1.89226	2.1
42218	1.89226	2.1
42219	1.91161	2.1
42220	2.45782	2.1
42221	1.39653	2.1

42222	2.44652	2.1
42223	4.35812	2.1
42224	2.73092	2.1
42225	1.89226	0.0830263
42226	1.89226	2.1
42227	1.89226	0.488745
42228	2.44652	2.1
42229	2.44652	0.0820804
42230	1.89226	2.1
42231	1.89226	1.02085
42232	1.89226	1.10695
42233	1.89226	2.1
42234	1.89226	1.11362
42235	1.89226	2.1
42236	2.64924	2.1
42237	1.31109	2.1
42238	1.31109	2.1
42239	1.49958	2.1
42240	1.95039	2.1
42241	2.14451	2.1
42242	0.825157	2.1
42243	0.634856	2.1
42244	0.816836	2.1
42245	0.816836	2.1
42246	0.556743	2.1
42247	0.639142	2.1
42248	0.985106	2.1
42249	0.795045	2.1
42250	0.967461	2.1
42251	0.552494	2.1
42252	0.676829	2.1
42253	0.98803	2.1
42254	2.28564	2.1
42255	2.34084	1.47787
42256	2.3502	2.1
42257	2.42908	2.1
42258	4.69104	2.1
42259	2.34084	2.1
42260	2.18077	2.1
42261	2.42908	2.1
42262	2.2947	1.106
42263	4.63554	2.1
42264	2.3502	2.1
42265	2.42908	2.1
42266	2.38681	2.1
42267	2.50438	1.58322
42268	4.85458	2.1
42269	2.42908	2.1
42270	2.2947	1.58398
42271	2.50438	1.24029
42272	2.40627	2.1
42273	4.79908	1.84722
42274	1.89226	0.138145
42275	2.20711	1.58587
42276	1.89226	0.0905392
42277	1.89226	1.92721
42278	2.02693	0.136865
42279	2.02693	1.96272

42280	1.2269	2.1
42281	2.20711	1.34746
42282	2.0815	0.828126
42283	1.11506	2.1
42284	3.22109	2.1
42285	0.545802	2.03231
42286	0.547489	2.1
42287	0.85914	1.29169
42288	1.11641	1.61709
42289	0.899974	1.83375
42290	0.849893	2.1
42291	0.747885	2.06549
42292	0.747885	0.751499
42293	0.557184	2.1
42294	0.887039	2.1
42295	1.08948	2.1
42296	0.59815	2.1
42297	0.974139	2.1
42298	1.33385	2.1
42299	1.02368	2.1
42300	1.02368	2.1
42301	1.00833	2.1
42302	1.00833	2.1
42303	1.89226	0.816002
42304	1.89226	0.548319
42305	1.89226	0.24251
42306	1.89226	0.05
42307	3.78453	0.05
42308	1.74651	0.315989
42309	1.44605	0.05
42310	1.63557	0.05
42311	1.31993	0.05
42312	3.08162	0.05
42313	1.89226	0.2144
42314	1.89226	0.335235
42315	1.89226	0.0916722
42316	1.89226	0.05
42317	3.78452	0.05
42318	1.63557	0.105383
42319	1.31992	0.05
42320	1.52615	0.116502
42321	1.18549	0.05
42322	2.82106	0.05
42323	1.89226	0.05
42324	2.58101	0.05
42325	2.73797	0.0757747
42326	2.73797	0.41498
42327	1.89226	0.05
42328	1.89226	0.05
42329	1.91762	0.353444
42330	1.44354	0.645195
42331	1.33622	0.504313
42332	1.33622	0.251863
42333	2.20248	0.871648
42334	2.20248	0.477174
42335	1.11712	2.1
42336	1.11712	2.1
42337	1.11421	2.1

42338	1.11421	2.1
42339	0.833871	2.1
42340	0.926528	2.1
42341	1.3048	2.1
42342	1.40335	2.1
42343	0.926527	2.1
42344	1.40335	2.1
42345	1.3048	2.1
42346	0.833871	2.1
42347	0.879657	2.1
42348	0.937695	2.1
42349	0.946129	2.1
42350	0.946129	2.1
42351	0.851541	2.1
42352	0.851541	2.1
42353	1.1126	2.1
42354	1.04519	2.1
42355	0.851486	2.1
42356	0.980501	2.1
42357	0.898251	2.1
42358	0.851528	2.1
42359	1.37756	2.1
42360	2.73081	2.1
42361	2.44311	2.1
42362	2.44311	2.1
42363	1.66428	2.1
42364	1.66428	2.1
42365	1.89226	2.1
42366	1.89226	2.1
42367	1.89226	2.1
42368	1.89226	2.1
42369	3.78452	2.1
42370	1.65781	2.1
42371	1.89837	2.1
42372	1.46713	2.1
42373	1.98027	2.1
42374	3.63808	2.1
42375	2.29054	2.1
42376	2.26369	0.238287
42377	1.98027	2.1
42378	2.15617	0.05
42379	4.24396	1.81954
42380	1.89226	2.1
42381	1.89226	0.213983
42382	1.89226	2.1
42383	1.89226	0.0755118
42384	3.78452	2.1
42385	0.946131	2.1
42386	0.946131	2.1
42387	0.946128	2.1
42388	0.946128	2.1
42389	0.946129	2.1
42390	0.946129	2.1
42391	0.94613	2.1
42392	0.94613	2.1
42393	0.94613	2.1
42394	0.94613	2.1
42395	0.94613	2.1

42396	0.94613	2.1
42397	0.812186	2.1
42398	0.812186	2.1
42399	0.819781	2.1
42400	0.819781	2.1
42401	0.913532	2.1
42402	0.990134	2.1
42403	0.733564	2.1
42404	0.641833	2.1
42405	0.641832	2.1
42406	0.913533	2.1
42407	0.733564	2.1
42408	0.990134	2.1
42409	0.994262	0.05
42410	0.994262	2.1
42411	0.997354	2.1
42412	0.997354	0.230049
42413	1.00775	0.05
42414	1.07808	0.05
42415	0.913533	2.1
42416	0.990135	2.1
42417	0.913532	1.4935
42418	1.00775	1.32984
42419	0.990134	1.33233
42420	1.07808	1.26992
42421	0.946131	2.1
42422	0.946131	0.943109
42423	0.946128	2.1
42424	0.946128	0.473358
42425	0.946128	0.0629706
42426	0.946128	0.084166
42427	0.94613	2.1
42428	0.94613	2.1
42429	0.946129	2.1
42430	0.946129	2.1
42431	0.946129	1.92019
42432	0.946129	2.1
42433	0.816232	2.1
42434	0.760038	2.1
42435	0.845999	2.1
42436	0.845999	2.1
42437	1.0631	2.1
42438	1.28131	2.1
42439	0.474065	2.1
42440	0.591905	2.1
42441	0.601756	2.1
42442	1.23903	2.1
42443	0.988592	2.1
42444	0.49051	2.1
42445	2.57021	2.1
42446	1.89226	2.1
42447	2.68172	2.1
42448	1.89226	1.84391
42449	4.46247	2.1
42450	1.89226	2.1
42451	1.89226	1.71079
42452	1.89226	1.94318
42453	1.89226	1.80091

42454	3.78452	1.97293
42455	1.12573	2.1
42456	1.65504	1.54693
42457	1.38848	2.1
42458	1.38848	2.1
42459	1.30567	1.30541
42460	1.30567	2.1
42461	2.82413	2.1
42462	1.89226	2.1
42463	2.68172	2.1
42464	2.68172	2.1
42465	1.89226	2.1
42466	1.89226	2.1
42467	1.89226	2.1
42468	1.89226	2.1
42469	1.89226	2.1
42470	1.89226	1.09669
42471	3.78452	2.04693
42472	1.30567	2.1
42473	1.65504	2.07125
42474	1.52724	2.1
42475	1.89249	2.1
42476	3.18228	2.1
42477	2.57386	0.05
42478	1.89226	0.0888021
42479	1.89226	0.0555169
42480	2.66753	0.05
42481	1.89226	0.05
42482	2.66753	0.05
42483	1.89226	0.05
42484	1.89226	0.129681
42485	2.2973	0.0708902
42486	3.12479	0.05
42487	1.89226	0.0528219
42488	2.57385	0.05
42489	0.489293	2.1
42490	0.672698	2.1
42491	0.59038	2.1
42492	0.738357	2.1
42493	0.62269	2.1
42494	0.62269	2.1
42495	0.640388	2.1
42496	0.640388	2.1
42497	0.59038	2.1
42498	0.489293	2.1
42499	0.738357	2.1
42500	0.672698	2.1
42501	1.89226	2.1
42502	1.89226	2.1
42503	1.89226	2.1
42504	1.89226	1.10218
42505	3.78452	2.1
42506	2.82858	2.1
42507	1.89226	1.14351
42508	2.5671	2.1
42509	1.89226	2.1
42510	2.5671	2.1
42511	1.89226	2.1

42512	2.06581	0.05
42513	2.47308	0.05
42514	1.89433	0.05
42515	1.67571	0.05
42516	3.87274	0.05
42517	2.55791	0.05
42518	2.10276	0.05
42519	2.47308	0.05
42520	2.47308	0.05
42521	1.83415	0.05
42522	1.90922	0.05
42523	1.18421	0.05
42524	2.03277	0.05
42525	1.18421	0.05
42526	2.01275	0.05
42527	1.42818	0.05
42528	2.25673	0.05
42529	0.882904	0.05
42530	0.958492	0.05
42531	0.905756	0.05
42532	0.905756	0.05
42533	1.06352	0.05
42534	0.797654	0.05
42535	1.11319	0.05
42536	0.716795	0.05
42537	0.820721	0.05
42538	0.731963	0.05
42539	1.13143	0.05
42540	1.10528	0.05
42541	1.89226	0.05
42542	1.89226	0.05
42543	1.89226	0.05
42544	1.89226	0.0579234
42545	1.89226	0.05
42546	1.89226	0.05
42547	1.01241	0.05
42548	0.703929	0.0518286
42549	0.946133	0.05
42550	0.946133	0.05
42551	1.04072	0.05
42552	1.163 0.05	
42553	0.639952	0.05
42554	0.851543	0.05
42555	0.946132	0.05
42556	0.920761	0.05
42557	0.711038	0.05
42558	0.711038	0.0951258
42559	1.89226	0.05
42560	1.89226	0.05
42561	1.89226	0.05
42562	1.89226	0.05
42563	1.89226	0.05
42564	1.89226	0.05
42565	1.89226	0.05
42566	1.89226	0.05
42567	2.41075	0.05
42568	1.89226	0.05
42569	3.78451	0.05

42570	0.968476	2.08174
42571	1.20674	1.20435
42572	1.13456	1.17712
42573	1.13456	2.1
42574	1.02074	1.13146
42575	1.02074	2.1
42576	1.02074	2.1
42577	1.20674	2.05938
42578	1.07866	1.27985
42579	1.28649	1.2744
42580	2.2854	2.1
42581	1.94221	0.0518356
42582	1.36717	0.05
42583	1.45535	0.0587846
42584	1.93771	0.05
42585	3.39756	0.0524475
42586	1.94222	0.140903
42587	2.25035	0.05
42588	2.41806	0.0506574
42589	2.42256	0.05
42590	1.93772	0.09873
42591	1.94222	0.0947995
42592	1.49906	0.05
42593	1.93771	0.05
42594	1.45535	0.05
42595	1.45535	0.0510282
42596	1.91484	0.05
42597	1.91484	0.05
42598	1.91484	0.05
42599	2.25035	0.05
42600	1.93772	0.05
42601	1.93772	0.05
42602	2.11465	0.05
42603	2.11465	0.05
42604	2.61735	0.05
42605	1.5082	0.05
42606	2.36481	0.05
42607	1.58597	0.05
42608	2.36481	0.05
42609	1.46653	0.05
42610	2.36482	0.0548095
42611	1.08133	0.0590956
42612	1.5082	0.05
42613	2.01868	0.05
42614	3.52688	0.0545055
42615	0.94613	0.05
42616	0.94613	0.05
42617	0.946131	0.05
42618	0.946131	0.05
42619	0.946129	0.05
42620	0.946129	0.05
42621	0.946132	0.05
42622	0.946132	0.05
42623	0.94613	0.05
42624	0.94613	0.05
42625	0.946131	0.05
42626	0.946131	0.05
42627	2.53725	0.05

42628	1.89227	0.05
42629	1.89227	0.05
42630	2.27073	0.05
42631	1.89227	0.05
42632	2.27073	0.05
42633	1.89227	0.05
42634	1.89227	0.05
42635	1.89227	0.05
42636	1.89227	0.05
42637	1.89227	0.05
42638	1.89227	0.05
42639	1.86766	2.1
42640	2.83487	2.1
42641	2.84318	2.1
42642	2.59401	2.1
42643	2.11684	2.1
42644	1.86766	2.1
42645	1.10638	2.1
42646	2.11684	2.1
42647	1.29111	2.1
42648	2.37439	2.1
42649	3.40796	2.1
42650	2.56843	2.1
42651	1.89226	2.1
42652	1.89226	2.1
42653	2.71932	2.1
42654	4.46069	2.1
42655	1.89226	2.1
42656	1.89226	2.1
42657	1.89226	2.1
42658	1.89226	2.1
42659	3.78452	2.1
42660	0.6416	2.1
42661	0.697585	2.1
42662	0.670706	2.1
42663	0.598927	2.1
42664	0.644313	2.1
42665	0.644313	2.1
42666	0.652199	2.1
42667	0.652199	2.1
42668	0.697587	2.1
42669	0.670705	2.1
42670	0.598926	2.1
42671	0.641601	2.1
42672	1.89226	2.1
42673	1.89226	2.1
42674	1.89226	2.1
42675	1.89226	2.1
42676	1.89226	2.1
42677	1.89226	2.1
42678	0.697585	2.1
42679	0.730617	2.1
42680	0.712082	2.1
42681	0.670706	2.1
42682	0.69854	2.1
42683	0.69854	2.1
42684	0.702783	2.1
42685	0.702783	2.1

42686	0.730618	2.1
42687	0.712081	2.1
42688	0.697586	2.1
42689	0.670705	2.1
42690	1.89226	2.1
42691	1.89226	2.1
42692	1.89226	0.696178
42693	1.89226	2.1
42694	1.89226	0.473207
42695	1.89226	2.1
42696	2.0815	2.1
42697	1.12076	2.1
42698	1.38662	2.1
42699	2.01244	2.1
42700	3.41508	2.1
42701	1.89226	2.1
42702	2.54324	2.1
42703	1.91981	2.1
42704	1.89226	2.1
42705	2.57079	2.1
42706	2.54324	2.1
42707	2.54325	2.1
42708	1.94779	2.1
42709	1.76879	2.1
42710	1.73811	2.1
42711	2.5708	2.1
42712	2.54325	2.1
42713	1.89226	2.1
42714	2.57079	2.1
42715	1.91894	2.1
42716	1.89226	2.1
42717	2.59747	2.1
42718	2.57079	2.1
42719	2.5708	2.1
42720	1.82268	2.1
42721	1.79738	2.1
42722	1.77071	2.1
42723	2.59747	2.1
42724	2.5708	2.1
42725	1.89226	0.561568
42726	1.89226	0.8932
42727	1.89226	0.05
42728	1.89226	1.24083
42729	3.78451	0.213948
42730	2.21582	0.0504816
42731	1.94412	0.87345
42732	2.13428	0.05
42733	1.86292	0.264512
42734	4.07839	0.0615386
42735	1.94412	0.0754584
42736	1.5854	2.1
42737	1.86293	0.205361
42738	1.50767	2.1
42739	3.45179	2.1
42740	1.89226	0.0854479
42741	1.89226	2.1
42742	1.89226	1.2187
42743	1.89226	2.1

42744	3.78452	2.1
42745	1.89226	0.734777
42746	1.89226	1.43091
42747	1.89226	0.05
42748	1.89226	0.909475
42749	3.78452	0.181137
42750	2.13428	0.246842
42751	1.86293	0.43174
42752	2.04737	0.05
42753	1.77122	0.864473
42754	3.9055	0.1672
42755	1.86293	0.05
42756	1.50767	2.1
42757	1.41077	0.88809
42758	1.41077	2.1
42759	1.77123	1.11123
42760	1.86813	2.1
42761	0.810943	0.409066
42762	0.810943	2.1
42763	0.810945	0.180204
42764	0.810945	0.881563
42765	0.810958	2.1
42766	0.810958	2.1
42767	1.89226	2.1
42768	1.35168	2.1
42769	1.08132	1.26047
42770	1.08132	2.1
42771	1.0813	2.1
42772	0.745979	2.1
42773	0.745979	2.1
42774	0.778781	2.1
42775	0.778781	2.1
42776	0.59549	2.1
42777	0.649658	2.1
42778	0.994784	2.1
42779	0.875102	2.1
42780	0.875102	2.1
42781	0.59549	2.1
42782	0.649658	2.1
42783	0.994784	2.1
42784	1.57887	2.1
42785	1.75021	2.1
42786	1.13341	2.1
42787	1.13341	2.1
42788	1.19098	2.1
42789	1.63644	2.1
42790	2.5761	0.05
42791	2.43404	0.367507
42792	2.44813	0.05
42793	2.31751	0.282706
42794	4.88218	0.05
42795	2.43405	0.05
42796	2.22575	2.1
42797	2.31751	0.070466
42798	2.10893	2.1
42799	4.54298	1.1816
42800	2.44813	0.05
42801	2.31751	0.268479

42802	2.30908	0.05
42803	2.19815	0.0862622
42804	4.64628	0.05
42805	2.31751	0.05
42806	2.10893	2.1
42807	2.19815	0.05
42808	1.98061	2.1
42809	4.30708	0.843857
42810	0.94613	2.1
42811	0.94613	2.1
42812	1.40372	2.1
42813	1.40372	2.1
42814	1.40372	2.1
42815	1.37167	2.1
42816	0.946132	2.1
42817	0.946132	2.1
42818	0.946131	2.1
42819	1.40372	2.1
42820	0.94613	2.1
42821	1.37167	2.1
42822	2.80744	2.1
42823	2.06984	2.1
42824	2.83418	2.1
42825	2.74335	2.1
42826	2.16067	2.1
42827	2.06984	2.1
42828	1.077	0.05
42829	0.809116	0.05
42830	0.600419	0.05
42831	0.790277	0.05
42832	0.927647	0.05
42833	0.758598	0.05
42834	0.85749	0.05
42835	0.85749	0.05
42836	1.02417	0.05
42837	0.600411	0.05
42838	0.751701	0.05
42839	1.07702	0.05
42840	1.89226	2.1
42841	1.89226	2.1
42842	1.89226	2.1
42843	1.89226	2.1
42844	1.89226	2.1
42845	1.89226	2.1
42846	1.89226	2.1
42847	1.89226	2.1
42848	1.89226	2.1
42849	1.89226	2.1
42850	3.78453	2.1
42851	1.89226	2.1
42852	1.89226	2.1
42853	1.89226	2.1
42854	1.89226	2.1
42855	1.89226	2.1
42856	1.89226	2.1
42857	0.946131	2.1
42858	0.946131	2.1
42859	0.946131	2.1

42860	0.946131	2.1
42861	0.946133	2.1
42862	0.946133	2.1
42863	0.946128	2.1
42864	0.946128	2.1
42865	0.946131	2.1
42866	0.946131	2.1
42867	0.94613	2.1
42868	0.94613	2.1
42869	2.17159	2.1
42870	2.00083	2.09862
42871	2.21305	2.1
42872	2.00551	2.1
42873	2.20837	2.1
42874	2.00083	2.1
42875	2.20837	2.1
42876	1.80328	1.24495
42877	2.22962	2.1
42878	2.00083	1.55856
42879	2.03207	1.11916
42880	1.80328	1.08572
42881	2.28564	2.1
42882	2.20837	1.59795
42883	2.30406	2.1
42884	2.17159	2.1
42885	2.34084	2.1
42886	2.20837	2.1
42887	1.09523	1.07487
42888	1.09523	2.06261
42889	1.09934	2.1
42890	1.09934	2.1
42891	1.01603	1.0366
42892	1.09038	1.98409
42893	1.10419	1.22081
42894	1.17042	2.1
42895	1.17042	2.1
42896	1.09038	1.90123
42897	1.10419	1.33652
42898	1.01603	2.1
42899	2.60152	0.0523812
42900	0.976334	0.389542
42901	2.22344	0.507059
42902	2.22344	0.09864
42903	1.36405	0.470484
42904	1.36405	0.05
42905	1.89226	2.1
42906	1.70302	2.1
42907	1.69019	2.1
42908	1.70302	2.1
42909	1.73933	2.1
42910	1.70303	1.66662
42911	1.89226	2.1
42912	1.89226	1.07882
42913	1.89226	2.1
42914	1.89226	2.1
42915	3.78452	2.1
42916	1.89226	2.1
42917	1.59036	2.1

42918	1.65683	2.1
42919	1.59036	2.1
42920	1.95873	2.1
42921	1.89226	1.11694
42922	0.946131	1.02449
42923	0.946131	1.14696
42924	0.946127	2.1
42925	0.946127	2.1
42926	0.946127	2.1
42927	0.946127	0.955012
42928	0.946131	2.1
42929	0.946131	1.16239
42930	0.946129	1.32713
42931	0.946129	1.44084
42932	0.94613	1.85576
42933	0.94613	1.61778
42934	0.851541	2.1
42935	0.851541	2.1
42936	0.851483	2.1
42937	1.15713	2.1
42938	0.851539	2.1
42939	0.851539	2.1
42940	0.942127	2.1
42941	1.14455	2.1
42942	0.838683	2.1
42943	0.946131	2.1
42944	0.957503	2.1
42945	0.957504	2.1
42946	1.89226	0.600909
42947	1.89226	0.05
42948	1.89226	0.599218
42949	1.89226	2.1
42950	3.78452	0.05
42951	1.89226	0.05
42952	1.89226	0.05
42953	1.89226	0.0633412
42954	1.89226	0.05
42955	1.89226	0.05
42956	1.89226	0.05
42957	1.89226	0.05
42958	1.89226	0.05
42959	1.89226	0.05
42960	1.89226	0.05
42961	3.78453	0.05
42962	1.89226	0.05
42963	1.89226	2.1
42964	1.89226	2.1
42965	1.89226	1.46136
42966	3.78453	2.1
42967	1.22575	0.618527
42968	1.05438	0.694056
42969	0.946131	0.943069
42970	0.946131	0.845653
42971	0.946129	1.60196
42972	1.22575	0.05
42973	1.05438	0.652762
42974	0.946132	0.0578561
42975	1.05438	0.855565

42976	1.05438	1.1572
42977	0.946131	0.156333
42978	0.946131	0.464196
42979	2.4515	0.05
42980	1.13726	0.05
42981	2.10875	0.05
42982	1.49515	0.05
42983	2.10875	0.05
42984	1.49515	0.05
42985	0.831477	0.05
42986	0.747575	0.05
42987	1.22575	0.0503659
42988	1.28174	0.05
42989	1.28174	0.05
42990	0.831476	0.0720553
42991	1.22575	0.05
42992	0.747575	0.05
42993	1.03561	0.05
42994	1.03561	0.0808964
42995	1.02162	0.05
42996	1.02162	0.0572447
42997	1.89227	2.1
42998	2.56348	0.305307
42999	1.89227	2.1
43000	2.45151	1.37035
43001	1.89227	2.1
43002	2.45151	0.138745
43003	1.40013	0.164903
43004	1.89226	1.66203
43005	1.89226	0.515071
43006	0.946643	0.0542288
43007	1.89226	1.21583
43008	0.946643	0.200202
43009	1.89226	0.05
43010	1.89226	0.05
43011	1.89226	0.05
43012	1.89226	0.05
43013	3.78453	0.05
43014	1.89226	0.05
43015	1.89226	0.05
43016	1.89226	0.05
43017	1.89226	0.0711015
43018	3.78453	0.05
43019	0.946131	0.05
43020	0.946131	0.05
43021	0.946128	0.05
43022	0.946128	0.05
43023	0.94613	0.05
43024	0.94613	0.05
43025	0.946129	0.05
43026	0.946129	0.05
43027	0.94613	0.05
43028	0.94613	0.05
43029	0.94613	0.05
43030	0.94613	0.05
43031	1.89226	0.05
43032	1.89226	0.05
43033	1.17244	0.05

43034	1.89226	0.05
43035	0.975291	0.05
43036	1.57408	0.05
43037	0.946132	0.05
43038	0.946132	0.05
43039	0.946131	0.05
43040	0.946131	0.05
43041	0.946132	0.05
43042	0.946132	0.05
43043	0.946131	0.05
43044	0.946131	0.05
43045	0.946131	0.05
43046	0.946131	0.05
43047	0.946132	0.05
43048	0.946132	0.05
43049	2.74744	0.05
43050	1.89226	0.0565539
43051	2.70805	0.05
43052	1.89226	0.05
43053	2.70805	0.05
43054	1.89226	0.05
43055	2.74744	0.05
43056	1.89226	0.05
43057	2.57465	0.05
43058	1.89226	0.0521683
43059	4.6397	0.05
43060	1.89226	0.05
43061	1.89226	0.0723479
43062	1.89226	0.125604
43063	1.89226	0.353487
43064	3.78452	0.0765791
43065	1.89226	0.0750533
43066	1.89226	0.315324
43067	1.89226	0.0571464
43068	1.89226	0.05
43069	1.89226	0.81294
43070	1.89226	0.509059
43071	2.16206	0.05
43072	2.67861	0.05
43073	3.11996	0.05
43074	1.70303	0.05
43075	2.89015	0.05
43076	1.486 0.05	
43077	2.12839	0.05
43078	2.83631	0.05
43079	3.08096	0.05
43080	1.84157	0.05
43081	3.08096	0.05
43082	1.84157	0.05
43083	1.89225	1.25461
43084	1.89225	0.732381
43085	1.89225	0.901746
43086	1.89225	1.24835
43087	1.89225	0.7175
43088	1.89225	1.22025
43089	1.89226	0.372609
43090	1.89226	0.54307
43091	1.89226	1.29299

43092	1.89226	0.229291
43093	3.78452	0.220052
43094	1.89226	0.0858711
43095	2.49041	0.0761611
43096	1.89226	0.0902044
43097	1.89226	0.05
43098	2.27073	0.05
43099	2.27073	0.0687907
43100	1.09904	0.05
43101	0.677147	0.05
43102	1.3365	0.05
43103	0.95759	0.05
43104	0.957591	0.05
43105	0.677148	0.05
43106	1.09904	0.05
43107	1.3365	0.05
43108	0.996015	0.05
43109	0.996015	0.05
43110	1.01764	0.05
43111	1.01764	0.05
43112	2.77349	0.05
43113	2.72723	0.05
43114	2.43156	0.05
43115	2.4564	0.05
43116	5.20505	0.05
43117	2.77349	0.05
43118	2.68508	0.05
43119	2.33761	0.05
43120	2.4564	0.05
43121	5.1111	0.05
43122	2.43156	0.05
43123	2.70208	0.05
43124	1.85779	0.05
43125	2.10695	0.05
43126	4.77572	0.05
43127	2.33761	0.05
43128	2.70208	0.05
43129	1.85779	0.05
43130	1.61597	0.05
43131	4.19542	0.05
43132	0.946132	1.17507
43133	0.946132	1.82133
43134	0.746729	1.70006
43135	0.746729	2.03607
43136	0.946132	2.07554
43137	0.915958	1.84921
43138	0.946133	0.993076
43139	0.74673	2.1
43140	0.946133	1.77703
43141	0.946133	1.77864
43142	0.915958	1.83768
43143	0.746729	1.84709
43144	1.50743	2.1
43145	2.45501	2.1
43146	2.29118	2.1
43147	2.29118	2.1
43148	1.75482	2.1
43149	1.75482	2.1

43150	2.45501	2.1
43151	2.76927	0.142175
43152	2.29118	2.1
43153	2.64523	0.0808793
43154	5.10024	1.85525
43155	1.25559	2.1
43156	2.29118	2.1
43157	2.0815	2.1
43158	2.26387	2.1
43159	1.35668	2.1
43160	1.47667	2.1
43161	2.28481	2.1
43162	2.64523	0.560427
43163	2.50057	2.1
43164	2.50057	0.397089
43165	2.29118	2.1
43166	2.29118	0.900349
43167	0.946129	0.05
43168	0.946129	0.05
43169	1.23929	0.05
43170	1.15246	0.05
43171	0.946131	0.0573003
43172	0.946131	0.070385
43173	1.15246	0.05
43174	1.15246	0.05
43175	0.946131	0.05
43176	1.15246	0.0774621
43177	0.946131	0.05
43178	1.23928	0.05
43179	0.946128	0.179328
43180	0.946128	0.127542
43181	1.15246	0.0600744
43182	0.942814	0.249694
43183	0.946131	0.128567
43184	0.946131	0.170761
43185	0.961347	0.0947945
43186	0.942811	0.05
43187	0.946131	0.107545
43188	0.961347	0.0553914
43189	0.946131	0.05
43190	1.15246	0.0501136
43191	2.32334	0.0758916
43192	1.57621	0.05
43193	1.90335	0.0533224
43194	1.24538	0.05
43195	3.47956	0.05
43196	0.965762	0.05
43197	0.965762	0.05
43198	1.00245	0.05
43199	1.00245	0.05
43200	0.92786	0.05
43201	0.705297	0.05
43202	0.856414	0.05
43203	1.28968	0.05
43204	1.00306	0.05
43205	0.705303	0.05
43206	1.28967	0.05
43207	0.975478	0.05

43208	3.05308	2.1
43209	1.76816	2.1
43210	1.76816	2.1
43211	1.69129	2.1
43212	2.58731	2.1
43213	2.51043	2.1
43214	1.89226	0.05
43215	2.71464	0.05
43216	2.34627	0.05
43217	1.89226	0.05
43218	2.34627	0.05
43219	1.89226	0.05
43220	2.34627	0.05
43221	2.71464	0.05
43222	1.98252	0.05
43223	1.98252	0.05
43224	1.51026	0.05
43225	2.34627	0.05
43226	2.38698	1.23113
43227	2.35142	2.1
43228	2.60343	1.24883
43229	2.6636	1.25001
43230	4.99041	1.4234
43231	1.07811	2.1
43232	1.1952	2.1
43233	1.17571	2.07331
43234	1.30171	1.16321
43235	1.18322	1.11335
43236	1.18322	2.1
43237	1.18769	2.1
43238	1.18769	1.23658
43239	1.1952	2.1
43240	1.30171	2.1
43241	1.17571	2.1
43242	1.07811	2.1
43243	1.89226	1.38631
43244	2.6636	1.97537
43245	1.89226	0.890544
43246	2.60343	1.33273
43247	1.89226	1.40863
43248	2.60343	2.1
43249	1.5344	0.05
43250	1.4516	0.05
43251	1.17816	0.05
43252	1.21056	0.05
43253	2.71257	0.05
43254	1.89226	2.08664
43255	1.89226	0.976677
43256	1.89226	1.15419
43257	1.89226	1.27589
43258	3.78452	1.11342
43259	1.89226	1.22173
43260	1.89226	0.218961
43261	1.89226	0.938484
43262	1.89226	0.796481
43263	1.65573	1.09607
43264	1.65573	0.953472
43265	1.89226	1.1332

43266	1.89226	1.33595
43267	1.89226	2.1
43268	2.64212	0.982858
43269	3.78452	1.41057
43270	1.89226	2.1
43271	1.89226	2.1
43272	1.89226	2.1
43273	1.89226	2.1
43274	3.78452	2.1
43275	2.7367	0.984658
43276	2.07947	2.1
43277	2.47319	2.1
43278	1.85057	2.1
43279	4.55267	2.1
43280	2.07948	2.1
43281	1.3294	2.1
43282	1.85057	2.1
43283	1.09962	2.1
43284	3.1791	2.1
43285	1.89226	2.1
43286	1.89226	2.1
43287	1.89226	2.1
43288	1.89226	2.1
43289	3.78453	2.1
43290	0.94613	2.1
43291	0.94613	1.23108
43292	0.94613	2.1
43293	0.94613	2.1
43294	0.946128	2.1
43295	0.946128	2.1
43296	0.946132	1.24569
43297	0.946132	1.3982
43298	0.94613	2.1
43299	0.94613	2.1
43300	0.946131	2.1
43301	0.946131	2.1
43302	1.00875	2.1
43303	1.00875	0.946252
43304	1.01676	2.1
43305	1.01676	0.948805
43306	0.925282	2.1
43307	0.804877	2.1
43308	1.2366	1.73639
43309	1.10023	1.85482
43310	0.804878	2.1
43311	1.10023	2.1
43312	1.2366	2.1
43313	0.925284	2.1
43314	1.89226	2.1
43315	1.89226	2.1
43316	1.89226	2.1
43317	1.89226	2.1
43318	3.78453	2.1
43319	1.89226	2.1
43320	1.89226	2.1
43321	1.89226	2.1
43322	1.89226	2.1
43323	1.89226	2.1

43324	1.89226	2.1
43325	1.09766	2.1
43326	1.14842	2.1
43327	1.02909	2.1
43328	1.07659	2.1
43329	1.16718	2.1
43330	0.954189	2.1
43331	1.06653	2.1
43332	1.06653	2.1
43333	1.20738	2.1
43334	1.02909	2.1
43335	1.09767	2.1
43336	0.815951	2.1
43337	2.00793	2.1
43338	1.14048	2.1
43339	1.19086	2.1
43340	1.13723	2.1
43341	2.00793	2.1
43342	2.11772	2.1
43343	1.89226	2.1
43344	1.89226	2.1
43345	1.89226	2.1
43346	1.89226	2.1
43347	1.89226	2.1
43348	1.89226	2.1
43349	1.89226	2.1
43350	1.89226	2.1
43351	1.89226	2.1
43352	1.89226	2.1
43353	1.89226	2.1
43354	1.89226	2.1
43355	1.1779	2.1
43356	1.26166	2.1
43357	1.0875	2.1
43358	1.00397	2.1
43359	1.13286	2.1
43360	1.13286	2.1
43361	1.13277	2.1
43362	1.13277	2.1
43363	1.26166	2.1
43364	1.08749	2.1
43365	1.1779	2.1
43366	1.00396	2.1
43367	1.0875	2.1
43368	1.00397	2.1
43369	0.685749	2.1
43370	0.595432	2.1
43371	0.83976	2.1
43372	0.83976	2.1
43373	0.843169	2.1
43374	0.843169	2.1
43375	1.0875	2.1
43376	0.685749	2.1
43377	1.00397	2.1
43378	0.595432	2.1
43379	1.89226	2.1
43380	1.86443	2.1
43381	2.4988	2.1

43382	2.0815	2.1
43383	2.28173	2.1
43384	1.86443	2.1
43385	1.89226	2.1
43386	1.89226	2.1
43387	1.89226	2.1
43388	1.89226	2.1
43389	1.89226	2.1
43390	1.89226	2.1
43391	0.946131	2.1
43392	0.946131	2.1
43393	0.969491	2.1
43394	0.949105	2.1
43395	0.949103	2.1
43396	0.949103	2.1
43397	0.946133	2.1
43398	0.946133	2.1
43399	0.946131	2.1
43400	0.969492	2.1
43401	0.946131	2.1
43402	0.949105	2.1
43403	1.89226	2.1
43404	2.07428	2.1
43405	1.93464	2.1
43406	1.89226	2.1
43407	2.11667	2.1
43408	2.07428	2.1
43409	0.946132	2.1
43410	0.946132	2.1
43411	0.946131	2.1
43412	0.946131	2.1
43413	0.946132	2.1
43414	0.946132	2.1
43415	0.946132	2.1
43416	0.946132	2.1
43417	0.946132	2.1
43418	0.946132	2.1
43419	0.946132	2.1
43420	0.946132	2.1
43421	1.97992	2.1
43422	1.89226	2.1
43423	1.89226	2.1
43424	1.93898	2.1
43425	1.93898	2.1
43426	1.89226	2.1
43427	2.12475	0.752535
43428	2.79029	0.85039
43429	2.7803	1.01708
43430	2.13052	0.747174
43431	4.92081	0.463677
43432	1.06238	0.906118
43433	1.06526	0.815426
43434	0.642901	0.545433
43435	0.655055	0.813045
43436	0.856391	0.627814
43437	0.856391	0.288403
43438	0.86104	0.977552
43439	0.86104	0.21667

43440	1.06526	0.834565
43441	0.655055	0.610321
43442	0.642901	0.629352
43443	1.06238	0.72102
43444	2.7803	0.942778
43445	2.13053	0.0900456
43446	2.14346	0.844391
43447	2.77937	0.826258
43448	4.9099	0.854501
43449	2.14346	0.88218
43450	1.31011	0.179428
43451	1.34124	0.771683
43452	1.31011	0.595515
43453	2.16166	0.726539
43454	2.13053	0.599655
43455	1.89226	2.1
43456	2.58176	2.1
43457	2.01422	2.1
43458	1.89226	2.1
43459	2.70373	2.1
43460	2.58176	2.1
43461	1.89226	2.1
43462	1.89226	2.1
43463	1.89226	2.1
43464	1.89226	2.1
43465	1.89226	2.1
43466	1.89226	2.1
43467	0.946128	2.1
43468	0.946128	2.1
43469	1.38521	2.1
43470	1.38521	2.1
43471	1.38521	2.1
43472	1.35186	2.1
43473	0.946131	2.1
43474	0.946131	2.1
43475	0.94613	2.1
43476	1.38521	2.1
43477	0.946131	2.1
43478	1.35186	2.1
43479	0.946133	2.1
43480	0.946133	2.1
43481	0.946132	2.1
43482	0.946132	2.1
43483	0.946132	2.1
43484	0.946132	2.1
43485	0.946132	2.1
43486	0.946132	2.1
43487	0.946132	2.1
43488	0.946132	2.1
43489	0.946133	2.1
43490	0.946133	2.1
43491	2.33374	2.1
43492	1.89226	2.1
43493	1.89226	2.1
43494	1.89226	2.1
43495	2.60377	2.1
43496	2.60377	2.1
43497	2.6771	0.05

43498	1.36416	0.05
43499	1.64983	0.05
43500	1.64983	0.05
43501	2.44131	0.05
43502	2.44131	0.05
43503	2.58321	0.182254
43504	1.89226	0.338245
43505	2.44283	0.05
43506	2.44283	0.10907
43507	1.89226	0.78275
43508	1.89226	0.903804
43509	2.58321	0.314397
43510	2.44283	0.111612
43511	1.49358	0.05
43512	1.57702	0.104488
43513	3.93641	0.05
43514	0.951913	0.05
43515	0.81556	0.05
43516	0.946132	0.05
43517	0.946132	0.05
43518	1.04072	0.05
43519	1.12182	0.05
43520	0.856745	0.05
43521	0.851542	0.05
43522	0.923163	0.05
43523	0.923163	0.05
43524	1.04304	0.05
43525	0.85098	0.05
43526	2.70125	1.33371
43527	2.37734	1.33611
43528	2.63495	1.09807
43529	2.30539	2.1
43530	5.01229	2.00232
43531	1.89226	1.44073
43532	1.89226	2.1
43533	1.89226	1.30857
43534	1.89226	2.1
43535	3.78452	1.11131
43536	1.89226	2.1
43537	1.89226	2.1
43538	1.89226	2.1
43539	1.89226	2.1
43540	3.78452	2.1
43541	2.37734	1.48639
43542	1.79346	2.1
43543	2.53594	1.84123
43544	1.70302	2.1
43545	4.27682	2.1
43546	2.63495	1.01601
43547	2.30539	1.56653
43548	2.49445	1.10537
43549	2.12089	1.09847
43550	4.75584	1.06116
43551	1.89226	1.15673
43552	1.89226	1.55259
43553	1.89226	1.04482
43554	1.89226	1.99303
43555	3.78452	2.1

43556	1.89226	1.4869
43557	1.89226	2.1
43558	1.89226	2.1
43559	1.89226	2.1
43560	1.89226	1.29586
43561	1.89226	1.31928
43562	2.53594	1.30517
43563	1.60017	1.36826
43564	1.57079	1.46498
43565	1.70302	1.14744
43566	2.15772	1.39363
43567	2.33299	1.09339
43568	2.09114	2.1
43569	2.34898	2.1
43570	2.30729	2.1
43571	2.06556	2.1
43572	4.41454	2.1
43573	2.09114	2.1
43574	1.59654	2.1
43575	1.57823	2.1
43576	2.06556	2.1
43577	3.6621	2.1
43578	2.30729	2.1
43579	2.06556	2.1
43580	2.03917	2.1
43581	2.26617	2.1
43582	4.33173	2.1
43583	2.03917	2.1
43584	2.06556	2.1
43585	2.045 2.1	
43586	1.55767	2.1
43587	1.57823	2.1
43588	1.57823	2.1
43589	1.89225	2.1
43590	1.09726	2.1
43591	1.61196	2.1
43592	1.28597	2.1
43593	1.28597	0.05
43594	1.45633	2.1
43595	1.45633	2.1
43596	1.9544	0.05
43597	1.61196	2.1
43598	1.45633	2.1
43599	1.53735	2.1
43600	3.14931	2.1
43601	1.89226	2.1
43602	1.89226	2.1
43603	1.89226	2.1
43604	1.89226	2.1
43605	3.78453	2.1
43606	0.810946	2.1
43607	0.810946	2.1
43608	0.810947	2.1
43609	0.810947	2.1
43610	0.810933	2.1
43611	0.810933	2.1
43612	1.89226	2.1
43613	1.3517	2.1

43614	1.08132	2.1
43615	1.08132	2.1
43616	1.08133	2.1
43617	0.851539	2.1
43618	0.851539	2.1
43619	0.972053	2.1
43620	1.05632	2.1
43621	0.94613	2.1
43622	0.94613	2.1
43623	1.0279	2.1
43624	0.94991	2.1
43625	1.0279	2.1
43626	0.946131	2.1
43627	0.865643	2.1
43628	0.94613	2.1
43629	1.89226	2.1
43630	1.89226	2.1
43631	1.89226	2.1
43632	1.89226	2.1
43633	3.78452	2.1
43634	1.03882	2.1
43635	1.91121	2.1
43636	1.41833	2.1
43637	2.34547	2.1
43638	3.32954	2.1
43639	2.3979	2.1
43640	1.89226	2.1
43641	1.89226	2.1
43642	1.89226	2.1
43643	2.0558	2.1
43644	2.0558	2.1
43645	1.89226	2.1
43646	1.89226	1.98236
43647	1.89226	2.1
43648	1.89226	2.1
43649	3.78452	2.1
43650	1.05816	2.1
43651	1.05816	2.1
43652	0.842045	2.1
43653	1.05252	2.1
43654	1.36057	2.1
43655	1.14339	2.1
43656	0.873397	2.1
43657	0.585073	2.1
43658	0.873388	2.1
43659	1.15129	2.1
43660	0.69143	2.1
43661	1.1434	2.1
43662	1.93338	2.1
43663	1.85883	2.1
43664	1.51714	2.1
43665	1.85883	2.1
43666	1.54795	2.1
43667	1.89226	2.1
43668	1.89226	2.1
43669	1.89226	2.1
43670	1.89226	2.1
43671	1.89226	2.1

43672	3.78452	2.1
43673	0.844726	2.1
43674	0.844726	2.1
43675	0.960524	2.1
43676	1.16288	2.1
43677	0.851541	2.1
43678	0.851541	2.1
43679	0.929147	2.1
43680	1.15533	2.1
43681	0.844697	2.1
43682	0.946131	2.1
43683	0.851632	2.1
43684	0.946131	2.1
43685	1.89226	2.1
43686	1.70303	2.1
43687	1.74006	2.1
43688	1.70303	2.1
43689	1.99471	2.1
43690	1.93338	2.1
43691	0.946131	2.1
43692	0.946131	2.1
43693	0.946131	2.1
43694	0.946131	2.1
43695	0.94613	2.1
43696	0.94613	2.1
43697	0.946132	2.1
43698	0.946132	2.1
43699	0.946131	2.1
43700	0.946131	2.1
43701	0.946131	2.1
43702	0.946131	2.1
43703	1.58158	2.1
43704	1.89226	2.1
43705	1.89226	2.1
43706	1.89226	2.1
43707	1.68939	2.1
43708	1.68939	2.1
43709	0.946132	0.05
43710	0.946132	0.05
43711	1.12988	0.05
43712	1.30783	0.05
43713	1.30783	0.05
43714	0.946133	0.05
43715	0.946132	0.05
43716	1.12988	0.05
43717	1.30783	0.05
43718	1.30783	0.05
43719	0.946133	0.05
43720	0.946133	0.05
43721	1.89227	0.05
43722	1.89227	0.05
43723	1.89227	0.05
43724	1.89227	0.05
43725	1.89227	0.05
43726	1.89227	0.05
43727	1.89226	0.05
43728	1.89226	0.05
43729	1.89226	0.05

43730	1.89226	0.05
43731	1.89226	0.05
43732	1.89226	0.05
43733	0.946131	0.05
43734	0.946131	0.05
43735	1.30783	0.05
43736	1.37715	0.05
43737	1.37715	0.05
43738	0.946133	0.05
43739	1.30783	0.05
43740	0.946129	0.05
43741	1.37715	0.05
43742	1.37715	0.05
43743	0.946131	0.05
43744	0.946131	0.05
43745	1.17056	0.05
43746	0.591757	0.05
43747	0.522025	0.05
43748	0.929727	0.05
43749	0.929728	0.05
43750	1.17056	0.05
43751	0.591756	0.05
43752	0.522025	0.05
43753	0.889054	0.05
43754	0.889054	0.05
43755	0.803535	0.05
43756	0.803535	0.05
43757	1.49814	2.1
43758	2.37439	2.1
43759	2.63923	2.1
43760	2.63923	2.1
43761	1.29111	2.1
43762	1.29111	2.1
43763	0.946132	2.1
43764	0.946132	2.1
43765	1.18719	2.1
43766	1.18719	2.1
43767	0.94613	2.1
43768	0.94613	2.1
43769	1.1872	2.1
43770	1.31961	2.1
43771	0.946131	2.1
43772	1.31961	2.1
43773	1.1872	2.1
43774	0.94613	2.1
43775	2.49445	0.831457
43776	2.12089	0.952184
43777	2.20843	0.798051
43778	1.82805	1.00555
43779	4.32932	0.896701
43780	1.89226	0.963926
43781	1.89226	1.41411
43782	1.89226	0.895355
43783	1.89226	0.933503
43784	3.78452	0.867521
43785	0.94613	0.995351
43786	0.94613	0.951635
43787	0.946128	0.942514

43788	0.946128	1.16427
43789	0.946131	1.10859
43790	0.946131	1.1574
43791	0.946127	1.1154
43792	0.946127	0.917752
43793	0.946129	0.996956
43794	0.946129	0.962503
43795	0.946129	1.05578
43796	0.946129	1.05275
43797	1.82804	0.937776
43798	1.60017	1.05205
43799	1.87679	1.04598
43800	2.12088	0.735946
43801	1.35608	1.11453
43802	1.60017	0.949618
43803	2.20843	0.740804
43804	1.3216	0.51505
43805	1.44426	0.613008
43806	1.82805	0.777234
43807	1.80399	0.571403
43808	2.20843	0.759596
43809	1.89226	0.790951
43810	1.89226	0.78199
43811	1.89226	0.898641
43812	1.89226	0.796235
43813	1.89226	0.841947
43814	1.89226	0.782377
43815	1.89226	0.922702
43816	1.89226	0.810952
43817	1.89226	0.807691
43818	1.89226	0.819911
43819	1.89226	0.887896
43820	1.89226	0.776663
43821	0.627705	0.0700323
43822	0.634941	0.268966
43823	0.629317	0.0949725
43824	0.783422	0.328452
43825	0.629328	0.324286
43826	0.448764	0.421488
43827	1.35608	0.548145
43828	1.00805	0.624735
43829	0.814295	0.356859
43830	0.598375	0.34687
43831	1.04461	0.314243
43832	1.09795	2.1
43833	1.00775	2.1
43834	1.61202	2.1
43835	1.63419	2.04514
43836	2.64194	1.89632
43837	1.44801	1.86888
43838	1.61201	2.1
43839	2.35614	1.99332
43840	2.27703	2.09384
43841	3.88904	2.06167
43842	2.35615	1.83445
43843	1.63419	1.70778
43844	2.29852	1.72189
43845	1.63419	1.63189

43846	2.27634	1.79613
43847	1.61202	2.09984
43848	0.973793	2.1
43849	2.2671	2.1
43850	2.32981	2.1
43851	2.32981	2.1
43852	0.990481	2.1
43853	0.990481	2.1
43854	1.50877	2.1
43855	1.11193	2.1
43856	2.29667	2.1
43857	1.94158	2.1
43858	3.45035	2.1
43859	2.29667	2.1
43860	1.89227	2.1
43861	1.94158	2.1
43862	1.94158	2.1
43863	1.89227	2.1
43864	1.89227	2.1
43865	2.51379	2.1
43866	1.30026	2.1
43867	1.84502	2.1
43868	2.72099	2.1
43869	4.35882	2.1
43870	1.89226	2.1
43871	2.721	2.1
43872	2.5138	2.1
43873	2.5138	2.1
43874	1.89226	2.1
43875	1.89226	2.1
43876	1.89226	0.05
43877	1.89226	0.05
43878	2.59101	0.05
43879	1.89226	0.05
43880	3.78453	0.05
43881	0.851541	0.05
43882	0.851541	0.05
43883	1.42502	0.05
43884	0.948679	0.05
43885	0.946132	0.05
43886	0.946132	0.05
43887	0.853617	0.05
43888	0.853618	0.05
43889	0.851475	0.05
43890	0.948666	0.05
43891	1.166	0.05
43892	0.851554	0.05
43893	2.51818	1.01466
43894	2.60723	2.1
43895	2.66532	0.251179
43896	2.76315	2.1
43897	5.27255	2.1
43898	2.60723	2.1
43899	2.45247	2.1
43900	2.76315	2.1
43901	2.55197	2.1
43902	5.1592	2.1
43903	1.23768	0.78808

43904	1.11433	0.877101
43905	1.32037	1.14801
43906	1.32037	0.861925
43907	1.0142	0.950784
43908	1.0142	0.709492
43909	0.886711	0.977028
43910	1.16806	0.941013
43911	0.999083	2.1
43912	0.710926	1.33038
43913	0.937757	1.54274
43914	0.937757	1.09299
43915	0.941224	0.851129
43916	0.941224	2.1
43917	1.16805	1.20153
43918	0.999084	2.1
43919	0.886709	1.74922
43920	0.710928	1.31676
43921	2.29332	2.1
43922	1.54904	2.1
43923	2.0815	2.1
43924	2.44199	2.1
43925	4.00418	2.1
43926	1.53873	0.05
43927	1.53422	0.0936078
43928	1.39661	0.05
43929	1.42387	0.106567
43930	2.9626	0.05
43931	1.53423	0.0553634
43932	1.44091	2.1
43933	1.42387	0.0763834
43934	1.35215	2.1
43935	2.86479	2.03798
43936	1.39661	0.05
43937	1.42387	0.211596
43938	1.28325	0.05
43939	1.33326	0.29911
43940	2.70713	0.05
43941	1.42387	0.05
43942	1.35215	2.1
43943	1.33327	0.226036
43944	1.27339	2.1
43945	2.69726	2.1
43946	2.58442	2.1
43947	1.1468	2.1
43948	1.18775	2.1
43949	2.60047	2.1
43950	3.74728	2.1
43951	2.58442	2.1
43952	1.89226	2.1
43953	1.89226	2.1
43954	2.60048	2.1
43955	4.49274	2.1
43956	1.89226	2.1
43957	1.89226	2.1
43958	1.89226	2.1
43959	1.89226	2.1
43960	3.78453	2.1
43961	1.89226	2.1

43962	1.89226	2.1
43963	1.89226	2.1
43964	1.89226	2.1
43965	3.78452	2.1
43966	1.22262	2.1
43967	2.60047	2.1
43968	1.18775	2.1
43969	1.18775	2.1
43970	2.61433	2.1
43971	2.61433	2.1
43972	1.89226	2.1
43973	2.60048	2.1
43974	2.61434	2.1
43975	2.60048	2.1
43976	1.90612	2.1
43977	1.89226	2.1
43978	1.89226	2.1
43979	1.89226	2.1
43980	1.89226	2.1
43981	1.89226	2.1
43982	3.78453	2.1
43983	1.89226	2.1
43984	1.89226	2.1
43985	1.89226	2.1
43986	1.89226	2.1
43987	3.78452	2.1
43988	2.12556	2.1
43989	0.991113	2.1
43990	1.27725	2.1
43991	2.31081	2.1
43992	3.40281	2.1
43993	1.89226	2.1
43994	2.31082	2.1
43995	2.12556	2.1
43996	2.12556	2.1
43997	1.89226	2.1
43998	1.89226	2.1
43999	1.22544	2.1
44000	1.12649	2.1
44001	0.775254	2.1
44002	0.643133	2.1
44003	0.917924	2.1
44004	0.934955	2.1
44005	0.975579	2.1
44006	0.975579	2.1
44007	1.23113	2.1
44008	0.685272	2.1
44009	0.651719	2.1
44010	1.1791	2.1
44011	1.89226	2.1
44012	2.5138	2.1
44013	2.31082	2.1
44014	2.31082	2.1
44015	1.89226	2.1
44016	1.89226	2.1
44017	1.89226	2.1
44018	1.89226	2.1
44019	1.89226	2.1

44020	1.89226	2.1
44021	3.78453	2.1
44022	0.922224	2.1
44023	0.922224	2.1
44024	1.23555	2.1
44025	1.19715	2.1
44026	1.01445	2.1
44027	1.16061	2.1
44028	0.946132	2.1
44029	0.946132	2.1
44030	0.946131	2.1
44031	1.2051	2.1
44032	1.13537	2.1
44033	1.01445	2.1
44034	0.946133	2.1
44035	0.946133	2.1
44036	1.20648	2.1
44037	1.20648	2.1
44038	1.06454	2.1
44039	1.20648	2.1
44040	0.946133	2.1
44041	0.946133	2.1
44042	1.20649	2.1
44043	0.946133	2.1
44044	1.06454	2.1
44045	0.946132	2.1
44046	0.946132	2.1
44047	0.946132	2.1
44048	0.946132	2.1
44049	0.946132	2.1
44050	0.946132	2.1
44051	0.946132	2.1
44052	0.946133	2.1
44053	0.946133	2.1
44054	0.946133	2.1
44055	0.946133	2.1
44056	0.946132	2.1
44057	0.946132	2.1
44058	1.33078	2.1
44059	1.37409	2.1
44060	1.31565	0.796781
44061	1.37114	2.1
44062	2.68973	2.1
44063	1.89225	1.98306
44064	1.89225	2.1
44065	1.89225	0.929519
44066	1.89225	1.94707
44067	3.7845	1.37909
44068	1.89225	1.78835
44069	1.89225	2.1
44070	1.89225	1.18893
44071	1.89225	2.1
44072	3.7845	2.1
44073	1.37409	2.05481
44074	1.20408	2.1
44075	1.18612	2.1
44076	1.18907	2.1
44077	1.37114	2.06711

44078	1.37409	2.1
44079	1.31566	2.1
44080	1.37115	2.1
44081	1.30318	0.83383
44082	1.37433	2.1
44083	2.68998	1.99611
44084	1.89226	2.1
44085	1.89226	2.1
44086	1.89226	1.0588
44087	1.89226	2.1
44088	3.78453	1.45273
44089	1.89226	1.38543
44090	1.89226	2.1
44091	1.89226	2.1
44092	1.89226	2.1
44093	1.89226	1.2305
44094	1.89226	2.1
44095	0.647001	2.1
44096	0.647001	2.1
44097	0.651851	1.15039
44098	0.651851	2.1
44099	0.602046	2.1
44100	0.613277	2.1
44101	0.685576	1.92989
44102	0.687164	2.1
44103	0.687164	2.1
44104	0.613276	2.1
44105	0.602046	2.1
44106	0.685576	2.1
44107	0.946128	1.0018
44108	0.946128	0.735698
44109	1.15307	2.00329
44110	1.19317	1.18737
44111	0.94613	1.32769
44112	0.94613	0.05
44113	1.15307	1.13725
44114	1.15307	1.6494
44115	0.946131	1.08084
44116	1.15307	1.07032
44117	0.946131	1.0009
44118	1.19317	0.799791
44119	1.18278	2.1
44120	2.38635	1.08664
44121	1.23042	2.1
44122	1.23042	2.09337
44123	2.30614	2.1
44124	2.30614	2.1
44125	0.946129	0.913371
44126	0.946129	0.858432
44127	1.12994	1.13997
44128	1.15307	1.03274
44129	0.946131	1.09389
44130	0.946131	0.05
44131	1.12994	0.944742
44132	1.12994	1.02304
44133	1.12994	1.00725
44134	0.946131	1.0261
44135	0.946131	0.881651

44136	1.15307	0.807922
44137	2.25988	1.42374
44138	2.30614	0.953137
44139	2.28696	2.1
44140	1.16361	2.1
44141	1.18278	2.1
44142	1.18278	2.02309
44143	2.3771	2.1
44144	1.90121	2.1
44145	1.81191	2.1
44146	2.46904	2.1
44147	4.37025	2.1
44148	1.15676	2.1
44149	1.23452	2.1
44150	0.823592	2.1
44151	0.737606	2.1
44152	0.988113	2.1
44153	0.988113	2.1
44154	0.984014	2.1
44155	0.984014	2.1
44156	1.15676	2.1
44157	0.737605	2.1
44158	0.823592	2.1
44159	1.23452	2.1
44160	1.30318	2.1
44161	1.37433	2.1
44162	1.30151	1.22944
44163	1.39067	2.1
44164	2.67584	1.98192
44165	1.89226	1.94542
44166	1.89226	2.1
44167	1.89226	1.22471
44168	1.89226	1.44993
44169	3.78453	1.35411
44170	1.89226	1.11626
44171	1.89226	2.1
44172	1.89226	0.993348
44173	1.89226	2.1
44174	1.89226	1.25746
44175	1.89226	2.1
44176	0.656819	2.1
44177	0.656819	1.21138
44178	0.661755	1.2781
44179	0.661755	2.1
44180	0.613277	2.1
44181	0.63141	2.1
44182	0.687164	1.25762
44183	0.695336	2.1
44184	0.695337	2.1
44185	0.63141	2.1
44186	0.687164	2.1
44187	0.613276	2.1
44188	1.30151	2.09703
44189	1.39067	2.1
44190	1.32143	1.25651
44191	1.43294	2.1
44192	2.73445	2.1
44193	1.89226	2.01628

44194	1.89226	2.1
44195	1.89226	1.08033
44196	1.89226	2.1
44197	3.78452	1.55395
44198	1.89226	1.53377
44199	1.89226	1.37799
44200	1.89226	1.25917
44201	1.89226	2.1
44202	1.89226	2.1
44203	1.89226	2.1
44204	1.43294	1.46264
44205	1.26282	2.1
44206	1.3268	1.27837
44207	1.3268	2.1
44208	1.39067	2.1
44209	1.39067	2.1
44210	1.5511	0.05
44211	1.00665	0.05
44212	1.28239	0.05
44213	1.88631	0.05
44214	2.89296	0.05
44215	1.89227	0.0538215
44216	1.89227	0.05
44217	1.89227	0.132141
44218	1.89227	0.082245
44219	1.89227	0.05
44220	1.89227	0.05
44221	2.08848	0.05
44222	1.89226	0.05
44223	1.89226	0.0537469
44224	2.37947	0.118161
44225	4.27173	0.05
44226	1.01456	0.05
44227	0.943157	0.05
44228	0.641198	0.0574447
44229	0.724757	0.05
44230	0.830923	0.0604306
44231	0.830923	0.05
44232	0.836991	0.0696296
44233	0.836991	0.05
44234	1.01456	0.0615925
44235	0.724757	0.107166
44236	0.641197	0.0657727
44237	0.943157	0.0613599
44238	1.89227	0.0522545
44239	1.89227	0.0851115
44240	1.89227	0.115335
44241	1.89227	0.139692
44242	3.78453	0.0917472
44243	0.946129	0.0743457
44244	0.946129	0.0704502
44245	1.28941	0.10873
44246	1.18974	0.0669309
44247	0.94613	0.0811555
44248	0.94613	0.0538326
44249	1.28941	0.0693337
44250	1.28941	0.05
44251	1.28941	0.069868

44252	0.946131	0.0823965
44253	0.94613	0.05
44254	1.18974	0.05
44255	0.710636	2.1
44256	0.780249	2.1
44257	0.579033	2.1
44258	0.478812	2.1
44259	0.621822	2.1
44260	0.621822	2.1
44261	0.63724	2.1
44262	0.63724	2.1
44263	0.780251	2.1
44264	0.579032	2.1
44265	0.710637	2.1
44266	0.478811	2.1
44267	1.05499	2.1
44268	1.00521	2.1
44269	1.24445	2.02515
44270	1.23082	1.12978
44271	2.28581	2.07719
44272	1.48751	2.1
44273	1.23083	1.61109
44274	1.4941	2.1
44275	1.24445	1.525
44276	1.48047	1.32302
44277	1.23083	1.35596
44278	0.621185	1.85491
44279	0.621185	2.08319
44280	0.620899	1.21994
44281	0.620899	2.1
44282	0.502515	1.53824
44283	0.626672	1.2536
44284	0.615412	1.17794
44285	0.740234	2.1
44286	0.740233	1.1507
44287	0.626672	1.09618
44288	0.615413	1.30938
44289	0.502516	1.46305
44290	1.95844	2.1
44291	2.6562	2.1
44292	1.95844	2.1
44293	2.20883	2.1
44294	2.38225	2.1
44295	2.63264	2.1
44296	1.89226	2.1
44297	1.89226	2.1
44298	1.89226	2.1
44299	1.89226	2.1
44300	3.78453	2.1
44301	1.95844	2.1
44302	2.38225	2.1
44303	1.71082	2.1
44304	2.14647	2.1
44305	4.1049	2.1
44306	0.946131	2.1
44307	0.946131	2.1
44308	0.946131	2.1
44309	0.946131	2.1

44310	0.946131	2.1
44311	0.946131	2.1
44312	0.946131	2.1
44313	0.946131	2.1
44314	0.946131	2.1
44315	0.946131	2.1
44316	0.946131	2.1
44317	0.946131	2.1
44318	2.12597	2.1
44319	1.2295	2.1
44320	1.28014	2.1
44321	2.19723	2.1
44322	3.40611	2.1
44323	2.12597	2.1
44324	2.79242	2.1
44325	2.73375	2.1
44326	2.66249	2.1
44327	2.19723	2.1
44328	2.12597	2.1
44329	1.13716	2.1
44330	1.09861	2.1
44331	0.666692	2.1
44332	0.640071	2.1
44333	0.891617	2.1
44334	0.891617	2.1
44335	0.885614	2.1
44336	0.885614	2.1
44337	1.13716	2.1
44338	0.666692	2.1
44339	0.640071	2.1
44340	1.09861	2.1
44341	1.89226	0.05
44342	1.70303	0.05
44343	1.89226	0.05
44344	2.64598	0.05
44345	1.81091	0.05
44346	2.53222	0.05
44347	2.32422	0.05
44348	1.89226	0.05
44349	2.78336	0.05
44350	2.78336	0.05
44351	1.89226	0.05
44352	1.89226	0.05
44353	0.946132	0.05
44354	0.946132	0.05
44355	0.946131	0.05
44356	0.946131	0.05
44357	0.946132	0.05
44358	0.946132	0.05
44359	0.946131	0.05
44360	0.946131	0.05
44361	0.946131	0.05
44362	0.946131	0.05
44363	0.946131	0.05
44364	0.946131	0.05
44365	0.946131	0.05
44366	0.946131	0.05
44367	0.946132	0.05

44368	0.946132	0.05
44369	0.946132	0.05
44370	0.946132	0.05
44371	0.946131	0.05
44372	0.946131	0.05
44373	0.946131	0.05
44374	0.946131	0.05
44375	0.946131	0.05
44376	0.946131	0.05
44377	2.24323	1.35196
44378	1.89226	2.04924
44379	2.75021	1.52771
44380	2.75021	1.69751
44381	1.89226	0.500136
44382	1.89226	0.961842
44383	2.30908	0.05
44384	2.19815	0.119403
44385	2.1609	0.05
44386	1.83796	0.101166
44387	4.35904	0.05
44388	2.19815	0.05
44389	2.02268	2.1
44390	2.17869	2.1
44391	1.9559	0.0854474
44392	2.0815	2.1
44393	1.83424	0.05
44394	2.1609	0.0689623
44395	1.88877	0.417911
44396	1.98413	0.05
44397	1.87355	0.271893
44398	4.03445	0.05
44399	1.87355	0.05
44400	2.02268	2.1
44401	1.87355	2.1
44402	2.10488	0.0520565
44403	1.68443	2.1
44404	1.89448	0.414568
44405	0.946131	0.05
44406	0.946131	0.05
44407	1.40574	0.05
44408	1.12373	0.05
44409	0.946129	0.05
44410	0.946129	0.05
44411	1.40574	0.05
44412	1.40574	0.05
44413	0.946131	0.05
44414	1.40574	0.05
44415	1.12373	0.05
44416	0.946131	0.05
44417	2.14277	0.05
44418	2.81148	0.0756872
44419	1.42762	0.05
44420	1.42762	0.05
44421	2.96261	0.05
44422	2.24746	0.05
44423	1.89226	0.72643
44424	1.89226	0.785859
44425	1.89226	0.742523

44426	1.89226	0.904975
44427	3.78453	0.837509
44428	1.89226	0.703315
44429	1.89226	0.809101
44430	1.89226	0.77687
44431	1.89226	0.91648
44432	3.78452	0.617014
44433	0.68433	0.672391
44434	0.946127	0.966674
44435	0.543921	0.888919
44436	0.946131	0.824145
44437	0.946131	0.797032
44438	0.946131	2.1
44439	0.543918	0.871655
44440	0.543918	0.96339
44441	0.684332	0.907248
44442	0.54392	2.1
44443	0.946129	0.28586
44444	0.946129	0.928462
44445	1.89226	0.799771
44446	1.08784	0.922586
44447	1.19352	0.352623
44448	1.89226	0.934408
44449	1.19352	2.05568
44450	1.89226	1.73665
44451	0.635711	0.561156
44452	0.946132	0.622594
44453	0.946132	0.853399
44454	0.56474	0.793841
44455	0.750045	0.886054
44456	1.25657	2.1
44457	0.635682	0.853609
44458	0.635682	0.718649
44459	0.891954	0.695626
44460	1.32751	2.03489
44461	0.564766	0.728644
44462	0.564766	0.924042
44463	0.858345	0.814668
44464	0.946128	1.06649
44465	0.635712	0.872587
44466	0.946133	0.813844
44467	0.977198	0.906227
44468	0.944521	2.1
44469	0.635682	1.01568
44470	0.635682	0.51837
44471	1.02924	0.900338
44472	0.705625	2.1
44473	0.851528	0.33248
44474	0.851528	1.62586
44475	2.18903	2.1
44476	2.10182	2.1
44477	2.10182	2.1
44478	1.89226	2.1
44479	1.89226	2.1
44480	1.64983	2.1
44481	1.89226	2.1
44482	1.83867	2.1
44483	1.83867	2.1

44484	1.89164	2.1
44485	2.0815	2.1
44486	2.17638	2.1
44487	2.57957	1.19742
44488	2.3506	2.08425
44489	2.69056	0.969632
44490	2.49439	1.55953
44491	5.04116	1.07779
44492	2.3506	1.00273
44493	1.8911	2.1
44494	2.49439	1.16215
44495	2.06689	2.1
44496	4.41748	2.1
44497	2.69056	1.1844
44498	2.49439	1.79708
44499	2.77227	0.992271
44500	2.63443	1.40055
44501	5.32499	1.73156
44502	2.49439	1.25668
44503	2.06689	2.1
44504	2.63443	0.158966
44505	2.26444	2.1
44506	4.70131	1.93039
44507	2.46327	2.1
44508	2.54347	2.1
44509	2.52869	2.1
44510	2.16534	2.1
44511	2.82661	2.1
44512	2.46327	2.1
44513	0.946131	2.1
44514	0.946131	2.1
44515	1.27174	2.1
44516	1.27174	2.1
44517	1.27174	2.1
44518	0.94613	2.1
44519	0.946128	2.1
44520	1.4133	2.1
44521	0.946129	2.1
44522	0.946129	2.1
44523	1.27174	2.1
44524	1.4133	2.1
44525	1.42757	2.1
44526	1.7457	2.1
44527	1.66047	2.1
44528	1.7457	2.1
44529	1.45754	2.1
44530	1.54278	2.1
44531	1.42757	2.1
44532	1.52996	2.1
44533	1.66047	2.1
44534	1.66047	2.1
44535	1.26397	2.1
44536	1.26397	2.1
44537	0.968536	2.1
44538	1.02461	2.1
44539	1.57575	2.1
44540	1.62391	2.1
44541	2.59245	2.1

44542	0.946131	2.1
44543	0.946131	2.1
44544	0.946132	2.1
44545	0.946132	2.1
44546	0.946132	2.1
44547	0.946132	2.1
44548	0.946132	2.1
44549	0.946132	2.1
44550	0.946132	2.1
44551	0.946132	2.1
44552	0.946132	2.1
44553	0.946132	2.1
44554	2.23765	2.1
44555	1.62391	2.1
44556	2.22154	2.1
44557	1.57575	2.1
44558	2.2697	2.1
44559	1.62391	2.1
44560	1.89226	2.1
44561	1.89226	2.1
44562	1.89226	2.1
44563	1.89226	2.1
44564	1.89226	2.1
44565	1.89226	2.1
44566	1.89226	2.1
44567	1.89226	2.1
44568	1.89226	2.1
44569	1.89226	2.1
44570	1.89226	2.1
44571	1.89226	2.1
44572	2.2697	2.1
44573	1.46129	2.1
44574	2.25873	2.1
44575	1.62391	2.1
44576	2.09611	2.1
44577	1.46129	2.1
44578	1.89226	2.1
44579	2.07088	2.1
44580	1.89226	2.1
44581	2.31884	2.1
44582	4.21111	2.1
44583	1.89226	2.1
44584	2.31884	2.1
44585	2.11713	2.1
44586	1.89226	2.1
44587	2.54371	2.1
44588	2.31884	2.1
44589	2.54371	2.1
44590	1.24041	2.1
44591	2.55208	2.1
44592	2.31884	2.1
44593	1.23712	2.1
44594	1.00388	2.1
44595	2.72769	0.05
44596	2.57507	0.05
44597	1.89226	0.05
44598	1.89226	0.05
44599	4.46733	0.05

44600	1.89226	0.05
44601	2.57506	0.05
44602	1.89226	0.05
44603	2.30076	0.05
44604	1.89226	0.05
44605	2.30076	0.05
44606	1.21171	0.05
44607	1.48589	0.05
44608	2.09166	0.05
44609	1.78381	0.05
44610	3.2697	0.05
44611	2.09166	0.05
44612	1.70302	0.05
44613	1.48589	0.05
44614	1.18027	0.05
44615	2.26571	0.05
44616	1.79969	0.05
44617	1.89226	0.05
44618	1.89226	0.05
44619	1.89226	0.05
44620	1.89226	0.05
44621	1.89226	0.05
44622	1.89226	0.05
44623	0.946131	0.05
44624	0.946131	0.05
44625	0.946128	0.05
44626	0.946128	0.05
44627	0.946129	0.05
44628	0.946129	0.05
44629	0.94613	0.05
44630	0.94613	0.05
44631	0.94613	0.05
44632	0.94613	0.05
44633	0.94613	0.05
44634	0.94613	0.05
44635	1.89226	1.72096
44636	1.89226	1.83722
44637	1.89226	2.1
44638	1.89226	1.55676
44639	1.89226	2.09618
44640	1.89226	1.50394
44641	1.89226	1.71871
44642	1.89226	1.74481
44643	1.89226	2.07135
44644	1.89226	1.50326
44645	1.89226	2.05044
44646	1.89226	1.47033
44647	1.89226	1.53697
44648	1.89226	1.61861
44649	1.89226	1.88987
44650	1.89226	1.35504
44651	1.89226	1.88714
44652	1.89226	1.33282
44653	1.79103	1.00702
44654	0.968249	2.1
44655	1.44807	1.98124
44656	2.17984	0.570784
44657	3.2391	2.1

44658	1.29735	1.3602
44659	1.08992	1.36575
44660	0.945147	2.1
44661	0.724033	2.1
44662	1.00723	2.1
44663	1.00723	1.71571
44664	1.01416	0.643736
44665	1.01416	2.1
44666	1.29735	1.99424
44667	0.94515	2.09527
44668	0.724034	2.0473
44669	1.08992	2.1
44670	1.39214	2.1
44671	1.39214	2.1
44672	0.946129	2.1
44673	0.946129	2.1
44674	1.33728	2.1
44675	1.39214	2.1
44676	0.94613	2.1
44677	0.94613	2.1
44678	0.94613	2.1
44679	1.33728	2.1
44680	0.94613	2.1
44681	1.39214	2.1
44682	2.78428	2.1
44683	2.28373	2.1
44684	2.67456	2.1
44685	2.20011	2.1
44686	4.95829	2.1
44687	1.90165	0.05
44688	2.24206	0.05
44689	1.66137	0.05
44690	2.07108	0.05
44691	3.90343	0.05
44692	1.89224	0.0729498
44693	1.89224	0.05
44694	1.89224	0.05
44695	1.89224	0.05
44696	3.78448	0.05
44697	2.82159	0.05
44698	1.89226	0.05
44699	2.50138	0.0535421
44700	1.89226	0.05
44701	4.39365	0.05
44702	1.66139	0.05
44703	1.86399	0.0555514
44704	1.56005	0.05
44705	1.90324	0.05
44706	3.56463	0.05
44707	0.946133	0.05
44708	0.946133	0.05
44709	0.946132	0.05
44710	0.946132	0.217943
44711	0.946133	0.0705466
44712	0.946133	0.05
44713	0.946132	0.05
44714	0.946132	0.05
44715	0.946133	0.05

44716	0.946133	0.05
44717	0.946132	0.05
44718	0.946132	0.124672
44719	1.5176	0.913862
44720	1.97964	0.525771
44721	2.32889	0.668058
44722	1.91762	0.787146
44723	3.84649	0.777665
44724	2.3376	0.837831
44725	1.97963	0.779947
44726	2.32889	0.879604
44727	2.58303	0.607257
44728	4.66649	0.807532
44729	2.32889	0.554225
44730	2.20248	0.756951
44731	1.91762	0.486052
44732	1.91762	0.241645
44733	2.5615	0.851284
44734	2.5615	0.589143
44735	2.32889	0.861675
44736	2.58303	0.435114
44737	2.71793	0.772898
44738	2.56149	0.758399
44739	5.04681	0.766798
44740	1.89226	0.679421
44741	2.53785	1.43617
44742	1.89226	0.460053
44743	2.60895	0.449537
44744	1.89226	0.686149
44745	2.60895	0.669512
44746	0.493248	0.151552
44747	1.30448	1.00996
44748	1.26893	1.2927
44749	0.653226	1.76711
44750	0.653227	0.856379
44751	0.493247	2.1
44752	1.26893	2.07048
44753	1.30448	0.675573
44754	0.832232	1.99505
44755	0.832233	2.08282
44756	0.929944	0.700853
44757	0.929944	0.785894
44758	1.89226	2.1
44759	1.47937	2.1
44760	1.50143	2.1
44761	1.47937	2.1
44762	1.91432	2.1
44763	1.89226	2.1
44764	1.89226	2.1
44765	1.89226	2.1
44766	1.89226	2.1
44767	1.89226	2.1
44768	1.89226	2.1
44769	1.89226	2.0541
44770	1.33419	2.1
44771	2.4542	2.1
44772	2.59834	2.1
44773	2.59834	2.1

44774	1.25716	2.1
44775	1.25716	2.1
44776	1.70303	2.1
44777	1.50143	2.1
44778	1.60878	2.1
44779	1.50143	2.1
44780	1.93599	2.1
44781	1.89226	2.1
44782	0.946131	1.87765
44783	0.946131	2.1
44784	0.946127	2.1
44785	0.946127	2.1
44786	0.946127	2.1
44787	0.946127	1.14372
44788	0.946131	2.1
44789	0.946131	2.1
44790	0.94613	2.1
44791	0.94613	2.1
44792	0.946129	2.1
44793	0.946129	2.1
44794	1.43912	2.1
44795	2.59834	2.1
44796	2.78093	2.1
44797	2.78093	2.1
44798	1.33419	2.1
44799	1.33419	2.1
44800	1.89226	2.1
44801	2.26322	2.1
44802	2.55682	2.1
44803	2.55682	2.1
44804	1.89226	2.1
44805	1.89226	2.1
44806	2.58032	0.178301
44807	2.28922	0.0790522
44808	1.88424	0.0743913
44809	2.32127	0.0588731
44810	4.46456	0.113288
44811	1.67265	0.206917
44812	2.28922	0.113013
44813	1.88424	0.05
44814	1.11314	0.109887
44815	3.55689	0.0639497
44816	1.43751	0.05
44817	2.32127	0.05
44818	1.92998	0.05
44819	2.37671	0.05
44820	1.43751	0.137126
44821	1.88424	0.123689
44822	2.36729	2.1
44823	1.56714	2.1
44824	1.56714	2.1
44825	1.62408	2.1
44826	2.28373	2.1
44827	2.34067	2.1
44828	0.937268	2.1
44829	0.937268	2.1
44830	0.94636	2.1
44831	0.94636	2.1

44832	0.76004	2.1
44833	0.783572	2.1
44834	1.14187	2.1
44835	1.10006	2.1
44836	1.10006	2.1
44837	0.76004	2.1
44838	0.783572	2.1
44839	1.14187	2.1
44840	1.28753	2.1
44841	1.36838	2.1
44842	1.62617	2.1
44843	1.82857	2.1
44844	3.1161	2.1
44845	1.82856	2.1
44846	1.17996	2.1
44847	1.53417	2.1
44848	1.56618	2.1
44849	1.36838	2.1
44850	1.36838	2.1
44851	1.35065	0.804098
44852	1.42659	0.05
44853	2.26675	0.235046
44854	2.26675	0.219376
44855	1.62137	0.118467
44856	2.53747	0.249083
44857	1.89226	0.05
44858	1.89226	0.05
44859	1.89226	0.05
44860	1.89226	0.05
44861	3.78452	0.05
44862	1.36569	0.05
44863	1.36569	0.05
44864	0.946131	0.05
44865	0.946131	0.05
44866	1.16041	0.05
44867	1.36569	0.05
44868	0.946129	0.05
44869	0.946129	0.05
44870	1.1604	0.05
44871	0.94613	0.05
44872	0.946131	0.05
44873	1.36569	0.05
44874	2.73139	0.05
44875	1.25265	0.05
44876	1.25265	0.05
44877	1.64799	0.05
44878	2.32081	0.05
44879	2.71615	0.05
44880	0.946133	0.05
44881	0.946133	0.05
44882	0.946132	0.05
44883	0.946132	0.05
44884	0.946132	0.05
44885	0.946132	0.05
44886	0.946133	0.05
44887	0.946133	0.05
44888	0.946132	0.05
44889	0.946132	0.05

44890	0.946133	0.05
44891	0.946133	0.05
44892	0.946131	0.05
44893	0.946131	0.05
44894	0.94613	0.05
44895	0.94613	0.05
44896	0.946132	0.05
44897	0.946132	0.05
44898	0.946129	0.05
44899	0.946129	0.05
44900	0.946131	0.05
44901	0.946131	0.05
44902	0.946131	0.05
44903	0.946131	0.05
44904	1.89226	0.144488
44905	2.48503	2.1
44906	2.64586	2.1
44907	2.64586	0.705695
44908	1.89226	2.1
44909	1.89226	0.05
44910	2.48504	2.1
44911	1.99985	2.1
44912	1.83584	2.1
44913	1.83584	2.1
44914	2.64586	2.1
44915	2.64586	2.1
44916	1.89225	0.844096
44917	2.33095	2.1
44918	2.48501	2.1
44919	2.48501	0.822295
44920	1.89225	2.1
44921	1.89225	0.108343
44922	2.33096	2.1
44923	1.83583	2.1
44924	1.68436	2.1
44925	1.68436	2.1
44926	2.48502	2.1
44927	2.48502	2.1
44928	1.14807	2.1
44929	1.76545	2.1
44930	1.21292	2.1
44931	1.83936	2.1
44932	2.97837	2.1
44933	1.89226	2.1
44934	1.89226	2.1
44935	1.89226	2.1
44936	1.89226	2.1
44937	3.78451	2.1
44938	1.89226	2.1
44939	1.89226	1.244
44940	1.89226	2.1
44941	1.89226	2.1
44942	3.78452	2.1
44943	1.76545	2.1
44944	2.12608	2.1
44945	1.83937	2.1
44946	2.22529	2.1
44947	3.99074	2.1

44948	0.780222	2.1
44949	0.780222	2.1
44950	0.784871	2.1
44951	0.784871	2.1
44952	0.95863	2.1
44953	0.919681	2.1
44954	0.636145	2.1
44955	0.606461	2.1
44956	0.636144	2.1
44957	0.958631	2.1
44958	0.60646	2.1
44959	0.919683	2.1
44960	1.89226	2.1
44961	1.89226	2.1
44962	1.89226	2.1
44963	1.89226	2.1
44964	3.78452	2.1
44965	1.89226	2.1
44966	1.89226	2.1
44967	1.89226	2.1
44968	1.89226	1.14126
44969	3.78452	2.1
44970	1.03789	2.1
44971	1.03789	2.1
44972	1.04231	2.1
44973	1.04231	2.1
44974	1.16052	2.1
44975	1.11265	1.25394
44976	0.958634	2.1
44977	0.919685	2.1
44978	0.958634	2.1
44979	1.16052	2.1
44980	0.919685	2.1
44981	1.11265	2.1
44982	1.43837	2.1
44983	2.57085	2.1
44984	1.39332	2.1
44985	2.58228	2.1
44986	3.96417	2.1
44987	1.89226	2.1
44988	1.89226	2.1
44989	1.89226	2.1
44990	1.89226	2.1
44991	1.89226	2.1
44992	1.89226	2.1
44993	1.89226	2.1
44994	1.89226	2.1
44995	1.89226	2.1
44996	1.89226	2.1
44997	3.78452	2.1
44998	2.57085	2.1
44999	1.89226	2.1
45000	2.58229	2.1
45001	1.89226	2.1
45002	4.46312	2.1
45003	1.32368	2.1
45004	2.58228	2.1
45005	2.55352	2.1

45006	2.55352	2.1
45007	1.39332	2.1
45008	1.39332	2.1
45009	1.89226	2.1
45010	1.89226	2.1
45011	1.89226	2.1
45012	1.89226	2.1
45013	1.89226	2.1
45014	1.89226	2.1
45015	1.89226	2.1
45016	1.89226	2.1
45017	1.89226	2.1
45018	1.89226	2.1
45019	1.89226	2.1
45020	1.89226	2.1
45021	2.55353	2.1
45022	1.89226	2.1
45023	2.58228	2.1
45024	1.89226	2.1
45025	1.89226	2.1
45026	2.58228	2.1
45027	1.40574	1.58913
45028	1.05175	1.22948
45029	1.71613	1.68109
45030	1.99961	1.27633
45031	3.12187	1.40504
45032	1.71613	1.66317
45033	2.50733	1.26735
45034	1.99961	1.94142
45035	1.99961	1.63312
45036	2.25746	1.28864
45037	2.25746	1.1947
45038	2.25746	1.17376
45039	1.89526	1.19064
45040	1.71613	1.59741
45041	1.71613	1.02983
45042	1.89526	1.2384
45043	1.27558	1.02888
45044	1.07536	2.1
45045	1.07536	2.1
45046	1.07034	2.1
45047	1.07034	2.1
45048	0.766879	2.1
45049	0.81527	2.1
45050	1.38896	2.1
45051	1.33043	2.1
45052	0.815264	2.1
45053	1.38895	2.1
45054	0.766884	2.1
45055	1.33044	2.1
45056	1.89225	2.1
45057	1.89225	2.1
45058	1.89225	2.1
45059	1.89225	2.1
45060	1.89225	2.1
45061	1.89225	2.1
45062	1.64059	2.1
45063	1.89225	2.1

45064	1.565	2.1
45065	1.565	2.1
45066	1.89225	2.1
45067	1.89225	2.1
45068	1.89226	2.1
45069	1.89226	2.1
45070	1.89226	2.1
45071	1.89226	2.1
45072	1.89226	2.1
45073	1.89226	2.1
45074	1.67906	2.1
45075	1.89226	2.1
45076	1.6406	2.1
45077	1.6406	2.1
45078	1.89226	2.1
45079	1.89226	2.1
45080	2.18106	1.65872
45081	2.20831	2.1
45082	2.64725	1.13915
45083	2.63102	1.41458
45084	4.82831	1.67623
45085	2.18106	1.36222
45086	2.09605	2.1
45087	2.5601	1.50504
45088	2.63102	1.42399
45089	4.74116	2.1
45090	2.64725	1.29118
45091	1.89226	1.42248
45092	1.87603	2.1
45093	2.63102	1.29894
45094	1.89226	1.7845
45095	2.64725	1.10038
45096	0.946132	1.54586
45097	1.28005	1.26164
45098	0.946132	2.1
45099	1.31551	1.15002
45100	1.28005	1.60899
45101	1.28005	2.1
45102	0.946132	1.16381
45103	0.946132	1.46367
45104	0.946132	1.20475
45105	0.946132	1.32057
45106	1.28005	1.64938
45107	1.31551	1.80592
45108	2.0815	2.1
45109	1.59318	2.1
45110	2.0815	2.1
45111	1.40161	2.1
45112	2.49782	2.1
45113	1.68553	2.1
45114	0.819885	2.1
45115	0.935082	2.1
45116	0.867363	2.1
45117	0.867363	2.1
45118	0.715431	2.1
45119	0.902777	2.1
45120	0.738933	2.1
45121	0.980053	2.1

45122	0.936716	2.1
45123	0.917651	2.1
45124	0.775853	2.1
45125	0.74037	2.1
45126	2.78977	2.1
45127	2.38239	2.1
45128	2.78977	2.1
45129	1.96468	2.1
45130	2.64107	2.1
45131	1.80733	2.1
45132	1.80141	2.1
45133	2.28512	2.1
45134	2.28512	2.1
45135	1.8519	2.1
45136	2.64107	2.1
45137	2.20785	2.1
45138	0.946133	0.404816
45139	0.946133	0.216904
45140	0.946132	0.154972
45141	0.946132	0.160054
45142	0.946132	0.160434
45143	0.946132	0.461739
45144	0.946132	0.18632
45145	0.946132	0.130773
45146	0.946132	0.192879
45147	0.946132	0.272113
45148	0.946133	0.159902
45149	0.946133	0.307185
45150	1.89226	0.071287
45151	1.89226	0.05
45152	1.89226	0.05
45153	1.89226	0.05
45154	1.89226	0.05
45155	1.89226	0.0897723
45156	1.89226	0.0686849
45157	1.89226	0.05
45158	1.89226	0.05
45159	1.89226	0.127119
45160	3.78453	0.0535738
45161	1.89226	0.295943
45162	1.89226	0.427743
45163	1.89226	0.0621669
45164	1.89226	0.60943
45165	1.89226	0.331445
45166	1.89226	0.798972
45167	2.12925	0.05
45168	2.51397	0.05
45169	2.22488	0.05
45170	1.86113	0.05
45171	4.3751	0.05
45172	2.82754	0.05
45173	2.51397	0.05
45174	2.22489	0.05
45175	2.47291	0.05
45176	4.98688	0.05
45177	1.89226	0.05
45178	1.89226	0.05
45179	1.89226	0.05

45180	1.89226	0.05
45181	3.78453	0.05
45182	1.89226	0.05
45183	1.89226	0.05
45184	1.89226	0.05
45185	1.89226	0.05
45186	3.78452	0.05
45187	0.90848	0.05
45188	1.11244	0.05
45189	0.930566	0.05
45190	0.73385	0.05
45191	0.921298	0.05
45192	0.921298	0.05
45193	0.924994	0.05
45194	0.924994	0.05
45195	0.908478	0.05
45196	0.733851	0.05
45197	1.11244	0.05
45198	0.930567	0.05
45199	2.22489	0.05
45200	2.0815	0.05
45201	2.0815	0.05
45202	2.30674	0.05
45203	1.99867	0.05
45204	2.33358	0.05
45205	1.89226	0.05
45206	1.89226	0.05
45207	1.89226	0.05
45208	1.89226	0.05
45209	1.89226	0.05
45210	1.89226	0.05
45211	0.946131	0.05
45212	0.946131	0.05
45213	0.946128	0.05
45214	0.946128	0.05
45215	0.946129	0.05
45216	0.946129	0.05
45217	0.94613	0.05
45218	0.94613	0.05
45219	0.94613	0.05
45220	0.94613	0.05
45221	0.94613	0.05
45222	0.94613	0.05
45223	2.28904	2.1
45224	1.89226	1.89059
45225	2.2846	2.08499
45226	2.2846	2.1
45227	1.89226	1.02732
45228	1.89226	2.1
45229	1.89226	1.31345
45230	1.89226	1.45343
45231	1.89226	2.1
45232	1.89226	2.1
45233	1.89226	1.10825
45234	1.89226	2.07395
45235	1.04037	1.1379
45236	1.34066	2.03218
45237	1.34955	1.15632

45238	1.34955	2.1
45239	1.03373	1.29167
45240	1.03373	2.1
45241	0.946131	0.205788
45242	0.946131	2.1
45243	1.14452	2.1
45244	1.14452	2.1
45245	0.946131	1.4822
45246	0.946131	1.31686
45247	1.14296	2.1
45248	1.14452	2.1
45249	1.14296	1.11192
45250	0.946131	2.1
45251	1.14452	2.1
45252	0.946132	2.09955
45253	1.89226	1.20082
45254	1.89226	2.1
45255	1.89226	1.91796
45256	1.89226	2.1
45257	1.89226	0.965315
45258	1.89226	2.1
45259	0.597454	1.17647
45260	0.597454	2.1
45261	0.597972	1.12807
45262	0.597972	2.1
45263	0.675238	2.1
45264	0.674776	1.34441
45265	0.519667	1.27003
45266	0.520188	1.41617
45267	0.519667	1.07897
45268	0.675238	1.15016
45269	0.520188	1.58971
45270	0.674777	1.57498
45271	1.89226	0.0505846
45272	1.82125	0.05
45273	1.89226	0.05
45274	1.84314	0.05
45275	1.89226	0.132797
45276	1.84314	0.0938775
45277	1.89226	0.05
45278	1.84314	0.05
45279	1.89226	0.05
45280	1.65794	0.05
45281	1.89226	0.05
45282	1.65794	0.05
45283	1.348	2.1
45284	1.43485	2.1
45285	1.79929	1.13222
45286	1.75309	1.90343
45287	3.14728	1.90258
45288	1.348	1.41188
45289	1.63892	2.03506
45290	1.59157	2.1
45291	1.18647	2.08046
45292	1.75309	1.48075
45293	1.348	1.91663
45294	1.79929	1.63743
45295	1.75309	2.1

45296	2.18106	1.35377
45297	2.20831	1.36819
45298	3.98034	2.04454
45299	2.18106	1.21041
45300	1.63892	2.1
45301	1.75309	1.23183
45302	1.75309	2.1
45303	2.09605	1.20455
45304	2.09605	2.1
45305	2.66076	0.05
45306	1.70302	0.05
45307	2.41575	0.05
45308	1.70302	0.05
45309	3.08598	0.05
45310	2.3661	0.05
45311	1.71053	0.05
45312	2.41575	0.05
45313	1.20143	0.05
45314	1.20143	0.05
45315	2.2123	0.05
45316	1.66739	0.05
45317	2.25633	0.05
45318	2.68302	0.05
45319	1.71051	0.05
45320	1.71051	0.05
45321	2.74192	0.05
45322	2.1961	0.05
45323	1.32553	1.63401
45324	2.34875	0.216399
45325	2.19139	2.1
45326	2.19139	0.05
45327	1.56172	2.1
45328	1.56172	0.05
45329	1.89226	2.1
45330	1.89226	0.308842
45331	1.89226	2.1
45332	1.89226	1.02463
45333	3.78453	1.93823
45334	2.667 2.1	
45335	1.26097	2.1
45336	1.26097	2.1
45337	1.5683	2.1
45338	2.42949	2.1
45339	2.73682	2.1
45340	1.13213	1.09055
45341	2.19139	0.0559463
45342	2.0815	2.09802
45343	1.77615	0.05
45344	1.37767	2.05807
45345	1.19297	0.05
45346	0.946132	0.928122
45347	0.946132	2.1
45348	0.946131	2.1
45349	0.946131	1.04141
45350	0.946131	1.01373
45351	0.946131	0.05
45352	0.946132	2.1
45353	0.946132	2.1

45354	0.946132	1.717
45355	0.946132	1.82341
45356	0.946131	1.17031
45357	0.946131	1.52551
45358	2.02064	2.1
45359	2.3606	2.1
45360	1.47478	2.1
45361	2.22997	2.1
45362	4.25061	2.1
45363	2.36059	2.1
45364	2.56952	2.1
45365	2.22997	2.1
45366	2.48631	2.1
45367	4.79949	2.1
45368	1.84261	2.1
45369	2.22997	2.1
45370	1.65706	2.1
45371	1.78837	2.1
45372	3.88703	2.1
45373	2.22997	2.1
45374	2.48631	2.1
45375	2.19528	2.1
45376	2.41175	2.1
45377	4.64172	2.1
45378	1.6466	2.1
45379	1.89226	2.1
45380	1.35972	2.1
45381	1.35972	2.1
45382	1.89226	2.1
45383	1.89226	2.1
45384	1.89226	2.1
45385	1.89226	2.1
45386	1.89226	2.1
45387	1.89226	2.1
45388	3.78453	2.1
45389	2.44797	2.1
45390	1.07214	2.1
45391	1.31589	2.1
45392	1.1211	2.1
45393	2.44797	2.1
45394	2.25318	2.1
45395	1.95954	2.1
45396	1.89226	2.1
45397	1.6466	2.1
45398	1.6466	2.1
45399	1.89226	2.1
45400	1.89226	2.1
45401	0.946133	2.1
45402	0.946133	2.1
45403	0.946132	2.1
45404	0.946132	2.1
45405	0.946132	2.1
45406	0.946132	2.1
45407	0.946133	2.1
45408	0.946133	2.1
45409	0.946132	2.1
45410	0.946132	2.1
45411	0.946133	2.1

45412	0.946133	2.1
45413	2.44797	2.1
45414	1.36904	2.1
45415	1.70303	2.1
45416	2.37586	2.1
45417	4.00507	2.1
45418	0.631553	0.05
45419	1.24489	0.05
45420	1.11597	0.05
45421	0.496799	0.05
45422	0.872272	0.05
45423	0.872272	0.05
45424	0.869415	0.05
45425	0.869415	0.05
45426	0.4968	0.05
45427	0.631553	0.05
45428	1.11597	0.05
45429	1.24489	0.05
45430	1.84253	2.1
45431	2.17384	2.1
45432	2.04818	2.1
45433	1.65925	2.1
45434	3.83309	2.1
45435	2.04818	2.1
45436	1.65925	2.1
45437	1.48162	2.1
45438	1.92264	2.1
45439	3.58189	2.1
45440	2.3305	0.608141
45441	1.89226	0.708296
45442	2.52753	0.687749
45443	2.52753	0.711749
45444	1.89226	0.702934
45445	1.89226	0.708654
45446	1.89226	0.707962
45447	1.89226	0.731783
45448	1.89226	0.720075
45449	1.89226	0.731543
45450	1.89226	0.708057
45451	1.89226	0.723454
45452	1.34563	0.705354
45453	2.1769	0.702684
45454	1.94955	0.678782
45455	1.94955	0.718127
45456	1.55063	0.702101
45457	1.55063	0.723331
45458	2.09455	0.163606
45459	1.89226	0.703752
45460	2.3305	0.476954
45461	2.3305	0.570902
45462	1.89226	0.607271
45463	1.89226	0.703197
45464	1.89226	0.702887
45465	1.89226	0.704333
45466	1.89226	0.711412
45467	1.89226	0.705118
45468	1.89226	0.708937
45469	1.89226	0.709112

45470	0.773392	0.05
45471	0.612765	0.704083
45472	0.79504	0.701912
45473	0.575323	0.698751
45474	1.0819	0.239826
45475	1.06106	0.701783
45476	1.04675	0.36065
45477	0.86858	0.708143
45478	0.796873	0.461402
45479	0.557469	0.705891
45480	0.807663	0.704089
45481	2.2024	2.1
45482	2.34106	2.1
45483	2.02634	2.1
45484	2.20125	2.1
45485	4.36739	2.1
45486	2.34106	2.1
45487	2.25195	0.21938
45488	2.20125	2.1
45489	2.13132	0.068349
45490	4.47237	2.1
45491	2.02635	2.1
45492	2.22465	2.1
45493	1.68504	2.1
45494	2.0815	2.1
45495	3.92673	2.1
45496	2.22465	2.1
45497	2.13133	0.353625
45498	2.0815	2.1
45499	2.23579	0.381265
45500	4.44862	2.1
45501	1.89226	1.19064
45502	1.89226	1.47171
45503	1.89226	1.80238
45504	1.89226	1.27199
45505	1.89226	1.56405
45506	1.89226	1.22564
45507	1.93967	0.05
45508	1.70302	0.05
45509	1.21883	0.05
45510	1.70302	0.05
45511	1.19889	0.05
45512	1.70302	0.05
45513	0.946131	0.05
45514	0.946131	0.05
45515	0.946131	0.05
45516	0.946131	0.05
45517	0.94613	0.05
45518	0.94613	0.05
45519	0.946131	0.05
45520	0.946131	0.05
45521	0.946131	0.05
45522	0.946131	0.05
45523	0.946131	0.05
45524	0.946131	0.05
45525	0.883128	0.05
45526	0.883128	0.05
45527	0.827519	0.05

45528	0.82752	0.05
45529	1.16234	0.05
45530	1.19063	0.05
45531	0.631294	0.05
45532	0.548311	0.05
45533	0.631293	0.05
45534	1.16234	0.05
45535	1.19063	0.05
45536	0.54831	0.0588556
45537	1.89226	0.05
45538	1.89226	0.05
45539	1.89226	0.05
45540	1.89226	0.05
45541	3.78453	0.05
45542	2.33328	0.05
45543	1.87991	0.05
45544	1.87991	0.05
45545	2.82938	0.05
45546	1.84457	0.05
45547	2.79403	0.05
45548	2.82937	0.05
45549	2.70881	0.05
45550	1.8799	0.05
45551	1.82739	0.05
45552	4.58874	0.05
45553	1.89226	0.05
45554	1.89226	0.05
45555	1.89226	0.05
45556	1.89226	0.05
45557	3.78452	0.05
45558	1.17897	0.05
45559	1.82739	0.05
45560	1.17897	0.05
45561	1.76199	0.05
45562	1.23885	0.05
45563	1.80989	0.05
45564	0.946131	0.05
45565	0.946131	0.05
45566	0.946131	0.05
45567	0.946131	0.05
45568	0.946129	0.05
45569	0.946129	0.05
45570	0.946133	0.05
45571	0.946133	0.05
45572	0.946131	0.05
45573	0.946131	0.05
45574	0.946131	0.05
45575	0.946131	0.05
45576	0.946126	0.967045
45577	0.946126	2.1
45578	1.24887	2.1
45579	1.24887	2.1
45580	0.946127	2.1
45581	0.946127	1.04519
45582	1.24887	2.1
45583	1.28511	2.1
45584	1.2851	2.1
45585	0.946128	2.1

45586	1.24887	2.1
45587	0.946128	2.1
45588	1.89226	2.1
45589	1.89226	2.09807
45590	1.89226	2.1
45591	1.89226	1.01364
45592	3.78452	1.49956
45593	0.592142	2.1
45594	0.592142	2.1
45595	0.60496	2.1
45596	0.60496	2.1
45597	0.694237	2.1
45598	0.608714	1.25075
45599	0.562863	2.1
45600	0.502864	2.1
45601	0.562863	2.1
45602	0.694237	2.1
45603	0.608714	2.1
45604	0.502864	2.1
45605	1.89226	1.04105
45606	1.89226	2.1
45607	1.89226	2.1
45608	1.89226	2.1
45609	3.78453	2.1
45610	1.55779	2.1
45611	1.57968	2.1
45612	1.47186	2.1
45613	1.51725	2.1
45614	3.05154	2.1
45615	1.57967	2.1
45616	1.474	2.1
45617	1.51725	2.1
45618	1.42895	2.1
45619	3.00863	2.1
45620	1.89226	2.1
45621	1.89226	2.1
45622	1.89226	2.1
45623	1.89226	2.1
45624	3.78452	2.1
45625	1.89226	2.1
45626	1.89226	2.1
45627	1.89226	0.890758
45628	1.89226	2.1
45629	3.78453	2.1
45630	1.47186	2.1
45631	1.51725	2.1
45632	1.38859	2.1
45633	1.45332	2.1
45634	2.92519	2.1
45635	1.51725	2.1
45636	1.42895	2.1
45637	1.45332	2.1
45638	1.38414	2.1
45639	2.88228	2.1
45640	1.89226	2.1
45641	1.89226	2.1
45642	1.89226	2.1
45643	1.89226	2.1

45644	3.78452	2.1
45645	1.34497	0.05
45646	1.36747	0.05
45647	1.18919	0.05
45648	2.08149	0.05
45649	1.23556	0.05
45650	2.13702	0.05
45651	1.10106	0.05
45652	2.08001	0.05
45653	1.34497	0.05
45654	2.31605	0.05
45655	1.34497	0.05
45656	2.31605	0.05
45657	2.31605	0.05
45658	2.0815	0.05
45659	1.9419	0.05
45660	2.46665	0.05
45661	4.52922	0.05
45662	1.20349	0.05
45663	2.08149	0.05
45664	1.36948	0.05
45665	1.34737	0.05
45666	2.08149	0.05
45667	2.08149	0.05
45668	0.946132	0.05
45669	0.946132	0.05
45670	1.37715	0.05
45671	0.823199	0.05
45672	1.21598	0.05
45673	0.946132	0.05
45674	1.37715	0.05
45675	0.946132	0.05
45676	1.21598	0.05
45677	0.823198	0.05
45678	0.946133	0.05
45679	0.946133	0.05
45680	1.89226	0.05
45681	1.89226	0.05
45682	1.89226	0.05
45683	1.89226	0.05
45684	3.78453	0.05
45685	1.89227	0.05
45686	1.89227	0.05
45687	1.89227	0.05
45688	1.89227	0.05
45689	3.78453	0.05
45690	0.946132	0.05
45691	0.946132	0.05
45692	1.21599	0.05
45693	1.21599	0.05
45694	0.966749	0.05
45695	0.946133	0.05
45696	1.21598	0.05
45697	0.946132	0.05
45698	0.966748	0.05
45699	1.21598	0.05
45700	0.946133	0.05
45701	0.946133	0.05

45702	0.946133	0.05
45703	0.946133	0.05
45704	1.1134	0.05
45705	1.11555	0.05
45706	1.11555	0.05
45707	0.946132	0.05
45708	0.946132	0.05
45709	1.1134	0.05
45710	1.11555	0.05
45711	1.11555	0.05
45712	0.946132	0.05
45713	0.946132	0.05
45714	0.946133	0.05
45715	0.946133	0.05
45716	1.11555	0.05
45717	0.978467	0.05
45718	0.978468	0.05
45719	0.946133	0.05
45720	1.11555	0.05
45721	0.946132	0.05
45722	0.978468	0.05
45723	0.978468	0.05
45724	0.946132	0.05
45725	0.946132	0.05
45726	0.851542	2.1
45727	0.851542	2.1
45728	1.14168	2.1
45729	1.27897	2.1
45730	1.02817	2.1
45731	1.13536	2.1
45732	0.946131	2.1
45733	0.861207	2.1
45734	0.94613	2.1
45735	0.94613	2.1
45736	0.868751	2.1
45737	1.15181	2.1
45738	0.946132	2.08253
45739	0.946132	1.99571
45740	0.946128	2.06222
45741	0.946128	1.23627
45742	0.94613	2.1
45743	0.94613	2.05228
45744	0.946131	1.75286
45745	0.946131	1.19167
45746	0.94613	1.12366
45747	0.94613	2.0405
45748	0.94613	1.48352
45749	0.94613	2.1
45750	2.53273	0.05
45751	1.89226	0.05
45752	1.89226	0.05
45753	1.89226	0.05
45754	1.89226	0.05
45755	1.89226	0.05
45756	1.89226	2.1
45757	1.89226	2.1
45758	1.89226	2.1
45759	1.89226	2.1

45760	3.78452	2.1
45761	1.89226	2.1
45762	2.54394	2.1
45763	1.89226	2.1
45764	2.42221	2.1
45765	4.43621	2.1
45766	2.54394	2.1
45767	1.62743	2.1
45768	2.42221	2.1
45769	1.5437	2.1
45770	4.08764	2.1
45771	1.89226	2.1
45772	1.89226	2.1
45773	1.89226	2.1
45774	1.89226	2.1
45775	3.78452	2.1
45776	1.89226	2.1
45777	1.89226	2.1
45778	1.89226	2.1
45779	1.89226	2.1
45780	3.78452	2.1
45781	1.89226	2.1
45782	2.30146	2.1
45783	2.42221	2.1
45784	1.89226	2.1
45785	1.89226	2.1
45786	2.42221	2.1
45787	2.30146	2.1
45788	2.42221	2.1
45789	1.39962	2.1
45790	1.39962	2.1
45791	1.5437	2.1
45792	2.44553	2.1
45793	1.89226	2.1
45794	1.89226	2.1
45795	1.89226	2.1
45796	1.89226	2.1
45797	3.78452	2.1
45798	1.62196	2.1
45799	2.00695	2.1
45800	1.95288	2.1
45801	1.70303	2.1
45802	3.53316	2.1
45803	1.80703	2.1
45804	2.00695	2.1
45805	1.92991	2.1
45806	2.00695	2.1
45807	1.85888	2.1
45808	1.93592	2.1
45809	1.89226	2.1
45810	1.89226	2.1
45811	1.89226	2.1
45812	1.89226	2.1
45813	1.89226	2.1
45814	1.89226	2.1
45815	1.89226	2.1
45816	1.89226	2.1
45817	1.89226	2.1

45818	1.89226	2.1
45819	3.78453	2.1
45820	1.95288	2.1
45821	1.50524	2.1
45822	1.83564	2.1
45823	1.70303	2.1
45824	2.00749	2.1
45825	1.84671	2.1
45826	1.62632	2.1
45827	1.82498	2.1
45828	1.92991	2.1
45829	1.92991	2.1
45830	1.47928	2.1
45831	1.47751	2.1
45832	0.946128	2.1
45833	0.946128	2.1
45834	0.946132	2.1
45835	0.946132	2.1
45836	0.94613	2.1
45837	0.94613	2.1
45838	0.946129	2.1
45839	0.946129	2.1
45840	0.94613	2.1
45841	0.94613	2.1
45842	0.94613	2.1
45843	0.94613	2.1
45844	1.89226	2.1
45845	1.89226	2.1
45846	1.89226	2.1
45847	1.89226	2.1
45848	1.89226	2.1
45849	1.89226	2.1
45850	2.27351	0.888025
45851	2.74068	0.143113
45852	2.72278	0.352233
45853	2.63356	0.642697
45854	2.36273	0.305749
45855	2.27351	0.599439
45856	2.36273	0.62992
45857	2.83112	0.128657
45858	2.81212	0.244848
45859	2.74068	0.436324
45860	2.43416	0.229412
45861	2.36273	0.433079
45862	1.89227	0.403553
45863	2.83112	0.113089
45864	2.74069	0.211276
45865	2.74069	0.0966737
45866	1.89227	0.668378
45867	1.89227	0.33109
45868	2.40887	2.1
45869	1.4494	2.1
45870	1.52164	2.1
45871	2.47534	2.1
45872	3.93051	2.1
45873	1.89226	2.1
45874	2.47534	2.1
45875	2.40887	2.1

45876	2.40887	2.1
45877	1.89226	2.1
45878	1.89226	2.1
45879	1.60861	2.1
45880	2.47534	2.1
45881	1.52164	2.1
45882	1.52164	2.1
45883	2.55301	2.1
45884	2.55301	2.1
45885	0.946128	2.1
45886	0.946128	2.1
45887	1.23767	2.1
45888	1.2765	2.1
45889	1.23767	2.1
45890	1.23767	2.1
45891	0.946131	2.1
45892	0.946131	2.1
45893	0.946131	2.1
45894	1.2765	2.1
45895	1.23767	2.1
45896	0.946131	2.1
45897	1.89226	2.1
45898	1.87511	2.1
45899	1.87511	2.1
45900	1.95852	2.1
45901	2.0815	2.1
45902	2.22169	2.1
45903	1.89226	2.1
45904	1.89226	2.1
45905	1.89226	2.1
45906	1.89226	2.1
45907	1.89226	2.1
45908	1.89226	2.1
45909	2.0815	2.1
45910	1.80067	2.1
45911	1.80067	2.1
45912	2.05518	2.1
45913	1.89226	2.1
45914	2.16509	2.1
45915	1.87153	2.1
45916	2.65897	2.1
45917	1.58724	2.1
45918	2.38924	2.1
45919	4.26077	2.1
45920	1.89226	2.1
45921	1.89226	2.1
45922	1.89226	2.1
45923	1.89226	2.1
45924	1.89226	2.1
45925	1.89226	2.1
45926	1.89226	0.927437
45927	2.21022	0.185009
45928	2.19769	0.979214
45929	2.21022	0.733091
45930	1.87973	0.280016
45931	1.89226	0.0907485
45932	1.18376	1.25158
45933	2.21022	0.0527794

45934	1.16239	1.16911
45935	1.16239	0.0957788
45936	2.19769	1.17688
45937	2.19769	0.102363
45938	0.946128	0.581704
45939	0.946128	0.251468
45940	1.09362	0.891965
45941	1.09884	0.314999
45942	0.94613	0.632733
45943	0.94613	0.05
45944	1.09362	0.87816
45945	1.09362	0.180454
45946	0.946131	0.697961
45947	1.09362	0.679826
45948	0.946131	0.290339
45949	1.09884	0.068372
45950	1.19323	1.09579
45951	2.19769	0.05
45952	1.18376	0.996094
45953	1.18376	0.100677
45954	2.18724	1.04155
45955	2.18724	0.201419
45956	2.0815	2.1
45957	0.922714	2.1
45958	2.0815	2.1
45959	1.84882	2.1
45960	1.20787	2.1
45961	1.08321	2.1
45962	2.6238	0.05
45963	1.89226	0.05
45964	1.89226	0.05
45965	2.4866	0.05
45966	1.89226	0.05
45967	2.4866	0.05
45968	2.66753	0.05
45969	1.89226	0.0590309
45970	1.89226	0.05
45971	2.6238	0.05
45972	1.89226	0.05
45973	2.6238	0.05
45974	0.946129	2.1
45975	0.946129	2.1
45976	1.04676	2.1
45977	1.04676	2.1
45978	0.946131	2.1
45979	0.946131	2.1
45980	1.08732	2.1
45981	1.04676	2.1
45982	1.08732	2.1
45983	0.946131	2.1
45984	1.04676	2.1
45985	0.946131	2.1
45986	1.20958	2.02216
45987	1.73802	0.87484
45988	1.39067	1.04335
45989	1.39067	0.829073
45990	1.50969	1.06452
45991	1.50969	1.05962

45992	1.00382	1.2262
45993	1.50969	0.0659074
45994	1.20958	1.12115
45995	1.20958	0.396039
45996	1.32039	1.23627
45997	1.32039	0.884714
45998	2.0815	2.1
45999	2.51396	2.1
46000	2.19826	2.1
46001	2.54175	2.1
46002	4.79599	2.1
46003	2.54174	2.1
46004	2.17978	2.1
46005	2.54174	2.1
46006	2.2854	2.1
46007	2.57212	2.1
46008	2.31578	2.1
46009	2.27103	2.1
46010	2.54175	2.1
46011	2.4255	2.1
46012	2.82251	2.1
46013	4.96725	2.1
46014	2.60614	0.05
46015	2.75374	0.05
46016	1.94002	0.05
46017	2.08392	0.05
46018	4.69006	0.05
46019	2.75373	0.05
46020	2.6875	0.05
46021	2.08392	0.05
46022	1.99944	0.05
46023	4.77142	0.05
46024	1.1211	0.05
46025	2.08392	0.05
46026	1.17787	0.05
46027	1.94002	0.05
46028	1.32178	0.05
46029	2.08392	0.05
46030	2.08392	0.05
46031	1.99944	0.05
46032	1.32178	0.05
46033	1.25929	0.05
46034	3.34321	0.05
46035	1.22266	0.05
46036	0.779473	0.05
46037	0.94613	0.05
46038	0.94613	0.05
46039	1.04072	0.05
46040	1.1407	0.05
46041	1.10042	0.05
46042	0.851542	0.05
46043	0.942046	0.05
46044	0.942046	0.05
46045	1.04114	0.05
46046	0.826602	0.05
46047	2.37468	2.1
46048	1.89226	2.1
46049	1.89226	2.1

46050	1.89226	2.1
46051	2.27917	2.1
46052	2.27917	2.1
46053	1.13959	2.1
46054	1.09451	2.1
46055	0.946131	2.1
46056	0.946131	2.1
46057	1.09451	2.1
46058	1.09451	2.1
46059	0.94613	2.1
46060	0.94613	2.1
46061	1.09451	2.1
46062	0.946131	2.1
46063	0.946131	2.1
46064	1.13959	2.1
46065	2.52454	1.11018
46066	2.12221	2.1
46067	2.67624	1.24009
46068	2.33865	2.1
46069	4.86319	2.1
46070	1.89226	2.07569
46071	1.89226	2.1
46072	1.89226	1.8527
46073	1.89226	2.1
46074	3.78452	2.1
46075	1.89226	2.1
46076	1.89226	2.1
46077	1.89226	2.1
46078	1.89226	2.1
46079	3.78452	2.1
46080	2.12221	2.1
46081	1.47769	2.1
46082	2.33865	2.1
46083	1.62898	2.1
46084	3.81634	2.1
46085	2.67624	2.1
46086	2.37734	1.71771
46087	2.33865	2.1
46088	2.33865	2.1
46089	2.70125	2.1
46090	2.70125	2.1
46091	1.89226	2.1
46092	1.89226	2.1
46093	1.89226	2.1
46094	1.89226	2.1
46095	1.89226	1.16077
46096	1.89226	1.22956
46097	1.89226	2.1
46098	1.89226	2.1
46099	1.89226	2.1
46100	1.89226	2.1
46101	1.89226	2.1
46102	1.89226	2.1
46103	2.37733	2.1
46104	1.95927	2.1
46105	1.82516	2.0622
46106	1.82516	2.1
46107	2.33865	2.1

46108	2.33865	2.1
46109	2.57466	2.1
46110	2.21715	2.1
46111	2.55653	2.1
46112	2.24775	2.1
46113	4.77367	2.1
46114	0.937095	2.1
46115	0.937095	2.1
46116	0.958028	2.1
46117	0.958028	2.1
46118	0.828386	2.1
46119	0.771244	2.1
46120	1.10858	2.1
46121	1.12388	2.1
46122	1.12388	2.1
46123	0.828386	2.1
46124	0.771244	2.1
46125	1.10858	2.1
46126	2.55653	2.1
46127	2.32606	2.1
46128	2.24775	2.1
46129	2.24775	2.1
46130	2.57928	2.1
46131	2.57928	2.1
46132	0.990481	2.1
46133	0.990481	2.1
46134	1.00094	2.1
46135	1.00094	2.1
46136	0.888385	2.1
46137	0.828386	2.1
46138	1.16303	2.1
46139	1.12388	2.1
46140	1.16303	2.1
46141	0.888385	2.1
46142	1.12388	2.1
46143	0.828387	2.1
46144	0.962524	2.1
46145	0.962524	2.1
46146	0.7976	2.1
46147	0.7976	2.1
46148	0.797628	2.1
46149	0.814	2.1
46150	0.946125	2.1
46151	0.946125	2.1
46152	0.946118	2.1
46153	0.797623	2.1
46154	0.814006	2.1
46155	0.946131	2.1
46156	1.8978	0.05
46157	2.23892	0.05
46158	1.71469	0.05
46159	4.13672	0.05
46160	2.64136	0.05
46161	2.23892	0.643879
46162	2.37578	0.850693
46163	1.77489	2.1
46164	4.6147	0.885532
46165	1.02284	2.1

46166	0.898796	0.873601
46167	0.948554	2.1
46168	0.948554	2.1
46169	0.878797	2.1
46170	0.796197	2.1
46171	1.09145	1.59922
46172	0.963109	1.10472
46173	1.12114	2.1
46174	0.782739	2.1
46175	1.11084	2.1
46176	0.850742	2.1
46177	2.48024	2.1
46178	2.405 2.1	
46179	2.24585	2.1
46180	2.34795	2.1
46181	4.75295	2.1
46182	1.93794	2.1
46183	2.405 2.1	
46184	2.24585	2.1
46185	1.67594	2.1
46186	4.08094	2.1
46187	1.89226	2.1
46188	1.89226	0.299564
46189	1.89226	2.1
46190	1.89226	2.00168
46191	3.78452	2.1
46192	1.89226	2.1
46193	1.89226	2.1
46194	1.89226	2.1
46195	1.89226	2.1
46196	3.78452	2.1
46197	2.24585	2.1
46198	2.34795	2.1
46199	2.21155	2.1
46200	2.06626	2.1
46201	4.4142	2.1
46202	2.24585	2.1
46203	1.41498	2.1
46204	1.41498	2.1
46205	1.67594	2.1
46206	2.06625	2.1
46207	2.32721	2.1
46208	1.89226	2.1
46209	1.89226	1.85245
46210	1.89226	2.1
46211	1.89226	1.07224
46212	1.89226	2.1
46213	1.89226	0.986716
46214	1.89226	2.1
46215	1.89226	2.09753
46216	1.89226	2.1
46217	1.89226	2.1
46218	3.78452	2.1
46219	1.89226	0.05
46220	1.89226	0.05
46221	1.89226	0.05
46222	1.89226	0.05
46223	3.78452	0.05

46224	1.89226	0.05
46225	2.53655	0.05
46226	1.89226	0.05
46227	1.89226	0.05
46228	1.89226	0.05
46229	1.89226	0.05
46230	1.89227	2.1
46231	1.89227	2.1
46232	1.89227	2.1
46233	1.89227	2.1
46234	1.89227	2.1
46235	1.89227	2.1
46236	0.91339	2.1
46237	0.91339	2.1
46238	1.2158	2.1
46239	1.20629	2.1
46240	1.00473	2.1
46241	1.15749	2.1
46242	0.946132	2.1
46243	0.946132	2.1
46244	0.946133	2.1
46245	1.17513	2.1
46246	1.00473	2.1
46247	0.946132	2.1
46248	1.89226	2.1
46249	1.89226	2.1
46250	1.89226	2.1
46251	1.89226	2.1
46252	1.89226	2.1
46253	1.89226	2.1
46254	1.89226	2.1
46255	2.6066	2.1
46256	2.35026	2.1
46257	2.35026	2.1
46258	1.89226	2.1
46259	1.89226	2.1
46260	2.04056	0.05
46261	2.77366	0.0934651
46262	1.24682	0.05
46263	1.24682	0.0563703
46264	2.93651	0.05
46265	2.14277	0.05
46266	2.68524	1.84659
46267	2.81847	1.6275
46268	1.89226	1.17387
46269	1.89226	1.62648
46270	4.57751	1.26894
46271	2.68524	1.43472
46272	1.89226	2.0065
46273	1.89226	1.98402
46274	2.50732	1.4639
46275	1.89226	1.74402
46276	2.50732	1.91048
46277	1.89226	1.23892
46278	1.89226	1.97334
46279	1.89226	1.48373
46280	1.89226	1.99624
46281	1.89226	1.23335

46282	1.89226	1.89454
46283	1.89226	1.99635
46284	1.89226	1.41956
46285	1.89226	1.60385
46286	1.89226	2.1
46287	1.89226	1.15347
46288	1.89226	1.2278
46289	1.61016	1.27953
46290	1.07467	1.55054
46291	1.24474	1.18416
46292	1.24474	2.03201
46293	1.45303	1.27506
46294	1.45303	2.04613
46295	1.61016	1.62751
46296	1.24474	2.1
46297	1.61016	1.7287
46298	1.77186	1.34366
46299	1.37813	1.29333
46300	1.37813	1.69555
46301	1.73305	2.1
46302	2.0815	2.1
46303	1.97658	2.1
46304	1.75791	2.1
46305	1.87228	2.1
46306	1.66515	2.1
46307	2.0815	2.1
46308	1.93938	0.221796
46309	2.03253	0.0963762
46310	2.2142	2.1
46311	1.97658	0.171197
46312	2.15324	2.1
46313	1.73305	2.1
46314	1.97658	2.1
46315	1.60428	2.1
46316	1.8589	2.1
46317	3.58085	2.1
46318	1.97657	2.1
46319	1.93938	0.456973
46320	1.51316	2.1
46321	1.85293	0.111787
46322	3.82951	2.1
46323	1.70303	0.05
46324	1.96087	0.0960654
46325	1.42308	0.0684064
46326	1.96087	0.116539
46327	1.25023	0.05
46328	1.70303	0.071238
46329	0.946132	0.166591
46330	0.946132	0.0661684
46331	0.946131	0.0603666
46332	0.946131	0.109102
46333	0.946132	0.114596
46334	0.946132	0.115997
46335	0.946132	0.0576442
46336	0.946132	0.063722
46337	0.946132	0.0884095
46338	0.946132	0.0808762
46339	0.946132	0.100879

46340	0.946132	0.104765
46341	1.89226	0.256914
46342	1.89226	0.583052
46343	1.89226	0.469481
46344	1.89226	0.527571
46345	3.78452	0.38825
46346	1.89226	0.0956321
46347	1.89226	0.203881
46348	1.89226	0.17769
46349	1.89226	0.432658
46350	1.89226	0.0742395
46351	1.89226	0.136985
46352	1.89226	0.165245
46353	1.89226	0.189828
46354	1.89226	0.456862
46355	1.89226	0.182688
46356	1.89226	0.22786
46357	1.89226	0.109655
46358	1.89226	0.05
46359	1.89226	0.138109
46360	2.48255	0.0735336
46361	1.89226	0.0604659
46362	3.78452	0.0510804
46363	1.89226	2.1
46364	2.42758	2.1
46365	1.9209	2.1
46366	1.89226	2.1
46367	2.45622	2.1
46368	2.42758	2.1
46369	2.42758	2.1
46370	1.49987	2.1
46371	1.61028	2.1
46372	1.58164	2.1
46373	2.45622	2.1
46374	2.42758	2.1
46375	0.94613	2.1
46376	0.94613	2.1
46377	1.24298	2.1
46378	1.24298	2.1
46379	1.24298	2.1
46380	1.22811	2.1
46381	0.946131	2.1
46382	0.946131	2.1
46383	0.94613	2.1
46384	1.24298	2.1
46385	0.94613	2.1
46386	1.22811	2.1
46387	2.48596	2.1
46388	2.45622	2.1
46389	1.63823	2.1
46390	1.4304	2.1
46391	1.45739	2.1
46392	2.45622	2.1
46393	2.4169	2.1
46394	2.55249	2.1
46395	2.51125	2.1
46396	2.35245	2.1
46397	4.90494	2.1

46398	2.45922	2.1
46399	2.55249	2.1
46400	2.51126	2.1
46401	2.43226	2.1
46402	4.98474	2.1
46403	2.51125	2.1
46404	2.35245	2.1
46405	2.28979	2.1
46406	2.47228	2.1
46407	4.82473	2.1
46408	2.51126	2.1
46409	2.43226	2.1
46410	2.40668	2.1
46411	2.47228	2.1
46412	4.90453	2.1
46413	2.8045	2.1
46414	1.93707	2.1
46415	2.14087	2.1
46416	2.14087	2.1
46417	2.61595	2.1
46418	2.61595	2.1
46419	1.89226	2.1
46420	1.89226	2.1
46421	1.89226	2.1
46422	1.89226	2.1
46423	1.89226	2.1
46424	1.89226	1.21645
46425	1.22368	2.1
46426	1.32339	2.1
46427	0.957125	2.1
46428	0.750074	2.1
46429	1.08438	2.1
46430	1.08438	2.1
46431	1.13094	2.1
46432	0.970797	2.1
46433	1.17568	2.1
46434	0.764392	2.1
46435	0.944286	2.1
46436	1.27523	2.1
46437	1.89226	2.1
46438	1.89226	2.1
46439	1.89226	2.1
46440	1.89226	2.1
46441	3.78453	2.1
46442	2.8239	2.1
46443	1.89227	2.1
46444	2.57276	2.1
46445	2.57276	2.1
46446	1.89227	2.1
46447	1.89227	2.1
46448	1.18139	2.1
46449	1.85195	2.1
46450	1.78595	2.1
46451	1.08172	2.1
46452	2.93367	2.1
46453	1.60735	2.1
46454	1.20356	2.1
46455	0.967034	2.1

46456	1.08172	2.1
46457	1.67324	2.1
46458	1.77293	2.1
46459	1.89226	2.1
46460	1.89226	2.1
46461	1.8213	2.1
46462	1.82129	2.1
46463	2.14171	2.1
46464	2.40192	2.1
46465	0.810943	2.1
46466	0.810943	2.1
46467	0.798469	2.1
46468	0.788336	2.1
46469	0.804015	2.1
46470	0.810945	2.1
46471	1.947	2.1
46472	1.36506	2.1
46473	1.08686	2.1
46474	1.07208	2.1
46475	1.08132	2.1
46476	1.13537	0.05
46477	1.14754	1.91021
46478	0.946131	0.194632
46479	0.946131	2.1
46480	0.94613	0.0920768
46481	0.94613	0.05
46482	1.25104	2.1
46483	1.14754	1.15095
46484	1.13537	0.541799
46485	0.946131	0.447614
46486	0.946131	1.05787
46487	1.25104	0.812902
46488	0.96542	0.05
46489	0.922284	1.04273
46490	0.946131	2.1
46491	0.946131	0.633868
46492	0.946131	0.383986
46493	0.946131	0.05
46494	1.13537	2.1
46495	0.924824	1.20912
46496	0.859476	0.362081
46497	0.842305	0.545003
46498	1.25996	0.879251
46499	1.04996	0.643128
46500	1.71007	0.05
46501	2.73283	0.05
46502	2.73283	0.05
46503	1.89226	0.05
46504	1.89226	0.05
46505	1.13262	0.05
46506	0.946131	0.05
46507	0.946131	0.05
46508	0.946131	0.05
46509	0.946131	0.05
46510	0.946132	0.05
46511	0.946132	0.05
46512	0.946131	0.05
46513	0.946131	0.05

46514	0.946131	0.05
46515	0.946131	0.05
46516	0.946132	0.05
46517	0.946132	0.05
46518	0.946131	0.05
46519	1.36642	0.05
46520	0.946125	0.05
46521	0.946125	0.05
46522	0.946128	0.05
46523	0.946128	0.05
46524	0.946126	0.05
46525	0.946128	0.05
46526	0.946128	0.05
46527	0.946128	0.05
46528	1.36641	0.05
46529	0.946128	0.05
46530	1.89226	0.05
46531	1.89226	0.05
46532	1.89226	0.05
46533	1.89226	0.05
46534	1.89226	0.05
46535	1.89226	0.05
46536	2.69784	2.1
46537	2.51651	2.1
46538	2.56999	2.1
46539	2.74623	2.1
46540	5.26783	2.1
46541	2.69784	2.1
46542	2.48718	2.1
46543	2.50161	2.1
46544	2.74623	2.1
46545	5.19945	2.1
46546	1.89226	2.1
46547	1.89226	2.1
46548	1.89226	2.1
46549	1.89226	2.1
46550	3.78453	2.1
46551	0.874264	2.1
46552	1.09879	2.1
46553	0.946131	2.1
46554	0.946131	2.1
46555	1.17062	2.1
46556	1.17062	2.1
46557	0.874293	2.1
46558	0.874293	2.1
46559	1.09879	2.1
46560	0.94613	2.1
46561	0.946131	2.1
46562	0.874265	2.1
46563	1.89226	2.1
46564	1.89226	2.1
46565	1.89226	2.1
46566	1.89226	2.1
46567	3.78453	2.1
46568	0.977382	2.1
46569	1.16424	2.1
46570	0.946133	2.1
46571	0.946133	2.1

46572	1.33012	2.1
46573	1.25589	2.1
46574	0.888498	2.1
46575	0.888498	2.1
46576	1.25375	2.1
46577	0.946131	2.1
46578	0.946131	2.1
46579	0.977384	2.1
46580	1.89226	2.1
46581	2.19757	2.1
46582	2.62906	2.1
46583	1.89226	2.1
46584	1.89226	2.1
46585	2.62906	2.1
46586	1.27944	1.03608
46587	1.27731	2.1
46588	0.615701	2.1
46589	0.615701	1.37766
46590	0.615673	0.05
46591	0.946133	2.03893
46592	0.948264	0.783806
46593	0.946132	0.98969
46594	0.94752	0.798484
46595	0.61706	0.361833
46596	0.946132	0.85293
46597	0.946132	0.703273
46598	2.76604	2.1
46599	1.66728	2.1
46600	1.66728	2.1
46601	1.83036	2.1
46602	2.54515	2.1
46603	2.70823	2.1
46604	2.54515	2.1
46605	1.49958	2.1
46606	1.49958	2.1
46607	1.66728	2.1
46608	2.34465	2.1
46609	2.51235	2.1
46610	1.21128	2.1
46611	1.21128	2.1
46612	0.946131	2.1
46613	0.946131	2.1
46614	0.946131	2.1
46615	0.946131	2.1
46616	1.31401	2.1
46617	1.21128	2.1
46618	1.21128	2.1
46619	0.946131	2.1
46620	0.946131	2.1
46621	1.31401	2.1
46622	1.67374	2.1
46623	2.29586	2.1
46624	1.50033	2.1
46625	2.16598	2.1
46626	3.83972	2.1
46627	1.89226	2.1
46628	1.89226	2.1
46629	1.89226	2.1

46630	1.89226	2.1
46631	3.78452	2.1
46632	1.13537	2.1
46633	1.02188	2.1
46634	0.946132	2.1
46635	0.946132	2.1
46636	0.946132	2.1
46637	0.946132	2.1
46638	1.21128	2.1
46639	1.02188	2.1
46640	1.04672	2.1
46641	1.04672	2.1
46642	1.21128	2.1
46643	0.946132	2.1
46644	0.815411	2.1
46645	0.870464	2.1
46646	0.885437	2.1
46647	0.885437	2.1
46648	1.06395	2.1
46649	1.00483	2.1
46650	0.757125	2.1
46651	0.673956	2.1
46652	0.684461	2.1
46653	1.06979	2.1
46654	0.731403	2.1
46655	1.0559	2.1
46656	1.89226	2.1
46657	1.89226	2.1
46658	1.89226	2.1
46659	1.89226	2.1
46660	1.89226	2.1
46661	1.89226	2.1
46662	1.76897	1.40083
46663	1.76932	1.53034
46664	1.97455	2.1
46665	1.94302	0.276436
46666	3.71233	0.84964
46667	1.83347	2.1
46668	1.76932	1.90983
46669	1.97455	2.1
46670	1.76932	2.1
46671	1.76522	2.1
46672	1.55999	2.1
46673	1.89227	1.87182
46674	1.89227	2.1
46675	1.89227	2.1
46676	1.89227	1.22072
46677	3.78453	1.46084
46678	1.89226	1.4733
46679	1.89226	0.05
46680	1.89226	0.108061
46681	1.89226	0.540881
46682	3.78452	0.548617
46683	1.97452	2.1
46684	1.94299	0.111636
46685	2.17831	0.627714
46686	2.23195	0.74947
46687	4.17495	1.12899

46688	2.23196	1.30124
46689	2.1499	2.1
46690	1.97452	2.1
46691	1.97452	2.1
46692	2.09088	2.1
46693	1.83345	2.1
46694	0.94612	2.1
46695	0.94612	2.1
46696	0.946119	0.393226
46697	0.946119	0.593295
46698	0.946119	0.721851
46699	0.946119	2.04402
46700	0.94612	1.28105
46701	0.94612	2.1
46702	0.946132	1.34479
46703	0.946132	0.895576
46704	0.946107	1.65266
46705	0.946107	1.53867
46706	1.89224	0.0584346
46707	1.89224	0.277555
46708	1.89224	0.324866
46709	1.89224	0.0685095
46710	3.78447	0.05
46711	1.70302	0.218058
46712	1.056	0.31856
46713	1.21829	0.603081
46714	1.89906	0.230865
46715	3.07892	0.210245
46716	2.20248	0.171767
46717	1.56498	0.320442
46718	1.46539	0.226296
46719	1.48787	0.127722
46720	2.37625	0.72563
46721	2.37625	0.284654
46722	1.14104	0.137428
46723	1.00675	0.199833
46724	0.700689	0.145206
46725	0.624389	0.158584
46726	0.782149	0.05
46727	0.940197	0.099906
46728	0.803437	0.158081
46729	0.803437	0.170074
46730	0.995423	0.0994797
46731	0.700699	0.0672082
46732	0.593895	0.342898
46733	1.00673	0.136876
46734	1.18097	0.168217
46735	1.15839	0.130284
46736	0.995407	0.137104
46737	0.865932	0.109685
46738	1.17299	0.150608
46739	0.957054	0.0802129
46740	1.08016	0.207043
46741	1.08016	0.281741
46742	0.995425	0.261765
46743	1.24161	0.0634919
46744	0.796771	0.0615473
46745	1.15837	0.169565

46746	2.57264	0.05
46747	1.89226	0.05
46748	2.41701	0.05
46749	1.89226	0.05
46750	4.30928	0.05
46751	1.69055	0.05
46752	2.39587	0.05
46753	1.53463	0.05
46754	2.2053	0.05
46755	3.89585	0.05
46756	0.946129	0.05
46757	0.946129	0.05
46758	0.94613	0.05
46759	0.94613	0.05
46760	0.946132	0.05
46761	0.946132	0.05
46762	0.946126	0.05
46763	0.946126	0.05
46764	0.94613	0.05
46765	0.94613	0.05
46766	0.946129	0.05
46767	0.946129	0.05
46768	2.41701	0.05
46769	1.89226	0.05
46770	1.89226	0.05
46771	1.89226	0.05
46772	2.32462	0.05
46773	2.32462	0.05
46774	1.41942	0.05
46775	2.2053	0.05
46776	2.02097	0.05
46777	2.02097	0.05
46778	1.53463	0.05
46779	1.53463	0.05
46780	1.89226	0.05
46781	1.89226	0.05
46782	1.89226	0.05
46783	1.89226	0.05
46784	1.89226	0.05
46785	1.89226	0.05
46786	1.89226	1.1232
46787	1.89226	2.1
46788	1.89226	2.1
46789	1.89226	1.58834
46790	1.89226	1.87936
46791	1.89226	1.24414
46792	1.02816	1.00406
46793	1.02816	1.63153
46794	0.595141	1.74522
46795	0.595141	1.93788
46796	0.946132	1.21685
46797	0.595113	1.98094
46798	0.677174	1.89163
46799	0.946132	0.848137
46800	0.946132	1.69856
46801	0.946132	1.72351
46802	0.595113	1.73088
46803	0.677174	1.80305

46804	1.89226	1.38349
46805	1.89226	2.1
46806	1.89226	2.00542
46807	1.89226	1.49324
46808	1.89226	2.0999
46809	1.89226	1.60885
46810	0.946133	1.0204
46811	0.946133	0.547606
46812	0.946132	1.95383
46813	0.946132	1.98416
46814	0.946132	1.95123
46815	0.946132	1.29978
46816	0.946132	1.61693
46817	0.946132	0.66841
46818	0.946132	1.56406
46819	0.946132	1.70195
46820	0.946133	1.33181
46821	0.946133	1.41165
46822	0.946131	1.62428
46823	0.946131	1.55966
46824	0.946131	2.1
46825	0.946131	2.1
46826	0.946128	2.1
46827	0.946128	1.62787
46828	0.946133	2.1
46829	0.946133	1.5126
46830	0.94613	1.93386
46831	0.94613	2.1
46832	0.946131	2.00377
46833	0.946131	1.74943
46834	2.0264	2.1
46835	1.67345	1.60789
46836	2.0264	1.68763
46837	1.70539	0.211839
46838	1.31611	1.38944
46839	1.31611	1.26015
46840	1.89226	0.290976
46841	1.89226	0.256354
46842	1.89226	0.625839
46843	1.89226	0.195139
46844	3.78452	0.223778
46845	1.89226	0.594827
46846	1.89226	0.366932
46847	1.89226	0.397526
46848	1.89226	0.658009
46849	1.89226	0.437979
46850	1.89226	0.757993
46851	1.02645	0.05
46852	0.810942	0.05
46853	0.882781	0.0512191
46854	0.882781	0.05
46855	2.80386	0.05
46856	1.56719	0.05
46857	1.22499	0.05
46858	1.08132	0.0523171
46859	1.36865	0.05
46860	0.810957	0.0513006
46861	0.810957	0.05

46862	0.946132	0.147211
46863	0.946132	0.20241
46864	0.946129	0.0622517
46865	0.946129	0.0925302
46866	0.946131	0.0587624
46867	0.946131	0.0747809
46868	0.946131	0.188353
46869	0.946131	0.119506
46870	0.94613	0.129906
46871	0.94613	0.129946
46872	0.946131	0.256451
46873	0.946131	0.109576
46874	1.89226	0.212871
46875	1.89226	0.0835115
46876	1.89226	0.24424
46877	1.89226	0.103773
46878	3.78453	0.0877042
46879	1.30462	0.05
46880	1.79906	0.701989
46881	1.01299	0.203632
46882	1.44185	0.103773
46883	1.3499	0.089914
46884	1.79906	0.119407
46885	1.89226	0.686942
46886	1.89226	0.704811
46887	1.89226	0.154752
46888	1.89226	0.704818
46889	1.89226	0.700076
46890	1.89226	0.713824
46891	1.89226	0.704468
46892	1.89226	0.707297
46893	1.89226	0.705449
46894	1.89226	0.705934
46895	1.89226	0.702178
46896	1.89226	0.706779
46897	1.3499	0.05
46898	1.79905	0.262607
46899	1.29001	0.103004
46900	1.36005	0.214827
46901	1.88294	0.38677
46902	1.79905	0.128791
46903	1.89226	0.118594
46904	2.75374	0.05
46905	2.60614	0.0911011
46906	1.89226	0.126934
46907	2.60614	0.05
46908	1.89226	0.05
46909	0.946131	0.109341
46910	0.946131	0.131215
46911	1.37687	0.0883309
46912	1.37687	0.0717449
46913	0.946131	0.180484
46914	1.34375	0.130558
46915	1.37686	0.0624736
46916	0.946128	0.0997369
46917	1.37687	0.0535863
46918	1.34375	0.129548
46919	0.94613	0.191744

46920	0.94613	0.0958104
46921	1.44247	2.1
46922	1.12665	2.1
46923	1.50044	2.1
46924	1.73651	2.1
46925	2.94291	2.1
46926	1.73652	2.1
46927	1.64926	2.1
46928	1.44247	2.1
46929	1.44247	2.1
46930	1.64926	2.1
46931	1.43872	2.1
46932	1.50044	2.1
46933	1.73651	2.1
46934	2.00955	2.1
46935	1.87875	2.1
46936	3.50999	2.1
46937	1.64926	2.1
46938	1.73652	2.1
46939	2.00955	2.1
46940	1.8493	2.1
46941	3.65881	2.1
46942	1.89226	0.05
46943	2.28188	0.05
46944	1.99182	0.05
46945	2.28188	0.05
46946	1.6022	0.05
46947	1.89226	0.05
46948	1.89226	0.05
46949	1.89226	0.05
46950	1.89226	0.05
46951	1.89226	0.05
46952	1.89226	0.05
46953	1.89226	0.05
46954	1.89226	0.05
46955	1.89226	0.05
46956	2.19578	0.05
46957	1.89226	0.05
46958	2.81422	0.05
46959	2.42521	0.05
46960	1.89224	0.05
46961	1.99179	0.05
46962	1.454	0.05
46963	1.99179	0.05
46964	1.35444	0.05
46965	1.89224	0.05
46966	1.89224	0.05
46967	1.89224	0.05
46968	1.89224	0.05
46969	1.89224	0.05
46970	1.89224	0.05
46971	1.89224	0.05
46972	1.703	0.05
46973	2.19575	0.05
46974	1.9911	0.05
46975	2.19575	0.05
46976	1.67811	0.05
46977	1.89223	0.05

46978	1.69586	2.1
46979	2.55301	2.1
46980	1.60861	2.1
46981	1.60861	2.1
46982	2.65233	2.1
46983	2.65233	2.1
46984	0.946129	2.1
46985	0.946129	2.1
46986	1.2765	2.1
46987	1.32617	2.1
46988	1.2765	2.1
46989	1.2765	2.1
46990	0.946131	2.1
46991	0.946131	2.1
46992	0.946131	2.1
46993	1.32616	2.1
46994	0.946131	2.1
46995	1.2765	2.1
46996	1.36757	2.1
46997	1.32617	2.1
46998	0.891503	2.1
46999	0.847932	2.1
47000	1.10722	2.1
47001	1.10722	2.1
47002	1.10829	2.1
47003	1.10829	2.1
47004	1.36757	2.1
47005	0.891504	2.1
47006	0.847932	2.1
47007	1.32617	2.1
47008	0.946129	2.1
47009	0.946129	2.1
47010	1.36757	2.1
47011	1.32617	2.1
47012	1.32616	2.1
47013	1.32616	2.1
47014	0.946131	2.1
47015	0.946131	2.1
47016	1.36757	2.1
47017	0.946131	2.1
47018	0.946131	2.1
47019	1.32616	2.1
47020	1.89226	0.05
47021	1.89226	0.05
47022	1.89226	0.05
47023	1.89226	0.05
47024	3.78452	0.05
47025	2.36336	0.05
47026	1.89226	0.05
47027	2.67831	0.05
47028	2.67831	0.05
47029	1.89226	0.05
47030	1.89226	0.05
47031	1.89227	0.05
47032	1.89227	0.05
47033	1.89227	0.05
47034	1.89227	0.05
47035	3.78453	0.05

47036	1.89226	0.05
47037	1.89226	0.05
47038	1.89226	0.05
47039	1.89226	0.05
47040	3.78452	0.05
47041	2.36336	0.05
47042	2.0815	0.05
47043	2.0815	0.05
47044	2.41048	0.05
47045	1.66267	0.05
47046	2.13452	0.05
47047	1.89226	0.05
47048	1.89226	0.05
47049	1.89226	0.05
47050	1.89226	0.05
47051	1.89226	0.05
47052	1.89226	0.05
47053	1.89226	0.59355
47054	1.89226	0.715336
47055	1.89226	0.575508
47056	1.89226	0.711274
47057	1.89226	0.623382
47058	1.89226	0.703505
47059	0.946128	0.736172
47060	0.946128	0.719262
47061	1.00122	0.688217
47062	1.00122	0.690207
47063	0.94613	0.738436
47064	1.01618	0.700117
47065	0.946131	0.714456
47066	1.00122	0.686878
47067	1.00122	0.695225
47068	1.01618	0.698297
47069	0.946131	0.713252
47070	0.946131	0.707183
47071	1.89226	1.87545
47072	1.89226	1.15052
47073	1.89226	1.13206
47074	1.89226	2.1
47075	3.78452	1.26214
47076	1.89226	1.4692
47077	1.89226	2.1
47078	1.89226	2.1
47079	1.89226	1.78239
47080	1.89226	2.1
47081	1.89226	1.62564
47082	1.89226	0.993874
47083	1.89226	1.90272
47084	1.89226	1.80741
47085	1.89226	1.10176
47086	1.89226	1.74312
47087	1.89226	1.11315
47088	1.89226	1.10749
47089	1.89226	2.1
47090	1.89226	2.1
47091	1.89226	1.20526
47092	1.89226	2.02477
47093	1.89226	1.22893

47094	2.71279	0.990686
47095	1.4155	1.00355
47096	2.28488	0.94763
47097	1.06122	0.958388
47098	3.70038	0.965079
47099	1.89226	1.0278
47100	1.89226	1.01402
47101	1.89226	0.974888
47102	1.89226	1.01388
47103	3.78452	1.07319
47104	0.851542	0.953083
47105	0.851542	0.760591
47106	0.851484	0.980596
47107	0.865567	1.06643
47108	0.981616	0.761199
47109	0.864767	0.979491
47110	0.851542	0.999707
47111	0.851542	0.984825
47112	0.851477	1.00457
47113	0.851477	1.03557
47114	0.865635	1.00755
47115	0.816472	0.609133
47116	1.68443	2.1
47117	1.44489	2.1
47118	1.54924	2.1
47119	1.34389	2.1
47120	3.02832	2.1
47121	1.44489	2.1
47122	1.03899	2.1
47123	1.03899	2.1
47124	1.12075	2.1
47125	1.34389	2.1
47126	1.42566	2.1
47127	1.54924	2.1
47128	1.34389	2.1
47129	1.44091	2.1
47130	1.25866	2.1
47131	2.7848	2.1
47132	0.573141	2.1
47133	0.573141	2.1
47134	0.575681	2.1
47135	0.575681	2.1
47136	0.481985	2.1
47137	0.519492	2.1
47138	0.671948	2.1
47139	0.629331	2.1
47140	0.629329	2.1
47141	0.481986	2.1
47142	0.519494	2.1
47143	0.671947	2.1
47144	1.89226	0.814625
47145	1.89226	0.960002
47146	1.89226	0.921639
47147	2.31925	0.872591
47148	3.78452	0.949146
47149	1.30916	2.1
47150	1.17426	2.1
47151	0.946129	2.1

47152	0.946129	2.1
47153	0.94613	2.1
47154	0.94613	2.1
47155	1.17426	2.1
47156	1.17426	2.1
47157	1.30916	2.1
47158	0.94613	2.1
47159	1.17426	2.1
47160	0.94613	2.1
47161	2.56346	2.1
47162	1.89226	1.03696
47163	1.89226	2.1
47164	1.89226	1.42865
47165	2.6074	2.1
47166	2.6074	1.45856
47167	1.89226	2.1
47168	1.89226	2.1
47169	1.89226	2.1
47170	1.89226	2.1
47171	3.78452	2.1
47172	1.33064	2.1
47173	1.84121	2.1
47174	0.946129	2.1
47175	0.946129	2.1
47176	0.946131	2.1
47177	0.946131	2.1
47178	1.30916	2.1
47179	1.8412	2.1
47180	1.33064	2.1
47181	0.94613	2.1
47182	1.30916	2.1
47183	0.946131	2.1
47184	1.19283	1.09316
47185	1.19283	0.836794
47186	0.946131	2.1
47187	0.946131	2.1
47188	0.946132	2.1
47189	0.946132	1.00678
47190	1.3037	2.1
47191	1.19283	0.881029
47192	1.19283	2.1
47193	0.946131	2.1
47194	0.946131	2.1
47195	1.3037	2.1
47196	1.89226	2.1
47197	1.89226	2.1
47198	1.89226	2.1
47199	1.89226	2.1
47200	1.89226	2.1
47201	1.89226	2.1
47202	2.17708	2.1
47203	1.70302	2.1
47204	2.28188	2.1
47205	2.13621	2.1
47206	4.28253	2.1
47207	1.89226	1.50817
47208	1.89226	2.1
47209	1.89226	2.1

47210	1.89226	2.1
47211	3.78452	2.1
47212	0.946132	2.1
47213	0.946132	2.1
47214	0.946132	2.1
47215	0.946132	2.1
47216	0.946131	2.1
47217	0.946131	2.1
47218	0.946132	2.1
47219	0.946132	2.1
47220	0.946132	2.1
47221	0.946132	2.1
47222	0.946132	2.1
47223	0.946132	2.1
47224	1.74933	2.1
47225	1.11833	2.1
47226	1.90074	2.1
47227	1.669	2.1
47228	1.335	2.1
47229	1.11833	2.1
47230	2.28188	1.55171
47231	2.31804	2.1
47232	2.42758	2.08725
47233	2.14895	2.1
47234	4.43083	2.1
47235	1.89226	1.71113
47236	1.89226	2.1
47237	1.89226	1.27983
47238	1.89226	2.1
47239	3.78452	2.1
47240	1.89226	2.1
47241	1.89226	2.1
47242	1.89226	2.1
47243	1.89226	2.1
47244	1.89226	2.1
47245	1.89226	2.1
47246	0.864133	2.1
47247	0.864133	2.1
47248	0.967939	2.1
47249	0.793739	2.1
47250	0.650797	2.1
47251	0.757546	2.1
47252	1.08941	2.1
47253	1.10136	2.1
47254	1.10138	2.1
47255	0.796445	2.1
47256	0.866907	2.1
47257	0.650788	2.1
47258	2.63062	0.157199
47259	1.89226	0.11279
47260	1.89226	0.615741
47261	1.89226	0.85894
47262	2.46557	0.1139
47263	2.46557	1.00588
47264	1.16777	0.164634
47265	1.38859	2.1
47266	1.05042	2.1
47267	1.28909	2.1

47268	2.45686	2.1
47269	1.89227	0.398442
47270	1.89227	1.06235
47271	1.89227	0.0514543
47272	1.89227	2.1
47273	3.78453	0.403781
47274	2.15114	2.1
47275	1.89226	2.1
47276	2.15005	2.1
47277	2.15005	2.1
47278	1.89226	2.1
47279	1.89226	2.1
47280	1.89226	2.1
47281	1.89226	1.31152
47282	1.89226	2.1
47283	1.89226	2.1
47284	1.89226	1.14218
47285	1.89226	2.1
47286	0.973447	2.1
47287	1.32143	2.1
47288	1.37444	2.1
47289	1.37444	2.1
47290	0.949134	2.1
47291	0.949134	2.1
47292	0.946131	1.15276
47293	0.946131	2.1
47294	1.07557	2.1
47295	1.07557	2.1
47296	0.946131	2.1
47297	0.946131	2.1
47298	1.07557	2.1
47299	1.08579	2.1
47300	1.08579	2.1
47301	0.946131	2.1
47302	1.07557	2.1
47303	0.946131	2.1
47304	0.946131	0.839081
47305	0.946131	2.1
47306	0.946131	2.0399
47307	0.946131	2.1
47308	0.94613	1.30306
47309	0.94613	1.46724
47310	0.946131	2.1
47311	0.946131	2.1
47312	0.946131	1.89034
47313	0.946131	1.59152
47314	0.946131	1.75243
47315	0.946131	2.09731
47316	0.606115	2.1
47317	0.606115	2.1
47318	0.616308	2.1
47319	0.616308	2.1
47320	0.68722	1.29903
47321	0.735699	2.1
47322	0.514789	2.1
47323	0.486724	2.1
47324	0.514788	1.498
47325	0.735699	1.9959

47326	0.486723	2.1
47327	0.687221	2.06099
47328	2.33761	0.867178
47329	2.51749	0.700695
47330	2.68092	0.789424
47331	2.58304	0.705642
47332	5.01853	0.78402
47333	2.50906	0.811986
47334	2.51749	0.791454
47335	2.68091	0.80295
47336	2.53176	0.712843
47337	5.18997	0.800897
47338	2.68092	0.791494
47339	2.58304	0.690648
47340	2.71793	0.799878
47341	2.67098	0.470697
47342	5.39885	0.677726
47343	2.68091	0.806104
47344	2.53175	0.547289
47345	2.36417	0.762456
47346	2.67098	0.546022
47347	5.04508	0.757656
47348	2.76309	2.1
47349	1.89226	2.1
47350	1.89226	2.1
47351	1.89226	2.1
47352	2.48573	2.1
47353	2.48573	2.1
47354	1.35879	2.1
47355	1.55161	0.156291
47356	1.10407	2.1
47357	1.32259	0.0768324
47358	2.68138	2.1
47359	1.89226	2.1
47360	1.89226	0.872932
47361	1.89226	2.1
47362	1.89226	0.0512268
47363	3.78452	1.46203
47364	1.19612	2.1
47365	1.24694	2.1
47366	1.24844	0.66373
47367	1.29747	2.1
47368	2.49358	2.1
47369	1.89226	2.1
47370	1.89226	2.1
47371	1.89226	0.16125
47372	1.89226	2.1
47373	3.78453	1.87904
47374	1.89226	2.1
47375	1.89226	2.1
47376	1.89226	2.1
47377	1.89226	2.1
47378	1.89226	2.1
47379	1.89226	2.1
47380	0.587463	2.1
47381	0.587463	2.1
47382	0.581977	2.1
47383	0.581977	2.1

47384	0.531709	2.1
47385	0.545972	2.1
47386	0.623469	2.1
47387	0.648733	2.1
47388	0.648733	2.1
47389	0.545971	2.1
47390	0.623469	2.1
47391	0.531709	2.1
47392	1.29417	0.320098
47393	1.29747	2.1
47394	1.27829	2.1
47395	1.24844	1.33818
47396	1.32732	2.1
47397	1.29747	1.19305
47398	1.89226	1.01836
47399	1.89226	2.1
47400	1.89226	0.21268
47401	1.89226	1.539
47402	1.89226	1.33602
47403	1.89226	2.1
47404	1.89226	2.1
47405	1.89226	2.1
47406	1.89226	2.1
47407	1.89226	2.1
47408	1.89226	2.1
47409	1.89226	1.83497
47410	0.60346	2.1
47411	0.60346	2.1
47412	0.60086	1.78514
47413	0.60086	2.1
47414	0.545971	2.1
47415	0.555587	2.1
47416	0.648733	2.1
47417	0.663661	2.1
47418	0.663661	2.1
47419	0.555587	2.1
47420	0.648733	2.1
47421	0.545971	2.1
47422	1.34066	2.0981
47423	1.36601	2.1
47424	1.34955	0.395037
47425	1.37623	2.1
47426	2.71556	2.1
47427	1.89226	1.40597
47428	1.89226	2.1
47429	1.89226	0.424172
47430	1.89226	2.01929
47431	3.78452	1.26292
47432	0.946132	2.1
47433	0.946132	2.1
47434	0.946132	0.957301
47435	0.946132	2.1
47436	0.946132	2.1
47437	0.946132	2.1
47438	0.946131	2.07182
47439	0.946131	1.65765
47440	0.946132	2.1
47441	0.946132	2.1

47442	0.946132	2.1
47443	0.946132	2.1
47444	0.631351	1.6853
47445	0.631351	2.1
47446	0.632421	2.1
47447	0.632421	2.1
47448	0.573604	2.1
47449	0.580765	2.1
47450	0.683006	1.13162
47451	0.688118	2.1
47452	0.688118	2.1
47453	0.580765	2.1
47454	0.683007	2.1
47455	0.573604	2.1
47456	1.34955	1.88897
47457	1.37624	2.1
47458	1.35048	0.600295
47459	1.37999	2.1
47460	2.72954	1.93391
47461	0.946131	1.78608
47462	0.946131	1.35745
47463	0.946131	2.1
47464	0.946131	2.1
47465	0.94613	2.1
47466	0.94613	2.05857
47467	0.946133	1.21948
47468	0.946133	1.44513
47469	0.946131	1.65486
47470	0.946131	1.23115
47471	0.946132	1.41768
47472	0.946132	1.34137
47473	1.89226	2.1
47474	1.89226	2.1
47475	1.89226	2.1
47476	1.89226	2.1
47477	1.89226	2.1
47478	1.89226	2.1
47479	0.532189	2.08305
47480	0.531141	2.1
47481	0.497784	2.1
47482	0.502536	2.1
47483	1.37999	2.1
47484	0.92433	2.1
47485	0.670081	2.1
47486	0.727155	2.1
47487	0.786438	2.05034
47488	0.589798	2.1
47489	0.497784	2.1
47490	1.89226	2.1
47491	2.72301	2.1
47492	2.4158	2.1
47493	2.4158	2.1
47494	1.89226	2.1
47495	1.89226	2.1
47496	1.89226	2.1
47497	1.89226	2.1
47498	1.89226	2.1
47499	1.89226	2.1

47500	1.89226	2.1
47501	1.89226	2.1
47502	1.36919	2.1
47503	1.12717	2.1
47504	1.38634	2.1
47505	1.56541	2.1
47506	2.75552	2.1
47507	1.89226	2.1
47508	1.89226	2.1
47509	1.89226	2.1
47510	1.89226	2.1
47511	1.89226	2.1
47512	1.89226	2.1
47513	0.907036	2.1
47514	0.782706	2.1
47515	0.838808	2.1
47516	0.693168	2.1
47517	0.794747	2.1
47518	0.794747	2.1
47519	0.805458	2.1
47520	0.805458	2.1
47521	0.907036	2.1
47522	0.838808	2.1
47523	0.693169	2.1
47524	0.782707	2.1
47525	0.94613	2.1
47526	0.94613	2.1
47527	1.188 2.1	
47528	1.28421	2.1
47529	1.18801	2.1
47530	1.18801	2.1
47531	0.946129	2.1
47532	0.946129	2.1
47533	1.28421	2.1
47534	0.94613	2.1
47535	1.188 2.1	
47536	0.94613	2.1
47537	1.89226	2.1
47538	1.89226	2.1
47539	1.89226	2.1
47540	1.89226	2.1
47541	1.89226	2.1
47542	1.89226	2.1
47543	1.16817	2.1
47544	1.06007	2.1
47545	1.19785	2.1
47546	1.2832	2.1
47547	2.36602	2.1
47548	0.960106	2.1
47549	2.0073	2.1
47550	1.51754	2.1
47551	2.56883	2.1
47552	3.52484	2.1
47553	1.06756	2.1
47554	1.57913	2.1
47555	1.87532	2.1
47556	1.38666	2.1
47557	2.94287	2.1

47558	2.15622	2.1
47559	1.38668	2.1
47560	1.87534	2.1
47561	1.87534	2.1
47562	1.70539	2.1
47563	1.70539	2.1
47564	0.986095	2.1
47565	1.36226	2.1
47566	1.3331	2.1
47567	1.3331	2.1
47568	1.07156	2.1
47569	1.07156	2.1
47570	0.682081	1.52732
47571	0.68748	1.23072
47572	0.650003	0.157477
47573	0.645873	0.890229
47574	0.666344	0.169296
47575	0.666344	1.07611
47576	0.66161	2.01207
47577	0.66161	0.155213
47578	0.68748	0.55789
47579	0.645873	0.621499
47580	0.650004	0.579255
47581	0.682082	0.617078
47582	0.946132	1.30136
47583	0.946132	0.694144
47584	0.946132	0.736561
47585	0.946132	0.10743
47586	0.946132	0.11889
47587	0.946132	0.735572
47588	0.946133	0.650476
47589	0.946133	1.2977
47590	0.946132	0.569573
47591	0.946132	0.555122
47592	0.946133	0.688622
47593	0.946133	0.545498
47594	1.89227	1.08215
47595	2.79312	0.145353
47596	2.77591	0.85914
47597	2.79312	0.714397
47598	1.87505	0.312017
47599	1.89227	0.351788
47600	0.68748	1.78659
47601	0.698626	1.34438
47602	0.645873	0.247697
47603	0.650148	1.05841
47604	0.670561	0.275341
47605	0.670561	1.32881
47606	0.667067	2.09008
47607	0.667067	0.13227
47608	0.698627	0.807787
47609	0.650148	1.1787
47610	0.68748	0.855336
47611	0.645873	0.952676
47612	0.946132	1.42714
47613	0.946132	0.929374
47614	0.946132	0.892947
47615	0.946132	0.229697

47616	0.946132	0.249252
47617	0.946132	1.0873
47618	0.946133	0.950294
47619	0.946133	1.43825
47620	0.946133	0.773529
47621	0.946133	0.892203
47622	0.946132	0.906082
47623	0.946132	0.92687
47624	0.946133	1.03249
47625	0.946133	0.400821
47626	1.38709	0.929772
47627	1.38795	0.213998
47628	0.946132	0.233264
47629	0.946132	0.32666
47630	1.38709	1.0673
47631	1.38709	0.260134
47632	1.38709	0.706929
47633	0.946133	0.821368
47634	0.946132	0.91112
47635	1.38795	0.414903
47636	1.70301	0.05
47637	2.07659	0.05
47638	1.89226	0.05
47639	1.89226	0.05
47640	1.76332	0.05
47641	1.76331	0.05
47642	1.89226	0.05
47643	1.89226	0.05
47644	1.89226	0.05
47645	1.89226	0.05
47646	1.89226	0.05
47647	1.89226	0.05
47648	1.89226	0.05
47649	1.89226	0.05
47650	1.89226	0.05
47651	1.89226	0.05
47652	3.78452	0.05
47653	1.89226	0.05
47654	2.12492	0.05
47655	1.89226	0.05
47656	1.89226	0.05
47657	2.07659	0.05
47658	2.07659	0.05
47659	0.94613	0.05
47660	0.94613	0.05
47661	1.17498	0.05
47662	1.32446	0.05
47663	1.32446	0.05
47664	0.94613	0.05
47665	0.946127	0.05
47666	1.17498	0.05
47667	1.32446	0.05
47668	1.32446	0.05
47669	0.946128	0.05
47670	0.946128	0.05
47671	1.89226	2.1
47672	1.86472	2.1
47673	2.11049	2.1

47674	1.89226	2.1
47675	2.08295	2.1
47676	1.86472	2.1
47677	1.89226	2.1
47678	2.08295	2.1
47679	2.14023	2.1
47680	1.89226	2.1
47681	2.33091	2.1
47682	2.08295	2.1
47683	0.809215	2.1
47684	0.809215	2.1
47685	0.810915	2.1
47686	0.810915	2.1
47687	1.09581	2.1
47688	1.08271	2.1
47689	0.520996	2.1
47690	0.537426	2.1
47691	0.520997	2.1
47692	1.08271	2.1
47693	0.537426	2.1
47694	1.09581	2.1
47695	0.79674	2.1
47696	0.79674	2.1
47697	0.799279	2.1
47698	0.799279	2.1
47699	1.07502	2.1
47700	1.0827	2.1
47701	0.508206	2.1
47702	0.520996	2.1
47703	0.508206	2.1
47704	1.07502	2.1
47705	0.520996	2.1
47706	1.0827	2.1
47707	2.15714	2.1
47708	1.70302	2.1
47709	1.78732	2.1
47710	2.02219	2.1
47711	3.98971	2.1
47712	2.0927	2.1
47713	2.162	2.1
47714	2.11563	2.1
47715	2.02219	2.1
47716	4.18419	2.1
47717	1.62483	2.1
47718	2.02219	2.1
47719	1.93617	2.1
47720	1.52008	2.1
47721	3.54227	2.1
47722	2.11563	2.1
47723	2.02219	2.1
47724	2.29218	2.1
47725	2.03975	2.1
47726	4.06194	2.1
47727	2.007	2.1
47728	1.89226	2.1
47729	1.89226	2.1
47730	1.89226	2.1
47731	2.06776	2.1

47732	2.06776	2.1
47733	0.946129	2.1
47734	0.946129	2.1
47735	1.0035	2.1
47736	1.0035	2.1
47737	0.946131	2.1
47738	0.946131	2.1
47739	0.968797	2.1
47740	1.0035	2.1
47741	0.968771	2.1
47742	0.946105	2.1
47743	1.00353	2.1
47744	0.946157	2.1
47745	1.79103	2.1
47746	2.53286	0.915412
47747	2.60781	2.1
47748	2.219	1.03382
47749	2.17985	1.4162
47750	1.79103	0.836395
47751	2.219	1.59281
47752	2.66359	1.53029
47753	2.70084	2.1
47754	2.38698	1.28168
47755	2.53285	1.13246
47756	2.219	0.948327
47757	1.33179	1.3813
47758	0.946128	1.03872
47759	0.946133	1.25844
47760	1.26643	1.96537
47761	1.3318	1.12308
47762	1.3318	1.96559
47763	0.946131	0.980614
47764	0.946131	1.41618
47765	0.946131	1.00032
47766	0.946131	0.923512
47767	1.33179	1.24659
47768	1.26643	2.1
47769	2.28979	2.1
47770	2.47227	2.1
47771	2.43451	2.1
47772	2.22627	2.1
47773	4.69855	2.1
47774	2.40667	2.1
47775	2.47228	2.1
47776	2.43451	2.1
47777	2.38069	2.1
47778	4.85296	2.1
47779	2.43451	2.1
47780	2.15715	2.1
47781	2.15715	2.1
47782	2.22627	2.1
47783	2.39489	2.1
47784	2.46402	2.1
47785	2.43451	2.1
47786	2.34899	2.1
47787	2.38069	2.1
47788	2.38069	2.1
47789	2.3949	2.1

47790	2.3949	2.1
47791	2.85425	1.37978
47792	1.70302	0.780829
47793	1.70302	0.0977461
47794	1.52254	0.185975
47795	2.34181	0.54265
47796	2.08499	0.878444
47797	1.89226	0.306714
47798	1.89226	0.76317
47799	1.89226	0.0656842
47800	1.89226	0.418764
47801	1.89226	0.466547
47802	1.89226	0.811521
47803	2.34181	0.951575
47804	1.38287	0.807628
47805	1.38287	0.869541
47806	1.84132	0.802839
47807	1.96416	0.84322
47808	2.4907	0.862896
47809	0.931432	0.857819
47810	0.946135	0.714268
47811	0.946133	0.711356
47812	0.946133	0.214876
47813	0.931464	0.7681
47814	0.931464	0.859508
47815	1.15573	0.715305
47816	0.96378	0.901031
47817	0.931434	0.800491
47818	0.946131	0.734536
47819	0.946131	0.713455
47820	1.13808	0.619417
47821	0.946132	0.827165
47822	0.946132	0.75304
47823	0.946132	0.826775
47824	0.946132	0.212513
47825	0.946133	0.807699
47826	0.946133	0.637838
47827	0.946132	0.819432
47828	0.946132	0.814104
47829	0.946133	0.788544
47830	0.946133	0.840807
47831	0.946132	0.708444
47832	0.946132	0.05034
47833	1.68074	2.1
47834	2.29459	2.1
47835	2.23361	2.1
47836	1.57234	2.1
47837	3.86693	2.1
47838	2.61243	2.1
47839	2.29458	2.1
47840	2.2336	2.1
47841	2.58234	2.1
47842	4.87692	2.1
47843	0.73506	2.1
47844	0.786166	2.1
47845	1.08729	2.1
47846	1.11679	2.1
47847	0.93134	2.1

47848	0.93134	2.1
47849	0.942113	2.1
47850	0.942113	2.1
47851	0.735065	2.1
47852	1.08729	2.1
47853	1.11679	2.1
47854	0.78616	2.1
47855	1.08729	2.1
47856	1.11679	2.1
47857	1.27502	2.1
47858	1.29116	2.1
47859	1.19259	2.1
47860	1.19259	2.1
47861	1.19923	2.1
47862	1.19923	2.1
47863	1.27503	2.1
47864	1.08729	2.1
47865	1.11679	2.1
47866	1.29115	2.1
47867	0.974715	0.988329
47868	1.43779	0.862986
47869	1.93915	0.865367
47870	1.53908	0.902152
47871	2.91387	0.878557
47872	1.53908	0.830942
47873	0.99845	0.904693
47874	1.5176	0.967541
47875	1.97963	0.777873
47876	3.05668	0.904079
47877	1.93915	0.851755
47878	1.97963	0.849797
47879	1.53908	0.858326
47880	1.53908	0.749204
47881	2.3376	0.918805
47882	2.3376	0.857711
47883	1.89226	2.1
47884	2.59747	2.1
47885	1.91802	2.1
47886	1.89226	2.1
47887	2.62322	2.1
47888	2.59747	2.1
47889	2.59747	2.1
47890	1.87119	2.1
47891	1.84844	2.1
47892	1.82268	2.1
47893	2.62323	2.1
47894	2.59747	2.1
47895	0.94613	2.1
47896	0.94613	2.1
47897	1.32697	2.1
47898	1.32697	2.1
47899	1.32697	2.1
47900	1.31161	2.1
47901	0.946132	2.1
47902	0.946132	2.1
47903	0.94613	2.1
47904	1.32697	2.1
47905	0.946131	2.1

47906	1.31161	2.1
47907	2.62323	2.1
47908	1.91704	2.1
47909	1.90191	2.1
47910	1.87119	2.1
47911	2.65395	2.1
47912	2.62323	2.1
47913	1.89226	2.1
47914	1.89226	2.1
47915	2.15958	2.1
47916	2.15958	2.1
47917	2.46558	2.1
47918	2.19826	2.1
47919	2.46558	2.1
47920	1.89226	2.1
47921	1.89226	2.1
47922	1.89226	2.1
47923	2.61064	2.1
47924	2.61064	2.1
47925	1.81759	0.908875
47926	1.89526	0.972407
47927	2.32219	1.13192
47928	1.881	0.887522
47929	1.81759	1.03817
47930	1.3764	0.448687
47931	1.81759	0.944762
47932	2.57079	0.874101
47933	2.32219	1.25822
47934	2.32219	0.88974
47935	1.99875	1.01932
47936	1.99875	0.391456
47937	0.536182	0.443357
47938	0.579206	0.0525977
47939	0.908796	0.931091
47940	0.999375	0.468962
47941	0.732091	0.663691
47942	0.732091	0.0622189
47943	0.755911	0.347992
47944	0.755911	0.818381
47945	0.999376	0.469174
47946	0.579206	0.624101
47947	0.908796	0.503944
47948	0.536182	0.446735
47949	0.94613	0.0796346
47950	0.94613	0.139684
47951	1.31112	0.175919
47952	1.18275	0.05
47953	0.946126	0.0587155
47954	0.946126	0.05
47955	1.31112	0.146765
47956	1.31112	0.05
47957	0.946128	0.0904815
47958	1.31112	0.10553
47959	0.946128	0.05
47960	1.18275	0.05
47961	2.64617	0.533789
47962	1.27737	0.0569025
47963	1.83076	0.180297

47964	1.27737	0.262834
47965	3.03764	0.123164
47966	2.3655	0.0587934
47967	1.12991	0.05
47968	1.22375	0.05
47969	1.35931	0.05
47970	1.04971	0.05
47971	1.0419	0.05
47972	1.15237	0.05
47973	1.19593	0.05
47974	1.30306	0.05
47975	1.12937	0.05
47976	1.31934	0.05
47977	1.15203	0.05
47978	1.15203	0.05
47979	2.65227	0.05
47980	1.46041	0.05
47981	2.22712	0.05
47982	2.04816	0.05
47983	2.22712	0.05
47984	2.04816	0.05
47985	1.89226	0.05
47986	1.89226	0.229963
47987	1.89226	0.101292
47988	1.89226	0.230549
47989	3.78452	0.140142
47990	1.24653	0.108591
47991	1.66554	0.100029
47992	1.53538	0.05
47993	1.53538	2.02094
47994	1.40915	0.05
47995	1.40915	2.07747
47996	1.66555	0.139871
47997	1.75835	2.1
47998	1.53538	2.1
47999	1.65313	2.1
48000	3.31868	2.1
48001	1.89226	0.237964
48002	1.89226	0.625761
48003	1.89226	0.231381
48004	1.89226	2.1
48005	3.78453	0.244502
48006	1.89226	0.05
48007	1.89226	0.0773701
48008	1.89226	0.0848259
48009	1.89226	0.909755
48010	3.78452	0.05
48011	0.658778	2.1
48012	0.658778	0.05
48013	0.661956	0.0767378
48014	0.661956	0.341413
48015	0.703822	0.154298
48016	0.767689	2.1
48017	0.623266	0.05
48018	0.553046	0.147793
48019	0.703804	0.0922495
48020	0.55303	0.0995079
48021	0.623282	0.11087

48022	0.767711	0.0621769
48023	1.53538	2.1
48024	1.65313	2.1
48025	1.40765	0.222906
48026	1.55779	2.1
48027	3.06078	2.1
48028	1.89227	0.0848151
48029	1.89227	2.1
48030	1.89227	0.973768
48031	1.89227	0.637614
48032	3.78453	1.07975
48033	1.10517	2.1
48034	2.16532	2.1
48035	1.94374	2.1
48036	1.94001	2.1
48037	1.37756	2.1
48038	1.37756	2.1
48039	1.7453	0.05
48040	1.83284	0.05
48041	2.28483	0.05
48042	1.77804	0.05
48043	1.7453	0.05
48044	1.23851	0.05
48045	1.7453	0.05
48046	2.54752	0.05
48047	2.28483	0.05
48048	2.28483	0.05
48049	2.0189	0.05
48050	2.0189	0.05
48051	1.12457	0.05
48052	2.0189	0.05
48053	1.7453	0.05
48054	1.7453	0.05
48055	1.40707	0.05
48056	1.40707	0.05
48057	2.41176	2.1
48058	2.59262	2.1
48059	2.34085	2.1
48060	2.55827	2.1
48061	4.97003	2.1
48062	2.59262	2.1
48063	2.61901	2.1
48064	2.55827	2.1
48065	2.64067	2.1
48066	5.17728	2.1
48067	2.34084	2.1
48068	2.55827	2.1
48069	2.24937	2.1
48070	2.49053	2.1
48071	4.80764	2.1
48072	2.55826	2.1
48073	2.64067	2.1
48074	2.49053	2.1
48075	2.58639	2.1
48076	5.14465	2.1
48077	2.27918	0.05
48078	2.44132	0.05
48079	1.89226	0.05

48080	1.89226	0.05
48081	4.33359	0.05
48082	1.89226	0.05
48083	2.44132	0.05
48084	2.6771	0.05
48085	2.6771	0.05
48086	1.89226	0.05
48087	1.89226	0.05
48088	1.60329	0.05
48089	1.38246	0.05
48090	1.98182	0.05
48091	2.2217	0.05
48092	3.60417	0.05
48093	2.05362	0.05
48094	1.02663	0.05
48095	1.43255	0.05
48096	1.52072	0.05
48097	1.70302	0.05
48098	1.7685	0.05
48099	0.946131	0.05
48100	0.946131	0.05
48101	0.946131	0.05
48102	0.946131	0.05
48103	0.946131	0.05
48104	0.946131	0.05
48105	0.946131	0.05
48106	0.946131	0.05
48107	0.946131	0.05
48108	0.946131	0.05
48109	0.946131	0.05
48110	0.946131	0.05
48111	1.89226	0.05
48112	1.89226	0.05
48113	1.89226	0.05
48114	1.89226	0.05
48115	1.89226	0.05
48116	1.89226	0.05
48117	1.92645	0.263146
48118	2.83045	0.161078
48119	1.92225	0.177303
48120	2.01873	0.0821071
48121	2.51732	0.509664
48122	2.51732	0.170194
48123	2.51732	0.0781366
48124	2.23857	0.0612027
48125	1.92645	0.112911
48126	1.92645	0.0532111
48127	2.23857	0.26398
48128	1.50865	0.05
48129	2.02687	2.1
48130	2.39604	2.1
48131	2.29198	2.1
48132	2.73046	2.1
48133	4.68803	2.1
48134	1.21148	0.0901356
48135	1.87957	0.903637
48136	2.03555	0.449253
48137	2.03555	0.823535

48138	1.09962	0.678707
48139	1.09962	0.902817
48140	1.92558	0.05
48141	2.05274	2.1
48142	2.2443	0.0565492
48143	2.24429	0.716042
48144	1.70869	0.05
48145	1.70868	2.00915
48146	0.818397	0.0635273
48147	0.818397	0.958269
48148	0.827911	0.0659665
48149	0.827908	0.967448
48150	1.00418	0.848322
48151	1.02054	0.862517
48152	0.692018	0.465473
48153	0.620899	0.556626
48154	0.65823	0.795339
48155	1.00418	0.05
48156	0.620899	0.87023
48157	1.0262	0.475612
48158	1.92558	0.95085
48159	2.51818	1.87581
48160	1.94816	0.546404
48161	2.01985	1.07999
48162	1.85389	0.05
48163	1.92558	1.07695
48164	0.903844	2.1
48165	0.903844	2.1
48166	0.946148	2.1
48167	0.946151	2.1
48168	0.946118	2.1
48169	0.903876	2.1
48170	0.946118	2.1
48171	1.10235	2.1
48172	0.946131	2.1
48173	0.946131	2.1
48174	1.10234	2.1
48175	0.903863	2.1
48176	1.63471	2.1
48177	1.08022	2.1
48178	1.56801	2.1
48179	1.03437	2.1
48180	2.66908	2.1
48181	1.56801	2.1
48182	0.97541	2.1
48183	0.97541	2.1
48184	1.03437	2.1
48185	1.49733	2.1
48186	1.55629	2.1
48187	1.89226	0.05
48188	1.89226	0.05
48189	1.89226	0.05
48190	1.89226	0.05
48191	3.78452	0.05
48192	0.946131	0.05
48193	1.16373	0.05
48194	0.946131	0.05
48195	0.734637	0.05

48196	0.946132	0.05
48197	0.946132	0.05
48198	1.16373	0.05
48199	0.734636	0.05
48200	0.946093	0.05
48201	0.946093	0.05
48202	0.734675	0.05
48203	0.734675	0.05
48204	1.98413	0.05
48205	1.87355	0.165516
48206	1.72774	0.05
48207	1.67865	0.0961857
48208	3.60128	0.05
48209	1.87355	0.05
48210	1.54924	2.1
48211	1.68443	2.1
48212	1.68443	0.0577294
48213	1.67866	2.1
48214	1.67866	0.0667179
48215	1.72774	0.05
48216	1.53422	0.0569917
48217	1.67865	0.05
48218	1.67865	0.05
48219	1.53873	0.05
48220	1.53873	0.05
48221	1.67866	0.05
48222	1.54924	2.1
48223	1.53423	0.05
48224	1.44091	2.1
48225	3.11957	1.82602
48226	1.89226	2.1
48227	1.89226	1.93974
48228	1.65573	2.1
48229	1.65573	2.1
48230	1.89226	2.1
48231	1.89226	2.1
48232	1.89226	2.1
48233	1.89614	1.5656
48234	1.7508	1.92549
48235	1.72666	2.0452
48236	1.89226	2.00768
48237	1.89226	2.1
48238	1.89223	2.1
48239	1.89223	1.60651
48240	1.89223	1.66408
48241	1.89223	2.06651
48242	1.89223	1.80957
48243	1.89223	2.1
48244	1.89224	2.1
48245	1.89224	1.29415
48246	1.89224	1.57154
48247	1.89224	1.63567
48248	1.89224	1.74856
48249	1.89224	2.05038
48250	2.09773	2.1
48251	2.06623	2.1
48252	2.43268	2.1
48253	2.33713	2.1

48254	1.703	2.1
48255	1.61372	2.1
48256	0.646929	2.1
48257	0.689776	2.1
48258	1.05897	2.1
48259	0.956364	2.1
48260	0.905594	2.1
48261	0.904315	2.1
48262	0.727014	2.1
48263	0.850757	2.1
48264	0.798445	2.1
48265	0.654101	2.1
48266	0.717539	2.1
48267	1.0478	2.1
48268	1.89223	2.1
48269	1.89223	1.31957
48270	1.89223	2.1
48271	1.6557	0.140076
48272	3.54794	1.91105
48273	0.946118	2.1
48274	0.946118	2.1
48275	0.946118	2.1
48276	0.946118	2.1
48277	0.946117	2.1
48278	0.946117	1.17637
48279	0.946119	2.1
48280	0.946119	2.1
48281	0.946105	2.1
48282	0.946105	2.1
48283	0.946131	2.1
48284	0.946131	2.1
48285	1.47671	2.1
48286	1.70303	2.1
48287	1.90705	2.1
48288	1.98945	2.1
48289	1.3454	2.1
48290	1.40353	2.1
48291	1.89226	2.1
48292	1.89226	2.1
48293	1.89226	2.1
48294	1.89226	2.1
48295	1.89226	2.1
48296	1.89226	0.944064
48297	2.39438	0.05
48298	2.69842	0.05
48299	2.45177	0.05
48300	2.28563	0.05
48301	4.84615	0.05
48302	1.28421	2.1
48303	1.35966	2.1
48304	0.961001	2.1
48305	0.860549	2.1
48306	1.1039	2.1
48307	1.1039	2.1
48308	1.11631	2.1
48309	1.11631	2.1
48310	1.35966	2.1
48311	0.961001	2.1

48312	0.860549	2.1
48313	1.28421	2.1
48314	1.89226	1.57979
48315	1.89226	2.03577
48316	1.89226	2.1
48317	1.89226	1.19345
48318	3.78452	1.61237
48319	1.48702	0.422579
48320	1.40656	0.489675
48321	0.626442	1.79248
48322	0.626442	1.68975
48323	0.626442	1.94349
48324	0.946132	0.458389
48325	1.05489	0.156328
48326	1.05012	1.99092
48327	0.946131	1.48776
48328	0.946131	1.66056
48329	0.626442	1.68923
48330	1.05012	1.31962
48331	1.89226	2.02781
48332	1.97212	1.16419
48333	1.89226	1.12802
48334	1.89226	1.73419
48335	3.78453	1.7814
48336	1.89226	2.1
48337	1.89226	1.38334
48338	1.89226	1.44285
48339	1.89226	1.90817
48340	3.78452	1.81907
48341	1.89226	1.10057
48342	1.89226	1.65818
48343	1.89226	1.88943
48344	1.89226	0.417782
48345	1.89226	1.89183
48346	1.89226	0.365445
48347	0.946161	0.336822
48348	0.946161	1.36708
48349	0.932156	1.66604
48350	0.932156	1.27032
48351	0.932184	1.65269
48352	0.946132	0.100906
48353	0.946133	1.65849
48354	0.946133	0.46523
48355	0.946132	0.831298
48356	0.946132	1.05107
48357	0.932185	0.924841
48358	0.946133	0.370221
48359	0.946132	1.173
48360	0.946132	0.306666
48361	0.946132	2.1
48362	0.946132	1.68815
48363	0.946132	1.62862
48364	0.946132	1.33603
48365	0.946133	1.38526
48366	0.946133	1.81686
48367	0.946132	1.5737
48368	0.946132	1.73589
48369	0.946133	1.14686

48370	0.946133	1.45708
48371	1.79397	0.0734194
48372	1.00636	0.05
48373	1.31658	0.327046
48374	2.02924	0.0607509
48375	3.0356	0.115539
48376	1.89226	0.086411
48377	1.89226	0.05
48378	1.89226	0.0522656
48379	1.89226	0.05
48380	1.89226	0.05
48381	1.89226	0.05
48382	0.946131	0.05
48383	0.946131	0.05
48384	1.24766	0.0657592
48385	1.03691	0.05
48386	0.946129	0.05
48387	0.946129	0.05
48388	1.24766	0.0754323
48389	1.24766	0.05
48390	0.946131	0.0557726
48391	1.24766	0.0579778
48392	1.03691	0.05
48393	0.946131	0.05
48394	1.44824	0.05
48395	2.02924	0.05
48396	1.7065	0.05
48397	1.38604	0.118657
48398	2.32017	0.05
48399	2.32017	0.05
48400	1.89226	0.05
48401	1.89226	0.05
48402	1.89226	0.05
48403	1.89226	0.05
48404	1.89226	0.0873573
48405	1.89226	0.05
48406	0.478349	1.05308
48407	0.612092	2.1
48408	0.606115	2.1
48409	0.506213	2.1
48410	0.550677	2.1
48411	0.550677	1.05086
48412	0.567628	2.1
48413	0.567628	2.1
48414	0.506213	2.1
48415	0.478349	2.1
48416	0.606116	2.1
48417	0.612092	2.1
48418	1.52226	2.1
48419	1.54241	2.1
48420	1.23167	2.1
48421	1.23167	2.1
48422	1.75555	2.1
48423	1.75555	2.1
48424	1.75557	2.1
48425	1.54243	2.1
48426	1.9943	2.1
48427	1.85871	2.1

48428	3.53673	2.1
48429	1.85871	2.1
48430	1.06936	2.1
48431	1.88926	2.1
48432	1.54243	2.1
48433	1.41619	2.1
48434	1.06936	2.1
48435	1.28649	1.9871
48436	1.28516	2.1
48437	1.38986	0.928985
48438	1.42399	1.67645
48439	2.67502	1.57032
48440	1.42399	1.14847
48441	1.07283	2.1
48442	1.46528	2.1
48443	1.28517	1.35533
48444	1.25294	2.1
48445	1.07283	1.14006
48446	1.38986	2.1
48447	1.42399	2.1
48448	1.52885	1.14316
48449	1.60058	1.3086
48450	2.99043	1.55908
48451	0.715316	2.1
48452	0.715316	1.74457
48453	0.719126	0.528708
48454	0.719126	2.1
48455	0.722447	2.1
48456	0.626469	2.1
48457	0.711995	1.1966
48458	0.800291	1.23451
48459	0.80029	1.85488
48460	0.722447	1.34376
48461	0.711995	1.95564
48462	0.62647	1.97562
48463	2.05632	0.05
48464	1.70303	0.05
48465	2.3923	0.05
48466	2.61277	0.05
48467	1.44836	0.05
48468	1.58184	0.05
48469	1.89226	0.05
48470	1.89226	0.05
48471	1.89226	0.05
48472	1.89226	0.05
48473	3.78453	0.05
48474	1.44091	2.1
48475	1.1822	2.1
48476	1.25866	2.1
48477	1.25866	2.1
48478	1.35215	2.1
48479	1.35215	2.1
48480	1.27338	2.1
48481	1.1822	2.1
48482	1.27338	2.1
48483	1.35215	2.1
48484	1.10817	2.1
48485	1.18694	2.1

48486	0.946132	0.0819627
48487	0.946132	0.05
48488	0.946132	0.05
48489	0.946132	0.05
48490	0.946133	0.05
48491	0.946133	0.0794413
48492	0.946132	0.05
48493	0.946132	0.05
48494	0.946132	0.05
48495	0.946132	0.05
48496	0.946133	0.05
48497	0.946133	0.05
48498	2.6875	0.05
48499	2.40558	0.05
48500	2.1994	0.05
48501	1.70303	0.05
48502	4.68305	0.05
48503	0.915523	0.05
48504	0.915523	0.05
48505	0.861057	0.05
48506	0.946796	0.05
48507	0.838652	0.05
48508	0.654182	0.05
48509	1.14044	0.05
48510	0.80252	0.05
48511	0.836435	0.05
48512	0.608659	0.05
48513	1.22198	0.05
48514	0.863472	0.05
48515	2.1994	0.05
48516	0.981586	0.05
48517	1.59619	0.05
48518	1.70303	0.05
48519	1.24816	0.05
48520	1.38523	0.05
48521	2.71141	2.1
48522	1.70302	2.1
48523	2.56527	2.1
48524	1.88649	2.1
48525	2.41279	2.1
48526	1.70302	2.1
48527	1.89226	2.1
48528	1.89226	2.1
48529	1.89226	2.1
48530	1.89226	2.1
48531	3.78453	2.1
48532	1.89226	2.1
48533	1.89226	2.1
48534	1.89226	2.1
48535	1.89226	2.1
48536	1.89226	2.1
48537	1.89226	2.1
48538	2.18867	2.1
48539	0.983723	2.1
48540	2.42263	2.1
48541	1.8014	2.1
48542	1.62992	2.1
48543	0.983723	2.1

48544	2.71141	2.1
48545	1.89226	2.1
48546	2.31157	2.1
48547	2.31157	2.1
48548	1.89226	2.1
48549	1.89226	2.1
48550	1.89227	2.1
48551	1.89227	1.98125
48552	1.89227	1.99311
48553	1.89227	2.1
48554	3.78453	2.1
48555	1.89226	2.1
48556	1.89226	2.1
48557	1.89226	2.1
48558	1.89226	2.06478
48559	1.89226	2.1
48560	1.89226	1.97676
48561	1.89226	1.50868
48562	1.89226	1.24183
48563	1.89226	2.1
48564	1.89226	1.01298
48565	1.89226	1.32723
48566	1.89226	0.862151
48567	1.1427	2.00343
48568	1.14956	2.1
48569	1.70302	1.22025
48570	1.81267	2.08509
48571	2.88983	2.05609
48572	1.89226	0.963122
48573	1.89226	2.1
48574	1.89226	1.0183
48575	1.89226	1.21648
48576	3.78453	1.24613
48577	1.89226	1.44754
48578	1.89226	1.86721
48579	1.89226	0.877604
48580	1.89226	1.33201
48581	3.78452	1.11313
48582	0.884197	2.1
48583	1.1125	1.34602
48584	1.13273	1.33446
48585	0.844557	1.76772
48586	0.981137	1.14328
48587	1.13971	1.94467
48588	0.980432	1.99362
48589	0.980432	1.20012
48590	1.15859	1.20882
48591	1.12206	0.973436
48592	0.898619	2.1
48593	0.94426	1.44443
48594	2.21016	1.14688
48595	1.44091	2.1
48596	1.98408	1.21511
48597	1.4218	1.08237
48598	2.21016	2.1
48599	1.64788	2.1
48600	1.07526	0.724541
48601	1.91531	2.1

48602	1.49155	2.1
48603	1.58551	0.193612
48604	1.49155	0.927368
48605	1.58551	2.08542
48606	1.98979	0.05
48607	2.21773	0.05
48608	2.30533	0.05
48609	2.55895	0.05
48610	4.52305	0.05
48611	2.55896	0.05
48612	2.27963	0.05
48613	2.60486	0.05
48614	2.60486	0.05
48615	2.21773	0.05
48616	2.21773	0.05
48617	2.30533	0.05
48618	2.55895	0.05
48619	2.29539	0.05
48620	2.47308	0.05
48621	4.7784	0.05
48622	2.55896	0.05
48623	2.55792	0.05
48624	2.55792	0.05
48625	2.60486	0.05
48626	2.47308	0.05
48627	2.52003	0.05
48628	0.946133	1.98209
48629	0.946133	2.1
48630	1.20851	1.56617
48631	1.20851	1.47394
48632	0.967411	1.49346
48633	1.20851	1.28541
48634	0.946132	2.1
48635	0.946132	1.9654
48636	0.946132	2.06405
48637	1.20851	1.49539
48638	0.967412	1.55439
48639	0.946133	1.93467
48640	1.89226	1.94931
48641	1.89226	1.35741
48642	1.89226	1.37677
48643	1.89226	1.37473
48644	1.89226	2.00495
48645	1.89226	2.01684
48646	1.89226	2.03674
48647	2.41701	1.8882
48648	2.71724	1.40893
48649	2.71724	1.75369
48650	1.89226	2.1
48651	1.89226	2.1
48652	0.946131	2.07195
48653	0.946131	2.1
48654	0.946128	1.64354
48655	0.946128	1.78898
48656	0.94613	1.23029
48657	0.94613	1.41746
48658	0.94613	2.09837
48659	0.94613	2.1

48660	0.94613	1.88227
48661	0.94613	1.3345
48662	0.94613	1.5565
48663	0.94613	2.1
48664	0.851541	0.05
48665	0.851541	0.0878253
48666	0.851481	0.0502051
48667	0.997219	0.06913
48668	0.997112	0.05
48669	0.992853	0.05
48670	0.85154	0.05
48671	0.85154	0.0550923
48672	0.894824	0.05
48673	0.894824	0.0765182
48674	0.94613	0.0871124
48675	0.906485	0.05
48676	0.802947	2.1
48677	0.845556	2.1
48678	0.582339	2.1
48679	0.519756	2.1
48680	0.714508	2.1
48681	0.714508	2.1
48682	0.628932	2.1
48683	0.708518	2.1
48684	0.801358	2.1
48685	0.476016	2.1
48686	0.569477	2.1
48687	0.878553	2.1
48688	1.89226	2.1
48689	1.89226	2.1
48690	1.89226	2.1
48691	1.89226	2.1
48692	1.89226	2.1
48693	1.89226	2.1
48694	0.946128	2.1
48695	0.946128	2.1
48696	1.01537	2.1
48697	0.9662	2.1
48698	0.946131	2.1
48699	0.946131	2.1
48700	0.966198	2.1
48701	0.966198	2.1
48702	0.94613	2.1
48703	0.966198	2.1
48704	0.946131	2.1
48705	1.01537	2.1
48706	1.89226	2.1
48707	1.89226	2.1
48708	1.91883	2.1
48709	1.91883	2.1
48710	2.15905	2.1
48711	2.13249	2.1
48712	1.07953	2.1
48713	1.2107	2.1
48714	0.946132	2.1
48715	0.946132	2.1
48716	1.2107	2.1
48717	1.2107	2.1

48718	0.946132	2.1
48719	0.946132	2.1
48720	1.2107	2.1
48721	0.946133	2.1
48722	0.946132	2.1
48723	1.07953	2.1
48724	2.46752	2.1
48725	2.53733	2.1
48726	2.26044	2.1
48727	2.33222	2.1
48728	4.79974	2.1
48729	2.53733	2.1
48730	2.37576	0.832172
48731	2.33222	2.1
48732	2.1845	2.1
48733	4.70798	2.1
48734	2.48652	2.1
48735	2.33224	2.1
48736	2.08149	2.1
48737	1.9839	2.1
48738	4.45623	2.1
48739	2.33223	2.1
48740	2.18452	1.76149
48741	2.16061	2.1
48742	2.01399	0.828242
48743	4.34623	2.07944
48744	1.4617	1.62611
48745	1.34701	2.1
48746	1.34701	1.92583
48747	1.25173	2.1
48748	1.5117	1.47887
48749	1.41642	2.1
48750	2.65164	2.1
48751	1.89227	2.1
48752	1.89227	2.1
48753	1.89227	2.1
48754	2.42504	2.1
48755	2.42504	2.1
48756	1.89226	1.16351
48757	1.89226	2.1
48758	1.89226	2.1
48759	1.89226	1.05679
48760	3.78452	1.47257
48761	1.5117	1.92651
48762	1.34701	2.1
48763	1.47001	2.1
48764	1.57961	1.23325
48765	2.92663	2.1
48766	1.89226	2.1
48767	1.89226	0.787422
48768	1.89226	1.22403
48769	1.89226	2.1
48770	3.78452	1.73105
48771	0.412773	0.05
48772	0.570839	0.05
48773	0.50721	0.05
48774	0.513638	0.05
48775	0.518805	0.05

48776	0.41278	0.05
48777	1.5344	0.05
48778	0.939282	0.05
48779	0.720051	0.05
48780	0.691767	0.05
48781	0.761152	0.05
48782	1.89226	2.1
48783	1.89226	2.1
48784	1.89226	2.1
48785	1.89226	2.1
48786	1.89226	2.1
48787	1.89226	2.1
48788	1.24618	2.1
48789	2.10585	2.1
48790	1.08134	2.1
48791	1.93032	2.1
48792	3.1765	2.1
48793	1.98474	2.1
48794	2.57777	2.1
48795	1.93031	2.1
48796	2.19041	2.1
48797	3.99251	2.1
48798	1.89226	2.1
48799	1.89226	2.1
48800	1.89226	2.1
48801	1.89226	2.1
48802	3.78452	2.1
48803	1.67374	2.1
48804	2.19042	2.1
48805	1.67374	2.1
48806	2.09769	2.1
48807	2.29586	2.1
48808	2.7198	2.1
48809	1.89226	2.1
48810	1.89226	2.1
48811	1.89226	2.1
48812	1.89226	2.1
48813	1.89226	2.1
48814	1.89226	2.1
48815	0.946132	0.700193
48816	0.946132	0.317169
48817	0.947763	0.375379
48818	0.909304	0.441086
48819	0.946127	0.246317
48820	0.947831	0.1303
48821	0.907673	0.243133
48822	0.907673	0.535767
48823	0.94613	0.318651
48824	0.947834	0.130792
48825	0.907671	0.720699
48826	0.94613	0.175661
48827	0.906519	0.185901
48828	0.906519	0.11803
48829	0.946097	0.102769
48830	0.946097	0.157976
48831	0.947834	0.130738
48832	0.906485	0.0801125
48833	0.946131	0.0749125

48834	0.946131	0.156486
48835	0.94613	0.255588
48836	0.906485	0.0637797
48837	0.946131	0.252598
48838	0.947835	0.120402
48839	2.58638	2.1
48840	2.51039	2.1
48841	2.47395	2.1
48842	2.41539	2.1
48843	5.00178	2.1
48844	2.51039	2.1
48845	2.16633	2.1
48846	2.4154	2.1
48847	2.13977	2.1
48848	4.58173	2.1
48849	2.47395	2.1
48850	2.41539	2.1
48851	2.2988	2.1
48852	2.2604	2.1
48853	4.7142	2.1
48854	2.4154	2.1
48855	2.13977	2.1
48856	2.26041	2.1
48857	1.98957	2.1
48858	4.40497	2.1
48859	2.15721	0.0507925
48860	2.31843	0.0801111
48861	1.93973	0.110439
48862	2.13243	0.05
48863	4.25816	0.05
48864	2.31844	0.567083
48865	2.30908	0.05
48866	2.13243	0.05
48867	2.1609	0.05
48868	4.47934	0.05
48869	1.93973	0.91922
48870	2.13243	0.0564092
48871	1.69661	0.0645006
48872	1.9339	0.05
48873	3.87363	0.05
48874	2.13243	0.146491
48875	2.1609	0.124755
48876	1.9339	0.05
48877	1.98414	0.0542264
48878	4.0948	0.05
48879	1.88857	0.05
48880	2.34107	0.05
48881	0.502431	0.05
48882	0.71416	0.05
48883	0.50243	0.05
48884	0.944284	0.05
48885	1.88856	2.1
48886	2.34107	2.1
48887	2.39662	2.1
48888	1.92293	2.1
48889	4.28519	2.1
48890	1.00486	2.1
48891	1.92293	2.1

48892	1.88857	2.1
48893	1.88857	2.1
48894	1.02605	2.1
48895	1.02605	2.1
48896	2.53728	2.1
48897	1.70303	2.1
48898	2.03734	2.1
48899	1.3624	2.1
48900	2.54706	2.1
48901	1.70303	2.1
48902	2.54706	2.1
48903	1.50876	2.1
48904	2.74067	2.1
48905	1.85391	2.1
48906	2.29667	2.1
48907	1.50876	2.1
48908	0.946133	2.1
48909	0.946133	2.1
48910	1.24191	2.1
48911	1.24191	2.1
48912	0.946132	2.1
48913	1.24191	2.1
48914	1.23779	2.1
48915	0.946132	2.1
48916	0.946132	2.1
48917	0.946132	2.1
48918	1.23779	2.1
48919	1.24191	2.1
48920	0.946131	2.1
48921	0.946131	2.1
48922	1.14833	2.1
48923	1.14833	2.1
48924	1.14834	2.1
48925	0.946132	2.1
48926	0.94613	2.1
48927	1.24191	2.1
48928	0.946131	2.1
48929	0.946131	2.1
48930	1.24191	2.1
48931	1.14833	2.1
48932	2.08149	2.1
48933	2.31116	2.1
48934	1.9701	1.58295
48935	2.18328	2.1
48936	4.49563	2.1
48937	2.08149	2.1
48938	2.14059	2.1
48939	1.72377	2.1
48940	2.18328	2.1
48941	3.88931	2.1
48942	2.14059	2.1
48943	2.40755	2.1
48944	2.31145	2.1
48945	2.23743	2.1
48946	2.31145	2.1
48947	2.23743	2.1
48948	2.31117	2.1
48949	2.40754	2.1

48950	2.41055	2.1
48951	2.31145	2.1
48952	2.41055	2.1
48953	2.31145	2.1
48954	2.5615	0.20726
48955	2.37625	0.323626
48956	2.20248	0.167187
48957	2.20248	0.110801
48958	2.65967	0.675868
48959	2.65967	0.350101
48960	2.71793	0.536973
48961	2.65966	0.307934
48962	2.56149	0.16352
48963	2.56149	0.155812
48964	2.71296	0.623823
48965	2.71296	0.420991
48966	1.31528	0.251574
48967	1.32983	0.144333
48968	1.21128	0.217967
48969	1.18812	0.141142
48970	1.24232	0.153315
48971	1.24232	0.115576
48972	1.26109	0.26859
48973	1.26109	0.348802
48974	1.21128	0.355142
48975	1.31528	0.05417
48976	1.18812	0.0695944
48977	1.32983	0.168951
48978	2.63056	0.123504
48979	2.58032	0.179508
48980	2.65966	0.05
48981	2.65966	0.165761
48982	2.58032	0.342357
48983	2.71296	0.37469
48984	1.92039	2.1
48985	1.06226	2.1
48986	1.09252	2.1
48987	1.96052	2.1
48988	3.01291	2.1
48989	1.92039	2.1
48990	2.46338	2.1
48991	2.47522	2.1
48992	2.43508	2.1
48993	1.96052	2.1
48994	1.92039	2.1
48995	0.985949	2.1
48996	0.980261	2.1
48997	0.553625	2.1
48998	0.546262	2.1
48999	0.765671	2.1
49000	0.765671	2.1
49001	0.766539	2.1
49002	0.766539	2.1
49003	0.985949	2.1
49004	0.553625	2.1
49005	0.546261	2.1
49006	0.980261	2.1
49007	1.96052	2.1

49008	2.46338	2.1
49009	1.97189	2.1
49010	1.96052	2.1
49011	2.47932	2.1
49012	2.47932	2.1
49013	0.964608	0.05
49014	0.691524	0.05
49015	1.25462	0.05
49016	0.691524	0.05
49017	1.89226	0.05
49018	1.89226	0.05
49019	1.44894	0.05
49020	2.77655	0.05
49021	1.4237	0.05
49022	2.77701	0.05
49023	2.77701	0.05
49024	1.38847	2.1
49025	1.46031	2.0464
49026	1.65504	1.37819
49027	1.75396	1.32942
49028	3.11535	1.73051
49029	1.89226	1.79889
49030	1.89226	2.1
49031	1.89226	1.49001
49032	1.89226	1.14945
49033	3.78452	1.79497
49034	1.89226	1.50503
49035	1.89226	2.1
49036	1.89226	1.00137
49037	1.89226	1.78125
49038	3.78452	1.14088
49039	1.46031	1.36299
49040	1.34851	2.1
49041	1.75395	1.17268
49042	1.62287	1.3894
49043	3.08318	1.89104
49044	1.65504	1.82759
49045	1.75396	1.97773
49046	1.89249	2.1
49047	1.9857	1.24726
49048	3.64073	1.8328
49049	1.89226	2.02985
49050	1.89226	1.78545
49051	1.89226	1.14161
49052	1.89226	1.30763
49053	3.78452	1.24595
49054	1.89226	1.25391
49055	1.89226	2.1
49056	1.89226	1.20887
49057	1.89226	1.64612
49058	3.78452	2.0403
49059	1.75395	1.6419
49060	1.62287	1.95486
49061	1.98569	1.12281
49062	1.86067	1.31865
49063	3.60856	1.2732
49064	1.35697	2.1
49065	2.02064	2.1

49066	1.89989	1.06536
49067	1.49571	2.1
49068	1.66753	2.07307
49069	1.36431	2.1
49070	0.744004	2.1
49071	0.743997	2.1
49072	0.717972	1.22016
49073	0.717972	2.1
49074	0.997982	2.1
49075	0.807796	2.1
49076	0.632235	1.79832
49077	0.488575	2.1
49078	0.849275	2.1
49079	0.488581	2.1
49080	0.724729	2.1
49081	0.997967	2.1
49082	2.5276	2.1
49083	1.70791	1.98035
49084	1.82872	0.216583
49085	1.56362	2.1
49086	2.5276	1.29116
49087	2.24781	2.1
49088	1.37591	2.1
49089	1.23217	2.05013
49090	1.00442	1.79012
49091	0.945282	1.0406
49092	1.15446	0.905033
49093	1.09123	2.1
49094	1.21506	2.1
49095	1.21506	1.24426
49096	1.39452	1.31964
49097	1.13108	1.83415
49098	1.27876	1.29784
49099	0.929006	1.18917
49100	1.46505	0.05
49101	1.91484	0.05
49102	1.49906	0.05
49103	1.49906	0.05
49104	1.90196	0.05
49105	1.90196	0.05
49106	1.90196	0.05
49107	2.11465	0.05
49108	1.91484	0.05
49109	1.91484	0.05
49110	2.04016	0.05
49111	2.04016	0.05
49112	0.912201	0.05
49113	0.950978	0.05
49114	0.665307	0.05
49115	0.732528	0.05
49116	0.829512	0.05
49117	0.829512	0.05
49118	0.815217	0.05
49119	0.815217	0.05
49120	0.912202	0.05
49121	0.665305	0.05
49122	0.732527	0.05
49123	0.95098	0.05

49124	1.90196	0.05
49125	2.08757	0.05
49126	1.9626	0.05
49127	2.04016	0.05
49128	1.82441	0.05
49129	1.90196	0.05
49130	1.13048	2.1
49131	0.821162	2.1
49132	0.946131	2.1
49133	0.946131	2.1
49134	1.04567	2.1
49135	0.882347	2.1
49136	0.897276	2.1
49137	0.897276	2.1
49138	0.863109	2.1
49139	1.01055	2.1
49140	0.940305	2.1
49141	0.942166	2.1
49142	2.23933	0.05
49143	0.976217	0.05
49144	2.78053	0.05
49145	2.78053	0.05
49146	0.976217	0.05
49147	1.65984	0.05
49148	1.54168	2.1
49149	2.01197	1.87877
49150	1.69136	2.1
49151	2.13284	1.09262
49152	3.67452	2.1
49153	0.988805	2.1
49154	1.78069	2.1
49155	1.69136	2.1
49156	1.69136	2.1
49157	1.05481	2.1
49158	1.05481	2.1
49159	1.69136	2.1
49160	2.13284	1.36297
49161	1.78069	2.1
49162	2.22044	1.83422
49163	3.91353	2.1
49164	2.69379	1.07904
49165	2.32219	1.14536
49166	2.25746	1.95329
49167	2.25746	1.03027
49168	2.32219	1.18639
49169	1.89526	1.00147
49170	1.4556	2.1
49171	1.85028	2.1
49172	2.4909	0.183073
49173	2.03799	2.1
49174	2.73495	1.997
49175	1.85028	2.1
49176	2.71938	0.331845
49177	1.85028	2.1
49178	3.36051	2.1
49179	2.73495	2.1
49180	2.47584	2.1
49181	1.85028	0.974095

49182	2.47584	2.1
49183	1.04413	2.1
49184	2.1461	2.1
49185	1.74586	2.1
49186	1.44437	2.1
49187	1.04413	2.1
49188	1.89226	2.1
49189	1.89226	1.3073
49190	1.89226	2.1
49191	1.89226	2.04651
49192	3.78452	2.1
49193	1.52724	2.1
49194	1.89249	2.1
49195	1.68143	2.1
49196	2.02433	1.80987
49197	3.55157	2.1
49198	1.07444	2.1
49199	1.76545	2.1
49200	1.68142	2.1
49201	1.68142	2.1
49202	1.14807	2.1
49203	1.14807	2.1
49204	1.89226	2.1
49205	1.89226	2.1
49206	1.89226	2.1
49207	1.89226	2.1
49208	1.89226	2.1
49209	1.89226	1.78599
49210	1.89226	2.1
49211	1.89226	2.1
49212	1.89226	2.1
49213	1.89226	1.09278
49214	3.78452	2.1
49215	1.68143	2.1
49216	2.02433	2.1
49217	1.76545	2.1
49218	2.12608	2.1
49219	3.78978	2.1
49220	2.62871	2.1
49221	1.49149	2.1
49222	1.36814	2.1
49223	2.44761	2.1
49224	3.9391	2.1
49225	1.89226	2.1
49226	1.89226	1.02683
49227	1.89226	2.1
49228	1.89226	2.1
49229	3.78452	2.1
49230	0.851542	2.1
49231	0.851542	2.1
49232	1.00897	2.1
49233	0.931224	2.1
49234	0.838952	2.1
49235	0.838952	2.1
49236	0.956848	2.1
49237	0.942474	2.1
49238	0.946132	2.1
49239	0.838952	2.1

49240	0.878743	2.1
49241	0.946132	2.1
49242	1.15541	2.1
49243	2.44761	1.20154
49244	1.36814	2.1
49245	1.36814	2.1
49246	2.19439	2.1
49247	2.19439	2.1
49248	1.89226	1.32506
49249	1.89226	2.1
49250	1.89226	2.1
49251	1.89226	1.09683
49252	3.78452	2.1
49253	1.89226	2.1
49254	1.67791	2.1
49255	1.5713	2.1
49256	1.67791	2.1
49257	1.78566	2.1
49258	1.89226	2.1
49259	1.89226	0.20057
49260	1.62911	2.1
49261	2.24602	1.99075
49262	1.89226	2.1
49263	1.98287	0.766429
49264	1.62911	0.744784
49265	1.89226	2.1
49266	1.89226	2.1
49267	1.89226	0.656168
49268	1.89226	2.1
49269	3.78452	1.63226
49270	1.26688	2.1
49271	1.11804	2.1
49272	0.852467	2.02061
49273	0.693314	2.1
49274	0.977459	2.1
49275	0.977459	2.1
49276	0.982741	1.85907
49277	0.982741	2.1
49278	1.26689	2.1
49279	0.852464	2.1
49280	0.693312	2.1
49281	1.11804	2.1
49282	0.946131	2.1
49283	0.946131	1.03606
49284	1.1794	1.02867
49285	1.1794	2.1
49286	0.991434	2.1
49287	1.1794	1.12624
49288	0.946131	0.410517
49289	0.946131	1.00195
49290	0.946131	2.04051
49291	1.1794	1.50508
49292	0.946131	1.99246
49293	0.991434	1.26582
49294	1.89226	1.0241
49295	1.89226	2.1
49296	1.89226	1.16543
49297	1.89226	1.41544

49298	3.78452	2.1
49299	2.53377	2.1
49300	1.53443	2.1
49301	2.0815	0.830526
49302	2.82979	2.1
49303	4.35462	2.1
49304	1.96192	0.906491
49305	1.23602	2.1
49306	2.16506	0.49642
49307	1.56841	1.86167
49308	3.40108	1.50749
49309	1.89226	2.0325
49310	1.89226	2.1
49311	1.89226	0.523391
49312	1.89226	2.1
49313	3.78452	1.79708
49314	0.946129	2.1
49315	0.946129	2.1
49316	1.13067	1.88142
49317	1.12952	2.1
49318	0.946129	2.1
49319	1.13089	2.1
49320	0.944978	1.91458
49321	0.944978	2.1
49322	0.946131	2.1
49323	1.13089	2.1
49324	0.944977	2.1
49325	0.946131	2.1
49326	0.710208	2.1
49327	0.710208	2.1
49328	0.718894	2.1
49329	0.718894	2.1
49330	0.559773	2.1
49331	0.579321	2.1
49332	0.886767	2.1
49333	0.849781	2.1
49334	0.849781	2.1
49335	0.559774	2.1
49336	0.579321	2.1
49337	0.886766	2.1
49338	1.63471	2.1
49339	1.69956	2.1
49340	1.08022	2.1
49341	1.08022	2.1
49342	1.11955	2.1
49343	1.67403	2.1
49344	1.03766	2.1
49345	2.02825	2.1
49346	2.02794	2.1
49347	2.0348	2.1
49348	1.07196	2.1
49349	1.07196	2.1
49350	1.9276	2.1
49351	2.1004	2.1
49352	2.1306	2.1
49353	2.1306	2.1
49354	1.92178	2.1
49355	1.92032	2.1

49356	0.986724	2.1
49357	2.03064	2.1
49358	2.01385	2.1
49359	2.02404	2.1
49360	1.03766	2.1
49361	1.03766	2.1
49362	1.92027	2.1
49363	2.1306	2.1
49364	2.15388	2.1
49365	2.15388	2.1
49366	1.92614	2.1
49367	1.9276	2.1
49368	1.09578	2.1
49369	0.963018	2.1
49370	0.782956	1.34371
49371	0.529693	2.1
49372	0.6994	1.62678
49373	0.862532	2.1
49374	0.898605	2.1
49375	0.898605	1.58951
49376	0.776756	2.1
49377	1.14805	2.1
49378	0.966521	2.1
49379	0.617676	2.1
49380	2.67185	2.1
49381	2.099	2.1
49382	2.80548	2.1
49383	2.2727	2.1
49384	4.94455	2.1
49385	1.89226	2.1
49386	1.89226	2.1
49387	1.89226	2.1
49388	1.89226	2.1
49389	3.78453	2.1
49390	1.89226	2.1
49391	1.89226	2.1
49392	1.89226	2.1
49393	1.89226	2.1
49394	1.89226	2.1
49395	1.89226	2.1
49396	0.884801	2.1
49397	0.884801	2.1
49398	0.895199	2.1
49399	0.895199	2.1
49400	0.622774	2.1
49401	0.730501	2.1
49402	1.0495	2.1
49403	1.13635	2.1
49404	1.13635	2.1
49405	0.730503	2.1
49406	1.0495	2.1
49407	0.622775	2.1
49408	1.89226	2.1
49409	1.89226	2.1
49410	1.89226	2.1
49411	1.89226	2.1
49412	1.89226	2.1
49413	1.89226	2.1

49414	1.89226	2.1
49415	1.89226	2.1
49416	1.89226	2.1
49417	1.89226	2.1
49418	1.89226	2.1
49419	1.89226	2.1
49420	2.2727	2.1
49421	1.89586	2.1
49422	1.66913	2.1
49423	1.46101	2.1
49424	2.48082	2.1
49425	2.2727	2.1
49426	1.89226	2.1
49427	1.89226	2.1
49428	1.84641	2.1
49429	1.89226	2.1
49430	1.30868	2.1
49431	1.34118	1.87815
49432	1.89226	1.13578
49433	1.89226	1.30361
49434	1.89226	1.43632
49435	1.89226	1.15852
49436	1.89226	1.22232
49437	1.89226	0.70001
49438	1.89226	1.33936
49439	1.89226	0.209165
49440	1.89226	1.10853
49441	1.89226	1.3036
49442	3.78452	1.17873
49443	0.946132	2.1
49444	0.946132	1.87955
49445	0.946131	2.1
49446	0.946131	2.1
49447	0.946132	2.1
49448	0.946132	1.42425
49449	0.946132	2.1
49450	0.946132	2.1
49451	0.946132	2.1
49452	0.946132	2.1
49453	0.946132	2.1
49454	0.946132	2.1
49455	1.89227	1.6513
49456	1.84641	2.1
49457	2.29517	2.1
49458	1.84641	1.60941
49459	2.34102	1.8963
49460	1.89227	1.22868
49461	1.89226	1.03055
49462	1.89226	0.56607
49463	1.89226	1.1856
49464	1.89226	0.873598
49465	1.89226	0.712717
49466	1.89226	0.173669
49467	1.89226	0.852701
49468	1.89226	0.745254
49469	1.89226	1.1453
49470	1.89226	0.844836
49471	1.89226	0.475792

49472	1.89226	0.098882
49473	1.89226	2.1
49474	1.89226	1.36666
49475	1.89226	2.1
49476	1.89226	1.29067
49477	1.89226	1.92238
49478	1.89226	1.25584
49479	0.851544	0.05
49480	0.757542	0.05
49481	1.13421	0.05
49482	1.04072	0.05
49483	0.946132	0.05
49484	0.946132	0.05
49485	0.841686	0.05
49486	0.834428	0.05
49487	1.13649	0.05
49488	0.937687	0.05
49489	0.841713	0.05
49490	0.841713	0.05
49491	1.89226	0.05
49492	1.89226	0.0556521
49493	1.89226	0.0579696
49494	1.89226	0.05
49495	1.89226	0.0657654
49496	1.89226	0.05
49497	2.31988	1.46072
49498	2.00082	2.1
49499	2.4594	0.906849
49500	2.17638	2.1
49501	4.46022	1.3173
49502	2.00082	1.26633
49503	1.41829	2.1
49504	1.61207	2.05209
49505	1.43652	2.1
49506	2.17637	0.692112
49507	2.00082	2.1
49508	2.4594	1.10305
49509	2.17638	2.1
49510	2.57957	1.05055
49511	2.3506	2.1
49512	4.81	1.45311
49513	2.3506	1.14772
49514	1.57368	2.1
49515	1.89111	0.151199
49516	1.89111	2.1
49517	2.17638	0.155756
49518	2.17638	2.1
49519	1.32813	2.1
49520	1.7237	2.1
49521	1.32461	2.1
49522	1.81656	2.1
49523	3.14468	2.1
49524	1.55132	2.1
49525	2.0815	2.1
49526	1.81656	2.1
49527	2.19826	2.1
49528	3.65818	2.1
49529	0.775951	2.1

49530	0.775951	2.1
49531	0.794601	2.1
49532	0.794601	2.1
49533	0.945641	2.1
49534	0.90828	2.1
49535	0.662302	2.1
49536	0.662273	2.1
49537	0.662274	2.1
49538	0.945639	2.1
49539	0.662304	2.1
49540	0.908279	2.1
49541	1.03062	2.1
49542	1.03062	2.1
49543	1.05054	2.1
49544	1.05054	2.1
49545	1.13551	2.1
49546	1.21275	2.1
49547	0.945641	2.1
49548	0.90828	2.1
49549	0.945641	2.1
49550	1.21275	2.1
49551	0.90828	2.1
49552	1.13552	2.1
49553	1.89226	2.1
49554	1.79883	2.1
49555	1.95341	2.1
49556	1.89226	2.1
49557	1.85998	2.1
49558	1.79883	2.1
49559	1.89226	2.1
49560	1.89226	2.1
49561	1.89226	2.1
49562	1.89226	2.1
49563	3.78453	2.1
49564	0.851541	2.1
49565	0.851541	2.1
49566	0.970398	2.1
49567	0.954365	2.1
49568	0.855356	2.1
49569	0.855357	2.1
49570	0.946133	2.1
49571	0.946133	2.1
49572	1.04072	2.1
49573	0.970397	2.1
49574	0.950003	2.1
49575	0.851538	2.1
49576	1.89226	2.1
49577	1.85998	2.1
49578	1.95296	2.1
49579	1.89226	2.1
49580	1.92067	2.1
49581	1.85998	2.1
49582	0.946133	2.1
49583	0.946133	2.1
49584	0.946132	2.1
49585	0.946132	2.1
49586	0.946132	2.1
49587	0.946132	2.1

49588	0.946132	2.1
49589	0.946132	2.1
49590	0.946132	2.1
49591	0.946132	2.1
49592	0.946133	2.1
49593	0.946133	2.1
49594	1.82152	2.1
49595	1.89226	2.1
49596	1.89226	2.1
49597	2.11152	2.1
49598	1.7472	2.1
49599	1.89226	2.1
49600	2.02687	2.1
49601	2.29199	2.1
49602	1.2734	2.1
49603	1.41077	2.1
49604	1.47381	2.1
49605	2.29199	2.1
49606	1.46889	2.1
49607	2.29199	2.1
49608	1.86766	2.1
49609	2.59401	2.1
49610	4.15965	2.1
49611	1.58995	2.1
49612	1.74246	2.1
49613	1.46643	2.1
49614	1.46643	2.1
49615	1.85097	2.1
49616	1.85097	2.1
49617	1.58995	2.1
49618	1.66584	2.1
49619	1.89214	2.1
49620	1.91787	2.1
49621	3.48208	2.1
49622	1.18474	2.1
49623	1.8144	2.1
49624	1.1486	2.1
49625	1.1486	2.1
49626	2.13071	2.1
49627	2.1257	2.1
49628	2.0815	2.1
49629	2.51651	2.1
49630	1.73681	2.1
49631	2.01601	2.1
49632	2.21406	2.1
49633	2.56998	2.1
49634	1.19995	2.1
49635	1.06256	2.1
49636	0.620677	2.1
49637	0.598084	2.1
49638	0.831426	2.1
49639	0.946056	2.1
49640	0.827692	2.1
49641	0.827692	2.1
49642	1.08958	2.1
49643	0.630132	2.1
49644	0.577547	2.1
49645	1.06104	2.1

49646	2.0815	2.1
49647	2.66249	2.1
49648	2.78966	2.1
49649	2.47108	2.1
49650	2.33858	2.1
49651	2.0815	2.1
49652	1.53735	2.1
49653	1.42278	2.1
49654	1.23654	2.1
49655	1.34678	2.1
49656	2.76955	2.1
49657	1.89226	2.1
49658	1.89226	2.1
49659	1.89226	2.1
49660	1.89226	2.1
49661	3.78452	2.1
49662	1.23654	2.1
49663	1.34678	2.1
49664	1.19 2.1	
49665	1.08576	2.1
49666	2.43254	2.1
49667	1.38776	2.1
49668	1.29366	2.1
49669	0.946131	2.1
49670	0.946131	2.1
49671	0.946131	2.1
49672	0.946131	2.1
49673	1.38775	2.1
49674	1.38775	2.1
49675	1.29366	2.1
49676	0.946132	2.1
49677	0.946131	2.1
49678	1.38775	2.1
49679	0.946131	2.1
49680	0.946131	2.1
49681	0.946129	2.1
49682	0.946129	2.1
49683	0.94613	2.1
49684	0.94613	2.1
49685	0.946131	2.1
49686	0.946131	2.1
49687	0.946131	2.1
49688	0.946131	2.1
49689	0.94613	2.1
49690	0.94613	2.1
49691	1.89226	0.396524
49692	1.89226	1.11788
49693	1.89226	0.384865
49694	1.89226	0.676425
49695	1.89226	0.845074
49696	1.89226	0.987216
49697	1.89226	0.338335
49698	1.89226	2.1
49699	1.89226	0.0896591
49700	1.89226	0.329342
49701	1.89226	0.693405
49702	1.89226	1.13664
49703	1.89226	0.781394

49704	1.89226	1.15465
49705	1.89226	0.755566
49706	1.89226	0.890913
49707	1.89226	0.831641
49708	1.89226	1.1559
49709	0.92338	2.1
49710	0.92338	0.396544
49711	0.94428	0.475862
49712	0.94428	0.119307
49713	1.19528	1.17555
49714	1.30598	2.1
49715	0.714073	0.151753
49716	0.561679	1.56923
49717	1.19528	1.1937
49718	0.561678	1.17855
49719	0.714072	1.31421
49720	1.30598	1.3074
49721	2.61197	2.1
49722	1.89226	1.94073
49723	1.89226	1.31133
49724	1.89226	2.1
49725	2.39056	0.768253
49726	2.39056	2.1
49727	0.946132	2.1
49728	0.946132	1.10209
49729	0.946132	0.179004
49730	0.946132	0.360107
49731	0.946131	0.320743
49732	0.946131	2.1
49733	0.946132	1.0721
49734	0.946132	0.820756
49735	0.946132	0.944048
49736	0.946132	0.692891
49737	0.946132	0.586612
49738	0.946132	1.01007
49739	1.89224	2.1
49740	1.89224	2.1
49741	1.89224	2.1
49742	1.89224	2.1
49743	3.78448	2.1
49744	1.97089	2.1
49745	1.57428	2.1
49746	1.84991	2.1
49747	1.49078	2.1
49748	3.42419	2.1
49749	0.621927	2.1
49750	0.621927	2.1
49751	0.630824	2.1
49752	0.630824	2.1
49753	0.483483	2.1
49754	0.507361	2.1
49755	0.787142	2.1
49756	0.745391	2.1
49757	0.745381	2.1
49758	0.483476	2.1
49759	0.507368	2.1
49760	0.787152	2.1
49761	1.89224	2.1

49762	1.89224	2.1
49763	1.89224	2.1
49764	1.89224	2.1
49765	1.89224	2.1
49766	1.89224	2.1
49767	1.89227	2.1
49768	1.89227	2.1
49769	1.89227	2.1
49770	1.89227	2.1
49771	1.89227	2.1
49772	1.89227	2.1
49773	1.84994	2.1
49774	1.41758	2.1
49775	1.4908	2.1
49776	1.4908	2.1
49777	1.74391	2.1
49778	1.74391	2.1
49779	1.89226	2.1
49780	1.89226	2.1
49781	1.89226	2.1
49782	1.89226	2.1
49783	3.78452	2.1
49784	1.01323	2.1
49785	1.01323	2.1
49786	1.01445	2.1
49787	1.01445	2.1
49788	0.839531	2.1
49789	0.875652	2.1
49790	1.19063	2.1
49791	1.15203	2.1
49792	1.15203	2.1
49793	0.839531	2.1
49794	0.875652	2.1
49795	1.19063	2.1
49796	1.89226	2.1
49797	2.75587	2.1
49798	2.81512	2.1
49799	2.81512	2.1
49800	1.89226	2.1
49801	1.89226	2.1
49802	1.89226	2.1
49803	1.89226	2.1
49804	1.89226	2.1
49805	1.89226	2.1
49806	3.78452	2.1
49807	0.974831	2.1
49808	0.974831	2.1
49809	0.976681	2.1
49810	0.976681	2.1
49811	0.803179	2.1
49812	0.83953	2.1
49813	1.11198	2.1
49814	1.15203	2.1
49815	1.11198	2.1
49816	0.803179	2.1
49817	1.15203	2.1
49818	0.83953	2.1
49819	1.37794	2.1

49820	1.37794	2.1
49821	0.946128	2.1
49822	0.946128	2.1
49823	1.3477	2.1
49824	1.37793	2.1
49825	0.946131	2.1
49826	0.946131	2.1
49827	0.946129	2.1
49828	1.3477	2.1
49829	1.37793	2.1
49830	0.946129	2.1
49831	2.38577	1.70084
49832	1.20247	2.1
49833	1.75182	2.1
49834	2.82546	1.75963
49835	4.1376	2.1
49836	2.82546	2.1
49837	1.89226	1.00934
49838	2.38577	2.1
49839	2.38577	1.72443
49840	1.89226	2.1
49841	1.89226	1.48298
49842	1.035 2.1	
49843	2.15429	2.1
49844	0.970998	2.1
49845	2.0815	2.1
49846	2.89672	2.1
49847	0.946131	0.172282
49848	0.946131	0.137977
49849	0.853249	0.0658875
49850	1.02715	0.248793
49851	0.851537	0.0752467
49852	0.851537	0.0924218
49853	1.02663	0.0900457
49854	1.13139	0.05
49855	0.946128	0.0635172
49856	0.932039	0.258112
49857	0.843985	0.0504653
49858	0.946129	0.0707647
49859	1.89226	0.0795813
49860	1.86408	0.0803374
49861	1.96548	0.283336
49862	1.86408	0.128748
49863	1.99366	0.0589007
49864	1.89226	0.05
49865	2.70702	2.1
49866	2.08981	2.1
49867	2.08693	2.1
49868	2.08693	2.1
49869	2.7188	2.1
49870	2.7188	2.1
49871	2.08982	2.1
49872	1.01964	2.1
49873	2.08693	2.1
49874	1.04615	2.1
49875	3.13597	2.1
49876	2.70702	2.1
49877	2.08693	2.1

49878	2.68784	2.1
49879	2.08831	2.1
49880	4.77477	2.1
49881	0.778233	2.1
49882	0.778233	2.1
49883	0.789001	2.1
49884	0.789001	2.1
49885	0.545307	2.1
49886	0.523075	2.1
49887	1.04347	2.1
49888	1.04416	2.1
49889	1.04416	2.1
49890	0.545308	2.1
49891	1.04347	2.1
49892	0.523075	2.1
49893	1.89226	0.743131
49894	1.89226	0.705035
49895	1.89226	0.700153
49896	1.89226	0.480332
49897	3.78453	0.7
49898	1.39237	0.65478
49899	1.17933	1.85592
49900	0.755796	1.00406
49901	0.755796	1.374
49902	0.755767	0.0820653
49903	0.94613	1.59671
49904	1.15918	0.6376
49905	0.94613	0.714334
49906	1.11632	0.6955
49907	0.92595	0.196941
49908	0.94613	0.716842
49909	0.94613	0.714797
49910	1.89226	0.710267
49911	1.89226	0.696009
49912	1.89226	0.200937
49913	1.89226	0.490677
49914	3.78452	0.425034
49915	0.950067	0.139908
49916	0.75071	0.326034
49917	0.906516	0.23521
49918	0.906516	0.05
49919	0.81586	0.05
49920	0.851541	0.7
49921	0.962101	0.05
49922	0.851476	0.408497
49923	0.962105	0.05
49924	0.815808	0.05
49925	0.851529	0.565522
49926	0.851529	0.251076
49927	1.05032	0.0967969
49928	0.972326	0.116732
49929	0.906515	0.0561032
49930	0.906515	0.05
49931	1.12245	0.05
49932	1.04072	0.0797399
49933	0.815857	0.05
49934	0.851539	0.279934
49935	1.04451	0.05

49936	0.815853	0.220192
49937	0.919795	0.270992
49938	0.919795	0.456622
49939	1.5855	2.1
49940	2.14058	2.1
49941	1.9153	2.1
49942	1.79922	2.1
49943	3.72608	2.1
49944	1.58551	2.04321
49945	1.24201	2.1
49946	0.960287	2.1
49947	1.79922	2.1
49948	2.82752	2.1
49949	0.679602	2.1
49950	0.621009	2.1
49951	0.899614	2.1
49952	0.940441	2.1
49953	0.679602	2.1
49954	0.94044	2.1
49955	0.621009	2.1
49956	0.899615	2.1
49957	0.794027	2.1
49958	0.794027	2.1
49959	0.785189	2.1
49960	0.785189	2.1
49961	2.14058	2.1
49962	1.79922	2.1
49963	2.23742	2.1
49964	2.23742	2.1
49965	1.78404	2.1
49966	1.88088	2.1
49967	1.15177	2.1
49968	2.0815	2.1
49969	1.93765	2.1
49970	1.78342	2.1
49971	1.39511	2.1
49972	1.28406	2.1
49973	1.97719	2.1
49974	2.50057	0.10815
49975	2.35349	2.1
49976	2.35349	0.0866222
49977	2.31686	2.1
49978	2.32857	0.52328
49979	1.93765	2.1
49980	2.35349	0.366169
49981	1.70048	2.1
49982	2.15721	0.05
49983	4.05397	0.903888
49984	0.850388	2.1
49985	0.850388	2.1
49986	0.840019	2.1
49987	0.840019	2.1
49988	1.13736	2.1
49989	1.13852	2.1
49990	0.573661	2.1
49991	0.551891	2.1
49992	0.573661	2.1
49993	1.13852	2.1

49994	0.551892	2.1
49995	1.13736	2.1
49996	0.860203	2.1
49997	0.860203	2.1
49998	0.855758	2.1
49999	0.855758	2.1
50000	1.1423	2.1
50001	1.13852	2.1
50002	0.58628	2.1
50003	0.573661	2.1
50004	0.586281	2.1
50005	1.1423	2.1
50006	0.573661	2.1
50007	1.13852	2.1
50008	1.28918	1.02857
50009	0.999086	0.956381
50010	1.0383	2.02243
50011	0.736019	1.4578
50012	1.00954	1.53326
50013	1.00954	1.04509
50014	1.01566	0.907925
50015	1.01566	1.97755
50016	1.28918	1.23754
50017	1.0383	2.08224
50018	0.999086	1.60405
50019	0.736019	1.30781
50020	2.20797	2.1
50021	2.64748	2.1
50022	2.50375	2.1
50023	2.50375	2.1
50024	2.40858	2.1
50025	2.40858	2.1
50026	2.64748	2.1
50027	2.74744	2.1
50028	2.50375	2.1
50029	2.6521	2.1
50030	5.29958	2.1
50031	2.20797	2.1
50032	2.50375	2.1
50033	2.02064	2.1
50034	2.3606	2.1
50035	4.52439	2.1
50036	2.36059	2.1
50037	2.6521	2.1
50038	2.56952	2.1
50039	2.56952	2.1
50040	2.50375	2.1
50041	2.50375	2.1
50042	2.49439	0.102828
50043	1.89226	0.441977
50044	2.81337	0.324633
50045	1.89226	0.481358
50046	4.38665	0.442636
50047	1.89226	0.533743
50048	1.89226	0.61143
50049	1.89226	0.637346
50050	1.89226	0.618903
50051	3.78452	0.601878

50052	1.89226	0.458362
50053	1.89226	0.534926
50054	1.89226	0.518716
50055	1.89226	0.581692
50056	3.78452	0.522963
50057	1.93948	0.671396
50058	1.98874	0.491787
50059	1.62494	0.603072
50060	2.14218	0.662645
50061	1.62494	0.328886
50062	2.14218	0.46454
50063	1.47354	0.273031
50064	1.98874	0.13225
50065	2.01865	0.387102
50066	1.62495	0.168761
50067	2.01865	0.505152
50068	1.62495	0.123216
50069	1.89226	0.97615
50070	1.89226	2.1
50071	1.89226	2.1
50072	1.89226	2.1
50073	3.78453	2.1
50074	2.05257	2.1
50075	1.94441	2.1
50076	1.88487	2.1
50077	1.82441	2.1
50078	3.82928	2.1
50079	1.94441	2.1
50080	1.74391	2.1
50081	1.82441	2.1
50082	1.65636	2.1
50083	3.60077	2.1
50084	1.89226	2.1
50085	1.89226	2.1
50086	1.89226	2.1
50087	1.89226	2.1
50088	3.78452	2.1
50089	1.89226	2.1
50090	1.89226	2.1
50091	1.89226	0.680927
50092	1.89226	2.1
50093	3.78453	2.1
50094	1.88487	2.1
50095	1.82441	2.1
50096	1.75835	2.1
50097	1.72866	2.1
50098	3.61354	2.1
50099	1.82441	2.1
50100	1.65636	2.1
50101	1.72866	2.1
50102	1.58401	2.1
50103	3.38503	2.1
50104	1.89226	2.1
50105	1.89226	2.1
50106	1.89226	2.1
50107	1.89226	2.1
50108	3.78452	2.1
50109	2.27432	2.1

50110	1.33339	2.1
50111	1.38905	2.1
50112	2.34471	2.1
50113	3.66337	2.1
50114	1.89226	2.1
50115	2.34471	2.1
50116	2.27432	2.1
50117	2.27432	2.1
50118	1.89226	2.1
50119	1.89226	2.1
50120	1.20443	2.1
50121	1.17235	2.1
50122	0.724695	2.1
50123	0.694522	2.1
50124	0.949994	2.1
50125	0.949993	2.1
50126	0.948955	2.1
50127	0.948955	2.1
50128	1.20443	2.1
50129	0.7247	2.1
50130	0.694517	2.1
50131	1.17234	2.1
50132	1.89225	2.1
50133	2.40885	2.1
50134	2.3447	2.1
50135	2.3447	2.1
50136	1.89225	2.1
50137	1.89225	2.1
50138	1.37138	2.1
50139	1.82597	2.1
50140	1.45027	2.1
50141	1.92772	2.1
50142	3.27624	2.1
50143	1.89226	2.1
50144	1.89226	2.1
50145	1.89226	2.1
50146	1.89226	2.1
50147	3.78452	2.1
50148	1.89226	2.1
50149	1.89226	1.55384
50150	1.89226	2.1
50151	1.89226	2.1
50152	3.78452	2.1
50153	1.82597	2.1
50154	2.02312	0.703187
50155	1.92772	2.1
50156	2.15888	2.1
50157	3.98485	2.1
50158	1.53446	2.1
50159	1.92772	2.1
50160	2.03975	2.1
50161	2.03975	2.1
50162	1.45027	2.1
50163	1.45027	2.1
50164	1.89226	2.1
50165	1.89226	2.1
50166	1.89226	2.1
50167	1.89226	2.1

50168	1.89226	2.1
50169	1.89226	2.1
50170	1.89226	2.1
50171	1.89226	2.1
50172	1.89226	2.1
50173	1.89226	2.1
50174	3.78452	2.1
50175	1.92772	2.1
50176	2.15888	2.1
50177	2.03975	2.1
50178	2.3111	2.1
50179	4.19863	2.1
50180	1.89226	2.1
50181	1.89226	2.1
50182	1.89226	2.1
50183	1.89226	2.1
50184	3.78452	2.1
50185	1.78376	2.1
50186	1.3201	2.1
50187	1.78376	2.1
50188	1.86102	2.1
50189	1.2679	2.1
50190	1.34517	2.1
50191	1.89226	2.1
50192	2.52442	2.1
50193	1.89226	2.1
50194	2.51691	2.1
50195	4.41667	2.1
50196	1.89226	2.1
50197	1.89226	2.1
50198	1.89226	2.1
50199	1.89226	2.1
50200	3.78452	2.1
50201	1.78376	2.1
50202	1.2679	2.1
50203	1.71839	2.1
50204	1.23504	2.1
50205	3.0188	2.1
50206	1.89226	2.1
50207	2.51795	2.1
50208	2.51691	2.1
50209	2.51691	2.1
50210	1.89226	2.1
50211	1.89226	2.1
50212	1.89226	0.971096
50213	1.89226	1.08968
50214	1.89226	1.06738
50215	1.89226	1.02619
50216	3.78452	1.01947
50217	1.89227	1.01184
50218	1.89227	1.1046
50219	3.26088	1.10427
50220	2.79543	1.0681
50221	2.20733	1.1113
50222	1.89227	1.06869
50223	1.89226	1.05998
50224	1.89226	1.03483
50225	1.89226	0.993124

50226	1.89226	1.04577
50227	1.89226	1.01268
50228	1.89226	1.0731
50229	1.89227	1.0202
50230	2.20733	1.07588
50231	1.46973	1.13488
50232	1.89227	1.03039
50233	1.7848	1.13326
50234	2.20733	1.02971
50235	1.89226	2.1
50236	2.55682	2.1
50237	2.81593	2.1
50238	2.81593	2.1
50239	1.89226	2.1
50240	1.89226	2.1
50241	1.0607	2.1
50242	1.0607	2.1
50243	1.12617	2.1
50244	0.986957	2.1
50245	0.699328	2.1
50246	0.785567	2.1
50247	1.28855	2.1
50248	1.41913	2.1
50249	0.766737	2.1
50250	1.34711	2.1
50251	1.24643	2.1
50252	0.687289	2.1
50253	3.11063	0.0623751
50254	1.89226	0.228233
50255	1.96536	0.113605
50256	1.89226	0.133251
50257	1.96536	0.094175
50258	1.89226	0.113164
50259	1.89226	0.964894
50260	1.89226	0.37581
50261	1.89226	0.596781
50262	1.89226	0.417447
50263	3.78452	0.956136
50264	1.89226	0.13802
50265	1.96256	0.05
50266	1.89226	0.05
50267	1.89226	0.0955042
50268	2.49192	0.0771604
50269	2.49192	0.05
50270	1.85716	0.0676081
50271	2.56871	0.0715072
50272	1.03043	0.05
50273	1.03043	0.05
50274	2.48125	0.05
50275	1.65452	0.0679606
50276	1.89226	2.1
50277	2.09622	2.1
50278	2.09622	2.1
50279	2.08508	2.1
50280	1.89226	2.1
50281	1.88112	2.1
50282	1.04811	2.1
50283	1.04811	2.1

50284	0.946131	2.1
50285	0.946131	2.1
50286	1.05014	2.1
50287	1.04811	2.1
50288	0.94613	2.1
50289	0.94613	2.1
50290	0.946131	2.1
50291	1.05013	2.1
50292	1.04811	2.1
50293	0.946131	2.1
50294	1.398	2.1
50295	2.10766	2.1
50296	2.3018	2.1
50297	2.3018	2.1
50298	1.34008	2.1
50299	1.34008	2.1
50300	1.1509	2.1
50301	1.1509	2.1
50302	0.946132	2.1
50303	0.946132	2.1
50304	0.946132	2.1
50305	0.946132	2.1
50306	1.05383	2.1
50307	1.1509	2.1
50308	1.1509	2.1
50309	0.946133	2.1
50310	0.946132	2.1
50311	1.05383	2.1
50312	0.931141	2.1
50313	0.931141	2.1
50314	0.971946	2.1
50315	0.971946	2.1
50316	1.28569	2.1
50317	1.1509	2.1
50318	0.699	2.1
50319	0.752185	2.1
50320	1.28568	2.1
50321	0.752186	2.1
50322	0.699001	2.1
50323	1.1509	2.1
50324	2.30181	2.1
50325	1.89226	2.1
50326	1.89226	2.1
50327	1.89226	2.1
50328	2.57137	2.1
50329	2.57137	2.1
50330	2.0815	2.1
50331	1.21962	2.1
50332	1.55173	2.1
50333	2.57849	2.1
50334	3.79001	2.1
50335	1.38779	2.1
50336	1.2923	2.1
50337	1.03974	2.1
50338	1.28832	2.1
50339	1.33566	2.1
50340	1.21116	2.1
50341	1.22343	2.1

50342	1.22343	2.1
50343	1.42288	2.1
50344	1.20138	2.1
50345	1.26025	2.1
50346	1.00644	2.1
50347	1.26066	2.1
50348	1.14271	2.1
50349	0.886635	2.1
50350	0.795277	2.1
50351	1.02966	2.1
50352	1.02967	2.1
50353	1.05216	2.1
50354	1.05216	2.1
50355	1.26068	2.1
50356	0.790042	2.1
50357	0.795269	2.1
50358	1.20135	2.1
50359	1.89226	0.189334
50360	1.89226	0.104193
50361	1.89226	0.136767
50362	1.89226	0.0669761
50363	1.89226	0.333451
50364	1.89226	0.156773
50365	1.89227	0.0784969
50366	1.89227	0.0587995
50367	1.89227	0.0738377
50368	1.89227	0.05
50369	1.89227	0.132596
50370	1.89227	0.069957
50371	0.563873	0.05
50372	0.50988	0.05
50373	0.598693	0.0621613
50374	0.558819	0.05
50375	0.564855	0.05
50376	0.564855	0.05
50377	0.557838	0.05
50378	0.557838	0.05
50379	0.563873	0.05
50380	0.598694	0.05
50381	0.558819	0.05
50382	0.50988	0.05
50383	0.598693	0.0546097
50384	0.558819	0.05
50385	0.572737	0.120546
50386	0.534248	0.0690468
50387	0.566788	0.0590927
50388	0.566788	0.05
50389	0.566153	0.080439
50390	0.566153	0.0637699
50391	0.598694	0.0600326
50392	0.572737	0.070698
50393	0.534248	0.0588372
50394	0.558819	0.05
50395	1.85217	2.1
50396	1.93111	2.1
50397	1.92875	2.1
50398	2.02391	2.1
50399	3.85986	2.1

50400	1.93112	2.1
50401	1.80425	2.1
50402	2.02392	2.1
50403	1.90981	2.1
50404	3.84092	2.1
50405	1.92876	1.18729
50406	2.02393	2.1
50407	2.05274	2.1
50408	2.16783	2.1
50409	4.09659	2.1
50410	2.02393	2.1
50411	1.90982	2.1
50412	2.16784	2.1
50413	2.05967	2.1
50414	4.07766	2.1
50415	1.89226	1.56928
50416	1.89226	1.29642
50417	1.89226	1.50912
50418	1.89226	1.40245
50419	3.78453	1.19135
50420	1.89226	1.19799
50421	1.89226	1.5586
50422	1.89226	2.1
50423	2.44829	1.14879
50424	3.78453	1.9969
50425	1.89226	1.50399
50426	1.89226	1.71706
50427	1.89226	1.32115
50428	2.60452	1.23117
50429	3.78453	2.1
50430	1.58723	0.05
50431	1.88071	0.05
50432	2.97376	0.05
50433	2.13255	0.05
50434	4.43982	0.05
50435	2.20641	0.05
50436	1.33575	0.05
50437	2.15824	0.05
50438	2.15824	0.05
50439	1.2951	0.05
50440	1.2951	0.05
50441	0.825383	0.05
50442	0.825383	0.05
50443	0.856104	0.05
50444	0.856104	0.05
50445	0.952196	0.05
50446	0.602363	0.05
50447	0.667877	0.05
50448	1.07913	0.05
50449	0.602363	0.05
50450	0.667876	0.05
50451	1.07913	0.0527655
50452	0.952197	0.05
50453	2.57657	1.27122
50454	1.44437	2.1
50455	2.65378	2.1
50456	2.25076	2.1
50457	1.84739	2.1

50458	1.44437	2.1
50459	2.0189	0.05
50460	2.54752	0.05
50461	2.63975	0.05
50462	2.31049	0.05
50463	4.65865	0.05
50464	1.40707	0.05
50465	1.89605	0.05
50466	2.0189	0.05
50467	2.0189	0.05
50468	1.50438	0.05
50469	1.50438	0.05
50470	2.63971	0.05
50471	2.38246	0.05
50472	2.13402	0.05
50473	2.57503	0.05
50474	4.77373	0.05
50475	1.50436	0.05
50476	1.96803	0.05
50477	2.13402	0.05
50478	1.45446	0.05
50479	3.63838	0.05
50480	2.30793	0.05
50481	2.11032	0.05
50482	1.24082	0.05
50483	1.05689	0.05
50484	3.36482	0.05
50485	1.89226	0.0915365
50486	1.89226	0.155593
50487	1.89226	0.170396
50488	1.89226	0.110637
50489	3.78453	0.112958
50490	0.812967	0.05
50491	0.812967	0.05
50492	1.00181	0.05
50493	1.08141	0.05
50494	0.946132	0.05
50495	0.812938	0.0523716
50496	0.932815	0.064037
50497	1.05577	0.05
50498	0.812938	0.05
50499	0.932812	0.05
50500	0.946133	0.05
50501	0.946133	0.05
50502	2.53897	2.1
50503	1.89226	2.1
50504	1.89226	2.1
50505	1.89226	2.1
50506	2.3245	2.1
50507	2.3245	2.1
50508	1.00255	1.50616
50509	1.06561	2.1
50510	1.05481	0.0528168
50511	1.11077	2.1
50512	2.12042	2.1
50513	1.89226	1.7305
50514	1.89226	2.1
50515	1.89226	0.108733

50516	1.89226	2.1
50517	3.78453	1.84339
50518	1.89226	2.1
50519	2.77314	2.1
50520	2.81276	2.1
50521	2.81276	2.1
50522	1.89226	2.1
50523	1.89226	2.1
50524	1.11077	2.1
50525	1.15114	2.1
50526	1.10274	1.10187
50527	1.1032	2.1
50528	1.05481	2.1
50529	1.1032	1.22588
50530	1.89226	1.3592
50531	1.89226	2.1
50532	1.89226	0.05
50533	1.89226	2.1
50534	3.78452	1.32078
50535	0.721957	0.245379
50536	0.721957	0.247253
50537	0.946103	0.228522
50538	0.946103	0.0668329
50539	0.814237	0.06776
50540	0.721929	0.0553124
50541	0.946132	0.29529
50542	0.946132	0.347046
50543	0.721928	0.290956
50544	0.946131	0.451622
50545	0.946132	0.617902
50546	0.814237	0.154418
50547	1.89226	0.9832
50548	1.89226	0.311821
50549	1.89226	0.299399
50550	1.89226	0.352291
50551	1.89226	0.902424
50552	1.89226	0.963741
50553	1.37196	0.139776
50554	2.83556	0.0603142
50555	1.5165	0.107665
50556	1.5165	0.148279
50557	2.74923	0.0691947
50558	2.74923	0.10503
50559	0.946128	0.288564
50560	0.946128	0.453968
50561	0.827866	0.108478
50562	0.946132	0.178904
50563	0.94613	0.093544
50564	0.94613	0.11003
50565	0.94613	0.480665
50566	0.94613	0.424286
50567	0.94613	0.373985
50568	0.827864	0.529944
50569	0.94613	0.286099
50570	0.94613	0.75218
50571	1.36208	0.05
50572	2.74923	0.05
50573	1.37196	0.05

50574	1.37196	0.05
50575	2.53256	0.05
50576	2.53256	0.05
50577	1.07705	2.1
50578	1.07705	2.1
50579	1.08253	2.1
50580	1.08253	2.1
50581	0.762985	2.1
50582	0.781638	2.1
50583	1.37794	2.1
50584	1.40756	2.1
50585	1.37794	2.1
50586	0.762986	2.1
50587	0.781638	2.1
50588	1.40756	2.1
50589	2.69541	2.1
50590	2.75587	2.1
50591	1.48572	2.1
50592	1.48572	2.1
50593	1.52597	2.1
50594	2.73566	2.1
50595	2.0554	1.76348
50596	1.00387	1.12146
50597	2.01001	1.03648
50598	0.994002	0.981627
50599	3.01389	0.767853
50600	1.89226	2.1
50601	2.41579	2.1
50602	2.12209	2.1
50603	2.12209	2.1
50604	1.89226	2.1
50605	1.89226	2.1
50606	1.89226	2.1
50607	1.89226	2.1
50608	1.89226	2.1
50609	1.89226	2.1
50610	1.89226	2.1
50611	1.89226	2.1
50612	0.88209	2.1
50613	0.899471	2.1
50614	0.651501	2.1
50615	0.533531	2.1
50616	0.724105	2.1
50617	0.711243	2.1
50618	0.692622	2.1
50619	0.692622	2.1
50620	0.890668	2.1
50621	0.738066	2.1
50622	0.778223	2.1
50623	0.531121	2.1
50624	1.89226	2.1
50625	2.77504	2.1
50626	2.4158	2.1
50627	2.4158	2.1
50628	1.89226	2.1
50629	1.89226	2.1
50630	1.89226	2.1
50631	1.89226	2.1

50632	1.89226	2.1
50633	1.89226	2.1
50634	1.89226	2.1
50635	1.89226	2.1
50636	1.70303	2.1
50637	1.39832	2.1
50638	1.51749	2.1
50639	1.68783	2.1
50640	3.24844	2.1
50641	1.89226	2.1
50642	2.38311	2.1
50643	1.98321	2.1
50644	1.89226	2.1
50645	2.47406	2.1
50646	2.38311	2.1
50647	1.89226	2.1
50648	1.89226	2.1
50649	1.89226	2.1
50650	1.89226	2.1
50651	1.89226	2.1
50652	1.89226	2.1
50653	0.946131	2.1
50654	0.946131	2.1
50655	1.25167	2.1
50656	1.19156	2.1
50657	1.19156	2.1
50658	1.19156	2.1
50659	0.946133	2.1
50660	0.946133	2.1
50661	0.946131	2.1
50662	1.25167	2.1
50663	0.946131	2.1
50664	1.19156	2.1
50665	1.89226	2.1
50666	2.47406	2.1
50667	1.99996	2.1
50668	1.89226	2.1
50669	2.58176	2.1
50670	2.47406	2.1
50671	1.89226	2.1
50672	1.89226	2.1
50673	1.89226	2.1
50674	1.89226	2.1
50675	1.89226	2.1
50676	1.89226	2.1
50677	0.946131	2.1
50678	0.946131	2.1
50679	1.32241	2.1
50680	1.25167	2.1
50681	1.25167	2.1
50682	1.25167	2.1
50683	0.946133	2.1
50684	0.946133	2.1
50685	0.946131	2.1
50686	1.32241	2.1
50687	0.946131	2.1
50688	1.25167	2.1
50689	1.65151	2.1

50690	1.92471	2.1
50691	1.81834	2.1
50692	2.13272	2.1
50693	3.74306	2.1
50694	1.92472	2.1
50695	1.9943	2.1
50696	2.13273	2.1
50697	2.24475	2.1
50698	4.16947	2.1
50699	1.81834	2.1
50700	2.13272	2.1
50701	2.02687	2.1
50702	2.39605	2.1
50703	4.21439	2.1
50704	2.13273	2.1
50705	2.24475	2.1
50706	2.39605	2.1
50707	2.54903	2.1
50708	4.6408	2.1
50709	1.10534	2.1
50710	1.22993	2.1
50711	0.946132	2.1
50712	0.946132	2.1
50713	0.946132	2.1
50714	0.946132	2.1
50715	1.22993	2.1
50716	1.22993	2.1
50717	1.10534	2.1
50718	0.946131	2.1
50719	0.946132	2.1
50720	1.22993	2.1
50721	1.47221	2.1
50722	2.14647	2.1
50723	1.95567	2.1
50724	1.95567	2.1
50725	1.71082	2.1
50726	1.71082	2.1
50727	0.946131	2.1
50728	0.946131	2.1
50729	0.946131	2.1
50730	0.946131	2.1
50731	0.946132	2.1
50732	0.946132	2.1
50733	0.946131	2.1
50734	0.946131	2.1
50735	0.946131	2.1
50736	0.946131	2.1
50737	0.946132	2.1
50738	0.946132	2.1
50739	0.998086	2.1
50740	1.09429	2.1
50741	0.946125	2.1
50742	0.946125	2.1
50743	0.90732	2.1
50744	0.90732	2.1
50745	1.14414	2.1
50746	1.16553	2.1
50747	1.05018	2.1

50748	0.907332	2.1
50749	0.99482	2.1
50750	0.851529	2.1
50751	0.80534	2.1
50752	0.80534	2.1
50753	0.812206	2.1
50754	0.812206	2.1
50755	0.895134	2.1
50756	0.977828	2.1
50757	0.736099	2.1
50758	0.639718	2.1
50759	0.639722	2.1
50760	0.89514	2.1
50761	0.736095	2.1
50762	0.977821	2.1
50763	0.946125	2.1
50764	0.946125	2.1
50765	0.946125	2.1
50766	0.946125	2.1
50767	0.946125	2.1
50768	0.946125	2.1
50769	0.946124	2.1
50770	0.946124	2.1
50771	0.946131	2.1
50772	0.946131	2.1
50773	0.946118	2.1
50774	0.946118	2.1
50775	2.22622	0.05
50776	2.43347	0.05
50777	2.34108	0.05
50778	1.24587	2.1
50779	1.21674	2.1
50780	1.14533	2.1
50781	1.11311	2.1
50782	1.17874	2.1
50783	1.17874	2.1
50784	1.18024	2.1
50785	1.18024	2.1
50786	1.14533	2.1
50787	1.24587	2.1
50788	1.21674	2.1
50789	1.11311	2.1
50790	2.43347	2.1
50791	2.34107	2.1
50792	2.39663	2.1
50793	2.49174	2.1
50794	4.8301	2.1
50795	1.86206	2.1
50796	1.41058	2.1
50797	1.70302	2.1
50798	2.42083	2.1
50799	3.68129	2.1
50800	1.70302	2.1
50801	2.43549	2.1
50802	2.20075	2.1
50803	2.50358	2.1
50804	2.04916	2.1
50805	2.33113	2.1

50806	1.07624	2.1
50807	2.12621	2.1
50808	1.57297	2.1
50809	1.60383	2.1
50810	1.30047	2.1
50811	1.30047	2.1
50812	2.33379	2.1
50813	2.56149	1.36115
50814	2.36734	1.44063
50815	2.62796	1.41921
50816	4.92883	1.86434
50817	2.5615	1.38123
50818	2.57957	1.7299
50819	2.62796	1.25172
50820	2.69056	1.21348
50821	5.25206	1.81806
50822	2.39322	2.1
50823	2.62796	2.1
50824	2.6849	2.1
50825	2.6849	2.1
50826	2.36734	2.1
50827	2.36734	2.06957
50828	2.62796	1.85172
50829	2.69056	1.91976
50830	2.6849	1.03753
50831	2.77227	1.06176
50832	5.37546	1.19297
50833	1.89226	0.05
50834	1.89226	0.05
50835	1.89226	0.05
50836	1.89226	0.05
50837	1.89226	0.05
50838	1.89226	0.05
50839	1.06974	0.05
50840	1.06974	0.05
50841	1.02299	0.05
50842	1.02299	0.05
50843	1.35299	0.05
50844	1.26227	0.05
50845	0.646252	0.05
50846	0.830464	0.05
50847	0.830463	0.05
50848	1.35299	0.05
50849	1.26227	0.05
50850	0.646252	0.05
50851	2.52454	0.05
50852	1.89226	0.05
50853	2.70598	0.05
50854	1.89226	0.05
50855	4.59824	0.05
50856	1.89226	0.05
50857	1.89226	0.05
50858	1.89226	0.05
50859	1.89226	0.05
50860	1.89226	0.05
50861	1.89226	0.05
50862	1.89226	0.05
50863	1.89226	0.05

50864	1.89226	0.05
50865	1.89226	0.05
50866	1.89226	0.05
50867	1.89226	0.05
50868	1.7666	0.05
50869	2.70598	0.05
50870	2.65378	0.05
50871	2.65378	0.05
50872	1.66093	0.05
50873	1.66093	0.05
50874	2.70598	0.05
50875	1.89226	0.05
50876	1.89226	0.05
50877	1.89226	0.05
50878	2.65377	0.05
50879	2.65377	0.05
50880	0.946132	0.05
50881	0.946132	0.05
50882	0.946131	0.05
50883	0.946131	0.05
50884	0.946131	0.05
50885	0.946131	0.05
50886	0.946132	0.05
50887	0.946132	0.05
50888	0.946132	0.05
50889	0.946132	0.05
50890	0.946131	0.05
50891	0.946131	0.05
50892	1.69858	2.1
50893	2.18238	2.1
50894	2.506	2.1
50895	1.93586	2.1
50896	4.20458	2.1
50897	2.21772	2.1
50898	2.18238	2.1
50899	2.506	2.1
50900	2.59215	2.1
50901	4.72372	2.1
50902	2.50596	2.1
50903	1.93584	2.1
50904	2.16728	2.1
50905	2.7679	2.1
50906	4.67325	2.1
50907	1.53296	2.1
50908	1.56299	2.1
50909	1.42624	2.1
50910	1.52961	2.1
50911	2.98923	2.1
50912	1.56299	2.1
50913	1.46568	2.1
50914	1.52961	2.1
50915	1.51916	2.1
50916	3.08215	2.1
50917	1.36226	2.1
50918	1.52961	2.1
50919	1.36226	2.1
50920	1.42624	2.1
50921	1.55369	2.1

50922	1.61768	2.1
50923	1.52961	2.1
50924	1.51916	2.1
50925	1.55369	2.1
50926	1.64892	2.1
50927	3.07285	2.1
50928	2.13405	0.05
50929	2.57507	0.05
50930	2.39438	0.05
50931	1.93408	0.05
50932	4.52843	0.05
50933	2.13405	0.05
50934	1.27778	0.05
50935	1.2545	0.05
50936	1.45448	0.05
50937	1.93408	0.05
50938	2.13405	0.05
50939	2.39438	0.05
50940	1.93407	0.05
50941	1.60578	0.05
50942	1.87985	0.05
50943	4.00016	0.05
50944	1.0525	2.1
50945	1.05758	2.1
50946	1.43484	1.11964
50947	1.38506	1.97444
50948	2.48734	2.08453
50949	1.20455	2.1
50950	1.67352	2.1
50951	1.52852	2.1
50952	2.05164	2.1
50953	3.20203	2.1
50954	1.89226	2.1
50955	1.89226	2.1
50956	1.89226	2.1
50957	1.89226	2.1
50958	3.78452	2.1
50959	1.89226	2.1
50960	1.89226	2.1
50961	1.89226	2.1
50962	1.89226	2.1
50963	1.89226	2.1
50964	1.89226	2.1
50965	2.05165	2.1
50966	1.94291	2.1
50967	2.09398	2.1
50968	1.67352	2.1
50969	2.36337	2.1
50970	1.94291	2.1
50971	1.52851	2.1
50972	2.52049	2.1
50973	2.43433	2.1
50974	2.05164	2.1
50975	1.9112	2.1
50976	1.52851	2.1
50977	1.89226	2.1
50978	1.89226	2.1
50979	1.89226	2.1

50980	1.89226	2.1
50981	3.78451	2.1
50982	1.89226	2.1
50983	1.89226	2.1
50984	1.89226	2.1
50985	1.89226	2.1
50986	3.78452	2.1
50987	2.5205	2.1
50988	2.36337	2.1
50989	2.4605	2.1
50990	2.05165	2.1
50991	2.77222	2.1
50992	2.36337	2.1
50993	1.89226	2.1
50994	1.89226	2.1
50995	1.89226	2.1
50996	1.89226	2.1
50997	1.89226	2.1
50998	1.89226	2.1
50999	1.89226	2.1
51000	1.89226	2.1
51001	1.89226	2.1
51002	1.89226	2.1
51003	3.78452	2.1
51004	1.38905	2.1
51005	1.26166	2.1
51006	1.19216	2.1
51007	1.0875	2.1
51008	1.24393	2.1
51009	1.24393	2.1
51010	1.23261	2.1
51011	1.23261	2.1
51012	1.38905	2.1
51013	1.19216	2.1
51014	1.26166	2.1
51015	1.0875	2.1
51016	1.16233	2.1
51017	1.06028	2.1
51018	0.777395	2.1
51019	0.705508	2.1
51020	0.940018	2.1
51021	0.936465	2.1
51022	0.954216	2.1
51023	0.954216	2.1
51024	1.16125	2.1
51025	0.685505	2.1
51026	1.1157	2.1
51027	0.703534	2.1
51028	1.89226	2.1
51029	1.89226	2.1
51030	1.89226	2.1
51031	1.89226	2.1
51032	1.89226	2.1
51033	1.89226	2.1
51034	1.89226	2.1
51035	1.89226	2.1
51036	1.89226	2.1
51037	1.89226	2.1

51038	1.89226	2.1
51039	1.89226	2.1
51040	2.38433	2.1
51041	1.43456	2.1
51042	1.93881	2.1
51043	2.36952	2.1
51044	4.32313	2.1
51045	1.8493	2.1
51046	1.5927	2.1
51047	1.45535	2.1
51048	1.38438	2.1
51049	1.89657	2.1
51050	1.8493	2.1
51051	2.0815	2.1
51052	1.6697	2.1
51053	2.0657	2.1
51054	1.5927	2.1
51055	2.59749	2.1
51056	2.0815	2.1
51057	2.76597	2.1
51058	1.89226	2.1
51059	1.89226	2.1
51060	1.89226	2.1
51061	2.6126	2.1
51062	2.6126	2.1
51063	2.62513	2.1
51064	1.89226	2.1
51065	1.89226	2.1
51066	1.89226	2.1
51067	2.76598	2.1
51068	2.76598	2.1
51069	2.54665	2.1
51070	2.6126	2.1
51071	2.76598	2.1
51072	2.76598	2.1
51073	2.34044	2.1
51074	2.34044	2.1
51075	2.62514	2.1
51076	2.76598	2.1
51077	2.54666	2.1
51078	2.45212	2.1
51079	5.2181	2.1
51080	2.14063	2.1
51081	1.47521	2.1
51082	1.27679	2.1
51083	1.47521	2.1
51084	2.11511	2.1
51085	2.31353	2.1
51086	0.93256	2.1
51087	1.07032	2.1
51088	0.638396	2.1
51089	0.521342	2.1
51090	0.790674	2.1
51091	0.790674	2.1
51092	0.80098	2.1
51093	0.800981	2.1
51094	0.932535	2.1
51095	0.521328	2.1

51096	1.07034	2.1
51097	0.638414	2.1
51098	1.89226	2.1
51099	1.89226	1.55668
51100	1.89226	2.04924
51101	1.89226	2.1
51102	3.78453	2.1
51103	1.89226	2.1
51104	1.9842	2.1
51105	1.89226	2.1
51106	1.89226	2.1
51107	2.79705	2.1
51108	2.79705	2.1
51109	0.469955	2.1
51110	0.791665	2.1
51111	0.989115	1.94481
51112	0.763235	2.1
51113	0.776356	2.1
51114	0.805461	2.1
51115	0.776759	2.1
51116	0.776759	2.1
51117	0.799151	2.1
51118	0.940869	2.1
51119	0.78449	2.1
51120	0.489635	2.1
51121	1.89226	1.60997
51122	1.89226	2.1
51123	1.89226	1.50392
51124	1.89226	2.1
51125	1.89226	1.45708
51126	1.89226	2.1
51127	2.46058	1.96552
51128	2.28991	2.1
51129	1.8525	2.1
51130	1.87697	2.1
51131	2.28991	2.02631
51132	1.62397	2.1
51133	0.946131	0.05
51134	0.974033	0.05
51135	1.31667	0.05
51136	0.946131	0.05
51137	0.885497	0.05
51138	0.885497	0.05
51139	1.0468	0.05
51140	1.04678	0.05
51141	1.31667	0.05
51142	0.974019	0.05
51143	0.946131	0.05
51144	0.946131	0.05
51145	1.89226	0.05
51146	1.89226	0.05
51147	1.89226	0.05
51148	1.89226	0.05
51149	3.78452	0.05
51150	0.946125	0.05
51151	0.946125	0.05
51152	0.946131	0.05
51153	0.946131	0.05

51154	0.946128	0.05
51155	0.946128	0.05
51156	0.946128	0.05
51157	0.946128	0.05
51158	0.946128	0.05
51159	0.946128	0.05
51160	0.946128	0.05
51161	0.946128	0.05
51162	0.946131	0.05
51163	0.98453	0.05
51164	1.32276	0.05
51165	0.946131	0.05
51166	0.946132	0.05
51167	0.946132	0.05
51168	0.98453	0.05
51169	1.32276	0.05
51170	1.32276	0.05
51171	1.32276	0.05
51172	0.946132	0.05
51173	0.946132	0.05
51174	1.89227	2.1
51175	1.89227	2.1
51176	1.89227	2.1
51177	1.89227	2.1
51178	1.89227	2.1
51179	1.89227	2.1
51180	1.89226	2.1
51181	1.89226	2.1
51182	1.89226	2.1
51183	1.89226	2.1
51184	1.89226	2.1
51185	1.89226	2.1
51186	1.05556	2.1
51187	1.38859	2.1
51188	1.46996	2.1
51189	1.25364	2.1
51190	2.52552	2.1
51191	0.694296	2.1
51192	0.73498	2.1
51193	0.561881	2.1
51194	0.512189	2.1
51195	0.621304	2.1
51196	0.621304	2.1
51197	0.625865	2.1
51198	0.625865	2.1
51199	0.734979	2.1
51200	0.561881	2.1
51201	0.51219	2.1
51202	0.694296	2.1
51203	0.946126	2.1
51204	0.946126	2.1
51205	0.946126	2.1
51206	0.946126	2.1
51207	0.946126	2.1
51208	0.946126	2.1
51209	0.946126	2.1
51210	0.946126	2.1
51211	0.946132	2.1

51212	0.946132	2.1
51213	0.94612	2.1
51214	0.94612	2.1
51215	1.89225	2.1
51216	1.89225	2.1
51217	1.89225	2.1
51218	1.89225	2.1
51219	1.89225	2.1
51220	1.89225	2.1
51221	0.626814	2.1
51222	0.632155	2.1
51223	0.746234	2.1
51224	0.734974	2.1
51225	0.68202	2.1
51226	0.68202	2.1
51227	0.685108	2.1
51228	0.685108	2.1
51229	0.746239	2.1
51230	0.632159	2.1
51231	0.734969	2.1
51232	0.62681	2.1
51233	0.746235	2.1
51234	0.734975	2.1
51235	0.595081	2.1
51236	0.561877	2.1
51237	0.648584	2.1
51238	0.648584	2.1
51239	0.659528	2.1
51240	0.659528	2.1
51241	0.746239	2.1
51242	0.595085	2.1
51243	0.734969	2.1
51244	0.561874	2.1
51245	1.89249	2.1
51246	2.2137	2.1
51247	1.33125	2.1
51248	1.06499	2.1
51249	2.12571	2.1
51250	1.78918	2.1
51251	0.946131	2.1
51252	0.946131	2.1
51253	0.94613	2.1
51254	0.94613	2.1
51255	0.946132	2.1
51256	0.946132	2.1
51257	0.946129	2.1
51258	0.946129	2.1
51259	0.946131	2.1
51260	0.946131	2.1
51261	0.946131	2.1
51262	0.946131	2.1
51263	1.89227	2.1
51264	1.89227	2.1
51265	1.89227	2.1
51266	1.89227	2.1
51267	3.78453	2.1
51268	1.89226	2.1
51269	1.89226	2.1

51270	1.89226	2.1
51271	1.89226	2.1
51272	1.89226	2.1
51273	1.89226	2.1
51274	2.01388	2.1
51275	1.38757	2.1
51276	1.24229	2.1
51277	1.3309	2.1
51278	1.88011	2.1
51279	2.01388	2.1
51280	2.01388	2.1
51281	2.01555	2.1
51282	2.13879	2.1
51283	1.80766	2.1
51284	4.15267	2.1
51285	0.946133	2.1
51286	0.946133	2.1
51287	0.946132	2.1
51288	0.946132	2.1
51289	0.946132	2.1
51290	0.946132	2.1
51291	0.946133	2.1
51292	0.946133	2.1
51293	0.946132	2.1
51294	0.946132	2.1
51295	0.946132	2.1
51296	0.946132	2.1
51297	1.89226	2.1
51298	1.89226	2.1
51299	1.89226	2.1
51300	1.89226	2.1
51301	1.89226	2.1
51302	1.89226	2.1
51303	0.676745	2.1
51304	0.68382	2.1
51305	1.07048	2.1
51306	1.1327	2.1
51307	0.929324	2.1
51308	0.871438	2.1
51309	0.867362	2.1
51310	0.867362	2.1
51311	1.10666	2.1
51312	0.695036	2.1
51313	0.744098	2.1
51314	1.10144	2.1
51315	2.25168	2.1
51316	2.22499	2.1
51317	2.15929	2.1
51318	2.13879	2.1
51319	2.15929	2.1
51320	2.13879	2.1
51321	2.15314	2.1
51322	1.70302	2.1
51323	2.24694	2.1
51324	1.65245	2.1
51325	3.82722	2.1
51326	1.65245	2.1
51327	1.00371	2.1

51328	1.7437	2.1
51329	1.65748	2.1
51330	1.10459	2.1
51331	1.00371	2.1
51332	2.24694	2.1
51333	1.65246	2.1
51334	2.69773	2.1
51335	1.88129	2.1
51336	4.12824	2.1
51337	1.65246	2.1
51338	1.10459	2.1
51339	1.88129	2.1
51340	1.05914	2.1
51341	2.76496	2.1
51342	1.89226	2.1
51343	1.89226	2.1
51344	1.89226	2.1
51345	1.89226	2.1
51346	3.78452	2.1
51347	0.718577	2.1
51348	0.718577	2.1
51349	0.725249	2.1
51350	0.725249	2.1
51351	0.597916	2.1
51352	0.61752	2.1
51353	0.826306	2.1
51354	0.859194	2.1
51355	0.826306	2.1
51356	0.597916	2.1
51357	0.859194	2.1
51358	0.61752	2.1
51359	1.25898	2.1
51360	1.25898	2.1
51361	0.946128	2.1
51362	0.946128	2.1
51363	1.25897	2.1
51364	1.25585	2.1
51365	0.946131	2.1
51366	0.946131	2.1
51367	0.946129	2.1
51368	1.25586	2.1
51369	1.25898	2.1
51370	0.946129	2.1
51371	1.89226	2.1
51372	1.89226	2.1
51373	1.89226	2.1
51374	1.89226	2.1
51375	3.78452	2.1
51376	0.693258	2.1
51377	0.693258	2.1
51378	0.697356	2.1
51379	0.697356	2.1
51380	0.572479	2.1
51381	0.597916	2.1
51382	0.792698	2.1
51383	0.826306	2.1
51384	0.572478	2.1
51385	0.792697	2.1

51386	0.826307	2.1
51387	0.597917	2.1
51388	1.25586	2.1
51389	1.25586	2.1
51390	0.946128	2.1
51391	0.946128	2.1
51392	1.25585	2.1
51393	1.24344	2.1
51394	0.946131	2.1
51395	0.946131	2.1
51396	0.946129	2.1
51397	1.24344	2.1
51398	0.94613	2.1
51399	1.25586	2.1
51400	1.65151	2.1
51401	1.81835	2.1
51402	1.12064	2.1
51403	1.12064	2.1
51404	1.24965	2.1
51405	1.81835	2.1
51406	1.24965	2.1
51407	1.81835	2.1
51408	1.41077	2.1
51409	2.02687	2.1
51410	3.22911	2.1
51411	1.89122	1.39398
51412	1.38776	2.1
51413	2.37014	1.09889
51414	1.80818	2.1
51415	3.6994	2.09927
51416	2.15466	1.15931
51417	2.00083	2.1
51418	1.80818	1.9673
51419	1.80818	1.21938
51420	2.31988	2.07362
51421	2.31988	1.14225
51422	1.80818	0.346247
51423	1.18041	2.1
51424	2.00082	2.1
51425	1.43652	2.1
51426	3.18123	2.1
51427	1.89226	1.16457
51428	1.89226	1.74554
51429	1.89226	1.23461
51430	1.89226	1.14054
51431	3.78453	1.20075
51432	1.89226	1.72289
51433	1.89226	1.70561
51434	1.89226	1.2431
51435	1.89226	1.21139
51436	3.78452	1.91259
51437	0.80759	1.31594
51438	0.686119	1.7383
51439	0.972147	1.46498
51440	1.00217	1.59259
51441	0.821334	2.03536
51442	0.821334	1.74429
51443	0.866957	1.39726

51444	0.866957	1.33159
51445	0.972145	1.89604
51446	0.686121	2.1
51447	0.807592	1.98695
51448	1.00217	2.1
51449	0.972146	1.46146
51450	1.00217	1.64085
51451	1.26654	1.90661
51452	1.1908	1.49117
51453	1.05501	1.54958
51454	1.05501	2.1
51455	1.10794	1.37995
51456	1.10794	1.09158
51457	0.972146	1.08079
51458	1.26654	1.65422
51459	1.00217	1.41242
51460	1.1908	2.09423
51461	1.89226	1.3909
51462	1.89226	1.11166
51463	1.89226	1.17408
51464	1.89226	1.9746
51465	1.89226	1.03953
51466	1.89226	1.25302
51467	0.972147	1.49231
51468	0.867854	1.82287
51469	1.26132	1.27953
51470	1.26654	1.55408
51471	1.0424	1.82879
51472	1.0424	2.1
51473	1.092	1.08644
51474	1.092	1.59563
51475	1.26132	1.79457
51476	0.867854	2.1
51477	0.972147	2.0236
51478	1.26654	2.1
51479	1.4375	0.054868
51480	1.92998	0.05
51481	1.66953	0.05
51482	1.0953	0.05
51483	3.10703	0.05
51484	1.89226	2.1
51485	1.89226	2.1
51486	1.89226	2.1
51487	1.89226	2.1
51488	3.78452	2.1
51489	2.74034	2.1
51490	2.08808	2.1
51491	2.63859	2.1
51492	2.00343	2.1
51493	4.72667	2.1
51494	0.799825	2.1
51495	0.799825	2.1
51496	0.80211	2.1
51497	0.80211	2.1
51498	0.562428	2.1
51499	0.600223	2.1
51500	1.04404	2.1
51501	1.00171	2.1

51502	1.00171	2.1
51503	0.56243	2.1
51504	0.600225	2.1
51505	1.04404	2.1
51506	0.94613	2.1
51507	0.94613	2.1
51508	0.946128	2.1
51509	0.946128	2.1
51510	0.946127	2.1
51511	0.946127	2.1
51512	0.946131	2.1
51513	0.946131	2.1
51514	0.946129	2.1
51515	0.946129	2.1
51516	0.946129	2.1
51517	0.946129	2.1
51518	1.89226	2.1
51519	1.89226	2.1
51520	1.89226	2.1
51521	1.89226	2.1
51522	3.78452	2.1
51523	2.63859	2.1
51524	2.00343	2.1
51525	2.54388	2.1
51526	1.91548	2.1
51527	4.55407	2.1
51528	1.91548	2.1
51529	2.00342	2.1
51530	1.04909	2.1
51531	1.04909	2.1
51532	1.12486	2.1
51533	1.99124	2.1
51534	0.810942	2.1
51535	0.810942	2.1
51536	0.810942	2.1
51537	0.810942	2.1
51538	1.89226	2.1
51539	1.35169	2.1
51540	1.08131	2.1
51541	1.08132	2.1
51542	1.08132	2.1
51543	0.810944	2.1
51544	0.810944	2.1
51545	1.89226	0.730404
51546	1.89226	0.875991
51547	1.89226	0.106349
51548	1.89226	0.866923
51549	3.78452	0.560508
51550	1.7968	0.412192
51551	1.49831	0.852944
51552	1.59244	0.05
51553	1.3395	0.113151
51554	3.09074	0.097836
51555	1.49831	0.107134
51556	1.16858	2.1
51557	1.33951	0.05
51558	1.0361	2.1
51559	2.53442	2.1

51560	1.89226	0.269667
51561	1.89226	0.10629
51562	1.89226	1.78351
51563	1.89226	2.1
51564	1.89226	2.1
51565	1.89226	2.1
51566	1.89223	0.728229
51567	1.89223	0.271954
51568	1.89223	0.782231
51569	1.89223	0.835712
51570	1.89223	0.05
51571	1.89223	0.530827
51572	1.59241	0.49296
51573	1.18952	0.145976
51574	1.33949	0.312479
51575	1.33949	0.585622
51576	1.40538	0.427803
51577	1.40538	0.679079
51578	1.89226	0.05
51579	1.89226	0.679129
51580	1.89226	0.625953
51581	1.89226	0.606199
51582	1.89226	0.690359
51583	1.89226	0.689195
51584	1.89226	2.1
51585	1.89226	2.1
51586	1.89226	2.1
51587	1.89226	2.1
51588	3.78452	2.1
51589	1.89226	2.1
51590	1.89226	2.1
51591	1.89226	2.1
51592	1.89226	2.1
51593	3.78452	2.1
51594	2.01867	2.1
51595	1.46955	2.1
51596	1.58476	2.1
51597	1.38189	2.1
51598	2.22153	2.1
51599	2.01867	2.1
51600	2.01867	2.1
51601	2.52881	2.1
51602	2.21732	2.1
51603	2.21732	2.1
51604	2.22154	2.1
51605	2.22154	2.1
51606	1.89226	2.1
51607	1.89226	2.1
51608	1.89226	2.1
51609	1.89226	2.1
51610	1.89226	2.1
51611	1.89226	2.1
51612	1.89226	2.1
51613	1.89226	2.1
51614	1.89226	2.1
51615	1.89226	2.1
51616	1.89226	2.1
51617	1.89226	2.1

51618	2.22153	2.1
51619	1.63361	2.1
51620	1.77336	2.1
51621	1.46955	2.1
51622	2.52534	2.1
51623	2.22153	2.1
51624	2.34431	2.1
51625	1.62905	2.1
51626	1.49693	2.1
51627	2.19197	2.1
51628	3.82103	2.1
51629	1.89226	2.1
51630	1.89226	2.1
51631	1.89226	2.1
51632	1.89226	2.1
51633	3.78453	2.1
51634	0.946128	2.1
51635	0.946128	2.1
51636	1.22372	2.1
51637	1.17331	2.1
51638	0.946131	2.1
51639	0.946131	2.1
51640	1.17331	2.1
51641	1.17331	2.1
51642	0.946131	2.1
51643	1.17331	2.1
51644	1.22372	2.1
51645	0.94613	2.1
51646	1.04041	2.1
51647	1.09599	2.1
51648	0.748462	2.1
51649	0.701976	2.1
51650	0.896701	2.1
51651	0.896701	2.1
51652	0.901262	2.1
51653	0.901262	2.1
51654	1.04041	2.1
51655	0.701977	2.1
51656	0.748463	2.1
51657	1.09599	2.1
51658	1.89226	2.1
51659	1.89226	2.1
51660	1.89226	2.1
51661	1.89226	2.1
51662	3.78453	2.1
51663	0.946128	2.1
51664	0.946128	2.1
51665	1.17331	2.1
51666	1.13785	2.1
51667	0.94613	2.1
51668	0.94613	2.1
51669	1.13785	2.1
51670	1.13785	2.1
51671	1.13785	2.1
51672	0.94613	2.1
51673	0.94613	2.1
51674	1.17331	2.1
51675	2.50381	2.1

51676	1.89226	2.1
51677	1.89226	2.1
51678	2.73387	2.1
51679	4.39607	2.1
51680	1.80045	2.1
51681	1.99777	0.982711
51682	2.45134	2.1
51683	2.12463	2.1
51684	1.70302	2.1
51685	1.42655	2.1
51686	2.20231	2.1
51687	1.70302	2.1
51688	1.93128	2.1
51689	1.66768	2.1
51690	1.24499	1.02837
51691	1.70302	0.648479
51692	2.55572	0.05
51693	1.22811	0.05
51694	1.82584	0.0720376
51695	1.22811	0.0541573
51696	2.62764	0.05
51697	1.93147	0.05
51698	2.14513	0.05
51699	1.89226	0.0773425
51700	2.66481	0.05
51701	1.89226	0.05
51702	2.66481	0.0909632
51703	1.89226	0.105884
51704	2.11649	2.1
51705	1.89226	2.1
51706	1.89226	2.1
51707	1.89226	2.1
51708	2.15426	2.1
51709	2.15426	2.1
51710	2.06776	2.1
51711	1.89226	2.1
51712	1.89226	2.1
51713	1.89226	2.1
51714	2.11649	2.1
51715	2.11649	2.1
51716	1.89226	2.1
51717	1.89226	2.1
51718	1.89226	2.1
51719	1.89226	2.1
51720	1.89226	2.1
51721	1.89226	2.1
51722	0.998134	2.1
51723	0.918904	2.1
51724	0.894708	2.1
51725	0.758742	2.1
51726	0.864276	2.1
51727	0.864276	2.1
51728	0.8926	2.1
51729	0.8926	2.1
51730	0.998134	2.1
51731	0.894709	2.1
51732	0.758742	2.1
51733	0.918904	2.1

51734	1.89226	2.1
51735	1.89226	2.1
51736	1.89226	2.1
51737	1.89226	2.1
51738	1.89226	2.1
51739	1.89226	2.1
51740	1.89226	2.1
51741	1.89226	2.1
51742	1.89226	2.1
51743	1.89226	2.1
51744	1.89226	2.1
51745	1.89226	2.1
51746	1.99627	2.1
51747	1.61047	2.1
51748	2.0815	2.1
51749	2.07569	2.1
51750	3.75063	2.1
51751	0.848077	2.1
51752	1.07748	2.1
51753	0.802498	2.1
51754	0.626808	2.1
51755	0.875552	2.1
51756	0.737527	2.1
51757	0.835602	2.1
51758	0.835602	2.1
51759	0.983245	2.1
51760	0.781108	2.1
51761	0.872306	2.1
51762	0.601497	2.1
51763	2.74745	2.1
51764	2.73988	2.1
51765	2.6521	2.1
51766	2.67792	2.1
51767	5.39198	2.1
51768	2.73988	2.1
51769	2.57466	2.1
51770	2.67791	2.1
51771	2.55653	2.1
51772	5.29641	2.1
51773	2.6521	2.1
51774	2.64148	2.1
51775	2.67792	2.1
51776	2.67792	2.1
51777	2.56953	2.1
51778	2.56953	2.1
51779	2.64148	2.1
51780	2.55653	2.1
51781	2.57929	2.1
51782	2.57929	2.1
51783	2.67792	2.1
51784	2.67792	2.1
51785	1.89226	0.766876
51786	1.89226	0.122476
51787	1.89226	0.217783
51788	1.89226	0.05
51789	3.78453	0.05
51790	1.68703	0.774387
51791	2.50385	0.05

51792	1.45958	0.05
51793	2.0815	0.05
51794	3.93806	0.05
51795	2.50385	0.05
51796	2.57414	0.05
51797	2.0815	0.05
51798	2.39921	0.05
51799	4.91181	0.05
51800	1.89226	0.794408
51801	1.89226	0.05
51802	1.89226	0.05
51803	1.89226	0.05
51804	3.78453	0.05
51805	1.89226	0.463852
51806	1.89226	0.05
51807	1.89226	0.178802
51808	1.89226	0.151612
51809	1.89226	0.05
51810	1.89226	0.705065
51811	1.23013	0.05
51812	2.0815	0.05
51813	1.90167	0.05
51814	1.71243	0.05
51815	1.45887	0.05
51816	1.31369	0.05
51817	2.0815	0.05
51818	2.39921	0.05
51819	1.7115	0.05
51820	2.2421	0.05
51821	4.09829	0.05
51822	1.89227	0.05
51823	1.89227	0.05
51824	1.89227	0.05
51825	1.89227	0.05
51826	3.78453	0.05
51827	2.39697	0.05
51828	1.85594	0.05
51829	1.91161	0.05
51830	2.49867	0.05
51831	4.35461	0.05
51832	1.12035	0.05
51833	1.85594	0.05
51834	1.91161	0.05
51835	1.15483	0.05
51836	3.01077	0.05
51837	1.89226	0.05
51838	1.89226	0.05
51839	1.89226	0.05
51840	1.89226	0.05
51841	1.89226	0.05
51842	1.89226	0.05
51843	1.89226	0.05
51844	1.89226	0.05
51845	1.89226	0.05
51846	1.89226	0.05
51847	1.89226	0.05
51848	1.89226	0.05
51849	0.936516	0.05

51850	1.03496	0.05
51851	1.22607	0.05
51852	1.28058	0.05
51853	1.06317	0.05
51854	1.06317	0.05
51855	1.19735	0.05
51856	1.0813	0.05
51857	0.858168	0.05
51858	1.32278	0.05
51859	0.957674	0.05
51860	1.25178	0.05
51861	1.92659	0.05
51862	0.998957	0.05
51863	0.998957	0.05
51864	1.15481	0.05
51865	1.6031	0.05
51866	1.74459	0.05
51867	0.946119	0.05
51868	0.946119	0.05
51869	0.946118	0.05
51870	0.946118	0.05
51871	0.946118	0.05
51872	0.946118	0.05
51873	0.946119	0.05
51874	0.946119	0.05
51875	0.946131	0.05
51876	0.946131	0.05
51877	0.946106	0.05
51878	0.946106	0.05
51879	0.946118	0.05
51880	0.946118	0.05
51881	0.946119	0.05
51882	0.946119	0.05
51883	0.946118	0.05
51884	0.946118	0.05
51885	0.946119	0.05
51886	0.946119	0.05
51887	0.946131	0.05
51888	0.946131	0.05
51889	0.946106	0.05
51890	0.946106	0.05
51891	2.47164	0.125118
51892	2.83689	0.05
51893	2.73872	0.182326
51894	2.41632	0.05
51895	5.21036	0.05
51896	0.946133	0.105193
51897	0.946133	0.17651
51898	1.41845	0.0537609
51899	1.36936	0.118543
51900	1.41845	0.05
51901	1.41845	0.05
51902	0.946132	0.16972
51903	0.946132	0.172722
51904	0.946132	0.104965
51905	1.36936	0.05
51906	1.41845	0.0670044
51907	0.946133	0.140133

51908	1.36936	0.05
51909	1.27581	0.05
51910	1.20816	0.05
51911	1.10464	0.103354
51912	1.24454	0.05
51913	1.24454	0.05
51914	1.23943	0.0549597
51915	1.23943	0.0844983
51916	1.10465	0.05
51917	1.27581	0.05
51918	1.36936	0.05
51919	1.20816	0.05
51920	0.946131	2.1
51921	0.946131	2.1
51922	1.37277	2.1
51923	1.37277	2.1
51924	1.37277	2.1
51925	1.27186	2.1
51926	0.946131	2.1
51927	0.946131	2.1
51928	0.946131	2.1
51929	1.37277	2.1
51930	0.946131	2.1
51931	1.27186	2.1
51932	2.74554	2.1
51933	1.23712	2.1
51934	2.80992	2.1
51935	2.54371	2.1
51936	1.50333	2.1
51937	1.23712	2.1
51938	2.77314	2.1
51939	2.33316	2.1
51940	2.28748	2.1
51941	2.28748	2.1
51942	2.81275	2.1
51943	2.81275	2.1
51944	2.28749	2.1
51945	1.45887	2.1
51946	2.33317	2.1
51947	1.50022	2.1
51948	3.79204	2.1
51949	2.37364	2.1
51950	2.33317	2.1
51951	1.5362	2.1
51952	1.5362	2.1
51953	1.50022	2.1
51954	2.33317	2.1
51955	1.89226	0.452613
51956	1.89226	0.453575
51957	1.89226	0.250355
51958	1.89226	0.431882
51959	1.89226	0.317501
51960	1.89226	0.613837
51961	0.946128	0.0758979
51962	0.946128	0.10482
51963	1.0644	0.0518351
51964	0.946133	0.0807148
51965	1.0644	0.05

51966	0.94613	0.0633038
51967	0.946131	0.125461
51968	0.946131	0.0537278
51969	1.0644	0.0654695
51970	1.0644	0.0692445
51971	0.946131	0.0914059
51972	0.946131	0.0784521
51973	1.89226	1.88658
51974	1.89226	0.77514
51975	1.89226	0.424953
51976	1.89226	0.05
51977	3.78452	0.172043
51978	1.14599	1.55894
51979	1.19993	0.738259
51980	1.00065	0.165735
51981	1.07497	0.05
51982	2.22096	0.350291
51983	0.546551	0.458354
51984	0.546551	0.762299
51985	0.54987	0.482257
51986	0.54987	0.761573
51987	0.503067	0.155622
51988	0.558937	0.228627
51989	0.599966	0.423499
51990	0.537484	0.72999
51991	0.503067	0.05
51992	0.537485	0.05
51993	0.599966	0.694582
51994	0.558936	0.174542
51995	0.946132	0.373283
51996	0.946132	0.739918
51997	0.94613	0.465907
51998	0.94613	0.749848
51999	0.946131	0.725671
52000	0.946131	0.525006
52001	0.946131	0.734029
52002	0.946131	0.732186
52003	0.946131	0.242377
52004	0.946131	0.690425
52005	0.946131	0.722228
52006	0.946131	0.733487
52007	2.19104	2.1
52008	2.5392	2.1
52009	2.53302	2.1
52010	2.19226	2.1
52011	4.73146	2.1
52012	1.09552	2.1
52013	1.09613	2.1
52014	0.802484	2.1
52015	0.807941	2.1
52016	0.95052	2.1
52017	0.95052	2.1
52018	0.952944	2.1
52019	0.952944	2.1
52020	1.09613	2.1
52021	0.807941	2.1
52022	0.802484	2.1
52023	1.09552	2.1

52024	2.533	2.1
52025	2.18531	2.1
52026	2.19224	2.1
52027	2.19224	2.1
52028	2.51603	2.1
52029	2.51603	2.1
52030	1.09266	2.1
52031	1.09612	2.1
52032	0.807935	2.1
52033	0.81091	2.1
52034	0.951897	2.1
52035	0.951897	2.1
52036	0.955134	2.1
52037	0.955134	2.1
52038	0.810915	2.1
52039	1.09266	2.1
52040	1.09611	2.1
52041	0.80793	2.1
52042	2.12741	0.70688
52043	2.79312	0.378443
52044	2.77591	1.02066
52045	2.10714	0.690943
52046	4.90026	0.22584
52047	2.12741	0.564236
52048	1.20859	0.06642
52049	1.21073	0.99056
52050	2.10714	0.811035
52051	3.31573	0.862269
52052	1.38708	0.945437
52053	1.38795	0.216542
52054	1.05331	0.841165
52055	1.05357	0.744134
52056	1.22049	0.802557
52057	1.22049	0.526356
52058	1.22078	1.04824
52059	1.22078	0.186955
52060	1.05331	0.878606
52061	1.38709	0.88489
52062	1.38795	0.54764
52063	1.05357	0.617722
52064	2.10662	0.937103
52065	2.10714	0.315191
52066	2.12499	0.481117
52067	1.22857	0.900997
52068	1.21073	0.42667
52069	1.21073	0.106307
52070	1.82014	0.05
52071	2.08757	0.05
52072	1.81292	0.05
52073	1.67485	0.05
52074	3.63306	0.05
52075	1.82013	0.05
52076	1.21748	0.05
52077	1.29891	0.05
52078	1.4442	0.05
52079	1.67484	0.05
52080	1.82013	0.05
52081	1.81292	0.05

52082	1.4516	0.05
52083	1.39633	0.05
52084	1.67485	0.05
52085	1.5344	0.05
52086	1.81292	0.05
52087	1.67484	0.05
52088	1.00982	0.05
52089	0.994235	0.05
52090	1.21748	0.05
52091	1.45159	0.05
52092	1.67484	0.05
52093	1.55216	2.1
52094	1.34407	2.1
52095	1.34407	2.1
52096	1.52008	2.1
52097	1.81783	2.1
52098	1.99384	2.1
52099	1.90817	2.1
52100	2.03975	2.1
52101	1.97317	2.1
52102	1.81783	2.1
52103	3.85758	2.1
52104	0.599049	2.1
52105	0.660673	2.1
52106	0.785063	2.1
52107	0.924278	2.1
52108	0.760416	2.1
52109	0.760416	2.1
52110	0.719088	2.1
52111	0.694532	2.1
52112	0.522441	2.1
52113	0.796789	2.1
52114	0.655207	2.1
52115	0.886155	2.1
52116	1.97317	2.1
52117	1.81783	2.1
52118	1.65098	2.1
52119	1.91767	2.1
52120	3.7355	2.1
52121	1.15038	1.32067
52122	1.28649	2.1
52123	1.38986	1.52447
52124	1.38986	2.1
52125	1.07866	1.79771
52126	1.07866	2.1
52127	0.664498	0.980976
52128	0.664498	2.1
52129	0.675122	2.1
52130	0.675122	2.1
52131	0.69493	1.18309
52132	0.764429	1.81204
52133	0.623438	1.57732
52134	0.575191	2.1
52135	0.764429	1.2882
52136	0.623438	1.19713
52137	0.575191	2.1
52138	0.69493	1.98529
52139	1.89226	0.789701

52140	1.89226	0.05
52141	1.89226	0.941079
52142	1.89226	0.05
52143	3.78453	0.05
52144	2.50075	0.157897
52145	2.15276	0.05
52146	2.24902	0.690792
52147	2.30422	0.05
52148	4.40178	0.05
52149	1.70424	0.138478
52150	1.34494	0.86658
52151	2.15276	0.05
52152	2.15276	0.153761
52153	2.0793	0.05
52154	1.63078	0.497874
52155	1.89226	0.05
52156	1.89226	0.816183
52157	1.89226	1.38695
52158	1.89226	0.05
52159	1.89226	0.824585
52160	1.89226	0.795775
52161	1.89226	0.446406
52162	1.89226	0.152734
52163	1.89226	0.05
52164	1.89226	0.05
52165	3.78452	0.05
52166	2.24902	0.269418
52167	1.91497	0.05
52168	1.97135	0.477502
52169	1.73957	0.05
52170	3.98859	0.05
52171	1.73957	0.05
52172	1.34494	0.783024
52173	1.73957	1.57255
52174	1.69006	0.05
52175	1.3126	0.137499
52176	1.34848	0.491714
52177	0.946132	0.05
52178	0.946132	0.833889
52179	0.946132	0.05
52180	0.946132	1.38221
52181	0.946132	0.541568
52182	0.946132	0.062551
52183	0.946131	0.05
52184	0.946131	0.05
52185	0.946132	0.05
52186	0.946132	0.05
52187	0.946132	0.0514777
52188	0.946132	0.728146
52189	1.89226	2.1
52190	1.89226	1.93179
52191	1.89226	2.1
52192	1.89226	2.01764
52193	3.78453	2.1
52194	1.89226	2.1
52195	2.82624	1.62584
52196	1.89226	2.1
52197	2.02536	1.78305

52198	1.89226	2.08215
52199	2.02536	1.53844
52200	2.25027	0.05
52201	1.1706	0.05
52202	2.02733	0.05
52203	2.02733	0.05
52204	1.61462	0.05
52205	1.69832	0.05
52206	1.89226	0.05
52207	1.89226	0.05
52208	1.89226	0.05
52209	1.89226	0.05
52210	3.78452	0.05
52211	0.946131	0.05
52212	0.946131	0.05
52213	1.18182	0.05
52214	1.18182	0.05
52215	0.973819	0.05
52216	0.94613	0.05
52217	1.18182	0.05
52218	0.94613	0.05
52219	0.973819	0.05
52220	1.18182	0.05
52221	0.94613	0.05
52222	0.94613	0.05
52223	2.23944	2.1
52224	1.52903	2.1
52225	1.35977	2.1
52226	2.04847	2.1
52227	3.5775	2.1
52228	2.23944	2.1
52229	2.34795	2.1
52230	2.34795	2.1
52231	2.48025	2.1
52232	2.04847	2.1
52233	2.18077	2.1
52234	1.89226	2.1
52235	1.89226	2.1
52236	1.89226	2.1
52237	1.89226	2.1
52238	3.78452	2.1
52239	0.946127	2.1
52240	0.946127	2.1
52241	0.946131	2.1
52242	0.946131	2.1
52243	0.946128	2.1
52244	0.946128	2.1
52245	0.94613	2.1
52246	0.94613	2.1
52247	0.946129	2.1
52248	0.946129	2.1
52249	0.946129	2.1
52250	0.946129	2.1
52251	1.26956	2.1
52252	2.04847	2.1
52253	1.49576	2.1
52254	1.22377	2.1
52255	1.91172	2.1

52256	1.50198	2.1
52257	1.48568	2.1
52258	2.34795	2.1
52259	2.04847	2.1
52260	2.04847	2.1
52261	2.21155	2.1
52262	2.21155	2.1
52263	1.89226	2.1
52264	1.89226	2.1
52265	1.89226	2.1
52266	1.89226	2.1
52267	1.89226	2.1
52268	1.89226	2.1
52269	0.810943	2.1
52270	0.810943	2.1
52271	0.810944	2.1
52272	0.810944	2.1
52273	0.810957	2.1
52274	0.810957	2.1
52275	1.89226	2.1
52276	1.35168	2.1
52277	1.08132	2.1
52278	1.08132	2.1
52279	1.0813	2.1
52280	0.946132	0.05
52281	1.33855	0.05
52282	0.946131	0.05
52283	1.38204	0.05
52284	0.946131	0.05
52285	0.946131	0.05
52286	1.38204	0.05
52287	1.38204	0.05
52288	0.946131	0.05
52289	0.946131	0.05
52290	1.33855	0.05
52291	1.38204	0.05
52292	1.89226	0.05
52293	2.76407	0.05
52294	1.89226	0.05
52295	2.72769	0.05
52296	1.89226	0.05
52297	2.72769	0.05
52298	2.03914	0.05
52299	1.67727	0.05
52300	1.94831	0.05
52301	1.81591	0.05
52302	2.32098	0.05
52303	2.32098	0.05
52304	0.801647	0.05
52305	1.11086	0.05
52306	0.838634	0.05
52307	1.16049	0.05
52308	0.977891	0.05
52309	0.977891	0.05
52310	0.984246	0.05
52311	0.984246	0.05
52312	1.11086	0.05
52313	1.16049	0.05

52314	0.801647	0.05
52315	0.838634	0.05
52316	0.946132	0.05
52317	0.946132	0.05
52318	0.946131	0.05
52319	0.946131	0.05
52320	0.946131	0.05
52321	0.946131	0.05
52322	0.946132	0.05
52323	0.946132	0.05
52324	0.946131	0.05
52325	0.946131	0.05
52326	0.946131	0.05
52327	0.946131	0.05
52328	0.946131	0.05
52329	0.946131	0.05
52330	0.946131	0.05
52331	0.946131	0.05
52332	0.946131	0.05
52333	0.946131	0.05
52334	0.946131	0.05
52335	0.946131	0.05
52336	0.946131	0.05
52337	0.946131	0.05
52338	0.946131	0.05
52339	0.946131	0.05
52340	2.0815	2.1
52341	1.20787	2.1
52342	1.92277	2.1
52343	1.3227	2.1
52344	3.29401	2.1
52345	2.16633	2.1
52346	2.13642	2.1
52347	1.40846	2.1
52348	1.40846	2.1
52349	1.3227	2.1
52350	2.08058	2.1
52351	0.938106	2.1
52352	0.790258	2.1
52353	0.742975	2.1
52354	0.742975	2.1
52355	0.874389	2.1
52356	1.17364	2.1
52357	0.525948	2.1
52358	0.52513	2.1
52359	0.553732	2.1
52360	0.876996	2.1
52361	0.530954	2.1
52362	1.095 2.1	
52363	1.00833	2.1
52364	1.70608	2.1
52365	1.33385	2.1
52366	1.33385	2.1
52367	1.07722	2.1
52368	1.07722	2.1
52369	0.953523	0.760028
52370	1.7457	0.408609
52371	1.03218	0.692791

52372	1.03218	0.546284
52373	1.79675	0.663372
52374	1.79675	0.526313
52375	1.13676	0.845594
52376	1.18136	0.346195
52377	0.87285	0.577023
52378	0.898375	0.904039
52379	1.03191	0.459117
52380	1.03191	0.238148
52381	1.0223	0.676486
52382	1.0223	0.782381
52383	1.18136	0.529138
52384	0.898374	0.363021
52385	0.87285	0.512239
52386	1.13676	0.4816
52387	2.36273	0.457868
52388	1.46965	0.32896
52389	1.79675	0.181776
52390	1.79675	0.111418
52391	2.43417	0.569554
52392	2.43417	0.366986
52393	1.4714	2.1
52394	1.65434	2.1
52395	1.62322	1.16875
52396	1.86068	2.01845
52397	3.27756	1.70074
52398	1.89226	2.1
52399	1.89226	2.1
52400	1.89226	1.19699
52401	1.89226	1.30873
52402	3.78453	1.46345
52403	1.89226	1.54584
52404	1.89226	2.1
52405	1.89226	1.17911
52406	1.89226	2.1
52407	3.78453	1.9363
52408	1.65434	1.5523
52409	1.60647	2.1
52410	1.86068	1.34341
52411	1.82649	2.1
52412	3.48083	2.1
52413	1.62322	2.1
52414	1.86068	2.1
52415	1.84368	1.14124
52416	2.18862	1.43446
52417	3.81184	1.65297
52418	1.89226	2.1
52419	1.89226	2.03291
52420	1.89226	1.06671
52421	1.89226	1.50551
52422	3.78453	1.56138
52423	1.89226	1.57077
52424	1.89226	2.1
52425	1.89226	1.27692
52426	1.89226	2.1
52427	3.78453	2.1
52428	1.86068	1.45919
52429	1.82649	2.1

52430	2.18862	1.24912
52431	2.1128	2.1
52432	4.01511	1.89124
52433	1.74351	2.1
52434	1.13854	2.1
52435	1.40233	2.1
52436	1.922 2.1	
52437	3.14584	2.1
52438	1.922 2.1	
52439	2.00775	2.1
52440	1.7435	2.1
52441	2.09224	2.1
52442	2.00775	2.1
52443	2.09295	2.1
52444	1.69858	2.1
52445	1.922 2.1	
52446	1.40233	2.1
52447	1.40233	2.1
52448	2.18238	2.1
52449	2.18238	2.1
52450	1.00387	2.1
52451	1.10886	2.1
52452	0.961001	2.1
52453	1.09119	2.1
52454	1.02868	2.1
52455	1.02868	2.1
52456	1.04118	2.1
52457	1.04118	2.1
52458	1.10886	2.1
52459	1.09119	2.1
52460	0.960999	2.1
52461	1.00388	2.1
52462	0.885958	0.802599
52463	0.885958	0.589935
52464	0.875826	0.244
52465	0.875826	0.835142
52466	0.913965	0.829387
52467	0.598609	0.420805
52468	1.16317	0.529823
52469	0.868115	0.597812
52470	0.868115	0.789991
52471	0.598609	0.35876
52472	1.16317	0.703763
52473	0.913965	0.650746
52474	0.946131	0.826654
52475	0.946131	0.805786
52476	0.946131	0.819613
52477	0.946131	0.78523
52478	0.946131	0.818245
52479	0.946131	0.82831
52480	0.946131	0.8041
52481	0.946131	0.783947
52482	0.946131	0.814291
52483	0.946131	0.813942
52484	0.946131	0.807971
52485	0.946131	0.810582
52486	0.946133	0.05
52487	0.946133	0.05

52488	1.06246	0.05
52489	1.06246	0.05
52490	0.973851	0.05
52491	0.946133	0.05
52492	1.06246	0.05
52493	0.946132	0.05
52494	0.97385	0.05
52495	1.06246	0.05
52496	0.946133	0.05
52497	0.946133	0.05
52498	1.89226	0.05
52499	1.89226	0.05
52500	1.89226	0.05
52501	1.89226	0.05
52502	1.89226	0.05
52503	1.89226	0.05
52504	0.946131	0.05
52505	0.946131	0.05
52506	0.946127	0.05
52507	0.946127	0.05
52508	0.946131	0.05
52509	0.946131	0.05
52510	0.946127	0.05
52511	0.946127	0.05
52512	0.946128	0.05
52513	0.946128	0.05
52514	0.946129	0.05
52515	0.946129	0.05
52516	0.946132	0.05
52517	0.946132	0.05
52518	1.32446	0.05
52519	0.794912	0.05
52520	1.32429	0.05
52521	0.946131	0.05
52522	1.32446	0.05
52523	0.94613	0.05
52524	1.32429	0.05
52525	0.794915	0.05
52526	0.94613	0.05
52527	0.94613	0.05
52528	1.89226	0.05
52529	2.36365	0.05
52530	2.64857	0.05
52531	1.89226	0.05
52532	2.64857	0.05
52533	1.89226	0.05
52534	1.18463	2.1
52535	1.27174	2.1
52536	0.946129	2.1
52537	0.946129	2.1
52538	0.946131	2.1
52539	0.946131	2.1
52540	1.27174	2.1
52541	1.27174	2.1
52542	1.18463	2.1
52543	0.94613	2.1
52544	0.946131	2.1
52545	1.27174	2.1

52546	2.57997	2.1
52547	1.89226	2.1
52548	1.89226	2.1
52549	1.89226	2.1
52550	2.41915	2.1
52551	2.41915	2.1
52552	1.89226	2.1
52553	1.89226	2.1
52554	1.89226	2.1
52555	1.89226	2.1
52556	3.78452	2.1
52557	1.08807	2.1
52558	1.18463	2.1
52559	0.946129	2.1
52560	0.946129	2.1
52561	0.946131	2.1
52562	0.946131	2.1
52563	1.18463	2.1
52564	1.18463	2.1
52565	0.946131	2.1
52566	1.08807	2.1
52567	1.18463	2.1
52568	0.94613	2.1
52569	1.07017	2.1
52570	1.07017	2.1
52571	0.946131	2.1
52572	0.946131	2.1
52573	0.946132	2.1
52574	0.946132	2.1
52575	1.20957	2.1
52576	1.07017	2.1
52577	1.07017	2.1
52578	0.946132	2.1
52579	0.946131	2.1
52580	1.20957	2.1
52581	1.89226	2.1
52582	1.89226	2.1
52583	1.89226	2.1
52584	1.89226	2.1
52585	1.89226	2.1
52586	1.89226	2.1
52587	1.16181	0.05
52588	1.0227	0.05
52589	1.0227	0.05
52590	0.970959	0.05
52591	1.24295	0.05
52592	1.19121	0.05
52593	1.12076	2.1
52594	2.53614	2.1
52595	1.28413	2.1
52596	1.28413	2.1
52597	2.36423	2.1
52598	2.36423	2.1
52599	1.89226	2.1
52600	1.89226	2.1
52601	1.89226	2.1
52602	1.89226	2.1
52603	3.78452	2.1

52604	1.22557	2.1
52605	2.771	2.1
52606	2.73012	2.1
52607	2.73012	2.1
52608	1.33356	2.1
52609	1.33356	2.1
52610	0.998682	2.1
52611	2.36423	2.1
52612	1.12076	2.1
52613	1.12076	2.1
52614	2.13757	2.1
52615	2.13757	2.1
52616	1.89226	2.1
52617	1.89226	2.1
52618	1.89226	2.1
52619	1.89226	2.1
52620	1.89226	2.1
52621	1.89226	2.1
52622	1.13987	2.1
52623	2.73011	2.1
52624	2.70875	2.1
52625	2.70875	2.1
52626	1.22557	2.1
52627	1.22557	2.1
52628	1.89227	2.1
52629	2.79243	2.1
52630	2.6625	2.1
52631	2.6625	2.1
52632	1.89227	2.1
52633	1.89227	2.1
52634	1.89226	2.1
52635	2.55971	2.1
52636	1.89226	2.1
52637	1.89226	2.1
52638	2.68704	2.1
52639	2.68704	2.1
52640	2.22043	2.1
52641	2.38975	1.84327
52642	2.28696	1.25259
52643	2.48682	1.10919
52644	4.67671	1.28351
52645	2.38976	1.29491
52646	2.31988	1.81196
52647	2.48683	1.10652
52648	2.4594	1.41266
52649	4.84915	1.47457
52650	2.28696	1.94608
52651	2.48682	1.71276
52652	2.33379	2.1
52653	2.56149	1.0441
52654	4.84845	1.48925
52655	2.48683	1.74347
52656	2.4594	1.63958
52657	2.5615	1.05564
52658	2.57957	1.30209
52659	5.0209	1.18857
52660	1.06659	2.1
52661	1.04291	0.290798

52662	1.36595	0.835493
52663	1.36595	2.1
52664	1.02761	2.1
52665	1.32697	0.794988
52666	0.946132	1.79552
52667	0.946132	1.62737
52668	1.36956	2.1
52669	1.1229	2.1
52670	1.1229	1.47612
52671	1.1229	2.1
52672	0.946132	1.17552
52673	0.946132	2.1
52674	0.946132	2.1
52675	1.36956	2.1
52676	1.1229	2.1
52677	0.946133	2.1
52678	0.946133	1.16908
52679	0.946133	1.12751
52680	0.946128	2.1
52681	0.946128	2.1
52682	0.946131	2.1
52683	0.946131	1.46287
52684	0.946131	0.929908
52685	0.946131	2.1
52686	0.94613	2.0004
52687	0.94613	1.90793
52688	0.946131	1.65021
52689	0.946131	1.62036
52690	0.966427	1.29366
52691	0.802265	2.1
52692	0.803714	2.1
52693	0.808476	1.75897
52694	1.94371	2.1
52695	1.36851	2.1
52696	0.853864	2.1
52697	1.26635	1.71676
52698	1.09085	1.417
52699	0.857653	2.1
52700	0.630243	1.87735
52701	1.20132	0.05
52702	1.24295	0.05
52703	1.24295	0.05
52704	1.16182	0.05
52705	1.28465	0.05
52706	1.20351	0.05
52707	0.600658	0.05
52708	0.642323	0.05
52709	0.54424	0.05
52710	0.576779	0.05
52711	0.590985	0.05
52712	0.590985	0.05
52713	0.586452	0.05
52714	0.586452	0.05
52715	0.642323	0.05
52716	0.576779	0.05
52717	0.600658	0.05
52718	0.54424	0.05
52719	1.28463	0.05

52720	1.24294	0.05
52721	1.12328	0.05
52722	1.15505	0.05
52723	2.39798	0.05
52724	1.98431	0.05
52725	2.18735	0.05
52726	1.98431	0.05
52727	1.70303	0.05
52728	1.70302	0.05
52729	1.37788	0.05
52730	1.89226	0.05
52731	2.37417	0.05
52732	1.89226	0.05
52733	2.18735	0.05
52734	1.89226	0.05
52735	2.18735	0.05
52736	1.89226	0.05
52737	1.89226	0.148155
52738	1.89226	0.05
52739	1.89226	0.0992289
52740	3.78452	0.172254
52741	1.8613	0.05
52742	2.03576	0.0863581
52743	1.61652	0.05
52744	1.83082	2.1
52745	3.65228	0.05
52746	2.03576	0.0778418
52747	2.05257	2.1
52748	1.83082	2.1
52749	1.88487	2.1
52750	3.92064	2.1
52751	1.89226	0.114095
52752	1.89226	0.977058
52753	1.89226	0.0832878
52754	1.89226	2.1
52755	3.78453	0.199252
52756	0.946131	0.103822
52757	0.946131	0.05
52758	0.94613	0.05
52759	0.94613	0.148867
52760	0.946129	0.05
52761	0.946129	0.142023
52762	0.946132	0.216621
52763	0.946132	0.05
52764	0.94613	0.0516108
52765	0.94613	0.113371
52766	0.946131	0.05
52767	0.946131	0.05
52768	0.804708	2.1
52769	0.804708	0.05
52770	0.815274	0.162578
52771	0.815274	0.05
52772	0.83277	0.0660447
52773	0.915407	2.1
52774	0.80826	0.05
52775	0.704574	0.05
52776	0.832771	0.05
52777	0.704573	0.05

52778	0.808259	0.0919372
52779	0.915409	0.05
52780	1.83082	2.1
52781	1.88487	2.1
52782	1.66555	0.0854506
52783	1.75835	2.1
52784	3.55042	2.1
52785	0.810947	0.05
52786	0.810947	0.800122
52787	0.810947	0.05
52788	0.810947	0.069733
52789	0.810934	0.4191
52790	0.810934	0.291607
52791	1.89227	2.1
52792	1.3517	0.6372
52793	1.08132	0.423341
52794	1.08132	0.697023
52795	1.08133	0.1327
52796	1.89226	2.1
52797	1.70303	2.1
52798	1.89226	2.1
52799	2.24712	2.1
52800	2.07363	2.1
52801	1.74617	2.1
52802	1.89226	2.1
52803	1.89226	1.41909
52804	1.89226	1.23251
52805	1.89226	2.1
52806	3.78452	1.73744
52807	1.50108	2.1
52808	1.43894	2.1
52809	1.8322	1.13085
52810	1.40967	2.1
52811	1.50108	2.1
52812	1.07854	2.1
52813	1.89226	1.17922
52814	1.89226	2.1
52815	1.89226	2.1
52816	1.89226	0.847605
52817	3.78452	1.98293
52818	1.89226	2.1
52819	1.89226	1.39762
52820	1.89226	1.45712
52821	1.89226	2.1
52822	3.78453	1.89224
52823	1.54457	2.1
52824	1.35097	1.86864
52825	1.70754	1.19221
52826	1.29629	2.1
52827	1.54457	2.1
52828	1.0305	2.1
52829	0.731763	2.1
52830	1.00043	2.1
52831	1.13883	1.27895
52832	0.939026	1.96347
52833	1.04003	1.25294
52834	0.853868	2.1
52835	0.927188	2.1

52836	0.927188	0.525536
52837	0.861565	1.60683
52838	1.13882	2.06618
52839	0.731769	2.01916
52840	0.89319	2.1
52841	1.46568	2.1
52842	1.20761	2.1
52843	1.51916	2.1
52844	1.36261	2.1
52845	2.72677	2.1
52846	1.51916	2.1
52847	1.60532	2.1
52848	1.36261	2.1
52849	1.36261	2.1
52850	1.64892	2.1
52851	1.64892	2.1
52852	1.3626	2.1
52853	0.98488	2.1
52854	1.60532	2.1
52855	1.32414	2.1
52856	2.68675	2.1
52857	1.78711	2.1
52858	1.14825	2.1
52859	2.16535	2.1
52860	1.6067	2.1
52861	3.3136	2.1
52862	1.89226	2.1
52863	1.89226	2.1
52864	1.89226	2.1
52865	1.89226	2.1
52866	3.78452	2.1
52867	2.34552	2.1
52868	1.53521	2.1
52869	1.53521	2.1
52870	1.60636	2.1
52871	1.92797	2.1
52872	1.99589	2.1
52873	1.34771	2.1
52874	1.34771	2.1
52875	0.946128	2.1
52876	0.946128	2.1
52877	1.3477	2.1
52878	1.31776	2.1
52879	0.946131	2.1
52880	0.946131	2.1
52881	0.94613	2.1
52882	1.31776	2.1
52883	0.946129	2.1
52884	1.3477	2.1
52885	1.89226	2.1
52886	1.89226	2.1
52887	1.89226	2.1
52888	1.89226	2.1
52889	3.78452	2.1
52890	0.92843	2.1
52891	0.672114	2.1
52892	0.89514	2.1
52893	0.89514	2.1

52894	0.720104	2.1
52895	0.755753	2.1
52896	0.789158	2.1
52897	0.967302	2.1
52898	1.06565	2.1
52899	0.7397	2.1
52900	0.758893	2.1
52901	0.94672	2.1
52902	1.89226	2.1
52903	2.588	2.1
52904	2.588	2.1
52905	2.63552	2.1
52906	1.89226	2.1
52907	1.93978	2.1
52908	1.49347	0.05
52909	2.67757	0.05
52910	1.80061	0.05
52911	2.27056	0.05
52912	4.07117	0.05
52913	1.1016	0.05
52914	2.27057	0.05
52915	1.49348	0.05
52916	1.81893	0.05
52917	1.49348	0.05
52918	1.81893	0.05
52919	1.81893	0.05
52920	2.82339	0.05
52921	2.27057	0.05
52922	2.25405	0.05
52923	4.52462	0.05
52924	1.89226	0.05
52925	1.89226	0.05
52926	1.89226	0.05
52927	1.89226	0.05
52928	1.89226	0.05
52929	1.89226	0.05
52930	1.89226	0.05
52931	1.89226	0.05
52932	1.89226	0.05
52933	1.89226	0.05
52934	3.78453	0.05
52935	1.89226	0.05
52936	1.89226	0.05
52937	1.89226	0.05
52938	1.89226	0.05
52939	3.78452	0.05
52940	1.89226	0.05
52941	1.89226	0.109096
52942	1.89226	0.05
52943	1.89226	0.05
52944	3.78451	0.05
52945	1.45633	2.1
52946	1.34678	2.1
52947	1.34678	2.1
52948	1.53735	2.1
52949	1.234	2.1
52950	1.42457	2.1
52951	1.89226	2.1

52952	1.89226	2.1
52953	1.89226	2.1
52954	1.89226	2.1
52955	1.89226	2.1
52956	1.89226	2.1
52957	1.09483	2.1
52958	1.34678	2.1
52959	1.234	2.1
52960	1.234	2.1
52961	1.19	2.1
52962	1.19	2.1
52963	0.946131	2.1
52964	0.946131	2.1
52965	0.946131	2.1
52966	0.946131	2.1
52967	0.946131	2.1
52968	0.946131	2.1
52969	0.946132	2.1
52970	0.946132	2.1
52971	0.946132	2.1
52972	0.946132	2.1
52973	0.946131	2.1
52974	0.946131	2.1
52975	2.82263	2.1
52976	1.89226	2.1
52977	2.71441	2.1
52978	2.71441	2.1
52979	1.89226	2.1
52980	1.89226	2.1
52981	1.1412	2.1
52982	1.1412	2.1
52983	1.14626	2.1
52984	1.14626	2.1
52985	1.28511	2.1
52986	1.34086	2.1
52987	0.992242	2.1
52988	0.946593	2.1
52989	0.992242	2.1
52990	1.34086	2.1
52991	0.946594	2.1
52992	1.28511	2.1
52993	1.18954	2.1
52994	1.18954	2.1
52995	1.21477	2.1
52996	1.21477	2.1
52997	1.41207	2.1
52998	1.34086	2.1
52999	1.01306	2.1
53000	0.992242	2.1
53001	1.01306	2.1
53002	1.41207	2.1
53003	0.992242	2.1
53004	1.34086	2.1
53005	1.09044	2.1
53006	1.44472	0.95745
53007	1.2955	2.1
53008	1.2955	1.78166
53009	1.22025	2.1

53010	1.22025	2.1
53011	0.810944	1.01494
53012	0.810944	2.1
53013	0.810946	1.04763
53014	0.810946	0.05
53015	0.810959	1.01928
53016	0.810959	0.997485
53017	1.89226	0.534237
53018	1.35167	1.03288
53019	1.08132	2.1
53020	1.08132	0.05
53021	1.0813	1.02834
53022	1.41894	0.787895
53023	1.28953	0.715209
53024	0.946132	0.766785
53025	0.946132	1.56977
53026	0.946133	0.831491
53027	0.946133	0.736718
53028	1.41894	0.905542
53029	1.41894	0.968364
53030	1.28953	0.767789
53031	0.946132	0.800336
53032	0.946132	0.849937
53033	1.41894	0.791549
53034	1.89226	2.1
53035	2.15444	2.1
53036	1.93239	2.1
53037	1.89226	2.1
53038	2.19457	2.1
53039	2.15444	2.1
53040	1.89226	2.1
53041	1.89226	2.1
53042	1.89226	2.1
53043	1.89226	2.1
53044	1.89226	2.1
53045	1.89226	2.1
53046	0.938507	2.1
53047	0.938507	2.1
53048	1.07871	2.1
53049	1.15801	2.1
53050	1.03236	2.1
53051	1.14766	2.1
53052	0.946133	2.1
53053	0.946133	2.1
53054	0.946131	2.1
53055	1.07009	2.1
53056	0.946131	2.1
53057	1.03237	2.1
53058	1.89226	2.1
53059	2.19457	2.1
53060	1.94492	2.1
53061	1.89226	2.1
53062	2.24723	2.1
53063	2.19457	2.1
53064	0.946132	2.1
53065	0.946132	2.1
53066	0.946131	2.1
53067	0.946131	2.1

53068	0.946132	2.1
53069	0.946132	2.1
53070	0.946132	2.1
53071	0.946132	2.1
53072	0.946132	2.1
53073	0.946132	2.1
53074	0.946132	2.1
53075	0.946132	2.1
53076	2.21345	2.1
53077	1.89226	2.1
53078	1.89226	2.1
53079	2.14017	2.1
53080	2.14017	2.1
53081	1.89226	2.1
53082	1.89226	0.189219
53083	1.89226	0.958045
53084	1.89226	0.372869
53085	1.85439	0.928402
53086	2.23961	0.141962
53087	2.19479	0.635949
53088	1.2916	0.372766
53089	1.2916	0.257256
53090	0.946128	0.612225
53091	0.946128	0.344411
53092	1.2916	0.153934
53093	0.946131	0.369672
53094	0.946126	0.908662
53095	1.1198	0.0671607
53096	1.2916	0.216435
53097	1.1198	0.691142
53098	0.946128	0.852723
53099	0.946128	0.646255
53100	1.89226	0.915974
53101	2.71262	0.447209
53102	1.89226	0.708972
53103	2.49959	0.741164
53104	4.39185	0.946238
53105	2.71262	0.433504
53106	1.34592	0.31405
53107	2.49959	0.556837
53108	1.14721	0.106281
53109	3.84551	0.180837
53110	0.946131	0.306663
53111	0.946131	0.383186
53112	1.11247	0.374979
53113	1.11247	0.253981
53114	1.24979	0.267507
53115	1.11247	0.236529
53116	0.946129	0.650756
53117	0.946129	0.456641
53118	0.946131	0.199361
53119	1.11247	0.05
53120	0.946131	0.180638
53121	1.24979	0.241443
53122	0.946131	2.1
53123	0.946131	2.1
53124	0.94613	2.1
53125	0.94613	2.1

53126	0.946132	2.1
53127	0.946132	2.1
53128	0.946129	2.1
53129	0.946129	2.1
53130	0.946131	2.1
53131	0.946131	2.1
53132	0.946131	2.1
53133	0.946131	2.1
53134	2.77816	2.1
53135	1.89226	2.1
53136	1.89226	2.1
53137	1.89226	2.1
53138	3.78453	2.1
53139	1.89226	2.1
53140	1.89226	2.1
53141	1.89226	2.1
53142	1.89226	2.1
53143	1.89226	2.1
53144	1.89226	2.1
53145	1.89226	2.1
53146	1.89226	2.1
53147	1.89226	2.1
53148	1.89226	2.1
53149	3.78452	2.1
53150	1.55698	2.1
53151	1.89226	2.1
53152	1.89226	2.1
53153	1.89226	2.1
53154	1.8575	2.1
53155	1.8575	2.1
53156	1.89226	2.1
53157	1.89226	2.1
53158	1.89226	2.1
53159	1.89226	2.1
53160	1.89226	2.1
53161	1.89226	2.1
53162	1.89227	2.1
53163	1.89227	2.1
53164	1.89227	2.1
53165	1.89227	2.1
53166	1.89227	2.1
53167	1.89227	2.1
53168	1.35509	2.1
53169	2.06238	2.1
53170	2.12244	2.1
53171	2.12244	2.1
53172	1.34817	2.1
53173	1.34817	2.1
53174	0.946126	2.1
53175	0.946126	2.1
53176	0.946131	2.1
53177	0.946131	2.1
53178	0.946127	2.1
53179	0.946127	2.1
53180	0.94613	2.1
53181	0.94613	2.1
53182	0.946128	2.1
53183	0.946128	2.1

53184	0.946128	2.1
53185	0.946128	2.1
53186	1.89226	2.1
53187	1.89226	1.64646
53188	1.89226	2.1
53189	1.89226	2.1
53190	1.89226	2.1
53191	1.89226	2.1
53192	1.16742	2.1
53193	1.16742	2.1
53194	1.18122	2.1
53195	1.18122	2.1
53196	1.31745	2.1
53197	1.2597	2.1
53198	1.03119	2.1
53199	1.06122	2.1
53200	1.06122	2.1
53201	1.31745	2.1
53202	1.03119	2.1
53203	1.2597	2.1
53204	1.36849	2.1
53205	2.12244	2.1
53206	2.20092	2.1
53207	2.20092	2.1
53208	1.35509	2.1
53209	1.35509	2.1
53210	1.89226	2.1
53211	1.89226	2.1
53212	1.89226	2.1
53213	1.89226	2.1
53214	1.89226	2.1
53215	1.89226	2.1
53216	1.89226	2.1
53217	1.89226	1.75548
53218	1.89226	2.1
53219	1.89226	2.1
53220	3.78452	2.1
53221	1.21731	2.1
53222	1.21731	2.1
53223	1.23412	2.1
53224	1.23412	2.1
53225	1.39021	2.1
53226	1.31745	2.1
53227	1.06122	2.1
53228	1.10046	2.1
53229	1.10046	2.1
53230	1.39021	2.1
53231	1.06122	2.1
53232	1.31745	2.1
53233	2.16503	0.712171
53234	1.78252	1.19966
53235	1.77293	1.13056
53236	1.56839	2.1
53237	2.36957	0.118151
53238	2.16503	2.1
53239	1.89223	0.716435
53240	1.89223	2.1
53241	1.89223	0.196595

53242	1.89223	1.29808
53243	3.78447	2.1
53244	2.3696	1.30408
53245	1.78254	1.56754
53246	2.63298	0.614425
53247	2.07312	2.1
53248	4.44272	1.40264
53249	1.89226	1.51323
53250	1.89226	2.01523
53251	1.89226	1.34435
53252	1.89226	2.1
53253	1.89226	0.0691987
53254	1.89226	2.1
53255	1.89226	0.946224
53256	1.89226	0.761225
53257	1.89226	0.619376
53258	1.89226	0.05
53259	3.78452	0.280413
53260	2.26369	0.941296
53261	2.2168	0.744813
53262	2.15617	0.05
53263	2.10864	0.05
53264	4.37234	0.091487
53265	2.2168	0.736641
53266	1.94499	0.285784
53267	1.94499	0.756236
53268	2.04737	0.704281
53269	2.10864	0.0755122
53270	2.21103	0.0517092
53271	1.89226	0.778876
53272	1.89226	0.606592
53273	1.89226	0.609552
53274	1.89226	0.74117
53275	1.89226	0.736387
53276	1.89226	0.735725
53277	1.89226	0.774341
53278	1.89226	0.769589
53279	1.89226	0.797847
53280	1.89226	0.928867
53281	1.89226	0.0548296
53282	1.89226	0.970113
53283	1.02995	0.05
53284	1.02995	0.939538
53285	1.03212	0.762865
53286	1.03212	0.551712
53287	0.988256	0.61073
53288	1.05432	0.0724263
53289	1.00775	0.72421
53290	1.07809	0.604231
53291	1.00775	0.05
53292	0.988257	0.0740787
53293	1.07808	0.794145
53294	1.05432	0.743521
53295	2.10864	0.754234
53296	1.94499	0.657107
53297	1.97652	0.05
53298	1.79681	0.316892
53299	3.9215	0.60027

53300	0.810943	0.314229
53301	0.810943	0.756047
53302	0.810943	0.51279
53303	0.810943	0.748879
53304	0.810958	0.096666
53305	0.810958	0.567475
53306	1.89226	0.731646
53307	1.35168	0.571888
53308	1.08132	0.283884
53309	1.08132	0.727107
53310	1.0813	0.713811
53311	0.657403	0.05
53312	1.35403	0.05
53313	1.24038	0.05
53314	0.499144	0.05
53315	1.24039	0.05
53316	1.35403	0.05
53317	0.657402	0.05
53318	0.499144	0.05
53319	0.915415	0.05
53320	0.915415	0.05
53321	0.937755	0.05
53322	0.937755	0.05
53323	1.72526	2.1
53324	1.17989	2.1
53325	1.96035	2.1
53326	1.47412	2.1
53327	3.14024	2.1
53328	1.89226	1.59062
53329	1.89226	2.1
53330	1.89226	2.1
53331	1.89226	1.06257
53332	3.78452	2.1
53333	0.946131	1.52425
53334	0.946131	1.57797
53335	1.25764	1.79409
53336	1.25764	2.1
53337	1.07087	1.19852
53338	1.25765	1.14784
53339	0.946128	1.17487
53340	0.946128	0.834372
53341	0.94613	1.06034
53342	1.25765	1.13342
53343	1.07086	1.27938
53344	0.946129	1.28993
53345	1.96035	2.1
53346	1.32668	2.1
53347	2.29301	2.1
53348	1.70303	2.1
53349	3.56428	2.1
53350	1.89226	2.1
53351	1.89226	1.62362
53352	1.89226	0.913297
53353	1.89226	2.1
53354	3.78452	0.90199
53355	0.897738	0.169127
53356	0.897738	0.183781
53357	0.946102	0.112378

53358	0.946102	0.05
53359	1.11247	0.05
53360	0.89771	0.05
53361	0.94613	0.114319
53362	0.94613	0.230808
53363	0.897709	0.160676
53364	0.946131	0.239452
53365	0.946131	0.39195
53366	1.11247	0.0666671
53367	1.89226	2.1
53368	1.89226	2.1
53369	1.89226	2.1
53370	1.89226	2.1
53371	1.89226	2.1
53372	1.89226	2.1
53373	2.15429	2.1
53374	1.89226	2.1
53375	1.89226	2.1
53376	1.89226	2.1
53377	2.27074	2.1
53378	2.27074	2.1
53379	0.993311	1.19272
53380	0.993311	1.95817
53381	0.904322	2.1
53382	0.904322	2.1
53383	0.803324	2.1
53384	1.36183	1.23388
53385	0.916274	2.1
53386	0.535808	1.65671
53387	0.535808	1.94466
53388	0.803322	2.09029
53389	0.916276	2.00465
53390	1.36183	2.08613
53391	1.89226	2.1
53392	1.89226	2.1
53393	1.89226	2.1
53394	1.89226	2.1
53395	1.89226	2.1
53396	1.89226	2.1
53397	2.11955	2.1
53398	2.62097	2.1
53399	1.85542	2.1
53400	2.39016	2.1
53401	4.50971	2.1
53402	2.39016	2.1
53403	2.20242	2.1
53404	2.39016	2.1
53405	1.64008	2.1
53406	1.85542	2.1
53407	1.64008	2.1
53408	1.79667	2.1
53409	1.28267	2.1
53410	1.28267	2.1
53411	1.40117	2.1
53412	1.74514	2.1
53413	1.83994	2.1
53414	0.575914	2.1
53415	0.625292	2.1

53416	0.949144	2.1
53417	0.882782	2.1
53418	0.773697	2.1
53419	0.773697	2.1
53420	0.786497	2.1
53421	0.786494	2.1
53422	0.605477	2.1
53423	0.949148	2.1
53424	0.625291	2.1
53425	0.862374	2.1
53426	1.89226	2.1
53427	2.44918	2.1
53428	2.4616	2.1
53429	2.44918	2.1
53430	1.90468	2.1
53431	1.89226	2.1
53432	1.89226	2.1
53433	1.89226	2.1
53434	1.89226	2.1
53435	1.89226	2.1
53436	3.78453	2.1
53437	0.946128	2.1
53438	0.946128	1.87031
53439	1.23631	2.1
53440	1.23631	2.1
53441	0.946131	2.1
53442	0.946131	2.1
53443	1.3147	2.1
53444	1.23631	2.1
53445	1.3147	2.1
53446	0.946131	2.1
53447	1.23631	2.1
53448	0.94613	2.1
53449	1.89226	2.1
53450	2.4616	2.1
53451	2.4717	2.1
53452	2.4616	2.1
53453	1.90237	2.1
53454	1.89226	2.1
53455	0.946132	2.1
53456	0.946132	2.1
53457	0.946131	2.1
53458	0.946131	2.1
53459	0.946132	2.1
53460	0.946132	2.1
53461	0.946131	2.1
53462	0.946131	2.1
53463	0.946131	2.1
53464	0.946131	2.1
53465	0.946131	2.1
53466	0.946131	2.1
53467	0.946128	2.1
53468	0.946128	2.1
53469	1.3147	2.1
53470	1.3147	2.1
53471	0.94613	2.1
53472	0.94613	2.1
53473	1.3419	2.1

53474	1.3147	2.1
53475	1.3419	2.1
53476	0.94613	2.1
53477	1.3147	2.1
53478	0.94613	2.1
53479	1.89226	2.1
53480	1.89226	2.1
53481	1.89226	2.1
53482	1.89226	2.1
53483	3.78452	2.1
53484	2.3333	2.1
53485	1.3864	2.1
53486	1.3864	2.1
53487	1.4628	2.1
53488	1.95763	2.1
53489	2.03403	2.1
53490	1.294 2.1	
53491	1.294 2.1	
53492	0.946128	2.1
53493	0.946128	2.1
53494	1.294 2.1	
53495	1.27474	2.1
53496	0.946131	2.1
53497	0.946131	2.1
53498	0.946129	2.1
53499	1.27474	2.1
53500	1.294 2.1	
53501	0.94613	2.1
53502	1.89226	2.1
53503	1.89226	2.1
53504	1.89226	2.1
53505	1.89226	2.1
53506	1.89226	2.1
53507	1.89226	2.1
53508	0.808088	2.1
53509	0.808088	2.1
53510	0.815623	2.1
53511	0.815623	2.1
53512	0.660053	2.1
53513	0.693199	2.1
53514	0.930512	2.1
53515	0.978817	2.1
53516	0.660053	2.1
53517	0.930513	2.1
53518	0.693198	2.1
53519	0.978817	2.1
53520	1.27474	2.1
53521	1.27474	2.1
53522	0.946128	2.1
53523	0.946128	2.1
53524	1.26221	2.1
53525	1.27474	2.1
53526	0.946131	2.1
53527	0.946131	2.1
53528	0.94613	2.1
53529	1.26221	2.1
53530	0.946129	2.1
53531	1.27474	2.1

53532	0.90207	0.05
53533	0.90207	0.05
53534	0.895719	0.05
53535	0.895719	0.05
53536	0.927855	0.05
53537	1.2493	0.05
53538	0.85723	0.05
53539	0.548484	0.05
53540	0.548485	0.05
53541	0.927855	0.05
53542	0.85723	0.05
53543	1.2493	0.05
53544	1.41319	0.238585
53545	1.22482	0.05
53546	1.46629	0.0678024
53547	1.92622	0.232363
53548	3.15104	0.174997
53549	1.02305	0.05
53550	1.22482	0.05
53551	1.80336	0.0972784
53552	1.44426	0.05
53553	2.66908	0.05
53554	1.3268	2.1
53555	0.999489	2.1
53556	1.44091	2.1
53557	1.14657	2.1
53558	2.4404	2.1
53559	1.89226	2.1
53560	1.89226	2.1
53561	1.89226	2.1
53562	1.89226	2.1
53563	3.78452	2.1
53564	1.89226	2.1
53565	2.28605	2.1
53566	2.43157	2.1
53567	2.43157	2.1
53568	1.89226	2.1
53569	1.89226	2.1
53570	1.60647	2.1
53571	1.44091	2.1
53572	1.31346	2.1
53573	1.31346	2.1
53574	1.14657	2.1
53575	1.44091	2.1
53576	1.89226	2.1
53577	1.89226	2.1
53578	1.89226	2.1
53579	1.89226	2.1
53580	3.78452	2.1
53581	0.946132	2.1
53582	0.946132	2.1
53583	1.3048	2.1
53584	1.3048	2.1
53585	1.3048	2.1
53586	1.21578	2.1
53587	0.946132	2.1
53588	0.946132	2.1
53589	1.3048	2.1

53590	0.946132	2.1
53591	0.946132	2.1
53592	1.21578	2.1
53593	1.89227	0.05
53594	1.89227	0.05
53595	1.89227	0.05
53596	1.89227	0.05
53597	3.78453	0.05
53598	1.6671	0.05
53599	1.87338	0.05
53600	1.6671	0.05
53601	2.02098	0.05
53602	1.38328	0.05
53603	1.73715	0.05
53604	1.89226	0.05
53605	1.89226	0.05
53606	1.89226	0.05
53607	1.89226	0.05
53608	3.78453	0.05
53609	1.6671	0.05
53610	1.38328	0.05
53611	1.35562	0.05
53612	1.01012	0.05
53613	2.67722	0.05
53614	0.819377	0.05
53615	0.647925	0.05
53616	1.17802	0.05
53617	1.33174	0.05
53618	0.98543	0.05
53619	0.98543	0.05
53620	0.994237	0.05
53621	0.994237	0.05
53622	1.17802	0.05
53623	0.647926	0.05
53624	1.33174	0.05
53625	0.819377	0.05
53626	1.27806	0.05
53627	1.68264	0.05
53628	1.44593	0.05
53629	1.878 0.05	
53630	3.15606	0.05
53631	1.878 0.05	
53632	1.9898	0.05
53633	1.878 0.05	
53634	1.68264	0.05
53635	2.30533	0.05
53636	2.10997	0.05
53637	0.814587	0.05
53638	0.814596	0.05
53639	0.82775	0.05
53640	0.82775	0.05
53641	1.0107	0.05
53642	0.91549	0.05
53643	0.741058	0.05
53644	0.714748	0.05
53645	0.714758	0.05
53646	1.03225	0.05
53647	0.704864	0.05

53648	0.915488	0.05
53649	1.90347	0.05
53650	2.06584	0.05
53651	1.878	0.05
53652	1.50236	0.05
53653	2.05749	0.05
53654	2.30533	0.05
53655	2.24475	2.1
53656	1.85871	2.1
53657	2.13711	2.1
53658	2.13711	2.1
53659	1.9943	2.1
53660	1.9943	2.1
53661	1.85871	2.1
53662	1.7871	2.1
53663	1.41619	2.1
53664	1.41619	2.1
53665	2.13711	2.1
53666	2.13711	2.1
53667	2.24475	2.1
53668	2.13711	2.1
53669	2.54902	2.1
53670	2.46327	2.1
53671	4.70802	2.1
53672	1.06913	2.1
53673	1.06913	2.1
53674	1.0821	2.1
53675	1.0821	2.1
53676	0.893552	2.1
53677	1.08267	2.1
53678	1.06855	2.1
53679	1.23163	2.1
53680	1.23163	2.1
53681	1.08267	2.1
53682	0.893551	2.1
53683	1.06855	2.1
53684	1.67046	0.902843
53685	2.4106	0.740894
53686	2.05835	1.01338
53687	2.4106	1.05472
53688	1.59562	0.781199
53689	1.94788	0.766629
53690	2.34737	1.03211
53691	2.4106	0.766926
53692	2.05834	0.991945
53693	1.99655	1.10022
53694	4.40715	0.892869
53695	1.67046	0.800387
53696	2.05835	0.634567
53697	1.588	0.916484
53698	1.34857	1.33172
53699	3.40692	0.81882
53700	1.73802	0.859926
53701	1.89668	0.958478
53702	1.99655	1.81761
53703	1.99655	0.790198
53704	1.79981	0.919728
53705	2.05834	0.683992

53706	1.89226	1.30524
53707	1.89226	1.09412
53708	1.89226	0.720133
53709	1.89226	1.9927
53710	1.89226	0.916067
53711	1.89226	2.1
53712	1.89226	1.11484
53713	1.89226	2.1
53714	1.89226	1.95219
53715	1.89226	0.644423
53716	3.78453	1.73114
53717	0.719473	2.1
53718	0.916103	1.79356
53719	1.02744	1.19326
53720	0.850328	2.08882
53721	0.888094	1.12473
53722	0.888094	2.1
53723	0.878337	2.1
53724	0.878337	0.436096
53725	0.916103	1.32887
53726	1.02744	1.42271
53727	0.719473	1.77034
53728	0.850328	2.01674
53729	1.02744	1.36242
53730	0.850329	2.1
53731	1.0875	1.18838
53732	0.919597	1.3959
53733	0.975841	1.02207
53734	0.975841	2.1
53735	0.971196	1.00278
53736	0.971196	2.05129
53737	1.02744	1.71479
53738	1.0875	1.15187
53739	0.850328	2.00075
53740	0.919598	1.66953
53741	1.89226	2.04734
53742	1.89226	1.58187
53743	1.89226	0.989815
53744	1.89226	1.74033
53745	3.78453	1.36313
53746	1.89226	1.55644
53747	1.89226	1.0877
53748	1.89226	0.414758
53749	1.89226	2.1
53750	1.89226	0.648718
53751	1.89226	2.1
53752	1.11103	1.64579
53753	0.916103	2.1
53754	1.21177	1.2733
53755	1.02744	1.35269
53756	1.07188	1.05993
53757	1.07188	2.1
53758	1.06659	1.03596
53759	1.06659	2.1
53760	1.11103	2.08713
53761	1.21177	1.53707
53762	0.916104	1.99325
53763	1.02744	2.08848

53764	2.54158	1.15866
53765	2.42354	1.40715
53766	2.17499	0.992891
53767	2.17499	2.1
53768	2.42354	1.93205
53769	2.05488	2.1
53770	1.89226	0.05
53771	1.89226	0.05
53772	1.89226	0.05
53773	1.89226	0.05
53774	3.78453	0.05
53775	1.89226	0.05
53776	1.89226	0.05
53777	1.89226	0.05
53778	1.89226	0.05
53779	1.89226	0.05
53780	1.89226	0.05
53781	1.89226	0.05
53782	1.89226	0.05
53783	1.89226	0.05
53784	1.89226	0.05
53785	1.89226	0.05
53786	1.89226	0.05
53787	1.89226	0.05
53788	1.89226	0.05
53789	1.89226	0.05
53790	1.89226	0.05
53791	3.78452	0.05
53792	0.802544	0.05
53793	0.946132	0.05
53794	0.946132	0.05
53795	0.787038	0.05
53796	0.787039	0.05
53797	0.787039	0.05
53798	0.961637	0.05
53799	0.961637	0.05
53800	0.787038	0.05
53801	0.802544	0.05
53802	0.946132	0.05
53803	0.946132	0.05
53804	1.89226	0.05
53805	1.60509	0.05
53806	1.89226	0.05
53807	1.89226	0.05
53808	1.3905	0.05
53809	1.3905	0.05
53810	0.946132	0.05
53811	0.946132	0.05
53812	0.946131	0.05
53813	0.946131	0.05
53814	0.946132	0.05
53815	0.946132	0.05
53816	0.946131	0.05
53817	0.946131	0.05
53818	0.946131	0.05
53819	0.946131	0.05
53820	0.946132	0.05
53821	0.946132	0.05

53822	0.946131	0.05
53823	0.946131	0.05
53824	0.946131	0.05
53825	0.946131	0.05
53826	0.946132	0.05
53827	0.946132	0.05
53828	0.946131	0.05
53829	0.946131	0.05
53830	0.946131	0.05
53831	0.946131	0.05
53832	0.946132	0.05
53833	0.946132	0.05
53834	1.42382	2.1
53835	1.32201	0.245679
53836	1.32096	0.953159
53837	1.42866	1.98425
53838	2.75067	1.2772
53839	1.89226	2.09515
53840	1.89226	0.12114
53841	1.89226	1.09347
53842	1.89226	1.25261
53843	1.89226	0.373471
53844	1.89226	0.645554
53845	1.89226	1.4046
53846	2.79029	0.455874
53847	2.7803	0.947723
53848	2.79029	0.974369
53849	1.88227	0.724856
53850	1.89226	0.863999
53851	0.716065	1.75039
53852	0.714333	2.1
53853	0.660483	0.471338
53854	0.659126	1.16167
53855	0.687538	0.476687
53856	0.687538	1.55018
53857	0.685921	2.1
53858	0.685921	0.401325
53859	0.659126	1.19447
53860	0.716065	1.19192
53861	0.660483	1.24544
53862	0.714333	1.24077
53863	1.89226	1.51628
53864	1.89226	0.132227
53865	1.89226	1.06807
53866	1.89226	1.22044
53867	3.78453	1.14839
53868	0.946133	0.747199
53869	0.946133	1.04926
53870	1.39015	0.502858
53871	1.38969	0.110422
53872	0.946132	0.146295
53873	0.946132	0.383152
53874	1.38969	1.06883
53875	1.38969	0.59767
53876	1.38969	0.428116
53877	0.946132	0.791052
53878	0.946133	0.967371
53879	1.39015	0.553761

53880	2.72157	0.05
53881	1.22176	0.05
53882	1.59492	0.05
53883	1.22176	0.0820964
53884	2.40743	0.05
53885	2.03426	0.052052
53886	0.946131	0.0658049
53887	0.946131	0.05
53888	1.36079	0.05
53889	1.01713	0.05
53890	0.94613	0.05
53891	0.94613	0.074942
53892	1.36079	0.05
53893	1.36079	0.05
53894	0.946131	0.05
53895	1.36079	0.053544
53896	1.01713	0.05
53897	0.946131	0.05
53898	1.89226	0.05
53899	1.89226	0.05
53900	1.89226	0.05
53901	1.89226	0.0883412
53902	3.78453	0.05
53903	1.89226	0.05
53904	1.89226	0.05
53905	1.89226	0.05
53906	1.89226	0.0915824
53907	1.89226	0.05
53908	1.89226	0.05
53909	0.946131	0.0585174
53910	0.946131	0.05
53911	0.946131	0.05
53912	1.36079	0.05
53913	0.94613	0.05
53914	0.94613	0.0794569
53915	0.946133	0.05
53916	0.946133	0.05
53917	0.946131	0.05
53918	0.946131	0.0595319
53919	0.946132	0.05
53920	1.36079	0.05
53921	1.89226	0.05
53922	1.89226	0.0780297
53923	1.89226	0.05
53924	1.89226	0.05
53925	1.89226	0.05
53926	1.89226	0.05
53927	1.89227	0.05
53928	1.89227	0.05
53929	1.89227	0.05
53930	1.89227	0.0905612
53931	1.89227	0.05
53932	1.89227	0.05
53933	1.60428	2.1
53934	2.2367	2.1
53935	1.50097	2.1
53936	1.80125	2.1
53937	3.40553	2.1

53938	1.89095	2.1
53939	1.85293	0.890724
53940	1.80125	2.1
53941	1.76268	0.0742059
53942	3.65418	2.1
53943	0.803177	2.1
53944	0.803177	2.1
53945	0.798644	2.1
53946	0.798644	2.1
53947	0.84233	2.1
53948	0.900624	2.1
53949	0.750483	2.1
53950	0.701197	2.1
53951	0.701197	2.1
53952	0.84233	2.1
53953	0.750483	2.1
53954	0.900625	2.1
53955	0.860925	0.092897
53956	0.860925	2.1
53957	0.862743	2.1
53958	0.862743	0.987372
53959	0.881338	0.176861
53960	0.826621	0.0620372
53961	0.84233	2.1
53962	0.900625	2.1
53963	0.84233	2.1
53964	0.826621	2.07169
53965	0.900624	2.1
53966	0.881339	1.94558
53967	1.37938	1.12035
53968	1.03319	1.07972
53969	1.00512	1.42777
53970	0.639582	1.50121
53971	1.00463	1.42628
53972	1.00463	1.11015
53973	1.01434	1.05979
53974	1.01434	1.5014
53975	1.37938	1.26863
53976	1.00512	1.47246
53977	1.03319	1.29604
53978	0.639582	1.29963
53979	0.728713	0.05
53980	0.728713	0.05
53981	0.73425	0.723391
53982	0.73425	1.94648
53983	0.525122	2.1
53984	0.605622	0.05
53985	0.948901	0.05
53986	0.857342	0.05
53987	0.85734	0.05
53988	0.525123	1.51474
53989	0.948899	0.05
53990	0.605624	0.05
53991	1.75449	0.845514
53992	1.4378	0.896325
53993	1.93916	0.940049
53994	2.22512	0.799054
53995	3.69364	0.897852

53996	1.75448	0.936846
53997	1.89186	0.797634
53998	2.29771	0.812043
53999	2.22512	0.802162
54000	4.05219	0.812261
54001	1.93916	0.926117
54002	2.51749	0.770776
54003	2.62357	0.837609
54004	2.22512	0.805467
54005	2.33761	0.869775
54006	1.93916	0.823609
54007	2.29771	0.833498
54008	2.51749	0.786523
54009	2.22512	0.780422
54010	2.22512	0.767939
54011	2.50905	0.844465
54012	2.50905	0.820589
54013	0.87382	0.05
54014	1.33662	0.05
54015	1.20756	0.05
54016	0.890467	0.05
54017	1.08859	0.05
54018	1.08859	0.05
54019	1.13084	0.05
54020	1.08793	0.05
54021	1.34722	0.05
54022	1.27184	0.05
54023	0.917678	0.05
54024	0.97821	0.05
54025	1.28396	0.05
54026	1.70302	0.05
54027	2.30076	0.05
54028	1.20417	0.05
54029	2.57506	0.05
54030	2.2656	0.05
54031	1.03642	1.08219
54032	1.03642	0.05
54033	1.07029	0.116441
54034	1.07029	0.0561304
54035	1.31531	0.955011
54036	1.23279	0.242902
54037	0.941743	0.228592
54038	0.791399	0.839665
54039	0.791399	0.16812
54040	1.23279	0.151643
54041	0.941744	0.348705
54042	1.31531	0.367037
54043	0.937354	1.08806
54044	0.937354	0.05
54045	0.954794	0.182808
54046	0.954794	0.05
54047	1.13563	0.350984
54048	1.23279	0.952227
54049	0.791398	0.606466
54050	0.659359	0.817715
54051	1.13563	0.175815
54052	0.659359	0.179198
54053	0.791399	0.303775

54054	1.23279	0.186483
54055	2.29852	0.547257
54056	1.89227	0.551097
54057	2.14485	0.311591
54058	2.14485	1.2242
54059	1.89227	0.194707
54060	1.89227	1.10104
54061	0.947863	0.601453
54062	1.19854	1.18345
54063	2.16841	0.771444
54064	1.76597	0.734694
54065	2.96451	0.6239
54066	1.89227	0.257125
54067	1.89227	0.386087
54068	1.89227	0.223736
54069	1.89227	1.04805
54070	1.89227	0.141589
54071	1.89227	0.792681
54072	2.46489	2.1
54073	1.98339	2.1
54074	1.81667	2.1
54075	2.36914	2.1
54076	4.35253	2.1
54077	2.46488	2.1
54078	2.70401	2.1
54079	2.64996	2.1
54080	2.36914	2.1
54081	5.07315	2.1
54082	1.81667	2.1
54083	2.29459	2.1
54084	2.36914	2.1
54085	2.36914	2.1
54086	1.68073	2.1
54087	1.68073	2.1
54088	2.64996	2.1
54089	2.29458	2.1
54090	2.29458	2.1
54091	2.36914	2.1
54092	2.61243	2.1
54093	2.68698	2.1
54094	1.89226	1.41739
54095	1.89226	0.993662
54096	1.89226	1.53103
54097	1.89226	1.2953
54098	1.89226	1.56637
54099	1.89226	1.28259
54100	1.89224	1.77405
54101	1.89224	1.02818
54102	1.65571	1.34757
54103	1.65571	1.20569
54104	1.89224	1.50009
54105	1.89224	1.3363
54106	1.89224	1.34076
54107	1.89224	1.18726
54108	1.89224	1.46279
54109	1.89224	0.979809
54110	1.89224	1.7813
54111	1.89224	1.22376

54112	1.98252	0.05
54113	2.20641	0.05
54114	1.05129	0.05
54115	1.2951	0.05
54116	3.27762	0.05
54117	1.85598	2.1
54118	1.88635	1.2479
54119	2.12346	1.04451
54120	2.10838	2.1
54121	3.97943	1.33555
54122	2.10838	1.91986
54123	1.87532	2.1
54124	1.85597	0.238868
54125	1.85597	2.1
54126	1.87532	2.01052
54127	1.57914	2.1
54128	2.12348	1.21082
54129	2.35141	2.1
54130	2.37191	1.39801
54131	2.10841	1.66104
54132	2.38698	1.13474
54133	2.12348	1.6643
54134	1.07811	2.1
54135	0.937672	2.1
54136	1.17571	2.1
54137	1.05421	0.471438
54138	1.07087	1.32915
54139	1.07087	2.1
54140	1.06145	2.1
54141	1.06145	1.23519
54142	1.17571	2.1
54143	1.07811	2.1
54144	1.05421	2.1
54145	0.937672	2.1
54146	1.67334	2.1
54147	2.76609	2.1
54148	2.74144	2.1
54149	2.74144	2.1
54150	1.71157	2.1
54151	1.71157	2.1
54152	2.76609	2.1
54153	1.89226	2.1
54154	1.89226	2.1
54155	1.89226	2.1
54156	2.74143	2.1
54157	2.74143	2.1
54158	1.10393	2.1
54159	1.10393	2.1
54160	1.10788	2.1
54161	1.10788	2.1
54162	1.38302	2.1
54163	1.37072	2.1
54164	0.836672	2.1
54165	0.841089	2.1
54166	1.38302	2.1
54167	0.84109	2.1
54168	0.836671	2.1
54169	1.37072	2.1

54170	1.38302	2.1
54171	1.38302	2.1
54172	0.946131	2.1
54173	0.946131	2.1
54174	0.946128	2.1
54175	0.946128	2.1
54176	1.38302	2.1
54177	1.37072	2.1
54178	1.38302	2.1
54179	0.946131	2.1
54180	0.94613	2.1
54181	1.37072	2.1
54182	1.28072	0.620371
54183	1.55779	2.1
54184	1.28072	2.1
54185	1.40765	2.1
54186	1.47186	2.1
54187	1.59879	2.1
54188	1.89227	1.07139
54189	1.89227	0.654998
54190	1.89227	0.0503665
54191	1.89227	2.1
54192	1.89227	0.05
54193	1.89227	2.1
54194	2.80057	0.147085
54195	1.89226	0.238553
54196	1.89226	0.825962
54197	1.89226	0.840984
54198	2.63061	0.0504791
54199	2.63061	1.06287
54200	1.28072	0.225004
54201	1.38859	2.1
54202	1.47186	2.1
54203	1.47186	2.1
54204	1.16777	2.1
54205	1.16777	2.1
54206	1.89226	0.356607
54207	1.89226	0.652947
54208	1.89226	0.285843
54209	1.89226	2.1
54210	1.89226	0.0633607
54211	1.89226	2.1
54212	1.83221	2.1
54213	2.0815	2.1
54214	2.0815	2.1
54215	2.08153	2.1
54216	1.86703	2.1
54217	1.87402	2.1
54218	1.86703	2.1
54219	2.08508	2.1
54220	2.08508	2.1
54221	2.29001	2.1
54222	1.89226	2.1
54223	2.08048	2.1
54224	2.7278	0.0828666
54225	1.10909	0.05
54226	1.50472	0.163834
54227	1.10909	0.0850446

54228	2.69881	0.05
54229	2.30319	0.093362
54230	1.23204	2.1
54231	1.92264	2.1
54232	1.48162	2.1
54233	1.48162	2.1
54234	1.72994	2.1
54235	1.72994	2.1
54236	0.864968	2.1
54237	0.696952	2.1
54238	0.61602	2.1
54239	0.479736	2.1
54240	0.66444	2.1
54241	0.66444	2.1
54242	0.680264	2.1
54243	0.680264	2.1
54244	0.696952	2.1
54245	0.479736	2.1
54246	0.61602	2.1
54247	0.864968	2.1
54248	1.89226	0.867026
54249	2.43219	2.1
54250	1.89226	1.27631
54251	2.82019	1.24906
54252	1.89226	1.91729
54253	2.82019	1.3729
54254	1.89226	0.973717
54255	2.83701	2.1
54256	2.2701	1.5826
54257	2.2701	1.13818
54258	1.89226	1.7082
54259	1.89226	1.2311
54260	1.89226	1.73915
54261	1.89226	0.880647
54262	1.89226	1.043
54263	1.89226	1.17878
54264	1.89226	1.23117
54265	1.89226	1.15492
54266	1.5795	1.84283
54267	1.44091	1.64793
54268	1.05267	2.1
54269	1.05267	1.26829
54270	1.98408	1.9592
54271	1.98408	1.19061
54272	2.20624	2.1
54273	1.08831	2.1
54274	2.49053	2.1
54275	1.42486	2.1
54276	3.57883	2.1
54277	1.89226	2.1
54278	1.89226	2.1
54279	1.89226	2.1
54280	1.89226	2.1
54281	3.78453	2.1
54282	0.946132	2.1
54283	0.946132	2.1
54284	0.853591	2.1
54285	0.642207	2.1

54286	0.94613	2.1
54287	0.94613	2.1
54288	0.642209	2.1
54289	0.642209	2.1
54290	0.946131	2.1
54291	0.853591	2.1
54292	0.642207	2.1
54293	0.946132	2.1
54294	1.0506	2.1
54295	1.0506	2.1
54296	1.0879	2.1
54297	0.960424	2.1
54298	0.708687	2.1
54299	0.782927	2.1
54300	1.40041	2.1
54301	1.2518	2.1
54302	0.767748	2.1
54303	1.31801	2.1
54304	1.21412	2.1
54305	0.69461	2.1
54306	1.89226	2.1
54307	1.89226	2.1
54308	1.89226	2.1
54309	1.89226	2.1
54310	1.89226	2.1
54311	1.89226	2.1
54312	0.922223	2.1
54313	0.922223	2.1
54314	1.04548	2.1
54315	0.768229	2.1
54316	0.948441	2.1
54317	0.806483	2.1
54318	0.85362	2.1
54319	0.85362	2.1
54320	1.05942	2.1
54321	0.922187	2.1
54322	0.851554	2.1
54323	0.768264	2.1
54324	2.16728	2.1
54325	0.95455	2.1
54326	1.21372	2.1
54327	0.98227	2.1
54328	2.16728	2.1
54329	1.93584	2.1
54330	2.08849	0.05
54331	1.08732	0.05
54332	1.3357	0.0654254
54333	2.37948	0.0886044
54334	3.4668	0.0558289
54335	2.57882	0.0511352
54336	2.37948	0.05
54337	2.53055	0.05
54338	1.48676	0.114989
54339	1.3357	0.119714
54340	1.3357	0.109937
54341	1.89226	2.1
54342	1.89226	2.1
54343	2.17471	2.1

54344	1.89226	2.1
54345	2.60085	2.1
54346	2.26306	2.1
54347	0.946131	2.1
54348	0.946131	2.1
54349	0.946131	2.1
54350	0.946131	2.1
54351	0.94613	2.1
54352	0.94613	2.1
54353	0.946132	2.1
54354	0.946132	2.1
54355	0.946131	2.1
54356	0.946131	2.1
54357	0.946131	2.1
54358	0.946131	2.1
54359	1.89226	2.1
54360	2.17471	2.1
54361	2.08196	2.1
54362	2.17471	2.1
54363	1.79952	2.1
54364	1.89226	2.1
54365	1.89226	2.1
54366	1.89226	2.1
54367	1.89226	2.1
54368	1.89226	2.1
54369	1.89226	2.1
54370	1.89226	2.1
54371	1.86016	2.1
54372	1.89226	2.1
54373	1.89226	2.1
54374	1.89226	2.1
54375	1.84826	2.1
54376	1.84826	2.1
54377	2.50136	2.1
54378	1.99781	2.1
54379	1.99729	2.1
54380	2.5214	2.1
54381	4.49865	2.1
54382	0.51724	2.1
54383	0.496076	2.1
54384	0.998906	2.1
54385	0.998645	2.1
54386	0.742249	2.1
54387	0.742249	2.1
54388	0.752734	2.1
54389	0.752734	2.1
54390	0.998639	2.1
54391	0.496073	2.1
54392	0.517243	2.1
54393	0.998913	2.1
54394	0.998653	2.1
54395	0.994293	2.1
54396	1.26071	2.1
54397	1.26504	2.1
54398	1.12529	2.1
54399	1.12529	2.1
54400	1.12971	2.1
54401	1.12971	2.1

54402	1.26504	2.1
54403	0.994292	2.1
54404	0.998651	2.1
54405	1.26071	2.1
54406	1.89226	2.1
54407	1.89226	2.1
54408	1.89226	2.1
54409	1.89226	2.1
54410	3.78452	2.1
54411	1.89226	2.1
54412	1.89226	2.1
54413	1.89226	2.1
54414	1.89226	2.1
54415	3.78452	2.1
54416	0.675872	2.1
54417	0.675641	2.1
54418	0.943072	2.1
54419	0.898317	2.1
54420	0.775717	2.1
54421	0.775717	2.1
54422	0.79824	2.1
54423	0.79824	2.1
54424	0.675642	2.1
54425	0.94307	2.1
54426	0.675874	2.1
54427	0.898315	2.1
54428	0.943071	2.1
54429	0.898316	2.1
54430	0.998133	2.1
54431	0.918903	2.1
54432	0.922369	2.1
54433	0.922369	2.1
54434	0.939606	2.1
54435	0.939606	2.1
54436	0.943071	2.1
54437	0.998133	2.1
54438	0.898316	2.1
54439	0.918903	2.1
54440	0.946131	2.1
54441	0.946131	2.1
54442	0.946131	2.1
54443	0.946131	2.1
54444	0.946132	2.1
54445	0.946132	2.1
54446	0.946129	2.1
54447	0.946129	2.1
54448	0.94613	2.1
54449	0.94613	2.1
54450	0.946131	2.1
54451	0.946131	2.1
54452	1.89226	2.1
54453	1.89226	2.1
54454	1.89226	2.1
54455	1.89226	2.1
54456	1.89226	2.1
54457	1.89226	2.1
54458	1.35129	2.1
54459	1.88614	2.1

54460	2.01867	2.1
54461	1.38189	2.1
54462	3.36995	2.1
54463	0.943071	2.1
54464	1.00934	2.1
54465	0.998134	2.1
54466	1.10866	2.1
54467	0.992617	2.1
54468	0.992617	2.1
54469	1.01485	2.1
54470	1.01485	2.1
54471	1.00934	2.1
54472	1.10866	2.1
54473	0.943072	2.1
54474	0.998134	2.1
54475	2.67272	2.1
54476	1.61859	2.1
54477	1.70303	2.1
54478	2.52175	2.1
54479	4.5326	2.1
54480	2.00954	2.1
54481	2.27801	2.1
54482	1.87875	2.1
54483	1.87875	2.1
54484	2.37837	2.1
54485	2.37837	2.1
54486	2.00955	2.1
54487	1.78	2.1
54488	2.21813	2.1
54489	1.8493	2.1
54490	2.37838	2.1
54491	2.00955	2.1
54492	2.37838	2.1
54493	2.27801	2.1
54494	2.58523	2.1
54495	2.71597	2.1
54496	4.9636	2.1
54497	2.37838	2.1
54498	2.71597	2.1
54499	1.72709	2.1
54500	2.0815	2.1
54501	2.59749	2.1
54502	2.78034	2.1
54503	0.94613	1.07279
54504	0.94613	1.10719
54505	0.946128	0.949049
54506	0.946128	0.851801
54507	0.94613	0.846251
54508	0.94613	0.966573
54509	0.946128	1.10117
54510	0.946128	0.587008
54511	0.946129	1.0555
54512	0.946129	1.03233
54513	0.946129	0.986203
54514	0.946129	1.00002
54515	1.89226	1.21557
54516	1.89226	1.13829
54517	1.89226	1.23489

54518	1.89226	1.03838
54519	3.78452	1.05801
54520	0.946133	0.518222
54521	0.946133	0.614398
54522	0.946132	0.229296
54523	0.946132	0.335197
54524	0.946132	0.30506
54525	0.946132	0.340886
54526	0.946133	0.599333
54527	0.946133	0.539845
54528	0.946132	0.497843
54529	0.946132	0.05
54530	0.946132	0.388155
54531	0.946132	0.393652
54532	1.89226	0.982992
54533	2.10495	0.260461
54534	1.89226	0.306433
54535	2.30932	0.326106
54536	1.89226	0.937508
54537	2.30932	1.02215
54538	2.30932	0.264234
54539	1.00366	0.05
54540	2.10496	0.05
54541	1.24728	0.0777599
54542	3.31298	0.0883182
54543	1.87557	2.1
54544	1.07167	2.1
54545	2.03045	2.1
54546	1.25185	2.1
54547	3.10212	2.1
54548	1.89226	2.1
54549	1.89226	2.1
54550	1.89226	2.1
54551	1.89226	2.1
54552	3.78453	2.1
54553	0.851541	2.1
54554	0.851541	2.1
54555	1.12236	2.1
54556	1.05783	2.1
54557	0.870663	2.1
54558	0.951856	2.1
54559	0.946132	2.1
54560	0.946132	2.1
54561	1.04073	2.1
54562	1.1296	2.1
54563	0.776062	2.1
54564	0.851527	2.1
54565	0.859395	2.1
54566	0.859395	2.1
54567	0.870165	2.1
54568	0.870165	2.1
54569	0.625924	2.1
54570	0.714333	2.1
54571	1.0821	2.1
54572	1.01523	2.1
54573	1.0821	2.1
54574	0.714333	2.1
54575	1.01523	2.1

54576	0.625924	2.1
54577	1.89226	2.1
54578	1.89226	2.1
54579	1.89226	2.1
54580	1.89226	2.1
54581	3.78453	2.1
54582	1.89226	2.1
54583	1.89226	2.1
54584	2.23164	2.1
54585	2.67802	2.1
54586	2.03663	2.1
54587	1.89226	2.1
54588	2.42221	2.1
54589	1.61933	2.1
54590	2.80896	2.1
54591	1.87043	2.1
54592	4.29262	2.1
54593	1.89226	2.1
54594	1.89226	2.1
54595	1.89226	2.1
54596	1.89226	2.1
54597	1.89226	2.1
54598	1.89226	2.1
54599	2.80892	2.1
54600	1.87041	2.1
54601	2.67182	2.1
54602	2.30891	2.1
54603	5.07665	2.1
54604	1.89224	2.1
54605	1.89224	2.1
54606	1.89224	2.1
54607	1.89224	2.1
54608	3.78448	2.1
54609	0.946118	2.1
54610	0.946118	2.1
54611	0.946118	2.1
54612	0.946118	2.1
54613	0.946115	2.1
54614	0.946115	2.1
54615	0.94612	2.1
54616	0.94612	2.1
54617	0.94613	2.1
54618	0.94613	2.1
54619	0.946105	2.1
54620	0.946105	2.1
54621	2.3089	2.1
54622	1.02783	2.1
54623	1.98533	2.1
54624	1.76762	2.1
54625	1.24553	2.1
54626	1.02783	2.1
54627	2.2049	0.05
54628	2.34673	0.05
54629	1.00763	0.05
54630	1.23746	0.05
54631	3.44236	0.05
54632	1.89226	0.05
54633	1.89226	0.139596

54634	1.89226	0.0664215
54635	1.89226	0.120927
54636	3.78452	0.126366
54637	1.89226	0.144985
54638	1.89226	0.0698548
54639	1.89226	0.122845
54640	1.89226	0.13668
54641	3.78453	0.129607
54642	0.891618	0.05
54643	0.891618	0.05
54644	0.902158	0.05
54645	0.902158	0.05
54646	1.15396	0.05
54647	0.620408	0.05
54648	1.17337	0.05
54649	0.618733	0.05
54650	0.620408	0.05
54651	0.618733	0.05
54652	1.17337	0.05
54653	1.15396	0.05
54654	0.69978	0.05
54655	0.69978	0.05
54656	0.946102	0.05
54657	0.946102	0.05
54658	0.831702	0.05
54659	0.946129	0.05
54660	0.699752	0.05
54661	0.946132	0.05
54662	0.831703	0.05
54663	0.699751	0.05
54664	0.946131	0.05
54665	0.946131	0.05
54666	0.831675	0.05
54667	0.831675	0.05
54668	0.946163	0.05
54669	0.864397	0.05
54670	0.853471	0.0517185
54671	0.962623	0.05
54672	0.748555	0.05
54673	0.851543	0.05
54674	0.864714	0.05
54675	0.760411	0.05
54676	0.946133	0.05
54677	0.946133	0.05
54678	1.89226	0.704472
54679	1.89226	0.818052
54680	1.89226	0.787762
54681	1.89226	0.708491
54682	3.78452	0.751049
54683	1.89226	0.860352
54684	1.89226	0.740963
54685	1.89226	0.769333
54686	1.89226	0.902276
54687	3.78452	0.803103
54688	0.946132	0.868982
54689	0.946132	0.915721
54690	0.946132	0.750157
54691	0.946132	0.786347

54692	0.946132	0.764609
54693	0.946132	0.945792
54694	0.946133	0.674483
54695	0.946133	0.908856
54696	0.946133	0.807027
54697	0.946133	0.799337
54698	0.946132	0.823444
54699	0.946132	0.864095
54700	1.89226	0.833302
54701	2.39715	0.785846
54702	1.89226	0.836737
54703	1.89226	0.951315
54704	1.89226	0.745554
54705	1.89226	0.79718
54706	0.94613	0.899426
54707	0.94613	0.886937
54708	0.94613	0.803607
54709	0.94613	0.776008
54710	0.946131	0.795788
54711	0.946131	0.909719
54712	0.946129	0.887693
54713	0.946129	0.787445
54714	0.94613	0.81321
54715	0.94613	0.826792
54716	0.94613	0.844369
54717	0.94613	0.843214
54718	2.54204	2.1
54719	2.3148	2.1
54720	2.55328	2.1
54721	2.39646	2.1
54722	4.71126	2.1
54723	1.62611	2.1
54724	2.3148	2.1
54725	2.16986	2.1
54726	1.43745	2.1
54727	3.75225	2.1
54728	1.89226	2.1
54729	1.89226	2.1
54730	1.89226	2.1
54731	1.89226	2.1
54732	3.78453	2.1
54733	1.89226	2.1
54734	1.89226	2.1
54735	1.89226	2.1
54736	1.89226	2.1
54737	3.78453	2.1
54738	2.12125	2.1
54739	2.39642	2.1
54740	2.2941	2.1
54741	1.9933	2.1
54742	4.38972	2.1
54743	1.73784	2.1
54744	1.26892	2.1
54745	1.26892	2.1
54746	1.43743	2.1
54747	1.9933	2.1
54748	2.1618	2.1
54749	0.946118	2.1

54750	0.946118	2.1
54751	0.946118	2.1
54752	0.946118	2.1
54753	0.946119	2.1
54754	0.946119	2.1
54755	0.946118	2.1
54756	0.946118	2.1
54757	0.946131	2.1
54758	0.946131	2.1
54759	0.946106	2.1
54760	0.946106	2.1
54761	1.89224	2.1
54762	1.89224	2.1
54763	1.89224	2.1
54764	1.89224	2.1
54765	3.78448	2.1
54766	2.45922	2.1
54767	2.15686	2.1
54768	2.14259	2.1
54769	2.43225	2.1
54770	4.58911	2.1
54771	1.62027	2.1
54772	2.15686	2.1
54773	1.62241	2.1
54774	1.62241	2.1
54775	2.1426	2.1
54776	2.1426	2.1
54777	2.14259	2.1
54778	2.43225	2.1
54779	2.40667	2.1
54780	2.1281	2.1
54781	4.56035	2.1
54782	1.0713	2.1
54783	1.06405	2.1
54784	0.810136	2.1
54785	0.808433	2.1
54786	0.938508	2.1
54787	0.938508	2.1
54788	0.941223	2.1
54789	0.941223	2.1
54790	1.06405	2.1
54791	0.808433	2.1
54792	0.810135	2.1
54793	1.0713	2.1
54794	1.89226	0.132835
54795	1.89226	0.05
54796	1.89226	0.131334
54797	1.89226	0.058089
54798	3.78453	0.0563821
54799	1.89227	0.05
54800	1.89227	0.05
54801	1.89227	0.0902099
54802	1.89227	0.05
54803	3.78453	0.0512094
54804	1.19	0.05
54805	1.12469	0.05
54806	1.01052	0.05
54807	1.08166	0.05

54808	2.20051	0.05
54809	0.594999	0.05
54810	0.540832	0.05
54811	0.514182	0.05
54812	0.567857	0.05
54813	0.554259	0.05
54814	0.554259	0.05
54815	0.55443	0.05
54816	0.55443	0.05
54817	0.540832	0.05
54818	0.514182	0.05
54819	0.567857	0.05
54820	0.594999	0.05
54821	1.38674	0.05
54822	0.946132	0.0605636
54823	1.36361	0.05
54824	0.946132	0.0506444
54825	1.36362	0.05
54826	1.36362	0.05
54827	0.946131	0.05949
54828	0.946131	0.0820432
54829	1.38674	0.05
54830	1.36361	0.05
54831	0.946132	0.0572398
54832	0.946132	0.05
54833	1.89226	0.05
54834	2.68508	0.05
54835	2.77349	0.05
54836	1.89226	0.05
54837	2.77349	0.05
54838	1.89226	0.05
54839	2.15315	0.05
54840	1.96254	0.078244
54841	1.51377	0.05
54842	1.89226	0.05
54843	1.47799	0.0682294
54844	1.89226	0.05
54845	2.49439	0.0511672
54846	1.89226	0.2508
54847	2.15315	0.141606
54848	1.89226	0.135116
54849	2.15315	0.186105
54850	1.89226	0.0814658
54851	1.89226	0.355444
54852	1.89226	0.467901
54853	1.89226	0.445161
54854	1.89226	0.221966
54855	1.89226	0.522907
54856	1.89226	0.409793
54857	1.89226	0.199941
54858	1.89226	0.142878
54859	1.89226	0.0994774
54860	1.89226	0.232925
54861	1.89226	0.0519174
54862	1.89226	0.367277
54863	1.375	0.05
54864	2.01865	0.051184
54865	2.12047	0.05

54866	1.47354	0.05
54867	2.12047	0.07712
54868	1.47354	0.05
54869	0.687499	0.05
54870	1.06023	0.05
54871	1.08019	0.05
54872	0.604229	0.05
54873	1.08018	0.05
54874	1.06023	0.05
54875	0.687502	0.05
54876	0.60423	0.05
54877	0.80646	0.05
54878	0.806461	0.05
54879	0.858001	0.05
54880	0.858001	0.05
54881	2.26414	2.1
54882	1.87342	2.1
54883	1.87342	2.1
54884	1.79667	2.1
54885	2.44045	2.1
54886	2.31924	2.1
54887	2.26414	2.1
54888	2.26616	2.1
54889	2.2355	2.1
54890	2.21858	2.1
54891	4.48474	2.1
54892	2.18516	2.1
54893	1.87344	2.1
54894	2.21859	2.1
54895	2.66233	2.1
54896	1.85196	2.1
54897	2.22236	2.1
54898	2.23551	2.1
54899	2.21859	2.1
54900	2.18516	2.1
54901	2.21189	2.1
54902	4.43049	2.1
54903	1.17171	2.1
54904	2.38924	2.1
54905	2.0815	2.1
54906	2.33348	2.1
54907	1.42851	2.1
54908	1.5959	2.1
54909	1.89226	2.1
54910	1.89226	2.1
54911	1.89226	2.1
54912	1.89226	2.1
54913	3.78452	2.1
54914	2.75425	2.1
54915	1.34366	2.1
54916	1.34366	2.1
54917	1.56512	2.1
54918	2.54186	2.1
54919	2.76332	2.1
54920	1.07751	2.1
54921	2.0815	2.1
54922	1.97259	2.1
54923	1.76455	2.1

54924	1.30191	2.1
54925	1.16461	2.1
54926	0.946131	2.1
54927	0.946131	2.1
54928	0.946129	2.1
54929	0.946129	2.1
54930	0.946131	2.1
54931	0.946131	2.1
54932	0.94613	2.1
54933	0.94613	2.1
54934	0.94613	2.1
54935	0.94613	2.1
54936	0.946131	2.1
54937	0.946131	2.1
54938	1.89226	0.245033
54939	1.89226	0.0636311
54940	1.89226	0.0806472
54941	1.89226	0.05
54942	3.78453	0.05
54943	1.89226	0.183654
54944	1.89226	0.106703
54945	1.89226	0.0928307
54946	1.89226	0.05
54947	3.78453	0.229589
54948	0.946132	0.05
54949	0.946132	0.10813
54950	1.30327	0.05
54951	1.33544	0.0611124
54952	1.33544	0.05
54953	1.33544	0.05
54954	0.946132	0.05
54955	0.946132	0.05
54956	0.946132	0.05
54957	1.30327	0.05
54958	1.33544	0.0507333
54959	0.946132	0.0796542
54960	1.30327	0.05
54961	1.33544	0.05
54962	0.709426	0.05
54963	0.790129	0.05
54964	1.05886	0.05
54965	1.05886	0.05
54966	1.03454	0.05
54967	1.03454	0.05
54968	1.30327	0.05
54969	0.709426	0.05
54970	1.33544	0.05
54971	0.790128	0.05
54972	0.946132	0.05
54973	0.946132	0.0583834
54974	0.827866	0.05
54975	0.946133	0.05
54976	0.946132	0.05
54977	0.946132	0.05
54978	0.946133	0.05
54979	0.946133	0.05
54980	0.946132	0.05
54981	0.827866	0.05

54982	0.946133	0.0725392
54983	0.946133	0.05
54984	1.89227	0.0806163
54985	1.89227	0.05
54986	1.89227	0.05
54987	1.89227	0.05
54988	1.89227	0.05
54989	1.89227	0.05
54990	0.946133	0.05
54991	0.946133	0.05
54992	1.21602	0.05
54993	1.30327	0.05
54994	1.30327	0.05
54995	1.30327	0.05
54996	0.946132	0.05
54997	0.946132	0.05
54998	0.946132	0.05
54999	1.21602	0.05
55000	0.946133	0.05
55001	1.30327	0.05
55002	1.04227	0.05
55003	1.11706	0.05
55004	0.968413	0.05
55005	0.945482	0.05
55006	1.3204	0.05
55007	1.23215	0.05
55008	0.810791	0.05
55009	1.38978	0.05
55010	1.07728	0.05
55011	1.11704	0.05
55012	0.608052	0.05
55013	1.89226	0.05
55014	1.89226	0.05
55015	1.89226	0.05
55016	1.89226	0.05
55017	3.78453	0.05
55018	1.89226	0.05
55019	1.89226	0.05
55020	1.89226	0.05
55021	1.89226	0.05
55022	3.78451	0.05
55023	2.58476	0.05
55024	1.56598	0.05
55025	1.55045	0.05
55026	1.5124	0.05
55027	2.62281	0.05
55028	2.58476	0.05
55029	2.58477	0.05
55030	1.89226	0.05
55031	1.89226	0.05
55032	1.89226	0.05
55033	2.62282	0.05
55034	2.62282	0.05
55035	1.89224	0.05
55036	1.89224	0.05
55037	1.89224	0.05
55038	1.89224	0.05
55039	3.78448	0.05

55040	1.89223	0.05
55041	1.89223	0.05
55042	1.89223	0.05
55043	1.89223	0.05
55044	1.89223	0.05
55045	1.89223	0.05
55046	2.62278	0.05
55047	1.45053	0.05
55048	1.49678	0.05
55049	1.56596	0.05
55050	2.5536	0.05
55051	2.62278	0.05
55052	1.31139	0.05
55053	1.2768	0.05
55054	0.946118	0.05
55055	0.946118	0.05
55056	1.2768	0.05
55057	1.2768	0.05
55058	0.946116	0.05
55059	0.946116	0.05
55060	1.27682	0.05
55061	0.94613	0.05
55062	0.946105	0.05
55063	1.31138	0.05
55064	1.38506	1.71002
55065	1.53348	2.1
55066	1.17042	1.81112
55067	1.17042	2.09448
55068	1.73669	1.81037
55069	1.73669	2.09208
55070	1.73669	1.45214
55071	1.79928	1.09033
55072	1.38506	1.33139
55073	1.38506	2.1
55074	1.79928	2.03266
55075	1.43484	2.1
55076	1.73669	1.31627
55077	1.93568	2.08142
55078	1.53347	1.25744
55079	1.53347	2.02149
55080	2.14058	1.28769
55081	2.14058	2.1
55082	0.89964	1.07437
55083	1.10415	2.1
55084	0.868346	1.41663
55085	1.07029	2.1
55086	0.98687	1.56128
55087	0.98687	2.1
55088	0.985627	0.922828
55089	0.985627	2.1
55090	1.07029	1.9443
55091	1.10415	1.55029
55092	0.868344	2.03259
55093	0.899642	2.07723
55094	2.40667	2.1
55095	2.1281	2.1
55096	2.11176	2.1
55097	2.38069	2.1

55098	4.50878	2.1
55099	2.11176	2.1
55100	1.61687	2.1
55101	1.60965	2.1
55102	1.61687	2.1
55103	2.12088	2.1
55104	2.1281	2.1
55105	2.11176	2.1
55106	2.34898	2.1
55107	2.34898	2.1
55108	2.38069	2.1
55109	2.09114	2.1
55110	2.12285	2.1
55111	1.05588	2.1
55112	1.04557	2.1
55113	0.804824	2.1
55114	0.798269	2.1
55115	0.92615	2.1
55116	0.92615	2.1
55117	0.927999	2.1
55118	0.927999	2.1
55119	1.04557	2.1
55120	0.798269	2.1
55121	1.05588	2.1
55122	0.804824	2.1
55123	1.43548	0.0940936
55124	1.53873	0.560003
55125	1.25063	0.05
55126	1.39662	0.05
55127	2.78937	0.05
55128	1.25063	0.605176
55129	1.39662	0.05
55130	1.10476	0.05
55131	1.28326	0.05
55132	2.53389	0.05
55133	1.51147	0.05
55134	1.86114	0.05
55135	1.86114	0.05
55136	2.12926	0.05
55137	1.3659	0.05
55138	1.63401	0.05
55139	1.89226	0.05
55140	1.89226	0.05
55141	1.89226	0.05
55142	1.89226	0.05
55143	1.89226	0.05
55144	1.89226	0.05
55145	1.89226	0.05
55146	1.89226	0.05
55147	2.46286	0.05
55148	1.89226	0.05
55149	3.78452	0.05
55150	1.89226	0.05
55151	1.89226	0.093021
55152	1.89226	0.05
55153	1.89226	0.05
55154	1.89226	0.05
55155	1.89226	0.05

55156	1.89226	0.05
55157	1.89226	0.05
55158	2.28992	0.05
55159	1.89226	0.05
55160	3.78452	0.05
55161	1.13537	0.05
55162	0.886595	0.0576818
55163	0.946131	0.05
55164	0.946131	0.05
55165	0.94613	0.141119
55166	0.94613	0.05
55167	1.25069	0.05
55168	0.841855	0.163976
55169	1.27436	0.05
55170	1.06196	0.05
55171	1.09758	0.05
55172	0.830306	0.05
55173	0.826931	0.698745
55174	0.826933	0.05
55175	0.764631	0.05
55176	0.764631	0.05
55177	1.00427	0.0874162
55178	0.788034	0.05
55179	0.726852	0.05
55180	0.604206	0.4936
55181	0.828475	0.05
55182	0.604198	0.05
55183	0.8332	0.05
55184	1.00428	0.05
55185	1.89226	0.0676771
55186	1.89226	0.05
55187	1.89226	0.05
55188	1.89226	0.195398
55189	1.89226	0.05
55190	1.89226	0.05
55191	0.768789	2.1
55192	0.849132	0.05
55193	0.666423	2.1
55194	0.688993	0.807059
55195	0.868671	0.05
55196	0.89117	2.1
55197	0.705632	2.1
55198	1.80425	2.1
55199	1.73883	2.1
55200	1.45159	2.1
55201	1.45159	2.1
55202	1.37681	2.1
55203	1.73883	2.1
55204	1.39679	0.05
55205	2.08149	0.05
55206	1.89226	0.05
55207	2.32763	0.05
55208	1.89226	0.05
55209	2.31274	0.05
55210	1.89226	0.05
55211	1.89226	0.05
55212	1.89226	0.05
55213	1.89226	0.05

55214	1.89226	0.05
55215	1.89226	0.05
55216	1.89226	0.05
55217	1.89226	0.05
55218	1.89226	0.05
55219	1.89226	0.05
55220	1.89226	0.05
55221	1.89226	0.05
55222	1.95181	0.05
55223	1.89226	0.05
55224	1.89226	0.05
55225	1.89226	0.05
55226	3.78451	0.05
55227	0.946131	0.05
55228	0.946131	0.05
55229	0.946125	0.05
55230	0.946125	0.05
55231	0.946127	0.05
55232	0.946127	0.05
55233	0.946128	0.05
55234	0.946128	0.05
55235	0.946128	0.05
55236	0.946128	0.05
55237	0.946128	0.05
55238	0.946128	0.05
55239	1.89226	0.05
55240	1.89226	0.05
55241	1.89226	0.05
55242	1.89226	0.05
55243	1.89226	0.05
55244	1.89226	0.05
55245	1.89226	0.825384
55246	1.89226	0.82305
55247	1.89226	0.779028
55248	1.89226	0.759917
55249	3.78453	0.795666
55250	1.89226	0.895612
55251	1.89226	0.830493
55252	1.89226	0.820862
55253	1.89226	0.825897
55254	3.78452	0.822181
55255	1.21387	0.823266
55256	0.8164	0.503013
55257	0.946133	0.786993
55258	0.946133	0.813387
55259	0.946102	0.53288
55260	0.946102	0.848793
55261	0.816431	0.877196
55262	0.816431	0.826752
55263	0.816401	0.786063
55264	0.94613	0.7029
55265	0.946131	0.946006
55266	1.21388	0.826643
55267	0.946132	0.703522
55268	0.946132	0.709075
55269	0.946132	0.773644
55270	0.946132	0.747784
55271	0.946132	0.493941

55272	0.946132	0.691675
55273	0.946132	0.802254
55274	0.946132	0.751646
55275	0.946131	0.438917
55276	0.946131	0.758937
55277	0.946132	0.799963
55278	0.946132	0.761069
55279	1.89226	0.812131
55280	1.89226	0.413891
55281	1.89226	0.479935
55282	1.89226	0.820341
55283	1.89226	0.701221
55284	1.89226	0.905529
55285	1.89226	0.867636
55286	1.89226	0.828839
55287	2.15871	0.324037
55288	2.15871	0.888649
55289	1.81454	0.183187
55290	1.54809	0.922368
55291	1.89226	0.930636
55292	1.89226	1.61301
55293	1.89226	0.994618
55294	1.89226	0.891443
55295	3.78453	1.1612
55296	1.89226	1.15564
55297	1.89226	0.86438
55298	1.89226	0.82588
55299	1.89226	1.05636
55300	1.89226	1.64709
55301	1.89226	1.18877
55302	2.29205	1.08604
55303	1.42633	1.05869
55304	1.69015	1.38721
55305	1.69015	1.32576
55306	2.10364	0.84552
55307	2.05344	0.84821
55308	2.29205	0.883381
55309	1.58147	1.03472
55310	2.16529	0.858815
55311	1.56339	0.825688
55312	2.29205	1.48084
55313	1.69015	1.21586
55314	1.89226	1.22635
55315	1.89226	0.9968
55316	1.89226	0.864894
55317	1.89226	1.00293
55318	3.78453	0.910752
55319	1.89226	0.963287
55320	1.89226	1.4737
55321	1.89226	1.06029
55322	1.89226	0.899754
55323	3.78452	1.5082
55324	2.29205	1.40504
55325	2.85335	0.798995
55326	2.55409	1.04335
55327	2.04345	0.862969
55328	2.76338	0.997451
55329	2.29205	0.909262

55330	2.29205	0.98428
55331	2.16529	1.25756
55332	2.72418	0.989533
55333	2.76338	0.779171
55334	5.01623	1.03222
55335	1.89227	0.05
55336	1.89227	0.05
55337	1.89227	0.05
55338	1.89227	0.05
55339	3.78453	0.05
55340	2.39588	0.05
55341	2.14918	0.05
55342	2.14918	0.05
55343	2.37907	0.05
55344	2.20531	0.05
55345	2.4352	0.05
55346	1.89226	0.05
55347	2.64327	0.05
55348	2.36175	0.05
55349	1.89226	0.05
55350	4.25402	0.05
55351	1.89226	0.05
55352	1.89226	0.05
55353	1.89226	0.05
55354	1.89226	0.05
55355	1.89226	0.05
55356	1.89226	0.05
55357	1.05395	0.05
55358	1.05395	0.05
55359	1.03114	0.05
55360	1.03114	0.05
55361	0.936689	0.05
55362	1.07459	0.05
55363	1.10265	0.05
55364	1.01049	0.05
55365	0.93669	0.05
55366	1.01049	0.05
55367	1.07459	0.05
55368	1.10265	0.05
55369	1.18088	0.05
55370	0.776509	0.05
55371	0.946132	0.05
55372	0.946132	0.05
55373	1.04225	0.05
55374	0.830392	0.05
55375	0.931352	0.05
55376	0.931352	0.05
55377	1.13006	0.05
55378	1.04074	0.05
55379	1.0628	0.05
55380	0.851529	0.05
55381	2.40032	2.1
55382	1.89226	2.1
55383	1.89226	2.1
55384	1.89226	2.1
55385	2.38918	2.1
55386	2.38918	2.1
55387	1.89226	2.1

55388	1.89226	2.1
55389	1.89226	2.1
55390	1.89226	2.1
55391	1.89226	2.1
55392	1.89226	2.1
55393	1.6585	2.1
55394	2.37364	2.1
55395	2.39622	2.1
55396	2.39622	2.1
55397	1.6488	2.1
55398	1.6488	2.1
55399	2.44603	2.1
55400	1.89224	2.1
55401	1.89224	2.1
55402	1.89224	2.1
55403	2.40029	2.1
55404	2.40029	2.1
55405	1.89224	2.1
55406	1.89224	2.1
55407	1.89224	2.1
55408	1.89224	2.1
55409	1.89224	2.1
55410	1.89224	2.1
55411	1.01946	2.1
55412	1.01946	2.1
55413	1.02497	2.1
55414	1.02497	2.1
55415	1.21519	2.1
55416	1.19809	2.1
55417	0.835303	2.1
55418	0.82924	2.1
55419	0.835314	2.1
55420	1.2152	2.1
55421	0.829228	2.1
55422	1.19808	2.1
55423	1.91027	2.1
55424	1.05809	2.1
55425	1.00659	2.1
55426	2.06727	2.1
55427	3.11819	2.1
55428	2.37166	2.1
55429	2.06726	2.1
55430	1.66994	2.1
55431	1.67623	2.1
55432	2.40308	2.1
55433	2.40308	2.1
55434	0.960194	2.1
55435	0.939664	2.1
55436	0.531131	2.1
55437	0.503298	2.1
55438	0.729927	2.1
55439	0.729927	2.1
55440	0.733565	2.1
55441	0.733565	2.1
55442	0.960195	2.1
55443	0.531132	2.1
55444	0.503297	2.1
55445	0.939663	2.1

55446	1.20154	2.1
55447	1.21754	2.1
55448	0.960195	2.1
55449	0.939665	2.1
55450	1.07745	2.1
55451	1.07745	2.1
55452	1.07976	2.1
55453	1.07976	2.1
55454	0.960194	2.1
55455	1.21754	2.1
55456	0.939662	2.1
55457	1.20155	2.1
55458	1.89224	2.1
55459	1.89224	2.1
55460	2.08609	2.1
55461	2.08609	2.1
55462	2.09527	2.1
55463	1.90142	2.1
55464	1.65815	2.1
55465	1.71398	2.1
55466	2.2001	2.1
55467	2.23791	2.1
55468	1.4502	2.1
55469	1.4502	2.1
55470	0.793587	2.1
55471	0.793587	2.1
55472	0.792408	2.1
55473	0.792408	2.1
55474	0.952221	2.1
55475	0.858585	2.1
55476	0.725101	2.1
55477	0.633774	2.1
55478	0.633775	2.1
55479	0.858585	2.1
55480	0.952221	2.1
55481	0.725101	2.1
55482	1.5735	2.1
55483	2.56975	2.1
55484	2.63166	2.1
55485	2.63166	2.1
55486	1.54711	2.1
55487	1.54711	2.1
55488	0.946131	2.1
55489	0.946131	2.1
55490	1.28487	2.1
55491	1.28487	2.1
55492	0.946127	2.1
55493	0.946127	2.1
55494	1.28488	2.1
55495	1.31583	2.1
55496	1.31583	2.1
55497	0.94613	2.1
55498	1.28487	2.1
55499	0.946129	2.1
55500	1.0596	2.1
55501	1.0596	2.1
55502	1.06939	2.1
55503	1.06939	2.1

55504	1.34225	2.1
55505	1.31583	2.1
55506	0.793563	2.1
55507	0.786748	2.1
55508	0.793563	2.1
55509	1.34225	2.1
55510	0.786748	2.1
55511	1.31583	2.1
55512	0.946131	2.1
55513	0.946131	2.1
55514	1.31582	2.1
55515	1.31582	2.1
55516	0.946127	2.1
55517	0.946127	2.1
55518	1.34225	2.1
55519	1.31583	2.1
55520	1.34225	2.1
55521	0.946129	2.1
55522	1.31583	2.1
55523	0.946129	2.1
55524	1.89226	2.1
55525	1.89226	2.1
55526	1.89226	2.1
55527	1.89226	2.1
55528	1.89226	2.1
55529	1.89226	2.1
55530	1.89226	2.1
55531	1.89226	2.1
55532	1.89226	2.1
55533	1.89226	2.1
55534	1.89226	2.1
55535	1.89226	2.1
55536	1.26992	2.1
55537	1.75003	2.1
55538	1.79883	2.1
55539	1.26732	2.1
55540	3.06875	2.1
55541	0.875017	2.1
55542	0.899414	2.1
55543	0.811147	2.1
55544	0.85053	2.1
55545	0.851513	2.1
55546	0.851513	2.1
55547	0.859048	2.1
55548	0.859048	2.1
55549	0.899413	2.1
55550	0.85053	2.1
55551	0.811147	2.1
55552	0.875016	2.1
55553	1.89227	2.1
55554	1.89227	2.1
55555	1.89227	2.1
55556	1.89227	2.1
55557	1.89227	2.1
55558	1.89227	2.1
55559	1.89226	2.1
55560	1.89226	2.1
55561	1.89226	2.1

55562	1.89226	2.1
55563	1.89226	2.1
55564	1.89226	2.1
55565	0.633661	2.1
55566	0.642005	2.1
55567	0.93285	2.1
55568	0.899413	2.1
55569	0.764487	2.1
55570	0.764487	2.1
55571	0.776931	2.1
55572	0.776931	2.1
55573	0.642004	2.1
55574	0.93285	2.1
55575	0.899414	2.1
55576	0.633661	2.1
55577	0.932851	2.1
55578	0.899414	2.1
55579	0.905419	2.1
55580	0.85053	2.1
55581	0.886336	2.1
55582	0.886336	2.1
55583	0.897045	2.1
55584	0.897045	2.1
55585	0.93285	2.1
55586	0.905419	2.1
55587	0.899414	2.1
55588	0.850531	2.1
55589	2.39322	2.1
55590	2.74149	1.11775
55591	2.70944	2.1
55592	2.6849	1.19479
55593	2.41776	2.1
55594	2.39322	0.967802
55595	2.41776	2.1
55596	2.80992	2.1
55597	2.77256	2.1
55598	2.74149	2.1
55599	2.44882	2.1
55600	2.41776	1.05004
55601	2.80992	2.1
55602	1.89226	1.73175
55603	2.74149	1.97748
55604	2.74149	2.1
55605	1.89226	1.14647
55606	1.89226	1.49693
55607	1.89226	0.05
55608	1.89226	0.05
55609	1.89226	0.05
55610	1.89226	0.05
55611	1.89226	0.05
55612	1.89226	0.05
55613	2.38796	0.05
55614	2.0815	0.05
55615	2.0815	0.05
55616	2.49452	0.05
55617	1.55994	0.05
55618	2.14721	0.05
55619	1.89227	0.05

55620	1.89227	0.05
55621	1.89227	0.05
55622	1.89227	0.05
55623	1.89227	0.05
55624	1.89227	0.05
55625	1.13387	0.05
55626	2.27494	0.05
55627	1.13387	0.05
55628	2.19489	0.05
55629	1.60867	0.05
55630	2.6994	0.05
55631	1.89226	0.05
55632	1.89226	0.05
55633	1.89226	0.05
55634	1.89226	0.05
55635	3.78453	0.05
55636	1.42381	1.91725
55637	1.10747	2.1
55638	1.10747	1.59852
55639	1.08911	2.1
55640	1.42866	1.20367
55641	1.4103	2.1
55642	1.89226	2.1
55643	1.88181	2.1
55644	1.88181	2.1
55645	1.76249	2.1
55646	2.0815	2.1
55647	1.97539	2.1
55648	0.946128	2.1
55649	0.946128	2.1
55650	0.946133	1.57397
55651	0.946133	0.403029
55652	0.94613	0.464969
55653	0.94613	2.1
55654	0.946131	2.1
55655	0.946131	0.646114
55656	0.946131	2.1
55657	0.946131	2.1
55658	0.946131	2.1
55659	0.946131	2.1
55660	1.42866	1.8795
55661	1.43213	1.62635
55662	1.10748	2.1
55663	1.10748	2.1
55664	1.13641	2.1
55665	1.43213	2.1
55666	2.0815	2.1
55667	2.10287	2.1
55668	2.10287	2.1
55669	2.0921	2.1
55670	1.89227	2.1
55671	1.83938	2.1
55672	0.810944	1.35293
55673	0.810944	0.796828
55674	0.810945	0.797139
55675	0.810945	2.1
55676	0.810933	1.91034
55677	0.810933	1.95183

55678	1.89226	2.1
55679	1.3517	2.1
55680	1.08132	1.98309
55681	1.08132	2.1
55682	1.08133	1.97929
55683	2.33097	2.1
55684	1.53822	2.1
55685	1.53822	2.1
55686	1.68437	2.1
55687	2.17996	2.1
55688	2.32612	2.1
55689	0.99189	2.1
55690	1.18948	0.406207
55691	1.19756	0.05
55692	1.19756	2.1
55693	0.978851	2.1
55694	0.926995	2.1
55695	1.26924	0.05
55696	1.34642	0.0597098
55697	1.3228	1.01787
55698	0.990899	1.16514
55699	1.0627	1.88171
55700	1.33473	1.48402
55701	1.96196	2.1
55702	1.39702	2.1
55703	1.39702	2.1
55704	1.53822	2.1
55705	2.17372	2.1
55706	2.31011	2.1
55707	1.89226	0.841864
55708	1.89226	0.473075
55709	1.89226	0.274889
55710	1.89226	0.05
55711	3.78453	0.078098
55712	2.38475	0.882047
55713	1.73957	0.671541
55714	1.88452	0.161848
55715	1.58695	0.05
55716	3.6241	0.05
55717	1.73958	0.229263
55718	1.3126	0.919625
55719	1.58695	0.05
55720	1.14946	0.0688289
55721	2.88903	0.05
55722	1.89226	0.395837
55723	1.89226	0.850547
55724	1.89226	0.05
55725	1.89226	0.199241
55726	3.78453	0.05
55727	1.89225	0.176707
55728	1.89225	0.075103
55729	1.89225	0.05
55730	1.89225	0.05
55731	3.7845	0.05
55732	1.88451	0.109303
55733	1.58694	0.05
55734	1.74649	0.300991
55735	1.44604	0.05

55736	3.33055	0.05
55737	1.58694	0.05
55738	0.995727	0.05
55739	0.995727	0.789338
55740	1.14945	0.0769574
55741	1.44604	0.05
55742	1.59976	0.383757
55743	1.89225	0.05
55744	1.89225	0.05
55745	1.89225	0.906432
55746	1.89225	0.300429
55747	1.89225	0.333261
55748	1.89225	0.225499
55749	2.01197	1.90182
55750	2.0961	1.79337
55751	2.13283	1.18339
55752	2.25399	1.0852
55753	4.22894	1.40193
55754	2.09611	1.26168
55755	2.37014	1.25694
55756	2.29031	1.69004
55757	2.10084	2.1
55758	2.25399	1.09783
55759	2.09611	2.01841
55760	2.13283	1.601
55761	2.25399	2.1
55762	2.22044	1.57118
55763	2.38975	1.10185
55764	4.52258	1.45641
55765	2.38976	0.976209
55766	2.15466	1.4197
55767	2.31988	0.442922
55768	2.31988	2.08214
55769	2.25399	0.992366
55770	2.25399	2.1
55771	2.58286	0.884372
55772	2.29193	0.428106
55773	2.40107	1.15983
55774	2.13815	0.05
55775	4.72101	0.05
55776	2.29193	0.621039
55777	1.90104	0.087369
55778	2.13815	0.05
55779	1.77249	0.157253
55780	4.03919	0.05
55781	2.25197	0.241274
55782	2.13815	0.15287
55783	2.00625	0.05
55784	2.00625	0.05
55785	2.40107	0.280777
55786	2.40107	0.42714
55787	2.13815	0.282591
55788	1.77249	0.231756
55789	2.00625	0.05
55790	1.65815	0.05
55791	3.7963	0.0587702
55792	2.43417	0.321742
55793	2.06653	0.203151

55794	1.64587	0.17864
55795	1.65284	0.140903
55796	2.46898	0.412416
55797	2.46898	0.284897
55798	2.46895	0.196592
55799	1.89016	0.0679542
55800	1.87861	0.0764841
55801	1.87861	0.0502254
55802	2.47161	0.258616
55803	2.47161	0.171629
55804	0.946132	1.09345
55805	0.946132	0.834229
55806	0.863311	1.1353
55807	1.34768	1.07631
55808	0.851542	1.0758
55809	0.851542	0.540353
55810	1.30451	1.03458
55811	1.45304	0.928095
55812	0.946132	0.849322
55813	1.20992	0.928887
55814	0.855162	1.09831
55815	0.946132	0.788109
55816	1.89226	0.534163
55817	1.89226	1.26976
55818	1.89226	1.10285
55819	1.89226	0.916574
55820	1.89226	1.12659
55821	1.89226	0.940781
55822	0.946132	0.907321
55823	0.946132	0.115112
55824	0.946133	0.17294
55825	0.946133	0.124649
55826	0.946132	0.143408
55827	0.946132	0.0591787
55828	0.946132	0.120344
55829	0.946132	0.155521
55830	0.946132	0.484676
55831	0.946132	0.129352
55832	0.946133	0.197812
55833	0.946133	0.149521
55834	2.35574	1.0512
55835	1.89226	0.875975
55836	1.89226	2.1
55837	1.89226	0.817003
55838	2.67456	1.10303
55839	2.67456	0.05
55840	0.583505	0.322948
55841	0.761972	0.733017
55842	1.17787	0.947213
55843	1.33728	0.05
55844	0.965128	0.823993
55845	0.965128	0.32829
55846	0.974714	0.817662
55847	0.974715	0.230862
55848	1.17787	0.793841
55849	0.583506	0.84087
55850	1.33728	0.05
55851	0.761974	0.223079

55852	1.89226	0.826577
55853	1.89226	0.139001
55854	1.89226	0.05
55855	1.89226	0.0505738
55856	1.89226	0.659675
55857	1.89226	0.792866
55858	1.89226	0.0838868
55859	1.89226	0.05
55860	1.89226	0.376218
55861	1.89226	0.572705
55862	3.78453	0.152826
55863	0.946132	0.10727
55864	0.946132	0.0836344
55865	0.946132	0.108084
55866	0.946132	0.05
55867	0.946133	0.781777
55868	0.946133	0.167688
55869	0.946132	0.816106
55870	0.946132	0.05
55871	0.946133	0.317237
55872	0.946133	0.82962
55873	0.946132	0.05
55874	0.946132	0.800187
55875	1.01779	2.1
55876	1.50685	2.1
55877	1.12064	2.1
55878	1.65151	2.1
55879	2.6275	2.1
55880	1.35733	2.1
55881	1.53507	2.1
55882	1.50683	2.1
55883	1.73626	2.1
55884	3.09359	2.1
55885	1.73626	2.1
55886	1.52226	2.1
55887	1.75555	2.1
55888	1.75555	2.1
55889	1.53507	2.1
55890	1.53507	2.1
55891	1.65151	2.1
55892	1.73628	2.1
55893	1.69528	2.1
55894	1.50685	2.1
55895	1.92471	2.1
55896	1.73628	2.1
55897	1.73629	2.1
55898	1.75558	2.1
55899	1.9943	2.1
55900	1.9943	2.1
55901	1.92471	2.1
55902	1.73629	2.1
55903	2.5601	1.16774
55904	1.97645	2.09967
55905	2.09605	1.07977
55906	2.09605	1.9652
55907	2.45853	1.1812
55908	2.45853	2.1
55909	2.33824	1.92726

55910	1.97646	1.59142
55911	2.45853	1.14744
55912	1.94984	2.1
55913	2.33824	1.87284
55914	1.82955	2.1
55915	1.22926	2.1
55916	0.946128	2.1
55917	0.946133	2.1
55918	1.28005	1.4158
55919	1.22927	1.42264
55920	1.22926	1.92296
55921	0.946131	1.86024
55922	0.946131	1.4635
55923	0.946131	1.52014
55924	0.946131	1.34721
55925	1.28005	1.61428
55926	1.22926	1.44982
55927	2.35349	0.200959
55928	2.61606	0.764274
55929	2.48209	0.287304
55930	2.48209	0.147331
55931	2.50057	0.491102
55932	2.50057	0.46561
55933	2.61607	0.72278
55934	2.57611	0.0546316
55935	2.4821	0.05
55936	2.44814	0.05
55937	5.06421	0.05
55938	2.15721	0.05
55939	2.48209	0.766615
55940	2.31844	0.479517
55941	2.31844	0.0927878
55942	2.35349	0.372905
55943	2.35349	0.05
55944	2.44814	0.05
55945	2.30908	0.05
55946	2.44814	0.05
55947	2.31844	0.05
55948	2.4821	0.34215
55949	2.31844	0.05
55950	0.91766	0.226972
55951	0.91766	0.21368
55952	0.912463	0.0670626
55953	0.912463	0.302153
55954	1.16525	0.186287
55955	1.26377	0.224251
55956	0.576692	0.202425
55957	0.664871	0.303468
55958	1.16525	0.121708
55959	0.576692	0.189038
55960	0.664871	0.331683
55961	1.26377	0.205229
55962	1.72901	2.1
55963	1.62414	2.1
55964	1.88971	2.1
55965	1.8411	1.67351
55966	3.57011	2.1
55967	1.89226	2.1

55968	1.89226	1.88376
55969	1.89226	1.1457
55970	1.89226	2.01059
55971	3.78452	1.21551
55972	1.89226	1.26182
55973	1.89226	2.1
55974	1.89226	2.1
55975	1.89226	1.25644
55976	3.78452	2.1
55977	1.62414	2.1
55978	1.30896	2.1
55979	1.8411	1.44989
55980	1.52776	2.1
55981	3.15006	2.03616
55982	1.51448	2.1
55983	1.8411	2.1
55984	2.02311	2.1
55985	1.99309	1.05383
55986	3.86421	1.83816
55987	1.89226	1.19859
55988	1.89226	2.1
55989	1.89226	2.1
55990	1.89226	1.29808
55991	3.78452	2.1
55992	1.89226	2.1
55993	1.89226	1.77359
55994	1.89226	1.58504
55995	1.89226	2.1
55996	3.78452	2.1
55997	1.8411	2.1
55998	1.52776	2.1
55999	1.99309	2.1
56000	1.72526	2.1
56001	3.56636	2.1
56002	1.89226	2.1
56003	1.89226	2.1
56004	1.89226	2.1
56005	1.89226	2.1
56006	3.78453	2.1
56007	1.0424	2.1
56008	1.12935	2.1
56009	0.929383	2.1
56010	0.929383	2.1
56011	1.24941	2.1
56012	1.12686	2.1
56013	0.946132	2.1
56014	0.946132	2.1
56015	1.12944	2.1
56016	0.851554	2.1
56017	1.05017	2.1
56018	0.929315	2.1
56019	0.898029	2.1
56020	0.973994	2.1
56021	0.946131	2.1
56022	0.946131	2.1
56023	1.0221	2.1
56024	1.0221	2.1
56025	0.898028	2.1

56026	0.898028	2.1
56027	0.973994	2.1
56028	0.946131	2.1
56029	0.946131	2.1
56030	0.898029	2.1
56031	1.89226	2.1
56032	1.89226	2.1
56033	1.89226	1.97474
56034	1.89226	2.1
56035	1.89226	2.01403
56036	1.89226	2.1
56037	1.13537	2.1
56038	0.879308	2.1
56039	0.946131	2.1
56040	0.946131	2.1
56041	1.13537	2.1
56042	0.85187	2.1
56043	0.94613	2.1
56044	0.94613	1.89664
56045	1.05777	2.1
56046	1.05777	2.1
56047	0.799318	2.1
56048	0.776452	2.1
56049	0.946116	2.1
56050	0.946116	2.1
56051	1.09531	2.1
56052	1.09531	2.1
56053	0.946118	2.1
56054	0.946118	2.1
56055	1.09135	2.1
56056	1.09531	2.1
56057	1.09134	2.1
56058	0.946105	2.1
56059	1.09532	2.1
56060	0.946131	2.1
56061	0.946129	2.1
56062	0.946129	2.1
56063	1.09137	2.1
56064	1.09137	2.1
56065	0.946131	2.1
56066	0.946131	2.1
56067	1.07713	2.1
56068	1.09137	2.1
56069	0.946131	2.1
56070	1.07713	2.1
56071	1.09137	2.1
56072	0.946131	2.1
56073	0.938916	2.1
56074	0.938916	2.1
56075	0.936995	2.1
56076	1.00447	2.1
56077	0.979819	2.1
56078	1.16821	2.1
56079	1.01263	2.1
56080	0.719167	2.1
56081	1.04536	2.1
56082	1.18901	2.1
56083	0.758057	2.1

56084	0.924111	2.1
56085	1.00532	2.1
56086	1.00532	2.1
56087	0.9925	2.1
56088	0.9925	2.1
56089	1.29322	2.1
56090	1.01985	2.1
56091	1.00365	2.1
56092	0.704601	2.1
56093	1.00365	2.1
56094	1.29322	2.1
56095	1.01985	2.1
56096	0.704602	2.1
56097	2.20011	2.1
56098	1.401	2.1
56099	1.401	2.1
56100	1.52008	2.1
56101	2.11084	2.1
56102	2.22991	2.1
56103	0.854068	2.1
56104	0.854068	2.1
56105	0.851357	2.1
56106	0.851357	2.1
56107	0.644681	2.1
56108	0.700493	2.1
56109	1.0554	2.1
56110	1.00493	2.1
56111	1.00495	2.1
56112	0.64469	2.1
56113	0.700483	2.1
56114	1.05539	2.1
56115	2.46665	0.05
56116	2.22713	0.05
56117	1.85877	0.05
56118	1.8967	0.05
56119	1.56002	0.05
56120	2.22713	0.05
56121	1.89225	2.1
56122	1.89225	2.1
56123	1.89225	2.1
56124	1.89225	2.1
56125	1.89225	2.1
56126	1.89225	2.1
56127	1.89225	2.1
56128	1.89225	2.1
56129	1.89225	2.1
56130	1.89225	2.1
56131	1.89225	2.1
56132	1.89225	2.1
56133	1.284	2.1
56134	1.86569	2.1
56135	1.95398	2.1
56136	1.31091	2.1
56137	3.23798	2.1
56138	0.932844	2.1
56139	0.976992	2.1
56140	0.960128	2.1
56141	0.905413	2.1

56142	0.938531	2.1
56143	0.938531	2.1
56144	0.943874	2.1
56145	0.943874	2.1
56146	0.976985	2.1
56147	0.960122	2.1
56148	0.93285	2.1
56149	0.905419	2.1
56150	1.89227	2.1
56151	1.89227	2.1
56152	1.89227	2.1
56153	1.89227	2.1
56154	3.78453	2.1
56155	1.89226	2.1
56156	1.89226	2.1
56157	1.89226	2.1
56158	1.89226	2.1
56159	1.89226	2.1
56160	1.89226	2.1
56161	0.65546	2.1
56162	0.665451	2.1
56163	1.00694	2.1
56164	0.976998	2.1
56165	0.816261	2.1
56166	0.816261	2.1
56167	0.826189	2.1
56168	0.826189	2.1
56169	1.00694	2.1
56170	0.665451	2.1
56171	0.976998	2.1
56172	0.65546	2.1
56173	2.01555	2.1
56174	2.01388	2.1
56175	1.92027	2.1
56176	1.92027	2.1
56177	2.01388	2.1
56178	1.954 2.1	
56179	2.75466	2.1
56180	1.89226	2.1
56181	1.89226	2.1
56182	2.54931	2.1
56183	4.44158	2.1
56184	2.47917	2.1
56185	1.92772	2.1
56186	1.80682	2.1
56187	2.54931	2.1
56188	4.47702	2.1
56189	0.946132	2.1
56190	0.946132	2.1
56191	1.17602	2.1
56192	1.27466	2.1
56193	0.946132	2.1
56194	0.946132	2.1
56195	1.17602	2.1
56196	1.17602	2.1
56197	0.946132	2.1
56198	1.17602	2.1
56199	0.946131	2.1

56200	1.27466	2.1
56201	2.35204	2.1
56202	2.54931	2.1
56203	2.40783	2.1
56204	1.50108	2.1
56205	1.64256	2.1
56206	1.64256	2.1
56207	1.89226	2.1
56208	1.89226	1.09932
56209	2.31012	2.1
56210	2.17534	2.1
56211	4.0676	2.1
56212	2.17533	2.1
56213	1.89226	0.952953
56214	2.17533	1.38562
56215	1.89226	1.23057
56216	1.66635	2.1
56217	1.38328	1.8623
56218	1.92027	2.1
56219	2.17597	2.1
56220	2.14619	2.1
56221	2.15388	2.1
56222	1.91087	2.1
56223	1.92027	2.1
56224	1.08622	2.1
56225	1.08622	2.1
56226	0.971184	2.1
56227	0.972761	2.1
56228	1.14845	2.1
56229	1.16321	2.1
56230	0.894769	2.1
56231	0.894967	2.1
56232	0.894775	2.1
56233	1.28563	2.1
56234	1.14844	2.1
56235	1.0167	2.1
56236	1.89226	1.31795
56237	1.89226	2.1
56238	1.89226	2.1
56239	1.89226	1.41434
56240	1.89226	2.1
56241	1.89226	1.54531
56242	0.937933	2.1
56243	0.937933	2.1
56244	1.26192	2.1
56245	1.26192	2.1
56246	0.937903	2.1
56247	1.25372	2.1
56248	0.946132	2.1
56249	0.946132	2.1
56250	0.946133	2.1
56251	1.25372	2.1
56252	0.946132	2.1
56253	0.937902	2.1
56254	0.946132	1.29381
56255	0.946132	1.15983
56256	0.946128	2.1
56257	0.946128	2.1

56258	0.94613	2.1
56259	0.94613	1.26045
56260	0.94613	1.10901
56261	0.94613	2.1
56262	0.946131	1.79695
56263	0.946131	1.94677
56264	0.94613	1.92811
56265	0.94613	1.89204
56266	0.946132	1.43207
56267	0.946132	1.24699
56268	1.2778	2.1
56269	1.00191	2.1
56270	1.00191	1.39048
56271	1.00191	2.1
56272	0.94613	2.1
56273	0.94613	1.39601
56274	0.946131	2.1
56275	1.2778	2.1
56276	1.00191	2.1
56277	0.94613	2.07036
56278	1.50847	0.05
56279	2.0815	0.05
56280	1.56806	0.05
56281	2.0815	0.05
56282	1.52043	0.05
56283	1.9224	0.05
56284	1.06882	0.05
56285	0.69567	0.05
56286	0.773112	0.05
56287	1.09473	0.05
56288	0.8941	0.05
56289	0.8941	0.05
56290	0.937064	0.05
56291	0.981131	0.05
56292	0.763762	0.05
56293	0.722535	0.05
56294	1.2153	0.05
56295	1.07279	0.05
56296	1.89226	0.05
56297	1.89226	0.05
56298	1.89226	0.05
56299	1.89226	0.05
56300	1.89226	0.05
56301	1.89226	0.05
56302	1.89226	0.05
56303	1.89226	0.05
56304	1.89226	0.05
56305	1.89226	0.05
56306	3.78453	0.05
56307	2.51518	0.05
56308	1.89226	0.05
56309	1.89226	0.05
56310	1.89226	0.05
56311	2.37584	0.05
56312	2.37584	0.05
56313	1.25759	0.05
56314	0.946131	0.05
56315	0.946131	0.05

56316	1.24489	0.05
56317	0.946131	0.05
56318	0.946131	0.05
56319	1.25759	0.05
56320	1.25759	0.05
56321	1.25759	0.05
56322	1.24489	0.05
56323	0.946131	0.05
56324	0.946131	0.05
56325	1.08028	0.573379
56326	1.09362	0.619702
56327	0.596616	0.282371
56328	0.606432	0.763774
56329	0.844265	0.873799
56330	0.844265	0.05
56331	0.855786	0.972097
56332	0.855786	0.167174
56333	1.08028	0.928308
56334	0.606433	0.219564
56335	0.596616	0.231857
56336	1.09362	0.351052
56337	2.837	0.733484
56338	2.16055	0.0774252
56339	2.12741	0.859172
56340	2.79312	0.608623
56341	4.95367	0.843249
56342	2.12741	0.806733
56343	1.21286	0.070879
56344	1.20859	0.949228
56345	1.21286	0.981818
56346	2.15628	0.503552
56347	2.16056	0.123252
56348	1.89226	2.1
56349	1.89226	2.1
56350	1.89226	2.1
56351	1.89226	2.1
56352	3.78453	2.1
56353	0.946102	2.1
56354	0.946102	2.1
56355	0.850039	2.1
56356	0.850039	2.1
56357	0.914891	2.1
56358	0.85001	2.1
56359	0.946131	2.1
56360	0.946131	2.1
56361	0.94613	2.1
56362	0.91489	2.1
56363	0.850011	2.1
56364	0.946131	2.1
56365	0.71107	2.1
56366	0.784813	2.1
56367	1.28453	2.1
56368	1.20328	2.1
56369	0.992128	2.1
56370	0.992128	2.1
56371	0.995965	2.1
56372	0.995965	2.1
56373	0.784813	2.1

56374	1.28453	2.1
56375	0.711071	2.1
56376	1.20328	2.1
56377	1.89226	2.1
56378	1.89226	2.1
56379	1.89226	2.1
56380	1.89226	2.1
56381	3.78453	2.1
56382	0.946132	2.1
56383	0.946132	2.1
56384	0.914889	2.1
56385	0.914889	2.1
56386	0.985255	2.1
56387	0.914891	2.1
56388	0.946131	2.1
56389	0.946131	2.1
56390	0.946131	2.1
56391	0.985255	2.1
56392	0.94613	2.1
56393	0.91489	2.1
56394	0.784813	2.1
56395	0.871551	2.1
56396	1.37791	2.1
56397	1.28453	2.1
56398	1.07644	2.1
56399	1.07644	2.1
56400	1.07964	2.1
56401	1.07964	2.1
56402	0.871551	2.1
56403	1.37791	2.1
56404	0.784813	2.1
56405	1.28453	2.1
56406	1.73075	1.51464
56407	2.10582	0.418797
56408	1.89319	0.420222
56409	1.38766	2.0992
56410	3.27777	0.548958
56411	1.08715	1.95774
56412	1.54185	1.89019
56413	1.73074	1.51053
56414	1.73074	2.1
56415	1.18948	0.555746
56416	1.18948	1.86987
56417	1.89319	0.313231
56418	1.41689	1.94399
56419	1.11619	0.754772
56420	1.38766	1.43826
56421	1.6052	0.469158
56422	1.89319	1.37979
56423	0.647894	1.68461
56424	0.594738	1.99769
56425	0.708445	1.77838
56426	0.770926	1.28929
56427	0.738326	1.83316
56428	0.738326	2.1
56429	0.680494	1.62771
56430	0.680494	0.509324
56431	0.708443	0.951988

56432	0.647895	0.871441
56433	0.770925	1.22899
56434	0.59474	0.270923
56435	1.89111	1.22579
56436	1.06994	2.1
56437	2.06689	2.1
56438	1.37063	2.1
56439	3.13683	2.1
56440	2.06689	2.1
56441	1.39295	2.1
56442	1.56818	2.1
56443	1.37063	2.1
56444	2.26444	2.07508
56445	2.06689	2.1
56446	1.49808	2.1
56447	1.50201	2.1
56448	2.57784	2.1
56449	2.58615	2.1
56450	4.08424	2.1
56451	0.946133	2.1
56452	0.946133	2.1
56453	0.946132	2.1
56454	0.946132	2.1
56455	0.946132	2.1
56456	0.946132	2.1
56457	0.946132	2.1
56458	0.946132	2.1
56459	0.946133	2.1
56460	0.946133	2.1
56461	0.946132	2.1
56462	0.946132	2.1
56463	1.89226	2.1
56464	1.89226	2.1
56465	1.89226	2.1
56466	1.89226	2.1
56467	3.78452	2.1
56468	0.977201	2.1
56469	0.977201	2.1
56470	0.980489	2.1
56471	0.980489	2.1
56472	0.657973	2.1
56473	1.20669	2.1
56474	0.751002	2.1
56475	1.29307	2.1
56476	1.29308	2.1
56477	1.20669	2.1
56478	0.751003	2.1
56479	0.657972	2.1
56480	0.946131	2.1
56481	0.946131	2.1
56482	1.29308	2.1
56483	1.29308	2.1
56484	0.946132	2.1
56485	1.29308	2.1
56486	0.946132	2.1
56487	1.28892	2.1
56488	0.946132	2.1
56489	0.946132	2.1

56490	1.28892	2.1
56491	1.29308	2.1
56492	0.946132	1.99464
56493	0.946132	1.51671
56494	0.946131	2.1
56495	0.946131	2.1
56496	0.946132	2.1
56497	0.946132	2.1
56498	0.946132	2.00049
56499	0.946132	1.48478
56500	0.946132	1.53903
56501	0.946132	2.1
56502	0.946132	2.1
56503	0.946132	2.1
56504	1.89226	2.1
56505	1.89226	2.1
56506	1.89226	2.1
56507	1.89226	2.0512
56508	1.89226	2.1
56509	1.89226	1.98075
56510	0.94613	2.1
56511	0.94613	2.1
56512	1.20669	2.1
56513	1.20669	2.1
56514	1.20669	2.1
56515	0.946132	2.1
56516	0.946129	2.1
56517	1.29307	2.1
56518	0.946131	2.1
56519	0.946131	2.1
56520	1.29308	2.1
56521	1.20669	2.1
56522	1.89226	0.05
56523	1.89226	0.05
56524	1.89226	0.05
56525	1.89226	0.05
56526	3.78453	0.05
56527	0.946132	0.05
56528	0.946132	0.05
56529	0.946126	0.05
56530	0.946126	0.05
56531	0.946129	0.05
56532	0.946129	0.05
56533	0.946129	0.05
56534	0.946129	0.05
56535	0.946128	0.05
56536	0.946128	0.05
56537	0.946129	0.05
56538	0.946129	0.05
56539	0.725277	0.05
56540	0.588155	0.05
56541	1.19403	0.05
56542	1.27681	0.05
56543	0.918903	0.05
56544	0.918903	0.05
56545	0.946068	0.05
56546	0.946068	0.05
56547	1.19404	0.05

56548	0.588153	0.05
56549	0.725275	0.05
56550	1.27682	0.05
56551	1.19404	0.05
56552	1.27682	0.05
56553	0.946131	0.05
56554	0.946131	0.05
56555	1.19404	0.05
56556	1.19404	0.05
56557	0.946129	0.05
56558	0.946129	0.05
56559	1.19404	0.05
56560	0.94613	0.05
56561	1.27682	0.05
56562	0.946131	0.05
56563	1.89226	0.05
56564	1.89226	0.05
56565	1.89226	0.05
56566	1.89226	0.05
56567	1.89226	0.05
56568	1.89226	0.05
56569	1.4263	1.81291
56570	1.67651	0.061696
56571	1.5955	0.862454
56572	1.31022	2.1
56573	2.98674	1.08333
56574	1.18824	2.1
56575	1.28802	2.1
56576	0.946132	0.833481
56577	0.946132	1.86605
56578	0.946132	1.37182
56579	0.946132	2.1
56580	1.28802	0.319153
56581	1.28802	2.1
56582	1.18824	2.1
56583	0.946132	2.1
56584	0.946132	2.1
56585	1.28802	2.1
56586	1.89226	0.272073
56587	1.89226	1.67329
56588	1.89226	2.1
56589	1.89226	0.787088
56590	3.78452	0.55593
56591	1.5955	0.791301
56592	1.52909	0.314954
56593	1.31022	0.176333
56594	1.31022	2.1
56595	1.2087	2.1
56596	1.52909	2.08112
56597	1.11825	2.1
56598	1.18824	2.1
56599	0.946132	1.88514
56600	0.946132	1.52299
56601	0.946132	1.58798
56602	0.946132	2.1
56603	1.18824	0.895033
56604	1.18824	2.1
56605	0.946132	2.1

56606	1.11825	2.1
56607	1.18824	2.1
56608	0.946133	2.1
56609	0.810943	2.03813
56610	0.810943	1.38937
56611	0.810945	0.71068
56612	0.810945	1.71825
56613	1.89226	0.471752
56614	1.35168	1.04296
56615	1.08132	0.74713
56616	1.0813	1.14204
56617	1.08132	1.89088
56618	0.810959	0.370716
56619	0.810959	1.0953
56620	1.89226	0.054017
56621	1.89226	0.05
56622	1.89226	0.05
56623	1.89226	0.05
56624	3.78453	0.05
56625	1.89226	0.05
56626	1.89226	0.05
56627	1.89226	0.05
56628	1.89226	0.05
56629	1.89226	0.05
56630	1.89226	0.05
56631	0.946131	0.05
56632	0.946131	0.05
56633	0.946126	0.05
56634	0.946126	0.05
56635	0.946128	0.05
56636	0.946128	0.05
56637	0.946128	0.05
56638	0.946128	0.05
56639	0.946128	0.05
56640	0.946128	0.05
56641	0.946128	0.05
56642	0.946128	0.05
56643	1.89226	0.063825
56644	1.89226	0.05
56645	1.89226	0.05
56646	1.89226	0.05
56647	1.89226	0.05
56648	1.89226	0.05
56649	1.89226	0.05
56650	1.89226	0.05
56651	1.89226	0.12549
56652	1.89226	0.05
56653	3.78453	0.05
56654	1.07176	0.05
56655	1.07176	0.05
56656	0.946126	0.05
56657	0.946126	0.05
56658	1.07176	0.05
56659	0.946129	0.05
56660	1.27474	0.05
56661	0.94613	0.05
56662	1.27474	0.05
56663	1.07176	0.05

56664	0.94613	0.05
56665	0.94613	0.05
56666	0.723486	0.05
56667	0.94613	0.05
56668	0.946126	0.05
56669	0.946126	0.05
56670	0.946129	0.05
56671	0.723484	0.05
56672	1.07176	0.05
56673	0.946129	0.05
56674	1.32398	0.05
56675	1.19835	0.05
56676	0.723471	0.05
56677	0.723471	0.05
56678	0.810946	0.0789823
56679	0.810946	0.05
56680	0.810946	0.0779065
56681	0.810946	0.05
56682	0.810933	0.05
56683	0.810933	0.05
56684	1.89226	0.05
56685	1.3517	0.05
56686	1.08132	0.05
56687	1.08132	0.05
56688	1.08133	0.05
56689	2.39923	2.1
56690	1.89226	2.1
56691	1.89226	2.1
56692	1.89226	2.1
56693	2.44606	2.1
56694	2.44606	2.1
56695	1.89226	2.1
56696	1.89226	2.1
56697	1.89226	2.1
56698	1.89226	2.1
56699	1.89226	2.1
56700	1.89226	2.1
56701	1.70302	2.1
56702	2.43041	2.1
56703	2.25275	2.1
56704	2.4747	2.1
56705	1.52079	2.1
56706	1.67063	2.1
56707	2.40644	2.1
56708	1.89226	2.1
56709	1.89226	2.1
56710	1.89226	2.1
56711	2.39923	2.1
56712	2.39923	2.1
56713	0.946131	1.64328
56714	0.946131	2.1
56715	0.946131	2.1
56716	0.946131	2.1
56717	0.94613	2.1
56718	0.94613	2.1
56719	0.946131	2.1
56720	0.946131	2.1
56721	0.946131	2.1

56722	0.946131	2.1
56723	0.946131	2.1
56724	0.946131	2.1
56725	1.04938	2.1
56726	1.04938	2.1
56727	1.111	2.1
56728	1.06978	2.1
56729	1.2748	2.1
56730	1.25472	2.1
56731	0.88782	2.1
56732	0.882987	2.1
56733	0.973779	2.1
56734	1.31997	2.1
56735	0.913905	2.1
56736	1.31312	2.1
56737	2.6536	2.1
56738	2.15287	2.1
56739	2.72238	2.1
56740	2.22887	2.1
56741	4.87525	2.1
56742	2.15288	2.1
56743	1.40719	2.1
56744	1.41839	2.1
56745	1.34239	2.1
56746	2.22887	2.1
56747	2.15288	2.1
56748	2.72237	2.1
56749	2.22887	2.1
56750	2.77314	2.1
56751	2.28748	2.1
56752	5.00985	2.1
56753	0.922802	2.1
56754	0.922802	2.1
56755	0.92107	2.1
56756	0.92107	2.1
56757	0.703593	2.1
56758	0.729436	2.1
56759	1.11444	2.1
56760	1.14374	2.1
56761	1.14374	2.1
56762	0.729436	2.1
56763	0.703593	2.1
56764	1.11444	2.1
56765	1.89226	2.1
56766	1.89226	2.1
56767	1.89226	2.1
56768	1.89226	2.1
56769	1.89226	2.1
56770	1.89226	2.1
56771	0.489927	2.1
56772	0.63008	2.1
56773	0.697385	2.1
56774	0.55524	2.1
56775	0.592141	2.1
56776	0.592141	2.1
56777	0.593179	2.1
56778	0.593179	2.1
56779	0.63008	2.1

56780	0.697385	2.1
56781	0.55524	2.1
56782	0.489927	2.1
56783	0.946133	2.1
56784	0.946133	2.1
56785	0.946132	1.72766
56786	0.946132	0.214852
56787	0.946133	1.00738
56788	0.946133	2.1
56789	0.946132	2.1
56790	0.946132	0.689905
56791	0.946132	1.85403
56792	0.946132	1.49125
56793	0.946132	1.20081
56794	0.946132	1.68357
56795	1.89226	2.1
56796	1.89226	2.1
56797	1.89226	2.1
56798	1.89226	2.1
56799	1.89226	2.1
56800	1.89226	2.1
56801	0.805746	2.1
56802	0.63008	2.1
56803	0.881598	2.1
56804	0.697385	2.1
56805	0.749417	2.1
56806	0.749417	2.1
56807	0.753714	2.1
56808	0.753714	2.1
56809	0.881598	2.1
56810	0.805746	2.1
56811	0.63008	2.1
56812	0.697385	2.1
56813	0.881599	2.1
56814	0.697385	2.1
56815	0.805746	2.1
56816	0.626731	1.28039
56817	0.755479	2.1
56818	0.755479	2.1
56819	0.752851	1.23935
56820	0.752851	2.1
56821	0.881598	2.1
56822	0.805747	2.1
56823	0.697385	2.1
56824	0.626731	2.1
56825	2.65348	2.1
56826	2.12314	2.1
56827	2.63899	2.1
56828	2.13898	2.1
56829	4.76213	2.1
56830	0.763518	2.1
56831	0.765889	2.1
56832	0.84086	2.1
56833	0.84086	2.1
56834	0.632078	2.1
56835	0.597565	2.1
56836	1.08813	2.1
56837	0.985643	2.1

56838	0.982464	2.1
56839	0.66404	2.1
56840	1.0358	2.1
56841	0.598023	2.1
56842	1.29293	2.1
56843	1.29628	2.1
56844	1.19266	2.1
56845	1.19266	2.1
56846	1.07109	2.1
56847	1.13093	2.1
56848	1.34467	2.1
56849	1.35251	2.1
56850	1.34369	2.1
56851	1.12525	2.1
56852	1.28746	2.1
56853	1.13511	2.1
56854	2.19717	2.1
56855	2.16547	2.1
56856	1.37392	2.1
56857	1.37392	2.1
56858	1.2966	2.1
56859	2.11985	2.1
56860	1.89226	2.1
56861	1.89226	2.1
56862	1.89226	2.1
56863	1.89226	2.1
56864	1.89226	2.1
56865	1.89226	2.1
56866	1.65637	2.1
56867	1.41758	2.1
56868	1.65637	2.1
56869	1.74391	2.1
56870	1.35504	2.1
56871	1.44258	2.1
56872	1.40774	2.1
56873	1.40774	2.1
56874	0.946131	2.1
56875	0.946131	2.1
56876	1.40774	2.1
56877	1.38882	2.1
56878	0.946132	2.1
56879	0.946132	2.1
56880	0.946132	2.1
56881	1.38882	2.1
56882	0.946132	2.1
56883	1.40774	2.1
56884	1.89227	2.1
56885	1.89227	2.1
56886	1.89227	2.1
56887	1.89227	2.1
56888	3.78453	2.1
56889	0.603148	2.1
56890	0.600177	2.1
56891	0.558615	2.1
56892	0.580714	2.1
56893	0.558606	2.1
56894	0.67883	2.1
56895	1.65637	2.1

56896	1.07933	2.1
56897	0.842649	2.1
56898	0.905168	2.1
56899	0.774334	2.1
56900	1.38882	2.1
56901	1.38882	2.1
56902	0.946132	2.1
56903	0.946132	2.1
56904	1.38882	2.1
56905	1.37381	2.1
56906	0.946132	2.1
56907	0.946132	2.1
56908	0.946132	2.1
56909	1.37381	2.1
56910	0.946133	2.1
56911	1.38882	2.1
56912	2.3771	2.1
56913	1.9339	2.1
56914	1.70782	2.1
56915	1.9339	2.1
56916	2.42693	2.1
56917	2.83279	2.1
56918	2.34901	0.586297
56919	1.45833	0.873462
56920	1.86695	0.7979
56921	1.03101	0.618892
56922	3.32528	0.65316
56923	1.89226	0.081372
56924	1.89226	0.101117
56925	1.89226	0.05
56926	1.89226	0.05
56927	3.78453	0.0810793
56928	2.71753	0.05
56929	1.89226	0.0731483
56930	2.76228	0.05
56931	2.76228	0.05
56932	1.89226	0.114966
56933	1.89226	0.280758
56934	0.946131	0.05
56935	0.946131	0.05
56936	0.946132	0.05
56937	0.946132	0.05
56938	0.946132	0.0734542
56939	0.946132	0.05
56940	0.946132	0.0627179
56941	0.946132	0.05
56942	0.946132	0.05
56943	0.946132	0.05
56944	0.946132	0.0523682
56945	0.946132	0.05
56946	1.89227	0.0631908
56947	1.89227	0.05
56948	1.89227	0.05
56949	1.89227	0.05
56950	1.89227	0.05
56951	1.89227	0.05
56952	2.71753	0.05
56953	2.37907	0.05

56954	2.37907	0.05
56955	2.76228	0.0672091
56956	2.39588	0.05
56957	2.77909	0.05
56958	1.6571	1.01863
56959	1.11682	1.72039
56960	1.47132	0.999382
56961	1.93763	0.270355
56962	3.12842	1.28224
56963	1.93762	0.898288
56964	2.12345	1.10676
56965	1.65709	0.664822
56966	1.65709	1.74202
56967	2.12345	1.11185
56968	1.88635	2.1
56969	1.47134	1.96419
56970	2.219 0.900254	
56971	2.25734	2.1
56972	1.93765	0.978371
56973	1.79103	1.26358
56974	1.47134	0.673604
56975	1.06174	1.06999
56976	1.19349	2.1
56977	1.1095	1.86479
56978	0.968827	0.985377
56979	1.07892	0.965679
56980	1.07892	2.1
56981	1.0834	0.781385
56982	1.0834	1.17267
56983	1.1095	1.2084
56984	1.19349	1.08363
56985	0.968825	1.18488
56986	1.06174	1.18036
56987	2.40627	2.1
56988	2.08288	1.43144
56989	2.5444	1.02376
56990	2.29285	1.4698
56991	4.62729	1.14431
56992	2.08289	1.06364
56993	1.65703	1.12013
56994	1.71105	2.1
56995	1.50108	1.96666
56996	2.29286	2.03731
56997	2.08289	2.06977
56998	2.5444	1.18866
56999	2.29285	2.1
57000	2.71429	2.1
57001	2.50288	0.999448
57002	5.04728	2.1
57003	2.50288	1.00108
57004	2.00927	2.08784
57005	2.02116	1.17375
57006	2.02116	2.1
57007	2.29286	1.0479
57008	2.29286	1.88919
57009	1.89226	2.1
57010	1.89226	2.1
57011	1.89226	1.06621

57012	1.89226	2.1
57013	3.78453	2.1
57014	1.17418	2.1
57015	1.29741	2.1
57016	1.04921	2.1
57017	1.19352	2.1
57018	2.3677	2.1
57019	1.29741	2.1
57020	1.27575	2.1
57021	1.19352	2.1
57022	1.19473	2.1
57023	2.46927	2.1
57024	1.89226	2.1
57025	1.89226	2.1
57026	1.89226	2.1
57027	1.89226	2.1
57028	1.89226	2.1
57029	1.89226	2.1
57030	0.561332	2.1
57031	0.561332	2.1
57032	0.567597	2.1
57033	0.567597	2.1
57034	0.597365	2.1
57035	0.544699	2.1
57036	0.596761	2.1
57037	0.531564	2.1
57038	0.5447	2.1
57039	0.531563	2.1
57040	0.596759	2.1
57041	0.597366	2.1
57042	0.946131	2.1
57043	0.946131	2.1
57044	0.946131	2.1
57045	0.946131	2.1
57046	0.946129	2.1
57047	0.946129	2.1
57048	0.946133	2.1
57049	0.946133	2.1
57050	0.946131	2.1
57051	0.946131	2.1
57052	0.946131	2.1
57053	0.946131	2.1
57054	1.10938	0.05
57055	1.17614	0.786023
57056	1.02478	0.05
57057	1.09056	0.218075
57058	2.19994	0.258793
57059	1.17614	0.653012
57060	1.11868	2.1
57061	1.03408	0.217132
57062	1.03408	2.1
57063	1.09056	0.549442
57064	1.17516	2.1
57065	1.89226	0.148684
57066	1.89226	0.0938195
57067	1.89226	0.104917
57068	1.89226	0.0598282
57069	1.89226	0.259839

57070	1.89226	0.141138
57071	1.89226	0.0582624
57072	1.89226	0.115935
57073	1.89226	0.0651739
57074	1.89226	0.05
57075	1.89226	0.153405
57076	1.89226	0.05
57077	0.579551	0.05
57078	0.563873	0.05
57079	0.611341	0.0508729
57080	0.598693	0.05
57081	0.589856	0.05
57082	0.589856	0.05
57083	0.588387	0.05
57084	0.588387	0.05
57085	0.57955	0.05
57086	0.611341	0.05
57087	0.563873	0.05
57088	0.598693	0.05
57089	0.611341	0.05
57090	0.598693	0.05
57091	0.586048	0.05
57092	0.572737	0.0580766
57093	0.591824	0.05
57094	0.591824	0.05
57095	0.592254	0.0605233
57096	0.592254	0.0510538
57097	0.611341	0.05
57098	0.586048	0.05
57099	0.598693	0.05
57100	0.572737	0.05
57101	0.946119	0.073546
57102	0.946119	0.167908
57103	0.946119	0.0713142
57104	0.946119	0.142342
57105	0.94612	0.0671788
57106	0.94612	0.0577934
57107	0.946119	0.154174
57108	0.946119	0.202111
57109	0.946132	0.0999579
57110	0.946132	0.0907374
57111	0.946107	0.116225
57112	0.946107	0.115332
57113	1.89224	0.0654957
57114	1.89224	0.0512428
57115	1.89224	0.0524101
57116	1.89224	0.05
57117	1.89224	0.139723
57118	1.89224	0.0722827
57119	0.562335	0.05
57120	0.579543	0.05
57121	0.59499	0.05
57122	0.611333	0.05
57123	0.586619	0.05
57124	0.586619	0.05
57125	0.587049	0.05
57126	0.587049	0.05
57127	0.594999	0.05

57128	0.562342	0.05
57129	0.579535	0.05
57130	0.611325	0.05
57131	0.59499	0.05
57132	0.611333	0.05
57133	0.567849	0.05
57134	0.58604	0.05
57135	0.590978	0.05
57136	0.590978	0.05
57137	0.590052	0.05
57138	0.590052	0.05
57139	0.567857	0.05
57140	0.594998	0.05
57141	0.611325	0.05
57142	0.586032	0.05
57143	1.69661	0.242409
57144	1.9339	0.05
57145	1.39379	0.05
57146	1.69107	0.05
57147	3.32768	0.05
57148	1.9339	0.0793109
57149	1.98414	0.05
57150	1.69107	0.05
57151	1.72774	0.05
57152	3.66164	0.05
57153	1.39379	0.965038
57154	1.69107	0.05
57155	1.14397	0.05
57156	1.43548	0.05
57157	2.82927	0.05
57158	1.69107	0.05
57159	1.72774	0.149966
57160	1.53873	0.05
57161	1.53873	0.109205
57162	1.43548	0.05
57163	1.62449	0.05
57164	2.58431	0.05
57165	1.34072	0.05
57166	1.80841	0.0866761
57167	1.34072	0.05
57168	2.51501	0.05
57169	2.04732	0.05
57170	1.89226	0.05
57171	1.89226	0.05
57172	1.89226	0.0797745
57173	1.89226	0.05
57174	1.89226	0.05
57175	1.89226	0.05
57176	0.946131	0.05
57177	0.946131	0.0981477
57178	1.3639	0.131147
57179	1.1516	0.05
57180	0.946129	0.05
57181	0.946129	0.0878673
57182	1.36391	0.0639492
57183	1.36391	0.094193
57184	0.946131	0.0937676
57185	1.3639	0.0531112

57186	1.1516	0.05
57187	0.946131	0.05
57188	0.946131	0.0624839
57189	0.946131	0.05
57190	0.94613	0.0815133
57191	0.94613	0.05
57192	0.946128	0.0971844
57193	0.946128	0.0660598
57194	0.946133	0.05
57195	0.946133	0.05
57196	0.946131	0.05
57197	0.946131	0.181295
57198	0.946131	0.05
57199	0.946131	0.059848
57200	1.89226	1.31359
57201	2.63634	2.1
57202	1.89226	1.36432
57203	1.83902	1.64842
57204	3.73128	1.32227
57205	1.47059	2.1
57206	2.02928	2.1
57207	1.47059	2.1
57208	1.65706	2.1
57209	1.98628	2.1
57210	2.17274	2.1
57211	2.43618	2.1
57212	2.41176	2.1
57213	1.98627	2.1
57214	2.34084	2.1
57215	4.39803	2.1
57216	1.47059	2.1
57217	1.82453	2.1
57218	1.98627	2.1
57219	1.98627	2.1
57220	1.26929	2.1
57221	1.26929	2.1
57222	1.98627	2.1
57223	2.34084	2.1
57224	1.47264	2.1
57225	2.24937	2.1
57226	4.23564	2.1
57227	1.33026	0.573904
57228	1.51171	1.74697
57229	1.51171	0.597681
57230	1.4617	1.92205
57231	1.35382	0.399233
57232	1.30382	2.1
57233	1.89226	0.114603
57234	1.89226	0.975467
57235	1.89226	0.577276
57236	1.89226	1.29371
57237	1.89226	0.19713
57238	1.89226	1.69217
57239	1.39263	1.0605
57240	0.962704	0.05
57241	1.0171	0.270888
57242	0.962704	0.303625
57243	1.40822	0.072686

57244	1.35382	0.0751857
57245	0.755853	2.1
57246	0.789808	1.04284
57247	0.696315	0.985071
57248	0.676912	0.107525
57249	0.729751	0.139582
57250	0.729751	1.03418
57251	0.722417	2.1
57252	0.722417	0.0549136
57253	0.696315	0.733692
57254	0.789808	0.860184
57255	0.755853	0.977183
57256	0.676912	0.865628
57257	0.946132	0.922914
57258	0.946132	0.512791
57259	0.946132	0.315293
57260	0.946132	0.0780019
57261	0.946131	0.0830779
57262	0.946131	0.645689
57263	0.946132	0.210943
57264	0.946132	0.560025
57265	0.946132	0.161266
57266	0.946132	0.273183
57267	0.946132	0.367298
57268	0.946132	0.326319
57269	0.946133	0.216829
57270	0.946133	0.0558935
57271	0.946132	0.05
57272	0.946132	0.0800936
57273	0.946132	0.05
57274	0.946132	0.0526854
57275	0.946133	0.05
57276	0.946133	0.295011
57277	0.946132	0.109275
57278	0.946132	0.123443
57279	0.946132	0.0590216
57280	0.946132	0.344605
57281	1.89227	1.93231
57282	2.53014	2.0268
57283	1.89227	2.1
57284	1.98241	1.79693
57285	1.89227	2.1
57286	1.98241	1.86158
57287	1.89226	1.55949
57288	1.89226	2.1
57289	1.89226	2.04231
57290	1.89226	1.96096
57291	1.89226	2.1
57292	1.89226	2.1
57293	1.89226	1.43989
57294	1.89226	2.1
57295	1.89226	1.76529
57296	1.89226	1.69768
57297	3.78452	1.80004
57298	2.45304	1.29966
57299	1.89226	2.09598
57300	1.89226	1.70462
57301	1.89226	1.68998

57302	1.89226	1.44646
57303	1.89226	1.37221
57304	1.89227	2.05023
57305	1.89227	1.81994
57306	1.89227	2.1
57307	1.89227	1.55245
57308	1.89227	2.1
57309	1.89227	1.59176
57310	1.89226	1.59892
57311	1.89226	2.1
57312	1.89226	2.04312
57313	1.89226	2.02677
57314	1.89226	2.1
57315	1.89226	2.1
57316	2.09871	0.12343
57317	2.71891	0.229421
57318	1.31126	0.114569
57319	1.31126	0.252895
57320	2.55341	0.123699
57321	1.76597	0.58741
57322	1.89226	0.25166
57323	1.89226	0.402504
57324	1.89226	0.0827646
57325	1.89226	0.833973
57326	1.89226	0.0839136
57327	1.89226	0.930017
57328	0.946128	0.339982
57329	0.946128	0.0726696
57330	0.946133	0.0972457
57331	0.946133	0.0574203
57332	0.946131	0.0906697
57333	0.946131	0.238395
57334	0.94613	0.05
57335	0.94613	0.0863665
57336	0.946131	0.0913954
57337	0.946131	0.103799
57338	0.946131	0.101921
57339	0.946131	0.0922162
57340	2.71891	0.0986254
57341	1.89226	0.085575
57342	2.09871	0.0621904
57343	2.09871	0.398279
57344	1.89226	0.05
57345	1.89226	0.237249
57346	1.89227	0.27355
57347	1.89227	0.271125
57348	1.89227	0.110391
57349	1.89227	0.688121
57350	1.89227	0.122755
57351	1.89227	0.882647
57352	0.946129	0.232712
57353	0.946129	0.0916021
57354	0.946133	0.0904079
57355	0.946133	0.05
57356	0.946131	0.084068
57357	0.946131	0.189943
57358	0.946131	0.05
57359	0.946131	0.105858

57360	0.946131	0.0814393
57361	0.946131	0.0831501
57362	0.946131	0.0757237
57363	0.946131	0.0878264
57364	2.32462	0.05
57365	1.89226	0.05
57366	1.89226	0.05
57367	1.89226	0.05
57368	2.2291	0.05
57369	2.2291	0.05
57370	0.847669	0.05
57371	0.847669	0.05
57372	0.796966	0.05
57373	0.796966	0.05
57374	0.833552	0.05
57375	1.01049	0.05
57376	0.709709	0.05
57377	0.634147	0.05
57378	0.634148	0.05
57379	0.833549	0.05
57380	0.709711	0.05
57381	1.01049	0.05
57382	1.89226	0.05
57383	1.89226	0.05
57384	1.89226	0.05
57385	1.89226	0.05
57386	1.89226	0.05
57387	1.89226	0.05
57388	1.03611	0.05
57389	0.936655	0.05
57390	0.946132	0.05
57391	0.946132	0.05
57392	0.941918	0.05
57393	0.941918	0.05
57394	1.00308	0.05
57395	1.04117	0.05
57396	1.1406	0.05
57397	1.04074	0.05
57398	0.851529	0.05
57399	1.00311	0.05
57400	1.01814	0.05
57401	1.6671	0.05
57402	1.35561	0.05
57403	1.35561	0.05
57404	1.2683	0.05
57405	1.2683	0.05
57406	0.946128	0.05
57407	0.946128	0.05
57408	0.946129	0.05
57409	0.946129	0.05
57410	0.946132	0.05
57411	0.946132	0.05
57412	0.946126	0.05
57413	0.946126	0.05
57414	0.946129	0.05
57415	0.946129	0.05
57416	0.946129	0.05
57417	0.946129	0.05

57418	2.23338	0.05
57419	1.96	0.05
57420	2.03438	0.05
57421	1.87835	0.05
57422	4.11172	0.05
57423	1.96	0.05
57424	1.52661	0.05
57425	1.87834	0.05
57426	1.53175	0.05
57427	3.40495	0.05
57428	2.03435	0.05
57429	1.65813	0.05
57430	1.65813	0.05
57431	1.87832	0.05
57432	1.72067	0.05
57433	1.94086	0.05
57434	1.65813	0.05
57435	1.40572	0.05
57436	1.87832	0.05
57437	1.87832	0.05
57438	1.31154	0.05
57439	1.53173	0.05
57440	1.86662	2.1
57441	2.0815	2.1
57442	2.0815	2.1
57443	2.0815	2.1
57444	1.87246	2.1
57445	1.87362	2.1
57446	1.0273	2.1
57447	0.996347	2.1
57448	0.946129	2.1
57449	0.946129	2.1
57450	1.04427	2.1
57451	1.04196	2.1
57452	0.910674	2.1
57453	0.910674	2.1
57454	1.04072	2.1
57455	1.10931	2.1
57456	1.03132	2.1
57457	0.933698	2.1
57458	2.63789	0.584666
57459	1.89226	0.810883
57460	2.3033	0.454255
57461	2.3033	0.797785
57462	1.89226	0.351394
57463	1.89226	0.803002
57464	1.89226	0.847043
57465	1.89226	0.899855
57466	1.89226	0.812016
57467	1.89226	0.846246
57468	3.78452	0.878257
57469	1.51898	0.78534
57470	1.36006	0.897895
57471	2.03143	0.888693
57472	2.03143	0.890079
57473	1.04026	0.835086
57474	1.04026	0.842708
57475	0.94613	0.375224

57476	0.94613	0.742624
57477	1.31894	0.299734
57478	1.31894	0.713813
57479	0.94613	0.270814
57480	0.94613	0.754539
57481	1.31894	0.7213
57482	1.41232	0.738248
57483	0.946129	0.718461
57484	1.41232	0.693526
57485	1.31895	0.721786
57486	0.946129	0.7316
57487	1.89226	0.735744
57488	1.89226	0.805649
57489	1.89226	0.502538
57490	1.89226	0.801352
57491	1.89226	0.439181
57492	1.89226	0.796592
57493	1.89804	0.691727
57494	2.03143	0.75689
57495	2.46286	0.758851
57496	2.46287	0.462682
57497	1.51898	0.733867
57498	1.51898	0.448521
57499	1.89226	2.1
57500	1.89226	2.1
57501	1.89226	2.1
57502	1.89226	2.1
57503	3.78452	2.1
57504	1.3864	2.1
57505	1.3864	2.1
57506	0.946132	2.1
57507	0.946132	2.1
57508	1.32905	2.1
57509	1.3864	2.1
57510	0.946132	2.1
57511	0.946132	2.1
57512	0.946132	2.1
57513	1.32905	2.1
57514	0.946133	2.1
57515	1.3864	2.1
57516	2.77279	2.1
57517	1.67883	2.1
57518	1.67883	2.1
57519	1.73415	2.1
57520	2.65811	2.1
57521	2.71343	2.1
57522	0.946131	2.1
57523	0.946131	2.1
57524	0.946128	2.1
57525	0.946128	2.1
57526	0.946127	2.1
57527	0.946127	2.1
57528	0.946131	2.1
57529	0.946131	2.1
57530	0.946129	2.1
57531	0.946129	2.1
57532	0.94613	2.1
57533	0.94613	2.1

57534	1.89226	2.1
57535	1.89226	2.1
57536	1.89226	2.1
57537	1.89226	2.1
57538	3.78452	2.1
57539	1.89226	2.1
57540	2.54394	2.1
57541	2.54394	2.1
57542	2.65811	2.1
57543	1.89226	2.1
57544	2.00643	2.1
57545	2.54394	2.1
57546	2.6581	2.1
57547	1.62743	2.1
57548	1.62743	2.1
57549	1.67883	2.1
57550	2.59533	2.1
57551	1.89226	2.1
57552	1.89226	2.1
57553	1.89226	2.1
57554	1.89226	2.1
57555	1.89226	2.1
57556	1.89226	2.1
57557	1.89226	1.5013
57558	1.89226	1.35346
57559	1.89226	2.1
57560	1.89226	0.871969
57561	1.89226	2.1
57562	1.89226	0.598495
57563	1.89226	1.61198
57564	1.89226	1.35768
57565	1.89226	2.1
57566	1.89226	1.21324
57567	1.89226	2.09595
57568	1.89226	0.992018
57569	1.89226	0.897125
57570	1.89226	0.382854
57571	1.89226	1.53289
57572	1.89226	0.2162
57573	1.89226	1.40523
57574	1.89226	0.162243
57575	2.68537	0.693462
57576	2.22575	0.499907
57577	2.47464	0.05
57578	1.79093	0.0520207
57579	4.70039	0.05
57580	2.81217	0.458466
57581	2.47464	0.140629
57582	2.58286	0.0797333
57583	2.29193	0.05
57584	5.0575	0.05
57585	2.47464	0.327975
57586	2.20365	0.140576
57587	2.29193	0.05
57588	1.90104	0.05
57589	4.37568	0.05
57590	1.89226	0.940328
57591	2.16957	2.1

57592	2.52823	1.22107
57593	2.52823	2.1
57594	1.89226	1.07244
57595	1.89226	1.72516
57596	1.36226	2.1
57597	1.55369	2.1
57598	1.33309	2.1
57599	1.62593	2.1
57600	2.98819	2.1
57601	0.802281	2.1
57602	0.802281	2.1
57603	0.835143	2.1
57604	0.835143	2.1
57605	0.824458	2.1
57606	0.92641	2.1
57607	0.776847	2.1
57608	0.812967	2.1
57609	0.812967	2.1
57610	0.926411	2.1
57611	0.776847	2.1
57612	0.824458	2.1
57613	1.33309	2.1
57614	1.62593	2.1
57615	1.32813	2.1
57616	1.7237	2.1
57617	2.95406	2.1
57618	0.814544	2.1
57619	0.875312	2.1
57620	0.910502	2.1
57621	0.910502	2.1
57622	0.886633	2.1
57623	0.975739	2.1
57624	0.792623	2.1
57625	0.840283	2.1
57626	0.796372	2.1
57627	1.06159	2.1
57628	0.81948	2.1
57629	0.933833	2.1
57630	2.63997	2.1
57631	1.29694	2.1
57632	1.28141	2.1
57633	2.62623	2.1
57634	3.92317	2.1
57635	2.63998	2.1
57636	1.89226	2.1
57637	1.89226	2.1
57638	2.62623	2.1
57639	4.5185	2.1
57640	1.89226	2.1
57641	1.89226	2.1
57642	1.89226	2.1
57643	1.89226	2.1
57644	3.78453	2.1
57645	1.89226	2.1
57646	1.89226	2.1
57647	1.89226	2.1
57648	1.89226	2.1
57649	1.89226	2.1

57650	1.89226	2.1
57651	1.25468	2.1
57652	2.62623	2.1
57653	1.28141	2.1
57654	1.28141	2.1
57655	2.60665	2.1
57656	2.60665	2.1
57657	1.89226	2.1
57658	2.62623	2.1
57659	2.60666	2.1
57660	2.62623	2.1
57661	1.87269	2.1
57662	1.89226	2.1
57663	1.89226	2.1
57664	1.89226	2.1
57665	1.89226	2.1
57666	1.89226	2.1
57667	3.78453	2.1
57668	1.89226	2.1
57669	1.89226	2.1
57670	1.89226	2.1
57671	1.89226	2.1
57672	1.89226	2.1
57673	1.89226	2.1
57674	2.30147	1.53245
57675	2.27703	2.1
57676	1.89227	1.22921
57677	1.89227	1.91272
57678	4.1693	1.72505
57679	1.38617	1.46192
57680	1.24613	1.57185
57681	2.59886	1.17178
57682	2.6238	1.62595
57683	3.86993	1.36045
57684	0.946133	1.60502
57685	0.946133	0.793848
57686	0.946132	0.930903
57687	0.946132	2.1
57688	0.946133	2.1
57689	0.946133	1.91323
57690	0.946132	0.861522
57691	0.946132	1.27641
57692	0.946133	1.45789
57693	0.946133	1.40292
57694	0.946132	1.29153
57695	0.946132	1.30744
57696	2.21773	2.1
57697	2.59216	2.1
57698	1.68541	2.1
57699	1.68541	2.1
57700	1.84814	2.1
57701	2.38046	2.1
57702	1.89966	2.1
57703	1.70303	2.1
57704	1.2028	2.1
57705	1.29897	2.1
57706	2.02873	2.1
57707	2.19093	2.1

57708	2.75218	2.1
57709	1.70303	2.1
57710	3.10254	2.1
57711	2.24783	2.1
57712	2.56839	2.1
57713	1.70303	2.1
57714	2.56839	2.1
57715	1.08276	2.1
57716	2.24061	2.1
57717	1.67491	2.1
57718	1.60948	2.1
57719	1.08276	2.1
57720	1.89226	0.878151
57721	1.89226	1.26336
57722	1.89226	0.747429
57723	1.89226	0.823601
57724	3.78453	0.627147
57725	1.89226	0.76759
57726	1.89226	1.14602
57727	1.89226	0.654966
57728	1.89226	0.822381
57729	3.78452	1.6539
57730	1.89226	1.04221
57731	2.7535	0.302579
57732	1.89226	1.38338
57733	1.89226	1.24926
57734	1.89226	0.358956
57735	1.89226	0.699132
57736	1.89226	1.10362
57737	1.89226	0.812777
57738	1.89226	0.365105
57739	1.89226	1.14413
57740	1.89226	0.481782
57741	1.89226	1.60736
57742	1.89227	0.927792
57743	1.89227	0.928553
57744	1.89227	0.717273
57745	1.89227	0.562094
57746	3.78453	1.75629
57747	1.89226	0.972303
57748	2.53412	0.136214
57749	1.89226	1.87149
57750	1.89226	0.210905
57751	1.89226	0.708935
57752	1.89226	0.138125
57753	2.21126	0.05
57754	1.89226	0.05
57755	2.57464	0.05
57756	1.89226	0.05
57757	2.57464	0.05
57758	1.89226	0.05
57759	1.89226	0.05
57760	1.89226	0.05
57761	1.89226	0.570006
57762	1.89226	0.05
57763	1.89226	0.210926
57764	1.89226	0.05
57765	1.1418	0.05

57766	0.653021	0.05
57767	1.37282	0.05
57768	0.931386	0.05
57769	1.34568	0.05
57770	1.17669	0.05
57771	0.666595	0.05
57772	0.786895	0.05
57773	0.933982	0.05
57774	1.05351	0.05
57775	1.00792	0.05
57776	1.00792	0.05
57777	1.89226	2.1
57778	1.89226	2.1
57779	1.89226	2.1
57780	1.89226	2.1
57781	3.78452	2.1
57782	1.89226	2.1
57783	1.89226	2.1
57784	1.89226	2.1
57785	1.89226	2.1
57786	1.89226	2.1
57787	1.89226	2.1
57788	1.65642	2.1
57789	1.30248	2.1
57790	1.38888	2.1
57791	1.25987	2.1
57792	1.70611	2.1
57793	1.65642	2.1
57794	1.65642	2.1
57795	1.81407	2.1
57796	2.19428	2.1
57797	1.92555	2.1
57798	3.8507	2.1
57799	1.89226	2.1
57800	1.89226	2.1
57801	1.89226	2.1
57802	1.89226	2.1
57803	3.78452	2.1
57804	1.89226	2.1
57805	1.89226	2.1
57806	1.89226	2.1
57807	1.89226	2.1
57808	1.89226	2.1
57809	1.89226	2.1
57810	0.705495	2.1
57811	0.809317	2.1
57812	1.1655	2.1
57813	1.09605	2.1
57814	0.983375	2.1
57815	0.797301	2.1
57816	0.906633	2.1
57817	0.906633	2.1
57818	0.85085	2.1
57819	1.16548	2.1
57820	0.705501	2.1
57821	1.00314	2.1
57822	2.19428	2.1
57823	2.31862	2.1

57824	2.27408	2.1
57825	2.45893	2.1
57826	4.46836	2.1
57827	1.89226	0.930817
57828	1.89226	1.10404
57829	1.89226	1.03582
57830	1.89226	0.9261
57831	1.89226	1.43832
57832	1.89226	1.02669
57833	2.0815	0.92903
57834	2.22136	1.0485
57835	1.5515	0.999691
57836	1.86323	0.90585
57837	2.74986	1.14046
57838	3.30238	0.95255
57839	1.89224	0.95533
57840	1.89224	1.07851
57841	1.89224	1.0787
57842	1.89224	0.939776
57843	1.89224	1.4138
57844	1.89224	1.00102
57845	1.89224	0.930415
57846	1.89224	1.0929
57847	1.89224	1.00125
57848	1.89224	0.912328
57849	1.89224	1.39724
57850	1.89224	1.00394
57851	1.17846	0.887607
57852	0.810935	0.88879
57853	0.93347	1.13731
57854	0.93347	0.919105
57855	0.810932	0.997389
57856	0.810932	0.938249
57857	2.22133	0.977625
57858	1.71919	0.972382
57859	1.32629	0.902102
57860	1.0813	1.02655
57861	1.57137	0.941386
57862	2.21149	2.1
57863	1.89226	2.1
57864	1.89226	2.1
57865	1.89226	2.1
57866	2.17159	2.1
57867	2.17159	2.1
57868	0.946131	0.271761
57869	0.946131	2.1
57870	0.946131	2.1
57871	0.946131	2.1
57872	0.94613	1.31567
57873	0.94613	1.92523
57874	0.946132	2.1
57875	0.946132	2.1
57876	0.946131	1.70228
57877	0.946131	1.68656
57878	0.946131	2.1
57879	0.946131	1.95027
57880	1.12266	2.1
57881	1.4714	2.1

57882	1.62322	2.1
57883	1.62322	1.97436
57884	1.02958	2.1
57885	1.02958	2.1
57886	2.2671	2.1
57887	1.89226	2.1
57888	1.89226	2.1
57889	1.89226	2.1
57890	2.21149	2.1
57891	2.21149	2.1
57892	1.89226	2.1
57893	1.89226	1.93319
57894	1.89226	2.1
57895	1.89226	1.44977
57896	1.89226	2.1
57897	1.89226	2.1
57898	0.736914	2.1
57899	0.736914	2.1
57900	0.822839	2.1
57901	0.822836	2.1
57902	0.791298	1.2301
57903	1.00003	2.1
57904	0.640254	2.1
57905	0.575379	2.1
57906	0.609001	1.87235
57907	1.00005	2.00451
57908	0.575371	2.1
57909	0.831908	1.7781
57910	1.89226	2.1
57911	1.89226	2.1
57912	1.89226	2.1
57913	1.89226	2.1
57914	1.89226	2.1
57915	1.89226	2.1
57916	1.89226	2.1
57917	1.89226	2.1
57918	1.89226	2.1
57919	1.89226	2.1
57920	1.89226	2.1
57921	1.89226	2.1
57922	0.730617	2.1
57923	0.71052	2.1
57924	0.7329	2.1
57925	0.712083	2.1
57926	0.701051	2.1
57927	0.701051	2.1
57928	0.721552	2.1
57929	0.721552	2.1
57930	0.710521	2.1
57931	0.732898	2.1
57932	0.730619	2.1
57933	0.712081	2.1
57934	0.7329	2.1
57935	0.712082	2.1
57936	0.559601	2.1
57937	0.495993	2.1
57938	0.603718	2.1
57939	0.603718	2.1

57940	0.625174	2.1
57941	0.625174	2.1
57942	0.732899	2.1
57943	0.559601	2.1
57944	0.712082	2.1
57945	0.495993	2.1
57946	0.94613	2.1
57947	0.94613	2.1
57948	0.94613	2.1
57949	0.94613	2.1
57950	0.946131	2.1
57951	0.946131	2.1
57952	0.946129	2.1
57953	0.946129	2.1
57954	0.94613	2.1
57955	0.94613	2.1
57956	0.94613	2.1
57957	0.94613	2.1
57958	1.89226	2.1
57959	1.89226	2.1
57960	1.89226	2.1
57961	1.89226	2.1
57962	1.89226	2.1
57963	1.89226	2.1
57964	1.42104	2.1
57965	1.4658	2.1
57966	1.51223	2.1
57967	1.42283	2.1
57968	2.93327	2.1
57969	0.732899	2.1
57970	0.756115	2.1
57971	0.629763	2.1
57972	0.559601	2.1
57973	0.646134	2.1
57974	0.646134	2.1
57975	0.669582	2.1
57976	0.669582	2.1
57977	0.756115	2.1
57978	0.629763	2.1
57979	0.732899	2.1
57980	0.559601	2.1
57981	2.566 2.1	
57982	1.20242	2.1
57983	2.64084	2.1
57984	2.18297	2.1
57985	1.66029	2.1
57986	1.20242	2.1
57987	0.946133	1.21197
57988	0.946133	1.4135
57989	0.946129	2.1
57990	0.946129	2.1
57991	0.946131	2.1
57992	0.946131	1.17387
57993	0.946131	0.294863
57994	0.946131	2.1
57995	0.946131	1.09598
57996	0.946131	2.04211
57997	0.946131	1.33116

57998	0.946131	1.81538
57999	1.89226	0.05
58000	1.89226	0.05
58001	1.89226	0.05
58002	1.89226	0.05
58003	3.78453	0.05
58004	1.03073	0.05
58005	1.03073	0.05
58006	1.04066	0.05
58007	1.04066	0.05
58008	1.20802	0.05
58009	1.32689	0.05
58010	0.883301	0.05
58011	0.744506	0.05
58012	1.20802	0.05
58013	0.744506	0.05
58014	0.883301	0.05
58015	1.32689	0.05
58016	1.20802	0.05
58017	1.20802	0.05
58018	0.946131	0.05
58019	0.946131	0.05
58020	0.94613	0.05
58021	0.94613	0.05
58022	1.20802	0.05
58023	1.32689	0.05
58024	1.20802	0.05
58025	0.946131	0.05
58026	0.946131	0.05
58027	1.32689	0.05
58028	1.89226	0.05
58029	1.89226	0.05
58030	1.89226	0.05
58031	1.89226	0.05
58032	1.89226	0.05
58033	1.89226	0.05
58034	0.946119	0.05
58035	0.946119	0.05
58036	0.946118	0.05
58037	0.946118	0.05
58038	0.946118	0.05
58039	0.946118	0.05
58040	0.946119	0.05
58041	0.946119	0.05
58042	0.946131	0.05
58043	0.946131	0.05
58044	0.946106	0.05
58045	0.946106	0.05
58046	1.3939	2.1
58047	1.49641	2.1
58048	1.15096	2.1
58049	1.10101	2.1
58050	2.59742	2.1
58051	1.22106	2.1
58052	2.60663	2.1
58053	1.25467	2.1
58054	1.25467	2.1
58055	2.58377	2.1

58056	2.58377	2.1
58057	1.89225	2.1
58058	2.60664	2.1
58059	2.58377	2.1
58060	2.60664	2.1
58061	1.86938	2.1
58062	1.89225	2.1
58063	1.89225	2.1
58064	1.89225	2.1
58065	1.89225	2.1
58066	1.89225	2.1
58067	1.89225	2.1
58068	1.89225	2.1
58069	0.946121	2.1
58070	0.946121	2.1
58071	0.946126	2.1
58072	0.946126	2.1
58073	0.946124	2.1
58074	0.946124	2.1
58075	0.946123	2.1
58076	0.946123	2.1
58077	0.946117	2.1
58078	0.946117	2.1
58079	0.94613	2.1
58080	0.94613	2.1
58081	1.28017	2.1
58082	1.29189	2.1
58083	0.610534	2.1
58084	0.592373	2.1
58085	0.943764	2.1
58086	0.943764	2.1
58087	0.940501	2.1
58088	0.940501	2.1
58089	1.28017	2.1
58090	0.592371	2.1
58091	0.610533	2.1
58092	1.29189	2.1
58093	0.946133	2.1
58094	0.946133	2.1
58095	1.28017	2.1
58096	1.29189	2.1
58097	0.946132	2.1
58098	0.946132	2.1
58099	1.28017	2.1
58100	1.28017	2.1
58101	1.28017	2.1
58102	0.946132	2.1
58103	0.946133	2.1
58104	1.2919	2.1
58105	1.89226	2.1
58106	1.89226	2.1
58107	1.89226	2.1
58108	1.89226	2.1
58109	1.89226	2.1
58110	1.89226	2.1
58111	1.89226	2.1
58112	1.89226	2.1
58113	1.89226	2.1

58114	1.89226	2.1
58115	3.78452	2.1
58116	1.89226	2.1
58117	2.08196	2.1
58118	1.97854	2.1
58119	2.08196	2.1
58120	1.78884	2.1
58121	1.89226	2.1
58122	1.89226	2.1
58123	1.89226	2.1
58124	1.89226	2.1
58125	1.89226	2.1
58126	1.89226	2.1
58127	1.89226	2.1
58128	1.83688	2.1
58129	1.89226	2.1
58130	1.89226	2.1
58131	1.89226	2.1
58132	1.86015	2.1
58133	1.86015	2.1
58134	1.89226	2.1
58135	1.97854	2.1
58136	1.85883	2.1
58137	1.97854	2.1
58138	1.77255	2.1
58139	1.89226	2.1
58140	0.946131	2.1
58141	0.946131	2.1
58142	0.946131	2.1
58143	0.946131	2.1
58144	0.94613	2.1
58145	0.94613	2.1
58146	0.946132	2.1
58147	0.946132	2.1
58148	0.946131	2.1
58149	0.946131	2.1
58150	0.946131	2.1
58151	0.946131	2.1
58152	0.918412	2.1
58153	0.918412	2.1
58154	0.94893	2.1
58155	0.750211	2.1
58156	0.851541	2.1
58157	0.851541	2.1
58158	0.970566	2.1
58159	0.826567	2.1
58160	0.970554	2.1
58161	1.04071	2.1
58162	0.826632	2.1
58163	0.851554	2.1
58164	1.89226	0.05
58165	1.89226	0.05
58166	1.89226	0.05
58167	1.89226	0.05
58168	3.78453	0.05
58169	0.946131	0.05
58170	0.946131	0.05
58171	0.811232	0.05

58172	0.811232	0.05
58173	0.94613	0.05
58174	0.94613	0.05
58175	0.959701	0.05
58176	0.811233	0.05
58177	0.94613	0.05
58178	0.811233	0.05
58179	0.959702	0.05
58180	0.946131	0.05
58181	1.89226	0.05
58182	1.89226	0.05
58183	1.89226	0.05
58184	1.89226	0.05
58185	1.89226	0.05
58186	1.89226	0.05
58187	1.89227	0.05
58188	1.89227	0.05
58189	1.89227	0.05
58190	1.89227	0.05
58191	1.89227	0.05
58192	1.89227	0.05
58193	1.89226	0.05
58194	1.89226	0.05
58195	1.89226	0.05
58196	1.89226	0.05
58197	3.78453	0.05
58198	1.89226	0.05
58199	1.89226	0.05
58200	1.89226	0.05
58201	1.89226	0.05
58202	1.89226	0.05
58203	1.89226	0.05
58204	1.89226	2.1
58205	2.36204	2.1
58206	2.39853	2.1
58207	2.36204	2.1
58208	1.92876	2.1
58209	1.89226	2.1
58210	1.89226	2.1
58211	1.89226	2.1
58212	1.89226	2.1
58213	1.89226	0.952212
58214	3.78453	2.1
58215	0.946128	2.1
58216	0.946128	0.933829
58217	1.13182	2.1
58218	1.13182	2.1
58219	0.94613	1.98537
58220	0.94613	2.1
58221	1.1749	2.1
58222	1.13182	2.1
58223	1.1749	2.1
58224	0.94613	2.1
58225	1.13182	2.1
58226	0.94613	2.1
58227	1.89226	2.1
58228	2.39853	2.1
58229	2.44918	2.1

58230	2.39853	2.1
58231	1.94291	2.1
58232	1.89226	2.1
58233	0.946132	2.1
58234	0.946132	2.1
58235	0.946131	2.1
58236	0.946131	2.1
58237	0.946132	2.1
58238	0.946132	2.1
58239	0.946131	2.1
58240	0.946131	2.1
58241	0.946131	2.1
58242	0.946131	2.1
58243	0.946132	2.1
58244	0.946132	2.1
58245	0.946128	2.1
58246	0.946128	2.1
58247	1.1749	2.1
58248	1.1749	2.1
58249	0.946131	2.1
58250	0.946131	2.1
58251	1.23631	2.1
58252	1.1749	2.1
58253	1.23631	2.1
58254	0.94613	2.1
58255	1.1749	2.1
58256	0.946131	2.1
58257	1.89226	1.16831
58258	1.89226	2.1
58259	1.89226	2.1
58260	1.89226	2.1
58261	3.78453	2.1
58262	1.38859	2.1
58263	1.45333	2.1
58264	1.28909	2.1
58265	1.38091	2.1
58266	2.74242	2.1
58267	1.45332	2.1
58268	1.38414	2.1
58269	1.38091	2.1
58270	1.33506	2.1
58271	2.78838	2.1
58272	1.89226	2.1
58273	1.89226	2.1
58274	1.89226	2.1
58275	1.89226	2.1
58276	3.78452	2.1
58277	1.89226	2.1
58278	1.89226	2.1
58279	1.89226	1.96634
58280	1.89226	2.1
58281	1.89226	2.1
58282	1.89226	2.1
58283	1.17418	2.1
58284	1.38091	2.1
58285	1.17418	2.1
58286	1.28909	2.1
58287	1.29741	2.1

58288	1.41232	2.1
58289	1.38091	2.1
58290	1.33506	2.1
58291	1.27575	2.1
58292	1.27575	2.1
58293	1.29741	2.1
58294	1.35671	2.1
58295	1.89226	2.1
58296	1.89226	2.1
58297	1.89226	2.1
58298	1.89226	2.1
58299	3.78452	2.1
58300	1.92737	0.05
58301	2.40223	0.059853
58302	1.56178	0.05
58303	1.97605	0.0566222
58304	3.90343	0.05
58305	1.89226	0.05
58306	1.89226	0.139977
58307	1.89226	0.0524503
58308	1.89226	0.140612
58309	3.78452	0.117942
58310	1.89226	0.137135
58311	1.89226	0.364528
58312	1.89226	0.310041
58313	1.89226	0.467406
58314	1.89226	0.13848
58315	1.89226	0.207234
58316	1.13641	0.0814589
58317	1.13641	0.055527
58318	1.12763	0.0520849
58319	1.12763	0.0890816
58320	1.29348	0.077407
58321	1.06293	0.1603
58322	1.20111	0.05
58323	0.988028	0.0601346
58324	0.988028	0.0681885
58325	1.06293	0.0876251
58326	1.29348	0.0745928
58327	1.20111	0.0617321
58328	1.56176	0.05
58329	1.46387	0.05
58330	1.97603	0.05
58331	1.97603	0.05
58332	1.08211	0.05
58333	1.08211	0.05
58334	1.89224	0.05
58335	1.89224	0.0751333
58336	1.89224	0.072124
58337	1.89224	0.0874067
58338	1.89224	0.05
58339	1.89224	0.05
58340	1.89224	0.0868008
58341	1.89224	0.198692
58342	1.89224	0.0817691
58343	1.89224	0.171805
58344	3.78448	0.090954
58345	0.820185	0.05

58346	0.818947	0.0710918
58347	0.911041	0.0791995
58348	0.695265	0.0781849
58349	1.46388	0.05
58350	1.23607	0.0510119
58351	0.927068	0.0555239
58352	1.02691	0.0754223
58353	1.12918	0.05
58354	0.846843	0.05
58355	0.911039	0.0633917
58356	1.29129	2.1
58357	1.45547	2.1
58358	1.67327	2.1
58359	1.66698	2.1
58360	1.20149	2.1
58361	1.20149	2.1
58362	0.946133	1.22672
58363	0.946133	2.1
58364	0.946129	2.1
58365	0.946129	2.1
58366	0.946131	2.1
58367	0.946131	1.20762
58368	0.946131	2.1
58369	0.946131	2.1
58370	0.946131	2.1
58371	0.946131	2.1
58372	0.946131	2.1
58373	0.946131	2.1
58374	1.89226	2.1
58375	1.89226	2.1
58376	1.89226	2.1
58377	1.89226	1.18587
58378	3.78452	2.1
58379	0.86875	2.1
58380	0.86875	2.1
58381	0.899002	2.1
58382	0.966245	2.1
58383	0.849364	1.30037
58384	1.01918	2.1
58385	0.764737	2.1
58386	0.89249	2.1
58387	0.764624	2.1
58388	0.967869	2.1
58389	0.808606	2.1
58390	0.864506	2.1
58391	0.773929	2.1
58392	0.773929	2.1
58393	0.781991	2.1
58394	0.836559	2.1
58395	0.983761	2.1
58396	0.834687	2.1
58397	0.680545	2.1
58398	0.640799	2.1
58399	0.70286	2.1
58400	0.935844	2.1
58401	0.629479	2.1
58402	0.823655	2.1
58403	0.946133	2.1

58404	0.946133	2.1
58405	0.946128	2.1
58406	0.946128	2.1
58407	0.94613	2.1
58408	0.94613	1.99912
58409	0.946131	2.1
58410	0.946131	2.1
58411	0.946131	2.1
58412	0.946131	2.1
58413	0.946131	2.1
58414	0.946131	2.1
58415	1.89226	2.1
58416	1.89226	1.24243
58417	1.89226	2.1
58418	1.89226	2.1
58419	1.89226	1.66382
58420	1.89226	2.1
58421	0.931638	2.1
58422	0.931638	2.1
58423	1.03559	2.1
58424	0.977442	2.1
58425	0.826487	1.44157
58426	0.993902	1.78626
58427	1.04922	2.1
58428	0.894324	2.1
58429	0.912984	2.1
58430	1.01156	2.1
58431	0.944692	2.1
58432	1.15456	2.1
58433	1.89226	0.05
58434	1.89226	0.05
58435	1.89226	0.05
58436	1.89226	0.05
58437	3.78453	0.05
58438	1.89226	0.05
58439	1.89226	0.05
58440	2.64262	0.05
58441	2.64262	0.05
58442	1.78052	0.05
58443	1.03015	0.05
58444	0.999721	0.05
58445	0.659011	0.05
58446	1.15564	0.05
58447	1.41388	0.05
58448	1.01583	0.05
58449	1.01583	0.05
58450	1.05706	0.05
58451	1.05706	0.05
58452	1.15564	0.05
58453	0.65901	0.05
58454	1.41388	0.05
58455	0.999721	0.05
58456	1.89226	2.1
58457	1.89226	2.1
58458	1.89226	2.1
58459	1.89226	2.1
58460	3.78453	2.1
58461	2.39022	2.1

58462	1.90734	2.1
58463	2.27634	2.1
58464	1.82666	2.1
58465	4.18368	2.1
58466	1.90734	2.1
58467	1.21564	2.1
58468	1.82666	2.1
58469	1.16938	2.1
58470	3.07672	2.1
58471	1.89226	2.1
58472	1.89226	2.1
58473	1.89226	2.1
58474	1.89226	2.1
58475	1.89226	2.1
58476	1.89226	2.1
58477	1.89227	2.1
58478	1.89227	2.1
58479	1.89227	2.1
58480	1.89227	2.1
58481	3.78453	2.1
58482	2.27634	2.1
58483	1.82666	2.1
58484	2.18042	2.1
58485	1.74738	2.1
58486	4.02372	2.1
58487	1.74738	2.1
58488	1.82666	2.1
58489	1.1181	2.1
58490	1.1181	2.1
58491	1.16938	2.1
58492	1.79865	2.1
58493	1.89226	2.1
58494	1.89226	2.1
58495	1.89226	2.1
58496	1.89226	2.1
58497	1.89226	2.1
58498	1.89226	2.1
58499	1.89226	1.49567
58500	1.89226	0.857092
58501	1.89226	0.944403
58502	1.89226	0.958314
58503	1.89226	1.49946
58504	1.89226	0.999132
58505	0.790736	0.956991
58506	1.08265	0.895919
58507	0.938909	1.39487
58508	0.638683	1.02331
58509	0.858588	1.07413
58510	0.858588	0.898824
58511	0.862743	1.55723
58512	0.862743	1.02099
58513	1.08265	1.33631
58514	0.938911	0.903402
58515	0.790735	1.20779
58516	0.638685	0.907897
58517	1.89226	0.903769
58518	1.89226	1.24058
58519	1.89226	0.987529

58520	1.89226	0.91228
58521	1.89226	1.48016
58522	1.89226	1.11519
58523	1.89226	0.895165
58524	1.89226	1.38361
58525	1.89226	0.918267
58526	1.89226	0.934533
58527	1.89226	1.12956
58528	1.89226	1.35482
58529	1.08265	0.899509
58530	1.36209	0.864916
58531	1.25233	0.928663
58532	0.938909	1.00043
58533	1.14195	0.895872
58534	1.14195	0.859058
58535	1.15904	1.3748
58536	1.15904	0.940277
58537	1.36209	1.03561
58538	1.25233	0.828357
58539	1.08265	1.0342
58540	0.938911	0.875156
58541	1.22077	0.879441
58542	0.915244	0.908996
58543	0.967835	1.12937
58544	0.742495	1.0855
58545	0.78648	1.08036
58546	0.985041	0.917523
58547	0.989098	1.66766
58548	0.989098	0.94669
58549	1.03206	0.986137
58550	0.967897	1.19348
58551	0.915404	1.08306
58552	0.706105	0.967084
58553	2.69662	0.05
58554	1.89226	0.05
58555	2.35499	0.05
58556	1.89226	0.05
58557	4.24725	0.05
58558	1.89227	0.05
58559	1.89227	0.05
58560	1.89227	0.05
58561	1.89227	0.05
58562	3.78453	0.05
58563	2.35499	0.05
58564	1.70302	0.05
58565	1.70302	0.05
58566	1.703 0.05	
58567	1.97374	0.05
58568	1.97372	0.05
58569	0.946133	0.05
58570	0.946133	0.05
58571	0.946132	0.05
58572	0.946132	0.05
58573	0.946133	0.05
58574	0.946133	0.05
58575	0.946132	0.05
58576	0.946132	0.05
58577	0.946133	0.05

58578	0.946133	0.05
58579	0.946132	0.05
58580	0.946132	0.05
58581	0.6794	2.1
58582	0.6794	2.1
58583	0.670812	2.1
58584	0.62182	2.1
58585	0.648326	2.1
58586	0.792154	2.1
58587	0.719167	2.1
58588	0.494515	2.1
58589	0.649421	2.1
58590	0.804869	2.1
58591	0.485585	2.1
58592	0.627666	2.1
58593	1.79347	2.1
58594	1.03968	2.1
58595	1.70303	2.1
58596	0.933265	1.80312
58597	2.62068	1.85799
58598	1.89226	2.1
58599	1.89226	2.1
58600	1.89226	2.1
58601	1.89226	1.45803
58602	3.78452	2.1
58603	1.89226	2.1
58604	2.24604	2.1
58605	2.0554	2.1
58606	2.0554	2.1
58607	1.89226	2.1
58608	1.89226	2.1
58609	1.89226	2.1
58610	1.89226	0.860579
58611	1.89226	1.07309
58612	1.89226	2.1
58613	1.89226	1.09204
58614	1.89226	2.1
58615	2.0815	2.1
58616	2.64424	0.05
58617	2.80807	0.05
58618	3.232	2.1
58619	1.89417	0.0599418
58620	2.18012	2.1
58621	0.946131	2.1
58622	0.946131	0.05
58623	0.946131	2.1
58624	0.946131	1.19783
58625	0.94613	1.10071
58626	0.94613	0.378557
58627	0.946132	2.1
58628	0.946132	2.1
58629	0.946131	2.1
58630	0.946131	2.1
58631	0.946131	2.1
58632	0.946131	2.1
58633	1.89227	0.05
58634	1.89227	0.05
58635	1.89227	0.05

58636	1.89227	0.05
58637	3.78453	0.05
58638	2.70881	0.05
58639	2.68687	0.05
58640	2.03046	0.05
58641	1.9682	0.05
58642	4.67701	0.05
58643	1.08518	0.05
58644	1.08518	0.05
58645	1.09033	0.05
58646	1.09033	0.05
58647	0.842273	0.05
58648	1.19141	0.05
58649	1.34343	0.05
58650	0.9841	0.05
58651	0.9841	0.05
58652	0.842273	0.05
58653	1.34344	0.05
58654	1.19141	0.05
58655	0.946133	0.05
58656	0.946133	0.05
58657	0.946132	0.05
58658	0.946132	0.05
58659	0.946132	0.05
58660	0.946132	0.05
58661	0.946132	0.05
58662	0.946132	0.05
58663	0.946132	0.05
58664	0.946132	0.05
58665	0.946133	0.05
58666	0.946133	0.05
58667	1.89227	0.05
58668	1.89227	0.05
58669	1.89227	0.05
58670	1.89227	0.05
58671	1.89227	0.05
58672	1.89227	0.05
58673	2.03046	0.05
58674	1.9682	0.05
58675	1.23886	0.05
58676	1.12971	0.05
58677	3.20706	0.05
58678	1.89226	0.16513
58679	1.89226	1.04066
58680	1.89226	0.0678191
58681	1.89226	0.597051
58682	3.78452	0.421991
58683	2.45569	0.0581763
58684	1.89226	0.103476
58685	2.22922	0.112976
58686	2.0815	0.420196
58687	1.85074	0.05
58688	1.70303	0.0688806
58689	2.72856	0.0556873
58690	1.89226	0.625472
58691	1.89226	0.05
58692	1.89226	0.22018
58693	3.78452	0.196695

58694	1.89226	0.207153
58695	1.89226	0.759188
58696	1.89226	0.0783667
58697	1.89226	0.739538
58698	3.78452	0.27663
58699	0.946131	0.711637
58700	0.946131	0.0665926
58701	0.946131	0.470763
58702	0.946131	0.05
58703	0.94613	0.367004
58704	0.94613	0.528313
58705	0.946132	0.062134
58706	0.946132	0.0743439
58707	0.946131	0.123746
58708	0.946131	0.185538
58709	0.946131	0.211282
58710	0.946131	0.140767
58711	2.0595	0.05
58712	2.58581	0.05
58713	1.48694	0.05
58714	1.48694	0.05
58715	2.60157	0.05
58716	2.02901	0.05
58717	1.89226	0.05
58718	1.89226	0.05
58719	1.89226	0.05
58720	1.89226	0.05
58721	1.89226	0.05
58722	1.89226	0.05
58723	2.04728	0.05
58724	2.64161	0.05
58725	1.47606	0.05
58726	1.47606	0.05
58727	2.63069	0.05
58728	2.05947	0.05
58729	1.89224	0.05
58730	1.89224	0.05
58731	1.89224	0.05
58732	1.89224	0.05
58733	1.89224	0.05
58734	1.89224	0.05
58735	0.946114	0.05
58736	0.946114	0.05
58737	0.946118	0.05
58738	0.946118	0.05
58739	0.946114	0.05
58740	0.946114	0.05
58741	0.946118	0.05
58742	0.946118	0.05
58743	0.946128	0.05
58744	0.946128	0.05
58745	0.946103	0.05
58746	0.946103	0.05
58747	1.1233	2.1
58748	1.15009	2.1
58749	2.36036	2.1
58750	2.38225	2.1
58751	3.50554	2.1

58752	2.38224	2.1
58753	0.977775	2.1
58754	2.20473	2.1
58755	2.20473	2.1
58756	1.15009	2.1
58757	1.15009	2.1
58758	0.946119	2.1
58759	0.946119	2.1
58760	1.19111	2.1
58761	1.19111	2.1
58762	0.946118	2.1
58763	1.19111	2.1
58764	1.18016	2.1
58765	0.946118	1.33891
58766	0.94613	0.950604
58767	0.94613	2.1
58768	1.18015	2.1
58769	1.19109	2.1
58770	0.946118	2.1
58771	0.946118	2.1
58772	1.10235	2.1
58773	1.10235	2.1
58774	0.946118	2.1
58775	1.10235	2.1
58776	0.946115	2.1
58777	1.1911	2.1
58778	0.946129	2.1
58779	0.946129	2.1
58780	1.19109	2.1
58781	1.10234	2.1
58782	1.89226	2.1
58783	2.23932	2.1
58784	2.11568	2.1
58785	2.23932	2.1
58786	1.76862	2.1
58787	1.89226	2.1
58788	1.89226	2.1
58789	1.89226	2.1
58790	1.89226	2.1
58791	1.89226	2.1
58792	1.89226	2.1
58793	1.89226	2.1
58794	2.07838	2.1
58795	1.89226	2.1
58796	1.89226	2.1
58797	1.89226	2.1
58798	2.10264	2.1
58799	2.10264	2.1
58800	1.89226	2.1
58801	2.11568	2.1
58802	1.99753	2.1
58803	2.11568	2.1
58804	1.77411	2.1
58805	1.89226	2.1
58806	0.946128	2.1
58807	0.946128	2.1
58808	0.946133	2.1
58809	0.946133	2.1

58810	0.94613	2.1
58811	0.94613	2.1
58812	0.946131	2.1
58813	0.946131	2.1
58814	0.946131	2.1
58815	0.946131	2.1
58816	0.946131	2.1
58817	0.946131	2.1
58818	0.946132	2.1
58819	0.946132	2.1
58820	1.03919	2.1
58821	1.03919	2.1
58822	0.946131	2.1
58823	0.946131	2.1
58824	1.02744	2.1
58825	1.03919	2.1
58826	1.02744	2.1
58827	0.946132	2.1
58828	1.03919	2.1
58829	0.946132	2.1
58830	1.64834	2.0827
58831	1.82955	1.68011
58832	2.33824	1.2734
58833	2.19301	2.1
58834	3.98658	1.65543
58835	1.40574	2.1
58836	2.19301	1.47727
58837	1.64834	1.83782
58838	1.64834	1.89039
58839	1.99961	1.35193
58840	1.99961	1.43432
58841	2.33824	1.31903
58842	2.19301	2.1
58843	2.68524	1.80429
58844	2.81847	1.42112
58845	5.02349	1.82736
58846	1.99961	2.1
58847	2.19301	1.33219
58848	2.68524	1.43898
58849	2.50732	1.77615
58850	4.68485	1.49437
58851	1.89226	2.1
58852	1.89226	2.1
58853	1.89226	2.1
58854	1.89226	2.1
58855	3.78453	2.1
58856	1.75142	2.1
58857	2.47026	2.1
58858	1.46811	2.1
58859	2.26652	2.1
58860	4.01794	2.1
58861	1.89226	2.1
58862	1.89226	2.1
58863	1.89226	2.1
58864	1.89226	2.1
58865	1.89226	2.1
58866	1.89226	2.1
58867	1.89226	2.1

58868	1.89226	2.1
58869	1.89226	2.1
58870	1.89226	2.1
58871	1.89226	2.1
58872	1.89226	2.1
58873	0.812082	2.1
58874	0.878759	2.1
58875	0.882782	2.1
58876	0.882782	2.1
58877	1.05572	2.1
58878	1.04037	2.1
58879	0.73849	2.1
58880	0.626857	2.1
58881	0.638677	2.1
58882	1.05581	2.1
58883	0.715695	2.1
58884	1.10492	2.1
58885	1.17058	2.1
58886	1.07863	2.1
58887	1.21113	2.1
58888	1.21113	2.1
58889	1.2811	2.1
58890	1.37319	2.1
58891	1.01788	2.1
58892	0.999492	2.1
58893	1.04352	2.1
58894	1.32112	2.1
58895	1.10492	2.1
58896	1.34699	2.1
58897	1.89226	2.1
58898	1.89226	2.1
58899	1.89226	2.1
58900	1.89226	2.1
58901	1.89226	2.1
58902	1.89226	2.1
58903	1.19813	0.05
58904	1.19813	0.05
58905	2.43432	0.05
58906	1.21716	0.05
58907	0.946124	0.05
58908	1.21715	0.05
58909	0.946118	0.05
58910	2.56627	2.1
58911	1.89226	2.1
58912	1.89226	2.1
58913	1.89226	2.1
58914	2.43434	2.1
58915	2.43434	2.1
58916	1.89227	0.05
58917	1.89227	0.05
58918	1.89227	0.05
58919	1.89227	0.05
58920	1.89227	0.05
58921	1.89227	0.05
58922	1.89226	0.05
58923	1.89226	0.05
58924	1.89226	0.05
58925	1.89226	0.05

58926	3.78453	0.05
58927	1.89226	0.05
58928	2.0764	0.05
58929	1.89226	0.05
58930	2.81674	0.05
58931	1.89226	0.05
58932	2.81674	0.05
58933	0.946132	0.05
58934	0.946132	0.05
58935	0.946131	0.05
58936	0.946131	0.05
58937	0.946132	0.05
58938	0.946132	0.05
58939	0.946132	0.05
58940	0.946132	0.05
58941	0.946132	0.05
58942	0.946132	0.05
58943	0.946132	0.05
58944	0.946132	0.05
58945	0.578684	1.45165
58946	0.578684	2.1
58947	0.589487	1.8449
58948	0.589487	2.1
58949	0.625461	2.1
58950	0.5378	2.1
58951	0.608714	2.1
58952	0.54271	1.78008
58953	0.608714	1.58026
58954	0.625461	1.29506
58955	0.54271	2.1
58956	0.537801	1.91163
58957	0.946128	1.20306
58958	0.946128	2.09651
58959	0.946133	1.19967
58960	0.946133	2.1
58961	0.946131	1.37519
58962	0.946131	1.19311
58963	0.946131	1.17275
58964	0.946131	1.42071
58965	0.94613	1.36093
58966	0.94613	1.16523
58967	0.946131	1.39918
58968	0.946131	1.53445
58969	1.21743	2.1
58970	1.25092	2.1
58971	1.38847	1.25082
58972	1.46031	1.24325
58973	2.67774	1.73447
58974	0.946128	1.15689
58975	0.946128	2.1
58976	0.946132	2.1
58977	0.946132	1.26406
58978	0.94613	1.54219
58979	0.94613	1.16561
58980	0.94613	1.38574
58981	0.94613	1.21637
58982	0.94613	1.1918
58983	0.94613	1.15515

58984	0.94613	1.5733
58985	0.94613	1.27207
58986	1.89226	1.10321
58987	1.89226	1.33023
58988	1.89226	0.944118
58989	1.89226	2.1
58990	1.89226	2.1
58991	1.89226	2.1
58992	0.539566	1.1432
58993	0.534046	2.1
58994	0.577914	1.98463
58995	0.471623	2.01035
58996	1.46031	1.0445
58997	0.945865	1.22039
58998	0.770603	1.08675
58999	0.737201	2.1
59000	0.714829	1.25267
59001	0.536085	1.57734
59002	0.471617	1.51026
59003	1.89226	2.1
59004	1.89226	1.16154
59005	1.89226	1.15629
59006	1.89226	1.48015
59007	1.89226	1.47972
59008	1.89226	2.1
59009	1.89225	1.69475
59010	1.89225	0.978926
59011	1.65572	1.00987
59012	1.65572	1.2279
59013	1.89225	1.19854
59014	1.89225	1.8629
59015	1.89226	1.51709
59016	1.89226	1.31501
59017	1.89226	1.30065
59018	1.89226	0.573372
59019	3.78453	1.63176
59020	1.89226	2.00478
59021	1.89226	0.973317
59022	1.89226	0.951638
59023	1.89226	1.31152
59024	1.89226	1.22904
59025	1.89226	2.1
59026	1.89225	1.4108
59027	1.89225	0.550102
59028	1.89225	0.816637
59029	1.89225	1.0918
59030	1.89225	0.993929
59031	1.89225	1.53158
59032	0.763592	0.99529
59033	0.763592	1.40796
59034	0.987417	1.13904
59035	0.987417	1.1726
59036	0.763592	0.818391
59037	0.804881	0.573433
59038	0.946126	1.15748
59039	0.946126	0.962162
59040	0.804879	0.5547
59041	0.94613	1.04558

59042	0.946129	1.23109
59043	0.76359	0.909301
59044	1.46055	2.1
59045	2.04945	2.1
59046	2.04587	2.1
59047	2.04587	2.1
59048	1.50714	2.1
59049	1.50714	2.1
59050	2.04587	2.1
59051	2.38681	2.1
59052	2.42082	2.1
59053	2.42082	2.1
59054	2.04945	2.1
59055	2.04945	2.1
59056	0.872964	2.1
59057	0.872964	2.1
59058	0.884806	2.1
59059	0.884806	2.1
59060	1.0275	2.1
59061	1.02293	2.1
59062	0.711243	2.1
59063	0.730273	2.1
59064	0.711244	2.1
59065	1.0275	2.1
59066	0.730273	2.1
59067	1.02293	2.1
59068	2.04587	2.1
59069	2.47753	2.1
59070	2.42994	2.1
59071	2.42082	2.1
59072	2.05499	2.1
59073	2.04587	2.1
59074	0.946132	2.1
59075	0.946132	2.1
59076	1.13078	2.1
59077	1.07817	2.1
59078	0.946132	2.1
59079	0.946132	2.1
59080	1.07817	2.1
59081	1.07817	2.1
59082	0.946132	2.1
59083	1.07817	2.1
59084	1.13078	2.1
59085	0.946132	2.1
59086	1.89226	2.1
59087	1.89226	2.1
59088	1.89226	2.1
59089	1.89226	2.1
59090	1.89226	2.1
59091	1.89226	2.1
59092	1.89226	2.1
59093	1.89226	2.1
59094	1.89226	2.1
59095	1.89226	2.1
59096	1.89226	2.1
59097	1.89226	2.1
59098	1.89226	2.1
59099	1.89226	2.1

59100	1.8173	2.1
59101	2.66885	2.1
59102	2.7438	2.1
59103	2.7438	2.1
59104	1.89226	0.786488
59105	1.89226	0.527952
59106	1.89226	0.725753
59107	1.89226	0.960796
59108	3.78453	0.759254
59109	1.89226	0.574214
59110	1.89226	0.588983
59111	1.89226	0.300143
59112	1.89226	1.42205
59113	1.89226	0.154452
59114	1.89226	0.788503
59115	1.89226	0.194301
59116	1.97385	0.891172
59117	1.7531	0.344269
59118	1.89226	1.75732
59119	1.7531	0.128457
59120	1.89226	0.506719
59121	0.946131	1.05611
59122	0.840904	0.41928
59123	0.684333	0.419197
59124	0.94613	0.856067
59125	0.972337	0.598369
59126	0.946278	1.86356
59127	0.684304	0.476507
59128	0.684304	0.909879
59129	0.999838	0.429537
59130	0.759605	1.95698
59131	0.851528	0.513081
59132	0.851528	1.20014
59133	1.89226	0.719664
59134	2.23818	1.01467
59135	1.79771	0.796217
59136	1.89226	1.16763
59137	1.79771	0.708934
59138	1.89226	0.822369
59139	2.79647	2.1
59140	2.01613	2.1
59141	1.46826	2.1
59142	2.54504	2.1
59143	4.56117	2.1
59144	1.89226	2.1
59145	1.89226	2.1
59146	1.89226	2.1
59147	1.89226	2.1
59148	1.89226	2.1
59149	1.89226	2.1
59150	1.89226	2.1
59151	2.66884	2.1
59152	2.56022	2.1
59153	2.66884	2.1
59154	1.78364	2.1
59155	1.89226	2.1
59156	1.14282	2.1
59157	1.24068	2.1

59158	0.880748	2.1
59159	0.834916	2.1
59160	1.05539	2.1
59161	1.05539	2.1
59162	1.04312	2.1
59163	1.04313	2.1
59164	1.2015	2.1
59165	0.83492	2.1
59166	0.789149	2.1
59167	1.24066	2.1
59168	1.89226	2.1
59169	1.89226	2.1
59170	1.89226	2.1
59171	1.89226	2.1
59172	3.78453	2.1
59173	0.946128	2.1
59174	0.946128	2.1
59175	1.28011	2.1
59176	1.22372	2.1
59177	0.946131	2.1
59178	0.946131	2.1
59179	1.22372	2.1
59180	1.22372	2.1
59181	1.22372	2.1
59182	0.94613	2.1
59183	0.946131	2.1
59184	1.28011	2.1
59185	2.28604	2.1
59186	1.34268	2.1
59187	2.43157	2.1
59188	1.50172	2.1
59189	3.77425	2.1
59190	2.6096	2.1
59191	2.43157	2.1
59192	1.66774	2.1
59193	1.66774	2.1
59194	1.50172	2.1
59195	2.43157	2.1
59196	2.16067	2.1
59197	1.08975	2.1
59198	2.19336	2.1
59199	2.06984	2.1
59200	1.21327	2.1
59201	1.08975	2.1
59202	2.28604	2.1
59203	1.21327	2.1
59204	2.29008	2.1
59205	2.16067	2.1
59206	1.34268	2.1
59207	1.21327	2.1
59208	1.89226	0.730975
59209	1.89226	0.257532
59210	1.89226	0.735108
59211	1.89226	0.708307
59212	3.78452	0.691313
59213	1.89804	0.714578
59214	2.21656	0.0885008
59215	1.70537	0.706373

59216	2.50023	0.335627
59217	3.8982	0.655254
59218	1.03136	0.631119
59219	1.89486	0.122058
59220	0.949364	0.631666
59221	1.82283	0.264601
59222	2.84422	0.585895
59223	1.89226	0.431695
59224	1.89226	0.396844
59225	1.89226	0.710168
59226	1.89226	0.708129
59227	3.78452	0.720975
59228	0.946132	0.742219
59229	0.946132	0.734484
59230	0.946129	0.731569
59231	0.946129	0.715096
59232	0.94613	0.715634
59233	0.94613	0.775425
59234	0.946131	0.716765
59235	0.946131	0.739106
59236	0.946131	0.749476
59237	0.946131	0.746706
59238	0.946131	0.628366
59239	0.946131	0.779021
59240	1.09466	0.717109
59241	1.09466	0.375327
59242	0.866512	0.701103
59243	1.08352	0.634943
59244	1.19189	0.674341
59245	1.21883	0.702739
59246	0.971139	0.715915
59247	0.873213	0.70147
59248	0.873202	0.702664
59249	1.00855	0.700681
59250	0.923735	0.668284
59251	1.21885	0.649996
59252	1.89225	0.05
59253	2.40019	0.05
59254	1.89225	0.05
59255	1.89225	0.05
59256	1.89225	0.05
59257	1.89225	0.05
59258	1.89225	0.05
59259	1.89225	0.05
59260	2.05088	0.05
59261	1.89225	0.05
59262	1.89225	0.05
59263	1.89225	0.05
59264	2.03956	0.05
59265	2.03956	0.05
59266	1.89226	2.1
59267	2.40021	2.1
59268	2.38473	0.578817
59269	2.40021	2.1
59270	1.87679	0.849468
59271	1.89226	2.1
59272	1.89226	2.1
59273	1.89226	0.807635

59274	1.89226	2.1
59275	1.89226	0.0625458
59276	1.89226	2.1
59277	1.89226	0.673193
59278	2.0692	2.1
59279	1.89226	0.838042
59280	1.89226	2.1
59281	1.89226	0.395809
59282	2.05089	2.1
59283	2.05089	0.383002
59284	1.89226	2.1
59285	1.89226	2.1
59286	1.89226	2.1
59287	1.89226	2.1
59288	3.78452	2.1
59289	1.89226	1.95903
59290	2.50051	2.1
59291	1.89226	2.1
59292	2.30076	2.1
59293	4.39277	2.1
59294	2.50052	2.1
59295	1.70377	2.1
59296	2.30076	2.1
59297	1.54231	2.1
59298	4.04283	2.1
59299	1.89226	2.1
59300	1.89226	2.1
59301	1.89226	2.1
59302	1.89226	2.1
59303	1.89226	2.1
59304	1.89226	2.1
59305	1.89226	2.07533
59306	1.89226	2.1
59307	1.89226	2.1
59308	1.89226	2.1
59309	3.78452	2.1
59310	2.07948	2.1
59311	2.30076	2.1
59312	1.3294	2.1
59313	1.3294	2.1
59314	1.54232	2.1
59315	2.29239	2.1
59316	0.810947	2.1
59317	0.810947	2.1
59318	0.810946	2.1
59319	0.810946	2.1
59320	1.89226	2.1
59321	1.35169	2.1
59322	1.08132	2.1
59323	1.08132	2.1
59324	1.08132	2.1
59325	0.810946	2.1
59326	0.810946	2.1
59327	1.34851	1.31503
59328	0.997471	2.1
59329	1.62287	1.04725
59330	1.27096	1.89266
59331	2.62034	1.26755

59332	1.89226	1.20117
59333	1.89226	2.1
59334	1.89226	1.15032
59335	1.89226	2.1
59336	3.78452	1.43044
59337	0.946128	2.1
59338	0.946128	2.1
59339	1.29331	0.175493
59340	1.29331	2.1
59341	1.29331	2.1
59342	1.10699	2.1
59343	0.946131	0.199072
59344	0.946131	2.1
59345	0.94613	2.1
59346	1.29331	2.1
59347	1.10699	2.1
59348	0.946131	2.1
59349	1.62287	1.20271
59350	1.27096	2.05777
59351	1.86067	1.05871
59352	1.58407	2.00109
59353	3.20694	1.68189
59354	1.89226	1.37258
59355	1.89226	2.1
59356	1.89226	0.837989
59357	1.89226	1.99844
59358	3.78452	1.3398
59359	2.62761	0.771602
59360	2.55849	0.967083
59361	2.12261	1.19404
59362	2.22939	0.899296
59363	4.78788	0.947054
59364	0.772522	2.01866
59365	0.839588	1.43757
59366	1.31126	0.238616
59367	1.08787	0.744658
59368	1.01318	0.511618
59369	1.01318	2.1
59370	0.900815	2.1
59371	1.10799	0.0584477
59372	0.772511	1.08854
59373	1.03473	1.15882
59374	1.31126	0.973493
59375	0.841464	1.16198
59376	1.89227	0.564813
59377	1.89227	2.1
59378	1.89227	0.683648
59379	1.89227	1.6364
59380	1.89227	0.439833
59381	1.89227	1.97592
59382	1.89226	0.903424
59383	1.89226	1.01873
59384	1.89226	0.853704
59385	1.89226	0.97938
59386	1.89226	0.859115
59387	1.89226	0.997265
59388	2.12261	1.04153
59389	2.22939	0.612724

59390	1.97004	0.871515
59391	1.8072	1.16656
59392	4.03658	0.898287
59393	1.5847	1.96851
59394	1.8072	0.221607
59395	2.12262	1.01132
59396	2.12262	1.95913
59397	1.23267	0.573524
59398	1.23267	1.96027
59399	1.89227	0.33557
59400	1.89227	2.1
59401	1.89227	0.563128
59402	1.89227	1.9031
59403	1.89227	0.289981
59404	1.89227	2.06318
59405	1.89226	0.711371
59406	1.89226	0.976992
59407	1.89226	1.17484
59408	1.89226	0.85214
59409	3.78452	0.917976
59410	2.49093	2.1
59411	2.67393	2.1
59412	2.47932	2.1
59413	2.47932	2.1
59414	2.67994	2.1
59415	2.67994	2.1
59416	2.49873	2.1
59417	2.67393	2.1
59418	2.67993	2.1
59419	2.69807	2.1
59420	2.49873	2.1
59421	2.51686	2.1
59422	2.49093	2.1
59423	2.67994	2.1
59424	2.69784	2.1
59425	2.51651	2.1
59426	5.18877	2.1
59427	1.24359	2.1
59428	1.24936	2.1
59429	1.33997	2.1
59430	1.34892	2.1
59431	1.28805	2.1
59432	1.28805	2.1
59433	1.2955	2.1
59434	1.2955	2.1
59435	1.34892	2.1
59436	1.24359	2.1
59437	1.24936	2.1
59438	1.33997	2.1
59439	1.15506	2.05612
59440	1.15506	2.1
59441	0.946128	2.1
59442	0.946128	2.1
59443	0.946131	2.1
59444	1.15506	2.07707
59445	1.06997	2.1
59446	0.946128	2.1
59447	1.06997	2.1

59448	1.15506	2.1
59449	0.946129	2.1
59450	0.946129	2.1
59451	1.70608	2.1
59452	2.13994	2.1
59453	1.33384	2.1
59454	1.43635	2.1
59455	3.14243	2.1
59456	2.13994	2.1
59457	2.31012	2.1
59458	1.43635	2.1
59459	1.23007	2.1
59460	3.74646	2.1
59461	2.46361	2.1
59462	1.89226	2.1
59463	1.89226	2.1
59464	1.89226	2.1
59465	2.39045	2.1
59466	2.39045	2.1
59467	1.89226	2.1
59468	1.89226	1.93586
59469	1.89226	2.1
59470	1.89226	1.23099
59471	3.78452	2.1
59472	1.52984	2.1
59473	2.25682	1.17221
59474	2.20831	1.13985
59475	2.20831	2.1
59476	1.61178	1.06507
59477	1.61178	2.1
59478	2.47395	2.1
59479	1.89226	2.1
59480	1.89226	2.1
59481	1.89226	2.1
59482	2.46362	2.1
59483	2.46362	2.1
59484	0.946131	0.764579
59485	0.946131	2.1
59486	0.946131	2.1
59487	0.946131	1.94348
59488	0.94613	1.33825
59489	0.94613	2.1
59490	0.946132	2.1
59491	0.946132	2.1
59492	0.946131	2.1
59493	0.946131	2.1
59494	0.946131	2.1
59495	0.946131	2.1
59496	0.982398	2.1
59497	0.982398	2.1
59498	0.931703	2.1
59499	0.97549	2.1
59500	1.11509	2.08866
59501	1.10017	1.26409
59502	0.774576	2.1
59503	0.789859	2.1
59504	0.716502	2.1
59505	1.12793	2.1

59506	1.14398	2.1
59507	0.817011	2.09887
59508	1.82499	2.1
59509	1.50524	2.1
59510	1.21304	2.1
59511	1.54191	2.1
59512	3.04715	2.1
59513	1.54191	2.1
59514	1.49823	2.1
59515	1.82499	2.1
59516	1.82499	2.1
59517	1.21147	2.1
59518	1.48977	2.1
59519	1.89226	2.1
59520	1.89226	2.1
59521	1.89226	2.1
59522	1.89226	2.1
59523	3.78452	2.1
59524	1.89226	2.1
59525	1.89226	2.1
59526	1.89226	2.1
59527	1.89226	2.1
59528	1.89226	2.1
59529	1.89226	2.1
59530	0.566674	2.1
59531	0.770957	2.1
59532	0.60652	2.1
59533	0.473557	2.1
59534	0.604441	2.1
59535	0.604441	2.1
59536	0.640073	2.1
59537	0.640073	2.1
59538	0.473557	2.1
59539	0.566674	2.1
59540	0.60652	2.1
59541	0.770956	2.1
59542	1.36203	2.1
59543	1.13335	2.1
59544	1.54191	2.1
59545	1.54191	2.1
59546	1.00965	2.1
59547	1.00965	2.1
59548	1.89226	2.1
59549	1.89226	2.1
59550	1.89226	2.1
59551	1.89226	2.1
59552	1.89226	2.1
59553	1.89226	2.1
59554	0.946131	2.1
59555	0.946131	2.1
59556	0.946131	2.1
59557	0.946131	2.1
59558	0.946132	2.1
59559	0.946132	2.1
59560	0.946131	2.1
59561	0.946131	2.1
59562	0.946132	2.1
59563	0.946132	2.1

59564	0.946131	2.1
59565	0.946131	2.1
59566	0.946132	0.05
59567	0.946132	0.05
59568	0.946133	0.05
59569	0.946133	0.05
59570	0.946132	0.05
59571	0.946132	0.05
59572	0.946132	0.05
59573	0.946132	0.05
59574	0.946132	0.05
59575	0.946132	0.05
59576	0.946133	0.05
59577	0.946133	0.05
59578	1.89226	0.05
59579	1.89226	0.05
59580	1.89226	0.05
59581	1.89226	0.05
59582	3.78453	0.05
59583	1.89226	0.05
59584	1.89226	0.05
59585	1.89226	0.05
59586	1.89226	0.05
59587	1.89226	0.05
59588	1.89226	0.05
59589	0.946132	0.05
59590	0.946132	0.05
59591	0.946131	0.05
59592	0.946131	0.05
59593	0.946132	0.05
59594	0.946132	0.05
59595	0.946132	0.05
59596	0.946132	0.05
59597	0.946132	0.05
59598	0.946132	0.05
59599	0.946132	0.05
59600	0.946132	0.05
59601	0.946129	0.05
59602	0.946129	0.05
59603	0.946128	0.05
59604	0.946128	0.05
59605	0.946132	0.05
59606	0.946132	0.05
59607	0.946126	0.05
59608	0.946126	0.05
59609	0.946129	0.05
59610	0.946129	0.05
59611	0.946129	0.05
59612	0.946129	0.05
59613	1.20674	0.05
59614	1.79877	0.05
59615	1.22586	0.05
59616	1.22586	0.05
59617	1.7919	0.05
59618	1.7919	0.05
59619	2.27622	0.05
59620	1.79877	0.05
59621	1.7919	0.05

59622	2.27102	0.05
59623	4.06979	0.05
59624	1.89226	0.05
59625	1.89226	0.05
59626	1.89226	0.05
59627	1.89226	0.05
59628	3.78452	0.05
59629	1.89226	0.05
59630	1.89226	0.05
59631	1.89226	0.05
59632	1.89226	0.05
59633	3.78453	0.05
59634	1.13259	0.05
59635	1.79187	0.05
59636	1.32741	0.05
59637	1.18784	0.05
59638	1.703 0.05	
59639	1.49575	0.05
59640	1.46201	0.05
59641	2.27099	0.05
59642	1.79187	0.05
59643	1.79187	0.05
59644	2.12923	0.05
59645	2.12923	0.05
59646	1.89224	0.05
59647	1.89224	0.05
59648	1.89224	0.05
59649	1.89224	0.05
59650	3.78447	0.05
59651	0.946119	0.05
59652	0.946119	0.05
59653	0.946118	0.05
59654	0.946118	0.05
59655	0.946118	0.05
59656	0.946118	0.05
59657	0.946119	0.05
59658	0.946119	0.05
59659	0.946131	0.05
59660	0.946131	0.05
59661	0.946106	0.05
59662	0.946106	0.05
59663	2.07526	0.228824
59664	1.89226	0.203674
59665	1.89226	0.897072
59666	2.13445	0.797228
59667	4.02671	0.178561
59668	1.89226	0.215226
59669	1.89226	0.427933
59670	1.89226	0.332636
59671	1.89226	0.05
59672	1.89226	0.914621
59673	1.89226	0.547262
59674	2.13446	0.331467
59675	1.00013	0.113577
59676	1.1475	1.08831
59677	2.20046	0.377492
59678	3.20059	0.896191
59679	0.946133	0.40791

59680	0.946133	0.576172
59681	1.06722	0.495519
59682	1.10023	0.392365
59683	0.946131	0.469516
59684	0.946131	0.0510502
59685	1.10023	0.835005
59686	1.10023	0.202243
59687	0.946131	0.822745
59688	1.10023	0.256562
59689	0.94613	0.772021
59690	1.06723	0.166977
59691	1.89226	0.102243
59692	1.89226	0.0520271
59693	1.89226	0.687791
59694	1.89226	0.503238
59695	3.78452	0.528864
59696	1.89226	0.422121
59697	1.89226	0.0561835
59698	1.89226	0.550731
59699	1.89226	0.412532
59700	1.89226	0.163624
59701	1.89226	0.103699
59702	1.89226	2.1
59703	1.89226	0.0561204
59704	1.89226	0.262248
59705	1.89226	0.0643519
59706	1.89226	2.1
59707	0.828588	0.05
59708	0.828588	0.05
59709	0.841316	0.05
59710	0.841316	2.1
59711	0.528387	2.1
59712	0.565893	0.05
59713	1.10401	0.05
59714	1.10401	2.1
59715	0.528387	2.1
59716	1.89226	2.1
59717	1.36047	0.262248
59718	1.33973	2.1
59719	1.33973	2.1
59720	1.89226	0.05
59721	2.28951	0.05
59722	1.89226	1.22338
59723	2.80796	2.06696
59724	2.01584	1.9863
59725	2.01584	1.395
59726	1.89226	1.7287
59727	1.89226	1.34901
59728	1.89226	1.00905
59729	1.89226	1.79996
59730	1.89226	1.38028
59731	1.89226	1.08908
59732	1.89226	1.39533
59733	1.89226	1.09839
59734	2.15223	1.10936
59735	1.30664	2.1
59736	1.96011	2.07537
59737	2.76516	1.19309

59738	4.11234	2.06015
59739	1.89226	0.964351
59740	1.89226	1.72803
59741	1.89226	1.23849
59742	1.89226	1.04918
59743	1.89226	1.2649
59744	1.89226	1.06555
59745	2.81143	2.1
59746	2.58517	2.1
59747	2.55318	2.1
59748	2.7542	2.1
59749	5.33937	2.1
59750	2.7777	2.1
59751	2.7542	2.1
59752	2.71672	2.1
59753	2.70401	2.1
59754	5.45821	2.1
59755	2.55318	2.1
59756	2.7542	2.1
59757	2.71672	2.1
59758	2.54198	2.1
59759	5.29618	2.1
59760	1.95416	2.1
59761	2.162 2.1	
59762	2.11563	2.1
59763	1.91813	2.1
59764	4.08013	2.1
59765	1.95417	2.1
59766	1.47712	2.1
59767	1.45182	2.1
59768	1.91813	2.1
59769	3.39525	2.1
59770	2.11562	2.1
59771	1.91813	2.1
59772	1.50979	2.1
59773	2.03974	2.1
59774	3.95787	2.1
59775	1.85605	2.1
59776	1.91813	2.1
59777	1.88885	2.1
59778	1.42255	2.1
59779	1.45182	2.1
59780	1.45182	2.1
59781	0.94613	0.675198
59782	0.94613	0.739597
59783	0.946128	0.05
59784	0.946128	0.911324
59785	0.946127	0.671164
59786	0.946127	0.44056
59787	0.946131	0.725757
59788	0.946131	0.503809
59789	0.946129	0.369379
59790	0.946129	0.385272
59791	0.946128	0.52572
59792	0.946128	0.771256
59793	0.543052	0.714878
59794	0.54608	0.7232
59795	0.602293	0.101387

59796	0.535325	0.720705
59797	1.05446	0.227724
59798	0.850496	0.424364
59799	0.699838	0.745828
59800	0.679751	0.702925
59801	0.713797	0.05
59802	0.509794	0.719916
59803	0.602304	0.376453
59804	1.40017	0.127321
59805	1.40017	2.1
59806	0.94613	0.05
59807	0.94613	2.1
59808	1.40017	2.1
59809	1.33997	2.1
59810	0.946131	0.0528847
59811	0.946131	0.05
59812	1.33997	2.01277
59813	0.946131	2.04331
59814	0.946131	1.85708
59815	1.40017	2.04877
59816	1.89226	0.05
59817	1.89226	0.05
59818	1.89226	0.05
59819	1.89226	0.05
59820	3.78452	0.05
59821	0.946133	0.05
59822	0.946133	0.05
59823	0.946132	0.05
59824	0.946132	0.05
59825	0.946132	0.05
59826	0.946132	0.05
59827	0.946133	0.05
59828	0.946133	0.05
59829	0.946132	0.05
59830	0.946132	0.05
59831	0.946132	0.05
59832	0.946132	0.05
59833	1.89224	0.05
59834	1.89224	0.05
59835	1.89224	0.05
59836	1.89224	0.05
59837	1.89224	0.05
59838	1.89224	0.05
59839	0.94612	0.05
59840	0.94612	0.05
59841	0.946119	0.05
59842	0.946119	0.05
59843	0.946119	0.05
59844	0.946119	0.05
59845	0.94612	0.05
59846	0.94612	0.05
59847	0.946132	0.05
59848	0.946132	0.05
59849	0.946107	0.05
59850	0.946107	0.05
59851	2.63939	2.1
59852	1.2862	2.1
59853	1.29891	2.1

59854	2.64419	2.1
59855	3.93039	2.1
59856	2.6394	2.1
59857	1.89226	2.1
59858	1.89226	2.1
59859	2.64419	2.1
59860	4.53646	2.1
59861	1.89226	2.1
59862	1.89226	2.1
59863	1.89226	2.1
59864	1.89226	2.1
59865	3.78453	2.1
59866	1.89226	2.1
59867	1.89226	2.1
59868	1.89226	2.1
59869	1.89226	2.1
59870	1.89226	2.1
59871	1.89226	2.1
59872	1.31998	2.1
59873	1.32209	2.1
59874	0.649456	2.1
59875	0.648474	2.1
59876	0.984995	2.1
59877	0.984995	2.1
59878	0.985573	2.1
59879	0.985573	2.1
59880	1.31999	2.1
59881	0.648472	2.1
59882	0.649455	2.1
59883	1.32209	2.1
59884	0.946133	2.1
59885	0.946133	2.1
59886	1.31999	2.1
59887	1.3221	2.1
59888	0.946132	2.1
59889	0.946132	2.1
59890	1.31999	2.1
59891	1.31999	2.1
59892	1.31999	2.1
59893	0.946132	2.1
59894	0.946133	2.1
59895	1.3221	2.1
59896	0.946132	2.1
59897	0.946132	2.1
59898	0.946131	2.1
59899	0.946131	2.1
59900	0.946132	2.1
59901	0.946132	2.1
59902	0.946131	2.1
59903	0.946131	2.1
59904	0.946131	2.1
59905	0.946131	2.1
59906	0.946132	2.1
59907	0.946132	2.1
59908	0.946128	2.1
59909	0.946128	2.1
59910	0.946132	2.1
59911	0.946132	2.1

59912	0.946131	2.1
59913	0.946131	2.1
59914	0.94613	2.1
59915	0.94613	2.1
59916	0.94613	2.1
59917	0.94613	2.1
59918	0.946131	2.1
59919	0.946131	2.1
59920	1.89226	0.05
59921	1.89226	0.473614
59922	1.89226	0.464131
59923	1.89226	0.275262
59924	1.89226	0.0956306
59925	1.89226	0.0651293
59926	1.89226	0.253514
59927	1.89226	1.11718
59928	1.89226	0.765421
59929	1.89226	1.07951
59930	3.78453	1.13971
59931	0.946133	0.344109
59932	0.946133	0.34477
59933	0.946132	0.114679
59934	0.946132	0.0792308
59935	0.946133	0.426911
59936	0.946133	0.55062
59937	0.946132	0.388662
59938	0.946132	0.0564441
59939	0.946132	0.296213
59940	0.946132	0.523786
59941	0.946132	0.12495
59942	0.946132	0.856223
59943	2.23638	0.05
59944	1.89226	0.141959
59945	2.66014	0.189337
59946	2.66014	0.32703
59947	1.89226	0.338051
59948	1.89226	0.129364
59949	1.11819	0.05
59950	1.33007	0.05
59951	0.660255	0.05
59952	0.91151	0.05
59953	0.911511	0.05
59954	1.33007	0.05
59955	1.11819	0.05
59956	0.660255	0.05
59957	0.985327	0.05
59958	0.985327	0.05
59959	1.005 0.05	
59960	1.005 0.05	
59961	1.89226	2.1
59962	1.89226	2.1
59963	1.89226	2.1
59964	1.89226	2.1
59965	3.78453	2.1
59966	1.59533	2.1
59967	2.68571	2.1
59968	1.28291	2.1
59969	2.46974	2.1

59970	4.06506	2.1
59971	2.68571	2.1
59972	1.89226	2.1
59973	1.89226	2.1
59974	1.89226	2.1
59975	2.46973	2.1
59976	2.46973	2.1
59977	1.89226	2.1
59978	1.89226	2.1
59979	1.89226	2.1
59980	1.89226	2.1
59981	1.89226	2.1
59982	1.89226	2.1
59983	1.89226	2.1
59984	1.89226	2.1
59985	1.89226	2.1
59986	1.89226	2.1
59987	1.89226	2.1
59988	1.89226	2.1
59989	0.883594	2.1
59990	0.883594	2.1
59991	0.876484	2.1
59992	0.876484	2.1
59993	1.10442	2.1
59994	1.23487	2.1
59995	0.641457	2.1
59996	0.52521	2.1
59997	1.10442	2.1
59998	0.52521	2.1
59999	0.641457	2.1
60000	1.23487	2.1
60001	0.946131	2.1
60002	0.946131	2.1
60003	0.946131	2.1
60004	0.946131	2.1
60005	0.946131	2.1
60006	0.946131	2.1
60007	0.946131	2.1
60008	0.946131	2.1
60009	0.946131	2.1
60010	0.946131	2.1
60011	0.946131	2.1
60012	0.946131	2.1
60013	2.04016	0.05
60014	2.13042	0.05
60015	2.11465	0.05
60016	2.11465	0.0559934
60017	1.95908	0.05
60018	1.95908	0.05
60019	1.66953	0.05
60020	2.13041	0.05
60021	1.95908	0.05
60022	2.21952	0.05
60023	1.66953	0.05
60024	1.92997	0.05
60025	2.04016	0.05
60026	1.95908	0.05
60027	1.82014	0.05

60028	2.08757	0.05
60029	3.86029	0.05
60030	0.834764	0.05
60031	0.722102	0.05
60032	0.979537	0.05
60033	0.910065	0.05
60034	0.840053	0.05
60035	0.840052	0.05
60036	0.861587	0.05
60037	0.861587	0.05
60038	0.910067	0.05
60039	0.722101	0.05
60040	0.834763	0.05
60041	0.979539	0.05
60042	1.89226	0.05
60043	1.89226	0.05
60044	1.89226	0.05
60045	1.89226	0.05
60046	1.89226	0.05
60047	1.89226	0.05
60048	1.89226	0.05
60049	1.89226	0.05
60050	1.89226	0.05
60051	1.89226	0.05
60052	3.78453	0.05
60053	1.89226	0.05
60054	1.89226	0.05
60055	1.89226	0.05
60056	1.89226	0.05
60057	1.89226	0.05
60058	1.89226	0.05
60059	1.20691	0.05
60060	2.27379	0.05
60061	2.58476	0.05
60062	1.5124	0.05
60063	3.79167	0.05
60064	1.89226	0.05
60065	2.2738	0.05
60066	2.58477	0.05
60067	1.89226	0.05
60068	4.47703	0.05
60069	0.952225	1.73628
60070	0.952225	2.03176
60071	0.909865	2.00364
60072	0.909865	2.1
60073	0.909895	2.1
60074	0.946132	1.77344
60075	0.946132	1.9654
60076	0.915958	1.75208
60077	0.946132	1.95408
60078	0.946132	1.93683
60079	0.909894	1.96317
60080	0.915958	2.04173
60081	0.983493	2.1
60082	2.40055	2.1
60083	1.02986	2.1
60084	1.02986	2.1
60085	2.34217	2.1

60086	2.34217	2.1
60087	1.89226	2.1
60088	2.40056	2.1
60089	2.34217	2.1
60090	2.40056	2.1
60091	1.83388	2.1
60092	1.89226	2.1
60093	1.89226	2.1
60094	1.89226	2.1
60095	1.89226	2.1
60096	1.89226	2.1
60097	1.89226	2.1
60098	1.89226	2.1
60099	1.89226	2.1
60100	1.89226	2.1
60101	1.89226	2.1
60102	1.89226	2.1
60103	1.89226	2.1
60104	1.89226	2.1
60105	0.946133	2.1
60106	0.946133	2.1
60107	1.17109	2.1
60108	1.13078	2.1
60109	0.946132	2.1
60110	0.946132	2.1
60111	1.13078	2.1
60112	1.13078	2.1
60113	1.13078	2.1
60114	0.946133	2.1
60115	0.946132	2.1
60116	1.17109	2.1
60117	1.89226	2.1
60118	1.89226	2.1
60119	1.89226	2.1
60120	1.89226	2.1
60121	1.89226	2.1
60122	1.89226	2.1
60123	2.33963	1.25446
60124	1.89227	0.969153
60125	1.74565	1.02821
60126	1.74565	1.0969
60127	1.89227	0.518789
60128	1.89227	0.799654
60129	1.89227	1.08246
60130	1.89227	0.957269
60131	1.89227	1.36689
60132	1.89227	1.01386
60133	3.78453	1.00098
60134	1.31398	0.815075
60135	1.10392	0.956947
60136	0.796878	0.89322
60137	0.524356	0.651708
60138	0.870614	0.922812
60139	0.945739	0.54738
60140	0.89208	0.961307
60141	0.89208	1.03448
60142	1.23425	0.638607
60143	0.774658	1.01533

60144	1.03182	0.624813
60145	0.557289	0.343367
60146	2.33963	1.18735
60147	1.89226	0.473171
60148	1.89226	1.13451
60149	2.77245	0.967065
60150	4.23189	1.07153
60151	1.89226	1.11553
60152	1.89226	0.787594
60153	1.89226	1.24441
60154	1.89226	1.1812
60155	1.89226	0.969459
60156	1.89226	0.682251
60157	1.89226	0.05
60158	1.89226	0.0810409
60159	1.89226	0.05
60160	1.89226	0.277644
60161	1.89226	0.05
60162	1.89226	0.228389
60163	1.89226	0.05
60164	1.89226	0.05
60165	1.89226	0.05
60166	1.89226	0.175173
60167	1.89226	0.05
60168	1.89226	0.0610197
60169	0.946128	0.05
60170	0.946128	0.05
60171	0.963918	0.05
60172	1.37543	0.05
60173	0.946129	0.05
60174	0.946129	0.05
60175	1.37542	0.05
60176	1.37542	0.05
60177	0.94613	0.05
60178	1.37542	0.05
60179	0.963916	0.05
60180	0.94613	0.05
60181	0.946132	0.05
60182	0.946132	0.05
60183	0.946131	0.05
60184	0.946131	0.05
60185	0.946131	0.05
60186	0.946131	0.0637775
60187	0.946132	0.05
60188	0.946132	0.05
60189	0.946131	0.05
60190	0.946131	0.05
60191	0.946131	0.05
60192	0.946131	0.05
60193	1.89226	0.0638719
60194	1.89226	0.103889
60195	1.89226	0.05
60196	1.89226	0.172851
60197	3.78452	0.0663194
60198	0.946127	0.113138
60199	0.946127	0.05
60200	0.946131	0.0504355
60201	0.946131	0.05

60202	0.946128	0.05
60203	0.946128	0.0826803
60204	0.94613	0.05
60205	0.94613	0.05
60206	0.946129	0.05
60207	0.946129	0.05
60208	0.946129	0.05
60209	0.946129	0.05
60210	1.89226	0.557272
60211	1.89226	1.1597
60212	1.89226	2.1
60213	1.89226	0.301631
60214	3.78453	0.183286
60215	1.89226	0.241636
60216	1.89226	0.05
60217	1.89226	0.0638139
60218	1.89226	0.154657
60219	3.78453	0.331451
60220	0.946131	0.05
60221	0.946131	0.05
60222	0.946132	1.3403
60223	0.946132	0.388409
60224	0.946132	1.18268
60225	0.946132	0.196746
60226	0.946132	1.18264
60227	0.946132	0.367961
60228	0.946132	0.381092
60229	0.946132	0.900015
60230	0.946132	1.07898
60231	0.946132	0.22048
60232	0.548169	0.05
60233	0.736603	0.05
60234	1.18322	0.05
60235	1.01139	0.05
60236	1.01139	0.05
60237	0.548168	0.05
60238	0.736603	0.05
60239	1.18322	0.05
60240	0.861589	0.05
60241	0.861589	0.05
60242	0.869798	0.05
60243	0.869798	0.05
60244	1.16037	0.05
60245	0.942012	0.05
60246	0.942012	0.05
60247	0.62356	0.05
60248	0.891932	0.05
60249	0.900255	0.05
60250	2.02279	0.05
60251	1.48677	0.05
60252	1.54724	0.05
60253	0.831456	0.05
60254	1.19775	0.05
60255	1.89226	1.44252
60256	2.76291	1.47731
60257	1.89226	1.62745
60258	1.89226	1.34137
60259	3.78452	1.53913

60260	1.89226	1.10382
60261	1.89226	1.39099
60262	1.89226	1.74825
60263	1.89226	1.16823
60264	1.89226	1.67361
60265	1.89226	1.15973
60266	1.89226	1.49105
60267	1.89226	1.20832
60268	1.89226	1.34511
60269	1.89226	1.32687
60270	3.78452	1.26185
60271	1.89226	1.04615
60272	1.89226	1.39686
60273	1.89226	1.64299
60274	1.89226	1.08241
60275	3.78453	1.28471
60276	0.946128	1.09922
60277	0.946128	1.13588
60278	0.946129	1.53859
60279	0.946129	1.32825
60280	0.946132	1.07906
60281	0.946132	1.29141
60282	0.946126	1.34286
60283	0.946126	1.09069
60284	0.946129	1.24034
60285	0.946129	1.24124
60286	0.946129	1.21177
60287	0.946129	1.18168
60288	2.80495	1.36784
60289	1.89225	1.21731
60290	1.89225	1.45745
60291	1.87794	1.44516
60292	1.89225	1.29448
60293	1.87794	1.27925
60294	2.25195	0.376787
60295	2.00624	0.60006
60296	2.13132	0.131748
60297	1.90239	0.05
60298	4.13756	0.05
60299	0.804661	0.05
60300	0.804663	0.05
60301	0.889591	0.05
60302	0.889591	0.577737
60303	0.808324	0.05
60304	0.761765	0.05
60305	1.02822	0.233805
60306	0.879624	0.05
60307	0.879617	0.05
60308	0.800864	0.05
60309	0.978023	0.150989
60310	0.808329	0.113857
60311	2.13133	0.558152
60312	2.00558	0.05
60313	2.09316	0.05
60314	2.09316	0.968235
60315	2.03253	0.05
60316	2.03253	1.11917
60317	0.84577	0.05

60318	0.845767	0.687453
60319	0.843917	0.05
60320	0.843917	0.781769
60321	0.76177	0.05
60322	0.719345	0.11182
60323	0.934435	0.0793061
60324	0.884197	0.102218
60325	0.934439	0.05
60326	0.75627	0.05
60327	0.849424	0.05
60328	0.76177	0.476907
60329	2.61855	0.0596341
60330	1.17793	0.0512744
60331	1.42965	0.148977
60332	1.17793	0.0674448
60333	2.50057	0.05
60334	2.24885	0.05
60335	0.946133	0.0931054
60336	0.946133	0.0685319
60337	1.30928	0.117172
60338	1.12443	0.05
60339	0.946132	0.0506474
60340	0.946132	0.05
60341	1.30928	0.05
60342	1.30928	0.05
60343	0.946132	0.07034
60344	1.30928	0.0564724
60345	1.12443	0.05
60346	0.946133	0.05
60347	1.89226	0.05
60348	1.89226	0.118703
60349	1.89226	0.258142
60350	1.89226	0.18228
60351	3.78452	0.14575
60352	1.89226	0.221064
60353	1.89226	0.100032
60354	1.89226	0.0501239
60355	1.89226	0.080817
60356	1.89226	0.0800234
60357	1.89226	0.05
60358	1.89226	0.0795133
60359	1.89226	0.0639389
60360	1.89226	0.0902941
60361	1.89226	0.506188
60362	3.78452	0.088782
60363	0.94613	0.0848482
60364	0.94613	0.125877
60365	0.946131	0.215479
60366	0.946131	0.0789557
60367	0.946128	0.0963954
60368	0.946128	0.0703835
60369	0.946133	0.075597
60370	0.946133	0.0552832
60371	0.946131	0.0899704
60372	0.946131	0.264533
60373	0.946131	0.071781
60374	0.946131	0.05
60375	1.89226	2.1

60376	1.89226	2.1
60377	1.89226	2.1
60378	1.89226	2.1
60379	3.78453	2.1
60380	1.0465	2.1
60381	2.73926	2.1
60382	2.03721	2.1
60383	2.03721	2.1
60384	1.44709	2.1
60385	1.44709	2.1
60386	0.946131	2.1
60387	0.946131	2.1
60388	0.946131	2.1
60389	0.946131	2.1
60390	0.946132	2.1
60391	0.946132	2.1
60392	0.94613	2.1
60393	0.94613	2.1
60394	0.946131	2.1
60395	0.946131	2.1
60396	0.946131	2.1
60397	0.946131	2.1
60398	0.946132	2.1
60399	0.946132	2.1
60400	0.946131	2.1
60401	0.946131	2.1
60402	0.946132	2.1
60403	0.946132	2.1
60404	0.946132	2.1
60405	0.946132	2.1
60406	0.946132	2.1
60407	0.946132	2.1
60408	0.946132	2.1
60409	0.946132	2.1
60410	1.89226	2.1
60411	1.89226	2.1
60412	1.89226	2.1
60413	1.89226	2.1
60414	3.78452	2.1
60415	1.61969	0.05
60416	1.79374	0.05
60417	1.29382	0.05
60418	1.29382	0.05
60419	2.05759	0.05
60420	2.05759	0.05
60421	1.89226	0.05
60422	1.89226	0.05
60423	1.89226	0.05
60424	1.89226	0.05
60425	1.89226	0.05
60426	1.89226	0.05
60427	1.89226	0.868279
60428	1.89226	0.977642
60429	1.89226	0.918589
60430	1.89226	0.96124
60431	1.89226	0.788923
60432	1.89226	0.873567
60433	1.89226	1.41092

60434	1.89226	1.07396
60435	1.89226	1.21102
60436	1.89226	1.28895
60437	3.78453	1.42073
60438	0.946131	1.04056
60439	0.946131	1.0695
60440	0.946131	0.975137
60441	0.946131	1.03516
60442	0.946131	0.977122
60443	0.946131	0.955489
60444	0.946131	1.05476
60445	0.946131	1.13739
60446	0.946131	1.02832
60447	0.946131	1.06269
60448	0.946131	1.03959
60449	0.946131	0.99399
60450	2.45177	0.05
60451	2.68507	0.05
60452	2.3376	0.05
60453	2.22086	0.05
60454	4.78937	0.05
60455	2.45178	0.05
60456	2.35552	0.05
60457	1.68062	0.05
60458	1.81902	0.05
60459	4.13239	0.05
60460	2.3376	0.05
60461	1.55612	0.05
60462	1.69811	0.05
60463	2.31527	0.05
60464	1.79553	0.05
60465	2.3376	0.05
60466	1.55613	0.05
60467	1.06336	0.05
60468	1.97516	0.05
60469	1.88966	0.05
60470	1.06336	0.05
60471	1.68062	0.05
60472	1.89226	1.07659
60473	1.91634	0.931547
60474	2.03709	1.48682
60475	1.91634	1.06074
60476	1.83185	1.02113
60477	1.70303	0.383532
60478	1.89226	0.975289
60479	1.89226	0.766449
60480	1.89226	1.30916
60481	1.89226	1.00404
60482	1.89226	0.521781
60483	1.89226	0.159342
60484	1.89226	2.08733
60485	2.03472	2.1
60486	2.22183	2.1
60487	2.03472	2.03194
60488	2.07936	2.1
60489	1.89226	1.11167
60490	1.89226	1.00813
60491	1.8519	0.40387

60492	1.92119	1.18582
60493	1.8519	0.991741
60494	1.96156	0.600262
60495	1.89226	0.197634
60496	1.89226	0.928502
60497	1.89226	0.781278
60498	1.89226	1.16066
60499	1.89226	0.96413
60500	1.89226	0.411372
60501	1.89226	0.105962
60502	1.89226	1.33113
60503	2.22183	1.9337
60504	2.39541	2.1
60505	2.22183	1.22023
60506	2.06585	1.72978
60507	1.89226	1.05078
60508	1.47001	2.1
60509	1.57962	0.820635
60510	1.65238	1.94005
60511	1.60502	2.1
60512	3.18464	2.1
60513	1.02415	2.1
60514	1.60503	2.1
60515	1.47001	2.1
60516	1.47001	2.1
60517	1.28228	2.1
60518	1.28228	2.1
60519	0.946133	2.1
60520	0.946133	2.1
60521	0.946132	2.1
60522	0.946132	2.07234
60523	0.946132	2.08974
60524	0.946132	2.1
60525	0.946133	2.1
60526	0.946133	2.1
60527	0.946132	2.1
60528	0.946132	2.1
60529	0.946133	2.1
60530	0.946133	2.1
60531	1.89226	0.59277
60532	1.89226	1.36028
60533	1.89226	2.1
60534	1.89226	0.229335
60535	3.78452	0.505301
60536	1.76897	1.57289
60537	1.60502	1.9793
60538	1.65238	1.17874
60539	1.65238	2.1
60540	1.76932	0.806026
60541	1.76932	2.1
60542	1.55998	2.1
60543	1.60503	2.1
60544	1.76932	2.1
60545	1.60503	2.1
60546	1.44657	2.1
60547	1.28228	2.1
60548	0.946133	2.1
60549	0.946133	2.1

60550	0.946132	2.1
60551	0.946132	1.26132
60552	0.946132	1.24071
60553	0.946132	2.1
60554	0.946133	2.1
60555	0.946133	2.1
60556	0.946132	2.1
60557	0.946132	2.1
60558	0.946132	2.1
60559	0.946132	2.1
60560	0.946128	1.99338
60561	0.946128	0.221992
60562	0.946133	0.0724958
60563	0.946133	0.255687
60564	0.94613	0.07985
60565	0.94613	0.466121
60566	0.946131	2.0048
60567	0.946131	0.498022
60568	0.946131	0.347191
60569	0.946131	0.397202
60570	0.946131	0.460382
60571	0.946131	0.707734
60572	1.85779	0.05
60573	1.00763	0.05
60574	1.74874	0.05
60575	1.74873	0.05
60576	1.14467	0.05
60577	1.14466	0.05
60578	1.85779	0.05
60579	1.61597	0.05
60580	1.00053	0.05
60581	1.03018	0.05
60582	2.65819	0.05
60583	1.89226	0.157963
60584	1.89226	0.0561663
60585	1.89226	0.195803
60586	1.89226	0.176033
60587	3.78451	0.139631
60588	1.89226	0.136784
60589	1.89226	0.05
60590	1.89226	0.05
60591	1.89226	0.05
60592	1.89226	0.05
60593	1.89226	0.05
60594	1.11983	0.05
60595	0.810945	0.05
60596	0.913906	0.05
60597	0.913906	0.05
60598	2.23303	0.05
60599	1.66057	0.05
60600	1.28724	0.05
60601	1.08132	0.05
60602	1.49316	0.05
60603	0.810958	0.05
60604	0.810958	0.05
60605	0.827861	0.05
60606	0.946126	0.0563302
60607	0.946132	0.131003

60608	0.946132	0.05
60609	0.827863	0.0612519
60610	0.827863	0.0557038
60611	0.946129	0.0632256
60612	0.946129	0.05
60613	0.827863	0.0569387
60614	0.946128	0.063814
60615	0.946129	0.05
60616	0.946129	0.0551442
60617	0.974718	0.05
60618	1.53671	0.05
60619	2.80542	0.05
60620	2.27259	0.05
60621	3.8093	0.05
60622	1.89226	0.05
60623	1.89226	0.164436
60624	1.89226	0.05
60625	1.89226	0.0823109
60626	3.78453	0.0843479
60627	0.946132	0.05
60628	0.946132	0.05
60629	0.946131	0.0782246
60630	0.946131	0.05
60631	0.946132	0.0781974
60632	0.946132	0.0998387
60633	0.946131	0.05
60634	0.946131	0.05
60635	0.946132	0.05
60636	0.946132	0.0885633
60637	0.946131	0.05
60638	0.946131	0.05
60639	2.80542	0.05
60640	1.89226	0.05
60641	2.27259	0.05
60642	2.27259	0.0608681
60643	1.89226	0.05
60644	1.89226	0.05
60645	1.89226	0.05
60646	1.89226	0.124921
60647	1.89226	0.05
60648	1.89226	0.121258
60649	1.89226	0.05
60650	1.89226	0.140367
60651	0.946132	0.129759
60652	0.946132	0.05
60653	0.946131	0.102266
60654	0.946131	0.0508505
60655	0.946132	0.102546
60656	0.946132	0.10345
60657	0.946131	0.05
60658	0.946131	0.05
60659	0.946131	0.05
60660	0.946131	0.0583539
60661	0.946131	0.05
60662	0.946131	0.0548401
60663	2.68213	2.1
60664	2.58234	2.1
60665	2.58234	2.1

60666	2.61243	2.1
60667	2.67035	2.1
60668	2.70044	2.1
60669	2.68213	2.1
60670	2.53921	2.1
60671	2.53303	2.1
60672	2.67035	2.1
60673	5.20956	2.1
60674	2.67033	2.1
60675	2.58232	2.1
60676	2.55004	2.1
60677	2.64567	2.1
60678	5.228 2.1	
60679	2.53301	2.1
60680	2.64567	2.1
60681	2.64567	2.1
60682	2.67033	2.1
60683	2.51603	2.1
60684	2.54069	2.1
60685	0.907497	2.1
60686	0.907497	2.1
60687	0.926641	2.1
60688	0.926641	2.1
60689	1.14989	2.1
60690	1.10046	2.1
60691	0.695334	2.1
60692	0.684247	2.1
60693	0.695333	2.1
60694	1.14989	2.1
60695	0.684246	2.1
60696	1.10046	2.1
60697	0.946126	2.1
60698	0.946126	2.1
60699	0.946131	2.1
60700	0.946131	2.1
60701	0.946127	2.1
60702	0.946127	2.1
60703	0.94613	2.1
60704	0.94613	2.1
60705	0.946128	2.1
60706	0.946128	2.1
60707	0.946128	2.1
60708	0.946128	2.1
60709	1.89226	2.1
60710	1.89226	2.1
60711	1.89226	2.1
60712	1.89226	2.1
60713	1.89226	2.1
60714	1.89226	1.14346
60715	1.42174	2.1
60716	2.29978	2.1
60717	2.41233	2.1
60718	2.41233	2.1
60719	1.39067	2.1
60720	1.39067	2.1
60721	1.89226	2.1
60722	1.89226	2.1
60723	1.89226	2.1

60724	1.89226	2.1
60725	3.78452	2.1
60726	1.89226	2.1
60727	1.89226	2.1
60728	1.89226	2.1
60729	1.89226	2.1
60730	1.89226	2.1
60731	1.89226	2.1
60732	2.41234	2.1
60733	1.89226	2.1
60734	2.29979	2.1
60735	1.89226	2.1
60736	1.89226	2.1
60737	2.29979	2.1
60738	1.09506	0.05
60739	2.82921	0.117096
60740	2.53251	0.05
60741	2.53251	0.112471
60742	1.42901	0.05
60743	1.42901	0.157811
60744	0.946132	0.323399
60745	0.946132	0.193231
60746	0.946128	0.05
60747	0.946128	0.201519
60748	0.94613	0.247907
60749	0.94613	0.358128
60750	0.946131	0.226573
60751	0.946131	0.131977
60752	0.94613	0.214517
60753	0.94613	0.137883
60754	0.94613	0.239802
60755	0.94613	0.421714
60756	1.89226	0.413935
60757	1.89226	0.537548
60758	1.89226	0.551478
60759	1.89226	0.622877
60760	3.78452	0.302547
60761	2.53251	0.0810063
60762	1.89226	0.140181
60763	2.82921	0.0743247
60764	1.89226	0.0827314
60765	1.89226	0.616829
60766	2.82921	0.675527
60767	1.89226	0.198526
60768	1.89226	0.084227
60769	1.89226	0.34164
60770	1.89226	0.314934
60771	1.89226	0.116229
60772	1.89226	0.117941
60773	2.53251	0.05
60774	1.89226	0.0838532
60775	2.06158	0.05
60776	1.89226	0.05
60777	4.42477	0.05
60778	1.23296	2.1
60779	2.55352	2.1
60780	2.47476	2.1
60781	2.47476	2.1

60782	1.32368	2.1
60783	1.32368	2.1
60784	1.89226	2.1
60785	1.89226	2.1
60786	1.89226	2.1
60787	1.89226	2.1
60788	1.89226	2.1
60789	1.89226	2.1
60790	1.89226	2.1
60791	1.89226	2.1
60792	1.89226	2.1
60793	1.89226	2.1
60794	1.89226	2.1
60795	1.89226	2.1
60796	2.47476	2.1
60797	1.89226	2.1
60798	1.89226	2.1
60799	1.89226	2.1
60800	2.55353	2.1
60801	2.55353	2.1
60802	1.05983	2.1
60803	2.47476	2.1
60804	2.37664	2.1
60805	2.37664	2.1
60806	1.23296	2.1
60807	1.23296	2.1
60808	0.946127	2.1
60809	0.946127	2.1
60810	0.946131	2.1
60811	0.946131	2.1
60812	0.946127	2.1
60813	0.946127	2.1
60814	0.946131	2.1
60815	0.946131	2.1
60816	0.946129	2.1
60817	0.946129	2.1
60818	0.946128	2.1
60819	0.946128	2.1
60820	1.89226	2.1
60821	1.89226	2.1
60822	1.89226	2.1
60823	1.89226	2.1
60824	3.78452	2.1
60825	2.37665	2.1
60826	1.89226	2.1
60827	1.89226	2.1
60828	1.89226	2.1
60829	2.47476	2.1
60830	2.47476	2.1
60831	1.89226	0.944277
60832	1.92119	0.165158
60833	1.99586	1.06854
60834	1.92119	0.952272
60835	1.96693	0.268168
60836	1.89226	0.12968
60837	1.89226	0.894571
60838	1.89226	0.454335
60839	1.89226	1.05741

60840	1.89226	0.914201
60841	1.89226	0.33656
60842	1.89226	0.0505405
60843	1.89226	1.13533
60844	2.39541	1.26906
60845	2.57183	1.8229
60846	2.39541	1.1011
60847	2.06868	1.05462
60848	1.89226	0.738666
60849	0.946133	0.761197
60850	0.946133	0.625944
60851	0.997928	0.344902
60852	1.03763	0.787218
60853	0.946131	0.80351
60854	0.946131	0.05
60855	1.03763	0.851949
60856	1.03763	0.160953
60857	1.03763	0.693275
60858	0.946131	0.836873
60859	0.94613	0.78571
60860	0.99793	0.110145
60861	1.89226	0.0949984
60862	1.89226	0.909139
60863	1.89226	0.905958
60864	1.89226	0.05
60865	1.89226	1.01684
60866	1.89226	0.243257
60867	0.946131	0.70844
60868	0.946131	0.720918
60869	1.36939	0.87994
60870	1.28592	1.17787
60871	0.946129	1.11148
60872	0.946129	0.05
60873	1.36939	0.858391
60874	1.36939	0.926539
60875	0.946131	0.697866
60876	1.36939	0.620823
60877	0.946131	0.977411
60878	1.28592	0.397036
60879	1.89226	0.760743
60880	1.89226	0.850014
60881	1.89226	0.812636
60882	1.89226	0.790195
60883	3.78453	0.80486
60884	1.89226	0.850149
60885	1.89226	0.821608
60886	1.89226	0.794248
60887	1.89226	0.873501
60888	3.78452	0.821277
60889	1.91005	0.811813
60890	1.89226	0.853608
60891	2.60215	0.763681
60892	2.60215	0.801565
60893	1.89226	0.803577
60894	1.89226	0.883394
60895	0.946132	0.837623
60896	0.946132	0.811381
60897	0.946132	0.809808

60898	0.946132	0.78141
60899	0.946132	0.811615
60900	0.946132	0.839075
60901	0.946132	0.814318
60902	0.946132	0.771735
60903	0.946132	0.807654
60904	0.946132	0.810596
60905	0.946131	0.8003
60906	0.946131	0.802264
60907	1.89226	0.812925
60908	1.89226	0.874364
60909	1.89226	0.868807
60910	1.89226	0.846776
60911	1.89226	0.844845
60912	1.89226	0.869817
60913	0.780483	0.837433
60914	0.78928	0.943439
60915	0.818566	0.889228
60916	0.460387	0.933485
60917	1.82793	0.813047
60918	1.32039	0.868226
60919	0.613889	0.808863
60920	1.0592	0.891612
60921	1.48698	0.847839
60922	0.818555	0.872278
60923	1.11515	0.86825
60924	1.2802	0.999197
60925	2.20046	0.209852
60926	1.1475	0.700007
60927	1.1475	0.189344
60928	2.27062	0.73349
60929	2.27062	0.170718
60930	1.89226	0.859168
60931	2.20046	0.242011
60932	2.27062	0.486895
60933	2.20046	0.237783
60934	1.96242	0.475671
60935	1.89226	0.261836
60936	1.89226	0.225821
60937	1.89226	0.0802384
60938	1.89226	0.798229
60939	1.89226	0.262642
60940	3.78452	0.119524
60941	1.89226	0.234733
60942	1.89226	0.05
60943	1.89226	0.780861
60944	1.89226	0.526514
60945	3.78452	0.411254
60946	1.17403	0.152874
60947	1.13531	0.633445
60948	0.6401	0.157271
60949	0.714566	0.751576
60950	0.915987	0.22959
60951	0.915987	0.05
60952	0.933891	0.897658
60953	0.933891	0.162672
60954	1.17403	0.67709
60955	0.714566	0.231686

60956	0.6401	0.614232
60957	1.13531	0.267422
60958	0.946133	0.265245
60959	0.946133	0.358113
60960	1.17403	0.169184
60961	1.13531	0.225408
60962	0.946131	0.177547
60963	0.946131	0.0696097
60964	1.17403	0.674045
60965	1.17403	0.05
60966	1.17403	0.182607
60967	0.94613	0.263483
60968	0.946131	0.664535
60969	1.13531	0.096307
60970	1.89226	0.0873723
60971	1.89226	0.174629
60972	1.89226	0.05
60973	1.89226	0.05
60974	1.89226	0.684113
60975	1.89226	0.202806
60976	0.946131	0.113686
60977	0.946131	0.0537443
60978	0.946131	0.176486
60979	0.946131	0.145687
60980	0.946129	0.262923
60981	0.946129	0.05
60982	0.946133	0.36141
60983	0.946133	0.05
60984	0.946131	0.0926169
60985	0.946131	0.142283
60986	0.946131	0.05
60987	0.946131	0.0690934
60988	2.29413	2.1
60989	1.99332	2.1
60990	1.84774	2.1
60991	2.21582	2.1
60992	4.20915	2.1
60993	0.517359	2.1
60994	0.634469	2.1
60995	0.996661	2.1
60996	0.92387	2.1
60997	0.768098	2.1
60998	0.768098	2.1
60999	0.790242	2.1
61000	0.790242	2.1
61001	0.923871	2.1
61002	0.517359	2.1
61003	0.634469	2.1
61004	0.996661	2.1
61005	1.89226	2.1
61006	1.89226	2.1
61007	1.89226	2.1
61008	1.89226	2.1
61009	3.78453	2.1
61010	0.946132	2.1
61011	0.946132	2.1
61012	0.946131	2.1
61013	0.946131	2.1

61014	0.946132	2.1
61015	0.946132	2.1
61016	0.946131	2.1
61017	0.946131	2.1
61018	0.946132	2.1
61019	0.946132	2.1
61020	0.946131	2.1
61021	0.946131	2.1
61022	1.62708	2.1
61023	2.21582	2.1
61024	2.13925	2.1
61025	2.21582	2.1
61026	1.77117	2.1
61027	1.84774	2.1
61028	0.946132	2.1
61029	0.946132	2.1
61030	0.946131	2.1
61031	0.946131	2.1
61032	0.946132	2.1
61033	0.946132	2.1
61034	0.946131	2.1
61035	0.946131	2.1
61036	0.946131	2.1
61037	0.946131	2.1
61038	0.946131	2.1
61039	0.946131	2.1
61040	1.89226	1.88738
61041	1.89226	0.908736
61042	1.89226	0.279576
61043	1.89226	1.19785
61044	3.78453	1.29351
61045	1.89226	0.65415
61046	1.89226	1.38603
61047	1.89226	1.28176
61048	1.89226	2.1
61049	1.89226	0.414921
61050	1.89226	1.20659
61051	1.89226	0.194153
61052	1.89226	2.02673
61053	1.89226	0.070555
61054	1.89226	1.76238
61055	1.89226	0.494647
61056	1.89226	2.1
61057	0.914369	0.818222
61058	0.914369	1.34037
61059	0.810945	2.1
61060	0.810945	1.06178
61061	2.26581	2.1
61062	1.66199	2.1
61063	1.49503	1.42405
61064	1.28816	2.1
61065	1.08133	1.92586
61066	0.810933	1.95296
61067	1.1212	1.91348
61068	1.89227	0.794784
61069	1.89227	0.737415
61070	1.89227	0.732392
61071	1.89227	0.829249

61072	3.78453	0.754807
61073	1.89226	0.739685
61074	1.89226	0.877052
61075	1.89226	0.861179
61076	1.89226	0.796764
61077	3.78453	0.835822
61078	1.89226	0.881396
61079	1.89226	0.801179
61080	1.89226	0.800713
61081	1.89226	0.91872
61082	3.78453	0.829322
61083	1.89226	0.74322
61084	1.89226	0.885052
61085	1.89226	0.834135
61086	2.25318	0.784613
61087	3.78453	0.846827
61088	1.89226	0.771873
61089	1.89226	0.880511
61090	1.89226	0.82127
61091	1.9637	0.793249
61092	3.78453	0.84155
61093	1.89226	0.872463
61094	1.89226	0.826838
61095	1.89226	0.798859
61096	1.89226	0.899392
61097	3.78453	0.832632
61098	1.89226	0.832858
61099	1.89226	0.959902
61100	1.89226	0.901107
61101	2.53384	0.86504
61102	3.78453	0.915983
61103	1.55405	0.781128
61104	1.03818	1.24013
61105	1.03818	0.7122
61106	1.34857	0.857915
61107	1.46338	0.792426
61108	1.77377	1.03134
61109	1.86273	0.796782
61110	1.73802	0.830996
61111	1.50969	1.6641
61112	1.46337	0.972969
61113	3.20139	0.876618
61114	1.2377	1.00638
61115	1.46337	0.799119
61116	1.27408	0.875966
61117	1.32039	1.28446
61118	1.50969	0.95534
61119	1.50969	0.79387
61120	1.89226	2.1
61121	1.89226	2.1
61122	1.89226	2.1
61123	1.89226	2.1
61124	1.89226	2.1
61125	1.89226	2.1
61126	1.89169	2.1
61127	1.89226	2.1
61128	1.89226	2.1
61129	1.89226	2.1

61130	1.96624	2.1
61131	1.96624	2.1
61132	2.62871	2.1
61133	1.6201	2.1
61134	1.49149	2.1
61135	1.6201	2.1
61136	2.62559	2.1
61137	2.75419	2.1
61138	0.946129	1.0843
61139	0.946129	2.1
61140	0.946133	2.1
61141	0.946133	2.1
61142	0.946131	2.1
61143	0.946131	1.02966
61144	0.946131	2.1
61145	0.946131	2.1
61146	0.946131	2.1
61147	0.946131	2.1
61148	0.946131	2.1
61149	0.946131	2.1
61150	0.851543	2.1
61151	0.851543	2.1
61152	1.04043	2.1
61153	0.997939	2.1
61154	0.946132	2.1
61155	0.946132	2.1
61156	0.894185	2.1
61157	0.893901	2.1
61158	0.998267	2.1
61159	1.04074	2.1
61160	0.85127	2.1
61161	0.85153	2.1
61162	1.89226	2.1
61163	1.89226	2.1
61164	1.89226	2.1
61165	1.89226	2.1
61166	1.89226	2.1
61167	1.89226	2.1
61168	0.928691	2.1
61169	0.928691	2.1
61170	0.935332	2.1
61171	0.935332	2.1
61172	1.398	2.1
61173	1.14418	2.1
61174	0.479331	2.1
61175	0.719848	2.1
61176	1.398	2.1
61177	0.719848	2.1
61178	1.14417	2.1
61179	0.47933	2.1
61180	2.28835	2.1
61181	1.89226	2.1
61182	1.89226	2.1
61183	1.89226	2.1
61184	2.796	2.1
61185	2.796	2.1
61186	0.946131	2.1
61187	0.946131	2.1

61188	0.946131	2.1
61189	0.946131	2.1
61190	0.946128	2.1
61191	0.946128	2.1
61192	0.946133	2.1
61193	0.946133	2.1
61194	0.946131	2.1
61195	0.946131	2.1
61196	0.94613	2.1
61197	0.94613	2.1
61198	0.946131	2.1
61199	0.946131	2.1
61200	0.946131	2.1
61201	0.946131	2.1
61202	0.946131	2.1
61203	0.946131	2.1
61204	0.946131	2.1
61205	0.946131	2.1
61206	0.946131	2.1
61207	0.946131	2.1
61208	0.946131	2.1
61209	0.946131	2.1
61210	1.89226	2.1
61211	1.89226	2.1
61212	1.89226	2.1
61213	1.89226	2.1
61214	3.78452	2.1
61215	2.05207	0.69006
61216	1.70302	0.944019
61217	1.33794	1.84639
61218	1.98194	0.909504
61219	3.36873	0.96211
61220	2.62761	0.820878
61221	1.98194	0.904361
61222	2.28009	1.17091
61223	2.28009	0.805737
61224	2.22939	0.96691
61225	2.22939	0.654043
61226	1.89226	0.914003
61227	1.89226	0.403751
61228	1.89226	0.849305
61229	1.89226	0.804465
61230	1.89226	0.891268
61231	1.89226	0.861499
61232	1.89227	0.917133
61233	1.89227	0.91811
61234	1.89227	1.67546
61235	1.89227	1.05663
61236	1.89227	0.293758
61237	1.89227	0.068172
61238	0.888355	0.772203
61239	0.990973	0.969144
61240	0.71133	0.976403
61241	0.634324	1.10219
61242	0.806209	1.03673
61243	0.806209	0.0836322
61244	0.819087	1.28935
61245	0.819087	1.07394

61246	0.888355	1.08642
61247	0.634324	1.07209
61248	0.71133	0.956821
61249	0.990972	0.940391
61250	1.11469	0.951394
61251	0.985023	0.905871
61252	0.888354	0.820201
61253	0.990972	0.999723
61254	0.994754	0.923961
61255	0.994754	0.82134
61256	1.0083	1.06207
61257	1.0083	0.785469
61258	0.888354	0.840603
61259	0.985024	0.901738
61260	0.990971	0.824139
61261	1.11469	0.769997
61262	0.946132	0.973974
61263	0.946132	0.83726
61264	0.946132	0.847013
61265	0.946132	0.825507
61266	0.946132	0.851244
61267	0.946132	0.702113
61268	0.946133	1.12646
61269	0.946133	0.870912
61270	0.946133	0.896323
61271	0.946133	0.889999
61272	0.946132	0.806565
61273	0.946132	0.815126
61274	0.810944	0.873409
61275	0.810944	0.808143
61276	0.810947	0.962567
61277	0.810947	0.101439
61278	1.89227	1.24676
61279	1.35168	0.940618
61280	1.08132	0.933133
61281	1.08131	0.958336
61282	1.08132	0.767385
61283	0.810959	0.684516
61284	0.810959	0.813347
61285	1.70303	2.1
61286	2.03975	2.1
61287	1.99324	2.1
61288	2.17615	2.1
61289	1.40549	2.1
61290	1.53446	2.1
61291	0.946133	2.1
61292	0.946133	2.1
61293	0.946129	2.1
61294	0.946129	2.1
61295	0.946131	2.1
61296	0.946131	2.1
61297	0.946131	2.1
61298	0.946131	2.1
61299	0.946131	2.1
61300	0.946131	2.1
61301	0.946131	2.1
61302	0.946131	2.1
61303	1.89226	2.1

61304	1.89226	2.1
61305	1.89226	2.1
61306	1.89226	2.1
61307	1.89226	2.1
61308	1.89226	2.1
61309	2.17615	2.1
61310	2.3111	2.1
61311	2.50288	2.1
61312	2.50288	2.1
61313	2.03975	2.1
61314	2.03975	2.1
61315	1.76401	2.1
61316	1.95852	2.1
61317	2.32134	2.1
61318	2.12306	2.1
61319	1.8513	2.1
61320	1.70303	2.1
61321	1.89226	2.1
61322	1.89226	2.1
61323	1.89226	2.1
61324	1.89226	2.1
61325	3.78452	2.1
61326	1.89226	2.1
61327	1.89226	1.18195
61328	1.89226	2.1
61329	1.89226	2.1
61330	3.78452	2.1
61331	1.2243	2.1
61332	1.2243	2.1
61333	1.24189	2.1
61334	1.24189	2.1
61335	1.37811	2.1
61336	1.25144	2.1
61337	1.08807	2.1
61338	1.17949	2.1
61339	1.17949	2.1
61340	1.37811	2.1
61341	1.08807	2.1
61342	1.25144	2.1
61343	1.89226	2.1
61344	1.89226	2.1
61345	1.89226	2.1
61346	1.89226	2.1
61347	1.89226	2.1
61348	1.89226	2.1
61349	1.89227	2.1
61350	1.89227	2.1
61351	1.89227	2.1
61352	1.89227	2.1
61353	1.89227	2.1
61354	1.89227	2.1
61355	1.89224	2.1
61356	1.89224	2.1
61357	1.89224	2.1
61358	1.89224	2.1
61359	3.78447	2.1
61360	1.89224	2.1
61361	1.89224	2.1

61362	1.89224	2.1
61363	1.89224	2.1
61364	1.89224	2.1
61365	1.89224	2.1
61366	1.89224	2.1
61367	1.89224	2.1
61368	1.89224	2.1
61369	1.89224	2.1
61370	3.78448	2.1
61371	1.89224	2.1
61372	1.703 2.1	
61373	1.89224	2.1
61374	1.72991	2.1
61375	1.07643	2.1
61376	1.36657	2.1
61377	1.44472	2.1
61378	2.41233	2.1
61379	2.51628	2.1
61380	2.51628	2.1
61381	1.42174	2.1
61382	1.42174	2.1
61383	1.89226	2.1
61384	1.89226	2.1
61385	1.89226	2.1
61386	1.89226	2.1
61387	1.89226	2.1
61388	1.89226	2.1
61389	1.89226	2.1
61390	1.89226	2.1
61391	1.89226	2.1
61392	1.89226	1.88677
61393	3.78452	2.1
61394	2.51628	2.1
61395	1.89226	2.1
61396	1.89226	2.1
61397	1.89226	2.1
61398	2.41234	2.1
61399	2.41234	2.1
61400	0.996254	2.1
61401	0.996254	2.1
61402	1.01153	2.1
61403	1.01153	2.1
61404	1.28542	2.1
61405	1.25814	2.1
61406	0.719184	2.1
61407	0.72236	2.1
61408	0.719183	2.1
61409	1.28542	2.1
61410	0.722359	2.1
61411	1.25814	2.1
61412	0.946126	2.1
61413	0.946126	2.1
61414	0.946131	2.1
61415	0.946131	2.1
61416	0.946127	2.1
61417	0.946127	2.1
61418	0.94613	2.1
61419	0.94613	2.1

61420	0.946128	2.1
61421	0.946128	2.1
61422	0.946128	2.1
61423	0.946128	2.1
61424	1.89226	2.1
61425	1.89226	2.1
61426	1.89226	2.1
61427	1.89226	2.1
61428	1.89226	2.1
61429	1.89226	2.1
61430	0.946132	2.1
61431	0.946132	2.1
61432	1.25814	2.1
61433	1.25814	2.1
61434	0.946131	2.1
61435	0.946131	2.1
61436	1.28543	2.1
61437	1.25814	2.1
61438	1.28543	2.1
61439	0.946132	2.1
61440	1.25814	2.1
61441	0.946132	2.1
61442	1.89226	1.39931
61443	1.89226	0.162699
61444	1.89226	1.18057
61445	1.89226	0.878008
61446	1.89226	0.365122
61447	1.89226	0.216741
61448	1.89226	1.20599
61449	2.27719	0.140952
61450	1.89226	0.180718
61451	1.89226	0.483504
61452	3.78452	0.376441
61453	1.89225	1.50602
61454	1.89225	0.291217
61455	1.89225	0.885523
61456	1.89225	1.21424
61457	3.78451	1.09772
61458	1.89226	0.116425
61459	1.89226	0.194811
61460	1.89226	0.610015
61461	1.89226	0.0552441
61462	3.78452	0.13371
61463	0.946132	0.05
61464	0.946132	0.168677
61465	0.946132	0.326853
61466	0.946132	0.137008
61467	0.946131	0.0952766
61468	0.946131	0.0628055
61469	0.946132	0.209417
61470	0.946132	0.0547125
61471	0.946132	0.0679284
61472	0.946132	0.108578
61473	0.946132	0.0973311
61474	0.946132	0.05
61475	0.946126	0.189341
61476	0.946126	0.079852
61477	0.946132	1.46688

61478	0.946132	0.647317
61479	0.946128	0.206803
61480	0.946128	0.434783
61481	0.946129	0.304897
61482	0.946129	1.05035
61483	0.946129	0.486814
61484	0.946129	0.775308
61485	0.946129	0.360026
61486	0.946129	0.41488
61487	1.39858	2.1
61488	1.39858	2.1
61489	0.94613	2.1
61490	0.94613	2.1
61491	0.946132	2.1
61492	0.946132	2.1
61493	1.39858	2.1
61494	1.25002	2.1
61495	1.39858	2.1
61496	0.946131	2.1
61497	1.25002	2.1
61498	0.946131	2.1
61499	2.0815	2.1
61500	1.89226	2.1
61501	1.89226	2.1
61502	2.05298	2.1
61503	2.5593	2.1
61504	2.77667	2.1
61505	0.946133	2.1
61506	0.946133	2.1
61507	0.946132	2.1
61508	0.946132	2.1
61509	0.946132	2.1
61510	0.946132	2.1
61511	0.946133	2.1
61512	0.946133	2.1
61513	0.946132	2.1
61514	0.946132	2.1
61515	0.946132	2.1
61516	0.946132	2.1
61517	1.38908	2.1
61518	1.38908	2.1
61519	0.946132	2.1
61520	0.946132	2.1
61521	0.946132	2.1
61522	0.946132	2.1
61523	1.38908	2.1
61524	1.27965	2.1
61525	1.38908	2.1
61526	0.946132	2.1
61527	0.946133	2.1
61528	1.27965	2.1
61529	0.946133	2.1
61530	0.946133	2.1
61531	0.946132	2.1
61532	0.946132	2.1
61533	0.946132	2.1
61534	0.946132	2.1
61535	0.946133	2.1

61536	0.946133	2.1
61537	0.946132	2.1
61538	0.946132	2.1
61539	0.946133	2.1
61540	0.946133	2.1
61541	1.89227	1.235
61542	1.89227	1.53306
61543	1.89227	1.0068
61544	1.89227	0.85869
61545	3.78453	1.02644
61546	1.33154	1.13451
61547	1.33154	1.28559
61548	0.946132	1.22707
61549	0.946132	1.04544
61550	1.19095	0.969573
61551	1.33154	1.37857
61552	0.946133	1.2009
61553	0.946133	1.27435
61554	0.946133	2.00009
61555	1.19095	1.02263
61556	0.946132	1.74601
61557	1.33154	1.13062
61558	1.0888	1.00328
61559	1.04745	1.01722
61560	0.946132	0.950398
61561	0.946132	0.979868
61562	1.04745	0.855215
61563	1.04745	1.39424
61564	0.946132	1.63132
61565	0.946132	0.8575
61566	1.04745	1.33172
61567	0.946133	0.877017
61568	0.946132	1.32835
61569	1.0888	1.00724
61570	1.89226	1.9988
61571	1.89226	0.848614
61572	1.89226	0.833279
61573	1.89226	0.978315
61574	1.89226	0.903252
61575	1.89226	1.17427
61576	1.89226	1.60424
61577	1.87592	0.866104
61578	1.87592	0.91665
61579	2.3819	1.0579
61580	1.89226	1.1423
61581	2.39825	1.42312
61582	1.89226	0.864261
61583	2.0949	1.15517
61584	1.88741	0.815102
61585	1.89226	0.822354
61586	1.89226	0.876932
61587	1.88741	1.21742
61588	1.07508	1.39944
61589	1.63419	1.7828
61590	1.63419	1.48776
61591	1.09795	1.91234
61592	1.56137	1.34232
61593	1.02513	2.0973

61594	0.967672	0.454685
61595	1.56137	0.982276
61596	1.56137	0.523557
61597	1.07508	1.1191
61598	1.41319	0.328793
61599	0.926902	1.44619
61600	1.56137	1.13846
61601	1.63419	1.42107
61602	2.29852	1.70051
61603	2.14485	0.965322
61604	3.77904	0.864733
61605	1.41319	0.223021
61606	1.92622	0.232488
61607	1.56137	0.165061
61608	1.56137	0.981933
61609	2.14485	0.836919
61610	1.92622	0.477314
61611	1.89227	2.1
61612	1.89227	2.1
61613	1.89227	2.1
61614	1.89227	2.1
61615	3.78453	2.1
61616	2.56618	2.1
61617	2.08612	2.1
61618	2.48048	2.1
61619	1.99644	2.1
61620	4.5666	2.1
61621	2.08612	2.1
61622	1.30659	2.1
61623	1.99644	2.1
61624	1.25404	2.1
61625	3.34016	2.1
61626	1.89226	2.1
61627	1.89226	2.1
61628	1.89226	2.1
61629	1.89226	2.1
61630	1.89226	2.1
61631	1.89226	2.1
61632	1.89227	2.1
61633	1.89227	2.1
61634	1.89227	2.1
61635	1.89227	2.1
61636	3.78453	2.1
61637	2.48048	2.1
61638	1.99644	2.1
61639	2.39022	2.1
61640	1.90734	2.1
61641	4.38782	2.1
61642	1.90734	2.1
61643	1.99644	2.1
61644	1.21564	2.1
61645	1.21564	2.1
61646	1.25404	2.1
61647	1.94574	2.1
61648	0.810946	2.1
61649	0.810946	2.1
61650	0.810947	2.1
61651	0.810947	2.1

61652	1.89226	2.1
61653	1.3517	2.1
61654	1.08133	2.1
61655	1.08132	2.1
61656	1.08132	2.1
61657	0.810934	2.1
61658	0.810934	2.1
61659	1.89226	2.1
61660	1.89226	1.00267
61661	1.89226	1.47454
61662	1.89226	2.1
61663	3.78453	2.1
61664	1.89226	0.05
61665	1.89226	0.05
61666	1.89226	1.40558
61667	1.89226	0.0669574
61668	1.89226	0.370339
61669	1.89226	0.05
61670	0.946131	0.221103
61671	0.946131	0.05
61672	0.94613	0.324751
61673	0.94613	0.177973
61674	0.946128	0.0716848
61675	0.946128	0.05
61676	0.946133	0.05
61677	0.946133	0.262907
61678	0.946131	0.0708454
61679	0.946131	0.0716467
61680	0.946131	0.13766
61681	0.946131	0.0613091
61682	1.89226	2.1
61683	1.89226	0.261446
61684	1.89226	1.99215
61685	1.89226	1.22452
61686	1.89226	0.971733
61687	1.89226	0.649105
61688	2.56348	0.359214
61689	1.89226	2.1
61690	1.89226	2.1
61691	2.53234	1.36587
61692	4.45574	1.14167
61693	2.53234	0.05
61694	1.65583	0.05
61695	2.56348	0.05
61696	2.56348	0.05
61697	1.66295	0.05
61698	1.65583	0.05
61699	0.736602	0.05
61700	0.827916	0.05
61701	1.26617	0.05
61702	1.18322	0.05
61703	0.7366	0.05
61704	1.18321	0.05
61705	1.26617	0.05
61706	0.827918	0.05
61707	0.99929	0.05
61708	0.99929	0.05
61709	1.00348	0.05

61710	1.00348	0.05
61711	1.26617	0.05
61712	1.18322	0.152756
61713	0.946131	2.1
61714	0.946131	1.04457
61715	0.946129	0.149588
61716	1.18321	0.0657251
61717	0.946133	2.1
61718	1.26617	1.52961
61719	1.26617	1.55434
61720	1.26617	0.05
61721	0.946131	2.1
61722	0.946131	0.38149
61723	2.23267	0.05
61724	1.89226	0.05
61725	2.07099	0.05
61726	1.89226	0.05
61727	2.07099	0.05
61728	1.89226	0.05
61729	0.946132	0.05
61730	1.35403	0.05
61731	1.24038	0.05
61732	0.946131	0.05
61733	0.946131	0.05
61734	0.946131	0.05
61735	1.24039	0.05
61736	1.35403	0.05
61737	1.24038	0.05
61738	1.24038	0.05
61739	0.946131	0.05
61740	0.946131	0.0534366
61741	1.89226	0.105558
61742	1.89226	0.27977
61743	1.89226	0.065926
61744	1.89226	0.0521643
61745	1.89226	0.503433
61746	1.89226	0.379905
61747	1.89226	0.05
61748	1.89226	0.295595
61749	1.89226	0.0581821
61750	1.89226	0.134163
61751	3.78452	0.207033
61752	1.56971	0.05
61753	2.80086	0.0540309
61754	2.59806	0.05
61755	1.93489	0.05
61756	2.59806	0.05
61757	1.93489	0.05
61758	0.827467	0.05
61759	1.26652	0.05
61760	1.13679	0.05
61761	0.639035	0.05
61762	1.11547	0.05
61763	1.30656	0.05
61764	0.869062	0.05
61765	0.696608	0.05
61766	1.07205	0.05
61767	0.953656	0.05

61768	0.970744	0.05
61769	0.970744	0.05
61770	2.38681	2.1
61771	2.50438	1.90772
61772	2.42082	2.1
61773	2.59106	2.1
61774	4.92521	2.1
61775	2.50438	1.47951
61776	2.40627	2.1
61777	2.59106	2.1
61778	2.5444	1.16749
61779	5.04879	2.1
61780	2.42082	2.1
61781	2.70575	2.1
61782	2.64778	2.1
61783	2.59106	2.1
61784	2.47753	2.1
61785	2.42082	1.91615
61786	2.70575	2.1
61787	2.5444	1.55038
61788	2.76095	2.1
61789	2.59106	2.1
61790	2.71429	2.1
61791	2.5444	2.1
61792	1.89226	2.1
61793	2.38473	2.1
61794	2.36689	2.1
61795	2.38473	2.1
61796	1.87442	2.1
61797	1.89226	2.1
61798	1.89226	2.1
61799	1.89226	1.40236
61800	1.89226	2.1
61801	1.89226	1.59985
61802	1.89226	2.1
61803	1.89226	2.1
61804	2.14952	2.1
61805	1.89226	0.859981
61806	1.89226	2.1
61807	1.89226	0.846885
61808	2.0692	2.1
61809	2.0692	2.1
61810	1.89226	2.1
61811	2.36689	2.1
61812	2.36204	2.1
61813	2.36689	2.1
61814	1.88742	2.1
61815	1.89226	2.1
61816	0.946132	0.901525
61817	0.946132	2.1
61818	0.946131	2.1
61819	0.946131	2.1
61820	0.946132	2.1
61821	0.946132	2.1
61822	0.946131	2.1
61823	0.946131	2.1
61824	0.946132	2.1
61825	0.946132	2.1

61826	0.946131	2.1
61827	0.946131	2.1
61828	0.946128	2.1
61829	0.946128	2.1
61830	1.07476	2.1
61831	1.07476	2.1
61832	0.946131	1.98639
61833	0.946131	2.1
61834	1.13182	2.1
61835	1.07476	2.1
61836	1.13182	2.1
61837	0.946131	2.1
61838	1.07476	2.1
61839	0.94613	1.62281
61840	1.89227	0.62331
61841	1.89227	0.649781
61842	1.89227	0.660965
61843	1.89227	0.650649
61844	3.78453	0.642431
61845	1.89226	0.64974
61846	1.89226	0.65629
61847	1.89226	0.64719
61848	1.89226	0.681239
61849	3.78452	0.651367
61850	1.89226	0.654865
61851	1.89226	0.678629
61852	1.89226	0.701196
61853	1.89226	0.687856
61854	3.78452	0.66328
61855	1.89226	0.649545
61856	1.89226	0.672341
61857	1.89226	0.661295
61858	1.89226	0.677281
61859	1.89226	0.656351
61860	1.89226	0.658292
61861	2.58344	0.707784
61862	2.00267	0.627422
61863	2.14218	0.689729
61864	2.22674	1.14844
61865	2.14218	0.509606
61866	2.22674	0.646923
61867	2.83531	1.22332
61868	1.56043	0.674486
61869	2.22674	1.01571
61870	1.90912	1.62467
61871	2.22674	0.643419
61872	1.90912	0.668572
61873	0.780217	0.670057
61874	1.11337	0.687794
61875	1.00133	0.901334
61876	0.804472	0.690606
61877	0.804472	0.821935
61878	0.780217	0.677072
61879	1.00133	0.623874
61880	1.11337	0.735178
61881	0.856668	0.744635
61882	0.856668	1.57877
61883	0.924883	0.675909

61884	0.924883	0.637301
61885	1.93107	2.1
61886	1.68657	2.1
61887	1.7237	2.1
61888	1.89226	2.1
61889	1.53436	2.1
61890	1.68657	2.1
61891	1.89226	2.1
61892	1.89226	2.1
61893	1.89226	2.1
61894	1.89226	2.1
61895	3.78453	2.1
61896	0.946131	2.1
61897	0.946131	2.1
61898	0.855092	2.1
61899	0.83953	2.1
61900	0.946131	2.1
61901	0.946131	2.1
61902	0.839531	2.1
61903	0.839531	2.1
61904	0.946131	2.1
61905	0.855093	2.1
61906	0.839531	2.1
61907	0.946131	2.1
61908	1.89226	2.1
61909	1.70302	2.1
61910	2.21419	2.1
61911	1.93107	2.1
61912	1.97872	2.1
61913	1.70302	2.1
61914	0.946133	2.1
61915	0.946133	2.1
61916	0.946132	2.1
61917	0.946132	2.1
61918	0.946132	2.1
61919	0.946132	2.1
61920	0.946132	2.1
61921	0.946132	2.1
61922	0.946133	2.1
61923	0.946133	2.1
61924	0.946132	2.1
61925	0.946132	2.1
61926	1.90081	2.1
61927	1.89226	2.1
61928	1.51379	2.1
61929	1.36813	2.1
61930	1.71019	2.1
61931	1.89226	2.1
61932	1.89227	1.30906
61933	1.89227	0.888431
61934	1.89227	0.866407
61935	1.89227	1.08348
61936	1.89227	1.36195
61937	1.89227	2.00553
61938	1.36428	1.21215
61939	1.37869	1.1047
61940	0.946132	0.963654
61941	0.946132	1.11975

61942	1.37869	0.950206
61943	1.37869	2.1
61944	0.946133	1.21954
61945	0.946133	1.42841
61946	1.37869	0.954005
61947	0.946132	1.053
61948	1.36428	1.28561
61949	0.946133	0.879019
61950	0.94612	1.07339
61951	0.94612	1.02499
61952	0.946119	1.01364
61953	0.946119	1.07862
61954	0.946119	0.865008
61955	0.946119	1.90765
61956	0.94612	1.24013
61957	0.94612	1.86244
61958	0.946132	0.994773
61959	0.946132	0.88054
61960	0.946107	0.906059
61961	0.946107	1.26379
61962	1.89225	0.05
61963	1.89225	2.1
61964	1.63564	0.05
61965	1.42278	2.1
61966	1.42278	2.1
61967	1.57534	0.05
61968	1.53735	2.1
61969	1.68991	0.05
61970	1.57535	0.05
61971	1.04442	2.1
61972	1.04442	2.1
61973	1.22031	0.05
61974	1.42278	2.1
61975	1.59868	0.05
61976	0.946132	2.1
61977	0.946132	2.1
61978	0.946131	2.1
61979	0.946131	2.1
61980	0.946131	2.1
61981	0.946131	2.1
61982	0.946132	2.1
61983	0.946132	2.1
61984	0.946132	2.1
61985	0.946132	2.1
61986	0.946131	2.1
61987	0.946131	2.1
61988	1.89226	2.1
61989	1.89226	2.1
61990	1.89226	2.1
61991	1.89226	2.1
61992	1.89226	2.1
61993	1.89226	2.1
61994	2.03143	0.991543
61995	1.47111	2.07403
61996	2.28685	1.89656
61997	2.28685	1.93665
61998	1.36006	1.08299
61999	1.36006	1.08513

62000	1.89226	0.902517
62001	1.89226	1.49063
62002	1.89226	1.1715
62003	1.89226	1.23457
62004	1.89226	0.92271
62005	1.89226	0.99714
62006	1.89226	1.20946
62007	1.89226	2.1
62008	1.89226	2.1
62009	1.89226	2.1
62010	1.89226	1.30019
62011	1.89226	2.1
62012	0.902821	2.1
62013	0.902821	2.1
62014	0.889218	2.1
62015	0.889218	2.1
62016	0.675759	2.1
62017	1.05649	2.1
62018	0.735554	2.1
62019	1.14342	2.1
62020	1.14343	2.08053
62021	1.05649	2.1
62022	0.735554	2.1
62023	0.675758	2.1
62024	2.46286	0.815068
62025	2.28685	0.960979
62026	2.67843	0.934037
62027	2.67843	0.895007
62028	2.03143	0.859009
62029	2.03143	0.842389
62030	1.89226	0.843172
62031	1.89226	0.907084
62032	1.89226	0.836223
62033	1.89226	0.83492
62034	3.78452	0.912793
62035	0.94613	1.06652
62036	0.94613	1.06018
62037	0.946128	1.94622
62038	0.946128	1.06605
62039	0.94613	2.1
62040	0.94613	1.07884
62041	0.946128	0.757366
62042	0.946128	1.02661
62043	0.946129	0.142872
62044	0.946129	1.15071
62045	0.946129	0.340631
62046	0.946129	2.1
62047	2.67843	0.989674
62048	2.11297	2.09162
62049	2.52453	1.44935
62050	2.52453	2.1
62051	2.28685	1.12616
62052	2.28685	1.07894
62053	1.89226	0.393722
62054	1.89226	1.8627
62055	1.89226	0.0959361
62056	1.89226	2.1
62057	3.78451	0.246762

62058	2.39524	0.0951579
62059	2.10188	1.53558
62060	2.29839	0.05
62061	2.02242	1.78024
62062	4.40028	0.0992942
62063	1.71839	2.1
62064	1.65261	2.1
62065	2.10189	0.908146
62066	2.10189	2.1
62067	2.02243	2.04336
62068	1.63893	1.89016
62069	0.946132	2.1
62070	0.946132	1.43088
62071	0.946129	2.07611
62072	0.946129	2.1
62073	0.94613	2.1
62074	0.94613	2.1
62075	0.946131	0.781747
62076	0.946131	1.71261
62077	0.946131	2.1
62078	0.946131	2.1
62079	0.946131	2.1
62080	0.946131	2.1
62081	1.89226	0.4853
62082	1.89226	2.1
62083	1.89226	0.05
62084	1.89226	0.889682
62085	3.78452	0.133987
62086	2.2984	0.0503649
62087	2.02242	2.08111
62088	2.21582	0.05
62089	1.94412	0.728524
62090	4.24251	0.126132
62091	1.94412	0.70403
62092	1.5854	2.1
62093	2.02243	2.1
62094	2.02243	2.03763
62095	1.57431	1.03316
62096	1.65261	2.1
62097	0.810943	1.20781
62098	0.810943	2.1
62099	0.810945	2.1
62100	0.810945	2.1
62101	1.89226	0.790605
62102	1.3517	2.1
62103	1.08133	2.1
62104	1.08132	2.1
62105	1.08132	0.295308
62106	0.810933	2.1
62107	0.810933	2.1
62108	1.89226	0.05
62109	1.89226	0.05
62110	1.89226	0.05
62111	1.89226	0.05
62112	1.89226	0.05
62113	1.89226	0.05
62114	1.89226	0.05
62115	1.89226	0.05

62116	1.89226	0.05
62117	1.89226	0.05
62118	3.78452	0.05
62119	1.89226	0.05
62120	1.89226	0.05
62121	1.89226	0.05
62122	1.89226	0.05
62123	1.89226	0.05
62124	1.89226	0.05
62125	0.946128	0.05
62126	0.946128	0.05
62127	0.946132	0.05
62128	0.946132	0.05
62129	0.94613	0.05
62130	0.94613	0.05
62131	0.946129	0.05
62132	0.946129	0.05
62133	0.94613	0.05
62134	0.94613	0.05
62135	0.94613	0.05
62136	0.94613	0.05
62137	1.89226	0.05
62138	1.89226	0.05
62139	1.89226	0.05
62140	1.89226	0.05
62141	1.89226	0.05
62142	1.89226	0.05
62143	2.81478	2.1
62144	1.89226	2.1
62145	2.65557	2.1
62146	1.89226	2.1
62147	4.54783	2.1
62148	1.89226	2.1
62149	1.89226	2.1
62150	1.89226	2.1
62151	1.89226	2.1
62152	3.78452	2.1
62153	2.65557	2.1
62154	1.89226	2.1
62155	1.89226	2.1
62156	1.89226	2.1
62157	2.57997	2.1
62158	2.57997	2.1
62159	0.946131	2.1
62160	0.946131	2.1
62161	0.946128	2.1
62162	0.946128	2.1
62163	0.94613	2.1
62164	0.94613	2.1
62165	0.946129	2.1
62166	0.946129	2.1
62167	0.94613	2.1
62168	0.94613	2.1
62169	0.94613	2.1
62170	0.94613	2.1
62171	1.89226	0.787474
62172	2.27703	1.97682
62173	2.35615	2.08605

62174	2.35615	1.28567
62175	1.89226	2.07203
62176	1.89226	1.3237
62177	0.946132	0.838219
62178	1.17807	2.1
62179	1.14926	0.862785
62180	0.946132	1.4564
62181	0.946133	1.39069
62182	0.946133	1.45268
62183	1.14926	0.970562
62184	1.14926	0.861945
62185	0.946132	1.40638
62186	0.946132	1.05039
62187	1.17807	1.514
62188	1.14926	1.20765
62189	2.16841	0.953149
62190	1.35487	1.37351
62191	1.19854	1.17952
62192	1.19854	1.06567
62193	2.46078	1.24069
62194	2.46078	1.22913
62195	0.69308	0.788669
62196	1.3119	1.4693
62197	0.677433	1.49435
62198	1.23039	1.26978
62199	0.978178	1.49621
62200	0.978178	1.4705
62201	0.945294	1.0457
62202	0.945294	1.278
62203	1.3119	1.33552
62204	1.23039	1.29146
62205	0.693081	1.31195
62206	0.677432	1.31696
62207	1.89226	0.856389
62208	1.89226	2.1
62209	1.89226	2.05065
62210	1.89226	1.19082
62211	1.89226	2.00476
62212	1.89226	1.17876
62213	0.946132	0.66434
62214	0.946132	2.1
62215	0.946132	0.449908
62216	0.946132	1.21014
62217	0.946133	1.20633
62218	0.946133	1.40548
62219	0.946132	0.665129
62220	0.946132	0.514957
62221	0.946133	1.06002
62222	0.946133	0.614575
62223	0.946132	1.22989
62224	0.946132	0.946347
62225	2.60637	2.1
62226	1.89226	2.1
62227	1.89226	2.1
62228	1.89226	1.61473
62229	3.78452	2.1
62230	1.89226	0.441984
62231	1.8396	1.68291

62232	1.89226	2.09533
62233	1.89226	0.54418
62234	1.60993	2.1
62235	1.60993	0.519713
62236	0.946131	1.32687
62237	0.946131	1.98039
62238	0.946128	2.1
62239	0.946128	2.1
62240	0.946131	1.16515
62241	0.946131	2.05088
62242	0.946127	2.1
62243	0.946127	1.75623
62244	0.946129	2.1
62245	0.946129	2.1
62246	0.94613	2.1
62247	0.94613	2.1
62248	1.89226	2.1
62249	1.89226	1.85322
62250	1.89226	2.1
62251	1.89226	2.1
62252	1.89226	2.1
62253	1.89226	2.1
62254	1.89226	0.411518
62255	1.89226	1.47693
62256	1.89226	2.06876
62257	1.89226	0.420703
62258	1.89226	2.1
62259	1.89226	0.273528
62260	0.946131	1.20373
62261	0.946131	2.00492
62262	0.946128	2.1
62263	0.946128	2.05587
62264	0.946131	1.10611
62265	0.946131	1.89862
62266	0.946127	1.96159
62267	0.946127	2.1
62268	0.94613	2.1
62269	0.94613	2.1
62270	0.946129	2.1
62271	0.946129	2.1
62272	0.946133	2.1
62273	0.946133	2.1
62274	1.28153	2.1
62275	1.26781	2.1
62276	0.946132	2.1
62277	0.946132	2.1
62278	1.28153	2.1
62279	1.28153	2.1
62280	0.946132	2.1
62281	1.28153	2.1
62282	0.946133	2.1
62283	1.26781	2.1
62284	1.89226	2.1
62285	1.89226	2.1
62286	1.89226	2.1
62287	1.89226	2.1
62288	1.89226	2.1
62289	1.89226	2.1

62290	2.5844	2.1
62291	1.09563	2.1
62292	1.1468	2.1
62293	1.09563	2.1
62294	2.6142	2.1
62295	2.56304	2.1
62296	0.946126	2.1
62297	0.946126	2.1
62298	1.2922	2.1
62299	1.28152	2.1
62300	0.946126	2.1
62301	0.946126	2.1
62302	1.2922	2.1
62303	1.2922	2.1
62304	1.29221	2.1
62305	0.946132	2.1
62306	0.946119	2.1
62307	1.28151	2.1
62308	1.89225	2.1
62309	1.89225	2.1
62310	1.89225	2.1
62311	1.89225	2.1
62312	1.89225	2.1
62313	1.89225	2.1
62314	0.946121	2.1
62315	0.946121	2.1
62316	0.946126	2.1
62317	0.946126	2.1
62318	0.946124	2.1
62319	0.946124	2.1
62320	0.946123	2.1
62321	0.946123	2.1
62322	0.94613	2.1
62323	0.94613	2.1
62324	0.946117	2.1
62325	0.946117	2.1
62326	1.89226	1.26331
62327	1.89226	1.19543
62328	1.89226	1.09823
62329	1.89226	2.1
62330	1.89226	0.0924345
62331	1.89226	2.1
62332	1.89226	2.1
62333	1.89226	2.04931
62334	1.89226	0.570993
62335	1.89226	2.1
62336	1.89226	0.0716923
62337	1.89226	2.1
62338	1.89226	2.1
62339	2.81281	2.1
62340	2.50369	1.01314
62341	2.50369	2.1
62342	1.89226	1.25667
62343	1.89226	2.1
62344	1.89226	2.1
62345	1.89226	2.1
62346	1.89226	2.1
62347	1.89226	2.1

62348	1.89226	2.1
62349	1.89226	2.1
62350	1.89226	2.1
62351	1.89226	2.1
62352	1.89226	2.1
62353	1.89226	2.1
62354	1.89226	2.1
62355	1.89226	2.1
62356	0.907036	2.1
62357	1.09714	2.1
62358	0.994609	2.1
62359	0.838808	2.1
62360	0.976536	2.1
62361	0.976536	2.1
62362	0.959412	2.1
62363	0.959412	2.1
62364	1.09714	2.1
62365	0.994609	2.1
62366	0.907036	2.1
62367	0.838808	2.1
62368	0.99461	2.1
62369	0.838808	2.1
62370	0.738212	2.1
62371	0.565634	2.1
62372	0.775921	2.1
62373	0.775921	2.1
62374	0.784322	2.1
62375	0.784322	2.1
62376	0.994609	2.1
62377	0.738212	2.1
62378	0.838808	2.1
62379	0.565635	2.1
62380	1.89226	2.1
62381	1.89226	2.1
62382	1.89226	2.1
62383	1.89226	2.1
62384	1.89226	2.1
62385	1.89226	2.1
62386	1.89226	2.1
62387	1.89226	2.1
62388	1.89226	2.1
62389	1.89226	2.1
62390	1.89226	2.1
62391	1.89226	2.1
62392	2.19428	2.1
62393	1.98922	2.1
62394	2.27322	2.1
62395	2.45893	2.1
62396	4.4675	2.1
62397	0.99461	2.1
62398	1.13661	2.1
62399	0.902272	2.1
62400	0.738212	2.1
62401	0.931874	2.1
62402	0.931874	2.1
62403	0.942953	2.1
62404	0.942953	2.1
62405	1.13658	2.1

62406	0.902248	2.1
62407	0.994637	2.1
62408	0.738233	2.1
62409	1.89226	2.1
62410	1.89226	2.09103
62411	1.89226	2.1
62412	1.89226	2.1
62413	1.89226	2.1
62414	1.89226	2.01875
62415	1.89227	2.1
62416	2.58246	1.46332
62417	1.89227	2.04126
62418	1.89227	2.04722
62419	3.78453	1.72269
62420	1.89226	2.1
62421	1.89226	2.02794
62422	1.65573	2.1
62423	1.65573	2.1
62424	1.89226	2.1
62425	1.89226	2.1
62426	1.89226	1.737
62427	1.89226	1.49428
62428	1.89226	1.66814
62429	1.89226	1.78857
62430	1.89226	1.46364
62431	1.89226	1.5689
62432	1.89226	1.47017
62433	1.89226	1.57792
62434	1.89226	1.44627
62435	1.89226	1.67139
62436	1.89226	1.43971
62437	1.89226	1.71141
62438	0.946132	2.1
62439	0.946132	2.1
62440	1.01023	1.55694
62441	1.13651	2.1
62442	1.01023	1.5254
62443	0.946133	1.98256
62444	1.0644	2.02016
62445	1.0644	2.1
62446	0.946132	2.1
62447	0.946132	2.0895
62448	1.13651	1.99057
62449	0.946132	2.01079
62450	1.89226	0.332584
62451	1.89226	1.77515
62452	1.89226	1.24738
62453	1.89226	0.099678
62454	3.78451	0.462033
62455	1.00934	0.0961054
62456	0.741561	0.0573814
62457	0.946131	0.127231
62458	0.946131	0.217665
62459	0.946131	0.734326
62460	0.741561	0.05
62461	1.00934	0.05
62462	0.946131	0.401043
62463	0.741586	0.110994

62464	0.741586	0.0908337
62465	0.946106	0.12847
62466	0.946106	0.05
62467	1.14895	0.164054
62468	0.957449	0.420266
62469	0.934806	1.46821
62470	0.934806	1.06293
62471	1.13536	0.0623173
62472	1.02829	1.78033
62473	0.946129	1.89163
62474	0.946129	0.176144
62475	0.946127	0.838266
62476	0.946127	0.948565
62477	0.946127	0.653292
62478	1.0283	0.889182
62479	1.89226	1.7773
62480	1.89226	0.36732
62481	1.89226	0.304694
62482	1.89226	1.50295
62483	3.78452	1.14869
62484	1.89226	0.111924
62485	1.89226	0.566373
62486	1.89226	0.404468
62487	1.89226	0.0685338
62488	3.78453	0.128848
62489	1.89226	0.618363
62490	2.69068	0.0963111
62491	1.89226	0.0763929
62492	1.89226	0.349008
62493	3.78452	0.26088
62494	1.89226	0.410724
62495	1.89226	1.65066
62496	1.89226	1.62006
62497	1.89226	0.847531
62498	1.89226	1.44486
62499	1.89226	0.453721
62500	1.89226	2.1
62501	1.89226	2.1
62502	1.89226	2.1
62503	1.89226	2.1
62504	1.89226	2.1
62505	1.89226	2.1
62506	1.89226	2.1
62507	1.89226	2.1
62508	1.89226	2.1
62509	1.89226	2.1
62510	1.89226	2.1
62511	1.89226	2.1
62512	0.985109	2.1
62513	1.31144	2.1
62514	1.45864	2.1
62515	1.10667	2.1
62516	2.44375	2.1
62517	0.655718	2.1
62518	0.729316	2.1
62519	0.684594	2.1
62520	0.782707	2.1
62521	0.700849	2.1

62522	0.700849	2.1
62523	0.71306	2.1
62524	0.71306	2.1
62525	0.729318	2.1
62526	0.782705	2.1
62527	0.684592	2.1
62528	0.655719	2.1
62529	0.946128	2.1
62530	0.946128	2.1
62531	0.946133	2.1
62532	0.946133	2.1
62533	0.946131	2.1
62534	0.946131	2.1
62535	0.946131	2.1
62536	0.946131	2.1
62537	0.94613	2.1
62538	0.94613	2.1
62539	0.946131	2.1
62540	0.946131	2.1
62541	1.89226	2.1
62542	1.89226	2.1
62543	1.89226	2.1
62544	1.89226	2.1
62545	1.89226	2.1
62546	1.89226	2.1
62547	0.553334	2.1
62548	0.629936	2.1
62549	0.828211	2.1
62550	0.729319	2.1
62551	0.674025	2.1
62552	0.674025	2.1
62553	0.68523	2.1
62554	0.68523	2.1
62555	0.828211	2.1
62556	0.629935	2.1
62557	0.72932	2.1
62558	0.553334	2.1
62559	0.828208	2.1
62560	0.729316	2.1
62561	0.907037	2.1
62562	0.782707	2.1
62563	0.799048	2.1
62564	0.799048	2.1
62565	0.811867	2.1
62566	0.811867	2.1
62567	0.828209	2.1
62568	0.907035	2.1
62569	0.729318	2.1
62570	0.782706	2.1
62571	2.32981	2.1
62572	1.89226	2.1
62573	1.89226	2.1
62574	1.89226	2.1
62575	2.2671	2.1
62576	2.2671	2.1
62577	1.89226	2.1
62578	1.89226	2.1
62579	1.89226	2.1

62580	1.89226	1.66292
62581	1.89226	2.1
62582	1.89226	2.1
62583	1.3991	2.1
62584	1.81793	2.05878
62585	2.07226	2.1
62586	2.07226	1.67024
62587	1.24924	2.1
62588	1.24924	2.1
62589	0.946131	2.1
62590	0.946131	2.1
62591	1.16491	2.1
62592	1.16491	2.1
62593	0.946131	2.1
62594	0.946131	2.1
62595	1.16491	2.1
62596	1.19523	2.1
62597	0.946132	2.1
62598	1.19523	2.1
62599	1.16491	2.1
62600	0.946131	2.1
62601	1.89226	2.1
62602	1.89226	1.94656
62603	1.89226	2.1
62604	1.89226	1.03233
62605	3.78452	1.64783
62606	0.901204	2.1
62607	0.901204	2.1
62608	0.902504	2.1
62609	0.902504	2.1
62610	1.10416	2.02241
62611	1.03613	1.2877
62612	0.764922	2.1
62613	0.699552	2.1
62614	0.764921	2.1
62615	1.10416	2.1
62616	0.699551	2.1
62617	1.03613	1.81318
62618	1.89226	0.51598
62619	1.89226	1.20434
62620	1.89226	1.21827
62621	1.89226	0.786311
62622	3.78453	1.03266
62623	1.89226	1.15654
62624	1.89226	1.34215
62625	1.89226	1.15598
62626	1.89226	1.44935
62627	1.89226	1.15838
62628	1.89226	1.45337
62629	0.946131	1.22877
62630	0.946131	1.222
62631	0.946131	1.04408
62632	0.946131	0.957387
62633	0.946131	1.09254
62634	0.946131	1.2295
62635	0.946131	1.22538
62636	0.946131	0.539987
62637	0.946131	1.03504

62638	0.946131	1.04555
62639	0.946131	0.957519
62640	0.946131	1.12704
62641	1.89226	1.15961
62642	1.89226	1.33636
62643	1.89226	1.23248
62644	1.89226	1.3556
62645	1.89226	1.23169
62646	1.89226	1.35587
62647	0.946127	1.26569
62648	0.946127	1.1954
62649	1.22788	0.951533
62650	1.35469	1.08218
62651	1.35469	1.2323
62652	1.35469	1.1144
62653	0.946128	1.22644
62654	0.946128	0.755817
62655	0.946129	1.06361
62656	1.22788	1.08152
62657	1.35469	1.11684
62658	0.946129	1.05832
62659	1.22788	1.22759
62660	1.35469	1.29927
62661	1.02602	0.834704
62662	0.794861	1.17611
62663	1.15301	1.24968
62664	1.15301	1.30195
62665	1.10089	0.820966
62666	1.10089	1.16567
62667	0.794861	1.14419
62668	1.22788	1.24684
62669	1.02602	1.22678
62670	1.35469	1.22471
62671	1.89226	0.406899
62672	1.89226	0.387035
62673	1.89226	0.978793
62674	1.89226	0.76636
62675	3.78452	0.80757
62676	1.13536	2.07765
62677	0.942524	0.05
62678	0.946131	0.18246
62679	0.946131	0.231513
62680	0.946127	0.354034
62681	0.946127	2.08876
62682	1.26282	0.183556
62683	0.917471	1.49544
62684	1.21023	0.502078
62685	1.00851	0.504547
62686	1.17955	0.45265
62687	0.883746	0.126929
62688	1.89226	0.260913
62689	1.89226	2.1
62690	1.89226	0.65763
62691	1.89226	1.27464
62692	1.89226	0.544432
62693	1.89226	1.99201
62694	1.89226	0.771396
62695	1.89226	1.06695

62696	1.89226	0.218595
62697	1.89226	0.327974
62698	1.89226	0.502327
62699	1.89226	0.987716
62700	0.94613	0.950936
62701	0.94613	0.798062
62702	0.94613	0.720784
62703	0.94613	0.762666
62704	0.946128	0.803585
62705	0.946128	0.97166
62706	0.946131	0.993975
62707	0.946131	0.704107
62708	0.94613	0.799827
62709	0.94613	0.852084
62710	0.94613	0.763791
62711	0.94613	0.773985
62712	0.883758	2.09862
62713	1.03041	0.595569
62714	0.946131	0.621436
62715	0.946131	0.394689
62716	0.851537	0.30621
62717	0.851537	2.09865
62718	1.23942	0.073041
62719	1.14906	1.73273
62720	0.851484	0.48087
62721	0.985518	0.230718
62722	1.0156	0.611815
62723	0.851526	0.646584
62724	1.89226	0.200303
62725	1.89226	2.1
62726	1.89226	0.386747
62727	1.89226	1.25713
62728	1.89226	0.343458
62729	1.89226	2.0996
62730	1.89226	0.802392
62731	1.89226	1.02301
62732	1.89226	0.627782
62733	1.89226	0.85407
62734	1.89226	0.522226
62735	1.89226	1.00635
62736	1.89227	0.543664
62737	1.89227	0.586352
62738	1.89227	0.580332
62739	1.89227	0.61704
62740	3.78453	0.570791
62741	1.89226	0.611818
62742	1.89226	0.60969
62743	1.89226	0.621122
62744	1.89226	0.651335
62745	3.78452	0.633884
62746	0.946132	0.645712
62747	0.946132	0.65399
62748	0.946128	0.658624
62749	0.946128	0.642069
62750	0.94613	0.639656
62751	0.94613	0.637697
62752	0.94613	0.603335
62753	0.94613	0.640995

62754	0.946131	0.63437
62755	0.946131	0.65923
62756	0.94613	0.626768
62757	0.94613	0.642167
62758	1.89226	0.620467
62759	1.89226	0.618892
62760	1.89226	0.625067
62761	1.89226	0.611391
62762	1.89226	0.653024
62763	1.89226	0.628099
62764	1.98874	0.115604
62765	2.33455	0.415825
62766	2.01865	0.486056
62767	2.07024	0.227774
62768	4.32329	0.529829
62769	1.98874	0.495089
62770	2.00266	0.576353
62771	2.14218	0.660552
62772	2.07024	0.655706
62773	3.9914	0.551513
62774	1.00133	0.656719
62775	0.804471	0.263272
62776	1.03512	0.659237
62777	0.995176	0.619078
62778	0.80447	0.754193
62779	0.995178	0.60922
62780	1.00133	0.659285
62781	1.03512	0.676856
62782	0.880587	0.678162
62783	0.880587	0.689215
62784	0.959002	0.184976
62785	0.959002	0.548172
62786	2.33455	0.399227
62787	1.99036	0.337587
62788	2.07024	0.162726
62789	2.58645	0.474197
62790	4.32491	0.140286
62791	1.89226	0.543223
62792	1.89226	0.371358
62793	1.89226	0.53525
62794	1.89226	0.201614
62795	3.78453	0.214287
62796	1.89226	0.982286
62797	1.89226	0.43754
62798	1.89226	0.769085
62799	1.89226	0.623925
62800	3.78453	0.996383
62801	1.89226	0.190337
62802	1.89226	0.0783151
62803	1.89226	0.10734
62804	1.89226	0.157244
62805	1.89226	0.103682
62806	1.89226	0.153964
62807	1.89226	0.154073
62808	2.1523	0.05
62809	2.53257	0.05
62810	2.53257	0.05
62811	1.89226	0.207284

62812	1.89226	0.187496
62813	0.946133	0.128178
62814	0.946133	0.229036
62815	0.946132	0.0780607
62816	0.946132	0.0979668
62817	0.946132	0.0712664
62818	0.946132	0.0821064
62819	0.946132	0.256141
62820	0.946132	0.062382
62821	0.946133	0.137218
62822	0.946133	0.158906
62823	0.946132	0.247144
62824	0.946132	0.141287
62825	0.946132	0.313976
62826	0.946132	0.577647
62827	0.946132	0.225571
62828	0.946132	0.403004
62829	0.946132	0.213186
62830	0.946132	0.240858
62831	0.946132	0.627374
62832	0.946132	0.371093
62833	0.946132	0.347536
62834	0.946132	0.331489
62835	0.946131	0.446873
62836	0.946131	0.394668
62837	1.62772	0.0854969
62838	1.70303	0.109892
62839	1.70303	0.167929
62840	1.70303	0.10239
62841	1.90116	0.0964039
62842	1.90116	0.0640739
62843	1.89226	0.240438
62844	1.89226	0.614524
62845	1.89226	0.678949
62846	1.89226	0.688286
62847	1.89226	0.272362
62848	1.89226	0.403249
62849	1.89226	0.706839
62850	1.89226	0.705456
62851	1.89226	0.631704
62852	1.89226	0.704571
62853	3.78452	0.703773
62854	1.89226	0.704433
62855	1.89226	0.703847
62856	1.89226	0.702699
62857	1.89226	0.70644
62858	1.89226	0.685408
62859	1.89226	0.691513
62860	0.946132	0.550391
62861	0.946132	0.457405
62862	0.946129	0.580847
62863	0.946129	0.46399
62864	0.94613	0.505677
62865	0.94613	0.579665
62866	0.946131	0.555521
62867	0.946131	0.346718
62868	0.94613	0.385869
62869	0.94613	0.534603

62870	0.946131	0.0871314
62871	0.946131	0.584255
62872	1.89226	0.591125
62873	1.89226	0.697082
62874	1.89226	0.702696
62875	1.89226	0.702134
62876	1.89226	0.562663
62877	1.89226	0.610693
62878	1.89226	0.0982461
62879	1.89226	0.945371
62880	1.89226	0.949028
62881	1.89226	0.149789
62882	3.78452	0.225122
62883	1.89226	0.420995
62884	1.89226	0.879074
62885	1.89226	0.147074
62886	1.89226	1.08549
62887	1.89226	0.152645
62888	1.89226	1.10701
62889	1.30165	0.472875
62890	0.946127	0.885581
62891	1.09659	0.307681
62892	0.946131	0.4124
62893	1.09659	0.271136
62894	1.09659	1.23182
62895	0.946128	1.33697
62896	0.946128	0.19108
62897	1.30165	0.383008
62898	1.09659	0.980845
62899	0.946129	0.543503
62900	0.946129	0.913182
62901	0.946128	0.951322
62902	0.946128	0.978715
62903	0.946133	0.710939
62904	0.946133	0.855215
62905	0.946131	0.815811
62906	0.946131	1.0267
62907	0.946131	0.977049
62908	0.946131	0.359963
62909	0.94613	0.628445
62910	0.94613	0.467843
62911	0.946131	0.269561
62912	0.946131	0.875601
62913	0.946133	0.992646
62914	0.946133	1.06883
62915	0.946132	0.466258
62916	0.946132	0.725572
62917	0.946132	0.470699
62918	0.946132	1.07902
62919	0.946132	1.07997
62920	0.946132	0.417759
62921	0.946132	0.628721
62922	0.946132	0.567294
62923	0.946133	0.847521
62924	0.946133	0.862903
62925	1.30165	1.06771
62926	0.972228	1.03042
62927	1.09659	0.891673

62928	0.656268	1.04979
62929	1.06217	0.894149
62930	1.06217	1.91313
62931	1.00665	0.852979
62932	1.00665	1.17151
62933	0.97223	0.871525
62934	0.656267	0.848512
62935	1.30165	0.876966
62936	1.09659	0.944899
62937	0.946132	1.76665
62938	0.946132	2.1
62939	1.19895	2.1
62940	1.19895	2.1
62941	0.94613	2.1
62942	0.94613	1.18491
62943	1.39356	2.1
62944	1.19895	2.1
62945	0.94613	2.1
62946	1.39356	2.1
62947	1.19895	2.1
62948	0.946131	2.1
62949	1.89226	1.33646
62950	1.89226	2.1
62951	1.89226	2.1
62952	1.89226	1.2676
62953	1.89226	2.1
62954	1.89226	1.33395
62955	0.946132	1.13192
62956	0.946132	1.1263
62957	0.946128	1.53942
62958	0.946128	2.1
62959	0.94613	1.54455
62960	0.94613	0.599319
62961	0.94613	1.16002
62962	0.94613	1.97999
62963	0.946131	1.17865
62964	0.946131	1.16568
62965	0.94613	1.23474
62966	0.94613	1.2843
62967	1.89226	0.05
62968	1.89226	0.05
62969	1.89226	0.05
62970	1.89226	0.05
62971	1.89226	0.05
62972	1.89226	0.05
62973	1.29881	0.05
62974	1.09985	0.05
62975	0.946126	0.05
62976	0.946126	0.05
62977	0.851539	0.05
62978	1.20421	0.05
62979	0.851483	0.05
62980	0.999426	0.05
62981	1.47172	0.05
62982	0.999413	0.05
62983	0.851552	0.05
62984	0.851552	0.05
62985	1.89226	0.05

62986	1.89226	0.05
62987	1.89226	0.05
62988	1.89226	0.05
62989	1.89226	0.05
62990	1.89226	0.05
62991	1.89226	0.05
62992	1.89226	0.05
62993	1.89226	0.05
62994	1.89226	0.05
62995	1.89226	0.05
62996	1.89226	0.05
62997	1.33915	2.1
62998	1.99297	2.1
62999	2.05216	2.1
63000	2.60163	2.1
63001	4.04513	2.1
63002	1.89226	2.1
63003	1.89226	2.1
63004	1.89226	2.1
63005	1.89226	2.1
63006	3.78452	2.1
63007	0.946132	2.1
63008	0.946132	2.1
63009	0.946133	2.1
63010	0.946133	2.1
63011	0.946132	2.1
63012	0.946132	1.78607
63013	0.946133	2.1
63014	0.946133	2.1
63015	0.946132	2.1
63016	0.946132	2.1
63017	0.946132	2.1
63018	0.946132	2.1
63019	1.89226	2.1
63020	1.89226	1.26113
63021	1.89226	1.38694
63022	1.89226	2.1
63023	3.78452	1.83781
63024	1.89227	1.3227
63025	1.89227	2.1
63026	1.89227	2.1
63027	1.89227	1.27117
63028	1.89227	2.1
63029	1.89227	1.49992
63030	2.29301	2.1
63031	1.70303	2.1
63032	2.46749	2.1
63033	2.30728	2.1
63034	4.53937	2.1
63035	1.89226	1.08604
63036	1.89226	2.1
63037	1.89226	2.1
63038	1.89226	1.09282
63039	3.78452	2.1
63040	1.89226	0.903979
63041	1.89226	2.1
63042	1.89226	2.1
63043	1.89226	2.1

63044	1.89226	0.980716
63045	1.89226	1.19888
63046	2.30728	2.1
63047	1.00757	1.76904
63048	2.0833	2.1
63049	1.65318	2.1
63050	1.417	2.1
63051	1.00757	2.1
63052	2.46749	2.1
63053	2.0975	2.1
63054	2.79653	2.1
63055	2.44785	2.1
63056	4.91534	2.1
63057	1.89226	2.1
63058	1.89226	2.1
63059	1.89226	1.06918
63060	1.89226	2.1
63061	3.78452	1.9157
63062	1.89226	1.20054
63063	1.89226	2.1
63064	1.89226	2.1
63065	1.89226	1.65992
63066	1.89226	2.1
63067	1.89226	1.54456
63068	0.970559	2.1
63069	0.970559	2.1
63070	0.979305	2.1
63071	0.979305	2.1
63072	0.708502	2.1
63073	0.901117	2.1
63074	1.04875	2.1
63075	1.22392	2.1
63076	1.22392	2.1
63077	0.901117	2.1
63078	1.04875	2.1
63079	0.7085	2.1
63080	1.89226	1.53025
63081	1.89226	1.07448
63082	1.89226	1.23314
63083	1.89226	1.3094
63084	3.78452	1.65184
63085	1.89226	1.35554
63086	1.89226	0.829454
63087	1.89226	1.3335
63088	1.89226	1.16537
63089	3.78452	1.14105
63090	1.89226	0.146564
63091	1.89226	0.05
63092	1.89226	0.208097
63093	1.89226	0.114037
63094	3.78453	0.161965
63095	1.89226	0.927964
63096	1.89226	0.65921
63097	1.89226	0.383308
63098	1.89226	0.349667
63099	1.89226	0.376952
63100	1.89226	0.152356
63101	1.89226	0.992868

63102	2.35441	0.990102
63103	1.89226	1.24075
63104	2.30931	0.174329
63105	4.24667	0.649374
63106	1.11612	0.128709
63107	0.946131	0.0812691
63108	1.1772	0.251572
63109	0.946131	0.678118
63110	1.17721	0.598415
63111	1.11612	0.225825
63112	0.94613	0.141061
63113	0.94613	0.789934
63114	1.11612	0.209072
63115	1.11612	0.283435
63116	0.946131	0.141464
63117	0.946131	0.182554
63118	1.11612	0.05
63119	0.582675	0.05
63120	1.17721	0.05
63121	0.651969	0.05
63122	0.651969	0.05
63123	0.582674	0.05
63124	1.17721	0.05
63125	1.11612	0.05
63126	0.877955	0.05
63127	0.877955	0.05
63128	0.881925	0.05
63129	0.881925	0.05
63130	1.17721	0.0700747
63131	0.651969	0.05
63132	1.15466	0.05167
63133	0.623642	0.0541698
63134	0.623642	0.0642748
63135	0.651969	0.05
63136	1.17721	0.0782284
63137	1.15466	0.0632825
63138	0.904714	0.0941266
63139	0.904714	0.05
63140	0.901914	0.0909483
63141	0.901914	0.0527112
63142	1.89226	1.15678
63143	1.89226	0.914429
63144	1.89226	0.866749
63145	1.89226	1.30183
63146	1.89226	0.372921
63147	1.89226	2.1
63148	1.89226	1.2053
63149	1.89226	0.401931
63150	1.89226	0.254052
63151	1.89226	2.1
63152	1.89226	0.243037
63153	1.89226	2.1
63154	2.33986	2.06503
63155	1.97819	1.0461
63156	2.39166	0.0980471
63157	2.70141	2.1
63158	4.73151	0.987364
63159	0.989095	1.06079

63160	1.19583	0.801499
63161	0.851194	0.808475
63162	0.581197	0.891544
63163	0.872704	0.398475
63164	0.872704	1.17646
63165	0.904322	0.816956
63166	0.904322	1.00614
63167	1.19583	0.646189
63168	0.851193	0.666375
63169	0.989095	0.958001
63170	0.581197	0.874554
63171	1.89226	0.999731
63172	1.89226	0.966242
63173	1.89226	0.962058
63174	1.89226	1.39459
63175	1.89226	0.88889
63176	1.89226	2.1
63177	1.89226	1.20761
63178	1.89226	0.70822
63179	1.89226	0.419839
63180	1.89226	2.1
63181	1.89226	0.255054
63182	1.89226	2.1
63183	2.39166	0.963346
63184	1.70239	1.04664
63185	2.13665	0.826194
63186	2.82684	0.896245
63187	4.52831	0.938028
63188	1.89226	0.05
63189	1.89226	0.05
63190	1.89226	0.05
63191	1.89226	0.05
63192	3.78453	0.05
63193	1.89226	0.05
63194	1.89226	0.05
63195	1.89226	0.05
63196	1.89226	0.05
63197	1.89226	0.05
63198	1.89226	0.05
63199	1.89226	0.05
63200	1.89226	0.05
63201	1.89226	0.05
63202	1.89226	0.05
63203	1.89226	0.05
63204	1.89226	0.05
63205	1.89226	0.05
63206	1.89226	0.05
63207	1.89226	0.102569
63208	1.89226	0.05
63209	1.89226	0.125789
63210	1.89226	0.05
63211	1.89226	0.05
63212	1.89226	0.05
63213	1.89226	0.05
63214	1.89226	0.05
63215	1.89226	0.0943839
63216	1.89226	0.05
63217	1.5169	0.05

63218	1.61969	0.05
63219	1.17262	0.05
63220	1.17262	0.05
63221	1.88868	0.05
63222	1.88868	0.05
63223	1.79981	0.05
63224	1.88868	0.05
63225	1.5169	0.05
63226	1.5169	0.05
63227	2.12891	0.05
63228	2.12891	0.0660961
63229	1.89226	0.0536822
63230	1.89226	0.05
63231	1.89226	0.05
63232	1.89226	0.05
63233	3.78452	0.05
63234	0.946132	0.05
63235	0.946132	0.05
63236	0.946132	0.05
63237	0.946132	0.05
63238	0.946132	0.05
63239	0.946132	0.05
63240	0.946133	0.05
63241	0.946133	0.05
63242	0.946133	0.05
63243	0.946133	0.05
63244	0.946132	0.05
63245	0.946132	0.05
63246	1.88868	0.05
63247	2.05759	0.05
63248	1.61969	0.05
63249	1.61969	0.05
63250	2.29747	0.05
63251	2.29747	0.05
63252	2.12891	0.0628943
63253	2.29747	0.05
63254	1.88868	0.05
63255	1.88868	0.05
63256	2.50894	0.05
63257	2.50894	0.05
63258	1.89226	0.05
63259	1.89226	0.05
63260	1.89226	0.05
63261	1.89226	0.05
63262	1.89226	0.05
63263	1.89226	0.064387
63264	0.946132	0.05
63265	0.946132	0.05
63266	0.946132	0.05
63267	0.946132	0.05
63268	0.946131	0.05
63269	0.946131	0.05
63270	0.946132	0.05
63271	0.946132	0.05
63272	0.946132	0.05
63273	0.946132	0.05
63274	0.946132	0.05
63275	0.946132	0.05

63276	2.42758	1.5744
63277	2.14895	2.1
63278	2.56975	1.35756
63279	2.28142	2.1
63280	4.71869	2.1
63281	1.89226	1.28454
63282	1.89226	2.1
63283	1.89226	1.39573
63284	1.89226	2.1
63285	3.78452	2.1
63286	1.89226	2.1
63287	1.89226	2.1
63288	1.89226	2.1
63289	1.89226	2.1
63290	1.89226	2.1
63291	1.89226	2.1
63292	0.960916	2.1
63293	0.960916	2.1
63294	0.978118	2.1
63295	0.875674	2.1
63296	0.775661	2.1
63297	0.773613	2.1
63298	1.07631	2.1
63299	1.14266	2.1
63300	1.16927	2.1
63301	0.898612	2.1
63302	1.04757	2.1
63303	0.757536	2.1
63304	2.56975	1.2825
63305	2.28142	2.1
63306	2.64384	1.47099
63307	2.40621	2.1
63308	4.97596	2.1
63309	1.89226	1.30713
63310	1.89226	2.1
63311	1.89226	0.20945
63312	1.89226	2.1
63313	3.78452	2.1
63314	1.89226	2.1
63315	1.89226	2.1
63316	1.89226	2.1
63317	1.89226	2.1
63318	1.89226	2.1
63319	1.89226	2.1
63320	1.03328	2.1
63321	1.03328	2.1
63322	1.04321	2.1
63323	1.15424	2.1
63324	0.936183	2.1
63325	0.944491	2.1
63326	1.14266	2.1
63327	1.20517	2.1
63328	1.23323	2.1
63329	0.961268	2.1
63330	1.11215	2.1
63331	0.827247	2.1
63332	1.89226	2.1
63333	1.89226	2.1

63334	1.89226	2.1
63335	1.89226	2.1
63336	3.78453	2.1
63337	2.14647	2.1
63338	2.20589	2.1
63339	1.95567	2.1
63340	2.03224	2.1
63341	4.17871	2.1
63342	2.06035	2.1
63343	1.88452	0.105082
63344	1.88452	2.1
63345	2.26997	0.05
63346	2.03224	2.1
63347	2.42272	0.368358
63348	1.89226	2.1
63349	1.89226	0.05
63350	1.89226	0.05
63351	1.89226	2.1
63352	1.89226	0.05
63353	1.89226	2.1
63354	1.89225	2.1
63355	1.89225	2.1
63356	1.89225	2.1
63357	1.89225	2.1
63358	1.89225	2.1
63359	1.89225	2.1
63360	0.952418	2.1
63361	0.952418	2.1
63362	0.95883	2.1
63363	0.95883	2.1
63364	0.946182	2.1
63365	1.01611	2.1
63366	0.895134	2.1
63367	0.977828	2.1
63368	0.89514	2.1
63369	0.946189	2.1
63370	0.977821	2.1
63371	1.01611	2.1
63372	2.03223	2.1
63373	1.88451	0.112094
63374	1.89236	2.1
63375	1.74649	0.05
63376	3.77687	0.38618
63377	0.81094	2.1
63378	0.81094	0.163418
63379	0.810939	2.1
63380	0.810939	2.1
63381	0.810945	1.04146
63382	0.810945	1.65788
63383	1.89225	0.05
63384	1.35167	0.753013
63385	1.08131	2.1
63386	1.08131	0.05
63387	1.0813	1.10422
63388	1.89226	0.05
63389	1.89226	0.05
63390	1.89226	0.05
63391	1.89226	0.05

63392	1.89226	0.05
63393	1.89226	0.05
63394	1.01842	0.05
63395	0.936656	0.05
63396	0.946131	0.05
63397	0.946131	0.05
63398	1.11157	0.05
63399	1.04281	0.05
63400	0.925803	0.05
63401	0.925803	0.05
63402	0.851553	0.05
63403	1.11158	0.05
63404	1.05017	0.05
63405	0.925798	0.05
63406	1.89226	0.05
63407	1.89226	0.05
63408	1.89226	0.05
63409	1.89226	0.05
63410	1.89226	0.05
63411	1.89226	0.05
63412	1.23504	0.05
63413	1.23504	0.05
63414	0.946131	0.05
63415	0.946131	0.05
63416	1.37358	0.05
63417	1.23504	0.05
63418	0.946129	0.05
63419	0.946129	0.05
63420	1.37358	0.05
63421	0.946131	0.05
63422	0.94613	0.05
63423	1.23504	0.05
63424	1.00261	0.05
63425	1.00261	0.05
63426	1.02952	0.05
63427	1.02952	0.05
63428	0.850964	0.05
63429	0.658553	0.05
63430	1.37358	0.05
63431	1.23504	0.05
63432	1.37358	0.05
63433	0.850965	0.05
63434	1.23504	0.05
63435	0.658553	0.05
63436	0.946133	0.05
63437	0.946133	0.05
63438	0.946132	0.05
63439	0.946132	0.05
63440	0.946132	0.05
63441	0.946132	0.05
63442	0.946133	0.05
63443	0.946133	0.05
63444	0.946133	0.05
63445	0.946133	0.05
63446	0.946132	0.05
63447	0.946132	0.05
63448	1.89226	0.0640862
63449	1.89226	0.0653949

63450	1.89226	0.05
63451	1.89226	0.05
63452	3.78452	0.0793657
63453	1.89226	0.0728806
63454	1.89226	0.138359
63455	1.89226	0.05
63456	1.89226	0.05
63457	3.78452	0.05
63458	1.89227	0.0595288
63459	1.89227	0.0509861
63460	1.89227	0.05
63461	1.89227	0.05
63462	1.89227	0.05
63463	1.89227	0.05
63464	0.94613	0.05
63465	0.94613	0.05
63466	0.946127	0.05
63467	0.946127	0.05
63468	0.94613	0.05
63469	0.94613	0.05
63470	0.946127	0.05
63471	0.946127	0.05
63472	0.946128	0.05
63473	0.946128	0.05
63474	0.946128	0.05
63475	0.946128	0.05
63476	0.946132	0.05
63477	0.946132	0.05
63478	0.946128	0.05
63479	0.946128	0.05
63480	0.94613	0.05
63481	0.94613	0.05
63482	0.946131	0.05
63483	0.946131	0.05
63484	0.94613	0.05
63485	0.94613	0.05
63486	0.94613	0.05
63487	0.94613	0.05
63488	1.89226	2.1
63489	1.89226	2.1
63490	1.89226	2.1
63491	1.89226	2.1
63492	3.78453	2.1
63493	2.72844	2.1
63494	1.89226	2.1
63495	2.26536	2.1
63496	2.26536	2.1
63497	1.89226	2.1
63498	1.89226	2.1
63499	1.89226	2.1
63500	1.89226	2.1
63501	1.89226	2.1
63502	1.89226	2.1
63503	1.89226	2.1
63504	1.89226	2.1
63505	1.89226	2.1
63506	1.89226	2.1
63507	1.89226	2.1

63508	1.89226	2.1
63509	1.89226	2.1
63510	1.89226	2.1
63511	2.72844	2.1
63512	2.26536	2.1
63513	2.28367	2.1
63514	1.5791	2.1
63515	4.54903	2.1
63516	0.810945	2.1
63517	0.810945	2.1
63518	0.810943	2.1
63519	0.810943	2.1
63520	1.89226	2.1
63521	1.3517	2.1
63522	1.08133	2.1
63523	1.08132	2.1
63524	1.08132	2.1
63525	0.810933	2.1
63526	0.810933	2.1
63527	1.89227	2.1
63528	1.89227	2.1
63529	1.89227	2.1
63530	1.89227	2.1
63531	3.78453	2.1
63532	2.71897	2.1
63533	2.27495	2.1
63534	2.648 2.1	
63535	2.20832	2.1
63536	4.92729	2.1
63537	2.27495	2.1
63538	1.38455	2.1
63539	1.38455	2.1
63540	1.46586	2.1
63541	2.20831	2.1
63542	2.28962	2.1
63543	0.946133	2.1
63544	0.946133	2.1
63545	0.946132	2.1
63546	0.946132	2.1
63547	0.946132	2.1
63548	0.946132	2.1
63549	0.946132	2.1
63550	0.946132	2.1
63551	0.946132	2.1
63552	0.946132	2.1
63553	0.946133	2.1
63554	0.946133	2.1
63555	1.89226	2.1
63556	1.89226	2.1
63557	1.89226	2.1
63558	1.89226	2.1
63559	1.89226	2.1
63560	1.89226	2.1
63561	2.648 2.1	
63562	2.08612	2.1
63563	2.08612	2.1
63564	2.20831	2.1
63565	2.56618	2.1

63566	2.68837	2.1
63567	2.08612	2.1
63568	2.20831	2.1
63569	1.30659	2.1
63570	1.30659	2.1
63571	1.38455	2.1
63572	2.16408	2.1
63573	0.810946	2.1
63574	0.810946	2.1
63575	0.810946	2.1
63576	0.810946	2.1
63577	1.89226	2.1
63578	1.3517	2.1
63579	1.08133	2.1
63580	1.08132	2.1
63581	1.08132	2.1
63582	0.810934	2.1
63583	0.810934	2.1
63584	1.89226	1.9163
63585	1.89226	1.441
63586	1.89226	1.10285
63587	1.89226	2.1
63588	3.78452	1.32207
63589	1.89226	1.13916
63590	1.89226	2.1
63591	1.89226	2.1
63592	1.89226	1.34912
63593	3.78453	2.0894
63594	1.08733	2.1
63595	0.856615	2.1
63596	1.25964	1.47313
63597	1.12378	1.4169
63598	1.23551	1.25414
63599	0.969894	2.1
63600	0.985977	2.1
63601	0.985977	1.29642
63602	1.16169	1.74166
63603	1.21072	1.9951
63604	0.765175	1.97076
63605	0.863419	1.8895
63606	2.39934	2.1
63607	2.44427	1.47643
63608	2.64003	1.11564
63609	2.39077	2.1
63610	2.39934	2.00035
63611	2.10194	2.1
63612	1.89226	1.13246
63613	1.89226	2.1
63614	1.89226	1.86732
63615	1.89226	0.986922
63616	3.78452	1.86342
63617	0.946132	2.1
63618	0.946132	2.1
63619	0.946131	1.22261
63620	0.946131	1.28223
63621	0.946132	1.22251
63622	0.946132	2.1
63623	0.946132	2.1

63624	0.946132	1.35276
63625	0.946132	1.72583
63626	0.946132	2.0061
63627	0.946132	1.56172
63628	0.946132	1.99695
63629	1.19967	2.1
63630	0.946128	1.83585
63631	0.946133	1.19695
63632	1.32002	1.5371
63633	1.19967	2.1
63634	1.19967	2.02597
63635	0.94613	2.1
63636	0.94613	0.8693
63637	0.946131	1.33327
63638	0.946131	1.47181
63639	1.19967	1.61146
63640	1.32001	1.62353
63641	1.89226	0.636369
63642	1.89226	2.1
63643	1.89226	0.10543
63644	1.89226	2.1
63645	3.78451	0.450741
63646	2.6402	0.598096
63647	2.28552	2.1
63648	2.50958	0.05
63649	2.18948	0.885875
63650	4.79509	0.336895
63651	2.28552	0.816447
63652	1.86102	2.1
63653	2.18949	0.877289
63654	1.78376	2.1
63655	4.06928	2.1
63656	1.89226	0.717423
63657	1.89226	2.1
63658	1.89226	2.1
63659	1.89226	2.1
63660	3.78452	2.1
63661	1.89226	0.149499
63662	1.89226	2.1
63663	1.89226	0.05
63664	1.89226	2.06641
63665	3.78452	0.113322
63666	2.50958	0.0578679
63667	2.18948	0.882746
63668	2.39524	0.05
63669	2.10188	2.0763
63670	4.61146	0.0691443
63671	2.18949	0.139053
63672	1.78376	2.1
63673	1.71839	2.09347
63674	1.71839	2.1
63675	2.10189	2.1
63676	2.16726	2.1
63677	0.810943	2.1
63678	0.810943	2.1
63679	0.810945	1.56453
63680	0.810945	1.8148
63681	0.810959	2.1

63682	0.810959	2.1
63683	1.89226	2.1
63684	1.35168	2.1
63685	1.08132	2.1
63686	1.08132	2.1
63687	1.0813	2.1
63688	1.89226	0.879352
63689	1.89226	1.84397
63690	1.89226	1.10334
63691	1.89226	1.078
63692	1.89226	1.24936
63693	1.89226	1.72436
63694	1.28421	1.36586
63695	0.992041	0.933447
63696	1.07707	2.1
63697	0.789746	1.91012
63698	1.03815	2.1
63699	1.03815	1.77126
63700	1.03581	1.96656
63701	1.03581	1.21752
63702	1.28421	1.63354
63703	1.07707	2.1
63704	0.789748	1.70085
63705	0.992038	1.22134
63706	1.89226	0.935512
63707	1.89226	1.92508
63708	1.89226	1.21143
63709	1.89226	1.01675
63710	1.89226	1.27134
63711	1.89226	1.05768
63712	1.89226	0.885749
63713	1.89226	1.25204
63714	1.89226	1.00621
63715	1.89226	1.03137
63716	1.89226	1.06713
63717	1.89226	1.3873
63718	0.92236	2.1
63719	0.655971	2.05826
63720	0.916772	2.1
63721	0.913834	1.26514
63722	1.18074	1.95174
63723	0.922346	2.1
63724	2.15414	1.02222
63725	1.53528	1.83958
63726	1.22748	2.08968
63727	1.57442	1.10723
63728	0.874683	1.94147
63729	1.25935	2.1
63730	2.61433	2.1
63731	1.22262	2.1
63732	1.22262	2.1
63733	2.62813	2.1
63734	2.62813	2.1
63735	1.89226	2.1
63736	2.61434	2.1
63737	2.62814	2.1
63738	2.61434	2.1
63739	1.90607	2.1

63740	1.89226	2.1
63741	1.89226	2.1
63742	1.89226	2.1
63743	1.89226	2.1
63744	1.89226	2.1
63745	1.89226	2.1
63746	1.89226	2.1
63747	1.89226	2.1
63748	1.89226	2.1
63749	1.89226	2.1
63750	1.89226	2.1
63751	1.89226	2.1
63752	1.89226	2.1
63753	1.31969	2.1
63754	1.31406	2.1
63755	0.629677	2.1
63756	0.643104	2.1
63757	0.976621	2.1
63758	0.976621	2.1
63759	0.98055	2.1
63760	0.98055	2.1
63761	1.3197	2.1
63762	0.643102	2.1
63763	0.629675	2.1
63764	1.31407	2.1
63765	0.946133	2.1
63766	0.946133	2.1
63767	1.3197	2.1
63768	1.31407	2.1
63769	0.946132	2.1
63770	0.946132	2.1
63771	1.3197	2.1
63772	1.3197	2.1
63773	1.3197	2.1
63774	0.946133	2.1
63775	0.946132	2.1
63776	1.31407	2.1
63777	1.89226	2.1
63778	1.89226	2.1
63779	1.89226	2.1
63780	1.89226	2.1
63781	1.89226	2.1
63782	1.89226	2.1
63783	1.89226	2.1
63784	1.89226	2.1
63785	1.89226	2.1
63786	1.89226	2.1
63787	1.89226	2.1
63788	1.89226	2.1
63789	1.89226	0.05
63790	1.89226	0.05
63791	1.89226	0.05
63792	1.89226	0.05
63793	3.78452	0.05
63794	1.89226	0.05
63795	1.89226	0.05
63796	1.89226	0.05
63797	1.89226	0.05

63798	1.89226	0.05
63799	1.89226	0.05
63800	0.946131	0.05
63801	0.946131	0.05
63802	0.946128	0.05
63803	0.946128	0.05
63804	0.94613	0.05
63805	0.94613	0.05
63806	0.94613	0.05
63807	0.94613	0.05
63808	0.94613	0.05
63809	0.94613	0.05
63810	0.94613	0.05
63811	0.94613	0.05
63812	1.89226	0.05
63813	1.89226	0.05
63814	1.89226	0.05
63815	1.89226	0.05
63816	1.89226	0.05
63817	1.89226	0.05
63818	0.924614	0.05
63819	0.914275	0.05
63820	0.906003	0.05
63821	0.62479	0.05
63822	2.02733	0.05
63823	1.48332	0.05
63824	0.833106	0.05
63825	1.19364	0.05
63826	1.57482	0.05
63827	1.18103	0.05
63828	0.905988	0.05
63829	1.89226	2.1
63830	1.89226	2.1
63831	1.89226	2.1
63832	1.89226	2.1
63833	3.78452	2.1
63834	1.89226	2.1
63835	1.89226	2.1
63836	1.89226	2.1
63837	1.89226	2.1
63838	1.89226	2.1
63839	1.89226	2.1
63840	0.816805	2.1
63841	0.928516	2.1
63842	1.4045	2.1
63843	1.26268	2.1
63844	1.0881	2.1
63845	1.0881	2.1
63846	1.10309	2.1
63847	1.10309	2.1
63848	0.928518	2.1
63849	1.4045	2.1
63850	0.816807	2.1
63851	1.26267	2.1
63852	1.89226	2.1
63853	2.52535	2.1
63854	1.89226	2.1
63855	1.89226	2.1

63856	2.809	2.1
63857	2.809	2.1
63858	1.89226	2.1
63859	1.89226	2.1
63860	1.89226	2.1
63861	1.89226	2.1
63862	3.78452	2.1
63863	0.946127	2.1
63864	0.946127	2.1
63865	0.946131	2.1
63866	0.946131	2.1
63867	0.94613	2.1
63868	0.94613	2.1
63869	0.946128	2.1
63870	0.946128	2.1
63871	0.946129	2.1
63872	0.946129	2.1
63873	0.946129	2.1
63874	0.946129	2.1
63875	1.89224	0.849278
63876	1.89224	2.1
63877	1.89224	2.1
63878	1.89224	2.1
63879	3.78448	2.1
63880	2.39244	2.1
63881	2.27036	2.1
63882	2.25945	2.1
63883	2.27398	2.1
63884	4.52981	2.1
63885	2.27036	2.1
63886	1.97088	2.1
63887	1.86302	2.1
63888	1.84991	2.1
63889	4.12027	2.1
63890	1.89224	2.1
63891	1.89224	2.1
63892	1.89224	2.1
63893	1.89224	2.1
63894	3.78447	2.1
63895	1.89226	2.1
63896	1.89226	2.1
63897	1.89226	0.861548
63898	1.89226	2.1
63899	3.78453	2.1
63900	2.25948	2.1
63901	2.33611	2.1
63902	2.05257	2.1
63903	1.94441	2.1
63904	4.20389	2.1
63905	1.92514	2.1
63906	1.84993	2.1
63907	1.94441	2.1
63908	1.74391	2.1
63909	3.79434	2.1
63910	1.89226	2.1
63911	1.89226	2.1
63912	1.89226	2.1
63913	1.89226	2.1

63914	3.78452	2.1
63915	15.1381	0.630487
63916	15.1381	0.746841
63917	15.1381	0.214827
63918	15.1381	0.970586
63919	30.2762	0.60992
63920	15.1381	0.699596
63921	15.1381	0.831952
63922	15.1381	0.735919
63923	15.1381	0.763232
63924	30.2762	0.761713
63925	15.1381	2.1
63926	15.1381	2.1
63927	15.1381	1.92
63928	15.1381	2.1
63929	30.2762	2.1
63930	15.1381	0.519898
63931	15.1381	0.763025
63932	15.1381	0.248447
63933	15.1381	0.718289
63934	30.2762	0.320349
63935	1.89225	2.1
63936	1.89225	2.1
63937	1.89225	2.1
63938	1.89225	2.1
63939	3.78449	2.1
63940	1.39909	2.1
63941	1.39909	2.1
63942	0.946123	2.1
63943	0.946123	2.1
63944	1.3291	2.1
63945	1.39908	2.1
63946	0.946126	2.1
63947	0.946126	2.1
63948	0.946118	2.1
63949	1.32909	2.1
63950	0.94613	2.1
63951	1.3991	2.1
63952	3.07801	2.1
63953	1.84322	2.1
63954	1.84322	2.1
63955	1.68788	2.1
63956	2.6582	2.1
63957	2.50287	2.1
63958	1.89225	2.1
63959	1.89225	2.1
63960	1.89225	2.1
63961	1.89225	2.1
63962	3.7845	2.1
63963	1.89226	2.1
63964	1.89226	2.1
63965	1.89226	2.1
63966	1.89226	2.1
63967	3.78452	2.1
63968	1.89226	2.1
63969	2.50051	2.1
63970	2.50051	2.1
63971	2.65822	2.1

63972	1.89226	2.1
63973	2.04997	2.1
63974	2.50052	2.1
63975	2.65822	2.1
63976	1.70377	2.1
63977	1.70377	2.1
63978	1.84324	2.1
63979	2.63998	2.1
63980	0.810947	2.1
63981	0.810947	2.1
63982	0.810946	2.1
63983	0.810946	2.1
63984	1.89226	2.1
63985	1.35169	2.1
63986	1.08132	2.1
63987	1.08132	2.1
63988	1.08132	2.1
63989	0.810946	2.1
63990	0.810946	2.1
63991	1.89226	2.1
63992	1.89226	2.1
63993	1.89226	2.1
63994	1.89226	2.1
63995	3.78453	2.1
63996	0.946164	2.1
63997	0.946164	2.1
63998	0.929842	2.1
63999	0.929842	2.1
64000	0.946132	2.1
64001	0.946132	2.1
64002	1.04101	2.1
64003	0.929874	2.1
64004	0.946132	2.1
64005	0.929874	2.1
64006	1.04101	2.1
64007	0.946132	2.1
64008	1.89226	2.1
64009	1.89226	2.1
64010	1.89226	2.1
64011	1.89226	2.1
64012	3.78453	2.1
64013	1.89226	2.1
64014	1.89226	2.1
64015	1.89226	2.1
64016	1.89226	2.1
64017	1.89226	2.1
64018	1.89226	2.1
64019	1.89226	2.1
64020	1.89226	2.1
64021	1.89226	2.1
64022	1.89226	2.1
64023	1.89226	2.1
64024	1.89226	2.1
64025	0.946131	2.1
64026	0.946131	2.1
64027	0.946131	2.1
64028	0.946131	2.1
64029	0.946132	2.1

64030	0.946132	2.1
64031	0.946131	2.1
64032	0.946131	2.1
64033	0.946131	2.1
64034	0.946131	2.1
64035	0.946132	2.1
64036	0.946132	2.1
64037	1.89226	1.23255
64038	1.89226	0.783699
64039	1.89226	0.05
64040	1.89226	0.788497
64041	3.78453	0.673263
64042	2.73497	0.05
64043	1.93823	0.482464
64044	1.93823	0.05
64045	2.20046	0.806073
64046	2.42279	0.05
64047	2.68501	2.1
64048	1.89226	0.124392
64049	1.89226	0.873435
64050	1.89226	2.1
64051	1.89226	0.05
64052	1.89226	2.1
64053	1.89226	0.05
64054	1.89226	0.05
64055	1.89226	0.109001
64056	1.89226	0.192841
64057	1.89226	1.27186
64058	1.89226	0.05
64059	1.89226	0.152615
64060	2.82984	0.656859
64061	2.15276	0.0509691
64062	2.15276	0.593685
64063	2.42278	0.05
64064	2.50074	0.05
64065	2.77077	0.345726
64066	2.42279	0.05
64067	1.93823	0.856738
64068	1.70423	0.05
64069	1.70423	0.0577643
64070	2.15276	0.05
64071	2.38676	0.528851
64072	0.810946	0.0866812
64073	0.810946	1.07632
64074	0.810944	0.0565783
64075	0.810944	0.0562252
64076	0.810946	0.630021
64077	0.810946	0.563346
64078	1.89226	0.879919
64079	1.35169	0.05
64080	1.08132	0.360566
64081	1.08132	0.143132
64082	1.08132	0.05
64083	1.89226	2.1
64084	1.89226	2.1
64085	1.89226	2.1
64086	1.89226	2.1
64087	3.78452	2.1

64088	1.89226	2.1
64089	1.89226	2.1
64090	1.89226	2.1
64091	1.89226	2.1
64092	3.78453	2.1
64093	1.04144	2.1
64094	1.04144	2.1
64095	0.938972	2.1
64096	0.938972	2.1
64097	0.969661	2.1
64098	1.03285	2.1
64099	0.946133	2.1
64100	0.946133	2.1
64101	0.946131	2.1
64102	0.969663	2.1
64103	0.946131	2.1
64104	1.03284	2.1
64105	0.946131	2.1
64106	0.946131	2.1
64107	0.946128	2.1
64108	0.946128	2.1
64109	0.946127	2.1
64110	0.946127	2.1
64111	0.946131	2.1
64112	0.946131	2.1
64113	0.94613	2.1
64114	0.94613	2.1
64115	0.946129	2.1
64116	0.946129	2.1
64117	1.89226	2.1
64118	1.89226	2.1
64119	1.89226	2.1
64120	1.89226	2.1
64121	3.78452	2.1
64122	1.89226	2.1
64123	1.89226	2.1
64124	1.89226	2.1
64125	1.89226	2.1
64126	3.78453	2.1
64127	1.89226	2.1
64128	1.89226	2.1
64129	1.85882	2.1
64130	1.85882	2.1
64131	1.93932	2.1
64132	1.97276	2.1
64133	0.94613	2.1
64134	0.94613	2.1
64135	0.946128	2.1
64136	0.946128	2.1
64137	0.946127	2.1
64138	0.946127	2.1
64139	0.946131	2.1
64140	0.946131	2.1
64141	0.946129	2.1
64142	0.946129	2.1
64143	0.946129	2.1
64144	0.946129	2.1
64145	1.89225	2.1

64146	1.89225	2.1
64147	1.89225	2.1
64148	1.89225	2.1
64149	3.7845	2.1
64150	1.0086	2.1
64151	1.09028	2.1
64152	0.946125	2.1
64153	0.946125	2.1
64154	0.916906	2.1
64155	0.916906	2.1
64156	1.13051	2.1
64157	1.1505	2.1
64158	1.11557	2.1
64159	1.04071	2.1
64160	0.851542	2.1
64161	0.991196	2.1
64162	1.89225	2.1
64163	1.89225	2.1
64164	1.89225	2.1
64165	1.89225	2.1
64166	3.7845	2.1
64167	1.89225	2.1
64168	1.89225	2.1
64169	1.89225	2.1
64170	1.89225	2.1
64171	1.89225	2.1
64172	1.89225	2.1
64173	1.89226	2.1
64174	1.89226	2.1
64175	1.89226	2.1
64176	1.89226	2.1
64177	3.78452	2.1
64178	0.890962	2.1
64179	0.952547	2.1
64180	0.946132	2.1
64181	0.946132	2.1
64182	0.851543	2.1
64183	0.851543	2.1
64184	1.11558	2.1
64185	1.06304	2.1
64186	0.994315	2.1
64187	1.04072	2.1
64188	0.900943	2.1
64189	0.851542	2.1
64190	1.89226	2.1
64191	1.89226	2.1
64192	1.89226	2.1
64193	1.89226	2.1
64194	1.89226	2.1
64195	1.89226	2.1
64196	1.89226	2.1
64197	1.89226	2.1
64198	1.89226	2.1
64199	1.89226	2.1
64200	3.78452	2.1
64201	1.89226	2.1
64202	1.89226	2.1
64203	1.89226	2.1

64204	1.89226	2.1
64205	3.78452	2.1
64206	0.782015	2.1
64207	0.929718	2.1
64208	0.946132	2.1
64209	0.946132	2.1
64210	0.782016	2.1
64211	0.782016	2.1
64212	1.06706	2.1
64213	1.11785	2.1
64214	0.782015	2.1
64215	0.946133	2.1
64216	0.878931	2.1
64217	0.946132	2.1
64218	1.89226	2.1
64219	1.89226	2.1
64220	1.89226	2.1
64221	1.89226	2.1
64222	1.89226	2.1
64223	1.89226	2.1
64224	0.946131	2.1
64225	0.946131	2.1
64226	0.946129	2.1
64227	0.946129	2.1
64228	0.946131	2.1
64229	0.946131	2.1
64230	0.94613	2.1
64231	0.94613	2.1
64232	0.946131	2.1
64233	0.946131	2.1
64234	0.94613	2.1
64235	0.94613	2.1
64236	0.827866	2.1
64237	0.946131	2.1
64238	0.946129	2.1
64239	0.946129	2.1
64240	0.94613	2.1
64241	0.94613	2.1
64242	0.94613	2.1
64243	0.94613	2.1
64244	0.827865	2.1
64245	0.94613	2.1
64246	0.94613	2.1
64247	0.94613	2.1
64248	1.89226	2.1
64249	1.89226	2.1
64250	1.89226	2.1
64251	1.89226	2.1
64252	3.78452	2.1
64253	1.89226	2.1
64254	1.89226	2.1
64255	1.89226	2.1
64256	1.89226	2.1
64257	1.89226	2.1
64258	1.89226	2.1
64259	1.89226	2.1
64260	1.89226	2.1
64261	1.89226	2.1

64262	1.89226	2.1
64263	3.78453	2.1
64264	1.89226	2.1
64265	1.89226	2.1
64266	1.89226	2.1
64267	1.89226	2.1
64268	3.78452	2.1
64269	0.744377	2.1
64270	0.541919	2.1
64271	0.652022	2.1
64272	0.544198	2.1
64273	0.764633	2.1
64274	0.673254	2.1
64275	1.89226	2.1
64276	1.31149	2.1
64277	1.05423	2.1
64278	1.02189	2.1
64279	0.870602	2.1
64280	1.89226	2.1
64281	1.89226	2.1
64282	1.89226	2.1
64283	1.89226	2.1
64284	1.89226	2.1
64285	1.89226	2.1
64286	1.89226	2.1
64287	1.89226	2.1
64288	1.89226	2.1
64289	1.89226	2.1
64290	1.89226	2.1
64291	1.89226	2.1
64292	1.89226	2.1
64293	1.89226	2.1
64294	1.89226	2.1
64295	1.89226	2.1
64296	1.89226	2.1
64297	1.89226	2.1
64298	0.815314	2.1
64299	0.815313	2.1
64300	0.714029	2.1
64301	0.782951	2.1
64302	0.810944	2.1
64303	0.782954	2.1
64304	1.89226	2.1
64305	1.30999	2.1
64306	1.08132	2.1
64307	1.03962	2.1
64308	0.95209	2.1
64309	1.89226	2.1
64310	1.89226	2.1
64311	1.89226	2.1
64312	1.89226	2.1
64313	1.89226	2.1
64314	1.89226	2.1
64315	1.89226	2.1
64316	1.89226	2.1
64317	1.89226	2.1
64318	1.89226	2.1
64319	1.89226	2.1

64320	1.89226	2.1
64321	1.89226	2.1
64322	1.89226	2.1
64323	1.89226	2.1
64324	1.89226	2.1
64325	1.89226	2.1
64326	1.89226	2.1
64327	0.871558	2.1
64328	0.713078	2.1
64329	0.756858	2.1
64330	0.703674	2.1
64331	0.838025	2.1
64332	0.862196	2.1
64333	1.89226	2.1
64334	1.33194	2.1
64335	1.10834	2.1
64336	1.06163	2.1
64337	1.01785	2.1
64338	1.89226	2.1
64339	1.89226	2.1
64340	1.89226	2.1
64341	1.89226	2.1
64342	3.78452	2.1
64343	1.89226	2.1
64344	1.89226	2.1
64345	1.89226	2.1
64346	1.89226	2.1
64347	1.89226	2.1
64348	1.89226	2.1
64349	1.89226	2.1
64350	1.89226	2.1
64351	1.89226	2.1
64352	1.89226	2.1
64353	1.89226	2.1
64354	1.89226	2.1
64355	0.958406	2.1
64356	1.1383	2.1
64357	0.865636	2.1
64358	1.10093	2.1
64359	0.838024	2.1
64360	0.975154	2.1
64361	1.89226	2.1
64362	1.38553	2.1
64363	1.10834	2.1
64364	1.11522	2.1
64365	1.15271	2.1
64366	1.89227	2.1
64367	1.89227	2.1
64368	1.89227	2.1
64369	1.89227	2.1
64370	3.78453	2.1
64371	0.946132	2.1
64372	0.946132	2.1
64373	0.946132	2.1
64374	0.946132	2.1
64375	0.946132	2.1
64376	0.946132	2.1
64377	0.946133	2.1

64378	0.946133	2.1
64379	0.946132	2.1
64380	0.946132	2.1
64381	0.946133	2.1
64382	0.946133	2.1
64383	0.719271	2.1
64384	0.774968	2.1
64385	1.28401	2.1
64386	1.17415	2.1
64387	0.960999	2.1
64388	0.960999	2.1
64389	0.988115	2.1
64390	0.988115	2.1
64391	0.774968	2.1
64392	1.28401	2.1
64393	0.719271	2.1
64394	1.17415	2.1
64395	2.77811	2.1
64396	2.34829	2.1
64397	2.56802	2.1
64398	2.52333	2.1
64399	2.56802	2.1
64400	2.52333	2.1
64401	1.89227	2.1
64402	1.89227	2.1
64403	1.89227	2.1
64404	1.89227	2.1
64405	3.78453	2.1
64406	1.89226	2.1
64407	1.89226	2.1
64408	1.89226	2.1
64409	1.89226	2.1
64410	1.89226	2.1
64411	1.89226	2.1
64412	1.89226	2.1
64413	2.77811	2.1
64414	1.89226	2.1
64415	2.56802	2.1
64416	1.89226	2.1
64417	2.56802	2.1
64418	1.89226	0.413555
64419	1.89226	2.1
64420	1.89226	0.05
64421	1.89226	2.1
64422	3.78452	0.170221
64423	2.41802	1.22184
64424	1.95764	2.1
64425	1.95764	2.1
64426	2.27727	2.1
64427	2.39311	2.1
64428	2.73961	2.1
64429	0.946132	2.1
64430	0.946132	2.1
64431	0.94613	2.1
64432	0.94613	2.1
64433	0.946131	2.1
64434	0.946131	2.1
64435	0.946131	2.1

64436	0.946131	2.1
64437	0.946131	2.1
64438	0.946131	2.1
64439	0.946131	2.1
64440	0.946131	2.1
64441	1.89226	0.148013
64442	1.89226	2.0846
64443	1.89226	2.1
64444	1.89226	0.339571
64445	1.89226	2.1
64446	1.89226	0.212115
64447	2.78856	0.627123
64448	2.28552	2.1
64449	2.28552	0.7051
64450	2.3931	1.13948
64451	2.6402	0.05
64452	2.74778	0.613581
64453	2.39311	0.333865
64454	1.95764	2.1
64455	2.28552	1.72545
64456	1.86103	2.1
64457	4.24316	2.1
64458	1.89226	1.87949
64459	1.89226	2.1
64460	1.89226	2.1
64461	1.89226	2.1
64462	1.89226	2.1
64463	1.89226	2.1
64464	1.47412	2.1
64465	2.18862	1.84327
64466	2.07226	1.19516
64467	2.33645	1.44711
64468	4.26088	1.47479
64469	1.89226	1.12933
64470	1.89226	1.97339
64471	1.89226	1.22264
64472	1.89226	2.05724
64473	1.89226	1.46027
64474	1.89226	2.1
64475	1.89226	1.46004
64476	1.89226	2.1
64477	1.89226	0.0713057
64478	1.89226	2.1
64479	3.78453	1.42275
64480	2.18862	1.43133
64481	2.1128	2.1
64482	2.33645	1.21926
64483	2.28188	2.1
64484	4.47051	2.1
64485	2.07226	2.1
64486	2.33645	2.05133
64487	2.20831	1.11498
64488	2.4646	1.25528
64489	4.53686	1.3984
64490	0.946131	1.09815
64491	0.946131	2.1
64492	0.946131	2.1
64493	0.946131	1.39398

64494	0.94613	2.1
64495	0.94613	1.28786
64496	0.946133	2.1
64497	0.946133	2.1
64498	0.946132	1.15517
64499	0.946132	1.11212
64500	0.946131	2.1
64501	0.946131	1.69675
64502	1.89226	1.95075
64503	1.89226	2.1
64504	1.89226	1.17666
64505	1.89226	2.1
64506	3.78453	1.64872
64507	2.33646	1.45655
64508	2.28189	2.1
64509	2.4646	1.14933
64510	2.42758	2.1
64511	4.74648	1.50024
64512	1.89226	2.1
64513	1.89226	2.1
64514	1.89226	2.1
64515	1.89226	2.1
64516	1.89226	2.1
64517	1.89226	2.1
64518	1.89226	2.1
64519	1.89226	2.1
64520	1.89226	2.1
64521	1.89226	2.1
64522	1.89226	2.1
64523	1.89226	2.1
64524	1.89226	2.1
64525	1.89226	2.1
64526	1.89226	2.1
64527	1.89226	2.1
64528	3.78453	2.1
64529	1.89226	2.1
64530	1.89226	2.1
64531	1.89226	2.1
64532	1.89226	2.1
64533	1.89226	2.1
64534	1.89226	2.1
64535	1.89226	2.1
64536	1.89226	2.1
64537	1.89226	2.1
64538	1.89226	2.1
64539	3.78452	2.1
64540	0.877238	2.1
64541	0.877238	2.1
64542	1.24746	2.1
64543	1.04764	2.1
64544	0.964969	2.1
64545	1.1521	2.1
64546	0.946132	2.1
64547	0.946132	2.1
64548	0.946132	2.1
64549	1.12659	2.1
64550	0.964969	2.1
64551	0.946131	2.1

64552	1.89226	2.1
64553	1.89226	2.1
64554	1.89226	2.1
64555	1.89226	2.1
64556	1.89226	2.1
64557	1.89226	2.1
64558	1.89226	2.1
64559	1.89226	2.1
64560	1.89226	2.1
64561	1.89226	2.1
64562	3.78453	2.1
64563	0.868144	2.1
64564	0.992762	2.1
64565	0.946131	2.1
64566	0.946131	2.1
64567	1.07072	2.1
64568	1.07072	2.1
64569	0.868171	2.1
64570	0.868171	2.1
64571	0.992762	2.1
64572	0.946131	2.1
64573	0.946131	2.1
64574	0.868144	2.1
64575	0.946132	2.1
64576	0.946132	2.1
64577	0.946131	2.1
64578	0.946131	2.1
64579	0.946132	2.1
64580	0.946132	2.1
64581	0.946132	2.1
64582	0.946132	2.1
64583	0.946132	2.1
64584	0.946132	2.1
64585	0.946132	2.1
64586	0.946132	2.1
64587	1.89227	2.1
64588	1.89227	2.1
64589	1.89227	2.1
64590	1.89227	2.1
64591	1.89227	2.1
64592	1.89227	2.1
64593	1.89226	2.1
64594	1.89226	2.1
64595	1.89226	2.1
64596	1.89226	2.1
64597	1.89226	2.1
64598	1.89226	2.1
64599	1.98552	2.1
64600	2.22298	2.1
64601	2.22298	2.1
64602	1.89226	2.1
64603	1.89226	2.1
64604	1.54525	2.1
64605	0.810946	2.1
64606	0.810946	2.1
64607	0.810947	2.1
64608	0.810947	2.1
64609	0.81096	2.1

64610	0.81096	2.1
64611	1.89226	2.1
64612	1.35168	2.1
64613	1.08132	2.1
64614	1.08132	2.1
64615	1.0813	2.1
64616	1.89226	2.1
64617	1.89226	2.1
64618	1.89226	2.1
64619	1.89226	2.1
64620	3.78452	2.1
64621	1.7753	2.1
64622	2.39703	2.1
64623	1.90577	2.1
64624	2.03445	2.1
64625	3.82335	2.1
64626	2.39703	2.1
64627	2.47949	0.183836
64628	2.26051	2.1
64629	2.36891	0.05
64630	4.76594	2.1
64631	1.89226	2.1
64632	1.89226	0.91453
64633	1.89226	2.1
64634	1.89226	0.05
64635	3.78452	1.52794
64636	1.89226	2.1
64637	1.89226	2.1
64638	1.89226	2.1
64639	1.89226	2.1
64640	1.89226	2.1
64641	1.89226	2.1
64642	0.883745	2.1
64643	0.973143	2.1
64644	0.988173	2.1
64645	0.988173	2.1
64646	0.941927	2.1
64647	1.02971	2.1
64648	0.854065	2.1
64649	0.82702	2.1
64650	0.847835	2.1
64651	0.981126	2.1
64652	1.02996	2.1
64653	1.10704	2.1
64654	2.28031	2.1
64655	2.26369	0.240354
64656	2.26369	2.1
64657	2.36891	0.897174
64658	2.30788	2.1
64659	2.42455	0.203773
64660	1.89226	2.1
64661	1.89226	0.249038
64662	1.89226	0.142553
64663	1.89226	2.1
64664	1.89226	0.412361
64665	1.89226	2.1
64666	2.71406	2.1
64667	1.83244	2.1

64668	1.83244	2.1
64669	2.07567	2.1
64670	2.52044	2.1
64671	2.76367	2.1
64672	1.89226	2.1
64673	1.89226	2.1
64674	1.89226	2.1
64675	1.89226	2.1
64676	3.78453	2.1
64677	1.89226	2.1
64678	1.89226	2.1
64679	1.89226	2.1
64680	1.89226	2.1
64681	1.89226	2.1
64682	1.89226	2.1
64683	2.70714	2.1
64684	2.3148	2.1
64685	2.3148	2.1
64686	2.52044	2.1
64687	2.54204	2.1
64688	2.74768	2.1
64689	2.52044	2.1
64690	2.3148	2.1
64691	1.83244	2.1
64692	1.83244	2.1
64693	1.62611	2.1
64694	2.3148	2.1
64695	0.946131	2.1
64696	0.946131	2.1
64697	0.946131	2.1
64698	0.946131	2.1
64699	0.946131	2.1
64700	0.946131	2.1
64701	0.946131	2.1
64702	0.946131	2.1
64703	0.946131	2.1
64704	0.946131	2.1
64705	0.946131	2.1
64706	0.946131	2.1
64707	1.89226	2.1
64708	1.89226	2.1
64709	1.89226	2.1
64710	1.89226	2.1
64711	3.78453	2.1
64712	1.89226	2.1
64713	1.89226	2.1
64714	1.89226	2.1
64715	1.89226	2.1
64716	1.89226	2.1
64717	1.89226	2.1
64718	1.89227	2.1
64719	1.89227	2.1
64720	1.89227	2.1
64721	1.89227	2.1
64722	3.78453	2.1
64723	1.89226	2.1
64724	1.89226	2.1
64725	1.89226	2.1

64726	1.89226	2.1
64727	3.78453	2.1
64728	0.915781	2.1
64729	0.863524	2.1
64730	0.717056	2.1
64731	0.830182	2.1
64732	0.865094	2.1
64733	0.918003	2.1
64734	1.89226	2.1
64735	1.29933	2.1
64736	1.11215	2.1
64737	1.05227	2.1
64738	0.966269	2.1
64739	1.89226	2.1
64740	1.89226	2.1
64741	1.89226	2.1
64742	1.89226	2.1
64743	1.89226	2.1
64744	1.89226	2.1
64745	1.89226	2.1
64746	1.89226	2.1
64747	1.89226	2.1
64748	1.89226	2.1
64749	1.89226	2.1
64750	1.89226	2.1
64751	1.89226	2.1
64752	1.89226	2.1
64753	1.89226	2.1
64754	1.89226	2.1
64755	1.89226	2.1
64756	1.89226	2.1
64757	1.01725	2.1
64758	0.997048	2.1
64759	0.818657	2.1
64760	0.960724	2.1
64761	0.838013	2.1
64762	1.04859	2.1
64763	1.89226	2.1
64764	1.41754	2.1
64765	1.17625	2.1
64766	1.0793	2.1
64767	1.18822	2.1
64768	2.16402	2.1
64769	1.89452	2.1
64770	1.50084	2.1
64771	2.09288	2.1
64772	3.9874	2.1
64773	2.16402	2.1
64774	2.13925	2.1
64775	2.04483	2.1
64776	2.09288	2.1
64777	4.23212	2.1
64778	1.89226	2.1
64779	1.89226	2.1
64780	1.89226	2.1
64781	1.89226	2.1
64782	3.78452	2.1
64783	1.89226	2.1

64784	1.89226	2.1
64785	1.89226	2.1
64786	1.89226	2.1
64787	1.89226	2.1
64788	1.89226	2.1
64789	1.9824	2.1
64790	2.09288	2.1
64791	1.69889	2.1
64792	1.6817	2.1
64793	2.00695	2.1
64794	2.00695	2.1
64795	1.93592	2.1
64796	2.09288	2.1
64797	2.00695	2.1
64798	2.09288	2.1
64799	1.9589	2.1
64800	2.04483	2.1
64801	0.946128	2.1
64802	0.946128	2.1
64803	0.946132	2.1
64804	0.946132	2.1
64805	0.94613	2.1
64806	0.94613	2.1
64807	0.946129	2.1
64808	0.946129	2.1
64809	0.94613	2.1
64810	0.94613	2.1
64811	0.94613	2.1
64812	0.94613	2.1
64813	0.946131	2.1
64814	0.946131	2.1
64815	0.946131	2.1
64816	0.946131	2.1
64817	0.946131	2.1
64818	0.946131	2.1
64819	0.946131	2.1
64820	0.946131	2.1
64821	0.946131	2.1
64822	0.946131	2.1
64823	0.946131	2.1
64824	0.946131	2.1
64825	1.89226	2.1
64826	2.48083	2.1
64827	2.6968	2.1
64828	2.6968	2.1
64829	1.89226	2.1
64830	1.89226	2.1
64831	1.89226	2.1
64832	1.89226	2.1
64833	1.89226	2.1
64834	1.89226	2.1
64835	3.78453	2.1
64836	1.89226	2.1
64837	1.89226	2.1
64838	1.89226	2.1
64839	1.89226	2.1
64840	1.89226	2.1
64841	1.89226	2.1

64842	1.1017	2.1
64843	1.1017	2.1
64844	1.10605	2.1
64845	1.01647	2.1
64846	0.868255	2.1
64847	0.883424	2.1
64848	1.24624	2.1
64849	1.35473	2.1
64850	0.942231	2.1
64851	1.31464	2.1
64852	0.910064	2.1
64853	1.27147	2.1
64854	1.89226	2.1
64855	1.89226	2.1
64856	1.89226	0.952088
64857	1.89226	2.1
64858	3.78453	2.1
64859	1.89226	2.1
64860	1.89226	2.1
64861	1.89226	2.1
64862	1.89226	2.1
64863	1.89226	2.1
64864	1.89226	2.1
64865	1.89226	2.1
64866	1.89226	2.1
64867	1.89226	2.1
64868	1.89226	2.1
64869	3.78453	2.1
64870	1.89226	2.1
64871	2.70015	2.1
64872	1.89226	2.1
64873	2.61064	2.1
64874	4.59241	2.1
64875	2.70015	2.1
64876	2.3372	2.1
64877	2.61064	2.1
64878	1.85184	2.1
64879	4.55198	2.1
64880	1.89226	2.1
64881	1.89226	2.1
64882	1.89226	2.1
64883	1.89226	2.1
64884	1.89226	2.1
64885	1.89226	2.1
64886	1.89227	2.1
64887	1.89227	2.1
64888	1.89227	2.1
64889	1.89227	2.1
64890	3.78453	2.1
64891	1.89226	2.1
64892	2.52194	2.1
64893	2.61064	2.1
64894	2.61064	2.1
64895	1.89226	2.1
64896	1.89226	2.1
64897	1.095 2.1	
64898	1.095 2.1	
64899	1.09189	2.1

64900	1.09189	2.1
64901	0.875334	2.1
64902	0.925919	2.1
64903	1.26097	2.1
64904	1.30532	2.1
64905	1.26097	2.1
64906	0.875334	2.1
64907	1.30532	2.1
64908	0.92592	2.1
64909	1.89226	2.1
64910	1.89226	2.1
64911	1.89226	2.1
64912	1.89226	2.1
64913	1.89226	2.1
64914	1.89226	2.1
64915	1.89226	2.1
64916	1.89226	2.1
64917	1.89226	2.1
64918	1.89226	2.1
64919	3.78453	2.1
64920	1.26097	2.1
64921	1.26097	2.1
64922	0.946132	2.1
64923	0.946132	2.1
64924	1.22176	2.1
64925	1.26097	2.1
64926	0.946132	2.1
64927	0.946132	2.1
64928	0.946132	2.1
64929	1.22176	2.1
64930	1.26097	2.1
64931	0.946132	2.1
64932	2.52194	2.1
64933	1.67381	2.1
64934	1.67381	2.1
64935	1.75067	2.1
64936	2.44352	2.1
64937	2.52038	2.1
64938	1.89226	2.1
64939	1.89226	2.1
64940	1.89226	2.1
64941	1.89226	2.1
64942	1.89226	2.1
64943	1.89226	2.1
64944	1.89227	2.1
64945	1.89227	2.1
64946	1.89227	2.1
64947	1.89227	2.1
64948	1.89227	2.1
64949	1.89227	2.1
64950	1.22176	2.1
64951	1.22176	2.1
64952	0.946132	2.1
64953	0.946132	2.1
64954	1.19095	2.1
64955	1.22176	2.1
64956	0.946133	2.1
64957	0.946133	2.1

64958	1.19095	2.1
64959	0.946132	2.1
64960	0.946133	2.1
64961	1.22176	2.1
64962	2.44352	2.1
64963	1.6287	2.1
64964	1.6287	2.1
64965	1.67381	2.1
64966	2.3819	2.1
64967	2.427	2.1
64968	1.89226	2.1
64969	1.89226	2.1
64970	1.89226	2.1
64971	1.89226	2.1
64972	1.89226	2.1
64973	1.89226	2.1
64974	1.89226	2.1
64975	1.89226	2.1
64976	1.89226	2.1
64977	1.89226	2.1
64978	3.78453	2.1
64979	1.89226	2.1
64980	1.89226	2.1
64981	1.89226	2.1
64982	1.89226	2.1
64983	1.89226	2.1
64984	1.89226	2.1
64985	1.89226	2.1
64986	1.89226	2.1
64987	1.89226	2.1
64988	1.89226	2.1
64989	1.89226	2.1
64990	1.89226	2.1
64991	0.851542	2.1
64992	0.851542	2.1
64993	1.1736	2.1
64994	1.29261	2.1
64995	0.852708	2.1
64996	1.18843	2.1
64997	0.946132	2.1
64998	0.946132	2.1
64999	0.946132	2.1
65000	1.07901	2.1
65001	1.04208	2.1
65002	0.936669	2.1
65003	1.89227	2.1
65004	1.89227	2.1
65005	1.89227	2.1
65006	1.89227	2.1
65007	3.78453	2.1
65008	1.89226	2.1
65009	1.89226	2.1
65010	1.89226	2.1
65011	1.89226	2.1
65012	3.78453	2.1
65013	1.89226	2.1
65014	1.89226	2.1
65015	1.89226	2.1

65016	1.89226	2.1
65017	1.89226	2.1
65018	1.89226	2.1
65019	0.946133	2.1
65020	0.946133	2.1
65021	1.28525	2.1
65022	1.28525	2.1
65023	1.07901	2.1
65024	1.28525	2.1
65025	0.946132	2.1
65026	0.946132	2.1
65027	0.946132	2.1
65028	1.28525	2.1
65029	0.946133	2.1
65030	1.07901	2.1
65031	1.89226	2.1
65032	1.89226	2.1
65033	1.89226	2.1
65034	1.89226	2.1
65035	1.89226	2.1
65036	1.89226	2.1
65037	1.89226	2.1
65038	1.89226	2.1
65039	1.89226	2.1
65040	1.89226	2.1
65041	1.89226	2.1
65042	1.89226	2.1
65043	1.24419	2.1
65044	1.09093	2.1
65045	1.22722	2.1
65046	1.18154	2.1
65047	1.27455	2.1
65048	1.08473	2.1
65049	1.15541	2.1
65050	1.15541	2.1
65051	1.29607	2.1
65052	1.23814	2.1
65053	1.13024	2.1
65054	1.12096	2.1
65055	1.18858	2.1
65056	1.13181	2.1
65057	0.988718	2.1
65058	0.802498	2.1
65059	0.994034	2.1
65060	1.15773	2.1
65061	1.0029	2.1
65062	1.0029	2.1
65063	1.23814	2.1
65064	0.997523	2.1
65065	1.11498	2.1
65066	0.899532	2.1
65067	1.89226	2.1
65068	1.89226	2.1
65069	1.89226	2.1
65070	1.89226	2.1
65071	1.89226	2.1
65072	1.89226	2.1
65073	1.89226	2.1

65074	1.89226	2.1
65075	1.89226	2.1
65076	1.89226	2.1
65077	1.89226	2.1
65078	1.89226	2.1
65079	0.993139	2.1
65080	0.834108	2.1
65081	1.01147	2.1
65082	1.01358	2.1
65083	1.20067	2.1
65084	0.993156	2.1
65085	2.4158	2.1
65086	1.69956	2.1
65087	1.3544	2.1
65088	1.60095	2.1
65089	1.11219	2.1
65090	1.89226	2.1
65091	1.89226	2.1
65092	1.89226	2.1
65093	1.89226	2.1
65094	3.78452	2.1
65095	1.89226	2.1
65096	1.89226	2.1
65097	1.89226	2.1
65098	1.89226	2.1
65099	1.89226	2.1
65100	1.89226	2.1
65101	1.89226	2.1
65102	2.83014	2.1
65103	2.32404	2.1
65104	2.32404	2.1
65105	1.89226	2.1
65106	1.89226	2.1
65107	1.37966	2.1
65108	1.13294	2.1
65109	0.881723	2.1
65110	0.582304	2.1
65111	0.977944	2.1
65112	0.977948	2.1
65113	1.0264	2.1
65114	1.0264	2.1
65115	1.37967	2.1
65116	1.03896	2.1
65117	1.19108	2.1
65118	0.582298	2.1
65119	1.89226	2.1
65120	1.89226	2.1
65121	1.89226	2.1
65122	1.89226	2.1
65123	3.78452	2.1
65124	0.946131	2.1
65125	0.946131	2.1
65126	0.946131	2.1
65127	0.946131	2.1
65128	0.946132	2.1
65129	0.946132	2.1
65130	0.946129	2.1
65131	0.946129	2.1

65132	0.946131	2.1
65133	0.946131	2.1
65134	0.94613	2.1
65135	0.94613	2.1
65136	1.89226	2.1
65137	1.89226	2.1
65138	1.89226	2.1
65139	1.89226	2.1
65140	3.78453	2.1
65141	1.89226	2.1
65142	1.89226	2.1
65143	1.89226	2.1
65144	1.89226	2.1
65145	1.89226	2.1
65146	1.89226	2.1
65147	2.65216	2.1
65148	1.93528	2.1
65149	1.78595	2.1
65150	2.48544	2.1
65151	4.42072	2.1
65152	1.89226	2.1
65153	1.89226	2.1
65154	1.89226	2.1
65155	1.89226	2.1
65156	1.89226	2.1
65157	1.89226	2.1
65158	1.89226	2.1
65159	1.89226	2.1
65160	1.89226	2.1
65161	1.89226	2.1
65162	3.78452	2.1
65163	1.66537	2.1
65164	2.48544	2.1
65165	1.78595	2.1
65166	1.78595	2.1
65167	2.36197	2.1
65168	2.36197	2.1
65169	2.80975	2.1
65170	2.48544	2.1
65171	2.36198	2.1
65172	2.65135	2.1
65173	5.13679	2.1
65174	1.89226	2.1
65175	1.89226	2.1
65176	1.89226	2.1
65177	1.89226	2.1
65178	1.89226	2.1
65179	1.89226	2.1
65180	0.946127	2.1
65181	0.946127	2.1
65182	0.946131	2.1
65183	0.946131	2.1
65184	0.946128	2.1
65185	0.946128	2.1
65186	0.94613	2.1
65187	0.94613	2.1
65188	0.946129	2.1
65189	0.946129	2.1

65190	0.946129	2.1
65191	0.946129	2.1
65192	1.11972	2.1
65193	1.18099	2.1
65194	0.832687	2.1
65195	0.764517	2.1
65196	0.974426	2.1
65197	0.974426	2.1
65198	0.971078	2.1
65199	0.971078	2.1
65200	1.11972	2.1
65201	0.764515	2.1
65202	0.832686	2.1
65203	1.18099	2.1
65204	2.36198	2.1
65205	2.48025	2.1
65206	2.48025	2.1
65207	2.65135	2.1
65208	2.23944	2.1
65209	2.41055	2.1
65210	0.946132	2.1
65211	0.946132	2.1
65212	0.946131	2.1
65213	0.946131	2.1
65214	0.946131	2.1
65215	0.946131	2.1
65216	0.946132	2.1
65217	0.946132	2.1
65218	0.946131	2.1
65219	0.946131	2.1
65220	0.946132	2.1
65221	0.946132	2.1
65222	0.810942	2.1
65223	0.810942	2.1
65224	0.810944	2.1
65225	0.810944	2.1
65226	1.89226	2.1
65227	1.3517	2.1
65228	1.08132	2.1
65229	1.08133	2.1
65230	1.08132	2.1
65231	0.810932	2.1
65232	0.810932	2.1
65233	1.89249	2.1
65234	1.9857	1.72612
65235	2.02433	1.84994
65236	2.15807	1.621
65237	4.01003	1.87024
65238	1.89226	1.40196
65239	1.89226	1.95354
65240	1.89226	2.1
65241	1.89226	1.13396
65242	3.78452	1.87834
65243	1.89226	1.76986
65244	1.89226	1.88759
65245	1.89226	1.0034
65246	1.89226	1.50245
65247	3.78452	1.14871

65248	1.98569	1.45463
65249	1.86067	1.92784
65250	2.15807	1.32326
65251	2.07965	1.30206
65252	4.06534	2.00181
65253	2.02433	2.1
65254	2.15807	2.1
65255	2.12608	2.1
65256	2.3025	1.18077
65257	4.32683	2.1
65258	1.89226	2.1
65259	1.89226	1.57212
65260	1.89226	1.18352
65261	1.89226	1.21887
65262	3.78452	1.28954
65263	1.89226	1.22794
65264	1.89226	2.1
65265	1.89226	1.20189
65266	1.89226	1.23343
65267	3.78452	1.98938
65268	2.15807	2.04601
65269	2.07965	1.8714
65270	2.3025	1.13022
65271	2.28384	1.31607
65272	4.38215	1.22631
65273	1.89226	1.05275
65274	1.89226	0.343726
65275	1.89226	0.276796
65276	1.89226	0.05
65277	3.78453	0.165826
65278	2.16905	0.05
65279	2.56369	0.402891
65280	1.88394	0.05
65281	2.41938	0.05
65282	4.44763	0.05
65283	2.56369	0.314547
65284	2.80663	0.05
65285	2.41938	0.05
65286	2.69045	0.05
65287	5.25414	0.05
65288	1.89226	0.261525
65289	1.89226	0.721678
65290	1.89226	0.0877785
65291	1.89226	0.05
65292	3.78453	0.0599879
65293	1.89226	0.153636
65294	1.89226	0.229638
65295	1.89226	1.00875
65296	1.89226	0.769074
65297	1.89226	0.570936
65298	1.89226	0.05
65299	1.01988	0.05
65300	1.01988	0.05
65301	1.03333	0.05
65302	1.03333	0.162175
65303	1.13811	0.05
65304	1.20969	0.05
65305	0.941974	0.05

65306	0.843515	0.05
65307	0.843514	0.05
65308	1.13811	0.05
65309	0.941973	0.0912577
65310	1.20969	0.05
65311	2.41938	0.05
65312	2.57414	0.05
65313	2.57414	0.780375
65314	2.69045	0.267078
65315	2.27623	0.05
65316	2.39253	0.05
65317	1.89227	0.738937
65318	1.89227	0.05
65319	1.89227	0.05
65320	1.89227	0.05
65321	1.89227	0.05
65322	1.89227	0.05
65323	2.28385	1.3989
65324	2.09114	1.66877
65325	2.46968	1.01691
65326	2.32438	1.38641
65327	4.56082	1.07427
65328	1.89226	1.11075
65329	1.89226	2.05746
65330	1.89226	1.1079
65331	1.89226	1.77137
65332	3.78452	1.55309
65333	1.89226	1.11002
65334	1.89226	2.1
65335	1.89226	0.599411
65336	1.89226	1.36445
65337	3.78452	1.25924
65338	2.09114	1.04556
65339	1.5249	2.1
65340	2.55684	1.00853
65341	1.86899	1.53013
65342	3.96011	1.3766
65343	2.46968	1.21617
65344	2.32439	1.94513
65345	2.62768	1.11878
65346	2.506	1.33904
65347	4.97568	2.04678
65348	1.89226	1.42125
65349	1.89226	1.65002
65350	1.89226	0.895745
65351	1.89226	1.35529
65352	3.78452	1.0619
65353	1.89226	1.0996
65354	1.89226	1.51857
65355	1.89226	1.22232
65356	1.89226	2.1
65357	1.89226	0.48089
65358	1.89226	2.1
65359	2.55684	1.19565
65360	2.16506	1.43354
65361	1.86901	1.93576
65362	1.86899	1.25827
65363	2.75664	1.17206

65364	2.75662	1.04407
65365	1.89224	0.05
65366	1.89224	0.164607
65367	1.89224	0.05
65368	1.89224	1.33915
65369	3.78447	0.05
65370	2.24206	0.05
65371	2.38862	1.83654
65372	2.07107	0.05
65373	2.23097	0.203206
65374	4.47303	0.0567829
65375	2.38863	1.90338
65376	2.25945	2.1
65377	2.25945	0.826313
65378	2.39244	2.1
65379	2.23098	0.331734
65380	2.36397	2.1
65381	1.89224	0.05
65382	1.89224	2.1
65383	1.89224	0.992211
65384	1.89224	0.466102
65385	1.89224	0.946588
65386	1.89224	1.45045
65387	1.89226	0.05
65388	1.89226	0.0727469
65389	1.89226	0.127139
65390	1.89226	0.05
65391	1.89226	0.05
65392	1.89226	0.109459
65393	0.920975	2.1
65394	0.920979	0.05
65395	1.03015	0.05
65396	1.03015	0.05
65397	0.992407	0.05
65398	1.08759	2.1
65399	0.854346	0.05
65400	0.87241	0.05
65401	0.872397	0.05
65402	1.04334	0.05
65403	1.00965	0.0855746
65404	1.0876	0.0575504
65405	2.23101	1.9086
65406	2.25948	2.1
65407	2.03577	0.0502639
65408	2.05257	2.1
65409	4.29524	2.1
65410	0.810947	0.05
65411	0.810947	0.842089
65412	0.810947	0.0516349
65413	0.810947	0.05
65414	0.810934	0.203223
65415	0.810934	0.137541
65416	1.89227	2.1
65417	1.3517	0.646215
65418	1.08132	0.32076
65419	1.08132	0.97446
65420	1.08133	0.0742549
65421	1.89226	1.68185

65422	1.89226	2.03741
65423	1.89226	1.16516
65424	1.89226	2.1
65425	1.89226	0.132804
65426	1.89226	2.1
65427	1.89226	2.1
65428	1.89226	2.1
65429	1.89226	1.06646
65430	1.89226	2.1
65431	1.89226	0.787662
65432	1.89226	2.1
65433	0.879432	2.1
65434	0.879432	1.422
65435	0.900223	0.202498
65436	0.900223	2.1
65437	0.54571	2.1
65438	0.743092	1.95135
65439	1.03656	2.1
65440	1.19234	0.936005
65441	1.19234	1.87137
65442	0.743092	2.1
65443	1.03656	2.1
65444	0.545711	2.1
65445	1.89226	0.943676
65446	2.38467	1.96451
65447	2.69293	0.968407
65448	2.69293	2.1
65449	1.89226	0.767597
65450	1.89226	1.82529
65451	1.89226	1.24025
65452	1.89226	1.84455
65453	1.89226	0.973812
65454	1.89226	2.1
65455	3.78452	1.31054
65456	0.946129	2.1
65457	0.946129	2.1
65458	0.946133	0.118076
65459	0.946133	2.1
65460	0.946131	2.04878
65461	0.946131	2.1
65462	0.946131	0.16749
65463	0.946131	2.1
65464	0.946131	2.09249
65465	0.946131	2.0678
65466	0.946131	1.98918
65467	0.946131	1.98636
65468	1.04846	0.05
65469	1.04846	2.1
65470	1.05594	0.484805
65471	1.05594	2.1
65472	0.743092	2.1
65473	0.912061	1.36642
65474	1.19234	0.603931
65475	1.34647	0.584954
65476	0.912061	0.204954
65477	1.34646	1.15132
65478	1.19234	0.988826
65479	0.743093	1.1181

65480	1.89226	1.10222
65481	1.89226	2.1
65482	1.89226	0.0634475
65483	1.89226	1.13515
65484	3.78452	1.14178
65485	0.946128	2.1
65486	0.946128	1.93666
65487	0.946133	1.99469
65488	0.946133	0.456358
65489	0.94613	2.1
65490	0.94613	2.1
65491	0.946131	2.08056
65492	0.946131	0.563279
65493	0.946131	2.1
65494	0.946131	2.1
65495	0.946131	1.97024
65496	0.946131	1.96959
65497	1.89226	1.37389
65498	1.89226	2.1
65499	1.89226	1.028
65500	1.89226	1.48553
65501	3.78452	1.82936
65502	1.89226	1.26103
65503	1.89226	1.34477
65504	1.89226	1.19234
65505	1.89226	2.1
65506	1.89226	0.230385
65507	1.89226	2.1
65508	1.89226	1.55557
65509	1.89226	1.98476
65510	1.89226	1.12304
65511	1.89226	2.1
65512	1.89226	0.460427
65513	1.89226	2.1
65514	0.946132	2.1
65515	0.946132	1.36987
65516	1.17758	1.01021
65517	1.17759	2.1
65518	1.04514	2.1
65519	1.17758	1.21495
65520	0.946133	0.981458
65521	0.946133	1.05082
65522	0.946107	1.26514
65523	1.17755	1.24978
65524	1.04517	1.30739
65525	0.946158	1.24885
65526	1.89226	2.1
65527	2.10171	2.1
65528	1.95064	2.1
65529	2.10171	2.1
65530	1.7412	2.1
65531	1.89226	2.1
65532	1.89226	2.1
65533	1.89226	2.1
65534	1.89226	2.1
65535	1.89226	2.1
65536	1.89226	2.1
65537	1.89226	2.1

65538	1.89226	2.1
65539	1.89226	2.1
65540	1.89226	2.1
65541	1.89226	2.1
65542	3.78453	2.1
65543	0.946133	2.1
65544	0.946133	2.1
65545	0.946132	2.1
65546	0.946132	2.1
65547	0.946133	2.1
65548	0.946133	2.1
65549	0.946132	2.1
65550	0.946132	2.1
65551	0.946132	2.1
65552	0.946132	2.1
65553	0.946132	2.1
65554	0.946132	2.1
65555	1.70303	2.1
65556	1.95064	2.1
65557	1.74039	2.1
65558	1.95064	2.1
65559	1.69368	2.1
65560	1.89226	2.1
65561	1.89226	2.1
65562	1.89226	2.1
65563	1.89226	2.1
65564	1.89226	2.1
65565	3.78453	2.1
65566	0.946132	1.21875
65567	0.946132	2.1
65568	0.946132	2.1
65569	0.946132	2.1
65570	0.946133	2.1
65571	0.946133	1.94422
65572	0.946132	2.1
65573	0.946132	2.1
65574	0.946107	2.1
65575	0.946107	2.1
65576	0.946158	2.1
65577	0.946158	2.1
65578	0.946133	2.1
65579	0.946133	2.1
65580	0.946132	2.1
65581	0.946132	2.1
65582	0.946133	2.1
65583	0.946133	2.1
65584	0.946132	2.1
65585	0.946132	2.1
65586	0.946107	2.1
65587	0.946107	2.1
65588	0.946158	2.1
65589	0.946158	2.1
65590	1.89226	2.1
65591	1.89226	2.1
65592	1.89226	2.1
65593	1.89226	2.1
65594	1.89226	2.1
65595	1.89226	2.1

65596	1.89226	2.1
65597	1.89226	2.1
65598	1.89226	2.1
65599	1.89226	2.1
65600	1.89226	2.1
65601	1.89226	2.1
65602	2.5198	2.1
65603	2.45893	2.1
65604	2.27322	2.1
65605	2.27322	2.1
65606	2.63576	2.1
65607	2.63576	2.1
65608	1.24045	2.1
65609	1.11906	2.1
65610	1.13578	2.1
65611	0.916205	2.1
65612	1.12539	2.1
65613	1.08404	2.1
65614	1.08484	2.1
65615	1.08484	2.1
65616	1.22836	2.1
65617	0.919363	2.1
65618	1.16507	2.1
65619	0.924864	2.1
65620	1.89226	2.1
65621	1.89226	2.1
65622	1.89226	2.1
65623	1.89226	2.1
65624	1.89226	2.1
65625	1.89226	2.1
65626	1.89226	2.1
65627	1.89226	2.1
65628	1.89226	2.1
65629	1.89226	2.1
65630	1.89226	2.1
65631	1.89226	2.1
65632	2.6946	2.1
65633	2.63576	2.1
65634	2.5198	2.1
65635	2.5198	2.1
65636	2.8362	2.1
65637	2.8362	2.1
65638	1.3265	2.1
65639	1.24045	2.1
65640	0.983223	2.1
65641	0.954429	2.1
65642	1.06046	2.1
65643	1.10594	2.1
65644	1.1915	2.1
65645	1.1915	2.1
65646	1.31357	2.1
65647	1.09005	2.1
65648	1.29145	2.1
65649	0.995825	2.1
65650	1.89226	2.1
65651	1.89226	2.1
65652	1.89226	2.1
65653	1.89226	2.1

65654	3.78452	2.1
65655	1.89226	2.1
65656	1.89226	2.1
65657	1.89226	2.1
65658	1.89226	2.1
65659	3.78453	2.1
65660	1.91802	2.1
65661	2.27409	2.1
65662	2.48048	2.1
65663	2.22264	2.1
65664	1.91802	2.1
65665	1.66018	2.1
65666	1.13704	2.1
65667	1.24023	2.1
65668	1.22947	2.1
65669	1.31788	2.1
65670	1.23859	2.1
65671	1.23859	2.1
65672	1.23111	2.1
65673	1.23111	2.1
65674	1.24024	2.1
65675	1.31788	2.1
65676	1.13704	2.1
65677	1.22947	2.1
65678	1.89226	2.1
65679	1.89226	2.1
65680	1.89226	2.1
65681	1.89226	2.1
65682	1.89226	2.1
65683	1.89226	2.1
65684	1.89226	2.1
65685	1.89226	2.1
65686	1.89226	2.1
65687	1.89226	2.1
65688	3.78453	2.1
65689	1.05568	2.1
65690	0.959008	2.1
65691	1.32068	2.1
65692	1.24024	2.1
65693	1.15206	2.1
65694	1.15206	2.1
65695	1.14386	2.1
65696	1.14386	2.1
65697	1.05568	2.1
65698	1.32068	2.1
65699	0.959008	2.1
65700	1.24024	2.1
65701	1.32068	2.1
65702	1.24023	2.1
65703	1.31788	2.1
65704	1.4181	2.1
65705	1.31436	2.1
65706	1.31436	2.1
65707	1.3242	2.1
65708	1.3242	2.1
65709	1.32068	2.1
65710	1.4181	2.1
65711	1.24024	2.1

65712	1.31788	2.1
65713	1.89226	2.03747
65714	1.89226	0.0513673
65715	1.89226	0.852303
65716	1.89226	0.0806195
65717	1.89226	0.795055
65718	1.89226	0.90034
65719	1.02925	0.05
65720	1.16678	0.0998258
65721	0.946132	0.05
65722	0.946132	0.710916
65723	0.935677	0.783408
65724	0.935677	0.05
65725	1.18902	0.109188
65726	1.21562	0.877202
65727	1.13433	0.0729485
65728	1.04071	0.285405
65729	0.851553	0.137035
65730	1.06075	0.05
65731	1.89226	0.793527
65732	1.89226	0.05
65733	1.89226	0.0500243
65734	1.89226	0.05
65735	1.89226	0.068156
65736	1.89226	0.718811
65737	1.89226	0.97394
65738	1.89226	0.05
65739	1.89226	0.0581044
65740	1.89226	0.0989699
65741	3.78453	0.05
65742	0.946128	0.819402
65743	0.946128	0.105611
65744	0.946133	0.0824896
65745	0.946133	0.05
65746	0.946131	0.105948
65747	0.946131	0.0533002
65748	0.94613	0.760933
65749	0.94613	0.052959
65750	0.946131	0.775275
65751	0.946131	0.679645
65752	0.946131	0.05
65753	0.946131	0.05
65754	0.946132	0.851594
65755	0.946132	0.0983901
65756	0.946132	0.549891
65757	0.946132	0.05
65758	0.946132	0.752921
65759	0.946132	0.05
65760	0.946131	0.748638
65761	0.946131	0.226446
65762	0.946132	0.0907766
65763	0.946132	0.860345
65764	0.946132	0.05
65765	0.946132	0.16176
65766	0.946131	2.1
65767	0.946131	1.32132
65768	0.946131	2.1
65769	0.946131	1.01771

65770	0.946131	1.09141
65771	0.946131	2.08766
65772	0.946131	1.00862
65773	0.946131	0.984791
65774	0.946131	1.18499
65775	0.946131	1.17175
65776	0.946131	1.24151
65777	0.946131	1.26037
65778	1.89226	1.28737
65779	1.89226	1.33105
65780	1.89226	1.05624
65781	1.89226	2.1
65782	1.89226	0.943048
65783	1.89226	2.1
65784	1.89226	1.17836
65785	1.89226	1.11152
65786	1.89226	1.14269
65787	1.89226	2.1
65788	1.89226	1.01059
65789	1.89226	2.1
65790	1.89226	1.38421
65791	1.89226	1.15057
65792	1.89226	1.00751
65793	1.89226	2.1
65794	1.89226	0.268969
65795	1.89226	2.1
65796	1.89226	2.1
65797	1.89226	2.1
65798	1.89226	0.560133
65799	1.89226	2.1
65800	3.78452	1.69126
65801	0.946131	2.1
65802	0.946131	0.935574
65803	1.17195	1.0133
65804	1.17195	2.1
65805	0.958489	2.1
65806	1.17195	1.41706
65807	0.946129	1.02474
65808	0.946129	1.41718
65809	0.946131	1.24307
65810	1.17195	1.24474
65811	0.958487	1.32376
65812	0.94613	1.33009
65813	2.69656	2.1
65814	2.17516	2.1
65815	2.41885	2.1
65816	2.41885	2.1
65817	2.46476	2.1
65818	2.46476	1.29976
65819	2.69657	2.1
65820	2.30233	0.897229
65821	2.30233	1.85963
65822	2.59476	1.1497
65823	2.46477	2.02807
65824	2.7572	1.94773
65825	1.89226	2.1
65826	1.89226	0.633568
65827	1.89226	0.229415

65828	1.89226	0.479364
65829	1.89226	0.305827
65830	1.89226	0.96396
65831	1.89226	2.1
65832	1.89226	1.21109
65833	1.89226	2.1
65834	1.89226	0.481453
65835	3.78452	1.69858
65836	2.46477	2.1
65837	2.17516	2.1
65838	1.80252	2.1
65839	2.09715	1.03105
65840	4.27231	2.1
65841	2.46477	2.1
65842	2.09715	1.01618
65843	2.30233	1.22503
65844	2.30233	1.08167
65845	1.96416	0.335456
65846	2.09715	0.159333
65847	1.89227	1.00088
65848	1.89227	0.05
65849	1.89227	0.207952
65850	1.89227	0.21199
65851	1.89227	0.888254
65852	1.89227	0.9613
65853	1.89226	2.1
65854	1.89226	0.0771479
65855	1.89226	1.01163
65856	1.89226	1.0557
65857	3.78452	1.01833
65858	0.946132	2.1
65859	0.946132	2.1
65860	0.946132	2.1
65861	0.946132	2.1
65862	0.946132	2.1
65863	0.946132	2.1
65864	0.946132	2.1
65865	0.946132	2.1
65866	0.946132	2.1
65867	0.946132	2.1
65868	0.946131	2.1
65869	0.946131	2.1
65870	1.89226	2.1
65871	1.89226	2.1
65872	1.89226	2.1
65873	1.89226	2.1
65874	3.78453	2.1
65875	1.89226	2.1
65876	1.89226	2.1
65877	1.89226	2.1
65878	1.89226	2.1
65879	1.89226	2.1
65880	1.89226	2.1
65881	1.89226	2.1
65882	1.63707	2.1
65883	1.63707	2.1
65884	1.38859	2.1
65885	1.89226	2.1

65886	1.64377	2.1
65887	0.94613	2.1
65888	0.94613	2.1
65889	0.94613	2.1
65890	0.94613	2.1
65891	0.946131	2.1
65892	0.946131	2.1
65893	0.946129	2.1
65894	0.946129	2.1
65895	0.94613	2.1
65896	0.94613	2.1
65897	0.94613	2.1
65898	0.94613	2.1
65899	2.15888	2.1
65900	1.99309	2.1
65901	2.16841	2.1
65902	2.16841	2.1
65903	2.02311	1.16797
65904	2.02311	1.07089
65905	1.89226	1.31609
65906	1.89226	1.99784
65907	1.89226	2.1
65908	1.89226	1.88872
65909	1.89226	2.1
65910	1.89226	1.08949
65911	1.89226	2.1
65912	1.89226	2.1
65913	1.89226	2.1
65914	1.89226	2.1
65915	1.89226	2.1
65916	1.89226	2.08205
65917	1.99309	2.1
65918	1.96035	2.1
65919	1.72526	2.1
65920	1.72526	2.1
65921	2.16841	2.1
65922	2.16841	2.1
65923	2.15888	2.1
65924	2.16841	2.1
65925	2.3111	2.1
65926	2.35938	2.1
65927	4.51826	2.1
65928	1.89226	2.1
65929	1.89226	1.22659
65930	1.89226	1.18923
65931	1.89226	0.05
65932	3.78452	2.1
65933	1.89226	2.1
65934	1.89226	2.1
65935	1.89226	2.1
65936	1.89226	0.883464
65937	3.78452	2.1
65938	2.16841	2.1
65939	1.96035	2.1
65940	2.35938	2.1
65941	2.18493	2.1
65942	4.31973	2.1
65943	1.89226	2.1

65944	1.89226	2.1
65945	1.89226	2.1
65946	1.89226	2.1
65947	3.78453	2.1
65948	1.89226	2.1
65949	1.89226	2.1
65950	1.89226	2.1
65951	1.89226	2.1
65952	3.78452	2.1
65953	0.946158	2.1
65954	0.946158	2.1
65955	0.818509	2.1
65956	0.818509	2.1
65957	0.933975	2.1
65958	0.818539	2.1
65959	0.946128	2.1
65960	0.946128	2.1
65961	0.94613	2.1
65962	0.933974	2.1
65963	0.818537	2.1
65964	0.946129	2.1
65965	0.946131	2.1
65966	0.946131	2.1
65967	0.946131	2.1
65968	0.946131	2.1
65969	0.946132	2.1
65970	0.946132	2.1
65971	0.946129	2.1
65972	0.946129	2.1
65973	0.946131	2.1
65974	0.946131	2.1
65975	0.94613	2.1
65976	0.94613	2.1
65977	1.89226	2.1
65978	1.89226	2.1
65979	1.89226	2.1
65980	1.89226	2.1
65981	3.78453	2.1
65982	1.89226	2.1
65983	1.89226	2.1
65984	1.89226	2.1
65985	1.89226	2.1
65986	3.78452	2.1
65987	1.89226	2.1
65988	1.8706	2.1
65989	1.8706	2.1
65990	1.68112	2.1
65991	2.0815	2.1
65992	1.88715	2.1
65993	0.94613	2.1
65994	0.94613	2.1
65995	0.94613	2.1
65996	0.94613	2.1
65997	0.946131	2.1
65998	0.946131	2.1
65999	0.946129	2.1
66000	0.946129	2.1
66001	0.94613	2.1

66002	0.94613	2.1
66003	0.94613	2.1
66004	0.94613	2.1
66005	1.76401	2.1
66006	2.60983	2.1
66007	2.50461	2.1
66008	2.35897	2.1
66009	1.90965	2.1
66010	1.76401	2.1
66011	1.89226	2.1
66012	1.89226	2.1
66013	1.89226	2.1
66014	1.89226	2.1
66015	3.78452	2.1
66016	1.89226	2.1
66017	1.89226	2.1
66018	1.89226	2.1
66019	1.89226	1.83496
66020	1.89226	2.1
66021	1.89226	2.1
66022	1.90965	2.1
66023	2.83834	2.1
66024	2.7538	2.1
66025	2.60983	2.1
66026	2.05361	2.1
66027	1.90965	2.1
66028	1.89226	2.1
66029	1.89226	2.1
66030	1.89226	2.1
66031	1.89226	2.1
66032	1.89226	2.1
66033	1.89226	2.1
66034	1.89226	2.1
66035	1.89226	2.1
66036	1.89226	2.1
66037	1.89226	2.07647
66038	3.78452	2.1
66039	2.83834	2.1
66040	1.89226	2.1
66041	2.60983	2.1
66042	2.60983	2.1
66043	1.89226	2.1
66044	1.89226	2.1
66045	1.89226	2.1
66046	1.89226	2.1
66047	1.89226	2.1
66048	1.89226	2.1
66049	1.89226	2.1
66050	1.89226	2.1
66051	1.89226	2.1
66052	1.89226	2.1
66053	1.89226	2.1
66054	1.89226	1.50602
66055	1.89226	2.1
66056	1.89226	1.81532
66057	2.44785	2.1
66058	1.80223	2.1
66059	2.80894	2.1

66060	2.18296	2.1
66061	4.63081	2.1
66062	1.89226	2.1
66063	1.89226	2.1
66064	1.89226	2.1
66065	1.89226	2.1
66066	1.89226	2.1
66067	1.89226	2.1
66068	0.946131	1.18798
66069	0.946131	2.1
66070	0.946127	2.1
66071	0.946127	2.1
66072	0.946131	2.08852
66073	0.946131	2.1
66074	0.946128	2.1
66075	0.946128	2.1
66076	0.94613	2.1
66077	0.94613	2.1
66078	0.946129	2.1
66079	0.946129	2.1
66080	1.89226	2.1
66081	1.89226	2.1
66082	1.89226	2.1
66083	1.89226	2.1
66084	3.78452	2.1
66085	1.89226	2.1
66086	1.89226	2.1
66087	1.89226	2.1
66088	1.89226	2.1
66089	3.78453	2.1
66090	2.41083	2.1
66091	2.93937	2.1
66092	1.68761	2.1
66093	1.68761	2.1
66094	1.67248	2.1
66095	2.3957	2.1
66096	1.89226	2.1
66097	1.89226	2.1
66098	1.89226	2.1
66099	1.89226	2.1
66100	1.89226	2.1
66101	1.89226	2.1
66102	1.89227	2.1
66103	1.89227	2.1
66104	1.89227	2.1
66105	1.89227	2.1
66106	1.89227	2.1
66107	1.89227	2.1
66108	1.89224	0.855876
66109	1.89224	0.278005
66110	1.89224	0.970468
66111	1.89224	0.864673
66112	3.78448	0.810045
66113	1.89224	1.22626
66114	1.89224	0.867991
66115	1.89224	1.13282
66116	1.89224	0.198227
66117	1.89224	1.20737

66118	1.89224	0.95693
66119	1.11152	0.821278
66120	1.29346	0.977059
66121	0.978596	1.0487
66122	0.918324	1.25368
66123	1.06821	1.14604
66124	1.06821	0.436405
66125	0.985654	1.36028
66126	1.15278	1.18295
66127	1.07693	1.10935
66128	0.902432	1.15664
66129	0.968939	1.0884
66130	1.34644	0.984927
66131	0.946132	0.758473
66132	0.946132	0.866081
66133	0.946132	0.862902
66134	0.946132	0.812082
66135	0.946131	0.827281
66136	0.946131	0.162046
66137	0.946132	1.00249
66138	0.946132	0.796025
66139	0.946132	0.857074
66140	0.946132	0.845687
66141	0.946132	0.653694
66142	0.946132	0.772117
66143	0.946133	0.920087
66144	0.946133	0.649995
66145	0.946132	0.953665
66146	0.946132	1.05813
66147	0.946132	0.983341
66148	0.946132	0.129877
66149	0.946133	1.93301
66150	0.946133	0.838256
66151	0.946132	1.01327
66152	0.946132	0.963669
66153	0.946132	0.78697
66154	0.946132	0.750222
66155	1.89224	0.345305
66156	1.89224	2.1
66157	1.89224	2.05133
66158	1.89224	2.1
66159	1.89224	1.93629
66160	1.89224	2.1
66161	1.89224	2.02354
66162	1.89224	2.1
66163	1.89224	2.1
66164	1.89224	2.1
66165	1.89224	2.1
66166	1.89224	2.1
66167	1.89226	1.2075
66168	1.89226	2.1
66169	1.89226	2.1
66170	1.89226	2.1
66171	3.78453	2.1
66172	1.11056	2.1
66173	1.21595	2.1
66174	1.12763	2.1
66175	1.12763	2.1

66176	1.23339	2.1
66177	1.35697	2.1
66178	1.03581	2.1
66179	0.95954	2.1
66180	0.89761	2.1
66181	1.26602	2.1
66182	1.12749	2.1
66183	1.45307	2.1
66184	2.71775	2.1
66185	1.89226	2.1
66186	1.89226	2.1
66187	1.89226	2.1
66188	2.47025	2.1
66189	2.47025	2.1
66190	0.94613	2.1
66191	0.94613	2.1
66192	0.94613	2.1
66193	0.94613	2.1
66194	0.946128	2.1
66195	0.946128	2.1
66196	0.946132	2.1
66197	0.946132	2.1
66198	0.94613	2.1
66199	0.94613	2.1
66200	0.94613	2.1
66201	0.94613	2.1
66202	1.89226	1.09529
66203	1.89226	2.1
66204	1.89226	2.1
66205	1.89226	1.22427
66206	1.89226	2.1
66207	1.89226	1.3675
66208	1.89226	1.12298
66209	1.89226	2.1
66210	1.89226	2.1
66211	1.89226	1.09549
66212	1.89226	2.1
66213	1.89226	1.20218
66214	0.946132	2.1
66215	0.946132	2.1
66216	0.946133	2.1
66217	0.946133	2.1
66218	0.946133	2.1
66219	0.946133	2.08072
66220	0.946132	2.00801
66221	0.946132	2.1
66222	0.946133	2.1
66223	0.946133	2.1
66224	0.946132	2.1
66225	0.946132	2.1
66226	1.89226	1.16226
66227	1.89226	2.1
66228	1.89226	2.1
66229	1.89226	1.46147
66230	1.89226	2.1
66231	1.89226	1.613
66232	0.946131	1.40971
66233	0.946131	1.1385

66234	0.946127	2.1
66235	0.946127	2.1
66236	0.946131	1.35759
66237	0.946131	2.1
66238	0.946127	2.1
66239	0.946127	1.38991
66240	0.94613	2.1
66241	0.94613	2.08143
66242	0.946129	2.01197
66243	0.946129	2.00824
66244	1.94291	2.1
66245	2.00118	2.1
66246	2.36337	2.1
66247	2.41217	2.1
66248	4.35508	2.1
66249	1.89226	2.1
66250	1.89226	2.1
66251	1.89226	2.1
66252	1.89226	2.1
66253	3.78452	2.1
66254	1.89226	2.1
66255	1.89226	2.1
66256	1.89226	2.1
66257	1.89226	2.1
66258	3.78452	2.1
66259	1.06209	2.1
66260	1.06209	2.1
66261	1.05987	2.1
66262	1.05987	2.1
66263	0.920378	2.1
66264	1.12137	2.1
66265	1.00059	2.1
66266	1.20608	2.1
66267	1.20608	2.1
66268	1.12137	2.1
66269	1.00059	2.1
66270	0.920376	2.1
66271	2.77222	2.1
66272	2.41216	2.1
66273	2.77002	2.1
66274	2.36337	2.1
66275	2.81882	2.1
66276	2.41216	2.1
66277	1.89226	2.1
66278	1.89226	2.1
66279	1.89226	2.1
66280	1.89226	2.1
66281	1.89226	2.1
66282	1.89226	2.1
66283	1.89226	2.1
66284	1.89226	2.1
66285	1.89226	2.1
66286	1.89226	2.1
66287	3.78451	2.1
66288	2.41216	2.1
66289	2.24273	2.1
66290	2.81882	2.1
66291	2.68473	2.1

66292	5.09689	2.1
66293	1.89226	2.1
66294	1.89226	2.1
66295	1.89226	2.1
66296	1.89226	2.1
66297	3.78453	2.1
66298	2.17614	2.1
66299	2.65326	2.1
66300	1.93101	2.1
66301	2.58552	2.1
66302	4.58427	2.1
66303	2.65326	2.1
66304	1.89226	2.1
66305	1.89226	2.1
66306	1.89226	2.1
66307	2.58552	2.1
66308	2.58552	2.1
66309	1.89226	2.1
66310	1.89226	2.1
66311	1.89226	2.1
66312	1.89226	2.1
66313	3.78453	2.1
66314	1.89226	2.1
66315	1.89226	2.1
66316	1.89226	2.1
66317	1.89226	2.1
66318	3.78453	2.1
66319	1.93101	2.1
66320	2.58552	2.1
66321	1.46507	2.1
66322	2.52893	2.1
66323	4.45994	2.1
66324	1.26446	2.1
66325	1.26446	2.1
66326	0.946132	2.1
66327	0.946132	2.1
66328	0.94613	2.1
66329	0.94613	2.1
66330	1.29276	2.1
66331	1.26446	2.1
66332	1.26446	2.1
66333	0.94613	2.1
66334	0.94613	2.1
66335	1.29276	2.1
66336	1.89226	2.1
66337	1.89226	2.1
66338	1.89226	2.1
66339	1.89226	2.1
66340	3.78453	2.1
66341	1.89226	2.1
66342	1.89226	2.1
66343	1.89226	2.1
66344	1.89226	2.1
66345	3.78453	2.1
66346	1.83378	2.1
66347	2.52893	2.1
66348	1.75592	2.1
66349	2.49844	2.1

66350	4.28485	2.1
66351	1.24922	2.1
66352	1.24922	2.1
66353	0.946133	2.1
66354	0.946133	2.1
66355	0.946131	2.1
66356	0.946131	2.1
66357	1.26446	2.1
66358	1.24922	2.1
66359	1.24922	2.1
66360	0.946131	2.1
66361	1.26446	2.1
66362	0.94613	2.1
66363	0.946132	2.1
66364	0.946132	2.1
66365	0.946132	2.1
66366	0.946132	2.1
66367	0.946132	2.1
66368	0.946132	2.1
66369	0.946132	2.1
66370	0.946132	2.1
66371	0.946132	2.1
66372	0.946132	2.1
66373	0.946131	2.1
66374	0.946131	2.1
66375	1.89226	2.1
66376	1.89226	2.1
66377	1.89226	2.1
66378	1.89226	2.1
66379	1.89226	2.1
66380	1.89226	2.1
66381	1.0465	2.1
66382	1.0465	2.1
66383	1.06047	2.1
66384	1.06047	2.1
66385	1.25687	2.1
66386	1.24922	2.1
66387	0.87796	2.1
66388	0.857749	2.1
66389	0.857749	2.1
66390	1.25687	2.1
66391	0.877959	2.1
66392	1.24922	2.1
66393	2.49843	2.1
66394	1.89226	2.1
66395	2.51374	2.1
66396	1.89226	2.1
66397	4.406 2.1	
66398	1.89226	2.1
66399	1.89226	2.1
66400	1.89226	2.1
66401	1.89226	2.1
66402	1.89226	2.1
66403	1.89226	2.1
66404	1.89226	1.8764
66405	1.89226	2.1
66406	1.89226	2.1
66407	1.89226	1.99241

66408	1.89226	2.1
66409	1.89226	2.1
66410	1.89226	2.1
66411	1.89226	2.06761
66412	1.89226	2.1
66413	1.89226	2.00667
66414	1.89226	2.1
66415	1.89226	2.1
66416	1.03835	2.1
66417	1.03835	2.1
66418	1.15388	2.1
66419	0.94267	2.1
66420	0.736433	2.1
66421	0.849239	2.1
66422	1.15014	2.1
66423	1.39058	2.1
66424	0.838639	2.1
66425	1.39056	2.1
66426	1.09399	2.1
66427	0.736444	2.1
66428	1.89226	1.56226
66429	1.89226	2.1
66430	1.89226	2.1
66431	1.89226	1.3895
66432	1.89226	2.1
66433	1.89226	1.52418
66434	0.94613	1.39599
66435	0.94613	1.54402
66436	0.946127	2.1
66437	0.946127	2.1
66438	0.946131	2.1
66439	0.946131	1.33891
66440	0.946126	2.1
66441	0.946126	1.83383
66442	0.946128	2.1
66443	0.946128	2.1
66444	0.946128	2.1
66445	0.946128	2.1
66446	1.89226	2.1
66447	1.89226	1.24421
66448	1.89226	1.07227
66449	1.89226	2.1
66450	3.78453	1.33006
66451	0.946133	1.59474
66452	0.946133	0.950741
66453	0.946128	2.1
66454	0.946128	1.54733
66455	0.946131	1.74359
66456	0.946131	1.47262
66457	0.946131	0.869245
66458	0.946131	1.51373
66459	0.946131	1.25247
66460	0.946131	1.22884
66461	0.94613	1.30606
66462	0.94613	1.3444
66463	1.89226	1.08442
66464	1.89226	2.1
66465	1.89226	2.1

66466	1.89226	1.18863
66467	1.89226	2.09222
66468	1.89226	1.26168
66469	1.89226	0.979333
66470	1.89226	1.98477
66471	1.89226	1.45372
66472	1.89226	1.11257
66473	1.89226	1.48214
66474	1.89226	1.32674
66475	1.89226	1.001
66476	1.89226	2.1
66477	1.89226	1.3367
66478	1.89226	1.3169
66479	1.89226	1.37054
66480	1.89226	1.68921
66481	1.89226	1.12178
66482	2.67388	2.1
66483	2.23684	2.1
66484	2.23684	1.41382
66485	1.89226	2.1
66486	1.89226	1.64455
66487	1.89226	2.1
66488	1.89226	2.1
66489	1.89226	2.1
66490	1.89226	2.1
66491	1.89226	2.1
66492	1.89226	2.1
66493	1.72197	2.1
66494	2.51374	2.1
66495	2.58004	2.1
66496	2.58004	2.1
66497	1.7155	2.1
66498	1.7155	2.1
66499	2.51374	2.1
66500	1.89226	2.1
66501	1.89226	2.1
66502	1.89226	2.1
66503	2.58003	2.1
66504	2.58003	2.1
66505	0.946132	2.1
66506	0.946132	2.1
66507	0.946132	2.1
66508	0.946132	2.1
66509	0.946132	2.1
66510	0.946132	2.1
66511	0.946132	2.1
66512	0.946132	2.1
66513	0.946131	2.1
66514	0.946131	2.1
66515	0.946132	2.1
66516	0.946132	2.1
66517	1.89226	2.1
66518	1.89226	2.1
66519	1.89226	2.1
66520	1.89226	2.1
66521	3.78453	2.1
66522	1.08262	2.1
66523	1.08262	2.1

66524	1.09674	2.1
66525	1.09674	2.1
66526	1.34652	2.1
66527	1.29002	2.1
66528	0.860984	2.1
66529	0.889338	2.1
66530	1.34652	2.1
66531	0.889338	2.1
66532	0.860983	2.1
66533	1.29002	2.1
66534	1.34652	2.1
66535	1.34652	2.1
66536	0.946133	2.1
66537	0.946133	2.1
66538	0.946131	2.1
66539	0.946131	2.1
66540	1.34652	2.1
66541	1.29002	2.1
66542	1.34652	2.1
66543	0.946131	2.1
66544	0.94613	2.1
66545	1.29002	2.1
66546	1.89226	2.1
66547	1.89226	2.1
66548	1.89226	2.1
66549	1.89226	2.1
66550	1.89226	2.1
66551	1.89226	2.1
66552	1.89226	2.1
66553	1.89226	2.1
66554	1.89226	2.1
66555	1.89226	2.1
66556	1.89226	2.1
66557	1.89226	2.1
66558	0.946131	2.1
66559	0.946131	2.1
66560	0.946131	2.1
66561	0.946131	2.1
66562	0.946131	2.1
66563	0.946131	2.1
66564	0.946131	2.1
66565	0.946131	2.1
66566	0.946131	2.1
66567	0.946131	2.1
66568	0.946131	2.1
66569	0.946131	2.1
66570	1.89226	2.1
66571	1.89226	2.1
66572	1.89226	2.1
66573	1.89226	2.1
66574	1.89226	2.1
66575	1.89226	2.1
66576	1.04103	2.1
66577	1.13849	2.1
66578	0.943014	2.1
66579	0.943014	2.1
66580	0.943591	2.1
66581	0.943591	2.1

66582	1.03757	2.1
66583	1.04098	2.1
66584	1.03761	2.1
66585	0.946131	2.1
66586	0.943041	2.1
66587	0.946131	2.1
66588	0.946128	2.1
66589	0.946128	2.1
66590	0.946133	2.1
66591	0.946133	2.1
66592	0.946131	2.1
66593	0.946131	2.1
66594	0.946131	2.1
66595	0.946131	2.1
66596	0.946131	2.1
66597	0.946131	2.1
66598	0.94613	2.1
66599	0.94613	2.1
66600	0.946132	2.1
66601	0.946132	2.1
66602	0.946132	2.1
66603	0.946132	2.1
66604	0.946132	2.1
66605	0.946132	2.1
66606	0.946132	2.1
66607	0.946132	2.1
66608	0.946132	2.1
66609	0.946132	2.1
66610	0.946131	2.1
66611	0.946131	2.1
66612	1.89226	1.02187
66613	1.89226	2.1
66614	1.89226	1.09688
66615	1.89226	1.04347
66616	3.78452	1.44098
66617	0.946132	1.26716
66618	0.946132	1.35127
66619	0.946131	1.07225
66620	0.946131	1.05877
66621	0.946132	1.08338
66622	0.946132	2.1
66623	0.946132	1.28279
66624	0.946132	1.20942
66625	0.946132	1.15277
66626	0.946132	1.18369
66627	0.946132	1.23277
66628	0.946132	1.37639
66629	0.935482	1.85867
66630	1.08999	1.67098
66631	1.24315	1.27527
66632	1.12559	1.69364
66633	1.21343	1.21442
66634	0.889397	2.1
66635	1.01265	1.0101
66636	1.01265	2.03563
66637	1.07332	1.12538
66638	1.34292	1.55642
66639	0.865649	1.5869

66640	0.840024	2.02535
66641	1.15433	1.48528
66642	1.02858	1.96604
66643	1.34082	1.38338
66644	1.08357	1.31723
66645	1.10938	1.04739
66646	1.33349	2.1
66647	1.12232	1.97352
66648	1.12232	0.976133
66649	1.21357	1.42022
66650	1.3408	1.69764
66651	1.02859	1.67545
66652	1.24211	2.03856
66653	1.89226	1.26681
66654	1.89226	0.902632
66655	1.89226	0.999323
66656	1.89226	2.1
66657	1.89226	0.890497
66658	1.89226	1.29362
66659	1.89226	1.00956
66660	1.89226	1.16982
66661	1.89226	1.04097
66662	1.89226	1.8408
66663	1.89226	1.18766
66664	1.89226	1.89099
66665	2.0815	1.54506
66666	2.79029	1.27975
66667	2.36793	1.21631
66668	2.58428	2.1
66669	2.38161	0.987288
66670	2.59922	1.73792
66671	1.89226	2.1
66672	1.89226	1.81351
66673	1.89226	2.1
66674	1.89226	2.1
66675	1.89226	2.1
66676	1.89226	2.1
66677	1.89226	1.86442
66678	1.89226	0.231202
66679	1.89226	0.981587
66680	1.89226	1.11434
66681	3.78452	0.980996
66682	1.89226	2.1
66683	1.89226	1.06768
66684	1.89226	1.94897
66685	1.89226	1.83026
66686	1.89226	1.87702
66687	1.89226	1.84427
66688	0.946132	1.27417
66689	0.946132	2.1
66690	0.94613	2.1
66691	0.94613	2.1
66692	0.946131	2.1
66693	0.946131	1.23452
66694	0.946131	2.1
66695	0.946131	2.1
66696	0.946131	2.1
66697	0.946131	2.1

66698	0.946131	2.1
66699	0.946131	2.1
66700	1.85178	2.07436
66701	1.89226	1.07482
66702	2.0815	0.46013
66703	2.27054	1.53797
66704	1.70303	0.316656
66705	1.5217	0.735317
66706	1.89226	2.1
66707	1.89226	1.05775
66708	1.89226	1.76408
66709	1.89226	1.26857
66710	1.89226	2.1
66711	1.89226	1.49522
66712	2.58823	0.169346
66713	2.02924	0.074951
66714	1.79398	0.05
66715	1.79398	0.05
66716	2.76708	0.458902
66717	2.76708	0.109188
66718	0.946133	0.0958618
66719	0.946133	0.0859525
66720	1.29411	0.0826965
66721	1.38353	0.0712
66722	0.946131	0.0592196
66723	0.946131	0.0588623
66724	1.38354	0.141488
66725	1.38354	0.05
66726	0.946131	0.067181
66727	1.38354	0.0691813
66728	0.94613	0.137625
66729	1.29411	0.05
66730	1.89226	0.05
66731	1.89226	0.05
66732	1.89226	0.152168
66733	1.89226	0.058341
66734	3.78452	0.060582
66735	0.946131	0.05
66736	0.946131	0.05
66737	0.946131	0.0954718
66738	0.946131	0.05
66739	0.946129	0.05
66740	0.946129	0.05
66741	0.946133	0.0832412
66742	0.946133	0.0506023
66743	0.946131	0.157993
66744	0.946131	0.05
66745	0.946131	0.05
66746	0.946131	0.05
66747	1.89226	0.05
66748	1.89226	0.05
66749	1.89226	0.05
66750	1.89226	0.0525238
66751	3.78452	0.05
66752	0.94613	0.05
66753	0.94613	0.05
66754	0.946131	0.05
66755	0.946131	0.05

66756	0.946128	0.05
66757	0.946128	0.05
66758	0.946133	0.05
66759	0.946133	0.05
66760	0.946131	0.0586561
66761	0.946131	0.05
66762	0.946131	0.05
66763	0.946131	0.05
66764	0.946129	0.828726
66765	0.946129	0.335527
66766	1.13486	0.319397
66767	0.859753	0.522641
66768	0.851541	0.453556
66769	0.851541	0.233359
66770	1.34346	0.35219
66771	1.26212	0.208591
66772	0.851553	0.238512
66773	1.12403	0.283738
66774	0.851474	0.0811641
66775	0.956163	0.181799
66776	0.946132	0.0984125
66777	0.946132	0.05
66778	0.946133	0.05
66779	0.946133	0.05
66780	0.946132	0.05
66781	0.946132	0.05
66782	0.946133	0.05
66783	0.946133	0.0673696
66784	0.946133	0.0660476
66785	0.946133	0.05
66786	0.946132	0.05
66787	0.946132	0.05
66788	1.89226	0.05
66789	1.89226	0.05
66790	1.89226	0.0762694
66791	1.89226	0.134338
66792	3.78453	0.05
66793	0.946128	0.800211
66794	0.946128	0.807805
66795	0.946132	0.498482
66796	0.946132	0.52951
66797	0.94613	0.520386
66798	0.94613	0.335118
66799	0.94613	1.0841
66800	0.94613	0.774378
66801	0.946131	1.08305
66802	0.946131	0.624581
66803	0.94613	0.368734
66804	0.94613	0.715022
66805	0.946132	0.05
66806	0.946132	0.05
66807	0.946133	0.05
66808	0.946133	0.05
66809	0.946132	0.05
66810	0.946132	0.05
66811	0.946133	0.05
66812	0.946133	0.05
66813	0.946132	0.05

66814	0.946132	0.05
66815	0.946132	0.05
66816	0.946132	0.05
66817	1.89226	0.05
66818	1.89226	0.05
66819	1.89226	0.0566566
66820	1.89226	0.05
66821	1.89226	0.177559
66822	1.89226	0.05
66823	1.89226	0.0856279
66824	1.89226	0.183624
66825	1.89226	0.05
66826	1.89226	0.0674133
66827	1.89226	0.146214
66828	1.89226	0.139564
66829	1.89226	2.1
66830	1.89226	2.1
66831	1.89226	2.1
66832	1.89226	2.1
66833	3.78452	2.1
66834	1.07652	2.1
66835	1.07652	2.1
66836	0.928749	2.1
66837	0.928749	2.1
66838	0.946132	2.1
66839	0.946132	2.1
66840	1.05914	2.1
66841	0.928746	2.1
66842	1.05914	2.1
66843	0.946131	2.1
66844	0.94613	2.1
66845	0.928747	2.1
66846	1.89226	2.1
66847	1.89226	2.1
66848	1.89226	2.1
66849	1.89226	2.1
66850	1.89226	2.1
66851	1.89226	2.1
66852	1.89227	2.1
66853	1.89227	2.1
66854	1.89227	2.1
66855	1.89227	2.1
66856	1.89227	2.1
66857	1.89227	2.1
66858	1.89226	2.1
66859	1.89226	2.1
66860	1.89226	2.1
66861	1.89226	2.1
66862	1.89226	2.1
66863	1.89226	2.1
66864	2.11828	2.1
66865	1.89226	2.1
66866	2.35845	2.1
66867	1.89226	2.1
66868	4.25071	2.1
66869	1.89226	2.1
66870	1.89226	2.1
66871	1.89226	2.1

66872	1.89226	2.1
66873	3.78453	2.1
66874	1.89226	2.1
66875	1.89226	2.1
66876	1.89226	2.1
66877	1.89226	2.1
66878	1.89226	2.1
66879	1.89226	2.1
66880	1.89226	1.05547
66881	1.89226	1.85442
66882	1.65573	1.57697
66883	1.65573	1.12228
66884	1.89226	1.50148
66885	1.89226	1.12684
66886	1.89226	0.93211
66887	1.89226	1.45915
66888	1.89226	1.16458
66889	1.89226	1.01381
66890	1.89226	1.20218
66891	1.89226	1.02774
66892	1.89226	1.00666
66893	1.89226	1.55026
66894	1.89226	1.36764
66895	1.89226	1.08531
66896	1.89226	1.41653
66897	1.89226	1.09058
66898	1.89226	0.906482
66899	1.89226	1.55708
66900	1.89226	1.11667
66901	1.89226	0.968469
66902	1.89226	1.1679
66903	1.89226	0.98809
66904	0.946128	1.00975
66905	0.946128	0.937828
66906	0.946125	1.34462
66907	0.946125	1.31647
66908	0.94613	1.01477
66909	0.94613	2.08753
66910	0.946124	1.67836
66911	0.946124	0.997091
66912	0.946127	1.10807
66913	0.946127	1.10932
66914	0.946127	1.1114
66915	0.946127	1.12494
66916	1.89226	0.938569
66917	1.89226	1.16421
66918	1.89226	0.947286
66919	1.89226	0.899307
66920	3.78452	0.96207
66921	1.89226	1.17631
66922	1.89226	1.20184
66923	1.89226	1.01087
66924	1.89226	1.21796
66925	3.78453	1.16563
66926	1.05926	1.81631
66927	0.891358	2.1
66928	0.946129	1.31133
66929	0.946129	1.96487

66930	0.946101	1.31924
66931	0.946101	2.1
66932	0.891386	1.34074
66933	0.891386	1.69481
66934	0.891357	1.70277
66935	0.946129	1.45666
66936	0.94613	1.54152
66937	1.05926	1.62459
66938	0.946126	1.0644
66939	0.946126	1.18964
66940	0.94613	1.0218
66941	0.94613	1.16674
66942	0.946131	1.13171
66943	0.946131	1.21286
66944	0.946125	1.18294
66945	0.946125	0.900851
66946	0.946127	1.02482
66947	0.946127	1.03209
66948	0.946128	1.17247
66949	0.946128	1.13846
66950	1.89226	1.02835
66951	1.89226	0.935376
66952	1.89226	0.876415
66953	1.89226	1.1707
66954	1.89226	0.910753
66955	1.89226	1.31125
66956	1.89226	1.1015
66957	1.89226	1.39993
66958	1.89226	1.2433
66959	1.89226	1.35938
66960	1.89226	1.09913
66961	1.89226	1.27997
66962	1.16109	2.1
66963	1.23243	2.1
66964	0.659819	1.47986
66965	0.659819	2.1
66966	0.697965	1.24057
66967	1.00087	2.1
66968	0.891386	2.08133
66969	0.891386	1.48759
66970	0.946129	1.99554
66971	0.65979	1.37045
66972	0.891357	1.92671
66973	0.946129	1.72561
66974	1.89225	0.963224
66975	1.89225	1.17455
66976	1.89225	1.32192
66977	1.89225	0.929854
66978	1.89225	2.03725
66979	1.89225	1.07465
66980	1.79981	0.05
66981	2.02514	0.05
66982	2.33247	0.145367
66983	2.12891	0.05
66984	4.15405	0.05
66985	2.48284	0.393392
66986	2.02514	0.05
66987	2.33247	0.0656849

66988	2.02514	0.162202
66989	2.48567	0.0552089
66990	2.17834	0.13147
66991	1.89226	0.05
66992	1.89226	0.12007
66993	1.89226	0.134394
66994	1.89226	0.05
66995	3.78453	0.05
66996	1.89227	0.05
66997	1.89227	0.05
66998	1.89227	0.05
66999	1.89227	0.0837038
67000	1.89227	0.05
67001	1.89227	0.123378
67002	2.50894	0.05
67003	2.70118	0.080331
67004	2.12891	0.05
67005	2.12891	0.05
67006	2.7125	0.05
67007	2.33247	0.05
67008	1.89226	0.115262
67009	1.89226	0.05
67010	1.89226	0.05
67011	1.89226	0.126111
67012	1.89226	0.05
67013	1.89226	0.0693783
67014	0.946133	0.05
67015	0.946133	0.0546092
67016	0.946132	0.05
67017	0.946132	0.05
67018	0.946132	0.05
67019	0.946132	0.0712003
67020	0.946133	0.05
67021	0.946133	0.0643009
67022	0.946132	0.05
67023	0.946132	0.05
67024	0.946132	0.05
67025	0.946132	0.05
67026	1.90098	0.135216
67027	1.36463	0.190269
67028	1.56656	0.338505
67029	1.36463	0.0819558
67030	2.04386	0.083923
67031	1.89227	0.0796424
67032	1.89226	0.154268
67033	1.89226	0.105829
67034	1.89226	0.427899
67035	1.89226	0.133011
67036	1.89226	0.0944293
67037	1.89226	0.0580471
67038	1.89226	0.246693
67039	1.89226	0.0987469
67040	1.89226	0.584776
67041	1.89226	0.141002
67042	1.89226	0.285865
67043	1.89226	0.105755
67044	1.89226	0.179513
67045	1.89226	0.359759

67046	1.89226	0.470994
67047	1.89226	0.108648
67048	1.89226	0.505297
67049	1.89226	0.10207
67050	1.89226	0.104405
67051	1.70303	0.143177
67052	2.1292	0.257282
67053	1.70303	0.0753139
67054	2.32302	0.0665013
67055	1.90098	0.0500114
67056	1.89226	0.0622222
67057	1.89226	0.100896
67058	1.89226	0.519006
67059	1.89226	0.112286
67060	1.89226	0.0875716
67061	1.89226	0.05
67062	1.89226	0.177438
67063	1.89226	0.23108
67064	1.89226	0.621257
67065	1.89226	0.132622
67066	1.89226	0.195945
67067	1.89226	0.0643315
67068	1.89226	0.142329
67069	1.89226	0.614829
67070	1.89226	0.643942
67071	1.89226	0.133508
67072	1.89226	0.349554
67073	1.89226	0.0767822
67074	1.89226	0.35678
67075	1.89226	1.30264
67076	1.89226	1.27379
67077	1.89226	1.02134
67078	3.78453	0.613473
67079	1.89226	0.981196
67080	1.89226	2.0613
67081	1.89226	0.28805
67082	1.89226	2.1
67083	1.89226	1.26167
67084	1.89226	2.1
67085	1.89226	1.30414
67086	1.89226	1.04708
67087	1.89226	0.868088
67088	1.89226	1.13477
67089	3.78452	1.21939
67090	1.89224	1.28374
67091	1.89224	0.984332
67092	1.89224	0.829356
67093	1.89224	1.33495
67094	1.89224	0.252942
67095	1.89224	1.10734
67096	1.89224	1.19599
67097	1.89224	2.1
67098	1.89224	1.51306
67099	1.89224	2.1
67100	1.89224	0.240776
67101	1.89224	2.1
67102	1.31307	2.1
67103	1.31307	2.1

67104	0.888948	1.48661
67105	0.888948	1.29328
67106	1.2559	1.53675
67107	0.888979	2.1
67108	0.946114	2.1
67109	0.946114	1.28162
67110	0.946129	1.18558
67111	0.888989	1.37253
67112	0.946104	1.06259
67113	1.25588	1.2757
67114	1.89224	0.662575
67115	1.89224	1.37276
67116	1.89224	0.992053
67117	1.89224	1.3313
67118	1.89224	1.09682
67119	1.89224	1.9549
67120	1.79375	0.05
67121	1.47689	0.05
67122	1.97732	0.05
67123	2.26687	0.05
67124	3.74375	0.05
67125	2.05759	0.05
67126	2.26686	0.05
67127	1.79374	0.05
67128	1.79374	0.05
67129	2.49889	0.05
67130	2.49889	0.05
67131	1.89226	0.05
67132	1.89226	0.05
67133	1.89226	0.05
67134	1.89226	0.05
67135	3.78452	0.05
67136	1.89227	0.05
67137	1.89227	0.05
67138	1.89227	0.05
67139	1.89227	0.05
67140	1.89227	0.05
67141	1.89227	0.05
67142	1.97732	0.05
67143	2.26687	0.05
67144	2.6607	0.05
67145	2.41464	0.05
67146	4.68151	0.05
67147	1.89226	0.05
67148	1.89226	0.05
67149	1.89226	0.05
67150	1.89226	0.05
67151	3.78452	0.05
67152	1.89226	0.05
67153	1.89226	0.05
67154	1.89226	0.05
67155	1.89226	0.05
67156	1.89226	0.05
67157	1.89226	0.05
67158	1.89226	0.937043
67159	1.89226	1.39075
67160	1.89226	1.01141
67161	1.89226	0.960308

67162	3.78452	0.965833
67163	1.89226	0.999338
67164	1.89226	1.5314
67165	1.89226	0.986103
67166	1.89226	1.22049
67167	1.89226	1.05353
67168	1.89226	1.70144
67169	1.89226	1.46164
67170	1.89226	0.992333
67171	1.89226	0.937113
67172	1.89226	1.25098
67173	1.89226	0.962036
67174	1.89226	1.32268
67175	1.89226	1.01449
67176	1.89226	0.958245
67177	1.89226	0.914377
67178	1.89226	1.24336
67179	1.89226	0.906664
67180	1.89226	1.41384
67181	1.89226	1.00442
67182	2.45282	1.24097
67183	1.89226	1.27742
67184	1.89226	1.70837
67185	1.89226	0.973968
67186	1.89226	1.13723
67187	1.89226	0.978464
67188	1.89226	1.38974
67189	1.89226	0.98753
67190	1.89226	1.03637
67191	1.89226	1.3928
67192	1.89226	2.00083
67193	1.87308	0.05
67194	1.88631	0.05
67195	1.63908	0.05
67196	1.70302	0.05
67197	2.08745	0.05
67198	2.16916	0.05
67199	2.41019	0.05
67200	2.733	0.05
67201	2.08121	0.05
67202	2.08121	0.05
67203	2.74362	0.05
67204	2.41464	0.05
67205	1.89226	0.05
67206	1.89226	0.05
67207	1.89226	0.05
67208	1.89226	0.05
67209	1.89226	0.05
67210	1.89226	0.05
67211	0.946133	0.05
67212	0.946133	0.05
67213	0.946132	0.0827529
67214	0.946132	0.0503016
67215	0.946132	0.0506188
67216	0.946132	0.05
67217	0.946133	0.05
67218	0.946133	0.05
67219	0.946132	0.05

67220	0.946132	0.110392
67221	0.946133	0.05
67222	0.946133	0.05
67223	2.02912	0.0745485
67224	2.57016	0.05
67225	1.88631	0.05
67226	1.88631	0.05
67227	2.55301	0.05
67228	2.4102	0.05
67229	1.89226	0.05
67230	1.89226	0.05
67231	1.89226	0.0742918
67232	1.89226	0.111502
67233	3.78452	0.0600537
67234	1.89227	0.05
67235	1.89227	0.205461
67236	1.89227	0.0874631
67237	1.89227	0.0820385
67238	1.89227	0.121928
67239	1.89227	0.0904614
67240	1.89226	2.1
67241	1.89226	2.1
67242	1.89226	2.1
67243	1.89226	2.1
67244	3.78452	2.1
67245	1.2515	2.1
67246	1.34788	2.1
67247	0.946132	2.1
67248	0.946132	2.1
67249	0.973043	2.1
67250	1.16766	2.1
67251	0.919222	2.1
67252	1.2159	2.1
67253	0.946132	2.1
67254	0.946132	2.1
67255	1.33746	2.1
67256	1.13537	2.1
67257	1.89226	2.1
67258	1.89226	2.1
67259	1.89226	2.1
67260	1.89226	2.1
67261	1.89226	2.1
67262	1.89226	2.1
67263	1.89226	2.1
67264	1.89226	2.1
67265	1.89226	2.1
67266	1.89226	2.1
67267	3.78452	2.1
67268	1.89227	2.1
67269	1.89227	1.85689
67270	1.89227	2.1
67271	1.89227	2.1
67272	1.89227	2.1
67273	1.89227	2.1
67274	0.946132	2.1
67275	0.946132	2.1
67276	0.946129	2.1
67277	0.946129	2.1

67278	0.946131	2.1
67279	0.946131	2.1
67280	0.946131	2.1
67281	0.946131	2.1
67282	0.946131	2.1
67283	0.946131	2.1
67284	0.94613	2.1
67285	0.94613	2.1
67286	1.89226	1.15645
67287	1.89226	1.0674
67288	1.89226	1.08622
67289	1.89226	0.944151
67290	3.78453	1.00962
67291	0.946132	1.80449
67292	0.946132	1.08153
67293	0.946132	1.15042
67294	0.946132	1.06671
67295	0.946132	0.953473
67296	0.946132	1.82341
67297	0.946132	1.09835
67298	0.946132	1.12877
67299	0.946131	2.07464
67300	0.946131	1.02028
67301	0.946132	1.34248
67302	0.946132	1.07518
67303	1.89226	1.23843
67304	1.89226	0.888969
67305	1.89226	0.85632
67306	1.89226	0.973541
67307	1.89226	1.00891
67308	1.89226	1.59123
67309	1.89226	1.61987
67310	1.89226	0.951687
67311	1.89226	0.907531
67312	1.89226	0.968756
67313	3.78453	0.963324
67314	0.96614	1.17769
67315	0.96614	1.07124
67316	0.946131	0.895641
67317	0.946131	0.999898
67318	0.946131	1.03912
67319	0.96614	1.02278
67320	1.37736	1.15404
67321	0.946131	1.23862
67322	1.37736	1.53555
67323	0.96614	0.989577
67324	0.946131	1.33542
67325	0.946131	0.9598
67326	0.946129	1.41976
67327	0.946129	1.01401
67328	0.946128	1.02366
67329	0.946128	1.29679
67330	0.946132	0.893058
67331	0.946132	1.87025
67332	0.946126	1.05258
67333	0.946126	0.994243
67334	0.946129	0.912531
67335	0.946129	0.956419

67336	0.946129	0.908995
67337	0.946129	1.0658
67338	1.89226	0.0719664
67339	1.65723	0.0567352
67340	1.83117	0.194232
67341	1.65723	0.164034
67342	2.0662	0.05
67343	1.89226	0.05
67344	1.89226	0.115126
67345	1.89226	0.100804
67346	1.89226	0.324983
67347	1.89226	0.34314
67348	1.89226	0.05
67349	1.89226	0.05
67350	1.89226	0.302598
67351	1.89226	0.277697
67352	1.89226	0.612128
67353	1.89226	0.560018
67354	1.89226	0.0937438
67355	1.89226	0.0646674
67356	1.89226	0.149045
67357	1.89226	0.0914599
67358	1.89226	0.461186
67359	1.89226	0.390088
67360	1.89226	0.0570654
67361	1.89226	0.0519288
67362	1.89226	0.176064
67363	1.83117	0.104158
67364	1.92807	0.251669
67365	1.83117	0.252875
67366	1.98916	0.0530759
67367	1.89226	0.05
67368	1.89226	0.0585991
67369	1.89226	0.180739
67370	1.89226	0.238828
67371	1.89226	0.243957
67372	3.78453	0.119321
67373	1.89226	0.489137
67374	1.89226	0.560101
67375	1.89226	0.622707
67376	1.89226	0.629492
67377	1.89226	0.15372
67378	1.89226	0.154256
67379	1.89226	0.245992
67380	1.89226	0.247418
67381	1.89226	0.582067
67382	1.89226	0.517595
67383	1.89226	0.102565
67384	1.89226	0.0842205
67385	1.88037	0.0719323
67386	1.89227	0.0861755
67387	1.89227	0.209303
67388	1.89227	0.0655393
67389	1.98599	0.112385
67390	1.98599	0.05
67391	1.89226	0.224205
67392	1.89226	0.248455
67393	1.89226	0.642992

67394	1.89226	0.487548
67395	1.89226	0.189497
67396	1.89226	0.113965
67397	1.89226	0.520725
67398	1.89226	0.462938
67399	1.89226	0.665171
67400	1.89226	0.719705
67401	3.78452	0.706303
67402	1.89226	0.558449
67403	1.89226	0.710807
67404	1.89226	0.699131
67405	1.89226	0.688143
67406	1.89226	0.641755
67407	1.89226	0.49507
67408	1.90116	0.0813041
67409	1.89227	0.0937051
67410	1.89226	0.185473
67411	1.89227	0.0803751
67412	1.88036	0.103313
67413	1.88037	0.0514927
67414	1.89226	0.359516
67415	1.89226	0.489807
67416	1.89226	0.669398
67417	1.89226	0.523679
67418	1.89226	0.291051
67419	1.89226	0.1456
67420	0.946132	0.70984
67421	0.946132	0.706413
67422	0.946128	0.709401
67423	0.946128	0.711124
67424	0.94613	0.711155
67425	0.94613	0.712307
67426	0.94613	0.708822
67427	0.94613	0.701555
67428	0.946131	0.713666
67429	0.946131	0.71317
67430	0.94613	0.358325
67431	0.94613	0.718925
67432	1.89226	0.703371
67433	1.89226	0.710277
67434	1.89226	0.705758
67435	1.89226	0.704542
67436	1.89226	0.707956
67437	1.89226	0.709609
67438	1.89226	0.196624
67439	1.89226	1.50677
67440	2.35239	1.48145
67441	2.27873	0.449539
67442	1.89226	1.40694
67443	1.83301	0.692528
67444	2.68449	0.899112
67445	1.89226	2.1
67446	1.89226	0.824448
67447	1.89226	1.21717
67448	3.78453	0.959725
67449	0.946132	2.07041
67450	0.946132	1.1866
67451	0.946129	0.420084

67452	0.946129	1.99846
67453	0.946131	2.0043
67454	0.946131	1.41431
67455	0.94613	1.08604
67456	0.94613	1.31257
67457	0.946131	1.43596
67458	0.946131	1.05549
67459	0.946131	1.30621
67460	0.946131	1.53055
67461	1.89226	0.301162
67462	1.89226	1.25219
67463	1.89226	1.44291
67464	1.89226	0.490526
67465	1.89226	1.36946
67466	1.89226	0.737193
67467	1.70303	0.492767
67468	1.89226	1.38325
67469	1.89226	0.279869
67470	1.89226	0.744414
67471	3.78453	0.467804
67472	1.89227	1.24188
67473	1.89227	1.35745
67474	1.89227	0.506885
67475	1.89227	1.91054
67476	3.78453	1.32128
67477	1.89226	1.14274
67478	1.89226	2.06118
67479	1.89226	1.18838
67480	1.89226	1.61056
67481	3.78452	1.38332
67482	1.89226	2.1
67483	1.89226	2.1
67484	1.89226	2.1
67485	1.89226	2.1
67486	3.78452	2.1
67487	0.946132	1.92379
67488	0.946132	2.1
67489	0.946129	2.1
67490	0.946129	2.1
67491	0.94613	2.1
67492	0.94613	2.1
67493	0.946131	2.1
67494	0.946131	2.1
67495	0.946131	2.1
67496	0.946131	2.1
67497	0.946131	2.1
67498	0.946131	2.0614
67499	1.89226	2.1
67500	1.89226	1.9907
67501	1.89226	2.1
67502	1.89226	2.1
67503	3.78452	2.1
67504	1.89226	1.64409
67505	1.89226	2.1
67506	1.89226	2.1
67507	1.89226	1.75935
67508	1.89226	2.1
67509	1.89226	1.71318

67510	1.89227	2.1
67511	2.48308	1.70653
67512	1.89227	1.76323
67513	1.89227	2.07903
67514	3.78453	2.1
67515	1.89226	1.9401
67516	1.89226	2.1
67517	1.89226	2.1
67518	1.89226	1.84072
67519	1.89226	2.1
67520	1.89226	1.73962
67521	1.89226	2.01178
67522	1.89226	1.94687
67523	1.89226	2.1
67524	1.89226	1.96959
67525	3.78453	2.1
67526	0.946131	1.98368
67527	0.946131	2.1
67528	0.946131	2.1
67529	0.946131	2.1
67530	0.946131	2.1
67531	0.946131	2.1
67532	0.946131	2.1
67533	0.946131	2.1
67534	0.946131	2.1
67535	0.946131	2.1
67536	0.946131	2.1
67537	0.946131	2.01623
67538	1.23719	2.1
67539	1.28439	2.02527
67540	0.94613	1.93968
67541	0.94613	2.1
67542	1.28439	2.1
67543	1.6431	2.1
67544	0.946129	1.93143
67545	0.946129	1.78903
67546	1.28439	1.60092
67547	0.946131	1.37835
67548	1.21038	1.08816
67549	1.21038	1.64689
67550	1.89226	2.06998
67551	1.89226	1.81908
67552	1.89226	1.94394
67553	1.89226	1.86019
67554	1.89226	1.8077
67555	1.89226	1.74644
67556	1.89226	1.95393
67557	1.89226	2.09318
67558	1.89226	2.1
67559	1.89226	1.78539
67560	1.89226	2.1
67561	1.89226	1.67786
67562	1.89226	1.21053
67563	1.89226	0.440604
67564	1.89226	0.643301
67565	1.89226	0.994197
67566	1.89226	0.963226
67567	1.89226	1.24404

67568	0.946128	0.738172
67569	0.946128	0.980183
67570	0.946125	0.139376
67571	0.946125	0.747246
67572	0.94613	0.311295
67573	0.94613	0.551338
67574	0.946124	0.878375
67575	0.946124	0.997319
67576	0.946127	0.4398
67577	0.946127	0.21823
67578	0.946127	0.888235
67579	0.946127	0.985477
67580	1.89226	0.222947
67581	2.78671	0.200203
67582	2.59302	0.190835
67583	2.59302	0.230555
67584	1.89226	0.552776
67585	1.89226	0.439266
67586	1.89226	0.364945
67587	1.89226	0.495973
67588	1.89226	0.251403
67589	1.89226	0.280729
67590	1.89226	0.776578
67591	1.89226	0.619893
67592	1.89225	0.280441
67593	1.89225	0.0543334
67594	1.89225	0.613927
67595	1.89225	0.373859
67596	1.89225	0.741224
67597	1.89225	0.204379
67598	0.979536	0.135631
67599	0.816597	0.196837
67600	1.15446	0.110172
67601	1.07423	0.0746363
67602	1.08452	0.176929
67603	0.603522	0.118429
67604	1.70302	0.10291
67605	1.29344	0.0824289
67606	1.21806	0.134589
67607	0.723758	0.0609694
67608	1.37562	0.163018
67609	1.89226	0.097064
67610	1.92807	0.143517
67611	1.90843	0.192008
67612	1.92807	0.280322
67613	1.87263	0.065303
67614	1.89226	0.0831313
67615	0.946132	0.229644
67616	0.946132	0.136368
67617	0.946131	0.198229
67618	0.946131	0.203652
67619	0.946132	0.212353
67620	0.946132	0.191174
67621	0.946132	0.0547284
67622	0.946132	0.114885
67623	0.946132	0.251895
67624	0.946132	0.460736
67625	0.946132	0.230174

67626	0.946132	0.28388
67627	1.89226	0.23741
67628	1.89226	0.601816
67629	1.89226	0.545684
67630	1.89226	0.584448
67631	3.78452	0.492486
67632	1.89226	0.312553
67633	1.89226	0.399049
67634	1.89226	0.567231
67635	1.89226	0.540928
67636	1.89226	0.151666
67637	1.89226	0.131854
67638	1.70303	0.11433
67639	1.90843	0.092287
67640	1.77002	0.13973
67641	1.90843	0.145053
67642	1.75709	0.0689432
67643	1.89226	0.0615574
67644	1.89226	0.0848933
67645	1.89226	0.174131
67646	1.89226	0.257829
67647	1.89226	0.382223
67648	3.78453	0.151815
67649	1.89226	0.40817
67650	1.89226	0.551429
67651	1.89226	0.462376
67652	1.89226	0.625376
67653	3.78452	0.5761
67654	0.946131	0.163275
67655	0.946131	0.364335
67656	0.946131	0.137651
67657	0.946131	0.271648
67658	0.946129	0.253953
67659	0.946129	0.267958
67660	0.946133	0.139729
67661	0.946133	0.154531
67662	0.946131	0.388655
67663	0.946131	0.274691
67664	0.946131	0.12787
67665	0.946131	0.33031
67666	1.89226	0.05
67667	2.10275	0.610246
67668	1.76377	0.334159
67669	1.76377	0.128802
67670	1.89226	0.222412
67671	1.89226	0.105475
67672	1.89226	0.05
67673	1.89226	0.56779
67674	2.10275	0.359874
67675	2.55889	0.104937
67676	1.89226	0.249602
67677	2.30274	0.0978664
67678	1.89226	0.137148
67679	1.89226	1.12622
67680	1.89226	1.05484
67681	1.89226	0.246845
67682	1.89226	0.933729
67683	1.89226	0.300429

67684	1.89226	0.106757
67685	1.89226	1.1179
67686	1.89226	1.03574
67687	1.89226	0.309606
67688	1.89226	0.88516
67689	1.89226	0.312024
67690	1.89226	0.05
67691	1.89226	0.615181
67692	1.89226	0.28765
67693	1.89226	0.0790865
67694	1.89226	0.237392
67695	1.89226	0.0965592
67696	1.89226	0.05
67697	1.89226	0.527175
67698	1.89226	0.339312
67699	1.89226	0.105612
67700	1.89226	0.244245
67701	1.89226	0.105735
67702	1.89226	0.15496
67703	1.89226	1.07489
67704	1.89226	0.955746
67705	1.89226	0.30814
67706	1.89226	0.906305
67707	1.89226	0.38934
67708	1.89226	0.847457
67709	1.89226	1.04196
67710	1.89226	0.162808
67711	1.89226	0.526563
67712	1.89226	0.169696
67713	1.89226	0.87821
67714	0.946131	0.0578718
67715	0.976399	0.05
67716	1.10124	0.05
67717	0.946131	0.132023
67718	0.887633	0.119723
67719	0.887633	0.0754217
67720	1.16933	0.0526506
67721	1.23163	0.05
67722	0.946131	0.0729008
67723	0.946131	0.05
67724	0.976399	0.05
67725	1.14992	0.0580902
67726	1.89226	0.114422
67727	1.89226	0.670049
67728	1.89226	0.0624403
67729	1.89226	0.12118
67730	3.78452	0.072182
67731	1.89226	0.05
67732	1.89226	0.246289
67733	1.89226	0.05
67734	1.89226	0.05
67735	3.78453	0.05
67736	0.946132	0.05
67737	0.946132	0.0931376
67738	0.946131	0.05
67739	0.946131	0.0587994
67740	0.946131	0.0541391
67741	0.946131	0.05

67742	0.946132	0.05
67743	0.946132	0.05
67744	0.946131	0.05
67745	0.946131	0.05
67746	0.946132	0.05
67747	0.946132	0.05
67748	0.946132	0.151404
67749	0.946132	0.05
67750	0.946131	0.05
67751	0.946131	0.136217
67752	0.946131	0.154321
67753	0.946131	0.0877039
67754	0.946132	0.05
67755	0.946132	0.05
67756	0.946131	0.0507709
67757	0.946131	0.0583448
67758	0.946132	0.0648664
67759	0.946132	0.0591055
67760	1.89226	0.189323
67761	1.89226	0.411322
67762	1.89226	0.05
67763	1.89226	0.465319
67764	3.78452	0.334504
67765	1.89226	0.869732
67766	1.89226	0.833157
67767	1.89226	0.774624
67768	1.89226	0.409812
67769	3.78453	0.568141
67770	1.89226	0.175531
67771	1.89226	0.869313
67772	1.89226	0.784631
67773	1.89226	0.88573
67774	1.89226	0.356975
67775	1.89226	0.784508
67776	1.89225	0.286181
67777	1.89225	0.698954
67778	1.89225	0.75594
67779	1.89225	0.847902
67780	1.89225	0.140218
67781	1.89225	0.763262
67782	1.89226	0.70029
67783	1.89226	0.794792
67784	1.89226	0.757473
67785	1.89226	0.81857
67786	1.89226	0.162925
67787	1.89226	0.621923
67788	1.89226	0.85021
67789	1.89226	0.416767
67790	1.89226	0.0891882
67791	1.89226	0.0930118
67792	3.78453	0.207693
67793	1.39491	0.165415
67794	1.39491	0.135496
67795	0.924649	0.178109
67796	0.924649	0.251882
67797	0.946131	0.398497
67798	1.37343	0.0817578
67799	0.924678	0.05

67800	0.946131	0.894387
67801	0.924678	0.0972837
67802	1.37343	0.0938288
67803	0.946131	0.552351
67804	0.946131	0.577853
67805	1.89226	0.739384
67806	1.89226	0.0699432
67807	1.89226	0.183084
67808	1.89226	0.615761
67809	1.89226	0.05
67810	1.89226	0.229396
67811	0.946126	0.05
67812	0.975461	0.05
67813	0.946132	0.05
67814	1.12674	0.05
67815	0.946129	0.05
67816	0.946129	0.05
67817	1.12673	0.05
67818	1.12673	0.05
67819	0.946129	0.05
67820	0.946129	0.05
67821	0.975464	0.05
67822	1.12673	0.05
67823	1.89226	0.05
67824	1.89226	0.05
67825	1.89226	0.05
67826	1.89226	0.05
67827	1.89226	0.05
67828	1.89226	0.05
67829	0.946126	0.05
67830	0.946126	0.05
67831	0.946131	0.05
67832	0.946131	0.05
67833	0.946129	0.05
67834	0.946129	0.05
67835	0.946128	0.05
67836	0.946128	0.05
67837	0.946129	0.05
67838	0.946129	0.05
67839	0.946128	0.05
67840	0.946128	0.05
67841	1.89226	0.05
67842	1.89226	0.05
67843	1.89226	0.05
67844	1.89226	0.05
67845	3.78451	0.05
67846	1.89226	0.05
67847	1.89226	0.05
67848	1.89226	0.05
67849	1.89226	0.05
67850	3.78452	0.05
67851	0.946125	0.05
67852	0.946125	0.05
67853	0.946131	0.05
67854	0.946131	0.05
67855	0.946128	0.05
67856	0.946128	0.05
67857	0.946128	0.05

67858	0.946128	0.05
67859	0.946128	0.05
67860	0.946128	0.05
67861	0.946128	0.05
67862	0.946128	0.05
67863	1.89226	0.0561653
67864	1.89226	0.264567
67865	1.89226	0.31578
67866	1.89226	0.369545
67867	3.78452	0.254906
67868	1.89226	0.153094
67869	1.89226	0.585936
67870	1.89226	0.53287
67871	1.89226	0.266383
67872	1.89226	0.549225
67873	1.89226	0.517362
67874	1.12492	0.441734
67875	1.41201	0.178448
67876	1.12082	0.267202
67877	0.771416	0.525431
67878	1.10725	0.574895
67879	1.10725	0.125671
67880	1.07618	0.624269
67881	1.07618	0.05
67882	1.12492	0.186254
67883	0.771415	0.331314
67884	1.12082	0.0698558
67885	1.41201	0.220145
67886	2.82401	0.0702898
67887	1.89226	0.510101
67888	1.89226	0.160297
67889	1.89226	0.080001
67890	2.24983	0.539146
67891	2.24983	0.187621
67892	0.946133	0.653231
67893	0.946133	0.233657
67894	0.946129	0.477338
67895	0.946129	0.604576
67896	0.946131	0.637985
67897	0.946131	0.592691
67898	0.946131	0.640068
67899	0.946131	0.0847972
67900	0.946131	0.17547
67901	0.946131	0.359358
67902	0.946131	0.187825
67903	0.946131	0.192044
67904	0.946132	0.540736
67905	0.946132	0.592304
67906	0.946133	0.586808
67907	0.946133	0.373501
67908	0.946132	0.606091
67909	0.946132	0.563163
67910	0.946133	0.631488
67911	0.946133	0.273476
67912	0.946132	0.589551
67913	0.946132	0.541737
67914	0.946133	0.586604
67915	0.946133	0.581626

67916	1.89227	0.05
67917	1.89227	0.0795304
67918	1.89227	0.0854978
67919	1.89227	0.05
67920	1.89227	0.0991438
67921	1.89227	0.05
67922	1.42982	0.05
67923	1.19944	0.05
67924	0.936311	0.05
67925	0.936311	0.05
67926	0.946132	0.05
67927	1.02993	0.05
67928	1.41513	0.05
67929	0.946133	0.05
67930	0.946133	0.05
67931	0.946133	0.05
67932	1.02993	0.05
67933	1.18181	0.05
67934	1.89226	0.0586791
67935	1.89226	0.140678
67936	1.89226	0.237059
67937	1.89226	0.05
67938	3.78453	0.0632905
67939	1.89226	0.05
67940	1.89226	0.05
67941	1.89226	0.05
67942	1.89226	0.05
67943	1.89226	0.05
67944	1.89226	0.05
67945	1.89226	0.05
67946	1.89226	0.05
67947	1.89226	0.05
67948	1.89226	0.05
67949	1.89226	0.05
67950	1.89226	0.05
67951	0.946131	0.05
67952	0.946131	0.0719142
67953	0.946132	0.157305
67954	0.946132	0.0971792
67955	0.946132	0.0519625
67956	0.946132	0.0770239
67957	0.946132	0.166576
67958	0.946132	0.05
67959	0.946132	0.0512837
67960	0.946132	0.0712966
67961	0.946132	0.0707198
67962	0.946132	0.05
67963	1.89226	1.2834
67964	1.89226	1.37064
67965	1.89226	1.54859
67966	1.89226	0.844805
67967	1.89226	1.54154
67968	1.89226	0.974616
67969	1.89226	1.52288
67970	2.22749	0.239067
67971	1.89226	0.381669
67972	1.89226	0.738876
67973	3.78453	0.73831

67974	1.89226	1.31354
67975	1.89226	1.42261
67976	1.89226	1.56256
67977	1.89226	0.934496
67978	3.78453	1.23805
67979	1.89226	0.689553
67980	1.89226	0.903093
67981	1.89226	1.26448
67982	1.89226	0.332536
67983	1.89226	1.33456
67984	1.89226	0.533465
67985	0.94613	0.358948
67986	0.94613	1.25617
67987	0.94613	1.47691
67988	0.94613	1.17597
67989	0.946131	0.788026
67990	0.946131	0.381645
67991	0.946129	1.41971
67992	0.946129	0.802056
67993	0.94613	0.864154
67994	0.94613	0.882241
67995	0.94613	0.910162
67996	0.94613	0.540895
67997	1.89226	0.05
67998	1.89226	0.05
67999	1.89226	0.05
68000	1.89226	0.05
68001	1.89226	0.05
68002	1.89226	0.05
68003	1.89226	0.05
68004	1.89226	0.05
68005	1.89226	0.05
68006	1.89226	0.05
68007	3.78452	0.05
68008	1.89225	0.05
68009	1.89225	0.05
68010	1.89225	0.05
68011	1.89225	0.05
68012	1.89225	0.05
68013	1.89225	0.05
68014	1.89226	0.05
68015	1.89226	0.05
68016	1.89226	0.05
68017	1.89226	0.05
68018	1.89226	0.05
68019	1.89226	0.05
68020	0.946132	0.05
68021	0.946132	0.05
68022	0.946132	0.05
68023	0.946132	0.05
68024	0.946131	0.05
68025	0.946131	0.05
68026	0.946132	0.05
68027	0.946132	0.05
68028	0.946132	0.05
68029	0.946132	0.05
68030	0.946132	0.05
68031	0.946132	0.05

68032	1.89226	0.05
68033	1.89226	0.0568196
68034	1.89226	0.05
68035	1.89226	0.102538
68036	1.89226	0.05
68037	1.89226	0.0761635
68038	1.89226	0.05
68039	1.89226	0.05
68040	1.89226	0.05
68041	1.89226	0.0633183
68042	1.89226	0.05
68043	1.89226	0.05
68044	1.89226	0.05
68045	1.89226	0.05
68046	1.89226	0.05
68047	1.89226	0.05
68048	1.89226	0.05
68049	1.89226	0.05
68050	1.89226	0.05
68051	1.89226	0.0555654
68052	1.89226	0.05
68053	1.89226	0.0649544
68054	3.78452	0.05
68055	1.89226	0.05
68056	1.89226	0.05
68057	1.89226	0.05
68058	1.89226	0.0798634
68059	1.89226	0.05
68060	1.89226	0.05
68061	1.89226	0.05
68062	1.89226	0.05
68063	1.89226	0.05
68064	1.89226	0.05
68065	3.78453	0.05
68066	1.89226	0.05
68067	2.48862	0.05
68068	1.89226	0.05
68069	2.22062	0.05
68070	1.89226	0.05
68071	2.22062	0.05
68072	1.89227	0.05
68073	1.89227	0.05
68074	1.89227	0.05
68075	1.89227	0.05
68076	1.89227	0.05
68077	1.89227	0.05
68078	0.946132	0.05
68079	0.946132	0.05
68080	0.946131	0.05
68081	0.946131	0.05
68082	0.94613	0.05
68083	0.94613	0.05
68084	0.946133	0.05
68085	0.946133	0.05
68086	0.946131	0.05
68087	0.946131	0.05
68088	0.946131	0.05
68089	0.946131	0.05

68090	1.89226	0.05
68091	1.89226	0.05
68092	1.89226	0.05
68093	1.89226	0.05
68094	3.78453	0.05
68095	1.89226	0.05
68096	1.89226	0.05
68097	1.89226	0.05
68098	1.89226	0.05
68099	3.78453	0.05
68100	1.89226	0.05
68101	1.89226	0.117935
68102	1.89226	0.0887818
68103	1.89226	0.140164
68104	1.89226	0.05
68105	1.89226	0.05
68106	1.89226	0.165999
68107	1.89226	0.20032
68108	1.89226	0.096729
68109	1.89226	0.611254
68110	3.78452	0.180224
68111	1.89226	0.0545535
68112	1.89226	0.417885
68113	1.89226	0.254066
68114	1.89226	0.635949
68115	3.78453	0.401667
68116	2.57454	0.278291
68117	1.89226	0.527057
68118	1.3663	0.407952
68119	1.89226	0.708447
68120	3.25856	0.640462
68121	1.89226	0.12825
68122	1.89226	0.239256
68123	1.89226	0.143333
68124	1.89226	0.384822
68125	3.78451	0.142444
68126	1.89226	0.192722
68127	1.89226	0.717396
68128	1.89226	0.216545
68129	1.89226	0.265753
68130	1.89226	0.522463
68131	1.89226	0.497959
68132	0.810946	0.686712
68133	0.810946	0.666304
68134	0.810946	0.696452
68135	0.810946	0.70424
68136	0.810959	0.699324
68137	0.810959	0.618977
68138	1.89226	0.517356
68139	1.35168	0.553365
68140	1.08132	0.660541
68141	1.08132	0.71207
68142	1.0813	0.636938
68143	1.89226	0.224183
68144	1.89226	0.630426
68145	1.89226	0.688028
68146	1.89226	0.631125
68147	3.78453	0.630061

68148	1.89226	0.674745
68149	1.89226	0.62215
68150	1.89226	0.648134
68151	1.89226	0.666795
68152	1.89226	0.148987
68153	1.89226	0.6497
68154	1.89226	0.619469
68155	1.89226	0.649249
68156	1.89226	0.674127
68157	1.89226	0.644046
68158	3.78452	0.644678
68159	1.89226	0.629234
68160	1.89226	0.622086
68161	1.89226	0.631078
68162	1.89226	0.661984
68163	3.78452	0.6334
68164	2.142	0.741984
68165	1.93949	0.376158
68166	1.39296	0.690835
68167	2.55711	0.710233
68168	1.39296	0.081341
68169	2.55711	0.674981
68170	1.89226	0.676137
68171	2.5571	0.525603
68172	2.142	0.697151
68173	1.89226	0.708483
68174	4.44936	0.690262
68175	0.946131	0.68139
68176	0.946131	0.675651
68177	1.27855	0.669123
68178	1.29171	0.662646
68179	1.29171	0.675281
68180	0.946127	0.666141
68181	1.27855	0.317228
68182	0.946131	0.691428
68183	1.29172	0.681362
68184	1.29172	0.674535
68185	0.946129	0.652571
68186	0.946129	0.65382
68187	1.27855	0.688011
68188	1.29172	0.670842
68189	0.969743	0.659536
68190	1.07109	0.648782
68191	1.29172	0.702978
68192	1.07109	0.625611
68193	0.969745	0.189533
68194	1.27855	0.698836
68195	1.10873	0.720532
68196	1.10873	0.690195
68197	1.15273	0.638854
68198	1.15273	0.648798
68199	1.89227	0.05
68200	1.89227	0.05
68201	1.89227	0.0577405
68202	1.89227	0.05
68203	2.1288	0.05
68204	2.1288	0.05
68205	1.89227	0.083031

68206	1.89227	0.149646
68207	1.89227	0.0939595
68208	1.89227	0.138961
68209	3.78453	0.179458
68210	1.89226	0.30072
68211	1.89226	0.510799
68212	1.89226	0.158727
68213	1.89226	0.281951
68214	3.78453	0.142861
68215	1.89226	0.0889436
68216	1.89226	0.163589
68217	1.89226	0.0751162
68218	1.89226	0.0648111
68219	1.89226	0.102314
68220	1.89226	0.168304
68221	1.94432	0.05
68222	1.89226	0.05
68223	2.07911	0.05
68224	1.89226	0.05
68225	2.07911	0.05
68226	1.89226	0.05
68227	1.89226	0.05
68228	1.89226	0.05
68229	1.89226	0.0696256
68230	1.89226	0.05
68231	1.89226	0.05
68232	1.89226	0.05
68233	1.89226	0.215329
68234	1.89226	0.314478
68235	1.89226	0.454053
68236	1.89226	0.119247
68237	1.89226	0.63626
68238	1.89226	0.167346
68239	1.89226	0.0531431
68240	1.89226	0.0564484
68241	1.89226	0.0761771
68242	1.89226	0.05
68243	1.89226	0.104602
68244	1.89226	0.0506099
68245	1.86778	0.05
68246	2.72348	0.05
68247	1.89226	0.0568093
68248	1.89226	0.05
68249	3.76004	0.05
68250	1.89226	0.271742
68251	1.89226	0.347993
68252	1.89226	0.983104
68253	1.89226	0.109325
68254	3.78451	0.276483
68255	1.89226	0.0561408
68256	1.89226	0.0734104
68257	1.89226	0.0866623
68258	1.89226	0.05
68259	1.89226	0.131747
68260	1.89226	0.0547624
68261	0.94613	0.05
68262	0.94613	0.0516182
68263	0.94613	0.16076

68264	0.94613	0.05
68265	0.946131	0.0627413
68266	0.946131	0.05
68267	0.946129	0.0646646
68268	0.946129	0.05
68269	0.94613	0.05
68270	0.94613	0.062618
68271	0.94613	0.05
68272	0.94613	0.05
68273	0.946124	0.103605
68274	0.946124	0.05
68275	0.94613	0.475011
68276	0.94613	0.111983
68277	0.946128	0.0612199
68278	0.946128	0.0771886
68279	0.946126	0.394813
68280	0.946126	0.103081
68281	0.946127	0.108759
68282	0.946127	0.119686
68283	0.946127	0.098591
68284	0.946127	0.0779196
68285	2.09166	0.05
68286	2.1285	0.05
68287	1.78381	0.05
68288	1.78381	0.05
68289	2.47689	0.05
68290	2.47689	0.05
68291	2.09167	0.05
68292	2.26572	0.05
68293	2.73393	0.05
68294	2.4769	0.05
68295	4.74261	0.05
68296	1.89226	0.05
68297	1.89226	0.05
68298	1.89226	0.05
68299	1.89226	0.05
68300	1.89226	0.05
68301	1.89226	0.05
68302	0.946131	0.05
68303	0.946131	0.05
68304	0.946129	0.05
68305	0.946129	0.05
68306	0.94613	0.05
68307	0.94613	0.05
68308	0.946131	0.05
68309	0.946131	0.05
68310	0.94613	0.05
68311	0.94613	0.05
68312	0.946131	0.05
68313	0.946131	0.05
68314	2.27622	0.05
68315	2.66786	0.05
68316	2.1285	0.05
68317	2.1285	0.05
68318	2.62461	0.05
68319	2.47689	0.05
68320	1.89226	0.05
68321	1.89226	0.05

68322	1.89226	0.05
68323	1.89226	0.05
68324	3.78453	0.05
68325	1.89226	0.05
68326	1.89226	0.05
68327	1.89226	0.05
68328	1.89226	0.05
68329	1.89226	0.05
68330	1.89226	0.05
68331	1.89226	0.892658
68332	1.89226	0.981024
68333	1.89226	0.895991
68334	1.89226	0.969277
68335	1.89226	0.898986
68336	1.89226	0.965842
68337	1.89226	1.08865
68338	1.89226	0.954328
68339	1.89226	0.955483
68340	1.89226	1.09814
68341	3.78452	1.01375
68342	1.89227	0.908199
68343	1.89227	1.09627
68344	1.89227	1.08712
68345	1.89227	1.02209
68346	3.78453	1.05746
68347	0.946127	1.02721
68348	0.946127	0.995011
68349	1.06438	0.995735
68350	0.946131	0.933429
68351	1.06438	0.987066
68352	0.94613	1.02756
68353	0.946128	0.99722
68354	0.946128	0.913799
68355	1.06438	0.995221
68356	1.06438	1.00305
68357	0.946129	0.989384
68358	0.946129	0.984574
68359	1.89226	0.966599
68360	1.89226	1.07956
68361	1.89226	1.00897
68362	1.89226	1.06165
68363	1.89226	1.01389
68364	1.89226	1.07007
68365	0.946133	1.08427
68366	0.946133	1.05831
68367	1.39771	1.08052
68368	0.946132	1.01014
68369	1.39771	1.08969
68370	1.39772	1.07997
68371	0.946133	1.06146
68372	0.946133	0.988698
68373	0.946132	1.07839
68374	1.39772	1.07039
68375	0.946132	1.03335
68376	0.946132	1.03964
68377	1.98182	0.05
68378	2.2217	0.05
68379	2.79889	0.05

68380	2.50097	0.05
68381	4.72267	0.05
68382	2.05362	0.05
68383	1.70302	0.05
68384	2.75109	0.05
68385	2.138 0.05	
68386	2.0815	0.05
68387	1.84751	0.05
68388	0.946131	0.05
68389	0.946131	0.05
68390	0.946131	0.05
68391	0.946131	0.05
68392	0.946131	0.05
68393	0.946131	0.05
68394	0.946132	0.05
68395	0.946132	0.05
68396	0.946131	0.05
68397	0.946131	0.05
68398	0.946132	0.05
68399	0.946132	0.05
68400	1.89226	0.05
68401	1.89226	0.05
68402	1.89226	0.05
68403	1.89226	0.05
68404	3.78451	0.05
68405	0.946126	0.05
68406	1.25048	0.05
68407	0.946131	0.05
68408	1.39945	0.05
68409	0.946127	0.05
68410	0.946127	0.05
68411	1.39944	0.05
68412	1.39944	0.05
68413	0.946129	0.05
68414	0.946129	0.05
68415	1.39944	0.05
68416	1.25048	0.05
68417	1.89226	0.05
68418	1.89226	0.05
68419	1.89226	0.05
68420	1.89226	0.05
68421	1.89226	0.05
68422	1.89226	0.05
68423	0.946126	0.05
68424	0.946126	0.05
68425	0.946131	0.05
68426	0.946131	0.05
68427	0.946128	0.05
68428	0.946128	0.05
68429	0.946129	0.05
68430	0.946129	0.05
68431	0.946129	0.05
68432	0.946129	0.05
68433	0.946128	0.05
68434	0.946128	0.05
68435	1.89226	0.05
68436	1.89226	0.05
68437	1.89226	0.05

68438	1.89226	0.05
68439	1.89226	0.05
68440	1.89226	0.05
68441	1.01393	0.05
68442	0.775245	0.05
68443	0.946126	0.05
68444	0.946126	0.05
68445	0.78305	0.05
68446	0.78305	0.05
68447	0.946159	0.05
68448	0.929267	0.05
68449	1.14956	0.05
68450	1.04073	0.05
68451	0.851526	0.05
68452	0.704781	0.05
68453	1.89226	0.05
68454	1.89226	0.05
68455	1.89226	0.05
68456	1.89226	0.05
68457	3.78451	0.05
68458	1.89226	0.05
68459	1.89226	0.05
68460	1.89226	0.05
68461	1.89226	0.05
68462	1.89226	0.05
68463	1.89226	0.05
68464	1.89226	0.05
68465	1.89226	0.05
68466	1.89226	0.05
68467	1.89226	0.05
68468	1.89226	0.05
68469	1.89226	0.05
68470	1.16317	0.05
68471	1.0292	0.05
68472	0.946126	0.05
68473	0.946126	0.05
68474	0.93562	0.05
68475	0.93562	0.05
68476	1.0418	0.05
68477	1.04181	0.05
68478	1.16317	0.05
68479	0.946129	0.05
68480	1.02919	0.05
68481	0.946128	0.05
68482	0.946131	0.05
68483	0.946131	0.05
68484	0.946126	0.05
68485	0.946126	0.05
68486	0.946129	0.05
68487	0.946129	0.05
68488	0.946128	0.05
68489	0.946128	0.05
68490	0.946129	0.05
68491	0.946129	0.05
68492	0.946128	0.05
68493	0.946128	0.05
68494	1.89226	0.05
68495	1.89226	0.05

68496	1.89226	0.05
68497	1.89226	0.05
68498	1.89226	0.05
68499	1.89226	0.05
68500	1.89226	0.05
68501	1.89226	0.05
68502	1.89226	0.05
68503	1.89226	0.05
68504	1.89226	0.05
68505	1.89226	0.05
68506	1.89226	0.05
68507	1.89226	0.05
68508	1.89226	0.05
68509	1.89226	0.05
68510	3.78453	0.05
68511	1.89226	0.05
68512	1.89226	0.05
68513	1.89226	0.05
68514	1.89226	0.05
68515	3.78452	0.05
68516	1.89225	0.05
68517	1.89225	0.05
68518	1.89225	0.05
68519	1.89225	0.05
68520	1.89225	0.05
68521	1.89225	0.05
68522	0.946132	0.05
68523	0.946132	0.05
68524	0.946133	0.05
68525	0.946133	0.05
68526	0.946132	0.05
68527	0.946132	0.05
68528	0.946132	0.05
68529	0.946132	0.05
68530	0.946133	0.05
68531	0.946133	0.05
68532	0.946132	0.05
68533	0.946132	0.05
68534	1.89226	0.05
68535	1.89226	0.05
68536	1.89226	0.05
68537	1.89226	0.05
68538	3.78453	0.05
68539	1.13537	0.05
68540	1.09222	0.05
68541	0.946126	0.05
68542	0.946126	0.05
68543	0.946128	0.05
68544	0.946128	0.05
68545	1.16317	0.05
68546	1.13034	0.05
68547	1.30155	0.05
68548	1.08462	0.05
68549	0.992915	0.05
68550	0.807642	0.05
68551	0.946131	0.05
68552	0.946131	0.05
68553	0.946125	0.05

68554	0.946125	0.05
68555	0.946128	0.05
68556	0.946128	0.05
68557	0.946128	0.05
68558	0.946128	0.05
68559	0.946128	0.05
68560	0.946128	0.05
68561	0.946128	0.05
68562	0.946128	0.05
68563	1.89226	0.05
68564	1.89226	0.05
68565	1.89226	0.05
68566	1.89226	0.05
68567	1.89226	0.05
68568	1.89226	0.05
68569	1.89224	0.05
68570	1.89224	0.05
68571	1.89224	0.05
68572	1.89224	0.05
68573	1.89224	0.05
68574	1.89224	0.05
68575	0.946086	0.05
68576	1.17919	0.05
68577	0.89738	0.05
68578	0.89738	0.05
68579	0.946116	0.05
68580	0.946116	0.05
68581	1.13048	0.05
68582	1.11844	0.05
68583	0.946128	0.05
68584	0.897362	0.05
68585	1.1282	0.05
68586	0.946103	0.05
68587	1.89223	0.05
68588	1.89223	0.05
68589	1.89223	0.05
68590	1.89223	0.05
68591	3.78446	0.05
68592	0.946118	0.05
68593	0.946118	0.05
68594	0.946118	0.05
68595	0.946118	0.05
68596	0.946119	0.05
68597	0.946119	0.05
68598	0.946118	0.05
68599	0.946118	0.05
68600	0.946131	0.05
68601	0.946131	0.05
68602	0.946106	0.05
68603	0.946106	0.05
68604	1.89227	0.921576
68605	1.89227	0.982307
68606	1.89227	0.977316
68607	1.89227	0.917739
68608	3.78453	0.914247
68609	1.89226	0.987092
68610	1.89226	0.841619
68611	1.89226	0.817141

68612	1.89226	0.891025
68613	1.89226	0.946401
68614	1.89226	0.978368
68615	1.89226	0.745418
68616	2.59378	0.193218
68617	2.30348	0.12857
68618	2.30348	0.39161
68619	1.89226	0.492892
68620	1.89226	0.850399
68621	1.89226	0.930074
68622	1.89226	0.868529
68623	1.89226	0.874304
68624	1.89226	0.89147
68625	3.78453	0.929961
68626	1.89226	0.891463
68627	1.89226	0.569351
68628	1.89226	0.340179
68629	1.89226	0.793467
68630	1.89226	0.880073
68631	1.89226	0.934107
68632	0.904406	0.31426
68633	1.01667	0.335873
68634	1.11974	0.202946
68635	0.994417	0.37391
68636	1.02013	0.263441
68637	0.628203	0.367121
68638	1.70302	0.279582
68639	1.49898	0.185826
68640	1.14559	0.304064
68641	0.900571	0.207105
68642	1.58886	0.306567
68643	2.81102	0.05
68644	2.3923	0.05
68645	2.05632	0.05
68646	2.43514	0.05
68647	4.82744	0.05
68648	1.89226	0.05
68649	1.89226	0.05
68650	1.89226	0.05
68651	1.89226	0.05
68652	1.89226	0.05
68653	1.89226	0.05
68654	1.89226	0.05
68655	1.89226	0.05
68656	1.89226	0.05
68657	1.89226	0.05
68658	3.78451	0.05
68659	2.50803	0.05
68660	1.903	0.05
68661	1.903	0.05
68662	2.19162	0.05
68663	2.09934	0.05
68664	2.39063	0.05
68665	1.00243	0.05
68666	0.731731	0.05
68667	0.876616	0.05
68668	1.00451	0.05
68669	0.986901	0.05

68670	0.986901	0.05
68671	0.887729	0.05
68672	0.887732	0.05
68673	0.876605	0.05
68674	0.769283	0.05
68675	1.00245	0.05
68676	1.18712	0.05
68677	0.946131	0.05
68678	0.946131	0.05
68679	0.946131	0.05
68680	0.946131	0.05
68681	0.946131	0.05
68682	0.946131	0.05
68683	0.946131	0.05
68684	0.946131	0.05
68685	0.946131	0.05
68686	0.946131	0.05
68687	0.946131	0.05
68688	0.946131	0.05
68689	1.89226	0.05
68690	1.89226	0.05
68691	1.89226	0.05
68692	1.89226	0.05
68693	1.89226	0.05
68694	1.89226	0.05
68695	1.89226	0.05
68696	1.89226	0.05
68697	1.89226	0.05
68698	1.89226	0.05
68699	3.78453	0.05
68700	1.89226	0.05
68701	1.89226	0.05
68702	1.89226	0.05
68703	1.89226	0.133934
68704	3.78453	0.05
68705	1.89226	0.05
68706	1.89226	0.0502526
68707	1.89226	0.05
68708	1.89226	0.05
68709	1.89226	0.05
68710	1.89226	0.05
68711	0.946132	0.05
68712	0.946132	0.05
68713	0.946131	0.05
68714	0.946131	0.05
68715	0.946131	0.05
68716	0.946131	0.05
68717	0.946132	0.05
68718	0.946132	0.05
68719	0.946131	0.05
68720	0.946131	0.05
68721	0.946131	0.05
68722	0.946131	0.05
68723	0.946131	0.05
68724	0.946131	0.05
68725	0.946131	0.05
68726	0.946131	0.05
68727	0.94613	0.0505166

68728	0.94613	0.0572142
68729	0.946132	0.05
68730	0.946132	0.05
68731	0.946131	0.05
68732	0.946131	0.05
68733	0.946131	0.05
68734	0.946131	0.05
68735	1.89226	0.05
68736	1.89226	0.05
68737	1.89226	0.05
68738	1.89226	0.05
68739	1.89226	0.05
68740	1.89226	0.05
68741	1.89226	0.05
68742	1.89226	0.05
68743	1.89226	0.05
68744	1.89226	0.05
68745	3.78453	0.05
68746	1.89226	0.05
68747	1.89226	0.05
68748	1.89226	0.05
68749	1.89226	0.05
68750	1.89226	0.05
68751	1.89226	0.05
68752	0.946132	0.05
68753	0.946132	0.05
68754	0.946131	0.05
68755	0.946131	0.05
68756	0.946132	0.05
68757	0.946132	0.05
68758	0.946131	0.05
68759	0.946131	0.05
68760	0.946132	0.05
68761	0.946132	0.05
68762	0.946131	0.05
68763	0.946131	0.05
68764	0.946131	0.05
68765	0.946131	0.05
68766	0.946132	0.05
68767	0.946132	0.05
68768	0.946132	0.05
68769	0.946132	0.05
68770	0.946131	0.05
68771	0.946131	0.05
68772	0.946132	0.05
68773	0.946132	0.05
68774	0.946131	0.05
68775	0.946131	0.05
68776	1.89226	0.576731
68777	1.89226	0.616419
68778	1.89226	0.779846
68779	1.89226	0.276646
68780	3.78452	0.304511
68781	1.89226	1.04795
68782	1.89226	0.645521
68783	1.89226	0.953522
68784	1.89226	0.974611
68785	3.78452	1.04983

68786	1.89226	0.622436
68787	1.89226	0.734701
68788	1.89226	0.948511
68789	1.89226	0.348108
68790	3.78452	0.35718
68791	1.89226	0.58868
68792	2.75945	0.0622095
68793	1.89226	0.190347
68794	2.35026	0.156933
68795	4.24252	0.607204
68796	0.946131	0.238432
68797	0.946131	0.356557
68798	0.946131	0.102429
68799	0.946131	0.152462
68800	0.946132	0.0983768
68801	0.946132	0.124277
68802	0.946129	0.432286
68803	0.946129	0.140177
68804	0.946131	0.260014
68805	0.946131	0.274615
68806	0.94613	0.482183
68807	0.94613	0.22493
68808	1.89226	0.781319
68809	1.89226	0.847455
68810	1.89226	0.543863
68811	1.89226	0.806864
68812	1.89226	0.748226
68813	1.89226	0.939904
68814	0.946129	0.225962
68815	0.946129	0.376093
68816	0.946133	0.298731
68817	0.946133	0.173077
68818	0.946131	0.133305
68819	0.946131	0.146541
68820	0.946131	0.390347
68821	0.946131	0.165289
68822	0.946131	0.330988
68823	0.946131	0.286585
68824	0.946131	0.445208
68825	0.946131	0.259136
68826	1.89226	0.0606498
68827	2.35026	0.05
68828	1.32126	0.05
68829	1.89226	0.0671998
68830	1.77925	0.05
68831	2.35026	0.0541807
68832	1.89226	0.05
68833	1.89226	0.05
68834	1.89226	0.05
68835	1.89226	0.05
68836	1.89226	0.05
68837	1.89226	0.05
68838	1.89226	0.05
68839	1.89226	0.0517143
68840	1.89226	0.05
68841	1.89226	0.05
68842	3.78453	0.0510815
68843	1.89226	0.05

68844	1.89226	0.05
68845	1.89226	0.05
68846	1.89226	0.05
68847	1.89226	0.05
68848	1.89226	0.05
68849	0.946131	0.05
68850	0.946131	0.05
68851	0.946131	0.05
68852	0.946131	0.05
68853	0.946132	0.05
68854	0.946132	0.05
68855	0.946131	0.05
68856	0.946131	0.05
68857	0.946132	0.05
68858	0.946132	0.05
68859	0.946131	0.05
68860	0.946131	0.05
68861	1.89226	0.05
68862	1.89226	0.05
68863	1.89226	0.05
68864	1.89226	0.05
68865	1.89226	0.05
68866	1.89226	0.05
68867	1.89226	0.05
68868	1.89226	0.129921
68869	1.89226	0.0527587
68870	1.89226	0.121975
68871	3.78453	0.117842
68872	1.89227	0.134922
68873	1.89227	0.184947
68874	1.89227	0.120716
68875	1.89227	0.342445
68876	3.78453	0.133954
68877	1.89226	0.05
68878	1.89226	0.100797
68879	1.89226	0.0506335
68880	1.89226	0.0903123
68881	3.78452	0.0872297
68882	0.946132	0.05
68883	0.946132	0.05
68884	0.946131	0.05
68885	0.946131	0.05
68886	0.946131	0.0560372
68887	0.946131	0.060463
68888	0.946133	0.05
68889	0.946133	0.05
68890	0.946132	0.05
68891	0.946132	0.05
68892	0.946131	0.05
68893	0.946131	0.05
68894	1.89227	0.0527506
68895	1.89227	0.173409
68896	1.89227	0.0542928
68897	1.89227	0.0565932
68898	1.89227	0.151287
68899	1.89227	0.155681
68900	2.20491	0.05
68901	1.81656	0.05

68902	1.81656	0.05
68903	1.89226	0.05
68904	1.89226	0.05
68905	1.89226	0.05
68906	1.89226	0.22106
68907	1.89226	0.329787
68908	1.89226	0.316146
68909	1.89226	0.182532
68910	3.78452	0.198433
68911	1.89226	0.867227
68912	1.89226	0.842746
68913	1.89226	0.835249
68914	1.89226	0.886699
68915	3.78453	0.866603
68916	1.89226	0.845919
68917	1.89226	0.86849
68918	1.89226	0.824726
68919	1.89226	0.863348
68920	1.89226	0.858745
68921	1.89226	0.884143
68922	1.89226	0.116227
68923	2.11032	0.05
68924	2.30793	0.0539283
68925	1.89226	0.0654292
68926	2.30793	0.0945542
68927	1.89226	0.117185
68928	1.89226	0.613092
68929	1.89226	0.618483
68930	1.89226	0.31411
68931	1.89226	0.465157
68932	1.89226	0.576685
68933	1.89226	0.8055
68934	0.810943	0.411222
68935	0.810943	0.342411
68936	0.810943	0.406487
68937	0.810943	0.309165
68938	0.810933	0.362721
68939	0.810933	0.425075
68940	1.89226	0.31226
68941	1.3517	0.441461
68942	1.08132	0.35207
68943	1.08132	0.666678
68944	1.08133	0.364037
68945	1.89226	0.05
68946	1.89226	0.05
68947	1.89226	0.05
68948	1.89226	0.05
68949	3.78453	0.05
68950	1.89226	0.05
68951	1.89226	0.05
68952	1.89226	0.05
68953	1.89226	0.05
68954	1.89226	0.05
68955	1.89226	0.05
68956	0.810945	0.05
68957	0.810945	0.05
68958	0.870829	0.05
68959	0.870829	0.05

68960	0.990502	0.05
68961	0.810933	0.05
68962	2.82777	0.05
68963	1.53126	0.05
68964	1.32076	0.05
68965	1.201	0.05
68966	1.08133	0.05
68967	1.89226	0.05
68968	1.89226	0.05
68969	1.89226	0.05
68970	1.89226	0.05
68971	1.89226	0.05
68972	1.89226	0.05
68973	0.946132	0.05
68974	0.946132	0.05
68975	0.946133	0.073449
68976	0.946133	0.05
68977	0.946132	0.05
68978	0.946132	0.05
68979	0.946132	0.05
68980	0.946132	0.05
68981	0.946133	0.05
68982	0.946133	0.05
68983	0.946132	0.05
68984	0.946132	0.05
68985	0.946133	0.05
68986	0.946133	0.05
68987	0.946132	0.05
68988	0.946132	0.05
68989	0.946132	0.05
68990	0.946132	0.05
68991	0.946132	0.05
68992	0.946132	0.05
68993	0.946133	0.05
68994	0.946133	0.05
68995	0.946132	0.05
68996	0.946132	0.05
68997	1.89226	0.05
68998	1.89226	0.109279
68999	1.89226	0.0735244
69000	1.89226	0.0564582
69001	1.89226	0.05
69002	1.89226	0.05
69003	1.89226	0.05
69004	1.89226	0.302275
69005	1.89226	0.0950523
69006	1.89226	0.278613
69007	3.78452	0.332237
69008	1.89226	0.286482
69009	1.89226	0.441636
69010	1.89226	0.283274
69011	1.89226	0.933702
69012	3.78452	0.27025
69013	1.89226	0.0612798
69014	1.89226	0.348859
69015	1.89226	0.0667305
69016	1.89226	0.0883621
69017	1.89226	0.372718

69018	1.89226	0.367652
69019	0.946128	0.0763627
69020	0.946128	0.0547508
69021	0.946132	0.0722606
69022	0.946132	0.0610868
69023	0.946129	0.118006
69024	0.946129	0.150995
69025	0.946131	0.057711
69026	0.946131	0.05
69027	0.94613	0.0773793
69028	0.94613	0.05
69029	0.946131	0.05
69030	0.946131	0.0706368
69031	0.946129	0.206099
69032	0.946129	0.0952784
69033	0.946133	0.172842
69034	0.946133	0.142991
69035	0.946131	0.219607
69036	0.946131	0.253831
69037	0.946131	0.104119
69038	0.946131	0.13598
69039	0.946131	0.20086
69040	0.946131	0.103854
69041	0.946131	0.15385
69042	0.946131	0.152111
69043	1.89226	0.05
69044	1.89226	0.05
69045	1.89226	0.05
69046	1.89226	0.05
69047	3.78452	0.05
69048	1.89225	0.05
69049	1.89225	0.05
69050	1.89225	0.05
69051	1.89225	0.05
69052	1.89225	0.05
69053	1.89225	0.05
69054	1.89226	0.05
69055	1.89226	0.05
69056	1.89226	0.05
69057	1.89226	0.05
69058	3.78452	0.05
69059	1.89226	0.05
69060	1.89226	0.0556718
69061	1.89226	0.05
69062	1.89226	0.05
69063	3.78453	0.05
69064	1.89226	0.05
69065	1.89226	0.05
69066	1.89226	0.05
69067	1.89226	0.05
69068	3.78452	0.05
69069	1.22164	0.05
69070	1.22164	0.05
69071	0.806032	0.05
69072	0.806032	0.05
69073	1.08154	0.05
69074	0.946126	0.05
69075	0.946129	0.05

69076	0.806065	0.05
69077	0.806063	0.05
69078	1.08155	0.05
69079	0.946128	0.05
69080	0.946128	0.05
69081	1.28554	0.05
69082	1.28554	0.05
69083	0.946126	0.05
69084	0.946126	0.05
69085	1.28554	0.05
69086	0.946129	0.05
69087	1.08155	0.05
69088	0.94613	0.05
69089	1.08155	0.05
69090	1.28554	0.05
69091	0.94613	0.05
69092	0.94613	0.05
69093	0.946132	0.05
69094	0.946132	0.05
69095	0.946132	0.05
69096	0.946132	0.05
69097	0.946132	0.0876605
69098	0.946132	0.05
69099	0.946133	0.05
69100	0.946133	0.05
69101	0.946132	0.05
69102	0.946132	0.05
69103	0.946133	0.05
69104	0.946133	0.05
69105	1.89226	0.746381
69106	1.89226	0.306134
69107	1.89226	0.878563
69108	1.89226	0.323837
69109	3.78453	0.773696
69110	1.89226	0.601642
69111	1.89226	0.158585
69112	1.89226	0.206463
69113	1.89226	0.265054
69114	1.89226	0.208577
69115	1.89226	0.26404
69116	1.89226	0.05
69117	1.89226	0.05
69118	1.89226	0.05
69119	1.89226	0.05
69120	1.89226	0.05
69121	1.89226	0.05
69122	1.89226	0.0797181
69123	1.89226	0.05
69124	1.89226	0.05
69125	1.89226	0.05
69126	1.89226	0.05
69127	1.89226	0.05
69128	0.946132	0.255807
69129	0.946132	0.32949
69130	0.946132	0.0865134
69131	0.946132	0.21183
69132	0.946132	0.12292
69133	0.946132	0.134955

69134	0.946132	0.243358
69135	0.946132	0.153953
69136	0.946131	0.05
69137	0.946131	0.213643
69138	0.946132	0.131564
69139	0.946132	0.149146
69140	1.89226	0.153386
69141	1.89226	0.05
69142	1.89226	0.106137
69143	1.89226	0.0853998
69144	3.78453	0.0936372
69145	0.851541	0.05
69146	0.72883	0.05
69147	0.991276	0.05
69148	0.851485	0.05
69149	1.00003	0.05
69150	1.00003	0.05
69151	0.809788	0.05
69152	0.809788	0.05
69153	0.890738	0.05
69154	0.991264	0.05
69155	0.851554	0.05
69156	0.851554	0.05
69157	0.8628	0.05
69158	0.78387	0.05
69159	0.830233	0.05
69160	0.830233	0.05
69161	0.808312	0.05
69162	0.817238	0.05
69163	1.70303	0.05
69164	1.24116	0.05
69165	0.995299	0.05
69166	1.0989	0.05
69167	1.02714	0.05
69168	1.89226	0.281604
69169	1.89226	0.284309
69170	1.89226	0.193235
69171	1.89226	1.57751
69172	3.78451	0.123225
69173	0.946131	0.403521
69174	0.946131	0.05
69175	0.946131	0.214825
69176	0.946131	0.212388
69177	0.94613	0.202723
69178	0.94613	0.05
69179	0.946132	0.05
69180	0.946132	0.05
69181	0.946131	0.0947152
69182	0.946131	0.342063
69183	0.946131	0.05
69184	0.946131	0.05
69185	1.89226	0.05
69186	1.89226	1.99874
69187	1.89226	1.32605
69188	1.89226	1.47046
69189	3.78453	1.45199
69190	1.89226	2.1
69191	1.89226	2.1

69192	1.89226	2.1
69193	1.89226	1.12848
69194	1.89226	2.1
69195	1.89226	1.15343
69196	0.946126	0.05
69197	1.17111	0.119719
69198	1.3393	0.0647532
69199	0.946131	0.05
69200	0.946127	0.05
69201	0.946127	0.05
69202	1.3393	0.05
69203	1.17111	0.148685
69204	1.3393	0.05
69205	1.3393	0.05
69206	0.946129	0.05
69207	0.946129	0.05
69208	0.946126	2.1
69209	0.946126	0.487937
69210	0.946132	2.1
69211	0.946132	2.1
69212	0.94613	2.1
69213	0.94613	0.172836
69214	0.946129	2.0647
69215	0.946129	1.29533
69216	0.946129	0.05
69217	0.946129	2.1
69218	0.94613	1.41812
69219	0.94613	1.15045
69220	0.946131	0.797513
69221	0.946131	0.396368
69222	0.94613	0.374872
69223	0.94613	0.410207
69224	0.946128	0.831391
69225	0.946128	0.957859
69226	0.946132	0.271503
69227	0.946132	0.384414
69228	0.94613	0.743052
69229	0.94613	0.211552
69230	0.94613	0.44953
69231	0.94613	0.758614
69232	0.94613	1.32288
69233	0.94613	1.1396
69234	0.94613	1.13213
69235	0.94613	1.07314
69236	0.946128	1.40827
69237	0.946128	1.41325
69238	0.946131	1.02084
69239	0.946131	0.976242
69240	0.94613	1.33869
69241	0.94613	0.993628
69242	0.94613	1.07118
69243	0.94613	1.29354
69244	1.89226	1.34656
69245	1.89226	1.12982
69246	1.89226	1.28751
69247	1.89226	2.1
69248	3.78453	1.27717
69249	1.89226	1.05127

69250	1.89226	1.04551
69251	1.89226	0.758506
69252	1.89226	1.05065
69253	1.89226	0.941165
69254	1.89226	1.03023
69255	1.89226	0.78032
69256	1.89226	1.40416
69257	1.89226	0.154266
69258	1.89226	0.660515
69259	1.89226	0.544478
69260	1.89226	1.0488
69261	1.89227	0.171498
69262	2.28622	0.328604
69263	2.16109	0.186711
69264	1.89227	0.319796
69265	4.17849	0.190817
69266	15.1381	0.707653
69267	15.1381	0.747155
69268	15.1381	0.10033
69269	15.1381	0.722843
69270	30.2762	0.705438
69271	15.1381	0.712711
69272	15.1381	0.740349
69273	15.1381	0.05
69274	15.1381	0.725738
69275	30.2762	0.703637
69276	15.1381	0.895166
69277	15.1381	0.801296
69278	15.1381	0.05
69279	15.1381	0.841096
69280	30.2762	0.808282
69281	15.1381	0.368156
69282	15.1381	0.753117
69283	15.1381	0.113974
69284	15.1381	0.714677
69285	30.2762	0.712358
69286	15.138	0.899268
69287	15.138	2.03811
69288	15.138	0.891104
69289	15.138	2.1
69290	30.276	1.67399
69291	15.138	2.07238
69292	15.138	2.1
69293	15.138	2.1
69294	15.138	2.1
69295	30.276	2.1
69296	15.138	1.75566
69297	15.138	1.36972
69298	15.138	0.863579
69299	15.138	2.1
69300	30.2759	1.96465
69301	15.138	1.66172
69302	15.138	2.1
69303	15.138	2.1
69304	15.138	1.38785
69305	30.2759	1.9835
69306	15.138	2.1
69307	15.138	2.1

69308	15.138	2.1
69309	15.138	2.1
69310	30.276	2.1
69311	15.1381	2.1
69312	15.1381	2.1
69313	15.1381	2.1
69314	15.1381	2.1
69315	30.2762	2.1
69316	15.1381	2.1
69317	15.1381	2.1
69318	15.1381	2.1
69319	15.1381	2.1
69320	30.2762	2.1
69321	15.138	1.92528
69322	15.138	2.1
69323	15.138	2.1
69324	15.138	2.1
69325	30.276	2.1
69326	15.138	2.1
69327	15.138	2.1
69328	15.138	2.1
69329	15.138	2.1
69330	30.276	2.1
69331	15.1381	1.21274
69332	15.1381	2.1
69333	15.1381	2.07594
69334	15.1381	1.61639
69335	30.2762	2.00257
69336	15.1381	2.1
69337	15.1381	2.1
69338	15.1381	2.04093
69339	15.1381	2.1
69340	30.2762	2.1
69341	15.1381	2.1
69342	15.1381	2.1
69343	15.1381	2.1
69344	15.1381	2.1
69345	30.2762	2.1
69346	15.138	0.724252
69347	15.138	0.718497
69348	15.138	0.0526882
69349	15.138	0.794303
69350	30.276	0.711791
69351	1.89226	2.1
69352	1.89226	2.1
69353	1.89226	2.1
69354	1.89226	2.1
69355	3.78453	2.1
69356	1.89226	2.1
69357	1.89226	2.1
69358	1.89226	2.1
69359	1.89226	2.1
69360	1.89226	2.1
69361	1.89226	2.1
69362	1.89226	2.1
69363	1.89226	2.1
69364	1.89226	2.1
69365	1.89226	2.1

69366	1.89226	2.1
69367	1.89226	2.1
69368	1.89226	2.1
69369	1.89226	2.1
69370	1.89226	2.1
69371	1.89226	2.1
69372	3.78453	2.1
69373	1.89226	2.1
69374	1.89226	2.1
69375	1.89226	2.1
69376	1.89226	2.1
69377	1.89226	2.1
69378	1.89226	2.1
69379	0.946127	2.1
69380	0.946127	2.1
69381	0.946131	2.1
69382	0.946131	2.1
69383	0.946131	2.1
69384	0.946131	2.1
69385	0.946128	2.1
69386	0.946128	2.1
69387	0.94613	2.1
69388	0.94613	2.1
69389	0.946129	2.1
69390	0.946129	2.1
69391	1.89226	2.1
69392	1.89226	2.1
69393	1.89226	2.1
69394	1.89226	2.1
69395	1.89226	2.1
69396	1.89226	2.1
69397	1.89226	2.1
69398	1.89226	2.1
69399	1.89226	2.1
69400	1.89226	2.1
69401	3.78452	2.1
69402	1.89226	2.1
69403	1.89226	2.1
69404	1.89226	2.1
69405	1.89226	2.1
69406	1.89226	2.1
69407	1.89226	2.1
69408	0.946129	2.1
69409	0.946129	2.1
69410	0.946133	2.1
69411	0.946133	2.1
69412	0.946131	2.1
69413	0.946131	2.1
69414	0.946131	2.1
69415	0.946131	2.1
69416	0.946131	2.1
69417	0.946131	2.1
69418	0.946131	2.1
69419	0.946131	2.1
69420	0.946132	2.1
69421	0.946132	2.1
69422	0.946132	2.1
69423	0.946132	2.1

69424	0.946132	2.1
69425	0.946132	2.1
69426	0.946133	2.1
69427	0.946133	2.1
69428	0.946132	2.1
69429	0.946132	2.1
69430	0.946133	2.1
69431	0.946133	2.1
69432	1.89226	2.1
69433	1.89226	2.1
69434	1.89226	2.1
69435	1.89226	2.1
69436	3.78452	2.1
69437	1.89226	2.1
69438	1.89226	2.1
69439	1.89226	2.1
69440	1.89226	2.1
69441	1.89226	2.1
69442	1.89226	2.1
69443	0.946129	2.1
69444	0.946129	2.1
69445	0.946133	2.1
69446	0.946133	2.1
69447	0.946131	2.1
69448	0.946131	2.1
69449	0.946131	2.1
69450	0.946131	2.1
69451	0.946131	2.1
69452	0.946131	2.1
69453	0.946131	2.1
69454	0.946131	2.1
69455	0.946132	2.1
69456	0.946132	2.1
69457	0.946132	2.1
69458	0.946132	2.1
69459	0.946132	2.1
69460	0.946132	2.1
69461	0.946133	2.1
69462	0.946133	2.1
69463	0.946133	2.1
69464	0.946133	2.1
69465	0.946132	2.1
69466	0.946132	2.1
69467	1.89226	2.1
69468	1.89226	2.1
69469	1.89226	2.1
69470	1.89226	2.1
69471	3.78453	2.1
69472	0.946128	2.1
69473	0.946128	2.1
69474	0.946132	2.1
69475	0.946132	2.1
69476	0.94613	2.1
69477	0.94613	2.1
69478	0.94613	2.1
69479	0.94613	2.1
69480	0.94613	2.1
69481	0.94613	2.1

69482	0.94613	2.1
69483	0.94613	2.1
69484	0.946127	2.1
69485	0.946127	2.1
69486	0.946131	2.1
69487	0.946131	2.1
69488	0.94613	2.1
69489	0.94613	2.1
69490	0.946128	2.1
69491	0.946128	2.1
69492	0.946129	2.1
69493	0.946129	2.1
69494	0.946129	2.1
69495	0.946129	2.1
69496	0.94613	2.1
69497	0.94613	2.1
69498	0.94613	2.1
69499	0.94613	2.1
69500	0.946131	2.1
69501	0.946131	2.1
69502	0.946129	2.1
69503	0.946129	2.1
69504	0.94613	2.1
69505	0.94613	2.1
69506	0.94613	2.1
69507	0.94613	2.1
69508	1.89226	2.1
69509	1.89226	2.1
69510	1.89226	2.1
69511	1.89226	2.1
69512	3.78453	2.1
69513	1.89226	2.1
69514	1.89226	2.1
69515	1.89226	2.1
69516	1.89226	2.1
69517	1.89226	2.1
69518	1.89226	2.1
69519	1.89226	2.1
69520	1.89226	2.1
69521	1.89226	2.1
69522	1.89226	2.1
69523	1.89226	2.1
69524	1.89226	2.1
69525	0.946131	2.1
69526	0.946131	2.1
69527	0.946131	2.1
69528	0.946131	2.1
69529	0.946132	2.1
69530	0.946132	2.1
69531	0.946129	2.1
69532	0.946129	2.1
69533	0.946131	2.1
69534	0.946131	2.1
69535	0.94613	2.1
69536	0.94613	2.1
69537	1.89224	2.1
69538	1.89224	2.1
69539	1.89224	2.1

69540	1.89224	2.1
69541	3.78448	2.1
69542	1.89224	2.1
69543	1.89224	2.1
69544	1.89224	2.1
69545	1.89224	2.1
69546	3.78447	2.1
69547	1.89223	2.1
69548	2.38995	2.1
69549	2.25315	2.1
69550	2.25315	2.1
69551	1.89223	2.1
69552	1.89223	2.1
69553	1.89224	2.1
69554	1.89224	2.1
69555	1.89224	2.1
69556	1.89224	2.1
69557	1.89224	2.1
69558	1.89224	2.1
69559	1.89226	2.1
69560	1.89226	2.1
69561	1.89226	2.1
69562	1.89226	2.1
69563	3.78453	2.1
69564	1.89226	2.1
69565	1.89226	2.1
69566	1.89226	2.1
69567	1.89226	2.1
69568	1.89226	2.1
69569	1.89226	2.1
69570	1.89226	2.1
69571	2.60272	2.1
69572	2.38998	2.1
69573	2.38998	2.1
69574	1.89226	2.1
69575	1.89226	2.1
69576	0.94613	2.1
69577	0.94613	2.1
69578	0.94613	2.1
69579	0.94613	2.1
69580	0.946131	2.1
69581	0.946131	2.1
69582	0.946129	2.1
69583	0.946129	2.1
69584	0.94613	2.1
69585	0.94613	2.1
69586	0.94613	2.1
69587	0.94613	2.1
69588	1.89226	2.1
69589	1.89226	2.1
69590	1.89226	2.1
69591	1.89226	2.1
69592	3.78452	2.1
69593	1.89226	2.1
69594	1.89226	2.1
69595	1.89226	2.1
69596	1.89226	2.1
69597	3.78452	2.1

69598	1.89226	2.1
69599	1.89226	2.1
69600	1.89226	2.1
69601	1.89226	2.1
69602	1.89226	2.1
69603	1.89226	2.1
69604	0.946132	2.1
69605	0.946132	2.1
69606	0.946132	2.1
69607	0.946132	2.1
69608	0.946131	2.1
69609	0.946131	2.1
69610	0.946132	2.1
69611	0.946132	2.1
69612	0.946132	2.1
69613	0.946132	2.1
69614	0.946132	2.1
69615	0.946132	2.1
69616	1.89226	2.1
69617	1.89226	2.1
69618	1.89226	2.1
69619	1.89226	2.1
69620	3.78452	2.1
69621	1.89226	2.1
69622	1.89226	2.1
69623	1.89226	2.1
69624	1.89226	2.1
69625	3.78452	2.1
69626	0.946129	2.1
69627	0.946129	2.1
69628	0.946133	2.1
69629	0.946133	2.1
69630	0.946131	2.1
69631	0.946131	2.1
69632	0.946131	2.1
69633	0.946131	2.1
69634	0.946131	2.1
69635	0.946131	2.1
69636	0.946131	2.1
69637	0.946131	2.1
69638	0.946132	2.1
69639	0.946132	2.1
69640	0.946132	2.1
69641	0.946132	2.1
69642	0.946132	2.1
69643	0.946132	2.1
69644	0.946133	2.1
69645	0.946133	2.1
69646	0.946133	2.1
69647	0.946133	2.1
69648	0.946132	2.1
69649	0.946132	2.1
69650	1.89224	2.1
69651	1.89224	2.1
69652	1.89224	2.1
69653	1.89224	2.1
69654	3.78447	2.1
69655	1.89223	2.1

69656	1.89223	2.1
69657	1.89223	2.1
69658	1.89223	2.1
69659	1.89223	2.1
69660	1.89223	2.1
69661	1.89224	2.1
69662	1.89224	2.1
69663	1.89224	2.1
69664	1.89224	2.1
69665	3.78447	2.1
69666	1.89224	2.1
69667	1.89224	2.1
69668	1.89224	2.1
69669	1.89224	2.1
69670	3.78448	2.1
69671	1.89226	2.1
69672	1.89226	2.1
69673	1.89226	2.1
69674	1.89226	2.1
69675	3.78452	2.1
69676	1.89226	2.1
69677	1.89226	2.1
69678	1.89226	2.1
69679	1.89226	2.1
69680	1.89226	2.1
69681	1.89226	2.1
69682	1.89226	2.1
69683	1.89226	2.1
69684	1.89226	2.1
69685	1.89226	2.1
69686	1.89226	2.1
69687	1.89226	2.1
69688	0.810946	2.1
69689	0.810946	2.1
69690	0.810946	2.1
69691	0.810946	2.1
69692	0.810933	2.1
69693	0.810933	2.1
69694	1.89226	2.1
69695	1.3517	2.1
69696	1.08132	2.1
69697	1.08132	2.1
69698	1.08133	2.1
69699	1.89226	2.1
69700	1.89226	2.1
69701	1.89226	2.1
69702	1.89226	2.1
69703	3.78453	2.1
69704	1.89226	2.1
69705	1.89226	2.1
69706	1.89226	2.1
69707	1.89226	2.1
69708	3.78452	2.1
69709	1.89226	2.1
69710	1.89226	2.1
69711	1.89226	2.1
69712	1.89226	2.1
69713	1.89226	2.1

69714	1.89226	2.1
69715	0.94613	2.1
69716	0.94613	2.1
69717	0.94613	2.1
69718	0.94613	2.1
69719	0.946131	2.1
69720	0.946131	2.1
69721	0.946129	2.1
69722	0.946129	2.1
69723	0.94613	2.1
69724	0.94613	2.1
69725	0.94613	2.1
69726	0.94613	2.1
69727	1.89226	2.1
69728	1.89226	2.1
69729	1.89226	2.1
69730	1.89226	2.1
69731	3.78453	2.1
69732	1.89226	2.1
69733	1.89226	2.1
69734	1.89226	2.1
69735	1.89226	2.1
69736	3.78452	2.1
69737	1.89226	2.1
69738	1.89226	2.1
69739	1.89226	2.1
69740	1.89226	2.1
69741	3.78452	2.1
69742	1.89226	2.1
69743	1.89226	2.1
69744	1.89226	2.1
69745	1.89226	2.1
69746	1.89226	2.1
69747	1.89226	2.1
69748	1.89226	2.1
69749	1.89226	2.1
69750	1.89226	2.1
69751	1.89226	2.1
69752	3.78452	2.1
69753	1.89226	2.1
69754	1.89226	2.1
69755	1.89226	2.1
69756	1.89226	2.1
69757	3.78452	2.1
69758	0.946128	2.1
69759	0.946128	2.1
69760	0.946133	2.1
69761	0.946133	2.1
69762	0.94613	2.1
69763	0.94613	2.1
69764	0.946131	2.1
69765	0.946131	2.1
69766	0.946131	2.1
69767	0.946131	2.1
69768	0.946131	2.1
69769	0.946131	2.1
69770	0.946132	2.1
69771	0.946132	2.1

69772	0.946132	2.1
69773	0.946132	2.1
69774	0.946131	2.1
69775	0.946131	2.1
69776	0.946132	2.1
69777	0.946132	2.1
69778	0.946132	2.1
69779	0.946132	2.1
69780	0.946132	2.1
69781	0.946132	2.1
69782	1.89226	2.1
69783	1.89226	2.1
69784	1.89226	2.1
69785	1.89226	2.1
69786	3.78452	2.1
69787	1.89226	2.1
69788	1.89226	2.1
69789	1.89226	2.1
69790	1.89226	2.1
69791	3.78452	2.1
69792	0.946129	2.1
69793	0.946129	2.1
69794	0.946133	2.1
69795	0.946133	2.1
69796	0.946131	2.1
69797	0.946131	2.1
69798	0.946131	2.1
69799	0.946131	2.1
69800	0.946131	2.1
69801	0.946131	2.1
69802	0.946131	2.1
69803	0.946131	2.1
69804	0.946132	2.1
69805	0.946132	2.1
69806	0.946132	2.1
69807	0.946132	2.1
69808	0.946132	2.1
69809	0.946132	2.1
69810	0.946133	2.1
69811	0.946133	2.1
69812	0.946132	2.1
69813	0.946132	2.1
69814	0.946133	2.1
69815	0.946133	2.1
69816	1.89226	2.1
69817	1.89226	2.1
69818	1.89226	2.1
69819	1.89226	2.1
69820	1.89226	2.1
69821	1.89226	2.1
69822	0.946128	2.1
69823	0.946128	2.1
69824	0.946133	2.1
69825	0.946133	2.1
69826	0.946131	2.1
69827	0.946131	2.1
69828	0.94613	2.1
69829	0.94613	2.1

69830	0.946131	2.1
69831	0.946131	2.1
69832	0.946131	2.1
69833	0.946131	2.1
69834	1.89226	2.1
69835	1.89226	2.1
69836	1.89226	2.1
69837	1.89226	2.1
69838	1.89226	2.1
69839	1.89226	2.1
69840	1.89226	2.1
69841	1.89226	2.1
69842	1.89226	2.1
69843	1.89226	2.1
69844	3.78453	2.1
69845	1.89226	2.1
69846	1.89226	2.1
69847	1.89226	2.1
69848	1.89226	2.1
69849	1.89226	2.1
69850	1.89226	2.1
69851	0.946127	2.1
69852	0.946127	2.1
69853	0.946131	2.1
69854	0.946131	2.1
69855	0.946131	2.1
69856	0.946131	2.1
69857	0.946127	2.1
69858	0.946127	2.1
69859	0.946129	2.1
69860	0.946129	2.1
69861	0.94613	2.1
69862	0.94613	2.1
69863	0.946131	2.1
69864	0.946131	2.1
69865	0.946131	2.1
69866	0.946131	2.1
69867	0.946133	2.1
69868	0.946133	2.1
69869	0.946128	2.1
69870	0.946128	2.1
69871	0.94613	2.1
69872	0.94613	2.1
69873	0.946131	2.1
69874	0.946131	2.1
69875	1.89226	2.1
69876	1.89226	2.1
69877	1.89226	2.1
69878	1.89226	2.1
69879	1.89226	2.1
69880	1.89226	2.1
69881	1.89226	2.1
69882	1.89226	2.1
69883	1.89226	2.1
69884	1.89226	2.1
69885	3.78452	2.1
69886	1.89226	2.1
69887	1.89226	2.1

69888	1.89226	2.1
69889	1.89226	2.1
69890	3.78452	2.1
69891	1.89226	2.1
69892	1.89226	2.1
69893	1.89226	2.1
69894	1.89226	2.1
69895	1.89226	2.1
69896	1.89226	2.1
69897	1.89226	2.1
69898	1.89226	2.1
69899	1.89226	2.1
69900	1.89226	2.1
69901	3.78453	2.1
69902	1.89226	2.1
69903	1.89226	2.1
69904	1.89226	2.1
69905	1.89226	2.1
69906	3.78452	2.1
69907	1.89226	2.1
69908	1.89226	2.1
69909	1.89226	2.1
69910	1.89226	2.1
69911	3.78452	2.1
69912	1.89226	2.1
69913	1.89226	2.1
69914	1.89226	2.1
69915	1.89226	2.1
69916	1.89226	2.1
69917	1.89226	2.1
69918	0.946131	2.1
69919	0.946131	2.1
69920	0.946131	2.1
69921	0.946131	2.1
69922	0.946131	2.1
69923	0.946131	2.1
69924	0.946131	2.1
69925	0.946131	2.1
69926	0.946131	2.1
69927	0.946131	2.1
69928	0.946131	2.1
69929	0.946131	2.1
69930	1.89226	2.1
69931	1.89226	2.1
69932	1.89226	2.1
69933	1.89226	2.1
69934	1.89226	2.1
69935	1.89226	2.1
69936	1.89226	2.1
69937	1.89226	2.1
69938	1.89226	2.1
69939	1.89226	2.1
69940	1.89226	2.1
69941	1.89226	2.1
69942	0.946131	2.1
69943	0.946131	2.1
69944	0.946131	2.1
69945	0.946131	2.1

69946	0.946131	2.1
69947	0.946131	2.1
69948	0.946131	2.1
69949	0.946131	2.1
69950	0.946131	2.1
69951	0.946131	2.1
69952	0.946131	2.1
69953	0.946131	2.1
69954	1.89226	2.1
69955	1.89226	2.1
69956	1.89226	2.1
69957	1.89226	2.1
69958	1.89226	2.1
69959	1.89226	2.1
69960	0.946128	2.1
69961	0.946128	2.1
69962	0.946133	2.1
69963	0.946133	2.1
69964	0.946131	2.1
69965	0.946131	2.1
69966	0.946131	2.1
69967	0.946131	2.1
69968	0.946131	2.1
69969	0.946131	2.1
69970	0.94613	2.1
69971	0.94613	2.1
69972	1.89226	2.1
69973	1.89226	2.1
69974	1.89226	2.1
69975	1.89226	2.1
69976	1.89226	2.1
69977	1.89226	2.1
69978	0.946131	2.1
69979	0.946131	2.1
69980	0.946131	2.1
69981	0.946131	2.1
69982	0.946132	2.1
69983	0.946132	2.1
69984	0.946131	2.1
69985	0.946131	2.1
69986	0.946132	2.1
69987	0.946132	2.1
69988	0.946131	2.1
69989	0.946131	2.1
69990	1.89226	2.1
69991	1.89226	2.1
69992	1.89226	2.1
69993	1.89226	2.1
69994	1.89226	2.1
69995	1.89226	2.1
69996	1.89226	2.1
69997	1.89226	2.1
69998	1.89226	2.1
69999	1.89226	2.1
70000	3.78452	2.1
70001	1.89226	2.1
70002	1.89226	2.1
70003	1.89226	2.1

70004	1.89226	2.1
70005	3.78452	2.1
70006	0.946131	2.1
70007	0.946131	2.1
70008	0.946131	2.1
70009	0.946131	2.1
70010	0.946132	2.1
70011	0.946132	2.1
70012	0.946131	2.1
70013	0.946131	2.1
70014	0.946132	2.1
70015	0.946132	2.1
70016	0.946131	2.1
70017	0.946131	2.1
70018	1.89226	2.1
70019	1.89226	2.1
70020	1.89226	2.1
70021	1.89226	2.1
70022	1.89226	2.1
70023	1.89226	2.1
70024	1.89226	2.1
70025	1.89226	2.1
70026	1.89226	2.1
70027	1.89226	2.1
70028	3.78452	2.1
70029	1.89226	2.1
70030	1.89226	2.1
70031	1.89226	2.1
70032	1.89226	2.1
70033	3.78452	2.1
70034	0.946131	2.1
70035	0.946131	2.1
70036	0.946131	2.1
70037	0.946131	2.1
70038	0.946131	2.1
70039	0.946131	2.1
70040	0.946131	2.1
70041	0.946131	2.1
70042	0.946131	2.1
70043	0.946131	2.1
70044	0.946131	2.1
70045	0.946131	2.1
70046	1.89226	2.1
70047	1.89226	2.1
70048	1.89226	2.1
70049	1.89226	2.1
70050	3.78453	2.1
70051	1.89226	2.1
70052	1.89226	2.1
70053	1.89226	2.1
70054	1.89226	2.1
70055	1.89226	2.1
70056	1.89226	2.1
70057	1.89226	2.1
70058	1.89226	2.1
70059	1.89226	2.1
70060	1.89226	2.1
70061	3.78453	2.1

70062	1.89226	2.1
70063	1.89226	2.1
70064	1.89226	2.1
70065	1.89226	2.1
70066	3.78453	2.1
70067	1.89226	2.1
70068	1.89226	2.1
70069	1.89226	2.1
70070	1.89226	2.1
70071	3.78453	2.1
70072	0.946132	2.1
70073	0.946132	2.1
70074	0.946132	2.1
70075	0.946132	2.1
70076	0.946132	2.1
70077	0.946132	2.1
70078	0.946132	2.1
70079	0.946132	2.1
70080	0.946132	2.1
70081	0.946132	2.1
70082	0.946131	2.1
70083	0.946131	2.1
70084	0.946131	2.1
70085	0.946131	2.1
70086	0.946131	2.1
70087	0.946131	2.1
70088	0.946132	2.1
70089	0.946132	2.1
70090	0.946131	2.1
70091	0.946131	2.1
70092	0.946132	2.1
70093	0.946132	2.1
70094	0.946131	2.1
70095	0.946131	2.1
70096	1.89226	2.1
70097	1.89226	2.1
70098	1.89226	2.1
70099	1.89226	2.1
70100	3.78453	2.1
70101	1.89226	2.1
70102	1.89226	2.1
70103	1.89226	2.1
70104	1.89226	2.1
70105	3.78453	2.1
70106	0.946131	2.1
70107	0.946131	2.1
70108	0.946131	2.1
70109	0.946131	2.1
70110	0.946131	2.1
70111	0.946131	2.1
70112	0.946131	2.1
70113	0.946131	2.1
70114	0.946131	2.1
70115	0.946131	2.1
70116	0.946131	2.1
70117	0.946131	2.1
70118	1.89226	2.1
70119	1.89226	2.1

70120	1.89226	2.1
70121	1.89226	2.1
70122	3.78452	2.1
70123	0.946132	2.1
70124	0.946132	2.1
70125	0.946131	2.1
70126	0.946131	2.1
70127	0.946132	2.1
70128	0.946132	2.1
70129	0.946131	2.1
70130	0.946131	2.1
70131	0.946131	2.1
70132	0.946131	2.1
70133	0.946131	2.1
70134	0.946131	2.1
70135	1.89226	2.1
70136	1.89226	2.1
70137	1.89226	2.1
70138	1.89226	2.1
70139	1.89226	2.1
70140	1.89226	2.1
70141	0.946132	2.1
70142	0.946132	2.1
70143	0.946132	2.1
70144	0.946132	2.1
70145	0.946132	2.1
70146	0.946132	2.1
70147	0.946132	2.1
70148	0.946132	2.1
70149	0.946132	2.1
70150	0.946132	2.1
70151	0.946131	2.1
70152	0.946131	2.1
70153	0.946131	2.1
70154	0.946131	2.1
70155	0.946131	2.1
70156	0.946131	2.1
70157	0.946132	2.1
70158	0.946132	2.1
70159	0.946131	2.1
70160	0.946131	2.1
70161	0.946132	2.1
70162	0.946132	2.1
70163	0.946131	2.1
70164	0.946131	2.1
70165	0.946132	2.1
70166	0.946132	2.1
70167	0.946131	2.1
70168	0.946131	2.1
70169	0.946132	2.1
70170	0.946132	2.1
70171	0.946131	2.1
70172	0.946131	2.1
70173	0.946132	2.1
70174	0.946132	2.1
70175	0.946131	2.1
70176	0.946131	2.1
70177	1.89226	2.1

70178	1.89226	2.1
70179	1.89226	2.1
70180	1.89226	2.1
70181	3.78453	2.1
70182	1.89226	2.1
70183	1.89226	2.1
70184	1.89226	2.1
70185	1.89226	2.1
70186	3.78453	2.1
70187	1.89226	2.1
70188	1.89226	2.1
70189	1.89226	2.1
70190	1.89226	2.1
70191	1.89226	2.1
70192	1.89226	2.1
70193	1.89226	2.1
70194	1.89226	2.1
70195	1.89226	2.1
70196	1.89226	2.1
70197	3.78453	2.1
70198	1.89226	2.1
70199	1.89226	2.1
70200	1.89226	2.1
70201	1.89226	2.1
70202	3.78453	2.1
70203	1.89226	2.1
70204	1.89226	2.1
70205	1.89226	2.1
70206	1.89226	2.1
70207	1.89226	2.1
70208	1.89226	2.1
70209	1.89226	2.1
70210	1.89226	2.1
70211	1.89226	2.1
70212	1.89226	2.1
70213	1.89226	2.1
70214	1.89226	2.1
70215	1.89226	2.1
70216	1.89226	2.1
70217	1.89226	2.1
70218	1.89226	2.1
70219	3.78453	2.1
70220	1.89226	2.1
70221	1.89226	2.1
70222	1.89226	2.1
70223	1.89226	2.1
70224	1.89226	2.1
70225	1.89226	2.1
70226	1.89226	2.1
70227	1.89226	2.1
70228	1.89226	2.1
70229	1.89226	2.1
70230	1.89226	2.1
70231	1.89226	2.1
70232	1.89226	2.1
70233	1.89226	1.37582
70234	1.89226	2.1
70235	1.89226	1.08593

70236	3.78453	1.98601
70237	1.89226	2.1
70238	1.89226	1.03763
70239	1.89226	0.614594
70240	1.89226	2.1
70241	1.89226	0.517835
70242	1.89226	2.1
70243	1.89226	2.1
70244	1.89226	2.1
70245	1.89226	2.1
70246	1.89226	2.1
70247	1.89226	2.1
70248	1.89226	2.1
70249	1.89226	2.1
70250	1.89226	2.1
70251	1.89226	2.1
70252	1.89226	2.1
70253	3.78453	2.1
70254	1.89226	2.1
70255	1.89226	1.57266
70256	1.89226	2.1
70257	1.89226	1.33768
70258	1.89226	2.1
70259	1.89226	1.47677
70260	1.89226	2.1
70261	1.89226	1.36694
70262	1.89226	2.1
70263	1.89226	2.1
70264	1.89226	1.9833
70265	1.89226	1.3195
70266	1.89226	2.1
70267	1.89226	2.1
70268	1.89226	2.1
70269	1.89226	2.1
70270	1.89226	2.1
70271	1.89226	2.1
70272	1.89226	2.1
70273	1.89226	2.1
70274	1.89226	2.1
70275	1.89226	2.1
70276	3.78452	2.1
70277	1.89226	2.1
70278	1.89226	1.17873
70279	1.89226	2.1
70280	1.89226	0.883132
70281	3.78452	2.09019
70282	0.946131	2.1
70283	0.946131	0.550383
70284	0.946131	1.70656
70285	0.946131	2.1
70286	0.94613	1.05884
70287	0.94613	1.77461
70288	0.946132	2.1
70289	0.946132	2.1
70290	0.946131	1.65846
70291	0.946131	1.16555
70292	0.946131	1.6655
70293	0.946131	1.28291

70294	1.89226	2.1
70295	1.89226	2.1
70296	1.89226	2.1
70297	1.89226	2.1
70298	1.89226	2.1
70299	1.89226	2.1
70300	1.89226	2.1
70301	1.89226	2.1
70302	1.89226	2.1
70303	1.89226	2.1
70304	3.78452	2.1
70305	1.89226	2.1
70306	1.89226	1.07115
70307	1.89226	2.1
70308	1.89226	0.0507312
70309	3.78452	1.07732
70310	0.946131	1.15259
70311	0.946131	2.1
70312	0.946131	2.1
70313	0.946131	1.29454
70314	0.94613	1.06661
70315	0.94613	0.541875
70316	0.946132	2.1
70317	0.946132	2.1
70318	0.946131	2.05946
70319	0.946131	2.03825
70320	0.946131	1.61431
70321	0.946131	1.23209
70322	1.89226	2.1
70323	1.89226	2.1
70324	1.89226	2.1
70325	1.89226	2.1
70326	3.78452	2.1
70327	1.89226	2.1
70328	1.89226	2.1
70329	1.89226	2.1
70330	1.89226	2.1
70331	3.78453	2.1
70332	1.89226	2.1
70333	1.89226	2.1
70334	1.89226	2.1
70335	1.89226	0.99581
70336	3.78452	1.91043
70337	1.89226	2.1
70338	1.89226	2.1
70339	1.89226	2.1
70340	1.89226	1.43656
70341	1.89226	2.1
70342	1.89226	2.1
70343	1.89226	2.1
70344	1.89226	2.1
70345	1.89226	2.1
70346	1.89226	2.1
70347	3.78452	2.1
70348	1.89226	2.1
70349	1.89226	2.1
70350	1.89226	2.1
70351	1.89226	2.1

70352	3.78453	2.1
70353	1.89226	2.1
70354	1.89226	1.12396
70355	1.89226	2.1
70356	1.89226	0.906784
70357	3.78453	2.1
70358	1.89226	2.1
70359	1.89226	1.05153
70360	1.89226	2.1
70361	1.89226	1.33121
70362	1.89226	2.1
70363	1.89226	0.358722
70364	1.89226	2.1
70365	1.89226	2.1
70366	1.89226	2.1
70367	1.89226	1.31693
70368	3.78452	2.1
70369	1.89226	2.1
70370	1.89226	2.1
70371	1.89226	2.1
70372	1.89226	1.93162
70373	3.78453	2.1
70374	1.89226	2.1
70375	1.89226	2.08658
70376	1.89226	2.1
70377	1.89226	0.97045
70378	3.78453	1.35597
70379	1.89226	2.1
70380	1.89226	1.40363
70381	1.89226	1.83761
70382	1.89226	1.27477
70383	1.89226	2.1
70384	1.89226	2.1
70385	0.946128	1.89225
70386	0.946128	2.1
70387	0.946132	2.1
70388	0.946132	2.1
70389	0.946129	2.1
70390	0.946129	2.1
70391	0.94613	2.1
70392	0.94613	2.1
70393	0.94613	2.1
70394	0.94613	2.1
70395	0.94613	2.1
70396	0.94613	2.1
70397	1.89226	2.1
70398	1.89226	2.1
70399	1.89226	2.1
70400	1.89226	2.1
70401	1.89226	2.1
70402	1.89226	1.98845
70403	1.89226	2.1
70404	1.89226	1.25508
70405	1.89226	1.44748
70406	1.89226	2.1
70407	1.89226	0.260745
70408	1.89226	2.1
70409	0.946127	2.1

70410	0.946127	1.48122
70411	0.946131	1.16226
70412	0.946131	2.1
70413	0.946128	2.03492
70414	0.946128	1.13388
70415	0.94613	2.1
70416	0.94613	2.1
70417	0.946129	2.00213
70418	0.946129	2.06223
70419	0.946129	2.1
70420	0.946129	2.1
70421	1.89226	2.1
70422	1.89226	2.1
70423	1.89226	2.1
70424	1.89226	2.1
70425	3.78452	2.1
70426	1.89226	2.1
70427	1.89226	2.1
70428	1.89226	2.1
70429	1.89226	2.1
70430	1.89226	2.1
70431	1.89226	2.1
70432	1.89226	2.1
70433	1.89226	1.06139
70434	1.89226	1.51393
70435	1.89226	2.1
70436	1.89226	0.664315
70437	1.89226	2.1
70438	1.89226	1.01516
70439	1.89226	2.1
70440	1.89226	2.1
70441	1.89226	1.29003
70442	1.89226	2.1
70443	1.89226	1.98452
70444	0.946131	2.1
70445	0.946131	2.1
70446	0.946131	2.1
70447	0.946131	2.1
70448	0.946131	2.1
70449	0.946131	2.1
70450	0.946132	2.1
70451	0.946132	2.1
70452	0.946132	2.1
70453	0.946132	2.1
70454	0.946131	2.1
70455	0.946131	2.1
70456	1.89226	2.1
70457	1.89226	2.1
70458	1.89226	2.1
70459	1.89226	2.1
70460	1.89226	2.1
70461	1.89226	2.1
70462	1.89226	2.1
70463	1.89226	0.978435
70464	1.89226	1.18295
70465	1.89226	2.1
70466	1.89226	0.05
70467	1.89226	2.1

70468	0.946131	2.1
70469	0.946131	1.104
70470	0.946131	1.10837
70471	0.946131	2.1
70472	0.94613	0.966799
70473	0.94613	1.00935
70474	0.946132	2.1
70475	0.946132	2.1
70476	0.946131	2.1
70477	0.946131	2.1
70478	0.946131	2.1
70479	0.946131	1.47864
70480	1.89226	2.1
70481	1.89226	2.1
70482	1.89226	2.1
70483	1.89226	2.1
70484	3.78453	2.1
70485	1.89226	2.1
70486	1.89226	2.1
70487	1.89226	2.1
70488	1.89226	2.1
70489	1.89226	2.1
70490	1.89226	2.1
70491	1.89226	2.1
70492	1.89226	1.61298
70493	1.89226	2.1
70494	1.89226	0.602562
70495	3.78452	1.15481
70496	1.89226	2.1
70497	1.89226	1.42378
70498	1.89226	2.1
70499	1.89226	1.0228
70500	3.78452	2.04263
70501	0.946131	1.10634
70502	0.946131	2.1
70503	0.946131	2.1
70504	0.946131	2.1
70505	0.946131	2.1
70506	0.946131	2.1
70507	0.946131	2.1
70508	0.946131	2.1
70509	0.946131	2.1
70510	0.946131	2.1
70511	0.946131	2.1
70512	0.946131	2.1
70513	0.946128	2.05038
70514	0.946128	2.1
70515	0.946132	2.1
70516	0.946132	2.1
70517	0.946129	2.1
70518	0.946129	2.1
70519	0.94613	2.1
70520	0.94613	2.1
70521	0.94613	2.1
70522	0.94613	2.1
70523	0.94613	2.1
70524	0.94613	2.1
70525	1.89226	2.1

70526	1.89226	0.993141
70527	1.89226	1.06127
70528	1.89226	2.1
70529	1.89226	0.0599257
70530	1.89226	2.1
70531	0.810945	2.1
70532	0.810945	1.15099
70533	0.810944	1.16432
70534	0.810944	1.16487
70535	1.89226	2.1
70536	1.35169	2.1
70537	1.08132	1.28982
70538	1.08132	1.94759
70539	1.08132	2.1
70540	0.810945	1.36329
70541	0.810945	2.1
70542	0.946131	2.1
70543	0.946131	2.1
70544	0.946131	2.1
70545	0.946131	2.1
70546	0.946131	2.1
70547	0.946131	2.1
70548	0.946131	2.1
70549	0.946131	2.1
70550	0.946131	2.1
70551	0.946131	2.1
70552	0.946131	2.1
70553	0.946131	2.1
70554	1.89226	2.1
70555	1.89226	2.1
70556	1.89226	2.1
70557	1.89226	2.1
70558	1.89226	2.1
70559	1.89226	2.1
70560	1.89226	2.1
70561	1.89226	2.1
70562	1.89226	2.1
70563	1.89226	2.1
70564	1.89226	2.1
70565	1.89226	2.1
70566	0.946131	2.1
70567	0.946131	2.1
70568	0.946131	2.1
70569	0.946131	2.1
70570	0.946131	2.1
70571	0.946131	2.1
70572	0.946131	2.1
70573	0.946131	2.1
70574	0.946131	2.1
70575	0.946131	2.1
70576	0.946131	2.1
70577	0.946131	2.1
70578	1.89226	2.1
70579	1.89226	2.1
70580	1.89226	2.1
70581	1.89226	2.1
70582	1.89226	2.1
70583	1.89226	2.1

70584	1.89226	2.1
70585	1.89226	2.1
70586	1.89226	2.1
70587	1.89226	2.1
70588	1.89226	2.1
70589	1.89226	2.1
70590	1.89226	2.1
70591	1.89226	2.1
70592	1.89226	2.1
70593	1.89226	2.1
70594	1.89226	2.1
70595	1.89226	2.1
70596	0.946132	2.1
70597	0.946132	2.1
70598	0.946131	2.1
70599	0.946131	2.1
70600	0.946132	2.1
70601	0.946132	2.1
70602	0.946132	2.1
70603	0.946132	2.1
70604	0.946132	2.1
70605	0.946132	2.1
70606	0.946132	2.1
70607	0.946132	2.1
70608	0.946131	2.1
70609	0.946131	2.1
70610	0.946131	2.1
70611	0.946131	2.1
70612	0.946131	2.1
70613	0.946131	2.1
70614	0.946131	2.1
70615	0.946131	2.1
70616	0.946131	2.1
70617	0.946131	2.1
70618	0.946131	2.1
70619	0.946131	2.1
70620	0.946132	2.1
70621	0.946132	2.1
70622	0.946131	2.1
70623	0.946131	2.1
70624	0.946132	2.1
70625	0.946132	2.1
70626	0.946131	2.1
70627	0.946131	2.1
70628	0.946131	2.1
70629	0.946131	2.1
70630	0.946131	2.1
70631	0.946131	2.1
70632	1.89226	2.1
70633	1.89226	2.1
70634	1.89226	2.1
70635	1.89226	2.1
70636	1.89226	2.1
70637	1.89226	2.1
70638	1.37713	2.1
70639	1.27093	2.1
70640	0.946132	2.1
70641	0.946132	2.1

70642	0.946132	2.1
70643	0.946132	2.1
70644	1.37713	2.1
70645	1.37713	2.1
70646	1.27093	2.1
70647	0.946131	2.1
70648	0.946132	2.1
70649	1.37713	2.1
70650	0.946131	2.1
70651	0.946131	2.1
70652	0.946131	2.1
70653	0.946131	2.1
70654	0.946132	2.1
70655	0.946132	2.1
70656	0.946131	2.1
70657	0.946131	2.1
70658	0.946131	2.1
70659	0.946131	2.1
70660	0.946132	2.1
70661	0.946132	2.1
70662	0.946132	2.1
70663	0.946132	2.1
70664	0.946131	2.1
70665	0.946131	2.1
70666	0.946132	2.1
70667	0.946132	2.1
70668	0.946131	2.1
70669	0.946131	2.1
70670	0.946131	2.1
70671	0.946131	2.1
70672	0.946132	2.1
70673	0.946132	2.1
70674	1.89226	2.1
70675	1.89226	2.1
70676	1.89226	2.1
70677	1.89226	2.1
70678	1.89226	2.1
70679	1.89226	2.1
70680	1.89226	2.1
70681	1.89226	2.1
70682	1.89226	2.1
70683	1.89226	2.1
70684	1.89226	2.1
70685	1.89226	2.1
70686	1.89226	2.1
70687	1.89226	2.1
70688	1.89226	2.1
70689	1.89226	1.07518
70690	3.78452	2.1
70691	1.89226	2.1
70692	1.89226	2.1
70693	1.89226	2.1
70694	1.89226	1.53518
70695	1.89226	2.1
70696	1.89226	2.1
70697	1.89226	2.1
70698	1.89226	2.1
70699	1.89226	2.1

70700	1.89226	2.1
70701	3.78452	2.1
70702	1.89226	2.1
70703	1.89226	2.1
70704	1.89226	2.1
70705	1.89226	2.1
70706	3.78453	2.1
70707	1.89226	2.1
70708	1.89226	1.18002
70709	1.89226	1.74306
70710	1.89226	2.1
70711	1.89226	0.861649
70712	1.89226	2.1
70713	1.89226	1.00993
70714	1.89226	2.1
70715	1.89226	2.1
70716	1.89226	1.75033
70717	1.89226	2.1
70718	1.89226	2.09393
70719	1.89226	2.1
70720	1.89226	2.1
70721	1.89226	2.1
70722	1.89226	2.1
70723	3.78452	2.1
70724	1.89226	2.1
70725	1.89226	2.1
70726	1.89226	2.1
70727	1.89226	2.1
70728	1.89226	2.1
70729	1.89226	2.1
70730	1.89226	2.1
70731	1.89226	2.053
70732	1.89226	1.85535
70733	1.89226	2.1
70734	1.89226	0.896331
70735	1.89226	2.1
70736	1.89226	1.24423
70737	1.89226	2.1
70738	1.89226	2.1
70739	1.89226	2.09395
70740	1.89226	2.1
70741	1.89226	2.1
70742	0.946131	2.1
70743	0.946131	2.1
70744	0.946131	2.1
70745	0.946131	2.1
70746	0.946131	2.1
70747	0.946131	2.1
70748	0.946132	2.1
70749	0.946132	2.1
70750	0.946131	2.1
70751	0.946131	2.1
70752	0.946132	2.1
70753	0.946132	2.1
70754	1.89226	2.1
70755	1.89226	2.1
70756	1.89226	2.1
70757	1.89226	2.1

70758	1.89226	2.1
70759	1.89226	2.1
70760	1.89226	2.1
70761	1.89226	1.1074
70762	1.89226	1.17843
70763	1.89226	2.1
70764	1.89226	0.113081
70765	1.89226	2.1
70766	0.946131	2.1
70767	0.946131	1.19878
70768	0.946131	1.25649
70769	0.946131	2.1
70770	0.94613	1.14257
70771	0.94613	1.07669
70772	0.946132	2.1
70773	0.946132	2.1
70774	0.946131	1.95235
70775	0.946131	2.1
70776	0.946131	2.1
70777	0.946131	2.00721
70778	1.89226	2.1
70779	1.89226	1.38833
70780	1.89226	0.519497
70781	1.89226	2.1
70782	3.78453	2.1
70783	2.81189	2.1
70784	1.89226	2.1
70785	1.89226	2.1
70786	1.89226	2.1
70787	2.56715	2.1
70788	2.56715	2.1
70789	1.89226	2.1
70790	1.89226	2.1
70791	1.89226	2.1
70792	1.89226	2.1
70793	3.78452	2.1
70794	1.89226	1.61539
70795	1.89226	2.1
70796	1.89226	2.1
70797	1.89226	2.1
70798	3.78452	2.1
70799	1.89226	0.810709
70800	1.89226	2.1
70801	1.89226	2.01951
70802	1.89226	2.1
70803	1.89226	1.68248
70804	1.89226	2.1
70805	1.1507	2.1
70806	1.1507	2.1
70807	0.946133	2.1
70808	0.946133	2.1
70809	0.946131	2.1
70810	0.946131	2.1
70811	1.28358	2.1
70812	1.1507	2.1
70813	1.1507	2.1
70814	0.946131	2.1
70815	0.94613	2.1

70816	1.28358	2.1
70817	1.89226	2.1
70818	1.89226	2.1
70819	1.89226	2.1
70820	1.89226	2.1
70821	1.89226	2.1
70822	1.89226	2.1
70823	0.946131	2.1
70824	0.946131	2.1
70825	0.946131	1.66457
70826	0.946131	2.1
70827	0.946128	2.1
70828	0.946128	2.1
70829	0.946133	2.1
70830	0.946133	2.09689
70831	0.946131	2.1
70832	0.946131	2.1
70833	0.94613	2.1
70834	0.94613	2.1
70835	1.89226	0.284879
70836	1.89226	2.1
70837	1.89226	2.1
70838	1.89226	1.06634
70839	3.78453	1.71759
70840	1.89226	2.1
70841	1.89226	2.1
70842	1.89226	1.64379
70843	1.89226	2.1
70844	3.78452	2.1
70845	1.89226	2.1
70846	1.89226	2.1
70847	1.89226	2.1
70848	1.89226	2.1
70849	3.78452	2.1
70850	1.89226	2.1
70851	1.89226	2.1
70852	1.89226	1.14093
70853	1.89226	2.1
70854	3.78452	2.1
70855	0.946132	2.1
70856	0.946132	2.1
70857	0.946132	0.064214
70858	0.946132	1.17781
70859	0.946132	1.0829
70860	0.946132	2.1
70861	0.946132	2.1
70862	0.946132	0.05
70863	0.946132	1.93009
70864	0.946132	2.03855
70865	0.946131	1.92361
70866	0.946131	2.02782
70867	1.89226	2.1
70868	1.89226	2.1
70869	1.89226	2.1
70870	1.89226	2.1
70871	1.89226	2.1
70872	1.89226	2.1
70873	1.89226	1.24391

70874	1.89226	2.1
70875	1.89226	2.1
70876	1.89226	2.1
70877	1.89226	2.1
70878	1.89226	2.1
70879	1.89226	2.1
70880	1.89226	2.1
70881	1.89226	2.1
70882	1.89226	2.1
70883	3.78453	2.1
70884	1.89226	2.1
70885	1.89226	2.1
70886	1.89226	2.1
70887	1.89226	2.1
70888	3.78452	2.1
70889	1.89226	2.1
70890	1.89226	2.1
70891	1.89226	2.1
70892	1.89226	2.1
70893	1.89226	2.1
70894	1.89226	2.1
70895	1.89226	2.1
70896	1.89226	2.1
70897	1.89226	2.1
70898	1.89226	2.1
70899	3.78453	2.1
70900	1.89226	2.1
70901	1.89226	2.1
70902	1.89226	2.1
70903	1.89226	2.1
70904	1.89226	2.1
70905	1.89226	2.1
70906	1.89226	2.1
70907	1.89226	2.1
70908	1.89226	2.1
70909	1.89226	2.1
70910	3.78452	2.1
70911	1.89226	2.1
70912	1.89226	2.1
70913	1.89226	2.1
70914	1.89226	2.1
70915	3.78452	2.1
70916	1.89226	2.1
70917	1.89226	2.1
70918	1.89226	2.1
70919	1.89226	2.1
70920	3.78453	2.1
70921	1.89224	2.1
70922	1.89224	2.1
70923	1.89224	2.1
70924	1.89224	2.1
70925	3.78448	2.1
70926	1.89224	2.1
70927	1.89224	2.1
70928	1.89224	2.1
70929	1.89224	2.1
70930	1.89224	2.1
70931	1.89224	2.1

70932	1.89224	2.1
70933	1.89224	2.1
70934	1.89224	2.1
70935	1.89224	2.1
70936	3.78447	2.1
70937	0.946119	2.1
70938	0.946119	2.1
70939	0.946119	2.1
70940	0.946119	2.1
70941	0.946119	2.1
70942	0.946119	2.1
70943	0.946119	2.1
70944	0.946119	2.1
70945	0.946106	2.1
70946	0.946106	2.1
70947	0.946131	2.1
70948	0.946131	2.1
70949	1.89226	2.1
70950	1.89226	2.1
70951	1.89226	2.1
70952	1.89226	2.1
70953	3.78453	2.1
70954	0.810943	2.1
70955	0.810943	2.1
70956	0.810945	2.1
70957	0.810945	2.1
70958	0.810932	2.1
70959	0.810932	2.1
70960	1.89226	2.1
70961	1.3517	2.1
70962	1.08132	2.1
70963	1.08132	2.1
70964	1.08133	2.1
70965	1.89226	2.1
70966	1.89226	2.1
70967	1.89226	2.1
70968	1.89226	2.1
70969	1.89226	2.1
70970	1.89226	2.1
70971	0.946131	2.1
70972	0.946131	2.1
70973	0.946131	2.1
70974	0.946131	2.1
70975	0.946131	2.1
70976	0.946131	2.1
70977	0.946131	2.1
70978	0.946131	2.1
70979	0.946131	2.1
70980	0.946131	2.1
70981	0.946131	2.1
70982	0.946131	2.1
70983	1.89226	2.1
70984	1.89226	2.1
70985	1.89226	2.1
70986	1.89226	2.1
70987	3.78453	2.1
70988	1.89226	2.1
70989	1.89226	2.1

70990	1.89226	2.1
70991	1.89226	2.1
70992	3.78452	2.1
70993	1.89226	2.1
70994	1.89226	2.1
70995	1.89226	2.1
70996	1.89226	2.1
70997	3.78452	2.1
70998	1.89226	2.1
70999	1.89226	2.1
71000	1.89226	2.1
71001	1.89226	2.1
71002	3.78453	2.1
71003	1.89226	2.1
71004	1.89226	2.1
71005	1.89226	2.1
71006	1.89226	2.1
71007	1.89226	2.1
71008	1.89226	2.1
71009	1.89226	2.1
71010	1.89226	2.1
71011	1.89226	2.1
71012	1.89226	2.1
71013	1.89226	2.1
71014	1.89226	2.1
71015	1.89226	2.1
71016	1.89226	2.1
71017	1.89226	2.1
71018	1.89226	2.1
71019	3.78452	2.1
71020	1.89226	2.1
71021	1.89226	2.1
71022	1.89226	2.1
71023	1.89226	2.1
71024	1.89226	2.1
71025	1.89226	2.1
71026	1.89226	2.1
71027	1.89226	2.1
71028	1.89226	2.1
71029	1.89226	2.1
71030	1.89226	2.1
71031	1.89226	2.1
71032	0.946128	2.1
71033	0.946128	2.1
71034	0.946133	2.1
71035	0.946133	2.1
71036	0.946131	2.1
71037	0.946131	2.1
71038	0.946131	2.1
71039	0.946131	2.1
71040	0.94613	2.1
71041	0.94613	2.1
71042	0.946131	2.1
71043	0.946131	2.1
71044	1.89226	2.1
71045	1.89226	2.1
71046	1.89226	2.1
71047	1.89226	2.1

71048	1.89226	2.1
71049	1.89226	2.1
71050	1.89226	2.1
71051	1.89226	2.1
71052	1.89226	2.1
71053	1.89226	2.1
71054	1.89226	2.1
71055	1.89226	2.1
71056	2.76309	2.1
71057	1.89226	2.1
71058	2.57778	2.1
71059	1.89226	2.1
71060	4.47004	2.1
71061	0.946128	2.1
71062	0.946128	2.1
71063	0.946133	2.1
71064	0.946133	2.1
71065	0.946131	2.1
71066	0.946131	2.1
71067	0.946131	2.1
71068	0.946131	2.1
71069	0.946131	2.1
71070	0.946131	2.1
71071	0.94613	2.1
71072	0.94613	2.1
71073	0.946132	2.1
71074	0.946132	2.1
71075	0.946132	2.1
71076	0.946132	2.1
71077	0.946132	2.1
71078	0.946132	2.1
71079	0.946132	2.1
71080	0.946132	2.1
71081	0.946132	2.1
71082	0.946132	2.1
71083	0.946131	2.1
71084	0.946131	2.1
71085	1.89227	2.05593
71086	1.89227	1.07349
71087	1.89227	1.02308
71088	1.89227	2.1
71089	3.78453	1.17717
71090	1.89226	0.866877
71091	1.89226	1.44088
71092	1.89226	1.07296
71093	1.89226	0.958916
71094	3.78452	1.03769
71095	1.89226	2.1
71096	1.89226	0.933802
71097	1.89226	1.37662
71098	1.89226	1.41805
71099	1.89226	0.923133
71100	1.89226	0.936069
71101	0.946131	1.0921
71102	0.946131	1.05501
71103	0.946131	2.1
71104	0.946131	2.1
71105	0.946133	2.1

71106	0.946133	1.15287
71107	0.946129	1.1554
71108	0.946129	2.1
71109	0.946131	1.29963
71110	0.946131	1.31261
71111	0.946131	1.28329
71112	0.946131	1.21383
71113	1.89226	1.00138
71114	1.89226	1.85383
71115	1.89226	1.96614
71116	1.89226	1.04502
71117	1.89226	1.69796
71118	1.89226	1.06941
71119	1.89226	1.09282
71120	1.89226	0.91307
71121	1.89226	0.850501
71122	1.89226	1.13976
71123	3.78452	0.930679
71124	1.89226	1.14016
71125	1.89226	0.999343
71126	1.89226	1.22479
71127	1.89226	0.892762
71128	1.89226	1.88453
71129	1.89226	0.962027
71130	0.94613	1.04411
71131	0.94613	1.07392
71132	0.946131	2.09503
71133	0.946131	1.96927
71134	0.946133	1.07149
71135	0.946133	1.8694
71136	0.946128	2.06404
71137	0.946128	1.07953
71138	0.946131	1.32168
71139	0.946131	1.32351
71140	0.946131	1.15569
71141	0.946131	1.16031
71142	1.89226	1.02287
71143	1.89226	2.1
71144	1.89226	2.028
71145	1.89226	1.11581
71146	1.89226	2.01499
71147	1.89226	1.1379
71148	1.89226	0.926907
71149	1.89226	1.82163
71150	1.89226	1.19078
71151	1.89226	1.07084
71152	1.89226	1.17744
71153	1.89226	1.1396
71154	0.946133	1.25126
71155	0.946133	0.898198
71156	0.946129	2.1
71157	0.946129	1.30382
71158	0.946131	2.1
71159	0.946131	1.10484
71160	0.946131	1.45627
71161	0.946131	1.06208
71162	0.946131	1.23916
71163	0.946131	1.21916

71164	0.946131	1.21286
71165	0.946131	1.26919
71166	1.89226	0.965597
71167	1.89226	2.06264
71168	1.89226	1.30361
71169	1.89226	1.04915
71170	1.89226	1.2987
71171	1.89226	1.07494
71172	1.89226	0.888113
71173	1.89226	1.68548
71174	1.89226	1.07347
71175	1.89226	0.986715
71176	1.89226	1.05714
71177	1.89226	1.04699
71178	1.89226	0.936861
71179	1.89226	2.05461
71180	1.89226	1.18589
[truncated: 424,029 more chars]
