# Supplementary material for: On the Use of Bone Remodelling Models to Estimate the Density Distribution of Bones. Uniqueness of the Solution
Source: PLoS One. 2016 Feb 9;11(2):e0148603. doi: 10.1371/journal.pone.0148603 (PMC4747586; doi:10.1371/journal.pone.0148603)
Supplement: S11 File — (PDF) [file pone.0148603.s011.pdf]

N° Elem	Volume (mm3)	Density (g/cm3)
1	1.32415	2.1
2	1.75823	2.1
3	0.984099	2.1
4	1.46442	2.1
5	1.53082	2.1
6	1.32068	2.1
7	1.91404	0.05
8	1.17036	0.05
9	0.929209	0.05
10	1.27254	0.05
11	2.0926	0.05
12	2.20407	0.05
13	1.21488	0.05
14	1.26024	0.05
15	0.794454	0.05
16	1.95087	0.05
17	0.953925	2.1
18	0.98949	2.1
19	1.27875	2.1
20	1.04598	2.1
21	0.594005	2.1
22	0.578861	2.1
23	1.09506	2.1
24	1.45327	2.1
25	2.58396	2.1
26	1.58581	2.1
27	1.5858	2.1
28	0.636946	2.1
29	0.94202	2.1
30	0.946119	2.1
31	0.636953	2.1
32	0.946107	2.1
33	1.09505	2.1
34	1.27389	2.1
35	1.00232	2.1
36	1.00231	2.1
37	1.83583	2.1
38	1.00231	2.1
39	1.28828	0.05
40	1.81696	0.05
41	1.28828	0.05
42	1.28828	0.05
43	1.0044	0.05
44	0.85148	0.05
45	0.851539	0.05
46	1.57767	0.05
47	1.52227	0.05
48	0.730657	0.05
49	0.94613	0.05
50	1.28228	2.1
51	1.64268	2.1
52	1.10101	2.1
53	1.3939	2.1
54	0.959471	2.1
55	1.10101	2.1

56	1.2873	2.1
57	1.59659	2.1
58	1.80841	0.500115
59	1.80841	1.96436
60	0.946129	0.709279
61	1.36991	0.217562
62	1.32262	2.1
63	1.32261	0.889633
64	1.38464	1.05021
65	0.962877	0.67939
66	0.962878	0.187048
67	1.82129	0.226769
68	1.82129	0.660234
69	0.94613	0.477787
70	1.39751	0.454349
71	0.946131	0.34559
72	1.34493	0.145176
73	1.39751	0.211414
74	0.837577	0.467072
75	0.823478	0.312415
76	1.10889	0.659615
77	1.01686	0.487824
78	1.1942	0.663761
79	0.836129	0.343244
80	0.937165	0.18324
81	0.969438	0.434716
82	0.895386	0.08178
83	1.30554	0.567195
84	1.15798	0.143613
85	1.22578	0.688669
86	1.15255	0.269848
87	0.9765	0.116548
88	2.02178	0.743845
89	1.63755	0.660016
90	0.727713	0.514978
91	0.810945	0.548687
92	1.00559	0.470636
93	0.879098	0.428655
94	1.07852	0.620562
95	0.657764	0.0953465
96	0.829406	0.442206
97	0.720201	0.0717807
98	0.946131	0.0725151
99	1.11827	0.069722
100	1.50754	0.05
101	1.64835	0.0505874
102	1.71903	0.102514
103	0.851554	0.05
104	1.75626	0.104247
105	2.1646	0.0842615
106	1.5563	0.0830301
107	1.5563	0.212323
108	2.10876	0.214371
109	1.05438	0.0932047
110	0.946133	0.182783
111	1.05438	1.01963
112	1.20256	0.15268
113	1.47879	1.13083

114	1.47879	1.37085
115	0.77815	1.41139
116	0.947114	2.1
117	1.31257	2.1
118	1.09235	2.1
119	0.946132	2.1
120	1.54246	2.1
121	1.54246	2.1
122	1.09235	2.1
123	0.946131	2.1
124	1.29507	2.1
125	1.0353	2.1
126	1.03529	2.1
127	1.28667	2.1
128	1.44777	2.1
129	1.61121	2.1
130	1.22176	0.0618102
131	0.946129	0.0954125
132	1.08848	0.05
133	0.946131	0.0905002
134	1.61201	0.05
135	1.61201	0.05
136	1.19993	0.073828
137	1.35822	0.0507943
138	1.1539	0.05
139	1.00765	0.05
140	1.28228	2.1
141	2.65339	2.1
142	1.28228	2.1
143	1.55998	2.1
144	1.00509	2.1
145	0.93845	2.1
146	1.27674	2.1
147	0.810958	2.1
148	1.91381	2.1
149	1.1861	2.1
150	1.24886	2.1
151	2.02842	2.1
152	0.991436	0.05
153	1.34588	0.05
154	2.02729	0.05
155	0.72904	0.05
156	1.06139	0.05
157	0.835368	0.05
158	1.63819	0.05
159	1.10745	0.05
160	1.79925	0.05
161	1.00085	0.05
162	0.946133	2.1
163	1.41123	2.1
164	1.8327	2.1
165	1.8327	2.1
166	0.946132	2.1
167	1.41123	2.1
168	1.3676	2.1
169	1.11048	2.1
170	1.11048	2.1
171	1.65815	2.1

172	1.9004	2.1
173	1.88691	2.1
174	1.82232	2.1
175	1.46591	2.1
176	1.09115	2.1
177	1.02051	1.7763
178	1.03373	1.9895
179	1.10307	2.1
180	1.05556	2.1
181	1.82533	1.33023
182	1.19496	1.26967
183	1.28597	2.1
184	1.20421	2.1
185	2.23644	2.1
186	1.08378	2.1
187	0.983724	2.1
188	0.602075	2.1
189	1.45876	2.1
190	1.45875	2.1
191	0.602103	2.1
192	1.11822	2.1
193	0.946133	2.1
194	0.602103	2.1
195	1.20421	2.1
196	1.26371	0.411504
197	1.55179	0.184302
198	1.14042	0.118208
199	1.56876	0.146112
200	2.27496	0.290799
201	1.13748	0.231423
202	1.02884	0.38954
203	1.25124	0.370739
204	0.82703	0.237155
205	0.607775	0.325239
206	1.49242	0.357883
207	1.35674	0.270311
208	1.6697	2.1
209	1.81655	2.1
210	0.900108	1.78323
211	0.851537	1.65605
212	0.703076	1.91134
213	0.639143	1.76429
214	1.42358	1.80189
215	1.4976	1.91348
216	0.86284	1.79724
217	1.2965	1.80708
218	1.27835	2.1
219	1.00012	1.5571
220	1.74332	1.93746
221	1.08576	2.1
222	1.19 2.1	
223	1.08576	2.1
224	1.20854	2.1
225	0.946131	2.1
226	0.946131	2.1
227	1.75985	2.1
228	1.75985	2.1
229	1.41232	2.1

230	1.29365	2.1
231	0.973559	2.1
232	1.23698	0.05
233	1.45055	0.05
234	1.26947	0.05
235	0.856218	0.05
236	1.75303	0.05
237	1.14938	0.05
238	0.984711	0.05
239	2.05771	0.05
240	0.625171	0.05
241	0.965675	0.05
242	1.17631	0.05
243	1.4677	0.05
244	1.35403	0.05
245	0.975149	0.05
246	0.946131	0.05
247	1.80893	0.05
248	1.80893	0.05
249	0.975149	0.05
250	0.946132	0.05
251	1.9503	0.05
252	2.80207	0.05
253	1.76411	0.05
254	2.77469	0.05
255	1.9503	0.05
256	1.26784	0.05
257	2.31093	0.05
258	0.851541	0.0604335
259	1.39521	0.05
260	1.45613	0.05
261	0.851541	0.0608875
262	0.996013	0.05
263	1.07989	0.0525708
264	0.946131	0.053273
265	1.08213	0.05
266	1.74289	0.05
267	1.75838	0.05
268	2.11163	0.0576939
269	1.23773	2.1
270	1.25398	2.1
271	1.0304	2.1
272	1.32368	2.1
273	1.11712	1.34926
274	1.07999	0.598621
275	1.1766	2.1
276	1.35735	2.1
277	1.089 0.05	
278	1.81193	0.05
279	1.11081	0.05
280	1.78069	0.05
281	0.717512	0.05
282	0.810946	0.05
283	0.703901	0.05
284	1.09028	0.05
285	0.958031	0.05
286	0.892585	0.05
287	0.873431	0.05

288	0.997755	0.05
289	1.85479	0.05
290	0.983029	0.147291
291	1.27922	0.185823
292	1.64346	0.408853
293	1.27922	0.279956
294	1.09795	2.1
295	1.07508	1.60308
296	1.02975	1.99419
297	1.01121	2.1
298	1.72369	0.05
299	1.12035	0.05
300	2.13511	0.05
301	0.9705	0.05
302	0.946131	0.05
303	0.86182	0.05
304	1.51379	0.05
305	1.51379	0.05
306	0.946132	0.05
307	0.861849	0.05
308	1.34591	0.262302
309	1.14721	0.17327
310	1.15539	2.1
311	1.90938	2.1
312	2.19436	2.1
313	1.41563	2.1
314	0.785641	2.1
315	0.736384	2.1
316	1.46489	2.1
317	1.46488	2.1
318	0.736372	2.1
319	0.946132	2.1
320	1.90941	2.1
321	1.18907	2.1
322	1.20409	2.1
323	0.973559	2.1
324	1.29461	2.1
325	0.946129	2.1
326	0.94613	2.1
327	1.78751	2.1
328	1.78751	2.1
329	1.38885	2.1
330	1.34479	2.1
331	1.07283	2.1
332	1.6869	2.1
333	1.52544	2.1
334	1.07797	2.1
335	1.13819	2.1
336	0.776179	2.1
337	1.61092	2.1
338	1.36155	2.1
339	0.77618	2.1
340	1.2933	2.1
341	1.15946	2.1
342	0.69857	2.1
343	1.87607	2.1
344	1.07612	2.1
345	1.31976	1.6043

346	1.01243	1.898
347	1.41279	1.89077
348	1.35146	0.0643934
349	1.90664	0.05
350	1.35145	0.0588855
351	1.22811	0.0891881
352	1.93147	0.0686519
353	1.42486	2.1
354	1.56558	2.1
355	1.0476	0.05
356	2.31605	0.05
357	1.89367	0.05
358	0.804706	0.05
359	0.783919	0.05
360	1.3116	0.05
361	1.03891	0.05
362	0.960473	0.05
363	1.08462	0.05
364	0.785994	0.05
365	1.1318	0.05
366	1.29576	0.05
367	1.3946	0.05
368	1.11924	0.05
369	0.834322	2.1
370	1.0256	2.1
371	1.74321	1.07329
372	1.41798	1.15136
373	0.871608	1.15197
374	1.47253	1.03315
375	1.47252	0.998078
376	0.817064	1.21741
377	0.817064	1.12952
378	0.946129	1.0444
379	1.63413	0.736818
380	1.9194	0.05
381	1.93562	0.05
382	2.06307	0.05
383	1.46021	0.05
384	0.955002	1.62157
385	0.833409	1.68362
386	0.838039	1.51714
387	1.48702	1.54443
388	1.1697	1.53348
389	1.23549	1.55476
390	1.18373	1.61621
391	2.02254	1.21094
392	1.68867	1.5533
393	0.875481	1.00537
394	0.821635	1.56951
395	0.858878	0.787107
396	1.30245	0.93202
397	1.07476	1.07402
398	1.11907	0.958388
399	1.10453	1.00533
400	1.70233	0.95376
401	1.70233	1.23122
402	2.6558	1.43466
403	1.7818	2.1

404	1.87059	2.1
405	1.34412	0.05
406	1.98068	0.05
407	2.56369	0.05
408	1.49512	0.05
409	1.35977	0.196295
410	1.34254	0.236875
411	1.75618	0.189396
412	1.75618	0.169654
413	0.946129	0.292874
414	0.946129	0.218058
415	1.17665	0.226383
416	1.01976	0.183624
417	1.27376	0.142843
418	1.35978	0.166879
419	1.68741	0.138098
420	1.68741	0.123327
421	0.946132	0.207071
422	0.946133	0.163414
423	1.14241	0.165632
424	0.996831	0.133899
425	0.996828	0.170649
426	1.12774	0.19477
427	1.13664	2.1
428	1.09627	2.1
429	2.8814	2.1
430	3.49751	2.1
431	1.79109	0.247246
432	1.54197	0.198478
433	2.75153	1.2127
434	1.93019	0.892356
435	1.51569	1.18328
436	1.55145	1.37499
437	1.78595	2.1
438	3.74452	2.1
439	1.66537	2.1
440	3.55161	2.1
441	1.35002	0.409582
442	1.19901	0.583556
443	1.61028	1.86643
444	1.35002	1.33817
445	1.65712	0.194538
446	1.10585	0.555501
447	0.961347	0.448354
448	0.810944	0.119804
449	1.16148	0.928949
450	1.08066	1.47786
451	0.934577	0.358337
452	1.05164	0.203448
453	0.79216	0.446675
454	2.99194	2.1
455	1.2832	2.1
456	2.97842	2.1
457	1.39517	2.1
458	1.03391	2.1
459	1.74416	2.1
460	1.05071	2.1
461	0.923782	2.1

462	1.55369	2.1
463	1.86444	2.1
464	1.01617	2.1
465	1.27174	2.1
466	1.20573	2.1
467	1.01616	2.1
468	1.13621	2.1
469	1.75366	2.1
470	2.27009	1.92183
471	1.6813	2.05757
472	1.47904	0.05
473	2.62714	0.05
474	1.80085	0.05
475	1.80085	0.05
476	2.74104	0.05
477	1.37052	0.05
478	0.946131	0.0597646
479	1.37052	0.05
480	1.31357	0.05
481	1.7837	0.0629421
482	1.7837	0.0688438
483	0.900397	0.0600443
484	1.74487	0.112365
485	2.88672	2.1
486	2.88249	2.1
487	1.0961	0.118764
488	1.20143	0.05
489	1.66739	0.0767231
490	1.85795	0.05
491	1.45789	0.05
492	2.11407	0.05
493	0.713524	0.05
494	1.58391	0.05
495	1.58391	0.05
496	1.04765	0.05
497	1.24979	0.05
498	0.713551	0.05
499	0.946127	0.05
500	1.36768	0.05
501	1.14433	0.05
502	1.66923	0.05
503	0.699479	0.05
504	0.70351	0.05
505	0.758416	0.05
506	1.07678	0.05
507	0.909874	0.05
508	0.940218	0.05
509	0.876604	0.05
510	1.27148	0.05
511	2.42348	0.05
512	1.19837	0.05
513	0.764764	0.05
514	0.829259	0.05
515	0.740584	0.05
516	1.2772	0.05
517	0.964465	0.05
518	1.06231	0.05
519	1.06924	0.05

520	1.17566	0.05
521	1.3063	0.05
522	2.51253	0.05
523	2.42267	0.05
524	1.89226	0.05
525	1.46492	0.05
526	0.946132	0.05
527	0.653151	0.05
528	0.653124	0.0507092
529	1.53995	0.05
530	1.53995	0.05
531	0.936806	0.05
532	1.25626	0.05
533	1.1893	0.0789762
534	1.62206	0.05
535	1.04921	2.1
536	1.22927	2.1
537	0.801765	2.1
538	0.774917	2.1
539	0.844623	2.1
540	0.737481	2.1
541	3.24379	2.1
542	2.48573	2.1
543	1.40967	1.57353
544	1.47203	1.51349
545	2.07659	1.40525
546	1.40967	1.52542
547	2.06638	1.37103
548	1.17418	2.1
549	1.04921	2.1
550	1.74593	2.1
551	1.74593	2.1
552	0.946132	2.1
553	1.38651	2.1
554	1.20761	1.77094
555	1.20761	2.1
556	1.30555	2.1
557	1.46812	0.543262
558	2.41602	0.859902
559	1.46812	0.889602
560	1.46812	0.528637
561	0.734059	0.655166
562	0.946132	1.5896
563	1.21261	1.40405
564	1.34225	0.986012
565	0.734084	1.32639
566	1.82077	1.2805
567	1.82077	0.812169
568	1.13751	0.320255
569	0.946131	0.773968
570	1.13751	0.574718
571	1.13748	0.375867
572	0.784392	0.563619
573	1.4906	0.492263
574	1.4906	0.248724
575	1.17879	1.5306
576	1.28291	1.49842
577	0.626495	2.1

578	1.45334	2.1
579	1.45334	2.1
580	0.626494	2.1
581	1.3852	2.1
582	0.946116	2.1
583	0.626499	2.1
584	1.47203	1.49494
585	1.42639	2.1
586	1.42639	2.1
587	0.795469	2.1
588	0.795469	2.1
589	0.946132	2.1
590	0.850877	2.1
591	1.37098	2.1
592	1.87194	2.1
593	1.76988	2.1
594	3.16295	0.05
595	3.26725	0.05
596	1.13322	2.1
597	1.20761	2.1
598	0.686933	0.477828
599	0.493248	0.134153
600	1.2053	1.29903
601	0.947149	0.359008
602	1.27591	0.251145
603	1.75732	0.490669
604	0.493261	0.462336
605	1.34337	1.23018
606	2.73207	0.409844
607	1.19857	0.320302
608	0.671688	0.0574763
609	0.946134	0.735863
610	1.36603	0.114874
611	2.07817	0.165901
612	1.82573	0.500223
613	0.671699	0.394006
614	1.47772	0.0925511
615	0.975349	0.887148
616	1.22615	2.1
617	1.47868	2.1
618	2.29351	2.1
619	1.47642	2.1
620	2.94443	2.1
621	1.80454	2.1
622	0.949135	2.1
623	0.949134	2.1
624	1.10476	0.0899137
625	0.982857	0.184671
626	1.10938	0.0945498
627	1.19052	0.80262
628	1.01012	0.05
629	1.35562	0.05
630	0.691742	0.05
631	2.39596	0.05
632	2.35604	0.05
633	0.974081	0.05
634	3.22444	0.05
635	1.3294	0.157748

636	1.37265	0.384261
637	1.3294	0.594705
638	1.51113	2.1
639	1.43643	2.1
640	1.72726	2.1
641	1.634	2.1
642	0.755566	2.1
643	1.29319	2.1
644	0.755567	2.1
645	0.946131	2.1
646	1.29319	2.1
647	0.973563	2.1
648	0.904398	2.1
649	0.904406	2.1
650	1.1538	2.1
651	1.10437	2.1
652	0.935418	2.1
653	1.16649	2.1
654	0.975667	2.1
655	1.17952	2.1
656	1.95053	2.1
657	0.948676	2.1
658	1.44287	2.1
659	0.746095	2.1
660	1.51224	2.1
661	1.27354	2.1
662	0.746066	2.1
663	1.20573	2.1
664	1.12442	2.1
665	0.671487	2.1
666	1.04355	2.1
667	0.948675	2.1
668	0.967673	0.941305
669	1.12931	0.05
670	1.38212	0.05
671	2.40113	0.05
672	1.08054	0.05
673	1.04072	0.05
674	0.691062	0.05
675	1.58052	0.05
676	1.33502	0.05
677	0.946131	0.05
678	0.691062	0.05
679	1.02663	0.05
680	1.80569	0.05
681	1.26062	0.05
682	1.18203	0.05
683	0.946131	0.05
684	0.795065	0.05
685	1.58753	0.05
686	1.58753	0.05
687	0.946131	0.05
688	0.795093	0.05
689	0.805057	2.1
690	0.812415	2.1
691	1.23868	0.0688977
692	1.34448	0.096841
693	1.67201	2.1

694	1.61202	2.1
695	1.41103	2.1
696	1.41103	2.1
697	0.841088	2.1
698	1.38302	2.1
699	0.841089	2.1
700	0.869097	2.1
701	1.38302	2.1
702	0.949941	2.1
703	0.94994	2.1
704	1.88608	2.1
705	0.854942	2.1
706	1.68893	2.1
707	1.70002	2.1
708	2.02107	0.142894
709	2.04982	0.154646
710	3.11063	0.204923
711	2.03805	0.146308
712	1.67638	2.1
713	0.967492	2.1
714	2.00209	2.1
715	0.967491	2.1
716	0.870742	2.1
717	1.30266	0.05
718	0.651334	0.05
719	1.69413	0.05
720	1.69413	0.05
721	0.946132	0.05
722	1.03079	0.05
723	1.31468	0.05
724	0.946132	0.05
725	0.919055	0.05
726	1.16815	0.05
727	2.79004	0.05
728	1.2136	0.05
729	2.62935	0.05
730	0.947692	0.167327
731	1.51835	0.143248
732	0.947693	0.10312
733	0.947692	0.118996
734	0.980841	2.1
735	1.43577	2.1
736	0.980841	2.1
737	1.67858	2.1
738	1.34124	0.287499
739	1.39818	0.167992
740	1.2065	2.1
741	0.946133	2.1
742	1.2079	2.1
743	1.65229	2.1
744	1.65229	2.1
745	0.946132	2.1
746	1.2079	2.1
747	1.39053	2.1
748	1.20651	2.1
749	1.36919	2.1
750	1.12717	2.1
751	1.09483	2.1

752	0.994294	2.1
753	0.97356	2.1
754	1.06827	2.1
755	1.40571	2.1
756	1.29259	2.1
757	0.946131	2.1
758	1.7787	2.1
759	1.7787	2.1
760	1.29259	2.1
761	0.946132	2.1
762	1.09408	0.92198
763	1.03824	0.270894
764	0.851541	0.262464
765	1.23608	0.253484
766	0.851539	0.256139
767	1.5021	0.260749
768	1.54839	0.261727
769	1.12373	0.244582
770	1.22617	0.236826
771	1.42762	0.407048
772	2.24746	0.372957
773	1.05475	0.0728506
774	1.46845	0.0662763
775	1.05475	0.0573926
776	0.929726	0.0606418
777	1.52072	0.067413
778	1.82488	0.0597348
779	1.02271	0.067173
780	1.19225	0.0527179
781	1.43071	0.05
782	0.94613	0.0518203
783	1.05475	0.05
784	1.39223	2.1
785	1.71255	2.1
786	0.946132	0.729824
787	1.10531	0.472723
788	1.46355	0.778956
789	1.46355	0.340306
790	0.78086	1.213
791	1.10531	0.60132
792	1.1391	0.866854
793	1.56166	0.88825
794	2.21062	0.549421
795	1.0361	2.1
796	1.73577	2.1
797	1.73577	2.1
798	0.946116	2.1
799	1.28174	2.1
800	0.946118	2.1
801	1.22883	2.1
802	1.28172	2.1
803	1.03609	2.1
804	0.553567	0.05
805	1.60581	0.05
806	1.60581	0.05
807	0.553594	0.05
808	0.966748	0.05
809	0.966748	0.05

810	0.946133	0.05
811	1.05602	0.05
812	2.38525	0.05
813	1.14503	0.05
814	1.24678	0.05
815	0.56262	0.05
816	1.24678	0.05
817	0.978467	0.05
818	1.66262	0.05
819	1.66262	0.05
820	0.562633	0.05
821	1.09522	0.05
822	2.49356	0.05
823	0.592547	0.05
824	1.04321	1.61761
825	1.25133	1.66509
826	1.70508	1.32738
827	1.81043	1.30482
828	0.494934	1.24716
829	0.601047	1.26721
830	0.993428	1.25373
831	0.946573	1.27273
832	1.35039	1.24388
833	0.816895	1.18389
834	1.5063	1.15327
835	0.816895	1.22301
836	0.946132	1.10192
837	1.9067	1.33687
838	2.14504	1.18686
839	0.816895	1.21247
840	2.29624	1.25237
841	2.76933	0.886748
842	1.254	1.24797
843	0.946128	2.1
844	1.38813	2.1
845	1.79998	2.1
846	1.79998	2.1
847	0.94613	2.1
848	1.38813	2.1
849	1.35798	2.1
850	1.27809	2.1
851	1.35798	2.1
852	1.68994	2.1
853	1.68994	2.1
854	0.946132	2.1
855	0.946132	2.1
856	1.18067	2.1
857	0.958256	2.1
858	0.958255	2.1
859	0.958257	2.1
860	0.958255	2.1
861	1.06457	2.1
862	1.14195	2.1
863	1.70423	2.1
864	1.14195	2.1
865	1.64285	2.1
866	2.02057	2.1
867	0.946132	2.1

868	1.44186	2.1
869	0.946133	2.1
870	1.16366	2.1
871	1.17233	2.1
872	1.31259	2.1
873	1.2016	2.1
874	1.17129	2.1
875	1.05724	0.356412
876	1.32697	2.1
877	1.05724	1.45937
878	1.04292	2.1
879	3.39037	2.1
880	2.57275	2.1
881	0.629212	2.1
882	2.32525	2.1
883	1.76949	0.497548
884	1.65809	0.609852
885	1.27665	2.1
886	1.19861	2.1
887	1.20045	2.1
888	1.1635	2.1
889	0.953633	0.145235
890	0.953634	0.0875105
891	1.21408	2.1
892	1.25716	2.1
893	2.01791	2.1
894	1.89318	2.1
895	0.947921	2.1
896	0.939049	2.1
897	0.947921	2.1
898	0.740753	2.1
899	0.916104	2.1
900	1.16941	2.1
901	1.72038	2.1
902	2.04441	2.1
903	0.973182	2.1
904	1.11751	2.1
905	0.611089	2.1
906	1.53584	0.0618662
907	0.767923	0.133746
908	1.38925	0.110323
909	1.38926	0.101819
910	0.946131	0.238957
911	0.961347	0.155848
912	1.19583	0.107951
913	0.946131	0.130854
914	1.3663	0.279851
915	1.21067	0.0578826
916	1.00166	0.0630727
917	1.40707	0.0547865
918	1.50435	0.056207
919	1.47954	1.3997
920	1.1233	2.1
921	2.36036	1.83551
922	1.04024	1.3818
923	0.739729	1.38019
924	1.45533	1.28692
925	1.45532	1.238

926	0.946118	1.3374
927	1.15163	0.545131
928	1.15165	1.37318
929	0.739748	1.30906
930	1.04025	1.67361
931	1.47608	0.112469
932	0.946131	0.165826
933	1.02028	0.140544
934	0.946129	0.157949
935	1.78223	0.109476
936	1.78223	0.104428
937	1.34153	0.136534
938	1.38683	0.138939
939	1.3407	0.0986264
940	1.3407	0.0863185
941	2.04729	0.104277
942	0.851529	0.140592
943	1.26671	0.104535
944	0.946116	0.134236
945	1.4869	0.0897781
946	1.29553	0.0841063
947	1.03645	0.125173
948	1.20731	0.118573
949	0.73665	2.1
950	0.693596	2.1
951	2.11152	2.1
952	1.73627	2.1
953	1.06191	2.1
954	1.52226	2.1
955	1.76869	2.1
956	1.1881	2.1
957	1.32349	2.1
958	1.51041	2.1
959	1.61591	2.1
960	0.946132	2.1
961	0.946132	2.1
962	1.13537	2.1
963	3.0048	2.1
964	0.985109	2.1
965	1.68899	2.1
966	3.23261	2.1
967	1.08831	2.1
968	2.20623	2.1
969	0.91749	2.1
970	1.15515	2.1
971	0.550438	2.1
972	1.66294	2.1
973	1.08257	2.1
974	0.87851	2.1
975	2.09046	2.1
976	0.738711	2.1
977	1.31242	0.05
978	1.26311	0.05
979	1.67652	0.05
980	0.968594	2.1
981	0.968594	2.1
982	0.981099	0.05
983	1.32418	0.05

984	2.67175	0.05
985	2.09678	0.05
986	1.2511	0.05
987	0.912698	0.05
988	0.810946	0.05
989	0.810959	0.05
990	1.67479	0.05
991	1.65677	0.05
992	1.28469	0.05
993	1.48817	0.05
994	1.08131	0.05
995	1.04349	1.10003
996	1.42492	0.703584
997	1.05724	0.394907
998	1.65723	0.0934205
999	1.95313	0.0776051
1000	1.9293	0.087886
1001	2.02729	0.0714803
1002	0.963393	1.63973
1003	0.963392	0.91179
1004	2.90171	2.1
1005	2.65447	2.1
1006	1.64158	2.1
1007	1.6488	2.1
1008	1.15991	0.116027
1009	0.946132	0.170766
1010	1.58199	0.128965
1011	1.58199	0.147826
1012	1.03333	0.188493
1013	1.25436	0.134605
1014	1.25436	0.157844
1015	0.946132	0.154613
1016	1.3771	0.217428
1017	0.687745	0.18714
1018	1.37709	0.180061
1019	0.812386	0.210207
1020	1.50172	0.214102
1021	1.50172	0.146735
1022	0.687758	0.227646
1023	1.17506	0.124176
1024	2.00539	0.173634
1025	2.2441	0.214365
1026	1.09667	0.132929
1027	1.43577	2.1
1028	2.24741	2.1
1029	1.26103	2.1
1030	0.851544	2.1
1031	1.5543	2.1
1032	1.59756	2.1
1033	0.851543	2.1
1034	1.44673	2.1
1035	1.31947	2.1
1036	1.1237	2.1
1037	1.26103	2.1
1038	1.089 0.05	
1039	0.946128	0.05
1040	0.965824	0.05
1041	0.94613	0.05

1042	1.51344	0.05
1043	1.51344	0.05
1044	1.13508	0.05
1045	1.32449	0.05
1046	1.089 0.05	
1047	3.33706	0.145871
1048	2.7542	0.181485
1049	1.27256	0.329142
1050	0.954172	0.255637
1051	1.55527	0.261376
1052	1.05765	0.365811
1053	1.57326	0.323046
1054	1.38251	0.171155
1055	0.94613	0.312472
1056	2.38829	0.171593
1057	2.00539	0.203294
1058	1.53798	0.172991
1059	1.20969	2.1
1060	1.15864	2.1
1061	1.7986	2.1
1062	1.74533	2.1
1063	0.944827	1.36277
1064	0.946131	1.16197
1065	0.851543	1.22868
1066	1.52816	0.97309
1067	1.5314	1.35706
1068	1.39266	0.777361
1069	0.851528	1.14685
1070	1.39589	1.23421
1071	1.7348	0.983604
1072	1.85439	1.0779
1073	1.86367	1.05583
1074	1.90905	0.667773
1075	1.22176	0.0582785
1076	1.59492	0.0679364
1077	2.97929	2.1
1078	2.1905	2.1
1079	1.24128	2.1
1080	0.994293	2.1
1081	2.49767	0.05
1082	2.37105	0.05
1083	1.10834	1.95937
1084	1.53214	0.332239
1085	1.417 2.1	
1086	2.13599	0.594561
1087	1.61727	2.1
1088	1.75004	2.1
1089	1.96301	2.1
1090	0.966631	2.1
1091	1.37791	2.1
1092	1.47302	2.1
1093	1.47302	2.1
1094	0.871519	2.1
1095	0.94613	2.1
1096	0.87155	2.1
1097	2.38731	2.1
1098	2.12399	1.19203
1099	1.74865	1.09964

1100	1.46724	0.430236
1101	2.38056	0.435621
1102	1.46724	0.435284
1103	0.818887	0.583429
1104	0.810944	1.51503
1105	0.805227	0.582509
1106	1.06558	0.76404
1107	0.841487	0.645429
1108	1.08456	0.789113
1109	0.903083	0.911662
1110	1.08929	0.05
1111	1.68454	0.05
1112	1.08929	0.05
1113	1.07658	0.05
1114	0.904439	0.05
1115	1.58501	0.05
1116	1.19729	0.05
1117	1.05427	0.05
1118	1.47764	0.05
1119	0.810959	0.05
1120	1.60946	1.15587
1121	2.28887	1.20202
1122	0.946133	1.09446
1123	1.14444	0.603934
1124	1.67964	0.430291
1125	1.67964	0.506555
1126	0.755334	1.12249
1127	1.14444	0.901015
1128	1.29054	0.686211
1129	1.37762	0.714573
1130	0.946132	1.06931
1131	0.533645	0.809017
1132	1.48361	0.491587
1133	1.48361	0.640913
1134	0.533645	0.812492
1135	0.872823	1.01502
1136	1.14444	0.700392
1137	1.37762	0.516071
1138	2.10101	0.713642
1139	1.02311	1.09245
1140	1.04083	2.1
1141	1.04084	2.1
1142	1.04084	2.1
1143	1.30116	2.1
1144	1.23477	0.05
1145	1.81893	0.05
1146	1.23477	0.05
1147	1.23477	0.05
1148	1.11967	0.05
1149	0.946131	0.05
1150	1.11966	0.05
1151	1.12628	0.05
1152	0.629641	0.05
1153	1.6163	0.05
1154	1.6163	0.05
1155	1.11117	2.1
1156	1.13006	2.1
1157	1.15149	2.1

1158	1.13179	2.1
1159	0.585368	0.730264
1160	0.986036	0.996372
1161	1.94788	0.159145
1162	1.34857	0.464864
1163	2.31806	2.1
1164	2.06363	2.1
1165	1.00423	2.1
1166	0.829065	2.1
1167	0.551816	2.1
1168	0.99406	2.1
1169	1.98339	2.1
1170	1.68073	2.1
1171	2.558	2.1
1172	0.711814	2.1
1173	2.97316	2.1
1174	2.41579	2.1
1175	1.15248	0.05
1176	1.14305	0.05
1177	1.15483	0.05
1178	1.90981	0.05
1179	0.998957	0.05
1180	1.83742	0.05
1181	1.27581	2.1
1182	0.94613	2.1
1183	1.6315	2.1
1184	1.6315	2.1
1185	0.946132	2.1
1186	1.28441	2.1
1187	1.28441	2.1
1188	1.00365	2.1
1189	0.968536	2.1
1190	1.57575	2.1
1191	1.27581	2.1
1192	1.99297	2.1
1193	1.32784	2.1
1194	1.4259	2.1
1195	2.27952	2.1
1196	1.5685	2.1
1197	1.54231	2.1
1198	3.54638	2.1
1199	1.3294	2.1
1200	2.98237	2.1
1201	2.01633	0.562408
1202	2.0985	0.609438
1203	2.26047	0.26521
1204	1.7913	0.21023
1205	1.09504	2.1
1206	1.74126	2.1
1207	1.05983	2.1
1208	2.37664	2.1
1209	2.28084	2.1
1210	2.19948	2.1
1211	1.33593	0.05
1212	1.31508	0.05
1213	1.30647	2.1
1214	1.28846	2.1
1215	1.2955	2.1

1216	1.09044	2.1
1217	1.4099	2.1
1218	1.14984	2.1
1219	0.946132	2.1
1220	1.70727	2.1
1221	1.70727	2.1
1222	1.14984	2.1
1223	0.946132	2.1
1224	0.911818	2.1
1225	2.12209	2.1
1226	2.4158	2.1
1227	1.20867	2.1
1228	2.84085	2.1
1229	1.42284	2.1
1230	2.45689	2.1
1231	1.41562	2.1
1232	0.918358	2.1
1233	1.11222	2.1
1234	0.999083	2.1
1235	0.696781	2.1
1236	1.30869	2.1
1237	1.66132	2.1
1238	0.696807	2.1
1239	0.745315	2.1
1240	1.23296	2.1
1241	0.780548	2.1
1242	1.93242	1.528
1243	1.27362	1.78118
1244	2.77828	1.81713
1245	1.02653	1.26432
1246	0.808576	1.05929
1247	0.744012	1.48093
1248	0.605174	0.717897
1249	1.16501	0.431031
1250	0.803432	0.523104
1251	0.961016	0.426205
1252	1.10425	0.851865
1253	1.3756	1.40837
1254	2.17765	0.476024
1255	1.84578	0.424927
1256	1.32801	0.223262
1257	1.76377	0.211899
1258	0.946131	0.216275
1259	1.3022	0.734048
1260	0.694814	0.516824
1261	1.53028	0.225956
1262	1.53028	0.193635
1263	1.3022	0.386013
1264	0.922889	0.497415
1265	1.57766	2.1
1266	2.06911	2.1
1267	1.57766	2.1
1268	1.37721	0.05
1269	1.72174	0.05
1270	1.4468	0.05
1271	1.37721	0.05
1272	1.73972	0.125247
1273	1.62324	0.129853

1274	0.946125	0.110181
1275	1.34259	0.0762898
1276	1.04187	0.0592575
1277	1.05019	0.0857409
1278	1.2361	0.101273
1279	1.69055	0.0671788
1280	2.25056	0.075044
1281	1.10574	1.72729
1282	1.21632	2.04838
1283	1.10574	2.1
1284	1.46181	2.1
1285	0.946131	1.57554
1286	1.74111	1.85401
1287	1.74111	1.7449
1288	0.94613	1.73511
1289	1.26891	1.31408
1290	1.26891	0.683729
1291	1.23452	1.70079
1292	1.00255	1.9177
1293	1.72286	0.156241
1294	1.12233	0.101492
1295	1.30682	0.210437
1296	1.97397	0.235164
1297	1.67425	2.1
1298	1.77775	2.1
1299	1.73571	1.86363
1300	0.426265	2.08443
1301	1.31817	1.83445
1302	1.81042	2.1
1303	1.85456	2.1
1304	0.466532	1.77032
1305	1.28025	1.81625
1306	0.964467	1.75905
1307	1.27921	2.1
1308	2.11851	1.77089
1309	2.37121	1.88185
1310	1.49357	0.169051
1311	1.42486	2.1
1312	1.00278	2.1
1313	1.00278	2.1
1314	1.12503	2.1
1315	0.568697	2.1
1316	0.555211	2.1
1317	0.982272	2.1
1318	1.57558	2.1
1319	0.851539	2.1
1320	1.07307	2.1
1321	1.58292	2.1
1322	1.50731	2.1
1323	0.851541	2.1
1324	1.00304	2.1
1325	1.36003	2.1
1326	1.13127	2.1
1327	0.672234	2.1
1328	0.763044	2.1
1329	0.966979	2.1
1330	1.31502	2.1
1331	1.67969	2.1

1332	1.62408	2.1
1333	0.661955	2.1
1334	1.35048	2.1
1335	1.40447	2.1
1336	1.40447	2.1
1337	0.607961	2.1
1338	0.946131	2.1
1339	0.607963	2.1
1340	1.01531	2.1
1341	1.01531	2.1
1342	1.91802	2.1
1343	0.777749	2.1
1344	1.22585	0.05
1345	1.20672	0.05
1346	1.03166	0.137038
1347	0.553805	2.1
1348	2.00551	2.1
1349	2.07203	2.1
1350	0.589479	2.1
1351	1.22352	2.1
1352	0.984227	2.1
1353	0.802455	2.1
1354	1.40942	2.1
1355	1.6662	2.1
1356	0.802485	2.1
1357	0.936669	2.1
1358	1.31127	0.213023
1359	0.946132	0.230761
1360	1.09729	0.145313
1361	0.946133	0.348671
1362	1.68703	0.16612
1363	1.68703	0.127838
1364	1.3689	0.194036
1365	1.26426	0.213024
1366	1.26923	2.1
1367	1.23614	2.1
1368	0.828371	2.1
1369	2.04556	2.1
1370	0.772648	2.1
1371	0.791059	2.1
1372	0.809958	2.1
1373	1.70012	2.1
1374	0.267032	2.1
1375	1.67604	2.1
1376	0.803823	2.1
1377	0.267305	2.1
1378	0.80246	2.1
1379	0.874582	2.1
1380	1.00188	2.1
1381	0.709588	2.1
1382	1.50679	2.1
1383	1.22456	2.1
1384	0.870622	2.1
1385	1.05754	2.1
1386	1.1689	2.1
1387	1.54556	2.1
1388	1.89226	2.1
1389	0.995311	2.1

1390	1.26195	2.1
1391	1.9	2.1
1392	0.845055	2.1
1393	0.737836	2.1
1394	0.823707	2.1
1395	0.626587	2.1
1396	1.16109	2.1
1397	0.85517	2.1
1398	0.932036	2.1
1399	1.08321	2.1
1400	1.43863	2.1
1401	2.72297	2.1
1402	2.90798	2.1
1403	1.47818	1.41216
1404	1.47819	1.56318
1405	0.694357	1.3517
1406	0.694382	1.42091
1407	0.946132	1.25992
1408	0.694382	1.33262
1409	1.22641	1.33346
1410	1.31155	1.535
1411	2.45282	1.41448
1412	0.787843	1.23101
1413	0.808984	1.1896
1414	1.14232	1.42013
1415	0.795037	1.34641
1416	0.887382	1.39605
1417	1.14493	1.29096
1418	0.78393	1.08917
1419	1.09129	1.2953
1420	0.791482	1.27259
1421	1.1137	1.47323
1422	1.11819	1.36618
1423	0.960173	1.35923
1424	1.09447	1.37501
1425	0.874365	1.31232
1426	1.2474	0.121724
1427	1.75038	0.240173
1428	1.75038	0.325506
1429	0.94613	0.519817
1430	1.30076	0.254663
1431	1.12817	0.826268
1432	0.94613	0.398252
1433	1.30076	0.365838
1434	1.51841	0.29031
1435	1.51841	0.373269
1436	0.946132	0.852596
1437	1.39575	0.333704
1438	0.970358	1.43538
1439	0.946133	0.601048
1440	1.39575	0.518407
1441	1.06486	0.165383
1442	1.06486	0.160382
1443	1.38622	0.18882
1444	1.04521	0.05
1445	1.00809	0.05
1446	1.28952	0.05
1447	0.849316	0.05

1448	0.946132	0.05
1449	0.849344	0.0619167
1450	1.53631	0.05
1451	1.53631	0.05
1452	1.77843	0.05
1453	1.69863	0.05
1454	1.91646	2.1
1455	1.8582	2.1
1456	2.8349	0.05
1457	1.36307	0.05
1458	1.23886	0.05
1459	1.12971	0.05
1460	1.59662	0.05
1461	1.59662	0.05
1462	0.640304	0.050354
1463	0.640333	0.05
1464	0.886276	0.05349
1465	0.886276	0.05
1466	1.35065	0.05
1467	2.09002	0.05
1468	1.35543	0.05
1469	1.94688	2.1
1470	1.97221	2.1
1471	0.719563	2.1
1472	1.20358	2.1
1473	0.874264	2.1
1474	1.27676	2.1
1475	1.46557	2.1
1476	1.46557	2.1
1477	0.68546	2.1
1478	0.94613	2.1
1479	0.685489	2.1
1480	1.39615	0.0931908
1481	1.05404	0.0709665
1482	1.06474	0.098017
1483	1.00713	0.0780356
1484	0.714535	0.0800603
1485	0.714563	0.0974178
1486	1.41646	0.0673068
1487	1.52582	0.0551061
1488	0.721694	0.0784566
1489	0.93665	0.0768281
1490	1.21067	0.146269
1491	1.21067	0.0666816
1492	1.73577	0.357423
1493	1.17799	0.05
1494	3.21432	0.213226
1495	2.41452	0.202856
1496	1.36208	0.173667
1497	2.53256	0.191426
1498	0.834593	0.142313
1499	2.5853	0.18858
1500	1.77259	0.161729
1501	0.615724	0.362048
1502	1.11374	0.550118
1503	0.615726	0.295544
1504	0.946129	0.722421
1505	0.615714	0.689775

1506	1.75489	0.56002
1507	1.75489	0.399647
1508	1.23146	0.247708
1509	2.2772	0.27245
1510	0.74463	0.251051
1511	1.3546	0.289864
1512	1.80252	0.193768
1513	2.38304	0.211867
1514	1.19151	0.333928
1515	1.1231	0.365267
1516	1.82345	0.272749
1517	1.79805	0.257021
1518	0.761337	0.471263
1519	0.946127	0.327775
1520	0.557726	0.331416
1521	1.05353	0.0859695
1522	1.05353	0.115579
1523	1.68599	0.247149
1524	1.05353	0.134393
1525	1.40588	2.1
1526	1.09373	2.1
1527	1.18382	1.47296
1528	1.46903	1.30375
1529	1.25103	2.1
1530	1.10378	2.1
1531	1.58941	2.1
1532	1.29639	2.1
1533	1.14874	0.05
1534	0.899817	0.05
1535	0.666898	0.05
1536	1.76519	0.05
1537	1.17289	0.05
1538	1.03526	0.05
1539	1.97708	0.05
1540	1.31711	0.05
1541	0.992517	0.05
1542	1.70193	0.05
1543	1.13852	0.05
1544	1.23943	1.42603
1545	1.40014	2.1
1546	1.04598	2.1
1547	1.1483	2.1
1548	1.11433	1.65805
1549	1.32037	0.353371
1550	1.00381	1.7017
1551	1.11435	0.215262
1552	0.973502	1.41795
1553	0.946128	1.87368
1554	1.83697	1.39762
1555	1.83697	2.1
1556	1.31721	1.8263
1557	1.38614	1.38997
1558	1.39696	1.35456
1559	1.31721	1.68024
1560	1.0756	1.68602
1561	1.08542	1.48117
1562	0.946127	2.1
1563	1.81052	0.471978

1564	1.81052	2.1
1565	1.24315	2.1
1566	1.39696	0.324133
1567	1.35969	1.8017
1568	1.24315	1.77265
1569	1.0756	0.206945
1570	1.20669	0.145126
1571	1.18508	0.179863
1572	1.71059	2.1
1573	1.66762	2.1
1574	2.50853	2.1
1575	1.8236	2.1
1576	1.78872	2.1
1577	1.41721	2.1
1578	1.01335	1.75725
1579	1.42439	1.29485
1580	0.946129	2.00902
1581	1.33266	1.22782
1582	1.79776	1.8836
1583	1.79776	2.1
1584	0.946131	0.649971
1585	1.33266	1.88503
1586	1.41123	2.02659
1587	1.25346	2.1
1588	0.946132	0.507903
1589	0.827287	1.30817
1590	1.57878	1.35544
1591	1.57878	1.56807
1592	0.827287	1.19698
1593	1.07341	1.2158
1594	1.33266	1.46536
1595	1.25346	1.61642
1596	1.61149	2.1
1597	1.15644	1.87881
1598	1.27558	1.34133
1599	1.3764	0.433736
1600	1.89526	1.55324
1601	1.37641	0.373855
1602	1.2064	2.1
1603	1.38426	2.1
1604	1.01595	2.1
1605	1.15067	2.1
1606	1.82276	2.1
1607	1.82276	2.1
1608	0.946131	2.1
1609	1.40609	2.1
1610	0.946132	2.1
1611	1.29143	2.1
1612	1.40609	2.1
1613	1.30272	2.1
1614	1.19548	2.1
1615	2.95451	2.1
1616	1.46124	2.1
1617	2.93215	2.1
1618	1.42105	2.1
1619	1.75477	0.618095
1620	2.31301	0.721004
1621	2.10275	0.229941

1622	1.80099	0.257053
1623	0.97541	2.1
1624	1.49733	2.1
1625	0.97541	2.1
1626	1.30751	2.1
1627	1.28614	0.0587456
1628	1.74759	0.05
1629	1.19756	0.05
1630	1.71053	0.0541903
1631	1.19757	0.0528006
1632	1.28954	0.05
1633	2.02703	0.05
1634	2.29453	0.05
1635	1.28265	0.05
1636	0.793267	0.0541032
1637	1.19974	0.0885329
1638	0.509551	0.05
1639	1.04072	0.0854443
1640	1.35302	0.05
1641	1.53133	0.0860302
1642	0.566153	0.0611036
1643	1.09634	0.05
1644	1.12792	0.05
1645	1.68908	2.1
1646	1.68908	2.1
1647	0.768212	2.1
1648	0.768213	2.1
1649	0.946132	2.1
1650	0.768212	2.1
1651	1.32461	2.1
1652	1.87291	2.1
1653	2.64921	2.1
1654	1.53642	2.1
1655	2.26537	2.1
1656	1.53642	2.1
1657	1.79874	2.1
1658	1.79874	2.1
1659	0.726635	2.0703
1660	0.726636	2.1
1661	0.946128	2.1
1662	0.726634	2.1
1663	1.23339	2.04984
1664	0.992674	2.1
1665	1.8023	2.1
1666	1.12324	0.05
1667	1.29163	0.05
1668	0.996831	0.130536
1669	1.0019	0.124066
1670	0.993831	0.0684114
1671	1.07283	2.1
1672	1.04788	2.1
1673	1.44489	2.1
1674	1.04788	2.1
1675	1.07652	2.1
1676	0.991406	2.1
1677	1.37	2.1
1678	1.14195	2.1
1679	1.47904	0.05

1680	1.26593	2.01344
1681	1.75366	1.91643
1682	1.57621	0.154586
1683	1.24538	0.0761994
1684	0.969118	2.1
1685	0.946133	2.1
1686	1.69387	2.1
1687	1.69387	2.1
1688	0.94613	2.1
1689	1.28713	2.1
1690	1.28713	2.1
1691	1.23877	2.1
1692	0.969118	2.1
1693	1.22615	2.1
1694	0.98084	2.1
1695	1.01843	2.1
1696	1.50876	2.1
1697	1.85391	2.1
1698	1.84322	2.1
1699	1.70377	2.1
1700	1.78919	2.1
1701	2.66787	2.1
1702	1.41549	2.1
1703	1.41549	2.1
1704	0.86707	2.1
1705	0.896172	2.1
1706	0.946126	2.1
1707	0.867074	2.1
1708	0.946118	2.1
1709	1.54468	0.05
1710	1.54468	0.05
1711	2.27259	0.0538896
1712	0.946131	0.0927007
1713	1.25837	0.0786918
1714	0.663171	0.117532
1715	1.68931	0.0582241
1716	1.68931	0.0528663
1717	1.25837	0.0818954
1718	1.09411	0.0783824
1719	1.73571	0.05
1720	2.34894	0.0505942
1721	0.946128	0.0643476
1722	1.30497	0.0573469
1723	0.772312	0.0807198
1724	1.66895	0.05
1725	1.66895	0.05
1726	1.30497	0.0554878
1727	1.13629	0.05
1728	1.22852	1.15281
1729	1.578	1.16091
1730	2.07781	2.1
1731	1.5478	2.1
1732	1.70048	2.1
1733	1.42329	2.1
1734	1.05481	2.1
1735	1.1032	1.92294
1736	1.19267	2.1
1737	1.77324	2.1

1738	1.01121	0.0809354
1739	0.991867	0.120392
1740	2.0744	2.1
1741	1.75142	2.1
1742	1.35418	2.1
1743	1.40041	2.1
1744	1.50045	2.1
1745	1.37704	2.1
1746	1.50045	2.1
1747	1.87875	2.1
1748	1.35308	1.05985
1749	1.29983	1.03982
1750	1.62784	2.1
1751	1.44807	2.1
1752	1.00965	2.1
1753	2.69264	2.1
1754	1.04075	2.1
1755	0.946131	2.1
1756	0.946132	2.1
1757	1.52704	2.1
1758	1.83246	2.1
1759	1.56757	2.1
1760	1.16687	2.1
1761	2.04857	2.1
1762	1.32707	2.1
1763	1.25519	0.349152
1764	1.32101	0.957607
1765	1.25055	2.08679
1766	0.827853	1.77229
1767	1.11835	0.853014
1768	1.07281	2.1
1769	1.54302	1.70205
1770	0.724064	1.98726
1771	1.32709	2.1
1772	3.44123	2.1
1773	2.46889	0.711385
1774	0.730076	0.0562594
1775	1.25809	0.154575
1776	0.730077	0.0919953
1777	0.946129	0.138181
1778	1.72777	0.05
1779	1.72777	0.135817
1780	0.73005	0.107511
1781	1.7522	0.0635645
1782	2.51618	0.063587
1783	0.628177	0.0561679
1784	1.1068	0.178032
1785	0.747435	0.0804469
1786	0.946133	0.162062
1787	1.84668	0.0535987
1788	1.8563	0.146594
1789	0.628177	0.142335
1790	1.45501	0.124028
1791	2.19809	0.107413
1792	1.45501	0.05
1793	1.48974	0.05
1794	1.7709	2.1
1795	2.63288	2.1

1796	1.5362	2.1
1797	1.56327	2.1
1798	3.03945	0.05
1799	0.899979	0.05
1800	0.869386	0.05
1801	1.85864	0.05
1802	1.78028	0.05
1803	1.28719	0.05
1804	1.09532	0.05
1805	0.881898	0.05
1806	1.28911	0.05
1807	1.39644	0.05
1808	0.688829	0.05
1809	2.14611	0.05
1810	1.80313	0.05
1811	0.68883	0.05
1812	0.946131	0.05
1813	1.25334	0.05
1814	1.88987	0.05
1815	1.94432	0.05
1816	1.37592	0.05
1817	1.11691	2.1
1818	1.02923	2.1
1819	0.45824	0.05
1820	0.646512	0.05
1821	2.46292	0.05
1822	0.493451	0.05
1823	2.00826	2.1
1824	2.49784	2.1
1825	1.17054	2.1
1826	1.01485	2.1
1827	1.26045	2.1
1828	1.17054	2.1
1829	1.44247	2.1
1830	2.02687	0.05
1831	1.66365	0.05
1832	1.64656	0.05
1833	1.56597	0.05
1834	1.21419	2.1
1835	1.5008	2.1
1836	1.10711	2.1
1837	1.44287	2.1
1838	1.44287	2.1
1839	0.750368	2.1
1840	1.25876	2.1
1841	0.82542	2.1
1842	1.04074	2.1
1843	1.25874	2.1
1844	1.04159	0.05
1845	1.2489	0.05
1846	0.980547	0.0853249
1847	1.62235	0.0670711
1848	1.15991	0.0890829
1849	0.980547	0.0731321
1850	0.94613	0.112514
1851	1.54662	0.0865107
1852	1.54661	0.0997648
1853	1.07912	0.125842

1854	1.1032	0.0890728
1855	1.1032	0.101099
1856	0.94613	0.0960857
1857	1.12468	2.1
1858	1.2724	2.1
1859	1.06521	2.1
1860	1.0752	2.1
1861	1.94936	0.0789829
1862	1.62772	0.0647174
1863	1.94733	0.0675182
1864	1.42901	0.0797914
1865	1.33022	2.1
1866	1.70839	2.1
1867	1.75048	2.1
1868	1.41201	2.1
1869	1.52314	2.1
1870	1.44039	2.1
1871	1.51113	2.1
1872	2.58638	2.1
1873	0.987247	0.078911
1874	1.38622	0.196916
1875	0.858359	0.104047
1876	1.19967	0.146164
1877	1.06414	0.102261
1878	0.946132	0.220334
1879	0.94613	0.101317
1880	0.687079	0.31242
1881	0.946132	0.204017
1882	0.822611	0.199705
1883	1.70242	0.118188
1884	1.70242	0.201102
1885	0.946132	0.380877
1886	1.11155	0.174271
1887	0.946132	0.451449
1888	1.11155	0.277424
1889	1.37981	0.305697
1890	1.18348	0.119373
1891	2.44179	0.192994
1892	2.00403	0.289576
1893	1.10961	2.1
1894	1.85415	2.1
1895	1.44825	2.1
1896	0.932272	2.1
1897	0.810942	2.1
1898	1.00189	2.1
1899	1.11779	2.1
1900	1.3359	2.1
1901	0.965279	2.1
1902	0.833779	2.1
1903	1.05337	2.1
1904	0.526656	2.1
1905	1.44599	2.1
1906	1.44599	2.1
1907	1.24853	2.1
1908	0.946132	2.1
1909	1.24853	2.1
1910	0.724122	2.1
1911	1.91547	2.1

1912	2.49705	2.1
1913	1.05931	2.1
1914	1.1729	2.1
1915	1.34526	2.1
1916	1.5379	2.1
1917	1.90855	2.1
1918	2.08597	2.1
1919	1.68165	2.1
1920	1.45887	2.1
1921	1.50022	2.1
1922	1.4748	1.9875
1923	2.63645	1.89559
1924	1.74431	1.96432
1925	1.25288	1.86576
1926	1.25289	1.77232
1927	0.970025	1.79026
1928	0.747542	2.03962
1929	0.810959	1.72074
1930	1.15369	1.80227
1931	0.977472	1.75557
1932	1.12695	1.80719
1933	0.996762	1.78878
1934	1.28249	0.819637
1935	0.946132	0.944067
1936	0.528621	0.993431
1937	1.59186	0.532768
1938	1.44072	0.494748
1939	0.633488	0.656052
1940	0.598506	0.99563
1941	1.51618	0.980423
1942	1.19999	0.507583
1943	1.46511	0.588938
1944	1.44056	0.459142
1945	0.912754	0.223917
1946	1.4725	0.159803
1947	1.4725	0.157476
1948	0.912753	0.245157
1949	1.23351	0.202146
1950	1.23351	0.220553
1951	0.946131	0.26177
1952	1.17129	0.177488
1953	1.82551	0.149615
1954	1.27575	1.95355
1955	0.946129	2.01759
1956	0.946129	2.1
1957	0.946128	1.99366
1958	0.792754	2.07342
1959	0.94613	1.89255
1960	0.792752	1.90053
1961	2.08054	2.05866
1962	1.20091	2.1
1963	2.1669	2.02524
1964	1.24383	1.82929
1965	0.904669	1.88725
1966	0.83804	1.74124
1967	1.90486	1.83521
1968	1.29162	1.77309
1969	1.37383	1.87532

1970	1.86533	1.80543
1971	1.76472	1.90399
1972	1.60428	1.81727
1973	2.43993	1.92538
1974	1.37833	2.1
1975	1.18632	2.1
1976	0.946132	2.1
1977	1.27544	2.1
1978	1.05085	2.1
1979	0.961178	2.1
1980	0.859844	2.1
1981	1.0499	2.1
1982	1.75592	2.1
1983	2.08668	2.1
1984	1.75592	2.1
1985	1.4402	2.1
1986	1.72285	2.1
1987	0.83312	0.05
1988	1.07295	0.05
1989	1.37607	0.05
1990	1.32097	0.05
1991	1.11332	0.05
1992	1.18568	0.05
1993	0.877482	0.05
1994	1.4458	0.05
1995	2.34112	0.05
1996	2.68021	0.05
1997	0.681677	0.05
1998	0.946131	0.05
1999	0.681649	0.05
2000	1.83115	0.05
2001	1.68029	0.05
2002	1.05482	0.05
2003	1.31485	0.05
2004	2.08247	0.985736
2005	1.33153	0.65372
2006	0.935062	1.19931
2007	0.806958	1.63019
2008	0.827683	1.09789
2009	1.01041	1.19704
2010	0.741832	1.05946
2011	1.15668	1.23733
2012	0.908649	1.24522
2013	0.59561	0.332876
2014	1.19028	0.543758
2015	0.59561	0.508442
2016	0.946132	0.602934
2017	0.595623	0.94344
2018	1.58164	0.57355
2019	1.58164	0.557532
2020	0.946131	2.1
2021	1.55201	2.1
2022	1.55201	2.1
2023	0.946127	2.1
2024	1.13312	2.1
2025	1.13312	2.1
2026	1.08108	2.1
2027	1.04973	2.1

2028	1.20149	2.1
2029	1.86902	1.55523
2030	0.613447	2.1
2031	1.22618	1.80981
2032	1.38663	1.98104
2033	1.38662	2.1
2034	0.613447	1.94535
2035	1.22617	2.1
2036	0.773899	2.1
2037	1.23515	1.93236
2038	1.87823	1.5743
2039	1.70303	0.537026
2040	1.4714	2.1
2041	1.74067	2.1
2042	0.735699	2.1
2043	1.73461	2.1
2044	1.47224	2.1
2045	1.22827	2.1
2046	1.1634	2.1
2047	1.36471	2.1
2048	0.735701	2.1
2049	2.8386	1.16795
2050	2.39056	2.1
2051	2.44128	2.1
2052	1.54102	2.1
2053	1.54102	2.1
2054	0.936141	2.1
2055	1.40594	2.1
2056	1.07121	2.09951
2057	0.936142	2.1
2058	1.07121	2.1
2059	1.22691	0.0687403
2060	1.42907	0.0585414
2061	2.18048	0.0575897
2062	0.947662	0.0534057
2063	2.18394	2.1
2064	1.21292	2.1
2065	2.18394	2.1
2066	2.19064	2.1
2067	1.27229	2.1
2068	1.20859	0.496499
2069	1.21073	0.439675
2070	1.73899	2.1
2071	1.3164	2.1
2072	1.61245	2.1
2073	1.41444	2.1
2074	2.55116	2.1
2075	1.26193	2.1
2076	1.89553	2.1
2077	0.916438	2.1
2078	1.26113	2.1
2079	0.810934	2.1
2080	2.00419	2.1
2081	1.27872	2.1
2082	1.35306	2.1
2083	1.96749	2.1
2084	1.07955	2.1
2085	1.31854	2.1

2086	1.29505	0.56008
2087	1.29504	0.889235
2088	0.612469	1.13961
2089	0.990053	0.819883
2090	1.04072	1.12218
2091	0.985266	0.780487
2092	0.990039	0.938413
2093	1.06892	1.16381
2094	1.57158	0.470294
2095	1.895	0.504487
2096	1.05698	0.400392
2097	0.925053	2.1
2098	0.624712	2.1
2099	1.42465	2.1
2100	1.49014	2.1
2101	0.687198	2.1
2102	1.22058	2.1
2103	0.851488	2.1
2104	0.588128	2.1
2105	1.12452	2.1
2106	1.02783	2.1
2107	1.7083	2.1
2108	1.06977	0.512715
2109	0.818569	0.189096
2110	1.14386	0.354342
2111	0.983791	0.297766
2112	1.12214	0.616471
2113	1.0915	0.124755
2114	0.810934	0.456885
2115	0.764114	0.645163
2116	1.87956	0.472744
2117	1.87956	0.655655
2118	0.764141	0.831613
2119	1.37675	0.338329
2120	0.764141	0.307946
2121	0.946132	0.430568
2122	1.9637	1.05682
2123	2.10107	0.669171
2124	1.67814	0.767321
2125	3.02886	0.582796
2126	0.908982	0.0881381
2127	0.621727	0.16491
2128	0.525963	0.118585
2129	0.946128	0.18255
2130	1.60979	0.0912769
2131	1.45596	0.124205
2132	0.556172	0.168697
2133	2.06779	0.0833392
2134	2.17911	0.0990884
2135	1.00205	0.05
2136	1.30685	0.0863991
2137	1.16534	0.0642069
2138	1.65532	2.1
2139	2.20346	2.1
2140	0.993889	2.1
2141	1.09328	2.1
2142	2.09372	2.1
2143	2.10036	2.1

2144	1.33727	0.996518
2145	2.53383	0.81195
2146	0.893379	1.24037
2147	1.52828	0.928127
2148	1.09599	0.05
2149	1.09599	0.05
2150	1.50524	2.1
2151	1.54711	2.1
2152	1.41469	2.1
2153	1.99459	1.9703
2154	1.79805	2.1
2155	1.79805	2.1
2156	0.707344	2.1
2157	1.30318	2.1
2158	0.707344	2.1
2159	0.946129	2.1
2160	1.30318	2.1
2161	2.40442	2.1
2162	1.89226	2.1
2163	1.40794	2.1
2164	0.898721	2.1
2165	0.791921	1.75281
2166	0.810933	2.1
2167	1.10904	1.97303
2168	0.991102	2.1
2169	0.808502	1.6255
2170	1.1398	2.04332
2171	1.31346	2.1
2172	1.11833	2.1
2173	1.669	2.1
2174	1.25715	2.1
2175	1.3199	2.1
2176	1.63092	2.1
2177	1.63092	2.1
2178	0.946131	2.1
2179	0.946131	2.1
2180	1.13534	2.1
2181	0.979853	2.1
2182	2.19994	2.1
2183	0.725844	2.1
2184	2.19994	2.1
2185	2.35055	2.1
2186	1.52204	2.1
2187	1.40118	2.1
2188	3.42285	2.1
2189	2.05217	2.1
2190	1.50319	2.1
2191	0.947516	1.92438
2192	0.903572	1.92511
2193	0.76996	1.97546
2194	1.90959	2.1
2195	1.18897	2.1
2196	1.24166	2.1
2197	2.26785	2.05715
2198	2.05217	2.1
2199	2.73306	2.1
2200	1.42729	0.0615917
2201	1.387	0.0697561

2202	1.59458	0.342692
2203	1.91051	0.369802
2204	1.68108	0.447132
2205	2.03357	0.703889
2206	0.928982	0.081017
2207	1.55687	0.0834295
2208	1.55687	0.05
2209	0.92898	0.0687664
2210	1.3495	0.0954206
2211	1.3495	0.0664813
2212	0.946133	0.0997888
2213	1.85795	0.0632203
2214	2.2727	0.0851687
2215	1.20324	2.06328
2216	1.07784	0.209899
2217	0.957792	0.139859
2218	1.2745	0.137633
2219	1.4659	0.148251
2220	0.946129	0.23005
2221	0.95778	0.214796
2222	0.851553	0.27127
2223	1.74103	0.156478
2224	2.28994	0.157538
2225	2.08213	0.139007
2226	2.1556	0.142554
2227	2.20372	0.117593
2228	0.838027	0.05
2229	1.11373	0.05
2230	1.2909	0.05
2231	0.998424	0.05
2232	1.40621	0.05
2233	1.19478	0.05
2234	1.11783	0.05
2235	1.6695	0.05
2236	1.7498	0.05
2237	0.477652	0.05
2238	1.77291	0.05
2239	0.590448	0.05
2240	1.07668	0.05
2241	0.810946	0.05
2242	1.1946	0.05
2243	2.03914	0.05
2244	1.25585	0.05
2245	2.02622	0.05
2246	1.39091	0.05
2247	1.31871	0.05
2248	1.15789	2.1
2249	1.15755	2.1
2250	1.82268	2.1
2251	1.87119	2.1
2252	3.10636	2.1
2253	2.96285	2.1
2254	1.73443	1.1579
2255	2.33812	1.10184
2256	1.59457	0.466561
2257	1.73443	1.40391
2258	2.00665	2.1
2259	1.99841	2.1

2260	1.19901	2.1
2261	1.16661	2.1
2262	2.15883	0.138774
2263	1.20951	0.138631
2264	2.49192	0.196526
2265	0.65112	0.116715
2266	2.45317	0.05
2267	1.73091	0.05
2268	2.0795	0.05
2269	1.45891	0.05
2270	1.6531	0.05
2271	1.78304	0.05
2272	0.775132	0.05
2273	1.35051	0.05
2274	1.0461	0.05
2275	1.37837	0.05
2276	1.00259	0.05
2277	1.25796	0.162126
2278	1.10719	0.209339
2279	1.51067	2.1
2280	2.65632	2.1
2281	1.30521	2.1
2282	2.456	2.1
2283	3.19218	2.1
2284	2.67387	2.09152
2285	0.988862	2.1
2286	1.917	2.1
2287	0.946132	2.1
2288	1.16202	2.1
2289	1.53871	2.1
2290	1.53871	2.1
2291	0.946132	2.1
2292	1.16202	2.1
2293	1.32282	2.1
2294	2.46356	2.1
2295	2.32404	2.1
2296	1.37562	0.0550301
2297	0.985546	0.05
2298	1.28983	0.05
2299	1.05566	0.05
2300	0.895312	0.05
2301	1.53325	0.05
2302	1.04342	0.05
2303	1.80881	0.05
2304	0.876865	0.05
2305	0.5689	0.05
2306	1.09506	0.05
2307	1.51789	2.1
2308	1.49872	2.1
2309	0.949364	0.0789605
2310	2.01233	0.092463
2311	1.55063	0.129796
2312	1.57783	0.248561
2313	1.57783	0.252335
2314	0.674605	0.223793
2315	0.98537	0.129329
2316	0.674633	0.0788445
2317	0.98537	0.104107

2318	1.26706	0.231275
2319	0.980664	0.349831
2320	1.03298	0.148764
2321	1.84936	0.306716
2322	1.51696	0.0936969
2323	1.97074	0.0940119
2324	1.03298	0.0968737
2325	1.51324	0.0661446
2326	1.51324	0.0660542
2327	0.758454	0.0667346
2328	1.28632	0.056132
2329	0.758482	0.05
2330	0.758482	0.05
2331	0.985372	0.0689674
2332	1.14105	0.05
2333	1.59787	0.253272
2334	2.46244	0.291764
2335	1.70367	0.135616
2336	2.13757	1.29747
2337	1.98142	1.2756
2338	1.35878	0.05
2339	2.39947	0.05
2340	1.24554	0.05
2341	1.7522	0.05
2342	1.49693	1.81596
2343	1.49652	1.86842
2344	1.6211	1.84236
2345	1.45938	1.89123
2346	0.737431	1.95408
2347	1.18643	1.89343
2348	1.04072	2.03416
2349	0.9459	1.87862
2350	0.66364	1.95557
2351	0.985109	1.72838
2352	0.638368	1.87154
2353	0.83804	1.65296
2354	0.998024	1.74309
2355	0.914471	1.61006
2356	1.14281	1.76269
2357	0.833972	1.73114
2358	0.991865	0.05
2359	1.42208	0.05
2360	1.70718	2.1
2361	1.79888	2.1
2362	1.16851	0.218118
2363	1.16851	0.190011
2364	1.51021	0.298019
2365	0.946132	0.143874
2366	1.14897	0.0831952
2367	0.946132	0.19547
2368	1.77177	0.149911
2369	1.77177	0.080315
2370	1.33038	0.123275
2371	1.38752	0.127358
2372	1.1726	0.0865729
2373	1.51688	0.144352
2374	1.06455	2.1
2375	0.98511	2.1

2376	1.31144	2.1
2377	1.79825	2.1
2378	1.79825	2.1
2379	1.73044	2.1
2380	1.59589	0.05
2381	1.59589	0.05
2382	2.78009	2.1
2383	1.68761	2.1
2384	3.05511	2.1
2385	2.25318	2.1
2386	0.578998	2.1
2387	1.03512	2.1
2388	2.26156	2.1
2389	1.44859	0.96398
2390	1.35897	1.11209
2391	1.18301	2.1
2392	1.25173	2.1
2393	1.98851	2.1
2394	2.70685	2.1
2395	0.682342	2.1
2396	1.52491	2.1
2397	1.37119	2.1
2398	0.682313	2.1
2399	1.35342	2.1
2400	0.947555	2.1
2401	0.614107	2.1
2402	1.23007	2.1
2403	1.5735	2.1
2404	1.58713	2.1
2405	1.57979	0.05
2406	1.30723	0.05
2407	0.935658	1.09841
2408	0.8952	1.0584
2409	1.27352	1.30775
2410	1.0985	1.24779
2411	1.07163	1.20105
2412	1.15823	1.07749
2413	0.836451	1.16142
2414	2.08051	0.387421
2415	1.92601	1.40074
2416	2.0644	0.852107
2417	1.37259	2.1
2418	1.37259	2.1
2419	0.752217	2.1
2420	1.28569	2.1
2421	0.752186	2.1
2422	0.839124	2.1
2423	1.28568	2.1
2424	1.04199	2.1
2425	1.04199	2.1
2426	2.59268	2.1
2427	1.04199	2.1
2428	1.5098	2.1
2429	1.5098	2.1
2430	0.520997	2.1
2431	1.29634	2.1
2432	0.520996	2.1
2433	0.73446	2.1

2434	1.29634	2.1
2435	0.983724	2.1
2436	1.23914	2.1
2437	2.65095	2.1
2438	1.52851	2.1
2439	2.399 2.1	
2440	1.68137	2.1
2441	1.11285	2.1
2442	1.04921	2.1
2443	1.30711	2.1
2444	1.04921	2.1
2445	1.07496	1.30936
2446	1.55831	0.507031
2447	1.07497	0.253763
2448	1.07497	1.446
2449	1.27391	2.1
2450	2.5615	2.1
2451	1.2739	2.1
2452	1.75253	2.1
2453	1.12907	0.05
2454	1.9194	0.0522959
2455	1.96617	0.05
2456	1.01225	2.1
2457	0.947979	2.1
2458	1.01225	2.1
2459	1.06561	2.1
2460	1.01225	2.1
2461	3.56876	2.1
2462	3.68767	2.1
2463	1.52301	2.1
2464	1.61013	2.1
2465	1.43726	2.1
2466	2.04275	2.1
2467	0.995734	2.1
2468	1.16199	2.1
2469	1.73704	0.05
2470	1.04019	0.05
2471	0.956294	2.1
2472	1.81488	2.1
2473	1.63719	2.1
2474	1.50103	0.05
2475	1.75289	0.05
2476	1.31491	0.05
2477	1.03567	0.05
2478	0.672257	0.05
2479	1.04072	0.05
2480	0.746903	0.05
2481	1.43317	0.05
2482	1.39986	0.05
2483	1.06385	0.05
2484	1.1624	0.05
2485	1.61667	0.05
2486	1.27258	0.05
2487	2.559 0.05	
2488	1.66093	0.05
2489	1.10719	0.233209
2490	2.03235	0.295907
2491	1.17105	0.430243

2492	1.10719	0.303389
2493	1.3028	2.1
2494	0.946131	2.1
2495	0.946127	2.1
2496	1.52912	2.1
2497	1.52912	2.1
2498	0.946128	2.1
2499	0.949997	2.1
2500	1.31565	2.1
2501	1.46643	2.1
2502	1.5482	2.1
2503	1.47102	2.1
2504	1.47102	2.1
2505	0.774101	2.1
2506	1.30583	2.1
2507	0.774103	2.1
2508	0.939287	2.1
2509	1.30584	2.1
2510	0.958661	2.1
2511	2.28835	2.1
2512	1.43213	0.05
2513	2.13255	0.05
2514	1.43213	0.05
2515	1.10476	0.05
2516	1.93165	0.05
2517	1.03235	1.48859
2518	1.31	1.48307
2519	1.22082	2.1
2520	1.35827	2.1
2521	0.790936	2.1
2522	0.716644	2.1
2523	1.43257	2.1
2524	1.43257	2.1
2525	0.716615	2.1
2526	0.946131	2.1
2527	0.730157	2.1
2528	1.47212	2.1
2529	1.47212	2.1
2530	0.946131	2.1
2531	0.790792	2.1
2532	1.41149	2.1
2533	0.790791	2.1
2534	1.01006	2.1
2535	1.53045	0.258807
2536	1.53045	0.372615
2537	0.946132	0.87984
2538	1.40779	0.31779
2539	1.06879	1.24504
2540	0.990713	0.577057
2541	1.06879	0.672403
2542	1.06486	0.178744
2543	1.18347	0.168158
2544	1.38622	0.186891
2545	1.18347	0.12762
2546	1.7304	0.194682
2547	1.7304	0.246518
2548	0.946131	0.502714
2549	1.26874	0.250741

2550	1.13337	1.30738
2551	1.13337	0.382498
2552	1.40779	0.397201
2553	1.695 2.1	
2554	1.77071	2.1
2555	0.649313	2.1
2556	0.692829	2.1
2557	1.40395	2.1
2558	1.34243	2.1
2559	0.813292	0.0890238
2560	0.632691	0.243585
2561	1.75766	0.301914
2562	2.25727	1.70824
2563	1.74206	0.0711989
2564	0.826073	0.0800843
2565	1.30022	0.0841673
2566	1.45641	0.0757522
2567	0.946131	0.0868493
2568	0.752245	0.0607305
2569	0.960952	0.113533
2570	0.743477	0.0835002
2571	1.04355	0.0563726
2572	1.6253	0.0719729
2573	0.960577	0.0725918
2574	1.34852	1.20051
2575	1.03235	1.30705
2576	1.78168	0.163001
2577	1.2966	0.05
2578	2.21116	0.05
2579	1.6395	0.05
2580	1.6395	0.05
2581	0.94613	0.05
2582	1.22187	0.05
2583	0.962737	0.05
2584	0.962735	0.05
2585	1.36376	0.05
2586	1.2966	0.05
2587	1.2966	0.05
2588	1.19669	0.992264
2589	2.37636	1.17768
2590	1.08894	1.36485
2591	1.62551	1.37811
2592	2.51472	1.45635
2593	1.89226	0.72057
2594	0.980038	1.21016
2595	1.10187	2.1
2596	1.01479	2.1
2597	1.81663	2.1
2598	1.83247	2.1
2599	0.921851	0.254664
2600	0.983148	2.1
2601	1.82816	2.1
2602	1.26103	2.1
2603	2.34818	2.1
2604	1.23152	2.1
2605	2.15738	2.1
2606	1.23152	2.1
2607	1.51042	2.1

2608	0.842995	2.1
2609	1.22257	2.1
2610	0.810959	2.1
2611	1.68242	2.1
2612	0.95701	2.1
2613	1.12389	2.1
2614	1.95255	2.1
2615	1.57974	2.1
2616	1.94632	2.1
2617	0.81534	2.1
2618	1.13996	2.1
2619	1.18958	0.05
2620	1.25929	0.05
2621	3.02933	1.60155
2622	2.37434	0.376385
2623	1.10609	2.02229
2624	1.64132	1.00695
2625	1.64132	1.1843
2626	0.946132	0.315997
2627	1.40029	0.953808
2628	1.08946	0.342346
2629	1.08946	0.383037
2630	1.18717	0.0927671
2631	2.71421	0.0700613
2632	1.08189	0.0802224
2633	0.94613	0.0711376
2634	1.37109	0.0909099
2635	1.17925	0.0596726
2636	0.851541	0.0806227
2637	0.942512	0.0840351
2638	1.18541	0.0644292
2639	0.851526	0.087055
2640	1.0819	0.0684254
2641	1.86009	0.110571
2642	0.946132	0.0508762
2643	1.67409	0.05
2644	1.67409	0.0696558
2645	0.946131	0.0642492
2646	1.31716	0.0646699
2647	1.30307	0.0565224
2648	0.946131	0.0705938
2649	1.18601	0.0602188
2650	3.15646	2.1
2651	0.985109	2.1
2652	3.37048	2.1
2653	1.10667	2.1
2654	1.77065	1.50021
2655	1.45837	1.43708
2656	1.77063	1.03807
2657	2.58104	1.17279
2658	1.37652	2.1
2659	1.54168	2.1
2660	1.00224	0.05
2661	1.05851	0.05
2662	1.39093	0.05
2663	1.43358	0.05
2664	0.575741	0.05
2665	0.518161	0.05

2666	1.04071	0.05
2667	1.13092	1.80096
2668	1.17042	1.86372
2669	1.53347	1.65514
2670	1.52938	1.79988
2671	1.00613	0.62925
2672	1.81618	2.1
2673	1.81618	0.670425
2674	0.946127	2.1
2675	1.36479	2.1
2676	0.94613	0.344851
2677	1.31724	1.30853
2678	1.3648	1.32322
2679	1.00613	0.672571
2680	0.781375	0.126711
2681	1.13431	0.146311
2682	1.03547	0.138112
2683	1.40668	0.238753
2684	0.94613	0.215599
2685	0.713285	0.170426
2686	1.72885	0.151442
2687	1.72885	0.178815
2688	0.713298	0.12528
2689	3.30075	0.100618
2690	2.81337	0.195875
2691	1.39296	0.237984
2692	0.830164	2.1
2693	1.50493	2.1
2694	1.50494	2.1
2695	0.830136	2.1
2696	1.28298	2.1
2697	1.0521	2.1
2698	0.830147	2.1
2699	2.0799	2.1
2700	1.52115	2.1
2701	2.5142	2.1
2702	1.31253	0.778276
2703	1.94446	1.28623
2704	1.18817	1.14181
2705	0.598348	0.819827
2706	1.69315	0.942888
2707	1.69315	1.26738
2708	0.598377	0.871976
2709	0.598347	0.911974
2710	0.94613	0.659098
2711	1.28418	1.3429
2712	2.05205	1.44863
2713	1.28418	1.36424
2714	1.0737	2.1
2715	1.81024	2.1
2716	2.73834	2.1
2717	1.71369	2.1
2718	1.71368	2.1
2719	0.946118	2.1
2720	1.29063	2.1
2721	1.36917	2.1
2722	0.94613	2.1
2723	1.29062	2.1

2724	0.947921	2.1
2725	1.58661	1.85319
2726	1.37658	1.17951
2727	1.2364	1.20196
2728	1.66685	1.03468
2729	1.66685	1.30742
2730	0.946133	0.985448
2731	0.946133	1.06847
2732	1.11978	1.175
2733	1.09194	1.02309
2734	1.21858	1.46111
2735	1.37658	1.23949
2736	1.64903	1.13834
2737	1.64903	1.67828
2738	0.946132	1.12904
2739	0.946132	1.21689
2740	1.05496	1.26045
2741	1.22851	1.09193
2742	1.22852	1.45823
2743	1.58482	0.964179
2744	1.60578	0.0786799
2745	1.06336	0.0768049
2746	1.68062	0.083202
2747	1.14721	0.0613393
2748	1.11924	0.05
2749	1.18421	0.05
2750	2.01276	0.05
2751	1.23502	1.76781
2752	1.34926	1.79863
2753	0.546751	1.58354
2754	0.790446	1.784
2755	1.04072	1.60436
2756	0.555868	1.56303
2757	0.930305	1.5793
2758	1.09344	1.56567
2759	1.18351	1.6379
2760	1.97215	1.59593
2761	1.07416	1.70862
2762	1.33204	1.51642
2763	1.22038	1.42557
2764	0.597449	1.32495
2765	0.821422	1.51421
2766	1.04071	1.44049
2767	0.608026	1.30124
2768	0.929427	1.4106
2769	1.64929	0.05
2770	1.68541	0.05
2771	0.496093	0.05
2772	1.3385	0.05
2773	0.790798	0.05
2774	1.22971	0.05
2775	0.898853	0.05
2776	2.14361	0.05
2777	2.10813	0.05
2778	1.05417	0.05
2779	1.80768	0.05
2780	1.09725	2.1
2781	1.41131	2.1

2782	1.1656	2.1
2783	0.946129	2.1
2784	1.68365	2.1
2785	1.68365	2.1
2786	1.1656	2.1
2787	0.94613	2.1
2788	1.21269	0.05
2789	1.48867	0.05
2790	1.27806	0.05
2791	1.68264	0.05
2792	1.37813	1.55835
2793	1.50743	1.4326
2794	1.04121	1.18952
2795	1.12434	0.310959
2796	1.39553	0.370023
2797	1.44721	1.65824
2798	0.946132	0.635629
2799	1.23943	0.521015
2800	1.05292	0.785983
2801	1.28607	0.293397
2802	1.16915	0.521028
2803	1.76306	1.99382
2804	1.42266	1.89061
2805	1.36844	2.1
2806	1.36844	2.1
2807	1.67352	2.1
2808	1.11256	1.44295
2809	1.03217	1.1914
2810	1.18422	0.05
2811	1.04159	0.05
2812	1.60139	0.05
2813	1.04159	0.05
2814	0.988805	2.1
2815	1.05481	2.1
2816	1.52938	1.94299
2817	1.94724	1.77788
2818	0.896336	0.0740065
2819	0.911484	0.0772657
2820	1.18315	0.05
2821	1.10515	0.0573025
2822	1.04067	0.05
2823	1.12758	0.0677022
2824	0.904106	0.0654837
2825	1.6901	0.05
2826	1.68671	0.05
2827	1.99908	0.05
2828	0.772426	0.376865
2829	0.810945	1.46493
2830	0.667562	0.898833
2831	0.496617	0.246941
2832	0.847689	0.589364
2833	0.914378	1.08555
2834	0.934003	0.541193
2835	1.07459	0.486273
2836	1.40225	2.1
2837	1.07043	2.1
2838	0.94613	2.1
2839	1.63072	2.1

2840	1.63072	2.1
2841	1.07043	2.1
2842	0.946131	2.1
2843	1.53082	2.1
2844	1.69916	2.1
2845	1.75211	1.02215
2846	1.96831	2.1
2847	1.75211	2.1
2848	0.946127	0.854283
2849	0.817065	0.756449
2850	1.41798	0.682942
2851	1.41798	0.360261
2852	0.791656	0.946601
2853	0.791656	0.955526
2854	1.41798	0.704512
2855	1.58331	0.457549
2856	1.41776	0.363667
2857	0.791647	0.394528
2858	1.4512	0.314465
2859	1.45119	0.316104
2860	0.75821	0.501641
2861	0.758248	0.440061
2862	0.946104	0.414044
2863	1.36482	0.320149
2864	1.29389	1.93138
2865	1.04164	1.8651
2866	1.40574	1.76301
2867	1.64833	1.79901
2868	1.71183	0.128326
2869	1.71183	0.17183
2870	0.946131	0.349908
2871	1.35721	0.222481
2872	0.946131	0.896309
2873	0.946131	0.408944
2874	1.30076	0.375335
2875	1.2474	0.127488
2876	2.85225	0.279677
2877	2.71441	0.130017
2878	1.2474	0.0995907
2879	1.38622	0.196564
2880	0.858359	0.0861083
2881	1.3682	0.112953
2882	1.57364	0.097989
2883	0.729695	0.208962
2884	0.729723	0.0912406
2885	0.822611	0.502952
2886	0.946133	0.221382
2887	0.729723	0.138302
2888	0.974704	0.36682
2889	0.974702	0.46977
2890	1.81603	2.1
2891	1.96348	2.1
2892	1.43612	1.25273
2893	1.43612	1.33332
2894	0.634295	1.21555
2895	0.93796	1.35089
2896	0.946133	1.29218
2897	0.634326	1.17929

2898	0.946133	1.25507
2899	1.2654	1.11053
2900	1.26865	1.20814
2901	2.2649	1.22936
2902	1.00864	1.20265
2903	1.09689	0.938609
2904	0.832021	1.10053
2905	1.53229	0.710225
2906	1.1145	0.883972
2907	0.929583	1.05023
2908	1.613	0.68611
2909	0.644729	1.01492
2910	1.03471	2.1
2911	2.7739	2.1
2912	1.36133	2.1
2913	2.50215	2.1
2914	2.52195	2.1
2915	1.0465	2.1
2916	1.42673	2.1
2917	1.42673	2.1
2918	0.946132	2.1
2919	1.31904	2.1
2920	0.986499	2.1
2921	0.946132	2.1
2922	1.31904	2.1
2923	0.966978	2.1
2924	1.42237	0.05
2925	2.60925	0.0550048
2926	0.753659	0.073386
2927	1.77241	0.05
2928	1.77241	0.0582443
2929	0.946129	0.0772278
2930	0.75366	0.05
2931	1.22144	0.0692291
2932	0.94613	0.0703375
2933	1.50732	0.0503681
2934	1.18514	0.0643839
2935	0.902187	0.0729804
2936	1.89858	0.05
2937	1.24294	0.0546762
2938	1.67211	0.0619299
2939	1.41786	0.0502562
2940	0.810959	0.0687638
2941	1.50732	0.0552511
2942	2.44288	0.0670272
2943	1.40969	0.05
2944	2.06654	0.05
2945	1.40969	0.05
2946	1.40882	0.05
2947	1.94063	0.0555375
2948	0.867921	0.05
2949	1.29289	0.05
2950	0.867918	0.05
2951	0.946128	0.05
2952	0.867944	0.05
2953	1.66589	0.05
2954	1.66589	0.05
2955	0.968595	2.1

2956	1.5534	2.1
2957	1.27793	2.1
2958	1.13433	2.1
2959	1.3439	0.05
2960	2.53709	0.05
2961	0.571998	0.05
2962	0.64837	0.05
2963	1.99755	0.05
2964	1.99854	0.05
2965	0.764229	2.1
2966	1.61842	2.1
2967	1.61842	2.1
2968	1.18315	2.1
2969	1.1995	2.1
2970	1.18315	2.1
2971	0.764256	2.1
2972	1.52364	2.1
2973	2.36631	2.1
2974	0.641912	2.09861
2975	1.7181	2.1
2976	1.459 2.1	
2977	0.634845	1.99135
2978	1.18315	2.1
2979	1.17615	2.1
2980	0.571367	2.1
2981	1.3324	0.145553
2982	0.798214	0.146331
2983	0.946128	0.156959
2984	0.627252	0.131328
2985	1.50337	0.136039
2986	1.50337	0.102409
2987	0.62725	0.160376
2988	1.07763	0.091905
2989	1.07763	0.127666
2990	2.16109	0.143545
2991	1.07763	0.118083
2992	1.12486	2.1
2993	1.11827	2.1
2994	1.0491	2.1
2995	1.09034	2.1
2996	1.09431	2.1
2997	0.946131	2.1
2998	0.748607	2.1
2999	1.44002	2.1
3000	1.44002	2.1
3001	0.748635	2.1
3002	0.782505	2.1
3003	1.40615	2.1
3004	1.19539	2.1
3005	1.29389	1.81869
3006	1.4867	1.59629
3007	1.50103	0.05
3008	1.63025	0.05
3009	2.72109	0.05
3010	0.915755	0.05
3011	0.946132	0.05
3012	0.915755	0.05
3013	1.70236	0.05

3014	1.70236	0.05
3015	1.26155	0.05
3016	1.35657	0.05
3017	1.83151	0.05
3018	1.83151	0.05
3019	2.52309	0.05
3020	0.983607	2.1
3021	1.06838	0.63032
3022	0.983609	2.1
3023	0.983596	2.05745
3024	2.74384	0.0877956
3025	1.6261	0.0703611
3026	1.15477	2.1
3027	1.02136	2.1
3028	1.83561	2.1
3029	1.83561	2.1
3030	0.946131	2.1
3031	1.39523	2.1
3032	1.29313	2.1
3033	1.29313	2.1
3034	1.38651	2.1
3035	1.06313	2.1
3036	0.974888	2.1
3037	1.0894	2.1
3038	0.974888	2.1
3039	1.82282	2.1
3040	1.82282	2.1
3041	0.946131	2.1
3042	1.37372	2.1
3043	1.32374	2.1
3044	1.32374	2.1
3045	1.39523	2.1
3046	1.40882	0.0521439
3047	0.861998	0.0576561
3048	1.24448	0.0633975
3049	0.861999	0.0566192
3050	0.946129	0.0693914
3051	0.862011	0.0718306
3052	1.67537	0.0631928
3053	1.67537	0.0503115
3054	1.72399	0.05
3055	2.58579	0.05
3056	1.72399	0.05
3057	1.50951	0.05
3058	2.48897	0.051636
3059	1.26755	2.1
3060	1.03824	2.1
3061	1.27875	2.1
3062	1.48901	2.1
3063	1.18902	2.1
3064	1.11764	2.1
3065	1.87436	2.1
3066	0.95345	2.07953
3067	0.724191	1.83131
3068	0.86668	1.80535
3069	0.811523	1.92615
3070	1.04074	1.63291
3071	0.843097	1.71593

3072	0.833434	1.86828
3073	0.965727	1.7812
3074	0.85346	1.80618
3075	1.00185	1.38248
3076	1.00184	1.7037
3077	0.980651	1.86662
3078	0.980664	1.71346
3079	0.475737	1.55758
3080	0.719696	1.55362
3081	0.946109	1.43913
3082	0.946116	1.47383
3083	0.719693	1.5098
3084	0.946117	1.48591
3085	1.14879	1.78158
3086	0.719683	1.67612
3087	0.900977	1.52633
3088	0.972364	1.54929
3089	1.22961	1.5806
3090	0.405384	1.48356
3091	1.34406	2.1
3092	1.2187	2.1
3093	0.963571	0.359523
3094	0.946131	0.922459
3095	1.57246	1.60918
3096	1.57246	2.1
3097	0.946131	1.5741
3098	1.1235	2.1
3099	1.1235	2.09372
3100	1.07439	2.1
3101	1.19612	1.62492
3102	1.24844	1.62911
3103	1.00007	1.94318
3104	1.02429	2.1
3105	1.10459	2.1
3106	1.06724	0.192062
3107	0.67519	0.456986
3108	0.49762	0.226482
3109	1.43435	0.16259
3110	0.833315	0.323776
3111	0.58135	0.25229
3112	1.14749	1.01756
3113	1.85091	1.75746
3114	1.2802	0.569101
3115	1.27737	1.58248
3116	2.3655	1.1677
3117	1.30584	2.1
3118	2.06424	2.1
3119	0.946131	0.0523352
3120	1.04257	0.0572573
3121	0.94613	0.0909959
3122	1.67201	0.0575081
3123	1.67201	0.05
3124	1.27696	0.0503172
3125	1.34118	0.05
3126	1.3413	0.0744022
3127	2.36238	0.0656284
3128	0.6036	0.10032
3129	1.11745	0.11263

3130	1.46126	0.0853772
3131	1.39612	0.0702255
3132	0.670678	0.102238
3133	1.03402	0.111502
3134	1.11271	0.0979604
3135	0.838028	0.0691242
3136	0.807019	0.0905093
3137	1.27787	0.0508391
3138	0.994705	0.0652929
3139	1.20014	0.055053
3140	0.964793	0.0852163
3141	0.863868	0.0723949
3142	1.3413	0.0541752
3143	1.79549	0.0654035
3144	1.35877	2.1
3145	1.63648	2.1
3146	1.08171	2.1
3147	1.27839	2.1
3148	1.52601	2.1
3149	1.27841	2.1
3150	1.71255	2.1
3151	2.16307	2.1
3152	1.91592	2.1
3153	1.78508	2.1
3154	0.946128	2.1
3155	1.41199	2.1
3156	0.946128	2.1
3157	0.901088	2.1
3158	1.55244	2.1
3159	1.59475	2.1
3160	1.40921	2.1
3161	0.960106	2.1
3162	1.40921	2.1
3163	2.0073	2.1
3164	2.2973	0.11078
3165	1.90705	0.112553
3166	1.53671	0.0688955
3167	1.86035	0.0679666
3168	1.33747	2.1
3169	1.14007	2.1
3170	1.3212	2.1
3171	1.66867	0.0538676
3172	1.30203	0.05
3173	1.27696	2.1
3174	1.05025	2.1
3175	1.05025	2.1
3176	1.3704	2.1
3177	1.15677	2.1
3178	0.946129	2.1
3179	1.65879	2.1
3180	1.65879	2.1
3181	1.15676	2.1
3182	0.94613	2.1
3183	0.867217	1.51224
3184	1.28684	1.50967
3185	1.58866	1.4614
3186	1.58866	1.37052
3187	0.867246	1.67304

3188	1.28684	1.47484
3189	1.16906	1.49534
3190	1.13438	1.45221
3191	1.37224	1.50002
3192	1.61519	1.55532
3193	0.883939	1.68068
3194	1.40092	1.61248
3195	1.80383	1.74921
3196	1.80383	1.61119
3197	0.883935	1.74983
3198	1.40092	1.59119
3199	1.28684	1.64895
3200	1.76788	1.47629
3201	2.57368	1.52026
3202	1.37224	1.73253
3203	1.65411	0.555208
3204	1.77042	2.1
3205	1.77042	2.1
3206	1.90446	2.1
3207	1.28667	2.1
3208	1.55635	2.1
3209	1.30646	0.601796
3210	2.31836	0.125631
3211	1.30645	0.34667
3212	1.86429	0.744691
3213	1.48676	0.0711467
3214	0.743379	0.079286
3215	1.41848	0.0835076
3216	1.41848	0.076744
3217	0.946131	0.0786251
3218	0.812649	0.0670956
3219	1.34921	0.0819601
3220	0.812649	0.0751774
3221	0.949366	0.0758694
3222	1.32974	0.0694782
3223	0.794204	0.05
3224	0.634352	2.1
3225	0.838025	2.1
3226	1.08802	2.08678
3227	0.838038	2.0324
3228	1.18314	2.1
3229	1.12495	2.1
3230	1.22774	2.1
3231	1.04343	2.1
3232	0.921111	0.05
3233	2.1609	0.05
3234	1.09987	0.05
3235	2.5043	0.05
3236	1.05492	0.05
3237	0.590344	0.05
3238	1.05492	0.05
3239	1.25215	0.05
3240	1.71674	0.05
3241	1.71674	0.05
3242	0.590331	0.05
3243	1.17078	0.05
3244	0.625542	0.05
3245	0.724673	2.1

3246	1.08011	2.1
3247	1.87598	2.1
3248	1.50738	2.1
3249	1.44951	0.105045
3250	1.50102	0.0983449
3251	1.8042	0.46297
3252	0.687769	1.35894
3253	1.66483	0.735405
3254	1.66483	1.18376
3255	0.946132	1.32672
3256	0.687798	0.784025
3257	1.20885	1.3246
3258	0.687799	1.14287
3259	0.886063	0.180446
3260	0.798751	0.222335
3261	0.584813	0.183017
3262	1.49483	0.118844
3263	0.888005	0.150632
3264	1.03589	0.124275
3265	1.70435	0.189484
3266	0.710414	2.1
3267	0.917609	2.00654
3268	0.810947	2.00193
3269	0.81096	1.92909
3270	1.48658	1.82921
3271	1.53653	1.88703
3272	1.19329	2.03247
3273	0.946153	1.87214
3274	1.50798	1.93221
3275	1.85017	2.1
3276	1.19768	2.1
3277	0.864304	2.1
3278	0.792906	2.01647
3279	0.989025	1.87068
3280	1.19768	1.78478
3281	1.54548	1.858
3282	1.18798	2.0376
3283	1.31875	1.77378
3284	1.05725	1.87267
3285	2.17365	0.05
3286	2.48106	0.05
3287	1.87451	0.05
3288	1.27189	1.53259
3289	2.79399	1.81535
3290	0.859565	1.50934
3291	0.926174	1.47119
3292	0.604744	1.47393
3293	1.41832	1.02918
3294	1.05494	1.40629
3295	0.874749	1.39045
3296	1.50067	1.32266
3297	0.917817	1.65283
3298	0.63591	1.70754
3299	1.72849	1.69484
3300	1.72849	1.50477
3301	0.635943	1.61252
3302	1.39699	1.70212
3303	0.967411	1.95062

3304	0.635943	1.57469
3305	1.39908	1.52628
3306	1.26971	1.62131
3307	1.02969	1.69481
3308	1.15326	1.89451
3309	2.30741	1.66267
3310	0.93545	1.50219
3311	0.802747	1.46597
3312	1.15466	1.59246
3313	0.859164	1.61688
3314	1.02616	1.67709
3315	1.23149	1.54787
3316	0.83804	1.53657
3317	1.13266	1.89202
3318	1.64142	1.73618
3319	1.21223	2.1
3320	1.43051	2.1
3321	1.32418	1.58024
3322	0.621319	1.97858
3323	1.47134	2.07611
3324	1.79103	0.957725
3325	3.25951	0.05
3326	3.16764	0.05
3327	1.65583	0.114644
3328	1.4732	0.05
3329	1.76476	2.1
3330	1.80114	2.1
3331	0.754121	2.1
3332	0.94613	1.29876
3333	0.754119	2.1
3334	1.45636	2.09192
3335	1.45636	0.804427
3336	1.30029	1.96151
3337	0.910183	1.82914
3338	1.29099	2.1
3339	1.08039	2.1
3340	1.05498	2.1
3341	1.24445	2.1
3342	1.2873	2.1
3343	0.987703	2.1
3344	0.964782	2.1
3345	1.73645	0.05
3346	0.871279	0.0661486
3347	0.884744	0.0752783
3348	1.85193	0.05
3349	1.68681	0.0550042
3350	1.33689	0.0632215
3351	1.23493	0.05
3352	0.892482	0.0704766
3353	2.95185	0.05
3354	2.10496	0.05
3355	1.87334	2.1
3356	0.94411	2.1
3357	0.754196	2.1
3358	1.43122	2.1
3359	1.58684	2.1
3360	0.838027	2.1
3361	1.1536	2.1

3362	1.24878	2.1
3363	1.23125	2.1
3364	2.86862	2.1
3365	1.57636	2.1
3366	0.998451	0.325191
3367	1.5176	0.259858
3368	1.13271	0.05
3369	1.60248	0.05
3370	1.22083	2.1
3371	1.79996	2.1
3372	1.19087	2.1
3373	2.00342	2.1
3374	1.3715	2.1
3375	2.79227	2.1
3376	2.7873	2.1
3377	0.987265	0.05
3378	1.62715	2.1
3379	1.70117	2.1
3380	1.27806	0.05
3381	1.45246	2.1
3382	1.45244	2.1
3383	1.87214	2.1
3384	0.995796	2.1
3385	1.68189	1.25716
3386	1.49155	2.1
3387	1.58054	2.1
3388	1.59169	2.1
3389	0.736364	0.05
3390	0.70236	0.05
3391	2.24367	0.05
3392	2.57085	0.05
3393	1.70952	0.05
3394	2.60516	0.05
3395	1.45158	2.1
3396	1.00371	2.1
3397	1.65748	2.1
3398	1.23706	0.104403
3399	1.55834	0.989033
3400	1.72671	1.01944
3401	0.597656	0.837081
3402	1.39137	1.14195
3403	0.855578	0.101141
3404	1.48398	0.463822
3405	0.745117	0.114707
3406	1.23706	0.394291
3407	1.57916	0.639819
3408	0.893025	0.18277
3409	3.22433	0.292142
3410	2.47324	1.79968
3411	1.63852	2.1
3412	1.63852	2.1
3413	0.946132	2.1
3414	1.19095	2.1
3415	0.946133	2.1
3416	1.16684	2.1
3417	1.19095	2.1
3418	1.06698	0.117159
3419	1.48692	0.0904069

3420	1.08334	1.96875
3421	1.07283	2.1
3422	1.28517	1.07473
3423	1.2746	0.05
3424	1.30723	0.05
3425	2.02069	0.05
3426	1.05579	0.05
3427	1.59475	2.1
3428	2.40113	2.1
3429	1.58875	2.1
3430	1.6881	2.1
3431	1.6881	2.1
3432	0.819953	2.1
3433	0.819954	2.1
3434	0.946131	2.1
3435	0.946131	2.1
3436	1.34782	2.1
3437	1.65619	2.1
3438	1.65619	2.06067
3439	0.79437	2.1
3440	1.25	2.1
3441	0.94613	2.1
3442	0.946131	2.06908
3443	0.794371	2.1
3444	1.58875	2.1
3445	2.5	2.1
3446	1.04147	2.1
3447	0.946131	1.41619
3448	1.79686	2.1
3449	1.79686	2.1
3450	1.25144	1.39648
3451	1.35714	2.05696
3452	1.38585	1.8323
3453	1.25144	2.08365
3454	1.15564	2.1
3455	1.34389	2.1
3456	0.946133	1.34956
3457	1.74353	1.98083
3458	1.74353	2.1
3459	1.14553	1.32892
3460	1.38585	1.73858
3461	1.30382	1.51312
3462	1.14553	1.80763
3463	1.15564	2.1
3464	1.90618	2.1
3465	2.11056	2.1
3466	1.032	2.1
3467	0.953636	2.1
3468	1.23131	1.61671
3469	1.39763	1.44173
3470	2.01024	1.14178
3471	1.9186	1.16097
3472	1.83658	0.0735513
3473	1.81656	0.0788516
3474	2.2049	0.093469
3475	2.16081	0.0847436
3476	1.12219	0.107811
3477	0.599771	0.124135

3478	1.61401	0.0812459
3479	1.05912	0.0998817
3480	0.951648	0.104729
3481	1.89328	0.0839837
3482	0.913682	0.118471
3483	1.00763	0.082695
3484	1.29129	2.1
3485	1.37138	2.1
3486	1.41986	0.05
3487	1.41986	0.05
3488	0.944166	0.05
3489	1.41789	0.05
3490	0.944167	0.0568505
3491	0.944166	0.05
3492	0.946132	0.05
3493	1.98713	0.05
3494	1.23626	0.05
3495	0.932791	0.05
3496	0.810251	0.05
3497	0.986748	0.05
3498	0.898155	0.05
3499	0.92624	0.05
3500	1.1349	0.05
3501	0.934572	0.05
3502	1.23104	0.742907
3503	1.23104	1.02444
3504	0.799994	0.398313
3505	0.94613	0.703179
3506	0.802822	0.345745
3507	1.40944	0.820303
3508	0.800035	0.678483
3509	1.41223	0.669276
3510	1.41223	0.513392
3511	1.6	0.366947
3512	1.60565	0.387613
3513	1.23042	2.1
3514	1.0645	2.1
3515	1.00319	2.1
3516	1.29435	2.1
3517	1.2243	2.1
3518	1.2243	2.1
3519	1.05655	1.72531
3520	1.29764	1.82364
3521	2.55252	2.1
3522	1.15676	2.1
3523	0.855295	2.1
3524	0.810959	2.1
3525	1.65857	2.1
3526	1.00849	2.1
3527	1.16021	2.1
3528	1.75869	2.1
3529	1.13775	2.1
3530	1.20091	2.1
3531	1.61582	2.1
3532	1.88031	2.1
3533	0.968536	2.1
3534	0.946131	2.1
3535	1.61481	2.1

3536	1.61481	2.1
3537	1.09547	2.1
3538	1.18486	2.1
3539	1.37609	2.1
3540	1.09546	2.1
3541	1.02461	2.1
3542	1.83223	2.1
3543	1.74002	2.1
3544	1.70939	2.1
3545	1.7094	2.1
3546	0.726287	2.1
3547	1.01712	2.1
3548	1.30318	2.1
3549	0.946131	2.1
3550	0.87155	2.1
3551	1.77093	1.96347
3552	2.02356	1.94362
3553	1.32449	2.1
3554	1.10003	2.1
3555	1.5161	2.1
3556	1.5161	2.1
3557	0.946132	2.1
3558	1.09206	2.1
3559	0.946132	2.1
3560	1.04404	2.1
3561	1.09206	2.1
3562	0.635628	1.43621
3563	1.14623	0.235499
3564	1.73197	1.25629
3565	1.73197	1.45139
3566	0.529655	0.591376
3567	1.14623	0.689454
3568	0.992085	0.562016
3569	1.16915	1.46687
3570	2.29245	0.258738
3571	0.584573	0.566647
3572	0.956756	0.159391
3573	1.52471	1.05215
3574	1.51409	1.5509
3575	0.584575	1.05681
3576	0.94613	0.147523
3577	1.14622	0.23758
3578	1.28096	1.49844
3579	1.34695	1.58788
3580	1.04193	2.1
3581	1.05378	2.1
3582	0.798209	2.1
3583	1.32007	2.09515
3584	1.64327	0.05
3585	2.64674	1.32864
3586	2.38936	0.05
3587	2.88475	0.05
3588	1.07745	2.1
3589	1.08911	2.1
3590	1.49127	2.1
3591	1.49127	2.1
3592	0.709714	2.1
3593	1.37199	2.1

3594	0.82899	2.1
3595	0.709714	2.1
3596	0.946132	2.1
3597	1.00133	2.1
3598	1.40182	2.1
3599	1.53278	2.1
3600	1.40182	2.1
3601	1.31764	0.05
3602	1.00691	0.05
3603	1.24094	0.05
3604	0.64868	0.05
3605	0.946133	0.05
3606	0.64868	0.05
3607	1.71762	0.05
3608	1.71762	0.05
3609	1.41662	0.05
3610	0.975477	0.05
3611	1.73584	0.05
3612	2.48188	0.05
3613	2.2045	0.36036
3614	2.08134	0.308674
3615	0.993366	1.62035
3616	0.993368	1.68629
3617	1.14187	0.291574
3618	0.946132	0.575579
3619	0.946133	1.3442
3620	1.68951	1.39164
3621	1.68951	1.47529
3622	1.34612	0.882456
3623	1.28953	0.384113
3624	1.34611	1.79001
3625	1.31892	1.79519
3626	0.946131	1.56292
3627	1.80988	1.77439
3628	1.80988	2.1
3629	1.31892	1.71256
3630	0.946131	1.74259
3631	2.13214	2.1
3632	1.94691	2.1
3633	1.52661	0.05
3634	1.53175	0.05
3635	0.956005	0.05
3636	0.955991	0.05
3637	1.40572	0.05
3638	0.955992	0.05
3639	1.51084	2.1
3640	1.67115	2.1
3641	1.73143	2.1
3642	1.55234	2.1
3643	1.69377	2.1
3644	2.51483	2.1
3645	1.25987	2.1
3646	1.4472	2.1
3647	1.83151	0.62098
3648	2.01001	1.23735
3649	0.993998	0.57979
3650	1.83151	0.38454
3651	1.40915	0.922623

3652	1.24653	0.948928
3653	1.74472	2.1
3654	1.74472	2.1
3655	1.74244	2.1
3656	1.04788	2.1
3657	1.28522	2.1
3658	1.03785	2.1
3659	0.896519	2.1
3660	1.60428	2.1
3661	1.73305	2.1
3662	1.39072	0.158426
3663	2.27501	0.305374
3664	1.39073	0.217825
3665	1.39073	0.244294
3666	1.06838	2.1
3667	0.968462	1.58509
3668	1.06837	1.79548
3669	1.13455	1.49346
3670	1.23527	1.35722
3671	2.71686	0.393681
3672	1.86881	0.628557
3673	1.10586	2.1
3674	1.03852	2.1
3675	1.03899	2.1
3676	0.963973	2.1
3677	1.00319	2.1
3678	1.00319	2.1
3679	0.987132	2.1
3680	0.974889	2.1
3681	0.987131	2.1
3682	1.7677	2.1
3683	1.7677	2.1
3684	0.946131	2.1
3685	1.31185	2.1
3686	0.946132	2.1
3687	1.30203	2.1
3688	1.31185	2.1
3689	1.82958	2.1
3690	1.82958	2.1
3691	0.946132	2.1
3692	1.40199	2.1
3693	0.946133	2.1
3694	1.36994	2.1
3695	1.40199	2.1
3696	0.97489	2.1
3697	1.05939	1.85507
3698	1.01922	1.89589
3699	0.810941	1.72343
3700	1.05372	1.98872
3701	0.754065	2.08793
3702	0.926131	1.91854
3703	1.35905	1.8184
3704	0.810931	1.7996
3705	1.75647	1.50313
3706	1.68265	2.1
3707	1.41995	2.1
3708	1.36457	1.62598
3709	0.933392	1.56742

3710	1.42255	2.1
3711	1.3868	2.1
3712	1.08074	2.1
3713	1.07196	2.1
3714	1.13537	1.01366
3715	1.01179	1.03926
3716	1.60196	0.355086
3717	1.72798	0.491736
3718	1.3715	0.568338
3719	1.56409	1.51473
3720	1.56409	0.139949
3721	1.97819	1.42381
3722	1.47954	1.2852
3723	0.798655	0.224168
3724	1.47952	0.674925
3725	2.30327	0.388789
3726	1.49111	2.1
3727	1.96013	2.07149
3728	1.54599	2.1
3729	2.06632	2.1
3730	1.54601	2.1
3731	1.10557	0.092543
3732	1.17624	0.196345
3733	1.60497	2.1
3734	1.61587	2.1
3735	1.65977	0.05
3736	1.69044	0.05
3737	1.00659	2.1
3738	1.06226	2.1
3739	1.15171	2.1
3740	1.50853	2.1
3741	1.80252	2.1
3742	1.41432	2.1
3743	1.04687	2.1
3744	0.940878	2.1
3745	1.52031	2.1
3746	1.52031	2.1
3747	0.940877	2.1
3748	0.946132	2.1
3749	1.50853	2.1
3750	0.963973	2.1
3751	1.70562	0.05
3752	2.43203	0.05
3753	1.36004	0.05
3754	1.06648	0.05
3755	1.66009	0.05
3756	1.66009	0.05
3757	0.766427	0.05
3758	0.766452	0.05
3759	0.946132	0.05
3760	1.98012	0.05
3761	1.20357	2.1
3762	1.20356	2.1
3763	1.67324	2.1
3764	1.52008	2.1
3765	1.19964	2.1
3766	1.36861	2.1
3767	1.19964	2.1

3768	2.85415	2.1
3769	1.43687	2.1
3770	0.935852	2.1
3771	1.09972	2.1
3772	0.810933	2.08674
3773	2.25974	2.1
3774	1.74149	2.1
3775	1.36984	2.1
3776	1.87602	2.1
3777	0.890748	2.1
3778	1.20587	1.94693
3779	0.810956	1.86136
3780	2.03174	1.93793
3781	1.34403	1.96094
3782	1.58825	1.95121
3783	1.63096	1.93898
3784	2.53735	1.99103
3785	1.98241	1.96635
3786	2.53014	2.1
3787	1.1775	1.9743
3788	1.06538	1.7499
3789	0.946132	1.25477
3790	1.4101	1.69698
3791	1.75162	2.05002
3792	1.75162	1.31965
3793	0.946132	1.49949
3794	1.4101	1.36641
3795	1.28765	1.36696
3796	1.1427	1.1054
3797	1.1427	1.24424
3798	1.3109	2.1
3799	1.02721	1.96112
3800	0.846447	1.90434
3801	0.862076	2.1
3802	0.644098	1.88511
3803	0.581308	1.97793
3804	1.02306	1.98087
3805	0.322641	1.10052
3806	0.589346	1.60206
3807	2.25572	1.83684
3808	1.31372	2.1
3809	1.18873	2.1
3810	1.78225	2.1
3811	1.764	2.1
3812	1.17503	2.1
3813	1.06821	2.1
3814	1.06821	1.90939
3815	1.7777	1.90666
3816	1.9096	0.571867
3817	1.70239	0.608214
3818	1.16182	0.05
3819	1.20132	0.05
3820	1.23475	2.1
3821	1.56859	2.1
3822	2.46263	2.1
3823	0.528744	2.1
3824	2.93337	2.1
3825	2.42504	2.1

3826	1.38646	0.257507
3827	1.61963	0.587505
3828	1.88016	0.188979
3829	0.622283	0.666382
3830	1.03243	0.588297
3831	0.710307	1.09722
3832	0.798124	0.92435
3833	0.931073	0.981952
3834	0.892862	0.954444
3835	1.06791	0.809056
3836	0.552787	0.583512
3837	0.946132	0.0751311
3838	1.36665	1.68931
3839	1.36665	0.830618
3840	0.506966	1.78431
3841	0.506964	1.2352
3842	1.36665	0.406428
3843	0.653469	0.749683
3844	1.36665	0.0567942
3845	1.21339	0.140383
3846	1.21339	1.0273
3847	0.552757	1.31454
3848	0.552786	0.348318
3849	1.21336	0.116302
3850	1.70744	1.40203
3851	1.0775	0.199657
3852	1.59527	0.61403
3853	1.48312	0.258636
3854	2.01868	0.214141
3855	1.18947	0.0803366
3856	1.48312	0.262316
3857	0.785626	0.102657
3858	1.19601	0.119836
3859	0.785626	0.116781
3860	0.946132	0.116993
3861	0.7856	0.174398
3862	1.59087	0.121137
3863	1.51158	0.114373
3864	1.74098	0.113135
3865	2.39202	0.111694
3866	0.870489	0.209476
3867	1.34534	0.259397
3868	0.870488	0.182478
3869	0.946133	0.269425
3870	0.870489	0.408768
3871	1.67086	0.274024
3872	1.67086	0.261002
3873	2.11311	2.1
3874	1.98765	1.89574
3875	0.946133	1.57778
3876	1.30881	1.95617
3877	1.72211	2.09485
3878	1.72211	1.69008
3879	0.94613	1.91121
3880	1.30881	1.73772
3881	1.35943	1.71755
3882	1.01294	0.432857
3883	1.01294	0.362605

3884	1.38963	2.1
3885	1.01294	0.763751
3886	1.60056	0.5528
3887	1.60056	1.99377
3888	0.946132	2.1
3889	1.204	1.66748
3890	0.946132	0.197106
3891	1.11288	1.08435
3892	1.204	0.680069
3893	1.14599	2.08588
3894	1.00065	2.00275
3895	1.74685	2.1
3896	1.74685	2.1
3897	0.946128	2.1
3898	1.38463	1.95359
3899	1.2275	2.1
3900	1.2275	2.1
3901	1.30835	2.1
3902	2.04407	2.1
3903	2.50425	2.1
3904	1.19647	0.565352
3905	1.19647	1.09451
3906	1.19645	1.11745
3907	1.75446	0.664518
3908	1.1315	1.13684
3909	1.1315	1.5548
3910	1.45569	0.78505
3911	1.1315	0.483287
3912	1.14357	2.1
3913	1.25793	2.1
3914	1.14357	2.1
3915	2.08181	2.0342
3916	1.4714	2.1
3917	0.71295	2.1
3918	1.68285	2.1
3919	1.68285	2.1
3920	1.25603	2.1
3921	1.13976	2.1
3922	1.25603	2.1
3923	0.71295	2.1
3924	1.61095	2.1
3925	2.51207	2.1
3926	1.63762	2.1
3927	2.74451	2.1
3928	0.671255	2.07424
3929	1.87601	2.1
3930	1.87601	2.1
3931	0.671226	2.02448
3932	1.25603	2.1
3933	1.29123	2.1
3934	0.671226	2.1
3935	1.63723	2.1
3936	0.872928	0.05
3937	1.47904	0.05
3938	1.18793	0.05
3939	2.57464	0.05
3940	1.14986	2.1
3941	1.19611	2.1

3942	0.96029	1.62757
3943	1.7961	2.1
3944	0.928721	2.1
3945	1.34352	2.1
3946	1.37935	2.1
3947	1.37935	2.1
3948	0.892888	2.1
3949	0.946131	2.1
3950	0.892888	2.1
3951	1.75422	2.1
3952	1.05556	2.1
3953	1.27362	0.251604
3954	1.27421	0.106031
3955	1.3003	1.14138
3956	1.31415	0.916752
3957	1.01431	2.1
3958	0.673951	2.1
3959	1.63384	2.1
3960	1.63661	2.1
3961	1.52245	2.1
3962	1.50714	2.1
3963	0.996778	0.05
3964	1.56091	0.05
3965	1.06543	2.1
3966	2.66689	2.1
3967	1.01474	2.1
3968	2.76422	2.1
3969	1.45321	0.103485
3970	0.974716	0.0661662
3971	0.810943	0.0771415
3972	0.909982	0.1426
3973	0.937793	0.087661
3974	1.05138	0.0683088
3975	1.25044	0.0701257
3976	0.92095	0.0782083
3977	0.830414	0.0994426
3978	2.1402	0.0839297
3979	1.75938	2.1
3980	1.05556	2.1
3981	1.78558	2.1
3982	1.379 2.1	
3983	0.970214	1.07778
3984	0.9702	1.06561
3985	1.08894	1.4124
3986	2.17251	1.30768
3987	1.51569	0.746
3988	1.06012	0.753721
3989	0.98966	0.564158
3990	1.55926	0.408275
3991	1.92645	0.491048
3992	1.37427	0.05
3993	2.16338	0.05
3994	1.5456	0.05
3995	1.5456	0.05
3996	1.94002	0.05
3997	1.33135	0.05
3998	1.31948	0.05
3999	2.23255	0.05

4000	2.34061	0.05
4001	1.52903	2.1
4002	3.38327	2.1
4003	1.35977	2.1
4004	2.22414	2.1
4005	1.6812	0.0740677
4006	2.33397	0.0768995
4007	1.16699	0.118479
4008	0.946131	0.108202
4009	1.58115	0.111508
4010	1.58115	0.106203
4011	0.519726	0.162033
4012	0.946131	0.131016
4013	0.519697	0.123911
4014	1.26868	0.0802689
4015	1.16699	0.0843261
4016	1.5951	0.070291
4017	1.5951	0.0667938
4018	0.840568	0.0967617
4019	0.946131	0.0833326
4020	0.840598	0.0799374
4021	1.30047	2.1
4022	1.5586	2.1
4023	1.31825	1.33907
4024	1.31924	1.17296
4025	1.15818	1.37032
4026	1.52782	1.1958
4027	1.15818	1.38881
4028	1.15818	1.48153
4029	0.99518	0.0500856
4030	0.94613	0.0784592
4031	0.922045	0.0747832
4032	1.29322	0.0601956
4033	1.29321	0.05
4034	1.29321	0.174583
4035	0.922056	0.05
4036	1.06848	0.0819934
4037	1.84409	0.0582899
4038	1.9186	1.292
4039	2.72244	1.15351
4040	0.946132	0.70323
4041	1.36122	0.729865
4042	1.79658	0.903751
4043	1.79658	0.549444
4044	0.946133	1.28532
4045	1.36122	1.02635
4046	1.38149	1.03363
4047	1.59106	0.527605
4048	0.946116	0.81066
4049	1.29052	0.546789
4050	1.76641	0.459375
4051	1.76641	0.480823
4052	0.885309	1.21658
4053	1.29054	0.844754
4054	1.36118	0.719168
4055	1.59103	1.21062
4056	2.27816	0.435746
4057	1.56164	0.540637

4058	1.41114	0.05
4059	2.04128	0.05
4060	0.986723	2.1
4061	1.03645	0.111684
4062	1.79224	0.115364
4063	1.2627	2.1
4064	1.23036	2.1
4065	1.45257	2.1
4066	1.41469	2.1
4067	1.41469	2.1
4068	2.60636	2.1
4069	1.45789	0.05
4070	0.851541	0.05
4071	1.57877	0.05
4072	1.53134	0.05
4073	0.851479	0.05
4074	1.08982	0.05
4075	0.716284	0.05
4076	0.946131	0.05
4077	1.73193	0.05
4078	2.57309	0.05
4079	1.36307	0.05
4080	2.42175	0.05
4081	1.72948	0.05
4082	0.978698	2.1
4083	0.978699	2.1
4084	2.08398	2.1
4085	0.978698	2.1
4086	1.44096	2.1
4087	1.44096	2.1
4088	0.638164	2.1
4089	0.681588	2.1
4090	0.946133	2.1
4091	0.638165	2.1
4092	0.94613	2.1
4093	1.55432	2.1
4094	0.901147	2.1
4095	0.85777	2.1
4096	1.36073	2.1
4097	1.44004	2.1
4098	0.946133	2.1
4099	1.09982	2.1
4100	1.27562	2.1
4101	1.55432	2.1
4102	1.62229	2.1
4103	1.0101	2.1
4104	2.28459	2.1
4105	2.50554	2.01867
4106	0.946131	1.6323
4107	1.25276	2.00836
4108	1.73721	2.0271
4109	1.73721	1.76754
4110	0.93406	1.91105
4111	1.25277	1.81044
4112	1.4185	1.77943
4113	1.53064	1.76149
4114	0.946133	1.80819
4115	0.609462	2.08909

4116	1.65123	2.08219
4117	1.65123	1.90345
4118	0.609466	2.09519
4119	1.00792	1.94999
4120	1.25277	1.90509
4121	1.53064	1.8234
4122	2.15223	1.65295
4123	1.30664	1.8995
4124	1.10674	2.1
4125	1.22862	2.1
4126	1.49358	0.108573
4127	2.0207	0.0823185
4128	2.09374	2.1
4129	1.88175	2.1
4130	1.10938	0.471908
4131	1.02478	1.35138
4132	1.13272	0.05
4133	1.13272	0.05
4134	1.03935	1.52818
4135	0.810945	1.62527
4136	0.527573	0.825177
4137	0.925011	1.51362
4138	1.03193	0.732314
4139	1.38587	0.880129
4140	0.703469	0.851053
4141	0.912282	1.50648
4142	1.75505	2.1
4143	1.78529	2.1
4144	1.60017	0.442907
4145	1.35608	0.152547
4146	1.01479	2.1
4147	1.47561	1.8811
4148	1.01479	2.1
4149	1.28418	1.89695
4150	2.62605	2.1
4151	1.71233	2.1
4152	3.22222	2.1
4153	1.97526	2.1
4154	0.949712	0.0950137
4155	1.78986	0.0794524
4156	2.72879	0.0729798
4157	2.66428	0.0780368
4158	1.68108	0.285007
4159	1.67265	0.206352
4160	2.24877	0.282535
4161	1.15558	0.340131
4162	1.39708	2.1
4163	1.39327	2.1
4164	1.69788	0.0644305
4165	1.1273	0.0502336
4166	1.17302	1.47511
4167	1.04349	0.841492
4168	0.979853	2.1
4169	1.19094	2.1
4170	1.39408	2.1
4171	1.61857	2.1
4172	1.66523	2.1
4173	0.946132	2.1

4174	0.838554	2.1
4175	1.05151	2.1
4176	1.18901	2.1
4177	1.18901	2.1
4178	1.61149	2.1
4179	1.22857	0.924293
4180	1.25848	0.951688
4181	1.09154	2.1
4182	2.20358	2.1
4183	1.23013	2.1
4184	1.55698	2.1
4185	1.55828	2.1
4186	1.52391	2.1
4187	0.60346	1.86957
4188	1.06336	2.1
4189	0.870714	1.80685
4190	0.543109	1.88359
4191	1.10367	1.82935
4192	1.20686	1.8385
4193	1.20684	2.06239
4194	2.5118	1.85282
4195	1.20684	2.1
4196	1.52468	1.8599
4197	1.52469	1.94043
4198	0.532308	1.73636
4199	0.903301	2.02618
4200	0.946119	1.68546
4201	0.532315	1.71079
4202	0.946107	1.73856
4203	1.46129	2.1
4204	0.804766	2.1
4205	1.4809	2.1
4206	1.4809	2.1
4207	1.11822	2.1
4208	1.16744	2.1
4209	1.11822	2.1
4210	0.804737	2.1
4211	1.04193	2.1
4212	0.983723	2.1
4213	1.8014	2.1
4214	1.1729	2.1
4215	1.03951	2.1
4216	1.03951	2.1
4217	1.17583	2.1
4218	1.42866	2.1
4219	1.78503	2.1
4220	1.17822	1.2057
4221	1.89121	2.1
4222	1.44472	2.1
4223	1.62143	2.1
4224	1.43837	2.1
4225	1.01121	0.0703674
4226	0.962703	1.41747
4227	0.946129	0.42957
4228	1.10941	0.253353
4229	0.946131	0.602457
4230	1.60326	0.568912
4231	1.60326	0.254364

4232	1.14215	0.383935
4233	1.40724	0.430413
4234	0.962703	0.487814
4235	1.53336	0.05
4236	1.40272	0.05
4237	1.64984	0.05
4238	1.67345	0.05
4239	1.08212	2.1
4240	1.40905	2.1
4241	0.946132	0.05
4242	0.946132	0.05
4243	0.81268	0.05
4244	0.946131	0.05
4245	0.812681	0.05
4246	0.946131	0.05
4247	0.946132	0.05
4248	0.997755	0.05
4249	1.94585	0.05
4250	1.2925	0.05
4251	2.52454	0.05
4252	1.69182	0.05
4253	1.69182	0.05
4254	0.92361	0.05
4255	1.35316	0.05
4256	1.26227	0.05
4257	0.946131	0.05
4258	1.35316	0.05
4259	1.48676	0.0953689
4260	1.33917	0.0829221
4261	0.757292	2.1
4262	1.02415	2.1
4263	1.48495	2.1
4264	0.97587	2.1
4265	0.946132	2.1
4266	0.747365	2.1
4267	1.55432	2.1
4268	1.55432	2.1
4269	0.946132	2.1
4270	0.747394	2.1
4271	1.17464	0.05
4272	1.0372	0.05
4273	2.16553	0.05
4274	2.00616	0.05
4275	1.12184	0.05
4276	1.33177	0.05
4277	0.936674	0.05
4278	0.946129	0.05
4279	0.851552	0.05
4280	1.60205	0.05
4281	1.77391	0.05
4282	1.03327	0.0631563
4283	1.41512	0.0692585
4284	1.03327	0.056215
4285	0.946133	0.0726822
4286	0.946131	0.0854247
4287	1.80077	0.071155
4288	1.80077	0.0626772
4289	1.54848	2.1

4290	1.29931	2.1
4291	1.04482	0.0994602
4292	2.08069	0.120843
4293	0.946127	0.0966029
4294	0.515539	0.129281
4295	0.515566	0.170485
4296	1.38423	0.0967218
4297	1.38423	0.0924122
4298	0.972058	0.127786
4299	0.927737	0.144167
4300	0.94971	0.0757178
4301	2.19682	0.0940703
4302	0.946132	0.137843
4303	1.09841	0.104612
4304	0.522382	0.206628
4305	1.5962	0.0869877
4306	1.72791	0.0742995
4307	1.07428	0.121604
4308	1.05215	0.114595
4309	1.54632	1.77808
4310	1.15693	1.81764
4311	1.41238	2.1
4312	1.41238	2.1
4313	1.77966	2.1
4314	1.77966	2.1
4315	0.946129	2.1
4316	1.40525	2.1
4317	0.946131	2.1
4318	0.94613	2.1
4319	1.32054	2.1
4320	1.41238	2.1
4321	1.94026	2.1
4322	1.0171	0.426968
4323	1.1211	0.353542
4324	0.993022	2.1
4325	0.773813	2.1
4326	1.4073	2.1
4327	1.4073	2.1
4328	0.94613	2.1
4329	0.787615	2.1
4330	0.94613	2.1
4331	0.787615	2.1
4332	1.764	2.1
4333	1.90964	2.1
4334	1.05787	2.1
4335	1.403	0.715339
4336	0.744189	0.983589
4337	2.32834	2.1
4338	2.34986	2.1
4339	1.17106	0.838491
4340	1.94826	0.05
4341	2.40606	0.05
4342	1.26387	0.0541437
4343	0.954013	0.057735
4344	0.946131	0.0639629
4345	1.71	0.05
4346	1.51926	0.05
4347	0.953997	0.05

4348	0.851554	0.0551959
4349	1.96502	0.05
4350	1.72954	0.0519899
4351	2.89117	0.05
4352	0.971958	0.0587257
4353	0.985575	0.0690702
4354	1.41943	0.05
4355	1.20021	0.05
4356	1.29599	0.0560327
4357	1.15687	0.05
4358	0.810958	0.0643165
4359	1.12919	0.05
4360	1.98859	2.1
4361	1.82044	2.1
4362	1.90488	2.1
4363	0.851542	2.1
4364	1.02973	2.1
4365	1.39286	2.1
4366	1.44873	2.1
4367	0.946132	2.1
4368	1.11826	2.1
4369	1.26842	2.1
4370	2.09461	2.1
4371	1.76952	0.05
4372	1.6473	0.05
4373	0.946131	0.05
4374	1.36486	0.05
4375	1.04243	0.05
4376	1.05017	0.05
4377	1.24059	0.05
4378	2.11646	0.05
4379	1.50368	0.05
4380	2.38796	0.05
4381	1.50368	0.05
4382	1.71844	0.05
4383	1.71844	0.05
4384	0.914836	0.05
4385	1.24416	0.05
4386	0.914805	0.05
4387	1.24416	0.05
4388	1.38911	0.05
4389	1.46859	0.05
4390	1.35348	0.05
4391	0.946131	0.05
4392	1.1226	0.05
4393	0.857078	0.05
4394	0.9746	0.05
4395	1.37624	0.05
4396	1.15131	0.05
4397	1.139	0.05
4398	1.90456	0.05
4399	2.09692	0.05
4400	1.16655	2.1
4401	1.23017	2.1
4402	2.14592	2.1
4403	1.23017	2.1
4404	1.0752	2.1
4405	1.52482	1.62245

4406	1.09252	2.1
4407	1.10725	2.1
4408	1.14305	0.05
4409	0.851541	0.05
4410	1.48073	0.05
4411	1.45607	0.05
4412	0.946131	0.0505116
4413	0.932823	0.05
4414	1.45828	0.05
4415	0.851528	0.05
4416	1.14305	0.05
4417	1.92737	0.05
4418	1.08211	0.05
4419	1.69831	0.05
4420	1.69831	0.05
4421	0.946131	0.05
4422	1.26868	0.05
4423	1.23377	0.05
4424	1.23377	0.05
4425	0.94723	0.05
4426	1.64322	0.05
4427	1.64322	0.05
4428	0.946131	1.66851
4429	1.32068	0.0592598
4430	0.946129	0.0592583
4431	1.18789	1.785
4432	1.32068	0.0909716
4433	1.32813	2.1
4434	1.32461	2.1
4435	1.42729	0.0511317
4436	2.47846	0.060604
4437	1.00024	0.106075
4438	1.23923	0.0637442
4439	0.946131	0.114234
4440	0.712304	0.05
4441	1.77501	0.0951317
4442	1.77501	0.05
4443	0.593593	0.155356
4444	1.23923	0.138265
4445	1.23175	0.095472
4446	0.94613	0.155972
4447	0.713646	0.102952
4448	1.75735	0.134209
4449	1.75735	0.0538247
4450	0.713633	0.217941
4451	1.37157	0.05
4452	1.47169	0.05
4453	2.21808	0.0714607
4454	1.47169	0.0578521
4455	1.04759	2.1
4456	0.987132	2.1
4457	1.11181	2.1
4458	2.61682	1.8504
4459	1.11181	2.1
4460	1.06461	1.63625
4461	1.69733	2.1
4462	2.79705	2.1
4463	1.08245	2.1

4464	1.40583	2.1
4465	1.39852	2.1
4466	2.0239	2.1
4467	2.0239	2.1
4468	0.780455	2.1
4469	0.946132	2.1
4470	0.780455	2.1
4471	1.01885	2.1
4472	0.564217	2.1
4473	0.733461	2.1
4474	0.537126	2.1
4475	0.683838	2.1
4476	0.779662	2.1
4477	0.634141	2.1
4478	0.685337	2.1
4479	1.58848	2.1
4480	1.17129	0.122506
4481	0.663168	0.203101
4482	1.55587	0.157688
4483	1.31342	0.150256
4484	0.596868	0.217597
4485	1.1666	0.216533
4486	0.867261	0.222077
4487	0.946132	0.240726
4488	1.28843	0.135609
4489	1.31248	0.189991
4490	2.59921	0.174666
4491	1.32634	0.13081
4492	1.35564	0.05
4493	1.52661	0.05
4494	1.12323	0.05
4495	1.17701	1.0803
4496	1.36405	0.941439
4497	1.19837	0.807649
4498	2.25636	0.693346
4499	1.0823	0.0708191
4500	1.31774	0.0664714
4501	1.0823	0.053104
4502	1.01121	0.0929095
4503	1.27408	0.0566205
4504	0.946131	0.250805
4505	0.566074	0.220678
4506	0.79576	0.229637
4507	1.24596	0.252425
4508	1.47567	0.252475
4509	1.47567	0.140627
4510	0.56605	0.229458
4511	1.6567	0.154126
4512	0.967792	0.144912
4513	1.00366	0.182324
4514	1.00366	0.174268
4515	1.56409	0.05
4516	1.56409	0.05
4517	0.774911	0.05
4518	1.09483	0.05
4519	0.774912	0.05
4520	1.09483	0.05
4521	1.24417	0.05

4522	1.13387	0.05
4523	1.13387	0.05
4524	1.30572	0.05
4525	2.18966	0.05
4526	0.98575	0.05
4527	1.54982	0.05
4528	2.48833	0.05
4529	1.54982	0.05
4530	1.28954	0.05
4531	1.64002	0.05
4532	1.64002	0.05
4533	1.85594	0.05
4534	1.12968	2.1
4535	1.23536	2.1
4536	1.4882	2.1
4537	1.4882	2.1
4538	0.876836	2.1
4539	0.946131	2.1
4540	0.876835	2.1
4541	1.43166	2.1
4542	2.25936	2.1
4543	1.0305	2.1
4544	0.992095	2.1
4545	1.12968	2.1
4546	1.53813	2.1
4547	1.53812	2.1
4548	0.583653	2.1
4549	0.94613	2.1
4550	0.583651	2.1
4551	0.964033	2.1
4552	0.964034	2.1
4553	1.54457	2.1
4554	3.54109	0.0578651
4555	0.99974	0.0805972
4556	0.78814	0.0913231
4557	1.65729	0.0603388
4558	1.1283	0.0713748
4559	1.09407	0.0796787
4560	1.55091	0.0618857
4561	0.870773	0.0842103
4562	1.19967	0.0623284
4563	1.56521	0.0703811
4564	1.30896	1.911
4565	1.17989	2.1
4566	1.72526	2.1
4567	0.851537	1.38506
4568	1.58944	2.07089
4569	1.56915	2.06232
4570	0.891039	1.37683
4571	1.4172	1.80337
4572	0.963818	1.65612
4573	0.851474	1.60252
4574	1.17989	2.1
4575	1.56261	2.1
4576	1.56261	2.1
4577	0.761574	2.1
4578	1.31323	2.1
4579	0.761573	2.1

4580	0.946133	2.1
4581	1.31323	2.1
4582	1.16759	2.1
4583	1.16759	2.1
4584	2.11829	2.1
4585	1.16759	2.1
4586	1.10748	0.05
4587	1.47688	0.05
4588	0.946133	0.05
4589	0.953319	0.0645861
4590	0.946132	0.05
4591	1.51168	0.05
4592	1.51168	0.0695579
4593	1.25797	0.05
4594	1.19984	0.0581127
4595	1.95729	2.1
4596	2.34853	2.1
4597	1.94343	2.1
4598	2.00551	2.1
4599	1.20067	1.04416
4600	1.87244	1.12818
4601	1.4178	2.1
4602	1.33538	2.1
4603	0.946131	2.1
4604	1.22895	2.1
4605	1.12433	2.1
4606	0.956757	2.1
4607	0.91826	2.1
4608	1.44221	2.1
4609	1.14206	2.1
4610	1.80514	0.810222
4611	0.890304	1.80703
4612	1.1362	0.778238
4613	1.32819	1.1107
4614	1.46311	2.0805
4615	0.8654	1.2007
4616	1.1699	1.645
4617	1.00267	1.58699
4618	1.02882	1.42695
4619	1.00832	2.1
4620	0.974137	2.1
4621	1.02368	2.1
4622	0.974138	2.1
4623	0.995734	1.99341
4624	1.44605	0.573148
4625	1.18549	0.554146
4626	1.33621	0.242503
4627	1.5176	0.359276
4628	1.91762	0.259563
4629	1.33621	0.236222
4630	1.6638	0.257185
4631	1.66774	2.1
4632	1.11833	2.1
4633	0.926499	2.1
4634	1.51229	2.1
4635	1.51229	2.1
4636	0.926528	2.1
4637	1.40335	2.1

4638	1.03544	2.1
4639	0.926527	2.1
4640	1.85305	2.1
4641	1.5812	2.1
4642	1.15529	2.1
4643	1.31086	2.1
4644	0.999206	2.1
4645	1.46714	2.1
4646	1.73087	2.1
4647	0.772299	2.1
4648	1.24341	2.1
4649	0.997867	2.1
4650	0.772311	2.1
4651	0.936657	2.1
4652	1.35986	2.1
4653	0.959414	2.1
4654	1.36541	2.1
4655	1.49268	2.1
4656	1.49268	2.1
4657	0.832137	2.1
4658	0.946132	2.1
4659	0.832138	2.1
4660	1.64523	2.1
4661	1.11764	2.1
4662	1.05776	2.1
4663	0.972465	2.1
4664	0.972464	2.1
4665	1.98425	2.1
4666	0.969822	2.1
4667	1.30566	2.1
4668	1.52724	2.1
4669	2.32237	0.109637
4670	2.30274	0.11828
4671	2.27761	0.05891
4672	3.33012	0.0619908
4673	1.24001	1.10962
4674	1.41821	1.01108
4675	0.978584	2.1
4676	1.18076	2.1
4677	1.07645	2.1
4678	0.877351	1.09471
4679	0.946132	2.1
4680	0.877353	1.87941
4681	1.58444	2.1
4682	1.49614	2.1
4683	1.28355	2.1
4684	1.13491	2.1
4685	1.56875	0.197522
4686	0.499592	0.05
4687	0.946131	0.05
4688	1.20538	0.05
4689	1.09913	0.05
4690	1.8049	0.05
4691	1.8049	0.05
4692	0.499606	0.05
4693	2.41076	0.05
4694	0.860202	0.05
4695	0.906798	0.05

4696	1.8626	0.05
4697	1.33986	0.05
4698	1.2992	0.05
4699	1.58702	0.05
4700	1.05939	0.05
4701	1.02074	2.1
4702	1.07866	2.1
4703	1.5082	0.0769645
4704	1.5522	0.0579149
4705	1.65903	0.118637
4706	1.86766	2.1
4707	2.11684	2.1
4708	0.946132	2.1
4709	1.6585	2.1
4710	1.6585	2.1
4711	0.94613	2.1
4712	1.1872	2.1
4713	1.1872	2.1
4714	1.05842	2.1
4715	1.20455	2.1
4716	1.34141	2.1
4717	1.34141	2.1
4718	1.42416	2.1
4719	1.75818	2.1
4720	2.63715	1.99007
4721	1.80454	2.1
4722	2.26198	2.1
4723	0.696255	2.1
4724	0.624075	2.1
4725	1.50905	1.0309
4726	1.50905	2.05872
4727	0.624075	2.1
4728	1.31857	0.879328
4729	0.946131	2.04823
4730	0.624075	1.97034
4731	0.914628	2.1
4732	1.14721	2.1
4733	1.16153	2.1
4734	1.47664	2.1
4735	1.54791	2.1
4736	1.19098	2.1
4737	1.13341	2.1
4738	1.00503	0.200969
4739	1.00503	1.28958
4740	1.22655	2.1
4741	0.999489	2.1
4742	1.3268	2.1
4743	0.946132	2.1
4744	1.60061	2.1
4745	1.60061	2.1
4746	1.08034	2.1
4747	1.40372	2.1
4748	1.14302	2.1
4749	1.08034	2.1
4750	0.999491	2.1
4751	1.17152	0.177109
4752	1.17152	0.12063
4753	1.17152	0.144562

4754	2.05423	0.151505
4755	1.40984	2.1
4756	1.2489	2.1
4757	0.885089	2.1
4758	0.810931	2.1
4759	1.84128	2.1
4760	1.11206	2.1
4761	1.21926	2.1
4762	1.99084	2.1
4763	0.976334	0.598183
4764	0.599363	0.54187
4765	1.02621	0.263275
4766	0.462959	0.437193
4767	1.42612	0.207218
4768	0.299682	0.340178
4769	1.51379	2.1
4770	0.719531	2.1
4771	1.3702	2.1
4772	1.33293	2.1
4773	0.851539	2.1
4774	0.62998	2.1
4775	0.851543	2.1
4776	0.629966	2.1
4777	1.43132	2.1
4778	1.13328	1.22819
4779	1.93212	1.08633
4780	1.62345	1.37303
4781	1.38511	1.49741
4782	1.40013	0.616217
4783	1.04132	0.534349
4784	0.83473	1.44166
4785	0.868732	1.22337
4786	0.810595	1.60304
4787	0.729919	1.18562
4788	0.992428	1.16794
4789	0.577107	0.945236
4790	1.18464	1.14415
4791	0.791199	1.2989
4792	0.810943	0.05
4793	0.860723	0.05
4794	1.33348	0.05
4795	1.57467	0.05
4796	0.981032	0.05
4797	0.731569	0.05
4798	0.770312	0.05
4799	1.46055	2.1
4800	1.42249	2.1
4801	1.75999	0.181164
4802	1.6868	0.22529
4803	1.17042	1.57981
4804	1.01485	2.1
4805	1.19952	0.05
4806	1.40592	0.05
4807	1.19952	0.05
4808	2.20145	0.05
4809	1.33649	0.270607
4810	1.11612	0.57602
4811	0.957588	0.392177

4812	0.946126	0.510866
4813	1.50648	0.160207
4814	1.50648	0.29714
4815	0.946128	0.366829
4816	2.10671	0.0635753
4817	1.91518	0.1489
4818	2.23223	0.132079
4819	1.53556	2.1
4820	1.56253	2.1
4821	1.27806	0.05
4822	1.44593	0.05
4823	1.03818	0.538281
4824	1.03818	0.451932
4825	1.38969	0.349296
4826	1.85548	0.153619
4827	2.6044	0.315757
4828	1.85548	0.152604
4829	1.49346	1.89001
4830	1.83192	1.84668
4831	0.834043	1.79523
4832	0.810943	1.41309
4833	0.887113	1.60303
4834	0.893827	1.48448
4835	0.991061	1.52643
4836	0.91939	1.6313
4837	0.793307	1.68466
4838	1.75291	1.65942
4839	1.35435	1.67104
4840	1.16989	0.196981
4841	1.36717	0.162351
4842	2.12276	0.0767665
4843	2.30493	0.118765
4844	2.47857	0.134251
4845	1.92269	0.0774931
4846	1.66426	0.10859
4847	0.956698	2.1
4848	0.956698	2.1
4849	0.857957	2.1
4850	1.4706	2.1
4851	1.65706	2.1
4852	1.47059	2.1
4853	1.04713	0.0732606
4854	0.946131	0.121125
4855	1.47374	0.122713
4856	1.47374	0.0807505
4857	0.951675	0.105692
4858	1.2582	0.132524
4859	1.2582	0.0902509
4860	0.94613	0.132369
4861	1.4468	0.05
4862	1.90335	0.07246
4863	1.14886	2.1
4864	2.59689	2.1
4865	1.02747	2.1
4866	0.946131	2.1
4867	0.934061	2.1
4868	1.63979	2.1
4869	1.65206	2.1

4870	1.48	2.1
4871	1.27467	2.1
4872	2.20449	2.1
4873	1.1333	0.05
4874	1.95334	2.1
4875	2.20984	2.1
4876	1.46389	0.05
4877	1.4639	0.05
4878	1.4639	0.0613773
4879	0.946132	0.0896954
4880	1.5144	0.0603576
4881	1.5144	0.0761993
4882	0.946132	0.0921714
4883	1.35732	0.0597807
4884	1.10321	0.0859854
4885	0.946132	0.0811758
4886	0.980549	0.0682296
4887	1.32819	2.1
4888	1.30171	1.70947
4889	1.68377	1.71519
4890	1.68377	2.1
4891	0.946132	1.03299
4892	0.946132	1.87916
4893	1.1952	1.9523
4894	0.990019	1.73953
4895	1.08195	1.83053
4896	1.2269	2.1
4897	1.19219	2.1
4898	1.01485	2.1
4899	1.57125	0.051739
4900	2.10696	0.0514536
4901	1.29666	0.05
4902	1.57125	0.0558355
4903	1.47712	2.1
4904	1.45182	2.1
4905	1.17816	0.05
4906	1.4516	0.05
4907	1.17816	0.05
4908	1.17816	0.05
4909	0.596954	1.02567
4910	1.65133	0.719605
4911	0.583859	0.511603
4912	1.04072	1.48249
4913	1.28795	0.533305
4914	1.59423	0.666805
4915	0.483559	0.653713
4916	1.74088	0.503783
4917	1.09545	1.49363
4918	1.02519	1.61457
4919	2.47579	1.14259
4920	1.54732	1.23046
4921	2.17663	1.34901
4922	1.75006	1.46075
4923	1.32354	1.45929
4924	1.09962	2.1
4925	1.85057	2.1
4926	1.91046	2.1
4927	2.17037	2.1

4928	1.99528	0.05
4929	1.97969	0.05
4930	3.82765	2.1
4931	1.85767	2.1
4932	2.77042	2.1
4933	1.37236	0.05
4934	2.23254	0.05
4935	1.10089	0.05
4936	0.994294	2.1
4937	1.81921	2.1
4938	1.00073	2.1
4939	0.681043	2.1
4940	0.946131	2.1
4941	0.681073	2.1
4942	1.51804	2.1
4943	1.60653	2.1
4944	1.30188	2.1
4945	0.919622	2.1
4946	1.00965	2.1
4947	1.93821	2.1
4948	2.60377	2.1
4949	1.36416	0.05
4950	1.36416	0.05
4951	1.36416	0.05
4952	2.44132	0.05
4953	2.15871	0.343383
4954	2.22171	0.239872
4955	1.70747	0.189827
4956	1.49515	0.150969
4957	0.773007	0.195642
4958	0.946125	0.222698
4959	0.946131	0.287159
4960	0.773034	0.147914
4961	1.15844	0.212507
4962	1.98104	0.137477
4963	2.44283	0.153169
4964	1.05417	0.05
4965	1.1596	0.05
4966	0.829921	0.05
4967	0.946131	0.05
4968	0.951911	0.05
4969	1.39027	0.05
4970	0.829946	0.05
4971	1.51223	0.05
4972	1.51223	0.05
4973	1.65984	0.05
4974	1.90382	0.05
4975	1.93228	1.28828
4976	2.47924	1.31169
4977	1.73904	1.30673
4978	1.40547	1.15608
4979	2.37589	1.15256
4980	0.662605	1.34995
4981	2.33293	0.90944
4982	1.72249	2.1
4983	1.68478	2.1
4984	1.00514	0.418273
4985	1.4537	2.1

4986	1.92698	2.1
4987	1.46041	0.05
4988	2.04817	0.05
4989	1.65954	2.1
4990	1.42342	2.1
4991	1.04405	0.05
4992	1.17056	0.05
4993	1.11339	0.05
4994	0.946133	0.05
4995	0.929724	0.05
4996	1.35424	0.05
4997	1.35424	0.05
4998	0.929713	0.05
4999	1.85945	0.05
5000	1.38905	2.1
5001	1.39056	2.1
5002	1.52852	2.1
5003	1.06472	2.1
5004	0.749096	2.1
5005	1.46076	2.1
5006	1.29263	2.1
5007	0.94613	2.1
5008	0.69097	2.1
5009	0.982284	2.1
5010	0.67419	2.1
5011	1.52852	2.1
5012	1.91121	2.1
5013	1.00775	2.1
5014	1.61201	2.09614
5015	0.529987	2.1
5016	0.506125	2.1
5017	1.44696	2.1
5018	2.32981	2.1
5019	1.32784	2.1
5020	1.94158	2.1
5021	1.31595	2.1
5022	0.663922	2.1
5023	1.44663	2.1
5024	1.44663	2.1
5025	1.13976	2.1
5026	0.970788	2.1
5027	1.13976	2.1
5028	0.663921	2.1
5029	0.966114	2.1
5030	1.05498	1.97487
5031	1.38086	2.1
5032	0.946127	2.1
5033	0.922513	2.1
5034	1.49903	2.1
5035	1.49903	2.1
5036	0.92251	2.1
5037	1.06105	2.1
5038	1.36049	2.1
5039	1.38086	2.1
5040	1.52534	2.1
5041	1.04101	2.1
5042	1.30861	0.05
5043	0.949112	0.05

5044	0.894275	0.05
5045	1.10232	0.05
5046	0.830889	0.05
5047	1.26554	0.05
5048	0.948347	0.05
5049	0.81096	0.05
5050	1.18338	0.05
5051	0.883764	0.05
5052	1.54793	0.05
5053	1.02867	0.05
5054	1.8573	0.05
5055	1.06065	0.05
5056	0.64842	0.05
5057	1.99319	0.05
5058	1.9476	0.05
5059	2.51436	0.05
5060	1.68337	0.05
5061	1.92321	0.05
5062	0.810942	0.05
5063	0.921001	0.05
5064	0.99758	0.05
5065	0.960785	0.05
5066	1.01565	0.05
5067	0.908624	0.05
5068	0.912835	0.05
5069	1.18351	0.05
5070	1.40239	2.1
5071	1.26091	2.1
5072	0.947979	2.1
5073	0.946131	2.1
5074	1.81959	2.1
5075	1.81959	2.1
5076	0.94613	2.1
5077	1.38415	2.04148
5078	1.38414	2.1
5079	1.33266	2.1
5080	0.946132	2.1
5081	1.76225	2.1
5082	1.76225	2.1
5083	1.27599	2.1
5084	1.38158	2.1
5085	1.3268	2.1
5086	1.27599	2.1
5087	0.947979	2.1
5088	1.0142	0.257742
5089	1.01422	0.198211
5090	1.11435	0.292924
5091	1.42186	1.40795
5092	1.24001	1.36663
5093	1.77342	1.27897
5094	1.42186	1.46431
5095	1.99817	1.34881
5096	1.27361	2.1
5097	1.68914	2.1
5098	1.30116	2.1
5099	1.32819	1.97187
5100	1.68322	2.01339
5101	1.68322	2.1

5102	0.94613	1.56993
5103	0.94613	2.1
5104	1.14666	2.1
5105	1.07612	1.98984
5106	1.31151	2.1
5107	1.22095	2.1
5108	1.57256	2.1
5109	1.6679	2.1
5110	0.946132	2.1
5111	0.946133	2.1
5112	1.13537	2.1
5113	1.07855	2.1
5114	1.07855	2.1
5115	1.43895	1.82829
5116	1.38481	0.05
5117	1.72385	0.05
5118	0.550441	0.05
5119	0.79807	0.05
5120	1.11627	0.05
5121	1.30349	0.05
5122	0.94613	0.05
5123	0.686176	0.05
5124	1.11627	0.05
5125	1.73363	0.05
5126	1.73363	0.05
5127	0.686139	0.05
5128	1.24932	0.05
5129	0.899582	0.05
5130	2.00472	0.05
5131	1.17121	0.05
5132	1.2874	0.05
5133	2.0194	0.05
5134	0.88601	0.05
5135	1.77409	0.05
5136	1.73293	0.05
5137	2.74045	0.05
5138	1.83787	0.05
5139	1.45448	0.060628
5140	1.27778	0.0553728
5141	1.1192	2.1
5142	1.25953	2.1
5143	1.93611	2.1
5144	1.92104	2.1
5145	1.15506	0.05
5146	1.15506	0.05
5147	1.23943	1.89953
5148	0.851543	2.1
5149	1.6061	2.1
5150	1.80058	2.1
5151	0.869471	2.1
5152	1.3931	2.1
5153	1.06454	2.1
5154	0.93667	2.1
5155	2.16916	2.1
5156	3.10246	2.1
5157	1.91539	2.1
5158	1.91539	2.06822
5159	0.620263	1.95913

5160	0.620265	2.1
5161	0.946132	1.94587
5162	0.620265	1.94655
5163	1.30226	1.88825
5164	1.87292	2.1
5165	1.18769	2.1
5166	2.60452	2.09654
5167	1.26574	2.1
5168	2.46679	2.1
5169	1.36459	2.1
5170	1.26865	1.78154
5171	1.1744	1.56536
5172	1.15841	0.288785
5173	1.02247	0.175533
5174	1.93649	2.1
5175	1.64718	2.1
5176	1.59654	2.1
5177	1.57823	2.1
5178	1.00665	0.0934371
5179	1.28239	0.104032
5180	0.887895	2.1
5181	1.22083	2.1
5182	1.42127	2.1
5183	1.07374	2.1
5184	1.00503	1.38867
5185	1.23082	1.34429
5186	2.78663	2.1
5187	2.20883	2.1
5188	2.54588	2.1
5189	0.946128	2.1
5190	1.13716	2.1
5191	1.58724	2.1
5192	1.58724	2.1
5193	0.946131	2.1
5194	1.13716	2.1
5195	1.39621	2.1
5196	1.02438	2.1
5197	1.49543	0.05
5198	2.78336	0.05
5199	1.27148	0.05
5200	1.0906	0.05
5201	1.04072	0.05
5202	0.63574	0.05
5203	1.45506	0.05
5204	1.5863	0.05
5205	0.928352	0.05
5206	0.647687	0.05
5207	2.2375	1.79727
5208	1.50998	1.76571
5209	2.75021	1.67329
5210	1.91912	1.49065
5211	1.99872	1.44749
5212	0.918127	1.73625
5213	1.03031	1.60791
5214	0.951169	1.48487
5215	1.27975	1.43887
5216	1.12735	1.53594
5217	1.15741	1.58053

5218	1.24998	1.59335
5219	1.65953	0.265629
5220	0.967555	0.399403
5221	0.967542	0.219456
5222	1.03824	0.152466
5223	1.48693	0.134277
5224	1.48693	0.100418
5225	2.02901	0.108215
5226	0.946127	0.18475
5227	1.07139	0.168785
5228	0.946126	0.170678
5229	1.84644	0.131388
5230	1.84644	0.127217
5231	1.38683	0.14954
5232	1.40574	0.149426
5233	1.36866	0.41303
5234	0.649325	0.573485
5235	1.93631	2.1
5236	1.50145	2.1
5237	1.6287	2.1
5238	1.31302	2.1
5239	1.18041	2.1
5240	1.18041	2.1
5241	0.974196	2.1
5242	0.946131	2.1
5243	1.74169	2.1
5244	1.74169	2.1
5245	1.23163	2.1
5246	1.27451	2.1
5247	1.41331	2.1
5248	1.23163	2.1
5249	1.28037	2.1
5250	1.28158	2.1
5251	1.28037	2.1
5252	2.28015	0.05
5253	2.06561	0.05
5254	2.61343	0.05
5255	1.11712	0.05
5256	1.63689	0.641594
5257	1.01393	0.631436
5258	0.996775	2.1
5259	1.54277	2.1
5260	1.26397	2.1
5261	1.46129	2.1
5262	1.62391	2.1
5263	1.08848	0.05
5264	1.11833	2.1
5265	1.24041	2.1
5266	2.31884	2.1
5267	1.335 2.1	
5268	1.48589	0.05
5269	1.77131	0.05
5270	1.8422	1.62796
5271	2.47453	1.74393
5272	1.23726	1.68662
5273	1.21996	1.71728
5274	1.65512	1.74812
5275	1.65512	1.72112

5276	0.802107	1.78235
5277	0.946129	1.60026
5278	0.802137	1.62587
5279	1.3279	1.50697
5280	1.23726	1.54516
5281	1.79759	1.41189
5282	1.79759	1.34697
5283	0.767577	1.63985
5284	0.946131	1.3888
5285	0.767577	1.39012
5286	1.03534	0.413701
5287	1.09962	1.99364
5288	0.929217	2.1
5289	1.70126	2.1
5290	0.991407	2.1
5291	1.41077	2.1
5292	0.991406	2.1
5293	1.62966	2.1
5294	1.62966	2.1
5295	0.946128	2.1
5296	1.18364	2.1
5297	0.946131	2.1
5298	1.14186	2.1
5299	1.18364	2.1
5300	1.16858	2.1
5301	1.66137	0.860112
5302	1.90165	0.312234
5303	1.66139	0.125491
5304	1.26893	0.156207
5305	1.15918	0.338946
5306	0.653228	0.414377
5307	0.946133	0.236811
5308	1.77488	0.642254
5309	1.77489	0.535936
5310	0.653228	0.319937
5311	1.56043	0.353291
5312	1.60894	0.420029
5313	1.56043	0.22439
5314	1.06848	0.365683
5315	1.04307	2.1
5316	1.14188	2.1
5317	1.09195	2.1
5318	0.615121	2.1
5319	1.50867	2.1
5320	1.27152	2.1
5321	0.615094	2.1
5322	1.07297	2.1
5323	1.1372	2.1
5324	0.553608	2.1
5325	1.23019	2.1
5326	0.992675	2.1
5327	1.40984	2.04153
5328	2.55682	2.1
5329	3.66069	2.1
5330	3.58726	2.1
5331	1.4375	0.0852885
5332	1.88424	0.113713
5333	1.56714	2.1

5334	1.6721	2.1
5335	1.18474	2.1
5336	1.2295	2.1
5337	1.28754	2.1
5338	1.36838	2.1
5339	2.78661	2.1
5340	2.64055	2.1
5341	1.35065	0.660335
5342	2.26675	0.34521
5343	1.35065	0.622357
5344	1.21558	0.441309
5345	1.49709	0.05
5346	1.4971	0.05
5347	0.85472	0.05
5348	0.854718	0.05
5349	0.946129	0.05
5350	0.854717	0.05
5351	1.1604	0.05
5352	1.25265	0.05
5353	2.32081	0.05
5354	1.15371	0.05
5355	1.14945	2.1
5356	0.995725	2.1
5357	1.00307	0.161611
5358	1.10907	0.110559
5359	2.30315	0.109012
5360	1.10908	0.136361
5361	1.27558	1.3527
5362	1.71613	1.71387
5363	1.32977	2.1
5364	0.815276	2.1
5365	1.41697	2.1
5366	1.41697	2.1
5367	0.815276	2.1
5368	1.38897	2.1
5369	0.946131	2.1
5370	0.815275	2.1
5371	1.44684	2.1
5372	1.21489	1.44074
5373	1.22476	1.35803
5374	0.870366	1.50685
5375	0.862572	1.41575
5376	0.835435	1.0814
5377	1.6993	0.667221
5378	1.23618	0.737736
5379	1.18624	0.771136
5380	1.53058	0.951241
5381	2.16108	0.977484
5382	2.21589	0.670679
5383	1.04037	2.1
5384	1.03933	2.1
5385	1.57753	0.104969
5386	1.87644	0.13788
5387	1.24728	0.152373
5388	1.30394	0.0918634
5389	1.3538	2.1
5390	1.69858	2.1
5391	1.1726	0.11818

5392	0.94612	0.0786353
5393	1.09805	0.05
5394	0.946118	0.163722
5395	1.72576	0.0743525
5396	1.72575	0.05
5397	1.34152	0.0677291
5398	1.33034	0.0637151
5399	0.784151	2.1
5400	0.946131	2.1
5401	0.784151	2.1
5402	1.47963	2.1
5403	1.47963	2.1
5404	1.3335	2.1
5405	0.930281	2.1
5406	1.29435	2.1
5407	0.883308	2.1
5408	1.31589	2.1
5409	1.67846	2.1
5410	0.993598	0.05
5411	2.23193	0.05
5412	0.9936	0.05
5413	0.615629	0.05
5414	1.65925	2.1
5415	2.05992	2.1
5416	1.65925	2.1
5417	4.08735	1.61414
5418	2.72856	1.54307
5419	0.869602	2.1
5420	3.42833	1.37115
5421	1.14397	1.53993
5422	1.34563	0.127581
5423	1.08189	0.103693
5424	0.782128	1.95647
5425	1.0022	2.1
5426	0.900427	1.99437
5427	1.51499	2.04245
5428	1.05588	1.99397
5429	1.13739	2.1
5430	1.37004	2.01652
5431	1.14603	2.0397
5432	0.851541	1.72177
5433	0.946799	1.94883
5434	1.14854	1.86962
5435	1.33793	1.763
5436	0.94613	1.84881
5437	1.24336	1.83673
5438	0.851473	1.84676
5439	2.08792	1.77546
5440	1.01869	1.86927
5441	0.986953	0.05
5442	0.896238	0.0691062
5443	0.906574	0.0657749
5444	0.852145	0.0550003
5445	2.14191	0.05
5446	1.15979	0.0562801
5447	1.18327	0.05
5448	2.32742	0.0555053
5449	2.35961	0.0804771

5450	2.38126	0.0519198
5451	1.13632	0.05
5452	1.17897	0.05
5453	1.76199	0.05
5454	1.00573	2.1
5455	0.946127	1.82553
5456	1.7077	2.1
5457	1.7077	2.1
5458	0.946127	2.1
5459	1.24887	2.1
5460	1.24887	2.1
5461	1.22441	2.1
5462	1.08542	2.1
5463	1.21743	1.99759
5464	1.12572	2.1
5465	1.05499	1.45524
5466	1.00521	1.42196
5467	2.86191	0.05
5468	2.27972	0.05
5469	1.01169	2.1
5470	1.03373	2.1
5471	1.39319	2.1
5472	0.636837	2.1
5473	1.63908	2.1
5474	1.45713	1.86874
5475	1.04072	2.1
5476	1.25025	1.92715
5477	1.38914	2.08302
5478	0.636807	2.1
5479	1.50211	2.1
5480	1.36555	2.07303
5481	2.77545	1.78782
5482	0.682805	2.1
5483	1.50267	1.91917
5484	1.50267	2.1
5485	0.797749	2.1
5486	1.38773	1.90898
5487	1.38773	2.1
5488	0.682776	2.1
5489	2.01245	0.05
5490	0.925918	0.05
5491	0.75689	0.05
5492	1.25823	0.05
5493	1.16505	0.05
5494	0.838779	0.05
5495	1.14722	0.05
5496	0.756849	0.05
5497	1.52298	0.05
5498	2.11034	0.05
5499	1.92174	0.05
5500	1.92174	0.05
5501	0.576083	0.05
5502	1.23146	0.05
5503	1.26637	0.05
5504	1.23146	0.05
5505	1.26637	0.05
5506	0.946133	0.842092
5507	1.37034	0.36426

5508	1.84145	0.528366
5509	1.84145	0.316422
5510	0.946132	1.23989
5511	1.37034	0.61322
5512	1.41724	0.592918
5513	1.0685	0.295624
5514	1.14547	0.317496
5515	1.24976	2.1
5516	1.30682	2.1
5517	2.12095	2.1
5518	1.46392	2.1
5519	1.46392	2.1
5520	0.935738	2.1
5521	1.32949	2.1
5522	1.07017	2.1
5523	0.935766	2.1
5524	1.07017	2.1
5525	1.78095	2.1
5526	1.05619	0.299391
5527	1.05793	0.177943
5528	1.77677	2.1
5529	2.04766	2.1
5530	1.02524	2.1
5531	1.18803	2.1
5532	1.70047	2.1
5533	1.556 2.1	
5534	1.24201	2.1
5535	1.3592	2.1
5536	2.11824	0.0553149
5537	2.20248	0.0917733
5538	3.43878	0.0511329
5539	3.12466	0.05
5540	2.09351	2.1
5541	0.946128	2.1
5542	1.51269	2.1
5543	1.51269	2.1
5544	0.946131	2.1
5545	1.01306	2.1
5546	1.04676	2.1
5547	1.01306	2.1
5548	1.07444	2.1
5549	2.17465	2.1
5550	1.14808	2.1
5551	1.39067	2.0676
5552	1.20958	1.96269
5553	0.662832	0.0736202
5554	1.93512	0.241993
5555	1.15471	2.1
5556	1.75119	2.1
5557	1.75118	2.1
5558	0.946131	2.1
5559	1.28606	2.1
5560	1.27087	2.1
5561	0.946131	2.1
5562	1.28606	2.1
5563	1.58916	0.05
5564	0.927822	0.077779
5565	2.24367	0.05

5566	1.8734	0.05
5567	3.51668	0.05
5568	2.68986	0.05
5569	2.32395	2.1
5570	1.39564	2.1
5571	2.10862	2.1
5572	1.36763	2.1
5573	0.970963	0.05
5574	0.97096	0.05
5575	1.11897	0.13512
5576	1.26885	0.212591
5577	1.11605	0.152495
5578	2.06612	0.122879
5579	1.717	0.132784
5580	0.59502	0.202129
5581	0.825755	0.10895
5582	0.709624	0.237485
5583	1.65741	0.110754
5584	0.307486	0.119003
5585	1.4785	0.141554
5586	0.983143	2.1
5587	1.08247	2.1
5588	1.42353	2.1
5589	1.42353	2.1
5590	0.781667	2.1
5591	0.797634	2.1
5592	0.946131	2.1
5593	0.781638	2.1
5594	0.946131	2.1
5595	0.98315	2.1
5596	1.083	2.1
5597	1.61975	0.05
5598	1.11946	0.05
5599	1.18977	0.0576058
5600	0.996484	0.064495
5601	2.24343	0.05
5602	1.4787	0.05
5603	1.66127	0.05
5604	1.86028	0.0504623
5605	1.09128	0.0504269
5606	1.03115	0.0529908
5607	0.838025	0.0511727
5608	1.3736	0.05
5609	1.44009	0.05
5610	0.92917	0.05
5611	1.03889	0.05
5612	0.956974	0.05
5613	3.23468	0.05
5614	0.953922	0.0763966
5615	0.957104	0.0789618
5616	1.66231	0.05
5617	1.26778	0.05
5618	1.47615	0.05
5619	1.28621	0.0668556
5620	1.08043	0.0636957
5621	2.35026	2.1
5622	2.35025	2.1
5623	2.60659	2.1

5624	1.57627	2.1
5625	1.5493	2.1
5626	1.24682	0.350916
5627	2.14277	0.335461
5628	1.24682	0.750681
5629	1.00307	0.283781
5630	2.04056	0.252535
5631	1.45408	2.1
5632	1.54249	2.1
5633	1.24474	1.58707
5634	1.37813	1.59685
5635	0.924158	0.0500903
5636	0.916623	0.0510319
5637	1.35344	0.05
5638	1.00859	0.05
5639	1.34786	0.05
5640	0.952457	0.0522101
5641	0.85404	0.0526376
5642	1.06341	2.1
5643	1.09194	2.1
5644	1.93707	2.1
5645	2.14087	2.1
5646	1.93707	2.1
5647	1.07519	2.1
5648	1.07519	2.1
5649	1.62518	2.1
5650	1.3592	2.1
5651	1.13183	2.1
5652	1.20357	2.1
5653	1.4858	2.1
5654	1.23694	2.1
5655	1.44011	2.1
5656	1.23989	2.1
5657	2.21604	2.1
5658	1.85574	0.71687
5659	1.55649	2.1
5660	1.48861	0.428364
5661	1.15968	0.05
5662	0.946132	0.05
5663	0.663056	0.05
5664	1.70837	0.05
5665	1.70837	0.05
5666	0.946131	0.05
5667	0.663026	0.05
5668	2.09604	0.05
5669	0.81094	0.05
5670	1.06559	0.05
5671	0.810956	0.05
5672	1.15474	0.05
5673	0.990051	0.05
5674	0.894971	0.05
5675	1.32536	0.05
5676	0.946133	2.1
5677	1.33125	2.1
5678	1.75823	2.1
5679	1.75823	2.1
5680	0.946133	2.1
5681	1.33125	2.1

5682	1.37312	2.1
5683	1.27985	2.1
5684	1.37312	2.1
5685	1.70684	2.1
5686	1.70684	2.1
5687	0.946132	2.1
5688	0.946132	2.1
5689	1.25081	2.1
5690	0.956293	2.1
5691	0.956294	2.1
5692	1.36035	2.1
5693	1.41944	2.1
5694	0.830638	2.1
5695	0.747568	2.1
5696	0.888498	2.1
5697	1.04071	2.1
5698	1.17128	2.1
5699	1.92339	2.1
5700	2.91208	2.1
5701	1.82208	0.906298
5702	1.89653	0.56651
5703	1.82588	1.18323
5704	1.26703	0.257399
5705	1.26703	0.417457
5706	0.6157	0.274787
5707	1.26706	1.25712
5708	0.61567	0.10417
5709	0.615671	0.136953
5710	1.26706	0.254323
5711	1.89158	0.197248
5712	1.23134	0.158829
5713	2.49497	0.05
5714	1.68103	0.05
5715	2.0554	2.1
5716	1.72647	2.1
5717	1.67375	2.1
5718	1.65037	2.1
5719	1.04349	1.03178
5720	1.69055	0.05
5721	1.53463	0.05
5722	1.19022	1.89765
5723	1.89226	1.60507
5724	1.15009	1.60043
5725	1.7054	1.11987
5726	1.31612	0.247268
5727	1.31611	1.37143
5728	1.67345	0.709782
5729	1.06577	0.237864
5730	1.40193	0.151759
5731	1.72411	0.130078
5732	1.46123	0.142681
5733	0.808808	0.22824
5734	0.897714	0.153251
5735	0.851554	0.267501
5736	1.3499	0.0678836
5737	1.29001	0.073866
5738	1.22083	2.1
5739	1.06191	2.1

5740	1.05758	1.31637
5741	1.348	1.60896
5742	1.43485	1.30988
5743	1.34799	1.55763
5744	1.36307	0.05
5745	2.28188	0.05
5746	1.7926	0.05
5747	1.38957	2.1
5748	1.47446	2.1
5749	0.953638	2.1
5750	1.11868	2.1
5751	0.953636	2.1
5752	1.03408	2.1
5753	1.52443	0.05
5754	1.38539	0.05
5755	0.946132	0.05
5756	1.12747	0.05
5757	1.04393	0.05
5758	1.05017	0.05
5759	1.20529	0.05
5760	1.3663	0.29848
5761	1.89226	0.299997
5762	1.17885	0.273573
5763	0.867881	0.233428
5764	1.32917	0.091671
5765	1.32917	0.0721943
5766	0.946127	0.492827
5767	1.19583	0.25397
5768	1.00122	0.159352
5769	0.946128	0.336202
5770	1.71405	0.541572
5771	0.871815	2.1
5772	1.16435	2.1
5773	0.686619	2.1
5774	1.63102	2.1
5775	1.06589	2.1
5776	1.05375	2.1
5777	1.9023	2.1
5778	1.27394	0.435987
5779	1.88345	0.39793
5780	2.05636	0.432899
5781	0.672977	0.640707
5782	1.41274	0.406669
5783	1.65606	0.422046
5784	0.869329	0.63502
5785	1.2207	0.537978
5786	1.35631	0.578552
5787	0.908776	0.764039
5788	0.955081	0.275893
5789	1.2243	2.1
5790	1.23169	2.1
5791	1.52228	2.1
5792	1.23169	2.1
5793	0.990892	0.526529
5794	1.08376	0.249115
5795	1.79348	0.476695
5796	1.50043	0.322336
5797	1.32599	1.16296

5798	1.37924	0.396346
5799	0.838014	0.346746
5800	0.87737	0.915436
5801	0.947086	0.520341
5802	1.27199	0.481711
5803	1.04918	0.365182
5804	1.04851	0.789432
5805	1.1699	0.615084
5806	0.810934	0.688095
5807	1.83602	1.34635
5808	2.21909	1.42404
5809	0.619441	0.751978
5810	2.03755	0.380124
5811	1.83472	0.766976
5812	1.20416	1.0726
5813	1.26691	0.297419
5814	0.655795	0.40712
5815	0.94613	0.556173
5816	2.35169	1.1069
5817	3.54705	2.1
5818	2.60741	2.1
5819	1.59019	0.05
5820	1.24526	0.05
5821	1.59019	0.05
5822	2.36406	0.05
5823	1.16776	2.1
5824	1.67311	2.1
5825	1.67311	1.97739
5826	0.94613	1.66432
5827	1.38645	2.1
5828	1.13563	0.479342
5829	1.13563	1.50697
5830	1.23279	1.02724
5831	1.05042	2.1
5832	1.17418	2.1
5833	1.28909	2.1
5834	0.973448	2.1
5835	1.02958	2.1
5836	3.3005	2.1
5837	2.86325	2.1
5838	1.35879	2.1
5839	1.69332	2.1
5840	1.69332	2.1
5841	0.946128	2.1
5842	1.39659	2.1
5843	1.12035	2.1
5844	1.12035	2.1
5845	1.24286	2.1
5846	1.10407	2.1
5847	1.14599	2.1
5848	1.32259	2.04979
5849	0.788592	0.05
5850	2.4588	2.1
5851	0.909581	1.97362
5852	1.35656	1.85147
5853	0.986895	1.90188
5854	2.30602	1.83866
5855	1.46873	1.8836

5856	1.55584	1.84463
5857	2.1547	1.84718
5858	1.87794	1.95451
5859	0.808286	2.03126
5860	0.928464	1.96835
5861	0.810959	1.93303
5862	1.50962	1.75359
5863	1.1053	1.84761
5864	1.0712	1.86645
5865	1.40504	1.86209
5866	1.87794	1.62665
5867	2.80496	1.65755
5868	1.41479	1.9455
5869	1.38634	2.1
5870	1.38634	2.1
5871	1.67762	2.1
5872	0.946131	2.1
5873	1.188	2.1
5874	1.62585	2.1
5875	1.62585	2.1
5876	0.946129	2.1
5877	1.18801	2.1
5878	1.38397	2.1
5879	1.04546	2.1
5880	1.16817	2.1
5881	1.06007	2.1
5882	1.19785	2.1
5883	1.43652	2.1
5884	1.06994	2.1
5885	1.89111	2.1
5886	1.07144	2.1
5887	1.51754	2.1
5888	2.0873	2.1
5889	0.758799	2.1
5890	1.59518	2.1
5891	1.59518	2.1
5892	0.946132	2.1
5893	0.758771	2.1
5894	1.06957	2.1
5895	0.758771	2.1
5896	1.33916	2.1
5897	2.03045	1.64484
5898	2.80184	1.72111
5899	1.76788	1.76519
5900	1.27442	1.99662
5901	1.06756	2.1
5902	0.966979	2.1
5903	1.7054	2.1
5904	1.07156	2.1
5905	0.986096	2.1
5906	1.32813	2.1
5907	1.3331	2.1
5908	2.08369	0.167746
5909	2.23818	1.38214
5910	1.10335	0.696812
5911	0.622314	0.494635
5912	1.72451	0.371146
5913	1.72451	0.751534

5914	0.622283	0.584146
5915	0.622314	0.479807
5916	0.946131	0.689636
5917	1.31254	1.77008
5918	1.15704	1.5521
5919	1.24463	0.466454
5920	2.2067	1.12163
5921	1.30001	0.638566
5922	1.29175	0.839306
5923	1.1233	0.05
5924	1.8689	0.05
5925	1.29494	0.05
5926	1.11895	0.05
5927	0.851539	0.05
5928	0.851474	0.05
5929	1.58352	0.05
5930	1.58228	0.05
5931	0.851527	0.05
5932	2.6389	0.05
5933	1.77548	0.05
5934	1.24555	2.1
5935	2.0512	2.1
5936	1.46101	2.1
5937	2.60635	2.1
5938	1.10476	0.05
5939	1.44103	0.05
5940	0.652391	2.1
5941	0.618322	2.1
5942	2.22915	2.1
5943	1.39067	2.1
5944	1.99737	2.1
5945	1.42174	2.1
5946	0.946132	1.16591
5947	1.29735	1.75415
5948	1.61765	2.1
5949	1.61765	1.31935
5950	0.946132	1.79433
5951	1.29735	1.49284
5952	1.26643	1.47657
5953	1.01335	1.3481
5954	1.01335	0.200151
5955	0.947669	2.1
5956	2.17167	2.1
5957	2.59733	2.01112
5958	1.50954	2.06638
5959	1.02638	1.8369
5960	0.555785	2.00774
5961	1.91101	1.9892
5962	2.09809	1.91157
5963	0.641974	2.04406
5964	1.2022	1.94793
5965	1.36359	1.91198
5966	1.8128	1.95959
5967	2.34348	0.126615
5968	1.66343	0.0808069
5969	0.974717	0.744752
5970	1.53908	0.340515
5971	1.27737	1.67569

5972	1.09408	0.961999
5973	2.07382	0.785994
5974	1.10466	1.78302
5975	1.05654	1.77395
5976	1.23498	1.53677
5977	1.48104	1.65474
5978	1.23498	1.39968
5979	1.17261	2.1
5980	1.18059	2.1
5981	2.43005	2.1
5982	1.04546	2.1
5983	2.81222	2.1
5984	1.16817	2.1
5985	1.75766	0.584229
5986	1.47021	1.02649
5987	1.07236	0.188049
5988	1.81759	0.63781
5989	1.42913	0.326376
5990	1.00636	0.476193
5991	1.00636	0.223441
5992	1.79397	0.23524
5993	0.946131	0.371135
5994	1.03691	0.40447
5995	0.851539	0.382893
5996	1.46001	0.446437
5997	1.37878	0.43231
5998	0.933258	0.368006
5999	1.32423	0.374574
6000	1.32614	0.05
6001	1.11356	0.05
6002	1.32614	0.05
6003	1.28895	0.05
6004	1.66895	0.05
6005	1.66895	0.05
6006	0.946132	0.05
6007	1.29576	0.05
6008	1.1318	0.05
6009	1.37621	1.57002
6010	1.94071	1.4909
6011	1.10517	2.1
6012	1.97098	2.1
6013	0.811 2.1	
6014	0.956127	2.1
6015	1.18647	1.9006
6016	0.988455	1.67562
6017	1.12457	0.0748835
6018	1.7453	0.0824083
6019	0.92492	2.1
6020	1.24353	2.1
6021	1.84536	0.440402
6022	1.67662	0.322363
6023	1.38247	0.05
6024	1.02663	0.05
6025	0.946132	0.515512
6026	1.41723	0.318234
6027	1.72976	0.460513
6028	1.72976	0.284484
6029	0.946131	0.971988

6030	1.41723	0.445062
6031	1.25866	0.484194
6032	1.34254	0.284135
6033	1.25866	0.358501
6034	1.65507	0.258839
6035	1.65507	0.232935
6036	0.946132	0.519213
6037	0.946132	0.327725
6038	1.11929	0.349859
6039	0.961779	0.252623
6040	0.961779	0.262575
6041	0.946132	2.1
6042	1.69361	2.1
6043	1.69361	2.1
6044	0.94613	2.1
6045	1.36523	2.1
6046	1.36523	2.1
6047	1.19802	2.1
6048	0.974196	2.1
6049	1.09518	2.1
6050	0.946131	2.1
6051	1.7161	2.1
6052	1.7161	2.1
6053	0.94613	2.1
6054	1.297	2.1
6055	1.297	2.1
6056	1.14599	2.1
6057	0.974197	2.1
6058	0.974197	2.1
6059	1.80778	2.1
6060	2.20473	2.1
6061	1.80777	2.1
6062	1.3515	2.1
6063	0.583237	2.1
6064	1.39359	2.1
6065	1.39359	2.1
6066	1.07296	2.1
6067	0.946114	2.1
6068	1.07297	2.1
6069	0.903862	2.1
6070	0.956572	2.1
6071	1.03437	2.1
6072	0.975409	2.1
6073	1.31167	0.0525225
6074	2.70246	0.05
6075	1.12018	0.0504829
6076	0.655836	0.0570437
6077	0.655837	0.0519129
6078	1.81557	0.05
6079	1.81557	0.05
6080	0.655836	0.0527037
6081	0.946131	0.0635283
6082	1.01847	0.05
6083	1.61334	0.05
6084	0.810946	0.0629544
6085	0.8311	0.0710782
6086	1.2466	0.05
6087	1.20286	0.05

6088	0.97508	0.0633254
6089	0.870805	0.05
6090	0.760087	0.0706444
6091	0.853389	2.1
6092	0.820894	2.1
6093	0.928291	2.1
6094	1.06922	2.1
6095	1.32829	2.1
6096	1.12173	2.1
6097	1.16615	2.1
6098	0.957748	2.1
6099	0.612491	2.07655
6100	0.548141	2.1
6101	0.979614	2.1
6102	0.658184	2.1
6103	0.678456	2.04666
6104	0.529544	2.04941
6105	0.739013	2.1
6106	0.694037	2.1
6107	0.475716	2.05804
6108	1.58794	2.00498
6109	1.58794	1.83317
6110	0.475743	1.91166
6111	1.08301	2.1
6112	0.980664	2.1
6113	0.475737	2.1
6114	1.32709	2.1
6115	1.59396	2.1
6116	1.19719	0.142431
6117	1.34921	0.148608
6118	1.69061	0.143517
6119	1.69061	0.104313
6120	0.946132	0.172646
6121	1.34921	0.148739
6122	1.28753	0.147597
6123	0.95895	0.111722
6124	1.01051	0.124041
6125	1.12468	0.139242
6126	1.22588	0.184271
6127	1.34254	0.167697
6128	1.74561	0.159037
6129	1.74561	0.128103
6130	0.946128	0.204391
6131	1.34253	0.170589
6132	1.3492	0.172522
6133	1.01051	0.11772
6134	1.922	2.1
6135	1.922	2.1
6136	2.12556	2.1
6137	0.946131	2.1
6138	1.06278	2.1
6139	1.47631	2.1
6140	1.47631	2.1
6141	0.946129	2.1
6142	1.06278	2.1
6143	1.35966	2.1
6144	0.991985	2.1
6145	0.738507	1.59641

6146	0.536935	1.77815
6147	0.799004	1.5944
6148	1.04281	1.20633
6149	0.879197	1.21981
6150	1.06538	1.21131
6151	0.715942	1.5978
6152	1.00636	0.247027
6153	1.03824	0.153951
6154	1.70147	0.166009
6155	1.21223	2.1
6156	1.01243	2.1
6157	1.01242	2.1
6158	1.06935	2.1
6159	1.54241	2.1
6160	0.998971	0.05
6161	1.60312	0.05
6162	1.44836	0.05
6163	2.05632	0.05
6164	1.44836	0.05
6165	0.973765	0.05
6166	0.946131	0.05
6167	0.851541	0.05
6168	1.73854	0.05
6169	1.91752	0.05
6170	1.49622	0.05
6171	1.23351	0.05
6172	3.33289	2.1
6173	1.86606	2.1
6174	2.71233	2.1
6175	0.788816	2.1
6176	0.963972	2.1
6177	1.25866	2.1
6178	1.10817	2.1
6179	1.41908	2.1
6180	1.48734	2.1
6181	1.40822	0.10281
6182	1.40822	0.102903
6183	2.09871	0.17424
6184	0.946133	0.117633
6185	1.14926	0.0637393
6186	0.946133	0.125812
6187	1.78098	0.0806548
6188	1.78098	0.0562455
6189	1.35822	0.0979665
6190	1.3689	0.103911
6191	1.22176	0.05
6192	2.03426	0.0904655
6193	0.946131	0.13985
6194	1.01713	0.0958891
6195	0.704112	0.149838
6196	1.36237	0.0971545
6197	1.36237	0.0809032
6198	1.01713	0.128701
6199	1.04936	0.135708
6200	1.87377	2.1
6201	1.60501	2.1
6202	0.981584	0.05
6203	1.69435	0.05

6204	0.85154	2.1
6205	1.47841	2.1
6206	1.4952	2.1
6207	1.31941	2.1
6208	1.04023	2.1
6209	1.1995	2.1
6210	0.914791	2.1
6211	1.1427	1.50194
6212	1.64787	1.2531
6213	1.07526	1.55077
6214	1.07527	1.03529
6215	1.07527	1.56874
6216	1.58551	1.72821
6217	2.77362	1.62077
6218	2.17974	1.30478
6219	2.71723	1.62128
6220	1.29868	0.0944016
6221	1.3594	0.0770141
6222	1.04786	0.0658528
6223	0.527876	0.0657669
6224	1.4085	0.0755377
6225	1.48538	0.05
6226	0.76829	0.0543863
6227	1.18514	0.0740963
6228	0.617954	0.05
6229	0.864161	0.0553921
6230	1.18835	0.05
6231	1.16817	2.1
6232	1.04263	2.1
6233	1.50524	2.1
6234	1.85355	2.1
6235	1.23963	2.1
6236	1.06279	2.1
6237	0.94613	2.1
6238	1.35229	2.1
6239	1.15947	2.1
6240	0.965593	2.1
6241	0.851553	2.1
6242	2.21296	2.1
6243	1.11764	2.1
6244	2.46399	2.1
6245	1.39292	2.1
6246	1.34701	2.1
6247	1.34701	2.1
6248	1.47001	2.1
6249	0.963166	0.05
6250	0.893445	0.05
6251	1.21056	0.05
6252	1.67375	2.1
6253	2.0977	2.1
6254	1.09154	2.1
6255	1.09154	2.1
6256	1.57122	0.926578
6257	1.60976	0.352074
6258	1.69533	0.200499
6259	1.6951	0.173323
6260	1.5831	0.127992
6261	1.20969	2.1

6262	1.12076	2.1
6263	1.49808	2.1
6264	1.50201	2.1
6265	1.00278	2.1
6266	1.22811	0.0807832
6267	1.11545	2.1
6268	1.09646	2.1
6269	1.03107	0.05
6270	1.44293	0.05
6271	1.66752	2.1
6272	1.11023	2.1
6273	2.57831	1.93848
6274	2.20191	1.45134
6275	2.18051	1.95423
6276	1.48035	0.212162
6277	1.0961	0.0966865
6278	1.63241	0.138893
6279	1.0961	0.177516
6280	1.20959	2.1
6281	2.86136	2.1
6282	1.64933	2.1
6283	1.47217	2.1
6284	0.915178	2.1
6285	1.19074	2.1
6286	0.897276	2.1
6287	0.915177	2.1
6288	0.987037	2.1
6289	1.04288	2.1
6290	2.39323	2.1
6291	0.976218	0.05
6292	2.23933	0.05
6293	0.976215	0.05
6294	1.07384	0.05
6295	1.07652	2.1
6296	1.55678	2.1
6297	1.54168	2.1
6298	1.69136	2.1
6299	1.64391	1.43973
6300	1.76477	1.50605
6301	0.611486	1.45252
6302	0.683421	1.36271
6303	0.946131	1.43586
6304	1.1019	1.42154
6305	1.12946	1.38703
6306	1.54226	1.71633
6307	1.19496	1.39487
6308	1.96199	1.40684
6309	2.0775	1.45436
6310	1.22297	1.41772
6311	1.3469	1.40498
6312	1.25366	1.56531
6313	1.65443	1.55153
6314	1.65443	1.53973
6315	0.946132	1.68405
6316	0.946132	1.63889
6317	1.12873	1.64009
6318	1.07468	1.53848
6319	1.2854	0.70969

6320	1.3469	1.49385
6321	1.68616	1.46392
6322	1.68616	1.36809
6323	0.946132	1.64032
6324	0.946132	1.538
6325	1.16109	1.53792
6326	1.17879	1.37358
6327	1.17879	1.45907
6328	1.45304	1.5702
6329	2.21058	2.1
6330	2.81552	2.1
6331	1.45535	0.105207
6332	1.49906	0.0742031
6333	1.04413	2.1
6334	1.74586	2.1
6335	1.34526	2.1
6336	1.17402	1.43481
6337	1.74564	1.17583
6338	1.52724	2.1
6339	1.68142	2.1
6340	1.09651	2.1
6341	1.49149	2.1
6342	1.36814	2.1
6343	1.46783	2.1
6344	2.18116	2.1
6345	1.23602	2.1
6346	1.74538	1.89193
6347	0.851539	2.1
6348	1.45775	2.1
6349	1.28919	2.1
6350	0.851483	2.1
6351	1.26102	2.1
6352	0.869889	2.1
6353	0.85151	2.1
6354	1.23451	2.1
6355	1.23602	2.1
6356	1.11955	2.1
6357	1.08022	2.1
6358	0.977776	2.1
6359	1.27896	2.1
6360	1.64268	2.1
6361	1.97548	2.1
6362	1.72527	2.07729
6363	0.946132	2.1
6364	1.69702	2.1
6365	1.69702	2.1
6366	1.13635	2.1
6367	1.40274	2.1
6368	1.24041	2.1
6369	1.13635	2.1
6370	1.78427	0.05
6371	1.68338	0.05
6372	1.00681	0.05
6373	1.12988	0.05
6374	0.946132	0.05
6375	0.957543	0.05
6376	1.40515	0.05
6377	1.40515	0.05

6378	0.797932	0.05
6379	1.30187	0.05
6380	1.14846	0.05
6381	0.665137	0.170112
6382	1.09575	0.215499
6383	0.596894	0.182822
6384	1.04072	0.24092
6385	0.663171	0.27809
6386	1.47218	0.225179
6387	1.32889	0.189533
6388	1.32639	0.0865138
6389	1.94887	0.104933
6390	1.32639	0.0923515
6391	1.19323	0.147644
6392	2.19794	0.181988
6393	0.754756	0.0900064
6394	1.11015	0.101847
6395	0.754755	0.0883068
6396	0.946133	0.111629
6397	0.754768	0.114028
6398	1.59986	0.103204
6399	1.59986	0.079651
6400	1.84082	2.1
6401	1.79044	2.1
6402	1.75922	2.1
6403	1.86766	2.1
6404	1.58995	2.1
6405	1.58995	2.1
6406	1.74351	2.1
6407	1.04442	2.1
6408	1.42278	2.1
6409	1.08576	2.1
6410	0.994294	2.1
6411	1.44884	2.1
6412	1.71036	2.1
6413	0.714101	1.18214
6414	1.17541	2.1
6415	1.00872	0.709582
6416	0.714072	1.70243
6417	0.936677	1.38158
6418	0.966979	2.1
6419	1.4908	2.1
6420	1.03108	0.12564
6421	0.949712	0.0767901
6422	0.810946	0.111818
6423	0.88209	0.112028
6424	0.646702	0.0772505
6425	0.972663	0.0547422
6426	0.862301	0.0682811
6427	0.868143	0.0711571
6428	1.11094	0.0765706
6429	1.35809	0.0709773
6430	1.32455	2.1
6431	1.32328	2.1
6432	1.69586	2.1
6433	1.78301	2.1
6434	1.67906	2.1
6435	1.60635	2.1

6436	1.57234	2.1
6437	1.47012	2.1
6438	1.22811	0.0970692
6439	1.08732	0.113597
6440	2.08849	0.09871
6441	1.08732	0.105468
6442	1.30635	0.05
6443	0.77315	0.05
6444	1.25732	0.05
6445	2.43845	2.1
6446	2.38577	2.1
6447	0.946132	1.65342
6448	1.19288	2.1
6449	1.54508	2.1
6450	1.54508	1.79226
6451	0.94515	2.1
6452	1.19288	1.9174
6453	1.29735	1.86685
6454	1.12087	1.60851
6455	1.54699	1.7048
6456	1.43635	2.1
6457	1.33385	2.1
6458	1.43635	2.1
6459	1.02368	2.1
6460	1.035 2.1	
6461	1.00128	2.1
6462	1.04009	2.1
6463	1.14052	2.1
6464	0.443552	2.1
6465	0.984351	2.1
6466	0.591443	2.1
6467	0.929561	2.1
6468	1.52079	2.1
6469	1.035 2.1	
6470	3.5282	2.1
6471	1.88288	2.1
6472	2.73877	1.91031
6473	0.906059	2.1
6474	1.23107	0.05
6475	0.906712	0.05
6476	0.841322	0.0548189
6477	1.53005	0.05
6478	1.10007	0.05
6479	1.38033	0.05
6480	1.03442	0.05
6481	0.787223	0.05
6482	1.42589	0.05
6483	2.35665	0.05
6484	1.17793	0.093716
6485	1.81356	0.091068
6486	1.42964	0.099966
6487	1.95641	0.0818433
6488	1.39139	0.100244
6489	1.39139	0.176793
6490	0.564533	0.0825173
6491	1.20016	0.61309
6492	0.755765	0.05856
6493	0.755766	0.0724758

6494	1.20016	0.0847153
6495	1.91011	0.0679081
6496	1.4695	0.0605586
6497	1.70048	2.1
6498	1.93765	2.1
6499	0.741307	2.1
6500	0.780371	2.1
6501	0.946133	1.12877
6502	1.28918	1.40206
6503	1.5111	1.43927
6504	1.5111	1.21493
6505	0.946131	1.33633
6506	1.28918	1.21725
6507	1.16805	1.16856
6508	1.01179	1.15429
6509	0.946132	1.20107
6510	1.35328	1.41444
6511	1.69633	1.42993
6512	1.69633	1.2601
6513	0.946133	1.35427
6514	1.35328	1.26517
6515	1.28918	1.23746
6516	1.01179	1.23001
6517	1.58147	1.18063
6518	1.27737	1.23621
6519	2.17223	0.05
6520	0.697168	0.05
6521	1.62495	0.127499
6522	1.62495	0.214106
6523	1.93948	0.396295
6524	0.601592	2.1
6525	1.93528	2.1
6526	2.13757	2.1
6527	1.67561	2.1
6528	2.70875	2.1
6529	1.93528	2.1
6530	3.98475	2.1
6531	1.12376	2.1
6532	1.19016	2.1
6533	1.67323	0.144909
6534	1.74019	0.130303
6535	1.84719	0.137848
6536	1.00764	0.11727
6537	1.23475	2.1
6538	1.06757	2.1
6539	1.57916	2.1
6540	1.06758	2.1
6541	1.28014	2.1
6542	1.33339	2.1
6543	1.2679	2.1
6544	1.23504	2.1
6545	1.50108	2.1
6546	1.3715	2.1
6547	0.58831	0.816056
6548	0.865971	0.627923
6549	0.813704	1.17425
6550	0.658856	0.739455
6551	0.495869	0.956457

6552	1.13845	1.07517
6553	0.780804	1.04613
6554	0.642023	0.973759
6555	0.707748	1.02584
6556	1.64476	0.440029
6557	1.64476	0.834971
6558	0.99611	0.992932
6559	1.35639	0.597565
6560	1.3564	0.91129
6561	0.946129	0.910354
6562	1.99222	0.551624
6563	1.4777	2.1
6564	1.03968	2.1
6565	1.82516	2.1
6566	0.851539	2.1
6567	1.63693	2.1
6568	1.59416	2.1
6569	0.878566	2.1
6570	1.40796	2.1
6571	1.13172	2.1
6572	0.851527	2.1
6573	1.14366	2.1
6574	0.823822	0.262901
6575	0.882001	0.269333
6576	1.09756	0.113294
6577	1.11857	0.160556
6578	1.0934	0.158378
6579	0.960104	0.24987
6580	0.982001	0.361875
6581	1.74319	0.191935
6582	1.03043	0.189056
6583	0.967194	0.151402
6584	1.16938	2.1
6585	2.48377	2.1
6586	1.1181	2.1
6587	2.57349	2.1
6588	1.0465	2.1
6589	1.44709	2.1
6590	0.946132	2.1
6591	1.29302	2.1
6592	1.73502	2.1
6593	1.73502	2.1
6594	0.946132	2.1
6595	1.29302	2.1
6596	1.38813	2.1
6597	1.03933	2.1
6598	1.01976	0.237751
6599	1.11764	0.260033
6600	0.961779	0.391902
6601	1.0685	0.272646
6602	1.08426	1.70099
6603	0.99695	1.77966
6604	2.25593	1.68851
6605	1.47992	1.76479
6606	1.88989	1.6059
6607	1.65813	1.66744
6608	1.20278	1.67184
6609	0.981842	1.87692

6610	0.802655	2.1
6611	0.953489	2.03061
6612	0.961719	2.00825
6613	0.975614	1.9176
6614	0.991617	1.87922
6615	0.940748	1.87092
6616	2.25019	2.037
6617	2.30712	1.77058
6618	1.98823	1.95117
6619	0.522844	1.83236
6620	0.946122	1.92803
6621	0.946132	1.84615
6622	0.593846	1.8212
6623	1.26501	1.77793
6624	1.21171	0.05
6625	0.851542	0.05
6626	0.940327	0.0535432
6627	0.940356	0.0554229
6628	1.52117	0.05
6629	1.58072	0.05
6630	1.35169	0.05
6631	1.13889	0.05
6632	1.35007	0.05
6633	1.35007	0.05
6634	1.78381	0.05
6635	0.946132	0.05
6636	1.06627	0.05
6637	0.946131	0.0533054
6638	1.73005	0.05
6639	1.73005	0.05
6640	1.32449	0.05
6641	1.35169	0.05
6642	1.42446	0.23208
6643	1.86697	0.19683
6644	1.13413	0.203325
6645	1.42446	0.178104
6646	1.33575	0.0857989
6647	1.20473	0.102309
6648	1.87517	0.05
6649	1.87517	0.05
6650	0.488712	0.05
6651	1.13242	0.05
6652	0.488713	0.05
6653	0.488713	0.05
6654	1.23146	0.05
6655	1.57932	0.05
6656	1.01698	0.05
6657	1.42085	0.05
6658	0.246725	0.05
6659	0.996283	0.05
6660	1.36965	0.05
6661	0.86361	0.05
6662	0.810945	0.05
6663	1.72938	0.05
6664	1.21649	0.05
6665	1.1865	0.05
6666	1.62387	0.05
6667	1.05302	0.05

6668	1.10049	0.532214
6669	0.946129	2.1
6670	1.70184	1.11983
6671	1.70184	2.1
6672	1.12538	2.1
6673	1.35969	0.897092
6674	1.28828	2.1
6675	1.12538	2.1
6676	0.99747	1.92959
6677	1.34851	0.620598
6678	0.923724	2.1
6679	1.47154	2.1
6680	1.47154	2.1
6681	1.10698	2.1
6682	1.28828	2.04939
6683	1.10698	2.1
6684	0.923695	2.1
6685	0.99747	1.91809
6686	1.65677	2.1
6687	1.77677	2.1
6688	1.05689	0.16599
6689	2.11031	0.148884
6690	1.07367	0.110712
6691	0.656239	0.181172
6692	1.4563	0.150122
6693	1.4563	0.141246
6694	0.656238	0.177632
6695	0.946133	0.162498
6696	0.812937	0.192824
6697	0.946131	0.201475
6698	1.05689	0.123023
6699	1.34009	2.1
6700	1.398 2.1	
6701	3.29047	2.1
6702	2.53897	2.1
6703	1.24509	2.1
6704	3.06162	2.1
6705	1.01595	2.1
6706	1.52661	2.1
6707	1.5266	2.1
6708	0.946131	2.1
6709	1.31049	2.1
6710	1.16225	2.1
6711	1.05978	2.1
6712	1.16225	2.1
6713	1.06561	2.1
6714	0.953927	2.1
6715	1.15114	2.1
6716	1.1807	0.244161
6717	0.507259	0.232113
6718	1.47709	0.212853
6719	1.36192	0.196205
6720	0.487293	0.209481
6721	1.65569	0.266207
6722	0.679725	0.175875
6723	0.721928	0.220599
6724	1.03931	0.200498
6725	1.0775	0.227729

6726	1.0775	0.461096
6727	2.30207	1.03886
6728	1.01814	0.935091
6729	2.33963	0.702906
6730	2.42589	1.03414
6731	1.33962	2.1
6732	1.27956	2.1
6733	2.14624	0.05
6734	1.6812	0.05
6735	1.25698	0.05
6736	1.35725	0.05
6737	1.86387	0.05
6738	1.86387	0.05
6739	0.750365	0.05
6740	0.946128	0.05
6741	0.750365	0.05
6742	1.72271	0.05
6743	1.72272	0.05
6744	2.53736	0.053246
6745	1.07327	2.1
6746	1.05376	2.1
6747	1.00387	1.62645
6748	2.0554	1.72544
6749	1.00387	1.48957
6750	1.00387	1.09751
6751	1.27119	2.1
6752	1.51748	2.1
6753	3.15271	2.1
6754	3.45801	2.1
6755	1.99871	2.1
6756	1.41186	2.1
6757	1.07624	2.1
6758	1.5954	2.1
6759	1.19323	1.59277
6760	1.21286	0.858457
6761	0.946114	2.1
6762	1.38395	2.1
6763	1.73389	2.1
6764	1.7339	2.1
6765	0.946115	2.1
6766	1.38397	2.1
6767	1.29604	2.1
6768	1.04544	2.1
6769	1.60578	0.0688102
6770	1.93407	0.0735737
6771	1.0525	1.36303
6772	1.0525	1.33665
6773	1.0525	1.76428
6774	1.38506	1.49071
6775	2.34737	0.05
6776	1.3531	0.05
6777	1.2925	0.05
6778	0.871466	0.05
6779	1.26623	0.05
6780	0.700631	0.05
6781	1.88774	0.05
6782	1.14167	0.05
6783	1.18606	0.05

6784	2.1238	0.05
6785	1.42762	1.14792
6786	1.67791	0.05
6787	1.47118	0.05
6788	0.946128	2.1
6789	1.31641	2.1
6790	1.75933	2.1
6791	1.75933	2.1
6792	0.946131	2.1
6793	1.31641	2.1
6794	1.38905	2.1
6795	1.38437	2.1
6796	1.51829	2.1
6797	0.947114	2.1
6798	1.13335	2.1
6799	1.47521	2.1
6800	1.14911	2.1
6801	1.53822	2.1
6802	1.39702	2.1
6803	0.739166	2.1
6804	0.706056	2.1
6805	0.964033	2.1
6806	1.58993	2.1
6807	2.51892	2.1
6808	0.595905	0.05
6809	1.18155	0.05
6810	1.73364	0.05
6811	0.952735	0.05
6812	2.10381	0.05
6813	1.12115	0.05
6814	1.27826	0.05
6815	2.74021	0.05
6816	1.78416	2.1
6817	1.78416	2.1
6818	0.946132	2.1
6819	1.41126	2.1
6820	1.21275	2.1
6821	0.946133	2.1
6822	1.41126	2.1
6823	1.33125	2.1
6824	1.33125	2.1
6825	1.78918	2.1
6826	1.0465	2.1
6827	1.99715	2.1
6828	1.17683	2.1
6829	1.25889	2.1
6830	0.860834	0.05
6831	0.946131	0.05
6832	1.05008	0.05
6833	0.970444	0.05
6834	1.19401	0.05
6835	1.25989	0.05
6836	0.702942	0.05
6837	1.40591	0.05
6838	1.72168	0.05
6839	0.446352	0.05
6840	1.04072	0.05
6841	0.711038	0.0649423

6842	0.980737	0.05
6843	1.05099	0.05
6844	1.26003	0.05
6845	0.495934	0.05
6846	1.03114	0.05
6847	1.09911	0.05
6848	1.24825	0.05
6849	2.19827	0.05
6850	2.0953	0.05
6851	2.30437	0.05
6852	0.775923	0.05
6853	2.2283	0.05
6854	1.19583	2.1
6855	1.14496	2.1
6856	1.62183	2.1
6857	1.62316	2.1
6858	1.02305	0.151068
6859	1.21136	0.0701228
6860	1.44426	0.120467
6861	1.21137	0.0519894
6862	1.20143	0.100254
6863	1.15009	0.211847
6864	1.24965	2.1
6865	1.41077	2.1
6866	1.00052	0.0997834
6867	1.79553	0.100543
6868	1.06336	0.0745337
6869	1.00053	0.0796457
6870	1.18041	2.1
6871	1.80818	2.1
6872	1.73571	1.58957
6873	1.94429	1.65717
6874	1.4375	0.0504414
6875	1.44421	0.05
6876	1.66953	0.0505764
6877	1.09529	0.05
6878	0.952395	2.1
6879	0.717337	2.1
6880	1.03609	2.1
6881	1.33949	2.1
6882	1.2774	0.167861
6883	2.24983	0.163616
6884	1.28727	0.135575
6885	0.583592	0.127515
6886	0.583591	0.240453
6887	1.82859	0.180002
6888	1.82859	0.233071
6889	0.583593	0.232577
6890	0.946128	0.116669
6891	0.692233	0.265715
6892	1.12226	0.322569
6893	2.57454	0.205918
6894	0.810944	0.174338
6895	0.810945	0.173549
6896	1.12226	0.218456
6897	1.45867	0.250228
6898	1.22403	0.265819
6899	1.15262	0.31208

6900	1.08132	0.169573
6901	0.846671	0.185461
6902	0.480954	0.21892
6903	0.917975	0.310127
6904	0.917975	0.501094
6905	0.720957	0.264826
6906	0.795998	0.289885
6907	0.69316	0.267719
6908	0.641306	0.255194
6909	0.690817	0.188411
6910	0.961899	2.1
6911	2.49579	2.1
6912	2.31106	2.1
6913	1.70482	0.076447
6914	1.93156	0.109431
6915	1.23868	0.134611
6916	1.73878	0.115927
6917	1.26267	2.1
6918	1.26441	2.1
6919	1.58095	2.1
6920	1.58095	2.1
6921	0.946132	2.1
6922	0.946132	2.1
6923	1.11077	2.1
6924	1.62905	2.1
6925	1.49692	2.1
6926	3.49966	2.1
6927	2.39213	2.1
6928	2.12603	2.1
6929	0.946129	2.1
6930	0.461661	2.1
6931	1.52841	2.1
6932	1.39091	2.1
6933	0.550679	2.1
6934	0.509894	2.1
6935	1.49508	2.1
6936	2.05862	2.1
6937	2.47851	2.1
6938	1.07213	2.1
6939	1.09148	1.80553
6940	1.99776	1.97293
6941	1.38333	1.95831
6942	1.04412	2.1
6943	1.04412	2.1
6944	1.1893	0.058117
6945	0.946132	0.067629
6946	0.965737	0.0913702
6947	0.946132	0.0690946
6948	1.54061	0.0693719
6949	1.54061	0.101409
6950	1.22878	0.0762663
6951	1.25797	0.0896384
6952	1.00665	0.0823672
6953	1.00665	0.0965122
6954	1.80369	0.0699035
6955	0.946128	0.0888789
6956	1.04424	0.105727
6957	0.85154	0.0855173

6958	1.39322	0.0883118
6959	1.35464	0.112774
6960	0.939857	0.0948833
6961	1.28272	0.106792
6962	1.19323	0.157885
6963	1.03498	0.0702615
6964	2.22029	0.0809814
6965	0.540005	0.0675381
6966	0.970382	0.103138
6967	2.14514	0.0871568
6968	1.31542	0.0884438
6969	1.05575	0.142911
6970	0.810943	0.163275
6971	1.31542	0.0706517
6972	1.70493	0.0907859
6973	1.22582	0.112563
6974	1.31681	0.131394
6975	1.40775	0.103484
6976	0.928633	0.196529
6977	1.2545	0.0944315
6978	2.66481	0.110289
6979	1.32051	0.0781122
6980	1.32051	0.127755
6981	2.66305	2.1
6982	1.3551	2.1
6983	2.45325	2.1
6984	1.3685	2.1
6985	1.51748	2.1
6986	1.78941	2.1
6987	1.02963	0.05
6988	1.28643	0.05
6989	0.86131	0.05
6990	0.851539	0.05
6991	1.46585	0.05
6992	1.38822	0.05
6993	0.996402	0.05
6994	0.851553	0.05
6995	1.73156	0.05
6996	1.91695	0.05
6997	1.92535	0.05
6998	2.57287	0.05
6999	1.77743	0.05
7000	1.02836	0.213603
7001	1.36362	0.257879
7002	1.36936	0.215326
7003	1.78684	0.170025
7004	1.78685	0.17864
7005	0.946132	0.33328
7006	1.27581	0.286622
7007	0.946132	0.405448
7008	1.02836	0.153635
7009	1.55398	2.1
7010	1.07167	2.1
7011	1.87557	2.1
7012	0.751639	2.1
7013	1.50884	2.1
7014	1.33793	2.1
7015	0.751664	2.1

7016	1.37277	2.1
7017	0.979515	2.1
7018	0.676508	2.1
7019	1.09578	2.1
7020	0.953925	2.1
7021	0.94613	2.1
7022	1.64707	2.1
7023	1.64707	2.1
7024	1.16658	2.1
7025	1.40638	2.1
7026	1.18682	2.1
7027	1.16658	2.1
7028	0.543765	0.164474
7029	1.57462	0.154233
7030	1.57462	0.135585
7031	1.00083	0.162132
7032	1.16834	0.20196
7033	1.16834	0.185979
7034	0.889626	0.180356
7035	1.25944	0.207542
7036	0.824061	0.214175
7037	1.9397	0.130421
7038	1.21893	0.150473
7039	1.91364	0.14905
7040	1.33672	0.199004
7041	0.928069	0.270243
7042	1.41484	0.205322
7043	0.538379	0.170882
7044	1.41484	0.184364
7045	1.35594	0.218783
7046	2.11414	0.198805
7047	2.11414	0.128758
7048	0.538378	0.203319
7049	1.38532	0.1238
7050	0.962877	1.03059
7051	1.07497	0.473347
7052	1.00613	0.582485
7053	0.962878	0.173126
7054	0.711447	2.1
7055	1.40008	0.0632911
7056	2.51674	0.0712359
7057	1.40008	0.0565684
7058	1.7263	0.05
7059	1.40008	0.0664488
7060	1.9216	0.05
7061	2.7013	0.05
7062	2.58584	0.05
7063	0.979296	0.05
7064	1.46459	0.821999
7065	0.899254	0.573274
7066	1.15038	2.1
7067	1.24687	2.1
7068	1.16738	2.1
7069	0.942323	2.1
7070	1.65573	1.98096
7071	0.844791	2.1
7072	0.54674	2.1
7073	0.795811	2.1

7074	1.08131	2.1
7075	1.03553	2.1
7076	0.758334	2.1
7077	0.708665	2.06137
7078	1.22626	2.0189
7079	0.835439	2.01283
7080	0.861131	1.93036
7081	0.68296	2.09889
7082	1.65224	2.01436
7083	2.82624	2.1
7084	1.0151	1.79626
7085	1.15905	1.89811
7086	1.71076	1.81959
7087	1.4529	1.70758
7088	0.494895	2.1
7089	0.946132	1.90773
7090	0.613132	1.85627
7091	0.863631	1.8778
7092	1.78391	2.1
7093	1.1706	0.05
7094	2.02734	0.05
7095	1.1706	0.05
7096	0.681532	0.05
7097	1.50316	0.05
7098	1.50316	0.05
7099	0.681534	0.05
7100	0.973819	0.05
7101	0.97382	0.05
7102	0.94613	0.05
7103	1.1706	0.05
7104	1.67727	0.05
7105	1.60329	0.05
7106	1.3227	2.1
7107	1.40846	2.1
7108	1.07889	2.1
7109	1.07721	2.1
7110	0.953523	0.700368
7111	1.79675	0.684016
7112	2.09859	1.4584
7113	1.21309	2.1
7114	1.54567	1.16774
7115	1.19722	0.331307
7116	1.82793	0.335272
7117	1.1599	0.14595
7118	1.73621	0.114046
7119	0.933474	0.268149
7120	1.51477	0.173568
7121	1.51477	0.206298
7122	0.93347	0.25481
7123	1.27374	0.183282
7124	1.27376	0.220284
7125	0.946105	0.311645
7126	1.1972	0.187848
7127	0.851541	0.05
7128	1.36971	0.05
7129	1.27832	0.05
7130	0.853489	0.05
7131	0.973847	0.05

7132	0.976308	0.05
7133	0.851528	0.05
7134	2.72654	0.05
7135	1.77867	0.05
7136	3.23032	2.1
7137	2.78208	2.1
7138	1.0227	0.05
7139	1.02269	0.05
7140	1.02269	0.05
7141	1.24293	0.05
7142	0.831695	2.1
7143	0.67513	2.1
7144	0.956294	2.1
7145	1.08623	2.1
7146	1.05556	2.1
7147	1.38859	2.1
7148	1.13179	2.1
7149	1.02438	2.1
7150	1.06659	2.1
7151	1.36595	2.1
7152	1.06659	2.1
7153	1.06659	2.1
7154	0.946131	1.96568
7155	0.875912	2.1
7156	1.65971	2.1
7157	1.65971	2.1
7158	0.875912	2.1
7159	1.1229	2.1
7160	1.41273	2.1
7161	1.54699	1.96049
7162	1.93655	1.73461
7163	1.42307	1.94212
7164	1.97907	1.97226
7165	0.996774	2.1
7166	1.15505	0.05
7167	1.28463	0.05
7168	1.86353	0.05
7169	2.71398	0.05
7170	1.56787	0.238188
7171	1.66295	0.137939
7172	1.73432	1.59387
7173	1.05019	0.05
7174	1.42117	0.05
7175	0.888299	0.05
7176	1.38127	0.05
7177	1.72993	0.05
7178	0.664129	0.05
7179	0.99302	2.1
7180	0.993023	2.1
7181	1.13491	2.1
7182	0.876316	2.1
7183	1.2588	2.1
7184	0.895613	2.1
7185	2.07526	2.1
7186	1.23369	2.1
7187	1.25146	2.1
7188	2.15282	2.1
7189	1.22239	2.1

7190	1.10962	2.1
7191	1.77402	2.1
7192	1.0305	2.1
7193	1.50108	2.04985
7194	1.20761	2.1
7195	1.3626	2.1
7196	1.14825	2.1
7197	1.78711	2.1
7198	1.17933	2.1
7199	1.14824	2.1
7200	1.09651	0.108095
7201	1.23851	0.105626
7202	1.83284	0.116879
7203	1.23851	0.0658423
7204	1.53521	2.1
7205	1.46279	2.1
7206	1.9233	0.05
7207	1.2966	0.05
7208	2.11443	0.05
7209	1.30265	0.05
7210	1.13528	0.152592
7211	0.946131	0.228434
7212	1.41169	0.190379
7213	1.33879	0.119032
7214	0.946128	0.175955
7215	1.80435	0.171535
7216	1.80435	0.0692987
7217	1.234 2.1	
7218	1.45633	2.1
7219	1.234 2.1	
7220	1.98448	2.1
7221	2.02612	2.1
7222	1.23527	1.68428
7223	1.44472	1.85603
7224	0.993366	2.1
7225	1.22025	2.0684
7226	0.993368	1.55728
7227	2.43669	2.1
7228	2.56707	2.1
7229	1.80348	1.01229
7230	1.74406	0.493133
7231	1.90937	0.831951
7232	1.49357	0.137208
7233	2.5832	0.258359
7234	1.82274	0.267361
7235	1.6957	0.231215
7236	2.22495	0.253456
7237	2.49959	0.248429
7238	2.01183	0.0684008
7239	1.37427	0.05
7240	1.15069	2.1
7241	1.39379	2.1
7242	1.69661	1.75314
7243	1.39379	1.88558
7244	1.39654	2.1
7245	1.93505	2.1
7246	1.5525	2.1
7247	1.5525	2.1

7248	0.615065	2.1
7249	1.38908	2.1
7250	0.615065	2.1
7251	0.77849	2.1
7252	1.38908	2.1
7253	1.56839	2.1
7254	1.09142	2.1
7255	2.07313	2.1
7256	0.998288	0.05
7257	1.31481	0.05
7258	1.88175	0.05
7259	2.23267	0.05
7260	1.06935	2.1
7261	1.06936	2.1
7262	1.17989	2.1
7263	1.00757	2.06983
7264	1.65318	2.1
7265	1.4185	0.188328
7266	2.22495	0.226663
7267	1.02011	0.136079
7268	0.59676	0.200864
7269	1.43606	0.166118
7270	1.43606	0.154404
7271	0.89771	0.180801
7272	1.13511	0.213753
7273	0.59676	0.138232
7274	0.897709	0.192333
7275	1.79542	0.168609
7276	2.38566	2.1
7277	1.63838	1.01179
7278	1.63837	1.45447
7279	0.916275	2.07522
7280	1.36183	1.38497
7281	1.19282	2.1
7282	0.916275	1.82521
7283	1.19283	1.97339
7284	2.01582	2.1
7285	1.00215	0.05
7286	2.60643	2.1
7287	2.15971	2.1
7288	1.28267	2.1
7289	1.29954	2.1
7290	3.40327	2.1
7291	3.49128	2.1
7292	1.3864	2.1
7293	1.3201	2.1
7294	1.45666	0.05
7295	1.55798	0.05
7296	0.857202	0.05
7297	1.24374	0.05
7298	1.04157	0.05
7299	0.85723	0.05
7300	1.02722	0.05
7301	1.01361	0.05
7302	0.974077	0.05
7303	1.01814	0.05
7304	0.974077	0.05
7305	1.43987	0.05

7306	1.43986	0.05
7307	0.927881	0.05
7308	0.946132	0.05
7309	0.927849	0.05
7310	0.927852	0.05
7311	1.2493	0.05
7312	1.22482	0.30838
7313	1.41319	0.598211
7314	1.22482	0.187693
7315	0.999492	2.1
7316	1.14657	2.1
7317	1.3617	0.05
7318	0.983269	0.05
7319	1.33174	0.05
7320	1.62816	0.05
7321	1.62817	0.05
7322	0.81938	0.05
7323	1.33174	0.05
7324	1.01332	0.05
7325	0.874788	0.05
7326	1.16725	2.1
7327	1.63991	2.1
7328	1.08848	0.05
7329	1.15354	0.05
7330	1.12665	2.1
7331	1.58742	2.1
7332	1.57523	2.1
7333	1.32201	1.39251
7334	1.32097	1.0597
7335	0.851542	0.101173
7336	0.877188	0.0611077
7337	0.797489	0.104914
7338	1.44721	0.0582475
7339	1.61761	0.05
7340	0.943799	0.0987339
7341	1.36079	0.0741482
7342	1.16361	2.1
7343	1.15852	2.1
7344	1.07624	2.1
7345	1.07624	2.1
7346	0.946132	1.23877
7347	1.37938	1.38681
7348	1.78653	1.39445
7349	1.78653	1.27979
7350	0.946131	1.35788
7351	1.37938	1.29193
7352	1.35328	1.26791
7353	1.44847	1.02584
7354	0.946133	0.842457
7355	1.38149	1.28162
7356	1.81474	1.34259
7357	1.81474	0.94722
7358	0.946133	1.34086
7359	1.38149	1.2492
7360	1.37938	1.23438
7361	1.44847	1.26811
7362	1.93665	1.0586
7363	1.5515	0.616389

7364	1.05025	2.1
7365	1.05025	2.1
7366	1.71468	2.1
7367	1.04759	2.1
7368	1.04646	0.05
7369	1.00739	0.05
7370	1.37086	0.05
7371	1.12547	0.05
7372	1.03853	0.05
7373	1.34824	0.05
7374	0.799786	0.05
7375	2.10105	0.05
7376	2.72007	0.05
7377	0.589918	0.05
7378	0.589918	0.05
7379	1.1509	0.05
7380	0.619832	0.05
7381	1.1509	0.05
7382	0.705903	0.05
7383	1.53703	0.05
7384	1.53703	0.05
7385	0.705904	0.05
7386	0.946133	0.05
7387	0.946132	0.05
7388	0.946132	0.05
7389	0.588467	0.05
7390	1.1078	0.05
7391	0.408061	0.05
7392	1.38703	0.05
7393	1.68328	0.05
7394	1.87031	0.05
7395	1.86268	0.0763098
7396	1.27472	0.0575159
7397	0.947862	1.00358
7398	1.19854	1.41156
7399	0.946129	0.749566
7400	0.963111	0.439674
7401	0.946131	1.18365
7402	1.39055	0.374036
7403	1.39055	0.26327
7404	1.26426	0.480412
7405	1.07242	0.531754
7406	1.31126	0.210538
7407	1.76597	0.616962
7408	0.94613	1.49689
7409	1.27214	1.25391
7410	1.90751	1.30817
7411	1.90751	0.951784
7412	0.767114	1.10334
7413	1.27214	1.47823
7414	1.40248	1.41629
7415	1.41479	1.56367
7416	2.22625	1.56905
7417	0.913373	1.48791
7418	1.0158	1.30979
7419	1.61259	1.3628
7420	1.69051	0.953401
7421	0.560949	1.36885

7422	0.898559	1.48362
7423	1.28011	1.41418
7424	1.51026	0.0544452
7425	1.98252	0.0692909
7426	1.05129	0.0665912
7427	1.2951	0.074243
7428	1.46041	0.05
7429	1.7155	2.1
7430	1.72196	2.1
7431	0.933885	2.1
7432	0.986723	2.1
7433	1.28072	1.27625
7434	1.40765	2.1
7435	1.28072	2.1
7436	1.61687	2.1
7437	1.60965	2.1
7438	1.21424	2.1
7439	2.07297	2.1
7440	1.19943	2.1
7441	2.38784	2.1
7442	1.10278	0.0930774
7443	1.43678	0.109718
7444	1.1142	0.0907576
7445	1.18034	0.0848877
7446	0.94613	0.101941
7447	0.752356	0.080224
7448	0.752329	0.0927077
7449	1.46634	0.0711847
7450	1.31789	0.0806611
7451	0.958159	0.108997
7452	1.22752	0.117417
7453	1.50472	0.0816842
7454	1.98974	2.1
7455	2.54186	2.1
7456	0.76512	2.1
7457	2.08777	2.1
7458	1.7753	2.1
7459	3.07372	2.1
7460	1.9756	2.1
7461	1.48162	2.1
7462	1.23204	2.1
7463	0.946131	1.5817
7464	1.13505	1.99258
7465	1.47432	2.07508
7466	1.47432	1.68123
7467	0.876831	1.85433
7468	1.13505	1.74065
7469	1.2161	1.71734
7470	1.05267	1.52134
7471	1.05267	1.67485
7472	1.5795	1.5932
7473	0.98186	2.1
7474	1.56735	2.1
7475	0.859099	2.1
7476	1.69858	2.1
7477	1.93586	2.1
7478	1.09233	2.1
7479	1.08732	0.102948

7480	1.3357	0.103541
7481	1.84826	2.1
7482	0.924132	2.1
7483	1.39188	2.1
7484	1.43685	2.1
7485	0.851541	2.1
7486	1.16251	2.1
7487	0.946131	2.1
7488	1.05685	2.1
7489	2.13781	2.1
7490	1.98859	2.1
7491	1.99731	2.1
7492	1.51379	2.1
7493	1.48796	2.1
7494	0.851542	2.1
7495	0.802558	2.1
7496	1.09244	2.1
7497	1.33891	2.1
7498	0.844958	2.1
7499	0.710864	2.1
7500	1.26087	2.1
7501	1.03679	2.1
7502	1.51026	0.0512456
7503	1.05248	0.617305
7504	0.827258	0.621951
7505	1.05248	0.631678
7506	1.28435	0.616461
7507	1.50953	0.612293
7508	1.50953	0.220179
7509	0.827298	0.673169
7510	1.00366	0.19506
7511	2.10495	0.316994
7512	1.17885	2.1
7513	1.25185	2.1
7514	1.02784	2.1
7515	1.76764	2.1
7516	1.23746	0.0908697
7517	1.24082	0.105042
7518	1.46506	0.0504104
7519	1.33061	0.05
7520	0.768407	0.563088
7521	0.972058	0.325013
7522	1.16536	0.415388
7523	1.13325	0.278213
7524	0.978906	1.28458
7525	1.02291	0.394345
7526	0.838015	0.364719
7527	1.01052	0.151105
7528	1.08166	0.168947
7529	1.42607	0.05
7530	1.47354	0.0607983
7531	1.21703	2.1
7532	1.29897	2.1
7533	1.83293	2.1
7534	1.29897	2.1
7535	1.24688	0.118073
7536	0.782563	2.1
7537	0.946129	2.1

7538	0.782591	2.1
7539	1.49487	2.1
7540	1.49487	2.1
7541	1.37713	2.1
7542	0.900337	2.1
7543	1.31284	2.1
7544	0.924468	2.1
7545	1.40602	2.1
7546	1.25669	2.1
7547	0.986901	2.1
7548	0.958565	2.1
7549	1.37266	2.1
7550	1.64146	2.1
7551	0.49345	2.1
7552	1.08276	2.1
7553	1.08276	2.1
7554	1.26929	2.1
7555	1.09847	2.1
7556	0.827081	0.165872
7557	1.07236	0.316012
7558	1.25063	0.846754
7559	1.43548	0.157923
7560	1.25063	0.167366
7561	1.3659	0.05
7562	1.72441	0.05
7563	1.3659	0.05
7564	1.4677	0.05
7565	1.86114	0.05
7566	1.40104	0.05
7567	0.962675	0.05
7568	0.946131	0.05
7569	1.74134	0.05
7570	1.74134	0.05
7571	0.962676	0.05
7572	0.946131	0.05
7573	1.05548	0.0765332
7574	0.902187	0.0891402
7575	2.14178	0.0537701
7576	1.74998	0.0664293
7577	1.38541	0.0751324
7578	1.64468	0.0553576
7579	0.949317	0.0821858
7580	2.06654	0.0618995
7581	0.839626	0.0850461
7582	0.810945	0.0928458
7583	1.29203	0.0509355
7584	1.18091	0.0653219
7585	1.06872	0.0811905
7586	1.11956	0.0586186
7587	0.926911	0.0975905
7588	0.901716	0.0904562
7589	0.909146	0.0967173
7590	1.33776	0.06397
7591	1.02048	0.0793782
7592	1.08365	0.0895548
7593	1.21042	0.0667994
7594	0.865634	0.0995082
7595	0.590354	2.1

7596	0.61173	2.1
7597	3.46825	2.1
7598	3.28404	2.1
7599	1.56004	0.0757322
7600	1.69344	0.249727
7601	1.7151	0.995888
7602	0.946131	0.300633
7603	1.40265	0.172683
7604	1.13537	0.884277
7605	0.965708	0.36509
7606	1.13537	0.384598
7607	1.29681	0.333259
7608	1.40915	0.270107
7609	1.61652	1.60276
7610	1.33171	2.1
7611	1.37681	2.1
7612	0.81094	0.05
7613	0.598606	0.05
7614	0.723111	0.05
7615	1.04562	0.05
7616	0.979052	0.05
7617	0.798185	0.05
7618	0.880117	0.05
7619	0.979341	0.306899
7620	0.800229	0.202047
7621	1.39126	0.288983
7622	1.13005	0.324049
7623	0.913877	0.35348
7624	1.26959	0.267447
7625	0.699741	0.337368
7626	1.19496	1.43713
7627	1.13392	0.05
7628	1.11913	0.05
7629	1.06866	0.05
7630	0.615546	0.05
7631	0.831006	0.05
7632	1.53286	0.05
7633	0.690977	0.05
7634	1.01956	0.05
7635	2.33818	0.05
7636	1.6585	2.1
7637	1.67061	2.1
7638	1.41414	2.1
7639	1.16756	2.1
7640	1.00898	0.05
7641	0.966964	2.1
7642	2.85443	2.1
7643	0.966977	2.1
7644	2.08427	2.1
7645	2.94901	2.1
7646	1.10735	0.23753
7647	1.65815	2.1
7648	1.4502	2.1
7649	1.45026	2.1
7650	1.53446	2.1
7651	0.946129	1.38885
7652	1.81075	2.1
7653	1.81075	1.55569

7654	0.946131	1.67936
7655	1.37075	2.1
7656	1.37075	1.57928
7657	1.34245	1.76939
7658	0.973502	1.55502
7659	1.08542	2.09165
7660	1.5814	0.05
7661	1.43836	0.05
7662	0.946132	0.05
7663	1.13747	0.05
7664	1.04429	0.05
7665	1.05017	0.05
7666	1.24732	0.05
7667	1.13387	0.05
7668	2.19489	0.05
7669	1.10748	2.1
7670	1.25006	2.1
7671	1.58834	2.1
7672	1.58834	2.1
7673	0.94613	2.1
7674	1.16548	2.1
7675	0.946132	2.1
7676	1.08998	2.1
7677	1.16548	2.1
7678	0.995732	2.0803
7679	0.657014	2.1
7680	0.957173	2.1
7681	1.01485	2.1
7682	1.12468	2.1
7683	1.43871	2.1
7684	1.23716	2.1
7685	1.89277	0.867543
7686	2.06653	0.335386
7687	0.967544	0.228309
7688	1.89016	0.301484
7689	2.22165	2.1
7690	0.83514	2.1
7691	1.76565	2.1
7692	1.76565	2.1
7693	0.94613	2.1
7694	0.835112	2.1
7695	1.23426	2.1
7696	1.36653	2.1
7697	0.895892	1.80362
7698	0.859278	1.78211
7699	0.791461	1.78596
7700	1.86249	1.7969
7701	1.14607	1.81708
7702	1.19904	1.77841
7703	1.84898	1.78725
7704	1.03452	1.86491
7705	0.946131	1.67218
7706	0.940472	0.704692
7707	1.53478	2.1
7708	1.84175	1.83223
7709	1.55687	1.74071
7710	1.17787	1.06785
7711	1.60589	0.388188

7712	2.35574	0.312156
7713	1.3305	0.399764
7714	1.15008	1.36127
7715	1.12457	0.05
7716	1.12457	0.0515747
7717	1.01778	2.1
7718	1.35733	2.1
7719	1.50683	2.1
7720	1.12064	2.1
7721	1.42716	1.14562
7722	1.42717	1.36069
7723	1.45927	1.51333
7724	1.04321	1.58907
7725	1.32634	0.188529
7726	2.27022	0.159805
7727	1.19352	0.127593
7728	1.32634	0.127619
7729	1.40924	1.60351
7730	1.22927	1.46555
7731	1.69237	1.54212
7732	1.69237	2.01636
7733	0.946132	1.49187
7734	0.946133	1.57006
7735	1.16912	1.59468
7736	1.0417	1.48655
7737	1.14605	1.49727
7738	1.1233	2.1
7739	1.96882	1.81148
7740	1.03804	0.0709497
7741	1.15338	0.056643
7742	1.15338	0.0612246
7743	2.33051	0.082054
7744	1.81507	2.1
7745	2.42949	2.1
7746	0.725879	2.1
7747	1.88394	2.1
7748	2.2699	0.692514
7749	2.78952	2.1
7750	1.88394	0.835433
7751	1.79606	2.1
7752	1.20091	2.1
7753	1.9614	2.1
7754	1.19901	2.07868
7755	3.07915	2.1
7756	1.32592	2.1
7757	2.86461	2.1
7758	1.34817	2.1
7759	1.44859	2.1
7760	2.03969	2.1
7761	0.946132	2.1
7762	1.50709	2.1
7763	1.50709	2.1
7764	0.946132	2.1
7765	1.29322	2.1
7766	1.29322	2.1
7767	1.01985	2.1
7768	0.968537	2.1
7769	1.401	2.1

7770	1.28936	2.1
7771	1.56002	0.05
7772	1.92548	0.05
7773	1.46041	0.05
7774	2.22712	0.05
7775	1.56001	0.05
7776	1.50524	2.1
7777	1.21304	2.1
7778	1.60665	1.82701
7779	1.85178	1.46147
7780	2.17534	2.1
7781	2.31012	2.1
7782	2.17533	1.90378
7783	0.986724	2.1
7784	2.01895	2.1
7785	2.00433	2.1
7786	1.45752	0.123934
7787	1.12695	0.128326
7788	0.652605	2.1
7789	1.5133	2.1
7790	1.5133	2.1
7791	0.652603	2.1
7792	1.228	2.1
7793	0.946132	2.1
7794	0.652604	2.1
7795	0.946132	1.94594
7796	0.716815	2.1
7797	1.62203	2.08856
7798	1.62203	2.1
7799	0.716814	2.1
7800	1.00191	2.1
7801	1.33694	2.1
7802	1.38216	2.1
7803	2.53338	2.1
7804	1.46932	0.05
7805	1.50848	0.05
7806	1.50108	1.39932
7807	1.18382	0.359562
7808	2.02115	0.277101
7809	0.921823	0.288157
7810	2.08356	0.05
7811	1.543	0.05
7812	1.00763	0.145075
7813	1.4185	0.159236
7814	1.08028	0.179677
7815	0.946131	1.0794
7816	1.56599	0.167655
7817	1.56599	0.913594
7818	1.08028	0.192366
7819	0.946131	0.0908265
7820	1.37518	2.1
7821	1.63453	2.1
7822	1.04627	1.61893
7823	1.04626	1.63345
7824	1.35111	1.62863
7825	1.22297	1.58365
7826	1.64499	2.1
7827	1.87598	2.1

7828	1.06994	2.1
7829	1.37063	2.1
7830	1.99943	0.05
7831	2.38807	0.05
7832	0.99972	0.0643505
7833	1.41388	0.071762
7834	1.66179	0.0646564
7835	1.66179	0.05
7836	0.946129	0.0796239
7837	1.41388	0.0690197
7838	1.19404	0.0673889
7839	1.31023	2.1
7840	1.20871	2.1
7841	1.69731	2.1
7842	1.67971	2.1
7843	0.998971	0.05
7844	1.32988	0.05
7845	0.701079	0.05
7846	0.946131	0.05
7847	0.582785	0.05
7848	1.48592	0.05
7849	1.48592	0.05
7850	1.23351	0.05
7851	0.835199	0.05
7852	0.92427	0.05
7853	1.68262	0.05
7854	2.46702	0.05
7855	1.08715	2.09782
7856	1.01606	1.90131
7857	1.29579	1.9662
7858	1.01606	2.01177
7859	1.63823	2.1
7860	1.67687	2.1
7861	1.11048	2.1
7862	1.39477	2.1
7863	0.991114	2.1
7864	2.90978	0.592472
7865	0.906811	2.02921
7866	0.872849	0.374327
7867	1.17091	1.31767
7868	0.685763	1.21074
7869	1.6386	1.69361
7870	1.06907	0.824514
7871	1.05516	1.81369
7872	1.9189	1.49781
7873	1.04615	0.0898329
7874	1.2966	2.1
7875	1.37392	2.1
7876	1.35504	2.1
7877	1.30347	2.1
7878	1.50877	2.1
7879	1.11193	2.1
7880	1.40984	2.1
7881	1.67682	2.1
7882	1.00371	2.1
7883	1.15412	2.1
7884	1.41638	2.1
7885	1.03796	2.1

7886	0.946117	2.1
7887	1.50823	2.1
7888	1.50822	2.1
7889	0.966923	2.1
7890	0.946131	2.1
7891	2.07593	2.1
7892	1.9339	2.1
7893	1.1817	0.316523
7894	0.729178	0.562396
7895	1.5079	0.390571
7896	1.26991	0.506175
7897	0.994049	0.435566
7898	1.17452	0.378917
7899	1.05711	0.477709
7900	1.04071	0.518572
7901	1.80299	0.326911
7902	1.60417	0.296093
7903	0.943405	0.05
7904	1.01129	0.05
7905	1.42972	0.05
7906	1.15904	0.05
7907	0.680409	0.05
7908	1.2816	0.05
7909	0.806176	0.05
7910	1.18954	0.05
7911	1.26055	0.05
7912	1.06313	2.1
7913	1.19352	2.1
7914	1.55766	2.1
7915	1.53729	2.1
7916	0.993021	1.27075
7917	1.09056	2.1
7918	1.03408	2.1
7919	0.993023	2.1
7920	0.659901	2.1
7921	0.632905	2.1
7922	1.34072	0.100078
7923	1.17793	0.0845538
7924	2.24885	0.0972937
7925	0.946132	0.0866289
7926	1.12443	0.101181
7927	0.904207	0.0835494
7928	1.51237	0.0787792
7929	1.51237	0.0998382
7930	1.12443	0.101248
7931	1.29215	0.111963
7932	1.6943	2.03078
7933	2.43858	2.07837
7934	2.0589	1.77431
7935	1.67204	2.1
7936	1.67204	1.99449
7937	0.555875	1.90911
7938	0.91951	2.1
7939	0.946131	1.77415
7940	0.555904	1.88795
7941	0.946131	1.88269
7942	1.6943	2.1
7943	1.04744	1.792

7944	1.50938	2.1
7945	1.60861	2.1
7946	1.92406	0.0533119
7947	1.18716	0.05
7948	0.962705	0.602841
7949	1.35382	1.44693
7950	1.43632	0.05
7951	1.09386	0.05
7952	1.92547	0.05
7953	0.994164	0.05
7954	0.903974	1.97797
7955	0.928829	1.845
7956	0.810958	1.76181
7957	1.92853	1.783
7958	1.598	1.75838
7959	1.28967	1.83598
7960	1.44993	1.80677
7961	0.946132	1.16808
7962	0.882984	0.977346
7963	0.882955	1.23348
7964	1.56071	0.279922
7965	1.56071	0.345675
7966	1.35946	0.926928
7967	1.08421	1.19022
7968	1.41943	0.05
7969	1.26265	0.05
7970	1.03235	0.309965
7971	1.50778	2.1
7972	1.70387	2.1
7973	1.65608	2.1
7974	1.39076	2.1
7975	1.6104	2.1
7976	1.37456	2.1
7977	1.51898	0.454161
7978	1.9726	0.128463
7979	2.13757	1.31637
7980	1.74856	1.38565
7981	0.635949	0.592947
7982	2.46448	1.16378
7983	1.23224	1.20216
7984	1.12786	1.68649
7985	1.82721	1.206
7986	1.82721	0.701434
7987	0.532892	1.87466
7988	0.946131	1.69206
7989	0.53292	1.65868
7990	1.41394	2.1
7991	1.45506	2.1
7992	1.39523	2.1
7993	1.09006	2.1
7994	1.80264	0.659788
7995	1.80264	2.08831
7996	0.946131	0.669377
7997	1.34268	0.0948998
7998	0.946132	1.91065
7999	1.23732	0.819044
8000	1.34268	0.880259
8001	1.2064	2.1

8002	1.58164	2.1
8003	1.73853	2.1
8004	1.01243	1.77041
8005	0.910672	1.51427
8006	1.47793	1.70076
8007	1.47793	1.9782
8008	0.910702	1.52733
8009	1.30382	1.55202
8010	1.08478	1.43938
8011	0.910702	1.79352
8012	2.60709	1.4037
8013	1.01243	1.73921
8014	1.85516	1.33447
8015	2.52823	1.06865
8016	1.24001	1.25495
8017	1.89793	0.815022
8018	1.73584	0.05
8019	1.24613	1.89346
8020	1.37074	1.69337
8021	1.23763	1.67409
8022	1.04072	2.01453
8023	0.75499	1.70456
8024	1.72329	1.67401
8025	1.46195	1.64511
8026	0.946132	1.66796
8027	0.747438	1.83537
8028	1.24613	1.61798
8029	2.59886	1.37327
8030	1.87279	2.1
8031	1.07977	2.1
8032	1.29606	2.1
8033	1.45178	2.1
8034	1.45177	2.1
8035	0.92406	2.1
8036	0.946131	2.1
8037	0.924047	2.1
8038	2.04529	2.1
8039	1.04544	2.1
8040	1.08276	2.1
8041	1.67491	2.1
8042	0.961826	1.27521
8043	0.810942	0.336445
8044	0.826622	1.22932
8045	0.914378	1.3506
8046	0.89022	0.947467
8047	0.922383	0.786534
8048	0.988165	1.12565
8049	1.31357	0.05
8050	0.946131	0.05
8051	1.31357	0.05
8052	1.10564	0.05
8053	1.6797	0.05
8054	1.6797	0.05
8055	0.739506	0.05
8056	1.33957	0.0899268
8057	1.33957	0.0690349
8058	1.33957	0.05
8059	2.34223	0.062778

8060	1.5515	1.24861
8061	2.22137	0.576563
8062	0.94612	0.549633
8063	1.13908	0.642804
8064	1.45423	0.452171
8065	1.45422	1.17998
8066	0.795519	1.21044
8067	1.1391	0.689737
8068	1.11065	0.939261
8069	1.12266	2.1
8070	1.24925	2.1
8071	1.20242	2.1
8072	2.18297	2.1
8073	3.00107	2.00131
8074	2.58297	0.713514
8075	0.884783	2.1
8076	0.851538	1.50179
8077	0.713587	2.03862
8078	1.47949	1.60522
8079	1.66673	2.1
8080	0.696199	2.1
8081	0.9383	1.85549
8082	1.29149	1.7321
8083	1.32266	2.1
8084	2.11428	0.05
8085	2.17439	0.05
8086	1.40489	0.05
8087	1.21071	0.05
8088	0.946118	0.05
8089	1.0746	0.05
8090	0.973119	0.05
8091	1.31333	0.05
8092	1.08723	0.05
8093	0.994292	2.1
8094	1.4964	2.1
8095	1.15096	2.1
8096	1.10101	2.1
8097	1.50438	2.1
8098	1.67825	2.1
8099	2.02103	2.1
8100	2.01978	2.1
8101	1.50931	0.05
8102	1.50931	0.05
8103	0.65023	0.05
8104	1.34831	0.05
8105	0.811233	0.05
8106	1.34831	0.05
8107	0.94613	0.05
8108	1.00754	1.67767
8109	0.714132	1.98532
8110	1.28877	1.6444
8111	1.39905	1.65557
8112	1.03282	1.60753
8113	0.997769	1.67013
8114	1.11743	1.63166
8115	1.86863	1.09631
8116	3.30723	1.66112
8117	1.66635	1.96363

8118	1.35609	0.05
8119	1.38212	0.05
8120	1.95597	2.1
8121	1.64257	2.1
8122	2.93316	2.1
8123	3.13417	2.1
8124	0.733947	2.1
8125	1.15259	2.1
8126	1.07976	2.1
8127	1.30237	2.1
8128	1.99943	0.05
8129	0.907431	0.0537039
8130	0.878161	0.0602568
8131	1.07544	0.05
8132	1.03401	0.05
8133	0.991975	0.05
8134	1.03635	0.05
8135	0.788549	0.05
8136	1.44984	0.05
8137	1.84007	0.0574801
8138	2.31127	0.0533361
8139	1.27362	2.1
8140	1.27737	1.27249
8141	1.87783	1.19132
8142	2.35499	0.05
8143	2.69662	0.05
8144	2.24065	0.05
8145	0.996082	2.1
8146	0.996083	2.1
8147	1.44859	2.1
8148	1.03968	2.1
8149	1.60018	1.13146
8150	1.73082	1.69814
8151	0.946132	1.65185
8152	1.49742	1.86719
8153	1.49742	0.972124
8154	1.00501	1.55848
8155	1.41585	1.94797
8156	1.41585	1.59334
8157	0.946131	1.86262
8158	1.03697	1.70418
8159	2.95786	2.1
8160	2.32185	2.1
8161	1.65783	1.97863
8162	1.772 2.1	
8163	0.946131	2.07501
8164	1.40404	1.68276
8165	1.13537	2.1
8166	1.04559	2.1
8167	1.16091	2.1
8168	2.63751	2.1
8169	1.68455	0.05
8170	1.9682	0.05
8171	2.16834	0.110237
8172	2.45569	0.183164
8173	0.946128	0.128686
8174	1.36428	0.205201
8175	0.663978	0.308888

8176	1.58218	0.139052
8177	1.58219	0.119579
8178	1.36428	0.184278
8179	0.881886	0.193775
8180	1.73849	2.1
8181	1.35668	2.1
8182	1.1233	2.1
8183	1.15009	2.1
8184	1.16482	1.04322
8185	1.29313	1.05225
8186	1.47608	0.0947615
8187	2.05949	0.105178
8188	0.946119	0.0637421
8189	1.02974	0.0832616
8190	0.946119	0.0699658
8191	1.66758	0.0506568
8192	1.66757	0.0611334
8193	1.32082	0.0637071
8194	1.29287	0.061635
8195	1.36005	2.1
8196	1.47109	2.1
8197	2.45958	2.1
8198	2.2975	2.1
8199	0.953521	0.972004
8200	0.953521	0.445087
8201	1.67054	2.1
8202	1.67054	2.1
8203	0.946131	2.1
8204	1.38154	2.1
8205	1.13326	2.1
8206	1.13326	2.1
8207	1.23513	2.1
8208	1.50998	2.00375
8209	2.47982	0.0698256
8210	1.45091	0.05
8211	0.958953	0.05
8212	0.79239	0.05
8213	2.11929	0.0621184
8214	1.26511	0.0685207
8215	1.27733	0.05
8216	2.18127	0.05
8217	1.30169	0.05
8218	1.19259	0.110099
8219	0.963395	1.77489
8220	0.999947	2.1
8221	1.62241	2.1
8222	1.62027	2.1
8223	0.981586	0.05
8224	0.981585	0.05
8225	0.721961	2.1
8226	0.891321	2.1
8227	1.04442	2.1
8228	1.66554	2.1
8229	2.43434	2.1
8230	1.73944	0.05
8231	1.88032	0.05
8232	0.711391	0.05
8233	1.40837	0.05

8234	0.946133	0.05
8235	0.92252	0.05
8236	1.13964	0.05
8237	1.83394	0.05
8238	2.81674	0.05
8239	1.10049	1.681
8240	1.25092	0.276283
8241	0.760299	1.41259
8242	1.48017	1.5637
8243	1.48017	1.57786
8244	0.760271	1.26367
8245	1.35861	1.53574
8246	0.946129	1.45767
8247	0.760269	1.31976
8248	2.26155	2.1
8249	2.15634	2.1
8250	1.39824	2.1
8251	1.07817	2.1
8252	0.946132	2.1
8253	1.53028	2.1
8254	1.53028	2.1
8255	1.00807	2.1
8256	0.946133	2.1
8257	2.06126	0.245249
8258	1.97385	1.59474
8259	1.75754	0.069551
8260	2.01613	2.1
8261	1.97995	2.1
8262	1.47696	2.1
8263	1.50172	2.1
8264	1.5177	0.05
8265	1.44146	0.05
8266	1.8807	0.05
8267	2.07068	0.05
8268	1.84267	0.05
8269	1.08975	2.1
8270	1.21327	2.1
8271	1.10106	0.05
8272	1.34496	0.05
8273	2.08452	0.122235
8274	1.70537	0.0740639
8275	2.62464	2.1
8276	1.5726	2.1
8277	1.5726	2.1
8278	0.94613	2.1
8279	1.15038	2.1
8280	0.946132	2.1
8281	1.03974	2.1
8282	1.15038	2.1
8283	0.99747	1.95411
8284	1.03472	2.1
8285	1.58407	1.23263
8286	1.21148	2.1
8287	1.35025	2.1
8288	1.3428	0.188898
8289	1.00775	2.1
8290	1.00775	2.1
8291	1.3879	2.1

8292	1.39958	2.1
8293	0.853042	2.1
8294	1.06997	2.1
8295	0.853042	2.1
8296	0.853042	2.1
8297	1.12491	2.1
8298	1.17879	2.1
8299	1.07162	2.1
8300	1.61179	2.1
8301	1.76016	2.1
8302	0.946132	0.663485
8303	1.23449	0.323359
8304	1.70392	0.376838
8305	1.70392	0.293531
8306	0.94613	0.983926
8307	1.23449	0.413101
8308	1.41556	0.424246
8309	1.14547	0.344068
8310	1.1721	0.236409
8311	0.96478	0.281836
8312	1.41843	0.36053
8313	1.2358	0.264391
8314	1.70678	0.250609
8315	1.70678	0.253294
8316	0.946118	0.664795
8317	1.23582	0.435773
8318	0.946105	0.607364
8319	0.964769	0.212764
8320	1.1357	0.230184
8321	0.964767	0.215861
8322	0.619856	0.05
8323	1.72366	0.05
8324	1.72367	0.05
8325	0.619831	0.05
8326	1.19262	0.05
8327	0.619829	0.05
8328	0.946129	0.05
8329	1.57459	2.1
8330	1.86512	2.1
8331	0.93841	2.1
8332	1.57459	2.1
8333	1.10476	0.05
8334	1.00013	1.36691
8335	2.13446	1.01784
8336	0.759781	2.1
8337	1.96011	1.73201
8338	0.946129	1.43884
8339	1.32056	1.64059
8340	1.75702	1.50216
8341	1.75702	1.64361
8342	0.946124	1.59661
8343	1.32056	1.52781
8344	1.38258	1.50039
8345	1.96011	1.71537
8346	2.31099	1.64708
8347	0.97356	2.1
8348	1.38885	2.1
8349	1.3771	2.1

8350	0.946131	2.1
8351	1.84843	2.1
8352	1.84843	2.1
8353	1.3771	2.1
8354	0.946131	2.1
8355	1.00278	2.1
8356	1.05447	2.1
8357	1.05446	2.1
8358	1.24913	0.532187
8359	1.05447	2.1
8360	1.80882	2.1
8361	1.80882	2.1
8362	0.946129	2.1
8363	1.33997	2.1
8364	0.946131	2.1
8365	1.28831	2.1
8366	1.33997	2.1
8367	1.44391	0.05
8368	1.44391	0.05
8369	0.850964	0.05
8370	0.850963	0.05
8371	0.946132	0.05
8372	0.921299	0.05
8373	1.37358	0.05
8374	1.46297	0.05
8375	1.46298	0.05
8376	0.823988	0.05
8377	0.921287	0.05
8378	0.94612	0.05
8379	0.823995	0.05
8380	0.946107	0.05
8381	1.44437	2.1
8382	1.06203	2.1
8383	2.21397	2.1
8384	0.637677	2.1
8385	1.8892	2.1
8386	2.075	2.1
8387	1.32051	0.0771058
8388	2.23637	0.119983
8389	1.10904	0.250496
8390	1.11819	0.154953
8391	0.94613	0.28952
8392	0.735845	0.172308
8393	1.4914	0.197546
8394	1.4914	0.0599834
8395	0.735831	0.373136
8396	1.61683	2.1
8397	1.61683	2.1
8398	0.946131	2.1
8399	1.3281	2.1
8400	1.10441	2.1
8401	1.10442	2.1
8402	1.23487	2.1
8403	1.09914	2.1
8404	1.20691	0.05
8405	2.27379	0.05
8406	1.1369	0.0527777
8407	1.26868	0.0571935

8408	1.54422	0.0504273
8409	1.54422	0.05
8410	0.861357	0.0646958
8411	0.94613	0.0553795
8412	0.861359	0.052738
8413	1.2858	0.286059
8414	1.31011	0.988067
8415	1.294 0.643102	
8416	1.03079	0.830079
8417	1.81979	1.94268
8418	1.57829	2.07809
8419	1.19023	1.80206
8420	1.19022	1.89334
8421	1.46458	1.85748
8422	1.46458	1.98375
8423	0.789173	2.04499
8424	1.34386	1.97826
8425	0.909896	2.0726
8426	0.946131	1.90193
8427	0.909893	1.92856
8428	1.27725	2.1
8429	1.68451	2.1
8430	2.26155	2.1
8431	2.34217	2.1
8432	1.55485	0.643023
8433	0.90854	0.441736
8434	0.858256	0.375933
8435	1.47042	0.264462
8436	1.62771	0.29454
8437	0.777398	0.46903
8438	0.982525	0.333894
8439	1.23865	0.311599
8440	2.09928	0.482706
8441	0.946128	0.105175
8442	0.474856	0.0971197
8443	0.474855	0.128633
8444	1.58747	0.0607518
8445	1.58747	0.0552948
8446	0.963917	0.097448
8447	1.09841	0.0913041
8448	2.23904	2.1
8449	2.60055	1.70015
8450	1.02599	2.1
8451	2.17837	2.1
8452	2.07122	2.1
8453	2.7309	2.01892
8454	2.08482	2.1
8455	1.18322	0.212677
8456	1.35381	1.40574
8457	1.01139	0.594225
8458	0.946133	1.51277
8459	1.59089	1.00979
8460	1.59089	0.187188
8461	0.946133	0.742562
8462	1.09634	0.0750495
8463	2.02279	0.12586
8464	1.03108	0.111743
8465	0.808978	1.62227

8466	1.07317	1.64499
8467	0.831695	1.61701
8468	1.22583	1.53398
8469	1.10215	1.6041
8470	1.07508	1.60937
8471	1.22204	1.60283
8472	1.04679	2.0126
8473	1.92535	0.05
8474	1.11081	0.05
8475	0.946131	0.10187
8476	0.714824	0.102873
8477	0.714795	0.0881467
8478	1.4231	0.100974
8479	1.4231	0.126501
8480	0.828617	0.0941377
8481	1.30928	0.123451
8482	1.49894	2.1
8483	1.55702	2.1
8484	0.946131	2.1
8485	1.10689	2.1
8486	0.946227	2.1
8487	0.946131	2.1
8488	1.21754	2.1
8489	1.504 2.1	
8490	1.504 2.1	
8491	0.723573	2.1
8492	1.24512	2.1
8493	0.723544	2.1
8494	0.946131	2.1
8495	1.24512	2.1
8496	1.29383	0.0734623
8497	1.29383	0.0552522
8498	1.61969	0.0750497
8499	0.946132	0.05
8500	1.12818	0.0537486
8501	0.946132	0.111754
8502	1.80922	0.05
8503	1.80922	0.05
8504	1.41383	0.05
8505	1.34153	0.05
8506	1.47689	0.0685927
8507	1.79375	0.05
8508	1.24333	0.431244
8509	0.768989	0.330105
8510	1.24333	0.332039
8511	1.0027	0.450031
8512	1.47705	0.417528
8513	1.47705	0.198428
8514	0.768977	0.419598
8515	1.28436	0.702844
8516	0.928583	0.858227
8517	1.28436	0.369506
8518	1.24333	0.762503
8519	1.5991	0.705105
8520	1.5991	0.169834
8521	0.928583	0.847835
8522	1.85717	0.235474
8523	2.48665	0.307602

8524	1.18423	0.05
8525	1.9493	2.09482
8526	1.8519	1.86894
8527	1.68802	2.1
8528	1.0302	0.110944
8529	0.900473	0.0972265
8530	1.99663	0.106529
8531	2.61301	0.0931043
8532	0.745772	0.158408
8533	0.946131	0.121427
8534	1.48881	0.117706
8535	1.48881	0.096317
8536	0.745745	0.146367
8537	0.809786	0.145138
8538	1.3065	0.134195
8539	0.946132	0.0690249
8540	0.768357	0.0776637
8541	0.768387	0.087611
8542	1.76897	0.05
8543	1.76896	0.05
8544	1.40271	0.0608004
8545	1.00777	0.0615224
8546	1.37427	0.05
8547	1.1967	1.64608
8548	0.946132	2.1
8549	1.59397	2.1
8550	1.59397	2.1
8551	0.946131	2.1
8552	1.14989	2.1
8553	1.14989	2.1
8554	1.10046	2.1
8555	1.09506	0.05
8556	2.53251	0.05
8557	1.9212	1.67381
8558	1.99586	1.41366
8559	0.913967	0.327899
8560	1.55028	0.089578
8561	1.55028	0.184278
8562	0.913966	0.259292
8563	1.16318	0.0774055
8564	1.16318	0.0903491
8565	0.946132	0.130745
8566	1.20478	2.1
8567	1.09662	2.1
8568	1.03472	2.1
8569	1.84774	2.1
8570	1.7984	2.1
8571	2.1221	2.1
8572	1.68223	2.1
8573	1.68224	2.1
8574	0.514872	1.87409
8575	0.888991	2.07059
8576	0.946127	1.77595
8577	0.514845	1.90839
8578	0.946129	1.90796
8579	1.10127	1.85295
8580	2.61623	1.83838
8581	0.983581	0.417797

8582	0.869663	0.12348
8583	1.1818	0.480311
8584	1.10406	0.223396
8585	1.07133	0.67867
8586	1.09405	0.139242
8587	0.838014	0.275937
8588	0.842998	1.39411
8589	1.03818	1.46543
8590	1.46338	0.119513
8591	1.903 2.1	
8592	1.23942	2.1
8593	0.965933	2.1
8594	0.810075	2.1
8595	1.40571	2.1
8596	1.66219	2.1
8597	0.810047	2.1
8598	0.936678	2.1
8599	1.45808	2.1
8600	1.6384	2.1
8601	1.54699	2.1
8602	0.719877	2.1
8603	1.33256	2.1
8604	0.753547	2.1
8605	1.04073	2.1
8606	1.25822	2.1
8607	0.851542	2.1
8608	0.714102	2.1
8609	1.45886	2.1
8610	1.80171	2.1
8611	0.597337	2.1
8612	0.957713	2.1
8613	1.3033	2.1
8614	0.596613	0.239921
8615	1.1386	0.358483
8616	0.596612	0.275246
8617	0.946133	0.418741
8618	0.596652	0.485294
8619	1.64092	0.374925
8620	1.64092	0.302867
8621	1.991 2.1	
8622	1.23013	2.1
8623	2.77816	2.1
8624	2.74107	1.18142
8625	2.07681	1.14408
8626	1.47764	0.05
8627	1.72677	0.05
8628	1.52161	2.1
8629	1.28526	2.1
8630	1.90502	2.1
8631	1.87359	0.0920453
8632	1.93489	0.099488
8633	2.70895	2.1
8634	2.83141	2.1
8635	1.41766	0.34135
8636	1.11337	0.338604
8637	1.41765	0.569542
8638	1.29172	0.386527
8639	1.76324	0.387359

8640	1.76324	0.488661
8641	0.946131	0.320973
8642	1.52428	2.1
8643	1.71459	2.1
8644	1.93852	1.13565
8645	1.07119	1.33466
8646	1.29698	1.21728
8647	1.42203	1.10049
8648	1.42203	1.36701
8649	0.946132	1.09858
8650	0.946132	1.16548
8651	0.96926	1.26367
8652	0.769913	1.62975
8653	0.946119	1.37657
8654	1.37867	1.29238
8655	1.94028	1.29976
8656	0.94612	1.20082
8657	0.946132	1.35647
8658	1.37865	1.30347
8659	2.14234	1.28256
8660	3.06459	1.33684
8661	1.44421	0.05
8662	1.33924	0.05
8663	1.33671	0.05
8664	1.10733	0.05
8665	0.68396	0.0542654
8666	1.76008	0.05
8667	1.76008	0.05
8668	0.68393	0.05
8669	0.946131	0.05
8670	3.79242	2.1
8671	2.46293	2.1
8672	3.56947	2.1
8673	1.35487	1.56896
8674	1.38616	1.73711
8675	0.954048	0.117634
8676	1.36663	0.109757
8677	0.909487	0.139986
8678	0.887288	1.96321
8679	0.810947	1.26463
8680	0.810934	1.70361
8681	1.07046	1.92151
8682	1.13979	1.96227
8683	0.927838	1.64857
8684	0.919995	1.85654
8685	1.61447	2.1
8686	2.56305	2.1
8687	0.943414	2.1
8688	1.17016	2.1
8689	1.7238	2.1
8690	1.89334	2.1
8691	0.946128	1.94814
8692	1.25184	2.1
8693	1.55639	2.1
8694	3.12098	2.06399
8695	3.57307	1.59119
8696	2.81281	2.1
8697	0.851542	2.1

8698	1.27689	2.1
8699	1.52568	2.1
8700	0.839689	2.1
8701	1.36471	2.1
8702	0.827865	2.1
8703	0.93667	2.1
8704	0.900861	2.1
8705	0.943152	2.1
8706	1.00667	2.1
8707	1.18141	2.1
8708	1.15298	2.1
8709	1.20335	2.1
8710	1.00713	2.1
8711	0.597537	2.1
8712	1.0577	2.1
8713	0.967342	0.558169
8714	0.838027	1.21394
8715	0.893587	0.466539
8716	1.06436	0.630446
8717	0.850048	0.474351
8718	1.22026	0.719879
8719	0.988077	0.58118
8720	1.18769	0.212596
8721	1.3991	2.1
8722	1.52984	2.1
8723	1.35469	1.60131
8724	1.25083	1.54146
8725	1.6594	1.73888
8726	1.6594	1.61919
8727	0.946129	1.29453
8728	1.02602	1.21915
8729	0.94613	0.658667
8730	1.29724	0.0709821
8731	1.14501	0.203343
8732	1.13078	0.163717
8733	1.48381	0.149062
8734	1.45921	0.139356
8735	0.851539	0.219389
8736	0.890836	0.21709
8737	0.946131	0.262433
8738	1.60428	2.1
8739	1.50097	2.1
8740	0.714507	0.0557111
8741	1.51396	0.0689064
8742	1.51396	0.0990592
8743	0.946132	0.0523371
8744	0.813862	0.0645458
8745	1.41461	0.0582028
8746	0.813862	0.0635385
8747	0.921311	2.1
8748	1.25084	1.49268
8749	1.30165	1.27296
8750	1.60636	1.52419
8751	1.60636	1.38638
8752	0.94613	1.20974
8753	0.972232	1.14029
8754	0.946131	0.553766
8755	2.35003	2.1

8756	0.92546	2.1
8757	1.00713	2.1
8758	0.810947	2.1
8759	0.810934	2.1
8760	1.056	2.1
8761	0.926811	2.1
8762	0.766408	2.1
8763	1.34292	2.1
8764	1.57932	0.05
8765	2.67595	0.05
8766	1.77737	0.05
8767	1.77738	0.05
8768	0.789633	0.05
8769	1.22903	0.05
8770	0.789663	0.05
8771	0.789662	0.05
8772	1.33797	0.05
8773	0.946128	2.1
8774	1.47351	2.1
8775	1.47351	2.1
8776	0.94613	2.1
8777	1.30082	2.1
8778	1.30082	2.1
8779	0.996486	2.1
8780	0.946127	2.1
8781	1.72122	2.1
8782	1.72122	2.1
8783	0.94613	2.1
8784	1.36653	2.1
8785	1.36653	2.1
8786	1.02608	2.1
8787	0.709123	1.58872
8788	0.946127	1.22729
8789	1.41342	0.393473
8790	1.818	1.76167
8791	1.818	1.51254
8792	0.946127	0.265497
8793	1.41342	0.826372
8794	1.35071	1.2549
8795	1.8993	0.627226
8796	1.4748	2.1
8797	1.18711	0.05
8798	0.946131	0.05
8799	1.18711	0.05
8800	1.20416	0.05
8801	1.91358	0.05
8802	1.91358	0.05
8803	0.477691	0.05
8804	2.16798	0.05
8805	2.22307	0.05
8806	0.758971	0.05
8807	1.80694	0.05
8808	1.80694	0.05
8809	0.946132	0.05
8810	0.75897	0.05
8811	1.25124	0.05
8812	0.946131	0.05
8813	1.76837	2.1

8814	1.76837	2.1
8815	0.946131	2.1
8816	1.36422	2.1
8817	0.946131	2.1
8818	1.14184	2.1
8819	1.36422	2.1
8820	0.793303	0.05
8821	1.79584	0.05
8822	1.35333	1.92303
8823	1.19967	1.6869
8824	1.55088	1.73758
8825	1.64246	2.00566
8826	0.946132	1.48673
8827	0.886293	1.85243
8828	1.25018	1.93418
8829	1.5795	1.69564
8830	2.15414	1.56206
8831	0.94613	1.23131
8832	1.37758	1.57237
8833	1.71567	1.32335
8834	1.71567	1.61512
8835	0.946125	1.45845
8836	1.37759	1.35045
8837	1.28421	1.32087
8838	0.933959	0.82196
8839	0.946133	0.05
8840	1.7813	0.05
8841	1.7813	0.05
8842	1.05704	0.05
8843	1.37794	0.05
8844	1.37794	0.05
8845	0.946132	0.05
8846	0.946132	0.05
8847	1.68197	0.05
8848	1.68197	0.05
8849	1.01367	0.05
8850	1.25017	0.05
8851	1.25017	0.05
8852	0.946132	0.05
8853	0.838953	2.1
8854	1.4045	2.1
8855	1.56388	2.1
8856	1.3182	2.1
8857	0.928518	2.1
8858	1.04073	2.1
8859	0.835676	2.1
8860	1.47507	2.1
8861	1.47506	2.1
8862	0.797664	2.1
8863	1.34285	2.1
8864	0.929875	2.1
8865	0.797663	2.1
8866	0.946133	2.1
8867	1.83628	0.916957
8868	1.83627	1.75735
8869	0.946131	1.75884
8870	1.36748	0.577211
8871	0.946133	0.847726

8872	1.21139	1.32333
8873	1.36748	0.322231
8874	1.06795	2.1
8875	1.08192	2.1
8876	0.827866	2.1
8877	1.02548	2.1
8878	0.95006	2.1
8879	1.53933	2.1
8880	0.586511	2.1
8881	0.868145	2.1
8882	1.28401	2.1
8883	1.37719	2.1
8884	1.37719	2.1
8885	0.77497	2.1
8886	0.946132	2.1
8887	0.774968	2.1
8888	1.7053	1.56151
8889	1.7053	2.1
8890	0.94613	2.1
8891	1.25715	2.1
8892	0.946132	0.973764
8893	1.19655	2.1
8894	1.25716	2.1
8895	0.851542	2.1
8896	0.80043	2.1
8897	1.27239	2.1
8898	1.02719	2.1
8899	0.946131	2.1
8900	0.981318	2.1
8901	1.06628	2.1
8902	1.26022	2.1
8903	0.946132	2.1
8904	0.946132	2.1
8905	1.76447	2.1
8906	1.76447	2.1
8907	1.35357	2.1
8908	1.35703	2.1
8909	0.946131	2.1
8910	1.50538	2.1
8911	1.50538	2.1
8912	1.10311	2.1
8913	1.3484	2.1
8914	1.10312	2.1
8915	0.966428	2.1
8916	0.946133	2.1
8917	1.4008	2.1
8918	1.71908	2.1
8919	1.71908	2.1
8920	0.946133	2.1
8921	1.40081	2.1
8922	1.26441	2.1
8923	0.851543	2.1
8924	0.961732	2.1
8925	1.54576	2.1
8926	1.35364	2.1
8927	0.946133	2.1
8928	1.02913	2.1
8929	1.2735	2.1

8930	1.32608	2.1
8931	0.967643	2.1
8932	0.94613	2.1
8933	1.44873	2.1
8934	1.44873	2.1
8935	0.967641	2.1
8936	0.946131	2.1
8937	1.40488	2.1
8938	1.24272	2.1
8939	0.946131	2.1
8940	1.78483	2.1
8941	1.78483	2.1
8942	1.24272	2.1
8943	0.946131	2.1
8944	0.94613	2.1
8945	1.5627	0.367635
8946	1.5627	1.82263
8947	1.03656	2.1
8948	1.31649	0.250535
8949	1.19233	1.70273
8950	1.03656	1.6905
8951	0.912059	2.1
8952	1.47954	0.800716
8953	1.47954	2.06264
8954	0.912061	2.1
8955	1.34647	0.615142
8956	1.04514	2.05908
8957	0.912061	1.87392
8958	0.761972	0.079659
8959	0.946132	0.500392
8960	0.761974	0.298955
8961	1.70225	1.07079
8962	1.70225	0.957711
8963	1.33728	0.595281
8964	1.01188	0.426965
8965	0.94613	1.82357
8966	0.746361	1.72672
8967	1.6369	2.05232
8968	1.62454	1.79092
8969	0.736738	1.36935
8970	0.958487	1.61664
8971	1.40641	1.85466
8972	1.2284	2.1
8973	0.92609	2.1
8974	0.595102	2.1
8975	1.80871	2.1
8976	1.1516	2.1
8977	0.962474	2.1
8978	2.24024	2.1
8979	0.946131	2.1
8980	1.73689	2.1
8981	1.73689	2.1
8982	0.946132	2.1
8983	1.30492	2.1
8984	1.30492	2.1
8985	1.17949	2.1
8986	0.946131	2.1
8987	1.8566	2.1

8988	1.8566	2.1
8989	1.22392	2.1
8990	1.39826	2.1
8991	1.40447	2.1
8992	1.22392	2.1
8993	0.946131	2.1
8994	1.74134	2.1
8995	1.74134	2.1
8996	1.09148	2.1
8997	1.40447	2.1
8998	1.283	2.1
8999	1.09148	2.1
9000	1.33607	2.1
9001	1.41022	2.1
9002	1.80016	2.1
9003	1.80015	2.1
9004	0.946127	2.1
9005	0.946129	2.1
9006	1.20541	2.1
9007	1.13078	2.1
9008	1.33607	2.1
9009	1.54624	2.1
9010	1.85553	2.1
9011	0.920606	2.1
9012	0.946131	2.1
9013	1.01271	2.1
9014	1.32664	1.87752
9015	0.954692	2.09487
9016	0.946116	1.80405
9017	1.47771	1.88059
9018	1.4777	2.1
9019	0.95468	1.92144
9020	0.94613	1.92981
9021	1.31381	1.48058
9022	1.14005	1.30674
9023	0.946128	1.3765
9024	1.69433	0.51513
9025	1.69433	1.79166
9026	1.14005	1.35136
9027	0.94613	0.851741
9028	1.521	2.1
9029	1.411	2.1
9030	0.94612	2.1
9031	1.35115	2.1
9032	1.04127	2.1
9033	1.05017	2.1
9034	1.03564	2.1
9035	0.607156	2.1
9036	1.54549	2.1
9037	1.36715	2.1
9038	0.607127	2.1
9039	1.2571	2.1
9040	0.998701	2.1
9041	0.546436	2.1
9042	0.946133	2.1
9043	1.75286	2.1
9044	1.75286	2.1
9045	1.12206	2.1

9046	1.34237	2.1
9047	1.35663	2.1
9048	1.12206	2.1
9049	0.919455	2.1
9050	1.66517	2.1
9051	1.99822	2.1
9052	1.228	2.1
9053	1.35662	2.1
9054	1.228	2.1
9055	1.01143	2.1
9056	0.851543	1.7556
9057	0.986856	2.1
9058	1.42762	1.8501
9059	1.23095	2.1
9060	0.946132	2.1
9061	1.00659	1.92983
9062	1.1779	1.91838
9063	1.40025	2.1
9064	1.40025	2.1
9065	0.889308	2.1
9066	1.34652	2.1
9067	0.943042	2.1
9068	0.943041	2.1
9069	1.34652	2.1
9070	1.39514	1.31368
9071	1.30809	1.20952
9072	1.75711	1.20888
9073	1.7571	1.70005
9074	0.946125	1.16486
9075	0.946127	1.25056
9076	1.18396	1.32798
9077	1.4844	1.17645
9078	1.4844	1.3661
9079	0.803322	1.80802
9080	0.946133	0.774223
9081	0.803324	2.1
9082	0.803324	1.73695
9083	1.36183	1.69123
9084	0.946131	0.0960306
9085	1.01462	0.0881603
9086	0.946132	0.234366
9087	1.59749	0.112935
9088	1.59749	0.0606373
9089	1.16009	0.0926419
9090	1.38354	0.0549724
9091	0.851539	1.351
9092	1.36005	1.65393
9093	1.5532	1.5621
9094	1.80402	1.65612
9095	0.851537	1.64868
9096	0.902632	1.59914
9097	1.44437	1.5618
9098	0.633098	2.1
9099	1.00307	0.427426
9100	0.94613	0.475589
9101	1.16623	0.312037
9102	0.946128	0.567273
9103	1.64588	0.467256

9104	1.64588	0.309096
9105	1.35059	0.422273
9106	1.24142	0.47684
9107	0.946131	0.0799071
9108	1.13343	0.0758682
9109	0.946127	0.0952587
9110	1.63367	0.0659787
9111	1.63367	0.0816301
9112	1.33035	0.0716315
9113	1.24945	0.0929767
9114	1.78733	1.52866
9115	1.70856	1.5709
9116	0.637995	1.45253
9117	0.712454	1.49146
9118	0.946132	1.43424
9119	0.637964	1.44648
9120	1.13021	1.4318
9121	0.94613	0.0813932
9122	1.20509	0.0826854
9123	0.946127	0.0778478
9124	1.70545	0.0720623
9125	1.70545	0.0853075
9126	1.28508	0.0733009
9127	1.3665	0.0887515
9128	1.58612	2.1
9129	1.51845	2.1
9130	0.797374	2.1
9131	1.20057	2.1
9132	0.946132	2.1
9133	0.946132	2.1
9134	0.899819	2.1
9135	1.50471	1.28762
9136	1.50472	1.27838
9137	0.59387	1.277
9138	0.593844	1.14908
9139	0.946126	1.27466
9140	0.966137	1.25618
9141	1.13245	1.24138
9142	1.20801	0.851989
9143	0.946131	1.34418
9144	1.47644	1.24307
9145	0.733223	0.84538
9146	0.563609	0.949274
9147	1.69218	1.16205
9148	1.6173	1.24723
9149	1.8126	1.93573
9150	1.8126	1.99871
9151	0.678938	1.95409
9152	0.678935	1.99223
9153	0.946133	2.07278
9154	0.946132	1.92803
9155	0.678936	2.09803
9156	1.21997	1.84284
9157	1.28439	1.88291
9158	1.65282	1.97044
9159	1.86735	1.95476
9160	0.851539	1.89151
9161	0.94613	1.73505

9162	0.87688	1.75189
9163	0.804877	0.528536
9164	1.40747	0.742608
9165	1.41312	0.292611
9166	0.885349	0.366481
9167	1.25405	0.729784
9168	1.34463	0.46513
9169	0.804878	0.381962
9170	0.810299	0.0902477
9171	1.04072	0.154685
9172	1.25601	0.133825
9173	1.04943	0.0908872
9174	0.900294	0.160272
9175	1.38777	0.130212
9176	1.33131	0.059715
9177	1.39168	0.05
9178	0.946132	0.05
9179	0.747712	0.05
9180	1.61944	0.05
9181	1.61944	0.05
9182	0.946132	0.05
9183	0.747712	0.05
9184	1.41201	0.106897
9185	1.12082	0.0846017
9186	0.946128	0.0938829
9187	1.79415	0.120845
9188	1.79415	0.102356
9189	1.12082	0.0797555
9190	0.94613	0.0919704
9191	0.711721	0.10296
9192	1.12316	0.12079
9193	0.707038	0.105484
9194	1.04072	0.129737
9195	0.704409	0.148103
9196	1.49036	0.125715
9197	1.27689	0.0996859
9198	0.793044	0.483316
9199	0.810886	0.96459
9200	0.885957	0.540289
9201	1.38254	0.532249
9202	1.35991	0.510517
9203	1.01375	0.567897
9204	1.03169	0.765557
9205	0.946131	0.86203
9206	1.3279	1.17408
9207	1.42284	0.702175
9208	1.42284	0.64848
9209	0.851192	1.47475
9210	0.946131	1.28116
9211	0.851165	1.00321
9212	0.748062	0.05
9213	1.27511	0.05
9214	0.74806	0.05
9215	0.94613	0.05
9216	0.748059	0.05
9217	1.86293	0.05
9218	1.86293	0.05
9219	0.851542	0.059188

9220	1.38186	0.0519526
9221	0.851542	0.0703696
9222	1.67869	0.05
9223	1.66579	0.05
9224	1.25626	0.05
9225	1.23046	0.05
9226	1.46023	0.05
9227	1.27083	0.05
9228	0.946131	0.05
9229	0.999304	0.05
9230	0.946803	0.05
9231	0.851554	0.05
9232	0.999227	0.05
9233	0.750097	0.05
9234	1.91815	0.05
9235	0.946132	0.05
9236	0.768117	0.05
9237	1.78003	0.05
9238	1.99572	0.05
9239	0.650453	0.05
9240	0.946132	0.05
9241	1.23845	0.05
9242	0.946131	0.05
9243	1.75477	0.05
9244	1.75477	0.05
9245	1.33393	0.05
9246	1.36697	0.05
9247	0.749018	1.18298
9248	1.02366	1.06849
9249	1.43924	1.24166
9250	1.00018	0.554129
9251	1.06438	0.706301
9252	0.946127	0.821687
9253	0.74906	0.809378
9254	0.946131	0.821023
9255	1.26488	0.05
9256	0.946131	0.05
9257	0.94613	0.05
9258	1.49051	0.05
9259	1.59837	0.05
9260	0.907797	0.05
9261	1.04455	0.05
9262	1.25214	0.05
9263	0.68452	0.05
9264	1.25215	0.05
9265	1.24677	0.05
9266	1.8144	0.05
9267	1.81441	0.05
9268	0.684518	0.05
9269	0.851542	0.307535
9270	1.52275	0.235275
9271	1.45223	0.254138
9272	1.0269	0.274062
9273	1.29689	0.27107
9274	1.32822	0.304492
9275	0.851553	0.378092
9276	1.21756	0.05
9277	0.946131	0.05

9278	0.946127	0.05
9279	1.78014	0.05
9280	1.78014	0.05
9281	1.32076	0.05
9282	1.40551	0.05
9283	0.711214	0.0560426
9284	1.82057	0.05
9285	1.82057	0.05
9286	0.946129	0.060184
9287	1.22716	0.05
9288	1.30463	0.0543574
9289	0.94613	0.0513741
9290	0.568927	0.05
9291	0.946132	0.05
9292	0.568927	0.05
9293	1.99266	0.05
9294	1.99266	0.05
9295	1.26201	0.05
9296	1.29957	0.05
9297	1.21614	0.0592774
9298	0.851484	0.0612262
9299	0.851543	0.0671283
9300	1.85815	0.05
9301	1.81937	0.05
9302	0.697705	0.05
9303	0.946132	0.0615209
9304	0.593295	0.0893555
9305	1.53642	0.0749084
9306	1.53642	0.0636764
9307	0.908276	0.101177
9308	0.593296	0.0622651
9309	0.593295	0.0746749
9310	0.94613	0.085264
9311	0.867593	0.205226
9312	1.42107	0.137582
9313	1.42107	0.141781
9314	0.867621	0.207365
9315	1.05516	0.177822
9316	0.867624	0.144152
9317	0.946129	0.195238
9318	0.766681	0.07664
9319	1.18119	0.0944553
9320	1.57018	0.073512
9321	1.57018	0.0611365
9322	0.766652	0.098578
9323	1.18119	0.0887258
9324	1.15564	0.080899
9325	0.946131	0.131868
9326	1.239	0.0991188
9327	0.851541	0.143774
9328	0.851487	0.0973803
9329	1.40021	0.129433
9330	1.38373	0.0809869
9331	0.851554	0.150818
9332	1.12266	0.127902
9333	0.946131	0.13752
9334	1.08234	0.102261
9335	1.2882	0.0609627

9336	1.49963	0.618308
9337	1.46188	0.324472
9338	0.851555	0.23549
9339	1.33007	0.303044
9340	1.08054	0.256307
9341	0.946129	0.383287
9342	0.91151	0.239163
9343	1.49914	0.270317
9344	1.49914	0.141582
9345	0.911471	0.526772
9346	0.946132	1.35899
9347	1.35943	1.86226
9348	1.68117	1.50731
9349	1.68117	2.08696
9350	0.946132	1.71962
9351	1.35943	1.53801
9352	1.26787	1.49783
9353	1.81612	2.1
9354	1.81612	1.38062
9355	0.946131	1.73435
9356	1.41492	0.862568
9357	0.946132	2.1
9358	1.25037	2.1
9359	1.41492	2.1
9360	1.30541	0.05
9361	1.23646	0.05
9362	0.94613	0.05
9363	1.77304	0.05
9364	1.77304	0.05
9365	1.23646	0.05
9366	0.946132	0.05
9367	0.946131	2.1
9368	1.26689	2.1
9369	1.66806	2.1
9370	1.66806	2.1
9371	0.94613	2.1
9372	1.26689	2.1
9373	1.3473	2.1
9374	1.39384	2.1
9375	1.4181	2.1
9376	1.86581	2.1
9377	1.86581	2.1
9378	0.946128	2.1
9379	0.946129	2.1
9380	1.32068	2.1
9381	1.20328	2.1
9382	1.39384	2.1
9383	1.651	2.1
9384	1.651	2.1
9385	0.946129	2.1
9386	0.946131	2.1
9387	1.12913	2.1
9388	1.21474	2.1
9389	0.946132	2.1
9390	0.94613	2.1
9391	1.79724	2.1
9392	1.79724	2.1
9393	1.40987	2.1

9394	1.3335	2.1
9395	1.65642	2.1
9396	1.65642	2.1
9397	0.946132	2.1
9398	1.19063	2.1
9399	0.946133	2.1
9400	1.15203	2.1
9401	1.19063	2.1
9402	0.946131	1.85295
9403	1.77815	2.1
9404	1.77815	2.1
9405	0.946131	2.1
9406	1.31745	2.1
9407	1.31745	2.1
9408	1.2597	2.1
9409	0.946131	0.92467
9410	1.85184	1.92336
9411	1.85184	2.1
9412	0.946128	1.72774
9413	1.40684	2.1
9414	1.40684	1.89859
9415	1.34049	1.95168
9416	0.946132	1.77065
9417	1.71333	1.26845
9418	1.71333	1.91374
9419	1.1848	1.75844
9420	1.34297	1.16861
9421	1.31649	1.26256
9422	1.1848	1.45831
9423	1.36604	0.268083
9424	0.954559	0.340592
9425	1.36604	0.253344
9426	1.41766	0.282987
9427	1.83756	0.3508
9428	1.83756	0.614971
9429	0.946131	0.219985
9430	0.946127	0.128848
9431	1.14874	0.0932758
9432	0.946127	0.18872
9433	1.66393	0.120835
9434	1.66393	0.0762045
9435	1.35559	0.129647
9436	1.25447	0.13846
9437	0.94613	0.0679957
9438	1.20732	0.0731957
9439	0.946129	0.0672757
9440	1.75073	0.0560814
9441	1.75073	0.0801589
9442	1.3665	0.0578124
9443	1.33035	0.0805542
9444	1.73256	2.1
9445	1.73256	2.1
9446	0.946131	2.1
9447	1.34921	2.1
9448	1.19462	2.1
9449	1.19462	2.1
9450	1.32949	2.1
9451	1.75754	0.380386

9452	1.75754	1.00822
9453	0.946132	1.01559
9454	1.3201	0.667322
9455	0.946132	0.25765
9456	1.25479	0.32205
9457	1.3201	0.351255
9458	1.8087	2.09249
9459	1.8087	0.373419
9460	0.946131	0.740668
9461	1.38357	0.299419
9462	0.946133	2.08045
9463	1.31034	0.958589
9464	1.38357	1.20958
9465	1.12702	0.139537
9466	0.946131	0.219244
9467	1.23923	0.195272
9468	1.4117	0.129895
9469	0.946131	0.205404
9470	1.70479	0.174561
9471	1.70479	0.0704944
9472	0.946119	2.1
9473	1.55309	2.1
9474	1.55309	2.1
9475	0.946119	2.1
9476	1.08006	2.1
9477	1.08005	2.1
9478	1.02681	2.1
9479	1.36581	2.1
9480	1.93075	2.1
9481	0.946133	2.1
9482	0.772538	2.1
9483	0.946131	2.1
9484	0.946131	2.1
9485	1.33748	2.1
9486	1.44991	2.1
9487	1.31183	2.1
9488	0.946132	2.1
9489	0.944104	2.1
9490	0.915236	2.1
9491	1.05017	2.1
9492	0.944148	2.1
9493	0.946132	0.0621845
9494	1.60171	0.05
9495	1.60171	0.05
9496	0.946131	0.201541
9497	1.16234	0.050385
9498	1.3855	0.05
9499	0.946132	0.0516507
9500	0.946133	0.100343
9501	1.73285	0.05
9502	1.73285	0.05
9503	0.946133	0.255496
9504	1.3855	0.0763767
9505	1.29348	0.0599043
9506	0.946132	0.0770026
9507	1.34828	2.1
9508	1.20943	2.1
9509	0.946129	2.1

9510	1.71013	2.1
9511	1.71013	2.1
9512	1.20943	2.1
9513	0.94613	2.1
9514	1.19613	0.05
9515	0.946118	0.05
9516	0.946117	0.05
9517	1.70868	0.05
9518	1.70869	0.05
9519	1.40551	0.05
9520	1.2493	0.05
9521	0.946131	2.1
9522	1.73123	2.1
9523	1.73123	2.1
9524	0.946132	2.1
9525	1.37811	2.1
9526	1.37811	2.1
9527	1.25144	2.1
9528	0.946132	2.1
9529	1.75139	2.1
9530	1.75139	2.1
9531	1.23374	2.1
9532	1.29926	2.1
9533	1.39827	2.1
9534	1.23374	2.1
9535	1.41375	0.05
9536	1.25697	0.05
9537	0.946118	0.05
9538	1.80377	0.05
9539	1.80377	0.05
9540	1.25699	0.05
9541	0.946106	0.05
9542	0.946133	1.95205
9543	1.81806	1.6175
9544	1.81806	2.1
9545	0.946133	2.07979
9546	1.37089	2.1
9547	1.37089	1.51757
9548	1.34999	1.87639
9549	0.946131	2.1
9550	1.80855	1.82703
9551	1.80855	2.1
9552	1.31184	2.1
9553	1.3933	1.78482
9554	1.36139	2.1
9555	1.31184	2.1
9556	1.01175	1.9885
9557	0.94613	0.695145
9558	0.919796	2.03221
9559	1.50484	0.478176
9560	1.80584	1.89299
9561	1.53512	1.10398
9562	1.14539	1.12184
9563	0.946133	2.1
9564	1.72797	2.1
9565	1.72797	2.1
9566	1.2111	2.1
9567	1.39731	2.1

9568	1.27679	2.1
9569	1.21111	2.1
9570	0.946119	1.13627
9571	1.67673	1.7897
9572	1.67673	2.1
9573	0.946118	1.74356
9574	1.21519	2.1
9575	1.2152	2.1
9576	1.19808	2.1
9577	0.946127	2.1
9578	1.54442	2.1
9579	1.54442	2.1
9580	1.04806	2.1
9581	1.13485	2.1
9582	1.3557	2.1
9583	1.04805	2.1
9584	0.946129	2.1
9585	1.32282	2.1
9586	1.77749	2.1
9587	1.77749	2.1
9588	0.946131	2.1
9589	1.32282	2.1
9590	1.4008	2.1
9591	0.946128	0.0736667
9592	1.02366	0.105681
9593	0.94613	0.0782387
9594	1.66684	0.0654717
9595	1.66684	0.0933704
9596	1.29215	0.0864941
9597	1.32082	0.0940292
9598	0.946133	0.05
9599	1.01451	0.0673542
9600	0.946132	0.0545756
9601	1.62374	0.05
9602	1.62374	0.05
9603	1.2929	0.0506099
9604	1.27697	0.05
9605	1.37869	2.1
9606	1.17215	2.1
9607	0.946132	2.1
9608	1.70508	2.1
9609	1.70508	2.1
9610	1.17215	2.1
9611	0.946132	2.1
9612	1.03784	2.1
9613	0.946132	2.1
9614	0.946132	2.1
9615	1.56718	2.1
9616	1.56717	2.1
9617	1.35703	2.1
9618	1.15628	2.1
9619	1.35356	2.1
9620	1.28976	2.1
9621	0.946131	2.1
9622	1.78612	2.1
9623	1.78612	2.1
9624	1.28976	2.1
9625	0.94613	2.1

9626	0.946132	2.1
9627	1.36373	2.1
9628	1.92382	2.1
9629	0.946131	2.1
9630	1.92382	2.1
9631	1.33471	2.1
9632	0.946132	2.1
9633	0.94613	0.237981
9634	1.29149	1.39856
9635	1.75878	1.14032
9636	1.75878	1.98012
9637	0.946128	1.57349
9638	1.29149	0.894785
9639	1.41342	1.16786
9640	1.41022	2.1
9641	1.37034	2.1
9642	1.83443	2.1
9643	1.83443	2.1
9644	0.946128	2.1
9645	0.946131	2.1
9646	1.26928	2.1
9647	0.946128	1.356
9648	1.38259	1.62946
9649	1.81405	1.43676
9650	1.81405	1.64105
9651	0.946126	1.55491
9652	1.38259	1.45454
9653	1.37759	1.43685
9654	1.68131	0.297209
9655	1.68131	1.06363
9656	0.946131	0.580704
9657	1.38896	0.228463
9658	1.17438	2.02166
9659	1.17438	0.667385
9660	1.23848	1.06745
9661	1.80088	0.802519
9662	1.80088	0.634753
9663	0.946132	0.486789
9664	1.40332	0.833617
9665	1.28185	0.596033
9666	1.28185	0.304761
9667	1.34369	0.151213
9668	1.71939	2.1
9669	1.71939	2.1
9670	0.946131	2.1
9671	1.28308	2.1
9672	0.946133	2.1
9673	1.24024	2.1
9674	1.28309	2.1
9675	1.8525	2.1
9676	1.8525	2.1
9677	0.946132	2.1
9678	1.38244	2.1
9679	0.946132	2.1
9680	1.33469	2.1
9681	1.38244	2.1
9682	1.26654	1.40622
9683	1.39514	1.36028

9684	1.71556	1.35818
9685	1.71556	1.60846
9686	0.946126	1.30732
9687	0.946129	1.41594
9688	1.1908	1.44996
9689	1.49995	2.1
9690	1.63875	2.1
9691	0.93608	2.1
9692	0.937208	2.1
9693	0.943708	2.1
9694	0.936134	2.1
9695	1.14341	2.1
9696	1.17802	0.05
9697	1.3542	0.05
9698	1.73981	0.05
9699	1.73981	0.05
9700	0.94613	0.05
9701	1.3542	0.05
9702	1.33174	0.05
9703	1.59452	2.1
9704	1.59452	2.1
9705	0.946132	2.1
9706	1.25	2.1
9707	0.975266	2.1
9708	0.946132	2.1
9709	1.25	2.1
9710	0.946131	2.1
9711	1.48685	2.1
9712	1.48685	2.1
9713	0.946132	2.1
9714	1.17273	2.1
9715	1.17273	2.1
9716	0.955602	2.1
9717	1.32827	0.0989275
9718	1.11166	0.0886409
9719	0.946127	0.0914415
9720	1.6136	0.112279
9721	1.6136	0.0858287
9722	1.11166	0.0831945
9723	0.946129	0.0952222
9724	0.851541	0.553424
9725	1.22107	0.390383
9726	1.64594	0.296214
9727	1.5271	0.331181
9728	0.94613	0.912586
9729	1.22112	0.673855
9730	1.25002	0.440109
9731	1.65795	1.32123
9732	1.65795	2.1
9733	0.946132	2.1
9734	1.2366	2.1
9735	0.946132	0.815967
9736	1.10023	2.02967
9737	1.2366	1.91238
9738	1.35856	0.05
9739	0.946132	0.05
9740	1.35856	0.05
9741	1.08019	0.05

9742	1.49261	0.05
9743	1.49261	0.05
9744	0.94613	0.05
9745	1.29322	0.05
9746	0.946132	0.05
9747	1.29322	0.05
9748	1.35856	0.05
9749	1.70565	0.05
9750	1.70565	0.079501
9751	0.946132	0.05
9752	1.66635	2.1
9753	1.66635	2.1
9754	0.946133	2.1
9755	1.39558	2.1
9756	1.14793	2.1
9757	1.14793	2.1
9758	1.21691	2.1
9759	1.35381	0.177874
9760	1.3365	1.51009
9761	1.09905	0.750912
9762	0.946132	1.48106
9763	1.74418	0.805807
9764	1.74418	0.180378
9765	0.946132	0.705253
9766	1.84759	2.1
9767	1.84759	1.50153
9768	0.94613	1.97453
9769	1.3904	2.1
9770	1.34522	0.4361
9771	1.34522	1.52156
9772	1.40331	1.04154
9773	1.86047	2.1
9774	1.86047	2.1
9775	0.946132	2.1
9776	1.41619	2.1
9777	0.946132	1.89535
9778	1.3659	2.1
9779	1.41619	2.1
9780	1.28464	2.1
9781	1.14496	1.89561
9782	1.57806	1.91021
9783	1.7753	1.98609
9784	0.851541	2.02904
9785	0.946131	2.00011
9786	0.872786	1.97742
9787	0.85062	1.08132
9788	1.52635	1.21959
9789	1.63785	1.02807
9790	1.69972	1.2802
9791	0.946132	1.22551
9792	1.37493	1.1193
9793	1.23854	1.14233
9794	1.41134	1.9249
9795	1.27079	1.50316
9796	1.736	1.3417
9797	1.736	2.1
9798	0.946132	1.20532
9799	0.946133	1.36946

9800	1.21177	1.56512
9801	0.946131	0.05
9802	0.946129	0.05
9803	0.94613	0.05
9804	0.946131	0.05
9805	0.946131	0.05
9806	1.35403	0.05
9807	0.946131	0.0522005
9808	1.37052	0.109389
9809	0.946132	0.433037
9810	0.94613	0.250471
9811	0.946133	0.129808
9812	0.946131	0.217492
9813	0.946131	0.287539
9814	0.946131	0.207381
9815	1.24249	0.0878899
9816	0.946132	0.131424
9817	0.946133	0.109016
9818	0.946132	0.061998
9819	0.946132	0.0714568
9820	0.946132	0.167002
9821	0.946132	0.104979
9822	0.946132	2.1
9823	0.946132	2.1
9824	0.946131	2.1
9825	0.946132	2.1
9826	0.946132	2.1
9827	0.946132	2.1
9828	1.20608	2.1
9829	1.41232	2.1
9830	0.946128	2.1
9831	0.946129	2.1
9832	0.94613	2.1
9833	0.94613	2.1
9834	0.94613	2.1
9835	0.94613	2.1
9836	0.946132	0.05
9837	0.946131	0.05
9838	0.946131	0.0550988
9839	0.946132	0.05
9840	0.946132	0.05
9841	0.946131	0.05
9842	0.946132	0.05
9843	0.946116	1.8517
9844	0.94612	2.1
9845	0.946118	2.1
9846	0.946124	2.1
9847	0.946118	2.1
9848	1.41561	2.1
9849	0.946131	2.1
9850	0.945846	2.1
9851	1.03508	2.1
9852	0.77579	2.1
9853	0.658908	2.1
9854	1.17474	2.1
9855	0.94303	2.1
9856	0.849189	2.1
9857	0.94298	2.1

9858	1.03963	2.1
9859	0.946131	1.95683
9860	0.946131	1.62608
9861	0.946131	2.1
9862	0.946131	1.60645
9863	0.946131	0.543089
9864	0.946131	1.77444
9865	1.32819	1.88067
9866	0.946133	1.40808
9867	0.946129	2.00151
9868	0.946128	1.95776
9869	0.94613	1.65287
9870	0.946131	2.1
9871	0.946131	1.6256
9872	0.946131	1.70989
9873	0.954072	0.05
9874	1.28895	0.05
9875	0.946133	0.05
9876	1.15803	0.05
9877	1.29689	0.05
9878	1.29689	0.05
9879	0.946132	0.05
9880	0.946132	1.42497
9881	0.946132	1.26767
9882	0.946132	1.24268
9883	0.946132	1.41553
9884	0.946132	1.59785
9885	0.946131	1.58523
9886	1.0264	1.57687
9887	0.946126	0.226885
9888	0.946132	0.199213
9889	0.946131	0.180279
9890	0.94613	0.204355
9891	0.946129	0.287478
9892	0.94613	0.221538
9893	1.35977	0.221909
9894	0.946132	0.449479
9895	0.946133	0.127755
9896	0.876632	1.47316
9897	0.946132	0.114232
9898	1.01566	0.41663
9899	1.01566	0.308704
9900	0.876606	0.415258
9901	0.946131	0.05
9902	0.946128	0.05
9903	0.946128	0.05
9904	0.94613	0.05
9905	0.94613	0.05
9906	1.24979	0.05
9907	0.94613	0.05
9908	0.946131	0.05
9909	0.946131	0.05
9910	0.921762	0.05
9911	0.9705	0.05
9912	0.9705	0.05
9913	0.946131	0.05
9914	0.921791	0.05
9915	0.810944	0.0769221

9916	0.810945	0.0846154
9917	0.810959	0.064597
9918	2.42267	0.05
9919	1.35168	0.0529682
9920	1.08132	0.0665261
9921	1.08132	0.0502752
9922	1.08131	0.0616136
9923	0.946133	0.0689295
9924	0.946132	0.0830928
9925	0.946132	0.0631218
9926	0.946133	0.0909111
9927	0.946133	0.0603208
9928	0.946132	0.0707829
9929	0.946133	0.101133
9930	0.946131	0.546396
9931	0.946131	1.22659
9932	0.94613	0.480862
9933	0.851514	0.448178
9934	0.946131	1.09208
9935	0.965268	0.933135
9936	0.851513	0.611993
9937	0.946122	2.1
9938	0.946123	2.1
9939	0.946126	2.1
9940	0.946124	2.1
9941	0.946124	2.1
9942	0.946118	2.1
9943	0.94613	2.1
9944	0.946131	0.0696695
9945	1.30448	0.0716949
9946	0.946132	0.382474
9947	0.946129	0.129656
9948	0.946131	0.392552
9949	0.946131	0.0506933
9950	0.94613	0.462472
9951	0.946133	2.1
9952	0.946128	2.1
9953	0.946128	2.1
9954	0.94613	2.1
9955	1.36503	2.1
9956	0.946131	2.1
9957	0.946131	2.1
9958	0.810947	0.05
9959	0.810947	0.05
9960	2.48233	0.05
9961	1.3517	0.05
9962	1.08132	0.05
9963	1.08132	0.05
9964	1.08133	0.05
9965	0.810934	0.05
9966	1.48874	0.05
9967	2.48233	0.05
9968	2.2369	0.05
9969	0.946133	0.57833
9970	0.946132	0.109053
9971	0.946132	1.29077
9972	0.946133	0.425024
9973	0.946133	1.15962

9974	1.41851	0.28607
9975	0.946132	0.389562
9976	1.03925	2.1
9977	0.946131	2.1
9978	0.94613	2.1
9979	0.946128	2.1
9980	0.946133	2.1
9981	0.944743	2.1
9982	1.13703	2.1
9983	0.946132	2.1
9984	0.946128	2.1
9985	0.946128	2.1
9986	0.946131	2.1
9987	0.94613	2.1
9988	0.94613	2.1
9989	0.94613	2.1
9990	0.946131	0.05
9991	0.946132	0.05
9992	0.946131	0.05
9993	0.946132	0.05
9994	0.946132	0.05
9995	0.946131	0.05
9996	1.20057	0.05
9997	0.946129	2.1
9998	0.94613	2.1
9999	0.94613	2.1
10000	0.946128	2.1
10001	0.946132	2.1
10002	0.94613	2.1
10003	1.38302	2.1
10004	0.856567	0.05
10005	1.14319	0.05
10006	0.716526	0.05
10007	1.20631	0.05
10008	0.626936	0.05
10009	0.946131	0.05
10010	0.94613	0.05
10011	1.39502	0.05
10012	0.881445	0.05
10013	0.946128	0.05
10014	0.94613	0.05
10015	0.881447	0.05
10016	0.946117	0.05
10017	0.946116	0.05
10018	0.88146	0.05
10019	0.946132	2.1
10020	1.39053	2.1
10021	0.946132	2.1
10022	0.946132	2.1
10023	0.946132	2.1
10024	0.946132	2.1
10025	0.946131	2.1
10026	0.946131	2.1
10027	0.946131	2.1
10028	0.946131	2.1
10029	0.946132	2.1
10030	0.946132	2.1
10031	1.40571	2.1

10032	0.946132	2.1
10033	0.94613	0.127732
10034	0.946127	0.156266
10035	0.946128	0.148405
10036	0.946131	0.160453
10037	0.946127	0.11796
10038	1.25819	0.161297
10039	0.946129	0.153794
10040	0.946129	0.603883
10041	1.29054	0.797953
10042	0.94613	0.390581
10043	0.94613	0.476025
10044	0.946131	1.29145
10045	0.946131	0.932094
10046	0.94613	0.788324
10047	0.810944	0.05
10048	0.810941	0.05
10049	1.61558	0.05
10050	1.3517	0.05
10051	1.08131	0.05
10052	1.08132	0.05
10053	1.08133	0.05
10054	0.810931	0.05
10055	1.02036	1.16424
10056	0.946132	1.12552
10057	1.02036	1.20332
10058	0.946132	1.04225
10059	1.1576	1.199
10060	0.964656	1.12241
10061	0.927608	1.18739
10062	0.946128	2.1
10063	1.38813	2.1
10064	0.946129	2.1
10065	0.946131	2.1
10066	0.946131	2.1
10067	0.94613	2.1
10068	0.946131	2.1
10069	0.946131	2.1
10070	0.94613	2.1
10071	0.94613	2.1
10072	1.41665	2.1
10073	0.946132	2.1
10074	0.946131	2.1
10075	0.94613	2.1
10076	0.946118	0.129417
10077	0.946118	0.10874
10078	0.946116	0.127309
10079	0.946126	0.0894666
10080	0.94612	0.0999918
10081	0.946131	0.105579
10082	0.946105	0.106819
10083	0.946132	2.1
10084	0.946132	2.1
10085	0.946132	2.1
10086	0.946131	2.1
10087	0.946132	2.1
10088	0.946132	2.1
10089	0.946132	2.1

10090	0.864997	0.05
10091	0.786573	0.05
10092	0.978561	0.05
10093	1.47385	0.05
10094	1.68652	0.05
10095	1.27803	0.05
10096	1.19771	0.05
10097	1.27595	0.05
10098	0.946127	0.50285
10099	1.3771	0.364101
10100	0.946127	0.370122
10101	0.946131	0.602962
10102	0.946129	0.328282
10103	0.946129	0.524924
10104	0.946129	0.468126
10105	0.95697	0.05
10106	1.48133	0.05
10107	1.24499	0.05
10108	1.5295	0.05
10109	0.938161	0.05
10110	0.808717	0.05
10111	1.3542	0.05
10112	0.946133	0.927567
10113	1.14444	0.63349
10114	0.946132	0.420564
10115	0.946132	0.520393
10116	0.946133	1.27155
10117	0.946132	0.999269
10118	0.946132	0.690718
10119	0.946131	0.05
10120	0.946132	0.0625444
10121	0.946131	0.05
10122	0.946132	0.05
10123	0.946131	0.0637371
10124	0.946132	0.05
10125	0.946132	0.052915
10126	0.946132	2.1
10127	0.946133	2.1
10128	0.946133	2.1
10129	0.946132	2.1
10130	1.29322	2.1
10131	0.946132	2.1
10132	0.946132	2.1
10133	0.946131	2.1
10134	0.946132	2.1
10135	0.946131	2.1
10136	0.946132	2.1
10137	1.28441	2.1
10138	0.946131	2.1
10139	0.946131	2.1
10140	0.946113	2.1
10141	0.946118	2.1
10142	0.946118	2.1
10143	1.28073	2.1
10144	0.946117	2.1
10145	0.946131	2.1
10146	0.946105	2.1
10147	0.810945	2.1

10148	0.810942	2.1
10149	2.75904	2.1
10150	1.3517	2.1
10151	1.08131	2.1
10152	1.08133	2.1
10153	1.08132	2.1
10154	0.81093	2.1
10155	0.946127	2.1
10156	1.37952	2.1
10157	1.86965	2.1
10158	0.946127	2.1
10159	1.86965	2.1
10160	1.28228	2.1
10161	1.37952	2.1
10162	0.946128	2.1
10163	0.946132	2.1
10164	1.14042	2.1
10165	0.946132	2.1
10166	1.14042	2.1
10167	1.09974	2.1
10168	1.09974	2.1
10169	0.946132	2.1
10170	0.946132	0.0836506
10171	0.946132	0.0566608
10172	0.946133	0.0790697
10173	0.946132	0.0553562
10174	0.946132	0.0724742
10175	0.946132	0.0584627
10176	0.946133	0.0765744
10177	1.31817	1.77968
10178	0.946131	1.64437
10179	0.946131	1.75935
10180	0.946131	1.724
10181	0.946131	1.55753
10182	0.946131	1.66923
10183	0.946131	1.65606
10184	0.946132	2.1
10185	0.946132	2.1
10186	0.946132	2.1
10187	0.946132	2.1
10188	0.946132	2.1
10189	0.946132	2.1
10190	1.35048	2.1
10191	0.946131	2.1
10192	0.946131	2.1
10193	0.94613	2.1
10194	0.946133	2.1
10195	0.946133	2.1
10196	1.35943	2.1
10197	0.946131	2.1
10198	0.946133	2.1
10199	0.946132	2.1
10200	0.946132	2.1
10201	0.946133	2.1
10202	0.946133	2.1
10203	0.946133	2.1
10204	0.990712	2.1
10205	0.965725	1.56931

10206	0.946129	1.3947
10207	0.946133	1.31909
10208	1.21721	1.38392
10209	0.877931	1.22211
10210	0.946131	1.36498
10211	0.946131	1.35648
10212	0.946132	0.299341
10213	0.946131	0.230107
10214	0.946131	0.565326
10215	0.946133	0.305623
10216	0.946128	1.16853
10217	0.946131	0.526702
10218	1.39575	0.470672
10219	0.810943	0.366717
10220	0.810942	0.392131
10221	2.58794	0.225558
10222	1.21652	0.238813
10223	0.946154	0.301393
10224	0.979952	0.298097
10225	1.0813	0.339793
10226	0.810959	0.344736
10227	0.924071	0.267648
10228	1.16453	0.174439
10229	1.28586	0.166888
10230	0.946131	0.290719
10231	1.1227	0.164943
10232	1.16462	0.200716
10233	0.851553	0.32375
10234	0.709578	0.210277
10235	0.810943	0.352159
10236	0.810959	0.25174
10237	1.82615	0.208694
10238	1.21651	0.209913
10239	1.08132	0.240264
10240	0.946155	0.222937
10241	0.979937	0.306696
10242	0.946128	1.69516
10243	0.946132	2.1
10244	0.946132	1.42397
10245	0.94613	1.88768
10246	0.94613	1.94445
10247	0.94613	1.23015
10248	0.94613	1.55701
10249	0.946131	1.52208
10250	1.29735	1.85317
10251	0.946131	2.1
10252	0.946131	1.40461
10253	0.946131	1.76309
10254	0.946131	1.45621
10255	0.946131	1.64347
10256	0.946128	2.1
10257	1.33266	1.00572
10258	0.94613	1.66218
10259	0.946131	2.1
10260	0.94613	0.581937
10261	0.946131	1.43569
10262	0.946131	1.49342
10263	0.946131	2.1

10264	0.946131	2.1
10265	0.946131	2.03116
10266	1.40608	2.1
10267	0.946131	2.1
10268	0.946131	2.1
10269	0.946131	2.1
10270	0.946131	1.83385
10271	0.946131	2.1
10272	0.946131	1.87754
10273	1.2334	1.91228
10274	0.946129	1.94716
10275	0.946131	1.92832
10276	0.946131	2.06397
10277	0.946133	2.1
10278	0.946133	2.1
10279	0.946132	2.05121
10280	1.29198	2.1
10281	0.946132	2.1
10282	0.946132	2.1
10283	0.946132	2.1
10284	0.946131	2.1
10285	0.946128	2.1
10286	0.946128	2.1
10287	0.946131	2.1
10288	0.946127	2.1
10289	0.94613	2.1
10290	0.946129	2.1
10291	0.946123	2.1
10292	0.946126	2.1
10293	0.946125	2.1
10294	1.38639	2.1
10295	0.946126	2.1
10296	0.946132	2.1
10297	0.946119	2.1
10298	0.946128	0.115355
10299	0.946132	0.106116
10300	0.946131	0.133358
10301	0.94613	0.0652022
10302	0.94613	0.0804133
10303	0.946131	0.100855
10304	0.94613	0.086705
10305	0.946131	0.0989133
10306	0.946131	0.0854569
10307	0.946131	0.118083
10308	0.946131	0.0508139
10309	0.946131	0.0621732
10310	0.946131	0.0826531
10311	0.946131	0.0682953
10312	0.810945	2.1
10313	0.810943	2.1
10314	0.810933	2.1
10315	2.24688	2.1
10316	1.3517	2.1
10317	1.08132	2.1
10318	1.08132	2.1
10319	1.08133	2.1
10320	1.25809	0.166244
10321	0.946131	0.577123

10322	1.25809	0.1814
10323	0.946129	0.487973
10324	0.94613	0.165418
10325	0.946131	0.486944
10326	0.946131	0.267023
10327	0.946131	0.05
10328	0.946131	0.05
10329	0.946129	0.086578
10330	1.28911	0.05
10331	0.946131	0.0537929
10332	0.946131	0.05
10333	0.946131	0.05
10334	0.94613	0.121323
10335	0.946131	0.0985838
10336	0.94613	0.0857888
10337	0.946128	0.119964
10338	0.946132	0.0845482
10339	1.38954	0.107688
10340	0.946131	0.110663
10341	0.946131	0.119051
10342	0.946131	0.246659
10343	0.946131	0.361313
10344	1.37981	0.202066
10345	0.946131	0.947104
10346	0.946131	0.274067
10347	0.946131	0.314826
10348	0.946131	2.1
10349	0.946127	2.1
10350	0.946127	2.1
10351	0.946131	2.1
10352	0.946127	2.1
10353	0.946129	2.1
10354	0.946128	2.1
10355	0.94613	2.1
10356	0.946126	2.1
10357	0.946127	2.1
10358	0.946131	2.1
10359	0.946126	2.1
10360	0.946128	2.1
10361	0.946128	2.1
10362	0.946133	0.282214
10363	0.946132	0.208981
10364	0.946132	0.227806
10365	0.946132	0.23854
10366	0.946132	0.272014
10367	1.13511	0.188158
10368	0.946132	0.22863
10369	0.946132	1.83484
10370	0.946129	1.84049
10371	0.94613	1.7692
10372	0.946131	1.88062
10373	0.946131	1.91508
10374	0.94613	2.09913
10375	1.27575	2.03449
10376	0.946129	2.1
10377	0.946131	2.1
10378	0.946131	2.1
10379	0.946128	2.1

10380	0.946133	2.1
10381	0.946131	2.1
10382	0.94613	2.1
10383	1.15581	0.05
10384	0.946132	0.05
10385	0.946131	0.05
10386	0.94613	0.05
10387	1.15581	0.05
10388	0.94613	0.05
10389	0.946131	0.05
10390	0.611407	1.58169
10391	0.946131	0.840887
10392	1.37335	1.53987
10393	1.15086	1.62963
10394	0.617549	1.20997
10395	0.851553	1.47253
10396	1.14592	1.63809
10397	1.22618	1.85689
10398	0.946132	0.695652
10399	0.946132	1.88443
10400	0.946132	1.36823
10401	0.946131	1.27424
10402	0.946132	1.6366
10403	0.946132	1.60588
10404	0.946131	2.1
10405	0.94613	2.1
10406	0.946131	2.1
10407	0.946133	2.1
10408	0.946129	2.1
10409	0.946131	2.1
10410	0.946131	2.1
10411	0.810945	0.970361
10412	0.810943	2.07615
10413	2.50715	1.78444
10414	1.3517	1.25099
10415	1.08133	1.17075
10416	1.08132	2.00972
10417	1.08132	0.20873
10418	0.810932	0.767345
10419	1.07811	2.06418
10420	0.94613	0.976594
10421	0.946128	2.0857
10422	1.25358	0.445475
10423	0.946131	1.18758
10424	1.25358	0.873657
10425	0.94613	0.911067
10426	0.946129	2.1
10427	1.09197	2.1
10428	0.946131	2.1
10429	1.09197	2.1
10430	1.09532	2.1
10431	1.09532	2.1
10432	0.946131	2.1
10433	1.58788	2.1
10434	1.34255	2.1
10435	0.594912	2.1
10436	0.58894	2.1
10437	0.946129	2.1

10438	1.16679	2.1
10439	1.04071	2.1
10440	0.535418	2.1
10441	0.946131	2.1
10442	0.946128	2.1
10443	0.946131	2.1
10444	1.20221	2.1
10445	0.946128	2.1
10446	0.94613	2.1
10447	1.20221	2.1
10448	0.946132	2.1
10449	0.946133	2.1
10450	0.946132	2.1
10451	0.946132	2.1
10452	0.946133	2.1
10453	0.946133	2.1
10454	0.946132	2.1
10455	0.946132	1.86651
10456	0.946132	2.06807
10457	0.946133	2.1
10458	0.946132	1.96997
10459	1.411	2.08182
10460	0.946132	2.04344
10461	0.946133	2.07003
10462	1.05575	0.081112
10463	0.563734	0.0748563
10464	1.05575	0.0894113
10465	0.773833	0.0547339
10466	0.751686	0.0627513
10467	0.75884	0.0740291
10468	0.815612	0.0653791
10469	0.793465	0.0752308
10470	0.768563	0.107172
10471	0.946129	0.0764306
10472	0.946125	0.065734
10473	0.946126	0.0863726
10474	0.94613	0.0663815
10475	0.946124	0.0929223
10476	1.31678	0.0590111
10477	0.946128	0.0656827
10478	0.946132	0.335716
10479	1.29914	0.23407
10480	0.946132	0.231837
10481	0.946132	0.249925
10482	0.946132	0.358136
10483	1.29914	0.176886
10484	0.946132	0.267054
10485	0.810946	0.336232
10486	0.810943	0.327966
10487	2.21082	0.188394
10488	1.35168	0.212907
10489	1.08132	0.249862
10490	1.08132	0.253897
10491	1.0813	0.334002
10492	0.81096	0.299157
10493	0.625813	0.05
10494	0.946132	0.05
10495	0.946131	0.05

10496	0.946132	0.05
10497	0.946131	0.05
10498	1.39301	0.05
10499	0.625812	0.05
10500	0.810946	2.01891
10501	0.810945	2.1
10502	0.810931	2.1
10503	2.70043	2.1
10504	1.3517	2.1
10505	1.08131	2.08892
10506	1.08131	2.1
10507	1.08133	2.1
10508	0.810943	2.1
10509	0.810944	2.1
10510	0.810958	2.1
10511	2.44802	2.1
10512	1.35168	2.1
10513	1.08132	2.1
10514	1.08132	2.1
10515	1.0813	2.1
10516	0.946131	0.121059
10517	0.946129	0.101371
10518	0.946128	0.0951496
10519	0.946131	0.199681
10520	1.3492	0.127862
10521	1.3492	0.128684
10522	0.94613	0.0928651
10523	0.946132	0.292667
10524	0.946132	0.159301
10525	0.946133	0.298042
10526	0.946133	0.140468
10527	0.946132	0.277325
10528	0.946132	0.143092
10529	0.946132	0.265138
10530	0.946129	0.214766
10531	0.946133	0.149242
10532	0.946131	0.222281
10533	0.94613	0.138663
10534	0.946131	0.248058
10535	0.946131	0.192512
10536	0.946131	0.181348
10537	0.946119	0.11697
10538	0.946119	0.076433
10539	0.946119	0.146644
10540	0.946125	0.065341
10541	0.94612	0.12815
10542	0.946132	0.0953017
10543	0.946107	0.0937523
10544	0.946133	2.1
10545	0.946132	2.1
10546	0.946132	2.1
10547	0.946132	2.1
10548	0.946132	2.1
10549	0.946133	2.1
10550	1.3868	2.1
10551	0.810947	2.1
10552	0.810946	2.1
10553	0.81096	2.1

10554	2.66094	2.1
10555	1.35168	2.1
10556	1.08132	2.1
10557	1.08132	2.1
10558	1.0813	2.1
10559	0.810946	2.1
10560	0.810946	2.1
10561	0.810933	2.1
10562	2.75737	2.1
10563	1.3517	2.1
10564	1.08132	2.1
10565	1.08132	2.1
10566	1.08133	2.1
10567	0.946133	2.1
10568	0.946132	2.1
10569	0.946132	2.1
10570	0.946132	2.1
10571	0.946133	2.1
10572	0.946132	2.1
10573	0.946133	2.1
10574	0.946133	2.1
10575	0.946133	2.1
10576	0.946132	2.1
10577	0.946132	2.1
10578	0.946133	2.1
10579	0.946132	2.1
10580	1.28569	2.1
10581	0.946132	2.0827
10582	0.946133	2.1
10583	0.946133	2.1
10584	0.946132	2.1
10585	0.946132	2.1
10586	0.946132	2.1
10587	0.946133	2.1
10588	0.946132	0.05
10589	0.946131	0.05
10590	0.94613	0.05
10591	0.946133	0.05
10592	0.946128	0.05
10593	0.946131	0.05
10594	0.946131	0.05
10595	0.946131	2.1
10596	0.94613	2.1
10597	0.946131	2.1
10598	0.94613	2.1
10599	1.41833	1.96363
10600	0.946131	2.1
10601	0.946131	2.1
10602	1.26155	0.05
10603	0.946131	0.05
10604	0.946131	0.05
10605	0.946132	0.05
10606	0.946132	0.05
10607	0.946131	0.05
10608	0.946132	0.05
10609	0.810946	2.1
10610	0.810946	2.1
10611	0.810959	2.1

10612	2.47938	2.1
10613	1.35168	2.1
10614	1.08132	2.1
10615	1.08132	2.1
10616	1.08131	2.1
10617	0.946131	0.25194
10618	0.94613	0.218042
10619	0.94613	0.527157
10620	0.946132	0.186573
10621	0.946128	0.881465
10622	0.94613	0.427905
10623	0.94613	0.487038
10624	0.94613	0.05
10625	0.946131	0.05
10626	0.94613	0.0687016
10627	0.946129	0.0544619
10628	0.946132	0.0532147
10629	0.94613	0.0552665
10630	0.946131	0.05
10631	0.946132	2.1
10632	0.946131	2.1
10633	0.946128	2.1
10634	1.40756	2.1
10635	0.946131	2.1
10636	0.946129	2.1
10637	0.94613	2.1
10638	0.946131	2.1
10639	0.946131	1.91815
10640	0.946131	2.1
10641	0.946132	2.1
10642	1.31947	2.08774
10643	0.946131	2.1
10644	0.946131	2.1
10645	0.946131	2.1
10646	1.25768	2.1
10647	0.946132	2.1
10648	0.946132	2.1
10649	0.946129	2.1
10650	0.946131	2.1
10651	0.94613	2.1
10652	0.946132	0.0816114
10653	0.946129	0.079691
10654	0.946129	0.148275
10655	0.94613	0.103691
10656	1.31716	0.109569
10657	1.31716	0.0920341
10658	0.946131	0.143844
10659	0.810943	1.61693
10660	0.810942	2.1
10661	0.81093	1.98137
10662	2.37052	2.1
10663	1.3517	2.06082
10664	1.08132	1.69486
10665	1.08132	2.1
10666	1.08133	1.86923
10667	0.946128	1.08418
10668	1.10335	0.490764
10669	0.94613	0.302476

10670	0.946131	1.13557
10671	0.946131	0.580656
10672	1.10335	1.22227
10673	0.94613	1.30031
10674	0.946132	1.36817
10675	1.18818	0.967714
10676	0.946132	0.663175
10677	0.946132	1.42996
10678	0.946132	0.909982
10679	1.18818	1.45841
10680	0.946132	1.4601
10681	0.946125	2.1
10682	0.94612	2.1
10683	0.946119	2.1
10684	0.946119	2.1
10685	0.946119	2.1
10686	0.946132	2.1
10687	1.31522	2.1
10688	1.28803	2.1
10689	0.946132	1.96668
10690	0.946132	2.1
10691	0.946133	2.1
10692	0.946133	1.86552
10693	0.946133	2.1
10694	0.946132	2.1
10695	0.946133	1.11634
10696	0.946132	1.20966
10697	0.946132	1.27863
10698	0.946132	1.04347
10699	0.946133	0.992892
10700	0.946132	1.01908
10701	1.37658	1.11886
10702	1.02239	1.54417
10703	0.946128	1.4452
10704	0.946132	1.4362
10705	1.02239	1.41556
10706	0.94613	1.3141
10707	0.96283	1.46221
10708	0.92943	1.40978
10709	0.946132	0.05
10710	0.946131	0.05
10711	0.946126	0.05
10712	0.946129	0.05
10713	0.946128	0.05
10714	0.946128	0.05
10715	0.946129	0.05
10716	0.810946	0.05
10717	0.810944	0.05
10718	2.22493	0.05
10719	1.21651	0.05
10720	1.0813	0.05
10721	0.946154	0.05
10722	0.979951	0.05
10723	0.70959	0.05
10724	0.946132	0.05
10725	0.946131	0.0753008
10726	0.946131	0.0520472
10727	0.946133	0.05

10728	0.946131	0.0815167
10729	0.946132	0.05
10730	0.946132	0.0655292
10731	0.946132	1.70769
10732	0.946131	1.49747
10733	0.946132	1.79734
10734	0.946132	1.43249
10735	0.946132	1.42922
10736	0.946132	1.46924
10737	1.40924	1.58113
10738	0.946131	2.1
10739	0.946128	2.1
10740	0.946127	2.1
10741	0.94613	2.1
10742	1.41743	2.1
10743	0.946128	2.1
10744	0.946129	2.1
10745	1.37675	0.902918
10746	0.946128	1.14932
10747	0.94613	0.881929
10748	0.94613	1.16841
10749	0.94613	0.910211
10750	0.946129	0.836805
10751	0.94613	1.13112
10752	0.946132	0.161415
10753	0.946132	0.223175
10754	0.946132	0.348397
10755	1.35721	0.147817
10756	0.946132	0.729702
10757	0.946132	0.345537
10758	1.35721	0.282566
10759	0.946133	1.15238
10760	0.946133	1.13204
10761	0.946132	1.12452
10762	1.10003	0.672429
10763	0.946133	1.06479
10764	0.946132	1.11598
10765	0.946132	1.12185
10766	0.946132	0.0752622
10767	0.946131	0.0528324
10768	0.946131	0.0609717
10769	0.946131	0.0832373
10770	1.0616	0.0520042
10771	1.0616	0.0588161
10772	0.946132	0.0755549
10773	0.946132	0.0954265
10774	0.946131	0.0671794
10775	0.946131	0.0768686
10776	0.946131	0.106638
10777	1.09402	0.0668574
10778	1.09402	0.0749211
10779	0.946131	0.10055
10780	0.946132	0.05
10781	0.946126	0.05
10782	1.24093	0.05
10783	0.946129	0.0521926
10784	0.946129	0.05
10785	0.946128	0.05

10786	0.946129	0.05
10787	0.946131	0.0736945
10788	0.946131	0.0683086
10789	1.2929	0.066261
10790	0.946131	0.0944179
10791	0.946131	0.080051
10792	0.946131	0.0817135
10793	0.946131	0.0785532
10794	0.810946	0.05
10795	0.810945	0.05
10796	0.810959	0.05
10797	2.41233	0.05
10798	1.35168	0.05
10799	1.08132	0.05
10800	1.08132	0.05
10801	1.0813	0.05
10802	0.810946	1.9272
10803	0.810943	2.1
10804	0.750103	2.1
10805	2.22778	2.1
10806	1.27059	2.1
10807	1.00021	2.01187
10808	1.02049	2.1
10809	1.08133	2.08087
10810	0.946128	2.1
10811	0.946132	2.1
10812	0.946132	2.1
10813	0.94613	2.1
10814	0.94613	2.1
10815	0.94613	2.1
10816	0.946131	2.1
10817	0.946126	0.309713
10818	0.946131	0.257411
10819	0.946127	0.380491
10820	1.3324	0.221296
10821	0.946127	0.239772
10822	0.946128	0.310848
10823	0.946128	0.445111
10824	0.94613	2.1
10825	0.946131	2.1
10826	0.94613	2.1
10827	0.946128	2.1
10828	0.946133	2.1
10829	0.946131	2.1
10830	0.946131	2.1
10831	0.946133	2.1
10832	0.946132	2.1
10833	0.946133	2.1
10834	0.946133	2.1
10835	0.946132	2.1
10836	0.946132	2.1
10837	1.36541	2.1
10838	0.946131	0.798785
10839	0.94613	2.1
10840	0.946131	1.7189
10841	0.94613	1.48112
10842	1.39509	1.79379
10843	0.946131	0.414241

10844	0.946131	1.83208
10845	0.946133	2.1
10846	0.946133	2.1
10847	0.946132	2.1
10848	0.946132	2.1
10849	0.946133	2.1
10850	0.946132	2.1
10851	1.29319	2.1
10852	1.01196	0.0754358
10853	0.946131	0.129276
10854	0.946132	0.0769139
10855	0.946132	0.0927175
10856	0.946132	0.129752
10857	0.946132	0.113538
10858	0.946132	0.101757
10859	0.946112	2.1
10860	0.946118	2.1
10861	0.946116	2.1
10862	0.946117	2.1
10863	0.946118	2.1
10864	0.94613	2.1
10865	0.946105	2.1
10866	1.28684	1.48278
10867	0.946131	1.54436
10868	0.946132	1.49867
10869	0.946132	1.58378
10870	0.946132	1.70254
10871	0.946132	1.54232
10872	0.946132	1.60564
10873	0.94613	2.05034
10874	0.94613	2.1
10875	0.946128	1.94416
10876	0.946131	1.8416
10877	0.946131	1.91344
10878	0.94613	1.91992
10879	1.10185	2.05375
10880	0.810947	0.05
10881	0.810945	0.05
10882	1.57348	0.05
10883	1.3517	0.05
10884	1.08131	0.05
10885	1.08132	0.05
10886	1.08133	0.05
10887	0.810932	0.05
10888	0.946119	0.170267
10889	0.946116	0.146646
10890	0.946117	0.188437
10891	0.946121	0.120285
10892	0.946118	0.136932
10893	0.94613	0.152771
10894	0.946105	0.159139
10895	0.94612	0.540978
10896	0.946122	0.21794
10897	0.946119	0.38752
10898	0.946119	0.571184
10899	1.20884	0.261983
10900	0.946132	0.445993
10901	0.946107	0.374705

10902	2.67849	1.72171
10903	1.17709	1.6817
10904	0.605418	1.74709
10905	0.286984	1.67227
10906	0.172654	1.67853
10907	0.810946	1.5496
10908	0.940878	1.48517
10909	0.941235	1.11606
10910	1.26696	0.869246
10911	1.56837	1.09294
10912	1.13737	1.12151
10913	1.34115	1.1986
10914	1.24889	0.980071
10915	0.946132	1.71493
10916	0.946129	1.39549
10917	0.946128	1.43498
10918	0.94613	1.48258
10919	0.94613	1.43781
10920	0.946131	1.52265
10921	0.94613	1.40134
10922	0.946116	2.1
10923	0.94612	2.1
10924	0.946118	2.1
10925	0.946123	2.1
10926	0.946118	2.1
10927	0.946131	2.1
10928	1.23836	2.1
10929	0.810946	0.05
10930	0.810946	0.05
10931	0.810959	0.05
10932	2.80677	0.05
10933	1.35168	0.05
10934	1.08132	0.05
10935	1.08132	0.05
10936	1.08131	0.05
10937	0.810947	0.128005
10938	0.810947	1.33437
10939	2.41574	1.1197
10940	1.21653	1.06755
10941	1.08133	0.271216
10942	0.946153	1.27579
10943	0.979949	0.477066
10944	0.709567	0.187316
10945	0.94613	2.1
10946	0.946131	2.1
10947	0.946128	2.1
10948	1.34782	2.1
10949	0.946128	2.1
10950	0.946129	2.1
10951	1.34782	2.1
10952	0.946132	2.02874
10953	0.946132	2.09596
10954	0.94613	2.1
10955	1.16023	2.1
10956	0.946131	2.1
10957	0.946131	2.1
10958	1.16023	2.1
10959	0.946131	1.48526

10960	0.946127	2.1
10961	0.946127	2.1
10962	0.946131	1.75121
10963	0.946128	2.1
10964	0.94613	1.82208
10965	0.946129	1.90386
10966	0.946133	0.150239
10967	0.946132	0.112088
10968	0.946132	0.124124
10969	0.946133	0.166852
10970	1.20045	0.11203
10971	1.20045	0.120267
10972	0.946132	0.157313
10973	0.918871	0.05
10974	0.971961	0.05
10975	1.37324	0.05
10976	1.78255	0.05
10977	1.25155	0.05
10978	1.42172	0.05
10979	1.40554	0.05
10980	1.08862	0.05
10981	1.03803	0.05
10982	0.946132	0.05
10983	0.946126	0.05
10984	0.943647	0.05
10985	0.948611	0.05
10986	0.946129	0.05
10987	0.946129	0.05
10988	1.1699	0.698053
10989	0.946132	0.317907
10990	0.946133	0.875041
10991	0.946133	0.4064
10992	0.946132	0.751254
10993	0.946133	0.65254
10994	0.946132	1.28078
10995	0.946132	2.1
10996	0.946131	2.1
10997	0.946131	2.1
10998	0.946132	2.1
10999	0.946131	2.1
11000	0.946131	2.1
11001	0.946132	2.1
11002	0.946132	1.08694
11003	1.38623	0.897736
11004	0.946132	0.468277
11005	0.946132	0.522367
11006	0.946133	0.853874
11007	0.946132	0.482825
11008	0.946133	1.25446
11009	0.946132	2.1
11010	1.40098	2.1
11011	0.946131	2.1
11012	0.946131	2.1
11013	0.946132	2.1
11014	0.946132	2.1
11015	0.946132	2.1
11016	1.01777	0.439397
11017	0.946132	0.243906

11018	0.946133	1.50489
11019	1.68068	0.289991
11020	1.68068	1.40098
11021	1.41894	0.422067
11022	1.20787	0.0660001
11023	0.946132	0.228044
11024	0.946133	2.1
11025	0.946133	2.1
11026	0.946132	2.1
11027	1.40199	2.1
11028	0.946133	2.1
11029	0.946132	2.1
11030	0.946132	2.1
11031	0.810946	1.58014
11032	0.810947	1.43249
11033	2.37827	1.71978
11034	1.3517	1.62556
11035	1.08132	1.61854
11036	1.08132	1.70211
11037	1.08133	1.58104
11038	0.810934	1.61745
11039	0.946124	1.8248
11040	0.94613	1.49552
11041	1.40248	1.52005
11042	0.946131	1.47386
11043	0.946125	1.3261
11044	0.946127	1.52105
11045	0.946128	1.45075
11046	1.25703	1.78223
11047	0.87821	1.53005
11048	1.18912	1.80639
11049	0.946119	1.48338
11050	0.87825	1.56693
11051	1.1891	1.6603
11052	0.946107	1.71424
11053	0.946132	1.93325
11054	1.28543	1.19761
11055	0.946132	1.41951
11056	0.946132	1.51398
11057	0.946131	1.38995
11058	0.946132	0.360218
11059	0.946132	0.729052
11060	0.946115	2.1
11061	0.946119	2.1
11062	0.946116	2.1
11063	0.946123	2.1
11064	0.946118	2.1
11065	1.41636	2.1
11066	0.946129	2.1
11067	0.946132	2.09532
11068	0.946132	2.1
11069	0.946132	2.1
11070	0.946131	2.1
11071	0.946131	2.1
11072	1.41432	2.1
11073	0.946132	2.1
11074	0.810947	0.05
11075	0.810947	0.05

11076	2.44433	0.05
11077	1.21654	0.05
11078	0.946154	0.05
11079	0.97995	0.05
11080	1.08133	0.05
11081	0.810934	0.05
11082	0.851542	0.05
11083	0.835698	0.05
11084	1.22216	0.05
11085	0.946133	0.05
11086	1.01217	0.05
11087	1.09996	0.05
11088	0.851529	0.05
11089	0.946132	1.86745
11090	0.946132	2.01684
11091	0.946129	2.1
11092	0.94613	1.93397
11093	0.946131	2.06531
11094	0.946131	2.03368
11095	1.2254	2.1
11096	0.800897	1.81263
11097	0.838029	2.1
11098	0.838013	1.85952
11099	0.93679	1.70386
11100	1.20217	1.70497
11101	1.16923	1.78066
11102	0.94948	1.82332
11103	0.958118	1.92685
11104	0.36751	1.74358
11105	0.800535	1.82536
11106	0.272087	1.04403
11107	0.428084	1.25942
11108	0.986335	1.82155
11109	0.946132	0.247091
11110	0.946133	0.284116
11111	1.19601	0.251
11112	0.946132	0.271369
11113	0.946132	0.292221
11114	0.946133	0.327627
11115	0.946133	0.245981
11116	0.946132	1.92796
11117	1.36956	2.1
11118	0.946132	2.1
11119	0.946132	1.95534
11120	0.946132	2.1
11121	0.946133	2.08802
11122	0.946132	2.1
11123	0.946132	0.751927
11124	0.946131	2.09539
11125	0.946131	1.05721
11126	1.34268	0.169818
11127	0.946133	1.98525
11128	0.946132	0.749656
11129	0.946131	0.933013
11130	0.946132	2.1
11131	0.946131	2.1
11132	0.946131	2.1
11133	0.946132	2.1

11134	0.946132	2.1
11135	0.946132	2.1
11136	0.946132	2.1
11137	0.810946	1.43303
11138	0.810943	1.36873
11139	2.51472	1.46046
11140	1.3517	1.44423
11141	1.08132	1.29916
11142	1.08132	1.44103
11143	1.08133	1.43069
11144	0.810933	1.44812
11145	0.902316	0.596982
11146	1.04517	0.546636
11147	0.974924	0.374854
11148	0.530063	0.959698
11149	1.1272	0.641595
11150	0.68207	0.692511
11151	0.477061	0.858055
11152	0.810946	0.0741209
11153	0.810943	0.120678
11154	0.810959	0.0928122
11155	1.99211	0.0824525
11156	1.35168	0.0776797
11157	1.08132	0.0849459
11158	1.08132	0.0812974
11159	1.08131	0.109912
11160	0.94613	0.128692
11161	0.946131	0.116198
11162	0.946132	0.111863
11163	0.946132	0.120343
11164	0.946129	0.178411
11165	0.946131	0.12537
11166	1.16699	0.126071
11167	0.810943	1.81162
11168	0.810942	1.72724
11169	1.6943	1.93415
11170	1.27059	1.9497
11171	1.00021	1.92316
11172	1.02049	1.87262
11173	1.08133	1.82257
11174	0.750103	1.79507
11175	0.957813	1.88468
11176	0.946128	1.9345
11177	0.946132	1.74681
11178	1.04832	1.97815
11179	0.870714	1.71181
11180	0.94613	1.80999
11181	0.94613	1.8543
11182	1.86374	2.1
11183	2.0815	1.76419
11184	1.15918	0.344426
11185	0.946129	0.257765
11186	1.15918	0.321751
11187	0.946131	0.294107
11188	0.946131	0.317053
11189	0.946131	0.289063
11190	0.94613	0.317059
11191	0.946119	0.47856

11192	1.3612	1.14388
11193	0.946125	0.845392
11194	0.946119	0.556949
11195	0.94612	1.27289
11196	0.946132	0.820854
11197	0.946107	0.968883
11198	0.810947	0.123363
11199	0.810947	0.230228
11200	2.50398	0.110752
11201	1.3517	0.117347
11202	1.08132	0.104089
11203	1.08133	0.125887
11204	1.08132	0.125337
11205	0.810934	0.116231
11206	0.946132	2.1
11207	0.946132	2.1
11208	0.946132	2.1
11209	1.39754	2.1
11210	0.946132	2.1
11211	0.946132	2.1
11212	0.946131	2.1
11213	0.946129	2.1
11214	1.41732	2.1
11215	0.946129	2.1
11216	0.946131	2.1
11217	0.946131	2.1
11218	0.946131	2.1
11219	0.946131	2.1
11220	0.946132	1.59917
11221	1.25276	1.79395
11222	0.946131	1.84848
11223	0.94613	1.63461
11224	0.94613	1.79441
11225	0.94613	1.67254
11226	0.946131	1.70881
11227	1.21261	1.17768
11228	0.94613	1.47613
11229	0.94613	1.68445
11230	0.946128	0.901536
11231	0.946131	1.54416
11232	0.94613	1.58389
11233	0.946129	1.58682
11234	0.946128	2.1
11235	0.946133	2.1
11236	0.946132	2.1
11237	0.946131	2.1
11238	0.94613	2.1
11239	0.946131	2.1
11240	1.39407	2.1
11241	0.946116	1.72907
11242	0.946119	1.81552
11243	0.946119	1.58259
11244	1.15369	1.73671
11245	0.946119	1.52799
11246	0.946131	1.6752
11247	0.946106	1.70297
11248	0.946131	2.1
11249	0.946131	2.1

11250	0.946131	2.1
11251	0.946133	2.1
11252	0.946129	2.1
11253	0.946131	2.1
11254	0.946131	2.1
11255	1.19564	0.05
11256	0.498881	0.05
11257	1.33964	0.05
11258	0.498878	0.05
11259	0.923583	0.05
11260	0.831259	0.05
11261	0.553735	0.05
11262	0.946132	0.05
11263	0.946132	0.05
11264	0.946131	0.05
11265	0.946131	0.05
11266	0.946132	0.05
11267	0.946131	0.05
11268	1.28673	0.05
11269	0.946132	0.0763706
11270	0.946132	0.0763585
11271	1.33178	0.0698673
11272	0.946132	0.0835092
11273	0.946132	0.0811586
11274	0.946131	0.082299
11275	0.946131	0.0743559
11276	0.946128	0.157938
11277	0.946132	0.116607
11278	0.946129	0.193659
11279	0.946131	0.075163
11280	0.946131	0.0909634
11281	0.946129	0.10769
11282	0.94613	0.0980132
11283	0.946131	2.1
11284	0.946131	2.1
11285	0.946129	2.1
11286	1.40525	2.1
11287	0.94613	2.1
11288	0.94613	2.1
11289	1.40525	2.1
11290	0.946131	2.1
11291	0.946126	2.1
11292	0.946127	2.1
11293	0.946127	2.1
11294	1.4073	2.1
11295	0.946129	2.1
11296	0.946129	2.1
11297	0.946131	0.0731339
11298	0.946131	0.0565039
11299	0.946132	0.0770379
11300	0.946131	0.05
11301	0.946131	0.0509011
11302	1.05016	0.0544236
11303	0.946132	0.0725951
11304	0.946128	0.0809632
11305	0.946132	0.0649583
11306	0.946131	0.0853972
11307	0.946129	0.0522067

11308	0.94613	0.0580389
11309	1.13398	0.0616494
11310	0.94613	0.088319
11311	0.946132	0.05
11312	0.946132	0.05
11313	0.946131	0.05
11314	0.946131	0.05
11315	0.946132	0.05
11316	0.946131	0.05
11317	0.946132	0.05
11318	0.946131	0.0605822
11319	0.94613	0.05
11320	0.946131	0.05
11321	0.94613	0.068191
11322	1.38645	0.05
11323	1.38645	0.05
11324	0.946131	0.0520839
11325	0.946131	0.256573
11326	0.946131	0.152687
11327	0.94613	0.279498
11328	1.23923	0.126537
11329	0.94613	0.107353
11330	0.946131	0.260322
11331	0.946131	0.361267
11332	0.946132	0.05
11333	0.946131	0.05
11334	0.946131	0.05
11335	0.946132	0.05
11336	0.946132	0.05
11337	0.946132	0.05
11338	1.40511	0.05
11339	0.946132	2.1
11340	0.946132	2.1
11341	0.946132	2.1
11342	0.946132	2.1
11343	0.946132	2.1
11344	0.946131	2.1
11345	1.12968	2.1
11346	0.946132	1.68492
11347	0.946128	2.1
11348	0.946128	2.1
11349	0.94613	1.87049
11350	0.94613	2.1
11351	0.94613	1.89387
11352	0.94613	1.89845
11353	0.946133	2.1
11354	0.946133	2.1
11355	0.946132	2.1
11356	0.946132	2.1
11357	0.946132	2.1
11358	0.946133	2.1
11359	1.31323	2.1
11360	0.94613	2.1
11361	0.946131	2.1
11362	0.946131	2.1
11363	0.94613	1.99186
11364	0.946131	2.1
11365	0.946131	2.1

11366	0.946131	2.1
11367	0.946132	2.1
11368	0.946132	0.701155
11369	0.946132	2.1
11370	1.36899	1.9614
11371	0.946132	0.191641
11372	0.946132	1.72648
11373	0.946133	1.09808
11374	1.03543	0.05
11375	0.946131	0.05
11376	0.946131	0.05
11377	0.946132	0.05
11378	0.94613	0.05
11379	0.950987	0.05
11380	0.941275	0.05
11381	0.946131	0.0936426
11382	0.94613	0.101705
11383	1.28952	0.106511
11384	0.946132	0.0886847
11385	0.94613	0.142352
11386	0.946131	0.124526
11387	0.946131	0.0981388
11388	0.94613	2.1
11389	1.35966	2.1
11390	0.946131	2.1
11391	0.946131	2.1
11392	0.946129	2.1
11393	0.94613	2.1
11394	0.94613	2.1
11395	0.810946	2.1
11396	0.810942	1.99549
11397	0.810932	2.1
11398	2.6655	2.1
11399	1.3517	2.1
11400	1.08132	2.1
11401	1.08131	2.02225
11402	1.08133	2.1
11403	0.94613	2.1
11404	0.946131	2.1
11405	0.94613	2.1
11406	0.946128	2.1
11407	0.946131	2.1
11408	0.94613	2.1
11409	0.94613	2.1
11410	0.946128	2.1
11411	0.946128	2.1
11412	0.946133	2.1
11413	1.25375	2.1
11414	0.94613	2.1
11415	0.946131	2.1
11416	1.25375	2.1
11417	0.94613	2.1
11418	0.94613	2.1
11419	0.946132	2.1
11420	0.946132	2.1
11421	0.946128	2.1
11422	0.94613	2.1
11423	1.31453	2.1

11424	0.946131	0.0630902
11425	0.946129	0.05
11426	0.94613	0.0640833
11427	0.94613	0.05
11428	0.94613	0.05
11429	1.12018	0.05
11430	0.94613	0.0643163
11431	0.946131	0.102625
11432	0.946129	0.0620808
11433	0.946131	0.103754
11434	1.16373	0.0533661
11435	0.94613	0.05
11436	0.94613	0.099013
11437	0.946131	0.12676
11438	1.36811	0.344317
11439	0.946132	0.541827
11440	1.36811	0.284521
11441	0.946129	0.464051
11442	0.94613	0.381588
11443	0.946129	0.480976
11444	0.946129	0.433692
11445	0.946132	0.0967402
11446	0.946133	0.0593785
11447	0.946132	0.0746021
11448	0.946133	0.0967679
11449	1.35732	0.0666451
11450	1.35732	0.0741788
11451	0.946133	0.0828334
11452	1.36399	0.643671
11453	0.946129	1.45193
11454	0.94613	1.01512
11455	0.946131	1.43559
11456	0.94613	1.6243
11457	0.946131	1.36176
11458	0.946131	1.47002
11459	0.946133	2.1
11460	0.946132	2.1
11461	0.946132	2.1
11462	1.38302	2.1
11463	0.946133	2.1
11464	0.946132	2.1
11465	0.946133	2.1
11466	0.810943	2.1
11467	0.810944	2.1
11468	0.810945	2.1
11469	2.80542	2.1
11470	1.35169	2.1
11471	1.08132	2.1
11472	1.08132	2.1
11473	1.08132	2.1
11474	1.30189	2.1
11475	0.946131	2.1
11476	0.946132	2.1
11477	0.946131	2.1
11478	0.946131	2.1
11479	0.946131	2.1
11480	0.946132	2.1
11481	0.810946	1.21122

11482	0.810947	1.16067
11483	2.33293	1.12432
11484	1.24357	1.16339
11485	1.08132	1.1667
11486	0.973183	1.20102
11487	1.00023	1.19652
11488	0.810934	1.18518
11489	1.06584	1.14466
11490	0.946129	1.15685
11491	0.84731	0.642608
11492	1.16646	1.1807
11493	0.946126	1.19313
11494	1.2831	1.03635
11495	0.851527	1.15163
11496	0.946131	2.1
11497	0.946128	2.1
11498	0.946128	2.1
11499	0.94613	2.1
11500	1.31961	2.1
11501	0.94613	2.1
11502	0.94613	2.1
11503	0.946131	2.1
11504	1.3605	2.1
11505	0.946133	2.1
11506	0.946132	2.1
11507	0.94613	2.1
11508	0.946131	2.1
11509	0.946131	2.1
11510	0.946128	2.1
11511	0.946133	1.84845
11512	0.946132	2.1
11513	0.946131	1.85335
11514	0.946131	1.12132
11515	0.946131	2.0443
11516	1.30116	2.1
11517	0.946132	0.05
11518	0.946129	0.05
11519	0.94613	0.05
11520	1.3035	0.05
11521	0.946131	0.05
11522	0.94613	0.05
11523	0.946131	0.05
11524	0.946132	2.1
11525	1.2051	2.1
11526	1.2051	2.1
11527	0.946132	2.1
11528	1.37869	2.1
11529	0.946132	2.1
11530	0.946131	2.1
11531	0.810946	2.1
11532	0.810945	2.1
11533	2.69247	2.1
11534	1.35169	2.1
11535	1.08132	2.1
11536	1.08132	2.1
11537	1.08132	2.1
11538	0.810946	2.1
11539	1.22993	2.1

11540	0.946131	2.1
11541	0.946131	2.1
11542	1.34623	2.1
11543	0.946131	2.1
11544	1.34623	2.1
11545	0.946131	2.1
11546	0.946131	0.05
11547	0.946132	0.05
11548	0.946132	0.05
11549	0.946132	0.05
11550	0.946131	0.05
11551	0.946132	0.05
11552	1.32299	0.05
11553	0.94613	0.118032
11554	0.946131	0.125639
11555	0.946128	0.117304
11556	0.946132	0.104069
11557	0.946133	0.11027
11558	0.946131	0.124301
11559	0.946131	0.11166
11560	0.946132	2.1
11561	0.946129	2.1
11562	0.946128	2.1
11563	0.94613	2.1
11564	0.94613	2.1
11565	0.946131	2.1
11566	0.94613	2.1
11567	0.94613	2.1
11568	0.94613	2.1
11569	0.946131	2.1
11570	0.946132	2.1
11571	0.946129	2.1
11572	0.946131	2.1
11573	0.946131	2.1
11574	0.946132	1.6563
11575	0.946131	1.6642
11576	1.13731	1.61432
11577	0.94775	1.70055
11578	0.944514	1.7758
11579	0.946132	1.8701
11580	1.039	1.83146
11581	0.946131	1.61328
11582	0.946128	1.58567
11583	0.94613	1.50133
11584	0.946131	1.62381
11585	0.946128	1.68676
11586	0.946129	1.77692
11587	1.23726	1.73894
11588	0.946132	2.1
11589	0.946132	2.1
11590	0.946129	2.1
11591	1.39214	2.1
11592	0.946131	2.1
11593	0.946131	2.1
11594	0.94613	2.1
11595	0.946125	1.26032
11596	0.946119	0.395141
11597	0.946118	0.511958

11598	0.946118	0.312525
11599	0.94612	1.0852
11600	0.946106	0.508933
11601	0.946131	0.584832
11602	1.26893	0.274412
11603	0.946131	0.258439
11604	1.26893	0.25712
11605	0.94613	0.336157
11606	0.946131	0.258705
11607	0.946131	0.286162
11608	0.946131	0.493749
11609	0.946133	0.05
11610	0.946133	0.05
11611	0.946132	0.0532835
11612	1.28924	0.05
11613	0.946133	0.0531601
11614	0.946132	0.05
11615	0.946132	0.05
11616	0.946133	0.478055
11617	0.946132	0.48856
11618	0.946132	0.526616
11619	0.946133	0.373056
11620	0.946133	0.425343
11621	0.946132	0.511619
11622	0.946133	0.433542
11623	0.946132	1.5697
11624	0.946133	0.652565
11625	0.946133	1.52421
11626	0.946132	1.57691
11627	1.15388	1.40541
11628	0.946132	1.53255
11629	0.946133	1.45689
11630	0.946132	0.40321
11631	0.946133	0.379103
11632	0.946132	0.456268
11633	0.946133	0.293828
11634	0.946133	0.341674
11635	0.946132	0.42385
11636	0.946132	0.35977
11637	1.0644	1.45349
11638	1.12486	1.82753
11639	1.08674	1.84943
11640	0.946132	1.6915
11641	0.946132	1.82676
11642	0.946133	1.74238
11643	0.946132	1.75476
11644	0.808978	1.38063
11645	0.911537	1.50713
11646	0.932832	1.5904
11647	1.14604	1.67484
11648	1.56953	1.57708
11649	1.25866	1.53575
11650	1.18488	1.64241
11651	1.341	1.42776
11652	0.946131	0.0943493
11653	0.946131	0.05632
11654	0.946131	0.0749179
11655	0.94613	0.0973441

11656	1.33922	0.0649783
11657	0.946131	0.0769919
11658	0.946131	0.069486
11659	0.946132	0.0543991
11660	0.946133	0.05
11661	0.946132	0.0615406
11662	0.946132	0.0531393
11663	0.946132	0.0630803
11664	0.946133	0.0575415
11665	0.946132	0.051334
11666	0.946129	1.2409
11667	0.946133	2.1
11668	0.946133	1.70737
11669	0.946131	1.63621
11670	1.40496	2.1
11671	0.946131	1.60661
11672	0.946131	2.1
11673	0.94613	1.73654
11674	0.946127	1.34993
11675	0.946127	1.63621
11676	0.94613	1.73621
11677	1.38913	1.49537
11678	0.946128	1.66379
11679	0.946128	1.54987
11680	1.98863	0.05
11681	2.59915	0.05
11682	1.40338	0.05
11683	0.946131	0.05
11684	1.22266	0.05
11685	1.40338	0.05
11686	0.946132	0.05
11687	0.946132	0.05
11688	1.22266	0.05
11689	0.94613	0.05
11690	0.94613	0.0501232
11691	0.946124	0.0896464
11692	1.21428	0.05
11693	0.946126	0.0873647
11694	0.946127	0.0803824
11695	0.946127	0.0614414
11696	1.03411	0.0565643
11697	0.946131	0.130859
11698	1.03411	0.0633785
11699	0.946129	0.13183
11700	1.14258	0.0552204
11701	0.952143	0.118681
11702	0.940118	0.088208
11703	0.810943	2.1
11704	0.810945	2.1
11705	0.810958	2.1
11706	2.82603	2.1
11707	1.35168	2.1
11708	1.08132	2.1
11709	1.08132	2.1
11710	1.0813	2.1
11711	0.946133	0.0591032
11712	0.946132	0.0572558
11713	0.946132	0.0626491

11714	0.946132	0.0526209
11715	0.946132	0.0605489
11716	1.11539	0.0594498
11717	0.946133	0.0643133
11718	0.946133	2.1
11719	0.946132	2.1
11720	0.946133	2.1
11721	0.946133	2.1
11722	0.946132	2.1
11723	1.41195	2.1
11724	0.946133	2.1
11725	0.946131	0.05
11726	0.946131	0.05
11727	0.946131	0.05
11728	0.946131	0.05
11729	0.946131	0.05
11730	0.946131	0.05
11731	1.15968	0.05
11732	0.946131	1.70329
11733	0.94613	1.70687
11734	0.94613	2.03439
11735	1.34386	1.77091
11736	0.946132	1.86152
11737	0.94613	2.0061
11738	1.34386	1.8337
11739	0.946131	1.56618
11740	0.935202	1.35788
11741	0.946131	1.7715
11742	1.25054	1.61035
11743	0.850155	1.69984
11744	0.946131	1.74281
11745	0.935205	1.69375
11746	0.946131	0.315134
11747	1.40193	0.229647
11748	0.946132	0.229847
11749	0.946132	0.239259
11750	0.946129	0.349499
11751	1.40193	0.193397
11752	0.946131	0.265083
11753	0.808779	0.212918
11754	0.561937	0.153935
11755	1.40193	0.154132
11756	1.40193	0.134914
11757	0.561936	0.186369
11758	0.561937	0.12889
11759	1.40193	0.144441
11760	0.946132	0.05
11761	0.946132	0.05
11762	0.946132	0.05
11763	0.946132	0.05
11764	0.946133	0.05
11765	0.946132	0.05
11766	0.946133	0.05
11767	0.946127	0.31487
11768	0.946131	0.268825
11769	0.946131	0.0789036
11770	0.946127	0.102056
11771	1.19583	0.0686919

11772	1.19583	0.0879066
11773	0.946129	0.141768
11774	0.589428	0.265308
11775	1.08737	0.142584
11776	1.10961	0.404386
11777	0.872901	0.11523
11778	0.541327	0.109228
11779	0.541312	0.102678
11780	0.85153	0.244434
11781	0.946132	2.1
11782	0.946131	2.1
11783	0.946131	2.1
11784	0.946132	2.1
11785	0.946132	2.1
11786	0.946132	2.1
11787	0.946132	2.1
11788	0.946132	1.82487
11789	1.2778	2.1
11790	0.946131	2.1
11791	0.946131	1.86085
11792	0.94613	2.1
11793	0.94613	1.91448
11794	0.94613	1.95551
11795	0.946118	1.09564
11796	0.946112	0.733579
11797	0.946118	0.563939
11798	0.946119	0.998197
11799	0.946117	0.74246
11800	1.35631	0.570957
11801	0.946105	0.750948
11802	0.946133	2.1
11803	0.946133	2.1
11804	0.946132	2.1
11805	0.946132	2.1
11806	0.946133	1.50584
11807	0.946132	2.1
11808	0.946132	2.1
11809	0.94613	2.1
11810	0.946131	2.1
11811	0.946128	2.1
11812	0.946128	2.05971
11813	0.94613	2.1
11814	0.946129	2.1
11815	0.946129	2.1
11816	0.946132	1.63703
11817	1.15139	1.6207
11818	0.946132	1.60289
11819	0.946132	1.66536
11820	0.946132	1.59255
11821	0.946132	1.68724
11822	0.946132	1.64531
11823	0.946133	2.1
11824	1.36151	2.1
11825	0.946132	2.1
11826	0.946132	2.1
11827	0.946132	2.1
11828	0.946132	2.1
11829	0.946133	2.1

11830	0.946127	2.1
11831	1.38397	2.1
11832	0.94613	2.1
11833	0.946131	2.1
11834	0.946128	2.1
11835	0.94613	2.1
11836	0.946129	2.1
11837	0.946131	0.199801
11838	0.946127	0.221618
11839	0.946127	0.210748
11840	0.946132	0.203454
11841	0.946131	0.218826
11842	0.946128	0.219652
11843	0.946129	0.205798
11844	1.0268	0.05
11845	1.23823	0.05
11846	1.2171	0.05
11847	0.883446	0.05
11848	1.23827	0.05
11849	1.32614	0.05
11850	0.94613	0.05
11851	0.946132	2.1
11852	0.946129	2.1
11853	0.946129	2.1
11854	0.94613	2.1
11855	1.36523	2.1
11856	0.946131	2.1
11857	0.94613	2.1
11858	0.946132	0.0918355
11859	0.946131	0.0664139
11860	0.946132	0.0981925
11861	0.946131	0.0534235
11862	0.946131	0.0602041
11863	1.01653	0.0590494
11864	0.946131	0.106981
11865	0.94612	2.1
11866	0.946121	2.1
11867	0.946119	2.1
11868	0.946119	2.1
11869	0.946119	2.1
11870	0.946132	2.1
11871	0.946107	2.1
11872	0.810942	2.1
11873	0.810944	1.95184
11874	2.60324	2.1
11875	1.21651	2.1
11876	1.0813	2.1
11877	0.946153	1.94862
11878	0.979948	2.1
11879	0.709588	2.1
11880	1.3492	0.172871
11881	0.946132	0.186473
11882	0.946131	0.150043
11883	0.94613	0.185939
11884	0.946129	0.242018
11885	0.946129	0.188525
11886	0.94613	0.199515
11887	0.946131	0.149709

11888	0.94613	0.166938
11889	0.946128	0.155083
11890	0.946133	0.152272
11891	0.946133	0.165346
11892	0.946131	0.167393
11893	0.946131	0.155708
11894	1.23351	0.05
11895	0.946131	0.05
11896	0.946131	0.05
11897	0.946132	0.05
11898	0.946132	0.05
11899	0.946131	0.05
11900	0.946132	0.05
11901	1.35657	0.05
11902	0.946132	0.05
11903	0.946131	0.05
11904	0.946132	0.05
11905	0.946132	0.05
11906	0.946131	0.05
11907	0.946131	0.05
11908	0.946132	0.181459
11909	0.946132	0.133648
11910	0.946132	0.181188
11911	0.946131	0.100372
11912	0.946131	0.141639
11913	0.946132	0.116512
11914	0.946132	0.122894
11915	0.946133	0.102924
11916	0.946132	0.0639719
11917	0.946133	0.101696
11918	0.946132	0.05
11919	0.946132	0.0765137
11920	0.946132	0.0604671
11921	0.946133	0.0636479
11922	0.946131	1.20911
11923	1.4101	1.48319
11924	0.946131	2.00369
11925	0.946131	1.19588
11926	0.946131	1.4096
11927	0.946131	1.22781
11928	0.946131	1.37903
11929	0.810944	1.55179
11930	0.810939	1.57956
11931	0.709586	1.42551
11932	2.71261	1.48671
11933	1.21651	1.53916
11934	0.946151	1.35297
11935	0.979943	1.55975
11936	1.0813	1.61592
11937	0.946132	2.1
11938	0.946131	2.1
11939	0.946132	2.1
11940	0.946131	2.1
11941	0.946131	2.1
11942	1.37733	2.1
11943	0.946131	2.1
11944	0.946133	2.1
11945	0.946132	2.1

11946	0.946132	2.1
11947	0.946133	2.1
11948	0.946133	2.1
11949	0.946132	2.1
11950	1.32582	2.1
11951	0.946132	2.1
11952	0.946132	2.1
11953	0.946132	2.1
11954	0.946132	2.1
11955	0.946132	2.1
11956	0.946132	2.1
11957	0.946131	2.1
11958	0.946133	1.67441
11959	0.946132	1.57876
11960	0.946132	1.51847
11961	0.946132	1.55138
11962	0.946132	1.62945
11963	0.946133	1.56048
11964	1.3469	1.57356
11965	0.946126	2.1
11966	0.94613	2.1
11967	0.946131	2.1
11968	0.946127	2.1
11969	1.41206	2.1
11970	0.946128	2.1
11971	0.946128	2.1
11972	0.946127	2.1
11973	0.94613	2.1
11974	0.946131	1.98728
11975	0.94613	2.1
11976	0.946128	1.95576
11977	0.946129	2.1
11978	0.946129	2.1
11979	0.946131	0.125845
11980	0.94613	0.105421
11981	1.24448	0.106193
11982	0.946129	0.166776
11983	0.946131	0.129577
11984	0.94613	0.139999
11985	0.946131	0.130112
11986	0.946132	0.185653
11987	0.946133	0.158137
11988	1.11015	0.160014
11989	0.946133	0.267585
11990	0.946132	0.205772
11991	0.946132	0.222388
11992	0.946132	0.200766
11993	1.38775	2.1
11994	0.946131	2.1
11995	0.946131	2.1
11996	0.946132	2.1
11997	0.946132	2.1
11998	0.946131	2.1
11999	0.946131	2.1
12000	0.946129	2.1
12001	0.946129	2.1
12002	0.946133	2.1
12003	0.946131	2.1

12004	0.946131	1.34149
12005	0.946131	2.1
12006	0.946131	2.1
12007	0.946132	2.1
12008	0.946133	2.1
12009	0.946132	2.1
12010	1.40774	2.1
12011	0.946132	2.1
12012	0.946133	2.1
12013	0.946132	2.1
12014	0.610968	0.05
12015	0.688233	0.05
12016	0.715226	0.05
12017	0.792409	0.05
12018	1.13418	0.05
12019	0.956667	0.05
12020	0.757321	0.05
12021	0.968845	0.05
12022	0.595679	0.05
12023	0.946133	1.1895
12024	1.28917	1.31741
12025	0.946131	1.40534
12026	0.94613	1.15913
12027	0.946131	1.28471
12028	0.946131	1.19053
12029	0.946131	1.26717
12030	0.946132	0.192058
12031	0.946128	0.360926
12032	0.94613	0.407535
12033	1.40668	0.306542
12034	0.946131	0.41366
12035	0.946131	0.189944
12036	0.94613	0.364877
12037	0.810946	2.1
12038	0.810945	2.1
12039	2.78163	2.1
12040	1.3517	2.1
12041	1.08132	2.1
12042	1.08133	2.1
12043	1.08132	2.1
12044	0.810933	2.1
12045	0.946131	2.1
12046	1.39081	2.1
12047	0.94613	2.1
12048	1.30339	2.1
12049	1.30339	2.1
12050	1.39081	2.1
12051	0.946131	2.1
12052	0.946127	0.979429
12053	0.821631	0.421839
12054	0.946131	0.328699
12055	0.946131	0.753599
12056	0.946128	0.436116
12057	0.996109	0.566971
12058	0.827876	0.874629
12059	0.946131	2.1
12060	0.946129	2.1
12061	0.946129	2.1

12062	0.94613	2.1
12063	0.94613	2.1
12064	0.94613	2.1
12065	0.94613	2.1
12066	0.946132	0.431648
12067	1.41723	0.415476
12068	0.946132	0.326197
12069	0.946133	0.263679
12070	0.946132	0.871072
12071	0.946132	0.439844
12072	0.946133	0.517796
12073	0.946133	0.323355
12074	0.946132	0.25611
12075	0.946133	0.251038
12076	0.946133	0.277029
12077	0.946132	0.480914
12078	0.946132	0.343291
12079	1.34254	0.33188
12080	1.26501	1.59819
12081	0.946132	1.68052
12082	0.946133	1.64951
12083	0.946133	1.75633
12084	0.946132	1.74873
12085	0.946133	1.6741
12086	0.946132	1.77879
12087	0.946131	0.0521363
12088	0.946131	0.0520118
12089	0.946131	0.0588649
12090	0.946131	0.05
12091	0.946131	0.05
12092	0.946131	0.05
12093	0.946131	0.05
12094	0.878843	0.05
12095	0.82561	0.05
12096	0.586974	0.05
12097	1.05343	0.05
12098	0.80631	0.05
12099	0.782663	0.05
12100	0.789405	0.05
12101	0.902491	0.05
12102	1.05344	0.05
12103	0.94613	0.05
12104	0.946131	0.05
12105	0.946131	0.0614695
12106	0.94613	0.05
12107	0.946131	0.05
12108	0.946131	0.05
12109	0.946131	0.05
12110	0.946128	2.1
12111	0.946133	2.1
12112	0.946133	0.846528
12113	0.94613	2.1
12114	0.946131	1.31846
12115	0.946131	1.91159
12116	0.946131	2.00207
12117	0.946131	0.284019
12118	0.946131	0.236868
12119	0.94613	0.229697

12120	0.946128	0.252997
12121	0.946133	0.259371
12122	1.2996	0.216746
12123	0.946131	0.244288
12124	0.946132	2.1
12125	0.946132	2.1
12126	0.946132	2.1
12127	0.946132	2.1
12128	0.946131	2.1
12129	0.946132	2.1
12130	0.946132	2.1
12131	1.99211	0.0530226
12132	2.55024	0.05
12133	0.946131	0.0602527
12134	0.946131	0.0556998
12135	0.946131	0.060807
12136	0.946131	0.0577421
12137	0.946131	0.075512
12138	0.946131	0.0618449
12139	1.35726	0.0613879
12140	0.946128	2.1
12141	0.946132	2.0956
12142	0.946131	2.1
12143	0.94613	2.1
12144	0.94613	2.1
12145	0.94613	2.1
12146	1.39852	2.1
12147	1.31666	0.05
12148	0.946131	0.05
12149	0.946128	0.05
12150	0.94613	0.05
12151	0.946128	0.05
12152	0.946128	0.05
12153	0.946128	0.05
12154	1.32275	0.05
12155	0.946131	0.0588518
12156	0.946129	0.05
12157	0.946128	0.05
12158	0.946129	0.05
12159	0.946128	0.05
12160	0.946129	0.05
12161	0.946132	2.1
12162	0.946131	2.1
12163	0.946131	2.1
12164	0.946132	2.1
12165	0.94613	2.1
12166	0.946131	2.1
12167	1.41125	2.1
12168	0.946132	2.1
12169	0.946132	2.1
12170	0.946131	2.1
12171	0.946132	2.1
12172	0.946132	2.1
12173	0.946132	2.1
12174	1.31904	2.1
12175	1.02425	0.05
12176	0.946132	0.05
12177	0.94613	0.05

12178	0.946131	0.05
12179	1.1534	0.05
12180	0.961161	0.05
12181	0.9311	0.05
12182	1.40092	1.55601
12183	0.946133	1.60863
12184	0.946132	1.58812
12185	0.946131	1.69659
12186	0.946131	1.61075
12187	0.94613	1.60041
12188	0.946131	1.68346
12189	0.946113	2.1
12190	0.946119	2.1
12191	0.946117	2.1
12192	1.40015	2.1
12193	0.946118	2.1
12194	0.946131	2.1
12195	0.946105	2.1
12196	0.946132	0.153584
12197	0.946133	0.290998
12198	0.946132	0.45539
12199	1.03547	0.195748
12200	0.946133	0.148956
12201	0.946133	0.344041
12202	0.946132	0.143975
12203	0.946133	2.1
12204	1.36694	2.1
12205	0.946133	2.1
12206	0.946132	2.1
12207	0.946133	2.1
12208	0.946133	2.1
12209	0.946132	2.1
12210	0.946132	0.0829213
12211	0.946133	0.0980281
12212	0.946132	0.0805441
12213	0.946133	0.106498
12214	0.946133	0.0815844
12215	0.946132	0.08647
12216	0.946132	0.10545
12217	0.491552	0.122641
12218	0.913078	0.160442
12219	0.946132	0.203537
12220	0.491552	0.107358
12221	0.946132	0.170809
12222	0.49154	0.181667
12223	1.53168	0.163774
12224	1.53168	0.119264
12225	0.946127	2.1
12226	1.38752	2.1
12227	0.946129	2.1
12228	0.94613	2.1
12229	0.946128	2.1
12230	0.946129	2.1
12231	0.946129	2.1
12232	0.946132	2.1
12233	0.946132	2.1
12234	0.946132	2.1
12235	0.946132	2.1

12236	0.946132	2.1
12237	0.946131	2.1
12238	0.946132	2.1
12239	0.94613	0.326557
12240	0.94613	0.231398
12241	0.94613	0.244365
12242	0.946131	0.25241
12243	0.946129	0.318536
12244	1.16834	0.177134
12245	0.94613	0.26343
12246	0.946127	0.396595
12247	1.35594	0.266627
12248	0.946131	0.296348
12249	0.946128	0.413768
12250	0.946127	0.206021
12251	0.946129	0.375926
12252	0.94613	0.3255
12253	0.94613	2.04489
12254	0.946131	0.57672
12255	0.946128	0.861691
12256	0.946128	0.191012
12257	0.946131	2.03996
12258	0.94613	0.961097
12259	0.94613	1.48958
12260	0.946132	0.596979
12261	0.946132	0.248356
12262	0.946129	0.425508
12263	1.39751	0.320884
12264	0.94613	1.03872
12265	0.946131	0.273047
12266	0.946131	0.401708
12267	0.946132	0.0773432
12268	0.700041	0.0723566
12269	0.700013	0.0900769
12270	1.7697	0.05
12271	1.7697	0.05
12272	1.21134	0.0643144
12273	0.946131	0.0609024
12274	1.25837	0.0600864
12275	0.946133	2.1
12276	0.946132	2.07129
12277	0.946132	2.05055
12278	0.946132	2.1
12279	0.946132	2.1
12280	0.946133	2.1
12281	1.40583	2.1
12282	0.946131	1.81415
12283	0.946131	2.02232
12284	0.946131	1.80666
12285	0.946131	2.1
12286	0.946131	2.05358
12287	0.827865	2.05397
12288	0.946131	2.07649
12289	0.946132	0.05
12290	0.946129	0.05
12291	0.946129	0.05
12292	0.946131	0.05
12293	0.94613	0.05

12294	1.21087	0.05
12295	0.946131	0.05
12296	0.94613	0.468316
12297	0.946131	0.357004
12298	0.94613	0.192717
12299	0.946128	0.423662
12300	0.946132	0.22518
12301	1.17452	0.198101
12302	0.94613	0.314309
12303	0.946119	0.214505
12304	0.946114	0.181642
12305	0.946119	0.136905
12306	0.946119	0.200676
12307	0.946119	0.143333
12308	1.27376	0.15639
12309	0.946106	0.181484
12310	0.946133	2.1
12311	1.39621	2.1
12312	0.946132	2.1
12313	0.946132	2.1
12314	0.946133	2.1
12315	0.946132	2.1
12316	0.946132	2.1
12317	0.946132	2.1
12318	0.946132	2.1
12319	0.946131	2.1
12320	0.946131	2.1
12321	0.946132	2.1
12322	0.946132	2.1
12323	1.34352	2.1
12324	0.946132	1.80439
12325	1.41272	2.1
12326	0.946131	2.1
12327	0.94613	1.77186
12328	0.94613	2.1
12329	0.94613	1.83931
12330	0.94613	1.92844
12331	0.946132	2.1
12332	0.946131	2.04394
12333	0.946132	2.1
12334	0.946132	2.08518
12335	0.946132	2.1
12336	0.946132	2.1
12337	1.23536	2.1
12338	0.946132	2.1
12339	0.946131	2.1
12340	0.946131	2.1
12341	0.946132	2.1
12342	0.946132	2.1
12343	1.41131	2.1
12344	0.946131	2.1
12345	1.41894	1.54371
12346	0.946132	0.926304
12347	0.946133	0.941105
12348	0.946132	1.52908
12349	0.946132	1.49918
12350	0.946133	1.38483
12351	0.946132	0.748413

12352	0.946128	0.475359
12353	0.946133	1.93568
12354	0.946131	1.65643
12355	0.946129	2.1
12356	0.94613	1.82919
12357	1.4099	1.93916
12358	0.946131	1.82778
12359	0.810947	2.1
12360	0.810945	2.1
12361	0.810933	2.1
12362	2.62883	2.1
12363	1.3517	2.1
12364	1.08132	2.1
12365	1.08132	2.1
12366	1.08133	2.1
12367	0.946131	1.10956
12368	0.946126	1.9626
12369	0.946127	2.02737
12370	0.94613	1.67119
12371	0.946128	2.1
12372	0.946129	1.70748
12373	0.946129	1.6975
12374	1.21347	2.1
12375	0.946129	2.1
12376	0.94613	2.1
12377	1.21347	2.1
12378	1.33064	2.1
12379	0.94613	2.1
12380	1.33064	2.1
12381	0.94613	0.05
12382	0.946129	0.05
12383	0.94613	0.05
12384	0.946132	0.05
12385	0.946126	0.05
12386	0.946129	0.05
12387	0.94613	0.05
12388	0.946132	1.19603
12389	1.37938	1.28496
12390	0.946131	1.29074
12391	0.94613	1.19889
12392	0.946131	1.31156
12393	0.94613	1.23157
12394	0.94613	1.24923
12395	0.946133	0.05
12396	0.946133	0.05
12397	1.2968	0.05
12398	0.946132	0.05
12399	0.946133	0.05
12400	1.2968	0.05
12401	0.946133	0.05
12402	1.43643	0.05
12403	0.946129	0.477841
12404	0.946133	0.381691
12405	0.946131	0.6796
12406	0.94613	0.190535
12407	0.946131	0.276505
12408	0.946131	0.258762
12409	0.946131	0.249328

12410	0.872447	1.65257
12411	0.946132	1.55373
12412	0.872447	1.5252
12413	0.827867	1.60075
12414	0.946132	1.61903
12415	0.827867	1.71315
12416	0.872447	1.68987
12417	0.946117	1.16538
12418	1.27213	0.913054
12419	0.946124	0.637894
12420	0.946119	1.00869
12421	0.946113	1.01704
12422	0.946129	1.20149
12423	0.946104	1.07042
12424	0.94613	1.79471
12425	0.946131	2.1
12426	0.94613	1.81281
12427	0.946129	2.1
12428	0.946132	0.318752
12429	0.946131	1.33334
12430	0.94613	1.2051
12431	0.810942	2.1
12432	0.810943	2.1
12433	2.80212	2.1
12434	1.3517	2.1
12435	1.08133	2.1
12436	1.08132	2.1
12437	1.08132	2.1
12438	0.810932	2.1
12439	1.29521	2.1
12440	0.946129	2.1
12441	0.946131	2.1
12442	1.40106	2.1
12443	0.94613	2.1
12444	1.40106	2.1
12445	0.946131	2.1
12446	0.946132	0.610384
12447	1.24596	0.409067
12448	0.94613	0.466871
12449	0.946131	0.591869
12450	0.946131	0.288728
12451	0.946131	0.59054
12452	0.946131	0.499326
12453	0.946131	2.1
12454	0.946131	2.1
12455	0.946131	2.1
12456	0.946128	2.1
12457	0.946133	2.1
12458	0.94613	2.1
12459	0.946131	2.1
12460	0.879548	0.181404
12461	1.21527	0.05
12462	0.967506	0.06804
12463	0.946132	0.390077
12464	0.946133	0.119794
12465	0.967511	0.31416
12466	0.946132	0.651063
12467	0.946132	0.664326

12468	0.946132	0.321292
12469	0.974253	0.307411
12470	0.974253	0.615932
12471	0.91801	0.399881
12472	0.918009	0.358516
12473	0.946132	0.581343
12474	0.946132	0.05
12475	0.946131	0.05
12476	0.946131	0.0505744
12477	0.946133	0.05
12478	0.946132	0.05
12479	1.28644	0.05
12480	0.946132	0.05
12481	0.946131	0.0548959
12482	0.946131	0.05
12483	0.946131	0.0582342
12484	0.946131	0.05
12485	0.946131	0.05
12486	1.40103	0.05
12487	0.946131	0.0518409
12488	0.946128	0.14751
12489	0.946132	0.103399
12490	0.946129	0.166904
12491	1.03327	0.0870114
12492	0.946129	0.0915893
12493	0.946129	0.143842
12494	0.94613	0.173937
12495	0.946134	0.655195
12496	0.946133	0.756205
12497	0.946132	0.231642
12498	0.946133	0.745284
12499	0.946132	0.101298
12500	0.946132	0.24462
12501	0.946133	0.20334
12502	0.856654	0.05
12503	0.946131	0.05
12504	0.946131	0.05
12505	0.946132	0.05
12506	0.856616	0.05
12507	1.03565	0.05
12508	1.03565	0.05
12509	1.15844	0.322662
12510	0.946132	0.452477
12511	0.946132	0.205966
12512	0.946132	0.295726
12513	0.946132	0.224433
12514	0.946132	0.337254
12515	0.946131	0.331377
12516	1.4582	2.1
12517	1.35067	2.1
12518	0.946131	2.1
12519	1.04106	2.1
12520	0.938748	2.1
12521	0.943015	2.1
12522	1.05017	2.1
12523	0.938793	2.1
12524	0.946131	0.05
12525	0.946132	0.05

12526	0.946131	0.0504699
12527	0.94613	0.05
12528	0.946133	0.05
12529	0.946131	0.05
12530	0.946131	0.05
12531	0.946132	0.79898
12532	0.946133	0.9965
12533	0.946132	0.881327
12534	0.946133	0.937054
12535	0.946133	1.05659
12536	0.946132	1.00142
12537	0.946133	1.04777
12538	0.946128	1.77762
12539	0.946132	2.00923
12540	0.946132	1.75801
12541	0.94613	1.84318
12542	1.23426	1.83811
12543	0.94613	1.86894
12544	0.946131	1.85005
12545	0.810943	0.276619
12546	0.810946	0.704997
12547	2.60388	0.893363
12548	1.3517	0.272275
12549	1.08133	0.207926
12550	1.08132	0.697691
12551	1.08132	0.174392
12552	0.810933	0.0754928
12553	0.810945	2.1
12554	0.810945	1.86572
12555	2.72377	2.1
12556	1.3517	2.1
12557	1.08133	2.1
12558	1.08132	1.91029
12559	1.08132	2.1
12560	0.810932	2.1
12561	1.25104	0.955207
12562	0.946131	2.00978
12563	0.946131	1.67649
12564	1.36188	2.1
12565	0.946131	2.1
12566	1.36188	2.1
12567	0.946132	2.1
12568	0.946132	2.08838
12569	0.946132	2.1
12570	0.946132	2.1
12571	0.946132	2.1
12572	0.946132	2.08353
12573	0.946132	2.1
12574	1.25488	2.1
12575	1.32132	0.05
12576	0.946126	0.0661686
12577	0.946127	0.05
12578	0.946128	0.0599678
12579	0.946128	0.0664632
12580	0.946128	0.0571873
12581	0.946128	0.058453
12582	0.946133	2.1
12583	1.41123	2.1

12584	0.946134	2.1
12585	0.946133	2.1
12586	0.946132	2.01148
12587	0.946133	2.1
12588	0.946132	2.1
12589	1.32163	0.05
12590	0.946133	0.05
12591	0.946133	0.05
12592	0.946132	0.05
12593	0.946132	0.05
12594	0.946132	0.05
12595	0.946133	0.051892
12596	0.946133	2.1
12597	0.946133	2.1
12598	0.946132	2.1
12599	0.946132	2.1
12600	0.946132	2.1
12601	0.946133	2.1
12602	0.946132	2.1
12603	0.94613	0.103749
12604	0.946131	0.0955526
12605	0.946128	0.0988864
12606	0.946133	0.0956387
12607	0.946133	0.0794052
12608	0.946131	0.0719564
12609	0.946131	0.0913866
12610	0.946133	0.860218
12611	0.946132	0.427085
12612	0.946132	1.1887
12613	0.946134	0.421001
12614	0.946133	1.39152
12615	0.946133	0.695149
12616	0.946132	0.654703
12617	0.94613	1.84587
12618	0.946128	2.01018
12619	0.946127	1.76697
12620	0.94613	1.69107
12621	0.946131	1.78379
12622	0.946128	1.79469
12623	1.21099	1.90859
12624	0.946132	0.859249
12625	0.946131	1.012
12626	0.946131	1.00901
12627	0.946133	0.18141
12628	0.946133	0.231125
12629	0.946131	0.343115
12630	0.946132	0.297362
12631	1.34794	0.764268
12632	0.517994	0.726881
12633	0.517965	0.554886
12634	0.946132	1.02088
12635	0.9198	1.78881
12636	0.946132	1.35334
12637	0.9198	1.42851
12638	0.946132	1.52431
12639	0.94613	1.54924
12640	0.946131	0.594325
12641	0.946131	1.56444

12642	0.946131	1.78285
12643	0.946131	1.56043
12644	1.23224	1.07094
12645	0.946132	1.71646
12646	0.946132	1.86432
12647	0.946133	1.65356
12648	0.946132	1.49071
12649	0.946132	1.54517
12650	0.946133	1.63414
12651	1.37511	1.63462
12652	0.946133	0.983218
12653	0.946129	0.991212
12654	0.851104	1.28134
12655	0.946131	0.197072
12656	1.0412	0.442019
12657	1.0412	0.455513
12658	0.851066	1.04825
12659	1.31357	0.0629454
12660	0.946131	0.191103
12661	0.94613	0.11049
12662	0.946131	0.109422
12663	0.94613	0.0549057
12664	0.946131	0.127442
12665	0.946131	0.132306
12666	0.946133	0.649881
12667	1.38149	1.25752
12668	0.946132	1.24656
12669	0.946132	0.703419
12670	0.946133	1.29936
12671	0.946132	1.16276
12672	0.946133	1.22469
12673	0.810944	1.69629
12674	0.810945	2.1
12675	0.810958	2.1
12676	2.65782	2.1
12677	1.35168	2.1
12678	1.08132	1.80925
12679	1.08132	2.1
12680	1.0813	1.91759
12681	0.946113	0.05
12682	0.946119	0.05
12683	0.946118	0.05
12684	0.946118	0.05
12685	0.946119	0.05
12686	0.946131	0.05
12687	0.946106	0.05
12688	0.946132	0.05
12689	0.946132	0.05
12690	0.946128	0.05
12691	0.946131	0.05
12692	0.94613	0.05
12693	0.94613	0.05
12694	0.94613	0.05
12695	1.00099	0.0606174
12696	0.946132	0.101019
12697	1.17876	0.061362
12698	0.982282	0.0742321
12699	0.909981	0.106771

12700	0.946132	0.090786
12701	0.946131	0.0820846
12702	0.946132	1.18676
12703	1.35328	1.30172
12704	0.946132	1.34963
12705	0.946132	1.18386
12706	0.946133	1.30289
12707	0.946132	1.21335
12708	0.946133	1.25354
12709	0.946133	0.05
12710	0.946133	0.05
12711	0.946132	0.05
12712	0.946132	0.05
12713	0.946132	0.05
12714	0.946132	0.05
12715	0.946133	0.05
12716	0.900195	0.05
12717	0.946132	0.05
12718	0.900167	0.05
12719	1.41963	0.05
12720	0.900195	0.05
12721	0.946133	0.05
12722	1.35385	0.05
12723	0.946132	1.72366
12724	0.946129	1.86792
12725	0.946129	1.91145
12726	0.946131	1.71656
12727	0.946131	1.93588
12728	1.41585	1.66261
12729	0.94613	1.8858
12730	0.810946	2.1
12731	0.810946	2.1
12732	2.68895	2.1
12733	1.3517	2.1
12734	1.08133	2.1
12735	1.08132	2.1
12736	1.08132	2.1
12737	0.810933	2.1
12738	1.19886	2.1
12739	0.946131	2.1
12740	0.946131	2.1
12741	1.34447	2.1
12742	0.946131	2.1
12743	1.34447	2.1
12744	0.946131	2.1
12745	0.946133	0.05
12746	0.946133	0.05
12747	0.946132	0.0669893
12748	1.35065	0.05
12749	0.946132	0.0684922
12750	0.946133	0.0622316
12751	0.946132	0.05
12752	0.946131	0.523683
12753	0.946131	0.671313
12754	0.94613	0.930004
12755	0.946131	0.164125
12756	0.946131	0.195302
12757	0.946131	0.305972

12758	0.946131	0.240854
12759	0.946127	0.128713
12760	0.946131	0.119205
12761	0.946127	0.126296
12762	0.94613	0.0993324
12763	0.946131	0.107638
12764	0.946128	0.118855
12765	0.946129	0.110368
12766	0.810946	0.05
12767	0.810947	0.05
12768	0.810959	0.05
12769	2.4391	0.05
12770	1.35168	0.05
12771	1.08132	0.05
12772	1.08132	0.05
12773	1.08131	0.05
12774	0.946132	0.05
12775	0.946132	0.05
12776	0.946132	0.05
12777	1.40837	0.05
12778	0.946132	0.05
12779	0.946132	0.05
12780	1.40837	0.05
12781	0.946127	2.1
12782	0.946132	2.1
12783	0.946131	0.386549
12784	0.946127	2.1
12785	0.946131	1.56712
12786	0.946129	1.52648
12787	0.94613	1.72537
12788	0.946133	1.90414
12789	0.946132	1.84448
12790	0.946132	1.828
12791	0.946132	1.67815
12792	0.946132	1.85594
12793	0.946132	1.61496
12794	0.946133	2.03731
12795	0.946132	0.657151
12796	1.24344	0.119223
12797	0.946132	0.089359
12798	0.946132	0.674065
12799	0.946132	0.237344
12800	1.24344	0.166665
12801	0.946132	1.01985
12802	0.946131	0.125642
12803	0.946128	0.149596
12804	0.946128	0.0862396
12805	0.946131	0.18736
12806	1.41232	0.0854203
12807	0.946129	0.0761232
12808	0.94613	0.123829
12809	0.946128	2.1
12810	0.946133	2.1
12811	0.946133	1.69233
12812	0.946131	2.1
12813	0.946131	1.68728
12814	0.946131	2.1
12815	0.94613	2.1

12816	0.946133	0.509292
12817	1.41556	0.358975
12818	0.946133	0.28174
12819	0.946133	0.264338
12820	0.946132	0.970159
12821	0.946132	0.370367
12822	0.946132	0.715418
12823	0.94612	0.381595
12824	1.41843	0.281479
12825	0.946114	0.278392
12826	0.94612	0.258649
12827	0.946119	0.688197
12828	1.41845	0.282648
12829	0.946107	0.568876
12830	0.946129	0.05
12831	0.946129	0.05
12832	0.94613	0.05
12833	0.946132	0.05
12834	0.946126	0.05
12835	1.1509	0.05
12836	0.94613	0.05
12837	0.946131	0.05
12838	0.946132	0.05
12839	0.946132	0.05
12840	0.946132	0.05
12841	0.946132	0.05
12842	1.19263	0.05
12843	0.946132	0.05
12844	0.94613	0.61883
12845	0.946131	0.887151
12846	0.946128	0.678577
12847	0.946133	1.05357
12848	0.946133	0.997391
12849	0.946131	0.756795
12850	0.946131	0.966301
12851	0.946133	1.39241
12852	1.22848	1.71511
12853	1.00336	1.7225
12854	0.827866	1.60072
12855	0.946132	1.75881
12856	0.827865	1.6669
12857	0.946133	1.62942
12858	0.946131	2.1
12859	0.94613	0.549096
12860	0.946127	2.1
12861	1.41498	2.1
12862	0.94613	0.473361
12863	0.946128	1.54597
12864	0.946128	1.12861
12865	0.94613	0.05
12866	0.94613	0.05
12867	0.94613	0.05
12868	1.37358	0.05
12869	0.946129	0.05
12870	0.94613	0.05
12871	1.37357	0.05
12872	0.946111	0.05
12873	0.946118	0.05

12874	0.946118	0.05
12875	1.36568	0.05
12876	0.946116	0.05
12877	0.94613	0.05
12878	0.946105	0.05
12879	0.946132	0.283579
12880	0.946131	0.182492
12881	0.94613	0.331336
12882	1.23175	0.149826
12883	0.946131	0.138362
12884	0.946131	0.287032
12885	0.946131	0.355881
12886	0.946132	0.05
12887	0.946126	0.05
12888	0.946128	0.05
12889	0.946129	0.05
12890	0.946129	0.05
12891	0.946128	0.05
12892	1.25698	0.05
12893	0.810943	2.1
12894	0.810944	2.1
12895	2.81171	2.1
12896	1.3517	2.1
12897	1.08132	2.1
12898	1.08133	2.1
12899	1.08132	2.1
12900	0.810932	2.1
12901	0.946132	0.131374
12902	0.946131	0.115735
12903	0.946131	0.146077
12904	0.946132	0.068797
12905	0.946132	0.0834141
12906	0.946132	0.101634
12907	0.946131	0.0917027
12908	0.810936	2.06203
12909	0.810935	2.1
12910	2.80914	2.1
12911	1.35167	2.1
12912	1.0813	2.1
12913	1.0813	2.1
12914	1.0813	1.70358
12915	0.810934	2.1
12916	1.23851	2.1
12917	0.946132	2.1
12918	0.946131	2.1
12919	1.40459	2.03628
12920	0.946132	2.1
12921	1.40459	2.1
12922	0.946132	2.1
12923	1.19974	0.107184
12924	0.946131	0.332946
12925	1.19974	0.119252
12926	0.946132	0.294462
12927	0.946132	0.0870015
12928	0.946132	0.324588
12929	0.946132	0.166312
12930	0.939141	0.80197
12931	1.14375	1.35676

12932	1.03306	1.18223
12933	0.946132	1.20864
12934	0.946126	1.39909
12935	0.946129	1.34743
12936	0.94613	1.11158
12937	0.946132	0.0971357
12938	0.946131	0.109557
12939	0.946132	0.0954154
12940	0.946132	0.114099
12941	0.946132	0.0940959
12942	0.946132	0.0908741
12943	0.946132	0.103451
12944	0.946131	2.1
12945	0.94613	2.1
12946	0.94613	2.1
12947	0.946131	2.1
12948	0.946129	2.1
12949	0.94613	2.1
12950	1.24512	2.1
12951	0.946133	0.070131
12952	0.946129	0.0628632
12953	0.946131	0.125166
12954	0.946132	0.05
12955	0.946131	0.0774883
12956	0.946131	0.0698141
12957	0.946131	0.123149
12958	0.946132	0.0706721
12959	0.946133	0.0818788
12960	0.946132	0.0767668
12961	0.946132	0.0809619
12962	0.946133	0.0761817
12963	0.946132	0.0641459
12964	0.946132	0.110187
12965	0.946132	0.82814
12966	1.41392	0.523818
12967	0.946132	0.555596
12968	0.946132	0.947255
12969	0.946132	0.395934
12970	0.946132	0.845626
12971	0.946132	0.721893
12972	0.946132	1.45886
12973	1.24333	0.96307
12974	0.946132	1.0293
12975	0.946133	1.51926
12976	0.946132	0.50285
12977	0.946132	1.50343
12978	0.946133	1.36316
12979	1.30649	0.165987
12980	0.94613	0.1936
12981	0.94613	0.155976
12982	0.946128	0.170393
12983	0.946126	0.218638
12984	0.946127	0.193891
12985	0.946127	0.190316
12986	0.709578	0.27863
12987	0.810943	0.260635
12988	2.53303	0.141698
12989	1.21651	0.142401

12990	0.979951	0.175534
12991	1.08132	0.162315
12992	0.946141	0.269441
12993	0.81096	0.223213
12994	0.946131	0.0772289
12995	0.946131	0.0856607
12996	0.946131	0.0849393
12997	0.946131	0.051032
12998	0.946131	0.061824
12999	0.946131	0.0858007
13000	0.946131	0.0699937
13001	0.946132	0.05
13002	0.946129	0.05
13003	0.946129	0.05
13004	0.94613	0.05
13005	1.20634	0.05
13006	1.20634	0.05
13007	0.946131	0.05
13008	0.946133	0.447994
13009	0.946132	0.0643213
13010	0.946132	0.060341
13011	0.946132	0.40744
13012	0.946132	0.0575601
13013	1.30107	0.127554
13014	0.946133	0.323288
13015	0.810946	2.1
13016	0.810944	2.1
13017	2.73936	2.1
13018	1.35168	2.1
13019	1.0813	2.1
13020	1.08132	2.1
13021	1.08132	2.1
13022	0.810958	2.1
13023	0.946129	2.1
13024	0.946129	2.1
13025	0.946133	1.76238
13026	1.30812	2.1
13027	0.946131	1.87981
13028	0.946131	2.05521
13029	0.946131	2.04769
13030	1.00901	1.67032
13031	0.94613	1.88364
13032	0.94613	1.42875
13033	1.17001	1.69582
13034	0.917259	0.849135
13035	0.94613	1.61075
13036	0.94613	1.65029
13037	0.946126	2.1
13038	0.946127	2.1
13039	0.946131	2.1
13040	0.946127	2.1
13041	0.946131	2.1
13042	0.946129	2.1
13043	1.398	2.1
13044	0.946133	2.1
13045	1.3033	2.1
13046	0.946132	2.1
13047	0.946132	2.1

13048	0.946132	2.1
13049	0.946132	2.1
13050	0.946133	2.1
13051	0.946131	0.436429
13052	0.946131	0.414271
13053	1.09897	0.360758
13054	0.946129	0.684887
13055	0.946131	0.468923
13056	0.94613	0.58464
13057	0.94613	0.442152
13058	0.946132	2.1
13059	0.946131	2.1
13060	0.946131	2.1
13061	0.946132	2.1
13062	0.94613	2.1
13063	0.946131	2.1
13064	1.39858	2.1
13065	1.07117	1.17296
13066	1.08878	1.22004
13067	0.946119	1.23326
13068	1.08878	1.35992
13069	0.946119	1.06322
13070	0.946132	1.19296
13071	1.07116	1.18144
13072	0.946132	0.0540388
13073	0.946132	0.05
13074	0.946132	0.057617
13075	0.946132	0.05
13076	0.946132	0.05
13077	1.10733	0.05
13078	0.946132	0.05
13079	0.946128	0.0657359
13080	0.946132	0.0513512
13081	0.94613	0.0705266
13082	0.946129	0.05
13083	0.94613	0.05
13084	1.3367	0.05
13085	0.94613	0.0616181
13086	0.810943	2.1
13087	0.810944	2.1
13088	2.70234	2.1
13089	1.21653	2.1
13090	1.08132	2.1
13091	0.946155	2.1
13092	0.979951	2.1
13093	0.709578	2.1
13094	1.27174	2.1
13095	0.946129	2.1
13096	0.94613	2.1
13097	1.35117	2.1
13098	0.94613	2.1
13099	1.35117	2.1
13100	0.94613	2.1
13101	0.946133	1.32551
13102	0.946133	1.57115
13103	0.946132	1.907
13104	0.946133	1.28002
13105	0.946132	2.1

13106	0.946133	1.86475
13107	0.997296	1.95215
13108	0.810943	2.1
13109	0.810944	2.1
13110	0.810945	2.1
13111	2.78833	2.1
13112	1.35169	2.1
13113	1.08132	2.1
13114	1.08132	2.1
13115	1.08132	2.1
13116	0.810946	2.1
13117	0.810943	1.39483
13118	0.810933	1.67356
13119	2.70049	1.75079
13120	1.3517	1.63371
13121	1.08132	2.1
13122	1.08132	0.266112
13123	1.08133	1.60992
13124	0.946127	1.5777
13125	1.35469	1.64806
13126	0.94613	1.63207
13127	0.946131	1.6
13128	0.946128	1.53892
13129	1.35469	1.72028
13130	0.946129	1.71333
13131	0.946131	0.259647
13132	1.2722	0.207821
13133	0.946132	0.201189
13134	0.946132	0.213087
13135	0.946129	0.292185
13136	1.2722	0.177504
13137	0.94613	0.225307
13138	0.946133	0.0671987
13139	0.946132	0.0758186
13140	0.946132	0.363526
13141	0.946132	0.104021
13142	1.41461	0.467899
13143	1.41461	0.0872515
13144	0.946133	0.102128
13145	0.946131	1.42917
13146	1.30165	0.670738
13147	0.946132	0.388716
13148	0.946132	1.47658
13149	0.946129	0.8072
13150	1.30165	1.39987
13151	0.946131	1.47206
13152	0.946132	1.86681
13153	0.946129	2.1
13154	0.94613	1.89991
13155	1.39356	2.1
13156	0.946131	1.92199
13157	0.946131	1.96124
13158	0.94613	2.1
13159	0.810946	2.1
13160	0.810947	2.1
13161	0.810934	2.1
13162	2.35003	2.1
13163	1.3517	2.1

13164	1.08132	2.1
13165	1.08132	2.1
13166	1.08133	2.1
13167	0.946131	0.05
13168	0.946131	0.05
13169	0.946125	0.0512458
13170	0.946127	0.05
13171	0.946129	0.05
13172	0.946128	0.05
13173	0.946128	0.05
13174	0.946131	0.05
13175	0.946131	0.05
13176	0.946131	0.05
13177	0.946131	0.05
13178	0.946132	0.05
13179	0.946132	0.05
13180	0.946131	0.05
13181	0.946129	2.1
13182	0.946132	2.1
13183	0.946132	2.1
13184	0.94613	2.1
13185	1.30082	2.1
13186	0.94613	2.1
13187	0.946131	2.1
13188	1.20416	0.05
13189	0.946132	0.0750955
13190	0.946131	0.05
13191	0.946132	0.05
13192	0.946131	0.05
13193	0.946131	0.0504418
13194	0.946132	0.05
13195	1.18711	0.05
13196	0.946131	0.0644061
13197	0.946131	0.05
13198	0.946132	0.05
13199	0.946131	0.05
13200	0.946131	0.0612613
13201	0.946132	0.0530517
13202	0.946133	0.05
13203	0.946133	0.05
13204	0.946132	0.05
13205	1.30212	0.05
13206	0.946133	0.05
13207	0.946132	0.05
13208	1.30212	0.05
13209	0.946131	0.05
13210	0.946131	0.05
13211	0.946131	0.05
13212	0.946132	0.05
13213	1.31467	0.05
13214	1.31467	0.05
13215	0.946131	0.05
13216	0.946133	0.05
13217	0.946132	0.05
13218	0.946132	0.05
13219	0.946132	0.05
13220	1.25124	0.05
13221	1.25124	0.05

13222	0.946132	0.05
13223	0.946132	2.1
13224	0.946132	2.1
13225	0.946132	2.1
13226	1.36422	2.1
13227	0.946132	2.1
13228	0.946131	2.1
13229	0.946132	2.1
13230	0.946131	1.48194
13231	1.41851	1.69867
13232	0.946132	1.80364
13233	0.946132	1.50419
13234	0.946132	1.70193
13235	0.946132	1.54581
13236	0.946132	1.60778
13237	0.946133	0.05
13238	0.946132	0.05
13239	0.946132	0.05
13240	0.946132	0.05
13241	0.946132	0.05
13242	1.37794	0.05
13243	0.946132	0.0509419
13244	0.946132	2.1
13245	0.946132	2.1
13246	0.946131	2.1
13247	0.946131	2.1
13248	0.946132	2.1
13249	0.946132	2.1
13250	1.4045	2.1
13251	0.946133	2.1
13252	0.946132	2.1
13253	0.946131	2.1
13254	0.946132	2.1
13255	0.946131	2.1
13256	0.946131	2.1
13257	0.946132	2.1
13258	0.946132	2.1
13259	0.946132	2.1
13260	0.946131	2.1
13261	0.946132	2.1
13262	0.946131	2.1
13263	0.946131	2.1
13264	0.946131	2.1
13265	0.946128	2.1
13266	1.31641	2.1
13267	0.946129	2.1
13268	0.94613	2.1
13269	0.94613	2.1
13270	0.94613	2.1
13271	0.94613	2.1
13272	0.946132	2.1
13273	0.946131	2.1
13274	0.946131	2.1
13275	0.946132	2.1
13276	0.946132	2.1
13277	0.946132	2.1
13278	0.946132	2.1
13279	0.946132	2.1

13280	1.41507	2.1
13281	0.946132	2.1
13282	0.946132	2.1
13283	0.946133	2.1
13284	0.946133	2.1
13285	0.946132	2.1
13286	0.946132	2.1
13287	0.946131	2.1
13288	0.946131	2.1
13289	0.946133	2.1
13290	0.946132	2.1
13291	1.32608	2.1
13292	0.946132	2.1
13293	0.94613	1.97034
13294	0.94613	2.08786
13295	0.94613	0.908571
13296	0.946131	2.05231
13297	0.946129	1.51742
13298	0.94613	1.33517
13299	0.94613	1.49932
13300	1.33728	1.94233
13301	0.946131	1.8895
13302	0.94613	1.51957
13303	0.946132	1.94809
13304	0.946132	2.1
13305	0.946131	1.77679
13306	0.946131	1.83278
13307	0.946127	2.1
13308	1.40641	2.1
13309	0.946129	2.1
13310	0.94613	2.1
13311	0.946128	1.84552
13312	0.946129	2.1
13313	0.946129	2.1
13314	0.946129	2.1
13315	0.946128	2.1
13316	0.946133	2.1
13317	1.37467	2.1
13318	0.946131	2.1
13319	0.946131	2.1
13320	1.37467	2.1
13321	0.946131	2.1
13322	0.946131	2.1
13323	0.946132	2.1
13324	0.946132	2.1
13325	0.946129	2.1
13326	0.946131	2.1
13327	1.34912	2.1
13328	0.946132	2.1
13329	0.946133	2.1
13330	0.946133	2.1
13331	0.946132	2.1
13332	0.946132	2.1
13333	0.946133	2.1
13334	0.946132	2.1
13335	0.946127	2.1
13336	0.946131	2.1
13337	0.946132	2.1

13338	0.946131	2.1
13339	0.946127	2.1
13340	0.94613	2.1
13341	1.33607	2.1
13342	0.946117	0.284122
13343	0.946117	1.55248
13344	0.946115	1.46313
13345	0.946124	2.1
13346	0.946119	0.610214
13347	1.32662	1.46991
13348	0.94613	0.483326
13349	0.946119	2.1
13350	0.946114	2.1
13351	0.946118	2.1
13352	0.946114	2.1
13353	0.946118	2.1
13354	0.946104	2.1
13355	0.946129	2.1
13356	0.946132	2.1
13357	0.946133	2.1
13358	0.946133	2.1
13359	0.946133	2.1
13360	0.946132	2.1
13361	0.946132	2.1
13362	0.946133	2.1
13363	0.946133	2.1
13364	0.946132	2.1
13365	0.946132	2.1
13366	0.946133	2.1
13367	0.946132	2.1
13368	0.946132	2.1
13369	0.946132	2.1
13370	0.946133	1.6302
13371	1.30881	1.87742
13372	0.946132	2.1
13373	0.946131	1.62164
13374	0.946131	1.8338
13375	0.946131	1.65824
13376	0.946131	1.75448
13377	0.946128	2.1
13378	0.946128	2.1
13379	0.946132	2.1
13380	0.94613	2.1
13381	0.94613	2.1
13382	0.94613	2.1
13383	1.34652	2.1
13384	0.946133	1.11391
13385	0.946133	1.14342
13386	0.946132	1.81022
13387	0.946133	0.995832
13388	0.946132	2.0301
13389	0.946132	1.55244
13390	0.946132	1.78626
13391	0.946132	0.107913
13392	0.946128	0.0746088
13393	0.94613	0.131761
13394	0.946131	0.05
13395	0.94613	0.137794

13396	0.94613	0.0946879
13397	0.94613	0.0910534
13398	0.946128	0.492022
13399	0.946132	0.525984
13400	0.94613	0.52629
13401	0.94613	0.422054
13402	0.946131	0.460776
13403	0.94613	0.52837
13404	0.94613	0.474032
13405	0.946129	1.3693
13406	1.32056	1.50069
13407	0.946131	1.53785
13408	0.94613	1.39784
13409	0.946124	1.49254
13410	0.946128	1.4232
13411	0.946127	1.44492
13412	0.946129	1.12072
13413	1.20351	1.50925
13414	0.946132	1.52009
13415	0.946132	1.42622
13416	0.946126	1.51156
13417	0.946129	1.46277
13418	0.946129	1.44217
13419	0.946127	1.96909
13420	0.946127	2.1
13421	0.946131	1.74414
13422	1.25592	1.97142
13423	0.946127	1.78167
13424	0.946129	1.88807
13425	0.94613	1.90749
13426	1.02332	1.73866
13427	0.946133	1.61052
13428	0.946132	1.46551
13429	1.02332	1.61088
13430	0.946132	1.37909
13431	0.96196	1.53801
13432	0.930306	1.5294
13433	0.946133	1.98136
13434	0.946133	1.99679
13435	0.946132	2.1
13436	1.20057	2.04394
13437	0.946132	2.1
13438	0.946133	2.1
13439	1.20057	2.1
13440	0.946128	1.41208
13441	0.946129	1.28493
13442	0.946129	1.26961
13443	1.13245	1.2476
13444	0.946126	1.17617
13445	0.946128	1.26827
13446	0.946129	1.26449
13447	0.946131	1.83123
13448	0.946131	1.9121
13449	0.94613	2.06905
13450	1.25	1.90422
13451	0.946129	2.06966
13452	0.946131	2.1
13453	1.25	2.0838

13454	0.540425	1.96299
13455	0.748493	1.99029
13456	1.46266	2.1
13457	1.20046	2.09477
13458	0.600454	2.00141
13459	1.41283	2.1
13460	0.582567	1.98397
13461	0.946131	1.85541
13462	0.946131	1.87512
13463	0.946132	1.82394
13464	0.946132	1.90425
13465	0.94613	1.81681
13466	0.946131	2.1
13467	1.28439	2.0745
13468	0.946132	0.937431
13469	0.946131	0.684804
13470	0.946131	1.00124
13471	0.946132	0.640337
13472	0.946132	1.1929
13473	1.39335	0.525594
13474	0.946132	0.606227
13475	0.946131	0.205561
13476	0.946131	0.417024
13477	0.94613	0.202621
13478	0.946132	0.185052
13479	0.946131	0.344067
13480	0.946131	0.231591
13481	0.946131	0.264
13482	0.946131	0.219691
13483	0.94613	0.189393
13484	0.946126	0.268005
13485	0.946131	0.141204
13486	0.946125	0.69526
13487	0.946127	0.157409
13488	0.946128	0.315972
13489	0.946131	0.05
13490	0.946131	0.0516138
13491	0.946131	0.05
13492	0.946131	0.05
13493	0.946131	0.05
13494	0.946131	0.05
13495	1.39168	0.05
13496	0.946131	0.102265
13497	0.946126	0.0600314
13498	0.946127	0.0790531
13499	0.946131	0.0712719
13500	0.94613	0.101366
13501	1.412	0.0755486
13502	0.946128	0.0863223
13503	0.948901	0.239546
13504	0.943364	0.22353
13505	1.03768	0.219644
13506	0.946132	0.332123
13507	0.946133	0.300489
13508	1.03767	0.308609
13509	0.946132	0.261414
13510	1.00902	0.53805
13511	0.88324	0.400923

13512	0.971584	0.399722
13513	0.946132	0.468662
13514	0.946129	1.15888
13515	0.946131	0.704419
13516	0.971572	0.826508
13517	0.946131	0.05
13518	0.946125	0.05
13519	1.33587	0.05
13520	0.946126	0.0536414
13521	0.946127	0.05
13522	0.946126	0.05
13523	0.946128	0.05
13524	0.946132	0.0615592
13525	0.946132	0.0566112
13526	1.27512	0.05
13527	0.946133	0.0837413
13528	0.946132	0.0650638
13529	0.946133	0.062631
13530	0.946133	0.0714383
13531	0.946128	0.0701561
13532	0.946133	0.0671698
13533	0.946131	0.0761914
13534	0.94613	0.05
13535	0.946131	0.05
13536	0.94613	0.061503
13537	0.946131	0.0536642
13538	0.946131	0.057539
13539	0.946131	0.0531205
13540	0.946131	0.0615581
13541	0.946131	0.05
13542	0.946131	0.05
13543	0.946131	0.0507644
13544	0.946131	0.05
13545	0.94613	0.05
13546	0.946131	0.05
13547	0.946131	0.05
13548	0.94613	0.05
13549	0.946132	0.05
13550	0.946131	0.05
13551	1.35316	0.05
13552	0.94613	0.115302
13553	0.94613	0.0778597
13554	0.946128	0.0945907
13555	0.946132	0.0881955
13556	0.946132	0.108502
13557	1.28727	0.0948147
13558	0.94613	0.0834108
13559	0.946131	0.05
13560	0.946132	0.05
13561	0.946132	0.05
13562	1.39502	0.05
13563	0.946132	0.05
13564	0.946132	0.05
13565	0.946132	0.05
13566	0.946125	0.340818
13567	0.946131	0.270875
13568	0.94613	0.261285
13569	0.946129	0.303818

13570	0.946126	0.565354
13571	0.946127	0.407356
13572	1.36173	0.416085
13573	0.946129	0.940861
13574	1.02363	0.446966
13575	0.94613	0.305838
13576	0.946131	0.9159
13577	0.946131	0.374975
13578	1.02363	1.0473
13579	0.946131	1.03615
13580	0.946128	0.05
13581	1.24678	0.05
13582	0.94613	0.05
13583	0.94613	0.05
13584	0.94613	0.05
13585	0.946131	0.05
13586	0.94613	0.05
13587	0.946129	0.05
13588	1.25215	0.05
13589	0.946132	0.05
13590	0.946126	0.05
13591	0.946129	0.05
13592	0.94613	0.05
13593	0.946129	0.05
13594	0.946129	0.366223
13595	0.946133	0.289595
13596	0.946133	0.234067
13597	0.946131	0.299196
13598	0.946131	0.283944
13599	1.29689	0.216879
13600	0.946131	0.264696
13601	0.946128	0.0615403
13602	0.946132	0.05
13603	0.946132	0.05
13604	0.94613	0.0694404
13605	1.22716	0.05
13606	1.22716	0.05
13607	0.946131	0.0571135
13608	0.946127	0.0788928
13609	0.946131	0.0527339
13610	0.946131	0.0645117
13611	0.946128	0.089446
13612	1.30463	0.0544509
13613	1.30463	0.0623849
13614	0.946129	0.0749803
13615	1.29958	0.05
13616	0.946132	0.05
13617	0.946132	0.05
13618	0.946132	0.05
13619	0.946132	0.05
13620	0.946132	0.05
13621	0.946133	0.05
13622	1.26201	0.05
13623	0.946133	0.05
13624	0.946132	0.05
13625	0.946132	0.05
13626	0.946132	0.05
13627	0.946133	0.05

13628	0.946132	0.05
13629	0.946133	0.0751359
13630	0.946132	0.0555078
13631	0.946132	0.0798513
13632	0.946133	0.05
13633	0.946132	0.05
13634	1.40427	0.0513871
13635	0.946133	0.0736007
13636	0.946132	0.0805886
13637	0.946132	0.0548598
13638	0.946132	0.0844037
13639	0.946132	0.05
13640	0.946132	0.05
13641	1.35123	0.05
13642	0.946131	0.077403
13643	0.946128	0.10262
13644	0.946132	0.0681734
13645	0.946132	0.0812557
13646	0.946129	0.119324
13647	1.22144	0.0699809
13648	1.22144	0.0781718
13649	0.946131	0.0971063
13650	0.946127	0.243476
13651	0.946132	0.19759
13652	0.946131	0.18662
13653	0.946127	0.218208
13654	0.946131	0.209271
13655	1.23351	0.178851
13656	0.94613	0.203835
13657	1.18119	0.0989441
13658	0.946131	0.140661
13659	0.946132	0.0958551
13660	0.946132	0.11683
13661	0.94613	0.148693
13662	0.946131	0.127548
13663	0.946131	0.12727
13664	0.946132	0.196358
13665	0.946132	0.154583
13666	0.946133	0.230905
13667	1.30563	0.131537
13668	0.946132	0.14776
13669	0.946132	0.189637
13670	0.946133	0.246802
13671	1.20256	0.348163
13672	0.946132	1.97885
13673	0.946133	1.63919
13674	0.946132	0.450402
13675	0.946132	0.276284
13676	0.946132	1.77872
13677	0.946133	1.01577
13678	0.94613	1.02942
13679	0.94613	0.672774
13680	0.946128	1.23931
13681	1.33007	0.484913
13682	0.946129	0.402338
13683	0.94613	1.05532
13684	0.946131	1.6541
13685	0.946127	2.1

13686	0.946127	2.1
13687	0.946131	2.1
13688	1.30136	2.1
13689	0.946128	2.1
13690	0.946129	2.1
13691	1.30136	2.1
13692	0.810942	2.1
13693	0.810942	2.1
13694	0.81093	2.1
13695	2.3208	2.1
13696	1.3517	2.1
13697	1.08132	2.1
13698	1.08132	2.1
13699	1.08133	2.1
13700	0.946132	2.1
13701	0.946132	2.1
13702	0.946132	2.1
13703	0.946132	2.1
13704	0.946132	2.1
13705	1.39823	2.1
13706	0.946132	2.1
13707	1.37713	2.1
13708	0.946131	2.1
13709	0.946131	2.1
13710	0.946131	2.1
13711	0.946131	2.1
13712	0.946131	2.1
13713	0.946131	2.1
13714	0.946128	2.1
13715	0.946128	2.1
13716	0.946133	2.1
13717	0.946131	2.1
13718	0.946131	2.1
13719	0.946131	2.1
13720	0.94613	2.1
13721	0.946129	2.1
13722	0.946129	2.1
13723	0.946133	2.1
13724	0.946131	2.1
13725	0.946131	2.1
13726	0.946131	2.1
13727	0.946131	2.1
13728	0.946132	1.69815
13729	1.33694	1.98607
13730	0.946133	2.1
13731	0.946133	1.71695
13732	0.946132	2.04396
13733	0.946132	1.78285
13734	0.946133	1.84959
13735	0.946131	2.1
13736	0.94613	2.1
13737	0.946131	2.1
13738	0.946133	2.1
13739	0.946129	2.1
13740	0.946131	2.1
13741	0.946131	2.1
13742	0.946132	1.54685
13743	1.35943	1.80204

13744	0.946132	2.1
13745	0.946132	1.43708
13746	0.946131	1.67657
13747	0.946132	1.47117
13748	0.946132	1.68708
13749	0.946132	0.05
13750	0.946132	0.05
13751	0.946133	0.05
13752	0.946133	0.05
13753	0.946132	0.05
13754	0.946132	0.05
13755	0.946132	0.05
13756	1.29322	0.05
13757	0.946131	0.05
13758	0.946132	0.05
13759	0.946129	0.05
13760	0.946131	0.05
13761	0.946131	0.05
13762	0.946131	0.05
13763	0.946132	1.8193
13764	0.946132	2.1
13765	0.946131	1.79007
13766	1.41492	0.828776
13767	0.946132	2.1
13768	0.946132	2.08078
13769	0.946131	2.1
13770	0.946132	2.1
13771	0.946132	2.1
13772	0.946132	2.1
13773	1.35028	2.1
13774	0.946132	2.1
13775	0.946132	2.1
13776	0.946132	2.1
13777	0.946132	1.87604
13778	0.946132	2.1
13779	0.946132	2.1
13780	1.32461	2.00581
13781	0.946131	2.1
13782	0.946132	2.09749
13783	0.946132	2.1
13784	0.946131	2.1
13785	0.946131	2.1
13786	0.946131	2.1
13787	0.946132	2.1
13788	0.946129	2.1
13789	0.94613	2.1
13790	1.40369	2.1
13791	0.946132	0.12464
13792	0.946131	0.0846824
13793	0.946131	0.134933
13794	0.979598	0.0704276
13795	0.946131	0.0735641
13796	0.946131	0.119725
13797	0.946132	0.138423
13798	0.946131	2.1
13799	1.41897	2.1
13800	0.946132	2.1
13801	0.946132	2.1

13802	0.94613	2.1
13803	0.946131	2.1
13804	0.946131	2.1
13805	0.946131	2.1
13806	0.94613	2.1
13807	0.946132	2.1
13808	0.946132	2.1
13809	0.946129	2.1
13810	0.946131	2.1
13811	1.37791	2.1
13812	0.946127	2.1
13813	0.946131	2.1
13814	0.946131	2.1
13815	0.94613	2.1
13816	0.946128	2.1
13817	0.946129	2.1
13818	1.39384	2.1
13819	0.810945	1.96479
13820	0.810944	1.4175
13821	0.810958	1.492
13822	2.70852	1.53666
13823	1.35168	1.50102
13824	1.08132	1.77602
13825	1.08132	1.12857
13826	1.0813	1.5273
13827	1.3335	2.1
13828	0.946132	2.1
13829	0.94613	2.1
13830	0.946132	2.1
13831	0.946131	2.1
13832	0.94613	2.1
13833	0.946131	2.1
13834	0.946132	2.1
13835	0.946131	1.93021
13836	0.94613	2.1
13837	1.41192	2.1
13838	0.946132	1.9592
13839	0.946131	2.1
13840	0.94613	2.1
13841	0.946133	1.63458
13842	0.946133	0.990792
13843	0.946132	1.08934
13844	1.39428	0.712842
13845	0.946132	0.188721
13846	0.946132	0.487913
13847	0.946133	1.15128
13848	0.94613	1.48114
13849	0.946129	2.1
13850	0.94613	1.91138
13851	0.946128	1.71129
13852	1.40684	2.1
13853	0.94613	1.79685
13854	0.94613	2.1
13855	0.946131	2.1
13856	0.946131	2.1
13857	0.946131	2.1
13858	0.946131	2.1
13859	1.39021	2.1

13860	0.946131	2.1
13861	0.946131	2.1
13862	0.946131	1.97173
13863	0.946131	1.73852
13864	0.946131	1.34656
13865	0.946131	2.025
13866	0.946131	2.02601
13867	0.946131	1.27866
13868	0.946131	1.41651
13869	0.810946	2.1
13870	0.810946	2.1
13871	2.69264	2.1
13872	1.35168	2.1
13873	1.0813	2.1
13874	1.08132	2.1
13875	1.08132	2.1
13876	0.810958	2.1
13877	1.16687	2.1
13878	0.946131	2.1
13879	0.946131	2.1
13880	1.34632	2.1
13881	0.946131	2.1
13882	1.34632	2.1
13883	0.946131	2.1
13884	0.946128	0.909768
13885	0.946133	0.408916
13886	0.946133	0.268468
13887	0.946131	0.704314
13888	0.946131	0.30753
13889	1.3564	0.475062
13890	0.94613	0.781533
13891	0.946128	2.1
13892	0.946132	2.1
13893	0.94613	2.1
13894	0.94613	2.1
13895	0.946131	2.1
13896	1.35827	2.1
13897	0.94613	2.1
13898	0.946133	1.00368
13899	0.946132	1.60093
13900	0.946132	1.2793
13901	0.946133	1.51078
13902	0.946133	1.42312
13903	0.946132	1.47316
13904	0.946133	1.5419
13905	0.946132	2.1
13906	0.946132	1.79272
13907	0.946132	0.404033
13908	0.946131	2.1
13909	0.946132	1.42596
13910	0.946132	1.61867
13911	0.946132	1.6422
13912	0.94613	0.370002
13913	0.94613	0.254906
13914	0.946128	0.394194
13915	0.946132	0.217333
13916	0.946132	0.389656
13917	0.94613	0.281384

13918	0.946131	0.273587
13919	0.946126	0.10997
13920	0.946131	0.0730087
13921	0.946127	0.143599
13922	0.946129	0.0623187
13923	0.94613	0.103349
13924	0.946128	0.0773044
13925	0.946128	0.085819
13926	0.946131	0.0687924
13927	0.946127	0.0668882
13928	0.946127	0.0705966
13929	0.946131	0.0695488
13930	0.946131	0.0572965
13931	0.946128	0.0576784
13932	0.946129	0.0863782
13933	0.94613	0.0752915
13934	0.946131	0.0765369
13935	0.946129	0.0703898
13936	0.946132	0.0872528
13937	0.946132	0.0667572
13938	0.946131	0.0672157
13939	0.94613	0.0953216
13940	0.94613	0.0975626
13941	0.946131	0.0720479
13942	0.946129	0.0884813
13943	0.946132	0.0647969
13944	0.946132	0.0942189
13945	1.32827	0.0767237
13946	0.946131	0.0886316
13947	0.946132	2.1
13948	0.946132	2.1
13949	0.946131	2.1
13950	0.946131	2.1
13951	0.946132	2.1
13952	0.946131	2.1
13953	0.946131	2.1
13954	0.946132	0.546697
13955	0.946132	2.1
13956	0.946131	0.602545
13957	1.38356	0.223207
13958	0.946133	2.07843
13959	0.946131	0.759675
13960	0.946131	1.26352
13961	0.94613	2.1
13962	0.946131	2.1
13963	0.946131	2.1
13964	0.946128	2.1
13965	0.946133	2.1
13966	0.946131	2.1
13967	1.36918	2.1
13968	0.946131	1.89433
13969	0.94613	2.1
13970	0.946129	2.1
13971	0.946133	2.1
13972	0.946132	2.1
13973	1.3771	2.1
13974	0.946131	2.1
13975	1.30029	1.63937

13976	0.94613	1.27434
13977	0.946129	1.88345
13978	0.946132	1.71114
13979	0.946132	0.110723
13980	0.946131	1.56676
13981	0.94613	0.514778
13982	0.946133	0.05
13983	0.946132	0.05
13984	0.946132	0.05
13985	0.946132	0.05
13986	0.946132	0.05
13987	0.946132	0.05
13988	1.21174	0.05
13989	0.810946	2.1
13990	0.810945	2.1
13991	2.63045	2.1
13992	1.35169	2.1
13993	1.08132	2.1
13994	1.08132	2.1
13995	1.08132	2.1
13996	0.810946	2.1
13997	1.1796	2.1
13998	0.946131	2.1
13999	0.946132	2.1
14000	1.31522	2.1
14001	0.946132	2.1
14002	1.31522	2.1
14003	0.946132	2.1
14004	0.946131	2.1
14005	1.40615	2.1
14006	0.946129	2.1
14007	0.946128	2.1
14008	0.946133	2.1
14009	0.946131	2.1
14010	0.94613	2.1
14011	0.810945	2.1
14012	0.810944	2.1
14013	2.80479	2.1
14014	1.35169	2.1
14015	1.08132	2.1
14016	1.08132	2.1
14017	1.08132	2.1
14018	0.810945	2.1
14019	1.35562	2.1
14020	0.94613	2.1
14021	1.35561	2.1
14022	0.946133	2.1
14023	0.946131	2.1
14024	1.40239	2.1
14025	0.946131	2.1
14026	0.946125	0.22509
14027	0.946131	0.365478
14028	0.946127	0.195665
14029	0.946129	0.21914
14030	0.946128	0.37388
14031	0.946129	0.31477
14032	0.946128	0.272385
14033	0.946119	2.1

14034	0.946113	2.1
14035	0.94612	2.1
14036	0.946119	2.1
14037	1.41915	2.1
14038	0.946107	2.1
14039	0.946132	2.1
14040	0.810946	1.90785
14041	0.810943	2.1
14042	0.810934	2.07534
14043	2.31411	2.1
14044	1.35171	2.1
14045	1.08132	1.97313
14046	1.08132	2.1
14047	1.08133	2.03299
14048	0.946127	2.1
14049	0.94613	2.1
14050	0.94613	2.1
14051	0.946131	2.1
14052	0.946126	2.1
14053	0.946128	2.1
14054	0.946128	2.1
14055	0.810944	2.1
14056	0.810947	2.1
14057	0.81096	2.1
14058	2.78019	2.1
14059	1.35168	2.1
14060	1.08132	2.1
14061	1.08132	2.1
14062	1.08131	2.1
14063	0.946133	0.0774954
14064	0.946132	0.0742339
14065	0.946132	0.0801196
14066	0.946133	0.0691241
14067	0.946132	0.0881851
14068	1.3495	0.0617574
14069	0.946133	0.0932545
14070	0.946133	2.1
14071	0.946132	2.1
14072	0.946132	2.1
14073	1.39529	2.1
14074	0.946133	2.1
14075	0.946132	2.1
14076	0.946133	2.1
14077	0.946133	2.1
14078	0.946132	2.1
14079	0.946132	2.1
14080	0.946132	2.1
14081	0.946132	2.1
14082	0.946133	2.1
14083	0.946132	2.1
14084	0.810946	2.1
14085	0.810945	2.1
14086	2.67861	2.1
14087	1.35169	2.1
14088	1.08132	2.1
14089	1.08132	2.1
14090	1.08132	2.1
14091	0.810946	2.1

14092	1.24616	2.1
14093	0.946132	2.1
14094	0.946132	2.1
14095	1.33931	2.1
14096	0.946132	2.1
14097	1.33931	2.1
14098	0.946133	2.1
14099	0.946133	0.183973
14100	0.946133	0.05
14101	0.946132	0.05
14102	0.946133	0.0792139
14103	1.3855	0.05
14104	1.3855	0.05
14105	0.946133	0.175812
14106	0.946128	2.1
14107	0.946132	2.1
14108	0.946128	2.1
14109	0.94613	2.1
14110	0.946131	2.1
14111	1.40225	2.1
14112	0.94613	2.1
14113	1.40548	0.05
14114	0.946118	0.05
14115	0.946114	0.05
14116	0.946112	0.05
14117	0.946117	0.05
14118	0.946128	0.05
14119	0.946103	0.05
14120	0.94613	2.1
14121	0.946132	2.1
14122	0.946131	2.1
14123	0.946132	2.1
14124	1.37811	2.1
14125	0.946131	2.1
14126	0.946131	2.1
14127	0.946132	2.1
14128	0.946133	2.1
14129	0.946133	2.1
14130	0.946132	2.1
14131	0.946133	2.1
14132	0.946132	2.1
14133	0.946132	2.1
14134	0.946118	2.1
14135	0.946113	2.1
14136	0.946118	2.1
14137	0.946119	2.1
14138	0.946117	2.1
14139	0.946105	2.1
14140	0.946131	2.1
14141	0.946118	0.05
14142	0.946118	0.05
14143	0.946117	0.05
14144	0.946113	0.05
14145	0.94612	0.05
14146	1.41377	0.05
14147	0.946106	0.05
14148	0.946132	1.56899
14149	1.28436	1.36228

14150	0.946131	1.40439
14151	0.946131	1.57113
14152	0.946131	0.458234
14153	0.94613	1.59254
14154	0.946131	1.45447
14155	0.810947	0.352926
14156	0.810946	0.391348
14157	2.68675	0.224055
14158	1.35168	0.261142
14159	1.08132	0.297654
14160	1.08132	0.303772
14161	1.0813	0.380784
14162	0.81096	0.333296
14163	0.946132	0.243391
14164	1.34338	0.206857
14165	0.946132	0.224569
14166	1.34338	0.250167
14167	0.946132	0.272315
14168	1.34337	0.229466
14169	0.946132	0.235615
14170	0.946127	2.1
14171	0.946131	2.1
14172	0.946127	2.1
14173	0.946131	1.97701
14174	0.946131	2.1
14175	0.946128	2.09004
14176	1.30281	2.1
14177	0.946132	0.567967
14178	0.946132	0.345287
14179	0.946132	0.398957
14180	0.946131	0.414707
14181	0.946132	0.553939
14182	1.37973	0.259227
14183	0.946132	0.380343
14184	0.946131	1.2457
14185	1.41798	0.747524
14186	0.946132	0.37645
14187	0.946132	0.7709
14188	0.946129	1.29356
14189	0.946131	0.926698
14190	0.946131	1.28292
14191	0.946118	1.08982
14192	1.41776	0.424371
14193	0.946113	0.42174
14194	0.946119	0.433877
14195	0.946117	1.24402
14196	1.41778	0.452178
14197	0.946105	1.24162
14198	0.946131	1.2941
14199	1.41798	1.33526
14200	0.946132	1.36882
14201	0.946132	1.1003
14202	0.94613	1.32851
14203	1.41799	1.06169
14204	0.946131	1.11144
14205	0.946133	2.1
14206	0.946132	2.1
14207	0.946132	1.62831

14208	0.946132	2.1
14209	0.946133	1.85904
14210	0.946132	2.1
14211	0.946132	2.1
14212	0.946131	2.1
14213	0.946131	2.1
14214	0.94613	0.992037
14215	0.946129	2.1
14216	0.946132	1.64449
14217	0.94613	1.88498
14218	0.946131	2.1
14219	0.94612	1.54415
14220	0.946126	2.1
14221	0.946119	1.59245
14222	0.94612	1.67084
14223	1.40766	1.98977
14224	0.946132	1.27786
14225	0.946107	2.1
14226	0.946128	2.1
14227	1.31213	2.1
14228	0.946129	2.1
14229	0.94613	2.1
14230	0.94613	2.1
14231	0.94613	2.1
14232	0.94613	2.1
14233	0.946127	2.1
14234	0.946131	2.1
14235	0.94613	2.1
14236	0.94613	2.1
14237	1.36653	2.1
14238	0.946128	2.1
14239	0.946128	2.1
14240	0.946127	2.1
14241	0.94613	2.1
14242	0.946131	2.1
14243	0.94613	2.1
14244	0.946128	2.1
14245	0.946128	2.1
14246	0.946129	2.1
14247	0.946133	2.1
14248	1.40081	2.1
14249	0.946132	2.1
14250	0.946132	2.1
14251	0.946133	2.1
14252	0.946132	2.1
14253	0.946133	2.1
14254	0.946128	2.1
14255	1.32282	2.1
14256	0.946129	2.1
14257	0.94613	2.1
14258	0.946131	2.1
14259	0.946131	2.1
14260	0.946131	2.1
14261	0.810945	2.1
14262	0.810943	2.1
14263	0.810933	2.1
14264	2.44097	2.1
14265	1.3517	2.1

14266	1.08132	2.1
14267	1.08132	2.1
14268	1.08133	2.1
14269	0.946131	2.1
14270	0.94613	2.1
14271	0.946133	2.1
14272	0.946133	2.1
14273	0.946128	2.1
14274	0.946131	2.1
14275	1.27676	2.1
14276	0.946126	1.61469
14277	0.946126	2.02326
14278	0.946131	1.53223
14279	0.946127	2.02002
14280	0.94613	0.184541
14281	0.946128	0.974537
14282	0.946128	0.78873
14283	0.946132	0.060889
14284	0.946133	0.0811018
14285	0.946132	0.0601967
14286	0.946132	0.0805592
14287	0.946132	0.0545524
14288	0.946133	0.0654842
14289	0.946132	0.0710856
14290	0.946128	0.0822769
14291	0.946131	0.098172
14292	0.94613	0.0797148
14293	0.946129	0.100115
14294	0.94613	0.0737941
14295	0.94613	0.0774676
14296	0.94613	0.0898857
14297	0.946132	0.05
14298	0.946128	0.05
14299	0.94613	0.0545693
14300	0.946131	0.05
14301	0.94613	0.05
14302	0.94613	0.0558848
14303	0.946131	0.05
14304	0.94612	0.05
14305	0.946119	0.0556221
14306	0.946119	0.05
14307	0.946124	0.05
14308	0.946119	0.05
14309	0.946132	0.05
14310	0.946107	0.05
14311	0.946128	2.1
14312	0.946133	2.1
14313	0.946131	2.1
14314	0.94613	2.1
14315	0.946131	2.1
14316	1.37869	2.1
14317	0.946131	2.1
14318	1.35703	2.1
14319	0.946132	2.1
14320	0.946132	2.1
14321	0.946132	2.1
14322	0.946132	2.1
14323	0.946132	2.1

14324	0.946133	2.1
14325	0.946133	2.1
14326	0.946133	2.1
14327	0.946132	2.1
14328	1.37017	2.1
14329	0.946133	2.1
14330	0.946132	2.1
14331	0.946133	2.1
14332	0.946114	0.05
14333	0.946119	0.05
14334	0.946119	0.05
14335	0.94612	0.05
14336	0.946119	0.05
14337	0.946132	0.05
14338	0.946107	0.05
14339	0.946131	0.05
14340	0.946131	0.05
14341	0.946126	0.0598273
14342	0.946127	0.0533689
14343	0.94613	0.05
14344	0.946129	0.051757
14345	0.946128	0.05
14346	0.946131	1.4684
14347	0.946131	1.24014
14348	0.946132	1.17079
14349	0.946132	1.40886
14350	0.94613	1.55582
14351	0.946131	1.57064
14352	1.3279	1.53938
14353	0.946132	2.1
14354	1.40932	2.1
14355	0.946132	2.1
14356	0.946132	2.1
14357	0.946132	2.1
14358	0.946131	2.1
14359	0.946132	2.1
14360	0.946131	0.0902139
14361	0.946131	0.0866525
14362	0.946132	0.0838786
14363	0.946132	0.0907562
14364	0.94613	0.117203
14365	0.946131	0.0982448
14366	1.26868	0.0946147
14367	0.810943	2.1
14368	0.810945	2.1
14369	0.810932	2.1
14370	2.82321	2.1
14371	1.3517	2.1
14372	1.08132	2.1
14373	1.08132	2.1
14374	1.08133	2.1
14375	0.810943	0.936138
14376	0.810944	0.565054
14377	2.63079	1.34356
14378	1.35168	0.773587
14379	1.0813	0.775167
14380	1.08132	0.397869
14381	1.08132	1.93697

14382	0.810957	0.924299
14383	1.17856	0.0982101
14384	0.946132	0.751451
14385	0.946128	0.459615
14386	1.3154	1.95386
14387	0.94613	0.641142
14388	1.31539	0.954683
14389	0.94613	1.71126
14390	0.94613	1.74874
14391	0.94613	1.74067
14392	0.946131	1.66983
14393	0.946131	1.77987
14394	0.946129	1.78927
14395	0.94613	2.02249
14396	1.21997	1.95488
14397	0.946131	1.59901
14398	1.25083	1.47257
14399	0.946132	1.44769
14400	0.946132	1.63335
14401	0.946129	1.22168
14402	1.25083	1.72758
14403	0.946131	1.7278
14404	0.946127	1.7357
14405	1.41342	1.17277
14406	0.94613	1.57232
14407	0.946131	1.67502
14408	0.946127	0.413707
14409	0.946129	0.632517
14410	0.946129	1.32648
14411	0.946131	1.48969
14412	1.29149	1.57375
14413	0.946133	2.1
14414	0.946133	1.32991
14415	0.946128	1.82136
14416	0.946131	1.22307
14417	0.94613	1.40383
14418	0.94613	2.1
14419	0.94613	2.1
14420	0.946132	2.1
14421	0.946132	2.1
14422	0.946128	2.0404
14423	0.94613	2.1
14424	1.41022	2.1
14425	0.946132	0.101658
14426	0.946132	0.0780981
14427	0.946128	0.102313
14428	0.94613	0.0789297
14429	0.94613	0.111372
14430	0.94613	0.0701734
14431	0.946131	0.113424
14432	0.946129	1.22703
14433	1.37758	1.43103
14434	0.946131	1.60761
14435	0.94613	1.2459
14436	0.946124	1.39471
14437	0.946127	1.28409
14438	0.946127	1.34109
14439	0.946129	1.30949

14440	1.38259	1.47106
14441	0.946132	1.55371
14442	0.946132	1.33507
14443	0.946126	1.45878
14444	0.946129	1.36871
14445	0.94613	1.40241
14446	0.946131	0.05
14447	0.946132	0.05
14448	0.946132	0.0747474
14449	0.946132	0.05
14450	0.946132	0.0547666
14451	0.946132	0.0566342
14452	0.946132	0.05
14453	0.946131	0.05
14454	0.94613	0.05
14455	0.94613	0.05
14456	0.946133	0.05
14457	0.946128	0.05
14458	1.25017	0.05
14459	0.946131	0.05
14460	0.946131	2.1
14461	0.946131	2.1
14462	0.946131	2.1
14463	1.3937	2.1
14464	0.946133	2.1
14465	0.946131	2.1
14466	0.94613	2.1
14467	0.946131	1.95216
14468	0.946131	0.638486
14469	0.946131	1.0067
14470	0.94613	0.360149
14471	0.946133	1.95623
14472	0.946132	0.923683
14473	0.946131	0.865218
14474	0.946132	1.46728
14475	0.946132	0.889512
14476	0.946132	0.554071
14477	0.946132	0.616253
14478	0.946131	0.226002
14479	0.946132	0.18889
14480	0.946132	0.251111
14481	0.946129	1.57281
14482	0.94613	2.1
14483	0.94613	1.96953
14484	0.946128	2.1
14485	0.946132	0.30404
14486	0.946131	1.32083
14487	0.94613	1.58315
14488	0.946132	2.1
14489	0.946132	2.1
14490	0.946132	2.1
14491	1.41619	2.1
14492	0.946133	1.72411
14493	0.946133	2.1
14494	0.946132	2.1
14495	0.946132	2.1
14496	0.946132	2.1
14497	0.946132	2.1

14498	1.38244	2.1
14499	0.946132	2.1
14500	0.946131	2.1
14501	0.946132	2.1
14502	0.810946	0.617561
14503	0.810943	1.43591
14504	0.810934	0.702476
14505	2.42741	0.273852
14506	1.35171	0.329615
14507	1.08132	0.416343
14508	1.08132	0.668614
14509	1.08133	0.780032
14510	0.810947	0.59024
14511	0.810943	1.38655
14512	2.44179	0.31318
14513	1.35171	0.414788
14514	1.08132	0.466854
14515	1.08132	0.478409
14516	1.08133	0.516749
14517	0.810934	0.495585
14518	0.946132	1.79176
14519	0.946132	1.74304
14520	0.946133	1.55082
14521	0.946133	1.74105
14522	0.946132	1.93613
14523	0.946132	1.77036
14524	1.12786	1.75115
14525	1.27806	1.42744
14526	0.946132	1.58755
14527	0.946133	1.47072
14528	0.946133	1.60289
14529	0.946132	1.74319
14530	0.946132	1.55849
14531	0.946133	1.66543
14532	0.946132	2.1
14533	0.946132	2.1
14534	0.946132	2.1
14535	1.37098	2.1
14536	0.946133	2.1
14537	0.946132	2.1
14538	1.37098	2.1
14539	0.946125	1.33072
14540	0.946129	1.23185
14541	0.946131	1.44613
14542	0.94613	1.188
14543	0.946124	1.15391
14544	0.946127	1.20098
14545	1.39514	1.28814
14546	0.946132	2.1
14547	0.946131	2.1
14548	0.94613	2.1
14549	1.39909	2.1
14550	0.946132	2.1
14551	0.946131	2.1
14552	0.94613	2.1
14553	0.946133	2.1
14554	0.946133	1.79042
14555	0.946132	2.1

14556	1.36835	2.1
14557	0.946132	1.85845
14558	0.946133	2.1
14559	0.946132	2.04541
14560	0.946132	2.1
14561	0.946133	2.1
14562	0.946133	2.1
14563	0.946132	2.1
14564	1.39047	2.1
14565	0.946132	2.1
14566	0.946132	2.1
14567	1.33174	0.05
14568	0.946128	0.05
14569	0.94613	0.05
14570	0.94613	0.05
14571	0.94613	0.05
14572	0.94613	0.05
14573	0.946131	0.05
14574	0.946128	0.05
14575	1.36004	0.05
14576	0.946129	0.05
14577	0.94613	0.05
14578	0.94613	0.05
14579	1.36003	0.05
14580	0.94613	0.05
14581	0.946124	2.1
14582	0.946119	2.1
14583	0.946119	2.1
14584	0.946119	2.1
14585	0.94612	2.1
14586	0.946132	2.1
14587	1.29062	2.1
14588	0.946128	2.1
14589	0.946128	2.1
14590	0.946133	2.1
14591	0.94613	2.1
14592	0.946131	2.1
14593	0.946131	2.1
14594	1.30584	2.1
14595	0.946131	2.1
14596	0.946131	2.1
14597	0.946131	2.1
14598	0.946132	2.1
14599	1.39549	2.1
14600	0.946131	2.1
14601	0.946131	2.1
14602	0.913386	1.36061
14603	1.17466	1.15292
14604	1.00473	0.8145
14605	0.946132	1.15028
14606	0.946126	1.39126
14607	0.94613	1.35872
14608	0.946129	1.3199
14609	0.946132	0.853327
14610	1.38894	1.00744
14611	0.946132	0.370107
14612	0.946132	0.34218
14613	0.946132	1.15343

14614	0.946132	0.827721
14615	0.946133	0.777147
14616	0.946128	0.526926
14617	1.23865	0.778381
14618	0.946129	0.23874
14619	0.94613	0.285098
14620	0.94613	0.466481
14621	0.94613	0.332098
14622	0.94613	0.419039
14623	0.946126	0.687236
14624	1.40248	0.844483
14625	0.946129	0.629442
14626	0.94613	0.746842
14627	0.946124	1.15787
14628	0.946127	1.01953
14629	0.946128	0.846481
14630	0.946133	0.483865
14631	1.1391	0.925161
14632	0.946132	1.05297
14633	0.946132	0.395739
14634	0.946132	1.20341
14635	0.946133	0.808857
14636	0.946132	0.985999
14637	1.17156	1.2229
14638	0.946133	1.1717
14639	1.33893	1.1871
14640	0.827864	1.13596
14641	0.995232	1.19262
14642	0.827864	1.17368
14643	0.94613	1.15637
14644	1.21387	0.925449
14645	0.946132	1.05445
14646	0.946131	0.454716
14647	0.94613	0.880991
14648	0.94613	0.994279
14649	0.94613	0.97939
14650	0.94613	1.03008
14651	0.810943	0.140155
14652	0.810945	0.0912213
14653	2.42463	0.0987631
14654	1.3517	0.0954674
14655	1.08132	0.116043
14656	1.08132	0.130853
14657	1.08133	0.106289
14658	0.810932	0.141499
14659	0.946133	1.83925
14660	0.946132	0.902575
14661	0.946131	1.68416
14662	1.36748	1.55801
14663	0.946133	1.21799
14664	0.946131	1.52764
14665	0.946132	0.263063
14666	1.35856	0.05
14667	0.946131	0.05
14668	0.94613	0.05
14669	0.94613	0.05
14670	0.946131	0.05
14671	0.94613	0.05

14672	0.94613	0.05
14673	0.946132	2.1
14674	0.946131	2.1
14675	0.946131	2.1
14676	0.946131	2.1
14677	1.41149	2.1
14678	1.41149	2.1
14679	0.946131	2.1
14680	0.946128	2.1
14681	0.946128	2.1
14682	0.946133	2.1
14683	0.946131	2.1
14684	0.946131	2.1
14685	0.946131	2.1
14686	0.94613	2.1
14687	1.41388	0.0807725
14688	0.94613	0.113064
14689	0.946132	0.0780435
14690	0.946132	0.100737
14691	0.946129	0.125861
14692	0.946131	0.102132
14693	0.946131	0.107369
14694	1.02798	0.0776483
14695	0.946132	0.16609
14696	1.02798	0.0827667
14697	0.946133	0.154338
14698	1.14931	0.0788255
14699	0.957746	0.14542
14700	0.93452	0.115563
14701	0.946129	2.1
14702	0.946134	2.1
14703	0.946133	2.1
14704	0.946131	2.1
14705	0.946131	2.1
14706	0.946131	2.1
14707	0.946131	2.1
14708	0.946133	2.04191
14709	0.946132	1.59339
14710	0.946132	1.77171
14711	0.946132	2.03276
14712	1.38773	1.63513
14713	0.946132	1.79527
14714	0.946133	1.68891
14715	0.946131	0.151013
14716	0.946131	0.143476
14717	1.41512	0.14125
14718	0.946133	0.188611
14719	0.946131	0.193813
14720	0.946131	0.184969
14721	0.946131	0.159436
14722	1.35381	1.58428
14723	0.946132	2.1
14724	1.35381	1.6076
14725	0.946133	2.1
14726	0.946132	1.56222
14727	0.946132	2.1
14728	0.946133	2.06239
14729	1.3365	1.60136

14730	0.946132	1.93886
14731	1.3365	1.35198
14732	0.946129	1.8239
14733	0.94613	1.64506
14734	0.946128	1.8607
14735	0.946129	1.7117
14736	0.939895	1.93883
14737	1.14285	2.1
14738	0.952366	2.1
14739	0.946131	2.05547
14740	0.94613	2.1
14741	0.946131	2.1
14742	0.94613	2.1
14743	0.946131	2.1
14744	0.946131	2.1
14745	0.946131	2.1
14746	0.946129	2.1
14747	0.946133	2.1
14748	0.946131	2.1
14749	0.946131	2.1
14750	0.946133	2.1
14751	0.946132	2.1
14752	0.946132	2.1
14753	0.946133	2.1
14754	0.946132	2.1
14755	0.946132	2.1
14756	0.946133	2.1
14757	0.946133	2.1
14758	0.946132	1.72761
14759	0.946132	2.1
14760	0.946133	2.02023
14761	0.946132	2.1
14762	0.946132	2.1
14763	0.946132	1.70547
14764	0.946132	2.1
14765	0.946131	2.1
14766	0.946131	2.1
14767	0.946131	2.1
14768	1.38969	2.1
14769	0.946131	2.1
14770	0.946132	2.1
14771	0.946128	2.0892
14772	0.946133	1.81945
14773	0.946132	1.83596
14774	0.946131	1.83375
14775	0.94613	1.9349
14776	0.946131	1.96975
14777	1.28464	1.94358
14778	0.946119	1.10271
14779	1.37492	1.17126
14780	0.946125	1.18086
14781	0.946119	0.876644
14782	0.946119	1.18554
14783	0.946132	1.07507
14784	0.946107	1.14759
14785	0.810942	2.1
14786	0.810943	2.1
14787	0.709587	2.1

14788	2.64488	2.1
14789	1.21651	2.1
14790	0.946151	2.1
14791	0.979948	2.1
14792	1.0813	2.1
14793	0.946133	1.56876
14794	0.946132	1.85488
14795	0.946132	2.1
14796	0.946133	1.25377
14797	0.946132	1.16706
14798	0.946132	1.25999
14799	1.41134	1.46436
14800	0.946133	2.00305
14801	0.946132	1.65532
14802	0.946133	2.00781
14803	0.946133	1.62632
14804	0.946132	1.29519
14805	0.946133	1.73257
14806	1.31338	1.8053
14807	2.07612	1.1167
14808	0.946102	0.05
14809	0.752448	0.05
14810	1.20617	0.05
14811	0.94613	0.05
14812	0.75242	0.05
14813	0.946131	0.05
14814	0.752419	0.05
14815	0.946132	1.39315
14816	1.25736	1.39854
14817	1.25736	1.35214
14818	0.946132	1.35359
14819	1.08054	1.2819
14820	1.25736	1.30721
14821	0.946132	1.38116
14822	0.946128	2.1
14823	1.1896	2.1
14824	1.18959	2.1
14825	0.946131	2.1
14826	1.33271	2.1
14827	0.946105	2.1
14828	0.946156	2.1
14829	0.741946	0.05
14830	1.33587	0.05
14831	0.741947	0.05
14832	1.21954	0.05
14833	0.94613	0.05
14834	0.741946	0.05
14835	1.81347	0.05
14836	1.81347	0.05
14837	0.653322	0.05
14838	1.36935	0.05
14839	0.661882	0.05
14840	1.04753	0.05
14841	0.507464	0.05
14842	0.945844	1.53354
14843	1.59507	2.1
14844	1.04043	1.55258
14845	0.946131	2.1

14846	1.32891	1.99992
14847	0.946131	1.89234
14848	0.946131	1.78593
14849	1.28866	1.48834
14850	1.12682	0.09343
14851	0.851543	0.13585
14852	1.04072	0.0878683
14853	0.946132	0.291981
14854	1.13437	0.0791055
14855	1.04167	0.0883192
14856	0.936652	0.0914625
14857	1.258	2.1
14858	0.946131	2.1
14859	0.946132	2.1
14860	1.36968	2.1
14861	0.946132	2.1
14862	1.36968	2.1
14863	0.946131	2.1
14864	2.30046	0.208771
14865	2.82969	0.239637
14866	0.946132	2.1
14867	1.35022	2.1
14868	1.12356	2.1
14869	0.94613	2.1
14870	1.35022	2.1
14871	0.946131	2.1
14872	0.94613	2.1
14873	0.946131	2.1
14874	1.22401	2.1
14875	0.952456	2.1
14876	0.94613	2.1
14877	1.22401	2.1
14878	0.946131	2.1
14879	0.946131	2.1
14880	1.33047	2.1
14881	0.946132	2.1
14882	1.33047	2.1
14883	0.946131	2.1
14884	1.33047	2.1
14885	0.946132	2.1
14886	1.2107	2.1
14887	0.61957	2.1
14888	1.80666	2.1
14889	1.53743	2.1
14890	0.619572	2.01459
14891	1.32547	2.1
14892	1.16166	2.1
14893	1.04074	2.1
14894	0.557621	2.1
14895	0.946128	2.1
14896	1.36607	2.1
14897	0.94613	2.1
14898	1.36607	2.1
14899	1.39416	2.1
14900	0.94613	2.1
14901	0.94613	2.1
14902	1.23969	2.1
14903	0.946131	2.1

14904	0.946131	2.1
14905	1.13537	2.1
14906	1.05719	2.1
14907	0.806848	2.1
14908	1.30251	2.1
14909	0.851526	1.68206
14910	1.14176	2.1
14911	0.93666	1.67241
14912	0.946114	2.1
14913	1.15832	2.00074
14914	0.924624	1.97725
14915	0.967609	1.85891
14916	0.771764	0.132682
14917	1.26652	0.145349
14918	0.946133	0.156505
14919	1.17957	0.086682
14920	0.771735	0.125494
14921	0.946122	0.149521
14922	0.946133	0.123085
14923	0.924325	1.71507
14924	0.656739	1.78991
14925	0.637876	1.80227
14926	0.530092	1.74287
14927	0.914415	1.71283
14928	0.821311	1.8388
14929	0.630589	1.81878
14930	0.738823	1.79255
14931	0.839109	1.80333
14932	1.55029	0.05
14933	1.13537	1.806
14934	0.946131	2.1
14935	0.946128	1.93642
14936	1.30162	2.1
14937	0.952476	2.1
14938	1.29289	2.1
14939	0.939783	2.1
14940	0.94612	2.1
14941	0.946131	2.1
14942	1.07998	2.1
14943	0.836985	2.1
14944	1.1604	2.1
14945	0.94613	2.1
14946	0.837016	2.1
14947	0.712461	0.572609
14948	1.36399	0.582806
14949	1.8724	0.392531
14950	1.8724	0.322714
14951	0.623426	1.35356
14952	1.36399	0.488928
14953	1.22089	0.473003
14954	0.946132	0.435703
14955	0.946131	2.1
14956	1.32241	2.1
14957	1.32241	2.1
14958	0.946133	2.1
14959	1.40271	2.1
14960	0.946131	2.1
14961	0.946131	2.1

14962	1.1989	1.67487
14963	0.954106	1.79922
14964	0.820068	1.60675
14965	0.563139	1.7318
14966	1.06448	1.73295
14967	0.892472	1.83404
14968	0.862598	1.68479
14969	0.745851	1.85097
14970	1.13336	1.76652
14971	0.946129	2.1
14972	1.28254	2.1
14973	1.28254	2.1
14974	0.946131	2.1
14975	1.41302	2.1
14976	0.946131	2.1
14977	0.946131	2.1
14978	0.851543	2.06591
14979	1.21961	2.1
14980	0.851484	1.98708
14981	0.851543	2.1
14982	1.15705	2.1
14983	0.946133	2.1
14984	0.909417	2.1
14985	1.13808	0.964303
14986	0.946133	0.14502
14987	0.946131	1.3243
14988	1.30194	0.129946
14989	0.946131	0.233786
14990	1.30194	0.138875
14991	0.946131	0.209202
14992	0.930941	1.50048
14993	1.20185	1.42156
14994	1.02404	1.42683
14995	0.946124	1.59133
14996	1.3563	1.47672
14997	0.946127	1.59187
14998	0.946128	1.51768
14999	0.946116	2.1
15000	1.3144	2.1
15001	1.13088	2.1
15002	0.946118	2.1
15003	1.31438	2.1
15004	0.946105	2.1
15005	0.946131	2.1
15006	1.76972	0.0762656
15007	1.42091	0.0500903
15008	0.946128	0.0760989
15009	0.946101	0.0966059
15010	0.863552	0.109415
15011	0.94613	0.0648988
15012	1.21232	0.0862555
15013	0.94613	0.121474
15014	1.2137	0.335778
15015	1.23224	0.991851
15016	1.86512	0.432801
15017	1.86512	0.28256
15018	0.580825	1.58942
15019	1.2137	1.064

15020	0.946133	0.957088
15021	0.580822	0.762987
15022	0.946128	2.1
15023	1.40586	2.1
15024	0.94613	2.1
15025	1.3719	2.1
15026	1.3719	2.1
15027	1.40586	2.1
15028	0.94613	2.1
15029	0.946132	2.1
15030	1.35025	1.76946
15031	1.1794	2.09403
15032	0.946132	0.62183
15033	1.35025	1.61221
15034	0.946131	1.60483
15035	0.946132	1.68632
15036	0.946133	2.1
15037	1.60149	2.1
15038	1.67058	2.1
15039	0.851542	2.1
15040	1.20296	2.1
15041	1.17501	2.1
15042	0.946132	2.1
15043	1.04993	2.1
15044	0.851541	2.1
15045	1.48219	2.1
15046	0.874565	2.1
15047	0.946129	2.1
15048	1.22049	2.1
15049	0.946131	2.1
15050	0.946131	2.1
15051	0.94613	2.1
15052	0.848719	1.96406
15053	1.17195	2.01803
15054	0.946129	1.35331
15055	1.35426	1.52425
15056	0.94613	1.55367
15057	0.94613	1.72689
15058	0.946133	2.1
15059	1.14342	2.1
15060	1.14342	2.1
15061	0.946133	2.1
15062	1.39009	2.1
15063	0.946132	2.1
15064	0.946132	2.1
15065	1.38819	2.1
15066	0.946133	2.1
15067	1.38819	2.1
15068	0.946131	2.1
15069	1.4116	2.1
15070	0.946131	2.1
15071	1.38819	2.1
15072	0.946127	2.1
15073	1.32244	2.1
15074	1.2176	2.1
15075	0.946131	2.1
15076	1.32244	2.1
15077	0.94613	2.1

15078	0.946129	2.1
15079	1.32415	2.1
15080	1.11239	2.1
15081	0.984099	2.1
15082	1.65532	2.1
15083	1.53082	2.1
15084	1.32068	2.1
15085	1.32068	2.1
15086	1.08172	2.1
15087	3.76293	0.05
15088	1.36335	0.05
15089	0.953925	2.1
15090	0.989497	2.1
15091	0.98949	2.1
15092	1.06341	2.1
15093	1.27875	2.1
15094	1.04598	2.1
15095	0.603154	2.1
15096	0.582457	2.1
15097	0.582457	2.1
15098	0.568994	2.1
15099	1.5791	2.1
15100	1.09506	2.1
15101	1.45327	2.1
15102	1.88406	2.1
15103	1.88404	2.1
15104	1.27389	2.1
15105	1.14206	2.1
15106	1.00232	2.1
15107	1.00231	2.1
15108	1.68436	2.1
15109	1.4677	0.05
15110	1.28828	0.05
15111	1.28828	0.05
15112	1.46932	0.05
15113	1.28228	2.1
15114	1.64268	2.1
15115	0.959473	2.1
15116	1.10101	2.1
15117	1.07976	2.1
15118	1.2873	2.1
15119	1.70303	0.104517
15120	1.40066	0.192387
15121	1.5563	0.05
15122	1.13727	0.233812
15123	1.03529	2.1
15124	1.11285	2.1
15125	1.03529	2.1
15126	1.28667	2.1
15127	1.32119	2.1
15128	1.44777	2.1
15129	1.44778	2.1
15130	1.61121	2.1
15131	1.09372	0.05
15132	1.17664	0.05
15133	1.17664	0.05
15134	0.872931	0.05
15135	1.02415	2.1

15136	1.28228	2.1
15137	2.14692	2.1
15138	3.90469	2.1
15139	1.00509	2.1
15140	1.28228	2.1
15141	3.32936	0.05
15142	1.1333	0.05
15143	1.11048	2.1
15144	0.979854	2.1
15145	1.65815	2.1
15146	1.00185	2.1
15147	1.84887	2.1
15148	1.59597	2.1
15149	1.78468	2.1
15150	1.87508	2.1
15151	1.82716	2.1
15152	1.33429	2.1
15153	1.34626	2.1
15154	0.797142	2.1
15155	1.00007	1.84526
15156	1.02051	2.1
15157	1.02051	1.99613
15158	1.03373	2.1
15159	1.05786	2.1
15160	0.887631	2.1
15161	0.992773	2.1
15162	1.28462	2.1
15163	2.35274	1.71247
15164	1.82533	1.33505
15165	1.82533	1.5705
15166	1.19496	1.25594
15167	1.28597	0.05
15168	1.09725	2.1
15169	1.60948	2.1
15170	1.20421	2.1
15171	1.16566	2.1
15172	1.88522	2.1
15173	1.81948	0.18166
15174	1.14042	0.147529
15175	1.21558	0.312541
15176	1.56876	0.211276
15177	1.6697	2.1
15178	1.81655	2.1
15179	1.81655	2.1
15180	1.5956	2.1
15181	0.906059	2.1
15182	1.63045	2.1
15183	0.9533	1.6763
15184	1.1475	1.56234
15185	1.72231	2.1
15186	1.94936	1.9547
15187	1.06826	2.1
15188	1.08576	2.1
15189	1.06826	2.1
15190	0.973559	2.1
15191	1.62737	0.05
15192	0.965676	0.05
15193	1.45055	0.05

15194	1.17631	0.05
15195	1.92535	0.05
15196	1.9503	0.05
15197	1.99828	0.05
15198	1.70302	0.05
15199	1.65139	0.05
15200	1.26784	0.05
15201	1.35923	0.0508766
15202	1.70302	0.0579097
15203	1.33033	2.1
15204	1.12481	2.1
15205	1.29452	2.1
15206	1.25399	2.1
15207	1.12481	2.1
15208	0.918358	2.1
15209	1.39332	2.1
15210	1.32368	2.1
15211	1.11712	1.39437
15212	1.07999	1.21113
15213	1.07999	0.85401
15214	1.05619	0.75217
15215	1.1766	2.1
15216	1.35735	2.1
15217	1.42456	0.05
15218	1.089	0.05
15219	1.11081	0.05
15220	2.88521	0.05
15221	1.09754	0.05
15222	1.85479	0.05
15223	1.27922	0.80076
15224	1.03556	0.374717
15225	1.07508	2.07896
15226	1.09795	2.1
15227	0.967672	1.4883
15228	1.07508	1.86265
15229	1.03933	2.09612
15230	1.02975	2.1
15231	1.02975	2.1
15232	1.01121	2.1
15233	1.24526	0.05
15234	1.72368	0.05
15235	1.89607	0.05
15236	2.13512	0.05
15237	1.34591	0.22425
15238	1.14721	0.256002
15239	1.15539	2.1
15240	1.90938	2.06832
15241	1.90941	2.1
15242	1.89242	2.1
15243	1.18907	2.1
15244	1.20408	2.1
15245	1.20409	2.1
15246	1.22655	2.1
15247	0.701447	2.1
15248	1.57567	2.1
15249	1.38806	2.1
15250	2.09407	2.1
15251	1.07612	1.61911

15252	0.990019	1.62018
15253	1.15564	2.1
15254	1.01243	1.9131
15255	1.28956	0.05
15256	1.35146	0.05
15257	1.35145	0.0638957
15258	1.22811	0.0706677
15259	1.09662	2.1
15260	1.42486	2.1
15261	1.10106	0.05
15262	1.90814	0.05
15263	1.3946	0.05
15264	1.11926	0.05
15265	0.725839	2.1
15266	0.925068	2.1
15267	0.925074	2.1
15268	1.43282	2.1
15269	1.91756	1.05976
15270	1.74321	1.34954
15271	1.74321	0.627322
15272	1.63413	1.03135
15273	1.9194	0.05
15274	1.62246	0.05
15275	1.90456	0.05
15276	1.06578	0.05
15277	1.46019	0.05
15278	1.17036	0.05
15279	1.70233	1.41921
15280	1.53515	1.10349
15281	2.03494	2.1
15282	1.78441	2.1
15283	1.75992	2.1
15284	1.94351	2.1
15285	1.51057	0.05
15286	0.991435	0.05
15287	1.98068	0.05
15288	1.64283	0.05
15289	2.96126	0.05
15290	2.20692	0.05
15291	1.95054	0.05
15292	1.21325	0.05
15293	0.996828	0.198943
15294	1.01976	0.174656
15295	1.17129	2.1
15296	1.13664	2.1
15297	1.13664	2.1
15298	1.09627	2.1
15299	2.65123	2.1
15300	3.23062	2.1
15301	3.23062	2.1
15302	3.92038	2.1
15303	1.89019	0.197343
15304	1.79109	0.228449
15305	1.79109	0.164538
15306	1.54197	0.180355
15307	3.41824	2.1
15308	1.93553	0.764951
15309	2.26215	2.1

15310	2.0103	0.62038
15311	1.51569	1.4024
15312	1.01683	1.09357
15313	1.78595	2.1
15314	1.66537	2.1
15315	3.73426	2.1
15316	3.53905	2.1
15317	1.66537	2.1
15318	1.52903	2.1
15319	3.53905	2.1
15320	3.3837	2.1
15321	1.19901	1.28862
15322	1.35002	1.85068
15323	1.35002	0.163459
15324	1.11683	0.265
15325	1.75326	1.56137
15326	0.712404	0.612752
15327	2.97768	2.1
15328	2.98949	2.1
15329	1.2832	2.1
15330	1.39518	2.1
15331	2.98949	2.1
15332	2.99339	2.1
15333	1.39517	2.1
15334	1.46124	2.1
15335	1.28534	2.1
15336	1.03391	2.1
15337	1.75366	1.8079
15338	1.26247	1.91834
15339	1.13621	2.09895
15340	2.43994	2.1
15341	1.47904	0.05
15342	1.80085	0.05
15343	1.80085	0.05
15344	1.64656	0.05
15345	2.87331	2.1
15346	2.82863	2.1
15347	2.82859	2.1
15348	2.84673	2.1
15349	1.0961	0.05
15350	1.39448	0.11178
15351	1.21366	0.113345
15352	1.20143	0.0795927
15353	1.85795	0.05
15354	1.36768	0.05
15355	1.36769	0.05
15356	1.45789	0.05
15357	1.32605	0.05
15358	1.27148	0.05
15359	1.70218	0.05
15360	1.3063	0.0500983
15361	1.45603	0.05
15362	1.89226	0.0682263
15363	1.2742	0.05
15364	2.10112	0.05
15365	1.10748	0.0513185
15366	1.1893	0.0557043
15367	1.04921	2.1

15368	1.22927	2.1
15369	1.22927	2.1
15370	1.46568	2.1
15371	0.811186	2.1
15372	0.792162	2.1
15373	0.792162	2.1
15374	0.758749	2.1
15375	1.03581	2.1
15376	0.737481	2.1
15377	2.24071	2.1
15378	1.34868	2.1
15379	1.47203	1.38028
15380	1.40967	1.40635
15381	1.40967	1.43532
15382	1.27916	1.41574
15383	1.17418	2.1
15384	1.04921	2.1
15385	1.28291	1.03675
15386	1.3213	0.493952
15387	1.46812	0.675139
15388	2.42522	0.987096
15389	1.17879	1.35714
15390	1.28291	1.40181
15391	1.28291	1.37467
15392	1.13438	1.41669
15393	1.75004	2.1
15394	1.78247	2.1
15395	1.78247	2.1
15396	1.76421	2.1
15397	2.25975	0.05
15398	3.66111	0.05
15399	3.6611	0.05
15400	3.44043	0.05
15401	1.13339	2.1
15402	1.13322	2.1
15403	1.13323	2.1
15404	1.20761	2.1
15405	1.34337	0.297868
15406	1.90912	1.22172
15407	1.04399	0.407432
15408	2.00578	1.21612
15409	1.04973	2.1
15410	1.22615	2.1
15411	1.93384	2.1
15412	2.62816	1.57638
15413	1.13127	2.1
15414	1.47642	2.1
15415	2.62816	2.1
15416	3.31755	1.26883
15417	1.47642	2.1
15418	1.80454	2.1
15419	0.964782	2.1
15420	0.949135	2.1
15421	0.949134	2.1
15422	0.949134	2.1
15423	1.10476	1.49316
15424	0.982858	0.172207
15425	1.10938	0.893324

15426	0.982858	0.787829
15427	1.01012	0.05
15428	0.974081	0.05
15429	1.82643	0.05
15430	1.29585	0.05
15431	1.00763	0.376282
15432	1.3294	0.502701
15433	1.3294	0.168363
15434	1.30001	0.691825
15435	1.59151	2.1
15436	1.99443	2.1
15437	1.07369	2.1
15438	1.30584	2.1
15439	1.05071	2.1
15440	0.948676	2.1
15441	1.13842	2.1
15442	1.42884	2.1
15443	1.59019	0.05
15444	1.38212	0.05
15445	1.34102	0.05
15446	1.02663	0.05
15447	0.797011	2.1
15448	0.81301	2.1
15449	0.81301	2.1
15450	0.811187	2.1
15451	0.991869	0.0936575
15452	1.23868	0.0956464
15453	1.67201	2.1
15454	1.61201	2.1
15455	1.61202	2.1
15456	1.57627	2.1
15457	0.94994	2.1
15458	1.77867	2.1
15459	0.854945	2.1
15460	0.973905	2.1
15461	1.64193	2.1
15462	1.68893	2.1
15463	1.68893	2.1
15464	1.70002	2.1
15465	2.08149	0.294263
15466	1.69456	0.191016
15467	1.67399	0.05
15468	1.83922	2.1
15469	1.91703	2.1
15470	0.967492	2.1
15471	1.13317	2.1
15472	0.980766	2.1
15473	2.06158	0.05
15474	0.986498	0.05
15475	1.76289	0.05
15476	1.17248	0.05
15477	1.51794	0.05
15478	1.30266	0.05
15479	1.03165	0.130631
15480	0.947692	0.162476
15481	1.20473	0.111848
15482	0.947693	0.110274
15483	1.54357	2.1

15484	0.980841	2.1
15485	1.34124	0.111671
15486	1.39818	0.376903
15487	1.39818	0.0840112
15488	1.48035	0.553912
15489	1.03932	2.1
15490	1.2065	2.1
15491	1.20651	2.1
15492	1.12717	2.1
15493	1.09483	2.1
15494	0.994293	2.1
15495	0.994293	2.1
15496	0.97356	2.1
15497	0.995252	0.811369
15498	1.66294	0.947064
15499	1.82775	0.434537
15500	1.42762	0.429009
15501	1.04713	0.0846315
15502	1.05475	0.0832122
15503	1.29002	0.05
15504	1.05475	0.05
15505	1.17585	2.1
15506	1.39223	2.1
15507	1.39223	2.1
15508	1.71255	2.1
15509	1.56166	0.813869
15510	1.37762	0.386642
15511	1.16858	2.1
15512	1.0361	2.1
15513	1.23966	0.05
15514	1.10718	0.05
15515	1.99957	0.05
15516	0.658392	0.05
15517	1.36904	0.05
15518	1.12524	0.05
15519	1.04321	1.69187
15520	1.04627	1.7021
15521	1.91351	1.18877
15522	1.63379	1.09984
15523	1.79718	1.33658
15524	0.59634	1.32187
15525	0.958257	2.1
15526	1.06457	2.1
15527	0.958255	2.1
15528	1.03933	2.1
15529	1.14195	2.1
15530	1.49438	2.1
15531	1.49438	2.1
15532	1.31259	2.1
15533	1.23036	2.1
15534	1.2016	2.1
15535	1.2016	2.1
15536	1.17129	2.1
15537	1.05724	2.1
15538	1.04291	2.1
15539	1.12341	2.1
15540	2.32525	2.1
15541	3.39038	2.1

15542	2.09374	2.1
15543	1.76185	0.459518
15544	1.7934	0.557431
15545	1.65809	0.554204
15546	1.54852	0.612395
15547	1.38766	2.1
15548	1.28765	2.1
15549	1.26599	2.1
15550	1.21397	2.1
15551	1.28765	2.1
15552	1.20045	2.1
15553	1.21398	2.1
15554	1.15234	2.1
15555	0.953634	0.266799
15556	1.39132	0.0635989
15557	1.17285	2.1
15558	1.21408	2.1
15559	1.21408	2.1
15560	0.982848	2.1
15561	2.00101	2.1
15562	2.00973	2.1
15563	2.20314	2.1
15564	1.89318	2.1
15565	1.23267	2.1
15566	0.947921	2.1
15567	1.18872	2.1
15568	0.740752	2.1
15569	1.92269	0.117348
15570	1.53584	0.0780177
15571	1.53584	0.10633
15572	1.21067	0.0544059
15573	1.40707	0.05
15574	1.50437	0.05
15575	1.50435	0.05
15576	1.45446	0.051026
15577	1.47954	1.33753
15578	2.08687	1.67564
15579	2.08687	2.07079
15580	1.1233	2.1
15581	1.04025	1.48811
15582	1.36005	1.95592
15583	1.47606	0.0953461
15584	1.3407	0.10067
15585	0.758743	2.1
15586	0.716917	2.1
15587	0.716922	2.1
15588	0.672964	2.1
15589	2.40014	2.1
15590	1.96848	2.1
15591	1.96848	2.1
15592	1.53555	2.1
15593	1.06191	2.1
15594	1.29579	2.1
15595	1.29579	2.1
15596	1.71289	2.1
15597	1.78584	2.1
15598	2.15211	2.1
15599	2.00427	2.1

15600	3.41615	2.1
15601	1.66408	2.1
15602	1.73954	2.1
15603	2.91663	2.1
15604	3.65818	2.1
15605	1.93286	2.1
15606	1.08831	2.1
15607	1.31242	0.05
15608	1.11712	0.05
15609	1.26311	0.05
15610	1.31242	0.05
15611	1.10378	2.1
15612	0.968594	2.1
15613	1.48389	0.05
15614	1.49613	0.05
15615	1.04349	1.20897
15616	1.26409	0.600316
15617	1.26409	0.529661
15618	1.05724	1.03486
15619	1.65723	0.109703
15620	1.83116	0.0931686
15621	2.06284	0.0954153
15622	2.00512	0.0758371
15623	1.83116	0.106983
15624	2.03139	0.0876151
15625	2.20564	0.0900284
15626	1.90048	0.0690264
15627	0.963392	2.1
15628	1.01346	0.866369
15629	2.98092	2.1
15630	2.70295	2.1
15631	2.70295	2.1
15632	2.48828	2.1
15633	1.63661	2.1
15634	1.64158	2.1
15635	1.64158	2.1
15636	1.6488	2.1
15637	1.17506	0.120021
15638	1.09667	0.110834
15639	2.00539	0.373357
15640	1.09667	0.175708
15641	1.54946	2.1
15642	1.43577	2.1
15643	1.26103	2.1
15644	1.49808	2.1
15645	1.089 0.05	
15646	1.22586	0.05
15647	2.35027	0.276643
15648	1.37549	0.166781
15649	3.33706	0.161807
15650	1.69894	0.155188
15651	2.00539	0.191919
15652	1.53798	0.154124
15653	1.28938	2.1
15654	1.20969	2.1
15655	1.20969	2.1
15656	1.15864	2.1
15657	1.7986	2.1

15658	1.75869	2.1
15659	1.58281	2.1
15660	1.7392	2.1
15661	1.30733	1.19044
15662	1.5419	1.25641
15663	1.50883	1.15166
15664	1.85439	0.926283
15665	1.73531	0.819516
15666	1.70225	0.624438
15667	1.22176	0.05
15668	1.59492	0.0790157
15669	1.59492	0.063028
15670	2.15542	0.093019
15671	3.24379	2.1
15672	2.64775	2.1
15673	3.16707	2.1
15674	1.93032	2.1
15675	1.50738	2.1
15676	1.24128	2.1
15677	1.24128	2.1
15678	0.994293	2.1
15679	2.90808	0.05
15680	2.45797	0.05
15681	1.85501	0.05
15682	2.90809	0.05
15683	0.892835	1.9045
15684	1.55871	2.1
15685	1.04615	1.1965
15686	1.66643	1.21584
15687	1.70044	2.1
15688	1.80224	2.1
15689	1.70186	1.1545
15690	3.30123	1.79395
15691	1.74789	2.1
15692	1.77446	2.1
15693	1.77901	2.1
15694	1.57995	2.1
15695	1.81518	2.1
15696	2.17716	2.1
15697	2.17716	2.1
15698	2.65123	2.1
15699	2.01899	1.25913
15700	1.77479	1.11133
15701	1.19122	0.486938
15702	1.46724	0.375368
15703	1.70944	0.05
15704	1.08929	0.05
15705	1.68454	0.05
15706	1.08929	0.05
15707	1.51067	1.37592
15708	1.06729	0.903339
15709	1.37762	0.7134
15710	1.13992	0.418869
15711	1.26397	2.1
15712	1.04083	2.1
15713	1.00278	2.1
15714	1.04084	2.1
15715	1.1016	0.05

15716	1.23477	0.05
15717	1.23477	0.05
15718	1.64985	0.05
15719	1.11117	2.1
15720	1.13006	2.1
15721	1.13006	2.1
15722	1.14721	2.1
15723	1.21946	2.1
15724	1.0186	2.1
15725	1.13178	2.1
15726	1.00133	2.1
15727	1.67046	0.05
15728	0.986036	0.173009
15729	1.67046	0.164935
15730	1.34857	1.84707
15731	2.46016	2.1
15732	2.07161	2.1
15733	2.10732	2.1
15734	2.08371	2.1
15735	1.35602	2.1
15736	0.829065	2.1
15737	1.81667	2.1
15738	0.99406	2.1
15739	1.81667	2.1
15740	1.68073	2.1
15741	2.05736	2.1
15742	2.97311	2.1
15743	2.14011	2.1
15744	1.23411	2.1
15745	1.13632	0.05
15746	1.40862	0.05
15747	1.28055	0.05
15748	1.14305	0.05
15749	1.12035	0.05
15750	1.15483	0.05
15751	1.94099	0.05
15752	1.82126	0.05
15753	1.15481	0.05
15754	0.998957	0.05
15755	2.10078	0.05
15756	1.81617	0.05
15757	1.27581	2.1
15758	0.968536	2.1
15759	1.33916	2.1
15760	1.27581	2.1
15761	1.32784	2.1
15762	1.4259	2.1
15763	1.70377	2.1
15764	1.54231	2.1
15765	3.77569	2.1
15766	3.42809	2.1
15767	1.54231	2.1
15768	1.51298	2.1
15769	3.55866	2.1
15770	2.86136	2.1
15771	1.83301	0.553594
15772	1.26371	0.520269
15773	1.98301	0.613621

15774	2.0609	0.528523
15775	2.26047	0.265099
15776	2.00537	0.228219
15777	1.7913	0.208692
15778	1.48864	0.180561
15779	1.09504	2.1
15780	1.39827	2.1
15781	2.28084	2.1
15782	1.05983	2.1
15783	1.33593	0.05
15784	1.31508	0.05
15785	1.31508	0.05
15786	1.09599	0.05
15787	1.30647	2.1
15788	1.28846	2.1
15789	1.28846	2.1
15790	1.26923	2.1
15791	1.2955	2.1
15792	1.09044	2.1
15793	2.12209	2.1
15794	1.26438	2.1
15795	0.911819	2.1
15796	1.38752	2.1
15797	2.57704	2.1
15798	2.71736	2.1
15799	1.42284	2.1
15800	1.41562	2.1
15801	2.69131	2.1
15802	2.36178	2.1
15803	1.41562	2.1
15804	1.39564	2.1
15805	1.32368	2.1
15806	0.908053	2.1
15807	0.908053	2.1
15808	0.629213	2.1
15809	1.8758	1.89102
15810	1.27362	1.93309
15811	1.3756	1.33115
15812	2.28749	1.59872
15813	1.84577	0.465081
15814	2.17765	0.483754
15815	1.38969	0.432832
15816	1.84578	0.451595
15817	1.32801	0.228619
15818	1.76377	0.207037
15819	1.22845	2.1
15820	1.57766	2.05312
15821	1.57766	2.1
15822	1.39067	2.1
15823	1.15743	0.05
15824	1.37721	0.05
15825	1.69055	0.0506717
15826	1.14858	0.05
15827	1.38333	2.1
15828	1.10575	1.44324
15829	1.22418	2.1
15830	1.10574	2.1
15831	1.91509	0.117753

15832	1.02001	0.104804
15833	1.03074	0.256175
15834	1.19122	0.193532
15835	1.67887	2.1
15836	1.67597	2.1
15837	1.84984	2.1
15838	1.77775	2.1
15839	1.73571	2.1
15840	1.27921	2.08824
15841	2.11851	1.77839
15842	1.27921	2.06901
15843	1.42486	2.1
15844	1.12503	2.1
15845	1.12503	2.1
15846	1.30116	2.1
15847	0.568995	2.1
15848	0.557348	2.1
15849	0.557348	2.1
15850	0.53907	2.1
15851	0.633575	2.1
15852	1.33756	2.1
15853	1.51074	2.1
15854	1.93384	1.99011
15855	0.655202	2.1
15856	0.731934	2.1
15857	0.731934	2.1
15858	1.09061	2.1
15859	0.966979	2.1
15860	1.31502	2.1
15861	1.31502	2.1
15862	1.72116	2.1
15863	1.70002	2.1
15864	1.67969	2.1
15865	1.67969	2.1
15866	1.62408	2.1
15867	1.01531	2.1
15868	1.66018	2.1
15869	1.01531	2.1
15870	0.886968	2.1
15871	1.22585	0.05
15872	1.20674	0.05
15873	1.20672	0.05
15874	1.02962	0.05
15875	0.956467	2.1
15876	2.00551	2.1
15877	0.553801	2.1
15878	0.617006	2.1
15879	1.26923	2.1
15880	1.23614	2.1
15881	1.23614	2.1
15882	1.16817	2.1
15883	2.14482	2.1
15884	1.72637	2.1
15885	1.09368	2.1
15886	1.26195	2.1
15887	0.760546	2.1
15888	1.84094	2.1
15889	2.62868	2.1

15890	2.79784	2.1
15891	2.79784	2.1
15892	3.00129	2.1
15893	1.27593	1.42675
15894	1.38877	1.61347
15895	1.06486	0.193452
15896	1.2474	0.112658
15897	1.76234	0.05
15898	1.8685	0.05
15899	1.69863	0.05
15900	1.29666	0.05
15901	1.91646	2.1
15902	1.8582	2.1
15903	1.8582	2.1
15904	1.7986	2.1
15905	3.44043	0.05
15906	1.94764	0.05
15907	1.94764	0.05
15908	1.36307	0.05
15909	1.32075	0.05
15910	1.12971	0.05
15911	2.06319	0.05
15912	1.94982	0.05
15913	1.77255	0.05
15914	0.979297	0.05
15915	1.59997	2.1
15916	1.93506	2.1
15917	1.94409	2.1
15918	1.9597	2.1
15919	0.57899	2.1
15920	0.953036	2.1
15921	0.953049	2.1
15922	1.53503	2.1
15923	1.39615	0.0774479
15924	1.06474	0.0837427
15925	0.947564	0.0823451
15926	1.45506	0.0977161
15927	1.17799	0.0916977
15928	1.21067	0.0942238
15929	1.35945	0.17493
15930	2.05648	0.232261
15931	1.36208	0.200126
15932	2.15229	0.165664
15933	2.89288	0.153678
15934	1.95719	0.169134
15935	2.25169	0.129336
15936	1.52756	0.151061
15937	1.19322	0.301898
15938	1.23146	0.233197
15939	2.45024	0.436335
15940	0.794122	0.263458
15941	1.6343	0.223606
15942	1.34161	0.168388
15943	1.05353	0.498251
15944	1.50795	0.119512
15945	1.05353	0.109856
15946	1.20669	0.112799
15947	1.59289	2.1

15948	0.989543	2.1
15949	1.40588	2.1
15950	0.991427	2.1
15951	0.921822	1.78558
15952	1.46903	0.548851
15953	1.46903	2.00797
15954	1.8214	0.248263
15955	1.39702	2.1
15956	1.25103	2.1
15957	1.25103	2.1
15958	1.10378	2.1
15959	1.71813	2.1
15960	1.39595	2.1
15961	1.386 2.1	
15962	1.13008	2.1
15963	1.31711	0.05
15964	1.70191	0.05
15965	0.893249	0.05
15966	1.42669	0.05
15967	1.23943	2.1
15968	1.1483	2.1
15969	1.04598	2.1
15970	1.1483	2.1
15971	1.00381	0.4325
15972	1.11433	0.170247
15973	0.973501	1.54669
15974	1.0756	1.48384
15975	1.20669	0.125128
15976	1.18508	0.116079
15977	1.18508	0.154944
15978	0.983027	0.118505
15979	1.43687	2.1
15980	1.71059	2.1
15981	1.71059	2.1
15982	1.66762	2.1
15983	2.8415	2.1
15984	2.40555	2.1
15985	1.91546	2.1
15986	1.61385	2.1
15987	2.01569	2.1
15988	1.33183	2.1
15989	1.23218	2.1
15990	1.23999	2.1
15991	1.01335	1.21631
15992	1.12088	1.30559
15993	1.25346	2.1
15994	1.15644	2.1
15995	1.3764	1.55616
15996	1.27558	1.21629
15997	1.2064	2.1
15998	1.15067	2.1
15999	1.01595	2.1
16000	1.15067	2.1
16001	1.41902	2.1
16002	1.30272	2.1
16003	1.30272	2.1
16004	1.19548	2.1
16005	2.99339	2.1

16006	2.98041	2.1
16007	1.46124	2.1
16008	1.42105	2.1
16009	2.98041	2.1
16010	2.86339	2.1
16011	1.42105	2.1
16012	1.42284	2.1
16013	2.31301	0.556159
16014	1.80273	0.5571
16015	2.17765	0.659966
16016	2.31301	0.535093
16017	1.76378	0.234186
16018	2.10275	0.214278
16019	2.10275	0.244508
16020	2.26047	0.240233
16021	0.97541	2.1
16022	1.40863	2.1
16023	1.40863	2.1
16024	1.30751	2.1
16025	1.20143	0.057326
16026	1.19756	0.0500354
16027	1.43707	0.05
16028	1.28954	0.05
16029	3.34254	0.05
16030	1.92916	0.05
16031	1.80089	0.05
16032	1.1323	0.05
16033	1.47321	0.103229
16034	1.23756	0.0823099
16035	1.87291	2.1
16036	1.53642	2.1
16037	1.53642	2.1
16038	1.5791	2.1
16039	1.0446	2.1
16040	0.992674	2.1
16041	1.12324	0.05
16042	1.29163	0.05
16043	1.29163	0.05
16044	1.15371	0.05
16045	0.996831	0.112331
16046	1.00192	0.124655
16047	1.0019	0.105305
16048	0.958937	0.119812
16049	1.07283	2.1
16050	1.25294	2.1
16051	1.25294	2.1
16052	1.04788	2.1
16053	1.14496	2.1
16054	1.07652	2.1
16055	1.07652	2.1
16056	0.991406	2.1
16057	1.60976	2.1
16058	1.37	2.1
16059	1.37	2.1
16060	1.14195	2.1
16061	1.26593	1.94575
16062	1.75366	2.1
16063	1.57621	0.147082

16064	1.24538	0.140874
16065	0.969118	2.1
16066	1.04147	2.1
16067	0.969118	2.1
16068	1.22615	2.1
16069	0.98084	2.1
16070	1.01843	2.1
16071	1.09435	2.1
16072	1.50876	2.1
16073	1.5976	2.1
16074	1.84322	2.1
16075	1.84324	2.1
16076	1.70377	2.1
16077	1.20709	2.1
16078	2.54729	2.1
16079	0.974716	0.05
16080	1.54468	0.0651087
16081	1.39021	0.05
16082	1.70303	0.0574836
16083	1.22852	1.49459
16084	1.578	1.23907
16085	1.578	1.56418
16086	1.93852	1.1602
16087	2.2506	2.1
16088	1.39302	2.1
16089	1.5478	2.1
16090	1.2269	2.1
16091	1.70048	2.1
16092	1.45477	2.1
16093	1.30929	2.1
16094	1.15069	2.1
16095	1.00255	2.1
16096	1.05481	0.42069
16097	1.05481	2.1
16098	1.1032	0.42219
16099	0.993888	2.1
16100	1.19294	2.1
16101	1.01121	0.0834693
16102	0.991866	0.0852327
16103	2.20052	1.96936
16104	1.7409	2.1
16105	1.75142	2.1
16106	1.46811	2.1
16107	1.7409	2.1
16108	1.23267	2.1
16109	1.46811	2.1
16110	1.18872	2.1
16111	1.12665	2.1
16112	1.50045	2.1
16113	1.50045	2.1
16114	1.37704	2.1
16115	1.35308	1.21996
16116	1.29983	0.936336
16117	1.29983	1.19554
16118	0.970212	1.00337
16119	1.82272	2.1
16120	1.62784	2.1
16121	1.62784	2.1

16122	1.87691	2.1
16123	3.65294	2.1
16124	2.33374	2.1
16125	2.12888	2.1
16126	1.62229	2.1
16127	1.41496	2.1
16128	1.32707	2.02253
16129	2.18871	1.86639
16130	1.4235	1.02134
16131	1.46015	0.0845336
16132	1.25635	0.0579962
16133	1.45501	0.211998
16134	1.3543	0.135724
16135	1.38139	2.1
16136	1.98159	2.1
16137	2.15868	2.1
16138	4.04308	2.1
16139	1.5362	2.1
16140	1.56328	2.1
16141	1.56327	2.1
16142	1.58163	2.1
16143	1.88987	0.05
16144	1.08761	0.05
16145	0.862154	0.05
16146	1.77688	0.05
16147	1.14238	2.1
16148	1.11691	2.1
16149	1.01537	2.1
16150	1.02923	2.1
16151	0.977425	0.05
16152	1.15211	0.05
16153	2.0815	2.1
16154	1.18621	2.1
16155	1.01485	2.1
16156	1.17054	2.1
16157	1.17054	2.1
16158	1.12665	2.1
16159	1.87213	0.05
16160	1.82244	0.05
16161	1.45039	0.05
16162	1.7226	0.05
16163	1.841	0.05
16164	1.79011	0.05
16165	1.8792	0.05
16166	1.45053	0.05
16167	1.08947	2.1
16168	1.5008	2.1
16169	1.5008	2.1
16170	1.83186	2.1
16171	1.04159	0.05
16172	1.2489	0.05
16173	1.2489	0.05
16174	1.33592	0.05
16175	1.51973	0.067346
16176	0.980547	0.0768492
16177	1.51974	0.0832112
16178	1.15991	0.106726
16179	1.34965	2.1

16180	1.2724	2.1
16181	1.2724	2.1
16182	1.38438	2.1
16183	1.02923	2.1
16184	1.06521	2.1
16185	1.06521	2.1
16186	1.0752	2.1
16187	2.03139	0.0875063
16188	1.92951	0.0795871
16189	1.74852	0.0649817
16190	1.62772	0.0656196
16191	1.73655	0.121583
16192	1.76809	0.0690685
16193	1.62772	0.0642685
16194	1.1432	0.0583212
16195	1.09235	2.1
16196	1.48081	2.1
16197	1.46712	2.1
16198	1.89321	2.1
16199	1.94548	2.1
16200	1.5636	2.1
16201	1.5636	2.1
16202	1.21124	2.1
16203	1.52314	2.1
16204	2.0219	2.1
16205	1.51113	2.1
16206	2.0219	2.1
16207	0.987247	0.226831
16208	1.18347	0.118702
16209	1.89226	0.346762
16210	1.61441	0.128492
16211	1.42884	2.1
16212	1.10961	2.1
16213	1.56991	2.1
16214	1.05337	2.1
16215	1.05337	2.1
16216	1.91547	2.1
16217	1.05931	2.1
16218	1.1729	2.1
16219	1.34526	2.1
16220	1.0593	2.1
16221	1.0856	2.1
16222	1.90854	2.1
16223	1.91674	2.1
16224	1.11345	2.1
16225	1.75067	2.1
16226	1.50022	2.1
16227	1.50022	2.1
16228	1.53619	2.1
16229	1.4748	2.06866
16230	1.74431	1.88165
16231	2.02581	1.75975
16232	1.25288	1.77026
16233	1.01814	0.866395
16234	1.2881	0.596582
16235	1.2881	0.591257
16236	1.54433	0.473686
16237	1.19999	0.480913

16238	1.44056	0.292853
16239	1.02011	0.13561
16240	1.17129	0.191085
16241	1.13775	1.99413
16242	3.34815	2.0339
16243	1.60428	1.88571
16244	1.85928	1.95414
16245	1.75592	2.1
16246	1.25989	2.1
16247	1.75592	2.1
16248	1.71549	2.1
16249	1.37634	2.1
16250	1.64693	2.1
16251	1.64693	2.1
16252	1.64193	2.1
16253	1.18351	0.05
16254	2.19398	0.05
16255	2.16272	0.716505
16256	1.19837	0.651804
16257	1.04973	2.1
16258	1.20149	2.1
16259	1.60574	2.1
16260	1.11164	1.55367
16261	1.87823	0.862243
16262	1.11164	1.68548
16263	0.772882	2.1
16264	1.4714	2.1
16265	2.14242	2.01105
16266	1.12336	2.1
16267	2.8386	1.41044
16268	2.13346	2.1
16269	1.06788	0.0721355
16270	0.947662	0.0673871
16271	1.05297	0.0579693
16272	1.42907	0.0634466
16273	2.17465	2.1
16274	2.18394	2.1
16275	1.14808	2.1
16276	1.21292	2.1
16277	1.21292	2.1
16278	1.27229	2.1
16279	1.20859	0.595916
16280	1.21073	0.706472
16281	1.21073	0.389422
16282	1.22857	1.12572
16283	1.79158	2.1
16284	1.73899	2.1
16285	1.34012	2.1
16286	1.39991	2.1
16287	1.73898	2.1
16288	1.61245	2.1
16289	1.40136	2.1
16290	1.57234	2.1
16291	1.82162	2.1
16292	3.09875	2.1
16293	1.10264	2.1
16294	1.02339	2.1
16295	1.02339	2.1

16296	1.23042	2.1
16297	2.82266	0.731384
16298	1.05698	0.338589
16299	1.96384	0.577711
16300	1.10244	0.432548
16301	0.929061	2.1
16302	1.47007	2.1
16303	0.985303	2.1
16304	1.24554	2.1
16305	1.05993	2.1
16306	2.02074	2.1
16307	2.10106	0.681149
16308	1.18171	0.169897
16309	1.68111	0.587765
16310	1.84226	0.51102
16311	1.82131	0.136685
16312	1.32371	0.0848612
16313	1.41902	0.0777924
16314	1.00205	0.0613889
16315	1.65532	2.1
16316	1.18977	2.1
16317	1.15471	2.1
16318	0.993889	2.1
16319	2.08521	2.1
16320	2.09885	2.1
16321	2.09497	2.1
16322	2.10604	2.1
16323	0.881028	0.953403
16324	1.52828	0.888294
16325	1.40574	0.05
16326	1.09599	0.05
16327	1.45506	2.1
16328	1.50524	2.1
16329	1.50524	2.1
16330	1.54711	2.1
16331	1.41469	2.1
16332	2.40442	2.1
16333	1.40794	2.1
16334	1.89226	2.1
16335	1.31346	2.1
16336	1.51067	2.1
16337	1.31363	2.1
16338	1.11833	2.1
16339	2.3373	2.1
16340	2.19994	2.1
16341	2.19994	2.1
16342	1.09616	2.1
16343	1.63719	2.1
16344	1.52204	2.1
16345	1.52205	2.1
16346	1.40118	2.1
16347	2.5946	2.1
16348	4.07065	2.1
16349	1.33916	2.1
16350	2.05217	2.1
16351	1.67023	2.1
16352	2.05217	2.1
16353	1.59458	0.484464

16354	1.02247	0.400186
16355	1.68108	0.839064
16356	1.03079	0.422739
16357	1.1643	0.0959973
16358	1.85795	0.084344
16359	1.70302	0.17503
16360	1.42076	0.142052
16361	1.91351	0.157973
16362	1.01148	0.139937
16363	2.52402	0.05
16364	0.752625	0.05
16365	0.886968	2.1
16366	1.28845	2.1
16367	1.28845	2.1
16368	1.27766	2.1
16369	1.82268	2.1
16370	1.87118	2.1
16371	1.87119	2.1
16372	1.91704	2.1
16373	3.13938	2.1
16374	2.97839	2.1
16375	2.97839	2.1
16376	2.87331	2.1
16377	1.73443	1.4382
16378	1.59457	1.06037
16379	2.01827	2.1
16380	2.04586	2.1
16381	2.04586	2.1
16382	2.00865	2.1
16383	1.19901	2.09194
16384	1.16661	2.1
16385	1.13215	0.245181
16386	1.96256	0.167747
16387	2.34043	0.05
16388	2.04154	0.05
16389	1.58991	0.05
16390	1.45893	0.05
16391	2.56818	0.05
16392	1.70532	0.05
16393	1.17798	0.05
16394	1.25796	0.17131
16395	1.25797	0.197219
16396	1.10719	0.207202
16397	1.51067	2.1
16398	1.60707	2.1
16399	2.06946	2.1
16400	3.19218	2.1
16401	2.19965	2.1
16402	1.30521	2.1
16403	2.23684	1.97584
16404	1.43363	2.09475
16405	1.51007	2.1
16406	2.46356	2.1
16407	1.90428	2.1
16408	1.13618	2.1
16409	2.13276	0.054047
16410	0.998345	0.0538121
16411	1.2861	0.05

16412	1.20457	0.05
16413	1.51789	2.1
16414	1.49872	2.1
16415	1.49872	2.1
16416	1.47712	2.1
16417	1.87129	0.0619311
16418	0.949364	0.0757748
16419	1.83142	0.0777841
16420	1.55063	0.106274
16421	1.03298	0.110009
16422	0.980664	0.212582
16423	1.51696	0.064957
16424	1.34927	0.15481
16425	1.70302	0.306311
16426	1.65952	0.219041
16427	1.76658	0.180468
16428	0.986848	0.13988
16429	2.13757	1.46031
16430	1.98142	0.418742
16431	1.98142	0.997398
16432	1.65411	0.45413
16433	1.1323	0.0693412
16434	1.46015	0.05
16435	2.15376	1.83486
16436	1.64622	1.86732
16437	0.991867	0.05
16438	1.58074	0.0511316
16439	1.69368	2.1
16440	1.76403	2.1
16441	1.76403	2.1
16442	1.83318	2.1
16443	1.28418	0.361795
16444	1.16851	0.25672
16445	1.1685	0.160488
16446	1.1726	0.134071
16447	1.06455	2.1
16448	1.18773	2.1
16449	0.98511	2.1
16450	1.18774	2.1
16451	1.76476	2.1
16452	1.79825	2.1
16453	1.79825	2.1
16454	0.961895	2.1
16455	1.59589	0.05
16456	2.25975	0.05
16457	2.76339	2.1
16458	3.0551	2.1
16459	1.68761	2.1
16460	1.59561	2.1
16461	2.17257	2.1
16462	1.05845	2.1
16463	1.03512	2.1
16464	2.19011	2.1
16465	1.44859	1.92821
16466	1.35897	0.888099
16467	1.35897	1.57335
16468	1.17822	1.07677
16469	1.13641	2.1

16470	1.18301	2.1
16471	1.18301	2.1
16472	1.25173	2.1
16473	2.41297	2.1
16474	1.36463	2.1
16475	1.54711	2.1
16476	1.5735	2.1
16477	1.5735	2.1
16478	1.58713	2.1
16479	1.69044	0.05
16480	1.57979	0.05
16481	1.57979	0.05
16482	1.14387	0.05
16483	1.04199	2.1
16484	1.96491	2.1
16485	1.04199	2.1
16486	1.46892	2.1
16487	0.983724	2.1
16488	1.70579	2.1
16489	1.88522	2.1
16490	1.23914	2.1
16491	1.79424	2.1
16492	1.52851	2.1
16493	1.04921	2.1
16494	1.11285	2.1
16495	1.25334	1.21704
16496	1.07496	0.237302
16497	1.07497	2.05672
16498	1.34852	0.767146
16499	1.27391	2.1
16500	2.20307	2.1
16501	1.81311	2.1
16502	2.10305	2.1
16503	1.21059	0.055561
16504	1.44253	0.0823216
16505	1.9194	0.05
16506	1.21159	0.0631935
16507	0.947979	2.1
16508	1.01225	1.96148
16509	0.947979	2.1
16510	1.01225	2.1
16511	1.00255	2.1
16512	1.01225	2.1
16513	1.01225	2.1
16514	1.06561	2.1
16515	3.50562	2.1
16516	3.61336	2.1
16517	3.61336	2.1
16518	3.73635	2.1
16519	1.61245	2.1
16520	1.52301	2.1
16521	1.57612	2.1
16522	1.83035	2.1
16523	1.52301	2.1
16524	1.24789	2.1
16525	1.85156	2.1
16526	1.89072	2.1
16527	0.995735	2.1

16528	1.57738	2.1
16529	1.87141	0.05
16530	1.73388	0.05
16531	1.61344	0.05
16532	0.922676	0.05
16533	1.56614	2.1
16534	0.956294	2.1
16535	1.82589	2.1
16536	1.63719	2.1
16537	1.12399	0.05
16538	1.50103	0.05
16539	1.56976	0.05
16540	1.03567	0.05
16541	1.21776	0.05
16542	1.89708	0.05
16543	1.16323	0.05
16544	1.63533	0.05
16545	2.29397	0.05
16546	3.01596	0.05
16547	1.80145	0.05
16548	1.7666	0.05
16549	1.91621	0.272463
16550	1.10719	0.115414
16551	1.57828	0.677554
16552	1.17105	0.384572
16553	1.15806	2.1
16554	1.31565	2.1
16555	1.31565	2.1
16556	1.46643	2.1
16557	1.47507	2.1
16558	1.87858	2.1
16559	0.958661	2.1
16560	1.87858	2.1
16561	1.44293	0.05
16562	1.43213	0.05
16563	1.43213	0.05
16564	1.10476	0.05
16565	1.03235	1.89567
16566	1.31	0.220515
16567	1.31	1.94924
16568	1.50108	0.869976
16569	1.32215	2.1
16570	1.10611	2.1
16571	1.10611	2.1
16572	0.904978	2.1
16573	1.06486	0.191789
16574	1.18347	0.123692
16575	1.70535	2.1
16576	1.94779	2.1
16577	1.77071	2.1
16578	1.82268	2.1
16579	0.61701	2.1
16580	0.672495	2.1
16581	0.672494	2.1
16582	0.713174	2.1
16583	1.40395	2.1
16584	1.34245	2.1
16585	1.34243	2.1

16586	1.30646	2.1
16587	1.06204	0.207045
16588	0.632691	0.491565
16589	1.75766	0.520853
16590	1.14287	1.64268
16591	1.52101	0.0795497
16592	1.71414	0.0637419
16593	1.86411	0.0886272
16594	1.6253	0.0637987
16595	1.04355	0.0892376
16596	1.0443	0.0683716
16597	1.34852	1.86468
16598	1.56016	0.177462
16599	1.56016	0.261033
16600	1.03235	0.147217
16601	1.2966	0.05
16602	1.82354	0.05
16603	1.2966	0.05
16604	1.67687	0.05
16605	1.19669	1.29914
16606	1.82979	1.32242
16607	1.58972	1.49003
16608	1.09219	1.33865
16609	1.42077	1.34878
16610	1.89226	1.09786
16611	1.19547	2.1
16612	1.10187	2.1
16613	1.10188	2.1
16614	1.01479	2.1
16615	1.81663	2.1
16616	1.81533	2.1
16617	1.83247	2.1
16618	1.51511	2.1
16619	0.983148	2.1
16620	1.7513	2.1
16621	1.85017	2.1
16622	1.26103	2.1
16623	1.23152	2.1
16624	1.65508	2.1
16625	1.23152	2.1
16626	2.15082	2.1
16627	1.57974	2.1
16628	1.07289	2.1
16629	0.81534	2.1
16630	1.51007	2.1
16631	1.00898	0.05
16632	1.18959	0.05
16633	1.32178	0.05
16634	1.25929	0.05
16635	2.17892	0.336288
16636	1.22938	0.30816
16637	3.02933	0.772343
16638	2.34619	0.372319
16639	1.47679	0.104391
16640	1.0819	0.128537
16641	3.10558	2.1
16642	3.31457	2.1
16643	0.985109	2.1

16644	1.10667	2.1
16645	3.31457	2.1
16646	3.54443	2.1
16647	1.10667	2.1
16648	1.25987	2.1
16649	1.77063	1.38629
16650	1.51065	0.866086
16651	1.24688	2.1
16652	1.37652	2.1
16653	1.37652	2.1
16654	1.54168	2.1
16655	1.17042	1.54639
16656	1.13092	1.65148
16657	1.13092	1.81066
16658	1.52938	2.1
16659	1.11787	0.672486
16660	1.00613	2.1
16661	0.781373	0.157056
16662	1.6885	0.169279
16663	2.49439	0.0839342
16664	1.42657	0.122468
16665	1.77903	2.1
16666	2.63606	2.1
16667	2.10425	2.1
16668	1.21425	2.1
16669	1.31253	1.47877
16670	1.28418	1.27483
16671	1.28418	1.48106
16672	1.58972	1.48271
16673	0.858948	2.1
16674	1.90196	2.1
16675	1.81024	2.1
16676	1.90194	2.1
16677	1.4263	1.52759
16678	0.947921	1.88032
16679	1.22852	1.05922
16680	1.09194	0.999247
16681	1.60578	0.0627045
16682	1.14721	0.0716982
16683	1.06336	0.100725
16684	1.14721	0.0831404
16685	1.11924	0.05
16686	1.53814	0.05
16687	1.18421	0.05
16688	1.8458	0.05
16689	1.7565	1.95836
16690	1.09344	1.51008
16691	1.09344	1.49509
16692	1.16971	2.00732
16693	1.71465	0.05
16694	1.19412	0.05
16695	1.64985	0.05
16696	1.05417	0.05
16697	1.2127	0.05
16698	1.48867	0.05
16699	1.27806	0.05
16700	1.21269	0.05
16701	1.37813	1.41501

16702	1.0417	1.41468
16703	1.88626	0.136902
16704	1.14977	0.726297
16705	1.78803	2.1
16706	1.33793	2.08462
16707	1.42266	2.1
16708	1.26865	1.99321
16709	1.09518	2.1
16710	1.36844	2.1
16711	1.20455	2.1
16712	1.36844	2.1
16713	1.22297	1.40392
16714	1.11256	1.60071
16715	1.11256	1.1025
16716	1.03217	1.43071
16717	1.18422	0.05
16718	1.4282	0.05
16719	1.04159	0.05
16720	1.4282	0.05
16721	0.988805	2.1
16722	1.05481	2.1
16723	1.52938	1.50505
16724	1.26593	1.77785
16725	1.53082	2.1
16726	1.09847	2.1
16727	1.75211	1.99207
16728	1.22222	2.1
16729	1.75211	0.977231
16730	1.51592	2.1
16731	1.63413	0.42049
16732	1.26662	0.490227
16733	1.42495	0.273397
16734	1.4356	0.324619
16735	1.29389	1.72677
16736	1.04164	1.97415
16737	1.40574	1.80555
16738	1.04164	1.72629
16739	2.22344	0.506112
16740	1.45945	0.106109
16741	0.987248	0.220794
16742	1.2474	0.127173
16743	0.974702	0.943216
16744	1.43777	0.417053
16745	1.71289	2.1
16746	1.91529	2.1
16747	1.91529	2.1
16748	2.01827	2.1
16749	1.87111	1.31404
16750	1.00864	0.783137
16751	1.26865	1.16885
16752	1.18769	1.30787
16753	1.24167	2.1
16754	1.36133	1.94287
16755	2.28168	2.1
16756	3.98524	2.1
16757	1.36133	2.1
16758	1.69436	1.50556
16759	2.21926	2.1

16760	1.70302	2.1
16761	1.50732	0.05
16762	1.42237	0.0531623
16763	1.18659	0.0616213
16764	1.50732	0.066234
16765	1.19785	0.05
16766	1.40969	0.05
16767	1.28339	0.0562396
16768	1.40882	0.0561339
16769	0.968595	2.1
16770	1.41903	2.1
16771	1.12567	2.1
16772	1.15898	2.1
16773	1.27241	2.1
16774	0.995796	2.1
16775	1.89983	0.05
16776	1.05417	0.05
16777	0.571999	0.05
16778	0.743597	0.05
16779	1.89353	0.05
16780	0.924068	0.05
16781	1.52851	2.1
16782	1.26969	2.1
16783	1.07763	0.201138
16784	1.82301	0.142122
16785	1.07763	0.100642
16786	1.17506	0.103667
16787	1.20045	2.1
16788	1.12486	2.1
16789	1.15234	2.1
16790	1.11598	2.1
16791	1.12486	2.1
16792	1.0491	2.1
16793	1.11598	2.1
16794	1.1047	2.1
16795	1.05902	2.1
16796	1.14361	2.1
16797	1.14361	2.1
16798	1.26851	2.1
16799	1.29389	1.60062
16800	0.988456	1.57297
16801	1.83688	0.05
16802	1.70302	0.05
16803	1.49386	0.05
16804	1.83151	0.05
16805	1.01346	0.742553
16806	0.983607	0.795327
16807	0.983596	2.1
16808	1.08332	1.87985
16809	2.36848	0.0938563
16810	2.97066	0.0951154
16811	1.12352	0.0631794
16812	2.5311	0.0665335
16813	1.09062	2.1
16814	0.962182	2.1
16815	0.962181	2.1
16816	1.2258	2.1
16817	0.974888	2.1

16818	1.06312	2.1
16819	1.73583	0.05
16820	1.72399	0.05
16821	1.724	0.0530782
16822	1.50951	0.0530338
16823	1.26755	2.1
16824	1.03824	2.1
16825	1.27875	2.1
16826	1.03824	2.1
16827	1.18902	2.1
16828	1.534	2.1
16829	1.11764	2.1
16830	1.534	2.1
16831	1.52008	2.1
16832	1.34406	2.1
16833	1.34406	2.1
16834	1.2187	2.1
16835	0.963571	1.60208
16836	1.19612	1.30485
16837	0.963571	2.01722
16838	1.00007	2.1
16839	0.903333	2.1
16840	1.12724	2.1
16841	1.23771	2.1
16842	1.17683	2.1
16843	1.20059	0.48298
16844	0.772023	0.253192
16845	1.14749	0.932504
16846	1.2802	1.25536
16847	1.85091	1.87606
16848	2.11193	1.73755
16849	1.2802	0.566375
16850	1.42913	1.01053
16851	2.11193	1.60725
16852	1.27737	1.37054
16853	1.30584	2.1
16854	1.60936	2.1
16855	1.3413	0.0918724
16856	1.53336	0.0623459
16857	2.60679	0.0665804
16858	1.3413	0.056434
16859	1.35877	2.1
16860	1.11024	2.1
16861	1.27839	2.1
16862	1.08171	2.1
16863	1.27841	2.1
16864	1.0577	2.1
16865	1.71255	2.1
16866	1.10611	2.1
16867	0.960106	2.1
16868	1.40921	2.1
16869	1.90705	0.111269
16870	2.79564	0.103733
16871	1.45321	0.114371
16872	1.90705	0.106543
16873	0.974718	0.0706238
16874	1.53671	0.0621529
16875	1.69039	0.0648998

16876	1.78229	0.0557869
16877	1.0101	2.1
16878	1.3167	2.1
16879	1.19699	2.1
16880	1.3212	2.1
16881	1.60811	0.0792916
16882	1.43224	0.0570723
16883	1.35401	0.0551122
16884	1.17464	0.05
16885	1.27696	2.1
16886	1.05025	2.1
16887	1.05025	2.1
16888	1.18076	2.1
16889	1.37224	1.59376
16890	1.13438	1.57915
16891	1.76788	1.60639
16892	1.73444	1.54034
16893	1.37704	2.1
16894	1.77042	2.1
16895	1.77042	2.1
16896	1.09778	2.1
16897	1.28667	2.1
16898	1.28228	2.1
16899	1.30646	0.577287
16900	1.68867	0.380242
16901	1.30645	1.3466
16902	0.986494	0.782601
16903	1.6253	0.0729657
16904	1.48676	0.0682333
16905	1.48676	0.0703624
16906	1.32974	0.0668499
16907	0.634347	0.05
16908	1.15142	0.05
16909	1.11395	0.05
16910	1.18068	0.05
16911	1.36904	0.05
16912	0.764563	0.05
16913	2.09213	0.05
16914	1.56631	2.1
16915	1.08011	2.1
16916	1.80283	2.1
16917	1.50738	2.1
16918	1.44951	0.104536
16919	1.50102	0.0975718
16920	1.50102	0.0913725
16921	1.39615	0.0925524
16922	1.02653	0.36625
16923	2.98258	0.397563
16924	2.0815	0.05
16925	1.39525	0.05
16926	1.82176	0.05
16927	0.977607	0.05
16928	1.96133	1.81236
16929	1.27189	1.45514
16930	0.826032	1.51656
16931	2.12831	1.4853
16932	1.77798	2.08274
16933	1.02969	1.70248

16934	1.06463	1.68231
16935	1.49219	2.01667
16936	1.10003	2.1
16937	1.21223	2.1
16938	1.00513	1.55459
16939	1.43788	2.1
16940	1.47134	0.803458
16941	0.968249	1.13234
16942	3.22585	0.05
16943	3.36933	0.05
16944	3.36932	0.05
16945	3.03865	0.05
16946	1.66295	0.174123
16947	1.65583	0.12103
16948	1.65583	0.233029
16949	1.4732	0.10417
16950	1.73704	0.05
16951	1.76476	2.1
16952	2.13345	1.89913
16953	1.55816	2.1
16954	1.55816	2.09141
16955	1.02601	2.1
16956	1.08039	2.1
16957	1.05498	1.88769
16958	1.08039	2.1
16959	1.2873	2.1
16960	1.01121	2.1
16961	0.987703	2.1
16962	0.98771	2.1
16963	0.964782	2.1
16964	1.35719	2.1
16965	2.61092	2.1
16966	2.6607	2.1
16967	3.41693	2.1
16968	1.23125	2.1
16969	1.73772	2.1
16970	0.99845	0.263605
16971	1.5176	0.36729
16972	1.13271	0.05
16973	1.332 0.05	
16974	1.63159	2.1
16975	1.61342	2.1
16976	1.03679	2.1
16977	1.19087	2.1
16978	1.97172	2.1
16979	2.68634	2.1
16980	1.19086	2.1
16981	1.50091	2.1
16982	2.84677	2.1
16983	2.77894	2.1
16984	2.77894	2.1
16985	2.74206	2.1
16986	1.3284	2.1
16987	1.83524	2.1
16988	1.65853	2.1
16989	1.56741	2.1
16990	1.00509	2.1
16991	1.45246	2.1

16992	1.26193	2.1
16993	1.45244	2.1
16994	1.61963	0.522402
16995	1.68189	0.240682
16996	1.68189	1.78566
16997	1.49155	1.48328
16998	1.58054	2.1
16999	1.59169	2.1
17000	1.59169	2.1
17001	1.60497	2.1
17002	0.743595	0.05
17003	0.702359	0.05
17004	1.15317	0.05
17005	2.24367	0.05
17006	1.15252	0.05
17007	2.60354	0.05
17008	1.45158	2.1
17009	1.56004	2.1
17010	1.64527	2.1
17011	1.00371	2.1
17012	1.38287	0.298009
17013	0.893024	1.28591
17014	1.23527	1.14595
17015	1.23706	0.486197
17016	2.20993	0.318687
17017	1.54858	1.76865
17018	0.798666	0.127584
17019	1.22487	0.113136
17020	1.33158	0.0921291
17021	1.85902	0.0963067
17022	1.08334	2.1
17023	1.17885	1.87074
17024	1.17885	1.04607
17025	1.07283	1.567
17026	1.30723	0.05
17027	1.62507	0.05
17028	1.62507	0.05
17029	1.08854	0.05
17030	1.59475	2.1
17031	1.58874	2.1
17032	1.58875	2.1
17033	1.35787	2.09706
17034	1.04147	2.1
17035	1.15564	2.1
17036	1.78301	2.1
17037	1.90618	2.1
17038	1.71555	2.1
17039	2.13143	2.1
17040	1.10817	2.1
17041	1.032	2.1
17042	1.032	2.1
17043	0.953636	2.1
17044	1.27916	1.42218
17045	1.11938	1.12866
17046	1.11937	1.2244
17047	1.9186	1.1112
17048	1.19967	0.066461
17049	1.63489	0.0761148

17050	1.00763	0.0892023
17051	1.81656	0.093174
17052	1.31814	0.0701047
17053	2.98183	0.0793612
17054	1.20149	2.1
17055	1.29129	2.1
17056	1.29129	2.1
17057	1.37138	2.1
17058	1.23104	0.665523
17059	1.75236	1.16617
17060	1.6	0.263571
17061	1.60565	0.441274
17062	1.60565	0.385446
17063	1.40013	0.475332
17064	1.23042	2.1
17065	1.18278	2.1
17066	1.10853	2.1
17067	1.04724	2.1
17068	1.1766	2.1
17069	1.00319	2.1
17070	1.29435	2.1
17071	1.00319	2.1
17072	1.05655	1.79582
17073	1.01606	1.85535
17074	1.80391	2.1
17075	2.7699	2.1
17076	1.52073	2.1
17077	2.06835	2.1
17078	2.00202	2.1
17079	2.04906	2.1
17080	0.968536	2.1
17081	1.02461	2.1
17082	1.59784	2.1
17083	1.78249	2.1
17084	1.81409	2.1
17085	1.79844	2.1
17086	1.77093	1.97648
17087	2.02356	1.82719
17088	1.32449	2.1
17089	1.10003	2.1
17090	1.16915	0.397517
17091	1.05937	0.294309
17092	1.28096	2.01989
17093	1.34696	1.70959
17094	1.34695	2.0898
17095	1.36457	1.78657
17096	1.06478	2.1
17097	1.06239	2.1
17098	1.06239	2.1
17099	1.09629	2.1
17100	0.798209	2.1
17101	1.8532	2.1
17102	2.10112	0.05
17103	2.8704	0.05
17104	2.8704	0.05
17105	2.92457	0.05
17106	1.0752	2.1
17107	1.07745	2.1

17108	1.07745	2.1
17109	1.08911	2.1
17110	1.40182	2.1
17111	1.00133	2.1
17112	1.40182	2.1
17113	1.24509	2.1
17114	2.22243	0.05
17115	1.07303	0.05
17116	1.29736	0.05
17117	1.73584	0.05
17118	2.0815	0.395788
17119	1.07999	0.213503
17120	2.1692	2.1
17121	1.99992	2.1
17122	1.99992	2.1
17123	1.78327	2.1
17124	1.52661	0.05
17125	0.956003	0.05
17126	1.53172	0.05
17127	0.955991	0.05
17128	1.35025	2.1
17129	1.51084	2.1
17130	1.51084	2.1
17131	1.67115	2.1
17132	2.00977	2.1
17133	1.63632	2.1
17134	1.63632	2.1
17135	2.66128	2.1
17136	1.13525	2.1
17137	2.37566	2.1
17138	1.25987	2.1
17139	1.4472	2.1
17140	1.4472	2.1
17141	1.66018	2.1
17142	0.994001	1.29766
17143	1.83151	0.985651
17144	1.83151	0.360591
17145	1.57621	0.321749
17146	1.40915	0.202633
17147	1.24653	1.72501
17148	1.24653	0.215929
17149	1.10609	2.08564
17150	1.61121	2.1
17151	1.74472	2.1
17152	1.74472	2.1
17153	1.0368	2.1
17154	1.04788	2.1
17155	1.28522	2.1
17156	1.28522	2.1
17157	1.54196	2.1
17158	1.23387	2.1
17159	0.896519	2.1
17160	1.60428	2.1
17161	1.08467	2.1
17162	1.56875	0.315568
17163	1.39072	0.205823
17164	1.39073	0.166511
17165	1.6	0.320184

17166	1.01346	2.1
17167	1.06838	1.5102
17168	0.968463	1.53601
17169	1.06837	1.52801
17170	3.22433	0.0572128
17171	2.05993	2.00825
17172	2.01836	0.19885
17173	1.66154	1.95376
17174	1.20871	2.1
17175	1.10586	2.1
17176	1.10586	2.1
17177	1.03853	2.1
17178	1.12076	2.1
17179	1.03899	2.1
17180	1.03899	2.1
17181	0.963973	2.1
17182	0.987132	2.1
17183	1.0894	2.1
17184	2.37823	1.98224
17185	1.75647	1.6082
17186	1.64625	2.1
17187	1.56195	2.1
17188	1.41995	2.1
17189	1.15171	2.1
17190	1.36457	2.1
17191	1.33847	1.70934
17192	1.33847	2.1
17193	0.869601	1.62253
17194	1.42255	2.1
17195	1.3868	2.1
17196	1.3868	2.1
17197	1.33962	2.1
17198	1.05481	2.1
17199	1.08074	2.1
17200	1.08074	2.1
17201	0.857557	2.1
17202	1.13537	1.37498
17203	1.09194	0.922304
17204	1.01179	1.4439
17205	1.13537	1.01906
17206	1.15645	1.87053
17207	1.56409	0.263939
17208	1.56409	0.462224
17209	1.16239	0.873326
17210	1.10746	0.416806
17211	1.47952	1.02971
17212	1.14733	2.1
17213	3.77068	2.1
17214	1.38497	2.1
17215	1.54599	2.1
17216	1.54601	2.1
17217	1.27697	2.1
17218	1.01393	0.572764
17219	1.10558	1.12798
17220	0.995012	0.308111
17221	1.17624	0.722124
17222	1.60497	2.1
17223	1.61588	2.1

17224	1.61587	2.1
17225	1.62182	2.1
17226	1.48866	0.05
17227	1.65977	0.05
17228	1.65977	0.05
17229	1.69044	0.05
17230	0.961892	2.1
17231	1.00659	2.1
17232	1.0066	2.1
17233	1.06226	2.1
17234	1.15171	2.1
17235	1.50853	2.1
17236	1.50853	2.1
17237	1.2955	2.1
17238	1.3204	0.05
17239	2.13295	0.05
17240	2.75524	0.05
17241	1.18156	0.05
17242	1.73729	2.1
17243	1.20356	2.1
17244	1.62483	2.1
17245	1.52008	2.1
17246	1.19964	2.1
17247	1.0577	2.1
17248	1.19964	2.1
17249	1.07156	2.1
17250	1.98241	2.1
17251	1.43687	2.04021
17252	1.27863	2.1
17253	1.19915	2.04677
17254	0.97269	2.02215
17255	1.0739	1.78921
17256	1.1427	1.74413
17257	1.22476	1.07889
17258	1.06584	1.83499
17259	1.22626	1.74246
17260	1.31388	2.1
17261	1.16932	2.1
17262	1.28819	2.1
17263	1.0499	2.1
17264	1.55894	2.1
17265	1.78709	2.1
17266	1.75986	2.1
17267	1.764	2.1
17268	1.44801	2.1
17269	1.28186	2.1
17270	1.20797	1.92083
17271	1.06821	2.00829
17272	1.87287	0.499938
17273	2.08014	0.591522
17274	1.63225	0.58283
17275	1.39501	0.640761
17276	1.20132	0.05
17277	1.16182	0.05
17278	1.08848	0.05
17279	1.20132	0.05
17280	1.23475	2.1
17281	1.16661	2.1

17282	2.31317	2.1
17283	2.50145	2.1
17284	1.04367	2.1
17285	2.23875	2.1
17286	0.953632	0.102296
17287	1.38646	0.188861
17288	1.38646	0.215704
17289	1.61963	0.240085
17290	1.62934	1.1842
17291	1.88626	1.53353
17292	1.0775	1.30345
17293	1.119	0.46243
17294	1.08133	0.227851
17295	1.48312	0.202689
17296	1.57125	0.114251
17297	1.74098	0.113783
17298	1.94372	1.88127
17299	1.22237	1.8918
17300	1.18549	0.924308
17301	1.01294	1.97802
17302	1.01294	2.07901
17303	1.2064	2.1
17304	1.14599	2.1
17305	1.00065	1.99211
17306	1.76357	2.1
17307	2.23468	2.1
17308	2.21735	2.1
17309	3.32769	2.1
17310	1.19647	1.1784
17311	1.35307	0.770934
17312	1.19645	0.720718
17313	1.43778	0.461407
17314	1.34412	1.44381
17315	1.1315	2.1
17316	1.1315	0.88151
17317	1.23943	0.786404
17318	1.57829	2.1
17319	1.22537	2.1
17320	1.14357	2.1
17321	1.32772	2.02529
17322	1.4259	2.1
17323	1.34245	2.1
17324	1.47953	2.1
17325	1.70303	2.1
17326	2.21127	0.05
17327	1.47904	0.05
17328	1.18793	0.05
17329	2.21126	0.05
17330	1.1032	2.1
17331	1.14986	0.713033
17332	1.14986	2.1
17333	1.19611	0.473004
17334	1.91883	2.1
17335	1.83019	2.1
17336	1.83019	2.1
17337	1.82462	2.1
17338	1.27362	0.117619
17339	1.27421	0.159869

17340	1.27421	0.0918009
17341	1.10736	0.211249
17342	1.3003	0.378443
17343	1.31415	1.49044
17344	1.31415	0.485445
17345	1.32201	1.62403
17346	1.21124	2.1
17347	0.81568	2.1
17348	0.81568	2.1
17349	0.551816	2.1
17350	1.67119	2.1
17351	1.63384	2.1
17352	1.63384	2.1
17353	1.63661	2.1
17354	1.48734	2.1
17355	1.52245	2.1
17356	1.52245	2.1
17357	1.50714	2.1
17358	0.996778	0.05
17359	1.56091	0.05
17360	1.56091	0.05
17361	1.90457	0.05
17362	1.1181	2.1
17363	1.06543	2.1
17364	2.66828	2.1
17365	2.76932	2.1
17366	1.06543	2.1
17367	1.01474	2.1
17368	2.76932	2.1
17369	2.86704	2.1
17370	1.29724	0.0784422
17371	1.96938	0.0693156
17372	1.82462	2.1
17373	1.84553	2.1
17374	1.05556	2.1
17375	1.25363	2.1
17376	1.84552	2.1
17377	1.53208	2.1
17378	1.12826	2.1
17379	1.39075	2.1
17380	0.9702	1.2535
17381	1.16481	1.08832
17382	1.08894	1.33808
17383	1.91756	1.44042
17384	1.50865	0.481852
17385	1.06012	0.384764
17386	1.5456	0.05
17387	1.37427	0.05
17388	1.1211	0.05
17389	1.5456	0.05
17390	1.33135	0.05
17391	1.07956	0.05
17392	2.23255	0.05
17393	1.21031	0.05
17394	1.52903	2.1
17395	1.35977	2.1
17396	3.3837	2.1
17397	3.20581	2.1

17398	1.35977	2.1
17399	1.15414	2.1
17400	3.20582	2.1
17401	2.07596	2.1
17402	1.6812	0.0752028
17403	1.03939	0.067632
17404	1.30047	2.1
17405	1.5586	2.1
17406	1.5586	2.1
17407	1.84214	2.1
17408	1.31825	0.523129
17409	1.31923	1.87719
17410	1.31924	0.559107
17411	1.33026	1.68924
17412	1.15818	1.18442
17413	1.17822	1.19567
17414	1.15818	1.75535
17415	1.28096	1.51052
17416	1.06848	0.0653197
17417	1.89639	0.0890547
17418	1.9186	1.31882
17419	1.77065	0.839155
17420	1.59103	0.743603
17421	1.56164	0.405191
17422	1.09697	0.05
17423	2.38719	0.05
17424	2.22123	0.05
17425	2.46059	0.05
17426	1.45506	0.122926
17427	1.03645	0.1271
17428	1.30347	2.1
17429	1.2627	2.1
17430	1.2627	2.1
17431	1.23036	2.1
17432	1.41469	2.1
17433	1.45257	2.1
17434	1.46907	0.05
17435	1.70302	0.05
17436	1.49196	0.05
17437	1.36307	0.05
17438	1.05677	2.1
17439	0.978698	2.1
17440	0.978699	2.1
17441	2.01822	2.1
17442	1.47446	2.1
17443	1.55432	2.1
17444	1.55432	2.1
17445	1.06053	2.1
17446	1.86813	1.92586
17447	1.21892	2.02807
17448	1.53064	1.61456
17449	1.30664	1.65281
17450	1.09629	2.1
17451	1.19244	2.1
17452	1.19244	2.1
17453	1.37635	2.1
17454	1.49358	0.129951
17455	1.57702	0.0767238

17456	2.09374	2.1
17457	1.88175	2.1
17458	1.88175	2.1
17459	1.51085	2.1
17460	1.10938	0.152709
17461	1.02478	1.20125
17462	1.33061	0.05
17463	1.13272	0.05
17464	1.75505	2.1
17465	1.78529	2.1
17466	1.6353	2.1
17467	1.59307	2.1
17468	1.60017	0.287851
17469	1.35608	0.353216
17470	1.35608	0.176728
17471	1.04713	0.150787
17472	1.01479	1.81872
17473	1.3868	1.28482
17474	1.3868	1.14543
17475	1.28418	2.1
17476	2.15949	2.1
17477	2.90429	2.1
17478	1.51902	2.1
17479	1.54755	2.1
17480	2.90429	2.1
17481	3.49966	2.1
17482	1.70842	2.1
17483	1.97526	2.1
17484	0.949712	0.069125
17485	1.92784	0.0655738
17486	1.34093	0.0650985
17487	2.53426	0.0860269
17488	1.92783	0.0628168
17489	3.89373	0.0566805
17490	2.53426	0.075322
17491	2.35961	0.0728403
17492	1.68108	0.375256
17493	1.15558	0.313546
17494	1.67265	0.290116
17495	1.27608	0.232026
17496	1.42249	2.1
17497	1.39708	2.1
17498	1.39708	2.1
17499	1.39327	2.1
17500	1.97046	0.0666912
17501	1.11688	0.0535792
17502	1.28418	0.837042
17503	1.17302	2.1
17504	1.17302	0.962376
17505	1.04349	2.1
17506	0.979853	2.1
17507	1.26016	2.1
17508	1.18901	2.1
17509	1.26016	2.1
17510	1.22857	0.274618
17511	1.25848	1.08692
17512	1.25848	0.23051
17513	1.2858	1.0958

17514	1.09154	2.1
17515	2.01915	2.1
17516	1.23013	2.1
17517	1.55698	2.1
17518	1.55698	2.1
17519	1.8575	2.1
17520	2.01854	2.1
17521	1.20686	2.04673
17522	1.20684	1.99046
17523	1.77796	2.1
17524	1.46129	2.1
17525	1.04193	2.1
17526	1.10051	2.1
17527	0.983723	2.1
17528	1.1729	2.1
17529	0.98505	2.1
17530	0.985052	2.1
17531	1.03951	2.1
17532	1.03949	2.1
17533	1.17583	2.1
17534	1.42866	2.1
17535	1.97261	2.1
17536	1.73274	2.1
17537	1.53629	2.1
17538	1.98182	2.1
17539	1.72511	2.1
17540	1.42174	2.1
17541	1.44472	2.1
17542	1.72511	2.1
17543	1.45411	2.1
17544	1.44472	2.1
17545	1.43836	2.1
17546	0.962703	0.305896
17547	1.0171	0.717084
17548	1.53336	0.05
17549	1.40274	0.05
17550	1.40272	0.05
17551	1.00213	0.05
17552	1.50892	0.05
17553	1.64984	0.05
17554	1.67464	0.05
17555	1.67481	0.05
17556	1.08212	2.1
17557	1.28095	2.1
17558	1.46956	2.1
17559	1.70303	2.1
17560	1.2925	0.05
17561	1.84716	0.05
17562	1.48676	0.101685
17563	1.48797	0.0944018
17564	1.33917	0.0843871
17565	1.22691	0.0808961
17566	0.528744	2.1
17567	1.13728	2.1
17568	1.13728	2.1
17569	1.99843	2.1
17570	1.17464	0.05
17571	1.0372	0.05

17572	2.0815	0.05
17573	0.933462	0.05
17574	1.54931	2.1
17575	1.5485	2.1
17576	1.4077	2.1
17577	1.29931	2.1
17578	1.14931	0.112548
17579	2.02223	0.107871
17580	1.04482	0.0790244
17581	0.94971	0.0911784
17582	1.54632	1.77229
17583	1.15693	1.77535
17584	1.66763	2.1
17585	1.41238	2.1
17586	1.41238	2.1
17587	1.48068	2.1
17588	1.0171	0.223418
17589	1.1211	0.551417
17590	1.1211	0.202339
17591	1.28418	0.404163
17592	0.993021	2.1
17593	1.764 2.1	
17594	0.993023	2.1
17595	1.05787	2.1
17596	1.90914	0.303622
17597	1.12229	2.09251
17598	1.02025	0.599688
17599	0.585368	2.08647
17600	1.99323	2.1
17601	2.34988	2.1
17602	2.34988	2.1
17603	2.36987	2.1
17604	1.23107	0.05
17605	1.90664	0.0512173
17606	1.53496	0.05
17607	1.70302	0.0577673
17608	1.98859	2.1
17609	1.61057	2.1
17610	1.79844	2.1
17611	2.10811	2.1
17612	1.4618	2.1
17613	1.96722	2.1
17614	1.95191	2.1
17615	2.13418	2.1
17616	1.50368	0.05
17617	1.61417	0.05
17618	1.15008	0.05
17619	1.19685	0.05
17620	1.90456	0.05
17621	1.90899	0.05
17622	2.08864	0.05
17623	2.10447	0.05
17624	1.16653	2.1
17625	1.23017	2.1
17626	1.0752	1.74134
17627	1.34412	1.44185
17628	1.06226	2.1
17629	1.09252	2.1

17630	1.09252	2.1
17631	0.88579	2.1
17632	1.56178	0.05
17633	1.14305	0.05
17634	1.56175	0.05
17635	1.08211	0.05
17636	1.32813	2.1
17637	1.32461	2.1
17638	1.32461	2.1
17639	1.32455	2.1
17640	1.42729	0.0847563
17641	1.18716	0.0603556
17642	1.47169	0.162969
17643	1.24687	0.0582018
17644	1.09627	2.1
17645	1.04759	2.1
17646	1.04759	2.1
17647	0.987132	2.1
17648	1.11181	1.83376
17649	1.80662	2.1
17650	1.8066	2.09957
17651	1.06461	1.74816
17652	1.56091	2.1
17653	1.9842	2.1
17654	1.73525	0.123755
17655	1.40557	0.124765
17656	2.26906	0.144074
17657	1.31248	0.200905
17658	1.66832	0.133853
17659	1.32634	0.125932
17660	1.03314	0.05
17661	1.24682	0.05
17662	1.38536	0.05
17663	1.52661	0.05
17664	1.177	1.15343
17665	1.36405	0.658833
17666	1.19837	0.911306
17667	1.17701	0.402618
17668	0.993831	0.05
17669	1.0823	0.0601574
17670	1.0823	0.0577494
17671	1.01121	0.0701082
17672	1.49653	0.105171
17673	1.89583	0.145047
17674	0.67242	0.103943
17675	1.36047	0.138312
17676	1.00366	0.137763
17677	1.24728	0.140984
17678	1.13387	0.05
17679	1.60867	0.05
17680	1.30572	0.05
17681	1.54982	0.05
17682	0.788592	0.05
17683	1.82961	0.05
17684	1.54982	0.05
17685	1.82961	0.05
17686	1.26062	0.05
17687	1.64002	0.05

17688	1.12035	0.05
17689	1.64002	0.05
17690	1.1673	2.1
17691	1.75367	2.1
17692	0.964034	2.1
17693	1.0305	2.1
17694	1.30896	2.01652
17695	1.52776	2.02914
17696	1.52776	2.1
17697	1.17989	2.1
17698	1.16759	2.1
17699	1.8575	2.1
17700	1.16759	2.1
17701	1.60936	2.1
17702	1.10747	0.05
17703	1.47688	0.0702074
17704	1.40794	2.1
17705	1.95729	1.86946
17706	1.95729	2.1
17707	3.18156	1.7967
17708	1.87598	2.1
17709	1.94343	2.1
17710	1.94343	2.1
17711	2.00551	2.1
17712	1.20067	1.29264
17713	1.70744	1.52609
17714	1.87244	1.35544
17715	1.32075	1.19054
17716	1.64955	2.1
17717	1.44221	2.1
17718	1.31109	2.1
17719	1.14206	2.1
17720	1.60452	1.96646
17721	1.12111	1.04606
17722	1.00832	2.1
17723	0.974137	2.1
17724	0.974137	2.1
17725	0.98505	2.1
17726	1.02368	2.1
17727	0.985051	2.1
17728	0.995734	0.622345
17729	1.31993	0.656885
17730	1.31993	0.560881
17731	1.18549	1.98643
17732	1.5176	0.308232
17733	1.33622	0.224587
17734	1.33621	0.256125
17735	1.02782	0.201476
17736	1.66774	2.1
17737	1.85305	2.1
17738	1.85305	2.1
17739	2.07088	2.1
17740	1.78873	2.1
17741	1.47533	2.1
17742	1.23152	2.1
17743	0.99922	2.1
17744	1.48493	2.1
17745	1.1394	2.1

17746	1.19179	2.1
17747	1.64289	2.1
17748	1.47858	2.1
17749	1.89687	2.1
17750	0.972464	2.1
17751	1.79473	2.1
17752	1.06971	2.1
17753	1.21089	2.1
17754	1.12572	2.1
17755	1.30566	2.1
17756	1.30566	2.1
17757	1.52724	2.1
17758	2.32237	0.107799
17759	2.20248	0.0928771
17760	2.79564	0.115643
17761	2.32237	0.105282
17762	1.59354	0.0585877
17763	3.098 0.0569534	
17764	3.09801	0.0588006
17765	3.45132	0.0540526
17766	1.24001	1.47686
17767	1.41822	1.06608
17768	1.41821	1.94855
17769	1.44859	0.883807
17770	1.34539	2.1
17771	1.06991	2.1
17772	1.18076	2.1
17773	0.978586	2.1
17774	0.999189	0.05
17775	2.41076	0.05
17776	0.968475	2.1
17777	1.02074	2.1
17778	1.02074	2.1
17779	1.07866	2.1
17780	0.817702	0.0569812
17781	1.5082	0.0641563
17782	1.48376	0.0758312
17783	1.18947	0.0782478
17784	1.10638	2.1
17785	1.86766	2.1
17786	1.19785	2.1
17787	1.34141	2.1
17788	1.34141	2.1
17789	0.991985	2.1
17790	2.35516	0.858659
17791	1.24815	1.98771
17792	1.80454	2.1
17793	1.22633	2.1
17794	0.696255	2.1
17795	1.19379	2.01298
17796	1.14721	2.1
17797	1.16153	2.1
17798	1.16153	2.1
17799	1.17261	2.1
17800	1.20814	2.1
17801	1.54791	2.1
17802	1.54791	2.1
17803	1.60477	2.1

17804	1.29931	2.1
17805	1.19098	2.1
17806	1.19098	2.1
17807	1.13341	2.1
17808	1.00503	1.77418
17809	1.25334	0.326416
17810	1.22655	2.1
17811	1.26282	2.1
17812	1.26282	2.1
17813	0.999489	2.1
17814	1.54197	0.14124
17815	1.17152	0.158268
17816	1.17152	0.15999
17817	1.49627	0.145568
17818	1.36405	0.647529
17819	0.976334	0.522047
17820	1.12495	2.1
17821	1.54096	2.1
17822	1.75263	2.1
17823	1.49114	2.1
17824	0.842998	1.58312
17825	1.60244	1.07053
17826	1.48911	1.32427
17827	2.18685	1.37723
17828	1.75236	1.04446
17829	1.54143	1.59654
17830	1.40013	0.440534
17831	0.946646	0.546674
17832	1.50714	2.1
17833	1.46055	2.1
17834	1.46055	2.1
17835	1.42249	2.1
17836	2.12839	0.05
17837	1.75211	0.0978764
17838	1.76297	0.12229
17839	1.70736	0.0875676
17840	1.19952	0.05
17841	1.56475	0.05
17842	1.74312	0.0836464
17843	1.48973	0.05
17844	1.91518	0.171664
17845	1.16534	0.108369
17846	1.53556	2.1
17847	1.56253	2.1
17848	1.56253	2.1
17849	1.58054	2.1
17850	1.27806	0.05
17851	1.44593	0.05
17852	1.44593	0.05
17853	1.3946	0.05
17854	1.34857	0.275074
17855	1.03818	1.95373
17856	1.38969	0.177555
17857	1.85548	0.234369
17858	1.03108	0.134015
17859	1.85548	0.151882
17860	1.49346	1.8369
17861	1.83192	2.03023

17862	1.75291	1.47657
17863	1.35435	1.84134
17864	0.921851	0.207568
17865	1.09646	0.191105
17866	1.2183	0.173499
17867	1.36717	0.154144
17868	2.14179	0.089333
17869	1.79805	0.111871
17870	1.3428	0.133469
17871	2.30493	0.130053
17872	1.81545	0.128781
17873	1.92269	0.0972008
17874	2.30492	0.221555
17875	1.3663	0.171719
17876	1.22418	2.1
17877	0.956698	1.96938
17878	1.4706	2.1
17879	0.953295	2.1
17880	1.47059	2.1
17881	1.26929	2.1
17882	1.24538	0.0694842
17883	1.4468	0.0629072
17884	2.86762	2.1
17885	2.32911	2.1
17886	2.10864	2.1
17887	1.94548	2.1
17888	1.83134	2.1
17889	2.19519	2.1
17890	2.19853	2.1
17891	2.63855	2.1
17892	1.08213	0.05
17893	1.46389	0.05
17894	1.62235	0.0641456
17895	0.980549	0.0734339
17896	1.08195	1.63605
17897	0.99002	2.06708
17898	1.2269	2.1
17899	1.0838	2.1
17900	0.889491	2.1
17901	1.19587	2.1
17902	1.18801	0.0515176
17903	1.57125	0.0523225
17904	1.47712	2.1
17905	1.45182	2.1
17906	1.45182	2.1
17907	1.42255	2.1
17908	1.17816	0.05
17909	1.00982	0.05
17910	1.21056	0.05
17911	1.17816	0.05
17912	3.80902	0.629693
17913	0.976991	0.511569
17914	1.09962	2.1
17915	1.60976	2.1
17916	1.87029	2.1
17917	2.33806	2.1
17918	2.12549	2.1
17919	2.21472	2.1

17920	2.16221	0.05
17921	1.99528	0.05
17922	1.99528	0.05
17923	1.97969	0.05
17924	3.60854	2.1
17925	4.00847	2.1
17926	2.70372	2.1
17927	1.253	2.1
17928	1.65739	2.1
17929	0.994294	2.1
17930	1.36203	2.1
17931	1.00965	2.1
17932	2.33374	2.1
17933	1.36209	2.1
17934	1.64983	0.05
17935	1.36416	0.05
17936	2.19766	0.05
17937	1.36416	0.05
17938	2.15871	0.412847
17939	1.81454	0.25433
17940	1.54607	0.115259
17941	1.57702	0.216703
17942	0.948748	0.05
17943	1.04522	0.05
17944	1.60994	0.05
17945	1.90382	0.05
17946	1.90382	0.05
17947	1.57999	0.05
17948	1.73905	1.192
17949	1.26535	1.35976
17950	2.12552	1.28762
17951	1.06891	1.18444
17952	1.13999	1.2302
17953	2.29812	1.19698
17954	2.83329	1.06509
17955	2.16272	0.694017
17956	1.71277	2.1
17957	1.68401	2.1
17958	1.68401	2.1
17959	1.69368	2.1
17960	1.06472	2.1
17961	1.56017	2.1
17962	1.2765	2.1
17963	1.99541	2.1
17964	1.46041	0.05
17965	2.04817	0.05
17966	2.04817	0.05
17967	2.33181	0.05
17968	2.06353	2.1
17969	1.35449	2.1
17970	1.52045	2.1
17971	1.45437	2.1
17972	1.04405	0.05
17973	1.85945	0.05
17974	1.85945	0.05
17975	2.22679	0.05
17976	1.33339	2.1
17977	1.19376	2.1

17978	1.33266	2.1
17979	1.44244	2.1
17980	1.20455	2.1
17981	1.52852	2.1
17982	1.03882	2.1
17983	1.52852	2.1
17984	1.44801	2.1
17985	1.00775	2.1
17986	0.53907	2.1
17987	0.506124	2.1
17988	2.39045	2.1
17989	0.973794	2.1
17990	1.11193	2.1
17991	1.32784	2.1
17992	1.31595	2.1
17993	0.966115	2.1
17994	1.25953	2.1
17995	1.38086	2.1
17996	1.38086	2.1
17997	1.04101	2.1
17998	1.99319	0.05
17999	1.23107	0.05
18000	3.3606	0.05
18001	1.23112	0.05
18002	1.40239	2.1
18003	1.26091	2.1
18004	1.26091	2.1
18005	1.05776	2.1
18006	1.23768	0.161806
18007	1.0142	1.62006
18008	1.11435	1.2944
18009	1.01422	0.242217
18010	1.24001	1.18713
18011	1.42186	1.27101
18012	1.42186	1.30057
18013	1.47204	1.34673
18014	1.27361	2.1
18015	1.56642	2.1
18016	1.71676	2.1
18017	2.16815	2.1
18018	1.07855	1.84654
18019	1.07612	1.80528
18020	1.703	0.05
18021	1.46853	0.05
18022	1.45448	0.05
18023	1.27778	0.0550804
18024	0.991986	2.1
18025	1.1192	2.1
18026	1.1192	2.1
18027	1.25953	2.1
18028	2.08506	2.1
18029	1.75162	2.1
18030	1.94523	2.1
18031	1.88405	1.42466
18032	1.15506	0.05
18033	1.1233	0.05
18034	2.09736	2.1
18035	2.98303	2.1

18036	2.71184	2.1
18037	3.51614	2.1
18038	1.18769	2.1
18039	1.24053	2.1
18040	1.24053	2.1
18041	1.45327	2.1
18042	1.26865	2.1
18043	1.1744	1.81436
18044	1.1744	1.90334
18045	1.11712	1.63893
18046	1.15841	0.137993
18047	1.02247	0.1692
18048	1.93649	2.1
18049	1.81191	2.1
18050	1.64718	2.1
18051	1.47521	2.1
18052	1.59654	2.1
18053	1.57823	2.1
18054	1.57823	2.1
18055	1.55768	2.1
18056	1.00665	0.108856
18057	1.28239	0.0919917
18058	1.28239	0.115056
18059	1.44951	0.0988237
18060	1.22083	2.1
18061	0.957622	2.1
18062	0.887896	2.1
18063	1.27387	2.1
18064	1.00521	1.5355
18065	1.00503	1.3655
18066	1.95844	2.1
18067	1.05042	2.1
18068	2.78662	2.1
18069	2.22399	2.1
18070	2.3356	0.05
18071	1.49543	0.05
18072	1.96854	0.05
18073	1.21681	0.05
18074	2.17803	1.71348
18075	1.50998	1.88593
18076	2.35239	1.46434
18077	2.26987	1.36618
18078	1.03824	0.140582
18079	1.51396	0.15591
18080	1.51396	0.101663
18081	1.48693	0.110208
18082	1.08784	1.42159
18083	1.36865	1.34083
18084	0.712405	1.69853
18085	1.08784	1.45154
18086	2.02522	2.1
18087	1.6738	2.1
18088	1.35175	2.1
18089	1.50094	2.1
18090	1.6738	2.1
18091	1.62871	2.1
18092	1.35084	2.1
18093	1.34276	2.1

18094	1.18041	2.1
18095	1.43652	2.1
18096	0.974196	2.1
18097	1.28037	2.1
18098	1.28037	2.1
18099	1.05071	2.1
18100	2.44737	0.05
18101	1.4672	0.05
18102	1.22883	0.05
18103	2.08149	0.05
18104	1.82208	1.57097
18105	1.59826	1.35211
18106	1.59826	0.547189
18107	1.01393	1.32426
18108	1.42757	2.1
18109	0.996775	2.1
18110	1.42757	2.1
18111	1.26397	2.1
18112	1.02461	2.1
18113	1.46129	2.1
18114	1.11833	2.1
18115	1.335 2.1	
18116	2.07087	2.1
18117	1.24041	2.1
18118	1.335 2.1	
18119	1.55398	2.1
18120	1.21171	0.05
18121	1.48589	0.05
18122	1.32886	0.05
18123	1.8027	0.05
18124	1.53515	1.7192
18125	1.60428	1.7264
18126	1.00035	0.05
18127	1.03534	0.25488
18128	1.03535	2.1
18129	1.09962	2.1
18130	0.62132	2.03199
18131	1.21033	2.1
18132	1.30326	2.1
18133	2.6823	2.1
18134	0.991407	2.1
18135	1.29729	2.1
18136	1.29729	2.1
18137	1.16858	2.1
18138	1.66137	0.390664
18139	1.23012	0.256609
18140	1.66139	0.653213
18141	1.41821	0.447393
18142	1.56043	0.391657
18143	1.06848	0.427778
18144	1.56043	0.745185
18145	1.90911	0.601844
18146	0.982847	2.1
18147	1.05863	2.1
18148	1.05863	2.1
18149	1.22352	2.1
18150	1.23019	2.1
18151	1.60509	2.1

18152	1.19122	2.1
18153	1.47769	2.1
18154	2.18868	2.1
18155	1.40984	2.1
18156	3.55197	2.1
18157	3.57227	2.1
18158	3.57227	2.1
18159	3.48926	2.1
18160	1.4375	0.111108
18161	1.11314	0.0999741
18162	1.62408	2.1
18163	1.74438	2.1
18164	1.72387	2.1
18165	1.50018	2.1
18166	1.1486	2.1
18167	1.18474	2.1
18168	1.18474	2.1
18169	1.2295	2.1
18170	1.28754	2.1
18171	1.36838	2.1
18172	1.36838	2.1
18173	1.21703	2.1
18174	2.74206	2.1
18175	2.70795	2.1
18176	2.70795	2.1
18177	2.55918	2.1
18178	1.35065	0.691548
18179	1.65411	0.347966
18180	1.48572	0.600433
18181	1.28393	0.470551
18182	1.25265	0.05
18183	1.70944	0.05
18184	1.31259	2.1
18185	1.14945	2.1
18186	1.14945	2.05057
18187	0.995725	2.1
18188	1.00307	0.157433
18189	2.05786	0.162875
18190	1.56357	0.0953256
18191	1.10907	0.125272
18192	1.27558	1.71727
18193	1.05175	1.6048
18194	1.2685	2.1
18195	1.41369	2.1
18196	1.4137	2.1
18197	1.49654	2.1
18198	1.21489	1.92316
18199	1.14605	1.38159
18200	1.22476	2.1
18201	1.21489	1.15661
18202	2.51472	1.23947
18203	2.52091	1.24609
18204	2.52091	0.861852
18205	1.79171	0.567641
18206	1.03373	2.1
18207	1.04037	2.1
18208	1.04038	2.1
18209	1.03933	2.1

18210	1.74123	0.0945589
18211	1.41902	0.0847814
18212	1.89777	0.10549
18213	1.89899	0.119583
18214	1.24728	0.105552
18215	1.30394	0.0968764
18216	1.30394	0.0748249
18217	1.16535	0.083438
18218	1.09914	2.1
18219	1.54259	2.1
18220	1.40233	2.1
18221	1.69858	2.1
18222	1.53555	2.1
18223	1.09859	2.1
18224	1.09859	2.1
18225	0.725879	2.1
18226	1.07213	2.1
18227	1.31589	2.1
18228	1.36904	2.1
18229	1.69711	2.1
18230	1.51028	0.05
18231	0.993598	0.05
18232	0.61563	0.05
18233	1.26311	0.05
18234	1.19496	2.1
18235	1.65925	2.1
18236	1.65925	2.1
18237	1.48162	2.1
18238	2.75738	1.52709
18239	1.24699	1.47898
18240	4.08729	1.72867
18241	2.35271	1.52621
18242	1.55063	0.12109
18243	1.34563	0.0939444
18244	1.34563	0.0905853
18245	1.08189	0.0795902
18246	1.09662	0.055887
18247	2.35961	0.0909567
18248	0.986952	0.05
18249	0.813251	0.05
18250	1.17897	0.05
18251	1.67685	0.05
18252	1.00573	1.98596
18253	1.08542	1.65202
18254	1.00573	2.1
18255	1.12572	2.1
18256	1.05499	2.1
18257	1.00521	1.51395
18258	2.22679	0.05
18259	2.86746	0.05
18260	2.86746	0.05
18261	1.99958	0.05
18262	0.996517	2.1
18263	1.01169	2.1
18264	1.01169	2.1
18265	1.26346	2.1
18266	1.68274	2.1
18267	1.75505	2.1

18268	2.28748	1.96813
18269	1.36555	2.08619
18270	3.47888	0.05
18271	0.692788	0.05
18272	1.0685	0.293509
18273	1.14547	0.346694
18274	1.19017	2.1
18275	1.24976	2.1
18276	1.24976	2.1
18277	1.30682	2.1
18278	2.2153	2.1
18279	1.85457	2.1
18280	2.03267	2.1
18281	1.51042	2.1
18282	1.05619	0.442244
18283	1.05793	0.336455
18284	1.05793	0.266456
18285	1.00763	0.213803
18286	1.77677	2.1
18287	1.02524	2.1
18288	1.02524	2.1
18289	1.18803	2.1
18290	1.68669	2.1
18291	1.71162	2.1
18292	1.556	2.1
18293	1.39523	2.1
18294	0.960288	2.00156
18295	1.24201	2.1
18296	1.24202	2.1
18297	1.3592	2.1
18298	2.14045	0.0500276
18299	1.62054	0.05
18300	2.20248	0.087164
18301	2.34699	0.0740608
18302	3.45132	0.05
18303	3.38241	0.05
18304	3.38241	0.05
18305	2.96127	0.05
18306	2.02612	2.1
18307	2.09351	2.1
18308	2.09351	2.1
18309	2.17465	2.1
18310	1.07445	2.1
18311	1.14808	2.1
18312	1.39067	2.1
18313	1.20958	2.1
18314	1.20958	2.1
18315	1.00382	1.99044
18316	0.986496	1.15499
18317	1.27142	1.26957
18318	1.39857	1.0747
18319	2.26482	1.12445
18320	2.24367	0.05
18321	1.9234	0.05
18322	1.59076	0.05
18323	1.185	0.05
18324	3.86179	0.05
18325	3.0638	0.05

18326	2.7574	0.05
18327	1.83266	0.05
18328	2.59418	2.1
18329	2.23048	2.1
18330	1.39564	2.1
18331	1.36763	2.1
18332	2.23048	2.1
18333	2.02522	2.1
18334	1.36763	2.1
18335	1.35175	2.1
18336	1.16181	0.05
18337	0.97096	0.05
18338	2.05665	0.126455
18339	0.483583	0.130058
18340	0.601928	0.101398
18341	3.88545	0.145452
18342	1.04909	2.1
18343	0.983143	2.1
18344	1.10469	2.1
18345	1.10516	2.1
18346	1.10517	2.1
18347	1.09951	2.1
18348	2.33985	2.1
18349	2.35026	2.1
18350	2.35025	2.1
18351	1.19462	2.1
18352	1.57627	2.1
18353	1.5493	2.1
18354	1.5493	2.1
18355	1.53556	2.1
18356	1.42763	0.343342
18357	1.24682	0.397016
18358	1.24682	0.24608
18359	1.00307	0.330211
18360	1.37392	2.1
18361	1.45408	2.1
18362	1.45408	2.1
18363	1.54249	2.1
18364	1.24474	1.58595
18365	1.07467	1.57019
18366	1.37813	1.54689
18367	1.24474	1.5773
18368	1.06341	2.1
18369	1.09194	2.1
18370	1.09194	2.1
18371	1.11117	2.1
18372	1.1658	2.1
18373	1.93707	2.1
18374	1.93707	2.1
18375	1.47729	2.1
18376	1.25669	2.1
18377	1.07519	2.1
18378	1.65417	2.1
18379	1.50956	2.1
18380	1.37231	2.1
18381	1.27285	2.1
18382	1.23612	2.1
18383	1.1899	2.1

18384	1.08172	2.1
18385	1.20357	2.1
18386	1.5664	2.1
18387	1.7472	2.1
18388	1.48434	2.1
18389	1.23989	2.1
18390	1.58836	2.1
18391	1.38766	2.1
18392	1.23989	2.1
18393	1.26599	2.1
18394	2.56248	2.1
18395	2.08673	2.1
18396	1.87216	2.1
18397	1.60476	0.566051
18398	1.84355	2.1
18399	1.25971	2.09725
18400	1.44141	2.04642
18401	1.25521	0.297444
18402	1.19837	0.05
18403	2.31936	0.05
18404	1.38153	2.1
18405	2.41734	2.1
18406	2.70856	2.1
18407	3.48385	2.1
18408	1.89226	1.24575
18409	1.64617	1.36651
18410	1.89112	0.677172
18411	1.23135	0.165752
18412	1.23134	0.253948
18413	1.34927	0.228404
18414	3.60096	0.05
18415	1.68103	0.05
18416	2.25644	2.1
18417	1.91955	2.1
18418	1.91955	2.1
18419	1.47688	2.1
18420	1.67375	2.1
18421	1.50033	2.1
18422	1.5098	2.1
18423	1.46908	2.1
18424	1.69055	0.05
18425	1.53463	0.05
18426	1.53463	0.05
18427	1.41943	0.05
18428	1.19022	1.81567
18429	1.95981	1.62968
18430	1.15009	1.89692
18431	1.31612	1.78242
18432	1.31611	0.522698
18433	1.10735	0.445291
18434	1.18601	0.0596935
18435	1.3499	0.0510399
18436	1.3499	0.0623136
18437	1.29001	0.0541664
18438	1.22083	2.1
18439	1.06191	2.1
18440	1.348	1.28503
18441	1.05758	1.40085

18442	1.34799	1.5204
18443	1.18647	1.58576
18444	2.92457	0.05
18445	1.99182	0.05
18446	2.25313	0.05
18447	1.30859	0.05
18448	1.30682	2.1
18449	1.38957	2.1
18450	1.38957	2.1
18451	1.47446	2.1
18452	0.953638	2.1
18453	1.03408	2.1
18454	1.17885	0.426453
18455	1.3663	0.128835
18456	1.08265	0.210583
18457	1.71948	0.528022
18458	1.29501	0.851092
18459	0.955093	0.505269
18460	0.955081	0.286154
18461	1.88345	0.399339
18462	1.2243	2.1
18463	1.23169	2.1
18464	2.04726	1.14575
18465	0.842869	0.87509
18466	3.18156	2.1
18467	3.60786	1.63599
18468	3.60786	2.1
18469	2.38566	1.38649
18470	1.24526	0.05
18471	1.59019	0.05
18472	1.16776	1.42786
18473	1.05042	2.1
18474	1.17418	2.1
18475	1.05042	2.1
18476	0.949135	2.1
18477	0.973448	2.1
18478	0.973447	2.1
18479	1.02958	2.1
18480	3.57768	2.1
18481	3.04246	2.1
18482	3.04246	2.1
18483	2.47824	2.1
18484	1.35879	2.1
18485	1.10407	2.1
18486	1.14599	2.1
18487	1.10407	2.1
18488	1.87794	1.68718
18489	1.53429	1.64741
18490	1.12717	2.1
18491	1.38634	2.1
18492	1.38634	2.1
18493	1.13127	2.1
18494	1.04546	2.1
18495	1.06007	2.1
18496	1.06007	2.1
18497	1.19785	2.1
18498	1.43652	2.1
18499	1.71741	2.1

18500	1.70921	2.1
18501	1.06994	2.1
18502	0.960106	2.1
18503	1.48077	2.1
18504	1.48077	2.1
18505	2.5946	2.1
18506	2.03045	1.75987
18507	1.76788	1.84299
18508	2.03045	2.1
18509	0.633098	2.1
18510	1.06756	2.1
18511	1.38667	2.1
18512	0.966979	2.1
18513	1.38669	2.1
18514	1.07156	2.1
18515	0.986095	2.1
18516	1.32813	2.1
18517	0.986095	2.1
18518	1.98697	1.1636
18519	1.12016	1.31423
18520	1.24463	1.50304
18521	1.1967	1.30595
18522	1.30001	0.316837
18523	1.29175	0.970191
18524	1.29175	0.343784
18525	1.3003	1.35566
18526	1.5606	0.05
18527	2.32098	0.05
18528	2.57888	0.05
18529	2.97463	0.05
18530	1.24555	2.1
18531	1.46101	2.1
18532	1.90723	2.1
18533	2.29126	2.1
18534	1.46101	2.1
18535	1.84089	2.1
18536	2.5519	2.1
18537	2.91663	2.1
18538	1.10476	0.05
18539	1.44103	0.05
18540	1.44103	0.05
18541	1.53336	0.05
18542	0.672964	2.1
18543	0.633884	2.1
18544	0.633884	2.1
18545	0.603154	2.1
18546	2.29663	2.1
18547	2.06892	2.1
18548	1.3685	2.1
18549	1.39067	2.1
18550	2.06892	2.1
18551	1.78208	2.1
18552	1.39067	2.1
18553	1.42174	2.1
18554	1.01335	1.51976
18555	1.08195	1.31791
18556	1.86463	1.99318
18557	1.39827	2.00455

18558	0.861515	2.1
18559	2.10074	2.1
18560	2.09511	1.49335
18561	2.04853	1.21019
18562	2.21488	1.1301
18563	1.38287	1.42097
18564	0.974717	0.389304
18565	0.998451	0.33374
18566	1.42281	1.72034
18567	1.82595	1.67542
18568	1.50458	1.0248
18569	1.09408	1.004
18570	1.23498	1.64376
18571	1.05654	1.58143
18572	1.17261	2.1
18573	1.18058	2.1
18574	1.18059	2.1
18575	1.18907	2.1
18576	2.38998	2.1
18577	2.77437	2.1
18578	1.04546	2.1
18579	1.16817	2.1
18580	2.77437	2.1
18581	2.97768	2.1
18582	1.16817	2.1
18583	1.2832	2.1
18584	1.75766	0.093092
18585	1.4934	1.9
18586	1.4934	0.203091
18587	1.20324	2.02053
18588	1.07236	0.719237
18589	1.37641	0.406863
18590	1.42913	0.326402
18591	1.93373	0.359379
18592	1.42629	0.358188
18593	1.00636	0.234551
18594	0.933959	1.78126
18595	2.22873	0.617577
18596	2.22873	1.48541
18597	2.13757	0.400512
18598	1.10517	2.1
18599	1.86737	2.1
18600	0.811 2.1	
18601	1.19179	2.1
18602	1.18647	1.66834
18603	0.988453	2.04884
18604	1.12457	0.0856614
18605	1.23851	0.0737897
18606	0.92492	2.1
18607	1.45656	2.1
18608	1.61251	0.566365
18609	1.56696	0.636009
18610	1.90245	0.320971
18611	1.46046	0.31183
18612	1.60329	0.05
18613	1.38247	0.05
18614	1.38247	0.05
18615	1.23198	0.05

18616	1.09518	2.1
18617	1.28159	2.1
18618	0.977777	2.1
18619	1.80778	2.1
18620	1.80777	2.1
18621	1.16655	2.1
18622	1.3515	2.1
18623	0.956573	2.1
18624	1.08022	2.1
18625	1.03437	2.1
18626	1.03437	2.1
18627	0.975409	2.1
18628	1.47369	0.05
18629	1.31167	0.0589114
18630	1.01847	0.05
18631	2.75562	0.05
18632	1.13443	2.1
18633	1.32709	2.1
18634	1.01051	0.168919
18635	0.958947	0.139044
18636	1.7211	2.1
18637	1.922 2.1	
18638	1.922 2.1	
18639	0.991113	2.1
18640	1.00636	0.237898
18641	1.31658	0.260521
18642	1.44627	0.175949
18643	1.03824	0.167053
18644	1.21223	2.1
18645	1.01242	2.1
18646	1.01243	2.1
18647	0.956698	2.1
18648	1.23167	2.1
18649	1.06935	2.1
18650	1.29215	0.05
18651	0.998971	0.05
18652	1.50103	0.05
18653	1.44836	0.05
18654	3.02513	2.1
18655	3.7335	2.1
18656	2.49988	2.1
18657	1.24948	2.1
18658	0.963972	2.1
18659	1.1822	2.1
18660	1.1822	2.1
18661	1.10817	2.1
18662	1.30237	2.1
18663	1.41908	2.1
18664	1.41908	2.1
18665	1.48734	2.1
18666	1.31127	0.152011
18667	1.40822	0.147054
18668	1.40822	0.0807636
18669	1.22176	0.0758951
18670	1.82525	2.1
18671	1.59394	2.1
18672	1.22731	0.05
18673	0.981584	0.05

18674	1.1427	1.12011
18675	1.44091	1.24786
18676	1.49154	1.70403
18677	1.07526	1.45534
18678	1.07527	0.937737
18679	0.96029	1.76203
18680	2.40568	1.56805
18681	3.46486	1.56459
18682	2.417	1.6274
18683	1.52054	1.37711
18684	1.51638	0.105981
18685	1.04786	0.0881329
18686	1.51404	0.0861713
18687	1.3461	0.0738886
18688	1.27696	0.05
18689	0.736457	0.05
18690	1.16817	2.1
18691	1.04263	2.1
18692	1.13449	2.1
18693	1.50524	2.1
18694	2.1182	2.1
18695	2.49001	2.1
18696	1.11764	2.1
18697	1.39292	2.1
18698	2.49001	2.1
18699	2.76339	2.1
18700	1.39292	2.1
18701	1.68761	2.1
18702	1.25173	2.1
18703	1.34701	2.1
18704	1.02415	2.1
18705	1.34701	2.1
18706	1.332	0.05
18707	0.989764	0.05
18708	0.963167	0.05
18709	1.21056	0.05
18710	1.67375	2.1
18711	1.17965	2.1
18712	1.09154	2.1
18713	1.52314	2.1
18714	1.54433	0.37703
18715	1.81969	0.308455
18716	1.3598	0.205159
18717	1.71041	0.179769
18718	1.70934	0.168979
18719	1.33871	0.155523
18720	1.79485	0.126338
18721	1.51404	0.108639
18722	1.30751	2.1
18723	1.20969	2.1
18724	1.20969	2.1
18725	1.12076	2.1
18726	1.49808	2.1
18727	1.50201	2.1
18728	1.50201	2.1
18729	1.31594	2.1
18730	1.2187	2.1
18731	1.11545	2.1

18732	1.227	2.1
18733	1.09645	2.1
18734	1.05902	0.05
18735	1.41595	1.15061
18736	1.35696	2.1
18737	1.29446	1.91213
18738	0.857957	2.1
18739	1.21125	0.05
18740	2.43115	2.1
18741	2.1151	0.192134
18742	2.0815	1.68355
18743	1.1723	1.92994
18744	1.48035	0.0881354
18745	1.59971	0.585942
18746	1.33668	0.173668
18747	1.0961	0.16799
18748	1.46235	2.1
18749	0.882195	2.1
18750	2.46624	2.1
18751	2.72161	2.1
18752	2.96094	2.1
18753	2.25644	2.1
18754	1.25923	0.05
18755	0.976218	0.05
18756	1.10319	0.05
18757	1.82583	0.05
18758	0.888955	2.1
18759	1.30817	2.1
18760	1.30817	2.1
18761	1.60787	2.1
18762	0.988805	2.1
18763	1.54168	2.1
18764	1.19496	1.29073
18765	1.12224	1.60437
18766	1.93597	1.44368
18767	1.31113	1.39185
18768	1.17879	1.53588
18769	1.07468	1.51891
18770	2.0523	2.1
18771	2.41385	2.1
18772	2.41385	2.1
18773	3.0355	2.1
18774	1.36717	0.120377
18775	1.45535	0.0953288
18776	1.45535	0.0783053
18777	1.49906	0.0673374
18778	1.37785	2.1
18779	1.04413	2.1
18780	1.06729	1.11176
18781	1.54305	0.758916
18782	1.07444	2.1
18783	1.52724	2.1
18784	1.49151	2.1
18785	1.36814	2.1
18786	1.36814	2.1
18787	1.15541	2.1
18788	1.19379	2.1
18789	1.78657	1.71155

18790	1.6079	2.1
18791	2.59469	1.13302
18792	1.35545	0.735877
18793	1.23602	1.87211
18794	1.23452	2.1
18795	1.56842	2.05099
18796	1.15864	2.1
18797	1.11955	2.1
18798	1.11955	2.1
18799	1.08022	2.1
18800	1.64268	2.1
18801	1.26247	2.1
18802	1.16912	2.1
18803	2.1095	1.19365
18804	1.75549	0.05
18805	1.7491	0.05
18806	1.59833	0.05
18807	1.68338	0.05
18808	1.14846	0.05
18809	1.06515	0.05
18810	1.2721	0.109217
18811	1.32639	0.0942297
18812	1.32639	0.193292
18813	1.19323	0.150089
18814	1.81034	2.1
18815	1.9034	2.1
18816	2.08245	2.1
18817	1.84887	2.1
18818	1.41448	2.1
18819	1.77326	2.1
18820	1.76872	2.1
18821	1.86766	2.1
18822	1.46643	2.1
18823	1.58995	2.1
18824	1.58995	2.1
18825	1.13854	2.1
18826	1.23654	2.1
18827	1.04442	2.1
18828	1.23654	2.1
18829	1.08576	2.1
18830	0.966979	2.1
18831	1.41758	2.1
18832	1.03108	0.0829201
18833	1.94412	0.0876615
18834	1.32455	2.1
18835	1.32328	2.1
18836	1.32328	2.1
18837	1.34008	2.1
18838	1.60861	2.1
18839	1.69586	2.1
18840	1.69586	2.1
18841	1.78301	2.1
18842	1.7513	2.1
18843	1.67906	2.1
18844	1.67906	2.1
18845	1.60635	2.1
18846	1.68073	2.1
18847	1.57234	2.1

18848	1.57233	2.1
18849	1.47012	2.1
18850	1.22811	0.11946
18851	1.79683	0.0858441
18852	1.53417	0.09705
18853	1.08732	0.0934508
18854	1.8903	2.1
18855	1.20247	2.1
18856	1.12087	1.72437
18857	1.54699	2.08789
18858	1.43635	2.1
18859	1.02368	2.1
18860	1.43635	2.1
18861	1.23007	2.1
18862	1.035 2.1	
18863	1.07889	2.1
18864	3.0355	2.1
18865	3.78805	2.1
18866	2.57183	2.1
18867	1.27835	1.96173
18868	1.29813	0.111282
18869	1.42965	0.0863613
18870	1.69832	0.101894
18871	2.18359	0.0772401
18872	1.42964	0.110926
18873	1.65723	0.100788
18874	1.98508	0.0996268
18875	2.06284	0.0763056
18876	1.50705	0.0535229
18877	1.51153	0.0804589
18878	1.36037	0.0624761
18879	1.09767	0.0907828
18880	1.70048	2.1
18881	1.15177	2.1
18882	0.713174	2.1
18883	0.763575	2.1
18884	0.763575	2.1
18885	0.797011	2.1
18886	1.01179	1.09563
18887	1.27737	1.14425
18888	3.16531	0.05
18889	1.0141	0.05
18890	1.47354	0.246926
18891	1.62495	0.142646
18892	1.62495	0.0966601
18893	1.39296	0.19678
18894	0.998682	2.1
18895	1.93528	2.1
18896	2.71383	2.1
18897	1.13987	2.1
18898	1.93528	2.1
18899	1.78595	2.1
18900	3.98933	2.1
18901	3.73426	2.1
18902	1.02438	2.1
18903	1.12376	2.1
18904	1.12375	2.1
18905	1.19016	2.1

18906	1.37157	0.116192
18907	1.60814	0.114938
18908	1.70303	0.145948
18909	1.01219	0.129822
18910	1.06757	2.1
18911	1.23475	2.1
18912	1.2295	2.1
18913	1.28014	2.1
18914	1.28014	2.1
18915	1.33339	2.1
18916	1.3201	2.1
18917	1.2679	2.1
18918	1.2679	2.1
18919	1.23504	2.1
18920	1.50108	2.1
18921	1.3715	2.1
18922	1.3715	2.1
18923	1.25075	2.1
18924	1.99222	1.16964
18925	1.4155	0.570886
18926	1.4777	2.1
18927	1.52613	2.1
18928	1.91031	2.1
18929	1.03968	2.1
18930	2.04289	0.200687
18931	0.967192	0.170996
18932	1.03043	0.135789
18933	1.65452	0.20933
18934	1.21564	2.1
18935	1.16938	2.1
18936	2.47056	2.1
18937	2.56853	2.1
18938	1.16938	2.1
18939	1.1181	2.1
18940	2.56853	2.1
18941	2.66828	2.1
18942	1.0465	2.1
18943	1.44709	2.1
18944	1.44709	2.1
18945	1.96491	2.1
18946	1.01976	0.273494
18947	0.961779	0.241575
18948	1.11764	0.263191
18949	0.961779	0.243063
18950	1.21171	0.05
18951	1.35007	0.05
18952	1.03102	0.183135
18953	1.42446	0.204123
18954	1.42446	0.190202
18955	1.03166	0.164321
18956	1.2951	0.083243
18957	1.33575	0.0933704
18958	1.33575	0.0987363
18959	1.20473	0.109488
18960	1.10049	0.394664
18961	0.99747	0.593591
18962	1.54249	2.1
18963	1.65677	2.1

18964	1.65677	2.1
18965	1.77677	2.1
18966	1.73525	0.126377
18967	1.05689	0.170695
18968	1.26051	0.116303
18969	1.16259	0.111881
18970	1.34009	2.1
18971	1.398	2.1
18972	1.398	2.1
18973	1.50437	2.1
18974	2.3245	2.1
18975	1.18237	2.1
18976	1.24509	2.1
18977	1.01595	2.1
18978	3.29047	2.1
18979	2.79168	2.1
18980	1.06561	2.1
18981	1.11077	2.1
18982	1.11077	2.1
18983	0.953927	2.1
18984	1.30509	0.207671
18985	1.04685	0.22916
18986	1.04685	0.170079
18987	0.834593	0.187199
18988	1.0775	0.180226
18989	1.27362	0.165069
18990	1.71762	0.89878
18991	2.5736	0.640955
18992	2.04717	1.06254
18993	1.45521	0.536633
18994	1.33962	2.1
18995	1.27956	2.1
18996	1.27956	2.1
18997	1.20478	2.1
18998	3.65841	0.05
18999	1.18656	0.05
19000	1.72272	0.0520274
19001	1.6812	0.05
19002	1.09951	2.1
19003	1.08231	2.1
19004	1.08231	2.1
19005	1.06478	2.1
19006	2.16196	1.87663
19007	1.00387	1.63327
19008	1.00387	1.09238
19009	0.993999	0.88101
19010	1.04101	2.1
19011	1.27119	2.1
19012	1.27119	2.1
19013	1.51748	2.1
19014	3.00129	2.1
19015	3.27303	2.1
19016	3.27303	2.1
19017	3.60855	2.1
19018	2.22399	2.1
19019	1.71995	2.1
19020	1.54793	2.1
19021	1.227	2.1

19022	1.07624	2.1
19023	1.264 2.1	
19024	1.19323	1.83376
19025	1.21286	1.61425
19026	1.21286	1.08031
19027	1.20859	1.28936
19028	1.60578	0.0949127
19029	1.27778	0.0740457
19030	1.0525	2.0761
19031	1.05758	1.28478
19032	1.0525	1.34519
19033	1.17042	1.48963
19034	1.62536	0.05
19035	3.71662	0.05
19036	1.78169	0.05
19037	1.91434	0.05
19038	2.12331	0.05
19039	1.33406	0.05
19040	1.38437	2.1
19041	1.51829	2.1
19042	1.51829	2.1
19043	1.6697	2.1
19044	0.947114	2.1
19045	1.13335	2.1
19046	1.13335	2.1
19047	1.00965	2.1
19048	1.47521	2.1
19049	1.27679	2.1
19050	1.14911	2.1
19051	0.93841	2.1
19052	1.68437	2.1
19053	1.53822	2.1
19054	1.53822	2.1
19055	1.39702	2.1
19056	0.726453	2.1
19057	0.754101	2.1
19058	0.754101	2.1
19059	0.69776	2.1
19060	0.964033	2.1
19061	1.62397	2.1
19062	1.70302	2.1
19063	1.78637	2.08249
19064	3.68158	0.05
19065	2.11685	0.05
19066	1.33125	2.1
19067	1.15471	2.1
19068	1.0465	2.1
19069	1.33125	2.1
19070	1.17683	2.1
19071	1.25889	2.1
19072	1.25889	2.1
19073	1.34239	2.1
19074	1.40591	0.05
19075	0.79348	0.05
19076	1.04019	0.05
19077	0.999187	0.05
19078	1.30218	0.05
19079	1.83339	0.05

19080	1.88574	0.05
19081	1.5177	0.05
19082	1.23504	2.1
19083	1.19583	2.1
19084	1.19583	2.1
19085	1.14496	2.1
19086	1.62183	2.1
19087	1.62316	2.1
19088	1.62316	2.1
19089	1.62241	2.1
19090	1.02305	0.123593
19091	1.21136	0.119351
19092	0.993832	0.05
19093	1.21137	0.0791994
19094	1.12064	2.1
19095	1.24965	2.1
19096	1.24965	2.1
19097	1.41077	2.1
19098	1.00052	0.125326
19099	1.49842	0.0993757
19100	1.49843	0.0810841
19101	1.10801	0.0716269
19102	1.54196	2.1
19103	1.18041	2.1
19104	1.73571	1.72293
19105	1.37224	1.83939
19106	1.4375	0.0649177
19107	1.09529	0.0606817
19108	1.44421	0.05
19109	1.0953	0.05
19110	1.0753	2.1
19111	0.815365	2.1
19112	0.815365	2.1
19113	0.597537	2.1
19114	1.03609	2.1
19115	1.18952	2.1
19116	1.54283	0.173971
19117	1.16719	0.156832
19118	1.16719	0.280729
19119	1.89226	0.210227
19120	2.55919	2.1
19121	2.37492	2.1
19122	2.37492	2.1
19123	2.07435	2.1
19124	1.36016	0.0993569
19125	1.70302	0.103202
19126	1.85252	0.115206
19127	1.2111	0.172889
19128	1.62905	2.1
19129	1.49692	2.1
19130	1.49692	2.1
19131	1.40395	2.1
19132	2.19083	2.1
19133	1.30542	2.1
19134	1.58736	2.1
19135	2.16579	2.1
19136	2.0815	2.1
19137	1.17935	2.1

19138	1.53803	2.1
19139	1.09148	1.97084
19140	1.81301	2.1
19141	1.38333	1.75309
19142	1.04412	2.1
19143	1.44437	2.1
19144	1.4675	0.0704366
19145	1.00665	0.0785326
19146	1.50951	0.0839776
19147	0.983105	0.0764988
19148	1.80252	0.124616
19149	0.540005	0.091317
19150	1.2545	0.1422
19151	2.14514	0.107552
19152	1.32051	0.0830405
19153	1.82302	0.0844473
19154	2.71057	2.1
19155	2.51107	2.1
19156	1.34817	2.1
19157	1.3551	2.1
19158	2.51107	2.1
19159	2.29663	2.1
19160	1.3551	2.1
19161	1.3685	2.1
19162	1.51748	2.1
19163	1.23389	2.1
19164	0.901928	0.05
19165	1.70302	0.05
19166	1.66787	0.05
19167	1.92535	0.05
19168	1.13571	0.187097
19169	1.02836	0.209501
19170	1.55398	2.1
19171	1.54179	2.1
19172	1.89608	2.1
19173	1.07167	2.1
19174	3.3488	0.132046
19175	0.550044	0.11483
19176	0.962877	0.294913
19177	1.00065	0.590949
19178	1.00613	0.311477
19179	1.07497	0.373404
19180	1.00486	0.05
19181	1.00486	0.05
19182	0.726453	2.1
19183	1.32634	0.0552932
19184	1.40008	0.0781131
19185	1.40008	0.05
19186	2.42267	0.05
19187	1.9216	0.05
19188	1.28066	0.05
19189	1.79102	2.1
19190	1.18219	0.651731
19191	1.18219	1.99082
19192	0.709123	0.533086
19193	1.07866	2.1
19194	1.15038	2.1
19195	1.15038	2.1

19196	1.24687	2.1
19197	1.83613	2.1
19198	1.99157	2.07699
19199	1.45789	0.05
19200	1.1706	0.05
19201	1.74746	0.05
19202	1.1706	0.05
19203	1.96383	0.05
19204	1.67727	0.05
19205	1.67727	0.05
19206	1.60329	0.05
19207	1.18803	2.1
19208	1.3227	2.1
19209	1.3227	2.1
19210	1.40844	2.1
19211	1.07889	2.1
19212	1.07721	2.1
19213	1.07721	2.1
19214	1.00832	2.1
19215	0.953523	0.69374
19216	1.62875	0.50032
19217	1.07427	0.24535
19218	1.19722	0.183113
19219	1.1972	0.120681
19220	1.1599	0.128011
19221	2.97463	0.05
19222	1.9477	0.05
19223	1.95222	0.05
19224	1.75083	0.05
19225	3.46787	2.1
19226	3.02896	2.1
19227	3.02896	2.1
19228	2.46145	2.1
19229	0.970962	0.05
19230	1.0227	0.05
19231	1.12328	0.05
19232	1.02269	0.05
19233	0.904978	2.1
19234	0.725887	2.1
19235	0.725887	2.1
19236	0.601592	2.1
19237	0.956294	2.1
19238	1.13179	2.1
19239	0.956294	2.1
19240	1.13179	2.1
19241	1.05556	2.1
19242	1.13179	2.1
19243	1.38859	2.1
19244	1.13179	2.1
19245	1.06659	2.1
19246	1.05776	2.1
19247	1.06659	2.1
19248	1.04291	2.1
19249	1.54699	1.65479
19250	1.42307	1.72072
19251	1.5654	1.97049
19252	1.97906	2.1
19253	1.15354	0.05

19254	1.15505	0.05
19255	1.26455	0.05
19256	2.50042	0.05
19257	2.50042	0.05
19258	3.22585	0.05
19259	1.19258	0.131561
19260	1.34562	0.138914
19261	1.49515	0.180168
19262	1.66295	0.111764
19263	1.02395	2.1
19264	1.51067	2.1
19265	1.35821	2.1
19266	2.10671	2.1
19267	1.0305	2.06522
19268	1.07855	2.00115
19269	1.20761	2.1
19270	0.98488	2.1
19271	1.4162	2.1
19272	1.14825	2.1
19273	1.29415	2.1
19274	1.6067	2.1
19275	1.23851	0.118363
19276	1.09651	0.101758
19277	1.60636	2.1
19278	1.53521	2.1
19279	1.53521	2.1
19280	1.46279	2.1
19281	1.28044	0.05
19282	1.64147	0.05
19283	1.18422	0.05
19284	1.68208	0.0531701
19285	1.09726	2.1
19286	1.234 2.1	
19287	1.234 2.1	
19288	1.09483	2.1
19289	1.89318	2.1
19290	1.98448	2.1
19291	1.98448	2.1
19292	2.02612	2.1
19293	1.22026	2.1
19294	1.23527	1.85736
19295	1.09044	2.07648
19296	0.993366	1.588
19297	1.22026	1.98519
19298	0.993368	1.58669
19299	2.61357	2.1
19300	2.48208	2.1
19301	2.48208	2.1
19302	2.62868	2.1
19303	1.85439	0.969851
19304	1.81776	0.57445
19305	1.81776	0.486883
19306	2.1587	0.460978
19307	1.8914	0.626793
19308	2.23961	0.401239
19309	1.49357	0.134923
19310	2.23961	0.326914
19311	1.78014	0.289635

19312	1.67635	0.244183
19313	1.66219	0.228979
19314	1.56036	0.209838
19315	1.14721	0.207231
19316	2.22495	0.295363
19317	1.88509	0.0651425
19318	1.95249	0.0559637
19319	2.11959	0.05
19320	1.37427	0.05
19321	1.39379	2.0389
19322	1.17614	1.9059
19323	1.39379	2.1
19324	1.14397	1.14945
19325	1.09651	2.1
19326	1.42843	2.1
19327	1.7069	2.1
19328	2.31293	2.1
19329	1.56839	2.1
19330	1.78252	1.62976
19331	1.78254	1.13406
19332	1.09142	2.1
19333	0.99829	0.05
19334	1.09372	0.05
19335	1.88175	0.0621399
19336	0.898452	0.05
19337	1.06936	2.1
19338	1.4162	2.1
19339	1.17989	2.1
19340	1.47411	2.1
19341	1.25376	2.1
19342	1.00757	2.1
19343	1.4185	0.160253
19344	1.02011	0.177927
19345	2.22495	0.197624
19346	1.79542	0.18442
19347	1.79542	0.153206
19348	1.19352	0.154536
19349	2.38566	2.1
19350	1.83255	1.61939
19351	1.62667	2.1
19352	1.17879	1.85584
19353	2.79168	2.1
19354	2.28162	2.1
19355	2.28162	2.1
19356	1.90905	2.1
19357	1.40117	2.1
19358	1.28268	2.1
19359	1.41096	2.1
19360	1.22978	2.1
19361	3.20034	2.1
19362	3.42052	2.1
19363	3.42052	2.1
19364	3.50562	2.1
19365	1.46279	2.1
19366	1.3864	2.1
19367	1.3864	2.1
19368	1.3201	2.1
19369	1.35561	0.05

19370	0.974077	0.05
19371	0.967673	0.661022
19372	1.22482	0.457778
19373	1.02305	0.166222
19374	1.22482	0.269238
19375	0.999492	2.1
19376	1.14657	2.1
19377	1.14657	2.1
19378	1.31346	2.1
19379	1.84417	0.05
19380	1.31777	0.05
19381	1.19796	0.05
19382	0.691742	0.05
19383	1.16725	2.1
19384	1.9679	2.1
19385	1.08848	0.05
19386	1.15356	0.05
19387	1.58713	2.1
19388	1.58742	2.1
19389	1.58742	2.1
19390	1.49662	2.1
19391	1.32201	0.50211
19392	1.32097	1.58715
19393	1.32097	0.523498
19394	1.31825	1.70297
19395	1.12028	2.1
19396	1.15852	2.1
19397	1.15852	2.1
19398	1.16239	2.1
19399	1.07624	2.1
19400	1.30047	2.1
19401	1.44847	1.12798
19402	1.55292	1.04408
19403	1.21125	0.05
19404	1.15262	2.1
19405	1.05025	2.1
19406	1.55351	2.1
19407	1.5329	0.05
19408	2.26758	0.05
19409	1.94968	0.05
19410	1.65747	0.05
19411	1.42549	0.05
19412	1.57656	0.05
19413	2.11157	0.484011
19414	1.56531	0.522263
19415	1.69279	0.342464
19416	1.06204	0.966115
19417	0.947863	1.38143
19418	1.19854	1.49974
19419	0.947862	0.426212
19420	1.31126	0.326869
19421	1.3425	1.66253
19422	1.29976	1.56103
19423	1.05129	0.0633388
19424	1.51026	0.0708858
19425	1.05129	0.0696138
19426	1.2951	0.0806892
19427	1.7155	2.1

19428	1.72196	2.1
19429	1.72196	2.1
19430	1.77867	2.1
19431	0.964755	2.1
19432	1.14142	2.1
19433	1.03766	2.1
19434	0.986723	2.1
19435	1.28072	2.1
19436	1.10609	2.1
19437	1.28072	1.5192
19438	1.16776	2.1
19439	1.61687	2.1
19440	1.60965	2.1
19441	1.60965	2.1
19442	1.59654	2.1
19443	1.26512	2.1
19444	1.23964	2.1
19445	2.07997	2.1
19446	2.60048	2.1
19447	1.23732	2.1
19448	1.3372	2.1
19449	2.62253	2.1
19450	2.47056	2.1
19451	0.92782	0.0818052
19452	1.40335	0.0971704
19453	1.26301	0.102897
19454	1.52668	0.104885
19455	0.99818	0.0800308
19456	1.51165	0.0940785
19457	1.66213	0.0794658
19458	1.55246	0.0695272
19459	2.31255	2.1
19460	1.34366	2.1
19461	1.90577	2.1
19462	0.969739	2.1
19463	3.4245	2.1
19464	2.8415	2.1
19465	1.97559	2.1
19466	1.82362	2.1
19467	1.48162	2.1
19468	1.23204	2.1
19469	1.23204	2.1
19470	0.95947	2.1
19471	1.05267	1.83019
19472	1.44091	1.43886
19473	1.05267	1.59677
19474	1.5795	1.83629
19475	0.738711	2.1
19476	1.28549	2.1
19477	1.15694	2.1
19478	1.72061	2.1
19479	1.69858	2.1
19480	0.954563	2.1
19481	0.859087	2.1
19482	0.788886	2.1
19483	1.08732	0.118326
19484	1.3357	0.10755
19485	1.3357	0.114245

19486	1.48676	0.0984029
19487	2.15079	2.1
19488	2.12414	2.1
19489	2.12414	2.1
19490	2.04664	2.1
19491	1.98859	2.1
19492	0.992159	2.1
19493	1.48064	2.1
19494	1.2163	2.1
19495	1.26337	2.1
19496	1.63159	2.1
19497	1.00366	0.439964
19498	1.65452	0.274813
19499	1.10408	2.1
19500	1.37704	2.1
19501	1.50224	2.1
19502	1.42866	2.1
19503	1.43939	2.1
19504	1.02784	2.1
19505	1.00763	0.0875685
19506	1.23746	0.0924081
19507	1.23747	0.0964975
19508	1.13642	0.110559
19509	1.49906	0.0530199
19510	1.46506	0.05
19511	1.46506	0.05
19512	1.33061	0.05
19513	1.08166	0.138332
19514	1.01052	0.125055
19515	1.02836	0.146371
19516	1.08166	0.136549
19517	1.25334	0.05
19518	1.23749	0.05
19519	1.37501	0.0510876
19520	1.47354	0.0778458
19521	1.30167	2.1
19522	1.29897	2.1
19523	1.51042	2.1
19524	1.07684	2.1
19525	1.07684	2.1
19526	0.765121	2.1
19527	1.5535	2.1
19528	1.40602	2.1
19529	1.40603	2.1
19530	1.25669	2.1
19531	1.08276	2.1
19532	1.60948	2.1
19533	1.26929	2.1
19534	1.09847	2.1
19535	0.827082	0.15892
19536	1.15841	0.153142
19537	1.25063	0.346951
19538	1.14397	0.33605
19539	1.25063	1.81717
19540	1.10476	0.202347
19541	1.14872	0.05
19542	1.3659	0.05
19543	1.36589	0.05

19544	1.4677	0.05
19545	0.590354	2.1
19546	0.655202	2.1
19547	3.48926	2.1
19548	3.32709	2.1
19549	3.32709	2.1
19550	3.13938	2.1
19551	1.24458	0.568247
19552	1.29681	0.625132
19553	1.40915	0.718791
19554	1.17891	1.59407
19555	1.33171	0.05
19556	1.37681	1.79356
19557	1.37681	2.1
19558	1.4516	2.1
19559	2.68241	0.05
19560	1.61031	0.05
19561	1.6488	2.1
19562	1.6585	2.1
19563	1.65848	2.1
19564	1.67061	2.1
19565	1.15152	2.1
19566	1.32548	2.1
19567	1.43677	2.1
19568	1.10264	2.1
19569	1.01472	2.1
19570	0.966964	2.1
19571	2.867	2.1
19572	2.94897	2.1
19573	1.65815	2.1
19574	1.4502	2.1
19575	1.4502	2.1
19576	1.26755	2.1
19577	1.37138	2.1
19578	1.45026	2.1
19579	1.45026	2.1
19580	1.53446	2.1
19581	0.973502	2.0846
19582	1.08542	2.1
19583	1.13387	0.05
19584	1.71031	0.05
19585	1.08911	2.1
19586	1.10748	2.1
19587	1.21823	2.1
19588	1.25005	2.1
19589	0.657014	2.1
19590	1.38139	2.1
19591	1.01485	2.1
19592	1.26045	2.1
19593	1.12468	2.1
19594	1.26045	2.1
19595	1.89278	0.481012
19596	1.06433	0.29695
19597	1.87861	0.328312
19598	0.967544	0.232805
19599	1.50319	2.1
19600	3.47245	2.1
19601	2.2354	0.753952

19602	1.16701	0.27381
19603	1.3592	2.1
19604	1.3305	1.76592
19605	1.3305	2.1
19606	1.15008	1.32103
19607	1.12457	0.05
19608	1.40707	0.05
19609	1.01778	2.1
19610	1.35733	2.1
19611	1.01779	2.1
19612	1.12064	2.1
19613	1.30694	0.714405
19614	1.42716	1.46004
19615	1.42717	1.53454
19616	1.04321	1.58301
19617	1.19352	0.1366
19618	1.32634	0.199747
19619	1.14605	1.5456
19620	1.0417	1.49908
19621	1.19676	0.0671066
19622	1.03804	0.077445
19623	2.09455	0.0809138
19624	1.15338	0.0889763
19625	2.21119	2.1
19626	1.26097	2.1
19627	1.88394	1.56984
19628	1.24106	0.664442
19629	2.98143	2.1
19630	2.56248	2.1
19631	1.88394	2.1
19632	1.68703	0.573052
19633	1.5846	2.1
19634	1.94799	2.1
19635	1.20091	2.1
19636	1.40393	2.1
19637	3.09031	2.1
19638	2.90243	2.1
19639	1.27227	2.1
19640	1.32592	2.1
19641	2.90247	2.1
19642	2.71057	2.1
19643	1.32594	2.1
19644	1.34817	2.1
19645	1.4092	2.1
19646	1.44859	2.1
19647	1.52008	2.1
19648	1.401	2.1
19649	1.40099	2.1
19650	1.28936	2.1
19651	1.56002	0.05
19652	1.08811	0.05
19653	1.46041	0.05
19654	1.56002	0.05
19655	1.50524	2.1
19656	1.21304	2.1
19657	1.21304	2.1
19658	0.947115	2.1
19659	1.60665	1.97959

19660	1.85179	1.77533
19661	1.85178	1.77433
19662	2.06277	1.2453
19663	2.17534	2.1
19664	1.23007	2.1
19665	2.17533	2.1
19666	1.66635	1.9651
19667	2.00869	2.1
19668	0.986724	2.1
19669	2.00475	2.1
19670	1.99394	2.1
19671	1.67244	0.119349
19672	1.39604	0.118013
19673	1.39604	0.126725
19674	0.954046	0.109586
19675	0.988863	2.1
19676	1.9864	2.1
19677	1.9864	2.1
19678	3.38645	2.1
19679	1.46932	0.05
19680	1.50847	0.05
19681	1.50848	0.05
19682	1.42705	0.05
19683	1.50108	2.1
19684	1.51621	0.689155
19685	1.94317	1.16007
19686	1.18382	0.143527
19687	2.05091	0.05
19688	1.88593	0.05
19689	1.54299	0.05
19690	0.987265	0.05
19691	1.27766	2.1
19692	1.5178	2.1
19693	1.5178	2.1
19694	1.81518	2.1
19695	1.04626	1.5955
19696	1.31057	1.63524
19697	1.31057	1.58577
19698	1.22297	1.68031
19699	1.67844	2.1
19700	1.62862	2.1
19701	1.62112	2.1
19702	1.87598	2.1
19703	1.17844	2.1
19704	1.37063	2.1
19705	1.37063	2.1
19706	1.68723	2.1
19707	1.99943	0.0611479
19708	1.17631	0.05
19709	1.4263	1.97982
19710	1.31023	2.1
19711	1.31023	2.1
19712	1.20871	2.1
19713	1.50356	2.1
19714	1.69311	2.1
19715	1.69599	2.1
19716	1.6801	2.1
19717	1.5843	0.05

19718	0.840233	0.05
19719	1.95358	0.05
19720	1.40216	0.05
19721	1.39078	2.1
19722	1.18948	2.1
19723	1.01606	1.89869
19724	1.18948	2.07938
19725	1.63823	2.1
19726	1.67687	2.1
19727	1.67687	2.1
19728	1.52855	2.1
19729	1.11048	2.1
19730	1.25346	2.1
19731	2.42984	1.44723
19732	3.41731	1.66836
19733	1.2258	2.1
19734	1.2966	2.1
19735	1.2966	2.1
19736	1.37392	2.1
19737	1.41758	2.1
19738	1.35504	2.1
19739	1.35504	2.1
19740	1.30347	2.1
19741	1.50877	2.1
19742	1.11193	2.1
19743	1.40984	2.1
19744	1.54537	2.1
19745	1.71638	2.1
19746	1.77795	2.05281
19747	2.07593	2.1
19748	1.93387	2.1
19749	1.9339	2.1
19750	1.75669	2.1
19751	1.80299	0.773364
19752	1.31251	0.396256
19753	1.31249	0.557758
19754	1.13411	0.292174
19755	1.06313	2.1
19756	1.04921	2.1
19757	1.55766	2.1
19758	1.53728	2.1
19759	1.53729	2.1
19760	1.51789	2.1
19761	0.993021	2.1
19762	1.02478	2.1
19763	1.03408	2.1
19764	1.09056	2.1
19765	0.697755	2.1
19766	0.632901	2.1
19767	1.34072	0.0953572
19768	1.80841	0.0868348
19769	1.80841	0.0881652
19770	1.17793	0.0765768
19771	1.75032	1.97759
19772	1.13076	2.1
19773	2.11851	2.1
19774	1.73255	2.05228
19775	1.73255	2.1

19776	1.11181	1.97103
19777	1.43772	2.1
19778	1.67381	2.1
19779	1.52164	2.1
19780	1.60861	2.1
19781	1.30588	0.05
19782	1.75241	0.05
19783	1.33026	1.84447
19784	0.962705	1.3439
19785	0.99678	0.05
19786	1.30572	0.05
19787	1.41943	0.05
19788	1.2683	0.05
19789	1.14145	0.05
19790	1.01361	0.05
19791	1.29943	2.1
19792	1.3483	2.1
19793	1.34832	2.1
19794	1.83488	2.1
19795	1.71065	2.1
19796	1.66178	2.1
19797	1.39076	2.1
19798	1.37456	2.1
19799	1.66126	2.1
19800	1.562	2.1
19801	1.37456	2.1
19802	1.36065	2.1
19803	1.04026	0.544569
19804	1.51898	0.441332
19805	1.67088	0.172911
19806	1.9726	0.111682
19807	1.74852	1.07783
19808	1.95512	1.42676
19809	1.16165	1.07891
19810	1.06584	0.878176
19811	1.39327	2.1
19812	1.41394	2.1
19813	1.41394	2.1
19814	1.45506	2.1
19815	1.39523	2.1
19816	1.21119	2.1
19817	1.09005	2.1
19818	0.921311	2.1
19819	1.58164	2.1
19820	1.77574	2.1
19821	1.73184	2.1
19822	1.59423	2.1
19823	1.8214	2.1
19824	3.10544	1.27989
19825	1.01243	1.78941
19826	1.30896	1.82312
19827	2.16956	1.44107
19828	1.29608	0.206749
19829	1.38617	1.78853
19830	1.49536	1.68162
19831	2.35239	1.25602
19832	1.24613	1.64655
19833	1.94351	2.1

19834	2.05361	2.1
19835	2.05358	2.1
19836	2.38994	2.1
19837	1.13533	2.1
19838	1.08276	2.1
19839	1.55611	0.108892
19840	1.33957	0.0967809
19841	1.33957	0.0761233
19842	1.69011	0.0846642
19843	1.5515	0.645889
19844	1.59106	0.577148
19845	1.02958	2.1
19846	1.12266	2.1
19847	1.12266	2.1
19848	1.24925	2.1
19849	1.80223	2.1
19850	1.20242	2.1
19851	2.13665	1.13591
19852	1.3924	1.10408
19853	0.904894	2.02193
19854	1.82633	2.1
19855	1.94138	0.05
19856	1.34009	0.05
19857	1.15096	2.1
19858	0.994292	2.1
19859	1.10101	2.1
19860	1.15096	2.1
19861	1.50438	2.1
19862	1.67825	2.1
19863	1.67825	2.1
19864	1.91894	2.1
19865	2.04664	2.1
19866	1.92063	2.1
19867	1.72857	2.1
19868	1.89757	2.1
19869	1.61843	0.05
19870	1.49431	0.05
19871	2.33925	2.1
19872	1.80683	2.1
19873	1.64257	2.1
19874	1.50108	2.1
19875	2.82561	2.1
19876	2.97847	2.1
19877	2.97848	2.1
19878	3.20034	2.1
19879	0.66055	2.1
19880	1.95919	2.1
19881	1.07976	2.1
19882	1.30237	2.1
19883	1.99943	0.05
19884	1.31802	0.05
19885	1.53336	0.0713002
19886	1.31802	0.0526667
19887	1.27737	1.15975
19888	1.44848	1.18087
19889	2.35499	0.05
19890	1.30041	0.05
19891	2.35499	0.05

19892	1.65381	0.05
19893	0.996083	2.1
19894	1.28754	2.1
19895	0.996081	2.1
19896	1.44859	2.1
19897	1.24763	2.1
19898	1.03697	2.1
19899	0.951273	1.66777
19900	1.60018	1.73778
19901	2.0815	2.1
19902	1.20488	2.1
19903	2.95785	2.1
19904	2.52245	2.1
19905	1.68455	0.05
19906	1.12971	0.05
19907	1.1952	0.118154
19908	2.0815	0.156896
19909	1.3963	2.1
19910	1.4934	2.1
19911	1.47973	2.1
19912	1.25559	2.1
19913	1.1233	2.1
19914	1.15009	2.1
19915	1.15009	2.1
19916	0.977775	2.1
19917	1.16482	1.26197
19918	1.29313	1.0489
19919	1.29313	1.14188
19920	1.294	0.940018
19921	1.48693	0.0958453
19922	1.47608	0.107008
19923	1.36005	1.97
19924	1.47109	2.1
19925	1.47109	2.1
19926	1.3515	2.1
19927	2.48828	2.1
19928	2.32378	2.1
19929	2.32378	2.1
19930	2.1692	2.1
19931	1.03217	0.713322
19932	0.953521	1.1709
19933	1.4515	0.05
19934	3.72406	0.05
19935	0.963395	2.00664
19936	1.01346	1.55774
19937	1.62241	2.1
19938	1.62027	2.1
19939	1.62027	2.1
19940	1.61687	2.1
19941	1.2593	0.05
19942	0.981586	0.05
19943	0.721961	2.1
19944	0.888955	2.1
19945	1.22031	0.05
19946	1.27566	2.1
19947	2.50066	2.1
19948	1.19814	2.1
19949	1.42278	0.05

19950	2.02657	0.05
19951	1.0756	0.339561
19952	1.10049	0.302489
19953	2.26155	2.1
19954	2.15634	2.1
19955	2.15634	2.1
19956	2.01613	2.1
19957	1.94233	1.796
19958	1.18127	1.30698
19959	1.36865	1.03878
19960	1.75755	0.805941
19961	2.01613	2.1
19962	1.78958	2.1
19963	1.97995	2.1
19964	1.79198	2.1
19965	1.20841	2.1
19966	1.6519	2.1
19967	1.80207	2.1
19968	1.66774	2.1
19969	1.17935	0.05
19970	1.88071	0.05
19971	1.58723	0.05
19972	1.7361	0.05
19973	0.794165	2.1
19974	1.21327	2.1
19975	1.21327	2.1
19976	1.34268	2.1
19977	1.34497	0.05
19978	0.793303	0.05
19979	1.12323	0.05
19980	1.34496	0.05
19981	0.928226	0.060628
19982	1.89804	0.100052
19983	2.60141	0.05
19984	2.60142	2.1
19985	2.60772	2.1
19986	0.99747	2.1
19987	1.27096	1.27395
19988	1.27096	0.849896
19989	1.03472	1.22763
19990	1.09962	2.1
19991	1.21148	2.1
19992	1.21148	2.1
19993	1.35025	2.1
19994	1.09795	2.1
19995	1.00775	2.1
19996	1.07162	2.1
19997	1.60665	1.97903
19998	1.52984	2.1
19999	1.61179	2.1
20000	1.70057	2.1
20001	1.76063	2.1
20002	1.14547	0.257581
20003	0.964782	0.232131
20004	1.17208	0.232774
20005	0.964769	0.207069
20006	1.04268	2.1
20007	1.57459	2.1

20008	1.57459	2.1
20009	1.32449	2.1
20010	2.07527	1.26654
20011	1.00013	1.25501
20012	0.813711	0.05
20013	1.05677	2.1
20014	1.30664	1.74917
20015	1.96011	1.89275
20016	1.7151	1.60793
20017	1.61309	1.65288
20018	1.18954	2.1
20019	1.05447	2.1
20020	1.05446	0.635228
20021	1.11787	0.615691
20022	1.44437	2.1
20023	1.84739	2.1
20024	1.84739	2.1
20025	1.06203	2.1
20026	1.78391	2.1
20027	1.89267	2.1
20028	1.9212	2.1
20029	2.02997	2.1
20030	1.32051	0.204333
20031	1.47169	0.11437
20032	1.20691	0.05
20033	1.72271	0.05
20034	1.2858	0.167872
20035	1.31011	0.983578
20036	1.31011	0.115009
20037	1.34124	1.19929
20038	1.294	1.15322
20039	1.03079	0.889964
20040	1.83192	1.97904
20041	1.81979	2.07571
20042	1.81979	2.04709
20043	1.57829	2.1
20044	1.35435	1.80107
20045	1.19023	1.92404
20046	0.991115	2.1
20047	1.27725	2.1
20048	1.38204	2.1
20049	1.48013	2.1
20050	0.983493	2.1
20051	2.26155	2.1
20052	1.02311	0.594227
20053	1.55485	0.469743
20054	1.71033	0.431111
20055	2.07319	0.364399
20056	2.29074	0.856281
20057	1.50822	1.70371
20058	2.08147	2.1
20059	1.1585	2.1
20060	3.74092	1.92714
20061	2.40799	2.1
20062	2.05349	2.1
20063	1.92656	2.1
20064	1.09634	0.137239
20065	1.45502	0.175401

20066	1.17261	0.0812957
20067	1.29383	0.0815051
20068	1.29383	0.0523535
20069	1.47689	0.0725784
20070	1.85717	0.581521
20071	1.53798	0.251677
20072	1.9493	2.1
20073	2.0371	2.09877
20074	1.8519	2.1
20075	1.92119	1.89312
20076	1.00764	0.102798
20077	1.0302	0.0972183
20078	1.0302	0.0887123
20079	0.90047	0.0911725
20080	1.49155	0.149773
20081	2.26403	0.0901502
20082	2.06158	0.05
20083	1.09506	0.05
20084	1.9212	1.85629
20085	1.99586	1.65381
20086	1.99586	1.72056
20087	2.07527	1.5186
20088	1.20478	2.1
20089	1.09662	2.1
20090	1.55961	2.1
20091	1.03472	2.1
20092	1.54983	2.1
20093	2.02953	2.1
20094	1.84502	2.1
20095	2.1221	2.1
20096	1.02969	1.99213
20097	2.23523	2.1
20098	1.2377	0.05
20099	1.03818	0.0762413
20100	1.978	2.1
20101	1.78159	2.1
20102	1.45762	2.1
20103	1.34234	2.1
20104	2.01624	2.1
20105	2.52072	2.1
20106	1.23013	2.1
20107	2.5593	2.1
20108	3.06464	1.36947
20109	2.63697	1.17808
20110	2.63697	1.3093
20111	1.87111	1.1309
20112	1.04574	0.05
20113	2.13428	0.05
20114	1.61715	2.1
20115	1.44137	2.1
20116	1.44867	2.1
20117	1.18283	2.1
20118	1.50584	0.05
20119	1.45466	0.0515568
20120	1.4959	0.05
20121	1.93489	0.05
20122	2.60772	2.1
20123	2.68868	2.1

20124	2.68868	2.1
20125	2.82561	2.1
20126	1.49654	2.1
20127	1.38672	2.1
20128	1.36813	2.1
20129	1.98396	2.1
20130	1.93852	1.47919
20131	2.14237	1.21314
20132	1.44421	0.05
20133	1.21749	0.05
20134	1.09572	0.05
20135	1.11081	0.05
20136	2.32363	2.1
20137	1.24375	2.1
20138	3.79242	2.1
20139	3.46787	2.1
20140	1.19854	1.58814
20141	1.35487	1.57146
20142	1.35486	1.67949
20143	1.38616	1.66107
20144	1.67265	0.163495
20145	0.909487	0.13651
20146	2.53562	2.1
20147	1.09564	2.1
20148	2.9068	2.1
20149	3.57307	0.660207
20150	2.50369	2.00825
20151	1.47348	2.1
20152	1.24925	2.1
20153	1.3991	2.1
20154	1.3991	2.1
20155	1.52984	2.1
20156	1.60428	2.1
20157	1.50097	2.1
20158	1.50097	2.1
20159	1.40239	2.1
20160	2.19621	2.1
20161	2.35003	2.1
20162	1.57932	0.05
20163	1.61194	0.05
20164	2.0815	0.05
20165	1.18539	0.05
20166	1.5795	1.4571
20167	1.53064	1.55907
20168	1.89227	0.05
20169	3.01037	0.05
20170	2.08149	2.06786
20171	2.06678	1.92458
20172	1.89226	0.05
20173	3.47888	0.05
20174	1.89226	0.0742954
20175	3.65842	0.05
20176	3.88546	0.143412
20177	1.89226	0.243204
20178	1.11239	2.1
20179	1.29133	0.05
20180	0.889309	0.05
20181	0.881934	0.05

20182	1.88406	2.1
20183	0.636946	2.1
20184	0.636945	2.1
20185	1.28073	2.1
20186	1.28075	2.1
20187	0.942007	2.1
20188	1.68436	2.1
20189	1.4677	0.05
20190	0.851542	0.05
20191	1.39063	0.05
20192	0.851539	0.05
20193	1.11597	0.05
20194	1.35403	0.05
20195	0.959473	2.1
20196	1.07976	2.1
20197	0.946129	2.1
20198	0.946129	0.330618
20199	1.38464	1.90042
20200	1.36991	0.745069
20201	0.946131	0.909691
20202	0.94613	1.58334
20203	1.34493	0.359535
20204	1.36991	0.254929
20205	1.36991	0.116491
20206	0.946131	0.211133
20207	0.78392	0.511909
20208	0.80282	0.372962
20209	1.10351	0.487051
20210	0.80846	0.480662
20211	0.838026	0.616907
20212	1.08558	0.413153
20213	0.718203	0.528613
20214	0.501866	0.0610623
20215	0.85426	0.445813
20216	1.24249	0.05
20217	1.37052	0.0726253
20218	0.851541	0.0509576
20219	1.0092	0.05
20220	0.851554	0.0821139
20221	1.49826	0.214853
20222	1.13727	0.129387
20223	0.778149	0.194519
20224	1.20257	0.240071
20225	0.946133	0.189981
20226	0.778149	0.179688
20227	0.77815	0.337486
20228	0.946132	2.1
20229	1.17602	2.1
20230	0.946132	2.1
20231	1.31257	2.1
20232	1.17602	2.1
20233	1.35821	0.0800525
20234	1.19993	0.05
20235	0.946131	0.05
20236	0.946131	0.0780777
20237	1.08848	0.127445
20238	1.00509	2.1
20239	1.19629	2.1

20240	0.867593	2.1
20241	0.757921	2.1
20242	0.729116	0.05
20243	0.89162	0.05
20244	1.16811	0.05
20245	1.3676	2.1
20246	1.2638	2.1
20247	0.946132	2.1
20248	0.946132	2.1
20249	1.2638	2.1
20250	1.00185	2.1
20251	1.60948	2.1
20252	0.602075	2.1
20253	0.942611	2.1
20254	0.946132	2.1
20255	0.942611	2.1
20256	1.11822	2.1
20257	1.21558	0.12514
20258	0.607788	0.200576
20259	0.827029	0.197762
20260	1.00691	0.405111
20261	0.607789	0.189588
20262	0.607775	0.320828
20263	1.23248	1.73651
20264	0.902653	1.95979
20265	0.639143	1.95135
20266	0.851545	1.7418
20267	0.605153	1.84459
20268	1.06826	2.1
20269	1.29365	2.1
20270	1.41232	2.1
20271	0.946131	2.1
20272	1.20854	2.1
20273	0.946131	2.1
20274	0.909005	0.05
20275	1.16672	0.05
20276	0.810959	0.05
20277	0.946131	0.05
20278	1.40104	0.05
20279	0.946131	0.05
20280	1.35403	0.05
20281	1.40104	0.05
20282	1.92535	0.05
20283	2.14534	0.05
20284	1.77532	0.05
20285	0.851541	0.05
20286	1.05585	0.052078
20287	1.27098	0.05
20288	0.870925	0.0526237
20289	1.15547	0.0526999
20290	1.47583	0.05
20291	1.35007	0.05
20292	0.740095	0.05
20293	0.644912	0.05
20294	0.819489	0.05
20295	0.983028	0.128949
20296	0.861849	0.05
20297	1.40511	0.05

20298	0.86182	0.05
20299	0.9705	0.05
20300	1.40511	0.05
20301	1.15539	2.1
20302	0.946119	2.1
20303	0.736382	2.1
20304	0.736384	2.1
20305	1.41561	2.1
20306	0.785651	2.1
20307	1.34479	2.1
20308	1.38885	2.1
20309	0.94613	2.1
20310	1.29461	2.1
20311	0.94613	2.1
20312	0.776179	2.1
20313	0.858611	2.1
20314	0.946128	2.1
20315	1.04073	2.1
20316	1.16399	2.1
20317	0.990019	2.1
20318	1.15564	2.08075
20319	1.28956	0.0689237
20320	1.35145	0.0958126
20321	1.09662	2.1
20322	1.8155	0.05
20323	0.811459	0.05
20324	0.731982	0.05
20325	0.900554	0.05
20326	0.946127	1.09143
20327	0.817066	1.29049
20328	0.817064	1.11983
20329	1.41798	1.07989
20330	0.871606	1.11209
20331	1.30265	0.05
20332	0.849402	1.73228
20333	0.868582	1.5956
20334	0.909137	1.60686
20335	0.807016	1.51624
20336	0.83393	0.613278
20337	0.9034	1.12938
20338	1.53515	1.05987
20339	1.17665	0.270317
20340	0.946132	0.413109
20341	0.946129	0.244871
20342	1.35977	0.227266
20343	1.34254	0.215943
20344	1.14241	0.17543
20345	0.946132	0.261759
20346	0.946132	0.169554
20347	1.27376	0.16713
20348	1.35978	0.155878
20349	1.01976	0.157166
20350	1.23498	0.97034
20351	1.35002	1.43083
20352	1.11683	1.45497
20353	0.88359	0.143642
20354	0.751374	1.34211
20355	0.791714	0.424846

20356	1.6067	2.1
20357	0.923782	2.1
20358	1.44689	2.1
20359	0.94613	2.1
20360	0.946131	2.1
20361	1.5261	2.1
20362	1.26247	2.1
20363	1.80085	0.05
20364	1.7693	0.052466
20365	0.900422	0.05
20366	1.31357	0.0595347
20367	0.94613	0.05
20368	0.900424	0.0555901
20369	0.900397	0.0621438
20370	1.39448	0.05
20371	1.36769	0.05
20372	0.713524	0.05
20373	0.713554	0.05
20374	0.946124	0.05
20375	1.24979	0.05
20376	1.04765	0.05
20377	0.70054	0.05
20378	0.776991	0.05
20379	0.738905	0.05
20380	1.32605	0.05
20381	0.784957	0.05
20382	0.803955	0.05
20383	0.819486	0.05
20384	1.79306	0.05
20385	1.18275	0.05
20386	1.25626	0.05
20387	0.936806	0.05
20388	0.653124	0.05
20389	0.946132	0.05
20390	0.653151	0.05
20391	1.10748	0.0801922
20392	1.95244	2.1
20393	1.40967	1.57094
20394	1.27916	1.54706
20395	0.946132	1.81773
20396	0.946132	2.1
20397	1.30555	2.1
20398	1.38651	2.1
20399	0.946132	2.1
20400	1.23571	0.485994
20401	1.21261	1.23515
20402	1.34225	1.27128
20403	0.946132	1.57336
20404	0.734059	0.584479
20405	0.734084	1.03629
20406	0.78438	0.274153
20407	1.13748	0.495205
20408	0.946131	0.723805
20409	0.78438	0.277854
20410	0.784392	0.421055
20411	0.626495	2.1
20412	0.694635	2.1
20413	0.946125	2.1

20414	0.694632	2.1
20415	1.38521	2.1
20416	0.795469	2.1
20417	0.850877	2.1
20418	1.37098	2.1
20419	0.946131	2.1
20420	0.79547	2.1
20421	0.946132	0.426435
20422	1.30448	0.295833
20423	0.628361	0.0757946
20424	0.686964	0.424608
20425	0.493261	0.283075
20426	1.90912	0.594473
20427	0.946127	0.472769
20428	1.36604	0.0998468
20429	0.671687	0.0687032
20430	0.946128	0.947943
20431	0.671699	0.334219
20432	1.04973	2.1
20433	0.982858	0.152486
20434	0.974081	0.05
20435	1.69993	0.05
20436	1.00763	0.129288
20437	0.755566	2.1
20438	0.946129	2.1
20439	1.04184	2.1
20440	1.13509	2.1
20441	0.755566	2.1
20442	1.114	2.1
20443	0.810946	2.1
20444	0.786259	2.1
20445	1.43455	2.1
20446	1.05071	2.1
20447	0.746095	2.1
20448	0.82737	2.1
20449	0.946126	2.1
20450	1.04071	2.1
20451	1.0852	2.1
20452	1.59019	0.05
20453	0.621972	0.05
20454	1.14475	0.05
20455	0.691062	0.05
20456	1.20057	0.05
20457	0.846868	0.05
20458	1.26062	0.05
20459	0.795093	0.05
20460	1.20056	0.05
20461	0.795065	0.05
20462	1.18203	0.05
20463	1.20057	0.05
20464	0.991869	0.0740841
20465	0.841088	2.1
20466	0.946131	2.1
20467	0.869097	2.1
20468	0.946131	2.1
20469	0.841089	2.1
20470	1.77867	2.1
20471	1.99296	0.129401

20472	1.91703	2.1
20473	0.651334	0.05
20474	1.31467	0.05
20475	0.651332	0.05
20476	0.651332	0.05
20477	1.03079	0.05
20478	0.808519	0.05
20479	1.41417	0.05
20480	1.03165	0.135826
20481	1.43577	2.1
20482	1.39053	2.1
20483	1.07006	2.1
20484	0.946132	2.1
20485	0.946133	2.1
20486	1.07006	2.1
20487	1.12717	2.1
20488	0.994293	2.1
20489	0.946132	2.1
20490	1.31912	2.1
20491	0.946131	2.1
20492	1.40571	2.1
20493	1.31912	2.1
20494	1.12292	0.238685
20495	1.07755	0.292479
20496	0.851539	0.244502
20497	0.946131	0.234094
20498	0.929834	0.244983
20499	1.83919	0.390147
20500	1.04713	0.0982524
20501	0.929726	0.0591188
20502	1.50985	0.0583795
20503	0.946127	0.0539481
20504	1.0227	0.0601552
20505	1.2582	0.0560676
20506	1.1391	0.352566
20507	0.780831	1.03821
20508	0.78086	0.371938
20509	0.946133	0.654611
20510	0.780831	0.420512
20511	1.37762	0.421934
20512	0.946116	2.1
20513	1.22882	2.1
20514	1.40015	2.1
20515	1.40017	2.1
20516	0.946105	2.1
20517	0.553567	0.05
20518	1.19263	0.05
20519	0.946132	0.05
20520	0.553594	0.05
20521	1.19263	0.05
20522	1.18237	0.05
20523	0.946133	0.05
20524	0.978467	0.05
20525	0.56262	0.05
20526	0.946132	0.05
20527	0.562633	0.05
20528	0.900175	0.05
20529	1.04627	1.7744

20530	0.549928	1.19096
20531	1.17765	1.24307
20532	1.11479	1.30443
20533	0.398261	1.37723
20534	0.927608	1.24269
20535	1.38467	1.17891
20536	1.04824	1.05053
20537	1.33893	1.14917
20538	1.55775	1.11077
20539	0.816895	1.10748
20540	1.96057	1.25945
20541	1.35798	2.1
20542	1.29262	2.1
20543	0.94613	2.1
20544	0.94613	2.1
20545	1.29261	2.1
20546	1.18067	2.1
20547	0.946131	2.1
20548	0.946132	2.1
20549	1.27809	2.1
20550	1.35799	2.1
20551	1.03933	2.1
20552	1.49438	2.1
20553	0.946132	2.1
20554	1.13537	2.1
20555	1.41666	2.1
20556	1.74237	2.1
20557	0.946132	2.1
20558	1.04291	0.58057
20559	1.63802	2.1
20560	1.24714	2.1
20561	0.842013	2.1
20562	0.810959	2.1
20563	0.767923	0.107599
20564	1.19583	0.103589
20565	0.767921	0.0868161
20566	0.767921	0.0645902
20567	0.961348	0.110055
20568	2.08687	1.82267
20569	0.739729	1.30778
20570	1.04342	1.27266
20571	0.739757	1.25922
20572	0.946129	1.31952
20573	1.04341	1.48111
20574	1.38683	0.137787
20575	1.34153	0.116967
20576	0.946129	0.140798
20577	0.946131	0.14471
20578	1.02028	0.139176
20579	1.47606	0.0875003
20580	1.20739	0.113734
20581	0.957684	0.0863638
20582	0.946116	0.117685
20583	0.851528	0.138214
20584	0.957685	0.130921
20585	1.04504	2.1
20586	0.902938	2.1
20587	0.946132	2.1

20588	1.07566	2.1
20589	1.4558	2.1
20590	1.93286	2.1
20591	1.27241	2.1
20592	0.828671	2.1
20593	0.810933	2.1
20594	1.31335	0.05
20595	1.40305	0.05
20596	0.912698	0.05
20597	0.810946	0.05
20598	1.11608	0.05
20599	1.26409	0.86804
20600	0.946132	0.154195
20601	1.27376	0.156357
20602	0.946132	0.151756
20603	1.03333	0.158591
20604	1.27376	0.151898
20605	0.94613	0.245972
20606	0.812384	0.213384
20607	0.687744	0.174417
20608	0.946132	0.241416
20609	0.687758	0.210999
20610	1.09667	0.159342
20611	1.56977	2.1
20612	0.851544	2.1
20613	1.01136	2.1
20614	0.851481	2.1
20615	0.946131	2.1
20616	0.705409	2.1
20617	1.32449	0.05
20618	1.13508	0.05
20619	0.94613	0.05
20620	0.94613	0.05
20621	0.965822	0.05
20622	1.95301	0.131035
20623	0.851538	0.355681
20624	1.29262	0.31125
20625	0.95417	0.236976
20626	0.851541	0.370713
20627	0.94613	0.319638
20628	1.02727	1.08622
20629	1.37675	1.2059
20630	0.955646	0.845407
20631	0.95114	1.20529
20632	0.851528	1.16522
20633	1.76513	1.2901
20634	0.87155	2.1
20635	0.94613	2.1
20636	0.871519	2.1
20637	0.966631	2.1
20638	1.37791	2.1
20639	1.19122	0.365276
20640	0.818971	0.847174
20641	0.628799	0.414857
20642	0.861027	0.715769
20643	0.800034	0.05
20644	0.91846	0.05
20645	0.765645	0.05

20646	1.13709	0.732841
20647	1.29054	0.960531
20648	0.755336	0.985579
20649	0.755334	0.647159
20650	0.946133	1.2952
20651	0.755336	0.436198
20652	1.14444	0.924668
20653	0.872823	0.897095
20654	0.533645	0.725683
20655	0.946132	0.685792
20656	0.533645	0.414636
20657	1.02311	0.426858
20658	1.00278	2.1
20659	1.1016	0.05
20660	0.629615	0.05
20661	1.12628	0.05
20662	0.946131	0.05
20663	0.629615	0.05
20664	0.629641	0.05
20665	1.67046	1.56845
20666	1.81667	2.1
20667	1.7145	2.1
20668	0.94613	2.1
20669	1.29322	2.1
20670	1.00365	2.1
20671	0.946131	2.1
20672	1.29322	2.1
20673	1.27581	2.1
20674	1.33916	2.1
20675	1.32784	2.1
20676	1.74787	2.1
20677	2.28084	2.1
20678	2.98092	2.1
20679	0.946132	2.0647
20680	1.2435	2.1
20681	0.946132	2.1
20682	1.4099	2.1
20683	1.2435	2.1
20684	1.26438	2.1
20685	0.745287	2.1
20686	0.696827	2.1
20687	0.696781	2.1
20688	1.41195	2.1
20689	0.946131	2.1
20690	1.8758	1.60675
20691	0.832979	0.732062
20692	0.75054	0.527426
20693	0.810958	0.486129
20694	0.922889	1.17409
20695	0.694844	0.258241
20696	0.694814	0.851881
20697	0.946131	0.395212
20698	0.694844	0.452723
20699	1.02369	2.1
20700	1.4468	0.05
20701	0.946125	0.128409
20702	0.93487	0.0924812
20703	1.23608	0.146348

20704	1.34257	0.0724261
20705	0.851525	0.0968955
20706	1.03298	0.0543775
20707	1.22418	2.1
20708	0.946131	2.06616
20709	1.41833	1.9006
20710	1.23452	1.51783
20711	0.946131	0.611976
20712	1.41833	1.67568
20713	1.13077	0.189921
20714	0.942874	1.81159
20715	0.946132	1.77281
20716	0.504366	1.77833
20717	0.593647	2.1
20718	0.898829	1.77859
20719	1.27921	2.07023
20720	1.12503	2.1
20721	1.22539	2.1
20722	1.10332	2.1
20723	0.851541	2.1
20724	0.94613	2.1
20725	0.945109	2.1
20726	0.607962	2.1
20727	0.946133	2.1
20728	0.607961	2.1
20729	0.661956	2.1
20730	1.35048	2.1
20731	1.66018	2.1
20732	0.956467	2.1
20733	0.851542	2.1
20734	0.890734	2.1
20735	0.802455	2.1
20736	1.35943	2.1
20737	1.04798	2.1
20738	1.26426	0.191122
20739	1.3689	0.114271
20740	0.946133	0.151294
20741	0.946133	0.196068
20742	1.09729	0.172392
20743	0.765259	2.1
20744	0.810945	2.1
20745	0.810957	2.1
20746	1.10284	2.1
20747	1.09368	2.1
20748	0.772601	2.1
20749	0.788011	2.1
20750	0.810934	2.1
20751	0.694357	1.41347
20752	1.22641	1.40075
20753	0.946132	1.64366
20754	0.946132	1.17303
20755	0.946133	1.28466
20756	1.42754	1.5419
20757	0.776414	1.28075
20758	0.806525	1.37039
20759	0.575339	1.50961
20760	0.97154	1.24661
20761	0.8217	1.3434

20762	0.838039	1.29531
20763	0.94613	0.839733
20764	0.946132	0.484045
20765	1.39575	0.272052
20766	1.39575	0.382208
20767	1.12818	0.456328
20768	0.946132	1.38762
20769	0.946133	0.792075
20770	1.06879	0.367944
20771	1.06879	0.594093
20772	0.970358	0.603105
20773	1.2474	0.175503
20774	1.01919	0.05
20775	0.94613	0.0565585
20776	1.28952	0.05
20777	1.00809	0.05
20778	0.849344	0.05
20779	0.640304	0.0620213
20780	1.35065	0.05
20781	0.946132	0.05
20782	0.640333	0.05
20783	0.946132	0.0510533
20784	0.685489	2.1
20785	0.94613	2.1
20786	0.68546	2.1
20787	0.874264	2.1
20788	1.27676	2.1
20789	0.946129	0.0870537
20790	1.09024	0.0709105
20791	0.714563	0.0678377
20792	1.12335	0.0736254
20793	1.07931	0.0710722
20794	1.17799	0.05
20795	1.8795	0.154312
20796	2.15229	0.160206
20797	1.13859	0.408711
20798	0.946132	1.20994
20799	1.11374	0.441208
20800	1.1386	0.580161
20801	0.615713	0.568027
20802	1.19322	0.215296
20803	1.3377	0.167974
20804	0.670801	0.215252
20805	0.94613	0.669616
20806	0.66617	0.39843
20807	1.36173	0.370029
20808	0.980473	0.345562
20809	1.39132	0.205865
20810	0.864653	0.05
20811	1.23837	0.05
20812	0.810959	0.05
20813	1.1483	1.67492
20814	1.00381	1.87971
20815	0.946128	1.72471
20816	1.39696	1.49822
20817	0.946131	1.361
20818	0.946131	1.43036
20819	1.38613	1.64831

20820	0.973501	1.79005
20821	0.946127	1.66923
20822	1.35968	1.7003
20823	0.946131	0.715975
20824	0.946129	1.81061
20825	1.39696	1.77058
20826	1.12088	2.08561
20827	1.41122	2.1
20828	1.14464	0.584593
20829	0.946131	2.1
20830	0.946131	1.95877
20831	1.14464	2.04504
20832	1.33266	1.67861
20833	1.07341	0.969395
20834	0.827287	1.71415
20835	0.946132	1.29867
20836	0.827287	1.72549
20837	1.15644	1.76015
20838	1.27558	0.595372
20839	1.15067	2.1
20840	0.946131	2.1
20841	1.29143	2.05305
20842	1.36281	2.1
20843	1.36281	2.1
20844	0.946131	2.1
20845	1.40863	2.1
20846	1.20143	0.05
20847	1.19755	0.05
20848	0.566153	0.0572001
20849	0.946131	0.113447
20850	1.07979	0.05
20851	1.00463	0.0952136
20852	0.566153	0.145526
20853	0.768212	2.1
20854	1.32461	2.1
20855	1.13268	2.1
20856	1.13268	2.1
20857	0.946133	2.1
20858	1.53642	2.1
20859	1.5791	2.1
20860	0.726635	2.1
20861	1.2334	2.1
20862	1.29198	2.1
20863	1.29198	2.03
20864	0.946131	1.93491
20865	0.956585	2.1
20866	1.25294	2.1
20867	0.946133	2.1
20868	1.35287	2.1
20869	1.23877	2.1
20870	0.946131	2.1
20871	1.35287	2.1
20872	1.01843	2.1
20873	3.77569	2.1
20874	0.86707	2.1
20875	0.867066	2.1
20876	1.38639	2.1
20877	1.38639	2.1

20878	0.896168	2.1
20879	0.974716	0.05
20880	1.09411	0.0743534
20881	0.663171	0.0541396
20882	0.663171	0.0873178
20883	0.946132	0.0819532
20884	0.66317	0.0655937
20885	1.47104	0.05
20886	1.13629	0.0534375
20887	0.772342	0.05
20888	0.772312	0.0606234
20889	0.94613	0.0562872
20890	0.77234	0.0565941
20891	1.43154	2.1
20892	1.37704	2.1
20893	1.40025	2.1
20894	1.3063	2.1
20895	0.946132	2.1
20896	1.04075	2.1
20897	0.946131	2.1
20898	0.946118	2.1
20899	0.750011	1.68654
20900	1.0971	1.99002
20901	0.889667	1.87343
20902	0.709598	1.98716
20903	2.39737	2.1
20904	1.19974	0.0810613
20905	0.94613	0.166375
20906	1.25809	0.070835
20907	1.19974	0.139541
20908	0.73005	0.243426
20909	1.50764	0.05
20910	1.25809	0.0907198
20911	0.946133	0.178737
20912	1.10357	0.106514
20913	1.25809	0.158923
20914	0.628177	0.237842
20915	1.3543	0.05
20916	0.793711	0.05
20917	1.18455	0.05
20918	1.03966	0.05
20919	0.946131	0.05
20920	0.767453	0.05
20921	0.612292	0.05
20922	0.946131	0.05
20923	1.24128	0.05
20924	1.08761	0.05
20925	0.762059	0.05
20926	1.005 2.1	
20927	1.17054	2.1
20928	1.12665	2.1
20929	0.750368	2.1
20930	0.851542	2.1
20931	0.975436	2.1
20932	0.975442	2.1
20933	0.675367	2.1
20934	1.51973	0.0789414
20935	0.94613	0.0990658

20936	1.38954	0.103172
20937	0.946131	0.0967374
20938	1.07912	0.103222
20939	1.38954	0.0959293
20940	2.0219	2.1
20941	1.18347	0.0943068
20942	0.946132	0.509914
20943	0.94613	0.230734
20944	1.00202	0.157774
20945	1.00201	0.179956
20946	0.946132	0.224099
20947	0.946132	0.694037
20948	1.37981	0.318381
20949	1.26874	0.246211
20950	1.26874	0.273297
20951	0.946132	0.30199
20952	2.8687	0.134636
20953	1.42884	2.1
20954	1.05188	2.1
20955	0.625307	2.1
20956	0.810956	2.1
20957	0.526656	2.1
20958	0.526684	2.1
20959	0.724122	2.1
20960	0.946132	2.1
20961	0.526684	2.1
20962	1.05337	2.1
20963	1.0593	2.1
20964	1.47981	2.1
20965	1.74431	1.9683
20966	0.885612	1.9583
20967	0.810946	1.69123
20968	0.845185	1.7621
20969	1.38622	0.602787
20970	0.723194	0.65811
20971	0.554302	0.939013
20972	0.709597	0.592966
20973	0.909507	0.599413
20974	0.912754	0.220408
20975	1.15174	0.171188
20976	0.946131	0.22625
20977	0.912753	0.213315
20978	1.15174	0.201773
20979	1.02011	0.133971
20980	0.792754	1.96406
20981	0.946129	2.1
20982	0.792754	1.94434
20983	1.27575	1.92629
20984	0.94613	1.94601
20985	1.60132	2.06347
20986	0.922455	1.96806
20987	1.18713	1.76537
20988	0.926957	1.80494
20989	1.66525	1.90149
20990	0.946132	2.1
20991	0.851541	2.1
20992	0.961191	2.1
20993	1.27534	2.1

20994	0.851554	2.1
20995	1.0499	2.1
20996	1.01776	0.05
20997	0.97622	0.05
20998	0.810934	0.05
20999	2.6802	0.05
21000	1.31485	0.05
21001	1.09699	0.05
21002	0.681649	0.05
21003	0.790369	0.05
21004	0.946131	0.05
21005	0.838026	1.52039
21006	0.567833	0.730131
21007	0.891492	1.26453
21008	0.986984	0.405766
21009	0.946132	1.3266
21010	1.19028	0.441465
21011	0.986984	0.499164
21012	0.595623	0.590372
21013	0.946131	2.1
21014	1.36502	2.1
21015	1.08109	2.1
21016	0.946129	2.1
21017	1.36503	2.1
21018	0.773897	2.1
21019	0.946133	1.91655
21020	0.613447	2.1
21021	0.613449	2.1
21022	0.946131	2.1
21023	1.11163	2.08026
21024	0.966114	2.1
21025	0.735699	2.1
21026	0.662147	2.1
21027	1.04072	2.1
21028	0.946131	2.1
21029	0.862978	2.1
21030	1.48839	2.00067
21031	0.936141	2.05187
21032	0.946131	2.1
21033	0.936142	2.1
21034	1.40595	2.1
21035	0.946131	2.1
21036	1.05297	0.0503715
21037	3.09035	2.1
21038	1.19952	2.1
21039	0.907198	2.1
21040	0.849144	2.1
21041	0.612469	0.721413
21042	0.551237	0.517018
21043	0.985256	1.09628
21044	0.551209	0.615047
21045	0.85153	1.13981
21046	0.624712	2.1
21047	0.900018	2.1
21048	0.851542	2.1
21049	0.864787	2.1
21050	1.27669	2.1
21051	0.946711	0.546276

21052	0.810947	0.776088
21053	0.841547	0.511686
21054	0.764114	0.732098
21055	1.26692	0.825505
21056	0.946132	0.787537
21057	1.37675	0.445685
21058	1.26692	0.659966
21059	1.37545	0.741779
21060	1.49637	0.11649
21061	0.709598	0.170737
21062	0.751252	0.162326
21063	1.36811	0.153306
21064	0.635625	0.178642
21065	1.31713	0.0606731
21066	1.18977	2.1
21067	0.893377	0.933094
21068	0.707344	2.1
21069	0.946131	2.1
21070	1.20221	2.1
21071	1.20221	2.1
21072	0.707345	2.1
21073	1.26786	2.1
21074	0.988048	2.1
21075	0.751293	2.1
21076	0.632008	2.1
21077	1.51067	2.1
21078	1.13534	2.1
21079	0.946133	2.1
21080	0.946131	2.1
21081	1.25716	2.1
21082	1.3199	2.1
21083	1.09616	2.1
21084	1.36677	1.96175
21085	1.34752	2.0897
21086	0.838041	1.92927
21087	1.67023	2.1
21088	1.02247	0.331393
21089	1.03079	0.34798
21090	0.928982	0.0981556
21091	1.13635	0.0689901
21092	0.946132	0.0955168
21093	0.92898	0.0772361
21094	1.13635	0.0860308
21095	1.1643	0.0540314
21096	0.851541	0.229895
21097	1.39939	0.183348
21098	0.946129	0.237917
21099	1.07775	0.199586
21100	1.14503	0.201992
21101	1.78573	0.13253
21102	1.29827	0.132954
21103	0.872373	0.05
21104	1.01162	0.05
21105	0.83804	0.05
21106	1.48851	0.05
21107	1.47385	0.05
21108	0.843858	0.05
21109	0.938626	0.05

21110	0.89567	0.05
21111	1.59457	1.0384
21112	2.09666	0.12659
21113	0.775132	0.05
21114	0.927005	0.05
21115	0.854224	0.05
21116	0.775142	0.05
21117	0.946106	0.05
21118	1.80784	2.1
21119	2.04593	2.1
21120	1.32282	2.1
21121	0.952138	2.1
21122	0.946132	2.1
21123	0.946132	2.1
21124	0.952138	2.1
21125	1.46988	2.1
21126	0.80465	0.05
21127	1.14155	0.05
21128	0.810931	0.05
21129	1.66203	0.105895
21130	0.674605	0.234952
21131	0.946132	0.278092
21132	1.26706	0.303576
21133	0.674633	0.145467
21134	0.946132	0.493583
21135	0.980664	0.26964
21136	1.34927	0.174364
21137	0.758454	0.102574
21138	0.946133	0.0775703
21139	0.985371	0.0616683
21140	1.28632	0.058534
21141	0.946133	0.156505
21142	1.46868	0.205582
21143	1.7522	0.05
21144	0.737431	2.05478
21145	0.663679	2.06126
21146	1.16541	1.84537
21147	1.3179	1.91834
21148	1.04081	1.93786
21149	0.887866	1.75293
21150	0.806961	1.49566
21151	0.929061	1.67893
21152	1.28418	0.193963
21153	1.38752	0.156795
21154	1.33038	0.101933
21155	0.946132	0.108102
21156	0.946132	0.125497
21157	1.14897	0.122492
21158	1.1685	0.0935397
21159	1.18774	2.1
21160	0.961899	2.1
21161	1.48841	2.1
21162	1.79105	2.1
21163	3.51614	2.1
21164	0.682342	2.1
21165	0.783184	2.1
21166	0.946132	2.1
21167	0.868396	2.1

21168	1.21813	2.1
21169	0.848305	0.967967
21170	0.94559	1.10478
21171	0.805691	1.06101
21172	0.752217	2.1
21173	0.946132	2.1
21174	0.839123	2.1
21175	0.946131	2.1
21176	0.752187	2.1
21177	1.96491	2.1
21178	0.520997	2.1
21179	0.946132	2.1
21180	0.73446	2.1
21181	0.946133	2.1
21182	0.520995	2.1
21183	1.88522	2.1
21184	1.8361	2.1
21185	1.11285	2.1
21186	1.25334	1.42542
21187	2.00756	2.1
21188	1.44252	0.0561089
21189	1.00255	2.1
21190	1.64516	2.1
21191	1.12399	0.05
21192	1.13542	0.05
21193	1.09038	0.05
21194	0.746903	0.05
21195	0.672241	0.05
21196	0.871774	0.05
21197	1.73513	0.378288
21198	0.949995	2.1
21199	1.17244	2.1
21200	0.946127	2.1
21201	1.3028	2.1
21202	1.17244	2.1
21203	0.774101	2.1
21204	0.946127	2.1
21205	0.939289	2.1
21206	0.946129	2.1
21207	0.774101	2.1
21208	1.87858	2.1
21209	1.44293	0.05
21210	1.43213	0.05
21211	0.946131	2.1
21212	0.716614	2.1
21213	0.716644	2.1
21214	1.35828	2.1
21215	0.790935	2.1
21216	0.730157	2.1
21217	1.41149	2.1
21218	0.730186	2.1
21219	0.730186	2.1
21220	0.946131	2.1
21221	0.946132	1.37379
21222	0.946132	0.989034
21223	0.990713	0.381642
21224	1.40779	0.566206
21225	0.946132	0.686371

21226	1.06486	0.203405
21227	0.946131	1.30546
21228	0.946133	0.444014
21229	1.40779	0.322762
21230	1.26874	0.358024
21231	0.946131	0.409967
21232	1.02588	0.576123
21233	0.826073	0.0664323
21234	1.41233	0.0930497
21235	0.861269	0.0621938
21236	1.04073	0.106436
21237	1.27111	0.123214
21238	1.56016	1.59083
21239	1.60866	0.05
21240	0.94613	0.05
21241	0.946129	0.05
21242	1.36376	0.05
21243	1.22187	0.05
21244	0.94613	0.05
21245	1.82581	1.02796
21246	1.19705	1.44345
21247	1.7513	2.1
21248	1.85017	2.1
21249	1.72116	2.1
21250	1.12058	2.1
21251	0.899991	2.1
21252	0.616288	2.1
21253	1.07289	2.1
21254	1.7092	0.606717
21255	0.946132	0.746343
21256	0.946133	0.305332
21257	1.18717	0.237886
21258	1.40029	0.844251
21259	0.946132	0.121379
21260	0.94613	0.0951537
21261	1.18547	0.0788601
21262	0.960326	0.0882992
21263	0.960345	0.0708557
21264	0.942557	0.0962145
21265	1.18601	0.0668845
21266	0.946132	0.0753089
21267	1.30307	0.0614957
21268	1.08169	0.0664071
21269	1.08169	0.054675
21270	1.31716	0.0791275
21271	1.51065	0.689791
21272	0.877642	0.05
21273	0.617384	0.05
21274	0.575741	0.05
21275	0.972448	0.05
21276	1.11334	0.05
21277	1.13092	2.07884
21278	0.946127	1.75841
21279	1.31724	2.1
21280	1.39751	0.380849
21281	1.39751	1.05107
21282	0.946128	1.1823
21283	0.946132	0.301197

21284	0.713284	0.123451
21285	1.40668	0.30393
21286	1.03547	0.148423
21287	0.713298	0.268432
21288	1.88774	0.127855
21289	0.830164	2.1
21290	1.05211	2.1
21291	0.946119	2.1
21292	0.946107	2.1
21293	1.283	2.1
21294	2.63609	2.1
21295	1.28418	0.68759
21296	0.946128	1.20102
21297	1.10335	0.917492
21298	0.598377	1.33833
21299	1.18818	0.476445
21300	1.10335	0.925799
21301	1.58972	1.44485
21302	1.90194	2.1
21303	0.946118	2.1
21304	0.946115	2.1
21305	0.950969	2.1
21306	1.36918	2.1
21307	0.950955	2.1
21308	1.4263	2.1
21309	1.11978	1.01134
21310	0.946132	1.0744
21311	0.946133	1.11656
21312	1.37658	0.999595
21313	1.2364	1.04106
21314	1.05496	1.22945
21315	0.946132	1.22542
21316	0.946132	1.28012
21317	1.21858	1.14041
21318	1.37658	1.26025
21319	1.09194	1.20604
21320	1.14721	0.0665535
21321	1.53814	0.05
21322	0.546751	1.44483
21323	0.492063	1.50484
21324	1.16889	1.822
21325	1.00349	1.67687
21326	0.863559	1.51619
21327	1.38102	1.60787
21328	0.597449	1.38199
21329	0.537748	1.2637
21330	1.16297	1.53948
21331	0.999366	1.40408
21332	0.896554	1.36073
21333	0.566962	0.05
21334	0.946129	0.05
21335	0.452729	0.05
21336	0.696552	0.05
21337	0.852524	0.05
21338	1.57992	0.05
21339	1.64985	0.05
21340	0.946128	2.1
21341	1.21847	2.1

21342	0.946129	2.1
21343	1.41131	2.1
21344	1.21847	2.1
21345	1.21269	0.05
21346	1.0417	1.63776
21347	1.15821	1.27118
21348	0.946132	0.439034
21349	0.946132	1.99221
21350	1.37967	0.592122
21351	0.851529	0.923814
21352	1.20455	2.1
21353	1.4282	0.05
21354	1.26593	2.1
21355	0.838026	0.0801345
21356	0.985096	0.0711835
21357	0.83804	0.0793224
21358	0.822395	0.796367
21359	0.420272	0.313347
21360	0.946131	2.1
21361	1.17461	2.1
21362	0.94613	2.1
21363	1.40225	2.1
21364	1.17461	2.1
21365	1.09847	2.1
21366	1.22222	2.08559
21367	1.41798	0.364437
21368	0.791658	1.10137
21369	0.791656	0.968494
21370	0.946129	0.839091
21371	0.817064	0.763231
21372	0.946114	0.553929
21373	0.75824	0.522831
21374	0.75821	0.628163
21375	1.41778	0.381121
21376	0.791635	0.534702
21377	1.04164	1.76859
21378	0.946131	0.93787
21379	1.11172	0.652772
21380	1.30076	0.348464
21381	1.35721	0.375445
21382	1.11172	0.379944
21383	1.87218	0.123799
21384	0.987248	0.14006
21385	0.729695	0.860988
21386	0.974389	0.214618
21387	1.18002	0.214695
21388	0.822612	0.197095
21389	1.35721	0.214288
21390	0.634295	1.17117
21391	0.634326	1.13877
21392	1.13245	1.35517
21393	1.13245	1.23977
21394	0.937961	1.16264
21395	1.18769	1.07876
21396	0.854949	0.954274
21397	0.967357	1.08113
21398	0.810934	0.731644
21399	3.91082	2.1

21400	0.946132	2.1
21401	0.946132	2.1
21402	1.05383	2.1
21403	1.05383	2.1
21404	0.986498	2.1
21405	1.50732	0.05
21406	0.753659	0.0593302
21407	1.22144	0.070833
21408	1.30463	0.0579096
21409	0.753659	0.0688276
21410	1.30463	0.066561
21411	0.886189	0.0731295
21412	1.04321	0.0676096
21413	0.909928	0.0618203
21414	1.18659	0.0545194
21415	1.19785	0.05
21416	1.40969	0.0529102
21417	1.24094	0.05
21418	0.946126	0.0501957
21419	1.2929	0.05
21420	1.24093	0.05
21421	0.867944	0.05
21422	1.41903	2.1
21423	2.47881	0.05
21424	1.12943	0.05
21425	0.764229	2.1
21426	0.764256	2.1
21427	0.94613	2.1
21428	0.94613	2.1
21429	1.1995	2.1
21430	1.83423	2.1
21431	0.634816	2.1
21432	0.877837	2.1
21433	0.94613	2.1
21434	1.04073	2.1
21435	1.06485	2.1
21436	0.94613	0.165107
21437	0.62725	0.137008
21438	0.798212	0.155622
21439	1.33241	0.1091
21440	0.62725	0.141264
21441	1.82301	0.0854952
21442	1.40615	2.1
21443	0.782504	2.1
21444	0.748635	2.1
21445	0.946131	2.1
21446	0.748607	2.1
21447	0.988456	1.90196
21448	1.64833	0.05
21449	1.35657	0.05
21450	1.26155	0.05
21451	0.915755	0.05
21452	0.915755	0.05
21453	0.946132	0.05
21454	1.49386	0.05
21455	1.01346	2.1
21456	0.946131	2.1
21457	0.946132	2.1

21458	1.38651	2.1
21459	1.39523	2.1
21460	0.946132	2.1
21461	1.06312	2.1
21462	0.946131	2.1
21463	0.946129	2.1
21464	1.39523	2.1
21465	1.37372	2.1
21466	0.946131	2.1
21467	1.2929	0.057544
21468	0.94613	0.0898614
21469	1.24448	0.0538051
21470	1.2929	0.0666848
21471	0.862011	0.0584145
21472	1.73583	0.05
21473	1.724	0.05
21474	1.03824	2.1
21475	1.534	2.1
21476	0.725492	1.88147
21477	0.771967	2.08594
21478	0.61731	1.59045
21479	0.475743	1.95496
21480	0.719694	1.66363
21481	1.14878	1.51245
21482	0.946117	1.93359
21483	0.94613	1.92915
21484	0.946105	1.97846
21485	0.482666	1.52711
21486	0.609172	1.53503
21487	0.946131	2.1
21488	1.39509	1.53496
21489	1.07439	2.1
21490	0.946131	2.1
21491	1.39509	2.1
21492	0.963571	1.73187
21493	0.591011	0.289735
21494	1.1357	0.18163
21495	2.11193	1.66242
21496	1.60936	2.1
21497	1.34118	0.05
21498	1.27696	0.05
21499	0.94613	0.065572
21500	0.94613	0.05
21501	1.04257	0.05
21502	1.53336	0.0519932
21503	1.0631	0.0743399
21504	1.04072	0.120284
21505	0.670678	0.0864281
21506	0.709531	0.104116
21507	0.891275	0.106171
21508	0.683583	0.110217
21509	0.902478	0.0835044
21510	0.807671	0.0916677
21511	1.11024	2.1
21512	1.08171	2.1
21513	1.16725	2.1
21514	0.946128	2.1
21515	1.13537	2.1

21516	1.40525	2.1
21517	1.28365	2.1
21518	1.04523	2.1
21519	0.960106	2.1
21520	0.946128	2.1
21521	1.23452	2.1
21522	0.946129	2.1
21523	1.3704	2.1
21524	1.23452	2.1
21525	1.16906	1.38766
21526	0.946131	1.6345
21527	0.867246	1.44219
21528	0.867217	1.58364
21529	0.946131	1.48854
21530	1.13438	1.51622
21531	1.28684	1.57695
21532	0.946132	1.72851
21533	0.883935	1.55985
21534	0.883938	2.03267
21535	0.946133	1.58489
21536	1.73444	1.48047
21537	1.21835	2.1
21538	1.28228	2.1
21539	1.8643	0.667724
21540	0.743379	0.0719388
21541	1.34921	0.0794686
21542	0.743381	0.0773854
21543	0.743381	0.084271
21544	0.946131	0.0848951
21545	0.915528	2.1
21546	0.982742	2.1
21547	0.975965	2.1
21548	0.891132	0.05
21549	1.27533	0.05
21550	0.946132	0.05
21551	1.25215	0.05
21552	0.590343	0.05
21553	0.946133	0.05
21554	0.590331	0.05
21555	1.62375	2.1
21556	0.687769	1.28398
21557	1.14374	1.27408
21558	1.20885	0.920283
21559	0.946132	1.31017
21560	1.14374	1.39657
21561	1.09308	0.172097
21562	1.02055	0.181517
21563	0.810934	0.115626
21564	0.917609	1.90577
21565	1.13094	2.05556
21566	0.70959	1.86312
21567	1.11223	2.1
21568	0.83051	1.84176
21569	0.810946	2.0269
21570	0.810959	1.85307
21571	1.5773	0.05
21572	1.96133	1.47886
21573	1.0214	1.46942

21574	0.762783	1.6484
21575	0.838036	1.38619
21576	0.63591	1.7723
21577	0.967411	1.58404
21578	0.946132	2.1
21579	0.946132	2.05838
21580	1.39699	1.8241
21581	1.61642	1.85589
21582	0.860954	1.52454
21583	0.957411	1.69158
21584	0.660856	1.68517
21585	1.10003	2.1
21586	0.968249	2.1
21587	0.910184	2.06959
21588	1.30029	0.675962
21589	0.754119	1.63649
21590	0.754121	1.93189
21591	0.94613	1.84373
21592	1.08039	2.1
21593	0.810942	0.0719288
21594	1.15153	0.0693846
21595	1.06968	0.0602503
21596	1.38456	2.1
21597	1.04118	2.1
21598	0.838027	2.1
21599	1.04073	2.1
21600	0.754209	2.1
21601	1.332 0.05	
21602	1.26193	2.1
21603	1.15317	0.05
21604	3.86179	0.05
21605	1.56005	2.1
21606	0.683035	0.201192
21607	0.931463	0.173473
21608	0.556019	0.701649
21609	0.929172	0.445448
21610	0.756874	0.122889
21611	1.23527	0.895197
21612	2.05961	1.06125
21613	0.946132	2.1
21614	1.16684	2.1
21615	1.3937	2.1
21616	1.3937	2.1
21617	0.946132	2.1
21618	1.17885	2.1
21619	1.62507	0.05
21620	1.58874	2.1
21621	0.819953	2.1
21622	1.16023	2.1
21623	1.34782	2.1
21624	1.16023	2.1
21625	0.819955	2.1
21626	0.79437	2.1
21627	0.794373	2.1
21628	1.20056	2.02833
21629	1.25 2.09216	
21630	1.20056	2.1
21631	1.35787	2.1

21632	0.946131	2.00583
21633	1.38585	1.98147
21634	0.946128	1.66197
21635	0.946129	1.82999
21636	1.35714	1.96467
21637	1.04147	2.1
21638	0.946133	1.58989
21639	1.30382	1.57486
21640	0.946131	0.830081
21641	0.946131	1.52037
21642	1.38585	1.73871
21643	1.22305	1.2458
21644	1.00763	0.0779025
21645	0.813199	0.123507
21646	0.810948	0.111104
21647	1.15928	0.104819
21648	0.944166	0.05
21649	0.94613	0.05
21650	0.946133	0.0600457
21651	1.4179	0.05
21652	0.946132	0.05
21653	1.36965	0.05
21654	1.05444	0.05
21655	0.690302	0.05
21656	0.838038	0.05
21657	0.802821	0.450389
21658	1.40944	0.635977
21659	0.946126	0.867794
21660	0.799998	0.339007
21661	0.800035	0.480364
21662	1.1766	2.1
21663	1.00319	2.1
21664	1.01606	1.79073
21665	0.872158	2.1
21666	1.04884	2.09877
21667	0.679494	2.1
21668	0.946131	2.1
21669	1.37609	2.1
21670	0.94613	2.1
21671	0.946131	2.1
21672	1.18486	2.1
21673	0.726287	2.1
21674	0.946131	2.1
21675	0.726286	2.1
21676	1.30318	2.1
21677	1.01712	2.1
21678	0.946132	2.1
21679	1.04404	2.1
21680	1.37017	2.1
21681	1.37017	2.1
21682	0.946133	2.1
21683	0.992084	1.26811
21684	0.946131	0.637631
21685	0.529655	1.98931
21686	0.529686	1.50147
21687	0.946131	0.59959
21688	1.05937	1.57074
21689	1.14622	0.331267

21690	0.946131	0.33806
21691	0.584575	1.98771
21692	0.584575	0.208922
21693	0.946129	0.149204
21694	0.709714	2.1
21695	0.946132	2.1
21696	0.709714	2.1
21697	1.37199	2.1
21698	0.82899	2.1
21699	1.00133	2.1
21700	0.778424	0.05
21701	0.946132	0.05
21702	1.24094	0.05
21703	1.00691	0.05
21704	0.64868	0.05
21705	1.29736	0.05
21706	1.24767	0.340999
21707	1.28953	0.551845
21708	1.34612	1.35408
21709	0.946133	0.692338
21710	1.14187	1.40526
21711	0.946132	1.10644
21712	0.946129	1.51221
21713	1.4099	2.1
21714	0.946131	1.85177
21715	1.34612	1.63321
21716	1.4099	1.75826
21717	0.956003	0.05
21718	1.40572	0.05
21719	3.54443	2.1
21720	0.994001	0.403126
21721	1.03679	2.1
21722	1.08467	2.1
21723	1.56875	0.160633
21724	0.968463	2.1
21725	0.974889	2.1
21726	0.946131	2.1
21727	1.30203	2.1
21728	1.40199	2.1
21729	1.40199	2.1
21730	0.946132	2.1
21731	0.946132	2.1
21732	1.36994	2.1
21733	1.37372	2.1
21734	1.37372	2.1
21735	0.946132	2.1
21736	0.928979	1.55575
21737	0.565509	1.6622
21738	1.60196	0.382811
21739	1.16239	0.171087
21740	1.10746	0.379941
21741	1.15412	2.1
21742	1.15171	2.1
21743	0.946133	2.1
21744	0.940877	2.1
21745	0.940878	2.1
21746	1.41432	2.1
21747	1.04687	2.1

21748	2.75524	0.05
21749	0.946132	0.05
21750	0.766452	0.05
21751	0.766427	0.05
21752	1.36004	0.05
21753	1.06648	0.05
21754	1.62483	2.1
21755	1.0577	2.1
21756	1.39158	2.1
21757	0.94255	2.1
21758	1.05029	2.1
21759	1.03735	1.95085
21760	0.931055	2.1
21761	1.03797	1.98272
21762	1.43687	1.9609
21763	1.28765	1.33654
21764	1.1811	1.4689
21765	0.946132	1.35445
21766	0.946132	1.38069
21767	1.1811	1.38975
21768	0.67805	1.53724
21769	1.50998	1.90718
21770	1.16661	2.1
21771	1.36749	2.1
21772	0.810945	0.999225
21773	1.14745	0.775818
21774	0.865588	0.423851
21775	1.36665	0.188085
21776	0.506964	1.1553
21777	0.506966	2.00248
21778	0.552786	0.101005
21779	0.946133	0.129277
21780	1.21336	0.309936
21781	0.552786	0.0815526
21782	0.552757	2.1
21783	0.653468	0.222401
21784	1.36665	2.0403
21785	1.27922	0.14391
21786	1.08133	0.211812
21787	1.05348	0.102464
21788	0.946132	0.1793
21789	1.19601	0.112173
21790	1.13276	0.113578
21791	0.7856	0.114416
21792	1.57125	0.107765
21793	1.19601	0.209432
21794	0.946131	0.555023
21795	1.34534	0.290334
21796	1.19601	0.240203
21797	0.870489	0.251771
21798	1.49404	2.1
21799	1.35943	1.64195
21800	1.10427	1.8805
21801	0.94613	1.66363
21802	0.946131	1.73666
21803	1.10427	1.74115
21804	1.2064	0.894859
21805	0.946132	1.06944

21806	1.11288	2.1
21807	1.34268	0.111173
21808	1.34268	0.572857
21809	0.946132	0.345043
21810	0.946128	2.1
21811	0.946128	2.07518
21812	1.30835	2.1
21813	1.38463	2.1
21814	0.946129	2.1
21815	1.43778	1.15708
21816	1.23943	1.6913
21817	1.4748	2.1
21818	0.71295	2.1
21819	0.712951	2.1
21820	0.946132	2.1
21821	0.946133	2.1
21822	1.13976	2.1
21823	1.71109	2.1
21824	1.32426	2.1
21825	0.671255	2.1
21826	1.29123	2.1
21827	0.946132	2.1
21828	0.946132	2.1
21829	1.25603	2.1
21830	2.21126	0.05
21831	0.892889	2.1
21832	0.946129	2.1
21833	0.892888	2.1
21834	0.92872	2.1
21835	1.34352	2.1
21836	0.622784	0.196052
21837	1.02947	0.0973821
21838	0.810957	0.0892114
21839	1.91756	1.52255
21840	1.06012	0.793984
21841	1.09569	0.364623
21842	1.5456	0.05
21843	1.1211	0.05
21844	1.21031	0.05
21845	1.03939	0.0678474
21846	0.519697	0.0901219
21847	0.933889	0.161163
21848	0.519726	0.147706
21849	1.16699	0.129587
21850	0.933889	0.146775
21851	0.840599	0.0573142
21852	0.94613	0.115199
21853	0.840568	0.0888109
21854	1.26868	0.0865392
21855	1.16699	0.0820955
21856	1.17822	1.43177
21857	0.922044	0.05
21858	1.29322	0.111145
21859	0.946132	0.0596798
21860	0.995178	0.05
21861	0.922056	0.05
21862	1.76232	0.0886604
21863	1.77065	0.726582

21864	1.38149	0.588426
21865	0.959301	1.27495
21866	0.946133	0.554428
21867	0.946132	1.01749
21868	0.959301	1.12245
21869	1.3612	0.790025
21870	0.885314	1.11801
21871	0.885309	0.54466
21872	0.94613	1.13732
21873	0.8853	0.446402
21874	1.56164	0.603561
21875	1.45506	0.109521
21876	1.45257	2.1
21877	0.851541	0.05
21878	1.29018	0.05
21879	0.851541	0.05
21880	1.21087	0.05
21881	1.24979	0.05
21882	1.56983	0.05
21883	1.60151	0.05
21884	1.78952	2.1
21885	0.638164	2.1
21886	0.638163	2.1
21887	1.39754	2.1
21888	1.39754	2.1
21889	0.681589	2.1
21890	1.34993	2.1
21891	1.04525	2.1
21892	0.946133	2.1
21893	1.04072	2.1
21894	0.857805	2.1
21895	1.06053	2.1
21896	1.49066	2.1
21897	1.4185	1.68504
21898	0.934059	1.87383
21899	0.93406	1.69424
21900	0.94613	1.79104
21901	0.93406	1.77838
21902	1.25277	1.81805
21903	1.00792	2.06752
21904	0.609466	1.84835
21905	0.946132	1.9429
21906	0.609463	1.94543
21907	1.30664	1.87543
21908	0.810943	1.65141
21909	1.05741	1.26728
21910	1.3868	2.1
21911	1.15558	0.187546
21912	1.13537	2.1
21913	0.946132	2.1
21914	0.946132	2.1
21915	1.08266	2.1
21916	1.54934	2.1
21917	1.26016	2.1
21918	2.01915	2.1
21919	0.60346	1.81251
21920	0.651529	1.81698
21921	1.15581	2.00725

21922	1.13037	1.79714
21923	1.04071	1.80403
21924	1.77796	1.9817
21925	0.532308	1.71816
21926	0.532308	1.60686
21927	1.15369	1.86178
21928	1.1537	1.6932
21929	0.903288	1.77272
21930	0.804766	2.1
21931	0.804739	2.1
21932	0.946128	2.1
21933	0.946131	2.1
21934	1.16745	2.1
21935	1.04193	2.1
21936	0.98505	2.1
21937	1.40724	0.547489
21938	1.14216	0.376879
21939	0.946131	0.274909
21940	0.946131	0.374671
21941	1.10941	0.365994
21942	0.81268	0.05
21943	0.946131	0.05
21944	1.28673	0.05
21945	1.28673	0.05
21946	0.812681	0.05
21947	1.84716	0.05
21948	1.84716	0.05
21949	0.92361	0.05
21950	0.94613	0.05
21951	0.923582	0.05
21952	1.26227	0.05
21953	0.923581	0.05
21954	0.747394	2.1
21955	1.32582	2.1
21956	0.747365	2.1
21957	0.97587	2.1
21958	1.32582	2.1
21959	1.04182	0.05
21960	0.961743	0.05
21961	0.946126	0.05
21962	1.47463	0.05
21963	1.12183	0.05
21964	0.851552	0.05
21965	1.33177	0.0600494
21966	0.946131	0.0972328
21967	1.41512	0.0712013
21968	1.33178	0.0694428
21969	0.946131	0.0650811
21970	0.927735	0.213229
21971	0.97206	0.0890364
21972	0.515566	0.143865
21973	0.946129	0.125075
21974	0.515538	0.10681
21975	1.04482	0.071318
21976	0.975535	0.111888
21977	0.522411	0.0763421
21978	0.522382	0.126192
21979	0.925346	0.120373

21980	0.575817	0.0911302
21981	0.946129	2.1
21982	1.14256	2.1
21983	1.32054	2.1
21984	1.40525	2.1
21985	1.14256	2.1
21986	1.59475	2.1
21987	0.773813	2.1
21988	1.4073	2.1
21989	0.773813	2.1
21990	0.773813	2.1
21991	1.4073	2.1
21992	0.993021	2.1
21993	1.50466	0.05
21994	0.851539	0.0629912
21995	1.20299	0.05
21996	0.946131	0.053597
21997	1.54464	0.0542425
21998	1.20309	0.05
21999	2.00701	0.05
22000	0.810946	0.0683831
22001	1.07678	0.0638337
22002	0.900128	0.0565118
22003	1.26842	2.1
22004	1.11206	2.1
22005	0.946132	2.1
22006	1.04074	2.1
22007	0.948809	2.1
22008	0.946131	0.05
22009	0.929617	0.05
22010	1.24057	0.05
22011	1.36485	0.05
22012	0.851554	0.05
22013	1.96823	0.05
22014	0.914836	0.05
22015	0.94613	0.05
22016	1.38911	0.05
22017	0.914805	0.05
22018	0.946131	0.05
22019	0.946131	0.05
22020	1.04072	0.05
22021	1.26837	0.05
22022	0.970054	0.05
22023	0.923958	0.05
22024	1.16653	2.1
22025	1.34412	2.1
22026	0.851541	0.05
22027	1.38645	0.05
22028	0.891637	0.05
22029	0.851487	0.05
22030	0.890871	0.05
22031	1.56178	0.05
22032	0.946128	0.05
22033	1.26868	0.05
22034	0.946131	0.0828456
22035	1.18789	0.335695
22036	1.26868	0.0592583
22037	1.26868	0.0909716

22038	0.946128	0.0909716
22039	1.18716	0.05
22040	0.946131	0.0984523
22041	0.59358	0.0609854
22042	1.23923	0.100242
22043	1.00024	0.0678434
22044	0.593593	0.0806648
22045	0.946131	0.135698
22046	0.713645	0.107165
22047	1.23175	0.149283
22048	1.23923	0.0795458
22049	0.713633	0.11557
22050	1.24687	0.05
22051	1.80662	2.09633
22052	2.15762	2.1
22053	0.780455	2.1
22054	0.946131	2.1
22055	0.780455	2.1
22056	1.40583	2.1
22057	1.39852	2.1
22058	0.565619	2.1
22059	1.1428	2.1
22060	0.663168	0.220525
22061	1.02163	0.173044
22062	1.04072	0.228114
22063	0.663169	0.202715
22064	1.13511	0.193248
22065	1.85277	0.128161
22066	1.17701	0.783827
22067	0.993831	0.0617008
22068	1.0823	0.0753706
22069	0.795761	0.234026
22070	1.24596	0.175034
22071	0.566075	0.236751
22072	0.94613	0.275268
22073	0.56605	0.26647
22074	0.774911	0.05
22075	0.946131	0.05
22076	1.24417	0.05
22077	0.774911	0.05
22078	0.946132	0.05
22079	1.54982	0.05
22080	1.82961	0.05
22081	1.12035	0.05
22082	0.876835	2.1
22083	0.946131	2.1
22084	0.876836	2.1
22085	1.12968	2.1
22086	1.23536	2.1
22087	2.15082	2.1
22088	0.58365	2.1
22089	0.946133	2.1
22090	0.583653	2.1
22091	0.992097	2.1
22092	1.12968	2.1
22093	1.0305	2.1
22094	0.82613	0.0886455
22095	0.879644	0.0769024

22096	1.00688	0.0753239
22097	1.52776	2.1
22098	0.851537	1.5487
22099	1.00851	1.54959
22100	0.946128	1.49877
22101	0.851552	1.54767
22102	1.4895	1.79681
22103	0.761574	2.1
22104	0.946132	2.1
22105	1.01095	2.1
22106	1.01095	2.1
22107	0.761572	2.1
22108	1.8575	2.1
22109	1.19984	0.05
22110	1.25797	0.0628763
22111	0.946132	0.0828631
22112	0.946132	0.0562018
22113	0.95332	0.101312
22114	0.946131	2.1
22115	0.851542	2.1
22116	1.03172	2.1
22117	1.22889	2.1
22118	1.0313	2.1
22119	1.15977	1.99862
22120	0.918875	1.24888
22121	0.8654	1.9729
22122	0.865374	1.36298
22123	0.946132	1.61181
22124	0.985051	2.1
22125	1.31993	1.96405
22126	1.33622	0.329296
22127	0.959992	0.239855
22128	0.926499	2.1
22129	1.03544	2.1
22130	0.946132	2.1
22131	0.946132	2.1
22132	1.40335	2.1
22133	0.772299	2.1
22134	0.851529	2.1
22135	0.857243	2.1
22136	1.38155	2.1
22137	1.05462	2.1
22138	0.832138	2.1
22139	0.946132	2.1
22140	0.832137	2.1
22141	0.959414	2.1
22142	1.36541	2.1
22143	1.91894	2.1
22144	1.04661	2.1
22145	1.28355	2.1
22146	0.877353	2.1
22147	0.877353	2.1
22148	0.94613	2.1
22149	1.20537	0.05
22150	1.09914	0.05
22151	0.94613	0.05
22152	0.499593	0.05
22153	0.499606	0.05

22154	1.0959	0.05
22155	0.838027	0.05
22156	0.883644	0.05
22157	0.95019	0.0687209
22158	1.10638	2.1
22159	0.946132	2.1
22160	1.41744	2.1
22161	1.05842	2.1
22162	0.94613	2.1
22163	1.41744	2.1
22164	0.991985	2.1
22165	3.31755	2.1
22166	1.05556	2.1
22167	0.624075	2.00969
22168	0.814554	1.92353
22169	0.946131	1.99542
22170	0.814554	2.0754
22171	1.31857	2.06441
22172	1.26282	2.1
22173	0.946132	2.1
22174	1.14302	2.1
22175	0.946132	2.1
22176	0.946132	2.1
22177	1.40372	2.1
22178	1.24688	0.137322
22179	0.89889	2.1
22180	1.17286	2.1
22181	0.71741	2.1
22182	0.491096	0.466417
22183	1.05432	0.223612
22184	0.719531	2.1
22185	1.25145	2.1
22186	0.864686	2.1
22187	0.867974	2.1
22188	1.21734	2.1
22189	0.405517	0.76322
22190	0.818878	1.2055
22191	0.502457	0.05
22192	1.05868	0.05
22193	0.912018	0.05
22194	1.40591	0.05
22195	0.957584	0.340538
22196	0.946131	0.510527
22197	1.11612	0.369693
22198	1.3365	0.368349
22199	0.946128	0.562989
22200	1.16534	0.103287
22201	1.38969	0.196938
22202	0.891597	1.68626
22203	0.640032	1.82376
22204	0.956758	1.67328
22205	1.3428	0.173249
22206	0.953295	2.1
22207	0.946131	0.127261
22208	1.16167	0.102607
22209	0.94613	0.131478
22210	0.951675	0.12011
22211	1.16167	0.117981

22212	1.24538	0.05
22213	1.13482	2.1
22214	1.38775	2.1
22215	0.934061	2.1
22216	0.91408	2.1
22217	1.06099	2.1
22218	0.980549	0.0584709
22219	0.946132	0.072971
22220	1.10321	0.0849818
22221	0.99126	0.0772794
22222	0.991261	0.0839836
22223	1.35732	0.0789895
22224	1.1952	2.1
22225	0.946132	1.72466
22226	0.946132	2.1
22227	1.32819	1.86145
22228	1.30171	1.90837
22229	1.29667	0.052652
22230	1.00982	0.05
22231	0.407589	0.540098
22232	1.18266	1.57083
22233	1.04037	0.491909
22234	0.956694	0.987203
22235	0.552639	0.633003
22236	0.810943	1.74513
22237	1.43144	1.44451
22238	0.918074	1.3361
22239	1.60976	2.1
22240	4.00847	2.1
22241	2.3995	0.05
22242	1.36203	2.1
22243	0.919619	2.1
22244	1.30188	2.1
22245	0.681073	2.1
22246	0.681043	2.1
22247	1.04687	2.1
22248	3.32085	2.1
22249	1.80396	0.05
22250	0.773007	0.187951
22251	0.773034	0.129915
22252	1.22141	0.217466
22253	1.22141	0.222149
22254	0.946131	0.316081
22255	2.02071	0.109654
22256	0.951912	0.05
22257	1.39027	0.05
22258	0.94613	0.05
22259	0.829922	0.05
22260	0.829946	0.05
22261	1.24902	1.3814
22262	2.8333	1.16197
22263	0.946131	0.05
22264	0.929725	0.05
22265	1.1134	0.05
22266	1.17056	0.05
22267	0.929713	0.05
22268	0.749096	2.1
22269	1.31961	2.1

22270	0.870191	2.1
22271	1.04071	2.1
22272	1.1877	2.1
22273	1.03882	2.1
22274	1.44801	2.1
22275	3.55198	2.1
22276	1.11193	2.1
22277	0.663922	2.1
22278	0.663922	2.1
22279	0.946129	2.1
22280	0.946131	2.1
22281	0.97079	2.1
22282	1.36049	2.1
22283	1.06105	2.1
22284	0.92251	2.1
22285	0.94613	2.1
22286	0.922511	2.1
22287	1.04101	2.1
22288	0.810944	0.05
22289	0.994109	0.05
22290	0.623143	0.05
22291	0.864244	0.05
22292	1.14627	0.05
22293	0.64586	0.05
22294	0.721422	0.05
22295	0.870885	0.05
22296	0.855175	0.05
22297	0.946131	2.09989
22298	1.38157	2.1
22299	1.33266	1.65358
22300	0.946131	2.1
22301	1.38158	2.1
22302	0.946132	2.1
22303	1.3268	2.1
22304	0.946132	2.1
22305	0.946132	2.1
22306	1.38158	2.1
22307	1.42186	1.55457
22308	1.47204	1.55965
22309	1.14665	2.1
22310	0.946132	1.95184
22311	0.94613	2.1
22312	1.30116	2.1
22313	1.32819	2.1
22314	1.25388	2.1
22315	0.887808	2.1
22316	0.946132	2.1
22317	1.23536	2.1
22318	1.34309	2.1
22319	1.07612	2.1
22320	0.728982	0.05
22321	1.3157	0.05
22322	0.946131	0.05
22323	0.686176	0.05
22324	1.3035	0.05
22325	0.686139	0.05
22326	0.906806	0.05
22327	0.794122	0.05

22328	1.17173	0.05
22329	1.8194	0.05
22330	0.851543	2.1
22331	1.19345	2.1
22332	0.946133	2.1
22333	0.946132	2.1
22334	1.56179	2.1
22335	0.620263	2.1
22336	1.30226	2.08272
22337	1.2334	1.91896
22338	1.2334	1.84817
22339	0.946133	1.82579
22340	1.24053	2.1
22341	1.4828	2.1
22342	0.957622	2.1
22343	1.00521	1.5805
22344	1.49463	2.1
22345	1.39621	2.1
22346	1.09861	2.1
22347	0.946131	2.1
22348	0.946131	2.1
22349	1.09861	2.1
22350	2.16987	0.05
22351	0.572182	0.05
22352	1.15802	0.05
22353	0.63574	0.05
22354	1.18897	0.05
22355	0.996733	0.05
22356	1.82402	1.73526
22357	0.969602	1.6006
22358	0.794777	1.72586
22359	1.02626	1.62392
22360	1.51396	0.0917663
22361	1.40574	0.150537
22362	1.38683	0.138497
22363	0.946126	0.159481
22364	0.946128	0.146102
22365	1.07138	0.144315
22366	0.946131	2.1
22367	1.41331	2.1
22368	0.94613	2.1
22369	0.94613	2.1
22370	1.27451	2.1
22371	0.974196	2.1
22372	1.2049	0.05
22373	1.42757	2.1
22374	1.02461	2.1
22375	2.07087	2.1
22376	1.92515	1.66256
22377	0.802138	1.62851
22378	0.946128	1.8416
22379	0.802107	1.67987
22380	1.23726	1.67886
22381	1.21997	1.6796
22382	0.767577	0.93949
22383	0.946131	1.69076
22384	0.767577	1.53433
22385	1.3279	1.51782

22386	1.23726	1.44787
22387	1.29729	2.1
22388	0.946128	2.1
22389	1.14186	2.1
22390	1.39214	2.1
22391	1.39214	2.1
22392	0.946129	2.1
22393	1.23012	0.274834
22394	0.653228	0.0810907
22395	0.946133	0.383395
22396	1.15918	0.250262
22397	1.26893	0.123293
22398	0.653228	0.305018
22399	1.06848	0.397829
22400	0.615121	2.1
22401	0.841488	2.1
22402	0.946131	2.1
22403	1.04071	2.1
22404	0.965717	2.1
22405	1.77893	2.03561
22406	1.11314	0.0793047
22407	1.65411	0.559273
22408	0.85472	0.05
22409	1.1604	0.05
22410	1.19141	0.05
22411	1.19141	0.05
22412	0.94613	0.05
22413	1.70944	0.05
22414	1.89082	0.0910723
22415	1.05175	1.50598
22416	0.815276	2.1
22417	0.843283	2.1
22418	0.946131	2.1
22419	0.843284	2.1
22420	1.38897	2.1
22421	1.01019	1.06513
22422	1.05197	0.919602
22423	0.810959	0.98181
22424	1.33037	0.078913
22425	1.3415	0.0545996
22426	0.946118	0.11605
22427	0.946131	0.0770619
22428	1.09803	0.0691153
22429	0.930281	2.1
22430	1.3335	2.1
22431	0.784151	2.1
22432	0.78415	2.1
22433	0.946132	2.1
22434	1.6299	0.05
22435	0.995795	2.1
22436	1.84846	1.55161
22437	0.929873	1.97511
22438	0.889245	1.98108
22439	0.808072	1.98033
22440	0.851541	1.8019
22441	1.51956	1.84773
22442	0.94613	1.80798
22443	0.851553	1.83344

22444	0.946787	1.85978
22445	1.15544	1.8184
22446	1.30289	0.0564773
22447	1.28323	0.0560494
22448	0.85024	0.05
22449	1.09662	0.0681188
22450	1.67687	0.05
22451	0.946127	2.1
22452	1.40496	2.1
22453	1.22442	2.1
22454	0.946128	2.1
22455	1.40496	2.1
22456	1.00573	2.1
22457	0.636837	2.1
22458	0.988642	2.1
22459	0.573141	2.0488
22460	0.946128	2.07675
22461	0.786538	2.09753
22462	2.28748	1.72004
22463	0.682805	2.1
22464	0.946132	2.1
22465	0.682776	2.1
22466	0.946132	2.1
22467	0.79775	2.1
22468	1.05403	0.05
22469	0.888273	0.05
22470	0.876373	0.05
22471	0.576083	0.05
22472	0.946132	0.05
22473	0.576057	0.05
22474	0.576058	0.05
22475	0.946131	0.05
22476	1.41724	0.323002
22477	1.31678	0.937626
22478	0.946132	1.12414
22479	0.946132	0.866719
22480	1.31678	0.696169
22481	0.935738	2.1
22482	0.946132	2.1
22483	0.935765	2.1
22484	1.32949	2.1
22485	0.946131	2.1
22486	1.02525	2.1
22487	0.946128	2.1
22488	1.41207	2.1
22489	1.04675	2.1
22490	0.946131	2.1
22491	1.41207	2.1
22492	0.946131	2.1
22493	0.946132	2.1
22494	1.41125	2.1
22495	1.41125	2.1
22496	1.27087	2.1
22497	0.924071	0.213978
22498	0.608893	0.165319
22499	0.528907	0.211831
22500	1.78249	0.202986
22501	0.720094	0.189671

22502	0.781667	2.1
22503	0.781638	2.1
22504	1.40756	2.1
22505	1.40756	2.1
22506	0.797635	2.1
22507	1.11946	0.05
22508	1.32068	0.05
22509	0.88757	0.0684701
22510	1.27891	0.0569678
22511	0.810957	0.0620066
22512	0.914656	0.0612916
22513	0.640819	0.05
22514	0.745643	0.0605866
22515	0.865108	0.0880368
22516	1.06883	0.0757069
22517	0.806001	0.0916668
22518	1.19462	2.1
22519	1.42763	0.311553
22520	1.24682	0.229004
22521	0.78392	0.0529799
22522	1.02874	0.0502808
22523	0.884512	0.0506364
22524	1.1658	2.1
22525	0.663026	0.05
22526	1.21174	0.05
22527	0.663056	0.05
22528	1.15968	0.05
22529	1.21174	0.05
22530	0.993976	0.05
22531	0.945495	0.05
22532	0.671202	0.05
22533	1.37312	2.1
22534	1.28499	2.1
22535	0.946133	2.1
22536	0.946132	2.1
22537	1.28499	2.1
22538	1.25081	2.1
22539	0.946132	2.1
22540	0.946132	2.1
22541	1.27985	2.1
22542	1.37312	2.1
22543	0.830638	2.1
22544	1.05358	2.1
22545	1.22209	2.1
22546	1.09874	2.1
22547	0.74758	2.1
22548	2.50011	1.39781
22549	0.6157	0.165028
22550	0.674634	0.168023
22551	0.946128	1.31026
22552	0.674633	0.20326
22553	0.946131	0.389998
22554	1.96192	1.83865
22555	1.31612	0.693974
22556	1.10735	0.392338
22557	0.936676	0.189609
22558	0.808779	0.220095
22559	0.808808	0.241567

22560	1.00245	0.22915
22561	1.26179	0.170049
22562	1.05758	1.98416
22563	1.03408	2.1
22564	0.946132	0.05
22565	0.914368	0.05
22566	1.20524	0.05
22567	1.12743	0.05
22568	0.851555	0.05
22569	1.17885	0.190273
22570	0.867881	0.192856
22571	1.00122	0.0960723
22572	0.867883	0.0787738
22573	0.867881	0.0531785
22574	1.19583	0.110193
22575	1.17743	2.1
22576	0.878684	2.1
22577	0.687087	2.1
22578	0.955079	0.285383
22579	0.672977	0.67316
22580	0.735937	0.546848
22581	0.851531	0.695233
22582	0.672957	0.586805
22583	0.923974	0.63459
22584	1.2243	2.1
22585	0.808463	0.595662
22586	0.971078	0.875749
22587	1.06161	0.52956
22588	0.810947	0.949622
22589	0.886074	1.17822
22590	0.786829	0.995178
22591	0.55062	0.813411
22592	0.574458	0.612513
22593	0.946129	0.844632
22594	1.42526	0.54399
22595	1.15962	0.822997
22596	1.24526	0.05
22597	0.94613	0.41261
22598	0.946128	1.21293
22599	1.23279	1.50362
22600	1.38645	1.67043
22601	0.94613	0.977102
22602	1.05042	2.1
22603	0.946128	2.1
22604	0.946129	2.1
22605	1.24286	2.1
22606	1.39659	2.1
22607	0.94613	2.1
22608	1.10407	2.1
22609	1.32809	1.88244
22610	0.993999	1.97798
22611	0.810959	1.86534
22612	0.983643	1.94694
22613	0.869552	2.0705
22614	0.747801	1.84983
22615	1.53429	1.60879
22616	1.13127	2.1
22617	1.38397	2.1

22618	1.08366	2.1
22619	0.946129	2.1
22620	0.946131	2.1
22621	1.08366	2.1
22622	1.06007	2.1
22623	1.79641	2.1
22624	0.758799	2.1
22625	1.28442	2.1
22626	1.06957	2.1
22627	0.946133	2.1
22628	1.28442	2.1
22629	1.76788	1.78354
22630	1.38669	2.1
22631	0.986095	2.1
22632	1.38478	0.351566
22633	0.946132	0.85607
22634	1.24344	0.378339
22635	0.622283	1.80243
22636	1.10335	0.443733
22637	1.24344	0.623376
22638	1.1967	0.453826
22639	0.946131	0.05
22640	0.901005	0.05
22641	1.05751	0.05
22642	1.37771	0.05
22643	0.851527	0.05
22644	1.26643	1.261
22645	1.08992	1.7528
22646	0.946132	1.43871
22647	0.946132	1.49394
22648	1.08992	1.49249
22649	1.53534	2.1
22650	1.58825	1.84931
22651	1.0447	1.98846
22652	0.593818	1.85873
22653	0.89883	1.9543
22654	0.734088	1.9129
22655	0.998451	0.66844
22656	1.64156	0.940808
22657	1.05654	1.49982
22658	1.37641	0.218407
22659	1.74553	0.226518
22660	1.31112	0.394572
22661	0.74518	0.496133
22662	0.851539	0.393276
22663	0.851554	0.352917
22664	0.851475	0.363653
22665	0.946132	0.05
22666	1.28895	0.05
22667	1.11356	0.05
22668	0.946132	0.05
22669	0.946132	0.05
22670	1.84214	2.1
22671	1.23851	0.0670658
22672	1.25866	0.294502
22673	1.28655	0.674958
22674	0.946131	0.741756
22675	0.946132	0.569748

22676	1.28655	0.445856
22677	1.11929	0.478036
22678	0.946132	0.767007
22679	0.946132	0.396811
22680	1.34254	0.341468
22681	1.25866	0.321507
22682	0.946132	2.1
22683	1.27451	2.1
22684	1.19803	2.1
22685	0.94613	2.1
22686	1.27451	2.1
22687	0.946131	2.1
22688	1.36523	2.1
22689	1.14599	2.1
22690	0.946129	2.1
22691	1.36523	2.1
22692	1.09518	2.1
22693	0.977777	2.1
22694	0.583237	2.1
22695	0.583267	2.1
22696	0.903872	2.1
22697	0.946129	2.1
22698	0.583258	2.1
22699	1.58179	0.05
22700	0.946131	0.0517491
22701	1.35123	0.05
22702	0.655837	0.0564447
22703	1.12018	0.05
22704	1.35123	0.05
22705	0.62967	0.0516872
22706	0.860497	0.0660278
22707	0.816639	0.0523479
22708	0.746716	2.1
22709	0.810947	2.1
22710	0.810959	2.1
22711	0.653422	2.1
22712	0.289127	1.90409
22713	0.475716	2.1
22714	0.980651	1.83016
22715	0.946119	2.1
22716	0.946132	2.1
22717	1.08299	2.1
22718	1.34539	2.1
22719	1.28753	0.100442
22720	0.946132	0.171324
22721	0.946132	0.142359
22722	1.19719	0.149641
22723	0.946133	0.145893
22724	0.958947	0.113203
22725	1.3492	0.124121
22726	0.946132	0.197102
22727	0.946128	0.18157
22728	1.22588	0.179822
22729	0.946129	0.173466
22730	0.991113	2.1
22731	1.35966	2.1
22732	0.961 2.1	
22733	0.946129	2.1

22734	0.946131	2.1
22735	0.961001	2.1
22736	0.727096	1.73005
22737	0.810946	1.10839
22738	0.810959	1.49948
22739	1.31658	0.154426
22740	1.23167	2.1
22741	1.44836	0.05
22742	1.50103	0.05
22743	1.23351	0.05
22744	1.35657	0.05
22745	0.851541	0.05
22746	0.93668	0.05
22747	0.946131	0.05
22748	3.7335	2.1
22749	1.1822	2.1
22750	1.31127	0.127381
22751	1.3689	0.1022
22752	1.35822	0.05
22753	0.946133	0.0692333
22754	0.946132	0.103463
22755	1.14926	0.0886396
22756	1.40822	0.0559185
22757	1.04935	0.122313
22758	0.704112	0.0801613
22759	0.704112	0.110568
22760	0.946131	0.130716
22761	0.704112	0.112474
22762	1.37721	0.05
22763	0.85154	2.1
22764	1.06949	2.1
22765	0.851541	2.1
22766	0.946128	2.1
22767	1.1175	2.1
22768	1.44091	2.08096
22769	0.96029	1.84125
22770	3.46486	1.56652
22771	0.603286	0.0852192
22772	0.432283	0.0524968
22773	0.851541	0.0728767
22774	1.09355	0.05
22775	0.851534	0.0763159
22776	1.04263	2.1
22777	0.851541	2.1
22778	0.965604	2.1
22779	0.94613	2.1
22780	1.23953	2.1
22781	0.869615	2.1
22782	1.02415	2.1
22783	0.983032	2.1
22784	1.35385	1.57319
22785	1.59971	0.0690697
22786	0.915178	2.1
22787	0.962435	2.1
22788	1.3116	2.1
22789	1.38302	2.1
22790	1.13227	2.1
22791	1.25923	0.05

22792	0.988805	2.1
22793	0.611486	1.37321
22794	1.1019	1.34391
22795	1.11124	1.57697
22796	0.716713	1.35278
22797	0.946131	1.42535
22798	1.22297	1.39129
22799	1.12873	1.64677
22800	0.946131	1.66852
22801	0.946132	1.65548
22802	1.3469	1.63572
22803	1.25366	1.70152
22804	1.1611	1.48328
22805	0.946132	1.60512
22806	0.946132	1.50893
22807	1.2854	1.53863
22808	1.3469	1.5957
22809	1.07468	1.49188
22810	1.79317	2.1
22811	1.07444	2.1
22812	1.69436	2.1
22813	0.851539	2.1
22814	0.870925	2.1
22815	0.851541	2.1
22816	0.870951	2.1
22817	1.26097	2.1
22818	1.26247	2.1
22819	0.946132	2.1
22820	1.24041	2.1
22821	0.946132	2.1
22822	0.946132	2.1
22823	1.40274	2.1
22824	0.946131	0.05
22825	0.797945	0.05
22826	1.12988	0.05
22827	1.00681	0.05
22828	0.797932	0.05
22829	0.977483	0.186098
22830	0.943362	0.358498
22831	0.9891	0.177234
22832	1.1703	0.223151
22833	0.663171	0.222394
22834	1.40882	0.0874596
22835	1.32639	0.143464
22836	1.24448	0.0896162
22837	0.946132	0.139334
22838	1.11015	0.0841083
22839	1.24448	0.107503
22840	0.754768	0.0984238
22841	1.13854	2.1
22842	1.23654	2.1
22843	0.714101	0.509575
22844	0.851538	1.26205
22845	0.79261	2.1
22846	1.30598	1.90012
22847	1.05338	1.26219
22848	1.41758	2.1
22849	0.83317	0.0869988

22850	1.00058	0.0868578
22851	0.810958	0.0876972
22852	1.63791	0.122787
22853	1.55116	2.1
22854	1.29735	1.7068
22855	0.945147	2.1
22856	0.94515	1.78857
22857	0.94613	1.91685
22858	0.945149	1.88496
22859	1.02368	2.1
22860	1.12787	2.1
22861	1.04007	2.1
22862	3.78805	2.1
22863	0.816891	0.0538357
22864	0.943996	0.0510454
22865	0.870002	0.05
22866	0.564533	0.0599935
22867	0.564536	0.076494
22868	0.946128	0.316275
22869	0.564535	0.112293
22870	0.946128	0.0812135
22871	1.15177	2.1
22872	1.16806	1.17221
22873	0.999083	1.32663
22874	0.946131	1.18301
22875	0.946131	1.20844
22876	0.999084	1.21607
22877	1.28918	1.20932
22878	1.0383	1.34777
22879	0.946133	1.24021
22880	0.946132	1.25136
22881	1.0383	1.25925
22882	1.27737	1.38285
22883	1.39296	0.22802
22884	0.998682	2.1
22885	3.98933	2.1
22886	1.05328	0.122291
22887	1.23475	2.1
22888	0.900856	1.17699
22889	0.94001	0.953013
22890	0.707748	0.851259
22891	0.707751	0.9818
22892	0.946128	0.864523
22893	0.70775	0.822032
22894	0.996109	0.861079
22895	1.68551	2.1
22896	0.851539	2.1
22897	0.962688	2.1
22898	0.94613	2.1
22899	1.04073	2.1
22900	1.44824	2.1
22901	0.794313	0.274991
22902	0.838031	0.286402
22903	1.11076	0.239815
22904	1.38813	2.1
22905	1.17204	2.1
22906	0.946132	2.1
22907	0.946131	2.1

22908	1.17204	2.1
22909	0.961779	0.218113
22910	1.0685	0.332924
22911	0.810947	1.93937
22912	1.2899	1.61494
22913	0.890404	1.59203
22914	0.909981	2.1
22915	0.810946	1.79678
22916	0.890402	1.76837
22917	0.522844	1.93477
22918	0.593846	1.93254
22919	1.30226	1.73864
22920	1.30226	1.78785
22921	0.946132	1.76486
22922	1.03537	0.05
22923	1.50035	0.05
22924	0.940356	0.05
22925	0.946131	0.05
22926	1.03441	0.05
22927	1.21171	0.05
22928	1.35169	0.05
22929	1.32449	0.05
22930	0.946131	0.05
22931	0.946131	0.05
22932	1.06627	0.05
22933	1.03102	0.201787
22934	0.488712	0.05
22935	0.946131	0.05
22936	1.23146	0.05
22937	1.13242	0.05
22938	0.946131	0.05
22939	0.409199	0.05
22940	0.429959	0.05
22941	1.08357	0.05
22942	0.80926	0.05
22943	0.879791	0.05
22944	0.946129	2.0619
22945	1.28828	2.1
22946	0.946131	1.99788
22947	0.946131	2.1
22948	1.35969	2.1
22949	1.10049	1.94536
22950	0.923724	2.1
22951	0.923695	2.1
22952	0.94613	2.1
22953	0.94613	2.1
22954	1.28828	2.1
22955	1.73525	0.123564
22956	0.656239	0.189525
22957	1.2996	0.151656
22958	0.812939	0.207973
22959	0.65624	0.182051
22960	1.2996	0.167807
22961	1.67372	2.1
22962	0.946131	2.1
22963	0.946132	2.1
22964	1.05978	2.1
22965	1.31049	2.1

22966	0.946132	2.1
22967	1.11077	2.1
22968	0.579725	0.283093
22969	0.373532	0.192243
22970	1.13537	0.303315
22971	1.20726	0.223245
22972	0.946131	0.223043
22973	1.45694	0.649543
22974	0.750367	0.05
22975	0.946126	0.05
22976	0.750365	0.05
22977	1.25698	0.05
22978	1.35726	0.05
22979	1.6812	0.05
22980	1.78665	1.95122
22981	1.46568	2.1
22982	1.29606	2.1
22983	1.25298	2.1
22984	0.946115	2.1
22985	0.946129	2.1
22986	1.25296	2.1
22987	1.27778	0.0591037
22988	1.17042	2.09948
22989	1.25858	0.05
22990	0.93106	0.05
22991	0.810959	0.05
22992	1.38905	2.1
22993	1.19216	2.1
22994	0.946131	2.1
22995	0.946131	2.1
22996	1.19216	2.1
22997	1.58339	2.1
22998	0.81094	0.05
22999	0.845129	0.05
23000	0.918535	0.05
23001	0.946132	2.1
23002	0.946132	2.1
23003	1.31904	2.1
23004	1.31904	2.1
23005	1.21275	2.1
23006	1.15471	2.1
23007	1.33125	2.1
23008	0.702954	0.05
23009	0.942973	0.05
23010	0.94613	0.05
23011	0.641772	0.05
23012	0.702942	0.05
23013	0.991865	0.056236
23014	0.639952	0.05
23015	1.17333	0.05
23016	0.946132	0.05
23017	0.490975	0.05
23018	0.495934	0.05
23019	1.19904	0.05
23020	1.30488	0.05
23021	1.02305	0.0768243
23022	1.49842	0.0834008
23023	1.54196	2.1

23024	1.37224	1.72467
23025	1.0953	0.05
23026	1.18952	2.1
23027	1.68851	0.274103
23028	0.946131	0.10488
23029	1.12491	0.118577
23030	0.583591	0.113146
23031	1.28727	0.137613
23032	1.12491	0.135163
23033	0.884641	0.436764
23034	0.810944	0.168321
23035	0.917973	0.294358
23036	0.846671	0.229054
23037	0.596968	0.143057
23038	0.716347	0.284672
23039	1.36255	0.0830346
23040	1.11077	2.1
23041	0.946132	2.1
23042	0.946132	2.1
23043	1.26267	2.1
23044	1.26441	2.1
23045	1.85238	2.1
23046	1.36693	2.1
23047	0.616123	2.1
23048	0.481844	2.1
23049	0.709599	2.1
23050	0.815889	2.1
23051	1.1502	2.1
23052	1.63937	1.88721
23053	1.25797	0.0707153
23054	1.22878	0.0861399
23055	0.946132	0.0973391
23056	0.946132	0.0813013
23057	0.965737	0.102738
23058	1.1893	0.0943512
23059	1.22877	0.0905934
23060	0.735217	0.107996
23061	0.85154	0.107596
23062	0.851553	0.0975715
23063	0.851473	0.111677
23064	1.58916	0.0699012
23065	1.25545	0.0693462
23066	0.810942	0.146048
23067	0.919314	0.149084
23068	0.928633	0.126327
23069	2.14514	0.0815492
23070	1.23389	2.1
23071	0.946128	0.05
23072	0.969504	0.05
23073	0.851539	0.05
23074	1.41503	0.05
23075	0.958517	0.05
23076	0.990615	0.05
23077	1.7943	0.05
23078	0.946133	0.326978
23079	1.27581	0.396595
23080	0.946132	0.342196
23081	1.36362	0.275083

23082	1.36936	0.25949
23083	1.71903	2.1
23084	0.751639	2.1
23085	0.77464	2.1
23086	0.946133	2.1
23087	1.04073	2.1
23088	1.23551	2.1
23089	0.94613	2.1
23090	1.18682	2.1
23091	0.946131	2.1
23092	0.94613	2.1
23093	1.40638	2.1
23094	0.483346	0.182916
23095	0.432527	0.132072
23096	0.946132	0.209236
23097	0.483312	0.170671
23098	0.946132	0.169762
23099	0.878798	0.226677
23100	0.810944	0.222395
23101	1.14604	0.181648
23102	0.946129	0.212843
23103	1.35594	0.176262
23104	0.538378	0.178123
23105	0.946131	0.222678
23106	0.538378	0.208582
23107	1.00065	1.88964
23108	0.962878	0.753986
23109	1.32634	0.0540656
23110	1.28066	0.05
23111	0.785651	2.1
23112	1.20327	2.1
23113	1.47058	1.98995
23114	0.551834	1.69176
23115	1.04072	2.08392
23116	0.506719	1.85061
23117	1.12786	1.91033
23118	1.45789	0.05
23119	0.681532	0.05
23120	1.21087	0.05
23121	0.94613	0.05
23122	0.681534	0.05
23123	1.21087	0.05
23124	1.98618	0.595535
23125	1.71742	1.46959
23126	1.07427	0.201486
23127	1.1972	0.17417
23128	0.933474	0.203418
23129	1.1745	0.243674
23130	0.94612	0.225914
23131	0.933485	0.219248
23132	1.17449	0.20722
23133	0.851541	0.05
23134	1.12774	0.05
23135	0.946129	0.05
23136	0.851486	0.05
23137	1.12517	0.05
23138	1.12328	0.05
23139	1.13179	2.1

23140	1.02438	2.1
23141	1.05776	2.1
23142	1.41273	1.98576
23143	1.1229	2.1
23144	0.875912	2.07584
23145	0.946132	2.1
23146	0.875912	2.1
23147	1.42307	2.1
23148	1.15354	0.05
23149	0.848382	0.05
23150	1.2685	2.1
23151	0.938104	2.1
23152	0.793166	2.1
23153	1.07855	2.1
23154	0.98488	2.1
23155	1.4162	2.1
23156	1.09651	0.0968617
23157	1.98502	0.05
23158	1.41168	0.130239
23159	1.33879	0.178417
23160	0.946127	0.227189
23161	1.13528	0.146644
23162	0.946128	0.172355
23163	1.09726	2.1
23164	1.22026	1.76904
23165	1.09044	2.1
23166	2.23961	0.381478
23167	1.14721	0.183196
23168	1.17614	2.1
23169	0.615065	2.1
23170	0.946131	2.1
23171	0.778488	2.1
23172	0.94613	2.1
23173	0.615066	2.1
23174	1.78254	2.1
23175	0.898452	0.05
23176	1.47411	2.1
23177	0.59676	0.220046
23178	0.59676	0.155475
23179	0.94613	0.233126
23180	1.13511	0.200558
23181	0.946131	0.181515
23182	0.916275	2.1
23183	0.946132	2.04202
23184	0.916274	1.4331
23185	1.36182	1.83402
23186	0.946131	1.97163
23187	0.857202	0.05
23188	0.941918	0.05
23189	0.943349	0.05
23190	1.2493	0.05
23191	0.946133	0.05
23192	1.01814	0.05
23193	0.927881	0.05
23194	1.11845	0.05
23195	1.2493	0.05
23196	1.11845	0.05
23197	0.946129	0.05

23198	0.967673	0.336818
23199	1.01332	0.05
23200	0.946133	0.05
23201	0.81938	0.05
23202	0.819377	0.05
23203	0.946133	0.05
23204	1.22474	0.0891609
23205	1.15321	0.0715624
23206	0.797489	0.0710373
23207	0.946131	0.0891182
23208	0.797461	0.0737177
23209	1.35328	1.23282
23210	1.03318	1.35206
23211	0.946131	1.26437
23212	0.94613	1.27689
23213	1.03319	1.27651
23214	1.37938	0.808681
23215	1.00512	1.3363
23216	0.946133	1.13544
23217	0.946132	1.20904
23218	1.00512	1.27816
23219	1.5515	1.30476
23220	1.55351	2.1
23221	0.94261	0.05
23222	0.828156	0.05
23223	0.916495	0.05
23224	3.06309	0.05
23225	0.619832	0.05
23226	0.705903	0.05
23227	1.2968	0.05
23228	0.946133	0.05
23229	0.705904	0.05
23230	1.2968	0.05
23231	1.01971	0.05
23232	0.60302	0.05
23233	1.07242	0.417534
23234	1.26426	0.244175
23235	0.946131	0.441304
23236	0.946131	0.465736
23237	0.963109	0.430714
23238	0.947862	0.321091
23239	1.40248	1.46076
23240	0.767143	1.47107
23241	0.767114	1.48631
23242	0.946129	1.49688
23243	0.767143	1.27497
23244	1.22637	1.51741
23245	1.2281	1.45678
23246	0.590909	1.45048
23247	0.490832	1.45474
23248	0.804174	1.49361
23249	0.747368	1.40216
23250	1.05129	0.0548124
23251	1.10609	2.05116
23252	1.3639	0.0871275
23253	0.776117	0.068659
23254	0.752329	0.0893166
23255	1.04073	0.119795

23256	0.67713	0.0956618
23257	3.4245	2.1
23258	1.18526	2.1
23259	1.2161	1.61931
23260	0.876834	1.81595
23261	0.876831	1.62692
23262	0.946132	1.72577
23263	0.876834	1.71326
23264	0.954563	2.1
23265	0.924132	2.1
23266	1.25075	2.1
23267	1.01652	2.1
23268	0.92413	2.1
23269	1.37579	2.1
23270	0.992159	2.1
23271	1.26092	2.1
23272	0.929435	2.1
23273	0.844958	2.1
23274	0.79598	2.1
23275	0.852559	2.1
23276	0.946131	0.698161
23277	1.28435	0.354195
23278	0.827261	0.541383
23279	0.946128	0.773398
23280	0.827298	0.794728
23281	1.65452	0.152785
23282	1.79929	2.1
23283	0.791947	0.482707
23284	0.902072	1.22297
23285	0.882672	0.490902
23286	1.21703	2.1
23287	0.900338	2.1
23288	1.37712	2.1
23289	0.782591	2.1
23290	0.782562	2.1
23291	0.94613	2.1
23292	1.34749	2.1
23293	0.61359	2.1
23294	1.14397	1.0962
23295	1.02963	0.05
23296	1.36589	0.05
23297	0.946132	0.05
23298	1.28644	0.05
23299	0.946131	0.05
23300	1.40104	0.05
23301	1.28644	0.05
23302	0.810946	0.0897374
23303	1.09178	0.0839642
23304	1.26956	0.0721498
23305	0.810943	0.0878746
23306	0.885633	0.0881534
23307	1.00844	0.0784408
23308	0.825671	0.0950963
23309	0.792093	0.0867174
23310	0.925037	0.0861409
23311	0.946131	1.88146
23312	0.946131	0.227262
23313	1.18052	0.584223

23314	1.40265	0.510351
23315	0.946132	1.39487
23316	1.17891	0.43975
23317	0.734248	0.05
23318	0.810943	0.05
23319	0.731621	0.05
23320	0.799857	0.287801
23321	0.834507	0.530668
23322	0.810932	0.27804
23323	1.01316	0.05
23324	1.23884	0.05
23325	1.88438	2.1
23326	0.946129	2.1
23327	1.38614	1.96115
23328	1.34245	1.81054
23329	0.946131	1.5962
23330	1.38614	1.78977
23331	0.946132	0.05
23332	0.910733	0.05
23333	1.24726	0.05
23334	1.13742	0.05
23335	0.851555	0.05
23336	1.50368	0.05
23337	0.94613	2.1
23338	1.08998	2.1
23339	1.36899	1.95723
23340	1.36898	2.1
23341	0.946131	2.1
23342	1.26045	2.1
23343	1.06432	0.923596
23344	1.89016	0.315838
23345	0.83514	2.1
23346	1.36653	2.1
23347	1.23426	2.1
23348	0.94613	2.1
23349	0.835112	2.1
23350	1.14567	1.77775
23351	1.12096	1.81167
23352	0.881106	1.80775
23353	1.17787	0.600691
23354	1.29738	2.1
23355	0.940472	1.40153
23356	1.03452	1.72037
23357	0.946131	1.18366
23358	2.7489	1.19407
23359	1.01778	2.1
23360	1.04321	1.41131
23361	1.19352	0.129136
23362	1.16912	1.63541
23363	0.946132	1.50209
23364	0.946132	1.66156
23365	1.40924	1.59561
23366	1.22927	1.71248
23367	2.09455	0.0683927
23368	3.00575	2.1
23369	1.08928	2.1
23370	1.94799	2.1
23371	1.4092	2.1

23372	0.946132	2.1
23373	1.16	2.1
23374	1.01985	2.1
23375	0.946133	2.1
23376	1.16	2.1
23377	1.15371	0.05
23378	1.56002	0.05
23379	1.23007	2.1
23380	1.9992	2.1
23381	0.652605	2.1
23382	0.937903	2.1
23383	0.946132	2.1
23384	0.937902	2.1
23385	1.228	2.1
23386	1.33694	1.9634
23387	1.00191	2.1
23388	0.716814	2.00667
23389	0.94613	2.1
23390	0.716816	2.1
23391	1.70388	1.5322
23392	0.946129	0.137996
23393	1.09362	0.112832
23394	0.946131	0.291498
23395	1.4185	0.230817
23396	1.09362	0.125373
23397	1.31057	1.72419
23398	1.17631	0.05
23399	1.19404	0.05
23400	0.94613	0.0846724
23401	0.946129	0.059332
23402	0.99972	0.0673749
23403	0.94613	0.068089
23404	0.835199	0.05
23405	1.23351	0.05
23406	0.582785	0.05
23407	0.701079	0.05
23408	0.946132	0.05
23409	2.17385	0.05
23410	1.18948	2.06785
23411	1.25346	2.1
23412	1.18586	0.355682
23413	0.880876	0.331855
23414	0.68632	0.731549
23415	0.946118	2.1
23416	0.966936	2.1
23417	0.946117	2.1
23418	1.41636	2.1
23419	1.03798	2.1
23420	0.729178	0.493247
23421	0.721888	0.463405
23422	0.946132	0.534713
23423	0.656286	0.372435
23424	1.13898	0.501072
23425	1.02351	0.05
23426	1.48803	0.05
23427	0.946131	0.05
23428	1.04921	2.1
23429	1.02478	1.36809

23430	0.993023	2.1
23431	1.80841	0.109344
23432	1.29215	0.0823493
23433	0.904206	0.0941525
23434	0.904207	0.102207
23435	0.946132	0.108427
23436	0.904207	0.0969866
23437	1.07347	2.1
23438	0.555875	1.76042
23439	0.555904	1.78709
23440	1.30841	1.90727
23441	1.30841	1.90391
23442	0.91951	1.94638
23443	1.33026	0.543906
23444	1.43289	0.05
23445	1.15703	1.79756
23446	0.886594	1.94087
23447	1.05129	1.83416
23448	1.08421	0.37178
23449	1.35946	0.221122
23450	0.882955	0.973279
23451	0.946131	0.939437
23452	0.882985	0.663563
23453	0.583501	0.685934
23454	0.532921	0.696372
23455	0.94613	1.83089
23456	0.532892	1.6538
23457	1.23224	1.69447
23458	1.12786	1.67956
23459	0.946131	2.1
23460	1.23732	0.284826
23461	1.40609	2.1
23462	1.40609	0.937864
23463	0.946131	0.780076
23464	0.910672	1.53541
23465	1.08478	1.38521
23466	0.94613	1.11745
23467	0.94613	1.51701
23468	1.30382	1.54621
23469	3.10544	1.46152
23470	0.679509	1.9283
23471	1.15966	1.71969
23472	0.75499	2.01056
23473	1.37511	1.74228
23474	1.01776	1.74651
23475	2.35239	1.27364
23476	0.92406	2.1
23477	0.946118	2.1
23478	0.92406	2.1
23479	1.07979	2.1
23480	1.29604	2.1
23481	1.29897	2.1
23482	0.902645	0.404263
23483	0.729493	1.30377
23484	0.906844	1.08664
23485	0.739518	0.05
23486	1.10564	0.0544994
23487	0.946131	0.05

23488	0.739519	0.05
23489	0.739506	0.05
23490	1.79907	0.1267
23491	1.59106	1.11676
23492	1.11067	0.43628
23493	0.795516	1.07483
23494	0.795519	0.726713
23495	0.946132	0.744677
23496	0.795506	0.694251
23497	1.80223	2.1
23498	1.95572	2.04636
23499	1.16237	1.499
23500	1.07518	2.1
23501	0.696199	1.72715
23502	0.94613	1.84888
23503	0.696199	1.79609
23504	1.6379	0.05
23505	0.946118	0.05
23506	0.851529	0.05
23507	1.08716	0.05
23508	0.973118	0.05
23509	0.851528	0.05
23510	1.15096	2.1
23511	0.65023	0.05
23512	0.94613	0.05
23513	0.650205	0.05
23514	0.650205	0.05
23515	0.811233	0.05
23516	0.778721	1.89439
23517	0.810945	1.50902
23518	0.997518	1.58472
23519	0.838028	0.0616242
23520	0.952893	0.05
23521	0.838037	0.0576201
23522	1.58166	0.05
23523	1.44848	1.35491
23524	1.30041	0.05
23525	1.03697	0.581536
23526	0.946132	1.81266
23527	1.0277	1.66434
23528	0.946131	1.90823
23529	1.00501	1.64578
23530	1.0277	1.78556
23531	1.64988	2.1
23532	0.946131	2.1
23533	0.94613	1.9995
23534	1.27702	2.1
23535	1.55163	2.1
23536	0.897905	2.1
23537	1.12971	0.05
23538	1.35715	0.113841
23539	0.881884	0.578883
23540	0.664007	0.138495
23541	0.663978	0.20744
23542	0.94613	0.185486
23543	0.664006	0.221408
23544	1.48693	0.0894055
23545	1.29289	0.0510844

23546	1.32081	0.0611005
23547	0.946119	0.0726911
23548	0.946132	0.0643318
23549	1.02972	0.0699529
23550	0.946131	2.1
23551	0.946127	2.1
23552	1.23513	2.1
23553	1.38154	2.1
23554	0.94613	2.1
23555	1.25933	0.05
23556	0.917954	0.05
23557	0.868589	0.05
23558	3.0957	2.1
23559	0.711391	0.05
23560	0.711391	0.05
23561	0.991303	0.05
23562	1.37322	0.05
23563	0.729145	0.05
23564	2.55648	0.05
23565	1.0756	1.84119
23566	0.760299	1.506
23567	0.763592	0.948472
23568	0.946126	1.42428
23569	0.76359	1.02209
23570	1.35862	1.58685
23571	0.946133	2.1
23572	1.00807	2.1
23573	0.946132	2.1
23574	1.39823	2.1
23575	1.07817	2.1
23576	1.44379	0.358031
23577	1.17934	0.05
23578	1.13451	0.0839382
23579	0.94613	2.1
23580	1.03974	2.1
23581	1.36835	2.1
23582	1.36835	2.1
23583	0.94613	2.1
23584	1.27096	2.1
23585	0.853042	2.1
23586	0.946131	2.1
23587	1.13658	2.1
23588	1.06997	2.1
23589	0.94613	2.1
23590	1.41556	0.292501
23591	1.21708	0.650914
23592	0.94613	0.902668
23593	0.946131	0.566816
23594	1.21708	0.51465
23595	0.964782	0.316817
23596	0.946119	0.66441
23597	1.23447	0.455312
23598	0.946118	0.653312
23599	1.41844	0.388949
23600	1.23446	0.470837
23601	1.1357	0.242569
23602	0.619856	0.05
23603	1.1509	0.05

23604	0.946126	0.05
23605	1.19262	0.05
23606	1.1509	0.05
23607	1.04268	2.1
23608	2.07527	1.04165
23609	1.38259	1.44529
23610	0.980054	1.58293
23611	0.946124	1.46536
23612	0.946127	1.51137
23613	0.980056	1.50907
23614	1.62916	1.78938
23615	0.946131	2.1
23616	1.40571	2.1
23617	0.946131	2.1
23618	1.38885	2.1
23619	1.40571	2.1
23620	1.11787	2.1
23621	0.946129	2.1
23622	1.28831	2.1
23623	1.41498	2.1
23624	1.41498	2.1
23625	0.946131	2.1
23626	0.850964	0.05
23627	0.921299	0.05
23628	1.37358	0.05
23629	0.946132	0.05
23630	0.850964	0.05
23631	0.823988	0.05
23632	0.823984	0.05
23633	1.36568	0.05
23634	1.36569	0.05
23635	0.921275	0.05
23636	1.84739	2.1
23637	1.47169	0.0612692
23638	0.946131	0.217041
23639	0.735844	0.143345
23640	1.11819	0.215987
23641	1.10904	0.106621
23642	0.735831	0.173054
23643	0.946131	2.1
23644	0.946132	2.1
23645	1.23487	2.1
23646	1.3281	2.1
23647	0.946131	2.1
23648	1.72271	0.05
23649	0.861359	0.05
23650	0.946131	0.0781907
23651	0.861357	0.0566734
23652	1.1369	0.0581425
23653	1.26868	0.0541558
23654	0.789173	1.99939
23655	0.789143	2.0869
23656	0.946133	1.81824
23657	1.34386	1.92205
23658	0.789144	1.94871
23659	0.983493	2.1
23660	1.18944	0.396815
23661	1.05121	0.463813

23662	0.777398	0.390171
23663	0.946132	0.920357
23664	0.777423	0.980374
23665	1.09841	0.0867725
23666	0.963919	0.0721233
23667	0.474855	0.0923524
23668	0.946131	0.0918405
23669	0.474855	0.0888727
23670	3.40074	2.1
23671	1.29453	2.1
23672	1.01139	0.502306
23673	0.946133	1.50956
23674	1.35381	0.112785
23675	1.18322	1.14655
23676	0.946133	1.64366
23677	1.45502	0.05
23678	0.970088	1.60338
23679	0.960333	1.62858
23680	0.838036	1.62694
23681	1.30927	0.0992544
23682	0.828617	0.10364
23683	0.714795	0.101189
23684	0.946132	0.106348
23685	0.714824	0.09918
23686	0.946131	2.1
23687	0.851542	2.1
23688	1.38204	2.1
23689	1.24512	2.1
23690	1.04086	2.1
23691	0.723573	2.1
23692	0.946131	2.1
23693	0.982454	2.1
23694	0.982454	2.1
23695	0.723544	2.1
23696	1.17261	0.056319
23697	1.34153	0.0540332
23698	1.41383	0.05
23699	0.946132	0.108864
23700	0.946133	0.0651362
23701	1.12818	0.132959
23702	1.29383	0.0721556
23703	0.946133	0.457469
23704	1.0027	0.410123
23705	0.768989	0.292581
23706	0.946133	0.465043
23707	0.768977	0.425138
23708	0.946132	0.795825
23709	1.24333	0.447668
23710	0.928583	0.792876
23711	0.946132	0.818181
23712	0.928583	0.871584
23713	1.53798	0.172007
23714	2.95436	0.0731168
23715	1.30649	0.10009
23716	0.809789	0.169757
23717	0.745744	0.152023
23718	0.745774	0.153467
23719	0.946128	0.144136

23720	1.00777	0.0583574
23721	1.40271	0.05
23722	0.768387	0.0685142
23723	0.946132	0.0637575
23724	0.922045	0.059732
23725	0.946132	2.1
23726	1.39021	2.1
23727	1.10046	2.1
23728	0.946132	2.1
23729	1.39021	2.1
23730	2.06158	0.05
23731	0.913967	0.352157
23732	1.30107	0.382793
23733	0.946132	0.460786
23734	0.913966	0.0939832
23735	1.30107	0.326755
23736	1.64117	2.1
23737	0.514872	1.87368
23738	0.514846	1.70584
23739	1.30811	2.1
23740	1.30812	1.77223
23741	0.888989	1.86847
23742	1.99279	1.96899
23743	0.910271	0.537189
23744	0.838027	0.839665
23745	0.928444	0.541604
23746	1.2377	1.48484
23747	0.851537	2.1
23748	0.899142	2.1
23749	0.810075	2.1
23750	1.3771	2.1
23751	1.03281	2.1
23752	0.719877	2.1
23753	0.901838	2.1
23754	1.01748	2.1
23755	1.12315	2.1
23756	0.64787	2.1
23757	1.173 2.1	
23758	0.876903	2.1
23759	0.597337	2.1
23760	1.00291	2.1
23761	0.597309	2.1
23762	1.09897	0.290001
23763	0.946132	0.75939
23764	1.1386	0.268364
23765	1.09897	0.370476
23766	0.596652	0.377179
23767	2.5593	2.1
23768	0.946133	0.389763
23769	1.29172	0.364879
23770	1.11337	0.208548
23771	0.946131	0.372424
23772	0.946131	0.281493
23773	0.96926	1.19437
23774	0.946132	1.17664
23775	0.946132	1.29075
23776	1.07118	1.09711
23777	1.29698	1.13977

23778	1.37867	1.32983
23779	0.946119	1.41269
23780	0.94612	1.53256
23781	1.33154	1.26792
23782	0.946107	1.34967
23783	2.17757	1.33772
23784	0.946131	0.0537929
23785	0.68393	0.05
23786	0.68396	0.05
23787	1.33671	0.05
23788	1.10733	0.05
23789	2.31367	2.1
23790	0.989326	1.87544
23791	0.689952	2.1
23792	0.854787	1.78774
23793	3.73635	2.1
23794	1.41489	2.1
23795	1.25544	1.99486
23796	0.946128	2.1
23797	0.946129	2.1
23798	1.13536	2.1
23799	2.10284	1.7715
23800	0.851542	2.1
23801	0.828523	2.1
23802	0.946132	2.1
23803	0.946132	2.1
23804	1.50873	2.1
23805	0.989048	2.1
23806	0.925055	2.1
23807	0.838041	2.1
23808	0.807714	0.688885
23809	0.656831	0.35199
23810	0.997603	0.566712
23811	0.946131	1.50606
23812	1.02602	1.38682
23813	0.946129	1.51115
23814	1.35469	1.22455
23815	1.25083	1.41569
23816	0.851541	0.227102
23817	0.851483	0.228258
23818	0.851539	0.234298
23819	1.19785	0.200879
23820	1.07615	0.194079
23821	0.714507	0.0663027
23822	1.41461	0.0651064
23823	0.714507	0.0689107
23824	0.714507	0.0707169
23825	0.946133	0.0673179
23826	0.946131	1.45382
23827	0.972232	1.31532
23828	0.94613	1.49705
23829	1.25083	0.831735
23830	1.30165	1.29946
23831	1.05332	2.1
23832	0.574768	2.1
23833	1.94541	0.05
23834	0.789633	0.05
23835	0.946127	0.05

23836	1.33798	0.05
23837	1.22903	0.05
23838	0.946128	0.05
23839	0.946128	2.1
23840	1.11882	2.1
23841	0.996487	2.1
23842	0.94613	2.1
23843	1.11883	2.1
23844	0.946127	2.1
23845	1.30082	2.1
23846	1.02608	2.1
23847	0.946128	2.1
23848	1.30081	2.1
23849	1.3507	1.83315
23850	1.19583	0.168892
23851	0.946127	1.74138
23852	0.94613	1.29069
23853	1.19583	1.63179
23854	0.477652	0.05
23855	1.20416	0.05
23856	0.946131	0.05
23857	0.477652	0.05
23858	0.477691	0.05
23859	1.34536	0.05
23860	0.758971	0.05
23861	1.25124	0.05
23862	1.31467	0.05
23863	0.758971	0.05
23864	1.31467	0.05
23865	0.946131	2.1
23866	1.14184	2.1
23867	1.35028	2.1
23868	1.35028	2.1
23869	0.946131	2.1
23870	1.13537	2.08405
23871	0.946132	1.72538
23872	0.946132	2.08715
23873	1.23031	1.85745
23874	1.27555	1.88972
23875	1.53064	1.82378
23876	1.28421	1.26555
23877	1.07707	1.44357
23878	0.946125	1.2964
23879	0.946127	1.33127
23880	1.07707	1.34968
23881	0.946133	0.0503204
23882	1.3495	0.05
23883	0.946132	0.05
23884	1.05704	0.05
23885	1.3495	0.05
23886	0.946132	0.05
23887	1.37794	0.05
23888	0.946132	0.05
23889	1.01367	0.05
23890	1.37794	0.05
23891	0.928516	2.1
23892	0.946133	2.1
23893	0.928518	2.1

23894	1.14105	2.1
23895	1.26406	2.1
23896	0.797664	2.1
23897	0.946132	2.1
23898	0.797663	2.1
23899	1.34285	2.1
23900	0.929875	2.1
23901	0.946131	2.1
23902	1.21139	0.757013
23903	1.41492	0.824766
23904	1.41492	1.3305
23905	0.946131	0.233512
23906	0.946131	2.1
23907	0.689388	2.1
23908	0.94365	2.1
23909	0.777345	2.1
23910	0.709599	2.1
23911	0.774968	2.1
23912	0.946132	2.1
23913	0.77497	2.1
23914	0.868145	2.1
23915	1.28401	2.1
23916	0.94613	2.0619
23917	1.19655	2.1
23918	1.39428	0.742609
23919	1.39428	2.1
23920	0.946131	2.1
23921	1.1848	2.1
23922	1.07591	2.1
23923	0.946131	2.1
23924	0.946131	2.1
23925	0.874788	2.1
23926	1.35703	2.1
23927	1.35357	2.1
23928	0.946132	2.1
23929	1.26022	2.1
23930	0.946132	2.1
23931	0.946131	2.1
23932	0.966426	2.1
23933	0.946133	2.1
23934	0.946131	2.1
23935	1.3484	2.1
23936	1.26441	2.1
23937	1.2079	2.1
23938	0.946133	2.1
23939	0.946132	2.1
23940	1.2079	2.1
23941	1.2736	2.1
23942	1.25774	2.1
23943	0.946133	2.1
23944	0.851555	2.1
23945	0.96172	2.1
23946	0.946128	2.1
23947	1.06879	2.1
23948	0.94613	2.1
23949	1.32608	2.1
23950	1.06879	2.1
23951	0.946132	2.1

23952	1.32608	2.1
23953	0.946131	2.1
23954	1.40488	2.1
23955	1.32608	2.1
23956	0.94613	1.71834
23957	1.19234	0.62858
23958	0.946129	0.839349
23959	0.94613	1.70555
23960	1.31649	1.76001
23961	0.912059	1.80413
23962	1.04514	1.43379
23963	0.946132	1.40771
23964	0.946132	2.08329
23965	1.34646	2.04002
23966	1.01187	0.320002
23967	1.33728	1.10183
23968	0.761974	0.520577
23969	0.761974	1.14559
23970	0.94613	0.500233
23971	1.40641	2.1
23972	0.955752	0.831232
23973	0.736738	1.92938
23974	0.94613	1.61811
23975	0.736739	1.80694
23976	0.861471	2.1
23977	1.34556	2.1
23978	0.810957	2.1
23979	0.946131	2.1
23980	1.37811	2.1
23981	1.17949	2.1
23982	0.946132	2.1
23983	1.37811	2.1
23984	0.946131	2.1
23985	1.40447	2.1
23986	0.946132	2.1
23987	0.946132	2.1
23988	1.39826	2.1
23989	0.946131	2.1
23990	1.283 2.1	
23991	0.94613	2.1
23992	0.946131	2.1
23993	1.40447	2.1
23994	1.20541	2.1
23995	0.946131	2.1
23996	0.946127	2.1
23997	1.33607	2.1
23998	1.41021	2.1
23999	1.01271	2.1
24000	0.946131	2.1
24001	0.920606	2.1
24002	1.13078	2.1
24003	1.60331	2.1
24004	0.946118	1.70093
24005	1.09718	2.1
24006	0.946116	2.1
24007	1.32662	1.86119
24008	1.0972	1.94642
24009	0.94613	1.32584

24010	1.32666	1.76234
24011	0.946128	1.26746
24012	1.31381	0.556338
24013	1.32666	0.655362
24014	0.94612	2.1
24015	0.940748	2.1
24016	1.03561	2.1
24017	1.35111	2.1
24018	0.851527	2.1
24019	0.607156	2.1
24020	0.789158	2.1
24021	0.946129	2.1
24022	1.04071	2.1
24023	1.13144	2.1
24024	0.946133	2.1
24025	1.35663	2.1
24026	0.946132	2.1
24027	0.946133	2.1
24028	1.34237	2.1
24029	0.919455	2.1
24030	1.01143	2.1
24031	0.946128	2.1
24032	0.946131	2.1
24033	1.62796	2.1
24034	1.17796	1.80061
24035	1.23024	2.1
24036	0.946132	1.8189
24037	0.85153	1.92884
24038	0.986867	1.91466
24039	0.889308	2.1
24040	0.94613	2.1
24041	0.889338	2.1
24042	0.946131	2.1
24043	0.889337	2.1
24044	1.18396	1.32589
24045	0.94613	1.18504
24046	0.946125	1.3956
24047	1.39514	1.22016
24048	1.30809	1.33655
24049	0.803322	2.0611
24050	0.925893	1.82119
24051	1.36183	1.50312
24052	0.925892	1.64174
24053	0.946133	1.64005
24054	1.38354	0.0928524
24055	1.16009	0.0877401
24056	0.946132	0.101432
24057	0.946132	0.0850132
24058	1.01462	0.0649133
24059	1.18854	1.40049
24060	0.888265	1.65176
24061	0.745095	1.52447
24062	0.709597	1.59397
24063	0.936541	1.58668
24064	1.24142	0.483713
24065	1.35059	0.330302
24066	0.946128	0.306142
24067	0.94613	0.414198

24068	1.16623	0.390799
24069	1.24945	0.0826362
24070	1.33035	0.082717
24071	0.946127	0.106113
24072	0.946128	0.0828852
24073	1.13343	0.132069
24074	0.637995	1.44273
24075	1.10455	1.45268
24076	1.22641	1.51135
24077	1.22641	1.45442
24078	0.946132	1.42167
24079	1.3665	0.0862001
24080	1.28507	0.0878696
24081	0.946127	0.101345
24082	0.946129	0.081336
24083	1.2051	0.121906
24084	0.797374	2.1
24085	0.797376	2.1
24086	1.13113	2.1
24087	1.20057	2.1
24088	1.12049	2.1
24089	0.59387	1.23337
24090	1.13245	1.21425
24091	0.966134	1.31548
24092	0.593842	1.18264
24093	0.946128	1.25936
24094	0.641455	0.696477
24095	0.916507	0.480553
24096	1.04072	1.4035
24097	0.415585	0.540647
24098	0.507244	1.3386
24099	0.678938	2.06998
24100	1.24154	2.05952
24101	1.25	1.89551
24102	1.24154	1.94927
24103	1.25	1.96823
24104	0.851009	1.87176
24105	0.946131	1.96945
24106	0.851539	1.81619
24107	1.21997	1.79311
24108	1.45109	1.8193
24109	0.804877	1.03841
24110	1.00062	0.329821
24111	0.851536	0.902383
24112	0.909673	0.368926
24113	0.946129	0.849809
24114	1.20494	0.0934508
24115	1.09088	0.14298
24116	0.887634	0.159575
24117	0.81027	0.083329
24118	0.900294	0.105548
24119	0.747713	0.05
24120	0.975467	0.05
24121	0.747712	0.05
24122	1.39168	0.05
24123	0.975467	0.05
24124	0.94613	0.0911326
24125	1.32827	0.0931669

24126	0.946128	0.083611
24127	1.41201	0.0943764
24128	1.32827	0.0883965
24129	0.981121	0.104071
24130	0.936311	0.17434
24131	1.00715	0.102399
24132	1.15914	0.127388
24133	0.704409	0.120389
24134	0.713444	0.730857
24135	0.954538	0.771256
24136	0.944508	0.609877
24137	0.851165	1.39745
24138	0.946131	1.53683
24139	0.851192	1.28694
24140	0.946131	1.13436
24141	1.3279	1.02362
24142	1.33587	0.05
24143	0.946126	0.05
24144	1.27512	0.05
24145	1.33587	0.05
24146	0.748059	0.05
24147	1.17451	0.05
24148	1.04299	0.05
24149	0.851542	0.0540695
24150	0.946131	0.05
24151	0.892109	0.05
24152	0.946131	0.05
24153	0.851541	0.05
24154	1.2178	0.05
24155	1.2179	0.05
24156	0.946793	0.05
24157	0.709599	0.05
24158	0.987426	0.05
24159	1.39502	0.05
24160	1.13624	0.05
24161	0.73176	0.05
24162	1.36697	0.05
24163	1.33393	0.05
24164	0.946131	0.05
24165	0.946132	0.05
24166	1.23845	0.05
24167	0.50702	1.07849
24168	1.06442	0.376723
24169	0.946131	1.1031
24170	1.02363	0.911845
24171	1.13537	0.05
24172	1.25049	0.05
24173	0.94613	0.05
24174	1.14993	0.05
24175	1.38056	0.05
24176	0.946126	0.05
24177	1.24678	0.05
24178	0.684521	0.05
24179	0.946124	0.05
24180	0.684518	0.05
24181	0.851542	0.291239
24182	0.925459	0.251145
24183	0.946131	0.29441

24184	0.851474	0.302902
24185	0.923305	0.289171
24186	1.4055	0.05
24187	1.32077	0.05
24188	0.946127	0.05
24189	1.21757	0.05
24190	0.946129	0.05
24191	0.711214	0.05
24192	1.30463	0.0542663
24193	0.711185	0.05
24194	0.711185	0.052776
24195	1.22716	0.0503164
24196	1.29958	0.05
24197	1.26201	0.05
24198	0.568927	0.05
24199	0.568927	0.05
24200	0.946132	0.05
24201	0.851542	0.0660133
24202	1.44968	0.05
24203	0.851543	0.0570749
24204	1.35123	0.059167
24205	1.40427	0.0502167
24206	0.593295	0.0775074
24207	0.946129	0.082351
24208	1.22144	0.0694755
24209	0.908278	0.0901149
24210	1.22144	0.0798435
24211	0.867593	0.194908
24212	1.23351	0.15079
24213	0.946131	0.194188
24214	1.05516	0.192728
24215	1.23351	0.161432
24216	1.15564	0.0603431
24217	0.946131	0.106032
24218	0.766652	0.0721007
24219	0.766681	0.0672645
24220	0.946131	0.0860393
24221	0.946131	0.119212
24222	0.897851	0.105403
24223	1.1751	0.139109
24224	1.00273	0.105791
24225	0.851554	0.11183
24226	0.883272	0.0917187
24227	1.17111	0.822877
24228	0.851541	0.125715
24229	1.01665	0.14836
24230	0.851555	0.125103
24231	0.946131	0.349167
24232	0.911508	0.218119
24233	1.08054	0.374037
24234	1.33007	0.166419
24235	0.911471	0.277794
24236	1.26787	1.55188
24237	1.16641	1.71106
24238	0.946132	1.49951
24239	0.946133	1.55224
24240	1.16641	1.59639
24241	0.946131	2.1

24242	1.25037	0.56438
24243	1.34733	2.1
24244	1.34733	2.1
24245	0.946131	2.1
24246	0.946131	0.05
24247	1.41377	0.05
24248	0.94613	0.05
24249	1.30541	0.05
24250	1.41377	0.05
24251	1.3473	2.1
24252	1.11804	2.1
24253	0.94613	2.1
24254	0.946131	2.1
24255	1.11805	2.1
24256	1.32068	2.1
24257	0.946131	2.1
24258	0.946128	2.1
24259	1.39384	2.1
24260	1.4181	2.1
24261	1.12913	2.1
24262	0.94613	2.1
24263	0.946129	2.1
24264	1.20328	2.1
24265	1.39385	2.1
24266	1.3335	2.1
24267	1.40987	2.1
24268	0.94613	2.1
24269	1.21474	2.1
24270	0.94613	2.1
24271	0.946132	2.1
24272	1.15203	2.1
24273	1.41192	2.1
24274	1.41192	2.1
24275	0.946132	2.1
24276	0.946131	2.1
24277	1.40684	2.1
24278	1.2597	2.1
24279	0.946131	2.1
24280	1.40684	2.1
24281	0.946131	2.1
24282	1.39113	2.1
24283	1.34049	2.05115
24284	0.946131	1.89643
24285	1.39113	1.94758
24286	0.946132	1.51864
24287	1.31649	0.360403
24288	0.946132	1.16987
24289	0.946132	1.309
24290	1.34297	1.44108
24291	0.946131	0.361844
24292	1.41766	0.38428
24293	0.954559	0.565171
24294	0.946131	0.441254
24295	0.946131	0.237001
24296	1.25447	0.130446
24297	1.35559	0.0799861
24298	0.946127	0.120108
24299	0.946128	0.15949

24300	1.14874	0.140663
24301	1.33035	0.0726084
24302	1.3665	0.0818811
24303	0.946129	0.0997612
24304	0.94613	0.0676019
24305	1.20732	0.118233
24306	0.946131	2.1
24307	0.946131	2.1
24308	1.32949	2.1
24309	1.34921	2.1
24310	0.946131	2.1
24311	0.946132	1.27151
24312	1.25479	0.91834
24313	1.38357	0.260502
24314	1.38357	0.254822
24315	0.946132	0.117387
24316	0.946131	2.1
24317	1.31034	0.354017
24318	1.37127	2.1
24319	1.37127	0.795286
24320	0.946131	1.43079
24321	1.23923	0.112716
24322	1.41169	0.175327
24323	0.94613	0.218175
24324	1.12703	0.123186
24325	0.946131	0.149822
24326	0.946119	2.1
24327	1.41915	2.1
24328	1.02679	2.1
24329	0.946107	2.1
24330	1.41917	2.1
24331	0.946133	2.1
24332	1.36581	2.1
24333	0.946131	2.1
24334	1.36581	2.1
24335	0.946131	2.1
24336	0.946132	2.1
24337	1.04384	2.1
24338	1.34973	2.1
24339	1.34967	2.1
24340	0.851555	2.1
24341	0.946132	0.053117
24342	1.3855	0.05
24343	1.08603	0.0529654
24344	1.08602	0.05
24345	1.16234	0.0562666
24346	0.946133	0.0543823
24347	1.29348	0.05
24348	1.20111	0.0522106
24349	1.20112	0.05
24350	1.3855	0.0594289
24351	0.946128	2.1
24352	1.30798	2.1
24353	0.946129	2.1
24354	1.34828	2.1
24355	1.30797	2.1
24356	1.24932	0.05
24357	1.40549	0.05

24358	0.946117	0.05
24359	1.19615	0.05
24360	0.946106	0.05
24361	0.946131	2.1
24362	1.29926	2.1
24363	1.25144	2.1
24364	0.946131	2.1
24365	1.29925	2.1
24366	0.946132	2.1
24367	1.39826	2.1
24368	0.946133	2.1
24369	0.946133	2.1
24370	1.29926	2.1
24371	0.946119	0.05
24372	1.33614	0.05
24373	0.946118	0.05
24374	1.41377	0.05
24375	1.33612	0.05
24376	0.946133	1.70348
24377	1.3933	2.1
24378	1.34999	1.40546
24379	0.946133	1.61217
24380	1.3933	1.69242
24381	0.946131	2.06131
24382	1.36139	2.1
24383	0.946132	1.80252
24384	0.946131	2.1
24385	1.39329	2.1
24386	1.37449	1.87069
24387	1.27925	0.328624
24388	0.919796	0.612683
24389	1.01176	1.39058
24390	0.94613	0.990492
24391	0.946133	2.1
24392	1.27679	2.1
24393	0.946133	2.1
24394	0.946132	2.1
24395	1.39731	2.1
24396	0.946119	2.1
24397	1.40765	1.88709
24398	1.1981	2.1
24399	0.946131	2.07246
24400	1.40764	2.1
24401	0.946127	2.1
24402	1.3557	2.1
24403	0.946127	2.1
24404	0.946128	2.1
24405	1.13485	2.1
24406	1.4008	2.1
24407	1.1587	2.1
24408	0.946131	2.1
24409	0.946131	2.1
24410	1.1587	2.1
24411	1.32082	0.0674223
24412	1.29215	0.0808958
24413	0.94613	0.0913837
24414	0.946131	0.0921256
24415	1.02366	0.0906556

24416	1.27697	0.05
24417	1.2929	0.05
24418	0.946132	0.0627076
24419	0.946132	0.05
24420	1.01451	0.051111
24421	0.946132	2.1
24422	1.27252	2.1
24423	0.946132	2.1
24424	1.37869	2.1
24425	1.27252	2.1
24426	1.15627	2.1
24427	1.35703	2.1
24428	0.946132	2.1
24429	1.03783	2.1
24430	0.946132	2.1
24431	0.946128	2.1
24432	1.37869	2.1
24433	0.946131	2.1
24434	1.35357	2.1
24435	1.37869	2.1
24436	0.946132	2.1
24437	0.946131	2.1
24438	1.33471	2.1
24439	0.946131	2.1
24440	1.36373	2.1
24441	1.41342	1.16558
24442	1.06832	1.49541
24443	0.946128	1.25316
24444	0.94613	0.439033
24445	1.06832	1.28977
24446	1.26929	2.1
24447	0.946131	2.1
24448	0.946128	2.1
24449	1.41022	2.1
24450	1.37034	2.1
24451	1.37759	1.3751
24452	1.07612	1.53673
24453	0.946126	1.40202
24454	0.946129	1.43727
24455	1.07612	1.45398
24456	0.946131	2.0873
24457	0.94613	0.41755
24458	1.23848	1.00729
24459	1.38897	1.03494
24460	0.946131	1.04823
24461	0.946132	1.12742
24462	0.946133	0.321341
24463	1.34369	0.139641
24464	1.40332	0.474893
24465	0.946132	0.127166
24466	0.946131	2.1
24467	1.24023	2.1
24468	1.38244	2.1
24469	1.38244	2.1
24470	0.946131	2.1
24471	0.946132	2.1
24472	1.33468	2.1
24473	1.41619	2.1

24474	1.41619	2.1
24475	0.946131	2.1
24476	1.1908	1.44066
24477	0.946128	1.34886
24478	0.946126	1.46912
24479	1.26654	1.43433
24480	1.39514	1.57482
24481	0.93608	2.1
24482	1.04105	2.1
24483	1.39551	2.1
24484	1.52471	2.1
24485	1.04072	2.1
24486	1.33174	0.05
24487	0.946128	0.05
24488	0.94613	0.05
24489	1.17801	0.05
24490	0.94613	0.05
24491	0.946132	2.1
24492	0.946132	2.1
24493	1.29065	2.1
24494	1.29065	2.1
24495	0.975266	2.1
24496	0.946131	2.1
24497	1.26025	2.1
24498	0.955601	2.1
24499	0.946131	2.1
24500	1.26025	2.1
24501	0.94613	0.0951249
24502	1.23146	0.0904928
24503	0.946127	0.0909318
24504	1.32827	0.0881184
24505	1.23146	0.093689
24506	1.25008	0.358193
24507	1.05018	0.491694
24508	0.94613	0.317846
24509	0.932744	1.09126
24510	1.04207	0.891599
24511	0.946132	1.16481
24512	1.10023	2.1
24513	1.36748	1.1585
24514	1.36748	1.93866
24515	0.946133	1.91426
24516	1.06023	0.05
24517	1.08019	0.05
24518	0.94613	0.05
24519	1.06023	0.05
24520	0.94613	0.05
24521	1.16727	0.05
24522	1.35856	0.05
24523	0.946132	0.05
24524	1.16727	0.05
24525	0.946132	0.05
24526	0.946133	2.1
24527	0.946131	2.1
24528	1.21691	2.1
24529	1.39558	2.1
24530	0.946131	2.1
24531	1.09905	0.47075

24532	0.946132	1.31265
24533	1.3365	0.395839
24534	1.35381	0.850048
24535	0.946132	1.67614
24536	0.94613	0.637619
24537	0.946128	1.99364
24538	1.40332	1.01696
24539	1.39041	1.56954
24540	0.94613	1.09663
24541	0.946132	2.09889
24542	1.3659	2.1
24543	1.39041	2.1
24544	1.39041	2.1
24545	0.946132	2.1
24546	0.840292	2.01441
24547	0.946133	2.02587
24548	0.851541	2.08922
24549	1.28464	2.01226
24550	1.28806	1.97467
24551	1.12591	1.08543
24552	1.05033	1.19959
24553	0.946132	1.13071
24554	0.946132	1.14736
24555	1.13537	1.14386
24556	1.21177	1.4961
24557	0.946132	1.32945
24558	0.946132	1.97463
24559	1.41134	1.28547
24560	1.27079	1.47887
24561	0.946131	0.05
24562	1.35403	0.05
24563	0.94613	0.0554358
24564	0.94613	0.05
24565	0.946131	0.05
24566	0.946131	0.110498
24567	0.946132	0.102278
24568	0.94613	0.304802
24569	1.37052	0.121861
24570	0.946131	0.162213
24571	0.946133	0.073605
24572	0.946132	0.148525
24573	0.946133	0.0997851
24574	1.24249	0.0762623
24575	0.946132	0.156607
24576	1.20608	2.1
24577	0.946132	2.1
24578	0.946131	2.1
24579	0.946132	2.1
24580	0.946132	2.1
24581	0.946131	2.1
24582	0.946128	2.1
24583	0.946129	2.1
24584	1.41232	2.1
24585	0.94613	2.1
24586	0.946132	0.05
24587	1.32449	0.05
24588	0.946131	0.05
24589	0.946131	0.05

24590	1.32449	0.05
24591	0.946116	2.1
24592	1.41563	2.1
24593	0.946118	2.1
24594	0.946105	2.1
24595	0.946131	2.1
24596	1.26563	2.1
24597	1.32819	2.02986
24598	0.946131	0.583558
24599	0.946131	2.0853
24600	0.946131	1.74201
24601	0.946131	1.91664
24602	0.946133	1.63306
24603	1.30382	2.1
24604	0.946131	1.68176
24605	0.946131	1.65485
24606	1.30382	1.6813
24607	0.946133	0.05
24608	1.15803	0.05
24609	1.28895	0.05
24610	0.954071	0.05
24611	0.946132	0.05
24612	1.0264	0.751183
24613	0.946132	1.60311
24614	0.946132	1.49784
24615	0.946131	1.53946
24616	0.946132	1.49578
24617	1.35977	0.159971
24618	0.946132	0.248104
24619	0.946129	0.249286
24620	0.94613	0.236123
24621	0.946129	0.213081
24622	0.876631	0.119253
24623	0.946133	0.127272
24624	0.946132	0.0993689
24625	0.946132	0.93006
24626	0.876606	0.312136
24627	0.946131	0.05
24628	1.24979	0.05
24629	0.94613	0.05
24630	0.94613	0.05
24631	0.94613	0.05
24632	0.921791	0.05
24633	0.946131	0.05
24634	0.921762	0.05
24635	0.946131	0.05
24636	0.946131	0.05
24637	0.810944	0.057965
24638	0.810945	0.0690185
24639	0.810959	0.0592704
24640	0.946133	0.0714815
24641	1.25797	0.0885705
24642	0.946132	0.0985171
24643	0.946132	0.0542824
24644	1.25797	0.0814416
24645	1.13748	0.369859
24646	0.851512	0.704547
24647	0.94613	1.2978

24648	1.02373	0.365303
24649	0.946131	0.714573
24650	0.946122	2.1
24651	1.3852	2.1
24652	0.946124	2.1
24653	0.946118	2.1
24654	1.38521	2.1
24655	0.946131	0.301696
24656	0.946131	0.244842
24657	1.30448	0.248675
24658	0.946129	0.39946
24659	0.94613	0.20885
24660	0.946133	2.1
24661	0.94613	2.1
24662	0.946131	2.1
24663	0.946131	2.1
24664	1.36503	2.1
24665	0.810947	0.05
24666	0.810947	0.05
24667	0.810934	0.05
24668	3.34066	0.05
24669	0.946133	0.610655
24670	1.4185	0.115294
24671	0.946132	0.330494
24672	0.946133	0.326442
24673	0.946132	0.328667
24674	0.94613	2.1
24675	0.946128	2.1
24676	1.03925	2.1
24677	0.944743	2.1
24678	0.947518	2.1
24679	0.946132	2.1
24680	1.20573	2.1
24681	0.94613	2.1
24682	0.94613	2.1
24683	1.20573	2.1
24684	1.20056	0.05
24685	0.946132	0.05
24686	0.946131	0.05
24687	0.946131	0.05
24688	0.946131	0.05
24689	0.94613	2.1
24690	0.946128	2.1
24691	1.38302	2.1
24692	0.94613	2.1
24693	0.94613	2.1
24694	0.827866	0.05
24695	0.913552	0.05
24696	0.716526	0.05
24697	0.82784	0.05
24698	1.20634	0.05
24699	0.946131	0.05
24700	0.94613	0.05
24701	1.39502	0.05
24702	0.88146	0.05
24703	0.946132	2.1
24704	0.946132	2.1
24705	0.946132	2.1

24706	0.946132	2.1
24707	1.39053	2.1
24708	0.946131	2.1
24709	1.40571	2.1
24710	0.946131	2.1
24711	0.946131	2.1
24712	0.946132	2.1
24713	0.94613	0.118536
24714	1.2582	0.162972
24715	0.946127	0.121383
24716	0.946129	0.135058
24717	0.946129	0.13635
24718	0.946129	1.16219
24719	0.946132	1.1831
24720	0.946131	0.428696
24721	0.946131	1.22461
24722	1.29054	0.51177
24723	0.810944	0.05
24724	0.810941	0.05
24725	0.810931	0.05
24726	0.946132	1.18991
24727	0.946132	1.15348
24728	0.946132	1.24571
24729	0.946132	1.20603
24730	0.927608	1.10141
24731	0.946128	2.1
24732	0.946133	2.1
24733	0.946131	2.1
24734	0.94613	2.1
24735	1.38813	2.1
24736	0.94613	2.1
24737	0.946128	2.1
24738	0.946132	2.1
24739	1.41666	2.1
24740	0.94613	2.1
24741	0.946118	0.114566
24742	1.34151	0.0861972
24743	0.946116	0.118921
24744	0.946131	0.102052
24745	1.34149	0.0961505
24746	0.946132	2.1
24747	1.31889	2.1
24748	0.946132	2.1
24749	0.946132	2.1
24750	1.31889	2.1
24751	0.864997	0.05
24752	0.928981	0.05
24753	0.872219	0.05
24754	0.946127	0.619095
24755	0.946131	0.462307
24756	1.37709	0.365687
24757	0.946131	0.587882
24758	0.946129	0.444121
24759	1.21882	0.05
24760	0.938168	0.05
24761	0.938161	0.05
24762	1.06327	0.05
24763	1.33457	0.05

24764	0.946133	1.17658
24765	0.946132	1.1476
24766	0.946133	0.546287
24767	0.946132	1.30832
24768	1.14444	0.452786
24769	1.12628	0.05
24770	0.946132	0.055347
24771	0.946131	0.0641873
24772	1.12628	0.05
24773	0.946131	0.05
24774	0.946132	2.1
24775	0.946132	2.1
24776	0.946133	2.1
24777	0.946132	2.1
24778	1.29322	2.1
24779	0.946131	2.1
24780	0.946132	2.1
24781	0.94613	2.1
24782	0.946131	2.1
24783	1.28441	2.1
24784	0.946118	2.1
24785	0.946119	2.1
24786	0.946117	2.1
24787	1.28075	2.1
24788	0.946105	2.1
24789	0.810945	2.1
24790	0.810942	2.1
24791	0.81093	2.1
24792	0.946127	2.1
24793	0.946127	2.1
24794	1.28228	2.1
24795	0.946129	2.1
24796	0.946132	2.1
24797	1.14042	2.1
24798	0.946132	2.1
24799	0.946133	2.1
24800	1.14042	2.1
24801	0.946133	0.0939361
24802	0.946132	0.0762274
24803	1.28632	0.0561704
24804	1.28632	0.05
24805	0.946133	0.065859
24806	0.946131	1.68216
24807	0.946131	1.59794
24808	0.946131	1.63147
24809	1.31817	1.71188
24810	0.946131	1.59184
24811	1.35048	2.1
24812	0.946132	2.1
24813	0.946132	2.1
24814	0.946132	2.1
24815	0.946131	2.1
24816	0.946131	2.1
24817	1.35943	2.1
24818	0.94613	2.1
24819	0.946132	2.1
24820	0.946131	2.1
24821	0.990713	2.1

24822	0.946132	2.1
24823	0.946132	2.1
24824	0.946133	2.1
24825	0.946132	2.1
24826	0.946133	1.33104
24827	1.01433	1.37696
24828	0.877931	1.46853
24829	0.96573	1.34009
24830	0.946131	1.32931
24831	0.946131	1.28616
24832	0.946133	0.751644
24833	1.39575	0.409728
24834	0.946131	0.496363
24835	0.94613	0.503761
24836	0.810943	0.443754
24837	0.810942	0.465978
24838	0.709589	0.34239
24839	0.924071	0.294798
24840	1.04293	0.220813
24841	1.15747	0.154663
24842	0.946131	0.304389
24843	1.04071	0.261954
24844	0.810946	0.322922
24845	0.810943	0.316574
24846	0.810959	0.265982
24847	0.946128	1.53428
24848	1.39696	1.70786
24849	0.94613	1.37508
24850	0.94613	1.30502
24851	1.39696	1.53528
24852	0.946131	1.33058
24853	0.946131	1.73968
24854	0.946131	1.53723
24855	0.946131	1.44894
24856	1.29735	1.61914
24857	0.946128	1.96002
24858	0.946133	0.455912
24859	0.94613	2.03971
24860	0.946131	1.75335
24861	1.33266	1.10281
24862	0.946131	2.1
24863	0.94613	1.89318
24864	0.946131	2.1
24865	1.40609	2.1
24866	0.946131	2.1
24867	0.946131	1.99369
24868	0.946133	1.97564
24869	0.946129	2.07394
24870	1.23339	2.09776
24871	0.946131	1.96259
24872	0.946132	2.1
24873	0.946133	2.1
24874	0.946132	2.1
24875	1.29198	2.1
24876	0.946132	2.1
24877	0.946131	2.1
24878	1.07297	2.1
24879	0.946127	2.1

24880	0.94613	2.1
24881	1.07297	2.1
24882	0.946125	2.1
24883	0.946126	2.1
24884	0.946126	2.1
24885	1.3864	2.1
24886	0.946119	2.1
24887	0.946128	0.0886511
24888	1.09411	0.0782162
24889	0.946131	0.111301
24890	0.946131	0.0974648
24891	1.09411	0.0858395
24892	0.946131	0.0704698
24893	1.25837	0.0601876
24894	0.946131	0.088659
24895	0.946131	0.0773676
24896	1.25837	0.0657393
24897	0.810945	2.1
24898	0.810943	2.1
24899	0.810933	2.1
24900	0.94613	0.194508
24901	0.946131	0.661537
24902	0.946132	0.229923
24903	0.946129	0.233852
24904	0.946131	0.473828
24905	0.946131	0.05
24906	1.28911	0.05
24907	0.946129	0.05
24908	0.946133	0.05
24909	0.946131	0.05
24910	0.94613	0.106058
24911	1.38954	0.103163
24912	0.946132	0.102758
24913	0.94613	0.107439
24914	0.946131	0.107324
24915	0.946131	1.07834
24916	0.946131	0.368685
24917	0.946131	0.277504
24918	1.37981	0.277696
24919	0.946131	0.325562
24920	0.946131	2.1
24921	1.26958	2.1
24922	0.946127	2.1
24923	0.946129	2.1
24924	1.26957	2.1
24925	0.94613	2.1
24926	1.16889	2.1
24927	0.946126	2.1
24928	0.946128	2.1
24929	1.16889	2.1
24930	0.946133	0.289838
24931	1.13511	0.204039
24932	0.946132	0.300305
24933	0.946133	0.24669
24934	0.946132	0.230919
24935	1.27575	1.70257
24936	0.946129	1.95964
24937	0.946131	1.81785

24938	0.94613	1.99243
24939	0.946131	1.89381
24940	0.946131	2.1
24941	0.946128	2.1
24942	1.41712	2.1
24943	1.41712	2.1
24944	0.94613	2.1
24945	0.946129	0.05
24946	0.946132	0.05
24947	0.946131	0.05
24948	0.946131	0.05
24949	0.946131	0.05
24950	0.979045	1.88263
24951	0.946131	1.35182
24952	0.617549	1.93677
24953	0.555844	2.05837
24954	1.04071	1.50183
24955	0.946132	1.94792
24956	0.946132	1.43543
24957	0.946131	2.07484
24958	1.22618	1.67033
24959	0.946132	1.08833
24960	0.946131	2.1
24961	1.13976	2.1
24962	0.946129	2.1
24963	0.946131	2.1
24964	1.13976	2.1
24965	0.810945	0.29714
24966	0.810943	0.554404
24967	0.810932	0.789869
24968	1.25358	1.47907
24969	0.94613	0.48185
24970	0.946128	0.869197
24971	1.25358	1.8391
24972	1.07811	1.21646
24973	0.946129	2.1
24974	1.09197	2.1
24975	0.946131	2.1
24976	0.946131	2.1
24977	1.09197	2.1
24978	0.594912	2.1
24979	0.993703	2.1
24980	1.10173	2.1
24981	0.9916	2.1
24982	0.946131	2.1
24983	0.946128	2.1
24984	0.946131	2.1
24985	0.94613	2.1
24986	0.946129	2.1
24987	0.946132	2.1
24988	1.40335	2.1
24989	0.946133	2.1
24990	0.946133	2.1
24991	1.40335	2.1
24992	0.946132	1.91042
24993	0.946132	2.1
24994	0.946132	1.9307
24995	0.946132	1.95418

24996	1.411	2.0867
24997	0.611675	0.0916864
24998	0.946129	0.108226
24999	1.31678	0.0620935
25000	0.946124	0.10815
25001	0.946127	0.065159
25002	0.946128	0.10007
25003	0.946132	0.406561
25004	0.946131	0.265371
25005	0.946132	0.422789
25006	0.946132	0.306294
25007	0.946132	0.291314
25008	0.810946	0.336686
25009	0.810943	0.354462
25010	0.81096	0.325914
25011	1.39301	0.05
25012	0.946132	0.05
25013	0.625812	0.05
25014	0.946132	0.05
25015	0.625812	0.05
25016	0.810946	1.96393
25017	0.810945	2.09384
25018	0.810931	2.1
25019	0.810943	2.1
25020	0.810944	2.1
25021	0.810958	2.1
25022	0.946131	0.13156
25023	0.946131	0.0979147
25024	0.946128	0.157211
25025	0.946129	0.12397
25026	0.94613	0.10398
25027	0.946133	0.201759
25028	0.946133	0.302865
25029	0.98537	0.14722
25030	0.985371	0.0761214
25031	0.946132	0.296144
25032	0.946129	0.242133
25033	1.38752	0.163422
25034	0.946131	0.156049
25035	0.946131	0.195544
25036	1.38752	0.177356
25037	0.946119	0.126949
25038	1.33036	0.0782818
25039	0.946119	0.10127
25040	0.946132	0.0967784
25041	1.33035	0.0835687
25042	1.3868	2.1
25043	0.946132	2.1
25044	0.946132	2.1
25045	0.946133	2.1
25046	0.946132	2.1
25047	0.810947	2.1
25048	0.810946	2.1
25049	0.81096	2.1
25050	0.810946	2.1
25051	0.810946	2.1
25052	0.810933	2.1
25053	0.946133	2.1

25054	1.35342	2.1
25055	0.946133	2.1
25056	0.946132	2.1
25057	1.35342	2.1
25058	0.946132	2.1
25059	0.946132	2.1
25060	1.28569	2.1
25061	0.946132	2.1
25062	0.946133	2.1
25063	0.946132	2.1
25064	1.32547	2.1
25065	0.946132	2.1
25066	0.946132	2.1
25067	1.32547	2.1
25068	0.94613	0.05
25069	0.946133	0.05
25070	1.39664	0.05
25071	1.39664	0.05
25072	0.946131	0.05
25073	0.946131	1.98071
25074	0.94613	2.1
25075	0.946132	1.80459
25076	0.946131	2.1
25077	1.41833	2.1
25078	0.946131	0.05
25079	0.946131	0.05
25080	0.946131	0.05
25081	1.26155	0.05
25082	0.946132	0.05
25083	0.810946	2.1
25084	0.810946	2.1
25085	0.810959	2.1
25086	0.94613	1.41115
25087	0.946132	0.52432
25088	1.40779	0.290303
25089	1.40779	0.415154
25090	0.94613	0.534545
25091	0.94613	0.057102
25092	0.946129	0.0658269
25093	1.36376	0.05
25094	1.36376	0.05
25095	0.946131	0.0545267
25096	0.946128	2.1
25097	0.946127	2.1
25098	0.946131	2.1
25099	1.40756	2.1
25100	0.94613	2.1
25101	0.946131	2.04283
25102	0.946132	2.1
25103	0.94613	2.05133
25104	0.946131	2.1
25105	1.31947	2.1
25106	0.946131	2.1
25107	0.946131	2.1
25108	0.946129	2.1
25109	0.946131	2.1
25110	1.25768	2.1
25111	0.946132	0.152128

25112	0.94613	0.113923
25113	0.946131	0.116298
25114	0.946131	0.0876037
25115	0.946131	0.151057
25116	0.810943	1.62287
25117	0.810942	1.83102
25118	0.81093	1.97945
25119	0.946128	1.16816
25120	0.946133	0.645036
25121	0.946131	1.32795
25122	0.946131	0.831375
25123	0.94613	1.34861
25124	0.946132	1.39074
25125	0.946131	1.18902
25126	0.946132	1.42468
25127	0.946132	1.32085
25128	0.946132	1.48057
25129	0.946119	2.1
25130	0.946119	2.1
25131	1.31523	2.1
25132	0.946132	2.1
25133	0.946107	2.1
25134	0.946133	2.1
25135	0.946132	1.61552
25136	0.946132	1.98313
25137	1.28803	2.1
25138	0.946132	2.1
25139	1.37658	1.15151
25140	0.946132	1.03642
25141	0.946133	1.23646
25142	0.946132	1.05698
25143	0.946132	1.20885
25144	0.946132	1.39191
25145	0.94613	1.39353
25146	0.94613	1.59852
25147	1.15541	1.37172
25148	0.92943	1.48161
25149	0.946126	0.05
25150	0.946129	0.05
25151	1.33849	0.05
25152	1.33849	0.05
25153	0.946129	0.05
25154	0.810946	0.05
25155	0.810944	0.05
25156	0.810959	0.05
25157	1.39027	0.05
25158	0.946131	0.0681736
25159	0.946131	0.0816709
25160	1.39027	0.05
25161	0.946131	0.0533655
25162	1.40924	1.63464
25163	0.946131	1.49235
25164	0.946132	1.66524
25165	0.946132	1.4569
25166	0.946132	1.55517
25167	0.946131	2.1
25168	0.94613	2.1
25169	0.946128	2.1

25170	0.946128	2.1
25171	1.41744	2.1
25172	0.946132	0.845694
25173	0.946128	0.812047
25174	0.94613	1.15005
25175	1.37675	0.79904
25176	0.94613	1.13848
25177	0.946132	1.27641
25178	0.946132	0.346742
25179	0.946132	0.209501
25180	0.946132	0.325926
25181	0.946131	0.325901
25182	0.946132	1.13384
25183	0.946132	1.07342
25184	0.946133	1.27487
25185	1.10003	0.726684
25186	0.946132	1.17694
25187	0.946132	0.0613655
25188	0.946131	0.0677908
25189	0.946132	0.0569262
25190	0.946131	0.0687363
25191	0.946132	0.0632556
25192	0.946132	0.0846462
25193	0.946131	0.0866047
25194	0.946132	0.0740426
25195	0.946132	0.0893846
25196	0.946131	0.0818983
25197	1.24094	0.05
25198	0.946126	0.0500093
25199	0.946128	0.05
25200	0.946129	0.0509764
25201	0.946129	0.05
25202	1.2929	0.0574007
25203	0.946131	0.0889425
25204	0.946131	0.0694422
25205	0.946131	0.0865036
25206	0.946131	0.0756097
25207	0.810946	0.05
25208	0.810945	0.05
25209	0.810959	0.05
25210	0.810946	1.96334
25211	0.810943	1.98785
25212	0.810933	2.1
25213	0.946128	2.1
25214	1.18315	2.1
25215	0.94613	2.1
25216	0.94613	2.1
25217	1.18316	2.1
25218	0.946126	0.30924
25219	1.3324	0.212085
25220	0.946127	0.333234
25221	0.94613	0.224081
25222	0.946128	0.264901
25223	0.94613	2.1
25224	0.946128	2.1
25225	1.39523	2.1
25226	1.39523	2.1
25227	0.946131	2.1

25228	1.36541	2.1
25229	0.946132	2.1
25230	0.946132	2.1
25231	0.946132	2.1
25232	0.946132	2.1
25233	0.946131	2.1
25234	0.94613	1.66771
25235	0.946132	1.84757
25236	0.946131	0.377456
25237	1.39509	1.68501
25238	0.946132	2.1
25239	0.946132	2.1
25240	1.29319	2.1
25241	0.946132	2.1
25242	0.946133	2.1
25243	0.946132	0.0854349
25244	0.946131	0.145451
25245	0.946132	0.0926859
25246	1.01196	0.0832563
25247	0.946132	0.129811
25248	0.946116	2.1
25249	0.946117	2.1
25250	1.38153	2.1
25251	1.38155	2.1
25252	0.946105	2.1
25253	0.946132	1.62456
25254	0.946131	1.74292
25255	0.946132	1.57871
25256	1.28684	1.61692
25257	0.946132	1.551
25258	1.10185	1.84424
25259	0.94613	1.89068
25260	0.946128	2.1
25261	0.94613	2.04627
25262	0.94613	1.90682
25263	0.810947	0.05
25264	0.810945	0.05
25265	0.810932	0.05
25266	0.946119	0.165405
25267	1.16717	0.121204
25268	0.946117	0.166475
25269	0.94613	0.112718
25270	1.16715	0.118965
25271	0.94612	0.413688
25272	0.946119	0.431346
25273	0.946119	0.330231
25274	0.946132	0.210659
25275	1.20882	0.28042
25276	1.07043	1.13777
25277	0.823268	1.04773
25278	0.810959	1.06002
25279	0.946132	1.57332
25280	1.14879	1.33371
25281	0.94613	1.56226
25282	0.946131	1.61537
25283	1.14879	1.18392
25284	1.23837	2.1
25285	0.94612	2.1

25286	0.946118	2.1
25287	0.946131	2.1
25288	0.946105	2.1
25289	0.810946	0.05
25290	0.810946	0.05
25291	0.810959	0.05
25292	0.810947	1.65177
25293	0.810947	0.226558
25294	0.810934	0.270428
25295	0.946128	2.1
25296	0.946131	2.1
25297	0.946128	2.1
25298	0.946129	2.1
25299	0.94613	2.1
25300	0.94613	2.1
25301	0.946131	2.1
25302	0.946131	2.08147
25303	0.946131	2.1
25304	0.946131	2.1
25305	0.946131	1.82879
25306	1.38585	2.1
25307	0.946128	1.87353
25308	0.94613	1.86739
25309	1.38585	1.9026
25310	0.946133	0.139898
25311	0.946133	0.135035
25312	0.946132	0.120269
25313	0.946132	0.14201
25314	0.946132	0.130394
25315	0.918871	0.05
25316	0.810248	0.05
25317	0.838037	0.05
25318	0.946126	0.0616385
25319	0.943647	0.05
25320	1.13836	0.05
25321	1.03802	0.05
25322	0.946129	0.0504409
25323	0.946133	1.3096
25324	0.946132	0.829126
25325	0.946132	2.09535
25326	1.1699	0.684729
25327	0.946132	0.713416
25328	0.946131	2.1
25329	0.946132	2.1
25330	1.37199	2.1
25331	1.37199	2.1
25332	0.946132	2.1
25333	0.946132	1.26247
25334	0.946133	0.825885
25335	0.946133	1.29735
25336	0.946132	0.799451
25337	1.38623	0.663013
25338	0.946132	2.1
25339	0.946132	2.1
25340	0.946132	2.1
25341	0.946132	2.1
25342	1.40098	2.1
25343	1.20787	0.152482

25344	1.41894	0.335921
25345	0.946133	0.273034
25346	1.01777	1.03465
25347	0.946132	2.1
25348	0.946132	2.1
25349	0.946133	2.1
25350	1.40199	2.1
25351	0.946132	2.1
25352	0.810946	1.54336
25353	0.810947	1.75132
25354	0.810934	1.62446
25355	0.946126	1.48301
25356	0.94613	1.46527
25357	0.946125	1.57253
25358	1.40247	1.43773
25359	0.946128	1.47199
25360	1.25703	1.68369
25361	0.87821	1.52053
25362	0.878238	1.47256
25363	0.946119	1.76071
25364	0.946132	1.81145
25365	0.946132	0.246422
25366	0.946132	0.254012
25367	0.946131	1.23794
25368	0.946132	0.173011
25369	1.28543	0.254147
25370	0.946115	2.1
25371	1.41638	2.1
25372	0.946116	2.1
25373	0.946104	2.1
25374	0.946129	2.1
25375	0.946132	2.1
25376	1.41432	2.1
25377	0.946132	2.1
25378	0.946132	2.1
25379	0.946132	2.1
25380	0.810947	0.05
25381	0.810947	0.05
25382	0.709567	0.05
25383	0.851542	0.05
25384	1.01217	0.05
25385	0.697993	0.05
25386	0.946133	0.05
25387	0.851488	0.05
25388	0.946129	2.05789
25389	0.94613	2.1
25390	1.2254	1.9041
25391	0.946131	2.09309
25392	0.946131	1.97472
25393	0.700785	2.1
25394	0.915893	1.95052
25395	0.838013	1.86237
25396	0.760188	1.45906
25397	1.19601	0.184569
25398	0.946133	0.277848
25399	0.946132	0.282335
25400	0.946132	0.242368
25401	0.946132	0.279541

25402	0.946132	1.87768
25403	0.946133	2.1
25404	0.946132	1.96472
25405	0.946133	2.09345
25406	1.36956	2.1
25407	0.946131	2.1
25408	0.94613	0.157164
25409	0.946133	2.1
25410	1.34268	0.302976
25411	0.946131	0.812968
25412	0.946132	2.1
25413	1.25603	2.1
25414	0.946132	2.1
25415	0.946132	2.1
25416	1.25603	2.1
25417	0.810946	1.41188
25418	0.810943	1.41183
25419	0.810933	1.44804
25420	0.496545	0.375899
25421	0.805767	0.848647
25422	0.936671	0.754885
25423	0.530063	0.750557
25424	1.05954	0.663633
25425	0.810946	0.110887
25426	0.810943	0.111234
25427	0.810959	0.0900259
25428	1.16699	0.0996853
25429	0.946131	0.156989
25430	0.946129	0.153976
25431	0.946131	0.134221
25432	0.946131	0.132544
25433	0.810943	1.77458
25434	0.810942	1.83629
25435	0.810933	1.78063
25436	0.946132	1.7791
25437	1.04832	1.76351
25438	0.870714	1.91095
25439	0.957819	1.91524
25440	0.94613	1.80187
25441	1.82931	2.1
25442	0.946132	0.311127
25443	0.946129	0.246045
25444	0.94613	0.390975
25445	0.946131	0.641148
25446	0.94613	0.246719
25447	0.946119	1.13101
25448	0.94612	1.22116
25449	0.94612	0.458111
25450	0.946132	0.945049
25451	1.36118	0.878931
25452	0.810947	0.121268
25453	0.810947	0.0988849
25454	0.810934	0.10849
25455	0.946132	2.1
25456	0.946132	2.1
25457	0.946132	2.1
25458	1.39754	2.1
25459	0.946131	2.1

25460	0.946129	2.1
25461	0.946133	2.1
25462	0.946131	2.1
25463	0.946131	2.1
25464	1.41732	2.1
25465	0.946132	1.25262
25466	0.946128	1.77547
25467	0.94613	1.61271
25468	0.94613	1.65456
25469	1.25277	1.72049
25470	0.94613	1.60056
25471	0.946128	1.74286
25472	1.21261	1.31064
25473	0.94613	1.58272
25474	0.94613	1.53836
25475	1.39407	2.1
25476	0.946133	2.1
25477	0.94613	2.1
25478	0.946131	2.1
25479	0.946131	2.1
25480	0.946119	1.62759
25481	0.946119	1.69587
25482	0.946119	1.82276
25483	1.1537	1.73683
25484	0.946106	1.65268
25485	0.946131	2.1
25486	1.11822	2.1
25487	0.946129	2.1
25488	0.946131	2.1
25489	1.11822	2.1
25490	1.46806	0.05
25491	0.498881	0.05
25492	0.996658	0.05
25493	1.2179	0.05
25494	0.991519	0.05
25495	0.946131	0.05
25496	0.946131	0.05
25497	1.28673	0.05
25498	0.946131	0.05
25499	0.946131	0.05
25500	1.33178	0.0576802
25501	0.946132	0.0798646
25502	0.946132	0.0733261
25503	0.946132	0.0796144
25504	0.946132	0.078093
25505	0.946128	0.10998
25506	0.972061	0.0794708
25507	0.946129	0.128269
25508	0.946129	0.11829
25509	0.972059	0.0843541
25510	0.946129	2.1
25511	0.94613	2.1
25512	0.94613	2.1
25513	0.94613	2.1
25514	0.94613	2.1
25515	0.946131	2.1
25516	0.946127	2.1
25517	0.946131	2.1

25518	0.946129	2.1
25519	1.4073	2.1
25520	0.946131	0.0562968
25521	1.05016	0.05
25522	0.946132	0.065942
25523	0.946131	0.0645783
25524	0.946132	0.0574032
25525	0.946128	0.0644876
25526	1.13398	0.0570023
25527	0.946131	0.0824292
25528	0.946131	0.0752298
25529	0.94613	0.0662406
25530	0.946131	0.05
25531	0.946131	0.05
25532	1.38911	0.05
25533	1.38911	0.05
25534	0.946132	0.05
25535	0.946131	0.0519148
25536	0.94613	0.05
25537	0.946131	0.0530349
25538	0.946131	0.05
25539	0.946131	0.0525835
25540	0.946131	0.20258
25541	1.23923	0.147491
25542	0.94613	0.253128
25543	0.946131	0.148651
25544	0.946131	0.164785
25545	1.40511	0.05
25546	0.946131	0.05
25547	0.946131	0.05
25548	0.946132	0.05
25549	0.946131	0.05
25550	1.12968	2.1
25551	0.946132	2.1
25552	0.946132	2.1
25553	0.946131	2.1
25554	0.946132	2.1
25555	0.946132	1.77084
25556	1.4172	2.1
25557	0.94613	1.83183
25558	0.94613	1.83718
25559	1.4172	1.92276
25560	0.946132	2.1
25561	0.946132	2.1
25562	1.31323	2.1
25563	0.946133	2.1
25564	0.946132	2.1
25565	0.946131	2.1
25566	0.94613	2.07677
25567	1.36546	2.1
25568	1.36546	2.1
25569	0.946131	2.1
25570	0.946132	1.82651
25571	0.946133	2.1
25572	0.946132	0.289076
25573	1.36899	1.16612
25574	0.946133	0.731294
25575	0.946131	0.05

25576	0.946132	0.05
25577	1.03543	0.05
25578	1.1412	0.05
25579	0.941275	0.05
25580	1.28952	0.0808806
25581	0.94613	0.104201
25582	0.946129	0.110165
25583	0.946132	0.0836646
25584	0.94613	0.0978122
25585	0.94613	2.1
25586	0.94613	2.1
25587	0.946129	2.1
25588	0.94613	2.1
25589	1.35966	2.1
25590	0.810946	2.1
25591	0.810942	2.1
25592	0.810932	2.1
25593	0.94613	2.1
25594	1.40372	2.1
25595	0.946131	2.1
25596	0.94613	2.1
25597	1.40372	2.1
25598	0.946133	2.1
25599	0.946131	2.1
25600	0.94613	2.1
25601	0.946131	2.1
25602	0.946131	2.1
25603	1.31453	2.1
25604	0.94613	2.1
25605	0.946128	2.1
25606	0.94613	2.1
25607	0.94613	2.1
25608	0.946131	0.05
25609	1.12018	0.05
25610	0.94613	0.0573184
25611	0.94613	0.0596155
25612	0.94613	0.05
25613	0.946131	0.0696237
25614	1.16373	0.0610894
25615	0.946131	0.107249
25616	0.94613	0.0620008
25617	0.946131	0.0652881
25618	0.946126	0.362106
25619	0.946132	0.533217
25620	0.946128	0.390266
25621	0.946129	0.588764
25622	0.946129	0.687136
25623	0.946132	0.0794346
25624	0.946133	0.079652
25625	0.946132	0.0790888
25626	0.946132	0.0774246
25627	0.946133	0.0825816
25628	0.946132	1.4235
25629	0.946129	1.58941
25630	0.94613	1.38844
25631	1.36399	1.45082
25632	0.946131	1.44541
25633	0.946132	2.1

25634	0.946132	2.1
25635	0.946133	2.1
25636	1.38302	2.1
25637	0.946133	2.1
25638	0.810943	2.1
25639	0.810944	2.1
25640	0.810945	2.1
25641	0.946132	2.1
25642	0.946131	2.1
25643	0.946132	2.1
25644	1.30188	2.1
25645	0.946132	2.1
25646	0.810946	1.19271
25647	0.72985	1.30221
25648	0.810934	1.22295
25649	0.856243	1.02005
25650	0.946129	1.14425
25651	0.946132	1.13665
25652	1.19686	0.826751
25653	0.851527	1.17813
25654	0.946131	2.1
25655	0.94613	2.1
25656	0.94613	2.1
25657	0.94613	2.1
25658	1.31961	2.1
25659	0.946131	2.1
25660	0.946131	2.1
25661	0.94613	2.1
25662	0.946131	2.1
25663	1.3605	2.1
25664	1.30116	2.1
25665	0.946133	1.25234
25666	0.946131	2.1
25667	0.946131	2.03338
25668	0.94613	2.1
25669	0.946132	0.05
25670	1.3035	0.05
25671	0.94613	0.05
25672	0.946131	0.05
25673	0.946131	0.05
25674	0.946132	2.1
25675	1.37869	2.1
25676	1.2051	2.1
25677	0.946132	2.1
25678	1.2051	2.1
25679	0.810946	2.1
25680	0.810945	2.1
25681	0.810946	2.1
25682	1.34623	2.1
25683	0.946131	2.1
25684	0.946131	2.1
25685	1.34623	2.1
25686	1.22993	2.1
25687	1.32299	0.05
25688	0.946132	0.05
25689	0.946132	0.05
25690	0.946132	0.05
25691	0.946131	0.05

25692	0.94613	0.107025
25693	1.40574	0.0965497
25694	0.946128	0.123638
25695	0.946131	0.128786
25696	1.40574	0.122693
25697	0.946132	2.1
25698	1.41331	2.1
25699	0.94613	2.1
25700	0.946131	2.1
25701	1.41331	2.1
25702	0.94613	2.1
25703	1.37609	2.1
25704	0.946129	2.1
25705	0.946131	2.1
25706	1.37609	2.1
25707	1.039 1.2408	
25708	0.946131	1.79801
25709	0.944514	1.66221
25710	0.946132	1.75898
25711	0.946132	1.69986
25712	1.23726	1.1237
25713	0.946128	1.69467
25714	0.946128	1.60883
25715	0.946129	1.70573
25716	0.94613	1.62816
25717	0.946129	2.1
25718	0.946131	2.1
25719	0.946131	2.1
25720	1.39214	2.1
25721	0.94613	2.1
25722	0.946118	1.95029
25723	0.946118	0.5892
25724	1.41078	0.781561
25725	1.41076	0.39479
25726	0.946131	0.777584
25727	0.946131	0.355997
25728	0.946131	0.294796
25729	0.946132	0.311424
25730	0.94613	0.412749
25731	0.946131	0.314764
25732	0.946132	0.0612136
25733	0.946132	0.05
25734	0.946133	0.05
25735	1.28924	0.05
25736	0.946132	0.05
25737	0.946133	0.447374
25738	1.09812	0.289806
25739	0.946132	0.500162
25740	0.946132	0.432364
25741	1.09812	0.478136
25742	0.946132	1.49716
25743	0.946132	1.53722
25744	0.946132	1.46716
25745	0.946132	1.47174
25746	1.15388	1.48963
25747	0.946132	0.381426
25748	0.974688	0.224902
25749	0.946132	0.402368

25750	0.946132	0.323432
25751	0.974687	0.378517
25752	1.0644	1.69162
25753	0.946133	1.81252
25754	0.946132	1.70085
25755	0.946133	1.71131
25756	1.26547	1.74138
25757	1.02905	1.39273
25758	0.911537	1.36462
25759	0.838038	1.45786
25760	0.946131	0.0747176
25761	0.94613	0.0752474
25762	0.946132	0.0712926
25763	0.946131	0.0665544
25764	1.33922	0.0706144
25765	0.946132	0.072214
25766	0.946132	0.057832
25767	1.40926	0.0568816
25768	1.40927	0.05
25769	0.946132	0.0544839
25770	0.946129	2.1
25771	0.946131	1.91339
25772	0.946131	1.94903
25773	0.946131	1.60101
25774	1.40496	2.1
25775	0.94613	1.62388
25776	0.94613	1.64817
25777	0.946127	1.58663
25778	0.946128	1.55161
25779	1.38914	1.5514
25780	2.675 0.05	
25781	1.22266	0.05
25782	0.946131	0.05
25783	0.946132	0.05
25784	0.946132	0.05
25785	1.22266	0.05
25786	0.946124	0.103948
25787	0.946128	0.0536054
25788	0.946126	0.0552864
25789	1.21428	0.05
25790	0.946127	0.0910602
25791	0.946131	0.074518
25792	0.946131	0.156906
25793	0.946132	0.0709441
25794	0.946129	0.0883358
25795	0.940118	0.142135
25796	0.810943	2.1
25797	0.810945	2.1
25798	0.810958	2.1
25799	0.946133	0.0601641
25800	1.11539	0.0534524
25801	0.946132	0.0576308
25802	0.946132	0.0583601
25803	0.946133	0.0594897
25804	0.946133	2.1
25805	1.41195	2.1
25806	0.946133	2.1
25807	0.946132	2.1

25808	0.946133	2.1
25809	1.15968	0.05
25810	0.946131	0.05
25811	0.946131	0.05
25812	0.946131	0.05
25813	0.946131	0.05
25814	0.94613	1.99157
25815	0.946128	2.03053
25816	0.946132	1.69255
25817	0.94613	1.90789
25818	0.94613	1.79599
25819	0.946131	1.75983
25820	1.04211	1.76668
25821	0.850155	1.56085
25822	0.946131	1.69609
25823	0.946131	1.63411
25824	0.946131	0.380554
25825	0.94613	0.268012
25826	0.946129	0.392145
25827	0.946131	0.307839
25828	0.946131	0.293466
25829	1.40193	0.150723
25830	1.19756	0.169444
25831	0.561936	0.201883
25832	0.808778	0.19679
25833	1.19756	0.182772
25834	0.946132	0.05
25835	0.946132	0.05
25836	1.33916	0.05
25837	1.33916	0.05
25838	0.946133	0.05
25839	0.946127	0.167875
25840	0.946127	0.113787
25841	0.946131	0.143776
25842	0.946128	0.092894
25843	0.946129	0.101372
25844	0.589428	0.354785
25845	0.851501	0.161473
25846	0.868924	0.177083
25847	0.877703	0.157642
25848	0.85153	0.185404
25849	0.946132	2.07515
25850	1.25372	2.1
25851	0.946132	2.1
25852	0.946132	2.1
25853	1.25372	2.1
25854	0.946132	1.67252
25855	0.946128	2.08997
25856	0.94613	1.81164
25857	0.94613	1.90479
25858	1.2778	1.95805
25859	0.946118	1.01346
25860	1.35629	0.680317
25861	0.946117	0.987925
25862	0.946131	0.686009
25863	0.946105	0.662074
25864	0.946132	1.72559
25865	0.946132	2.1

25866	1.38646	2.1
25867	1.38646	2.1
25868	0.946132	2.1
25869	0.946128	2.1
25870	0.946128	2.1
25871	1.39659	2.1
25872	1.39659	2.1
25873	0.946129	2.1
25874	0.946132	1.68088
25875	0.946131	1.67759
25876	0.946132	1.68505
25877	0.946132	1.68475
25878	1.15139	1.67731
25879	0.946133	2.1
25880	0.946132	2.1
25881	0.946132	2.1
25882	0.946132	2.1
25883	1.36151	2.1
25884	0.946127	2.1
25885	0.946131	2.1
25886	0.946128	2.1
25887	0.94613	2.1
25888	1.38397	2.1
25889	0.946131	0.207002
25890	1.31112	0.210513
25891	0.946127	0.220508
25892	0.946128	0.224887
25893	1.31112	0.210842
25894	1.29778	0.05
25895	0.883417	0.05
25896	1.32613	0.05
25897	0.94613	0.05
25898	0.94613	0.05
25899	0.946132	2.1
25900	0.94613	2.1
25901	0.946131	2.1
25902	0.946131	2.1
25903	1.36523	2.1
25904	0.946132	0.0683848
25905	1.01653	0.061105
25906	0.946132	0.0961528
25907	0.946131	0.0872486
25908	0.946131	0.0686717
25909	0.94612	2.1
25910	1.08301	2.1
25911	0.946119	2.1
25912	0.946132	2.1
25913	1.08299	2.1
25914	0.810942	2.1
25915	0.810944	2.1
25916	0.810957	2.1
25917	0.946126	0.202399
25918	0.946132	0.230797
25919	0.946129	0.203817
25920	1.3492	0.188008
25921	0.94613	0.203242
25922	0.946131	0.155817
25923	1.24766	0.142739

25924	0.946128	0.164545
25925	0.946131	0.174236
25926	1.24766	0.167126
25927	0.946132	0.05
25928	0.946131	0.05
25929	0.946131	0.05
25930	1.23351	0.05
25931	0.946132	0.05
25932	0.946131	0.05
25933	0.946132	0.05
25934	0.946131	0.05
25935	1.35657	0.05
25936	0.946131	0.05
25937	0.946132	0.155857
25938	1.3689	0.105982
25939	0.946132	0.139407
25940	0.946132	0.141483
25941	1.3689	0.109723
25942	0.946133	0.0914825
25943	1.35822	0.05
25944	0.946133	0.0699424
25945	0.946132	0.0727229
25946	1.35822	0.0501042
25947	0.946131	1.09566
25948	0.946131	1.38794
25949	0.946131	1.26147
25950	0.946131	1.23152
25951	1.4101	1.35199
25952	0.810944	1.63047
25953	0.810939	1.634
25954	0.810955	1.52334
25955	0.946132	2.1
25956	1.37733	2.1
25957	0.946132	2.1
25958	0.946131	2.1
25959	0.946131	2.1
25960	1.32582	2.1
25961	0.946132	2.1
25962	0.946132	2.1
25963	0.946132	2.1
25964	0.946132	2.1
25965	0.946132	2.1
25966	0.946132	2.1
25967	1.31401	2.1
25968	1.31401	2.1
25969	0.946131	2.1
25970	1.3469	1.56478
25971	0.946132	1.70449
25972	0.946132	1.66276
25973	0.946133	1.54109
25974	0.946132	1.57446
25975	0.946126	2.1
25976	0.946127	2.1
25977	0.94613	2.1
25978	0.946128	2.1
25979	1.41206	2.1
25980	0.946127	2.1
25981	1.40109	2.1

25982	0.946128	2.1
25983	0.946129	2.1
25984	1.40109	2.1
25985	1.24448	0.0909603
25986	0.94613	0.151879
25987	0.946132	0.111714
25988	0.946129	0.15055
25989	0.946131	0.131086
25990	1.11015	0.137133
25991	0.946133	0.242584
25992	0.946132	0.171353
25993	0.946133	0.227404
25994	0.946132	0.202847
25995	0.946132	2.1
25996	0.946131	2.1
25997	0.946131	2.1
25998	1.38775	2.1
25999	0.946131	2.1
26000	0.946133	1.49814
26001	0.946131	2.1
26002	1.30598	2.1
26003	1.30598	2.1
26004	0.946131	2.1
26005	0.946132	2.1
26006	0.946132	2.1
26007	0.946132	2.1
26008	1.40774	2.1
26009	0.946132	2.1
26010	0.798396	0.05
26011	0.946133	1.09392
26012	0.946128	1.29448
26013	0.946131	1.1729
26014	0.946131	1.19741
26015	1.28918	1.24359
26016	0.946132	0.198672
26017	1.40668	0.105353
26018	0.94613	0.199272
26019	0.94613	0.192318
26020	0.94613	0.343778
26021	0.810946	2.1
26022	0.810945	2.1
26023	0.810933	2.1
26024	0.946131	2.1
26025	1.30338	2.1
26026	0.94613	2.1
26027	1.30339	2.1
26028	0.946131	2.1
26029	0.946127	0.944952
26030	0.87161	0.374617
26031	0.946128	1.04224
26032	0.946129	0.807071
26033	0.827876	0.961629
26034	0.946131	2.1
26035	1.40796	2.1
26036	0.94613	2.1
26037	0.94613	2.1
26038	1.40796	2.1
26039	0.946132	0.786331

26040	0.946132	1.11191
26041	0.946132	0.678986
26042	0.946132	0.519863
26043	1.41724	0.392835
26044	1.34254	0.216631
26045	0.946132	0.37917
26046	0.946132	0.397703
26047	0.946132	0.34893
26048	0.946133	0.319763
26049	0.946133	1.78931
26050	0.946132	1.7514
26051	0.946132	1.69998
26052	1.26501	1.84738
26053	0.946132	1.69424
26054	0.946131	0.05
26055	1.35169	0.05
26056	0.946131	0.0530664
26057	0.946131	0.05
26058	1.35169	0.05
26059	0.676844	0.05
26060	0.946131	0.05
26061	0.94613	0.0542991
26062	1.13242	0.05
26063	1.13242	0.05
26064	0.946131	0.05
26065	0.946128	1.85086
26066	1.28828	2.1
26067	0.946131	1.65715
26068	0.946131	1.91801
26069	1.28828	1.99581
26070	0.946131	0.289627
26071	1.2996	0.233047
26072	0.946133	0.280495
26073	0.946131	0.267226
26074	0.946131	0.239112
26075	0.946132	2.1
26076	1.40638	2.1
26077	0.946131	2.1
26078	0.946132	2.1
26079	1.40638	2.1
26080	2.84909	0.05
26081	1.35726	0.05
26082	0.946131	0.0684877
26083	0.946131	0.0675181
26084	0.946131	0.0603616
26085	0.946131	0.0608588
26086	1.39852	2.1
26087	0.946132	2.1
26088	0.94613	2.1
26089	0.94613	2.1
26090	0.94613	2.1
26091	0.946126	0.05
26092	0.946131	0.05
26093	0.946128	0.05
26094	1.31666	0.05
26095	0.946128	0.05
26096	0.946126	0.05
26097	0.946131	0.05

26098	0.946129	0.0584955
26099	1.32275	0.05
26100	0.946129	0.05
26101	0.946131	2.1
26102	0.946132	2.1
26103	1.41125	2.1
26104	0.946131	2.1
26105	0.946131	2.1
26106	0.946131	2.1
26107	0.946132	2.1
26108	1.31904	2.1
26109	0.946132	2.1
26110	0.946132	2.1
26111	0.946129	0.05
26112	0.946132	0.05
26113	0.94613	0.05
26114	1.02426	0.05
26115	0.9311	0.05
26116	0.946128	1.67896
26117	0.946133	1.62078
26118	0.946131	1.60709
26119	1.40092	1.72184
26120	0.946131	1.60928
26121	0.946117	2.1
26122	0.946118	2.1
26123	0.946118	2.1
26124	1.40017	2.1
26125	0.946105	2.1
26126	0.946132	0.146401
26127	1.03547	0.121867
26128	0.946132	0.107625
26129	0.946133	0.15789
26130	0.946132	0.204958
26131	0.946133	2.1
26132	0.946132	2.1
26133	0.946133	2.1
26134	0.946133	2.1
26135	1.36694	2.1
26136	0.946132	0.0871603
26137	1.22878	0.103274
26138	0.946132	0.105021
26139	0.946132	0.0757614
26140	1.22878	0.0970788
26141	1.11014	0.127808
26142	0.913078	0.128746
26143	1.11015	0.166502
26144	0.49154	0.162878
26145	0.946127	2.1
26146	0.946131	2.1
26147	0.946128	2.1
26148	0.946129	2.1
26149	1.38752	2.1
26150	0.946132	2.1
26151	1.37277	2.1
26152	0.946132	2.1
26153	0.946131	2.1
26154	1.37277	2.1
26155	0.94613	0.371705

26156	1.16834	0.218686
26157	0.946129	0.367348
26158	0.94613	0.287335
26159	0.94613	0.2585
26160	0.946127	0.461446
26161	0.946131	0.310184
26162	1.35594	0.265363
26163	0.946128	0.470361
26164	0.94613	0.332984
26165	0.946128	2.1
26166	0.946128	0.39917
26167	1.38464	1.89656
26168	1.38463	0.980792
26169	0.94613	1.2464
26170	0.946129	1.54311
26171	0.946131	0.385535
26172	0.94613	0.25883
26173	1.39751	0.208709
26174	0.946131	0.739039
26175	1.25837	0.0595395
26176	1.21134	0.0533294
26177	0.700013	0.0661609
26178	0.700041	0.06577
26179	1.40583	2.1
26180	0.946132	2.1
26181	0.946132	2.1
26182	0.946133	2.1
26183	0.946132	2.1
26184	0.827865	2.1
26185	0.946131	2.1
26186	0.946131	2.03243
26187	0.946131	2.1
26188	0.946131	2.04237
26189	0.946132	0.05
26190	1.21087	0.05
26191	0.94613	0.05
26192	0.94613	0.05
26193	0.946131	0.05
26194	0.94613	0.355753
26195	1.17452	0.249749
26196	0.946132	0.434245
26197	0.94613	0.344385
26198	0.94613	0.423121
26199	0.946119	0.177964
26200	1.27374	0.166985
26201	0.946119	0.185637
26202	0.946131	0.176291
26203	0.946106	0.188687
26204	0.946133	2.1
26205	0.946132	2.1
26206	0.946133	2.1
26207	0.946132	2.1
26208	1.39621	2.1
26209	1.34352	2.1
26210	0.946132	2.1
26211	0.946132	2.1
26212	0.946132	2.1
26213	0.946132	2.1

26214	0.946132	1.61924
26215	0.946128	2.06157
26216	0.94613	1.76757
26217	0.94613	1.85051
26218	1.41273	1.9273
26219	1.23536	2.1
26220	0.946131	2.1
26221	0.946132	2.1
26222	0.946132	2.1
26223	0.946132	2.1
26224	0.946132	2.1
26225	1.41131	2.1
26226	0.946131	2.1
26227	0.946131	2.1
26228	0.946131	2.1
26229	0.946133	0.627769
26230	0.946132	2.07407
26231	0.946133	0.790588
26232	1.41894	0.542659
26233	0.946132	1.13023
26234	0.946128	1.82754
26235	1.4099	2.1
26236	0.946131	1.94166
26237	0.946131	1.97074
26238	0.946131	1.83362
26239	0.810947	2.1
26240	0.810945	2.1
26241	0.810933	2.1
26242	0.946131	0.849346
26243	1.25765	2.06154
26244	0.946128	1.60999
26245	0.946129	1.63383
26246	1.25765	1.76032
26247	1.21347	2.1
26248	0.946129	2.1
26249	0.94613	2.1
26250	1.21347	2.1
26251	0.94613	2.1
26252	0.94613	0.05
26253	0.946132	0.05
26254	1.2493	0.05
26255	1.2493	0.05
26256	0.94613	0.05
26257	0.946132	1.1955
26258	0.946128	1.30215
26259	0.946131	1.21002
26260	0.94613	1.23034
26261	1.37938	1.25246
26262	0.946133	0.05
26263	0.946132	0.05
26264	0.946133	0.05
26265	0.946132	0.05
26266	0.946133	0.05
26267	0.946129	0.297679
26268	1.26426	0.213058
26269	0.946131	0.391686
26270	0.946131	0.314774
26271	1.26426	0.235625

26272	0.872447	1.7979
26273	0.946132	1.65542
26274	0.946132	1.91925
26275	0.946132	1.85612
26276	0.946133	1.74339
26277	0.946117	1.39891
26278	0.946116	1.11416
26279	0.946113	1.26031
26280	0.946129	1.39865
26281	1.27211	0.914505
26282	0.94613	0.640093
26283	0.946129	1.55308
26284	1.40029	1.16878
26285	1.40028	1.27192
26286	0.94613	1.17808
26287	0.810942	2.1
26288	0.810943	2.1
26289	0.810932	2.1
26290	1.29521	2.1
26291	0.946129	2.1
26292	0.946131	2.1
26293	1.29521	2.1
26294	1.29521	2.1
26295	0.946132	0.68026
26296	0.946129	0.437283
26297	1.24596	0.380057
26298	0.946131	0.698893
26299	0.946131	0.528576
26300	0.946131	2.1
26301	1.35616	2.1
26302	0.946133	2.1
26303	0.94613	2.1
26304	1.35616	2.1
26305	0.879548	0.509222
26306	1.01272	0.0964568
26307	0.946133	1.28029
26308	0.946133	0.0860951
26309	0.946132	0.389869
26310	0.946132	0.821982
26311	0.918009	0.413332
26312	0.91801	1.33815
26313	0.946132	0.260989
26314	0.946132	0.660271
26315	0.946132	0.05
26316	1.28644	0.05
26317	0.946131	0.0503406
26318	0.946131	0.05
26319	0.946132	0.05
26320	0.946131	0.05
26321	1.40103	0.05
26322	0.946131	0.0545712
26323	0.946131	0.05
26324	0.946131	0.05
26325	0.946128	0.118696
26326	1.03327	0.0942879
26327	0.946129	0.137686
26328	0.946131	0.0955372
26329	0.94613	0.109538

26330	0.946132	1.01211
26331	0.946133	0.321902
26332	1.40265	0.294825
26333	1.40265	0.221192
26334	0.946133	0.190937
26335	0.946131	0.05
26336	0.946131	0.05
26337	0.946131	0.05
26338	0.856655	0.05
26339	0.856616	0.05
26340	0.946132	0.289529
26341	0.946132	0.356891
26342	0.946132	0.776856
26343	1.15844	0.204172
26344	0.946131	0.750759
26345	0.946131	2.1
26346	1.4031	2.1
26347	1.40304	2.1
26348	0.851554	2.1
26349	0.946131	0.05
26350	0.94613	0.05
26351	1.24417	0.05
26352	1.24416	0.05
26353	0.946131	0.05
26354	0.946132	0.961482
26355	1.20992	0.945618
26356	0.946132	0.968441
26357	0.946132	1.17823
26358	1.20992	1.1098
26359	0.946128	1.79557
26360	0.94613	1.98522
26361	0.94613	1.80775
26362	0.94613	1.83804
26363	1.23426	1.8911
26364	0.810943	0.768477
26365	0.810946	0.102694
26366	0.810933	0.0866107
26367	0.810945	2.1
26368	0.810945	2.09082
26369	0.810932	2.1
26370	1.25104	2.1
26371	0.946131	2.1
26372	0.946131	1.97608
26373	1.25104	2.1
26374	1.25104	2.09133
26375	1.25488	2.1
26376	0.946132	2.1
26377	0.946132	2.07355
26378	0.946132	2.1
26379	0.946131	2.1
26380	0.946131	0.05
26381	0.946126	0.0732744
26382	0.946128	0.05
26383	1.32131	0.0558386
26384	0.946128	0.0629069
26385	0.946133	2.1
26386	0.946132	2.02157
26387	0.946132	2.1

26388	0.946133	2.1
26389	1.41123	2.1
26390	0.946132	0.05
26391	0.946133	0.0529676
26392	0.946132	0.0660159
26393	1.32163	0.05
26394	0.946133	0.0578499
26395	0.946132	2.1
26396	0.946132	2.1
26397	1.38651	2.1
26398	1.38651	2.1
26399	0.946132	2.1
26400	0.94613	0.0908951
26401	1.3639	0.0834855
26402	0.946128	0.107475
26403	0.946131	0.0806066
26404	1.3639	0.0750119
26405	0.946133	1.21957
26406	1.40724	0.540699
26407	0.946132	0.457216
26408	0.946133	0.728289
26409	1.40724	0.639102
26410	1.21099	1.69581
26411	0.946128	1.77873
26412	0.946127	2.03038
26413	0.946128	1.88719
26414	0.946129	1.76629
26415	0.946132	0.27037
26416	1.35946	0.228132
26417	0.946131	1.04039
26418	0.946131	0.423794
26419	1.35946	0.267028
26420	1.34794	0.60642
26421	0.517994	1.70322
26422	0.946132	0.642863
26423	0.946132	0.915266
26424	0.517965	1.33807
26425	1.23224	0.846204
26426	0.94613	1.83097
26427	0.946131	1.62405
26428	0.946131	1.33643
26429	0.946131	1.52135
26430	1.37511	1.51071
26431	0.946132	1.54561
26432	0.946133	1.86292
26433	0.946133	1.7034
26434	0.946132	1.55813
26435	0.851106	0.870669
26436	0.946129	0.781893
26437	0.946131	0.281386
26438	0.946131	1.42208
26439	0.851066	1.15294
26440	0.946131	0.0777039
26441	0.946131	0.148817
26442	0.94613	0.158101
26443	1.31357	0.0738388
26444	0.946131	0.121565
26445	0.946133	1.17759

26446	0.946132	1.28466
26447	0.946133	0.87205
26448	0.946132	1.20878
26449	1.38149	1.20606
26450	0.810944	1.73734
26451	0.810945	1.89572
26452	0.810958	2.09795
26453	0.946118	0.05
26454	0.946118	0.05
26455	1.37835	0.05
26456	1.37837	0.05
26457	0.946106	0.05
26458	0.946128	0.05
26459	0.946131	0.05
26460	1.34831	0.05
26461	1.34831	0.05
26462	0.94613	0.05
26463	0.946132	0.0701981
26464	0.946132	0.118047
26465	0.909981	0.0729415
26466	1.00099	0.0706817
26467	0.946131	0.0958644
26468	0.946132	1.14018
26469	0.946133	1.30228
26470	0.946133	1.18627
26471	0.946132	1.21032
26472	1.35328	1.24834
26473	0.946132	0.05
26474	0.946132	0.05
26475	1.35386	0.05
26476	1.35385	0.05
26477	0.946133	0.05
26478	0.946121	0.05
26479	0.946132	0.05
26480	0.900167	0.05
26481	1.01189	0.05
26482	0.946132	0.05
26483	0.946132	2.0281
26484	1.41585	1.74516
26485	0.946131	2.01112
26486	0.946131	1.7337
26487	0.94613	1.90986
26488	0.810946	2.1
26489	0.810946	2.1
26490	0.810933	2.1
26491	1.34447	2.1
26492	0.946131	2.1
26493	0.946131	2.1
26494	1.34447	2.1
26495	1.19886	2.1
26496	0.946132	0.0788742
26497	0.946132	0.05
26498	0.946132	0.05
26499	1.35065	0.05
26500	0.946132	0.0717126
26501	0.946131	0.223478
26502	1.3022	0.207999
26503	0.94613	0.763932

26504	0.946131	0.330228
26505	1.3022	0.223914
26506	0.946127	0.112232
26507	1.38683	0.0948716
26508	0.946127	0.122163
26509	0.946128	0.118031
26510	1.38683	0.113575
26511	0.810946	0.05
26512	0.810947	0.05
26513	0.810959	0.05
26514	0.946132	0.05
26515	0.946131	0.05
26516	0.946132	0.05
26517	0.946132	0.05
26518	0.946132	0.05
26519	0.946127	1.63329
26520	1.35968	1.90888
26521	0.946131	1.53269
26522	0.946129	1.11202
26523	1.35969	1.74079
26524	0.946133	2.01822
26525	1.35862	1.78876
26526	0.946132	2.08754
26527	0.946132	1.68568
26528	1.35862	1.84381
26529	0.946132	0.840512
26530	0.946132	0.279408
26531	0.946132	1.67371
26532	0.946131	0.158472
26533	0.946132	0.600353
26534	0.946131	0.141064
26535	0.946131	0.119576
26536	0.946128	0.192569
26537	0.946129	0.0576001
26538	1.41232	0.0753302
26539	0.946128	2.1
26540	1.29331	2.1
26541	0.946131	2.1
26542	0.946131	2.1
26543	1.29331	2.1
26544	0.946133	0.866146
26545	0.946132	0.962889
26546	0.946132	0.847434
26547	0.946132	0.593545
26548	1.41556	0.370794
26549	0.94612	0.612811
26550	0.946119	0.393355
26551	0.946119	0.621754
26552	0.946132	0.427514
26553	0.946107	0.348381
26554	0.946129	0.05
26555	1.1509	0.05
26556	0.946126	0.05
26557	0.946129	0.05
26558	0.94613	0.05
26559	0.946131	0.05
26560	1.19263	0.05
26561	0.946132	0.05

26562	0.946132	0.05
26563	0.946132	0.05
26564	0.94613	0.767097
26565	1.36939	1.42855
26566	0.946128	0.896063
26567	0.946131	1.13621
26568	1.36939	1.0105
26569	0.946133	1.30213
26570	0.827865	1.73196
26571	0.946132	1.48809
26572	0.946132	1.64957
26573	1.40398	1.68999
26574	0.946127	0.97925
26575	0.946127	2.1
26576	0.94613	0.412488
26577	1.41498	1.53638
26578	0.946128	1.02137
26579	0.94613	0.05
26580	0.946131	0.05
26581	0.946129	0.05
26582	0.94613	0.05
26583	0.94613	0.05
26584	0.946118	0.05
26585	0.946119	0.05
26586	0.946116	0.05
26587	1.36569	0.05
26588	0.946105	0.05
26589	0.946132	0.23009
26590	1.23175	0.17083
26591	0.94613	0.24399
26592	0.946133	0.165554
26593	0.946131	0.187571
26594	1.25698	0.05
26595	0.946126	0.05
26596	0.946129	0.05
26597	0.946128	0.05
26598	0.946129	0.05
26599	0.810943	2.1
26600	0.810944	2.1
26601	0.810932	2.1
26602	0.946132	0.0958818
26603	1.09841	0.0800907
26604	0.946131	0.119893
26605	0.946132	0.104917
26606	1.09841	0.0886002
26607	0.810936	1.42766
26608	0.810935	2.07801
26609	0.810934	2.1
26610	1.23851	2.1
26611	0.946132	1.93192
26612	0.946131	2.1
26613	1.23851	2.1
26614	1.23851	2.1
26615	0.946132	0.140158
26616	0.946131	0.35651
26617	0.946132	0.126126
26618	0.946132	0.221554
26619	0.946132	0.403708

26620	0.939141	1.31814
26621	0.953118	1.44073
26622	0.946126	0.96934
26623	0.946129	1.18019
26624	1.03306	1.32157
26625	0.946132	0.0944825
26626	1.30927	0.10773
26627	0.946132	0.110763
26628	0.946132	0.0913007
26629	1.30928	0.101222
26630	0.94613	2.1
26631	0.946131	2.1
26632	1.24511	2.1
26633	0.94613	2.1
26634	0.94613	2.1
26635	0.946133	0.0942358
26636	1.34152	0.0781085
26637	0.946131	0.116431
26638	0.946131	0.05
26639	1.34152	0.0505768
26640	0.946132	0.0840686
26641	1.41383	0.0919675
26642	0.946132	0.100146
26643	0.946132	0.05
26644	1.41383	0.0781059
26645	0.946132	1.03873
26646	0.946131	0.67587
26647	1.41392	0.511124
26648	0.946132	1.05964
26649	0.946132	0.737338
26650	0.946132	1.47872
26651	0.946132	1.25037
26652	1.24333	0.947729
26653	0.946133	1.52175
26654	0.946133	1.37939
26655	0.946124	0.187954
26656	0.94613	0.218346
26657	0.946126	0.211916
26658	1.3065	0.171614
26659	0.946127	0.213387
26660	0.810946	0.291342
26661	0.810943	0.213559
26662	0.81096	0.275876
26663	0.946131	0.0641143
26664	1.40271	0.0659632
26665	0.946131	0.0875751
26666	0.946131	0.0742017
26667	1.40271	0.0709401
26668	0.946132	0.05
26669	0.94613	0.05
26670	0.946131	0.05
26671	0.94613	0.05
26672	0.946131	0.05
26673	0.946133	0.300402
26674	1.30107	0.0549015
26675	0.946132	0.614011
26676	0.946132	0.0811933
26677	0.946133	0.53067

26678	0.810946	2.1
26679	0.810944	2.1
26680	0.810958	2.1
26681	0.946133	1.88584
26682	0.946131	1.87548
26683	0.946131	2.1
26684	1.30812	2.09706
26685	0.946131	1.97337
26686	0.94613	1.30516
26687	0.975002	1.57743
26688	0.917259	1.94182
26689	1.00902	1.74414
26690	0.94613	1.06701
26691	0.946131	2.1
26692	0.946127	2.1
26693	1.398	2.1
26694	0.946129	2.1
26695	0.946129	2.1
26696	0.946133	2.1
26697	0.946132	2.1
26698	0.946132	2.1
26699	0.946132	2.1
26700	1.3033	2.1
26701	1.09897	0.277869
26702	0.946131	0.570961
26703	0.946132	0.422749
26704	0.946129	0.529564
26705	0.946131	0.522406
26706	0.946131	2.1
26707	0.946132	2.1
26708	1.39858	2.1
26709	0.946131	2.1
26710	0.946131	2.1
26711	0.946119	1.10845
26712	1.08878	1.13469
26713	0.946119	1.33118
26714	1.0888	1.13853
26715	0.946107	1.29037
26716	0.946132	0.05
26717	1.10733	0.05
26718	0.946132	0.05
26719	0.946131	0.05
26720	0.946132	0.05
26721	0.946128	0.05
26722	1.33671	0.05
26723	0.94613	0.0593089
26724	0.94613	0.0564628
26725	0.94613	0.0510232
26726	0.810943	2.1
26727	0.810944	2.1
26728	0.810945	2.1
26729	1.35117	2.1
26730	0.946129	2.1
26731	0.94613	2.1
26732	1.35117	2.1
26733	1.27174	2.1
26734	0.946132	2.1
26735	0.946133	2.05611

26736	0.997296	1.76604
26737	0.946133	1.93715
26738	0.946132	1.85663
26739	0.810943	2.1
26740	0.810944	2.1
26741	0.810945	2.1
26742	0.810946	1.70091
26743	0.810943	1.82849
26744	0.810933	1.65261
26745	0.946127	1.58176
26746	0.946131	1.56907
26747	0.946128	1.57255
26748	0.94613	1.66831
26749	0.946129	1.6887
26750	0.946131	0.305465
26751	0.946131	0.240528
26752	0.946129	0.315307
26753	0.946131	0.249304
26754	0.94613	0.248189
26755	0.946133	0.0796644
26756	0.946132	0.113453
26757	0.946132	0.090592
26758	0.946132	0.08138
26759	0.946133	0.438704
26760	0.946131	1.47179
26761	0.94613	1.18064
26762	0.946129	1.49583
26763	0.946131	1.02358
26764	0.946131	1.47113
26765	0.946132	1.89456
26766	0.946129	2.1
26767	0.94613	2.1
26768	0.946131	2.00853
26769	1.39356	2.1
26770	0.810946	2.1
26771	0.810947	2.1
26772	0.810934	2.1
26773	0.946125	0.05
26774	0.946127	0.0509608
26775	1.33798	0.05
26776	1.33797	0.05
26777	0.946128	0.05
26778	0.946131	0.05
26779	0.946131	0.05
26780	1.23146	0.05
26781	1.23146	0.05
26782	0.946131	0.05
26783	0.946129	2.1
26784	0.94613	2.1
26785	0.946131	2.1
26786	0.94613	2.1
26787	1.30082	2.1
26788	0.946131	0.05
26789	0.946132	0.05
26790	0.946131	0.0593411
26791	1.20416	0.05
26792	0.946132	0.05
26793	0.946132	0.05

26794	0.946131	0.05
26795	0.946131	0.0582566
26796	1.18711	0.05
26797	0.946132	0.05
26798	0.946132	0.05
26799	0.946132	0.05
26800	0.946133	0.05
26801	0.946132	0.05
26802	0.946133	0.05
26803	0.946131	0.05
26804	0.946132	0.05
26805	0.94613	0.05
26806	0.946131	0.05
26807	0.946131	0.05
26808	0.946133	0.05
26809	0.946132	0.05
26810	0.946133	0.05
26811	0.946132	0.05
26812	0.946132	0.05
26813	0.946132	2.1
26814	0.946132	2.1
26815	0.946132	2.1
26816	1.36422	2.1
26817	0.946132	2.1
26818	0.946131	1.09685
26819	0.946132	1.67246
26820	0.946132	1.50205
26821	0.946132	1.53846
26822	1.4185	1.61231
26823	0.946133	0.0526106
26824	1.37794	0.05
26825	0.946132	0.05
26826	0.946133	0.05
26827	0.946132	0.05
26828	1.4045	2.1
26829	0.946132	2.1
26830	0.946132	2.1
26831	0.946132	2.1
26832	0.946132	2.1
26833	0.946131	2.1
26834	0.946132	2.1
26835	1.34285	2.1
26836	1.34285	2.1
26837	0.946132	2.1
26838	0.946131	2.1
26839	0.946132	2.1
26840	1.40739	2.1
26841	1.40739	2.1
26842	0.946131	2.1
26843	0.946128	2.1
26844	0.946132	2.1
26845	0.94613	2.1
26846	0.94613	2.1
26847	1.3164	2.1
26848	0.946132	2.1
26849	1.3484	2.1
26850	0.946132	2.1
26851	0.946132	2.1

26852	1.3484	2.1
26853	0.946132	2.1
26854	0.946132	2.1
26855	0.946133	2.1
26856	0.946133	2.1
26857	1.41507	2.1
26858	0.946132	2.1
26859	1.32608	2.1
26860	0.946131	2.1
26861	0.946131	2.1
26862	0.946132	2.1
26863	0.94613	1.45259
26864	1.34646	1.7595
26865	0.946129	1.45176
26866	0.94613	0.998079
26867	1.34646	1.50706
26868	0.946131	1.36594
26869	0.946131	2.1
26870	0.94613	1.90869
26871	1.33728	1.6002
26872	0.946131	1.8458
26873	0.946127	2.1
26874	0.946131	1.84002
26875	0.946128	2.1
26876	0.946129	2.1
26877	1.4064	2.1
26878	0.946133	2.1
26879	0.946131	2.1
26880	0.946131	2.1
26881	0.946131	2.1
26882	0.94613	2.1
26883	1.34912	2.1
26884	0.946131	2.1
26885	0.946129	2.1
26886	0.946131	2.1
26887	0.94613	2.1
26888	0.946132	2.1
26889	1.40447	2.1
26890	0.946132	2.1
26891	0.946133	2.1
26892	1.40447	2.1
26893	1.33607	2.1
26894	0.946131	2.1
26895	0.946127	2.1
26896	0.94613	2.1
26897	0.946129	2.1
26898	0.946117	0.541706
26899	1.32664	1.66247
26900	0.946115	1.59472
26901	0.946105	1.48762
26902	0.94613	0.608234
26903	0.946118	2.1
26904	0.946114	2.1
26905	1.35886	2.1
26906	1.35884	2.1
26907	0.946129	2.1
26908	0.946132	2.1
26909	1.2571	2.1

26910	0.946132	2.1
26911	0.946132	2.1
26912	1.2571	2.1
26913	0.946133	2.1
26914	1.35663	2.1
26915	0.946132	2.1
26916	0.946132	2.1
26917	1.35663	2.1
26918	0.946133	1.50438
26919	0.946129	1.80389
26920	0.946131	1.64727
26921	0.946131	1.66186
26922	1.30881	1.74827
26923	0.946132	2.1
26924	0.94613	2.1
26925	1.34652	2.1
26926	0.94613	2.1
26927	0.94613	2.1
26928	0.946132	2.05844
26929	0.946133	1.75489
26930	1.36183	1.37116
26931	1.36183	1.64345
26932	0.946132	1.78783
26933	0.946132	0.127667
26934	1.38353	0.0702537
26935	0.94613	0.100748
26936	0.94613	0.100972
26937	1.38354	0.0845525
26938	0.946128	0.469379
26939	1.24887	0.373466
26940	0.94613	0.527429
26941	0.94613	0.48043
26942	1.24887	0.491688
26943	0.946129	1.35295
26944	0.946126	1.49072
26945	0.946124	1.38622
26946	0.946128	1.40553
26947	1.32056	1.45419
26948	0.946129	1.30296
26949	0.946128	1.51045
26950	0.946126	1.25536
26951	0.946129	1.40455
26952	1.20351	1.48058
26953	0.946131	1.85788
26954	0.946131	1.86045
26955	0.946127	2.09955
26956	1.25591	1.97531
26957	0.94613	1.8218
26958	0.946132	1.51394
26959	0.946133	1.52732
26960	0.946132	1.67456
26961	1.15436	1.5167
26962	0.930306	1.4949
26963	0.946132	2.1
26964	0.946132	2.1
26965	0.946132	1.98334
26966	0.946133	2.1
26967	0.946132	2.1

26968	0.946129	1.28488
26969	0.946132	1.24934
26970	0.946126	1.42578
26971	1.13245	1.22335
26972	0.946129	1.28565
26973	0.94613	2.0998
26974	0.946132	2.08548
26975	0.946129	1.89729
26976	0.946131	2.1
26977	0.946131	2.001
26978	1.41279	2.04347
26979	1.76254	2.1
26980	0.600454	1.9716
26981	0.858991	1.99694
26982	0.940722	2.02808
26983	1.28439	1.76812
26984	0.946131	1.85953
26985	0.94613	1.82601
26986	0.946131	2.04689
26987	0.946131	1.99709
26988	0.946132	1.33892
26989	1.39335	0.506025
26990	0.946132	1.33992
26991	0.946132	0.689486
26992	0.946132	1.30372
26993	1.33879	0.161895
26994	0.946131	0.289148
26995	0.94613	0.474592
26996	1.33879	0.15978
26997	0.946131	0.25061
26998	0.946126	0.27897
26999	0.946131	0.237053
27000	1.37342	0.194985
27001	1.37343	0.162383
27002	0.946128	0.723876
27003	1.39168	0.05
27004	0.946131	0.05
27005	0.946131	0.0515632
27006	0.946131	0.05
27007	0.946131	0.05
27008	0.946131	0.100205
27009	1.412	0.076251
27010	0.946127	0.0751918
27011	0.946128	0.0716482
27012	0.946128	0.0649207
27013	1.1387	0.173904
27014	0.943364	0.32296
27015	0.946132	0.241051
27016	0.946132	0.280082
27017	0.946133	0.266991
27018	1.21085	0.329056
27019	0.88324	0.787779
27020	0.946129	0.789677
27021	0.946131	0.712108
27022	0.946131	0.587719
27023	1.33587	0.05
27024	0.946125	0.0505534
27025	0.946129	0.05

27026	0.946126	0.0504911
27027	0.946127	0.05
27028	1.27512	0.0525565
27029	0.946132	0.0811953
27030	0.946132	0.0795747
27031	0.946133	0.0761528
27032	0.946132	0.0616938
27033	0.946128	0.0541849
27034	1.30498	0.05
27035	0.946131	0.0685133
27036	0.94613	0.0575964
27037	1.30497	0.0535163
27038	0.946131	0.05
27039	1.25626	0.05
27040	0.946131	0.0535088
27041	0.946131	0.05
27042	1.25626	0.05
27043	0.946131	0.05
27044	0.94613	0.05
27045	1.35316	0.05
27046	0.946131	0.05
27047	0.946131	0.05
27048	0.94613	0.118284
27049	1.28727	0.121645
27050	0.946128	0.0775068
27051	0.946131	0.0966491
27052	0.94613	0.0754926
27053	0.946131	0.05
27054	1.39502	0.05
27055	0.946132	0.05
27056	0.946132	0.05
27057	0.946132	0.05
27058	1.36173	0.223465
27059	0.946131	0.437529
27060	0.946126	0.418717
27061	0.946127	0.401345
27062	0.946128	0.336769
27063	0.946129	0.990645
27064	0.946133	0.381942
27065	0.946131	1.11266
27066	0.946131	0.808073
27067	0.946131	1.14711
27068	0.946128	0.05
27069	0.946132	0.05
27070	1.24678	0.05
27071	0.94613	0.05
27072	0.94613	0.05
27073	0.946129	0.05
27074	0.94613	0.05
27075	1.25215	0.05
27076	0.946126	0.05
27077	0.946129	0.05
27078	0.946129	0.377645
27079	1.29689	0.271526
27080	0.946131	0.405853
27081	0.946131	0.2991
27082	0.946131	0.2561
27083	0.946128	0.0500462

27084	0.94613	0.0507124
27085	0.946131	0.05
27086	0.94613	0.0524762
27087	0.946131	0.0524098
27088	0.946127	0.0646015
27089	0.946128	0.0671814
27090	0.94613	0.0617171
27091	0.946129	0.0688496
27092	0.946129	0.0681596
27093	0.946133	0.05
27094	0.946132	0.05
27095	0.946132	0.05
27096	1.29957	0.05
27097	0.946133	0.05
27098	0.946132	0.05
27099	0.946133	0.05
27100	0.946132	0.05
27101	1.26201	0.05
27102	0.946132	0.05
27103	0.946133	0.052173
27104	1.40427	0.05
27105	0.946132	0.0684207
27106	0.946132	0.0662244
27107	0.946133	0.0558135
27108	0.946132	0.0527264
27109	1.35123	0.05
27110	0.946132	0.072503
27111	0.946132	0.0727612
27112	0.946131	0.054804
27113	0.946128	0.0847607
27114	0.946129	0.0867517
27115	0.946131	0.0775755
27116	0.94613	0.0920102
27117	0.946131	0.0857112
27118	0.946127	0.238881
27119	1.23351	0.19397
27120	0.946131	0.235908
27121	0.946129	0.217382
27122	0.94613	0.198716
27123	0.946131	0.113842
27124	0.946131	0.158396
27125	0.94613	0.117362
27126	1.18119	0.113002
27127	0.946131	0.135855
27128	0.946132	0.173928
27129	1.30563	0.131544
27130	0.946133	0.195409
27131	0.946132	0.138736
27132	0.946133	0.161819
27133	0.946132	0.282665
27134	0.946132	0.509408
27135	0.946133	1.78731
27136	1.20256	0.368114
27137	0.946133	0.503281
27138	0.94613	1.31235
27139	1.33007	0.579585
27140	0.946128	1.29622
27141	0.946132	0.623787

27142	0.946131	0.78415
27143	0.946131	2.1
27144	0.946131	2.1
27145	0.946128	2.1
27146	0.946129	2.1
27147	0.94613	2.1
27148	0.810942	2.1
27149	0.810942	2.1
27150	0.81093	2.1
27151	0.946132	2.1
27152	1.39823	2.1
27153	0.946132	2.1
27154	0.946131	2.1
27155	0.946132	2.1
27156	0.946131	2.1
27157	0.946131	2.1
27158	0.946131	2.1
27159	1.37713	2.1
27160	0.946131	2.1
27161	0.946133	2.1
27162	0.946131	2.1
27163	1.40594	2.1
27164	1.40594	2.1
27165	0.94613	2.1
27166	0.946133	2.1
27167	0.946131	2.1
27168	1.38155	2.1
27169	1.38155	2.1
27170	0.946131	2.1
27171	0.946132	1.45528
27172	0.946133	1.95423
27173	0.946132	1.73574
27174	0.946132	1.78189
27175	1.33694	1.84866
27176	0.946131	2.1
27177	1.228	2.1
27178	0.946129	2.1
27179	0.946131	2.1
27180	1.228	2.1
27181	0.946132	1.36849
27182	0.946132	1.66827
27183	0.946131	1.62036
27184	0.946132	1.48781
27185	1.35943	1.65694
27186	0.946133	0.05
27187	0.946133	0.05
27188	1.02377	0.05
27189	1.02377	0.05
27190	0.946132	0.05
27191	0.94613	0.05
27192	0.946131	0.05
27193	0.946132	0.05
27194	1.29322	0.05
27195	0.946131	0.05
27196	0.946131	2.1
27197	0.946131	0.871762
27198	0.946132	2.1
27199	1.41492	1.97758

27200	0.946131	2.1
27201	0.946132	2.1
27202	0.946131	2.1
27203	0.946132	2.1
27204	1.35028	2.1
27205	0.946132	2.1
27206	0.946132	2.1
27207	0.946132	2.1
27208	0.946131	2.1
27209	1.32461	2.1
27210	0.946132	2.1
27211	0.946131	2.1
27212	0.946132	2.1
27213	1.40369	2.1
27214	0.94613	2.1
27215	0.946131	2.1
27216	0.946132	0.0902184
27217	0.979597	0.0806692
27218	0.946131	0.11307
27219	0.946132	0.0798213
27220	0.946132	0.0889906
27221	0.946131	2.1
27222	0.946131	2.1
27223	0.94613	2.1
27224	0.946131	2.1
27225	1.41897	2.1
27226	1.37791	2.1
27227	0.94613	2.1
27228	0.946129	2.1
27229	0.946131	2.1
27230	0.946131	2.1
27231	1.39384	2.1
27232	0.946131	2.1
27233	0.946128	2.1
27234	0.946129	2.1
27235	0.946129	2.1
27236	0.810945	1.61768
27237	0.810944	2.09224
27238	0.810958	1.43952
27239	0.946128	2.1
27240	0.946132	2.1
27241	0.94613	2.1
27242	1.3335	2.1
27243	0.946131	2.1
27244	0.94613	2.04234
27245	0.946129	2.1
27246	0.946132	1.40073
27247	1.41192	2.1
27248	0.94613	2.1
27249	0.946132	1.45527
27250	0.946133	1.0412
27251	0.946132	0.33222
27252	1.39428	0.40681
27253	0.946133	0.31522
27254	0.94613	2.04921
27255	0.946128	2.0888
27256	0.946132	1.93203
27257	0.94613	1.85618

27258	1.40684	2.1
27259	0.946131	2.1
27260	0.946131	2.1
27261	0.946131	2.1
27262	0.946131	2.1
27263	1.39021	2.1
27264	0.946131	1.37315
27265	1.31649	1.68415
27266	0.946131	1.2843
27267	0.946131	1.24657
27268	1.31649	1.38141
27269	0.810946	2.1
27270	0.810946	2.1
27271	0.810958	2.1
27272	1.16687	2.1
27273	0.946131	2.1
27274	0.946131	2.1
27275	1.16687	2.1
27276	1.16687	2.1
27277	0.946128	0.89523
27278	1.3564	0.376779
27279	0.946131	0.933179
27280	0.946131	0.59696
27281	0.94613	0.803926
27282	0.946128	2.1
27283	1.35828	2.1
27284	0.94613	2.1
27285	0.946131	2.1
27286	0.94613	2.1
27287	0.946133	1.35078
27288	1.36477	1.60209
27289	0.946132	1.57859
27290	0.946132	1.38493
27291	1.36477	1.56119
27292	0.946132	1.62574
27293	1.31857	1.78348
27294	0.946132	1.63242
27295	0.946132	1.6448
27296	1.31857	1.63495
27297	0.94613	0.380837
27298	1.35059	0.252669
27299	0.946128	0.251923
27300	0.94613	0.319395
27301	1.35059	0.27145
27302	0.946126	0.111239
27303	1.35559	0.0720797
27304	0.946127	0.0904887
27305	0.946128	0.0868898
27306	1.35559	0.0723761
27307	0.946131	0.0704748
27308	1.33035	0.0705086
27309	0.946127	0.081329
27310	0.946128	0.05
27311	1.33035	0.0681664
27312	0.94613	0.0795281
27313	1.3665	0.0811013
27314	0.946129	0.0905127
27315	0.946131	0.0575151

27316	1.3665	0.0817677
27317	0.94613	0.0928223
27318	1.32828	0.0759829
27319	0.946129	0.0840435
27320	0.94613	0.0801942
27321	0.946131	0.0756131
27322	0.946131	2.1
27323	0.946131	2.1
27324	1.32949	2.1
27325	1.32949	2.1
27326	0.946131	2.1
27327	0.946131	2.1
27328	0.94613	0.191695
27329	0.946133	2.1
27330	1.38357	0.636643
27331	0.946131	1.34007
27332	0.946131	2.1
27333	0.946128	2.1
27334	1.36919	2.1
27335	0.946131	2.1
27336	0.94613	2.1
27337	0.946131	2.1
27338	1.3771	2.1
27339	0.946129	2.1
27340	0.94613	2.1
27341	0.946131	2.1
27342	0.946131	1.85488
27343	0.94613	1.20382
27344	0.946129	0.338727
27345	1.30029	1.55364
27346	0.94613	0.533262
27347	1.21174	0.05
27348	0.946132	0.05
27349	0.946132	0.05
27350	0.946132	0.05
27351	0.946133	0.05
27352	0.810946	2.1
27353	0.810945	2.1
27354	0.810946	2.1
27355	1.1796	2.1
27356	0.946131	2.1
27357	0.946132	2.1
27358	1.1796	2.1
27359	1.1796	2.1
27360	0.946131	2.1
27361	0.946131	2.1
27362	0.946133	2.1
27363	0.946131	2.1
27364	1.40615	2.1
27365	0.810945	2.1
27366	0.810944	2.1
27367	0.810945	2.1
27368	1.40239	2.1
27369	0.94613	2.1
27370	1.35561	2.1
27371	0.946133	2.1
27372	1.35562	2.1
27373	1.41169	0.179572

27374	0.946131	0.306325
27375	0.946127	0.426789
27376	1.41169	0.184476
27377	0.946128	0.282059
27378	0.946119	2.1
27379	0.946119	2.1
27380	0.946119	2.1
27381	0.946107	2.1
27382	1.41917	2.1
27383	0.810946	1.88294
27384	0.810943	1.92425
27385	0.810934	2.0669
27386	0.946127	2.1
27387	1.1995	2.1
27388	0.946126	2.1
27389	0.946128	2.1
27390	1.1995	2.1
27391	0.810944	2.1
27392	0.810947	2.1
27393	0.81096	2.1
27394	0.946133	0.123745
27395	1.3495	0.0669772
27396	0.946132	0.0992099
27397	0.946132	0.0711806
27398	0.946133	0.0797795
27399	0.946132	2.1
27400	0.946132	2.1
27401	0.946133	2.1
27402	1.39529	2.1
27403	0.946133	2.1
27404	0.946133	2.1
27405	1.28525	2.1
27406	0.946132	2.1
27407	0.946133	2.1
27408	1.28525	2.1
27409	0.810946	2.1
27410	0.810945	2.1
27411	0.810946	2.1
27412	1.33931	2.1
27413	0.946132	2.1
27414	0.946132	2.1
27415	1.33931	2.1
27416	1.24616	2.1
27417	0.946133	0.0790561
27418	0.946133	0.0508973
27419	0.946132	0.106996
27420	0.946132	0.05
27421	0.946133	0.110222
27422	0.946128	2.1
27423	1.40225	2.1
27424	0.946128	2.1
27425	0.94613	2.1
27426	0.94613	2.1
27427	0.946113	0.05
27428	0.946118	0.05
27429	0.946114	0.05
27430	1.40551	0.05
27431	0.946103	0.05

27432	0.94613	2.1
27433	0.946132	2.1
27434	0.946129	2.1
27435	0.946131	2.1
27436	1.37811	2.1
27437	0.946132	2.1
27438	1.39826	2.1
27439	0.946133	2.1
27440	0.946132	2.1
27441	1.39826	2.1
27442	0.946118	2.1
27443	1.28298	2.1
27444	0.946117	2.1
27445	0.946105	2.1
27446	1.283 2.1	
27447	0.946118	0.05
27448	1.41375	0.05
27449	0.946117	0.05
27450	0.946131	0.05
27451	0.946106	0.05
27452	0.946132	1.57175
27453	0.946129	1.39969
27454	1.28436	1.33264
27455	0.946131	1.58124
27456	0.946131	1.46139
27457	0.810947	0.369433
27458	0.810946	0.384939
27459	0.81096	0.329392
27460	0.946132	0.257559
27461	1.34338	0.227497
27462	0.961435	0.211323
27463	0.961435	0.24149
27464	0.946132	0.239591
27465	1.3028	2.00518
27466	0.946131	2.03985
27467	0.946127	2.1
27468	0.946128	2.1
27469	0.946129	2.0686
27470	0.946132	0.696111
27471	1.37973	0.320439
27472	0.946132	0.726527
27473	0.946132	0.428764
27474	0.946132	0.401256
27475	0.946131	1.29813
27476	0.94613	1.26912
27477	0.946129	1.29796
27478	0.946131	1.25098
27479	1.41798	0.973241
27480	0.946118	1.24551
27481	0.946118	0.873317
27482	0.946117	1.24688
27483	0.946131	1.08619
27484	0.946105	0.901453
27485	0.946131	1.3444
27486	0.946131	1.29906
27487	0.94613	1.34151
27488	0.946131	1.30017
27489	0.946131	1.26031

27490	0.946133	1.92317
27491	1.40274	2.1
27492	0.946133	1.38742
27493	0.946132	2.1
27494	1.40274	2.1
27495	0.946131	1.80724
27496	1.39731	2.1
27497	0.946132	1.52619
27498	0.94613	1.89344
27499	1.39731	2.09524
27500	0.94612	2.1
27501	0.94612	1.71833
27502	0.946119	1.78033
27503	0.946132	1.50939
27504	1.40764	1.95585
27505	0.946128	2.1
27506	0.946132	2.1
27507	0.94613	2.1
27508	0.94613	2.1
27509	1.31213	2.1
27510	0.946127	2.1
27511	0.94613	2.1
27512	0.946127	2.1
27513	0.946128	2.1
27514	1.36653	2.1
27515	0.946127	2.1
27516	1.3557	2.1
27517	0.946128	2.1
27518	0.946128	2.1
27519	1.3557	2.1
27520	0.946133	2.1
27521	0.946132	2.1
27522	0.946133	2.1
27523	0.946132	2.1
27524	1.40081	2.1
27525	0.946128	2.1
27526	0.946133	2.1
27527	0.946131	2.1
27528	0.946131	2.1
27529	1.32282	2.1
27530	0.810945	2.1
27531	0.810943	2.1
27532	0.810933	2.1
27533	1.27676	2.1
27534	0.94613	2.1
27535	0.946128	2.1
27536	0.946131	2.1
27537	0.946131	2.1
27538	0.946131	0.58356
27539	0.946127	0.867504
27540	1.41328	1.18131
27541	1.41327	0.94881
27542	0.946128	0.834529
27543	0.946132	0.0544691
27544	1.32083	0.0878444
27545	0.946132	0.082362
27546	0.946133	0.0611091
27547	1.32082	0.0659728

27548	0.946128	0.0759333
27549	1.29215	0.101037
27550	0.94613	0.100159
27551	0.94613	0.0756339
27552	1.29215	0.0842972
27553	0.946132	0.05
27554	1.27696	0.05
27555	0.94613	0.05
27556	0.94613	0.05
27557	1.27696	0.05
27558	0.94612	0.05
27559	1.29289	0.0579692
27560	0.946119	0.052812
27561	0.946132	0.05
27562	1.29287	0.05
27563	0.946128	2.1
27564	1.37869	2.1
27565	0.946131	2.1
27566	0.94613	2.1
27567	0.946131	2.1
27568	0.946133	2.1
27569	0.946132	2.1
27570	0.946132	2.1
27571	1.35703	2.1
27572	0.946133	2.1
27573	0.946132	2.1
27574	0.946132	2.1
27575	0.946133	2.1
27576	1.37017	2.1
27577	0.946133	2.1
27578	0.946119	0.05
27579	0.94612	0.05
27580	1.38583	0.05
27581	1.38585	0.05
27582	0.946107	0.05
27583	0.946126	0.05
27584	0.946127	0.0611014
27585	1.22904	0.05
27586	1.22904	0.05
27587	0.946128	0.05
27588	1.3279	0.734272
27589	0.946131	1.55224
27590	0.94613	1.47664
27591	0.946131	1.53512
27592	0.946131	1.4576
27593	0.946132	2.1
27594	0.946132	2.1
27595	0.946132	2.1
27596	0.946131	2.1
27597	1.40932	2.1
27598	1.26868	0.0687812
27599	0.946131	0.107371
27600	0.94613	0.0992969
27601	0.946131	0.0965272
27602	0.946131	0.0906567
27603	0.810943	2.1
27604	0.810945	2.1
27605	0.810932	2.1

27606	0.810943	2.1
27607	0.810944	0.795078
27608	0.810957	0.955924
27609	1.31539	0.751847
27610	0.946132	2.1
27611	0.946128	0.713414
27612	1.3154	1.7423
27613	1.17856	0.634597
27614	1.21997	1.50381
27615	0.94613	1.81794
27616	0.946129	1.72759
27617	0.94613	1.88856
27618	0.94613	1.80921
27619	0.946131	1.56096
27620	0.946131	1.4418
27621	0.946129	1.58176
27622	0.94613	1.61166
27623	0.946131	1.72094
27624	0.946127	0.735934
27625	0.946131	0.158662
27626	0.946127	1.65656
27627	0.946129	0.971092
27628	1.41342	1.17433
27629	0.946131	0.979537
27630	0.946131	1.59154
27631	0.946128	1.48997
27632	0.946131	1.23961
27633	1.29149	1.37192
27634	1.41022	2.1
27635	0.94613	2.05576
27636	0.946128	2.1
27637	0.94613	2.1
27638	0.94613	2.1
27639	0.946128	0.0770633
27640	0.94613	0.129834
27641	1.27864	0.0645422
27642	1.27864	0.0519419
27643	0.946131	0.0902532
27644	0.946129	1.14377
27645	0.946125	1.39553
27646	0.946124	1.24197
27647	0.946127	1.27085
27648	1.37759	1.3398
27649	0.946129	1.27337
27650	0.94613	1.45725
27651	0.946126	1.32889
27652	0.946129	1.35315
27653	1.38259	1.40898
27654	0.946131	0.05
27655	1.34118	0.05
27656	0.946132	0.0520852
27657	0.946132	0.05
27658	1.34118	0.05
27659	0.946131	0.05
27660	1.25017	0.05
27661	0.946128	0.05
27662	0.946131	0.05
27663	0.946131	0.05

27664	0.946131	2.1
27665	0.946128	2.1
27666	0.946133	2.1
27667	1.3937	2.1
27668	0.94613	2.1
27669	0.946131	2.1
27670	0.94613	0.596349
27671	1.40404	1.49946
27672	1.40404	0.752371
27673	0.946131	1.08321
27674	0.946132	1.68311
27675	0.946132	0.318709
27676	1.38897	0.273282
27677	1.38897	0.148209
27678	0.946132	0.437532
27679	0.94613	0.821769
27680	0.946128	1.8851
27681	1.40332	0.921305
27682	1.40331	1.22352
27683	0.94613	1.51301
27684	0.946132	1.94931
27685	0.946132	2.1
27686	0.946133	2.1
27687	1.41619	2.1
27688	0.946132	2.1
27689	0.946132	2.1
27690	0.946132	2.1
27691	0.946132	2.1
27692	1.38244	2.1
27693	0.946132	2.1
27694	0.810946	1.54471
27695	0.810943	0.945643
27696	0.810934	0.50217
27697	0.810947	1.46395
27698	0.810943	0.476316
27699	0.810934	0.560389
27700	1.12786	1.65726
27701	0.946132	2.00995
27702	0.946132	1.83769
27703	0.946132	1.76036
27704	0.946133	1.76447
27705	0.946132	1.64024
27706	0.946132	1.72161
27707	0.946132	1.58526
27708	1.27806	1.68796
27709	0.946133	1.61921
27710	0.946132	2.1
27711	0.946132	2.1
27712	0.946133	2.1
27713	0.946132	2.1
27714	0.946133	2.1
27715	1.39514	1.33652
27716	0.946129	1.23155
27717	0.946124	1.35072
27718	0.946127	1.1724
27719	0.946127	1.31639
27720	0.94613	2.1
27721	0.946129	2.1

27722	0.946132	2.1
27723	1.3991	2.1
27724	0.94613	2.1
27725	0.946132	1.59061
27726	0.946133	2.1
27727	0.946132	2.08844
27728	1.36835	2.1
27729	0.946132	2.1
27730	0.946132	2.1
27731	0.946132	2.1
27732	0.946133	2.1
27733	0.946132	2.1
27734	1.39047	2.1
27735	0.946132	0.05
27736	0.946128	0.05
27737	0.94613	0.05
27738	1.33174	0.05
27739	0.946131	0.05
27740	0.946128	0.05
27741	0.946131	0.05
27742	0.94613	0.05
27743	0.94613	0.05
27744	0.94613	0.05
27745	0.946119	2.1
27746	0.946119	2.1
27747	1.29064	2.1
27748	0.946132	2.1
27749	0.946107	2.1
27750	0.946133	2.1
27751	0.94613	2.1
27752	1.30584	2.1
27753	0.946131	2.1
27754	0.946131	2.1
27755	0.946131	2.1
27756	0.946132	2.1
27757	0.94613	2.1
27758	0.946131	2.1
27759	1.39549	2.1
27760	0.913386	1.41138
27761	0.978873	1.30891
27762	0.946126	1.39162
27763	0.94613	1.46058
27764	1.00473	1.02059
27765	0.946132	1.08775
27766	0.946133	1.12887
27767	0.946132	0.324081
27768	0.946132	1.10909
27769	1.38894	0.425455
27770	0.946128	0.897615
27771	0.946131	0.54669
27772	0.94613	0.301599
27773	0.94613	0.522455
27774	1.23865	0.288025
27775	0.946126	1.35113
27776	0.946129	1.05056
27777	0.946124	0.794674
27778	0.946127	1.21197
27779	1.40248	0.685133

27780	0.946133	1.08321
27781	0.946132	1.18116
27782	0.946132	0.533112
27783	0.946133	1.06265
27784	1.1391	0.835938
27785	0.827862	1.15547
27786	0.946133	1.12258
27787	0.946131	1.21694
27788	0.946131	1.17482
27789	0.94613	1.13761
27790	0.946128	0.951583
27791	0.946132	1.07385
27792	0.94613	1.08841
27793	1.21388	0.482881
27794	0.94613	1.0564
27795	0.810943	0.136584
27796	0.810945	0.195377
27797	0.810932	0.183462
27798	0.946131	1.82035
27799	0.946131	1.59596
27800	0.946133	1.34752
27801	1.36748	1.01335
27802	0.946132	0.193932
27803	0.946128	0.05
27804	0.946131	0.05
27805	0.94613	0.05
27806	1.35856	0.05
27807	0.94613	0.05
27808	0.946132	2.1
27809	0.946131	2.1
27810	0.946133	2.1
27811	0.946132	2.1
27812	0.946131	2.1
27813	0.946133	2.1
27814	0.946131	2.1
27815	1.39558	2.1
27816	1.39558	2.1
27817	0.94613	2.1
27818	0.946131	0.0956909
27819	0.94613	0.13191
27820	0.946129	0.0985513
27821	1.41388	0.0917173
27822	0.946131	0.11278
27823	0.946133	0.0955568
27824	0.946132	0.18276
27825	0.946132	0.0970135
27826	0.946133	0.110705
27827	0.93452	0.1742
27828	0.946129	2.1
27829	1.38897	2.1
27830	0.946131	2.1
27831	0.946131	2.1
27832	1.38897	2.1
27833	0.946133	1.81232
27834	0.946132	1.78588
27835	0.946133	1.75187
27836	0.946132	1.56019
27837	1.38773	1.653

27838	1.41512	0.115275
27839	0.946131	0.189367
27840	0.946129	0.153338
27841	0.946133	0.166208
27842	0.946131	0.166969
27843	0.946132	1.67202
27844	0.946132	2.1
27845	0.946132	1.75988
27846	0.946133	2.1
27847	0.946133	2.1
27848	0.946126	1.56951
27849	0.946132	1.91581
27850	0.946129	1.67647
27851	0.946129	1.87738
27852	0.946129	2.04402
27853	0.939895	2.03605
27854	1.03386	2.1
27855	0.94613	2.03211
27856	0.946131	2.04945
27857	1.03386	2.08715
27858	0.946131	2.1
27859	1.39309	2.1
27860	0.946133	2.1
27861	0.946131	2.1
27862	1.39309	2.1
27863	0.946133	2.1
27864	1.24853	2.1
27865	0.946132	2.1
27866	0.946132	2.1
27867	1.24853	2.1
27868	0.946133	2.04366
27869	1.39699	1.55727
27870	0.946132	1.8838
27871	0.946132	2.1
27872	1.39699	1.57948
27873	0.946132	2.1
27874	0.946131	2.1
27875	0.946133	2.1
27876	0.946131	2.1
27877	1.38969	2.1
27878	1.28464	1.9224
27879	0.946133	2.03062
27880	0.94613	2.07005
27881	0.946131	1.94557
27882	0.946131	1.93246
27883	0.946119	1.09896
27884	0.94612	1.17145
27885	0.946119	1.11375
27886	0.946132	1.10832
27887	1.3749	1.1111
27888	0.810942	2.1
27889	0.810943	2.1
27890	0.810957	2.1
27891	1.41134	1.89689
27892	0.946132	1.23192
27893	0.946132	1.75506
27894	0.946132	1.2787
27895	0.946132	1.59141

27896	1.31338	1.91622
27897	0.946132	1.65678
27898	0.946132	1.95297
27899	0.946133	1.72647
27900	0.946132	1.8073
27901	0.946103	0.05
27902	0.752448	0.05
27903	0.946132	0.05
27904	0.946131	0.05
27905	1.20617	0.05
27906	0.946132	1.36125
27907	1.08054	1.14869
27908	1.25736	1.41136
27909	0.946132	1.36228
27910	0.946131	1.36873
27911	0.946128	2.1
27912	1.33275	2.1
27913	1.1896	2.1
27914	0.946131	2.1
27915	1.18962	2.1
27916	1.21955	0.05
27917	0.946131	0.05
27918	1.33587	0.05
27919	0.741946	0.05
27920	1.07387	0.05
27921	0.945844	1.60351
27922	1.3292	2.1
27923	1.59472	2.01648
27924	0.946131	1.74488
27925	1.04043	1.86019
27926	1.13529	0.0754147
27927	0.946132	0.0781179
27928	1.24893	0.0946133
27929	1.12675	0.0940291
27930	0.936652	0.0947754
27931	1.36968	2.1
27932	0.946131	2.1
27933	0.946132	2.1
27934	1.36968	2.1
27935	1.258 2.1	
27936	3.17458	0.125941
27937	0.946132	2.1
27938	1.35022	2.1
27939	1.35022	2.1
27940	0.94613	2.1
27941	1.12356	2.1
27942	0.946131	2.1
27943	1.22401	2.1
27944	1.22401	2.1
27945	0.94613	2.1
27946	0.952455	2.1
27947	1.2107	2.1
27948	0.946132	2.1
27949	1.33047	2.1
27950	0.946131	2.1
27951	0.946132	2.1
27952	0.61957	2.1
27953	0.8636	2.1

27954	0.946132	2.1
27955	1.19294	2.1
27956	0.946128	2.1
27957	1.36607	2.1
27958	0.94613	2.1
27959	1.39416	2.1
27960	1.36607	2.1
27961	1.21474	2.1
27962	0.946131	2.1
27963	0.946131	2.1
27964	1.16288	2.1
27965	1.08542	2.1
27966	0.851526	1.62339
27967	1.30374	2.1
27968	1.01708	2.1
27969	0.946114	1.78102
27970	0.96976	1.88716
27971	0.946132	0.0922468
27972	1.49997	0.106238
27973	0.771735	0.100027
27974	0.771764	0.125812
27975	0.946133	0.150501
27976	0.700863	1.80928
27977	1.42222	2.1
27978	0.946131	2.1
27979	0.946128	2.1
27980	1.42509	2.1
27981	1.14298	2.1
27982	0.837014	2.1
27983	0.946131	2.1
27984	1.29426	2.1
27985	0.836985	2.1
27986	0.946129	2.1
27987	1.22089	0.379122
27988	0.946132	1.39627
27989	0.712486	0.378824
27990	0.6234	0.418542
27991	0.946131	2.1
27992	1.40271	2.1
27993	1.32241	2.1
27994	0.946133	2.1
27995	1.32241	2.1
27996	0.769373	1.65728
27997	0.946129	2.1
27998	1.41302	2.1
27999	1.28254	2.1
28000	0.946131	2.1
28001	1.28254	2.1
28002	0.851543	2.01972
28003	1.23056	2.1
28004	1.24356	2.1
28005	0.851543	2.06404
28006	0.909417	2.1
28007	1.13808	0.102461
28008	0.946133	0.473119
28009	0.946131	0.222016
28010	1.13808	1.35597
28011	1.13808	0.218365

28012	0.930941	1.56497
28013	1.37808	1.52392
28014	1.17573	1.25838
28015	0.946124	1.55429
28016	1.02404	1.39882
28017	0.946116	2.1
28018	1.3144	2.1
28019	1.3144	2.1
28020	0.946118	2.1
28021	1.13089	2.1
28022	0.946128	0.0747005
28023	0.946101	0.059184
28024	1.2606	0.083936
28025	1.26055	0.0624718
28026	0.580823	0.675231
28027	0.946132	1.55026
28028	0.580825	0.782708
28029	1.23224	0.984057
28030	0.946128	2.1
28031	1.3719	2.1
28032	0.94613	2.1
28033	1.3719	2.1
28034	0.94613	2.1
28035	0.946132	1.75846
28036	1.35025	0.349898
28037	1.35025	1.00869
28038	0.946132	1.79472
28039	1.1794	1.68121
28040	0.946133	2.1
28041	1.25598	2.1
28042	1.29249	2.1
28043	1.38154	2.1
28044	0.851541	2.1
28045	1.31508	2.1
28046	1.37558	2.1
28047	0.946129	2.1
28048	0.867663	2.1
28049	0.94613	1.67633
28050	1.35426	1.2707
28051	0.84872	0.546005
28052	0.946129	1.6427
28053	1.17195	1.71345
28054	0.946133	2.1
28055	1.39009	2.1
28056	1.14342	2.1
28057	0.946133	2.1
28058	1.14343	2.1
28059	1.38819	2.1
28060	0.946133	2.1
28061	1.4116	2.1
28062	0.946131	2.1
28063	0.946131	2.1
28064	0.946127	2.1
28065	1.32244	2.1
28066	1.32244	2.1
28067	0.946131	2.1
28068	1.2176	2.1
28069	1.75823	2.1

28070	1.11239	2.1
28071	2.17979	2.1
28072	1.65533	2.1
28073	3.41356	2.1
28074	1.94841	2.1
28075	1.91767	2.1
28076	1.76726	2.1
28077	1.70302	2.1
28078	3.76137	2.1
28079	1.94841	2.1
28080	1.33962	2.1
28081	1.31365	2.1
28082	1.70302	2.1
28083	3.2123	2.1
28084	1.76726	2.1
28085	1.70302	2.1
28086	1.47581	2.1
28087	1.7457	2.1
28088	3.22764	2.1
28089	1.20478	2.1
28090	1.70302	2.1
28091	1.27956	2.1
28092	1.18569	2.1
28093	1.63979	2.1
28094	1.51949	2.1
28095	1.53082	2.1
28096	1.92911	2.1
28097	1.32068	2.1
28098	1.74454	2.1
28099	3.27536	2.1
28100	1.08172	2.1
28101	1.74454	2.1
28102	1.52603	2.1
28103	1.52603	2.1
28104	1.32068	2.1
28105	1.32068	2.1
28106	1.89226	2.1
28107	2.37364	2.1
28108	1.89226	2.1
28109	2.42769	2.1
28110	4.31995	2.1
28111	2.37365	2.1
28112	1.5362	2.1
28113	2.4277	2.1
28114	1.56328	2.1
28115	3.9639	2.1
28116	1.89225	2.1
28117	2.42768	2.1
28118	1.89213	2.1
28119	1.89225	2.1
28120	2.42756	2.1
28121	2.42768	2.1
28122	2.42768	2.1
28123	1.58163	2.1
28124	1.56315	2.1
28125	1.56327	2.1
28126	2.42757	2.1
28127	2.42768	2.1

28128	1.01641	2.1
28129	2.15005	2.1
28130	1.00099	2.1
28131	2.15114	2.1
28132	3.15104	2.1
28133	0.994813	2.1
28134	2.15114	2.1
28135	2.17159	2.1
28136	2.17159	2.1
28137	1.00099	2.1
28138	1.00099	2.1
28139	1.29608	2.1
28140	1.29608	2.1
28141	0.942035	2.1
28142	0.942035	2.1
28143	1.29198	2.1
28144	0.942033	2.1
28145	0.946132	2.1
28146	0.946132	2.1
28147	0.946132	2.1
28148	0.942033	2.1
28149	0.946133	2.1
28150	1.29198	2.1
28151	1.99985	2.1
28152	1.14207	2.1
28153	1.83584	2.1
28154	1.00232	2.1
28155	3.00217	2.1
28156	1.99867	0.05
28157	2.0815	0.05
28158	1.56806	0.05
28159	1.28828	0.05
28160	3.58519	0.05
28161	1.89226	0.05
28162	1.89226	0.0515421
28163	1.89226	0.05
28164	1.89226	0.0516841
28165	1.89226	0.05
28166	1.89226	0.05
28167	1.89226	0.05
28168	1.89226	0.05
28169	2.23193	0.05
28170	2.48978	0.05
28171	4.38204	0.05
28172	1.85293	2.1
28173	1.64279	0.334456
28174	1.76268	2.1
28175	1.54214	0.31244
28176	3.39507	0.948162
28177	1.6428	0.334959
28178	1.17302	1.02271
28179	1.17302	1.94192
28180	1.28419	0.799761
28181	1.54214	0.296493
28182	1.65331	0.918645
28183	1.76268	2.1
28184	1.54214	0.71654
28185	1.65324	2.09709

28186	1.42491	0.623188
28187	3.19538	0.713387
28188	0.651043	1.91057
28189	0.651043	1.5227
28190	0.647925	0.500482
28191	0.647925	1.83502
28192	0.521747	0.779919
28193	0.58651	0.93068
28194	0.77107	0.16168
28195	0.712457	0.273742
28196	0.712457	0.377947
28197	0.521747	0.320161
28198	0.58651	0.31298
28199	0.77107	0.462491
28200	1.2873	2.1
28201	1.48751	2.1
28202	1.59659	2.1
28203	1.77596	2.1
28204	3.06326	2.1
28205	1.07976	2.1
28206	1.59659	2.1
28207	1.30237	2.1
28208	1.77723	2.1
28209	2.89897	2.1
28210	1.77723	2.1
28211	1.77596	2.1
28212	1.82614	2.1
28213	1.59659	2.1
28214	2.0055	2.1
28215	1.77596	2.1
28216	2.64523	2.09351
28217	2.73982	0.453514
28218	2.50057	2.02989
28219	2.61606	0.344378
28220	5.26129	0.996909
28221	2.73982	0.244426
28222	2.68987	0.515622
28223	2.61607	0.389143
28224	2.57611	0.560136
28225	5.30593	0.05
28226	1.89226	0.105394
28227	1.89226	0.394772
28228	1.89226	0.108599
28229	2.02178	0.0646112
28230	3.78452	0.102939
28231	1.89226	0.527205
28232	1.89226	0.507139
28233	1.89226	1.68976
28234	1.89226	0.378114
28235	1.89226	1.71018
28236	1.89226	0.281537
28237	2.35204	2.1
28238	1.50108	2.1
28239	1.3715	2.1
28240	2.18469	2.1
28241	3.68577	2.1
28242	2.62514	2.1
28243	2.1847	2.1

28244	2.0487	2.1
28245	2.45213	2.1
28246	4.63682	2.1
28247	1.25075	2.1
28248	2.18469	2.1
28249	1.3715	2.1
28250	1.3715	2.1
28251	2.0487	2.1
28252	2.0487	2.1
28253	1.28667	2.1
28254	1.11285	2.1
28255	1.03529	2.1
28256	1.03529	2.1
28257	1.30711	2.1
28258	1.30711	2.1
28259	2.35053	2.1
28260	1.89225	2.1
28261	1.89225	2.1
28262	2.51216	2.1
28263	4.24278	2.1
28264	1.89225	2.1
28265	1.89225	2.1
28266	1.89225	2.1
28267	1.89225	2.1
28268	3.7845	2.1
28269	1.92025	2.1
28270	1.32119	2.1
28271	1.44777	2.1
28272	1.33834	2.1
28273	1.92025	2.1
28274	1.81082	2.1
28275	1.89226	2.1
28276	2.67272	2.1
28277	2.51218	2.1
28278	2.51218	2.1
28279	1.89226	2.1
28280	1.89226	2.1
28281	1.89226	2.1
28282	1.89226	2.1
28283	1.89226	2.1
28284	1.89226	2.1
28285	3.78452	2.1
28286	1.92027	2.1
28287	1.44778	2.1
28288	1.61121	2.1
28289	2.01555	2.1
28290	3.53147	2.1
28291	1.90727	0.05
28292	2.29851	0.0791391
28293	2.71643	0.0515835
28294	2.17695	0.05
28295	4.47547	0.05
28296	1.90728	0.058543
28297	1.56339	0.05
28298	1.58143	0.05
28299	2.17696	0.0645598
28300	3.74035	0.0538162
28301	1.91368	0.05

28302	2.17696	0.0682513
28303	2.39986	0.05
28304	1.75904	0.05
28305	3.936	0.05
28306	1.18793	0.05
28307	2.74744	0.05
28308	1.38716	0.05
28309	2.57465	0.05
28310	3.93537	0.05
28311	1.3148	0.05
28312	2.74744	0.0522364
28313	2.70805	0.05
28314	1.38716	0.05
28315	2.70805	0.05
28316	1.38716	0.05
28317	1.20608	2.1
28318	0.975871	2.1
28319	0.946132	2.1
28320	0.946132	2.1
28321	0.946132	2.1
28322	0.946132	2.1
28323	0.975871	2.1
28324	0.975871	2.1
28325	1.20608	2.1
28326	0.946132	2.1
28327	0.946133	2.1
28328	0.975871	2.1
28329	2.8198	2.1
28330	2.29967	2.1
28331	2.09542	2.1
28332	2.63784	1.96465
28333	4.93751	2.1
28334	2.09542	2.1
28335	1.39523	2.1
28336	1.21119	2.1
28337	1.39523	2.1
28338	2.11563	2.1
28339	2.29967	2.1
28340	1.88584	2.1
28341	2.63784	1.82274
28342	2.11262	2.1
28343	1.91132	2.1
28344	4.02153	2.1
28345	1.88584	2.1
28346	1.21119	2.1
28347	1.12606	2.1
28348	1.91132	2.1
28349	3.1225	2.1
28350	2.18077	2.0841
28351	1.78167	1.70347
28352	2.2947	1.42631
28353	1.93139	1.61212
28354	4.07637	1.48914
28355	1.5709	1.42625
28356	1.03235	1.71683
28357	2.05933	1.55132
28358	1.78168	1.36192
28359	1.31	0.259802

28360	1.03235	1.02065
28361	2.2947	1.62917
28362	2.31785	1.90782
28363	2.40627	2.1
28364	2.08288	1.34011
28365	4.37758	1.89316
28366	1.95737	1.7034
28367	1.31	1.69186
28368	2.08289	0.492891
28369	1.50108	0.595375
28370	3.39289	1.14458
28371	2.49676	2.1
28372	2.24781	2.1
28373	2.5276	2.1
28374	2.7352	2.1
28375	5.02436	2.1
28376	2.49676	2.1
28377	2.43549	2.1
28378	2.6398	2.1
28379	2.7352	2.1
28380	5.13656	2.1
28381	1.89226	2.1
28382	2.7352	2.1
28383	1.89226	2.1
28384	2.6398	2.1
28385	1.89226	2.1
28386	2.6398	2.1
28387	2.16843	0.05
28388	1.00185	0.05
28389	1.90039	2.1
28390	3.1547	0.05
28391	2.54064	0.05
28392	2.46752	2.1
28393	1.90039	2.1
28394	2.26044	2.1
28395	4.36791	1.96902
28396	1.65815	2.1
28397	2.48652	2.1
28398	1.65815	2.1
28399	1.9004	2.1
28400	2.21711	2.1
28401	2.5034	2.1
28402	2.37167	2.1
28403	2.59965	2.1
28404	2.62812	2.1
28405	2.40308	2.1
28406	4.99979	2.1
28407	2.50137	2.1
28408	2.59965	2.1
28409	2.62812	2.1
28410	2.5214	2.1
28411	5.12948	2.1
28412	2.62814	2.1
28413	2.4031	2.1
28414	2.43509	2.1
28415	2.65066	2.1
28416	5.06323	2.1
28417	2.62814	2.1

28418	2.52142	2.1
28419	2.53008	2.1
28420	2.65066	2.1
28421	5.15822	2.1
28422	2.0815	2.1
28423	1.92067	2.1
28424	1.59611	2.1
28425	1.89226	2.1
28426	1.62453	2.1
28427	1.92067	2.1
28428	1.89226	2.1
28429	1.89226	2.1
28430	1.89226	2.1
28431	1.89226	2.1
28432	3.78453	2.1
28433	0.946131	2.1
28434	0.946131	2.1
28435	0.929501	2.1
28436	0.910761	2.1
28437	0.9461	2.1
28438	0.9461	2.1
28439	0.910792	2.1
28440	0.910792	2.1
28441	0.946131	2.1
28442	0.929501	2.1
28443	0.910761	2.1
28444	0.946131	2.1
28445	2.0815	2.1
28446	1.81661	2.1
28447	2.1341	2.1
28448	2.0815	2.1
28449	1.86921	2.1
28450	1.81661	2.1
28451	1.89226	2.1
28452	1.89226	2.1
28453	1.89226	2.1
28454	1.89226	2.1
28455	1.89226	2.1
28456	1.89226	2.1
28457	0.946131	2.1
28458	0.946131	2.1
28459	0.949104	2.1
28460	0.9295	2.1
28461	0.946132	2.1
28462	0.946132	2.1
28463	0.929499	2.1
28464	0.929499	2.1
28465	0.946131	2.1
28466	0.949105	2.1
28467	0.946131	2.1
28468	0.929501	2.1
28469	1.66104	2.1
28470	2.34044	2.1
28471	2.08889	2.1
28472	1.33264	2.1
28473	3.67308	2.1
28474	2.0889	2.1
28475	1.08011	2.1

28476	1.08011	2.1
28477	1.33264	2.1
28478	1.87598	2.1
28479	2.12851	2.1
28480	2.27472	2.1
28481	1.89226	1.8941
28482	2.27703	2.1
28483	1.89226	1.83091
28484	4.16699	2.1
28485	1.89226	1.96775
28486	1.89226	1.69206
28487	1.89226	1.88377
28488	1.89226	1.58384
28489	3.78452	1.65517
28490	1.00007	1.9562
28491	1.32316	1.49607
28492	1.29417	1.32919
28493	1.29417	1.90399
28494	1.02051	1.68116
28495	1.02051	1.84302
28496	2.2846	2.1
28497	1.89226	2.08474
28498	2.27703	2.06733
28499	2.27703	2.1
28500	1.89226	1.8163
28501	1.89226	2.1
28502	1.89226	2.1
28503	1.89226	2.02698
28504	1.89226	1.77703
28505	1.89226	0.651412
28506	3.78452	1.51412
28507	1.02051	2.1
28508	1.32316	1.95831
28509	1.03373	1.76574
28510	1.34066	1.68818
28511	2.35689	2.04615
28512	1.703 2.1	
28513	1.89224	2.1
28514	1.52266	2.1
28515	1.89224	2.1
28516	1.23344	2.1
28517	1.53282	2.1
28518	1.89223	2.1
28519	1.89223	2.1
28520	1.89223	2.1
28521	1.89223	2.1
28522	3.78447	2.1
28523	0.796845	2.1
28524	0.796845	2.1
28525	0.812146	2.1
28526	0.812146	2.1
28527	1.08006	2.1
28528	1.02679	2.1
28529	0.551538	2.1
28530	0.528929	2.1
28531	0.551529	2.1
28532	1.08005	2.1
28533	0.528935	2.1

28534	1.0268	2.1
28535	1.5001	2.1
28536	1.70302	2.1
28537	2.0726	2.1
28538	1.70302	2.1
28539	1.99601	2.1
28540	1.70303	2.1
28541	1.89226	2.1
28542	1.89226	2.1
28543	1.89226	2.1
28544	1.89226	2.1
28545	1.89226	2.1
28546	1.89226	2.1
28547	1.17285	2.1
28548	2.16015	2.1
28549	2.23563	2.1
28550	2.23563	2.1
28551	1.10309	2.1
28552	1.10309	2.1
28553	0.946128	2.1
28554	0.946128	2.1
28555	1.21847	2.1
28556	1.2884	2.1
28557	0.94613	2.1
28558	0.94613	2.1
28559	1.21847	2.1
28560	1.21847	2.1
28561	0.946131	2.1
28562	1.21847	2.1
28563	0.94613	2.1
28564	1.2884	2.1
28565	1.83922	2.1
28566	2.5768	2.1
28567	1.83488	2.1
28568	1.82846	0.05
28569	2.43693	2.1
28570	2.43693	2.1
28571	2.36134	2.1
28572	1.81656	2.1
28573	1.86454	2.1
28574	1.6697	2.1
28575	2.55618	2.1
28576	2.36134	2.1
28577	2.55618	2.1
28578	1.9323	2.1
28579	1.9302	2.1
28580	1.81656	2.1
28581	2.66983	2.1
28582	2.55618	2.1
28583	1.89226	1.54776
28584	1.70302	1.66193
28585	1.94935	1.75463
28586	1.70302	1.62319
28587	1.95569	1.54514
28588	1.70302	0.904561
28589	2.82465	2.1
28590	2.41707	2.1
28591	2.24481	2.1

28592	2.68958	2.1
28593	5.10665	2.1
28594	1.41232	2.1
28595	1.34479	2.1
28596	0.946129	2.1
28597	0.946129	2.1
28598	0.94613	2.1
28599	0.94613	2.1
28600	1.41232	2.1
28601	1.41232	2.1
28602	1.34479	2.1
28603	0.94613	2.1
28604	0.946131	2.1
28605	1.41232	2.1
28606	1.86859	0.05
28607	1.89226	0.05
28608	1.89226	0.05
28609	1.89226	0.05
28610	1.45875	0.05
28611	1.45875	0.05
28612	2.1769	0.356973
28613	2.50259	0.469983
28614	1.94955	0.194376
28615	2.25959	0.219439
28616	4.45214	0.547798
28617	1.89226	1.01152
28618	1.89226	1.04321
28619	1.89226	0.363134
28620	1.89226	1.01244
28621	3.78452	0.427601
28622	1.89226	1.13063
28623	1.89226	1.22154
28624	1.89226	0.948229
28625	1.89226	1.18239
28626	3.78452	1.30261
28627	2.50259	1.05483
28628	2.49445	0.396094
28629	2.25959	0.210087
28630	2.20843	0.661095
28631	4.71102	0.300636
28632	1.94955	0.205264
28633	2.25959	0.159232
28634	1.24689	0.091761
28635	1.79906	0.187338
28636	3.74861	0.132425
28637	1.89226	0.458439
28638	1.89226	0.459808
28639	1.89226	0.173555
28640	1.89226	0.235718
28641	3.78452	0.938676
28642	1.89226	0.392211
28643	1.89226	0.531427
28644	1.89226	0.211016
28645	1.89226	0.291233
28646	3.78452	0.199477
28647	2.25959	0.157362
28648	2.20843	0.270219
28649	1.79906	0.166827

28650	1.98391	0.167961
28651	4.00748	0.217865
28652	1.89226	2.1
28653	1.89226	2.1
28654	1.89226	2.1
28655	1.89226	2.1
28656	3.78452	2.1
28657	1.89226	2.1
28658	1.89226	2.1
28659	1.89226	2.1
28660	1.89226	2.1
28661	3.78452	2.1
28662	1.64718	2.1
28663	1.23085	2.1
28664	1.46028	2.1
28665	1.39564	2.1
28666	1.68372	2.1
28667	1.64718	2.1
28668	1.64718	2.1
28669	1.64981	2.1
28670	1.70303	2.1
28671	1.6991	2.1
28672	3.3199	2.1
28673	1.89226	2.1
28674	1.89226	2.1
28675	1.89226	2.1
28676	1.89226	2.1
28677	3.78452	2.1
28678	1.89226	2.1
28679	1.89226	2.1
28680	1.89226	2.1
28681	1.89226	2.1
28682	3.78452	2.1
28683	0.666701	2.1
28684	0.65896	2.1
28685	0.920797	2.1
28686	0.895441	2.1
28687	0.775871	2.1
28688	0.687493	2.1
28689	0.777393	2.1
28690	0.777393	2.1
28691	0.677856	2.1
28692	0.90095	2.1
28693	0.621435	2.1
28694	0.858124	2.1
28695	1.70303	2.1
28696	1.70731	2.1
28697	1.79663	2.1
28698	1.68783	2.1
28699	3.17337	2.1
28700	1.22724	2.1
28701	2.0021	2.1
28702	1.90427	2.1
28703	1.08698	2.1
28704	3.08907	2.1
28705	2.41689	2.1
28706	2.00209	2.1
28707	1.90427	2.1

28708	2.35245	2.1
28709	4.35454	2.1
28710	1.90427	2.1
28711	0.956294	2.1
28712	0.956294	2.1
28713	1.08698	2.1
28714	1.81488	2.1
28715	1.94556	2.1
28716	1.90427	2.1
28717	2.35245	2.1
28718	2.28979	2.1
28719	1.81488	2.1
28720	4.16733	2.1
28721	1.6272	2.1
28722	1.70302	2.1
28723	1.70303	2.1
28724	1.70302	2.1
28725	1.91167	2.1
28726	1.91167	2.1
28727	1.49063	2.1
28728	1.89226	2.1
28729	1.89226	2.1
28730	1.89226	2.1
28731	1.6272	2.1
28732	1.6272	2.1
28733	2.25988	1.7925
28734	1.89226	0.397112
28735	1.89226	1.46688
28736	2.23152	1.62255
28737	4.12379	0.899184
28738	2.25988	1.85558
28739	1.16361	2.1
28740	1.15853	2.1
28741	2.23153	1.74514
28742	3.39513	2.1
28743	1.89226	1.27693
28744	2.23153	0.769792
28745	2.21022	1.62151
28746	2.23153	1.39171
28747	1.87095	0.523896
28748	1.89226	0.247692
28749	1.16239	2.1
28750	2.23153	1.15967
28751	1.15853	2.01762
28752	1.15853	1.6078
28753	2.21022	2.02802
28754	2.21022	1.58284
28755	1.78381	0.05
28756	1.70302	0.05
28757	1.15453	0.05
28758	1.35007	0.05
28759	1.82021	0.05
28760	2.12851	0.05
28761	1.89226	0.05
28762	1.89226	0.0555599
28763	1.89226	0.05
28764	1.89226	0.0652149
28765	1.89226	0.05

28766	1.89226	0.05
28767	1.89258	0.05
28768	1.089 0.05	
28769	1.22586	0.05
28770	1.79877	0.05
28771	2.88777	0.05
28772	1.91564	0.05
28773	1.79877	0.05
28774	1.96356	0.05
28775	1.70302	0.05
28776	2.37828	0.05
28777	2.04859	0.05
28778	1.89226	0.05
28779	1.89226	0.0561816
28780	1.89226	0.05
28781	1.89226	0.0629845
28782	3.78453	0.05
28783	1.89226	0.05
28784	1.89226	0.0520424
28785	1.89226	0.05
28786	1.89226	0.0561924
28787	1.89226	0.05
28788	1.89226	0.05
28789	2.28593	2.1
28790	1.89226	2.1
28791	2.2731	2.1
28792	1.89226	1.92648
28793	4.17819	2.1
28794	1.89226	2.1
28795	1.89226	2.08816
28796	1.89226	1.97279
28797	1.89226	1.59817
28798	3.78452	1.75851
28799	1.03933	2.1
28800	1.34355	1.52135
28801	1.35048	1.49116
28802	1.35048	2.1
28803	1.02975	1.54966
28804	1.02975	2.03701
28805	2.25105	2.1
28806	1.89226	2.1
28807	2.27311	2.1
28808	2.27311	2.1
28809	1.89226	2.1
28810	1.89226	2.1
28811	1.89226	2.1
28812	1.89226	2.04572
28813	1.89226	2.06782
28814	1.89226	0.43163
28815	3.78452	1.6098
28816	1.02975	2.1
28817	1.34355	1.99585
28818	1.01121	1.98226
28819	1.33079	1.59791
28820	2.35476	2.1
28821	1.24526	0.05
28822	2.36406	0.05
28823	2.81021	0.05

28824	1.7237	0.05
28825	4.08776	0.05
28826	1.89226	2.1
28827	1.89226	2.1
28828	1.89226	2.1
28829	1.89226	2.1
28830	3.78453	2.1
28831	1.79028	2.1
28832	1.89238	2.1
28833	1.65533	2.1
28834	1.77821	2.1
28835	3.5477	2.1
28836	1.89238	2.1
28837	1.7465	2.1
28838	1.77821	2.1
28839	1.63557	2.02335
28840	3.52795	2.1
28841	1.89226	2.1
28842	1.89226	1.88375
28843	1.89226	2.1
28844	1.89226	2.00605
28845	3.78452	2.1
28846	1.89226	2.1
28847	1.89226	2.1
28848	1.89226	2.1
28849	1.89226	2.1
28850	3.78452	2.1
28851	1.65532	2.1
28852	1.77821	2.1
28853	1.53278	2.1
28854	1.67571	2.1
28855	3.33103	2.1
28856	1.77821	2.1
28857	1.63557	2.1
28858	1.67571	2.1
28859	1.52615	2.1
28860	3.31128	2.1
28861	1.89226	2.1
28862	1.89226	2.1
28863	1.89226	2.1
28864	1.89226	1.82574
28865	3.78452	2.1
28866	2.83129	2.1
28867	1.89226	1.7547
28868	1.89226	2.1
28869	2.6491	2.1
28870	4.54136	2.1
28871	2.83129	2.1
28872	1.47278	2.1
28873	1.3909	2.1
28874	2.6491	2.1
28875	4.12188	2.1
28876	1.89225	2.1
28877	2.65393	2.1
28878	1.89225	2.1
28879	2.69591	2.1
28880	4.58816	2.1
28881	1.91703	2.1

28882	2.00209	2.1
28883	2.65393	2.1
28884	2.65393	2.1
28885	2.69591	2.1
28886	2.00209	2.1
28887	1.89226	2.1
28888	2.69593	2.1
28889	1.93967	2.1
28890	1.89226	2.1
28891	2.74334	2.1
28892	2.69593	2.1
28893	2.74335	2.1
28894	2.0021	2.1
28895	2.76367	2.1
28896	2.69593	2.1
28897	2.06984	2.1
28898	2.0021	2.1
28899	2.24481	2.1
28900	2.58921	2.1
28901	2.68958	2.1
28902	2.68958	2.1
28903	2.11984	2.1
28904	2.11984	2.1
28905	2.11984	2.1
28906	2.58921	2.1
28907	2.46488	2.1
28908	1.98339	2.1
28909	4.5726	2.1
28910	2.7777	2.1
28911	2.58921	2.1
28912	2.46488	2.1
28913	2.70401	2.1
28914	5.29322	2.1
28915	2.17741	2.1
28916	2.54197	2.1
28917	2.54018	2.1
28918	2.18608	2.1
28919	4.72805	2.1
28920	2.17742	2.1
28921	1.58054	2.1
28922	1.59169	2.1
28923	2.18608	2.1
28924	3.76662	2.1
28925	2.54018	2.1
28926	2.18608	2.1
28927	2.19104	2.1
28928	2.5392	2.1
28929	4.72528	2.1
28930	1.60497	2.1
28931	2.18608	2.1
28932	1.59169	2.1
28933	1.59169	2.1
28934	2.19105	2.1
28935	2.19105	2.1
28936	2.58661	2.1
28937	1.06203	2.1
28938	2.7043	2.1
28939	2.21397	2.1

28940	1.55236	2.1
28941	1.06203	2.1
28942	1.89226	1.66307
28943	1.89226	1.54513
28944	1.89226	1.11111
28945	1.89226	1.81716
28946	3.78453	1.52056
28947	1.08195	1.50804
28948	0.990019	2.1
28949	1.31976	1.80988
28950	1.4539	1.30403
28951	2.40171	1.82029
28952	1.89226	1.31266
28953	1.89226	2.02949
28954	1.89226	1.55307
28955	1.89226	0.583436
28956	3.78453	1.46445
28957	1.89226	1.8966
28958	1.89226	1.63579
28959	1.89226	1.48207
28960	1.89226	2.00464
28961	3.78453	1.70799
28962	1.43895	1.98673
28963	1.31976	1.73983
28964	1.70066	1.53407
28965	1.33783	2.1
28966	1.43895	1.83551
28967	1.07612	2.1
28968	0.65988	1.80588
28969	0.850329	1.68216
28970	0.919598	1.19198
28971	0.726952	1.44673
28972	0.788106	1.14757
28973	0.788106	2.1
28974	0.789175	1.9631
28975	0.789175	0.524588
28976	0.850329	1.28195
28977	0.919598	1.28499
28978	0.659879	1.62718
28979	0.726952	1.59565
28980	1.34389	2.1
28981	1.41279	1.96115
28982	1.15564	2.1
28983	1.15564	2.1
28984	1.55998	2.1
28985	1.55998	2.1
28986	1.89226	1.86656
28987	1.89226	1.96281
28988	1.89226	2.1
28989	1.89226	1.67826
28990	1.89226	2.1
28991	1.89226	1.6347
28992	1.55998	2.1
28993	1.41279	2.06534
28994	1.72901	2.1
28995	1.62414	2.1
28996	3.1418	2.1
28997	1.89226	1.95428

28998	1.89226	2.1
28999	1.89226	2.1
29000	1.89226	1.61907
29001	3.78452	1.99376
29002	1.89226	1.58708
29003	1.89226	2.00749
29004	1.89226	1.86504
29005	1.89226	1.85512
29006	1.89226	1.69673
29007	1.89226	1.6287
29008	1.62414	2.1
29009	1.01243	1.96613
29010	1.70932	2.1
29011	1.41279	1.93874
29012	1.30896	1.99913
29013	1.01243	1.87774
29014	1.9719	2.1
29015	1.10725	2.1
29016	1.12411	2.1
29017	1.98691	2.1
29018	3.09601	2.1
29019	1.97189	2.1
29020	2.47932	2.1
29021	2.49092	2.1
29022	1.98691	2.1
29023	4.46282	2.1
29024	1.1486	2.1
29025	1.98691	2.1
29026	1.12411	2.1
29027	1.12411	2.1
29028	2.01602	2.1
29029	2.01602	2.1
29030	2.49093	2.1
29031	1.98691	2.1
29032	2.01601	2.1
29033	2.51651	2.1
29034	4.50694	2.1
29035	1.93147	0.0826198
29036	2.51593	0.05
29037	1.35146	0.0867071
29038	1.35146	0.081778
29039	2.48665	0.0804266
29040	1.90664	0.0636807
29041	1.63979	2.1
29042	1.7457	2.1
29043	1.66046	2.1
29044	1.56558	2.1
29045	3.31128	2.1
29046	0.819895	2.1
29047	0.782792	2.1
29048	0.60239	2.1
29049	0.54831	2.1
29050	0.688347	2.1
29051	0.688347	2.1
29052	0.679857	2.1
29053	0.679857	2.1
29054	0.782792	2.1
29055	0.54831	2.1

29056	0.60239	2.1
29057	0.819895	2.1
29058	1.66046	2.1
29059	1.42485	2.1
29060	1.56558	2.1
29061	1.56558	2.1
29062	1.52995	2.1
29063	1.52995	2.1
29064	2.58101	2.1
29065	2.09803	2.1
29066	2.408	1.98514
29067	1.86893	2.1
29068	4.44994	2.1
29069	2.36062	2.1
29070	1.25104	2.1
29071	1.25104	2.1
29072	1.39703	2.1
29073	1.86893	2.1
29074	2.01492	2.1
29075	2.408	1.94419
29076	1.86893	2.1
29077	2.22575	2.1
29078	1.70677	2.1
29079	4.09468	2.1
29080	0.737605	2.1
29081	0.737605	2.1
29082	0.741302	2.1
29083	0.741302	2.1
29084	0.55189	2.1
29085	0.625517	2.1
29086	0.934468	2.1
29087	0.853389	2.1
29088	0.853389	2.1
29089	0.55189	2.1
29090	0.625517	2.1
29091	0.934467	2.1
29092	2.35053	2.1
29093	1.09615	2.1
29094	1.25998	2.1
29095	2.51216	2.1
29096	3.61051	2.1
29097	1.33636	2.1
29098	1.25609	2.1
29099	0.698455	2.1
29100	0.629995	2.1
29101	0.986092	2.1
29102	0.986092	2.1
29103	0.980263	2.1
29104	0.980263	2.1
29105	1.33636	2.1
29106	0.698455	2.1
29107	0.629995	2.1
29108	1.25609	2.1
29109	1.89226	1.37465
29110	1.89226	1.38999
29111	1.89226	1.40955
29112	1.89226	1.40718
29113	3.78452	1.39464

29114	1.74321	1.38118
29115	1.89226	1.21805
29116	1.91755	1.41825
29117	1.91755	1.30808
29118	1.89226	1.38062
29119	1.89226	1.24985
29120	0.946132	1.33776
29121	0.946132	1.32426
29122	0.949798	1.05009
29123	1.08054	1.25144
29124	1.08054	1.14672
29125	1.08054	1.27196
29126	0.946132	1.32501
29127	0.946132	1.19123
29128	0.946132	1.32166
29129	0.949798	1.11624
29130	1.08054	1.13849
29131	0.946131	1.34147
29132	1.89226	1.37616
29133	1.89226	1.3424
29134	1.89226	1.33184
29135	1.89226	1.32269
29136	1.89226	1.35534
29137	1.89226	1.34429
29138	2.39378	0.05
29139	2.29234	0.05
29140	2.29234	0.05
29141	2.7933	0.05
29142	1.18422	0.05
29143	1.68518	0.05
29144	2.79329	0.05
29145	1.89226	0.05
29146	2.29233	0.05
29147	1.89226	0.05
29148	4.18459	0.05
29149	0.857046	0.05
29150	0.857037	0.05
29151	0.94613	0.05
29152	0.94613	0.05
29153	0.851539	0.05
29154	0.851539	0.05
29155	1.0315	0.05
29156	0.952867	0.05
29157	0.851486	0.05
29158	0.952869	0.05
29159	1.03156	0.05
29160	0.851528	0.05
29161	1.61981	2.1
29162	1.93096	2.1
29163	2.00776	2.1
29164	1.62218	2.1
29165	3.62757	2.1
29166	2.00776	2.1
29167	1.68541	2.1
29168	1.83215	2.1
29169	1.62218	2.1
29170	2.21773	2.1
29171	2.00776	2.1

29172	1.89188	0.05
29173	1.70303	0.05
29174	1.89226	0.05
29175	1.72465	0.05
29176	1.89226	0.05
29177	1.70552	0.05
29178	2.48659	0.0518103
29179	2.0815	0.0664937
29180	1.89226	0.0700648
29181	2.08111	0.0565996
29182	1.89226	0.0672594
29183	2.08111	0.0596237
29184	2.35332	0.227532
29185	1.83284	0.13328
29186	2.28483	0.153892
29187	2.71955	0.137668
29188	4.63815	0.138136
29189	1.89227	2.1
29190	1.89227	2.1
29191	1.89227	2.1
29192	1.89227	2.1
29193	3.78453	2.1
29194	1.33506	2.1
29195	1.17129	2.1
29196	1.33506	2.1
29197	1.38415	2.1
29198	1.13664	2.1
29199	1.18573	2.1
29200	1.89226	2.1
29201	2.68785	2.1
29202	1.89226	2.1
29203	2.6692	2.1
29204	4.58011	2.1
29205	1.89226	2.1
29206	1.89226	2.1
29207	1.89226	2.1
29208	1.89226	2.1
29209	3.78453	2.1
29210	1.33506	2.1
29211	1.13664	2.1
29212	1.27575	2.1
29213	1.09627	2.1
29214	2.43133	2.1
29215	1.89226	2.1
29216	2.65349	2.1
29217	2.66919	2.1
29218	2.66919	2.1
29219	1.89226	2.1
29220	1.89226	2.1
29221	1.89226	2.1
29222	1.89226	2.1
29223	1.89226	2.1
29224	1.89226	2.1
29225	3.78453	2.1
29226	1.16834	2.1
29227	1.16834	2.1
29228	0.946132	2.1
29229	0.946132	2.1

29230	1.26978	2.1
29231	1.16834	2.1
29232	0.94613	2.1
29233	0.94613	2.1
29234	0.94613	2.1
29235	1.26978	2.1
29236	0.94613	2.1
29237	1.16834	2.1
29238	2.14698	2.1
29239	1.89226	2.1
29240	1.89226	2.1
29241	1.89226	2.1
29242	2.40719	2.1
29243	2.40719	2.1
29244	1.89226	2.1
29245	1.89226	2.1
29246	1.89226	2.1
29247	1.89226	2.1
29248	3.78453	2.1
29249	1.26977	2.1
29250	1.26977	2.1
29251	0.946133	2.1
29252	0.946133	2.1
29253	1.37467	2.1
29254	1.26978	2.1
29255	0.946131	2.1
29256	0.946131	2.1
29257	1.37467	2.1
29258	0.94613	2.1
29259	0.946131	2.1
29260	1.26978	2.1
29261	1.2036	2.1
29262	1.34912	2.1
29263	0.946131	2.1
29264	0.946131	2.1
29265	1.34912	2.1
29266	1.34912	2.1
29267	0.946129	2.1
29268	0.946129	2.1
29269	1.34912	2.1
29270	0.94613	2.1
29271	0.946131	2.1
29272	1.2036	2.1
29273	2.47164	0.362187
29274	1.89019	0.193766
29275	1.79109	0.342662
29276	2.41632	0.200575
29277	4.26273	0.34682
29278	1.54197	0.286443
29279	2.41632	0.164576
29280	1.79109	0.232559
29281	1.79109	0.268341
29282	2.20929	0.187328
29283	2.20929	0.228315
29284	1.70302	1.31461
29285	1.48104	1.53405
29286	1.99365	1.56784
29287	1.32897	1.03984

29288	1.70302	1.57498
29289	1.14034	1.24557
29290	1.70302	1.40317
29291	1.99365	1.32124
29292	1.99297	1.48456
29293	2.36337	0.789931
29294	4.04804	1.3757
29295	1.51569	1.1383
29296	2.49093	0.752783
29297	1.6026	0.798988
29298	1.43229	0.644873
29299	2.0815	1.29233
29300	1.83402	1.02114
29301	2.54665	2.1
29302	2.34044	2.1
29303	2.08889	2.1
29304	2.37653	2.1
29305	4.71697	2.1
29306	2.54665	2.1
29307	2.45212	2.1
29308	2.31012	2.1
29309	2.37653	2.1
29310	4.82865	2.1
29311	2.08889	2.1
29312	2.37653	2.1
29313	2.23366	2.1
29314	1.87598	2.1
29315	4.25251	2.1
29316	2.31012	2.1
29317	2.37653	2.1
29318	2.23367	2.1
29319	2.17384	2.1
29320	4.55037	2.1
29321	0.946131	2.1
29322	0.946131	2.1
29323	1.30338	2.1
29324	1.23186	2.1
29325	0.94613	2.1
29326	0.94613	2.1
29327	1.23187	2.1
29328	1.23187	2.1
29329	0.946131	2.1
29330	1.23186	2.1
29331	1.30338	2.1
29332	0.946131	2.1
29333	1.89226	2.1
29334	2.46373	2.1
29335	2.34725	2.1
29336	2.46373	2.1
29337	1.77579	2.1
29338	1.89226	2.1
29339	2.61901	2.1
29340	2.45899	2.1
29341	2.64067	2.1
29342	2.52708	2.1
29343	5.14609	2.1
29344	2.41795	2.1
29345	2.0815	2.1

29346	2.52708	2.1
29347	1.92277	2.1
29348	4.31368	2.1
29349	2.64067	2.1
29350	2.52708	2.1
29351	2.58638	2.1
29352	2.51039	2.1
29353	5.11346	2.1
29354	2.52708	2.1
29355	2.13642	2.1
29356	2.51039	2.1
29357	2.16633	2.1
29358	4.69341	2.1
29359	1.61028	1.70641
29360	1.65712	0.396563
29361	1.35002	0.229676
29362	1.35002	1.77427
29363	1.88637	0.31928
29364	1.88637	1.99994
29365	2.57663	2.1
29366	2.24227	2.1
29367	2.48354	2.1
29368	2.14317	2.1
29369	4.7198	2.1
29370	2.24227	2.1
29371	1.77353	2.1
29372	2.14317	2.1
29373	1.69956	2.1
29374	3.9167	2.1
29375	2.48353	2.1
29376	2.14317	2.1
29377	2.39282	2.1
29378	1.79824	2.1
29379	4.53599	2.1
29380	2.14317	2.1
29381	1.69956	2.1
29382	1.91427	2.1
29383	1.6347	2.1
29384	3.77787	2.1
29385	1.89226	0.472208
29386	1.89226	0.483816
29387	1.89226	0.12354
29388	1.89226	0.125586
29389	3.78453	0.358824
29390	1.89226	0.386189
29391	1.89226	0.237451
29392	1.89226	0.403881
29393	1.89226	0.152994
29394	3.78452	0.150148
29395	1.89226	0.253382
29396	1.89652	0.362657
29397	1.89226	0.177434
29398	1.89226	1.45104
29399	1.19352	0.229845
29400	1.19352	1.11741
29401	1.89226	0.359742
29402	1.89653	0.188355
29403	1.89226	0.812256

29404	1.89226	1.49177
29405	1.89226	0.108013
29406	1.89226	0.138345
29407	1.89226	0.544385
29408	1.75326	0.518207
29409	1.89226	1.16243
29410	1.12948	0.624328
29411	1.89226	0.348816
29412	1.12948	0.257248
29413	2.61064	2.1
29414	1.89226	2.1
29415	1.89226	2.1
29416	1.89226	2.1
29417	2.6085	2.1
29418	2.6085	2.1
29419	2.6085	2.1
29420	1.89226	2.1
29421	1.89226	2.1
29422	1.89226	2.1
29423	2.62161	2.1
29424	2.62161	2.1
29425	2.16535	2.1
29426	1.44603	2.1
29427	2.79784	2.1
29428	1.84756	2.1
29429	4.01292	2.1
29430	2.65262	2.1
29431	1.03391	2.1
29432	2.20239	2.1
29433	1.74416	2.1
29434	1.49213	2.1
29435	1.03391	2.1
29436	2.29066	2.1
29437	2.49174	2.1
29438	2.56502	2.1
29439	2.33802	2.1
29440	4.85568	2.1
29441	2.39663	2.1
29442	2.49174	2.1
29443	2.56502	2.1
29444	2.45929	2.1
29445	4.96165	2.1
29446	2.56502	2.1
29447	2.33802	2.1
29448	2.37168	2.1
29449	2.59967	2.1
29450	4.9367	2.1
29451	2.56502	2.1
29452	2.45929	2.1
29453	2.50138	2.1
29454	2.59967	2.1
29455	5.0664	2.1
29456	2.27009	1.73054
29457	1.26247	2.1
29458	1.86812	2.05646
29459	2.837	1.81046
29460	4.13822	1.96032
29461	1.89226	2.1

29462	1.89226	2.1
29463	1.89226	2.1
29464	1.89226	2.1
29465	3.78453	2.1
29466	1.75835	2.1
29467	1.72866	2.1
29468	1.65313	2.1
29469	1.64822	2.1
29470	3.3818	2.1
29471	1.72866	2.1
29472	1.58401	2.1
29473	1.64822	2.1
29474	1.52441	2.1
29475	3.25307	2.1
29476	1.89226	2.1
29477	1.89226	2.1
29478	1.89226	2.1
29479	1.89226	2.1
29480	3.78452	2.1
29481	1.89226	2.1
29482	1.89226	2.1
29483	1.89226	2.1
29484	1.89226	2.1
29485	3.78453	2.1
29486	1.65313	2.1
29487	1.64822	2.1
29488	1.55779	2.1
29489	1.57968	2.1
29490	3.23281	2.1
29491	1.64822	2.1
29492	1.52441	2.1
29493	1.57967	2.1
29494	1.474 2.1	
29495	3.10408	2.1
29496	1.89226	2.1
29497	1.89226	2.1
29498	1.89226	2.1
29499	1.89226	2.1
29500	3.78452	2.1
29501	1.86067	1.49442
29502	1.58407	2.1
29503	2.07965	0.447279
29504	1.83123	1.49126
29505	3.66372	1.39575
29506	1.89226	1.41035
29507	1.89226	2.1
29508	1.89226	1.20126
29509	1.89226	2.05744
29510	3.78452	1.72111
29511	1.89226	2.00896
29512	1.89226	2.1
29513	1.89226	1.15568
29514	1.89226	2.1
29515	1.89226	1.29648
29516	1.89226	2.1
29517	1.83123	0.532648
29518	1.03472	2.1
29519	1.91068	1.93581

29520	1.58406	1.81971
29521	1.36133	1.70802
29522	1.03472	1.38215
29523	2.07965	1.3131
29524	1.83123	1.63816
29525	2.28385	1.36097
29526	2.09114	1.61247
29527	4.1708	1.43894
29528	1.89226	1.3823
29529	1.89226	2.07215
29530	1.89226	0.530692
29531	1.89226	1.60194
29532	3.78452	1.50692
29533	1.89226	1.89417
29534	1.89226	2.1
29535	1.89226	0.753933
29536	1.89226	2.1
29537	3.78452	2.01521
29538	1.83123	1.28455
29539	1.36133	2.1
29540	2.09114	0.582215
29541	1.69436	1.78946
29542	3.45248	1.58585
29543	1.89226	2.1
29544	1.89226	2.1
29545	1.89226	2.1
29546	1.89226	2.1
29547	3.78453	2.1
29548	1.53278	2.1
29549	1.67571	2.1
29550	1.40182	2.1
29551	1.55686	2.1
29552	3.07752	2.1
29553	1.67571	2.1
29554	1.52615	2.1
29555	1.55686	2.1
29556	1.38963	1.8568
29557	3.06534	2.1
29558	1.89226	2.1
29559	1.89226	1.97285
29560	1.89226	2.1
29561	1.89226	1.89609
29562	3.78452	2.1
29563	1.89226	2.1
29564	1.89226	2.1
29565	1.89226	2.1
29566	1.89226	2.1
29567	3.78453	2.1
29568	1.40182	2.1
29569	1.55686	2.1
29570	1.24509	2.1
29571	1.38426	2.1
29572	2.78608	2.1
29573	1.55686	2.1
29574	1.38963	2.1
29575	1.2064	2.1
29576	1.2064	2.1
29577	1.38426	2.1

29578	1.56749	2.1
29579	0.810946	2.1
29580	0.810946	2.0913
29581	0.810945	2.1
29582	0.810945	2.1
29583	0.810945	2.1
29584	0.810945	2.1
29585	1.89226	2.1
29586	1.35169	2.1
29587	1.08132	2.1
29588	1.08132	1.99614
29589	1.08132	2.1
29590	1.28418	0.336547
29591	1.59382	0.462217
29592	1.78289	0.617688
29593	1.51021	0.354298
29594	3.10403	0.391257
29595	1.76897	1.88021
29596	1.59382	0.489086
29597	1.78289	0.881039
29598	1.94302	1.32105
29599	3.53684	1.34832
29600	1.89226	0.475192
29601	1.89226	0.764886
29602	1.89226	1.03716
29603	1.89226	0.505996
29604	3.78453	0.592435
29605	1.89227	0.450245
29606	1.89227	0.250992
29607	1.89227	0.263702
29608	1.89227	0.342895
29609	1.89227	0.339088
29610	1.89227	0.40757
29611	1.78286	0.465963
29612	1.51019	0.256827
29613	1.79979	0.296803
29614	2.02511	0.389577
29615	3.5353	0.361629
29616	1.78286	0.621534
29617	1.94299	0.861259
29618	2.17831	1.59958
29619	2.02511	0.491185
29620	3.9681	0.555838
29621	1.89224	0.731381
29622	1.89224	0.353058
29623	1.89224	0.450445
29624	1.89224	0.683824
29625	3.78448	0.585646
29626	1.89224	0.357068
29627	1.89224	0.217621
29628	1.89224	0.243626
29629	1.89224	0.344938
29630	1.89224	0.233278
29631	1.89224	0.342387
29632	1.83198	0.132473
29633	1.0961	0.0552948
29634	1.39448	0.092084
29635	1.99568	0.132588

29636	3.09178	0.112505
29637	1.25503	0.05
29638	1.99568	0.101736
29639	2.3661	0.149092
29640	1.70302	0.0785768
29641	3.55833	0.0660814
29642	2.69899	0.05
29643	1.85796	0.05
29644	2.11407	0.05
29645	1.36769	0.05
29646	3.97203	0.05
29647	1.89226	0.0604645
29648	1.89226	0.0551483
29649	1.89226	0.05
29650	1.89226	0.05
29651	3.78452	0.0656014
29652	1.89225	0.05
29653	2.63355	0.05
29654	2.09529	0.05
29655	2.09529	0.05
29656	1.89225	0.05
29657	1.89225	0.05
29658	1.89226	0.052062
29659	1.89226	0.0615218
29660	1.89226	0.05
29661	1.89226	0.0785029
29662	1.89226	0.05
29663	1.89226	0.0721619
29664	2.12204	0.05
29665	1.89226	0.0577852
29666	2.05798	0.05
29667	2.01954	0.05
29668	1.70302	0.05
29669	1.3865	0.05
29670	1.28326	0.588692
29671	1.33326	2.1
29672	1.19052	0.90371
29673	1.25369	2.1
29674	2.53694	1.95022
29675	1.33327	2.1
29676	1.27339	2.1
29677	1.25369	2.1
29678	1.19733	2.1
29679	2.52708	2.1
29680	1.19052	0.71656
29681	1.25369	2.1
29682	1.10938	0.682789
29683	1.17614	2.1
29684	2.36307	1.84413
29685	1.25369	2.1
29686	1.19733	2.1
29687	1.17614	2.1
29688	1.11868	2.1
29689	2.37237	2.1
29690	1.10747	0.0729925
29691	1.47688	0.0996157
29692	1.97732	0.05
29693	1.62206	0.0543148

29694	3.09894	0.0744887
29695	1.89226	0.101286
29696	1.89226	0.077059
29697	1.89226	0.0893545
29698	1.89226	0.121514
29699	1.89226	0.0506599
29700	1.89226	0.06113
29701	1.62206	0.0862176
29702	1.70302	0.0747813
29703	1.22962	0.0870004
29704	1.1893	0.101913
29705	2.15177	0.0660454
29706	2.08121	0.0768731
29707	1.97732	0.0670051
29708	2.08121	0.065452
29709	1.62206	0.0782787
29710	1.62206	0.113224
29711	2.41464	0.0503401
29712	2.41464	0.0753939
29713	1.89226	0.107295
29714	1.89226	0.125589
29715	1.89226	0.0636268
29716	1.89226	0.0724487
29717	3.78452	0.077026
29718	0.946133	0.0867534
29719	0.946133	0.0925149
29720	0.946132	0.0969263
29721	0.946132	0.103356
29722	0.946132	0.0929219
29723	0.946132	0.111222
29724	0.946133	0.0695972
29725	0.946133	0.0908076
29726	0.946132	0.0666145
29727	0.946132	0.085611
29728	0.946132	0.114791
29729	0.946132	0.0890477
29730	1.83157	1.85801
29731	2.19989	1.71321
29732	2.20664	0.672988
29733	1.81487	0.694393
29734	4.03146	1.27246
29735	2.41055	2.1
29736	2.19989	2.1
29737	2.20665	2.1
29738	2.38023	1.98788
29739	4.61044	2.1
29740	2.19989	2.1
29741	2.22558	2.1
29742	2.38023	2.1
29743	2.07065	1.38839
29744	4.42547	2.1
29745	2.19988	1.83749
29746	1.70303	1.49495
29747	1.89939	1.0067
29748	1.86357	0.467478
29749	3.78536	1.29683
29750	1.04921	2.1
29751	1.30711	2.1

29752	1.53076	2.1
29753	1.22927	2.1
29754	2.57997	2.1
29755	1.53076	2.1
29756	1.46568	2.1
29757	1.56056	2.1
29758	1.22927	2.1
29759	1.86206	2.1
29760	1.53076	2.1
29761	1.18353	2.1
29762	2.28593	2.1
29763	1.16979	2.1
29764	2.2731	2.1
29765	3.45571	2.1
29766	0.854776	2.1
29767	0.854776	2.1
29768	0.855641	2.1
29769	0.855641	2.1
29770	1.12552	2.1
29771	1.13655	2.1
29772	0.57216	2.1
29773	0.584893	2.1
29774	0.57216	2.1
29775	1.12552	2.1
29776	0.584893	2.1
29777	1.13655	2.1
29778	1.54462	2.1
29779	2.76309	2.1
29780	1.34869	2.1
29781	2.48573	2.1
29782	4.03035	2.1
29783	1.35328	1.34679
29784	1.0383	1.29785
29785	1.03318	1.44772
29786	0.704834	1.43254
29787	1.0257	1.44876
29788	1.0257	1.34787
29789	1.03241	1.29009
29790	1.03241	1.41789
29791	1.35328	1.40795
29792	1.03319	1.41925
29793	1.0383	1.40201
29794	0.704835	1.41156
29795	2.27126	0.392755
29796	2.77291	2.1
29797	1.81771	1.86844
29798	2.6111	1.73357
29799	4.88236	1.91682
29800	2.77291	2.1
29801	1.89227	2.1
29802	1.89227	1.95238
29803	1.89227	2.1
29804	2.6111	1.84404
29805	2.6111	2.1
29806	2.22061	1.53794
29807	2.41522	1.8476
29808	2.41522	0.938725
29809	2.6111	2.1

29810	1.75765	0.829547
29811	1.95353	2.1
29812	2.27502	0.830169
29813	1.89227	1.59295
29814	1.89227	0.709176
29815	2.8189	0.470614
29816	4.71116	0.494508
29817	1.89226	0.535929
29818	1.89226	1.5644
29819	1.89226	1.33368
29820	1.89226	0.774261
29821	1.89226	1.40251
29822	1.89226	1.17105
29823	1.89226	1.64725
29824	1.89226	1.57595
29825	1.89226	1.63134
29826	1.89226	1.47054
29827	3.78453	1.60345
29828	1.89226	1.63722
29829	2.57079	1.35488
29830	2.5932	1.51778
29831	1.89226	1.47696
29832	4.48546	1.47555
29833	0.589396	1.48511
29834	0.641454	1.40073
29835	0.814062	1.55557
29836	0.726518	1.47973
29837	0.675126	1.52108
29838	0.675125	1.45557
29839	0.692847	1.55667
29840	0.692847	1.48893
29841	0.641454	1.43513
29842	0.814062	1.48598
29843	0.726518	1.54599
29844	0.589396	1.51276
29845	1.89226	1.65762
29846	1.89226	1.47036
29847	1.89226	1.59322
29848	1.89226	1.62927
29849	1.89226	1.56588
29850	1.89226	1.61883
29851	1.16906	0.702785
29852	1.2966	1.47111
29853	0.946132	1.46514
29854	0.946132	1.67082
29855	1.16906	1.46521
29856	1.16906	1.25298
29857	0.946132	1.48907
29858	0.946132	1.58675
29859	1.16906	1.47443
29860	0.946131	1.46434
29861	1.2966	1.51438
29862	0.946132	1.58786
29863	0.641455	1.47531
29864	0.567191	1.41525
29865	0.807594	1.60018
29866	0.814062	1.50113
29867	0.673735	1.57132

29868	0.673734	1.44492
29869	0.707519	1.48545
29870	0.707519	1.59616
29871	0.807594	1.52372
29872	0.56719	1.56742
29873	0.641455	1.54686
29874	0.814063	1.61506
29875	1.89225	2.1
29876	1.89225	2.1
29877	1.89225	2.1
29878	1.89225	2.1
29879	3.7845	2.1
29880	1.89225	2.1
29881	1.89225	2.1
29882	1.89225	2.1
29883	1.89225	2.1
29884	1.89225	2.1
29885	1.89225	2.1
29886	1.89226	2.1
29887	1.89226	2.1
29888	1.89226	2.1
29889	1.89226	2.1
29890	3.78452	2.1
29891	1.89226	2.1
29892	1.89226	2.1
29893	1.89226	2.1
29894	1.89226	2.1
29895	1.89226	2.1
29896	1.89226	2.1
29897	1.89226	2.1
29898	1.89226	2.1
29899	1.89226	2.1
29900	1.89226	2.1
29901	3.78452	2.1
29902	1.89226	2.1
29903	1.38928	2.1
29904	1.89226	2.1
29905	1.51378	2.1
29906	1.89061	2.1
29907	1.89226	2.1
29908	1.89226	2.1
29909	1.89226	2.1
29910	1.89226	2.1
29911	1.89226	2.1
29912	3.78453	2.1
29913	1.04572	2.1
29914	0.994213	2.1
29915	0.912466	2.1
29916	0.912466	2.1
29917	0.947743	2.1
29918	1.00388	2.1
29919	0.900913	2.1
29920	0.900913	2.1
29921	0.900915	2.1
29922	0.946131	2.1
29923	0.946131	2.1
29924	0.999196	2.1
29925	1.89226	2.1

29926	1.89226	2.1
29927	1.89226	2.1
29928	1.89226	2.1
29929	3.78453	2.1
29930	0.946161	2.1
29931	1.1354	2.1
29932	0.850847	2.1
29933	0.850847	2.1
29934	1.01445	2.1
29935	1.02106	2.1
29936	0.946131	2.1
29937	0.946131	2.1
29938	1.13537	2.1
29939	1.01444	2.1
29940	0.850877	2.1
29941	0.946131	2.1
29942	1.80183	2.1
29943	1.89226	2.1
29944	1.89226	2.1
29945	1.89226	2.1
29946	1.79606	2.1
29947	1.79606	2.1
29948	1.24001	1.41034
29949	1.41822	1.11667
29950	1.95665	0.992511
29951	1.77342	1.37961
29952	3.19666	1.1463
29953	1.95665	1.19424
29954	1.44859	1.03602
29955	1.41821	1.47843
29956	1.41821	1.06613
29957	1.95543	1.13585
29958	1.95543	0.975919
29959	1.95665	1.01392
29960	1.77342	1.35821
29961	2.33611	1.25317
29962	2.49694	1.06122
29963	4.29276	1.27572
29964	1.95665	1.25827
29965	1.95544	1.03967
29966	2.4728	0.84626
29967	2.49693	1.20197
29968	4.42945	0.981738
29969	2.24937	2.1
29970	2.49053	2.1
29971	2.16323	2.1
29972	2.36377	2.1
29973	4.61314	2.1
29974	2.49053	2.1
29975	2.58639	2.1
29976	2.36377	2.1
29977	2.47395	2.1
29978	4.95016	2.1
29979	2.45054	2.1
29980	2.36377	2.1
29981	1.92911	2.1
29982	2.181 2.1	
29983	4.29288	2.1

29984	2.36377	2.1
29985	2.47395	2.1
29986	2.181 2.1	
29987	2.29881	2.1
29988	4.66258	2.1
29989	1.57885	2.1
29990	1.13339	2.1
29991	1.48594	2.1
29992	1.13322	2.1
29993	2.71207	2.1
29994	1.48596	2.1
29995	1.20761	2.1
29996	1.20761	2.1
29997	1.13323	2.1
29998	1.46568	2.1
29999	1.3913	2.1
30000	1.89226	0.05
30001	1.89226	0.395467
30002	1.89226	0.248574
30003	1.89226	0.902691
30004	1.89226	0.05
30005	1.89226	0.140565
30006	1.11712	0.287067
30007	0.946129	0.720526
30008	0.686934	0.05
30009	0.946133	0.05
30010	0.9461	0.119392
30011	1.10729	1.21121
30012	0.686963	0.37058
30013	0.686963	0.05
30014	1.10945	0.248564
30015	0.80397	1.00096
30016	0.946131	0.0784057
30017	0.946131	0.352356
30018	1.89226	2.1
30019	1.89226	1.91899
30020	1.89226	2.1
30021	1.89226	2.1
30022	3.78452	2.1
30023	1.22615	2.1
30024	1.3439	2.1
30025	1.47868	2.1
30026	1.55998	2.1
30027	2.78613	2.1
30028	1.04973	2.1
30029	1.47868	2.1
30030	1.20149	2.1
30031	1.61721	2.1
30032	2.68017	2.1
30033	1.89226	2.1
30034	1.89226	2.1
30035	1.89226	2.1
30036	1.89226	2.07042
30037	3.78452	2.1
30038	0.946133	1.78047
30039	0.946133	2.1
30040	0.946128	2.1
30041	0.946128	2.1

30042	0.946131	1.74416
30043	0.946131	2.1
30044	0.946131	2.1
30045	0.946131	2.01607
30046	0.94613	2.1
30047	0.94613	2.1
30048	0.946131	2.1
30049	0.946131	2.1
30050	0.798126	2.1
30051	0.798126	2.1
30052	0.805719	2.1
30053	0.805719	2.1
30054	0.779992	2.1
30055	0.864507	2.1
30056	0.739338	2.1
30057	0.808607	2.1
30058	0.808606	2.1
30059	0.864506	2.1
30060	0.739339	2.1
30061	0.779993	2.1
30062	0.946128	2.1
30063	0.946128	2.1
30064	1.18959	2.1
30065	1.00304	2.1
30066	1.00304	2.1
30067	1.00304	2.1
30068	0.94613	2.1
30069	0.94613	2.1
30070	0.94613	2.1
30071	1.18959	2.1
30072	1.00304	2.1
30073	0.94613	2.1
30074	2.19163	2.1
30075	1.89226	2.1
30076	2.16541	2.1
30077	1.89226	2.1
30078	4.08389	2.1
30079	1.89226	2.1
30080	1.89226	1.907
30081	1.89226	2.1
30082	1.89226	1.56159
30083	3.78452	2.1
30084	0.964782	2.1
30085	1.30151	1.51376
30086	1.30318	1.50096
30087	1.30318	2.1
30088	0.949134	1.61071
30089	0.949134	2.1
30090	2.15005	2.1
30091	1.89226	2.1
30092	2.16541	2.1
30093	2.16541	2.1
30094	1.89226	2.1
30095	1.89226	2.1
30096	1.89226	2.1
30097	1.89226	2.06819
30098	1.89226	2.1
30099	1.89226	1.11857

30100	3.78452	1.87879
30101	0.949134	2.1
30102	1.30151	2.04459
30103	0.949134	2.1
30104	1.32143	1.5452
30105	2.25064	2.1
30106	0.982859	0.19828
30107	1.28326	0.536787
30108	1.19052	0.249509
30109	1.19052	0.793446
30110	1.10476	0.185366
30111	1.10476	0.768286
30112	1.37265	0.479557
30113	1.38247	1.24494
30114	1.36636	1.56245
30115	1.3294	0.456513
30116	2.71187	0.826208
30117	1.89226	1.60967
30118	1.89226	0.547554
30119	1.89226	0.566474
30120	1.89226	1.39132
30121	1.89226	0.543483
30122	1.89226	1.40714
30123	1.36636	1.75177
30124	1.3294	0.634929
30125	1.30001	0.678983
30126	1.36416	1.60294
30127	2.69356	1.15412
30128	1.89226	1.82144
30129	1.89226	0.60057
30130	1.89226	0.834245
30131	1.89226	1.87598
30132	1.89226	0.629633
30133	1.89226	1.63235
30134	2.83701	0.185781
30135	1.89226	0.587289
30136	1.89226	1.29431
30137	2.79312	0.233009
30138	4.68539	0.425663
30139	1.44288	2.1
30140	0.948677	2.1
30141	1.84076	2.1
30142	1.42884	2.1
30143	2.78943	2.1
30144	1.89226	2.1
30145	1.89226	2.1
30146	1.89226	2.1
30147	1.89226	2.1
30148	3.78452	2.1
30149	0.909402	2.1
30150	0.909402	2.1
30151	1.3063	2.1
30152	1.56757	2.1
30153	1.00038	2.1
30154	1.5235	2.1
30155	0.946127	2.1
30156	0.946127	2.1
30157	0.946128	2.1

30158	1.26957	2.1
30159	1.00038	2.1
30160	0.946129	2.1
30161	1.17256	2.1
30162	2.2846	2.1
30163	1.1844	2.1
30164	2.28904	2.1
30165	3.469	2.1
30166	0.867886	2.1
30167	0.867886	2.1
30168	0.867277	2.1
30169	0.867277	2.1
30170	1.14296	2.1
30171	1.14452	2.1
30172	0.591766	2.1
30173	0.592199	2.1
30174	0.591766	2.1
30175	1.14296	2.1
30176	0.5922	2.1
30177	1.14452	2.1
30178	1.27408	0.0625539
30179	1.56339	0.106071
30180	1.52522	0.0549919
30181	1.34447	0.05
30182	2.90786	0.0501659
30183	1.27408	0.0953339
30184	1.01121	0.0745743
30185	0.991868	0.0528064
30186	1.34447	0.0649908
30187	2.35569	0.0704485
30188	0.944818	0.0757487
30189	0.883008	0.0714089
30190	0.66957	0.0525024
30191	0.616883	0.05
30192	0.755679	0.05
30193	0.755679	0.121964
30194	0.791912	0.0707052
30195	0.701943	0.05
30196	0.901541	0.0610578
30197	0.634847	0.0642103
30198	0.82781	0.068214
30199	0.655405	0.0881661
30200	1.73819	2.1
30201	1.70303	2.1
30202	1.70303	2.1
30203	1.70303	2.1
30204	1.94516	2.1
30205	1.94517	2.1
30206	0.94994	2.1
30207	2.07469	2.1
30208	1.08506	2.1
30209	2.0815	2.1
30210	3.12902	2.1
30211	0.946131	2.1
30212	0.946131	2.1
30213	0.946131	2.1
30214	0.946131	2.1
30215	0.946128	2.1

30216	0.946128	2.1
30217	0.946133	2.1
30218	0.946133	2.1
30219	0.946131	2.1
30220	0.946131	2.1
30221	0.94613	2.1
30222	0.94613	2.1
30223	2.51796	2.1
30224	1.64193	2.1
30225	2.51172	2.1
30226	1.68893	2.1
30227	4.20689	2.1
30228	2.51172	2.1
30229	1.70002	2.1
30230	1.70002	2.1
30231	1.68893	2.1
30232	2.48689	2.1
30233	2.4758	2.1
30234	2.27963	0.05
30235	2.21582	0.05
30236	2.60486	0.05
30237	2.5105	0.05
30238	4.79013	0.05
30239	2.21582	0.05
30240	2.02069	0.05
30241	2.5105	0.05
30242	2.28615	0.05
30243	4.53119	0.05
30244	2.60486	0.05
30245	2.5105	0.05
30246	2.55791	0.05
30247	2.47141	0.05
30248	5.06842	0.05
30249	2.5105	0.05
30250	2.28615	0.05
30251	2.47141	0.05
30252	2.23337	0.05
30253	4.74387	0.05
30254	2.06984	2.1
30255	0.967499	2.1
30256	2.12435	2.1
30257	2.0021	2.1
30258	1.08975	2.1
30259	0.967499	2.1
30260	1.89226	0.05
30261	1.89226	0.05
30262	1.89226	0.05
30263	1.89226	0.05
30264	1.89226	0.05
30265	1.89226	0.05
30266	1.89226	2.1
30267	1.89226	2.1
30268	1.89226	2.1
30269	1.89226	2.1
30270	3.78452	2.1
30271	1.89227	2.1
30272	1.89227	2.1
30273	1.89227	2.1

30274	1.89227	2.1
30275	3.78453	2.1
30276	0.55882	2.1
30277	0.696462	2.1
30278	1.07003	2.1
30279	0.937179	2.1
30280	0.818077	2.1
30281	0.818077	2.1
30282	0.815565	2.1
30283	0.815565	2.1
30284	0.696462	2.1
30285	1.07003	2.1
30286	0.93718	2.1
30287	0.55882	2.1
30288	2.53857	2.1
30289	1.87436	2.1
30290	2.27541	2.1
30291	2.27541	2.1
30292	2.14005	2.1
30293	2.14005	2.1
30294	1.89226	2.1
30295	1.89226	2.1
30296	1.89226	2.1
30297	1.89226	2.1
30298	3.78452	2.1
30299	1.89227	2.1
30300	1.89227	2.1
30301	1.89227	2.1
30302	1.89227	2.1
30303	3.78453	2.1
30304	1.39292	2.1
30305	2.14006	2.1
30306	2.41083	2.1
30307	1.68761	2.1
30308	3.80376	2.1
30309	2.53857	2.1
30310	2.14005	2.1
30311	2.41083	2.1
30312	2.82043	2.1
30313	4.9494	2.1
30314	1.90439	0.135202
30315	1.51835	0.133686
30316	1.20473	0.114609
30317	0.947693	0.156533
30318	2.85208	0.134892
30319	2.586	2.1
30320	1.46262	2.1
30321	2.14009	2.1
30322	2.78101	2.1
30323	4.72609	2.1
30324	1.89224	2.1
30325	2.78101	2.1
30326	2.586	2.1
30327	2.586	2.1
30328	1.89224	2.1
30329	1.89224	2.1
30330	0.994293	2.1
30331	1.19	2.1

30332	1.09483	2.1
30333	1.09483	2.1
30334	1.06827	2.1
30335	1.06827	2.1
30336	1.89226	2.1
30337	1.89226	2.1
30338	1.89226	2.1
30339	1.89226	2.1
30340	3.78452	2.1
30341	0.946132	2.1
30342	0.946132	2.1
30343	1.31912	2.1
30344	1.3572	2.1
30345	0.946131	2.1
30346	0.946131	2.1
30347	1.31912	2.1
30348	1.31912	2.1
30349	0.946131	2.1
30350	1.31912	2.1
30351	1.35721	2.1
30352	0.946131	2.1
30353	2.24577	0.406443
30354	1.09408	0.422761
30355	1.60239	0.444613
30356	1.09408	0.455439
30357	2.39404	0.382478
30358	2.07382	0.389278
30359	1.70303	0.0992309
30360	1.3216	0.138018
30361	1.23104	0.0581422
30362	1.05475	0.115407
30363	2.60408	0.0743516
30364	1.89226	0.164255
30365	1.89226	0.214996
30366	1.89226	0.125034
30367	1.89226	0.166092
30368	3.78452	0.23356
30369	1.89226	0.0771257
30370	2.3845	0.0784415
30371	2.6875	0.0980327
30372	1.89226	0.0998439
30373	2.6875	0.0554858
30374	1.89226	0.0590831
30375	1.46759	2.1
30376	1.17585	2.1
30377	1.85189	2.1
30378	1.39223	2.1
30379	2.85981	2.1
30380	1.85189	2.1
30381	1.71255	2.1
30382	1.71255	2.1
30383	1.39223	2.1
30384	2.28511	2.1
30385	1.96479	2.1
30386	1.89227	0.563741
30387	1.89227	1.17715
30388	1.89227	1.23879
30389	1.89227	0.420433

30390	1.89227	1.27316
30391	1.89227	0.933056
30392	1.89226	0.776124
30393	1.89226	1.18843
30394	1.89226	1.24446
30395	1.89226	0.352536
30396	1.89226	1.30868
30397	1.89226	0.785303
30398	1.89226	0.787973
30399	1.89226	1.18128
30400	1.89226	1.20812
30401	1.89226	0.355449
30402	1.89226	1.27499
30403	1.89226	0.993676
30404	1.89226	0.439954
30405	2.77788	0.988227
30406	2.21062	1.14513
30407	2.21062	0.343392
30408	1.89226	1.18746
30409	1.89226	0.850498
30410	0.868548	0.355695
30411	0.610104	0.481649
30412	0.796678	0.998659
30413	0.865021	0.321498
30414	1.0883	0.337714
30415	0.904938	0.529095
30416	2.21062	0.393998
30417	1.49548	0.417146
30418	1.2068	1.11904
30419	1.54764	0.469782
30420	0.790005	0.294725
30421	1.89226	2.1
30422	2.67457	2.1
30423	1.89226	2.1
30424	2.56352	2.1
30425	4.56683	2.1
30426	2.67456	2.1
30427	2.20011	2.1
30428	2.56351	2.1
30429	2.11083	2.1
30430	4.78539	2.1
30431	2.56348	2.1
30432	2.1108	2.1
30433	2.45763	2.1
30434	2.00986	2.1
30435	4.56843	2.1
30436	0.946133	0.05
30437	0.946133	0.05
30438	0.946132	0.05
30439	0.946132	0.05
30440	0.946132	0.05
30441	0.946132	0.05
30442	0.946132	0.05
30443	0.946132	0.05
30444	0.946132	0.05
30445	0.946132	0.05
30446	0.946133	0.05
30447	0.946133	0.05

30448	1.45927	1.46468
30449	1.25133	1.79809
30450	1.00082	1.72345
30451	1.41689	1.71936
30452	1.0432	1.59594
30453	1.45927	1.70632
30454	1.41689	1.68466
30455	1.29579	1.85167
30456	1.34874	1.61097
30457	1.25133	1.87169
30458	2.76563	1.98065
30459	0.675555	1.74682
30460	0.674371	1.69841
30461	0.625664	1.71468
30462	0.523133	1.75163
30463	0.676509	1.81
30464	0.676509	1.82202
30465	0.62471	1.67403
30466	0.62471	1.62773
30467	0.675556	1.62707
30468	0.523132	1.46275
30469	0.674373	1.36337
30470	0.625663	1.63175
30471	1.89226	1.24013
30472	1.89226	1.19494
30473	1.89226	1.08661
30474	1.89226	1.18312
30475	3.78453	1.17579
30476	0.851517	1.27327
30477	0.946133	1.21738
30478	0.946132	1.22574
30479	0.946132	1.24243
30480	0.946133	1.16534
30481	0.851517	1.36106
30482	0.946132	1.23902
30483	0.946132	1.22452
30484	0.851516	1.24651
30485	0.851516	1.18545
30486	0.946133	1.28578
30487	0.946133	1.21657
30488	0.569995	1.22342
30489	0.816895	1.3415
30490	1.33893	1.32488
30491	1.18795	1.24671
30492	1.18795	1.1999
30493	0.569995	1.26853
30494	0.816895	1.36417
30495	1.33893	1.2952
30496	0.859572	1.38655
30497	0.859572	1.23042
30498	1.04936	1.25803
30499	1.04936	1.19561
30500	1.89226	2.1
30501	1.89226	2.1
30502	2.55618	2.1
30503	2.55618	2.1
30504	2.66983	2.1
30505	2.00591	2.1

30506	1.89226	2.1
30507	2.64586	2.1
30508	2.83331	2.1
30509	2.83331	2.1
30510	1.89226	2.1
30511	1.89226	2.1
30512	2.64586	2.1
30513	2.0815	2.1
30514	1.99985	2.1
30515	1.94005	2.1
30516	2.83332	2.1
30517	2.74859	2.1
30518	1.98061	2.1
30519	1.68356	2.1
30520	1.83879	2.1
30521	1.55987	2.1
30522	3.54048	2.1
30523	1.68356	2.1
30524	1.20969	2.1
30525	1.20969	2.1
30526	1.30751	2.1
30527	1.55987	2.1
30528	1.65769	2.1
30529	1.83879	2.1
30530	1.55987	2.1
30531	1.68443	2.1
30532	1.4449	2.1
30533	3.2443	2.1
30534	0.660368	2.1
30535	0.660368	2.1
30536	0.666922	2.1
30537	0.666922	2.1
30538	0.560377	2.1
30539	0.604842	2.1
30540	0.779935	2.1
30541	0.722448	2.1
30542	0.722446	2.1
30543	0.560377	2.1
30544	0.604844	2.1
30545	0.779934	2.1
30546	1.89227	2.1
30547	1.89227	2.1
30548	1.89227	2.1
30549	1.89227	2.1
30550	3.78453	2.1
30551	1.42896	2.1
30552	1.23037	2.1
30553	1.42896	2.1
30554	1.474 2.1	
30555	1.2016	2.1
30556	1.24665	2.1
30557	1.89226	2.1
30558	2.7188	2.1
30559	1.89226	2.1
30560	2.70702	2.1
30561	4.61106	2.1
30562	1.89226	2.1
30563	1.89226	2.1

30564	1.89226	2.1
30565	1.89226	2.1
30566	3.78453	2.1
30567	0.644603	2.1
30568	0.644603	2.1
30569	0.648272	2.1
30570	0.648272	2.1
30571	0.585644	2.1
30572	0.600802	2.1
30573	0.692073	2.1
30574	0.714478	2.1
30575	0.585644	2.1
30576	0.692073	2.1
30577	0.714478	2.1
30578	0.600802	2.1
30579	1.35351	2.1
30580	1.35351	2.1
30581	0.946131	2.1
30582	0.946131	2.1
30583	1.34392	2.1
30584	1.35351	2.1
30585	0.946132	2.1
30586	0.946132	2.1
30587	0.946132	2.1
30588	1.34392	2.1
30589	0.946132	2.1
30590	1.35351	2.1
30591	1.87609	2.1
30592	1.79155	2.1
30593	1.79155	2.1
30594	1.89226	2.1
30595	1.89226	2.1
30596	1.99211	2.1
30597	1.89226	2.1
30598	1.71706	2.1
30599	1.71706	2.1
30600	1.79155	2.1
30601	1.89226	2.1
30602	1.96675	2.1
30603	1.46869	2.1
30604	1.89226	2.1
30605	1.89226	2.1
30606	1.89226	2.1
30607	1.5001	2.1
30608	1.5001	2.1
30609	1.89226	2.1
30610	1.89226	2.1
30611	1.89226	2.1
30612	1.89226	2.1
30613	1.89226	2.1
30614	1.89226	2.1
30615	1.21408	2.1
30616	2.23563	2.1
30617	2.34021	2.1
30618	2.34021	2.1
30619	1.17285	2.1
30620	1.17285	2.1
30621	1.89226	2.1

30622	1.89226	2.1
30623	1.47937	2.1
30624	1.89226	2.1
30625	1.14822	2.1
30626	1.46869	2.1
30627	1.89226	2.1
30628	1.89226	2.1
30629	1.89226	2.1
30630	1.89226	2.1
30631	1.89226	2.1
30632	1.89226	2.1
30633	1.25716	2.1
30634	2.34021	2.1
30635	2.4542	2.1
30636	2.4542	2.1
30637	1.21408	2.1
30638	1.21408	2.1
30639	1.66824	2.1
30640	2.44883	2.1
30641	1.93003	2.1
30642	2.24795	2.1
30643	3.88907	2.1
30644	1.89319	2.1
30645	2.24795	2.1
30646	2.82724	2.1
30647	2.31318	2.1
30648	2.30868	2.1
30649	1.93003	2.1
30650	1.89226	2.1
30651	1.42589	2.1
30652	1.42589	2.1
30653	1.80501	2.1
30654	1.89226	2.1
30655	2.27137	2.1
30656	1.21067	0.0536468
30657	2.39166	0.211529
30658	1.73577	0.0648119
30659	1.73577	0.0589862
30660	1.53584	0.110658
30661	1.53584	0.0804243
30662	1.35025	2.1
30663	2.0599	1.74455
30664	1.51084	2.1
30665	2.27218	0.649059
30666	3.57074	2.01645
30667	2.0599	1.69857
30668	2.51818	2.1
30669	2.27218	0.556072
30670	2.66532	2.068
30671	4.72522	1.65817
30672	1.67115	2.1
30673	2.27218	1.62378
30674	2.39605	2.1
30675	2.39605	1.52432
30676	1.51084	2.1
30677	1.51084	1.88276
30678	2.27218	1.505
30679	2.66532	2.1

30680	2.39605	0.407405
30681	2.76829	2.04875
30682	5.06137	1.54595
30683	2.08684	1.5131
30684	2.36033	1.81432
30685	1.89224	1.45083
30686	1.89224	1.81049
30687	4.25257	1.49966
30688	2.41473	0.117915
30689	1.00307	0.154953
30690	2.01365	0.126807
30691	1.00307	0.167133
30692	3.05113	0.11691
30693	2.04056	0.180236
30694	1.11099	2.1
30695	2.25103	2.1
30696	2.22227	2.1
30697	2.22227	2.1
30698	1.14431	2.1
30699	1.14431	2.1
30700	1.07485	2.1
30701	2.22229	2.1
30702	2.19163	2.1
30703	2.19163	2.1
30704	1.111 2.1	
30705	1.111 2.1	
30706	1.0547	2.1
30707	1.19886	2.1
30708	0.946131	2.1
30709	0.946131	2.1
30710	0.946131	2.1
30711	0.946131	2.1
30712	1.19886	2.1
30713	1.19886	2.1
30714	1.0547	2.1
30715	0.946131	2.1
30716	0.946132	2.1
30717	1.19886	2.1
30718	1.89417	2.1
30719	2.64424	1.08358
30720	1.76585	2.1
30721	2.47695	2.03359
30722	4.37112	2.1
30723	1.89226	2.1
30724	1.89226	2.1
30725	1.89226	2.1
30726	1.89226	2.09114
30727	3.78453	2.1
30728	0.930281	2.1
30729	1.0547	2.1
30730	0.946131	2.1
30731	0.946131	2.1
30732	0.930309	2.1
30733	0.930309	2.1
30734	1.07052	2.1
30735	1.07052	2.1
30736	0.93028	2.1
30737	0.946131	2.1

30738	1.0547	2.1
30739	0.946131	2.1
30740	1.00015	0.78786
30741	1.00015	2.1
30742	1.01918	2.1
30743	1.01918	1.93174
30744	1.17437	1.98138
30745	1.23847	0.599888
30746	0.882926	2.1
30747	0.780859	2.1
30748	0.780859	2.03555
30749	1.17438	2.1
30750	0.882925	2.1
30751	1.23848	2.1
30752	1.89226	2.1
30753	1.89226	1.97341
30754	1.89226	2.1
30755	1.89226	2.1
30756	1.89226	2.1
30757	1.89226	2.1
30758	1.22083	2.1
30759	1.29579	2.1
30760	1.26236	2.1
30761	1.06191	2.1
30762	1.42128	2.1
30763	1.22083	2.1
30764	1.42128	2.1
30765	1.45806	2.1
30766	1.43502	2.1
30767	1.29579	2.1
30768	1.5605	2.1
30769	1.42128	2.1
30770	1.02813	2.1
30771	1.12121	2.1
30772	1.40557	2.1
30773	1.35298	2.1
30774	1.1314	2.1
30775	1.28679	2.1
30776	1.1715	2.1
30777	1.1715	2.1
30778	0.975656	2.1
30779	1.24908	2.1
30780	0.98956	2.1
30781	1.2681	2.1
30782	0.946132	2.1
30783	0.946132	2.1
30784	1.31889	2.1
30785	1.31889	2.1
30786	1.16546	2.1
30787	1.31889	2.1
30788	0.946133	2.1
30789	0.946133	2.1
30790	0.946133	2.1
30791	1.31889	2.1
30792	0.946132	2.1
30793	1.16546	2.1
30794	2.51518	0.05
30795	2.37584	0.05

30796	1.11712	0.05
30797	1.31242	0.05
30798	3.68826	0.05
30799	2.51518	0.05
30800	1.26311	0.05
30801	2.48977	0.05
30802	2.48977	0.05
30803	1.31242	0.05
30804	1.31242	0.05
30805	1.89226	0.879643
30806	1.89226	2.1
30807	1.89226	0.71013
30808	1.89226	2.1
30809	3.78452	1.90346
30810	2.04737	0.507376
30811	1.77122	2.1
30812	1.94498	0.66117
30813	1.65174	2.1
30814	3.71621	1.98728
30815	1.41077	2.1
30816	1.29729	2.1
30817	1.77123	2.1
30818	1.77123	2.1
30819	1.65175	2.1
30820	1.29129	2.1
30821	1.89226	2.1
30822	1.89226	2.1
30823	1.89226	2.1
30824	1.89226	2.1
30825	1.89226	2.1
30826	1.89226	2.1
30827	1.89226	0.508124
30828	1.89226	2.1
30829	1.89226	0.713836
30830	1.89226	2.1
30831	3.78451	1.4341
30832	1.94498	0.476025
30833	1.65174	2.1
30834	1.7968	0.989212
30835	1.49831	2.1
30836	3.44329	1.80454
30837	1.49831	2.1
30838	1.29729	2.1
30839	1.49831	2.1
30840	1.65175	2.1
30841	1.16858	2.1
30842	1.32201	2.1
30843	1.89226	2.1
30844	1.89226	2.1
30845	1.89226	2.1
30846	1.89226	2.1
30847	3.78452	2.1
30848	1.89226	0.05
30849	1.89226	0.05
30850	1.89226	0.05
30851	1.89226	0.05
30852	1.89226	0.05
30853	1.89226	0.05

30854	1.65324	2.07276
30855	1.26409	0.285475
30856	1.42491	0.293222
30857	1.42491	2.08435
30858	1.52268	0.677626
30859	1.52268	2.03198
30860	1.52268	2.1
30861	1.26409	0.706788
30862	1.32697	2.06781
30863	1.05724	0.435124
30864	2.57991	1.74538
30865	1.89226	0.0827617
30866	1.96548	0.0805999
30867	2.0178	0.0831266
30868	1.96548	0.0780297
30869	1.94458	0.0977866
30870	1.89226	0.0905456
30871	1.99366	0.0814314
30872	2.0178	0.0772506
30873	1.89226	0.134767
30874	2.0178	0.0771468
30875	1.75999	0.0962366
30876	1.89226	0.0832221
30877	0.946126	2.1
30878	0.946126	2.1
30879	1.28228	2.1
30880	1.19444	2.1
30881	0.946127	2.1
30882	0.946127	2.1
30883	1.19444	2.1
30884	1.19444	2.1
30885	0.946128	2.1
30886	1.19444	2.1
30887	1.28228	2.1
30888	0.946128	2.1
30889	1.89226	2.1
30890	1.89226	2.1
30891	1.89226	2.1
30892	1.89226	2.1
30893	3.78452	2.1
30894	2.14104	2.1
30895	1.89226	2.1
30896	2.19948	2.1
30897	1.89226	2.1
30898	1.89226	2.1
30899	2.19948	2.1
30900	1.89226	2.1
30901	2.38888	2.1
30902	2.23932	2.1
30903	2.38888	2.1
30904	1.74269	2.1
30905	1.89226	2.1
30906	1.89226	2.1
30907	1.89226	2.1
30908	1.89226	2.1
30909	1.89226	2.1
30910	3.78452	2.1
30911	0.946132	2.1

30912	0.946132	2.1
30913	1.07052	2.1
30914	1.07052	2.1
30915	0.946132	2.1
30916	0.946132	2.1
30917	1.05132	2.1
30918	1.07052	2.1
30919	1.05132	2.1
30920	0.946132	2.1
30921	1.07052	2.1
30922	0.946133	2.1
30923	2.40171	2.1
30924	1.89226	2.1
30925	1.89226	2.1
30926	1.89226	2.1
30927	2.42723	2.1
30928	2.42723	2.1
30929	1.89226	2.1
30930	1.89226	2.1
30931	1.89226	2.1
30932	1.89226	2.1
30933	3.78452	2.1
30934	1.63661	2.1
30935	2.3532	2.1
30936	2.33287	2.1
30937	2.33287	2.1
30938	1.64158	2.1
30939	1.64158	2.1
30940	2.38918	2.1
30941	1.89226	2.1
30942	1.89226	2.1
30943	1.89226	2.1
30944	2.40171	2.1
30945	2.40171	2.1
30946	1.89226	2.1
30947	1.89226	2.1
30948	1.89226	2.1
30949	1.89226	2.1
30950	1.89226	2.1
30951	1.89226	2.1
30952	1.64158	2.1
30953	2.3532	2.1
30954	1.6488	2.1
30955	2.37364	2.1
30956	4.002 2.1	
30957	2.54752	0.150889
30958	1.42445	0.199891
30959	1.86697	0.194034
30960	1.86697	0.173415
30961	2.06666	0.19085
30962	2.06666	0.17342
30963	2.50872	0.122046
30964	2.06666	0.176242
30965	1.90439	0.142541
30966	1.51835	0.14284
30967	3.97105	0.142395
30968	0.755161	0.183966
30969	0.755161	0.16297

30970	0.793994	0.137207
30971	0.793994	0.159802
30972	0.712225	0.176011
30973	0.515826	0.164379
30974	1.03333	0.168192
30975	0.759175	0.15291
30976	0.759173	0.166637
30977	0.515827	0.146466
30978	0.712226	0.156657
30979	1.03333	0.159215
30980	1.69633	0.201545
30981	1.89226	0.21733
30982	1.62477	0.19362
30983	1.89226	0.215791
30984	1.62477	0.201705
30985	1.89226	0.216359
30986	1.89226	0.372308
30987	1.89226	0.555802
30988	1.89226	0.506968
30989	1.89226	0.482217
30990	3.78452	0.478485
30991	2.28622	0.200494
30992	1.09667	0.146035
30993	1.17506	0.146719
30994	2.24411	0.180384
30995	1.17506	0.171667
30996	2.24411	0.221596
30997	2.24741	2.1
30998	1.70303	2.1
30999	2.49168	2.1
31000	1.57936	2.1
31001	2.53729	2.1
31002	1.70303	2.1
31003	2.24741	2.1
31004	2.47557	2.1
31005	1.89227	2.1
31006	1.89227	2.1
31007	4.36784	2.1
31008	2.27016	0.05
31009	1.10476	0.05
31010	1.44103	0.05
31011	1.10476	0.05
31012	2.26791	0.05
31013	1.93165	0.05
31014	2.27016	0.05
31015	1.89226	0.05
31016	1.89226	0.05
31017	2.4065	0.05
31018	4.29876	0.05
31019	2.27016	0.05
31020	1.44103	0.05
31021	1.53336	0.05
31022	2.4065	0.05
31023	3.84753	0.05
31024	2.7542	0.318604
31025	1.89226	0.439574
31026	2.35027	0.385307
31027	1.89226	0.394327

31028	2.35027	0.373399
31029	1.89226	0.383123
31030	2.3301	2.1
31031	2.36071	2.1
31032	2.07629	2.1
31033	2.01105	2.1
31034	4.37176	2.1
31035	1.86512	2.1
31036	2.01105	2.1
31037	2.01105	2.1
31038	2.3301	2.1
31039	1.57459	2.1
31040	1.89364	2.1
31041	2.52671	2.1
31042	2.01105	2.1
31043	1.78251	2.1
31044	1.70302	2.1
31045	3.98362	2.1
31046	1.227	2.1
31047	2.01105	2.1
31048	1.45869	2.1
31049	1.41713	2.1
31050	1.70302	2.1
31051	1.64994	2.1
31052	2.00989	2.1
31053	1.28938	2.1
31054	1.8769	2.1
31055	1.20969	2.1
31056	3.21958	2.1
31057	1.8769	2.1
31058	1.15864	2.1
31059	1.15864	2.1
31060	1.20969	2.1
31061	1.77353	2.1
31062	1.82458	2.1
31063	1.7986	2.1
31064	2.48717	2.1
31065	2.50161	2.1
31066	1.75869	2.1
31067	4.30021	2.1
31068	2.50161	2.1
31069	1.75251	2.1
31070	1.81678	2.1
31071	1.75869	2.1
31072	2.5597	2.1
31073	2.50161	2.1
31074	1.91334	0.54344
31075	1.85439	1.12149
31076	1.91334	0.640414
31077	1.70303	1.18144
31078	1.70303	0.469417
31079	1.49692	1.07561
31080	2.39986	0.05
31081	1.89226	0.0967006
31082	1.89226	0.0727237
31083	2.50756	0.05
31084	4.39982	0.05
31085	2.39986	0.0769622

31086	1.70303	0.05
31087	1.75564	0.05
31088	1.75904	0.0935901
31089	2.50272	0.0604084
31090	2.50756	0.0889119
31091	1.89226	0.0812153
31092	2.50756	0.05
31093	2.47857	0.071972
31094	2.50756	0.0791611
31095	1.86327	0.0807208
31096	1.89226	0.0724737
31097	1.22241	0.0768696
31098	1.20827	0.103229
31099	0.772416	0.0615679
31100	0.555208	0.11516
31101	1.004	0.0592416
31102	1.004	0.0973692
31103	0.93504	0.112525
31104	0.744364	0.100047
31105	1.2089	0.107313
31106	0.654935	0.108188
31107	0.773927	0.086962
31108	1.03618	0.0968132
31109	1.89227	0.05
31110	1.89227	0.05
31111	1.89227	0.05
31112	1.89227	0.05
31113	3.78453	0.05
31114	1.89226	0.05
31115	1.89226	0.05
31116	1.89226	0.05
31117	1.89226	0.05
31118	3.78452	0.05
31119	1.82643	0.05
31120	2.7084	0.05
31121	2.12654	0.05
31122	2.65614	0.05
31123	1.82643	0.05
31124	2.35603	0.05
31125	1.29385	0.05
31126	0.946133	0.05
31127	1.05492	0.05
31128	0.946132	0.05
31129	1.05492	0.05
31130	1.29385	0.05
31131	0.946133	0.05
31132	0.946133	0.05
31133	1.29385	0.05
31134	1.29385	0.05
31135	0.946132	0.05
31136	0.946132	0.05
31137	0.946132	0.05
31138	1.27329	0.05
31139	1.29385	0.05
31140	0.946129	0.05
31141	1.29385	0.05
31142	1.27328	0.05
31143	0.946131	0.05

31144	0.946131	0.05
31145	1.27328	0.05
31146	1.27328	0.05
31147	0.94613	0.05
31148	0.94613	0.05
31149	1.89226	1.54453
31150	1.89226	1.58377
31151	1.89226	1.16618
31152	1.89226	1.28778
31153	3.78453	1.36526
31154	1.89226	1.46437
31155	1.89226	1.50173
31156	1.89226	1.18127
31157	1.89226	1.37745
31158	3.78453	1.28595
31159	1.21488	1.46326
31160	1.14605	1.91111
31161	1.62974	1.34027
31162	1.52399	1.22571
31163	2.84463	1.45544
31164	0.577078	1.2252
31165	0.81251	1.79219
31166	0.821003	1.28191
31167	0.543413	1.43774
31168	0.683929	1.27439
31169	0.722433	2.1
31170	0.730674	1.58839
31171	0.730674	1.25357
31172	0.857766	1.24272
31173	0.862119	1.19565
31174	0.600938	1.52484
31175	0.596094	1.30807
31176	1.89226	1.25753
31177	1.89226	1.35149
31178	1.89226	1.04838
31179	1.89226	1.18225
31180	3.78453	1.12144
31181	1.89226	1.22926
31182	1.89226	1.73909
31183	1.89226	1.27136
31184	1.89226	1.17427
31185	3.78453	1.34722
31186	1.62974	1.54134
31187	1.56958	1.73428
31188	2.2362	1.13379
31189	2.11424	1.22867
31190	3.86595	1.26434
31191	2.2362	1.11689
31192	1.85414	1.75224
31193	2.28211	1.03104
31194	1.78367	1.06899
31195	2.2362	1.34469
31196	1.73256	1.63236
31197	0.851539	1.28513
31198	0.851539	1.22448
31199	1.14454	1.94336
31200	0.938988	1.85442
31201	0.851485	1.28662

31202	0.920593	1.67384
31203	0.851541	0.329387
31204	0.851541	1.82073
31205	1.04073	0.845823
31206	1.16342	1.49287
31207	0.825315	1.61966
31208	0.825316	1.37095
31209	1.89226	2.1
31210	1.89226	2.1
31211	1.89226	2.1
31212	1.89226	2.1
31213	3.78453	2.1
31214	2.57016	2.1
31215	1.74789	2.1
31216	2.57016	2.1
31217	2.522	2.1
31218	1.61727	2.1
31219	1.56911	2.1
31220	1.28075	2.1
31221	1.406	2.1
31222	0.946131	2.1
31223	0.946131	2.1
31224	1.28075	2.1
31225	1.00333	2.1
31226	0.94613	2.1
31227	0.94613	2.1
31228	1.041	2.1
31229	1.041	2.1
31230	0.943484	2.1
31231	1.27717	2.1
31232	1.89226	2.1
31233	1.89226	2.1
31234	1.89226	2.1
31235	1.89226	2.1
31236	1.89226	2.1
31237	1.89226	2.1
31238	2.57016	2.1
31239	1.61727	2.1
31240	2.74196	2.1
31241	1.59094	2.1
31242	4.16109	2.1
31243	1.89226	2.1
31244	2.0815	2.1
31245	2.2034	2.1
31246	1.82493	2.1
31247	1.87847	2.1
31248	2.16142	2.1
31249	1.89226	2.1
31250	1.89226	2.1
31251	1.89226	2.1
31252	1.89226	2.1
31253	3.78453	2.1
31254	0.985252	2.1
31255	0.985252	2.1
31256	0.946132	2.1
31257	0.946132	2.1
31258	1.0655	2.1
31259	0.985255	2.1

31260	0.94613	2.1
31261	0.94613	2.1
31262	0.94613	2.1
31263	1.0655	2.1
31264	0.94613	2.1
31265	0.985255	2.1
31266	1.89226	2.1
31267	1.89226	2.1
31268	1.89226	2.1
31269	1.89226	2.1
31270	3.78453	2.1
31271	1.06549	2.1
31272	1.06549	2.1
31273	0.946133	2.1
31274	0.946133	2.1
31275	1.16834	2.1
31276	1.0655	2.1
31277	0.946131	2.1
31278	0.946131	2.1
31279	0.946131	2.1
31280	1.16834	2.1
31281	0.94613	2.1
31282	1.0655	2.1
31283	0.966631	2.1
31284	1.07349	2.1
31285	0.946131	2.1
31286	0.946131	2.1
31287	1.07349	2.1
31288	1.07349	2.1
31289	0.946129	2.1
31290	0.946129	2.1
31291	1.07349	2.1
31292	0.946131	2.1
31293	0.94613	2.1
31294	0.966631	2.1
31295	2.38055	0.888128
31296	1.89226	1.46888
31297	1.89226	1.34866
31298	1.46724	0.398901
31299	3.35949	0.957847
31300	1.42249	2.1
31301	2.05499	2.1
31302	1.39708	2.1
31303	2.09033	2.1
31304	3.45207	2.1
31305	2.05499	2.1
31306	2.57425	2.1
31307	2.51287	2.1
31308	2.47753	2.1
31309	2.09033	2.1
31310	2.05499	2.1
31311	1.39327	2.1
31312	2.09033	2.1
31313	2.16217	2.1
31314	2.16217	2.1
31315	1.39708	2.1
31316	1.39708	2.1
31317	2.16217	2.1

31318	2.57426	2.1
31319	2.73006	2.1
31320	2.73006	2.1
31321	2.09033	2.1
31322	2.09033	2.1
31323	1.89226	0.0698163
31324	1.89226	0.05
31325	1.89226	0.05
31326	1.89226	0.0641278
31327	3.78452	0.062962
31328	0.838749	0.05
31329	0.838749	0.05
31330	0.85824	0.05
31331	0.85824	0.05
31332	0.544644	0.05
31333	0.854718	0.05
31334	0.842271	0.05
31335	1.19141	0.05
31336	0.842272	0.05
31337	0.544643	0.05
31338	0.854718	0.05
31339	1.19141	0.05
31340	1.34214	0.05
31341	1.34214	0.05
31342	0.893236	0.0510411
31343	0.893236	0.05
31344	0.893265	0.05
31345	1.28924	0.05
31346	0.946133	0.05
31347	0.946133	0.05
31348	0.946132	0.05
31349	0.893265	0.05
31350	0.946133	0.05
31351	1.28924	0.05
31352	2.7013	0.05
31353	1.89226	0.0568478
31354	1.9216	0.0580424
31355	1.9216	0.05
31356	1.89226	0.0547345
31357	1.89226	0.05
31358	1.26397	2.1
31359	1.52995	2.1
31360	1.30116	2.1
31361	1.04084	2.1
31362	2.57079	2.1
31363	1.47013	2.1
31364	2.17459	2.1
31365	2.10668	2.1
31366	1.38328	2.1
31367	3.55787	2.1
31368	2.55006	2.1
31369	2.17459	2.1
31370	2.10668	2.1
31371	2.49168	2.1
31372	4.66626	2.1
31373	0.613622	2.1
31374	0.691639	2.1
31375	1.00105	2.1

31376	1.05334	2.1
31377	0.839874	2.1
31378	0.839874	2.1
31379	0.852814	2.1
31380	0.852814	2.1
31381	0.613622	2.1
31382	1.00105	2.1
31383	1.05334	2.1
31384	0.691639	2.1
31385	1.00105	2.1
31386	1.05334	2.1
31387	1.20844	2.1
31388	1.24584	2.1
31389	1.12717	2.1
31390	1.12717	2.1
31391	1.13462	2.1
31392	1.13462	2.1
31393	1.20845	2.1
31394	1.00105	2.1
31395	1.05334	2.1
31396	1.24584	2.1
31397	2.22558	2.1
31398	1.85981	2.1
31399	2.18943	1.96538
31400	1.89142	0.769418
31401	4.0854	2.1
31402	2.02641	2.1
31403	1.85981	2.1
31404	2.18943	1.93456
31405	1.67346	1.99058
31406	3.88622	1.92814
31407	1.85981	0.580663
31408	1.10736	0.320449
31409	1.27421	0.598952
31410	1.67346	0.483832
31411	1.27421	0.362393
31412	1.67346	1.91029
31413	1.85981	1.43611
31414	1.27362	0.901783
31415	1.89142	0.40717
31416	1.27421	0.345737
31417	3.13343	0.468076
31418	1.81893	0.05
31419	1.64985	0.050726
31420	1.23477	0.05
31421	2.25405	0.0696025
31422	3.48882	0.0590492
31423	2.23933	0.05
31424	1.89226	0.0522064
31425	1.89226	0.0520899
31426	2.78053	0.05
31427	1.89226	0.05
31428	2.78053	0.05
31429	0.946131	0.0584481
31430	0.946131	0.056701
31431	0.946132	0.0804523
31432	0.946132	0.0985617
31433	0.946131	0.106046

31434	0.946131	0.0952745
31435	0.946132	0.0599083
31436	0.946132	0.0844902
31437	0.946131	0.066519
31438	0.946131	0.100062
31439	0.946132	0.05
31440	0.946132	0.0905999
31441	1.89226	2.1
31442	2.48596	2.1
31443	1.89226	2.1
31444	2.51506	2.1
31445	4.40732	2.1
31446	2.48596	2.1
31447	1.63823	2.1
31448	2.51507	2.1
31449	1.67687	2.1
31450	4.1533	2.1
31451	1.89226	2.1
31452	2.51506	2.1
31453	1.92044	2.1
31454	1.89226	2.1
31455	2.54325	2.1
31456	2.51506	2.1
31457	2.51507	2.1
31458	1.5704	2.1
31459	1.70505	2.1
31460	1.67687	2.1
31461	2.54325	2.1
31462	2.51507	2.1
31463	2.0815	2.1
31464	1.70303	2.1
31465	1.70303	2.1
31466	1.80354	2.1
31467	1.98172	2.1
31468	2.09868	2.1
31469	1.27944	2.1
31470	1.79028	2.1
31471	1.13179	2.1
31472	1.65533	2.1
31473	2.93477	2.1
31474	1.89226	2.1
31475	1.89226	2.1
31476	1.89226	2.1
31477	1.89226	2.1
31478	1.89226	2.1
31479	1.89226	2.1
31480	1.98171	2.1
31481	1.89226	2.1
31482	1.89226	2.1
31483	1.89226	2.1
31484	1.65798	2.1
31485	1.65797	2.1
31486	1.00133	2.1
31487	1.65532	2.1
31488	1.53278	2.1
31489	1.53278	2.1
31490	1.13179	2.1
31491	1.13179	2.1

31492	0.810946	2.1
31493	0.810946	2.1
31494	0.810944	2.1
31495	0.810944	2.1
31496	0.810946	2.1
31497	0.810946	2.1
31498	1.89226	2.1
31499	1.35169	2.1
31500	1.08132	2.1
31501	1.08132	2.1
31502	1.08132	2.1
31503	1.99528	0.05
31504	1.70303	0.05
31505	2.08152	0.05
31506	1.70303	0.0562413
31507	2.33877	0.05
31508	1.93967	0.0543931
31509	1.89226	0.05
31510	1.89226	0.0846396
31511	1.89226	0.0502089
31512	1.89226	0.051077
31513	3.78452	0.0579116
31514	1.26259	0.05
31515	2.17205	0.05
31516	2.32468	0.05
31517	2.32468	0.05
31518	1.28055	0.05
31519	1.28055	0.05
31520	0.946131	0.05
31521	0.946131	0.05
31522	0.997642	0.05
31523	0.997642	0.05
31524	0.946131	0.05
31525	0.946131	0.0533078
31526	0.989846	0.05
31527	0.997642	0.05
31528	0.946132	0.05
31529	0.989846	0.05
31530	0.997641	0.05
31531	0.946131	0.05
31532	1.89226	0.0536335
31533	1.89226	0.05
31534	1.89226	0.05
31535	1.89226	0.0719511
31536	3.78452	0.05
31537	1.28055	0.05
31538	2.17205	0.05
31539	1.14305	0.05
31540	1.92737	0.05
31541	3.3151	0.05
31542	0.954908	0.05
31543	0.9705	0.05
31544	0.946131	0.05
31545	0.946131	0.05
31546	0.946131	0.05
31547	0.946131	0.05
31548	0.9705	0.05
31549	0.9705	0.05

31550	0.954908	0.05
31551	0.946131	0.05
31552	0.946131	0.05
31553	0.9705	0.05
31554	0.835187	0.05
31555	0.954896	0.05
31556	0.946118	0.05
31557	0.946118	0.05
31558	0.835186	0.05
31559	0.835186	0.05
31560	1.06583	0.05
31561	1.06583	0.05
31562	0.835198	0.05
31563	0.946131	0.05
31564	0.954883	0.05
31565	0.946106	0.05
31566	1.89227	2.1
31567	1.89227	2.1
31568	1.89227	2.1
31569	1.89227	2.1
31570	1.89227	2.1
31571	1.89227	2.1
31572	0.885265	2.1
31573	0.885265	2.1
31574	0.871467	2.1
31575	0.871467	2.1
31576	1.11883	2.1
31577	0.787875	2.1
31578	0.996487	2.1
31579	0.637907	2.1
31580	0.996487	2.1
31581	1.11883	2.1
31582	0.787875	2.1
31583	0.637906	2.1
31584	1.89226	2.1
31585	2.39258	2.1
31586	2.39258	2.1
31587	2.54729	2.1
31588	1.89226	2.1
31589	2.04697	2.1
31590	1.89226	2.1
31591	2.0815	2.1
31592	2.0815	2.1
31593	2.48371	2.1
31594	2.0815	2.1
31595	2.51469	2.1
31596	1.00035	0.05
31597	1.55755	0.529914
31598	1.03534	1.49964
31599	1.61333	1.63543
31600	2.59289	0.377021
31601	1.55755	0.529914
31602	1.85217	2.1
31603	1.61333	2.1
31604	1.92875	2.1
31605	3.48629	2.1
31606	1.09962	1.38319
31607	1.61334	0.71755

31608	1.70868	2.1
31609	1.70868	1.8038
31610	1.03535	2.1
31611	1.03535	1.31619
31612	1.61334	0.748516
31613	1.92876	2.1
31614	1.70868	1.58367
31615	2.05274	2.1
31616	3.63744	1.89864
31617	1.82233	0.27895
31618	2.05768	0.726279
31619	1.89226	0.598253
31620	1.65406	0.363919
31621	1.89226	0.585176
31622	1.65406	0.394051
31623	2.26047	0.318615
31624	2.0815	1.13232
31625	1.89226	0.906246
31626	2.01156	0.446107
31627	1.89226	0.873135
31628	2.01156	0.458199
31629	1.89226	2.1
31630	1.89226	2.1
31631	1.89226	2.1
31632	1.89226	2.1
31633	3.78453	2.1
31634	2.70055	2.1
31635	2.28367	2.1
31636	2.56456	2.1
31637	2.33565	2.1
31638	4.84823	2.1
31639	2.28367	2.1
31640	1.5791	2.1
31641	1.96966	2.1
31642	1.09506	2.1
31643	3.37873	2.1
31644	1.89226	2.1
31645	1.89226	2.1
31646	1.89226	2.1
31647	1.89226	2.1
31648	3.78452	2.1
31649	0.94612	2.1
31650	0.94612	2.1
31651	0.946119	2.1
31652	0.946119	2.1
31653	0.946119	2.1
31654	0.946119	2.1
31655	0.946119	2.1
31656	0.946119	2.1
31657	0.946132	2.1
31658	0.946132	2.1
31659	0.946107	2.1
31660	0.946107	2.1
31661	2.56452	2.1
31662	1.57307	2.1
31663	2.10299	2.1
31664	2.1115	2.1
31665	2.52196	2.1

31666	2.52196	2.1
31667	1.89226	2.1
31668	1.89226	2.1
31669	1.89226	2.1
31670	1.89226	2.1
31671	3.78452	2.1
31672	2.37665	2.1
31673	1.89226	2.1
31674	2.28084	2.1
31675	1.89226	2.1
31676	4.26891	2.1
31677	0.946129	2.1
31678	0.946129	2.1
31679	0.946133	2.1
31680	0.946133	2.1
31681	0.946131	2.1
31682	0.946131	2.1
31683	0.946131	2.1
31684	0.946131	2.1
31685	0.946131	2.1
31686	0.946131	2.1
31687	0.946131	2.1
31688	0.946131	2.1
31689	1.7207	0.05
31690	1.65815	0.05
31691	1.33593	0.05
31692	1.31508	0.05
31693	2.99408	0.05
31694	0.676047	0.05
31695	0.676047	0.05
31696	0.684362	0.05
31697	0.684362	0.05
31698	0.547994	0.05
31699	0.702871	0.05
31700	0.829076	0.05
31701	0.657538	0.05
31702	0.657538	0.05
31703	0.547994	0.05
31704	0.829077	0.05
31705	0.702871	0.05
31706	1.9518	2.1
31707	1.30648	2.1
31708	1.28846	2.1
31709	1.92033	2.1
31710	3.22681	2.1
31711	1.89226	2.1
31712	1.89226	2.1
31713	1.89226	2.1
31714	1.89226	2.1
31715	3.78453	2.1
31716	0.946128	2.1
31717	0.946128	2.1
31718	1.10113	2.1
31719	1.09587	2.1
31720	0.946131	2.1
31721	0.946131	2.1
31722	1.09587	2.1
31723	1.09587	2.1

31724	0.946131	2.1
31725	1.09587	2.1
31726	1.10112	2.1
31727	0.94613	2.1
31728	1.26923	2.1
31729	1.92033	2.1
31730	1.28846	2.1
31731	1.28846	2.1
31732	1.89452	2.1
31733	1.89452	2.1
31734	1.89226	2.1
31735	1.89226	2.1
31736	1.89226	2.1
31737	1.89226	2.1
31738	3.78453	2.1
31739	1.89226	2.1
31740	2.19174	2.1
31741	2.15616	2.1
31742	2.19174	2.1
31743	1.85669	2.1
31744	1.89226	2.1
31745	1.89226	2.1
31746	1.89226	2.1
31747	1.89226	2.1
31748	1.89226	2.1
31749	3.78452	2.1
31750	1.89226	2.1
31751	1.89226	2.1
31752	1.89226	2.1
31753	1.89226	2.1
31754	3.78452	2.1
31755	1.51223	2.1
31756	1.41562	2.1
31757	1.5139	2.1
31758	1.42284	2.1
31759	1.6033	2.1
31760	1.51223	2.1
31761	1.51223	2.1
31762	1.25953	2.1
31763	1.38085	2.1
31764	1.6033	2.1
31765	2.89309	2.1
31766	0.946131	2.1
31767	0.946131	2.1
31768	0.946131	2.1
31769	0.946131	2.1
31770	0.946132	2.1
31771	0.946132	2.1
31772	0.946129	2.1
31773	0.946129	2.1
31774	0.946131	2.1
31775	0.946131	2.1
31776	0.94613	2.1
31777	0.94613	2.1
31778	1.89226	2.1
31779	1.89226	2.1
31780	1.89226	2.1
31781	1.89226	2.1

31782	3.78452	2.1
31783	0.707806	2.1
31784	0.697816	2.1
31785	0.823593	2.1
31786	0.801649	2.1
31787	0.74182	2.1
31788	0.74182	2.1
31789	0.757645	2.1
31790	0.757645	2.1
31791	0.823592	2.1
31792	0.697818	2.1
31793	0.707807	2.1
31794	0.801647	2.1
31795	1.52534	2.1
31796	1.6033	2.1
31797	1.64719	2.1
31798	1.38085	2.1
31799	1.64719	2.1
31800	1.38085	2.1
31801	2.67041	2.1
31802	1.89226	2.09121
31803	1.89226	2.1
31804	1.89226	2.1
31805	2.487	2.1
31806	2.487	2.1
31807	2.70097	2.1
31808	1.70303	2.1
31809	1.71163	2.1
31810	2.487	2.1
31811	4.46723	2.1
31812	1.14984	2.1
31813	1.2435	2.1
31814	0.778	2.1
31815	0.697616	2.1
31816	0.967211	2.1
31817	0.967211	2.1
31818	0.973907	2.1
31819	0.973907	2.1
31820	0.697616	2.1
31821	1.14984	2.1
31822	0.778	2.1
31823	1.2435	2.1
31824	2.4158	2.1
31825	1.26438	2.1
31826	1.676	2.1
31827	2.77505	2.1
31828	4.0918	2.1
31829	2.58258	2.1
31830	1.89226	2.1
31831	1.89226	2.1
31832	1.89226	2.1
31833	2.47032	2.1
31834	2.47032	2.1
31835	1.23516	2.1
31836	1.18734	2.1
31837	0.946131	2.1
31838	0.946131	2.1
31839	1.18734	2.1

31840	1.18734	2.1
31841	0.94613	2.1
31842	0.94613	2.1
31843	1.18734	2.1
31844	0.946131	2.1
31845	0.946131	2.1
31846	1.23516	2.1
31847	2.8239	2.1
31848	1.39361	2.1
31849	1.12342	2.1
31850	2.57275	2.1
31851	3.96637	2.1
31852	0.937929	1.56078
31853	0.937929	1.43341
31854	1.39734	1.348
31855	1.39734	1.23062
31856	1.38914	1.41769
31857	0.94613	1.48938
31858	0.937902	1.45368
31859	0.94613	0.448302
31860	0.94613	1.29003
31861	0.94613	1.37622
31862	1.38914	1.40072
31863	0.937902	1.34332
31864	1.89226	0.215689
31865	2.17765	1.18866
31866	1.84577	1.03213
31867	1.84577	0.427746
31868	1.89226	0.898314
31869	1.89226	0.319654
31870	2.11262	2.04572
31871	1.57766	2.1
31872	2.22689	2.1
31873	1.91132	1.72321
31874	2.19624	2.1
31875	1.79689	0.860813
31876	1.99656	1.61066
31877	1.57766	2.1
31878	1.39067	2.1
31879	1.73802	1.64236
31880	3.31568	2.00045
31881	1.89226	0.248868
31882	1.89226	0.113724
31883	1.89226	0.170236
31884	1.89226	0.485995
31885	1.89226	0.0693054
31886	1.89226	0.181042
31887	1.84936	0.276825
31888	2.0815	0.0696697
31889	2.0815	0.147194
31890	2.47217	0.171028
31891	1.04528	0.0554516
31892	1.62774	0.136156
31893	1.89226	0.179464
31894	1.89226	0.0850532
31895	1.89226	0.114284
31896	1.89226	0.226638
31897	1.89226	0.0682705

31898	1.89226	0.143798
31899	1.69055	0.05
31900	2.68518	0.0615444
31901	1.69055	0.05
31902	2.25055	0.07464
31903	2.39587	0.05
31904	3.01801	0.0654581
31905	1.89226	0.0760526
31906	1.89226	0.0922026
31907	1.89226	0.05
31908	1.89226	0.05
31909	3.78452	0.0752675
31910	1.24499	2.1
31911	1.70302	2.1
31912	1.41207	2.1
31913	1.10574	1.91794
31914	2.66056	2.1
31915	1.70303	2.1
31916	2.39605	1.85488
31917	2.72674	2.1
31918	2.46904	1.64776
31919	1.84557	2.1
31920	1.67115	1.94125
31921	2.46904	0.327144
31922	2.76829	2.1
31923	2.83666	0.385738
31924	2.83666	1.86827
31925	2.39605	1.27537
31926	2.39605	2.1
31927	1.07323	0.442973
31928	1.07323	2.1
31929	1.06715	2.1
31930	1.18052	1.81939
31931	1.30067	0.74604
31932	1.26542	1.48551
31933	0.914516	2.1
31934	0.839633	2.1
31935	0.936958	1.95348
31936	1.26751	1.94552
31937	0.847764	2.06061
31938	1.35395	2.05187
31939	2.69069	0.291246
31940	1.03074	0.253655
31941	1.74098	0.178772
31942	1.97397	0.234194
31943	3.71495	0.231554
31944	2.44554	2.1
31945	1.89226	2.1
31946	1.89226	2.1
31947	1.89226	2.1
31948	2.40645	2.1
31949	2.40645	2.1
31950	1.89226	2.1
31951	1.89226	2.1
31952	1.89226	2.1
31953	1.89226	2.1
31954	3.78452	2.1
31955	1.82117	2.1

31956	2.55786	2.1
31957	2.51431	2.1
31958	2.51431	2.1
31959	1.85054	2.1
31960	1.84375	2.1
31961	2.38467	2.1
31962	1.89226	2.1
31963	1.89226	2.1
31964	1.89226	2.1
31965	2.44554	2.1
31966	2.44554	2.1
31967	1.89226	2.1
31968	1.89226	2.1
31969	1.89226	2.1
31970	1.89226	2.1
31971	1.89226	2.1
31972	1.89226	2.1
31973	2.02397	2.1
31974	2.55786	2.1
31975	1.77775	2.1
31976	2.61404	2.1
31977	4.33561	2.1
31978	1.89226	1.63056
31979	1.89226	1.61499
31980	1.89226	1.51685
31981	1.89226	1.56565
31982	3.78453	1.63432
31983	1.89226	1.72786
31984	2.80184	1.71067
31985	1.89226	1.64678
31986	1.89226	1.68928
31987	2.03045	1.78709
31988	2.03045	1.80044
31989	2.52263	1.62595
31990	1.27921	1.98422
31991	1.58429	2.03725
31992	1.73571	1.78926
31993	2.37121	1.74343
31994	2.52263	1.63159
31995	0.764974	2.1
31996	0.650577	2.1
31997	0.712428	2.1
31998	0.562516	2.1
31999	0.67264	2.1
32000	0.67264	2.1
32001	0.654851	2.1
32002	0.654851	2.1
32003	0.562515	2.1
32004	0.650579	2.1
32005	0.712427	2.1
32006	0.764976	2.1
32007	1.89227	2.1
32008	1.89227	2.1
32009	1.89227	2.1
32010	1.89227	2.1
32011	3.78453	2.1
32012	1.89226	2.1
32013	1.89226	2.1

32014	1.89226	2.1
32015	1.89226	2.1
32016	3.78453	2.1
32017	1.49248	2.1
32018	1.24959	2.1
32019	1.28211	2.1
32020	1.26432	2.1
32021	1.51027	2.1
32022	1.49248	2.1
32023	1.49248	2.1
32024	1.19017	2.1
32025	1.24977	2.1
32026	1.51027	2.1
32027	2.74225	2.1
32028	1.89226	2.1
32029	1.89226	2.1
32030	1.89226	2.1
32031	1.89226	2.1
32032	3.78453	2.1
32033	1.89226	2.1
32034	1.89226	2.1
32035	1.89226	2.1
32036	1.89226	2.1
32037	3.78453	2.1
32038	0.624796	2.1
32039	0.618472	2.1
32040	0.770147	2.1
32041	0.755132	2.1
32042	0.681445	2.1
32043	0.681445	2.1
32044	0.69216	2.1
32045	0.69216	2.1
32046	0.618472	2.1
32047	0.770147	2.1
32048	0.624796	2.1
32049	0.755132	2.1
32050	1.54029	2.1
32051	1.51027	2.1
32052	1.54029	2.1
32053	1.30682	2.1
32054	1.24977	2.1
32055	1.24977	2.1
32056	0.994293	2.1
32057	2.17159	2.1
32058	2.21149	2.1
32059	2.21149	2.1
32060	0.994814	2.1
32061	0.994814	2.1
32062	0.990481	2.1
32063	2.21149	2.1
32064	2.2671	2.1
32065	2.2671	2.1
32066	0.994293	2.1
32067	0.994293	2.1
32068	2.41579	2.1
32069	1.09847	2.1
32070	1.70302	2.1
32071	2.45069	2.1

32072	3.61169	2.1
32073	2.48689	2.1
32074	1.67969	2.1
32075	1.67969	2.1
32076	1.70002	2.1
32077	2.43826	2.1
32078	2.45859	2.1
32079	2.43827	2.1
32080	1.62408	2.1
32081	1.62408	2.1
32082	1.67969	2.1
32083	2.36729	2.1
32084	2.4229	2.1
32085	2.47199	2.1
32086	1.0823	2.1
32087	1.0607	2.1
32088	2.44276	2.1
32089	3.52506	2.1
32090	2.47199	2.1
32091	1.89226	2.1
32092	1.89226	2.1
32093	2.44277	2.1
32094	4.33503	2.1
32095	1.89226	2.1
32096	1.89226	2.1
32097	1.89226	2.1
32098	1.89226	2.1
32099	3.78453	2.1
32100	1.89226	2.1
32101	1.89226	2.1
32102	1.89226	2.1
32103	1.89226	2.1
32104	1.89226	2.1
32105	1.89226	2.1
32106	1.02986	2.1
32107	2.44276	2.1
32108	1.0607	2.1
32109	1.0607	2.1
32110	2.40055	2.1
32111	2.40055	2.1
32112	1.89226	2.1
32113	2.44277	2.1
32114	2.40056	2.1
32115	2.44277	2.1
32116	1.85005	2.1
32117	1.89226	2.1
32118	1.89226	2.1
32119	1.89226	2.1
32120	1.89226	2.1
32121	1.89226	2.1
32122	3.78453	2.1
32123	1.89226	2.1
32124	1.89226	2.1
32125	1.89226	2.1
32126	1.89226	2.1
32127	3.78452	2.1
32128	2.79502	0.474725
32129	2.63448	2.1

32130	2.68986	0.414179
32131	2.53868	2.1
32132	5.32434	1.1463
32133	2.63449	2.1
32134	2.39282	2.1
32135	2.53869	2.1
32136	2.3085	2.1
32137	4.94299	2.1
32138	2.68986	0.35273
32139	2.53868	2.1
32140	2.5761	0.646058
32141	2.43404	2.1
32142	5.1239	1.08358
32143	2.53869	2.1
32144	2.3085	2.1
32145	2.43405	2.1
32146	2.22575	2.1
32147	4.74255	2.1
32148	1.89226	2.1
32149	1.89226	2.1
32150	1.89226	2.1
32151	1.89226	2.1
32152	3.78453	2.1
32153	1.32391	2.1
32154	1.44951	2.1
32155	1.44951	2.1
32156	1.89226	2.1
32157	1.89226	2.1
32158	2.20203	2.1
32159	0.507656	2.1
32160	0.578945	2.1
32161	1.05568	2.1
32162	0.959007	2.1
32163	0.76264	2.1
32164	0.76264	2.1
32165	0.775313	2.1
32166	0.775313	2.1
32167	0.578945	2.1
32168	1.05568	2.1
32169	0.959007	2.1
32170	0.507656	2.1
32171	1.89226	0.05
32172	2.4065	0.05
32173	2.36561	0.05
32174	2.4065	0.05
32175	1.85138	0.05
32176	1.89226	0.05
32177	1.40274	0.05
32178	2.4065	0.05
32179	1.53336	0.05
32180	1.53336	0.05
32181	2.36562	0.05
32182	2.36562	0.05
32183	0.946115	0.05
32184	0.946115	0.05
32185	1.06496	0.05
32186	1.18279	0.05
32187	0.946117	0.05

32188	0.946117	0.05
32189	1.06496	0.05
32190	1.06496	0.05
32191	0.94613	0.05
32192	1.06497	0.05
32193	0.946105	0.05
32194	1.18278	0.05
32195	1.00213	0.05
32196	2.36559	0.05
32197	1.40272	0.05
32198	1.40272	0.05
32199	2.12992	0.05
32200	2.12992	0.05
32201	0.95646	2.1
32202	2.07201	2.1
32203	1.01808	2.1
32204	2.14876	2.1
32205	3.09009	2.1
32206	1.89226	2.1
32207	1.89226	2.1
32208	1.89226	2.1
32209	1.89226	2.1
32210	3.78453	2.1
32211	1.89227	2.1
32212	1.89227	2.1
32213	1.89227	2.1
32214	1.89227	2.1
32215	1.89227	2.1
32216	1.89227	2.1
32217	2.71886	2.1
32218	1.60497	2.1
32219	1.43935	2.1
32220	2.55151	2.1
32221	4.15648	2.1
32222	2.71886	2.1
32223	1.89226	2.1
32224	1.89226	2.1
32225	2.55151	2.1
32226	4.44377	2.1
32227	1.89226	2.1
32228	1.89226	2.1
32229	1.89226	2.1
32230	1.89226	2.1
32231	3.78453	2.1
32232	1.89226	2.1
32233	1.89226	2.1
32234	1.89226	2.1
32235	1.89226	2.1
32236	3.78453	2.1
32237	1.58942	0.182808
32238	1.92622	0.28305
32239	2.52852	0.246056
32240	2.19457	0.170877
32241	4.12079	0.17112
32242	1.92649	0.145552
32243	1.44426	0.0952718
32244	1.87529	0.103424
32245	2.19457	0.151431

32246	3.63883	0.136734
32247	1.87529	0.0776729
32248	2.19457	0.132564
32249	2.7378	0.114547
32250	2.29852	0.0782783
32251	4.49309	0.0780223
32252	1.35976	2.1
32253	1.89452	2.1
32254	1.39616	2.1
32255	1.1423	2.1
32256	1.8996	2.1
32257	1.52068	2.1
32258	1.89226	2.1
32259	1.89226	2.1
32260	1.89226	2.1
32261	1.89226	2.1
32262	1.89226	2.1
32263	1.89226	2.1
32264	1.89226	2.1
32265	2.15617	2.1
32266	2.0953	2.1
32267	2.15617	2.1
32268	1.83139	2.1
32269	1.89226	2.1
32270	1.16817	2.1
32271	1.8996	2.1
32272	1.23614	2.1
32273	1.48338	2.1
32274	1.80218	2.1
32275	2.16263	2.1
32276	1.89226	2.1
32277	1.89226	2.1
32278	1.89226	2.1
32279	1.89226	2.1
32280	1.89226	2.1
32281	1.89226	2.1
32282	1.89226	2.1
32283	2.0953	2.1
32284	2.03074	2.1
32285	2.0953	2.1
32286	1.8277	2.1
32287	1.89226	2.1
32288	1.74455	2.1
32289	2.181	2.1
32290	1.74455	2.1
32291	1.92911	2.1
32292	1.98195	2.1
32293	2.16651	2.1
32294	2.181	2.1
32295	2.29881	2.1
32296	1.98195	2.1
32297	1.80729	2.1
32298	4.28075	2.1
32299	1.74455	2.1
32300	1.98195	2.1
32301	1.52603	2.1
32302	1.76603	2.1
32303	3.51058	2.1

32304	1.76603	2.1
32305	1.94506	2.1
32306	1.76603	2.1
32307	1.98195	2.1
32308	1.86795	2.1
32309	2.08387	2.1
32310	0.984305	2.1
32311	1.9	2.1
32312	2.86619	2.1
32313	1.86189	2.1
32314	3.76189	2.1
32315	1.4621	2.1
32316	1.89226	2.1
32317	2.81374	2.1
32318	1.89226	2.1
32319	3.35437	2.1
32320	0.946133	2.1
32321	0.946133	2.1
32322	0.946132	2.1
32323	0.946132	2.1
32324	0.946132	2.1
32325	0.946132	2.1
32326	0.946133	2.1
32327	0.946133	2.1
32328	0.946132	2.1
32329	0.946132	2.1
32330	0.946132	2.1
32331	0.946132	2.1
32332	1.64268	2.1
32333	1.93673	2.1
32334	2.25284	2.1
32335	1.97548	2.1
32336	3.89552	2.1
32337	1.86206	2.1
32338	1.93673	2.1
32339	2.25284	2.1
32340	2.20075	2.1
32341	4.1149	2.1
32342	2.25284	2.1
32343	2.24781	2.1
32344	1.97548	2.1
32345	1.97548	2.1
32346	2.49676	2.1
32347	2.49676	2.1
32348	2.25284	2.1
32349	2.43549	2.1
32350	2.44467	2.1
32351	2.20075	2.1
32352	2.49676	2.1
32353	2.25284	2.1
32354	2.57928	2.1
32355	2.32606	2.1
32356	2.59522	2.1
32357	2.39022	2.1
32358	4.9695	2.1
32359	2.32606	2.1
32360	1.77677	2.1
32361	2.39022	2.1

32362	2.28669	2.1
32363	4.16699	2.1
32364	2.59522	2.1
32365	2.39022	2.1
32366	2.61901	2.1
32367	2.45899	2.1
32368	5.00923	2.1
32369	2.39022	2.1
32370	2.11575	2.1
32371	2.41795	2.1
32372	2.0815	2.1
32373	4.67085	2.1
32374	1.89226	2.1
32375	2.24723	2.1
32376	1.95405	2.1
32377	1.89226	2.1
32378	2.30902	2.1
32379	2.24723	2.1
32380	1.89226	2.1
32381	1.89226	2.1
32382	1.89226	2.1
32383	1.89226	2.1
32384	3.78453	2.1
32385	0.946131	2.1
32386	0.946131	2.1
32387	1.14643	2.1
32388	1.10673	2.1
32389	1.10673	2.1
32390	1.10673	2.1
32391	0.946133	2.1
32392	0.946133	2.1
32393	0.946131	2.1
32394	1.14643	2.1
32395	1.10673	2.1
32396	0.946131	2.1
32397	1.89226	2.1
32398	2.30902	2.1
32399	1.96635	2.1
32400	1.89226	2.1
32401	2.38311	2.1
32402	2.30902	2.1
32403	1.89226	2.1
32404	1.89226	2.1
32405	1.89226	2.1
32406	1.89226	2.1
32407	1.89226	2.1
32408	1.89226	2.1
32409	0.946131	2.1
32410	0.946131	2.1
32411	1.19156	2.1
32412	1.14643	2.1
32413	1.14643	2.1
32414	1.14643	2.1
32415	0.946133	2.1
32416	0.946133	2.1
32417	0.946131	2.1
32418	1.19156	2.1
32419	0.946131	2.1

32420	1.14643	2.1
32421	1.89226	1.41289
32422	1.89226	1.21606
32423	1.89226	1.2989
32424	1.89226	1.44416
32425	1.89226	1.18223
32426	1.89226	1.27009
32427	1.89226	1.24361
32428	1.89226	1.23362
32429	1.89226	1.09113
32430	1.9645	1.22495
32431	3.78452	1.29479
32432	2.39489	2.1
32433	2.15715	2.1
32434	2.07821	2.1
32435	2.33541	2.1
32436	4.49256	2.1
32437	2.39489	2.1
32438	2.34898	2.1
32439	2.30729	2.1
32440	2.33541	2.1
32441	4.6844	2.1
32442	2.07821	2.1
32443	2.33541	2.1
32444	2.26415	2.1
32445	1.99632	2.1
32446	4.33174	2.1
32447	2.30729	2.1
32448	2.33542	2.1
32449	2.26416	2.1
32450	2.26618	2.1
32451	4.60159	2.1
32452	1.19837	1.63719
32453	2.79151	0.269014
32454	2.25636	0.821496
32455	2.25636	0.564665
32456	1.94072	0.746235
32457	1.94072	0.58016
32458	2.53725	0.05
32459	1.16699	0.05
32460	1.69864	0.05
32461	2.0815	0.0543839
32462	3.58273	0.05
32463	1.91646	2.1
32464	2.51686	2.1
32465	2.49872	2.1
32466	1.85819	2.1
32467	4.41518	2.1
32468	0.929098	2.1
32469	0.8993	2.1
32470	1.24359	2.1
32471	1.24936	2.1
32472	1.06832	2.1
32473	1.06832	2.1
32474	1.08034	2.1
32475	1.08034	2.1
32476	0.899298	2.1
32477	1.24359	2.1

32478	1.24936	2.1
32479	0.929097	2.1
32480	2.0815	0.051414
32481	1.89226	0.05
32482	2.0815	0.05
32483	1.82811	0.05
32484	1.94982	0.05
32485	1.71246	0.05
32486	2.3256	2.1
32487	1.89226	2.1
32488	1.89226	2.1
32489	1.89226	2.1
32490	2.38467	2.1
32491	2.38467	2.1
32492	1.89226	2.1
32493	1.89226	2.1
32494	1.89226	2.1
32495	1.89226	2.1
32496	1.89226	2.1
32497	1.89226	2.1
32498	1.89763	2.1
32499	2.61405	2.1
32500	2.15035	2.1
32501	2.68796	2.1
32502	1.42219	2.1
32503	1.77775	2.1
32504	2.26306	2.1
32505	1.89226	2.1
32506	1.89226	2.1
32507	1.89226	2.1
32508	2.3256	2.1
32509	2.3256	2.1
32510	1.89226	2.1
32511	1.89226	2.1
32512	1.89226	2.1
32513	1.89226	2.1
32514	1.89226	2.1
32515	1.89226	2.1
32516	1.92887	2.1
32517	2.41915	2.1
32518	2.50142	2.1
32519	2.50142	2.1
32520	1.90388	2.1
32521	1.89763	2.1
32522	1.89224	2.1
32523	1.89224	2.1
32524	1.89224	2.1
32525	1.89224	2.1
32526	3.78448	2.1
32527	2.25315	2.1
32528	1.05843	2.1
32529	2.38995	2.1
32530	1.31541	2.1
32531	3.56856	2.1
32532	0.517552	2.1
32533	0.68548	2.1
32534	1.27675	2.1
32535	1.13076	2.1

32536	0.913611	2.1
32537	0.913612	2.1
32538	0.902633	2.1
32539	0.902633	2.1
32540	0.68547	2.1
32541	1.27673	2.1
32542	1.13078	2.1
32543	0.517559	2.1
32544	1.89226	2.1
32545	1.89226	2.1
32546	1.89226	2.1
32547	1.89226	2.1
32548	3.78453	2.1
32549	0.962936	2.1
32550	0.962936	2.1
32551	0.996141	2.1
32552	0.996141	2.1
32553	0.830609	2.1
32554	0.657714	2.1
32555	1.30136	2.1
32556	1.19499	2.1
32557	1.30136	2.1
32558	0.830609	2.1
32559	1.19499	2.1
32560	0.657713	2.1
32561	1.60057	0.100429
32562	1.39615	0.0898467
32563	1.06474	0.0841794
32564	1.39615	0.111004
32565	1.64655	0.0835928
32566	2.04424	0.106963
32567	1.89226	0.0862373
32568	1.89226	0.118026
32569	1.89226	0.0925965
32570	1.89226	0.112048
32571	1.89226	0.0932816
32572	1.89226	0.114484
32573	2.56322	0.07599
32574	1.89226	0.111185
32575	1.89226	0.0839824
32576	1.89226	0.101775
32577	2.18047	0.0780203
32578	2.18047	0.0939872
32579	1.89226	0.539662
32580	1.89226	0.481189
32581	1.89226	0.538773
32582	1.65573	0.392402
32583	1.89226	0.699629
32584	1.65573	0.49752
32585	1.05353	0.349784
32586	1.83158	0.418729
32587	1.50282	0.151634
32588	1.20669	0.189542
32589	2.88511	0.203332
32590	1.44807	2.1
32591	1.93592	2.1
32592	1.80703	2.1
32593	1.27806	2.1

32594	3.21398	2.1
32595	2.0815	2.1
32596	1.87465	2.1
32597	1.87465	2.1
32598	1.50566	2.1
32599	2.0815	2.1
32600	1.69263	2.1
32601	1.89226	2.1
32602	1.89226	2.1
32603	1.89226	2.1
32604	1.89226	2.1
32605	1.89226	2.1
32606	1.89226	2.1
32607	1.62632	2.1
32608	0.994294	2.1
32609	0.994294	2.1
32610	1.27806	2.1
32611	1.81921	2.1
32612	2.10776	2.1
32613	2.0815	2.1
32614	1.70303	2.1
32615	1.70303	2.1
32616	1.85749	2.1
32617	1.90451	2.1
32618	2.07724	2.1
32619	1.89226	2.1
32620	1.89226	2.1
32621	1.89226	2.1
32622	1.89226	2.1
32623	1.89226	2.1
32624	1.89226	2.1
32625	2.69773	2.1
32626	1.88129	2.1
32627	2.55196	2.1
32628	2.26469	2.1
32629	4.94367	2.1
32630	1.88129	2.1
32631	1.25889	2.1
32632	1.26614	2.1
32633	1.05914	2.1
32634	2.26469	2.1
32635	1.88129	2.1
32636	2.55196	2.1
32637	2.0588	2.1
32638	2.6536	2.1
32639	2.15287	2.1
32640	4.70484	2.1
32641	0.8516	2.1
32642	0.8516	2.1
32643	0.849002	2.1
32644	0.849002	2.1
32645	0.629445	2.1
32646	0.671197	2.1
32647	1.0294	2.1
32648	1.07644	2.1
32649	1.07644	2.1
32650	0.671197	2.1
32651	0.629446	2.1

32652	1.0294	2.1
32653	2.02115	1.83421
32654	1.18382	1.66583
32655	2.29107	0.250502
32656	1.46903	1.23094
32657	3.47488	1.19191
32658	2.60763	1.63595
32659	2.29107	1.45784
32660	1.82141	1.51204
32661	1.82141	1.59555
32662	1.46903	1.54787
32663	2.29107	1.5908
32664	1.64007	2.1
32665	2.2024	2.1
32666	1.44044	2.1
32667	2.02634	2.1
32668	3.6664	2.1
32669	1.25565	2.1
32670	2.02635	2.1
32671	1.87228	2.1
32672	1.87228	2.1
32673	1.44045	2.1
32674	1.44045	2.1
32675	1.89226	0.05
32676	1.78503	0.05
32677	1.78503	0.05
32678	1.40048	0.05
32679	2.0815	0.05
32680	1.62972	0.05
32681	1.27875	2.1
32682	1.1483	2.1
32683	1.40014	2.1
32684	1.40014	2.1
32685	1.04598	2.1
32686	1.04598	2.1
32687	2.63443	1.80937
32688	2.26444	2.08845
32689	2.79392	0.466813
32690	2.4863	2.1
32691	5.12073	1.96291
32692	1.83158	0.607246
32693	1.18508	0.20854
32694	1.20669	0.194815
32695	1.81488	0.340115
32696	1.20669	0.219266
32697	1.81488	0.373754
32698	1.08134	0.22198
32699	1.70303	0.531311
32700	1.24027	0.311395
32701	1.30359	0.224609
32702	1.89939	0.464486
32703	1.70303	0.458797
32704	1.89226	2.1
32705	2.69027	2.1
32706	1.89226	2.1
32707	2.2078	2.1
32708	1.89226	2.1
32709	2.2078	2.1

32710	1.89226	2.1
32711	2.78977	2.1
32712	1.89226	2.1
32713	2.69027	2.1
32714	1.89226	2.1
32715	2.69027	2.1
32716	1.89226	2.1
32717	1.89226	2.1
32718	1.89226	1.94005
32719	1.89226	2.1
32720	3.78452	2.1
32721	1.43687	2.0423
32722	1.71059	2.1
32723	2.53014	2.07611
32724	2.6521	2.1
32725	4.24073	2.1
32726	2.6521	2.1
32727	1.66762	2.1
32728	2.50299	2.1
32729	2.50299	2.1
32730	1.71059	2.1
32731	1.71059	2.1
32732	1.89226	2.1
32733	1.89226	2.1
32734	1.89226	2.1
32735	1.89226	2.1
32736	1.89226	2.1
32737	1.89226	2.1
32738	2.10662	0.187817
32739	2.77417	0.371361
32740	2.78873	1.30027
32741	2.11661	0.246618
32742	4.89078	0.286647
32743	2.10662	0.819613
32744	1.22857	0.088361
32745	1.25849	1.03382
32746	2.11661	0.660141
32747	3.34518	0.818371
32748	2.78873	1.1511
32749	2.11661	0.190726
32750	2.12475	0.284265
32751	2.79029	0.589597
32752	4.9069	0.317395
32753	1.2858	0.816515
32754	2.11661	0.826401
32755	1.25849	0.427384
32756	1.25849	0.664865
32757	2.12475	0.566054
32758	2.12475	0.823469
32759	1.13537	2.1
32760	1.20714	2.1
32761	0.946129	2.1
32762	0.946129	2.1
32763	0.946131	2.1
32764	0.946131	2.1
32765	1.29521	2.1
32766	1.20713	2.1
32767	1.13537	2.1

32768	0.946131	2.1
32769	0.94613	2.1
32770	1.29521	2.1
32771	1.00533	2.1
32772	0.876055	2.1
32773	0.946128	2.1
32774	0.946128	2.1
32775	0.913908	2.1
32776	0.913908	2.1
32777	1.17081	2.1
32778	0.896413	2.1
32779	0.955905	2.1
32780	0.893715	2.1
32781	1.20327	2.1
32782	0.998544	2.1
32783	1.89226	1.58521
32784	1.89226	1.6762
32785	1.89226	1.08289
32786	1.89226	1.33533
32787	3.78452	1.1922
32788	0.540975	1.44467
32789	0.726949	2.0854
32790	0.712198	1.64215
32791	0.506675	1.32323
32792	0.611916	1.33882
32793	0.611916	2.1
32794	0.621708	1.61499
32795	0.621708	1.35459
32796	0.726951	1.29247
32797	0.712196	1.26772
32798	0.506674	1.4935
32799	0.540976	1.50875
32800	1.89226	1.4245
32801	1.89226	2.06034
32802	1.89226	1.70023
32803	1.89226	1.35395
32804	3.78453	1.66195
32805	1.89226	1.28134
32806	1.89226	1.39834
32807	1.89226	1.25378
32808	1.89226	1.6977
32809	1.89226	1.24504
32810	1.89226	2.08312
32811	0.919594	1.30034
32812	0.726949	1.49665
32813	0.898318	1.36943
32814	0.712198	1.49283
32815	0.817559	1.32002
32816	0.817559	2.1
32817	0.814234	1.34233
32818	0.814234	1.574
32819	0.919597	1.1864
32820	0.898316	1.14764
32821	0.726951	1.5392
32822	0.712196	1.44244
32823	1.79663	1.2626
32824	1.12088	2.02553
32825	1.54699	1.7048

32826	1.17475	1.5986
32827	1.79663	1.846
32828	1.42439	1.70556
32829	2.28928	1.59138
32830	1.23077	1.50333
32831	1.65458	0.43235
32832	2.66533	1.8499
32833	3.94385	1.47267
32834	1.89226	2.1
32835	1.89226	2.1
32836	1.89226	2.1
32837	1.89226	2.1
32838	3.78452	2.1
32839	1.38426	2.1
32840	1.15067	2.1
32841	1.38426	2.1
32842	1.01595	2.1
32843	1.24509	2.1
32844	1.01595	2.1
32845	2.62097	2.1
32846	1.89226	2.1
32847	2.72561	2.1
32848	2.72561	2.1
32849	1.89226	2.1
32850	1.89226	2.1
32851	1.90103	1.86571
32852	1.41902	2.1
32853	1.77249	1.93991
32854	1.30272	2.1
32855	3.20375	2.1
32856	1.77249	1.82669
32857	1.19548	2.1
32858	1.19548	2.1
32859	1.30272	2.1
32860	1.65815	2.00797
32861	1.76539	2.1
32862	2.62161	2.1
32863	1.89226	2.1
32864	1.89226	2.1
32865	1.89226	2.1
32866	2.6446	2.1
32867	2.6446	2.1
32868	2.64459	2.1
32869	1.89226	2.1
32870	1.89226	2.1
32871	1.89226	2.1
32872	2.58257	2.1
32873	2.58257	2.1
32874	1.89226	0.309154
32875	1.89226	1.29665
32876	2.31301	1.15255
32877	2.76309	0.491874
32878	1.89226	1.12437
32879	2.26047	0.493802
32880	1.89226	0.27799
32881	2.31301	1.24925
32882	2.17765	1.1128
32883	2.17765	0.526349

32884	1.89226	1.04075
32885	1.89226	0.445575
32886	2.18516	2.1
32887	1.85196	2.1
32888	1.78595	2.1
32889	2.14606	2.1
32890	3.99802	2.1
32891	2.18516	2.1
32892	2.21189	2.1
32893	2.18937	2.1
32894	2.14606	2.1
32895	4.35796	2.1
32896	1.60735	2.1
32897	2.14606	2.1
32898	2.15714	2.1
32899	1.70302	2.1
32900	3.70055	2.1
32901	2.18937	2.1
32902	2.14606	2.1
32903	2.0927	2.1
32904	2.162	2.1
32905	4.30806	2.1
32906	2.17326	2.1
32907	2.58517	2.1
32908	2.55318	2.1
32909	2.1665	2.1
32910	4.75167	2.1
32911	2.17326	2.1
32912	1.53556	2.1
32913	1.56253	2.1
32914	2.1665	2.1
32915	3.70206	2.1
32916	2.55317	2.1
32917	2.1665	2.1
32918	2.17741	2.1
32919	2.54197	2.1
32920	4.70847	2.1
32921	2.17742	2.1
32922	2.1665	2.1
32923	2.18452	2.1
32924	1.58054	2.1
32925	1.56253	2.1
32926	1.56253	2.1
32927	1.71051	0.05
32928	1.89807	0.05
32929	1.19755	0.0561917
32930	1.19755	0.05
32931	2.25632	0.052859
32932	2.25632	0.05
32933	0.876046	0.0802133
32934	1.27293	0.05
32935	0.946132	0.128082
32936	0.946132	0.125179
32937	0.946131	0.0730091
32938	0.869312	0.0720102
32939	1.12767	0.106668
32940	0.946132	0.110045
32941	1.22225	0.108552

32942	1.37968	0.05
32943	0.851555	0.158011
32944	0.851555	0.0816077
32945	0.946133	2.04558
32946	0.946133	2.1
32947	0.946132	2.09058
32948	0.946132	1.86817
32949	0.946133	1.99466
32950	0.946133	2.07919
32951	0.946132	2.1
32952	0.946132	2.08571
32953	0.946132	2.1
32954	0.946132	2.1
32955	0.946133	2.05691
32956	0.946133	2.1
32957	1.35152	2.1
32958	0.948936	2.1
32959	1.90166	2.1
32960	1.70303	2.1
32961	2.90858	2.1
32962	1.89226	2.1
32963	1.89226	2.1
32964	1.89226	2.1
32965	1.89226	2.1
32966	3.78452	2.1
32967	1.90166	2.1
32968	1.70303	2.1
32969	2.52454	2.1
32970	2.13919	2.1
32971	3.99529	2.1
32972	1.89226	2.1
32973	1.89226	2.1
32974	1.89226	2.1
32975	1.89226	2.1
32976	3.78452	2.1
32977	1.89226	2.1
32978	1.89226	2.1
32979	1.89226	2.1
32980	1.89226	2.1
32981	1.89226	2.1
32982	1.89226	2.1
32983	2.13918	2.1
32984	0.992674	2.1
32985	2.3397	2.1
32986	1.8023	2.1
32987	1.47769	2.1
32988	0.992674	2.1
32989	1.89226	0.229526
32990	1.89226	0.216685
32991	1.89226	0.311518
32992	1.89226	0.164979
32993	3.78452	0.237738
32994	1.89226	0.217952
32995	2.54752	0.124652
32996	2.63974	0.168951
32997	1.89226	0.158375
32998	4.53201	0.155055
32999	0.498412	0.165318

33000	0.500955	0.151276
33001	0.57955	0.200725
33002	0.563873	0.175137
33003	0.529107	0.167007
33004	0.529107	0.150103
33005	0.53572	0.218192
33006	0.53572	0.156349
33007	0.500956	0.165143
33008	0.579548	0.159147
33009	0.563871	0.166829
33010	0.498414	0.170365
33011	1.89223	0.261865
33012	1.89223	0.162131
33013	1.89223	0.207429
33014	1.89223	0.224628
33015	1.89223	0.204225
33016	1.89223	0.221087
33017	1.28752	0.126548
33018	1.31985	0.154921
33019	0.946119	0.151796
33020	0.946119	0.188897
33021	1.28752	0.154319
33022	1.28752	0.126567
33023	0.946119	0.147277
33024	0.946119	0.284487
33025	1.28753	0.153395
33026	0.946132	0.151174
33027	1.31984	0.167696
33028	0.946107	0.161913
33029	0.500948	0.148269
33030	0.479465	0.139696
33031	0.562335	0.185368
33032	0.579542	0.159511
33033	0.528436	0.153798
33034	0.528436	0.139961
33035	0.530571	0.146004
33036	0.530571	0.195701
33037	0.56234	0.152857
33038	0.479473	0.153744
33039	0.500943	0.160941
33040	0.579533	0.154492
33041	1.89226	2.1
33042	1.89226	2.1
33043	1.89226	2.1
33044	1.89226	2.1
33045	3.78452	2.1
33046	1.50767	2.1
33047	1.14496	2.1
33048	1.50767	2.1
33049	1.58539	2.1
33050	1.07653	2.1
33051	1.15425	2.1
33052	1.89226	2.1
33053	2.43826	2.1
33054	2.43826	2.1
33055	2.48688	2.1
33056	1.89226	2.1
33057	1.94088	2.1

33058	1.89226	2.1
33059	1.89226	2.1
33060	1.89226	2.1
33061	1.89226	2.1
33062	3.78452	2.1
33063	1.50767	2.1
33064	1.07653	2.1
33065	1.41077	2.1
33066	0.991407	2.1
33067	2.49907	2.1
33068	1.21913	2.1
33069	1.21913	2.1
33070	0.946128	2.1
33071	0.946128	2.1
33072	1.21913	2.1
33073	1.18364	2.1
33074	0.946131	2.1
33075	0.946131	2.1
33076	1.18364	2.1
33077	0.946129	2.1
33078	1.21913	2.1
33079	0.94613	2.1
33080	1.89226	2.1
33081	1.89226	2.1
33082	1.89226	2.1
33083	1.89226	2.1
33084	3.78452	2.1
33085	1.93823	2.1
33086	1.60976	2.1
33087	1.93823	2.1
33088	2.20045	2.1
33089	1.36999	2.1
33090	1.63222	2.1
33091	1.38302	2.1
33092	1.38302	2.1
33093	0.946132	2.1
33094	0.946132	2.1
33095	1.27258	2.1
33096	1.38302	2.1
33097	0.946133	2.1
33098	0.946133	2.1
33099	0.946133	2.1
33100	1.27258	2.1
33101	0.946132	2.1
33102	1.38302	2.1
33103	1.89226	2.1
33104	1.89226	2.1
33105	1.89226	2.1
33106	1.89226	2.1
33107	3.78452	2.1
33108	1.93823	2.1
33109	1.36999	2.1
33110	1.70423	2.1
33111	1.14195	2.1
33112	3.08018	2.1
33113	1.27258	2.1
33114	1.27258	2.1
33115	0.946132	2.1

33116	0.946132	2.1
33117	1.17233	2.1
33118	1.27258	2.1
33119	0.946132	2.1
33120	0.946132	2.1
33121	1.17233	2.1
33122	0.946132	2.1
33123	0.946132	2.1
33124	1.27258	2.1
33125	2.70575	2.1
33126	1.89226	1.75923
33127	2.71429	2.1
33128	2.71429	2.1
33129	1.89226	2.1
33130	1.89226	2.07679
33131	0.946133	2.09727
33132	0.946133	2.1
33133	1.28713	2.1
33134	1.28713	2.1
33135	0.946131	2.1
33136	0.946131	2.05469
33137	1.28713	2.1
33138	1.36503	2.1
33139	0.946131	2.1
33140	1.36503	2.1
33141	1.28713	2.1
33142	0.946131	2.1
33143	1.89226	2.1
33144	1.89226	1.88999
33145	1.89226	2.1
33146	1.89226	2.1
33147	1.89226	2.1
33148	1.89226	2.1
33149	1.22615	2.1
33150	1.04147	2.1
33151	1.3439	2.1
33152	1.3439	2.1
33153	0.969118	2.1
33154	0.969118	2.1
33155	0.980841	2.1
33156	0.992505	2.1
33157	1.34289	2.1
33158	1.70303	2.1
33159	2.56081	2.1
33160	1.89225	2.1
33161	2.66785	2.1
33162	2.79506	2.1
33163	2.79506	2.1
33164	1.89225	2.1
33165	1.89225	2.1
33166	0.978778	2.1
33167	0.978778	2.1
33168	0.993303	2.1
33169	0.993303	2.1
33170	0.603538	2.1
33171	0.638158	2.1
33172	1.33393	2.1
33173	1.39753	2.1

33174	1.33391	2.1
33175	0.603535	2.1
33176	0.638164	2.1
33177	1.39754	2.1
33178	1.33393	2.1
33179	1.33393	2.1
33180	0.946132	2.1
33181	0.946132	2.1
33182	1.33393	2.1
33183	1.27365	2.1
33184	0.946132	2.1
33185	0.946132	2.1
33186	0.946132	2.1
33187	1.27365	2.1
33188	1.33393	2.1
33189	0.946131	2.1
33190	1.89226	2.1
33191	1.89226	2.1
33192	1.89226	2.1
33193	1.89226	2.1
33194	3.78452	2.1
33195	1.89226	2.1
33196	1.89226	2.1
33197	1.89226	2.1
33198	1.89226	2.1
33199	3.78453	2.1
33200	0.979364	2.1
33201	0.979364	2.1
33202	0.89618	2.1
33203	0.89618	2.1
33204	0.896178	2.1
33205	0.929411	2.1
33206	0.946133	2.1
33207	0.946133	2.1
33208	0.946131	2.1
33209	0.89618	2.1
33210	0.946131	2.1
33211	0.929413	2.1
33212	1.89226	2.1
33213	1.89226	2.1
33214	1.89226	2.1
33215	1.89226	2.1
33216	1.89226	2.1
33217	1.89226	2.1
33218	1.89225	2.1
33219	1.89225	2.1
33220	1.89225	2.1
33221	1.89225	2.1
33222	3.7845	2.1
33223	1.89225	2.1
33224	1.89225	2.1
33225	1.89225	2.1
33226	1.89225	2.1
33227	1.89225	2.1
33228	1.89225	2.1
33229	0.946128	0.131513
33230	0.946128	0.0922057
33231	0.946132	0.114716

33232	0.946132	0.0762701
33233	0.94613	0.116443
33234	0.94613	0.138288
33235	0.946129	0.0833729
33236	0.946129	0.064017
33237	0.94613	0.100103
33238	0.94613	0.099487
33239	0.94613	0.0951443
33240	0.94613	0.0922221
33241	1.89227	1.26405
33242	1.89227	1.23931
33243	1.89227	1.1075
33244	1.89227	1.13289
33245	3.78453	1.13847
33246	1.89226	1.2857
33247	2.43717	1.51509
33248	2.63259	1.32318
33249	1.89226	1.1703
33250	4.52485	1.35765
33251	0.616808	1.18809
33252	0.792277	1.27049
33253	1.04986	1.09531
33254	0.72303	1.19964
33255	0.702675	1.00273
33256	0.76862	1.61139
33257	0.809154	1.3194
33258	0.809154	1.16043
33259	0.808757	1.17328
33260	1.07789	1.03602
33261	0.772568	1.16601
33262	0.598878	1.30573
33263	1.89226	1.24034
33264	1.89226	1.32675
33265	1.89226	1.19784
33266	1.89226	1.13995
33267	3.78453	1.25024
33268	1.36428	1.54616
33269	1.31629	1.38177
33270	0.946132	1.29416
33271	0.946132	1.27499
33272	1.36428	1.22101
33273	1.36428	1.67177
33274	0.946132	1.39446
33275	0.946132	1.42492
33276	1.36428	1.44958
33277	0.946132	1.27458
33278	1.31629	1.4056
33279	0.946132	1.29953
33280	1.578	1.34395
33281	2.85336	1.07703
33282	2.11368	1.02158
33283	2.14375	1.24046
33284	1.93852	1.1448
33285	1.93852	1.35651
33286	2.34806	0.160763
33287	1.42913	0.163118
33288	1.61539	0.283443
33289	2.45322	0.229731

33290	3.88235	0.205283
33291	2.34806	0.109958
33292	1.89226	0.150223
33293	1.89226	0.539473
33294	2.45322	0.504333
33295	4.34548	0.139404
33296	1.89226	0.131664
33297	1.89226	0.100354
33298	1.89226	0.169058
33299	1.89226	0.388441
33300	3.78452	0.251272
33301	1.89226	0.165294
33302	1.89226	0.128431
33303	1.89226	0.238657
33304	1.89226	0.243625
33305	3.78452	0.162734
33306	1.25972	0.114486
33307	2.45322	0.123277
33308	2.58823	0.330109
33309	1.79398	0.272293
33310	4.2472	0.131002
33311	1.89226	0.222752
33312	2.45322	0.0964123
33313	2.58823	0.150615
33314	2.45322	0.192739
33315	2.02727	0.0722112
33316	1.89226	0.105025
33317	1.89226	0.0807204
33318	1.89226	0.124173
33319	1.89226	0.180968
33320	1.89226	0.173359
33321	3.78452	0.08988
33322	1.89226	0.123187
33323	1.89226	0.155263
33324	1.89226	0.135991
33325	1.89226	0.0822721
33326	1.89226	0.196493
33327	1.89226	0.129219
33328	1.70048	2.1
33329	2.1572	1.42041
33330	1.45477	2.1
33331	1.93973	1.97825
33332	3.64021	2.1
33333	1.17614	2.1
33334	1.93973	1.91133
33335	1.69661	2.1
33336	1.69661	1.85314
33337	1.45477	2.1
33338	1.45477	1.90861
33339	1.94343	2.1
33340	2.53782	1.90501
33341	2.59903	2.1
33342	2.59903	1.68122
33343	1.87598	2.1
33344	1.87598	1.84276
33345	0.946131	0.987729
33346	0.946131	1.87384
33347	1.26891	2.1

33348	1.26891	0.435009
33349	0.94613	2.01816
33350	0.94613	1.74837
33351	1.26891	1.75767
33352	1.29952	1.55967
33353	1.29952	0.20884
33354	0.946131	0.392171
33355	1.26891	1.55465
33356	0.946131	1.55816
33357	2.00551	2.1
33358	2.59903	1.94843
33359	2.65811	2.1
33360	2.65811	1.83738
33361	1.94343	2.1
33362	1.94343	2.06087
33363	2.65811	1.30086
33364	1.89226	2.1
33365	1.89226	0.573194
33366	1.89226	1.69038
33367	2.59903	1.46338
33368	2.59903	2.05088
33369	1.89227	2.1
33370	1.89227	2.1
33371	1.89227	2.1
33372	1.89227	2.1
33373	3.78453	2.1
33374	1.15471	2.1
33375	0.993889	2.1
33376	1.78919	2.1
33377	1.77325	2.1
33378	2.78308	2.1
33379	1.89226	2.1
33380	1.89226	2.1
33381	1.89226	2.1
33382	1.89226	2.1
33383	3.78453	2.1
33384	1.78919	2.1
33385	1.77325	2.1
33386	2.45969	2.1
33387	2.70835	2.1
33388	4.49753	2.1
33389	0.939314	2.1
33390	0.939315	2.1
33391	1.01133	2.1
33392	1.01133	2.1
33393	0.596471	2.1
33394	1.20813	2.1
33395	0.886619	2.1
33396	1.35417	2.1
33397	1.35417	2.1
33398	1.20813	2.1
33399	0.886621	2.1
33400	0.59647	2.1
33401	0.946131	2.1
33402	0.946131	2.1
33403	0.94613	2.1
33404	0.94613	2.1
33405	0.946133	2.1

33406	0.946133	2.1
33407	0.946128	2.1
33408	0.946128	2.1
33409	0.946131	2.1
33410	0.946131	2.1
33411	0.946131	2.1
33412	0.946131	2.1
33413	1.03939	2.1
33414	1.03939	2.1
33415	0.946132	2.1
33416	0.946132	2.1
33417	0.946132	2.1
33418	0.946132	2.1
33419	1.23851	2.1
33420	1.03939	2.1
33421	1.03939	2.1
33422	0.946132	2.1
33423	0.946133	2.1
33424	1.23851	2.1
33425	0.902503	2.1
33426	0.946129	2.1
33427	0.946132	2.1
33428	0.946132	2.1
33429	0.902472	2.1
33430	0.902472	2.1
33431	1.08736	2.1
33432	0.994093	2.1
33433	0.946133	2.1
33434	0.902504	2.1
33435	1.03939	2.1
33436	0.946132	2.1
33437	1.35306	1.21456
33438	1.29982	1.10404
33439	1.7216	1.02049
33440	1.89183	1.05323
33441	3.07466	1.05315
33442	0.485098	1.07797
33443	0.582401	1.1014
33444	0.649908	1.15362
33445	0.8608	1.08872
33446	0.587739	0.990875
33447	0.58774	1.1807
33448	0.644567	1.07849
33449	0.644567	1.23125
33450	0.58241	1.09285
33451	0.86081	1.00758
33452	0.649898	1.18419
33453	0.485093	1.10116
33454	2.25936	2.1
33455	2.71441	2.1
33456	2.63824	2.1
33457	2.20652	2.1
33458	4.92092	2.1
33459	2.25936	2.1
33460	1.57627	2.1
33461	1.5493	2.1
33462	2.20652	2.1
33463	3.78279	2.1

33464	2.63824	2.1
33465	2.20652	2.1
33466	2.17326	2.1
33467	2.58517	2.1
33468	4.79169	2.1
33469	2.17326	2.1
33470	2.20652	2.1
33471	2.19278	2.1
33472	1.53556	2.1
33473	1.5493	2.1
33474	1.5493	2.1
33475	1.97465	2.1
33476	2.13925	2.1
33477	2.04483	2.1
33478	1.62784	2.1
33479	3.76709	2.1
33480	1.89226	2.1
33481	1.89226	2.1
33482	1.89226	2.1
33483	1.89226	2.1
33484	1.89226	2.1
33485	1.89226	2.1
33486	0.724037	2.1
33487	0.81392	2.1
33488	0.96796	2.1
33489	1.02242	2.1
33490	0.882091	2.1
33491	0.882091	2.1
33492	0.899789	2.1
33493	0.899789	2.1
33494	0.724036	2.1
33495	0.96796	2.1
33496	1.02242	2.1
33497	0.81392	2.1
33498	1.13537	2.1
33499	1.63054	2.1
33500	0.946131	2.1
33501	0.946131	2.1
33502	0.946131	2.1
33503	0.946131	2.1
33504	1.258	2.1
33505	1.63054	2.1
33506	1.22632	2.1
33507	1.22632	2.1
33508	0.946131	2.1
33509	1.258	2.1
33510	1.89226	2.1
33511	1.89226	2.1
33512	1.89226	2.1
33513	1.89226	2.1
33514	1.89226	2.1
33515	1.89226	2.1
33516	0.809739	2.1
33517	0.862674	2.1
33518	1.1995	2.1
33519	1.33899	2.1
33520	1.06324	2.1
33521	1.06324	2.1

33522	0.981932	2.1
33523	1.19563	2.1
33524	0.809727	2.1
33525	1.14092	2.1
33526	1.33902	2.1
33527	0.863494	2.1
33528	2.23742	2.1
33529	1.84902	2.1
33530	1.88088	2.1
33531	2.20392	2.1
33532	4.08644	2.1
33533	1.3592	2.1
33534	1.84902	2.1
33535	1.88088	2.1
33536	1.3305	2.1
33537	1.88088	2.1
33538	1.3305	2.1
33539	1.84902	2.1
33540	1.15008	1.00193
33541	1.3305	2.1
33542	1.70539	2.1
33543	2.9991	2.1
33544	1.84901	2.1
33545	2.0264	2.1
33546	2.20391	2.1
33547	1.70539	2.1
33548	3.87541	2.1
33549	1.03852	2.1
33550	1.38247	1.43659
33551	1.36635	1.58571
33552	1.01537	2.1
33553	2.39784	2.07138
33554	2.0815	2.1
33555	1.91744	2.1
33556	1.91744	2.1
33557	2.06496	2.1
33558	1.89227	2.1
33559	2.03474	2.1
33560	1.89226	1.79009
33561	1.89226	2.1
33562	1.89226	1.79883
33563	1.89226	2.1
33564	1.89226	1.18414
33565	1.89226	2.1
33566	1.36635	1.79267
33567	1.02923	2.1
33568	1.02923	1.96032
33569	1.01537	2.1
33570	1.36416	1.61494
33571	1.3503	2.1
33572	1.89226	2.1
33573	1.89359	2.1
33574	1.89359	2.1
33575	1.91744	2.1
33576	1.89226	2.1
33577	1.91611	2.1
33578	0.946128	2.1
33579	0.946128	2.1

33580	0.946133	1.81115
33581	0.946133	1.45637
33582	0.94613	1.30519
33583	0.94613	2.1
33584	0.946131	2.1
33585	0.946131	1.6294
33586	0.946131	2.1
33587	0.946131	2.1
33588	0.946131	2.1
33589	0.946131	2.1
33590	2.689	2.1
33591	2.0815	2.1
33592	2.72786	2.1
33593	2.48295	2.1
33594	5.02397	2.1
33595	2.72786	2.1
33596	1.86424	2.1
33597	2.7188	2.1
33598	2.08981	2.1
33599	4.81768	2.1
33600	2.08982	2.1
33601	0.986987	2.1
33602	2.08982	2.1
33603	1.87249	2.1
33604	1.01964	2.1
33605	0.906115	2.1
33606	1.44247	2.1
33607	1.43872	2.1
33608	1.17054	2.1
33609	1.17054	2.1
33610	1.43872	2.1
33611	1.26045	2.1
33612	2.0815	0.05
33613	2.0815	0.05
33614	2.0815	0.05
33615	1.81468	0.05
33616	1.89065	0.05
33617	1.55625	0.05
33618	1.89062	0.05
33619	1.89224	0.05
33620	1.89224	0.05
33621	2.27073	0.05
33622	1.86856	0.05
33623	2.24232	0.05
33624	1.21419	2.1
33625	2.79716	2.1
33626	2.50005	2.1
33627	2.50005	2.1
33628	1.5008	2.1
33629	1.5008	2.1
33630	1.60139	0.05
33631	1.7103	0.05
33632	1.04159	0.05
33633	1.2489	0.05
33634	2.75189	0.05
33635	0.732812	0.05
33636	0.732812	0.05
33637	0.751986	0.05

33638	0.751986	0.05
33639	0.860346	0.05
33640	0.667961	0.05
33641	0.85515	0.05
33642	0.624452	0.05
33643	0.62445	0.05
33644	0.667962	0.05
33645	0.855148	0.05
33646	0.860348	0.05
33647	1.89226	0.129513
33648	2.50872	0.123123
33649	2.77909	0.120465
33650	1.89226	0.146609
33651	2.77909	0.109978
33652	1.89226	0.138511
33653	2.15825	0.129308
33654	2.50872	0.118459
33655	1.90439	0.100191
33656	1.90439	0.145201
33657	2.77909	0.100402
33658	2.77909	0.141908
33659	1.12468	2.1
33660	1.43872	2.1
33661	1.64926	2.1
33662	1.2724	2.1
33663	2.77395	2.1
33664	1.64926	2.1
33665	1.38438	2.1
33666	1.47244	2.1
33667	1.2724	2.1
33668	1.8493	2.1
33669	1.64926	2.1
33670	1.36416	1.90265
33671	1.0652	2.1
33672	1.0652	2.00143
33673	1.02923	2.1
33674	1.37496	1.82264
33675	1.33898	2.1
33676	1.89227	2.1
33677	1.90433	2.1
33678	1.90433	2.1
33679	1.89359	2.1
33680	1.89227	2.1
33681	1.88153	2.1
33682	0.946129	2.1
33683	0.946129	2.1
33684	0.946133	1.92015
33685	0.946133	1.83356
33686	0.946131	1.77998
33687	0.946131	2.1
33688	0.946131	2.1
33689	0.946131	1.7495
33690	0.946131	2.1
33691	0.946131	2.1
33692	0.946131	2.07249
33693	0.946131	2.08695
33694	1.37496	1.94333
33695	1.07519	2.1

33696	1.07519	2.04236
33697	1.0652	2.1
33698	1.39725	1.91822
33699	1.38726	2.1
33700	1.89227	2.1
33701	1.9052	2.1
33702	1.9052	2.1
33703	1.90433	2.1
33704	1.89227	2.1
33705	1.8914	2.1
33706	0.946129	2.1
33707	0.946129	2.1
33708	0.946133	1.9684
33709	0.946133	1.88722
33710	0.946131	1.85662
33711	0.946131	2.1
33712	0.946131	2.1
33713	0.946131	1.85126
33714	0.946131	2.1
33715	0.946131	2.1
33716	0.946131	2.09999
33717	0.946131	2.1
33718	1.91316	0.0782742
33719	1.89226	0.0849304
33720	1.89226	0.176725
33721	1.89226	0.0897022
33722	1.99366	0.0826866
33723	1.99366	0.0752205
33724	1.93083	0.0678377
33725	1.89226	0.160738
33726	1.51379	0.0688732
33727	1.89226	0.0600415
33728	1.53051	0.0936077
33729	1.91316	0.0635395
33730	2.16731	2.1
33731	1.21373	2.1
33732	1.47804	2.1
33733	2.37601	2.1
33734	3.64535	2.1
33735	1.7211	2.1
33736	2.37601	2.1
33737	1.47804	2.1
33738	1.47804	2.1
33739	2.56843	2.1
33740	2.56843	2.1
33741	2.58731	2.1
33742	1.59094	2.1
33743	1.59094	2.1
33744	1.76816	2.1
33745	2.41707	2.1
33746	2.5943	2.1
33747	0.718346	2.1
33748	0.795468	2.1
33749	1.20854	2.1
33750	1.1224	2.1
33751	0.961203	2.1
33752	0.961203	2.1
33753	0.95667	2.1

33754	0.95667	2.1
33755	0.718346	2.1
33756	1.1224	2.1
33757	0.795469	2.1
33758	1.20854	2.1
33759	1.84076	2.1
33760	1.42884	2.1
33761	2.24274	2.1
33762	1.85415	2.1
33763	3.69491	2.1
33764	1.89226	2.1
33765	1.89226	2.1
33766	1.89226	2.1
33767	1.89226	2.1
33768	3.78452	2.1
33769	2.24273	2.1
33770	2.24412	2.1
33771	1.85415	2.1
33772	1.85415	2.1
33773	2.68473	2.1
33774	2.68473	2.1
33775	1.89226	2.1
33776	1.89226	2.1
33777	1.89226	2.1
33778	1.89226	2.1
33779	1.89226	2.1
33780	1.89226	2.1
33781	1.89226	2.1
33782	1.89226	2.1
33783	1.89226	2.1
33784	1.89226	2.1
33785	3.78451	2.1
33786	1.85415	2.1
33787	1.10961	2.1
33788	2.24411	2.1
33789	1.51067	2.1
33790	3.35373	2.1
33791	1.70302	2.1
33792	1.89226	2.1
33793	1.76399	2.1
33794	1.43577	2.1
33795	2.08149	2.1
33796	1.70302	2.1
33797	1.89226	2.1
33798	1.89226	2.1
33799	1.89226	2.1
33800	1.89226	2.1
33801	1.89226	2.1
33802	1.89226	2.1
33803	1.0593	2.1
33804	1.17289	2.1
33805	1.5379	2.1
33806	1.46757	2.1
33807	2.71079	2.1
33808	0.931235	2.1
33809	1.1151	2.1
33810	0.975236	2.1
33811	0.975236	2.1

33812	1.31147	2.1
33813	0.762882	2.1
33814	1.13152	2.1
33815	0.505799	2.1
33816	1.31149	2.1
33817	1.07629	2.1
33818	0.884817	2.1
33819	0.505793	2.1
33820	2.69027	2.1
33821	1.60441	2.1
33822	2.69027	2.1
33823	1.64771	2.1
33824	2.78977	2.1
33825	1.72731	2.1
33826	0.77878	0.05
33827	0.843226	0.05
33828	0.845997	0.05
33829	0.926091	0.05
33830	0.77878	0.05
33831	0.926093	0.05
33832	1.3922	1.72495
33833	1.3922	1.72106
33834	0.872156	1.95791
33835	0.872156	1.88858
33836	0.872157	2.01632
33837	0.946132	1.6804
33838	0.946129	1.64233
33839	1.31822	2.06899
33840	0.94613	1.82948
33841	0.94613	1.87171
33842	1.31823	1.83137
33843	0.872157	1.91021
33844	1.81534	0.729923
33845	1.89226	0.421803
33846	1.59602	0.754812
33847	1.59602	1.04435
33848	1.89226	0.303443
33849	1.89226	0.375026
33850	1.89227	0.328977
33851	1.89227	0.471308
33852	1.89227	0.506072
33853	1.89227	0.331563
33854	3.78453	0.34175
33855	1.89226	0.431301
33856	1.89226	0.233118
33857	1.89226	0.309213
33858	1.89226	0.394723
33859	3.78453	0.423105
33860	2.30348	0.150818
33861	1.4185	0.193498
33862	1.82551	0.225141
33863	1.02011	0.164919
33864	3.24401	0.166989
33865	2.46702	0.145413
33866	1.17129	0.196776
33867	1.82551	0.159924
33868	1.82551	0.171283
33869	1.73525	0.156231

33870	1.73525	0.184802
33871	1.89227	0.37758
33872	1.89227	0.263071
33873	1.89227	0.271312
33874	1.89227	0.297282
33875	1.89227	0.340763
33876	1.89227	0.365277
33877	2.80034	2.1
33878	2.45767	2.1
33879	2.67994	2.1
33880	2.35395	2.1
33881	5.1376	2.1
33882	2.45766	2.1
33883	1.8769	2.1
33884	2.00989	2.1
33885	2.00989	2.1
33886	2.35395	2.1
33887	2.35395	2.1
33888	2.57662	2.1
33889	2.35395	2.1
33890	2.57662	2.1
33891	2.67993	2.1
33892	2.24227	2.1
33893	2.34558	2.1
33894	1.02874	2.1
33895	1.02874	2.1
33896	1.03084	2.1
33897	1.03084	2.1
33898	0.938448	2.1
33899	0.886762	2.1
33900	1.17698	2.1
33901	1.12114	2.1
33902	1.12113	2.1
33903	0.886764	2.1
33904	0.93845	2.1
33905	1.17697	2.1
33906	1.91969	2.1
33907	2.5508	2.1
33908	1.88273	1.82622
33909	2.76609	2.1
33910	4.68578	2.1
33911	2.83423	2.1
33912	1.89226	2.1
33913	2.76609	2.1
33914	1.89226	2.1
33915	4.65835	2.1
33916	1.56685	0.171083
33917	1.11712	0.290016
33918	1.07999	1.41047
33919	1.51448	0.526579
33920	2.6316	1.06605
33921	1.56685	0.0731775
33922	1.67652	0.636369
33923	1.5955	1.37282
33924	1.51448	0.363187
33925	3.19099	0.385755
33926	1.89226	1.33324
33927	1.89226	0.248815

33928	1.89226	0.341323
33929	1.89226	1.61785
33930	3.78453	1.28102
33931	1.89226	0.453318
33932	1.89226	0.339862
33933	1.89226	1.36623
33934	1.89226	1.52997
33935	1.89226	0.17264
33936	1.89226	0.283656
33937	1.0562	0.942623
33938	1.51448	0.188095
33939	1.07999	0.484231
33940	1.07999	0.183118
33941	1.46441	1.2202
33942	1.46441	0.152381
33943	1.5955	1.13907
33944	1.51448	0.177203
33945	1.46441	0.442808
33946	1.52909	1.37511
33947	3.04357	0.625818
33948	1.89226	0.234774
33949	1.89226	1.50918
33950	1.89226	1.45257
33951	1.89226	0.384708
33952	3.78453	0.470267
33953	1.89227	0.250911
33954	1.89227	0.21619
33955	1.89227	1.2134
33956	1.89227	1.11402
33957	3.78453	0.915247
33958	1.89994	1.0521
33959	1.70303	0.874546
33960	1.68576	0.262219
33961	1.89608	0.32105
33962	3.8237	0.530796
33963	1.89994	1.63175
33964	2.31116	2.1
33965	1.9386	1.07369
33966	1.88653	1.79378
33967	3.74885	1.70159
33968	2.31117	2.1
33969	2.20665	1.97913
33970	2.09617	1.95084
33971	2.41055	2.1
33972	2.09617	1.93738
33973	2.41055	2.1
33974	1.10777	0.636081
33975	1.0523	0.873098
33976	0.912098	0.727701
33977	0.757533	0.666512
33978	0.89287	1.5995
33979	1.07571	0.294417
33980	0.77485	0.806924
33981	0.98645	0.405629
33982	0.989171	0.250689
33983	0.875089	0.318549
33984	0.979686	0.754089
33985	0.979686	1.36983

33986	2.52442	2.1
33987	1.49998	2.1
33988	2.51692	2.1
33989	1.56957	2.1
33990	4.09399	2.1
33991	1.01298	2.1
33992	1.01298	2.1
33993	1.03079	2.1
33994	1.03079	2.1
33995	0.820964	2.1
33996	0.784786	2.1
33997	1.25898	2.1
33998	1.25846	2.1
33999	1.25898	2.1
34000	0.820964	2.1
34001	1.25846	2.1
34002	0.784786	2.1
34003	1.89226	0.05
34004	1.89226	0.05
34005	2.6297	0.05
34006	1.89226	0.05
34007	3.78452	0.05
34008	1.89226	0.05
34009	1.89226	0.05
34010	1.89226	0.05
34011	1.89226	0.05
34012	1.89226	0.05
34013	1.89226	0.05
34014	2.19397	0.05
34015	1.89226	0.05
34016	2.34112	0.05
34017	2.34112	0.05
34018	1.89226	0.05
34019	1.89226	0.05
34020	2.23366	2.1
34021	1.87598	2.1
34022	1.64716	2.1
34023	2.06048	2.1
34024	3.93645	2.1
34025	2.23367	2.1
34026	2.17384	2.1
34027	2.04818	2.1
34028	2.06048	2.1
34029	4.23432	2.1
34030	1.9855	2.1
34031	2.06048	2.1
34032	1.93163	2.1
34033	1.50737	2.1
34034	3.56785	2.1
34035	2.04818	2.1
34036	2.06048	2.1
34037	2.27465	2.1
34038	1.92264	2.1
34039	3.98312	2.1
34040	2.08149	0.623674
34041	1.89226	1.61601
34042	1.89226	1.51656
34043	1.33153	0.564524

34044	1.68038	1.70159
34045	1.18244	0.817453
34046	1.89226	1.27276
34047	1.89226	1.63771
34048	1.89226	1.55389
34049	1.89226	0.847503
34050	3.78452	1.33914
34051	2.69069	0.485197
34052	1.89226	0.633396
34053	1.89226	0.616543
34054	1.97397	0.510677
34055	1.89226	0.615219
34056	1.97397	0.494876
34057	1.89226	1.63111
34058	2.38056	0.887578
34059	1.89226	0.958398
34060	1.89226	1.36982
34061	3.78452	1.30459
34062	0.904435	2.1
34063	0.904435	2.1
34064	0.925315	2.1
34065	0.925315	2.1
34066	1.13312	2.1
34067	1.08109	2.1
34068	0.70697	2.1
34069	0.696633	2.1
34070	0.70697	2.1
34071	1.13312	2.1
34072	0.696633	2.1
34073	1.08109	2.1
34074	1.45506	2.1
34075	2.26623	2.1
34076	2.38363	2.1
34077	2.38363	2.1
34078	1.41394	2.1
34079	1.41394	2.1
34080	0.946131	2.1
34081	0.946131	2.1
34082	1.13311	2.1
34083	1.13311	2.1
34084	0.946127	2.1
34085	0.946127	2.1
34086	1.13312	2.1
34087	1.19182	2.1
34088	1.19181	2.1
34089	0.946129	2.1
34090	1.13312	2.1
34091	0.94613	2.1
34092	1.89226	0.786797
34093	1.89226	1.93511
34094	1.89226	0.860081
34095	1.89226	1.98177
34096	1.89226	1.57915
34097	1.89226	1.99228
34098	1.05168	2.1
34099	0.696527	2.1
34100	0.907442	1.75181
34101	0.907442	1.90726

34102	0.953145	1.5989
34103	0.782285	2.1
34104	0.773928	2.1
34105	0.773928	1.81125
34106	0.696538	1.88628
34107	0.851554	1.87582
34108	1.04071	1.93278
34109	1.12335	1.93114
34110	1.89226	1.12415
34111	1.89226	0.258889
34112	1.89226	0.557945
34113	1.89226	0.201073
34114	3.78453	0.210061
34115	1.89226	1.06771
34116	1.89226	0.239029
34117	1.89226	0.716159
34118	1.89226	0.195455
34119	3.78453	0.920954
34120	2.50912	0.20073
34121	1.89226	0.139755
34122	1.89226	0.214072
34123	2.63195	0.134123
34124	4.40139	0.136626
34125	2.50912	0.227183
34126	1.3598	0.175438
34127	1.70303	0.1912
34128	2.36874	0.120658
34129	3.82055	0.211758
34130	1.89227	0.256871
34131	1.89227	0.180334
34132	1.89227	0.197124
34133	1.89227	0.121384
34134	3.78453	0.265433
34135	1.89227	0.295883
34136	1.89227	0.161254
34137	1.89227	0.20256
34138	1.89227	0.12665
34139	3.78453	0.133977
34140	1.89226	0.166291
34141	2.67088	0.0900338
34142	2.63195	0.0913776
34143	2.63195	0.122503
34144	1.89226	0.112986
34145	1.89226	0.173353
34146	1.30202	0.121209
34147	1.28304	0.137436
34148	0.809902	0.124424
34149	0.888801	0.123826
34150	1.13262	0.0892472
34151	0.968276	0.10522
34152	1.0733	0.143856
34153	1.0733	0.134199
34154	1.35419	0.107818
34155	0.801222	0.0793783
34156	0.899732	0.145336
34157	1.23967	0.106135
34158	1.31595	2.1
34159	0.869501	2.1

34160	2.65473	2.1
34161	1.88782	2.1
34162	3.20377	2.1
34163	1.89227	2.1
34164	1.89227	2.1
34165	1.89227	2.1
34166	1.89227	2.1
34167	3.78453	2.1
34168	2.65473	2.1
34169	1.89226	2.1
34170	1.88781	2.1
34171	1.88782	2.1
34172	2.0815	2.1
34173	2.0815	2.1
34174	1.89226	2.1
34175	1.89226	2.1
34176	1.89226	2.1
34177	1.89226	2.1
34178	1.89226	2.1
34179	1.89226	2.1
34180	1.89226	2.1
34181	1.89226	2.1
34182	1.89226	2.1
34183	1.89226	2.1
34184	1.89226	2.1
34185	1.89226	2.1
34186	2.39057	2.1
34187	1.89226	2.1
34188	1.89226	2.04384
34189	1.89226	2.1
34190	2.14243	2.0464
34191	2.14243	2.1
34192	1.89226	0.617318
34193	1.89226	2.1
34194	1.89226	1.65155
34195	1.89226	1.96531
34196	1.89226	1.84624
34197	1.89226	2.1
34198	1.89226	0.836099
34199	1.89226	2.1
34200	1.89226	1.95167
34201	1.89226	2.04144
34202	1.89226	1.77293
34203	1.89226	2.1
34204	2.56323	0.0794146
34205	1.36324	0.0763529
34206	1.05296	0.0699916
34207	2.18048	0.0836892
34208	3.54372	0.0708221
34209	0.946128	2.1
34210	0.946128	2.1
34211	1.08733	2.1
34212	1.08733	2.1
34213	0.946131	2.1
34214	0.946131	2.1
34215	1.09197	2.1
34216	1.08732	2.1
34217	0.946131	2.1

34218	1.09197	2.1
34219	1.08732	2.1
34220	0.946131	2.1
34221	1.35111	1.62109
34222	1.51327	1.54496
34223	1.64493	1.38294
34224	1.31058	1.65696
34225	2.99605	1.69896
34226	1.8124	1.39717
34227	1.51327	1.61866
34228	1.64493	1.62159
34229	1.31358	1.31394
34230	1.8124	1.60747
34231	1.48105	1.36194
34232	1.64493	1.46785
34233	1.31058	1.63323
34234	1.22297	1.55715
34235	1.70101	1.38924
34236	2.8679	1.42159
34237	1.70101	1.49442
34238	1.99297	1.40992
34239	1.64493	1.47066
34240	1.64493	1.26236
34241	1.99297	1.55764
34242	1.8124	1.04961
34243	2.0815	2.1
34244	1.80258	2.1
34245	1.80258	2.1
34246	1.83506	2.1
34247	2.0815	2.1
34248	2.1212	2.1
34249	2.0815	2.1
34250	1.82129	2.1
34251	1.82129	2.1
34252	1.80445	2.1
34253	2.0815	2.1
34254	2.06092	2.1
34255	1.89224	2.1
34256	1.703 2.1	
34257	1.97839	2.1
34258	1.703 2.1	
34259	1.97402	2.1
34260	1.703 2.1	
34261	1.89226	0.166038
34262	1.89226	0.535328
34263	1.89226	0.392335
34264	2.75351	0.168188
34265	3.78453	0.230963
34266	1.89226	0.412374
34267	3.02887	0.560573
34268	2.03149	0.197631
34269	2.03149	0.433377
34270	1.89226	0.211955
34271	1.89226	0.476878
34272	2.17979	2.1
34273	1.65533	2.1
34274	2.57212	2.1
34275	2.20346	2.1

34276	4.22745	2.1
34277	1.89227	2.1
34278	2.20347	2.1
34279	1.89227	2.1
34280	2.57212	2.1
34281	1.89227	2.1
34282	2.57212	2.1
34283	2.05274	2.1
34284	2.16783	2.1
34285	2.24429	2.1
34286	2.38404	2.1
34287	4.41212	2.1
34288	2.16783	2.1
34289	2.05967	2.1
34290	2.38404	2.1
34291	2.24695	2.1
34292	4.41478	2.1
34293	2.24429	2.1
34294	2.38404	2.1
34295	2.51819	2.1
34296	2.60723	2.1
34297	4.85152	2.1
34298	2.38404	2.1
34299	2.24695	2.1
34300	2.60723	2.1
34301	2.45247	2.1
34302	4.85418	2.1
34303	1.89227	2.1
34304	1.89227	2.1
34305	1.89227	2.1
34306	1.89227	2.1
34307	3.78453	2.1
34308	1.89226	2.1
34309	1.89226	2.1
34310	1.89226	2.1
34311	1.89226	2.1
34312	3.78453	2.1
34313	0.618473	2.1
34314	0.619945	2.1
34315	0.797692	2.1
34316	0.770147	2.1
34317	0.68852	2.1
34318	0.68852	2.1
34319	0.701573	2.1
34320	0.701573	2.1
34321	0.619945	2.1
34322	0.797691	2.1
34323	0.618473	2.1
34324	0.770147	2.1
34325	0.797692	2.1
34326	0.770148	2.1
34327	0.694787	2.1
34328	0.653411	2.1
34329	0.722102	2.1
34330	0.722102	2.1
34331	0.729001	2.1
34332	0.729001	2.1
34333	0.797692	2.1

34334	0.694787	2.1
34335	0.770148	2.1
34336	0.653412	2.1
34337	1.89227	2.1
34338	1.89227	2.1
34339	1.89227	2.1
34340	1.89227	2.1
34341	3.78453	2.1
34342	1.89226	2.1
34343	1.89226	2.1
34344	1.89226	2.1
34345	1.89226	2.1
34346	3.78453	2.1
34347	1.23989	2.1
34348	1.59538	2.1
34349	1.66647	2.1
34350	1.26599	2.1
34351	2.90636	2.1
34352	1.66647	2.1
34353	1.38957	2.1
34354	1.47446	2.1
34355	1.40337	2.1
34356	1.66647	2.1
34357	1.59538	2.1
34358	2.80619	2.1
34359	2.0815	2.1
34360	2.0815	2.1
34361	2.0815	2.1
34362	2.73721	2.1
34363	2.74192	2.1
34364	2.73721	2.1
34365	2.0815	2.1
34366	2.0815	2.1
34367	2.0815	2.1
34368	2.689	2.1
34369	2.69166	2.1
34370	2.62369	2.1
34371	2.19717	2.1
34372	2.60405	2.1
34373	2.20566	2.1
34374	4.80122	2.1
34375	2.19717	2.1
34376	1.37392	2.1
34377	2.20566	2.1
34378	1.45408	2.1
34379	3.65125	2.1
34380	2.60405	2.1
34381	2.20566	2.1
34382	2.57466	2.1
34383	2.21715	2.1
34384	4.8212	2.1
34385	2.20566	2.1
34386	1.54249	2.1
34387	1.54249	2.1
34388	1.45408	2.1
34389	2.21715	2.1
34390	2.12874	2.1
34391	1.60647	2.1

34392	1.31346	2.1
34393	1.82649	2.1
34394	1.51067	2.1
34395	3.13995	2.1
34396	1.89226	2.1
34397	1.89226	2.1
34398	1.89226	2.1
34399	1.89226	2.1
34400	3.78452	2.1
34401	1.89226	2.1
34402	2.6096	2.1
34403	2.8067	2.1
34404	2.8067	2.1
34405	1.89226	2.1
34406	1.89226	2.1
34407	1.82649	2.1
34408	1.3596	2.1
34409	2.17708	2.1
34410	1.70302	2.1
34411	3.40243	2.1
34412	1.89226	2.1
34413	1.89226	2.1
34414	1.89226	2.1
34415	1.89226	2.1
34416	3.78452	2.1
34417	2.27067	2.1
34418	2.43549	2.1
34419	2.63979	2.1
34420	2.41828	2.1
34421	2.27067	2.1
34422	2.04916	2.1
34423	1.30047	2.1
34424	2.04916	2.1
34425	2.27068	2.1
34426	1.5586	2.1
34427	3.57115	2.1
34428	2.27068	2.1
34429	1.84214	2.1
34430	1.80224	2.1
34431	1.5586	2.1
34432	2.51432	2.1
34433	2.27068	2.1
34434	1.63719	2.1
34435	2.15714	2.1
34436	2.07821	2.1
34437	1.52204	2.1
34438	3.67919	2.1
34439	2.07821	2.1
34440	1.40118	2.1
34441	1.40118	2.1
34442	1.52204	2.1
34443	1.99632	2.1
34444	2.11718	2.1
34445	0.946131	2.1
34446	0.946131	2.1
34447	1.06957	2.1
34448	1.06957	2.1
34449	0.946132	2.1

34450	0.946132	2.03187
34451	1.411	2.1
34452	1.06957	2.1
34453	0.946132	2.1
34454	1.411	2.1
34455	1.06957	2.1
34456	0.946132	2.1
34457	1.99875	1.18476
34458	2.57079	0.893487
34459	2.59319	1.51769
34460	1.9105	0.548945
34461	4.59194	0.982808
34462	1.99875	1.27404
34463	1.02247	0.250298
34464	1.07017	0.315936
34465	1.15841	0.336464
34466	1.91051	0.321322
34467	1.99875	0.37895
34468	2.59319	1.4614
34469	1.9105	0.609721
34470	2.33812	1.1699
34471	2.59319	1.3125
34472	1.59457	1.17992
34473	1.59457	1.34768
34474	2.50906	1.03055
34475	2.0467	0.523108
34476	2.03357	0.820952
34477	2.53176	0.43035
34478	4.54263	0.923096
34479	1.294	1.08143
34480	1.66636	0.753575
34481	2.03357	0.71211
34482	1.03079	0.49586
34483	3.32757	0.732913
34484	1.68108	0.51595
34485	2.53176	0.395616
34486	2.36417	0.330529
34487	2.71666	0.280231
34488	1.68108	1.05419
34489	2.03357	0.857192
34490	1.89226	0.153966
34491	2.59302	0.164163
34492	2.2727	0.138865
34493	2.2727	0.142019
34494	1.89226	0.175999
34495	1.89226	0.160695
34496	1.89226	0.230841
34497	1.89226	0.194802
34498	1.89226	0.173567
34499	1.89226	0.11628
34500	3.78452	0.2491
34501	1.89225	0.328421
34502	1.89225	0.0863697
34503	1.89225	0.121139
34504	1.89225	0.148397
34505	1.89225	0.133951
34506	1.89225	0.165718
34507	0.934114	0.166068

34508	0.934114	0.107875
34509	0.944544	0.145384
34510	0.944544	0.166355
34511	0.721493	0.102023
34512	0.58215	0.100591
34513	1.2965	0.139847
34514	1.13634	0.107273
34515	1.13635	0.0991603
34516	0.582148	0.0944902
34517	1.29651	0.138461
34518	0.721491	0.11214
34519	1.89226	0.121741
34520	1.89226	0.0897707
34521	1.89226	0.081701
34522	1.89226	0.0680133
34523	3.78452	0.0664049
34524	0.946131	0.437905
34525	0.946131	0.493079
34526	0.946131	0.379571
34527	0.946131	0.491309
34528	0.946131	0.369002
34529	0.946131	0.404057
34530	0.946131	0.496261
34531	0.946131	0.799021
34532	0.946131	0.410444
34533	0.946131	0.428196
34534	0.946131	0.480597
34535	0.946131	0.432948
34536	1.89226	0.05
34537	1.89226	0.05
34538	2.78602	0.05
34539	1.89226	0.05
34540	3.78453	0.05
34541	1.52601	2.1
34542	1.76601	2.1
34543	1.27839	2.1
34544	1.56095	2.1
34545	3.0444	2.1
34546	1.76601	2.1
34547	1.86793	2.1
34548	1.56095	2.1
34549	1.68152	2.1
34550	3.44753	2.1
34551	1.27841	2.1
34552	1.56097	2.1
34553	1.0577	2.1
34554	1.36861	2.1
34555	2.64702	2.1
34556	1.56097	2.1
34557	1.68154	2.1
34558	1.36861	2.1
34559	1.53296	2.1
34560	3.05015	2.1
34561	1.89226	2.1
34562	1.89226	2.1
34563	1.89226	2.1
34564	1.89226	2.1
34565	3.78453	2.1

34566	0.983075	2.1
34567	0.788361	2.1
34568	0.724726	2.1
34569	0.724726	2.1
34570	0.983027	2.1
34571	0.724734	2.1
34572	0.788364	2.1
34573	0.788364	2.1
34574	0.788335	2.1
34575	0.94613	2.1
34576	0.724757	2.1
34577	0.946131	2.1
34578	1.15789	2.1
34579	2.11135	2.1
34580	2.25826	2.1
34581	1.28845	2.1
34582	3.41615	2.1
34583	1.89226	2.1
34584	1.89226	2.1
34585	1.89226	2.1
34586	1.89226	2.1
34587	3.78453	2.1
34588	1.57667	2.1
34589	1.70002	2.1
34590	1.70002	2.1
34591	1.89226	2.1
34592	1.89226	2.1
34593	2.05244	2.1
34594	2.25826	2.1
34595	1.42214	2.1
34596	1.43675	2.1
34597	1.28845	2.1
34598	2.40656	2.1
34599	2.25826	2.1
34600	1.58066	2.1
34601	1.74247	2.1
34602	1.85097	2.1
34603	1.61155	2.1
34604	3.43163	2.1
34605	0.785612	2.1
34606	0.789638	2.1
34607	0.882561	2.1
34608	0.763529	2.1
34609	0.686921	2.1
34610	0.86048	2.1
34611	0.871812	2.1
34612	0.871812	2.1
34613	0.830183	2.1
34614	0.882572	2.1
34615	0.902315	2.1
34616	0.785602	2.1
34617	1.01972	0.05
34618	0.936657	0.05
34619	0.946131	0.05
34620	0.946131	0.05
34621	0.927018	0.05
34622	0.927018	0.05
34623	1.1376	0.05

34624	0.953663	0.05
34625	1.1257	0.05
34626	1.04073	0.05
34627	1.00358	0.05
34628	0.851528	0.05
34629	1.73576	0.146782
34630	2.00244	0.316194
34631	1.17798	0.05
34632	1.25796	0.05
34633	2.99373	0.05
34634	2.00244	0.242119
34635	1.10719	0.05
34636	2.03236	0.29251
34637	2.03236	0.388937
34638	1.25797	0.05
34639	1.25797	0.237556
34640	1.89226	2.1
34641	1.89226	2.1
34642	1.89226	2.1
34643	1.89226	2.1
34644	3.78452	2.1
34645	2.3174	2.1
34646	1.50005	2.1
34647	1.90428	2.1
34648	2.64564	2.1
34649	4.22168	2.1
34650	1.89226	2.1
34651	1.89226	2.1
34652	1.89226	2.1
34653	1.89226	2.1
34654	1.89226	2.1
34655	1.89226	2.1
34656	1.32749	0.0524396
34657	1.70302	0.0508519
34658	2.08149	0.05484
34659	1.70302	0.05
34660	2.19634	0.0540399
34661	1.93083	0.0530292
34662	1.89226	1.75002
34663	1.89226	1.5714
34664	1.89226	1.23295
34665	1.89226	1.88286
34666	3.78453	1.47815
34667	1.89226	1.44144
34668	1.89226	1.74069
34669	1.89226	1.3023
34670	1.89226	1.25796
34671	3.78452	1.44733
34672	2.17499	1.17528
34673	1.79663	1.7507
34674	2.1538	1.2398
34675	1.818	1.37047
34676	2.17499	1.59043
34677	1.83919	2.1
34678	0.898316	1.54111
34679	1.0769	1.379
34680	0.968276	2.04355
34681	0.773495	1.77093

34682	0.921126	1.84159
34683	0.921126	1.78536
34684	0.92927	1.54211
34685	0.92927	1.7854
34686	1.0769	1.6504
34687	0.968276	1.61294
34688	0.898316	1.66052
34689	0.773495	1.63445
34690	1.89226	1.36729
34691	1.89226	2.0183
34692	1.89226	1.6706
34693	1.89226	1.39883
34694	3.78453	1.68315
34695	1.89226	1.50182
34696	1.89226	1.96175
34697	1.89226	1.15719
34698	1.89226	1.55985
34699	3.78452	1.30427
34700	2.17499	1.55866
34701	2.1538	2.1
34702	2.53574	1.38574
34703	2.54157	1.20805
34704	4.71073	1.52929
34705	1.0769	1.51606
34706	1.26787	1.39866
34707	1.16641	1.722
34708	0.968276	1.90152
34709	1.11625	1.73229
34710	1.11625	1.67436
34711	1.11989	1.79153
34712	1.11989	1.27777
34713	1.26787	1.54281
34714	1.16641	1.65082
34715	1.0769	1.7746
34716	0.968277	1.70924
34717	1.89226	0.247401
34718	1.89226	0.340962
34719	1.89226	0.344238
34720	1.89226	0.325524
34721	3.78452	0.272876
34722	1.91739	0.194281
34723	2.20045	0.0759202
34724	1.79005	0.0917649
34725	2.33802	0.145733
34726	4.25539	0.147124
34727	2.52754	0.121439
34728	1.89226	0.148824
34729	2.69841	0.12384
34730	2.69841	0.155737
34731	1.89226	0.112811
34732	1.89226	0.132447
34733	1.89226	0.330518
34734	1.89226	0.529121
34735	1.89226	0.256294
34736	1.89226	0.864505
34737	3.78452	0.365524
34738	1.93544	0.1589
34739	2.33802	0.219683

34740	1.55063	0.160974
34741	2.1769	0.094782
34742	3.88865	0.149627
34743	2.20929	0.223976
34744	2.55162	0.151609
34745	2.27807	0.251338
34746	1.48831	0.160389
34747	4.48736	0.169625
34748	2.27807	0.272626
34749	2.72723	0.159816
34750	2.55162	0.210159
34751	2.55162	0.243503
34752	2.43157	0.170661
34753	2.43157	0.21302
34754	2.27807	0.19896
34755	1.51612	0.146385
34756	1.96048	0.142781
34757	1.73022	0.137371
34758	1.70302	0.229033
34759	1.61016	0.212876
34760	2.27807	0.211333
34761	2.08048	0.136216
34762	1.98522	0.150885
34763	2.43157	0.172969
34764	1.83798	0.157749
34765	2.27807	0.184024
34766	1.70302	0.259017
34767	2.68507	0.192792
34768	2.23856	0.218927
34769	2.52174	0.186296
34770	2.35331	0.278358
34771	2.65099	0.235754
34772	1.70303	0.237914
34773	0.986848	0.149809
34774	2.41566	0.152374
34775	1.70303	0.146402
34776	1.88139	0.148811
34777	1.64955	0.148613
34778	1.89226	2.01849
34779	2.34978	1.84912
34780	1.89226	1.97549
34781	2.68773	1.76778
34782	1.89226	2.03457
34783	2.68773	1.82726
34784	1.89226	2.02287
34785	1.89226	1.71577
34786	1.89226	1.68511
34787	1.89226	1.89556
34788	3.78453	1.84673
34789	1.00825	1.46768
34790	1.09847	1.53203
34791	0.939987	1.59058
34792	0.783516	1.7344
34793	0.838041	1.64195
34794	0.838041	1.77665
34795	1.49693	1.846
34796	1.26602	1.65094
34797	1.10875	1.38965

34798	1.05187	1.94808
34799	1.28743	1.67616
34800	0.946132	1.63402
34801	0.946132	1.71897
34802	0.946132	2.0454
34803	0.946132	1.87802
34804	0.946132	1.51283
34805	1.31823	2.03604
34806	0.946132	1.70979
34807	0.946132	1.85196
34808	0.946131	1.89088
34809	0.946131	2.07468
34810	0.946132	1.8187
34811	1.31823	1.89591
34812	1.51688	0.146338
34813	1.79978	0.242151
34814	1.1685	0.153184
34815	1.1685	0.209754
34816	1.85857	0.146257
34817	1.51018	0.236316
34818	0.946116	0.19883
34819	0.946116	0.211725
34820	0.94612	0.122951
34821	0.94612	0.134041
34822	0.946118	0.129303
34823	0.946118	0.197444
34824	0.946118	0.145688
34825	0.946118	0.212853
34826	0.946131	0.157439
34827	0.946131	0.183006
34828	0.946105	0.178646
34829	0.946105	0.176338
34830	1.89224	2.1
34831	1.89224	2.1
34832	1.89224	2.1
34833	1.89224	2.1
34834	1.89224	2.1
34835	1.89224	2.1
34836	1.38678	2.1
34837	1.3349	2.1
34838	0.946119	2.1
34839	0.946119	2.1
34840	1.38678	2.1
34841	1.38678	2.1
34842	0.946119	2.1
34843	0.946119	2.1
34844	1.38676	2.1
34845	0.946107	2.1
34846	0.946132	2.1
34847	1.33491	2.1
34848	1.06455	2.1
34849	1.03932	2.1
34850	1.20649	2.1
34851	1.18773	2.1
34852	2.27105	2.1
34853	1.89226	2.1
34854	1.89226	2.1
34855	1.89226	2.1

34856	1.89226	2.1
34857	1.89226	2.1
34858	1.89226	2.1
34859	1.36918	2.1
34860	1.18774	2.1
34861	1.31144	2.1
34862	1.20651	2.1
34863	1.31144	2.1
34864	1.20651	2.1
34865	2.29066	2.1
34866	1.76476	2.1
34867	1.79825	2.1
34868	2.33801	2.1
34869	4.08891	2.1
34870	1.79825	2.1
34871	2.33801	2.1
34872	2.37168	2.1
34873	1.49325	2.1
34874	4.16993	2.1
34875	1.13641	2.1
34876	1.43213	1.97944
34877	1.44085	2.1
34878	1.18301	2.1
34879	2.61513	2.1
34880	1.89226	2.1
34881	2.23875	2.1
34882	2.23875	2.1
34883	2.10287	2.1
34884	1.89226	2.1
34885	1.75639	2.1
34886	1.89226	2.01822
34887	1.89226	2.1
34888	1.89226	2.06056
34889	1.89226	2.1
34890	1.89226	2.02159
34891	1.89226	2.1
34892	1.44085	2.1
34893	1.25173	2.1
34894	1.25173	2.1
34895	1.18301	2.1
34896	1.4617	2.1
34897	1.39298	2.1
34898	1.89227	2.1
34899	2.23875	2.1
34900	2.42504	2.1
34901	1.89227	2.1
34902	4.13101	2.1
34903	1.89226	2.06053
34904	1.89226	2.1
34905	1.89226	1.99449
34906	1.89226	2.1
34907	1.89226	1.98836
34908	1.89226	2.1
34909	1.89226	2.1
34910	2.41297	2.1
34911	2.70685	2.1
34912	2.70685	2.1
34913	1.89226	2.1

34914	1.89226	2.1
34915	1.89226	2.1
34916	1.89226	2.1
34917	1.89226	2.1
34918	1.89226	2.1
34919	1.89226	2.1
34920	1.89226	2.1
34921	1.89226	2.1
34922	1.89226	2.1
34923	1.89226	2.1
34924	1.89226	2.1
34925	3.78453	2.1
34926	1.89226	2.1
34927	1.89226	2.1
34928	1.89226	2.1
34929	1.89226	2.1
34930	1.89226	2.1
34931	1.89226	2.1
34932	1.69044	0.05
34933	1.57979	0.05
34934	2.27963	0.05
34935	2.21582	0.05
34936	3.85942	0.05
34937	2.21582	0.05
34938	1.30722	0.05
34939	2.02069	0.05
34940	2.02069	0.05
34941	1.57979	0.05
34942	1.57979	0.05
34943	1.67825	2.1
34944	1.89226	2.1
34945	1.89226	2.1
34946	1.89226	2.1
34947	1.91894	2.1
34948	1.91894	2.1
34949	0.946133	2.1
34950	0.946133	2.1
34951	0.946132	2.1
34952	0.946132	2.1
34953	0.946132	2.1
34954	0.946132	2.1
34955	0.946133	2.1
34956	0.946133	2.1
34957	0.946133	2.1
34958	0.946133	2.1
34959	0.946132	2.1
34960	0.946132	2.1
34961	0.942609	2.1
34962	0.942609	2.1
34963	1.329	2.1
34964	1.329	2.1
34965	1.32547	2.1
34966	0.94261	2.1
34967	0.946132	2.1
34968	0.946132	2.1
34969	0.946132	2.1
34970	1.32547	2.1
34971	0.946132	2.1

34972	0.94261	2.1
34973	1.55831	1.87684
34974	1.07496	1.97953
34975	1.80327	1.0842
34976	1.34851	1.16432
34977	2.87824	1.28188
34978	2.14346	0.329943
34979	2.77937	0.444858
34980	2.79173	1.32859
34981	2.17129	0.325736
34982	4.95066	0.352297
34983	2.14346	1.14053
34984	1.34124	0.121559
34985	1.39818	0.253114
34986	2.1713	0.67533
34987	3.51253	0.679524
34988	2.79173	0.834966
34989	2.21882	0.307796
34990	2.17129	0.324524
34991	2.17129	0.439576
34992	2.81448	0.382545
34993	2.81448	0.464901
34994	1.10941	0.285217
34995	1.08565	0.343193
34996	0.699089	0.133186
34997	0.740176	0.24748
34998	0.908594	0.14742
34999	0.908594	0.240231
35000	0.91723	0.87411
35001	0.91723	0.135889
35002	1.10941	0.235307
35003	0.740176	0.23064
35004	0.699089	0.1951
35005	1.08565	0.213143
35006	1.89227	0.0542627
35007	1.89227	0.0784717
35008	1.89227	0.0529557
35009	1.89227	0.05
35010	3.78453	0.0671579
35011	2.40032	0.151983
35012	2.19526	0.0658715
35013	1.12907	0.05
35014	1.44253	0.0976298
35015	3.32433	0.065411
35016	2.19526	0.0534021
35017	2.71093	0.118024
35018	1.29826	0.0665695
35019	2.0815	0.05
35020	4.18911	0.0850744
35021	1.89226	0.0604173
35022	1.89226	0.0644526
35023	1.89226	0.05
35024	1.89226	0.05
35025	3.78452	0.05
35026	2.30822	0.05
35027	1.89226	0.05
35028	2.79329	0.05
35029	2.79329	0.05

35030	1.89226	0.0655335
35031	1.89226	0.0551343
35032	0.946131	2.1
35033	0.946131	1.96448
35034	1.38414	2.1
35035	1.38414	1.89745
35036	0.94613	2.1
35037	0.94613	2.1
35038	1.38415	1.98107
35039	1.41833	1.05398
35040	0.946132	2.1
35041	1.41833	2.1
35042	1.38414	2.1
35043	0.946131	2.1
35044	1.89226	2.1
35045	2.6536	2.1
35046	1.89226	2.1
35047	2.72238	2.1
35048	4.61465	2.1
35049	0.946132	2.1
35050	0.946132	2.1
35051	1.38657	2.1
35052	1.38657	2.1
35053	1.38657	2.1
35054	1.36119	2.1
35055	0.946131	2.1
35056	0.946131	2.1
35057	0.946132	2.1
35058	1.38657	2.1
35059	0.946132	2.1
35060	1.36119	2.1
35061	1.89226	2.1
35062	2.4717	2.1
35063	2.5026	2.1
35064	2.4717	2.1
35065	1.92316	2.1
35066	1.89226	2.1
35067	1.89226	2.1
35068	1.89226	2.1
35069	1.89226	2.1
35070	1.89226	2.1
35071	3.78453	2.1
35072	0.946128	2.1
35073	0.946128	2.1
35074	1.3419	2.1
35075	1.3419	2.1
35076	0.946131	2.1
35077	0.946131	2.1
35078	1.36607	2.1
35079	1.3419	2.1
35080	0.946131	2.1
35081	1.36607	2.1
35082	1.3419	2.1
35083	0.94613	2.1
35084	1.89226	2.1
35085	2.50259	2.1
35086	2.53563	2.1
35087	2.50259	2.1

35088	1.92529	2.1
35089	1.89226	2.1
35090	0.946132	2.1
35091	0.946132	2.1
35092	0.946131	2.1
35093	0.946131	2.1
35094	0.946132	2.1
35095	0.946132	2.1
35096	0.946131	2.1
35097	0.946131	2.1
35098	0.946131	2.1
35099	0.946131	2.1
35100	0.946131	2.1
35101	0.946131	2.1
35102	2.0815	2.1
35103	1.86506	2.1
35104	1.86506	2.1
35105	1.82567	2.1
35106	2.0815	2.1
35107	2.03335	2.1
35108	2.0815	2.1
35109	2.0815	2.1
35110	2.0815	2.1
35111	1.88668	2.1
35112	1.83221	2.1
35113	1.57275	2.1
35114	2.34061	0.05
35115	1.89226	0.05
35116	2.23254	0.05
35117	1.89226	0.05
35118	2.23254	0.05
35119	1.89226	0.05
35120	1.8619	0.05
35121	1.89226	0.05
35122	2.34061	0.05
35123	1.89226	0.05
35124	2.34061	0.05
35125	1.89226	0.05
35126	1.89227	0.05
35127	1.89227	0.05
35128	1.89227	0.05
35129	1.89227	0.05
35130	1.89227	0.05
35131	1.89227	0.05
35132	1.89227	0.05
35133	1.89227	0.05
35134	1.89227	0.05
35135	1.89227	0.05
35136	1.89227	0.05
35137	1.89227	0.05
35138	1.06365	0.05
35139	0.503305	0.05
35140	0.794912	0.05
35141	1.42752	0.05
35142	1.34826	0.05
35143	1.07163	0.05
35144	0.533113	0.05
35145	0.761277	0.05

35146	0.848403	0.05
35147	0.876191	0.05
35148	0.963372	0.05
35149	0.963372	0.05
35150	1.55571	0.05
35151	2.76574	0.05
35152	2.92453	0.05
35153	1.70301	0.05
35154	2.33089	0.05
35155	1.06905	0.05
35156	2.28979	2.1
35157	1.81488	2.1
35158	1.63722	2.1
35159	2.22627	2.1
35160	4.04115	2.1
35161	1.98246	2.1
35162	2.15715	2.1
35163	2.22627	2.1
35164	2.22627	2.1
35165	1.63719	2.1
35166	1.63719	2.1
35167	2.1644	0.05
35168	1.903 0.05	
35169	1.23639	0.05
35170	1.42704	0.05
35171	3.61546	0.05
35172	1.89226	0.05
35173	1.89226	0.05
35174	1.89226	0.05
35175	1.89226	0.05
35176	3.78451	0.05
35177	2.61343	0.05
35178	1.89226	0.05
35179	2.08149	0.05
35180	1.74357	0.05
35181	2.08149	0.05
35182	1.74357	0.05
35183	1.63483	0.05
35184	1.8631	0.05
35185	1.8631	0.05
35186	1.70301	0.05
35187	2.0815	0.05
35188	1.94319	0.05
35189	2.0815	0.05
35190	1.89226	0.05
35191	1.89226	0.05
35192	2.05236	0.05
35193	2.23014	0.05
35194	2.41882	0.05
35195	2.14993	2.1
35196	1.87217	2.1
35197	2.34488	2.1
35198	2.5254	2.1
35199	4.39757	2.1
35200	0.949995	2.1
35201	0.630972	2.1
35202	0.936084	2.1
35203	1.17244	2.1

35204	0.922394	2.1
35205	0.922394	2.1
35206	0.88102	2.1
35207	0.88102	2.1
35208	0.949998	2.1
35209	1.17244	2.1
35210	0.936081	2.1
35211	0.630973	2.1
35212	1.89226	2.1
35213	1.89226	2.1
35214	1.89226	2.1
35215	1.89226	2.1
35216	3.78452	2.1
35217	1.89226	2.1
35218	1.89226	2.1
35219	1.89226	2.1
35220	1.89226	2.1
35221	1.89226	2.1
35222	1.89226	2.1
35223	2.34488	2.1
35224	2.5254	2.09588
35225	1.89226	2.09725
35226	1.89226	2.1
35227	4.41766	2.1
35228	1.89226	2.1
35229	1.89226	2.1
35230	1.89226	2.1
35231	1.89226	2.1
35232	3.78452	2.1
35233	1.89226	2.05305
35234	1.89226	2.1
35235	1.89226	2.06956
35236	1.89226	2.1
35237	1.89226	2.1
35238	1.89226	2.1
35239	1.5605	2.1
35240	1.15806	2.1
35241	1.31565	2.1
35242	1.65642	2.1
35243	2.87615	2.1
35244	1.46643	2.1
35245	1.65642	2.1
35246	1.31565	2.1
35247	1.31565	2.1
35248	1.74246	2.1
35249	1.74246	2.1
35250	1.89226	2.1
35251	1.89226	2.1
35252	1.89226	2.1
35253	1.89226	2.1
35254	1.89226	2.1
35255	1.89226	2.1
35256	1.15099	2.1
35257	1.15099	2.1
35258	0.939316	2.1
35259	0.939316	2.1
35260	0.946127	2.1
35261	0.946127	2.1

35262	1.14418	2.1
35263	0.939289	2.1
35264	1.14417	2.1
35265	0.946129	2.1
35266	0.939287	2.1
35267	0.94613	2.1
35268	1.89226	2.1
35269	1.89226	2.1
35270	1.89226	2.1
35271	1.89226	2.1
35272	3.78452	2.1
35273	1.93164	0.05
35274	2.64898	0.05
35275	1.43213	0.05
35276	1.43213	0.05
35277	2.63206	0.05
35278	2.13254	0.05
35279	1.89226	2.1
35280	1.89226	2.1
35281	1.89226	2.1
35282	1.89226	2.1
35283	3.78452	2.1
35284	2.71655	2.1
35285	1.43323	2.1
35286	1.28413	2.1
35287	2.53614	2.1
35288	3.96937	2.1
35289	1.89226	2.1
35290	1.89226	2.1
35291	1.89226	2.1
35292	1.89226	2.1
35293	1.89226	2.1
35294	1.89226	2.1
35295	1.33356	2.1
35296	2.82297	2.1
35297	2.771	2.1
35298	2.771	2.1
35299	1.46037	2.1
35300	1.46037	2.1
35301	2.56034	2.1
35302	1.18474	2.1
35303	1.15113	2.1
35304	2.53724	2.1
35305	3.72198	2.1
35306	2.56034	2.1
35307	1.89226	2.1
35308	1.89226	2.1
35309	2.53725	2.1
35310	4.42951	2.1
35311	1.89226	2.1
35312	1.89226	2.1
35313	1.89226	2.1
35314	1.89226	2.1
35315	3.78453	2.1
35316	1.89226	2.1
35317	1.89226	2.1
35318	1.89226	2.1
35319	1.89226	2.1

35320	3.78452	2.1
35321	1.1229	2.1
35322	2.53724	2.1
35323	1.15113	2.1
35324	1.15113	2.1
35325	2.51559	2.1
35326	2.51559	2.1
35327	1.89226	2.1
35328	2.53724	2.1
35329	2.51559	2.1
35330	2.53724	2.1
35331	1.87061	2.1
35332	1.89226	2.1
35333	1.89226	2.1
35334	1.89226	2.1
35335	1.89226	2.1
35336	1.89226	2.1
35337	3.78453	2.1
35338	1.89226	2.1
35339	1.89226	2.1
35340	1.89226	2.1
35341	1.89226	2.1
35342	3.78452	2.1
35343	1.98143	1.32173
35344	2.81559	0.249786
35345	1.65411	0.420897
35346	2.26675	1.01736
35347	4.24818	0.566788
35348	0.810656	2.1
35349	0.810656	2.1
35350	0.821888	2.1
35351	0.821888	2.1
35352	1.1235	2.1
35353	1.07439	2.1
35354	0.535693	2.1
35355	0.509042	2.1
35356	0.535693	2.1
35357	1.1235	2.1
35358	0.509043	2.1
35359	1.07439	2.1
35360	1.10378	2.1
35361	2.247	2.1
35362	2.27472	2.1
35363	2.27472	2.1
35364	1.07139	2.1
35365	1.07139	2.1
35366	2.08081	2.1
35367	1.40395	2.1
35368	1.34245	2.1
35369	2.00239	2.1
35370	3.40634	2.1
35371	1.89226	2.1
35372	1.89226	2.1
35373	1.89226	2.1
35374	1.89226	2.1
35375	3.78453	2.1
35376	0.946128	2.1
35377	0.946128	2.1

35378	1.13785	2.1
35379	1.11496	2.1
35380	0.946131	2.1
35381	0.946131	2.1
35382	1.11495	2.1
35383	1.11495	2.1
35384	0.94613	2.1
35385	1.11495	2.1
35386	1.13785	2.1
35387	0.946131	2.1
35388	0.975888	2.1
35389	1.00118	2.1
35390	0.671218	2.1
35391	0.653229	2.1
35392	0.825372	2.1
35393	0.825372	2.1
35394	0.829038	2.1
35395	0.829038	2.1
35396	0.975901	2.1
35397	0.653238	2.1
35398	0.671209	2.1
35399	1.00117	2.1
35400	1.89224	2.1
35401	1.89224	2.1
35402	1.89224	2.1
35403	1.89224	2.1
35404	3.78448	2.1
35405	0.946115	2.1
35406	0.946115	2.1
35407	1.11494	2.1
35408	1.10111	2.1
35409	0.946118	2.1
35410	0.946118	2.1
35411	1.10111	2.1
35412	1.10111	2.1
35413	1.10112	2.1
35414	0.94613	2.1
35415	0.946105	2.1
35416	1.11492	2.1
35417	1.04028	0.490584
35418	2.27126	0.34766
35419	2.0815	0.303614
35420	1.82249	1.47999
35421	1.33723	0.360528
35422	1.18684	1.10182
35423	0.812616	0.0723692
35424	0.812616	0.0674532
35425	0.997777	0.0747621
35426	1.08122	0.0712203
35427	0.94613	0.0828473
35428	0.94613	0.0744824
35429	0.812648	0.0692544
35430	0.932779	0.0682376
35431	0.812648	0.0636274
35432	0.94613	0.0593749
35433	0.849335	0.05
35434	0.94613	0.0614856
35435	1.80327	1.34384

35436	1.56016	1.21739
35437	1.57731	1.87846
35438	1.34851	1.51189
35439	2.03207	1.57875
35440	1.80327	1.60946
35441	2.03207	1.65468
35442	1.56016	1.608
35443	2.18076	1.8001
35444	1.78167	1.26684
35445	3.81374	1.5804
35446	1.37597	2.00695
35447	1.60058	2.1
35448	1.70302	1.35603
35449	1.85448	1.42911
35450	3.15028	1.47189
35451	1.60058	1.60993
35452	1.4054	2.1
35453	1.66202	1.22829
35454	1.70302	2.1
35455	3.09979	1.81172
35456	1.70302	1.74399
35457	1.85448	1.73015
35458	2.21317	1.7078
35459	2.0961	1.34656
35460	4.01757	1.53269
35461	1.66202	1.49529
35462	1.70302	2.1
35463	2.09611	0.746715
35464	2.00892	1.99209
35465	3.6468	1.56679
35466	1.2966	0.05
35467	2.25495	0.05
35468	1.2966	0.05
35469	2.21117	0.05
35470	1.67687	0.05
35471	2.6167	0.05
35472	1.89227	0.0696154
35473	1.89227	0.05
35474	1.89227	0.0518615
35475	1.89227	0.0862311
35476	3.78453	0.0579941
35477	2.44375	0.05
35478	1.89227	0.0611795
35479	2.81853	0.0562068
35480	2.81853	0.0542576
35481	1.89227	0.05
35482	1.89227	0.05
35483	1.89226	1.2899
35484	1.75853	1.37488
35485	2.37636	1.31371
35486	2.37636	1.46598
35487	1.89226	0.540988
35488	1.89226	1.3554
35489	1.65814	1.80088
35490	1.10187	2.1
35491	1.10187	2.1
35492	1.19547	2.1
35493	1.56263	1.97557

35494	1.65624	2.1
35495	1.56264	1.63049
35496	1.01479	2.1
35497	1.01479	2.1
35498	1.10187	2.1
35499	1.47561	1.78955
35500	1.5627	2.1
35501	1.89225	2.1
35502	1.89225	2.1
35503	1.89225	2.1
35504	1.89225	2.1
35505	3.7845	2.1
35506	2.54386	2.1
35507	1.91546	2.1
35508	2.46008	2.1
35509	1.82815	2.1
35510	4.37554	2.1
35511	1.91546	2.1
35512	1.04909	2.1
35513	1.82815	2.1
35514	0.983141	2.1
35515	2.8986	2.1
35516	1.89225	2.1
35517	1.89225	2.1
35518	1.89225	2.1
35519	1.89225	2.1
35520	3.78449	2.1
35521	1.89226	2.1
35522	1.89226	2.1
35523	1.89226	2.1
35524	1.89226	2.1
35525	1.89226	2.1
35526	1.89226	2.1
35527	2.46009	2.1
35528	1.7513	2.1
35529	1.82817	2.1
35530	1.82817	2.1
35531	2.38127	2.1
35532	2.38127	2.1
35533	2.55006	2.1
35534	2.64569	2.1
35535	2.60128	2.1
35536	2.49168	2.1
35537	5.13737	2.1
35538	2.51605	2.1
35539	2.64569	2.1
35540	2.60128	2.1
35541	2.48891	2.1
35542	5.1346	2.1
35543	2.60128	2.1
35544	2.49168	2.1
35545	2.41689	2.1
35546	2.55248	2.1
35547	5.04416	2.1
35548	2.60128	2.1
35549	2.48891	2.1
35550	2.45922	2.1
35551	2.55249	2.1

35552	5.04139	2.1
35553	1.89227	2.1
35554	1.89227	2.1
35555	1.89227	2.1
35556	1.89227	2.1
35557	1.89227	2.1
35558	1.89227	2.1
35559	0.96063	2.1
35560	0.96063	2.1
35561	0.958806	2.1
35562	0.958806	2.1
35563	1.28892	2.1
35564	0.749042	2.1
35565	1.17409	2.1
35566	0.630516	2.1
35567	1.17409	2.1
35568	1.28892	2.1
35569	0.630515	2.1
35570	0.749042	2.1
35571	0.92511	2.1
35572	0.92511	2.1
35573	1.19511	2.1
35574	1.19511	2.06767
35575	0.946132	2.1
35576	1.17409	2.1
35577	0.925081	2.1
35578	0.946129	2.1
35579	0.946131	2.1
35580	0.946131	2.1
35581	1.17409	2.1
35582	0.925083	2.1
35583	1.89226	1.65651
35584	1.89226	1.87502
35585	1.89226	1.63579
35586	1.89226	1.75193
35587	1.89226	1.68206
35588	1.89226	1.79097
35589	1.89226	1.78131
35590	1.89226	2.02967
35591	1.89226	1.87979
35592	1.89226	1.99003
35593	3.78453	1.90526
35594	0.946132	2.1
35595	0.946132	2.1
35596	1.28892	2.1
35597	1.28892	2.1
35598	0.946132	2.1
35599	1.28892	2.1
35600	0.946133	2.1
35601	1.17409	2.1
35602	0.946132	2.1
35603	0.946132	2.1
35604	1.17409	2.1
35605	1.28892	2.1
35606	0.625229	2.1
35607	0.890321	2.1
35608	1.21636	2.1
35609	1.13022	2.1

35610	1.07094	2.1
35611	1.03541	2.1
35612	0.942207	2.1
35613	0.942207	2.1
35614	0.898766	2.1
35615	1.26626	2.1
35616	1.0853	2.1
35617	0.600358	2.1
35618	0.719012	2.1
35619	0.719012	2.1
35620	1.17957	2.1
35621	1.17957	2.1
35622	0.719012	2.1
35623	0.952456	2.1
35624	0.946129	2.1
35625	0.946129	2.1
35626	0.946131	2.1
35627	0.952454	2.1
35628	0.946131	2.1
35629	0.719011	2.1
35630	0.94613	2.1
35631	0.94613	2.1
35632	0.94613	2.1
35633	0.94613	2.1
35634	0.946131	2.1
35635	0.946131	2.1
35636	0.946129	2.1
35637	0.946129	2.1
35638	0.94613	2.1
35639	0.94613	2.1
35640	0.94613	2.1
35641	0.94613	2.1
35642	1.1587	2.1
35643	0.973158	2.1
35644	0.750023	2.1
35645	0.536443	2.1
35646	0.84054	2.1
35647	0.84054	2.1
35648	0.854602	2.1
35649	0.854602	2.1
35650	1.1587	2.1
35651	0.750023	2.1
35652	0.973158	2.1
35653	0.536443	2.1
35654	1.89227	1.22382
35655	1.89227	2.1
35656	1.89227	2.1
35657	1.89227	1.82366
35658	3.78453	2.1
35659	1.89226	2.1
35660	1.89226	2.1
35661	1.89226	2.1
35662	1.89226	2.1
35663	3.78453	2.1
35664	1.7632	2.1
35665	1.61149	2.1
35666	1.98749	2.1
35667	2.11615	2.1

35668	3.75068	2.1
35669	1.7632	2.1
35670	1.61149	2.1
35671	1.97733	1.98634
35672	2.11615	2.1
35673	3.74052	2.1
35674	1.89226	2.1
35675	1.89226	1.64778
35676	1.89226	1.12286
35677	1.89226	2.1
35678	3.78453	2.02427
35679	1.89226	2.1
35680	1.89226	2.1
35681	1.89226	2.1
35682	1.89226	2.1
35683	3.78453	2.1
35684	1.98749	2.1
35685	2.11615	2.1
35686	2.43151	2.1
35687	2.27541	2.1
35688	4.41899	2.1
35689	1.97733	1.87668
35690	2.11615	2.1
35691	2.43151	2.1
35692	2.33986	1.97937
35693	4.40883	2.1
35694	2.17892	0.220981
35695	2.80057	1.76728
35696	1.88349	1.25112
35697	2.63062	0.428254
35698	4.80954	0.491461
35699	1.89226	2.1
35700	1.89226	0.799762
35701	1.89226	2.09137
35702	1.89226	0.32132
35703	3.78452	1.71973
35704	2.0155	2.1
35705	1.97652	0.949344
35706	1.8247	2.05023
35707	1.80645	0.282145
35708	3.80122	0.779448
35709	1.97652	0.41327
35710	1.79681	1.49998
35711	1.80645	0.280159
35712	1.59244	0.498653
35713	3.56896	0.178987
35714	1.89226	0.528141
35715	1.89226	1.62565
35716	1.89226	0.257846
35717	1.89226	0.527465
35718	3.78452	0.122696
35719	1.89223	2.09322
35720	1.89223	0.67774
35721	1.89223	2.09482
35722	1.89223	0.289242
35723	3.78447	0.764009
35724	1.82467	2.05925
35725	1.80643	0.359309

35726	1.55159	2.01145
35727	1.53969	0.223902
35728	3.36437	0.926208
35729	1.80643	0.356627
35730	1.59242	1.38477
35731	1.5397	0.255354
35732	1.40538	0.693496
35733	3.13211	0.221656
35734	1.89224	0.472692
35735	1.89224	1.45109
35736	1.89224	0.292632
35737	1.89224	0.588408
35738	3.78447	0.257299
35739	1.37427	0.05
35740	2.37086	0.0556002
35741	2.37972	0.05
35742	1.94703	0.061157
35743	2.19299	0.05
35744	1.91813	0.0618517
35745	1.07566	2.1
35746	1.12954	2.1
35747	0.946131	2.1
35748	0.946131	2.1
35749	1.12954	2.1
35750	1.12954	2.1
35751	0.946131	2.1
35752	0.946131	2.1
35753	1.12954	2.1
35754	0.946131	2.1
35755	0.946131	2.1
35756	1.07566	2.1
35757	2.25908	2.1
35758	1.89226	2.1
35759	1.89226	2.1
35760	1.89226	2.1
35761	2.37567	2.1
35762	2.37567	2.1
35763	1.37597	1.95159
35764	1.70302	1.48928
35765	1.24162	2.1
35766	1.12219	2.01767
35767	1.37072	2.1
35768	1.70302	2.1
35769	1.37072	2.1
35770	1.70302	1.77932
35771	1.54168	2.1
35772	2.21318	1.73471
35773	3.39238	2.1
35774	1.89226	0.05
35775	1.89226	0.05
35776	1.89226	0.05
35777	1.89226	0.05
35778	3.78452	0.05
35779	1.89227	0.05
35780	1.89227	0.05
35781	1.89227	0.05
35782	1.89227	0.05
35783	3.78453	0.05

35784	1.89226	0.05
35785	1.89226	0.05
35786	1.89226	0.05
35787	1.89226	0.05
35788	3.78453	0.05
35789	1.89226	0.05
35790	2.21654	0.05
35791	2.72007	0.05
35792	2.72007	0.05
35793	1.89226	0.05
35794	1.89226	0.05
35795	0.946128	0.05
35796	0.946128	0.05
35797	0.946132	0.05
35798	0.946132	0.05
35799	0.94613	0.05
35800	0.94613	0.05
35801	0.946131	0.05
35802	0.946131	0.05
35803	0.94613	0.05
35804	0.94613	0.05
35805	0.94613	0.05
35806	0.94613	0.05
35807	1.89226	0.05
35808	1.89226	0.05
35809	1.89226	0.05
35810	1.89226	0.05
35811	3.78453	0.05
35812	1.33457	0.05
35813	0.946131	0.05
35814	1.08045	0.05
35815	0.946132	0.05
35816	1.08045	0.05
35817	1.08045	0.05
35818	0.946132	0.05
35819	0.946132	0.05
35820	1.33457	0.05
35821	1.08045	0.05
35822	0.946132	0.05
35823	0.946132	0.05
35824	1.53347	1.55233
35825	1.13092	2.03212
35826	1.52938	1.81631
35827	1.93568	1.58814
35828	3.06285	1.78052
35829	1.89226	0.638947
35830	2.72959	2.1
35831	2.82996	2.1
35832	2.82996	0.800957
35833	1.89226	2.1
35834	1.89226	0.59243
35835	2.7296	2.1
35836	2.57663	2.1
35837	2.48354	2.1
35838	2.48354	2.1
35839	2.82996	2.1
35840	2.82996	2.1
35841	2.63449	2.1

35842	2.7296	2.1
35843	2.39282	2.1
35844	2.39282	2.1
35845	2.48354	2.1
35846	2.72521	2.1
35847	1.89224	2.1
35848	1.89224	1.88026
35849	1.89224	1.85211
35850	1.89224	2.1
35851	3.78447	1.98695
35852	1.89226	2.1
35853	2.10425	2.1
35854	2.5142	2.1
35855	2.5142	2.1
35856	1.89226	2.1
35857	1.89226	2.1
35858	0.946133	1.68952
35859	0.946133	1.87718
35860	0.946128	2.1
35861	0.946128	2.1
35862	0.946131	2.1
35863	0.946131	1.69256
35864	0.94613	1.68264
35865	0.94613	2.1
35866	0.946131	2.07872
35867	0.946131	2.1
35868	0.946131	2.1
35869	0.946131	2.1
35870	1.89226	1.0295
35871	1.89226	1.41633
35872	1.89226	0.842943
35873	1.89226	1.39809
35874	1.89226	0.796904
35875	1.89226	1.41041
35876	1.94446	1.22578
35877	2.50167	1.53123
35878	1.28418	1.21182
35879	1.94446	1.46482
35880	2.05205	1.39293
35881	2.05205	1.46095
35882	1.27835	2.1
35883	1.08341	2.1
35884	0.946132	2.1
35885	0.946132	2.1
35886	0.851542	2.1
35887	0.851542	2.1
35888	0.977356	2.1
35889	1.18368	2.1
35890	1.18378	2.1
35891	0.851555	2.1
35892	0.977346	2.1
35893	0.851476	2.1
35894	1.07369	2.1
35895	2.5813	2.1
35896	1.95053	2.1
35897	1.95053	2.1
35898	1.90196	2.1
35899	1.90196	2.1

35900	1.89226	2.1
35901	1.89226	2.1
35902	1.89226	2.1
35903	1.89226	2.1
35904	3.78452	2.1
35905	1.89224	2.1
35906	1.89224	2.1
35907	1.89224	2.1
35908	1.89224	2.1
35909	1.89224	2.1
35910	1.89224	2.1
35911	1.97004	0.181082
35912	1.8072	1.72892
35913	1.5866	1.73486
35914	1.79478	0.39556
35915	3.60198	0.913838
35916	1.23267	2.1
35917	1.8072	1.50618
35918	1.58661	0.898985
35919	0.947921	2.1
35920	2.75512	2.08496
35921	1.89226	1.67066
35922	1.89226	2.1
35923	1.89226	1.98706
35924	1.89226	2.1
35925	1.89226	1.80807
35926	1.89226	2.1
35927	1.89226	1.82626
35928	1.89226	0.602879
35929	1.89226	0.522629
35930	1.89226	1.98746
35931	3.78452	1.30984
35932	1.4263	1.86619
35933	1.79478	0.457753
35934	1.67651	0.540999
35935	1.79478	1.89646
35936	1.46834	0.564215
35937	1.5866	1.46313
35938	1.89226	0.601358
35939	1.89226	2.01479
35940	1.89226	1.34154
35941	1.89226	1.66452
35942	1.89226	0.724925
35943	1.89226	1.8793
35944	2.23956	1.23016
35945	1.81847	1.35574
35946	2.10991	1.19084
35947	2.75317	1.15335
35948	4.34947	1.23608
35949	1.60578	0.0894085
35950	1.94974	0.0894821
35951	1.68062	0.124219
35952	1.14721	0.0803461
35953	3.2864	0.0872304
35954	1.3946	0.05
35955	1.692	0.05
35956	2.0154	0.05
35957	2.0154	0.05

35958	1.11926	0.05
35959	1.11926	0.05
35960	1.90347	0.05
35961	1.83626	0.05
35962	1.84583	0.05
35963	1.89434	0.05
35964	3.79781	0.05
35965	1.89226	1.57657
35966	1.89226	1.41379
35967	1.89226	1.33185
35968	1.89226	1.49843
35969	3.78452	1.39875
35970	1.20986	1.58737
35971	1.20986	1.51562
35972	0.912635	1.40101
35973	0.912635	1.45892
35974	0.912667	1.34081
35975	1.17637	1.67996
35976	0.946125	1.53828
35977	0.946125	1.6288
35978	0.946128	1.55348
35979	0.912664	1.43815
35980	0.946127	1.65186
35981	1.17637	1.5207
35982	1.89226	0.05
35983	1.65573	0.05
35984	1.65573	0.05
35985	1.89226	0.05
35986	1.89226	0.05
35987	2.12879	0.05
35988	1.89226	0.05
35989	1.89226	0.05
35990	1.89226	0.05
35991	1.89226	0.05
35992	1.89226	0.05
35993	1.89226	0.05
35994	2.40928	0.05
35995	1.89226	0.05
35996	1.95959	0.05
35997	1.70302	0.05
35998	3.47338	0.05
35999	1.89226	0.05
36000	1.89226	0.05
36001	1.89226	0.05
36002	1.89226	0.05
36003	3.78452	0.05
36004	1.64985	0.0540616
36005	2.47846	0.0935654
36006	2.25405	0.0775717
36007	1.80768	0.0740788
36008	4.06173	0.0621758
36009	1.98846	0.05
36010	0.912729	0.0539852
36011	1.05417	0.05
36012	1.70303	0.0613233
36013	2.82107	0.0600747
36014	2.47846	0.101154
36015	1.70303	0.115286

36016	2.86918	0.126512
36017	2.05973	0.12405
36018	2.51808	0.0988837
36019	1.80768	0.0954575
36020	0.946131	0.101207
36021	0.946131	0.0918421
36022	0.946132	0.0995658
36023	0.946132	0.145754
36024	0.946131	0.157479
36025	0.946131	0.138337
36026	0.946133	0.0979152
36027	0.946133	0.0877307
36028	0.946131	0.120154
36029	0.946131	0.156088
36030	0.946132	0.128293
36031	0.946132	0.137817
36032	0.946133	0.0638124
36033	0.946133	0.0621225
36034	0.946132	0.0955509
36035	0.946132	0.0884222
36036	0.946133	0.0935154
36037	0.946133	0.0886638
36038	0.946132	0.0598694
36039	0.946132	0.0911669
36040	0.946132	0.0697449
36041	0.946132	0.101796
36042	0.946133	0.0550896
36043	0.946133	0.0865222
36044	1.89227	0.161453
36045	1.89227	0.180191
36046	1.89227	0.174811
36047	1.89227	0.147979
36048	1.89227	0.208847
36049	1.89227	0.183535
36050	1.1656	2.1
36051	1.21847	2.1
36052	0.836006	2.1
36053	0.806007	2.1
36054	1.00653	2.1
36055	1.00653	2.1
36056	1.01794	2.1
36057	1.01794	2.1
36058	1.1656	2.1
36059	0.806007	2.1
36060	0.836006	2.1
36061	1.21847	2.1
36062	2.82263	2.1
36063	2.3312	2.1
36064	2.25936	2.1
36065	2.71441	2.1
36066	5.0456	2.1
36067	2.25936	2.1
36068	1.61201	2.1
36069	1.57627	2.1
36070	1.61201	2.1
36071	2.29545	2.1
36072	2.3312	2.1
36073	1.21269	0.05

36074	1.48867	0.05
36075	1.68264	0.05
36076	1.9898	0.05
36077	3.17131	0.05
36078	2.0487	2.1
36079	2.45212	2.1
36080	2.31012	2.1
36081	1.94095	2.1
36082	4.39307	2.1
36083	1.02435	2.1
36084	0.970469	2.1
36085	0.625375	2.1
36086	0.567166	2.1
36087	0.796854	2.1
36088	0.796854	2.1
36089	0.79466	2.1
36090	0.79466	2.1
36091	0.970471	2.1
36092	0.567165	2.1
36093	0.625374	2.1
36094	1.02435	2.1
36095	2.31012	2.1
36096	1.94095	2.1
36097	1.4825	2.1
36098	2.17384	2.1
36099	4.11479	2.1
36100	0.946187	2.1
36101	0.803763	2.1
36102	0.581357	2.1
36103	0.510356	2.1
36104	0.745632	2.1
36105	0.745632	2.1
36106	0.734566	2.1
36107	0.734563	2.1
36108	0.896142	2.1
36109	0.510366	2.1
36110	0.946173	2.1
36111	0.552965	2.1
36112	1.89226	1.37603
36113	1.89226	1.44294
36114	1.89226	1.46638
36115	1.89226	1.56738
36116	1.89226	1.36206
36117	1.89226	1.54449
36118	1.50743	1.43333
36119	1.0417	1.65676
36120	1.50743	1.48576
36121	1.62974	1.33189
36122	1.14605	1.32675
36123	1.14605	1.56782
36124	1.89226	1.28687
36125	1.89226	1.50047
36126	1.89226	1.2288
36127	1.89226	1.5095
36128	1.89226	1.25683
36129	1.89226	1.58602
36130	1.89226	1.60057
36131	1.89226	1.45956

36132	1.89226	1.37075
36133	1.89226	1.50908
36134	3.78453	1.44851
36135	1.77186	1.5021
36136	1.35668	1.52929
36137	1.70254	1.41945
36138	1.38756	1.53675
36139	1.77186	1.48648
36140	1.37814	1.65352
36141	0.734857	1.56051
36142	1.00549	1.48338
36143	0.835261	1.30478
36144	1.08357	1.40462
36145	0.97665	1.2961
36146	0.795559	1.99686
36147	0.891385	1.46876
36148	0.891385	1.24629
36149	0.874231	1.3057
36150	1.08356	1.31427
36151	0.734863	1.42815
36152	0.79449	1.45474
36153	1.89226	1.46535
36154	1.89226	0.109898
36155	2.42671	0.209178
36156	1.98783	1.46559
36157	4.31897	1.02735
36158	0.579854	1.19943
36159	0.965582	1.21182
36160	0.634403	1.36505
36161	1.17796	0.405478
36162	1.00592	0.155759
36163	0.797259	2.1
36164	0.905246	1.2573
36165	0.905246	0.393341
36166	0.695715	0.260454
36167	0.637106	0.240514
36168	1.24374	1.12434
36169	0.95617	0.666705
36170	2.0815	0.623671
36171	1.60521	1.61731
36172	1.29556	1.2944
36173	2.32205	1.57934
36174	1.28445	1.14542
36175	2.0815	1.53453
36176	2.3085	2.1
36177	2.21147	2.1
36178	2.3085	2.1
36179	2.39282	2.1
36180	1.98899	2.1
36181	2.0733	2.1
36182	2.3275	2.1
36183	1.63471	2.1
36184	1.98899	2.1
36185	1.56801	2.1
36186	3.62369	2.1
36187	2.3085	2.1
36188	1.98899	2.1
36189	2.22575	2.1

36190	1.90502	2.1
36191	4.21353	2.1
36192	1.90502	2.1
36193	1.56801	2.1
36194	1.90502	2.1
36195	1.98899	2.1
36196	1.49733	2.1
36197	1.58129	2.1
36198	1.89226	2.00717
36199	2.6491	2.1
36200	2.49266	2.1
36201	2.6491	2.06099
36202	1.73582	2.1
36203	1.89226	1.73819
36204	1.31854	2.1
36205	2.6491	2.1
36206	1.3909	2.1
36207	1.3909	2.1
36208	2.49266	2.1
36209	2.49266	2.1
36210	1.89226	1.89951
36211	2.49266	2.09994
36212	2.38634	2.1
36213	2.49266	1.91587
36214	1.78595	2.09356
36215	1.89226	1.67468
36216	1.23042	2.1
36217	2.49266	2.1
36218	1.31854	2.1
36219	1.31854	2.1
36220	2.38635	2.1
36221	2.38635	2.1
36222	2.83487	2.1
36223	1.89226	2.1
36224	2.594	2.1
36225	2.594	2.1
36226	1.89226	2.1
36227	1.89226	2.1
36228	1.89226	2.1
36229	1.89226	2.1
36230	1.89226	2.1
36231	1.89226	2.1
36232	3.78452	2.1
36233	1.09518	2.1
36234	1.28158	2.1
36235	1.36844	2.1
36236	1.59972	2.1
36237	2.65003	2.1
36238	1.89226	2.1
36239	1.89226	2.1
36240	1.89226	2.1
36241	1.89226	2.1
36242	3.78452	2.1
36243	1.36844	2.1
36244	1.59972	2.1
36245	1.67352	2.1
36246	1.94291	2.1
36247	3.31135	2.1

36248	1.52938	1.77408
36249	1.93568	1.62194
36250	2.36221	1.44665
36251	1.94723	1.91724
36252	3.89158	1.68026
36253	1.94724	1.63737
36254	1.26593	2.0742
36255	1.75367	1.92821
36256	2.4322	1.71176
36257	3.7009	1.86567
36258	2.36221	1.42289
36259	2.43219	1.77112
36260	1.94723	1.66494
36261	1.94723	1.5544
36262	2.8202	1.66313
36263	2.8202	1.54092
36264	2.51604	2.1
36265	2.18533	2.1
36266	2.17228	2.1
36267	2.4889	2.1
36268	4.67423	2.1
36269	2.17228	2.1
36270	1.62183	2.1
36271	1.62316	2.1
36272	1.62183	2.1
36273	2.18666	2.1
36274	2.18533	2.1
36275	2.17227	2.1
36276	2.4889	2.1
36277	2.45922	2.1
36278	2.15686	2.1
36279	4.64576	2.1
36280	2.17228	2.1
36281	1.62316	2.1
36282	1.62241	2.1
36283	2.15686	2.1
36284	3.78002	2.1
36285	1.89226	1.156
36286	1.89226	1.29541
36287	1.89226	1.01507
36288	1.89226	1.04379
36289	3.78453	1.17426
36290	1.89226	1.13105
36291	1.89226	1.13236
36292	1.89226	1.07583
36293	1.89226	0.365477
36294	3.78452	1.06332
36295	1.89226	1.26554
36296	1.89226	0.636793
36297	1.89226	1.10498
36298	1.89226	1.1177
36299	1.89226	0.995257
36300	1.89226	1.02642
36301	1.89226	1.00204
36302	1.89226	1.15447
36303	2.53413	0.263925
36304	1.89226	0.25466
36305	3.78453	0.745255

36306	1.38048	0.429693
36307	1.38048	0.507949
36308	0.490332	0.454998
36309	0.490332	0.411734
36310	0.924679	0.170936
36311	0.946131	1.30962
36312	0.946131	1.21828
36313	0.490332	0.103088
36314	0.924678	0.195107
36315	0.490332	0.137666
36316	0.946131	0.558978
36317	0.946131	0.393204
36318	1.89227	0.218277
36319	1.89227	0.688765
36320	1.89227	0.323236
36321	1.89227	1.1028
36322	1.89227	0.238009
36323	1.89227	0.544562
36324	1.30803	2.1
36325	2.55151	2.1
36326	1.43935	2.1
36327	1.43935	2.1
36328	2.34922	2.1
36329	2.34922	2.1
36330	0.946131	2.1
36331	0.946131	2.1
36332	1.27575	2.1
36333	1.17461	2.1
36334	0.94613	2.1
36335	0.94613	2.1
36336	1.17461	2.1
36337	1.17461	2.1
36338	0.946131	2.1
36339	1.17461	2.1
36340	0.946132	2.1
36341	1.27575	2.1
36342	1.89226	2.1
36343	1.89226	2.1
36344	1.89226	2.1
36345	1.89226	2.1
36346	1.89226	2.1
36347	1.89226	2.1
36348	0.946133	2.1
36349	0.946133	2.1
36350	0.946132	2.1
36351	0.946132	2.1
36352	0.946133	2.1
36353	0.946133	2.1
36354	0.946132	2.1
36355	0.946132	2.1
36356	0.946132	2.1
36357	0.946132	2.1
36358	0.946133	2.1
36359	0.946133	2.1
36360	1.07043	2.1
36361	1.17461	2.1
36362	0.654016	2.1
36363	0.5829	2.1

36364	0.870481	2.1
36365	0.870481	2.1
36366	0.887027	2.1
36367	0.887027	2.1
36368	1.07043	2.1
36369	0.5829	2.1
36370	0.654016	2.1
36371	1.17461	2.1
36372	1.89226	2.1
36373	1.89226	2.1
36374	1.89226	2.1
36375	1.89226	2.1
36376	3.78453	2.1
36377	1.89226	2.1
36378	1.89226	2.1
36379	1.89226	2.1
36380	1.89226	2.1
36381	3.78453	2.1
36382	1.26929	2.1
36383	2.19313	2.1
36384	1.09847	2.1
36385	1.69916	2.1
36386	2.96844	2.1
36387	1.84124	2.1
36388	2.24937	2.1
36389	1.69915	2.1
36390	1.75691	2.1
36391	3.94852	2.1
36392	1.53082	2.1
36393	2.04422	2.1
36394	1.53082	2.1
36395	1.69915	2.1
36396	1.92911	2.1
36397	2.09744	2.1
36398	1.39263	0.910856
36399	1.0171	0.364753
36400	1.1211	0.598117
36401	1.46482	0.810773
36402	2.48192	0.71155
36403	1.39263	1.20269
36404	1.57962	1.84127
36405	1.65238	2.1
36406	1.46482	1.20891
36407	3.04443	1.38423
36408	1.89226	1.80224
36409	1.89226	0.577106
36410	1.89226	1.00545
36411	1.89226	1.43785
36412	3.78453	1.37587
36413	1.89227	0.36082
36414	1.89227	0.515226
36415	1.89227	0.753538
36416	1.89227	0.390929
36417	3.78453	0.437913
36418	1.28418	0.471241
36419	1.46482	0.631352
36420	1.1211	0.457327
36421	1.1211	0.786636

36422	1.59382	0.4331
36423	1.59382	0.783432
36424	1.76897	1.97216
36425	1.46482	0.799253
36426	1.59382	1.36491
36427	1.46482	1.75064
36428	1.78138	0.958725
36429	1.65238	1.34707
36430	1.89226	0.730895
36431	1.89226	0.994813
36432	1.89226	0.636098
36433	1.89226	1.26859
36434	1.89226	0.673393
36435	1.89226	1.58545
36436	1.89226	0.60067
36437	1.89226	0.323703
36438	1.89226	0.341213
36439	1.89226	0.458773
36440	1.89226	0.420911
36441	1.89226	0.510531
36442	1.96832	2.1
36443	2.40858	2.1
36444	1.75211	2.03957
36445	2.20797	2.1
36446	4.1607	2.1
36447	1.75211	2.1
36448	2.20797	2.1
36449	1.51592	2.1
36450	2.02064	2.1
36451	3.77275	2.1
36452	2.71793	0.572664
36453	2.67099	0.204879
36454	2.55105	0.311897
36455	2.71297	0.160716
36456	5.26898	0.325947
36457	2.36417	0.552886
36458	2.67098	0.258363
36459	2.55104	0.355981
36460	1.83418	0.188922
36461	4.91522	0.244879
36462	2.55105	0.247305
36463	2.71297	0.171449
36464	2.58032	0.201063
36465	2.28922	0.125391
36466	5.13137	0.161179
36467	2.55104	0.298825
36468	2.23787	0.182091
36469	1.67265	0.19515
36470	2.28922	0.1512
36471	4.22369	0.214607
36472	0.851542	1.26264
36473	0.851542	1.25892
36474	0.981419	0.705027
36475	0.854774	0.858795
36476	0.949431	0.800644
36477	0.753763	0.885164
36478	0.946131	1.27014
36479	0.946131	0.918659

36480	1.04073	1.20322
36481	0.981433	0.612196
36482	0.851528	1.27548
36483	0.854828	0.923355
36484	1.89226	1.3321
36485	1.89226	1.27975
36486	1.89226	1.27874
36487	1.89226	1.27434
36488	1.89226	1.31607
36489	1.89226	1.31134
36490	0.814227	0.746739
36491	0.814227	0.899457
36492	0.946117	0.370744
36493	1.07285	0.379415
36494	0.879132	0.356483
36495	0.923293	0.363824
36496	0.946119	0.945496
36497	0.946119	0.563189
36498	0.946131	0.653439
36499	0.814236	0.299231
36500	0.946106	0.882531
36501	0.870405	0.434819
36502	1.89224	1.2832
36503	1.89224	1.17144
36504	1.89224	1.18399
36505	1.89224	1.21044
36506	1.89224	1.26523
36507	1.89224	1.26462
36508	1.29388	1.69849
36509	1.04164	1.80667
36510	1.64834	1.83454
36511	1.82955	1.62596
36512	2.94222	1.78207
36513	1.4934	1.86351
36514	2.41522	1.57279
36515	2.20387	0.761396
36516	2.20387	2.1
36517	1.75765	1.39577
36518	1.75765	2.1
36519	2.41522	1.6511
36520	2.77303	2.1
36521	2.20387	2.1
36522	2.58627	2.1
36523	5.00149	2.1
36524	1.22222	1.92899
36525	2.20387	2.07962
36526	1.96831	0.910138
36527	1.96831	2.1
36528	1.4934	1.51453
36529	1.4934	2.1
36530	1.96832	2.1
36531	2.58627	2.1
36532	2.40858	2.08818
36533	2.40858	2.1
36534	2.20387	2.1
36535	2.20387	2.1
36536	1.5605	2.1
36537	1.53775	2.1

36538	1.55398	2.1
36539	1.45806	2.1
36540	1.65642	2.1
36541	1.5605	2.1
36542	0.768876	2.1
36543	0.79033	2.1
36544	0.828211	2.1
36545	0.871233	2.1
36546	0.803843	2.1
36547	0.803843	2.1
36548	0.814698	2.1
36549	0.814698	2.1
36550	0.79033	2.1
36551	0.871233	2.1
36552	0.768876	2.1
36553	0.828212	2.1
36554	1.80252	2.1
36555	2.09715	2.09581
36556	1.9394	2.1
36557	1.50853	2.1
36558	3.60567	2.1
36559	1.96416	1.8409
36560	2.09715	2.00205
36561	1.88484	2.1
36562	1.57916	1.57472
36563	3.67631	1.99462
36564	1.89226	1.94873
36565	1.89226	0.34269
36566	1.89226	1.58504
36567	1.89226	1.68411
36568	3.78453	1.14738
36569	1.89226	2.01223
36570	1.89226	2.1
36571	1.89226	2.1
36572	1.89226	1.70277
36573	1.89226	2.1
36574	1.89226	0.848735
36575	1.5852	2.1
36576	1.50853	2.1
36577	1.2955	2.1
36578	1.44472	2.02215
36579	2.95325	2.1
36580	1.53065	2.1
36581	1.57916	0.869455
36582	1.44472	1.95107
36583	1.78861	1.9115
36584	1.23527	1.94597
36585	1.23527	1.86715
36586	1.89227	0.791256
36587	1.89227	1.3215
36588	1.89227	1.96393
36589	1.89227	0.81133
36590	3.78453	1.19352
36591	1.89226	2.0601
36592	1.89226	2.0945
36593	1.89226	2.1
36594	1.89226	1.94695
36595	1.89226	2.1

36596	1.89226	1.55154
36597	1.89227	1.2599
36598	1.89227	1.13757
36599	1.89227	1.12025
36600	1.89227	1.13259
36601	3.78453	1.11515
36602	0.943708	1.14438
36603	0.752219	1.01871
36604	0.946132	1.09341
36605	0.946132	1.09635
36606	0.946133	0.810389
36607	0.946133	1.30197
36608	0.752218	1.18118
36609	0.752218	1.15357
36610	0.752218	1.11208
36611	0.946132	1.07512
36612	0.946133	1.16587
36613	0.943708	1.14235
36614	0.943992	2.1
36615	0.943992	2.1
36616	1.23507	2.1
36617	1.48209	2.1
36618	1.0384	2.1
36619	1.47953	2.1
36620	0.946128	2.1
36621	0.946128	2.1
36622	0.946129	2.1
36623	1.23293	2.1
36624	1.0384	2.1
36625	0.946129	2.1
36626	1.89226	2.1
36627	2.46586	2.1
36628	2.80218	2.1
36629	2.80218	2.1
36630	1.89226	2.1
36631	1.89226	2.1
36632	1.32455	2.1
36633	1.89128	2.1
36634	1.32328	2.1
36635	1.97299	2.1
36636	3.29754	2.1
36637	1.89128	2.1
36638	2.4255	2.1
36639	1.973	2.1
36640	2.63807	2.1
36641	4.3985	2.1
36642	1.34008	2.1
36643	1.97299	2.1
36644	2.10766	2.1
36645	2.10766	2.1
36646	1.32328	2.1
36647	1.32328	2.1
36648	2.41055	2.1
36649	2.37491	2.1
36650	2.40754	2.1
36651	2.38023	2.1
36652	4.78546	2.1
36653	2.23743	2.1

36654	2.37491	2.1
36655	2.40754	2.1
36656	2.20392	2.1
36657	4.61234	2.1
36658	2.37491	2.1
36659	2.02641	2.1
36660	2.20392	2.1
36661	2.18943	2.1
36662	4.40132	2.1
36663	2.37491	2.1
36664	2.22558	2.1
36665	2.38023	2.1
36666	2.18943	2.1
36667	4.60049	2.1
36668	1.89226	0.0710588
36669	1.89226	0.133907
36670	1.89226	0.0971834
36671	1.89226	0.0950249
36672	1.89226	0.0902717
36673	1.89226	0.0886522
36674	1.03599	0.0666975
36675	0.706698	0.0621932
36676	1.41141	0.081236
36677	1.17573	0.0695635
36678	1.11957	0.0560829
36679	0.765034	0.0583575
36680	1.05913	0.0575401
36681	1.45054	0.0720111
36682	1.04847	0.0579987
36683	1.04847	0.0722688
36684	1.15323	0.0654746
36685	1.05825	0.053771
36686	1.89226	0.09252
36687	1.89226	0.0989588
36688	1.89226	0.0981436
36689	1.89226	0.087483
36690	1.89226	0.102181
36691	1.89226	0.0920009
36692	2.22575	2.07068
36693	1.70677	2.1
36694	1.90157	2.00592
36695	1.5534	2.1
36696	3.77914	2.1
36697	1.70678	2.1
36698	0.968594	2.1
36699	0.968594	2.1
36700	1.10378	2.1
36701	1.5534	2.1
36702	1.68859	2.1
36703	2.31429	1.88474
36704	1.5534	2.1
36705	1.90103	2.04541
36706	1.41902	2.1
36707	3.45443	2.1
36708	2.53709	0.05
36709	1.209 0.05	
36710	1.05417	0.05
36711	2.6513	0.05

36712	3.70547	0.05
36713	1.02675	0.05
36714	1.16384	0.05
36715	2.22062	0.05
36716	2.48862	0.05
36717	3.38446	0.05
36718	1.89226	0.05
36719	1.89226	0.05
36720	1.89226	0.05
36721	1.89226	0.05
36722	1.89226	0.05
36723	1.89226	0.05
36724	1.89226	1.83969
36725	1.89226	2.1
36726	1.89226	2.1
36727	1.89226	1.90915
36728	1.89226	2.1
36729	1.89226	1.9009
36730	1.89226	1.74788
36731	1.89226	1.97472
36732	1.89226	2.03377
36733	1.89226	1.78957
36734	1.89226	2.00657
36735	1.89226	1.76611
36736	1.89227	1.77718
36737	1.89227	1.98353
36738	1.75032	2.0016
36739	1.75032	1.80454
36740	1.89227	1.97873
36741	1.89227	1.7836
36742	1.89226	0.323099
36743	1.89226	0.461284
36744	1.89226	0.326791
36745	1.89226	0.360667
36746	1.89226	0.378317
36747	1.89226	0.40751
36748	0.946132	0.191167
36749	0.946132	0.186499
36750	0.798215	0.165334
36751	0.848163	0.158667
36752	0.848163	0.186454
36753	0.946131	0.193807
36754	0.946132	0.164314
36755	0.798216	0.157986
36756	0.996105	0.187375
36757	0.996105	0.15084
36758	0.79819	0.165577
36759	0.79819	0.208567
36760	1.07763	0.190788
36761	2.28622	0.177298
36762	2.16109	0.133484
36763	1.17506	0.143481
36764	3.36385	0.158185
36765	1.89226	2.1
36766	1.6663	2.1
36767	1.6663	2.1
36768	1.71706	2.1
36769	1.89226	2.1

36770	1.94302	2.1
36771	1.89226	2.1
36772	1.62801	2.1
36773	1.62801	2.1
36774	1.6663	2.1
36775	1.89226	2.1
36776	1.93055	2.1
36777	1.48734	2.1
36778	1.99609	2.1
36779	1.52245	2.1
36780	2.04137	2.1
36781	3.51854	2.1
36782	1.99609	2.1
36783	2.28563	2.1
36784	2.04136	2.1
36785	2.35019	2.1
36786	4.34628	2.1
36787	1.50714	2.1
36788	2.04137	2.1
36789	2.04945	2.1
36790	2.04945	2.1
36791	1.52245	2.1
36792	1.52245	2.1
36793	2.04136	2.1
36794	2.35019	2.1
36795	2.04945	2.1
36796	2.38681	2.1
36797	4.39964	2.1
36798	2.50924	0.05
36799	1.38305	2.1
36800	2.56918	2.1
36801	1.44535	2.1
36802	3.95222	2.1
36803	1.89226	2.1
36804	1.89226	2.1
36805	1.89226	2.1
36806	1.89226	2.1
36807	3.78453	2.1
36808	1.40615	2.1
36809	1.38851	2.1
36810	0.748607	2.1
36811	0.724468	2.1
36812	1.06362	2.1
36813	1.06362	2.1
36814	1.067 2.1	
36815	1.067 2.1	
36816	1.40615	2.1
36817	0.748607	2.1
36818	0.724468	2.1
36819	1.38851	2.1
36820	2.56918	2.1
36821	1.53377	2.1
36822	1.53706	2.1
36823	1.44535	2.1
36824	2.66088	2.1
36825	2.56918	2.1
36826	1.89226	2.1
36827	1.89226	2.1

36828	1.89226	2.1
36829	1.89226	2.1
36830	3.78453	2.1
36831	1.40239	2.1
36832	1.68466	2.1
36833	1.26091	2.1
36834	1.54641	2.1
36835	2.94881	2.1
36836	1.68466	2.1
36837	1.65324	2.1
36838	1.54641	2.1
36839	1.52268	2.1
36840	3.19965	2.1
36841	1.05776	2.1
36842	1.54641	2.1
36843	1.36595	2.1
36844	1.36595	2.1
36845	1.26091	2.1
36846	1.26091	2.1
36847	1.54641	2.1
36848	1.32697	2.07483
36849	1.52268	2.1
36850	1.52268	2.1
36851	1.36595	2.1
36852	1.36595	2.1
36853	1.18647	1.53411
36854	0.988454	2.08277
36855	1.4867	1.74397
36856	1.63892	1.44355
36857	2.67317	1.87076
36858	1.4867	1.89417
36859	1.63892	1.49064
36860	2.09605	1.37941
36861	1.97645	1.66822
36862	3.58275	1.49604
36863	1.29389	1.80632
36864	1.97645	1.53849
36865	1.4867	1.64501
36866	1.4867	1.8131
36867	1.82955	1.55233
36868	1.82955	1.74484
36869	1.13454	1.48494
36870	0.983594	2.1
36871	1.16809	2.1
36872	1.06836	2.03979
36873	1.08332	2.1
36874	0.983594	1.84339
36875	1.48751	2.1
36876	1.48047	1.82924
36877	1.77596	2.09445
36878	1.75506	1.98273
36879	3.25643	2.0845
36880	1.48047	1.7518
36881	1.25334	1.8509
36882	1.75505	1.90347
36883	1.55831	0.993104
36884	3.03878	1.70742
36885	1.77596	2.1

36886	1.75506	2.1
36887	2.00551	2.1
36888	2.00083	1.6922
36889	3.77679	2.1
36890	1.75506	2.06712
36891	1.80328	1.26152
36892	1.55831	1.81973
36893	1.55831	1.94269
36894	2.00083	1.52595
36895	2.00083	1.70767
36896	2.58627	2.1
36897	2.79047	2.1
36898	2.40858	2.1
36899	2.64748	2.1
36900	5.23375	2.1
36901	1.26755	2.1
36902	1.71717	2.1
36903	1.03824	2.1
36904	1.48901	2.1
36905	2.75656	2.1
36906	1.89226	2.1
36907	1.89226	2.1
36908	1.89226	2.1
36909	1.89226	2.1
36910	1.89226	2.1
36911	1.89226	2.1
36912	1.36541	2.1
36913	1.22156	2.1
36914	0.946132	2.1
36915	0.946132	2.1
36916	1.36541	2.1
36917	1.36541	2.1
36918	0.946132	2.1
36919	0.946132	2.1
36920	1.36541	2.1
36921	0.946133	2.1
36922	0.946132	2.1
36923	1.22156	2.1
36924	1.18901	2.1
36925	1.61149	2.1
36926	1.98748	2.1
36927	1.534 2.1	
36928	3.1765	2.1
36929	1.89226	2.1
36930	1.89226	2.1
36931	1.89226	2.1
36932	1.89226	2.1
36933	3.78452	2.1
36934	1.98748	2.1
36935	1.534 2.1	
36936	2.27541	2.1
36937	1.98748	2.1
36938	1.87436	2.1
36939	1.87436	2.1
36940	2.2988	2.1
36941	2.2604	2.1
36942	2.20831	2.1
36943	2.02802	2.1

36944	4.32683	2.1
36945	2.26041	2.1
36946	1.98957	2.1
36947	2.02803	2.1
36948	1.75021	2.1
36949	4.0176	2.1
36950	2.34607	2.1
36951	2.02803	2.1
36952	1.86795	2.1
36953	1.81448	2.1
36954	3.89597	2.1
36955	2.02803	2.1
36956	1.75021	2.1
36957	1.81448	2.1
36958	1.57887	2.1
36959	3.6069	2.1
36960	0.946131	1.64537
36961	0.946131	2.1
36962	1.1235	1.99875
36963	1.1235	2.1
36964	0.946131	1.81016
36965	0.946131	1.71709
36966	1.1235	2.1
36967	1.13736	2.1
36968	1.13736	2.1
36969	0.946131	2.1
36970	1.1235	2.1
36971	0.946131	2.1
36972	1.89226	2.09397
36973	1.89226	2.1
36974	1.89226	1.55038
36975	1.89226	0.642568
36976	3.78452	1.30129
36977	0.563266	2.1
36978	0.563266	1.75545
36979	0.565606	1.11107
36980	0.565606	2.1
36981	0.647086	2.09252
36982	0.624219	2.03067
36983	0.500035	1.63683
36984	0.481785	1.56528
36985	0.500034	0.633826
36986	0.647087	0.530018
36987	0.624219	1.84556
36988	0.481785	1.70951
36989	0.915788	0.177955
36990	0.915788	0.223843
36991	1.02732	0.549666
36992	1.02732	0.371662
36993	0.660831	0.492947
36994	1.11155	0.182422
36995	0.713295	0.214264
36996	1.26874	0.477473
36997	0.713295	0.3099
36998	0.660831	0.27896
36999	1.11155	0.296044
37000	1.26874	0.28263
37001	1.89226	1.10474

37002	1.85091	0.89573
37003	2.11193	1.35412
37004	1.85091	1.05877
37005	2.15328	0.732613
37006	1.89226	0.597935
37007	0.946131	0.398816
37008	0.946131	0.517201
37009	1.18275	0.511526
37010	1.05596	0.713646
37011	0.946127	0.54761
37012	0.946127	0.294638
37013	1.18275	0.570499
37014	1.18275	0.566105
37015	0.946128	0.522825
37016	1.18275	0.493412
37017	0.946129	0.508853
37018	1.05596	0.502725
37019	1.60936	2.1
37020	2.35846	2.1
37021	2.06424	2.1
37022	2.57651	2.1
37023	4.42269	2.1
37024	1.89226	2.1
37025	1.89226	2.1
37026	1.89226	2.1
37027	1.89226	2.1
37028	3.78453	2.1
37029	1.89226	2.1
37030	1.89226	2.1
37031	1.89226	2.1
37032	1.89226	2.1
37033	1.89226	2.1
37034	1.89226	2.1
37035	1.95053	2.1
37036	2.06424	2.1
37037	1.95053	2.1
37038	1.30584	2.1
37039	2.5	2.1
37040	1.8553	2.1
37041	2.49999	2.1
37042	2.57651	2.1
37043	2.49999	2.1
37044	2.06423	2.1
37045	2.80738	2.1
37046	2.37162	2.1
37047	1.89226	2.1
37048	1.89226	2.1
37049	1.89226	2.1
37050	1.89226	2.1
37051	1.89226	2.1
37052	1.89226	2.1
37053	1.49295	0.0923963
37054	1.03824	0.0979145
37055	1.51396	0.110915
37056	2.08514	0.108129
37057	3.12338	0.10116
37058	1.49798	0.0569824
37059	2.32017	0.05

37060	2.68236	0.0935792
37061	2.08514	0.0817858
37062	4.4053	0.0611112
37063	1.89226	0.05
37064	1.89226	0.0714403
37065	1.89226	0.0749758
37066	1.89226	0.0752562
37067	3.78452	0.0662225
37068	1.89226	0.10806
37069	1.89226	0.118057
37070	1.89226	0.107338
37071	1.89226	0.117477
37072	1.89226	0.102334
37073	1.89226	0.105141
37074	1.51396	0.0653445
37075	2.08514	0.062828
37076	2.55393	0.0794258
37077	2.02901	0.0873544
37078	4.11415	0.06916
37079	1.89226	0.05
37080	1.89226	0.05
37081	1.89226	0.0646748
37082	1.89226	0.0741083
37083	3.78451	0.05
37084	1.89226	0.0626972
37085	1.89226	0.0940102
37086	1.89226	0.0751158
37087	1.89226	0.0635028
37088	1.89226	0.0832634
37089	1.89226	0.0773521
37090	0.946131	0.127436
37091	0.946131	0.146911
37092	0.946132	0.262696
37093	0.946132	0.168502
37094	0.946132	0.192779
37095	0.946132	0.151779
37096	0.946132	0.196056
37097	0.946132	0.246724
37098	0.946132	0.146705
37099	0.946132	0.187361
37100	0.946132	0.198949
37101	0.946132	0.167579
37102	1.89226	2.1
37103	1.89226	2.1
37104	1.89226	2.1
37105	1.89226	2.1
37106	3.78452	2.1
37107	1.28367	2.1
37108	1.82707	2.1
37109	1.11025	2.1
37110	1.6365	2.1
37111	2.92016	2.1
37112	1.82707	2.1
37113	1.8247	2.0614
37114	1.8247	2.1
37115	2.01549	2.1
37116	1.6365	2.1
37117	1.82729	2.1

37118	1.89226	2.1
37119	1.89226	2.08134
37120	1.89226	1.99332
37121	1.89226	2.1
37122	1.89226	2.04447
37123	1.89226	2.1
37124	1.63648	2.1
37125	1.82467	2.09319
37126	1.35877	2.1
37127	1.55159	1.99231
37128	3.18345	2.1
37129	1.89223	2.1
37130	1.89223	2.1
37131	1.89223	2.1
37132	1.89223	2.1
37133	3.78447	2.1
37134	1.59972	2.1
37135	1.28037	2.1
37136	1.63702	2.1
37137	1.28158	2.1
37138	1.63581	2.1
37139	1.28037	2.1
37140	1.89226	2.1
37141	1.89226	2.1
37142	1.89226	2.1
37143	1.89226	2.1
37144	1.89226	2.1
37145	1.89226	2.1
37146	1.89226	2.1
37147	1.89226	2.1
37148	1.89226	2.1
37149	1.89226	2.1
37150	3.78451	2.1
37151	1.28037	2.1
37152	1.05071	2.1
37153	1.63581	2.1
37154	1.44287	2.1
37155	2.72324	2.1
37156	1.59972	2.1
37157	1.63581	2.1
37158	1.94291	2.1
37159	2.00118	2.1
37160	3.57872	2.1
37161	1.89226	2.1
37162	1.89226	2.1
37163	1.89226	2.1
37164	1.89226	2.1
37165	3.78452	2.1
37166	0.946131	2.1
37167	0.946131	2.1
37168	0.946127	2.1
37169	0.946127	2.1
37170	0.94613	2.1
37171	0.94613	2.1
37172	0.946128	2.1
37173	0.946128	2.1
37174	0.946128	2.1
37175	0.946128	2.1

37176	0.946129	2.1
37177	0.946129	2.1
37178	0.865056	2.1
37179	0.865056	2.1
37180	0.873224	2.1
37181	0.873224	2.1
37182	0.721437	2.1
37183	0.920378	2.1
37184	0.817903	2.1
37185	1.00059	2.1
37186	1.00059	2.1
37187	0.920376	2.1
37188	0.817904	2.1
37189	0.721437	2.1
37190	3.09157	2.1
37191	1.71255	2.1
37192	2.0815	2.1
37193	1.9918	2.1
37194	2.51364	2.1
37195	2.39396	2.1
37196	2.0815	2.1
37197	1.05051	2.1
37198	2.56724	2.1
37199	1.63991	2.1
37200	3.47929	2.1
37201	1.89226	0.085986
37202	2.2973	0.178549
37203	1.90705	0.150906
37204	1.90705	0.109822
37205	1.89226	0.129204
37206	1.89226	0.0903652
37207	1.89226	0.0800295
37208	1.90705	0.163749
37209	1.45321	0.156689
37210	1.45321	0.111743
37211	1.89226	0.128089
37212	1.89226	0.0898345
37213	0.901146	2.1
37214	0.901146	2.1
37215	1.15227	2.1
37216	1.21279	2.1
37217	0.991265	2.1
37218	1.15003	2.1
37219	0.946132	2.1
37220	0.946132	2.1
37221	0.946132	2.1
37222	1.09997	2.1
37223	0.991266	2.1
37224	0.946132	2.1
37225	1.89226	2.1
37226	1.89226	2.1
37227	1.89226	2.1
37228	1.89226	2.1
37229	1.89226	2.1
37230	1.89226	2.1
37231	1.62229	2.1
37232	1.06053	2.1
37233	1.19698	2.1

37234	1.70106	2.1
37235	2.81928	2.1
37236	0.946132	2.1
37237	0.946132	2.1
37238	1.17527	2.1
37239	1.09997	2.1
37240	1.09997	2.1
37241	1.09997	2.1
37242	0.946133	2.1
37243	0.946133	2.1
37244	1.17527	2.1
37245	0.946132	2.1
37246	0.946133	2.1
37247	1.09997	2.1
37248	1.89226	2.1
37249	1.89226	2.1
37250	1.89226	2.1
37251	1.89226	2.1
37252	1.89226	2.1
37253	1.89226	2.1
37254	0.905416	2.1
37255	0.850527	2.1
37256	0.660601	2.1
37257	0.598494	2.1
37258	0.750137	2.1
37259	0.750137	2.1
37260	0.753774	2.1
37261	0.753774	2.1
37262	0.660599	2.1
37263	0.905418	2.1
37264	0.598493	2.1
37265	0.850529	2.1
37266	2.43509	2.1
37267	2.65066	2.1
37268	2.66602	2.1
37269	2.46339	2.1
37270	5.10111	2.1
37271	2.53008	2.1
37272	2.65066	2.1
37273	2.66602	2.1
37274	2.52852	2.1
37275	5.1961	2.1
37276	2.66602	2.1
37277	2.46339	2.1
37278	2.47932	2.1
37279	2.67393	2.1
37280	5.14535	2.1
37281	2.66602	2.1
37282	2.52853	2.1
37283	2.51686	2.1
37284	2.67393	2.1
37285	5.18288	2.1
37286	1.89226	2.1
37287	1.89226	0.566118
37288	1.89226	2.08497
37289	1.89226	0.438005
37290	3.78452	1.57967
37291	1.55161	2.1

37292	1.53972	0.50704
37293	1.32259	2.04079
37294	1.35082	0.268708
37295	2.8623	0.834415
37296	1.53972	0.447474
37297	1.4054	1.38735
37298	1.35083	0.278862
37299	1.24913	0.409451
37300	2.78885	0.318242
37301	1.89226	0.517658
37302	1.89226	1.56934
37303	1.89226	0.437766
37304	1.89226	0.390977
37305	3.78452	0.157407
37306	1.89226	2.04894
37307	1.89226	0.902065
37308	1.89226	2.1
37309	1.89226	0.651732
37310	1.89226	2.09316
37311	1.89226	0.972552
37312	1.14599	2.03398
37313	1.35082	0.482427
37314	1.14599	1.26617
37315	1.32259	2.03887
37316	1.19993	0.362582
37317	1.37652	2.05613
37318	1.35083	0.347678
37319	1.24913	1.50586
37320	1.19993	0.520808
37321	1.11787	0.756701
37322	2.44906	0.263265
37323	1.89226	1.06532
37324	1.89226	1.50164
37325	1.89226	0.632877
37326	1.89226	0.362067
37327	3.78452	0.3292
37328	1.51697	0.05
37329	2.57264	0.0524828
37330	1.30203	0.05
37331	2.41701	0.05
37332	3.93398	0.05
37333	1.17464	0.05
37334	2.41701	0.05
37335	2.32462	0.05
37336	2.32462	0.05
37337	1.30203	0.05
37338	1.30203	0.05
37339	2.08149	2.1
37340	1.9839	2.1
37341	1.71398	2.1
37342	1.99182	2.1
37343	3.71121	2.1
37344	2.16061	2.1
37345	2.01399	0.785204
37346	1.99182	2.1
37347	1.83895	2.04958
37348	4.00581	2.09433
37349	1.90444	2.1

37350	1.99182	2.1
37351	1.71717	2.1
37352	1.805	2.1
37353	3.70899	2.1
37354	1.99182	2.1
37355	1.83896	1.54061
37356	1.805	2.1
37357	1.65243	1.59372
37358	3.64425	2.06048
37359	1.18076	2.1
37360	1.27697	2.1
37361	1.05025	2.1
37362	1.27697	2.1
37363	1.25	2.1
37364	1.47671	2.1
37365	1.89226	2.1
37366	1.89226	2.1
37367	1.89226	2.1
37368	1.89226	2.1
37369	3.78452	2.1
37370	2.64124	2.1
37371	1.89226	2.1
37372	1.89226	2.1
37373	1.89226	2.1
37374	2.46904	2.1
37375	2.46904	2.1
37376	1.89226	2.1
37377	1.89226	2.1
37378	2.7408	2.1
37379	2.82858	2.1
37380	4.72084	2.1
37381	1.87875	2.1
37382	1.37704	2.1
37383	1.77042	2.1
37384	2.27801	2.1
37385	3.64916	2.1
37386	1.77042	2.1
37387	2.27801	2.1
37388	2.58523	2.1
37389	1.90178	2.1
37390	4.35565	2.1
37391	1.28667	2.1
37392	1.30711	2.1
37393	1.53077	2.1
37394	1.55635	2.1
37395	2.81743	2.1
37396	1.55635	2.1
37397	1.28228	2.1
37398	1.64268	2.1
37399	1.93673	2.1
37400	3.19903	2.1
37401	1.53077	2.1
37402	1.93673	2.1
37403	1.55635	2.1
37404	1.55635	2.1
37405	1.86206	2.1
37406	1.86206	2.1
37407	1.64892	2.1

37408	1.60532	2.1
37409	1.85282	2.1
37410	1.96716	2.1
37411	3.61608	2.1
37412	1.60531	2.1
37413	1.32414	2.1
37414	1.96716	2.1
37415	1.75823	2.1
37416	3.2913	2.1
37417	1.91219	2.1
37418	1.96716	2.1
37419	2.0815	2.1
37420	2.51396	2.1
37421	4.30843	2.1
37422	1.96716	2.1
37423	2.17979	2.1
37424	1.75823	2.1
37425	1.75823	2.1
37426	2.28541	2.1
37427	2.28541	2.1
37428	1.89226	1.55058
37429	1.89226	1.57541
37430	1.89226	1.56741
37431	1.89226	1.46025
37432	3.78453	1.53213
37433	1.89226	1.61666
37434	1.89226	1.56968
37435	1.89226	1.60888
37436	1.89226	1.56792
37437	1.89226	1.59863
37438	1.89226	1.55828
37439	1.61016	1.56786
37440	1.45303	1.53429
37441	1.62812	1.54143
37442	1.86379	1.51835
37443	3.23828	1.52469
37444	1.61016	1.57267
37445	1.78716	1.57147
37446	2.0815	1.46202
37447	1.6774	1.49892
37448	3.54454	1.53338
37449	1.89226	1.58527
37450	1.89226	1.51183
37451	1.89226	1.46882
37452	1.89226	1.49982
37453	3.78453	1.53372
37454	1.89226	1.63918
37455	1.89226	1.56507
37456	1.89226	1.6521
37457	1.89226	1.63903
37458	1.89226	1.54245
37459	1.89226	1.49821
37460	0.807591	1.61208
37461	0.814059	1.51672
37462	1.00217	1.63816
37463	0.931896	1.57012
37464	0.850586	1.60046
37465	0.850586	1.55516

37466	0.8889	1.54903
37467	0.8889	1.59775
37468	0.807592	1.4511
37469	1.00217	1.57218
37470	0.814061	1.57892
37471	0.931894	1.67312
37472	2.0815	1.5139
37473	1.6774	1.60236
37474	2.00435	1.50573
37475	2.36104	1.49758
37476	3.86809	1.48479
37477	1.00532	0.0755863
37478	1.00532	0.0818777
37479	1.00183	0.100533
37480	1.00183	0.0804116
37481	1.26377	0.0872609
37482	1.34921	0.0753691
37483	0.664872	0.0850639
37484	0.743381	0.0770676
37485	0.664872	0.0851351
37486	1.26377	0.0942748
37487	1.34921	0.0859874
37488	0.743381	0.0886445
37489	1.26826	0.05
37490	0.946131	2.1
37491	1.02404	2.04612
37492	0.94613	2.1
37493	1.30281	2.08837
37494	0.930941	2.1
37495	0.930941	2.1
37496	1.318	2.0866
37497	1.58161	2.06284
37498	0.94613	2.1
37499	0.94613	2.00769
37500	1.56338	2.06704
37501	1.02404	2.05129
37502	2.81374	2.0871
37503	1.89226	2.1
37504	1.89226	2.1
37505	1.89226	2.1
37506	3.78453	2.1
37507	0.946133	2.1
37508	0.946133	2.1
37509	0.946132	2.1
37510	0.946132	2.1
37511	0.946132	2.1
37512	0.946132	2.1
37513	0.946133	2.1
37514	0.946133	2.1
37515	0.946132	2.1
37516	0.946132	2.1
37517	0.946133	2.1
37518	0.946133	2.1
37519	1.89226	1.86034
37520	1.89226	2.1
37521	1.89226	1.91908
37522	1.89226	2.01847
37523	3.78452	2.04006

37524	1.89226	2.1
37525	1.89226	2.08584
37526	1.89226	2.1
37527	1.89226	1.95511
37528	1.89226	2.1
37529	1.89226	1.96166
37530	0.946133	1.99944
37531	0.946133	2.1
37532	0.946132	1.9955
37533	0.946132	2.1
37534	0.946132	2.1
37535	0.946132	2.1
37536	0.946133	2.1
37537	0.946133	2.00617
37538	0.946132	2.1
37539	0.946132	2.04996
37540	0.946132	2.1
37541	0.946132	2.08617
37542	2.56953	2.1
37543	2.64148	2.1
37544	2.48631	2.1
37545	2.62213	2.1
37546	5.19166	2.1
37547	2.64148	2.1
37548	2.57929	2.1
37549	2.62213	2.1
37550	2.59522	2.1
37551	5.20142	2.1
37552	2.48631	2.1
37553	2.62213	2.1
37554	2.41176	2.1
37555	2.59262	2.1
37556	5.03389	2.1
37557	2.62213	2.1
37558	2.59522	2.1
37559	2.59262	2.1
37560	2.61901	2.1
37561	5.24114	2.1
37562	0.946132	0.05
37563	0.946132	0.05
37564	0.946133	0.05
37565	0.946133	0.05
37566	0.946132	0.05
37567	0.946132	0.05
37568	0.946133	0.05
37569	0.946133	0.05
37570	0.946132	0.05
37571	0.946132	0.05
37572	0.946133	0.05
37573	0.946133	0.05
37574	1.5681	0.05
37575	1.01248	0.05
37576	1.10749	0.05
37577	0.570913	0.05
37578	1.15589	0.05
37579	1.15588	0.05
37580	0.977549	0.05
37581	0.977549	0.05

37582	1.01249	0.05
37583	0.654445	0.05
37584	1.36795	0.05
37585	1.10748	0.05
37586	1.73883	0.05
37587	1.87638	0.05
37588	1.85219	0.05
37589	1.93113	2.1
37590	3.7407	0.05
37591	1.87638	0.05
37592	1.73883	2.1
37593	1.93113	2.1
37594	1.80426	2.1
37595	3.66996	2.1
37596	2.02912	0.120009
37597	1.44951	0.108074
37598	1.50103	0.0913599
37599	2.02248	0.104427
37600	3.47199	0.0977397
37601	1.89226	0.102986
37602	1.89226	0.122174
37603	1.89226	0.100591
37604	1.89226	0.0975316
37605	3.78453	0.105038
37606	2.57881	0.100226
37607	1.89226	0.102311
37608	1.89226	0.100932
37609	1.89226	0.111637
37610	2.6428	0.0929359
37611	2.6428	0.10415
37612	1.01379	0.102716
37613	0.985937	0.101837
37614	0.769295	0.0962176
37615	0.715547	0.0918907
37616	0.853042	0.0970658
37617	0.853042	0.123205
37618	0.864117	0.104198
37619	0.864114	0.0928555
37620	0.715556	0.098488
37621	1.03047	0.0935294
37622	0.731721	0.107599
37623	0.985921	0.106234
37624	1.89227	0.115003
37625	1.89227	0.106713
37626	1.89227	0.0890318
37627	1.89227	0.114357
37628	3.78453	0.100616
37629	0.946129	0.10825
37630	0.946129	0.104243
37631	1.28161	0.0868289
37632	1.3214	0.0891541
37633	0.94613	0.0937321
37634	0.94613	0.117447
37635	1.28161	0.0949768
37636	1.28161	0.0880799
37637	1.28161	0.0874539
37638	0.94613	0.0945824
37639	0.946131	0.0990853

37640	1.3214	0.0954916
37641	0.946132	0.370546
37642	0.946132	0.33202
37643	0.706045	0.260178
37644	1.16718	0.224426
37645	0.706017	0.249733
37646	0.706017	0.674207
37647	1.4073	0.213294
37648	1.4073	0.192629
37649	0.94613	0.278529
37650	1.16719	0.254176
37651	0.706046	0.343224
37652	0.946131	0.287708
37653	1.89226	0.800736
37654	1.89226	0.522809
37655	1.89226	0.278498
37656	1.89226	1.29165
37657	1.89226	0.266547
37658	1.89226	1.16903
37659	0.946119	0.309224
37660	0.946119	0.294475
37661	0.946115	0.301386
37662	0.946115	0.221595
37663	0.946117	0.308523
37664	0.946117	0.833427
37665	0.946117	0.245749
37666	0.946117	0.204576
37667	0.94613	0.183426
37668	0.94613	0.319305
37669	0.946105	0.406241
37670	0.946105	0.249009
37671	0.946132	1.63077
37672	0.946132	1.54019
37673	0.946131	1.63569
37674	0.946131	1.23326
37675	0.946132	1.63678
37676	0.946132	1.65171
37677	0.946132	1.51792
37678	0.827865	1.46126
37679	0.946132	1.52743
37680	0.946132	1.58783
37681	0.946132	1.53111
37682	0.827865	1.54458
37683	1.89226	1.61172
37684	1.89226	1.57144
37685	1.89226	1.70808
37686	1.89226	1.50037
37687	1.89226	1.79965
37688	1.89226	1.61379
37689	0.93094	1.42285
37690	0.93094	1.43854
37691	1.04649	1.45419
37692	0.614468	1.35242
37693	0.950358	1.33427
37694	0.799012	1.1194
37695	0.682742	1.36585
37696	0.682742	1.44042
37697	1.04073	1.57592

37698	1.15596	1.52079
37699	0.614478	1.53621
37700	0.851524	1.43304
37701	2.22575	2.1
37702	1.90502	2.1
37703	2.10893	2.1
37704	1.80258	2.1
37705	4.01395	2.1
37706	1.90502	2.1
37707	1.40863	2.1
37708	1.49733	2.1
37709	1.49733	2.1
37710	1.80258	2.1
37711	1.80258	2.1
37712	2.10893	2.1
37713	1.68356	2.1
37714	1.80258	2.1
37715	1.80258	2.1
37716	1.98061	2.1
37717	1.98061	2.1
37718	1.80258	2.1
37719	1.40863	2.1
37720	1.68356	2.1
37721	1.30751	2.1
37722	3.11009	2.1
37723	1.35374	1.7964
37724	1.35374	1.72861
37725	0.746096	1.61584
37726	0.746096	1.55187
37727	1.1537	1.62585
37728	0.746095	1.72341
37729	0.946132	1.72082
37730	0.946132	1.58174
37731	0.946132	1.48144
37732	0.746095	1.58306
37733	0.946131	1.5197
37734	1.1537	1.57545
37735	2.15247	2.1
37736	1.70303	2.1
37737	1.41207	2.1
37738	1.43052	2.1
37739	3.22793	2.1
37740	0.940246	2.1
37741	0.728442	2.1
37742	0.650037	2.1
37743	0.539874	2.1
37744	0.694756	2.1
37745	0.694756	2.1
37746	0.776346	2.1
37747	0.656772	2.1
37748	0.733146	2.1
37749	0.563769	2.1
37750	0.66316	2.1
37751	0.899692	2.1
37752	1.21223	2.1
37753	1.22418	2.1
37754	1.43052	2.1
37755	1.43052	2.1

37756	1.24353	2.1
37757	1.46181	2.1
37758	1.89226	0.13636
37759	2.39894	0.0760032
37760	1.89226	0.145495
37761	2.37417	0.0835057
37762	1.89226	0.133021
37763	2.37417	0.0702734
37764	1.12767	0.0774918
37765	1.19947	0.0686884
37766	0.94613	0.198876
37767	0.94613	0.11535
37768	1.12766	0.075147
37769	0.946128	0.0663556
37770	1.19947	0.0922623
37771	0.946132	0.152419
37772	1.19947	0.161624
37773	1.19947	0.05
37774	0.94613	0.16704
37775	0.94613	0.067676
37776	2.22621	0.05
37777	1.14533	2.1
37778	1.1131	2.1
37779	0.88238	2.1
37780	0.868521	2.1
37781	1.01153	2.1
37782	1.01153	2.1
37783	1.00232	2.1
37784	1.00232	2.1
37785	1.14533	2.1
37786	0.882378	2.1
37787	0.868519	2.1
37788	1.11311	2.1
37789	0.910183	2.1
37790	1.07811	1.76716
37791	0.94613	0.486575
37792	0.94613	1.02202
37793	0.910212	1.05815
37794	0.910212	2.1
37795	1.11403	0.587323
37796	1.11403	1.95565
37797	0.910183	1.53046
37798	0.94613	1.83062
37799	0.94613	1.7618
37800	1.07811	1.68544
37801	1.87228	2.1
37802	2.81189	2.1
37803	1.6197	2.1
37804	2.56716	2.1
37805	4.43944	2.1
37806	0.946132	2.1
37807	0.946132	2.1
37808	0.946132	0.952331
37809	0.946132	1.92605
37810	0.946131	2.01031
37811	0.946131	2.1
37812	0.946132	1.09318
37813	0.946132	2.1

37814	0.946132	2.1
37815	0.946132	2.1
37816	0.946132	2.1
37817	0.946132	2.1
37818	1.28724	2.1
37819	2.56716	2.1
37820	2.3014	2.1
37821	2.3014	2.1
37822	1.6197	2.1
37823	1.6197	2.1
37824	0.946132	2.1
37825	0.946132	2.1
37826	0.946132	1.18711
37827	0.946132	2.0968
37828	0.946132	2.07246
37829	0.946132	2.1
37830	0.946133	2.1
37831	0.946133	1.69674
37832	0.946133	2.1
37833	0.946133	2.09196
37834	0.946132	2.1
37835	0.946132	2.1
37836	1.08039	2.1
37837	1.24445	2.1
37838	1.2873	2.1
37839	1.48751	2.1
37840	2.53175	2.1
37841	0.946125	1.92407
37842	0.946125	2.1
37843	1.12552	2.1
37844	1.12552	2.1
37845	0.946124	2.1
37846	0.946124	2.1
37847	1.12552	2.1
37848	1.11114	2.1
37849	1.11113	2.1
37850	0.946118	2.1
37851	1.12552	2.1
37852	0.946131	2.1
37853	1.89225	1.87918
37854	1.89225	1.71794
37855	1.89225	2.1
37856	1.89225	2.1
37857	1.89225	1.55417
37858	1.89225	2.1
37859	0.987703	1.83587
37860	1.33078	2.00181
37861	1.31565	1.86017
37862	1.31565	1.8724
37863	1.01121	1.87575
37864	1.01121	2.1
37865	2.19163	2.1
37866	1.89226	2.1
37867	1.89226	2.1
37868	1.89226	2.1
37869	2.22229	2.1
37870	2.22229	2.1
37871	1.89226	2.1

37872	1.89226	1.08858
37873	1.89226	0.647467
37874	1.89226	2.1
37875	1.89226	1.52022
37876	1.89226	2.1
37877	0.57141	2.1
37878	0.57141	1.65754
37879	0.574036	1.97288
37880	0.574036	2.1
37881	0.651591	1.98288
37882	0.657827	1.69686
37883	0.482391	2.05738
37884	0.493855	2.1
37885	0.482391	1.53109
37886	0.651591	1.52281
37887	0.493855	1.96666
37888	0.657828	1.95116
37889	1.88428	2.1
37890	2.03975	2.1
37891	1.97316	2.1
37892	1.8266	2.1
37893	3.86635	2.1
37894	2.23053	2.1
37895	1.42255	2.1
37896	1.3868	2.1
37897	1.8266	2.1
37898	3.24915	2.1
37899	1.97316	2.1
37900	1.8266	2.1
37901	1.77128	2.1
37902	1.91767	2.1
37903	3.74427	2.1
37904	1.77128	2.1
37905	1.8266	2.1
37906	1.77942	2.1
37907	1.33962	2.1
37908	1.3868	2.1
37909	1.3868	2.1
37910	0.944111	2.1
37911	0.944111	2.1
37912	1.26036	2.1
37913	1.16968	2.1
37914	1.03853	2.1
37915	1.16646	2.1
37916	0.946129	2.1
37917	0.946129	2.1
37918	0.94613	2.1
37919	1.25768	2.1
37920	1.03853	2.1
37921	0.946131	2.1
37922	1.82441	0.05
37923	1.33061	0.05
37924	1.13272	0.05
37925	1.60249	0.05
37926	2.95712	0.05
37927	1.82441	0.05
37928	2.08757	0.05
37929	1.81292	0.05

37930	1.60249	0.05
37931	3.63733	0.05
37932	1.81292	0.05
37933	1.332 0.05	
37934	1.60249	0.05
37935	1.60249	0.05
37936	1.5344	0.05
37937	1.5344	0.05
37938	1.10107	2.1
37939	2.51559	2.1
37940	1.1229	2.1
37941	1.1229	2.1
37942	2.49456	2.1
37943	2.49456	2.1
37944	1.89226	2.1
37945	2.51559	2.1
37946	2.49457	2.1
37947	2.51559	2.1
37948	1.87124	2.1
37949	1.89226	2.1
37950	1.89226	2.1
37951	1.89226	2.1
37952	1.89226	2.1
37953	1.89226	2.1
37954	1.89226	2.1
37955	1.89226	2.1
37956	1.89226	2.1
37957	1.89226	2.1
37958	1.89226	2.1
37959	1.89226	2.1
37960	1.89226	2.1
37961	1.89226	2.1
37962	1.23599	2.1
37963	1.24728	2.1
37964	0.550534	2.1
37965	0.541151	2.1
37966	0.893754	2.1
37967	0.893754	2.1
37968	0.894678	2.1
37969	0.894678	2.1
37970	1.23599	2.1
37971	0.541149	2.1
37972	0.550533	2.1
37973	1.24728	2.1
37974	0.946132	2.1
37975	0.946132	2.1
37976	1.23599	2.1
37977	1.24728	2.1
37978	0.946132	2.1
37979	0.946132	2.1
37980	1.23599	2.1
37981	1.23599	2.1
37982	1.23599	2.1
37983	0.946132	2.1
37984	0.946132	2.1
37985	1.24728	2.1
37986	1.89226	2.1
37987	1.89226	2.1

37988	1.89226	2.1
37989	1.89226	2.1
37990	1.89226	2.1
37991	1.89226	2.1
37992	0.946128	2.1
37993	0.946128	2.1
37994	0.946132	2.1
37995	0.946132	2.1
37996	0.94613	2.1
37997	0.94613	2.1
37998	0.946129	2.1
37999	0.946129	2.1
38000	0.94613	2.1
38001	0.94613	2.1
38002	0.94613	2.1
38003	0.94613	2.1
38004	2.58696	0.0600081
38005	2.57073	0.0966093
38006	2.12585	0.0698788
38007	2.07607	0.0766847
38008	4.69658	0.0762803
38009	1.89226	0.150534
38010	1.89226	0.188215
38011	1.89226	0.0747682
38012	1.89226	0.164912
38013	3.78452	0.103
38014	1.89226	0.191788
38015	1.89226	0.270191
38016	1.89226	0.176692
38017	1.89226	0.10998
38018	3.78453	0.150627
38019	2.57074	0.101417
38020	2.32635	0.104711
38021	2.07608	0.100658
38022	1.73623	0.105965
38023	4.30697	0.0800966
38024	2.12582	0.0743001
38025	1.51971	0.0702479
38026	1.57255	0.0648308
38027	2.07604	0.0860706
38028	1.62233	0.0599515
38029	2.12582	0.0800285
38030	1.89223	0.0770635
38031	1.89223	0.141051
38032	1.89223	0.0914942
38033	1.89223	0.104655
38034	1.89223	0.0926201
38035	1.89223	0.108426
38036	0.946119	0.145514
38037	0.946119	0.135459
38038	0.946119	0.0928128
38039	0.946119	0.131144
38040	0.946119	0.124356
38041	0.946119	0.15487
38042	0.94612	0.140459
38043	0.94612	0.0898536
38044	0.946132	0.123507
38045	0.946132	0.124644

38046	0.946107	0.129838
38047	0.946107	0.128734
38048	0.811456	0.0982574
38049	0.811456	0.118742
38050	0.806521	0.0696061
38051	0.806521	0.10606
38052	0.868104	0.103542
38053	0.579949	0.122427
38054	0.759859	0.0783497
38055	1.03802	0.092412
38056	0.579957	0.0969087
38057	0.759868	0.0951293
38058	1.03801	0.0981689
38059	0.868093	0.101918
38060	2.7408	2.1
38061	2.82858	2.1
38062	2.57368	2.1
38063	2.52633	2.1
38064	5.3549	2.1
38065	2.7408	2.1
38066	2.31353	2.1
38067	2.14063	2.1
38068	2.52632	2.1
38069	4.83985	2.1
38070	2.57368	2.1
38071	2.52633	2.1
38072	2.3301	2.1
38073	2.36071	2.1
38074	4.88704	2.1
38075	1.86512	2.1
38076	2.52632	2.1
38077	2.14063	2.1
38078	2.14063	2.1
38079	2.3301	2.1
38080	2.3301	2.1
38081	1.86431	2.1
38082	1.89227	2.1
38083	1.68992	2.1
38084	1.68992	2.1
38085	1.89227	2.1
38086	1.89227	2.1
38087	2.0815	2.1
38088	1.89226	2.1
38089	2.04022	2.1
38090	1.86431	2.1
38091	2.07081	2.1
38092	1.89226	2.1
38093	1.91686	2.04144
38094	1.65683	2.1
38095	1.86871	2.1
38096	1.65683	2.1
38097	2.13075	2.1
38098	1.89226	1.78988
38099	1.89226	2.1
38100	1.89226	1.79475
38101	1.89226	1.93487
38102	1.89226	2.1
38103	3.78452	2.05692

38104	0.851541	2.1
38105	0.851541	2.1
38106	0.952803	2.1
38107	0.851573	2.1
38108	0.946129	2.1
38109	0.946129	2.1
38110	0.929184	2.1
38111	0.757482	2.1
38112	0.836274	2.1
38113	0.851528	2.1
38114	0.952816	2.1
38115	1.04073	2.1
38116	1.70303	1.81063
38117	1.70302	2.0125
38118	1.5544	2.1
38119	1.70302	1.87947
38120	1.74957	1.95933
38121	1.91686	1.63282
38122	1.89226	1.77341
38123	1.89226	2.07579
38124	1.89226	2.1
38125	1.89226	1.8785
38126	1.89226	1.94164
38127	1.89226	1.58919
38128	1.89226	2.1
38129	1.89226	2.1
38130	2.03472	2.1
38131	1.89226	2.1
38132	1.99827	2.1
38133	1.85836	2.1
38134	1.00509	2.1
38135	1.55998	2.1
38136	1.83347	2.1
38137	1.45246	2.1
38138	3.01244	2.1
38139	1.23839	2.1
38140	0.884251	2.1
38141	0.946133	2.1
38142	0.946133	2.1
38143	0.884226	2.1
38144	0.884226	2.1
38145	0.946158	2.1
38146	0.946159	2.1
38147	1.23839	2.1
38148	0.946131	2.1
38149	0.946131	2.1
38150	0.884254	2.1
38151	1.89226	2.1
38152	1.89226	2.1
38153	1.89226	2.1
38154	1.89226	2.1
38155	1.89226	2.1
38156	1.89226	2.1
38157	2.1499	2.1
38158	1.45244	2.1
38159	1.83345	2.1
38160	1.83345	2.1
38161	1.87214	2.1

38162	1.87214	2.1
38163	1.89224	2.1
38164	1.89224	2.1
38165	1.89224	2.1
38166	1.89224	2.1
38167	3.78448	2.1
38168	1.49155	1.99161
38169	2.0815	2.1
38170	1.77515	1.99065
38171	1.72377	2.1
38172	3.50659	1.94434
38173	1.69876	0.352623
38174	2.0815	2.1
38175	1.9133	1.60013
38176	1.68189	0.708688
38177	2.17628	2.07945
38178	1.94335	2.1
38179	2.65129	0.05
38180	1.15317	0.05
38181	2.57085	0.05
38182	1.209 0.05	
38183	2.57085	0.05
38184	1.209 0.05	
38185	1.89226	0.05
38186	1.89226	0.05
38187	1.89226	0.05
38188	1.89226	0.05
38189	3.78452	0.05
38190	2.48861	0.05
38191	1.15252	0.05
38192	2.60515	0.05
38193	2.60515	0.05
38194	1.16384	0.05
38195	1.16384	0.05
38196	1.59903	0.61025
38197	1.26865	0.869317
38198	1.30486	1.75947
38199	1.70302	1.31377
38200	2.98573	1.50205
38201	1.59903	0.372688
38202	1.79478	1.42512
38203	1.79478	1.37826
38204	1.97005	0.271443
38205	1.50365	1.19071
38206	1.64192	1.33396
38207	1.89226	0.26515
38208	1.89226	0.989187
38209	1.89226	1.42383
38210	1.89226	0.55746
38211	3.78453	0.258814
38212	1.89226	1.54078
38213	1.89226	1.04133
38214	1.89226	1.59205
38215	1.89226	0.37205
38216	1.89226	1.49823
38217	1.89226	1.32691
38218	0.761672	0.255286
38219	0.896406	0.39316

38220	0.603509	0.589458
38221	0.574071	0.910759
38222	0.695159	1.48067
38223	0.695159	0.194099
38224	0.762564	1.43348
38225	0.770159	0.555825
38226	0.803021	1.33699
38227	0.572529	1.34883
38228	0.696466	0.405238
38229	0.899733	0.386516
38230	1.66034	0.140492
38231	1.79478	1.10676
38232	1.56685	0.383039
38233	1.68512	1.57663
38234	1.67652	0.951058
38235	1.67652	0.874181
38236	1.89226	1.37392
38237	1.89226	1.07865
38238	1.89226	1.22595
38239	1.89226	0.934864
38240	1.89226	1.24598
38241	1.89226	0.898069
38242	0.946133	0.267566
38243	0.946133	0.350975
38244	0.946132	1.07312
38245	0.946132	1.01704
38246	0.946132	1.34289
38247	0.946132	0.139686
38248	0.946133	1.68043
38249	0.946133	0.521321
38250	0.946133	1.25473
38251	0.946133	1.27417
38252	0.946132	0.289094
38253	0.946132	0.348837
38254	1.80424	2.1
38255	1.45158	2.1
38256	1.9098	2.1
38257	1.56003	2.1
38258	3.36138	2.1
38259	1.90982	2.1
38260	1.71606	2.1
38261	2.15314	2.1
38262	1.70302	2.1
38263	3.89051	2.1
38264	1.96416	1.82608
38265	1.23706	1.2193
38266	1.00053	1.14719
38267	1.38287	0.390174
38268	1.57916	0.746495
38269	1.9615	1.04627
38270	0.827865	0.279125
38271	0.946132	0.189647
38272	0.946132	0.440377
38273	0.946132	0.465218
38274	0.946132	1.18403
38275	0.946132	0.303923
38276	0.946131	1.23049
38277	0.946131	0.525816

38278	0.827865	1.31798
38279	0.946132	0.298175
38280	0.946132	0.2458
38281	0.946132	0.20634
38282	1.89227	2.1
38283	1.89227	2.1
38284	1.89227	2.1
38285	1.89227	2.1
38286	3.78453	2.1
38287	2.3819	2.1
38288	1.58089	2.1
38289	1.58089	2.1
38290	1.6287	2.1
38291	2.33368	2.1
38292	2.3815	2.1
38293	0.946133	2.1
38294	0.946133	2.1
38295	0.946132	2.1
38296	0.946132	2.1
38297	0.946132	2.1
38298	0.946132	2.1
38299	0.946132	2.1
38300	0.946132	2.1
38301	0.946133	2.1
38302	0.946133	2.1
38303	0.946132	2.1
38304	0.946132	2.1
38305	1.89226	2.1
38306	1.89226	2.1
38307	1.89226	2.1
38308	1.89226	2.1
38309	3.78453	2.1
38310	2.7874	2.1
38311	2.33368	2.1
38312	2.71897	2.1
38313	2.27495	2.1
38314	5.05266	2.1
38315	0.970991	2.1
38316	0.970991	2.1
38317	0.956929	2.1
38318	0.956929	2.1
38319	0.732928	2.1
38320	0.790444	2.1
38321	1.16684	2.1
38322	1.13748	2.1
38323	1.13748	2.1
38324	0.732928	2.1
38325	0.790444	2.1
38326	1.16684	2.1
38327	0.946132	2.1
38328	0.946132	2.1
38329	0.946131	2.1
38330	0.946131	2.1
38331	0.946132	2.1
38332	0.946132	2.1
38333	0.946132	2.1
38334	0.946132	2.1
38335	0.946132	2.1

38336	0.946132	2.1
38337	0.946132	2.1
38338	0.946132	2.1
38339	1.10747	0.173763
38340	2.30331	0.299526
38341	1.47954	0.130503
38342	2.63789	0.143306
38343	3.78285	0.187514
38344	1.65215	0.082275
38345	2.63789	0.119864
38346	2.82465	0.11306
38347	2.82465	0.127195
38348	1.47954	0.0887538
38349	1.47954	0.106259
38350	1.13456	2.04527
38351	1.08334	2.1
38352	1.20674	1.23799
38353	1.17885	2.1
38354	2.29008	1.95895
38355	1.20674	1.71925
38356	1.17885	2.1
38357	1.28649	1.37709
38358	1.28516	1.98901
38359	2.4919	1.83128
38360	1.89226	2.1
38361	1.89226	2.1
38362	1.89226	2.1
38363	1.89226	2.09561
38364	1.89226	2.1
38365	1.89226	2.07796
38366	2.50288	1.63874
38367	2.02115	1.81602
38368	2.7717	2.02431
38369	2.29107	1.27193
38370	4.79395	1.76052
38371	1.27339	2.1
38372	1.10817	2.1
38373	1.19733	2.1
38374	1.032	2.1
38375	2.30539	2.1
38376	1.19733	2.1
38377	0.953637	2.1
38378	0.953637	2.1
38379	1.032	2.1
38380	1.11868	2.1
38381	1.19704	2.1
38382	2.01024	0.981034
38383	1.11938	0.991613
38384	1.9186	1.3208
38385	2.76298	0.918762
38386	3.92884	0.840242
38387	0.851541	0.117491
38388	0.851541	0.0994348
38389	0.957577	0.091513
38390	0.848952	0.0956484
38391	1.08043	0.117342
38392	0.851541	0.12428
38393	0.851484	0.098014

38394	0.957578	0.0826007
38395	1.20045	0.112511
38396	0.726289	0.0809724
38397	0.946131	0.0970155
38398	0.946131	0.114042
38399	1.50524	2.1
38400	2.38363	2.1
38401	2.49018	2.1
38402	2.49018	2.1
38403	1.45506	2.1
38404	1.45506	2.1
38405	2.49017	2.1
38406	1.89226	2.1
38407	1.89226	2.1
38408	1.89226	2.1
38409	2.38363	2.1
38410	2.38363	2.1
38411	1.54711	2.1
38412	2.49018	2.1
38413	2.56975	2.1
38414	2.56975	2.1
38415	1.50524	2.1
38416	1.50524	2.1
38417	2.56975	2.1
38418	1.89226	2.1
38419	1.89226	2.1
38420	1.89226	2.1
38421	2.49017	2.1
38422	2.49017	2.1
38423	1.89226	0.0850605
38424	1.89226	0.0854931
38425	1.89226	0.128262
38426	1.89226	0.0730308
38427	1.89226	0.130662
38428	1.89226	0.0569376
38429	1.89226	0.138519
38430	1.89226	0.118923
38431	1.89226	0.22829
38432	1.89226	0.0983417
38433	1.89226	0.233936
38434	1.89226	0.0946292
38435	1.89226	0.0689548
38436	1.89226	0.0712965
38437	2.8358	0.05
38438	1.89226	0.164968
38439	3.78453	0.05
38440	1.60565	0.334869
38441	1.89226	1.35149
38442	1.89226	1.24556
38443	1.40013	0.621792
38444	1.89226	0.936886
38445	1.40013	0.616716
38446	1.1766	2.1
38447	1.29435	2.1
38448	1.35735	2.1
38449	1.53509	2.1
38450	2.65169	2.1
38451	0.69701	2.1

38452	0.69701	2.1
38453	0.711303	2.1
38454	0.711303	2.1
38455	0.761138	2.1
38456	0.612149	2.1
38457	0.647174	2.1
38458	0.767548	2.1
38459	0.767548	2.1
38460	0.761139	2.1
38461	0.612149	2.1
38462	0.647174	2.1
38463	1.29764	1.70868
38464	1.29579	1.9315
38465	1.34875	1.85208
38466	1.06717	1.85504
38467	1.29764	1.79928
38468	1.01606	1.77286
38469	0.756635	1.66365
38470	0.648821	1.68157
38471	0.675557	1.85306
38472	0.674374	1.78534
38473	0.74214	1.87692
38474	0.74214	1.72593
38475	0.688868	1.61294
38476	0.688868	1.76902
38477	0.675557	1.59084
38478	0.756634	1.66001
38479	0.648821	1.49901
38480	0.674374	1.65536
38481	1.51327	1.71851
38482	1.48105	1.55347
38483	1.29764	1.73012
38484	1.29764	1.64292
38485	1.48105	1.73923
38486	1.05655	1.55656
38487	1.13537	2.1
38488	1.07326	2.1
38489	0.94613	2.1
38490	0.94613	2.1
38491	0.946132	2.1
38492	0.946132	2.1
38493	1.13537	2.1
38494	1.08927	2.1
38495	1.13537	2.1
38496	0.946131	2.1
38497	0.946131	2.1
38498	0.95947	2.1
38499	1.46892	2.1
38500	1.89226	2.1
38501	1.89226	2.1
38502	1.89226	2.1
38503	1.88031	2.1
38504	1.88031	2.1
38505	1.89226	2.1
38506	1.89226	2.1
38507	1.89226	2.1
38508	1.89226	2.1
38509	3.78453	2.1

38510	2.18254	2.1
38511	1.89226	2.1
38512	2.50005	2.1
38513	1.89226	2.1
38514	4.39231	2.1
38515	1.88031	2.1
38516	2.0815	2.1
38517	2.0815	2.1
38518	1.92078	2.1
38519	2.0815	2.1
38520	1.9218	2.1
38521	1.89227	2.1
38522	1.89227	2.1
38523	1.89227	2.1
38524	1.89227	2.1
38525	1.89227	2.1
38526	1.89227	2.1
38527	1.98693	2.1
38528	1.82857	2.1
38529	2.32	2.1
38530	2.36972	2.1
38531	4.14857	2.1
38532	1.82857	2.1
38533	1.58688	2.1
38534	2.36971	2.1
38535	2.19093	2.1
38536	4.0195	2.1
38537	0.946132	2.1
38538	0.946132	2.1
38539	1.18486	2.1
38540	1.18486	2.1
38541	1.18486	2.1
38542	0.946132	2.1
38543	0.946132	2.1
38544	1.16	2.1
38545	0.946133	2.1
38546	0.946133	2.1
38547	1.16	2.1
38548	1.18486	2.1
38549	1.07798	2.00256
38550	1.08101	1.88348
38551	0.910786	2.1
38552	0.910786	2.1
38553	0.851537	2.1
38554	0.901377	1.8789
38555	1.14652	2.1
38556	1.04072	2.1
38557	0.946129	2.1
38558	0.946129	2.1
38559	1.0019	2.0962
38560	1.04263	2.1
38561	1.89226	2.1
38562	1.89226	2.1
38563	1.89226	2.1
38564	1.89226	2.1
38565	3.78452	2.1
38566	1.89226	2.1
38567	2.30146	2.1

38568	1.89226	2.1
38569	2.18412	2.1
38570	4.19372	2.1
38571	2.30146	2.1
38572	1.39962	2.1
38573	2.18411	2.1
38574	1.28765	2.1
38575	3.5891	2.1
38576	1.89226	2.1
38577	1.89226	2.1
38578	1.89226	2.1
38579	1.89226	2.1
38580	1.89226	2.1
38581	1.89226	2.1
38582	1.89226	2.1
38583	1.89226	2.1
38584	1.89226	2.1
38585	1.89226	2.1
38586	3.78452	2.1
38587	2.18412	2.1
38588	1.20045	2.1
38589	1.20045	2.1
38590	1.28765	2.1
38591	2.08808	2.1
38592	2.17528	2.1
38593	0.946131	2.1
38594	0.946131	2.1
38595	0.946128	2.1
38596	0.946128	2.1
38597	0.946127	2.1
38598	0.946127	2.1
38599	0.946131	2.1
38600	0.946131	2.1
38601	0.946129	2.1
38602	0.946129	2.1
38603	0.94613	2.1
38604	0.94613	2.1
38605	1.89226	0.229316
38606	1.89226	1.45567
38607	1.89226	1.79203
38608	1.89226	0.257847
38609	3.78452	0.215988
38610	1.89227	0.410699
38611	1.89227	1.88651
38612	1.89227	0.322962
38613	1.89227	1.43715
38614	1.89227	0.383601
38615	1.89227	1.74537
38616	1.89226	1.46004
38617	1.89226	1.11735
38618	1.89226	0.211022
38619	2.29245	1.13074
38620	3.78452	1.35859
38621	1.89226	1.55851
38622	1.89226	0.135865
38623	1.89226	0.194174
38624	1.89226	1.42212
38625	1.89226	0.234701

38626	1.89226	0.287853
38627	1.28095	1.47111
38628	1.81846	1.49047
38629	2.10991	1.29215
38630	1.34695	1.47978
38631	3.39086	1.44315
38632	2.10991	1.39003
38633	1.36457	1.55198
38634	1.67421	1.60883
38635	1.34695	1.56822
38636	2.43717	1.42709
38637	2.10991	1.50408
38638	2.69541	2.1
38639	1.48572	2.1
38640	2.63553	2.1
38641	1.45385	2.1
38642	4.14926	2.1
38643	2.63553	2.1
38644	1.44207	2.1
38645	1.44207	2.1
38646	1.45385	2.1
38647	2.588	2.1
38648	2.59978	2.1
38649	1.89227	2.1
38650	1.89227	2.1
38651	1.89227	2.1
38652	1.89227	2.1
38653	3.78453	2.1
38654	1.89226	2.1
38655	1.89226	2.1
38656	1.89226	2.1
38657	1.89226	2.1
38658	3.78453	2.1
38659	2.15929	2.1
38660	1.38276	2.1
38661	1.43617	2.1
38662	1.35614	2.1
38663	2.23932	2.1
38664	2.15929	2.1
38665	2.15929	2.1
38666	2.25168	2.1
38667	2.3558	2.1
38668	2.23932	2.1
38669	4.51509	2.1
38670	1.89226	2.1
38671	1.89226	2.1
38672	1.89226	2.1
38673	1.89226	2.1
38674	3.78453	2.1
38675	1.89226	2.1
38676	1.89226	2.1
38677	1.89226	2.1
38678	1.89226	2.1
38679	1.89226	2.1
38680	1.89226	2.1
38681	2.23932	2.1
38682	1.43854	2.1
38683	1.49173	2.1

38684	1.38276	2.1
38685	2.34829	2.1
38686	2.23932	2.1
38687	2.3558	2.1
38688	2.34829	2.1
38689	2.40685	2.1
38690	2.23932	2.1
38691	2.52332	2.1
38692	2.3558	2.1
38693	1.89226	0.05
38694	1.87361	0.05
38695	2.25082	0.05
38696	1.87361	0.05
38697	2.26947	0.05
38698	1.89226	0.05
38699	1.89226	0.05
38700	1.89226	0.0618873
38701	1.89226	0.05
38702	1.89226	0.0771072
38703	1.89226	0.05
38704	1.89226	0.0738746
38705	2.41314	0.05
38706	1.89226	0.05
38707	1.89226	0.05
38708	1.89226	0.0561822
38709	2.12204	0.05
38710	2.12204	0.0536509
38711	1.89226	0.05
38712	2.25082	0.05
38713	2.28189	0.05
38714	2.25082	0.05
38715	1.92333	0.05
38716	1.89226	0.05
38717	1.89226	0.05
38718	1.89226	0.0551872
38719	1.89226	0.05
38720	1.89226	0.0760671
38721	1.89226	0.05
38722	1.89226	0.0691442
38723	2.42521	0.05
38724	1.89226	0.05
38725	1.89226	0.05
38726	1.89226	0.05
38727	2.41314	0.05
38728	2.41314	0.05
38729	1.39725	2.01004
38730	1.07745	2.1
38731	1.07745	2.06863
38732	1.07519	2.1
38733	1.41336	1.96284
38734	1.41111	2.1
38735	1.89226	2.1
38736	1.92039	2.1
38737	1.92039	2.1
38738	1.9052	2.1
38739	1.89226	2.1
38740	1.87708	2.1
38741	0.946128	2.1

38742	0.946128	2.1
38743	0.946133	1.99279
38744	0.946133	1.91913
38745	0.94613	1.91997
38746	0.94613	2.1
38747	0.946131	2.1
38748	0.946131	1.88717
38749	0.946131	2.1
38750	0.946131	2.1
38751	0.946131	2.1
38752	0.946131	2.1
38753	1.41336	2.05396
38754	1.08911	2.1
38755	1.08911	2.1
38756	1.07745	2.1
38757	1.42381	2.02961
38758	1.41215	2.1
38759	1.89227	2.1
38760	1.95833	2.1
38761	1.95833	2.1
38762	1.92039	2.1
38763	1.89227	2.1
38764	1.85433	2.1
38765	0.946129	2.1
38766	0.946129	2.1
38767	0.946133	2.07045
38768	0.946133	1.95567
38769	0.946131	1.95523
38770	0.946131	2.1
38771	0.946131	2.1
38772	0.946131	1.99194
38773	0.946131	2.1
38774	0.946131	2.1
38775	0.946131	2.1
38776	0.946131	2.1
38777	1.41943	2.1
38778	2.74398	2.1
38779	1.18238	2.1
38780	2.53897	2.1
38781	3.9584	2.1
38782	2.74398	2.1
38783	1.89226	2.1
38784	1.89226	2.1
38785	1.89226	2.1
38786	2.53897	2.1
38787	2.53897	2.1
38788	1.89227	1.21462
38789	1.89227	0.684075
38790	1.89227	1.21603
38791	1.89227	0.479242
38792	3.78453	0.713067
38793	1.89226	0.489574
38794	2.08134	0.252145
38795	2.00632	0.259494
38796	2.00632	0.27181
38797	1.89226	0.659191
38798	1.89226	0.616954
38799	1.89227	1.12316

38800	1.89227	0.4262
38801	1.89227	0.915922
38802	1.89227	0.322294
38803	3.78453	0.806786
38804	1.89227	1.16784
38805	1.89227	0.295709
38806	1.89227	0.297775
38807	1.89227	0.455546
38808	1.89227	1.03744
38809	1.89227	1.16454
38810	2.50912	0.257813
38811	1.89226	0.479637
38812	1.89226	0.193444
38813	2.31261	0.279295
38814	2.31261	0.246377
38815	1.89226	0.689329
38816	2.50912	0.299113
38817	1.19999	0.230639
38818	1.44056	0.207714
38819	1.24405	0.19077
38820	2.50912	0.293715
38821	2.31261	0.300537
38822	2.69224	1.54506
38823	2.28373	0.21464
38824	1.94788	1.33933
38825	2.4106	1.1726
38826	4.69434	1.21093
38827	2.69223	0.99094
38828	2.63783	1.73431
38829	2.34737	2.08774
38830	2.4106	1.03236
38831	5.04843	1.71665
38832	1.89226	2.1
38833	1.99753	2.1
38834	1.87366	2.1
38835	1.99753	2.1
38836	1.76839	2.1
38837	1.89226	2.1
38838	1.89226	2.1
38839	1.89226	2.1
38840	1.89226	2.1
38841	1.89226	2.1
38842	3.78452	2.1
38843	0.946132	2.1
38844	0.946132	2.1
38845	1.02744	2.1
38846	1.02744	2.1
38847	0.946132	2.1
38848	0.946132	2.1
38849	1.00989	2.1
38850	1.02744	2.1
38851	1.00989	2.1
38852	0.946133	2.1
38853	1.02744	2.1
38854	0.946132	2.1
38855	1.70302	2.1
38856	1.87366	2.1
38857	1.71618	2.1

38858	1.87366	2.1
38859	1.73105	2.1
38860	1.89226	2.1
38861	1.89226	2.1
38862	1.89226	2.1
38863	1.89226	2.1
38864	1.89226	2.1
38865	1.89226	2.1
38866	1.89226	2.1
38867	1.96624	2.1
38868	1.89226	2.1
38869	1.89226	2.1
38870	1.89226	2.1
38871	2.01977	2.1
38872	2.01977	2.1
38873	1.1032	2.1
38874	1.15114	2.1
38875	1.14986	0.791297
38876	1.19242	2.1
38877	2.301 2.1	
38878	1.89226	1.83951
38879	1.89226	2.1
38880	1.89226	0.858023
38881	1.89226	2.1
38882	3.78453	2.1
38883	0.946132	2.1
38884	0.946132	2.1
38885	0.946132	2.1
38886	0.946132	2.1
38887	0.946133	2.1
38888	0.946133	2.1
38889	0.946132	2.1
38890	0.946132	2.1
38891	0.946132	2.1
38892	0.946132	2.1
38893	0.946133	2.1
38894	0.946133	2.1
38895	1.19243	2.1
38896	0.953928	2.1
38897	1.18671	2.1
38898	1.15114	2.1
38899	0.9895	2.1
38900	0.953928	2.1
38901	1.19611	0.82991
38902	1.19242	2.1
38903	1.24693	1.77457
38904	1.24693	2.1
38905	1.14986	1.60748
38906	1.14986	2.1
38907	1.89225	0.898336
38908	1.89225	2.1
38909	1.89225	2.1
38910	1.89225	1.87467
38911	1.89225	2.1
38912	1.89225	2.08718
38913	1.89225	2.1
38914	1.89225	2.1
38915	1.89225	2.1

38916	1.89225	2.1
38917	1.89225	2.1
38918	1.89225	2.1
38919	0.561532	2.1
38920	0.561532	2.1
38921	0.566382	2.1
38922	0.566382	2.1
38923	0.494747	2.1
38924	0.531705	2.1
38925	0.596208	2.1
38926	0.623464	2.1
38927	0.623469	2.1
38928	0.531708	2.1
38929	0.596205	2.1
38930	0.494743	2.1
38931	1.25742	2.1
38932	1.18783	2.1
38933	0.946131	2.1
38934	0.946131	2.1
38935	1.25742	2.1
38936	1.25742	2.1
38937	0.946131	2.1
38938	0.946131	2.1
38939	1.25742	2.1
38940	0.946131	2.1
38941	0.946131	2.1
38942	1.18783	2.1
38943	1.35048	2.1
38944	1.25742	2.1
38945	0.946132	2.1
38946	0.946132	2.1
38947	1.35048	2.1
38948	1.35048	2.1
38949	0.946132	2.1
38950	0.946132	2.1
38951	1.35044	2.1
38952	0.946106	2.1
38953	1.25745	2.1
38954	0.946158	2.1
38955	1.13525	2.1
38956	2.70095	2.1
38957	2.51483	2.1
38958	2.51483	2.1
38959	1.21593	2.1
38960	1.21593	2.1
38961	1.93142	1.1634
38962	2.8053	0.265514
38963	1.559	1.35656
38964	2.60359	0.160814
38965	4.53501	0.387185
38966	2.80531	0.178194
38967	1.89226	0.261278
38968	2.60359	0.2666
38969	1.89226	1.38014
38970	4.49586	0.249193
38971	1.22938	0.509314
38972	2.60359	0.173524
38973	2.37434	1.58598

38974	2.37434	0.587195
38975	1.55901	1.03882
38976	1.55901	0.170768
38977	1.18717	1.13841
38978	1.18717	0.114682
38979	0.946132	0.0913038
38980	0.946132	0.418967
38981	0.946133	0.335682
38982	0.946133	0.98127
38983	1.3018	0.0955558
38984	1.18717	1.27939
38985	1.18714	0.27617
38986	0.946107	0.325773
38987	1.30183	0.343188
38988	0.946158	0.304407
38989	1.89226	2.1
38990	2.80196	2.1
38991	2.67272	2.1
38992	2.67272	2.1
38993	1.89226	2.1
38994	1.89226	2.1
38995	1.89226	2.1
38996	1.89226	2.1
38997	1.89226	2.1
38998	1.89226	2.1
38999	1.89226	2.1
39000	1.89226	2.1
39001	1.74471	2.1
39002	2.01555	2.1
39003	1.61121	2.1
39004	1.61121	2.1
39005	2.13878	2.1
39006	2.13878	2.1
39007	1.89226	2.1
39008	1.89226	2.1
39009	1.89226	2.1
39010	1.89226	2.1
39011	1.89226	2.1
39012	1.89226	2.1
39013	1.09766	2.1
39014	1.04263	2.1
39015	0.881794	2.1
39016	0.894188	2.1
39017	0.795402	2.1
39018	0.99524	2.1
39019	1.00647	2.1
39020	1.00647	2.1
39021	1.15401	2.1
39022	0.881789	2.1
39023	0.850526	2.1
39024	1.04263	2.1
39025	1.4054	1.98295
39026	1.04788	2.1
39027	1.70302	2.08237
39028	1.41375	2.1
39029	2.82706	2.1
39030	1.70302	1.97042
39031	1.38776	2.1

39032	1.59457	2.1
39033	1.41375	2.1
39034	1.77261	1.6556
39035	1.70302	2.1
39036	1.89226	2.1
39037	1.89226	2.1
39038	1.89226	2.1
39039	1.89226	2.1
39040	3.78453	2.1
39041	2.18042	2.1
39042	1.74738	2.1
39043	2.08433	2.1
39044	1.66347	2.1
39045	3.83171	2.1
39046	1.74738	2.1
39047	1.1181	2.1
39048	1.66347	2.1
39049	1.06543	2.1
39050	2.81281	2.1
39051	1.89226	2.1
39052	1.89226	2.1
39053	1.89226	2.1
39054	1.89226	2.1
39055	3.78453	2.1
39056	1.89227	2.1
39057	1.89227	2.1
39058	1.89227	2.1
39059	1.89227	2.1
39060	3.78453	2.1
39061	2.08433	2.1
39062	1.66347	2.1
39063	1.97092	2.1
39064	1.57431	2.1
39065	3.65864	2.1
39066	1.66347	2.1
39067	1.01474	2.1
39068	1.01474	2.1
39069	1.06544	2.1
39070	1.57431	2.1
39071	1.625 2.1	
39072	1.89226	2.1
39073	1.89226	2.1
39074	1.89226	2.1
39075	1.89226	2.1
39076	1.89226	2.1
39077	1.89226	2.1
39078	1.08467	2.1
39079	1.87228	2.1
39080	1.73305	2.1
39081	1.73305	2.1
39082	1.25565	2.1
39083	1.25565	2.1
39084	0.695361	0.29265
39085	0.799996	0.337454
39086	1.13751	0.513247
39087	1.40945	0.494104
39088	1.40944	0.567897
39089	0.799999	0.254905

39090	1.13751	0.342834
39091	0.695363	0.252538
39092	1.01059	0.302063
39093	1.01059	0.448323
39094	0.926908	0.714764
39095	0.926909	0.24076
39096	1.05481	2.1
39097	1.78069	2.1
39098	1.08074	2.1
39099	1.83284	2.1
39100	2.86143	2.1
39101	1.78069	2.1
39102	2.22044	2.1
39103	1.83284	2.1
39104	2.28696	1.93071
39105	4.06766	2.1
39106	1.07196	2.1
39107	1.64955	2.1
39108	2.02665	2.1
39109	2.0284	2.1
39110	1.08074	2.1
39111	1.08074	2.1
39112	1.64955	2.1
39113	2.28696	2.1
39114	1.92033	2.1
39115	2.1004	2.1
39116	3.74988	2.1
39117	2.83787	1.18317
39118	1.70303	1.52047
39119	1.70303	0.332094
39120	1.83195	0.115516
39121	2.64075	0.818942
39122	2.70231	1.20643
39123	1.2087	2.1
39124	1.52909	0.931155
39125	1.43063	0.858951
39126	1.10586	2.1
39127	2.63494	1.9177
39128	1.89227	2.1
39129	2.10732	2.1
39130	2.10732	2.1
39131	2.2365	2.1
39132	1.89227	2.1
39133	2.02145	2.1
39134	1.89226	1.13218
39135	1.89226	2.1
39136	1.89226	1.88114
39137	1.89226	2.1
39138	1.89226	1.37281
39139	1.89226	2.1
39140	0.51926	2.1
39141	0.552927	2.1
39142	0.691235	0.861788
39143	0.715314	1.04567
39144	0.619684	1.05203
39145	0.619684	2.1
39146	0.624477	2.1
39147	0.624477	1.03627

39148	0.519262	2.05561
39149	0.691234	2.05816
39150	0.715313	2.05512
39151	0.552928	2.04339
39152	1.89226	2.1
39153	1.87321	2.1
39154	1.87321	2.1
39155	1.89658	2.1
39156	2.0815	2.1
39157	2.14789	2.1
39158	0.946128	2.1
39159	0.946128	2.1
39160	0.946133	1.23712
39161	0.946133	1.70904
39162	0.94613	1.10012
39163	0.94613	2.1
39164	0.946131	1.758
39165	0.946131	2.1
39166	0.946131	1.94037
39167	0.946131	1.92435
39168	0.946131	2.09611
39169	0.946131	2.1
39170	1.99297	1.44953
39171	2.19514	0.657643
39172	2.46153	1.24321
39173	2.187	1.02427
39174	4.45451	1.00199
39175	2.0815	1.04538
39176	2.33402	0.611469
39177	2.46154	1.29607
39178	2.83046	0.41539
39179	4.94496	1.01824
39180	2.46153	1.17531
39181	2.187	0.74888
39182	2.27351	1.37146
39183	2.63356	0.441514
39184	4.73504	1.17258
39185	2.46154	1.23066
39186	2.83447	0.36628
39187	2.74513	0.648852
39188	2.5731	1.02872
39189	2.63357	0.542503
39190	2.46154	0.927982
39191	2.80398	2.1
39192	2.60406	2.1
39193	2.73988	2.1
39194	2.57466	2.1
39195	5.34394	2.1
39196	1.89226	1.57272
39197	1.89226	1.33289
39198	1.89226	1.27417
39199	1.89226	1.58381
39200	1.89226	1.30929
39201	1.89226	1.67674
39202	1.89224	1.62563
39203	1.89224	1.32678
39204	1.89224	1.30332
39205	1.89224	1.66477

39206	1.89224	1.32914
39207	1.89224	1.67395
39208	1.40245	1.54068
39209	1.40246	1.66227
39210	0.946117	1.44702
39211	0.946117	1.53589
39212	1.17635	1.378
39213	1.40246	1.76039
39214	0.946112	1.34363
39215	0.946112	1.68388
39216	0.946127	1.46581
39217	1.17637	1.39778
39218	0.946102	1.65323
39219	1.40244	1.52888
39220	2.53269	2.1
39221	1.70302	2.1
39222	1.56194	2.1
39223	2.17516	2.1
39224	4.19807	2.1
39225	1.89226	2.1
39226	1.89226	2.1
39227	1.89226	2.1
39228	1.89226	2.1
39229	3.78453	2.1
39230	2.57276	2.1
39231	1.89226	2.1
39232	1.89226	2.1
39233	1.89226	2.1
39234	2.32525	2.1
39235	2.32525	2.1
39236	1.15171	2.1
39237	2.17517	2.1
39238	1.41995	2.1
39239	1.41995	2.1
39240	1.80252	2.1
39241	1.80252	2.1
39242	1.89226	2.1
39243	1.89226	2.1
39244	1.89226	2.1
39245	1.89226	2.1
39246	3.78453	2.1
39247	0.946133	2.1
39248	0.946133	2.1
39249	1.16263	2.1
39250	1.04687	2.1
39251	0.946133	2.1
39252	0.946133	2.1
39253	1.04687	2.1
39254	1.04687	2.1
39255	0.946133	2.1
39256	1.04687	2.1
39257	0.946132	2.1
39258	1.16263	2.1
39259	0.682281	1.61996
39260	0.669233	1.72518
39261	1.21859	1.62621
39262	1.31629	1.46498
39263	0.916158	1.37424

39264	0.916158	1.99926
39265	0.971658	1.60987
39266	0.971658	1.57706
39267	0.669234	1.58448
39268	1.31629	1.49512
39269	0.682283	1.61081
39270	1.21858	1.62549
39271	0.669232	1.58758
39272	0.623495	1.65482
39273	1.36428	1.52343
39274	1.31629	1.41621
39275	0.946475	1.36436
39276	0.946476	1.90961
39277	0.99331	1.61243
39278	0.99331	1.55891
39279	1.36428	1.51746
39280	0.623497	1.42552
39281	0.669234	1.54685
39282	1.31629	1.50223
39283	2.02069	0.05
39284	1.46253	0.05
39285	2.51479	0.05
39286	1.87136	0.05
39287	3.89203	0.05
39288	1.46253	0.05
39289	1.38536	0.05
39290	1.38536	0.05
39291	1.05579	0.05
39292	1.73281	0.05
39293	1.46916	0.05
39294	2.51479	0.05
39295	1.87136	0.05
39296	2.23338	0.05
39297	2.15603	0.05
39298	4.55146	0.05
39299	0.822553	0.05
39300	0.822563	0.05
39301	0.842935	0.05
39302	0.842935	0.05
39303	0.744203	0.05
39304	0.675346	0.05
39305	0.874795	0.05
39306	1.00453	0.05
39307	1.00454	0.05
39308	0.782417	0.05
39309	0.675337	0.05
39310	0.858007	0.05
39311	0.946132	1.07634
39312	1.24847	0.967273
39313	0.946132	1.22518
39314	1.16806	1.30217
39315	1.24847	1.03236
39316	1.24847	1.45552
39317	0.946133	0.953802
39318	0.946133	1.26004
39319	0.946133	1.02987
39320	0.946133	1.02117
39321	1.16805	1.15106

39322	1.24847	1.06732
39323	1.2364	1.10652
39324	0.946129	0.955132
39325	0.946133	1.20992
39326	1.24847	1.08141
39327	1.2364	0.973013
39328	1.2364	1.3533
39329	0.946131	0.894899
39330	0.946131	1.51024
39331	0.946131	1.01482
39332	0.946131	0.960234
39333	1.2364	1.26307
39334	1.24847	1.10426
39335	1.89226	1.02957
39336	1.89226	1.28255
39337	1.89226	1.11087
39338	1.89226	1.06686
39339	1.89226	1.17443
39340	1.89226	1.15707
39341	1.89226	1.07234
39342	1.89226	1.06892
39343	1.89226	0.971318
39344	1.89226	1.11799
39345	1.89226	1.04128
39346	1.89226	1.29045
39347	1.69014	1.09538
39348	1.09194	1.10032
39349	1.13537	1.25193
39350	1.13537	1.11211
39351	1.58481	1.0894
39352	1.58481	1.02191
39353	1.58147	1.27153
39354	1.13537	1.11633
39355	1.69015	1.24183
39356	1.01179	1.1757
39357	1.69015	1.07928
39358	1.01179	1.11435
39359	0.946132	1.53664
39360	0.946132	1.20914
39361	1.28543	0.196765
39362	1.07341	1.35631
39363	1.07341	1.51757
39364	1.07341	0.05
39365	0.946132	0.338089
39366	0.946132	0.370121
39367	0.946133	0.14492
39368	1.28543	0.759338
39369	1.07341	0.495493
39370	0.946132	1.14657
39371	1.89226	1.39454
39372	1.89226	0.465128
39373	1.89226	0.629831
39374	1.89226	1.96159
39375	1.89226	0.335178
39376	1.89226	2.1
39377	0.805744	2.1
39378	0.988659	1.98761
39379	0.782044	0.54215

39380	0.578223	0.970818
39381	0.77823	1.0131
39382	0.778231	2.1
39383	0.788653	0.488595
39384	0.788653	1.95432
39385	0.988662	1.39893
39386	0.782042	1.30144
39387	0.805745	1.9085
39388	0.578222	1.6276
39389	1.89226	0.50545
39390	1.89226	1.02694
39391	1.89226	1.49933
39392	1.89226	1.91003
39393	1.89226	0.459958
39394	1.89226	1.99618
39395	1.97732	1.90719
39396	1.97819	0.477961
39397	1.56408	0.511815
39398	1.56408	2.03117
39399	2.33986	0.584983
39400	2.33986	2.09186
39401	1.84516	2.1
39402	2.21152	2.1
39403	1.90702	2.1
39404	1.54599	2.1
39405	3.75751	2.1
39406	1.89224	2.1
39407	1.89224	2.1
39408	1.89224	2.1
39409	1.89224	2.1
39410	1.89224	2.1
39411	1.89224	2.1
39412	1.47672	2.1
39413	1.54601	2.1
39414	1.27697	2.1
39415	1.54601	2.1
39416	1.638 2.1	
39417	1.90705	2.1
39418	0.946132	2.1
39419	0.946132	2.1
39420	0.946131	2.1
39421	0.946131	2.1
39422	0.946131	2.1
39423	0.946131	2.1
39424	0.946132	2.1
39425	0.946132	2.1
39426	0.946132	2.1
39427	0.946132	2.1
39428	0.946131	2.1
39429	0.946131	2.1
39430	0.946128	2.1
39431	0.946128	2.1
39432	1.32062	2.1
39433	1.4164	2.1
39434	0.946129	2.1
39435	0.946129	2.1
39436	1.32062	2.1
39437	1.32062	2.1

39438	1.32062	2.1
39439	0.94613	2.1
39440	1.41639	2.1
39441	0.946129	2.1
39442	1.98979	0.05
39443	1.65977	0.05
39444	2.21772	0.05
39445	2.21772	0.05
39446	1.48866	0.05
39447	1.48866	0.05
39448	0.973129	0.05
39449	0.973129	0.05
39450	0.980954	0.05
39451	0.980954	0.05
39452	0.845218	0.05
39453	1.13981	0.05
39454	0.829886	0.05
39455	1.10886	0.05
39456	1.10886	0.05
39457	1.13982	0.05
39458	0.845219	0.05
39459	0.829886	0.05
39460	0.946132	2.1
39461	0.946132	2.1
39462	1.3352	2.1
39463	1.41432	2.1
39464	0.946133	2.1
39465	0.946133	2.01614
39466	1.3352	2.1
39467	1.3352	2.1
39468	1.3352	2.1
39469	0.946132	2.1
39470	1.41432	2.1
39471	0.946133	2.1
39472	2.70097	2.1
39473	1.88175	2.1
39474	1.51482	2.1
39475	1.88175	2.1
39476	2.37784	2.1
39477	2.82864	2.1
39478	1.89227	0.0599562
39479	1.89227	0.05
39480	1.89227	0.05
39481	1.89227	0.05
39482	3.78453	0.05
39483	1.89226	0.05
39484	2.13295	0.05
39485	2.43203	0.05
39486	2.43203	0.05
39487	1.89226	0.05
39488	1.89226	0.05
39489	0.946133	0.05
39490	0.946133	0.05
39491	0.946132	0.05
39492	0.946132	0.05
39493	0.946132	0.05
39494	0.946132	0.05
39495	0.946133	0.05

39496	0.946133	0.05
39497	0.946132	0.05
39498	0.946132	0.05
39499	0.946132	0.05
39500	0.946132	0.05
39501	1.54987	2.1
39502	1.24128	2.1
39503	1.24128	2.1
39504	1.50738	2.1
39505	1.75702	2.1
39506	2.02312	2.1
39507	1.89289	2.1
39508	1.92264	2.1
39509	1.72994	2.1
39510	1.75702	2.1
39511	3.67966	2.1
39512	1.75702	2.1
39513	0.994291	2.1
39514	0.994291	2.1
39515	1.24128	2.1
39516	1.4964	2.1
39517	1.74339	2.1
39518	1.72993	2.1
39519	1.49641	2.1
39520	1.49641	2.1
39521	1.75702	2.1
39522	1.3939	2.1
39523	1.65452	2.1
39524	1.19964	2.1
39525	1.53296	2.1
39526	1.19964	2.1
39527	1.36861	2.1
39528	1.42624	2.1
39529	1.59521	2.1
39530	1.19964	2.1
39531	1.42624	2.1
39532	1.07156	2.1
39533	1.36226	2.1
39534	2.5619	2.1
39535	2.14058	1.35818
39536	1.93569	1.93327
39537	2.36221	1.5561
39538	2.5753	1.3963
39539	4.50279	1.58824
39540	2.14057	1.62954
39541	2.20831	1.91107
39542	2.64725	1.14598
39543	2.57529	1.63857
39544	4.78782	1.52024
39545	2.64725	1.19827
39546	2.57529	1.63696
39547	1.89226	1.29183
39548	1.89226	1.07931
39549	4.53951	1.36525
39550	2.0815	2.1
39551	1.86137	2.1
39552	1.86137	2.1
39553	1.86159	2.1

39554	2.0815	2.1
39555	2.10235	2.1
39556	1.33552	2.1
39557	2.32417	2.1
39558	1.18872	2.1
39559	1.93101	2.1
39560	3.26653	2.1
39561	1.89226	2.1
39562	1.89226	2.1
39563	1.89226	2.1
39564	1.89226	2.1
39565	1.89226	2.1
39566	1.89226	2.1
39567	0.955191	2.1
39568	1.03372	2.1
39569	0.946131	2.1
39570	0.946131	2.1
39571	0.930654	2.1
39572	0.930654	2.1
39573	1.04233	2.1
39574	1.05247	2.1
39575	0.955341	2.1
39576	0.946131	2.1
39577	1.02375	2.1
39578	1.13537	2.1
39579	1.1549	2.1
39580	1.93101	2.1
39581	1.91108	2.1
39582	1.52487	2.1
39583	1.3076	2.1
39584	1.06985	2.1
39585	1.89226	2.1
39586	1.89226	2.1
39587	1.89226	2.1
39588	1.89226	2.1
39589	1.89226	2.1
39590	1.89226	2.1
39591	1.58713	2.1
39592	2.6845	2.1
39593	1.58742	2.1
39594	2.74201	2.1
39595	4.27191	2.1
39596	2.68449	2.1
39597	1.89226	2.1
39598	2.74201	2.1
39599	1.89226	2.1
39600	4.57675	2.1
39601	1.57523	2.1
39602	2.74201	2.1
39603	2.8146	2.1
39604	2.8146	2.1
39605	1.58742	2.1
39606	1.58742	2.1
39607	0.946131	2.1
39608	0.946131	2.1
39609	1.371 2.1	
39610	1.371 2.1	
39611	0.946127	2.1

39612	0.946127	2.1
39613	1.37101	2.1
39614	1.4073	2.1
39615	0.94613	2.1
39616	1.4073	2.1
39617	1.371	2.1
39618	0.946129	2.1
39619	1.44801	1.93581
39620	1.06821	1.96063
39621	2.00542	1.89937
39622	2.27703	2.04199
39623	3.34524	1.85879
39624	1.70301	0.163951
39625	1.89226	0.310096
39626	1.65809	0.238781
39627	1.89226	0.339696
39628	1.62758	0.11785
39629	1.85744	0.139926
39630	1.89226	0.100213
39631	1.89226	0.345848
39632	1.89226	0.0952277
39633	1.89226	0.0848735
39634	3.78452	0.0906173
39635	1.89226	0.187916
39636	1.89226	0.102388
39637	1.89226	0.0778096
39638	1.89226	0.0990014
39639	3.78452	0.10713
39640	0.851541	0.375199
39641	0.851541	0.361071
39642	0.91193	0.246215
39643	0.824837	0.2325
39644	0.854559	0.259858
39645	0.851534	0.316571
39646	0.81851	0.288233
39647	0.717219	0.237033
39648	0.871743	0.197382
39649	0.837985	0.147009
39650	0.82902	0.302294
39651	0.82902	0.359146
39652	1.89226	0.431191
39653	1.92659	0.0508194
39654	2.03235	0.066144
39655	1.89226	0.548944
39656	2.03235	0.05
39657	1.89226	0.426094
39658	0.585557	0.512538
39659	0.585557	0.271571
39660	1.22612	0.126321
39661	1.09436	0.363759
39662	0.851537	0.526943
39663	0.526987	0.389175
39664	0.983879	0.0681551
39665	1.04072	0.614081
39666	0.585526	0.05
39667	0.792325	0.0501289
39668	0.946129	0.399946
39669	0.946129	0.333946

39670	1.68152	2.1
39671	1.81445	2.1
39672	1.68152	2.1
39673	1.86792	2.1
39674	1.65888	2.1
39675	1.84529	2.1
39676	1.81445	2.1
39677	1.57885	2.1
39678	1.65889	2.1
39679	1.48594	2.1
39680	3.23773	2.1
39681	1.68154	2.1
39682	1.65891	2.1
39683	1.53296	2.1
39684	1.56299	2.1
39685	3.24453	2.1
39686	1.56299	2.1
39687	1.48596	2.1
39688	1.56299	2.1
39689	1.65891	2.1
39690	1.46568	2.1
39691	1.5616	2.1
39692	2.68785	2.1
39693	2.08831	2.1
39694	2.66919	2.1
39695	2.1024	2.1
39696	4.79025	2.1
39697	2.08832	2.1
39698	1.15478	2.1
39699	1.15478	2.1
39700	1.09062	2.1
39701	2.10241	2.1
39702	2.03825	2.1
39703	2.65348	2.1
39704	2.1024	2.1
39705	2.12313	2.1
39706	2.12313	2.1
39707	2.66919	2.1
39708	2.66919	2.1
39709	0.813169	2.1
39710	0.813169	2.1
39711	0.825788	2.1
39712	0.825788	2.1
39713	0.612902	2.1
39714	0.577387	2.1
39715	1.0512	2.1
39716	1.06157	2.1
39717	1.06157	2.1
39718	0.612902	2.1
39719	0.577387	2.1
39720	1.0512	2.1
39721	1.27227	2.1
39722	1.91724	2.1
39723	1.32591	2.1
39724	1.99837	2.1
39725	3.24315	2.1
39726	1.89223	2.1
39727	1.89223	2.1

39728	1.89223	2.1
39729	1.89223	2.1
39730	3.78446	2.1
39731	1.89224	2.1
39732	1.89224	2.1
39733	1.89224	2.1
39734	1.89224	2.1
39735	3.78447	2.1
39736	1.91724	2.1
39737	2.321	2.1
39738	1.99838	2.1
39739	2.4149	2.1
39740	4.33214	2.1
39741	1.34817	2.1
39742	1.9984	2.1
39743	2.06238	2.1
39744	2.06238	2.1
39745	1.32593	2.1
39746	1.32593	2.1
39747	1.89226	2.1
39748	1.89226	2.1
39749	1.89226	2.1
39750	1.89226	2.1
39751	1.89226	2.1
39752	1.89226	2.1
39753	1.89226	2.1
39754	1.89226	2.1
39755	1.89226	2.1
39756	1.89226	2.1
39757	3.78452	2.1
39758	1.9984	2.1
39759	2.41494	2.1
39760	2.06239	2.1
39761	2.5194	2.1
39762	4.47732	2.1
39763	1.19901	1.75083
39764	1.16661	2.1
39765	1.56859	2.1
39766	1.61029	1.70704
39767	2.7676	2.1
39768	1.56859	2.1
39769	1.88637	0.6536
39770	1.61029	0.600392
39771	1.61029	2.1
39772	1.856	0.662399
39773	1.856	2.1
39774	1.856	2.02186
39775	1.57916	2.1
39776	1.56859	2.1
39777	1.56859	2.1
39778	1.57916	2.1
39779	1.23475	2.1
39780	0.890924	0.35958
39781	1.07608	0.342134
39782	0.72877	0.608304
39783	0.910106	0.974604
39784	1.00196	1.12778
39785	1.09519	0.926353

39786	0.839967	0.489182
39787	0.719043	0.269696
39788	0.893859	0.30193
39789	0.942408	1.04818
39790	0.873497	0.854194
39791	0.873497	1.83442
39792	1.53046	0.181875
39793	1.89994	0.501575
39794	1.049	0.270615
39795	1.049	0.167689
39796	1.52512	0.188973
39797	1.89994	0.640119
39798	1.65573	1.60194
39799	1.89226	0.483105
39800	1.89226	0.217844
39801	2.73329	1.20293
39802	3.548	1.11625
39803	1.65573	0.121068
39804	2.42671	1.05145
39805	2.73329	0.140903
39806	1.89226	0.136485
39807	2.73329	0.492505
39808	1.89226	0.158528
39809	0.941078	0.509169
39810	1.01599	1.13525
39811	0.818936	0.500497
39812	0.651554	1.12237
39813	0.904729	0.967207
39814	0.665756	0.79334
39815	0.843423	1.66821
39816	0.918649	0.810741
39817	0.855806	1.11554
39818	0.814384	0.191897
39819	0.844437	1.549
39820	0.844437	1.50218
39821	2.22558	1.95407
39822	1.95785	0.53127
39823	2.07065	1.509
39824	1.89142	1.53291
39825	2.07065	1.8684
39826	1.89142	1.85128
39827	0.640813	1.09173
39828	0.951654	1.00458
39829	0.797442	0.377682
39830	0.535364	1.03777
39831	0.525269	1.31948
39832	0.620874	0.481028
39833	0.916953	0.77285
39834	0.969374	1.00402
39835	0.788925	1.38637
39836	0.69596	0.248205
39837	0.743154	1.39432
39838	0.743154	1.42249
39839	1.89226	1.95468
39840	1.89226	1.70565
39841	1.89226	1.57415
39842	1.89226	2.00123
39843	3.78452	1.72686

39844	1.32541	1.70407
39845	1.13722	1.60144
39846	0.91103	2.1
39847	0.881123	1.95418
39848	1.03363	2.08451
39849	1.03364	1.77386
39850	1.1505	1.72015
39851	1.1505	1.97407
39852	1.32539	1.8864
39853	1.07662	1.93254
39854	0.881121	1.80628
39855	0.962303	1.80939
39856	1.89226	1.82267
39857	1.89226	2.1
39858	1.89226	2.1
39859	1.89226	1.85852
39860	3.78453	2.09141
39861	1.89226	1.549
39862	1.89226	1.91557
39863	1.89226	1.83473
39864	1.89226	1.65075
39865	1.89226	1.83119
39866	1.89226	1.66882
39867	1.27606	1.88156
39868	1.07664	1.82831
39869	0.905895	2.1
39870	0.696099	2.1
39871	0.984902	2.1
39872	0.984905	1.90859
39873	1.01878	2.1
39874	1.01878	1.75257
39875	1.27604	2.03312
39876	1.06044	2.01312
39877	1.13192	2.02007
39878	0.696111	2.01798
39879	1.89226	1.93644
39880	1.89226	0.656898
39881	1.89226	0.230861
39882	1.89226	1.96654
39883	1.89226	0.59971
39884	1.89226	1.80702
39885	1.52615	2.1
39886	1.01294	0.912588
39887	1.01294	2.1
39888	1.18549	0.241415
39889	1.38963	1.67445
39890	1.56218	0.643979
39891	1.89226	1.50339
39892	2.40801	1.61174
39893	2.58101	2.07598
39894	2.58101	1.3478
39895	1.89226	2.1
39896	1.89226	0.451486
39897	2.24071	2.1
39898	2.79317	2.1
39899	1.99136	2.1
39900	2.6167	2.1
39901	4.85741	2.1

39902	2.79317	2.1
39903	1.89226	2.1
39904	1.89226	2.1
39905	1.89226	2.1
39906	2.6167	2.1
39907	2.6167	2.09508
39908	1.08354	2.1
39909	1.08354	2.1
39910	1.10222	2.1
39911	1.10222	2.1
39912	1.2275	2.1
39913	1.30835	2.1
39914	0.995678	2.1
39915	0.877412	2.1
39916	0.877411	2.1
39917	1.2275	2.1
39918	0.995678	2.1
39919	1.30835	2.1
39920	1.05793	0.556869
39921	1.46441	0.227332
39922	1.0562	0.277363
39923	1.0562	0.244119
39924	1.41787	0.931238
39925	1.41787	0.207343
39926	1.41787	0.313869
39927	1.52909	1.19962
39928	1.46441	0.305694
39929	1.46441	0.876489
39930	1.43063	0.344016
39931	1.43063	0.748675
39932	1.89226	1.32443
39933	1.89226	0.396038
39934	1.89226	0.412136
39935	1.89226	1.83107
39936	3.78453	1.24418
39937	1.89227	1.02272
39938	1.89227	0.887166
39939	1.89227	0.31467
39940	1.89227	0.351559
39941	1.89227	0.978685
39942	1.89227	0.281791
39943	1.00763	0.161543
39944	1.41787	0.320675
39945	1.05793	0.163566
39946	1.05793	0.318106
39947	1.37265	0.671096
39948	1.37265	0.298001
39949	1.37265	0.295373
39950	1.43063	1.00757
39951	1.41787	0.312262
39952	1.41787	0.897897
39953	1.38247	0.342918
39954	1.38247	0.884355
39955	0.946132	1.29791
39956	0.946132	1.42186
39957	0.946132	0.330007
39958	0.946132	0.432879
39959	0.946131	0.382775

39960	0.946131	1.41486
39961	0.946132	1.59543
39962	0.946132	0.706499
39963	0.946132	0.90605
39964	0.946132	1.14209
39965	0.946132	0.95542
39966	0.946132	0.954977
39967	1.89226	0.498752
39968	1.89226	0.52108
39969	1.89226	0.199434
39970	1.89226	0.543834
39971	1.89226	0.234143
39972	1.89226	0.595575
39973	1.19645	1.17576
39974	1.89183	0.915829
39975	1.91106	1.13337
39976	1.35306	1.04963
39977	1.75446	1.11735
39978	1.19645	1.03237
39979	2.37576	1.92905
39980	1.90139	2.1
39981	2.1845	2.1
39982	1.86568	2.1
39983	4.24145	2.1
39984	2.29435	2.1
39985	1.40602	2.1
39986	1.40602	2.1
39987	1.55351	2.1
39988	1.86568	2.1
39989	2.01317	2.1
39990	2.18452	2.1
39991	1.8657	1.7246
39992	2.01399	1.93057
39993	1.70141	2.1
39994	3.87969	2.08055
39995	0.774999	2.1
39996	0.774999	2.1
39997	0.778721	2.1
39998	0.778721	2.1
39999	0.628348	2.1
40000	0.703016	2.1
40001	0.932848	2.1
40002	0.850704	2.1
40003	0.850704	2.1
40004	0.628348	2.1
40005	0.703015	2.1
40006	0.932848	2.1
40007	1.65243	0.719803
40008	1.34412	0.667098
40009	1.45569	1.85664
40010	1.1315	1.84986
40011	2.78393	1.46151
40012	1.48901	2.1
40013	1.805 2.1	
40014	1.48901	2.1
40015	1.71717	2.1
40016	1.60635	2.1
40017	1.83451	2.1

40018	1.805	2.1
40019	1.65243	0.636741
40020	1.60635	2.1
40021	1.45569	1.77141
40022	3.25879	2.00217
40023	1.27875	2.1
40024	1.60635	2.1
40025	1.27875	2.1
40026	1.48901	2.1
40027	1.40014	2.1
40028	1.6104	2.1
40029	0.715199	1.98081
40030	0.715199	2.1
40031	0.712714	2.1
40032	0.712714	0.9701
40033	0.727843	0.933006
40034	0.619713	1.44133
40035	0.803177	2.1
40036	0.70007	2.1
40037	0.700069	2.1
40038	0.619713	2.1
40039	0.803177	2.06538
40040	0.727844	2.09871
40041	1.14357	2.1
40042	2.3506	2.03803
40043	1.14357	2.09913
40044	2.68773	1.99571
40045	1.57829	2.08851
40046	2.68773	1.98187
40047	2.00551	2.1
40048	2.65811	2.0484
40049	2.07203	2.1
40050	2.72117	0.752205
40051	4.72667	2.1
40052	0.946131	1.43767
40053	0.946131	2.1
40054	1.32905	2.1
40055	1.32905	0.560559
40056	0.94613	1.58103
40057	0.94613	1.90188
40058	1.32905	1.829
40059	1.36058	1.7369
40060	1.36058	0.36973
40061	0.946131	0.259004
40062	1.32905	1.69883
40063	0.946131	1.7454
40064	1.21649	0.872994
40065	1.21649	2.1
40066	1.21459	2.1
40067	1.21459	2.1
40068	1.39508	1.76435
40069	1.36057	1.77246
40070	1.03601	2.1
40071	1.07438	2.1
40072	1.07439	2.1
40073	1.39509	2.1
40074	1.036	2.1
40075	1.36056	2.1

40076	2.79015	0.931888
40077	2.72114	2.1
40078	1.89225	1.49478
40079	1.89225	2.1
40080	1.89225	1.93249
40081	2.72114	1.77654
40082	0.876258	2.1
40083	0.892889	2.1
40084	1.34352	2.1
40085	1.27985	2.1
40086	1.07462	2.1
40087	1.07462	2.1
40088	1.09812	2.1
40089	1.09812	2.1
40090	0.892888	2.1
40091	1.34352	2.1
40092	0.876256	2.1
40093	1.27985	2.1
40094	1.39725	2.0178
40095	1.30029	0.785198
40096	1.31415	1.05491
40097	1.41336	1.97283
40098	2.71366	1.67468
40099	1.89226	0.916392
40100	1.89226	1.91619
40101	1.89226	1.99861
40102	1.89226	1.24184
40103	3.78453	1.65264
40104	0.946133	1.46307
40105	0.946133	1.18422
40106	1.38709	0.360301
40107	1.39437	0.46434
40108	0.946132	0.364823
40109	0.946132	0.990788
40110	1.39437	1.5101
40111	1.39437	0.380094
40112	0.946132	0.720394
40113	1.39437	0.665096
40114	1.38709	0.742349
40115	0.946132	0.775304
40116	0.706681	2.07164
40117	0.711908	2.01627
40118	0.657077	0.91355
40119	0.661003	1.45208
40120	0.684188	0.972463
40121	0.684188	2.01296
40122	0.683496	2.1
40123	0.683496	0.994029
40124	0.711908	1.76391
40125	0.661003	1.87651
40126	0.657076	1.65169
40127	0.706681	1.6516
40128	0.946132	2.04838
40129	0.946132	1.94393
40130	0.946132	1.61622
40131	0.946132	0.901249
40132	0.946132	0.902599
40133	0.946132	1.96414

40134	0.946133	1.58845
40135	0.946133	2.06182
40136	0.946133	1.75905
40137	0.946133	1.82812
40138	0.946132	1.48167
40139	0.946132	1.81496
40140	2.79029	0.427568
40141	1.89227	1.69066
40142	2.78873	0.619256
40143	2.78873	0.798021
40144	1.89383	0.711856
40145	1.89227	0.888583
40146	1.43669	2.1
40147	2.11984	2.1
40148	2.24481	2.1
40149	2.24481	2.1
40150	1.20311	2.1
40151	1.20311	2.1
40152	0.49703	2.1
40153	0.601553	2.1
40154	1.05992	2.1
40155	0.991694	2.1
40156	0.787558	2.1
40157	0.787558	2.1
40158	0.805689	2.1
40159	0.805689	2.1
40160	0.991694	2.1
40161	0.49703	2.1
40162	1.05992	2.1
40163	0.601553	2.1
40164	2.45905	2.1
40165	1.89226	2.1
40166	1.89226	2.1
40167	1.89226	2.1
40168	2.47395	2.1
40169	2.47395	2.1
40170	1.89226	2.1
40171	1.89226	2.1
40172	1.89226	2.1
40173	1.89226	2.04686
40174	3.78452	2.1
40175	1.38427	2.1
40176	2.31203	1.95381
40177	2.28743	1.97996
40178	2.28743	2.1
40179	1.63384	1.96347
40180	1.63384	2.1
40181	2.42723	2.1
40182	1.89226	2.1
40183	1.89226	2.1
40184	1.89226	2.1
40185	2.45905	2.1
40186	2.45905	2.1
40187	0.946131	1.87371
40188	0.946131	2.1
40189	0.946131	2.1
40190	0.946131	2.1
40191	0.94613	2.08129

40192	0.94613	2.1
40193	0.946132	2.1
40194	0.946132	2.1
40195	0.946131	2.1
40196	0.946131	2.1
40197	0.946131	2.1
40198	0.946131	2.1
40199	0.989441	2.1
40200	0.989441	2.1
40201	0.993918	2.1
40202	0.993918	2.1
40203	1.16644	2.1
40204	1.15601	2.1
40205	0.818308	2.1
40206	0.816922	2.1
40207	0.818307	2.1
40208	1.16644	2.1
40209	1.15601	2.1
40210	0.816922	2.1
40211	0.996778	0.05
40212	2.44375	0.05
40213	0.996778	0.05
40214	1.92547	0.05
40215	1.56091	0.05
40216	2.4896	0.05
40217	1.56091	0.05
40218	2.44375	0.05
40219	1.90456	0.05
40220	1.90456	0.05
40221	2.81853	0.05
40222	2.47487	0.05
40223	1.32143	2.05357
40224	1.43294	2.1
40225	1.37444	1.31746
40226	1.51691	2.1
40227	2.80738	1.91961
40228	1.89226	1.70092
40229	1.89226	2.1
40230	1.89226	1.44724
40231	1.89226	1.80716
40232	3.78453	1.57455
40233	1.89226	2.1
40234	1.89226	2.1
40235	1.89226	1.70875
40236	1.89226	2.1
40237	3.78453	2.1
40238	1.43294	2.08841
40239	1.3268	2.1
40240	1.51691	2.00142
40241	1.44091	2.1
40242	2.87385	2.1
40243	1.37444	2.01345
40244	1.51691	2.1
40245	1.4714	1.36284
40246	1.65434	1.88902
40247	3.02878	1.74282
40248	1.89226	2.03734
40249	1.89226	2.1

40250	1.89226	1.46349
40251	1.89226	1.92629
40252	3.78452	1.72511
40253	1.89226	1.89029
40254	1.89226	2.1
40255	1.89226	1.7509
40256	1.89226	2.1
40257	3.78453	2.1
40258	1.51691	2.1
40259	1.44091	2.1
40260	1.65434	1.75606
40261	1.60647	2.1
40262	3.09526	2.1
40263	1.05013	2.1
40264	1.05013	2.1
40265	0.94613	2.1
40266	0.94613	2.1
40267	1.04959	2.1
40268	1.05014	2.1
40269	0.946129	2.1
40270	0.946129	2.1
40271	0.946131	2.1
40272	1.04958	2.1
40273	0.946131	2.1
40274	1.05013	2.1
40275	1.04959	2.1
40276	1.04959	2.1
40277	0.946131	2.1
40278	0.946131	2.1
40279	1.04765	2.1
40280	1.04959	2.1
40281	0.94613	2.1
40282	0.94613	2.1
40283	0.946131	2.1
40284	1.04765	2.1
40285	0.946131	2.1
40286	1.04959	2.1
40287	1.85744	2.1
40288	1.89226	2.1
40289	1.89226	2.1
40290	1.89226	2.1
40291	1.94839	2.1
40292	1.94839	2.1
40293	0.97419	2.1
40294	0.822054	2.1
40295	0.946123	2.1
40296	0.946123	2.1
40297	1.04202	2.1
40298	0.84898	2.1
40299	0.933307	2.1
40300	0.933307	2.1
40301	0.933285	2.1
40302	1.05018	2.1
40303	0.851528	2.1
40304	0.876787	2.1
40305	1.87528	0.0810781
40306	1.21137	0.0535262
40307	1.57893	0.0518067

40308	1.21137	0.118584
40309	1.81182	0.0533464
40310	1.44426	0.103004
40311	0.993833	0.05
40312	1.21137	0.116943
40313	1.57893	0.0684209
40314	1.31774	0.05
40315	2.5291	0.051718
40316	1.87528	0.072886
40317	1.57893	0.05
40318	1.90728	0.05
40319	2.29851	0.077519
40320	3.87744	0.0675246
40321	1.90728	0.05
40322	1.31774	0.0667721
40323	1.56339	0.05
40324	1.31774	0.0756115
40325	1.82458	0.05
40326	1.57893	0.0919623
40327	1.89226	1.39028
40328	1.89226	1.46653
40329	1.89226	1.42626
40330	1.89226	1.44422
40331	1.89226	1.42627
40332	1.89226	1.44399
40333	1.89226	1.44741
40334	1.89226	1.47724
40335	1.89226	1.51039
40336	1.89226	1.48216
40337	3.78452	1.5179
40338	1.89226	1.46188
40339	2.17251	1.54553
40340	2.45575	1.44916
40341	2.45575	1.47066
40342	1.89226	1.45677
40343	1.89226	1.46784
40344	1.08625	1.53545
40345	1.22788	1.46305
40346	0.54447	1.4027
40347	0.794861	1.48383
40348	0.967729	1.56147
40349	0.967729	1.53642
40350	0.913386	1.40955
40351	0.913386	1.3197
40352	1.08625	1.52829
40353	0.544469	1.36866
40354	0.794862	1.5424
40355	1.22788	1.50747
40356	0.946128	1.43578
40357	0.946128	1.45297
40358	0.946132	1.451
40359	0.946132	1.43028
40360	0.94613	1.44948
40361	0.94613	1.44342
40362	0.94613	1.45274
40363	0.94613	1.41172
40364	0.94613	1.41851
40365	0.94613	1.41946

40366	0.94613	1.4083
40367	0.94613	1.42781
40368	0.946127	1.43336
40369	0.946127	1.41966
40370	1.08625	1.39232
40371	0.958779	1.45199
40372	1.08625	1.33743
40373	1.08625	1.44624
40374	0.946128	1.42814
40375	0.946128	1.42077
40376	0.946129	1.40341
40377	0.958777	1.41793
40378	1.08625	1.38073
40379	0.946129	1.41448
40380	2.16338	0.05
40381	1.94002	0.05
40382	1.98836	0.05
40383	2.60614	0.05
40384	1.5456	0.05
40385	2.16338	0.05
40386	1.19952	0.05
40387	2.34061	0.05
40388	1.21031	0.05
40389	2.1711	0.05
40390	3.54013	0.05
40391	1.13624	2.1
40392	1.70302	2.1
40393	1.8267	2.1
40394	1.11545	2.1
40395	2.85455	2.1
40396	1.82671	2.1
40397	1.91767	2.1
40398	1.81563	2.1
40399	1.91767	2.1
40400	1.71362	2.1
40401	1.83277	2.1
40402	1.66064	2.1
40403	0.996776	2.1
40404	0.996776	2.1
40405	1.11545	2.1
40406	1.54278	2.1
40407	1.66145	2.1
40408	1.66064	2.1
40409	1.7457	2.1
40410	1.81563	2.1
40411	1.81563	2.1
40412	1.54278	2.1
40413	1.54278	2.1
40414	2.71672	2.1
40415	2.70402	2.1
40416	2.64996	2.1
40417	2.69443	2.1
40418	5.39845	2.1
40419	2.71672	2.1
40420	2.54198	2.1
40421	2.54019	2.1
40422	2.69443	2.1
40423	5.23641	2.1

40424	2.64996	2.1
40425	2.69443	2.1
40426	2.68213	2.1
40427	2.61243	2.1
40428	5.30686	2.1
40429	2.54019	2.1
40430	2.69443	2.1
40431	2.68213	2.1
40432	2.5392	2.1
40433	5.23363	2.1
40434	1.89226	2.1
40435	2.34725	2.1
40436	2.22414	2.1
40437	2.34725	2.1
40438	1.76914	2.1
40439	1.89226	2.1
40440	0.946131	2.1
40441	0.946131	2.1
40442	1.11207	2.1
40443	1.03798	2.1
40444	0.94613	2.1
40445	0.94613	2.1
40446	1.03798	2.1
40447	1.03798	2.1
40448	0.946131	2.1
40449	1.03798	2.1
40450	0.946131	2.1
40451	1.11207	2.1
40452	1.89226	0.182671
40453	1.89226	0.159068
40454	1.89226	0.172625
40455	1.89226	0.145717
40456	1.89226	0.202116
40457	1.89226	0.173395
40458	1.89226	0.193176
40459	1.89226	0.187164
40460	1.89226	0.203024
40461	1.89226	0.158785
40462	1.89226	0.234272
40463	1.89226	0.186952
40464	1.89226	0.113965
40465	1.89226	0.104836
40466	1.89226	0.115839
40467	1.89226	0.098396
40468	1.89226	0.131207
40469	1.89226	0.112711
40470	1.44085	2.1
40471	1.31825	1.00803
40472	1.31923	1.85544
40473	1.31825	2.09545
40474	1.43311	1.42496
40475	1.43213	1.9776
40476	1.89226	1.14882
40477	1.89226	2.01121
40478	1.89226	1.67371
40479	1.89226	2.02584
40480	1.89226	1.24516
40481	1.89226	2.06428

40482	0.946133	1.70048
40483	0.946133	1.25914
40484	1.38969	0.482093
40485	1.39587	0.72175
40486	0.946132	0.515088
40487	0.946132	1.14458
40488	1.39587	1.71785
40489	1.39587	0.524632
40490	0.946132	0.854778
40491	1.39587	0.764577
40492	1.38969	0.938097
40493	0.946132	1.14601
40494	1.44085	2.1
40495	1.31923	0.952443
40496	1.33026	1.85039
40497	1.4617	2.09883
40498	2.78093	1.96638
40499	1.89226	1.08803
40500	1.89226	1.97334
40501	1.89226	1.6523
40502	1.89226	2.06363
40503	1.89226	1.29766
40504	1.89226	2.1
40505	0.946133	1.59301
40506	0.946133	1.10989
40507	1.39587	0.652014
40508	1.40724	0.445206
40509	0.946132	0.496506
40510	0.946132	0.979148
40511	1.40724	1.62144
40512	1.40724	0.576882
40513	0.946132	0.777606
40514	1.40724	0.764175
40515	0.946133	1.21737
40516	1.39587	0.836225
40517	1.15818	1.40718
40518	1.81846	1.32848
40519	1.52782	1.21469
40520	1.52782	1.6113
40521	1.28095	1.20909
40522	1.28095	1.44792
40523	2.56709	2.1
40524	1.4349	1.73711
40525	1.4349	2.1
40526	1.75471	2.1
40527	2.29909	2.1
40528	2.61889	2.1
40529	2.5671	2.1
40530	2.82858	2.1
40531	2.57368	2.1
40532	2.29909	2.1
40533	5.12767	2.1
40534	2.29909	2.1
40535	1.09148	1.07343
40536	1.09148	2.1
40537	1.4349	1.96147
40538	1.99776	2.01939
40539	2.34119	2.09962

40540	2.57368	2.1
40541	1.99777	2.1
40542	1.99777	2.1
40543	2.29909	2.1
40544	2.36071	2.1
40545	2.66203	2.1
40546	1.89226	1.53856
40547	1.89226	1.65919
40548	1.89226	1.57265
40549	1.89226	1.52249
40550	3.78453	1.54003
40551	0.946128	1.77479
40552	0.946128	1.8545
40553	0.946133	1.6776
40554	0.946133	1.73636
40555	0.946131	1.72668
40556	0.946131	1.82851
40557	0.946131	1.81624
40558	0.946131	1.74138
40559	0.94613	1.82708
40560	0.94613	1.76102
40561	0.946131	1.71657
40562	0.946131	1.78081
40563	1.89226	1.66193
40564	1.89226	1.66826
40565	1.89226	1.52529
40566	1.89226	1.72116
40567	3.78453	1.70151
40568	1.89226	1.70029
40569	1.89226	1.60857
40570	1.89226	1.50335
40571	1.89226	1.66568
40572	3.78453	1.67098
40573	1.89226	1.6141
40574	2.61682	1.80694
40575	1.89226	1.74469
40576	1.89226	1.79792
40577	1.89226	1.60099
40578	1.89226	1.74008
40579	1.89226	0.338278
40580	1.89226	0.118659
40581	1.89226	0.220912
40582	1.89226	0.243931
40583	3.78452	0.317102
40584	1.89226	0.338772
40585	1.89226	0.254267
40586	1.89226	0.226117
40587	1.89226	0.322874
40588	1.89226	0.314545
40589	1.89226	0.316842
40590	1.89227	0.178723
40591	1.89227	0.0539674
40592	1.89227	0.248232
40593	1.89227	0.321115
40594	1.89227	0.05
40595	1.89227	0.142468
40596	0.80447	0.287069
40597	0.534241	0.246854

40598	0.995175	0.338462
40599	0.92204	0.118106
40600	0.922042	0.0875351
40601	0.534241	0.123751
40602	0.804468	0.269475
40603	0.995176	0.12702
40604	0.71543	0.309845
40605	0.71543	0.0668856
40606	0.813988	0.157559
40607	0.813988	0.16372
40608	0.922045	0.05
40609	0.942899	0.181744
40610	0.946132	0.123937
40611	0.946132	0.05
40612	1.05169	0.05
40613	0.936661	0.141656
40614	0.922045	0.05
40615	0.946133	0.05
40616	1.01665	0.05
40617	1.13008	0.05
40618	0.851529	0.05
40619	0.851529	0.05
40620	1.41908	2.1
40621	1.77723	2.1
40622	1.90485	2.1
40623	1.90485	2.1
40624	1.30237	2.1
40625	1.30237	2.1
40626	1.77723	2.1
40627	2.0055	2.1
40628	1.90485	2.1
40629	2.17158	2.1
40630	3.91035	2.1
40631	0.850906	2.1
40632	0.850906	2.1
40633	0.85668	2.1
40634	0.85668	2.1
40635	0.998045	2.1
40636	0.952425	2.1
40637	0.74367	2.1
40638	0.709541	2.1
40639	0.74367	2.1
40640	0.998045	2.1
40641	0.709541	2.1
40642	0.952425	2.1
40643	1.90485	2.1
40644	2.28563	2.1
40645	2.26282	2.1
40646	2.17158	2.1
40647	1.99609	2.1
40648	1.90485	2.1
40649	2.00098	0.126232
40650	1.90462	0.092685
40651	1.60057	0.0994221
40652	1.79224	0.120796
40653	3.69686	0.104456
40654	2.65654	0.127758
40655	1.79224	0.0888801

40656	2.22332	0.092474
40657	2.22332	0.110543
40658	2.24163	0.104217
40659	2.24163	0.113174
40660	1.89226	0.113236
40661	1.89226	0.109318
40662	1.89226	0.0884098
40663	1.89226	0.113783
40664	3.78452	0.110978
40665	1.89226	0.0903864
40666	1.89226	0.216345
40667	1.89226	0.0989626
40668	1.89226	0.132553
40669	1.89226	0.100496
40670	1.89226	0.131975
40671	2.24163	0.147286
40672	1.03645	0.117181
40673	1.79224	0.121372
40674	1.79224	0.131339
40675	1.54283	0.138856
40676	1.54283	0.141805
40677	0.946132	0.117253
40678	0.946132	0.117752
40679	0.946129	0.143098
40680	0.946129	0.122835
40681	0.94613	0.103883
40682	0.94613	0.125754
40683	0.946131	0.125882
40684	0.946131	0.135023
40685	0.94613	0.112824
40686	0.94613	0.16091
40687	0.946131	0.122771
40688	0.946131	0.10256
40689	1.89226	2.1
40690	1.89226	2.1
40691	1.89226	2.1
40692	1.89226	2.1
40693	3.78453	2.1
40694	1.52441	2.1
40695	1.30347	2.1
40696	1.52441	2.1
40697	1.58401	2.1
40698	1.2627	2.1
40699	1.3223	2.1
40700	1.89226	2.1
40701	2.72787	2.1
40702	2.72787	2.1
40703	2.74762	2.1
40704	1.89226	2.1
40705	1.91202	2.1
40706	1.89227	2.1
40707	1.89227	2.1
40708	1.89227	2.1
40709	1.89227	2.1
40710	3.78453	2.1
40711	0.56414	2.1
40712	0.561557	2.1
40713	0.527284	2.1

40714	0.541143	2.1
40715	0.527276	2.1
40716	0.631685	2.1
40717	1.52441	2.1
40718	1.00232	2.1
40719	0.784495	2.1
40720	0.842304	2.1
40721	0.721569	2.1
40722	1.36393	2.1
40723	1.36393	2.1
40724	0.946132	2.1
40725	0.946132	2.1
40726	1.36393	2.1
40727	1.3594	2.1
40728	0.946132	2.1
40729	0.946132	2.1
40730	0.946107	2.1
40731	1.35936	2.1
40732	1.36397	2.1
40733	0.946158	2.1
40734	1.89226	2.1
40735	1.89226	2.1
40736	1.89226	2.1
40737	1.89226	2.1
40738	3.78453	2.1
40739	2.20802	2.1
40740	1.05677	2.1
40741	2.30479	2.1
40742	0.978699	2.1
40743	3.18672	2.1
40744	0.94613	2.1
40745	0.94613	2.1
40746	0.669866	2.1
40747	0.669866	2.1
40748	0.681588	2.1
40749	0.669865	2.1
40750	0.946133	2.1
40751	0.946133	2.1
40752	0.94613	2.1
40753	0.681589	2.1
40754	0.946131	2.1
40755	0.669867	2.1
40756	2.73514	2.1
40757	1.78301	2.1
40758	1.90618	2.1
40759	2.83464	2.1
40760	4.64132	2.1
40761	2.73514	2.1
40762	1.89226	2.1
40763	1.89226	2.1
40764	2.83464	2.1
40765	4.6274	2.1
40766	2.03915	2.1
40767	2.26616	2.1
40768	2.23549	2.1
40769	2.01704	2.1
40770	4.2832	2.1
40771	2.03916	2.1

40772	1.55766	2.1
40773	1.53728	2.1
40774	2.01704	2.1
40775	3.5747	2.1
40776	2.23551	2.1
40777	2.01705	2.1
40778	1.99823	2.1
40779	2.21189	2.1
40780	4.22894	2.1
40781	1.51788	2.1
40782	2.01705	2.1
40783	1.53729	2.1
40784	1.53729	2.1
40785	1.99823	2.1
40786	1.99823	2.1
40787	2.588	2.1
40788	1.45581	2.1
40789	1.45581	2.1
40790	1.44207	2.1
40791	2.54949	2.1
40792	2.53574	2.1
40793	0.98641	2.1
40794	0.98641	2.1
40795	1.00371	2.1
40796	1.00371	2.1
40797	0.749992	2.1
40798	0.727904	2.1
40799	1.26221	2.1
40800	1.27474	2.1
40801	1.26221	2.1
40802	0.749993	2.1
40803	0.727904	2.1
40804	1.27474	2.1
40805	1.23105	0.851397
40806	1.35435	1.75694
40807	1.89226	1.58916
40808	1.89226	1.23157
40809	1.89226	1.66165
40810	1.7043	1.41448
40811	1.89226	1.81311
40812	1.89226	1.6247
40813	1.89226	1.02502
40814	1.89226	1.77656
40815	3.78453	1.69158
40816	1.89226	0.730554
40817	1.89226	1.74733
40818	1.89226	1.45937
40819	1.89226	1.41766
40820	1.89226	1.53431
40821	1.89226	1.55754
40822	0.946131	0.978638
40823	1.40945	0.813514
40824	0.94613	1.29455
40825	0.94613	1.56926
40826	0.946129	1.57579
40827	0.946129	1.41468
40828	0.946132	1.64946
40829	1.40945	0.641595

40830	0.94613	1.23813
40831	0.94613	1.38044
40832	0.946131	1.41248
40833	0.94613	0.932712
40834	0.946133	1.27477
40835	0.946133	1.29074
40836	0.946132	1.54402
40837	0.946132	1.67275
40838	0.946132	1.66768
40839	0.946132	1.49835
40840	0.946133	1.52063
40841	0.946133	1.32398
40842	0.946132	1.50712
40843	0.946132	1.52156
40844	0.946133	1.36275
40845	0.946133	1.50309
40846	1.89226	1.42299
40847	1.89226	1.85723
40848	1.89226	1.6203
40849	1.89226	1.56685
40850	1.89226	1.71726
40851	1.89226	1.63069
40852	1.89226	2.1
40853	1.89226	2.1
40854	1.89226	2.1
40855	1.89226	2.1
40856	1.89226	2.1
40857	1.89226	2.1
40858	1.89226	2.1
40859	1.89226	2.1
40860	1.89226	2.1
40861	1.89226	2.1
40862	3.78453	2.1
40863	1.66647	2.1
40864	1.15399	2.1
40865	1.25443	2.1
40866	1.26599	2.1
40867	1.58697	2.1
40868	1.66647	2.1
40869	1.66647	2.1
40870	1.47446	2.1
40871	1.55433	2.1
40872	1.74241	2.1
40873	3.2208	2.1
40874	1.89227	2.1
40875	1.89227	2.1
40876	1.89227	2.1
40877	1.89227	2.1
40878	3.78453	2.1
40879	0.946132	2.1
40880	0.946132	2.1
40881	0.946132	2.1
40882	0.946132	2.1
40883	0.946132	2.1
40884	0.946132	2.1
40885	0.946133	2.1
40886	0.946133	2.1
40887	0.946132	2.1

40888	0.946132	2.1
40889	0.946133	2.1
40890	0.946133	2.1
40891	0.625067	2.1
40892	0.61907	2.1
40893	0.896913	2.1
40894	0.946199	2.1
40895	0.806287	2.1
40896	0.677685	2.1
40897	0.750657	2.1
40898	0.750657	2.1
40899	0.646004	2.1
40900	0.890236	2.1
40901	0.61049	2.1
40902	0.888979	2.1
40903	1.6223	2.1
40904	1.98034	2.1
40905	1.75003	2.1
40906	1.55433	2.1
40907	1.75003	2.1
40908	1.55433	2.1
40909	1.88602	2.1
40910	2.08149	2.1
40911	2.07991	2.1
40912	1.88473	2.1
40913	2.08149	2.1
40914	1.88602	2.1
40915	1.88458	2.1
40916	2.08149	2.1
40917	1.7069	2.1
40918	1.56284	2.1
40919	2.08149	2.1
40920	1.88458	2.1
40921	1.5955	2.1
40922	1.89226	2.1
40923	1.91975	2.1
40924	1.62299	2.1
40925	1.89226	2.1
40926	1.5955	2.1
40927	1.89226	2.1
40928	1.89226	2.1
40929	1.89226	1.99586
40930	1.89226	2.1
40931	1.89226	1.908
40932	1.89226	2.1
40933	1.89226	2.1
40934	1.89226	2.1
40935	1.89226	2.1
40936	1.89226	2.1
40937	1.89226	2.1
40938	1.89226	2.1
40939	1.62299	2.1
40940	1.89226	2.1
40941	1.71752	2.1
40942	1.44824	2.1
40943	1.89226	2.1
40944	1.62299	2.1
40945	1.89226	1.38784

40946	2.8317	0.719367
40947	1.89226	1.07518
40948	2.64055	0.630714
40949	4.53281	1.32085
40950	2.8317	1.19965
40951	1.83151	0.523308
40952	2.01001	0.773293
40953	2.01001	1.39477
40954	2.64055	0.464438
40955	2.64055	0.981227
40956	0.946132	0.251261
40957	0.946132	0.3784
40958	1.16167	0.281354
40959	1.16167	0.296555
40960	1.16167	0.235444
40961	1.32027	0.245886
40962	0.946131	0.286262
40963	0.946131	0.332318
40964	0.94613	0.237131
40965	1.16167	0.210021
40966	0.946131	0.299331
40967	1.32027	0.274944
40968	2.32335	0.305846
40969	1.83151	0.318434
40970	2.38525	0.297384
40971	2.64055	0.341238
40972	1.57621	0.231845
40973	1.83151	0.248802
40974	2.25677	2.1
40975	1.88158	2.1
40976	1.86856	2.1
40977	2.0815	2.1
40978	1.70303	2.1
40979	1.88158	2.1
40980	2.50381	2.1
40981	1.89227	2.1
40982	2.25677	2.1
40983	2.25677	2.1
40984	1.89227	2.1
40985	1.89227	2.1
40986	1.89226	0.0848225
40987	1.50899	0.0788417
40988	2.13659	0.0845768
40989	1.50899	0.116412
40990	2.51986	0.0696221
40991	1.89226	0.101945
40992	0.946131	0.117749
40993	0.946131	0.0884343
40994	1.06829	0.0780251
40995	0.895375	0.0758811
40996	0.94613	0.070652
40997	0.94613	0.112167
40998	1.33922	0.0803359
40999	0.895376	0.073608
41000	0.946131	0.0837296
41001	1.33922	0.08836
41002	0.946131	0.0914138
41003	1.06829	0.097693

41004	0.841467	0.34484
41005	0.578428	0.410875
41006	1.18079	0.274632
41007	0.920237	0.432319
41008	0.772317	0.22817
41009	0.856555	0.76141
41010	0.910271	0.34437
41011	0.910271	0.7146
41012	0.975752	0.328717
41013	0.536929	0.255972
41014	0.81952	0.414355
41015	1.15252	0.275975
41016	1.89226	2.1
41017	2.51431	2.1
41018	1.89226	2.1
41019	1.89226	2.1
41020	2.78815	2.1
41021	2.78815	2.1
41022	2.0815	2.1
41023	2.0516	2.1
41024	2.0815	2.1
41025	3.06699	2.1
41026	2.76576	2.1
41027	2.8002	2.1
41028	1.94112	2.1
41029	1.89226	2.1
41030	2.20358	2.1
41031	1.89226	2.1
41032	4.09584	2.1
41033	1.09154	2.1
41034	2.62647	2.1
41035	2.20358	2.1
41036	2.20358	2.1
41037	1.52314	2.1
41038	1.52314	2.1
41039	1.31323	2.1
41040	1.31323	2.1
41041	0.946132	2.1
41042	0.946132	2.1
41043	0.946132	2.1
41044	0.946132	2.1
41045	1.31323	2.1
41046	1.10179	2.1
41047	1.31323	2.1
41048	0.946132	2.1
41049	1.10179	2.1
41050	0.946133	2.1
41051	1.89226	1.56684
41052	1.89226	1.7502
41053	1.89226	1.68849
41054	1.89226	1.80637
41055	1.89226	1.52762
41056	1.89226	1.70978
41057	1.35126	1.85477
41058	1.35126	2.05047
41059	0.903285	1.72424
41060	0.903285	1.77374
41061	1.30841	1.79236

41062	0.903313	1.98866
41063	0.946132	1.79808
41064	0.946132	1.69026
41065	0.946132	1.69695
41066	0.903312	1.7728
41067	1.30841	1.74176
41068	0.946133	1.70394
41069	2.62097	2.1
41070	2.72561	2.1
41071	2.39016	2.1
41072	2.51062	2.1
41073	5.11577	2.1
41074	2.51062	2.1
41075	2.58286	2.1
41076	2.40107	2.1
41077	2.40107	2.1
41078	2.72561	2.1
41079	2.72561	2.1
41080	2.39016	2.1
41081	2.51062	2.1
41082	2.20242	2.1
41083	2.34107	2.1
41084	4.73124	2.1
41085	2.51062	2.1
41086	2.40107	2.1
41087	2.34107	2.1
41088	2.25197	2.1
41089	4.74215	2.1
41090	2.75218	2.1
41091	1.89226	2.1
41092	2.3349	2.1
41093	2.3349	2.1
41094	1.89226	2.1
41095	1.89226	2.1
41096	1.46129	2.1
41097	0.996308	2.1
41098	1.88649	2.1
41099	1.70302	2.1
41100	2.95419	2.1
41101	1.89227	2.1
41102	1.89227	2.1
41103	1.89227	2.1
41104	1.89227	2.1
41105	3.78453	2.1
41106	0.646558	2.1
41107	0.646557	2.1
41108	0.60697	2.1
41109	0.60697	2.1
41110	0.587917	2.1
41111	0.519747	2.1
41112	0.586443	2.1
41113	0.733782	2.1
41114	0.733793	2.1
41115	0.587924	2.1
41116	0.519739	2.1
41117	0.586436	2.1
41118	2.1642	2.1
41119	1.42867	2.1

41120	2.28847	2.1
41121	1.68299	2.1
41122	3.71714	2.1
41123	1.89226	2.1
41124	1.89226	2.1
41125	1.89226	2.1
41126	1.89226	2.1
41127	3.78453	2.1
41128	0.946131	2.1
41129	0.946131	2.1
41130	1.24994	2.1
41131	1.24994	2.1
41132	1.11582	2.1
41133	1.24994	2.1
41134	0.946133	2.1
41135	0.946133	2.1
41136	0.946131	2.1
41137	1.24994	2.1
41138	0.94613	2.1
41139	1.11582	2.1
41140	2.28847	2.1
41141	1.53629	2.1
41142	1.57498	2.1
41143	1.47417	2.1
41144	2.42221	2.1
41145	2.28847	2.1
41146	1.89226	2.1
41147	1.89226	2.1
41148	1.89226	2.1
41149	1.89226	2.1
41150	3.78453	2.1
41151	1.89226	2.1
41152	2.49988	2.1
41153	2.71233	2.1
41154	2.71233	2.1
41155	1.89226	2.1
41156	1.89226	2.1
41157	0.924534	2.1
41158	0.924534	2.1
41159	0.990395	2.1
41160	0.990395	2.1
41161	0.946131	2.1
41162	0.946131	2.1
41163	0.924505	2.1
41164	0.968797	2.1
41165	0.924505	2.1
41166	0.946131	2.1
41167	0.968797	2.1
41168	0.946131	2.1
41169	0.924533	2.1
41170	0.924533	2.1
41171	0.948264	2.1
41172	0.945345	2.1
41173	0.851541	2.1
41174	0.851541	2.1
41175	0.958934	2.1
41176	1.02617	2.1
41177	0.936668	2.1

41178	1.04829	2.1
41179	0.924505	2.1
41180	0.946131	2.1
41181	2.28431	0.590146
41182	1.48035	0.123816
41183	1.59971	0.236361
41184	1.48035	0.29013
41185	2.33818	0.166672
41186	2.21882	0.212731
41187	2.28431	0.286715
41188	1.89226	0.34455
41189	1.89226	0.548389
41190	2.3968	0.264705
41191	4.28906	0.298773
41192	2.28431	0.284486
41193	1.70303	0.130716
41194	1.5428	0.133067
41195	1.59971	0.186266
41196	2.31152	0.190322
41197	2.3968	0.235532
41198	1.99823	2.1
41199	2.21189	2.1
41200	2.18937	2.1
41201	1.979 2.1	
41202	4.19089	2.1
41203	0.999114	2.1
41204	0.9895	2.1
41205	0.758942	2.1
41206	0.749358	2.1
41207	0.874236	2.1
41208	0.874236	2.1
41209	0.874236	2.1
41210	0.874236	2.1
41211	0.9895	2.1
41212	0.749358	2.1
41213	0.758942	2.1
41214	0.999114	2.1
41215	2.18937	2.1
41216	1.979 2.1	
41217	1.95416	2.1
41218	2.162 2.1	
41219	4.14099	2.1
41220	1.95416	2.1
41221	1.49871	2.1
41222	1.47712	2.1
41223	1.49871	2.1
41224	1.9574	2.1
41225	1.979 2.1	
41226	1.68508	0.05
41227	1.64984	0.05
41228	2.67711	0.05
41229	2.76407	0.05
41230	4.41391	0.05
41231	1.84227	0.05
41232	2.76407	0.05
41233	2.76407	0.05
41234	1.85007	0.05
41235	2.72769	0.05

41236	1.80812	0.05
41237	1.94516	2.1
41238	1.89226	2.1
41239	1.89226	2.1
41240	1.89226	2.1
41241	1.98853	2.1
41242	1.98852	2.1
41243	1.28095	2.1
41244	2.0815	2.1
41245	2.29123	2.1
41246	2.06833	2.1
41247	1.08212	2.1
41248	0.976848	2.1
41249	1.89226	2.1
41250	1.89226	2.1
41251	1.89226	2.1
41252	1.89226	2.1
41253	1.89226	2.1
41254	1.89226	2.1
41255	1.13537	2.1
41256	1.18233	2.1
41257	0.946132	2.1
41258	0.946132	2.1
41259	0.946132	2.1
41260	0.946132	2.1
41261	0.994264	2.1
41262	1.18233	2.1
41263	1.13537	2.1
41264	0.946131	2.1
41265	0.946132	2.1
41266	0.994264	2.1
41267	0.953204	2.1
41268	0.953204	2.1
41269	0.966515	2.1
41270	0.966515	2.1
41271	1.30583	2.1
41272	1.14561	2.1
41273	0.640475	2.1
41274	0.774103	2.1
41275	1.30584	2.1
41276	0.774101	2.1
41277	0.640474	2.1
41278	1.14562	2.1
41279	0.946131	2.1
41280	0.946131	2.1
41281	0.946131	2.1
41282	0.946131	2.1
41283	0.946128	2.1
41284	0.946128	2.1
41285	0.946133	2.1
41286	0.946133	2.1
41287	0.94613	2.1
41288	0.94613	2.1
41289	0.946131	2.1
41290	0.946131	2.1
41291	0.946132	0.05
41292	0.946132	0.05
41293	0.946131	0.05

41294	0.946131	0.05
41295	0.946131	0.05
41296	0.946131	0.05
41297	0.946132	0.05
41298	0.946132	0.05
41299	0.946131	0.05
41300	0.946131	0.05
41301	0.946132	0.05
41302	0.946132	0.05
41303	0.946131	0.05
41304	0.946131	0.05
41305	0.946131	0.05
41306	0.946131	0.05
41307	0.94613	0.05
41308	0.94613	0.05
41309	0.946132	0.05
41310	0.946132	0.05
41311	0.946131	0.05
41312	0.946131	0.05
41313	0.946131	0.05
41314	0.946131	0.05
41315	2.57882	0.100148
41316	1.48676	0.102781
41317	1.48797	0.09351
41318	2.64281	0.0973826
41319	4.12957	0.0953956
41320	2.56323	0.0867603
41321	2.64281	0.0937237
41322	2.51807	0.0856978
41323	1.36324	0.0846739
41324	1.48797	0.0869699
41325	1.48797	0.0915328
41326	1.04367	2.1
41327	2.42504	2.1
41328	2.65164	2.1
41329	1.49479	2.1
41330	3.91983	2.1
41331	2.15622	2.1
41332	1.70539	2.1
41333	2.02369	2.1
41334	2.39039	2.1
41335	4.17992	2.1
41336	0.966979	2.1
41337	1.7054	2.1
41338	2.0237	2.1
41339	1.31502	2.1
41340	2.99067	2.1
41341	2.02369	2.1
41342	2.65637	2.1
41343	2.39039	2.1
41344	2.39039	2.1
41345	2.29332	2.1
41346	2.29332	2.1
41347	2.0237	2.1
41348	1.72116	2.1
41349	1.58465	2.1
41350	1.31502	2.1
41351	2.29332	2.1

41352	2.0237	2.1
41353	1.0372	0.05
41354	2.32462	0.05
41355	2.2291	0.05
41356	2.2291	0.05
41357	1.17464	0.05
41358	1.17464	0.05
41359	2.68289	0.05
41360	1.07805	0.05
41361	1.70302	0.05
41362	2.06653	0.05
41363	3.5779	0.05
41364	1.89226	0.102757
41365	1.89226	0.14971
41366	1.89226	0.126554
41367	1.89226	0.0808511
41368	3.78453	0.106481
41369	1.89226	0.05
41370	1.89226	0.0637812
41371	1.89226	0.0634586
41372	1.89226	0.0575106
41373	1.89226	0.056529
41374	1.89226	0.0509568
41375	1.89225	0.0518105
41376	1.89225	0.0559711
41377	1.89225	0.0573645
41378	1.89225	0.051975
41379	1.89225	0.0582869
41380	1.89225	0.0534332
41381	0.94613	0.0868347
41382	0.94613	0.107641
41383	0.94613	0.15112
41384	0.94613	0.118081
41385	0.946128	0.0984368
41386	0.946128	0.107031
41387	0.946132	0.154526
41388	0.946132	0.0849454
41389	0.94613	0.0973253
41390	0.94613	0.116877
41391	0.94613	0.112161
41392	0.94613	0.0916487
41393	1.29417	2.1
41394	1.32732	2.1
41395	1.32316	1.51074
41396	1.34973	2.1
41397	2.65048	2.1
41398	1.89226	1.70762
41399	1.89226	2.1
41400	1.89226	1.58305
41401	1.89226	2.1
41402	3.78453	2.09695
41403	1.89226	2.1
41404	1.89226	2.1
41405	1.89226	2.1
41406	1.89226	2.1
41407	3.78453	2.1
41408	1.32732	2.1
41409	1.13006	2.1

41410	1.13358	2.1
41411	1.11117	2.1
41412	1.34973	2.1
41413	1.32732	2.1
41414	1.32316	1.96094
41415	1.34973	2.1
41416	1.34066	1.73464
41417	1.36601	2.1
41418	2.68918	2.1
41419	1.89226	1.98185
41420	1.89226	2.1
41421	1.89226	0.644941
41422	1.89226	2.1
41423	3.78453	2.1
41424	1.89226	2.1
41425	1.89226	2.1
41426	1.89226	2.1
41427	1.89226	2.1
41428	1.89226	2.1
41429	1.89226	2.1
41430	0.624134	2.1
41431	0.624134	2.1
41432	0.624334	2.1
41433	0.624334	2.1
41434	0.56503	2.1
41435	0.573605	2.1
41436	0.674864	2.1
41437	0.683007	2.1
41438	0.683007	2.1
41439	0.573605	2.1
41440	0.565029	2.1
41441	0.674864	2.1
41442	2.16633	2.1
41443	1.40846	2.1
41444	2.13977	2.1
41445	1.40771	2.1
41446	3.57404	2.1
41447	2.13977	2.1
41448	1.29932	2.1
41449	1.29932	2.1
41450	1.40771	2.1
41451	1.98957	2.1
41452	2.09796	2.1
41453	2.6044	0.168231
41454	1.85548	0.242436
41455	1.89226	0.104603
41456	1.89226	0.297856
41457	3.74774	0.186118
41458	1.89226	0.114126
41459	1.89226	0.403857
41460	1.89226	0.143107
41461	1.89226	0.183343
41462	3.78453	0.151126
41463	1.89226	2.1
41464	2.64107	2.1
41465	1.89226	2.1
41466	2.78977	2.1
41467	1.89226	2.1

41468	2.78977	2.1
41469	1.89226	2.1
41470	1.89226	2.1
41471	1.89226	2.1
41472	1.89226	2.1
41473	1.89226	2.1
41474	1.89226	2.1
41475	2.43173	2.1
41476	1.41238	2.1
41477	2.0815	2.1
41478	1.98048	2.1
41479	1.62014	2.1
41480	1.50086	2.1
41481	0.946129	0.379883
41482	0.946129	0.32841
41483	1.27636	0.278877
41484	1.1984	0.203699
41485	0.946131	0.214009
41486	0.946131	0.305778
41487	1.27636	0.398772
41488	1.27636	0.216318
41489	0.946131	0.284652
41490	1.27636	0.279454
41491	0.946131	0.274479
41492	1.1984	0.272761
41493	1.99569	0.140156
41494	2.55272	0.276237
41495	1.77403	0.13972
41496	1.70303	0.168532
41497	3.05772	0.144352
41498	2.55121	0.185438
41499	0.946128	0.272978
41500	0.946128	0.31359
41501	1.38752	0.23838
41502	1.27636	0.170056
41503	0.94613	0.177275
41504	0.94613	0.258077
41505	1.38752	0.322952
41506	1.38752	0.180276
41507	0.946131	0.235426
41508	1.38752	0.234405
41509	0.946131	0.235628
41510	1.27636	0.232322
41511	2.77504	0.215021
41512	1.99569	0.10903
41513	2.29794	0.126936
41514	1.99569	0.168832
41515	2.85497	0.1077
41516	2.55272	0.143457
41517	1.53284	2.1
41518	1.89226	2.1
41519	1.89226	2.1
41520	1.89226	2.1
41521	1.54763	2.1
41522	1.54763	2.1
41523	0.946131	2.1
41524	0.946131	2.1
41525	0.946127	2.1

41526	0.946127	2.1
41527	0.946127	2.1
41528	0.946127	2.1
41529	0.946131	2.1
41530	0.946131	2.1
41531	0.94613	2.1
41532	0.94613	2.1
41533	0.946129	2.1
41534	0.946129	2.1
41535	0.751754	2.1
41536	0.751754	2.1
41537	0.771562	2.1
41538	0.771561	2.1
41539	1.0268	2.1
41540	0.95482	2.1
41541	0.528937	2.1
41542	0.496512	2.1
41543	0.528936	2.1
41544	1.0268	2.1
41545	0.954821	2.1
41546	0.49651	2.1
41547	2.64075	0.208406
41548	1.35181	1.53069
41549	1.35181	0.264608
41550	1.83823	0.234429
41551	2.28373	1.14432
41552	2.88566	1.39145
41553	0.493018	0.855118
41554	0.675903	0.69207
41555	1.14187	0.243171
41556	0.973937	0.0512668
41557	0.821166	0.27949
41558	0.821166	1.19032
41559	0.828674	1.70343
41560	0.828674	0.187412
41561	0.973937	1.27615
41562	0.493018	0.495088
41563	0.675903	0.290381
41564	1.14187	0.339318
41565	1.89226	2.1
41566	2.11667	2.1
41567	1.91184	2.1
41568	1.89226	2.1
41569	2.13625	2.1
41570	2.11667	2.1
41571	1.89226	2.1
41572	1.89226	2.1
41573	1.89226	2.1
41574	1.89226	2.1
41575	3.78453	2.1
41576	0.907729	2.1
41577	0.907729	2.1
41578	1.04459	2.1
41579	0.890955	2.1
41580	0.988181	2.1
41581	0.841856	2.1
41582	0.946133	2.1
41583	0.946133	2.1

41584	1.04072	2.1
41585	1.1064	2.1
41586	0.89099	2.1
41587	0.851541	2.1
41588	1.89225	2.1
41589	2.13623	2.1
41590	1.91044	2.1
41591	1.89225	2.1
41592	2.15442	2.1
41593	2.13623	2.1
41594	1.89225	2.1
41595	1.89225	2.1
41596	1.89225	2.1
41597	1.89225	2.1
41598	1.89225	2.1
41599	1.89225	2.1
41600	0.907723	2.1
41601	0.907723	2.1
41602	1.07536	2.1
41603	1.07814	2.1
41604	0.998499	2.1
41605	1.03575	2.1
41606	0.946126	2.1
41607	0.946126	2.1
41608	1.04072	2.1
41609	1.14025	2.1
41610	0.851528	2.1
41611	0.898284	2.1
41612	1.89226	0.0566362
41613	1.89226	0.124875
41614	1.89226	0.0857099
41615	1.89226	0.0800058
41616	1.89226	0.0767522
41617	1.89226	0.0699789
41618	2.53008	2.1
41619	1.94534	2.1
41620	1.98702	2.1
41621	1.98858	2.1
41622	2.52852	2.1
41623	2.53008	2.1
41624	1.03267	2.1
41625	1.12591	2.1
41626	1.2959	2.1
41627	1.28992	2.1
41628	1.10851	2.1
41629	1.33078	2.1
41630	1.10661	2.1
41631	1.10661	2.1
41632	0.982208	2.1
41633	1.28992	2.1
41634	1.03267	2.1
41635	1.23262	2.1
41636	2.2712	2.1
41637	1.89226	2.1
41638	1.95954	2.1
41639	1.95954	2.1
41640	1.89226	2.1
41641	1.89226	2.1

41642	1.89226	2.1
41643	1.89226	2.1
41644	1.89226	2.1
41645	1.89226	2.1
41646	3.78453	2.1
41647	1.33887	2.1
41648	1.25395	2.1
41649	0.88327	2.1
41650	0.676665	2.1
41651	0.84881	2.1
41652	0.987503	2.1
41653	1.13243	2.1
41654	1.13243	2.1
41655	1.15338	2.1
41656	0.849966	2.1
41657	0.793074	2.1
41658	1.11957	2.1
41659	0.946129	2.1
41660	0.946129	2.1
41661	1.28254	2.1
41662	1.1356	2.1
41663	1.1356	2.1
41664	1.1356	2.1
41665	0.946131	2.1
41666	0.946131	2.1
41667	0.946131	2.1
41668	1.28254	2.1
41669	1.1356	2.1
41670	0.946131	2.1
41671	1.89227	2.1
41672	1.89227	2.1
41673	1.89227	2.1
41674	1.89227	2.1
41675	3.78453	2.1
41676	0.936287	0.05
41677	1.01399	0.05
41678	0.959415	0.05
41679	0.959415	0.05
41680	0.910786	0.05
41681	1.38301	0.05
41682	1.0286	0.05
41683	0.535742	0.05
41684	0.505202	0.05
41685	0.937685	0.05
41686	1.11066	0.05
41687	1.46907	0.05
41688	1.00546	0.05
41689	1.01524	0.05
41690	0.94613	0.05
41691	0.94613	0.05
41692	1.05018	0.05
41693	0.914025	0.05
41694	0.913512	0.05
41695	0.851542	0.05
41696	1.05385	0.05
41697	1.05494	0.05
41698	0.91404	0.0506741
41699	0.91404	0.05

41700	2.03253	2.09522
41701	1.82325	0.356368
41702	1.93938	2.1
41703	1.73756	0.32553
41704	3.77009	1.33747
41705	1.82325	0.310056
41706	1.47562	1.91496
41707	1.73756	0.345675
41708	1.3868	1.61442
41709	3.21317	0.427433
41710	1.93938	2.1
41711	1.73756	0.458157
41712	1.85293	2.1
41713	1.64279	0.335851
41714	3.59049	0.734477
41715	1.73756	0.299341
41716	1.3868	1.71458
41717	1.64279	0.434525
41718	1.28419	1.72987
41719	3.02174	0.490964
41720	1.35048	2.1
41721	1.37999	2.1
41722	1.34355	1.4765
41723	1.37854	2.1
41724	2.72354	2.1
41725	1.89226	1.73838
41726	1.89226	2.1
41727	1.89226	1.45073
41728	1.89226	2.1
41729	3.78452	1.88262
41730	1.89226	2.1
41731	1.89226	2.1
41732	1.89226	2.0838
41733	1.89226	2.1
41734	3.78453	2.1
41735	1.37999	2.1
41736	1.18059	2.1
41737	1.17117	2.1
41738	1.17262	2.1
41739	1.37855	2.1
41740	1.37999	2.1
41741	1.34355	1.93177
41742	1.37854	2.1
41743	1.33079	1.68622
41744	1.3741	2.1
41745	2.71765	2.1
41746	1.89226	1.89175
41747	1.89226	2.1
41748	1.89226	0.864142
41749	1.89226	2.1
41750	3.78453	1.96953
41751	1.89226	2.1
41752	1.89226	2.1
41753	1.89226	2.1
41754	1.89226	2.1
41755	3.78453	2.1
41756	0.640305	2.1
41757	0.640305	2.1

41758	0.643507	2.1
41759	0.643507	2.1
41760	0.590294	2.1
41761	0.594539	2.1
41762	0.689273	2.1
41763	0.687048	2.1
41764	0.687048	2.1
41765	0.594538	2.1
41766	0.590294	2.1
41767	0.689273	2.1
41768	2.01399	1.467
41769	1.70141	1.76563
41770	1.83896	2.1
41771	1.52482	2.1
41772	3.53881	1.81795
41773	1.70141	2.05518
41774	1.0752	2.1
41775	1.0752	1.95532
41776	1.25669	2.1
41777	1.52482	2.1
41778	1.70632	2.1
41779	1.83896	1.79156
41780	1.52482	1.00566
41781	1.65244	1.67587
41782	1.34412	2.00594
41783	3.17725	1.68854
41784	1.26782	0.05
41785	2.90353	0.05
41786	2.54203	0.05
41787	2.41979	0.05
41788	1.77007	0.05
41789	1.703 0.05	
41790	0.946118	0.0601724
41791	0.946118	0.05
41792	1.38643	0.05
41793	1.38644	0.05
41794	0.946117	0.050003
41795	0.946117	0.0617943
41796	1.38644	0.05
41797	1.15545	0.05
41798	0.946131	0.05
41799	1.15546	0.05
41800	1.38642	0.05
41801	0.946105	0.05
41802	2.42256	0.186783
41803	2.63056	0.105155
41804	2.43174	0.111523
41805	2.25035	0.0945788
41806	4.8543	0.10526
41807	2.58031	0.169199
41808	2.63056	0.124466
41809	2.43174	0.120245
41810	2.32127	0.0957413
41811	5.01205	0.125262
41812	2.43174	0.108868
41813	2.25035	0.0839683
41814	2.11465	0.0777352
41815	2.13042	0.068232

41816	4.54639	0.0824193
41817	2.43174	0.118418
41818	2.32127	0.0861778
41819	1.92997	0.077355
41820	2.13041	0.0740673
41821	4.36171	0.086825
41822	1.89227	2.1
41823	1.89227	2.1
41824	1.89227	2.1
41825	1.89227	2.1
41826	3.78453	2.1
41827	0.568724	2.1
41828	0.568724	2.1
41829	0.57678	2.1
41830	0.57678	2.1
41831	0.523796	2.1
41832	0.548137	2.1
41833	0.597368	2.1
41834	0.637877	2.1
41835	0.597367	2.1
41836	0.523796	2.1
41837	0.637877	2.1
41838	0.548137	2.1
41839	1.32674	2.1
41840	1.32674	2.1
41841	0.946132	2.1
41842	0.946132	2.1
41843	1.3195	2.1
41844	1.32674	2.1
41845	0.946132	2.1
41846	0.946132	2.1
41847	0.946132	2.1
41848	1.3195	2.1
41849	1.32674	2.1
41850	0.946133	2.1
41851	1.89227	2.1
41852	1.89227	2.1
41853	1.89227	2.1
41854	1.89227	2.1
41855	3.78453	2.1
41856	1.19473	2.1
41857	0.987132	2.1
41858	0.987132	2.1
41859	1.04759	2.1
41860	1.0894	2.1
41861	1.14986	2.1
41862	1.3195	2.1
41863	1.3195	2.1
41864	0.946132	2.1
41865	0.946132	2.1
41866	1.31185	2.1
41867	1.3195	2.1
41868	0.946132	2.1
41869	0.946132	2.1
41870	1.31185	2.1
41871	0.946133	2.1
41872	0.946132	2.1
41873	1.3195	2.1

41874	1.11256	1.45697
41875	1.70101	1.15061
41876	1.22297	1.45619
41877	1.22297	1.37494
41878	1.7252	1.44592
41879	1.7252	1.34979
41880	1.99298	1.39933
41881	1.70101	1.29593
41882	1.7252	1.47222
41883	2.18701	0.890857
41884	3.71818	1.40041
41885	1.03218	1.36892
41886	1.7252	0.891415
41887	1.11256	1.35481
41888	1.11256	1.21261
41889	1.7457	1.30159
41890	1.7457	1.15943
41891	1.7457	1.40498
41892	2.187	0.682745
41893	1.7252	1.32599
41894	1.7252	1.30563
41895	2.27351	1.03148
41896	2.27351	1.02657
41897	1.95544	0.953258
41898	1.35897	1.63819
41899	1.44859	0.990803
41900	1.44859	1.14674
41901	1.80709	0.97129
41902	1.80709	1.13631
41903	1.80709	0.995788
41904	1.17822	1.21066
41905	1.35897	1.02477
41906	1.35897	1.2869
41907	1.52782	0.986785
41908	1.52782	1.22986
41909	1.11978	1.10169
41910	0.903546	0.993906
41911	0.977719	1.18106
41912	1.2364	1.00788
41913	1.03812	0.967855
41914	1.03812	1.57193
41915	1.05938	0.954066
41916	1.05938	1.36002
41917	1.11978	0.96931
41918	1.2364	0.898011
41919	0.97772	1.12152
41920	0.903546	1.04857
41921	1.80709	1.24499
41922	1.81847	1.17629
41923	1.96029	1.27776
41924	1.52782	1.21757
41925	2.23956	1.12547
41926	1.80709	1.19157
41927	1.89226	0.300514
41928	2.32962	0.187273
41929	2.5992	0.188044
41930	1.89226	0.204803
41931	2.5992	0.283335

41932	1.89226	0.301103
41933	2.55791	0.05
41934	2.47141	0.05
41935	2.13515	0.05
41936	2.10939	0.05
41937	4.66731	0.05
41938	2.47141	0.05
41939	2.23337	0.05
41940	2.10939	0.05
41941	2.03438	0.05
41942	4.34276	0.05
41943	2.13512	0.05
41944	1.71027	0.05
41945	1.71027	0.05
41946	2.10936	0.05
41947	1.60137	0.05
41948	2.00046	0.05
41949	1.71027	0.05
41950	2.03435	0.05
41951	1.71027	0.05
41952	2.10936	0.05
41953	1.72067	0.05
41954	2.11976	0.05
41955	1.177	0.400803
41956	1.36405	0.781854
41957	2.25635	0.781887
41958	2.60151	0.239749
41959	3.6204	0.628534
41960	0.658869	0.0641809
41961	0.781697	0.075748
41962	0.637037	0.059332
41963	0.541148	0.0577747
41964	0.654682	0.05
41965	0.654682	0.119304
41966	0.641224	0.0578154
41967	0.641224	0.0713408
41968	0.637039	0.05
41969	0.781696	0.054844
41970	0.658867	0.0702316
41971	0.541149	0.0641515
41972	1.89226	0.318992
41973	1.59152	0.204192
41974	1.89226	0.204166
41975	1.82125	0.198917
41976	1.89226	0.315149
41977	1.82125	0.297272
41978	2.18967	0.05
41979	1.89227	0.0529022
41980	2.72752	0.0511931
41981	2.72752	0.0534864
41982	1.89227	0.05
41983	1.89227	0.05
41984	2.18967	0.05
41985	1.92547	0.0512493
41986	1.92547	0.05
41987	2.72752	0.05
41988	1.30572	0.05
41989	2.10777	0.05

41990	2.39663	2.1
41991	1.92293	2.1
41992	1.96889	2.1
41993	2.45928	2.1
41994	4.36552	2.1
41995	1.02605	2.1
41996	1.92293	2.1
41997	1.9689	2.1
41998	1.04679	2.1
41999	2.99495	2.1
42000	1.96889	2.1
42001	2.45928	2.1
42002	2.50138	2.1
42003	1.99782	2.1
42004	4.47027	2.1
42005	1.9689	2.1
42006	1.03449	2.1
42007	1.07572	2.1
42008	1.04679	2.1
42009	1.99783	2.1
42010	1.9689	2.1
42011	1.72162	1.13323
42012	1.89186	1.07063
42013	2.29771	1.04432
42014	1.80149	0.559856
42015	4.01933	1.05512
42016	1.16482	1.17109
42017	1.97003	0.893126
42018	1.72162	0.937563
42019	1.72162	1.07057
42020	1.29313	1.0419
42021	1.29313	1.17326
42022	2.29771	1.08738
42023	2.84159	0.391837
42024	2.19622	0.781611
42025	1.97366	0.982992
42026	2.50906	0.929812
42027	2.29771	1.00825
42028	1.29313	1.15738
42029	2.4483	0.729366
42030	2.14223	0.909611
42031	2.14228	1.05757
42032	1.294	1.01205
42033	1.294	1.10682
42034	1.21495	0.05
42035	1.70303	0.05
42036	1.7924	0.05
42037	1.64002	0.05
42038	3.11963	0.05
42039	2.81021	0.05
42040	1.89226	0.05
42041	1.89226	0.05
42042	1.89226	0.05
42043	2.36406	0.05
42044	2.36406	0.05
42045	1.89226	0.05
42046	1.89226	0.05
42047	1.89226	0.05

42048	1.89226	0.05
42049	1.89226	0.05
42050	1.89226	0.05
42051	2.29976	0.05
42052	1.64002	0.05
42053	1.8959	0.05
42054	1.90873	0.05
42055	1.85594	0.05
42056	1.85594	0.05
42057	1.89226	0.05
42058	1.89226	0.05
42059	1.89226	0.05
42060	1.89226	0.05
42061	3.78453	0.05
42062	1.89226	1.52584
42063	2.52823	2.1
42064	2.83441	1.81291
42065	2.83441	2.04463
42066	1.89226	1.65775
42067	1.89226	1.7682
42068	1.04522	1.40193
42069	1.04522	1.39994
42070	1.12831	1.80271
42071	1.18364	2.02689
42072	0.640525	1.40243
42073	0.924882	1.42126
42074	1.27877	1.74946
42075	1.43363	1.42099
42076	0.93474	1.36613
42077	1.58926	1.59
42078	1.29573	1.71702
42079	0.664247	1.6575
42080	2.02191	2.1
42081	1.89226	2.1
42082	1.89226	2.1
42083	1.89226	2.1
42084	2.58639	2.1
42085	2.58639	2.1
42086	1.16759	2.1
42087	2.11828	2.1
42088	1.60936	2.1
42089	2.35846	2.1
42090	3.52605	2.1
42091	1.89226	2.1
42092	1.89226	2.1
42093	1.89226	2.1
42094	1.89226	2.1
42095	3.78453	2.1
42096	2.82765	0.05
42097	1.89809	0.0738162
42098	2.15971	0.05
42099	1.89809	0.0514987
42100	2.29233	0.05
42101	2.03071	0.05
42102	1.90664	0.0610666
42103	2.15971	0.05
42104	1.28956	0.069589
42105	1.28956	0.05

42106	2.64414	0.0581763
42107	2.02706	0.05
42108	2.82765	0.0617196
42109	1.89226	0.0570491
42110	2.39968	0.0718807
42111	2.39968	0.101508
42112	1.89226	0.05
42113	1.89226	0.05
42114	0.978643	2.1
42115	0.703968	2.1
42116	0.946129	2.1
42117	0.946129	2.1
42118	0.703939	2.1
42119	0.703939	2.1
42120	0.946159	2.1
42121	0.946159	2.1
42122	0.978642	2.1
42123	0.94613	2.1
42124	0.94613	2.1
42125	0.703968	2.1
42126	1.89226	2.1
42127	2.17309	1.32716
42128	2.17309	2.1
42129	1.47475	1.84739
42130	1.89226	2.1
42131	1.19393	1.7421
42132	1.89226	2.1
42133	1.89226	2.1
42134	1.89226	2.1
42135	1.89226	2.09279
42136	3.78452	2.1
42137	1.17426	2.1
42138	0.978644	2.1
42139	0.946129	2.1
42140	0.946129	2.1
42141	0.946131	2.1
42142	0.946131	2.1
42143	0.978643	2.1
42144	0.978642	2.1
42145	1.17426	2.1
42146	0.946131	2.1
42147	0.978642	2.1
42148	0.94613	2.1
42149	1.89226	2.1
42150	1.89226	1.17059
42151	1.89226	2.09469
42152	2.17309	1.42763
42153	2.56346	2.07118
42154	2.94389	1.64385
42155	0.946131	2.1
42156	0.946131	2.08469
42157	0.946128	2.1
42158	0.946128	2.1
42159	0.946131	2.1
42160	0.946131	2.08072
42161	0.946128	2.1
42162	0.946128	2.1
42163	0.94613	2.1

42164	0.94613	2.1
42165	0.946129	2.1
42166	0.946129	2.1
42167	1.89226	1.00126
42168	1.89226	1.05523
42169	1.89226	1.21884
42170	1.89226	1.06612
42171	3.78453	0.998123
42172	1.89226	1.23802
42173	1.89226	1.0231
42174	1.89226	1.04842
42175	1.89226	1.11819
42176	1.89226	0.985582
42177	1.89226	0.982621
42178	1.89226	0.880595
42179	1.30733	1.12269
42180	1.89226	1.16097
42181	1.92403	1.18107
42182	3.38635	1.05821
42183	1.89226	1.01453
42184	3.02887	1.23955
42185	2.0815	0.885962
42186	1.88422	1.12562
42187	2.0815	1.09664
42188	1.88422	1.2011
42189	1.89226	1.27429
42190	1.70221	1.09867
42191	1.89226	1.17072
42192	1.20068	1.197
42193	1.89226	1.13538
42194	1.20068	0.979837
42195	1.01393	1.46434
42196	1.20067	0.989505
42197	1.89226	0.890863
42198	2.44958	1.45649
42199	1.01393	0.59436
42200	1.89226	0.432159
42201	1.89226	2.1
42202	1.89226	2.1
42203	1.89226	2.1
42204	1.89226	2.1
42205	1.89226	2.1
42206	1.89226	2.1
42207	1.89226	2.1
42208	1.89226	2.1
42209	1.89226	2.1
42210	1.89226	2.1
42211	1.89226	2.1
42212	1.89226	2.1
42213	1.89226	2.1
42214	1.89226	2.1
42215	1.89226	2.1
42216	1.89226	2.1
42217	1.89226	2.1
42218	1.89226	2.1
42219	1.91161	2.1
42220	2.45782	2.1
42221	1.39653	2.1

42222	2.44652	2.1
42223	4.35812	2.1
42224	2.73092	2.1
42225	1.89226	2.1
42226	1.89226	2.1
42227	1.89226	2.04308
42228	2.44652	2.1
42229	2.44652	1.90553
42230	1.89226	2.1
42231	1.89226	2.1
42232	1.89226	2.1
42233	1.89226	2.1
42234	1.89226	2.1
42235	1.89226	2.1
42236	2.64924	2.1
42237	1.31109	2.1
42238	1.31109	2.1
42239	1.49958	2.1
42240	1.95039	2.1
42241	2.14451	2.1
42242	0.825157	2.1
42243	0.634856	2.1
42244	0.816836	2.1
42245	0.816836	2.1
42246	0.556743	2.1
42247	0.639142	2.1
42248	0.985106	2.1
42249	0.795045	2.1
42250	0.967461	2.1
42251	0.552494	2.1
42252	0.676829	2.1
42253	0.98803	2.1
42254	2.28564	2.1
42255	2.34084	2.00843
42256	2.3502	2.1
42257	2.42908	2.1
42258	4.69104	2.1
42259	2.34084	2.03993
42260	2.18077	2.1
42261	2.42908	2.1
42262	2.2947	1.53351
42263	4.63554	2.08651
42264	2.3502	2.1
42265	2.42908	2.1
42266	2.38681	2.1
42267	2.50438	2.06992
42268	4.85458	2.1
42269	2.42908	2.1
42270	2.2947	1.71627
42271	2.50438	1.9163
42272	2.40627	2.1
42273	4.79908	1.94757
42274	1.89226	1.04805
42275	2.20711	1.86403
42276	1.89226	0.825723
42277	1.89226	1.92888
42278	2.02693	0.93008
42279	2.02693	1.90588

42280	1.2269	2.1
42281	2.20711	1.8877
42282	2.0815	1.89583
42283	1.11506	2.1
42284	3.22109	2.1
42285	0.545802	2.03215
42286	0.547489	2.1
42287	0.85914	1.89271
42288	1.11641	2.02523
42289	0.899974	2.06705
42290	0.849893	2.1
42291	0.747885	2.0381
42292	0.747885	1.76068
42293	0.557184	2.1
42294	0.887039	2.1
42295	1.08948	2.1
42296	0.59815	2.1
42297	0.974139	2.1
42298	1.33385	2.1
42299	1.02368	2.1
42300	1.02368	2.1
42301	1.00833	2.1
42302	1.00833	2.1
42303	1.89226	1.85434
42304	1.89226	0.461022
42305	1.89226	2.06307
42306	1.89226	0.348481
42307	3.78453	0.561734
42308	1.74651	2.1
42309	1.44605	0.248467
42310	1.63557	2.03333
42311	1.31993	0.463188
42312	3.08162	0.569268
42313	1.89226	2.1
42314	1.89226	0.765838
42315	1.89226	1.85807
42316	1.89226	0.826273
42317	3.78452	0.424124
42318	1.63557	2.09857
42319	1.31992	0.316081
42320	1.52615	2.1
42321	1.18549	0.759908
42322	2.82106	1.16414
42323	1.89226	0.300941
42324	2.58101	1.86358
42325	2.73797	2.1
42326	2.73797	0.84133
42327	1.89226	2.1
42328	1.89226	0.418132
42329	1.91762	0.209821
42330	1.44354	0.235809
42331	1.33622	0.199562
42332	1.33622	0.172544
42333	2.20248	0.415418
42334	2.20248	0.239534
42335	1.11712	2.1
42336	1.11712	2.1
42337	1.11421	2.1

42338	1.11421	2.1
42339	0.833871	2.1
42340	0.926528	2.1
42341	1.3048	2.1
42342	1.40335	2.1
42343	0.926527	2.1
42344	1.40335	2.1
42345	1.3048	2.1
42346	0.833871	2.1
42347	0.879657	2.1
42348	0.937695	2.1
42349	0.946129	2.1
42350	0.946129	2.1
42351	0.851541	2.1
42352	0.851541	2.1
42353	1.1126	2.1
42354	1.04519	2.1
42355	0.851486	2.1
42356	0.980501	2.1
42357	0.898251	2.1
42358	0.851528	2.1
42359	1.37756	2.1
42360	2.73081	2.1
42361	2.44311	2.1
42362	2.44311	2.1
42363	1.66428	2.1
42364	1.66428	2.1
42365	1.89226	2.1
42366	1.89226	2.1
42367	1.89226	2.1
42368	1.89226	2.1
42369	3.78452	2.1
42370	1.65781	2.1
42371	1.89837	2.1
42372	1.46713	2.1
42373	1.98027	2.1
42374	3.63808	2.1
42375	2.29054	2.1
42376	2.26369	2.1
42377	1.98027	2.1
42378	2.15617	2.01002
42379	4.24396	2.1
42380	1.89226	2.1
42381	1.89226	2.1
42382	1.89226	2.1
42383	1.89226	2.09772
42384	3.78452	2.1
42385	0.946131	2.1
42386	0.946131	2.1
42387	0.946128	2.1
42388	0.946128	2.1
42389	0.946129	2.1
42390	0.946129	2.1
42391	0.94613	2.1
42392	0.94613	2.1
42393	0.94613	2.1
42394	0.94613	2.1
42395	0.94613	2.1

42396	0.94613	2.1
42397	0.812186	2.1
42398	0.812186	2.1
42399	0.819781	2.1
42400	0.819781	2.1
42401	0.913532	2.1
42402	0.990134	2.1
42403	0.733564	2.1
42404	0.641833	2.1
42405	0.641832	2.1
42406	0.913533	2.1
42407	0.733564	2.1
42408	0.990134	2.1
42409	0.994262	2.1
42410	0.994262	2.1
42411	0.997354	2.1
42412	0.997354	2.08194
42413	1.00775	1.87584
42414	1.07808	1.97698
42415	0.913533	2.1
42416	0.990135	2.1
42417	0.913532	2.1
42418	1.00775	2.1
42419	0.990134	2.1
42420	1.07808	2.1
42421	0.946131	2.1
42422	0.946131	2.1
42423	0.946128	2.1
42424	0.946128	2.1
42425	0.946128	2.08497
42426	0.946128	1.93781
42427	0.94613	2.1
42428	0.94613	2.1
42429	0.946129	2.1
42430	0.946129	2.1
42431	0.946129	2.1
42432	0.946129	2.1
42433	0.816232	2.1
42434	0.760038	2.1
42435	0.845999	2.1
42436	0.845999	2.1
42437	1.0631	2.1
42438	1.28131	2.1
42439	0.474065	2.1
42440	0.591905	2.1
42441	0.601756	2.1
42442	1.23903	2.1
42443	0.988592	2.1
42444	0.49051	2.1
42445	2.57021	2.1
42446	1.89226	2.1
42447	2.68172	2.1
42448	1.89226	2.1
42449	4.46247	2.1
42450	1.89226	2.1
42451	1.89226	1.83778
42452	1.89226	2.1
42453	1.89226	1.99932

42454	3.78452	2.1
42455	1.12573	2.1
42456	1.65504	1.9656
42457	1.38848	2.1
42458	1.38848	2.1
42459	1.30567	2.1
42460	1.30567	2.1
42461	2.82413	2.1
42462	1.89226	2.1
42463	2.68172	2.1
42464	2.68172	2.1
42465	1.89226	2.1
42466	1.89226	2.1
42467	1.89226	2.1
42468	1.89226	2.1
42469	1.89226	2.1
42470	1.89226	1.72727
42471	3.78452	2.1
42472	1.30567	2.1
42473	1.65504	2.1
42474	1.52724	2.1
42475	1.89249	2.1
42476	3.18228	2.1
42477	2.57386	0.0813493
42478	1.89226	0.161951
42479	1.89226	0.148287
42480	2.66753	0.099018
42481	1.89226	0.127031
42482	2.66753	0.0950932
42483	1.89226	0.0777263
42484	1.89226	0.185777
42485	2.2973	0.152239
42486	3.12479	0.104074
42487	1.89226	0.136387
42488	2.57385	0.0957212
42489	0.489293	2.1
42490	0.672698	2.1
42491	0.59038	2.1
42492	0.738357	2.1
42493	0.62269	2.1
42494	0.62269	2.1
42495	0.640388	2.1
42496	0.640388	2.1
42497	0.59038	2.1
42498	0.489293	2.1
42499	0.738357	2.1
42500	0.672698	2.1
42501	1.89226	2.1
42502	1.89226	2.1
42503	1.89226	2.1
42504	1.89226	2.1
42505	3.78452	2.1
42506	2.82858	2.1
42507	1.89226	2.1
42508	2.5671	2.1
42509	1.89226	2.1
42510	2.5671	2.1
42511	1.89226	2.1

42512	2.06581	0.05
42513	2.47308	0.05
42514	1.89433	0.05
42515	1.67571	0.05
42516	3.87274	0.05
42517	2.55791	0.05
42518	2.10276	0.05
42519	2.47308	0.05
42520	2.47308	0.05
42521	1.83415	0.05
42522	1.90922	0.05
42523	1.18421	0.05
42524	2.03277	0.05
42525	1.18421	0.05
42526	2.01275	0.05
42527	1.42818	0.05
42528	2.25673	0.05
42529	0.882904	0.05
42530	0.958492	0.05
42531	0.905756	0.05
42532	0.905756	0.05
42533	1.06352	0.05
42534	0.797654	0.05
42535	1.11319	0.05
42536	0.716795	0.05
42537	0.820721	0.05
42538	0.731963	0.05
42539	1.13143	0.05
42540	1.10528	0.05
42541	1.89226	0.05
42542	1.89226	0.05
42543	1.89226	0.05
42544	1.89226	0.05
42545	1.89226	0.05
42546	1.89226	0.05
42547	1.01241	0.05
42548	0.703929	0.05
42549	0.946133	0.05
42550	0.946133	0.05
42551	1.04072	0.05
42552	1.163 0.05	
42553	0.639952	0.05
42554	0.851543	0.05
42555	0.946132	0.05
42556	0.920761	0.05
42557	0.711038	0.05
42558	0.711038	0.05
42559	1.89226	0.05
42560	1.89226	0.05
42561	1.89226	0.05
42562	1.89226	0.05
42563	1.89226	0.05
42564	1.89226	0.05
42565	1.89226	0.05
42566	1.89226	0.05
42567	2.41075	0.05
42568	1.89226	0.05
42569	3.78451	0.05

42570	0.968476	2.0973
42571	1.20674	1.34215
42572	1.13456	1.33447
42573	1.13456	2.1
42574	1.02074	1.46947
42575	1.02074	2.1
42576	1.02074	2.1
42577	1.20674	1.80499
42578	1.07866	1.80152
42579	1.28649	1.44654
42580	2.2854	2.00415
42581	1.94221	0.11722
42582	1.36717	0.100939
42583	1.45535	0.119537
42584	1.93771	0.0850614
42585	3.39756	0.113748
42586	1.94222	0.164787
42587	2.25035	0.089024
42588	2.41806	0.104304
42589	2.42256	0.0997762
42590	1.93772	0.117706
42591	1.94222	0.118669
42592	1.49906	0.0795988
42593	1.93771	0.0782382
42594	1.45535	0.0876261
42595	1.45535	0.102098
42596	1.91484	0.0637493
42597	1.91484	0.0693827
42598	1.91484	0.077241
42599	2.25035	0.0812892
42600	1.93772	0.0865528
42601	1.93772	0.0989776
42602	2.11465	0.0639373
42603	2.11465	0.0676987
42604	2.61735	0.149932
42605	1.5082	0.143538
42606	2.36481	0.177246
42607	1.58597	0.168266
42608	2.36481	0.147538
42609	1.46653	0.134718
42610	2.36482	0.191621
42611	1.08133	0.223211
42612	1.5082	0.132754
42613	2.01868	0.226046
42614	3.52688	0.228425
42615	0.94613	0.183841
42616	0.94613	0.180111
42617	0.946131	0.235379
42618	0.946131	0.251659
42619	0.946129	0.251532
42620	0.946129	0.216687
42621	0.946132	0.18089
42622	0.946132	0.210913
42623	0.94613	0.219057
42624	0.94613	0.23589
42625	0.946131	0.227975
42626	0.946131	0.220363
42627	2.53725	0.0918076

42628	1.89227	0.124831
42629	1.89227	0.124482
42630	2.27073	0.119688
42631	1.89227	0.101998
42632	2.27073	0.0980978
42633	1.89227	0.193682
42634	1.89227	0.290353
42635	1.89227	0.291094
42636	1.89227	0.250028
42637	1.89227	0.223697
42638	1.89227	0.192728
42639	1.86766	2.1
42640	2.83487	2.1
42641	2.84318	2.1
42642	2.59401	2.1
42643	2.11684	2.1
42644	1.86766	2.1
42645	1.10638	2.1
42646	2.11684	2.1
42647	1.29111	2.1
42648	2.37439	2.1
42649	3.40796	2.1
42650	2.56843	2.1
42651	1.89226	2.1
42652	1.89226	2.1
42653	2.71932	2.1
42654	4.46069	2.1
42655	1.89226	2.1
42656	1.89226	2.1
42657	1.89226	2.1
42658	1.89226	2.1
42659	3.78452	2.1
42660	0.6416	2.1
42661	0.697585	2.1
42662	0.670706	2.1
42663	0.598927	2.1
42664	0.644313	2.1
42665	0.644313	2.1
42666	0.652199	2.1
42667	0.652199	2.1
42668	0.697587	2.1
42669	0.670705	2.1
42670	0.598926	2.1
42671	0.641601	2.1
42672	1.89226	2.1
42673	1.89226	2.1
42674	1.89226	2.1
42675	1.89226	2.1
42676	1.89226	2.1
42677	1.89226	2.1
42678	0.697585	2.1
42679	0.730617	2.1
42680	0.712082	2.1
42681	0.670706	2.1
42682	0.69854	2.1
42683	0.69854	2.1
42684	0.702783	2.1
42685	0.702783	2.1

42686	0.730618	2.1
42687	0.712081	2.1
42688	0.697586	2.1
42689	0.670705	2.1
42690	1.89226	2.1
42691	1.89226	2.1
42692	1.89226	2.02921
42693	1.89226	2.1
42694	1.89226	1.98955
42695	1.89226	2.1
42696	2.0815	2.1
42697	1.12076	2.1
42698	1.38662	2.1
42699	2.01244	2.1
42700	3.41508	2.1
42701	1.89226	2.1
42702	2.54324	2.1
42703	1.91981	2.1
42704	1.89226	2.1
42705	2.57079	2.1
42706	2.54324	2.1
42707	2.54325	2.1
42708	1.94779	2.1
42709	1.76879	2.1
42710	1.73811	2.1
42711	2.5708	2.1
42712	2.54325	2.1
42713	1.89226	2.1
42714	2.57079	2.1
42715	1.91894	2.1
42716	1.89226	2.1
42717	2.59747	2.1
42718	2.57079	2.1
42719	2.5708	2.1
42720	1.82268	2.1
42721	1.79738	2.1
42722	1.77071	2.1
42723	2.59747	2.1
42724	2.5708	2.1
42725	1.89226	0.937271
42726	1.89226	2.1
42727	1.89226	0.819579
42728	1.89226	2.1
42729	3.78451	1.84693
42730	2.21582	0.650393
42731	1.94412	2.1
42732	2.13428	0.733521
42733	1.86292	2.1
42734	4.07839	1.81861
42735	1.94412	2.1
42736	1.5854	2.1
42737	1.86293	2.1
42738	1.50767	2.1
42739	3.45179	2.1
42740	1.89226	2.1
42741	1.89226	2.1
42742	1.89226	2.1
42743	1.89226	2.1

42744	3.78452	2.1
42745	1.89226	0.644815
42746	1.89226	2.1
42747	1.89226	0.839607
42748	1.89226	2.1
42749	3.78452	1.99719
42750	2.13428	0.550483
42751	1.86293	2.1
42752	2.04737	0.882614
42753	1.77122	2.1
42754	3.9055	1.68917
42755	1.86293	2.1
42756	1.50767	2.1
42757	1.41077	2.1
42758	1.41077	2.1
42759	1.77123	2.1
42760	1.86813	2.1
42761	0.810943	2.1
42762	0.810943	2.1
42763	0.810945	2.1
42764	0.810945	2.1
42765	0.810958	2.1
42766	0.810958	2.1
42767	1.89226	2.1
42768	1.35168	2.1
42769	1.08132	2.1
42770	1.08132	2.1
42771	1.0813	2.1
42772	0.745979	2.1
42773	0.745979	2.1
42774	0.778781	2.1
42775	0.778781	2.1
42776	0.59549	2.1
42777	0.649658	2.1
42778	0.994784	2.1
42779	0.875102	2.1
42780	0.875102	2.1
42781	0.59549	2.1
42782	0.649658	2.1
42783	0.994784	2.1
42784	1.57887	2.1
42785	1.75021	2.1
42786	1.13341	2.1
42787	1.13341	2.1
42788	1.19098	2.1
42789	1.63644	2.1
42790	2.5761	0.510579
42791	2.43404	2.1
42792	2.44813	0.423133
42793	2.31751	2.1
42794	4.88218	1.08834
42795	2.43405	2.1
42796	2.22575	2.1
42797	2.31751	2.1
42798	2.10893	2.1
42799	4.54298	2.1
42800	2.44813	0.305048
42801	2.31751	2.1

42802	2.30908	0.541074
42803	2.19815	2.08643
42804	4.64628	0.975793
42805	2.31751	2.1
42806	2.10893	2.1
42807	2.19815	2.1
42808	1.98061	2.1
42809	4.30708	2.1
42810	0.94613	2.1
42811	0.94613	2.1
42812	1.40372	2.1
42813	1.40372	2.1
42814	1.40372	2.1
42815	1.37167	2.1
42816	0.946132	2.1
42817	0.946132	2.1
42818	0.946131	2.1
42819	1.40372	2.1
42820	0.94613	2.1
42821	1.37167	2.1
42822	2.80744	2.1
42823	2.06984	2.1
42824	2.83418	2.1
42825	2.74335	2.1
42826	2.16067	2.1
42827	2.06984	2.1
42828	1.077	0.202144
42829	0.809116	0.171201
42830	0.600419	0.259887
42831	0.790277	0.178143
42832	0.927647	0.159829
42833	0.758598	0.157616
42834	0.85749	0.213014
42835	0.85749	0.224942
42836	1.02417	0.177573
42837	0.600411	0.194174
42838	0.751701	0.192398
42839	1.07702	0.172797
42840	1.89226	2.1
42841	1.89226	2.1
42842	1.89226	2.1
42843	1.89226	2.1
42844	1.89226	2.1
42845	1.89226	2.1
42846	1.89226	2.1
42847	1.89226	2.1
42848	1.89226	2.1
42849	1.89226	2.1
42850	3.78453	2.1
42851	1.89226	2.1
42852	1.89226	2.1
42853	1.89226	2.1
42854	1.89226	2.1
42855	1.89226	2.1
42856	1.89226	2.1
42857	0.946131	2.1
42858	0.946131	2.1
42859	0.946131	2.1

42860	0.946131	2.1
42861	0.946133	2.1
42862	0.946133	2.1
42863	0.946128	2.1
42864	0.946128	2.1
42865	0.946131	2.1
42866	0.946131	2.1
42867	0.94613	2.1
42868	0.94613	2.1
42869	2.17159	2.1
42870	2.00083	1.92415
42871	2.21305	2.1
42872	2.00551	2.1
42873	2.20837	2.1
42874	2.00083	2.1
42875	2.20837	2.1
42876	1.80328	1.48115
42877	2.22962	1.92402
42878	2.00083	1.83319
42879	2.03207	1.55813
42880	1.80328	1.67008
42881	2.28564	2.1
42882	2.20837	1.92057
42883	2.30406	2.1
42884	2.17159	2.1
42885	2.34084	2.1
42886	2.20837	2.1
42887	1.09523	1.43487
42888	1.09523	1.87382
42889	1.09934	2.1
42890	1.09934	2.1
42891	1.01603	1.38745
42892	1.09038	1.88784
42893	1.10419	1.82142
42894	1.17042	2.1
42895	1.17042	1.94848
42896	1.09038	1.92854
42897	1.10419	1.84754
42898	1.01603	1.93055
42899	2.60152	0.247149
42900	0.976334	0.497131
42901	2.22344	0.500313
42902	2.22344	0.297419
42903	1.36405	0.625173
42904	1.36405	0.216921
42905	1.89226	2.1
42906	1.70302	2.1
42907	1.69019	2.1
42908	1.70302	2.1
42909	1.73933	2.1
42910	1.70303	2.1
42911	1.89226	2.1
42912	1.89226	2.00311
42913	1.89226	2.1
42914	1.89226	2.1
42915	3.78452	2.1
42916	1.89226	2.1
42917	1.59036	2.1

42918	1.65683	2.1
42919	1.59036	2.1
42920	1.95873	2.1
42921	1.89226	1.9608
42922	0.946131	1.87145
42923	0.946131	2.03246
42924	0.946127	2.1
42925	0.946127	2.1
42926	0.946127	2.1
42927	0.946127	1.80371
42928	0.946131	2.1
42929	0.946131	2.05318
42930	0.946129	2.1
42931	0.946129	2.1
42932	0.94613	2.1
42933	0.94613	2.1
42934	0.851541	2.1
42935	0.851541	2.1
42936	0.851483	2.1
42937	1.15713	2.1
42938	0.851539	2.1
42939	0.851539	2.1
42940	0.942127	2.1
42941	1.14455	2.1
42942	0.838683	2.1
42943	0.946131	2.1
42944	0.957503	2.1
42945	0.957504	2.1
42946	1.89226	1.63963
42947	1.89226	0.743591
42948	1.89226	1.12061
42949	1.89226	2.1
42950	3.78452	0.658265
42951	1.89226	0.0886611
42952	1.89226	0.353549
42953	1.89226	0.423497
42954	1.89226	0.440462
42955	1.89226	0.08269
42956	1.89226	0.0831113
42957	1.89226	0.368144
42958	1.89226	0.317142
42959	1.89226	0.260252
42960	1.89226	0.54637
42961	3.78453	0.256836
42962	1.89226	0.816598
42963	1.89226	2.1
42964	1.89226	2.1
42965	1.89226	2.04474
42966	3.78453	2.1
42967	1.22575	0.416156
42968	1.05438	1.38633
42969	0.946131	0.983766
42970	0.946131	1.16086
42971	0.946129	1.8463
42972	1.22575	0.341796
42973	1.05438	0.491362
42974	0.946132	0.238184
42975	1.05438	0.873829

42976	1.05438	1.28982
42977	0.946131	0.430401
42978	0.946131	0.980622
42979	2.4515	0.345083
42980	1.13726	0.166271
42981	2.10875	0.112455
42982	1.49515	0.155648
42983	2.10875	0.238482
42984	1.49515	0.121007
42985	0.831477	0.05
42986	0.747575	0.108591
42987	1.22575	0.169644
42988	1.28174	0.18759
42989	1.28174	0.112458
42990	0.831476	0.172949
42991	1.22575	0.239862
42992	0.747575	0.0796947
42993	1.03561	0.200179
42994	1.03561	0.226117
42995	1.02162	0.0879051
42996	1.02162	0.138719
42997	1.89227	2.1
42998	2.56348	1.54766
42999	1.89227	2.1
43000	2.45151	2.09372
43001	1.89227	2.1
43002	2.45151	1.07642
43003	1.40013	0.628536
43004	1.89226	1.58852
43005	1.89226	1.24297
43006	0.946643	0.560394
43007	1.89226	1.29402
43008	0.946643	0.770989
43009	1.89226	0.05
43010	1.89226	0.0676498
43011	1.89226	0.05
43012	1.89226	0.0648202
43013	3.78453	0.0614191
43014	1.89226	0.102856
43015	1.89226	0.143869
43016	1.89226	0.105312
43017	1.89226	0.20494
43018	3.78453	0.110279
43019	0.946131	0.159403
43020	0.946131	0.140202
43021	0.946128	0.09004
43022	0.946128	0.0973539
43023	0.94613	0.149983
43024	0.94613	0.168759
43025	0.946129	0.099945
43026	0.946129	0.0923308
43027	0.94613	0.13634
43028	0.94613	0.0765688
43029	0.94613	0.104249
43030	0.94613	0.159287
43031	1.89226	0.05
43032	1.89226	0.05
43033	1.17244	0.05

43034	1.89226	0.05
43035	0.975291	0.05
43036	1.57408	0.05
43037	0.946132	0.102421
43038	0.946132	0.0641006
43039	0.946131	0.110845
43040	0.946131	0.0690371
43041	0.946132	0.0943247
43042	0.946132	0.114718
43043	0.946131	0.0628632
43044	0.946131	0.0713815
43045	0.946131	0.0865417
43046	0.946131	0.0915755
43047	0.946132	0.0733928
43048	0.946132	0.1018
43049	2.74744	0.0795234
43050	1.89226	0.175736
43051	2.70805	0.10735
43052	1.89226	0.139817
43053	2.70805	0.114795
43054	1.89226	0.153995
43055	2.74744	0.0619962
43056	1.89226	0.0941429
43057	2.57465	0.071636
43058	1.89226	0.146937
43059	4.6397	0.0760637
43060	1.89226	0.222889
43061	1.89226	0.448907
43062	1.89226	0.375609
43063	1.89226	0.612206
43064	3.78452	0.396794
43065	1.89226	0.323662
43066	1.89226	0.772156
43067	1.89226	0.266287
43068	1.89226	0.241256
43069	1.89226	0.916365
43070	1.89226	0.793341
43071	2.16206	0.0834799
43072	2.67861	0.315536
43073	3.11996	0.112521
43074	1.70303	0.0914588
43075	2.89015	0.0952383
43076	1.486	0.0705104
43077	2.12839	0.05
43078	2.83631	0.269335
43079	3.08096	0.0804311
43080	1.84157	0.112454
43081	3.08096	0.141422
43082	1.84157	0.0852655
43083	1.89225	1.76541
43084	1.89225	1.31656
43085	1.89225	1.50579
43086	1.89225	1.6474
43087	1.89225	1.33377
43088	1.89225	1.62804
43089	1.89226	0.732564
43090	1.89226	1.12483
43091	1.89226	1.62406

43092	1.89226	0.533979
43093	3.78452	0.572826
43094	1.89226	0.34242
43095	2.49041	0.305932
43096	1.89226	0.34475
43097	1.89226	0.206135
43098	2.27073	0.184876
43099	2.27073	0.299955
43100	1.09904	0.188251
43101	0.677147	0.0933276
43102	1.3365	0.0740346
43103	0.95759	0.357269
43104	0.957591	0.158863
43105	0.677148	0.15188
43106	1.09904	0.221616
43107	1.3365	0.135476
43108	0.996015	0.0782164
43109	0.996015	0.129405
43110	1.01764	0.373417
43111	1.01764	0.185523
43112	2.77349	0.248756
43113	2.72723	0.150401
43114	2.43156	0.285728
43115	2.4564	0.147905
43116	5.20505	0.23354
43117	2.77349	0.227537
43118	2.68508	0.135738
43119	2.33761	0.205937
43120	2.4564	0.144626
43121	5.1111	0.16053
43122	2.43156	0.189754
43123	2.70208	0.131185
43124	1.85779	0.220037
43125	2.10695	0.130667
43126	4.77572	0.142274
43127	2.33761	0.162354
43128	2.70208	0.128257
43129	1.85779	0.190591
43130	1.61597	0.120779
43131	4.19542	0.152404
43132	0.946132	1.63905
43133	0.946132	1.78454
43134	0.746729	1.70761
43135	0.746729	1.8555
43136	0.946132	1.87547
43137	0.915958	1.78919
43138	0.946133	1.60314
43139	0.74673	1.89913
43140	0.946133	1.71305
43141	0.946133	1.71505
43142	0.915958	1.78385
43143	0.746729	1.76833
43144	1.50743	2.1
43145	2.45501	2.1
43146	2.29118	2.1
43147	2.29118	2.1
43148	1.75482	2.1
43149	1.75482	2.1

43150	2.45501	2.1
43151	2.76927	2.01019
43152	2.29118	2.1
43153	2.64523	2.09556
43154	5.10024	2.1
43155	1.25559	2.1
43156	2.29118	2.1
43157	2.0815	2.1
43158	2.26387	2.1
43159	1.35668	2.1
43160	1.47667	2.1
43161	2.28481	2.1
43162	2.64523	2.1
43163	2.50057	2.1
43164	2.50057	2.08241
43165	2.29118	2.1
43166	2.29118	2.094
43167	0.946129	0.0946062
43168	0.946129	0.0820538
43169	1.23929	0.0670158
43170	1.15246	0.083386
43171	0.946131	0.0823289
43172	0.946131	0.113206
43173	1.15246	0.0740452
43174	1.15246	0.0896678
43175	0.946131	0.0793203
43176	1.15246	0.0927754
43177	0.946131	0.0779529
43178	1.23928	0.0899269
43179	0.946128	0.122526
43180	0.946128	0.112559
43181	1.15246	0.0965314
43182	0.942814	0.15789
43183	0.946131	0.0930522
43184	0.946131	0.130415
43185	0.961347	0.0947426
43186	0.942811	0.145701
43187	0.946131	0.104918
43188	0.961347	0.102036
43189	0.946131	0.0915947
43190	1.15246	0.120527
43191	2.32334	0.150745
43192	1.57621	0.129446
43193	1.90335	0.127826
43194	1.24538	0.102779
43195	3.47956	0.0984159
43196	0.965762	0.0510673
43197	0.965762	0.0679356
43198	1.00245	0.05
43199	1.00245	0.05
43200	0.92786	0.051688
43201	0.705297	0.05
43202	0.856414	0.058264
43203	1.28968	0.05
43204	1.00306	0.0501898
43205	0.705303	0.05
43206	1.28967	0.05
43207	0.975478	0.0641177

43208	3.05308	2.1
43209	1.76816	2.1
43210	1.76816	2.1
43211	1.69129	2.1
43212	2.58731	2.1
43213	2.51043	2.1
43214	1.89226	0.064209
43215	2.71464	0.0632953
43216	2.34627	0.0613763
43217	1.89226	0.0768203
43218	2.34627	0.0533543
43219	1.89226	0.070613
43220	2.34627	0.0602137
43221	2.71464	0.0589948
43222	1.98252	0.0532805
43223	1.98252	0.0793796
43224	1.51026	0.0536597
43225	2.34627	0.0661678
43226	2.38698	1.42597
43227	2.35142	2.1
43228	2.60343	1.78197
43229	2.6636	1.41278
43230	4.99041	1.67331
43231	1.07811	2.1
43232	1.1952	2.1
43233	1.17571	1.92381
43234	1.30171	1.74986
43235	1.18322	1.83099
43236	1.18322	2.1
43237	1.18769	2.1
43238	1.18769	1.85185
43239	1.1952	2.1
43240	1.30171	2.1
43241	1.17571	2.1
43242	1.07811	2.1
43243	1.89226	1.70984
43244	2.6636	1.58173
43245	1.89226	0.604476
43246	2.60343	1.60774
43247	1.89226	1.49597
43248	2.60343	2.1
43249	1.5344	0.05
43250	1.4516	0.05
43251	1.17816	0.05
43252	1.21056	0.05
43253	2.71257	0.05
43254	1.89226	1.84664
43255	1.89226	1.24218
43256	1.89226	1.54744
43257	1.89226	1.59074
43258	3.78452	1.57247
43259	1.89226	1.54107
43260	1.89226	0.385332
43261	1.89226	1.07324
43262	1.89226	0.687008
43263	1.65573	1.23785
43264	1.65573	0.949991
43265	1.89226	1.52932

43266	1.89226	1.60847
43267	1.89226	1.9273
43268	2.64212	1.4233
43269	3.78452	1.57699
43270	1.89226	2.1
43271	1.89226	2.1
43272	1.89226	2.1
43273	1.89226	2.1
43274	3.78452	2.1
43275	2.7367	2.1
43276	2.07947	2.1
43277	2.47319	2.1
43278	1.85057	2.1
43279	4.55267	2.1
43280	2.07948	2.1
43281	1.3294	2.1
43282	1.85057	2.1
43283	1.09962	2.1
43284	3.1791	2.1
43285	1.89226	2.1
43286	1.89226	2.1
43287	1.89226	2.1
43288	1.89226	2.1
43289	3.78453	2.1
43290	0.94613	2.1
43291	0.94613	2.1
43292	0.94613	2.1
43293	0.94613	2.1
43294	0.946128	2.1
43295	0.946128	2.1
43296	0.946132	2.1
43297	0.946132	2.1
43298	0.94613	2.1
43299	0.94613	2.1
43300	0.946131	2.1
43301	0.946131	2.1
43302	1.00875	2.1
43303	1.00875	2.1
43304	1.01676	2.1
43305	1.01676	2.1
43306	0.925282	2.1
43307	0.804877	2.1
43308	1.2366	2.1
43309	1.10023	2.1
43310	0.804878	2.1
43311	1.10023	2.1
43312	1.2366	2.1
43313	0.925284	2.1
43314	1.89226	2.1
43315	1.89226	2.1
43316	1.89226	2.1
43317	1.89226	2.1
43318	3.78453	2.1
43319	1.89226	2.1
43320	1.89226	2.1
43321	1.89226	2.1
43322	1.89226	2.1
43323	1.89226	2.1

43324	1.89226	2.1
43325	1.09766	2.1
43326	1.14842	2.1
43327	1.02909	2.1
43328	1.07659	2.1
43329	1.16718	2.1
43330	0.954189	2.1
43331	1.06653	2.1
43332	1.06653	2.1
43333	1.20738	2.1
43334	1.02909	2.1
43335	1.09767	2.1
43336	0.815951	2.1
43337	2.00793	2.1
43338	1.14048	2.1
43339	1.19086	2.1
43340	1.13723	2.1
43341	2.00793	2.1
43342	2.11772	2.1
43343	1.89226	2.1
43344	1.89226	2.1
43345	1.89226	2.1
43346	1.89226	2.1
43347	1.89226	2.1
43348	1.89226	2.1
43349	1.89226	2.1
43350	1.89226	2.1
43351	1.89226	2.1
43352	1.89226	2.1
43353	1.89226	2.1
43354	1.89226	2.1
43355	1.1779	2.1
43356	1.26166	2.1
43357	1.0875	2.1
43358	1.00397	2.1
43359	1.13286	2.1
43360	1.13286	2.1
43361	1.13277	2.1
43362	1.13277	2.1
43363	1.26166	2.1
43364	1.08749	2.1
43365	1.1779	2.1
43366	1.00396	2.1
43367	1.0875	2.1
43368	1.00397	2.1
43369	0.685749	2.1
43370	0.595432	2.1
43371	0.83976	2.1
43372	0.83976	2.1
43373	0.843169	2.1
43374	0.843169	2.1
43375	1.0875	2.1
43376	0.685749	2.1
43377	1.00397	2.1
43378	0.595432	2.1
43379	1.89226	2.1
43380	1.86443	2.1
43381	2.4988	2.1

43382	2.0815	2.1
43383	2.28173	2.1
43384	1.86443	2.1
43385	1.89226	2.1
43386	1.89226	2.1
43387	1.89226	2.1
43388	1.89226	2.1
43389	1.89226	2.1
43390	1.89226	2.1
43391	0.946131	2.1
43392	0.946131	2.1
43393	0.969491	2.1
43394	0.949105	2.1
43395	0.949103	2.1
43396	0.949103	2.1
43397	0.946133	2.1
43398	0.946133	2.1
43399	0.946131	2.1
43400	0.969492	2.1
43401	0.946131	2.1
43402	0.949105	2.1
43403	1.89226	2.1
43404	2.07428	2.1
43405	1.93464	2.1
43406	1.89226	2.1
43407	2.11667	2.1
43408	2.07428	2.1
43409	0.946132	2.1
43410	0.946132	2.1
43411	0.946131	2.1
43412	0.946131	2.1
43413	0.946132	2.1
43414	0.946132	2.1
43415	0.946132	2.1
43416	0.946132	2.1
43417	0.946132	2.1
43418	0.946132	2.1
43419	0.946132	2.1
43420	0.946132	2.1
43421	1.97992	2.1
43422	1.89226	2.1
43423	1.89226	2.1
43424	1.93898	2.1
43425	1.93898	2.1
43426	1.89226	2.1
43427	2.12475	0.238762
43428	2.79029	0.726796
43429	2.7803	1.50088
43430	2.13052	0.303308
43431	4.92081	0.353286
43432	1.06238	1.24535
43433	1.06526	0.376052
43434	0.642901	0.153418
43435	0.655055	0.947015
43436	0.856391	0.229399
43437	0.856391	0.244936
43438	0.86104	1.34031
43439	0.86104	0.118213

43440	1.06526	0.316854
43441	0.655055	0.299104
43442	0.642901	0.290135
43443	1.06238	0.275619
43444	2.7803	1.14718
43445	2.13053	0.204849
43446	2.14346	0.326689
43447	2.77937	0.691756
43448	4.9099	0.405749
43449	2.14346	0.432975
43450	1.31011	0.110925
43451	1.34124	0.374571
43452	1.31011	0.569314
43453	2.16166	0.1443
43454	2.13053	0.337052
43455	1.89226	2.1
43456	2.58176	2.1
43457	2.01422	2.1
43458	1.89226	2.1
43459	2.70373	2.1
43460	2.58176	2.1
43461	1.89226	2.1
43462	1.89226	2.1
43463	1.89226	2.1
43464	1.89226	2.1
43465	1.89226	2.1
43466	1.89226	2.1
43467	0.946128	2.1
43468	0.946128	2.1
43469	1.38521	2.1
43470	1.38521	2.1
43471	1.38521	2.1
43472	1.35186	2.1
43473	0.946131	2.1
43474	0.946131	2.1
43475	0.94613	2.1
43476	1.38521	2.1
43477	0.946131	2.1
43478	1.35186	2.1
43479	0.946133	2.1
43480	0.946133	2.1
43481	0.946132	2.1
43482	0.946132	2.1
43483	0.946132	2.1
43484	0.946132	2.1
43485	0.946132	2.1
43486	0.946132	2.1
43487	0.946132	2.1
43488	0.946132	2.1
43489	0.946133	2.1
43490	0.946133	2.1
43491	2.33374	2.1
43492	1.89226	2.1
43493	1.89226	2.1
43494	1.89226	2.1
43495	2.60377	2.1
43496	2.60377	2.1
43497	2.6771	0.05

43498	1.36416	0.05
43499	1.64983	0.05
43500	1.64983	0.05
43501	2.44131	0.05
43502	2.44131	0.05
43503	2.58321	0.240646
43504	1.89226	0.257657
43505	2.44283	0.129691
43506	2.44283	0.165128
43507	1.89226	0.67989
43508	1.89226	0.937839
43509	2.58321	0.25326
43510	2.44283	0.14181
43511	1.49358	0.109187
43512	1.57702	0.146942
43513	3.93641	0.123223
43514	0.951913	0.05
43515	0.81556	0.05
43516	0.946132	0.05
43517	0.946132	0.05
43518	1.04072	0.05
43519	1.12182	0.05
43520	0.856745	0.05
43521	0.851542	0.05
43522	0.923163	0.05
43523	0.923163	0.0528884
43524	1.04304	0.05
43525	0.85098	0.05
43526	2.70125	1.65689
43527	2.37734	1.90791
43528	2.63495	1.50521
43529	2.30539	1.8607
43530	5.01229	1.86527
43531	1.89226	1.79085
43532	1.89226	2.1
43533	1.89226	1.64358
43534	1.89226	2.1
43535	3.78452	1.68036
43536	1.89226	2.1
43537	1.89226	2.1
43538	1.89226	2.1
43539	1.89226	2.1
43540	3.78452	2.1
43541	2.37734	2.1
43542	1.79346	2.1
43543	2.53594	1.74902
43544	1.70302	2.1
43545	4.27682	2.1
43546	2.63495	1.4258
43547	2.30539	1.57542
43548	2.49445	0.606064
43549	2.12089	1.51311
43550	4.75584	1.30741
43551	1.89226	1.55317
43552	1.89226	1.78336
43553	1.89226	1.38666
43554	1.89226	1.7609
43555	3.78452	1.90571

43556	1.89226	1.80917
43557	1.89226	1.97182
43558	1.89226	2.06077
43559	1.89226	2.1
43560	1.89226	1.62842
43561	1.89226	1.67866
43562	2.53594	0.965362
43563	1.60017	1.73472
43564	1.57079	1.45644
43565	1.70302	1.59238
43566	2.15772	1.28598
43567	2.33299	1.53037
43568	2.09114	2.1
43569	2.34898	2.1
43570	2.30729	2.1
43571	2.06556	2.1
43572	4.41454	2.1
43573	2.09114	2.1
43574	1.59654	2.1
43575	1.57823	2.1
43576	2.06556	2.1
43577	3.6621	2.1
43578	2.30729	2.1
43579	2.06556	2.1
43580	2.03917	2.1
43581	2.26617	2.1
43582	4.33173	2.1
43583	2.03917	2.1
43584	2.06556	2.1
43585	2.045	2.1
43586	1.55767	2.1
43587	1.57823	2.1
43588	1.57823	2.1
43589	1.89225	2.1
43590	1.09726	2.1
43591	1.61196	2.1
43592	1.28597	2.1
43593	1.28597	0.05
43594	1.45633	2.1
43595	1.45633	2.1
43596	1.9544	0.05
43597	1.61196	2.1
43598	1.45633	2.1
43599	1.53735	2.1
43600	3.14931	2.1
43601	1.89226	2.1
43602	1.89226	2.1
43603	1.89226	2.1
43604	1.89226	2.1
43605	3.78453	2.1
43606	0.810946	2.1
43607	0.810946	2.1
43608	0.810947	2.1
43609	0.810947	2.1
43610	0.810933	2.1
43611	0.810933	2.1
43612	1.89226	2.1
43613	1.3517	2.1

43614	1.08132	2.1
43615	1.08132	2.1
43616	1.08133	2.1
43617	0.851539	2.1
43618	0.851539	2.1
43619	0.972053	2.1
43620	1.05632	2.1
43621	0.94613	2.1
43622	0.94613	2.1
43623	1.0279	2.1
43624	0.94991	2.1
43625	1.0279	2.1
43626	0.946131	2.1
43627	0.865643	2.1
43628	0.94613	2.1
43629	1.89226	2.1
43630	1.89226	2.1
43631	1.89226	2.1
43632	1.89226	2.1
43633	3.78452	2.1
43634	1.03882	2.1
43635	1.91121	2.1
43636	1.41833	2.1
43637	2.34547	2.1
43638	3.32954	2.1
43639	2.3979	2.1
43640	1.89226	2.1
43641	1.89226	2.1
43642	1.89226	2.1
43643	2.0558	2.1
43644	2.0558	2.1
43645	1.89226	2.1
43646	1.89226	2.1
43647	1.89226	2.1
43648	1.89226	2.1
43649	3.78452	2.1
43650	1.05816	2.1
43651	1.05816	2.1
43652	0.842045	2.1
43653	1.05252	2.1
43654	1.36057	2.1
43655	1.14339	2.1
43656	0.873397	2.1
43657	0.585073	2.1
43658	0.873388	2.1
43659	1.15129	2.1
43660	0.69143	2.1
43661	1.1434	2.1
43662	1.93338	2.1
43663	1.85883	2.1
43664	1.51714	2.1
43665	1.85883	2.1
43666	1.54795	2.1
43667	1.89226	2.1
43668	1.89226	2.1
43669	1.89226	2.1
43670	1.89226	2.1
43671	1.89226	2.1

43672	3.78452	2.1
43673	0.844726	2.1
43674	0.844726	2.1
43675	0.960524	2.1
43676	1.16288	2.1
43677	0.851541	2.1
43678	0.851541	2.1
43679	0.929147	2.1
43680	1.15533	2.1
43681	0.844697	2.1
43682	0.946131	2.1
43683	0.851632	2.1
43684	0.946131	2.1
43685	1.89226	2.1
43686	1.70303	2.1
43687	1.74006	2.1
43688	1.70303	2.1
43689	1.99471	2.1
43690	1.93338	2.1
43691	0.946131	2.1
43692	0.946131	2.1
43693	0.946131	2.1
43694	0.946131	2.1
43695	0.94613	2.1
43696	0.94613	2.1
43697	0.946132	2.1
43698	0.946132	2.1
43699	0.946131	2.1
43700	0.946131	2.1
43701	0.946131	2.1
43702	0.946131	2.1
43703	1.58158	2.1
43704	1.89226	2.1
43705	1.89226	2.1
43706	1.89226	2.1
43707	1.68939	2.1
43708	1.68939	2.1
43709	0.946132	0.05
43710	0.946132	0.05
43711	1.12988	0.05
43712	1.30783	0.05
43713	1.30783	0.05
43714	0.946133	0.05
43715	0.946132	0.05
43716	1.12988	0.05
43717	1.30783	0.05
43718	1.30783	0.05
43719	0.946133	0.05
43720	0.946133	0.05
43721	1.89227	0.05
43722	1.89227	0.05
43723	1.89227	0.05
43724	1.89227	0.05
43725	1.89227	0.05
43726	1.89227	0.05
43727	1.89226	0.05
43728	1.89226	0.05
43729	1.89226	0.05

43730	1.89226	0.05
43731	1.89226	0.05
43732	1.89226	0.05
43733	0.946131	0.05
43734	0.946131	0.05
43735	1.30783	0.05
43736	1.37715	0.05
43737	1.37715	0.05
43738	0.946133	0.05
43739	1.30783	0.05
43740	0.946129	0.05
43741	1.37715	0.05
43742	1.37715	0.05
43743	0.946131	0.05
43744	0.946131	0.05
43745	1.17056	0.05
43746	0.591757	0.05
43747	0.522025	0.05
43748	0.929727	0.05
43749	0.929728	0.05
43750	1.17056	0.05
43751	0.591756	0.05
43752	0.522025	0.05
43753	0.889054	0.05
43754	0.889054	0.05
43755	0.803535	0.05
43756	0.803535	0.05
43757	1.49814	2.1
43758	2.37439	2.1
43759	2.63923	2.1
43760	2.63923	2.1
43761	1.29111	2.1
43762	1.29111	2.1
43763	0.946132	2.1
43764	0.946132	2.1
43765	1.18719	2.1
43766	1.18719	2.1
43767	0.94613	2.1
43768	0.94613	2.1
43769	1.1872	2.1
43770	1.31961	2.1
43771	0.946131	2.1
43772	1.31961	2.1
43773	1.1872	2.1
43774	0.94613	2.1
43775	2.49445	0.272005
43776	2.12089	0.99823
43777	2.20843	0.422166
43778	1.82805	0.35945
43779	4.32932	0.593258
43780	1.89226	1.23002
43781	1.89226	1.52376
43782	1.89226	1.11583
43783	1.89226	1.25161
43784	3.78452	0.839792
43785	0.94613	1.2912
43786	0.94613	1.28968
43787	0.946128	0.892671

43788	0.946128	1.46754
43789	0.946131	1.47817
43790	0.946131	1.46236
43791	0.946127	1.38463
43792	0.946127	1.26082
43793	0.946129	1.20083
43794	0.946129	1.03778
43795	0.946129	1.44157
43796	0.946129	1.37719
43797	1.82804	0.246362
43798	1.60017	1.0401
43799	1.87679	0.326218
43800	2.12088	0.429539
43801	1.35608	0.39902
43802	1.60017	0.612998
43803	2.20843	0.196175
43804	1.3216	0.161441
43805	1.44426	0.194269
43806	1.82805	0.253121
43807	1.80399	0.11706
43808	2.20843	0.182391
43809	1.89226	0.341576
43810	1.89226	0.435772
43811	1.89226	0.716732
43812	1.89226	0.742988
43813	1.89226	0.378976
43814	1.89226	0.4233
43815	1.89226	0.4733
43816	1.89226	0.362748
43817	1.89226	0.325919
43818	1.89226	0.30567
43819	1.89226	0.540654
43820	1.89226	0.307951
43821	0.627705	0.0827912
43822	0.634941	0.180349
43823	0.629317	0.0987585
43824	0.783422	0.131653
43825	0.629328	0.144689
43826	0.448764	0.218167
43827	1.35608	0.231515
43828	1.00805	0.233694
43829	0.814295	0.147231
43830	0.598375	0.199558
43831	1.04461	0.186801
43832	1.09795	2.03445
43833	1.00775	2.1
43834	1.61202	2.07963
43835	1.63419	2.01609
43836	2.64194	1.96905
43837	1.44801	1.81663
43838	1.61201	2.0648
43839	2.35614	1.85564
43840	2.27703	2.00096
43841	3.88904	1.96168
43842	2.35615	1.7642
43843	1.63419	1.83207
43844	2.29852	1.74188
43845	1.63419	1.82899

43846	2.27634	1.79775
43847	1.61202	2.03065
43848	0.973793	2.1
43849	2.2671	2.1
43850	2.32981	2.1
43851	2.32981	2.1
43852	0.990481	2.1
43853	0.990481	2.1
43854	1.50877	2.1
43855	1.11193	2.1
43856	2.29667	2.1
43857	1.94158	2.1
43858	3.45035	2.1
43859	2.29667	2.1
43860	1.89227	2.1
43861	1.94158	2.1
43862	1.94158	2.1
43863	1.89227	2.1
43864	1.89227	2.1
43865	2.51379	2.1
43866	1.30026	2.1
43867	1.84502	2.1
43868	2.72099	2.1
43869	4.35882	2.1
43870	1.89226	2.1
43871	2.721	2.1
43872	2.5138	2.1
43873	2.5138	2.1
43874	1.89226	2.1
43875	1.89226	2.1
43876	1.89226	0.05
43877	1.89226	0.0547258
43878	2.59101	0.05
43879	1.89226	0.0732396
43880	3.78453	0.05
43881	0.851541	0.0543169
43882	0.851541	0.05
43883	1.42502	0.05
43884	0.948679	0.05
43885	0.946132	0.0526592
43886	0.946132	0.0590921
43887	0.853617	0.05
43888	0.853618	0.05
43889	0.851475	0.0511828
43890	0.948666	0.0552345
43891	1.166	0.05
43892	0.851554	0.0506486
43893	2.51818	2.1
43894	2.60723	2.1
43895	2.66532	2.06078
43896	2.76315	2.1
43897	5.27255	2.1
43898	2.60723	2.1
43899	2.45247	2.1
43900	2.76315	2.1
43901	2.55197	2.1
43902	5.1592	2.1
43903	1.23768	0.17843

43904	1.11433	1.16742
43905	1.32037	1.51498
43906	1.32037	0.524995
43907	1.0142	1.38623
43908	1.0142	0.337439
43909	0.886711	1.2096
43910	1.16806	1.16833
43911	0.999083	1.51308
43912	0.710926	1.38194
43913	0.937757	1.43389
43914	0.937757	1.22979
43915	0.941224	1.12148
43916	0.941224	1.38542
43917	1.16805	1.3303
43918	0.999084	1.41541
43919	0.886709	1.34323
43920	0.710928	1.33741
43921	2.29332	2.1
43922	1.54904	2.1
43923	2.0815	2.1
43924	2.44199	2.1
43925	4.00418	2.1
43926	1.53873	0.513825
43927	1.53422	2.1
43928	1.39661	0.915612
43929	1.42387	2.1
43930	2.9626	1.79265
43931	1.53423	2.1
43932	1.44091	2.1
43933	1.42387	2.1
43934	1.35215	2.1
43935	2.86479	2.1
43936	1.39661	0.860362
43937	1.42387	2.1
43938	1.28325	0.567252
43939	1.33326	2.1
43940	2.70713	1.70295
43941	1.42387	2.1
43942	1.35215	2.1
43943	1.33327	2.1
43944	1.27339	2.1
43945	2.69726	2.1
43946	2.58442	2.1
43947	1.1468	2.1
43948	1.18775	2.1
43949	2.60047	2.1
43950	3.74728	2.1
43951	2.58442	2.1
43952	1.89226	2.1
43953	1.89226	2.1
43954	2.60048	2.1
43955	4.49274	2.1
43956	1.89226	2.1
43957	1.89226	2.1
43958	1.89226	2.1
43959	1.89226	2.1
43960	3.78453	2.1
43961	1.89226	2.1

43962	1.89226	2.1
43963	1.89226	2.1
43964	1.89226	2.1
43965	3.78452	2.1
43966	1.22262	2.1
43967	2.60047	2.1
43968	1.18775	2.1
43969	1.18775	2.1
43970	2.61433	2.1
43971	2.61433	2.1
43972	1.89226	2.1
43973	2.60048	2.1
43974	2.61434	2.1
43975	2.60048	2.1
43976	1.90612	2.1
43977	1.89226	2.1
43978	1.89226	2.1
43979	1.89226	2.1
43980	1.89226	2.1
43981	1.89226	2.1
43982	3.78453	2.1
43983	1.89226	2.1
43984	1.89226	2.1
43985	1.89226	2.1
43986	1.89226	2.1
43987	3.78452	2.1
43988	2.12556	2.1
43989	0.991113	2.1
43990	1.27725	2.1
43991	2.31081	2.1
43992	3.40281	2.1
43993	1.89226	2.1
43994	2.31082	2.1
43995	2.12556	2.1
43996	2.12556	2.1
43997	1.89226	2.1
43998	1.89226	2.1
43999	1.22544	2.1
44000	1.12649	2.1
44001	0.775254	2.1
44002	0.643133	2.1
44003	0.917924	2.1
44004	0.934955	2.1
44005	0.975579	2.1
44006	0.975579	2.1
44007	1.23113	2.1
44008	0.685272	2.1
44009	0.651719	2.1
44010	1.1791	2.1
44011	1.89226	2.1
44012	2.5138	2.1
44013	2.31082	2.1
44014	2.31082	2.1
44015	1.89226	2.1
44016	1.89226	2.1
44017	1.89226	2.1
44018	1.89226	2.1
44019	1.89226	2.1

44020	1.89226	2.1
44021	3.78453	2.1
44022	0.922224	2.1
44023	0.922224	2.1
44024	1.23555	2.1
44025	1.19715	2.1
44026	1.01445	2.1
44027	1.16061	2.1
44028	0.946132	2.1
44029	0.946132	2.1
44030	0.946131	2.1
44031	1.2051	2.1
44032	1.13537	2.1
44033	1.01445	2.1
44034	0.946133	2.1
44035	0.946133	2.1
44036	1.20648	2.1
44037	1.20648	2.1
44038	1.06454	2.1
44039	1.20648	2.1
44040	0.946133	2.1
44041	0.946133	2.1
44042	1.20649	2.1
44043	0.946133	2.1
44044	1.06454	2.1
44045	0.946132	2.1
44046	0.946132	2.1
44047	0.946132	2.1
44048	0.946132	2.1
44049	0.946132	2.1
44050	0.946132	2.1
44051	0.946132	2.1
44052	0.946133	2.1
44053	0.946133	2.1
44054	0.946133	2.1
44055	0.946133	2.1
44056	0.946132	2.1
44057	0.946132	2.1
44058	1.33078	2.08829
44059	1.37409	2.1
44060	1.31565	0.495455
44061	1.37114	2.1
44062	2.68973	2.1
44063	1.89225	1.83269
44064	1.89225	2.1
44065	1.89225	0.576272
44066	1.89225	2.1
44067	3.7845	1.71381
44068	1.89225	2.1
44069	1.89225	2.1
44070	1.89225	2.0448
44071	1.89225	2.1
44072	3.7845	2.1
44073	1.37409	2.1
44074	1.20408	2.1
44075	1.18612	2.1
44076	1.18907	2.1
44077	1.37114	2.1

44078	1.37409	2.1
44079	1.31566	2.05867
44080	1.37115	2.1
44081	1.30318	0.90276
44082	1.37433	2.1
44083	2.68998	2.02525
44084	1.89226	2.07287
44085	1.89226	2.1
44086	1.89226	1.14225
44087	1.89226	2.1
44088	3.78453	1.82438
44089	1.89226	2.1
44090	1.89226	2.1
44091	1.89226	2.1
44092	1.89226	2.1
44093	1.89226	2.1
44094	1.89226	2.1
44095	0.647001	2.1
44096	0.647001	2.1
44097	0.651851	2.1
44098	0.651851	2.1
44099	0.602046	2.1
44100	0.613277	2.1
44101	0.685576	2.1
44102	0.687164	2.1
44103	0.687164	2.1
44104	0.613276	2.1
44105	0.602046	2.1
44106	0.685576	2.1
44107	0.946128	1.71837
44108	0.946128	0.794858
44109	1.15307	2.1
44110	1.19317	2.03395
44111	0.94613	2.03068
44112	0.94613	0.50552
44113	1.15307	1.79369
44114	1.15307	2.1
44115	0.946131	1.84524
44116	1.15307	1.87519
44117	0.946131	1.82193
44118	1.19317	1.81509
44119	1.18278	2.1
44120	2.38635	2.06211
44121	1.23042	2.1
44122	1.23042	2.1
44123	2.30614	2.1
44124	2.30614	2.1
44125	0.946129	1.23633
44126	0.946129	1.18903
44127	1.12994	1.93679
44128	1.15307	1.84594
44129	0.946131	1.84588
44130	0.946131	0.356225
44131	1.12994	1.54727
44132	1.12994	1.88655
44133	1.12994	1.71054
44134	0.946131	1.71898
44135	0.946131	1.60191

44136	1.15307	1.67009
44137	2.25988	2.1
44138	2.30614	1.84698
44139	2.28696	2.1
44140	1.16361	2.1
44141	1.18278	2.1
44142	1.18278	2.1
44143	2.3771	2.1
44144	1.90121	2.1
44145	1.81191	2.1
44146	2.46904	2.1
44147	4.37025	2.1
44148	1.15676	2.1
44149	1.23452	2.1
44150	0.823592	2.1
44151	0.737606	2.1
44152	0.988113	2.1
44153	0.988113	2.1
44154	0.984014	2.1
44155	0.984014	2.1
44156	1.15676	2.1
44157	0.737605	2.1
44158	0.823592	2.1
44159	1.23452	2.1
44160	1.30318	2.1
44161	1.37433	2.1
44162	1.30151	1.34558
44163	1.39067	2.1
44164	2.67584	2.04465
44165	1.89226	1.83068
44166	1.89226	2.1
44167	1.89226	1.33695
44168	1.89226	1.92423
44169	3.78453	1.6942
44170	1.89226	1.84658
44171	1.89226	2.1
44172	1.89226	2.06445
44173	1.89226	2.1
44174	1.89226	2.07069
44175	1.89226	2.1
44176	0.656819	2.1
44177	0.656819	2.1
44178	0.661755	2.1
44179	0.661755	2.1
44180	0.613277	2.1
44181	0.63141	2.1
44182	0.687164	2.1
44183	0.695336	2.1
44184	0.695337	2.1
44185	0.63141	2.1
44186	0.687164	2.1
44187	0.613276	2.1
44188	1.30151	1.9168
44189	1.39067	2.1
44190	1.32143	1.43793
44191	1.43294	2.1
44192	2.73445	2.05347
44193	1.89226	1.87807

44194	1.89226	2.1
44195	1.89226	1.20518
44196	1.89226	2.1
44197	3.78452	1.76881
44198	1.89226	2.00051
44199	1.89226	2.07842
44200	1.89226	2.1
44201	1.89226	2.1
44202	1.89226	2.1
44203	1.89226	2.1
44204	1.43294	1.98387
44205	1.26282	2.1
44206	1.3268	2.1
44207	1.3268	2.1
44208	1.39067	2.1
44209	1.39067	2.1
44210	1.5511	0.100442
44211	1.00665	0.108372
44212	1.28239	0.0856641
44213	1.88631	0.0880626
44214	2.89296	0.092851
44215	1.89227	0.0856531
44216	1.89227	0.11821
44217	1.89227	0.0983897
44218	1.89227	0.0929076
44219	1.89227	0.107895
44220	1.89227	0.100363
44221	2.08848	0.11103
44222	1.89226	0.108127
44223	1.89226	0.0873255
44224	2.37947	0.104037
44225	4.27173	0.104796
44226	1.01456	0.0970849
44227	0.943157	0.0961795
44228	0.641198	0.0996136
44229	0.724757	0.0972861
44230	0.830923	0.100841
44231	0.830923	0.11335
44232	0.836991	0.0892988
44233	0.836991	0.101901
44234	1.01456	0.0901963
44235	0.724757	0.0973767
44236	0.641197	0.0991279
44237	0.943157	0.105378
44238	1.89227	0.107337
44239	1.89227	0.119877
44240	1.89227	0.0934184
44241	1.89227	0.0968005
44242	3.78453	0.099455
44243	0.946129	0.107718
44244	0.946129	0.104979
44245	1.28941	0.103067
44246	1.18974	0.103928
44247	0.94613	0.105562
44248	0.94613	0.112329
44249	1.28941	0.0985016
44250	1.28941	0.109642
44251	1.28941	0.0999996

44252	0.946131	0.103814
44253	0.94613	0.108363
44254	1.18974	0.112494
44255	0.710636	2.1
44256	0.780249	2.1
44257	0.579033	2.1
44258	0.478812	2.1
44259	0.621822	2.1
44260	0.621822	2.1
44261	0.63724	2.1
44262	0.63724	2.1
44263	0.780251	2.1
44264	0.579032	2.1
44265	0.710637	2.1
44266	0.478811	2.1
44267	1.05499	2.1
44268	1.00521	1.96173
44269	1.24445	2.1
44270	1.23082	1.50881
44271	2.28581	1.98059
44272	1.48751	2.1
44273	1.23083	1.72561
44274	1.4941	2.08773
44275	1.24445	2.05332
44276	1.48047	1.8455
44277	1.23083	2.03334
44278	0.621185	1.49569
44279	0.621185	1.71761
44280	0.620899	1.77379
44281	0.620899	2.04807
44282	0.502515	1.36429
44283	0.626672	1.38595
44284	0.615412	1.48964
44285	0.740234	2.07747
44286	0.740233	1.42021
44287	0.626672	1.42558
44288	0.615413	1.50719
44289	0.502516	1.56147
44290	1.95844	2.1
44291	2.6562	2.1
44292	1.95844	2.1
44293	2.20883	2.1
44294	2.38225	2.1
44295	2.63264	2.1
44296	1.89226	2.1
44297	1.89226	2.1
44298	1.89226	2.1
44299	1.89226	2.1
44300	3.78453	2.1
44301	1.95844	2.1
44302	2.38225	2.1
44303	1.71082	2.1
44304	2.14647	2.1
44305	4.1049	2.1
44306	0.946131	2.1
44307	0.946131	2.1
44308	0.946131	2.1
44309	0.946131	2.1

44310	0.946131	2.1
44311	0.946131	2.1
44312	0.946131	2.1
44313	0.946131	2.1
44314	0.946131	2.1
44315	0.946131	2.1
44316	0.946131	2.1
44317	0.946131	2.1
44318	2.12597	2.1
44319	1.2295	2.1
44320	1.28014	2.1
44321	2.19723	2.1
44322	3.40611	2.1
44323	2.12597	2.1
44324	2.79242	2.1
44325	2.73375	2.1
44326	2.66249	2.1
44327	2.19723	2.1
44328	2.12597	2.1
44329	1.13716	2.1
44330	1.09861	2.1
44331	0.666692	2.1
44332	0.640071	2.1
44333	0.891617	2.1
44334	0.891617	2.1
44335	0.885614	2.1
44336	0.885614	2.1
44337	1.13716	2.1
44338	0.666692	2.1
44339	0.640071	2.1
44340	1.09861	2.1
44341	1.89226	0.05
44342	1.70303	0.05
44343	1.89226	0.05
44344	2.64598	0.05
44345	1.81091	0.05
44346	2.53222	0.05
44347	2.32422	0.05
44348	1.89226	0.05
44349	2.78336	0.05
44350	2.78336	0.05
44351	1.89226	0.05
44352	1.89226	0.05
44353	0.946132	0.05
44354	0.946132	0.05
44355	0.946131	0.05
44356	0.946131	0.05
44357	0.946132	0.05
44358	0.946132	0.05
44359	0.946131	0.05
44360	0.946131	0.05
44361	0.946131	0.05
44362	0.946131	0.05
44363	0.946131	0.05
44364	0.946131	0.05
44365	0.946131	0.05
44366	0.946131	0.05
44367	0.946132	0.05

44368	0.946132	0.0580609
44369	0.946132	0.0592555
44370	0.946132	0.054018
44371	0.946131	0.05
44372	0.946131	0.05
44373	0.946131	0.05
44374	0.946131	0.0548955
44375	0.946131	0.05
44376	0.946131	0.05
44377	2.24323	1.53948
44378	1.89226	1.75508
44379	2.75021	1.62527
44380	2.75021	1.69344
44381	1.89226	1.27966
44382	1.89226	1.50664
44383	2.30908	0.534986
44384	2.19815	2.08973
44385	2.1609	0.501657
44386	1.83796	2.1
44387	4.35904	1.15622
44388	2.19815	2.08549
44389	2.02268	2.1
44390	2.17869	2.1
44391	1.9559	2.1
44392	2.0815	2.1
44393	1.83424	2.1
44394	2.1609	0.439957
44395	1.88877	2.1
44396	1.98413	0.750122
44397	1.87355	2.1
44398	4.03445	1.34588
44399	1.87355	2.1
44400	2.02268	2.1
44401	1.87355	2.1
44402	2.10488	2.1
44403	1.68443	2.1
44404	1.89448	2.1
44405	0.946131	0.161562
44406	0.946131	0.13788
44407	1.40574	0.157934
44408	1.12373	0.189819
44409	0.946129	0.174129
44410	0.946129	0.164182
44411	1.40574	0.157091
44412	1.40574	0.151671
44413	0.946131	0.150732
44414	1.40574	0.161827
44415	1.12373	0.177731
44416	0.946131	0.161221
44417	2.14277	0.241563
44418	2.81148	0.213766
44419	1.42762	0.217877
44420	1.42762	0.266597
44421	2.96261	0.210858
44422	2.24746	0.244326
44423	1.89226	0.506657
44424	1.89226	0.178748
44425	1.89226	0.253478

44426	1.89226	0.446689
44427	3.78453	0.519733
44428	1.89226	0.20618
44429	1.89226	1.35966
44430	1.89226	0.499156
44431	1.89226	0.05
44432	3.78452	0.213005
44433	0.68433	0.0808917
44434	0.946127	1.05282
44435	0.543921	1.07198
44436	0.946131	0.0605933
44437	0.946131	1.39787
44438	0.946131	1.23449
44439	0.543918	1.66957
44440	0.543918	0.592638
44441	0.684332	0.297367
44442	0.54392	1.33262
44443	0.946129	0.165171
44444	0.946129	0.561108
44445	1.89226	0.19479
44446	1.08784	1.24838
44447	1.19352	0.49383
44448	1.89226	0.7182
44449	1.19352	1.24864
44450	1.89226	0.93582
44451	0.635711	0.0661386
44452	0.946132	0.414163
44453	0.946132	0.564431
44454	0.56474	0.0957688
44455	0.750045	0.398441
44456	1.25657	1.3377
44457	0.635682	0.131126
44458	0.635682	0.350641
44459	0.891954	0.0844296
44460	1.32751	0.82934
44461	0.564766	0.300871
44462	0.564766	0.460267
44463	0.858345	0.259315
44464	0.946128	1.21918
44465	0.635712	0.19302
44466	0.946133	0.104437
44467	0.977198	0.385555
44468	0.944521	1.18614
44469	0.635682	1.12974
44470	0.635682	0.0715302
44471	1.02924	0.323751
44472	0.705625	1.1854
44473	0.851528	0.0736635
44474	0.851528	1.86078
44475	2.18903	2.1
44476	2.10182	2.1
44477	2.10182	2.1
44478	1.89226	2.1
44479	1.89226	2.1
44480	1.64983	2.1
44481	1.89226	2.1
44482	1.83867	2.1
44483	1.83867	2.1

44484	1.89164	2.1
44485	2.0815	2.1
44486	2.17638	2.1
44487	2.57957	1.42456
44488	2.3506	2.09597
44489	2.69056	0.345778
44490	2.49439	1.71378
44491	5.04116	1.5076
44492	2.3506	1.96873
44493	1.8911	2.1
44494	2.49439	1.42403
44495	2.06689	2.1
44496	4.41748	2.1
44497	2.69056	1.41101
44498	2.49439	1.8933
44499	2.77227	0.859142
44500	2.63443	1.82285
44501	5.32499	1.57932
44502	2.49439	1.67342
44503	2.06689	2.1
44504	2.63443	1.03239
44505	2.26444	2.1
44506	4.70131	2.1
44507	2.46327	2.1
44508	2.54347	2.1
44509	2.52869	2.1
44510	2.16534	2.1
44511	2.82661	2.1
44512	2.46327	2.1
44513	0.946131	2.1
44514	0.946131	2.1
44515	1.27174	2.1
44516	1.27174	2.1
44517	1.27174	2.1
44518	0.94613	2.1
44519	0.946128	2.1
44520	1.4133	2.1
44521	0.946129	2.1
44522	0.946129	2.1
44523	1.27174	2.1
44524	1.4133	2.1
44525	1.42757	2.1
44526	1.7457	2.1
44527	1.66047	2.1
44528	1.7457	2.1
44529	1.45754	2.1
44530	1.54278	2.1
44531	1.42757	2.1
44532	1.52996	2.1
44533	1.66047	2.1
44534	1.66047	2.1
44535	1.26397	2.1
44536	1.26397	2.1
44537	0.968536	2.1
44538	1.02461	2.1
44539	1.57575	2.1
44540	1.62391	2.1
44541	2.59245	2.1

44542	0.946131	2.1
44543	0.946131	2.1
44544	0.946132	2.1
44545	0.946132	2.1
44546	0.946132	2.1
44547	0.946132	2.1
44548	0.946132	2.1
44549	0.946132	2.1
44550	0.946132	2.1
44551	0.946132	2.1
44552	0.946132	2.1
44553	0.946132	2.1
44554	2.23765	2.1
44555	1.62391	2.1
44556	2.22154	2.1
44557	1.57575	2.1
44558	2.2697	2.1
44559	1.62391	2.1
44560	1.89226	2.1
44561	1.89226	2.1
44562	1.89226	2.1
44563	1.89226	2.1
44564	1.89226	2.1
44565	1.89226	2.1
44566	1.89226	2.1
44567	1.89226	2.1
44568	1.89226	2.1
44569	1.89226	2.1
44570	1.89226	2.1
44571	1.89226	2.1
44572	2.2697	2.1
44573	1.46129	2.1
44574	2.25873	2.1
44575	1.62391	2.1
44576	2.09611	2.1
44577	1.46129	2.1
44578	1.89226	2.1
44579	2.07088	2.1
44580	1.89226	2.1
44581	2.31884	2.1
44582	4.21111	2.1
44583	1.89226	2.1
44584	2.31884	2.1
44585	2.11713	2.1
44586	1.89226	2.1
44587	2.54371	2.1
44588	2.31884	2.1
44589	2.54371	2.1
44590	1.24041	2.1
44591	2.55208	2.1
44592	2.31884	2.1
44593	1.23712	2.1
44594	1.00388	2.1
44595	2.72769	0.05
44596	2.57507	0.0554188
44597	1.89226	0.05
44598	1.89226	0.0542976
44599	4.46733	0.05

44600	1.89226	0.0525203
44601	2.57506	0.0543982
44602	1.89226	0.05
44603	2.30076	0.05
44604	1.89226	0.05
44605	2.30076	0.0509756
44606	1.21171	0.05
44607	1.48589	0.05
44608	2.09166	0.05
44609	1.78381	0.05
44610	3.2697	0.05
44611	2.09166	0.05
44612	1.70302	0.05
44613	1.48589	0.05
44614	1.18027	0.05
44615	2.26571	0.05
44616	1.79969	0.05
44617	1.89226	0.05
44618	1.89226	0.0591524
44619	1.89226	0.0576936
44620	1.89226	0.05
44621	1.89226	0.0599138
44622	1.89226	0.05
44623	0.946131	0.05
44624	0.946131	0.0721172
44625	0.946128	0.0577673
44626	0.946128	0.05
44627	0.946129	0.05937
44628	0.946129	0.0610248
44629	0.94613	0.05
44630	0.94613	0.05
44631	0.94613	0.05
44632	0.94613	0.0504551
44633	0.94613	0.0511294
44634	0.94613	0.05
44635	1.89226	1.73227
44636	1.89226	1.78957
44637	1.89226	1.89484
44638	1.89226	1.67331
44639	1.89226	1.84929
44640	1.89226	1.65503
44641	1.89226	1.6833
44642	1.89226	1.70785
44643	1.89226	1.81828
44644	1.89226	1.61091
44645	1.89226	1.79782
44646	1.89226	1.6032
44647	1.89226	1.59554
44648	1.89226	1.62875
44649	1.89226	1.72188
44650	1.89226	1.51927
44651	1.89226	1.72074
44652	1.89226	1.5206
44653	1.79103	1.67045
44654	0.968249	2.1
44655	1.44807	2.1
44656	2.17984	0.882339
44657	3.2391	1.96753

44658	1.29735	1.69768
44659	1.08992	1.70048
44660	0.945147	2.1
44661	0.724033	2.1
44662	1.00723	2.1
44663	1.00723	1.79903
44664	1.01416	1.10615
44665	1.01416	2.1
44666	1.29735	1.91191
44667	0.94515	1.92177
44668	0.724034	1.98912
44669	1.08992	2.02818
44670	1.39214	2.1
44671	1.39214	2.1
44672	0.946129	2.1
44673	0.946129	2.1
44674	1.33728	2.1
44675	1.39214	2.1
44676	0.94613	2.1
44677	0.94613	2.1
44678	0.94613	2.1
44679	1.33728	2.1
44680	0.94613	2.1
44681	1.39214	2.1
44682	2.78428	2.1
44683	2.28373	2.1
44684	2.67456	2.1
44685	2.20011	2.1
44686	4.95829	2.1
44687	1.90165	0.14974
44688	2.24206	0.511142
44689	1.66137	0.215105
44690	2.07108	1.59006
44691	3.90343	0.237166
44692	1.89224	0.550921
44693	1.89224	0.570119
44694	1.89224	0.312601
44695	1.89224	0.259463
44696	3.78448	0.240642
44697	2.82159	0.63788
44698	1.89226	0.665803
44699	2.50138	1.64582
44700	1.89226	0.37859
44701	4.39365	0.507535
44702	1.66139	0.142523
44703	1.86399	1.62278
44704	1.56005	0.387883
44705	1.90324	0.65649
44706	3.56463	0.380566
44707	0.946133	0.0747697
44708	0.946133	0.680375
44709	0.946132	0.208878
44710	0.946132	0.922367
44711	0.946133	0.302838
44712	0.946133	0.611944
44713	0.946132	0.189689
44714	0.946132	0.405809
44715	0.946133	0.172547

44716	0.946133	0.239405
44717	0.946132	0.127387
44718	0.946132	0.234824
44719	1.5176	0.852564
44720	1.97964	0.259686
44721	2.32889	0.26964
44722	1.91762	0.373196
44723	3.84649	0.325955
44724	2.3376	0.568122
44725	1.97963	0.402958
44726	2.32889	0.566347
44727	2.58303	0.270622
44728	4.66649	0.439005
44729	2.32889	0.290494
44730	2.20248	0.274346
44731	1.91762	0.204971
44732	1.91762	0.20259
44733	2.5615	0.630903
44734	2.5615	0.315587
44735	2.32889	1.03726
44736	2.58303	0.240911
44737	2.71793	0.409191
44738	2.56149	0.281761
44739	5.04681	0.415246
44740	1.89226	0.425968
44741	2.53785	0.45963
44742	1.89226	0.227531
44743	2.60895	0.364696
44744	1.89226	0.278337
44745	2.60895	0.288439
44746	0.493248	0.1194
44747	1.30448	0.480697
44748	1.26893	1.01141
44749	0.653226	0.319248
44750	0.653227	0.427057
44751	0.493247	1.47588
44752	1.26893	1.10758
44753	1.30448	0.493715
44754	0.832232	0.868543
44755	0.832233	1.03479
44756	0.929944	0.347374
44757	0.929944	0.414249
44758	1.89226	2.1
44759	1.47937	2.1
44760	1.50143	2.1
44761	1.47937	2.1
44762	1.91432	2.1
44763	1.89226	2.1
44764	1.89226	2.1
44765	1.89226	2.1
44766	1.89226	2.1
44767	1.89226	2.1
44768	1.89226	2.1
44769	1.89226	2.1
44770	1.33419	2.1
44771	2.4542	2.1
44772	2.59834	2.1
44773	2.59834	2.1

44774	1.25716	2.1
44775	1.25716	2.1
44776	1.70303	2.1
44777	1.50143	2.1
44778	1.60878	2.1
44779	1.50143	2.1
44780	1.93599	2.1
44781	1.89226	2.1
44782	0.946131	2.1
44783	0.946131	2.1
44784	0.946127	2.1
44785	0.946127	2.1
44786	0.946127	2.1
44787	0.946127	2.09526
44788	0.946131	2.1
44789	0.946131	2.1
44790	0.94613	2.1
44791	0.94613	2.1
44792	0.946129	2.1
44793	0.946129	2.1
44794	1.43912	2.1
44795	2.59834	2.1
44796	2.78093	2.1
44797	2.78093	2.1
44798	1.33419	2.1
44799	1.33419	2.1
44800	1.89226	2.1
44801	2.26322	2.1
44802	2.55682	2.1
44803	2.55682	2.1
44804	1.89226	2.1
44805	1.89226	2.1
44806	2.58032	0.20374
44807	2.28922	0.121783
44808	1.88424	0.131161
44809	2.32127	0.109387
44810	4.46456	0.138611
44811	1.67265	0.215897
44812	2.28922	0.150522
44813	1.88424	0.110462
44814	1.11314	0.128006
44815	3.55689	0.129838
44816	1.43751	0.0873026
44817	2.32127	0.0990128
44818	1.92998	0.0711929
44819	2.37671	0.0808162
44820	1.43751	0.149673
44821	1.88424	0.141018
44822	2.36729	2.1
44823	1.56714	2.1
44824	1.56714	2.1
44825	1.62408	2.1
44826	2.28373	2.1
44827	2.34067	2.1
44828	0.937268	2.1
44829	0.937268	2.1
44830	0.94636	2.1
44831	0.94636	2.1

44832	0.76004	2.1
44833	0.783572	2.1
44834	1.14187	2.1
44835	1.10006	2.1
44836	1.10006	2.1
44837	0.76004	2.1
44838	0.783572	2.1
44839	1.14187	2.1
44840	1.28753	2.1
44841	1.36838	2.1
44842	1.62617	2.1
44843	1.82857	2.1
44844	3.1161	2.1
44845	1.82856	2.1
44846	1.17996	2.1
44847	1.53417	2.1
44848	1.56618	2.1
44849	1.36838	2.1
44850	1.36838	2.1
44851	1.35065	0.723608
44852	1.42659	0.222218
44853	2.26675	0.491245
44854	2.26675	0.34428
44855	1.62137	0.324004
44856	2.53747	0.475127
44857	1.89226	0.0705871
44858	1.89226	0.05
44859	1.89226	0.05
44860	1.89226	0.0654064
44861	3.78452	0.0607888
44862	1.36569	0.05
44863	1.36569	0.05
44864	0.946131	0.05
44865	0.946131	0.05
44866	1.16041	0.05
44867	1.36569	0.05
44868	0.946129	0.05
44869	0.946129	0.05
44870	1.1604	0.05
44871	0.94613	0.05
44872	0.946131	0.05
44873	1.36569	0.05
44874	2.73139	0.05
44875	1.25265	0.05
44876	1.25265	0.05
44877	1.64799	0.05
44878	2.32081	0.05
44879	2.71615	0.05
44880	0.946133	0.05
44881	0.946133	0.05
44882	0.946132	0.0622865
44883	0.946132	0.0533137
44884	0.946132	0.05
44885	0.946132	0.0534509
44886	0.946133	0.05
44887	0.946133	0.0534604
44888	0.946132	0.05
44889	0.946132	0.0514415

44890	0.946133	0.05
44891	0.946133	0.05
44892	0.946131	0.05
44893	0.946131	0.05
44894	0.94613	0.0856362
44895	0.94613	0.0605282
44896	0.946132	0.0732125
44897	0.946132	0.0504922
44898	0.946129	0.0593619
44899	0.946129	0.0823227
44900	0.946131	0.0609715
44901	0.946131	0.05
44902	0.946131	0.05
44903	0.946131	0.0618493
44904	1.89226	2.1
44905	2.48503	2.1
44906	2.64586	2.1
44907	2.64586	2.1
44908	1.89226	2.1
44909	1.89226	2.1
44910	2.48504	2.1
44911	1.99985	2.1
44912	1.83584	2.1
44913	1.83584	2.1
44914	2.64586	2.1
44915	2.64586	2.1
44916	1.89225	2.1
44917	2.33095	2.1
44918	2.48501	2.1
44919	2.48501	2.1
44920	1.89225	2.1
44921	1.89225	2.1
44922	2.33096	2.1
44923	1.83583	2.1
44924	1.68436	2.1
44925	1.68436	2.1
44926	2.48502	2.1
44927	2.48502	2.1
44928	1.14807	2.1
44929	1.76545	2.1
44930	1.21292	2.1
44931	1.83936	2.1
44932	2.97837	2.1
44933	1.89226	2.1
44934	1.89226	2.1
44935	1.89226	2.1
44936	1.89226	2.1
44937	3.78451	2.1
44938	1.89226	2.1
44939	1.89226	1.99681
44940	1.89226	2.1
44941	1.89226	2.1
44942	3.78452	2.1
44943	1.76545	2.1
44944	2.12608	2.1
44945	1.83937	2.1
44946	2.22529	2.1
44947	3.99074	2.1

44948	0.780222	2.1
44949	0.780222	2.1
44950	0.784871	2.1
44951	0.784871	2.1
44952	0.95863	2.1
44953	0.919681	2.1
44954	0.636145	2.1
44955	0.606461	2.1
44956	0.636144	2.1
44957	0.958631	2.1
44958	0.60646	2.1
44959	0.919683	2.1
44960	1.89226	2.1
44961	1.89226	2.1
44962	1.89226	2.1
44963	1.89226	2.1
44964	3.78452	2.1
44965	1.89226	2.1
44966	1.89226	2.1
44967	1.89226	2.1
44968	1.89226	2.01562
44969	3.78452	2.1
44970	1.03789	2.1
44971	1.03789	2.1
44972	1.04231	2.1
44973	1.04231	2.1
44974	1.16052	2.1
44975	1.11265	2.1
44976	0.958634	2.1
44977	0.919685	2.1
44978	0.958634	2.1
44979	1.16052	2.1
44980	0.919685	2.1
44981	1.11265	2.1
44982	1.43837	2.1
44983	2.57085	2.1
44984	1.39332	2.1
44985	2.58228	2.1
44986	3.96417	2.1
44987	1.89226	2.1
44988	1.89226	2.1
44989	1.89226	2.1
44990	1.89226	2.1
44991	1.89226	2.1
44992	1.89226	2.1
44993	1.89226	2.1
44994	1.89226	2.1
44995	1.89226	2.1
44996	1.89226	2.1
44997	3.78452	2.1
44998	2.57085	2.1
44999	1.89226	2.1
45000	2.58229	2.1
45001	1.89226	2.1
45002	4.46312	2.1
45003	1.32368	2.1
45004	2.58228	2.1
45005	2.55352	2.1

45006	2.55352	2.1
45007	1.39332	2.1
45008	1.39332	2.1
45009	1.89226	2.1
45010	1.89226	2.1
45011	1.89226	2.1
45012	1.89226	2.1
45013	1.89226	2.1
45014	1.89226	2.1
45015	1.89226	2.1
45016	1.89226	2.1
45017	1.89226	2.1
45018	1.89226	2.1
45019	1.89226	2.1
45020	1.89226	2.1
45021	2.55353	2.1
45022	1.89226	2.1
45023	2.58228	2.1
45024	1.89226	2.1
45025	1.89226	2.1
45026	2.58228	2.1
45027	1.40574	1.80486
45028	1.05175	1.61751
45029	1.71613	1.71888
45030	1.99961	1.66503
45031	3.12187	1.68037
45032	1.71613	1.69138
45033	2.50733	1.60232
45034	1.99961	1.7917
45035	1.99961	1.60991
45036	2.25746	1.6754
45037	2.25746	1.54674
45038	2.25746	1.56296
45039	1.89526	1.57406
45040	1.71613	1.70303
45041	1.71613	1.29865
45042	1.89526	1.62072
45043	1.27558	1.34193
45044	1.07536	2.1
45045	1.07536	2.1
45046	1.07034	2.1
45047	1.07034	2.1
45048	0.766879	2.1
45049	0.81527	2.1
45050	1.38896	2.1
45051	1.33043	2.1
45052	0.815264	2.1
45053	1.38895	2.1
45054	0.766884	2.1
45055	1.33044	2.1
45056	1.89225	2.1
45057	1.89225	2.1
45058	1.89225	2.1
45059	1.89225	2.1
45060	1.89225	2.1
45061	1.89225	2.1
45062	1.64059	2.1
45063	1.89225	2.1

45064	1.565	2.1
45065	1.565	2.1
45066	1.89225	2.1
45067	1.89225	2.1
45068	1.89226	2.1
45069	1.89226	2.1
45070	1.89226	2.1
45071	1.89226	2.1
45072	1.89226	2.1
45073	1.89226	2.1
45074	1.67906	2.1
45075	1.89226	2.1
45076	1.6406	2.1
45077	1.6406	2.1
45078	1.89226	2.1
45079	1.89226	2.1
45080	2.18106	1.50555
45081	2.20831	2.01767
45082	2.64725	1.22283
45083	2.63102	1.27519
45084	4.82831	1.33661
45085	2.18106	1.38894
45086	2.09605	2.1
45087	2.5601	1.47496
45088	2.63102	1.3439
45089	4.74116	1.68959
45090	2.64725	1.27451
45091	1.89226	1.35303
45092	1.87603	1.87922
45093	2.63102	1.28898
45094	1.89226	1.53026
45095	2.64725	1.19025
45096	0.946132	1.39625
45097	1.28005	1.3992
45098	0.946132	1.76369
45099	1.31551	1.29945
45100	1.28005	1.44721
45101	1.28005	1.63182
45102	0.946132	1.38356
45103	0.946132	1.41935
45104	0.946132	1.35987
45105	0.946132	1.38527
45106	1.28005	1.45216
45107	1.31551	1.43817
45108	2.0815	2.1
45109	1.59318	2.1
45110	2.0815	2.1
45111	1.40161	2.1
45112	2.49782	2.1
45113	1.68553	2.1
45114	0.819885	2.1
45115	0.935082	2.1
45116	0.867363	2.1
45117	0.867363	2.1
45118	0.715431	2.1
45119	0.902777	2.1
45120	0.738933	2.1
45121	0.980053	2.1

45122	0.936716	2.1
45123	0.917651	2.1
45124	0.775853	2.1
45125	0.74037	2.1
45126	2.78977	2.1
45127	2.38239	2.1
45128	2.78977	2.1
45129	1.96468	2.1
45130	2.64107	2.1
45131	1.80733	2.1
45132	1.80141	2.1
45133	2.28512	2.1
45134	2.28512	2.1
45135	1.8519	2.1
45136	2.64107	2.1
45137	2.20785	2.1
45138	0.946133	1.04663
45139	0.946133	0.642798
45140	0.946132	0.842215
45141	0.946132	0.603366
45142	0.946132	0.865508
45143	0.946132	1.29718
45144	0.946132	0.595146
45145	0.946132	0.561407
45146	0.946132	0.65506
45147	0.946132	0.98913
45148	0.946133	0.637635
45149	0.946133	1.07504
45150	1.89226	0.256692
45151	1.89226	0.25512
45152	1.89226	0.19889
45153	1.89226	0.308275
45154	1.89226	0.20711
45155	1.89226	0.299255
45156	1.89226	0.341364
45157	1.89226	0.388048
45158	1.89226	0.287164
45159	1.89226	0.498089
45160	3.78453	0.381715
45161	1.89226	0.931253
45162	1.89226	0.621539
45163	1.89226	0.404218
45164	1.89226	1.25535
45165	1.89226	0.577855
45166	1.89226	1.09686
45167	2.12925	0.05
45168	2.51397	0.05
45169	2.22488	0.05
45170	1.86113	0.05
45171	4.3751	0.05
45172	2.82754	0.05
45173	2.51397	0.05
45174	2.22489	0.05
45175	2.47291	0.05
45176	4.98688	0.05
45177	1.89226	0.05
45178	1.89226	0.0675281
45179	1.89226	0.05

45180	1.89226	0.0676325
45181	3.78453	0.0591755
45182	1.89226	0.05
45183	1.89226	0.0651346
45184	1.89226	0.05
45185	1.89226	0.0670859
45186	3.78452	0.05
45187	0.90848	0.05
45188	1.11244	0.05
45189	0.930566	0.05
45190	0.73385	0.05
45191	0.921298	0.05
45192	0.921298	0.05
45193	0.924994	0.05
45194	0.924994	0.05
45195	0.908478	0.05
45196	0.733851	0.05
45197	1.11244	0.05
45198	0.930567	0.05
45199	2.22489	0.05
45200	2.0815	0.05
45201	2.0815	0.05
45202	2.30674	0.05
45203	1.99867	0.05
45204	2.33358	0.05
45205	1.89226	0.05
45206	1.89226	0.0644515
45207	1.89226	0.05
45208	1.89226	0.0546838
45209	1.89226	0.05
45210	1.89226	0.05
45211	0.946131	0.064454
45212	0.946131	0.050011
45213	0.946128	0.0623285
45214	0.946128	0.05
45215	0.946129	0.0608308
45216	0.946129	0.0676977
45217	0.94613	0.05
45218	0.94613	0.05
45219	0.94613	0.0530266
45220	0.94613	0.05
45221	0.94613	0.05
45222	0.94613	0.0535255
45223	2.28904	2.1
45224	1.89226	2.1
45225	2.2846	2.1
45226	2.2846	2.1
45227	1.89226	1.87244
45228	1.89226	2.1
45229	1.89226	1.87248
45230	1.89226	1.69486
45231	1.89226	2.1
45232	1.89226	2.1
45233	1.89226	1.36446
45234	1.89226	2.1
45235	1.04037	1.76556
45236	1.34066	2.08434
45237	1.34955	1.68927

45238	1.34955	2.00438
45239	1.03373	1.72942
45240	1.03373	2.1
45241	0.946131	1.80458
45242	0.946131	2.1
45243	1.14452	2.1
45244	1.14452	2.1
45245	0.946131	1.93547
45246	0.946131	1.97493
45247	1.14296	2.1
45248	1.14452	2.1
45249	1.14296	2.1
45250	0.946131	2.1
45251	1.14452	2.1
45252	0.946132	2.1
45253	1.89226	1.73996
45254	1.89226	2.00312
45255	1.89226	1.95296
45256	1.89226	2.1
45257	1.89226	0.870433
45258	1.89226	2.1
45259	0.597454	1.7425
45260	0.597454	1.9674
45261	0.597972	1.78312
45262	0.597972	2.1
45263	0.675238	2.06436
45264	0.674776	1.72269
45265	0.519667	1.76619
45266	0.520188	1.82462
45267	0.519667	1.4416
45268	0.675238	1.4665
45269	0.520188	1.78232
45270	0.674777	1.7852
45271	1.89226	0.302137
45272	1.82125	0.19807
45273	1.89226	0.211467
45274	1.84314	0.174689
45275	1.89226	0.41141
45276	1.84314	0.346025
45277	1.89226	0.195852
45278	1.84314	0.213729
45279	1.89226	0.188814
45280	1.65794	0.159032
45281	1.89226	0.228809
45282	1.65794	0.198175
45283	1.348	1.84868
45284	1.43485	2.1
45285	1.79929	1.20668
45286	1.75309	1.49772
45287	3.14728	1.57547
45288	1.348	1.41217
45289	1.63892	1.62333
45290	1.59157	1.89919
45291	1.18647	1.73041
45292	1.75309	1.424
45293	1.348	1.64744
45294	1.79929	1.45657
45295	1.75309	2.1

45296	2.18106	1.34285
45297	2.20831	1.35568
45298	3.98034	1.609
45299	2.18106	1.30348
45300	1.63892	1.71292
45301	1.75309	1.29538
45302	1.75309	1.91095
45303	2.09605	1.34062
45304	2.09605	1.9063
45305	2.66076	0.106682
45306	1.70302	0.0579003
45307	2.41575	0.0625998
45308	1.70302	0.0989913
45309	3.08598	0.0503953
45310	2.3661	0.0777107
45311	1.71053	0.05
45312	2.41575	0.0873151
45313	1.20143	0.05
45314	1.20143	0.05
45315	2.2123	0.05
45316	1.66739	0.05
45317	2.25633	0.0533465
45318	2.68302	0.0555991
45319	1.71051	0.05
45320	1.71051	0.0721439
45321	2.74192	0.05
45322	2.1961	0.05
45323	1.32553	2.1
45324	2.34875	1.85987
45325	2.19139	2.1
45326	2.19139	1.53233
45327	1.56172	2.1
45328	1.56172	0.81631
45329	1.89226	2.1
45330	1.89226	1.87817
45331	1.89226	2.1
45332	1.89226	2.1
45333	3.78453	2.1
45334	2.667 2.1	
45335	1.26097	2.1
45336	1.26097	2.1
45337	1.5683	2.1
45338	2.42949	2.1
45339	2.73682	2.1
45340	1.13213	2.1
45341	2.19139	1.66211
45342	2.0815	2.1
45343	1.77615	1.13024
45344	1.37767	2.1
45345	1.19297	0.74195
45346	0.946132	1.92158
45347	0.946132	2.1
45348	0.946131	2.1
45349	0.946131	2.1
45350	0.946131	2.1
45351	0.946131	1.58881
45352	0.946132	2.1
45353	0.946132	2.1

45354	0.946132	2.1
45355	0.946132	2.1
45356	0.946131	2.1
45357	0.946131	2.1
45358	2.02064	2.1
45359	2.3606	2.1
45360	1.47478	2.1
45361	2.22997	2.1
45362	4.25061	2.1
45363	2.36059	2.1
45364	2.56952	2.1
45365	2.22997	2.1
45366	2.48631	2.1
45367	4.79949	2.1
45368	1.84261	2.1
45369	2.22997	2.1
45370	1.65706	2.1
45371	1.78837	2.1
45372	3.88703	2.1
45373	2.22997	2.1
45374	2.48631	2.1
45375	2.19528	2.1
45376	2.41175	2.1
45377	4.64172	2.1
45378	1.6466	2.1
45379	1.89226	2.1
45380	1.35972	2.1
45381	1.35972	2.1
45382	1.89226	2.1
45383	1.89226	2.1
45384	1.89226	2.1
45385	1.89226	2.1
45386	1.89226	2.1
45387	1.89226	2.1
45388	3.78453	2.1
45389	2.44797	2.1
45390	1.07214	2.1
45391	1.31589	2.1
45392	1.1211	2.1
45393	2.44797	2.1
45394	2.25318	2.1
45395	1.95954	2.1
45396	1.89226	2.1
45397	1.6466	2.1
45398	1.6466	2.1
45399	1.89226	2.1
45400	1.89226	2.1
45401	0.946133	2.1
45402	0.946133	2.1
45403	0.946132	2.1
45404	0.946132	2.1
45405	0.946132	2.1
45406	0.946132	2.1
45407	0.946133	2.1
45408	0.946133	2.1
45409	0.946132	2.1
45410	0.946132	2.1
45411	0.946133	2.1

45412	0.946133	2.1
45413	2.44797	2.1
45414	1.36904	2.1
45415	1.70303	2.1
45416	2.37586	2.1
45417	4.00507	2.1
45418	0.631553	0.05
45419	1.24489	0.05
45420	1.11597	0.05
45421	0.496799	0.05
45422	0.872272	0.05
45423	0.872272	0.05
45424	0.869415	0.05
45425	0.869415	0.05
45426	0.4968	0.05
45427	0.631553	0.05
45428	1.11597	0.05
45429	1.24489	0.05
45430	1.84253	2.1
45431	2.17384	2.1
45432	2.04818	2.1
45433	1.65925	2.1
45434	3.83309	2.1
45435	2.04818	2.1
45436	1.65925	2.1
45437	1.48162	2.1
45438	1.92264	2.1
45439	3.58189	2.1
45440	2.3305	0.113629
45441	1.89226	0.147585
45442	2.52753	0.126408
45443	2.52753	0.182113
45444	1.89226	0.115884
45445	1.89226	0.206297
45446	1.89226	0.179295
45447	1.89226	0.895982
45448	1.89226	0.31627
45449	1.89226	0.897074
45450	1.89226	0.232023
45451	1.89226	0.870158
45452	1.34563	0.129494
45453	2.1769	0.171845
45454	1.94955	0.134186
45455	1.94955	0.382839
45456	1.55063	0.126264
45457	1.55063	0.262588
45458	2.09455	0.0895135
45459	1.89226	0.146033
45460	2.3305	0.104
45461	2.3305	0.133015
45462	1.89226	0.0879443
45463	1.89226	0.113513
45464	1.89226	0.139293
45465	1.89226	0.210215
45466	1.89226	0.242739
45467	1.89226	0.165682
45468	1.89226	0.185284
45469	1.89226	0.229234

45470	0.773392	0.0939114
45471	0.612765	0.270403
45472	0.79504	0.134236
45473	0.575323	0.144255
45474	1.0819	0.101722
45475	1.06106	0.127552
45476	1.04675	0.0963357
45477	0.86858	0.173794
45478	0.796873	0.115588
45479	0.557469	0.1344
45480	0.807663	0.147171
45481	2.2024	2.1
45482	2.34106	2.1
45483	2.02634	2.1
45484	2.20125	2.1
45485	4.36739	2.1
45486	2.34106	2.1
45487	2.25195	2.1
45488	2.20125	2.1
45489	2.13132	2.1
45490	4.47237	2.1
45491	2.02635	2.1
45492	2.22465	2.1
45493	1.68504	2.1
45494	2.0815	2.1
45495	3.92673	2.1
45496	2.22465	2.1
45497	2.13133	2.1
45498	2.0815	2.1
45499	2.23579	2.1
45500	4.44862	2.1
45501	1.89226	1.24234
45502	1.89226	1.34108
45503	1.89226	1.68347
45504	1.89226	1.39673
45505	1.89226	1.51511
45506	1.89226	1.03287
45507	1.93967	0.05
45508	1.70302	0.0552137
45509	1.21883	0.05
45510	1.70302	0.0569025
45511	1.19889	0.05
45512	1.70302	0.0566151
45513	0.946131	0.0830998
45514	0.946131	0.0647342
45515	0.946131	0.0533068
45516	0.946131	0.0595491
45517	0.94613	0.0566401
45518	0.94613	0.0963458
45519	0.946131	0.0602345
45520	0.946131	0.0620297
45521	0.946131	0.0590886
45522	0.946131	0.057019
45523	0.946131	0.0743809
45524	0.946131	0.0642467
45525	0.883128	0.0505918
45526	0.883128	0.058409
45527	0.827519	0.05

45528	0.82752	0.0515902
45529	1.16234	0.05
45530	1.19063	0.0673739
45531	0.631294	0.05
45532	0.548311	0.0564141
45533	0.631293	0.05
45534	1.16234	0.05
45535	1.19063	0.0543766
45536	0.54831	0.057819
45537	1.89226	0.05
45538	1.89226	0.0829465
45539	1.89226	0.0926602
45540	1.89226	0.0572738
45541	3.78453	0.063717
45542	2.33328	0.05
45543	1.87991	0.05
45544	1.87991	0.05
45545	2.82938	0.05
45546	1.84457	0.05
45547	2.79403	0.05
45548	2.82937	0.05
45549	2.70881	0.05
45550	1.8799	0.05
45551	1.82739	0.05
45552	4.58874	0.05
45553	1.89226	0.0916665
45554	1.89226	0.05
45555	1.89226	0.0622088
45556	1.89226	0.0918823
45557	3.78452	0.0798709
45558	1.17897	0.05
45559	1.82739	0.05
45560	1.17897	0.05
45561	1.76199	0.05
45562	1.23885	0.05
45563	1.80989	0.05
45564	0.946131	0.05
45565	0.946131	0.05
45566	0.946131	0.0742061
45567	0.946131	0.0683683
45568	0.946129	0.0749288
45569	0.946129	0.05
45570	0.946133	0.0636964
45571	0.946133	0.071162
45572	0.946131	0.0667794
45573	0.946131	0.05
45574	0.946131	0.0504749
45575	0.946131	0.0610678
45576	0.946126	1.93167
45577	0.946126	2.1
45578	1.24887	2.1
45579	1.24887	2.1
45580	0.946127	2.1
45581	0.946127	1.99629
45582	1.24887	2.1
45583	1.28511	2.1
45584	1.2851	2.1
45585	0.946128	2.1

45586	1.24887	2.1
45587	0.946128	2.1
45588	1.89226	2.1
45589	1.89226	2.03225
45590	1.89226	2.1
45591	1.89226	1.47721
45592	3.78452	1.98146
45593	0.592142	2.1
45594	0.592142	2.1
45595	0.60496	2.1
45596	0.60496	2.1
45597	0.694237	2.1
45598	0.608714	1.83875
45599	0.562863	2.1
45600	0.502864	2.1
45601	0.562863	2.1
45602	0.694237	2.1
45603	0.608714	2.1
45604	0.502864	2.1
45605	1.89226	2.1
45606	1.89226	2.1
45607	1.89226	2.1
45608	1.89226	2.1
45609	3.78453	2.1
45610	1.55779	2.1
45611	1.57968	2.1
45612	1.47186	2.1
45613	1.51725	2.1
45614	3.05154	2.1
45615	1.57967	2.1
45616	1.474	2.1
45617	1.51725	2.1
45618	1.42895	2.1
45619	3.00863	2.1
45620	1.89226	2.1
45621	1.89226	2.1
45622	1.89226	2.1
45623	1.89226	2.1
45624	3.78452	2.1
45625	1.89226	2.1
45626	1.89226	2.1
45627	1.89226	2.1
45628	1.89226	2.1
45629	3.78453	2.1
45630	1.47186	2.1
45631	1.51725	2.1
45632	1.38859	2.1
45633	1.45332	2.1
45634	2.92519	2.1
45635	1.51725	2.1
45636	1.42895	2.1
45637	1.45332	2.1
45638	1.38414	2.1
45639	2.88228	2.1
45640	1.89226	2.1
45641	1.89226	2.1
45642	1.89226	2.1
45643	1.89226	2.1

45644	3.78452	2.1
45645	1.34497	0.05
45646	1.36747	0.05
45647	1.18919	0.05
45648	2.08149	0.05
45649	1.23556	0.05
45650	2.13702	0.05
45651	1.10106	0.05
45652	2.08001	0.05
45653	1.34497	0.05
45654	2.31605	0.05
45655	1.34497	0.05
45656	2.31605	0.05
45657	2.31605	0.05
45658	2.0815	0.05
45659	1.9419	0.05
45660	2.46665	0.05
45661	4.52922	0.05
45662	1.20349	0.05
45663	2.08149	0.05
45664	1.36948	0.05
45665	1.34737	0.05
45666	2.08149	0.05
45667	2.08149	0.05
45668	0.946132	0.05
45669	0.946132	0.05
45670	1.37715	0.05
45671	0.823199	0.05
45672	1.21598	0.05
45673	0.946132	0.05
45674	1.37715	0.05
45675	0.946132	0.05
45676	1.21598	0.05
45677	0.823198	0.05
45678	0.946133	0.05
45679	0.946133	0.05
45680	1.89226	0.05
45681	1.89226	0.05
45682	1.89226	0.05
45683	1.89226	0.05
45684	3.78453	0.05
45685	1.89227	0.05
45686	1.89227	0.05
45687	1.89227	0.05
45688	1.89227	0.05
45689	3.78453	0.05
45690	0.946132	0.05
45691	0.946132	0.05
45692	1.21599	0.05
45693	1.21599	0.05
45694	0.966749	0.05
45695	0.946133	0.05
45696	1.21598	0.05
45697	0.946132	0.05
45698	0.966748	0.05
45699	1.21598	0.05
45700	0.946133	0.05
45701	0.946133	0.05

45702	0.946133	0.05
45703	0.946133	0.05
45704	1.1134	0.05
45705	1.11555	0.05
45706	1.11555	0.05
45707	0.946132	0.05
45708	0.946132	0.05
45709	1.1134	0.05
45710	1.11555	0.05
45711	1.11555	0.05
45712	0.946132	0.05
45713	0.946132	0.05
45714	0.946133	0.05
45715	0.946133	0.05
45716	1.11555	0.05
45717	0.978467	0.05
45718	0.978468	0.05
45719	0.946133	0.05
45720	1.11555	0.05
45721	0.946132	0.05
45722	0.978468	0.05
45723	0.978468	0.05
45724	0.946132	0.05
45725	0.946132	0.05
45726	0.851542	2.1
45727	0.851542	2.1
45728	1.14168	2.1
45729	1.27897	2.1
45730	1.02817	2.1
45731	1.13536	2.1
45732	0.946131	2.1
45733	0.861207	2.1
45734	0.94613	2.1
45735	0.94613	2.1
45736	0.868751	2.1
45737	1.15181	2.1
45738	0.946132	2.1
45739	0.946132	1.86465
45740	0.946128	2.04541
45741	0.946128	1.619
45742	0.94613	2.1
45743	0.94613	2.04451
45744	0.946131	1.77719
45745	0.946131	1.67353
45746	0.94613	1.71018
45747	0.94613	2.01502
45748	0.94613	1.8067
45749	0.94613	2.02743
45750	2.53273	0.05
45751	1.89226	0.05
45752	1.89226	0.05
45753	1.89226	0.05
45754	1.89226	0.05
45755	1.89226	0.05
45756	1.89226	2.1
45757	1.89226	2.1
45758	1.89226	2.1
45759	1.89226	2.1

45760	3.78452	2.1
45761	1.89226	2.1
45762	2.54394	2.1
45763	1.89226	2.1
45764	2.42221	2.1
45765	4.43621	2.1
45766	2.54394	2.1
45767	1.62743	2.1
45768	2.42221	2.1
45769	1.5437	2.1
45770	4.08764	2.1
45771	1.89226	2.1
45772	1.89226	2.1
45773	1.89226	2.1
45774	1.89226	2.1
45775	3.78452	2.1
45776	1.89226	2.1
45777	1.89226	2.1
45778	1.89226	2.1
45779	1.89226	2.1
45780	3.78452	2.1
45781	1.89226	2.1
45782	2.30146	2.1
45783	2.42221	2.1
45784	1.89226	2.1
45785	1.89226	2.1
45786	2.42221	2.1
45787	2.30146	2.1
45788	2.42221	2.1
45789	1.39962	2.1
45790	1.39962	2.1
45791	1.5437	2.1
45792	2.44553	2.1
45793	1.89226	2.1
45794	1.89226	2.1
45795	1.89226	2.1
45796	1.89226	2.1
45797	3.78452	2.1
45798	1.62196	2.1
45799	2.00695	2.1
45800	1.95288	2.1
45801	1.70303	2.1
45802	3.53316	2.1
45803	1.80703	2.1
45804	2.00695	2.1
45805	1.92991	2.1
45806	2.00695	2.1
45807	1.85888	2.1
45808	1.93592	2.1
45809	1.89226	2.1
45810	1.89226	2.1
45811	1.89226	2.1
45812	1.89226	2.1
45813	1.89226	2.1
45814	1.89226	2.1
45815	1.89226	2.1
45816	1.89226	2.1
45817	1.89226	2.1

45818	1.89226	2.1
45819	3.78453	2.1
45820	1.95288	2.1
45821	1.50524	2.1
45822	1.83564	2.1
45823	1.70303	2.1
45824	2.00749	2.1
45825	1.84671	2.1
45826	1.62632	2.1
45827	1.82498	2.1
45828	1.92991	2.1
45829	1.92991	2.1
45830	1.47928	2.1
45831	1.47751	2.1
45832	0.946128	2.1
45833	0.946128	2.1
45834	0.946132	2.1
45835	0.946132	2.1
45836	0.94613	2.1
45837	0.94613	2.1
45838	0.946129	2.1
45839	0.946129	2.1
45840	0.94613	2.1
45841	0.94613	2.1
45842	0.94613	2.1
45843	0.94613	2.1
45844	1.89226	2.1
45845	1.89226	2.1
45846	1.89226	2.1
45847	1.89226	2.1
45848	1.89226	2.1
45849	1.89226	2.1
45850	2.27351	1.30922
45851	2.74068	0.401604
45852	2.72278	0.682301
45853	2.63356	1.09188
45854	2.36273	0.613829
45855	2.27351	1.04342
45856	2.36273	1.16937
45857	2.83112	0.356955
45858	2.81212	0.525175
45859	2.74068	0.797028
45860	2.43416	0.521721
45861	2.36273	0.887818
45862	1.89227	0.975668
45863	2.83112	0.340791
45864	2.74069	0.445477
45865	2.74069	0.328481
45866	1.89227	1.20966
45867	1.89227	0.892923
45868	2.40887	2.1
45869	1.4494	2.1
45870	1.52164	2.1
45871	2.47534	2.1
45872	3.93051	2.1
45873	1.89226	2.1
45874	2.47534	2.1
45875	2.40887	2.1

45876	2.40887	2.1
45877	1.89226	2.1
45878	1.89226	2.1
45879	1.60861	2.1
45880	2.47534	2.1
45881	1.52164	2.1
45882	1.52164	2.1
45883	2.55301	2.1
45884	2.55301	2.1
45885	0.946128	2.1
45886	0.946128	2.1
45887	1.23767	2.1
45888	1.2765	2.1
45889	1.23767	2.1
45890	1.23767	2.1
45891	0.946131	2.1
45892	0.946131	2.1
45893	0.946131	2.1
45894	1.2765	2.1
45895	1.23767	2.1
45896	0.946131	2.1
45897	1.89226	2.1
45898	1.87511	2.1
45899	1.87511	2.1
45900	1.95852	2.1
45901	2.0815	2.1
45902	2.22169	2.1
45903	1.89226	2.1
45904	1.89226	2.1
45905	1.89226	2.1
45906	1.89226	2.1
45907	1.89226	2.1
45908	1.89226	2.1
45909	2.0815	2.1
45910	1.80067	2.1
45911	1.80067	2.1
45912	2.05518	2.1
45913	1.89226	2.1
45914	2.16509	2.1
45915	1.87153	2.1
45916	2.65897	2.1
45917	1.58724	2.1
45918	2.38924	2.1
45919	4.26077	2.1
45920	1.89226	2.1
45921	1.89226	2.1
45922	1.89226	2.1
45923	1.89226	2.1
45924	1.89226	2.1
45925	1.89226	2.1
45926	1.89226	1.04225
45927	2.21022	0.531129
45928	2.19769	1.33457
45929	2.21022	0.826977
45930	1.87973	0.288491
45931	1.89226	0.158189
45932	1.18376	1.87965
45933	2.21022	0.657023

45934	1.16239	1.8199
45935	1.16239	0.845128
45936	2.19769	1.82454
45937	2.19769	0.748134
45938	0.946128	0.142683
45939	0.946128	0.193668
45940	1.09362	0.397051
45941	1.09884	0.325415
45942	0.94613	0.382326
45943	0.94613	0.106099
45944	1.09362	0.228381
45945	1.09362	0.253594
45946	0.946131	0.233183
45947	1.09362	0.267117
45948	0.946131	0.133159
45949	1.09884	0.116056
45950	1.19323	1.32373
45951	2.19769	0.349867
45952	1.18376	1.03989
45953	1.18376	0.517266
45954	2.18724	1.10569
45955	2.18724	0.524345
45956	2.0815	2.1
45957	0.922714	2.1
45958	2.0815	2.1
45959	1.84882	2.1
45960	1.20787	2.1
45961	1.08321	2.1
45962	2.6238	0.0610009
45963	1.89226	0.098326
45964	1.89226	0.0916486
45965	2.4866	0.0660175
45966	1.89226	0.0858026
45967	2.4866	0.0700867
45968	2.66753	0.0758452
45969	1.89226	0.133743
45970	1.89226	0.122449
45971	2.6238	0.0858891
45972	1.89226	0.110027
45973	2.6238	0.0881755
45974	0.946129	2.1
45975	0.946129	2.1
45976	1.04676	2.1
45977	1.04676	2.1
45978	0.946131	2.1
45979	0.946131	2.1
45980	1.08732	2.1
45981	1.04676	2.1
45982	1.08732	2.1
45983	0.946131	2.1
45984	1.04676	2.1
45985	0.946131	2.1
45986	1.20958	2.1
45987	1.73802	1.55768
45988	1.39067	1.91769
45989	1.39067	1.70252
45990	1.50969	1.90267
45991	1.50969	1.78049

45992	1.00382	1.93732
45993	1.50969	0.460773
45994	1.20958	1.89644
45995	1.20958	0.960207
45996	1.32039	1.9136
45997	1.32039	1.41532
45998	2.0815	2.1
45999	2.51396	2.1
46000	2.19826	2.1
46001	2.54175	2.1
46002	4.79599	2.1
46003	2.54174	2.1
46004	2.17978	2.1
46005	2.54174	2.1
46006	2.2854	2.1
46007	2.57212	2.1
46008	2.31578	2.1
46009	2.27103	2.1
46010	2.54175	2.1
46011	2.4255	2.1
46012	2.82251	2.1
46013	4.96725	2.1
46014	2.60614	0.05
46015	2.75374	0.05
46016	1.94002	0.05
46017	2.08392	0.05
46018	4.69006	0.05
46019	2.75373	0.05
46020	2.6875	0.0555401
46021	2.08392	0.05
46022	1.99944	0.05
46023	4.77142	0.05
46024	1.1211	0.05
46025	2.08392	0.05
46026	1.17787	0.05
46027	1.94002	0.05
46028	1.32178	0.05
46029	2.08392	0.05
46030	2.08392	0.05
46031	1.99944	0.05
46032	1.32178	0.05
46033	1.25929	0.05
46034	3.34321	0.05
46035	1.22266	0.05
46036	0.779473	0.05
46037	0.94613	0.05
46038	0.94613	0.05
46039	1.04072	0.05
46040	1.1407	0.05
46041	1.10042	0.05
46042	0.851542	0.05
46043	0.942046	0.05
46044	0.942046	0.05
46045	1.04114	0.05
46046	0.826602	0.05
46047	2.37468	2.1
46048	1.89226	2.1
46049	1.89226	2.1

46050	1.89226	2.1
46051	2.27917	2.1
46052	2.27917	2.1
46053	1.13959	2.1
46054	1.09451	2.1
46055	0.946131	2.1
46056	0.946131	2.1
46057	1.09451	2.1
46058	1.09451	2.1
46059	0.94613	2.1
46060	0.94613	2.1
46061	1.09451	2.1
46062	0.946131	2.1
46063	0.946131	2.1
46064	1.13959	2.1
46065	2.52454	1.94902
46066	2.12221	2.1
46067	2.67624	1.76864
46068	2.33865	2.1
46069	4.86319	2.1
46070	1.89226	2.1
46071	1.89226	2.1
46072	1.89226	2.09438
46073	1.89226	2.1
46074	3.78452	2.1
46075	1.89226	2.1
46076	1.89226	2.1
46077	1.89226	2.1
46078	1.89226	2.1
46079	3.78452	2.1
46080	2.12221	2.1
46081	1.47769	2.1
46082	2.33865	2.1
46083	1.62898	2.1
46084	3.81634	2.1
46085	2.67624	2.1
46086	2.37734	2.1
46087	2.33865	2.1
46088	2.33865	2.1
46089	2.70125	2.1
46090	2.70125	2.1
46091	1.89226	2.1
46092	1.89226	2.1
46093	1.89226	2.1
46094	1.89226	2.1
46095	1.89226	1.69475
46096	1.89226	1.79175
46097	1.89226	2.1
46098	1.89226	2.1
46099	1.89226	2.1
46100	1.89226	2.1
46101	1.89226	2.1
46102	1.89226	2.1
46103	2.37733	2.1
46104	1.95927	2.1
46105	1.82516	2.1
46106	1.82516	2.1
46107	2.33865	2.1

46108	2.33865	2.1
46109	2.57466	2.1
46110	2.21715	2.1
46111	2.55653	2.1
46112	2.24775	2.1
46113	4.77367	2.1
46114	0.937095	2.1
46115	0.937095	2.1
46116	0.958028	2.1
46117	0.958028	2.1
46118	0.828386	2.1
46119	0.771244	2.1
46120	1.10858	2.1
46121	1.12388	2.1
46122	1.12388	2.1
46123	0.828386	2.1
46124	0.771244	2.1
46125	1.10858	2.1
46126	2.55653	2.1
46127	2.32606	2.1
46128	2.24775	2.1
46129	2.24775	2.1
46130	2.57928	2.1
46131	2.57928	2.1
46132	0.990481	2.1
46133	0.990481	2.1
46134	1.00094	2.1
46135	1.00094	2.1
46136	0.888385	2.1
46137	0.828386	2.1
46138	1.16303	2.1
46139	1.12388	2.1
46140	1.16303	2.1
46141	0.888385	2.1
46142	1.12388	2.1
46143	0.828387	2.1
46144	0.962524	2.1
46145	0.962524	2.1
46146	0.7976	2.1
46147	0.7976	2.1
46148	0.797628	2.1
46149	0.814 2.1	
46150	0.946125	2.1
46151	0.946125	2.1
46152	0.946118	2.1
46153	0.797623	2.1
46154	0.814006	2.1
46155	0.946131	2.1
46156	1.8978	0.05
46157	2.23892	0.05
46158	1.71469	0.05
46159	4.13672	0.05
46160	2.64136	0.05
46161	2.23892	1.7503
46162	2.37578	1.91498
46163	1.77489	2.1
46164	4.6147	2.0873
46165	1.02284	2.1

46166	0.898796	2.05712
46167	0.948554	2.1
46168	0.948554	2.1
46169	0.878797	2.1
46170	0.796197	2.1
46171	1.09145	2.1
46172	0.963109	2.1
46173	1.12114	2.1
46174	0.782739	2.1
46175	1.11084	2.1
46176	0.850742	2.1
46177	2.48024	2.1
46178	2.405	2.1
46179	2.24585	2.1
46180	2.34795	2.1
46181	4.75295	2.1
46182	1.93794	2.1
46183	2.405	2.1
46184	2.24585	2.1
46185	1.67594	2.1
46186	4.08094	2.1
46187	1.89226	2.1
46188	1.89226	1.97736
46189	1.89226	2.1
46190	1.89226	2.1
46191	3.78452	2.1
46192	1.89226	2.1
46193	1.89226	2.1
46194	1.89226	2.1
46195	1.89226	2.1
46196	3.78452	2.1
46197	2.24585	2.1
46198	2.34795	2.1
46199	2.21155	2.1
46200	2.06626	2.1
46201	4.4142	2.1
46202	2.24585	2.1
46203	1.41498	2.1
46204	1.41498	2.1
46205	1.67594	2.1
46206	2.06625	2.1
46207	2.32721	2.1
46208	1.89226	2.1
46209	1.89226	2.1
46210	1.89226	2.1
46211	1.89226	2.1
46212	1.89226	2.1
46213	1.89226	2.00347
46214	1.89226	2.1
46215	1.89226	2.1
46216	1.89226	2.1
46217	1.89226	2.1
46218	3.78452	2.1
46219	1.89226	0.121798
46220	1.89226	0.184232
46221	1.89226	0.23379
46222	1.89226	0.101935
46223	3.78452	0.108425

46224	1.89226	0.130144
46225	2.53655	0.0675031
46226	1.89226	0.0646979
46227	1.89226	0.0763741
46228	1.89226	0.132176
46229	1.89226	0.138889
46230	1.89227	2.1
46231	1.89227	2.1
46232	1.89227	2.1
46233	1.89227	2.1
46234	1.89227	2.1
46235	1.89227	2.1
46236	0.91339	2.1
46237	0.91339	2.1
46238	1.2158	2.1
46239	1.20629	2.1
46240	1.00473	2.1
46241	1.15749	2.1
46242	0.946132	2.1
46243	0.946132	2.1
46244	0.946133	2.1
46245	1.17513	2.1
46246	1.00473	2.1
46247	0.946132	2.1
46248	1.89226	2.1
46249	1.89226	2.1
46250	1.89226	2.1
46251	1.89226	2.1
46252	1.89226	2.1
46253	1.89226	2.1
46254	1.89226	2.1
46255	2.6066	2.1
46256	2.35026	2.1
46257	2.35026	2.1
46258	1.89226	2.1
46259	1.89226	2.1
46260	2.04056	0.198048
46261	2.77366	0.169635
46262	1.24682	0.168182
46263	1.24682	0.240958
46264	2.93651	0.170411
46265	2.14277	0.218995
46266	2.68524	1.6897
46267	2.81847	1.57251
46268	1.89226	1.54674
46269	1.89226	1.56051
46270	4.57751	1.561
46271	2.68524	1.66665
46272	1.89226	1.70847
46273	1.89226	1.67263
46274	2.50732	1.60997
46275	1.89226	1.64791
46276	2.50732	1.62442
46277	1.89226	1.55321
46278	1.89226	1.53917
46279	1.89226	1.52997
46280	1.89226	1.64386
46281	1.89226	1.50433

46282	1.89226	1.64375
46283	1.89226	1.70517
46284	1.89226	1.53707
46285	1.89226	1.67436
46286	1.89226	1.6628
46287	1.89226	1.55166
46288	1.89226	1.52581
46289	1.61016	1.53589
46290	1.07467	1.56112
46291	1.24474	1.50751
46292	1.24474	1.65315
46293	1.45303	1.53551
46294	1.45303	1.65313
46295	1.61016	1.53745
46296	1.24474	1.61576
46297	1.61016	1.54095
46298	1.77186	1.51329
46299	1.37813	1.49082
46300	1.37813	1.52553
46301	1.73305	2.1
46302	2.0815	2.1
46303	1.97658	2.1
46304	1.75791	2.1
46305	1.87228	2.1
46306	1.66515	2.1
46307	2.0815	2.1
46308	1.93938	2.1
46309	2.03253	2.1
46310	2.2142	2.1
46311	1.97658	2.1
46312	2.15324	2.1
46313	1.73305	2.1
46314	1.97658	2.1
46315	1.60428	2.1
46316	1.8589	2.1
46317	3.58085	2.1
46318	1.97657	2.1
46319	1.93938	2.1
46320	1.51316	2.1
46321	1.85293	2.1
46322	3.82951	2.1
46323	1.70303	0.0651118
46324	1.96087	0.0697674
46325	1.42308	0.070156
46326	1.96087	0.0933192
46327	1.25023	0.0647917
46328	1.70303	0.0639888
46329	0.946132	0.0834568
46330	0.946132	0.101416
46331	0.946131	0.0733781
46332	0.946131	0.0707792
46333	0.946132	0.0762289
46334	0.946132	0.221725
46335	0.946132	0.0726598
46336	0.946132	0.117004
46337	0.946132	0.0793677
46338	0.946132	0.0713904
46339	0.946132	0.0868853

46340	0.946132	0.10016
46341	1.89226	0.276052
46342	1.89226	0.351964
46343	1.89226	0.250926
46344	1.89226	0.2724
46345	3.78452	0.30506
46346	1.89226	0.0676551
46347	1.89226	0.0728301
46348	1.89226	0.157643
46349	1.89226	0.286381
46350	1.89226	0.0620607
46351	1.89226	0.0680847
46352	1.89226	0.144587
46353	1.89226	0.061057
46354	1.89226	0.05
46355	1.89226	0.179169
46356	1.89226	0.0598798
46357	1.89226	0.289402
46358	1.89226	0.05
46359	1.89226	0.0639329
46360	2.48255	0.05
46361	1.89226	0.05
46362	3.78452	0.0528007
46363	1.89226	2.1
46364	2.42758	2.1
46365	1.9209	2.1
46366	1.89226	2.1
46367	2.45622	2.1
46368	2.42758	2.1
46369	2.42758	2.1
46370	1.49987	2.1
46371	1.61028	2.1
46372	1.58164	2.1
46373	2.45622	2.1
46374	2.42758	2.1
46375	0.94613	2.1
46376	0.94613	2.1
46377	1.24298	2.1
46378	1.24298	2.1
46379	1.24298	2.1
46380	1.22811	2.1
46381	0.946131	2.1
46382	0.946131	2.1
46383	0.94613	2.1
46384	1.24298	2.1
46385	0.94613	2.1
46386	1.22811	2.1
46387	2.48596	2.1
46388	2.45622	2.1
46389	1.63823	2.1
46390	1.4304	2.1
46391	1.45739	2.1
46392	2.45622	2.1
46393	2.4169	2.1
46394	2.55249	2.1
46395	2.51125	2.1
46396	2.35245	2.1
46397	4.90494	2.1

46398	2.45922	2.1
46399	2.55249	2.1
46400	2.51126	2.1
46401	2.43226	2.1
46402	4.98474	2.1
46403	2.51125	2.1
46404	2.35245	2.1
46405	2.28979	2.1
46406	2.47228	2.1
46407	4.82473	2.1
46408	2.51126	2.1
46409	2.43226	2.1
46410	2.40668	2.1
46411	2.47228	2.1
46412	4.90453	2.1
46413	2.8045	2.1
46414	1.93707	2.1
46415	2.14087	2.1
46416	2.14087	2.1
46417	2.61595	2.1
46418	2.61595	2.1
46419	1.89226	2.1
46420	1.89226	2.1
46421	1.89226	2.1
46422	1.89226	2.1
46423	1.89226	2.1
46424	1.89226	2.1
46425	1.22368	2.1
46426	1.32339	2.1
46427	0.957125	2.1
46428	0.750074	2.1
46429	1.08438	2.1
46430	1.08438	2.1
46431	1.13094	2.1
46432	0.970797	2.1
46433	1.17568	2.1
46434	0.764392	2.1
46435	0.944286	2.1
46436	1.27523	2.1
46437	1.89226	2.1
46438	1.89226	2.1
46439	1.89226	2.1
46440	1.89226	2.1
46441	3.78453	2.1
46442	2.8239	2.1
46443	1.89227	2.1
46444	2.57276	2.1
46445	2.57276	2.1
46446	1.89227	2.1
46447	1.89227	2.1
46448	1.18139	2.1
46449	1.85195	2.1
46450	1.78595	2.1
46451	1.08172	2.1
46452	2.93367	2.1
46453	1.60735	2.1
46454	1.20356	2.1
46455	0.967034	2.1

46456	1.08172	2.1
46457	1.67324	2.1
46458	1.77293	2.1
46459	1.89226	2.1
46460	1.89226	2.1
46461	1.8213	2.1
46462	1.82129	2.1
46463	2.14171	2.1
46464	2.40192	2.1
46465	0.810943	2.1
46466	0.810943	2.1
46467	0.798469	2.1
46468	0.788336	2.1
46469	0.804015	2.1
46470	0.810945	2.1
46471	1.947 2.1	
46472	1.36506	2.1
46473	1.08686	2.1
46474	1.07208	2.1
46475	1.08132	2.1
46476	1.13537	0.781632
46477	1.14754	2.1
46478	0.946131	1.94184
46479	0.946131	2.1
46480	0.94613	1.92785
46481	0.94613	0.820632
46482	1.25104	2.1
46483	1.14754	2.1
46484	1.13537	2.02561
46485	0.946131	2.04701
46486	0.946131	2.1
46487	1.25104	2.09207
46488	0.96542	0.498781
46489	0.922284	2.02087
46490	0.946131	2.1
46491	0.946131	1.96878
46492	0.946131	1.94353
46493	0.946131	0.515622
46494	1.13537	2.1
46495	0.924824	2.09648
46496	0.859476	1.9921
46497	0.842305	1.99034
46498	1.25996	1.98391
46499	1.04996	2.00126
46500	1.71007	0.05
46501	2.73283	0.05
46502	2.73283	0.05
46503	1.89226	0.05
46504	1.89226	0.05
46505	1.13262	0.05
46506	0.946131	0.05
46507	0.946131	0.05
46508	0.946131	0.05
46509	0.946131	0.05
46510	0.946132	0.05
46511	0.946132	0.05
46512	0.946131	0.05
46513	0.946131	0.05

46514	0.946131	0.05
46515	0.946131	0.05
46516	0.946132	0.05
46517	0.946132	0.05
46518	0.946131	0.05
46519	1.36642	0.05
46520	0.946125	0.05
46521	0.946125	0.05
46522	0.946128	0.05
46523	0.946128	0.05
46524	0.946126	0.05
46525	0.946128	0.05
46526	0.946128	0.05
46527	0.946128	0.05
46528	1.36641	0.05
46529	0.946128	0.05
46530	1.89226	0.05
46531	1.89226	0.05
46532	1.89226	0.05
46533	1.89226	0.05
46534	1.89226	0.05
46535	1.89226	0.05
46536	2.69784	2.1
46537	2.51651	2.1
46538	2.56999	2.1
46539	2.74623	2.1
46540	5.26783	2.1
46541	2.69784	2.1
46542	2.48718	2.1
46543	2.50161	2.1
46544	2.74623	2.1
46545	5.19945	2.1
46546	1.89226	2.1
46547	1.89226	2.1
46548	1.89226	2.1
46549	1.89226	2.1
46550	3.78453	2.1
46551	0.874264	2.1
46552	1.09879	2.1
46553	0.946131	2.1
46554	0.946131	2.1
46555	1.17062	2.1
46556	1.17062	2.1
46557	0.874293	2.1
46558	0.874293	2.1
46559	1.09879	2.1
46560	0.94613	2.1
46561	0.946131	2.1
46562	0.874265	2.1
46563	1.89226	2.1
46564	1.89226	2.1
46565	1.89226	2.1
46566	1.89226	2.1
46567	3.78453	2.1
46568	0.977382	2.1
46569	1.16424	2.1
46570	0.946133	2.1
46571	0.946133	2.1

46572	1.33012	2.1
46573	1.25589	2.1
46574	0.888498	2.1
46575	0.888498	2.1
46576	1.25375	2.1
46577	0.946131	2.1
46578	0.946131	2.1
46579	0.977384	2.1
46580	1.89226	2.1
46581	2.19757	2.1
46582	2.62906	2.1
46583	1.89226	2.1
46584	1.89226	2.1
46585	2.62906	2.1
46586	1.27944	0.542648
46587	1.27731	1.75433
46588	0.615701	1.51054
46589	0.615701	0.889555
46590	0.615673	0.11524
46591	0.946133	1.78645
46592	0.948264	0.181582
46593	0.946132	0.663266
46594	0.94752	0.160296
46595	0.61706	0.178998
46596	0.946132	0.462196
46597	0.946132	0.833314
46598	2.76604	2.1
46599	1.66728	2.1
46600	1.66728	2.1
46601	1.83036	2.1
46602	2.54515	2.1
46603	2.70823	2.1
46604	2.54515	2.1
46605	1.49958	2.1
46606	1.49958	2.1
46607	1.66728	2.1
46608	2.34465	2.1
46609	2.51235	2.1
46610	1.21128	2.1
46611	1.21128	2.1
46612	0.946131	2.1
46613	0.946131	2.1
46614	0.946131	2.1
46615	0.946131	2.1
46616	1.31401	2.1
46617	1.21128	2.1
46618	1.21128	2.1
46619	0.946131	2.1
46620	0.946131	2.1
46621	1.31401	2.1
46622	1.67374	2.1
46623	2.29586	2.1
46624	1.50033	2.1
46625	2.16598	2.1
46626	3.83972	2.1
46627	1.89226	2.1
46628	1.89226	2.1
46629	1.89226	2.1

46630	1.89226	2.1
46631	3.78452	2.1
46632	1.13537	2.1
46633	1.02188	2.1
46634	0.946132	2.1
46635	0.946132	2.1
46636	0.946132	2.1
46637	0.946132	2.1
46638	1.21128	2.1
46639	1.02188	2.1
46640	1.04672	2.1
46641	1.04672	2.1
46642	1.21128	2.1
46643	0.946132	2.1
46644	0.815411	2.1
46645	0.870464	2.1
46646	0.885437	2.1
46647	0.885437	2.1
46648	1.06395	2.1
46649	1.00483	2.1
46650	0.757125	2.1
46651	0.673956	2.1
46652	0.684461	2.1
46653	1.06979	2.1
46654	0.731403	2.1
46655	1.0559	2.1
46656	1.89226	2.1
46657	1.89226	2.1
46658	1.89226	2.1
46659	1.89226	2.1
46660	1.89226	2.1
46661	1.89226	2.1
46662	1.76897	2.00241
46663	1.76932	2.06424
46664	1.97455	2.1
46665	1.94302	1.78011
46666	3.71233	2.0065
46667	1.83347	2.1
46668	1.76932	2.1
46669	1.97455	2.1
46670	1.76932	2.1
46671	1.76522	2.1
46672	1.55999	2.1
46673	1.89227	2.1
46674	1.89227	2.1
46675	1.89227	2.1
46676	1.89227	1.99842
46677	3.78453	2.1
46678	1.89226	2.06803
46679	1.89226	1.12191
46680	1.89226	1.73488
46681	1.89226	1.89119
46682	3.78452	1.91346
46683	1.97452	2.1
46684	1.94299	1.5226
46685	2.17831	1.83433
46686	2.23195	1.90968
46687	4.17495	1.97978

46688	2.23196	2.06511
46689	2.1499	2.1
46690	1.97452	2.1
46691	1.97452	2.1
46692	2.09088	2.1
46693	1.83345	2.1
46694	0.94612	2.1
46695	0.94612	2.1
46696	0.946119	1.9049
46697	0.946119	1.88452
46698	0.946119	1.90794
46699	0.946119	2.1
46700	0.94612	2.04799
46701	0.94612	2.1
46702	0.946132	2.1
46703	0.946132	2.02797
46704	0.946107	2.1
46705	0.946107	2.1
46706	1.89224	1.18166
46707	1.89224	1.64701
46708	1.89224	1.87647
46709	1.89224	0.845287
46710	3.78447	0.973265
46711	1.70302	0.187954
46712	1.056	0.172989
46713	1.21829	0.349143
46714	1.89906	0.159663
46715	3.07892	0.176307
46716	2.20248	0.184347
46717	1.56498	0.172949
46718	1.46539	0.159131
46719	1.48787	0.145894
46720	2.37625	0.310375
46721	2.37625	0.195639
46722	1.14104	0.142523
46723	1.00675	0.181768
46724	0.700689	0.16055
46725	0.624389	0.166237
46726	0.782149	0.120307
46727	0.940197	0.130527
46728	0.803437	0.16422
46729	0.803437	0.182003
46730	0.995423	0.138566
46731	0.700699	0.115468
46732	0.593895	0.236383
46733	1.00673	0.141884
46734	1.18097	0.146203
46735	1.15839	0.154301
46736	0.995407	0.142731
46737	0.865932	0.150596
46738	1.17299	0.128951
46739	0.957054	0.13027
46740	1.08016	0.178884
46741	1.08016	0.261203
46742	0.995425	0.172665
46743	1.24161	0.114004
46744	0.796771	0.126524
46745	1.15837	0.176979

46746	2.57264	0.05
46747	1.89226	0.05
46748	2.41701	0.05
46749	1.89226	0.05
46750	4.30928	0.05
46751	1.69055	0.05
46752	2.39587	0.05
46753	1.53463	0.05
46754	2.2053	0.05
46755	3.89585	0.05
46756	0.946129	0.05
46757	0.946129	0.05
46758	0.94613	0.05
46759	0.94613	0.05
46760	0.946132	0.05
46761	0.946132	0.05
46762	0.946126	0.05
46763	0.946126	0.05
46764	0.94613	0.05
46765	0.94613	0.05
46766	0.946129	0.05
46767	0.946129	0.05
46768	2.41701	0.05
46769	1.89226	0.05
46770	1.89226	0.05
46771	1.89226	0.05
46772	2.32462	0.05
46773	2.32462	0.05
46774	1.41942	0.05
46775	2.2053	0.05
46776	2.02097	0.05
46777	2.02097	0.05
46778	1.53463	0.05
46779	1.53463	0.05
46780	1.89226	0.05
46781	1.89226	0.05
46782	1.89226	0.05
46783	1.89226	0.05
46784	1.89226	0.05
46785	1.89226	0.05
46786	1.89226	1.6202
46787	1.89226	1.89692
46788	1.89226	1.84931
46789	1.89226	1.71303
46790	1.89226	1.75167
46791	1.89226	1.64734
46792	1.02816	1.51866
46793	1.02816	1.67223
46794	0.595141	1.71547
46795	0.595141	1.7788
46796	0.946132	1.59271
46797	0.595113	1.81779
46798	0.677174	1.75502
46799	0.946132	1.4536
46800	0.946132	1.66311
46801	0.946132	1.67489
46802	0.595113	1.71309
46803	0.677174	1.69529

46804	1.89226	1.56048
46805	1.89226	2.00159
46806	1.89226	1.7596
46807	1.89226	1.63076
46808	1.89226	1.86281
46809	1.89226	1.66194
46810	0.946133	1.50968
46811	0.946133	1.41384
46812	0.946132	1.69602
46813	0.946132	1.74479
46814	0.946132	1.73761
46815	0.946132	1.58414
46816	0.946132	1.60299
46817	0.946132	1.44731
46818	0.946132	1.59026
46819	0.946132	1.6593
46820	0.946133	1.55284
46821	0.946133	1.58742
46822	0.946131	1.68237
46823	0.946131	1.6603
46824	0.946131	1.99346
46825	0.946131	1.96414
46826	0.946128	2.01141
46827	0.946128	1.68888
46828	0.946133	1.98919
46829	0.946133	1.64147
46830	0.94613	1.76104
46831	0.94613	1.87528
46832	0.946131	1.79679
46833	0.946131	1.72637
46834	2.0264	2.1
46835	1.67345	2.01708
46836	2.0264	2.03751
46837	1.70539	1.18277
46838	1.31611	1.78777
46839	1.31611	1.83899
46840	1.89226	0.832981
46841	1.89226	0.578771
46842	1.89226	1.17204
46843	1.89226	0.458441
46844	3.78452	0.529607
46845	1.89226	1.16697
46846	1.89226	0.749487
46847	1.89226	0.881746
46848	1.89226	1.21056
46849	1.89226	0.909617
46850	1.89226	1.27378
46851	1.02645	0.216115
46852	0.810942	0.261057
46853	0.882781	0.286534
46854	0.882781	0.271011
46855	2.80386	0.21937
46856	1.56719	0.262183
46857	1.22499	0.215112
46858	1.08132	0.282086
46859	1.36865	0.25755
46860	0.810957	0.27896
46861	0.810957	0.243858

46862	0.946132	0.469445
46863	0.946132	0.535272
46864	0.946129	0.327246
46865	0.946129	0.37305
46866	0.946131	0.31497
46867	0.946131	0.342377
46868	0.946131	0.540572
46869	0.946131	0.446503
46870	0.94613	0.43845
46871	0.94613	0.464074
46872	0.946131	0.668554
46873	0.946131	0.402158
46874	1.89226	0.629003
46875	1.89226	0.366371
46876	1.89226	0.751438
46877	1.89226	0.37666
46878	3.78453	0.392423
46879	1.30462	0.0709711
46880	1.79906	0.129542
46881	1.01299	0.146447
46882	1.44185	0.1115
46883	1.3499	0.0800332
46884	1.79906	0.0856954
46885	1.89226	0.0919807
46886	1.89226	0.166915
46887	1.89226	0.0858831
46888	1.89226	0.14498
46889	1.89226	0.112786
46890	1.89226	0.366981
46891	1.89226	0.141236
46892	1.89226	0.151331
46893	1.89226	0.132106
46894	1.89226	0.156827
46895	1.89226	0.127467
46896	1.89226	0.155621
46897	1.3499	0.0694023
46898	1.79905	0.113921
46899	1.29001	0.0783953
46900	1.36005	0.115621
46901	1.88294	0.123855
46902	1.79905	0.0924621
46903	1.89226	0.0798564
46904	2.75374	0.0600194
46905	2.60614	0.0885641
46906	1.89226	0.091253
46907	2.60614	0.05
46908	1.89226	0.0510733
46909	0.946131	0.0688053
46910	0.946131	0.0872271
46911	1.37687	0.0917126
46912	1.37687	0.0762434
46913	0.946131	0.0855953
46914	1.34375	0.0898096
46915	1.37686	0.073364
46916	0.946128	0.0760466
46917	1.37687	0.0645646
46918	1.34375	0.0718406
46919	0.94613	0.0894694

46920	0.94613	0.090266
46921	1.44247	2.1
46922	1.12665	2.1
46923	1.50044	2.1
46924	1.73651	2.1
46925	2.94291	2.1
46926	1.73652	2.1
46927	1.64926	2.1
46928	1.44247	2.1
46929	1.44247	2.1
46930	1.64926	2.1
46931	1.43872	2.1
46932	1.50044	2.1
46933	1.73651	2.1
46934	2.00955	2.1
46935	1.87875	2.1
46936	3.50999	2.1
46937	1.64926	2.1
46938	1.73652	2.1
46939	2.00955	2.1
46940	1.8493	2.1
46941	3.65881	2.1
46942	1.89226	0.05
46943	2.28188	0.05
46944	1.99182	0.05
46945	2.28188	0.05
46946	1.6022	0.05
46947	1.89226	0.05
46948	1.89226	0.05
46949	1.89226	0.0560757
46950	1.89226	0.05
46951	1.89226	0.0792428
46952	1.89226	0.05
46953	1.89226	0.0695636
46954	1.89226	0.05
46955	1.89226	0.05
46956	2.19578	0.05
46957	1.89226	0.0565008
46958	2.81422	0.05
46959	2.42521	0.05
46960	1.89224	0.05
46961	1.99179	0.05
46962	1.454 0.05	
46963	1.99179	0.05
46964	1.35444	0.05
46965	1.89224	0.05
46966	1.89224	0.05
46967	1.89224	0.0539557
46968	1.89224	0.05
46969	1.89224	0.0740661
46970	1.89224	0.05
46971	1.89224	0.0633768
46972	1.703 0.05	
46973	2.19575	0.05
46974	1.9911	0.05
46975	2.19575	0.0503671
46976	1.67811	0.05
46977	1.89223	0.05

46978	1.69586	2.1
46979	2.55301	2.1
46980	1.60861	2.1
46981	1.60861	2.1
46982	2.65233	2.1
46983	2.65233	2.1
46984	0.946129	2.1
46985	0.946129	2.1
46986	1.2765	2.1
46987	1.32617	2.1
46988	1.2765	2.1
46989	1.2765	2.1
46990	0.946131	2.1
46991	0.946131	2.1
46992	0.946131	2.1
46993	1.32616	2.1
46994	0.946131	2.1
46995	1.2765	2.1
46996	1.36757	2.1
46997	1.32617	2.1
46998	0.891503	2.1
46999	0.847932	2.1
47000	1.10722	2.1
47001	1.10722	2.1
47002	1.10829	2.1
47003	1.10829	2.1
47004	1.36757	2.1
47005	0.891504	2.1
47006	0.847932	2.1
47007	1.32617	2.1
47008	0.946129	2.1
47009	0.946129	2.1
47010	1.36757	2.1
47011	1.32617	2.1
47012	1.32616	2.1
47013	1.32616	2.1
47014	0.946131	2.1
47015	0.946131	2.1
47016	1.36757	2.1
47017	0.946131	2.1
47018	0.946131	2.1
47019	1.32616	2.1
47020	1.89226	0.0653116
47021	1.89226	0.05
47022	1.89226	0.05
47023	1.89226	0.07435
47024	3.78452	0.0535352
47025	2.36336	0.05
47026	1.89226	0.05
47027	2.67831	0.05
47028	2.67831	0.05
47029	1.89226	0.05
47030	1.89226	0.05
47031	1.89227	0.05
47032	1.89227	0.0813273
47033	1.89227	0.0774079
47034	1.89227	0.0511569
47035	3.78453	0.0626901

47036	1.89226	0.05
47037	1.89226	0.0719792
47038	1.89226	0.0766243
47039	1.89226	0.05
47040	3.78452	0.0608641
47041	2.36336	0.05
47042	2.0815	0.05
47043	2.0815	0.05
47044	2.41048	0.05
47045	1.66267	0.05
47046	2.13452	0.05
47047	1.89226	0.0754233
47048	1.89226	0.0527582
47049	1.89226	0.0699791
47050	1.89226	0.05
47051	1.89226	0.0825706
47052	1.89226	0.0621554
47053	1.89226	0.111553
47054	1.89226	0.0959255
47055	1.89226	0.108699
47056	1.89226	0.180663
47057	1.89226	0.0760349
47058	1.89226	0.111381
47059	0.946128	0.429211
47060	0.946128	0.20945
47061	1.00122	0.0758137
47062	1.00122	0.0740048
47063	0.94613	0.496582
47064	1.01618	0.0700995
47065	0.946131	0.399386
47066	1.00122	0.051911
47067	1.00122	0.0541105
47068	1.01618	0.0571123
47069	0.946131	0.267781
47070	0.946131	0.32701
47071	1.89226	1.98479
47072	1.89226	1.74248
47073	1.89226	1.76939
47074	1.89226	2.08319
47075	3.78452	1.86071
47076	1.89226	1.9113
47077	1.89226	2.1
47078	1.89226	2.1
47079	1.89226	1.97359
47080	1.89226	2.1
47081	1.89226	1.97991
47082	1.89226	1.42005
47083	1.89226	1.97115
47084	1.89226	1.91385
47085	1.89226	1.65918
47086	1.89226	1.91729
47087	1.89226	1.66687
47088	1.89226	1.6176
47089	1.89226	2.05134
47090	1.89226	1.99339
47091	1.89226	1.7319
47092	1.89226	1.97078
47093	1.89226	1.74448

47094	2.71279	0.415553
47095	1.4155	0.945229
47096	2.28488	0.644927
47097	1.06122	0.40289
47098	3.70038	0.511731
47099	1.89226	1.09064
47100	1.89226	0.91926
47101	1.89226	0.563482
47102	1.89226	1.25079
47103	3.78452	1.17312
47104	0.851542	1.04241
47105	0.851542	0.815102
47106	0.851484	0.577363
47107	0.865567	0.916337
47108	0.981616	0.911436
47109	0.864767	0.972203
47110	0.851542	0.451917
47111	0.851542	0.827042
47112	0.851477	0.964771
47113	0.851477	0.925976
47114	0.865635	0.848758
47115	0.816472	0.899185
47116	1.68443	2.1
47117	1.44489	2.1
47118	1.54924	2.1
47119	1.34389	2.1
47120	3.02832	2.1
47121	1.44489	2.1
47122	1.03899	2.1
47123	1.03899	2.1
47124	1.12075	2.1
47125	1.34389	2.1
47126	1.42566	2.1
47127	1.54924	2.1
47128	1.34389	2.1
47129	1.44091	2.1
47130	1.25866	2.1
47131	2.7848	2.1
47132	0.573141	2.1
47133	0.573141	2.1
47134	0.575681	2.1
47135	0.575681	2.1
47136	0.481985	2.1
47137	0.519492	2.1
47138	0.671948	2.1
47139	0.629331	2.1
47140	0.629329	2.1
47141	0.481986	2.1
47142	0.519494	2.1
47143	0.671947	2.1
47144	1.89226	0.194685
47145	1.89226	0.530977
47146	1.89226	0.599282
47147	2.31925	0.228682
47148	3.78452	0.473947
47149	1.30916	2.1
47150	1.17426	2.1
47151	0.946129	2.1

47152	0.946129	2.1
47153	0.94613	2.1
47154	0.94613	2.1
47155	1.17426	2.1
47156	1.17426	2.1
47157	1.30916	2.1
47158	0.94613	2.1
47159	1.17426	2.1
47160	0.94613	2.1
47161	2.56346	2.1
47162	1.89226	1.22339
47163	1.89226	2.07542
47164	1.89226	1.40439
47165	2.6074	2.06128
47166	2.6074	1.37677
47167	1.89226	2.1
47168	1.89226	2.1
47169	1.89226	2.1
47170	1.89226	2.06055
47171	3.78452	2.1
47172	1.33064	2.1
47173	1.84121	2.1
47174	0.946129	2.1
47175	0.946129	2.1
47176	0.946131	2.1
47177	0.946131	2.1
47178	1.30916	2.1
47179	1.8412	2.1
47180	1.33064	2.1
47181	0.94613	2.1
47182	1.30916	2.1
47183	0.946131	2.1
47184	1.19283	1.32453
47185	1.19283	0.935219
47186	0.946131	2.1
47187	0.946131	2.0522
47188	0.946132	2.0765
47189	0.946132	1.19207
47190	1.3037	2.1
47191	1.19283	1.08769
47192	1.19283	1.91592
47193	0.946131	1.91352
47194	0.946131	1.88245
47195	1.3037	1.94537
47196	1.89226	2.1
47197	1.89226	2.05369
47198	1.89226	2.1
47199	1.89226	2.1
47200	1.89226	2.1
47201	1.89226	2.1
47202	2.17708	2.1
47203	1.70302	2.1
47204	2.28188	2.1
47205	2.13621	2.1
47206	4.28253	2.1
47207	1.89226	2.1
47208	1.89226	2.1
47209	1.89226	2.1

47210	1.89226	2.1
47211	3.78452	2.1
47212	0.946132	2.1
47213	0.946132	2.1
47214	0.946132	2.1
47215	0.946132	2.1
47216	0.946131	2.1
47217	0.946131	2.1
47218	0.946132	2.1
47219	0.946132	2.1
47220	0.946132	2.1
47221	0.946132	2.1
47222	0.946132	2.1
47223	0.946132	2.1
47224	1.74933	2.1
47225	1.11833	2.1
47226	1.90074	2.1
47227	1.669 2.1	
47228	1.335 2.1	
47229	1.11833	2.1
47230	2.28188	2.1
47231	2.31804	2.1
47232	2.42758	2.1
47233	2.14895	2.1
47234	4.43083	2.1
47235	1.89226	2.1
47236	1.89226	2.1
47237	1.89226	2.1
47238	1.89226	2.1
47239	3.78452	2.1
47240	1.89226	2.1
47241	1.89226	2.1
47242	1.89226	2.1
47243	1.89226	2.1
47244	1.89226	2.1
47245	1.89226	2.1
47246	0.864133	2.1
47247	0.864133	2.1
47248	0.967939	2.1
47249	0.793739	2.1
47250	0.650797	2.1
47251	0.757546	2.1
47252	1.08941	2.1
47253	1.10136	2.1
47254	1.10138	2.1
47255	0.796445	2.1
47256	0.866907	2.1
47257	0.650788	2.1
47258	2.63062	1.49862
47259	1.89226	1.14772
47260	1.89226	0.908575
47261	1.89226	2.1
47262	2.46557	0.614548
47263	2.46557	2.1
47264	1.16777	2.1
47265	1.38859	2.1
47266	1.05042	2.1
47267	1.28909	2.1

47268	2.45686	2.1
47269	1.89227	2.1
47270	1.89227	2.1
47271	1.89227	1.41019
47272	1.89227	2.1
47273	3.78453	2.1
47274	2.15114	2.1
47275	1.89226	2.1
47276	2.15005	2.1
47277	2.15005	2.1
47278	1.89226	2.1
47279	1.89226	2.1
47280	1.89226	2.1
47281	1.89226	1.74672
47282	1.89226	2.1
47283	1.89226	2.1
47284	1.89226	1.63275
47285	1.89226	2.1
47286	0.973447	2.1
47287	1.32143	2.08371
47288	1.37444	2.1
47289	1.37444	2.06502
47290	0.949134	2.1
47291	0.949134	2.1
47292	0.946131	2.1
47293	0.946131	2.1
47294	1.07557	2.1
47295	1.07557	2.1
47296	0.946131	2.1
47297	0.946131	2.1
47298	1.07557	2.1
47299	1.08579	2.1
47300	1.08579	2.1
47301	0.946131	2.1
47302	1.07557	2.1
47303	0.946131	2.1
47304	0.946131	1.59441
47305	0.946131	2.1
47306	0.946131	2.1
47307	0.946131	2.08586
47308	0.94613	1.6199
47309	0.94613	1.72295
47310	0.946131	2.1
47311	0.946131	2.1
47312	0.946131	2.08822
47313	0.946131	1.99127
47314	0.946131	2.1
47315	0.946131	2.1
47316	0.606115	2.1
47317	0.606115	2.1
47318	0.616308	2.1
47319	0.616308	2.1
47320	0.68722	1.68339
47321	0.735699	1.93553
47322	0.514789	2.1
47323	0.486724	2.1
47324	0.514788	2.05036
47325	0.735699	2.1

47326	0.486723	2.1
47327	0.687221	2.1
47328	2.33761	1.05922
47329	2.51749	0.361315
47330	2.68092	0.691432
47331	2.58304	0.356661
47332	5.01853	0.800403
47333	2.50906	1.01003
47334	2.51749	0.514858
47335	2.68091	0.955178
47336	2.53176	0.34181
47337	5.18997	0.679467
47338	2.68092	0.77655
47339	2.58304	0.364242
47340	2.71793	0.521537
47341	2.67098	0.237654
47342	5.39885	0.367519
47343	2.68091	1.01055
47344	2.53175	0.307595
47345	2.36417	0.561966
47346	2.67098	0.292473
47347	5.04508	0.606574
47348	2.76309	2.1
47349	1.89226	2.1
47350	1.89226	2.1
47351	1.89226	2.1
47352	2.48573	2.1
47353	2.48573	2.1
47354	1.35879	2.1
47355	1.55161	2.1
47356	1.10407	2.1
47357	1.32259	2.0349
47358	2.68138	2.1
47359	1.89226	2.1
47360	1.89226	2.1
47361	1.89226	2.1
47362	1.89226	2.07309
47363	3.78452	2.1
47364	1.19612	2.06301
47365	1.24694	2.1
47366	1.24844	0.892909
47367	1.29747	2.1
47368	2.49358	2.1
47369	1.89226	2.0956
47370	1.89226	2.1
47371	1.89226	0.480303
47372	1.89226	2.1
47373	3.78453	2.1
47374	1.89226	2.1
47375	1.89226	2.1
47376	1.89226	2.1
47377	1.89226	2.1
47378	1.89226	2.1
47379	1.89226	2.1
47380	0.587463	2.1
47381	0.587463	2.1
47382	0.581977	2.1
47383	0.581977	2.1

47384	0.531709	2.1
47385	0.545972	2.1
47386	0.623469	2.1
47387	0.648733	2.1
47388	0.648733	2.1
47389	0.545971	2.1
47390	0.623469	2.1
47391	0.531709	2.1
47392	1.29417	1.68193
47393	1.29747	2.1
47394	1.27829	2.1
47395	1.24844	1.95263
47396	1.32732	2.1
47397	1.29747	2.04649
47398	1.89226	0.721677
47399	1.89226	2.1
47400	1.89226	1.55976
47401	1.89226	2.1
47402	1.89226	1.76105
47403	1.89226	2.1
47404	1.89226	2.1
47405	1.89226	2.1
47406	1.89226	2.1
47407	1.89226	2.1
47408	1.89226	2.1
47409	1.89226	2.1
47410	0.60346	2.1
47411	0.60346	2.1
47412	0.60086	2.1
47413	0.60086	2.1
47414	0.545971	2.1
47415	0.555587	2.1
47416	0.648733	2.1
47417	0.663661	2.1
47418	0.663661	2.1
47419	0.555587	2.1
47420	0.648733	2.1
47421	0.545971	2.1
47422	1.34066	2.09814
47423	1.36601	2.1
47424	1.34955	0.605455
47425	1.37623	2.1
47426	2.71556	2.1
47427	1.89226	1.68695
47428	1.89226	2.1
47429	1.89226	0.585676
47430	1.89226	2.1
47431	3.78452	1.80381
47432	0.946132	2.1
47433	0.946132	2.1
47434	0.946132	2.1
47435	0.946132	2.1
47436	0.946132	2.1
47437	0.946132	2.1
47438	0.946131	2.1
47439	0.946131	2.1
47440	0.946132	2.1
47441	0.946132	2.1

47442	0.946132	2.1
47443	0.946132	2.1
47444	0.631351	2.1
47445	0.631351	2.1
47446	0.632421	2.1
47447	0.632421	2.1
47448	0.573604	2.1
47449	0.580765	2.1
47450	0.683006	2.1
47451	0.688118	2.1
47452	0.688118	2.1
47453	0.580765	2.1
47454	0.683007	2.1
47455	0.573604	2.1
47456	1.34955	1.9111
47457	1.37624	2.1
47458	1.35048	1.12304
47459	1.37999	2.1
47460	2.72954	2.09196
47461	0.946131	2.1
47462	0.946131	1.76311
47463	0.946131	2.1
47464	0.946131	2.1
47465	0.94613	2.1
47466	0.94613	2.1
47467	0.946133	1.50973
47468	0.946133	1.59272
47469	0.946131	2.07473
47470	0.946131	2.06648
47471	0.946132	2.01493
47472	0.946132	1.92655
47473	1.89226	2.1
47474	1.89226	2.1
47475	1.89226	2.1
47476	1.89226	2.1
47477	1.89226	2.1
47478	1.89226	2.1
47479	0.532189	2.1
47480	0.531141	2.1
47481	0.497784	2.1
47482	0.502536	2.1
47483	1.37999	2.1
47484	0.92433	2.1
47485	0.670081	2.1
47486	0.727155	2.1
47487	0.786438	2.1
47488	0.589798	2.1
47489	0.497784	2.1
47490	1.89226	2.1
47491	2.72301	2.1
47492	2.4158	2.1
47493	2.4158	2.1
47494	1.89226	2.1
47495	1.89226	2.1
47496	1.89226	2.1
47497	1.89226	2.1
47498	1.89226	2.1
47499	1.89226	2.1

47500	1.89226	2.1
47501	1.89226	2.1
47502	1.36919	2.1
47503	1.12717	2.1
47504	1.38634	2.1
47505	1.56541	2.1
47506	2.75552	2.1
47507	1.89226	2.1
47508	1.89226	2.1
47509	1.89226	2.1
47510	1.89226	2.1
47511	1.89226	2.1
47512	1.89226	2.1
47513	0.907036	2.1
47514	0.782706	2.1
47515	0.838808	2.1
47516	0.693168	2.1
47517	0.794747	2.1
47518	0.794747	2.1
47519	0.805458	2.1
47520	0.805458	2.1
47521	0.907036	2.1
47522	0.838808	2.1
47523	0.693169	2.1
47524	0.782707	2.1
47525	0.94613	2.1
47526	0.94613	2.1
47527	1.188	2.1
47528	1.28421	2.1
47529	1.18801	2.1
47530	1.18801	2.1
47531	0.946129	2.1
47532	0.946129	2.1
47533	1.28421	2.1
47534	0.94613	2.1
47535	1.188	2.1
47536	0.94613	2.1
47537	1.89226	2.1
47538	1.89226	2.1
47539	1.89226	2.1
47540	1.89226	2.1
47541	1.89226	2.1
47542	1.89226	2.1
47543	1.16817	2.1
47544	1.06007	2.1
47545	1.19785	2.1
47546	1.2832	2.1
47547	2.36602	2.1
47548	0.960106	2.1
47549	2.0073	2.1
47550	1.51754	2.1
47551	2.56883	2.1
47552	3.52484	2.1
47553	1.06756	2.1
47554	1.57913	2.1
47555	1.87532	2.1
47556	1.38666	2.1
47557	2.94287	2.1

47558	2.15622	2.1
47559	1.38668	2.1
47560	1.87534	2.1
47561	1.87534	2.1
47562	1.70539	2.1
47563	1.70539	2.1
47564	0.986095	2.1
47565	1.36226	2.1
47566	1.3331	2.1
47567	1.3331	2.1
47568	1.07156	2.1
47569	1.07156	2.1
47570	0.682081	1.8872
47571	0.68748	1.78276
47572	0.650003	0.624796
47573	0.645873	0.768897
47574	0.666344	0.662246
47575	0.666344	1.7597
47576	0.66161	1.92483
47577	0.66161	0.765239
47578	0.68748	1.13565
47579	0.645873	1.24477
47580	0.650004	1.09405
47581	0.682082	1.10876
47582	0.946132	1.9138
47583	0.946132	1.83673
47584	0.946132	0.78415
47585	0.946132	0.626889
47586	0.946132	0.58067
47587	0.946132	1.87593
47588	0.946133	0.890449
47589	0.946133	1.94234
47590	0.946132	1.1643
47591	0.946132	1.65485
47592	0.946133	1.18571
47593	0.946133	1.4088
47594	1.89227	1.31137
47595	2.79312	0.233745
47596	2.77591	0.42255
47597	2.79312	1.50146
47598	1.87505	0.308349
47599	1.89227	0.644021
47600	0.68748	1.95189
47601	0.698626	1.8815
47602	0.645873	0.756047
47603	0.650148	0.853983
47604	0.670561	0.770061
47605	0.670561	1.90072
47606	0.667067	1.99536
47607	0.667067	0.779224
47608	0.698627	1.36701
47609	0.650148	1.47765
47610	0.68748	1.22932
47611	0.645873	1.35212
47612	0.946132	1.9536
47613	0.946132	1.86972
47614	0.946132	1.21064
47615	0.946132	0.888243

47616	0.946132	0.787242
47617	0.946132	1.89356
47618	0.946133	1.4183
47619	0.946133	1.99619
47620	0.946133	1.3493
47621	0.946133	1.27594
47622	0.946132	1.11925
47623	0.946132	1.44545
47624	0.946133	1.37839
47625	0.946133	0.932785
47626	1.38709	0.424959
47627	1.38795	0.305021
47628	0.946132	0.303629
47629	0.946132	0.992894
47630	1.38709	1.16973
47631	1.38709	0.342276
47632	1.38709	0.591938
47633	0.946133	0.607272
47634	0.946132	0.51785
47635	1.38795	0.581085
47636	1.70301	0.05
47637	2.07659	0.05
47638	1.89226	0.05
47639	1.89226	0.05
47640	1.76332	0.05
47641	1.76331	0.05
47642	1.89226	0.05
47643	1.89226	0.05
47644	1.89226	0.05
47645	1.89226	0.05
47646	1.89226	0.05
47647	1.89226	0.05
47648	1.89226	0.05
47649	1.89226	0.05
47650	1.89226	0.05
47651	1.89226	0.05
47652	3.78452	0.05
47653	1.89226	0.05
47654	2.12492	0.05
47655	1.89226	0.05
47656	1.89226	0.05
47657	2.07659	0.05
47658	2.07659	0.05
47659	0.94613	0.05
47660	0.94613	0.05
47661	1.17498	0.05
47662	1.32446	0.05
47663	1.32446	0.05
47664	0.94613	0.05
47665	0.946127	0.05
47666	1.17498	0.05
47667	1.32446	0.05
47668	1.32446	0.05
47669	0.946128	0.05
47670	0.946128	0.05
47671	1.89226	2.1
47672	1.86472	2.1
47673	2.11049	2.1

47674	1.89226	2.1
47675	2.08295	2.1
47676	1.86472	2.1
47677	1.89226	2.1
47678	2.08295	2.1
47679	2.14023	2.1
47680	1.89226	2.1
47681	2.33091	2.1
47682	2.08295	2.1
47683	0.809215	2.1
47684	0.809215	2.1
47685	0.810915	2.1
47686	0.810915	2.1
47687	1.09581	2.1
47688	1.08271	2.1
47689	0.520996	2.1
47690	0.537426	2.1
47691	0.520997	2.1
47692	1.08271	2.1
47693	0.537426	2.1
47694	1.09581	2.1
47695	0.79674	2.1
47696	0.79674	2.1
47697	0.799279	2.1
47698	0.799279	2.1
47699	1.07502	2.1
47700	1.0827	2.1
47701	0.508206	2.1
47702	0.520996	2.1
47703	0.508206	2.1
47704	1.07502	2.1
47705	0.520996	2.1
47706	1.0827	2.1
47707	2.15714	2.1
47708	1.70302	2.1
47709	1.78732	2.1
47710	2.02219	2.1
47711	3.98971	2.1
47712	2.0927	2.1
47713	2.162	2.1
47714	2.11563	2.1
47715	2.02219	2.1
47716	4.18419	2.1
47717	1.62483	2.1
47718	2.02219	2.1
47719	1.93617	2.1
47720	1.52008	2.1
47721	3.54227	2.1
47722	2.11563	2.1
47723	2.02219	2.1
47724	2.29218	2.1
47725	2.03975	2.1
47726	4.06194	2.1
47727	2.007	2.1
47728	1.89226	2.1
47729	1.89226	2.1
47730	1.89226	2.1
47731	2.06776	2.1

47732	2.06776	2.1
47733	0.946129	2.1
47734	0.946129	2.1
47735	1.0035	2.1
47736	1.0035	2.1
47737	0.946131	2.1
47738	0.946131	2.1
47739	0.968797	2.1
47740	1.0035	2.1
47741	0.968771	2.1
47742	0.946105	2.1
47743	1.00353	2.1
47744	0.946157	2.1
47745	1.79103	1.81865
47746	2.53286	0.560558
47747	2.60781	2.02483
47748	2.219	1.33051
47749	2.17985	1.54101
47750	1.79103	0.208433
47751	2.219	1.4422
47752	2.66359	1.5326
47753	2.70084	1.93722
47754	2.38698	1.43146
47755	2.53285	1.13504
47756	2.219	1.27065
47757	1.33179	1.39225
47758	0.946128	1.26239
47759	0.946133	1.33242
47760	1.26643	1.51277
47761	1.3318	1.22433
47762	1.3318	1.6346
47763	0.946131	1.27919
47764	0.946131	1.40694
47765	0.946131	1.05976
47766	0.946131	0.963902
47767	1.33179	1.34766
47768	1.26643	1.59613
47769	2.28979	2.1
47770	2.47227	2.1
47771	2.43451	2.1
47772	2.22627	2.1
47773	4.69855	2.1
47774	2.40667	2.1
47775	2.47228	2.1
47776	2.43451	2.1
47777	2.38069	2.1
47778	4.85296	2.1
47779	2.43451	2.1
47780	2.15715	2.1
47781	2.15715	2.1
47782	2.22627	2.1
47783	2.39489	2.1
47784	2.46402	2.1
47785	2.43451	2.1
47786	2.34899	2.1
47787	2.38069	2.1
47788	2.38069	2.1
47789	2.3949	2.1

47790	2.3949	2.1
47791	2.85425	2.1
47792	1.70302	0.500304
47793	1.70302	1.08834
47794	1.52254	0.824317
47795	2.34181	1.39802
47796	2.08499	1.52032
47797	1.89226	1.17115
47798	1.89226	0.907961
47799	1.89226	0.500124
47800	1.89226	0.289269
47801	1.89226	0.393787
47802	1.89226	1.14841
47803	2.34181	1.89059
47804	1.38287	1.26367
47805	1.38287	1.53545
47806	1.84132	0.58712
47807	1.96416	1.28342
47808	2.4907	1.32275
47809	0.931432	1.19706
47810	0.946135	0.113765
47811	0.946133	0.062577
47812	0.946133	0.178711
47813	0.931464	0.391022
47814	0.931464	1.09111
47815	1.15573	0.10054
47816	0.96378	1.43914
47817	0.931434	0.839912
47818	0.946131	0.229621
47819	0.946131	0.142398
47820	1.13808	0.102664
47821	0.946132	1.28397
47822	0.946132	0.469032
47823	0.946132	0.737232
47824	0.946132	0.435159
47825	0.946133	1.26953
47826	0.946133	0.192846
47827	0.946132	1.31213
47828	0.946132	0.62084
47829	0.946133	0.610951
47830	0.946133	1.31352
47831	0.946132	0.221974
47832	0.946132	0.259144
47833	1.68074	2.1
47834	2.29459	2.1
47835	2.23361	2.1
47836	1.57234	2.1
47837	3.86693	2.1
47838	2.61243	2.1
47839	2.29458	2.1
47840	2.2336	2.1
47841	2.58234	2.1
47842	4.87692	2.1
47843	0.73506	2.1
47844	0.786166	2.1
47845	1.08729	2.1
47846	1.11679	2.1
47847	0.93134	2.1

47848	0.93134	2.1
47849	0.942113	2.1
47850	0.942113	2.1
47851	0.735065	2.1
47852	1.08729	2.1
47853	1.11679	2.1
47854	0.78616	2.1
47855	1.08729	2.1
47856	1.11679	2.1
47857	1.27502	2.1
47858	1.29116	2.1
47859	1.19259	2.1
47860	1.19259	2.1
47861	1.19923	2.1
47862	1.19923	2.1
47863	1.27503	2.1
47864	1.08729	2.1
47865	1.11679	2.1
47866	1.29115	2.1
47867	0.974715	1.14817
47868	1.43779	0.49608
47869	1.93915	0.485501
47870	1.53908	0.669204
47871	2.91387	0.661411
47872	1.53908	0.371991
47873	0.99845	0.881018
47874	1.5176	1.11415
47875	1.97963	0.280579
47876	3.05668	0.483846
47877	1.93915	0.70831
47878	1.97963	0.396284
47879	1.53908	0.66551
47880	1.53908	0.328858
47881	2.3376	1.07909
47882	2.3376	0.568003
47883	1.89226	2.1
47884	2.59747	2.1
47885	1.91802	2.1
47886	1.89226	2.1
47887	2.62322	2.1
47888	2.59747	2.1
47889	2.59747	2.1
47890	1.87119	2.1
47891	1.84844	2.1
47892	1.82268	2.1
47893	2.62323	2.1
47894	2.59747	2.1
47895	0.94613	2.1
47896	0.94613	2.1
47897	1.32697	2.1
47898	1.32697	2.1
47899	1.32697	2.1
47900	1.31161	2.1
47901	0.946132	2.1
47902	0.946132	2.1
47903	0.94613	2.1
47904	1.32697	2.1
47905	0.946131	2.1

47906	1.31161	2.1
47907	2.62323	2.1
47908	1.91704	2.1
47909	1.90191	2.1
47910	1.87119	2.1
47911	2.65395	2.1
47912	2.62323	2.1
47913	1.89226	2.1
47914	1.89226	2.1
47915	2.15958	2.1
47916	2.15958	2.1
47917	2.46558	2.1
47918	2.19826	2.1
47919	2.46558	2.1
47920	1.89226	2.1
47921	1.89226	2.1
47922	1.89226	2.1
47923	2.61064	2.1
47924	2.61064	2.1
47925	1.81759	0.807248
47926	1.89526	1.03744
47927	2.32219	1.50247
47928	1.881	0.67952
47929	1.81759	1.15613
47930	1.3764	0.448389
47931	1.81759	1.12669
47932	2.57079	0.878552
47933	2.32219	1.53275
47934	2.32219	1.03437
47935	1.99875	1.28334
47936	1.99875	0.339393
47937	0.536182	0.348397
47938	0.579206	0.185899
47939	0.908796	1.0512
47940	0.999375	0.399689
47941	0.732091	0.500618
47942	0.732091	0.203121
47943	0.755911	0.289435
47944	0.755911	0.658033
47945	0.999376	0.384058
47946	0.579206	0.4454
47947	0.908796	0.410006
47948	0.536182	0.407855
47949	0.94613	0.306943
47950	0.94613	0.32265
47951	1.31112	0.361725
47952	1.18275	0.346771
47953	0.946126	0.370446
47954	0.946126	0.223333
47955	1.31112	0.314708
47956	1.31112	0.31051
47957	0.946128	0.320659
47958	1.31112	0.327078
47959	0.946128	0.287968
47960	1.18275	0.282445
47961	2.64617	0.745975
47962	1.27737	0.685306
47963	1.83076	0.824631

47964	1.27737	0.810773
47965	3.03764	0.642399
47966	2.3655	0.554459
47967	1.12991	0.05
47968	1.22375	0.05
47969	1.35931	0.05
47970	1.04971	0.05
47971	1.0419	0.05
47972	1.15237	0.05
47973	1.19593	0.05
47974	1.30306	0.05
47975	1.12937	0.05
47976	1.31934	0.05
47977	1.15203	0.05
47978	1.15203	0.05
47979	2.65227	0.05
47980	1.46041	0.05
47981	2.22712	0.05
47982	2.04816	0.05
47983	2.22712	0.05
47984	2.04816	0.05
47985	1.89226	0.285574
47986	1.89226	2.1
47987	1.89226	1.34702
47988	1.89226	1.07855
47989	3.78452	1.1582
47990	1.24653	1.86404
47991	1.66554	2.1
47992	1.53538	0.986177
47993	1.53538	2.1
47994	1.40915	1.35399
47995	1.40915	2.1
47996	1.66555	2.1
47997	1.75835	2.1
47998	1.53538	2.1
47999	1.65313	2.1
48000	3.31868	2.1
48001	1.89226	2.1
48002	1.89226	2.1
48003	1.89226	1.39198
48004	1.89226	2.1
48005	3.78453	2.1
48006	1.89226	0.685283
48007	1.89226	1.27104
48008	1.89226	0.456655
48009	1.89226	2.1
48010	3.78452	0.755509
48011	0.658778	2.1
48012	0.658778	1.89846
48013	0.661956	1.05188
48014	0.661956	2.1
48015	0.703822	2.1
48016	0.767689	2.1
48017	0.623266	0.890198
48018	0.553046	2.01359
48019	0.703804	2.1
48020	0.55303	2.1
48021	0.623282	1.94059

48022	0.767711	1.98085
48023	1.53538	2.1
48024	1.65313	2.1
48025	1.40765	2.1
48026	1.55779	2.1
48027	3.06078	2.1
48028	1.89227	1.23713
48029	1.89227	2.1
48030	1.89227	2.1
48031	1.89227	2.1
48032	3.78453	2.1
48033	1.10517	2.1
48034	2.16532	2.1
48035	1.94374	2.1
48036	1.94001	2.1
48037	1.37756	2.1
48038	1.37756	2.1
48039	1.7453	0.0951223
48040	1.83284	0.11171
48041	2.28483	0.127886
48042	1.77804	0.0885256
48043	1.7453	0.15332
48044	1.23851	0.100398
48045	1.7453	0.115231
48046	2.54752	0.10218
48047	2.28483	0.112414
48048	2.28483	0.0941613
48049	2.0189	0.129594
48050	2.0189	0.1087
48051	1.12457	0.078724
48052	2.0189	0.07042
48053	1.7453	0.0813175
48054	1.7453	0.0681808
48055	1.40707	0.09241
48056	1.40707	0.0784124
48057	2.41176	2.1
48058	2.59262	2.1
48059	2.34085	2.1
48060	2.55827	2.1
48061	4.97003	2.1
48062	2.59262	2.1
48063	2.61901	2.1
48064	2.55827	2.1
48065	2.64067	2.1
48066	5.17728	2.1
48067	2.34084	2.1
48068	2.55827	2.1
48069	2.24937	2.1
48070	2.49053	2.1
48071	4.80764	2.1
48072	2.55826	2.1
48073	2.64067	2.1
48074	2.49053	2.1
48075	2.58639	2.1
48076	5.14465	2.1
48077	2.27918	0.05
48078	2.44132	0.05
48079	1.89226	0.05

48080	1.89226	0.05
48081	4.33359	0.05
48082	1.89226	0.05
48083	2.44132	0.05
48084	2.6771	0.05
48085	2.6771	0.05
48086	1.89226	0.05
48087	1.89226	0.05
48088	1.60329	0.05
48089	1.38246	0.05
48090	1.98182	0.05
48091	2.2217	0.05
48092	3.60417	0.05
48093	2.05362	0.05
48094	1.02663	0.05
48095	1.43255	0.05
48096	1.52072	0.05
48097	1.70302	0.05
48098	1.7685	0.05
48099	0.946131	0.05
48100	0.946131	0.05
48101	0.946131	0.05
48102	0.946131	0.05
48103	0.946131	0.05
48104	0.946131	0.05
48105	0.946131	0.05
48106	0.946131	0.05
48107	0.946131	0.05
48108	0.946131	0.05
48109	0.946131	0.05
48110	0.946131	0.05
48111	1.89226	0.05
48112	1.89226	0.05
48113	1.89226	0.05
48114	1.89226	0.05
48115	1.89226	0.05
48116	1.89226	0.05
48117	1.92645	0.642393
48118	2.83045	0.427289
48119	1.92225	0.459264
48120	2.01873	0.389144
48121	2.51732	0.869715
48122	2.51732	0.539614
48123	2.51732	0.308464
48124	2.23857	0.332269
48125	1.92645	0.347754
48126	1.92645	0.29096
48127	2.23857	0.581316
48128	1.50865	0.283439
48129	2.02687	2.1
48130	2.39604	2.1
48131	2.29198	2.1
48132	2.73046	2.1
48133	4.68803	2.1
48134	1.21148	1.55994
48135	1.87957	1.87593
48136	2.03555	1.92167
48137	2.03555	1.56752

48138	1.09962	1.80021
48139	1.09962	1.83369
48140	1.92558	0.609733
48141	2.05274	2.1
48142	2.2443	1.70476
48143	2.24429	2.1
48144	1.70869	0.490836
48145	1.70868	2.1
48146	0.818397	0.292232
48147	0.818397	2.1
48148	0.827911	2.1
48149	0.827908	1.8651
48150	1.00418	1.44215
48151	1.02054	1.46035
48152	0.692018	1.97444
48153	0.620899	1.90165
48154	0.65823	1.57442
48155	1.00418	0.36672
48156	0.620899	1.54052
48157	1.0262	1.0202
48158	1.92558	1.7349
48159	2.51818	2.1
48160	1.94816	0.672016
48161	2.01985	2.1
48162	1.85389	0.60594
48163	1.92558	2.1
48164	0.903844	2.1
48165	0.903844	2.1
48166	0.946148	2.1
48167	0.946151	2.1
48168	0.946118	2.1
48169	0.903876	2.1
48170	0.946118	2.1
48171	1.10235	2.1
48172	0.946131	2.1
48173	0.946131	2.1
48174	1.10234	2.1
48175	0.903863	2.1
48176	1.63471	2.1
48177	1.08022	2.1
48178	1.56801	2.1
48179	1.03437	2.1
48180	2.66908	2.1
48181	1.56801	2.1
48182	0.97541	2.1
48183	0.97541	2.1
48184	1.03437	2.1
48185	1.49733	2.1
48186	1.55629	2.1
48187	1.89226	0.0664689
48188	1.89226	0.128384
48189	1.89226	0.0836518
48190	1.89226	0.118678
48191	3.78452	0.122158
48192	0.946131	0.0652695
48193	1.16373	0.0668576
48194	0.946131	0.0773692
48195	0.734637	0.0649782

48196	0.946132	0.0703311
48197	0.946132	0.0819011
48198	1.16373	0.0546023
48199	0.734636	0.05
48200	0.946093	0.0691819
48201	0.946093	0.0584486
48202	0.734675	0.0569218
48203	0.734675	0.0768346
48204	1.98413	0.634569
48205	1.87355	2.1
48206	1.72774	0.567079
48207	1.67865	2.1
48208	3.60128	1.28439
48209	1.87355	2.1
48210	1.54924	2.1
48211	1.68443	2.1
48212	1.68443	2.1
48213	1.67866	2.1
48214	1.67866	2.1
48215	1.72774	0.371081
48216	1.53422	2.1
48217	1.67865	2.01396
48218	1.67865	0.552068
48219	1.53873	1.99741
48220	1.53873	0.639304
48221	1.67866	2.05061
48222	1.54924	2.1
48223	1.53423	2.1
48224	1.44091	2.1
48225	3.11957	2.1
48226	1.89226	2.1
48227	1.89226	2.00375
48228	1.65573	2.1
48229	1.65573	2.1
48230	1.89226	2.1
48231	1.89226	2.1
48232	1.89226	2.1
48233	1.89614	1.78787
48234	1.7508	2.02761
48235	1.72666	2.09427
48236	1.89226	2.00213
48237	1.89226	2.07907
48238	1.89223	2.1
48239	1.89223	1.7375
48240	1.89223	1.79952
48241	1.89223	2.05901
48242	1.89223	1.84842
48243	1.89223	2.1
48244	1.89224	2.02715
48245	1.89224	1.50458
48246	1.89224	1.68515
48247	1.89224	1.86765
48248	1.89224	1.73441
48249	1.89224	2.051
48250	2.09773	2.1
48251	2.06623	2.1
48252	2.43268	2.1
48253	2.33713	2.1

48254	1.703	2.1
48255	1.61372	2.1
48256	0.646929	2.1
48257	0.689776	2.1
48258	1.05897	2.1
48259	0.956364	2.1
48260	0.905594	2.1
48261	0.904315	2.1
48262	0.727014	2.1
48263	0.850757	2.1
48264	0.798445	2.1
48265	0.654101	2.1
48266	0.717539	2.1
48267	1.0478	2.1
48268	1.89223	2.1
48269	1.89223	2.1
48270	1.89223	2.1
48271	1.6557	1.89011
48272	3.54794	2.1
48273	0.946118	2.1
48274	0.946118	2.1
48275	0.946118	2.1
48276	0.946118	2.1
48277	0.946117	2.1
48278	0.946117	2.1
48279	0.946119	2.1
48280	0.946119	2.1
48281	0.946105	2.1
48282	0.946105	2.1
48283	0.946131	2.1
48284	0.946131	2.1
48285	1.47671	2.1
48286	1.70303	2.1
48287	1.90705	2.1
48288	1.98945	2.1
48289	1.3454	2.1
48290	1.40353	2.1
48291	1.89226	2.1
48292	1.89226	2.1
48293	1.89226	2.1
48294	1.89226	2.1
48295	1.89226	2.1
48296	1.89226	2.1
48297	2.39438	0.131222
48298	2.69842	0.11827
48299	2.45177	0.171132
48300	2.28563	0.103695
48301	4.84615	0.12914
48302	1.28421	2.1
48303	1.35966	2.1
48304	0.961001	2.1
48305	0.860549	2.1
48306	1.1039	2.1
48307	1.1039	2.1
48308	1.11631	2.1
48309	1.11631	2.1
48310	1.35966	2.1
48311	0.961001	2.1

48312	0.860549	2.1
48313	1.28421	2.1
48314	1.89226	1.64963
48315	1.89226	1.83867
48316	1.89226	1.97556
48317	1.89226	1.57039
48318	3.78452	1.66642
48319	1.48702	1.29804
48320	1.40656	1.19045
48321	0.626442	1.67788
48322	0.626442	1.64195
48323	0.626442	1.77572
48324	0.946132	1.08581
48325	1.05489	0.942989
48326	1.05012	1.78884
48327	0.946131	1.57736
48328	0.946131	1.59326
48329	0.626442	1.64725
48330	1.05012	1.59472
48331	1.89226	1.82544
48332	1.97212	1.58964
48333	1.89226	1.55083
48334	1.89226	1.67878
48335	3.78453	1.68787
48336	1.89226	1.94352
48337	1.89226	1.53592
48338	1.89226	1.5465
48339	1.89226	1.72507
48340	3.78452	1.69463
48341	1.89226	1.41798
48342	1.89226	1.60252
48343	1.89226	1.6817
48344	1.89226	1.06217
48345	1.89226	1.73091
48346	1.89226	1.29122
48347	0.946161	1.04866
48348	0.946161	1.47752
48349	0.932156	1.59516
48350	0.932156	1.4981
48351	0.932184	1.57695
48352	0.946132	0.617142
48353	0.946133	1.59938
48354	0.946133	1.13814
48355	0.946132	1.30216
48356	0.946132	1.38207
48357	0.932185	1.41575
48358	0.946133	1.15225
48359	0.946132	1.51
48360	0.946132	1.11134
48361	0.946132	1.96303
48362	0.946132	1.63009
48363	0.946132	1.6015
48364	0.946132	1.49506
48365	0.946133	1.53263
48366	0.946133	1.68757
48367	0.946132	1.59365
48368	0.946132	1.65946
48369	0.946133	1.48577

48370	0.946133	1.54538
48371	1.79397	0.128489
48372	1.00636	0.131303
48373	1.31658	0.1805
48374	2.02924	0.149084
48375	3.0356	0.146914
48376	1.89226	0.147608
48377	1.89226	0.150349
48378	1.89226	0.156887
48379	1.89226	0.160445
48380	1.89226	0.127429
48381	1.89226	0.119887
48382	0.946131	0.225019
48383	0.946131	0.20563
48384	1.24766	0.250439
48385	1.03691	0.265826
48386	0.946129	0.260596
48387	0.946129	0.210138
48388	1.24766	0.238453
48389	1.24766	0.230448
48390	0.946131	0.226567
48391	1.24766	0.241435
48392	1.03691	0.244644
48393	0.946131	0.229314
48394	1.44824	0.0965283
48395	2.02924	0.0935592
48396	1.7065	0.115717
48397	1.38604	0.145685
48398	2.32017	0.113802
48399	2.32017	0.0918894
48400	1.89226	0.0933265
48401	1.89226	0.111833
48402	1.89226	0.0960486
48403	1.89226	0.071969
48404	1.89226	0.116649
48405	1.89226	0.0973858
48406	0.478349	2.07477
48407	0.612092	2.1
48408	0.606115	2.1
48409	0.506213	2.1
48410	0.550677	2.1
48411	0.550677	2.06718
48412	0.567628	2.1
48413	0.567628	2.1
48414	0.506213	2.1
48415	0.478349	2.1
48416	0.606116	2.1
48417	0.612092	2.1
48418	1.52226	2.1
48419	1.54241	2.1
48420	1.23167	2.1
48421	1.23167	2.1
48422	1.75555	2.1
48423	1.75555	2.1
48424	1.75557	2.1
48425	1.54243	2.1
48426	1.9943	2.1
48427	1.85871	2.1

48428	3.53673	2.1
48429	1.85871	2.1
48430	1.06936	2.1
48431	1.88926	2.1
48432	1.54243	2.1
48433	1.41619	2.1
48434	1.06936	2.1
48435	1.28649	1.8571
48436	1.28516	2.1
48437	1.38986	0.474399
48438	1.42399	1.81976
48439	2.67502	1.72912
48440	1.42399	1.21663
48441	1.07283	2.1
48442	1.46528	2.1
48443	1.28517	1.85174
48444	1.25294	2.1
48445	1.07283	1.91643
48446	1.38986	1.93983
48447	1.42399	2.1
48448	1.52885	1.521
48449	1.60058	1.5451
48450	2.99043	1.57979
48451	0.715316	2.1
48452	0.715316	1.79812
48453	0.719126	0.739318
48454	0.719126	2.1
48455	0.722447	2.1
48456	0.626469	2.1
48457	0.711995	1.13048
48458	0.800291	1.32874
48459	0.80029	1.90671
48460	0.722447	1.8799
48461	0.711995	2.00536
48462	0.62647	1.98922
48463	2.05632	0.05
48464	1.70303	0.05
48465	2.3923	0.05
48466	2.61277	0.05
48467	1.44836	0.05
48468	1.58184	0.05
48469	1.89226	0.05
48470	1.89226	0.05
48471	1.89226	0.05
48472	1.89226	0.05
48473	3.78453	0.05
48474	1.44091	2.1
48475	1.1822	2.1
48476	1.25866	2.1
48477	1.25866	2.1
48478	1.35215	2.1
48479	1.35215	2.1
48480	1.27338	2.1
48481	1.1822	2.1
48482	1.27338	2.1
48483	1.35215	2.1
48484	1.10817	2.1
48485	1.18694	2.1

48486	0.946132	0.163395
48487	0.946132	0.138838
48488	0.946132	0.106578
48489	0.946132	0.0878886
48490	0.946133	0.103079
48491	0.946133	0.143359
48492	0.946132	0.128835
48493	0.946132	0.0933362
48494	0.946132	0.134247
48495	0.946132	0.110393
48496	0.946133	0.116169
48497	0.946133	0.132377
48498	2.6875	0.05
48499	2.40558	0.05
48500	2.1994	0.05
48501	1.70303	0.05
48502	4.68305	0.05
48503	0.915523	0.05
48504	0.915523	0.05
48505	0.861057	0.05
48506	0.946796	0.05
48507	0.838652	0.05
48508	0.654182	0.05
48509	1.14044	0.05
48510	0.80252	0.05
48511	0.836435	0.05
48512	0.608659	0.05
48513	1.22198	0.05
48514	0.863472	0.05
48515	2.1994	0.05
48516	0.981586	0.05
48517	1.59619	0.05
48518	1.70303	0.05
48519	1.24816	0.05
48520	1.38523	0.05
48521	2.71141	2.1
48522	1.70302	2.1
48523	2.56527	2.1
48524	1.88649	2.1
48525	2.41279	2.1
48526	1.70302	2.1
48527	1.89226	2.1
48528	1.89226	2.1
48529	1.89226	2.1
48530	1.89226	2.1
48531	3.78453	2.1
48532	1.89226	2.1
48533	1.89226	2.1
48534	1.89226	2.1
48535	1.89226	2.1
48536	1.89226	2.1
48537	1.89226	2.1
48538	2.18867	2.1
48539	0.983723	2.1
48540	2.42263	2.1
48541	1.8014	2.1
48542	1.62992	2.1
48543	0.983723	2.1

48544	2.71141	2.1
48545	1.89226	2.1
48546	2.31157	2.1
48547	2.31157	2.1
48548	1.89226	2.1
48549	1.89226	2.1
48550	1.89227	2.1
48551	1.89227	2.0602
48552	1.89227	2.1
48553	1.89227	2.1
48554	3.78453	2.1
48555	1.89226	2.1
48556	1.89226	2.1
48557	1.89226	2.1
48558	1.89226	2.04787
48559	1.89226	2.1
48560	1.89226	2.05361
48561	1.89226	1.46883
48562	1.89226	1.15472
48563	1.89226	1.81466
48564	1.89226	1.12243
48565	1.89226	1.44857
48566	1.89226	1.00627
48567	1.1427	1.57856
48568	1.14956	2.09546
48569	1.70302	1.13638
48570	1.81267	1.68705
48571	2.88983	1.45178
48572	1.89226	1.11607
48573	1.89226	1.48935
48574	1.89226	1.31634
48575	1.89226	1.17653
48576	3.78453	1.29888
48577	1.89226	1.36857
48578	1.89226	1.20126
48579	1.89226	0.945525
48580	1.89226	1.44167
48581	3.78452	1.14435
48582	0.884197	2.02105
48583	1.1125	1.13872
48584	1.13273	1.26663
48585	0.844557	1.38415
48586	0.981137	1.30003
48587	1.13971	1.21427
48588	0.980432	1.25125
48589	0.980432	1.30509
48590	1.15859	1.15943
48591	1.12206	1.07936
48592	0.898619	1.90325
48593	0.94426	1.23051
48594	2.21016	1.16845
48595	1.44091	1.87318
48596	1.98408	1.38483
48597	1.4218	1.25567
48598	2.21016	1.46601
48599	1.64788	1.37858
48600	1.07526	1.3774
48601	1.91531	2.1

48602	1.49155	2.1
48603	1.58551	1.92561
48604	1.49155	1.81282
48605	1.58551	2.1
48606	1.98979	0.05
48607	2.21773	0.05
48608	2.30533	0.05
48609	2.55895	0.05
48610	4.52305	0.05
48611	2.55896	0.05
48612	2.27963	0.05
48613	2.60486	0.05
48614	2.60486	0.05
48615	2.21773	0.05
48616	2.21773	0.05
48617	2.30533	0.05
48618	2.55895	0.05
48619	2.29539	0.05
48620	2.47308	0.05
48621	4.7784	0.05
48622	2.55896	0.05
48623	2.55792	0.05
48624	2.55792	0.05
48625	2.60486	0.05
48626	2.47308	0.05
48627	2.52003	0.05
48628	0.946133	1.91518
48629	0.946133	2.04373
48630	1.20851	1.9091
48631	1.20851	1.81405
48632	0.967411	1.76721
48633	1.20851	1.72358
48634	0.946132	2.1
48635	0.946132	1.99344
48636	0.946132	2.01059
48637	1.20851	1.84331
48638	0.967412	1.84482
48639	0.946133	1.95384
48640	1.89226	1.82382
48641	1.89226	1.64888
48642	1.89226	1.71249
48643	1.89226	1.62489
48644	1.89226	1.8868
48645	1.89226	1.82285
48646	1.89226	2.0288
48647	2.41701	1.89831
48648	2.71724	1.84944
48649	2.71724	1.89904
48650	1.89226	2.08908
48651	1.89226	2.09391
48652	0.946131	1.8696
48653	0.946131	2.0297
48654	0.946128	1.80086
48655	0.946128	1.76729
48656	0.94613	1.60014
48657	0.94613	1.63671
48658	0.94613	1.94438
48659	0.94613	1.9734

48660	0.94613	1.84295
48661	0.94613	1.63923
48662	0.94613	1.74652
48663	0.94613	1.95392
48664	0.851541	0.103898
48665	0.851541	0.122242
48666	0.851481	0.119921
48667	0.997219	0.129882
48668	0.997112	0.0891301
48669	0.992853	0.0829362
48670	0.85154	0.0944916
48671	0.85154	0.121573
48672	0.894824	0.0870826
48673	0.894824	0.136856
48674	0.94613	0.130933
48675	0.906485	0.0813481
48676	0.802947	2.1
48677	0.845556	2.1
48678	0.582339	2.1
48679	0.519756	2.1
48680	0.714508	2.1
48681	0.714508	2.1
48682	0.628932	2.1
48683	0.708518	2.1
48684	0.801358	2.1
48685	0.476016	2.1
48686	0.569477	2.1
48687	0.878553	2.1
48688	1.89226	2.1
48689	1.89226	2.1
48690	1.89226	2.1
48691	1.89226	2.1
48692	1.89226	2.1
48693	1.89226	2.1
48694	0.946128	2.1
48695	0.946128	2.1
48696	1.01537	2.1
48697	0.9662	2.1
48698	0.946131	2.1
48699	0.946131	2.1
48700	0.966198	2.1
48701	0.966198	2.1
48702	0.94613	2.1
48703	0.966198	2.1
48704	0.946131	2.1
48705	1.01537	2.1
48706	1.89226	2.1
48707	1.89226	2.1
48708	1.91883	2.1
48709	1.91883	2.1
48710	2.15905	2.1
48711	2.13249	2.1
48712	1.07953	2.1
48713	1.2107	2.1
48714	0.946132	2.1
48715	0.946132	2.1
48716	1.2107	2.1
48717	1.2107	2.1

48718	0.946132	2.1
48719	0.946132	2.1
48720	1.2107	2.1
48721	0.946133	2.1
48722	0.946132	2.1
48723	1.07953	2.1
48724	2.46752	2.1
48725	2.53733	2.1
48726	2.26044	2.1
48727	2.33222	2.1
48728	4.79974	2.1
48729	2.53733	2.1
48730	2.37576	1.96389
48731	2.33222	2.1
48732	2.1845	2.1
48733	4.70798	2.1
48734	2.48652	2.1
48735	2.33224	2.1
48736	2.08149	2.1
48737	1.9839	2.1
48738	4.45623	2.1
48739	2.33223	2.1
48740	2.18452	2.1
48741	2.16061	2.1
48742	2.01399	1.84672
48743	4.34623	2.1
48744	1.4617	2.1
48745	1.34701	2.1
48746	1.34701	2.1
48747	1.25173	2.1
48748	1.5117	2.1
48749	1.41642	2.1
48750	2.65164	2.1
48751	1.89227	2.1
48752	1.89227	2.1
48753	1.89227	2.1
48754	2.42504	2.1
48755	2.42504	2.1
48756	1.89226	2.02045
48757	1.89226	2.1
48758	1.89226	2.1
48759	1.89226	1.89853
48760	3.78452	2.1
48761	1.5117	2.1
48762	1.34701	2.1
48763	1.47001	2.1
48764	1.57961	2.07702
48765	2.92663	2.1
48766	1.89226	2.1
48767	1.89226	1.85234
48768	1.89226	2.0718
48769	1.89226	2.1
48770	3.78452	2.1
48771	0.412773	0.05
48772	0.570839	0.05
48773	0.50721	0.05
48774	0.513638	0.05
48775	0.518805	0.05

48776	0.41278	0.05
48777	1.5344	0.05
48778	0.939282	0.05
48779	0.720051	0.05
48780	0.691767	0.05
48781	0.761152	0.05
48782	1.89226	2.1
48783	1.89226	2.1
48784	1.89226	2.1
48785	1.89226	2.1
48786	1.89226	2.1
48787	1.89226	2.1
48788	1.24618	2.1
48789	2.10585	2.1
48790	1.08134	2.1
48791	1.93032	2.1
48792	3.1765	2.1
48793	1.98474	2.1
48794	2.57777	2.1
48795	1.93031	2.1
48796	2.19041	2.1
48797	3.99251	2.1
48798	1.89226	2.1
48799	1.89226	2.1
48800	1.89226	2.1
48801	1.89226	2.1
48802	3.78452	2.1
48803	1.67374	2.1
48804	2.19042	2.1
48805	1.67374	2.1
48806	2.09769	2.1
48807	2.29586	2.1
48808	2.7198	2.1
48809	1.89226	2.1
48810	1.89226	2.1
48811	1.89226	2.1
48812	1.89226	2.1
48813	1.89226	2.1
48814	1.89226	2.1
48815	0.946132	0.40038
48816	0.946132	0.30253
48817	0.947763	0.329772
48818	0.909304	0.346837
48819	0.946127	0.256123
48820	0.947831	0.226609
48821	0.907673	0.270591
48822	0.907673	0.368445
48823	0.94613	0.271647
48824	0.947834	0.228837
48825	0.907671	0.421931
48826	0.94613	0.230147
48827	0.906519	0.159226
48828	0.906519	0.157745
48829	0.946097	0.178073
48830	0.946097	0.186488
48831	0.947834	0.15673
48832	0.906485	0.136811
48833	0.946131	0.156284

48834	0.946131	0.194353
48835	0.94613	0.27859
48836	0.906485	0.145922
48837	0.946131	0.203922
48838	0.947835	0.18488
48839	2.58638	2.1
48840	2.51039	2.1
48841	2.47395	2.1
48842	2.41539	2.1
48843	5.00178	2.1
48844	2.51039	2.1
48845	2.16633	2.1
48846	2.4154	2.1
48847	2.13977	2.1
48848	4.58173	2.1
48849	2.47395	2.1
48850	2.41539	2.1
48851	2.2988	2.1
48852	2.2604	2.1
48853	4.7142	2.1
48854	2.4154	2.1
48855	2.13977	2.1
48856	2.26041	2.1
48857	1.98957	2.1
48858	4.40497	2.1
48859	2.15721	1.65595
48860	2.31843	0.6138
48861	1.93973	1.97972
48862	2.13243	0.137146
48863	4.25816	0.479714
48864	2.31844	0.346029
48865	2.30908	1.08444
48866	2.13243	0.292864
48867	2.1609	0.36734
48868	4.47934	0.234874
48869	1.93973	1.93854
48870	2.13243	0.247354
48871	1.69661	1.76327
48872	1.9339	0.23973
48873	3.87363	0.677947
48874	2.13243	0.120109
48875	2.1609	0.433792
48876	1.9339	0.267358
48877	1.98414	0.676039
48878	4.0948	0.0989841
48879	1.88857	0.05
48880	2.34107	0.05
48881	0.502431	0.05
48882	0.71416	0.05
48883	0.50243	0.05
48884	0.944284	0.05
48885	1.88856	2.1
48886	2.34107	2.1
48887	2.39662	2.1
48888	1.92293	2.1
48889	4.28519	2.1
48890	1.00486	2.1
48891	1.92293	2.1

48892	1.88857	2.1
48893	1.88857	2.1
48894	1.02605	2.1
48895	1.02605	2.1
48896	2.53728	2.1
48897	1.70303	2.1
48898	2.03734	2.1
48899	1.3624	2.1
48900	2.54706	2.1
48901	1.70303	2.1
48902	2.54706	2.1
48903	1.50876	2.1
48904	2.74067	2.1
48905	1.85391	2.1
48906	2.29667	2.1
48907	1.50876	2.1
48908	0.946133	2.1
48909	0.946133	2.1
48910	1.24191	2.1
48911	1.24191	2.1
48912	0.946132	2.1
48913	1.24191	2.1
48914	1.23779	2.1
48915	0.946132	2.1
48916	0.946132	2.1
48917	0.946132	2.1
48918	1.23779	2.1
48919	1.24191	2.1
48920	0.946131	2.1
48921	0.946131	2.1
48922	1.14833	2.1
48923	1.14833	2.1
48924	1.14834	2.1
48925	0.946132	2.1
48926	0.94613	2.1
48927	1.24191	2.1
48928	0.946131	2.1
48929	0.946131	2.1
48930	1.24191	2.1
48931	1.14833	2.1
48932	2.08149	2.1
48933	2.31116	2.1
48934	1.9701	1.98414
48935	2.18328	2.1
48936	4.49563	2.1
48937	2.08149	2.1
48938	2.14059	2.1
48939	1.72377	2.1
48940	2.18328	2.1
48941	3.88931	2.1
48942	2.14059	2.1
48943	2.40755	2.1
48944	2.31145	2.1
48945	2.23743	2.1
48946	2.31145	2.1
48947	2.23743	2.1
48948	2.31117	2.1
48949	2.40754	2.1

48950	2.41055	2.1
48951	2.31145	2.1
48952	2.41055	2.1
48953	2.31145	2.1
48954	2.5615	0.208751
48955	2.37625	0.164618
48956	2.20248	0.155278
48957	2.20248	0.151004
48958	2.65967	0.305752
48959	2.65967	0.226956
48960	2.71793	0.400442
48961	2.65966	0.176689
48962	2.56149	0.157092
48963	2.56149	0.17386
48964	2.71296	0.33281
48965	2.71296	0.263481
48966	1.31528	0.155957
48967	1.32983	0.148781
48968	1.21128	0.153828
48969	1.18812	0.15874
48970	1.24232	0.126667
48971	1.24232	0.132473
48972	1.26109	0.17624
48973	1.26109	0.329253
48974	1.21128	0.179309
48975	1.31528	0.109173
48976	1.18812	0.124122
48977	1.32983	0.183672
48978	2.63056	0.133214
48979	2.58032	0.170444
48980	2.65966	0.10612
48981	2.65966	0.158113
48982	2.58032	0.277404
48983	2.71296	0.246488
48984	1.92039	2.1
48985	1.06226	2.1
48986	1.09252	2.1
48987	1.96052	2.1
48988	3.01291	2.1
48989	1.92039	2.1
48990	2.46338	2.1
48991	2.47522	2.1
48992	2.43508	2.1
48993	1.96052	2.1
48994	1.92039	2.1
48995	0.985949	2.1
48996	0.980261	2.1
48997	0.553625	2.1
48998	0.546262	2.1
48999	0.765671	2.1
49000	0.765671	2.1
49001	0.766539	2.1
49002	0.766539	2.1
49003	0.985949	2.1
49004	0.553625	2.1
49005	0.546261	2.1
49006	0.980261	2.1
49007	1.96052	2.1

49008	2.46338	2.1
49009	1.97189	2.1
49010	1.96052	2.1
49011	2.47932	2.1
49012	2.47932	2.1
49013	0.964608	0.05
49014	0.691524	0.05
49015	1.25462	0.05
49016	0.691524	0.05
49017	1.89226	0.05
49018	1.89226	0.05
49019	1.44894	0.05
49020	2.77655	0.05
49021	1.4237	0.05
49022	2.77701	0.05
49023	2.77701	0.05
49024	1.38847	2.1
49025	1.46031	1.72285
49026	1.65504	1.84916
49027	1.75396	1.63175
49028	3.11535	1.84138
49029	1.89226	1.80974
49030	1.89226	1.71928
49031	1.89226	1.87889
49032	1.89226	1.35994
49033	3.78452	1.63046
49034	1.89226	1.52706
49035	1.89226	2.06605
49036	1.89226	0.575122
49037	1.89226	1.53329
49038	3.78452	1.3587
49039	1.46031	1.46129
49040	1.34851	1.84976
49041	1.75395	1.4603
49042	1.62287	1.52485
49043	3.08318	1.5145
49044	1.65504	2.05826
49045	1.75396	1.9202
49046	1.89249	2.1
49047	1.9857	1.51233
49048	3.64073	1.92533
49049	1.89226	2.093
49050	1.89226	1.60324
49051	1.89226	1.62584
49052	1.89226	1.53152
49053	3.78452	1.63087
49054	1.89226	1.39285
49055	1.89226	1.78986
49056	1.89226	1.39544
49057	1.89226	1.74514
49058	3.78452	1.54621
49059	1.75395	1.84328
49060	1.62287	1.74741
49061	1.98569	1.3164
49062	1.86067	1.34496
49063	3.60856	1.40448
49064	1.35697	2.1
49065	2.02064	2.1

49066	1.89989	2.1
49067	1.49571	2.1
49068	1.66753	2.1
49069	1.36431	2.1
49070	0.744004	2.1
49071	0.743997	2.1
49072	0.717972	2.1
49073	0.717972	2.1
49074	0.997982	2.1
49075	0.807796	2.1
49076	0.632235	2.1
49077	0.488575	2.1
49078	0.849275	2.1
49079	0.488581	2.1
49080	0.724729	2.1
49081	0.997967	2.1
49082	2.5276	2.1
49083	1.70791	2.1
49084	1.82872	1.26057
49085	1.56362	2.1
49086	2.5276	1.89132
49087	2.24781	2.1
49088	1.37591	2.1
49089	1.23217	2.1
49090	1.00442	1.8879
49091	0.945282	1.0336
49092	1.15446	1.57301
49093	1.09123	2.1
49094	1.21506	2.1
49095	1.21506	1.75224
49096	1.39452	1.99173
49097	1.13108	2.00641
49098	1.27876	2.00379
49099	0.929006	1.9981
49100	1.46505	0.0528681
49101	1.91484	0.0543733
49102	1.49906	0.0565091
49103	1.49906	0.0683879
49104	1.90196	0.05
49105	1.90196	0.05
49106	1.90196	0.0511692
49107	2.11465	0.0584127
49108	1.91484	0.0581869
49109	1.91484	0.0736977
49110	2.04016	0.05
49111	2.04016	0.0515398
49112	0.912201	0.05
49113	0.950978	0.05
49114	0.665307	0.05
49115	0.732528	0.05
49116	0.829512	0.05
49117	0.829512	0.05
49118	0.815217	0.05
49119	0.815217	0.05
49120	0.912202	0.05
49121	0.665305	0.05
49122	0.732527	0.05
49123	0.95098	0.05

49124	1.90196	0.05
49125	2.08757	0.05
49126	1.9626	0.05
49127	2.04016	0.05
49128	1.82441	0.05
49129	1.90196	0.05
49130	1.13048	2.1
49131	0.821162	2.1
49132	0.946131	2.1
49133	0.946131	2.1
49134	1.04567	2.1
49135	0.882347	2.1
49136	0.897276	2.1
49137	0.897276	2.1
49138	0.863109	2.1
49139	1.01055	2.1
49140	0.940305	2.1
49141	0.942166	2.1
49142	2.23933	0.05
49143	0.976217	0.05
49144	2.78053	0.05
49145	2.78053	0.05
49146	0.976217	0.05
49147	1.65984	0.05
49148	1.54168	2.1
49149	2.01197	2.02238
49150	1.69136	2.1
49151	2.13284	1.76158
49152	3.67452	2.1
49153	0.988805	2.1
49154	1.78069	2.1
49155	1.69136	2.1
49156	1.69136	2.1
49157	1.05481	2.1
49158	1.05481	2.1
49159	1.69136	2.1
49160	2.13284	1.96642
49161	1.78069	2.1
49162	2.22044	2.05903
49163	3.91353	2.1
49164	2.69379	1.48249
49165	2.32219	1.52772
49166	2.25746	1.70041
49167	2.25746	1.44013
49168	2.32219	1.5809
49169	1.89526	1.31744
49170	1.4556	2.1
49171	1.85028	2.1
49172	2.4909	1.89419
49173	2.03799	2.1
49174	2.73495	2.1
49175	1.85028	2.1
49176	2.71938	1.17175
49177	1.85028	2.1
49178	3.36051	2.1
49179	2.73495	2.1
49180	2.47584	2.1
49181	1.85028	2.1

49182	2.47584	2.1
49183	1.04413	2.1
49184	2.1461	2.1
49185	1.74586	2.1
49186	1.44437	2.1
49187	1.04413	2.1
49188	1.89226	2.1
49189	1.89226	1.88616
49190	1.89226	2.1
49191	1.89226	2.1
49192	3.78452	2.1
49193	1.52724	2.1
49194	1.89249	2.1
49195	1.68143	2.1
49196	2.02433	2.1
49197	3.55157	2.1
49198	1.07444	2.1
49199	1.76545	2.1
49200	1.68142	2.1
49201	1.68142	2.1
49202	1.14807	2.1
49203	1.14807	2.1
49204	1.89226	2.1
49205	1.89226	2.1
49206	1.89226	2.1
49207	1.89226	2.1
49208	1.89226	2.1
49209	1.89226	2.1
49210	1.89226	2.1
49211	1.89226	2.1
49212	1.89226	2.1
49213	1.89226	1.87074
49214	3.78452	2.1
49215	1.68143	2.1
49216	2.02433	2.1
49217	1.76545	2.1
49218	2.12608	2.1
49219	3.78978	2.1
49220	2.62871	2.1
49221	1.49149	2.1
49222	1.36814	2.1
49223	2.44761	2.1
49224	3.9391	2.1
49225	1.89226	2.1
49226	1.89226	2.01313
49227	1.89226	2.1
49228	1.89226	2.1
49229	3.78452	2.1
49230	0.851542	2.1
49231	0.851542	2.1
49232	1.00897	2.1
49233	0.931224	2.1
49234	0.838952	2.1
49235	0.838952	2.1
49236	0.956848	2.1
49237	0.942474	2.1
49238	0.946132	2.1
49239	0.838952	2.1

49240	0.878743	2.1
49241	0.946132	2.1
49242	1.15541	2.1
49243	2.44761	2.1
49244	1.36814	2.1
49245	1.36814	2.1
49246	2.19439	2.1
49247	2.19439	2.1
49248	1.89226	2.1
49249	1.89226	2.1
49250	1.89226	2.1
49251	1.89226	2.1
49252	3.78452	2.1
49253	1.89226	2.1
49254	1.67791	2.1
49255	1.5713	2.1
49256	1.67791	2.1
49257	1.78566	2.1
49258	1.89226	2.1
49259	1.89226	0.442043
49260	1.62911	2.1
49261	2.24602	1.85137
49262	1.89226	2.01765
49263	1.98287	1.29479
49264	1.62911	1.58131
49265	1.89226	2.1
49266	1.89226	2.1
49267	1.89226	1.68797
49268	1.89226	2.1
49269	3.78452	2.1
49270	1.26688	2.1
49271	1.11804	2.1
49272	0.852467	2.1
49273	0.693314	2.1
49274	0.977459	2.1
49275	0.977459	2.1
49276	0.982741	2.1
49277	0.982741	2.1
49278	1.26689	2.1
49279	0.852464	2.1
49280	0.693312	2.1
49281	1.11804	2.1
49282	0.946131	2.1
49283	0.946131	1.19651
49284	1.1794	0.63849
49285	1.1794	1.81898
49286	0.991434	2.1
49287	1.1794	1.07252
49288	0.946131	1.09709
49289	0.946131	0.766679
49290	0.946131	2.06513
49291	1.1794	1.89167
49292	0.946131	2.08682
49293	0.991434	1.85688
49294	1.89226	1.30527
49295	1.89226	2.1
49296	1.89226	2.1
49297	1.89226	2.1

49298	3.78452	2.1
49299	2.53377	2.1
49300	1.53443	2.1
49301	2.0815	2.1
49302	2.82979	2.1
49303	4.35462	2.1
49304	1.96192	0.669442
49305	1.23602	2.1
49306	2.16506	1.21829
49307	1.56841	2.01934
49308	3.40108	1.97235
49309	1.89226	2.1
49310	1.89226	2.1
49311	1.89226	1.38693
49312	1.89226	2.1
49313	3.78452	2.1
49314	0.946129	2.1
49315	0.946129	2.1
49316	1.13067	2.1
49317	1.12952	2.1
49318	0.946129	2.1
49319	1.13089	2.1
49320	0.944978	2.1
49321	0.944978	2.1
49322	0.946131	2.1
49323	1.13089	2.1
49324	0.944977	2.1
49325	0.946131	2.1
49326	0.710208	2.1
49327	0.710208	2.1
49328	0.718894	2.1
49329	0.718894	2.1
49330	0.559773	2.1
49331	0.579321	2.1
49332	0.886767	2.1
49333	0.849781	2.1
49334	0.849781	2.1
49335	0.559774	2.1
49336	0.579321	2.1
49337	0.886766	2.1
49338	1.63471	2.1
49339	1.69956	2.1
49340	1.08022	2.1
49341	1.08022	2.1
49342	1.11955	2.1
49343	1.67403	2.1
49344	1.03766	2.1
49345	2.02825	2.1
49346	2.02794	2.1
49347	2.0348	2.1
49348	1.07196	2.1
49349	1.07196	2.1
49350	1.9276	2.1
49351	2.1004	2.1
49352	2.1306	2.1
49353	2.1306	2.1
49354	1.92178	2.1
49355	1.92032	2.1

49356	0.986724	2.1
49357	2.03064	2.1
49358	2.01385	2.1
49359	2.02404	2.1
49360	1.03766	2.1
49361	1.03766	2.1
49362	1.92027	2.1
49363	2.1306	2.1
49364	2.15388	2.1
49365	2.15388	2.1
49366	1.92614	2.1
49367	1.9276	2.1
49368	1.09578	2.1
49369	0.963018	2.1
49370	0.782956	1.98812
49371	0.529693	2.1
49372	0.6994	2.1
49373	0.862532	2.1
49374	0.898605	2.1
49375	0.898605	2.05535
49376	0.776756	2.1
49377	1.14805	2.1
49378	0.966521	2.1
49379	0.617676	2.1
49380	2.67185	2.1
49381	2.099	2.1
49382	2.80548	2.1
49383	2.2727	2.1
49384	4.94455	2.1
49385	1.89226	2.1
49386	1.89226	2.1
49387	1.89226	2.1
49388	1.89226	2.1
49389	3.78453	2.1
49390	1.89226	2.1
49391	1.89226	2.1
49392	1.89226	2.1
49393	1.89226	2.1
49394	1.89226	2.1
49395	1.89226	2.1
49396	0.884801	2.1
49397	0.884801	2.1
49398	0.895199	2.1
49399	0.895199	2.1
49400	0.622774	2.1
49401	0.730501	2.1
49402	1.0495	2.1
49403	1.13635	2.1
49404	1.13635	2.1
49405	0.730503	2.1
49406	1.0495	2.1
49407	0.622775	2.1
49408	1.89226	2.1
49409	1.89226	2.1
49410	1.89226	2.1
49411	1.89226	2.1
49412	1.89226	2.1
49413	1.89226	2.1

49414	1.89226	2.1
49415	1.89226	2.1
49416	1.89226	2.1
49417	1.89226	2.1
49418	1.89226	2.1
49419	1.89226	2.1
49420	2.2727	2.1
49421	1.89586	2.1
49422	1.66913	2.1
49423	1.46101	2.1
49424	2.48082	2.1
49425	2.2727	2.1
49426	1.89226	2.1
49427	1.89226	2.1
49428	1.84641	2.1
49429	1.89226	2.1
49430	1.30868	2.1
49431	1.34118	2.09567
49432	1.89226	1.75638
49433	1.89226	1.92388
49434	1.89226	2.02961
49435	1.89226	1.83065
49436	1.89226	1.84061
49437	1.89226	1.67428
49438	1.89226	1.95209
49439	1.89226	1.61478
49440	1.89226	1.77301
49441	1.89226	1.90843
49442	3.78452	1.81351
49443	0.946132	2.1
49444	0.946132	2.06988
49445	0.946131	2.1
49446	0.946131	2.1
49447	0.946132	2.1
49448	0.946132	1.96495
49449	0.946132	2.1
49450	0.946132	2.1
49451	0.946132	2.1
49452	0.946132	2.1
49453	0.946132	2.1
49454	0.946132	2.1
49455	1.89227	2.00269
49456	1.84641	2.1
49457	2.29517	2.1
49458	1.84641	2.08498
49459	2.34102	2.04364
49460	1.89227	1.84436
49461	1.89226	1.58947
49462	1.89226	1.70997
49463	1.89226	1.78307
49464	1.89226	1.64606
49465	1.89226	1.58884
49466	1.89226	0.999564
49467	1.89226	1.53271
49468	1.89226	1.71818
49469	1.89226	1.78739
49470	1.89226	1.66051
49471	1.89226	1.43476

49472	1.89226	0.968814
49473	1.89226	2.1
49474	1.89226	1.96733
49475	1.89226	2.1
49476	1.89226	1.9367
49477	1.89226	2.08246
49478	1.89226	1.87141
49479	0.851544	0.05
49480	0.757542	0.05
49481	1.13421	0.05
49482	1.04072	0.05
49483	0.946132	0.05
49484	0.946132	0.05
49485	0.841686	0.05
49486	0.834428	0.05
49487	1.13649	0.05
49488	0.937687	0.05
49489	0.841713	0.05
49490	0.841713	0.05
49491	1.89226	0.260009
49492	1.89226	0.272515
49493	1.89226	0.27807
49494	1.89226	0.229162
49495	1.89226	0.301923
49496	1.89226	0.248284
49497	2.31988	1.67626
49498	2.00082	2.1
49499	2.4594	0.606711
49500	2.17638	2.1
49501	4.46022	1.88767
49502	2.00082	2.1
49503	1.41829	2.1
49504	1.61207	2.1
49505	1.43652	2.1
49506	2.17637	2.1
49507	2.00082	2.1
49508	2.4594	1.40837
49509	2.17638	2.1
49510	2.57957	1.16425
49511	2.3506	2.1
49512	4.81	1.76631
49513	2.3506	2.07057
49514	1.57368	2.1
49515	1.89111	1.72387
49516	1.89111	2.1
49517	2.17638	1.60635
49518	2.17638	2.1
49519	1.32813	2.1
49520	1.7237	2.1
49521	1.32461	2.1
49522	1.81656	2.1
49523	3.14468	2.1
49524	1.55132	2.1
49525	2.0815	2.1
49526	1.81656	2.1
49527	2.19826	2.1
49528	3.65818	2.1
49529	0.775951	2.1

49530	0.775951	2.1
49531	0.794601	2.1
49532	0.794601	2.1
49533	0.945641	2.1
49534	0.90828	2.1
49535	0.662302	2.1
49536	0.662273	2.1
49537	0.662274	2.1
49538	0.945639	2.1
49539	0.662304	2.1
49540	0.908279	2.1
49541	1.03062	2.1
49542	1.03062	2.1
49543	1.05054	2.1
49544	1.05054	2.1
49545	1.13551	2.1
49546	1.21275	2.1
49547	0.945641	2.1
49548	0.90828	2.1
49549	0.945641	2.1
49550	1.21275	2.1
49551	0.90828	2.1
49552	1.13552	2.1
49553	1.89226	2.1
49554	1.79883	2.1
49555	1.95341	2.1
49556	1.89226	2.1
49557	1.85998	2.1
49558	1.79883	2.1
49559	1.89226	2.1
49560	1.89226	2.1
49561	1.89226	2.1
49562	1.89226	2.1
49563	3.78453	2.1
49564	0.851541	2.1
49565	0.851541	2.1
49566	0.970398	2.1
49567	0.954365	2.1
49568	0.855356	2.1
49569	0.855357	2.1
49570	0.946133	2.1
49571	0.946133	2.1
49572	1.04072	2.1
49573	0.970397	2.1
49574	0.950003	2.1
49575	0.851538	2.1
49576	1.89226	2.1
49577	1.85998	2.1
49578	1.95296	2.1
49579	1.89226	2.1
49580	1.92067	2.1
49581	1.85998	2.1
49582	0.946133	2.1
49583	0.946133	2.1
49584	0.946132	2.1
49585	0.946132	2.1
49586	0.946132	2.1
49587	0.946132	2.1

49588	0.946132	2.1
49589	0.946132	2.1
49590	0.946132	2.1
49591	0.946132	2.1
49592	0.946133	2.1
49593	0.946133	2.1
49594	1.82152	2.1
49595	1.89226	2.1
49596	1.89226	2.1
49597	2.11152	2.1
49598	1.7472	2.1
49599	1.89226	2.1
49600	2.02687	2.1
49601	2.29199	2.1
49602	1.2734	2.1
49603	1.41077	2.1
49604	1.47381	2.1
49605	2.29199	2.1
49606	1.46889	2.1
49607	2.29199	2.1
49608	1.86766	2.1
49609	2.59401	2.1
49610	4.15965	2.1
49611	1.58995	2.1
49612	1.74246	2.1
49613	1.46643	2.1
49614	1.46643	2.1
49615	1.85097	2.1
49616	1.85097	2.1
49617	1.58995	2.1
49618	1.66584	2.1
49619	1.89214	2.1
49620	1.91787	2.1
49621	3.48208	2.1
49622	1.18474	2.1
49623	1.8144	2.1
49624	1.1486	2.1
49625	1.1486	2.1
49626	2.13071	2.1
49627	2.1257	2.1
49628	2.0815	2.1
49629	2.51651	2.1
49630	1.73681	2.1
49631	2.01601	2.1
49632	2.21406	2.1
49633	2.56998	2.1
49634	1.19995	2.1
49635	1.06256	2.1
49636	0.620677	2.1
49637	0.598084	2.1
49638	0.831426	2.1
49639	0.946056	2.1
49640	0.827692	2.1
49641	0.827692	2.1
49642	1.08958	2.1
49643	0.630132	2.1
49644	0.577547	2.1
49645	1.06104	2.1

49646	2.0815	2.1
49647	2.66249	2.1
49648	2.78966	2.1
49649	2.47108	2.1
49650	2.33858	2.1
49651	2.0815	2.1
49652	1.53735	2.1
49653	1.42278	2.1
49654	1.23654	2.1
49655	1.34678	2.1
49656	2.76955	2.1
49657	1.89226	2.1
49658	1.89226	2.1
49659	1.89226	2.1
49660	1.89226	2.1
49661	3.78452	2.1
49662	1.23654	2.1
49663	1.34678	2.1
49664	1.19 2.1	
49665	1.08576	2.1
49666	2.43254	2.1
49667	1.38776	2.1
49668	1.29366	2.1
49669	0.946131	2.1
49670	0.946131	2.1
49671	0.946131	2.1
49672	0.946131	2.1
49673	1.38775	2.1
49674	1.38775	2.1
49675	1.29366	2.1
49676	0.946132	2.1
49677	0.946131	2.1
49678	1.38775	2.1
49679	0.946131	2.1
49680	0.946131	2.1
49681	0.946129	2.1
49682	0.946129	2.1
49683	0.94613	2.1
49684	0.94613	2.1
49685	0.946131	2.1
49686	0.946131	2.1
49687	0.946131	2.1
49688	0.946131	2.1
49689	0.94613	2.1
49690	0.94613	2.1
49691	1.89226	0.14795
49692	1.89226	1.82483
49693	1.89226	0.567945
49694	1.89226	0.523229
49695	1.89226	1.3481
49696	1.89226	1.56648
49697	1.89226	0.487678
49698	1.89226	2.1
49699	1.89226	1.15889
49700	1.89226	1.27931
49701	1.89226	1.05113
49702	1.89226	1.85609
49703	1.89226	0.289117

49704	1.89226	1.99006
49705	1.89226	0.686029
49706	1.89226	0.799145
49707	1.89226	0.58354
49708	1.89226	1.74166
49709	0.92338	2.1
49710	0.92338	1.99109
49711	0.94428	2.01104
49712	0.94428	0.925502
49713	1.19528	2.05712
49714	1.30598	2.1
49715	0.714073	1.21706
49716	0.561679	2.1
49717	1.19528	2.1
49718	0.561678	2.1
49719	0.714072	2.1
49720	1.30598	2.1
49721	2.61197	2.1
49722	1.89226	2.1
49723	1.89226	2.1
49724	1.89226	2.1
49725	2.39056	2.05538
49726	2.39056	2.1
49727	0.946132	2.1
49728	0.946132	1.98451
49729	0.946132	0.465639
49730	0.946132	1.36579
49731	0.946131	1.37682
49732	0.946131	2.1
49733	0.946132	1.97911
49734	0.946132	1.0759
49735	0.946132	1.80995
49736	0.946132	1.72178
49737	0.946132	1.76011
49738	0.946132	1.7617
49739	1.89224	2.1
49740	1.89224	2.1
49741	1.89224	2.1
49742	1.89224	2.1
49743	3.78448	2.1
49744	1.97089	2.1
49745	1.57428	2.1
49746	1.84991	2.1
49747	1.49078	2.1
49748	3.42419	2.1
49749	0.621927	2.1
49750	0.621927	2.1
49751	0.630824	2.1
49752	0.630824	2.1
49753	0.483483	2.1
49754	0.507361	2.1
49755	0.787142	2.1
49756	0.745391	2.1
49757	0.745381	2.1
49758	0.483476	2.1
49759	0.507368	2.1
49760	0.787152	2.1
49761	1.89224	2.1

49762	1.89224	2.1
49763	1.89224	2.1
49764	1.89224	2.1
49765	1.89224	2.1
49766	1.89224	2.1
49767	1.89227	2.1
49768	1.89227	2.1
49769	1.89227	2.1
49770	1.89227	2.1
49771	1.89227	2.1
49772	1.89227	2.1
49773	1.84994	2.1
49774	1.41758	2.1
49775	1.4908	2.1
49776	1.4908	2.1
49777	1.74391	2.1
49778	1.74391	2.1
49779	1.89226	2.1
49780	1.89226	2.1
49781	1.89226	2.1
49782	1.89226	2.1
49783	3.78452	2.1
49784	1.01323	2.1
49785	1.01323	2.1
49786	1.01445	2.1
49787	1.01445	2.1
49788	0.839531	2.1
49789	0.875652	2.1
49790	1.19063	2.1
49791	1.15203	2.1
49792	1.15203	2.1
49793	0.839531	2.1
49794	0.875652	2.1
49795	1.19063	2.1
49796	1.89226	2.1
49797	2.75587	2.1
49798	2.81512	2.1
49799	2.81512	2.1
49800	1.89226	2.1
49801	1.89226	2.1
49802	1.89226	2.1
49803	1.89226	2.1
49804	1.89226	2.1
49805	1.89226	2.1
49806	3.78452	2.1
49807	0.974831	2.1
49808	0.974831	2.1
49809	0.976681	2.1
49810	0.976681	2.1
49811	0.803179	2.1
49812	0.83953	2.1
49813	1.11198	2.1
49814	1.15203	2.1
49815	1.11198	2.1
49816	0.803179	2.1
49817	1.15203	2.1
49818	0.83953	2.1
49819	1.37794	2.1

49820	1.37794	2.1
49821	0.946128	2.1
49822	0.946128	2.1
49823	1.3477	2.1
49824	1.37793	2.1
49825	0.946131	2.1
49826	0.946131	2.1
49827	0.946129	2.1
49828	1.3477	2.1
49829	1.37793	2.1
49830	0.946129	2.1
49831	2.38577	2.04385
49832	1.20247	2.1
49833	1.75182	2.1
49834	2.82546	2.04394
49835	4.1376	2.1
49836	2.82546	2.1
49837	1.89226	1.64306
49838	2.38577	2.0572
49839	2.38577	1.76938
49840	1.89226	1.96927
49841	1.89226	1.77153
49842	1.035 2.1	
49843	2.15429	2.1
49844	0.970998	2.1
49845	2.0815	2.1
49846	2.89672	2.1
49847	0.946131	0.0884436
49848	0.946131	0.0799336
49849	0.853249	0.0920906
49850	1.02715	0.0781448
49851	0.851537	0.097137
49852	0.851537	0.0850928
49853	1.02663	0.0764696
49854	1.13139	0.0927883
49855	0.946128	0.0816637
49856	0.932039	0.066288
49857	0.843985	0.0634783
49858	0.946129	0.0795005
49859	1.89226	0.0868054
49860	1.86408	0.0840678
49861	1.96548	0.0802923
49862	1.86408	0.077833
49863	1.99366	0.0991408
49864	1.89226	0.0948804
49865	2.70702	2.1
49866	2.08981	2.1
49867	2.08693	2.1
49868	2.08693	2.1
49869	2.7188	2.1
49870	2.7188	2.1
49871	2.08982	2.1
49872	1.01964	2.1
49873	2.08693	2.1
49874	1.04615	2.1
49875	3.13597	2.1
49876	2.70702	2.1
49877	2.08693	2.1

49878	2.68784	2.1
49879	2.08831	2.1
49880	4.77477	2.1
49881	0.778233	2.1
49882	0.778233	2.1
49883	0.789001	2.1
49884	0.789001	2.1
49885	0.545307	2.1
49886	0.523075	2.1
49887	1.04347	2.1
49888	1.04416	2.1
49889	1.04416	2.1
49890	0.545308	2.1
49891	1.04347	2.1
49892	0.523075	2.1
49893	1.89226	0.0655895
49894	1.89226	0.270214
49895	1.89226	0.210701
49896	1.89226	0.05
49897	3.78453	0.05
49898	1.39237	0.102418
49899	1.17933	0.545417
49900	0.755796	0.0704281
49901	0.755796	0.329016
49902	0.755767	0.0557582
49903	0.94613	0.802312
49904	1.15918	0.0701759
49905	0.94613	0.24854
49906	1.11632	0.079544
49907	0.92595	0.106943
49908	0.94613	0.0682241
49909	0.94613	0.0762199
49910	1.89226	0.365427
49911	1.89226	0.0601299
49912	1.89226	0.05
49913	1.89226	0.0667184
49914	3.78452	0.0505472
49915	0.950067	0.05
49916	0.75071	0.0686378
49917	0.906516	0.05
49918	0.906516	0.05
49919	0.81586	0.05
49920	0.851541	0.0908174
49921	0.962101	0.05
49922	0.851476	0.05
49923	0.962105	0.05
49924	0.815808	0.05
49925	0.851529	0.0556484
49926	0.851529	0.05
49927	1.05032	0.0563888
49928	0.972326	0.0546878
49929	0.906515	0.05
49930	0.906515	0.05
49931	1.12245	0.05
49932	1.04072	0.05
49933	0.815857	0.05
49934	0.851539	0.0570945
49935	1.04451	0.05

49936	0.815853	0.0585594
49937	0.919795	0.05
49938	0.919795	0.0592504
49939	1.5855	2.1
49940	2.14058	2.1
49941	1.9153	2.1
49942	1.79922	2.1
49943	3.72608	2.1
49944	1.58551	2.1
49945	1.24201	2.1
49946	0.960287	2.1
49947	1.79922	2.1
49948	2.82752	2.1
49949	0.679602	2.1
49950	0.621009	2.1
49951	0.899614	2.1
49952	0.940441	2.1
49953	0.679602	2.1
49954	0.94044	2.1
49955	0.621009	2.1
49956	0.899615	2.1
49957	0.794027	2.1
49958	0.794027	2.1
49959	0.785189	2.1
49960	0.785189	2.1
49961	2.14058	2.1
49962	1.79922	2.1
49963	2.23742	2.1
49964	2.23742	2.1
49965	1.78404	2.1
49966	1.88088	2.1
49967	1.15177	2.1
49968	2.0815	2.1
49969	1.93765	2.1
49970	1.78342	2.1
49971	1.39511	2.1
49972	1.28406	2.1
49973	1.97719	2.1
49974	2.50057	2.0245
49975	2.35349	2.1
49976	2.35349	1.9862
49977	2.31686	2.1
49978	2.32857	2.03576
49979	1.93765	2.1
49980	2.35349	2.04578
49981	1.70048	2.1
49982	2.15721	1.62804
49983	4.05397	2.1
49984	0.850388	2.1
49985	0.850388	2.1
49986	0.840019	2.1
49987	0.840019	2.1
49988	1.13736	2.1
49989	1.13852	2.1
49990	0.573661	2.1
49991	0.551891	2.1
49992	0.573661	2.1
49993	1.13852	2.1

49994	0.551892	2.1
49995	1.13736	2.1
49996	0.860203	2.1
49997	0.860203	2.1
49998	0.855758	2.1
49999	0.855758	2.1
50000	1.1423	2.1
50001	1.13852	2.1
50002	0.58628	2.1
50003	0.573661	2.1
50004	0.586281	2.1
50005	1.1423	2.1
50006	0.573661	2.1
50007	1.13852	2.1
50008	1.28918	1.3033
50009	0.999086	1.24485
50010	1.0383	1.48019
50011	0.736019	1.41865
50012	1.00954	1.4569
50013	1.00954	1.31273
50014	1.01566	1.23185
50015	1.01566	1.41
50016	1.28918	1.39199
50017	1.0383	1.42821
50018	0.999086	1.3873
50019	0.736019	1.38992
50020	2.20797	2.1
50021	2.64748	2.1
50022	2.50375	2.1
50023	2.50375	2.1
50024	2.40858	2.1
50025	2.40858	2.1
50026	2.64748	2.1
50027	2.74744	2.1
50028	2.50375	2.1
50029	2.6521	2.1
50030	5.29958	2.1
50031	2.20797	2.1
50032	2.50375	2.1
50033	2.02064	2.1
50034	2.3606	2.1
50035	4.52439	2.1
50036	2.36059	2.1
50037	2.6521	2.1
50038	2.56952	2.1
50039	2.56952	2.1
50040	2.50375	2.1
50041	2.50375	2.1
50042	2.49439	0.0829939
50043	1.89226	0.212104
50044	2.81337	0.144047
50045	1.89226	0.175319
50046	4.38665	0.169187
50047	1.89226	0.140787
50048	1.89226	0.381376
50049	1.89226	0.36055
50050	1.89226	0.304874
50051	3.78452	0.290408

50052	1.89226	0.202633
50053	1.89226	0.245107
50054	1.89226	0.227828
50055	1.89226	0.270275
50056	3.78452	0.248577
50057	1.93948	0.372564
50058	1.98874	0.212019
50059	1.62494	0.193524
50060	2.14218	0.360934
50061	1.62494	0.081994
50062	2.14218	0.203785
50063	1.47354	0.21028
50064	1.98874	0.161955
50065	2.01865	0.222987
50066	1.62495	0.137801
50067	2.01865	0.210381
50068	1.62495	0.107081
50069	1.89226	2.1
50070	1.89226	2.1
50071	1.89226	2.1
50072	1.89226	2.1
50073	3.78453	2.1
50074	2.05257	2.1
50075	1.94441	2.1
50076	1.88487	2.1
50077	1.82441	2.1
50078	3.82928	2.1
50079	1.94441	2.1
50080	1.74391	2.1
50081	1.82441	2.1
50082	1.65636	2.1
50083	3.60077	2.1
50084	1.89226	2.1
50085	1.89226	2.1
50086	1.89226	2.1
50087	1.89226	2.1
50088	3.78452	2.1
50089	1.89226	2.1
50090	1.89226	2.1
50091	1.89226	2.1
50092	1.89226	2.1
50093	3.78453	2.1
50094	1.88487	2.1
50095	1.82441	2.1
50096	1.75835	2.1
50097	1.72866	2.1
50098	3.61354	2.1
50099	1.82441	2.1
50100	1.65636	2.1
50101	1.72866	2.1
50102	1.58401	2.1
50103	3.38503	2.1
50104	1.89226	2.1
50105	1.89226	2.1
50106	1.89226	2.1
50107	1.89226	2.1
50108	3.78452	2.1
50109	2.27432	2.1

50110	1.33339	2.1
50111	1.38905	2.1
50112	2.34471	2.1
50113	3.66337	2.1
50114	1.89226	2.1
50115	2.34471	2.1
50116	2.27432	2.1
50117	2.27432	2.1
50118	1.89226	2.1
50119	1.89226	2.1
50120	1.20443	2.1
50121	1.17235	2.1
50122	0.724695	2.1
50123	0.694522	2.1
50124	0.949994	2.1
50125	0.949993	2.1
50126	0.948955	2.1
50127	0.948955	2.1
50128	1.20443	2.1
50129	0.7247	2.1
50130	0.694517	2.1
50131	1.17234	2.1
50132	1.89225	2.1
50133	2.40885	2.1
50134	2.3447	2.1
50135	2.3447	2.1
50136	1.89225	2.1
50137	1.89225	2.1
50138	1.37138	2.1
50139	1.82597	2.1
50140	1.45027	2.1
50141	1.92772	2.1
50142	3.27624	2.1
50143	1.89226	2.1
50144	1.89226	2.1
50145	1.89226	2.1
50146	1.89226	2.1
50147	3.78452	2.1
50148	1.89226	2.1
50149	1.89226	2.1
50150	1.89226	2.1
50151	1.89226	2.1
50152	3.78452	2.1
50153	1.82597	2.1
50154	2.02312	1.74216
50155	1.92772	2.1
50156	2.15888	2.1
50157	3.98485	2.1
50158	1.53446	2.1
50159	1.92772	2.1
50160	2.03975	2.1
50161	2.03975	2.1
50162	1.45027	2.1
50163	1.45027	2.1
50164	1.89226	2.1
50165	1.89226	2.1
50166	1.89226	2.1
50167	1.89226	2.1

50168	1.89226	2.1
50169	1.89226	2.1
50170	1.89226	2.1
50171	1.89226	2.1
50172	1.89226	2.1
50173	1.89226	2.1
50174	3.78452	2.1
50175	1.92772	2.1
50176	2.15888	2.1
50177	2.03975	2.1
50178	2.3111	2.1
50179	4.19863	2.1
50180	1.89226	2.1
50181	1.89226	2.1
50182	1.89226	2.1
50183	1.89226	2.1
50184	3.78452	2.1
50185	1.78376	2.1
50186	1.3201	2.1
50187	1.78376	2.1
50188	1.86102	2.1
50189	1.2679	2.1
50190	1.34517	2.1
50191	1.89226	2.1
50192	2.52442	2.1
50193	1.89226	2.1
50194	2.51691	2.1
50195	4.41667	2.1
50196	1.89226	2.1
50197	1.89226	2.1
50198	1.89226	2.1
50199	1.89226	2.1
50200	3.78452	2.1
50201	1.78376	2.1
50202	1.2679	2.1
50203	1.71839	2.1
50204	1.23504	2.1
50205	3.0188	2.1
50206	1.89226	2.1
50207	2.51795	2.1
50208	2.51691	2.1
50209	2.51691	2.1
50210	1.89226	2.1
50211	1.89226	2.1
50212	1.89226	0.513704
50213	1.89226	1.16358
50214	1.89226	1.25099
50215	1.89226	0.912354
50216	3.78452	1.00476
50217	1.89227	0.659126
50218	1.89227	1.28208
50219	3.26088	1.05644
50220	2.79543	1.2556
50221	2.20733	1.20236
50222	1.89227	1.26658
50223	1.89226	1.19543
50224	1.89226	1.05444
50225	1.89226	0.818548

50226	1.89226	1.22072
50227	1.89226	0.832471
50228	1.89226	1.23186
50229	1.89227	0.590831
50230	2.20733	1.23585
50231	1.46973	1.12876
50232	1.89227	1.05397
50233	1.7848	1.106
50234	2.20733	1.097
50235	1.89226	2.1
50236	2.55682	2.1
50237	2.81593	2.1
50238	2.81593	2.1
50239	1.89226	2.1
50240	1.89226	2.1
50241	1.0607	2.1
50242	1.0607	2.1
50243	1.12617	2.1
50244	0.986957	2.1
50245	0.699328	2.1
50246	0.785567	2.1
50247	1.28855	2.1
50248	1.41913	2.1
50249	0.766737	2.1
50250	1.34711	2.1
50251	1.24643	2.1
50252	0.687289	2.1
50253	3.11063	0.318448
50254	1.89226	0.482461
50255	1.96536	0.388873
50256	1.89226	0.411195
50257	1.96536	0.350941
50258	1.89226	0.368068
50259	1.89226	1.32638
50260	1.89226	0.691388
50261	1.89226	0.925303
50262	1.89226	0.77667
50263	3.78452	1.319
50264	1.89226	0.448491
50265	1.96256	0.208064
50266	1.89226	0.284624
50267	1.89226	0.419742
50268	2.49192	0.399236
50269	2.49192	0.252856
50270	1.85716	0.407509
50271	2.56871	0.258177
50272	1.03043	0.279995
50273	1.03043	0.330102
50274	2.48125	0.193199
50275	1.65452	0.357466
50276	1.89226	2.1
50277	2.09622	2.1
50278	2.09622	2.1
50279	2.08508	2.1
50280	1.89226	2.1
50281	1.88112	2.1
50282	1.04811	2.1
50283	1.04811	2.1

50284	0.946131	2.1
50285	0.946131	2.1
50286	1.05014	2.1
50287	1.04811	2.1
50288	0.94613	2.1
50289	0.94613	2.1
50290	0.946131	2.1
50291	1.05013	2.1
50292	1.04811	2.1
50293	0.946131	2.1
50294	1.398	2.1
50295	2.10766	2.1
50296	2.3018	2.1
50297	2.3018	2.1
50298	1.34008	2.1
50299	1.34008	2.1
50300	1.1509	2.1
50301	1.1509	2.1
50302	0.946132	2.1
50303	0.946132	2.1
50304	0.946132	2.1
50305	0.946132	2.1
50306	1.05383	2.1
50307	1.1509	2.1
50308	1.1509	2.1
50309	0.946133	2.1
50310	0.946132	2.1
50311	1.05383	2.1
50312	0.931141	2.1
50313	0.931141	2.1
50314	0.971946	2.1
50315	0.971946	2.1
50316	1.28569	2.1
50317	1.1509	2.1
50318	0.699	2.1
50319	0.752185	2.1
50320	1.28568	2.1
50321	0.752186	2.1
50322	0.699001	2.1
50323	1.1509	2.1
50324	2.30181	2.1
50325	1.89226	2.1
50326	1.89226	2.1
50327	1.89226	2.1
50328	2.57137	2.1
50329	2.57137	2.1
50330	2.0815	2.1
50331	1.21962	2.1
50332	1.55173	2.1
50333	2.57849	2.1
50334	3.79001	2.1
50335	1.38779	2.1
50336	1.2923	2.1
50337	1.03974	2.1
50338	1.28832	2.1
50339	1.33566	2.1
50340	1.21116	2.1
50341	1.22343	2.1

50342	1.22343	2.1
50343	1.42288	2.1
50344	1.20138	2.1
50345	1.26025	2.1
50346	1.00644	2.1
50347	1.26066	2.1
50348	1.14271	2.1
50349	0.886635	2.1
50350	0.795277	2.1
50351	1.02966	2.1
50352	1.02967	2.1
50353	1.05216	2.1
50354	1.05216	2.1
50355	1.26068	2.1
50356	0.790042	2.1
50357	0.795269	2.1
50358	1.20135	2.1
50359	1.89226	0.584229
50360	1.89226	0.352098
50361	1.89226	0.392381
50362	1.89226	0.294299
50363	1.89226	0.850017
50364	1.89226	0.542828
50365	1.89227	0.353539
50366	1.89227	0.283717
50367	1.89227	0.311691
50368	1.89227	0.242632
50369	1.89227	0.479651
50370	1.89227	0.337848
50371	0.563873	0.219058
50372	0.50988	0.193753
50373	0.598693	0.273537
50374	0.558819	0.230215
50375	0.564855	0.21451
50376	0.564855	0.1999
50377	0.557838	0.260484
50378	0.557838	0.236578
50379	0.563873	0.233082
50380	0.598694	0.222211
50381	0.558819	0.219032
50382	0.50988	0.229459
50383	0.598693	0.265613
50384	0.558819	0.224382
50385	0.572737	0.357026
50386	0.534248	0.289489
50387	0.566788	0.270174
50388	0.566788	0.242928
50389	0.566153	0.31857
50390	0.566153	0.286628
50391	0.598694	0.284331
50392	0.572737	0.277907
50393	0.534248	0.265885
50394	0.558819	0.26456
50395	1.85217	2.1
50396	1.93111	2.1
50397	1.92875	2.1
50398	2.02391	2.1
50399	3.85986	2.1

50400	1.93112	2.1
50401	1.80425	2.1
50402	2.02392	2.1
50403	1.90981	2.1
50404	3.84092	2.1
50405	1.92876	2.1
50406	2.02393	2.1
50407	2.05274	2.1
50408	2.16783	2.1
50409	4.09659	2.1
50410	2.02393	2.1
50411	1.90982	2.1
50412	2.16784	2.1
50413	2.05967	2.1
50414	4.07766	2.1
50415	1.89226	1.82283
50416	1.89226	1.67924
50417	1.89226	1.76326
50418	1.89226	1.74824
50419	3.78453	1.67342
50420	1.89226	1.64928
50421	1.89226	1.77895
50422	1.89226	2.00281
50423	2.44829	1.61511
50424	3.78453	1.79203
50425	1.89226	1.83637
50426	1.89226	1.84568
50427	1.89226	1.83412
50428	2.60452	1.73648
50429	3.78453	1.91678
50430	1.58723	0.05
50431	1.88071	0.0537684
50432	2.97376	0.05
50433	2.13255	0.05
50434	4.43982	0.05
50435	2.20641	0.0808561
50436	1.33575	0.120335
50437	2.15824	0.0959543
50438	2.15824	0.0946201
50439	1.2951	0.0969661
50440	1.2951	0.100594
50441	0.825383	0.132409
50442	0.825383	0.113096
50443	0.856104	0.0972822
50444	0.856104	0.105952
50445	0.952196	0.126716
50446	0.602363	0.110402
50447	0.667877	0.123775
50448	1.07913	0.0980647
50449	0.602363	0.122794
50450	0.667876	0.119961
50451	1.07913	0.119253
50452	0.952197	0.11038
50453	2.57657	2.1
50454	1.44437	2.1
50455	2.65378	2.1
50456	2.25076	2.1
50457	1.84739	2.1

50458	1.44437	2.1
50459	2.0189	0.110402
50460	2.54752	0.111348
50461	2.63975	0.144356
50462	2.31049	0.0852526
50463	4.65865	0.112274
50464	1.40707	0.0753826
50465	1.89605	0.073729
50466	2.0189	0.0808135
50467	2.0189	0.0652116
50468	1.50438	0.0899042
50469	1.50438	0.072786
50470	2.63971	0.135307
50471	2.38246	0.0879202
50472	2.13402	0.104592
50473	2.57503	0.0966984
50474	4.77373	0.101575
50475	1.50436	0.0744723
50476	1.96803	0.0750814
50477	2.13402	0.0960106
50478	1.45446	0.0606099
50479	3.63838	0.0754463
50480	2.30793	0.145228
50481	2.11032	0.126275
50482	1.24082	0.126081
50483	1.05689	0.157012
50484	3.36482	0.143118
50485	1.89226	0.192189
50486	1.89226	0.272408
50487	1.89226	0.28727
50488	1.89226	0.230194
50489	3.78453	0.236482
50490	0.812967	0.181834
50491	0.812967	0.187736
50492	1.00181	0.166897
50493	1.08141	0.164299
50494	0.946132	0.16133
50495	0.812938	0.207012
50496	0.932815	0.200858
50497	1.05577	0.152765
50498	0.812938	0.185949
50499	0.932812	0.182211
50500	0.946133	0.180351
50501	0.946133	0.170449
50502	2.53897	2.1
50503	1.89226	2.1
50504	1.89226	2.1
50505	1.89226	2.1
50506	2.3245	2.1
50507	2.3245	2.1
50508	1.00255	2.1
50509	1.06561	2.1
50510	1.05481	0.911948
50511	1.11077	2.1
50512	2.12042	2.1
50513	1.89226	1.96001
50514	1.89226	2.1
50515	1.89226	1.1436

50516	1.89226	2.1
50517	3.78453	2.1
50518	1.89226	2.1
50519	2.77314	2.1
50520	2.81276	2.1
50521	2.81276	2.1
50522	1.89226	2.1
50523	1.89226	2.1
50524	1.11077	2.1
50525	1.15114	2.1
50526	1.10274	2.1
50527	1.1032	2.1
50528	1.05481	2.1
50529	1.1032	1.91707
50530	1.89226	1.94043
50531	1.89226	2.1
50532	1.89226	0.477192
50533	1.89226	2.1
50534	3.78452	2.1
50535	0.721957	0.346934
50536	0.721957	0.375982
50537	0.946103	0.332106
50538	0.946103	0.254183
50539	0.814237	0.26188
50540	0.721929	0.242417
50541	0.946132	0.4105
50542	0.946132	0.355399
50543	0.721928	0.369886
50544	0.946131	0.439397
50545	0.946132	0.571998
50546	0.814237	0.338512
50547	1.89226	1.23517
50548	1.89226	0.545683
50549	1.89226	0.582635
50550	1.89226	0.638592
50551	1.89226	1.20758
50552	1.89226	1.20848
50553	1.37196	0.334783
50554	2.83556	0.214325
50555	1.5165	0.283196
50556	1.5165	0.33864
50557	2.74923	0.2449
50558	2.74923	0.299428
50559	0.946128	0.500471
50560	0.946128	0.759885
50561	0.827866	0.320513
50562	0.946132	0.421608
50563	0.94613	0.304489
50564	0.94613	0.335275
50565	0.94613	0.792422
50566	0.94613	0.652146
50567	0.94613	0.569434
50568	0.827864	0.706654
50569	0.94613	0.537054
50570	0.94613	0.983047
50571	1.36208	0.247413
50572	2.74923	0.175366
50573	1.37196	0.212929

50574	1.37196	0.238334
50575	2.53256	0.193668
50576	2.53256	0.223129
50577	1.07705	2.1
50578	1.07705	2.1
50579	1.08253	2.1
50580	1.08253	2.1
50581	0.762985	2.1
50582	0.781638	2.1
50583	1.37794	2.1
50584	1.40756	2.1
50585	1.37794	2.1
50586	0.762986	2.1
50587	0.781638	2.1
50588	1.40756	2.1
50589	2.69541	2.1
50590	2.75587	2.1
50591	1.48572	2.1
50592	1.48572	2.1
50593	1.52597	2.1
50594	2.73566	2.1
50595	2.0554	1.68134
50596	1.00387	1.30133
50597	2.01001	1.43488
50598	0.994002	1.16857
50599	3.01389	1.02271
50600	1.89226	2.1
50601	2.41579	2.1
50602	2.12209	2.1
50603	2.12209	2.1
50604	1.89226	2.1
50605	1.89226	2.1
50606	1.89226	2.1
50607	1.89226	2.1
50608	1.89226	2.1
50609	1.89226	2.1
50610	1.89226	2.1
50611	1.89226	2.1
50612	0.88209	2.1
50613	0.899471	2.1
50614	0.651501	2.1
50615	0.533531	2.1
50616	0.724105	2.1
50617	0.711243	2.1
50618	0.692622	2.1
50619	0.692622	2.1
50620	0.890668	2.1
50621	0.738066	2.1
50622	0.778223	2.1
50623	0.531121	2.1
50624	1.89226	2.1
50625	2.77504	2.1
50626	2.4158	2.1
50627	2.4158	2.1
50628	1.89226	2.1
50629	1.89226	2.1
50630	1.89226	2.1
50631	1.89226	2.1

50632	1.89226	2.1
50633	1.89226	2.1
50634	1.89226	2.1
50635	1.89226	2.1
50636	1.70303	2.1
50637	1.39832	2.1
50638	1.51749	2.1
50639	1.68783	2.1
50640	3.24844	2.1
50641	1.89226	2.1
50642	2.38311	2.1
50643	1.98321	2.1
50644	1.89226	2.1
50645	2.47406	2.1
50646	2.38311	2.1
50647	1.89226	2.1
50648	1.89226	2.1
50649	1.89226	2.1
50650	1.89226	2.1
50651	1.89226	2.1
50652	1.89226	2.1
50653	0.946131	2.1
50654	0.946131	2.1
50655	1.25167	2.1
50656	1.19156	2.1
50657	1.19156	2.1
50658	1.19156	2.1
50659	0.946133	2.1
50660	0.946133	2.1
50661	0.946131	2.1
50662	1.25167	2.1
50663	0.946131	2.1
50664	1.19156	2.1
50665	1.89226	2.1
50666	2.47406	2.1
50667	1.99996	2.1
50668	1.89226	2.1
50669	2.58176	2.1
50670	2.47406	2.1
50671	1.89226	2.1
50672	1.89226	2.1
50673	1.89226	2.1
50674	1.89226	2.1
50675	1.89226	2.1
50676	1.89226	2.1
50677	0.946131	2.1
50678	0.946131	2.1
50679	1.32241	2.1
50680	1.25167	2.1
50681	1.25167	2.1
50682	1.25167	2.1
50683	0.946133	2.1
50684	0.946133	2.1
50685	0.946131	2.1
50686	1.32241	2.1
50687	0.946131	2.1
50688	1.25167	2.1
50689	1.65151	2.1

50690	1.92471	2.1
50691	1.81834	2.1
50692	2.13272	2.1
50693	3.74306	2.1
50694	1.92472	2.1
50695	1.9943	2.1
50696	2.13273	2.1
50697	2.24475	2.1
50698	4.16947	2.1
50699	1.81834	2.1
50700	2.13272	2.1
50701	2.02687	2.1
50702	2.39605	2.1
50703	4.21439	2.1
50704	2.13273	2.1
50705	2.24475	2.1
50706	2.39605	2.1
50707	2.54903	2.1
50708	4.6408	2.1
50709	1.10534	2.1
50710	1.22993	2.1
50711	0.946132	2.1
50712	0.946132	2.1
50713	0.946132	2.1
50714	0.946132	2.1
50715	1.22993	2.1
50716	1.22993	2.1
50717	1.10534	2.1
50718	0.946131	2.1
50719	0.946132	2.1
50720	1.22993	2.1
50721	1.47221	2.1
50722	2.14647	2.1
50723	1.95567	2.1
50724	1.95567	2.1
50725	1.71082	2.1
50726	1.71082	2.1
50727	0.946131	2.1
50728	0.946131	2.1
50729	0.946131	2.1
50730	0.946131	2.1
50731	0.946132	2.1
50732	0.946132	2.1
50733	0.946131	2.1
50734	0.946131	2.1
50735	0.946131	2.1
50736	0.946131	2.1
50737	0.946132	2.1
50738	0.946132	2.1
50739	0.998086	2.1
50740	1.09429	2.1
50741	0.946125	2.1
50742	0.946125	2.1
50743	0.90732	2.1
50744	0.90732	2.1
50745	1.14414	2.1
50746	1.16553	2.1
50747	1.05018	2.1

50748	0.907332	2.1
50749	0.99482	2.1
50750	0.851529	2.1
50751	0.80534	2.1
50752	0.80534	2.1
50753	0.812206	2.1
50754	0.812206	2.1
50755	0.895134	2.1
50756	0.977828	2.1
50757	0.736099	2.1
50758	0.639718	2.1
50759	0.639722	2.1
50760	0.89514	2.1
50761	0.736095	2.1
50762	0.977821	2.1
50763	0.946125	2.1
50764	0.946125	2.1
50765	0.946125	2.1
50766	0.946125	2.1
50767	0.946125	2.1
50768	0.946125	2.1
50769	0.946124	2.1
50770	0.946124	2.1
50771	0.946131	2.1
50772	0.946131	2.1
50773	0.946118	2.1
50774	0.946118	2.1
50775	2.22622	0.05
50776	2.43347	0.05
50777	2.34108	0.05
50778	1.24587	2.1
50779	1.21674	2.1
50780	1.14533	2.1
50781	1.11311	2.1
50782	1.17874	2.1
50783	1.17874	2.1
50784	1.18024	2.1
50785	1.18024	2.1
50786	1.14533	2.1
50787	1.24587	2.1
50788	1.21674	2.1
50789	1.11311	2.1
50790	2.43347	2.1
50791	2.34107	2.1
50792	2.39663	2.1
50793	2.49174	2.1
50794	4.8301	2.1
50795	1.86206	2.1
50796	1.41058	2.1
50797	1.70302	2.1
50798	2.42083	2.1
50799	3.68129	2.1
50800	1.70302	2.1
50801	2.43549	2.1
50802	2.20075	2.1
50803	2.50358	2.1
50804	2.04916	2.1
50805	2.33113	2.1

50806	1.07624	2.1
50807	2.12621	2.1
50808	1.57297	2.1
50809	1.60383	2.1
50810	1.30047	2.1
50811	1.30047	2.1
50812	2.33379	2.1
50813	2.56149	1.70515
50814	2.36734	2.09821
50815	2.62796	1.84017
50816	4.92883	2.04812
50817	2.5615	1.62187
50818	2.57957	1.67099
50819	2.62796	1.81542
50820	2.69056	1.38393
50821	5.25206	1.55514
50822	2.39322	2.1
50823	2.62796	2.1
50824	2.6849	2.1
50825	2.6849	2.09157
50826	2.36734	2.1
50827	2.36734	2.0313
50828	2.62796	2.1
50829	2.69056	1.73937
50830	2.6849	1.55969
50831	2.77227	1.35636
50832	5.37546	1.55781
50833	1.89226	0.05
50834	1.89226	0.05
50835	1.89226	0.05
50836	1.89226	0.05
50837	1.89226	0.05
50838	1.89226	0.05
50839	1.06974	0.05
50840	1.06974	0.05
50841	1.02299	0.05
50842	1.02299	0.05
50843	1.35299	0.05
50844	1.26227	0.05
50845	0.646252	0.05
50846	0.830464	0.05
50847	0.830463	0.05
50848	1.35299	0.05
50849	1.26227	0.05
50850	0.646252	0.05
50851	2.52454	0.05
50852	1.89226	0.05
50853	2.70598	0.05
50854	1.89226	0.05
50855	4.59824	0.05
50856	1.89226	0.05
50857	1.89226	0.05
50858	1.89226	0.05
50859	1.89226	0.05
50860	1.89226	0.05
50861	1.89226	0.05
50862	1.89226	0.05
50863	1.89226	0.05

50864	1.89226	0.05
50865	1.89226	0.05
50866	1.89226	0.05
50867	1.89226	0.05
50868	1.7666	0.05
50869	2.70598	0.05
50870	2.65378	0.05
50871	2.65378	0.05
50872	1.66093	0.05
50873	1.66093	0.05
50874	2.70598	0.05
50875	1.89226	0.05
50876	1.89226	0.05
50877	1.89226	0.05
50878	2.65377	0.05
50879	2.65377	0.05
50880	0.946132	0.05
50881	0.946132	0.05
50882	0.946131	0.05
50883	0.946131	0.05
50884	0.946131	0.05
50885	0.946131	0.05
50886	0.946132	0.05
50887	0.946132	0.05
50888	0.946132	0.05
50889	0.946132	0.05
50890	0.946131	0.05
50891	0.946131	0.05
50892	1.69858	2.1
50893	2.18238	2.1
50894	2.506	2.1
50895	1.93586	2.1
50896	4.20458	2.1
50897	2.21772	2.1
50898	2.18238	2.1
50899	2.506	2.1
50900	2.59215	2.1
50901	4.72372	2.1
50902	2.50596	2.1
50903	1.93584	2.1
50904	2.16728	2.1
50905	2.7679	2.1
50906	4.67325	2.1
50907	1.53296	2.1
50908	1.56299	2.1
50909	1.42624	2.1
50910	1.52961	2.1
50911	2.98923	2.1
50912	1.56299	2.1
50913	1.46568	2.1
50914	1.52961	2.1
50915	1.51916	2.1
50916	3.08215	2.1
50917	1.36226	2.1
50918	1.52961	2.1
50919	1.36226	2.1
50920	1.42624	2.1
50921	1.55369	2.1

50922	1.61768	2.1
50923	1.52961	2.1
50924	1.51916	2.1
50925	1.55369	2.1
50926	1.64892	2.1
50927	3.07285	2.1
50928	2.13405	0.0996204
50929	2.57507	0.099421
50930	2.39438	0.132856
50931	1.93408	0.0825857
50932	4.52843	0.101497
50933	2.13405	0.0914529
50934	1.27778	0.0634985
50935	1.2545	0.0760641
50936	1.45448	0.0737783
50937	1.93408	0.0734038
50938	2.13405	0.0732144
50939	2.39438	0.121484
50940	1.93407	0.0830319
50941	1.60578	0.097436
50942	1.87985	0.0950659
50943	4.00016	0.0972427
50944	1.0525	2.0785
50945	1.05758	2.07292
50946	1.43484	1.24816
50947	1.38506	1.70617
50948	2.48734	1.72869
50949	1.20455	2.1
50950	1.67352	2.1
50951	1.52852	2.1
50952	2.05164	2.1
50953	3.20203	2.1
50954	1.89226	2.1
50955	1.89226	2.1
50956	1.89226	2.1
50957	1.89226	2.1
50958	3.78452	2.1
50959	1.89226	2.1
50960	1.89226	2.1
50961	1.89226	2.1
50962	1.89226	2.1
50963	1.89226	2.1
50964	1.89226	2.1
50965	2.05165	2.1
50966	1.94291	2.1
50967	2.09398	2.1
50968	1.67352	2.1
50969	2.36337	2.1
50970	1.94291	2.1
50971	1.52851	2.1
50972	2.52049	2.1
50973	2.43433	2.1
50974	2.05164	2.1
50975	1.9112	2.1
50976	1.52851	2.1
50977	1.89226	2.1
50978	1.89226	2.1
50979	1.89226	2.1

50980	1.89226	2.1
50981	3.78451	2.1
50982	1.89226	2.1
50983	1.89226	2.1
50984	1.89226	2.1
50985	1.89226	2.1
50986	3.78452	2.1
50987	2.5205	2.1
50988	2.36337	2.1
50989	2.4605	2.1
50990	2.05165	2.1
50991	2.77222	2.1
50992	2.36337	2.1
50993	1.89226	2.1
50994	1.89226	2.1
50995	1.89226	2.1
50996	1.89226	2.1
50997	1.89226	2.1
50998	1.89226	2.1
50999	1.89226	2.1
51000	1.89226	2.1
51001	1.89226	2.1
51002	1.89226	2.1
51003	3.78452	2.1
51004	1.38905	2.1
51005	1.26166	2.1
51006	1.19216	2.1
51007	1.0875	2.1
51008	1.24393	2.1
51009	1.24393	2.1
51010	1.23261	2.1
51011	1.23261	2.1
51012	1.38905	2.1
51013	1.19216	2.1
51014	1.26166	2.1
51015	1.0875	2.1
51016	1.16233	2.1
51017	1.06028	2.1
51018	0.777395	2.1
51019	0.705508	2.1
51020	0.940018	2.1
51021	0.936465	2.1
51022	0.954216	2.1
51023	0.954216	2.1
51024	1.16125	2.1
51025	0.685505	2.1
51026	1.1157	2.1
51027	0.703534	2.1
51028	1.89226	2.1
51029	1.89226	2.1
51030	1.89226	2.1
51031	1.89226	2.1
51032	1.89226	2.1
51033	1.89226	2.1
51034	1.89226	2.1
51035	1.89226	2.1
51036	1.89226	2.1
51037	1.89226	2.1

51038	1.89226	2.1
51039	1.89226	2.1
51040	2.38433	2.1
51041	1.43456	2.1
51042	1.93881	2.1
51043	2.36952	2.1
51044	4.32313	2.1
51045	1.8493	2.1
51046	1.5927	2.1
51047	1.45535	2.1
51048	1.38438	2.1
51049	1.89657	2.1
51050	1.8493	2.1
51051	2.0815	2.1
51052	1.6697	2.1
51053	2.0657	2.1
51054	1.5927	2.1
51055	2.59749	2.1
51056	2.0815	2.1
51057	2.76597	2.1
51058	1.89226	2.1
51059	1.89226	2.1
51060	1.89226	2.1
51061	2.6126	2.1
51062	2.6126	2.1
51063	2.62513	2.1
51064	1.89226	2.1
51065	1.89226	2.1
51066	1.89226	2.1
51067	2.76598	2.1
51068	2.76598	2.1
51069	2.54665	2.1
51070	2.6126	2.1
51071	2.76598	2.1
51072	2.76598	2.1
51073	2.34044	2.1
51074	2.34044	2.1
51075	2.62514	2.1
51076	2.76598	2.1
51077	2.54666	2.1
51078	2.45212	2.1
51079	5.2181	2.1
51080	2.14063	2.1
51081	1.47521	2.1
51082	1.27679	2.1
51083	1.47521	2.1
51084	2.11511	2.1
51085	2.31353	2.1
51086	0.93256	2.1
51087	1.07032	2.1
51088	0.638396	2.1
51089	0.521342	2.1
51090	0.790674	2.1
51091	0.790674	2.1
51092	0.80098	2.1
51093	0.800981	2.1
51094	0.932535	2.1
51095	0.521328	2.1

51096	1.07034	2.1
51097	0.638414	2.1
51098	1.89226	2.1
51099	1.89226	2.02996
51100	1.89226	2.1
51101	1.89226	2.1
51102	3.78453	2.1
51103	1.89226	2.1
51104	1.9842	2.1
51105	1.89226	2.1
51106	1.89226	2.1
51107	2.79705	2.1
51108	2.79705	2.1
51109	0.469955	2.1
51110	0.791665	2.1
51111	0.989115	2.08317
51112	0.763235	2.1
51113	0.776356	2.08242
51114	0.805461	2.1
51115	0.776759	2.1
51116	0.776759	2.1
51117	0.799151	2.1
51118	0.940869	2.1
51119	0.78449	2.1
51120	0.489635	2.1
51121	1.89226	2.03374
51122	1.89226	2.09903
51123	1.89226	1.93642
51124	1.89226	2.1
51125	1.89226	1.9318
51126	1.89226	2.1
51127	2.46058	1.87901
51128	2.28991	2.1
51129	1.8525	2.05742
51130	1.87697	2.1
51131	2.28991	2.01582
51132	1.62397	2.09429
51133	0.946131	0.05
51134	0.974033	0.05
51135	1.31667	0.05
51136	0.946131	0.05
51137	0.885497	0.05
51138	0.885497	0.05
51139	1.0468	0.05
51140	1.04678	0.05
51141	1.31667	0.05
51142	0.974019	0.05
51143	0.946131	0.05
51144	0.946131	0.05
51145	1.89226	0.05
51146	1.89226	0.075829
51147	1.89226	0.0622769
51148	1.89226	0.0526467
51149	3.78452	0.0765365
51150	0.946125	0.0806521
51151	0.946125	0.074035
51152	0.946131	0.0865357
51153	0.946131	0.0932184

51154	0.946128	0.093482
51155	0.946128	0.0844025
51156	0.946128	0.0529832
51157	0.946128	0.0739896
51158	0.946128	0.0868945
51159	0.946128	0.0705023
51160	0.946128	0.0769481
51161	0.946128	0.0785493
51162	0.946131	0.05
51163	0.98453	0.05
51164	1.32276	0.05
51165	0.946131	0.05
51166	0.946132	0.05
51167	0.946132	0.05
51168	0.98453	0.05
51169	1.32276	0.05
51170	1.32276	0.05
51171	1.32276	0.05
51172	0.946132	0.05
51173	0.946132	0.05
51174	1.89227	2.1
51175	1.89227	2.1
51176	1.89227	2.1
51177	1.89227	2.1
51178	1.89227	2.1
51179	1.89227	2.1
51180	1.89226	2.1
51181	1.89226	2.1
51182	1.89226	2.1
51183	1.89226	2.1
51184	1.89226	2.1
51185	1.89226	2.1
51186	1.05556	2.1
51187	1.38859	2.1
51188	1.46996	2.1
51189	1.25364	2.1
51190	2.52552	2.1
51191	0.694296	2.1
51192	0.73498	2.1
51193	0.561881	2.1
51194	0.512189	2.1
51195	0.621304	2.1
51196	0.621304	2.1
51197	0.625865	2.1
51198	0.625865	2.1
51199	0.734979	2.1
51200	0.561881	2.1
51201	0.51219	2.1
51202	0.694296	2.1
51203	0.946126	2.1
51204	0.946126	2.1
51205	0.946126	2.1
51206	0.946126	2.1
51207	0.946126	2.1
51208	0.946126	2.1
51209	0.946126	2.1
51210	0.946126	2.1
51211	0.946132	2.1

51212	0.946132	2.1
51213	0.94612	2.1
51214	0.94612	2.1
51215	1.89225	2.1
51216	1.89225	2.1
51217	1.89225	2.1
51218	1.89225	2.1
51219	1.89225	2.1
51220	1.89225	2.1
51221	0.626814	2.1
51222	0.632155	2.1
51223	0.746234	2.1
51224	0.734974	2.1
51225	0.68202	2.1
51226	0.68202	2.1
51227	0.685108	2.1
51228	0.685108	2.1
51229	0.746239	2.1
51230	0.632159	2.1
51231	0.734969	2.1
51232	0.62681	2.1
51233	0.746235	2.1
51234	0.734975	2.1
51235	0.595081	2.1
51236	0.561877	2.1
51237	0.648584	2.1
51238	0.648584	2.1
51239	0.659528	2.1
51240	0.659528	2.1
51241	0.746239	2.1
51242	0.595085	2.1
51243	0.734969	2.1
51244	0.561874	2.1
51245	1.89249	2.1
51246	2.2137	2.1
51247	1.33125	2.1
51248	1.06499	2.1
51249	2.12571	2.1
51250	1.78918	2.1
51251	0.946131	2.1
51252	0.946131	2.1
51253	0.94613	2.1
51254	0.94613	2.1
51255	0.946132	2.1
51256	0.946132	2.1
51257	0.946129	2.1
51258	0.946129	2.1
51259	0.946131	2.1
51260	0.946131	2.1
51261	0.946131	2.1
51262	0.946131	2.1
51263	1.89227	2.1
51264	1.89227	2.1
51265	1.89227	2.1
51266	1.89227	2.1
51267	3.78453	2.1
51268	1.89226	2.1
51269	1.89226	2.1

51270	1.89226	2.1
51271	1.89226	2.1
51272	1.89226	2.1
51273	1.89226	2.1
51274	2.01388	2.1
51275	1.38757	2.1
51276	1.24229	2.1
51277	1.3309	2.1
51278	1.88011	2.1
51279	2.01388	2.1
51280	2.01388	2.1
51281	2.01555	2.1
51282	2.13879	2.1
51283	1.80766	2.1
51284	4.15267	2.1
51285	0.946133	2.1
51286	0.946133	2.1
51287	0.946132	2.1
51288	0.946132	2.1
51289	0.946132	2.1
51290	0.946132	2.1
51291	0.946133	2.1
51292	0.946133	2.1
51293	0.946132	2.1
51294	0.946132	2.1
51295	0.946132	2.1
51296	0.946132	2.1
51297	1.89226	2.1
51298	1.89226	2.1
51299	1.89226	2.1
51300	1.89226	2.1
51301	1.89226	2.1
51302	1.89226	2.1
51303	0.676745	2.1
51304	0.68382	2.1
51305	1.07048	2.1
51306	1.1327	2.1
51307	0.929324	2.1
51308	0.871438	2.1
51309	0.867362	2.1
51310	0.867362	2.1
51311	1.10666	2.1
51312	0.695036	2.1
51313	0.744098	2.1
51314	1.10144	2.1
51315	2.25168	2.1
51316	2.22499	2.1
51317	2.15929	2.1
51318	2.13879	2.1
51319	2.15929	2.1
51320	2.13879	2.1
51321	2.15314	2.1
51322	1.70302	2.1
51323	2.24694	2.1
51324	1.65245	2.1
51325	3.82722	2.1
51326	1.65245	2.1
51327	1.00371	2.1

51328	1.7437	2.1
51329	1.65748	2.1
51330	1.10459	2.1
51331	1.00371	2.1
51332	2.24694	2.1
51333	1.65246	2.1
51334	2.69773	2.1
51335	1.88129	2.1
51336	4.12824	2.1
51337	1.65246	2.1
51338	1.10459	2.1
51339	1.88129	2.1
51340	1.05914	2.1
51341	2.76496	2.1
51342	1.89226	2.1
51343	1.89226	2.1
51344	1.89226	2.1
51345	1.89226	2.1
51346	3.78452	2.1
51347	0.718577	2.1
51348	0.718577	2.1
51349	0.725249	2.1
51350	0.725249	2.1
51351	0.597916	2.1
51352	0.61752	2.1
51353	0.826306	2.1
51354	0.859194	2.1
51355	0.826306	2.1
51356	0.597916	2.1
51357	0.859194	2.1
51358	0.61752	2.1
51359	1.25898	2.1
51360	1.25898	2.1
51361	0.946128	2.1
51362	0.946128	2.1
51363	1.25897	2.1
51364	1.25585	2.1
51365	0.946131	2.1
51366	0.946131	2.1
51367	0.946129	2.1
51368	1.25586	2.1
51369	1.25898	2.1
51370	0.946129	2.1
51371	1.89226	2.1
51372	1.89226	2.1
51373	1.89226	2.1
51374	1.89226	2.1
51375	3.78452	2.1
51376	0.693258	2.1
51377	0.693258	2.1
51378	0.697356	2.1
51379	0.697356	2.1
51380	0.572479	2.1
51381	0.597916	2.1
51382	0.792698	2.1
51383	0.826306	2.1
51384	0.572478	2.1
51385	0.792697	2.1

51386	0.826307	2.1
51387	0.597917	2.1
51388	1.25586	2.1
51389	1.25586	2.1
51390	0.946128	2.1
51391	0.946128	2.1
51392	1.25585	2.1
51393	1.24344	2.1
51394	0.946131	2.1
51395	0.946131	2.1
51396	0.946129	2.1
51397	1.24344	2.1
51398	0.94613	2.1
51399	1.25586	2.1
51400	1.65151	2.1
51401	1.81835	2.1
51402	1.12064	2.1
51403	1.12064	2.1
51404	1.24965	2.1
51405	1.81835	2.1
51406	1.24965	2.1
51407	1.81835	2.1
51408	1.41077	2.1
51409	2.02687	2.1
51410	3.22911	2.1
51411	1.89122	1.89031
51412	1.38776	2.1
51413	2.37014	1.15735
51414	1.80818	2.1
51415	3.6994	2.1
51416	2.15466	1.59103
51417	2.00083	2.1
51418	1.80818	2.07165
51419	1.80818	1.79074
51420	2.31988	2.08703
51421	2.31988	1.63599
51422	1.80818	2.04647
51423	1.18041	2.1
51424	2.00082	2.1
51425	1.43652	2.1
51426	3.18123	2.1
51427	1.89226	1.50759
51428	1.89226	1.5485
51429	1.89226	1.51888
51430	1.89226	1.47555
51431	3.78453	1.47776
51432	1.89226	1.61268
51433	1.89226	1.61827
51434	1.89226	1.5841
51435	1.89226	1.50075
51436	3.78452	1.66264
51437	0.80759	1.53168
51438	0.686119	1.57721
51439	0.972147	1.62897
51440	1.00217	1.60631
51441	0.821334	1.77488
51442	0.821334	1.56315
51443	0.866957	1.60041

51444	0.866957	1.53115
51445	0.972145	1.64123
51446	0.686121	2.02558
51447	0.807592	1.68547
51448	1.00217	2.00136
51449	0.972146	1.5665
51450	1.00217	1.53402
51451	1.26654	1.60655
51452	1.1908	1.59843
51453	1.05501	1.57984
51454	1.05501	1.7007
51455	1.10794	1.5498
51456	1.10794	1.46828
51457	0.972146	1.41649
51458	1.26654	1.55001
51459	1.00217	1.5597
51460	1.1908	1.67455
51461	1.89226	1.54845
51462	1.89226	1.49968
51463	1.89226	1.52689
51464	1.89226	1.6349
51465	1.89226	1.45142
51466	1.89226	1.52808
51467	0.972147	1.58887
51468	0.867854	1.66888
51469	1.26132	1.55743
51470	1.26654	1.56187
51471	1.0424	1.68642
51472	1.0424	1.67543
51473	1.092	1.49206
51474	1.092	1.61136
51475	1.26132	1.62692
51476	0.867854	2.1
51477	0.972147	1.7182
51478	1.26654	2.03504
51479	1.4375	0.0927274
51480	1.92998	0.0732312
51481	1.66953	0.05
51482	1.0953	0.0607309
51483	3.10703	0.0594612
51484	1.89226	2.1
51485	1.89226	2.1
51486	1.89226	2.1
51487	1.89226	2.1
51488	3.78452	2.1
51489	2.74034	2.1
51490	2.08808	2.1
51491	2.63859	2.1
51492	2.00343	2.1
51493	4.72667	2.1
51494	0.799825	2.1
51495	0.799825	2.1
51496	0.80211	2.1
51497	0.80211	2.1
51498	0.562428	2.1
51499	0.600223	2.1
51500	1.04404	2.1
51501	1.00171	2.1

51502	1.00171	2.1
51503	0.56243	2.1
51504	0.600225	2.1
51505	1.04404	2.1
51506	0.94613	2.1
51507	0.94613	2.1
51508	0.946128	2.1
51509	0.946128	2.1
51510	0.946127	2.1
51511	0.946127	2.1
51512	0.946131	2.1
51513	0.946131	2.1
51514	0.946129	2.1
51515	0.946129	2.1
51516	0.946129	2.1
51517	0.946129	2.1
51518	1.89226	2.1
51519	1.89226	2.1
51520	1.89226	2.1
51521	1.89226	2.1
51522	3.78452	2.1
51523	2.63859	2.1
51524	2.00343	2.1
51525	2.54388	2.1
51526	1.91548	2.1
51527	4.55407	2.1
51528	1.91548	2.1
51529	2.00342	2.1
51530	1.04909	2.1
51531	1.04909	2.1
51532	1.12486	2.1
51533	1.99124	2.1
51534	0.810942	2.1
51535	0.810942	2.1
51536	0.810942	2.1
51537	0.810942	2.1
51538	1.89226	2.1
51539	1.35169	2.1
51540	1.08131	2.1
51541	1.08132	2.1
51542	1.08132	2.1
51543	0.810944	2.1
51544	0.810944	2.1
51545	1.89226	0.629345
51546	1.89226	2.1
51547	1.89226	0.743366
51548	1.89226	2.1
51549	3.78452	1.5925
51550	1.7968	0.594623
51551	1.49831	2.1
51552	1.59244	0.615285
51553	1.3395	2.1
51554	3.09074	1.82115
51555	1.49831	2.1
51556	1.16858	2.1
51557	1.33951	2.1
51558	1.0361	2.1
51559	2.53442	2.1

51560	1.89226	2.1
51561	1.89226	2.1
51562	1.89226	2.1
51563	1.89226	2.1
51564	1.89226	2.1
51565	1.89226	2.1
51566	1.89223	0.579304
51567	1.89223	2.1
51568	1.89223	1.53133
51569	1.89223	2.1
51570	1.89223	0.670196
51571	1.89223	2.1
51572	1.59241	0.457641
51573	1.18952	2.1
51574	1.33949	2.1
51575	1.33949	0.951491
51576	1.40538	2.1
51577	1.40538	0.550713
51578	1.89226	0.0846748
51579	1.89226	0.104699
51580	1.89226	0.0975927
51581	1.89226	0.116899
51582	1.89226	0.129692
51583	1.89226	0.130482
51584	1.89226	2.1
51585	1.89226	2.1
51586	1.89226	2.1
51587	1.89226	2.1
51588	3.78452	2.1
51589	1.89226	2.1
51590	1.89226	2.1
51591	1.89226	2.1
51592	1.89226	2.1
51593	3.78452	2.1
51594	2.01867	2.1
51595	1.46955	2.1
51596	1.58476	2.1
51597	1.38189	2.1
51598	2.22153	2.1
51599	2.01867	2.1
51600	2.01867	2.1
51601	2.52881	2.1
51602	2.21732	2.1
51603	2.21732	2.1
51604	2.22154	2.1
51605	2.22154	2.1
51606	1.89226	2.1
51607	1.89226	2.1
51608	1.89226	2.1
51609	1.89226	2.1
51610	1.89226	2.1
51611	1.89226	2.1
51612	1.89226	2.1
51613	1.89226	2.1
51614	1.89226	2.1
51615	1.89226	2.1
51616	1.89226	2.1
51617	1.89226	2.1

51618	2.22153	2.1
51619	1.63361	2.1
51620	1.77336	2.1
51621	1.46955	2.1
51622	2.52534	2.1
51623	2.22153	2.1
51624	2.34431	2.1
51625	1.62905	2.1
51626	1.49693	2.1
51627	2.19197	2.1
51628	3.82103	2.1
51629	1.89226	2.1
51630	1.89226	2.1
51631	1.89226	2.1
51632	1.89226	2.1
51633	3.78453	2.1
51634	0.946128	2.1
51635	0.946128	2.1
51636	1.22372	2.1
51637	1.17331	2.1
51638	0.946131	2.1
51639	0.946131	2.1
51640	1.17331	2.1
51641	1.17331	2.1
51642	0.946131	2.1
51643	1.17331	2.1
51644	1.22372	2.1
51645	0.94613	2.1
51646	1.04041	2.1
51647	1.09599	2.1
51648	0.748462	2.1
51649	0.701976	2.1
51650	0.896701	2.1
51651	0.896701	2.1
51652	0.901262	2.1
51653	0.901262	2.1
51654	1.04041	2.1
51655	0.701977	2.1
51656	0.748463	2.1
51657	1.09599	2.1
51658	1.89226	2.1
51659	1.89226	2.1
51660	1.89226	2.1
51661	1.89226	2.1
51662	3.78453	2.1
51663	0.946128	2.1
51664	0.946128	2.1
51665	1.17331	2.1
51666	1.13785	2.1
51667	0.94613	2.1
51668	0.94613	2.1
51669	1.13785	2.1
51670	1.13785	2.1
51671	1.13785	2.1
51672	0.94613	2.1
51673	0.94613	2.1
51674	1.17331	2.1
51675	2.50381	2.1

51676	1.89226	2.1
51677	1.89226	2.1
51678	2.73387	2.1
51679	4.39607	2.1
51680	1.80045	2.1
51681	1.99777	2.0502
51682	2.45134	2.1
51683	2.12463	2.1
51684	1.70302	2.1
51685	1.42655	2.1
51686	2.20231	2.1
51687	1.70302	2.1
51688	1.93128	2.1
51689	1.66768	2.1
51690	1.24499	2.00149
51691	1.70302	1.98206
51692	2.55572	0.0814776
51693	1.22811	0.113108
51694	1.82584	0.0954137
51695	1.22811	0.0802465
51696	2.62764	0.105763
51697	1.93147	0.0919511
51698	2.14513	0.131588
51699	1.89226	0.252822
51700	2.66481	0.135255
51701	1.89226	0.160328
51702	2.66481	0.262535
51703	1.89226	0.288807
51704	2.11649	2.1
51705	1.89226	2.1
51706	1.89226	2.1
51707	1.89226	2.1
51708	2.15426	2.1
51709	2.15426	2.1
51710	2.06776	2.1
51711	1.89226	2.1
51712	1.89226	2.1
51713	1.89226	2.1
51714	2.11649	2.1
51715	2.11649	2.1
51716	1.89226	2.1
51717	1.89226	2.1
51718	1.89226	2.1
51719	1.89226	2.1
51720	1.89226	2.1
51721	1.89226	2.1
51722	0.998134	2.1
51723	0.918904	2.1
51724	0.894708	2.1
51725	0.758742	2.1
51726	0.864276	2.1
51727	0.864276	2.1
51728	0.8926	2.1
51729	0.8926	2.1
51730	0.998134	2.1
51731	0.894709	2.1
51732	0.758742	2.1
51733	0.918904	2.1

51734	1.89226	2.1
51735	1.89226	2.1
51736	1.89226	2.1
51737	1.89226	2.1
51738	1.89226	2.1
51739	1.89226	2.1
51740	1.89226	2.1
51741	1.89226	2.1
51742	1.89226	2.1
51743	1.89226	2.1
51744	1.89226	2.1
51745	1.89226	2.1
51746	1.99627	2.1
51747	1.61047	2.1
51748	2.0815	2.1
51749	2.07569	2.1
51750	3.75063	2.1
51751	0.848077	2.1
51752	1.07748	2.1
51753	0.802498	2.1
51754	0.626808	2.1
51755	0.875552	2.1
51756	0.737527	2.1
51757	0.835602	2.1
51758	0.835602	2.1
51759	0.983245	2.1
51760	0.781108	2.1
51761	0.872306	2.1
51762	0.601497	2.1
51763	2.74745	2.1
51764	2.73988	2.1
51765	2.6521	2.1
51766	2.67792	2.1
51767	5.39198	2.1
51768	2.73988	2.1
51769	2.57466	2.1
51770	2.67791	2.1
51771	2.55653	2.1
51772	5.29641	2.1
51773	2.6521	2.1
51774	2.64148	2.1
51775	2.67792	2.1
51776	2.67792	2.1
51777	2.56953	2.1
51778	2.56953	2.1
51779	2.64148	2.1
51780	2.55653	2.1
51781	2.57929	2.1
51782	2.57929	2.1
51783	2.67792	2.1
51784	2.67792	2.1
51785	1.89226	1.46931
51786	1.89226	0.949887
51787	1.89226	1.93565
51788	1.89226	0.288226
51789	3.78453	0.719253
51790	1.68703	1.51959
51791	2.50385	0.227908

51792	1.45958	0.609434
51793	2.0815	0.158512
51794	3.93806	0.352209
51795	2.50385	0.168812
51796	2.57414	0.427059
51797	2.0815	0.263083
51798	2.39921	1.21545
51799	4.91181	0.241934
51800	1.89226	1.21322
51801	1.89226	0.509799
51802	1.89226	0.23871
51803	1.89226	0.212704
51804	3.78453	0.332157
51805	1.89226	1.9435
51806	1.89226	0.209406
51807	1.89226	1.14425
51808	1.89226	0.90035
51809	1.89226	0.471114
51810	1.89226	1.48925
51811	1.23013	0.31397
51812	2.0815	0.120804
51813	1.90167	0.648687
51814	1.71243	0.359317
51815	1.45887	0.500377
51816	1.31369	0.203562
51817	2.0815	0.218452
51818	2.39921	1.31515
51819	1.7115	0.288638
51820	2.2421	0.434155
51821	4.09829	0.343792
51822	1.89227	0.141282
51823	1.89227	0.262283
51824	1.89227	0.496657
51825	1.89227	0.677205
51826	3.78453	0.0895938
51827	2.39697	0.05
51828	1.85594	0.05
51829	1.91161	0.05
51830	2.49867	0.05
51831	4.35461	0.05
51832	1.12035	0.05
51833	1.85594	0.05
51834	1.91161	0.05
51835	1.15483	0.05
51836	3.01077	0.05
51837	1.89226	0.05
51838	1.89226	0.05
51839	1.89226	0.05
51840	1.89226	0.05
51841	1.89226	0.05
51842	1.89226	0.05
51843	1.89226	0.05
51844	1.89226	0.05
51845	1.89226	0.05
51846	1.89226	0.05
51847	1.89226	0.05
51848	1.89226	0.05
51849	0.936516	0.05

51850	1.03496	0.05
51851	1.22607	0.05
51852	1.28058	0.05
51853	1.06317	0.05
51854	1.06317	0.05
51855	1.19735	0.05
51856	1.0813	0.05
51857	0.858168	0.05
51858	1.32278	0.05
51859	0.957674	0.05
51860	1.25178	0.05
51861	1.92659	0.05
51862	0.998957	0.05
51863	0.998957	0.05
51864	1.15481	0.05
51865	1.6031	0.05
51866	1.74459	0.05
51867	0.946119	0.05
51868	0.946119	0.05
51869	0.946118	0.05
51870	0.946118	0.05
51871	0.946118	0.05
51872	0.946118	0.05
51873	0.946119	0.05
51874	0.946119	0.05
51875	0.946131	0.05
51876	0.946131	0.05
51877	0.946106	0.05
51878	0.946106	0.05
51879	0.946118	0.05
51880	0.946118	0.05
51881	0.946119	0.05
51882	0.946119	0.05
51883	0.946118	0.05
51884	0.946118	0.05
51885	0.946119	0.05
51886	0.946119	0.05
51887	0.946131	0.05
51888	0.946131	0.05
51889	0.946106	0.05
51890	0.946106	0.05
51891	2.47164	0.443935
51892	2.83689	0.206258
51893	2.73872	0.472421
51894	2.41632	0.21355
51895	5.21036	0.223639
51896	0.946133	0.36034
51897	0.946133	0.48452
51898	1.41845	0.248446
51899	1.36936	0.400346
51900	1.41845	0.214528
51901	1.41845	0.210108
51902	0.946132	0.482177
51903	0.946132	0.51898
51904	0.946132	0.383148
51905	1.36936	0.248518
51906	1.41845	0.274165
51907	0.946133	0.429357

51908	1.36936	0.156819
51909	1.27581	0.260358
51910	1.20816	0.20018
51911	1.10464	0.397579
51912	1.24454	0.172259
51913	1.24454	0.172073
51914	1.23943	0.293471
51915	1.23943	0.339987
51916	1.10465	0.260803
51917	1.27581	0.23489
51918	1.36936	0.256383
51919	1.20816	0.212181
51920	0.946131	2.1
51921	0.946131	2.1
51922	1.37277	2.1
51923	1.37277	2.1
51924	1.37277	2.1
51925	1.27186	2.1
51926	0.946131	2.1
51927	0.946131	2.1
51928	0.946131	2.1
51929	1.37277	2.1
51930	0.946131	2.1
51931	1.27186	2.1
51932	2.74554	2.1
51933	1.23712	2.1
51934	2.80992	2.1
51935	2.54371	2.1
51936	1.50333	2.1
51937	1.23712	2.1
51938	2.77314	2.1
51939	2.33316	2.1
51940	2.28748	2.1
51941	2.28748	2.1
51942	2.81275	2.1
51943	2.81275	2.1
51944	2.28749	2.1
51945	1.45887	2.1
51946	2.33317	2.1
51947	1.50022	2.1
51948	3.79204	2.1
51949	2.37364	2.1
51950	2.33317	2.1
51951	1.5362	2.1
51952	1.5362	2.1
51953	1.50022	2.1
51954	2.33317	2.1
51955	1.89226	0.578567
51956	1.89226	0.727833
51957	1.89226	0.517324
51958	1.89226	0.701957
51959	1.89226	0.530087
51960	1.89226	0.779009
51961	0.946128	0.312313
51962	0.946128	0.352737
51963	1.0644	0.253751
51964	0.946133	0.304528
51965	1.0644	0.245311

51966	0.94613	0.277338
51967	0.946131	0.377523
51968	0.946131	0.291891
51969	1.0644	0.294964
51970	1.0644	0.303145
51971	0.946131	0.310366
51972	0.946131	0.289441
51973	1.89226	2.1
51974	1.89226	1.24021
51975	1.89226	2.07972
51976	1.89226	0.22072
51977	3.78452	0.801024
51978	1.14599	2.1
51979	1.19993	0.720961
51980	1.00065	2.0042
51981	1.07497	0.203159
51982	2.22096	1.80677
51983	0.546551	1.28817
51984	0.546551	0.648273
51985	0.54987	0.513921
51986	0.54987	1.72371
51987	0.503067	0.444387
51988	0.558937	0.466215
51989	0.599966	0.124908
51990	0.537484	0.241935
51991	0.503067	0.146337
51992	0.537485	0.157838
51993	0.599966	0.267577
51994	0.558936	0.241321
51995	0.946132	0.434218
51996	0.946132	1.56265
51997	0.94613	0.225739
51998	0.94613	1.54849
51999	0.946131	0.450494
52000	0.946131	0.513084
52001	0.946131	0.206884
52002	0.946131	0.168081
52003	0.946131	0.275617
52004	0.946131	0.110826
52005	0.946131	0.445655
52006	0.946131	0.643481
52007	2.19104	2.1
52008	2.5392	2.1
52009	2.53302	2.1
52010	2.19226	2.1
52011	4.73146	2.1
52012	1.09552	2.1
52013	1.09613	2.1
52014	0.802484	2.1
52015	0.807941	2.1
52016	0.95052	2.1
52017	0.95052	2.1
52018	0.952944	2.1
52019	0.952944	2.1
52020	1.09613	2.1
52021	0.807941	2.1
52022	0.802484	2.1
52023	1.09552	2.1

52024	2.533	2.1
52025	2.18531	2.1
52026	2.19224	2.1
52027	2.19224	2.1
52028	2.51603	2.1
52029	2.51603	2.1
52030	1.09266	2.1
52031	1.09612	2.1
52032	0.807935	2.1
52033	0.81091	2.1
52034	0.951897	2.1
52035	0.951897	2.1
52036	0.955134	2.1
52037	0.955134	2.1
52038	0.810915	2.1
52039	1.09266	2.1
52040	1.09611	2.1
52041	0.80793	2.1
52042	2.12741	0.111107
52043	2.79312	0.256883
52044	2.77591	1.2286
52045	2.10714	0.265635
52046	4.90026	0.173442
52047	2.12741	0.166854
52048	1.20859	0.173659
52049	1.21073	1.25732
52050	2.10714	0.678088
52051	3.31573	0.798443
52052	1.38708	0.652902
52053	1.38795	0.332179
52054	1.05331	0.176609
52055	1.05357	0.176746
52056	1.22049	0.267811
52057	1.22049	0.902686
52058	1.22078	1.37314
52059	1.22078	0.116054
52060	1.05331	0.303086
52061	1.38709	0.33627
52062	1.38795	0.184545
52063	1.05357	0.21703
52064	2.10662	0.973981
52065	2.10714	0.14502
52066	2.12499	0.114584
52067	1.22857	1.02943
52068	1.21073	0.313863
52069	1.21073	0.152846
52070	1.82014	0.05
52071	2.08757	0.05
52072	1.81292	0.05
52073	1.67485	0.05
52074	3.63306	0.05
52075	1.82013	0.05
52076	1.21748	0.05
52077	1.29891	0.05
52078	1.4442	0.05
52079	1.67484	0.05
52080	1.82013	0.05
52081	1.81292	0.05

52082	1.4516	0.05
52083	1.39633	0.05
52084	1.67485	0.05
52085	1.5344	0.05
52086	1.81292	0.05
52087	1.67484	0.05
52088	1.00982	0.05
52089	0.994235	0.05
52090	1.21748	0.05
52091	1.45159	0.05
52092	1.67484	0.05
52093	1.55216	2.1
52094	1.34407	2.1
52095	1.34407	2.1
52096	1.52008	2.1
52097	1.81783	2.1
52098	1.99384	2.1
52099	1.90817	2.1
52100	2.03975	2.1
52101	1.97317	2.1
52102	1.81783	2.1
52103	3.85758	2.1
52104	0.599049	2.1
52105	0.660673	2.1
52106	0.785063	2.1
52107	0.924278	2.1
52108	0.760416	2.1
52109	0.760416	2.1
52110	0.719088	2.1
52111	0.694532	2.1
52112	0.522441	2.1
52113	0.796789	2.1
52114	0.655207	2.1
52115	0.886155	2.1
52116	1.97317	2.1
52117	1.81783	2.1
52118	1.65098	2.1
52119	1.91767	2.1
52120	3.7355	2.1
52121	1.15038	1.98847
52122	1.28649	1.89684
52123	1.38986	2.07031
52124	1.38986	1.95461
52125	1.07866	2.0591
52126	1.07866	2.06523
52127	0.664498	1.26848
52128	0.664498	2.1
52129	0.675122	2.1
52130	0.675122	2.1
52131	0.69493	1.53336
52132	0.764429	1.82076
52133	0.623438	2.1
52134	0.575191	2.1
52135	0.764429	1.81936
52136	0.623438	1.70789
52137	0.575191	2.05649
52138	0.69493	2.03557
52139	1.89226	1.40406

52140	1.89226	0.375484
52141	1.89226	1.83893
52142	1.89226	0.715213
52143	3.78453	0.316349
52144	2.50075	1.89124
52145	2.15276	0.57945
52146	2.24902	1.83557
52147	2.30422	0.557025
52148	4.40178	0.169488
52149	1.70424	2.1
52150	1.34494	2.1
52151	2.15276	1.12676
52152	2.15276	2.1
52153	2.0793	0.92215
52154	1.63078	1.77152
52155	1.89226	0.85215
52156	1.89226	2.1
52157	1.89226	2.1
52158	1.89226	0.431672
52159	1.89226	2.1
52160	1.89226	1.7686
52161	1.89226	1.80426
52162	1.89226	0.832862
52163	1.89226	0.989496
52164	1.89226	0.30755
52165	3.78452	0.231902
52166	2.24902	1.65227
52167	1.91497	0.566366
52168	1.97135	2.1
52169	1.73957	0.408816
52170	3.98859	0.532895
52171	1.73957	0.40145
52172	1.34494	2.1
52173	1.73957	2.1
52174	1.69006	0.460697
52175	1.3126	2.1
52176	1.34848	1.599
52177	0.946132	0.468183
52178	0.946132	2.1
52179	0.946132	1.62164
52180	0.946132	2.1
52181	0.946132	2.1
52182	0.946132	2.1
52183	0.946131	0.280646
52184	0.946131	0.113197
52185	0.946132	0.864488
52186	0.946132	1.73667
52187	0.946132	1.12879
52188	0.946132	1.86227
52189	1.89226	2.1
52190	1.89226	2.05296
52191	1.89226	2.1
52192	1.89226	1.9949
52193	3.78453	2.1
52194	1.89226	2.04975
52195	2.82624	1.88369
52196	1.89226	2.07181
52197	2.02536	1.85789

52198	1.89226	2.06317
52199	2.02536	1.83222
52200	2.25027	0.05
52201	1.1706	0.05
52202	2.02733	0.05
52203	2.02733	0.05
52204	1.61462	0.05
52205	1.69832	0.05
52206	1.89226	0.05
52207	1.89226	0.05
52208	1.89226	0.05
52209	1.89226	0.05
52210	3.78452	0.05
52211	0.946131	0.05
52212	0.946131	0.05
52213	1.18182	0.05
52214	1.18182	0.05
52215	0.973819	0.05
52216	0.94613	0.05
52217	1.18182	0.05
52218	0.94613	0.05
52219	0.973819	0.05
52220	1.18182	0.05
52221	0.94613	0.05
52222	0.94613	0.05
52223	2.23944	2.1
52224	1.52903	2.1
52225	1.35977	2.1
52226	2.04847	2.1
52227	3.5775	2.1
52228	2.23944	2.1
52229	2.34795	2.1
52230	2.34795	2.1
52231	2.48025	2.1
52232	2.04847	2.1
52233	2.18077	2.1
52234	1.89226	2.1
52235	1.89226	2.1
52236	1.89226	2.1
52237	1.89226	2.1
52238	3.78452	2.1
52239	0.946127	2.1
52240	0.946127	2.1
52241	0.946131	2.1
52242	0.946131	2.1
52243	0.946128	2.1
52244	0.946128	2.1
52245	0.94613	2.1
52246	0.94613	2.1
52247	0.946129	2.1
52248	0.946129	2.1
52249	0.946129	2.1
52250	0.946129	2.1
52251	1.26956	2.1
52252	2.04847	2.1
52253	1.49576	2.1
52254	1.22377	2.1
52255	1.91172	2.1

52256	1.50198	2.1
52257	1.48568	2.1
52258	2.34795	2.1
52259	2.04847	2.1
52260	2.04847	2.1
52261	2.21155	2.1
52262	2.21155	2.1
52263	1.89226	2.1
52264	1.89226	2.1
52265	1.89226	2.1
52266	1.89226	2.1
52267	1.89226	2.1
52268	1.89226	2.1
52269	0.810943	2.1
52270	0.810943	2.1
52271	0.810944	2.1
52272	0.810944	2.1
52273	0.810957	2.1
52274	0.810957	2.1
52275	1.89226	2.1
52276	1.35168	2.1
52277	1.08132	2.1
52278	1.08132	2.1
52279	1.0813	2.1
52280	0.946132	0.05
52281	1.33855	0.05
52282	0.946131	0.05
52283	1.38204	0.05
52284	0.946131	0.0514009
52285	0.946131	0.05
52286	1.38204	0.05
52287	1.38204	0.05
52288	0.946131	0.05
52289	0.946131	0.05
52290	1.33855	0.05
52291	1.38204	0.05
52292	1.89226	0.051465
52293	2.76407	0.052769
52294	1.89226	0.05
52295	2.72769	0.05
52296	1.89226	0.05
52297	2.72769	0.05
52298	2.03914	0.05
52299	1.67727	0.05
52300	1.94831	0.05
52301	1.81591	0.05
52302	2.32098	0.05
52303	2.32098	0.05
52304	0.801647	0.05
52305	1.11086	0.05
52306	0.838634	0.05
52307	1.16049	0.05
52308	0.977891	0.05
52309	0.977891	0.05
52310	0.984246	0.05
52311	0.984246	0.05
52312	1.11086	0.05
52313	1.16049	0.05

52314	0.801647	0.05
52315	0.838634	0.05
52316	0.946132	0.05
52317	0.946132	0.0501383
52318	0.946131	0.05
52319	0.946131	0.0529804
52320	0.946131	0.0555529
52321	0.946131	0.0507083
52322	0.946132	0.05
52323	0.946132	0.05
52324	0.946131	0.05
52325	0.946131	0.05
52326	0.946131	0.05
52327	0.946131	0.05
52328	0.946131	0.05
52329	0.946131	0.0595963
52330	0.946131	0.0518004
52331	0.946131	0.05
52332	0.946131	0.0545343
52333	0.946131	0.0533476
52334	0.946131	0.05
52335	0.946131	0.05
52336	0.946131	0.05
52337	0.946131	0.05
52338	0.946131	0.05
52339	0.946131	0.05
52340	2.0815	2.1
52341	1.20787	2.1
52342	1.92277	2.1
52343	1.3227	2.1
52344	3.29401	2.1
52345	2.16633	2.1
52346	2.13642	2.1
52347	1.40846	2.1
52348	1.40846	2.1
52349	1.3227	2.1
52350	2.08058	2.1
52351	0.938106	2.1
52352	0.790258	2.1
52353	0.742975	2.1
52354	0.742975	2.1
52355	0.874389	2.1
52356	1.17364	2.1
52357	0.525948	2.1
52358	0.52513	2.1
52359	0.553732	2.1
52360	0.876996	2.1
52361	0.530954	2.1
52362	1.095 2.1	
52363	1.00833	2.1
52364	1.70608	2.1
52365	1.33385	2.1
52366	1.33385	2.1
52367	1.07722	2.1
52368	1.07722	2.1
52369	0.953523	1.28887
52370	1.7457	0.678579
52371	1.03218	1.14697

52372	1.03218	0.98183
52373	1.79675	1.03502
52374	1.79675	0.875123
52375	1.13676	1.2807
52376	1.18136	0.728436
52377	0.87285	0.996091
52378	0.898375	1.34416
52379	1.03191	0.732616
52380	1.03191	0.541196
52381	1.0223	1.09013
52382	1.0223	1.33822
52383	1.18136	0.933218
52384	0.898374	0.662851
52385	0.87285	0.937594
52386	1.13676	0.83975
52387	2.36273	0.87621
52388	1.46965	0.556546
52389	1.79675	0.454926
52390	1.79675	0.375071
52391	2.43417	1.08176
52392	2.43417	0.785201
52393	1.4714	2.1
52394	1.65434	2.1
52395	1.62322	1.39563
52396	1.86068	2.00733
52397	3.27756	1.72748
52398	1.89226	2.1
52399	1.89226	2.1
52400	1.89226	1.45025
52401	1.89226	1.63669
52402	3.78453	1.55179
52403	1.89226	1.89654
52404	1.89226	2.1
52405	1.89226	1.52619
52406	1.89226	2.1
52407	3.78453	2.1
52408	1.65434	1.89759
52409	1.60647	2.1
52410	1.86068	1.74382
52411	1.82649	2.1
52412	3.48083	2.1
52413	1.62322	2.09412
52414	1.86068	2.1
52415	1.84368	1.52218
52416	2.18862	1.64389
52417	3.81184	1.61093
52418	1.89226	2.1
52419	1.89226	1.98185
52420	1.89226	1.30534
52421	1.89226	1.68585
52422	3.78453	1.6307
52423	1.89226	1.78879
52424	1.89226	2.1
52425	1.89226	1.55593
52426	1.89226	2.1
52427	3.78453	2.1
52428	1.86068	1.83741
52429	1.82649	2.1

52430	2.18862	1.53412
52431	2.1128	2.1
52432	4.01511	2.08331
52433	1.74351	2.1
52434	1.13854	2.1
52435	1.40233	2.1
52436	1.922 2.1	
52437	3.14584	2.1
52438	1.922 2.1	
52439	2.00775	2.1
52440	1.7435	2.1
52441	2.09224	2.1
52442	2.00775	2.1
52443	2.09295	2.1
52444	1.69858	2.1
52445	1.922 2.1	
52446	1.40233	2.1
52447	1.40233	2.1
52448	2.18238	2.1
52449	2.18238	2.1
52450	1.00387	2.1
52451	1.10886	2.1
52452	0.961001	2.1
52453	1.09119	2.1
52454	1.02868	2.1
52455	1.02868	2.1
52456	1.04118	2.1
52457	1.04118	2.1
52458	1.10886	2.1
52459	1.09119	2.1
52460	0.960999	2.1
52461	1.00388	2.1
52462	0.885958	0.252226
52463	0.885958	0.191996
52464	0.875826	0.0971885
52465	0.875826	0.308603
52466	0.913965	0.293948
52467	0.598609	0.160279
52468	1.16317	0.124334
52469	0.868115	0.119906
52470	0.868115	0.202091
52471	0.598609	0.267966
52472	1.16317	0.208557
52473	0.913965	0.248025
52474	0.946131	0.361288
52475	0.946131	0.286248
52476	0.946131	0.159132
52477	0.946131	0.13664
52478	0.946131	0.146492
52479	0.946131	0.288979
52480	0.946131	0.393477
52481	0.946131	0.155285
52482	0.946131	0.163539
52483	0.946131	0.167536
52484	0.946131	0.343064
52485	0.946131	0.243662
52486	0.946133	0.05
52487	0.946133	0.05

52488	1.06246	0.05
52489	1.06246	0.05
52490	0.973851	0.05
52491	0.946133	0.05
52492	1.06246	0.05
52493	0.946132	0.05
52494	0.97385	0.05
52495	1.06246	0.05
52496	0.946133	0.05
52497	0.946133	0.05
52498	1.89226	0.05
52499	1.89226	0.05
52500	1.89226	0.05
52501	1.89226	0.05
52502	1.89226	0.05
52503	1.89226	0.05
52504	0.946131	0.05
52505	0.946131	0.05
52506	0.946127	0.05
52507	0.946127	0.05
52508	0.946131	0.05
52509	0.946131	0.05
52510	0.946127	0.05
52511	0.946127	0.05
52512	0.946128	0.05
52513	0.946128	0.05
52514	0.946129	0.05
52515	0.946129	0.05
52516	0.946132	0.05
52517	0.946132	0.05
52518	1.32446	0.05
52519	0.794912	0.05
52520	1.32429	0.05
52521	0.946131	0.05
52522	1.32446	0.05
52523	0.94613	0.05
52524	1.32429	0.05
52525	0.794915	0.05
52526	0.94613	0.05
52527	0.94613	0.05
52528	1.89226	0.05
52529	2.36365	0.05
52530	2.64857	0.05
52531	1.89226	0.05
52532	2.64857	0.05
52533	1.89226	0.05
52534	1.18463	2.1
52535	1.27174	2.1
52536	0.946129	2.1
52537	0.946129	2.1
52538	0.946131	2.1
52539	0.946131	2.1
52540	1.27174	2.1
52541	1.27174	2.1
52542	1.18463	2.1
52543	0.94613	2.1
52544	0.946131	2.1
52545	1.27174	2.1

52546	2.57997	2.1
52547	1.89226	2.1
52548	1.89226	2.1
52549	1.89226	2.1
52550	2.41915	2.1
52551	2.41915	2.1
52552	1.89226	2.1
52553	1.89226	2.1
52554	1.89226	2.1
52555	1.89226	2.1
52556	3.78452	2.1
52557	1.08807	2.1
52558	1.18463	2.1
52559	0.946129	2.1
52560	0.946129	2.1
52561	0.946131	2.1
52562	0.946131	2.1
52563	1.18463	2.1
52564	1.18463	2.1
52565	0.946131	2.1
52566	1.08807	2.1
52567	1.18463	2.1
52568	0.94613	2.1
52569	1.07017	2.1
52570	1.07017	2.1
52571	0.946131	2.1
52572	0.946131	2.1
52573	0.946132	2.1
52574	0.946132	2.1
52575	1.20957	2.1
52576	1.07017	2.1
52577	1.07017	2.1
52578	0.946132	2.1
52579	0.946131	2.1
52580	1.20957	2.1
52581	1.89226	2.1
52582	1.89226	2.1
52583	1.89226	2.1
52584	1.89226	2.1
52585	1.89226	2.1
52586	1.89226	2.1
52587	1.16181	0.05
52588	1.0227	0.05
52589	1.0227	0.05
52590	0.970959	0.05
52591	1.24295	0.05
52592	1.19121	0.05
52593	1.12076	2.1
52594	2.53614	2.1
52595	1.28413	2.1
52596	1.28413	2.1
52597	2.36423	2.1
52598	2.36423	2.1
52599	1.89226	2.1
52600	1.89226	2.1
52601	1.89226	2.1
52602	1.89226	2.1
52603	3.78452	2.1

52604	1.22557	2.1
52605	2.771	2.1
52606	2.73012	2.1
52607	2.73012	2.1
52608	1.33356	2.1
52609	1.33356	2.1
52610	0.998682	2.1
52611	2.36423	2.1
52612	1.12076	2.1
52613	1.12076	2.1
52614	2.13757	2.1
52615	2.13757	2.1
52616	1.89226	2.1
52617	1.89226	2.1
52618	1.89226	2.1
52619	1.89226	2.1
52620	1.89226	2.1
52621	1.89226	2.1
52622	1.13987	2.1
52623	2.73011	2.1
52624	2.70875	2.1
52625	2.70875	2.1
52626	1.22557	2.1
52627	1.22557	2.1
52628	1.89227	2.1
52629	2.79243	2.1
52630	2.6625	2.1
52631	2.6625	2.1
52632	1.89227	2.1
52633	1.89227	2.1
52634	1.89226	2.1
52635	2.55971	2.1
52636	1.89226	2.1
52637	1.89226	2.1
52638	2.68704	2.1
52639	2.68704	2.1
52640	2.22043	2.1
52641	2.38975	1.61928
52642	2.28696	1.84333
52643	2.48682	1.51424
52644	4.67671	1.74436
52645	2.38976	1.40014
52646	2.31988	1.84687
52647	2.48683	1.4067
52648	2.4594	1.63014
52649	4.84915	1.3921
52650	2.28696	2.1
52651	2.48682	1.86686
52652	2.33379	2.1
52653	2.56149	1.43982
52654	4.84845	1.89013
52655	2.48683	1.84239
52656	2.4594	1.7785
52657	2.5615	1.26663
52658	2.57957	1.39869
52659	5.0209	1.36991
52660	1.06659	2.1
52661	1.04291	2.1

52662	1.36595	2.1
52663	1.36595	2.1
52664	1.02761	2.1
52665	1.32697	2.1
52666	0.946132	1.98864
52667	0.946132	1.97754
52668	1.36956	2.1
52669	1.1229	2.1
52670	1.1229	1.96314
52671	1.1229	2.1
52672	0.946132	1.91367
52673	0.946132	2.1
52674	0.946132	2.1
52675	1.36956	2.1
52676	1.1229	2.1
52677	0.946133	2.1
52678	0.946133	1.67469
52679	0.946133	1.66116
52680	0.946128	2.1
52681	0.946128	2.05905
52682	0.946131	1.95656
52683	0.946131	1.70499
52684	0.946131	1.24978
52685	0.946131	2.04276
52686	0.94613	1.84022
52687	0.94613	1.83155
52688	0.946131	1.85379
52689	0.946131	1.86439
52690	0.966427	1.66761
52691	0.802265	1.76487
52692	0.803714	2.01295
52693	0.808476	1.78814
52694	1.94371	1.99623
52695	1.36851	1.92999
52696	0.853864	2.07978
52697	1.26635	1.81939
52698	1.09085	1.7299
52699	0.857653	1.8664
52700	0.630243	1.85332
52701	1.20132	0.05
52702	1.24295	0.05
52703	1.24295	0.05
52704	1.16182	0.05
52705	1.28465	0.05
52706	1.20351	0.05
52707	0.600658	0.05
52708	0.642323	0.05
52709	0.54424	0.05
52710	0.576779	0.05
52711	0.590985	0.05
52712	0.590985	0.05
52713	0.586452	0.05
52714	0.586452	0.05
52715	0.642323	0.05
52716	0.576779	0.05
52717	0.600658	0.05
52718	0.54424	0.05
52719	1.28463	0.05

52720	1.24294	0.05
52721	1.12328	0.05
52722	1.15505	0.05
52723	2.39798	0.05
52724	1.98431	0.05
52725	2.18735	0.05
52726	1.98431	0.05
52727	1.70303	0.0694966
52728	1.70302	0.0912718
52729	1.37788	0.0635518
52730	1.89226	0.0681887
52731	2.37417	0.0602117
52732	1.89226	0.158284
52733	2.18735	0.0728365
52734	1.89226	0.126475
52735	2.18735	0.0690658
52736	1.89226	0.252031
52737	1.89226	2.1
52738	1.89226	0.903125
52739	1.89226	1.08593
52740	3.78452	1.01832
52741	1.8613	1.39765
52742	2.03576	2.1
52743	1.61652	0.771707
52744	1.83082	2.1
52745	3.65228	1.79956
52746	2.03576	2.1
52747	2.05257	2.1
52748	1.83082	2.1
52749	1.88487	2.1
52750	3.92064	2.1
52751	1.89226	2.09338
52752	1.89226	2.1
52753	1.89226	1.34446
52754	1.89226	2.1
52755	3.78453	2.1
52756	0.946131	2.1
52757	0.946131	0.616156
52758	0.94613	0.202669
52759	0.94613	1.8015
52760	0.946129	1.09096
52761	0.946129	2.1
52762	0.946132	1.25784
52763	0.946132	0.193508
52764	0.94613	0.947494
52765	0.94613	1.094
52766	0.946131	0.714851
52767	0.946131	0.778386
52768	0.804708	2.1
52769	0.804708	1.57776
52770	0.815274	2.1
52771	0.815274	0.743811
52772	0.83277	2.1
52773	0.915407	2.1
52774	0.80826	1.51538
52775	0.704574	0.696378
52776	0.832771	2.07365
52777	0.704573	2.08628

52778	0.808259	1.97265
52779	0.915409	2.00308
52780	1.83082	2.1
52781	1.88487	2.1
52782	1.66555	2.1
52783	1.75835	2.1
52784	3.55042	2.1
52785	0.810947	1.23674
52786	0.810947	2.1
52787	0.810947	1.14431
52788	0.810947	2.1
52789	0.810934	2.1
52790	0.810934	2.1
52791	1.89227	2.1
52792	1.3517	2.1
52793	1.08132	2.1
52794	1.08132	2.1
52795	1.08133	2.1
52796	1.89226	2.1
52797	1.70303	2.1
52798	1.89226	2.1
52799	2.24712	2.1
52800	2.07363	2.1
52801	1.74617	2.1
52802	1.89226	2.1
52803	1.89226	1.80735
52804	1.89226	1.67131
52805	1.89226	2.1
52806	3.78452	1.90585
52807	1.50108	2.1
52808	1.43894	1.90219
52809	1.8322	1.76371
52810	1.40967	2.1
52811	1.50108	1.95364
52812	1.07854	2.1
52813	1.89226	1.70775
52814	1.89226	2.09586
52815	1.89226	2.06722
52816	1.89226	1.10474
52817	3.78452	1.9026
52818	1.89226	2.1
52819	1.89226	1.93601
52820	1.89226	1.97317
52821	1.89226	2.1
52822	3.78453	2.05541
52823	1.54457	2.1
52824	1.35097	1.96832
52825	1.70754	1.92122
52826	1.29629	2.1
52827	1.54457	2.09448
52828	1.0305	2.1
52829	0.731763	2.1
52830	1.00043	2.04594
52831	1.13883	1.63812
52832	0.939026	1.67142
52833	1.04003	1.6153
52834	0.853868	2.1
52835	0.927188	2.07805

52836	0.927188	0.85257
52837	0.861565	1.75693
52838	1.13882	1.79407
52839	0.731769	1.91687
52840	0.89319	1.93674
52841	1.46568	2.1
52842	1.20761	2.1
52843	1.51916	2.1
52844	1.36261	2.1
52845	2.72677	2.1
52846	1.51916	2.1
52847	1.60532	2.1
52848	1.36261	2.1
52849	1.36261	2.1
52850	1.64892	2.1
52851	1.64892	2.1
52852	1.3626	2.1
52853	0.98488	2.1
52854	1.60532	2.1
52855	1.32414	2.1
52856	2.68675	2.1
52857	1.78711	2.1
52858	1.14825	2.1
52859	2.16535	2.1
52860	1.6067	2.1
52861	3.3136	2.1
52862	1.89226	2.1
52863	1.89226	2.1
52864	1.89226	2.1
52865	1.89226	2.1
52866	3.78452	2.1
52867	2.34552	2.1
52868	1.53521	2.1
52869	1.53521	2.1
52870	1.60636	2.1
52871	1.92797	2.1
52872	1.99589	2.1
52873	1.34771	2.1
52874	1.34771	2.1
52875	0.946128	2.1
52876	0.946128	2.1
52877	1.3477	2.1
52878	1.31776	2.1
52879	0.946131	2.1
52880	0.946131	2.1
52881	0.94613	2.1
52882	1.31776	2.1
52883	0.946129	2.1
52884	1.3477	2.1
52885	1.89226	2.1
52886	1.89226	2.1
52887	1.89226	2.1
52888	1.89226	2.1
52889	3.78452	2.1
52890	0.92843	2.1
52891	0.672114	2.1
52892	0.89514	2.1
52893	0.89514	2.1

52894	0.720104	2.1
52895	0.755753	2.1
52896	0.789158	2.1
52897	0.967302	2.1
52898	1.06565	2.1
52899	0.7397	2.1
52900	0.758893	2.1
52901	0.94672	2.1
52902	1.89226	2.1
52903	2.588	2.1
52904	2.588	2.1
52905	2.63552	2.1
52906	1.89226	2.1
52907	1.93978	2.1
52908	1.49347	0.05
52909	2.67757	0.0904647
52910	1.80061	0.0699924
52911	2.27056	0.0821304
52912	4.07117	0.0555304
52913	1.1016	0.05
52914	2.27057	0.060817
52915	1.49348	0.0664243
52916	1.81893	0.0686727
52917	1.49348	0.05
52918	1.81893	0.05
52919	1.81893	0.0556925
52920	2.82339	0.111145
52921	2.27057	0.0820208
52922	2.25405	0.0821264
52923	4.52462	0.05843
52924	1.89226	0.0815698
52925	1.89226	0.134864
52926	1.89226	0.1297
52927	1.89226	0.0982638
52928	1.89226	0.134552
52929	1.89226	0.113685
52930	1.89226	0.0549676
52931	1.89226	0.105061
52932	1.89226	0.0624917
52933	1.89226	0.0741918
52934	3.78453	0.0524781
52935	1.89226	0.128124
52936	1.89226	0.156838
52937	1.89226	0.0836057
52938	1.89226	0.175041
52939	3.78452	0.1864
52940	1.89226	0.160077
52941	1.89226	0.33422
52942	1.89226	0.159812
52943	1.89226	0.194861
52944	3.78451	0.14605
52945	1.45633	2.1
52946	1.34678	2.1
52947	1.34678	2.1
52948	1.53735	2.1
52949	1.234	2.1
52950	1.42457	2.1
52951	1.89226	2.1

52952	1.89226	2.1
52953	1.89226	2.1
52954	1.89226	2.1
52955	1.89226	2.1
52956	1.89226	2.1
52957	1.09483	2.1
52958	1.34678	2.1
52959	1.234	2.1
52960	1.234	2.1
52961	1.19	2.1
52962	1.19	2.1
52963	0.946131	2.1
52964	0.946131	2.1
52965	0.946131	2.1
52966	0.946131	2.1
52967	0.946131	2.1
52968	0.946131	2.1
52969	0.946132	2.1
52970	0.946132	2.1
52971	0.946132	2.1
52972	0.946132	2.1
52973	0.946131	2.1
52974	0.946131	2.1
52975	2.82263	2.1
52976	1.89226	2.1
52977	2.71441	2.1
52978	2.71441	2.1
52979	1.89226	2.1
52980	1.89226	2.1
52981	1.1412	2.1
52982	1.1412	2.1
52983	1.14626	2.1
52984	1.14626	2.1
52985	1.28511	2.1
52986	1.34086	2.1
52987	0.992242	2.1
52988	0.946593	2.1
52989	0.992242	2.1
52990	1.34086	2.1
52991	0.946594	2.1
52992	1.28511	2.1
52993	1.18954	2.1
52994	1.18954	2.1
52995	1.21477	2.1
52996	1.21477	2.1
52997	1.41207	2.1
52998	1.34086	2.1
52999	1.01306	2.1
53000	0.992242	2.1
53001	1.01306	2.1
53002	1.41207	2.1
53003	0.992242	2.1
53004	1.34086	2.1
53005	1.09044	2.1
53006	1.44472	1.89475
53007	1.2955	2.1
53008	1.2955	2.1
53009	1.22025	2.1

53010	1.22025	2.1
53011	0.810944	2.00617
53012	0.810944	2.1
53013	0.810946	2.00867
53014	0.810946	0.570346
53015	0.810959	1.95582
53016	0.810959	1.9589
53017	1.89226	1.9228
53018	1.35167	1.9819
53019	1.08132	2.1
53020	1.08132	0.729054
53021	1.0813	1.97376
53022	1.41894	0.431547
53023	1.28953	0.131063
53024	0.946132	0.568533
53025	0.946132	1.93507
53026	0.946133	1.37199
53027	0.946133	0.211064
53028	1.41894	1.54058
53029	1.41894	1.56932
53030	1.28953	0.446709
53031	0.946132	1.21445
53032	0.946132	1.40266
53033	1.41894	0.538012
53034	1.89226	2.1
53035	2.15444	2.1
53036	1.93239	2.1
53037	1.89226	2.1
53038	2.19457	2.1
53039	2.15444	2.1
53040	1.89226	2.1
53041	1.89226	2.1
53042	1.89226	2.1
53043	1.89226	2.1
53044	1.89226	2.1
53045	1.89226	2.1
53046	0.938507	2.1
53047	0.938507	2.1
53048	1.07871	2.1
53049	1.15801	2.1
53050	1.03236	2.1
53051	1.14766	2.1
53052	0.946133	2.1
53053	0.946133	2.1
53054	0.946131	2.1
53055	1.07009	2.1
53056	0.946131	2.1
53057	1.03237	2.1
53058	1.89226	2.1
53059	2.19457	2.1
53060	1.94492	2.1
53061	1.89226	2.1
53062	2.24723	2.1
53063	2.19457	2.1
53064	0.946132	2.1
53065	0.946132	2.1
53066	0.946131	2.1
53067	0.946131	2.1

53068	0.946132	2.1
53069	0.946132	2.1
53070	0.946132	2.1
53071	0.946132	2.1
53072	0.946132	2.1
53073	0.946132	2.1
53074	0.946132	2.1
53075	0.946132	2.1
53076	2.21345	2.1
53077	1.89226	2.1
53078	1.89226	2.1
53079	2.14017	2.1
53080	2.14017	2.1
53081	1.89226	2.1
53082	1.89226	0.357394
53083	1.89226	1.14529
53084	1.89226	0.360412
53085	1.85439	1.17147
53086	2.23961	0.266176
53087	2.19479	0.734512
53088	1.2916	0.335704
53089	1.2916	0.334936
53090	0.946128	0.500521
53091	0.946128	0.316941
53092	1.2916	0.234092
53093	0.946131	0.339923
53094	0.946126	1.09698
53095	1.1198	0.207876
53096	1.2916	0.27741
53097	1.1198	0.637868
53098	0.946128	0.871274
53099	0.946128	0.499435
53100	1.89226	0.636555
53101	2.71262	0.311034
53102	1.89226	0.349651
53103	2.49959	0.634135
53104	4.39185	0.766064
53105	2.71262	0.281068
53106	1.34592	0.335378
53107	2.49959	0.408639
53108	1.14721	0.259837
53109	3.84551	0.27242
53110	0.946131	0.374349
53111	0.946131	0.403418
53112	1.11247	0.27449
53113	1.11247	0.252887
53114	1.24979	0.282463
53115	1.11247	0.346361
53116	0.946129	0.425976
53117	0.946129	0.302534
53118	0.946131	0.315577
53119	1.11247	0.210253
53120	0.946131	0.238766
53121	1.24979	0.287283
53122	0.946131	2.1
53123	0.946131	2.1
53124	0.94613	2.1
53125	0.94613	2.1

53126	0.946132	2.1
53127	0.946132	2.1
53128	0.946129	2.1
53129	0.946129	2.1
53130	0.946131	2.1
53131	0.946131	2.1
53132	0.946131	2.1
53133	0.946131	2.1
53134	2.77816	2.1
53135	1.89226	2.1
53136	1.89226	2.1
53137	1.89226	2.1
53138	3.78453	2.1
53139	1.89226	2.1
53140	1.89226	2.1
53141	1.89226	2.1
53142	1.89226	2.1
53143	1.89226	2.1
53144	1.89226	2.1
53145	1.89226	2.1
53146	1.89226	2.1
53147	1.89226	2.1
53148	1.89226	2.1
53149	3.78452	2.1
53150	1.55698	2.1
53151	1.89226	2.1
53152	1.89226	2.1
53153	1.89226	2.1
53154	1.8575	2.1
53155	1.8575	2.1
53156	1.89226	2.1
53157	1.89226	2.1
53158	1.89226	2.1
53159	1.89226	2.1
53160	1.89226	2.1
53161	1.89226	2.1
53162	1.89227	2.1
53163	1.89227	2.1
53164	1.89227	2.1
53165	1.89227	2.1
53166	1.89227	2.1
53167	1.89227	2.1
53168	1.35509	2.1
53169	2.06238	2.1
53170	2.12244	2.1
53171	2.12244	2.1
53172	1.34817	2.1
53173	1.34817	2.1
53174	0.946126	2.1
53175	0.946126	2.1
53176	0.946131	2.1
53177	0.946131	2.1
53178	0.946127	2.1
53179	0.946127	2.1
53180	0.94613	2.1
53181	0.94613	2.1
53182	0.946128	2.1
53183	0.946128	2.1

53184	0.946128	2.1
53185	0.946128	2.1
53186	1.89226	2.1
53187	1.89226	2.1
53188	1.89226	2.1
53189	1.89226	2.1
53190	1.89226	2.1
53191	1.89226	2.1
53192	1.16742	2.1
53193	1.16742	2.1
53194	1.18122	2.1
53195	1.18122	2.1
53196	1.31745	2.1
53197	1.2597	2.1
53198	1.03119	2.1
53199	1.06122	2.1
53200	1.06122	2.1
53201	1.31745	2.1
53202	1.03119	2.1
53203	1.2597	2.1
53204	1.36849	2.1
53205	2.12244	2.1
53206	2.20092	2.1
53207	2.20092	2.1
53208	1.35509	2.1
53209	1.35509	2.1
53210	1.89226	2.1
53211	1.89226	2.1
53212	1.89226	2.1
53213	1.89226	2.1
53214	1.89226	2.1
53215	1.89226	2.1
53216	1.89226	2.1
53217	1.89226	2.1
53218	1.89226	2.1
53219	1.89226	2.1
53220	3.78452	2.1
53221	1.21731	2.1
53222	1.21731	2.1
53223	1.23412	2.1
53224	1.23412	2.1
53225	1.39021	2.1
53226	1.31745	2.1
53227	1.06122	2.1
53228	1.10046	2.1
53229	1.10046	2.1
53230	1.39021	2.1
53231	1.06122	2.1
53232	1.31745	2.1
53233	2.16503	1.02117
53234	1.78252	1.56191
53235	1.77293	1.61806
53236	1.56839	2.1
53237	2.36957	0.386957
53238	2.16503	2.1
53239	1.89223	1.10141
53240	1.89223	2.1
53241	1.89223	2.06458

53242	1.89223	2.1
53243	3.78447	2.1
53244	2.3696	1.75904
53245	1.78254	1.8022
53246	2.63298	0.277404
53247	2.07312	2.1
53248	4.44272	1.73447
53249	1.89226	2.1
53250	1.89226	2.1
53251	1.89226	1.89899
53252	1.89226	2.1
53253	1.89226	0.817048
53254	1.89226	2.1
53255	1.89226	2.1
53256	1.89226	0.691492
53257	1.89226	2.09683
53258	1.89226	0.277309
53259	3.78452	0.741138
53260	2.26369	2.1
53261	2.2168	0.441459
53262	2.15617	2.00409
53263	2.10864	0.216137
53264	4.37234	0.891904
53265	2.2168	0.326301
53266	1.94499	0.611356
53267	1.94499	1.35952
53268	2.04737	0.511879
53269	2.10864	0.13606
53270	2.21103	0.845307
53271	1.89226	1.36889
53272	1.89226	0.460924
53273	1.89226	0.558093
53274	1.89226	0.390863
53275	1.89226	0.63619
53276	1.89226	0.257546
53277	1.89226	2.0701
53278	1.89226	0.770356
53279	1.89226	1.12455
53280	1.89226	2.0036
53281	1.89226	0.381145
53282	1.89226	2.1
53283	1.02995	0.440435
53284	1.02995	2.08875
53285	1.03212	0.643873
53286	1.03212	1.86387
53287	0.988256	0.326638
53288	1.05432	0.215601
53289	1.00775	1.8486
53290	1.07809	1.97712
53291	1.00775	0.737719
53292	0.988257	0.777935
53293	1.07808	1.23715
53294	1.05432	1.018
53295	2.10864	0.750328
53296	1.94499	1.02267
53297	1.97652	0.355149
53298	1.79681	0.628341
53299	3.9215	0.211569

53300	0.810943	0.441692
53301	0.810943	1.57956
53302	0.810943	0.363696
53303	0.810943	0.405889
53304	0.810958	0.156115
53305	0.810958	0.303184
53306	1.89226	0.642396
53307	1.35168	0.235633
53308	1.08132	0.672503
53309	1.08132	0.499595
53310	1.0813	0.235533
53311	0.657403	0.05
53312	1.35403	0.05
53313	1.24038	0.05
53314	0.499144	0.05
53315	1.24039	0.05
53316	1.35403	0.0593113
53317	0.657402	0.05
53318	0.499144	0.05
53319	0.915415	0.05
53320	0.915415	0.05
53321	0.937755	0.05
53322	0.937755	0.0626275
53323	1.72526	2.1
53324	1.17989	2.1
53325	1.96035	2.1
53326	1.47412	2.1
53327	3.14024	2.1
53328	1.89226	1.87295
53329	1.89226	2.1
53330	1.89226	2.1
53331	1.89226	1.60283
53332	3.78452	2.1
53333	0.946131	1.37638
53334	0.946131	1.57159
53335	1.25764	2.04154
53336	1.25764	2.09565
53337	1.07087	1.38254
53338	1.25765	1.72636
53339	0.946128	1.89147
53340	0.946128	0.681038
53341	0.94613	1.55517
53342	1.25765	1.69207
53343	1.07086	1.7228
53344	0.946129	1.66392
53345	1.96035	2.1
53346	1.32668	2.1
53347	2.29301	2.1
53348	1.70303	2.1
53349	3.56428	2.1
53350	1.89226	2.1
53351	1.89226	1.66218
53352	1.89226	1.77261
53353	1.89226	2.1
53354	3.78452	1.66927
53355	0.897738	0.275449
53356	0.897738	0.286077
53357	0.946102	0.231892

53358	0.946102	0.17402
53359	1.11247	0.216549
53360	0.89771	0.176157
53361	0.94613	0.225885
53362	0.94613	0.302808
53363	0.897709	0.273157
53364	0.946131	0.282837
53365	0.946131	0.353962
53366	1.11247	0.240214
53367	1.89226	2.1
53368	1.89226	2.04555
53369	1.89226	2.1
53370	1.89226	2.1
53371	1.89226	2.1
53372	1.89226	2.1
53373	2.15429	2.1
53374	1.89226	2.1
53375	1.89226	2.1
53376	1.89226	2.1
53377	2.27074	2.1
53378	2.27074	2.1
53379	0.993311	1.37866
53380	0.993311	1.83729
53381	0.904322	2.1
53382	0.904322	2.03407
53383	0.803324	2.1
53384	1.36183	1.36326
53385	0.916274	2.1
53386	0.535808	1.82831
53387	0.535808	1.89011
53388	0.803322	1.89901
53389	0.916276	1.87023
53390	1.36183	1.87579
53391	1.89226	2.1
53392	1.89226	1.91454
53393	1.89226	2.1
53394	1.89226	2.1
53395	1.89226	2.1
53396	1.89226	2.1
53397	2.11955	2.1
53398	2.62097	2.1
53399	1.85542	2.1
53400	2.39016	2.1
53401	4.50971	2.1
53402	2.39016	2.1
53403	2.20242	2.1
53404	2.39016	2.1
53405	1.64008	2.1
53406	1.85542	2.1
53407	1.64008	2.1
53408	1.79667	2.1
53409	1.28267	2.1
53410	1.28267	2.1
53411	1.40117	2.1
53412	1.74514	2.1
53413	1.83994	2.1
53414	0.575914	2.1
53415	0.625292	2.1

53416	0.949144	2.1
53417	0.882782	2.1
53418	0.773697	2.1
53419	0.773697	2.1
53420	0.786497	2.1
53421	0.786494	2.1
53422	0.605477	2.1
53423	0.949148	2.1
53424	0.625291	2.1
53425	0.862374	2.1
53426	1.89226	2.1
53427	2.44918	2.1
53428	2.4616	2.1
53429	2.44918	2.1
53430	1.90468	2.1
53431	1.89226	2.1
53432	1.89226	2.1
53433	1.89226	2.1
53434	1.89226	2.1
53435	1.89226	2.1
53436	3.78453	2.1
53437	0.946128	2.1
53438	0.946128	2.1
53439	1.23631	2.1
53440	1.23631	2.1
53441	0.946131	2.1
53442	0.946131	2.1
53443	1.3147	2.1
53444	1.23631	2.1
53445	1.3147	2.1
53446	0.946131	2.1
53447	1.23631	2.1
53448	0.94613	2.1
53449	1.89226	2.1
53450	2.4616	2.1
53451	2.4717	2.1
53452	2.4616	2.1
53453	1.90237	2.1
53454	1.89226	2.1
53455	0.946132	2.1
53456	0.946132	2.1
53457	0.946131	2.1
53458	0.946131	2.1
53459	0.946132	2.1
53460	0.946132	2.1
53461	0.946131	2.1
53462	0.946131	2.1
53463	0.946131	2.1
53464	0.946131	2.1
53465	0.946131	2.1
53466	0.946131	2.1
53467	0.946128	2.1
53468	0.946128	2.1
53469	1.3147	2.1
53470	1.3147	2.1
53471	0.94613	2.1
53472	0.94613	2.1
53473	1.3419	2.1

53474	1.3147	2.1
53475	1.3419	2.1
53476	0.94613	2.1
53477	1.3147	2.1
53478	0.94613	2.1
53479	1.89226	2.1
53480	1.89226	2.1
53481	1.89226	2.1
53482	1.89226	2.1
53483	3.78452	2.1
53484	2.3333	2.1
53485	1.3864	2.1
53486	1.3864	2.1
53487	1.4628	2.1
53488	1.95763	2.1
53489	2.03403	2.1
53490	1.294	2.1
53491	1.294	2.1
53492	0.946128	2.1
53493	0.946128	2.1
53494	1.294	2.1
53495	1.27474	2.1
53496	0.946131	2.1
53497	0.946131	2.1
53498	0.946129	2.1
53499	1.27474	2.1
53500	1.294	2.1
53501	0.94613	2.1
53502	1.89226	2.1
53503	1.89226	2.1
53504	1.89226	2.1
53505	1.89226	2.1
53506	1.89226	2.1
53507	1.89226	2.1
53508	0.808088	2.1
53509	0.808088	2.1
53510	0.815623	2.1
53511	0.815623	2.1
53512	0.660053	2.1
53513	0.693199	2.1
53514	0.930512	2.1
53515	0.978817	2.1
53516	0.660053	2.1
53517	0.930513	2.1
53518	0.693198	2.1
53519	0.978817	2.1
53520	1.27474	2.1
53521	1.27474	2.1
53522	0.946128	2.1
53523	0.946128	2.1
53524	1.26221	2.1
53525	1.27474	2.1
53526	0.946131	2.1
53527	0.946131	2.1
53528	0.94613	2.1
53529	1.26221	2.1
53530	0.946129	2.1
53531	1.27474	2.1

53532	0.90207	0.05
53533	0.90207	0.05
53534	0.895719	0.05
53535	0.895719	0.05
53536	0.927855	0.05
53537	1.2493	0.05
53538	0.85723	0.05
53539	0.548484	0.05
53540	0.548485	0.05
53541	0.927855	0.05
53542	0.85723	0.05
53543	1.2493	0.05
53544	1.41319	0.338585
53545	1.22482	0.18876
53546	1.46629	0.229005
53547	1.92622	0.375268
53548	3.15104	0.314443
53549	1.02305	0.128648
53550	1.22482	0.191348
53551	1.80336	0.19129
53552	1.44426	0.126739
53553	2.66908	0.131276
53554	1.3268	2.1
53555	0.999489	2.1
53556	1.44091	2.1
53557	1.14657	2.1
53558	2.4404	2.1
53559	1.89226	2.1
53560	1.89226	2.1
53561	1.89226	2.1
53562	1.89226	2.1
53563	3.78452	2.1
53564	1.89226	2.1
53565	2.28605	2.1
53566	2.43157	2.1
53567	2.43157	2.1
53568	1.89226	2.1
53569	1.89226	2.1
53570	1.60647	2.1
53571	1.44091	2.1
53572	1.31346	2.1
53573	1.31346	2.1
53574	1.14657	2.1
53575	1.44091	2.1
53576	1.89226	2.1
53577	1.89226	2.1
53578	1.89226	2.1
53579	1.89226	2.1
53580	3.78452	2.1
53581	0.946132	2.1
53582	0.946132	2.1
53583	1.3048	2.1
53584	1.3048	2.1
53585	1.3048	2.1
53586	1.21578	2.1
53587	0.946132	2.1
53588	0.946132	2.1
53589	1.3048	2.1

53590	0.946132	2.1
53591	0.946132	2.1
53592	1.21578	2.1
53593	1.89227	0.05
53594	1.89227	0.05
53595	1.89227	0.05
53596	1.89227	0.05
53597	3.78453	0.05
53598	1.6671	0.05
53599	1.87338	0.05
53600	1.6671	0.05
53601	2.02098	0.05
53602	1.38328	0.05
53603	1.73715	0.05
53604	1.89226	0.05
53605	1.89226	0.05
53606	1.89226	0.05
53607	1.89226	0.05
53608	3.78453	0.05
53609	1.6671	0.05
53610	1.38328	0.05
53611	1.35562	0.05
53612	1.01012	0.05
53613	2.67722	0.05
53614	0.819377	0.05
53615	0.647925	0.05
53616	1.17802	0.05
53617	1.33174	0.05
53618	0.98543	0.05
53619	0.98543	0.05
53620	0.994237	0.05
53621	0.994237	0.05
53622	1.17802	0.05
53623	0.647926	0.05
53624	1.33174	0.05
53625	0.819377	0.05
53626	1.27806	0.05
53627	1.68264	0.05
53628	1.44593	0.05
53629	1.878 0.05	
53630	3.15606	0.05
53631	1.878 0.05	
53632	1.9898	0.05
53633	1.878 0.05	
53634	1.68264	0.05
53635	2.30533	0.05
53636	2.10997	0.05
53637	0.814587	0.05
53638	0.814596	0.05
53639	0.82775	0.05
53640	0.82775	0.05
53641	1.0107	0.05
53642	0.91549	0.05
53643	0.741058	0.05
53644	0.714748	0.05
53645	0.714758	0.05
53646	1.03225	0.05
53647	0.704864	0.05

53648	0.915488	0.05
53649	1.90347	0.05
53650	2.06584	0.05
53651	1.878	0.05
53652	1.50236	0.05
53653	2.05749	0.05
53654	2.30533	0.05
53655	2.24475	2.1
53656	1.85871	2.1
53657	2.13711	2.1
53658	2.13711	2.1
53659	1.9943	2.1
53660	1.9943	2.1
53661	1.85871	2.1
53662	1.7871	2.1
53663	1.41619	2.1
53664	1.41619	2.1
53665	2.13711	2.1
53666	2.13711	2.1
53667	2.24475	2.1
53668	2.13711	2.1
53669	2.54902	2.1
53670	2.46327	2.1
53671	4.70802	2.1
53672	1.06913	2.1
53673	1.06913	2.1
53674	1.0821	2.1
53675	1.0821	2.1
53676	0.893552	2.1
53677	1.08267	2.1
53678	1.06855	2.1
53679	1.23163	2.1
53680	1.23163	2.1
53681	1.08267	2.1
53682	0.893551	2.1
53683	1.06855	2.1
53684	1.67046	1.16682
53685	2.4106	0.209589
53686	2.05835	1.45738
53687	2.4106	1.45932
53688	1.59562	0.403846
53689	1.94788	0.215325
53690	2.34737	1.93466
53691	2.4106	0.393069
53692	2.05834	1.5402
53693	1.99655	1.78805
53694	4.40715	1.53609
53695	1.67046	0.230871
53696	2.05835	0.17146
53697	1.588	1.37847
53698	1.34857	1.61578
53699	3.40692	1.08849
53700	1.73802	1.45369
53701	1.89668	1.51409
53702	1.99655	2.01186
53703	1.99655	0.397809
53704	1.79981	1.55086
53705	2.05834	0.322571

53706	1.89226	1.58086
53707	1.89226	1.17773
53708	1.89226	0.837249
53709	1.89226	1.72646
53710	1.89226	1.09671
53711	1.89226	2.1
53712	1.89226	1.48182
53713	1.89226	2.05163
53714	1.89226	1.84179
53715	1.89226	0.585771
53716	3.78453	1.71917
53717	0.719473	2.07767
53718	0.916103	1.8762
53719	1.02744	1.43791
53720	0.850328	1.57094
53721	0.888094	1.41758
53722	0.888094	2.1
53723	0.878337	2.00758
53724	0.878337	0.458312
53725	0.916103	1.57757
53726	1.02744	1.61222
53727	0.719473	1.79407
53728	0.850328	1.83166
53729	1.02744	1.55226
53730	0.850329	1.85176
53731	1.0875	1.20081
53732	0.919597	1.35457
53733	0.975841	1.16705
53734	0.975841	2.1
53735	0.971196	1.13226
53736	0.971196	1.75275
53737	1.02744	1.32611
53738	1.0875	1.22189
53739	0.850328	1.61094
53740	0.919598	1.59413
53741	1.89226	1.89477
53742	1.89226	1.49261
53743	1.89226	1.12054
53744	1.89226	1.51417
53745	3.78453	1.4082
53746	1.89226	1.80349
53747	1.89226	1.34007
53748	1.89226	0.585855
53749	1.89226	1.82968
53750	1.89226	0.617558
53751	1.89226	2.07997
53752	1.11103	1.75139
53753	0.916103	1.96371
53754	1.21177	1.38546
53755	1.02744	1.39563
53756	1.07188	1.31762
53757	1.07188	2.1
53758	1.06659	1.33197
53759	1.06659	1.88964
53760	1.11103	1.61852
53761	1.21177	1.53964
53762	0.916104	1.70104
53763	1.02744	1.77131

53764	2.54158	1.23384
53765	2.42354	1.48624
53766	2.17499	1.21379
53767	2.17499	2.02465
53768	2.42354	1.56138
53769	2.05488	2.1
53770	1.89226	0.05
53771	1.89226	0.0640803
53772	1.89226	0.05
53773	1.89226	0.0598825
53774	3.78453	0.05
53775	1.89226	0.05
53776	1.89226	0.05
53777	1.89226	0.05
53778	1.89226	0.05
53779	1.89226	0.05
53780	1.89226	0.05
53781	1.89226	0.100332
53782	1.89226	0.118526
53783	1.89226	0.0996823
53784	1.89226	0.0701877
53785	1.89226	0.15083
53786	1.89226	0.113965
53787	1.89226	0.101586
53788	1.89226	0.154924
53789	1.89226	0.108161
53790	1.89226	0.177092
53791	3.78452	0.140448
53792	0.802544	0.05
53793	0.946132	0.05
53794	0.946132	0.05
53795	0.787038	0.05
53796	0.787039	0.05
53797	0.787039	0.05
53798	0.961637	0.05
53799	0.961637	0.05
53800	0.787038	0.05
53801	0.802544	0.05
53802	0.946132	0.05
53803	0.946132	0.05
53804	1.89226	0.05
53805	1.60509	0.05
53806	1.89226	0.05
53807	1.89226	0.05
53808	1.3905	0.05
53809	1.3905	0.05
53810	0.946132	0.067545
53811	0.946132	0.05
53812	0.946131	0.0673138
53813	0.946131	0.066537
53814	0.946132	0.064477
53815	0.946132	0.0710331
53816	0.946131	0.0511238
53817	0.946131	0.0616202
53818	0.946131	0.0504879
53819	0.946131	0.0634208
53820	0.946132	0.0552093
53821	0.946132	0.0563923

53822	0.946131	0.0881306
53823	0.946131	0.0949126
53824	0.946131	0.101702
53825	0.946131	0.0601728
53826	0.946132	0.0905871
53827	0.946132	0.111496
53828	0.946131	0.0683567
53829	0.946131	0.0670669
53830	0.946131	0.0938926
53831	0.946131	0.0532526
53832	0.946132	0.0694988
53833	0.946132	0.0863305
53834	1.42382	2.1
53835	1.32201	0.976542
53836	1.32096	1.50315
53837	1.42866	2.08356
53838	2.75067	1.89976
53839	1.89226	2.1
53840	1.89226	0.834899
53841	1.89226	1.76452
53842	1.89226	2.01033
53843	1.89226	0.964186
53844	1.89226	1.76603
53845	1.89226	1.76974
53846	2.79029	0.447461
53847	2.7803	0.70034
53848	2.79029	1.55016
53849	1.88227	0.490419
53850	1.89226	0.792481
53851	0.716065	2.09196
53852	0.714333	2.1
53853	0.660483	1.14279
53854	0.659126	1.73713
53855	0.687538	1.2613
53856	0.687538	2.08468
53857	0.685921	2.1
53858	0.685921	1.19102
53859	0.659126	1.91383
53860	0.716065	1.91732
53861	0.660483	1.86805
53862	0.714333	1.86621
53863	1.89226	2.08729
53864	1.89226	1.03675
53865	1.89226	1.76266
53866	1.89226	1.95739
53867	3.78453	1.85395
53868	0.946133	1.08287
53869	0.946133	1.70667
53870	1.39015	0.58776
53871	1.38969	0.41096
53872	0.946132	0.430216
53873	0.946132	0.880897
53874	1.38969	1.61771
53875	1.38969	0.542963
53876	1.38969	0.702394
53877	0.946132	0.751567
53878	0.946133	1.21195
53879	1.39015	0.706719

53880	2.72157	0.0746026
53881	1.22176	0.0525472
53882	1.59492	0.0553865
53883	1.22176	0.0976825
53884	2.40743	0.05
53885	2.03426	0.094715
53886	0.946131	0.131971
53887	0.946131	0.109354
53888	1.36079	0.0741678
53889	1.01713	0.0971239
53890	0.94613	0.0921941
53891	0.94613	0.120209
53892	1.36079	0.0888752
53893	1.36079	0.0689227
53894	0.946131	0.0946066
53895	1.36079	0.0859205
53896	1.01713	0.078389
53897	0.946131	0.0907272
53898	1.89226	0.136447
53899	1.89226	0.091975
53900	1.89226	0.092239
53901	1.89226	0.164321
53902	3.78453	0.146347
53903	1.89226	0.0923708
53904	1.89226	0.0539398
53905	1.89226	0.0545985
53906	1.89226	0.0914381
53907	1.89226	0.0533818
53908	1.89226	0.0936354
53909	0.946131	0.136304
53910	0.946131	0.113781
53911	0.946131	0.0952378
53912	1.36079	0.0695178
53913	0.94613	0.0887645
53914	0.94613	0.115671
53915	0.946133	0.101473
53916	0.946133	0.0705018
53917	0.946131	0.118473
53918	0.946131	0.0987948
53919	0.946132	0.103316
53920	1.36079	0.0852061
53921	1.89226	0.0946023
53922	1.89226	0.159872
53923	1.89226	0.109703
53924	1.89226	0.119523
53925	1.89226	0.107545
53926	1.89226	0.124922
53927	1.89227	0.0984872
53928	1.89227	0.0553209
53929	1.89227	0.0628751
53930	1.89227	0.110043
53931	1.89227	0.0598307
53932	1.89227	0.106718
53933	1.60428	2.1
53934	2.2367	2.1
53935	1.50097	2.1
53936	1.80125	2.1
53937	3.40553	2.1

53938	1.89095	2.1
53939	1.85293	2.1
53940	1.80125	2.1
53941	1.76268	2.1
53942	3.65418	2.1
53943	0.803177	2.1
53944	0.803177	2.1
53945	0.798644	2.1
53946	0.798644	2.1
53947	0.84233	2.1
53948	0.900624	2.1
53949	0.750483	2.1
53950	0.701197	2.1
53951	0.701197	2.1
53952	0.84233	2.1
53953	0.750483	2.1
53954	0.900625	2.1
53955	0.860925	2.1
53956	0.860925	2.1
53957	0.862743	2.1
53958	0.862743	2.1
53959	0.881338	2.1
53960	0.826621	2.1
53961	0.84233	2.1
53962	0.900625	2.1
53963	0.84233	2.1
53964	0.826621	2.1
53965	0.900624	2.1
53966	0.881339	2.1
53967	1.37938	1.16738
53968	1.03319	1.30896
53969	1.00512	1.40638
53970	0.639582	1.42406
53971	1.00463	1.41319
53972	1.00463	1.14129
53973	1.01434	1.1137
53974	1.01434	1.40656
53975	1.37938	1.3055
53976	1.00512	1.39814
53977	1.03319	1.38998
53978	0.639582	1.40255
53979	0.728713	0.931141
53980	0.728713	0.87292
53981	0.73425	1.72813
53982	0.73425	2.1
53983	0.525122	2.1
53984	0.605622	0.28944
53985	0.948901	0.28944
53986	0.857342	0.28944
53987	0.85734	0.278268
53988	0.525123	2.1
53989	0.948899	0.278268
53990	0.605624	0.278268
53991	1.75449	1.01084
53992	1.4378	1.03727
53993	1.93916	1.11363
53994	2.22512	0.429497
53995	3.69364	0.997961

53996	1.75448	1.14338
53997	1.89186	0.865808
53998	2.29771	0.997236
53999	2.22512	0.805251
54000	4.05219	1.01095
54001	1.93916	1.12025
54002	2.51749	0.358977
54003	2.62357	0.736147
54004	2.22512	0.748274
54005	2.33761	1.00115
54006	1.93916	0.890097
54007	2.29771	1.03105
54008	2.51749	0.4916
54009	2.22512	0.742594
54010	2.22512	0.47829
54011	2.50905	1.03343
54012	2.50905	1.02972
54013	0.87382	0.05
54014	1.33662	0.05
54015	1.20756	0.05
54016	0.890467	0.0502419
54017	1.08859	0.0503778
54018	1.08859	0.05
54019	1.13084	0.05
54020	1.08793	0.05
54021	1.34722	0.05
54022	1.27184	0.05
54023	0.917678	0.05
54024	0.97821	0.05
54025	1.28396	0.05
54026	1.70302	0.0547791
54027	2.30076	0.05
54028	1.20417	0.05
54029	2.57506	0.05
54030	2.2656	0.05
54031	1.03642	1.97554
54032	1.03642	0.309331
54033	1.07029	0.109704
54034	1.07029	0.453324
54035	1.31531	1.92578
54036	1.23279	0.497619
54037	0.941743	0.177391
54038	0.791399	1.49307
54039	0.791399	0.450654
54040	1.23279	0.42982
54041	0.941744	0.38344
54042	1.31531	0.366611
54043	0.937354	1.99609
54044	0.937354	0.256866
54045	0.954794	0.136009
54046	0.954794	0.452709
54047	1.13563	0.470945
54048	1.23279	1.8642
54049	0.791398	0.21517
54050	0.659359	1.29528
54051	1.13563	0.405103
54052	0.659359	0.432037
54053	0.791399	0.317184

54054	1.23279	0.30793
54055	2.29852	1.41154
54056	1.89227	1.32322
54057	2.14485	1.0786
54058	2.14485	1.47573
54059	1.89227	0.827502
54060	1.89227	1.4801
54061	0.947863	0.805768
54062	1.19854	1.34823
54063	2.16841	0.703508
54064	1.76597	1.10436
54065	2.96451	1.10105
54066	1.89227	0.967705
54067	1.89227	1.13429
54068	1.89227	0.604572
54069	1.89227	1.3918
54070	1.89227	0.496961
54071	1.89227	1.38814
54072	2.46489	2.1
54073	1.98339	2.1
54074	1.81667	2.1
54075	2.36914	2.1
54076	4.35253	2.1
54077	2.46488	2.1
54078	2.70401	2.1
54079	2.64996	2.1
54080	2.36914	2.1
54081	5.07315	2.1
54082	1.81667	2.1
54083	2.29459	2.1
54084	2.36914	2.1
54085	2.36914	2.1
54086	1.68073	2.1
54087	1.68073	2.1
54088	2.64996	2.1
54089	2.29458	2.1
54090	2.29458	2.1
54091	2.36914	2.1
54092	2.61243	2.1
54093	2.68698	2.1
54094	1.89226	1.55988
54095	1.89226	1.01547
54096	1.89226	1.51225
54097	1.89226	1.50976
54098	1.89226	1.38869
54099	1.89226	1.44135
54100	1.89224	1.74613
54101	1.89224	1.21397
54102	1.65571	1.1228
54103	1.65571	1.44564
54104	1.89224	1.44908
54105	1.89224	1.54281
54106	1.89224	1.43909
54107	1.89224	0.834842
54108	1.89224	1.26781
54109	1.89224	1.23984
54110	1.89224	1.39544
54111	1.89224	1.25482

54112	1.98252	0.0724541
54113	2.20641	0.07737
54114	1.05129	0.0655112
54115	1.2951	0.0992474
54116	3.27762	0.070906
54117	1.85598	2.1
54118	1.88635	1.57004
54119	2.12346	0.699663
54120	2.10838	2.1
54121	3.97943	1.73657
54122	2.10838	2.06016
54123	1.87532	2.1
54124	1.85597	1.9165
54125	1.85597	2.1
54126	1.87532	2.1
54127	1.57914	2.1
54128	2.12348	1.44927
54129	2.35141	2.04344
54130	2.37191	1.7171
54131	2.10841	1.84577
54132	2.38698	1.59203
54133	2.12348	1.86151
54134	1.07811	2.1
54135	0.937672	2.1
54136	1.17571	1.98734
54137	1.05421	1.31314
54138	1.07087	1.93622
54139	1.07087	2.1
54140	1.06145	2.1
54141	1.06145	1.88707
54142	1.17571	2.1
54143	1.07811	2.1
54144	1.05421	2.1
54145	0.937672	2.1
54146	1.67334	2.1
54147	2.76609	2.1
54148	2.74144	2.1
54149	2.74144	2.1
54150	1.71157	2.1
54151	1.71157	2.1
54152	2.76609	2.1
54153	1.89226	2.1
54154	1.89226	2.1
54155	1.89226	2.1
54156	2.74143	2.1
54157	2.74143	2.1
54158	1.10393	2.1
54159	1.10393	2.1
54160	1.10788	2.1
54161	1.10788	2.1
54162	1.38302	2.1
54163	1.37072	2.1
54164	0.836672	2.1
54165	0.841089	2.1
54166	1.38302	2.1
54167	0.84109	2.1
54168	0.836671	2.1
54169	1.37072	2.1

54170	1.38302	2.1
54171	1.38302	2.1
54172	0.946131	2.1
54173	0.946131	2.1
54174	0.946128	2.1
54175	0.946128	2.1
54176	1.38302	2.1
54177	1.37072	2.1
54178	1.38302	2.1
54179	0.946131	2.1
54180	0.94613	2.1
54181	1.37072	2.1
54182	1.28072	2.1
54183	1.55779	2.1
54184	1.28072	2.1
54185	1.40765	2.1
54186	1.47186	2.1
54187	1.59879	2.1
54188	1.89227	2.1
54189	1.89227	2.1
54190	1.89227	1.63692
54191	1.89227	2.1
54192	1.89227	1.92189
54193	1.89227	2.1
54194	2.80057	1.5326
54195	1.89226	1.75476
54196	1.89226	1.37079
54197	1.89226	2.1
54198	2.63061	0.572319
54199	2.63061	2.1
54200	1.28072	2.1
54201	1.38859	2.1
54202	1.47186	2.1
54203	1.47186	2.1
54204	1.16777	2.1
54205	1.16777	2.1
54206	1.89226	2.1
54207	1.89226	2.1
54208	1.89226	1.93098
54209	1.89226	2.1
54210	1.89226	1.73405
54211	1.89226	2.1
54212	1.83221	2.1
54213	2.0815	2.1
54214	2.0815	2.1
54215	2.08153	2.1
54216	1.86703	2.1
54217	1.87402	2.1
54218	1.86703	2.1
54219	2.08508	2.1
54220	2.08508	2.1
54221	2.29001	2.1
54222	1.89226	2.1
54223	2.08048	2.1
54224	2.7278	0.0813314
54225	1.10909	0.0888515
54226	1.50472	0.0754222
54227	1.10909	0.10282

54228	2.69881	0.0742773
54229	2.30319	0.116355
54230	1.23204	2.1
54231	1.92264	2.1
54232	1.48162	2.1
54233	1.48162	2.1
54234	1.72994	2.1
54235	1.72994	2.1
54236	0.864968	2.1
54237	0.696952	2.1
54238	0.61602	2.1
54239	0.479736	2.1
54240	0.66444	2.1
54241	0.66444	2.1
54242	0.680264	2.1
54243	0.680264	2.1
54244	0.696952	2.1
54245	0.479736	2.1
54246	0.61602	2.1
54247	0.864968	2.1
54248	1.89226	0.741182
54249	2.43219	1.69996
54250	1.89226	1.5882
54251	2.82019	1.52695
54252	1.89226	1.56948
54253	2.82019	1.43987
54254	1.89226	1.1951
54255	2.83701	1.83427
54256	2.2701	1.7465
54257	2.2701	1.57346
54258	1.89226	1.74191
54259	1.89226	1.58771
54260	1.89226	1.60525
54261	1.89226	1.24115
54262	1.89226	1.4762
54263	1.89226	1.35059
54264	1.89226	1.45154
54265	1.89226	1.34107
54266	1.5795	1.60941
54267	1.44091	1.43138
54268	1.05267	1.61493
54269	1.05267	1.38291
54270	1.98408	1.62207
54271	1.98408	1.37834
54272	2.20624	2.1
54273	1.08831	2.1
54274	2.49053	2.1
54275	1.42486	2.1
54276	3.57883	2.1
54277	1.89226	2.1
54278	1.89226	2.1
54279	1.89226	2.1
54280	1.89226	2.1
54281	3.78453	2.1
54282	0.946132	2.1
54283	0.946132	2.1
54284	0.853591	2.1
54285	0.642207	2.1

54286	0.94613	2.1
54287	0.94613	2.1
54288	0.642209	2.1
54289	0.642209	2.1
54290	0.946131	2.1
54291	0.853591	2.1
54292	0.642207	2.1
54293	0.946132	2.1
54294	1.0506	2.1
54295	1.0506	2.1
54296	1.0879	2.1
54297	0.960424	2.1
54298	0.708687	2.1
54299	0.782927	2.1
54300	1.40041	2.1
54301	1.2518	2.1
54302	0.767748	2.1
54303	1.31801	2.1
54304	1.21412	2.1
54305	0.69461	2.1
54306	1.89226	2.1
54307	1.89226	2.1
54308	1.89226	2.1
54309	1.89226	2.1
54310	1.89226	2.1
54311	1.89226	2.1
54312	0.922223	2.1
54313	0.922223	2.1
54314	1.04548	2.1
54315	0.768229	2.1
54316	0.948441	2.1
54317	0.806483	2.1
54318	0.85362	2.1
54319	0.85362	2.1
54320	1.05942	2.1
54321	0.922187	2.1
54322	0.851554	2.1
54323	0.768264	2.1
54324	2.16728	2.1
54325	0.95455	2.1
54326	1.21372	2.1
54327	0.98227	2.1
54328	2.16728	2.1
54329	1.93584	2.1
54330	2.08849	0.108485
54331	1.08732	0.112951
54332	1.3357	0.11025
54333	2.37948	0.10112
54334	3.4668	0.109037
54335	2.57882	0.098279
54336	2.37948	0.107559
54337	2.53055	0.10756
54338	1.48676	0.102235
54339	1.3357	0.1075
54340	1.3357	0.103061
54341	1.89226	2.1
54342	1.89226	2.1
54343	2.17471	2.1

54344	1.89226	2.1
54345	2.60085	2.1
54346	2.26306	2.1
54347	0.946131	2.1
54348	0.946131	2.1
54349	0.946131	2.1
54350	0.946131	2.1
54351	0.94613	2.1
54352	0.94613	2.1
54353	0.946132	2.1
54354	0.946132	2.1
54355	0.946131	2.1
54356	0.946131	2.1
54357	0.946131	2.1
54358	0.946131	2.1
54359	1.89226	2.1
54360	2.17471	2.1
54361	2.08196	2.1
54362	2.17471	2.1
54363	1.79952	2.1
54364	1.89226	2.1
54365	1.89226	2.1
54366	1.89226	2.1
54367	1.89226	2.1
54368	1.89226	2.1
54369	1.89226	2.1
54370	1.89226	2.1
54371	1.86016	2.1
54372	1.89226	2.1
54373	1.89226	2.1
54374	1.89226	2.1
54375	1.84826	2.1
54376	1.84826	2.1
54377	2.50136	2.1
54378	1.99781	2.1
54379	1.99729	2.1
54380	2.5214	2.1
54381	4.49865	2.1
54382	0.51724	2.1
54383	0.496076	2.1
54384	0.998906	2.1
54385	0.998645	2.1
54386	0.742249	2.1
54387	0.742249	2.1
54388	0.752734	2.1
54389	0.752734	2.1
54390	0.998639	2.1
54391	0.496073	2.1
54392	0.517243	2.1
54393	0.998913	2.1
54394	0.998653	2.1
54395	0.994293	2.1
54396	1.26071	2.1
54397	1.26504	2.1
54398	1.12529	2.1
54399	1.12529	2.1
54400	1.12971	2.1
54401	1.12971	2.1

54402	1.26504	2.1
54403	0.994292	2.1
54404	0.998651	2.1
54405	1.26071	2.1
54406	1.89226	2.1
54407	1.89226	2.1
54408	1.89226	2.1
54409	1.89226	2.1
54410	3.78452	2.1
54411	1.89226	2.1
54412	1.89226	2.1
54413	1.89226	2.1
54414	1.89226	2.1
54415	3.78452	2.1
54416	0.675872	2.1
54417	0.675641	2.1
54418	0.943072	2.1
54419	0.898317	2.1
54420	0.775717	2.1
54421	0.775717	2.1
54422	0.79824	2.1
54423	0.79824	2.1
54424	0.675642	2.1
54425	0.94307	2.1
54426	0.675874	2.1
54427	0.898315	2.1
54428	0.943071	2.1
54429	0.898316	2.1
54430	0.998133	2.1
54431	0.918903	2.1
54432	0.922369	2.1
54433	0.922369	2.1
54434	0.939606	2.1
54435	0.939606	2.1
54436	0.943071	2.1
54437	0.998133	2.1
54438	0.898316	2.1
54439	0.918903	2.1
54440	0.946131	2.1
54441	0.946131	2.1
54442	0.946131	2.1
54443	0.946131	2.1
54444	0.946132	2.1
54445	0.946132	2.1
54446	0.946129	2.1
54447	0.946129	2.1
54448	0.94613	2.1
54449	0.94613	2.1
54450	0.946131	2.1
54451	0.946131	2.1
54452	1.89226	2.1
54453	1.89226	2.1
54454	1.89226	2.1
54455	1.89226	2.1
54456	1.89226	2.1
54457	1.89226	2.1
54458	1.35129	2.1
54459	1.88614	2.1

54460	2.01867	2.1
54461	1.38189	2.1
54462	3.36995	2.1
54463	0.943071	2.1
54464	1.00934	2.1
54465	0.998134	2.1
54466	1.10866	2.1
54467	0.992617	2.1
54468	0.992617	2.1
54469	1.01485	2.1
54470	1.01485	2.1
54471	1.00934	2.1
54472	1.10866	2.1
54473	0.943072	2.1
54474	0.998134	2.1
54475	2.67272	2.1
54476	1.61859	2.1
54477	1.70303	2.1
54478	2.52175	2.1
54479	4.5326	2.1
54480	2.00954	2.1
54481	2.27801	2.1
54482	1.87875	2.1
54483	1.87875	2.1
54484	2.37837	2.1
54485	2.37837	2.1
54486	2.00955	2.1
54487	1.78	2.1
54488	2.21813	2.1
54489	1.8493	2.1
54490	2.37838	2.1
54491	2.00955	2.1
54492	2.37838	2.1
54493	2.27801	2.1
54494	2.58523	2.1
54495	2.71597	2.1
54496	4.9636	2.1
54497	2.37838	2.1
54498	2.71597	2.1
54499	1.72709	2.1
54500	2.0815	2.1
54501	2.59749	2.1
54502	2.78034	2.1
54503	0.94613	1.4786
54504	0.94613	1.49904
54505	0.946128	1.37217
54506	0.946128	1.34565
54507	0.94613	1.33794
54508	0.94613	1.38932
54509	0.946128	1.49858
54510	0.946128	1.18915
54511	0.946129	1.45674
54512	0.946129	1.45055
54513	0.946129	1.40398
54514	0.946129	1.39692
54515	1.89226	1.62792
54516	1.89226	1.54924
54517	1.89226	1.69882

54518	1.89226	1.47121
54519	3.78452	1.50014
54520	0.946133	1.03195
54521	0.946133	1.12532
54522	0.946132	0.614622
54523	0.946132	0.736336
54524	0.946132	0.678779
54525	0.946132	0.742293
54526	0.946133	1.15567
54527	0.946133	1.04983
54528	0.946132	1.03218
54529	0.946132	0.334234
54530	0.946132	0.910554
54531	0.946132	0.752196
54532	1.89226	1.45223
54533	2.10495	0.455901
54534	1.89226	0.6135
54535	2.30932	0.724656
54536	1.89226	1.31013
54537	2.30932	1.43493
54538	2.30932	0.541761
54539	1.00366	0.246423
54540	2.10496	0.222134
54541	1.24728	0.256229
54542	3.31298	0.286877
54543	1.87557	2.1
54544	1.07167	2.1
54545	2.03045	2.1
54546	1.25185	2.1
54547	3.10212	2.1
54548	1.89226	2.1
54549	1.89226	2.1
54550	1.89226	2.1
54551	1.89226	2.1
54552	3.78453	2.1
54553	0.851541	2.1
54554	0.851541	2.1
54555	1.12236	2.1
54556	1.05783	2.1
54557	0.870663	2.1
54558	0.951856	2.1
54559	0.946132	2.1
54560	0.946132	2.1
54561	1.04073	2.1
54562	1.1296	2.1
54563	0.776062	2.1
54564	0.851527	2.1
54565	0.859395	2.1
54566	0.859395	2.1
54567	0.870165	2.1
54568	0.870165	2.1
54569	0.625924	2.1
54570	0.714333	2.1
54571	1.0821	2.1
54572	1.01523	2.1
54573	1.0821	2.1
54574	0.714333	2.1
54575	1.01523	2.1

54576	0.625924	2.1
54577	1.89226	2.1
54578	1.89226	2.1
54579	1.89226	2.1
54580	1.89226	2.1
54581	3.78453	2.1
54582	1.89226	2.1
54583	1.89226	2.1
54584	2.23164	2.1
54585	2.67802	2.1
54586	2.03663	2.1
54587	1.89226	2.1
54588	2.42221	2.1
54589	1.61933	2.1
54590	2.80896	2.1
54591	1.87043	2.1
54592	4.29262	2.1
54593	1.89226	2.1
54594	1.89226	2.1
54595	1.89226	2.1
54596	1.89226	2.1
54597	1.89226	2.1
54598	1.89226	2.1
54599	2.80892	2.1
54600	1.87041	2.1
54601	2.67182	2.1
54602	2.30891	2.1
54603	5.07665	2.1
54604	1.89224	2.1
54605	1.89224	2.1
54606	1.89224	2.1
54607	1.89224	2.1
54608	3.78448	2.1
54609	0.946118	2.1
54610	0.946118	2.1
54611	0.946118	2.1
54612	0.946118	2.1
54613	0.946115	2.1
54614	0.946115	2.1
54615	0.94612	2.1
54616	0.94612	2.1
54617	0.94613	2.1
54618	0.94613	2.1
54619	0.946105	2.1
54620	0.946105	2.1
54621	2.3089	2.1
54622	1.02783	2.1
54623	1.98533	2.1
54624	1.76762	2.1
54625	1.24553	2.1
54626	1.02783	2.1
54627	2.2049	0.101211
54628	2.34673	0.0965405
54629	1.00763	0.0937249
54630	1.23746	0.127252
54631	3.44236	0.0986439
54632	1.89226	0.138653
54633	1.89226	0.199237

54634	1.89226	0.174586
54635	1.89226	0.185048
54636	3.78452	0.19241
54637	1.89226	0.205561
54638	1.89226	0.180529
54639	1.89226	0.18915
54640	1.89226	0.268297
54641	3.78453	0.198292
54642	0.891618	0.138575
54643	0.891618	0.13047
54644	0.902158	0.102904
54645	0.902158	0.113584
54646	1.15396	0.131085
54647	0.620408	0.120104
54648	1.17337	0.103595
54649	0.618733	0.130237
54650	0.620408	0.125847
54651	0.618733	0.116397
54652	1.17337	0.120972
54653	1.15396	0.127516
54654	0.69978	0.134046
54655	0.69978	0.125072
54656	0.946102	0.12701
54657	0.946102	0.131694
54658	0.831702	0.14292
54659	0.946129	0.145957
54660	0.699752	0.129143
54661	0.946132	0.115294
54662	0.831703	0.138116
54663	0.699751	0.117431
54664	0.946131	0.118978
54665	0.946131	0.126425
54666	0.831675	0.161563
54667	0.831675	0.157539
54668	0.946163	0.144041
54669	0.864397	0.154449
54670	0.853471	0.189697
54671	0.962623	0.148008
54672	0.748555	0.142903
54673	0.851543	0.147658
54674	0.864714	0.163715
54675	0.760411	0.153893
54676	0.946133	0.148307
54677	0.946133	0.159477
54678	1.89226	0.0725839
54679	1.89226	0.572188
54680	1.89226	0.308354
54681	1.89226	0.0684345
54682	3.78452	0.0923819
54683	1.89226	0.702743
54684	1.89226	0.410879
54685	1.89226	0.153472
54686	1.89226	0.669446
54687	3.78452	0.437566
54688	0.946132	0.607652
54689	0.946132	0.810472
54690	0.946132	0.152928
54691	0.946132	0.40981

54692	0.946132	0.163621
54693	0.946132	0.616818
54694	0.946133	0.377264
54695	0.946133	0.754451
54696	0.946133	0.176335
54697	0.946133	0.218834
54698	0.946132	0.549923
54699	0.946132	0.542639
54700	1.89226	0.580299
54701	2.39715	0.100044
54702	1.89226	0.0981827
54703	1.89226	0.292834
54704	1.89226	0.128488
54705	1.89226	0.370124
54706	0.94613	0.529913
54707	0.94613	0.626714
54708	0.94613	0.328428
54709	0.94613	0.136528
54710	0.946131	0.130057
54711	0.946131	0.54855
54712	0.946129	0.677689
54713	0.946129	0.248885
54714	0.94613	0.186339
54715	0.94613	0.169414
54716	0.94613	0.497297
54717	0.94613	0.44503
54718	2.54204	2.1
54719	2.3148	2.1
54720	2.55328	2.1
54721	2.39646	2.1
54722	4.71126	2.1
54723	1.62611	2.1
54724	2.3148	2.1
54725	2.16986	2.1
54726	1.43745	2.1
54727	3.75225	2.1
54728	1.89226	2.1
54729	1.89226	2.1
54730	1.89226	2.1
54731	1.89226	2.1
54732	3.78453	2.1
54733	1.89226	2.1
54734	1.89226	2.1
54735	1.89226	2.1
54736	1.89226	2.1
54737	3.78453	2.1
54738	2.12125	2.1
54739	2.39642	2.1
54740	2.2941	2.1
54741	1.9933	2.1
54742	4.38972	2.1
54743	1.73784	2.1
54744	1.26892	2.1
54745	1.26892	2.1
54746	1.43743	2.1
54747	1.9933	2.1
54748	2.1618	2.1
54749	0.946118	2.1

54750	0.946118	2.1
54751	0.946118	2.1
54752	0.946118	2.1
54753	0.946119	2.1
54754	0.946119	2.1
54755	0.946118	2.1
54756	0.946118	2.1
54757	0.946131	2.1
54758	0.946131	2.1
54759	0.946106	2.1
54760	0.946106	2.1
54761	1.89224	2.1
54762	1.89224	2.1
54763	1.89224	2.1
54764	1.89224	2.1
54765	3.78448	2.1
54766	2.45922	2.1
54767	2.15686	2.1
54768	2.14259	2.1
54769	2.43225	2.1
54770	4.58911	2.1
54771	1.62027	2.1
54772	2.15686	2.1
54773	1.62241	2.1
54774	1.62241	2.1
54775	2.1426	2.1
54776	2.1426	2.1
54777	2.14259	2.1
54778	2.43225	2.1
54779	2.40667	2.1
54780	2.1281	2.1
54781	4.56035	2.1
54782	1.0713	2.1
54783	1.06405	2.1
54784	0.810136	2.1
54785	0.808433	2.1
54786	0.938508	2.1
54787	0.938508	2.1
54788	0.941223	2.1
54789	0.941223	2.1
54790	1.06405	2.1
54791	0.808433	2.1
54792	0.810135	2.1
54793	1.0713	2.1
54794	1.89226	0.470978
54795	1.89226	0.23839
54796	1.89226	0.401268
54797	1.89226	0.270842
54798	3.78453	0.276746
54799	1.89227	0.280435
54800	1.89227	0.239601
54801	1.89227	0.366508
54802	1.89227	0.198387
54803	3.78453	0.277649
54804	1.19	0.200918
54805	1.12469	0.147422
54806	1.01052	0.174096
54807	1.08166	0.160902

54808	2.20051	0.160313
54809	0.594999	0.199348
54810	0.540832	0.180398
54811	0.514182	0.234559
54812	0.567857	0.186461
54813	0.554259	0.177142
54814	0.554259	0.167586
54815	0.55443	0.201136
54816	0.55443	0.212882
54817	0.540832	0.193181
54818	0.514182	0.173524
54819	0.567857	0.183661
54820	0.594999	0.178202
54821	1.38674	0.239657
54822	0.946132	0.299823
54823	1.36361	0.228731
54824	0.946132	0.277359
54825	1.36362	0.222126
54826	1.36362	0.173577
54827	0.946131	0.302756
54828	0.946131	0.370051
54829	1.38674	0.260385
54830	1.36361	0.20113
54831	0.946132	0.298507
54832	0.946132	0.253981
54833	1.89226	0.28311
54834	2.68508	0.170213
54835	2.77349	0.200186
54836	1.89226	0.221787
54837	2.77349	0.21233
54838	1.89226	0.230178
54839	2.15315	0.0664515
54840	1.96254	0.056553
54841	1.51377	0.0673417
54842	1.89226	0.0818257
54843	1.47799	0.0576097
54844	1.89226	0.0599979
54845	2.49439	0.0849382
54846	1.89226	0.124621
54847	2.15315	0.192138
54848	1.89226	0.204486
54849	2.15315	0.0893018
54850	1.89226	0.080509
54851	1.89226	0.151327
54852	1.89226	0.259984
54853	1.89226	0.220855
54854	1.89226	0.226069
54855	1.89226	0.192059
54856	1.89226	0.228397
54857	1.89226	0.0692893
54858	1.89226	0.220832
54859	1.89226	0.0541299
54860	1.89226	0.174611
54861	1.89226	0.0621537
54862	1.89226	0.187836
54863	1.375	0.0531837
54864	2.01865	0.0619809
54865	2.12047	0.05

54866	1.47354	0.0599483
54867	2.12047	0.05
54868	1.47354	0.063232
54869	0.687499	0.05
54870	1.06023	0.05
54871	1.08019	0.05
54872	0.604229	0.05
54873	1.08018	0.05
54874	1.06023	0.05
54875	0.687502	0.05
54876	0.60423	0.05
54877	0.80646	0.05
54878	0.806461	0.05
54879	0.858001	0.05
54880	0.858001	0.05
54881	2.26414	2.1
54882	1.87342	2.1
54883	1.87342	2.1
54884	1.79667	2.1
54885	2.44045	2.1
54886	2.31924	2.1
54887	2.26414	2.1
54888	2.26616	2.1
54889	2.2355	2.1
54890	2.21858	2.1
54891	4.48474	2.1
54892	2.18516	2.1
54893	1.87344	2.1
54894	2.21859	2.1
54895	2.66233	2.1
54896	1.85196	2.1
54897	2.22236	2.1
54898	2.23551	2.1
54899	2.21859	2.1
54900	2.18516	2.1
54901	2.21189	2.1
54902	4.43049	2.1
54903	1.17171	2.1
54904	2.38924	2.1
54905	2.0815	2.1
54906	2.33348	2.1
54907	1.42851	2.1
54908	1.5959	2.1
54909	1.89226	2.1
54910	1.89226	2.1
54911	1.89226	2.1
54912	1.89226	2.1
54913	3.78452	2.1
54914	2.75425	2.1
54915	1.34366	2.1
54916	1.34366	2.1
54917	1.56512	2.1
54918	2.54186	2.1
54919	2.76332	2.1
54920	1.07751	2.1
54921	2.0815	2.1
54922	1.97259	2.1
54923	1.76455	2.1

54924	1.30191	2.1
54925	1.16461	2.1
54926	0.946131	2.1
54927	0.946131	2.1
54928	0.946129	2.1
54929	0.946129	2.1
54930	0.946131	2.1
54931	0.946131	2.1
54932	0.94613	2.1
54933	0.94613	2.1
54934	0.94613	2.1
54935	0.94613	2.1
54936	0.946131	2.1
54937	0.946131	2.1
54938	1.89226	0.178779
54939	1.89226	0.104162
54940	1.89226	0.100185
54941	1.89226	0.0840232
54942	3.78453	0.0816035
54943	1.89226	0.155899
54944	1.89226	0.112851
54945	1.89226	0.107441
54946	1.89226	0.070773
54947	3.78453	0.139653
54948	0.946132	0.0800716
54949	0.946132	0.105049
54950	1.30327	0.0731135
54951	1.33544	0.0817748
54952	1.33544	0.0664291
54953	1.33544	0.0608129
54954	0.946132	0.0847372
54955	0.946132	0.0672716
54956	0.946132	0.0627493
54957	1.30327	0.0546014
54958	1.33544	0.0803054
54959	0.946132	0.0895496
54960	1.30327	0.0778565
54961	1.33544	0.0769404
54962	0.709426	0.0750489
54963	0.790129	0.0760301
54964	1.05886	0.0582121
54965	1.05886	0.0682911
54966	1.03454	0.0989047
54967	1.03454	0.0801313
54968	1.30327	0.0696245
54969	0.709426	0.0567432
54970	1.33544	0.0977511
54971	0.790128	0.0794137
54972	0.946132	0.0590281
54973	0.946132	0.0722818
54974	0.827866	0.05
54975	0.946133	0.0676817
54976	0.946132	0.05
54977	0.946132	0.0542243
54978	0.946133	0.0535634
54979	0.946133	0.0825024
54980	0.946132	0.05
54981	0.827866	0.0738096

54982	0.946133	0.087195
54983	0.946133	0.074257
54984	1.89227	0.0912014
54985	1.89227	0.05
54986	1.89227	0.0626227
54987	1.89227	0.0635406
54988	1.89227	0.0569757
54989	1.89227	0.0576791
54990	0.946133	0.0515179
54991	0.946133	0.0614074
54992	1.21602	0.05
54993	1.30327	0.0520296
54994	1.30327	0.05
54995	1.30327	0.05
54996	0.946132	0.05
54997	0.946132	0.0558668
54998	0.946132	0.05
54999	1.21602	0.05
55000	0.946133	0.059139
55001	1.30327	0.0511763
55002	1.04227	0.05
55003	1.11706	0.05
55004	0.968413	0.05
55005	0.945482	0.05
55006	1.3204	0.05
55007	1.23215	0.05
55008	0.810791	0.05
55009	1.38978	0.05
55010	1.07728	0.05244
55011	1.11704	0.0538626
55012	0.608052	0.05
55013	1.89226	0.114349
55014	1.89226	0.06791
55015	1.89226	0.079058
55016	1.89226	0.0945524
55017	3.78453	0.0886317
55018	1.89226	0.05
55019	1.89226	0.0576473
55020	1.89226	0.0699003
55021	1.89226	0.05
55022	3.78451	0.0525409
55023	2.58476	0.0501959
55024	1.56598	0.05
55025	1.55045	0.05
55026	1.5124	0.05
55027	2.62281	0.05
55028	2.58476	0.05
55029	2.58477	0.0601913
55030	1.89226	0.0704665
55031	1.89226	0.0744051
55032	1.89226	0.0564747
55033	2.62282	0.0759914
55034	2.62282	0.0575691
55035	1.89224	0.0838252
55036	1.89224	0.099534
55037	1.89224	0.126681
55038	1.89224	0.0735451
55039	3.78448	0.088093

55040	1.89223	0.075174
55041	1.89223	0.05
55042	1.89223	0.05856
55043	1.89223	0.0523678
55044	1.89223	0.0633167
55045	1.89223	0.0585585
55046	2.62278	0.0550928
55047	1.45053	0.05
55048	1.49678	0.05
55049	1.56596	0.05
55050	2.5536	0.05
55051	2.62278	0.05
55052	1.31139	0.0607775
55053	1.2768	0.05
55054	0.946118	0.0966038
55055	0.946118	0.0720387
55056	1.2768	0.073842
55057	1.2768	0.0590824
55058	0.946116	0.091552
55059	0.946116	0.0603688
55060	1.27682	0.0595637
55061	0.94613	0.0734956
55062	0.946105	0.0711
55063	1.31138	0.064279
55064	1.38506	1.48371
55065	1.53348	1.97549
55066	1.17042	1.73638
55067	1.17042	1.65792
55068	1.73669	1.71655
55069	1.73669	1.59611
55070	1.73669	1.56205
55071	1.79928	1.21761
55072	1.38506	1.48759
55073	1.38506	1.67702
55074	1.79928	1.74822
55075	1.43484	1.78806
55076	1.73669	1.42717
55077	1.93568	1.75735
55078	1.53347	1.59933
55079	1.53347	1.61656
55080	2.14058	1.58541
55081	2.14058	1.60114
55082	0.89964	1.20084
55083	1.10415	1.99319
55084	0.868346	1.5429
55085	1.07029	1.84611
55086	0.98687	1.61013
55087	0.98687	2.1
55088	0.985627	1.10448
55089	0.985627	1.78342
55090	1.07029	1.60727
55091	1.10415	1.50733
55092	0.868344	1.60528
55093	0.899642	1.69434
55094	2.40667	2.1
55095	2.1281	2.1
55096	2.11176	2.1
55097	2.38069	2.1

55098	4.50878	2.1
55099	2.11176	2.1
55100	1.61687	2.1
55101	1.60965	2.1
55102	1.61687	2.1
55103	2.12088	2.1
55104	2.1281	2.1
55105	2.11176	2.1
55106	2.34898	2.1
55107	2.34898	2.1
55108	2.38069	2.1
55109	2.09114	2.1
55110	2.12285	2.1
55111	1.05588	2.1
55112	1.04557	2.1
55113	0.804824	2.1
55114	0.798269	2.1
55115	0.92615	2.1
55116	0.92615	2.1
55117	0.927999	2.1
55118	0.927999	2.1
55119	1.04557	2.1
55120	0.798269	2.1
55121	1.05588	2.1
55122	0.804824	2.1
55123	1.43548	0.30493
55124	1.53873	0.712496
55125	1.25063	0.331486
55126	1.39662	0.974591
55127	2.78937	0.264323
55128	1.25063	0.311547
55129	1.39662	0.933232
55130	1.10476	0.261565
55131	1.28326	0.521385
55132	2.53389	0.395416
55133	1.51147	0.05
55134	1.86114	0.05
55135	1.86114	0.05
55136	2.12926	0.05
55137	1.3659	0.05
55138	1.63401	0.05
55139	1.89226	0.05
55140	1.89226	0.060837
55141	1.89226	0.05
55142	1.89226	0.05
55143	1.89226	0.0588191
55144	1.89226	0.0539318
55145	1.89226	0.0855774
55146	1.89226	0.117638
55147	2.46286	0.0767379
55148	1.89226	0.144647
55149	3.78452	0.0776762
55150	1.89226	0.128093
55151	1.89226	0.246846
55152	1.89226	0.188422
55153	1.89226	0.181575
55154	1.89226	0.138337
55155	1.89226	0.136614

55156	1.89226	0.0947853
55157	1.89226	0.113396
55158	2.28992	0.0877082
55159	1.89226	0.147401
55160	3.78452	0.0868567
55161	1.13537	0.127998
55162	0.886595	0.453685
55163	0.946131	1.12823
55164	0.946131	0.64124
55165	0.94613	0.499294
55166	0.94613	0.106784
55167	1.25069	1.18011
55168	0.841855	1.00692
55169	1.27436	0.33638
55170	1.06196	0.52995
55171	1.09758	0.639874
55172	0.830306	0.329588
55173	0.826931	2.01335
55174	0.826933	0.342618
55175	0.764631	0.100387
55176	0.764631	0.535466
55177	1.00427	1.8252
55178	0.788034	0.622063
55179	0.726852	0.0704512
55180	0.604206	0.819263
55181	0.828475	0.505031
55182	0.604198	0.508157
55183	0.8332	0.389058
55184	1.00428	0.391241
55185	1.89226	0.947376
55186	1.89226	0.366424
55187	1.89226	0.150489
55188	1.89226	1.1016
55189	1.89226	0.413955
55190	1.89226	0.720534
55191	0.768789	2.1
55192	0.849132	0.05
55193	0.666423	2.1
55194	0.688993	1.91944
55195	0.868671	0.05
55196	0.89117	2.1
55197	0.705632	2.1
55198	1.80425	2.1
55199	1.73883	2.1
55200	1.45159	2.1
55201	1.45159	2.1
55202	1.37681	2.1
55203	1.73883	2.1
55204	1.39679	0.05
55205	2.08149	0.05
55206	1.89226	0.05
55207	2.32763	0.05
55208	1.89226	0.05
55209	2.31274	0.05
55210	1.89226	0.05
55211	1.89226	0.0511209
55212	1.89226	0.05
55213	1.89226	0.05

55214	1.89226	0.05
55215	1.89226	0.05
55216	1.89226	0.05
55217	1.89226	0.05
55218	1.89226	0.05
55219	1.89226	0.05
55220	1.89226	0.05
55221	1.89226	0.05
55222	1.95181	0.05
55223	1.89226	0.05
55224	1.89226	0.05
55225	1.89226	0.05
55226	3.78451	0.05
55227	0.946131	0.05
55228	0.946131	0.05
55229	0.946125	0.05
55230	0.946125	0.05
55231	0.946127	0.05
55232	0.946127	0.05
55233	0.946128	0.05
55234	0.946128	0.05
55235	0.946128	0.05
55236	0.946128	0.05
55237	0.946128	0.05
55238	0.946128	0.05
55239	1.89226	0.05
55240	1.89226	0.0598281
55241	1.89226	0.05
55242	1.89226	0.05
55243	1.89226	0.05
55244	1.89226	0.05
55245	1.89226	1.04359
55246	1.89226	1.03504
55247	1.89226	0.979427
55248	1.89226	0.846846
55249	3.78453	1.01051
55250	1.89226	1.12375
55251	1.89226	0.960614
55252	1.89226	1.02665
55253	1.89226	0.626629
55254	3.78452	0.848524
55255	1.21387	0.697128
55256	0.8164	0.362699
55257	0.946133	0.585816
55258	0.946133	0.727684
55259	0.946102	0.329734
55260	0.946102	0.724621
55261	0.816431	1.07454
55262	0.816431	0.940015
55263	0.816401	0.630583
55264	0.94613	0.425111
55265	0.946131	1.1187
55266	1.21388	0.804178
55267	0.946132	0.463195
55268	0.946132	0.589035
55269	0.946132	0.929988
55270	0.946132	0.647438
55271	0.946132	0.294145

55272	0.946132	0.543872
55273	0.946132	1.01139
55274	0.946132	0.764692
55275	0.946131	0.281046
55276	0.946131	0.674387
55277	0.946132	0.996109
55278	0.946132	0.732777
55279	1.89226	0.940825
55280	1.89226	0.252775
55281	1.89226	0.45191
55282	1.89226	0.837343
55283	1.89226	0.490773
55284	1.89226	0.729814
55285	1.89226	1.00641
55286	1.89226	0.657363
55287	2.15871	0.299541
55288	2.15871	0.858186
55289	1.81454	0.241579
55290	1.54809	0.890611
55291	1.89226	1.03198
55292	1.89226	1.30128
55293	1.89226	1.15835
55294	1.89226	1.03828
55295	3.78453	1.19614
55296	1.89226	1.17965
55297	1.89226	1.00604
55298	1.89226	0.969608
55299	1.89226	1.12837
55300	1.89226	1.28432
55301	1.89226	1.24451
55302	2.29205	1.15779
55303	1.42633	1.16139
55304	1.69015	1.23509
55305	1.69015	1.1381
55306	2.10364	1.09678
55307	2.05344	1.03177
55308	2.29205	1.03165
55309	1.58147	1.26018
55310	2.16529	1.11719
55311	1.56339	1.05832
55312	2.29205	1.26045
55313	1.69015	1.2272
55314	1.89226	1.21808
55315	1.89226	1.12064
55316	1.89226	1.00942
55317	1.89226	1.17465
55318	3.78453	1.07287
55319	1.89226	1.13885
55320	1.89226	1.30223
55321	1.89226	1.14064
55322	1.89226	1.08344
55323	3.78452	1.23085
55324	2.29205	1.2778
55325	2.85335	1.0021
55326	2.55409	1.20029
55327	2.04345	1.09924
55328	2.76338	1.12255
55329	2.29205	1.08684

55330	2.29205	1.10642
55331	2.16529	1.2698
55332	2.72418	1.16194
55333	2.76338	0.968778
55334	5.01623	1.21635
55335	1.89227	0.05
55336	1.89227	0.05
55337	1.89227	0.05
55338	1.89227	0.05
55339	3.78453	0.05
55340	2.39588	0.05
55341	2.14918	0.05
55342	2.14918	0.05
55343	2.37907	0.05
55344	2.20531	0.05
55345	2.4352	0.05
55346	1.89226	0.05
55347	2.64327	0.05
55348	2.36175	0.05
55349	1.89226	0.05
55350	4.25402	0.05
55351	1.89226	0.05
55352	1.89226	0.05
55353	1.89226	0.05
55354	1.89226	0.05
55355	1.89226	0.05
55356	1.89226	0.05
55357	1.05395	0.05
55358	1.05395	0.05
55359	1.03114	0.05
55360	1.03114	0.05
55361	0.936689	0.05
55362	1.07459	0.05
55363	1.10265	0.05
55364	1.01049	0.05
55365	0.93669	0.05
55366	1.01049	0.05
55367	1.07459	0.05
55368	1.10265	0.05
55369	1.18088	0.05
55370	0.776509	0.05
55371	0.946132	0.05
55372	0.946132	0.05
55373	1.04225	0.05
55374	0.830392	0.05
55375	0.931352	0.05
55376	0.931352	0.05
55377	1.13006	0.05
55378	1.04074	0.05
55379	1.0628	0.05
55380	0.851529	0.05
55381	2.40032	2.1
55382	1.89226	2.1
55383	1.89226	2.1
55384	1.89226	2.1
55385	2.38918	2.1
55386	2.38918	2.1
55387	1.89226	2.1

55388	1.89226	2.1
55389	1.89226	2.1
55390	1.89226	2.1
55391	1.89226	2.1
55392	1.89226	2.1
55393	1.6585	2.1
55394	2.37364	2.1
55395	2.39622	2.1
55396	2.39622	2.1
55397	1.6488	2.1
55398	1.6488	2.1
55399	2.44603	2.1
55400	1.89224	2.1
55401	1.89224	2.1
55402	1.89224	2.1
55403	2.40029	2.1
55404	2.40029	2.1
55405	1.89224	2.1
55406	1.89224	2.1
55407	1.89224	2.1
55408	1.89224	2.1
55409	1.89224	2.1
55410	1.89224	2.1
55411	1.01946	2.1
55412	1.01946	2.1
55413	1.02497	2.1
55414	1.02497	2.1
55415	1.21519	2.1
55416	1.19809	2.1
55417	0.835303	2.1
55418	0.82924	2.1
55419	0.835314	2.1
55420	1.2152	2.1
55421	0.829228	2.1
55422	1.19808	2.1
55423	1.91027	2.1
55424	1.05809	2.1
55425	1.00659	2.1
55426	2.06727	2.1
55427	3.11819	2.1
55428	2.37166	2.1
55429	2.06726	2.1
55430	1.66994	2.1
55431	1.67623	2.1
55432	2.40308	2.1
55433	2.40308	2.1
55434	0.960194	2.1
55435	0.939664	2.1
55436	0.531131	2.1
55437	0.503298	2.1
55438	0.729927	2.1
55439	0.729927	2.1
55440	0.733565	2.1
55441	0.733565	2.1
55442	0.960195	2.1
55443	0.531132	2.1
55444	0.503297	2.1
55445	0.939663	2.1

55446	1.20154	2.1
55447	1.21754	2.1
55448	0.960195	2.1
55449	0.939665	2.1
55450	1.07745	2.1
55451	1.07745	2.1
55452	1.07976	2.1
55453	1.07976	2.1
55454	0.960194	2.1
55455	1.21754	2.1
55456	0.939662	2.1
55457	1.20155	2.1
55458	1.89224	2.1
55459	1.89224	2.1
55460	2.08609	2.1
55461	2.08609	2.1
55462	2.09527	2.1
55463	1.90142	2.1
55464	1.65815	2.1
55465	1.71398	2.1
55466	2.2001	2.1
55467	2.23791	2.1
55468	1.4502	2.1
55469	1.4502	2.1
55470	0.793587	2.1
55471	0.793587	2.1
55472	0.792408	2.1
55473	0.792408	2.1
55474	0.952221	2.1
55475	0.858585	2.1
55476	0.725101	2.1
55477	0.633774	2.1
55478	0.633775	2.1
55479	0.858585	2.1
55480	0.952221	2.1
55481	0.725101	2.1
55482	1.5735	2.1
55483	2.56975	2.1
55484	2.63166	2.1
55485	2.63166	2.1
55486	1.54711	2.1
55487	1.54711	2.1
55488	0.946131	2.1
55489	0.946131	2.1
55490	1.28487	2.1
55491	1.28487	2.1
55492	0.946127	2.1
55493	0.946127	2.1
55494	1.28488	2.1
55495	1.31583	2.1
55496	1.31583	2.1
55497	0.94613	2.1
55498	1.28487	2.1
55499	0.946129	2.1
55500	1.0596	2.1
55501	1.0596	2.1
55502	1.06939	2.1
55503	1.06939	2.1

55504	1.34225	2.1
55505	1.31583	2.1
55506	0.793563	2.1
55507	0.786748	2.1
55508	0.793563	2.1
55509	1.34225	2.1
55510	0.786748	2.1
55511	1.31583	2.1
55512	0.946131	2.1
55513	0.946131	2.1
55514	1.31582	2.1
55515	1.31582	2.1
55516	0.946127	2.1
55517	0.946127	2.1
55518	1.34225	2.1
55519	1.31583	2.1
55520	1.34225	2.1
55521	0.946129	2.1
55522	1.31583	2.1
55523	0.946129	2.1
55524	1.89226	2.1
55525	1.89226	2.1
55526	1.89226	2.1
55527	1.89226	2.1
55528	1.89226	2.1
55529	1.89226	2.1
55530	1.89226	2.1
55531	1.89226	2.1
55532	1.89226	2.1
55533	1.89226	2.1
55534	1.89226	2.1
55535	1.89226	2.1
55536	1.26992	2.1
55537	1.75003	2.1
55538	1.79883	2.1
55539	1.26732	2.1
55540	3.06875	2.1
55541	0.875017	2.1
55542	0.899414	2.1
55543	0.811147	2.1
55544	0.85053	2.1
55545	0.851513	2.1
55546	0.851513	2.1
55547	0.859048	2.1
55548	0.859048	2.1
55549	0.899413	2.1
55550	0.85053	2.1
55551	0.811147	2.1
55552	0.875016	2.1
55553	1.89227	2.1
55554	1.89227	2.1
55555	1.89227	2.1
55556	1.89227	2.1
55557	1.89227	2.1
55558	1.89227	2.1
55559	1.89226	2.1
55560	1.89226	2.1
55561	1.89226	2.1

55562	1.89226	2.1
55563	1.89226	2.1
55564	1.89226	2.1
55565	0.633661	2.1
55566	0.642005	2.1
55567	0.93285	2.1
55568	0.899413	2.1
55569	0.764487	2.1
55570	0.764487	2.1
55571	0.776931	2.1
55572	0.776931	2.1
55573	0.642004	2.1
55574	0.93285	2.1
55575	0.899414	2.1
55576	0.633661	2.1
55577	0.932851	2.1
55578	0.899414	2.1
55579	0.905419	2.1
55580	0.85053	2.1
55581	0.886336	2.1
55582	0.886336	2.1
55583	0.897045	2.1
55584	0.897045	2.1
55585	0.93285	2.1
55586	0.905419	2.1
55587	0.899414	2.1
55588	0.850531	2.1
55589	2.39322	2.1
55590	2.74149	2.1
55591	2.70944	2.1
55592	2.6849	1.97049
55593	2.41776	2.1
55594	2.39322	1.7726
55595	2.41776	2.1
55596	2.80992	2.1
55597	2.77256	2.1
55598	2.74149	2.1
55599	2.44882	2.1
55600	2.41776	1.92602
55601	2.80992	2.1
55602	1.89226	1.66868
55603	2.74149	1.96537
55604	2.74149	2.1
55605	1.89226	1.6015
55606	1.89226	2.04008
55607	1.89226	0.05
55608	1.89226	0.05
55609	1.89226	0.0548215
55610	1.89226	0.05
55611	1.89226	0.0555695
55612	1.89226	0.05
55613	2.38796	0.05
55614	2.0815	0.05
55615	2.0815	0.05
55616	2.49452	0.05
55617	1.55994	0.05
55618	2.14721	0.05
55619	1.89227	0.0523821

55620	1.89227	0.05
55621	1.89227	0.0639285
55622	1.89227	0.05
55623	1.89227	0.0672043
55624	1.89227	0.05
55625	1.13387	0.05
55626	2.27494	0.05
55627	1.13387	0.05
55628	2.19489	0.05
55629	1.60867	0.05
55630	2.6994	0.05
55631	1.89226	0.0531924
55632	1.89226	0.05
55633	1.89226	0.05
55634	1.89226	0.0724628
55635	3.78453	0.05
55636	1.42381	2.1
55637	1.10747	2.1
55638	1.10747	2.1
55639	1.08911	2.1
55640	1.42866	2.05961
55641	1.4103	2.1
55642	1.89226	2.1
55643	1.88181	2.1
55644	1.88181	2.1
55645	1.76249	2.1
55646	2.0815	2.1
55647	1.97539	2.1
55648	0.946128	2.1
55649	0.946128	2.1
55650	0.946133	2.09917
55651	0.946133	1.89485
55652	0.94613	1.92279
55653	0.94613	2.1
55654	0.946131	2.1
55655	0.946131	2.03019
55656	0.946131	2.1
55657	0.946131	2.1
55658	0.946131	2.1
55659	0.946131	2.1
55660	1.42866	2.1
55661	1.43213	2.1
55662	1.10748	2.1
55663	1.10748	2.1
55664	1.13641	2.1
55665	1.43213	2.1
55666	2.0815	2.1
55667	2.10287	2.1
55668	2.10287	2.1
55669	2.0921	2.1
55670	1.89227	2.1
55671	1.83938	2.1
55672	0.810944	2.1
55673	0.810944	1.94382
55674	0.810945	1.95808
55675	0.810945	2.1
55676	0.810933	2.1
55677	0.810933	2.1

55678	1.89226	2.1
55679	1.3517	2.1
55680	1.08132	2.1
55681	1.08132	2.1
55682	1.08133	2.1
55683	2.33097	2.1
55684	1.53822	2.1
55685	1.53822	2.1
55686	1.68437	2.1
55687	2.17996	2.1
55688	2.32612	2.1
55689	0.99189	2.1
55690	1.18948	2.1
55691	1.19756	2.1
55692	1.19756	2.1
55693	0.978851	2.1
55694	0.926995	2.1
55695	1.26924	1.8362
55696	1.34642	2.1
55697	1.3228	2.1
55698	0.990899	2.1
55699	1.0627	2.1
55700	1.33473	2.1
55701	1.96196	2.1
55702	1.39702	2.1
55703	1.39702	2.1
55704	1.53822	2.1
55705	2.17372	2.1
55706	2.31011	2.1
55707	1.89226	1.9078
55708	1.89226	0.406972
55709	1.89226	1.88441
55710	1.89226	0.469361
55711	3.78453	0.58897
55712	2.38475	2.1
55713	1.73957	0.563773
55714	1.88452	1.88548
55715	1.58695	0.386473
55716	3.6241	0.532033
55717	1.73958	0.601871
55718	1.3126	2.1
55719	1.58695	0.396149
55720	1.14946	2.1
55721	2.88903	1.67718
55722	1.89226	0.397607
55723	1.89226	2.1
55724	1.89226	0.404397
55725	1.89226	2.1
55726	3.78453	1.6671
55727	1.89225	1.94561
55728	1.89225	0.55575
55729	1.89225	1.6913
55730	1.89225	0.748185
55731	3.7845	0.263565
55732	1.88451	1.94338
55733	1.58694	0.193395
55734	1.74649	2.1
55735	1.44604	0.780387

55736	3.33055	0.567885
55737	1.58694	0.237805
55738	0.995727	2.1
55739	0.995727	1.76518
55740	1.14945	1.96666
55741	1.44604	0.415716
55742	1.59976	2.05159
55743	1.89225	0.415053
55744	1.89225	2.1
55745	1.89225	2.1
55746	1.89225	0.770447
55747	1.89225	2.1
55748	1.89225	0.426286
55749	2.01197	2.08506
55750	2.0961	1.6228
55751	2.13283	1.66658
55752	2.25399	1.24435
55753	4.22894	1.57905
55754	2.09611	1.43953
55755	2.37014	1.65601
55756	2.29031	1.68852
55757	2.10084	2.04526
55758	2.25399	1.2644
55759	2.09611	2.00347
55760	2.13283	1.93597
55761	2.25399	1.95405
55762	2.22044	1.96469
55763	2.38975	1.24758
55764	4.52258	1.64826
55765	2.38976	0.27376
55766	2.15466	1.74197
55767	2.31988	0.393127
55768	2.31988	2.03263
55769	2.25399	0.253274
55770	2.25399	2.1
55771	2.58286	2.1
55772	2.29193	0.399643
55773	2.40107	2.1
55774	2.13815	0.198013
55775	4.72101	0.900893
55776	2.29193	0.373415
55777	1.90104	1.95463
55778	2.13815	0.216018
55779	1.77249	1.78118
55780	4.03919	0.48254
55781	2.25197	2.1
55782	2.13815	0.440325
55783	2.00625	1.93358
55784	2.00625	0.355156
55785	2.40107	1.83777
55786	2.40107	1.40415
55787	2.13815	0.276166
55788	1.77249	1.88969
55789	2.00625	0.895792
55790	1.65815	2.00493
55791	3.7963	0.597236
55792	2.43417	0.660968
55793	2.06653	0.355011

55794	1.64587	0.339061
55795	1.65284	0.311597
55796	2.46898	0.828578
55797	2.46898	0.605312
55798	2.46895	0.460791
55799	1.89016	0.253662
55800	1.87861	0.26709
55801	1.87861	0.229806
55802	2.47161	0.587484
55803	2.47161	0.430976
55804	0.946132	1.67628
55805	0.946132	1.63948
55806	0.863311	1.77708
55807	1.34768	1.73978
55808	0.851542	1.77871
55809	0.851542	1.57578
55810	1.30451	1.66608
55811	1.45304	1.70667
55812	0.946132	1.69476
55813	1.20992	1.70845
55814	0.855162	1.72306
55815	0.946132	1.68643
55816	1.89226	1.60919
55817	1.89226	1.86517
55818	1.89226	1.82965
55819	1.89226	1.64142
55820	1.89226	1.82121
55821	1.89226	1.64753
55822	0.946132	1.50404
55823	0.946132	0.921973
55824	0.946133	1.21296
55825	0.946133	1.25817
55826	0.946132	1.23369
55827	0.946132	0.772723
55828	0.946132	0.92558
55829	0.946132	1.02884
55830	0.946132	1.41594
55831	0.946132	1.22804
55832	0.946133	1.32963
55833	0.946133	1.02024
55834	2.35574	1.9555
55835	1.89226	1.71511
55836	1.89226	2.1
55837	1.89226	1.64278
55838	2.67456	2.1
55839	2.67456	0.513695
55840	0.583505	0.110967
55841	0.761972	0.285903
55842	1.17787	1.54901
55843	1.33728	0.531853
55844	0.965128	1.36608
55845	0.965128	0.155058
55846	0.974714	1.26955
55847	0.974715	1.46843
55848	1.17787	0.602563
55849	0.583506	1.3868
55850	1.33728	0.392879
55851	0.761974	0.446389

55852	1.89226	1.39291
55853	1.89226	0.385566
55854	1.89226	0.458175
55855	1.89226	0.130803
55856	1.89226	0.138195
55857	1.89226	1.10309
55858	1.89226	1.58085
55859	1.89226	0.225539
55860	1.89226	0.630741
55861	1.89226	0.581089
55862	3.78453	0.477798
55863	0.946132	0.125231
55864	0.946132	0.253563
55865	0.946132	0.744624
55866	0.946132	0.514418
55867	0.946133	0.573115
55868	0.946133	0.16924
55869	0.946132	0.702722
55870	0.946132	0.693454
55871	0.946133	0.566387
55872	0.946133	1.39281
55873	0.946132	0.350736
55874	0.946132	1.20391
55875	1.01779	2.1
55876	1.50685	2.1
55877	1.12064	2.1
55878	1.65151	2.1
55879	2.6275	2.1
55880	1.35733	2.1
55881	1.53507	2.1
55882	1.50683	2.1
55883	1.73626	2.1
55884	3.09359	2.1
55885	1.73626	2.1
55886	1.52226	2.1
55887	1.75555	2.1
55888	1.75555	2.1
55889	1.53507	2.1
55890	1.53507	2.1
55891	1.65151	2.1
55892	1.73628	2.1
55893	1.69528	2.1
55894	1.50685	2.1
55895	1.92471	2.1
55896	1.73628	2.1
55897	1.73629	2.1
55898	1.75558	2.1
55899	1.9943	2.1
55900	1.9943	2.1
55901	1.92471	2.1
55902	1.73629	2.1
55903	2.5601	1.3822
55904	1.97645	1.70759
55905	2.09605	1.32509
55906	2.09605	1.64906
55907	2.45853	1.37147
55908	2.45853	1.86022
55909	2.33824	1.6833

55910	1.97646	1.55888
55911	2.45853	1.53332
55912	1.94984	1.73589
55913	2.33824	1.58346
55914	1.82955	1.8792
55915	1.22926	1.69969
55916	0.946128	1.7075
55917	0.946133	1.72303
55918	1.28005	1.43182
55919	1.22927	1.46591
55920	1.22926	1.47121
55921	0.946131	1.57556
55922	0.946131	1.44642
55923	0.946131	1.46869
55924	0.946131	1.45767
55925	1.28005	1.47178
55926	1.22926	1.4544
55927	2.35349	1.97236
55928	2.61606	0.53101
55929	2.48209	1.9744
55930	2.48209	0.508742
55931	2.50057	1.94399
55932	2.50057	0.567689
55933	2.61607	0.442154
55934	2.57611	0.56459
55935	2.4821	1.03931
55936	2.44814	0.320665
55937	5.06421	0.173403
55938	2.15721	1.30526
55939	2.48209	0.671372
55940	2.31844	2.01064
55941	2.31844	0.435058
55942	2.35349	1.97574
55943	2.35349	0.383205
55944	2.44814	0.34687
55945	2.30908	1.00568
55946	2.44814	0.368238
55947	2.31844	0.469279
55948	2.4821	0.307447
55949	2.31844	0.159815
55950	0.91766	0.0700113
55951	0.91766	0.0890777
55952	0.912463	0.0882661
55953	0.912463	0.0909065
55954	1.16525	0.094155
55955	1.26377	0.0823911
55956	0.576692	0.0934719
55957	0.664871	0.0825203
55958	1.16525	0.0858324
55959	0.576692	0.0848766
55960	0.664871	0.103068
55961	1.26377	0.101575
55962	1.72901	2.1
55963	1.62414	2.1
55964	1.88971	2.1
55965	1.8411	1.76312
55966	3.57011	2.1
55967	1.89226	2.1

55968	1.89226	1.80903
55969	1.89226	1.87811
55970	1.89226	1.78824
55971	3.78452	1.83975
55972	1.89226	1.75644
55973	1.89226	2.1
55974	1.89226	2.06325
55975	1.89226	1.65815
55976	3.78452	2.06562
55977	1.62414	2.1
55978	1.30896	2.09363
55979	1.8411	1.98363
55980	1.52776	2.1
55981	3.15006	2.05933
55982	1.51448	1.94113
55983	1.8411	1.89285
55984	2.02311	2.1
55985	1.99309	1.83322
55986	3.86421	1.88648
55987	1.89226	1.9776
55988	1.89226	1.97245
55989	1.89226	2.1
55990	1.89226	1.94033
55991	3.78452	2.07285
55992	1.89226	2.1
55993	1.89226	1.88972
55994	1.89226	2.04767
55995	1.89226	2.1
55996	3.78452	2.07698
55997	1.8411	2.08411
55998	1.52776	2.1
55999	1.99309	2.1
56000	1.72526	2.1
56001	3.56636	2.1
56002	1.89226	2.1
56003	1.89226	2.07098
56004	1.89226	2.07388
56005	1.89226	2.1
56006	3.78453	2.1
56007	1.0424	2.1
56008	1.12935	2.1
56009	0.929383	2.1
56010	0.929383	2.1
56011	1.24941	2.1
56012	1.12686	2.1
56013	0.946132	2.1
56014	0.946132	2.1
56015	1.12944	2.1
56016	0.851554	2.1
56017	1.05017	2.1
56018	0.929315	2.1
56019	0.898029	2.1
56020	0.973994	2.1
56021	0.946131	2.08382
56022	0.946131	2.1
56023	1.0221	2.1
56024	1.0221	2.1
56025	0.898028	2.06489

56026	0.898028	2.1
56027	0.973994	2.1
56028	0.946131	2.1
56029	0.946131	2.1
56030	0.898029	2.1
56031	1.89226	2.02262
56032	1.89226	2.06769
56033	1.89226	1.9768
56034	1.89226	2.05252
56035	1.89226	1.97476
56036	1.89226	2.0488
56037	1.13537	2.06532
56038	0.879308	2.1
56039	0.946131	2.04719
56040	0.946131	2.0833
56041	1.13537	2.09279
56042	0.85187	2.1
56043	0.94613	2.05931
56044	0.94613	1.95048
56045	1.05777	2.1
56046	1.05777	2.1
56047	0.799318	2.1
56048	0.776452	2.1
56049	0.946116	2.1
56050	0.946116	2.1
56051	1.09531	2.1
56052	1.09531	2.1
56053	0.946118	2.1
56054	0.946118	2.1
56055	1.09135	2.1
56056	1.09531	2.1
56057	1.09134	2.1
56058	0.946105	2.1
56059	1.09532	2.1
56060	0.946131	2.1
56061	0.946129	2.1
56062	0.946129	2.1
56063	1.09137	2.1
56064	1.09137	2.1
56065	0.946131	2.1
56066	0.946131	2.1
56067	1.07713	2.1
56068	1.09137	2.1
56069	0.946131	2.1
56070	1.07713	2.1
56071	1.09137	2.1
56072	0.946131	2.1
56073	0.938916	2.1
56074	0.938916	2.1
56075	0.936995	2.1
56076	1.00447	2.1
56077	0.979819	2.1
56078	1.16821	2.1
56079	1.01263	2.1
56080	0.719167	2.1
56081	1.04536	2.1
56082	1.18901	2.1
56083	0.758057	2.1

56084	0.924111	2.1
56085	1.00532	2.1
56086	1.00532	2.1
56087	0.9925	2.1
56088	0.9925	2.1
56089	1.29322	2.1
56090	1.01985	2.1
56091	1.00365	2.1
56092	0.704601	2.1
56093	1.00365	2.1
56094	1.29322	2.1
56095	1.01985	2.1
56096	0.704602	2.1
56097	2.20011	2.1
56098	1.401	2.1
56099	1.401	2.1
56100	1.52008	2.1
56101	2.11084	2.1
56102	2.22991	2.1
56103	0.854068	2.1
56104	0.854068	2.1
56105	0.851357	2.1
56106	0.851357	2.1
56107	0.644681	2.1
56108	0.700493	2.1
56109	1.0554	2.1
56110	1.00493	2.1
56111	1.00495	2.1
56112	0.64469	2.1
56113	0.700483	2.1
56114	1.05539	2.1
56115	2.46665	0.05
56116	2.22713	0.05
56117	1.85877	0.05
56118	1.8967	0.05
56119	1.56002	0.05
56120	2.22713	0.05
56121	1.89225	2.1
56122	1.89225	2.1
56123	1.89225	2.1
56124	1.89225	2.1
56125	1.89225	2.1
56126	1.89225	2.1
56127	1.89225	2.1
56128	1.89225	2.1
56129	1.89225	2.1
56130	1.89225	2.1
56131	1.89225	2.1
56132	1.89225	2.1
56133	1.284	2.1
56134	1.86569	2.1
56135	1.95398	2.1
56136	1.31091	2.1
56137	3.23798	2.1
56138	0.932844	2.1
56139	0.976992	2.1
56140	0.960128	2.1
56141	0.905413	2.1

56142	0.938531	2.1
56143	0.938531	2.1
56144	0.943874	2.1
56145	0.943874	2.1
56146	0.976985	2.1
56147	0.960122	2.1
56148	0.93285	2.1
56149	0.905419	2.1
56150	1.89227	2.1
56151	1.89227	2.1
56152	1.89227	2.1
56153	1.89227	2.1
56154	3.78453	2.1
56155	1.89226	2.1
56156	1.89226	2.1
56157	1.89226	2.1
56158	1.89226	2.1
56159	1.89226	2.1
56160	1.89226	2.1
56161	0.65546	2.1
56162	0.665451	2.1
56163	1.00694	2.1
56164	0.976998	2.1
56165	0.816261	2.1
56166	0.816261	2.1
56167	0.826189	2.1
56168	0.826189	2.1
56169	1.00694	2.1
56170	0.665451	2.1
56171	0.976998	2.1
56172	0.65546	2.1
56173	2.01555	2.1
56174	2.01388	2.1
56175	1.92027	2.1
56176	1.92027	2.1
56177	2.01388	2.1
56178	1.954 2.1	
56179	2.75466	2.1
56180	1.89226	2.1
56181	1.89226	2.1
56182	2.54931	2.1
56183	4.44158	2.1
56184	2.47917	2.1
56185	1.92772	2.1
56186	1.80682	2.1
56187	2.54931	2.1
56188	4.47702	2.1
56189	0.946132	2.1
56190	0.946132	2.1
56191	1.17602	2.1
56192	1.27466	2.1
56193	0.946132	2.1
56194	0.946132	2.1
56195	1.17602	2.1
56196	1.17602	2.1
56197	0.946132	2.1
56198	1.17602	2.1
56199	0.946131	2.1

56200	1.27466	2.1
56201	2.35204	2.1
56202	2.54931	2.1
56203	2.40783	2.1
56204	1.50108	2.1
56205	1.64256	2.1
56206	1.64256	2.1
56207	1.89226	2.1
56208	1.89226	1.65021
56209	2.31012	1.97561
56210	2.17534	2.1
56211	4.0676	2.07019
56212	2.17533	2.09797
56213	1.89226	1.35285
56214	2.17533	1.78843
56215	1.89226	1.66512
56216	1.66635	1.95354
56217	1.38328	1.80105
56218	1.92027	2.1
56219	2.17597	2.1
56220	2.14619	2.1
56221	2.15388	2.1
56222	1.91087	2.1
56223	1.92027	2.1
56224	1.08622	2.1
56225	1.08622	2.1
56226	0.971184	2.1
56227	0.972761	2.1
56228	1.14845	2.1
56229	1.16321	2.1
56230	0.894769	2.1
56231	0.894967	2.1
56232	0.894775	2.1
56233	1.28563	2.1
56234	1.14844	2.1
56235	1.0167	2.1
56236	1.89226	1.90526
56237	1.89226	2.1
56238	1.89226	2.1
56239	1.89226	1.9465
56240	1.89226	2.1
56241	1.89226	1.96279
56242	0.937933	2.1
56243	0.937933	2.1
56244	1.26192	2.1
56245	1.26192	2.1
56246	0.937903	2.1
56247	1.25372	2.1
56248	0.946132	2.1
56249	0.946132	2.1
56250	0.946133	2.1
56251	1.25372	2.1
56252	0.946132	2.1
56253	0.937902	2.1
56254	0.946132	1.85885
56255	0.946132	1.80949
56256	0.946128	2.1
56257	0.946128	2.1

56258	0.94613	2.1
56259	0.94613	1.80768
56260	0.94613	1.78795
56261	0.94613	2.1
56262	0.946131	1.99641
56263	0.946131	2.0204
56264	0.94613	2.07536
56265	0.94613	2.06599
56266	0.946132	1.9073
56267	0.946132	1.82194
56268	1.2778	2.1
56269	1.00191	2.1
56270	1.00191	1.87524
56271	1.00191	2.1
56272	0.94613	2.1
56273	0.94613	1.889
56274	0.946131	2.1
56275	1.2778	2.1
56276	1.00191	2.06172
56277	0.94613	2.04584
56278	1.50847	0.05
56279	2.0815	0.05
56280	1.56806	0.05
56281	2.0815	0.05
56282	1.52043	0.05
56283	1.9224	0.05
56284	1.06882	0.05
56285	0.69567	0.05
56286	0.773112	0.05
56287	1.09473	0.05
56288	0.8941	0.05
56289	0.8941	0.05
56290	0.937064	0.05
56291	0.981131	0.05
56292	0.763762	0.05
56293	0.722535	0.05
56294	1.2153	0.05
56295	1.07279	0.05
56296	1.89226	0.05
56297	1.89226	0.05
56298	1.89226	0.05
56299	1.89226	0.05
56300	1.89226	0.05
56301	1.89226	0.05
56302	1.89226	0.05
56303	1.89226	0.0514349
56304	1.89226	0.05
56305	1.89226	0.0535415
56306	3.78453	0.05
56307	2.51518	0.05
56308	1.89226	0.05
56309	1.89226	0.05
56310	1.89226	0.05
56311	2.37584	0.05
56312	2.37584	0.05
56313	1.25759	0.05
56314	0.946131	0.05
56315	0.946131	0.05

56316	1.24489	0.05
56317	0.946131	0.05
56318	0.946131	0.05
56319	1.25759	0.05
56320	1.25759	0.05
56321	1.25759	0.05
56322	1.24489	0.05
56323	0.946131	0.05
56324	0.946131	0.05
56325	1.08028	0.325037
56326	1.09362	0.218029
56327	0.596616	0.55597
56328	0.606432	0.561011
56329	0.844265	0.668432
56330	0.844265	0.127232
56331	0.855786	0.436478
56332	0.855786	0.48245
56333	1.08028	0.585429
56334	0.606433	0.410999
56335	0.596616	0.437379
56336	1.09362	0.424353
56337	2.837	0.578335
56338	2.16055	0.0812524
56339	2.12741	0.271578
56340	2.79312	1.08104
56341	4.95367	0.17855
56342	2.12741	0.265305
56343	1.21286	0.281303
56344	1.20859	0.942835
56345	1.21286	1.31441
56346	2.15628	0.274113
56347	2.16056	0.0944671
56348	1.89226	2.1
56349	1.89226	2.1
56350	1.89226	2.1
56351	1.89226	2.1
56352	3.78453	2.1
56353	0.946102	2.1
56354	0.946102	2.1
56355	0.850039	2.1
56356	0.850039	2.1
56357	0.914891	2.1
56358	0.85001	2.1
56359	0.946131	2.1
56360	0.946131	2.1
56361	0.94613	2.1
56362	0.91489	2.1
56363	0.850011	2.1
56364	0.946131	2.1
56365	0.71107	2.1
56366	0.784813	2.1
56367	1.28453	2.1
56368	1.20328	2.1
56369	0.992128	2.1
56370	0.992128	2.1
56371	0.995965	2.1
56372	0.995965	2.1
56373	0.784813	2.1

56374	1.28453	2.1
56375	0.711071	2.1
56376	1.20328	2.1
56377	1.89226	2.1
56378	1.89226	2.1
56379	1.89226	2.1
56380	1.89226	2.1
56381	3.78453	2.1
56382	0.946132	2.1
56383	0.946132	2.1
56384	0.914889	2.1
56385	0.914889	2.1
56386	0.985255	2.1
56387	0.914891	2.1
56388	0.946131	2.1
56389	0.946131	2.1
56390	0.946131	2.1
56391	0.985255	2.1
56392	0.94613	2.1
56393	0.91489	2.1
56394	0.784813	2.1
56395	0.871551	2.1
56396	1.37791	2.1
56397	1.28453	2.1
56398	1.07644	2.1
56399	1.07644	2.1
56400	1.07964	2.1
56401	1.07964	2.1
56402	0.871551	2.1
56403	1.37791	2.1
56404	0.784813	2.1
56405	1.28453	2.1
56406	1.73075	1.76003
56407	2.10582	1.35942
56408	1.89319	1.42783
56409	1.38766	2.02995
56410	3.27777	1.5308
56411	1.08715	1.94174
56412	1.54185	2.03862
56413	1.73074	1.92997
56414	1.73074	2.09676
56415	1.18948	1.65866
56416	1.18948	1.9639
56417	1.89319	1.28332
56418	1.41689	1.96313
56419	1.11619	1.66119
56420	1.38766	1.76521
56421	1.6052	1.53036
56422	1.89319	1.74231
56423	0.647894	1.87108
56424	0.594738	2.00577
56425	0.708445	1.97553
56426	0.770926	1.83121
56427	0.738326	2.0042
56428	0.738326	2.05849
56429	0.680494	1.82416
56430	0.680494	1.57925
56431	0.708443	1.70661

56432	0.647895	1.74915
56433	0.770925	1.83526
56434	0.59474	1.39899
56435	1.89111	2.1
56436	1.06994	2.1
56437	2.06689	2.1
56438	1.37063	2.1
56439	3.13683	2.1
56440	2.06689	2.1
56441	1.39295	2.1
56442	1.56818	2.1
56443	1.37063	2.1
56444	2.26444	2.1
56445	2.06689	2.1
56446	1.49808	2.1
56447	1.50201	2.1
56448	2.57784	2.1
56449	2.58615	2.1
56450	4.08424	2.1
56451	0.946133	2.1
56452	0.946133	2.1
56453	0.946132	2.1
56454	0.946132	2.1
56455	0.946132	2.1
56456	0.946132	2.1
56457	0.946132	2.1
56458	0.946132	2.1
56459	0.946133	2.1
56460	0.946133	2.1
56461	0.946132	2.1
56462	0.946132	2.1
56463	1.89226	2.1
56464	1.89226	2.1
56465	1.89226	2.1
56466	1.89226	2.1
56467	3.78452	2.1
56468	0.977201	2.1
56469	0.977201	2.1
56470	0.980489	2.1
56471	0.980489	2.1
56472	0.657973	2.1
56473	1.20669	2.1
56474	0.751002	2.1
56475	1.29307	2.1
56476	1.29308	2.1
56477	1.20669	2.1
56478	0.751003	2.1
56479	0.657972	2.1
56480	0.946131	2.1
56481	0.946131	2.1
56482	1.29308	2.1
56483	1.29308	2.1
56484	0.946132	2.1
56485	1.29308	2.1
56486	0.946132	2.1
56487	1.28892	2.1
56488	0.946132	2.1
56489	0.946132	2.1

56490	1.28892	2.1
56491	1.29308	2.1
56492	0.946132	2.1
56493	0.946132	1.93284
56494	0.946131	2.1
56495	0.946131	2.1
56496	0.946132	2.1
56497	0.946132	2.08154
56498	0.946132	2.08106
56499	0.946132	1.9239
56500	0.946132	2.06058
56501	0.946132	2.1
56502	0.946132	2.09634
56503	0.946132	2.1
56504	1.89226	2.1
56505	1.89226	2.1
56506	1.89226	2.1
56507	1.89226	2.04782
56508	1.89226	2.1
56509	1.89226	1.99245
56510	0.94613	2.1
56511	0.94613	2.1
56512	1.20669	2.1
56513	1.20669	2.1
56514	1.20669	2.1
56515	0.946132	2.1
56516	0.946129	2.1
56517	1.29307	2.1
56518	0.946131	2.1
56519	0.946131	2.1
56520	1.29308	2.1
56521	1.20669	2.1
56522	1.89226	0.129035
56523	1.89226	0.0745925
56524	1.89226	0.0853449
56525	1.89226	0.110528
56526	3.78453	0.109809
56527	0.946132	0.05
56528	0.946132	0.0548276
56529	0.946126	0.0631132
56530	0.946126	0.0834419
56531	0.946129	0.0667824
56532	0.946129	0.0538576
56533	0.946129	0.06163
56534	0.946129	0.0822661
56535	0.946128	0.0580712
56536	0.946128	0.0625379
56537	0.946129	0.0592099
56538	0.946129	0.0625354
56539	0.725277	0.05
56540	0.588155	0.05
56541	1.19403	0.0564811
56542	1.27681	0.05
56543	0.918903	0.05
56544	0.918903	0.05
56545	0.946068	0.05
56546	0.946068	0.05
56547	1.19404	0.05

56548	0.588153	0.05
56549	0.725275	0.05
56550	1.27682	0.05
56551	1.19404	0.063367
56552	1.27682	0.05
56553	0.946131	0.0894292
56554	0.946131	0.0721395
56555	1.19404	0.0745918
56556	1.19404	0.0600273
56557	0.946129	0.061286
56558	0.946129	0.074781
56559	1.19404	0.067144
56560	0.94613	0.0676273
56561	1.27682	0.0590664
56562	0.946131	0.0717665
56563	1.89226	0.0776059
56564	1.89226	0.116966
56565	1.89226	0.124811
56566	1.89226	0.0862117
56567	1.89226	0.112937
56568	1.89226	0.0686802
56569	1.4263	1.98634
56570	1.67651	0.565369
56571	1.5955	0.725559
56572	1.31022	2.1
56573	2.98674	1.54419
56574	1.18824	2.1
56575	1.28802	2.1
56576	0.946132	2.1
56577	0.946132	2.1
56578	0.946132	2.1
56579	0.946132	2.1
56580	1.28802	2.1
56581	1.28802	2.1
56582	1.18824	2.1
56583	0.946132	2.1
56584	0.946132	2.1
56585	1.28802	2.1
56586	1.89226	0.699857
56587	1.89226	2.1
56588	1.89226	2.1
56589	1.89226	0.92066
56590	3.78452	1.68893
56591	1.5955	0.94505
56592	1.52909	0.853912
56593	1.31022	1.21609
56594	1.31022	2.1
56595	1.2087	2.1
56596	1.52909	2.1
56597	1.11825	2.1
56598	1.18824	2.1
56599	0.946132	2.1
56600	0.946132	2.1
56601	0.946132	2.1
56602	0.946132	2.1
56603	1.18824	2.1
56604	1.18824	2.1
56605	0.946132	2.1

56606	1.11825	2.1
56607	1.18824	2.1
56608	0.946133	2.1
56609	0.810943	2.1
56610	0.810943	2.1
56611	0.810945	0.987996
56612	0.810945	2.1
56613	1.89226	0.998816
56614	1.35168	1.77119
56615	1.08132	1.06307
56616	1.0813	1.90402
56617	1.08132	2.1
56618	0.810959	1.75505
56619	0.810959	1.83838
56620	1.89226	0.369496
56621	1.89226	0.170248
56622	1.89226	0.230306
56623	1.89226	0.344422
56624	3.78453	0.35256
56625	1.89226	0.0868192
56626	1.89226	0.123944
56627	1.89226	0.126852
56628	1.89226	0.0784084
56629	1.89226	0.112805
56630	1.89226	0.0697482
56631	0.946131	0.0593463
56632	0.946131	0.0685019
56633	0.946126	0.120359
56634	0.946126	0.0894775
56635	0.946128	0.128336
56636	0.946128	0.0826726
56637	0.946128	0.116886
56638	0.946128	0.0960976
56639	0.946128	0.0881876
56640	0.946128	0.100505
56641	0.946128	0.101837
56642	0.946128	0.0849654
56643	1.89226	0.325467
56644	1.89226	0.115617
56645	1.89226	0.232376
56646	1.89226	0.222783
56647	1.89226	0.163684
56648	1.89226	0.157052
56649	1.89226	0.159912
56650	1.89226	0.2485
56651	1.89226	0.499604
56652	1.89226	0.14321
56653	3.78453	0.143437
56654	1.07176	0.0569081
56655	1.07176	0.05
56656	0.946126	0.0970226
56657	0.946126	0.0767064
56658	1.07176	0.0638045
56659	0.946129	0.05
56660	1.27474	0.0765342
56661	0.94613	0.0839251
56662	1.27474	0.077286
56663	1.07176	0.05

56664	0.94613	0.0518071
56665	0.94613	0.0699351
56666	0.723486	0.05
56667	0.94613	0.05
56668	0.946126	0.0669148
56669	0.946126	0.0575695
56670	0.946129	0.0560862
56671	0.723484	0.0519956
56672	1.07176	0.0528767
56673	0.946129	0.05
56674	1.32398	0.0548058
56675	1.19835	0.05
56676	0.723471	0.05
56677	0.723471	0.0712168
56678	0.810946	0.282573
56679	0.810946	0.164884
56680	0.810946	0.267584
56681	0.810946	0.215379
56682	0.810933	0.0771343
56683	0.810933	0.220038
56684	1.89226	0.0794326
56685	1.3517	0.171861
56686	1.08132	0.0933567
56687	1.08132	0.153465
56688	1.08133	0.189063
56689	2.39923	2.1
56690	1.89226	2.1
56691	1.89226	2.1
56692	1.89226	2.1
56693	2.44606	2.1
56694	2.44606	2.1
56695	1.89226	2.1
56696	1.89226	2.1
56697	1.89226	2.1
56698	1.89226	2.1
56699	1.89226	2.1
56700	1.89226	2.1
56701	1.70302	2.1
56702	2.43041	2.1
56703	2.25275	2.1
56704	2.4747	2.1
56705	1.52079	2.1
56706	1.67063	2.1
56707	2.40644	2.1
56708	1.89226	2.1
56709	1.89226	2.1
56710	1.89226	2.1
56711	2.39923	2.1
56712	2.39923	2.1
56713	0.946131	2.1
56714	0.946131	2.1
56715	0.946131	2.1
56716	0.946131	2.1
56717	0.94613	2.1
56718	0.94613	2.1
56719	0.946131	2.1
56720	0.946131	2.1
56721	0.946131	2.1

56722	0.946131	2.1
56723	0.946131	2.1
56724	0.946131	2.1
56725	1.04938	2.1
56726	1.04938	2.1
56727	1.111	2.1
56728	1.06978	2.1
56729	1.2748	2.1
56730	1.25472	2.1
56731	0.88782	2.1
56732	0.882987	2.1
56733	0.973779	2.1
56734	1.31997	2.1
56735	0.913905	2.1
56736	1.31312	2.1
56737	2.6536	2.1
56738	2.15287	2.1
56739	2.72238	2.1
56740	2.22887	2.1
56741	4.87525	2.1
56742	2.15288	2.1
56743	1.40719	2.1
56744	1.41839	2.1
56745	1.34239	2.1
56746	2.22887	2.1
56747	2.15288	2.1
56748	2.72237	2.1
56749	2.22887	2.1
56750	2.77314	2.1
56751	2.28748	2.1
56752	5.00985	2.1
56753	0.922802	2.1
56754	0.922802	2.1
56755	0.92107	2.1
56756	0.92107	2.1
56757	0.703593	2.1
56758	0.729436	2.1
56759	1.11444	2.1
56760	1.14374	2.1
56761	1.14374	2.1
56762	0.729436	2.1
56763	0.703593	2.1
56764	1.11444	2.1
56765	1.89226	2.1
56766	1.89226	2.1
56767	1.89226	2.1
56768	1.89226	2.1
56769	1.89226	2.1
56770	1.89226	2.1
56771	0.489927	2.1
56772	0.63008	2.1
56773	0.697385	2.1
56774	0.55524	2.1
56775	0.592141	2.1
56776	0.592141	2.1
56777	0.593179	2.1
56778	0.593179	2.1
56779	0.63008	2.1

56780	0.697385	2.1
56781	0.55524	2.1
56782	0.489927	2.1
56783	0.946133	2.1
56784	0.946133	2.1
56785	0.946132	2.1
56786	0.946132	1.83371
56787	0.946133	2.1
56788	0.946133	2.1
56789	0.946132	2.1
56790	0.946132	1.98921
56791	0.946132	2.1
56792	0.946132	2.1
56793	0.946132	2.1
56794	0.946132	2.1
56795	1.89226	2.1
56796	1.89226	2.1
56797	1.89226	2.1
56798	1.89226	2.1
56799	1.89226	2.1
56800	1.89226	2.1
56801	0.805746	2.1
56802	0.63008	2.1
56803	0.881598	2.1
56804	0.697385	2.1
56805	0.749417	2.1
56806	0.749417	2.1
56807	0.753714	2.1
56808	0.753714	2.1
56809	0.881598	2.1
56810	0.805746	2.1
56811	0.63008	2.1
56812	0.697385	2.1
56813	0.881599	2.1
56814	0.697385	2.1
56815	0.805746	2.1
56816	0.626731	2.1
56817	0.755479	2.1
56818	0.755479	2.1
56819	0.752851	2.1
56820	0.752851	2.1
56821	0.881598	2.1
56822	0.805747	2.1
56823	0.697385	2.1
56824	0.626731	2.1
56825	2.65348	2.1
56826	2.12314	2.1
56827	2.63899	2.1
56828	2.13898	2.1
56829	4.76213	2.1
56830	0.763518	2.1
56831	0.765889	2.1
56832	0.84086	2.1
56833	0.84086	2.1
56834	0.632078	2.1
56835	0.597565	2.1
56836	1.08813	2.1
56837	0.985643	2.1

56838	0.982464	2.1
56839	0.66404	2.1
56840	1.0358	2.1
56841	0.598023	2.1
56842	1.29293	2.1
56843	1.29628	2.1
56844	1.19266	2.1
56845	1.19266	2.1
56846	1.07109	2.1
56847	1.13093	2.1
56848	1.34467	2.1
56849	1.35251	2.1
56850	1.34369	2.1
56851	1.12525	2.1
56852	1.28746	2.1
56853	1.13511	2.1
56854	2.19717	2.1
56855	2.16547	2.1
56856	1.37392	2.1
56857	1.37392	2.1
56858	1.2966	2.1
56859	2.11985	2.1
56860	1.89226	2.1
56861	1.89226	2.1
56862	1.89226	2.1
56863	1.89226	2.1
56864	1.89226	2.1
56865	1.89226	2.1
56866	1.65637	2.1
56867	1.41758	2.1
56868	1.65637	2.1
56869	1.74391	2.1
56870	1.35504	2.1
56871	1.44258	2.1
56872	1.40774	2.1
56873	1.40774	2.1
56874	0.946131	2.1
56875	0.946131	2.1
56876	1.40774	2.1
56877	1.38882	2.1
56878	0.946132	2.1
56879	0.946132	2.1
56880	0.946132	2.1
56881	1.38882	2.1
56882	0.946132	2.1
56883	1.40774	2.1
56884	1.89227	2.1
56885	1.89227	2.1
56886	1.89227	2.1
56887	1.89227	2.1
56888	3.78453	2.1
56889	0.603148	2.1
56890	0.600177	2.1
56891	0.558615	2.1
56892	0.580714	2.1
56893	0.558606	2.1
56894	0.67883	2.1
56895	1.65637	2.1

56896	1.07933	2.1
56897	0.842649	2.1
56898	0.905168	2.1
56899	0.774334	2.1
56900	1.38882	2.1
56901	1.38882	2.1
56902	0.946132	2.1
56903	0.946132	2.1
56904	1.38882	2.1
56905	1.37381	2.1
56906	0.946132	2.1
56907	0.946132	2.1
56908	0.946132	2.1
56909	1.37381	2.1
56910	0.946133	2.1
56911	1.38882	2.1
56912	2.3771	2.1
56913	1.9339	2.1
56914	1.70782	2.1
56915	1.9339	2.1
56916	2.42693	2.1
56917	2.83279	2.1
56918	2.34901	0.244741
56919	1.45833	0.396731
56920	1.86695	0.231302
56921	1.03101	0.248226
56922	3.32528	0.274663
56923	1.89226	0.131095
56924	1.89226	0.116875
56925	1.89226	0.0804092
56926	1.89226	0.0639193
56927	3.78453	0.11644
56928	2.71753	0.0677463
56929	1.89226	0.108132
56930	2.76228	0.0538414
56931	2.76228	0.0673605
56932	1.89226	0.11296
56933	1.89226	0.169618
56934	0.946131	0.0709796
56935	0.946131	0.0712228
56936	0.946132	0.0724698
56937	0.946132	0.0740405
56938	0.946132	0.100248
56939	0.946132	0.0594714
56940	0.946132	0.0998855
56941	0.946132	0.0553292
56942	0.946132	0.0579649
56943	0.946132	0.0639912
56944	0.946132	0.0891834
56945	0.946132	0.0799958
56946	1.89227	0.0870919
56947	1.89227	0.05
56948	1.89227	0.0567885
56949	1.89227	0.078422
56950	1.89227	0.05
56951	1.89227	0.05
56952	2.71753	0.05
56953	2.37907	0.05

56954	2.37907	0.05
56955	2.76228	0.0613639
56956	2.39588	0.05
56957	2.77909	0.05
56958	1.6571	1.16658
56959	1.11682	1.63464
56960	1.47132	1.47648
56961	1.93763	0.144266
56962	3.12842	1.5121
56963	1.93762	0.424187
56964	2.12345	1.45843
56965	1.65709	0.204191
56966	1.65709	1.76381
56967	2.12345	1.27605
56968	1.88635	1.89635
56969	1.47134	1.71752
56970	2.219	0.788119
56971	2.25734	2.04158
56972	1.93765	1.26605
56973	1.79103	1.57212
56974	1.47134	0.475549
56975	1.06174	1.39896
56976	1.19349	1.87578
56977	1.1095	1.43904
56978	0.968827	1.15307
56979	1.07892	0.947255
56980	1.07892	2.1
56981	1.0834	1.47538
56982	1.0834	1.36776
56983	1.1095	1.23208
56984	1.19349	1.1626
56985	0.968825	1.36424
56986	1.06174	1.35038
56987	2.40627	2.1
56988	2.08288	1.52413
56989	2.5444	1.58186
56990	2.29285	1.75944
56991	4.62729	1.62048
56992	2.08289	1.29939
56993	1.65703	1.27024
56994	1.71105	2.1
56995	1.50108	1.55267
56996	2.29286	1.88621
56997	2.08289	1.59233
56998	2.5444	1.7635
56999	2.29285	2.1
57000	2.71429	2.1
57001	2.50288	1.40416
57002	5.04728	2.06325
57003	2.50288	1.35197
57004	2.00927	1.72465
57005	2.02116	1.76031
57006	2.02116	1.98078
57007	2.29286	1.63616
57008	2.29286	1.6951
57009	1.89226	2.1
57010	1.89226	2.1
57011	1.89226	2.1

57012	1.89226	2.1
57013	3.78453	2.1
57014	1.17418	2.1
57015	1.29741	2.1
57016	1.04921	2.1
57017	1.19352	2.1
57018	2.3677	2.1
57019	1.29741	2.1
57020	1.27575	2.1
57021	1.19352	2.1
57022	1.19473	2.1
57023	2.46927	2.1
57024	1.89226	2.1
57025	1.89226	2.1
57026	1.89226	2.1
57027	1.89226	2.1
57028	1.89226	2.1
57029	1.89226	2.1
57030	0.561332	2.1
57031	0.561332	2.1
57032	0.567597	2.1
57033	0.567597	2.1
57034	0.597365	2.1
57035	0.544699	2.1
57036	0.596761	2.1
57037	0.531564	2.1
57038	0.5447	2.1
57039	0.531563	2.1
57040	0.596759	2.1
57041	0.597366	2.1
57042	0.946131	2.1
57043	0.946131	2.1
57044	0.946131	2.1
57045	0.946131	2.1
57046	0.946129	2.1
57047	0.946129	2.1
57048	0.946133	2.1
57049	0.946133	2.1
57050	0.946131	2.1
57051	0.946131	2.1
57052	0.946131	2.1
57053	0.946131	2.1
57054	1.10938	0.581988
57055	1.17614	2.1
57056	1.02478	1.23729
57057	1.09056	2.1
57058	2.19994	2.0312
57059	1.17614	2.1
57060	1.11868	2.1
57061	1.03408	2.1
57062	1.03408	2.1
57063	1.09056	2.1
57064	1.17516	2.1
57065	1.89226	0.500309
57066	1.89226	0.334693
57067	1.89226	0.352456
57068	1.89226	0.273479
57069	1.89226	0.734831

57070	1.89226	0.491697
57071	1.89226	0.270872
57072	1.89226	0.388803
57073	1.89226	0.285621
57074	1.89226	0.228743
57075	1.89226	0.53847
57076	1.89226	0.23976
57077	0.579551	0.205717
57078	0.563873	0.179849
57079	0.611341	0.25274
57080	0.598693	0.2142
57081	0.589856	0.202608
57082	0.589856	0.18674
57083	0.588387	0.238087
57084	0.588387	0.216998
57085	0.57955	0.215272
57086	0.611341	0.204662
57087	0.563873	0.21275
57088	0.598693	0.202709
57089	0.611341	0.245179
57090	0.598693	0.20999
57091	0.586048	0.241629
57092	0.572737	0.263095
57093	0.591824	0.239636
57094	0.591824	0.221125
57095	0.592254	0.28468
57096	0.592254	0.260513
57097	0.611341	0.256688
57098	0.586048	0.235492
57099	0.598693	0.249378
57100	0.572737	0.242223
57101	0.946119	0.310024
57102	0.946119	0.489309
57103	0.946119	0.297754
57104	0.946119	0.436905
57105	0.94612	0.287706
57106	0.94612	0.263722
57107	0.946119	0.470175
57108	0.946119	0.624669
57109	0.946132	0.383835
57110	0.946132	0.351847
57111	0.946107	0.378642
57112	0.946107	0.431836
57113	1.89224	0.314504
57114	1.89224	0.252966
57115	1.89224	0.272435
57116	1.89224	0.202776
57117	1.89224	0.481224
57118	1.89224	0.317478
57119	0.562335	0.18539
57120	0.579543	0.160988
57121	0.59499	0.218108
57122	0.611333	0.189293
57123	0.586619	0.181005
57124	0.586619	0.169007
57125	0.587049	0.208831
57126	0.587049	0.19109
57127	0.594999	0.198471

57128	0.562342	0.19476
57129	0.579535	0.194159
57130	0.611325	0.184731
57131	0.59499	0.20858
57132	0.611333	0.183925
57133	0.567849	0.206947
57134	0.58604	0.223207
57135	0.590978	0.203184
57136	0.590978	0.192014
57137	0.590052	0.235225
57138	0.590052	0.220913
57139	0.567857	0.224389
57140	0.594998	0.216428
57141	0.611325	0.225462
57142	0.586032	0.209623
57143	1.69661	1.75527
57144	1.9339	0.432244
57145	1.39379	1.89221
57146	1.69107	0.244431
57147	3.32768	0.689844
57148	1.9339	0.233089
57149	1.98414	0.698822
57150	1.69107	0.246904
57151	1.72774	0.379252
57152	3.66164	0.220799
57153	1.39379	2.03442
57154	1.69107	0.241772
57155	1.14397	1.58779
57156	1.43548	0.193102
57157	2.82927	1.02692
57158	1.69107	0.133937
57159	1.72774	0.369375
57160	1.53873	0.244779
57161	1.53873	0.724833
57162	1.43548	0.175061
57163	1.62449	0.94227
57164	2.58431	0.080358
57165	1.34072	0.0963045
57166	1.80841	0.0824757
57167	1.34072	0.0906955
57168	2.51501	0.0918283
57169	2.04732	0.104061
57170	1.89226	0.0826159
57171	1.89226	0.100272
57172	1.89226	0.0816467
57173	1.89226	0.10912
57174	1.89226	0.0953017
57175	1.89226	0.112533
57176	0.946131	0.115334
57177	0.946131	0.0969281
57178	1.3639	0.0795622
57179	1.1516	0.097625
57180	0.946129	0.103989
57181	0.946129	0.12076
57182	1.36391	0.0826971
57183	1.36391	0.0858585
57184	0.946131	0.111882
57185	1.3639	0.0909129

57186	1.1516	0.0881236
57187	0.946131	0.105225
57188	0.946131	0.097125
57189	0.946131	0.085399
57190	0.94613	0.109047
57191	0.94613	0.0947836
57192	0.946128	0.118133
57193	0.946128	0.0914321
57194	0.946133	0.0846763
57195	0.946133	0.109682
57196	0.946131	0.0982335
57197	0.946131	0.0904877
57198	0.946131	0.10704
57199	0.946131	0.0924657
57200	1.89226	1.68154
57201	2.63634	1.96164
57202	1.89226	1.74139
57203	1.83902	1.75921
57204	3.73128	1.70196
57205	1.47059	2.1
57206	2.02928	2.1
57207	1.47059	2.1
57208	1.65706	2.1
57209	1.98628	2.1
57210	2.17274	2.1
57211	2.43618	2.1
57212	2.41176	2.1
57213	1.98627	2.1
57214	2.34084	2.1
57215	4.39803	2.1
57216	1.47059	2.1
57217	1.82453	2.1
57218	1.98627	2.1
57219	1.98627	2.1
57220	1.26929	2.1
57221	1.26929	2.1
57222	1.98627	2.1
57223	2.34084	2.1
57224	1.47264	2.1
57225	2.24937	2.1
57226	4.23564	2.1
57227	1.33026	1.45255
57228	1.51171	2.07045
57229	1.51171	1.70943
57230	1.4617	2.09467
57231	1.35382	1.59166
57232	1.30382	2.1
57233	1.89226	1.01953
57234	1.89226	1.8544
57235	1.89226	1.34503
57236	1.89226	2.01605
57237	1.89226	1.09819
57238	1.89226	2.06411
57239	1.39263	1.38062
57240	0.962704	0.440188
57241	1.0171	0.715076
57242	0.962704	1.12743
57243	1.40822	0.533248

57244	1.35382	0.720185
57245	0.755853	2.1
57246	0.789808	1.95758
57247	0.696315	1.73575
57248	0.676912	0.971212
57249	0.729751	1.03406
57250	0.729751	1.93
57251	0.722417	2.1
57252	0.722417	0.998384
57253	0.696315	1.87846
57254	0.789808	1.86353
57255	0.755853	1.79075
57256	0.676912	1.77201
57257	0.946132	1.71694
57258	0.946132	1.92968
57259	0.946132	1.27535
57260	0.946132	0.808453
57261	0.946131	0.875136
57262	0.946131	1.68502
57263	0.946132	1.42568
57264	0.946132	1.93061
57265	0.946132	1.44352
57266	0.946132	1.51862
57267	0.946132	1.48434
57268	0.946132	1.44394
57269	0.946133	0.802001
57270	0.946133	0.60921
57271	0.946132	0.407829
57272	0.946132	0.55828
57273	0.946132	0.424906
57274	0.946132	0.59133
57275	0.946133	0.432151
57276	0.946133	0.862445
57277	0.946132	0.599491
57278	0.946132	0.605278
57279	0.946132	0.548297
57280	0.946132	0.568781
57281	1.89227	1.85251
57282	2.53014	1.92243
57283	1.89227	2.04136
57284	1.98241	1.81916
57285	1.89227	2.06046
57286	1.98241	1.84624
57287	1.89226	1.76816
57288	1.89226	2.1
57289	1.89226	1.87973
57290	1.89226	1.89787
57291	1.89226	2.08819
57292	1.89226	2.05827
57293	1.89226	1.63862
57294	1.89226	2.04312
57295	1.89226	1.69675
57296	1.89226	1.74383
57297	3.78452	1.72544
57298	2.45304	1.59887
57299	1.89226	1.82903
57300	1.89226	1.72818
57301	1.89226	1.7368

57302	1.89226	1.61345
57303	1.89226	1.64052
57304	1.89227	1.81105
57305	1.89227	1.82486
57306	1.89227	2.00452
57307	1.89227	1.71205
57308	1.89227	2.01875
57309	1.89227	1.73617
57310	1.89226	1.73387
57311	1.89226	2.1
57312	1.89226	1.86458
57313	1.89226	1.87267
57314	1.89226	2.0394
57315	1.89226	2.02487
57316	2.09871	0.184854
57317	2.71891	0.230031
57318	1.31126	0.167886
57319	1.31126	0.32188
57320	2.55341	0.17041
57321	1.76597	0.715639
57322	1.89226	0.682199
57323	1.89226	1.03185
57324	1.89226	0.253944
57325	1.89226	1.25305
57326	1.89226	0.330929
57327	1.89226	1.38021
57328	0.946128	0.686322
57329	0.946128	0.232433
57330	0.946133	0.210667
57331	0.946133	0.166077
57332	0.946131	0.204968
57333	0.946131	0.329205
57334	0.94613	0.192681
57335	0.94613	0.249025
57336	0.946131	0.245459
57337	0.946131	0.202651
57338	0.946131	0.218529
57339	0.946131	0.257968
57340	2.71891	0.197283
57341	1.89226	0.154519
57342	2.09871	0.13214
57343	2.09871	0.380553
57344	1.89226	0.128955
57345	1.89226	0.290467
57346	1.89227	0.602635
57347	1.89227	0.626881
57348	1.89227	0.257879
57349	1.89227	1.12434
57350	1.89227	0.473092
57351	1.89227	1.32215
57352	0.946129	0.481721
57353	0.946129	0.229283
57354	0.946133	0.184357
57355	0.946133	0.146255
57356	0.946131	0.177272
57357	0.946131	0.3093
57358	0.946131	0.174229
57359	0.946131	0.253173

57360	0.946131	0.229022
57361	0.946131	0.190253
57362	0.946131	0.240433
57363	0.946131	0.202262
57364	2.32462	0.05
57365	1.89226	0.05
57366	1.89226	0.05
57367	1.89226	0.05
57368	2.2291	0.05
57369	2.2291	0.05
57370	0.847669	0.05
57371	0.847669	0.05
57372	0.796966	0.05
57373	0.796966	0.05
57374	0.833552	0.05
57375	1.01049	0.05
57376	0.709709	0.05
57377	0.634147	0.05
57378	0.634148	0.05
57379	0.833549	0.05
57380	0.709711	0.05
57381	1.01049	0.05
57382	1.89226	0.05
57383	1.89226	0.05
57384	1.89226	0.05
57385	1.89226	0.05
57386	1.89226	0.05
57387	1.89226	0.05
57388	1.03611	0.05
57389	0.936655	0.05
57390	0.946132	0.05
57391	0.946132	0.05
57392	0.941918	0.05
57393	0.941918	0.05
57394	1.00308	0.05
57395	1.04117	0.05
57396	1.1406	0.05
57397	1.04074	0.05
57398	0.851529	0.05
57399	1.00311	0.05
57400	1.01814	0.05
57401	1.6671	0.05
57402	1.35561	0.05
57403	1.35561	0.05
57404	1.2683	0.05
57405	1.2683	0.05
57406	0.946128	0.05
57407	0.946128	0.05
57408	0.946129	0.05
57409	0.946129	0.05
57410	0.946132	0.05
57411	0.946132	0.05
57412	0.946126	0.05
57413	0.946126	0.05
57414	0.946129	0.05
57415	0.946129	0.05
57416	0.946129	0.05
57417	0.946129	0.05

57418	2.23338	0.05
57419	1.96	0.05
57420	2.03438	0.05
57421	1.87835	0.05
57422	4.11172	0.05
57423	1.96	0.05
57424	1.52661	0.05
57425	1.87834	0.05
57426	1.53175	0.05
57427	3.40495	0.05
57428	2.03435	0.05
57429	1.65813	0.05
57430	1.65813	0.05
57431	1.87832	0.05
57432	1.72067	0.05
57433	1.94086	0.05
57434	1.65813	0.05
57435	1.40572	0.05
57436	1.87832	0.05
57437	1.87832	0.05
57438	1.31154	0.05
57439	1.53173	0.05
57440	1.86662	2.1
57441	2.0815	2.1
57442	2.0815	2.1
57443	2.0815	2.1
57444	1.87246	2.1
57445	1.87362	2.1
57446	1.0273	2.1
57447	0.996347	2.1
57448	0.946129	2.1
57449	0.946129	2.1
57450	1.04427	2.1
57451	1.04196	2.1
57452	0.910674	2.1
57453	0.910674	2.1
57454	1.04072	2.1
57455	1.10931	2.1
57456	1.03132	2.1
57457	0.933698	2.1
57458	2.63789	0.24764
57459	1.89226	1.18733
57460	2.3033	0.285057
57461	2.3033	0.76161
57462	1.89226	0.289104
57463	1.89226	0.801426
57464	1.89226	1.27074
57465	1.89226	1.41422
57466	1.89226	0.785165
57467	1.89226	1.29221
57468	3.78452	1.33642
57469	1.51898	0.947902
57470	1.36006	1.45313
57471	2.03143	1.44155
57472	2.03143	1.4608
57473	1.04026	1.27151
57474	1.04026	1.30006
57475	0.94613	0.125877

57476	0.94613	0.390704
57477	1.31894	0.128487
57478	1.31894	0.133696
57479	0.94613	0.132537
57480	0.94613	0.375101
57481	1.31894	0.190418
57482	1.41232	0.321919
57483	0.946129	0.208655
57484	1.41232	0.20079
57485	1.31895	0.17419
57486	0.946129	0.237978
57487	1.89226	0.192063
57488	1.89226	1.23567
57489	1.89226	0.161708
57490	1.89226	1.04124
57491	1.89226	0.157634
57492	1.89226	1.12353
57493	1.89804	0.148256
57494	2.03143	0.615057
57495	2.46286	0.375058
57496	2.46287	0.358169
57497	1.51898	0.334714
57498	1.51898	0.317713
57499	1.89226	2.1
57500	1.89226	2.1
57501	1.89226	2.1
57502	1.89226	2.1
57503	3.78452	2.1
57504	1.3864	2.1
57505	1.3864	2.1
57506	0.946132	2.1
57507	0.946132	2.1
57508	1.32905	2.1
57509	1.3864	2.1
57510	0.946132	2.1
57511	0.946132	2.1
57512	0.946132	2.1
57513	1.32905	2.1
57514	0.946133	2.1
57515	1.3864	2.1
57516	2.77279	2.1
57517	1.67883	2.1
57518	1.67883	2.1
57519	1.73415	2.1
57520	2.65811	2.1
57521	2.71343	2.1
57522	0.946131	2.1
57523	0.946131	2.1
57524	0.946128	2.1
57525	0.946128	2.1
57526	0.946127	2.1
57527	0.946127	2.1
57528	0.946131	2.1
57529	0.946131	2.1
57530	0.946129	2.1
57531	0.946129	2.1
57532	0.94613	2.1
57533	0.94613	2.1

57534	1.89226	2.1
57535	1.89226	2.1
57536	1.89226	2.1
57537	1.89226	2.1
57538	3.78452	2.1
57539	1.89226	2.1
57540	2.54394	2.1
57541	2.54394	2.1
57542	2.65811	2.1
57543	1.89226	2.1
57544	2.00643	2.1
57545	2.54394	2.1
57546	2.6581	2.1
57547	1.62743	2.1
57548	1.62743	2.1
57549	1.67883	2.1
57550	2.59533	2.1
57551	1.89226	2.1
57552	1.89226	2.1
57553	1.89226	2.1
57554	1.89226	2.1
57555	1.89226	2.1
57556	1.89226	2.1
57557	1.89226	1.78931
57558	1.89226	1.77094
57559	1.89226	2.07244
57560	1.89226	1.59475
57561	1.89226	2.03554
57562	1.89226	1.54452
57563	1.89226	1.7515
57564	1.89226	1.73949
57565	1.89226	2.03205
57566	1.89226	1.62802
57567	1.89226	1.97914
57568	1.89226	1.52141
57569	1.89226	1.15508
57570	1.89226	0.57101
57571	1.89226	1.6961
57572	1.89226	0.617853
57573	1.89226	1.67492
57574	1.89226	0.596911
57575	2.68537	0.513175
57576	2.22575	2.1
57577	2.47464	0.261821
57578	1.79093	1.85681
57579	4.70039	0.616757
57580	2.81217	2.1
57581	2.47464	0.405972
57582	2.58286	2.01666
57583	2.29193	0.276886
57584	5.0575	0.531248
57585	2.47464	0.345752
57586	2.20365	1.90647
57587	2.29193	0.484556
57588	1.90104	2.00274
57589	4.37568	0.454681
57590	1.89226	1.39129
57591	2.16957	2.01687

57592	2.52823	1.65108
57593	2.52823	1.86848
57594	1.89226	1.51503
57595	1.89226	1.61681
57596	1.36226	2.1
57597	1.55369	2.1
57598	1.33309	2.1
57599	1.62593	2.1
57600	2.98819	2.1
57601	0.802281	2.1
57602	0.802281	2.1
57603	0.835143	2.1
57604	0.835143	2.1
57605	0.824458	2.1
57606	0.92641	2.1
57607	0.776847	2.1
57608	0.812967	2.1
57609	0.812967	2.1
57610	0.926411	2.1
57611	0.776847	2.1
57612	0.824458	2.1
57613	1.33309	2.1
57614	1.62593	2.1
57615	1.32813	2.1
57616	1.7237	2.1
57617	2.95406	2.1
57618	0.814544	2.1
57619	0.875312	2.1
57620	0.910502	2.1
57621	0.910502	2.1
57622	0.886633	2.1
57623	0.975739	2.1
57624	0.792623	2.1
57625	0.840283	2.1
57626	0.796372	2.1
57627	1.06159	2.1
57628	0.81948	2.1
57629	0.933833	2.1
57630	2.63997	2.1
57631	1.29694	2.1
57632	1.28141	2.1
57633	2.62623	2.1
57634	3.92317	2.1
57635	2.63998	2.1
57636	1.89226	2.1
57637	1.89226	2.1
57638	2.62623	2.1
57639	4.5185	2.1
57640	1.89226	2.1
57641	1.89226	2.1
57642	1.89226	2.1
57643	1.89226	2.1
57644	3.78453	2.1
57645	1.89226	2.1
57646	1.89226	2.1
57647	1.89226	2.1
57648	1.89226	2.1
57649	1.89226	2.1

57650	1.89226	2.1
57651	1.25468	2.1
57652	2.62623	2.1
57653	1.28141	2.1
57654	1.28141	2.1
57655	2.60665	2.1
57656	2.60665	2.1
57657	1.89226	2.1
57658	2.62623	2.1
57659	2.60666	2.1
57660	2.62623	2.1
57661	1.87269	2.1
57662	1.89226	2.1
57663	1.89226	2.1
57664	1.89226	2.1
57665	1.89226	2.1
57666	1.89226	2.1
57667	3.78453	2.1
57668	1.89226	2.1
57669	1.89226	2.1
57670	1.89226	2.1
57671	1.89226	2.1
57672	1.89226	2.1
57673	1.89226	2.1
57674	2.30147	1.66024
57675	2.27703	1.98336
57676	1.89227	1.56741
57677	1.89227	1.80402
57678	4.1693	1.72768
57679	1.38617	1.38302
57680	1.24613	1.5978
57681	2.59886	1.33232
57682	2.6238	1.54216
57683	3.86993	1.45274
57684	0.946133	1.67093
57685	0.946133	1.47789
57686	0.946132	1.41232
57687	0.946132	1.99717
57688	0.946133	2.00016
57689	0.946133	1.76319
57690	0.946132	1.47316
57691	0.946132	1.49706
57692	0.946133	1.60748
57693	0.946133	1.59011
57694	0.946132	1.55423
57695	0.946132	1.555
57696	2.21773	2.1
57697	2.59216	2.1
57698	1.68541	2.1
57699	1.68541	2.1
57700	1.84814	2.1
57701	2.38046	2.1
57702	1.89966	2.1
57703	1.70303	2.1
57704	1.2028	2.1
57705	1.29897	2.1
57706	2.02873	2.1
57707	2.19093	2.1

57708	2.75218	2.1
57709	1.70303	2.1
57710	3.10254	2.1
57711	2.24783	2.1
57712	2.56839	2.1
57713	1.70303	2.1
57714	2.56839	2.1
57715	1.08276	2.1
57716	2.24061	2.1
57717	1.67491	2.1
57718	1.60948	2.1
57719	1.08276	2.1
57720	1.89226	1.07232
57721	1.89226	1.19158
57722	1.89226	1.07754
57723	1.89226	1.0591
57724	3.78453	1.00943
57725	1.89226	1.04941
57726	1.89226	1.1077
57727	1.89226	0.939703
57728	1.89226	1.02254
57729	3.78452	1.27959
57730	1.89226	1.08122
57731	2.7535	1.07399
57732	1.89226	1.28253
57733	1.89226	1.90461
57734	1.89226	0.802978
57735	1.89226	1.02005
57736	1.89226	1.09572
57737	1.89226	1.05732
57738	1.89226	0.927771
57739	1.89226	1.37434
57740	1.89226	1.06065
57741	1.89226	1.41936
57742	1.89227	1.2517
57743	1.89227	1.06196
57744	1.89227	0.246783
57745	1.89227	0.890826
57746	3.78453	1.02595
57747	1.89226	1.08243
57748	2.53412	0.502497
57749	1.89226	1.63848
57750	1.89226	0.620464
57751	1.89226	1.0622
57752	1.89226	0.361978
57753	2.21126	0.05
57754	1.89226	0.0646269
57755	2.57464	0.0720137
57756	1.89226	0.0669957
57757	2.57464	0.05
57758	1.89226	0.05
57759	1.89226	0.19778
57760	1.89226	0.208194
57761	1.89226	0.606118
57762	1.89226	0.220948
57763	1.89226	0.500987
57764	1.89226	0.169264
57765	1.1418	0.192045

57766	0.653021	0.05
57767	1.37282	0.104973
57768	0.931386	0.230708
57769	1.34568	0.286022
57770	1.17669	0.103103
57771	0.666595	0.0696257
57772	0.786895	0.083814
57773	0.933982	0.122686
57774	1.05351	0.101806
57775	1.00792	0.131382
57776	1.00792	0.0746142
57777	1.89226	2.1
57778	1.89226	2.1
57779	1.89226	2.1
57780	1.89226	2.1
57781	3.78452	2.1
57782	1.89226	2.1
57783	1.89226	2.1
57784	1.89226	2.1
57785	1.89226	2.1
57786	1.89226	2.1
57787	1.89226	2.1
57788	1.65642	2.1
57789	1.30248	2.1
57790	1.38888	2.1
57791	1.25987	2.1
57792	1.70611	2.1
57793	1.65642	2.1
57794	1.65642	2.1
57795	1.81407	2.1
57796	2.19428	2.1
57797	1.92555	2.1
57798	3.8507	2.1
57799	1.89226	2.1
57800	1.89226	2.1
57801	1.89226	2.1
57802	1.89226	2.1
57803	3.78452	2.1
57804	1.89226	2.1
57805	1.89226	2.1
57806	1.89226	2.1
57807	1.89226	2.1
57808	1.89226	2.1
57809	1.89226	2.1
57810	0.705495	2.1
57811	0.809317	2.1
57812	1.1655	2.1
57813	1.09605	2.1
57814	0.983375	2.1
57815	0.797301	2.1
57816	0.906633	2.1
57817	0.906633	2.1
57818	0.85085	2.1
57819	1.16548	2.1
57820	0.705501	2.1
57821	1.00314	2.1
57822	2.19428	2.1
57823	2.31862	2.1

57824	2.27408	2.1
57825	2.45893	2.1
57826	4.46836	2.1
57827	1.89226	1.14536
57828	1.89226	1.22318
57829	1.89226	1.22045
57830	1.89226	1.09014
57831	1.89226	1.25393
57832	1.89226	1.17034
57833	2.0815	1.14025
57834	2.22136	1.13221
57835	1.5515	1.03117
57836	1.86323	0.808283
57837	2.74986	1.23109
57838	3.30238	1.05751
57839	1.89224	0.944639
57840	1.89224	1.11397
57841	1.89224	1.22499
57842	1.89224	0.690884
57843	1.89224	1.25655
57844	1.89224	1.099
57845	1.89224	1.12846
57846	1.89224	1.19941
57847	1.89224	1.18333
57848	1.89224	1.10552
57849	1.89224	1.23276
57850	1.89224	1.15418
57851	1.17846	0.721458
57852	0.810935	0.949322
57853	0.93347	1.21051
57854	0.93347	1.05321
57855	0.810932	1.15343
57856	0.810932	1.1181
57857	2.22133	1.08682
57858	1.71919	0.982785
57859	1.32629	0.586289
57860	1.0813	1.19566
57861	1.57137	1.07795
57862	2.21149	2.1
57863	1.89226	2.1
57864	1.89226	2.1
57865	1.89226	2.1
57866	2.17159	2.1
57867	2.17159	2.1
57868	0.946131	1.01307
57869	0.946131	2.1
57870	0.946131	2.1
57871	0.946131	2.1
57872	0.94613	1.71603
57873	0.94613	1.83831
57874	0.946132	2.1
57875	0.946132	2.1
57876	0.946131	2.03137
57877	0.946131	2.05925
57878	0.946131	2.1
57879	0.946131	2.1
57880	1.12266	2.1
57881	1.4714	2.03894

57882	1.62322	2.1
57883	1.62322	1.96806
57884	1.02958	2.1
57885	1.02958	2.1
57886	2.2671	2.1
57887	1.89226	2.1
57888	1.89226	2.1
57889	1.89226	2.1
57890	2.21149	2.1
57891	2.21149	2.1
57892	1.89226	2.1
57893	1.89226	1.97912
57894	1.89226	2.1
57895	1.89226	1.85966
57896	1.89226	2.1
57897	1.89226	2.05922
57898	0.736914	2.1
57899	0.736914	2.1
57900	0.822839	2.1
57901	0.822836	2.1
57902	0.791298	1.66423
57903	1.00003	1.94131
57904	0.640254	2.1
57905	0.575379	2.1
57906	0.609001	2.1
57907	1.00005	2.1
57908	0.575371	2.1
57909	0.831908	2.1
57910	1.89226	2.1
57911	1.89226	2.1
57912	1.89226	2.1
57913	1.89226	2.1
57914	1.89226	2.1
57915	1.89226	2.1
57916	1.89226	2.1
57917	1.89226	2.1
57918	1.89226	2.1
57919	1.89226	2.1
57920	1.89226	2.1
57921	1.89226	2.1
57922	0.730617	2.1
57923	0.71052	2.1
57924	0.7329	2.1
57925	0.712083	2.1
57926	0.701051	2.1
57927	0.701051	2.1
57928	0.721552	2.1
57929	0.721552	2.1
57930	0.710521	2.1
57931	0.732898	2.1
57932	0.730619	2.1
57933	0.712081	2.1
57934	0.7329	2.1
57935	0.712082	2.1
57936	0.559601	2.1
57937	0.495993	2.1
57938	0.603718	2.1
57939	0.603718	2.1

57940	0.625174	2.1
57941	0.625174	2.1
57942	0.732899	2.1
57943	0.559601	2.1
57944	0.712082	2.1
57945	0.495993	2.1
57946	0.94613	2.1
57947	0.94613	2.1
57948	0.94613	2.1
57949	0.94613	2.1
57950	0.946131	2.1
57951	0.946131	2.1
57952	0.946129	2.1
57953	0.946129	2.1
57954	0.94613	2.1
57955	0.94613	2.1
57956	0.94613	2.1
57957	0.94613	2.1
57958	1.89226	2.1
57959	1.89226	2.1
57960	1.89226	2.1
57961	1.89226	2.1
57962	1.89226	2.1
57963	1.89226	2.1
57964	1.42104	2.1
57965	1.4658	2.1
57966	1.51223	2.1
57967	1.42283	2.1
57968	2.93327	2.1
57969	0.732899	2.1
57970	0.756115	2.1
57971	0.629763	2.1
57972	0.559601	2.1
57973	0.646134	2.1
57974	0.646134	2.1
57975	0.669582	2.1
57976	0.669582	2.1
57977	0.756115	2.1
57978	0.629763	2.1
57979	0.732899	2.1
57980	0.559601	2.1
57981	2.566 2.1	
57982	1.20242	2.1
57983	2.64084	2.1
57984	2.18297	2.1
57985	1.66029	2.1
57986	1.20242	2.1
57987	0.946133	1.73998
57988	0.946133	1.83594
57989	0.946129	2.1
57990	0.946129	2.1
57991	0.946131	2.1
57992	0.946131	1.70507
57993	0.946131	1.57014
57994	0.946131	2.1
57995	0.946131	1.97725
57996	0.946131	2.04747
57997	0.946131	2.06237

57998	0.946131	2.09577
57999	1.89226	0.05
58000	1.89226	0.05
58001	1.89226	0.05
58002	1.89226	0.05
58003	3.78453	0.05
58004	1.03073	0.05
58005	1.03073	0.05
58006	1.04066	0.05
58007	1.04066	0.05
58008	1.20802	0.05
58009	1.32689	0.05
58010	0.883301	0.05
58011	0.744506	0.05
58012	1.20802	0.05
58013	0.744506	0.05
58014	0.883301	0.05
58015	1.32689	0.05
58016	1.20802	0.05
58017	1.20802	0.05
58018	0.946131	0.05
58019	0.946131	0.05
58020	0.94613	0.05
58021	0.94613	0.05
58022	1.20802	0.05
58023	1.32689	0.05
58024	1.20802	0.05
58025	0.946131	0.05
58026	0.946131	0.05
58027	1.32689	0.05
58028	1.89226	0.05
58029	1.89226	0.05
58030	1.89226	0.05
58031	1.89226	0.05
58032	1.89226	0.05
58033	1.89226	0.05
58034	0.946119	0.05
58035	0.946119	0.05
58036	0.946118	0.05
58037	0.946118	0.05
58038	0.946118	0.05
58039	0.946118	0.05
58040	0.946119	0.05
58041	0.946119	0.05
58042	0.946131	0.05
58043	0.946131	0.05
58044	0.946106	0.05
58045	0.946106	0.05
58046	1.3939	2.1
58047	1.49641	2.1
58048	1.15096	2.1
58049	1.10101	2.1
58050	2.59742	2.1
58051	1.22106	2.1
58052	2.60663	2.1
58053	1.25467	2.1
58054	1.25467	2.1
58055	2.58377	2.1

58056	2.58377	2.1
58057	1.89225	2.1
58058	2.60664	2.1
58059	2.58377	2.1
58060	2.60664	2.1
58061	1.86938	2.1
58062	1.89225	2.1
58063	1.89225	2.1
58064	1.89225	2.1
58065	1.89225	2.1
58066	1.89225	2.1
58067	1.89225	2.1
58068	1.89225	2.1
58069	0.946121	2.1
58070	0.946121	2.1
58071	0.946126	2.1
58072	0.946126	2.1
58073	0.946124	2.1
58074	0.946124	2.1
58075	0.946123	2.1
58076	0.946123	2.1
58077	0.946117	2.1
58078	0.946117	2.1
58079	0.94613	2.1
58080	0.94613	2.1
58081	1.28017	2.1
58082	1.29189	2.1
58083	0.610534	2.1
58084	0.592373	2.1
58085	0.943764	2.1
58086	0.943764	2.1
58087	0.940501	2.1
58088	0.940501	2.1
58089	1.28017	2.1
58090	0.592371	2.1
58091	0.610533	2.1
58092	1.29189	2.1
58093	0.946133	2.1
58094	0.946133	2.1
58095	1.28017	2.1
58096	1.29189	2.1
58097	0.946132	2.1
58098	0.946132	2.1
58099	1.28017	2.1
58100	1.28017	2.1
58101	1.28017	2.1
58102	0.946132	2.1
58103	0.946133	2.1
58104	1.2919	2.1
58105	1.89226	2.1
58106	1.89226	2.1
58107	1.89226	2.1
58108	1.89226	2.1
58109	1.89226	2.1
58110	1.89226	2.1
58111	1.89226	2.1
58112	1.89226	2.1
58113	1.89226	2.1

58114	1.89226	2.1
58115	3.78452	2.1
58116	1.89226	2.1
58117	2.08196	2.1
58118	1.97854	2.1
58119	2.08196	2.1
58120	1.78884	2.1
58121	1.89226	2.1
58122	1.89226	2.1
58123	1.89226	2.1
58124	1.89226	2.1
58125	1.89226	2.1
58126	1.89226	2.1
58127	1.89226	2.1
58128	1.83688	2.1
58129	1.89226	2.1
58130	1.89226	2.1
58131	1.89226	2.1
58132	1.86015	2.1
58133	1.86015	2.1
58134	1.89226	2.1
58135	1.97854	2.1
58136	1.85883	2.1
58137	1.97854	2.1
58138	1.77255	2.1
58139	1.89226	2.1
58140	0.946131	2.1
58141	0.946131	2.1
58142	0.946131	2.1
58143	0.946131	2.1
58144	0.94613	2.1
58145	0.94613	2.1
58146	0.946132	2.1
58147	0.946132	2.1
58148	0.946131	2.1
58149	0.946131	2.1
58150	0.946131	2.1
58151	0.946131	2.1
58152	0.918412	2.1
58153	0.918412	2.1
58154	0.94893	2.1
58155	0.750211	2.1
58156	0.851541	2.1
58157	0.851541	2.1
58158	0.970566	2.1
58159	0.826567	2.1
58160	0.970554	2.1
58161	1.04071	2.1
58162	0.826632	2.1
58163	0.851554	2.1
58164	1.89226	0.05
58165	1.89226	0.05
58166	1.89226	0.05
58167	1.89226	0.05
58168	3.78453	0.05
58169	0.946131	0.05
58170	0.946131	0.05
58171	0.811232	0.05

58172	0.811232	0.05
58173	0.94613	0.05
58174	0.94613	0.05
58175	0.959701	0.05
58176	0.811233	0.05
58177	0.94613	0.05
58178	0.811233	0.05
58179	0.959702	0.05
58180	0.946131	0.05
58181	1.89226	0.05
58182	1.89226	0.05
58183	1.89226	0.05
58184	1.89226	0.05
58185	1.89226	0.05
58186	1.89226	0.05
58187	1.89227	0.05
58188	1.89227	0.05
58189	1.89227	0.05
58190	1.89227	0.05
58191	1.89227	0.05
58192	1.89227	0.05
58193	1.89226	0.05
58194	1.89226	0.05
58195	1.89226	0.05
58196	1.89226	0.05
58197	3.78453	0.05
58198	1.89226	0.05
58199	1.89226	0.05
58200	1.89226	0.05
58201	1.89226	0.05
58202	1.89226	0.05
58203	1.89226	0.05
58204	1.89226	2.1
58205	2.36204	2.1
58206	2.39853	2.1
58207	2.36204	2.1
58208	1.92876	2.1
58209	1.89226	2.1
58210	1.89226	2.1
58211	1.89226	2.1
58212	1.89226	2.1
58213	1.89226	2.1
58214	3.78453	2.1
58215	0.946128	2.1
58216	0.946128	2.1
58217	1.13182	2.1
58218	1.13182	2.1
58219	0.94613	2.1
58220	0.94613	2.1
58221	1.1749	2.1
58222	1.13182	2.1
58223	1.1749	2.1
58224	0.94613	2.1
58225	1.13182	2.1
58226	0.94613	2.1
58227	1.89226	2.1
58228	2.39853	2.1
58229	2.44918	2.1

58230	2.39853	2.1
58231	1.94291	2.1
58232	1.89226	2.1
58233	0.946132	2.1
58234	0.946132	2.1
58235	0.946131	2.1
58236	0.946131	2.1
58237	0.946132	2.1
58238	0.946132	2.1
58239	0.946131	2.1
58240	0.946131	2.1
58241	0.946131	2.1
58242	0.946131	2.1
58243	0.946132	2.1
58244	0.946132	2.1
58245	0.946128	2.1
58246	0.946128	2.1
58247	1.1749	2.1
58248	1.1749	2.1
58249	0.946131	2.1
58250	0.946131	2.1
58251	1.23631	2.1
58252	1.1749	2.1
58253	1.23631	2.1
58254	0.94613	2.1
58255	1.1749	2.1
58256	0.946131	2.1
58257	1.89226	2.1
58258	1.89226	2.1
58259	1.89226	2.1
58260	1.89226	2.1
58261	3.78453	2.1
58262	1.38859	2.1
58263	1.45333	2.1
58264	1.28909	2.1
58265	1.38091	2.1
58266	2.74242	2.1
58267	1.45332	2.1
58268	1.38414	2.1
58269	1.38091	2.1
58270	1.33506	2.1
58271	2.78838	2.1
58272	1.89226	2.1
58273	1.89226	2.1
58274	1.89226	2.1
58275	1.89226	2.1
58276	3.78452	2.1
58277	1.89226	2.1
58278	1.89226	2.1
58279	1.89226	2.1
58280	1.89226	2.1
58281	1.89226	2.1
58282	1.89226	2.1
58283	1.17418	2.1
58284	1.38091	2.1
58285	1.17418	2.1
58286	1.28909	2.1
58287	1.29741	2.1

58288	1.41232	2.1
58289	1.38091	2.1
58290	1.33506	2.1
58291	1.27575	2.1
58292	1.27575	2.1
58293	1.29741	2.1
58294	1.35671	2.1
58295	1.89226	2.1
58296	1.89226	2.1
58297	1.89226	2.1
58298	1.89226	2.1
58299	3.78452	2.1
58300	1.92737	0.05
58301	2.40223	0.05
58302	1.56178	0.05
58303	1.97605	0.0584311
58304	3.90343	0.05
58305	1.89226	0.05
58306	1.89226	0.0870931
58307	1.89226	0.0677505
58308	1.89226	0.0629016
58309	3.78452	0.0672155
58310	1.89226	0.0904009
58311	1.89226	0.0711465
58312	1.89226	0.0684517
58313	1.89226	0.178278
58314	1.89226	0.0657759
58315	1.89226	0.101371
58316	1.13641	0.0632385
58317	1.13641	0.0582638
58318	1.12763	0.05
58319	1.12763	0.0637886
58320	1.29348	0.0581552
58321	1.06293	0.0730756
58322	1.20111	0.05
58323	0.988028	0.0592452
58324	0.988028	0.0534279
58325	1.06293	0.0536607
58326	1.29348	0.05
58327	1.20111	0.05
58328	1.56176	0.05
58329	1.46387	0.0588128
58330	1.97603	0.05
58331	1.97603	0.05
58332	1.08211	0.05
58333	1.08211	0.05
58334	1.89224	0.0641368
58335	1.89224	0.0631106
58336	1.89224	0.0577155
58337	1.89224	0.0783498
58338	1.89224	0.0516147
58339	1.89224	0.0740021
58340	1.89224	0.0830027
58341	1.89224	0.0750495
58342	1.89224	0.0649961
58343	1.89224	0.108736
58344	3.78448	0.0794727
58345	0.820185	0.05

58346	0.818947	0.0590738
58347	0.911041	0.069564
58348	0.695265	0.0577076
58349	1.46388	0.0609958
58350	1.23607	0.0579285
58351	0.927068	0.0580264
58352	1.02691	0.0758211
58353	1.12918	0.05
58354	0.846843	0.0571927
58355	0.911039	0.0591478
58356	1.29129	2.1
58357	1.45547	2.1
58358	1.67327	2.1
58359	1.66698	2.1
58360	1.20149	2.1
58361	1.20149	2.1
58362	0.946133	2.1
58363	0.946133	2.1
58364	0.946129	2.1
58365	0.946129	2.1
58366	0.946131	2.1
58367	0.946131	2.1
58368	0.946131	2.1
58369	0.946131	2.1
58370	0.946131	2.1
58371	0.946131	2.1
58372	0.946131	2.1
58373	0.946131	2.1
58374	1.89226	2.1
58375	1.89226	2.1
58376	1.89226	2.1
58377	1.89226	1.92867
58378	3.78452	2.1
58379	0.86875	2.1
58380	0.86875	2.1
58381	0.899002	2.1
58382	0.966245	2.1
58383	0.849364	2.00373
58384	1.01918	2.1
58385	0.764737	2.1
58386	0.89249	2.1
58387	0.764624	2.1
58388	0.967869	2.1
58389	0.808606	2.1
58390	0.864506	2.1
58391	0.773929	2.1
58392	0.773929	2.1
58393	0.781991	2.1
58394	0.836559	2.1
58395	0.983761	2.1
58396	0.834687	2.1
58397	0.680545	2.1
58398	0.640799	2.1
58399	0.70286	2.1
58400	0.935844	2.1
58401	0.629479	2.1
58402	0.823655	2.1
58403	0.946133	2.1

58404	0.946133	2.1
58405	0.946128	2.1
58406	0.946128	2.1
58407	0.94613	2.1
58408	0.94613	2.1
58409	0.946131	2.1
58410	0.946131	2.1
58411	0.946131	2.1
58412	0.946131	2.1
58413	0.946131	2.1
58414	0.946131	2.1
58415	1.89226	2.1
58416	1.89226	2.04468
58417	1.89226	2.1
58418	1.89226	2.1
58419	1.89226	2.1
58420	1.89226	2.1
58421	0.931638	2.1
58422	0.931638	2.1
58423	1.03559	2.1
58424	0.977442	1.96995
58425	0.826487	1.47151
58426	0.993902	2.06086
58427	1.04922	2.1
58428	0.894324	2.1
58429	0.912984	2.1
58430	1.01156	2.1
58431	0.944692	2.1
58432	1.15456	2.1
58433	1.89226	0.0735048
58434	1.89226	0.106206
58435	1.89226	0.141882
58436	1.89226	0.0701938
58437	3.78453	0.075796
58438	1.89226	0.0673232
58439	1.89226	0.0627223
58440	2.64262	0.0653215
58441	2.64262	0.05
58442	1.78052	0.05
58443	1.03015	0.0600275
58444	0.999721	0.0528654
58445	0.659011	0.05
58446	1.15564	0.0888883
58447	1.41388	0.0614406
58448	1.01583	0.0687816
58449	1.01583	0.0512608
58450	1.05706	0.0880422
58451	1.05706	0.0618742
58452	1.15564	0.0610931
58453	0.65901	0.0562945
58454	1.41388	0.0594164
58455	0.999721	0.05843
58456	1.89226	2.1
58457	1.89226	2.1
58458	1.89226	2.1
58459	1.89226	2.1
58460	3.78453	2.1
58461	2.39022	2.1

58462	1.90734	2.1
58463	2.27634	2.1
58464	1.82666	2.1
58465	4.18368	2.1
58466	1.90734	2.1
58467	1.21564	2.1
58468	1.82666	2.1
58469	1.16938	2.1
58470	3.07672	2.1
58471	1.89226	2.1
58472	1.89226	2.1
58473	1.89226	2.1
58474	1.89226	2.1
58475	1.89226	2.1
58476	1.89226	2.1
58477	1.89227	2.1
58478	1.89227	2.1
58479	1.89227	2.1
58480	1.89227	2.1
58481	3.78453	2.1
58482	2.27634	2.1
58483	1.82666	2.1
58484	2.18042	2.1
58485	1.74738	2.1
58486	4.02372	2.1
58487	1.74738	2.1
58488	1.82666	2.1
58489	1.1181	2.1
58490	1.1181	2.1
58491	1.16938	2.1
58492	1.79865	2.1
58493	1.89226	2.1
58494	1.89226	2.1
58495	1.89226	2.1
58496	1.89226	2.1
58497	1.89226	2.1
58498	1.89226	2.1
58499	1.89226	1.28076
58500	1.89226	1.04449
58501	1.89226	1.17089
58502	1.89226	1.11837
58503	1.89226	1.24569
58504	1.89226	1.143
58505	0.790736	1.12586
58506	1.08265	1.08794
58507	0.938909	1.30256
58508	0.638683	1.23513
58509	0.858588	1.26811
58510	0.858588	1.11283
58511	0.862743	1.27187
58512	0.862743	1.12597
58513	1.08265	1.20876
58514	0.938911	1.15577
58515	0.790735	1.17606
58516	0.638685	1.14825
58517	1.89226	1.11795
58518	1.89226	1.25065
58519	1.89226	1.21185

58520	1.89226	1.1357
58521	1.89226	1.24765
58522	1.89226	1.1687
58523	1.89226	1.06267
58524	1.89226	1.23272
58525	1.89226	1.14566
58526	1.89226	1.13386
58527	1.89226	1.20736
58528	1.89226	1.20633
58529	1.08265	1.11077
58530	1.36209	1.07426
58531	1.25233	1.17504
58532	0.938909	1.21993
58533	1.14195	1.16607
58534	1.14195	1.09776
58535	1.15904	1.21454
58536	1.15904	1.12751
58537	1.36209	1.14932
58538	1.25233	1.06814
58539	1.08265	1.18454
58540	0.938911	1.12858
58541	1.22077	1.12267
58542	0.915244	1.14452
58543	0.967835	1.23484
58544	0.742495	1.24984
58545	0.78648	1.2491
58546	0.985041	1.14948
58547	0.989098	1.28216
58548	0.989098	1.1498
58549	1.03206	1.21915
58550	0.967897	1.23334
58551	0.915404	1.20637
58552	0.706105	1.19734
58553	2.69662	0.05
58554	1.89226	0.05
58555	2.35499	0.05
58556	1.89226	0.05
58557	4.24725	0.05
58558	1.89227	0.05
58559	1.89227	0.05
58560	1.89227	0.05
58561	1.89227	0.05
58562	3.78453	0.05
58563	2.35499	0.05
58564	1.70302	0.05
58565	1.70302	0.05
58566	1.703 0.05	
58567	1.97374	0.05
58568	1.97372	0.05
58569	0.946133	0.05
58570	0.946133	0.05
58571	0.946132	0.05
58572	0.946132	0.05
58573	0.946133	0.05
58574	0.946133	0.05
58575	0.946132	0.05
58576	0.946132	0.05
58577	0.946133	0.05

58578	0.946133	0.05
58579	0.946132	0.05
58580	0.946132	0.05
58581	0.6794	2.1
58582	0.6794	2.1
58583	0.670812	2.1
58584	0.62182	2.1
58585	0.648326	2.1
58586	0.792154	2.1
58587	0.719167	2.1
58588	0.494515	2.1
58589	0.649421	2.1
58590	0.804869	2.1
58591	0.485585	2.1
58592	0.627666	2.1
58593	1.79347	2.1
58594	1.03968	2.1
58595	1.70303	2.1
58596	0.933265	2.0476
58597	2.62068	2.1
58598	1.89226	2.1
58599	1.89226	2.1
58600	1.89226	2.1
58601	1.89226	2.1
58602	3.78452	2.1
58603	1.89226	2.1
58604	2.24604	2.1
58605	2.0554	2.1
58606	2.0554	2.1
58607	1.89226	2.1
58608	1.89226	2.1
58609	1.89226	2.1
58610	1.89226	2.0765
58611	1.89226	2.1
58612	1.89226	2.1
58613	1.89226	2.1
58614	1.89226	2.1
58615	2.0815	2.1
58616	2.64424	1.09211
58617	2.80807	1.95103
58618	3.232	2.1
58619	1.89417	1.98825
58620	2.18012	2.1
58621	0.946131	2.1
58622	0.946131	1.96289
58623	0.946131	2.1
58624	0.946131	2.1
58625	0.94613	2.1
58626	0.94613	2.04498
58627	0.946132	2.1
58628	0.946132	2.1
58629	0.946131	2.1
58630	0.946131	2.1
58631	0.946131	2.1
58632	0.946131	2.1
58633	1.89227	0.05
58634	1.89227	0.0798213
58635	1.89227	0.0961517

58636	1.89227	0.0513805
58637	3.78453	0.057459
58638	2.70881	0.05
58639	2.68687	0.05
58640	2.03046	0.05
58641	1.9682	0.05
58642	4.67701	0.05
58643	1.08518	0.05
58644	1.08518	0.05
58645	1.09033	0.05
58646	1.09033	0.05
58647	0.842273	0.05
58648	1.19141	0.05
58649	1.34343	0.05
58650	0.9841	0.05
58651	0.9841	0.05
58652	0.842273	0.05
58653	1.34344	0.05
58654	1.19141	0.05
58655	0.946133	0.05
58656	0.946133	0.05
58657	0.946132	0.0933803
58658	0.946132	0.0684877
58659	0.946132	0.0569861
58660	0.946132	0.05
58661	0.946132	0.0889326
58662	0.946132	0.0686793
58663	0.946132	0.05
58664	0.946132	0.0692148
58665	0.946133	0.0729942
58666	0.946133	0.0561607
58667	1.89227	0.0766381
58668	1.89227	0.05
58669	1.89227	0.0708329
58670	1.89227	0.0557218
58671	1.89227	0.0601475
58672	1.89227	0.05
58673	2.03046	0.05
58674	1.9682	0.05
58675	1.23886	0.05
58676	1.12971	0.05
58677	3.20706	0.05
58678	1.89226	0.306001
58679	1.89226	1.22971
58680	1.89226	0.226714
58681	1.89226	0.857112
58682	3.78452	0.606141
58683	2.45569	0.133945
58684	1.89226	0.159962
58685	2.22922	0.138411
58686	2.0815	0.377859
58687	1.85074	0.0906584
58688	1.70303	0.162105
58689	2.72856	0.140746
58690	1.89226	0.510388
58691	1.89226	0.125387
58692	1.89226	0.233413
58693	3.78452	0.208396

58694	1.89226	0.43776
58695	1.89226	1.06276
58696	1.89226	0.200854
58697	1.89226	1.13065
58698	3.78452	0.611065
58699	0.946131	0.913019
58700	0.946131	0.181201
58701	0.946131	0.542188
58702	0.946131	0.141274
58703	0.94613	0.45478
58704	0.94613	0.667299
58705	0.946132	0.194638
58706	0.946132	0.201543
58707	0.946131	0.288648
58708	0.946131	0.274899
58709	0.946131	0.27789
58710	0.946131	0.301951
58711	2.0595	0.0903967
58712	2.58581	0.0677672
58713	1.48694	0.0753476
58714	1.48694	0.0761382
58715	2.60157	0.0716417
58716	2.02901	0.0758816
58717	1.89226	0.0764372
58718	1.89226	0.103793
58719	1.89226	0.0850548
58720	1.89226	0.0847583
58721	1.89226	0.0856525
58722	1.89226	0.0946959
58723	2.04728	0.0945844
58724	2.64161	0.069298
58725	1.47606	0.0834041
58726	1.47606	0.0853456
58727	2.63069	0.0798754
58728	2.05947	0.0881311
58729	1.89224	0.0646916
58730	1.89224	0.0967015
58731	1.89224	0.0742689
58732	1.89224	0.086189
58733	1.89224	0.0739064
58734	1.89224	0.0742903
58735	0.946114	0.103091
58736	0.946114	0.0765815
58737	0.946118	0.103454
58738	0.946118	0.0905771
58739	0.946114	0.108238
58740	0.946114	0.105225
58741	0.946118	0.0897194
58742	0.946118	0.0794163
58743	0.946128	0.0980503
58744	0.946128	0.0989595
58745	0.946103	0.0926881
58746	0.946103	0.0955278
58747	1.1233	2.1
58748	1.15009	2.1
58749	2.36036	2.1
58750	2.38225	2.1
58751	3.50554	2.1

58752	2.38224	2.1
58753	0.977775	2.1
58754	2.20473	2.1
58755	2.20473	2.1
58756	1.15009	2.1
58757	1.15009	2.1
58758	0.946119	2.1
58759	0.946119	2.09935
58760	1.19111	2.1
58761	1.19111	2.07256
58762	0.946118	2.1
58763	1.19111	2.1
58764	1.18016	2.08525
58765	0.946118	1.76144
58766	0.94613	1.61703
58767	0.94613	2.1
58768	1.18015	2.00886
58769	1.19109	2.06278
58770	0.946118	2.1
58771	0.946118	2.1
58772	1.10235	2.1
58773	1.10235	2.1
58774	0.946118	2.1
58775	1.10235	2.1
58776	0.946115	2.1
58777	1.1911	2.1
58778	0.946129	2.1
58779	0.946129	2.1
58780	1.19109	2.1
58781	1.10234	2.1
58782	1.89226	2.1
58783	2.23932	2.1
58784	2.11568	2.1
58785	2.23932	2.1
58786	1.76862	2.1
58787	1.89226	2.1
58788	1.89226	2.1
58789	1.89226	2.1
58790	1.89226	2.1
58791	1.89226	2.1
58792	1.89226	2.1
58793	1.89226	2.1
58794	2.07838	2.1
58795	1.89226	2.1
58796	1.89226	2.1
58797	1.89226	2.1
58798	2.10264	2.1
58799	2.10264	2.1
58800	1.89226	2.1
58801	2.11568	2.1
58802	1.99753	2.1
58803	2.11568	2.1
58804	1.77411	2.1
58805	1.89226	2.1
58806	0.946128	2.1
58807	0.946128	2.1
58808	0.946133	2.1
58809	0.946133	2.1

58810	0.94613	2.1
58811	0.94613	2.1
58812	0.946131	2.1
58813	0.946131	2.1
58814	0.946131	2.1
58815	0.946131	2.1
58816	0.946131	2.1
58817	0.946131	2.1
58818	0.946132	2.1
58819	0.946132	2.1
58820	1.03919	2.1
58821	1.03919	2.1
58822	0.946131	2.1
58823	0.946131	2.1
58824	1.02744	2.1
58825	1.03919	2.1
58826	1.02744	2.1
58827	0.946132	2.1
58828	1.03919	2.1
58829	0.946132	2.1
58830	1.64834	1.81114
58831	1.82955	1.63655
58832	2.33824	1.62268
58833	2.19301	1.71397
58834	3.98658	1.66274
58835	1.40574	1.84916
58836	2.19301	1.65029
58837	1.64834	1.81441
58838	1.64834	1.69688
58839	1.99961	1.72859
58840	1.99961	1.66251
58841	2.33824	1.60848
58842	2.19301	1.6915
58843	2.68524	1.69787
58844	2.81847	1.56809
58845	5.02349	1.65182
58846	1.99961	1.77739
58847	2.19301	1.61211
58848	2.68524	1.67937
58849	2.50732	1.62791
58850	4.68485	1.65147
58851	1.89226	2.1
58852	1.89226	2.1
58853	1.89226	2.1
58854	1.89226	2.1
58855	3.78453	2.1
58856	1.75142	2.1
58857	2.47026	2.1
58858	1.46811	2.1
58859	2.26652	2.1
58860	4.01794	2.1
58861	1.89226	2.1
58862	1.89226	2.1
58863	1.89226	2.1
58864	1.89226	2.1
58865	1.89226	2.1
58866	1.89226	2.1
58867	1.89226	2.1

58868	1.89226	2.1
58869	1.89226	2.1
58870	1.89226	2.1
58871	1.89226	2.1
58872	1.89226	2.1
58873	0.812082	2.1
58874	0.878759	2.1
58875	0.882782	2.1
58876	0.882782	2.1
58877	1.05572	2.1
58878	1.04037	2.1
58879	0.73849	2.1
58880	0.626857	2.1
58881	0.638677	2.1
58882	1.05581	2.1
58883	0.715695	2.1
58884	1.10492	2.1
58885	1.17058	2.1
58886	1.07863	2.1
58887	1.21113	2.1
58888	1.21113	2.1
58889	1.2811	2.1
58890	1.37319	2.1
58891	1.01788	2.1
58892	0.999492	2.1
58893	1.04352	2.1
58894	1.32112	2.1
58895	1.10492	2.1
58896	1.34699	2.1
58897	1.89226	2.1
58898	1.89226	2.1
58899	1.89226	2.1
58900	1.89226	2.1
58901	1.89226	2.1
58902	1.89226	2.1
58903	1.19813	0.05
58904	1.19813	0.05
58905	2.43432	0.05
58906	1.21716	0.05
58907	0.946124	0.05
58908	1.21715	0.05
58909	0.946118	0.05
58910	2.56627	2.1
58911	1.89226	2.1
58912	1.89226	2.1
58913	1.89226	2.1
58914	2.43434	2.1
58915	2.43434	2.1
58916	1.89227	0.05
58917	1.89227	0.05
58918	1.89227	0.05
58919	1.89227	0.05
58920	1.89227	0.05
58921	1.89227	0.05
58922	1.89226	0.05
58923	1.89226	0.0521814
58924	1.89226	0.0502375
58925	1.89226	0.05

58926	3.78453	0.05
58927	1.89226	0.05
58928	2.0764	0.05
58929	1.89226	0.05
58930	2.81674	0.05
58931	1.89226	0.05
58932	2.81674	0.05
58933	0.946132	0.05
58934	0.946132	0.05
58935	0.946131	0.05
58936	0.946131	0.05
58937	0.946132	0.05
58938	0.946132	0.05
58939	0.946132	0.050065
58940	0.946132	0.05
58941	0.946132	0.05
58942	0.946132	0.05
58943	0.946132	0.05
58944	0.946132	0.05
58945	0.578684	1.55801
58946	0.578684	2.1
58947	0.589487	1.96708
58948	0.589487	2.1
58949	0.625461	1.73197
58950	0.5378	1.81884
58951	0.608714	2.1
58952	0.54271	1.82364
58953	0.608714	1.60507
58954	0.625461	1.55497
58955	0.54271	1.87486
58956	0.537801	1.80412
58957	0.946128	1.51774
58958	0.946128	1.83647
58959	0.946133	1.37854
58960	0.946133	2.1
58961	0.946131	1.41766
58962	0.946131	1.5247
58963	0.946131	1.49382
58964	0.946131	1.68048
58965	0.94613	1.40303
58966	0.94613	1.34214
58967	0.946131	1.53828
58968	0.946131	1.62337
58969	1.21743	2.03252
58970	1.25092	2.05111
58971	1.38847	1.91806
58972	1.46031	1.37806
58973	2.67774	1.7688
58974	0.946128	1.40336
58975	0.946128	1.90413
58976	0.946132	2.1
58977	0.946132	1.39421
58978	0.94613	1.46452
58979	0.94613	1.39892
58980	0.94613	1.72963
58981	0.94613	1.52968
58982	0.94613	1.33066
58983	0.94613	1.34851

58984	0.94613	1.61217
58985	0.94613	1.54669
58986	1.89226	1.14839
58987	1.89226	1.52974
58988	1.89226	0.609247
58989	1.89226	2.03642
58990	1.89226	2.1
58991	1.89226	2.01315
58992	0.539566	1.38678
58993	0.534046	2.1
58994	0.577914	1.8215
58995	0.471623	1.78102
58996	1.46031	1.00693
58997	0.945865	1.2969
58998	0.770603	1.16208
58999	0.737201	1.96764
59000	0.714829	1.36167
59001	0.536085	1.64495
59002	0.471617	1.6083
59003	1.89226	1.9248
59004	1.89226	1.50381
59005	1.89226	1.51867
59006	1.89226	1.61422
59007	1.89226	1.71911
59008	1.89226	1.92669
59009	1.89225	1.55256
59010	1.89225	1.03416
59011	1.65572	1.22163
59012	1.65572	1.41514
59013	1.89225	1.46652
59014	1.89225	1.54951
59015	1.89226	1.58027
59016	1.89226	1.49281
59017	1.89226	1.54862
59018	1.89226	0.979763
59019	3.78453	1.6024
59020	1.89226	1.67001
59021	1.89226	1.23674
59022	1.89226	1.29052
59023	1.89226	1.48188
59024	1.89226	1.49636
59025	1.89226	1.72976
59026	1.89225	1.43597
59027	1.89225	0.53597
59028	1.89225	0.856301
59029	1.89225	1.30321
59030	1.89225	1.30405
59031	1.89225	1.45156
59032	0.763592	1.11751
59033	0.763592	1.45538
59034	0.987417	1.35088
59035	0.987417	1.36106
59036	0.763592	0.742825
59037	0.804881	0.596171
59038	0.946126	1.37203
59039	0.946126	1.31418
59040	0.804879	0.658577
59041	0.94613	1.3448

59042	0.946129	1.43726
59043	0.76359	0.842491
59044	1.46055	2.1
59045	2.04945	2.1
59046	2.04587	2.1
59047	2.04587	2.1
59048	1.50714	2.1
59049	1.50714	2.1
59050	2.04587	2.1
59051	2.38681	2.1
59052	2.42082	2.1
59053	2.42082	2.1
59054	2.04945	2.1
59055	2.04945	2.1
59056	0.872964	2.1
59057	0.872964	2.1
59058	0.884806	2.1
59059	0.884806	2.1
59060	1.0275	2.1
59061	1.02293	2.1
59062	0.711243	2.1
59063	0.730273	2.1
59064	0.711244	2.1
59065	1.0275	2.1
59066	0.730273	2.1
59067	1.02293	2.1
59068	2.04587	2.1
59069	2.47753	2.1
59070	2.42994	2.1
59071	2.42082	2.1
59072	2.05499	2.1
59073	2.04587	2.1
59074	0.946132	2.1
59075	0.946132	2.1
59076	1.13078	2.1
59077	1.07817	2.1
59078	0.946132	2.1
59079	0.946132	2.1
59080	1.07817	2.1
59081	1.07817	2.1
59082	0.946132	2.1
59083	1.07817	2.1
59084	1.13078	2.1
59085	0.946132	2.1
59086	1.89226	2.1
59087	1.89226	2.1
59088	1.89226	2.1
59089	1.89226	2.1
59090	1.89226	2.1
59091	1.89226	2.1
59092	1.89226	2.1
59093	1.89226	2.1
59094	1.89226	2.1
59095	1.89226	2.1
59096	1.89226	2.1
59097	1.89226	2.1
59098	1.89226	2.1
59099	1.89226	2.1

59100	1.8173	2.1
59101	2.66885	2.1
59102	2.7438	2.1
59103	2.7438	2.1
59104	1.89226	1.72924
59105	1.89226	0.05
59106	1.89226	0.0874883
59107	1.89226	0.53394
59108	3.78453	0.772803
59109	1.89226	0.671446
59110	1.89226	0.646787
59111	1.89226	0.602075
59112	1.89226	1.75755
59113	1.89226	0.12741
59114	1.89226	0.672669
59115	1.89226	0.309235
59116	1.97385	0.608406
59117	1.7531	1.39005
59118	1.89226	1.99551
59119	1.7531	0.180088
59120	1.89226	0.431675
59121	0.946131	1.21466
59122	0.840904	0.329276
59123	0.684333	0.0745039
59124	0.94613	0.0676615
59125	0.972337	0.0905352
59126	0.946278	1.19992
59127	0.684304	0.0584207
59128	0.684304	0.261791
59129	0.999838	0.122823
59130	0.759605	0.220387
59131	0.851528	0.05
59132	0.851528	1.14485
59133	1.89226	0.18957
59134	2.23818	0.393359
59135	1.79771	0.389468
59136	1.89226	1.78499
59137	1.79771	0.05
59138	1.89226	0.139703
59139	2.79647	2.1
59140	2.01613	2.1
59141	1.46826	2.1
59142	2.54504	2.1
59143	4.56117	2.1
59144	1.89226	2.1
59145	1.89226	2.1
59146	1.89226	2.1
59147	1.89226	2.1
59148	1.89226	2.1
59149	1.89226	2.1
59150	1.89226	2.1
59151	2.66884	2.1
59152	2.56022	2.1
59153	2.66884	2.1
59154	1.78364	2.1
59155	1.89226	2.1
59156	1.14282	2.1
59157	1.24068	2.1

59158	0.880748	2.1
59159	0.834916	2.1
59160	1.05539	2.1
59161	1.05539	2.1
59162	1.04312	2.1
59163	1.04313	2.1
59164	1.2015	2.1
59165	0.83492	2.1
59166	0.789149	2.1
59167	1.24066	2.1
59168	1.89226	2.1
59169	1.89226	2.1
59170	1.89226	2.1
59171	1.89226	2.1
59172	3.78453	2.1
59173	0.946128	2.1
59174	0.946128	2.1
59175	1.28011	2.1
59176	1.22372	2.1
59177	0.946131	2.1
59178	0.946131	2.1
59179	1.22372	2.1
59180	1.22372	2.1
59181	1.22372	2.1
59182	0.94613	2.1
59183	0.946131	2.1
59184	1.28011	2.1
59185	2.28604	2.1
59186	1.34268	2.1
59187	2.43157	2.1
59188	1.50172	2.1
59189	3.77425	2.1
59190	2.6096	2.1
59191	2.43157	2.1
59192	1.66774	2.1
59193	1.66774	2.1
59194	1.50172	2.1
59195	2.43157	2.1
59196	2.16067	2.1
59197	1.08975	2.1
59198	2.19336	2.1
59199	2.06984	2.1
59200	1.21327	2.1
59201	1.08975	2.1
59202	2.28604	2.1
59203	1.21327	2.1
59204	2.29008	2.1
59205	2.16067	2.1
59206	1.34268	2.1
59207	1.21327	2.1
59208	1.89226	0.341694
59209	1.89226	0.289821
59210	1.89226	0.264976
59211	1.89226	0.151629
59212	3.78452	0.126344
59213	1.89804	0.165505
59214	2.21656	0.212965
59215	1.70537	0.0604412

59216	2.50023	0.0771463
59217	3.8982	0.080276
59218	1.03136	0.0754703
59219	1.89486	0.084727
59220	0.949364	0.0838297
59221	1.82283	0.0807467
59222	2.84422	0.0676836
59223	1.89226	0.0858306
59224	1.89226	0.0908166
59225	1.89226	0.100046
59226	1.89226	0.107188
59227	3.78452	0.167417
59228	0.946132	0.223607
59229	0.946132	0.208548
59230	0.946129	0.210451
59231	0.946129	0.140964
59232	0.94613	0.160305
59233	0.94613	0.522148
59234	0.946131	0.103482
59235	0.946131	0.294197
59236	0.946131	0.434713
59237	0.946131	0.611889
59238	0.946131	0.200633
59239	0.946131	0.729808
59240	1.09466	0.142183
59241	1.09466	0.0889506
59242	0.866512	0.0698336
59243	1.08352	0.0598722
59244	1.19189	0.05006
59245	1.21883	0.096153
59246	0.971139	0.132641
59247	0.873213	0.0653527
59248	0.873202	0.0770389
59249	1.00855	0.0508923
59250	0.923735	0.0596339
59251	1.21885	0.0734547
59252	1.89225	0.05
59253	2.40019	0.05
59254	1.89225	0.264229
59255	1.89225	0.304621
59256	1.89225	0.194728
59257	1.89225	0.397723
59258	1.89225	0.264229
59259	1.89225	0.437481
59260	2.05088	0.191026
59261	1.89225	0.112268
59262	1.89225	0.0972052
59263	1.89225	0.280619
59264	2.03956	0.191026
59265	2.03956	0.304621
59266	1.89226	2.1
59267	2.40021	2.1
59268	2.38473	2.1
59269	2.40021	2.1
59270	1.87679	2.1
59271	1.89226	2.1
59272	1.89226	2.1
59273	1.89226	2.05878

59274	1.89226	2.1
59275	1.89226	2.00903
59276	1.89226	2.1
59277	1.89226	2.1
59278	2.0692	2.1
59279	1.89226	2.1
59280	1.89226	2.1
59281	1.89226	1.95866
59282	2.05089	2.1
59283	2.05089	2.05539
59284	1.89226	2.1
59285	1.89226	2.1
59286	1.89226	2.1
59287	1.89226	2.1
59288	3.78452	2.1
59289	1.89226	2.1
59290	2.50051	2.1
59291	1.89226	2.1
59292	2.30076	2.1
59293	4.39277	2.1
59294	2.50052	2.1
59295	1.70377	2.1
59296	2.30076	2.1
59297	1.54231	2.1
59298	4.04283	2.1
59299	1.89226	2.1
59300	1.89226	2.1
59301	1.89226	2.1
59302	1.89226	2.1
59303	1.89226	2.1
59304	1.89226	2.1
59305	1.89226	2.1
59306	1.89226	2.1
59307	1.89226	2.1
59308	1.89226	2.1
59309	3.78452	2.1
59310	2.07948	2.1
59311	2.30076	2.1
59312	1.3294	2.1
59313	1.3294	2.1
59314	1.54232	2.1
59315	2.29239	2.1
59316	0.810947	2.1
59317	0.810947	2.1
59318	0.810946	2.1
59319	0.810946	2.1
59320	1.89226	2.1
59321	1.35169	2.1
59322	1.08132	2.1
59323	1.08132	2.1
59324	1.08132	2.1
59325	0.810946	2.1
59326	0.810946	2.1
59327	1.34851	1.45824
59328	0.997471	2.1
59329	1.62287	0.482428
59330	1.27096	1.79638
59331	2.62034	1.67025

59332	1.89226	1.81972
59333	1.89226	2.1
59334	1.89226	1.3773
59335	1.89226	2.1
59336	3.78452	1.92264
59337	0.946128	2.1
59338	0.946128	2.1
59339	1.29331	1.95323
59340	1.29331	2.1
59341	1.29331	2.1
59342	1.10699	2.1
59343	0.946131	1.93971
59344	0.946131	2.1
59345	0.94613	2.1
59346	1.29331	2.1
59347	1.10699	2.1
59348	0.946131	2.1
59349	1.62287	1.44873
59350	1.27096	1.8346
59351	1.86067	1.15234
59352	1.58407	1.82644
59353	3.20694	1.64285
59354	1.89226	1.4981
59355	1.89226	2.1
59356	1.89226	0.803085
59357	1.89226	1.83581
59358	3.78452	1.81996
59359	2.62761	0.138263
59360	2.55849	0.974649
59361	2.12261	1.4742
59362	2.22939	1.16043
59363	4.78788	1.16565
59364	0.772522	2.1
59365	0.839588	1.98576
59366	1.31126	0.766906
59367	1.08787	0.369054
59368	1.01318	0.907101
59369	1.01318	2.1
59370	0.900815	2.1
59371	1.10799	0.479132
59372	0.772511	1.53798
59373	1.03473	1.51966
59374	1.31126	1.31069
59375	0.841464	1.3992
59376	1.89227	0.835479
59377	1.89227	2.1
59378	1.89227	1.77608
59379	1.89227	2.1
59380	1.89227	1.31978
59381	1.89227	2.1
59382	1.89226	1.17036
59383	1.89226	1.7458
59384	1.89226	0.552801
59385	1.89226	1.5827
59386	1.89226	0.496752
59387	1.89226	1.48314
59388	2.12261	1.40085
59389	2.22939	0.185578

59390	1.97004	0.305587
59391	1.8072	1.77533
59392	4.03658	0.482866
59393	1.5847	2.1
59394	1.8072	1.01492
59395	2.12262	1.08599
59396	2.12262	2.1
59397	1.23267	0.97483
59398	1.23267	2.1
59399	1.89227	1.10306
59400	1.89227	2.1
59401	1.89227	1.72063
59402	1.89227	2.1
59403	1.89227	1.40937
59404	1.89227	2.1
59405	1.89226	0.216764
59406	1.89226	1.67759
59407	1.89226	1.78258
59408	1.89226	0.454848
59409	3.78452	0.711338
59410	2.49093	2.1
59411	2.67393	2.1
59412	2.47932	2.1
59413	2.47932	2.1
59414	2.67994	2.1
59415	2.67994	2.1
59416	2.49873	2.1
59417	2.67393	2.1
59418	2.67993	2.1
59419	2.69807	2.1
59420	2.49873	2.1
59421	2.51686	2.1
59422	2.49093	2.1
59423	2.67994	2.1
59424	2.69784	2.1
59425	2.51651	2.1
59426	5.18877	2.1
59427	1.24359	2.1
59428	1.24936	2.1
59429	1.33997	2.1
59430	1.34892	2.1
59431	1.28805	2.1
59432	1.28805	2.1
59433	1.2955	2.1
59434	1.2955	2.1
59435	1.34892	2.1
59436	1.24359	2.1
59437	1.24936	2.1
59438	1.33997	2.1
59439	1.15506	1.96472
59440	1.15506	2.1
59441	0.946128	2.1
59442	0.946128	2.1
59443	0.946131	2.1
59444	1.15506	1.9647
59445	1.06997	2.1
59446	0.946128	2.1
59447	1.06997	2.1

59448	1.15506	2.1
59449	0.946129	2.1
59450	0.946129	2.1
59451	1.70608	2.1
59452	2.13994	2.1
59453	1.33384	2.1
59454	1.43635	2.1
59455	3.14243	2.1
59456	2.13994	2.1
59457	2.31012	2.1
59458	1.43635	2.1
59459	1.23007	2.1
59460	3.74646	2.1
59461	2.46361	2.1
59462	1.89226	2.1
59463	1.89226	2.1
59464	1.89226	2.1
59465	2.39045	2.1
59466	2.39045	2.1
59467	1.89226	2.1
59468	1.89226	2.1
59469	1.89226	2.1
59470	1.89226	1.86395
59471	3.78452	2.1
59472	1.52984	2.1
59473	2.25682	1.80999
59474	2.20831	1.79307
59475	2.20831	2.1
59476	1.61178	1.77391
59477	1.61178	2.1
59478	2.47395	2.1
59479	1.89226	2.1
59480	1.89226	2.1
59481	1.89226	2.1
59482	2.46362	2.1
59483	2.46362	2.1
59484	0.946131	1.80776
59485	0.946131	2.1
59486	0.946131	2.1
59487	0.946131	2.1
59488	0.94613	1.92809
59489	0.94613	2.07307
59490	0.946132	2.1
59491	0.946132	2.1
59492	0.946131	2.1
59493	0.946131	2.1
59494	0.946131	2.1
59495	0.946131	2.1
59496	0.982398	2.1
59497	0.982398	2.1
59498	0.931703	2.1
59499	0.97549	2.1
59500	1.11509	2.1
59501	1.10017	1.9417
59502	0.774576	2.1
59503	0.789859	2.1
59504	0.716502	2.1
59505	1.12793	2.1

59506	1.14398	2.1
59507	0.817011	2.1
59508	1.82499	2.1
59509	1.50524	2.1
59510	1.21304	2.1
59511	1.54191	2.1
59512	3.04715	2.1
59513	1.54191	2.1
59514	1.49823	2.1
59515	1.82499	2.1
59516	1.82499	2.1
59517	1.21147	2.1
59518	1.48977	2.1
59519	1.89226	2.1
59520	1.89226	2.1
59521	1.89226	2.1
59522	1.89226	2.1
59523	3.78452	2.1
59524	1.89226	2.1
59525	1.89226	2.1
59526	1.89226	2.1
59527	1.89226	2.1
59528	1.89226	2.1
59529	1.89226	2.1
59530	0.566674	2.1
59531	0.770957	2.1
59532	0.60652	2.1
59533	0.473557	2.1
59534	0.604441	2.1
59535	0.604441	2.1
59536	0.640073	2.1
59537	0.640073	2.1
59538	0.473557	2.1
59539	0.566674	2.1
59540	0.60652	2.1
59541	0.770956	2.1
59542	1.36203	2.1
59543	1.13335	2.1
59544	1.54191	2.1
59545	1.54191	2.1
59546	1.00965	2.1
59547	1.00965	2.1
59548	1.89226	2.1
59549	1.89226	2.1
59550	1.89226	2.1
59551	1.89226	2.1
59552	1.89226	2.1
59553	1.89226	2.1
59554	0.946131	2.1
59555	0.946131	2.1
59556	0.946131	2.1
59557	0.946131	2.1
59558	0.946132	2.1
59559	0.946132	2.1
59560	0.946131	2.1
59561	0.946131	2.1
59562	0.946132	2.1
59563	0.946132	2.1

59564	0.946131	2.1
59565	0.946131	2.1
59566	0.946132	0.05
59567	0.946132	0.05
59568	0.946133	0.05
59569	0.946133	0.05
59570	0.946132	0.05
59571	0.946132	0.05
59572	0.946132	0.05
59573	0.946132	0.05
59574	0.946132	0.05
59575	0.946132	0.05
59576	0.946133	0.05
59577	0.946133	0.05
59578	1.89226	0.05
59579	1.89226	0.05
59580	1.89226	0.05
59581	1.89226	0.05
59582	3.78453	0.05
59583	1.89226	0.05
59584	1.89226	0.05
59585	1.89226	0.05
59586	1.89226	0.05
59587	1.89226	0.05
59588	1.89226	0.05
59589	0.946132	0.05
59590	0.946132	0.05
59591	0.946131	0.05
59592	0.946131	0.05
59593	0.946132	0.05
59594	0.946132	0.05
59595	0.946132	0.05
59596	0.946132	0.05
59597	0.946132	0.05
59598	0.946132	0.05
59599	0.946132	0.05
59600	0.946132	0.05
59601	0.946129	0.05
59602	0.946129	0.05
59603	0.946128	0.05
59604	0.946128	0.05
59605	0.946132	0.05
59606	0.946132	0.05
59607	0.946126	0.05
59608	0.946126	0.05
59609	0.946129	0.05
59610	0.946129	0.05
59611	0.946129	0.05
59612	0.946129	0.05
59613	1.20674	0.05
59614	1.79877	0.05
59615	1.22586	0.05
59616	1.22586	0.05
59617	1.7919	0.05
59618	1.7919	0.05
59619	2.27622	0.05
59620	1.79877	0.05
59621	1.7919	0.05

59622	2.27102	0.05
59623	4.06979	0.05
59624	1.89226	0.05
59625	1.89226	0.0650932
59626	1.89226	0.05
59627	1.89226	0.0605892
59628	3.78452	0.0545424
59629	1.89226	0.05
59630	1.89226	0.05
59631	1.89226	0.05
59632	1.89226	0.0556025
59633	3.78453	0.05
59634	1.13259	0.05
59635	1.79187	0.05
59636	1.32741	0.05
59637	1.18784	0.05
59638	1.703 0.05	
59639	1.49575	0.05
59640	1.46201	0.05
59641	2.27099	0.05
59642	1.79187	0.05
59643	1.79187	0.05
59644	2.12923	0.05
59645	2.12923	0.05
59646	1.89224	0.05
59647	1.89224	0.06082
59648	1.89224	0.05
59649	1.89224	0.0618417
59650	3.78447	0.05
59651	0.946119	0.0588397
59652	0.946119	0.05
59653	0.946118	0.0500459
59654	0.946118	0.05
59655	0.946118	0.0518551
59656	0.946118	0.0581255
59657	0.946119	0.05
59658	0.946119	0.05
59659	0.946131	0.05
59660	0.946131	0.05
59661	0.946106	0.05
59662	0.946106	0.05
59663	2.07526	0.292781
59664	1.89226	0.17323
59665	1.89226	1.14466
59666	2.13445	0.673957
59667	4.02671	0.241014
59668	1.89226	0.209688
59669	1.89226	0.448891
59670	1.89226	0.473099
59671	1.89226	0.17433
59672	1.89226	1.30963
59673	1.89226	0.212685
59674	2.13446	0.356397
59675	1.00013	0.579062
59676	1.1475	1.41703
59677	2.20046	0.509656
59678	3.20059	0.813795
59679	0.946133	0.281132

59680	0.946133	0.33139
59681	1.06722	0.264944
59682	1.10023	0.25223
59683	0.946131	0.355675
59684	0.946131	0.0919191
59685	1.10023	0.471855
59686	1.10023	0.205232
59687	0.946131	0.464198
59688	1.10023	0.22813
59689	0.94613	0.197909
59690	1.06723	0.169065
59691	1.89226	0.115857
59692	1.89226	0.251052
59693	1.89226	0.400422
59694	1.89226	0.257039
59695	3.78452	0.304291
59696	1.89226	0.596771
59697	1.89226	0.532749
59698	1.89226	0.748238
59699	1.89226	0.611913
59700	1.89226	0.49817
59701	1.89226	0.387805
59702	1.89226	2.1
59703	1.89226	1.57435
59704	1.89226	2.1
59705	1.89226	1.55884
59706	1.89226	2.1
59707	0.828588	0.05
59708	0.828588	0.05
59709	0.841316	0.05
59710	0.841316	2.1
59711	0.528387	2.1
59712	0.565893	0.05
59713	1.10401	0.05
59714	1.10401	2.1
59715	0.528387	2.1
59716	1.89226	2.1
59717	1.36047	2.1
59718	1.33973	2.1
59719	1.33973	2.1
59720	1.89226	0.05
59721	2.28951	1.50774
59722	1.89226	1.6002
59723	2.80796	1.89686
59724	2.01584	1.87735
59725	2.01584	1.72324
59726	1.89226	1.85942
59727	1.89226	1.71086
59728	1.89226	1.22305
59729	1.89226	1.70706
59730	1.89226	1.66235
59731	1.89226	1.51956
59732	1.89226	1.65401
59733	1.89226	1.51785
59734	2.15223	1.52255
59735	1.30664	1.75554
59736	1.96011	1.72315
59737	2.76516	1.58291

59738	4.11234	1.68683
59739	1.89226	1.14266
59740	1.89226	1.58059
59741	1.89226	1.55954
59742	1.89226	1.43603
59743	1.89226	1.54997
59744	1.89226	1.43283
59745	2.81143	2.1
59746	2.58517	2.1
59747	2.55318	2.1
59748	2.7542	2.1
59749	5.33937	2.1
59750	2.7777	2.1
59751	2.7542	2.1
59752	2.71672	2.1
59753	2.70401	2.1
59754	5.45821	2.1
59755	2.55318	2.1
59756	2.7542	2.1
59757	2.71672	2.1
59758	2.54198	2.1
59759	5.29618	2.1
59760	1.95416	2.1
59761	2.162 2.1	
59762	2.11563	2.1
59763	1.91813	2.1
59764	4.08013	2.1
59765	1.95417	2.1
59766	1.47712	2.1
59767	1.45182	2.1
59768	1.91813	2.1
59769	3.39525	2.1
59770	2.11562	2.1
59771	1.91813	2.1
59772	1.50979	2.1
59773	2.03974	2.1
59774	3.95787	2.1
59775	1.85605	2.1
59776	1.91813	2.1
59777	1.88885	2.1
59778	1.42255	2.1
59779	1.45182	2.1
59780	1.45182	2.1
59781	0.94613	2.1
59782	0.94613	1.02563
59783	0.946128	0.553379
59784	0.946128	2.1
59785	0.946127	2.1
59786	0.946127	2.1
59787	0.946131	0.37613
59788	0.946131	0.381511
59789	0.946129	1.39704
59790	0.946129	1.46292
59791	0.946128	1.46431
59792	0.946128	1.84194
59793	0.543052	2.1
59794	0.54608	0.907707
59795	0.602293	0.399521

59796	0.535325	0.594107
59797	1.05446	2.1
59798	0.850496	1.95072
59799	0.699838	1.42762
59800	0.679751	2.1
59801	0.713797	1.34786
59802	0.509794	1.14686
59803	0.602304	0.906243
59804	1.40017	2.1
59805	1.40017	2.1
59806	0.94613	2.1
59807	0.94613	2.1
59808	1.40017	2.1
59809	1.33997	2.1
59810	0.946131	2.1
59811	0.946131	2.1
59812	1.33997	2.1
59813	0.946131	2.1
59814	0.946131	2.1
59815	1.40017	2.1
59816	1.89226	0.05
59817	1.89226	0.060394
59818	1.89226	0.0636762
59819	1.89226	0.05
59820	3.78452	0.05
59821	0.946133	0.05
59822	0.946133	0.05
59823	0.946132	0.0633237
59824	0.946132	0.05
59825	0.946132	0.05
59826	0.946132	0.05
59827	0.946133	0.0611072
59828	0.946133	0.05
59829	0.946132	0.05
59830	0.946132	0.05
59831	0.946132	0.05
59832	0.946132	0.05
59833	1.89224	0.065497
59834	1.89224	0.05
59835	1.89224	0.05
59836	1.89224	0.05
59837	1.89224	0.058625
59838	1.89224	0.0568308
59839	0.94612	0.05
59840	0.94612	0.05
59841	0.946119	0.063939
59842	0.946119	0.05
59843	0.946119	0.05
59844	0.946119	0.0501573
59845	0.94612	0.05
59846	0.94612	0.06038
59847	0.946132	0.05
59848	0.946132	0.05
59849	0.946107	0.05
59850	0.946107	0.05
59851	2.63939	2.1
59852	1.2862	2.1
59853	1.29891	2.1

59854	2.64419	2.1
59855	3.93039	2.1
59856	2.6394	2.1
59857	1.89226	2.1
59858	1.89226	2.1
59859	2.64419	2.1
59860	4.53646	2.1
59861	1.89226	2.1
59862	1.89226	2.1
59863	1.89226	2.1
59864	1.89226	2.1
59865	3.78453	2.1
59866	1.89226	2.1
59867	1.89226	2.1
59868	1.89226	2.1
59869	1.89226	2.1
59870	1.89226	2.1
59871	1.89226	2.1
59872	1.31998	2.1
59873	1.32209	2.1
59874	0.649456	2.1
59875	0.648474	2.1
59876	0.984995	2.1
59877	0.984995	2.1
59878	0.985573	2.1
59879	0.985573	2.1
59880	1.31999	2.1
59881	0.648472	2.1
59882	0.649455	2.1
59883	1.32209	2.1
59884	0.946133	2.1
59885	0.946133	2.1
59886	1.31999	2.1
59887	1.3221	2.1
59888	0.946132	2.1
59889	0.946132	2.1
59890	1.31999	2.1
59891	1.31999	2.1
59892	1.31999	2.1
59893	0.946132	2.1
59894	0.946133	2.1
59895	1.3221	2.1
59896	0.946132	2.1
59897	0.946132	2.1
59898	0.946131	2.1
59899	0.946131	2.1
59900	0.946132	2.1
59901	0.946132	2.1
59902	0.946131	2.1
59903	0.946131	2.1
59904	0.946131	2.1
59905	0.946131	2.1
59906	0.946132	2.1
59907	0.946132	2.1
59908	0.946128	2.1
59909	0.946128	2.1
59910	0.946132	2.1
59911	0.946132	2.1

59912	0.946131	2.1
59913	0.946131	2.1
59914	0.94613	2.1
59915	0.94613	2.1
59916	0.94613	2.1
59917	0.94613	2.1
59918	0.946131	2.1
59919	0.946131	2.1
59920	1.89226	0.261358
59921	1.89226	0.702795
59922	1.89226	0.674092
59923	1.89226	0.597144
59924	1.89226	0.312394
59925	1.89226	0.32159
59926	1.89226	0.569351
59927	1.89226	1.5112
59928	1.89226	1.03225
59929	1.89226	1.38415
59930	3.78453	1.53282
59931	0.946133	0.432155
59932	0.946133	0.481957
59933	0.946132	0.373786
59934	0.946132	0.336893
59935	0.946133	0.580118
59936	0.946133	0.644696
59937	0.946132	0.468443
59938	0.946132	0.320223
59939	0.946132	0.482398
59940	0.946132	0.597108
59941	0.946132	0.400406
59942	0.946132	0.880675
59943	2.23638	0.154841
59944	1.89226	0.431254
59945	2.66014	0.49095
59946	2.66014	0.500839
59947	1.89226	0.609117
59948	1.89226	0.361467
59949	1.11819	0.111725
59950	1.33007	0.156079
59951	0.660255	0.19723
59952	0.91151	0.152962
59953	0.911511	0.254178
59954	1.33007	0.160533
59955	1.11819	0.0934154
59956	0.660255	0.166574
59957	0.985327	0.0908934
59958	0.985327	0.164722
59959	1.005	0.196992
59960	1.005	0.202249
59961	1.89226	2.1
59962	1.89226	2.1
59963	1.89226	2.1
59964	1.89226	2.1
59965	3.78453	2.1
59966	1.59533	2.1
59967	2.68571	2.1
59968	1.28291	2.1
59969	2.46974	2.1

59970	4.06506	2.1
59971	2.68571	2.1
59972	1.89226	2.1
59973	1.89226	2.1
59974	1.89226	2.1
59975	2.46973	2.1
59976	2.46973	2.1
59977	1.89226	2.1
59978	1.89226	2.1
59979	1.89226	2.1
59980	1.89226	2.1
59981	1.89226	2.1
59982	1.89226	2.1
59983	1.89226	2.1
59984	1.89226	2.1
59985	1.89226	2.1
59986	1.89226	2.1
59987	1.89226	2.1
59988	1.89226	2.1
59989	0.883594	2.1
59990	0.883594	2.1
59991	0.876484	2.1
59992	0.876484	2.1
59993	1.10442	2.1
59994	1.23487	2.1
59995	0.641457	2.1
59996	0.52521	2.1
59997	1.10442	2.1
59998	0.52521	2.1
59999	0.641457	2.1
60000	1.23487	2.1
60001	0.946131	2.1
60002	0.946131	2.1
60003	0.946131	2.1
60004	0.946131	2.1
60005	0.946131	2.1
60006	0.946131	2.1
60007	0.946131	2.1
60008	0.946131	2.1
60009	0.946131	2.1
60010	0.946131	2.1
60011	0.946131	2.1
60012	0.946131	2.1
60013	2.04016	0.0522791
60014	2.13042	0.0605833
60015	2.11465	0.0602957
60016	2.11465	0.0855078
60017	1.95908	0.05
60018	1.95908	0.0565601
60019	1.66953	0.0533047
60020	2.13041	0.0652453
60021	1.95908	0.05
60022	2.21952	0.054738
60023	1.66953	0.0772638
60024	1.92997	0.0807809
60025	2.04016	0.05
60026	1.95908	0.05
60027	1.82014	0.05

60028	2.08757	0.05
60029	3.86029	0.05
60030	0.834764	0.05
60031	0.722102	0.05
60032	0.979537	0.05
60033	0.910065	0.05
60034	0.840053	0.05
60035	0.840052	0.0565972
60036	0.861587	0.05
60037	0.861587	0.05
60038	0.910067	0.05
60039	0.722101	0.05
60040	0.834763	0.05
60041	0.979539	0.05
60042	1.89226	0.072906
60043	1.89226	0.0736228
60044	1.89226	0.0761642
60045	1.89226	0.0646209
60046	1.89226	0.0847232
60047	1.89226	0.072263
60048	1.89226	0.0786498
60049	1.89226	0.090755
60050	1.89226	0.116904
60051	1.89226	0.0625968
60052	3.78453	0.0869759
60053	1.89226	0.05
60054	1.89226	0.0518925
60055	1.89226	0.0565375
60056	1.89226	0.05
60057	1.89226	0.0581448
60058	1.89226	0.05
60059	1.20691	0.05
60060	2.27379	0.05
60061	2.58476	0.05
60062	1.5124	0.05
60063	3.79167	0.05
60064	1.89226	0.0852534
60065	2.2738	0.050879
60066	2.58477	0.0542399
60067	1.89226	0.0647912
60068	4.47703	0.0615581
60069	0.952225	1.75476
60070	0.952225	1.87785
60071	0.909865	1.84968
60072	0.909865	1.95699
60073	0.909895	2.03408
60074	0.946132	1.80487
60075	0.946132	1.83685
60076	0.915958	1.75547
60077	0.946132	1.83189
60078	0.946132	1.84182
60079	0.909894	1.87305
60080	0.915958	1.89102
60081	0.983493	2.1
60082	2.40055	2.1
60083	1.02986	2.1
60084	1.02986	2.1
60085	2.34217	2.1

60086	2.34217	2.1
60087	1.89226	2.1
60088	2.40056	2.1
60089	2.34217	2.1
60090	2.40056	2.1
60091	1.83388	2.1
60092	1.89226	2.1
60093	1.89226	2.1
60094	1.89226	2.1
60095	1.89226	2.1
60096	1.89226	2.1
60097	1.89226	2.1
60098	1.89226	2.1
60099	1.89226	2.1
60100	1.89226	2.1
60101	1.89226	2.1
60102	1.89226	2.1
60103	1.89226	2.1
60104	1.89226	2.1
60105	0.946133	2.1
60106	0.946133	2.1
60107	1.17109	2.1
60108	1.13078	2.1
60109	0.946132	2.1
60110	0.946132	2.1
60111	1.13078	2.1
60112	1.13078	2.1
60113	1.13078	2.1
60114	0.946133	2.1
60115	0.946132	2.1
60116	1.17109	2.1
60117	1.89226	2.1
60118	1.89226	2.1
60119	1.89226	2.1
60120	1.89226	2.1
60121	1.89226	2.1
60122	1.89226	2.1
60123	2.33963	1.31626
60124	1.89227	0.631664
60125	1.74565	0.993028
60126	1.74565	1.03732
60127	1.89227	0.448907
60128	1.89227	0.59893
60129	1.89227	1.15588
60130	1.89227	0.525648
60131	1.89227	1.35535
60132	1.89227	0.80243
60133	3.78453	0.950826
60134	1.31398	0.421838
60135	1.10392	0.637145
60136	0.796878	0.437192
60137	0.524356	0.394295
60138	0.870614	0.443918
60139	0.945739	0.324492
60140	0.89208	0.542165
60141	0.89208	1.16947
60142	1.23425	0.355976
60143	0.774658	1.04102

60144	1.03182	0.378511
60145	0.557289	0.298303
60146	2.33963	1.32346
60147	1.89226	0.411872
60148	1.89226	1.32162
60149	2.77245	0.586065
60150	4.23189	0.896298
60151	1.89226	1.26965
60152	1.89226	0.513687
60153	1.89226	1.32389
60154	1.89226	1.29263
60155	1.89226	1.01693
60156	1.89226	0.593054
60157	1.89226	0.145864
60158	1.89226	0.148657
60159	1.89226	0.119444
60160	1.89226	0.313525
60161	1.89226	0.111085
60162	1.89226	0.282783
60163	1.89226	0.114615
60164	1.89226	0.100951
60165	1.89226	0.087932
60166	1.89226	0.176799
60167	1.89226	0.0777406
60168	1.89226	0.143413
60169	0.946128	0.100853
60170	0.946128	0.0740406
60171	0.963918	0.0743132
60172	1.37543	0.05893
60173	0.946129	0.068623
60174	0.946129	0.0938639
60175	1.37542	0.0620103
60176	1.37542	0.0523329
60177	0.94613	0.0606141
60178	1.37542	0.0697436
60179	0.963916	0.0650663
60180	0.94613	0.069762
60181	0.946132	0.130899
60182	0.946132	0.0955754
60183	0.946131	0.100322
60184	0.946131	0.068762
60185	0.946131	0.0990591
60186	0.946131	0.1251
60187	0.946132	0.0858726
60188	0.946132	0.0659465
60189	0.946131	0.0966706
60190	0.946131	0.0965413
60191	0.946131	0.0917935
60192	0.946131	0.0930762
60193	1.89226	0.153999
60194	1.89226	0.161399
60195	1.89226	0.106986
60196	1.89226	0.257019
60197	3.78452	0.165113
60198	0.946127	0.167977
60199	0.946127	0.103799
60200	0.946131	0.103332
60201	0.946131	0.0747652

60202	0.946128	0.101132
60203	0.946128	0.130453
60204	0.94613	0.0855857
60205	0.94613	0.117987
60206	0.946129	0.103129
60207	0.946129	0.0999069
60208	0.946129	0.109181
60209	0.946129	0.106808
60210	1.89226	1.53691
60211	1.89226	1.70577
60212	1.89226	2.1
60213	1.89226	0.976244
60214	3.78453	1.05814
60215	1.89226	0.751892
60216	1.89226	0.186369
60217	1.89226	0.331766
60218	1.89226	0.576314
60219	3.78453	0.726515
60220	0.946131	0.401999
60221	0.946131	0.447285
60222	0.946132	1.65947
60223	0.946132	1.1144
60224	0.946132	1.56163
60225	0.946132	0.741022
60226	0.946132	1.57983
60227	0.946132	1.05685
60228	0.946132	1.02995
60229	0.946132	1.53293
60230	0.946132	1.6088
60231	0.946132	0.805668
60232	0.548169	0.214478
60233	0.736603	0.0779662
60234	1.18322	0.305576
60235	1.01139	0.167817
60236	1.01139	0.109534
60237	0.548168	0.130284
60238	0.736603	0.139728
60239	1.18322	0.0536765
60240	0.861589	0.0527822
60241	0.861589	0.320373
60242	0.869798	0.12311
60243	0.869798	0.184742
60244	1.16037	0.171263
60245	0.942012	0.310471
60246	0.942012	0.157268
60247	0.62356	0.225326
60248	0.891932	0.132198
60249	0.900255	0.180722
60250	2.02279	0.231924
60251	1.48677	0.186495
60252	1.54724	0.0776587
60253	0.831456	0.137766
60254	1.19775	0.334497
60255	1.89226	1.62979
60256	2.76291	1.55805
60257	1.89226	1.64635
60258	1.89226	1.58687
60259	3.78452	1.51061

60260	1.89226	0.868859
60261	1.89226	1.29389
60262	1.89226	1.46061
60263	1.89226	0.961092
60264	1.89226	1.35734
60265	1.89226	0.801851
60266	1.89226	1.25225
60267	1.89226	1.08103
60268	1.89226	1.38304
60269	1.89226	1.05587
60270	3.78452	1.31338
60271	1.89226	0.700412
60272	1.89226	1.24765
60273	1.89226	1.34075
60274	1.89226	0.796184
60275	3.78453	1.10113
60276	0.946128	0.587922
60277	0.946128	1.10299
60278	0.946129	1.32213
60279	0.946129	1.18876
60280	0.946132	0.832164
60281	0.946132	1.33515
60282	0.946126	1.2768
60283	0.946126	1.04523
60284	0.946129	1.12511
60285	0.946129	1.16826
60286	0.946129	1.05514
60287	0.946129	0.881652
60288	2.80495	1.2075
60289	1.89225	1.05488
60290	1.89225	1.44262
60291	1.87794	1.40337
60292	1.89225	1.34073
60293	1.87794	1.43924
60294	2.25195	2.1
60295	2.00624	1.41737
60296	2.13132	2.1
60297	1.90239	1.29195
60298	4.13756	0.758466
60299	0.804661	1.91448
60300	0.804663	0.413467
60301	0.889591	0.640829
60302	0.889591	2.02104
60303	0.808324	1.24369
60304	0.761765	1.75144
60305	1.02822	0.129707
60306	0.879624	0.142822
60307	0.879617	0.696031
60308	0.800864	0.614371
60309	0.978023	0.938279
60310	0.808329	0.607914
60311	2.13133	2.1
60312	2.00558	0.610001
60313	2.09316	0.325987
60314	2.09316	2.1
60315	2.03253	0.673657
60316	2.03253	2.1
60317	0.84577	1.6389

60318	0.845767	0.836652
60319	0.843917	0.553354
60320	0.843917	2.00975
60321	0.76177	0.955315
60322	0.719345	1.5988
60323	0.934435	0.0895374
60324	0.884197	0.0979527
60325	0.934439	0.498369
60326	0.75627	0.499333
60327	0.849424	0.387598
60328	0.76177	0.494821
60329	2.61855	0.0957294
60330	1.17793	0.0964963
60331	1.42965	0.0887659
60332	1.17793	0.0849751
60333	2.50057	0.113538
60334	2.24885	0.112662
60335	0.946133	0.0891831
60336	0.946133	0.0842315
60337	1.30928	0.0968439
60338	1.12443	0.115223
60339	0.946132	0.120714
60340	0.946132	0.098964
60341	1.30928	0.0826024
60342	1.30928	0.107989
60343	0.946132	0.10105
60344	1.30928	0.0976296
60345	1.12443	0.0929504
60346	0.946133	0.0976341
60347	1.89226	0.0915589
60348	1.89226	0.115553
60349	1.89226	0.0972585
60350	1.89226	0.0955218
60351	3.78452	0.106426
60352	1.89226	0.0940032
60353	1.89226	0.0845114
60354	1.89226	0.0844855
60355	1.89226	0.0811189
60356	1.89226	0.0981187
60357	1.89226	0.0999683
60358	1.89226	0.106972
60359	1.89226	0.104965
60360	1.89226	0.0915941
60361	1.89226	0.113984
60362	3.78452	0.0963871
60363	0.94613	0.0881222
60364	0.94613	0.0927325
60365	0.946131	0.0838954
60366	0.946131	0.092625
60367	0.946128	0.0958362
60368	0.946128	0.0802874
60369	0.946133	0.0901263
60370	0.946133	0.104041
60371	0.946131	0.0949576
60372	0.946131	0.0814028
60373	0.946131	0.0897954
60374	0.946131	0.108735
60375	1.89226	2.1

60376	1.89226	2.1
60377	1.89226	2.1
60378	1.89226	2.1
60379	3.78453	2.1
60380	1.0465	2.1
60381	2.73926	2.1
60382	2.03721	2.1
60383	2.03721	2.1
60384	1.44709	2.1
60385	1.44709	2.1
60386	0.946131	2.1
60387	0.946131	2.1
60388	0.946131	2.1
60389	0.946131	2.1
60390	0.946132	2.1
60391	0.946132	2.1
60392	0.94613	2.1
60393	0.94613	2.1
60394	0.946131	2.1
60395	0.946131	2.1
60396	0.946131	2.1
60397	0.946131	2.1
60398	0.946132	2.1
60399	0.946132	2.1
60400	0.946131	2.1
60401	0.946131	2.1
60402	0.946132	2.1
60403	0.946132	2.1
60404	0.946132	2.1
60405	0.946132	2.1
60406	0.946132	2.1
60407	0.946132	2.1
60408	0.946132	2.1
60409	0.946132	2.1
60410	1.89226	2.1
60411	1.89226	2.1
60412	1.89226	2.1
60413	1.89226	2.1
60414	3.78452	2.1
60415	1.61969	0.106999
60416	1.79374	0.05
60417	1.29382	0.0718035
60418	1.29382	0.116778
60419	2.05759	0.05
60420	2.05759	0.0772791
60421	1.89226	0.116952
60422	1.89226	0.0853419
60423	1.89226	0.100821
60424	1.89226	0.156409
60425	1.89226	0.05
60426	1.89226	0.0711815
60427	1.89226	1.10404
60428	1.89226	1.3891
60429	1.89226	1.15064
60430	1.89226	1.22118
60431	1.89226	1.07511
60432	1.89226	1.15244
60433	1.89226	1.75574

60434	1.89226	1.51794
60435	1.89226	1.65186
60436	1.89226	1.71603
60437	3.78453	1.77542
60438	0.946131	1.47632
60439	0.946131	1.50144
60440	0.946131	1.39808
60441	0.946131	1.4531
60442	0.946131	1.39983
60443	0.946131	1.37459
60444	0.946131	1.46479
60445	0.946131	1.554
60446	0.946131	1.46394
60447	0.946131	1.4871
60448	0.946131	1.45647
60449	0.946131	1.41546
60450	2.45177	0.1595
60451	2.68507	0.128701
60452	2.3376	0.196919
60453	2.22086	0.120329
60454	4.78937	0.154215
60455	2.45178	0.141771
60456	2.35552	0.0996032
60457	1.68062	0.133927
60458	1.81902	0.110548
60459	4.13239	0.117833
60460	2.3376	0.154491
60461	1.55612	0.106501
60462	1.69811	0.113199
60463	2.31527	0.125002
60464	1.79553	0.11454
60465	2.3376	0.129369
60466	1.55613	0.0977059
60467	1.06336	0.108174
60468	1.97516	0.108109
60469	1.88966	0.0918977
60470	1.06336	0.1441
60471	1.68062	0.0930658
60472	1.89226	1.65187
60473	1.91634	1.82907
60474	2.03709	1.93961
60475	1.91634	1.71664
60476	1.83185	1.81416
60477	1.70303	0.966314
60478	1.89226	1.62323
60479	1.89226	1.79885
60480	1.89226	1.93113
60481	1.89226	1.71285
60482	1.89226	1.77063
60483	1.89226	1.00009
60484	1.89226	2.1
60485	2.03472	2.1
60486	2.22183	2.1
60487	2.03472	2.1
60488	2.07936	2.1
60489	1.89226	1.87033
60490	1.89226	1.46457
60491	1.8519	1.46013

60492	1.92119	1.74236
60493	1.8519	1.56914
60494	1.96156	1.03508
60495	1.89226	0.598655
60496	1.89226	1.25679
60497	1.89226	1.56678
60498	1.89226	1.77505
60499	1.89226	1.59115
60500	1.89226	1.0224
60501	1.89226	0.622336
60502	1.89226	1.90978
60503	2.22183	2.1
60504	2.39541	2.1
60505	2.22183	1.9616
60506	2.06585	2.04252
60507	1.89226	1.76121
60508	1.47001	2.1
60509	1.57962	1.92098
60510	1.65238	2.1
60511	1.60502	2.1
60512	3.18464	2.1
60513	1.02415	2.1
60514	1.60503	2.1
60515	1.47001	2.1
60516	1.47001	2.1
60517	1.28228	2.1
60518	1.28228	2.1
60519	0.946133	2.1
60520	0.946133	2.1
60521	0.946132	2.1
60522	0.946132	2.1
60523	0.946132	2.1
60524	0.946132	2.1
60525	0.946133	2.1
60526	0.946133	2.1
60527	0.946132	2.1
60528	0.946132	2.1
60529	0.946133	2.1
60530	0.946133	2.1
60531	1.89226	1.91599
60532	1.89226	2.1
60533	1.89226	2.1
60534	1.89226	1.7971
60535	3.78452	1.96664
60536	1.76897	2.09969
60537	1.60502	2.1
60538	1.65238	1.91872
60539	1.65238	2.1
60540	1.76932	1.87929
60541	1.76932	2.1
60542	1.55998	2.1
60543	1.60503	2.1
60544	1.76932	2.1
60545	1.60503	2.1
60546	1.44657	2.1
60547	1.28228	2.1
60548	0.946133	2.1
60549	0.946133	2.1

60550	0.946132	2.1
60551	0.946132	2.1
60552	0.946132	2.1
60553	0.946132	2.1
60554	0.946133	2.1
60555	0.946133	2.1
60556	0.946132	2.1
60557	0.946132	2.1
60558	0.946132	2.1
60559	0.946132	2.1
60560	0.946128	2.1
60561	0.946128	1.94675
60562	0.946133	1.28798
60563	0.946133	1.79468
60564	0.94613	1.55881
60565	0.94613	1.95464
60566	0.946131	2.1
60567	0.946131	1.88243
60568	0.946131	1.88152
60569	0.946131	1.86104
60570	0.946131	1.94404
60571	0.946131	1.94882
60572	1.85779	0.150745
60573	1.00763	0.121209
60574	1.74874	0.124554
60575	1.74873	0.115738
60576	1.14467	0.165312
60577	1.14466	0.148424
60578	1.85779	0.130452
60579	1.61597	0.107414
60580	1.00053	0.134869
60581	1.03018	0.10569
60582	2.65819	0.112865
60583	1.89226	0.612835
60584	1.89226	0.33538
60585	1.89226	0.624667
60586	1.89226	0.513499
60587	3.78451	0.526302
60588	1.89226	0.549375
60589	1.89226	0.185053
60590	1.89226	0.297315
60591	1.89226	0.318361
60592	1.89226	0.262216
60593	1.89226	0.28319
60594	1.11983	0.1536
60595	0.810945	0.170412
60596	0.913906	0.136502
60597	0.913906	0.16081
60598	2.23303	0.111823
60599	1.66057	0.114327
60600	1.28724	0.124488
60601	1.08132	0.127772
60602	1.49316	0.149438
60603	0.810958	0.152339
60604	0.810958	0.144811
60605	0.827861	0.28896
60606	0.946126	0.319172
60607	0.946132	0.533673

60608	0.946132	0.27427
60609	0.827863	0.325405
60610	0.827863	0.314654
60611	0.946129	0.35296
60612	0.946129	0.350369
60613	0.827863	0.3134
60614	0.946128	0.337002
60615	0.946129	0.270645
60616	0.946129	0.305261
60617	0.974718	0.05
60618	1.53671	0.0766215
60619	2.80542	0.05
60620	2.27259	0.0629296
60621	3.8093	0.0526361
60622	1.89226	0.100219
60623	1.89226	0.205192
60624	1.89226	0.0865213
60625	1.89226	0.163142
60626	3.78453	0.143014
60627	0.946132	0.0933017
60628	0.946132	0.131233
60629	0.946131	0.136842
60630	0.946131	0.0987705
60631	0.946132	0.13983
60632	0.946132	0.159668
60633	0.946131	0.084882
60634	0.946131	0.0853426
60635	0.946132	0.111453
60636	0.946132	0.141473
60637	0.946131	0.10847
60638	0.946131	0.110332
60639	2.80542	0.0598222
60640	1.89226	0.0732374
60641	2.27259	0.0619818
60642	2.27259	0.102029
60643	1.89226	0.05
60644	1.89226	0.0726036
60645	1.89226	0.0867241
60646	1.89226	0.19188
60647	1.89226	0.100412
60648	1.89226	0.197007
60649	1.89226	0.0908617
60650	1.89226	0.202191
60651	0.946132	0.183915
60652	0.946132	0.0904358
60653	0.946131	0.162929
60654	0.946131	0.105457
60655	0.946132	0.167001
60656	0.946132	0.171054
60657	0.946131	0.0881537
60658	0.946131	0.0989912
60659	0.946131	0.114556
60660	0.946131	0.125796
60661	0.946131	0.104869
60662	0.946131	0.128332
60663	2.68213	2.1
60664	2.58234	2.1
60665	2.58234	2.1

60666	2.61243	2.1
60667	2.67035	2.1
60668	2.70044	2.1
60669	2.68213	2.1
60670	2.53921	2.1
60671	2.53303	2.1
60672	2.67035	2.1
60673	5.20956	2.1
60674	2.67033	2.1
60675	2.58232	2.1
60676	2.55004	2.1
60677	2.64567	2.1
60678	5.228 2.1	
60679	2.53301	2.1
60680	2.64567	2.1
60681	2.64567	2.1
60682	2.67033	2.1
60683	2.51603	2.1
60684	2.54069	2.1
60685	0.907497	2.1
60686	0.907497	2.1
60687	0.926641	2.1
60688	0.926641	2.1
60689	1.14989	2.1
60690	1.10046	2.1
60691	0.695334	2.1
60692	0.684247	2.1
60693	0.695333	2.1
60694	1.14989	2.1
60695	0.684246	2.1
60696	1.10046	2.1
60697	0.946126	2.1
60698	0.946126	2.1
60699	0.946131	2.1
60700	0.946131	2.1
60701	0.946127	2.1
60702	0.946127	2.1
60703	0.94613	2.1
60704	0.94613	2.1
60705	0.946128	2.1
60706	0.946128	2.1
60707	0.946128	2.1
60708	0.946128	2.1
60709	1.89226	2.1
60710	1.89226	2.1
60711	1.89226	2.1
60712	1.89226	2.1
60713	1.89226	2.1
60714	1.89226	2.1
60715	1.42174	2.1
60716	2.29978	2.1
60717	2.41233	2.1
60718	2.41233	2.1
60719	1.39067	2.1
60720	1.39067	2.1
60721	1.89226	2.1
60722	1.89226	2.1
60723	1.89226	2.1

60724	1.89226	2.1
60725	3.78452	2.1
60726	1.89226	2.1
60727	1.89226	2.1
60728	1.89226	2.1
60729	1.89226	2.1
60730	1.89226	2.1
60731	1.89226	2.1
60732	2.41234	2.1
60733	1.89226	2.1
60734	2.29979	2.1
60735	1.89226	2.1
60736	1.89226	2.1
60737	2.29979	2.1
60738	1.09506	0.0520292
60739	2.82921	0.0512794
60740	2.53251	0.05
60741	2.53251	0.0544208
60742	1.42901	0.051439
60743	1.42901	0.055592
60744	0.946132	0.05
60745	0.946132	0.0517691
60746	0.946128	0.0563128
60747	0.946128	0.0622807
60748	0.94613	0.0539581
60749	0.94613	0.0501612
60750	0.946131	0.0607912
60751	0.946131	0.0615246
60752	0.94613	0.05
60753	0.94613	0.06211
60754	0.94613	0.0676133
60755	0.94613	0.05
60756	1.89226	0.0637529
60757	1.89226	0.0841086
60758	1.89226	0.487902
60759	1.89226	0.0653856
60760	3.78452	0.194814
60761	2.53251	0.0547275
60762	1.89226	0.0686134
60763	2.82921	0.0527493
60764	1.89226	0.0627979
60765	1.89226	0.272591
60766	2.82921	0.140619
60767	1.89226	0.121962
60768	1.89226	0.05
60769	1.89226	0.05
60770	1.89226	0.0531109
60771	1.89226	0.05
60772	1.89226	0.0677116
60773	2.53251	0.0501498
60774	1.89226	0.0564419
60775	2.06158	0.05
60776	1.89226	0.05
60777	4.42477	0.05
60778	1.23296	2.1
60779	2.55352	2.1
60780	2.47476	2.1
60781	2.47476	2.1

60782	1.32368	2.1
60783	1.32368	2.1
60784	1.89226	2.1
60785	1.89226	2.1
60786	1.89226	2.1
60787	1.89226	2.1
60788	1.89226	2.1
60789	1.89226	2.1
60790	1.89226	2.1
60791	1.89226	2.1
60792	1.89226	2.1
60793	1.89226	2.1
60794	1.89226	2.1
60795	1.89226	2.1
60796	2.47476	2.1
60797	1.89226	2.1
60798	1.89226	2.1
60799	1.89226	2.1
60800	2.55353	2.1
60801	2.55353	2.1
60802	1.05983	2.1
60803	2.47476	2.1
60804	2.37664	2.1
60805	2.37664	2.1
60806	1.23296	2.1
60807	1.23296	2.1
60808	0.946127	2.1
60809	0.946127	2.1
60810	0.946131	2.1
60811	0.946131	2.1
60812	0.946127	2.1
60813	0.946127	2.1
60814	0.946131	2.1
60815	0.946131	2.1
60816	0.946129	2.1
60817	0.946129	2.1
60818	0.946128	2.1
60819	0.946128	2.1
60820	1.89226	2.1
60821	1.89226	2.1
60822	1.89226	2.1
60823	1.89226	2.1
60824	3.78452	2.1
60825	2.37665	2.1
60826	1.89226	2.1
60827	1.89226	2.1
60828	1.89226	2.1
60829	2.47476	2.1
60830	2.47476	2.1
60831	1.89226	0.993754
60832	1.92119	0.827323
60833	1.99586	1.51576
60834	1.92119	1.41847
60835	1.96693	0.500605
60836	1.89226	0.330495
60837	1.89226	0.931823
60838	1.89226	0.939114
60839	1.89226	1.62364

60840	1.89226	1.27525
60841	1.89226	0.655759
60842	1.89226	0.408559
60843	1.89226	1.71672
60844	2.39541	1.93485
60845	2.57183	2.08012
60846	2.39541	1.798
60847	2.06868	1.77908
60848	1.89226	1.571
60849	0.946133	0.460384
60850	0.946133	0.318915
60851	0.997928	0.545613
60852	1.03763	0.567796
60853	0.946131	0.657093
60854	0.946131	0.146468
60855	1.03763	0.355621
60856	1.03763	0.386011
60857	1.03763	0.462701
60858	0.946131	0.451503
60859	0.94613	0.360773
60860	0.99793	0.365573
60861	1.89226	0.247811
60862	1.89226	0.80365
60863	1.89226	0.809851
60864	1.89226	0.310263
60865	1.89226	1.42187
60866	1.89226	0.359145
60867	0.946131	1.2543
60868	0.946131	1.29007
60869	1.36939	1.68687
60870	1.28592	1.83989
60871	0.946129	1.71036
60872	0.946129	0.601599
60873	1.36939	1.3926
60874	1.36939	1.70176
60875	0.946131	1.48241
60876	1.36939	1.50828
60877	0.946131	1.58549
60878	1.28592	1.63283
60879	1.89226	0.111517
60880	1.89226	0.551322
60881	1.89226	0.23689
60882	1.89226	0.11006
60883	3.78453	0.131389
60884	1.89226	0.572317
60885	1.89226	0.136808
60886	1.89226	0.124454
60887	1.89226	0.244757
60888	3.78452	0.16944
60889	1.91005	0.075406
60890	1.89226	0.520824
60891	2.60215	0.0672474
60892	2.60215	0.074993
60893	1.89226	0.568813
60894	1.89226	0.434262
60895	0.946132	0.395974
60896	0.946132	0.433532
60897	0.946132	0.161905

60898	0.946132	0.155692
60899	0.946132	0.119222
60900	0.946132	0.312191
60901	0.946132	0.402643
60902	0.946132	0.185111
60903	0.946132	0.156253
60904	0.946132	0.133216
60905	0.946131	0.398391
60906	0.946131	0.336359
60907	1.89226	0.160769
60908	1.89226	0.281995
60909	1.89226	0.371415
60910	1.89226	0.639201
60911	1.89226	0.23215
60912	1.89226	0.25937
60913	0.780483	0.409159
60914	0.78928	0.635592
60915	0.818566	0.301251
60916	0.460387	0.148863
60917	1.82793	0.283688
60918	1.32039	0.449937
60919	0.613889	0.280367
60920	1.0592	0.499102
60921	1.48698	0.365795
60922	0.818555	0.49746
60923	1.11515	0.43256
60924	1.2802	1.10878
60925	2.20046	0.274588
60926	1.1475	0.66614
60927	1.1475	0.243707
60928	2.27062	0.5912
60929	2.27062	0.210776
60930	1.89226	1.02831
60931	2.20046	0.178076
60932	2.27062	0.553982
60933	2.20046	0.431527
60934	1.96242	0.14262
60935	1.89226	0.173788
60936	1.89226	0.203067
60937	1.89226	0.142574
60938	1.89226	0.94848
60939	1.89226	0.382507
60940	3.78452	0.172637
60941	1.89226	0.335267
60942	1.89226	0.353513
60943	1.89226	0.601615
60944	1.89226	0.422186
60945	3.78452	0.431655
60946	1.17403	0.254642
60947	1.13531	0.312931
60948	0.6401	0.291798
60949	0.714566	0.326435
60950	0.915987	0.347213
60951	0.915987	0.127734
60952	0.933891	1.09488
60953	0.933891	0.249381
60954	1.17403	0.319259
60955	0.714566	0.301129

60956	0.6401	0.278088
60957	1.13531	0.238478
60958	0.946133	0.227477
60959	0.946133	0.268288
60960	1.17403	0.186644
60961	1.13531	0.16541
60962	0.946131	0.230372
60963	0.946131	0.119107
60964	1.17403	0.359749
60965	1.17403	0.111048
60966	1.17403	0.202958
60967	0.94613	0.209551
60968	0.946131	0.163936
60969	1.13531	0.138341
60970	1.89226	0.112032
60971	1.89226	0.256059
60972	1.89226	0.181293
60973	1.89226	0.110914
60974	1.89226	0.271134
60975	1.89226	0.13086
60976	0.946131	0.227859
60977	0.946131	0.212738
60978	0.946131	0.278951
60979	0.946131	0.301945
60980	0.946129	0.297698
60981	0.946129	0.148829
60982	0.946133	0.440338
60983	0.946133	0.196309
60984	0.946131	0.251498
60985	0.946131	0.252126
60986	0.946131	0.210161
60987	0.946131	0.216795
60988	2.29413	2.1
60989	1.99332	2.1
60990	1.84774	2.1
60991	2.21582	2.1
60992	4.20915	2.1
60993	0.517359	2.1
60994	0.634469	2.1
60995	0.996661	2.1
60996	0.92387	2.1
60997	0.768098	2.1
60998	0.768098	2.1
60999	0.790242	2.1
61000	0.790242	2.1
61001	0.923871	2.1
61002	0.517359	2.1
61003	0.634469	2.1
61004	0.996661	2.1
61005	1.89226	2.1
61006	1.89226	2.1
61007	1.89226	2.1
61008	1.89226	2.1
61009	3.78453	2.1
61010	0.946132	2.1
61011	0.946132	2.1
61012	0.946131	2.1
61013	0.946131	2.1

61014	0.946132	2.1
61015	0.946132	2.1
61016	0.946131	2.1
61017	0.946131	2.1
61018	0.946132	2.1
61019	0.946132	2.1
61020	0.946131	2.1
61021	0.946131	2.1
61022	1.62708	2.1
61023	2.21582	2.1
61024	2.13925	2.1
61025	2.21582	2.1
61026	1.77117	2.1
61027	1.84774	2.1
61028	0.946132	2.1
61029	0.946132	2.1
61030	0.946131	2.1
61031	0.946131	2.1
61032	0.946132	2.1
61033	0.946132	2.1
61034	0.946131	2.1
61035	0.946131	2.1
61036	0.946131	2.1
61037	0.946131	2.1
61038	0.946131	2.1
61039	0.946131	2.1
61040	1.89226	1.90356
61041	1.89226	1.38044
61042	1.89226	0.881574
61043	1.89226	1.75868
61044	3.78453	1.79981
61045	1.89226	1.12596
61046	1.89226	1.74157
61047	1.89226	1.66994
61048	1.89226	1.93334
61049	1.89226	0.743958
61050	1.89226	1.72404
61051	1.89226	0.929214
61052	1.89226	1.94471
61053	1.89226	0.61719
61054	1.89226	1.91362
61055	1.89226	1.34355
61056	1.89226	1.98941
61057	0.914369	1.7878
61058	0.914369	1.88634
61059	0.810945	2.1
61060	0.810945	1.85712
61061	2.26581	2.1
61062	1.66199	2.1
61063	1.49503	1.89379
61064	1.28816	2.1
61065	1.08133	2.04616
61066	0.810933	2.00832
61067	1.1212	2.0092
61068	1.89227	0.43071
61069	1.89227	0.0815469
61070	1.89227	0.098492
61071	1.89227	0.172371

61072	3.78453	0.118192
61073	1.89226	0.144238
61074	1.89226	0.457023
61075	1.89226	0.245586
61076	1.89226	0.14624
61077	3.78453	0.179446
61078	1.89226	0.523977
61079	1.89226	0.138944
61080	1.89226	0.167177
61081	1.89226	0.492887
61082	3.78453	0.28433
61083	1.89226	0.0928351
61084	1.89226	0.44509
61085	1.89226	0.235889
61086	2.25318	0.0910044
61087	3.78453	0.119437
61088	1.89226	0.22512
61089	1.89226	0.503619
61090	1.89226	0.201686
61091	1.9637	0.0874923
61092	3.78453	0.117179
61093	1.89226	0.481214
61094	1.89226	0.150852
61095	1.89226	0.145257
61096	1.89226	0.420715
61097	3.78453	0.223975
61098	1.89226	0.174076
61099	1.89226	0.581733
61100	1.89226	0.434714
61101	2.53384	0.1745
61102	3.78453	0.275892
61103	1.55405	1.04886
61104	1.03818	1.59804
61105	1.03818	0.489798
61106	1.34857	0.297284
61107	1.46338	0.251116
61108	1.77377	1.30182
61109	1.86273	0.494148
61110	1.73802	1.19332
61111	1.50969	1.89109
61112	1.46337	1.25944
61113	3.20139	1.39219
61114	1.2377	1.28312
61115	1.46337	0.209156
61116	1.27408	1.41344
61117	1.32039	1.63904
61118	1.50969	1.42307
61119	1.50969	0.662484
61120	1.89226	2.1
61121	1.89226	2.1
61122	1.89226	2.1
61123	1.89226	2.1
61124	1.89226	2.1
61125	1.89226	2.1
61126	1.89169	2.1
61127	1.89226	2.1
61128	1.89226	2.1
61129	1.89226	2.1

61130	1.96624	2.1
61131	1.96624	2.1
61132	2.62871	2.1
61133	1.6201	2.1
61134	1.49149	2.1
61135	1.6201	2.1
61136	2.62559	2.1
61137	2.75419	2.1
61138	0.946129	2.07032
61139	0.946129	2.1
61140	0.946133	2.1
61141	0.946133	2.1
61142	0.946131	2.1
61143	0.946131	2.00619
61144	0.946131	2.1
61145	0.946131	2.1
61146	0.946131	2.1
61147	0.946131	2.1
61148	0.946131	2.1
61149	0.946131	2.1
61150	0.851543	2.1
61151	0.851543	2.1
61152	1.04043	2.1
61153	0.997939	2.1
61154	0.946132	2.1
61155	0.946132	2.1
61156	0.894185	2.1
61157	0.893901	2.1
61158	0.998267	2.1
61159	1.04074	2.1
61160	0.85127	2.1
61161	0.85153	2.1
61162	1.89226	2.1
61163	1.89226	2.1
61164	1.89226	2.1
61165	1.89226	2.1
61166	1.89226	2.1
61167	1.89226	2.1
61168	0.928691	2.1
61169	0.928691	2.1
61170	0.935332	2.1
61171	0.935332	2.1
61172	1.398	2.1
61173	1.14418	2.1
61174	0.479331	2.1
61175	0.719848	2.1
61176	1.398	2.1
61177	0.719848	2.1
61178	1.14417	2.1
61179	0.47933	2.1
61180	2.28835	2.1
61181	1.89226	2.1
61182	1.89226	2.1
61183	1.89226	2.1
61184	2.796	2.1
61185	2.796	2.1
61186	0.946131	2.1
61187	0.946131	2.1

61188	0.946131	2.1
61189	0.946131	2.1
61190	0.946128	2.1
61191	0.946128	2.1
61192	0.946133	2.1
61193	0.946133	2.1
61194	0.946131	2.1
61195	0.946131	2.1
61196	0.94613	2.1
61197	0.94613	2.1
61198	0.946131	2.1
61199	0.946131	2.1
61200	0.946131	2.1
61201	0.946131	2.1
61202	0.946131	2.1
61203	0.946131	2.1
61204	0.946131	2.1
61205	0.946131	2.1
61206	0.946131	2.1
61207	0.946131	2.1
61208	0.946131	2.1
61209	0.946131	2.1
61210	1.89226	2.1
61211	1.89226	2.1
61212	1.89226	2.1
61213	1.89226	2.1
61214	3.78452	2.1
61215	2.05207	0.673825
61216	1.70302	1.77876
61217	1.33794	2.07932
61218	1.98194	1.47005
61219	3.36873	1.70427
61220	2.62761	0.221487
61221	1.98194	1.31117
61222	2.28009	1.63967
61223	2.28009	0.353639
61224	2.22939	1.43838
61225	2.22939	0.288618
61226	1.89226	1.39063
61227	1.89226	0.351783
61228	1.89226	0.763491
61229	1.89226	0.340267
61230	1.89226	1.34668
61231	1.89226	0.47667
61232	1.89227	1.48141
61233	1.89227	1.70593
61234	1.89227	1.93435
61235	1.89227	1.68316
61236	1.89227	0.946315
61237	1.89227	0.38154
61238	0.888355	0.440429
61239	0.990973	1.52404
61240	0.71133	1.72792
61241	0.634324	1.81776
61242	0.806209	1.73594
61243	0.806209	0.212651
61244	0.819087	1.71111
61245	0.819087	1.83872

61246	0.888355	1.71621
61247	0.634324	1.66313
61248	0.71133	1.68788
61249	0.990972	1.65769
61250	1.11469	1.2277
61251	0.985023	0.419372
61252	0.888354	0.368062
61253	0.990972	1.46313
61254	0.994754	1.27235
61255	0.994754	0.474556
61256	1.0083	1.31538
61257	1.0083	0.344983
61258	0.888354	0.320649
61259	0.985024	1.06081
61260	0.990971	0.27224
61261	1.11469	0.134253
61262	0.946132	1.2649
61263	0.946132	0.38646
61264	0.946132	1.1675
61265	0.946132	1.1832
61266	0.946132	1.21916
61267	0.946132	0.340005
61268	0.946133	1.57726
61269	0.946133	0.658816
61270	0.946133	1.15528
61271	0.946133	1.15555
61272	0.946132	0.372076
61273	0.946132	0.191815
61274	0.810944	1.3405
61275	0.810944	0.568821
61276	0.810947	1.63193
61277	0.810947	0.187841
61278	1.89227	1.83439
61279	1.35168	1.50936
61280	1.08132	1.61159
61281	1.08131	1.51738
61282	1.08132	0.634159
61283	0.810959	0.60705
61284	0.810959	0.705596
61285	1.70303	2.1
61286	2.03975	2.1
61287	1.99324	2.1
61288	2.17615	2.1
61289	1.40549	2.1
61290	1.53446	2.1
61291	0.946133	2.1
61292	0.946133	2.1
61293	0.946129	2.1
61294	0.946129	2.1
61295	0.946131	2.1
61296	0.946131	2.1
61297	0.946131	2.1
61298	0.946131	2.1
61299	0.946131	2.1
61300	0.946131	2.1
61301	0.946131	2.1
61302	0.946131	2.1
61303	1.89226	2.1

61304	1.89226	2.1
61305	1.89226	2.1
61306	1.89226	2.1
61307	1.89226	2.1
61308	1.89226	2.1
61309	2.17615	2.1
61310	2.3111	2.1
61311	2.50288	2.1
61312	2.50288	2.1
61313	2.03975	2.1
61314	2.03975	2.1
61315	1.76401	2.1
61316	1.95852	2.1
61317	2.32134	2.1
61318	2.12306	2.1
61319	1.8513	2.1
61320	1.70303	2.1
61321	1.89226	2.1
61322	1.89226	2.1
61323	1.89226	2.1
61324	1.89226	2.1
61325	3.78452	2.1
61326	1.89226	2.1
61327	1.89226	2.1
61328	1.89226	2.1
61329	1.89226	2.1
61330	3.78452	2.1
61331	1.2243	2.1
61332	1.2243	2.1
61333	1.24189	2.1
61334	1.24189	2.1
61335	1.37811	2.1
61336	1.25144	2.1
61337	1.08807	2.1
61338	1.17949	2.1
61339	1.17949	2.1
61340	1.37811	2.1
61341	1.08807	2.1
61342	1.25144	2.1
61343	1.89226	2.1
61344	1.89226	2.1
61345	1.89226	2.1
61346	1.89226	2.1
61347	1.89226	2.1
61348	1.89226	2.1
61349	1.89227	2.1
61350	1.89227	2.1
61351	1.89227	2.1
61352	1.89227	2.1
61353	1.89227	2.1
61354	1.89227	2.1
61355	1.89224	2.1
61356	1.89224	2.1
61357	1.89224	2.1
61358	1.89224	2.1
61359	3.78447	2.1
61360	1.89224	2.1
61361	1.89224	2.1

61362	1.89224	2.1
61363	1.89224	2.1
61364	1.89224	2.1
61365	1.89224	2.1
61366	1.89224	2.1
61367	1.89224	2.1
61368	1.89224	2.1
61369	1.89224	2.1
61370	3.78448	2.1
61371	1.89224	2.1
61372	1.703 2.1	
61373	1.89224	2.1
61374	1.72991	2.1
61375	1.07643	2.1
61376	1.36657	2.1
61377	1.44472	2.1
61378	2.41233	2.1
61379	2.51628	2.1
61380	2.51628	2.1
61381	1.42174	2.1
61382	1.42174	2.1
61383	1.89226	2.1
61384	1.89226	2.1
61385	1.89226	2.1
61386	1.89226	2.1
61387	1.89226	2.1
61388	1.89226	2.1
61389	1.89226	2.1
61390	1.89226	2.1
61391	1.89226	2.1
61392	1.89226	2.1
61393	3.78452	2.1
61394	2.51628	2.1
61395	1.89226	2.1
61396	1.89226	2.1
61397	1.89226	2.1
61398	2.41234	2.1
61399	2.41234	2.1
61400	0.996254	2.1
61401	0.996254	2.1
61402	1.01153	2.1
61403	1.01153	2.1
61404	1.28542	2.1
61405	1.25814	2.1
61406	0.719184	2.1
61407	0.72236	2.1
61408	0.719183	2.1
61409	1.28542	2.1
61410	0.722359	2.1
61411	1.25814	2.1
61412	0.946126	2.1
61413	0.946126	2.1
61414	0.946131	2.1
61415	0.946131	2.1
61416	0.946127	2.1
61417	0.946127	2.1
61418	0.94613	2.1
61419	0.94613	2.1

61420	0.946128	2.1
61421	0.946128	2.1
61422	0.946128	2.1
61423	0.946128	2.1
61424	1.89226	2.1
61425	1.89226	2.1
61426	1.89226	2.1
61427	1.89226	2.1
61428	1.89226	2.1
61429	1.89226	2.1
61430	0.946132	2.1
61431	0.946132	2.1
61432	1.25814	2.1
61433	1.25814	2.1
61434	0.946131	2.1
61435	0.946131	2.1
61436	1.28543	2.1
61437	1.25814	2.1
61438	1.28543	2.1
61439	0.946132	2.1
61440	1.25814	2.1
61441	0.946132	2.1
61442	1.89226	1.45908
61443	1.89226	0.669933
61444	1.89226	1.36202
61445	1.89226	1.30615
61446	1.89226	1.00543
61447	1.89226	0.834058
61448	1.89226	1.35109
61449	2.27719	0.570259
61450	1.89226	0.703074
61451	1.89226	0.785635
61452	3.78452	0.723943
61453	1.89225	1.49405
61454	1.89225	0.968048
61455	1.89225	1.27221
61456	1.89225	1.38797
61457	3.78451	1.36734
61458	1.89226	0.533391
61459	1.89226	0.5644
61460	1.89226	0.941752
61461	1.89226	0.34588
61462	3.78452	0.556816
61463	0.946132	0.313637
61464	0.946132	0.568566
61465	0.946132	0.702869
61466	0.946132	0.450405
61467	0.946131	0.363305
61468	0.946131	0.317602
61469	0.946132	0.588631
61470	0.946132	0.342498
61471	0.946132	0.35919
61472	0.946132	0.37549
61473	0.946132	0.401939
61474	0.946132	0.323821
61475	0.946126	0.751851
61476	0.946126	0.505297
61477	0.946132	1.45684

61478	0.946132	1.07196
61479	0.946128	0.582684
61480	0.946128	0.766817
61481	0.946129	0.996609
61482	0.946129	1.35705
61483	0.946129	1.09591
61484	0.946129	1.23229
61485	0.946129	1.05754
61486	0.946129	0.842587
61487	1.39858	2.1
61488	1.39858	2.1
61489	0.94613	2.1
61490	0.94613	2.1
61491	0.946132	2.1
61492	0.946132	2.1
61493	1.39858	2.1
61494	1.25002	2.1
61495	1.39858	2.1
61496	0.946131	2.1
61497	1.25002	2.1
61498	0.946131	2.1
61499	2.0815	2.1
61500	1.89226	2.1
61501	1.89226	2.1
61502	2.05298	2.1
61503	2.5593	2.1
61504	2.77667	2.1
61505	0.946133	2.1
61506	0.946133	2.1
61507	0.946132	2.1
61508	0.946132	2.1
61509	0.946132	2.1
61510	0.946132	2.1
61511	0.946133	2.1
61512	0.946133	2.1
61513	0.946132	2.1
61514	0.946132	2.1
61515	0.946132	2.1
61516	0.946132	2.1
61517	1.38908	2.1
61518	1.38908	2.1
61519	0.946132	2.1
61520	0.946132	2.1
61521	0.946132	2.1
61522	0.946132	2.1
61523	1.38908	2.1
61524	1.27965	2.1
61525	1.38908	2.1
61526	0.946132	2.1
61527	0.946133	2.1
61528	1.27965	2.1
61529	0.946133	2.1
61530	0.946133	2.1
61531	0.946132	2.1
61532	0.946132	2.1
61533	0.946132	2.1
61534	0.946132	2.1
61535	0.946133	2.1

61536	0.946133	2.1
61537	0.946132	2.1
61538	0.946132	2.1
61539	0.946133	2.1
61540	0.946133	2.1
61541	1.89227	1.23508
61542	1.89227	1.41837
61543	1.89227	1.20452
61544	1.89227	1.10315
61545	3.78453	1.23353
61546	1.33154	1.33912
61547	1.33154	1.34348
61548	0.946132	1.29214
61549	0.946132	1.24263
61550	1.19095	1.20744
61551	1.33154	1.41606
61552	0.946133	1.36489
61553	0.946133	1.36717
61554	0.946133	1.48562
61555	1.19095	1.24746
61556	0.946132	1.44388
61557	1.33154	1.3272
61558	1.0888	1.22125
61559	1.04745	1.20892
61560	0.946132	1.14743
61561	0.946132	1.15642
61562	1.04745	1.09231
61563	1.04745	1.40402
61564	0.946132	1.38546
61565	0.946132	1.09326
61566	1.04745	1.23659
61567	0.946133	1.10813
61568	0.946132	1.2963
61569	1.0888	1.22071
61570	1.89226	1.47552
61571	1.89226	1.08014
61572	1.89226	1.08879
61573	1.89226	1.23062
61574	1.89226	1.12377
61575	1.89226	1.25891
61576	1.89226	1.51242
61577	1.87592	1.12393
61578	1.87592	1.17695
61579	2.3819	1.27364
61580	1.89226	1.25409
61581	2.39825	1.35105
61582	1.89226	1.08407
61583	2.0949	1.29827
61584	1.88741	1.05084
61585	1.89226	1.05326
61586	1.89226	1.13756
61587	1.88741	1.26682
61588	1.07508	1.61474
61589	1.63419	1.88895
61590	1.63419	1.72285
61591	1.09795	1.86907
61592	1.56137	1.71565
61593	1.02513	2.02688

61594	0.967672	1.04516
61595	1.56137	1.58316
61596	1.56137	1.27123
61597	1.07508	1.51508
61598	1.41319	1.17496
61599	0.926902	1.63563
61600	1.56137	1.4947
61601	1.63419	1.65982
61602	2.29852	1.72316
61603	2.14485	1.47726
61604	3.77904	1.48772
61605	1.41319	0.462212
61606	1.92622	0.756457
61607	1.56137	0.406028
61608	1.56137	1.30016
61609	2.14485	1.34061
61610	1.92622	1.10879
61611	1.89227	2.1
61612	1.89227	2.1
61613	1.89227	2.1
61614	1.89227	2.1
61615	3.78453	2.1
61616	2.56618	2.1
61617	2.08612	2.1
61618	2.48048	2.1
61619	1.99644	2.1
61620	4.5666	2.1
61621	2.08612	2.1
61622	1.30659	2.1
61623	1.99644	2.1
61624	1.25404	2.1
61625	3.34016	2.1
61626	1.89226	2.1
61627	1.89226	2.1
61628	1.89226	2.1
61629	1.89226	2.1
61630	1.89226	2.1
61631	1.89226	2.1
61632	1.89227	2.1
61633	1.89227	2.1
61634	1.89227	2.1
61635	1.89227	2.1
61636	3.78453	2.1
61637	2.48048	2.1
61638	1.99644	2.1
61639	2.39022	2.1
61640	1.90734	2.1
61641	4.38782	2.1
61642	1.90734	2.1
61643	1.99644	2.1
61644	1.21564	2.1
61645	1.21564	2.1
61646	1.25404	2.1
61647	1.94574	2.1
61648	0.810946	2.1
61649	0.810946	2.1
61650	0.810947	2.1
61651	0.810947	2.1

61652	1.89226	2.1
61653	1.3517	2.1
61654	1.08133	2.1
61655	1.08132	2.1
61656	1.08132	2.1
61657	0.810934	2.1
61658	0.810934	2.1
61659	1.89226	2.1
61660	1.89226	1.70194
61661	1.89226	2.0332
61662	1.89226	2.1
61663	3.78453	2.1
61664	1.89226	0.532941
61665	1.89226	0.228771
61666	1.89226	1.29157
61667	1.89226	0.4786
61668	1.89226	0.782022
61669	1.89226	0.224094
61670	0.946131	0.625961
61671	0.946131	0.434613
61672	0.94613	1.00274
61673	0.94613	0.688301
61674	0.946128	0.415856
61675	0.946128	0.399945
61676	0.946133	0.274527
61677	0.946133	0.878121
61678	0.946131	0.60132
61679	0.946131	0.650895
61680	0.946131	0.778494
61681	0.946131	0.464468
61682	1.89226	2.1
61683	1.89226	1.32668
61684	1.89226	2.09416
61685	1.89226	1.92404
61686	1.89226	1.79598
61687	1.89226	1.65081
61688	2.56348	1.60936
61689	1.89226	2.1
61690	1.89226	2.1
61691	2.53234	1.81333
61692	4.45574	1.71438
61693	2.53234	0.196938
61694	1.65583	0.103713
61695	2.56348	0.133018
61696	2.56348	0.42949
61697	1.66295	0.148363
61698	1.65583	0.120239
61699	0.736602	0.202682
61700	0.827916	0.1438
61701	1.26617	0.474912
61702	1.18322	0.0962826
61703	0.7366	0.177275
61704	1.18321	0.150506
61705	1.26617	0.0868182
61706	0.827918	0.179122
61707	0.99929	0.0942128
61708	0.99929	0.37086
61709	1.00348	0.135022

61710	1.00348	0.231227
61711	1.26617	0.446309
61712	1.18322	1.04654
61713	0.946131	2.1
61714	0.946131	1.79674
61715	0.946129	0.883644
61716	1.18321	0.638392
61717	0.946133	2.1
61718	1.26617	2.02555
61719	1.26617	2.04767
61720	1.26617	0.165364
61721	0.946131	2.1
61722	0.946131	1.6443
61723	2.23267	0.052435
61724	1.89226	0.122609
61725	2.07099	0.0920518
61726	1.89226	0.100528
61727	2.07099	0.10105
61728	1.89226	0.0909257
61729	0.946132	0.160419
61730	1.35403	0.087243
61731	1.24038	0.0902352
61732	0.946131	0.0808287
61733	0.946131	0.144126
61734	0.946131	0.171257
61735	1.24039	0.0834995
61736	1.35403	0.095208
61737	1.24038	0.140046
61738	1.24038	0.133933
61739	0.946131	0.0923939
61740	0.946131	0.220801
61741	1.89226	0.304779
61742	1.89226	0.652518
61743	1.89226	0.237718
61744	1.89226	0.24819
61745	1.89226	0.773044
61746	1.89226	0.695951
61747	1.89226	0.176266
61748	1.89226	0.536058
61749	1.89226	0.258526
61750	1.89226	0.371256
61751	3.78452	0.448014
61752	1.56971	0.05
61753	2.80086	0.273862
61754	2.59806	0.0779131
61755	1.93489	0.142174
61756	2.59806	0.131986
61757	1.93489	0.113806
61758	0.827467	0.0625127
61759	1.26652	0.0983864
61760	1.13679	0.0846464
61761	0.639035	0.0882315
61762	1.11547	0.106722
61763	1.30656	0.187785
61764	0.869062	0.0784155
61765	0.696608	0.0570146
61766	1.07205	0.0502162
61767	0.953656	0.11665

61768	0.970744	0.105489
61769	0.970744	0.201402
61770	2.38681	2.1
61771	2.50438	2.1
61772	2.42082	2.1
61773	2.59106	2.1
61774	4.92521	2.1
61775	2.50438	1.98058
61776	2.40627	2.1
61777	2.59106	2.1
61778	2.5444	1.78464
61779	5.04879	2.1
61780	2.42082	2.1
61781	2.70575	2.1
61782	2.64778	2.1
61783	2.59106	2.1
61784	2.47753	2.1
61785	2.42082	2.1
61786	2.70575	2.1
61787	2.5444	1.91661
61788	2.76095	2.1
61789	2.59106	2.1
61790	2.71429	2.1
61791	2.5444	2.1
61792	1.89226	2.1
61793	2.38473	2.1
61794	2.36689	2.1
61795	2.38473	2.1
61796	1.87442	2.1
61797	1.89226	2.1
61798	1.89226	2.1
61799	1.89226	2.1
61800	1.89226	2.1
61801	1.89226	2.1
61802	1.89226	2.1
61803	1.89226	2.1
61804	2.14952	2.1
61805	1.89226	2.1
61806	1.89226	2.1
61807	1.89226	2.1
61808	2.0692	2.1
61809	2.0692	2.1
61810	1.89226	2.1
61811	2.36689	2.1
61812	2.36204	2.1
61813	2.36689	2.1
61814	1.88742	2.1
61815	1.89226	2.1
61816	0.946132	2.1
61817	0.946132	2.1
61818	0.946131	2.1
61819	0.946131	2.1
61820	0.946132	2.1
61821	0.946132	2.1
61822	0.946131	2.1
61823	0.946131	2.1
61824	0.946132	2.1
61825	0.946132	2.1

61826	0.946131	2.1
61827	0.946131	2.1
61828	0.946128	2.1
61829	0.946128	2.1
61830	1.07476	2.1
61831	1.07476	2.1
61832	0.946131	2.1
61833	0.946131	2.1
61834	1.13182	2.1
61835	1.07476	2.1
61836	1.13182	2.1
61837	0.946131	2.1
61838	1.07476	2.1
61839	0.94613	2.1
61840	1.89227	0.256147
61841	1.89227	0.349757
61842	1.89227	0.432317
61843	1.89227	0.309079
61844	3.78453	0.273999
61845	1.89226	0.24365
61846	1.89226	0.258618
61847	1.89226	0.231935
61848	1.89226	0.345946
61849	3.78452	0.275481
61850	1.89226	0.244382
61851	1.89226	0.404182
61852	1.89226	0.379665
61853	1.89226	0.312673
61854	3.78452	0.264223
61855	1.89226	0.33444
61856	1.89226	0.27563
61857	1.89226	0.313395
61858	1.89226	0.394332
61859	1.89226	0.357739
61860	1.89226	0.356879
61861	2.58344	0.476334
61862	2.00267	0.13148
61863	2.14218	0.332202
61864	2.22674	0.50997
61865	2.14218	0.0709442
61866	2.22674	0.266419
61867	2.83531	0.563293
61868	1.56043	0.281787
61869	2.22674	0.469877
61870	1.90912	0.587437
61871	2.22674	0.0631726
61872	1.90912	0.272995
61873	0.780217	0.368641
61874	1.11337	0.363863
61875	1.00133	0.47734
61876	0.804472	0.334325
61877	0.804472	0.300137
61878	0.780217	0.260877
61879	1.00133	0.124674
61880	1.11337	0.431942
61881	0.856668	0.43858
61882	0.856668	0.454338
61883	0.924883	0.241894

61884	0.924883	0.314019
61885	1.93107	2.1
61886	1.68657	2.1
61887	1.7237	2.1
61888	1.89226	2.1
61889	1.53436	2.1
61890	1.68657	2.1
61891	1.89226	2.1
61892	1.89226	2.1
61893	1.89226	2.1
61894	1.89226	2.1
61895	3.78453	2.1
61896	0.946131	2.1
61897	0.946131	2.1
61898	0.855092	2.1
61899	0.83953	2.1
61900	0.946131	2.1
61901	0.946131	2.1
61902	0.839531	2.1
61903	0.839531	2.1
61904	0.946131	2.1
61905	0.855093	2.1
61906	0.839531	2.1
61907	0.946131	2.1
61908	1.89226	2.1
61909	1.70302	2.1
61910	2.21419	2.1
61911	1.93107	2.1
61912	1.97872	2.1
61913	1.70302	2.1
61914	0.946133	2.1
61915	0.946133	2.1
61916	0.946132	2.1
61917	0.946132	2.1
61918	0.946132	2.1
61919	0.946132	2.1
61920	0.946132	2.1
61921	0.946132	2.1
61922	0.946133	2.1
61923	0.946133	2.1
61924	0.946132	2.1
61925	0.946132	2.1
61926	1.90081	2.1
61927	1.89226	2.1
61928	1.51379	2.1
61929	1.36813	2.1
61930	1.71019	2.1
61931	1.89226	2.1
61932	1.89227	1.35502
61933	1.89227	1.11779
61934	1.89227	1.10141
61935	1.89227	1.33521
61936	1.89227	1.24085
61937	1.89227	1.52894
61938	1.36428	1.38752
61939	1.37869	1.37317
61940	0.946132	1.2429
61941	0.946132	1.32244

61942	1.37869	1.23606
61943	1.37869	1.67936
61944	0.946133	1.39014
61945	0.946133	1.39553
61946	1.37869	1.2509
61947	0.946132	1.28949
61948	1.36428	1.38726
61949	0.946133	1.19548
61950	0.94612	1.23847
61951	0.94612	1.26315
61952	0.946119	1.19199
61953	0.946119	1.21629
61954	0.946119	1.11714
61955	0.946119	1.55615
61956	0.94612	1.28725
61957	0.94612	1.39087
61958	0.946132	1.17675
61959	0.946132	1.13513
61960	0.946107	1.18128
61961	0.946107	1.35491
61962	1.89225	0.05
61963	1.89225	2.1
61964	1.63564	0.05
61965	1.42278	2.1
61966	1.42278	2.1
61967	1.57534	0.05
61968	1.53735	2.1
61969	1.68991	0.05
61970	1.57535	0.05
61971	1.04442	2.1
61972	1.04442	2.1
61973	1.22031	0.05
61974	1.42278	2.1
61975	1.59868	0.05
61976	0.946132	2.1
61977	0.946132	2.1
61978	0.946131	2.1
61979	0.946131	2.1
61980	0.946131	2.1
61981	0.946131	2.1
61982	0.946132	2.1
61983	0.946132	2.1
61984	0.946132	2.1
61985	0.946132	2.1
61986	0.946131	2.1
61987	0.946131	2.1
61988	1.89226	2.1
61989	1.89226	2.1
61990	1.89226	2.1
61991	1.89226	2.1
61992	1.89226	2.1
61993	1.89226	2.1
61994	2.03143	1.60534
61995	1.47111	1.95577
61996	2.28685	1.91795
61997	2.28685	1.97644
61998	1.36006	1.64667
61999	1.36006	1.6862

62000	1.89226	1.35726
62001	1.89226	1.88311
62002	1.89226	1.73303
62003	1.89226	1.83599
62004	1.89226	1.40939
62005	1.89226	1.54013
62006	1.89226	1.74649
62007	1.89226	2.1
62008	1.89226	2.1
62009	1.89226	2.1
62010	1.89226	1.79237
62011	1.89226	2.1
62012	0.902821	2.1
62013	0.902821	2.1
62014	0.889218	2.1
62015	0.889218	2.1
62016	0.675759	2.1
62017	1.05649	2.1
62018	0.735554	2.1
62019	1.14342	2.1
62020	1.14343	2.1
62021	1.05649	2.1
62022	0.735554	2.1
62023	0.675758	2.1
62024	2.46286	0.94561
62025	2.28685	1.54657
62026	2.67843	1.43812
62027	2.67843	1.46635
62028	2.03143	1.25841
62029	2.03143	1.33175
62030	1.89226	1.30189
62031	1.89226	1.46123
62032	1.89226	0.761042
62033	1.89226	0.587783
62034	3.78452	1.45416
62035	0.94613	0.582985
62036	0.94613	1.64554
62037	0.946128	1.99734
62038	0.946128	1.77681
62039	0.94613	2.1
62040	0.94613	1.70917
62041	0.946128	1.2472
62042	0.946128	1.53772
62043	0.946129	0.113541
62044	0.946129	1.74092
62045	0.946129	1.41125
62046	0.946129	2.1
62047	2.67843	1.56821
62048	2.11297	2.1
62049	2.52453	1.94512
62050	2.52453	2.1
62051	2.28685	1.64173
62052	2.28685	1.79184
62053	1.89226	1.23702
62054	1.89226	2.1
62055	1.89226	0.876052
62056	1.89226	2.1
62057	3.78451	2.1

62058	2.39524	0.748184
62059	2.10188	2.1
62060	2.29839	0.836501
62061	2.02242	2.1
62062	4.40028	2.1
62063	1.71839	2.1
62064	1.65261	2.1
62065	2.10189	2.1
62066	2.10189	2.1
62067	2.02243	2.1
62068	1.63893	2.1
62069	0.946132	2.1
62070	0.946132	2.1
62071	0.946129	2.1
62072	0.946129	2.1
62073	0.94613	2.1
62074	0.94613	2.1
62075	0.946131	2.1
62076	0.946131	2.1
62077	0.946131	2.1
62078	0.946131	2.1
62079	0.946131	2.1
62080	0.946131	2.1
62081	1.89226	0.698446
62082	1.89226	2.1
62083	1.89226	1.09311
62084	1.89226	2.1
62085	3.78452	2.05582
62086	2.2984	0.631418
62087	2.02242	2.1
62088	2.21582	1.35257
62089	1.94412	2.1
62090	4.24251	2.02779
62091	1.94412	2.1
62092	1.5854	2.1
62093	2.02243	2.1
62094	2.02243	2.1
62095	1.57431	2.1
62096	1.65261	2.1
62097	0.810943	2.1
62098	0.810943	2.1
62099	0.810945	2.1
62100	0.810945	2.1
62101	1.89226	2.1
62102	1.3517	2.1
62103	1.08133	2.1
62104	1.08132	2.1
62105	1.08132	2.1
62106	0.810933	2.1
62107	0.810933	2.1
62108	1.89226	0.05
62109	1.89226	0.0796958
62110	1.89226	0.0656122
62111	1.89226	0.0549667
62112	1.89226	0.057655
62113	1.89226	0.05
62114	1.89226	0.0672184
62115	1.89226	0.127231

62116	1.89226	0.0821447
62117	1.89226	0.111532
62118	3.78452	0.112734
62119	1.89226	0.05292
62120	1.89226	0.0940586
62121	1.89226	0.0745148
62122	1.89226	0.0645739
62123	1.89226	0.0723985
62124	1.89226	0.0620628
62125	0.946128	0.108874
62126	0.946128	0.0701345
62127	0.946132	0.0710717
62128	0.946132	0.0905838
62129	0.94613	0.111538
62130	0.94613	0.120344
62131	0.946129	0.0795609
62132	0.946129	0.0682503
62133	0.94613	0.071548
62134	0.94613	0.089645
62135	0.94613	0.075708
62136	0.94613	0.0903893
62137	1.89226	0.05
62138	1.89226	0.0857865
62139	1.89226	0.0631206
62140	1.89226	0.0603616
62141	1.89226	0.0813367
62142	1.89226	0.0808257
62143	2.81478	2.1
62144	1.89226	2.1
62145	2.65557	2.1
62146	1.89226	2.1
62147	4.54783	2.1
62148	1.89226	2.1
62149	1.89226	2.1
62150	1.89226	2.1
62151	1.89226	2.1
62152	3.78452	2.1
62153	2.65557	2.1
62154	1.89226	2.1
62155	1.89226	2.1
62156	1.89226	2.1
62157	2.57997	2.1
62158	2.57997	2.1
62159	0.946131	2.1
62160	0.946131	2.1
62161	0.946128	2.1
62162	0.946128	2.1
62163	0.94613	2.1
62164	0.94613	2.1
62165	0.946129	2.1
62166	0.946129	2.1
62167	0.94613	2.1
62168	0.94613	2.1
62169	0.94613	2.1
62170	0.94613	2.1
62171	1.89226	1.4348
62172	2.27703	1.89581
62173	2.35615	1.94818

62174	2.35615	1.56869
62175	1.89226	1.95611
62176	1.89226	1.56348
62177	0.946132	1.46218
62178	1.17807	2.086
62179	1.14926	1.43179
62180	0.946132	1.70175
62181	0.946133	1.61655
62182	0.946133	1.6841
62183	1.14926	1.46443
62184	1.14926	1.47777
62185	0.946132	1.57287
62186	0.946132	1.50508
62187	1.17807	1.63474
62188	1.14926	1.52195
62189	2.16841	1.03838
62190	1.35487	1.48136
62191	1.19854	1.35821
62192	1.19854	1.18394
62193	2.46078	1.35306
62194	2.46078	1.3088
62195	0.69308	0.915645
62196	1.3119	1.47262
62197	0.677433	1.53104
62198	1.23039	1.21404
62199	0.978178	1.52273
62200	0.978178	1.52004
62201	0.945294	1.07639
62202	0.945294	1.26916
62203	1.3119	1.39733
62204	1.23039	1.39537
62205	0.693081	1.40619
62206	0.677432	1.41256
62207	1.89226	1.39827
62208	1.89226	1.99805
62209	1.89226	1.92348
62210	1.89226	1.44823
62211	1.89226	1.929
62212	1.89226	1.45401
62213	0.946132	1.3767
62214	0.946132	2.05934
62215	0.946132	1.16827
62216	0.946132	1.56239
62217	0.946133	1.49485
62218	0.946133	1.58433
62219	0.946132	1.37265
62220	0.946132	1.23924
62221	0.946133	1.43183
62222	0.946133	1.23139
62223	0.946132	1.45443
62224	0.946132	1.4073
62225	2.60637	2.1
62226	1.89226	2.1
62227	1.89226	2.1
62228	1.89226	1.89249
62229	3.78452	2.1
62230	1.89226	1.06958
62231	1.8396	1.81282

62232	1.89226	1.89849
62233	1.89226	1.3587
62234	1.60993	2.07981
62235	1.60993	1.43149
62236	0.946131	1.76929
62237	0.946131	1.94304
62238	0.946128	2.1
62239	0.946128	2.1
62240	0.946131	1.75924
62241	0.946131	2.01917
62242	0.946127	2.1
62243	0.946127	1.88838
62244	0.946129	2.0937
62245	0.946129	2.07814
62246	0.94613	2.0961
62247	0.94613	2.082
62248	1.89226	2.1
62249	1.89226	1.87318
62250	1.89226	2.1
62251	1.89226	2.1
62252	1.89226	2.1
62253	1.89226	2.1
62254	1.89226	0.955271
62255	1.89226	1.72006
62256	1.89226	1.90488
62257	1.89226	1.19512
62258	1.89226	2.00411
62259	1.89226	1.08656
62260	0.946131	1.71673
62261	0.946131	1.93941
62262	0.946128	2.1
62263	0.946128	1.99796
62264	0.946131	1.69455
62265	0.946131	1.92294
62266	0.946127	1.89736
62267	0.946127	2.1
62268	0.94613	2.04463
62269	0.94613	2.03645
62270	0.946129	2.03689
62271	0.946129	2.04003
62272	0.946133	2.1
62273	0.946133	2.1
62274	1.28153	2.1
62275	1.26781	2.1
62276	0.946132	2.1
62277	0.946132	2.1
62278	1.28153	2.1
62279	1.28153	2.1
62280	0.946132	2.1
62281	1.28153	2.1
62282	0.946133	2.1
62283	1.26781	2.1
62284	1.89226	2.1
62285	1.89226	2.1
62286	1.89226	2.1
62287	1.89226	2.1
62288	1.89226	2.1
62289	1.89226	2.1

62290	2.5844	2.1
62291	1.09563	2.1
62292	1.1468	2.1
62293	1.09563	2.1
62294	2.6142	2.1
62295	2.56304	2.1
62296	0.946126	2.1
62297	0.946126	2.1
62298	1.2922	2.1
62299	1.28152	2.1
62300	0.946126	2.1
62301	0.946126	2.1
62302	1.2922	2.1
62303	1.2922	2.1
62304	1.29221	2.1
62305	0.946132	2.1
62306	0.946119	2.1
62307	1.28151	2.1
62308	1.89225	2.1
62309	1.89225	2.1
62310	1.89225	2.1
62311	1.89225	2.1
62312	1.89225	2.1
62313	1.89225	2.1
62314	0.946121	2.1
62315	0.946121	2.1
62316	0.946126	2.1
62317	0.946126	2.1
62318	0.946124	2.1
62319	0.946124	2.1
62320	0.946123	2.1
62321	0.946123	2.1
62322	0.94613	2.1
62323	0.94613	2.1
62324	0.946117	2.1
62325	0.946117	2.1
62326	1.89226	2.1
62327	1.89226	1.9542
62328	1.89226	1.83203
62329	1.89226	2.1
62330	1.89226	0.859527
62331	1.89226	2.1
62332	1.89226	2.1
62333	1.89226	1.99759
62334	1.89226	1.69754
62335	1.89226	2.1
62336	1.89226	0.829595
62337	1.89226	2.1
62338	1.89226	2.1
62339	2.81281	2.1
62340	2.50369	1.96291
62341	2.50369	2.1
62342	1.89226	1.98867
62343	1.89226	2.1
62344	1.89226	2.1
62345	1.89226	2.1
62346	1.89226	2.1
62347	1.89226	2.1

62348	1.89226	2.1
62349	1.89226	2.1
62350	1.89226	2.1
62351	1.89226	2.1
62352	1.89226	2.1
62353	1.89226	2.1
62354	1.89226	2.1
62355	1.89226	2.1
62356	0.907036	2.1
62357	1.09714	2.1
62358	0.994609	2.1
62359	0.838808	2.1
62360	0.976536	2.1
62361	0.976536	2.1
62362	0.959412	2.1
62363	0.959412	2.1
62364	1.09714	2.1
62365	0.994609	2.1
62366	0.907036	2.1
62367	0.838808	2.1
62368	0.99461	2.1
62369	0.838808	2.1
62370	0.738212	2.1
62371	0.565634	2.1
62372	0.775921	2.1
62373	0.775921	2.1
62374	0.784322	2.1
62375	0.784322	2.1
62376	0.994609	2.1
62377	0.738212	2.1
62378	0.838808	2.1
62379	0.565635	2.1
62380	1.89226	2.1
62381	1.89226	2.1
62382	1.89226	2.1
62383	1.89226	2.1
62384	1.89226	2.1
62385	1.89226	2.1
62386	1.89226	2.1
62387	1.89226	2.1
62388	1.89226	2.1
62389	1.89226	2.1
62390	1.89226	2.1
62391	1.89226	2.1
62392	2.19428	2.1
62393	1.98922	2.1
62394	2.27322	2.1
62395	2.45893	2.1
62396	4.4675	2.1
62397	0.99461	2.1
62398	1.13661	2.1
62399	0.902272	2.1
62400	0.738212	2.1
62401	0.931874	2.1
62402	0.931874	2.1
62403	0.942953	2.1
62404	0.942953	2.1
62405	1.13658	2.1

62406	0.902248	2.1
62407	0.994637	2.1
62408	0.738233	2.1
62409	1.89226	1.99135
62410	1.89226	2.1
62411	1.89226	2.1
62412	1.89226	2.06148
62413	1.89226	2.1
62414	1.89226	1.93312
62415	1.89227	2.01948
62416	2.58246	1.85738
62417	1.89227	2.061
62418	1.89227	1.91448
62419	3.78453	1.90393
62420	1.89226	2.1
62421	1.89226	2.07188
62422	1.65573	2.1
62423	1.65573	2.1
62424	1.89226	2.1
62425	1.89226	2.1
62426	1.89226	1.75085
62427	1.89226	1.77551
62428	1.89226	1.81209
62429	1.89226	1.79862
62430	1.89226	1.76467
62431	1.89226	1.72618
62432	1.89226	1.73159
62433	1.89226	1.69109
62434	1.89226	1.6743
62435	1.89226	1.73303
62436	1.89226	1.61855
62437	1.89226	1.71964
62438	0.946132	2.0813
62439	0.946132	2.02003
62440	1.01023	1.91198
62441	1.13651	2.1
62442	1.01023	1.89349
62443	0.946133	2.02693
62444	1.0644	2.05766
62445	1.0644	2.08343
62446	0.946132	2.06307
62447	0.946132	2.07253
62448	1.13651	2.08593
62449	0.946132	2.02393
62450	1.89226	0.948757
62451	1.89226	1.62581
62452	1.89226	1.44141
62453	1.89226	0.677109
62454	3.78451	1.02571
62455	1.00934	0.391013
62456	0.741561	0.349994
62457	0.946131	0.543743
62458	0.946131	0.603924
62459	0.946131	0.896391
62460	0.741561	0.276588
62461	1.00934	0.290395
62462	0.946131	0.66507
62463	0.741586	0.420653

62464	0.741586	0.487577
62465	0.946106	0.562167
62466	0.946106	0.373231
62467	1.14895	0.852479
62468	0.957449	1.25585
62469	0.934806	1.53947
62470	0.934806	1.47205
62471	1.13536	0.618786
62472	1.02829	1.61906
62473	0.946129	1.65285
62474	0.946129	0.921774
62475	0.946127	1.42965
62476	0.946127	1.46753
62477	0.946127	1.3773
62478	1.0283	1.42602
62479	1.89226	1.57676
62480	1.89226	1.133
62481	1.89226	0.992883
62482	1.89226	1.51427
62483	3.78452	1.46332
62484	1.89226	0.476378
62485	1.89226	0.827761
62486	1.89226	0.801594
62487	1.89226	0.424637
62488	3.78453	0.490533
62489	1.89226	0.881974
62490	2.69068	0.486729
62491	1.89226	0.443875
62492	1.89226	0.677714
62493	3.78452	0.650141
62494	1.89226	1.20592
62495	1.89226	1.5568
62496	1.89226	1.55164
62497	1.89226	1.35509
62498	1.89226	1.49748
62499	1.89226	1.17795
62500	1.89226	2.1
62501	1.89226	2.1
62502	1.89226	2.1
62503	1.89226	2.1
62504	1.89226	2.1
62505	1.89226	2.1
62506	1.89226	2.1
62507	1.89226	2.1
62508	1.89226	2.1
62509	1.89226	2.1
62510	1.89226	2.1
62511	1.89226	2.1
62512	0.985109	2.1
62513	1.31144	2.1
62514	1.45864	2.1
62515	1.10667	2.1
62516	2.44375	2.1
62517	0.655718	2.1
62518	0.729316	2.1
62519	0.684594	2.1
62520	0.782707	2.1
62521	0.700849	2.1

62522	0.700849	2.1
62523	0.71306	2.1
62524	0.71306	2.1
62525	0.729318	2.1
62526	0.782705	2.1
62527	0.684592	2.1
62528	0.655719	2.1
62529	0.946128	2.1
62530	0.946128	2.1
62531	0.946133	2.1
62532	0.946133	2.1
62533	0.946131	2.1
62534	0.946131	2.1
62535	0.946131	2.1
62536	0.946131	2.1
62537	0.94613	2.1
62538	0.94613	2.1
62539	0.946131	2.1
62540	0.946131	2.1
62541	1.89226	2.1
62542	1.89226	2.1
62543	1.89226	2.1
62544	1.89226	2.1
62545	1.89226	2.1
62546	1.89226	2.1
62547	0.553334	2.1
62548	0.629936	2.1
62549	0.828211	2.1
62550	0.729319	2.1
62551	0.674025	2.1
62552	0.674025	2.1
62553	0.68523	2.1
62554	0.68523	2.1
62555	0.828211	2.1
62556	0.629935	2.1
62557	0.72932	2.1
62558	0.553334	2.1
62559	0.828208	2.1
62560	0.729316	2.1
62561	0.907037	2.1
62562	0.782707	2.1
62563	0.799048	2.1
62564	0.799048	2.1
62565	0.811867	2.1
62566	0.811867	2.1
62567	0.828209	2.1
62568	0.907035	2.1
62569	0.729318	2.1
62570	0.782706	2.1
62571	2.32981	2.1
62572	1.89226	2.1
62573	1.89226	2.1
62574	1.89226	2.1
62575	2.2671	2.1
62576	2.2671	2.1
62577	1.89226	2.1
62578	1.89226	2.1
62579	1.89226	2.1

62580	1.89226	1.99946
62581	1.89226	2.1
62582	1.89226	2.1
62583	1.3991	2.1
62584	1.81793	2.02789
62585	2.07226	2.1
62586	2.07226	1.95111
62587	1.24924	2.1
62588	1.24924	2.1
62589	0.946131	2.1
62590	0.946131	2.1
62591	1.16491	2.1
62592	1.16491	2.1
62593	0.946131	2.1
62594	0.946131	2.1
62595	1.16491	2.1
62596	1.19523	2.1
62597	0.946132	2.1
62598	1.19523	2.1
62599	1.16491	2.1
62600	0.946131	2.1
62601	1.89226	2.1
62602	1.89226	2.08227
62603	1.89226	2.1
62604	1.89226	1.73892
62605	3.78452	2.1
62606	0.901204	2.1
62607	0.901204	2.1
62608	0.902504	2.1
62609	0.902504	2.1
62610	1.10416	2.00486
62611	1.03613	1.85373
62612	0.764922	2.1
62613	0.699552	2.1
62614	0.764921	2.1
62615	1.10416	2.1
62616	0.699551	2.1
62617	1.03613	2.1
62618	1.89226	1.39806
62619	1.89226	1.52138
62620	1.89226	1.50275
62621	1.89226	1.46087
62622	3.78453	1.52461
62623	1.89226	1.53444
62624	1.89226	1.57496
62625	1.89226	1.54336
62626	1.89226	1.61341
62627	1.89226	1.54833
62628	1.89226	1.61505
62629	0.946131	1.49255
62630	0.946131	1.49236
62631	0.946131	1.43807
62632	0.946131	1.43127
62633	0.946131	1.43774
62634	0.946131	1.49738
62635	0.946131	1.49245
62636	0.946131	1.38395
62637	0.946131	1.4107

62638	0.946131	1.40853
62639	0.946131	1.33077
62640	0.946131	1.4269
62641	1.89226	1.51191
62642	1.89226	1.53629
62643	1.89226	1.51627
62644	1.89226	1.55546
62645	1.89226	1.51289
62646	1.89226	1.55077
62647	0.946127	1.53262
62648	0.946127	1.51619
62649	1.22788	1.5249
62650	1.35469	1.58594
62651	1.35469	1.56524
62652	1.35469	1.58993
62653	0.946128	1.5175
62654	0.946128	1.49097
62655	0.946129	1.52641
62656	1.22788	1.55589
62657	1.35469	1.54164
62658	0.946129	1.49993
62659	1.22788	1.52392
62660	1.35469	1.5847
62661	1.02602	1.4211
62662	0.794861	1.49071
62663	1.15301	1.55837
62664	1.15301	1.58107
62665	1.10089	1.40442
62666	1.10089	1.50987
62667	0.794861	1.42736
62668	1.22788	1.53607
62669	1.02602	1.55979
62670	1.35469	1.54799
62671	1.89226	0.248309
62672	1.89226	1.01352
62673	1.89226	1.49706
62674	1.89226	0.410203
62675	3.78452	0.598994
62676	1.13536	2.1
62677	0.942524	1.0253
62678	0.946131	1.1413
62679	0.946131	0.357982
62680	0.946127	1.15415
62681	0.946127	2.1
62682	1.26282	0.384822
62683	0.917471	1.97345
62684	1.21023	1.50128
62685	1.00851	1.45258
62686	1.17955	1.19743
62687	0.883746	1.19168
62688	1.89226	1.08348
62689	1.89226	2.1
62690	1.89226	2.1
62691	1.89226	2.1
62692	1.89226	2.1
62693	1.89226	2.1
62694	1.89226	0.50448
62695	1.89226	2.1

62696	1.89226	1.08431
62697	1.89226	1.03502
62698	1.89226	1.11839
62699	1.89226	1.81391
62700	0.94613	1.4851
62701	0.94613	0.512561
62702	0.94613	0.093032
62703	0.94613	0.574483
62704	0.946128	0.522901
62705	0.946128	1.7307
62706	0.946131	1.56877
62707	0.946131	0.05
62708	0.94613	1.14995
62709	0.94613	1.13841
62710	0.94613	0.696057
62711	0.94613	0.422484
62712	0.883758	2.1
62713	1.03041	1.81797
62714	0.946131	1.13106
62715	0.946131	0.516079
62716	0.851537	1.30619
62717	0.851537	2.1
62718	1.23942	0.604546
62719	1.14906	1.97236
62720	0.851484	1.4106
62721	0.985518	1.06203
62722	1.0156	1.2736
62723	0.851526	1.20214
62724	1.89226	1.18681
62725	1.89226	2.1
62726	1.89226	2.1
62727	1.89226	2.1
62728	1.89226	2.1
62729	1.89226	2.1
62730	1.89226	0.613294
62731	1.89226	2.1
62732	1.89226	1.36564
62733	1.89226	1.58244
62734	1.89226	1.03387
62735	1.89226	1.78959
62736	1.89227	0.239488
62737	1.89227	0.320667
62738	1.89227	0.265752
62739	1.89227	0.229655
62740	3.78453	0.236562
62741	1.89226	0.352838
62742	1.89226	0.241543
62743	1.89226	0.30626
62744	1.89226	0.343951
62745	3.78452	0.361472
62746	0.946132	0.274464
62747	0.946132	0.329718
62748	0.946128	0.430832
62749	0.946128	0.22982
62750	0.94613	0.323441
62751	0.94613	0.204236
62752	0.94613	0.24062
62753	0.94613	0.344958

62754	0.946131	0.289563
62755	0.946131	0.28771
62756	0.94613	0.248519
62757	0.94613	0.243376
62758	1.89226	0.288221
62759	1.89226	0.245149
62760	1.89226	0.272498
62761	1.89226	0.321813
62762	1.89226	0.203747
62763	1.89226	0.310112
62764	1.98874	0.116025
62765	2.33455	0.0673516
62766	2.01865	0.224718
62767	2.07024	0.0888484
62768	4.32329	0.221771
62769	1.98874	0.199914
62770	2.00266	0.138028
62771	2.14218	0.375912
62772	2.07024	0.301071
62773	3.9914	0.139954
62774	1.00133	0.126356
62775	0.804471	0.0676028
62776	1.03512	0.199015
62777	0.995176	0.222382
62778	0.80447	0.425289
62779	0.995178	0.209051
62780	1.00133	0.269719
62781	1.03512	0.405782
62782	0.880587	0.393941
62783	0.880587	0.0677647
62784	0.959002	0.159303
62785	0.959002	0.211156
62786	2.33455	0.117991
62787	1.99036	0.05
62788	2.07024	0.117901
62789	2.58645	0.0661158
62790	4.32491	0.05
62791	1.89226	1.00772
62792	1.89226	0.691272
62793	1.89226	0.995535
62794	1.89226	0.424126
62795	3.78453	0.465317
62796	1.89226	1.28157
62797	1.89226	0.943877
62798	1.89226	1.23534
62799	1.89226	1.11506
62800	3.78453	1.27348
62801	1.89226	0.500325
62802	1.89226	0.341116
62803	1.89226	0.339632
62804	1.89226	0.445022
62805	1.89226	0.354788
62806	1.89226	0.478752
62807	1.89226	0.378538
62808	2.1523	0.166183
62809	2.53257	0.184273
62810	2.53257	0.178011
62811	1.89226	0.434535

62812	1.89226	0.393417
62813	0.946133	0.412026
62814	0.946133	0.543189
62815	0.946132	0.294837
62816	0.946132	0.334462
62817	0.946132	0.286725
62818	0.946132	0.313644
62819	0.946132	0.580712
62820	0.946132	0.318699
62821	0.946133	0.41042
62822	0.946133	0.437296
62823	0.946132	0.541601
62824	0.946132	0.396484
62825	0.946132	0.706311
62826	0.946132	1.0703
62827	0.946132	0.560654
62828	0.946132	0.828992
62829	0.946132	0.540521
62830	0.946132	0.595892
62831	0.946132	1.12223
62832	0.946132	0.862952
62833	0.946132	0.76504
62834	0.946132	0.720251
62835	0.946131	0.904018
62836	0.946131	0.786448
62837	1.62772	0.072739
62838	1.70303	0.0683148
62839	1.70303	0.101908
62840	1.70303	0.106021
62841	1.90116	0.0683955
62842	1.90116	0.0653348
62843	1.89226	0.0734535
62844	1.89226	0.0786368
62845	1.89226	0.36124
62846	1.89226	0.498673
62847	1.89226	0.0731188
62848	1.89226	0.0713074
62849	1.89226	0.112194
62850	1.89226	0.111643
62851	1.89226	0.0893416
62852	1.89226	0.669793
62853	3.78452	0.105172
62854	1.89226	0.165489
62855	1.89226	0.424529
62856	1.89226	0.100031
62857	1.89226	0.129388
62858	1.89226	0.231517
62859	1.89226	0.287717
62860	0.946132	0.0646874
62861	0.946132	0.0651102
62862	0.946129	0.0720555
62863	0.946129	0.0793694
62864	0.94613	0.0689624
62865	0.94613	0.0658114
62866	0.946131	0.0738617
62867	0.946131	0.0748665
62868	0.94613	0.646285
62869	0.94613	0.101737

62870	0.946131	0.0737015
62871	0.946131	0.596318
62872	1.89226	0.071018
62873	1.89226	0.123156
62874	1.89226	0.600211
62875	1.89226	0.467772
62876	1.89226	0.0697872
62877	1.89226	0.0648412
62878	1.89226	0.237216
62879	1.89226	1.1564
62880	1.89226	1.16632
62881	1.89226	0.197653
62882	3.78452	0.447462
62883	1.89226	0.774006
62884	1.89226	1.30388
62885	1.89226	0.454441
62886	1.89226	1.44342
62887	1.89226	0.430915
62888	1.89226	1.46174
62889	1.30165	1.02278
62890	0.946127	1.24901
62891	1.09659	0.566782
62892	0.946131	0.641488
62893	1.09659	0.67244
62894	1.09659	1.1795
62895	0.946128	1.9031
62896	0.946128	0.696898
62897	1.30165	0.768068
62898	1.09659	0.988858
62899	0.946129	1.0073
62900	0.946129	1.50118
62901	0.946128	1.0154
62902	0.946128	1.18908
62903	0.946133	0.386284
62904	0.946133	0.656519
62905	0.946131	0.273558
62906	0.946131	1.10241
62907	0.946131	1.1704
62908	0.946131	0.499075
62909	0.94613	0.284809
62910	0.94613	0.364477
62911	0.946131	0.630451
62912	0.946131	0.842318
62913	0.946133	1.35074
62914	0.946133	1.39099
62915	0.946132	0.677932
62916	0.946132	1.06976
62917	0.946132	0.453956
62918	0.946132	1.33144
62919	0.946132	1.43168
62920	0.946132	1.02561
62921	0.946132	1.14867
62922	0.946132	0.889251
62923	0.946133	1.24047
62924	0.946133	1.17353
62925	1.30165	1.2356
62926	0.972228	1.28925
62927	1.09659	0.709195

62928	0.656268	0.611775
62929	1.06217	0.456059
62930	1.06217	1.45797
62931	1.00665	0.859843
62932	1.00665	1.69259
62933	0.97223	0.779289
62934	0.656267	0.799161
62935	1.30165	1.00596
62936	1.09659	1.13785
62937	0.946132	1.99343
62938	0.946132	2.1
62939	1.19895	2.1
62940	1.19895	2.1
62941	0.94613	2.1
62942	0.94613	1.86742
62943	1.39356	2.1
62944	1.19895	2.1
62945	0.94613	2.1
62946	1.39356	2.1
62947	1.19895	2.1
62948	0.946131	2.1
62949	1.89226	1.94514
62950	1.89226	2.1
62951	1.89226	2.1
62952	1.89226	1.94869
62953	1.89226	2.1
62954	1.89226	1.93271
62955	0.946132	1.81877
62956	0.946132	1.8674
62957	0.946128	2.1
62958	0.946128	2.1
62959	0.94613	2.1
62960	0.94613	1.70512
62961	0.94613	1.89599
62962	0.94613	2.1
62963	0.946131	1.95851
62964	0.946131	1.95276
62965	0.94613	2.06528
62966	0.94613	2.01308
62967	1.89226	0.05
62968	1.89226	0.0518701
62969	1.89226	0.0681382
62970	1.89226	0.05
62971	1.89226	0.0715577
62972	1.89226	0.05
62973	1.29881	0.05
62974	1.09985	0.05
62975	0.946126	0.05
62976	0.946126	0.05
62977	0.851539	0.05
62978	1.20421	0.05
62979	0.851483	0.05
62980	0.999426	0.05
62981	1.47172	0.05
62982	0.999413	0.05
62983	0.851552	0.05
62984	0.851552	0.05
62985	1.89226	0.0749557

62986	1.89226	0.0734375
62987	1.89226	0.117646
62988	1.89226	0.0606496
62989	1.89226	0.116212
62990	1.89226	0.0615926
62991	1.89226	0.0582281
62992	1.89226	0.0552192
62993	1.89226	0.0818434
62994	1.89226	0.0511786
62995	1.89226	0.0824408
62996	1.89226	0.05
62997	1.33915	2.1
62998	1.99297	2.1
62999	2.05216	2.1
63000	2.60163	2.1
63001	4.04513	2.1
63002	1.89226	2.1
63003	1.89226	2.1
63004	1.89226	2.1
63005	1.89226	2.1
63006	3.78452	2.1
63007	0.946132	2.1
63008	0.946132	2.1
63009	0.946133	2.1
63010	0.946133	2.1
63011	0.946132	2.1
63012	0.946132	2.06044
63013	0.946133	2.1
63014	0.946133	2.1
63015	0.946132	2.1
63016	0.946132	2.1
63017	0.946132	2.1
63018	0.946132	2.1
63019	1.89226	2.1
63020	1.89226	1.8719
63021	1.89226	2.01386
63022	1.89226	2.1
63023	3.78452	2.07217
63024	1.89227	1.90738
63025	1.89227	2.1
63026	1.89227	2.1
63027	1.89227	1.8989
63028	1.89227	2.1
63029	1.89227	2.0043
63030	2.29301	2.1
63031	1.70303	2.1
63032	2.46749	2.1
63033	2.30728	2.1
63034	4.53937	2.1
63035	1.89226	2.04097
63036	1.89226	2.1
63037	1.89226	2.1
63038	1.89226	1.87562
63039	3.78452	2.1
63040	1.89226	1.70835
63041	1.89226	2.1
63042	1.89226	2.1
63043	1.89226	2.0764

63044	1.89226	1.99221
63045	1.89226	1.72195
63046	2.30728	2.1
63047	1.00757	2.1
63048	2.0833	2.1
63049	1.65318	2.1
63050	1.417	2.1
63051	1.00757	2.1
63052	2.46749	2.1
63053	2.0975	2.1
63054	2.79653	2.1
63055	2.44785	2.1
63056	4.91534	2.1
63057	1.89226	2.1
63058	1.89226	2.1
63059	1.89226	2.09474
63060	1.89226	2.1
63061	3.78452	2.1
63062	1.89226	1.91378
63063	1.89226	2.1
63064	1.89226	2.1
63065	1.89226	1.95647
63066	1.89226	2.1
63067	1.89226	1.90251
63068	0.970559	2.1
63069	0.970559	2.1
63070	0.979305	2.1
63071	0.979305	2.1
63072	0.708502	2.1
63073	0.901117	2.1
63074	1.04875	2.1
63075	1.22392	2.1
63076	1.22392	2.1
63077	0.901117	2.1
63078	1.04875	2.1
63079	0.7085	2.1
63080	1.89226	1.81515
63081	1.89226	1.52882
63082	1.89226	1.7114
63083	1.89226	1.6906
63084	3.78452	1.80425
63085	1.89226	1.76092
63086	1.89226	1.54717
63087	1.89226	1.85761
63088	1.89226	1.63177
63089	3.78452	1.65662
63090	1.89226	0.741108
63091	1.89226	0.419277
63092	1.89226	0.871696
63093	1.89226	0.571323
63094	3.78453	0.656932
63095	1.89226	1.35563
63096	1.89226	1.29882
63097	1.89226	0.913121
63098	1.89226	0.770445
63099	1.89226	0.830885
63100	1.89226	0.604224
63101	1.89226	1.44828

63102	2.35441	1.42612
63103	1.89226	1.479
63104	2.30931	0.50674
63105	4.24667	1.089
63106	1.11612	0.696306
63107	0.946131	0.740163
63108	1.1772	0.732172
63109	0.946131	0.814442
63110	1.17721	0.782424
63111	1.11612	0.743903
63112	0.94613	0.776468
63113	0.94613	1.24591
63114	1.11612	0.734908
63115	1.11612	0.624635
63116	0.946131	0.987732
63117	0.946131	0.699619
63118	1.11612	0.169518
63119	0.582675	0.135926
63120	1.17721	0.13674
63121	0.651969	0.210669
63122	0.651969	0.19393
63123	0.582674	0.162733
63124	1.17721	0.14276
63125	1.11612	0.220996
63126	0.877955	0.129245
63127	0.877955	0.145818
63128	0.881925	0.229962
63129	0.881925	0.189003
63130	1.17721	0.257622
63131	0.651969	0.319282
63132	1.15466	0.233568
63133	0.623642	0.216962
63134	0.623642	0.271827
63135	0.651969	0.16076
63136	1.17721	0.35786
63137	1.15466	0.207467
63138	0.904714	0.215966
63139	0.904714	0.163814
63140	0.901914	0.40821
63141	0.901914	0.252434
63142	1.89226	1.27591
63143	1.89226	0.504181
63144	1.89226	1.5399
63145	1.89226	1.4087
63146	1.89226	0.56381
63147	1.89226	1.52103
63148	1.89226	1.69401
63149	1.89226	0.308496
63150	1.89226	0.490322
63151	1.89226	1.96045
63152	1.89226	0.313665
63153	1.89226	2.07227
63154	2.33986	1.89416
63155	1.97819	0.691135
63156	2.39166	0.233763
63157	2.70141	2.06759
63158	4.73151	0.678476
63159	0.989095	1.38133

63160	1.19583	0.392603
63161	0.851194	0.165836
63162	0.581197	0.212104
63163	0.872704	0.101619
63164	0.872704	1.53912
63165	0.904322	0.307463
63166	0.904322	1.24965
63167	1.19583	0.05
63168	0.851193	0.184319
63169	0.989095	1.20105
63170	0.581197	0.302049
63171	1.89226	1.23904
63172	1.89226	1.40302
63173	1.89226	1.6715
63174	1.89226	1.40914
63175	1.89226	1.54759
63176	1.89226	1.51797
63177	1.89226	1.62967
63178	1.89226	0.313108
63179	1.89226	1.11413
63180	1.89226	1.83747
63181	1.89226	0.380774
63182	1.89226	2.02828
63183	2.39166	1.0912
63184	1.70239	1.27805
63185	2.13665	0.317222
63186	2.82684	0.440724
63187	4.52831	1.11911
63188	1.89226	0.0558013
63189	1.89226	0.124169
63190	1.89226	0.0912075
63191	1.89226	0.0989332
63192	3.78453	0.125448
63193	1.89226	0.05
63194	1.89226	0.05
63195	1.89226	0.064651
63196	1.89226	0.05
63197	1.89226	0.0736174
63198	1.89226	0.05
63199	1.89226	0.0940405
63200	1.89226	0.0947001
63201	1.89226	0.132122
63202	1.89226	0.0828961
63203	1.89226	0.138923
63204	1.89226	0.066848
63205	1.89226	0.180492
63206	1.89226	0.192818
63207	1.89226	0.346327
63208	1.89226	0.133274
63209	1.89226	0.389795
63210	1.89226	0.128405
63211	1.89226	0.11161
63212	1.89226	0.118729
63213	1.89226	0.202978
63214	1.89226	0.103947
63215	1.89226	0.22984
63216	1.89226	0.0791236
63217	1.5169	0.128756

63218	1.61969	0.0710209
63219	1.17262	0.0744529
63220	1.17262	0.125374
63221	1.88868	0.0790276
63222	1.88868	0.119463
63223	1.79981	0.200073
63224	1.88868	0.124686
63225	1.5169	0.12946
63226	1.5169	0.188668
63227	2.12891	0.140197
63228	2.12891	0.193357
63229	1.89226	0.179608
63230	1.89226	0.117289
63231	1.89226	0.120206
63232	1.89226	0.195582
63233	3.78452	0.166114
63234	0.946132	0.106161
63235	0.946132	0.122175
63236	0.946132	0.0820919
63237	0.946132	0.148763
63238	0.946132	0.0720246
63239	0.946132	0.164653
63240	0.946133	0.0759807
63241	0.946133	0.145109
63242	0.946133	0.0830864
63243	0.946133	0.105892
63244	0.946132	0.136089
63245	0.946132	0.0922869
63246	1.88868	0.127688
63247	2.05759	0.0628929
63248	1.61969	0.0684035
63249	1.61969	0.163797
63250	2.29747	0.0730914
63251	2.29747	0.113924
63252	2.12891	0.184119
63253	2.29747	0.104072
63254	1.88868	0.108364
63255	1.88868	0.150152
63256	2.50894	0.119991
63257	2.50894	0.157832
63258	1.89226	0.0976383
63259	1.89226	0.1767
63260	1.89226	0.0947359
63261	1.89226	0.144677
63262	1.89226	0.0993108
63263	1.89226	0.16571
63264	0.946132	0.111277
63265	0.946132	0.0998862
63266	0.946132	0.134196
63267	0.946132	0.0908863
63268	0.946131	0.0667155
63269	0.946131	0.205416
63270	0.946132	0.0666543
63271	0.946132	0.149613
63272	0.946132	0.0759192
63273	0.946132	0.103661
63274	0.946132	0.0883096
63275	0.946132	0.167744

63276	2.42758	2.1
63277	2.14895	2.1
63278	2.56975	2.0734
63279	2.28142	2.1
63280	4.71869	2.1
63281	1.89226	2.1
63282	1.89226	2.1
63283	1.89226	2.08211
63284	1.89226	2.1
63285	3.78452	2.1
63286	1.89226	2.1
63287	1.89226	2.1
63288	1.89226	2.1
63289	1.89226	2.1
63290	1.89226	2.1
63291	1.89226	2.1
63292	0.960916	2.1
63293	0.960916	2.1
63294	0.978118	2.1
63295	0.875674	2.1
63296	0.775661	2.1
63297	0.773613	2.1
63298	1.07631	2.1
63299	1.14266	2.1
63300	1.16927	2.1
63301	0.898612	2.1
63302	1.04757	2.1
63303	0.757536	2.1
63304	2.56975	2.0055
63305	2.28142	2.1
63306	2.64384	2.00394
63307	2.40621	2.1
63308	4.97596	2.1
63309	1.89226	2.02178
63310	1.89226	2.1
63311	1.89226	1.60608
63312	1.89226	2.1
63313	3.78452	2.1
63314	1.89226	2.1
63315	1.89226	2.1
63316	1.89226	2.1
63317	1.89226	2.1
63318	1.89226	2.1
63319	1.89226	2.1
63320	1.03328	2.1
63321	1.03328	2.1
63322	1.04321	2.1
63323	1.15424	2.1
63324	0.936183	2.1
63325	0.944491	2.1
63326	1.14266	2.1
63327	1.20517	2.1
63328	1.23323	2.1
63329	0.961268	2.1
63330	1.11215	2.1
63331	0.827247	2.1
63332	1.89226	2.1
63333	1.89226	2.1

63334	1.89226	2.1
63335	1.89226	2.1
63336	3.78453	2.1
63337	2.14647	2.1
63338	2.20589	2.1
63339	1.95567	2.1
63340	2.03224	2.1
63341	4.17871	2.1
63342	2.06035	2.1
63343	1.88452	1.94553
63344	1.88452	2.1
63345	2.26997	2.1
63346	2.03224	2.1
63347	2.42272	2.1
63348	1.89226	2.1
63349	1.89226	1.65972
63350	1.89226	1.13216
63351	1.89226	2.1
63352	1.89226	1.34768
63353	1.89226	2.1
63354	1.89225	2.1
63355	1.89225	2.1
63356	1.89225	2.1
63357	1.89225	2.1
63358	1.89225	2.1
63359	1.89225	2.1
63360	0.952418	2.1
63361	0.952418	2.1
63362	0.95883	2.1
63363	0.95883	2.1
63364	0.946182	2.1
63365	1.01611	2.1
63366	0.895134	2.1
63367	0.977828	2.1
63368	0.89514	2.1
63369	0.946189	2.1
63370	0.977821	2.1
63371	1.01611	2.1
63372	2.03223	2.1
63373	1.88451	1.99304
63374	1.89236	2.1
63375	1.74649	2.06745
63376	3.77687	2.1
63377	0.81094	2.1
63378	0.81094	2.01757
63379	0.810939	2.1
63380	0.810939	2.1
63381	0.810945	2.1
63382	0.810945	2.1
63383	1.89225	1.94966
63384	1.35167	2.1
63385	1.08131	2.1
63386	1.08131	1.81272
63387	1.0813	2.1
63388	1.89226	0.05
63389	1.89226	0.05
63390	1.89226	0.05
63391	1.89226	0.05

63392	1.89226	0.05
63393	1.89226	0.05
63394	1.01842	0.05
63395	0.936656	0.05
63396	0.946131	0.05
63397	0.946131	0.05
63398	1.11157	0.05
63399	1.04281	0.05
63400	0.925803	0.05
63401	0.925803	0.05
63402	0.851553	0.05
63403	1.11158	0.05
63404	1.05017	0.05
63405	0.925798	0.05
63406	1.89226	0.05
63407	1.89226	0.0530418
63408	1.89226	0.0561262
63409	1.89226	0.05
63410	1.89226	0.0516211
63411	1.89226	0.05
63412	1.23504	0.05
63413	1.23504	0.05
63414	0.946131	0.05
63415	0.946131	0.05
63416	1.37358	0.05
63417	1.23504	0.05
63418	0.946129	0.05
63419	0.946129	0.05
63420	1.37358	0.05
63421	0.946131	0.05
63422	0.94613	0.05
63423	1.23504	0.05
63424	1.00261	0.05
63425	1.00261	0.05
63426	1.02952	0.05
63427	1.02952	0.05
63428	0.850964	0.05
63429	0.658553	0.05
63430	1.37358	0.05
63431	1.23504	0.05
63432	1.37358	0.05
63433	0.850965	0.05
63434	1.23504	0.05
63435	0.658553	0.05
63436	0.946133	0.05
63437	0.946133	0.05
63438	0.946132	0.0539191
63439	0.946132	0.05
63440	0.946132	0.05
63441	0.946132	0.05
63442	0.946133	0.05
63443	0.946133	0.05
63444	0.946133	0.05
63445	0.946133	0.05
63446	0.946132	0.05
63447	0.946132	0.05
63448	1.89226	0.0543663
63449	1.89226	0.05

63450	1.89226	0.05
63451	1.89226	0.05
63452	3.78452	0.05
63453	1.89226	0.05
63454	1.89226	0.0598415
63455	1.89226	0.05
63456	1.89226	0.05
63457	3.78452	0.05
63458	1.89227	0.0637075
63459	1.89227	0.053475
63460	1.89227	0.05
63461	1.89227	0.0604035
63462	1.89227	0.05
63463	1.89227	0.05
63464	0.94613	0.05
63465	0.94613	0.05
63466	0.946127	0.05
63467	0.946127	0.05
63468	0.94613	0.05
63469	0.94613	0.05
63470	0.946127	0.05
63471	0.946127	0.05
63472	0.946128	0.05
63473	0.946128	0.05
63474	0.946128	0.05
63475	0.946128	0.05
63476	0.946132	0.05
63477	0.946132	0.05
63478	0.946128	0.05
63479	0.946128	0.05
63480	0.94613	0.05
63481	0.94613	0.05
63482	0.946131	0.05
63483	0.946131	0.05
63484	0.94613	0.05
63485	0.94613	0.05
63486	0.94613	0.05
63487	0.94613	0.05
63488	1.89226	2.1
63489	1.89226	2.1
63490	1.89226	2.1
63491	1.89226	2.1
63492	3.78453	2.1
63493	2.72844	2.1
63494	1.89226	2.1
63495	2.26536	2.1
63496	2.26536	2.1
63497	1.89226	2.1
63498	1.89226	2.1
63499	1.89226	2.1
63500	1.89226	2.09639
63501	1.89226	2.1
63502	1.89226	2.1
63503	1.89226	2.1
63504	1.89226	2.1
63505	1.89226	2.1
63506	1.89226	2.1
63507	1.89226	2.1

63508	1.89226	2.1
63509	1.89226	2.1
63510	1.89226	2.1
63511	2.72844	2.1
63512	2.26536	2.1
63513	2.28367	2.1
63514	1.5791	2.1
63515	4.54903	2.1
63516	0.810945	2.1
63517	0.810945	2.1
63518	0.810943	2.1
63519	0.810943	2.1
63520	1.89226	2.1
63521	1.3517	2.1
63522	1.08133	2.1
63523	1.08132	2.1
63524	1.08132	2.1
63525	0.810933	2.1
63526	0.810933	2.1
63527	1.89227	2.1
63528	1.89227	2.1
63529	1.89227	2.1
63530	1.89227	2.1
63531	3.78453	2.1
63532	2.71897	2.1
63533	2.27495	2.1
63534	2.648	2.1
63535	2.20832	2.1
63536	4.92729	2.1
63537	2.27495	2.1
63538	1.38455	2.1
63539	1.38455	2.1
63540	1.46586	2.1
63541	2.20831	2.1
63542	2.28962	2.1
63543	0.946133	2.1
63544	0.946133	2.1
63545	0.946132	2.1
63546	0.946132	2.1
63547	0.946132	2.1
63548	0.946132	2.1
63549	0.946132	2.1
63550	0.946132	2.1
63551	0.946132	2.1
63552	0.946132	2.1
63553	0.946133	2.1
63554	0.946133	2.1
63555	1.89226	2.1
63556	1.89226	2.1
63557	1.89226	2.1
63558	1.89226	2.1
63559	1.89226	2.1
63560	1.89226	2.1
63561	2.648	2.1
63562	2.08612	2.1
63563	2.08612	2.1
63564	2.20831	2.1
63565	2.56618	2.1

63566	2.68837	2.1
63567	2.08612	2.1
63568	2.20831	2.1
63569	1.30659	2.1
63570	1.30659	2.1
63571	1.38455	2.1
63572	2.16408	2.1
63573	0.810946	2.1
63574	0.810946	2.1
63575	0.810946	2.1
63576	0.810946	2.1
63577	1.89226	2.1
63578	1.3517	2.1
63579	1.08133	2.1
63580	1.08132	2.1
63581	1.08132	2.1
63582	0.810934	2.1
63583	0.810934	2.1
63584	1.89226	1.93149
63585	1.89226	1.65236
63586	1.89226	1.58959
63587	1.89226	1.93845
63588	3.78452	1.70294
63589	1.89226	1.79522
63590	1.89226	2.1
63591	1.89226	2.1
63592	1.89226	1.79647
63593	3.78453	2.0625
63594	1.08733	2.1
63595	0.856615	2.1
63596	1.25964	1.80644
63597	1.12378	1.7748
63598	1.23551	1.7748
63599	0.969894	2.1
63600	0.985977	2.09175
63601	0.985977	1.77414
63602	1.16169	1.85531
63603	1.21072	1.85958
63604	0.765175	1.96976
63605	0.863419	1.96247
63606	2.39934	1.91792
63607	2.44427	1.60315
63608	2.64003	1.55957
63609	2.39077	1.86672
63610	2.39934	1.72883
63611	2.10194	2.01746
63612	1.89226	1.57596
63613	1.89226	1.99381
63614	1.89226	1.84764
63615	1.89226	1.48277
63616	3.78452	1.75908
63617	0.946132	2.08291
63618	0.946132	2.04965
63619	0.946131	1.71731
63620	0.946131	1.79584
63621	0.946132	1.73025
63622	0.946132	2.1
63623	0.946132	2.09036

63624	0.946132	1.83527
63625	0.946132	1.89668
63626	0.946132	1.89733
63627	0.946132	1.90987
63628	0.946132	1.91309
63629	1.19967	1.90949
63630	0.946128	1.8379
63631	0.946133	1.52902
63632	1.32002	1.55634
63633	1.19967	1.75719
63634	1.19967	1.62514
63635	0.94613	1.90364
63636	0.94613	1.31317
63637	0.946131	1.60982
63638	0.946131	1.6374
63639	1.19967	1.70543
63640	1.32001	1.6644
63641	1.89226	1.88472
63642	1.89226	2.1
63643	1.89226	1.00889
63644	1.89226	2.1
63645	3.78451	2.1
63646	2.6402	0.848579
63647	2.28552	2.1
63648	2.50958	0.912566
63649	2.18948	2.1
63650	4.79509	2.1
63651	2.28552	2.1
63652	1.86102	2.1
63653	2.18949	2.1
63654	1.78376	2.1
63655	4.06928	2.1
63656	1.89226	2.1
63657	1.89226	2.1
63658	1.89226	2.1
63659	1.89226	2.1
63660	3.78452	2.1
63661	1.89226	0.927953
63662	1.89226	2.1
63663	1.89226	1.01482
63664	1.89226	2.1
63665	3.78452	2.1
63666	2.50958	0.830733
63667	2.18948	2.1
63668	2.39524	0.917346
63669	2.10188	2.1
63670	4.61146	2.071
63671	2.18949	2.1
63672	1.78376	2.1
63673	1.71839	2.1
63674	1.71839	2.1
63675	2.10189	2.1
63676	2.16726	2.1
63677	0.810943	2.1
63678	0.810943	2.1
63679	0.810945	2.1
63680	0.810945	2.1
63681	0.810959	2.1

63682	0.810959	2.1
63683	1.89226	2.1
63684	1.35168	2.1
63685	1.08132	2.1
63686	1.08132	2.1
63687	1.0813	2.1
63688	1.89226	1.20271
63689	1.89226	1.52719
63690	1.89226	1.4462
63691	1.89226	1.31208
63692	1.89226	1.4327
63693	1.89226	1.31975
63694	1.28421	1.36862
63695	0.992041	1.2628
63696	1.07707	1.71233
63697	0.789746	1.53194
63698	1.03815	1.64937
63699	1.03815	1.41449
63700	1.03581	1.54639
63701	1.03581	1.34944
63702	1.28421	1.49907
63703	1.07707	1.52793
63704	0.789748	1.43915
63705	0.992038	1.43707
63706	1.89226	1.35267
63707	1.89226	1.61916
63708	1.89226	1.56766
63709	1.89226	1.41839
63710	1.89226	1.56641
63711	1.89226	1.42471
63712	1.89226	1.16829
63713	1.89226	1.45219
63714	1.89226	1.37148
63715	1.89226	1.25816
63716	1.89226	1.36705
63717	1.89226	1.2598
63718	0.92236	1.70422
63719	0.655971	1.63612
63720	0.916772	1.68709
63721	0.913834	1.50468
63722	1.18074	1.61829
63723	0.922346	1.64258
63724	2.15414	1.41598
63725	1.53528	1.57785
63726	1.22748	1.64452
63727	1.57442	1.48926
63728	0.874683	1.5845
63729	1.25935	2.1
63730	2.61433	2.1
63731	1.22262	2.1
63732	1.22262	2.1
63733	2.62813	2.1
63734	2.62813	2.1
63735	1.89226	2.1
63736	2.61434	2.1
63737	2.62814	2.1
63738	2.61434	2.1
63739	1.90607	2.1

63740	1.89226	2.1
63741	1.89226	2.1
63742	1.89226	2.1
63743	1.89226	2.1
63744	1.89226	2.1
63745	1.89226	2.1
63746	1.89226	2.1
63747	1.89226	2.1
63748	1.89226	2.1
63749	1.89226	2.1
63750	1.89226	2.1
63751	1.89226	2.1
63752	1.89226	2.1
63753	1.31969	2.1
63754	1.31406	2.1
63755	0.629677	2.1
63756	0.643104	2.1
63757	0.976621	2.1
63758	0.976621	2.1
63759	0.98055	2.1
63760	0.98055	2.1
63761	1.3197	2.1
63762	0.643102	2.1
63763	0.629675	2.1
63764	1.31407	2.1
63765	0.946133	2.1
63766	0.946133	2.1
63767	1.3197	2.1
63768	1.31407	2.1
63769	0.946132	2.1
63770	0.946132	2.1
63771	1.3197	2.1
63772	1.3197	2.1
63773	1.3197	2.1
63774	0.946133	2.1
63775	0.946132	2.1
63776	1.31407	2.1
63777	1.89226	2.1
63778	1.89226	2.1
63779	1.89226	2.1
63780	1.89226	2.1
63781	1.89226	2.1
63782	1.89226	2.1
63783	1.89226	2.1
63784	1.89226	2.1
63785	1.89226	2.1
63786	1.89226	2.1
63787	1.89226	2.1
63788	1.89226	2.1
63789	1.89226	0.0750719
63790	1.89226	0.0525524
63791	1.89226	0.05
63792	1.89226	0.05
63793	3.78452	0.05
63794	1.89226	0.0572355
63795	1.89226	0.05
63796	1.89226	0.05
63797	1.89226	0.05

63798	1.89226	0.0506932
63799	1.89226	0.0572635
63800	0.946131	0.05
63801	0.946131	0.05
63802	0.946128	0.05
63803	0.946128	0.05
63804	0.94613	0.05
63805	0.94613	0.05
63806	0.94613	0.05
63807	0.94613	0.05
63808	0.94613	0.05
63809	0.94613	0.05
63810	0.94613	0.05
63811	0.94613	0.05
63812	1.89226	0.05
63813	1.89226	0.05
63814	1.89226	0.05
63815	1.89226	0.05
63816	1.89226	0.05
63817	1.89226	0.05
63818	0.924614	0.05
63819	0.914275	0.05
63820	0.906003	0.05
63821	0.62479	0.05
63822	2.02733	0.05
63823	1.48332	0.05
63824	0.833106	0.05
63825	1.19364	0.05
63826	1.57482	0.05
63827	1.18103	0.05
63828	0.905988	0.05
63829	1.89226	2.1
63830	1.89226	2.1
63831	1.89226	2.1
63832	1.89226	2.1
63833	3.78452	2.1
63834	1.89226	2.1
63835	1.89226	2.1
63836	1.89226	2.1
63837	1.89226	2.1
63838	1.89226	2.1
63839	1.89226	2.1
63840	0.816805	2.1
63841	0.928516	2.1
63842	1.4045	2.1
63843	1.26268	2.1
63844	1.0881	2.1
63845	1.0881	2.1
63846	1.10309	2.1
63847	1.10309	2.1
63848	0.928518	2.1
63849	1.4045	2.1
63850	0.816807	2.1
63851	1.26267	2.1
63852	1.89226	2.1
63853	2.52535	2.1
63854	1.89226	2.1
63855	1.89226	2.1

63856	2.809	2.1
63857	2.809	2.1
63858	1.89226	2.1
63859	1.89226	2.1
63860	1.89226	2.1
63861	1.89226	2.1
63862	3.78452	2.1
63863	0.946127	2.1
63864	0.946127	2.1
63865	0.946131	2.1
63866	0.946131	2.1
63867	0.94613	2.1
63868	0.94613	2.1
63869	0.946128	2.1
63870	0.946128	2.1
63871	0.946129	2.1
63872	0.946129	2.1
63873	0.946129	2.1
63874	0.946129	2.1
63875	1.89224	2.1
63876	1.89224	2.1
63877	1.89224	2.1
63878	1.89224	2.1
63879	3.78448	2.1
63880	2.39244	2.1
63881	2.27036	2.1
63882	2.25945	2.1
63883	2.27398	2.1
63884	4.52981	2.1
63885	2.27036	2.1
63886	1.97088	2.1
63887	1.86302	2.1
63888	1.84991	2.1
63889	4.12027	2.1
63890	1.89224	2.1
63891	1.89224	2.1
63892	1.89224	2.1
63893	1.89224	2.1
63894	3.78447	2.1
63895	1.89226	2.1
63896	1.89226	2.1
63897	1.89226	2.1
63898	1.89226	2.1
63899	3.78453	2.1
63900	2.25948	2.1
63901	2.33611	2.1
63902	2.05257	2.1
63903	1.94441	2.1
63904	4.20389	2.1
63905	1.92514	2.1
63906	1.84993	2.1
63907	1.94441	2.1
63908	1.74391	2.1
63909	3.79434	2.1
63910	1.89226	2.1
63911	1.89226	2.1
63912	1.89226	2.1
63913	1.89226	2.1

63914	3.78452	2.1
63915	15.1381	1.08017
63916	15.1381	0.978143
63917	15.1381	0.18717
63918	15.1381	1.39412
63919	30.2762	0.569581
63920	15.1381	0.940772
63921	15.1381	1.39734
63922	15.1381	0.681795
63923	15.1381	0.949487
63924	30.2762	0.996682
63925	15.1381	1.99156
63926	15.1381	1.97866
63927	15.1381	1.70255
63928	15.1381	2.08879
63929	30.2762	2.08523
63930	15.1381	0.505592
63931	15.1381	1.14214
63932	15.1381	0.668403
63933	15.1381	0.757712
63934	30.2762	0.626753
63935	1.89225	2.1
63936	1.89225	2.1
63937	1.89225	2.1
63938	1.89225	2.1
63939	3.78449	2.1
63940	1.39909	2.1
63941	1.39909	2.1
63942	0.946123	2.1
63943	0.946123	2.1
63944	1.3291	2.1
63945	1.39908	2.1
63946	0.946126	2.1
63947	0.946126	2.1
63948	0.946118	2.1
63949	1.32909	2.1
63950	0.94613	2.1
63951	1.3991	2.1
63952	3.07801	2.1
63953	1.84322	2.1
63954	1.84322	2.1
63955	1.68788	2.1
63956	2.6582	2.1
63957	2.50287	2.1
63958	1.89225	2.1
63959	1.89225	2.1
63960	1.89225	2.1
63961	1.89225	2.1
63962	3.7845	2.1
63963	1.89226	2.1
63964	1.89226	2.1
63965	1.89226	2.1
63966	1.89226	2.1
63967	3.78452	2.1
63968	1.89226	2.1
63969	2.50051	2.1
63970	2.50051	2.1
63971	2.65822	2.1

63972	1.89226	2.1
63973	2.04997	2.1
63974	2.50052	2.1
63975	2.65822	2.1
63976	1.70377	2.1
63977	1.70377	2.1
63978	1.84324	2.1
63979	2.63998	2.1
63980	0.810947	2.1
63981	0.810947	2.1
63982	0.810946	2.1
63983	0.810946	2.1
63984	1.89226	2.1
63985	1.35169	2.1
63986	1.08132	2.1
63987	1.08132	2.1
63988	1.08132	2.1
63989	0.810946	2.1
63990	0.810946	2.1
63991	1.89226	2.1
63992	1.89226	2.1
63993	1.89226	2.1
63994	1.89226	2.1
63995	3.78453	2.1
63996	0.946164	2.1
63997	0.946164	2.1
63998	0.929842	2.1
63999	0.929842	2.1
64000	0.946132	2.1
64001	0.946132	2.1
64002	1.04101	2.1
64003	0.929874	2.1
64004	0.946132	2.1
64005	0.929874	2.1
64006	1.04101	2.1
64007	0.946132	2.1
64008	1.89226	2.1
64009	1.89226	2.1
64010	1.89226	2.1
64011	1.89226	2.1
64012	3.78453	2.1
64013	1.89226	2.1
64014	1.89226	2.1
64015	1.89226	2.1
64016	1.89226	2.1
64017	1.89226	2.1
64018	1.89226	2.1
64019	1.89226	2.1
64020	1.89226	2.1
64021	1.89226	2.1
64022	1.89226	2.1
64023	1.89226	2.1
64024	1.89226	2.1
64025	0.946131	2.1
64026	0.946131	2.1
64027	0.946131	2.1
64028	0.946131	2.1
64029	0.946132	2.1

64030	0.946132	2.1
64031	0.946131	2.1
64032	0.946131	2.1
64033	0.946131	2.1
64034	0.946131	2.1
64035	0.946132	2.1
64036	0.946132	2.1
64037	1.89226	1.92459
64038	1.89226	0.925441
64039	1.89226	0.640756
64040	1.89226	1.81236
64041	3.78453	0.750468
64042	2.73497	0.565084
64043	1.93823	2.1
64044	1.93823	0.931432
64045	2.20046	2.1
64046	2.42279	1.66592
64047	2.68501	2.1
64048	1.89226	0.746462
64049	1.89226	2.1
64050	1.89226	2.1
64051	1.89226	1.10814
64052	1.89226	2.1
64053	1.89226	0.642855
64054	1.89226	0.741307
64055	1.89226	0.455761
64056	1.89226	0.539526
64057	1.89226	1.74946
64058	1.89226	0.626491
64059	1.89226	0.500996
64060	2.82984	1.76742
64061	2.15276	0.6491
64062	2.15276	1.2664
64063	2.42278	0.05
64064	2.50074	0.602428
64065	2.77077	0.551699
64066	2.42279	0.43597
64067	1.93823	2.1
64068	1.70423	0.765349
64069	1.70423	2.1
64070	2.15276	0.98427
64071	2.38676	2.1
64072	0.810946	1.35265
64073	0.810946	2.1
64074	0.810944	0.599632
64075	0.810944	0.535391
64076	0.810946	1.55518
64077	0.810946	1.81807
64078	1.89226	2.1
64079	1.35169	1.66466
64080	1.08132	1.47437
64081	1.08132	2.1
64082	1.08132	1.80385
64083	1.89226	2.1
64084	1.89226	2.1
64085	1.89226	2.1
64086	1.89226	2.1
64087	3.78452	2.1

64088	1.89226	2.1
64089	1.89226	2.1
64090	1.89226	2.1
64091	1.89226	2.1
64092	3.78453	2.1
64093	1.04144	2.1
64094	1.04144	2.1
64095	0.938972	2.1
64096	0.938972	2.1
64097	0.969661	2.1
64098	1.03285	2.1
64099	0.946133	2.1
64100	0.946133	2.1
64101	0.946131	2.1
64102	0.969663	2.1
64103	0.946131	2.1
64104	1.03284	2.1
64105	0.946131	2.1
64106	0.946131	2.1
64107	0.946128	2.1
64108	0.946128	2.1
64109	0.946127	2.1
64110	0.946127	2.1
64111	0.946131	2.1
64112	0.946131	2.1
64113	0.94613	2.1
64114	0.94613	2.1
64115	0.946129	2.1
64116	0.946129	2.1
64117	1.89226	2.1
64118	1.89226	2.1
64119	1.89226	2.1
64120	1.89226	2.1
64121	3.78452	2.1
64122	1.89226	2.1
64123	1.89226	2.1
64124	1.89226	2.1
64125	1.89226	2.1
64126	3.78453	2.1
64127	1.89226	2.1
64128	1.89226	2.1
64129	1.85882	2.1
64130	1.85882	2.1
64131	1.93932	2.1
64132	1.97276	2.1
64133	0.94613	2.1
64134	0.94613	2.1
64135	0.946128	2.1
64136	0.946128	2.1
64137	0.946127	2.1
64138	0.946127	2.1
64139	0.946131	2.1
64140	0.946131	2.1
64141	0.946129	2.1
64142	0.946129	2.1
64143	0.946129	2.1
64144	0.946129	2.1
64145	1.89225	2.1

64146	1.89225	2.1
64147	1.89225	2.1
64148	1.89225	2.1
64149	3.7845	2.1
64150	1.0086	2.1
64151	1.09028	2.1
64152	0.946125	2.1
64153	0.946125	2.1
64154	0.916906	2.1
64155	0.916906	2.1
64156	1.13051	2.1
64157	1.1505	2.1
64158	1.11557	2.1
64159	1.04071	2.1
64160	0.851542	2.1
64161	0.991196	2.1
64162	1.89225	2.1
64163	1.89225	2.1
64164	1.89225	2.1
64165	1.89225	2.1
64166	3.7845	2.1
64167	1.89225	2.1
64168	1.89225	2.1
64169	1.89225	2.1
64170	1.89225	2.1
64171	1.89225	2.1
64172	1.89225	2.1
64173	1.89226	2.1
64174	1.89226	2.1
64175	1.89226	2.1
64176	1.89226	2.1
64177	3.78452	2.1
64178	0.890962	2.1
64179	0.952547	2.1
64180	0.946132	2.1
64181	0.946132	2.1
64182	0.851543	2.1
64183	0.851543	2.1
64184	1.11558	2.1
64185	1.06304	2.1
64186	0.994315	2.1
64187	1.04072	2.1
64188	0.900943	2.1
64189	0.851542	2.1
64190	1.89226	2.1
64191	1.89226	2.1
64192	1.89226	2.1
64193	1.89226	2.1
64194	1.89226	2.1
64195	1.89226	2.1
64196	1.89226	2.1
64197	1.89226	2.1
64198	1.89226	2.1
64199	1.89226	2.1
64200	3.78452	2.1
64201	1.89226	2.1
64202	1.89226	2.1
64203	1.89226	2.1

64204	1.89226	2.1
64205	3.78452	2.1
64206	0.782015	2.1
64207	0.929718	2.1
64208	0.946132	2.1
64209	0.946132	2.1
64210	0.782016	2.1
64211	0.782016	2.1
64212	1.06706	2.1
64213	1.11785	2.1
64214	0.782015	2.1
64215	0.946133	2.1
64216	0.878931	2.1
64217	0.946132	2.1
64218	1.89226	2.1
64219	1.89226	2.1
64220	1.89226	2.1
64221	1.89226	2.1
64222	1.89226	2.1
64223	1.89226	2.1
64224	0.946131	2.1
64225	0.946131	2.1
64226	0.946129	2.1
64227	0.946129	2.1
64228	0.946131	2.1
64229	0.946131	2.1
64230	0.94613	2.1
64231	0.94613	2.1
64232	0.946131	2.1
64233	0.946131	2.1
64234	0.94613	2.1
64235	0.94613	2.1
64236	0.827866	2.1
64237	0.946131	2.1
64238	0.946129	2.1
64239	0.946129	2.1
64240	0.94613	2.1
64241	0.94613	2.1
64242	0.94613	2.1
64243	0.94613	2.1
64244	0.827865	2.1
64245	0.94613	2.1
64246	0.94613	2.1
64247	0.94613	2.1
64248	1.89226	2.1
64249	1.89226	2.1
64250	1.89226	2.1
64251	1.89226	2.1
64252	3.78452	2.1
64253	1.89226	2.1
64254	1.89226	2.1
64255	1.89226	2.1
64256	1.89226	2.1
64257	1.89226	2.1
64258	1.89226	2.1
64259	1.89226	2.1
64260	1.89226	2.1
64261	1.89226	2.1

64262	1.89226	2.1
64263	3.78453	2.1
64264	1.89226	2.1
64265	1.89226	2.1
64266	1.89226	2.1
64267	1.89226	2.1
64268	3.78452	2.1
64269	0.744377	2.1
64270	0.541919	2.1
64271	0.652022	2.1
64272	0.544198	2.1
64273	0.764633	2.1
64274	0.673254	2.1
64275	1.89226	2.1
64276	1.31149	2.1
64277	1.05423	2.1
64278	1.02189	2.1
64279	0.870602	2.1
64280	1.89226	2.1
64281	1.89226	2.1
64282	1.89226	2.1
64283	1.89226	2.1
64284	1.89226	2.1
64285	1.89226	2.1
64286	1.89226	2.1
64287	1.89226	2.1
64288	1.89226	2.1
64289	1.89226	2.1
64290	1.89226	2.1
64291	1.89226	2.1
64292	1.89226	2.1
64293	1.89226	2.1
64294	1.89226	2.1
64295	1.89226	2.1
64296	1.89226	2.1
64297	1.89226	2.1
64298	0.815314	2.1
64299	0.815313	2.1
64300	0.714029	2.1
64301	0.782951	2.1
64302	0.810944	2.1
64303	0.782954	2.1
64304	1.89226	2.1
64305	1.30999	2.1
64306	1.08132	2.1
64307	1.03962	2.1
64308	0.95209	2.1
64309	1.89226	2.1
64310	1.89226	2.1
64311	1.89226	2.1
64312	1.89226	2.1
64313	1.89226	2.1
64314	1.89226	2.1
64315	1.89226	2.1
64316	1.89226	2.1
64317	1.89226	2.1
64318	1.89226	2.1
64319	1.89226	2.1

64320	1.89226	2.1
64321	1.89226	2.1
64322	1.89226	2.1
64323	1.89226	2.1
64324	1.89226	2.1
64325	1.89226	2.1
64326	1.89226	2.1
64327	0.871558	2.1
64328	0.713078	2.1
64329	0.756858	2.1
64330	0.703674	2.1
64331	0.838025	2.1
64332	0.862196	2.1
64333	1.89226	2.1
64334	1.33194	2.1
64335	1.10834	2.1
64336	1.06163	2.1
64337	1.01785	2.1
64338	1.89226	2.1
64339	1.89226	2.1
64340	1.89226	2.1
64341	1.89226	2.1
64342	3.78452	2.1
64343	1.89226	2.1
64344	1.89226	2.1
64345	1.89226	2.1
64346	1.89226	2.1
64347	1.89226	2.1
64348	1.89226	2.1
64349	1.89226	2.1
64350	1.89226	2.1
64351	1.89226	2.1
64352	1.89226	2.1
64353	1.89226	2.1
64354	1.89226	2.1
64355	0.958406	2.1
64356	1.1383	2.1
64357	0.865636	2.1
64358	1.10093	2.1
64359	0.838024	2.1
64360	0.975154	2.1
64361	1.89226	2.1
64362	1.38553	2.1
64363	1.10834	2.1
64364	1.11522	2.1
64365	1.15271	2.1
64366	1.89227	2.1
64367	1.89227	2.1
64368	1.89227	2.1
64369	1.89227	2.1
64370	3.78453	2.1
64371	0.946132	2.1
64372	0.946132	2.1
64373	0.946132	2.1
64374	0.946132	2.1
64375	0.946132	2.1
64376	0.946132	2.1
64377	0.946133	2.1

64378	0.946133	2.1
64379	0.946132	2.1
64380	0.946132	2.1
64381	0.946133	2.1
64382	0.946133	2.1
64383	0.719271	2.1
64384	0.774968	2.1
64385	1.28401	2.1
64386	1.17415	2.1
64387	0.960999	2.1
64388	0.960999	2.1
64389	0.988115	2.1
64390	0.988115	2.1
64391	0.774968	2.1
64392	1.28401	2.1
64393	0.719271	2.1
64394	1.17415	2.1
64395	2.77811	2.1
64396	2.34829	2.1
64397	2.56802	2.1
64398	2.52333	2.1
64399	2.56802	2.1
64400	2.52333	2.1
64401	1.89227	2.1
64402	1.89227	2.1
64403	1.89227	2.1
64404	1.89227	2.1
64405	3.78453	2.1
64406	1.89226	2.1
64407	1.89226	2.1
64408	1.89226	2.1
64409	1.89226	2.1
64410	1.89226	2.1
64411	1.89226	2.1
64412	1.89226	2.1
64413	2.77811	2.1
64414	1.89226	2.1
64415	2.56802	2.1
64416	1.89226	2.1
64417	2.56802	2.1
64418	1.89226	1.79195
64419	1.89226	2.1
64420	1.89226	1.53985
64421	1.89226	2.1
64422	3.78452	2.1
64423	2.41802	2.1
64424	1.95764	2.1
64425	1.95764	2.1
64426	2.27727	2.1
64427	2.39311	2.1
64428	2.73961	2.1
64429	0.946132	2.1
64430	0.946132	2.1
64431	0.94613	2.1
64432	0.94613	2.1
64433	0.946131	2.1
64434	0.946131	2.1
64435	0.946131	2.1

64436	0.946131	2.1
64437	0.946131	2.1
64438	0.946131	2.1
64439	0.946131	2.1
64440	0.946131	2.1
64441	1.89226	1.78816
64442	1.89226	2.1
64443	1.89226	2.1
64444	1.89226	1.74177
64445	1.89226	2.1
64446	1.89226	1.53171
64447	2.78856	0.85027
64448	2.28552	2.1
64449	2.28552	1.63774
64450	2.3931	2.1
64451	2.6402	1.37663
64452	2.74778	2.1
64453	2.39311	2.1
64454	1.95764	2.1
64455	2.28552	2.1
64456	1.86103	2.1
64457	4.24316	2.1
64458	1.89226	2.1
64459	1.89226	2.1
64460	1.89226	2.1
64461	1.89226	2.1
64462	1.89226	2.1
64463	1.89226	2.1
64464	1.47412	2.07245
64465	2.18862	1.86612
64466	2.07226	1.52403
64467	2.33645	1.6909
64468	4.26088	1.59042
64469	1.89226	1.5535
64470	1.89226	1.90404
64471	1.89226	1.58026
64472	1.89226	1.81574
64473	1.89226	1.71155
64474	1.89226	2.1
64475	1.89226	1.71338
64476	1.89226	2.1
64477	1.89226	0.276588
64478	1.89226	2.1
64479	3.78453	1.87691
64480	2.18862	1.6999
64481	2.1128	2.1
64482	2.33645	1.51732
64483	2.28188	2.1
64484	4.47051	2.1
64485	2.07226	2.1
64486	2.33645	2.01445
64487	2.20831	1.61
64488	2.4646	1.42518
64489	4.53686	1.5387
64490	0.946131	1.55145
64491	0.946131	2.1
64492	0.946131	2.1
64493	0.946131	1.57661

64494	0.94613	2.03831
64495	0.94613	1.47915
64496	0.946133	2.02776
64497	0.946133	2.1
64498	0.946132	1.43946
64499	0.946132	1.31844
64500	0.946131	1.92254
64501	0.946131	1.79031
64502	1.89226	1.77631
64503	1.89226	2.1
64504	1.89226	1.21255
64505	1.89226	2.1
64506	3.78453	1.90173
64507	2.33646	1.74762
64508	2.28189	2.1
64509	2.4646	1.0502
64510	2.42758	2.1
64511	4.74648	1.87009
64512	1.89226	2.1
64513	1.89226	2.1
64514	1.89226	2.1
64515	1.89226	2.1
64516	1.89226	2.1
64517	1.89226	2.1
64518	1.89226	2.1
64519	1.89226	2.1
64520	1.89226	2.1
64521	1.89226	2.1
64522	1.89226	2.1
64523	1.89226	2.1
64524	1.89226	2.1
64525	1.89226	2.1
64526	1.89226	2.1
64527	1.89226	2.1
64528	3.78453	2.1
64529	1.89226	2.1
64530	1.89226	2.1
64531	1.89226	2.1
64532	1.89226	2.1
64533	1.89226	2.1
64534	1.89226	2.1
64535	1.89226	2.1
64536	1.89226	2.1
64537	1.89226	2.1
64538	1.89226	2.1
64539	3.78452	2.1
64540	0.877238	2.1
64541	0.877238	2.1
64542	1.24746	2.1
64543	1.04764	2.1
64544	0.964969	2.1
64545	1.1521	2.1
64546	0.946132	2.1
64547	0.946132	2.1
64548	0.946132	2.1
64549	1.12659	2.1
64550	0.964969	2.1
64551	0.946131	2.1

64552	1.89226	2.1
64553	1.89226	2.1
64554	1.89226	2.1
64555	1.89226	2.1
64556	1.89226	2.1
64557	1.89226	2.1
64558	1.89226	2.1
64559	1.89226	2.1
64560	1.89226	2.1
64561	1.89226	2.1
64562	3.78453	2.1
64563	0.868144	2.1
64564	0.992762	2.1
64565	0.946131	2.1
64566	0.946131	2.1
64567	1.07072	2.1
64568	1.07072	2.1
64569	0.868171	2.1
64570	0.868171	2.1
64571	0.992762	2.1
64572	0.946131	2.1
64573	0.946131	2.1
64574	0.868144	2.1
64575	0.946132	2.1
64576	0.946132	2.1
64577	0.946131	2.1
64578	0.946131	2.1
64579	0.946132	2.1
64580	0.946132	2.1
64581	0.946132	2.1
64582	0.946132	2.1
64583	0.946132	2.1
64584	0.946132	2.1
64585	0.946132	2.1
64586	0.946132	2.1
64587	1.89227	2.1
64588	1.89227	2.1
64589	1.89227	2.1
64590	1.89227	2.1
64591	1.89227	2.1
64592	1.89227	2.1
64593	1.89226	2.1
64594	1.89226	2.1
64595	1.89226	2.1
64596	1.89226	2.1
64597	1.89226	2.1
64598	1.89226	2.1
64599	1.98552	2.1
64600	2.22298	2.1
64601	2.22298	2.1
64602	1.89226	2.1
64603	1.89226	2.1
64604	1.54525	2.1
64605	0.810946	2.1
64606	0.810946	2.1
64607	0.810947	2.1
64608	0.810947	2.1
64609	0.81096	2.1

64610	0.81096	2.1
64611	1.89226	2.1
64612	1.35168	2.1
64613	1.08132	2.1
64614	1.08132	2.1
64615	1.0813	2.1
64616	1.89226	2.1
64617	1.89226	2.1
64618	1.89226	2.1
64619	1.89226	2.1
64620	3.78452	2.1
64621	1.7753	2.1
64622	2.39703	2.1
64623	1.90577	2.1
64624	2.03445	2.1
64625	3.82335	2.1
64626	2.39703	2.1
64627	2.47949	2.1
64628	2.26051	2.1
64629	2.36891	2.1
64630	4.76594	2.1
64631	1.89226	2.1
64632	1.89226	2.1
64633	1.89226	2.1
64634	1.89226	2.1
64635	3.78452	2.1
64636	1.89226	2.1
64637	1.89226	2.1
64638	1.89226	2.1
64639	1.89226	2.1
64640	1.89226	2.1
64641	1.89226	2.1
64642	0.883745	2.1
64643	0.973143	2.1
64644	0.988173	2.1
64645	0.988173	2.1
64646	0.941927	2.1
64647	1.02971	2.1
64648	0.854065	2.1
64649	0.82702	2.1
64650	0.847835	2.1
64651	0.981126	2.1
64652	1.02996	2.1
64653	1.10704	2.1
64654	2.28031	2.1
64655	2.26369	2.1
64656	2.26369	2.1
64657	2.36891	2.1
64658	2.30788	2.1
64659	2.42455	2.1
64660	1.89226	2.1
64661	1.89226	2.1
64662	1.89226	2.02249
64663	1.89226	2.1
64664	1.89226	2.07709
64665	1.89226	2.1
64666	2.71406	2.1
64667	1.83244	2.1

64668	1.83244	2.1
64669	2.07567	2.1
64670	2.52044	2.1
64671	2.76367	2.1
64672	1.89226	2.1
64673	1.89226	2.1
64674	1.89226	2.1
64675	1.89226	2.1
64676	3.78453	2.1
64677	1.89226	2.1
64678	1.89226	2.1
64679	1.89226	2.1
64680	1.89226	2.1
64681	1.89226	2.1
64682	1.89226	2.1
64683	2.70714	2.1
64684	2.3148	2.1
64685	2.3148	2.1
64686	2.52044	2.1
64687	2.54204	2.1
64688	2.74768	2.1
64689	2.52044	2.1
64690	2.3148	2.1
64691	1.83244	2.1
64692	1.83244	2.1
64693	1.62611	2.1
64694	2.3148	2.1
64695	0.946131	2.1
64696	0.946131	2.1
64697	0.946131	2.1
64698	0.946131	2.1
64699	0.946131	2.1
64700	0.946131	2.1
64701	0.946131	2.1
64702	0.946131	2.1
64703	0.946131	2.1
64704	0.946131	2.1
64705	0.946131	2.1
64706	0.946131	2.1
64707	1.89226	2.1
64708	1.89226	2.1
64709	1.89226	2.1
64710	1.89226	2.1
64711	3.78453	2.1
64712	1.89226	2.1
64713	1.89226	2.1
64714	1.89226	2.1
64715	1.89226	2.1
64716	1.89226	2.1
64717	1.89226	2.1
64718	1.89227	2.1
64719	1.89227	2.1
64720	1.89227	2.1
64721	1.89227	2.1
64722	3.78453	2.1
64723	1.89226	2.1
64724	1.89226	2.1
64725	1.89226	2.1

64726	1.89226	2.1
64727	3.78453	2.1
64728	0.915781	2.1
64729	0.863524	2.1
64730	0.717056	2.1
64731	0.830182	2.1
64732	0.865094	2.1
64733	0.918003	2.1
64734	1.89226	2.1
64735	1.29933	2.1
64736	1.11215	2.1
64737	1.05227	2.1
64738	0.966269	2.1
64739	1.89226	2.1
64740	1.89226	2.1
64741	1.89226	2.1
64742	1.89226	2.1
64743	1.89226	2.1
64744	1.89226	2.1
64745	1.89226	2.1
64746	1.89226	2.1
64747	1.89226	2.1
64748	1.89226	2.1
64749	1.89226	2.1
64750	1.89226	2.1
64751	1.89226	2.1
64752	1.89226	2.1
64753	1.89226	2.1
64754	1.89226	2.1
64755	1.89226	2.1
64756	1.89226	2.1
64757	1.01725	2.1
64758	0.997048	2.1
64759	0.818657	2.1
64760	0.960724	2.1
64761	0.838013	2.1
64762	1.04859	2.1
64763	1.89226	2.1
64764	1.41754	2.1
64765	1.17625	2.1
64766	1.0793	2.1
64767	1.18822	2.1
64768	2.16402	2.1
64769	1.89452	2.1
64770	1.50084	2.1
64771	2.09288	2.1
64772	3.9874	2.1
64773	2.16402	2.1
64774	2.13925	2.1
64775	2.04483	2.1
64776	2.09288	2.1
64777	4.23212	2.1
64778	1.89226	2.1
64779	1.89226	2.1
64780	1.89226	2.1
64781	1.89226	2.1
64782	3.78452	2.1
64783	1.89226	2.1

64784	1.89226	2.1
64785	1.89226	2.1
64786	1.89226	2.1
64787	1.89226	2.1
64788	1.89226	2.1
64789	1.9824	2.1
64790	2.09288	2.1
64791	1.69889	2.1
64792	1.6817	2.1
64793	2.00695	2.1
64794	2.00695	2.1
64795	1.93592	2.1
64796	2.09288	2.1
64797	2.00695	2.1
64798	2.09288	2.1
64799	1.9589	2.1
64800	2.04483	2.1
64801	0.946128	2.1
64802	0.946128	2.1
64803	0.946132	2.1
64804	0.946132	2.1
64805	0.94613	2.1
64806	0.94613	2.1
64807	0.946129	2.1
64808	0.946129	2.1
64809	0.94613	2.1
64810	0.94613	2.1
64811	0.94613	2.1
64812	0.94613	2.1
64813	0.946131	2.1
64814	0.946131	2.1
64815	0.946131	2.1
64816	0.946131	2.1
64817	0.946131	2.1
64818	0.946131	2.1
64819	0.946131	2.1
64820	0.946131	2.1
64821	0.946131	2.1
64822	0.946131	2.1
64823	0.946131	2.1
64824	0.946131	2.1
64825	1.89226	2.1
64826	2.48083	2.1
64827	2.6968	2.1
64828	2.6968	2.1
64829	1.89226	2.1
64830	1.89226	2.1
64831	1.89226	2.1
64832	1.89226	2.1
64833	1.89226	2.1
64834	1.89226	2.1
64835	3.78453	2.1
64836	1.89226	2.1
64837	1.89226	2.1
64838	1.89226	2.1
64839	1.89226	2.1
64840	1.89226	2.1
64841	1.89226	2.1

64842	1.1017	2.1
64843	1.1017	2.1
64844	1.10605	2.1
64845	1.01647	2.1
64846	0.868255	2.1
64847	0.883424	2.1
64848	1.24624	2.1
64849	1.35473	2.1
64850	0.942231	2.1
64851	1.31464	2.1
64852	0.910064	2.1
64853	1.27147	2.1
64854	1.89226	2.1
64855	1.89226	2.1
64856	1.89226	2.1
64857	1.89226	2.1
64858	3.78453	2.1
64859	1.89226	2.1
64860	1.89226	2.1
64861	1.89226	2.1
64862	1.89226	2.1
64863	1.89226	2.1
64864	1.89226	2.1
64865	1.89226	2.1
64866	1.89226	2.1
64867	1.89226	2.1
64868	1.89226	2.1
64869	3.78453	2.1
64870	1.89226	2.1
64871	2.70015	2.1
64872	1.89226	2.1
64873	2.61064	2.1
64874	4.59241	2.1
64875	2.70015	2.1
64876	2.3372	2.1
64877	2.61064	2.1
64878	1.85184	2.1
64879	4.55198	2.1
64880	1.89226	2.1
64881	1.89226	2.1
64882	1.89226	2.1
64883	1.89226	2.1
64884	1.89226	2.1
64885	1.89226	2.1
64886	1.89227	2.1
64887	1.89227	2.1
64888	1.89227	2.1
64889	1.89227	2.1
64890	3.78453	2.1
64891	1.89226	2.1
64892	2.52194	2.1
64893	2.61064	2.1
64894	2.61064	2.1
64895	1.89226	2.1
64896	1.89226	2.1
64897	1.095 2.1	
64898	1.095 2.1	
64899	1.09189	2.1

64900	1.09189	2.1
64901	0.875334	2.1
64902	0.925919	2.1
64903	1.26097	2.1
64904	1.30532	2.1
64905	1.26097	2.1
64906	0.875334	2.1
64907	1.30532	2.1
64908	0.92592	2.1
64909	1.89226	2.1
64910	1.89226	2.1
64911	1.89226	2.1
64912	1.89226	2.1
64913	1.89226	2.1
64914	1.89226	2.1
64915	1.89226	2.1
64916	1.89226	2.1
64917	1.89226	2.1
64918	1.89226	2.1
64919	3.78453	2.1
64920	1.26097	2.1
64921	1.26097	2.1
64922	0.946132	2.1
64923	0.946132	2.1
64924	1.22176	2.1
64925	1.26097	2.1
64926	0.946132	2.1
64927	0.946132	2.1
64928	0.946132	2.1
64929	1.22176	2.1
64930	1.26097	2.1
64931	0.946132	2.1
64932	2.52194	2.1
64933	1.67381	2.1
64934	1.67381	2.1
64935	1.75067	2.1
64936	2.44352	2.1
64937	2.52038	2.1
64938	1.89226	2.1
64939	1.89226	2.1
64940	1.89226	2.1
64941	1.89226	2.1
64942	1.89226	2.1
64943	1.89226	2.1
64944	1.89227	2.1
64945	1.89227	2.1
64946	1.89227	2.1
64947	1.89227	2.1
64948	1.89227	2.1
64949	1.89227	2.1
64950	1.22176	2.1
64951	1.22176	2.1
64952	0.946132	2.1
64953	0.946132	2.1
64954	1.19095	2.1
64955	1.22176	2.1
64956	0.946133	2.1
64957	0.946133	2.1

64958	1.19095	2.1
64959	0.946132	2.1
64960	0.946133	2.1
64961	1.22176	2.1
64962	2.44352	2.1
64963	1.6287	2.1
64964	1.6287	2.1
64965	1.67381	2.1
64966	2.3819	2.1
64967	2.427	2.1
64968	1.89226	2.1
64969	1.89226	2.1
64970	1.89226	2.1
64971	1.89226	2.1
64972	1.89226	2.1
64973	1.89226	2.1
64974	1.89226	2.1
64975	1.89226	2.1
64976	1.89226	2.1
64977	1.89226	2.1
64978	3.78453	2.1
64979	1.89226	2.1
64980	1.89226	2.1
64981	1.89226	2.1
64982	1.89226	2.1
64983	1.89226	2.1
64984	1.89226	2.1
64985	1.89226	2.1
64986	1.89226	2.1
64987	1.89226	2.1
64988	1.89226	2.1
64989	1.89226	2.1
64990	1.89226	2.1
64991	0.851542	2.1
64992	0.851542	2.1
64993	1.1736	2.1
64994	1.29261	2.1
64995	0.852708	2.1
64996	1.18843	2.1
64997	0.946132	2.1
64998	0.946132	2.1
64999	0.946132	2.1
65000	1.07901	2.1
65001	1.04208	2.1
65002	0.936669	2.1
65003	1.89227	2.1
65004	1.89227	2.1
65005	1.89227	2.1
65006	1.89227	2.1
65007	3.78453	2.1
65008	1.89226	2.1
65009	1.89226	2.1
65010	1.89226	2.1
65011	1.89226	2.1
65012	3.78453	2.1
65013	1.89226	2.1
65014	1.89226	2.1
65015	1.89226	2.1

65016	1.89226	2.1
65017	1.89226	2.1
65018	1.89226	2.1
65019	0.946133	2.1
65020	0.946133	2.1
65021	1.28525	2.1
65022	1.28525	2.1
65023	1.07901	2.1
65024	1.28525	2.1
65025	0.946132	2.1
65026	0.946132	2.1
65027	0.946132	2.1
65028	1.28525	2.1
65029	0.946133	2.1
65030	1.07901	2.1
65031	1.89226	2.1
65032	1.89226	2.1
65033	1.89226	2.1
65034	1.89226	2.1
65035	1.89226	2.1
65036	1.89226	2.1
65037	1.89226	2.1
65038	1.89226	2.1
65039	1.89226	2.1
65040	1.89226	2.1
65041	1.89226	2.1
65042	1.89226	2.1
65043	1.24419	2.1
65044	1.09093	2.1
65045	1.22722	2.1
65046	1.18154	2.1
65047	1.27455	2.1
65048	1.08473	2.1
65049	1.15541	2.1
65050	1.15541	2.1
65051	1.29607	2.1
65052	1.23814	2.1
65053	1.13024	2.1
65054	1.12096	2.1
65055	1.18858	2.1
65056	1.13181	2.1
65057	0.988718	2.1
65058	0.802498	2.1
65059	0.994034	2.1
65060	1.15773	2.1
65061	1.0029	2.1
65062	1.0029	2.1
65063	1.23814	2.1
65064	0.997523	2.1
65065	1.11498	2.1
65066	0.899532	2.1
65067	1.89226	2.1
65068	1.89226	2.1
65069	1.89226	2.1
65070	1.89226	2.1
65071	1.89226	2.1
65072	1.89226	2.1
65073	1.89226	2.1

65074	1.89226	2.1
65075	1.89226	2.1
65076	1.89226	2.1
65077	1.89226	2.1
65078	1.89226	2.1
65079	0.993139	2.1
65080	0.834108	2.1
65081	1.01147	2.1
65082	1.01358	2.1
65083	1.20067	2.1
65084	0.993156	2.1
65085	2.4158	2.1
65086	1.69956	2.1
65087	1.3544	2.1
65088	1.60095	2.1
65089	1.11219	2.1
65090	1.89226	2.1
65091	1.89226	2.1
65092	1.89226	2.1
65093	1.89226	2.1
65094	3.78452	2.1
65095	1.89226	2.1
65096	1.89226	2.1
65097	1.89226	2.1
65098	1.89226	2.1
65099	1.89226	2.1
65100	1.89226	2.1
65101	1.89226	2.1
65102	2.83014	2.1
65103	2.32404	2.1
65104	2.32404	2.1
65105	1.89226	2.1
65106	1.89226	2.1
65107	1.37966	2.1
65108	1.13294	2.1
65109	0.881723	2.1
65110	0.582304	2.1
65111	0.977944	2.1
65112	0.977948	2.1
65113	1.0264	2.1
65114	1.0264	2.1
65115	1.37967	2.1
65116	1.03896	2.1
65117	1.19108	2.1
65118	0.582298	2.1
65119	1.89226	2.1
65120	1.89226	2.1
65121	1.89226	2.1
65122	1.89226	2.1
65123	3.78452	2.1
65124	0.946131	2.1
65125	0.946131	2.1
65126	0.946131	2.1
65127	0.946131	2.1
65128	0.946132	2.1
65129	0.946132	2.1
65130	0.946129	2.1
65131	0.946129	2.1

65132	0.946131	2.1
65133	0.946131	2.1
65134	0.94613	2.1
65135	0.94613	2.1
65136	1.89226	2.1
65137	1.89226	2.1
65138	1.89226	2.1
65139	1.89226	2.1
65140	3.78453	2.1
65141	1.89226	2.1
65142	1.89226	2.1
65143	1.89226	2.1
65144	1.89226	2.1
65145	1.89226	2.1
65146	1.89226	2.1
65147	2.65216	2.1
65148	1.93528	2.1
65149	1.78595	2.1
65150	2.48544	2.1
65151	4.42072	2.1
65152	1.89226	2.1
65153	1.89226	2.1
65154	1.89226	2.1
65155	1.89226	2.1
65156	1.89226	2.1
65157	1.89226	2.1
65158	1.89226	2.1
65159	1.89226	2.1
65160	1.89226	2.1
65161	1.89226	2.1
65162	3.78452	2.1
65163	1.66537	2.1
65164	2.48544	2.1
65165	1.78595	2.1
65166	1.78595	2.1
65167	2.36197	2.1
65168	2.36197	2.1
65169	2.80975	2.1
65170	2.48544	2.1
65171	2.36198	2.1
65172	2.65135	2.1
65173	5.13679	2.1
65174	1.89226	2.1
65175	1.89226	2.1
65176	1.89226	2.1
65177	1.89226	2.1
65178	1.89226	2.1
65179	1.89226	2.1
65180	0.946127	2.1
65181	0.946127	2.1
65182	0.946131	2.1
65183	0.946131	2.1
65184	0.946128	2.1
65185	0.946128	2.1
65186	0.94613	2.1
65187	0.94613	2.1
65188	0.946129	2.1
65189	0.946129	2.1

65190	0.946129	2.1
65191	0.946129	2.1
65192	1.11972	2.1
65193	1.18099	2.1
65194	0.832687	2.1
65195	0.764517	2.1
65196	0.974426	2.1
65197	0.974426	2.1
65198	0.971078	2.1
65199	0.971078	2.1
65200	1.11972	2.1
65201	0.764515	2.1
65202	0.832686	2.1
65203	1.18099	2.1
65204	2.36198	2.1
65205	2.48025	2.1
65206	2.48025	2.1
65207	2.65135	2.1
65208	2.23944	2.1
65209	2.41055	2.1
65210	0.946132	2.1
65211	0.946132	2.1
65212	0.946131	2.1
65213	0.946131	2.1
65214	0.946131	2.1
65215	0.946131	2.1
65216	0.946132	2.1
65217	0.946132	2.1
65218	0.946131	2.1
65219	0.946131	2.1
65220	0.946132	2.1
65221	0.946132	2.1
65222	0.810942	2.1
65223	0.810942	2.1
65224	0.810944	2.1
65225	0.810944	2.1
65226	1.89226	2.1
65227	1.3517	2.1
65228	1.08132	2.1
65229	1.08133	2.1
65230	1.08132	2.1
65231	0.810932	2.1
65232	0.810932	2.1
65233	1.89249	2.1
65234	1.9857	1.75493
65235	2.02433	2.06233
65236	2.15807	1.91709
65237	4.01003	2.00729
65238	1.89226	1.82926
65239	1.89226	1.76775
65240	1.89226	2.1
65241	1.89226	1.3757
65242	3.78452	1.73947
65243	1.89226	1.70485
65244	1.89226	1.82387
65245	1.89226	1.1502
65246	1.89226	1.37006
65247	3.78452	1.30317

65248	1.98569	1.63571
65249	1.86067	1.69891
65250	2.15807	1.84018
65251	2.07965	1.37239
65252	4.06534	1.64536
65253	2.02433	2.1
65254	2.15807	2.1
65255	2.12608	2.1
65256	2.3025	1.6887
65257	4.32683	2.1
65258	1.89226	2.1
65259	1.89226	1.61321
65260	1.89226	1.81322
65261	1.89226	1.70976
65262	3.78452	1.80239
65263	1.89226	1.42822
65264	1.89226	1.59777
65265	1.89226	1.60442
65266	1.89226	1.50734
65267	3.78452	1.49225
65268	2.15807	2.1
65269	2.07965	1.58337
65270	2.3025	1.53519
65271	2.28384	1.55803
65272	4.38215	1.54127
65273	1.89226	2.1
65274	1.89226	0.622647
65275	1.89226	1.85232
65276	1.89226	0.631196
65277	3.78453	1.67649
65278	2.16905	0.439966
65279	2.56369	0.570402
65280	1.88394	1.11681
65281	2.41938	0.398974
65282	4.44763	0.376014
65283	2.56369	0.306767
65284	2.80663	1.06212
65285	2.41938	0.373432
65286	2.69045	0.379173
65287	5.25414	0.309768
65288	1.89226	0.31179
65289	1.89226	0.454977
65290	1.89226	0.567149
65291	1.89226	0.457692
65292	3.78453	0.120659
65293	1.89226	1.52055
65294	1.89226	0.791402
65295	1.89226	2.05978
65296	1.89226	1.43418
65297	1.89226	1.93315
65298	1.89226	0.468506
65299	1.01988	0.651776
65300	1.01988	0.362933
65301	1.03333	0.785414
65302	1.03333	0.771296
65303	1.13811	0.0978585
65304	1.20969	0.266322
65305	0.941974	0.801362

65306	0.843515	0.440683
65307	0.843514	0.374038
65308	1.13811	0.526898
65309	0.941973	0.681653
65310	1.20969	0.309244
65311	2.41938	0.166746
65312	2.57414	0.352511
65313	2.57414	1.3602
65314	2.69045	1.15741
65315	2.27623	0.181606
65316	2.39253	1.21521
65317	1.89227	0.566972
65318	1.89227	0.513612
65319	1.89227	0.205753
65320	1.89227	0.228715
65321	1.89227	0.216256
65322	1.89227	0.259056
65323	2.28385	1.67939
65324	2.09114	1.71113
65325	2.46968	1.1236
65326	2.32438	1.34219
65327	4.56082	1.18273
65328	1.89226	1.42336
65329	1.89226	1.71039
65330	1.89226	1.25787
65331	1.89226	1.89303
65332	3.78452	1.56669
65333	1.89226	1.45206
65334	1.89226	2.1
65335	1.89226	1.49261
65336	1.89226	1.84846
65337	3.78452	1.8082
65338	2.09114	0.837441
65339	1.5249	1.84753
65340	2.55684	0.85626
65341	1.86899	1.91632
65342	3.96011	1.60635
65343	2.46968	1.38984
65344	2.32439	1.55851
65345	2.62768	1.66955
65346	2.506	1.46401
65347	4.97568	1.55165
65348	1.89226	1.49578
65349	1.89226	1.90492
65350	1.89226	0.32895
65351	1.89226	1.43508
65352	3.78452	1.39903
65353	1.89226	1.54714
65354	1.89226	1.82145
65355	1.89226	1.63244
65356	1.89226	2.1
65357	1.89226	0.567611
65358	1.89226	2.1
65359	2.55684	1.37177
65360	2.16506	1.81481
65361	1.86901	1.59988
65362	1.86899	1.51804
65363	2.75664	1.47377

65364	2.75662	1.34006
65365	1.89224	0.473584
65366	1.89224	1.07168
65367	1.89224	0.287383
65368	1.89224	2.1
65369	3.78447	0.718661
65370	2.24206	0.645053
65371	2.38862	2.1
65372	2.07107	1.37903
65373	2.23097	2.1
65374	4.47303	1.88633
65375	2.38863	2.1
65376	2.25945	2.1
65377	2.25945	2.1
65378	2.39244	2.1
65379	2.23098	2.1
65380	2.36397	2.1
65381	1.89224	1.02815
65382	1.89224	2.1
65383	1.89224	2.1
65384	1.89224	2.1
65385	1.89224	2.1
65386	1.89224	2.1
65387	1.89226	0.419568
65388	1.89226	2.1
65389	1.89226	1.31765
65390	1.89226	0.669761
65391	1.89226	1.13287
65392	1.89226	1.45501
65393	0.920975	2.1
65394	0.920979	1.59903
65395	1.03015	2.1
65396	1.03015	0.753012
65397	0.992407	2.07727
65398	1.08759	2.1
65399	0.854346	1.55017
65400	0.87241	0.655795
65401	0.872397	1.87359
65402	1.04334	1.86493
65403	1.00965	1.91725
65404	1.0876	1.90484
65405	2.23101	2.1
65406	2.25948	2.1
65407	2.03577	2.1
65408	2.05257	2.1
65409	4.29524	2.1
65410	0.810947	1.17111
65411	0.810947	2.1
65412	0.810947	2.08108
65413	0.810947	1.16297
65414	0.810934	2.07848
65415	0.810934	2.08757
65416	1.89227	2.1
65417	1.3517	2.1
65418	1.08132	2.1
65419	1.08132	2.1
65420	1.08133	2.1
65421	1.89226	2.0951

65422	1.89226	2.1
65423	1.89226	1.77971
65424	1.89226	2.1
65425	1.89226	0.764367
65426	1.89226	2.1
65427	1.89226	2.1
65428	1.89226	2.1
65429	1.89226	2.1
65430	1.89226	2.1
65431	1.89226	2.09444
65432	1.89226	2.1
65433	0.879432	2.1
65434	0.879432	2.1
65435	0.900223	1.00039
65436	0.900223	2.1
65437	0.54571	2.1
65438	0.743092	2.1
65439	1.03656	2.1
65440	1.19234	1.09761
65441	1.19234	2.05677
65442	0.743092	2.07497
65443	1.03656	2.1
65444	0.545711	2.1
65445	1.89226	0.498766
65446	2.38467	1.6666
65447	2.69293	0.443832
65448	2.69293	2.1
65449	1.89226	0.192882
65450	1.89226	1.95038
65451	1.89226	1.884
65452	1.89226	1.93628
65453	1.89226	0.996276
65454	1.89226	2.1
65455	3.78452	1.901
65456	0.946129	2.1
65457	0.946129	2.1
65458	0.946133	1.64346
65459	0.946133	2.1
65460	0.946131	2.1
65461	0.946131	2.1
65462	0.946131	1.51952
65463	0.946131	2.1
65464	0.946131	2.1
65465	0.946131	2.1
65466	0.946131	2.1
65467	0.946131	2.1
65468	1.04846	1.21171
65469	1.04846	2.1
65470	1.05594	0.596955
65471	1.05594	2.05095
65472	0.743092	2.1
65473	0.912061	1.97317
65474	1.19234	0.556774
65475	1.34647	1.42823
65476	0.912061	1.09034
65477	1.34646	1.79008
65478	1.19234	1.84397
65479	0.743093	1.82523

65480	1.89226	1.48051
65481	1.89226	2.1
65482	1.89226	0.883785
65483	1.89226	1.81156
65484	3.78452	1.86906
65485	0.946128	2.1
65486	0.946128	2.1
65487	0.946133	2.1
65488	0.946133	1.59831
65489	0.94613	2.1
65490	0.94613	2.1
65491	0.946131	2.1
65492	0.946131	1.88646
65493	0.946131	2.1
65494	0.946131	2.1
65495	0.946131	2.1
65496	0.946131	2.1
65497	1.89226	1.57179
65498	1.89226	1.96734
65499	1.89226	1.14761
65500	1.89226	1.81536
65501	3.78452	1.58172
65502	1.89226	1.83719
65503	1.89226	1.80759
65504	1.89226	1.68208
65505	1.89226	2.1
65506	1.89226	0.514297
65507	1.89226	2.1
65508	1.89226	2.1
65509	1.89226	2.1
65510	1.89226	1.84328
65511	1.89226	2.1
65512	1.89226	1.27045
65513	1.89226	2.1
65514	0.946132	2.1
65515	0.946132	1.86879
65516	1.17758	0.682093
65517	1.17759	1.92985
65518	1.04514	2.1
65519	1.17758	1.58814
65520	0.946133	0.816128
65521	0.946133	1.46378
65522	0.946107	1.76503
65523	1.17755	1.7543
65524	1.04517	1.80516
65525	0.946158	1.80206
65526	1.89226	2.1
65527	2.10171	2.1
65528	1.95064	2.1
65529	2.10171	2.1
65530	1.7412	2.1
65531	1.89226	2.1
65532	1.89226	2.1
65533	1.89226	2.1
65534	1.89226	2.1
65535	1.89226	2.1
65536	1.89226	2.1
65537	1.89226	2.1

65538	1.89226	2.1
65539	1.89226	2.1
65540	1.89226	2.1
65541	1.89226	2.1
65542	3.78453	2.1
65543	0.946133	2.1
65544	0.946133	2.1
65545	0.946132	2.1
65546	0.946132	2.1
65547	0.946133	2.1
65548	0.946133	2.1
65549	0.946132	2.1
65550	0.946132	2.1
65551	0.946132	2.1
65552	0.946132	2.1
65553	0.946132	2.1
65554	0.946132	2.1
65555	1.70303	2.1
65556	1.95064	2.1
65557	1.74039	2.1
65558	1.95064	2.1
65559	1.69368	2.1
65560	1.89226	2.1
65561	1.89226	2.1
65562	1.89226	2.1
65563	1.89226	2.1
65564	1.89226	2.1
65565	3.78453	2.1
65566	0.946132	2.1
65567	0.946132	2.1
65568	0.946132	2.1
65569	0.946132	2.1
65570	0.946133	2.1
65571	0.946133	2.1
65572	0.946132	2.1
65573	0.946132	2.1
65574	0.946107	2.1
65575	0.946107	2.1
65576	0.946158	2.1
65577	0.946158	2.1
65578	0.946133	2.1
65579	0.946133	2.1
65580	0.946132	2.1
65581	0.946132	2.1
65582	0.946133	2.1
65583	0.946133	2.1
65584	0.946132	2.1
65585	0.946132	2.1
65586	0.946107	2.1
65587	0.946107	2.1
65588	0.946158	2.1
65589	0.946158	2.1
65590	1.89226	2.1
65591	1.89226	2.1
65592	1.89226	2.1
65593	1.89226	2.1
65594	1.89226	2.1
65595	1.89226	2.1

65596	1.89226	2.1
65597	1.89226	2.1
65598	1.89226	2.1
65599	1.89226	2.1
65600	1.89226	2.1
65601	1.89226	2.1
65602	2.5198	2.1
65603	2.45893	2.1
65604	2.27322	2.1
65605	2.27322	2.1
65606	2.63576	2.1
65607	2.63576	2.1
65608	1.24045	2.1
65609	1.11906	2.1
65610	1.13578	2.1
65611	0.916205	2.1
65612	1.12539	2.1
65613	1.08404	2.1
65614	1.08484	2.1
65615	1.08484	2.1
65616	1.22836	2.1
65617	0.919363	2.1
65618	1.16507	2.1
65619	0.924864	2.1
65620	1.89226	2.1
65621	1.89226	2.1
65622	1.89226	2.1
65623	1.89226	2.1
65624	1.89226	2.1
65625	1.89226	2.1
65626	1.89226	2.1
65627	1.89226	2.1
65628	1.89226	2.1
65629	1.89226	2.1
65630	1.89226	2.1
65631	1.89226	2.1
65632	2.6946	2.1
65633	2.63576	2.1
65634	2.5198	2.1
65635	2.5198	2.1
65636	2.8362	2.1
65637	2.8362	2.1
65638	1.3265	2.1
65639	1.24045	2.1
65640	0.983223	2.1
65641	0.954429	2.1
65642	1.06046	2.1
65643	1.10594	2.1
65644	1.1915	2.1
65645	1.1915	2.1
65646	1.31357	2.1
65647	1.09005	2.1
65648	1.29145	2.1
65649	0.995825	2.1
65650	1.89226	2.1
65651	1.89226	2.1
65652	1.89226	2.1
65653	1.89226	2.1

65654	3.78452	2.1
65655	1.89226	2.1
65656	1.89226	2.1
65657	1.89226	2.1
65658	1.89226	2.1
65659	3.78453	2.1
65660	1.91802	2.1
65661	2.27409	2.1
65662	2.48048	2.1
65663	2.22264	2.1
65664	1.91802	2.1
65665	1.66018	2.1
65666	1.13704	2.1
65667	1.24023	2.1
65668	1.22947	2.1
65669	1.31788	2.1
65670	1.23859	2.1
65671	1.23859	2.1
65672	1.23111	2.1
65673	1.23111	2.1
65674	1.24024	2.1
65675	1.31788	2.1
65676	1.13704	2.1
65677	1.22947	2.1
65678	1.89226	2.1
65679	1.89226	2.1
65680	1.89226	2.1
65681	1.89226	2.1
65682	1.89226	2.1
65683	1.89226	2.1
65684	1.89226	2.1
65685	1.89226	2.1
65686	1.89226	2.1
65687	1.89226	2.1
65688	3.78453	2.1
65689	1.05568	2.1
65690	0.959008	2.1
65691	1.32068	2.1
65692	1.24024	2.1
65693	1.15206	2.1
65694	1.15206	2.1
65695	1.14386	2.1
65696	1.14386	2.1
65697	1.05568	2.1
65698	1.32068	2.1
65699	0.959008	2.1
65700	1.24024	2.1
65701	1.32068	2.1
65702	1.24023	2.1
65703	1.31788	2.1
65704	1.4181	2.1
65705	1.31436	2.1
65706	1.31436	2.1
65707	1.3242	2.1
65708	1.3242	2.1
65709	1.32068	2.1
65710	1.4181	2.1
65711	1.24024	2.1

65712	1.31788	2.1
65713	1.89226	2.1
65714	1.89226	0.732917
65715	1.89226	1.88148
65716	1.89226	1.89964
65717	1.89226	1.85703
65718	1.89226	1.95227
65719	1.02925	0.093997
65720	1.16678	0.46202
65721	0.946132	0.501553
65722	0.946132	1.96973
65723	0.935677	0.919441
65724	0.935677	0.152258
65725	1.18902	1.75681
65726	1.21562	1.36542
65727	1.13433	0.371735
65728	1.04071	0.413949
65729	0.851553	0.588666
65730	1.06075	0.602426
65731	1.89226	1.2908
65732	1.89226	0.411831
65733	1.89226	0.505194
65734	1.89226	0.133636
65735	1.89226	0.296651
65736	1.89226	1.3289
65737	1.89226	1.93614
65738	1.89226	0.289761
65739	1.89226	0.746226
65740	1.89226	0.659374
65741	3.78453	0.572288
65742	0.946128	1.30715
65743	0.946128	0.498813
65744	0.946133	0.259254
65745	0.946133	0.404095
65746	0.946131	0.200699
65747	0.946131	0.352556
65748	0.94613	0.521119
65749	0.94613	0.228465
65750	0.946131	1.22669
65751	0.946131	0.205374
65752	0.946131	0.197957
65753	0.946131	0.100132
65754	0.946132	1.39797
65755	0.946132	0.555829
65756	0.946132	1.70956
65757	0.946132	0.838986
65758	0.946132	0.613094
65759	0.946132	0.110312
65760	0.946131	1.1046
65761	0.946131	1.57641
65762	0.946132	0.846837
65763	0.946132	1.08993
65764	0.946132	0.5621
65765	0.946132	0.751062
65766	0.946131	2.00055
65767	0.946131	1.67223
65768	0.946131	1.72671
65769	0.946131	1.12983

65770	0.946131	0.890831
65771	0.946131	1.95282
65772	0.946131	1.08329
65773	0.946131	0.709782
65774	0.946131	1.48748
65775	0.946131	1.48395
65776	0.946131	1.58178
65777	0.946131	1.59101
65778	1.89226	1.95642
65779	1.89226	1.82928
65780	1.89226	1.64846
65781	1.89226	2.1
65782	1.89226	0.767551
65783	1.89226	2.1
65784	1.89226	1.52837
65785	1.89226	1.27151
65786	1.89226	1.41542
65787	1.89226	2.03186
65788	1.89226	1.23618
65789	1.89226	2.1
65790	1.89226	1.93033
65791	1.89226	1.53245
65792	1.89226	1.59143
65793	1.89226	2.1
65794	1.89226	0.503673
65795	1.89226	2.1
65796	1.89226	2.1
65797	1.89226	2.06867
65798	1.89226	0.840433
65799	1.89226	2.1
65800	3.78452	2.04738
65801	0.946131	2.04464
65802	0.946131	1.24538
65803	1.17195	0.459085
65804	1.17195	1.80124
65805	0.958489	2.07847
65806	1.17195	1.58718
65807	0.946129	0.857018
65808	0.946129	1.76042
65809	0.946131	1.61325
65810	1.17195	1.60762
65811	0.958487	1.71166
65812	0.94613	1.72558
65813	2.69656	2.1
65814	2.17516	2.1
65815	2.41885	2.1
65816	2.41885	2.1
65817	2.46476	2.1
65818	2.46476	2.1
65819	2.69657	2.1
65820	2.30233	1.77797
65821	2.30233	2.1
65822	2.59476	2.1
65823	2.46477	2.1
65824	2.7572	2.1
65825	1.89226	2.1
65826	1.89226	0.669985
65827	1.89226	1.69295

65828	1.89226	1.69385
65829	1.89226	1.61216
65830	1.89226	1.81151
65831	1.89226	2.1
65832	1.89226	2.1
65833	1.89226	2.1
65834	1.89226	1.69233
65835	3.78452	2.1
65836	2.46477	2.1
65837	2.17516	2.1
65838	1.80252	2.1
65839	2.09715	2.1
65840	4.27231	2.1
65841	2.46477	2.1
65842	2.09715	2.0085
65843	2.30233	2.1
65844	2.30233	2.05532
65845	1.96416	1.6004
65846	2.09715	1.05339
65847	1.89227	1.96886
65848	1.89227	0.435175
65849	1.89227	1.21785
65850	1.89227	1.54851
65851	1.89227	1.5347
65852	1.89227	1.74317
65853	1.89226	2.1
65854	1.89226	1.60825
65855	1.89226	2.07669
65856	1.89226	2.1
65857	3.78452	2.09731
65858	0.946132	2.1
65859	0.946132	2.1
65860	0.946132	2.1
65861	0.946132	2.1
65862	0.946132	2.1
65863	0.946132	2.1
65864	0.946132	2.1
65865	0.946132	2.1
65866	0.946132	2.1
65867	0.946132	2.1
65868	0.946131	2.1
65869	0.946131	2.1
65870	1.89226	2.1
65871	1.89226	2.1
65872	1.89226	2.1
65873	1.89226	2.1
65874	3.78453	2.1
65875	1.89226	2.1
65876	1.89226	2.1
65877	1.89226	2.1
65878	1.89226	2.1
65879	1.89226	2.1
65880	1.89226	2.1
65881	1.89226	2.1
65882	1.63707	2.1
65883	1.63707	2.1
65884	1.38859	2.1
65885	1.89226	2.1

65886	1.64377	2.1
65887	0.94613	2.1
65888	0.94613	2.1
65889	0.94613	2.1
65890	0.94613	2.1
65891	0.946131	2.1
65892	0.946131	2.1
65893	0.946129	2.1
65894	0.946129	2.1
65895	0.94613	2.1
65896	0.94613	2.1
65897	0.94613	2.1
65898	0.94613	2.1
65899	2.15888	2.1
65900	1.99309	2.1
65901	2.16841	2.1
65902	2.16841	2.1
65903	2.02311	2.1
65904	2.02311	1.8775
65905	1.89226	2.1
65906	1.89226	2.07314
65907	1.89226	2.1
65908	1.89226	2.1
65909	1.89226	2.1
65910	1.89226	2.00806
65911	1.89226	2.1
65912	1.89226	2.1
65913	1.89226	2.1
65914	1.89226	2.1
65915	1.89226	2.1
65916	1.89226	2.1
65917	1.99309	2.1
65918	1.96035	2.1
65919	1.72526	2.1
65920	1.72526	2.1
65921	2.16841	2.1
65922	2.16841	2.1
65923	2.15888	2.1
65924	2.16841	2.1
65925	2.3111	2.1
65926	2.35938	2.1
65927	4.51826	2.1
65928	1.89226	2.1
65929	1.89226	2.1
65930	1.89226	2.1
65931	1.89226	1.91127
65932	3.78452	2.1
65933	1.89226	2.1
65934	1.89226	2.1
65935	1.89226	2.1
65936	1.89226	1.83543
65937	3.78452	2.1
65938	2.16841	2.1
65939	1.96035	2.1
65940	2.35938	2.1
65941	2.18493	2.1
65942	4.31973	2.1
65943	1.89226	2.1

65944	1.89226	2.1
65945	1.89226	2.1
65946	1.89226	2.1
65947	3.78453	2.1
65948	1.89226	2.1
65949	1.89226	2.1
65950	1.89226	2.1
65951	1.89226	2.1
65952	3.78452	2.1
65953	0.946158	2.1
65954	0.946158	2.1
65955	0.818509	2.1
65956	0.818509	2.1
65957	0.933975	2.1
65958	0.818539	2.1
65959	0.946128	2.1
65960	0.946128	2.1
65961	0.94613	2.1
65962	0.933974	2.1
65963	0.818537	2.1
65964	0.946129	2.1
65965	0.946131	2.1
65966	0.946131	2.1
65967	0.946131	2.1
65968	0.946131	2.1
65969	0.946132	2.1
65970	0.946132	2.1
65971	0.946129	2.1
65972	0.946129	2.1
65973	0.946131	2.1
65974	0.946131	2.1
65975	0.94613	2.1
65976	0.94613	2.1
65977	1.89226	2.1
65978	1.89226	2.1
65979	1.89226	2.1
65980	1.89226	2.1
65981	3.78453	2.1
65982	1.89226	2.1
65983	1.89226	2.1
65984	1.89226	2.1
65985	1.89226	2.1
65986	3.78452	2.1
65987	1.89226	2.1
65988	1.8706	2.1
65989	1.8706	2.1
65990	1.68112	2.1
65991	2.0815	2.1
65992	1.88715	2.1
65993	0.94613	2.1
65994	0.94613	2.1
65995	0.94613	2.1
65996	0.94613	2.1
65997	0.946131	2.1
65998	0.946131	2.1
65999	0.946129	2.1
66000	0.946129	2.1
66001	0.94613	2.1

66002	0.94613	2.1
66003	0.94613	2.1
66004	0.94613	2.1
66005	1.76401	2.1
66006	2.60983	2.1
66007	2.50461	2.1
66008	2.35897	2.1
66009	1.90965	2.1
66010	1.76401	2.1
66011	1.89226	2.1
66012	1.89226	2.1
66013	1.89226	2.1
66014	1.89226	2.1
66015	3.78452	2.1
66016	1.89226	2.1
66017	1.89226	2.1
66018	1.89226	2.1
66019	1.89226	2.1
66020	1.89226	2.1
66021	1.89226	2.1
66022	1.90965	2.1
66023	2.83834	2.1
66024	2.7538	2.1
66025	2.60983	2.1
66026	2.05361	2.1
66027	1.90965	2.1
66028	1.89226	2.1
66029	1.89226	2.1
66030	1.89226	2.1
66031	1.89226	2.1
66032	1.89226	2.1
66033	1.89226	2.1
66034	1.89226	2.1
66035	1.89226	2.1
66036	1.89226	2.1
66037	1.89226	2.1
66038	3.78452	2.1
66039	2.83834	2.1
66040	1.89226	2.1
66041	2.60983	2.1
66042	2.60983	2.1
66043	1.89226	2.1
66044	1.89226	2.1
66045	1.89226	2.1
66046	1.89226	2.1
66047	1.89226	2.1
66048	1.89226	2.1
66049	1.89226	2.1
66050	1.89226	2.1
66051	1.89226	2.1
66052	1.89226	2.1
66053	1.89226	2.1
66054	1.89226	2.06166
66055	1.89226	2.1
66056	1.89226	1.9893
66057	2.44785	2.1
66058	1.80223	2.1
66059	2.80894	2.1

66060	2.18296	2.1
66061	4.63081	2.1
66062	1.89226	2.1
66063	1.89226	2.1
66064	1.89226	2.1
66065	1.89226	2.1
66066	1.89226	2.1
66067	1.89226	2.1
66068	0.946131	1.87117
66069	0.946131	2.1
66070	0.946127	2.1
66071	0.946127	2.1
66072	0.946131	2.1
66073	0.946131	2.1
66074	0.946128	2.1
66075	0.946128	2.1
66076	0.94613	2.1
66077	0.94613	2.1
66078	0.946129	2.1
66079	0.946129	2.1
66080	1.89226	2.1
66081	1.89226	2.1
66082	1.89226	2.1
66083	1.89226	2.1
66084	3.78452	2.1
66085	1.89226	2.1
66086	1.89226	2.1
66087	1.89226	2.1
66088	1.89226	2.1
66089	3.78453	2.1
66090	2.41083	2.1
66091	2.93937	2.1
66092	1.68761	2.1
66093	1.68761	2.1
66094	1.67248	2.1
66095	2.3957	2.1
66096	1.89226	2.1
66097	1.89226	2.1
66098	1.89226	2.1
66099	1.89226	2.1
66100	1.89226	2.1
66101	1.89226	2.1
66102	1.89227	2.1
66103	1.89227	2.1
66104	1.89227	2.1
66105	1.89227	2.1
66106	1.89227	2.1
66107	1.89227	2.1
66108	1.89224	1.46623
66109	1.89224	0.357092
66110	1.89224	1.35804
66111	1.89224	1.411
66112	3.78448	0.523598
66113	1.89224	2.1
66114	1.89224	1.49603
66115	1.89224	1.98266
66116	1.89224	1.44658
66117	1.89224	1.9473

66118	1.89224	1.70014
66119	1.11152	0.838128
66120	1.29346	1.70934
66121	0.978596	1.95571
66122	0.918324	2.08944
66123	1.06821	1.96629
66124	1.06821	0.559263
66125	0.985654	1.88688
66126	1.15278	2.072
66127	1.07693	1.88496
66128	0.902432	1.88774
66129	0.968939	1.89116
66130	1.34644	1.81966
66131	0.946132	0.357866
66132	0.946132	0.816051
66133	0.946132	0.794092
66134	0.946132	0.693785
66135	0.946131	1.06574
66136	0.946131	0.233129
66137	0.946132	1.35118
66138	0.946132	0.427433
66139	0.946132	0.819909
66140	0.946132	0.621314
66141	0.946132	0.282159
66142	0.946132	0.296967
66143	0.946133	1.60368
66144	0.946133	1.00922
66145	0.946132	1.77297
66146	0.946132	1.91066
66147	0.946132	1.76267
66148	0.946132	0.297811
66149	0.946133	2.1
66150	0.946133	1.37984
66151	0.946132	1.73458
66152	0.946132	1.71759
66153	0.946132	1.22651
66154	0.946132	1.3947
66155	1.89224	2.08527
66156	1.89224	2.1
66157	1.89224	2.1
66158	1.89224	2.1
66159	1.89224	2.1
66160	1.89224	2.1
66161	1.89224	2.1
66162	1.89224	2.1
66163	1.89224	2.1
66164	1.89224	2.1
66165	1.89224	2.1
66166	1.89224	2.1
66167	1.89226	2.1
66168	1.89226	2.1
66169	1.89226	2.1
66170	1.89226	2.1
66171	3.78453	2.1
66172	1.11056	2.1
66173	1.21595	2.1
66174	1.12763	2.1
66175	1.12763	2.1

66176	1.23339	2.1
66177	1.35697	2.1
66178	1.03581	2.1
66179	0.95954	2.1
66180	0.89761	2.1
66181	1.26602	2.1
66182	1.12749	2.1
66183	1.45307	2.1
66184	2.71775	2.1
66185	1.89226	2.1
66186	1.89226	2.1
66187	1.89226	2.1
66188	2.47025	2.1
66189	2.47025	2.1
66190	0.94613	2.1
66191	0.94613	2.1
66192	0.94613	2.1
66193	0.94613	2.1
66194	0.946128	2.1
66195	0.946128	2.1
66196	0.946132	2.1
66197	0.946132	2.1
66198	0.94613	2.1
66199	0.94613	2.1
66200	0.94613	2.1
66201	0.94613	2.1
66202	1.89226	1.84952
66203	1.89226	2.1
66204	1.89226	2.1
66205	1.89226	1.88283
66206	1.89226	2.1
66207	1.89226	1.87977
66208	1.89226	1.7906
66209	1.89226	2.1
66210	1.89226	2.1
66211	1.89226	1.73243
66212	1.89226	2.1
66213	1.89226	1.74047
66214	0.946132	2.1
66215	0.946132	2.1
66216	0.946133	2.1
66217	0.946133	2.1
66218	0.946133	2.1
66219	0.946133	2.1
66220	0.946132	2.1
66221	0.946132	2.1
66222	0.946133	2.1
66223	0.946133	2.1
66224	0.946132	2.1
66225	0.946132	2.1
66226	1.89226	1.8617
66227	1.89226	2.1
66228	1.89226	2.1
66229	1.89226	1.88984
66230	1.89226	2.1
66231	1.89226	1.89492
66232	0.946131	1.79729
66233	0.946131	1.79822

66234	0.946127	2.1
66235	0.946127	2.1
66236	0.946131	1.86135
66237	0.946131	2.1
66238	0.946127	2.1
66239	0.946127	1.91074
66240	0.94613	1.98284
66241	0.94613	1.98048
66242	0.946129	2.01064
66243	0.946129	1.99931
66244	1.94291	2.1
66245	2.00118	2.1
66246	2.36337	2.1
66247	2.41217	2.1
66248	4.35508	2.1
66249	1.89226	2.1
66250	1.89226	2.1
66251	1.89226	2.1
66252	1.89226	2.1
66253	3.78452	2.1
66254	1.89226	2.1
66255	1.89226	2.1
66256	1.89226	2.1
66257	1.89226	2.1
66258	3.78452	2.1
66259	1.06209	2.1
66260	1.06209	2.1
66261	1.05987	2.1
66262	1.05987	2.1
66263	0.920378	2.1
66264	1.12137	2.1
66265	1.00059	2.1
66266	1.20608	2.1
66267	1.20608	2.1
66268	1.12137	2.1
66269	1.00059	2.1
66270	0.920376	2.1
66271	2.77222	2.1
66272	2.41216	2.1
66273	2.77002	2.1
66274	2.36337	2.1
66275	2.81882	2.1
66276	2.41216	2.1
66277	1.89226	2.1
66278	1.89226	2.1
66279	1.89226	2.1
66280	1.89226	2.1
66281	1.89226	2.1
66282	1.89226	2.1
66283	1.89226	2.1
66284	1.89226	2.1
66285	1.89226	2.1
66286	1.89226	2.1
66287	3.78451	2.1
66288	2.41216	2.1
66289	2.24273	2.1
66290	2.81882	2.1
66291	2.68473	2.1

66292	5.09689	2.1
66293	1.89226	2.1
66294	1.89226	2.1
66295	1.89226	2.1
66296	1.89226	2.1
66297	3.78453	2.1
66298	2.17614	2.1
66299	2.65326	2.1
66300	1.93101	2.1
66301	2.58552	2.1
66302	4.58427	2.1
66303	2.65326	2.1
66304	1.89226	2.1
66305	1.89226	2.1
66306	1.89226	2.1
66307	2.58552	2.1
66308	2.58552	2.1
66309	1.89226	2.1
66310	1.89226	2.1
66311	1.89226	2.1
66312	1.89226	2.1
66313	3.78453	2.1
66314	1.89226	2.1
66315	1.89226	2.1
66316	1.89226	2.1
66317	1.89226	2.1
66318	3.78453	2.1
66319	1.93101	2.1
66320	2.58552	2.1
66321	1.46507	2.1
66322	2.52893	2.1
66323	4.45994	2.1
66324	1.26446	2.1
66325	1.26446	2.1
66326	0.946132	2.1
66327	0.946132	2.1
66328	0.94613	2.1
66329	0.94613	2.1
66330	1.29276	2.1
66331	1.26446	2.1
66332	1.26446	2.1
66333	0.94613	2.1
66334	0.94613	2.1
66335	1.29276	2.1
66336	1.89226	2.1
66337	1.89226	2.1
66338	1.89226	2.1
66339	1.89226	2.1
66340	3.78453	2.1
66341	1.89226	2.1
66342	1.89226	2.1
66343	1.89226	2.1
66344	1.89226	2.1
66345	3.78453	2.1
66346	1.83378	2.1
66347	2.52893	2.1
66348	1.75592	2.1
66349	2.49844	2.1

66350	4.28485	2.1
66351	1.24922	2.1
66352	1.24922	2.1
66353	0.946133	2.1
66354	0.946133	2.1
66355	0.946131	2.1
66356	0.946131	2.1
66357	1.26446	2.1
66358	1.24922	2.1
66359	1.24922	2.1
66360	0.946131	2.1
66361	1.26446	2.1
66362	0.94613	2.1
66363	0.946132	2.1
66364	0.946132	2.1
66365	0.946132	2.1
66366	0.946132	2.1
66367	0.946132	2.1
66368	0.946132	2.1
66369	0.946132	2.1
66370	0.946132	2.1
66371	0.946132	2.1
66372	0.946132	2.1
66373	0.946131	2.1
66374	0.946131	2.1
66375	1.89226	2.1
66376	1.89226	2.1
66377	1.89226	2.1
66378	1.89226	2.1
66379	1.89226	2.1
66380	1.89226	2.1
66381	1.0465	2.1
66382	1.0465	2.1
66383	1.06047	2.1
66384	1.06047	2.1
66385	1.25687	2.1
66386	1.24922	2.1
66387	0.87796	2.1
66388	0.857749	2.1
66389	0.857749	2.1
66390	1.25687	2.1
66391	0.877959	2.1
66392	1.24922	2.1
66393	2.49843	2.1
66394	1.89226	2.1
66395	2.51374	2.1
66396	1.89226	2.1
66397	4.406 2.1	
66398	1.89226	2.1
66399	1.89226	2.1
66400	1.89226	2.1
66401	1.89226	2.1
66402	1.89226	2.1
66403	1.89226	2.1
66404	1.89226	2.1
66405	1.89226	2.1
66406	1.89226	2.1
66407	1.89226	2.1

66408	1.89226	2.1
66409	1.89226	2.1
66410	1.89226	2.1
66411	1.89226	2.1
66412	1.89226	2.1
66413	1.89226	2.1
66414	1.89226	2.1
66415	1.89226	2.1
66416	1.03835	2.1
66417	1.03835	2.1
66418	1.15388	2.1
66419	0.94267	2.1
66420	0.736433	2.1
66421	0.849239	2.1
66422	1.15014	2.1
66423	1.39058	2.1
66424	0.838639	2.1
66425	1.39056	2.1
66426	1.09399	2.1
66427	0.736444	2.1
66428	1.89226	1.99734
66429	1.89226	2.1
66430	1.89226	2.1
66431	1.89226	1.98587
66432	1.89226	2.1
66433	1.89226	2.01574
66434	0.94613	1.98062
66435	0.94613	2.00354
66436	0.946127	2.1
66437	0.946127	2.1
66438	0.946131	2.1
66439	0.946131	1.94062
66440	0.946126	2.1
66441	0.946126	2.06402
66442	0.946128	2.1
66443	0.946128	2.1
66444	0.946128	2.1
66445	0.946128	2.1
66446	1.89226	2.1
66447	1.89226	1.79232
66448	1.89226	1.75018
66449	1.89226	2.1
66450	3.78453	1.88411
66451	0.946133	1.57846
66452	0.946133	1.48791
66453	0.946128	1.82473
66454	0.946128	1.78953
66455	0.946131	1.76647
66456	0.946131	1.6097
66457	0.946131	0.684265
66458	0.946131	1.7548
66459	0.946131	1.64845
66460	0.946131	1.65386
66461	0.94613	1.6984
66462	0.94613	1.69697
66463	1.89226	1.68412
66464	1.89226	2.05657
66465	1.89226	1.99173

66466	1.89226	1.74128
66467	1.89226	1.99515
66468	1.89226	1.75893
66469	1.89226	1.44726
66470	1.89226	1.77905
66471	1.89226	1.71931
66472	1.89226	1.52092
66473	1.89226	1.70397
66474	1.89226	1.53459
66475	1.89226	1.47376
66476	1.89226	1.84574
66477	1.89226	1.73191
66478	1.89226	1.57593
66479	1.89226	1.73407
66480	1.89226	1.58164
66481	1.89226	1.70624
66482	2.67388	2.1
66483	2.23684	2.00787
66484	2.23684	1.79802
66485	1.89226	2.02409
66486	1.89226	1.8116
66487	1.89226	2.1
66488	1.89226	2.1
66489	1.89226	2.1
66490	1.89226	2.1
66491	1.89226	2.1
66492	1.89226	2.1
66493	1.72197	2.1
66494	2.51374	2.1
66495	2.58004	2.1
66496	2.58004	2.1
66497	1.7155	2.1
66498	1.7155	2.1
66499	2.51374	2.1
66500	1.89226	2.1
66501	1.89226	2.1
66502	1.89226	2.1
66503	2.58003	2.1
66504	2.58003	2.1
66505	0.946132	2.1
66506	0.946132	2.1
66507	0.946132	2.1
66508	0.946132	2.1
66509	0.946132	2.1
66510	0.946132	2.1
66511	0.946132	2.1
66512	0.946132	2.1
66513	0.946131	2.1
66514	0.946131	2.1
66515	0.946132	2.1
66516	0.946132	2.1
66517	1.89226	2.1
66518	1.89226	2.1
66519	1.89226	2.1
66520	1.89226	2.1
66521	3.78453	2.1
66522	1.08262	2.1
66523	1.08262	2.1

66524	1.09674	2.1
66525	1.09674	2.1
66526	1.34652	2.1
66527	1.29002	2.1
66528	0.860984	2.1
66529	0.889338	2.1
66530	1.34652	2.1
66531	0.889338	2.1
66532	0.860983	2.1
66533	1.29002	2.1
66534	1.34652	2.1
66535	1.34652	2.1
66536	0.946133	2.1
66537	0.946133	2.1
66538	0.946131	2.1
66539	0.946131	2.1
66540	1.34652	2.1
66541	1.29002	2.1
66542	1.34652	2.1
66543	0.946131	2.1
66544	0.94613	2.1
66545	1.29002	2.1
66546	1.89226	2.1
66547	1.89226	2.1
66548	1.89226	2.1
66549	1.89226	2.1
66550	1.89226	2.1
66551	1.89226	2.1
66552	1.89226	2.1
66553	1.89226	2.1
66554	1.89226	2.1
66555	1.89226	2.1
66556	1.89226	2.1
66557	1.89226	2.1
66558	0.946131	2.1
66559	0.946131	2.1
66560	0.946131	2.1
66561	0.946131	2.1
66562	0.946131	2.1
66563	0.946131	2.1
66564	0.946131	2.1
66565	0.946131	2.1
66566	0.946131	2.1
66567	0.946131	2.1
66568	0.946131	2.1
66569	0.946131	2.1
66570	1.89226	2.1
66571	1.89226	2.1
66572	1.89226	2.1
66573	1.89226	2.1
66574	1.89226	2.1
66575	1.89226	2.1
66576	1.04103	2.1
66577	1.13849	2.1
66578	0.943014	2.1
66579	0.943014	2.1
66580	0.943591	2.1
66581	0.943591	2.1

66582	1.03757	2.1
66583	1.04098	2.1
66584	1.03761	2.1
66585	0.946131	2.1
66586	0.943041	2.1
66587	0.946131	2.1
66588	0.946128	2.1
66589	0.946128	2.1
66590	0.946133	2.1
66591	0.946133	2.1
66592	0.946131	2.1
66593	0.946131	2.1
66594	0.946131	2.1
66595	0.946131	2.1
66596	0.946131	2.1
66597	0.946131	2.1
66598	0.94613	2.1
66599	0.94613	2.1
66600	0.946132	2.1
66601	0.946132	2.1
66602	0.946132	2.1
66603	0.946132	2.1
66604	0.946132	2.1
66605	0.946132	2.1
66606	0.946132	2.1
66607	0.946132	2.1
66608	0.946132	2.1
66609	0.946132	2.1
66610	0.946131	2.1
66611	0.946131	2.1
66612	1.89226	1.30379
66613	1.89226	1.47139
66614	1.89226	1.37271
66615	1.89226	1.24492
66616	3.78452	1.38001
66617	0.946132	1.48517
66618	0.946132	1.42863
66619	0.946131	1.35486
66620	0.946131	1.38851
66621	0.946132	1.37046
66622	0.946132	1.60583
66623	0.946132	1.48642
66624	0.946132	1.44335
66625	0.946132	1.38338
66626	0.946132	1.40427
66627	0.946132	1.43347
66628	0.946132	1.46135
66629	0.935482	1.51398
66630	1.08999	1.50353
66631	1.24315	1.39563
66632	1.12559	1.39674
66633	1.21343	1.37777
66634	0.889397	1.89648
66635	1.01265	1.35609
66636	1.01265	1.53172
66637	1.07332	1.36725
66638	1.34292	1.39782
66639	0.865649	1.50843

66640	0.840024	1.58791
66641	1.15433	1.4148
66642	1.02858	1.42899
66643	1.34082	1.25061
66644	1.08357	1.24519
66645	1.10938	1.21784
66646	1.33349	1.75602
66647	1.12232	1.44897
66648	1.12232	1.22478
66649	1.21357	1.27353
66650	1.3408	1.27486
66651	1.02859	1.39549
66652	1.24211	1.4208
66653	1.89226	1.39585
66654	1.89226	1.17541
66655	1.89226	1.25756
66656	1.89226	1.47702
66657	1.89226	1.18496
66658	1.89226	1.31769
66659	1.89226	1.32897
66660	1.89226	1.43509
66661	1.89226	1.38921
66662	1.89226	1.49981
66663	1.89226	1.41287
66664	1.89226	1.4642
66665	2.0815	1.48849
66666	2.79029	1.38259
66667	2.36793	1.4146
66668	2.58428	1.55977
66669	2.38161	1.32366
66670	2.59922	1.44123
66671	1.89226	2.1
66672	1.89226	1.77976
66673	1.89226	2.04091
66674	1.89226	2.02391
66675	1.89226	2.04811
66676	1.89226	2.04318
66677	1.89226	1.75776
66678	1.89226	0.545794
66679	1.89226	1.23723
66680	1.89226	1.38424
66681	3.78452	1.51287
66682	1.89226	2.03977
66683	1.89226	1.52995
66684	1.89226	1.82517
66685	1.89226	1.74616
66686	1.89226	1.85816
66687	1.89226	1.77281
66688	0.946132	1.63074
66689	0.946132	1.94308
66690	0.94613	2.1
66691	0.94613	1.97089
66692	0.946131	2.0871
66693	0.946131	1.59515
66694	0.946131	1.92632
66695	0.946131	2.1
66696	0.946131	1.99992
66697	0.946131	2.01169

66698	0.946131	2.00754
66699	0.946131	1.98841
66700	1.85178	1.81783
66701	1.89226	1.35587
66702	2.0815	0.800305
66703	2.27054	1.45112
66704	1.70303	0.56817
66705	1.5217	0.975677
66706	1.89226	2.08077
66707	1.89226	1.5127
66708	1.89226	1.77068
66709	1.89226	1.638
66710	1.89226	1.91272
66711	1.89226	1.69994
66712	2.58823	0.146931
66713	2.02924	0.177382
66714	1.79398	0.101781
66715	1.79398	0.0717826
66716	2.76708	0.45876
66717	2.76708	0.112233
66718	0.946133	0.143915
66719	0.946133	0.172065
66720	1.29411	0.115359
66721	1.38353	0.0852293
66722	0.946131	0.0965105
66723	0.946131	0.118375
66724	1.38354	0.183666
66725	1.38354	0.0794283
66726	0.946131	0.117781
66727	1.38354	0.110938
66728	0.94613	0.104822
66729	1.29411	0.118346
66730	1.89226	0.098946
66731	1.89226	0.0599341
66732	1.89226	0.0984043
66733	1.89226	0.153937
66734	3.78452	0.105451
66735	0.946131	0.0861062
66736	0.946131	0.0885976
66737	0.946131	0.0931666
66738	0.946131	0.101999
66739	0.946129	0.107522
66740	0.946129	0.0609777
66741	0.946133	0.11461
66742	0.946133	0.0843054
66743	0.946131	0.106876
66744	0.946131	0.102889
66745	0.946131	0.0690096
66746	0.946131	0.0738337
66747	1.89226	0.05
66748	1.89226	0.0808053
66749	1.89226	0.0988205
66750	1.89226	0.0914114
66751	3.78452	0.0550271
66752	0.94613	0.0786842
66753	0.94613	0.0708138
66754	0.946131	0.0656639
66755	0.946131	0.08052

66756	0.946128	0.0793048
66757	0.946128	0.05
66758	0.946133	0.0910619
66759	0.946133	0.05
66760	0.946131	0.0807407
66761	0.946131	0.0757657
66762	0.946131	0.0501285
66763	0.946131	0.052343
66764	0.946129	1.54908
66765	0.946129	1.19333
66766	1.13486	1.02267
66767	0.859753	1.36186
66768	0.851541	1.34365
66769	0.851541	0.94631
66770	1.34346	1.07381
66771	1.26212	0.919004
66772	0.851553	1.01538
66773	1.12403	0.994007
66774	0.851474	0.763696
66775	0.956163	0.959649
66776	0.946132	0.460984
66777	0.946132	0.468151
66778	0.946133	0.537087
66779	0.946133	0.425203
66780	0.946132	0.566057
66781	0.946132	0.410215
66782	0.946133	0.450933
66783	0.946133	0.472818
66784	0.946133	0.449875
66785	0.946133	0.514229
66786	0.946132	0.430028
66787	0.946132	0.4551
66788	1.89226	0.468063
66789	1.89226	0.582092
66790	1.89226	0.660177
66791	1.89226	0.614888
66792	3.78453	0.575636
66793	0.946128	1.55514
66794	0.946128	1.46159
66795	0.946132	1.63877
66796	0.946132	1.45858
66797	0.94613	1.64755
66798	0.94613	1.56163
66799	0.94613	1.58811
66800	0.94613	1.46891
66801	0.946131	1.58218
66802	0.946131	1.59516
66803	0.94613	1.56764
66804	0.94613	1.51195
66805	0.946132	0.291494
66806	0.946132	0.266594
66807	0.946133	0.300792
66808	0.946133	0.254268
66809	0.946132	0.307395
66810	0.946132	0.283533
66811	0.946133	0.265935
66812	0.946133	0.257663
66813	0.946132	0.267059

66814	0.946132	0.292022
66815	0.946132	0.265335
66816	0.946132	0.277556
66817	1.89226	0.339824
66818	1.89226	0.340059
66819	1.89226	0.429416
66820	1.89226	0.296646
66821	1.89226	0.42221
66822	1.89226	0.271709
66823	1.89226	0.81342
66824	1.89226	0.620758
66825	1.89226	0.51172
66826	1.89226	0.642017
66827	1.89226	0.565096
66828	1.89226	0.623028
66829	1.89226	2.1
66830	1.89226	2.1
66831	1.89226	2.1
66832	1.89226	2.1
66833	3.78452	2.1
66834	1.07652	2.1
66835	1.07652	2.1
66836	0.928749	2.1
66837	0.928749	2.1
66838	0.946132	2.1
66839	0.946132	2.1
66840	1.05914	2.1
66841	0.928746	2.1
66842	1.05914	2.1
66843	0.946131	2.1
66844	0.94613	2.1
66845	0.928747	2.1
66846	1.89226	2.1
66847	1.89226	2.1
66848	1.89226	2.1
66849	1.89226	2.1
66850	1.89226	2.1
66851	1.89226	2.1
66852	1.89227	2.1
66853	1.89227	2.1
66854	1.89227	2.1
66855	1.89227	2.1
66856	1.89227	2.1
66857	1.89227	2.1
66858	1.89226	2.1
66859	1.89226	2.1
66860	1.89226	2.1
66861	1.89226	2.1
66862	1.89226	2.1
66863	1.89226	2.1
66864	2.11828	2.1
66865	1.89226	2.1
66866	2.35845	2.1
66867	1.89226	2.1
66868	4.25071	2.1
66869	1.89226	2.1
66870	1.89226	2.1
66871	1.89226	2.1

66872	1.89226	2.1
66873	3.78453	2.1
66874	1.89226	2.1
66875	1.89226	2.1
66876	1.89226	2.1
66877	1.89226	2.1
66878	1.89226	2.1
66879	1.89226	2.1
66880	1.89226	1.04262
66881	1.89226	1.63669
66882	1.65573	1.61896
66883	1.65573	1.22613
66884	1.89226	1.59442
66885	1.89226	1.16793
66886	1.89226	1.22476
66887	1.89226	1.48278
66888	1.89226	1.45888
66889	1.89226	1.36215
66890	1.89226	1.45529
66891	1.89226	1.36046
66892	1.89226	0.897963
66893	1.89226	1.50173
66894	1.89226	1.5059
66895	1.89226	1.24088
66896	1.89226	1.49038
66897	1.89226	1.12125
66898	1.89226	1.24899
66899	1.89226	1.41554
66900	1.89226	1.38311
66901	1.89226	1.2837
66902	1.89226	1.38108
66903	1.89226	1.28202
66904	0.946128	1.29269
66905	0.946128	1.26333
66906	0.946125	1.41387
66907	0.946125	1.41398
66908	0.94613	1.31747
66909	0.94613	1.43855
66910	0.946124	1.41687
66911	0.946124	1.29916
66912	0.946127	1.34397
66913	0.946127	1.35398
66914	0.946127	1.36571
66915	0.946127	1.35705
66916	1.89226	1.31458
66917	1.89226	1.49665
66918	1.89226	1.35224
66919	1.89226	1.26581
66920	3.78452	1.3453
66921	1.89226	1.58655
66922	1.89226	1.56843
66923	1.89226	1.44498
66924	1.89226	1.61141
66925	3.78453	1.56376
66926	1.05926	1.71871
66927	0.891358	2.04479
66928	0.946129	1.60624
66929	0.946129	1.69904

66930	0.946101	1.59711
66931	0.946101	2.1
66932	0.891386	1.61758
66933	0.891386	1.69126
66934	0.891357	1.65236
66935	0.946129	1.64828
66936	0.94613	1.67163
66937	1.05926	1.68779
66938	0.946126	1.44842
66939	0.946126	1.50882
66940	0.94613	1.36452
66941	0.94613	1.43219
66942	0.946131	1.46799
66943	0.946131	1.46349
66944	0.946125	1.50457
66945	0.946125	1.26876
66946	0.946127	1.40002
66947	0.946127	1.38962
66948	0.946128	1.48991
66949	0.946128	1.47634
66950	1.89226	1.45851
66951	1.89226	1.30378
66952	1.89226	1.26067
66953	1.89226	1.56582
66954	1.89226	1.29736
66955	1.89226	1.60829
66956	1.89226	1.53844
66957	1.89226	1.6903
66958	1.89226	1.59724
66959	1.89226	1.72672
66960	1.89226	1.49838
66961	1.89226	1.68097
66962	1.16109	1.80943
66963	1.23243	2.00493
66964	0.659819	1.63068
66965	0.659819	1.71442
66966	0.697965	1.54868
66967	1.00087	2.1
66968	0.891386	1.79699
66969	0.891386	1.68332
66970	0.946129	1.70841
66971	0.65979	1.62438
66972	0.891357	1.73172
66973	0.946129	1.72859
66974	1.89225	1.32602
66975	1.89225	1.46139
66976	1.89225	1.61622
66977	1.89225	1.3074
66978	1.89225	1.68491
66979	1.89225	1.42299
66980	1.79981	0.223473
66981	2.02514	0.323236
66982	2.33247	0.417697
66983	2.12891	0.21851
66984	4.15405	0.238232
66985	2.48284	0.995798
66986	2.02514	0.378472
66987	2.33247	0.460755

66988	2.02514	0.709987
66989	2.48567	0.427159
66990	2.17834	0.637498
66991	1.89226	0.343555
66992	1.89226	0.509352
66993	1.89226	0.623284
66994	1.89226	0.35466
66995	3.78453	0.38719
66996	1.89227	0.293899
66997	1.89227	0.174611
66998	1.89227	0.181856
66999	1.89227	0.295712
67000	1.89227	0.217004
67001	1.89227	0.27758
67002	2.50894	0.179103
67003	2.70118	0.297251
67004	2.12891	0.186325
67005	2.12891	0.24124
67006	2.7125	0.180285
67007	2.33247	0.255621
67008	1.89226	0.475973
67009	1.89226	0.26911
67010	1.89226	0.27983
67011	1.89226	0.423492
67012	1.89226	0.244615
67013	1.89226	0.372419
67014	0.946133	0.240516
67015	0.946133	0.234791
67016	0.946132	0.155113
67017	0.946132	0.15581
67018	0.946132	0.159497
67019	0.946132	0.253547
67020	0.946133	0.179346
67021	0.946133	0.24073
67022	0.946132	0.191319
67023	0.946132	0.179017
67024	0.946132	0.201205
67025	0.946132	0.209709
67026	1.90098	0.0887532
67027	1.36463	0.122457
67028	1.56656	0.0912588
67029	1.36463	0.113552
67030	2.04386	0.085663
67031	1.89227	0.124066
67032	1.89226	0.10164
67033	1.89226	0.116935
67034	1.89226	0.0943789
67035	1.89226	0.121525
67036	1.89226	0.0970093
67037	1.89226	0.123505
67038	1.89226	0.129383
67039	1.89226	0.186356
67040	1.89226	0.140113
67041	1.89226	0.202821
67042	1.89226	0.129493
67043	1.89226	0.187213
67044	1.89226	0.134208
67045	1.89226	0.208832

67046	1.89226	0.14235
67047	1.89226	0.184194
67048	1.89226	0.147923
67049	1.89226	0.203561
67050	1.89226	0.0680151
67051	1.70303	0.0899502
67052	2.1292	0.0703045
67053	1.70303	0.0892242
67054	2.32302	0.0702858
67055	1.90098	0.0946349
67056	1.89226	0.0852393
67057	1.89226	0.0952392
67058	1.89226	0.0752099
67059	1.89226	0.103758
67060	1.89226	0.08315
67061	1.89226	0.112104
67062	1.89226	0.100122
67063	1.89226	0.120865
67064	1.89226	0.100904
67065	1.89226	0.139872
67066	1.89226	0.0939426
67067	1.89226	0.129466
67068	1.89226	0.0919233
67069	1.89226	0.112734
67070	1.89226	0.0871935
67071	1.89226	0.113835
67072	1.89226	0.0900491
67073	1.89226	0.121924
67074	1.89226	0.933379
67075	1.89226	1.80686
67076	1.89226	1.81886
67077	1.89226	1.38367
67078	3.78453	1.13894
67079	1.89226	1.83114
67080	1.89226	2.1
67081	1.89226	1.27193
67082	1.89226	2.1
67083	1.89226	1.81276
67084	1.89226	2.1
67085	1.89226	1.79598
67086	1.89226	1.57571
67087	1.89226	1.10327
67088	1.89226	1.68583
67089	3.78452	1.71404
67090	1.89224	1.85971
67091	1.89224	1.34352
67092	1.89224	1.58139
67093	1.89224	1.83021
67094	1.89224	1.11263
67095	1.89224	1.75803
67096	1.89224	1.87235
67097	1.89224	2.1
67098	1.89224	1.93398
67099	1.89224	2.1
67100	1.89224	1.51172
67101	1.89224	2.1
67102	1.31307	2.1
67103	1.31307	2.1

67104	0.888948	1.8632
67105	0.888948	1.79274
67106	1.2559	1.88286
67107	0.888979	2.1
67108	0.946114	2.1
67109	0.946114	1.83598
67110	0.946129	1.79609
67111	0.888989	1.86276
67112	0.946104	1.76446
67113	1.25588	1.85288
67114	1.89224	0.936435
67115	1.89224	1.82219
67116	1.89224	1.28351
67117	1.89224	1.82064
67118	1.89224	1.56603
67119	1.89224	1.90499
67120	1.79375	0.0983593
67121	1.47689	0.091082
67122	1.97732	0.05
67123	2.26687	0.0649837
67124	3.74375	0.0644967
67125	2.05759	0.10991
67126	2.26686	0.0722508
67127	1.79374	0.0780226
67128	1.79374	0.132438
67129	2.49889	0.053106
67130	2.49889	0.104855
67131	1.89226	0.136981
67132	1.89226	0.135194
67133	1.89226	0.0658286
67134	1.89226	0.111289
67135	3.78452	0.10267
67136	1.89227	0.0621113
67137	1.89227	0.0610336
67138	1.89227	0.110503
67139	1.89227	0.121409
67140	1.89227	0.0983586
67141	1.89227	0.102535
67142	1.97732	0.0727254
67143	2.26687	0.0975417
67144	2.6607	0.0532806
67145	2.41464	0.0655328
67146	4.68151	0.0714564
67147	1.89226	0.100392
67148	1.89226	0.158128
67149	1.89226	0.0842769
67150	1.89226	0.0755827
67151	3.78452	0.1011
67152	1.89226	0.0930653
67153	1.89226	0.0757907
67154	1.89226	0.106649
67155	1.89226	0.159319
67156	1.89226	0.0636652
67157	1.89226	0.0989758
67158	1.89226	1.29823
67159	1.89226	1.54357
67160	1.89226	1.40027
67161	1.89226	1.29116

67162	3.78452	1.32041
67163	1.89226	1.37568
67164	1.89226	1.63668
67165	1.89226	1.3069
67166	1.89226	1.59953
67167	1.89226	1.38407
67168	1.89226	1.70996
67169	1.89226	1.57267
67170	1.89226	1.3169
67171	1.89226	1.30434
67172	1.89226	1.53297
67173	1.89226	1.28599
67174	1.89226	1.49004
67175	1.89226	1.37538
67176	1.89226	1.24561
67177	1.89226	1.28629
67178	1.89226	1.50144
67179	1.89226	1.19739
67180	1.89226	1.36171
67181	1.89226	1.35972
67182	2.45282	1.44073
67183	1.89226	1.45081
67184	1.89226	1.61721
67185	1.89226	1.28149
67186	1.89226	1.46541
67187	1.89226	1.25614
67188	1.89226	1.52192
67189	1.89226	1.32549
67190	1.89226	1.29914
67191	1.89226	1.57412
67192	1.89226	1.59133
67193	1.87308	0.0803157
67194	1.88631	0.0840784
67195	1.63908	0.0881717
67196	1.70302	0.108939
67197	2.08745	0.0691595
67198	2.16916	0.079013
67199	2.41019	0.0760925
67200	2.733	0.0614881
67201	2.08121	0.07837
67202	2.08121	0.107117
67203	2.74362	0.0759672
67204	2.41464	0.078573
67205	1.89226	0.0746417
67206	1.89226	0.117062
67207	1.89226	0.0680378
67208	1.89226	0.106119
67209	1.89226	0.0781752
67210	1.89226	0.12737
67211	0.946133	0.0935227
67212	0.946133	0.0889454
67213	0.946132	0.0955701
67214	0.946132	0.0957186
67215	0.946132	0.0948038
67216	0.946132	0.126951
67217	0.946133	0.0778054
67218	0.946133	0.0993676
67219	0.946132	0.0776203

67220	0.946132	0.0899071
67221	0.946133	0.0946351
67222	0.946133	0.122049
67223	2.02912	0.0831692
67224	2.57016	0.0790272
67225	1.88631	0.0851854
67226	1.88631	0.111157
67227	2.55301	0.0860781
67228	2.4102	0.0911452
67229	1.89226	0.105245
67230	1.89226	0.122852
67231	1.89226	0.0767004
67232	1.89226	0.0788598
67233	3.78452	0.0994186
67234	1.89227	0.104659
67235	1.89227	0.0885409
67236	1.89227	0.0954773
67237	1.89227	0.121904
67238	1.89227	0.0790998
67239	1.89227	0.0925949
67240	1.89226	2.1
67241	1.89226	2.1
67242	1.89226	2.09872
67243	1.89226	2.1
67244	3.78452	2.1
67245	1.2515	2.05716
67246	1.34788	2.1
67247	0.946132	2.1
67248	0.946132	2.1
67249	0.973043	2.1
67250	1.16766	2.1
67251	0.919222	1.98744
67252	1.2159	2.1
67253	0.946132	2.1
67254	0.946132	2.1
67255	1.33746	2.1
67256	1.13537	2.1
67257	1.89226	2.1
67258	1.89226	2.1
67259	1.89226	2.1
67260	1.89226	2.1
67261	1.89226	2.1
67262	1.89226	2.1
67263	1.89226	2.06958
67264	1.89226	2.1
67265	1.89226	2.1
67266	1.89226	2.07573
67267	3.78452	2.1
67268	1.89227	2.1
67269	1.89227	1.8874
67270	1.89227	2.1
67271	1.89227	1.99868
67272	1.89227	2.1
67273	1.89227	1.98187
67274	0.946132	2.09963
67275	0.946132	2.08614
67276	0.946129	2.1
67277	0.946129	2.1

67278	0.946131	2.1
67279	0.946131	2.1
67280	0.946131	2.1
67281	0.946131	2.08162
67282	0.946131	2.1
67283	0.946131	2.1
67284	0.94613	2.1
67285	0.94613	2.1
67286	1.89226	1.26717
67287	1.89226	1.27183
67288	1.89226	1.24961
67289	1.89226	1.15835
67290	3.78453	1.26124
67291	0.946132	1.37353
67292	0.946132	1.31355
67293	0.946132	1.23996
67294	0.946132	1.26555
67295	0.946132	1.19538
67296	0.946132	1.52641
67297	0.946132	1.33975
67298	0.946132	1.34159
67299	0.946131	1.43151
67300	0.946131	1.25655
67301	0.946132	1.31524
67302	0.946132	1.30503
67303	1.89226	1.32419
67304	1.89226	1.11642
67305	1.89226	1.10544
67306	1.89226	1.20642
67307	1.89226	1.19503
67308	1.89226	1.29062
67309	1.89226	1.30088
67310	1.89226	1.1799
67311	1.89226	1.16333
67312	1.89226	1.18589
67313	3.78453	1.17427
67314	0.96614	1.31495
67315	0.96614	1.34649
67316	0.946131	1.18879
67317	0.946131	1.22588
67318	0.946131	1.23454
67319	0.96614	1.23557
67320	1.37736	1.34422
67321	0.946131	1.32921
67322	1.37736	1.40549
67323	0.96614	1.22971
67324	0.946131	1.31491
67325	0.946131	1.21019
67326	0.946129	1.25417
67327	0.946129	1.20976
67328	0.946128	1.17664
67329	0.946128	1.18977
67330	0.946132	1.10792
67331	0.946132	1.45639
67332	0.946126	1.23268
67333	0.946126	1.21746
67334	0.946129	1.1389
67335	0.946129	1.16134

67336	0.946129	1.14312
67337	0.946129	1.2225
67338	1.89226	0.0941036
67339	1.65723	0.09929
67340	1.83117	0.100005
67341	1.65723	0.0913926
67342	2.0662	0.114293
67343	1.89226	0.104588
67344	1.89226	0.0955845
67345	1.89226	0.112433
67346	1.89226	0.100977
67347	1.89226	0.0990277
67348	1.89226	0.108703
67349	1.89226	0.103939
67350	1.89226	0.0931093
67351	1.89226	0.113394
67352	1.89226	0.111949
67353	1.89226	0.104985
67354	1.89226	0.108887
67355	1.89226	0.107081
67356	1.89226	0.087077
67357	1.89226	0.0915242
67358	1.89226	0.0886372
67359	1.89226	0.0932087
67360	1.89226	0.0958382
67361	1.89226	0.096839
67362	1.89226	0.0929347
67363	1.83117	0.0963087
67364	1.92807	0.0967312
67365	1.83117	0.091238
67366	1.98916	0.106303
67367	1.89226	0.099194
67368	1.89226	0.102704
67369	1.89226	0.107734
67370	1.89226	0.0969623
67371	1.89226	0.0981329
67372	3.78453	0.102659
67373	1.89226	0.0969196
67374	1.89226	0.149183
67375	1.89226	0.219536
67376	1.89226	0.177244
67377	1.89226	0.104152
67378	1.89226	0.105086
67379	1.89226	0.0881579
67380	1.89226	0.0894782
67381	1.89226	0.158207
67382	1.89226	0.149285
67383	1.89226	0.0932601
67384	1.89226	0.0909996
67385	1.88037	0.072303
67386	1.89227	0.0682446
67387	1.89227	0.0727132
67388	1.89227	0.077721
67389	1.98599	0.0792829
67390	1.98599	0.0788453
67391	1.89226	0.0790508
67392	1.89226	0.0791068
67393	1.89226	0.084046

67394	1.89226	0.206855
67395	1.89226	0.0818126
67396	1.89226	0.0872218
67397	1.89226	0.0868458
67398	1.89226	0.0937345
67399	1.89226	0.0988814
67400	1.89226	0.315725
67401	3.78452	0.0764869
67402	1.89226	0.0768261
67403	1.89226	0.149402
67404	1.89226	0.106295
67405	1.89226	0.0746482
67406	1.89226	0.114049
67407	1.89226	0.0919058
67408	1.90116	0.0735503
67409	1.89227	0.0656088
67410	1.89226	0.0750397
67411	1.89227	0.0865549
67412	1.88036	0.0786566
67413	1.88037	0.0699781
67414	1.89226	0.0794371
67415	1.89226	0.0766059
67416	1.89226	0.10018
67417	1.89226	0.173451
67418	1.89226	0.0780565
67419	1.89226	0.0802572
67420	0.946132	0.113208
67421	0.946132	0.0931609
67422	0.946128	0.109463
67423	0.946128	0.12123
67424	0.94613	0.146596
67425	0.94613	0.106644
67426	0.94613	0.0885395
67427	0.94613	0.156084
67428	0.946131	0.415148
67429	0.946131	0.694853
67430	0.94613	0.132132
67431	0.94613	0.3496
67432	1.89226	0.0900097
67433	1.89226	0.0886012
67434	1.89226	0.140564
67435	1.89226	0.114884
67436	1.89226	0.178196
67437	1.89226	0.0863977
67438	1.89226	0.627649
67439	1.89226	1.52747
67440	2.35239	1.48783
67441	2.27873	1.02759
67442	1.89226	1.46451
67443	1.83301	1.20534
67444	2.68449	1.38327
67445	1.89226	1.79956
67446	1.89226	1.34346
67447	1.89226	1.49633
67448	3.78453	1.36875
67449	0.946132	1.75067
67450	0.946132	1.41913
67451	0.946129	1.19299

67452	0.946129	1.70071
67453	0.946131	1.7049
67454	0.946131	1.58875
67455	0.94613	1.41673
67456	0.94613	1.4679
67457	0.946131	1.53835
67458	0.946131	1.47905
67459	0.946131	1.5406
67460	0.946131	1.59555
67461	1.89226	0.873548
67462	1.89226	1.42374
67463	1.89226	1.47945
67464	1.89226	1.20223
67465	1.89226	1.46281
67466	1.89226	1.2521
67467	1.70303	0.946448
67468	1.89226	1.47268
67469	1.89226	0.659407
67470	1.89226	1.1894
67471	3.78453	0.829283
67472	1.89227	1.44727
67473	1.89227	1.49803
67474	1.89227	1.16981
67475	1.89227	1.6404
67476	3.78453	1.51172
67477	1.89226	1.4049
67478	1.89226	1.80896
67479	1.89226	1.42343
67480	1.89226	1.57969
67481	3.78452	1.48979
67482	1.89226	2.03468
67483	1.89226	2.1
67484	1.89226	2.1
67485	1.89226	1.99699
67486	3.78452	2.01181
67487	0.946132	1.90901
67488	0.946132	2.02524
67489	0.946129	2.1
67490	0.946129	2.1
67491	0.94613	2.1
67492	0.94613	1.96592
67493	0.946131	2.1
67494	0.946131	1.9785
67495	0.946131	2.00013
67496	0.946131	2.07615
67497	0.946131	2.03884
67498	0.946131	1.93199
67499	1.89226	2.1
67500	1.89226	1.86195
67501	1.89226	1.93461
67502	1.89226	2.07026
67503	3.78452	2.07021
67504	1.89226	1.74941
67505	1.89226	2.01409
67506	1.89226	2.09588
67507	1.89226	1.77799
67508	1.89226	2.08229
67509	1.89226	1.77506

67510	1.89227	1.99056
67511	2.48308	1.76331
67512	1.89227	1.77323
67513	1.89227	1.90055
67514	3.78453	1.95191
67515	1.89226	1.84696
67516	1.89226	1.98528
67517	1.89226	2.07081
67518	1.89226	1.83196
67519	1.89226	2.03846
67520	1.89226	1.8084
67521	1.89226	1.92539
67522	1.89226	1.91926
67523	1.89226	1.95565
67524	1.89226	1.92749
67525	3.78453	2.03119
67526	0.946131	1.93484
67527	0.946131	2.00486
67528	0.946131	2.02536
67529	0.946131	2.1
67530	0.946131	2.02145
67531	0.946131	1.98004
67532	0.946131	1.98604
67533	0.946131	2.02271
67534	0.946131	1.97884
67535	0.946131	2.06726
67536	0.946131	2.00834
67537	0.946131	1.92429
67538	1.23719	2.08873
67539	1.28439	1.95192
67540	0.94613	1.94612
67541	0.94613	2.02553
67542	1.28439	2.07077
67543	1.6431	2.1
67544	0.946129	1.92031
67545	0.946129	1.90674
67546	1.28439	1.87822
67547	0.946131	1.80658
67548	1.21038	1.75993
67549	1.21038	1.91454
67550	1.89226	1.88072
67551	1.89226	1.8399
67552	1.89226	1.87573
67553	1.89226	1.84152
67554	1.89226	1.84138
67555	1.89226	1.82008
67556	1.89226	1.85095
67557	1.89226	1.92675
67558	1.89226	2.02687
67559	1.89226	1.82603
67560	1.89226	1.96591
67561	1.89226	1.79327
67562	1.89226	1.3648
67563	1.89226	0.487183
67564	1.89226	0.600573
67565	1.89226	1.20763
67566	1.89226	1.15405
67567	1.89226	1.39171

67568	0.946128	0.641039
67569	0.946128	1.166
67570	0.946125	0.276669
67571	0.946125	0.577174
67572	0.94613	0.342918
67573	0.94613	0.447917
67574	0.946124	0.836643
67575	0.946124	1.20944
67576	0.946127	0.412349
67577	0.946127	0.319655
67578	0.946127	0.756455
67579	0.946127	1.16547
67580	1.89226	0.275263
67581	2.78671	0.30724
67582	2.59302	0.279764
67583	2.59302	0.299807
67584	1.89226	0.413695
67585	1.89226	0.406296
67586	1.89226	0.373865
67587	1.89226	0.435038
67588	1.89226	0.291863
67589	1.89226	0.292153
67590	1.89226	0.765968
67591	1.89226	0.740074
67592	1.89225	0.275343
67593	1.89225	0.158129
67594	1.89225	0.384535
67595	1.89225	0.31653
67596	1.89225	0.561865
67597	1.89225	0.229892
67598	0.979536	0.236441
67599	0.816597	0.284385
67600	1.15446	0.226882
67601	1.07423	0.196954
67602	1.08452	0.240918
67603	0.603522	0.214429
67604	1.70302	0.199703
67605	1.29344	0.177601
67606	1.21806	0.201853
67607	0.723758	0.176492
67608	1.37562	0.258132
67609	1.89226	0.0872859
67610	1.92807	0.0876496
67611	1.90843	0.102538
67612	1.92807	0.105505
67613	1.87263	0.0940934
67614	1.89226	0.0901939
67615	0.946132	0.0926371
67616	0.946132	0.0953954
67617	0.946131	0.092626
67618	0.946131	0.0934976
67619	0.946132	0.0949591
67620	0.946132	0.105443
67621	0.946132	0.0959379
67622	0.946132	0.0973029
67623	0.946132	0.0844763
67624	0.946132	0.0916644
67625	0.946132	0.0954349

67626	0.946132	0.0961988
67627	1.89226	0.102514
67628	1.89226	0.248693
67629	1.89226	0.130879
67630	1.89226	0.118901
67631	3.78452	0.176135
67632	1.89226	0.0896547
67633	1.89226	0.119966
67634	1.89226	0.278044
67635	1.89226	0.148428
67636	1.89226	0.0857622
67637	1.89226	0.0845334
67638	1.70303	0.0754084
67639	1.90843	0.119535
67640	1.77002	0.0979024
67641	1.90843	0.110075
67642	1.75709	0.0788331
67643	1.89226	0.0798477
67644	1.89226	0.088895
67645	1.89226	0.0938054
67646	1.89226	0.0797811
67647	1.89226	0.122335
67648	3.78453	0.0844213
67649	1.89226	0.20627
67650	1.89226	0.332345
67651	1.89226	0.133754
67652	1.89226	0.377743
67653	3.78452	0.190901
67654	0.946131	0.159379
67655	0.946131	0.0787997
67656	0.946131	0.104302
67657	0.946131	0.0774553
67658	0.946129	0.0881452
67659	0.946129	0.101063
67660	0.946133	0.130844
67661	0.946133	0.251165
67662	0.946131	0.118466
67663	0.946131	0.0670587
67664	0.946131	0.0881007
67665	0.946131	0.374176
67666	1.89226	0.132314
67667	2.10275	0.459616
67668	1.76377	0.335647
67669	1.76377	0.203401
67670	1.89226	0.267622
67671	1.89226	0.166153
67672	1.89226	0.148373
67673	1.89226	0.536141
67674	2.10275	0.341331
67675	2.55889	0.20507
67676	1.89226	0.302172
67677	2.30274	0.188026
67678	1.89226	0.343317
67679	1.89226	1.2932
67680	1.89226	1.24965
67681	1.89226	0.5719
67682	1.89226	1.21596
67683	1.89226	0.586413

67684	1.89226	0.285718
67685	1.89226	1.27744
67686	1.89226	1.22931
67687	1.89226	0.608191
67688	1.89226	1.18029
67689	1.89226	0.550365
67690	1.89226	0.136759
67691	1.89226	0.567537
67692	1.89226	0.331251
67693	1.89226	0.190975
67694	1.89226	0.293953
67695	1.89226	0.166625
67696	1.89226	0.151583
67697	1.89226	0.561026
67698	1.89226	0.424938
67699	1.89226	0.22182
67700	1.89226	0.378678
67701	1.89226	0.202186
67702	1.89226	0.401544
67703	1.89226	1.28905
67704	1.89226	1.23711
67705	1.89226	0.696919
67706	1.89226	1.22305
67707	1.89226	0.789287
67708	1.89226	1.18506
67709	1.89226	1.25538
67710	1.89226	0.33718
67711	1.89226	0.928746
67712	1.89226	0.36731
67713	1.89226	1.1527
67714	0.946131	0.15335
67715	0.976399	0.135917
67716	1.10124	0.110947
67717	0.946131	0.251718
67718	0.887633	0.236532
67719	0.887633	0.19888
67720	1.16933	0.150818
67721	1.23163	0.130978
67722	0.946131	0.180662
67723	0.946131	0.191512
67724	0.976399	0.184065
67725	1.14992	0.182998
67726	1.89226	0.269412
67727	1.89226	0.861581
67728	1.89226	0.255484
67729	1.89226	0.354534
67730	3.78452	0.247393
67731	1.89226	0.154715
67732	1.89226	0.322288
67733	1.89226	0.140061
67734	1.89226	0.215792
67735	3.78453	0.151146
67736	0.946132	0.107656
67737	0.946132	0.23328
67738	0.946131	0.174621
67739	0.946131	0.232177
67740	0.946131	0.232648
67741	0.946131	0.194713

67742	0.946132	0.100884
67743	0.946132	0.113263
67744	0.946131	0.138648
67745	0.946131	0.180805
67746	0.946132	0.166325
67747	0.946132	0.109416
67748	0.946132	0.313144
67749	0.946132	0.195654
67750	0.946131	0.24607
67751	0.946131	0.406732
67752	0.946131	0.422172
67753	0.946131	0.34162
67754	0.946132	0.217051
67755	0.946132	0.1849
67756	0.946131	0.249256
67757	0.946131	0.341493
67758	0.946132	0.309457
67759	0.946132	0.217691
67760	1.89226	0.394474
67761	1.89226	0.681613
67762	1.89226	0.236221
67763	1.89226	0.737653
67764	3.78452	0.680707
67765	1.89226	1.08148
67766	1.89226	1.0643
67767	1.89226	0.834471
67768	1.89226	0.44182
67769	3.78453	0.98513
67770	1.89226	0.42457
67771	1.89226	1.0653
67772	1.89226	0.830626
67773	1.89226	1.11512
67774	1.89226	0.361395
67775	1.89226	1.05527
67776	1.89225	0.29778
67777	1.89225	0.497725
67778	1.89225	0.475644
67779	1.89225	1.07347
67780	1.89225	0.22998
67781	1.89225	0.797354
67782	1.89226	0.621393
67783	1.89226	0.770214
67784	1.89226	0.570033
67785	1.89226	1.05089
67786	1.89226	0.241547
67787	1.89226	0.526952
67788	1.89226	0.860695
67789	1.89226	0.401428
67790	1.89226	0.193857
67791	1.89226	0.188285
67792	3.78453	0.232008
67793	1.39491	0.274181
67794	1.39491	0.284903
67795	0.924649	0.322951
67796	0.924649	0.37567
67797	0.946131	0.394032
67798	1.37343	0.179301
67799	0.924678	0.110622

67800	0.946131	1.14821
67801	0.924678	0.229364
67802	1.37343	0.195775
67803	0.946131	0.765204
67804	0.946131	0.445941
67805	1.89226	0.509573
67806	1.89226	0.141073
67807	1.89226	0.227675
67808	1.89226	0.381273
67809	1.89226	0.130971
67810	1.89226	0.219143
67811	0.946126	0.05
67812	0.975461	0.05
67813	0.946132	0.05
67814	1.12674	0.0501618
67815	0.946129	0.0539032
67816	0.946129	0.05
67817	1.12673	0.05
67818	1.12673	0.05
67819	0.946129	0.05
67820	0.946129	0.0505561
67821	0.975464	0.05
67822	1.12673	0.05
67823	1.89226	0.0531052
67824	1.89226	0.0532743
67825	1.89226	0.067671
67826	1.89226	0.0514314
67827	1.89226	0.0605713
67828	1.89226	0.0534411
67829	0.946126	0.0555235
67830	0.946126	0.0654664
67831	0.946131	0.0543272
67832	0.946131	0.0990104
67833	0.946129	0.101685
67834	0.946129	0.089579
67835	0.946128	0.0611248
67836	0.946128	0.0527376
67837	0.946129	0.0765245
67838	0.946129	0.0991946
67839	0.946128	0.0813082
67840	0.946128	0.0818262
67841	1.89226	0.05
67842	1.89226	0.079355
67843	1.89226	0.05
67844	1.89226	0.0515554
67845	3.78451	0.05
67846	1.89226	0.0649614
67847	1.89226	0.072613
67848	1.89226	0.05
67849	1.89226	0.0823188
67850	3.78452	0.0801063
67851	0.946125	0.0643994
67852	0.946125	0.0776425
67853	0.946131	0.0715874
67854	0.946131	0.115801
67855	0.946128	0.120109
67856	0.946128	0.110033
67857	0.946128	0.0762824

67858	0.946128	0.0641582
67859	0.946128	0.0796427
67860	0.946128	0.10392
67861	0.946128	0.0713226
67862	0.946128	0.0950426
67863	1.89226	0.071797
67864	1.89226	0.115162
67865	1.89226	0.0810725
67866	1.89226	0.05
67867	3.78452	0.0656088
67868	1.89226	0.0831311
67869	1.89226	0.0954484
67870	1.89226	0.0985276
67871	1.89226	0.131453
67872	1.89226	0.0762199
67873	1.89226	0.0934139
67874	1.12492	0.129598
67875	1.41201	0.143867
67876	1.12082	0.11881
67877	0.771416	0.115109
67878	1.10725	0.103732
67879	1.10725	0.121106
67880	1.07618	0.219726
67881	1.07618	0.104021
67882	1.12492	0.128776
67883	0.771415	0.167591
67884	1.12082	0.127548
67885	1.41201	0.147779
67886	2.82401	0.0959197
67887	1.89226	0.0948969
67888	1.89226	0.089834
67889	1.89226	0.113519
67890	2.24983	0.0879248
67891	2.24983	0.105043
67892	0.946133	0.0883165
67893	0.946133	0.0957656
67894	0.946129	0.0750421
67895	0.946129	0.0708944
67896	0.946131	0.0677223
67897	0.946131	0.140316
67898	0.946131	0.086134
67899	0.946131	0.0678764
67900	0.946131	0.0809085
67901	0.946131	0.0800337
67902	0.946131	0.0857409
67903	0.946131	0.0924021
67904	0.946132	0.114196
67905	0.946132	0.114348
67906	0.946133	0.10782
67907	0.946133	0.104634
67908	0.946132	0.097721
67909	0.946132	0.142492
67910	0.946133	0.106512
67911	0.946133	0.12358
67912	0.946132	0.105753
67913	0.946132	0.113776
67914	0.946133	0.107052
67915	0.946133	0.110655

67916	1.89227	0.30045
67917	1.89227	0.342677
67918	1.89227	0.353208
67919	1.89227	0.283048
67920	1.89227	0.376764
67921	1.89227	0.303172
67922	1.42982	0.122096
67923	1.19944	0.0844948
67924	0.936311	0.157137
67925	0.936311	0.125057
67926	0.946132	0.129543
67927	1.02993	0.126593
67928	1.41513	0.140799
67929	0.946133	0.152508
67930	0.946133	0.128344
67931	0.946133	0.147318
67932	1.02993	0.147045
67933	1.18181	0.111311
67934	1.89226	0.354626
67935	1.89226	0.470572
67936	1.89226	0.542469
67937	1.89226	0.261077
67938	3.78453	0.363714
67939	1.89226	0.165206
67940	1.89226	0.193445
67941	1.89226	0.203339
67942	1.89226	0.166913
67943	1.89226	0.198936
67944	1.89226	0.164885
67945	1.89226	0.161113
67946	1.89226	0.171603
67947	1.89226	0.181556
67948	1.89226	0.152121
67949	1.89226	0.185515
67950	1.89226	0.156778
67951	0.946131	0.266743
67952	0.946131	0.361177
67953	0.946132	0.492912
67954	0.946132	0.358926
67955	0.946132	0.271148
67956	0.946132	0.312006
67957	0.946132	0.498159
67958	0.946132	0.268526
67959	0.946132	0.304584
67960	0.946132	0.326153
67961	0.946132	0.330268
67962	0.946132	0.288617
67963	1.89226	1.44649
67964	1.89226	1.46573
67965	1.89226	1.52937
67966	1.89226	1.32422
67967	1.89226	1.53751
67968	1.89226	1.39375
67969	1.89226	1.5061
67970	2.22749	0.742442
67971	1.89226	0.967222
67972	1.89226	1.01773
67973	3.78453	1.05076

67974	1.89226	1.42918
67975	1.89226	1.47022
67976	1.89226	1.53787
67977	1.89226	1.31482
67978	3.78453	1.4129
67979	1.89226	1.27735
67980	1.89226	1.24685
67981	1.89226	1.35005
67982	1.89226	0.823522
67983	1.89226	1.41438
67984	1.89226	1.24922
67985	0.94613	0.963597
67986	0.94613	1.39329
67987	0.94613	1.48577
67988	0.94613	1.33778
67989	0.946131	0.988869
67990	0.946131	0.75432
67991	0.946129	1.46971
67992	0.946129	1.26768
67993	0.94613	1.22837
67994	0.94613	1.20479
67995	0.94613	1.22372
67996	0.94613	1.10225
67997	1.89226	0.0830013
67998	1.89226	0.0879547
67999	1.89226	0.0864112
68000	1.89226	0.0788688
68001	1.89226	0.0883327
68002	1.89226	0.0806097
68003	1.89226	0.0983613
68004	1.89226	0.130679
68005	1.89226	0.131368
68006	1.89226	0.0758668
68007	3.78452	0.10706
68008	1.89225	0.0525027
68009	1.89225	0.0567886
68010	1.89225	0.0583887
68011	1.89225	0.05
68012	1.89225	0.0623817
68013	1.89225	0.0536757
68014	1.89226	0.05
68015	1.89226	0.05
68016	1.89226	0.0504339
68017	1.89226	0.05
68018	1.89226	0.05
68019	1.89226	0.05
68020	0.946132	0.072473
68021	0.946132	0.0961855
68022	0.946132	0.125388
68023	0.946132	0.08946
68024	0.946131	0.0778952
68025	0.946131	0.0694967
68026	0.946132	0.129168
68027	0.946132	0.0730968
68028	0.946132	0.0800858
68029	0.946132	0.0850352
68030	0.946132	0.0874783
68031	0.946132	0.0830824

68032	1.89226	0.080877
68033	1.89226	0.111962
68034	1.89226	0.0796384
68035	1.89226	0.162195
68036	1.89226	0.0719387
68037	1.89226	0.141665
68038	1.89226	0.0839215
68039	1.89226	0.0698409
68040	1.89226	0.062964
68041	1.89226	0.108209
68042	1.89226	0.0531547
68043	1.89226	0.0995306
68044	1.89226	0.05
68045	1.89226	0.0645064
68046	1.89226	0.05
68047	1.89226	0.0777062
68048	1.89226	0.05
68049	1.89226	0.0639988
68050	1.89226	0.0846649
68051	1.89226	0.129305
68052	1.89226	0.0715966
68053	1.89226	0.14706
68054	3.78452	0.0959815
68055	1.89226	0.0665245
68056	1.89226	0.0792644
68057	1.89226	0.0606808
68058	1.89226	0.128486
68059	1.89226	0.058625
68060	1.89226	0.114451
68061	1.89226	0.05
68062	1.89226	0.05
68063	1.89226	0.05
68064	1.89226	0.05
68065	3.78453	0.05
68066	1.89226	0.05
68067	2.48862	0.05
68068	1.89226	0.05
68069	2.22062	0.05
68070	1.89226	0.05
68071	2.22062	0.05
68072	1.89227	0.05
68073	1.89227	0.05
68074	1.89227	0.05
68075	1.89227	0.05
68076	1.89227	0.05
68077	1.89227	0.05
68078	0.946132	0.05
68079	0.946132	0.05
68080	0.946131	0.05
68081	0.946131	0.05
68082	0.94613	0.05
68083	0.94613	0.05
68084	0.946133	0.05
68085	0.946133	0.05
68086	0.946131	0.05
68087	0.946131	0.05
68088	0.946131	0.05
68089	0.946131	0.05

68090	1.89226	0.05
68091	1.89226	0.05
68092	1.89226	0.05
68093	1.89226	0.05
68094	3.78453	0.05
68095	1.89226	0.05
68096	1.89226	0.05
68097	1.89226	0.05
68098	1.89226	0.05
68099	3.78453	0.05
68100	1.89226	0.107563
68101	1.89226	0.0679522
68102	1.89226	0.05
68103	1.89226	0.0997408
68104	1.89226	0.0761115
68105	1.89226	0.114449
68106	1.89226	0.125627
68107	1.89226	0.05
68108	1.89226	0.0639682
68109	1.89226	0.0939367
68110	3.78452	0.089427
68111	1.89226	0.0642774
68112	1.89226	0.211162
68113	1.89226	0.0937013
68114	1.89226	0.0687657
68115	3.78453	0.0600645
68116	2.57454	0.142335
68117	1.89226	0.125524
68118	1.3663	0.114697
68119	1.89226	0.147291
68120	3.25856	0.118088
68121	1.89226	0.112166
68122	1.89226	0.0916674
68123	1.89226	0.0908296
68124	1.89226	0.104843
68125	3.78451	0.115998
68126	1.89226	0.0873547
68127	1.89226	0.188784
68128	1.89226	0.0894244
68129	1.89226	0.109842
68130	1.89226	0.0726418
68131	1.89226	0.0829818
68132	0.810946	0.101632
68133	0.810946	0.100601
68134	0.810946	0.110415
68135	0.810946	0.166176
68136	0.810959	0.101494
68137	0.810959	0.099771
68138	1.89226	0.122686
68139	1.35168	0.120458
68140	1.08132	0.105686
68141	1.08132	0.132053
68142	1.0813	0.124184
68143	1.89226	0.0895274
68144	1.89226	0.341356
68145	1.89226	0.402341
68146	1.89226	0.266899
68147	3.78453	0.176861

68148	1.89226	0.0941513
68149	1.89226	0.23398
68150	1.89226	0.0935705
68151	1.89226	0.317175
68152	1.89226	0.0724225
68153	1.89226	0.104421
68154	1.89226	0.156759
68155	1.89226	0.267768
68156	1.89226	0.31413
68157	1.89226	0.183214
68158	3.78452	0.102565
68159	1.89226	0.297634
68160	1.89226	0.275053
68161	1.89226	0.271738
68162	1.89226	0.405242
68163	3.78452	0.328473
68164	2.142	0.417049
68165	1.93949	0.116153
68166	1.39296	0.319272
68167	2.55711	0.494116
68168	1.39296	0.109093
68169	2.55711	0.234852
68170	1.89226	0.164349
68171	2.5571	0.0893281
68172	2.142	0.282237
68173	1.89226	0.425053
68174	4.44936	0.334789
68175	0.946131	0.306552
68176	0.946131	0.204536
68177	1.27855	0.301947
68178	1.29171	0.291555
68179	1.29171	0.327249
68180	0.946127	0.31328
68181	1.27855	0.0875374
68182	0.946131	0.391241
68183	1.29172	0.344813
68184	1.29172	0.258748
68185	0.946129	0.267871
68186	0.946129	0.229017
68187	1.27855	0.224423
68188	1.29172	0.350563
68189	0.969743	0.350222
68190	1.07109	0.355039
68191	1.29172	0.503599
68192	1.07109	0.148806
68193	0.969745	0.0974597
68194	1.27855	0.439698
68195	1.10873	0.439049
68196	1.10873	0.346099
68197	1.15273	0.271337
68198	1.15273	0.196321
68199	1.89227	0.05
68200	1.89227	0.05
68201	1.89227	0.05
68202	1.89227	0.05
68203	2.1288	0.05
68204	2.1288	0.05
68205	1.89227	0.05

68206	1.89227	0.05
68207	1.89227	0.05
68208	1.89227	0.0551016
68209	3.78453	0.05
68210	1.89226	0.0954176
68211	1.89226	0.05
68212	1.89226	0.0519777
68213	1.89226	0.05
68214	3.78453	0.118797
68215	1.89226	0.0533367
68216	1.89226	0.05
68217	1.89226	0.05
68218	1.89226	0.05
68219	1.89226	0.05
68220	1.89226	0.05
68221	1.94432	0.05
68222	1.89226	0.05
68223	2.07911	0.05
68224	1.89226	0.05
68225	2.07911	0.05
68226	1.89226	0.05
68227	1.89226	0.05
68228	1.89226	0.05
68229	1.89226	0.05
68230	1.89226	0.05
68231	1.89226	0.05
68232	1.89226	0.05
68233	1.89226	0.661335
68234	1.89226	0.638836
68235	1.89226	0.720279
68236	1.89226	0.429054
68237	1.89226	1.03391
68238	1.89226	0.630793
68239	1.89226	0.321984
68240	1.89226	0.262035
68241	1.89226	0.304483
68242	1.89226	0.22925
68243	1.89226	0.419055
68244	1.89226	0.314956
68245	1.86778	0.228888
68246	2.72348	0.210145
68247	1.89226	0.254459
68248	1.89226	0.135625
68249	3.76004	0.232422
68250	1.89226	0.791406
68251	1.89226	0.732238
68252	1.89226	1.28522
68253	1.89226	0.428748
68254	3.78451	0.832448
68255	1.89226	0.341581
68256	1.89226	0.305431
68257	1.89226	0.33044
68258	1.89226	0.241647
68259	1.89226	0.468816
68260	1.89226	0.33247
68261	0.94613	0.190405
68262	0.94613	0.265291
68263	0.94613	0.449129

68264	0.94613	0.228155
68265	0.946131	0.239345
68266	0.946131	0.151997
68267	0.946129	0.285704
68268	0.946129	0.235945
68269	0.94613	0.22398
68270	0.94613	0.24393
68271	0.94613	0.221839
68272	0.94613	0.229168
68273	0.946124	0.393655
68274	0.946124	0.223876
68275	0.94613	0.947414
68276	0.94613	0.36994
68277	0.946128	0.264546
68278	0.946128	0.292596
68279	0.946126	0.839378
68280	0.946126	0.409146
68281	0.946127	0.405107
68282	0.946127	0.379918
68283	0.946127	0.337487
68284	0.946127	0.342778
68285	2.09166	0.05
68286	2.1285	0.05
68287	1.78381	0.05
68288	1.78381	0.05
68289	2.47689	0.05
68290	2.47689	0.05
68291	2.09167	0.05
68292	2.26572	0.05
68293	2.73393	0.05
68294	2.4769	0.05
68295	4.74261	0.05
68296	1.89226	0.05
68297	1.89226	0.0670825
68298	1.89226	0.0563294
68299	1.89226	0.05
68300	1.89226	0.0636364
68301	1.89226	0.05
68302	0.946131	0.05
68303	0.946131	0.0703888
68304	0.946129	0.05
68305	0.946129	0.0610918
68306	0.94613	0.0613031
68307	0.94613	0.0640625
68308	0.946131	0.05
68309	0.946131	0.05
68310	0.94613	0.05
68311	0.94613	0.0510502
68312	0.946131	0.05
68313	0.946131	0.052637
68314	2.27622	0.05
68315	2.66786	0.05
68316	2.1285	0.05
68317	2.1285	0.05
68318	2.62461	0.05
68319	2.47689	0.05
68320	1.89226	0.05
68321	1.89226	0.0691601

68322	1.89226	0.05
68323	1.89226	0.0671597
68324	3.78453	0.0562268
68325	1.89226	0.05
68326	1.89226	0.0644735
68327	1.89226	0.0516536
68328	1.89226	0.0696793
68329	1.89226	0.05
68330	1.89226	0.0525293
68331	1.89226	0.291582
68332	1.89226	0.69853
68333	1.89226	0.261317
68334	1.89226	0.878632
68335	1.89226	0.256777
68336	1.89226	0.857161
68337	1.89226	1.31369
68338	1.89226	0.516254
68339	1.89226	0.869647
68340	1.89226	1.32056
68341	3.78452	1.03134
68342	1.89227	0.645713
68343	1.89227	1.26258
68344	1.89227	1.31402
68345	1.89227	0.873426
68346	3.78453	1.18692
68347	0.946127	0.978612
68348	0.946127	0.985911
68349	1.06438	0.434008
68350	0.946131	0.467328
68351	1.06438	0.317888
68352	0.94613	0.813663
68353	0.946128	0.990105
68354	0.946128	0.305779
68355	1.06438	0.377824
68356	1.06438	0.558667
68357	0.946129	0.761382
68358	0.946129	0.688388
68359	1.89226	0.601736
68360	1.89226	1.31192
68361	1.89226	0.912884
68362	1.89226	1.28154
68363	1.89226	0.830031
68364	1.89226	1.26637
68365	0.946133	1.22709
68366	0.946133	1.26121
68367	1.39771	1.21227
68368	0.946132	1.10577
68369	1.39771	1.19778
68370	1.39772	1.26753
68371	0.946133	1.2636
68372	0.946133	0.503298
68373	0.946132	0.793926
68374	1.39772	0.997175
68375	0.946132	0.463892
68376	0.946132	1.03975
68377	1.98182	0.05
68378	2.2217	0.05
68379	2.79889	0.05

68380	2.50097	0.05
68381	4.72267	0.05
68382	2.05362	0.05
68383	1.70302	0.05
68384	2.75109	0.05
68385	2.138 0.05	
68386	2.0815	0.05
68387	1.84751	0.05
68388	0.946131	0.05
68389	0.946131	0.05
68390	0.946131	0.05
68391	0.946131	0.05
68392	0.946131	0.05
68393	0.946131	0.05
68394	0.946132	0.05
68395	0.946132	0.05
68396	0.946131	0.05
68397	0.946131	0.05
68398	0.946132	0.05
68399	0.946132	0.05
68400	1.89226	0.05
68401	1.89226	0.05
68402	1.89226	0.05
68403	1.89226	0.05
68404	3.78451	0.05
68405	0.946126	0.05
68406	1.25048	0.05
68407	0.946131	0.05
68408	1.39945	0.05
68409	0.946127	0.05
68410	0.946127	0.05
68411	1.39944	0.05
68412	1.39944	0.05
68413	0.946129	0.05
68414	0.946129	0.05
68415	1.39944	0.05
68416	1.25048	0.05
68417	1.89226	0.05
68418	1.89226	0.05
68419	1.89226	0.05
68420	1.89226	0.05
68421	1.89226	0.05
68422	1.89226	0.05
68423	0.946126	0.05
68424	0.946126	0.05
68425	0.946131	0.05
68426	0.946131	0.05
68427	0.946128	0.0521056
68428	0.946128	0.05
68429	0.946129	0.05
68430	0.946129	0.05
68431	0.946129	0.05
68432	0.946129	0.05
68433	0.946128	0.05
68434	0.946128	0.05
68435	1.89226	0.05
68436	1.89226	0.05
68437	1.89226	0.05

68438	1.89226	0.05
68439	1.89226	0.05
68440	1.89226	0.05
68441	1.01393	0.05
68442	0.775245	0.05
68443	0.946126	0.05
68444	0.946126	0.05
68445	0.78305	0.05
68446	0.78305	0.05
68447	0.946159	0.05
68448	0.929267	0.05
68449	1.14956	0.05
68450	1.04073	0.05
68451	0.851526	0.05
68452	0.704781	0.05
68453	1.89226	0.05
68454	1.89226	0.05
68455	1.89226	0.05
68456	1.89226	0.05
68457	3.78451	0.05
68458	1.89226	0.05
68459	1.89226	0.05
68460	1.89226	0.05
68461	1.89226	0.05
68462	1.89226	0.05
68463	1.89226	0.05
68464	1.89226	0.05
68465	1.89226	0.05
68466	1.89226	0.05
68467	1.89226	0.05
68468	1.89226	0.05
68469	1.89226	0.05
68470	1.16317	0.05
68471	1.0292	0.05
68472	0.946126	0.05
68473	0.946126	0.05
68474	0.93562	0.05
68475	0.93562	0.05
68476	1.0418	0.05
68477	1.04181	0.05
68478	1.16317	0.05
68479	0.946129	0.05
68480	1.02919	0.05
68481	0.946128	0.05
68482	0.946131	0.05
68483	0.946131	0.05
68484	0.946126	0.05
68485	0.946126	0.05
68486	0.946129	0.05
68487	0.946129	0.05
68488	0.946128	0.05
68489	0.946128	0.05
68490	0.946129	0.05
68491	0.946129	0.05
68492	0.946128	0.05
68493	0.946128	0.05
68494	1.89226	0.05
68495	1.89226	0.05

68496	1.89226	0.05
68497	1.89226	0.05
68498	1.89226	0.05
68499	1.89226	0.05
68500	1.89226	0.05
68501	1.89226	0.05
68502	1.89226	0.05
68503	1.89226	0.05
68504	1.89226	0.05
68505	1.89226	0.05
68506	1.89226	0.05
68507	1.89226	0.05
68508	1.89226	0.05
68509	1.89226	0.05
68510	3.78453	0.05
68511	1.89226	0.05
68512	1.89226	0.05
68513	1.89226	0.05
68514	1.89226	0.05
68515	3.78452	0.05
68516	1.89225	0.05
68517	1.89225	0.05
68518	1.89225	0.05
68519	1.89225	0.05
68520	1.89225	0.05
68521	1.89225	0.05
68522	0.946132	0.05
68523	0.946132	0.05
68524	0.946133	0.05
68525	0.946133	0.05
68526	0.946132	0.05
68527	0.946132	0.05
68528	0.946132	0.05
68529	0.946132	0.05
68530	0.946133	0.05
68531	0.946133	0.05
68532	0.946132	0.05
68533	0.946132	0.05
68534	1.89226	0.05
68535	1.89226	0.05
68536	1.89226	0.05
68537	1.89226	0.05
68538	3.78453	0.05
68539	1.13537	0.05
68540	1.09222	0.05
68541	0.946126	0.05
68542	0.946126	0.05
68543	0.946128	0.05
68544	0.946128	0.05
68545	1.16317	0.05
68546	1.13034	0.05
68547	1.30155	0.05
68548	1.08462	0.05
68549	0.992915	0.05
68550	0.807642	0.05
68551	0.946131	0.05
68552	0.946131	0.05
68553	0.946125	0.05

68554	0.946125	0.05
68555	0.946128	0.05
68556	0.946128	0.05
68557	0.946128	0.05
68558	0.946128	0.05
68559	0.946128	0.05
68560	0.946128	0.05
68561	0.946128	0.05
68562	0.946128	0.05
68563	1.89226	0.05
68564	1.89226	0.05
68565	1.89226	0.05
68566	1.89226	0.05
68567	1.89226	0.05
68568	1.89226	0.05
68569	1.89224	0.05
68570	1.89224	0.05
68571	1.89224	0.05
68572	1.89224	0.05
68573	1.89224	0.05
68574	1.89224	0.05
68575	0.946086	0.05
68576	1.17919	0.05
68577	0.89738	0.05
68578	0.89738	0.05
68579	0.946116	0.05
68580	0.946116	0.05
68581	1.13048	0.05
68582	1.11844	0.05
68583	0.946128	0.05
68584	0.897362	0.05
68585	1.1282	0.05
68586	0.946103	0.05
68587	1.89223	0.05
68588	1.89223	0.05
68589	1.89223	0.05
68590	1.89223	0.05
68591	3.78446	0.05
68592	0.946118	0.05
68593	0.946118	0.05
68594	0.946118	0.05
68595	0.946118	0.05
68596	0.946119	0.05
68597	0.946119	0.05
68598	0.946118	0.05
68599	0.946118	0.05
68600	0.946131	0.05
68601	0.946131	0.05
68602	0.946106	0.05
68603	0.946106	0.05
68604	1.89227	0.418394
68605	1.89227	0.959815
68606	1.89227	0.823589
68607	1.89227	0.616612
68608	3.78453	0.576989
68609	1.89226	1.03875
68610	1.89226	0.445429
68611	1.89226	0.435223

68612	1.89226	0.667058
68613	1.89226	0.734385
68614	1.89226	1.00903
68615	1.89226	0.289437
68616	2.59378	0.198052
68617	2.30348	0.19716
68618	2.30348	0.280618
68619	1.89226	0.236726
68620	1.89226	0.364849
68621	1.89226	0.605485
68622	1.89226	0.403907
68623	1.89226	0.397806
68624	1.89226	0.636856
68625	3.78453	0.628787
68626	1.89226	0.544714
68627	1.89226	0.353769
68628	1.89226	0.325953
68629	1.89226	0.552464
68630	1.89226	0.396832
68631	1.89226	0.905427
68632	0.904406	0.20003
68633	1.01667	0.219624
68634	1.11974	0.18889
68635	0.994417	0.247093
68636	1.02013	0.224679
68637	0.628203	0.244083
68638	1.70302	0.261939
68639	1.49898	0.222383
68640	1.14559	0.25301
68641	0.900571	0.219764
68642	1.58886	0.222991
68643	2.81102	0.05
68644	2.3923	0.05
68645	2.05632	0.05
68646	2.43514	0.05
68647	4.82744	0.05
68648	1.89226	0.05
68649	1.89226	0.05
68650	1.89226	0.05
68651	1.89226	0.05
68652	1.89226	0.05
68653	1.89226	0.05
68654	1.89226	0.05
68655	1.89226	0.05
68656	1.89226	0.05
68657	1.89226	0.05
68658	3.78451	0.05
68659	2.50803	0.05
68660	1.903	0.05
68661	1.903	0.05
68662	2.19162	0.05
68663	2.09934	0.05
68664	2.39063	0.05
68665	1.00243	0.05
68666	0.731731	0.05
68667	0.876616	0.05
68668	1.00451	0.05
68669	0.986901	0.05

68670	0.986901	0.05
68671	0.887729	0.05
68672	0.887732	0.05
68673	0.876605	0.05
68674	0.769283	0.05
68675	1.00245	0.05
68676	1.18712	0.05
68677	0.946131	0.05
68678	0.946131	0.05
68679	0.946131	0.05
68680	0.946131	0.05
68681	0.946131	0.05
68682	0.946131	0.05
68683	0.946131	0.05
68684	0.946131	0.05
68685	0.946131	0.05
68686	0.946131	0.05
68687	0.946131	0.05
68688	0.946131	0.05
68689	1.89226	0.05
68690	1.89226	0.05
68691	1.89226	0.05
68692	1.89226	0.05
68693	1.89226	0.05
68694	1.89226	0.05
68695	1.89226	0.0570302
68696	1.89226	0.0975927
68697	1.89226	0.0748496
68698	1.89226	0.0831428
68699	3.78453	0.0884052
68700	1.89226	0.100379
68701	1.89226	0.107447
68702	1.89226	0.0864158
68703	1.89226	0.162178
68704	3.78453	0.096529
68705	1.89226	0.0548321
68706	1.89226	0.0950165
68707	1.89226	0.0719242
68708	1.89226	0.0734404
68709	1.89226	0.079467
68710	1.89226	0.0806916
68711	0.946132	0.0841335
68712	0.946132	0.0603426
68713	0.946131	0.0786497
68714	0.946131	0.0601087
68715	0.946131	0.0811041
68716	0.946131	0.0914085
68717	0.946132	0.0514444
68718	0.946132	0.0588119
68719	0.946131	0.0702205
68720	0.946131	0.0586996
68721	0.946131	0.0568486
68722	0.946131	0.0670859
68723	0.946131	0.133387
68724	0.946131	0.0908016
68725	0.946131	0.10447
68726	0.946131	0.0868704
68727	0.94613	0.11447

68728	0.94613	0.13819
68729	0.946132	0.0810246
68730	0.946132	0.0916905
68731	0.946131	0.0929435
68732	0.946131	0.108302
68733	0.946131	0.0995895
68734	0.946131	0.101384
68735	1.89226	0.05
68736	1.89226	0.05
68737	1.89226	0.05
68738	1.89226	0.05
68739	1.89226	0.05
68740	1.89226	0.05
68741	1.89226	0.05
68742	1.89226	0.05
68743	1.89226	0.05
68744	1.89226	0.057059
68745	3.78453	0.05
68746	1.89226	0.0555744
68747	1.89226	0.0638472
68748	1.89226	0.0978448
68749	1.89226	0.0562571
68750	1.89226	0.0891517
68751	1.89226	0.05
68752	0.946132	0.05
68753	0.946132	0.05
68754	0.946131	0.05
68755	0.946131	0.05
68756	0.946132	0.05
68757	0.946132	0.05
68758	0.946131	0.05
68759	0.946131	0.05
68760	0.946132	0.05
68761	0.946132	0.05
68762	0.946131	0.05
68763	0.946131	0.05
68764	0.946131	0.05
68765	0.946131	0.05
68766	0.946132	0.0595349
68767	0.946132	0.0721035
68768	0.946132	0.0654694
68769	0.946132	0.05
68770	0.946131	0.05
68771	0.946131	0.05
68772	0.946132	0.054267
68773	0.946132	0.0518131
68774	0.946131	0.05
68775	0.946131	0.0535001
68776	1.89226	0.471929
68777	1.89226	0.682494
68778	1.89226	0.772497
68779	1.89226	0.400123
68780	3.78452	0.413579
68781	1.89226	1.32087
68782	1.89226	1.02405
68783	1.89226	1.24559
68784	1.89226	1.27149
68785	3.78452	1.30954

68786	1.89226	0.928439
68787	1.89226	0.991989
68788	1.89226	1.25793
68789	1.89226	0.557888
68790	3.78452	0.582448
68791	1.89226	0.533978
68792	2.75945	0.21009
68793	1.89226	0.327645
68794	2.35026	0.318748
68795	4.24252	0.578326
68796	0.946131	0.408197
68797	0.946131	0.491557
68798	0.946131	0.290071
68799	0.946131	0.3347
68800	0.946132	0.286186
68801	0.946132	0.322704
68802	0.946129	0.516249
68803	0.946129	0.334779
68804	0.946131	0.41618
68805	0.946131	0.413219
68806	0.94613	0.509647
68807	0.94613	0.405606
68808	1.89226	0.919772
68809	1.89226	1.1373
68810	1.89226	0.757979
68811	1.89226	1.11572
68812	1.89226	0.834113
68813	1.89226	1.22543
68814	0.946129	0.479837
68815	0.946129	0.668247
68816	0.946133	0.617423
68817	0.946133	0.385469
68818	0.946131	0.357301
68819	0.946131	0.378401
68820	0.946131	0.689971
68821	0.946131	0.413432
68822	0.946131	0.56419
68823	0.946131	0.532809
68824	0.946131	0.680557
68825	0.946131	0.503973
68826	1.89226	0.236652
68827	2.35026	0.217369
68828	1.32126	0.191062
68829	1.89226	0.238988
68830	1.77925	0.1941
68831	2.35026	0.236331
68832	1.89226	0.0515193
68833	1.89226	0.109534
68834	1.89226	0.0959047
68835	1.89226	0.0743456
68836	1.89226	0.0733204
68837	1.89226	0.0555244
68838	1.89226	0.0914568
68839	1.89226	0.185207
68840	1.89226	0.12451
68841	1.89226	0.168005
68842	3.78453	0.174036
68843	1.89226	0.0725521

68844	1.89226	0.155359
68845	1.89226	0.126477
68846	1.89226	0.106914
68847	1.89226	0.107346
68848	1.89226	0.0875916
68849	0.946131	0.153101
68850	0.946131	0.0986995
68851	0.946131	0.102463
68852	0.946131	0.11724
68853	0.946132	0.155727
68854	0.946132	0.166241
68855	0.946131	0.11333
68856	0.946131	0.0976231
68857	0.946132	0.0897664
68858	0.946132	0.13323
68859	0.946131	0.102422
68860	0.946131	0.124046
68861	1.89226	0.0633775
68862	1.89226	0.123643
68863	1.89226	0.0817116
68864	1.89226	0.0769226
68865	1.89226	0.12695
68866	1.89226	0.12916
68867	1.89226	0.0997943
68868	1.89226	0.162859
68869	1.89226	0.134394
68870	1.89226	0.14607
68871	3.78453	0.153439
68872	1.89227	0.166953
68873	1.89227	0.160505
68874	1.89227	0.14812
68875	1.89227	0.24344
68876	3.78453	0.156293
68877	1.89226	0.0897609
68878	1.89226	0.133668
68879	1.89226	0.123209
68880	1.89226	0.121336
68881	3.78452	0.125263
68882	0.946132	0.130548
68883	0.946132	0.106373
68884	0.946131	0.122009
68885	0.946131	0.107345
68886	0.946131	0.132471
68887	0.946131	0.142913
68888	0.946133	0.114059
68889	0.946133	0.0933516
68890	0.946132	0.0987869
68891	0.946132	0.119574
68892	0.946131	0.0975211
68893	0.946131	0.111611
68894	1.89227	0.132216
68895	1.89227	0.194481
68896	1.89227	0.135377
68897	1.89227	0.141087
68898	1.89227	0.177517
68899	1.89227	0.182739
68900	2.20491	0.107318
68901	1.81656	0.106185

68902	1.81656	0.105906
68903	1.89226	0.113913
68904	1.89226	0.0898192
68905	1.89226	0.108757
68906	1.89226	0.170083
68907	1.89226	0.239844
68908	1.89226	0.219844
68909	1.89226	0.190144
68910	3.78452	0.193252
68911	1.89226	0.38996
68912	1.89226	0.327812
68913	1.89226	0.303555
68914	1.89226	0.536206
68915	3.78453	0.388675
68916	1.89226	0.326152
68917	1.89226	0.464213
68918	1.89226	0.359316
68919	1.89226	0.432447
68920	1.89226	0.382225
68921	1.89226	0.480139
68922	1.89226	0.184084
68923	2.11032	0.155039
68924	2.30793	0.156884
68925	1.89226	0.174053
68926	2.30793	0.165897
68927	1.89226	0.181808
68928	1.89226	0.255448
68929	1.89226	0.375849
68930	1.89226	0.27455
68931	1.89226	0.320945
68932	1.89226	0.306562
68933	1.89226	0.381316
68934	0.810943	0.340111
68935	0.810943	0.310987
68936	0.810943	0.328654
68937	0.810943	0.292883
68938	0.810933	0.319126
68939	0.810933	0.31805
68940	1.89226	0.254556
68941	1.3517	0.31459
68942	1.08132	0.291951
68943	1.08132	0.363671
68944	1.08133	0.313924
68945	1.89226	0.33097
68946	1.89226	0.159635
68947	1.89226	0.210014
68948	1.89226	0.27925
68949	3.78453	0.295242
68950	1.89226	0.145339
68951	1.89226	0.149978
68952	1.89226	0.197901
68953	1.89226	0.11972
68954	1.89226	0.188259
68955	1.89226	0.111238
68956	0.810945	0.131742
68957	0.810945	0.101305
68958	0.870829	0.130116
68959	0.870829	0.110498

68960	0.990502	0.092617
68961	0.810933	0.109367
68962	2.82777	0.0744373
68963	1.53126	0.104712
68964	1.32076	0.0970056
68965	1.201	0.101963
68966	1.08133	0.105386
68967	1.89226	0.196314
68968	1.89226	0.11422
68969	1.89226	0.143335
68970	1.89226	0.136585
68971	1.89226	0.149094
68972	1.89226	0.14217
68973	0.946132	0.172279
68974	0.946132	0.199759
68975	0.946133	0.370459
68976	0.946133	0.230259
68977	0.946132	0.282844
68978	0.946132	0.2074
68979	0.946132	0.250223
68980	0.946132	0.296839
68981	0.946133	0.23224
68982	0.946133	0.194337
68983	0.946132	0.171058
68984	0.946132	0.219097
68985	0.946133	0.0989437
68986	0.946133	0.118259
68987	0.946132	0.167283
68988	0.946132	0.194289
68989	0.946132	0.164046
68990	0.946132	0.123582
68991	0.946132	0.149287
68992	0.946132	0.203338
68993	0.946133	0.111407
68994	0.946133	0.140382
68995	0.946132	0.122119
68996	0.946132	0.142868
68997	1.89226	0.116976
68998	1.89226	0.227327
68999	1.89226	0.168625
69000	1.89226	0.170419
69001	1.89226	0.140509
69002	1.89226	0.144559
69003	1.89226	0.179542
69004	1.89226	0.363619
69005	1.89226	0.260686
69006	1.89226	0.330076
69007	3.78452	0.381873
69008	1.89226	0.368924
69009	1.89226	0.398368
69010	1.89226	0.342491
69011	1.89226	0.771948
69012	3.78452	0.333631
69013	1.89226	0.166109
69014	1.89226	0.296406
69015	1.89226	0.191538
69016	1.89226	0.219447
69017	1.89226	0.294542

69018	1.89226	0.303874
69019	0.946128	0.23612
69020	0.946128	0.198201
69021	0.946132	0.212303
69022	0.946132	0.19131
69023	0.946129	0.259105
69024	0.946129	0.283956
69025	0.946131	0.192817
69026	0.946131	0.161546
69027	0.94613	0.230087
69028	0.94613	0.13618
69029	0.946131	0.162461
69030	0.946131	0.19704
69031	0.946129	0.376545
69032	0.946129	0.290629
69033	0.946133	0.311499
69034	0.946133	0.269268
69035	0.946131	0.371529
69036	0.946131	0.400703
69037	0.946131	0.243308
69038	0.946131	0.267591
69039	0.946131	0.357932
69040	0.946131	0.258963
69041	0.946131	0.288431
69042	0.946131	0.335923
69043	1.89226	0.189058
69044	1.89226	0.134305
69045	1.89226	0.13795
69046	1.89226	0.290988
69047	3.78452	0.127181
69048	1.89225	0.05
69049	1.89225	0.0937363
69050	1.89225	0.0958602
69051	1.89225	0.101177
69052	1.89225	0.05
69053	1.89225	0.05
69054	1.89226	0.092013
69055	1.89226	0.0629224
69056	1.89226	0.0771703
69057	1.89226	0.110442
69058	3.78452	0.0771893
69059	1.89226	0.132973
69060	1.89226	0.323781
69061	1.89226	0.298279
69062	1.89226	0.219177
69063	3.78453	0.269755
69064	1.89226	0.0858466
69065	1.89226	0.156622
69066	1.89226	0.144814
69067	1.89226	0.122749
69068	3.78452	0.15909
69069	1.22164	0.05
69070	1.22164	0.05
69071	0.806032	0.05
69072	0.806032	0.05
69073	1.08154	0.05
69074	0.946126	0.05
69075	0.946129	0.05

69076	0.806065	0.05
69077	0.806063	0.05
69078	1.08155	0.05
69079	0.946128	0.05
69080	0.946128	0.05
69081	1.28554	0.05
69082	1.28554	0.05
69083	0.946126	0.05
69084	0.946126	0.0723286
69085	1.28554	0.0699511
69086	0.946129	0.05
69087	1.08155	0.05
69088	0.94613	0.05
69089	1.08155	0.05
69090	1.28554	0.05
69091	0.94613	0.05
69092	0.94613	0.0518833
69093	0.946132	0.110635
69094	0.946132	0.171042
69095	0.946132	0.190734
69096	0.946132	0.25534
69097	0.946132	0.341272
69098	0.946132	0.14863
69099	0.946133	0.13827
69100	0.946133	0.133445
69101	0.946132	0.168062
69102	0.946132	0.178755
69103	0.946133	0.16492
69104	0.946133	0.170426
69105	1.89226	1.25679
69106	1.89226	0.671652
69107	1.89226	1.35008
69108	1.89226	0.769738
69109	3.78453	1.2406
69110	1.89226	1.10994
69111	1.89226	0.528084
69112	1.89226	0.613044
69113	1.89226	0.7577
69114	1.89226	0.683761
69115	1.89226	0.821686
69116	1.89226	0.301277
69117	1.89226	0.203108
69118	1.89226	0.223608
69119	1.89226	0.245598
69120	1.89226	0.246585
69121	1.89226	0.268811
69122	1.89226	0.397664
69123	1.89226	0.242715
69124	1.89226	0.288893
69125	1.89226	0.358915
69126	1.89226	0.311987
69127	1.89226	0.255268
69128	0.946132	0.604196
69129	0.946132	0.707141
69130	0.946132	0.389078
69131	0.946132	0.682836
69132	0.946132	0.414819
69133	0.946132	0.43777

69134	0.946132	0.640442
69135	0.946132	0.564832
69136	0.946131	0.329999
69137	0.946131	0.6143
69138	0.946132	0.487997
69139	0.946132	0.451472
69140	1.89226	0.520642
69141	1.89226	0.280495
69142	1.89226	0.536472
69143	1.89226	0.392363
69144	3.78453	0.417584
69145	0.851541	0.128717
69146	0.72883	0.176641
69147	0.991276	0.185911
69148	0.851485	0.150517
69149	1.00003	0.15002
69150	1.00003	0.124126
69151	0.809788	0.169443
69152	0.809788	0.167711
69153	0.890738	0.171965
69154	0.991264	0.149901
69155	0.851554	0.153073
69156	0.851554	0.164004
69157	0.8628	0.22551
69158	0.78387	0.169701
69159	0.830233	0.213266
69160	0.830233	0.233646
69161	0.808312	0.214388
69162	0.817238	0.150526
69163	1.70303	0.160489
69164	1.24116	0.185714
69165	0.995299	0.149346
69166	1.0989	0.194544
69167	1.02714	0.221819
69168	1.89226	1.1907
69169	1.89226	0.935226
69170	1.89226	0.800401
69171	1.89226	1.95647
69172	3.78451	0.689802
69173	0.946131	0.98077
69174	0.946131	0.392534
69175	0.946131	0.703772
69176	0.946131	0.734605
69177	0.94613	0.626602
69178	0.94613	0.397002
69179	0.946132	0.374157
69180	0.946132	0.463343
69181	0.946131	0.496648
69182	0.946131	0.695336
69183	0.946131	0.483544
69184	0.946131	0.40583
69185	1.89226	0.780117
69186	1.89226	2.1
69187	1.89226	1.86765
69188	1.89226	1.88094
69189	3.78453	2.07665
69190	1.89226	2.1
69191	1.89226	2.1

69192	1.89226	2.1
69193	1.89226	1.90503
69194	1.89226	2.1
69195	1.89226	1.87787
69196	0.946126	0.450841
69197	1.17111	0.148995
69198	1.3393	0.689829
69199	0.946131	0.756745
69200	0.946127	0.774753
69201	0.946127	0.30589
69202	1.3393	0.345956
69203	1.17111	0.36194
69204	1.3393	0.492843
69205	1.3393	0.107209
69206	0.946129	0.352162
69207	0.946129	0.542706
69208	0.946126	2.1
69209	0.946126	1.34777
69210	0.946132	2.1
69211	0.946132	2.1
69212	0.94613	2.1
69213	0.94613	1.93238
69214	0.946129	2.1
69215	0.946129	1.95361
69216	0.946129	0.820725
69217	0.946129	2.1
69218	0.94613	1.91703
69219	0.94613	1.89602
69220	0.946131	0.891306
69221	0.946131	0.694261
69222	0.94613	0.568957
69223	0.94613	0.562125
69224	0.946128	0.895364
69225	0.946128	1.01051
69226	0.946132	0.494395
69227	0.946132	0.548969
69228	0.94613	0.843349
69229	0.94613	0.467241
69230	0.94613	0.600657
69231	0.94613	0.883988
69232	0.94613	1.81952
69233	0.94613	1.62647
69234	0.94613	1.58707
69235	0.94613	1.437
69236	0.946128	1.80439
69237	0.946128	1.84769
69238	0.946131	1.39105
69239	0.946131	1.28357
69240	0.94613	1.77468
69241	0.94613	1.35473
69242	0.94613	1.5148
69243	0.94613	1.72704
69244	1.89226	1.85567
69245	1.89226	1.68474
69246	1.89226	1.7436
69247	1.89226	2.1
69248	3.78453	1.74943
69249	1.89226	1.22325

69250	1.89226	1.27493
69251	1.89226	0.860038
69252	1.89226	1.22973
69253	1.89226	0.979037
69254	1.89226	1.35697
69255	1.89226	1.11699
69256	1.89226	1.7113
69257	1.89226	1.26639
69258	1.89226	1.67064
69259	1.89226	0.842188
69260	1.89226	1.23979
69261	1.89227	0.40639
69262	2.28622	0.454309
69263	2.16109	0.343057
69264	1.89227	0.350601
69265	4.17849	0.356583
69266	15.1381	0.425415
69267	15.1381	1.02809
69268	15.1381	0.160857
69269	15.1381	0.856116
69270	30.2762	0.768127
69271	15.1381	0.866933
69272	15.1381	1.00471
69273	15.1381	0.153123
69274	15.1381	0.952008
69275	30.2762	0.185031
69276	15.1381	0.839653
69277	15.1381	1.15612
69278	15.1381	0.152678
69279	15.1381	1.29292
69280	30.2762	1.0083
69281	15.1381	0.256065
69282	15.1381	1.12373
69283	15.1381	0.0557455
69284	15.1381	0.883044
69285	30.2762	0.841843
69286	15.138	1.35649
69287	15.138	1.82524
69288	15.138	1.33878
69289	15.138	2.01182
69290	30.276	1.84692
69291	15.138	1.86794
69292	15.138	2.1
69293	15.138	2.04164
69294	15.138	2.1
69295	30.276	1.93179
69296	15.138	1.64485
69297	15.138	1.53517
69298	15.138	1.37686
69299	15.138	2.05093
69300	30.2759	1.91587
69301	15.138	1.58542
69302	15.138	1.92909
69303	15.138	2.09298
69304	15.138	1.5259
69305	30.2759	1.68149
69306	15.138	2.1
69307	15.138	2.03043

69308	15.138	2.1
69309	15.138	2.09878
69310	30.276	2.1
69311	15.1381	2.09105
69312	15.1381	2.1
69313	15.1381	2.08892
69314	15.1381	2.1
69315	30.2762	2.1
69316	15.1381	2.1
69317	15.1381	2.1
69318	15.1381	2.1
69319	15.1381	2.1
69320	30.2762	2.1
69321	15.138	1.97051
69322	15.138	2.1
69323	15.138	2.08815
69324	15.138	2.08587
69325	30.276	2.09277
69326	15.138	2.1
69327	15.138	2.05211
69328	15.138	2.1
69329	15.138	2.1
69330	30.276	2.08405
69331	15.1381	1.59253
69332	15.1381	2.04317
69333	15.1381	1.94364
69334	15.1381	1.87641
69335	30.2762	1.96206
69336	15.1381	1.98632
69337	15.1381	2.1
69338	15.1381	2.01921
69339	15.1381	2.0855
69340	30.2762	2.01644
69341	15.1381	2.1
69342	15.1381	2.07406
69343	15.1381	2.1
69344	15.1381	2.0847
69345	30.2762	2.1
69346	15.138	0.98741
69347	15.138	0.862333
69348	15.138	0.0584283
69349	15.138	1.16068
69350	30.276	0.676446
69351	1.89226	2.1
69352	1.89226	2.1
69353	1.89226	2.1
69354	1.89226	2.1
69355	3.78453	2.1
69356	1.89226	2.1
69357	1.89226	2.1
69358	1.89226	2.1
69359	1.89226	2.1
69360	1.89226	2.1
69361	1.89226	2.1
69362	1.89226	2.1
69363	1.89226	2.1
69364	1.89226	2.1
69365	1.89226	2.1

69366	1.89226	2.1
69367	1.89226	2.1
69368	1.89226	2.1
69369	1.89226	2.1
69370	1.89226	2.1
69371	1.89226	2.1
69372	3.78453	2.1
69373	1.89226	2.1
69374	1.89226	2.1
69375	1.89226	2.1
69376	1.89226	2.1
69377	1.89226	2.1
69378	1.89226	2.1
69379	0.946127	2.1
69380	0.946127	2.1
69381	0.946131	2.1
69382	0.946131	2.1
69383	0.946131	2.1
69384	0.946131	2.1
69385	0.946128	2.1
69386	0.946128	2.1
69387	0.94613	2.1
69388	0.94613	2.1
69389	0.946129	2.1
69390	0.946129	2.1
69391	1.89226	2.1
69392	1.89226	2.1
69393	1.89226	2.1
69394	1.89226	2.1
69395	1.89226	2.1
69396	1.89226	2.1
69397	1.89226	2.1
69398	1.89226	2.1
69399	1.89226	2.1
69400	1.89226	2.1
69401	3.78452	2.1
69402	1.89226	2.1
69403	1.89226	2.1
69404	1.89226	2.1
69405	1.89226	2.1
69406	1.89226	2.1
69407	1.89226	2.1
69408	0.946129	2.1
69409	0.946129	2.1
69410	0.946133	2.1
69411	0.946133	2.1
69412	0.946131	2.1
69413	0.946131	2.1
69414	0.946131	2.1
69415	0.946131	2.1
69416	0.946131	2.1
69417	0.946131	2.1
69418	0.946131	2.1
69419	0.946131	2.1
69420	0.946132	2.1
69421	0.946132	2.1
69422	0.946132	2.1
69423	0.946132	2.1

69424	0.946132	2.1
69425	0.946132	2.1
69426	0.946133	2.1
69427	0.946133	2.1
69428	0.946132	2.1
69429	0.946132	2.1
69430	0.946133	2.1
69431	0.946133	2.1
69432	1.89226	2.1
69433	1.89226	2.1
69434	1.89226	2.1
69435	1.89226	2.1
69436	3.78452	2.1
69437	1.89226	2.1
69438	1.89226	2.1
69439	1.89226	2.1
69440	1.89226	2.1
69441	1.89226	2.1
69442	1.89226	2.1
69443	0.946129	2.1
69444	0.946129	2.1
69445	0.946133	2.1
69446	0.946133	2.1
69447	0.946131	2.1
69448	0.946131	2.1
69449	0.946131	2.1
69450	0.946131	2.1
69451	0.946131	2.1
69452	0.946131	2.1
69453	0.946131	2.1
69454	0.946131	2.1
69455	0.946132	2.1
69456	0.946132	2.1
69457	0.946132	2.1
69458	0.946132	2.1
69459	0.946132	2.1
69460	0.946132	2.1
69461	0.946133	2.1
69462	0.946133	2.1
69463	0.946133	2.1
69464	0.946133	2.1
69465	0.946132	2.1
69466	0.946132	2.1
69467	1.89226	2.1
69468	1.89226	2.1
69469	1.89226	2.1
69470	1.89226	2.1
69471	3.78453	2.1
69472	0.946128	2.1
69473	0.946128	2.1
69474	0.946132	2.1
69475	0.946132	2.1
69476	0.94613	2.1
69477	0.94613	2.1
69478	0.94613	2.1
69479	0.94613	2.1
69480	0.94613	2.1
69481	0.94613	2.1

69482	0.94613	2.1
69483	0.94613	2.1
69484	0.946127	2.1
69485	0.946127	2.1
69486	0.946131	2.1
69487	0.946131	2.1
69488	0.94613	2.1
69489	0.94613	2.1
69490	0.946128	2.1
69491	0.946128	2.1
69492	0.946129	2.1
69493	0.946129	2.1
69494	0.946129	2.1
69495	0.946129	2.1
69496	0.94613	2.1
69497	0.94613	2.1
69498	0.94613	2.1
69499	0.94613	2.1
69500	0.946131	2.1
69501	0.946131	2.1
69502	0.946129	2.1
69503	0.946129	2.1
69504	0.94613	2.1
69505	0.94613	2.1
69506	0.94613	2.1
69507	0.94613	2.1
69508	1.89226	2.1
69509	1.89226	2.1
69510	1.89226	2.1
69511	1.89226	2.1
69512	3.78453	2.1
69513	1.89226	2.1
69514	1.89226	2.1
69515	1.89226	2.1
69516	1.89226	2.1
69517	1.89226	2.1
69518	1.89226	2.1
69519	1.89226	2.1
69520	1.89226	2.1
69521	1.89226	2.1
69522	1.89226	2.1
69523	1.89226	2.1
69524	1.89226	2.1
69525	0.946131	2.1
69526	0.946131	2.1
69527	0.946131	2.1
69528	0.946131	2.1
69529	0.946132	2.1
69530	0.946132	2.1
69531	0.946129	2.1
69532	0.946129	2.1
69533	0.946131	2.1
69534	0.946131	2.1
69535	0.94613	2.1
69536	0.94613	2.1
69537	1.89224	2.1
69538	1.89224	2.1
69539	1.89224	2.1

69540	1.89224	2.1
69541	3.78448	2.1
69542	1.89224	2.1
69543	1.89224	2.1
69544	1.89224	2.1
69545	1.89224	2.1
69546	3.78447	2.1
69547	1.89223	2.1
69548	2.38995	2.1
69549	2.25315	2.1
69550	2.25315	2.1
69551	1.89223	2.1
69552	1.89223	2.1
69553	1.89224	2.1
69554	1.89224	2.1
69555	1.89224	2.1
69556	1.89224	2.1
69557	1.89224	2.1
69558	1.89224	2.1
69559	1.89226	2.1
69560	1.89226	2.1
69561	1.89226	2.1
69562	1.89226	2.1
69563	3.78453	2.1
69564	1.89226	2.1
69565	1.89226	2.1
69566	1.89226	2.1
69567	1.89226	2.1
69568	1.89226	2.1
69569	1.89226	2.1
69570	1.89226	2.1
69571	2.60272	2.1
69572	2.38998	2.1
69573	2.38998	2.1
69574	1.89226	2.1
69575	1.89226	2.1
69576	0.94613	2.1
69577	0.94613	2.1
69578	0.94613	2.1
69579	0.94613	2.1
69580	0.946131	2.1
69581	0.946131	2.1
69582	0.946129	2.1
69583	0.946129	2.1
69584	0.94613	2.1
69585	0.94613	2.1
69586	0.94613	2.1
69587	0.94613	2.1
69588	1.89226	2.1
69589	1.89226	2.1
69590	1.89226	2.1
69591	1.89226	2.1
69592	3.78452	2.1
69593	1.89226	2.1
69594	1.89226	2.1
69595	1.89226	2.1
69596	1.89226	2.1
69597	3.78452	2.1

69598	1.89226	2.1
69599	1.89226	2.1
69600	1.89226	2.1
69601	1.89226	2.1
69602	1.89226	2.1
69603	1.89226	2.1
69604	0.946132	2.1
69605	0.946132	2.1
69606	0.946132	2.1
69607	0.946132	2.1
69608	0.946131	2.1
69609	0.946131	2.1
69610	0.946132	2.1
69611	0.946132	2.1
69612	0.946132	2.1
69613	0.946132	2.1
69614	0.946132	2.1
69615	0.946132	2.1
69616	1.89226	2.1
69617	1.89226	2.1
69618	1.89226	2.1
69619	1.89226	2.1
69620	3.78452	2.1
69621	1.89226	2.1
69622	1.89226	2.1
69623	1.89226	2.1
69624	1.89226	2.1
69625	3.78452	2.1
69626	0.946129	2.1
69627	0.946129	2.1
69628	0.946133	2.1
69629	0.946133	2.1
69630	0.946131	2.1
69631	0.946131	2.1
69632	0.946131	2.1
69633	0.946131	2.1
69634	0.946131	2.1
69635	0.946131	2.1
69636	0.946131	2.1
69637	0.946131	2.1
69638	0.946132	2.1
69639	0.946132	2.1
69640	0.946132	2.1
69641	0.946132	2.1
69642	0.946132	2.1
69643	0.946132	2.1
69644	0.946133	2.1
69645	0.946133	2.1
69646	0.946133	2.1
69647	0.946133	2.1
69648	0.946132	2.1
69649	0.946132	2.1
69650	1.89224	2.1
69651	1.89224	2.1
69652	1.89224	2.1
69653	1.89224	2.1
69654	3.78447	2.1
69655	1.89223	2.1

69656	1.89223	2.1
69657	1.89223	2.1
69658	1.89223	2.1
69659	1.89223	2.1
69660	1.89223	2.1
69661	1.89224	2.1
69662	1.89224	2.1
69663	1.89224	2.1
69664	1.89224	2.1
69665	3.78447	2.1
69666	1.89224	2.1
69667	1.89224	2.1
69668	1.89224	2.1
69669	1.89224	2.1
69670	3.78448	2.1
69671	1.89226	2.1
69672	1.89226	2.1
69673	1.89226	2.1
69674	1.89226	2.1
69675	3.78452	2.1
69676	1.89226	2.1
69677	1.89226	2.1
69678	1.89226	2.1
69679	1.89226	2.1
69680	1.89226	2.1
69681	1.89226	2.1
69682	1.89226	2.1
69683	1.89226	2.1
69684	1.89226	2.1
69685	1.89226	2.1
69686	1.89226	2.1
69687	1.89226	2.1
69688	0.810946	2.1
69689	0.810946	2.1
69690	0.810946	2.1
69691	0.810946	2.1
69692	0.810933	2.1
69693	0.810933	2.1
69694	1.89226	2.1
69695	1.3517	2.1
69696	1.08132	2.1
69697	1.08132	2.1
69698	1.08133	2.1
69699	1.89226	2.1
69700	1.89226	2.1
69701	1.89226	2.1
69702	1.89226	2.1
69703	3.78453	2.1
69704	1.89226	2.1
69705	1.89226	2.1
69706	1.89226	2.1
69707	1.89226	2.1
69708	3.78452	2.1
69709	1.89226	2.1
69710	1.89226	2.1
69711	1.89226	2.1
69712	1.89226	2.1
69713	1.89226	2.1

69714	1.89226	2.1
69715	0.94613	2.1
69716	0.94613	2.1
69717	0.94613	2.1
69718	0.94613	2.1
69719	0.946131	2.1
69720	0.946131	2.1
69721	0.946129	2.1
69722	0.946129	2.1
69723	0.94613	2.1
69724	0.94613	2.1
69725	0.94613	2.1
69726	0.94613	2.1
69727	1.89226	2.1
69728	1.89226	2.1
69729	1.89226	2.1
69730	1.89226	2.1
69731	3.78453	2.1
69732	1.89226	2.1
69733	1.89226	2.1
69734	1.89226	2.1
69735	1.89226	2.1
69736	3.78452	2.1
69737	1.89226	2.1
69738	1.89226	2.1
69739	1.89226	2.1
69740	1.89226	2.1
69741	3.78452	2.1
69742	1.89226	2.1
69743	1.89226	2.1
69744	1.89226	2.1
69745	1.89226	2.1
69746	1.89226	2.1
69747	1.89226	2.1
69748	1.89226	2.1
69749	1.89226	2.1
69750	1.89226	2.1
69751	1.89226	2.1
69752	3.78452	2.1
69753	1.89226	2.1
69754	1.89226	2.1
69755	1.89226	2.1
69756	1.89226	2.1
69757	3.78452	2.1
69758	0.946128	2.1
69759	0.946128	2.1
69760	0.946133	2.1
69761	0.946133	2.1
69762	0.94613	2.1
69763	0.94613	2.1
69764	0.946131	2.1
69765	0.946131	2.1
69766	0.946131	2.1
69767	0.946131	2.1
69768	0.946131	2.1
69769	0.946131	2.1
69770	0.946132	2.1
69771	0.946132	2.1

69772	0.946132	2.1
69773	0.946132	2.1
69774	0.946131	2.1
69775	0.946131	2.1
69776	0.946132	2.1
69777	0.946132	2.1
69778	0.946132	2.1
69779	0.946132	2.1
69780	0.946132	2.1
69781	0.946132	2.1
69782	1.89226	2.1
69783	1.89226	2.1
69784	1.89226	2.1
69785	1.89226	2.1
69786	3.78452	2.1
69787	1.89226	2.1
69788	1.89226	2.1
69789	1.89226	2.1
69790	1.89226	2.1
69791	3.78452	2.1
69792	0.946129	2.1
69793	0.946129	2.1
69794	0.946133	2.1
69795	0.946133	2.1
69796	0.946131	2.1
69797	0.946131	2.1
69798	0.946131	2.1
69799	0.946131	2.1
69800	0.946131	2.1
69801	0.946131	2.1
69802	0.946131	2.1
69803	0.946131	2.1
69804	0.946132	2.1
69805	0.946132	2.1
69806	0.946132	2.1
69807	0.946132	2.1
69808	0.946132	2.1
69809	0.946132	2.1
69810	0.946133	2.1
69811	0.946133	2.1
69812	0.946132	2.1
69813	0.946132	2.1
69814	0.946133	2.1
69815	0.946133	2.1
69816	1.89226	2.1
69817	1.89226	2.1
69818	1.89226	2.1
69819	1.89226	2.1
69820	1.89226	2.1
69821	1.89226	2.1
69822	0.946128	2.1
69823	0.946128	2.1
69824	0.946133	2.1
69825	0.946133	2.1
69826	0.946131	2.1
69827	0.946131	2.1
69828	0.94613	2.1
69829	0.94613	2.1

69830	0.946131	2.1
69831	0.946131	2.1
69832	0.946131	2.1
69833	0.946131	2.1
69834	1.89226	2.1
69835	1.89226	2.1
69836	1.89226	2.1
69837	1.89226	2.1
69838	1.89226	2.1
69839	1.89226	2.1
69840	1.89226	2.1
69841	1.89226	2.1
69842	1.89226	2.1
69843	1.89226	2.1
69844	3.78453	2.1
69845	1.89226	2.1
69846	1.89226	2.1
69847	1.89226	2.1
69848	1.89226	2.1
69849	1.89226	2.1
69850	1.89226	2.1
69851	0.946127	2.1
69852	0.946127	2.1
69853	0.946131	2.1
69854	0.946131	2.1
69855	0.946131	2.1
69856	0.946131	2.1
69857	0.946127	2.1
69858	0.946127	2.1
69859	0.946129	2.1
69860	0.946129	2.1
69861	0.94613	2.1
69862	0.94613	2.1
69863	0.946131	2.1
69864	0.946131	2.1
69865	0.946131	2.1
69866	0.946131	2.1
69867	0.946133	2.1
69868	0.946133	2.1
69869	0.946128	2.1
69870	0.946128	2.1
69871	0.94613	2.1
69872	0.94613	2.1
69873	0.946131	2.1
69874	0.946131	2.1
69875	1.89226	2.1
69876	1.89226	2.1
69877	1.89226	2.1
69878	1.89226	2.1
69879	1.89226	2.1
69880	1.89226	2.1
69881	1.89226	2.1
69882	1.89226	2.1
69883	1.89226	2.1
69884	1.89226	2.1
69885	3.78452	2.1
69886	1.89226	2.1
69887	1.89226	2.1

69888	1.89226	2.1
69889	1.89226	2.1
69890	3.78452	2.1
69891	1.89226	2.1
69892	1.89226	2.1
69893	1.89226	2.1
69894	1.89226	2.1
69895	1.89226	2.1
69896	1.89226	2.1
69897	1.89226	2.1
69898	1.89226	2.1
69899	1.89226	2.1
69900	1.89226	2.1
69901	3.78453	2.1
69902	1.89226	2.1
69903	1.89226	2.1
69904	1.89226	2.1
69905	1.89226	2.1
69906	3.78452	2.1
69907	1.89226	2.1
69908	1.89226	2.1
69909	1.89226	2.1
69910	1.89226	2.1
69911	3.78452	2.1
69912	1.89226	2.1
69913	1.89226	2.1
69914	1.89226	2.1
69915	1.89226	2.1
69916	1.89226	2.1
69917	1.89226	2.1
69918	0.946131	2.1
69919	0.946131	2.1
69920	0.946131	2.1
69921	0.946131	2.1
69922	0.946131	2.1
69923	0.946131	2.1
69924	0.946131	2.1
69925	0.946131	2.1
69926	0.946131	2.1
69927	0.946131	2.1
69928	0.946131	2.1
69929	0.946131	2.1
69930	1.89226	2.1
69931	1.89226	2.1
69932	1.89226	2.1
69933	1.89226	2.1
69934	1.89226	2.1
69935	1.89226	2.1
69936	1.89226	2.1
69937	1.89226	2.1
69938	1.89226	2.1
69939	1.89226	2.1
69940	1.89226	2.1
69941	1.89226	2.1
69942	0.946131	2.1
69943	0.946131	2.1
69944	0.946131	2.1
69945	0.946131	2.1

69946	0.946131	2.1
69947	0.946131	2.1
69948	0.946131	2.1
69949	0.946131	2.1
69950	0.946131	2.1
69951	0.946131	2.1
69952	0.946131	2.1
69953	0.946131	2.1
69954	1.89226	2.1
69955	1.89226	2.1
69956	1.89226	2.1
69957	1.89226	2.1
69958	1.89226	2.1
69959	1.89226	2.1
69960	0.946128	2.1
69961	0.946128	2.1
69962	0.946133	2.1
69963	0.946133	2.1
69964	0.946131	2.1
69965	0.946131	2.1
69966	0.946131	2.1
69967	0.946131	2.1
69968	0.946131	2.1
69969	0.946131	2.1
69970	0.94613	2.1
69971	0.94613	2.1
69972	1.89226	2.1
69973	1.89226	2.1
69974	1.89226	2.1
69975	1.89226	2.1
69976	1.89226	2.1
69977	1.89226	2.1
69978	0.946131	2.1
69979	0.946131	2.1
69980	0.946131	2.1
69981	0.946131	2.1
69982	0.946132	2.1
69983	0.946132	2.1
69984	0.946131	2.1
69985	0.946131	2.1
69986	0.946132	2.1
69987	0.946132	2.1
69988	0.946131	2.1
69989	0.946131	2.1
69990	1.89226	2.1
69991	1.89226	2.1
69992	1.89226	2.1
69993	1.89226	2.1
69994	1.89226	2.1
69995	1.89226	2.1
69996	1.89226	2.1
69997	1.89226	2.1
69998	1.89226	2.1
69999	1.89226	2.1
70000	3.78452	2.1
70001	1.89226	2.1
70002	1.89226	2.1
70003	1.89226	2.1

70004	1.89226	2.1
70005	3.78452	2.1
70006	0.946131	2.1
70007	0.946131	2.1
70008	0.946131	2.1
70009	0.946131	2.1
70010	0.946132	2.1
70011	0.946132	2.1
70012	0.946131	2.1
70013	0.946131	2.1
70014	0.946132	2.1
70015	0.946132	2.1
70016	0.946131	2.1
70017	0.946131	2.1
70018	1.89226	2.1
70019	1.89226	2.1
70020	1.89226	2.1
70021	1.89226	2.1
70022	1.89226	2.1
70023	1.89226	2.1
70024	1.89226	2.1
70025	1.89226	2.1
70026	1.89226	2.1
70027	1.89226	2.1
70028	3.78452	2.1
70029	1.89226	2.1
70030	1.89226	2.1
70031	1.89226	2.1
70032	1.89226	2.1
70033	3.78452	2.1
70034	0.946131	2.1
70035	0.946131	2.1
70036	0.946131	2.1
70037	0.946131	2.1
70038	0.946131	2.1
70039	0.946131	2.1
70040	0.946131	2.1
70041	0.946131	2.1
70042	0.946131	2.1
70043	0.946131	2.1
70044	0.946131	2.1
70045	0.946131	2.1
70046	1.89226	2.1
70047	1.89226	2.1
70048	1.89226	2.1
70049	1.89226	2.1
70050	3.78453	2.1
70051	1.89226	2.1
70052	1.89226	2.1
70053	1.89226	2.1
70054	1.89226	2.1
70055	1.89226	2.1
70056	1.89226	2.1
70057	1.89226	2.1
70058	1.89226	2.1
70059	1.89226	2.1
70060	1.89226	2.1
70061	3.78453	2.1

70062	1.89226	2.1
70063	1.89226	2.1
70064	1.89226	2.1
70065	1.89226	2.1
70066	3.78453	2.1
70067	1.89226	2.1
70068	1.89226	2.1
70069	1.89226	2.1
70070	1.89226	2.1
70071	3.78453	2.1
70072	0.946132	2.1
70073	0.946132	2.1
70074	0.946132	2.1
70075	0.946132	2.1
70076	0.946132	2.1
70077	0.946132	2.1
70078	0.946132	2.1
70079	0.946132	2.1
70080	0.946132	2.1
70081	0.946132	2.1
70082	0.946131	2.1
70083	0.946131	2.1
70084	0.946131	2.1
70085	0.946131	2.1
70086	0.946131	2.1
70087	0.946131	2.1
70088	0.946132	2.1
70089	0.946132	2.1
70090	0.946131	2.1
70091	0.946131	2.1
70092	0.946132	2.1
70093	0.946132	2.1
70094	0.946131	2.1
70095	0.946131	2.1
70096	1.89226	2.1
70097	1.89226	2.1
70098	1.89226	2.1
70099	1.89226	2.1
70100	3.78453	2.1
70101	1.89226	2.1
70102	1.89226	2.1
70103	1.89226	2.1
70104	1.89226	2.1
70105	3.78453	2.1
70106	0.946131	2.1
70107	0.946131	2.1
70108	0.946131	2.1
70109	0.946131	2.1
70110	0.946131	2.1
70111	0.946131	2.1
70112	0.946131	2.1
70113	0.946131	2.1
70114	0.946131	2.1
70115	0.946131	2.1
70116	0.946131	2.1
70117	0.946131	2.1
70118	1.89226	2.1
70119	1.89226	2.1

70120	1.89226	2.1
70121	1.89226	2.1
70122	3.78452	2.1
70123	0.946132	2.1
70124	0.946132	2.1
70125	0.946131	2.1
70126	0.946131	2.1
70127	0.946132	2.1
70128	0.946132	2.1
70129	0.946131	2.1
70130	0.946131	2.1
70131	0.946131	2.1
70132	0.946131	2.1
70133	0.946131	2.1
70134	0.946131	2.1
70135	1.89226	2.1
70136	1.89226	2.1
70137	1.89226	2.1
70138	1.89226	2.1
70139	1.89226	2.1
70140	1.89226	2.1
70141	0.946132	2.1
70142	0.946132	2.1
70143	0.946132	2.1
70144	0.946132	2.1
70145	0.946132	2.1
70146	0.946132	2.1
70147	0.946132	2.1
70148	0.946132	2.1
70149	0.946132	2.1
70150	0.946132	2.1
70151	0.946131	2.1
70152	0.946131	2.1
70153	0.946131	2.1
70154	0.946131	2.1
70155	0.946131	2.1
70156	0.946131	2.1
70157	0.946132	2.1
70158	0.946132	2.1
70159	0.946131	2.1
70160	0.946131	2.1
70161	0.946132	2.1
70162	0.946132	2.1
70163	0.946131	2.1
70164	0.946131	2.1
70165	0.946132	2.1
70166	0.946132	2.1
70167	0.946131	2.1
70168	0.946131	2.1
70169	0.946132	2.1
70170	0.946132	2.1
70171	0.946131	2.1
70172	0.946131	2.1
70173	0.946132	2.1
70174	0.946132	2.1
70175	0.946131	2.1
70176	0.946131	2.1
70177	1.89226	2.1

70178	1.89226	2.1
70179	1.89226	2.1
70180	1.89226	2.1
70181	3.78453	2.1
70182	1.89226	2.1
70183	1.89226	2.1
70184	1.89226	2.1
70185	1.89226	2.1
70186	3.78453	2.1
70187	1.89226	2.1
70188	1.89226	2.1
70189	1.89226	2.1
70190	1.89226	2.1
70191	1.89226	2.1
70192	1.89226	2.1
70193	1.89226	2.1
70194	1.89226	2.1
70195	1.89226	2.1
70196	1.89226	2.1
70197	3.78453	2.1
70198	1.89226	2.1
70199	1.89226	2.1
70200	1.89226	2.1
70201	1.89226	2.1
70202	3.78453	2.1
70203	1.89226	2.1
70204	1.89226	2.1
70205	1.89226	2.1
70206	1.89226	2.1
70207	1.89226	2.1
70208	1.89226	2.1
70209	1.89226	2.1
70210	1.89226	2.1
70211	1.89226	2.1
70212	1.89226	2.1
70213	1.89226	2.1
70214	1.89226	2.1
70215	1.89226	2.1
70216	1.89226	2.1
70217	1.89226	2.1
70218	1.89226	2.1
70219	3.78453	2.1
70220	1.89226	2.1
70221	1.89226	2.1
70222	1.89226	2.1
70223	1.89226	2.1
70224	1.89226	2.1
70225	1.89226	2.1
70226	1.89226	2.1
70227	1.89226	2.1
70228	1.89226	2.1
70229	1.89226	2.1
70230	1.89226	2.1
70231	1.89226	2.1
70232	1.89226	2.1
70233	1.89226	1.94622
70234	1.89226	2.1
70235	1.89226	1.61568

70236	3.78453	2.1
70237	1.89226	2.1
70238	1.89226	1.55347
70239	1.89226	1.3688
70240	1.89226	2.1
70241	1.89226	1.34461
70242	1.89226	2.1
70243	1.89226	2.1
70244	1.89226	2.1
70245	1.89226	2.1
70246	1.89226	2.1
70247	1.89226	2.1
70248	1.89226	2.1
70249	1.89226	2.1
70250	1.89226	2.1
70251	1.89226	2.1
70252	1.89226	2.1
70253	3.78453	2.1
70254	1.89226	2.1
70255	1.89226	1.94729
70256	1.89226	2.1
70257	1.89226	1.91607
[truncated: 385,090 more chars]
