# Supplementary material for: On the Use of Bone Remodelling Models to Estimate the Density Distribution of Bones. Uniqueness of the Solution
Source: PLoS One. 2016 Feb 9;11(2):e0148603. doi: 10.1371/journal.pone.0148603 (PMC4747586; doi:10.1371/journal.pone.0148603)
Supplement: S12 File — (PDF) [file pone.0148603.s012.pdf]

N° Elem	Volume (mm3)	Density (g/cm3)
1	1.32415	2.1
2	1.75823	2.1
3	0.984099	2.1
4	1.46442	2.1
5	1.53082	2.1
6	1.32068	2.1
7	1.91404	0.05
8	1.17036	0.05
9	0.929209	0.05
10	1.27254	0.05
11	2.0926	0.05
12	2.20407	0.05
13	1.21488	0.05
14	1.26024	0.05
15	0.794454	0.05
16	1.95087	0.05
17	0.953925	2.1
18	0.98949	2.1
19	1.27875	2.1
20	1.04598	2.1
21	0.594005	2.1
22	0.578861	2.1
23	1.09506	2.1
24	1.45327	2.1
25	2.58396	2.1
26	1.58581	2.1
27	1.5858	2.1
28	0.636946	2.1
29	0.94202	2.1
30	0.946119	2.1
31	0.636953	2.1
32	0.946107	2.1
33	1.09505	2.1
34	1.27389	2.1
35	1.00232	2.1
36	1.00231	2.1
37	1.83583	2.1
38	1.00231	2.1
39	1.28828	0.05
40	1.81696	0.05
41	1.28828	0.05
42	1.28828	0.05
43	1.0044	0.0570326
44	0.85148	0.0574322
45	0.851539	0.0576915
46	1.57767	0.05
47	1.52227	0.05
48	0.730657	0.05
49	0.94613	0.058205
50	1.28228	2.1
51	1.64268	2.1
52	1.10101	2.1
53	1.3939	2.1
54	0.959471	2.1
55	1.10101	2.1

56	1.2873	2.1
57	1.59659	2.1
58	1.80841	0.562134
59	1.80841	1.9777
60	0.946129	0.791415
61	1.36991	0.245559
62	1.32262	2.1
63	1.32261	0.981579
64	1.38464	1.15837
65	0.962877	0.751637
66	0.962878	0.21039
67	1.82129	0.255224
68	1.82129	0.734257
69	0.94613	0.535425
70	1.39751	0.509464
71	0.946131	0.388117
72	1.34493	0.161554
73	1.39751	0.243183
74	0.837577	0.477679
75	0.823478	0.325347
76	1.10889	0.677845
77	1.01686	0.501341
78	1.1942	0.679146
79	0.836129	0.343904
80	0.937165	0.184841
81	0.969438	0.443439
82	0.895386	0.092948
83	1.30554	0.570833
84	1.15798	0.145427
85	1.22578	0.689049
86	1.15255	0.277797
87	0.9765	0.125621
88	2.02178	0.758535
89	1.63755	0.672457
90	0.727713	0.522356
91	0.810945	0.558277
92	1.00559	0.470859
93	0.879098	0.433751
94	1.07852	0.626892
95	0.657764	0.105443
96	0.829406	0.454039
97	0.720201	0.0873376
98	0.946131	0.0847438
99	1.11827	0.082415
100	1.50754	0.05
101	1.64835	0.0582299
102	1.71903	0.119676
103	0.851554	0.0578887
104	1.75626	0.121182
105	2.1646	0.100402
106	1.5563	0.103775
107	1.5563	0.249484
108	2.10876	0.252212
109	1.05438	0.112173
110	0.946133	0.204686
111	1.05438	1.06959
112	1.20256	0.160932
113	1.47879	1.18363

114	1.47879	1.38118
115	0.77815	1.38203
116	0.947114	2.1
117	1.31257	2.1
118	1.09235	2.1
119	0.946132	2.1
120	1.54246	2.1
121	1.54246	2.1
122	1.09235	2.1
123	0.946131	2.1
124	1.29507	2.1
125	1.0353	2.1
126	1.03529	2.1
127	1.28667	2.1
128	1.44777	2.1
129	1.61121	2.1
130	1.22176	0.0625304
131	0.946129	0.10116
132	1.08848	0.05
133	0.946131	0.0956289
134	1.61201	0.05
135	1.61201	0.05
136	1.19993	0.0697266
137	1.35822	0.0574379
138	1.1539	0.0533572
139	1.00765	0.0521393
140	1.28228	2.1
141	2.65339	2.1
142	1.28228	2.1
143	1.55998	2.1
144	1.00509	2.1
145	0.93845	2.1
146	1.27674	2.1
147	0.810958	2.1
148	1.91381	2.1
149	1.1861	2.1
150	1.24886	2.1
151	2.02842	2.1
152	0.991436	0.05
153	1.34588	0.05
154	2.02729	0.05
155	0.72904	0.0535921
156	1.06139	0.0566719
157	0.835368	0.0607108
158	1.63819	0.05
159	1.10745	0.05
160	1.79925	0.05
161	1.00085	0.0550858
162	0.946133	2.1
163	1.41123	2.1
164	1.8327	2.1
165	1.8327	2.1
166	0.946132	2.1
167	1.41123	2.1
168	1.3676	2.1
169	1.11048	2.1
170	1.11048	2.1
171	1.65815	2.1

172	1.9004	2.1
173	1.88691	2.1
174	1.82232	2.1
175	1.46591	2.1
176	1.09115	2.1
177	1.02051	1.79101
178	1.03373	2.00932
179	1.10307	2.1
180	1.05556	2.1
181	1.82533	1.33972
182	1.19496	1.27933
183	1.28597	2.1
184	1.20421	2.1
185	2.23644	2.1
186	1.08378	2.1
187	0.983724	2.1
188	0.602075	2.1
189	1.45876	2.1
190	1.45875	2.1
191	0.602103	2.1
192	1.11822	2.1
193	0.946133	2.1
194	0.602103	2.1
195	1.20421	2.1
196	1.26371	0.46752
197	1.55179	0.219436
198	1.14042	0.146887
199	1.56876	0.177542
200	2.27496	0.331972
201	1.13748	0.262663
202	1.02884	0.431822
203	1.25124	0.413415
204	0.82703	0.268101
205	0.607775	0.35587
206	1.49242	0.400486
207	1.35674	0.314252
208	1.6697	2.1
209	1.81655	2.1
210	0.900108	1.79404
211	0.851537	1.66733
212	0.703076	1.92392
213	0.639143	1.77685
214	1.42358	1.81311
215	1.4976	1.92511
216	0.86284	1.80895
217	1.2965	1.81798
218	1.27835	2.1
219	1.00012	1.56898
220	1.74332	1.95023
221	1.08576	2.1
222	1.19 2.1	
223	1.08576	2.1
224	1.20854	2.1
225	0.946131	2.1
226	0.946131	2.1
227	1.75985	2.1
228	1.75985	2.1
229	1.41232	2.1

230	1.29365	2.1
231	0.973559	2.1
232	1.23698	0.05
233	1.45055	0.0528699
234	1.26947	0.0596489
235	0.856218	0.0589989
236	1.75303	0.0534136
237	1.14938	0.0588288
238	0.984711	0.05
239	2.05771	0.0563398
240	0.625171	0.05
241	0.965675	0.05
242	1.17631	0.0538757
243	1.4677	0.05
244	1.35403	0.0623086
245	0.975149	0.0647388
246	0.946131	0.0656143
247	1.80893	0.05
248	1.80893	0.05
249	0.975149	0.05
250	0.946132	0.0642016
251	1.9503	0.0617189
252	2.80207	0.055106
253	1.76411	0.0610402
254	2.77469	0.0558205
255	1.9503	0.0537547
256	1.26784	0.05
257	2.31093	0.0570427
258	0.851541	0.0673947
259	1.39521	0.0506377
260	1.45613	0.0573737
261	0.851541	0.0695881
262	0.996013	0.0516942
263	1.07989	0.0632922
264	0.946131	0.0630933
265	1.08213	0.0535322
266	1.74289	0.056757
267	1.75838	0.0582556
268	2.11163	0.0675147
269	1.23773	2.1
270	1.25398	2.1
271	1.0304	2.1
272	1.32368	2.1
273	1.11712	1.38999
274	1.07999	0.670094
275	1.1766	2.1
276	1.35735	2.1
277	1.089 0.05	
278	1.81193	0.05
279	1.11081	0.05
280	1.78069	0.05
281	0.717512	0.05
282	0.810946	0.05
283	0.703901	0.05
284	1.09028	0.05
285	0.958031	0.05
286	0.892585	0.05
287	0.873431	0.05

288	0.997755	0.05
289	1.85479	0.05
290	0.983029	0.16582
291	1.27922	0.207153
292	1.64346	0.458994
293	1.27922	0.31282
294	1.09795	2.1
295	1.07508	1.61526
296	1.02975	2.01427
297	1.01121	2.1
298	1.72369	0.05
299	1.12035	0.05
300	2.13511	0.05
301	0.9705	0.05
302	0.946131	0.05
303	0.86182	0.05
304	1.51379	0.05
305	1.51379	0.05
306	0.946132	0.05
307	0.861849	0.05
308	1.34591	0.293786
309	1.14721	0.195217
310	1.15539	2.1
311	1.90938	2.1
312	2.19436	2.1
313	1.41563	2.1
314	0.785641	2.1
315	0.736384	2.1
316	1.46489	2.1
317	1.46488	2.1
318	0.736372	2.1
319	0.946132	2.1
320	1.90941	2.1
321	1.18907	2.1
322	1.20409	2.1
323	0.973559	2.1
324	1.29461	2.1
325	0.946129	2.1
326	0.94613	2.1
327	1.78751	2.1
328	1.78751	2.1
329	1.38885	2.1
330	1.34479	2.1
331	1.07283	2.1
332	1.6869	2.1
333	1.52544	2.1
334	1.07797	2.1
335	1.13819	2.1
336	0.776179	2.1
337	1.61092	2.1
338	1.36155	2.1
339	0.77618	2.1
340	1.2933	2.1
341	1.15946	2.1
342	0.69857	2.1
343	1.87607	2.1
344	1.07612	2.1
345	1.31976	1.61581

346	1.01243	1.90851
347	1.41279	1.90201
348	1.35146	0.0777016
349	1.90664	0.0564126
350	1.35145	0.0695261
351	1.22811	0.103934
352	1.93147	0.079573
353	1.42486	2.1
354	1.56558	2.1
355	1.0476	0.05
356	2.31605	0.05
357	1.89367	0.05
358	0.804706	0.05
359	0.783919	0.05
360	1.3116	0.05
361	1.03891	0.05
362	0.960473	0.05
363	1.08462	0.05
364	0.785994	0.05
365	1.1318	0.05
366	1.29576	0.05
367	1.3946	0.05
368	1.11924	0.05
369	0.834322	2.1
370	1.0256	2.1
371	1.74321	1.12374
372	1.41798	1.17331
373	0.871608	1.17291
374	1.47253	1.06982
375	1.47252	1.04611
376	0.817064	1.22619
377	0.817064	1.15645
378	0.946129	1.07407
379	1.63413	0.801534
380	1.9194	0.05
381	1.93562	0.05
382	2.06307	0.05
383	1.46021	0.05
384	0.955002	1.64194
385	0.833409	1.69558
386	0.838039	1.55381
387	1.48702	1.59435
388	1.1697	1.56975
389	1.23549	1.60646
390	1.18373	1.63416
391	2.02254	1.31036
392	1.68867	1.6067
393	0.875481	1.09561
394	0.821635	1.58049
395	0.858878	0.863184
396	1.30245	1.02165
397	1.07476	1.14259
398	1.11907	1.04885
399	1.10453	1.09672
400	1.70233	1.04333
401	1.70233	1.29883
402	2.6558	1.47773
403	1.7818	2.1

404	1.87059	2.1
405	1.34412	0.0549188
406	1.98068	0.0676898
407	2.56369	0.0580519
408	1.49512	0.05
409	1.35977	0.220394
410	1.34254	0.265915
411	1.75618	0.213924
412	1.75618	0.192111
413	0.946129	0.325788
414	0.946129	0.244264
415	1.17665	0.254068
416	1.01976	0.207451
417	1.27376	0.161832
418	1.35978	0.188319
419	1.68741	0.156868
420	1.68741	0.141254
421	0.946132	0.231396
422	0.946133	0.184061
423	1.14241	0.186891
424	0.996831	0.152148
425	0.996828	0.192859
426	1.12774	0.219624
427	1.13664	2.1
428	1.09627	2.1
429	2.8814	2.1
430	3.49751	2.1
431	1.79109	0.276324
432	1.54197	0.222058
433	2.75153	1.3195
434	1.93019	0.978977
435	1.51569	1.24912
436	1.55145	1.4144
437	1.78595	2.1
438	3.74452	2.1
439	1.66537	2.1
440	3.55161	2.1
441	1.35002	0.454067
442	1.19901	0.644488
443	1.61028	1.87385
444	1.35002	1.34665
445	1.65712	0.207455
446	1.10585	0.556443
447	0.961347	0.44073
448	0.810944	0.144533
449	1.16148	0.911842
450	1.08066	1.40784
451	0.934577	0.371102
452	1.05164	0.238044
453	0.79216	0.453824
454	2.99194	2.1
455	1.2832	2.1
456	2.97842	2.1
457	1.39517	2.1
458	1.03391	2.1
459	1.74416	2.1
460	1.05071	2.1
461	0.923782	2.1

462	1.55369	2.1
463	1.86444	2.1
464	1.01617	2.1
465	1.27174	2.1
466	1.20573	2.1
467	1.01616	2.1
468	1.13621	2.1
469	1.75366	2.1
470	2.27009	1.93692
471	1.6813	2.06806
472	1.47904	0.05
473	2.62714	0.05
474	1.80085	0.0506329
475	1.80085	0.0628783
476	2.74104	0.05
477	1.37052	0.05
478	0.946131	0.0717333
479	1.37052	0.0555363
480	1.31357	0.05
481	1.7837	0.0667287
482	1.7837	0.0828954
483	0.900397	0.0705889
484	1.74487	0.138045
485	2.88672	2.1
486	2.88249	2.1
487	1.0961	0.132145
488	1.20143	0.0604135
489	1.66739	0.0867469
490	1.85795	0.0712524
491	1.45789	0.05
492	2.11407	0.0567807
493	0.713524	0.0520816
494	1.58391	0.0591871
495	1.58391	0.05
496	1.04765	0.05
497	1.24979	0.0633175
498	0.713551	0.05
499	0.946127	0.0646072
500	1.36768	0.056222
501	1.14433	0.05
502	1.66923	0.05
503	0.699479	0.05
504	0.70351	0.05
505	0.758416	0.05
506	1.07678	0.05
507	0.909874	0.05
508	0.940218	0.05
509	0.876604	0.05
510	1.27148	0.05
511	2.42348	0.05
512	1.19837	0.05
513	0.764764	0.05
514	0.829259	0.05
515	0.740584	0.05
516	1.2772	0.05
517	0.964465	0.05
518	1.06231	0.05
519	1.06924	0.05

520	1.17566	0.05
521	1.3063	0.057748
522	2.51253	0.0571308
523	2.42267	0.0599483
524	1.89226	0.055247
525	1.46492	0.0527711
526	0.946132	0.0561531
527	0.653151	0.0585875
528	0.653124	0.0604855
529	1.53995	0.05
530	1.53995	0.05
531	0.936806	0.0532609
532	1.25626	0.0520772
533	1.1893	0.0910631
534	1.62206	0.0622509
535	1.04921	2.1
536	1.22927	2.1
537	0.801765	2.1
538	0.774917	2.1
539	0.844623	2.1
540	0.737481	2.1
541	3.24379	2.1
542	2.48573	2.1
543	1.40967	1.58758
544	1.47203	1.52622
545	2.07659	1.41806
546	1.40967	1.53875
547	2.06638	1.39235
548	1.17418	2.1
549	1.04921	2.1
550	1.74593	2.1
551	1.74593	2.1
552	0.946132	2.1
553	1.38651	2.1
554	1.20761	1.83397
555	1.20761	2.1
556	1.30555	2.1
557	1.46812	0.609736
558	2.41602	0.943447
559	1.46812	0.984836
560	1.46812	0.585854
561	0.734059	0.722688
562	0.946132	1.60132
563	1.21261	1.44705
564	1.34225	1.07935
565	0.734084	1.3613
566	1.82077	1.35051
567	1.82077	0.89517
568	1.13751	0.358484
569	0.946131	0.832914
570	1.13751	0.635067
571	1.13748	0.432308
572	0.784392	0.608099
573	1.4906	0.547652
574	1.4906	0.288641
575	1.17879	1.53785
576	1.28291	1.5063
577	0.626495	2.1

578	1.45334	2.1
579	1.45334	2.1
580	0.626494	2.1
581	1.3852	2.1
582	0.946116	2.1
583	0.626499	2.1
584	1.47203	1.50903
585	1.42639	2.1
586	1.42639	2.1
587	0.795469	2.1
588	0.795469	2.1
589	0.946132	2.1
590	0.850877	2.1
591	1.37098	2.1
592	1.87194	2.1
593	1.76988	2.1
594	3.16295	0.05
595	3.26725	0.05
596	1.13322	2.1
597	1.20761	2.1
598	0.686933	0.512353
599	0.493248	0.245842
600	1.2053	1.18145
601	0.947149	0.363602
602	1.27591	0.268896
603	1.75732	0.46048
604	0.493261	0.381045
605	1.34337	1.13806
606	2.73207	0.410595
607	1.19857	0.341014
608	0.671688	0.0710923
609	0.946134	0.702632
610	1.36603	0.140601
611	2.07817	0.195757
612	1.82573	0.484749
613	0.671699	0.406994
614	1.47772	0.11253
615	0.975349	0.786591
616	1.22615	2.1
617	1.47868	2.1
618	2.29351	2.1
619	1.47642	2.1
620	2.94443	2.1
621	1.80454	2.1
622	0.949135	2.1
623	0.949134	2.1
624	1.10476	0.102283
625	0.982857	0.209714
626	1.10938	0.108008
627	1.19052	0.888829
628	1.01012	0.05
629	1.35562	0.05
630	0.691742	0.05
631	2.39596	0.05
632	2.35604	0.05
633	0.974081	0.05
634	3.22444	0.05
635	1.3294	0.17816

636	1.37265	0.431997
637	1.3294	0.659643
638	1.51113	2.1
639	1.43643	2.1
640	1.72726	2.1
641	1.634 2.1	
642	0.755566	2.1
643	1.29319	2.1
644	0.755567	2.1
645	0.946131	2.1
646	1.29319	2.1
647	0.973563	2.1
648	0.904398	2.1
649	0.904406	2.1
650	1.1538	2.1
651	1.10437	2.1
652	0.935418	2.1
653	1.16649	2.1
654	0.975667	2.1
655	1.17952	2.1
656	1.95053	2.1
657	0.948676	2.1
658	1.44287	2.1
659	0.746095	2.1
660	1.51224	2.1
661	1.27354	2.1
662	0.746066	2.1
663	1.20573	2.1
664	1.12442	2.1
665	0.671487	2.1
666	1.04355	2.1
667	0.948675	2.1
668	0.967673	1.03384
669	1.12931	0.05
670	1.38212	0.05
671	2.40113	0.05
672	1.08054	0.05
673	1.04072	0.05
674	0.691062	0.05
675	1.58052	0.05
676	1.33502	0.05
677	0.946131	0.05
678	0.691062	0.05
679	1.02663	0.05
680	1.80569	0.05
681	1.26062	0.05
682	1.18203	0.05
683	0.946131	0.05
684	0.795065	0.05
685	1.58753	0.05
686	1.58753	0.05
687	0.946131	0.05
688	0.795093	0.05
689	0.805057	2.1
690	0.812415	2.1
691	1.23868	0.0821034
692	1.34448	0.110787
693	1.67201	2.1

694	1.61202	2.1
695	1.41103	2.1
696	1.41103	2.1
697	0.841088	2.1
698	1.38302	2.1
699	0.841089	2.1
700	0.869097	2.1
701	1.38302	2.1
702	0.949941	2.1
703	0.94994	2.1
704	1.88608	2.1
705	0.854942	2.1
706	1.68893	2.1
707	1.70002	2.1
708	2.02107	0.160561
709	2.04982	0.172628
710	3.11063	0.230397
711	2.03805	0.164004
712	1.67638	2.1
713	0.967492	2.1
714	2.00209	2.1
715	0.967491	2.1
716	0.870742	2.1
717	1.30266	0.0503607
718	0.651334	0.05
719	1.69413	0.05
720	1.69413	0.0561392
721	0.946132	0.05079
722	1.03079	0.0522735
723	1.31468	0.05
724	0.946132	0.0552453
725	0.919055	0.05
726	1.16815	0.05
727	2.79004	0.05
728	1.2136	0.05
729	2.62935	0.05
730	0.947692	0.188928
731	1.51835	0.162437
732	0.947693	0.117659
733	0.947692	0.135228
734	0.980841	2.1
735	1.43577	2.1
736	0.980841	2.1
737	1.67858	2.1
738	1.34124	0.330407
739	1.39818	0.194869
740	1.2065	2.1
741	0.946133	2.1
742	1.2079	2.1
743	1.65229	2.1
744	1.65229	2.1
745	0.946132	2.1
746	1.2079	2.1
747	1.39053	2.1
748	1.20651	2.1
749	1.36919	2.1
750	1.12717	2.1
751	1.09483	2.1

752	0.994294	2.1
753	0.97356	2.1
754	1.06827	2.1
755	1.40571	2.1
756	1.29259	2.1
757	0.946131	2.1
758	1.7787	2.1
759	1.7787	2.1
760	1.29259	2.1
761	0.946132	2.1
762	1.09408	1.02257
763	1.03824	0.310701
764	0.851541	0.297418
765	1.23608	0.289029
766	0.851539	0.293077
767	1.5021	0.299667
768	1.54839	0.298916
769	1.12373	0.279627
770	1.22617	0.270916
771	1.42762	0.459933
772	2.24746	0.422467
773	1.05475	0.090738
774	1.46845	0.0962918
775	1.05475	0.0931487
776	0.929726	0.0774324
777	1.52072	0.0961526
778	1.82488	0.0859867
779	1.02271	0.0844578
780	1.19225	0.0750795
781	1.43071	0.0701177
782	0.94613	0.073738
783	1.05475	0.0704959
784	1.39223	2.1
785	1.71255	2.1
786	0.946132	0.730913
787	1.10531	0.526332
788	1.46355	0.784204
789	1.46355	0.379648
790	0.78086	1.2204
791	1.10531	0.658946
792	1.1391	0.865889
793	1.56166	0.889639
794	2.21062	0.60924
795	1.0361	2.1
796	1.73577	2.1
797	1.73577	2.1
798	0.946116	2.1
799	1.28174	2.1
800	0.946118	2.1
801	1.22883	2.1
802	1.28172	2.1
803	1.03609	2.1
804	0.553567	0.05
805	1.60581	0.05
806	1.60581	0.05
807	0.553594	0.05
808	0.966748	0.05
809	0.966748	0.05

810	0.946133	0.05
811	1.05602	0.05
812	2.38525	0.05
813	1.14503	0.05
814	1.24678	0.05
815	0.56262	0.05
816	1.24678	0.05
817	0.978467	0.05
818	1.66262	0.05
819	1.66262	0.05
820	0.562633	0.05
821	1.09522	0.05
822	2.49356	0.05
823	0.592547	0.05
824	1.04321	1.62979
825	1.25133	1.67783
826	1.70508	1.33612
827	1.81043	1.31257
828	0.494934	1.25605
829	0.601047	1.27613
830	0.993428	1.26352
831	0.946573	1.28115
832	1.35039	1.2537
833	0.816895	1.1837
834	1.5063	1.15866
835	0.816895	1.22769
836	0.946132	1.10844
837	1.9067	1.33886
838	2.14504	1.18873
839	0.816895	1.21926
840	2.29624	1.2573
841	2.76933	0.946836
842	1.254 1.25641	
843	0.946128	2.1
844	1.38813	2.1
845	1.79998	2.1
846	1.79998	2.1
847	0.94613	2.1
848	1.38813	2.1
849	1.35798	2.1
850	1.27809	2.1
851	1.35798	2.1
852	1.68994	2.1
853	1.68994	2.1
854	0.946132	2.1
855	0.946132	2.1
856	1.18067	2.1
857	0.958256	2.1
858	0.958255	2.1
859	0.958257	2.1
860	0.958255	2.1
861	1.06457	2.1
862	1.14195	2.1
863	1.70423	2.1
864	1.14195	2.1
865	1.64285	2.1
866	2.02057	2.1
867	0.946132	2.1

868	1.44186	2.1
869	0.946133	2.1
870	1.16366	2.1
871	1.17233	2.1
872	1.31259	2.1
873	1.2016	2.1
874	1.17129	2.1
875	1.05724	0.40059
876	1.32697	2.1
877	1.05724	1.47021
878	1.04292	2.1
879	3.39037	2.1
880	2.57275	2.1
881	0.629212	2.1
882	2.32525	2.1
883	1.76949	0.534916
884	1.65809	0.625567
885	1.27665	2.1
886	1.19861	2.1
887	1.20045	2.1
888	1.1635	2.1
889	0.953633	0.158933
890	0.953634	0.0989143
891	1.21408	2.1
892	1.25716	2.1
893	2.01791	2.1
894	1.89318	2.1
895	0.947921	2.1
896	0.939049	2.1
897	0.947921	2.1
898	0.740753	2.1
899	0.916104	2.1
900	1.16941	2.1
901	1.72038	2.1
902	2.04441	2.1
903	0.973182	2.1
904	1.11751	2.1
905	0.611089	2.1
906	1.53584	0.071461
907	0.767923	0.140565
908	1.38925	0.12523
909	1.38926	0.115073
910	0.946131	0.241345
911	0.961347	0.175857
912	1.19583	0.119297
913	0.946131	0.142312
914	1.3663	0.296668
915	1.21067	0.0679897
916	1.00166	0.07365
917	1.40707	0.0675375
918	1.50435	0.0684419
919	1.47954	1.41308
920	1.1233	2.1
921	2.36036	1.85692
922	1.04024	1.39641
923	0.739729	1.39001
924	1.45533	1.29731
925	1.45532	1.25264

926	0.946118	1.34963
927	1.15163	0.617721
928	1.15165	1.38534
929	0.739748	1.32116
930	1.04025	1.69207
931	1.47608	0.131809
932	0.946131	0.189681
933	1.02028	0.162785
934	0.946129	0.182269
935	1.78223	0.127988
936	1.78223	0.122087
937	1.34153	0.157606
938	1.38683	0.159966
939	1.3407	0.115301
940	1.3407	0.100733
941	2.04729	0.119868
942	0.851529	0.161739
943	1.26671	0.122541
944	0.946116	0.156117
945	1.4869	0.105701
946	1.29553	0.0993685
947	1.03645	0.144984
948	1.20731	0.137196
949	0.73665	2.1
950	0.693596	2.1
951	2.11152	2.1
952	1.73627	2.1
953	1.06191	2.1
954	1.52226	2.1
955	1.76869	2.1
956	1.1881	2.1
957	1.32349	2.1
958	1.51041	2.1
959	1.61591	2.1
960	0.946132	2.1
961	0.946132	2.1
962	1.13537	2.1
963	3.0048	2.1
964	0.985109	2.1
965	1.68899	2.1
966	3.23261	2.1
967	1.08831	2.1
968	2.20623	2.1
969	0.91749	2.1
970	1.15515	2.1
971	0.550438	2.1
972	1.66294	2.1
973	1.08257	2.1
974	0.87851	2.1
975	2.09046	2.1
976	0.738711	2.1
977	1.31242	0.05
978	1.26311	0.05
979	1.67652	0.05
980	0.968594	2.1
981	0.968594	2.1
982	0.981099	0.05
983	1.32418	0.05

984	2.67175	0.05
985	2.09678	0.05
986	1.2511	0.05
987	0.912698	0.05
988	0.810946	0.05
989	0.810959	0.05
990	1.67479	0.05
991	1.65677	0.05
992	1.28469	0.05
993	1.48817	0.05
994	1.08131	0.0501384
995	1.04349	1.18341
996	1.42492	0.77184
997	1.05724	0.44184
998	1.65723	0.10768
999	1.95313	0.0943389
1000	1.9293	0.101407
1001	2.02729	0.0881045
1002	0.963393	1.67848
1003	0.963392	0.98315
1004	2.90171	2.1
1005	2.65447	2.1
1006	1.64158	2.1
1007	1.6488	2.1
1008	1.15991	0.132504
1009	0.946132	0.193031
1010	1.58199	0.146615
1011	1.58199	0.167765
1012	1.03333	0.212406
1013	1.25436	0.152771
1014	1.25436	0.17891
1015	0.946132	0.175528
1016	1.3771	0.244106
1017	0.687745	0.210143
1018	1.37709	0.202356
1019	0.812386	0.236393
1020	1.50172	0.240706
1021	1.50172	0.165525
1022	0.687758	0.255038
1023	1.17506	0.140039
1024	2.00539	0.195171
1025	2.2441	0.241482
1026	1.09667	0.149698
1027	1.43577	2.1
1028	2.24741	2.1
1029	1.26103	2.1
1030	0.851544	2.1
1031	1.5543	2.1
1032	1.59756	2.1
1033	0.851543	2.1
1034	1.44673	2.1
1035	1.31947	2.1
1036	1.1237	2.1
1037	1.26103	2.1
1038	1.089 0.05	
1039	0.946128	0.052763
1040	0.965824	0.0543089
1041	0.94613	0.0575873

1042	1.51344	0.05
1043	1.51344	0.05
1044	1.13508	0.0513274
1045	1.32449	0.05
1046	1.089	0.05
1047	3.33706	0.163789
1048	2.7542	0.20423
1049	1.27256	0.368102
1050	0.954172	0.285198
1051	1.55527	0.291925
1052	1.05765	0.40879
1053	1.57326	0.360746
1054	1.38251	0.192084
1055	0.94613	0.348793
1056	2.38829	0.192234
1057	2.00539	0.227762
1058	1.53798	0.194484
1059	1.20969	2.1
1060	1.15864	2.1
1061	1.7986	2.1
1062	1.74533	2.1
1063	0.944827	1.37268
1064	0.946131	1.16243
1065	0.851543	1.24046
1066	1.52816	1.05113
1067	1.5314	1.38431
1068	1.39266	0.849648
1069	0.851528	1.15602
1070	1.39589	1.2478
1071	1.7348	1.05935
1072	1.85439	1.15804
1073	1.86367	1.08176
1074	1.90905	0.730992
1075	1.22176	0.0625715
1076	1.59492	0.0772898
1077	2.97929	2.1
1078	2.1905	2.1
1079	1.24128	2.1
1080	0.994293	2.1
1081	2.49767	0.05
1082	2.37105	0.05
1083	1.10834	1.97318
1084	1.53214	0.370807
1085	1.417	2.1
1086	2.13599	0.65838
1087	1.61727	2.1
1088	1.75004	2.1
1089	1.96301	2.1
1090	0.966631	2.1
1091	1.37791	2.1
1092	1.47302	2.1
1093	1.47302	2.1
1094	0.871519	2.1
1095	0.94613	2.1
1096	0.87155	2.1
1097	2.38731	2.1
1098	2.12399	1.27919
1099	1.74865	1.1942

1100	1.46724	0.482273
1101	2.38056	0.485485
1102	1.46724	0.488135
1103	0.818887	0.647605
1104	0.810944	1.53423
1105	0.805227	0.643257
1106	1.06558	0.843883
1107	0.841487	0.714906
1108	1.08456	0.869554
1109	0.903083	0.997723
1110	1.08929	0.05
1111	1.68454	0.05
1112	1.08929	0.05
1113	1.07658	0.0576785
1114	0.904439	0.0580796
1115	1.58501	0.05
1116	1.19729	0.0542342
1117	1.05427	0.05
1118	1.47764	0.0563272
1119	0.810959	0.0508547
1120	1.60946	1.21805
1121	2.28887	1.24157
1122	0.946133	1.11146
1123	1.14444	0.667325
1124	1.67964	0.47529
1125	1.67964	0.56016
1126	0.755334	1.13325
1127	1.14444	0.947654
1128	1.29054	0.749958
1129	1.37762	0.78536
1130	0.946132	1.1087
1131	0.533645	0.859854
1132	1.48361	0.539407
1133	1.48361	0.699658
1134	0.533645	0.873122
1135	0.872823	1.0694
1136	1.14444	0.765251
1137	1.37762	0.565546
1138	2.10101	0.767759
1139	1.02311	1.12053
1140	1.04083	2.1
1141	1.04084	2.1
1142	1.04084	2.1
1143	1.30116	2.1
1144	1.23477	0.05
1145	1.81893	0.0587248
1146	1.23477	0.05
1147	1.23477	0.055408
1148	1.11967	0.05
1149	0.946131	0.055697
1150	1.11966	0.0584293
1151	1.12628	0.05
1152	0.629641	0.0625532
1153	1.6163	0.0560335
1154	1.6163	0.05
1155	1.11117	2.1
1156	1.13006	2.1
1157	1.15149	2.1

1158	1.13179	2.1
1159	0.585368	0.808279
1160	0.986036	1.07524
1161	1.94788	0.180878
1162	1.34857	0.520431
1163	2.31806	2.1
1164	2.06363	2.1
1165	1.00423	2.1
1166	0.829065	2.1
1167	0.551816	2.1
1168	0.99406	2.1
1169	1.98339	2.1
1170	1.68073	2.1
1171	2.558 2.1	
1172	0.711814	2.1
1173	2.97316	2.1
1174	2.41579	2.1
1175	1.15248	0.0502415
1176	1.14305	0.05
1177	1.15483	0.05
1178	1.90981	0.05
1179	0.998957	0.05
1180	1.83742	0.05
1181	1.27581	2.1
1182	0.94613	2.1
1183	1.6315	2.1
1184	1.6315	2.1
1185	0.946132	2.1
1186	1.28441	2.1
1187	1.28441	2.1
1188	1.00365	2.1
1189	0.968536	2.1
1190	1.57575	2.1
1191	1.27581	2.1
1192	1.99297	2.1
1193	1.32784	2.1
1194	1.4259	2.1
1195	2.27952	2.1
1196	1.5685	2.1
1197	1.54231	2.1
1198	3.54638	2.1
1199	1.3294	2.1
1200	2.98237	2.1
1201	2.01633	0.628674
1202	2.0985	0.678041
1203	2.26047	0.301331
1204	1.7913	0.242061
1205	1.09504	2.1
1206	1.74126	2.1
1207	1.05983	2.1
1208	2.37664	2.1
1209	2.28084	2.1
1210	2.19948	2.1
1211	1.33593	0.05
1212	1.31508	0.05
1213	1.30647	2.1
1214	1.28846	2.1
1215	1.2955	2.1

1216	1.09044	2.1
1217	1.4099	2.1
1218	1.14984	2.1
1219	0.946132	2.1
1220	1.70727	2.1
1221	1.70727	2.1
1222	1.14984	2.1
1223	0.946132	2.1
1224	0.911818	2.1
1225	2.12209	2.1
1226	2.4158	2.1
1227	1.20867	2.1
1228	2.84085	2.1
1229	1.42284	2.1
1230	2.45689	2.1
1231	1.41562	2.1
1232	0.918358	2.1
1233	1.11222	2.1
1234	0.999083	2.1
1235	0.696781	2.1
1236	1.30869	2.1
1237	1.66132	2.1
1238	0.696807	2.1
1239	0.745315	2.1
1240	1.23296	2.1
1241	0.780548	2.1
1242	1.93242	1.54438
1243	1.27362	1.79891
1244	2.77828	1.83568
1245	1.02653	1.29477
1246	0.808576	1.13881
1247	0.744012	1.49389
1248	0.605174	0.810881
1249	1.16501	0.488582
1250	0.803432	0.586655
1251	0.961016	0.483229
1252	1.10425	0.952987
1253	1.3756	1.42024
1254	2.17765	0.530608
1255	1.84578	0.476215
1256	1.32801	0.257503
1257	1.76377	0.241827
1258	0.946131	0.243375
1259	1.3022	0.795378
1260	0.694814	0.573109
1261	1.53028	0.255092
1262	1.53028	0.219947
1263	1.3022	0.427059
1264	0.922889	0.555175
1265	1.57766	2.1
1266	2.06911	2.1
1267	1.57766	2.1
1268	1.37721	0.05
1269	1.72174	0.0563466
1270	1.4468	0.0728748
1271	1.37721	0.05
1272	1.73972	0.156104
1273	1.62324	0.1544

1274	0.946125	0.110501
1275	1.34259	0.0884454
1276	1.04187	0.068032
1277	1.05019	0.0936696
1278	1.2361	0.108296
1279	1.69055	0.0768873
1280	2.25056	0.111421
1281	1.10574	1.78053
1282	1.21632	2.06012
1283	1.10574	2.1
1284	1.46181	2.1
1285	0.946131	1.63939
1286	1.74111	1.8684
1287	1.74111	1.75297
1288	0.94613	1.74599
1289	1.26891	1.33457
1290	1.26891	0.730441
1291	1.23452	1.71774
1292	1.00255	1.92591
1293	1.72286	0.181015
1294	1.12233	0.120057
1295	1.30682	0.240032
1296	1.97397	0.264754
1297	1.67425	2.1
1298	1.77775	2.1
1299	1.73571	1.8736
1300	0.426265	2.0837
1301	1.31817	1.84719
1302	1.81042	2.1
1303	1.85456	2.1
1304	0.466532	1.7822
1305	1.28025	1.82848
1306	0.964467	1.77131
1307	1.27921	2.1
1308	2.11851	1.78135
1309	2.37121	1.89277
1310	1.49357	0.268689
1311	1.42486	2.1
1312	1.00278	2.1
1313	1.00278	2.1
1314	1.12503	2.1
1315	0.568697	2.1
1316	0.555211	2.1
1317	0.982272	2.1
1318	1.57558	2.1
1319	0.851539	2.1
1320	1.07307	2.1
1321	1.58292	2.1
1322	1.50731	2.1
1323	0.851541	2.1
1324	1.00304	2.1
1325	1.36003	2.1
1326	1.13127	2.1
1327	0.672234	2.1
1328	0.763044	2.1
1329	0.966979	2.1
1330	1.31502	2.1
1331	1.67969	2.1

1332	1.62408	2.1
1333	0.661955	2.1
1334	1.35048	2.1
1335	1.40447	2.1
1336	1.40447	2.1
1337	0.607961	2.1
1338	0.946131	2.1
1339	0.607963	2.1
1340	1.01531	2.1
1341	1.01531	2.1
1342	1.91802	2.1
1343	0.777749	2.1
1344	1.22585	0.05
1345	1.20672	0.05
1346	1.03166	0.155259
1347	0.553805	2.1
1348	2.00551	2.1
1349	2.07203	2.1
1350	0.589479	2.1
1351	1.22352	2.1
1352	0.984227	2.1
1353	0.802455	2.1
1354	1.40942	2.1
1355	1.6662	2.1
1356	0.802485	2.1
1357	0.936669	2.1
1358	1.31127	0.242014
1359	0.946132	0.259873
1360	1.09729	0.165276
1361	0.946133	0.383483
1362	1.68703	0.189035
1363	1.68703	0.145997
1364	1.3689	0.219773
1365	1.26426	0.241411
1366	1.26923	2.1
1367	1.23614	2.1
1368	0.828371	2.1
1369	2.04556	2.1
1370	0.772648	2.1
1371	0.791059	2.1
1372	0.809958	2.1
1373	1.70012	2.1
1374	0.267032	2.1
1375	1.67604	2.1
1376	0.803823	2.1
1377	0.267305	2.1
1378	0.80246	2.1
1379	0.874582	2.1
1380	1.00188	2.1
1381	0.709588	2.1
1382	1.50679	2.1
1383	1.22456	2.1
1384	0.870622	2.1
1385	1.05754	2.1
1386	1.1689	2.1
1387	1.54556	2.1
1388	1.89226	2.1
1389	0.995311	2.1

1390	1.26195	2.1
1391	1.9	2.1
1392	0.845055	2.1
1393	0.737836	2.1
1394	0.823707	2.1
1395	0.626587	2.1
1396	1.16109	2.1
1397	0.85517	2.1
1398	0.932036	2.1
1399	1.08321	2.1
1400	1.43863	2.1
1401	2.72297	2.1
1402	2.90798	2.1
1403	1.47818	1.42308
1404	1.47819	1.5756
1405	0.694357	1.36164
1406	0.694382	1.43104
1407	0.946132	1.27082
1408	0.694382	1.3428
1409	1.22641	1.34498
1410	1.31155	1.54889
1411	2.45282	1.42545
1412	0.787843	1.24065
1413	0.808984	1.20072
1414	1.14232	1.43098
1415	0.795037	1.35763
1416	0.887382	1.40752
1417	1.14493	1.30149
1418	0.78393	1.12098
1419	1.09129	1.30464
1420	0.791482	1.28124
1421	1.1137	1.48146
1422	1.11819	1.37664
1423	0.960173	1.3655
1424	1.09447	1.38093
1425	0.874365	1.32137
1426	1.2474	0.138961
1427	1.75038	0.27194
1428	1.75038	0.364716
1429	0.94613	0.580142
1430	1.30076	0.288954
1431	1.12817	0.906338
1432	0.94613	0.447237
1433	1.30076	0.410129
1434	1.51841	0.328235
1435	1.51841	0.417251
1436	0.946132	0.946614
1437	1.39575	0.377505
1438	0.970358	1.48309
1439	0.946133	0.669162
1440	1.39575	0.575956
1441	1.06486	0.188498
1442	1.06486	0.182688
1443	1.38622	0.213584
1444	1.04521	0.05
1445	1.00809	0.0606547
1446	1.28952	0.0606392
1447	0.849316	0.0578865

1448	0.946132	0.0583093
1449	0.849344	0.0685633
1450	1.53631	0.0599783
1451	1.53631	0.0631358
1452	1.77843	0.05
1453	1.69863	0.05
1454	1.91646	2.1
1455	1.8582	2.1
1456	2.8349	0.05
1457	1.36307	0.05
1458	1.23886	0.05
1459	1.12971	0.05
1460	1.59662	0.05
1461	1.59662	0.05
1462	0.640304	0.0691637
1463	0.640333	0.05
1464	0.886276	0.0701534
1465	0.886276	0.0696827
1466	1.35065	0.0531457
1467	2.09002	0.05
1468	1.35543	0.0528665
1469	1.94688	2.1
1470	1.97221	2.1
1471	0.719563	2.1
1472	1.20358	2.1
1473	0.874264	2.1
1474	1.27676	2.1
1475	1.46557	2.1
1476	1.46557	2.1
1477	0.68546	2.1
1478	0.94613	2.1
1479	0.685489	2.1
1480	1.39615	0.102629
1481	1.05404	0.0896125
1482	1.06474	0.109466
1483	1.00713	0.0927098
1484	0.714535	0.0918685
1485	0.714563	0.0986097
1486	1.41646	0.0872596
1487	1.52582	0.0766347
1488	0.721694	0.0918419
1489	0.93665	0.0907338
1490	1.21067	0.18359
1491	1.21067	0.0802729
1492	1.73577	0.331928
1493	1.17799	0.05
1494	3.21432	0.238824
1495	2.41452	0.227214
1496	1.36208	0.19552
1497	2.53256	0.214256
1498	0.834593	0.160649
1499	2.5853	0.211184
1500	1.77259	0.18128
1501	0.615724	0.405278
1502	1.11374	0.612444
1503	0.615726	0.333183
1504	0.946129	0.789721
1505	0.615714	0.763571

1506	1.75489	0.623911
1507	1.75489	0.44939
1508	1.23146	0.282411
1509	2.2772	0.307402
1510	0.74463	0.285817
1511	1.3546	0.326862
1512	1.80252	0.219387
1513	2.38304	0.240749
1514	1.19151	0.374744
1515	1.1231	0.409073
1516	1.82345	0.308212
1517	1.79805	0.290862
1518	0.761337	0.522442
1519	0.946127	0.36638
1520	0.557726	0.374279
1521	1.05353	0.098975
1522	1.05353	0.133327
1523	1.68599	0.269964
1524	1.05353	0.151832
1525	1.40588	2.1
1526	1.09373	2.1
1527	1.18382	1.48118
1528	1.46903	1.31445
1529	1.25103	2.1
1530	1.10378	2.1
1531	1.58941	2.1
1532	1.29639	2.1
1533	1.14874	0.05
1534	0.899817	0.05
1535	0.666898	0.05
1536	1.76519	0.05
1537	1.17289	0.05
1538	1.03526	0.05
1539	1.97708	0.05
1540	1.31711	0.05
1541	0.992517	0.05
1542	1.70193	0.05
1543	1.13852	0.05
1544	1.23943	1.5298
1545	1.40014	2.1
1546	1.04598	2.1
1547	1.1483	2.1
1548	1.11433	1.67034
1549	1.32037	0.401187
1550	1.00381	1.71448
1551	1.11435	0.245499
1552	0.973502	1.42184
1553	0.946128	1.8821
1554	1.83697	1.40342
1555	1.83697	2.1
1556	1.31721	1.83355
1557	1.38614	1.39949
1558	1.39696	1.35856
1559	1.31721	1.69139
1560	1.0756	1.69596
1561	1.08542	1.48719
1562	0.946127	2.1
1563	1.81052	0.522818

1564	1.81052	2.1
1565	1.24315	2.1
1566	1.39696	0.360834
1567	1.35969	1.81323
1568	1.24315	1.78646
1569	1.0756	0.225232
1570	1.20669	0.16266
1571	1.18508	0.20111
1572	1.71059	2.1
1573	1.66762	2.1
1574	2.50853	2.1
1575	1.8236	2.1
1576	1.78872	2.1
1577	1.41721	2.1
1578	1.01335	1.76309
1579	1.42439	1.29765
1580	0.946129	2.03096
1581	1.33266	1.34424
1582	1.79776	1.89928
1583	1.79776	2.1
1584	0.946131	0.725602
1585	1.33266	1.91429
1586	1.41123	2.04689
1587	1.25346	2.1
1588	0.946132	0.56717
1589	0.827287	1.32144
1590	1.57878	1.36645
1591	1.57878	1.57799
1592	0.827287	1.20267
1593	1.07341	1.2281
1594	1.33266	1.47848
1595	1.25346	1.68742
1596	1.61149	2.1
1597	1.15644	1.8904
1598	1.27558	1.37764
1599	1.3764	0.484007
1600	1.89526	1.5631
1601	1.37641	0.41552
1602	1.2064	2.1
1603	1.38426	2.1
1604	1.01595	2.1
1605	1.15067	2.1
1606	1.82276	2.1
1607	1.82276	2.1
1608	0.946131	2.1
1609	1.40609	2.1
1610	0.946132	2.1
1611	1.29143	2.1
1612	1.40609	2.1
1613	1.30272	2.1
1614	1.19548	2.1
1615	2.95451	2.1
1616	1.46124	2.1
1617	2.93215	2.1
1618	1.42105	2.1
1619	1.75477	0.686223
1620	2.31301	0.795237
1621	2.10275	0.261227

1622	1.80099	0.291987
1623	0.97541	2.1
1624	1.49733	2.1
1625	0.97541	2.1
1626	1.30751	2.1
1627	1.28614	0.0778976
1628	1.74759	0.0574741
1629	1.19756	0.05
1630	1.71053	0.0637195
1631	1.19757	0.0665352
1632	1.28954	0.05
1633	2.02703	0.050775
1634	2.29453	0.0557409
1635	1.28265	0.05
1636	0.793267	0.0672084
1637	1.19974	0.103095
1638	0.509551	0.0628221
1639	1.04072	0.0983387
1640	1.35302	0.05
1641	1.53133	0.101563
1642	0.566153	0.0702679
1643	1.09634	0.05
1644	1.12792	0.0584477
1645	1.68908	2.1
1646	1.68908	2.1
1647	0.768212	2.1
1648	0.768213	2.1
1649	0.946132	2.1
1650	0.768212	2.1
1651	1.32461	2.1
1652	1.87291	2.1
1653	2.64921	2.1
1654	1.53642	2.1
1655	2.26537	2.1
1656	1.53642	2.1
1657	1.79874	2.1
1658	1.79874	2.1
1659	0.726635	2.08032
1660	0.726636	2.1
1661	0.946128	2.1
1662	0.726634	2.1
1663	1.23339	2.0637
1664	0.992674	2.1
1665	1.8023	2.1
1666	1.12324	0.05
1667	1.29163	0.05
1668	0.996831	0.148321
1669	1.0019	0.141302
1670	0.993831	0.070871
1671	1.07283	2.1
1672	1.04788	2.1
1673	1.44489	2.1
1674	1.04788	2.1
1675	1.07652	2.1
1676	0.991406	2.1
1677	1.37	2.1
1678	1.14195	2.1
1679	1.47904	0.0507312

1680	1.26593	2.0331
1681	1.75366	1.93197
1682	1.57621	0.194927
1683	1.24538	0.112968
1684	0.969118	2.1
1685	0.946133	2.1
1686	1.69387	2.1
1687	1.69387	2.1
1688	0.94613	2.1
1689	1.28713	2.1
1690	1.28713	2.1
1691	1.23877	2.1
1692	0.969118	2.1
1693	1.22615	2.1
1694	0.98084	2.1
1695	1.01843	2.1
1696	1.50876	2.1
1697	1.85391	2.1
1698	1.84322	2.1
1699	1.70377	2.1
1700	1.78919	2.1
1701	2.66787	2.1
1702	1.41549	2.1
1703	1.41549	2.1
1704	0.86707	2.1
1705	0.896172	2.1
1706	0.946126	2.1
1707	0.867074	2.1
1708	0.946118	2.1
1709	1.54468	0.0600916
1710	1.54468	0.0630778
1711	2.27259	0.0654219
1712	0.946131	0.10579
1713	1.25837	0.0922768
1714	0.663171	0.133774
1715	1.68931	0.0698503
1716	1.68931	0.0666177
1717	1.25837	0.0945513
1718	1.09411	0.0910368
1719	1.73571	0.0596993
1720	2.34894	0.0623188
1721	0.946128	0.0725809
1722	1.30497	0.0675691
1723	0.772312	0.0908738
1724	1.66895	0.0607406
1725	1.66895	0.0547201
1726	1.30497	0.0668696
1727	1.13629	0.061055
1728	1.22852	1.16039
1729	1.578	1.16853
1730	2.07781	2.1
1731	1.5478	2.1
1732	1.70048	2.1
1733	1.42329	2.1
1734	1.05481	2.1
1735	1.1032	1.93413
1736	1.19267	2.1
1737	1.77324	2.1

1738	1.01121	0.0930613
1739	0.991867	0.139402
1740	2.0744	2.1
1741	1.75142	2.1
1742	1.35418	2.1
1743	1.40041	2.1
1744	1.50045	2.1
1745	1.37704	2.1
1746	1.50045	2.1
1747	1.87875	2.1
1748	1.35308	1.06542
1749	1.29983	1.04441
1750	1.62784	2.1
1751	1.44807	2.1
1752	1.00965	2.1
1753	2.69264	2.1
1754	1.04075	2.1
1755	0.946131	2.1
1756	0.946132	2.1
1757	1.52704	2.1
1758	1.83246	2.1
1759	1.56757	2.1
1760	1.16687	2.1
1761	2.04857	2.1
1762	1.32707	2.1
1763	1.25519	0.39468
1764	1.32101	1.05732
1765	1.25055	2.08736
1766	0.827853	1.80485
1767	1.11835	0.940708
1768	1.07281	2.1
1769	1.54302	1.75167
1770	0.724064	1.99891
1771	1.32709	2.1
1772	3.44123	2.1
1773	2.46889	0.78768
1774	0.730076	0.0676464
1775	1.25809	0.17554
1776	0.730077	0.104158
1777	0.946129	0.156201
1778	1.72777	0.0538788
1779	1.72777	0.154198
1780	0.73005	0.120033
1781	1.7522	0.0729867
1782	2.51618	0.0739425
1783	0.628177	0.0699654
1784	1.1068	0.203139
1785	0.747435	0.0910623
1786	0.946133	0.183407
1787	1.84668	0.0671934
1788	1.8563	0.165852
1789	0.628177	0.160766
1790	1.45501	0.134271
1791	2.19809	0.122435
1792	1.45501	0.0544917
1793	1.48974	0.05
1794	1.7709	2.1
1795	2.63288	2.1

1796	1.5362	2.1
1797	1.56327	2.1
1798	3.03945	0.0574169
1799	0.899979	0.0569333
1800	0.869386	0.0685586
1801	1.85864	0.060542
1802	1.78028	0.0610779
1803	1.28719	0.0566078
1804	1.09532	0.069724
1805	0.881898	0.0623491
1806	1.28911	0.05
1807	1.39644	0.05
1808	0.688829	0.05
1809	2.14611	0.05
1810	1.80313	0.05
1811	0.68883	0.05
1812	0.946131	0.05
1813	1.25334	0.05
1814	1.88987	0.05
1815	1.94432	0.05
1816	1.37592	0.05
1817	1.11691	2.1
1818	1.02923	2.1
1819	0.45824	0.05
1820	0.646512	0.05
1821	2.46292	0.05
1822	0.493451	0.05
1823	2.00826	2.1
1824	2.49784	2.1
1825	1.17054	2.1
1826	1.01485	2.1
1827	1.26045	2.1
1828	1.17054	2.1
1829	1.44247	2.1
1830	2.02687	0.05
1831	1.66365	0.05
1832	1.64656	0.05
1833	1.56597	0.0517865
1834	1.21419	2.1
1835	1.5008	2.1
1836	1.10711	2.1
1837	1.44287	2.1
1838	1.44287	2.1
1839	0.750368	2.1
1840	1.25876	2.1
1841	0.82542	2.1
1842	1.04074	2.1
1843	1.25874	2.1
1844	1.04159	0.05
1845	1.2489	0.05
1846	0.980547	0.0995084
1847	1.62235	0.0791514
1848	1.15991	0.102303
1849	0.980547	0.0853887
1850	0.94613	0.127889
1851	1.54662	0.0992262
1852	1.54661	0.114147
1853	1.07912	0.142249

1854	1.1032	0.10225
1855	1.1032	0.116102
1856	0.94613	0.110842
1857	1.12468	2.1
1858	1.2724	2.1
1859	1.06521	2.1
1860	1.0752	2.1
1861	1.94936	0.0916744
1862	1.62772	0.0803983
1863	1.94733	0.0792538
1864	1.42901	0.120425
1865	1.33022	2.1
1866	1.70839	2.1
1867	1.75048	2.1
1868	1.41201	2.1
1869	1.52314	2.1
1870	1.44039	2.1
1871	1.51113	2.1
1872	2.58638	2.1
1873	0.987247	0.0897661
1874	1.38622	0.22236
1875	0.858359	0.117685
1876	1.19967	0.165042
1877	1.06414	0.115455
1878	0.946132	0.248432
1879	0.94613	0.114021
1880	0.687079	0.348859
1881	0.946132	0.229684
1882	0.822611	0.224755
1883	1.70242	0.134429
1884	1.70242	0.226914
1885	0.946132	0.429547
1886	1.11155	0.197407
1887	0.946132	0.502359
1888	1.11155	0.311639
1889	1.37981	0.342567
1890	1.18348	0.135087
1891	2.44179	0.21748
1892	2.00403	0.323616
1893	1.10961	2.1
1894	1.85415	2.1
1895	1.44825	2.1
1896	0.932272	2.1
1897	0.810942	2.1
1898	1.00189	2.1
1899	1.11779	2.1
1900	1.3359	2.1
1901	0.965279	2.1
1902	0.833779	2.1
1903	1.05337	2.1
1904	0.526656	2.1
1905	1.44599	2.1
1906	1.44599	2.1
1907	1.24853	2.1
1908	0.946132	2.1
1909	1.24853	2.1
1910	0.724122	2.1
1911	1.91547	2.1

1912	2.49705	2.1
1913	1.05931	2.1
1914	1.1729	2.1
1915	1.34526	2.1
1916	1.5379	2.1
1917	1.90855	2.1
1918	2.08597	2.1
1919	1.68165	2.1
1920	1.45887	2.1
1921	1.50022	2.1
1922	1.4748	1.99899
1923	2.63645	1.90755
1924	1.74431	1.97598
1925	1.25288	1.87687
1926	1.25289	1.78489
1927	0.970025	1.80332
1928	0.747542	2.04176
1929	0.810959	1.73328
1930	1.15369	1.81469
1931	0.977472	1.76814
1932	1.12695	1.81944
1933	0.996762	1.80202
1934	1.28249	0.88454
1935	0.946132	0.972111
1936	0.528621	1.03314
1937	1.59186	0.58139
1938	1.44072	0.543267
1939	0.633488	0.713818
1940	0.598506	1.05354
1941	1.51618	1.02576
1942	1.19999	0.569271
1943	1.46511	0.634785
1944	1.44056	0.509216
1945	0.912754	0.251234
1946	1.4725	0.180516
1947	1.4725	0.177927
1948	0.912753	0.274266
1949	1.23351	0.227577
1950	1.23351	0.247623
1951	0.946131	0.293199
1952	1.17129	0.199971
1953	1.82551	0.168906
1954	1.27575	1.96618
1955	0.946129	2.02931
1956	0.946129	2.1
1957	0.946128	2.00525
1958	0.792754	2.07549
1959	0.94613	1.90566
1960	0.792752	1.91463
1961	2.08054	2.0652
1962	1.20091	2.1
1963	2.1669	2.03631
1964	1.24383	1.84197
1965	0.904669	1.89918
1966	0.83804	1.75408
1967	1.90486	1.84767
1968	1.29162	1.78613
1969	1.37383	1.88724

1970	1.86533	1.81852
1971	1.76472	1.9156
1972	1.60428	1.83029
1973	2.43993	1.93745
1974	1.37833	2.1
1975	1.18632	2.1
1976	0.946132	2.1
1977	1.27544	2.1
1978	1.05085	2.1
1979	0.961178	2.1
1980	0.859844	2.1
1981	1.0499	2.1
1982	1.75592	2.1
1983	2.08668	2.1
1984	1.75592	2.1
1985	1.4402	2.1
1986	1.72285	2.1
1987	0.83312	0.05
1988	1.07295	0.05
1989	1.37607	0.05
1990	1.32097	0.05
1991	1.11332	0.05
1992	1.18568	0.05
1993	0.877482	0.05
1994	1.4458	0.05
1995	2.34112	0.05
1996	2.68021	0.05
1997	0.681677	0.05
1998	0.946131	0.05
1999	0.681649	0.05
2000	1.83115	0.05
2001	1.68029	0.05
2002	1.05482	0.05
2003	1.31485	0.05
2004	2.08247	1.06282
2005	1.33153	0.72349
2006	0.935062	1.2884
2007	0.806958	1.64164
2008	0.827683	1.17171
2009	1.01041	1.28574
2010	0.741832	1.14793
2011	1.15668	1.32008
2012	0.908649	1.32833
2013	0.59561	0.372599
2014	1.19028	0.602078
2015	0.59561	0.568144
2016	0.946132	0.664043
2017	0.595623	1.02716
2018	1.58164	0.635565
2019	1.58164	0.619386
2020	0.946131	2.1
2021	1.55201	2.1
2022	1.55201	2.1
2023	0.946127	2.1
2024	1.13312	2.1
2025	1.13312	2.1
2026	1.08108	2.1
2027	1.04973	2.1

2028	1.20149	2.1
2029	1.86902	1.62888
2030	0.613447	2.1
2031	1.22618	1.83958
2032	1.38663	1.9986
2033	1.38662	2.1
2034	0.613447	1.96715
2035	1.22617	2.1
2036	0.773899	2.1
2037	1.23515	1.95184
2038	1.87823	1.62068
2039	1.70303	0.59251
2040	1.4714	2.1
2041	1.74067	2.1
2042	0.735699	2.1
2043	1.73461	2.1
2044	1.47224	2.1
2045	1.22827	2.1
2046	1.1634	2.1
2047	1.36471	2.1
2048	0.735701	2.1
2049	2.8386	1.27591
2050	2.39056	2.1
2051	2.44128	2.1
2052	1.54102	2.1
2053	1.54102	2.1
2054	0.936141	2.1
2055	1.40594	2.1
2056	1.07121	2.1
2057	0.936142	2.1
2058	1.07121	2.1
2059	1.22691	0.0821533
2060	1.42907	0.0755674
2061	2.18048	0.0766078
2062	0.947662	0.0720945
2063	2.18394	2.1
2064	1.21292	2.1
2065	2.18394	2.1
2066	2.19064	2.1
2067	1.27229	2.1
2068	1.20859	0.556379
2069	1.21073	0.478412
2070	1.73899	2.1
2071	1.3164	2.1
2072	1.61245	2.1
2073	1.41444	2.1
2074	2.55116	2.1
2075	1.26193	2.1
2076	1.89553	2.1
2077	0.916438	2.1
2078	1.26113	2.1
2079	0.810934	2.1
2080	2.00419	2.1
2081	1.27872	2.1
2082	1.35306	2.1
2083	1.96749	2.1
2084	1.07955	2.1
2085	1.31854	2.1

2086	1.29505	0.614993
2087	1.29504	0.929837
2088	0.612469	1.15032
2089	0.990053	0.877157
2090	1.04072	1.13181
2091	0.985266	0.821922
2092	0.990039	0.970703
2093	1.06892	1.20255
2094	1.57158	0.51482
2095	1.895	0.562777
2096	1.05698	0.448771
2097	0.925053	2.1
2098	0.624712	2.1
2099	1.42465	2.1
2100	1.49014	2.1
2101	0.687198	2.1
2102	1.22058	2.1
2103	0.851488	2.1
2104	0.588128	2.1
2105	1.12452	2.1
2106	1.02783	2.1
2107	1.7083	2.1
2108	1.06977	0.522225
2109	0.818569	0.20464
2110	1.14386	0.35968
2111	0.983791	0.305863
2112	1.12214	0.628087
2113	1.0915	0.138883
2114	0.810934	0.463697
2115	0.764114	0.65241
2116	1.87956	0.459596
2117	1.87956	0.652915
2118	0.764141	0.829123
2119	1.37675	0.321438
2120	0.764141	0.348106
2121	0.946132	0.433634
2122	1.9637	1.0416
2123	2.10107	0.67818
2124	1.67814	0.779857
2125	3.02886	0.591881
2126	0.908982	0.100759
2127	0.621727	0.186466
2128	0.525963	0.134952
2129	0.946128	0.20721
2130	1.60979	0.104594
2131	1.45596	0.140851
2132	0.556172	0.190749
2133	2.06779	0.0940199
2134	2.17911	0.112073
2135	1.00205	0.0583969
2136	1.30685	0.0979754
2137	1.16534	0.0735604
2138	1.65532	2.1
2139	2.20346	2.1
2140	0.993889	2.1
2141	1.09328	2.1
2142	2.09372	2.1
2143	2.10036	2.1

2144	1.33727	1.01122
2145	2.53383	0.802411
2146	0.893379	1.25452
2147	1.52828	0.94163
2148	1.09599	0.05
2149	1.09599	0.05
2150	1.50524	2.1
2151	1.54711	2.1
2152	1.41469	2.1
2153	1.99459	1.98612
2154	1.79805	2.1
2155	1.79805	2.1
2156	0.707344	2.1
2157	1.30318	2.1
2158	0.707344	2.1
2159	0.946129	2.1
2160	1.30318	2.1
2161	2.40442	2.1
2162	1.89226	2.1
2163	1.40794	2.1
2164	0.898721	2.1
2165	0.791921	1.7736
2166	0.810933	2.1
2167	1.10904	1.99946
2168	0.991102	2.1
2169	0.808502	1.69267
2170	1.1398	2.05457
2171	1.31346	2.1
2172	1.11833	2.1
2173	1.669	2.1
2174	1.25715	2.1
2175	1.3199	2.1
2176	1.63092	2.1
2177	1.63092	2.1
2178	0.946131	2.1
2179	0.946131	2.1
2180	1.13534	2.1
2181	0.979853	2.1
2182	2.19994	2.1
2183	0.725844	2.1
2184	2.19994	2.1
2185	2.35055	2.1
2186	1.52204	2.1
2187	1.40118	2.1
2188	3.42285	2.1
2189	2.05217	2.1
2190	1.50319	2.1
2191	0.947516	1.93608
2192	0.903572	1.93844
2193	0.76996	1.99548
2194	1.90959	2.1
2195	1.18897	2.1
2196	1.24166	2.1
2197	2.26785	2.06239
2198	2.05217	2.1
2199	2.73306	2.1
2200	1.42729	0.0752606
2201	1.387	0.0813656

2202	1.59458	0.383235
2203	1.91051	0.409778
2204	1.68108	0.481524
2205	2.03357	0.752648
2206	0.928982	0.115891
2207	1.55687	0.117541
2208	1.55687	0.0816479
2209	0.92898	0.105256
2210	1.3495	0.134074
2211	1.3495	0.0958232
2212	0.946133	0.133655
2213	1.85795	0.0956149
2214	2.2727	0.124704
2215	1.20324	2.06845
2216	1.07784	0.235459
2217	0.957792	0.157298
2218	1.2745	0.154736
2219	1.4659	0.166639
2220	0.946129	0.25752
2221	0.95778	0.240527
2222	0.851553	0.302768
2223	1.74103	0.17592
2224	2.28994	0.176679
2225	2.08213	0.156204
2226	2.1556	0.160312
2227	2.20372	0.132429
2228	0.838027	0.05
2229	1.11373	0.05
2230	1.2909	0.05
2231	0.998424	0.05
2232	1.40621	0.05
2233	1.19478	0.05
2234	1.11783	0.05
2235	1.6695	0.05
2236	1.7498	0.05
2237	0.477652	0.05
2238	1.77291	0.05
2239	0.590448	0.05
2240	1.07668	0.05
2241	0.810946	0.05
2242	1.1946	0.05
2243	2.03914	0.05
2244	1.25585	0.05
2245	2.02622	0.05
2246	1.39091	0.05
2247	1.31871	0.05
2248	1.15789	2.1
2249	1.15755	2.1
2250	1.82268	2.1
2251	1.87119	2.1
2252	3.10636	2.1
2253	2.96285	2.1
2254	1.73443	1.19808
2255	2.33812	1.14254
2256	1.59457	0.517404
2257	1.73443	1.42075
2258	2.00665	2.1
2259	1.99841	2.1

2260	1.19901	2.1
2261	1.16661	2.1
2262	2.15883	0.155721
2263	1.20951	0.155323
2264	2.49192	0.220553
2265	0.65112	0.131237
2266	2.45317	0.05
2267	1.73091	0.05
2268	2.0795	0.05
2269	1.45891	0.05
2270	1.6531	0.05
2271	1.78304	0.05
2272	0.775132	0.05
2273	1.35051	0.05
2274	1.0461	0.05
2275	1.37837	0.05
2276	1.00259	0.05
2277	1.25796	0.206297
2278	1.10719	0.256455
2279	1.51067	2.1
2280	2.65632	2.1
2281	1.30521	2.1
2282	2.456 2.1	
2283	3.19218	2.1
2284	2.67387	2.09829
2285	0.988862	2.1
2286	1.917 2.1	
2287	0.946132	2.1
2288	1.16202	2.1
2289	1.53871	2.1
2290	1.53871	2.1
2291	0.946132	2.1
2292	1.16202	2.1
2293	1.32282	2.1
2294	2.46356	2.1
2295	2.32404	2.1
2296	1.37562	0.0693549
2297	0.985546	0.0599599
2298	1.28983	0.0559488
2299	1.05566	0.052755
2300	0.895312	0.0541507
2301	1.53325	0.0526114
2302	1.04342	0.0542387
2303	1.80881	0.0511615
2304	0.876865	0.0555635
2305	0.5689	0.0596248
2306	1.09506	0.0516634
2307	1.51789	2.1
2308	1.49872	2.1
2309	0.949364	0.090982
2310	2.01233	0.0980168
2311	1.55063	0.143947
2312	1.57783	0.263253
2313	1.57783	0.260489
2314	0.674605	0.221766
2315	0.98537	0.134283
2316	0.674633	0.0789241
2317	0.98537	0.0997382

2318	1.26706	0.235174
2319	0.980664	0.380597
2320	1.03298	0.13182
2321	1.84936	0.355769
2322	1.51696	0.0883591
2323	1.97074	0.104994
2324	1.03298	0.127982
2325	1.51324	0.080273
2326	1.51324	0.0745713
2327	0.758454	0.0787504
2328	1.28632	0.0698719
2329	0.758482	0.0534993
2330	0.758482	0.0615198
2331	0.985372	0.0820484
2332	1.14105	0.05
2333	1.59787	0.284447
2334	2.46244	0.327942
2335	1.70367	0.154584
2336	2.13757	1.36923
2337	1.98142	1.35211
2338	1.35878	0.0646762
2339	2.39947	0.0643055
2340	1.24554	0.0520251
2341	1.7522	0.0598413
2342	1.49693	1.82863
2343	1.49652	1.88068
2344	1.6211	1.8551
2345	1.45938	1.90333
2346	0.737431	1.96556
2347	1.18643	1.90547
2348	1.04072	2.03624
2349	0.9459	1.8903
2350	0.66364	1.96773
2351	0.985109	1.74112
2352	0.638368	1.88295
2353	0.83804	1.66471
2354	0.998024	1.75584
2355	0.914471	1.63471
2356	1.14281	1.77577
2357	0.833972	1.74406
2358	0.991865	0.0644695
2359	1.42208	0.140411
2360	1.70718	2.1
2361	1.79888	2.1
2362	1.16851	0.250622
2363	1.16851	0.217763
2364	1.51021	0.340211
2365	0.946132	0.166593
2366	1.14897	0.0970411
2367	0.946132	0.222853
2368	1.77177	0.173388
2369	1.77177	0.0936828
2370	1.33038	0.14317
2371	1.38752	0.146935
2372	1.1726	0.101303
2373	1.51688	0.167174
2374	1.06455	2.1
2375	0.98511	2.1

2376	1.31144	2.1
2377	1.79825	2.1
2378	1.79825	2.1
2379	1.73044	2.1
2380	1.59589	0.05
2381	1.59589	0.05
2382	2.78009	2.1
2383	1.68761	2.1
2384	3.05511	2.1
2385	2.25318	2.1
2386	0.578998	2.1
2387	1.03512	2.1
2388	2.26156	2.1
2389	1.44859	0.96535
2390	1.35897	1.11341
2391	1.18301	2.1
2392	1.25173	2.1
2393	1.98851	2.1
2394	2.70685	2.1
2395	0.682342	2.1
2396	1.52491	2.1
2397	1.37119	2.1
2398	0.682313	2.1
2399	1.35342	2.1
2400	0.947555	2.1
2401	0.614107	2.1
2402	1.23007	2.1
2403	1.5735	2.1
2404	1.58713	2.1
2405	1.57979	0.05
2406	1.30723	0.05
2407	0.935658	1.10575
2408	0.8952	1.08716
2409	1.27352	1.31234
2410	1.0985	1.25674
2411	1.07163	1.20956
2412	1.15823	1.08467
2413	0.836451	1.16935
2414	2.08051	0.42643
2415	1.92601	1.4093
2416	2.0644	0.932696
2417	1.37259	2.1
2418	1.37259	2.1
2419	0.752217	2.1
2420	1.28569	2.1
2421	0.752186	2.1
2422	0.839124	2.1
2423	1.28568	2.1
2424	1.04199	2.1
2425	1.04199	2.1
2426	2.59268	2.1
2427	1.04199	2.1
2428	1.5098	2.1
2429	1.5098	2.1
2430	0.520997	2.1
2431	1.29634	2.1
2432	0.520996	2.1
2433	0.73446	2.1

2434	1.29634	2.1
2435	0.983724	2.1
2436	1.23914	2.1
2437	2.65095	2.1
2438	1.52851	2.1
2439	2.399	2.1
2440	1.68137	2.1
2441	1.11285	2.1
2442	1.04921	2.1
2443	1.30711	2.1
2444	1.04921	2.1
2445	1.07496	1.31888
2446	1.55831	0.54845
2447	1.07497	0.281128
2448	1.07497	1.45604
2449	1.27391	2.1
2450	2.5615	2.1
2451	1.2739	2.1
2452	1.75253	2.1
2453	1.12907	0.0719019
2454	1.9194	0.062319
2455	1.96617	0.0545951
2456	1.01225	2.1
2457	0.947979	2.1
2458	1.01225	2.1
2459	1.06561	2.1
2460	1.01225	2.1
2461	3.56876	2.1
2462	3.68767	2.1
2463	1.52301	2.1
2464	1.61013	2.1
2465	1.43726	2.1
2466	2.04275	2.1
2467	0.995734	2.1
2468	1.16199	2.1
2469	1.73704	0.0566063
2470	1.04019	0.0547305
2471	0.956294	2.1
2472	1.81488	2.1
2473	1.63719	2.1
2474	1.50103	0.05
2475	1.75289	0.05
2476	1.31491	0.05
2477	1.03567	0.05
2478	0.672257	0.05
2479	1.04072	0.05
2480	0.746903	0.05
2481	1.43317	0.05
2482	1.39986	0.05
2483	1.06385	0.05
2484	1.1624	0.05
2485	1.61667	0.05
2486	1.27258	0.05
2487	2.559	0.05
2488	1.66093	0.05
2489	1.10719	0.281271
2490	2.03235	0.3232
2491	1.17105	0.457135

2492	1.10719	0.327185
2493	1.3028	2.1
2494	0.946131	2.1
2495	0.946127	2.1
2496	1.52912	2.1
2497	1.52912	2.1
2498	0.946128	2.1
2499	0.949997	2.1
2500	1.31565	2.1
2501	1.46643	2.1
2502	1.5482	2.1
2503	1.47102	2.1
2504	1.47102	2.1
2505	0.774101	2.1
2506	1.30583	2.1
2507	0.774103	2.1
2508	0.939287	2.1
2509	1.30584	2.1
2510	0.958661	2.1
2511	2.28835	2.1
2512	1.43213	0.05
2513	2.13255	0.0519804
2514	1.43213	0.05
2515	1.10476	0.05
2516	1.93165	0.05
2517	1.03235	1.49964
2518	1.31	1.4921
2519	1.22082	2.1
2520	1.35827	2.1
2521	0.790936	2.1
2522	0.716644	2.1
2523	1.43257	2.1
2524	1.43257	2.1
2525	0.716615	2.1
2526	0.946131	2.1
2527	0.730157	2.1
2528	1.47212	2.1
2529	1.47212	2.1
2530	0.946131	2.1
2531	0.790792	2.1
2532	1.41149	2.1
2533	0.790791	2.1
2534	1.01006	2.1
2535	1.53045	0.29359
2536	1.53045	0.417821
2537	0.946132	0.981731
2538	1.40779	0.358696
2539	1.06879	1.30755
2540	0.990713	0.645662
2541	1.06879	0.742566
2542	1.06486	0.203356
2543	1.18347	0.1912
2544	1.38622	0.21122
2545	1.18347	0.145237
2546	1.7304	0.221109
2547	1.7304	0.279088
2548	0.946131	0.563643
2549	1.26874	0.28407

2550	1.13337	1.35012
2551	1.13337	0.429313
2552	1.40779	0.443779
2553	1.695 2.1	
2554	1.77071	2.1
2555	0.649313	2.1
2556	0.692829	2.1
2557	1.40395	2.1
2558	1.34243	2.1
2559	0.813292	0.101981
2560	0.632691	0.277033
2561	1.75766	0.340474
2562	2.25727	1.75242
2563	1.74206	0.0859598
2564	0.826073	0.093741
2565	1.30022	0.0995384
2566	1.45641	0.0894746
2567	0.946131	0.09973
2568	0.752245	0.0736474
2569	0.960952	0.129519
2570	0.743477	0.0981141
2571	1.04355	0.0678577
2572	1.6253	0.0889713
2573	0.960577	0.0856962
2574	1.34852	1.20994
2575	1.03235	1.31751
2576	1.78168	0.177554
2577	1.2966	0.05
2578	2.21116	0.05
2579	1.6395	0.05
2580	1.6395	0.05
2581	0.94613	0.0524736
2582	1.22187	0.0572018
2583	0.962737	0.063527
2584	0.962735	0.0548921
2585	1.36376	0.0529159
2586	1.2966	0.05
2587	1.2966	0.05
2588	1.19669	1.0038
2589	2.37636	1.18877
2590	1.08894	1.38004
2591	1.62551	1.38915
2592	2.51472	1.46692
2593	1.89226	0.756782
2594	0.980038	1.26561
2595	1.10187	2.1
2596	1.01479	2.1
2597	1.81663	2.1
2598	1.83247	2.1
2599	0.921851	0.287321
2600	0.983148	2.1
2601	1.82816	2.1
2602	1.26103	2.1
2603	2.34818	2.1
2604	1.23152	2.1
2605	2.15738	2.1
2606	1.23152	2.1
2607	1.51042	2.1

2608	0.842995	2.1
2609	1.22257	2.1
2610	0.810959	2.1
2611	1.68242	2.1
2612	0.95701	2.1
2613	1.12389	2.1
2614	1.95255	2.1
2615	1.57974	2.1
2616	1.94632	2.1
2617	0.81534	2.1
2618	1.13996	2.1
2619	1.18958	0.05
2620	1.25929	0.05
2621	3.02933	1.61129
2622	2.37434	0.422333
2623	1.10609	2.04025
2624	1.64132	1.09976
2625	1.64132	1.27468
2626	0.946132	0.355961
2627	1.40029	1.04592
2628	1.08946	0.380538
2629	1.08946	0.422733
2630	1.18717	0.107609
2631	2.71421	0.0806729
2632	1.08189	0.100064
2633	0.94613	0.0904608
2634	1.37109	0.106486
2635	1.17925	0.0713408
2636	0.851541	0.104399
2637	0.942512	0.10232
2638	1.18541	0.0842335
2639	0.851526	0.105286
2640	1.0819	0.0879513
2641	1.86009	0.132569
2642	0.946132	0.0757049
2643	1.67409	0.0603966
2644	1.67409	0.0919848
2645	0.946131	0.0926571
2646	1.31716	0.0871346
2647	1.30307	0.0777602
2648	0.946131	0.0979231
2649	1.18601	0.0851675
2650	3.15646	2.1
2651	0.985109	2.1
2652	3.37048	2.1
2653	1.10667	2.1
2654	1.77065	1.51261
2655	1.45837	1.45058
2656	1.77063	1.11995
2657	2.58104	1.20486
2658	1.37652	2.1
2659	1.54168	2.1
2660	1.00224	0.05
2661	1.05851	0.05
2662	1.39093	0.05
2663	1.43358	0.05
2664	0.575741	0.05
2665	0.518161	0.05

2666	1.04071	0.05
2667	1.13092	1.81465
2668	1.17042	1.87444
2669	1.53347	1.66626
2670	1.52938	1.81391
2671	1.00613	0.702835
2672	1.81618	2.1
2673	1.81618	0.743565
2674	0.946127	2.1
2675	1.36479	2.1
2676	0.94613	0.388034
2677	1.31724	1.4346
2678	1.3648	1.45202
2679	1.00613	0.744579
2680	0.781375	0.14341
2681	1.13431	0.16564
2682	1.03547	0.156307
2683	1.40668	0.222741
2684	0.94613	0.22148
2685	0.713285	0.185357
2686	1.72885	0.173096
2687	1.72885	0.193842
2688	0.713298	0.134003
2689	3.30075	0.107015
2690	2.81337	0.192275
2691	1.39296	0.247833
2692	0.830164	2.1
2693	1.50493	2.1
2694	1.50494	2.1
2695	0.830136	2.1
2696	1.28298	2.1
2697	1.0521	2.1
2698	0.830147	2.1
2699	2.0799	2.1
2700	1.52115	2.1
2701	2.5142	2.1
2702	1.31253	0.790749
2703	1.94446	1.2966
2704	1.18817	1.15185
2705	0.598348	0.815227
2706	1.69315	0.957147
2707	1.69315	1.2747
2708	0.598377	0.881709
2709	0.598347	0.918741
2710	0.94613	0.655762
2711	1.28418	1.36152
2712	2.05205	1.45987
2713	1.28418	1.36941
2714	1.0737	2.1
2715	1.81024	2.1
2716	2.73834	2.1
2717	1.71369	2.1
2718	1.71368	2.1
2719	0.946118	2.1
2720	1.29063	2.1
2721	1.36917	2.1
2722	0.94613	2.1
2723	1.29062	2.1

2724	0.947921	2.1
2725	1.58661	1.88531
2726	1.37658	1.18065
2727	1.2364	1.2077
2728	1.66685	1.03973
2729	1.66685	1.3062
2730	0.946133	0.990603
2731	0.946133	1.07231
2732	1.11978	1.18174
2733	1.09194	1.02798
2734	1.21858	1.46411
2735	1.37658	1.24781
2736	1.64903	1.1468
2737	1.64903	1.66991
2738	0.946132	1.13827
2739	0.946132	1.22439
2740	1.05496	1.27042
2741	1.22851	1.09957
2742	1.22852	1.45501
2743	1.58482	0.968946
2744	1.60578	0.0905306
2745	1.06336	0.0887083
2746	1.68062	0.0954439
2747	1.14721	0.0727028
2748	1.11924	0.05
2749	1.18421	0.05
2750	2.01276	0.05
2751	1.23502	1.78254
2752	1.34926	1.81376
2753	0.546751	1.59675
2754	0.790446	1.79918
2755	1.04072	1.61797
2756	0.555868	1.57469
2757	0.930305	1.5924
2758	1.09344	1.57728
2759	1.18351	1.65285
2760	1.97215	1.61014
2761	1.07416	1.72547
2762	1.33204	1.52731
2763	1.22038	1.43614
2764	0.597449	1.33505
2765	0.821422	1.52462
2766	1.04071	1.45212
2767	0.608026	1.31079
2768	0.929427	1.42152
2769	1.64929	0.05
2770	1.68541	0.05
2771	0.496093	0.05
2772	1.3385	0.05
2773	0.790798	0.05
2774	1.22971	0.05
2775	0.898853	0.05
2776	2.14361	0.05
2777	2.10813	0.05
2778	1.05417	0.05
2779	1.80768	0.0693918
2780	1.09725	2.1
2781	1.41131	2.1

2782	1.1656	2.1
2783	0.946129	2.1
2784	1.68365	2.1
2785	1.68365	2.1
2786	1.1656	2.1
2787	0.94613	2.1
2788	1.21269	0.05
2789	1.48867	0.05
2790	1.27806	0.05
2791	1.68264	0.05
2792	1.37813	1.56182
2793	1.50743	1.43615
2794	1.04121	1.29778
2795	1.12434	0.354687
2796	1.39553	0.422236
2797	1.44721	1.67163
2798	0.946132	0.715245
2799	1.23943	0.585909
2800	1.05292	0.875295
2801	1.28607	0.33538
2802	1.16915	0.568953
2803	1.76306	2.01303
2804	1.42266	1.9037
2805	1.36844	2.1
2806	1.36844	2.1
2807	1.67352	2.1
2808	1.11256	1.47729
2809	1.03217	1.24768
2810	1.18422	0.05
2811	1.04159	0.05
2812	1.60139	0.05
2813	1.04159	0.05
2814	0.988805	2.1
2815	1.05481	2.1
2816	1.52938	1.95761
2817	1.94724	1.79188
2818	0.896336	0.0845515
2819	0.911484	0.0863777
2820	1.18315	0.0626947
2821	1.10515	0.0691467
2822	1.04067	0.0645815
2823	1.12758	0.0785645
2824	0.904106	0.0742999
2825	1.6901	0.0582917
2826	1.68671	0.0624516
2827	1.99908	0.063903
2828	0.772426	0.37248
2829	0.810945	1.54871
2830	0.667562	0.932334
2831	0.496617	0.251624
2832	0.847689	0.621289
2833	0.914378	1.12027
2834	0.934003	0.57179
2835	1.07459	0.50969
2836	1.40225	2.1
2837	1.07043	2.1
2838	0.94613	2.1
2839	1.63072	2.1

2840	1.63072	2.1
2841	1.07043	2.1
2842	0.946131	2.1
2843	1.53082	2.1
2844	1.69916	2.1
2845	1.75211	1.11499
2846	1.96831	2.1
2847	1.75211	2.1
2848	0.946127	0.896163
2849	0.817065	0.803039
2850	1.41798	0.738595
2851	1.41798	0.401383
2852	0.791656	0.981596
2853	0.791656	0.994472
2854	1.41798	0.751579
2855	1.58331	0.509423
2856	1.41776	0.404652
2857	0.791647	0.438289
2858	1.4512	0.349856
2859	1.45119	0.353055
2860	0.75821	0.553205
2861	0.758248	0.488014
2862	0.946104	0.456816
2863	1.36482	0.357912
2864	1.29389	1.93714
2865	1.04164	1.87568
2866	1.40574	1.77269
2867	1.64833	1.80847
2868	1.71183	0.146059
2869	1.71183	0.194525
2870	0.946131	0.39232
2871	1.35721	0.252003
2872	0.946131	0.944796
2873	0.946131	0.456985
2874	1.30076	0.418929
2875	1.2474	0.144216
2876	2.85225	0.314179
2877	2.71441	0.146877
2878	1.2474	0.113342
2879	1.38622	0.222109
2880	0.858359	0.0975426
2881	1.3682	0.127762
2882	1.57364	0.110888
2883	0.729695	0.235474
2884	0.729723	0.102814
2885	0.822611	0.552605
2886	0.946133	0.248436
2887	0.729723	0.156597
2888	0.974704	0.40827
2889	0.974702	0.517533
2890	1.81603	2.1
2891	1.96348	2.1
2892	1.43612	1.26168
2893	1.43612	1.34204
2894	0.634295	1.22441
2895	0.93796	1.35869
2896	0.946133	1.30144
2897	0.634326	1.18693

2898	0.946133	1.26451
2899	1.2654	1.12231
2900	1.26865	1.22747
2901	2.2649	1.2399
2902	1.00864	1.21527
2903	1.09689	0.967856
2904	0.832021	1.10831
2905	1.53229	0.763325
2906	1.1145	0.926165
2907	0.929583	1.06589
2908	1.613	0.734863
2909	0.644729	1.03415
2910	1.03471	2.1
2911	2.7739	2.1
2912	1.36133	2.1
2913	2.50215	2.1
2914	2.52195	2.1
2915	1.0465	2.1
2916	1.42673	2.1
2917	1.42673	2.1
2918	0.946132	2.1
2919	1.31904	2.1
2920	0.986499	2.1
2921	0.946132	2.1
2922	1.31904	2.1
2923	0.966978	2.1
2924	1.42237	0.0620782
2925	2.60925	0.067175
2926	0.753659	0.0838356
2927	1.77241	0.0637856
2928	1.77241	0.0697278
2929	0.946129	0.0889574
2930	0.75366	0.0634482
2931	1.22144	0.0804202
2932	0.94613	0.0800606
2933	1.50732	0.0646494
2934	1.18514	0.0772081
2935	0.902187	0.0852375
2936	1.89858	0.0645636
2937	1.24294	0.068367
2938	1.67211	0.0752332
2939	1.41786	0.0668215
2940	0.810959	0.0806688
2941	1.50732	0.0676243
2942	2.44288	0.0782684
2943	1.40969	0.05
2944	2.06654	0.0558364
2945	1.40969	0.052495
2946	1.40882	0.0668306
2947	1.94063	0.0704365
2948	0.867921	0.0550036
2949	1.29289	0.058209
2950	0.867918	0.0576982
2951	0.946128	0.0559377
2952	0.867944	0.0582584
2953	1.66589	0.0583526
2954	1.66589	0.0537021
2955	0.968595	2.1

2956	1.5534	2.1
2957	1.27793	2.1
2958	1.13433	2.1
2959	1.3439	0.05
2960	2.53709	0.05
2961	0.571998	0.05
2962	0.64837	0.05
2963	1.99755	0.05
2964	1.99854	0.05
2965	0.764229	2.1
2966	1.61842	2.1
2967	1.61842	2.1
2968	1.18315	2.1
2969	1.1995	2.1
2970	1.18315	2.1
2971	0.764256	2.1
2972	1.52364	2.1
2973	2.36631	2.1
2974	0.641912	2.1
2975	1.7181	2.1
2976	1.459 2.1	
2977	0.634845	2.01217
2978	1.18315	2.1
2979	1.17615	2.1
2980	0.571367	2.1
2981	1.3324	0.164203
2982	0.798214	0.165295
2983	0.946128	0.177623
2984	0.627252	0.149075
2985	1.50337	0.153781
2986	1.50337	0.116663
2987	0.62725	0.181348
2988	1.07763	0.104027
2989	1.07763	0.145222
2990	2.16109	0.162492
2991	1.07763	0.133596
2992	1.12486	2.1
2993	1.11827	2.1
2994	1.0491	2.1
2995	1.09034	2.1
2996	1.09431	2.1
2997	0.946131	2.1
2998	0.748607	2.1
2999	1.44002	2.1
3000	1.44002	2.1
3001	0.748635	2.1
3002	0.782505	2.1
3003	1.40615	2.1
3004	1.19539	2.1
3005	1.29389	1.82737
3006	1.4867	1.60377
3007	1.50103	0.05
3008	1.63025	0.05
3009	2.72109	0.05
3010	0.915755	0.05
3011	0.946132	0.05
3012	0.915755	0.05
3013	1.70236	0.05

3014	1.70236	0.05
3015	1.26155	0.05
3016	1.35657	0.05
3017	1.83151	0.05
3018	1.83151	0.05
3019	2.52309	0.05
3020	0.983607	2.1
3021	1.06838	0.684111
3022	0.983609	2.1
3023	0.983596	2.06981
3024	2.74384	0.0997824
3025	1.6261	0.0894825
3026	1.15477	2.1
3027	1.02136	2.1
3028	1.83561	2.1
3029	1.83561	2.1
3030	0.946131	2.1
3031	1.39523	2.1
3032	1.29313	2.1
3033	1.29313	2.1
3034	1.38651	2.1
3035	1.06313	2.1
3036	0.974888	2.1
3037	1.0894	2.1
3038	0.974888	2.1
3039	1.82282	2.1
3040	1.82282	2.1
3041	0.946131	2.1
3042	1.37372	2.1
3043	1.32374	2.1
3044	1.32374	2.1
3045	1.39523	2.1
3046	1.40882	0.0696851
3047	0.861998	0.0722065
3048	1.24448	0.0770489
3049	0.861999	0.0734593
3050	0.946129	0.0793977
3051	0.862011	0.0831914
3052	1.67537	0.0782543
3053	1.67537	0.0691739
3054	1.72399	0.0501072
3055	2.58579	0.0546563
3056	1.72399	0.0515906
3057	1.50951	0.0630995
3058	2.48897	0.0691761
3059	1.26755	2.1
3060	1.03824	2.1
3061	1.27875	2.1
3062	1.48901	2.1
3063	1.18902	2.1
3064	1.11764	2.1
3065	1.87436	2.1
3066	0.95345	2.08036
3067	0.724191	1.85069
3068	0.86668	1.82645
3069	0.811523	1.94653
3070	1.04074	1.65188
3071	0.843097	1.73619

3072	0.833434	1.88893
3073	0.965727	1.802
3074	0.85346	1.82648
3075	1.00185	1.39541
3076	1.00184	1.72091
3077	0.980651	1.88772
3078	0.980664	1.73236
3079	0.475737	1.57642
3080	0.719696	1.56792
3081	0.946109	1.45445
3082	0.946116	1.48925
3083	0.719693	1.52227
3084	0.946117	1.5014
3085	1.14879	1.79793
3086	0.719683	1.68934
3087	0.900977	1.53947
3088	0.972364	1.5644
3089	1.22961	1.59488
3090	0.405384	1.49552
3091	1.34406	2.1
3092	1.2187	2.1
3093	0.963571	0.40306
3094	0.946131	1.01104
3095	1.57246	1.62559
3096	1.57246	2.1
3097	0.946131	1.6082
3098	1.1235	2.1
3099	1.1235	2.09512
3100	1.07439	2.1
3101	1.19612	1.64077
3102	1.24844	1.63645
3103	1.00007	1.95495
3104	1.02429	2.1
3105	1.10459	2.1
3106	1.06724	0.216866
3107	0.67519	0.509346
3108	0.49762	0.257048
3109	1.43435	0.183996
3110	0.833315	0.366525
3111	0.58135	0.286065
3112	1.14749	1.10147
3113	1.85091	1.76902
3114	1.2802	0.63998
3115	1.27737	1.62152
3116	2.3655	1.28045
3117	1.30584	2.1
3118	2.06424	2.1
3119	0.946131	0.0647774
3120	1.04257	0.0685935
3121	0.94613	0.0927048
3122	1.67201	0.0689364
3123	1.67201	0.0523398
3124	1.27696	0.06379
3125	1.34118	0.0512632
3126	1.3413	0.0858245
3127	2.36238	0.0757127
3128	0.6036	0.11358
3129	1.11745	0.127574

3130	1.46126	0.0975859
3131	1.39612	0.0807823
3132	0.670678	0.11556
3133	1.03402	0.12617
3134	1.11271	0.110949
3135	0.838028	0.0790335
3136	0.807019	0.102456
3137	1.27787	0.0660699
3138	0.994705	0.0743789
3139	1.20014	0.0679877
3140	0.964793	0.0967313
3141	0.863868	0.0804106
3142	1.3413	0.0659017
3143	1.79549	0.0740907
3144	1.35877	2.1
3145	1.63648	2.1
3146	1.08171	2.1
3147	1.27839	2.1
3148	1.52601	2.1
3149	1.27841	2.1
3150	1.71255	2.1
3151	2.16307	2.1
3152	1.91592	2.1
3153	1.78508	2.1
3154	0.946128	2.1
3155	1.41199	2.1
3156	0.946128	2.1
3157	0.901088	2.1
3158	1.55244	2.1
3159	1.59475	2.1
3160	1.40921	2.1
3161	0.960106	2.1
3162	1.40921	2.1
3163	2.0073	2.1
3164	2.2973	0.12937
3165	1.90705	0.130608
3166	1.53671	0.0833639
3167	1.86035	0.082442
3168	1.33747	2.1
3169	1.14007	2.1
3170	1.3212	2.1
3171	1.66867	0.063547
3172	1.30203	0.05
3173	1.27696	2.1
3174	1.05025	2.1
3175	1.05025	2.1
3176	1.3704	2.1
3177	1.15677	2.1
3178	0.946129	2.1
3179	1.65879	2.1
3180	1.65879	2.1
3181	1.15676	2.1
3182	0.94613	2.1
3183	0.867217	1.5216
3184	1.28684	1.51922
3185	1.58866	1.47049
3186	1.58866	1.37945
3187	0.867246	1.68067

3188	1.28684	1.48422
3189	1.16906	1.50356
3190	1.13438	1.4619
3191	1.37224	1.50829
3192	1.61519	1.56345
3193	0.883939	1.69157
3194	1.40092	1.62367
3195	1.80383	1.75511
3196	1.80383	1.61927
3197	0.883935	1.75948
3198	1.40092	1.60187
3199	1.28684	1.65798
3200	1.76788	1.48719
3201	2.57368	1.53022
3202	1.37224	1.74359
3203	1.65411	0.622296
3204	1.77042	2.1
3205	1.77042	2.1
3206	1.90446	2.1
3207	1.28667	2.1
3208	1.55635	2.1
3209	1.30646	0.467962
3210	2.31836	0.132389
3211	1.30645	0.350827
3212	1.86429	0.735871
3213	1.48676	0.0883245
3214	0.743379	0.0929782
3215	1.41848	0.101587
3216	1.41848	0.0898034
3217	0.946131	0.0902213
3218	0.812649	0.0841028
3219	1.34921	0.0965218
3220	0.812649	0.0913573
3221	0.949366	0.0898085
3222	1.32974	0.0865933
3223	0.794204	0.05
3224	0.634352	2.1
3225	0.838025	2.1
3226	1.08802	2.09517
3227	0.838038	2.04957
3228	1.18314	2.1
3229	1.12495	2.1
3230	1.22774	2.1
3231	1.04343	2.1
3232	0.921111	0.05
3233	2.1609	0.05
3234	1.09987	0.05
3235	2.5043	0.05
3236	1.05492	0.05
3237	0.590344	0.05
3238	1.05492	0.05
3239	1.25215	0.05
3240	1.71674	0.05
3241	1.71674	0.05
3242	0.590331	0.05
3243	1.17078	0.05
3244	0.625542	0.05
3245	0.724673	2.1

3246	1.08011	2.1
3247	1.87598	2.1
3248	1.50738	2.1
3249	1.44951	0.114596
3250	1.50102	0.1075
3251	1.8042	0.522492
3252	0.687769	1.36808
3253	1.66483	0.819263
3254	1.66483	1.21681
3255	0.946132	1.33849
3256	0.687798	0.869807
3257	1.20885	1.33647
3258	0.687799	1.19379
3259	0.886063	0.205413
3260	0.798751	0.253636
3261	0.584813	0.209418
3262	1.49483	0.13666
3263	0.888005	0.170114
3264	1.03589	0.14439
3265	1.70435	0.216736
3266	0.710414	2.1
3267	0.917609	2.01869
3268	0.810947	2.01392
3269	0.81096	1.94856
3270	1.48658	1.84569
3271	1.53653	1.90202
3272	1.19329	2.04118
3273	0.946153	1.88657
3274	1.50798	1.95214
3275	1.85017	2.1
3276	1.19768	2.1
3277	0.864304	2.1
3278	0.792906	2.02483
3279	0.989025	1.88794
3280	1.19768	1.80311
3281	1.54548	1.87338
3282	1.18798	2.04551
3283	1.31875	1.78969
3284	1.05725	1.88889
3285	2.17365	0.05
3286	2.48106	0.05
3287	1.87451	0.05
3288	1.27189	1.5431
3289	2.79399	1.83726
3290	0.859565	1.51899
3291	0.926174	1.48436
3292	0.604744	1.48503
3293	1.41832	1.08272
3294	1.05494	1.41502
3295	0.874749	1.40151
3296	1.50067	1.3299
3297	0.917817	1.67032
3298	0.63591	1.71691
3299	1.72849	1.70253
3300	1.72849	1.51435
3301	0.635943	1.61944
3302	1.39699	1.70887
3303	0.967411	1.95813

3304	0.635943	1.57736
3305	1.39908	1.53186
3306	1.26971	1.64091
3307	1.02969	1.70894
3308	1.15326	1.91345
3309	2.30741	1.67643
3310	0.93545	1.51339
3311	0.802747	1.47864
3312	1.15466	1.60494
3313	0.859164	1.62947
3314	1.02616	1.69023
3315	1.23149	1.55964
3316	0.83804	1.54961
3317	1.13266	1.9062
3318	1.64142	1.7503
3319	1.21223	2.1
3320	1.43051	2.1
3321	1.32418	1.59072
3322	0.621319	1.99274
3323	1.47134	2.08073
3324	1.79103	1.04199
3325	3.25951	0.05
3326	3.16764	0.05
3327	1.65583	0.125494
3328	1.4732	0.05
3329	1.76476	2.1
3330	1.80114	2.1
3331	0.754121	2.1
3332	0.94613	1.41692
3333	0.754119	2.1
3334	1.45636	2.09915
3335	1.45636	0.890898
3336	1.30029	1.97482
3337	0.910183	1.88123
3338	1.29099	2.1
3339	1.08039	2.1
3340	1.05498	2.1
3341	1.24445	2.1
3342	1.2873	2.1
3343	0.987703	2.1
3344	0.964782	2.1
3345	1.73645	0.0642193
3346	0.871279	0.0813618
3347	0.884744	0.0883055
3348	1.85193	0.0669622
3349	1.68681	0.0715196
3350	1.33689	0.0791627
3351	1.23493	0.0688531
3352	0.892482	0.0833521
3353	2.95185	0.0677983
3354	2.10496	0.0688873
3355	1.87334	2.1
3356	0.94411	2.1
3357	0.754196	2.1
3358	1.43122	2.1
3359	1.58684	2.1
3360	0.838027	2.1
3361	1.1536	2.1

3362	1.24878	2.1
3363	1.23125	2.1
3364	2.86862	2.1
3365	1.57636	2.1
3366	0.998451	0.362862
3367	1.5176	0.290298
3368	1.13271	0.05
3369	1.60248	0.05
3370	1.22083	2.1
3371	1.79996	2.1
3372	1.19087	2.1
3373	2.00342	2.1
3374	1.3715	2.1
3375	2.79227	2.1
3376	2.7873	2.1
3377	0.987265	0.05
3378	1.62715	2.1
3379	1.70117	2.1
3380	1.27806	0.05
3381	1.45246	2.1
3382	1.45244	2.1
3383	1.87214	2.1
3384	0.995796	2.1
3385	1.68189	1.31394
3386	1.49155	2.1
3387	1.58054	2.1
3388	1.59169	2.1
3389	0.736364	0.05
3390	0.70236	0.05
3391	2.24367	0.05
3392	2.57085	0.05
3393	1.70952	0.05
3394	2.60516	0.05
3395	1.45158	2.1
3396	1.00371	2.1
3397	1.65748	2.1
3398	1.23706	0.11925
3399	1.55834	1.06753
3400	1.72671	1.05443
3401	0.597656	0.833938
3402	1.39137	1.2041
3403	0.855578	0.115017
3404	1.48398	0.517397
3405	0.745117	0.130199
3406	1.23706	0.421236
3407	1.57916	0.709883
3408	0.893025	0.207867
3409	3.22433	0.326786
3410	2.47324	1.81046
3411	1.63852	2.1
3412	1.63852	2.1
3413	0.946132	2.1
3414	1.19095	2.1
3415	0.946133	2.1
3416	1.16684	2.1
3417	1.19095	2.1
3418	1.06698	0.135002
3419	1.48692	0.106202

3420	1.08334	1.98219
3421	1.07283	2.1
3422	1.28517	1.16703
3423	1.2746	0.05
3424	1.30723	0.05
3425	2.02069	0.05
3426	1.05579	0.05
3427	1.59475	2.1
3428	2.40113	2.1
3429	1.58875	2.1
3430	1.6881	2.1
3431	1.6881	2.1
3432	0.819953	2.1
3433	0.819954	2.1
3434	0.946131	2.1
3435	0.946131	2.1
3436	1.34782	2.1
3437	1.65619	2.1
3438	1.65619	2.07095
3439	0.79437	2.1
3440	1.25	2.1
3441	0.94613	2.1
3442	0.946131	2.07595
3443	0.794371	2.1
3444	1.58875	2.1
3445	2.5	2.1
3446	1.04147	2.1
3447	0.946131	1.42589
3448	1.79686	2.1
3449	1.79686	2.1
3450	1.25144	1.40622
3451	1.35714	2.06747
3452	1.38585	1.84473
3453	1.25144	2.07941
3454	1.15564	2.1
3455	1.34389	2.1
3456	0.946133	1.35522
3457	1.74353	1.99463
3458	1.74353	2.1
3459	1.14553	1.33323
3460	1.38585	1.74857
3461	1.30382	1.52117
3462	1.14553	1.80916
3463	1.15564	2.1
3464	1.90618	2.1
3465	2.11056	2.1
3466	1.032	2.1
3467	0.953636	2.1
3468	1.23131	1.6305
3469	1.39763	1.46278
3470	2.01024	1.19536
3471	1.9186	1.22446
3472	1.83658	0.0853433
3473	1.81656	0.0907536
3474	2.2049	0.106803
3475	2.16081	0.0975776
3476	1.12219	0.122557
3477	0.599771	0.14066

3478	1.61401	0.0929579
3479	1.05912	0.113798
3480	0.951648	0.119119
3481	1.89328	0.0960557
3482	0.913682	0.134355
3483	1.00763	0.0944514
3484	1.29129	2.1
3485	1.37138	2.1
3486	1.41986	0.0507024
3487	1.41986	0.0531136
3488	0.944166	0.0633549
3489	1.41789	0.0632014
3490	0.944167	0.0716944
3491	0.944166	0.0589916
3492	0.946132	0.059276
3493	1.98713	0.05
3494	1.23626	0.05
3495	0.932791	0.05
3496	0.810251	0.05
3497	0.986748	0.05
3498	0.898155	0.0568378
3499	0.92624	0.05
3500	1.1349	0.05
3501	0.934572	0.05
3502	1.23104	0.819669
3503	1.23104	1.11768
3504	0.799994	0.441945
3505	0.94613	0.772214
3506	0.802822	0.384136
3507	1.40944	0.906697
3508	0.800035	0.743038
3509	1.41223	0.738693
3510	1.41223	0.57531
3511	1.6	0.407778
3512	1.60565	0.43116
3513	1.23042	2.1
3514	1.0645	2.1
3515	1.00319	2.1
3516	1.29435	2.1
3517	1.2243	2.1
3518	1.2243	2.1
3519	1.05655	1.73743
3520	1.29764	1.83613
3521	2.55252	2.1
3522	1.15676	2.1
3523	0.855295	2.1
3524	0.810959	2.1
3525	1.65857	2.1
3526	1.00849	2.1
3527	1.16021	2.1
3528	1.75869	2.1
3529	1.13775	2.1
3530	1.20091	2.1
3531	1.61582	2.1
3532	1.88031	2.1
3533	0.968536	2.1
3534	0.946131	2.1
3535	1.61481	2.1

3536	1.61481	2.1
3537	1.09547	2.1
3538	1.18486	2.1
3539	1.37609	2.1
3540	1.09546	2.1
3541	1.02461	2.1
3542	1.83223	2.1
3543	1.74002	2.1
3544	1.70939	2.1
3545	1.7094	2.1
3546	0.726287	2.1
3547	1.01712	2.1
3548	1.30318	2.1
3549	0.946131	2.1
3550	0.87155	2.1
3551	1.77093	1.97867
3552	2.02356	1.95802
3553	1.32449	2.1
3554	1.10003	2.1
3555	1.5161	2.1
3556	1.5161	2.1
3557	0.946132	2.1
3558	1.09206	2.1
3559	0.946132	2.1
3560	1.04404	2.1
3561	1.09206	2.1
3562	0.635628	1.45034
3563	1.14623	0.267061
3564	1.73197	1.27115
3565	1.73197	1.46533
3566	0.529655	0.671627
3567	1.14623	0.719955
3568	0.992085	0.625454
3569	1.16915	1.48038
3570	2.29245	0.294872
3571	0.584573	0.603826
3572	0.956756	0.167526
3573	1.52471	1.0626
3574	1.51409	1.54978
3575	0.584575	1.14109
3576	0.94613	0.164483
3577	1.14622	0.269627
3578	1.28096	1.50727
3579	1.34695	1.59705
3580	1.04193	2.1
3581	1.05378	2.1
3582	0.798209	2.1
3583	1.32007	2.1
3584	1.64327	0.05
3585	2.64674	1.25363
3586	2.38936	0.05
3587	2.88475	0.05
3588	1.07745	2.1
3589	1.08911	2.1
3590	1.49127	2.1
3591	1.49127	2.1
3592	0.709714	2.1
3593	1.37199	2.1

3594	0.82899	2.1
3595	0.709714	2.1
3596	0.946132	2.1
3597	1.00133	2.1
3598	1.40182	2.1
3599	1.53278	2.1
3600	1.40182	2.1
3601	1.31764	0.05
3602	1.00691	0.05
3603	1.24094	0.05
3604	0.64868	0.05
3605	0.946133	0.05
3606	0.64868	0.05
3607	1.71762	0.05
3608	1.71762	0.05
3609	1.41662	0.05
3610	0.975477	0.05
3611	1.73584	0.05
3612	2.48188	0.05
3613	2.2045	0.399232
3614	2.08134	0.353679
3615	0.993366	1.67489
3616	0.993368	1.69795
3617	1.14187	0.319351
3618	0.946132	0.638394
3619	0.946133	1.35476
3620	1.68951	1.41125
3621	1.68951	1.48868
3622	1.34612	0.961887
3623	1.28953	0.423919
3624	1.34611	1.80014
3625	1.31892	1.80681
3626	0.946131	1.57536
3627	1.80988	1.7862
3628	1.80988	2.1
3629	1.31892	1.7251
3630	0.946131	1.75312
3631	2.13214	2.1
3632	1.94691	2.1
3633	1.52661	0.05
3634	1.53175	0.05
3635	0.956005	0.05
3636	0.955991	0.05
3637	1.40572	0.05
3638	0.955992	0.05
3639	1.51084	2.1
3640	1.67115	2.1
3641	1.73143	2.1
3642	1.55234	2.1
3643	1.69377	2.1
3644	2.51483	2.1
3645	1.25987	2.1
3646	1.4472	2.1
3647	1.83151	0.658007
3648	2.01001	1.27885
3649	0.993998	0.633232
3650	1.83151	0.429278
3651	1.40915	1.01251

3652	1.24653	1.04494
3653	1.74472	2.1
3654	1.74472	2.1
3655	1.74244	2.1
3656	1.04788	2.1
3657	1.28522	2.1
3658	1.03785	2.1
3659	0.896519	2.1
3660	1.60428	2.1
3661	1.73305	2.1
3662	1.39072	0.190005
3663	2.27501	0.345938
3664	1.39073	0.264878
3665	1.39073	0.271689
3666	1.06838	2.1
3667	0.968462	1.59248
3668	1.06837	1.80773
3669	1.13455	1.4959
3670	1.23527	1.35729
3671	2.71686	0.441634
3672	1.86881	0.699735
3673	1.10586	2.1
3674	1.03852	2.1
3675	1.03899	2.1
3676	0.963973	2.1
3677	1.00319	2.1
3678	1.00319	2.1
3679	0.987132	2.1
3680	0.974889	2.1
3681	0.987131	2.1
3682	1.7677	2.1
3683	1.7677	2.1
3684	0.946131	2.1
3685	1.31185	2.1
3686	0.946132	2.1
3687	1.30203	2.1
3688	1.31185	2.1
3689	1.82958	2.1
3690	1.82958	2.1
3691	0.946132	2.1
3692	1.40199	2.1
3693	0.946133	2.1
3694	1.36994	2.1
3695	1.40199	2.1
3696	0.97489	2.1
3697	1.05939	1.8682
3698	1.01922	1.91106
3699	0.810941	1.73728
3700	1.05372	1.99495
3701	0.754065	2.08483
3702	0.926131	1.93585
3703	1.35905	1.83099
3704	0.810931	1.81296
3705	1.75647	1.51509
3706	1.68265	2.1
3707	1.41995	2.1
3708	1.36457	1.63584
3709	0.933392	1.57769

3710	1.42255	2.1
3711	1.3868	2.1
3712	1.08074	2.1
3713	1.07196	2.1
3714	1.13537	1.01891
3715	1.01179	1.04738
3716	1.60196	0.420236
3717	1.72798	0.478145
3718	1.3715	0.595316
3719	1.56409	1.52737
3720	1.56409	0.158069
3721	1.97819	1.43521
3722	1.47954	1.29581
3723	0.798655	0.251772
3724	1.47952	0.764427
3725	2.30327	0.436643
3726	1.49111	2.1
3727	1.96013	2.07709
3728	1.54599	2.1
3729	2.06632	2.1
3730	1.54601	2.1
3731	1.10557	0.106137
3732	1.17624	0.242079
3733	1.60497	2.1
3734	1.61587	2.1
3735	1.65977	0.05
3736	1.69044	0.05
3737	1.00659	2.1
3738	1.06226	2.1
3739	1.15171	2.1
3740	1.50853	2.1
3741	1.80252	2.1
3742	1.41432	2.1
3743	1.04687	2.1
3744	0.940878	2.1
3745	1.52031	2.1
3746	1.52031	2.1
3747	0.940877	2.1
3748	0.946132	2.1
3749	1.50853	2.1
3750	0.963973	2.1
3751	1.70562	0.0594811
3752	2.43203	0.0519765
3753	1.36004	0.05
3754	1.06648	0.05
3755	1.66009	0.05
3756	1.66009	0.05
3757	0.766427	0.05
3758	0.766452	0.05
3759	0.946132	0.05
3760	1.98012	0.05
3761	1.20357	2.1
3762	1.20356	2.1
3763	1.67324	2.1
3764	1.52008	2.1
3765	1.19964	2.1
3766	1.36861	2.1
3767	1.19964	2.1

3768	2.85415	2.1
3769	1.43687	2.1
3770	0.935852	2.1
3771	1.09972	2.1
3772	0.810933	2.09503
3773	2.25974	2.1
3774	1.74149	2.1
3775	1.36984	2.1
3776	1.87602	2.1
3777	0.890748	2.1
3778	1.20587	1.96104
3779	0.810956	1.87592
3780	2.03174	1.95118
3781	1.34403	1.97369
3782	1.58825	1.96437
3783	1.63096	1.95338
3784	2.53735	2.00301
3785	1.98241	1.97975
3786	2.53014	2.1
3787	1.1775	1.99486
3788	1.06538	1.76177
3789	0.946132	1.26507
3790	1.4101	1.70364
3791	1.75162	2.02955
3792	1.75162	1.33087
3793	0.946132	1.51063
3794	1.4101	1.37834
3795	1.28765	1.37784
3796	1.1427	1.11398
3797	1.1427	1.25598
3798	1.3109	2.1
3799	1.02721	1.976
3800	0.846447	1.91884
3801	0.862076	2.1
3802	0.644098	1.89959
3803	0.581308	1.99255
3804	1.02306	1.9961
3805	0.322641	1.20319
3806	0.589346	1.63431
3807	2.25572	1.84978
3808	1.31372	2.1
3809	1.18873	2.1
3810	1.78225	2.1
3811	1.764 2.1	
3812	1.17503	2.1
3813	1.06821	2.1
3814	1.06821	1.92446
3815	1.7777	1.92073
3816	1.9096	0.594036
3817	1.70239	0.617279
3818	1.16182	0.05
3819	1.20132	0.05
3820	1.23475	2.1
3821	1.56859	2.1
3822	2.46263	2.1
3823	0.528744	2.1
3824	2.93337	2.1
3825	2.42504	2.1

3826	1.38646	0.287277
3827	1.61963	0.666014
3828	1.88016	0.211628
3829	0.622283	0.726547
3830	1.03243	0.616675
3831	0.710307	1.10302
3832	0.798124	0.958289
3833	0.931073	1.00093
3834	0.892862	0.988186
3835	1.06791	0.836809
3836	0.552787	0.613157
3837	0.946132	0.0769691
3838	1.36665	1.67083
3839	1.36665	0.81574
3840	0.506966	1.69191
3841	0.506964	1.14218
3842	1.36665	0.437924
3843	0.653469	0.754055
3844	1.36665	0.0664244
3845	1.21339	0.153329
3846	1.21339	0.969844
3847	0.552757	1.36483
3848	0.552786	0.410433
3849	1.21336	0.136328
3850	1.70744	1.41544
3851	1.0775	0.223482
3852	1.59527	0.675851
3853	1.48312	0.291865
3854	2.01868	0.239733
3855	1.18947	0.0978974
3856	1.48312	0.296267
3857	0.785626	0.114835
3858	1.19601	0.133727
3859	0.785626	0.135983
3860	0.946132	0.128706
3861	0.7856	0.193912
3862	1.59087	0.139505
3863	1.51158	0.132563
3864	1.74098	0.131973
3865	2.39202	0.125717
3866	0.870489	0.232783
3867	1.34534	0.28905
3868	0.870488	0.200456
3869	0.946133	0.299005
3870	0.870489	0.451518
3871	1.67086	0.306294
3872	1.67086	0.293433
3873	2.11311	2.1
3874	1.98765	1.90884
3875	0.946133	1.58916
3876	1.30881	1.96716
3877	1.72211	2.09067
3878	1.72211	1.70183
3879	0.94613	1.9234
3880	1.30881	1.75014
3881	1.35943	1.72945
3882	1.01294	0.491415
3883	1.01294	0.411684

3884	1.38963	2.1
3885	1.01294	0.834298
3886	1.60056	0.608761
3887	1.60056	2.00676
3888	0.946132	2.1
3889	1.204	1.76499
3890	0.946132	0.227629
3891	1.11288	1.18906
3892	1.204	0.777136
3893	1.14599	2.09716
3894	1.00065	2.02816
3895	1.74685	2.1
3896	1.74685	2.1
3897	0.946128	2.1
3898	1.38463	1.96712
3899	1.2275	2.1
3900	1.2275	2.1
3901	1.30835	2.1
3902	2.04407	2.1
3903	2.50425	2.1
3904	1.19647	0.617587
3905	1.19647	1.10078
3906	1.19645	1.12433
3907	1.75446	0.709831
3908	1.1315	1.22214
3909	1.1315	1.56094
3910	1.45569	0.868356
3911	1.1315	0.52784
3912	1.14357	2.1
3913	1.25793	2.1
3914	1.14357	2.1
3915	2.08181	2.04483
3916	1.4714	2.1
3917	0.71295	2.1
3918	1.68285	2.1
3919	1.68285	2.1
3920	1.25603	2.1
3921	1.13976	2.1
3922	1.25603	2.1
3923	0.71295	2.1
3924	1.61095	2.1
3925	2.51207	2.1
3926	1.63762	2.1
3927	2.74451	2.1
3928	0.671255	2.08442
3929	1.87601	2.1
3930	1.87601	2.1
3931	0.671226	2.03992
3932	1.25603	2.1
3933	1.29123	2.1
3934	0.671226	2.1
3935	1.63723	2.1
3936	0.872928	0.05
3937	1.47904	0.05
3938	1.18793	0.05
3939	2.57464	0.05
3940	1.14986	2.1
3941	1.19611	2.1

3942	0.96029	1.67143
3943	1.7961	2.1
3944	0.928721	2.1
3945	1.34352	2.1
3946	1.37935	2.1
3947	1.37935	2.1
3948	0.892888	2.1
3949	0.946131	2.1
3950	0.892888	2.1
3951	1.75422	2.1
3952	1.05556	2.1
3953	1.27362	0.287574
3954	1.27421	0.122456
3955	1.3003	1.20913
3956	1.31415	1.00719
3957	1.01431	2.1
3958	0.673951	2.1
3959	1.63384	2.1
3960	1.63661	2.1
3961	1.52245	2.1
3962	1.50714	2.1
3963	0.996778	0.0511303
3964	1.56091	0.05
3965	1.06543	2.1
3966	2.66689	2.1
3967	1.01474	2.1
3968	2.76422	2.1
3969	1.45321	0.121038
3970	0.974716	0.0807617
3971	0.810943	0.0899104
3972	0.909982	0.163678
3973	0.937793	0.102886
3974	1.05138	0.0822605
3975	1.25044	0.0838167
3976	0.92095	0.092037
3977	0.830414	0.116334
3978	2.1402	0.100146
3979	1.75938	2.1
3980	1.05556	2.1
3981	1.78558	2.1
3982	1.379 2.1	
3983	0.970214	1.08415
3984	0.9702	1.07053
3985	1.08894	1.43467
3986	2.17251	1.34449
3987	1.51569	0.819906
3988	1.06012	0.832437
3989	0.98966	0.627283
3990	1.55926	0.456574
3991	1.92645	0.54637
3992	1.37427	0.05
3993	2.16338	0.0646702
3994	1.5456	0.0535697
3995	1.5456	0.05
3996	1.94002	0.0566568
3997	1.33135	0.05
3998	1.31948	0.05
3999	2.23255	0.0515538

4000	2.34061	0.05
4001	1.52903	2.1
4002	3.38327	2.1
4003	1.35977	2.1
4004	2.22414	2.1
4005	1.6812	0.0891508
4006	2.33397	0.0938175
4007	1.16699	0.13489
4008	0.946131	0.124924
4009	1.58115	0.129548
4010	1.58115	0.123021
4011	0.519726	0.183876
4012	0.946131	0.148217
4013	0.519697	0.143254
4014	1.26868	0.0932385
4015	1.16699	0.0992737
4016	1.5951	0.085316
4017	1.5951	0.08209
4018	0.840568	0.10901
4019	0.946131	0.0951439
4020	0.840598	0.0940881
4021	1.30047	2.1
4022	1.5586	2.1
4023	1.31825	1.43214
4024	1.31924	1.28804
4025	1.15818	1.37333
4026	1.52782	1.20098
4027	1.15818	1.39452
4028	1.15818	1.48861
4029	0.99518	0.0694466
4030	0.94613	0.151497
4031	0.922045	0.0612588
4032	1.29322	0.147337
4033	1.29321	0.05
4034	1.29321	0.185039
4035	0.922056	0.0555573
4036	1.06848	0.0888338
4037	1.84409	0.0756052
4038	1.9186	1.30703
4039	2.72244	1.17123
4040	0.946132	0.735381
4041	1.36122	0.799106
4042	1.79658	0.926656
4043	1.79658	0.608999
4044	0.946133	1.29504
4045	1.36122	1.05306
4046	1.38149	1.03686
4047	1.59106	0.587043
4048	0.946116	0.838355
4049	1.29052	0.607553
4050	1.76641	0.510226
4051	1.76641	0.535139
4052	0.885309	1.22681
4053	1.29054	0.891923
4054	1.36118	0.75436
4055	1.59103	1.21809
4056	2.27816	0.48493
4057	1.56164	0.603544

4058	1.41114	0.05
4059	2.04128	0.05
4060	0.986723	2.1
4061	1.03645	0.126038
4062	1.79224	0.13196
4063	1.2627	2.1
4064	1.23036	2.1
4065	1.45257	2.1
4066	1.41469	2.1
4067	1.41469	2.1
4068	2.60636	2.1
4069	1.45789	0.05
4070	0.851541	0.05
4071	1.57877	0.05
4072	1.53134	0.05
4073	0.851479	0.05
4074	1.08982	0.05
4075	0.716284	0.05
4076	0.946131	0.05
4077	1.73193	0.05
4078	2.57309	0.05
4079	1.36307	0.05
4080	2.42175	0.05
4081	1.72948	0.05
4082	0.978698	2.1
4083	0.978699	2.1
4084	2.08398	2.1
4085	0.978698	2.1
4086	1.44096	2.1
4087	1.44096	2.1
4088	0.638164	2.1
4089	0.681588	2.1
4090	0.946133	2.1
4091	0.638165	2.1
4092	0.94613	2.1
4093	1.55432	2.1
4094	0.901147	2.1
4095	0.85777	2.1
4096	1.36073	2.1
4097	1.44004	2.1
4098	0.946133	2.1
4099	1.09982	2.1
4100	1.27562	2.1
4101	1.55432	2.1
4102	1.62229	2.1
4103	1.0101	2.1
4104	2.28459	2.1
4105	2.50554	2.03721
4106	0.946131	1.65626
4107	1.25276	2.02307
4108	1.73721	2.03674
4109	1.73721	1.78272
4110	0.93406	1.92615
4111	1.25277	1.82558
4112	1.4185	1.79405
4113	1.53064	1.77641
4114	0.946133	1.82273
4115	0.609462	2.09729

4116	1.65123	2.09118
4117	1.65123	1.91938
4118	0.609466	2.1
4119	1.00792	1.966
4120	1.25277	1.92058
4121	1.53064	1.83744
4122	2.15223	1.66724
4123	1.30664	1.91464
4124	1.10674	2.1
4125	1.22862	2.1
4126	1.49358	0.177964
4127	2.0207	0.122376
4128	2.09374	2.1
4129	1.88175	2.1
4130	1.10938	0.52845
4131	1.02478	1.47526
4132	1.13272	0.05
4133	1.13272	0.05
4134	1.03935	1.56152
4135	0.810945	1.63744
4136	0.527573	0.905331
4137	0.925011	1.52799
4138	1.03193	0.809902
4139	1.38587	0.965069
4140	0.703469	0.934038
4141	0.912282	1.54808
4142	1.75505	2.1
4143	1.78529	2.1
4144	1.60017	0.47897
4145	1.35608	0.186427
4146	1.01479	2.1
4147	1.47561	1.89588
4148	1.01479	2.1
4149	1.28418	1.91158
4150	2.62605	2.1
4151	1.71233	2.1
4152	3.22222	2.1
4153	1.97526	2.1
4154	0.949712	0.108767
4155	1.78986	0.0905124
4156	2.72879	0.0844823
4157	2.66428	0.0890456
4158	1.68108	0.311849
4159	1.67265	0.223074
4160	2.24877	0.318355
4161	1.15558	0.386302
4162	1.39708	2.1
4163	1.39327	2.1
4164	1.69788	0.0712585
4165	1.1273	0.0649718
4166	1.17302	1.57239
4167	1.04349	0.936688
4168	0.979853	2.1
4169	1.19094	2.1
4170	1.39408	2.1
4171	1.61857	2.1
4172	1.66523	2.1
4173	0.946132	2.1

4174	0.838554	2.1
4175	1.05151	2.1
4176	1.18901	2.1
4177	1.18901	2.1
4178	1.61149	2.1
4179	1.22857	0.9552
4180	1.25848	0.992529
4181	1.09154	2.1
4182	2.20358	2.1
4183	1.23013	2.1
4184	1.55698	2.1
4185	1.55828	2.1
4186	1.52391	2.1
4187	0.60346	1.88405
4188	1.06336	2.1
4189	0.870714	1.82146
4190	0.543109	1.89877
4191	1.10367	1.84453
4192	1.20686	1.8532
4193	1.20684	2.06463
4194	2.5118	1.86839
4195	1.20684	2.1
4196	1.52468	1.87538
4197	1.52469	1.95718
4198	0.532308	1.75096
4199	0.903301	2.03629
4200	0.946119	1.69937
4201	0.532315	1.72439
4202	0.946107	1.75296
4203	1.46129	2.1
4204	0.804766	2.1
4205	1.4809	2.1
4206	1.4809	2.1
4207	1.11822	2.1
4208	1.16744	2.1
4209	1.11822	2.1
4210	0.804737	2.1
4211	1.04193	2.1
4212	0.983723	2.1
4213	1.8014	2.1
4214	1.1729	2.1
4215	1.03951	2.1
4216	1.03951	2.1
4217	1.17583	2.1
4218	1.42866	2.1
4219	1.78503	2.1
4220	1.17822	1.2092
4221	1.89121	2.1
4222	1.44472	2.1
4223	1.62143	2.1
4224	1.43837	2.1
4225	1.01121	0.0845437
4226	0.962703	1.47658
4227	0.946129	0.48279
4228	1.10941	0.287468
4229	0.946131	0.667451
4230	1.60326	0.635817
4231	1.60326	0.290902

4232	1.14215	0.433678
4233	1.40724	0.479964
4234	0.962703	0.546911
4235	1.53336	0.05
4236	1.40272	0.05
4237	1.64984	0.05
4238	1.67345	0.05
4239	1.08212	2.1
4240	1.40905	2.1
4241	0.946132	0.05
4242	0.946132	0.05
4243	0.81268	0.05
4244	0.946131	0.05
4245	0.812681	0.05
4246	0.946131	0.05
4247	0.946132	0.05
4248	0.997755	0.05
4249	1.94585	0.05
4250	1.2925	0.05
4251	2.52454	0.05
4252	1.69182	0.05
4253	1.69182	0.05
4254	0.92361	0.05
4255	1.35316	0.05
4256	1.26227	0.05
4257	0.946131	0.05
4258	1.35316	0.05
4259	1.48676	0.102493
4260	1.33917	0.0913616
4261	0.757292	2.1
4262	1.02415	2.1
4263	1.48495	2.1
4264	0.97587	2.1
4265	0.946132	2.1
4266	0.747365	2.1
4267	1.55432	2.1
4268	1.55432	2.1
4269	0.946132	2.1
4270	0.747394	2.1
4271	1.17464	0.05
4272	1.0372	0.05
4273	2.16553	0.05
4274	2.00616	0.05
4275	1.12184	0.0518655
4276	1.33177	0.0511363
4277	0.936674	0.05
4278	0.946129	0.050477
4279	0.851552	0.0594079
4280	1.60205	0.0506015
4281	1.77391	0.0512197
4282	1.03327	0.0726323
4283	1.41512	0.0796442
4284	1.03327	0.0664341
4285	0.946133	0.0789755
4286	0.946131	0.0932792
4287	1.80077	0.0842385
4288	1.80077	0.0765823
4289	1.54848	2.1

4290	1.29931	2.1
4291	1.04482	0.115915
4292	2.08069	0.139741
4293	0.946127	0.110473
4294	0.515539	0.147291
4295	0.515566	0.192921
4296	1.38423	0.110797
4297	1.38423	0.105355
4298	0.972058	0.145401
4299	0.927737	0.164448
4300	0.94971	0.0882099
4301	2.19682	0.109203
4302	0.946132	0.156082
4303	1.09841	0.12074
4304	0.522382	0.232254
4305	1.5962	0.100146
4306	1.72791	0.0863161
4307	1.07428	0.138668
4308	1.05215	0.131196
4309	1.54632	1.7894
4310	1.15693	1.82858
4311	1.41238	2.1
4312	1.41238	2.1
4313	1.77966	2.1
4314	1.77966	2.1
4315	0.946129	2.1
4316	1.40525	2.1
4317	0.946131	2.1
4318	0.94613	2.1
4319	1.32054	2.1
4320	1.41238	2.1
4321	1.94026	2.1
4322	1.0171	0.48112
4323	1.1211	0.401281
4324	0.993022	2.1
4325	0.773813	2.1
4326	1.4073	2.1
4327	1.4073	2.1
4328	0.94613	2.1
4329	0.787615	2.1
4330	0.94613	2.1
4331	0.787615	2.1
4332	1.764	2.1
4333	1.90964	2.1
4334	1.05787	2.1
4335	1.403	0.793896
4336	0.744189	1.08109
4337	2.32834	2.1
4338	2.34986	2.1
4339	1.17106	0.836319
4340	1.94826	0.067032
4341	2.40606	0.0598302
4342	1.26387	0.0721691
4343	0.954013	0.0735147
4344	0.946131	0.0757129
4345	1.71	0.059117
4346	1.51926	0.0549835
4347	0.953997	0.0581907

4348	0.851554	0.0702982
4349	1.96502	0.0617624
4350	1.72954	0.0717012
4351	2.89117	0.0654573
4352	0.971958	0.0746353
4353	0.985575	0.0811357
4354	1.41943	0.0636474
4355	1.20021	0.0676393
4356	1.29599	0.0726859
4357	1.15687	0.0657592
4358	0.810958	0.0776759
4359	1.12919	0.05
4360	1.98859	2.1
4361	1.82044	2.1
4362	1.90488	2.1
4363	0.851542	2.1
4364	1.02973	2.1
4365	1.39286	2.1
4366	1.44873	2.1
4367	0.946132	2.1
4368	1.11826	2.1
4369	1.26842	2.1
4370	2.09461	2.1
4371	1.76952	0.05
4372	1.6473	0.05
4373	0.946131	0.05
4374	1.36486	0.05
4375	1.04243	0.05
4376	1.05017	0.05
4377	1.24059	0.05
4378	2.11646	0.05
4379	1.50368	0.05
4380	2.38796	0.05
4381	1.50368	0.05
4382	1.71844	0.05
4383	1.71844	0.05
4384	0.914836	0.0512561
4385	1.24416	0.0504864
4386	0.914805	0.05
4387	1.24416	0.05
4388	1.38911	0.05
4389	1.46859	0.05
4390	1.35348	0.05
4391	0.946131	0.0517879
4392	1.1226	0.0524954
4393	0.857078	0.055215
4394	0.9746	0.0552482
4395	1.37624	0.0505902
4396	1.15131	0.05
4397	1.139 0.05	
4398	1.90456	0.05
4399	2.09692	0.05
4400	1.16655	2.1
4401	1.23017	2.1
4402	2.14592	2.1
4403	1.23017	2.1
4404	1.0752	2.1
4405	1.52482	1.64024

4406	1.09252	2.1
4407	1.10725	2.1
4408	1.14305	0.05
4409	0.851541	0.0550741
4410	1.48073	0.05
4411	1.45607	0.05
4412	0.946131	0.0587637
4413	0.932823	0.05
4414	1.45828	0.0506834
4415	0.851528	0.0521227
4416	1.14305	0.05
4417	1.92737	0.0523316
4418	1.08211	0.0575315
4419	1.69831	0.05
4420	1.69831	0.05
4421	0.946131	0.05
4422	1.26868	0.05
4423	1.23377	0.05
4424	1.23377	0.05
4425	0.94723	0.05
4426	1.64322	0.062914
4427	1.64322	0.05
4428	0.946131	1.69277
4429	1.32068	0.0920989
4430	0.946129	0.0920973
4431	1.18789	1.79248
4432	1.32068	0.0876769
4433	1.32813	2.1
4434	1.32461	2.1
4435	1.42729	0.0677128
4436	2.47846	0.0743529
4437	1.00024	0.122569
4438	1.23923	0.0766843
4439	0.946131	0.131203
4440	0.712304	0.0648952
4441	1.77501	0.111289
4442	1.77501	0.0570797
4443	0.593593	0.177392
4444	1.23923	0.15791
4445	1.23175	0.108718
4446	0.94613	0.177674
4447	0.713646	0.117406
4448	1.75735	0.153086
4449	1.75735	0.0680872
4450	0.713633	0.246163
4451	1.37157	0.054362
4452	1.47169	0.0615262
4453	2.21808	0.0816749
4454	1.47169	0.0690621
4455	1.04759	2.1
4456	0.987132	2.1
4457	1.11181	2.1
4458	2.61682	1.86569
4459	1.11181	2.1
4460	1.06461	1.64965
4461	1.69733	2.1
4462	2.79705	2.1
4463	1.08245	2.1

4464	1.40583	2.1
4465	1.39852	2.1
4466	2.0239	2.1
4467	2.0239	2.1
4468	0.780455	2.1
4469	0.946132	2.1
4470	0.780455	2.1
4471	1.01885	2.1
4472	0.564217	2.1
4473	0.733461	2.1
4474	0.537126	2.1
4475	0.683838	2.1
4476	0.779662	2.1
4477	0.634141	2.1
4478	0.685337	2.1
4479	1.58848	2.1
4480	1.17129	0.138407
4481	0.663168	0.228417
4482	1.55587	0.177383
4483	1.31342	0.169114
4484	0.596868	0.244085
4485	1.1666	0.243261
4486	0.867261	0.2492
4487	0.946132	0.269736
4488	1.28843	0.153039
4489	1.31248	0.213695
4490	2.59921	0.196335
4491	1.32634	0.146697
4492	1.35564	0.05
4493	1.52661	0.05
4494	1.12323	0.05
4495	1.17701	1.19608
4496	1.36405	1.03634
4497	1.19837	0.896095
4498	2.25636	0.770803
4499	1.0823	0.0889438
4500	1.31774	0.0694324
4501	1.0823	0.0697268
4502	1.01121	0.0979414
4503	1.27408	0.0768721
4504	0.946131	0.28126
4505	0.566074	0.247279
4506	0.79576	0.256243
4507	1.24596	0.28299
4508	1.47567	0.282706
4509	1.47567	0.15779
4510	0.56605	0.257221
4511	1.6567	0.173506
4512	0.967792	0.163305
4513	1.00366	0.204118
4514	1.00366	0.197001
4515	1.56409	0.05
4516	1.56409	0.05
4517	0.774911	0.0551302
4518	1.09483	0.0575384
4519	0.774912	0.05
4520	1.09483	0.0559321
4521	1.24417	0.05

4522	1.13387	0.05
4523	1.13387	0.05
4524	1.30572	0.05
4525	2.18966	0.05
4526	0.98575	0.05
4527	1.54982	0.05
4528	2.48833	0.05
4529	1.54982	0.05
4530	1.28954	0.0569691
4531	1.64002	0.05
4532	1.64002	0.05
4533	1.85594	0.05
4534	1.12968	2.1
4535	1.23536	2.1
4536	1.4882	2.1
4537	1.4882	2.1
4538	0.876836	2.1
4539	0.946131	2.1
4540	0.876835	2.1
4541	1.43166	2.1
4542	2.25936	2.1
4543	1.0305	2.1
4544	0.992095	2.1
4545	1.12968	2.1
4546	1.53813	2.1
4547	1.53812	2.1
4548	0.583653	2.1
4549	0.94613	2.1
4550	0.583651	2.1
4551	0.964033	2.1
4552	0.964034	2.1
4553	1.54457	2.1
4554	3.54109	0.0714312
4555	0.99974	0.0938094
4556	0.78814	0.104709
4557	1.65729	0.0725437
4558	1.1283	0.0835176
4559	1.09407	0.0927889
4560	1.55091	0.0740143
4561	0.870773	0.0974672
4562	1.19967	0.0729576
4563	1.56521	0.0821958
4564	1.30896	1.91715
4565	1.17989	2.1
4566	1.72526	2.1
4567	0.851537	1.39028
4568	1.58944	2.07131
4569	1.56915	2.06242
4570	0.891039	1.38172
4571	1.4172	1.81023
4572	0.963818	1.66235
4573	0.851474	1.60918
4574	1.17989	2.1
4575	1.56261	2.1
4576	1.56261	2.1
4577	0.761574	2.1
4578	1.31323	2.1
4579	0.761573	2.1

4580	0.946133	2.1
4581	1.31323	2.1
4582	1.16759	2.1
4583	1.16759	2.1
4584	2.11829	2.1
4585	1.16759	2.1
4586	1.10748	0.05
4587	1.47688	0.0541158
4588	0.946133	0.0559574
4589	0.953319	0.0789447
4590	0.946132	0.0616302
4591	1.51168	0.0588495
4592	1.51168	0.0807995
4593	1.25797	0.0644103
4594	1.19984	0.0754006
4595	1.95729	2.1
4596	2.34853	2.1
4597	1.94343	2.1
4598	2.00551	2.1
4599	1.20067	1.05012
4600	1.87244	1.13215
4601	1.4178	2.1
4602	1.33538	2.1
4603	0.946131	2.1
4604	1.22895	2.1
4605	1.12433	2.1
4606	0.956757	2.1
4607	0.91826	2.1
4608	1.44221	2.1
4609	1.14206	2.1
4610	1.80514	0.894207
4611	0.890304	1.84326
4612	1.1362	0.865669
4613	1.32819	1.23165
4614	1.46311	2.08232
4615	0.8654	1.30231
4616	1.1699	1.69166
4617	1.00267	1.6496
4618	1.02882	1.48851
4619	1.00832	2.1
4620	0.974137	2.1
4621	1.02368	2.1
4622	0.974138	2.1
4623	0.995734	2.00677
4624	1.44605	0.647719
4625	1.18549	0.619837
4626	1.33621	0.274432
4627	1.5176	0.395875
4628	1.91762	0.292117
4629	1.33621	0.266232
4630	1.6638	0.293415
4631	1.66774	2.1
4632	1.11833	2.1
4633	0.926499	2.1
4634	1.51229	2.1
4635	1.51229	2.1
4636	0.926528	2.1
4637	1.40335	2.1

4638	1.03544	2.1
4639	0.926527	2.1
4640	1.85305	2.1
4641	1.5812	2.1
4642	1.15529	2.1
4643	1.31086	2.1
4644	0.999206	2.1
4645	1.46714	2.1
4646	1.73087	2.1
4647	0.772299	2.1
4648	1.24341	2.1
4649	0.997867	2.1
4650	0.772311	2.1
4651	0.936657	2.1
4652	1.35986	2.1
4653	0.959414	2.1
4654	1.36541	2.1
4655	1.49268	2.1
4656	1.49268	2.1
4657	0.832137	2.1
4658	0.946132	2.1
4659	0.832138	2.1
4660	1.64523	2.1
4661	1.11764	2.1
4662	1.05776	2.1
4663	0.972465	2.1
4664	0.972464	2.1
4665	1.98425	2.1
4666	0.969822	2.1
4667	1.30566	2.1
4668	1.52724	2.1
4669	2.32237	0.13043
4670	2.30274	0.137901
4671	2.27761	0.077021
4672	3.33012	0.0779971
4673	1.24001	1.12199
4674	1.41821	1.01843
4675	0.978584	2.1
4676	1.18076	2.1
4677	1.07645	2.1
4678	0.877351	1.19794
4679	0.946132	2.1
4680	0.877353	1.89219
4681	1.58444	2.1
4682	1.49614	2.1
4683	1.28355	2.1
4684	1.13491	2.1
4685	1.56875	0.237246
4686	0.499592	0.0596996
4687	0.946131	0.05
4688	1.20538	0.053916
4689	1.09913	0.05
4690	1.8049	0.0580229
4691	1.8049	0.0587528
4692	0.499606	0.0536917
4693	2.41076	0.0513533
4694	0.860202	0.05
4695	0.906798	0.0543853

4696	1.8626	0.0596208
4697	1.33986	0.0560415
4698	1.2992	0.0590572
4699	1.58702	0.05
4700	1.05939	0.05
4701	1.02074	2.1
4702	1.07866	2.1
4703	1.5082	0.0970588
4704	1.5522	0.0752468
4705	1.65903	0.137807
4706	1.86766	2.1
4707	2.11684	2.1
4708	0.946132	2.1
4709	1.6585	2.1
4710	1.6585	2.1
4711	0.94613	2.1
4712	1.1872	2.1
4713	1.1872	2.1
4714	1.05842	2.1
4715	1.20455	2.1
4716	1.34141	2.1
4717	1.34141	2.1
4718	1.42416	2.1
4719	1.75818	2.1
4720	2.63715	2.00684
4721	1.80454	2.1
4722	2.26198	2.1
4723	0.696255	2.1
4724	0.624075	2.1
4725	1.50905	1.13101
4726	1.50905	2.06752
4727	0.624075	2.1
4728	1.31857	0.969203
4729	0.946131	2.05976
4730	0.624075	1.98445
4731	0.914628	2.1
4732	1.14721	2.1
4733	1.16153	2.1
4734	1.47664	2.1
4735	1.54791	2.1
4736	1.19098	2.1
4737	1.13341	2.1
4738	1.00503	0.216348
4739	1.00503	1.29432
4740	1.22655	2.1
4741	0.999489	2.1
4742	1.3268	2.1
4743	0.946132	2.1
4744	1.60061	2.1
4745	1.60061	2.1
4746	1.08034	2.1
4747	1.40372	2.1
4748	1.14302	2.1
4749	1.08034	2.1
4750	0.999491	2.1
4751	1.17152	0.198304
4752	1.17152	0.136407
4753	1.17152	0.162974

4754	2.05423	0.170454
4755	1.40984	2.1
4756	1.2489	2.1
4757	0.885089	2.1
4758	0.810931	2.1
4759	1.84128	2.1
4760	1.11206	2.1
4761	1.21926	2.1
4762	1.99084	2.1
4763	0.976334	0.66551
4764	0.599363	0.602796
4765	1.02621	0.296579
4766	0.462959	0.489534
4767	1.42612	0.234446
4768	0.299682	0.385486
4769	1.51379	2.1
4770	0.719531	2.1
4771	1.3702	2.1
4772	1.33293	2.1
4773	0.851539	2.1
4774	0.62998	2.1
4775	0.851543	2.1
4776	0.629966	2.1
4777	1.43132	2.1
4778	1.13328	1.2388
4779	1.93212	1.09303
4780	1.62345	1.45154
4781	1.38511	1.56247
4782	1.40013	0.682493
4783	1.04132	0.59455
4784	0.83473	1.49911
4785	0.868732	1.31937
4786	0.810595	1.62694
4787	0.729919	1.27017
4788	0.992428	1.26499
4789	0.577107	1.03491
4790	1.18464	1.23957
4791	0.791199	1.38553
4792	0.810943	0.0600471
4793	0.860723	0.0661078
4794	1.33348	0.05
4795	1.57467	0.05
4796	0.981032	0.0577035
4797	0.731569	0.05
4798	0.770312	0.0638845
4799	1.46055	2.1
4800	1.42249	2.1
4801	1.75999	0.199753
4802	1.6868	0.243769
4803	1.17042	1.59162
4804	1.01485	2.1
4805	1.19952	0.05
4806	1.40592	0.0562114
4807	1.19952	0.05027
4808	2.20145	0.05
4809	1.33649	0.300554
4810	1.11612	0.651636
4811	0.957588	0.442499

4812	0.946126	0.577274
4813	1.50648	0.18476
4814	1.50648	0.338215
4815	0.946128	0.414953
4816	2.10671	0.0682528
4817	1.91518	0.166864
4818	2.23223	0.150017
4819	1.53556	2.1
4820	1.56253	2.1
4821	1.27806	0.05
4822	1.44593	0.05
4823	1.03818	0.603234
4824	1.03818	0.508344
4825	1.38969	0.395569
4826	1.85548	0.175764
4827	2.6044	0.354892
4828	1.85548	0.174708
4829	1.49346	1.90233
4830	1.83192	1.86028
4831	0.834043	1.80758
4832	0.810943	1.49849
4833	0.887113	1.63083
4834	0.893827	1.56068
4835	0.991061	1.58754
4836	0.91939	1.66565
4837	0.793307	1.69847
4838	1.75291	1.69874
4839	1.35435	1.68698
4840	1.16989	0.223822
4841	1.36717	0.184327
4842	2.12276	0.0881699
4843	2.30493	0.134157
4844	2.47857	0.150741
4845	1.92269	0.0889901
4846	1.66426	0.122641
4847	0.956698	2.1
4848	0.956698	2.1
4849	0.857957	2.1
4850	1.4706	2.1
4851	1.65706	2.1
4852	1.47059	2.1
4853	1.04713	0.105435
4854	0.946131	0.144061
4855	1.47374	0.159972
4856	1.47374	0.113846
4857	0.951675	0.125615
4858	1.2582	0.162166
4859	1.2582	0.115771
4860	0.94613	0.162563
4861	1.4468	0.0843508
4862	1.90335	0.11793
4863	1.14886	2.1
4864	2.59689	2.1
4865	1.02747	2.1
4866	0.946131	2.1
4867	0.934061	2.1
4868	1.63979	2.1
4869	1.65206	2.1

4870	1.48	2.1
4871	1.27467	2.1
4872	2.20449	2.1
4873	1.1333	0.05
4874	1.95334	2.1
4875	2.20984	2.1
4876	1.46389	0.0563997
4877	1.4639	0.0600611
4878	1.4639	0.0732921
4879	0.946132	0.102685
4880	1.5144	0.0711629
4881	1.5144	0.0881298
4882	0.946132	0.105416
4883	1.35732	0.0706205
4884	1.10321	0.0971806
4885	0.946132	0.0923964
4886	0.980549	0.0797209
4887	1.32819	2.1
4888	1.30171	1.72193
4889	1.68377	1.72899
4890	1.68377	2.1
4891	0.946132	1.13073
4892	0.946132	1.89135
4893	1.1952	1.96634
4894	0.990019	1.75285
4895	1.08195	1.83916
4896	1.2269	2.1
4897	1.19219	2.1
4898	1.01485	2.1
4899	1.57125	0.068207
4900	2.10696	0.0656627
4901	1.29666	0.05
4902	1.57125	0.0706418
4903	1.47712	2.1
4904	1.45182	2.1
4905	1.17816	0.05
4906	1.4516	0.05
4907	1.17816	0.05
4908	1.17816	0.05
4909	0.596954	1.08047
4910	1.65133	0.799628
4911	0.583859	0.571578
4912	1.04072	1.5078
4913	1.28795	0.5964
4914	1.59423	0.743419
4915	0.483559	0.723091
4916	1.74088	0.56253
4917	1.09545	1.50485
4918	1.02519	1.62514
4919	2.47579	1.21223
4920	1.54732	1.29185
4921	2.17663	1.37176
4922	1.75006	1.47189
4923	1.32354	1.46938
4924	1.09962	2.1
4925	1.85057	2.1
4926	1.91046	2.1
4927	2.17037	2.1

4928	1.99528	0.0520822
4929	1.97969	0.05
4930	3.82765	2.1
4931	1.85767	2.1
4932	2.77042	2.1
4933	1.37236	0.05
4934	2.23254	0.05
4935	1.10089	0.05
4936	0.994294	2.1
4937	1.81921	2.1
4938	1.00073	2.1
4939	0.681043	2.1
4940	0.946131	2.1
4941	0.681073	2.1
4942	1.51804	2.1
4943	1.60653	2.1
4944	1.30188	2.1
4945	0.919622	2.1
4946	1.00965	2.1
4947	1.93821	2.1
4948	2.60377	2.1
4949	1.36416	0.05
4950	1.36416	0.05
4951	1.36416	0.05
4952	2.44132	0.05
4953	2.15871	0.396223
4954	2.22171	0.280218
4955	1.70747	0.209599
4956	1.49515	0.169734
4957	0.773007	0.216712
4958	0.946125	0.276738
4959	0.946131	0.308773
4960	0.773034	0.159169
4961	1.15844	0.233943
4962	1.98104	0.17979
4963	2.44283	0.180986
4964	1.05417	0.05
4965	1.1596	0.05
4966	0.829921	0.05
4967	0.946131	0.0563098
4968	0.951911	0.0563941
4969	1.39027	0.05
4970	0.829946	0.0620991
4971	1.51223	0.0516984
4972	1.51223	0.05
4973	1.65984	0.05
4974	1.90382	0.05
4975	1.93228	1.30147
4976	2.47924	1.32198
4977	1.73904	1.32047
4978	1.40547	1.16745
4979	2.37589	1.19214
4980	0.662605	1.35995
4981	2.33293	0.977122
4982	1.72249	2.1
4983	1.68478	2.1
4984	1.00514	0.465912
4985	1.4537	2.1

4986	1.92698	2.1
4987	1.46041	0.05
4988	2.04817	0.05
4989	1.65954	2.1
4990	1.42342	2.1
4991	1.04405	0.05
4992	1.17056	0.05
4993	1.11339	0.05
4994	0.946133	0.05
4995	0.929724	0.05
4996	1.35424	0.05
4997	1.35424	0.05
4998	0.929713	0.05
4999	1.85945	0.05
5000	1.38905	2.1
5001	1.39056	2.1
5002	1.52852	2.1
5003	1.06472	2.1
5004	0.749096	2.1
5005	1.46076	2.1
5006	1.29263	2.1
5007	0.94613	2.1
5008	0.69097	2.1
5009	0.982284	2.1
5010	0.67419	2.1
5011	1.52852	2.1
5012	1.91121	2.1
5013	1.00775	2.1
5014	1.61201	2.09772
5015	0.529987	2.1
5016	0.506125	2.1
5017	1.44696	2.1
5018	2.32981	2.1
5019	1.32784	2.1
5020	1.94158	2.1
5021	1.31595	2.1
5022	0.663922	2.1
5023	1.44663	2.1
5024	1.44663	2.1
5025	1.13976	2.1
5026	0.970788	2.1
5027	1.13976	2.1
5028	0.663921	2.1
5029	0.966114	2.1
5030	1.05498	1.98893
5031	1.38086	2.1
5032	0.946127	2.1
5033	0.922513	2.1
5034	1.49903	2.1
5035	1.49903	2.1
5036	0.92251	2.1
5037	1.06105	2.1
5038	1.36049	2.1
5039	1.38086	2.1
5040	1.52534	2.1
5041	1.04101	2.1
5042	1.30861	0.05
5043	0.949112	0.054929

5044	0.894275	0.0599193
5045	1.10232	0.05
5046	0.830889	0.0501174
5047	1.26554	0.0531753
5048	0.948347	0.0522386
5049	0.81096	0.0530463
5050	1.18338	0.0550008
5051	0.883764	0.0596187
5052	1.54793	0.05
5053	1.02867	0.0521399
5054	1.8573	0.0538738
5055	1.06065	0.0505983
5056	0.64842	0.0533066
5057	1.99319	0.0604892
5058	1.9476	0.0620771
5059	2.51436	0.05
5060	1.68337	0.0581617
5061	1.92321	0.0569046
5062	0.810942	0.05
5063	0.921001	0.05
5064	0.99758	0.0541761
5065	0.960785	0.0514289
5066	1.01565	0.0525084
5067	0.908624	0.05
5068	0.912835	0.05
5069	1.18351	0.0517968
5070	1.40239	2.1
5071	1.26091	2.1
5072	0.947979	2.1
5073	0.946131	2.1
5074	1.81959	2.1
5075	1.81959	2.1
5076	0.94613	2.1
5077	1.38415	2.05601
5078	1.38414	2.1
5079	1.33266	2.1
5080	0.946132	2.1
5081	1.76225	2.1
5082	1.76225	2.1
5083	1.27599	2.1
5084	1.38158	2.1
5085	1.3268	2.1
5086	1.27599	2.1
5087	0.947979	2.1
5088	1.0142	0.291993
5089	1.01422	0.225465
5090	1.11435	0.313902
5091	1.42186	1.42182
5092	1.24001	1.37883
5093	1.77342	1.29095
5094	1.42186	1.47673
5095	1.99817	1.36143
5096	1.27361	2.1
5097	1.68914	2.1
5098	1.30116	2.1
5099	1.32819	1.98623
5100	1.68322	2.03623
5101	1.68322	2.1

5102	0.94613	1.68514
5103	0.94613	2.1
5104	1.14666	2.1
5105	1.07612	2.01023
5106	1.31151	2.1
5107	1.22095	2.1
5108	1.57256	2.1
5109	1.6679	2.1
5110	0.946132	2.1
5111	0.946133	2.1
5112	1.13537	2.1
5113	1.07855	2.1
5114	1.07855	2.1
5115	1.43895	1.8416
5116	1.38481	0.05
5117	1.72385	0.05
5118	0.550441	0.05
5119	0.79807	0.05
5120	1.11627	0.05
5121	1.30349	0.05
5122	0.94613	0.05
5123	0.686176	0.05
5124	1.11627	0.05
5125	1.73363	0.05
5126	1.73363	0.05
5127	0.686139	0.05
5128	1.24932	0.05
5129	0.899582	0.05
5130	2.00472	0.05
5131	1.17121	0.05
5132	1.2874	0.05
5133	2.0194	0.05
5134	0.88601	0.05
5135	1.77409	0.0537699
5136	1.73293	0.05
5137	2.74045	0.0513151
5138	1.83787	0.05
5139	1.45448	0.0720268
5140	1.27778	0.0681691
5141	1.1192	2.1
5142	1.25953	2.1
5143	1.93611	2.1
5144	1.92104	2.1
5145	1.15506	0.05
5146	1.15506	0.05
5147	1.23943	1.9166
5148	0.851543	2.1
5149	1.6061	2.1
5150	1.80058	2.1
5151	0.869471	2.1
5152	1.3931	2.1
5153	1.06454	2.1
5154	0.93667	2.1
5155	2.16916	2.1
5156	3.10246	2.1
5157	1.91539	2.1
5158	1.91539	2.07677
5159	0.620263	1.97458

5160	0.620265	2.1
5161	0.946132	1.96197
5162	0.620265	1.96218
5163	1.30226	1.90395
5164	1.87292	2.1
5165	1.18769	2.1
5166	2.60452	2.09947
5167	1.26574	2.1
5168	2.46679	2.1
5169	1.36459	2.1
5170	1.26865	1.79506
5171	1.1744	1.57849
5172	1.15841	0.320587
5173	1.02247	0.197239
5174	1.93649	2.1
5175	1.64718	2.1
5176	1.59654	2.1
5177	1.57823	2.1
5178	1.00665	0.104434
5179	1.28239	0.114693
5180	0.887895	2.1
5181	1.22083	2.1
5182	1.42127	2.1
5183	1.07374	2.1
5184	1.00503	1.39862
5185	1.23082	1.35172
5186	2.78663	2.1
5187	2.20883	2.1
5188	2.54588	2.1
5189	0.946128	2.1
5190	1.13716	2.1
5191	1.58724	2.1
5192	1.58724	2.1
5193	0.946131	2.1
5194	1.13716	2.1
5195	1.39621	2.1
5196	1.02438	2.1
5197	1.49543	0.05
5198	2.78336	0.05
5199	1.27148	0.05
5200	1.0906	0.05
5201	1.04072	0.05
5202	0.63574	0.05
5203	1.45506	0.05
5204	1.5863	0.05
5205	0.928352	0.05
5206	0.647687	0.05
5207	2.2375	1.81026
5208	1.50998	1.78022
5209	2.75021	1.68729
5210	1.91912	1.52439
5211	1.99872	1.5135
5212	0.918127	1.74839
5213	1.03031	1.62138
5214	0.951169	1.5228
5215	1.27975	1.5074
5216	1.12735	1.57076
5217	1.15741	1.60042

5218	1.24998	1.6072
5219	1.65953	0.30004
5220	0.967555	0.443005
5221	0.967542	0.246918
5222	1.03824	0.177657
5223	1.48693	0.157463
5224	1.48693	0.1174
5225	2.02901	0.126069
5226	0.946127	0.210532
5227	1.07139	0.194084
5228	0.946126	0.196682
5229	1.84644	0.153444
5230	1.84644	0.148063
5231	1.38683	0.172756
5232	1.40574	0.172173
5233	1.36866	0.413531
5234	0.649325	0.531707
5235	1.93631	2.1
5236	1.50145	2.1
5237	1.6287	2.1
5238	1.31302	2.1
5239	1.18041	2.1
5240	1.18041	2.1
5241	0.974196	2.1
5242	0.946131	2.1
5243	1.74169	2.1
5244	1.74169	2.1
5245	1.23163	2.1
5246	1.27451	2.1
5247	1.41331	2.1
5248	1.23163	2.1
5249	1.28037	2.1
5250	1.28158	2.1
5251	1.28037	2.1
5252	2.28015	0.05
5253	2.06561	0.05
5254	2.61343	0.05
5255	1.11712	0.05
5256	1.63689	0.632896
5257	1.01393	0.668591
5258	0.996775	2.1
5259	1.54277	2.1
5260	1.26397	2.1
5261	1.46129	2.1
5262	1.62391	2.1
5263	1.08848	0.05
5264	1.11833	2.1
5265	1.24041	2.1
5266	2.31884	2.1
5267	1.335 2.1	
5268	1.48589	0.05
5269	1.77131	0.05
5270	1.8422	1.64055
5271	2.47453	1.75588
5272	1.23726	1.69896
5273	1.21996	1.72962
5274	1.65512	1.76021
5275	1.65512	1.73309

5276	0.802107	1.79482
5277	0.946129	1.63146
5278	0.802137	1.66234
5279	1.3279	1.52669
5280	1.23726	1.5664
5281	1.79759	1.46997
5282	1.79759	1.41072
5283	0.767577	1.65125
5284	0.946131	1.43151
5285	0.767577	1.4366
5286	1.03534	0.466837
5287	1.09962	2.02059
5288	0.929217	2.1
5289	1.70126	2.1
5290	0.991407	2.1
5291	1.41077	2.1
5292	0.991406	2.1
5293	1.62966	2.1
5294	1.62966	2.1
5295	0.946128	2.1
5296	1.18364	2.1
5297	0.946131	2.1
5298	1.14186	2.1
5299	1.18364	2.1
5300	1.16858	2.1
5301	1.66137	0.877724
5302	1.90165	0.352249
5303	1.66139	0.142179
5304	1.26893	0.151126
5305	1.15918	0.314548
5306	0.653228	0.393226
5307	0.946133	0.269551
5308	1.77488	0.595833
5309	1.77489	0.525597
5310	0.653228	0.308086
5311	1.56043	0.359936
5312	1.60894	0.42554
5313	1.56043	0.221456
5314	1.06848	0.265568
5315	1.04307	2.1
5316	1.14188	2.1
5317	1.09195	2.1
5318	0.615121	2.1
5319	1.50867	2.1
5320	1.27152	2.1
5321	0.615094	2.1
5322	1.07297	2.1
5323	1.1372	2.1
5324	0.553608	2.1
5325	1.23019	2.1
5326	0.992675	2.1
5327	1.40984	2.04196
5328	2.55682	2.1
5329	3.66069	2.1
5330	3.58726	2.1
5331	1.4375	0.0924887
5332	1.88424	0.140268
5333	1.56714	2.1

5334	1.6721	2.1
5335	1.18474	2.1
5336	1.2295	2.1
5337	1.28754	2.1
5338	1.36838	2.1
5339	2.78661	2.1
5340	2.64055	2.1
5341	1.35065	0.736076
5342	2.26675	0.390447
5343	1.35065	0.694337
5344	1.21558	0.496839
5345	1.49709	0.05
5346	1.4971	0.05
5347	0.85472	0.0565885
5348	0.854718	0.05
5349	0.946129	0.0561032
5350	0.854717	0.0560225
5351	1.1604	0.05
5352	1.25265	0.0535594
5353	2.32081	0.05
5354	1.15371	0.05
5355	1.14945	2.1
5356	0.995725	2.1
5357	1.00307	0.188734
5358	1.10907	0.130076
5359	2.30315	0.128538
5360	1.10908	0.158526
5361	1.27558	1.39496
5362	1.71613	1.72364
5363	1.32977	2.1
5364	0.815276	2.1
5365	1.41697	2.1
5366	1.41697	2.1
5367	0.815276	2.1
5368	1.38897	2.1
5369	0.946131	2.1
5370	0.815275	2.1
5371	1.44684	2.1
5372	1.21489	1.43981
5373	1.22476	1.35742
5374	0.870366	1.5185
5375	0.862572	1.42971
5376	0.835435	1.13633
5377	1.6993	0.74706
5378	1.23618	0.820574
5379	1.18624	0.861564
5380	1.53058	1.04707
5381	2.16108	1.0211
5382	2.21589	0.723894
5383	1.04037	2.1
5384	1.03933	2.1
5385	1.57753	0.118858
5386	1.87644	0.155347
5387	1.24728	0.172028
5388	1.30394	0.104744
5389	1.3538	2.1
5390	1.69858	2.1
5391	1.1726	0.136119

5392	0.94612	0.092245
5393	1.09805	0.0567447
5394	0.946118	0.195607
5395	1.72576	0.0875218
5396	1.72575	0.05
5397	1.34152	0.0788048
5398	1.33034	0.0739407
5399	0.784151	2.1
5400	0.946131	2.1
5401	0.784151	2.1
5402	1.47963	2.1
5403	1.47963	2.1
5404	1.3335	2.1
5405	0.930281	2.1
5406	1.29435	2.1
5407	0.883308	2.1
5408	1.31589	2.1
5409	1.67846	2.1
5410	0.993598	0.0503217
5411	2.23193	0.05
5412	0.9936	0.05
5413	0.615629	0.05
5414	1.65925	2.1
5415	2.05992	2.1
5416	1.65925	2.1
5417	4.08735	1.62206
5418	2.72856	1.55285
5419	0.869602	2.1
5420	3.42833	1.38055
5421	1.14397	1.63666
5422	1.34563	0.143094
5423	1.08189	0.120691
5424	0.782128	1.97189
5425	1.0022	2.1
5426	0.900427	2.0168
5427	1.51499	2.06096
5428	1.05588	2.0164
5429	1.13739	2.1
5430	1.37004	2.03828
5431	1.14603	2.05362
5432	0.851541	1.73667
5433	0.946799	1.96727
5434	1.14854	1.88571
5435	1.33793	1.77822
5436	0.94613	1.86556
5437	1.24336	1.85261
5438	0.851473	1.86246
5439	2.08792	1.79026
5440	1.01869	1.88585
5441	0.986953	0.0603851
5442	0.896238	0.0787326
5443	0.906574	0.0748603
5444	0.852145	0.065486
5445	2.14191	0.0631957
5446	1.15979	0.067628
5447	1.18327	0.0569947
5448	2.32742	0.0671412
5449	2.35961	0.0937209

5450	2.38126	0.0634008
5451	1.13632	0.0545475
5452	1.17897	0.05
5453	1.76199	0.05
5454	1.00573	2.1
5455	0.946127	1.83855
5456	1.7077	2.1
5457	1.7077	2.1
5458	0.946127	2.1
5459	1.24887	2.1
5460	1.24887	2.1
5461	1.22441	2.1
5462	1.08542	2.1
5463	1.21743	2.01132
5464	1.12572	2.1
5465	1.05499	1.46703
5466	1.00521	1.42917
5467	2.86191	0.05
5468	2.27972	0.05
5469	1.01169	2.1
5470	1.03373	2.1
5471	1.39319	2.1
5472	0.636837	2.1
5473	1.63908	2.1
5474	1.45713	1.89439
5475	1.04072	2.1
5476	1.25025	1.94178
5477	1.38914	2.08374
5478	0.636807	2.1
5479	1.50211	2.1
5480	1.36555	2.08028
5481	2.77545	1.80771
5482	0.682805	2.1
5483	1.50267	1.93555
5484	1.50267	2.1
5485	0.797749	2.1
5486	1.38773	1.92376
5487	1.38773	2.1
5488	0.682776	2.1
5489	2.01245	0.05
5490	0.925918	0.05
5491	0.75689	0.05
5492	1.25823	0.05
5493	1.16505	0.05
5494	0.838779	0.05
5495	1.14722	0.05
5496	0.756849	0.05
5497	1.52298	0.05
5498	2.11034	0.05
5499	1.92174	0.05
5500	1.92174	0.05
5501	0.576083	0.05
5502	1.23146	0.05
5503	1.26637	0.05
5504	1.23146	0.05
5505	1.26637	0.05
5506	0.946133	0.909514
5507	1.37034	0.406643

5508	1.84145	0.583503
5509	1.84145	0.354079
5510	0.946132	1.2623
5511	1.37034	0.675373
5512	1.41724	0.654837
5513	1.0685	0.331467
5514	1.14547	0.355149
5515	1.24976	2.1
5516	1.30682	2.1
5517	2.12095	2.1
5518	1.46392	2.1
5519	1.46392	2.1
5520	0.935738	2.1
5521	1.32949	2.1
5522	1.07017	2.1
5523	0.935766	2.1
5524	1.07017	2.1
5525	1.78095	2.1
5526	1.05619	0.340053
5527	1.05793	0.200883
5528	1.77677	2.1
5529	2.04766	2.1
5530	1.02524	2.1
5531	1.18803	2.1
5532	1.70047	2.1
5533	1.556 2.1	
5534	1.24201	2.1
5535	1.3592	2.1
5536	2.11824	0.0798632
5537	2.20248	0.110808
5538	3.43878	0.0713941
5539	3.12466	0.0653297
5540	2.09351	2.1
5541	0.946128	2.1
5542	1.51269	2.1
5543	1.51269	2.1
5544	0.946131	2.1
5545	1.01306	2.1
5546	1.04676	2.1
5547	1.01306	2.1
5548	1.07444	2.1
5549	2.17465	2.1
5550	1.14808	2.1
5551	1.39067	2.07871
5552	1.20958	1.97642
5553	0.662832	0.0991738
5554	1.93512	0.313095
5555	1.15471	2.1
5556	1.75119	2.1
5557	1.75118	2.1
5558	0.946131	2.1
5559	1.28606	2.1
5560	1.27087	2.1
5561	0.946131	2.1
5562	1.28606	2.1
5563	1.58916	0.0662332
5564	0.927822	0.0888695
5565	2.24367	0.05

5566	1.8734	0.05
5567	3.51668	0.05
5568	2.68986	0.05
5569	2.32395	2.1
5570	1.39564	2.1
5571	2.10862	2.1
5572	1.36763	2.1
5573	0.970963	0.05
5574	0.97096	0.05
5575	1.11897	0.151811
5576	1.26885	0.238221
5577	1.11605	0.171308
5578	2.06612	0.138289
5579	1.717	0.149266
5580	0.59502	0.227026
5581	0.825755	0.122494
5582	0.709624	0.26538
5583	1.65741	0.125156
5584	0.307486	0.133546
5585	1.4785	0.15903
5586	0.983143	2.1
5587	1.08247	2.1
5588	1.42353	2.1
5589	1.42353	2.1
5590	0.781667	2.1
5591	0.797634	2.1
5592	0.946131	2.1
5593	0.781638	2.1
5594	0.946131	2.1
5595	0.98315	2.1
5596	1.083	2.1
5597	1.61975	0.05
5598	1.11946	0.05
5599	1.18977	0.0704007
5600	0.996484	0.0731519
5601	2.24343	0.0550185
5602	1.4787	0.0590291
5603	1.66127	0.0596778
5604	1.86028	0.0687699
5605	1.09128	0.0635474
5606	1.03115	0.0694862
5607	0.838025	0.0678646
5608	1.3736	0.05
5609	1.44009	0.0503708
5610	0.92917	0.0513214
5611	1.03889	0.0685247
5612	0.956974	0.0562929
5613	3.23468	0.057053
5614	0.953922	0.0875908
5615	0.957104	0.0894715
5616	1.66231	0.0583359
5617	1.26778	0.0634957
5618	1.47615	0.0667741
5619	1.28621	0.0784684
5620	1.08043	0.0715304
5621	2.35026	2.1
5622	2.35025	2.1
5623	2.60659	2.1

5624	1.57627	2.1
5625	1.5493	2.1
5626	1.24682	0.397956
5627	2.14277	0.381182
5628	1.24682	0.840758
5629	1.00307	0.322426
5630	2.04056	0.289728
5631	1.45408	2.1
5632	1.54249	2.1
5633	1.24474	1.593
5634	1.37813	1.60014
5635	0.924158	0.068375
5636	0.916623	0.0682476
5637	1.35344	0.065188
5638	1.00859	0.0661812
5639	1.34786	0.0656505
5640	0.952457	0.0700659
5641	0.85404	0.0694998
5642	1.06341	2.1
5643	1.09194	2.1
5644	1.93707	2.1
5645	2.14087	2.1
5646	1.93707	2.1
5647	1.07519	2.1
5648	1.07519	2.1
5649	1.62518	2.1
5650	1.3592	2.1
5651	1.13183	2.1
5652	1.20357	2.1
5653	1.4858	2.1
5654	1.23694	2.1
5655	1.44011	2.1
5656	1.23989	2.1
5657	2.21604	2.1
5658	1.85574	0.790147
5659	1.55649	2.1
5660	1.48861	0.477027
5661	1.15968	0.05
5662	0.946132	0.05
5663	0.663056	0.05
5664	1.70837	0.05
5665	1.70837	0.05
5666	0.946131	0.05
5667	0.663026	0.05
5668	2.09604	0.05
5669	0.81094	0.05
5670	1.06559	0.05
5671	0.810956	0.05
5672	1.15474	0.05
5673	0.990051	0.05
5674	0.894971	0.05
5675	1.32536	0.05
5676	0.946133	2.1
5677	1.33125	2.1
5678	1.75823	2.1
5679	1.75823	2.1
5680	0.946133	2.1
5681	1.33125	2.1

5682	1.37312	2.1
5683	1.27985	2.1
5684	1.37312	2.1
5685	1.70684	2.1
5686	1.70684	2.1
5687	0.946132	2.1
5688	0.946132	2.1
5689	1.25081	2.1
5690	0.956293	2.1
5691	0.956294	2.1
5692	1.36035	2.1
5693	1.41944	2.1
5694	0.830638	2.1
5695	0.747568	2.1
5696	0.888498	2.1
5697	1.04071	2.1
5698	1.17128	2.1
5699	1.92339	2.1
5700	2.91208	2.1
5701	1.82208	0.922589
5702	1.89653	0.485545
5703	1.82588	1.1881
5704	1.26703	0.272596
5705	1.26703	0.402015
5706	0.6157	0.227712
5707	1.26706	1.29741
5708	0.61567	0.10493
5709	0.615671	0.140789
5710	1.26706	0.262416
5711	1.89158	0.237795
5712	1.23134	0.198946
5713	2.49497	0.0629964
5714	1.68103	0.0507374
5715	2.0554	2.1
5716	1.72647	2.1
5717	1.67375	2.1
5718	1.65037	2.1
5719	1.04349	1.13548
5720	1.69055	0.05
5721	1.53463	0.05
5722	1.19022	1.90909
5723	1.89226	1.63416
5724	1.15009	1.6102
5725	1.7054	1.2256
5726	1.31612	0.287008
5727	1.31611	1.41076
5728	1.67345	0.799396
5729	1.06577	0.266818
5730	1.40193	0.170171
5731	1.72411	0.146134
5732	1.46123	0.160474
5733	0.808808	0.256015
5734	0.897714	0.172587
5735	0.851554	0.299511
5736	1.3499	0.0948039
5737	1.29001	0.103783
5738	1.22083	2.1
5739	1.06191	2.1

5740	1.05758	1.31529
5741	1.348	1.61512
5742	1.43485	1.31001
5743	1.34799	1.55826
5744	1.36307	0.05
5745	2.28188	0.05
5746	1.7926	0.0503224
5747	1.38957	2.1
5748	1.47446	2.1
5749	0.953638	2.1
5750	1.11868	2.1
5751	0.953636	2.1
5752	1.03408	2.1
5753	1.52443	0.05
5754	1.38539	0.05
5755	0.946132	0.05
5756	1.12747	0.05
5757	1.04393	0.0544521
5758	1.05017	0.0509453
5759	1.20529	0.05
5760	1.3663	0.314669
5761	1.89226	0.246157
5762	1.17885	0.380934
5763	0.867881	0.192981
5764	1.32917	0.101781
5765	1.32917	0.0833755
5766	0.946127	0.484286
5767	1.19583	0.254093
5768	1.00122	0.15079
5769	0.946128	0.27852
5770	1.71405	0.571122
5771	0.871815	2.1
5772	1.16435	2.1
5773	0.686619	2.1
5774	1.63102	2.1
5775	1.06589	2.1
5776	1.05375	2.1
5777	1.9023	2.1
5778	1.27394	0.46885
5779	1.88345	0.435045
5780	2.05636	0.46284
5781	0.672977	0.666644
5782	1.41274	0.454874
5783	1.65606	0.469328
5784	0.869329	0.67108
5785	1.2207	0.58251
5786	1.35631	0.619045
5787	0.908776	0.77546
5788	0.955081	0.31002
5789	1.2243	2.1
5790	1.23169	2.1
5791	1.52228	2.1
5792	1.23169	2.1
5793	0.990892	0.53216
5794	1.08376	0.24942
5795	1.79348	0.481573
5796	1.50043	0.339633
5797	1.32599	1.03838

5798	1.37924	0.397627
5799	0.838014	0.338417
5800	0.87737	0.927974
5801	0.947086	0.519218
5802	1.27199	0.477189
5803	1.04918	0.356201
5804	1.04851	0.793014
5805	1.1699	0.619289
5806	0.810934	0.693383
5807	1.83602	1.2635
5808	2.21909	1.3472
5809	0.619441	0.759908
5810	2.03755	0.357726
5811	1.83472	0.768164
5812	1.20416	1.05916
5813	1.26691	0.291268
5814	0.655795	0.45846
5815	0.94613	0.552719
5816	2.35169	1.08515
5817	3.54705	2.1
5818	2.60741	2.1
5819	1.59019	0.05
5820	1.24526	0.05
5821	1.59019	0.05
5822	2.36406	0.05
5823	1.16776	2.1
5824	1.67311	2.1
5825	1.67311	1.99182
5826	0.94613	1.70132
5827	1.38645	2.1
5828	1.13563	0.539795
5829	1.13563	1.57978
5830	1.23279	1.13053
5831	1.05042	2.1
5832	1.17418	2.1
5833	1.28909	2.1
5834	0.973448	2.1
5835	1.02958	2.1
5836	3.3005	2.1
5837	2.86325	2.1
5838	1.35879	2.1
5839	1.69332	2.1
5840	1.69332	2.1
5841	0.946128	2.1
5842	1.39659	2.1
5843	1.12035	2.1
5844	1.12035	2.1
5845	1.24286	2.1
5846	1.10407	2.1
5847	1.14599	2.1
5848	1.32259	2.06347
5849	0.788592	0.05
5850	2.4588	2.1
5851	0.909581	1.98218
5852	1.35656	1.86994
5853	0.986895	1.92094
5854	2.30602	1.85788
5855	1.46873	1.9014

5856	1.55584	1.86395
5857	2.1547	1.86507
5858	1.87794	1.97547
5859	0.808286	2.03653
5860	0.928464	1.98876
5861	0.810959	1.95352
5862	1.50962	1.77322
5863	1.1053	1.8678
5864	1.0712	1.88732
5865	1.40504	1.88214
5866	1.87794	1.64176
5867	2.80496	1.67144
5868	1.41479	1.96819
5869	1.38634	2.1
5870	1.38634	2.1
5871	1.67762	2.1
5872	0.946131	2.1
5873	1.188 2.1	
5874	1.62585	2.1
5875	1.62585	2.1
5876	0.946129	2.1
5877	1.18801	2.1
5878	1.38397	2.1
5879	1.04546	2.1
5880	1.16817	2.1
5881	1.06007	2.1
5882	1.19785	2.1
5883	1.43652	2.1
5884	1.06994	2.1
5885	1.89111	2.1
5886	1.07144	2.1
5887	1.51754	2.1
5888	2.0873	2.1
5889	0.758799	2.1
5890	1.59518	2.1
5891	1.59518	2.1
5892	0.946132	2.1
5893	0.758771	2.1
5894	1.06957	2.1
5895	0.758771	2.1
5896	1.33916	2.1
5897	2.03045	1.65653
5898	2.80184	1.73238
5899	1.76788	1.77715
5900	1.27442	2.00741
5901	1.06756	2.1
5902	0.966979	2.1
5903	1.7054	2.1
5904	1.07156	2.1
5905	0.986096	2.1
5906	1.32813	2.1
5907	1.3331	2.1
5908	2.08369	0.196255
5909	2.23818	1.30237
5910	1.10335	0.701529
5911	0.622314	0.457547
5912	1.72451	0.380304
5913	1.72451	0.756076

5914	0.622283	0.594542
5915	0.622314	0.475796
5916	0.946131	0.638097
5917	1.31254	1.69535
5918	1.15704	1.47082
5919	1.24463	0.471101
5920	2.2067	1.11427
5921	1.30001	0.708093
5922	1.29175	0.917019
5923	1.1233	0.05
5924	1.8689	0.05
5925	1.29494	0.05
5926	1.11895	0.05
5927	0.851539	0.05
5928	0.851474	0.05
5929	1.58352	0.05
5930	1.58228	0.05
5931	0.851527	0.05
5932	2.6389	0.05
5933	1.77548	0.0509265
5934	1.24555	2.1
5935	2.0512	2.1
5936	1.46101	2.1
5937	2.60635	2.1
5938	1.10476	0.05
5939	1.44103	0.05
5940	0.652391	2.1
5941	0.618322	2.1
5942	2.22915	2.1
5943	1.39067	2.1
5944	1.99737	2.1
5945	1.42174	2.1
5946	0.946132	1.17046
5947	1.29735	1.76256
5948	1.61765	2.1
5949	1.61765	1.32697
5950	0.946132	1.80308
5951	1.29735	1.50085
5952	1.26643	1.48512
5953	1.01335	1.3497
5954	1.01335	0.215438
5955	0.947669	2.1
5956	2.17167	2.1
5957	2.59733	2.02916
5958	1.50954	2.07522
5959	1.02638	1.85132
5960	0.555785	2.02796
5961	1.91101	2.00692
5962	2.09809	1.92728
5963	0.641974	2.05927
5964	1.2022	1.96373
5965	1.36359	1.92747
5966	1.8128	1.97533
5967	2.34348	0.144235
5968	1.66343	0.0926526
5969	0.974717	0.788265
5970	1.53908	0.37794
5971	1.27737	1.68881

5972	1.09408	1.06189
5973	2.07382	0.871652
5974	1.10466	1.79781
5975	1.05654	1.78564
5976	1.23498	1.56068
5977	1.48104	1.66695
5978	1.23498	1.46184
5979	1.17261	2.1
5980	1.18059	2.1
5981	2.43005	2.1
5982	1.04546	2.1
5983	2.81222	2.1
5984	1.16817	2.1
5985	1.75766	0.650458
5986	1.47021	1.12857
5987	1.07236	0.212374
5988	1.81759	0.691386
5989	1.42913	0.373148
5990	1.00636	0.538009
5991	1.00636	0.25662
5992	1.79397	0.270683
5993	0.946131	0.418479
5994	1.03691	0.456298
5995	0.851539	0.43435
5996	1.46001	0.505613
5997	1.37878	0.486893
5998	0.933258	0.416753
5999	1.32423	0.423242
6000	1.32614	0.05
6001	1.11356	0.05
6002	1.32614	0.05
6003	1.28895	0.05
6004	1.66895	0.05
6005	1.66895	0.05
6006	0.946132	0.05
6007	1.29576	0.05
6008	1.1318	0.05
6009	1.37621	1.62439
6010	1.94071	1.53693
6011	1.10517	2.1
6012	1.97098	2.1
6013	0.811 2.1	
6014	0.956127	2.1
6015	1.18647	1.89376
6016	0.988455	1.68542
6017	1.12457	0.0895259
6018	1.7453	0.0979502
6019	0.92492	2.1
6020	1.24353	2.1
6021	1.84536	0.492046
6022	1.67662	0.360913
6023	1.38247	0.05
6024	1.02663	0.05
6025	0.946132	0.569521
6026	1.41723	0.356896
6027	1.72976	0.511301
6028	1.72976	0.319489
6029	0.946131	1.02771

6030	1.41723	0.494075
6031	1.25866	0.535726
6032	1.34254	0.318511
6033	1.25866	0.400127
6034	1.65507	0.291121
6035	1.65507	0.263002
6036	0.946132	0.572133
6037	0.946132	0.3655
6038	1.11929	0.389837
6039	0.961779	0.283734
6040	0.961779	0.29506
6041	0.946132	2.1
6042	1.69361	2.1
6043	1.69361	2.1
6044	0.94613	2.1
6045	1.36523	2.1
6046	1.36523	2.1
6047	1.19802	2.1
6048	0.974196	2.1
6049	1.09518	2.1
6050	0.946131	2.1
6051	1.7161	2.1
6052	1.7161	2.1
6053	0.94613	2.1
6054	1.297	2.1
6055	1.297	2.1
6056	1.14599	2.1
6057	0.974197	2.1
6058	0.974197	2.1
6059	1.80778	2.1
6060	2.20473	2.1
6061	1.80777	2.1
6062	1.3515	2.1
6063	0.583237	2.1
6064	1.39359	2.1
6065	1.39359	2.1
6066	1.07296	2.1
6067	0.946114	2.1
6068	1.07297	2.1
6069	0.903862	2.1
6070	0.956572	2.1
6071	1.03437	2.1
6072	0.975409	2.1
6073	1.31167	0.0730799
6074	2.70246	0.0679857
6075	1.12018	0.0703734
6076	0.655836	0.0743167
6077	0.655837	0.069312
6078	1.81557	0.0560959
6079	1.81557	0.0531542
6080	0.655836	0.069873
6081	0.946131	0.0782129
6082	1.01847	0.062534
6083	1.61334	0.0620487
6084	0.810946	0.0788715
6085	0.8311	0.0860094
6086	1.2466	0.0631891
6087	1.20286	0.0666294

6088	0.97508	0.079808
6089	0.870805	0.0662858
6090	0.760087	0.0839325
6091	0.853389	2.1
6092	0.820894	2.1
6093	0.928291	2.1
6094	1.06922	2.1
6095	1.32829	2.1
6096	1.12173	2.1
6097	1.16615	2.1
6098	0.957748	2.1
6099	0.612491	2.07839
6100	0.548141	2.1
6101	0.979614	2.1
6102	0.658184	2.1
6103	0.678456	2.05574
6104	0.529544	2.05684
6105	0.739013	2.1
6106	0.694037	2.1
6107	0.475716	2.06362
6108	1.58794	2.02487
6109	1.58794	1.85367
6110	0.475743	1.93261
6111	1.08301	2.1
6112	0.980664	2.1
6113	0.475737	2.1
6114	1.32709	2.1
6115	1.59396	2.1
6116	1.19719	0.160084
6117	1.34921	0.1676
6118	1.69061	0.162618
6119	1.69061	0.119361
6120	0.946132	0.193072
6121	1.34921	0.167715
6122	1.28753	0.166391
6123	0.95895	0.127841
6124	1.01051	0.140984
6125	1.12468	0.157891
6126	1.22588	0.206466
6127	1.34254	0.188668
6128	1.74561	0.179585
6129	1.74561	0.145225
6130	0.946128	0.228667
6131	1.34253	0.191417
6132	1.3492	0.193586
6133	1.01051	0.134139
6134	1.922 2.1	
6135	1.922 2.1	
6136	2.12556	2.1
6137	0.946131	2.1
6138	1.06278	2.1
6139	1.47631	2.1
6140	1.47631	2.1
6141	0.946129	2.1
6142	1.06278	2.1
6143	1.35966	2.1
6144	0.991985	2.1
6145	0.738507	1.62239

6146	0.536935	1.78937
6147	0.799004	1.6228
6148	1.04281	1.30583
6149	0.879197	1.2977
6150	1.06538	1.31063
6151	0.715942	1.62297
6152	1.00636	0.284521
6153	1.03824	0.177997
6154	1.70147	0.192
6155	1.21223	2.1
6156	1.01243	2.1
6157	1.01242	2.1
6158	1.06935	2.1
6159	1.54241	2.1
6160	0.998971	0.05
6161	1.60312	0.05
6162	1.44836	0.05
6163	2.05632	0.05
6164	1.44836	0.05
6165	0.973765	0.05
6166	0.946131	0.05
6167	0.851541	0.05
6168	1.73854	0.05
6169	1.91752	0.05
6170	1.49622	0.05
6171	1.23351	0.05
6172	3.33289	2.1
6173	1.86606	2.1
6174	2.71233	2.1
6175	0.788816	2.1
6176	0.963972	2.1
6177	1.25866	2.1
6178	1.10817	2.1
6179	1.41908	2.1
6180	1.48734	2.1
6181	1.40822	0.116786
6182	1.40822	0.116975
6183	2.09871	0.197588
6184	0.946133	0.133408
6185	1.14926	0.0727157
6186	0.946133	0.143125
6187	1.78098	0.0925252
6188	1.78098	0.0648642
6189	1.35822	0.111322
6190	1.3689	0.118407
6191	1.22176	0.052103
6192	2.03426	0.103912
6193	0.946131	0.158199
6194	1.01713	0.109293
6195	0.704112	0.169427
6196	1.36237	0.110959
6197	1.36237	0.0928292
6198	1.01713	0.146364
6199	1.04936	0.154533
6200	1.87377	2.1
6201	1.60501	2.1
6202	0.981584	0.05
6203	1.69435	0.05

6204	0.85154	2.1
6205	1.47841	2.1
6206	1.4952	2.1
6207	1.31941	2.1
6208	1.04023	2.1
6209	1.1995	2.1
6210	0.914791	2.1
6211	1.1427	1.50977
6212	1.64787	1.26287
6213	1.07526	1.55783
6214	1.07527	1.15303
6215	1.07527	1.58252
6216	1.58551	1.73507
6217	2.77362	1.62604
6218	2.17974	1.34183
6219	2.71723	1.62707
6220	1.29868	0.109771
6221	1.3594	0.0919869
6222	1.04786	0.0992483
6223	0.527876	0.0896207
6224	1.4085	0.103307
6225	1.48538	0.0728117
6226	0.76829	0.0808806
6227	1.18514	0.104529
6228	0.617954	0.0719481
6229	0.864161	0.0823619
6230	1.18835	0.0671531
6231	1.16817	2.1
6232	1.04263	2.1
6233	1.50524	2.1
6234	1.85355	2.1
6235	1.23963	2.1
6236	1.06279	2.1
6237	0.94613	2.1
6238	1.35229	2.1
6239	1.15947	2.1
6240	0.965593	2.1
6241	0.851553	2.1
6242	2.21296	2.1
6243	1.11764	2.1
6244	2.46399	2.1
6245	1.39292	2.1
6246	1.34701	2.1
6247	1.34701	2.1
6248	1.47001	2.1
6249	0.963166	0.05
6250	0.893445	0.05
6251	1.21056	0.05
6252	1.67375	2.1
6253	2.0977	2.1
6254	1.09154	2.1
6255	1.09154	2.1
6256	1.57122	1.00387
6257	1.60976	0.381512
6258	1.69533	0.224388
6259	1.6951	0.18584
6260	1.5831	0.140757
6261	1.20969	2.1

6262	1.12076	2.1
6263	1.49808	2.1
6264	1.50201	2.1
6265	1.00278	2.1
6266	1.22811	0.0932256
6267	1.11545	2.1
6268	1.09646	2.1
6269	1.03107	0.05
6270	1.44293	0.05
6271	1.66752	2.1
6272	1.11023	2.1
6273	2.57831	1.95216
6274	2.20191	1.46145
6275	2.18051	1.96756
6276	1.48035	0.244461
6277	1.0961	0.113882
6278	1.63241	0.161514
6279	1.0961	0.20522
6280	1.20959	2.1
6281	2.86136	2.1
6282	1.64933	2.1
6283	1.47217	2.1
6284	0.915178	2.1
6285	1.19074	2.1
6286	0.897276	2.1
6287	0.915177	2.1
6288	0.987037	2.1
6289	1.04288	2.1
6290	2.39323	2.1
6291	0.976218	0.05
6292	2.23933	0.05
6293	0.976215	0.05
6294	1.07384	0.05
6295	1.07652	2.1
6296	1.55678	2.1
6297	1.54168	2.1
6298	1.69136	2.1
6299	1.64391	1.45196
6300	1.76477	1.51667
6301	0.611486	1.46467
6302	0.683421	1.37361
6303	0.946131	1.44859
6304	1.1019	1.43215
6305	1.12946	1.39892
6306	1.54226	1.73284
6307	1.19496	1.40582
6308	1.96199	1.41908
6309	2.0775	1.46855
6310	1.22297	1.42957
6311	1.3469	1.41035
6312	1.25366	1.57248
6313	1.65443	1.55898
6314	1.65443	1.5475
6315	0.946132	1.69198
6316	0.946132	1.64605
6317	1.12873	1.64805
6318	1.07468	1.54494
6319	1.2854	0.754134

6320	1.3469	1.50062
6321	1.68616	1.47071
6322	1.68616	1.37612
6323	0.946132	1.64758
6324	0.946132	1.54606
6325	1.16109	1.54549
6326	1.17879	1.39152
6327	1.17879	1.46595
6328	1.45304	1.5761
6329	2.21058	2.1
6330	2.81552	2.1
6331	1.45535	0.123354
6332	1.49906	0.0867047
6333	1.04413	2.1
6334	1.74586	2.1
6335	1.34526	2.1
6336	1.17402	1.44527
6337	1.74564	1.22796
6338	1.52724	2.1
6339	1.68142	2.1
6340	1.09651	2.1
6341	1.49149	2.1
6342	1.36814	2.1
6343	1.46783	2.1
6344	2.18116	2.1
6345	1.23602	2.1
6346	1.74538	1.92966
6347	0.851539	2.1
6348	1.45775	2.1
6349	1.28919	2.1
6350	0.851483	2.1
6351	1.26102	2.1
6352	0.869889	2.1
6353	0.85151	2.1
6354	1.23451	2.1
6355	1.23602	2.1
6356	1.11955	2.1
6357	1.08022	2.1
6358	0.977776	2.1
6359	1.27896	2.1
6360	1.64268	2.1
6361	1.97548	2.1
6362	1.72527	2.08392
6363	0.946132	2.1
6364	1.69702	2.1
6365	1.69702	2.1
6366	1.13635	2.1
6367	1.40274	2.1
6368	1.24041	2.1
6369	1.13635	2.1
6370	1.78427	0.05
6371	1.68338	0.0558109
6372	1.00681	0.05
6373	1.12988	0.05
6374	0.946132	0.05
6375	0.957543	0.05
6376	1.40515	0.05
6377	1.40515	0.05

6378	0.797932	0.05
6379	1.30187	0.05
6380	1.14846	0.05
6381	0.665137	0.19083
6382	1.09575	0.243543
6383	0.596894	0.208985
6384	1.04072	0.267714
6385	0.663171	0.315895
6386	1.47218	0.254793
6387	1.32889	0.217783
6388	1.32639	0.104844
6389	1.94887	0.120905
6390	1.32639	0.112141
6391	1.19323	0.171972
6392	2.19794	0.206862
6393	0.754756	0.103257
6394	1.11015	0.119459
6395	0.754755	0.10658
6396	0.946133	0.126149
6397	0.754768	0.13574
6398	1.59986	0.121628
6399	1.59986	0.0973113
6400	1.84082	2.1
6401	1.79044	2.1
6402	1.75922	2.1
6403	1.86766	2.1
6404	1.58995	2.1
6405	1.58995	2.1
6406	1.74351	2.1
6407	1.04442	2.1
6408	1.42278	2.1
6409	1.08576	2.1
6410	0.994294	2.1
6411	1.44884	2.1
6412	1.71036	2.1
6413	0.714101	1.29154
6414	1.17541	2.1
6415	1.00872	0.789075
6416	0.714072	1.7788
6417	0.936677	1.50805
6418	0.966979	2.1
6419	1.4908	2.1
6420	1.03108	0.143157
6421	0.949712	0.0898131
6422	0.810946	0.128617
6423	0.88209	0.12788
6424	0.646702	0.0889215
6425	0.972663	0.0636001
6426	0.862301	0.0789852
6427	0.868143	0.0817955
6428	1.11094	0.0881721
6429	1.35809	0.0823307
6430	1.32455	2.1
6431	1.32328	2.1
6432	1.69586	2.1
6433	1.78301	2.1
6434	1.67906	2.1
6435	1.60635	2.1

6436	1.57234	2.1
6437	1.47012	2.1
6438	1.22811	0.111381
6439	1.08732	0.12979
6440	2.08849	0.11195
6441	1.08732	0.118634
6442	1.30635	0.05
6443	0.77315	0.05
6444	1.25732	0.05
6445	2.43845	2.1
6446	2.38577	2.1
6447	0.946132	1.664
6448	1.19288	2.1
6449	1.54508	2.1
6450	1.54508	1.80375
6451	0.94515	2.1
6452	1.19288	1.92898
6453	1.29735	1.87822
6454	1.12087	1.61899
6455	1.54699	1.71535
6456	1.43635	2.1
6457	1.33385	2.1
6458	1.43635	2.1
6459	1.02368	2.1
6460	1.035 2.1	
6461	1.00128	2.1
6462	1.04009	2.1
6463	1.14052	2.1
6464	0.443552	2.1
6465	0.984351	2.1
6466	0.591443	2.1
6467	0.929561	2.1
6468	1.52079	2.1
6469	1.035 2.1	
6470	3.5282	2.1
6471	1.88288	2.1
6472	2.73877	1.92179
6473	0.906059	2.1
6474	1.23107	0.058277
6475	0.906712	0.0657504
6476	0.841322	0.0679593
6477	1.53005	0.0600221
6478	1.10007	0.0634517
6479	1.38033	0.0655683
6480	1.03442	0.0604513
6481	0.787223	0.0614053
6482	1.42589	0.059218
6483	2.35665	0.0638707
6484	1.17793	0.108381
6485	1.81356	0.108216
6486	1.42964	0.115419
6487	1.95641	0.0985276
6488	1.39139	0.114606
6489	1.39139	0.324557
6490	0.564533	0.106027
6491	1.20016	0.656731
6492	0.755765	0.069076
6493	0.755766	0.0901072

6494	1.20016	0.0965329
6495	1.91011	0.119728
6496	1.4695	0.0864449
6497	1.70048	2.1
6498	1.93765	2.1
6499	0.741307	2.1
6500	0.780371	2.1
6501	0.946133	1.13875
6502	1.28918	1.41273
6503	1.5111	1.44902
6504	1.5111	1.22604
6505	0.946131	1.34568
6506	1.28918	1.22856
6507	1.16805	1.18026
6508	1.01179	1.16613
6509	0.946132	1.21088
6510	1.35328	1.42548
6511	1.69633	1.44093
6512	1.69633	1.27114
6513	0.946133	1.36417
6514	1.35328	1.2766
6515	1.28918	1.24885
6516	1.01179	1.2401
6517	1.58147	1.19
6518	1.27737	1.24696
6519	2.17223	0.05
6520	0.697168	0.05
6521	1.62495	0.110767
6522	1.62495	0.133859
6523	1.93948	0.39569
6524	0.601592	2.1
6525	1.93528	2.1
6526	2.13757	2.1
6527	1.67561	2.1
6528	2.70875	2.1
6529	1.93528	2.1
6530	3.98475	2.1
6531	1.12376	2.1
6532	1.19016	2.1
6533	1.67323	0.163147
6534	1.74019	0.146996
6535	1.84719	0.155302
6536	1.00764	0.132243
6537	1.23475	2.1
6538	1.06757	2.1
6539	1.57916	2.1
6540	1.06758	2.1
6541	1.28014	2.1
6542	1.33339	2.1
6543	1.2679	2.1
6544	1.23504	2.1
6545	1.50108	2.1
6546	1.3715	2.1
6547	0.58831	0.845092
6548	0.865971	0.64253
6549	0.813704	1.18748
6550	0.658856	0.742368
6551	0.495869	0.979919

6552	1.13845	1.08472
6553	0.780804	1.05119
6554	0.642023	0.992792
6555	0.707748	1.03483
6556	1.64476	0.472757
6557	1.64476	0.833939
6558	0.99611	1.00401
6559	1.35639	0.591729
6560	1.3564	0.918843
6561	0.946129	0.917277
6562	1.99222	0.589073
6563	1.4777	2.1
6564	1.03968	2.1
6565	1.82516	2.1
6566	0.851539	2.1
6567	1.63693	2.1
6568	1.59416	2.1
6569	0.878566	2.1
6570	1.40796	2.1
6571	1.13172	2.1
6572	0.851527	2.1
6573	1.14366	2.1
6574	0.823822	0.294293
6575	0.882001	0.301429
6576	1.09756	0.126374
6577	1.11857	0.179749
6578	1.0934	0.176895
6579	0.960104	0.278532
6580	0.982001	0.403023
6581	1.74319	0.215254
6582	1.03043	0.213225
6583	0.967194	0.169912
6584	1.16938	2.1
6585	2.48377	2.1
6586	1.1181	2.1
6587	2.57349	2.1
6588	1.0465	2.1
6589	1.44709	2.1
6590	0.946132	2.1
6591	1.29302	2.1
6592	1.73502	2.1
6593	1.73502	2.1
6594	0.946132	2.1
6595	1.29302	2.1
6596	1.38813	2.1
6597	1.03933	2.1
6598	1.01976	0.267301
6599	1.11764	0.292133
6600	0.961779	0.436723
6601	1.0685	0.305895
6602	1.08426	1.71381
6603	0.99695	1.7919
6604	2.25593	1.70021
6605	1.47992	1.77683
6606	1.88989	1.61823
6607	1.65813	1.67981
6608	1.20278	1.68358
6609	0.981842	1.89184

6610	0.802655	2.1
6611	0.953489	2.0404
6612	0.961719	2.02217
6613	0.975614	1.9325
6614	0.991617	1.89428
6615	0.940748	1.88551
6616	2.25019	2.044
6617	2.30712	1.78427
6618	1.98823	1.96741
6619	0.522844	1.84611
6620	0.946122	1.94274
6621	0.946132	1.85969
6622	0.593846	1.83455
6623	1.26501	1.79174
6624	1.21171	0.05
6625	0.851542	0.0565438
6626	0.940327	0.0649512
6627	0.940356	0.0650138
6628	1.52117	0.0511142
6629	1.58072	0.05
6630	1.35169	0.054763
6631	1.13889	0.0520726
6632	1.35007	0.05
6633	1.35007	0.05
6634	1.78381	0.05
6635	0.946132	0.0550927
6636	1.06627	0.0551849
6637	0.946131	0.0623405
6638	1.73005	0.05
6639	1.73005	0.05
6640	1.32449	0.0522014
6641	1.35169	0.0516314
6642	1.42446	0.261582
6643	1.86697	0.221686
6644	1.13413	0.229509
6645	1.42446	0.200865
6646	1.33575	0.0988622
6647	1.20473	0.116714
6648	1.87517	0.05
6649	1.87517	0.05
6650	0.488712	0.05
6651	1.13242	0.0526259
6652	0.488713	0.05
6653	0.488713	0.05
6654	1.23146	0.05
6655	1.57932	0.05
6656	1.01698	0.05
6657	1.42085	0.05
6658	0.246725	0.05
6659	0.996283	0.05
6660	1.36965	0.05
6661	0.86361	0.05
6662	0.810945	0.05
6663	1.72938	0.05
6664	1.21649	0.0515102
6665	1.1865	0.05
6666	1.62387	0.05
6667	1.05302	0.05

6668	1.10049	0.594142
6669	0.946129	2.1
6670	1.70184	1.22359
6671	1.70184	2.1
6672	1.12538	2.1
6673	1.35969	0.982123
6674	1.28828	2.1
6675	1.12538	2.1
6676	0.99747	1.94223
6677	1.34851	0.684398
6678	0.923724	2.1
6679	1.47154	2.1
6680	1.47154	2.1
6681	1.10698	2.1
6682	1.28828	2.06658
6683	1.10698	2.1
6684	0.923695	2.1
6685	0.99747	1.94433
6686	1.65677	2.1
6687	1.77677	2.1
6688	1.05689	0.187589
6689	2.11031	0.168084
6690	1.07367	0.125586
6691	0.656239	0.204255
6692	1.4563	0.169359
6693	1.4563	0.159264
6694	0.656238	0.200617
6695	0.946133	0.183212
6696	0.812937	0.217171
6697	0.946131	0.226794
6698	1.05689	0.139279
6699	1.34009	2.1
6700	1.398 2.1	
6701	3.29047	2.1
6702	2.53897	2.1
6703	1.24509	2.1
6704	3.06162	2.1
6705	1.01595	2.1
6706	1.52661	2.1
6707	1.5266	2.1
6708	0.946131	2.1
6709	1.31049	2.1
6710	1.16225	2.1
6711	1.05978	2.1
6712	1.16225	2.1
6713	1.06561	2.1
6714	0.953927	2.1
6715	1.15114	2.1
6716	1.1807	0.273401
6717	0.507259	0.259862
6718	1.47709	0.238827
6719	1.36192	0.220388
6720	0.487293	0.234675
6721	1.65569	0.297637
6722	0.679725	0.198139
6723	0.721928	0.246605
6724	1.03931	0.224633
6725	1.0775	0.255565

6726	1.0775	0.516284
6727	2.30207	1.10332
6728	1.01814	1.0077
6729	2.33963	0.758747
6730	2.42589	1.09179
6731	1.33962	2.1
6732	1.27956	2.1
6733	2.14624	0.0519585
6734	1.6812	0.0608834
6735	1.25698	0.0543837
6736	1.35725	0.0588992
6737	1.86387	0.0531869
6738	1.86387	0.0514726
6739	0.750365	0.0559589
6740	0.946128	0.0548171
6741	0.750365	0.0513738
6742	1.72271	0.05
6743	1.72272	0.0689379
6744	2.53736	0.0692992
6745	1.07327	2.1
6746	1.05376	2.1
6747	1.00387	1.63103
6748	2.0554	1.72964
6749	1.00387	1.48933
6750	1.00387	1.13663
6751	1.27119	2.1
6752	1.51748	2.1
6753	3.15271	2.1
6754	3.45801	2.1
6755	1.99871	2.1
6756	1.41186	2.1
6757	1.07624	2.1
6758	1.5954	2.1
6759	1.19323	1.61315
6760	1.21286	0.941291
6761	0.946114	2.1
6762	1.38395	2.1
6763	1.73389	2.1
6764	1.7339	2.1
6765	0.946115	2.1
6766	1.38397	2.1
6767	1.29604	2.1
6768	1.04544	2.1
6769	1.60578	0.0806168
6770	1.93407	0.0855508
6771	1.0525	1.37375
6772	1.0525	1.33873
6773	1.0525	1.77001
6774	1.38506	1.49722
6775	2.34737	0.05
6776	1.3531	0.05
6777	1.2925	0.05
6778	0.871466	0.05
6779	1.26623	0.05
6780	0.700631	0.05
6781	1.88774	0.05
6782	1.14167	0.05
6783	1.18606	0.05

6784	2.1238	0.05
6785	1.42762	1.22568
6786	1.67791	0.050418
6787	1.47118	0.0511788
6788	0.946128	2.1
6789	1.31641	2.1
6790	1.75933	2.1
6791	1.75933	2.1
6792	0.946131	2.1
6793	1.31641	2.1
6794	1.38905	2.1
6795	1.38437	2.1
6796	1.51829	2.1
6797	0.947114	2.1
6798	1.13335	2.1
6799	1.47521	2.1
6800	1.14911	2.1
6801	1.53822	2.1
6802	1.39702	2.1
6803	0.739166	2.1
6804	0.706056	2.1
6805	0.964033	2.1
6806	1.58993	2.1
6807	2.51892	2.1
6808	0.595905	0.05
6809	1.18155	0.05
6810	1.73364	0.05
6811	0.952735	0.05
6812	2.10381	0.05
6813	1.12115	0.05
6814	1.27826	0.05
6815	2.74021	0.05
6816	1.78416	2.1
6817	1.78416	2.1
6818	0.946132	2.1
6819	1.41126	2.1
6820	1.21275	2.1
6821	0.946133	2.1
6822	1.41126	2.1
6823	1.33125	2.1
6824	1.33125	2.1
6825	1.78918	2.1
6826	1.0465	2.1
6827	1.99715	2.1
6828	1.17683	2.1
6829	1.25889	2.1
6830	0.860834	0.05
6831	0.946131	0.05
6832	1.05008	0.05
6833	0.970444	0.05
6834	1.19401	0.05
6835	1.25989	0.05
6836	0.702942	0.05
6837	1.40591	0.0544691
6838	1.72168	0.0555303
6839	0.446352	0.05
6840	1.04072	0.05
6841	0.711038	0.128782

6842	0.980737	0.05
6843	1.05099	0.05
6844	1.26003	0.0514666
6845	0.495934	0.0724067
6846	1.03114	0.0544495
6847	1.09911	0.056634
6848	1.24825	0.05
6849	2.19827	0.0548211
6850	2.0953	0.0535911
6851	2.30437	0.0678117
6852	0.775923	0.0559537
6853	2.2283	0.05
6854	1.19583	2.1
6855	1.14496	2.1
6856	1.62183	2.1
6857	1.62316	2.1
6858	1.02305	0.172919
6859	1.21136	0.0816801
6860	1.44426	0.138666
6861	1.21137	0.0611237
6862	1.20143	0.10007
6863	1.15009	0.255557
6864	1.24965	2.1
6865	1.41077	2.1
6866	1.00052	0.113826
6867	1.79553	0.113871
6868	1.06336	0.0852138
6869	1.00053	0.0909834
6870	1.18041	2.1
6871	1.80818	2.1
6872	1.73571	1.59894
6873	1.94429	1.66647
6874	1.4375	0.0686933
6875	1.44421	0.0587311
6876	1.66953	0.0749312
6877	1.09529	0.0777458
6878	0.952395	2.1
6879	0.717337	2.1
6880	1.03609	2.1
6881	1.33949	2.1
6882	1.2774	0.187059
6883	2.24983	0.184849
6884	1.28727	0.152964
6885	0.583592	0.148054
6886	0.583591	0.25139
6887	1.82859	0.201593
6888	1.82859	0.251707
6889	0.583593	0.250142
6890	0.946128	0.126606
6891	0.692233	0.304811
6892	1.12226	0.30193
6893	2.57454	0.224876
6894	0.810944	0.174802
6895	0.810945	0.174271
6896	1.12226	0.225847
6897	1.45867	0.236005
6898	1.22403	0.236863
6899	1.15262	0.272231

6900	1.08132	0.186751
6901	0.846671	0.183733
6902	0.480954	0.2467
6903	0.917975	0.276488
6904	0.917975	0.504478
6905	0.720957	0.298268
6906	0.795998	0.266133
6907	0.69316	0.285127
6908	0.641306	0.299455
6909	0.690817	0.190803
6910	0.961899	2.1
6911	2.49579	2.1
6912	2.31106	2.1
6913	1.70482	0.0831185
6914	1.93156	0.124315
6915	1.23868	0.15668
6916	1.73878	0.131917
6917	1.26267	2.1
6918	1.26441	2.1
6919	1.58095	2.1
6920	1.58095	2.1
6921	0.946132	2.1
6922	0.946132	2.1
6923	1.11077	2.1
6924	1.62905	2.1
6925	1.49692	2.1
6926	3.49966	2.1
6927	2.39213	2.1
6928	2.12603	2.1
6929	0.946129	2.1
6930	0.461661	2.1
6931	1.52841	2.1
6932	1.39091	2.1
6933	0.550679	2.1
6934	0.509894	2.1
6935	1.49508	2.1
6936	2.05862	2.1
6937	2.47851	2.1
6938	1.07213	2.1
6939	1.09148	1.81736
6940	1.99776	1.98617
6941	1.38333	1.97045
6942	1.04412	2.1
6943	1.04412	2.1
6944	1.1893	0.0730333
6945	0.946132	0.0800468
6946	0.965737	0.106328
6947	0.946132	0.0800604
6948	1.54061	0.0820666
6949	1.54061	0.115961
6950	1.22878	0.0877308
6951	1.25797	0.101688
6952	1.00665	0.0931667
6953	1.00665	0.110067
6954	1.80369	0.0822155
6955	0.946128	0.100928
6956	1.04424	0.121159
6957	0.85154	0.0968119

6958	1.39322	0.100069
6959	1.35464	0.129429
6960	0.939857	0.107927
6961	1.28272	0.121623
6962	1.19323	0.183443
6963	1.03498	0.0873614
6964	2.22029	0.0967924
6965	0.540005	0.085209
6966	0.970382	0.119154
6967	2.14514	0.0997098
6968	1.31542	0.100723
6969	1.05575	0.161873
6970	0.810943	0.184755
6971	1.31542	0.0813104
6972	1.70493	0.103597
6973	1.22582	0.128208
6974	1.31681	0.148902
6975	1.40775	0.117368
6976	0.928633	0.220737
6977	1.2545	0.107704
6978	2.66481	0.125113
6979	1.32051	0.0892309
6980	1.32051	0.145111
6981	2.66305	2.1
6982	1.3551	2.1
6983	2.45325	2.1
6984	1.3685	2.1
6985	1.51748	2.1
6986	1.78941	2.1
6987	1.02963	0.05
6988	1.28643	0.0563259
6989	0.86131	0.0595791
6990	0.851539	0.0602941
6991	1.46585	0.05
6992	1.38822	0.05
6993	0.996402	0.05
6994	0.851553	0.0551854
6995	1.73156	0.0536187
6996	1.91695	0.05
6997	1.92535	0.0584866
6998	2.57287	0.0520058
6999	1.77743	0.050261
7000	1.02836	0.239317
7001	1.36362	0.288128
7002	1.36936	0.2417
7003	1.78684	0.191738
7004	1.78685	0.2012
7005	0.946132	0.370155
7006	1.27581	0.319526
7007	0.946132	0.446895
7008	1.02836	0.173621
7009	1.55398	2.1
7010	1.07167	2.1
7011	1.87557	2.1
7012	0.751639	2.1
7013	1.50884	2.1
7014	1.33793	2.1
7015	0.751664	2.1

7016	1.37277	2.1
7017	0.979515	2.1
7018	0.676508	2.1
7019	1.09578	2.1
7020	0.953925	2.1
7021	0.94613	2.1
7022	1.64707	2.1
7023	1.64707	2.1
7024	1.16658	2.1
7025	1.40638	2.1
7026	1.18682	2.1
7027	1.16658	2.1
7028	0.543765	0.18522
7029	1.57462	0.173267
7030	1.57462	0.152527
7031	1.00083	0.181561
7032	1.16834	0.226558
7033	1.16834	0.2089
7034	0.889626	0.20151
7035	1.25944	0.232853
7036	0.824061	0.240337
7037	1.9397	0.146218
7038	1.21893	0.168691
7039	1.91364	0.1669
7040	1.33672	0.223023
7041	0.928069	0.301734
7042	1.41484	0.230204
7043	0.538379	0.190785
7044	1.41484	0.206008
7045	1.35594	0.245234
7046	2.11414	0.222397
7047	2.11414	0.144536
7048	0.538378	0.226505
7049	1.38532	0.139057
7050	0.962877	1.12802
7051	1.07497	0.531262
7052	1.00613	0.647923
7053	0.962878	0.196418
7054	0.711447	2.1
7055	1.40008	0.0766599
7056	2.51674	0.0844499
7057	1.40008	0.0696857
7058	1.7263	0.0564833
7059	1.40008	0.0772589
7060	1.9216	0.0522731
7061	2.7013	0.05
7062	2.58584	0.05
7063	0.979296	0.05
7064	1.46459	0.904176
7065	0.899254	0.635552
7066	1.15038	2.1
7067	1.24687	2.1
7068	1.16738	2.1
7069	0.942323	2.1
7070	1.65573	1.99517
7071	0.844791	2.1
7072	0.54674	2.1
7073	0.795811	2.1

7074	1.08131	2.1
7075	1.03553	2.1
7076	0.758334	2.1
7077	0.708665	2.06834
7078	1.22626	2.03431
7079	0.835439	2.02872
7080	0.861131	1.94439
7081	0.68296	2.1
7082	1.65224	2.02787
7083	2.82624	2.1
7084	1.0151	1.80926
7085	1.15905	1.91058
7086	1.71076	1.83157
7087	1.4529	1.7204
7088	0.494895	2.1
7089	0.946132	1.92069
7090	0.613132	1.86872
7091	0.863631	1.89021
7092	1.78391	2.1
7093	1.1706	0.05
7094	2.02734	0.05
7095	1.1706	0.05
7096	0.681532	0.05
7097	1.50316	0.05
7098	1.50316	0.05
7099	0.681534	0.05
7100	0.973819	0.05
7101	0.97382	0.05
7102	0.94613	0.05
7103	1.1706	0.05
7104	1.67727	0.05
7105	1.60329	0.05
7106	1.3227	2.1
7107	1.40846	2.1
7108	1.07889	2.1
7109	1.07721	2.1
7110	0.953523	0.766961
7111	1.79675	0.752383
7112	2.09859	1.4714
7113	1.21309	2.1
7114	1.54567	1.24059
7115	1.19722	0.341718
7116	1.82793	0.338294
7117	1.1599	0.166023
7118	1.73621	0.128232
7119	0.933474	0.271831
7120	1.51477	0.189062
7121	1.51477	0.225464
7122	0.93347	0.278305
7123	1.27374	0.191914
7124	1.27376	0.230134
7125	0.946105	0.294878
7126	1.1972	0.195569
7127	0.851541	0.05
7128	1.36971	0.05
7129	1.27832	0.05
7130	0.853489	0.05
7131	0.973847	0.05

7132	0.976308	0.05
7133	0.851528	0.05
7134	2.72654	0.05
7135	1.77867	0.05
7136	3.23032	2.1
7137	2.78208	2.1
7138	1.0227	0.05
7139	1.02269	0.05
7140	1.02269	0.05
7141	1.24293	0.05
7142	0.831695	2.1
7143	0.67513	2.1
7144	0.956294	2.1
7145	1.08623	2.1
7146	1.05556	2.1
7147	1.38859	2.1
7148	1.13179	2.1
7149	1.02438	2.1
7150	1.06659	2.1
7151	1.36595	2.1
7152	1.06659	2.1
7153	1.06659	2.1
7154	0.946131	1.97895
7155	0.875912	2.1
7156	1.65971	2.1
7157	1.65971	2.1
7158	0.875912	2.1
7159	1.1229	2.1
7160	1.41273	2.1
7161	1.54699	1.9729
7162	1.93655	1.7449
7163	1.42307	1.95513
7164	1.97907	1.98582
7165	0.996774	2.1
7166	1.15505	0.05
7167	1.28463	0.05
7168	1.86353	0.0654212
7169	2.71398	0.064722
7170	1.56787	0.274136
7171	1.66295	0.157912
7172	1.73432	1.64836
7173	1.05019	0.05
7174	1.42117	0.05
7175	0.888299	0.05
7176	1.38127	0.05
7177	1.72993	0.05
7178	0.664129	0.05
7179	0.99302	2.1
7180	0.993023	2.1
7181	1.13491	2.1
7182	0.876316	2.1
7183	1.2588	2.1
7184	0.895613	2.1
7185	2.07526	2.1
7186	1.23369	2.1
7187	1.25146	2.1
7188	2.15282	2.1
7189	1.22239	2.1

7190	1.10962	2.1
7191	1.77402	2.1
7192	1.0305	2.1
7193	1.50108	2.06481
7194	1.20761	2.1
7195	1.3626	2.1
7196	1.14825	2.1
7197	1.78711	2.1
7198	1.17933	2.1
7199	1.14824	2.1
7200	1.09651	0.12618
7201	1.23851	0.121741
7202	1.83284	0.134776
7203	1.23851	0.0815119
7204	1.53521	2.1
7205	1.46279	2.1
7206	1.9233	0.0586687
7207	1.2966	0.0585779
7208	2.11443	0.0547921
7209	1.30265	0.0613729
7210	1.13528	0.170654
7211	0.946131	0.253576
7212	1.41169	0.213043
7213	1.33879	0.14762
7214	0.946128	0.180095
7215	1.80435	0.194871
7216	1.80435	0.0931873
7217	1.234	2.1
7218	1.45633	2.1
7219	1.234	2.1
7220	1.98448	2.1
7221	2.02612	2.1
7222	1.23527	1.69692
7223	1.44472	1.8678
7224	0.993366	2.1
7225	1.22025	2.07587
7226	0.993368	1.6142
7227	2.43669	2.1
7228	2.56707	2.1
7229	1.80348	1.11529
7230	1.74406	0.578386
7231	1.90937	0.943103
7232	1.49357	0.140442
7233	2.5832	0.326058
7234	1.82274	0.300346
7235	1.6957	0.259242
7236	2.22495	0.283763
7237	2.49959	0.278543
7238	2.01183	0.0861322
7239	1.37427	0.0656348
7240	1.15069	2.1
7241	1.39379	2.1
7242	1.69661	1.82151
7243	1.39379	1.93056
7244	1.39654	2.1
7245	1.93505	2.1
7246	1.5525	2.1
7247	1.5525	2.1

7248	0.615065	2.1
7249	1.38908	2.1
7250	0.615065	2.1
7251	0.77849	2.1
7252	1.38908	2.1
7253	1.56839	2.1
7254	1.09142	2.1
7255	2.07313	2.1
7256	0.998288	0.05
7257	1.31481	0.0612113
7258	1.88175	0.05
7259	2.23267	0.05
7260	1.06935	2.1
7261	1.06936	2.1
7262	1.17989	2.1
7263	1.00757	2.07641
7264	1.65318	2.1
7265	1.4185	0.211523
7266	2.22495	0.253741
7267	1.02011	0.153615
7268	0.59676	0.225383
7269	1.43606	0.186912
7270	1.43606	0.174007
7271	0.89771	0.20261
7272	1.13511	0.239772
7273	0.59676	0.155816
7274	0.897709	0.215052
7275	1.79542	0.189339
7276	2.38566	2.1
7277	1.63838	1.12389
7278	1.63837	1.53762
7279	0.916275	2.07418
7280	1.36183	1.45198
7281	1.19282	2.1
7282	0.916275	1.83888
7283	1.19283	1.97854
7284	2.01582	2.1
7285	1.00215	0.05
7286	2.60643	2.1
7287	2.15971	2.1
7288	1.28267	2.1
7289	1.29954	2.1
7290	3.40327	2.1
7291	3.49128	2.1
7292	1.3864	2.1
7293	1.3201	2.1
7294	1.45666	0.05
7295	1.55798	0.05
7296	0.857202	0.05
7297	1.24374	0.05
7298	1.04157	0.05
7299	0.85723	0.05
7300	1.02722	0.05
7301	1.01361	0.05
7302	0.974077	0.05
7303	1.01814	0.05
7304	0.974077	0.05
7305	1.43987	0.05

7306	1.43986	0.05
7307	0.927881	0.05
7308	0.946132	0.05
7309	0.927849	0.05
7310	0.927852	0.05
7311	1.2493	0.05
7312	1.22482	0.347829
7313	1.41319	0.665272
7314	1.22482	0.215788
7315	0.999492	2.1
7316	1.14657	2.1
7317	1.3617	0.05
7318	0.983269	0.05
7319	1.33174	0.05
7320	1.62816	0.05
7321	1.62817	0.05
7322	0.81938	0.05
7323	1.33174	0.05
7324	1.01332	0.05
7325	0.874788	0.05
7326	1.16725	2.1
7327	1.63991	2.1
7328	1.08848	0.05
7329	1.15354	0.05
7330	1.12665	2.1
7331	1.58742	2.1
7332	1.57523	2.1
7333	1.32201	1.46863
7334	1.32097	1.15987
7335	0.851542	0.11677
7336	0.877188	0.070146
7337	0.797489	0.119279
7338	1.44721	0.0665676
7339	1.61761	0.0581209
7340	0.943799	0.112175
7341	1.36079	0.0848442
7342	1.16361	2.1
7343	1.15852	2.1
7344	1.07624	2.1
7345	1.07624	2.1
7346	0.946132	1.24868
7347	1.37938	1.39847
7348	1.78653	1.40629
7349	1.78653	1.29114
7350	0.946131	1.36845
7351	1.37938	1.30336
7352	1.35328	1.27927
7353	1.44847	1.07734
7354	0.946133	0.882666
7355	1.38149	1.30478
7356	1.81474	1.35473
7357	1.81474	0.999784
7358	0.946133	1.35192
7359	1.38149	1.26045
7360	1.37938	1.24334
7361	1.44847	1.27829
7362	1.93665	1.08948
7363	1.5515	0.682282

7364	1.05025	2.1
7365	1.05025	2.1
7366	1.71468	2.1
7367	1.04759	2.1
7368	1.04646	0.05
7369	1.00739	0.05
7370	1.37086	0.05
7371	1.12547	0.05
7372	1.03853	0.05
7373	1.34824	0.05
7374	0.799786	0.05
7375	2.10105	0.05
7376	2.72007	0.05
7377	0.589918	0.05
7378	0.589918	0.05
7379	1.1509	0.05
7380	0.619832	0.05
7381	1.1509	0.05
7382	0.705903	0.05
7383	1.53703	0.05
7384	1.53703	0.05
7385	0.705904	0.05
7386	0.946133	0.05
7387	0.946132	0.05
7388	0.946132	0.05
7389	0.588467	0.05
7390	1.1078	0.05
7391	0.408061	0.05
7392	1.38703	0.05
7393	1.68328	0.05
7394	1.87031	0.0516376
7395	1.86268	0.0876691
7396	1.27472	0.0680037
7397	0.947862	1.09716
7398	1.19854	1.42335
7399	0.946129	0.822023
7400	0.963111	0.489127
7401	0.946131	1.24941
7402	1.39055	0.419882
7403	1.39055	0.29835
7404	1.26426	0.532603
7405	1.07242	0.590631
7406	1.31126	0.238812
7407	1.76597	0.679909
7408	0.94613	1.50719
7409	1.27214	1.3059
7410	1.90751	1.35668
7411	1.90751	1.04512
7412	0.767114	1.18069
7413	1.27214	1.49017
7414	1.40248	1.44038
7415	1.41479	1.5782
7416	2.22625	1.58341
7417	0.913373	1.49886
7418	1.0158	1.34297
7419	1.61259	1.37942
7420	1.69051	1.03514
7421	0.560949	1.40181

7422	0.898559	1.49482
7423	1.28011	1.42444
7424	1.51026	0.0654294
7425	1.98252	0.0801431
7426	1.05129	0.0771064
7427	1.2951	0.0859789
7428	1.46041	0.05
7429	1.7155	2.1
7430	1.72196	2.1
7431	0.933885	2.1
7432	0.986723	2.1
7433	1.28072	1.39464
7434	1.40765	2.1
7435	1.28072	2.1
7436	1.61687	2.1
7437	1.60965	2.1
7438	1.21424	2.1
7439	2.07297	2.1
7440	1.19943	2.1
7441	2.38784	2.1
7442	1.10278	0.106043
7443	1.43678	0.124386
7444	1.1142	0.107981
7445	1.18034	0.0987929
7446	0.94613	0.118312
7447	0.752356	0.0956744
7448	0.752329	0.108963
7449	1.46634	0.0845517
7450	1.31789	0.095394
7451	0.958159	0.126472
7452	1.22752	0.135914
7453	1.50472	0.0974571
7454	1.98974	2.1
7455	2.54186	2.1
7456	0.76512	2.1
7457	2.08777	2.1
7458	1.7753	2.1
7459	3.07372	2.1
7460	1.9756	2.1
7461	1.48162	2.1
7462	1.23204	2.1
7463	0.946131	1.59553
7464	1.13505	2.00931
7465	1.47432	2.07501
7466	1.47432	1.69576
7467	0.876831	1.86846
7468	1.13505	1.75516
7469	1.2161	1.73148
7470	1.05267	1.53455
7471	1.05267	1.68903
7472	1.5795	1.60698
7473	0.98186	2.1
7474	1.56735	2.1
7475	0.859099	2.1
7476	1.69858	2.1
7477	1.93586	2.1
7478	1.09233	2.1
7479	1.08732	0.113882

7480	1.3357	0.114033
7481	1.84826	2.1
7482	0.924132	2.1
7483	1.39188	2.1
7484	1.43685	2.1
7485	0.851541	2.1
7486	1.16251	2.1
7487	0.946131	2.1
7488	1.05685	2.1
7489	2.13781	2.1
7490	1.98859	2.1
7491	1.99731	2.1
7492	1.51379	2.1
7493	1.48796	2.1
7494	0.851542	2.1
7495	0.802558	2.1
7496	1.09244	2.1
7497	1.33891	2.1
7498	0.844958	2.1
7499	0.710864	2.1
7500	1.26087	2.1
7501	1.03679	2.1
7502	1.51026	0.0630188
7503	1.05248	0.683567
7504	0.827258	0.685137
7505	1.05248	0.69415
7506	1.28435	0.680041
7507	1.50953	0.676405
7508	1.50953	0.247364
7509	0.827298	0.745482
7510	1.00366	0.218181
7511	2.10495	0.354743
7512	1.17885	2.1
7513	1.25185	2.1
7514	1.02784	2.1
7515	1.76764	2.1
7516	1.23746	0.103923
7517	1.24082	0.119354
7518	1.46506	0.0608153
7519	1.33061	0.05
7520	0.768407	0.574438
7521	0.972058	0.355323
7522	1.16536	0.417804
7523	1.13325	0.288177
7524	0.978906	1.19523
7525	1.02291	0.410244
7526	0.838015	0.371565
7527	1.01052	0.170611
7528	1.08166	0.190237
7529	1.42607	0.05
7530	1.47354	0.0814246
7531	1.21703	2.1
7532	1.29897	2.1
7533	1.83293	2.1
7534	1.29897	2.1
7535	1.24688	0.133217
7536	0.782563	2.1
7537	0.946129	2.1

7538	0.782591	2.1
7539	1.49487	2.1
7540	1.49487	2.1
7541	1.37713	2.1
7542	0.900337	2.1
7543	1.31284	2.1
7544	0.924468	2.1
7545	1.40602	2.1
7546	1.25669	2.1
7547	0.986901	2.1
7548	0.958565	2.1
7549	1.37266	2.1
7550	1.64146	2.1
7551	0.49345	2.1
7552	1.08276	2.1
7553	1.08276	2.1
7554	1.26929	2.1
7555	1.09847	2.1
7556	0.827081	0.18593
7557	1.07236	0.350791
7558	1.25063	0.931521
7559	1.43548	0.18063
7560	1.25063	0.188588
7561	1.3659	0.05
7562	1.72441	0.05
7563	1.3659	0.05
7564	1.4677	0.05
7565	1.86114	0.05
7566	1.40104	0.0600866
7567	0.962675	0.0633614
7568	0.946131	0.0646003
7569	1.74134	0.05
7570	1.74134	0.05
7571	0.962676	0.05
7572	0.946131	0.0588808
7573	1.05548	0.0900755
7574	0.902187	0.10348
7575	2.14178	0.0704656
7576	1.74998	0.0796034
7577	1.38541	0.0888876
7578	1.64468	0.0712182
7579	0.949317	0.0958258
7580	2.06654	0.075103
7581	0.839626	0.0998922
7582	0.810945	0.108309
7583	1.29203	0.0686641
7584	1.18091	0.0780446
7585	1.06872	0.0956066
7586	1.11956	0.0737468
7587	0.926911	0.113695
7588	0.901716	0.104739
7589	0.909146	0.111006
7590	1.33776	0.0755381
7591	1.02048	0.0919565
7592	1.08365	0.103609
7593	1.21042	0.0783411
7594	0.865634	0.114638
7595	0.590354	2.1

7596	0.61173	2.1
7597	3.46825	2.1
7598	3.28404	2.1
7599	1.56004	0.0875521
7600	1.69344	0.288166
7601	1.7151	1.09621
7602	0.946131	0.33806
7603	1.40265	0.199042
7604	1.13537	0.96743
7605	0.965708	0.41273
7606	1.13537	0.430666
7607	1.29681	0.378029
7608	1.40915	0.305908
7609	1.61652	1.67831
7610	1.33171	2.1
7611	1.37681	2.1
7612	0.81094	0.05
7613	0.598606	0.05
7614	0.723111	0.05
7615	1.04562	0.05
7616	0.979052	0.05
7617	0.798185	0.05
7618	0.880117	0.05
7619	0.979341	0.345248
7620	0.800229	0.224406
7621	1.39126	0.324123
7622	1.13005	0.366868
7623	0.913877	0.390379
7624	1.26959	0.298061
7625	0.699741	0.37337
7626	1.19496	1.44777
7627	1.13392	0.05
7628	1.11913	0.05
7629	1.06866	0.05
7630	0.615546	0.05
7631	0.831006	0.0581725
7632	1.53286	0.05
7633	0.690977	0.0522503
7634	1.01956	0.05
7635	2.33818	0.05
7636	1.6585	2.1
7637	1.67061	2.1
7638	1.41414	2.1
7639	1.16756	2.1
7640	1.00898	0.05
7641	0.966964	2.1
7642	2.85443	2.1
7643	0.966977	2.1
7644	2.08427	2.1
7645	2.94901	2.1
7646	1.10735	0.27105
7647	1.65815	2.1
7648	1.4502	2.1
7649	1.45026	2.1
7650	1.53446	2.1
7651	0.946129	1.39315
7652	1.81075	2.1
7653	1.81075	1.56422

7654	0.946131	1.69063
7655	1.37075	2.1
7656	1.37075	1.58824
7657	1.34245	1.78166
7658	0.973502	1.564
7659	1.08542	2.0931
7660	1.5814	0.05
7661	1.43836	0.05
7662	0.946132	0.05
7663	1.13747	0.05
7664	1.04429	0.05
7665	1.05017	0.05
7666	1.24732	0.05
7667	1.13387	0.05
7668	2.19489	0.05
7669	1.10748	2.1
7670	1.25006	2.1
7671	1.58834	2.1
7672	1.58834	2.1
7673	0.94613	2.1
7674	1.16548	2.1
7675	0.946132	2.1
7676	1.08998	2.1
7677	1.16548	2.1
7678	0.995732	2.09236
7679	0.657014	2.1
7680	0.957173	2.1
7681	1.01485	2.1
7682	1.12468	2.1
7683	1.43871	2.1
7684	1.23716	2.1
7685	1.89277	0.903114
7686	2.06653	0.373981
7687	0.967544	0.256126
7688	1.89016	0.336532
7689	2.22165	2.1
7690	0.83514	2.1
7691	1.76565	2.1
7692	1.76565	2.1
7693	0.94613	2.1
7694	0.835112	2.1
7695	1.23426	2.1
7696	1.36653	2.1
7697	0.895892	1.81585
7698	0.859278	1.79594
7699	0.791461	1.80014
7700	1.86249	1.8115
7701	1.14607	1.83235
7702	1.19904	1.79358
7703	1.84898	1.80111
7704	1.03452	1.87686
7705	0.946131	1.72122
7706	0.940472	0.785979
7707	1.53478	2.1
7708	1.84175	1.84529
7709	1.55687	1.75495
7710	1.17787	1.17305
7711	1.60589	0.437522

7712	2.35574	0.353883
7713	1.3305	0.424395
7714	1.15008	1.4701
7715	1.12457	0.0647514
7716	1.12457	0.0657823
7717	1.01778	2.1
7718	1.35733	2.1
7719	1.50683	2.1
7720	1.12064	2.1
7721	1.42716	1.13487
7722	1.42717	1.37231
7723	1.45927	1.52658
7724	1.04321	1.59924
7725	1.32634	0.211791
7726	2.27022	0.179621
7727	1.19352	0.143718
7728	1.32634	0.143739
7729	1.40924	1.60672
7730	1.22927	1.4699
7731	1.69237	1.54625
7732	1.69237	1.9972
7733	0.946132	1.49844
7734	0.946133	1.5746
7735	1.16912	1.60064
7736	1.0417	1.48991
7737	1.14605	1.4978
7738	1.1233	2.1
7739	1.96882	1.82378
7740	1.03804	0.0875849
7741	1.15338	0.0741568
7742	1.15338	0.0752952
7743	2.33051	0.100644
7744	1.81507	2.1
7745	2.42949	2.1
7746	0.725879	2.1
7747	1.88394	2.1
7748	2.2699	0.766803
7749	2.78952	2.1
7750	1.88394	0.918497
7751	1.79606	2.1
7752	1.20091	2.1
7753	1.9614	2.1
7754	1.19901	2.07856
7755	3.07915	2.1
7756	1.32592	2.1
7757	2.86461	2.1
7758	1.34817	2.1
7759	1.44859	2.1
7760	2.03969	2.1
7761	0.946132	2.1
7762	1.50709	2.1
7763	1.50709	2.1
7764	0.946132	2.1
7765	1.29322	2.1
7766	1.29322	2.1
7767	1.01985	2.1
7768	0.968537	2.1
7769	1.401 2.1	

7770	1.28936	2.1
7771	1.56002	0.05
7772	1.92548	0.05
7773	1.46041	0.05
7774	2.22712	0.05
7775	1.56001	0.05
7776	1.50524	2.1
7777	1.21304	2.1
7778	1.60665	1.84138
7779	1.85178	1.52711
7780	2.17534	2.1
7781	2.31012	2.1
7782	2.17533	1.91921
7783	0.986724	2.1
7784	2.01895	2.1
7785	2.00433	2.1
7786	1.45752	0.139965
7787	1.12695	0.144258
7788	0.652605	2.1
7789	1.5133	2.1
7790	1.5133	2.1
7791	0.652603	2.1
7792	1.228 2.1	
7793	0.946132	2.1
7794	0.652604	2.1
7795	0.946132	1.96105
7796	0.716815	2.1
7797	1.62203	2.09663
7798	1.62203	2.1
7799	0.716814	2.1
7800	1.00191	2.1
7801	1.33694	2.1
7802	1.38216	2.1
7803	2.53338	2.1
7804	1.46932	0.05
7805	1.50848	0.05
7806	1.50108	1.40831
7807	1.18382	0.392318
7808	2.02115	0.299632
7809	0.921823	0.322039
7810	2.08356	0.05
7811	1.543 0.05	
7812	1.00763	0.165101
7813	1.4185	0.178667
7814	1.08028	0.201445
7815	0.946131	1.07095
7816	1.56599	0.190355
7817	1.56599	0.922681
7818	1.08028	0.215386
7819	0.946131	0.102299
7820	1.37518	2.1
7821	1.63453	2.1
7822	1.04627	1.63021
7823	1.04626	1.64448
7824	1.35111	1.64048
7825	1.22297	1.59434
7826	1.64499	2.1
7827	1.87598	2.1

7828	1.06994	2.1
7829	1.37063	2.1
7830	1.99943	0.0606839
7831	2.38807	0.0591763
7832	0.99972	0.073096
7833	1.41388	0.081843
7834	1.66179	0.075674
7835	1.66179	0.0635727
7836	0.946129	0.0880062
7837	1.41388	0.0792556
7838	1.19404	0.0763784
7839	1.31023	2.1
7840	1.20871	2.1
7841	1.69731	2.1
7842	1.67971	2.1
7843	0.998971	0.05
7844	1.32988	0.05
7845	0.701079	0.05
7846	0.946131	0.05
7847	0.582785	0.05
7848	1.48592	0.05
7849	1.48592	0.05
7850	1.23351	0.05
7851	0.835199	0.05
7852	0.92427	0.05
7853	1.68262	0.05
7854	2.46702	0.05
7855	1.08715	2.09953
7856	1.01606	1.91464
7857	1.29579	1.98296
7858	1.01606	2.02286
7859	1.63823	2.1
7860	1.67687	2.1
7861	1.11048	2.1
7862	1.39477	2.1
7863	0.991114	2.1
7864	2.90978	0.633418
7865	0.906811	2.04075
7866	0.872849	0.427246
7867	1.17091	1.39123
7868	0.685763	1.26562
7869	1.6386	1.70553
7870	1.06907	0.886778
7871	1.05516	1.82483
7872	1.9189	1.51037
7873	1.04615	0.103436
7874	1.2966	2.1
7875	1.37392	2.1
7876	1.35504	2.1
7877	1.30347	2.1
7878	1.50877	2.1
7879	1.11193	2.1
7880	1.40984	2.1
7881	1.67682	2.1
7882	1.00371	2.1
7883	1.15412	2.1
7884	1.41638	2.1
7885	1.03796	2.1

7886	0.946117	2.1
7887	1.50823	2.1
7888	1.50822	2.1
7889	0.966923	2.1
7890	0.946131	2.1
7891	2.07593	2.1
7892	1.9339	2.1
7893	1.1817	0.340708
7894	0.729178	0.566097
7895	1.5079	0.381927
7896	1.26991	0.499787
7897	0.994049	0.449332
7898	1.17452	0.368877
7899	1.05711	0.472243
7900	1.04071	0.520571
7901	1.80299	0.350589
7902	1.60417	0.308809
7903	0.943405	0.0846301
7904	1.01129	0.0639126
7905	1.42972	0.0664708
7906	1.15904	0.0695738
7907	0.680409	0.0632263
7908	1.2816	0.05
7909	0.806176	0.0618193
7910	1.18954	0.0606995
7911	1.26055	0.072414
7912	1.06313	2.1
7913	1.19352	2.1
7914	1.55766	2.1
7915	1.53729	2.1
7916	0.993021	1.39668
7917	1.09056	2.1
7918	1.03408	2.1
7919	0.993023	2.1
7920	0.659901	2.1
7921	0.632905	2.1
7922	1.34072	0.115536
7923	1.17793	0.0983691
7924	2.24885	0.111796
7925	0.946132	0.0986424
7926	1.12443	0.116662
7927	0.904207	0.0958486
7928	1.51237	0.090991
7929	1.51237	0.115834
7930	1.12443	0.115792
7931	1.29215	0.128596
7932	1.6943	2.03907
7933	2.43858	2.08013
7934	2.0589	1.78659
7935	1.67204	2.1
7936	1.67204	2.0062
7937	0.555875	1.92394
7938	0.91951	2.1
7939	0.946131	1.7881
7940	0.555904	1.90149
7941	0.946131	1.89775
7942	1.6943	2.1
7943	1.04744	1.80528

7944	1.50938	2.1
7945	1.60861	2.1
7946	1.92406	0.0713659
7947	1.18716	0.0675095
7948	0.962705	0.672742
7949	1.35382	1.5476
7950	1.43632	0.05
7951	1.09386	0.05
7952	1.92547	0.05
7953	0.994164	0.05
7954	0.903974	1.98865
7955	0.928829	1.85857
7956	0.810958	1.77593
7957	1.92853	1.7965
7958	1.598	1.77224
7959	1.28967	1.84875
7960	1.44993	1.82093
7961	0.946132	1.20564
7962	0.882984	1.04278
7963	0.882955	1.25971
7964	1.56071	0.31506
7965	1.56071	0.394162
7966	1.35946	0.972163
7967	1.08421	1.21926
7968	1.41943	0.05
7969	1.26265	0.05
7970	1.03235	0.347929
7971	1.50778	2.1
7972	1.70387	2.1
7973	1.65608	2.1
7974	1.39076	2.1
7975	1.6104	2.1
7976	1.37456	2.1
7977	1.51898	0.518881
7978	1.9726	0.144565
7979	2.13757	1.40669
7980	1.74856	1.47116
7981	0.635949	0.661341
7982	2.46448	1.22389
7983	1.23224	1.26394
7984	1.12786	1.69802
7985	1.82721	1.27792
7986	1.82721	0.777093
7987	0.532892	1.88728
7988	0.946131	1.70384
7989	0.53292	1.66936
7990	1.41394	2.1
7991	1.45506	2.1
7992	1.39523	2.1
7993	1.09006	2.1
7994	1.80264	0.721889
7995	1.80264	2.09633
7996	0.946131	0.744405
7997	1.34268	0.107586
7998	0.946132	1.92473
7999	1.23732	0.899967
8000	1.34268	0.974922
8001	1.2064	2.1

8002	1.58164	2.1
8003	1.73853	2.1
8004	1.01243	1.77825
8005	0.910672	1.52045
8006	1.47793	1.70874
8007	1.47793	1.97106
8008	0.910702	1.53163
8009	1.30382	1.56058
8010	1.08478	1.44285
8011	0.910702	1.79537
8012	2.60709	1.4129
8013	1.01243	1.74656
8014	1.85516	1.34404
8015	2.52823	1.03123
8016	1.24001	1.26865
8017	1.89793	0.897318
8018	1.73584	0.05
8019	1.24613	1.90561
8020	1.37074	1.70701
8021	1.23763	1.68801
8022	1.04072	2.01348
8023	0.75499	1.71857
8024	1.72329	1.68783
8025	1.46195	1.65909
8026	0.946132	1.68179
8027	0.747438	1.84925
8028	1.24613	1.63146
8029	2.59886	1.40827
8030	1.87279	2.1
8031	1.07977	2.1
8032	1.29606	2.1
8033	1.45178	2.1
8034	1.45177	2.1
8035	0.92406	2.1
8036	0.946131	2.1
8037	0.924047	2.1
8038	2.04529	2.1
8039	1.04544	2.1
8040	1.08276	2.1
8041	1.67491	2.1
8042	0.961826	1.27319
8043	0.810942	0.345928
8044	0.826622	1.27338
8045	0.914378	1.34842
8046	0.89022	0.992396
8047	0.922383	0.801346
8048	0.988165	1.12806
8049	1.31357	0.05
8050	0.946131	0.05
8051	1.31357	0.0501937
8052	1.10564	0.05
8053	1.6797	0.05
8054	1.6797	0.0519786
8055	0.739506	0.0538994
8056	1.33957	0.0913719
8057	1.33957	0.0876467
8058	1.33957	0.0596486
8059	2.34223	0.0765533

8060	1.5515	1.25899
8061	2.22137	0.635823
8062	0.94612	0.583544
8063	1.13908	0.7038
8064	1.45423	0.500187
8065	1.45422	1.19007
8066	0.795519	1.21847
8067	1.1391	0.736799
8068	1.11065	0.95525
8069	1.12266	2.1
8070	1.24925	2.1
8071	1.20242	2.1
8072	2.18297	2.1
8073	3.00107	2.0163
8074	2.58297	0.793686
8075	0.884783	2.1
8076	0.851538	1.52489
8077	0.713587	2.05318
8078	1.47949	1.61735
8079	1.66673	2.1
8080	0.696199	2.1
8081	0.9383	1.86726
8082	1.29149	1.74432
8083	1.32266	2.1
8084	2.11428	0.05
8085	2.17439	0.05
8086	1.40489	0.05
8087	1.21071	0.05
8088	0.946118	0.05
8089	1.0746	0.05
8090	0.973119	0.05
8091	1.31333	0.05
8092	1.08723	0.05
8093	0.994292	2.1
8094	1.4964	2.1
8095	1.15096	2.1
8096	1.10101	2.1
8097	1.50438	2.1
8098	1.67825	2.1
8099	2.02103	2.1
8100	2.01978	2.1
8101	1.50931	0.05
8102	1.50931	0.05
8103	0.65023	0.05
8104	1.34831	0.05
8105	0.811233	0.05
8106	1.34831	0.05
8107	0.94613	0.05
8108	1.00754	1.69129
8109	0.714132	1.99702
8110	1.28877	1.65765
8111	1.39905	1.66876
8112	1.03282	1.62024
8113	0.997769	1.68353
8114	1.11743	1.64426
8115	1.86863	1.20032
8116	3.30723	1.67344
8117	1.66635	1.97915

8118	1.35609	0.05
8119	1.38212	0.05
8120	1.95597	2.1
8121	1.64257	2.1
8122	2.93316	2.1
8123	3.13417	2.1
8124	0.733947	2.1
8125	1.15259	2.1
8126	1.07976	2.1
8127	1.30237	2.1
8128	1.99943	0.0631616
8129	0.907431	0.0678082
8130	0.878161	0.0679113
8131	1.07544	0.0566194
8132	1.03401	0.0640866
8133	0.991975	0.0555858
8134	1.03635	0.0646222
8135	0.788549	0.0582301
8136	1.44984	0.0573168
8137	1.84007	0.0704555
8138	2.31127	0.0659014
8139	1.27362	2.1
8140	1.27737	1.28235
8141	1.87783	1.2008
8142	2.35499	0.05
8143	2.69662	0.05
8144	2.24065	0.05
8145	0.996082	2.1
8146	0.996083	2.1
8147	1.44859	2.1
8148	1.03968	2.1
8149	1.60018	1.21968
8150	1.73082	1.70475
8151	0.946132	1.65421
8152	1.49742	1.87069
8153	1.49742	1.04989
8154	1.00501	1.56226
8155	1.41585	1.95102
8156	1.41585	1.59397
8157	0.946131	1.86828
8158	1.03697	1.71192
8159	2.95786	2.1
8160	2.32185	2.1
8161	1.65783	1.99355
8162	1.772 2.1	
8163	0.946131	2.08647
8164	1.40404	1.75823
8165	1.13537	2.1
8166	1.04559	2.1
8167	1.16091	2.1
8168	2.63751	2.1
8169	1.68455	0.0505063
8170	1.9682	0.05
8171	2.16834	0.128001
8172	2.45569	0.208317
8173	0.946128	0.145356
8174	1.36428	0.232196
8175	0.663978	0.347732

8176	1.58218	0.158467
8177	1.58219	0.137506
8178	1.36428	0.208528
8179	0.881886	0.219285
8180	1.73849	2.1
8181	1.35668	2.1
8182	1.1233	2.1
8183	1.15009	2.1
8184	1.16482	1.04874
8185	1.29313	1.05733
8186	1.47608	0.110453
8187	2.05949	0.122253
8188	0.946119	0.0726083
8189	1.02974	0.0965533
8190	0.946119	0.0809535
8191	1.66758	0.0610701
8192	1.66757	0.0721392
8193	1.32082	0.0746174
8194	1.29287	0.072109
8195	1.36005	2.1
8196	1.47109	2.1
8197	2.45958	2.1
8198	2.2975	2.1
8199	0.953521	1.0292
8200	0.953521	0.495957
8201	1.67054	2.1
8202	1.67054	2.1
8203	0.946131	2.1
8204	1.38154	2.1
8205	1.13326	2.1
8206	1.13326	2.1
8207	1.23513	2.1
8208	1.50998	2.01404
8209	2.47982	0.083078
8210	1.45091	0.0623995
8211	0.958953	0.0512781
8212	0.79239	0.0515207
8213	2.11929	0.0753195
8214	1.26511	0.0838246
8215	1.27733	0.0545216
8216	2.18127	0.0685473
8217	1.30169	0.06592
8218	1.19259	0.133922
8219	0.963395	1.78034
8220	0.999947	2.1
8221	1.62241	2.1
8222	1.62027	2.1
8223	0.981586	0.05
8224	0.981585	0.05
8225	0.721961	2.1
8226	0.891321	2.1
8227	1.04442	2.1
8228	1.66554	2.1
8229	2.43434	2.1
8230	1.73944	0.05
8231	1.88032	0.05
8232	0.711391	0.05
8233	1.40837	0.05

8234	0.946133	0.05
8235	0.92252	0.05
8236	1.13964	0.05
8237	1.83394	0.05
8238	2.81674	0.05
8239	1.10049	1.69093
8240	1.25092	0.303159
8241	0.760299	1.41669
8242	1.48017	1.56633
8243	1.48017	1.58121
8244	0.760271	1.29569
8245	1.35861	1.53898
8246	0.946129	1.46151
8247	0.760269	1.34156
8248	2.26155	2.1
8249	2.15634	2.1
8250	1.39824	2.1
8251	1.07817	2.1
8252	0.946132	2.1
8253	1.53028	2.1
8254	1.53028	2.1
8255	1.00807	2.1
8256	0.946133	2.1
8257	2.06126	0.283936
8258	1.97385	1.53293
8259	1.75754	0.0836241
8260	2.01613	2.1
8261	1.97995	2.1
8262	1.47696	2.1
8263	1.50172	2.1
8264	1.5177	0.05
8265	1.44146	0.05
8266	1.8807	0.0513358
8267	2.07068	0.0517817
8268	1.84267	0.0505681
8269	1.08975	2.1
8270	1.21327	2.1
8271	1.10106	0.05
8272	1.34496	0.05
8273	2.08452	0.138978
8274	1.70537	0.0847075
8275	2.62464	2.1
8276	1.5726	2.1
8277	1.5726	2.1
8278	0.94613	2.1
8279	1.15038	2.1
8280	0.946132	2.1
8281	1.03974	2.1
8282	1.15038	2.1
8283	0.99747	1.96797
8284	1.03472	2.1
8285	1.58407	1.34312
8286	1.21148	2.1
8287	1.35025	2.1
8288	1.3428	0.212405
8289	1.00775	2.1
8290	1.00775	2.1
8291	1.3879	2.1

8292	1.39958	2.1
8293	0.853042	2.1
8294	1.06997	2.1
8295	0.853042	2.1
8296	0.853042	2.1
8297	1.12491	2.1
8298	1.17879	2.1
8299	1.07162	2.1
8300	1.61179	2.1
8301	1.76016	2.1
8302	0.946132	0.725412
8303	1.23449	0.361046
8304	1.70392	0.419032
8305	1.70392	0.327887
8306	0.94613	1.02605
8307	1.23449	0.457516
8308	1.41556	0.469392
8309	1.14547	0.383166
8310	1.1721	0.265257
8311	0.96478	0.315184
8312	1.41843	0.401031
8313	1.2358	0.295656
8314	1.70678	0.280303
8315	1.70678	0.2838
8316	0.946118	0.725698
8317	1.23582	0.483122
8318	0.946105	0.665965
8319	0.964769	0.239108
8320	1.1357	0.258345
8321	0.964767	0.242669
8322	0.619856	0.05
8323	1.72366	0.05
8324	1.72367	0.05
8325	0.619831	0.05
8326	1.19262	0.05
8327	0.619829	0.05
8328	0.946129	0.05
8329	1.57459	2.1
8330	1.86512	2.1
8331	0.93841	2.1
8332	1.57459	2.1
8333	1.10476	0.05
8334	1.00013	1.41213
8335	2.13446	1.09055
8336	0.759781	2.1
8337	1.96011	1.74656
8338	0.946129	1.44902
8339	1.32056	1.65438
8340	1.75702	1.51616
8341	1.75702	1.65724
8342	0.946124	1.60991
8343	1.32056	1.54168
8344	1.38258	1.51397
8345	1.96011	1.73007
8346	2.31099	1.66141
8347	0.97356	2.1
8348	1.38885	2.1
8349	1.3771	2.1

8350	0.946131	2.1
8351	1.84843	2.1
8352	1.84843	2.1
8353	1.3771	2.1
8354	0.946131	2.1
8355	1.00278	2.1
8356	1.05447	2.1
8357	1.05446	2.1
8358	1.24913	0.594762
8359	1.05447	2.1
8360	1.80882	2.1
8361	1.80882	2.1
8362	0.946129	2.1
8363	1.33997	2.1
8364	0.946131	2.1
8365	1.28831	2.1
8366	1.33997	2.1
8367	1.44391	0.05
8368	1.44391	0.05
8369	0.850964	0.05
8370	0.850963	0.05
8371	0.946132	0.05
8372	0.921299	0.05
8373	1.37358	0.05
8374	1.46297	0.05
8375	1.46298	0.05
8376	0.823988	0.05
8377	0.921287	0.0511171
8378	0.94612	0.0510262
8379	0.823995	0.05
8380	0.946107	0.05
8381	1.44437	2.1
8382	1.06203	2.1
8383	2.21397	2.1
8384	0.637677	2.1
8385	1.8892	2.1
8386	2.075 2.1	
8387	1.32051	0.0871883
8388	2.23637	0.135708
8389	1.10904	0.281847
8390	1.11819	0.175013
8391	0.94613	0.325932
8392	0.735845	0.194526
8393	1.4914	0.224031
8394	1.4914	0.0690126
8395	0.735831	0.417915
8396	1.61683	2.1
8397	1.61683	2.1
8398	0.946131	2.1
8399	1.3281	2.1
8400	1.10441	2.1
8401	1.10442	2.1
8402	1.23487	2.1
8403	1.09914	2.1
8404	1.20691	0.0541747
8405	2.27379	0.0563975
8406	1.1369	0.0698796
8407	1.26868	0.0720306

8408	1.54422	0.0673529
8409	1.54422	0.0667607
8410	0.861357	0.0736625
8411	0.94613	0.0685914
8412	0.861359	0.069685
8413	1.2858	0.327838
8414	1.31011	1.03418
8415	1.294 0.688606	
8416	1.03079	0.88424
8417	1.81979	1.95562
8418	1.57829	2.08459
8419	1.19023	1.81527
8420	1.19022	1.90574
8421	1.46458	1.87059
8422	1.46458	1.99487
8423	0.789173	2.05292
8424	1.34386	1.98948
8425	0.909896	2.07155
8426	0.946131	1.91396
8427	0.909893	1.94175
8428	1.27725	2.1
8429	1.68451	2.1
8430	2.26155	2.1
8431	2.34217	2.1
8432	1.55485	0.704641
8433	0.90854	0.476468
8434	0.858256	0.41615
8435	1.47042	0.297467
8436	1.62771	0.329442
8437	0.777398	0.514466
8438	0.982525	0.373874
8439	1.23865	0.346646
8440	2.09928	0.533754
8441	0.946128	0.120702
8442	0.474856	0.112309
8443	0.474855	0.145573
8444	1.58747	0.0706699
8445	1.58747	0.0662994
8446	0.963917	0.111655
8447	1.09841	0.105681
8448	2.23904	2.1
8449	2.60055	1.76333
8450	1.02599	2.1
8451	2.17837	2.1
8452	2.07122	2.1
8453	2.7309	2.03923
8454	2.08482	2.1
8455	1.18322	0.241191
8456	1.35381	1.4796
8457	1.01139	0.665493
8458	0.946133	1.5266
8459	1.59089	1.12968
8460	1.59089	0.219737
8461	0.946133	0.829161
8462	1.09634	0.0791348
8463	2.02279	0.14255
8464	1.03108	0.128082
8465	0.808978	1.6346

8466	1.07317	1.65917
8467	0.831695	1.63072
8468	1.22583	1.58404
8469	1.10215	1.61985
8470	1.07508	1.64308
8471	1.22204	1.62311
8472	1.04679	2.01598
8473	1.92535	0.0531831
8474	1.11081	0.05
8475	0.946131	0.115686
8476	0.714824	0.119359
8477	0.714795	0.0995939
8478	1.4231	0.114386
8479	1.4231	0.146444
8480	0.828617	0.108566
8481	1.30928	0.141279
8482	1.49894	2.1
8483	1.55702	2.1
8484	0.946131	2.1
8485	1.10689	2.1
8486	0.946227	2.1
8487	0.946131	2.1
8488	1.21754	2.1
8489	1.504 2.1	
8490	1.504 2.1	
8491	0.723573	2.1
8492	1.24512	2.1
8493	0.723544	2.1
8494	0.946131	2.1
8495	1.24512	2.1
8496	1.29383	0.0702008
8497	1.29383	0.0664319
8498	1.61969	0.0865416
8499	0.946132	0.0562404
8500	1.12818	0.0629863
8501	0.946132	0.113938
8502	1.80922	0.0502154
8503	1.80922	0.0503222
8504	1.41383	0.0545002
8505	1.34153	0.0512504
8506	1.47689	0.0917827
8507	1.79375	0.0521813
8508	1.24333	0.480923
8509	0.768989	0.36862
8510	1.24333	0.371002
8511	1.0027	0.504162
8512	1.47705	0.467232
8513	1.47705	0.223614
8514	0.768977	0.469382
8515	1.28436	0.774813
8516	0.928583	0.93722
8517	1.28436	0.411677
8518	1.24333	0.840473
8519	1.5991	0.774961
8520	1.5991	0.191537
8521	0.928583	0.934288
8522	1.85717	0.263219
8523	2.48665	0.344198

8524	1.18423	0.05
8525	1.9493	2.09519
8526	1.8519	1.88006
8527	1.68802	2.1
8528	1.0302	0.125281
8529	0.900473	0.110202
8530	1.99663	0.120929
8531	2.61301	0.105306
8532	0.745772	0.179326
8533	0.946131	0.136926
8534	1.48881	0.133574
8535	1.48881	0.109423
8536	0.745745	0.165446
8537	0.809786	0.163754
8538	1.3065	0.151504
8539	0.946132	0.0792643
8540	0.768357	0.0916613
8541	0.768387	0.101985
8542	1.76897	0.0668763
8543	1.76896	0.0618128
8544	1.40271	0.0726696
8545	1.00777	0.0734918
8546	1.37427	0.0550798
8547	1.1967	1.56458
8548	0.946132	2.1
8549	1.59397	2.1
8550	1.59397	2.1
8551	0.946131	2.1
8552	1.14989	2.1
8553	1.14989	2.1
8554	1.10046	2.1
8555	1.09506	0.0528791
8556	2.53251	0.0565705
8557	1.9212	1.68473
8558	1.99586	1.46782
8559	0.913967	0.35707
8560	1.55028	0.0904704
8561	1.55028	0.148512
8562	0.913966	0.242161
8563	1.16318	0.0883156
8564	1.16318	0.105028
8565	0.946132	0.219952
8566	1.20478	2.1
8567	1.09662	2.1
8568	1.03472	2.1
8569	1.84774	2.1
8570	1.7984	2.1
8571	2.1221	2.1
8572	1.68223	2.1
8573	1.68224	2.1
8574	0.514872	1.89036
8575	0.888991	2.07561
8576	0.946127	1.79072
8577	0.514845	1.93146
8578	0.946129	1.92396
8579	1.10127	1.86965
8580	2.61623	1.85369
8581	0.983581	0.40923

8582	0.869663	0.141378
8583	1.1818	0.483683
8584	1.10406	0.229147
8585	1.07133	0.687003
8586	1.09405	0.155041
8587	0.838014	0.273506
8588	0.842998	1.40444
8589	1.03818	1.47815
8590	1.46338	0.136264
8591	1.903 2.1	
8592	1.23942	2.1
8593	0.965933	2.1
8594	0.810075	2.1
8595	1.40571	2.1
8596	1.66219	2.1
8597	0.810047	2.1
8598	0.936678	2.1
8599	1.45808	2.1
8600	1.6384	2.1
8601	1.54699	2.1
8602	0.719877	2.1
8603	1.33256	2.1
8604	0.753547	2.1
8605	1.04073	2.1
8606	1.25822	2.1
8607	0.851542	2.1
8608	0.714102	2.1
8609	1.45886	2.1
8610	1.80171	2.1
8611	0.597337	2.1
8612	0.957713	2.1
8613	1.3033	2.1
8614	0.596613	0.269926
8615	1.1386	0.402223
8616	0.596612	0.312134
8617	0.946133	0.464232
8618	0.596652	0.542822
8619	1.64092	0.420981
8620	1.64092	0.342888
8621	1.991 2.1	
8622	1.23013	2.1
8623	2.77816	2.1
8624	2.74107	1.1885
8625	2.07681	1.15162
8626	1.47764	0.0536608
8627	1.72677	0.05312
8628	1.52161	2.1
8629	1.28526	2.1
8630	1.90502	2.1
8631	1.87359	0.106059
8632	1.93489	0.113996
8633	2.70895	2.1
8634	2.83141	2.1
8635	1.41766	0.329646
8636	1.11337	0.334022
8637	1.41765	0.592482
8638	1.29172	0.389083
8639	1.76324	0.391762

8640	1.76324	0.51052
8641	0.946131	0.310569
8642	1.52428	2.1
8643	1.71459	2.1
8644	1.93852	1.14303
8645	1.07119	1.3393
8646	1.29698	1.22388
8647	1.42203	1.10705
8648	1.42203	1.371
8649	0.946132	1.1053
8650	0.946132	1.17149
8651	0.96926	1.2731
8652	0.769913	1.63684
8653	0.946119	1.38437
8654	1.37867	1.30038
8655	1.94028	1.30673
8656	0.94612	1.20978
8657	0.946132	1.36228
8658	1.37865	1.31219
8659	2.14234	1.28955
8660	3.06459	1.34111
8661	1.44421	0.05
8662	1.33924	0.05
8663	1.33671	0.0643962
8664	1.10733	0.0613916
8665	0.68396	0.0672117
8666	1.76008	0.0558209
8667	1.76008	0.0537488
8668	0.68393	0.0561841
8669	0.946131	0.063002
8670	3.79242	2.1
8671	2.46293	2.1
8672	3.56947	2.1
8673	1.35487	1.5812
8674	1.38616	1.75139
8675	0.954048	0.13299
8676	1.36663	0.121285
8677	0.909487	0.191209
8678	0.887288	1.984
8679	0.810947	1.36807
8680	0.810934	1.7463
8681	1.07046	1.93678
8682	1.13979	1.97758
8683	0.927838	1.6977
8684	0.919995	1.87319
8685	1.61447	2.1
8686	2.56305	2.1
8687	0.943414	2.1
8688	1.17016	2.1
8689	1.7238	2.1
8690	1.89334	2.1
8691	0.946128	1.98389
8692	1.25184	2.1
8693	1.55639	2.1
8694	3.12098	2.07187
8695	3.57307	1.60521
8696	2.81281	2.1
8697	0.851542	2.1

8698	1.27689	2.1
8699	1.52568	2.1
8700	0.839689	2.1
8701	1.36471	2.1
8702	0.827865	2.1
8703	0.93667	2.1
8704	0.900861	2.1
8705	0.943152	2.1
8706	1.00667	2.1
8707	1.18141	2.1
8708	1.15298	2.1
8709	1.20335	2.1
8710	1.00713	2.1
8711	0.597537	2.1
8712	1.0577	2.1
8713	0.967342	0.618976
8714	0.838027	1.27762
8715	0.893587	0.517308
8716	1.06436	0.698864
8717	0.850048	0.528206
8718	1.22026	0.794476
8719	0.988077	0.644686
8720	1.18769	0.243005
8721	1.3991	2.1
8722	1.52984	2.1
8723	1.35469	1.61199
8724	1.25083	1.55506
8725	1.6594	1.7528
8726	1.6594	1.63135
8727	0.946129	1.34401
8728	1.02602	1.27395
8729	0.94613	0.734369
8730	1.29724	0.0866518
8731	1.14501	0.227833
8732	1.13078	0.184012
8733	1.48381	0.167483
8734	1.45921	0.156748
8735	0.851539	0.245514
8736	0.890836	0.242962
8737	0.946131	0.291931
8738	1.60428	2.1
8739	1.50097	2.1
8740	0.714507	0.0653346
8741	1.51396	0.0836586
8742	1.51396	0.116246
8743	0.946132	0.066379
8744	0.813862	0.0799966
8745	1.41461	0.0732701
8746	0.813862	0.0783498
8747	0.921311	2.1
8748	1.25084	1.50429
8749	1.30165	1.28538
8750	1.60636	1.53556
8751	1.60636	1.40034
8752	0.94613	1.22183
8753	0.972232	1.14039
8754	0.946131	0.589368
8755	2.35003	2.1

8756	0.92546	2.1
8757	1.00713	2.1
8758	0.810947	2.1
8759	0.810934	2.1
8760	1.056	2.1
8761	0.926811	2.1
8762	0.766408	2.1
8763	1.34292	2.1
8764	1.57932	0.05
8765	2.67595	0.05
8766	1.77737	0.05
8767	1.77738	0.05
8768	0.789633	0.05
8769	1.22903	0.0533724
8770	0.789663	0.0536117
8771	0.789662	0.05
8772	1.33797	0.05
8773	0.946128	2.1
8774	1.47351	2.1
8775	1.47351	2.1
8776	0.94613	2.1
8777	1.30082	2.1
8778	1.30082	2.1
8779	0.996486	2.1
8780	0.946127	2.1
8781	1.72122	2.1
8782	1.72122	2.1
8783	0.94613	2.1
8784	1.36653	2.1
8785	1.36653	2.1
8786	1.02608	2.1
8787	0.709123	1.65307
8788	0.946127	1.31579
8789	1.41342	0.437486
8790	1.818	1.77362
8791	1.818	1.52575
8792	0.946127	0.301627
8793	1.41342	0.880075
8794	1.35071	1.31146
8795	1.8993	0.643816
8796	1.4748	2.1
8797	1.18711	0.05
8798	0.946131	0.05
8799	1.18711	0.05
8800	1.20416	0.05
8801	1.91358	0.05
8802	1.91358	0.05
8803	0.477691	0.0518793
8804	2.16798	0.05
8805	2.22307	0.05
8806	0.758971	0.05
8807	1.80694	0.052643
8808	1.80694	0.05
8809	0.946132	0.05
8810	0.75897	0.05
8811	1.25124	0.05
8812	0.946131	0.0544524
8813	1.76837	2.1

8814	1.76837	2.1
8815	0.946131	2.1
8816	1.36422	2.1
8817	0.946131	2.1
8818	1.14184	2.1
8819	1.36422	2.1
8820	0.793303	0.05
8821	1.79584	0.05
8822	1.35333	1.93295
8823	1.19967	1.69718
8824	1.55088	1.74823
8825	1.64246	2.00374
8826	0.946132	1.53435
8827	0.886293	1.86293
8828	1.25018	1.94594
8829	1.5795	1.70909
8830	2.15414	1.57571
8831	0.94613	1.24374
8832	1.37758	1.5841
8833	1.71567	1.33658
8834	1.71567	1.62498
8835	0.946125	1.47047
8836	1.37759	1.36405
8837	1.28421	1.33401
8838	0.933959	0.911929
8839	0.946133	0.070102
8840	1.7813	0.0701828
8841	1.7813	0.0535968
8842	1.05704	0.0647786
8843	1.37794	0.0752318
8844	1.37794	0.0599174
8845	0.946132	0.0802468
8846	0.946132	0.05
8847	1.68197	0.05
8848	1.68197	0.05
8849	1.01367	0.05
8850	1.25017	0.05
8851	1.25017	0.05
8852	0.946132	0.05
8853	0.838953	2.1
8854	1.4045	2.1
8855	1.56388	2.1
8856	1.3182	2.1
8857	0.928518	2.1
8858	1.04073	2.1
8859	0.835676	2.1
8860	1.47507	2.1
8861	1.47506	2.1
8862	0.797664	2.1
8863	1.34285	2.1
8864	0.929875	2.1
8865	0.797663	2.1
8866	0.946133	2.1
8867	1.83628	1.0143
8868	1.83627	1.76177
8869	0.946131	1.76776
8870	1.36748	0.617249
8871	0.946133	0.939029

8872	1.21139	1.32621
8873	1.36748	0.368305
8874	1.06795	2.1
8875	1.08192	2.1
8876	0.827866	2.1
8877	1.02548	2.1
8878	0.95006	2.1
8879	1.53933	2.1
8880	0.586511	2.1
8881	0.868145	2.1
8882	1.28401	2.1
8883	1.37719	2.1
8884	1.37719	2.1
8885	0.77497	2.1
8886	0.946132	2.1
8887	0.774968	2.1
8888	1.7053	1.65362
8889	1.7053	2.1
8890	0.94613	2.1
8891	1.25715	2.1
8892	0.946132	1.06788
8893	1.19655	2.1
8894	1.25716	2.1
8895	0.851542	2.1
8896	0.80043	2.1
8897	1.27239	2.1
8898	1.02719	2.1
8899	0.946131	2.1
8900	0.981318	2.1
8901	1.06628	2.1
8902	1.26022	2.1
8903	0.946132	2.1
8904	0.946132	2.1
8905	1.76447	2.1
8906	1.76447	2.1
8907	1.35357	2.1
8908	1.35703	2.1
8909	0.946131	2.1
8910	1.50538	2.1
8911	1.50538	2.1
8912	1.10311	2.1
8913	1.3484	2.1
8914	1.10312	2.1
8915	0.966428	2.1
8916	0.946133	2.1
8917	1.4008	2.1
8918	1.71908	2.1
8919	1.71908	2.1
8920	0.946133	2.1
8921	1.40081	2.1
8922	1.26441	2.1
8923	0.851543	2.1
8924	0.961732	2.1
8925	1.54576	2.1
8926	1.35364	2.1
8927	0.946133	2.1
8928	1.02913	2.1
8929	1.2735	2.1

8930	1.32608	2.1
8931	0.967643	2.1
8932	0.94613	2.1
8933	1.44873	2.1
8934	1.44873	2.1
8935	0.967641	2.1
8936	0.946131	2.1
8937	1.40488	2.1
8938	1.24272	2.1
8939	0.946131	2.1
8940	1.78483	2.1
8941	1.78483	2.1
8942	1.24272	2.1
8943	0.946131	2.1
8944	0.94613	2.1
8945	1.5627	0.410098
8946	1.5627	1.83452
8947	1.03656	2.1
8948	1.31649	0.28096
8949	1.19233	1.71037
8950	1.03656	1.70295
8951	0.912059	2.1
8952	1.47954	0.884563
8953	1.47954	2.06076
8954	0.912061	2.1
8955	1.34647	0.684241
8956	1.04514	2.05983
8957	0.912061	1.88709
8958	0.761972	0.0897341
8959	0.946132	0.563454
8960	0.761974	0.339361
8961	1.70225	1.16437
8962	1.70225	1.02255
8963	1.33728	0.663487
8964	1.01188	0.476566
8965	0.94613	1.8417
8966	0.746361	1.73798
8967	1.6369	2.06517
8968	1.62454	1.80435
8969	0.736738	1.40374
8970	0.958487	1.62833
8971	1.40641	1.86701
8972	1.2284	2.1
8973	0.92609	2.1
8974	0.595102	2.1
8975	1.80871	2.1
8976	1.1516	2.1
8977	0.962474	2.1
8978	2.24024	2.1
8979	0.946131	2.1
8980	1.73689	2.1
8981	1.73689	2.1
8982	0.946132	2.1
8983	1.30492	2.1
8984	1.30492	2.1
8985	1.17949	2.1
8986	0.946131	2.1
8987	1.8566	2.1

8988	1.8566	2.1
8989	1.22392	2.1
8990	1.39826	2.1
8991	1.40447	2.1
8992	1.22392	2.1
8993	0.946131	2.1
8994	1.74134	2.1
8995	1.74134	2.1
8996	1.09148	2.1
8997	1.40447	2.1
8998	1.283 2.1	
8999	1.09148	2.1
9000	1.33607	2.1
9001	1.41022	2.1
9002	1.80016	2.1
9003	1.80015	2.1
9004	0.946127	2.1
9005	0.946129	2.1
9006	1.20541	2.1
9007	1.13078	2.1
9008	1.33607	2.1
9009	1.54624	2.1
9010	1.85553	2.1
9011	0.920606	2.1
9012	0.946131	2.1
9013	1.01271	2.1
9014	1.32664	1.88916
9015	0.954692	2.1
9016	0.946116	1.81714
9017	1.47771	1.89361
9018	1.4777	2.1
9019	0.95468	1.93445
9020	0.94613	1.9409
9021	1.31381	1.48969
9022	1.14005	1.32425
9023	0.946128	1.39023
9024	1.69433	0.570932
9025	1.69433	1.80256
9026	1.14005	1.36251
9027	0.94613	0.896715
9028	1.521 2.1	
9029	1.411 2.1	
9030	0.94612	2.1
9031	1.35115	2.1
9032	1.04127	2.1
9033	1.05017	2.1
9034	1.03564	2.1
9035	0.607156	2.1
9036	1.54549	2.1
9037	1.36715	2.1
9038	0.607127	2.1
9039	1.2571	2.1
9040	0.998701	2.1
9041	0.546436	2.1
9042	0.946133	2.1
9043	1.75286	2.1
9044	1.75286	2.1
9045	1.12206	2.1

9046	1.34237	2.1
9047	1.35663	2.1
9048	1.12206	2.1
9049	0.919455	2.1
9050	1.66517	2.1
9051	1.99822	2.1
9052	1.228 2.1	
9053	1.35662	2.1
9054	1.228 2.1	
9055	1.01143	2.1
9056	0.851543	1.76921
9057	0.986856	2.1
9058	1.42762	1.8645
9059	1.23095	2.1
9060	0.946132	2.1
9061	1.00659	1.94451
9062	1.1779	1.93222
9063	1.40025	2.1
9064	1.40025	2.1
9065	0.889308	2.1
9066	1.34652	2.1
9067	0.943042	2.1
9068	0.943041	2.1
9069	1.34652	2.1
9070	1.39514	1.31719
9071	1.30809	1.21368
9072	1.75711	1.21307
9073	1.7571	1.65401
9074	0.946125	1.17004
9075	0.946127	1.25449
9076	1.18396	1.33287
9077	1.4844	1.26683
9078	1.4844	1.45432
9079	0.803322	1.82325
9080	0.946133	0.859743
9081	0.803324	2.1
9082	0.803324	1.75075
9083	1.36183	1.70714
9084	0.946131	0.109049
9085	1.01462	0.10179
9086	0.946132	0.263242
9087	1.59749	0.130301
9088	1.59749	0.072029
9089	1.16009	0.107496
9090	1.38354	0.071237
9091	0.851539	1.37127
9092	1.36005	1.66832
9093	1.5532	1.57638
9094	1.80402	1.67054
9095	0.851537	1.66233
9096	0.902632	1.61304
9097	1.44437	1.57555
9098	0.633098	2.1
9099	1.00307	0.484113
9100	0.94613	0.53241
9101	1.16623	0.353482
9102	0.946128	0.631657
9103	1.64588	0.5234

9104	1.64588	0.350264
9105	1.35059	0.474909
9106	1.24142	0.534069
9107	0.946131	0.0901922
9108	1.13343	0.0897362
9109	0.946127	0.104042
9110	1.63367	0.0719339
9111	1.63367	0.097223
9112	1.33035	0.0726774
9113	1.24945	0.103929
9114	1.78733	1.53885
9115	1.70856	1.58346
9116	0.637995	1.46424
9117	0.712454	1.5034
9118	0.946132	1.44519
9119	0.637964	1.45756
9120	1.13021	1.44347
9121	0.94613	0.0938171
9122	1.20509	0.093994
9123	0.946127	0.0911842
9124	1.70545	0.0859895
9125	1.70545	0.0957209
9126	1.28508	0.0874064
9127	1.3665	0.0994393
9128	1.58612	2.1
9129	1.51845	2.1
9130	0.797374	2.1
9131	1.20057	2.1
9132	0.946132	2.1
9133	0.946132	2.1
9134	0.899819	2.1
9135	1.50471	1.29746
9136	1.50472	1.28976
9137	0.59387	1.28753
9138	0.593844	1.18715
9139	0.946126	1.28526
9140	0.966137	1.26647
9141	1.13245	1.25227
9142	1.20801	0.935566
9143	0.946131	1.3923
9144	1.47644	1.30048
9145	0.733223	0.925841
9146	0.563609	1.01969
9147	1.69218	1.23599
9148	1.6173	1.30185
9149	1.8126	1.94807
9150	1.8126	2.00985
9151	0.678938	1.96626
9152	0.678935	2.0037
9153	0.946133	2.07351
9154	0.946132	1.9399
9155	0.678936	2.09825
9156	1.21997	1.85599
9157	1.28439	1.89605
9158	1.65282	1.98281
9159	1.86735	1.96652
9160	0.851539	1.90544
9161	0.94613	1.75903

9162	0.87688	1.78445
9163	0.804877	0.569337
9164	1.40747	0.788182
9165	1.41312	0.313861
9166	0.885349	0.400669
9167	1.25405	0.772939
9168	1.34463	0.48914
9169	0.804878	0.415936
9170	0.810299	0.103392
9171	1.04072	0.172633
9172	1.25601	0.150646
9173	1.04943	0.103237
9174	0.900294	0.173057
9175	1.38777	0.149095
9176	1.33131	0.0877338
9177	1.39168	0.05
9178	0.946132	0.0559566
9179	0.747712	0.0545448
9180	1.61944	0.05
9181	1.61944	0.05
9182	0.946132	0.0616911
9183	0.747712	0.0552874
9184	1.41201	0.122623
9185	1.12082	0.0964259
9186	0.946128	0.105122
9187	1.79415	0.138341
9188	1.79415	0.114463
9189	1.12082	0.0913668
9190	0.94613	0.105752
9191	0.711721	0.115594
9192	1.12316	0.137804
9193	0.707038	0.122029
9194	1.04072	0.144326
9195	0.704409	0.169413
9196	1.49036	0.144326
9197	1.27689	0.117927
9198	0.793044	0.539114
9199	0.810886	1.03022
9200	0.885957	0.598717
9201	1.38254	0.593977
9202	1.35991	0.569575
9203	1.01375	0.631714
9204	1.03169	0.844024
9205	0.946131	0.945233
9206	1.3279	1.23362
9207	1.42284	0.774522
9208	1.42284	0.718631
9209	0.851192	1.48918
9210	0.946131	1.32758
9211	0.851165	1.07757
9212	0.748062	0.059028
9213	1.27511	0.0590932
9214	0.74806	0.0553169
9215	0.94613	0.054936
9216	0.748059	0.0578383
9217	1.86293	0.059086
9218	1.86293	0.0538363
9219	0.851542	0.0661531

9220	1.38186	0.0628113
9221	0.851542	0.0783242
9222	1.67869	0.0539435
9223	1.66579	0.0516853
9224	1.25626	0.0617046
9225	1.23046	0.0585797
9226	1.46023	0.05
9227	1.27083	0.05
9228	0.946131	0.05
9229	0.999304	0.05
9230	0.946803	0.05
9231	0.851554	0.05
9232	0.999227	0.05
9233	0.750097	0.05
9234	1.91815	0.05
9235	0.946132	0.05
9236	0.768117	0.05
9237	1.78003	0.05
9238	1.99572	0.05
9239	0.650453	0.05
9240	0.946132	0.05
9241	1.23845	0.05
9242	0.946131	0.0500966
9243	1.75477	0.05
9244	1.75477	0.05
9245	1.33393	0.05
9246	1.36697	0.05
9247	0.749018	1.1951
9248	1.02366	1.07463
9249	1.43924	1.25413
9250	1.00018	0.573042
9251	1.06438	0.699737
9252	0.946127	0.821678
9253	0.74906	0.821197
9254	0.946131	0.821865
9255	1.26488	0.05
9256	0.946131	0.05
9257	0.94613	0.05
9258	1.49051	0.05
9259	1.59837	0.05
9260	0.907797	0.05
9261	1.04455	0.05
9262	1.25214	0.05
9263	0.68452	0.05
9264	1.25215	0.05
9265	1.24677	0.05
9266	1.8144	0.05
9267	1.81441	0.05
9268	0.684518	0.05
9269	0.851542	0.344598
9270	1.52275	0.265023
9271	1.45223	0.285816
9272	1.0269	0.307734
9273	1.29689	0.304761
9274	1.32822	0.341576
9275	0.851553	0.415965
9276	1.21756	0.05
9277	0.946131	0.05

9278	0.946127	0.05
9279	1.78014	0.05
9280	1.78014	0.05
9281	1.32076	0.05
9282	1.40551	0.05
9283	0.711214	0.0662322
9284	1.82057	0.0599954
9285	1.82057	0.0561986
9286	0.946129	0.0704973
9287	1.22716	0.0559674
9288	1.30463	0.067145
9289	0.94613	0.063581
9290	0.568927	0.05
9291	0.946132	0.05
9292	0.568927	0.05
9293	1.99266	0.05
9294	1.99266	0.05
9295	1.26201	0.05
9296	1.29957	0.05
9297	1.21614	0.0763953
9298	0.851484	0.0765194
9299	0.851543	0.0789277
9300	1.85815	0.0600316
9301	1.81937	0.0560108
9302	0.697705	0.0571339
9303	0.946132	0.0761546
9304	0.593295	0.101839
9305	1.53642	0.0869072
9306	1.53642	0.0752993
9307	0.908276	0.114687
9308	0.593296	0.0736837
9309	0.593295	0.0865773
9310	0.94613	0.0975235
9311	0.867593	0.230643
9312	1.42107	0.155807
9313	1.42107	0.160198
9314	0.867621	0.232213
9315	1.05516	0.200643
9316	0.867624	0.162876
9317	0.946129	0.219967
9318	0.766681	0.0866489
9319	1.18119	0.107107
9320	1.57018	0.0849074
9321	1.57018	0.0715765
9322	0.766652	0.11106
9323	1.18119	0.100442
9324	1.15564	0.0916758
9325	0.946131	0.149458
9326	1.239	0.113381
9327	0.851541	0.163046
9328	0.851487	0.111472
9329	1.40021	0.146891
9330	1.38373	0.0931523
9331	0.851554	0.170044
9332	1.12266	0.146257
9333	0.946131	0.155926
9334	1.08234	0.124904
9335	1.2882	0.0744544

9336	1.49963	0.720354
9337	1.46188	0.374791
9338	0.851555	0.258192
9339	1.33007	0.339472
9340	1.08054	0.290389
9341	0.946129	0.430437
9342	0.91151	0.269184
9343	1.49914	0.304553
9344	1.49914	0.160857
9345	0.911471	0.583825
9346	0.946132	1.36755
9347	1.35943	1.86736
9348	1.68117	1.51635
9349	1.68117	2.07514
9350	0.946132	1.72814
9351	1.35943	1.54739
9352	1.26787	1.50696
9353	1.81612	2.1
9354	1.81612	1.51346
9355	0.946131	1.75286
9356	1.41492	0.959365
9357	0.946132	2.1
9358	1.25037	2.1
9359	1.41492	2.1
9360	1.30541	0.05
9361	1.23646	0.05
9362	0.94613	0.05
9363	1.77304	0.05
9364	1.77304	0.05
9365	1.23646	0.05
9366	0.946132	0.05
9367	0.946131	2.1
9368	1.26689	2.1
9369	1.66806	2.1
9370	1.66806	2.1
9371	0.94613	2.1
9372	1.26689	2.1
9373	1.3473	2.1
9374	1.39384	2.1
9375	1.4181	2.1
9376	1.86581	2.1
9377	1.86581	2.1
9378	0.946128	2.1
9379	0.946129	2.1
9380	1.32068	2.1
9381	1.20328	2.1
9382	1.39384	2.1
9383	1.651	2.1
9384	1.651	2.1
9385	0.946129	2.1
9386	0.946131	2.1
9387	1.12913	2.1
9388	1.21474	2.1
9389	0.946132	2.1
9390	0.94613	2.1
9391	1.79724	2.1
9392	1.79724	2.1
9393	1.40987	2.1

9394	1.3335	2.1
9395	1.65642	2.1
9396	1.65642	2.1
9397	0.946132	2.1
9398	1.19063	2.1
9399	0.946133	2.1
9400	1.15203	2.1
9401	1.19063	2.1
9402	0.946131	1.86733
9403	1.77815	2.1
9404	1.77815	2.1
9405	0.946131	2.1
9406	1.31745	2.1
9407	1.31745	2.1
9408	1.2597	2.1
9409	0.946131	1.0077
9410	1.85184	1.93572
9411	1.85184	2.1
9412	0.946128	1.73989
9413	1.40684	2.1
9414	1.40684	1.91143
9415	1.34049	1.96431
9416	0.946132	1.77792
9417	1.71333	1.27368
9418	1.71333	1.91312
9419	1.1848	1.76547
9420	1.34297	1.17548
9421	1.31649	1.26512
9422	1.1848	1.46737
9423	1.36604	0.254648
9424	0.954559	0.339448
9425	1.36604	0.255772
9426	1.41766	0.282522
9427	1.83756	0.35414
9428	1.83756	0.617168
9429	0.946131	0.223584
9430	0.946127	0.147758
9431	1.14874	0.107158
9432	0.946127	0.207689
9433	1.66393	0.139213
9434	1.66393	0.0891248
9435	1.35559	0.146508
9436	1.25447	0.158513
9437	0.94613	0.0860278
9438	1.20732	0.0854129
9439	0.946129	0.0824501
9440	1.75073	0.0740101
9441	1.75073	0.0862973
9442	1.3665	0.0764262
9443	1.33035	0.0889385
9444	1.73256	2.1
9445	1.73256	2.1
9446	0.946131	2.1
9447	1.34921	2.1
9448	1.19462	2.1
9449	1.19462	2.1
9450	1.32949	2.1
9451	1.75754	0.422419

9452	1.75754	1.10831
9453	0.946132	1.11522
9454	1.3201	0.743644
9455	0.946132	0.289442
9456	1.25479	0.361124
9457	1.3201	0.402065
9458	1.8087	2.0995
9459	1.8087	0.419329
9460	0.946131	0.826277
9461	1.38357	0.335105
9462	0.946133	2.09237
9463	1.31034	1.04527
9464	1.38357	1.33367
9465	1.12702	0.155499
9466	0.946131	0.244406
9467	1.23923	0.220255
9468	1.4117	0.162033
9469	0.946131	0.214087
9470	1.70479	0.198804
9471	1.70479	0.0919828
9472	0.946119	2.1
9473	1.55309	2.1
9474	1.55309	2.1
9475	0.946119	2.1
9476	1.08006	2.1
9477	1.08005	2.1
9478	1.02681	2.1
9479	1.36581	2.1
9480	1.93075	2.1
9481	0.946133	2.1
9482	0.772538	2.1
9483	0.946131	2.1
9484	0.946131	2.1
9485	1.33748	2.1
9486	1.44991	2.1
9487	1.31183	2.1
9488	0.946132	2.1
9489	0.944104	2.1
9490	0.915236	2.1
9491	1.05017	2.1
9492	0.944148	2.1
9493	0.946132	0.0685255
9494	1.60171	0.053683
9495	1.60171	0.05
9496	0.946131	0.154861
9497	1.16234	0.0612394
9498	1.3855	0.0546641
9499	0.946132	0.0605496
9500	0.946133	0.0771503
9501	1.73285	0.05
9502	1.73285	0.05
9503	0.946133	0.238774
9504	1.3855	0.0763144
9505	1.29348	0.0812199
9506	0.946132	0.0745879
9507	1.34828	2.1
9508	1.20943	2.1
9509	0.946129	2.1

9510	1.71013	2.1
9511	1.71013	2.1
9512	1.20943	2.1
9513	0.94613	2.1
9514	1.19613	0.05
9515	0.946118	0.05
9516	0.946117	0.05
9517	1.70868	0.05
9518	1.70869	0.05
9519	1.40551	0.05
9520	1.2493	0.05
9521	0.946131	2.1
9522	1.73123	2.1
9523	1.73123	2.1
9524	0.946132	2.1
9525	1.37811	2.1
9526	1.37811	2.1
9527	1.25144	2.1
9528	0.946132	2.1
9529	1.75139	2.1
9530	1.75139	2.1
9531	1.23374	2.1
9532	1.29926	2.1
9533	1.39827	2.1
9534	1.23374	2.1
9535	1.41375	0.05
9536	1.25697	0.0547206
9537	0.946118	0.0511614
9538	1.80377	0.05
9539	1.80377	0.05
9540	1.25699	0.05
9541	0.946106	0.05
9542	0.946133	1.95792
9543	1.81806	1.62355
9544	1.81806	2.1
9545	0.946133	2.07635
9546	1.37089	2.1
9547	1.37089	1.51903
9548	1.34999	1.88614
9549	0.946131	2.1
9550	1.80855	1.83927
9551	1.80855	2.1
9552	1.31184	2.1
9553	1.3933	1.79474
9554	1.36139	2.1
9555	1.31184	2.1
9556	1.01175	2.0039
9557	0.94613	0.770164
9558	0.919796	2.04284
9559	1.50484	0.537626
9560	1.80584	1.90687
9561	1.53512	1.21703
9562	1.14539	1.19481
9563	0.946133	2.1
9564	1.72797	2.1
9565	1.72797	2.1
9566	1.2111	2.1
9567	1.39731	2.1

9568	1.27679	2.1
9569	1.21111	2.1
9570	0.946119	1.22903
9571	1.67673	1.8
9572	1.67673	2.1
9573	0.946118	1.75421
9574	1.21519	2.1
9575	1.2152	2.09898
9576	1.19808	2.1
9577	0.946127	2.1
9578	1.54442	2.1
9579	1.54442	2.1
9580	1.04806	2.1
9581	1.13485	2.1
9582	1.3557	2.1
9583	1.04805	2.1
9584	0.946129	2.1
9585	1.32282	2.1
9586	1.77749	2.1
9587	1.77749	2.1
9588	0.946131	2.1
9589	1.32282	2.1
9590	1.4008	2.1
9591	0.946128	0.0840209
9592	1.02366	0.121942
9593	0.94613	0.089953
9594	1.66684	0.0766791
9595	1.66684	0.108966
9596	1.29215	0.0996423
9597	1.32082	0.108613
9598	0.946133	0.0602746
9599	1.01451	0.0797031
9600	0.946132	0.067063
9601	1.62374	0.0618639
9602	1.62374	0.0561707
9603	1.2929	0.0620111
9604	1.27697	0.0536199
9605	1.37869	2.1
9606	1.17215	2.1
9607	0.946132	2.1
9608	1.70508	2.1
9609	1.70508	2.1
9610	1.17215	2.1
9611	0.946132	2.1
9612	1.03784	2.1
9613	0.946132	2.1
9614	0.946132	2.1
9615	1.56718	2.1
9616	1.56717	2.1
9617	1.35703	2.1
9618	1.15628	2.1
9619	1.35356	2.1
9620	1.28976	2.1
9621	0.946131	2.1
9622	1.78612	2.1
9623	1.78612	2.1
9624	1.28976	2.1
9625	0.94613	2.1

9626	0.946132	2.1
9627	1.36373	2.1
9628	1.92382	2.1
9629	0.946131	2.1
9630	1.92382	2.1
9631	1.33471	2.1
9632	0.946132	2.1
9633	0.94613	0.255866
9634	1.29149	1.40695
9635	1.75878	1.15126
9636	1.75878	1.96608
9637	0.946128	1.58478
9638	1.29149	0.899588
9639	1.41342	1.17658
9640	1.41022	2.1
9641	1.37034	2.1
9642	1.83443	2.1
9643	1.83443	2.1
9644	0.946128	2.1
9645	0.946131	2.1
9646	1.26928	2.1
9647	0.946128	1.36858
9648	1.38259	1.64194
9649	1.81405	1.45033
9650	1.81405	1.6531
9651	0.946126	1.56747
9652	1.38259	1.46829
9653	1.37759	1.4502
9654	1.68131	0.339491
9655	1.68131	1.16685
9656	0.946131	0.651192
9657	1.38896	0.260591
9658	1.17438	2.03592
9659	1.17438	0.742237
9660	1.23848	1.14316
9661	1.80088	0.884505
9662	1.80088	0.705929
9663	0.946132	0.543041
9664	1.40332	0.91879
9665	1.28185	0.648719
9666	1.28185	0.340973
9667	1.34369	0.175699
9668	1.71939	2.1
9669	1.71939	2.1
9670	0.946131	2.1
9671	1.28308	2.1
9672	0.946133	2.1
9673	1.24024	2.1
9674	1.28309	2.1
9675	1.8525	2.1
9676	1.8525	2.1
9677	0.946132	2.1
9678	1.38244	2.1
9679	0.946132	2.1
9680	1.33469	2.1
9681	1.38244	2.1
9682	1.26654	1.41302
9683	1.39514	1.36746

9684	1.71556	1.36536
9685	1.71556	1.60362
9686	0.946126	1.31544
9687	0.946129	1.42282
9688	1.1908	1.45736
9689	1.49995	2.1
9690	1.63875	2.1
9691	0.93608	2.1
9692	0.937208	2.1
9693	0.943708	2.1
9694	0.936134	2.1
9695	1.14341	2.1
9696	1.17802	0.05
9697	1.3542	0.05
9698	1.73981	0.05
9699	1.73981	0.05
9700	0.94613	0.05
9701	1.3542	0.05
9702	1.33174	0.05
9703	1.59452	2.1
9704	1.59452	2.1
9705	0.946132	2.1
9706	1.25	2.1
9707	0.975266	2.1
9708	0.946132	2.1
9709	1.25	2.1
9710	0.946131	2.1
9711	1.48685	2.1
9712	1.48685	2.1
9713	0.946132	2.1
9714	1.17273	2.1
9715	1.17273	2.1
9716	0.955602	2.1
9717	1.32827	0.11363
9718	1.11166	0.102084
9719	0.946127	0.104468
9720	1.6136	0.128967
9721	1.6136	0.0975586
9722	1.11166	0.0960694
9723	0.946129	0.109928
9724	0.851541	0.581406
9725	1.22107	0.442308
9726	1.64594	0.327383
9727	1.5271	0.365405
9728	0.94613	0.910794
9729	1.22112	0.704965
9730	1.25002	0.473722
9731	1.65795	1.40283
9732	1.65795	2.1
9733	0.946132	2.1
9734	1.2366	2.1
9735	0.946132	0.87565
9736	1.10023	2.04803
9737	1.2366	1.92401
9738	1.35856	0.05
9739	0.946132	0.05
9740	1.35856	0.05
9741	1.08019	0.05

9742	1.49261	0.05
9743	1.49261	0.05
9744	0.94613	0.05
9745	1.29322	0.05
9746	0.946132	0.0519671
9747	1.29322	0.0694686
9748	1.35856	0.077529
9749	1.70565	0.05
9750	1.70565	0.081099
9751	0.946132	0.05
9752	1.66635	2.1
9753	1.66635	2.1
9754	0.946133	2.1
9755	1.39558	2.1
9756	1.14793	2.1
9757	1.14793	2.1
9758	1.21691	2.1
9759	1.35381	0.194321
9760	1.3365	1.58858
9761	1.09905	0.836512
9762	0.946132	1.52191
9763	1.74418	0.897765
9764	1.74418	0.207877
9765	0.946132	0.78604
9766	1.84759	2.1
9767	1.84759	1.61911
9768	0.94613	1.98827
9769	1.3904	2.1
9770	1.34522	0.492045
9771	1.34522	1.61034
9772	1.40331	1.14201
9773	1.86047	2.1
9774	1.86047	2.1
9775	0.946132	2.1
9776	1.41619	2.1
9777	0.946132	1.94197
9778	1.3659	2.1
9779	1.41619	2.1
9780	1.28464	2.1
9781	1.14496	1.90742
9782	1.57806	1.92202
9783	1.7753	2.00089
9784	0.851541	2.0404
9785	0.946131	2.0134
9786	0.872786	1.99327
9787	0.85062	1.0875
9788	1.52635	1.22839
9789	1.63785	1.04519
9790	1.69972	1.28938
9791	0.946132	1.23339
9792	1.37493	1.12715
9793	1.23854	1.1497
9794	1.41134	1.91805
9795	1.27079	1.50813
9796	1.736	1.34171
9797	1.736	2.1
9798	0.946132	1.20722
9799	0.946133	1.36823

9800	1.21177	1.5743
9801	0.946131	0.0640109
9802	0.946129	0.0534478
9803	0.94613	0.0639036
9804	0.946131	0.05
9805	0.946131	0.0502184
9806	1.35403	0.0511333
9807	0.946131	0.0666835
9808	1.37052	0.128477
9809	0.946132	0.485884
9810	0.94613	0.289763
9811	0.946133	0.147237
9812	0.946131	0.212932
9813	0.946131	0.334184
9814	0.946131	0.232467
9815	1.24249	0.107879
9816	0.946132	0.156532
9817	0.946133	0.128793
9818	0.946132	0.0745329
9819	0.946132	0.074843
9820	0.946132	0.192455
9821	0.946132	0.121138
9822	0.946132	2.1
9823	0.946132	2.1
9824	0.946131	2.1
9825	0.946132	2.1
9826	0.946132	2.1
9827	0.946132	2.1
9828	1.20608	2.1
9829	1.41232	2.1
9830	0.946128	2.1
9831	0.946129	2.1
9832	0.94613	2.1
9833	0.94613	2.1
9834	0.94613	2.1
9835	0.94613	2.1
9836	0.946132	0.0571111
9837	0.946131	0.059579
9838	0.946131	0.0628019
9839	0.946132	0.05
9840	0.946132	0.0502829
9841	0.946131	0.0584739
9842	0.946132	0.0526555
9843	0.946116	1.86576
9844	0.94612	2.1
9845	0.946118	2.1
9846	0.946124	2.1
9847	0.946118	2.1
9848	1.41561	2.1
9849	0.946131	2.1
9850	0.945846	2.1
9851	1.03508	2.1
9852	0.77579	2.1
9853	0.658908	2.1
9854	1.17474	2.1
9855	0.94303	2.1
9856	0.849189	2.1
9857	0.94298	2.1

9858	1.03963	2.1
9859	0.946131	1.97149
9860	0.946131	1.63874
9861	0.946131	2.1
9862	0.946131	1.61869
9863	0.946131	0.609591
9864	0.946131	1.78798
9865	1.32819	1.89484
9866	0.946133	1.41362
9867	0.946129	2.00537
9868	0.946128	1.96906
9869	0.94613	1.66353
9870	0.946131	2.1
9871	0.946131	1.63303
9872	0.946131	1.71859
9873	0.954072	0.05
9874	1.28895	0.05
9875	0.946133	0.05
9876	1.15803	0.05
9877	1.29689	0.05
9878	1.29689	0.05
9879	0.946132	0.05
9880	0.946132	1.45213
9881	0.946132	1.32633
9882	0.946132	1.30542
9883	0.946132	1.45175
9884	0.946132	1.60869
9885	0.946131	1.59612
9886	1.0264	1.58848
9887	0.946126	0.25445
9888	0.946132	0.224064
9889	0.946131	0.204207
9890	0.94613	0.229669
9891	0.946129	0.319461
9892	0.94613	0.249284
9893	1.35977	0.249723
9894	0.946132	0.426784
9895	0.946133	0.175611
9896	0.876632	1.21135
9897	0.946132	0.15548
9898	1.01566	0.411461
9899	1.01566	0.319305
9900	0.876606	0.398868
9901	0.946131	0.05
9902	0.946128	0.05
9903	0.946128	0.0552304
9904	0.94613	0.05
9905	0.94613	0.0568337
9906	1.24979	0.05
9907	0.94613	0.0590696
9908	0.946131	0.05
9909	0.946131	0.05
9910	0.921762	0.05
9911	0.9705	0.05
9912	0.9705	0.05
9913	0.946131	0.05
9914	0.921791	0.05
9915	0.810944	0.0839435

9916	0.810945	0.0942744
9917	0.810959	0.0743747
9918	2.42267	0.0616712
9919	1.35168	0.0628794
9920	1.08132	0.0745565
9921	1.08132	0.0613324
9922	1.08131	0.0702586
9923	0.946133	0.0819363
9924	0.946132	0.0955536
9925	0.946132	0.0783191
9926	0.946133	0.0998328
9927	0.946133	0.077785
9928	0.946132	0.0828753
9929	0.946133	0.109582
9930	0.946131	0.597705
9931	0.946131	1.27336
9932	0.94613	0.529147
9933	0.851514	0.492276
9934	0.946131	1.16198
9935	0.965268	1.01052
9936	0.851513	0.671257
9937	0.946122	2.1
9938	0.946123	2.1
9939	0.946126	2.1
9940	0.946124	2.1
9941	0.946124	2.1
9942	0.946118	2.1
9943	0.94613	2.1
9944	0.946131	0.127201
9945	1.30448	0.192187
9946	0.946132	0.361884
9947	0.946129	0.165556
9948	0.946131	0.403733
9949	0.946131	0.0657379
9950	0.94613	0.398175
9951	0.946133	2.1
9952	0.946128	2.1
9953	0.946128	2.1
9954	0.94613	2.1
9955	1.36503	2.1
9956	0.946131	2.1
9957	0.946131	2.1
9958	0.810947	0.05
9959	0.810947	0.05
9960	2.48233	0.05
9961	1.3517	0.05
9962	1.08132	0.05
9963	1.08132	0.05
9964	1.08133	0.05
9965	0.810934	0.05
9966	1.48874	0.05
9967	2.48233	0.05
9968	2.2369	0.05
9969	0.946133	0.638665
9970	0.946132	0.122645
9971	0.946132	1.30467
9972	0.946133	0.481042
9973	0.946133	1.18441

9974	1.41851	0.320392
9975	0.946132	0.440627
9976	1.03925	2.1
9977	0.946131	2.1
9978	0.94613	2.1
9979	0.946128	2.1
9980	0.946133	2.1
9981	0.944743	2.1
9982	1.13703	2.1
9983	0.946132	2.1
9984	0.946128	2.1
9985	0.946128	2.1
9986	0.946131	2.1
9987	0.94613	2.1
9988	0.94613	2.1
9989	0.94613	2.1
9990	0.946131	0.05
9991	0.946132	0.05
9992	0.946131	0.05
9993	0.946132	0.05
9994	0.946132	0.05
9995	0.946131	0.05
9996	1.20057	0.05
9997	0.946129	2.1
9998	0.94613	2.1
9999	0.94613	2.1
10000	0.946128	2.1
10001	0.946132	2.1
10002	0.94613	2.1
10003	1.38302	2.1
10004	0.856567	0.05
10005	1.14319	0.0508967
10006	0.716526	0.05
10007	1.20631	0.05
10008	0.626936	0.0504941
10009	0.946131	0.05
10010	0.94613	0.05
10011	1.39502	0.05
10012	0.881445	0.05
10013	0.946128	0.0509391
10014	0.94613	0.05
10015	0.881447	0.05
10016	0.946117	0.05
10017	0.946116	0.05
10018	0.88146	0.05
10019	0.946132	2.1
10020	1.39053	2.1
10021	0.946132	2.1
10022	0.946132	2.1
10023	0.946132	2.1
10024	0.946132	2.1
10025	0.946131	2.1
10026	0.946131	2.1
10027	0.946131	2.1
10028	0.946131	2.1
10029	0.946132	2.1
10030	0.946132	2.1
10031	1.40571	2.1

10032	0.946132	2.1
10033	0.94613	0.142244
10034	0.946127	0.191603
10035	0.946128	0.17765
10036	0.946131	0.178144
10037	0.946127	0.139987
10038	1.25819	0.180074
10039	0.946129	0.170463
10040	0.946129	0.644491
10041	1.29054	0.824637
10042	0.94613	0.434171
10043	0.94613	0.523161
10044	0.946131	1.29032
10045	0.946131	0.952529
10046	0.94613	0.789961
10047	0.810944	0.05
10048	0.810941	0.05
10049	1.61558	0.05
10050	1.3517	0.05
10051	1.08131	0.05
10052	1.08132	0.05
10053	1.08133	0.05
10054	0.810931	0.05
10055	1.02036	1.16824
10056	0.946132	1.13249
10057	1.02036	1.20933
10058	0.946132	1.07802
10059	1.1576	1.20532
10060	0.964656	1.12864
10061	0.927608	1.19439
10062	0.946128	2.1
10063	1.38813	2.1
10064	0.946129	2.1
10065	0.946131	2.1
10066	0.946131	2.1
10067	0.94613	2.1
10068	0.946131	2.1
10069	0.946131	2.1
10070	0.94613	2.1
10071	0.94613	2.1
10072	1.41665	2.1
10073	0.946132	2.1
10074	0.946131	2.1
10075	0.94613	2.1
10076	0.946118	0.146978
10077	0.946118	0.125592
10078	0.946116	0.145798
10079	0.946126	0.103852
10080	0.94612	0.115853
10081	0.946131	0.12241
10082	0.946105	0.123445
10083	0.946132	2.1
10084	0.946132	2.1
10085	0.946132	2.1
10086	0.946131	2.1
10087	0.946132	2.1
10088	0.946132	2.1
10089	0.946132	2.1

10090	0.864997	0.05
10091	0.786573	0.05
10092	0.978561	0.05
10093	1.47385	0.05
10094	1.68652	0.05
10095	1.27803	0.05
10096	1.19771	0.05
10097	1.27595	0.05
10098	0.946127	0.55967
10099	1.3771	0.405979
10100	0.946127	0.413641
10101	0.946131	0.667356
10102	0.946129	0.367799
10103	0.946129	0.583874
10104	0.946129	0.522066
10105	0.95697	0.05
10106	1.48133	0.05
10107	1.24499	0.05
10108	1.5295	0.05
10109	0.938161	0.05
10110	0.808717	0.05
10111	1.3542	0.05
10112	0.946133	0.953131
10113	1.14444	0.682977
10114	0.946132	0.465036
10115	0.946132	0.570857
10116	0.946133	1.27363
10117	0.946132	1.01607
10118	0.946132	0.736184
10119	0.946131	0.0608138
10120	0.946132	0.0815792
10121	0.946131	0.0651749
10122	0.946132	0.0614697
10123	0.946131	0.0787153
10124	0.946132	0.054937
10125	0.946132	0.0782344
10126	0.946132	2.1
10127	0.946133	2.1
10128	0.946133	2.1
10129	0.946132	2.1
10130	1.29322	2.1
10131	0.946132	2.1
10132	0.946132	2.1
10133	0.946131	2.1
10134	0.946132	2.1
10135	0.946131	2.1
10136	0.946132	2.1
10137	1.28441	2.1
10138	0.946131	2.1
10139	0.946131	2.1
10140	0.946113	2.1
10141	0.946118	2.1
10142	0.946118	2.1
10143	1.28073	2.1
10144	0.946117	2.1
10145	0.946131	2.1
10146	0.946105	2.1
10147	0.810945	2.1

10148	0.810942	2.1
10149	2.75904	2.1
10150	1.3517	2.1
10151	1.08131	2.1
10152	1.08133	2.1
10153	1.08132	2.1
10154	0.81093	2.1
10155	0.946127	2.1
10156	1.37952	2.1
10157	1.86965	2.1
10158	0.946127	2.1
10159	1.86965	2.1
10160	1.28228	2.1
10161	1.37952	2.1
10162	0.946128	2.1
10163	0.946132	2.1
10164	1.14042	2.1
10165	0.946132	2.1
10166	1.14042	2.1
10167	1.09974	2.1
10168	1.09974	2.1
10169	0.946132	2.1
10170	0.946132	0.102651
10171	0.946132	0.0774129
10172	0.946133	0.0913329
10173	0.946132	0.0723168
10174	0.946132	0.0871365
10175	0.946132	0.0732801
10176	0.946133	0.087821
10177	1.31817	1.79006
10178	0.946131	1.65599
10179	0.946131	1.76996
10180	0.946131	1.73571
10181	0.946131	1.56914
10182	0.946131	1.68039
10183	0.946131	1.668
10184	0.946132	2.1
10185	0.946132	2.1
10186	0.946132	2.1
10187	0.946132	2.1
10188	0.946132	2.1
10189	0.946132	2.1
10190	1.35048	2.1
10191	0.946131	2.1
10192	0.946131	2.1
10193	0.94613	2.1
10194	0.946133	2.1
10195	0.946133	2.1
10196	1.35943	2.1
10197	0.946131	2.1
10198	0.946133	2.1
10199	0.946132	2.1
10200	0.946132	2.1
10201	0.946133	2.1
10202	0.946133	2.1
10203	0.946133	2.1
10204	0.990712	2.1
10205	0.965725	1.58023

10206	0.946129	1.40463
10207	0.946133	1.32946
10208	1.21721	1.39368
10209	0.877931	1.23252
10210	0.946131	1.37343
10211	0.946131	1.36685
10212	0.946132	0.336405
10213	0.946131	0.261152
10214	0.946131	0.628894
10215	0.946133	0.344865
10216	0.946128	1.22468
10217	0.946131	0.584487
10218	1.39575	0.52402
10219	0.810943	0.406536
10220	0.810942	0.434576
10221	2.58794	0.252187
10222	1.21652	0.267443
10223	0.946154	0.335654
10224	0.979952	0.331103
10225	1.0813	0.378477
10226	0.810959	0.380734
10227	0.924071	0.298729
10228	1.16453	0.195807
10229	1.28586	0.187258
10230	0.946131	0.323979
10231	1.1227	0.184978
10232	1.16462	0.22453
10233	0.851553	0.359698
10234	0.709578	0.23693
10235	0.810943	0.390411
10236	0.810959	0.285019
10237	1.82615	0.236659
10238	1.21651	0.236888
10239	1.08132	0.26766
10240	0.946155	0.251369
10241	0.979937	0.343565
10242	0.946128	1.70072
10243	0.946132	2.1
10244	0.946132	1.42889
10245	0.94613	1.89133
10246	0.94613	1.95057
10247	0.94613	1.23272
10248	0.94613	1.56818
10249	0.946131	1.5276
10250	1.29735	1.86045
10251	0.946131	2.1
10252	0.946131	1.41292
10253	0.946131	1.77061
10254	0.946131	1.46444
10255	0.946131	1.65235
10256	0.946128	2.1
10257	1.33266	1.09776
10258	0.94613	1.68784
10259	0.946131	2.1
10260	0.94613	0.654658
10261	0.946131	1.53228
10262	0.946131	1.574
10263	0.946131	2.1

10264	0.946131	2.1
10265	0.946131	2.05272
10266	1.40608	2.1
10267	0.946131	2.1
10268	0.946131	2.1
10269	0.946131	2.1
10270	0.946131	1.84972
10271	0.946131	2.1
10272	0.946131	1.89284
10273	1.2334	1.92841
10274	0.946129	1.96999
10275	0.946131	1.94432
10276	0.946131	2.07147
10277	0.946133	2.1
10278	0.946133	2.1
10279	0.946132	2.06122
10280	1.29198	2.1
10281	0.946132	2.1
10282	0.946132	2.1
10283	0.946132	2.1
10284	0.946131	2.1
10285	0.946128	2.1
10286	0.946128	2.1
10287	0.946131	2.1
10288	0.946127	2.1
10289	0.94613	2.1
10290	0.946129	2.1
10291	0.946123	2.1
10292	0.946126	2.1
10293	0.946125	2.1
10294	1.38639	2.1
10295	0.946126	2.1
10296	0.946132	2.1
10297	0.946119	2.1
10298	0.946128	0.128755
10299	0.946132	0.11933
10300	0.946131	0.150575
10301	0.94613	0.0747308
10302	0.94613	0.0905358
10303	0.946131	0.116063
10304	0.94613	0.0987239
10305	0.946131	0.108857
10306	0.946131	0.0957049
10307	0.946131	0.132808
10308	0.946131	0.0612745
10309	0.946131	0.0709625
10310	0.946131	0.0968829
10311	0.946131	0.0780268
10312	0.810945	2.1
10313	0.810943	2.1
10314	0.810933	2.1
10315	2.24688	2.1
10316	1.3517	2.1
10317	1.08132	2.1
10318	1.08132	2.1
10319	1.08133	2.1
10320	1.25809	0.186624
10321	0.946131	0.643635

10322	1.25809	0.203768
10323	0.946129	0.54371
10324	0.94613	0.186636
10325	0.946131	0.546794
10326	0.946131	0.300157
10327	0.946131	0.05
10328	0.946131	0.0565802
10329	0.946129	0.0934218
10330	1.28911	0.05
10331	0.946131	0.0665929
10332	0.946131	0.0503367
10333	0.946131	0.0532624
10334	0.94613	0.137448
10335	0.946131	0.112543
10336	0.94613	0.0978773
10337	0.946128	0.135737
10338	0.946132	0.0966631
10339	1.38954	0.121101
10340	0.946131	0.124633
10341	0.946131	0.134638
10342	0.946131	0.277616
10343	0.946131	0.4055
10344	1.37981	0.228155
10345	0.946131	1.00462
10346	0.946131	0.308638
10347	0.946131	0.353403
10348	0.946131	2.1
10349	0.946127	2.1
10350	0.946127	2.1
10351	0.946131	2.1
10352	0.946127	2.1
10353	0.946129	2.1
10354	0.946128	2.1
10355	0.94613	2.1
10356	0.946126	2.1
10357	0.946127	2.1
10358	0.946131	2.1
10359	0.946126	2.1
10360	0.946128	2.1
10361	0.946128	2.1
10362	0.946133	0.315219
10363	0.946132	0.234725
10364	0.946132	0.255305
10365	0.946132	0.26648
10366	0.946132	0.304716
10367	1.13511	0.211464
10368	0.946132	0.255964
10369	0.946132	1.84765
10370	0.946129	1.85293
10371	0.94613	1.78235
10372	0.946131	1.89298
10373	0.946131	1.92804
10374	0.94613	2.09499
10375	1.27575	2.03752
10376	0.946129	2.1
10377	0.946131	2.1
10378	0.946131	2.1
10379	0.946128	2.1

10380	0.946133	2.1
10381	0.946131	2.1
10382	0.94613	2.1
10383	1.15581	0.05
10384	0.946132	0.05
10385	0.946131	0.05
10386	0.94613	0.05
10387	1.15581	0.05
10388	0.94613	0.05
10389	0.946131	0.05
10390	0.611407	1.5965
10391	0.946131	0.917017
10392	1.37335	1.55385
10393	1.15086	1.6438
10394	0.617549	1.27935
10395	0.851553	1.50784
10396	1.14592	1.65181
10397	1.22618	1.87295
10398	0.946132	0.770858
10399	0.946132	1.90394
10400	0.946132	1.43459
10401	0.946131	1.35775
10402	0.946132	1.66922
10403	0.946132	1.6472
10404	0.946131	2.1
10405	0.94613	2.1
10406	0.946131	2.1
10407	0.946133	2.1
10408	0.946129	2.1
10409	0.946131	2.1
10410	0.946131	2.1
10411	0.810945	1.01981
10412	0.810943	2.07886
10413	2.50715	1.7964
10414	1.3517	1.33945
10415	1.08133	1.25482
10416	1.08132	2.02626
10417	1.08132	0.236795
10418	0.810932	0.845444
10419	1.07811	2.07587
10420	0.94613	1.07112
10421	0.946128	2.09191
10422	1.25358	0.499967
10423	0.946131	1.298
10424	1.25358	0.961714
10425	0.94613	1.00513
10426	0.946129	2.1
10427	1.09197	2.1
10428	0.946131	2.1
10429	1.09197	2.1
10430	1.09532	2.1
10431	1.09532	2.1
10432	0.946131	2.1
10433	1.58788	2.1
10434	1.34255	2.1
10435	0.594912	2.1
10436	0.58894	2.1
10437	0.946129	2.1

10438	1.16679	2.1
10439	1.04071	2.1
10440	0.535418	2.1
10441	0.946131	2.1
10442	0.946128	2.1
10443	0.946131	2.1
10444	1.20221	2.1
10445	0.946128	2.1
10446	0.94613	2.1
10447	1.20221	2.1
10448	0.946132	2.1
10449	0.946133	2.1
10450	0.946132	2.1
10451	0.946132	2.1
10452	0.946133	2.1
10453	0.946133	2.1
10454	0.946132	2.1
10455	0.946132	1.87876
10456	0.946132	2.07244
10457	0.946133	2.1
10458	0.946132	1.98941
10459	1.411	2.0826
10460	0.946132	2.04976
10461	0.946133	2.07211
10462	1.05575	0.094009
10463	0.563734	0.0872857
10464	1.05575	0.103175
10465	0.773833	0.0675545
10466	0.751686	0.0735757
10467	0.75884	0.0859833
10468	0.815612	0.0762894
10469	0.793465	0.0866514
10470	0.768563	0.123269
10471	0.946129	0.0952316
10472	0.946125	0.0914828
10473	0.946126	0.111136
10474	0.94613	0.0903874
10475	0.946124	0.114854
10476	1.31678	0.0868658
10477	0.946128	0.0885486
10478	0.946132	0.373418
10479	1.29914	0.261066
10480	0.946132	0.258198
10481	0.946132	0.278567
10482	0.946132	0.397465
10483	1.29914	0.198485
10484	0.946132	0.297428
10485	0.810946	0.375748
10486	0.810943	0.366526
10487	2.21082	0.211179
10488	1.35168	0.238513
10489	1.08132	0.278932
10490	1.08132	0.28359
10491	1.0813	0.372691
10492	0.81096	0.333131
10493	0.625813	0.05
10494	0.946132	0.05
10495	0.946131	0.05

10496	0.946132	0.05
10497	0.946131	0.05
10498	1.39301	0.05
10499	0.625812	0.05
10500	0.810946	2.03884
10501	0.810945	2.1
10502	0.810931	2.1
10503	2.70043	2.1
10504	1.3517	2.1
10505	1.08131	2.09513
10506	1.08131	2.1
10507	1.08133	2.1
10508	0.810943	2.1
10509	0.810944	2.1
10510	0.810958	2.1
10511	2.44802	2.1
10512	1.35168	2.1
10513	1.08132	2.1
10514	1.08132	2.1
10515	1.0813	2.1
10516	0.946131	0.133392
10517	0.946129	0.117523
10518	0.946128	0.104423
10519	0.946131	0.221389
10520	1.3492	0.148318
10521	1.3492	0.144742
10522	0.94613	0.104917
10523	0.946132	0.274443
10524	0.946132	0.162082
10525	0.946133	0.264904
10526	0.946133	0.137431
10527	0.946132	0.27194
10528	0.946132	0.135725
10529	0.946132	0.272473
10530	0.946129	0.245918
10531	0.946133	0.172506
10532	0.946131	0.254843
10533	0.94613	0.159908
10534	0.946131	0.283246
10535	0.946131	0.221473
10536	0.946131	0.208582
10537	0.946119	0.135072
10538	0.946119	0.0897732
10539	0.946119	0.165704
10540	0.946125	0.0759461
10541	0.94612	0.147977
10542	0.946132	0.111089
10543	0.946107	0.10935
10544	0.946133	2.1
10545	0.946132	2.1
10546	0.946132	2.1
10547	0.946132	2.1
10548	0.946132	2.1
10549	0.946133	2.1
10550	1.3868	2.1
10551	0.810947	2.1
10552	0.810946	2.1
10553	0.81096	2.1

10554	2.66094	2.1
10555	1.35168	2.1
10556	1.08132	2.1
10557	1.08132	2.1
10558	1.0813	2.1
10559	0.810946	2.1
10560	0.810946	2.1
10561	0.810933	2.1
10562	2.75737	2.1
10563	1.3517	2.1
10564	1.08132	2.1
10565	1.08132	2.1
10566	1.08133	2.1
10567	0.946133	2.1
10568	0.946132	2.1
10569	0.946132	2.1
10570	0.946132	2.1
10571	0.946133	2.1
10572	0.946132	2.1
10573	0.946133	2.1
10574	0.946133	2.1
10575	0.946133	2.1
10576	0.946132	2.1
10577	0.946132	2.1
10578	0.946133	2.1
10579	0.946132	2.1
10580	1.28569	2.1
10581	0.946132	2.08963
10582	0.946133	2.1
10583	0.946133	2.1
10584	0.946132	2.1
10585	0.946132	2.1
10586	0.946132	2.1
10587	0.946133	2.1
10588	0.946132	0.05
10589	0.946131	0.05
10590	0.94613	0.05
10591	0.946133	0.05
10592	0.946128	0.05
10593	0.946131	0.05
10594	0.946131	0.05
10595	0.946131	2.1
10596	0.94613	2.1
10597	0.946131	2.1
10598	0.94613	2.1
10599	1.41833	1.97497
10600	0.946131	2.1
10601	0.946131	2.1
10602	1.26155	0.05
10603	0.946131	0.05
10604	0.946131	0.05
10605	0.946132	0.05
10606	0.946132	0.05
10607	0.946131	0.05
10608	0.946132	0.05
10609	0.810946	2.1
10610	0.810946	2.1
10611	0.810959	2.1

10612	2.47938	2.1
10613	1.35168	2.1
10614	1.08132	2.1
10615	1.08132	2.1
10616	1.08131	2.1
10617	0.946131	0.284624
10618	0.94613	0.246951
10619	0.94613	0.589151
10620	0.946132	0.212303
10621	0.946128	0.96044
10622	0.94613	0.479398
10623	0.94613	0.542137
10624	0.94613	0.057809
10625	0.946131	0.0584651
10626	0.94613	0.0753255
10627	0.946129	0.0671013
10628	0.946132	0.0612652
10629	0.94613	0.0674224
10630	0.946131	0.058092
10631	0.946132	2.1
10632	0.946131	2.1
10633	0.946128	2.1
10634	1.40756	2.1
10635	0.946131	2.1
10636	0.946129	2.1
10637	0.94613	2.1
10638	0.946131	2.1
10639	0.946131	1.94439
10640	0.946131	2.1
10641	0.946132	2.1
10642	1.31947	2.08749
10643	0.946131	2.1
10644	0.946131	2.1
10645	0.946131	2.1
10646	1.25768	2.1
10647	0.946132	2.1
10648	0.946132	2.1
10649	0.946129	2.1
10650	0.946131	2.1
10651	0.94613	2.1
10652	0.946132	0.108317
10653	0.946129	0.0944053
10654	0.946129	0.173172
10655	0.94613	0.119943
10656	1.31716	0.121751
10657	1.31716	0.112443
10658	0.946131	0.21897
10659	0.810943	1.62854
10660	0.810942	2.1
10661	0.81093	1.9983
10662	2.37052	2.1
10663	1.3517	2.07367
10664	1.08132	1.70704
10665	1.08132	2.1
10666	1.08133	1.88189
10667	0.946128	1.09011
10668	1.10335	0.485489
10669	0.94613	0.322353

10670	0.946131	1.12201
10671	0.946131	0.584693
10672	1.10335	1.23409
10673	0.94613	1.26989
10674	0.946132	1.37862
10675	1.18818	0.990005
10676	0.946132	0.731556
10677	0.946132	1.44076
10678	0.946132	0.945694
10679	1.18818	1.46955
10680	0.946132	1.47115
10681	0.946125	2.1
10682	0.94612	2.1
10683	0.946119	2.1
10684	0.946119	2.1
10685	0.946119	2.1
10686	0.946132	2.1
10687	1.31522	2.1
10688	1.28803	2.1
10689	0.946132	1.98128
10690	0.946132	2.1
10691	0.946133	2.1
10692	0.946133	1.89352
10693	0.946133	2.1
10694	0.946132	2.1
10695	0.946133	1.12301
10696	0.946132	1.21899
10697	0.946132	1.28358
10698	0.946132	1.04985
10699	0.946133	0.999175
10700	0.946132	1.02265
10701	1.37658	1.12546
10702	1.02239	1.55577
10703	0.946128	1.45606
10704	0.946132	1.44705
10705	1.02239	1.42604
10706	0.94613	1.32479
10707	0.96283	1.47169
10708	0.92943	1.42121
10709	0.946132	0.05
10710	0.946131	0.05
10711	0.946126	0.05
10712	0.946129	0.05
10713	0.946128	0.05
10714	0.946128	0.05
10715	0.946129	0.05
10716	0.810946	0.05
10717	0.810944	0.05
10718	2.22493	0.05
10719	1.21651	0.05
10720	1.0813	0.05
10721	0.946154	0.05
10722	0.979951	0.05
10723	0.70959	0.05
10724	0.946132	0.0690307
10725	0.946131	0.0855156
10726	0.946131	0.0674238
10727	0.946133	0.0681977

10728	0.946131	0.0837472
10729	0.946132	0.0686128
10730	0.946132	0.0804626
10731	0.946132	1.7136
10732	0.946131	1.50241
10733	0.946132	1.78144
10734	0.946132	1.43694
10735	0.946132	1.43534
10736	0.946132	1.47462
10737	1.40924	1.58638
10738	0.946131	2.1
10739	0.946128	2.1
10740	0.946127	2.1
10741	0.94613	2.1
10742	1.41743	2.1
10743	0.946128	2.1
10744	0.946129	2.1
10745	1.37675	0.964077
10746	0.946128	1.16085
10747	0.94613	0.942142
10748	0.94613	1.18368
10749	0.94613	0.956227
10750	0.946129	0.92366
10751	0.94613	1.13599
10752	0.946132	0.182815
10753	0.946132	0.251615
10754	0.946132	0.389616
10755	1.35721	0.167416
10756	0.946132	0.786509
10757	0.946132	0.38573
10758	1.35721	0.316036
10759	0.946133	1.16201
10760	0.946133	1.1404
10761	0.946132	1.13181
10762	1.10003	0.719454
10763	0.946133	1.07007
10764	0.946132	1.12271
10765	0.946132	1.13026
10766	0.946132	0.0858846
10767	0.946131	0.0678005
10768	0.946131	0.0725722
10769	0.946131	0.094396
10770	1.0616	0.0675248
10771	1.0616	0.0714777
10772	0.946132	0.0845575
10773	0.946132	0.108905
10774	0.946131	0.0794538
10775	0.946131	0.0894805
10776	0.946131	0.120796
10777	1.09402	0.0789521
10778	1.09402	0.0872703
10779	0.946131	0.113231
10780	0.946132	0.0599606
10781	0.946126	0.0528409
10782	1.24093	0.0585566
10783	0.946129	0.0624769
10784	0.946129	0.0588879
10785	0.946128	0.0648278

10786	0.946129	0.0617079
10787	0.946131	0.0811761
10788	0.946131	0.0752836
10789	1.2929	0.0784649
10790	0.946131	0.102615
10791	0.946131	0.0895981
10792	0.946131	0.0962232
10793	0.946131	0.0895218
10794	0.810946	0.05
10795	0.810945	0.05
10796	0.810959	0.05
10797	2.41233	0.05
10798	1.35168	0.05
10799	1.08132	0.05
10800	1.08132	0.05
10801	1.0813	0.05
10802	0.810946	1.94309
10803	0.810943	2.1
10804	0.750103	2.1
10805	2.22778	2.1
10806	1.27059	2.1
10807	1.00021	2.03442
10808	1.02049	2.1
10809	1.08133	2.08731
10810	0.946128	2.1
10811	0.946132	2.1
10812	0.946132	2.1
10813	0.94613	2.1
10814	0.94613	2.1
10815	0.94613	2.1
10816	0.946131	2.1
10817	0.946126	0.346196
10818	0.946131	0.289739
10819	0.946127	0.426121
10820	1.3324	0.249625
10821	0.946127	0.270084
10822	0.946128	0.348465
10823	0.946128	0.493827
10824	0.94613	2.1
10825	0.946131	2.1
10826	0.94613	2.1
10827	0.946128	2.1
10828	0.946133	2.1
10829	0.946131	2.1
10830	0.946131	2.1
10831	0.946133	2.1
10832	0.946132	2.1
10833	0.946133	2.1
10834	0.946133	2.1
10835	0.946132	2.1
10836	0.946132	2.1
10837	1.36541	2.1
10838	0.946131	0.872707
10839	0.94613	2.1
10840	0.946131	1.72725
10841	0.94613	1.49244
10842	1.39509	1.80686
10843	0.946131	0.448079

10844	0.946131	1.84638
10845	0.946133	2.1
10846	0.946133	2.1
10847	0.946132	2.1
10848	0.946132	2.1
10849	0.946133	2.1
10850	0.946132	2.1
10851	1.29319	2.1
10852	1.01196	0.084142
10853	0.946131	0.145623
10854	0.946132	0.0867132
10855	0.946132	0.104289
10856	0.946132	0.145207
10857	0.946132	0.127625
10858	0.946132	0.113946
10859	0.946112	2.1
10860	0.946118	2.1
10861	0.946116	2.1
10862	0.946117	2.1
10863	0.946118	2.1
10864	0.94613	2.1
10865	0.946105	2.1
10866	1.28684	1.4909
10867	0.946131	1.55439
10868	0.946132	1.50608
10869	0.946132	1.59326
10870	0.946132	1.71068
10871	0.946132	1.55175
10872	0.946132	1.61398
10873	0.94613	2.05246
10874	0.94613	2.1
10875	0.946128	1.95883
10876	0.946131	1.85633
10877	0.946131	1.92824
10878	0.94613	1.93503
10879	1.10185	2.05768
10880	0.810947	0.05
10881	0.810945	0.05
10882	1.57348	0.05
10883	1.3517	0.05
10884	1.08131	0.05
10885	1.08132	0.05
10886	1.08133	0.05
10887	0.810932	0.05
10888	0.946119	0.196104
10889	0.946116	0.168858
10890	0.946117	0.216678
10891	0.946121	0.140419
10892	0.946118	0.160175
10893	0.94613	0.177095
10894	0.946105	0.18499
10895	0.94612	0.594604
10896	0.946122	0.251713
10897	0.946119	0.432998
10898	0.946119	0.640999
10899	1.20884	0.296336
10900	0.946132	0.503817
10901	0.946107	0.42731

10902	2.67849	1.73752
10903	1.17709	1.69975
10904	0.605418	1.7648
10905	0.286984	1.68942
10906	0.172654	1.69602
10907	0.810946	1.56146
10908	0.940878	1.50266
10909	0.941235	1.19857
10910	1.26696	0.9651
10911	1.56837	1.17226
10912	1.13737	1.2071
10913	1.34115	1.26077
10914	1.24889	1.0833
10915	0.946132	1.72558
10916	0.946129	1.40209
10917	0.946128	1.44402
10918	0.94613	1.49176
10919	0.94613	1.44653
10920	0.946131	1.53044
10921	0.94613	1.40891
10922	0.946116	2.1
10923	0.94612	2.1
10924	0.946118	2.1
10925	0.946123	2.1
10926	0.946118	2.1
10927	0.946131	2.1
10928	1.23836	2.1
10929	0.810946	0.05
10930	0.810946	0.05
10931	0.810959	0.05
10932	2.80677	0.05
10933	1.35168	0.05
10934	1.08132	0.05
10935	1.08132	0.05
10936	1.08131	0.05
10937	0.810947	0.14447
10938	0.810947	1.34646
10939	2.41574	1.13028
10940	1.21653	1.06562
10941	1.08133	0.305534
10942	0.946153	1.28595
10943	0.979949	0.522475
10944	0.709567	0.206816
10945	0.94613	2.1
10946	0.946131	2.1
10947	0.946128	2.1
10948	1.34782	2.1
10949	0.946128	2.1
10950	0.946129	2.1
10951	1.34782	2.1
10952	0.946132	2.04365
10953	0.946132	2.09968
10954	0.94613	2.1
10955	1.16023	2.1
10956	0.946131	2.1
10957	0.946131	2.1
10958	1.16023	2.1
10959	0.946131	1.49539

10960	0.946127	2.1
10961	0.946127	2.1
10962	0.946131	1.76438
10963	0.946128	2.1
10964	0.94613	1.83328
10965	0.946129	1.91533
10966	0.946133	0.169308
10967	0.946132	0.127563
10968	0.946132	0.140903
10969	0.946133	0.187605
10970	1.20045	0.127356
10971	1.20045	0.136536
10972	0.946132	0.176599
10973	0.918871	0.0594835
10974	0.971961	0.0624051
10975	1.37324	0.05
10976	1.78255	0.05
10977	1.25155	0.0608476
10978	1.42172	0.05
10979	1.40554	0.05
10980	1.08862	0.05
10981	1.03803	0.0535735
10982	0.946132	0.0552536
10983	0.946126	0.0591871
10984	0.943647	0.0626456
10985	0.948611	0.0544565
10986	0.946129	0.0652158
10987	0.946129	0.0593509
10988	1.1699	0.777586
10989	0.946132	0.359704
10990	0.946133	0.966333
10991	0.946133	0.459841
10992	0.946132	0.851073
10993	0.946133	0.716366
10994	0.946132	1.31008
10995	0.946132	2.1
10996	0.946131	2.1
10997	0.946131	2.1
10998	0.946132	2.1
10999	0.946131	2.1
11000	0.946131	2.1
11001	0.946132	2.1
11002	0.946132	1.11805
11003	1.38623	0.919021
11004	0.946132	0.514792
11005	0.946132	0.5676
11006	0.946133	0.889744
11007	0.946132	0.525808
11008	0.946133	1.25263
11009	0.946132	2.1
11010	1.40098	2.1
11011	0.946131	2.1
11012	0.946131	2.1
11013	0.946132	2.1
11014	0.946132	2.1
11015	0.946132	2.1
11016	1.01777	0.479952
11017	0.946132	0.280334

11018	0.946133	1.51283
11019	1.68068	0.324451
11020	1.68068	1.40883
11021	1.41894	0.466585
11022	1.20787	0.0758352
11023	0.946132	0.249409
11024	0.946133	2.1
11025	0.946133	2.1
11026	0.946132	2.1
11027	1.40199	2.1
11028	0.946133	2.1
11029	0.946132	2.1
11030	0.946132	2.1
11031	0.810946	1.59282
11032	0.810947	1.44536
11033	2.37827	1.73209
11034	1.3517	1.63773
11035	1.08132	1.63211
11036	1.08132	1.71464
11037	1.08133	1.59237
11038	0.810934	1.63029
11039	0.946124	1.83446
11040	0.94613	1.50489
11041	1.40248	1.52927
11042	0.946131	1.48477
11043	0.946125	1.33695
11044	0.946127	1.52883
11045	0.946128	1.46179
11046	1.25703	1.79707
11047	0.87821	1.54235
11048	1.18912	1.82181
11049	0.946119	1.49539
11050	0.87825	1.57899
11051	1.1891	1.67401
11052	0.946107	1.72682
11053	0.946132	1.90286
11054	1.28543	1.20787
11055	0.946132	1.42783
11056	0.946132	1.52412
11057	0.946131	1.39647
11058	0.946132	0.388774
11059	0.946132	0.748653
11060	0.946115	2.1
11061	0.946119	2.1
11062	0.946116	2.1
11063	0.946123	2.1
11064	0.946118	2.1
11065	1.41636	2.1
11066	0.946129	2.1
11067	0.946132	2.1
11068	0.946132	2.1
11069	0.946132	2.1
11070	0.946131	2.1
11071	0.946131	2.1
11072	1.41432	2.1
11073	0.946132	2.1
11074	0.810947	0.0667826
11075	0.810947	0.0740991

11076	2.44433	0.0534844
11077	1.21654	0.0511878
11078	0.946154	0.0704806
11079	0.97995	0.0617324
11080	1.08133	0.0533854
11081	0.810934	0.0581095
11082	0.851542	0.05
11083	0.835698	0.0513154
11084	1.22216	0.05
11085	0.946133	0.05
11086	1.01217	0.05
11087	1.09996	0.0511818
11088	0.851529	0.0531532
11089	0.946132	1.88216
11090	0.946132	2.03064
11091	0.946129	2.1
11092	0.94613	1.94795
11093	0.946131	2.0682
11094	0.946131	2.04192
11095	1.2254	2.1
11096	0.800897	1.82621
11097	0.838029	2.1
11098	0.838013	1.87333
11099	0.93679	1.71801
11100	1.20217	1.73988
11101	1.16923	1.79424
11102	0.94948	1.83663
11103	0.958118	1.94129
11104	0.36751	1.75822
11105	0.800535	1.8413
11106	0.272087	1.13979
11107	0.428084	1.36279
11108	0.986335	1.83671
11109	0.946132	0.273118
11110	0.946133	0.314988
11111	1.19601	0.279845
11112	0.946132	0.299788
11113	0.946132	0.324856
11114	0.946133	0.365317
11115	0.946133	0.272774
11116	0.946132	1.94211
11117	1.36956	2.1
11118	0.946132	2.1
11119	0.946132	1.96956
11120	0.946132	2.1
11121	0.946133	2.09333
11122	0.946132	2.1
11123	0.946132	0.829693
11124	0.946131	2.1
11125	0.946131	1.17303
11126	1.34268	0.189793
11127	0.946133	1.99737
11128	0.946132	0.834297
11129	0.946131	1.02856
11130	0.946132	2.1
11131	0.946131	2.1
11132	0.946131	2.1
11133	0.946132	2.1

11134	0.946132	2.1
11135	0.946132	2.1
11136	0.946132	2.1
11137	0.810946	1.44295
11138	0.810943	1.37878
11139	2.51472	1.47097
11140	1.3517	1.45412
11141	1.08132	1.31746
11142	1.08132	1.45135
11143	1.08133	1.44036
11144	0.810933	1.4583
11145	0.902316	0.659863
11146	1.04517	0.606638
11147	0.974924	0.420554
11148	0.530063	1.03067
11149	1.1272	0.708953
11150	0.68207	0.763265
11151	0.477061	0.941195
11152	0.810946	0.0869689
11153	0.810943	0.134523
11154	0.810959	0.110451
11155	1.99211	0.0993658
11156	1.35168	0.0917658
11157	1.08132	0.0957234
11158	1.08132	0.0962625
11159	1.08131	0.127357
11160	0.94613	0.144872
11161	0.946131	0.132443
11162	0.946132	0.128792
11163	0.946132	0.137311
11164	0.946129	0.198693
11165	0.946131	0.143278
11166	1.16699	0.144849
11167	0.810943	1.82572
11168	0.810942	1.74108
11169	1.6943	1.94843
11170	1.27059	1.96444
11171	1.00021	1.93747
11172	1.02049	1.88671
11173	1.08133	1.83639
11174	0.750103	1.80894
11175	0.957813	1.89946
11176	0.946128	1.95063
11177	0.946132	1.76102
11178	1.04832	1.99967
11179	0.870714	1.72597
11180	0.94613	1.82428
11181	0.94613	1.86991
11182	1.86374	2.1
11183	2.0815	1.77859
11184	1.15918	0.336604
11185	0.946129	0.256681
11186	1.15918	0.254215
11187	0.946131	0.280073
11188	0.946131	0.283542
11189	0.946131	0.271338
11190	0.94613	0.293404
11191	0.946119	0.528164

11192	1.3612	1.14993
11193	0.946125	0.851357
11194	0.946119	0.608564
11195	0.94612	1.28167
11196	0.946132	0.857912
11197	0.946107	0.969091
11198	0.810947	0.13905
11199	0.810947	0.269491
11200	2.50398	0.125157
11201	1.3517	0.133495
11202	1.08132	0.115742
11203	1.08133	0.142302
11204	1.08132	0.143158
11205	0.810934	0.129425
11206	0.946132	2.1
11207	0.946132	2.1
11208	0.946132	2.1
11209	1.39754	2.1
11210	0.946132	2.1
11211	0.946132	2.1
11212	0.946131	2.1
11213	0.946129	2.1
11214	1.41732	2.1
11215	0.946129	2.1
11216	0.946131	2.1
11217	0.946131	2.1
11218	0.946131	2.1
11219	0.946131	2.1
11220	0.946132	1.61358
11221	1.25276	1.80916
11222	0.946131	1.86325
11223	0.94613	1.64927
11224	0.94613	1.80844
11225	0.94613	1.68722
11226	0.946131	1.72283
11227	1.21261	1.2577
11228	0.94613	1.50256
11229	0.94613	1.69704
11230	0.946128	0.991892
11231	0.946131	1.55615
11232	0.94613	1.5965
11233	0.946129	1.6002
11234	0.946128	2.1
11235	0.946133	2.1
11236	0.946132	2.1
11237	0.946131	2.1
11238	0.94613	2.1
11239	0.946131	2.1
11240	1.39407	2.1
11241	0.946116	1.74155
11242	0.946119	1.8302
11243	0.946119	1.59447
11244	1.15369	1.75023
11245	0.946119	1.54105
11246	0.946131	1.68747
11247	0.946106	1.71694
11248	0.946131	2.1
11249	0.946131	2.1

11250	0.946131	2.1
11251	0.946133	2.1
11252	0.946129	2.1
11253	0.946131	2.1
11254	0.946131	2.1
11255	1.19564	0.05
11256	0.498881	0.05
11257	1.33964	0.05
11258	0.498878	0.05
11259	0.923583	0.05
11260	0.831259	0.05
11261	0.553735	0.05
11262	0.946132	0.05
11263	0.946132	0.05
11264	0.946131	0.05
11265	0.946131	0.05
11266	0.946132	0.05
11267	0.946131	0.05
11268	1.28673	0.05
11269	0.946132	0.0834847
11270	0.946132	0.0821156
11271	1.33178	0.079042
11272	0.946132	0.0877992
11273	0.946132	0.0861279
11274	0.946131	0.0951545
11275	0.946131	0.0817188
11276	0.946128	0.177937
11277	0.946132	0.131752
11278	0.946129	0.219547
11279	0.946131	0.086022
11280	0.946131	0.103243
11281	0.946129	0.123356
11282	0.94613	0.112267
11283	0.946131	2.1
11284	0.946131	2.1
11285	0.946129	2.1
11286	1.40525	2.1
11287	0.94613	2.1
11288	0.94613	2.1
11289	1.40525	2.1
11290	0.946131	2.1
11291	0.946126	2.1
11292	0.946127	2.1
11293	0.946127	2.1
11294	1.4073	2.1
11295	0.946129	2.1
11296	0.946129	2.1
11297	0.946131	0.0832885
11298	0.946131	0.0698135
11299	0.946132	0.0868001
11300	0.946131	0.0656721
11301	0.946131	0.0672729
11302	1.05016	0.0700685
11303	0.946132	0.081951
11304	0.946128	0.0928542
11305	0.946132	0.0768234
11306	0.946131	0.097331
11307	0.946129	0.0707342

11308	0.94613	0.0728063
11309	1.13398	0.0760124
11310	0.94613	0.0988514
11311	0.946132	0.05
11312	0.946132	0.05
11313	0.946131	0.053371
11314	0.946131	0.0501588
11315	0.946132	0.05
11316	0.946131	0.05
11317	0.946132	0.05
11318	0.946131	0.0645551
11319	0.94613	0.05
11320	0.946131	0.0556733
11321	0.94613	0.0743914
11322	1.38645	0.0521717
11323	1.38645	0.0571252
11324	0.946131	0.0595529
11325	0.946131	0.288973
11326	0.946131	0.172739
11327	0.94613	0.314303
11328	1.23923	0.142423
11329	0.94613	0.121974
11330	0.946131	0.293833
11331	0.946131	0.402681
11332	0.946132	0.05
11333	0.946131	0.05
11334	0.946131	0.05
11335	0.946132	0.05
11336	0.946132	0.05
11337	0.946132	0.05
11338	1.40511	0.05
11339	0.946132	2.1
11340	0.946132	2.1
11341	0.946132	2.1
11342	0.946132	2.1
11343	0.946132	2.1
11344	0.946131	2.1
11345	1.12968	2.1
11346	0.946132	1.69042
11347	0.946128	2.1
11348	0.946128	2.1
11349	0.94613	1.87745
11350	0.94613	2.1
11351	0.94613	1.90008
11352	0.94613	1.90562
11353	0.946133	2.1
11354	0.946133	2.1
11355	0.946132	2.1
11356	0.946132	2.1
11357	0.946132	2.1
11358	0.946133	2.1
11359	1.31323	2.1
11360	0.94613	2.1
11361	0.946131	2.1
11362	0.946131	2.1
11363	0.94613	2.00464
11364	0.946131	2.1
11365	0.946131	2.1

11366	0.946131	2.1
11367	0.946132	2.1
11368	0.946132	0.774977
11369	0.946132	2.1
11370	1.36899	1.97519
11371	0.946132	0.213627
11372	0.946132	1.76293
11373	0.946133	1.20311
11374	1.03543	0.0503646
11375	0.946131	0.0505304
11376	0.946131	0.05
11377	0.946132	0.05
11378	0.94613	0.05
11379	0.950987	0.05
11380	0.941275	0.0512563
11381	0.946131	0.102771
11382	0.94613	0.112481
11383	1.28952	0.121176
11384	0.946132	0.0942093
11385	0.94613	0.155101
11386	0.946131	0.139865
11387	0.946131	0.108026
11388	0.94613	2.1
11389	1.35966	2.1
11390	0.946131	2.1
11391	0.946131	2.1
11392	0.946129	2.1
11393	0.94613	2.1
11394	0.94613	2.1
11395	0.810946	2.1
11396	0.810942	2.02035
11397	0.810932	2.1
11398	2.6655	2.1
11399	1.3517	2.1
11400	1.08132	2.1
11401	1.08131	2.04221
11402	1.08133	2.1
11403	0.94613	2.1
11404	0.946131	2.1
11405	0.94613	2.1
11406	0.946128	2.1
11407	0.946131	2.1
11408	0.94613	2.1
11409	0.94613	2.1
11410	0.946128	2.1
11411	0.946128	2.1
11412	0.946133	2.1
11413	1.25375	2.1
11414	0.94613	2.1
11415	0.946131	2.1
11416	1.25375	2.1
11417	0.94613	2.1
11418	0.94613	2.1
11419	0.946132	2.1
11420	0.946132	2.1
11421	0.946128	2.1
11422	0.94613	2.1
11423	1.31453	2.1

11424	0.946131	0.0765029
11425	0.946129	0.060209
11426	0.94613	0.0760005
11427	0.94613	0.0554728
11428	0.94613	0.0600065
11429	1.12018	0.0580439
11430	0.94613	0.0756479
11431	0.946131	0.12026
11432	0.946129	0.0767415
11433	0.946131	0.119343
11434	1.16373	0.0709647
11435	0.94613	0.0674526
11436	0.94613	0.115379
11437	0.946131	0.142744
11438	1.36811	0.387167
11439	0.946132	0.605725
11440	1.36811	0.320287
11441	0.946129	0.518918
11442	0.94613	0.4286
11443	0.946129	0.539282
11444	0.946129	0.485453
11445	0.946132	0.109082
11446	0.946133	0.0690715
11447	0.946132	0.0857191
11448	0.946133	0.109496
11449	1.35732	0.0767507
11450	1.35732	0.0853822
11451	0.946133	0.0937568
11452	1.36399	0.709979
11453	0.946129	1.46223
11454	0.94613	1.0719
11455	0.946131	1.44487
11456	0.94613	1.63395
11457	0.946131	1.38142
11458	0.946131	1.4796
11459	0.946133	2.1
11460	0.946132	2.1
11461	0.946132	2.1
11462	1.38302	2.1
11463	0.946133	2.1
11464	0.946132	2.1
11465	0.946133	2.1
11466	0.810943	2.1
11467	0.810944	2.1
11468	0.810945	2.1
11469	2.80542	2.1
11470	1.35169	2.1
11471	1.08132	2.1
11472	1.08132	2.1
11473	1.08132	2.1
11474	1.30189	2.1
11475	0.946131	2.1
11476	0.946132	2.1
11477	0.946131	2.1
11478	0.946131	2.1
11479	0.946131	2.1
11480	0.946132	2.1
11481	0.810946	1.21925

11482	0.810947	1.16952
11483	2.33293	1.15163
11484	1.24357	1.17283
11485	1.08132	1.17601
11486	0.973183	1.20944
11487	1.00023	1.20555
11488	0.810934	1.19431
11489	1.06584	1.15452
11490	0.946129	1.16706
11491	0.84731	0.697038
11492	1.16646	1.18987
11493	0.946126	1.20237
11494	1.2831	1.05814
11495	0.851527	1.16208
11496	0.946131	2.1
11497	0.946128	2.1
11498	0.946128	2.1
11499	0.94613	2.1
11500	1.31961	2.1
11501	0.94613	2.1
11502	0.94613	2.1
11503	0.946131	2.1
11504	1.3605	2.1
11505	0.946133	2.1
11506	0.946132	2.1
11507	0.94613	2.1
11508	0.946131	2.1
11509	0.946131	2.1
11510	0.946128	2.1
11511	0.946133	1.86222
11512	0.946132	2.1
11513	0.946131	1.86699
11514	0.946131	1.22951
11515	0.946131	2.05401
11516	1.30116	2.1
11517	0.946132	0.05
11518	0.946129	0.05
11519	0.94613	0.05
11520	1.3035	0.05
11521	0.946131	0.05
11522	0.94613	0.05
11523	0.946131	0.05
11524	0.946132	2.1
11525	1.2051	2.1
11526	1.2051	2.1
11527	0.946132	2.1
11528	1.37869	2.1
11529	0.946132	2.1
11530	0.946131	2.1
11531	0.810946	2.1
11532	0.810945	2.1
11533	2.69247	2.1
11534	1.35169	2.1
11535	1.08132	2.1
11536	1.08132	2.1
11537	1.08132	2.1
11538	0.810946	2.1
11539	1.22993	2.1

11540	0.946131	2.1
11541	0.946131	2.1
11542	1.34623	2.1
11543	0.946131	2.1
11544	1.34623	2.1
11545	0.946131	2.1
11546	0.946131	0.0522787
11547	0.946132	0.067822
11548	0.946132	0.0598247
11549	0.946132	0.0502862
11550	0.946131	0.0530711
11551	0.946132	0.0673959
11552	1.32299	0.05
11553	0.94613	0.134875
11554	0.946131	0.144059
11555	0.946128	0.1355
11556	0.946132	0.121023
11557	0.946133	0.129023
11558	0.946131	0.143411
11559	0.946131	0.128912
11560	0.946132	2.1
11561	0.946129	2.1
11562	0.946128	2.1
11563	0.94613	2.1
11564	0.94613	2.1
11565	0.946131	2.1
11566	0.94613	2.1
11567	0.94613	2.1
11568	0.94613	2.1
11569	0.946131	2.1
11570	0.946132	2.1
11571	0.946129	2.1
11572	0.946131	2.1
11573	0.946131	2.1
11574	0.946132	1.66852
11575	0.946131	1.67623
11576	1.13731	1.62684
11577	0.94775	1.71247
11578	0.944514	1.78741
11579	0.946132	1.87972
11580	1.039	1.84294
11581	0.946131	1.62486
11582	0.946128	1.60243
11583	0.94613	1.52573
11584	0.946131	1.63527
11585	0.946128	1.69799
11586	0.946129	1.78713
11587	1.23726	1.75008
11588	0.946132	2.1
11589	0.946132	2.1
11590	0.946129	2.1
11591	1.39214	2.1
11592	0.946131	2.1
11593	0.946131	2.1
11594	0.94613	2.1
11595	0.946125	1.332
11596	0.946119	0.449559
11597	0.946118	0.569106

11598	0.946118	0.35575
11599	0.94612	1.18152
11600	0.946106	0.567095
11601	0.946131	0.650874
11602	1.26893	0.273651
11603	0.946131	0.262506
11604	1.26893	0.268998
11605	0.94613	0.3254
11606	0.946131	0.248841
11607	0.946131	0.2705
11608	0.946131	0.363144
11609	0.946133	0.0548694
11610	0.946133	0.0537947
11611	0.946132	0.0659412
11612	1.28924	0.0538089
11613	0.946133	0.0625724
11614	0.946132	0.0634177
11615	0.946132	0.0572529
11616	0.946133	0.534114
11617	0.946132	0.546487
11618	0.946132	0.588487
11619	0.946133	0.422346
11620	0.946133	0.479781
11621	0.946132	0.573426
11622	0.946133	0.48832
11623	0.946132	1.58063
11624	0.946133	0.734625
11625	0.946133	1.53756
11626	0.946132	1.58985
11627	1.15388	1.41982
11628	0.946132	1.54596
11629	0.946133	1.47096
11630	0.946132	0.452748
11631	0.946133	0.42644
11632	0.946132	0.512444
11633	0.946133	0.334159
11634	0.946133	0.388448
11635	0.946132	0.478209
11636	0.946132	0.408405
11637	1.0644	1.52824
11638	1.12486	1.84342
11639	1.08674	1.86516
11640	0.946132	1.70639
11641	0.946132	1.84158
11642	0.946133	1.7572
11643	0.946132	1.76925
11644	0.808978	1.42392
11645	0.911537	1.547
11646	0.932832	1.61288
11647	1.14604	1.69011
11648	1.56953	1.61201
11649	1.25866	1.57413
11650	1.18488	1.6585
11651	1.341	1.47219
11652	0.946131	0.104876
11653	0.946131	0.0666426
11654	0.946131	0.084931
11655	0.94613	0.108288

11656	1.33922	0.075348
11657	0.946131	0.0886086
11658	0.946131	0.0801988
11659	0.946132	0.0680915
11660	0.946133	0.0624243
11661	0.946132	0.0693443
11662	0.946132	0.0646219
11663	0.946132	0.0705884
11664	0.946133	0.0680411
11665	0.946132	0.0611421
11666	0.946129	1.27086
11667	0.946133	2.1
11668	0.946133	1.7178
11669	0.946131	1.6477
11670	1.40496	2.1
11671	0.946131	1.61543
11672	0.946131	2.1
11673	0.94613	1.74948
11674	0.946127	1.36468
11675	0.946127	1.65066
11676	0.94613	1.7508
11677	1.38913	1.50914
11678	0.946128	1.67875
11679	0.946128	1.56462
11680	1.98863	0.05
11681	2.59915	0.05
11682	1.40338	0.05
11683	0.946131	0.05
11684	1.22266	0.05
11685	1.40338	0.05
11686	0.946132	0.05
11687	0.946132	0.05
11688	1.22266	0.05
11689	0.94613	0.0588192
11690	0.94613	0.0679803
11691	0.946124	0.10067
11692	1.21428	0.0646228
11693	0.946126	0.0984108
11694	0.946127	0.0915386
11695	0.946127	0.0710377
11696	1.03411	0.0712278
11697	0.946131	0.148014
11698	1.03411	0.0752887
11699	0.946129	0.148199
11700	1.14258	0.0707107
11701	0.952143	0.133604
11702	0.940118	0.0966929
11703	0.810943	2.1
11704	0.810945	2.1
11705	0.810958	2.1
11706	2.82603	2.1
11707	1.35168	2.1
11708	1.08132	2.1
11709	1.08132	2.1
11710	1.0813	2.1
11711	0.946133	0.0772542
11712	0.946132	0.0738339
11713	0.946132	0.0791111

11714	0.946132	0.0678487
11715	0.946132	0.078224
11716	1.11539	0.0768952
11717	0.946133	0.078972
11718	0.946133	2.1
11719	0.946132	2.1
11720	0.946133	2.1
11721	0.946133	2.1
11722	0.946132	2.1
11723	1.41195	2.1
11724	0.946133	2.1
11725	0.946131	0.05
11726	0.946131	0.05
11727	0.946131	0.05
11728	0.946131	0.05
11729	0.946131	0.05
11730	0.946131	0.05
11731	1.15968	0.05
11732	0.946131	1.73006
11733	0.94613	1.72019
11734	0.94613	2.03231
11735	1.34386	1.78445
11736	0.946132	1.87341
11737	0.94613	2.00772
11738	1.34386	1.84691
11739	0.946131	1.57862
11740	0.935202	1.41598
11741	0.946131	1.78291
11742	1.25054	1.62317
11743	0.850155	1.71161
11744	0.946131	1.75515
11745	0.935205	1.70713
11746	0.946131	0.352048
11747	1.40193	0.256939
11748	0.946132	0.257069
11749	0.946132	0.267931
11750	0.946129	0.388569
11751	1.40193	0.217137
11752	0.946131	0.296284
11753	0.808779	0.238918
11754	0.561937	0.172304
11755	1.40193	0.172695
11756	1.40193	0.15198
11757	0.561936	0.209721
11758	0.561937	0.144544
11759	1.40193	0.162387
11760	0.946132	0.05
11761	0.946132	0.05
11762	0.946132	0.05
11763	0.946132	0.05
11764	0.946133	0.05
11765	0.946132	0.05
11766	0.946133	0.05
11767	0.946127	0.270942
11768	0.946131	0.241092
11769	0.946131	0.0896103
11770	0.946127	0.105186
11771	1.19583	0.0787251

11772	1.19583	0.100131
11773	0.946129	0.142996
11774	0.589428	0.23435
11775	1.08737	0.153071
11776	1.10961	0.36299
11777	0.872901	0.11925
11778	0.541327	0.120192
11779	0.541312	0.117971
11780	0.85153	0.186942
11781	0.946132	2.1
11782	0.946131	2.1
11783	0.946131	2.1
11784	0.946132	2.1
11785	0.946132	2.1
11786	0.946132	2.1
11787	0.946132	2.1
11788	0.946132	1.84003
11789	1.2778	2.1
11790	0.946131	2.1
11791	0.946131	1.87638
11792	0.94613	2.1
11793	0.94613	1.93038
11794	0.94613	1.97081
11795	0.946118	1.10001
11796	0.946112	0.771235
11797	0.946118	0.613065
11798	0.946119	1.00653
11799	0.946117	0.781829
11800	1.35631	0.615718
11801	0.946105	0.779832
11802	0.946133	2.1
11803	0.946133	2.1
11804	0.946132	2.1
11805	0.946132	2.1
11806	0.946133	1.61549
11807	0.946132	2.1
11808	0.946132	2.1
11809	0.94613	2.1
11810	0.946131	2.1
11811	0.946128	2.1
11812	0.946128	2.07189
11813	0.94613	2.1
11814	0.946129	2.1
11815	0.946129	2.1
11816	0.946132	1.65005
11817	1.15139	1.63613
11818	0.946132	1.62347
11819	0.946132	1.67922
11820	0.946132	1.64322
11821	0.946132	1.70136
11822	0.946132	1.65947
11823	0.946133	2.1
11824	1.36151	2.1
11825	0.946132	2.1
11826	0.946132	2.1
11827	0.946132	2.1
11828	0.946132	2.1
11829	0.946133	2.1

11830	0.946127	2.1
11831	1.38397	2.1
11832	0.94613	2.1
11833	0.946131	2.1
11834	0.946128	2.1
11835	0.94613	2.1
11836	0.946129	2.1
11837	0.946131	0.229558
11838	0.946127	0.253301
11839	0.946127	0.242053
11840	0.946132	0.233218
11841	0.946131	0.251634
11842	0.946128	0.25206
11843	0.946129	0.235688
11844	1.0268	0.05
11845	1.23823	0.05
11846	1.2171	0.05
11847	0.883446	0.05
11848	1.23827	0.05
11849	1.32614	0.05
11850	0.94613	0.05
11851	0.946132	2.1
11852	0.946129	2.1
11853	0.946129	2.1
11854	0.94613	2.1
11855	1.36523	2.1
11856	0.946131	2.1
11857	0.94613	2.1
11858	0.946132	0.106273
11859	0.946131	0.0789548
11860	0.946132	0.112356
11861	0.946131	0.0721453
11862	0.946131	0.0755657
11863	1.01653	0.0751398
11864	0.946131	0.120043
11865	0.94612	2.1
11866	0.946121	2.1
11867	0.946119	2.1
11868	0.946119	2.1
11869	0.946119	2.1
11870	0.946132	2.1
11871	0.946107	2.1
11872	0.810942	2.1
11873	0.810944	1.96425
11874	2.60324	2.1
11875	1.21651	2.1
11876	1.0813	2.1
11877	0.946153	1.96122
11878	0.979948	2.1
11879	0.709588	2.1
11880	1.3492	0.194942
11881	0.946132	0.209482
11882	0.946131	0.169733
11883	0.94613	0.209045
11884	0.946129	0.269406
11885	0.946129	0.211743
11886	0.94613	0.223698
11887	0.946131	0.171993

11888	0.94613	0.191471
11889	0.946128	0.178977
11890	0.946133	0.176202
11891	0.946133	0.191731
11892	0.946131	0.192909
11893	0.946131	0.179342
11894	1.23351	0.05
11895	0.946131	0.05
11896	0.946131	0.05
11897	0.946132	0.05
11898	0.946132	0.05
11899	0.946131	0.05
11900	0.946132	0.05
11901	1.35657	0.05
11902	0.946132	0.05
11903	0.946131	0.05
11904	0.946132	0.05
11905	0.946132	0.05
11906	0.946131	0.05
11907	0.946131	0.05
11908	0.946132	0.204799
11909	0.946132	0.151901
11910	0.946132	0.204696
11911	0.946131	0.115228
11912	0.946131	0.160791
11913	0.946132	0.133004
11914	0.946132	0.140595
11915	0.946133	0.11721
11916	0.946132	0.0724793
11917	0.946133	0.116199
11918	0.946132	0.0546109
11919	0.946132	0.0875368
11920	0.946132	0.0700915
11921	0.946133	0.0728012
11922	0.946131	1.21968
11923	1.4101	1.49392
11924	0.946131	1.99209
11925	0.946131	1.20737
11926	0.946131	1.42091
11927	0.946131	1.2397
11928	0.946131	1.38792
11929	0.810944	1.55738
11930	0.810939	1.5836
11931	0.709586	1.42962
11932	2.71261	1.49421
11933	1.21651	1.54273
11934	0.946151	1.36436
11935	0.979943	1.56333
11936	1.0813	1.62
11937	0.946132	2.1
11938	0.946131	2.1
11939	0.946132	2.1
11940	0.946131	2.1
11941	0.946131	2.1
11942	1.37733	2.1
11943	0.946131	2.1
11944	0.946133	2.1
11945	0.946132	2.1

11946	0.946132	2.1
11947	0.946133	2.1
11948	0.946133	2.1
11949	0.946132	2.1
11950	1.32582	2.1
11951	0.946132	2.1
11952	0.946132	2.1
11953	0.946132	2.1
11954	0.946132	2.1
11955	0.946132	2.1
11956	0.946132	2.1
11957	0.946131	2.1
11958	0.946133	1.68349
11959	0.946132	1.58636
11960	0.946132	1.5258
11961	0.946132	1.55786
11962	0.946132	1.63659
11963	0.946133	1.568
11964	1.3469	1.5813
11965	0.946126	2.1
11966	0.94613	2.1
11967	0.946131	2.1
11968	0.946127	2.1
11969	1.41206	2.1
11970	0.946128	2.1
11971	0.946128	2.1
11972	0.946127	2.1
11973	0.94613	2.1
11974	0.946131	2.00644
11975	0.94613	2.1
11976	0.946128	1.97526
11977	0.946129	2.1
11978	0.946129	2.1
11979	0.946131	0.138506
11980	0.94613	0.116294
11981	1.24448	0.120788
11982	0.946129	0.183049
11983	0.946131	0.148454
11984	0.94613	0.16472
11985	0.946131	0.144858
11986	0.946132	0.206444
11987	0.946133	0.177017
11988	1.11015	0.181232
11989	0.946133	0.296185
11990	0.946132	0.2334
11991	0.946132	0.255043
11992	0.946132	0.22502
11993	1.38775	2.1
11994	0.946131	2.1
11995	0.946131	2.1
11996	0.946132	2.1
11997	0.946132	2.1
11998	0.946131	2.1
11999	0.946131	2.1
12000	0.946129	2.1
12001	0.946129	2.1
12002	0.946133	2.1
12003	0.946131	2.1

12004	0.946131	1.47242
12005	0.946131	2.1
12006	0.946131	2.1
12007	0.946132	2.1
12008	0.946133	2.1
12009	0.946132	2.1
12010	1.40774	2.1
12011	0.946132	2.1
12012	0.946133	2.1
12013	0.946132	2.1
12014	0.610968	0.05
12015	0.688233	0.05
12016	0.715226	0.05
12017	0.792409	0.05
12018	1.13418	0.05
12019	0.956667	0.05
12020	0.757321	0.05
12021	0.968845	0.05
12022	0.595679	0.05
12023	0.946133	1.20038
12024	1.28917	1.32794
12025	0.946131	1.41295
12026	0.94613	1.16874
12027	0.946131	1.29218
12028	0.946131	1.19994
12029	0.946131	1.27636
12030	0.946132	0.189217
12031	0.946128	0.354247
12032	0.94613	0.414846
12033	1.40668	0.303946
12034	0.946131	0.412116
12035	0.946131	0.182589
12036	0.94613	0.361638
12037	0.810946	2.1
12038	0.810945	2.1
12039	2.78163	2.1
12040	1.3517	2.1
12041	1.08132	2.1
12042	1.08133	2.1
12043	1.08132	2.1
12044	0.810933	2.1
12045	0.946131	2.1
12046	1.39081	2.1
12047	0.94613	2.1
12048	1.30339	2.1
12049	1.30339	2.1
12050	1.39081	2.1
12051	0.946131	2.1
12052	0.946127	0.987027
12053	0.821631	0.440776
12054	0.946131	0.363561
12055	0.946131	0.746959
12056	0.946128	0.443984
12057	0.996109	0.605511
12058	0.827876	0.870336
12059	0.946131	2.1
12060	0.946129	2.1
12061	0.946129	2.1

12062	0.94613	2.1
12063	0.94613	2.1
12064	0.94613	2.1
12065	0.94613	2.1
12066	0.946132	0.47811
12067	1.41723	0.462293
12068	0.946132	0.364578
12069	0.946133	0.296187
12070	0.946132	0.935299
12071	0.946132	0.488423
12072	0.946133	0.57131
12073	0.946133	0.360357
12074	0.946132	0.287142
12075	0.946133	0.28266
12076	0.946133	0.310119
12077	0.946132	0.529625
12078	0.946132	0.383807
12079	1.34254	0.371127
12080	1.26501	1.61014
12081	0.946132	1.69266
12082	0.946133	1.66095
12083	0.946133	1.76881
12084	0.946132	1.76127
12085	0.946133	1.68584
12086	0.946132	1.79033
12087	0.946131	0.0592889
12088	0.946131	0.0612061
12089	0.946131	0.065555
12090	0.946131	0.05
12091	0.946131	0.0525779
12092	0.946131	0.0596486
12093	0.946131	0.0552015
12094	0.878843	0.05
12095	0.82561	0.05
12096	0.586974	0.05
12097	1.05343	0.05
12098	0.80631	0.05
12099	0.782663	0.05
12100	0.789405	0.05
12101	0.902491	0.05
12102	1.05344	0.05
12103	0.94613	0.05
12104	0.946131	0.0505839
12105	0.946131	0.0732949
12106	0.94613	0.068901
12107	0.946131	0.0510665
12108	0.946131	0.0700605
12109	0.946131	0.0620934
12110	0.946128	2.1
12111	0.946133	2.1
12112	0.946133	0.930705
12113	0.94613	2.1
12114	0.946131	1.39997
12115	0.946131	1.93244
12116	0.946131	2.01943
12117	0.946131	0.317604
12118	0.946131	0.265593
12119	0.94613	0.258181

12120	0.946128	0.283108
12121	0.946133	0.291267
12122	1.2996	0.243206
12123	0.946131	0.273684
12124	0.946132	2.1
12125	0.946132	2.1
12126	0.946132	2.1
12127	0.946132	2.1
12128	0.946131	2.1
12129	0.946132	2.1
12130	0.946132	2.1
12131	1.99211	0.0720773
12132	2.55024	0.0642784
12133	0.946131	0.073274
12134	0.946131	0.0684883
12135	0.946131	0.0772796
12136	0.946131	0.0716201
12137	0.946131	0.0833374
12138	0.946131	0.0743805
12139	1.35726	0.077486
12140	0.946128	2.1
12141	0.946132	2.1
12142	0.946131	2.1
12143	0.94613	2.1
12144	0.94613	2.1
12145	0.94613	2.1
12146	1.39852	2.1
12147	1.31666	0.05
12148	0.946131	0.0598475
12149	0.946128	0.05
12150	0.94613	0.05
12151	0.946128	0.05
12152	0.946128	0.05
12153	0.946128	0.05
12154	1.32275	0.0532509
12155	0.946131	0.0733542
12156	0.946129	0.058314
12157	0.946128	0.0581938
12158	0.946129	0.055734
12159	0.946128	0.0566224
12160	0.946129	0.0608446
12161	0.946132	2.1
12162	0.946131	2.1
12163	0.946131	2.1
12164	0.946132	2.1
12165	0.94613	2.1
12166	0.946131	2.1
12167	1.41125	2.1
12168	0.946132	2.1
12169	0.946132	2.1
12170	0.946131	2.1
12171	0.946132	2.1
12172	0.946132	2.1
12173	0.946132	2.1
12174	1.31904	2.1
12175	1.02425	0.05
12176	0.946132	0.05
12177	0.94613	0.05

12178	0.946131	0.05
12179	1.1534	0.05
12180	0.961161	0.05
12181	0.9311	0.05
12182	1.40092	1.56564
12183	0.946133	1.61888
12184	0.946132	1.59667
12185	0.946131	1.70673
12186	0.946131	1.62005
12187	0.94613	1.61028
12188	0.946131	1.69229
12189	0.946113	2.1
12190	0.946119	2.1
12191	0.946117	2.1
12192	1.40015	2.1
12193	0.946118	2.1
12194	0.946131	2.1
12195	0.946105	2.1
12196	0.946132	0.163386
12197	0.946133	0.270414
12198	0.946132	0.455899
12199	1.03547	0.206485
12200	0.946133	0.166489
12201	0.946133	0.321445
12202	0.946132	0.154953
12203	0.946133	2.1
12204	1.36694	2.1
12205	0.946133	2.1
12206	0.946132	2.1
12207	0.946133	2.1
12208	0.946133	2.1
12209	0.946132	2.1
12210	0.946132	0.0936527
12211	0.946133	0.111862
12212	0.946132	0.0907808
12213	0.946133	0.121465
12214	0.946133	0.0930957
12215	0.946132	0.098332
12216	0.946132	0.119843
12217	0.491552	0.13887
12218	0.913078	0.184521
12219	0.946132	0.231143
12220	0.491552	0.123707
12221	0.946132	0.192162
12222	0.49154	0.211002
12223	1.53168	0.188321
12224	1.53168	0.138523
12225	0.946127	2.1
12226	1.38752	2.1
12227	0.946129	2.1
12228	0.94613	2.1
12229	0.946128	2.1
12230	0.946129	2.1
12231	0.946129	2.1
12232	0.946132	2.1
12233	0.946132	2.1
12234	0.946132	2.1
12235	0.946132	2.1

12236	0.946132	2.1
12237	0.946131	2.1
12238	0.946132	2.1
12239	0.94613	0.363882
12240	0.94613	0.258697
12241	0.94613	0.273117
12242	0.946131	0.281242
12243	0.946129	0.355776
12244	1.16834	0.198982
12245	0.94613	0.293388
12246	0.946127	0.44135
12247	1.35594	0.297255
12248	0.946131	0.330051
12249	0.946128	0.460425
12250	0.946127	0.230994
12251	0.946129	0.419699
12252	0.94613	0.362988
12253	0.94613	2.06336
12254	0.946131	0.649619
12255	0.946128	0.983112
12256	0.946128	0.216702
12257	0.946131	2.05639
12258	0.94613	1.05394
12259	0.94613	1.54637
12260	0.946132	0.665949
12261	0.946132	0.28151
12262	0.946129	0.475307
12263	1.39751	0.359667
12264	0.94613	1.01535
12265	0.946131	0.30378
12266	0.946131	0.454839
12267	0.946132	0.0877403
12268	0.700041	0.0863098
12269	0.700013	0.102413
12270	1.7697	0.0619705
12271	1.7697	0.0606624
12272	1.21134	0.0739838
12273	0.946131	0.0694224
12274	1.25837	0.071046
12275	0.946133	2.1
12276	0.946132	2.07986
12277	0.946132	2.06453
12278	0.946132	2.1
12279	0.946132	2.1
12280	0.946133	2.1
12281	1.40583	2.1
12282	0.946131	1.8277
12283	0.946131	2.03288
12284	0.946131	1.82003
12285	0.946131	2.1
12286	0.946131	2.05839
12287	0.827865	2.06247
12288	0.946131	2.08258
12289	0.946132	0.05
12290	0.946129	0.05
12291	0.946129	0.05
12292	0.946131	0.05
12293	0.94613	0.05

12294	1.21087	0.05
12295	0.946131	0.05
12296	0.94613	0.466971
12297	0.946131	0.356255
12298	0.94613	0.2055
12299	0.946128	0.42618
12300	0.946132	0.228498
12301	1.17452	0.224232
12302	0.94613	0.334438
12303	0.946119	0.221049
12304	0.946114	0.188991
12305	0.946119	0.149817
12306	0.946119	0.210879
12307	0.946119	0.154283
12308	1.27376	0.17463
12309	0.946106	0.190773
12310	0.946133	2.1
12311	1.39621	2.1
12312	0.946132	2.1
12313	0.946132	2.1
12314	0.946133	2.1
12315	0.946132	2.1
12316	0.946132	2.1
12317	0.946132	2.1
12318	0.946132	2.1
12319	0.946131	2.1
12320	0.946131	2.1
12321	0.946132	2.1
12322	0.946132	2.1
12323	1.34352	2.1
12324	0.946132	1.81682
12325	1.41272	2.1
12326	0.946131	2.1
12327	0.94613	1.78309
12328	0.94613	2.1
12329	0.94613	1.85098
12330	0.94613	1.93976
12331	0.946132	2.1
12332	0.946131	2.05946
12333	0.946132	2.1
12334	0.946132	2.09381
12335	0.946132	2.1
12336	0.946132	2.1
12337	1.23536	2.1
12338	0.946132	2.1
12339	0.946131	2.1
12340	0.946131	2.1
12341	0.946132	2.1
12342	0.946132	2.1
12343	1.41131	2.1
12344	0.946131	2.1
12345	1.41894	1.55292
12346	0.946132	1.02
12347	0.946133	0.975747
12348	0.946132	1.54435
12349	0.946132	1.58723
12350	0.946133	1.39302
12351	0.946132	0.821959

12352	0.946128	0.528313
12353	0.946133	1.9491
12354	0.946131	1.67017
12355	0.946129	2.1
12356	0.94613	1.84175
12357	1.4099	1.95148
12358	0.946131	1.83963
12359	0.810947	2.1
12360	0.810945	2.1
12361	0.810933	2.1
12362	2.62883	2.1
12363	1.3517	2.1
12364	1.08132	2.1
12365	1.08132	2.1
12366	1.08133	2.1
12367	0.946131	1.18977
12368	0.946126	1.9774
12369	0.946127	2.04655
12370	0.94613	1.6865
12371	0.946128	2.1
12372	0.946129	1.72087
12373	0.946129	1.70974
12374	1.21347	2.1
12375	0.946129	2.1
12376	0.94613	2.1
12377	1.21347	2.1
12378	1.33064	2.1
12379	0.94613	2.1
12380	1.33064	2.1
12381	0.94613	0.05
12382	0.946129	0.05
12383	0.94613	0.05
12384	0.946132	0.05
12385	0.946126	0.05
12386	0.946129	0.05
12387	0.94613	0.05
12388	0.946132	1.20613
12389	1.37938	1.29611
12390	0.946131	1.30107
12391	0.94613	1.20798
12392	0.946131	1.32045
12393	0.94613	1.24136
12394	0.94613	1.25933
12395	0.946133	0.05
12396	0.946133	0.05
12397	1.2968	0.05
12398	0.946132	0.05
12399	0.946133	0.05
12400	1.2968	0.05
12401	0.946133	0.05
12402	1.43643	0.05
12403	0.946129	0.524
12404	0.946133	0.421337
12405	0.946131	0.742526
12406	0.94613	0.216356
12407	0.946131	0.310778
12408	0.946131	0.291815
12409	0.946131	0.282094

12410	0.872447	1.66635
12411	0.946132	1.56907
12412	0.872447	1.53943
12413	0.827867	1.61466
12414	0.946132	1.63579
12415	0.827867	1.72774
12416	0.872447	1.70434
12417	0.946117	1.19655
12418	1.27213	0.974536
12419	0.946124	0.702319
12420	0.946119	1.07368
12421	0.946113	1.07847
12422	0.946129	1.23979
12423	0.946104	1.11561
12424	0.94613	1.80945
12425	0.946131	2.1
12426	0.94613	1.85566
12427	0.946129	2.1
12428	0.946132	0.361463
12429	0.946131	1.45684
12430	0.94613	1.32295
12431	0.810942	2.1
12432	0.810943	2.1
12433	2.80212	2.1
12434	1.3517	2.1
12435	1.08133	2.1
12436	1.08132	2.1
12437	1.08132	2.1
12438	0.810932	2.1
12439	1.29521	2.1
12440	0.946129	2.1
12441	0.946131	2.1
12442	1.40106	2.1
12443	0.94613	2.1
12444	1.40106	2.1
12445	0.946131	2.1
12446	0.946132	0.674339
12447	1.24596	0.453837
12448	0.94613	0.517351
12449	0.946131	0.654498
12450	0.946131	0.323412
12451	0.946131	0.654114
12452	0.946131	0.556294
12453	0.946131	2.1
12454	0.946131	2.1
12455	0.946131	2.1
12456	0.946128	2.1
12457	0.946133	2.1
12458	0.94613	2.1
12459	0.946131	2.1
12460	0.879548	0.204838
12461	1.21527	0.0597292
12462	0.967506	0.077545
12463	0.946132	0.402685
12464	0.946133	0.132635
12465	0.967511	0.35566
12466	0.946132	0.622873
12467	0.946132	0.665859

12468	0.946132	0.31847
12469	0.974253	0.307074
12470	0.974253	0.615067
12471	0.91801	0.407577
12472	0.918009	0.353221
12473	0.946132	0.583752
12474	0.946132	0.0616652
12475	0.946131	0.0552064
12476	0.946131	0.0638039
12477	0.946133	0.05
12478	0.946132	0.0522785
12479	1.28644	0.0543318
12480	0.946132	0.0633574
12481	0.946131	0.0669448
12482	0.946131	0.0573751
12483	0.946131	0.0698695
12484	0.946131	0.0509877
12485	0.946131	0.0545913
12486	1.40103	0.0559459
12487	0.946131	0.0664872
12488	0.946128	0.167218
12489	0.946132	0.118433
12490	0.946129	0.188508
12491	1.03327	0.10025
12492	0.946129	0.104882
12493	0.946129	0.163752
12494	0.94613	0.195028
12495	0.946134	0.728025
12496	0.946133	0.834964
12497	0.946132	0.262086
12498	0.946133	0.823513
12499	0.946132	0.114417
12500	0.946132	0.278689
12501	0.946133	0.233319
12502	0.856654	0.05
12503	0.946131	0.0521606
12504	0.946131	0.05
12505	0.946132	0.05
12506	0.856616	0.0523701
12507	1.03565	0.05
12508	1.03565	0.05
12509	1.15844	0.354725
12510	0.946132	0.490991
12511	0.946132	0.222415
12512	0.946132	0.32586
12513	0.946132	0.232003
12514	0.946132	0.353957
12515	0.946131	0.357712
12516	1.4582	2.1
12517	1.35067	2.1
12518	0.946131	2.1
12519	1.04106	2.1
12520	0.938748	2.1
12521	0.943015	2.1
12522	1.05017	2.1
12523	0.938793	2.1
12524	0.946131	0.05
12525	0.946132	0.05

12526	0.946131	0.0603705
12527	0.94613	0.0591987
12528	0.946133	0.05
12529	0.946131	0.0585144
12530	0.946131	0.0502724
12531	0.946132	0.879409
12532	0.946133	1.09421
12533	0.946132	0.972508
12534	0.946133	1.02865
12535	0.946133	1.14902
12536	0.946132	1.10857
12537	0.946133	1.14054
12538	0.946128	1.78901
12539	0.946132	2.02046
12540	0.946132	1.7746
12541	0.94613	1.85644
12542	1.23426	1.85264
12543	0.94613	1.88244
12544	0.946131	1.86333
12545	0.810943	0.30218
12546	0.810946	0.785344
12547	2.60388	0.891863
12548	1.3517	0.308899
12549	1.08133	0.23648
12550	1.08132	0.749084
12551	1.08132	0.195703
12552	0.810933	0.0873513
12553	0.810945	2.1
12554	0.810945	1.87831
12555	2.72377	2.1
12556	1.3517	2.1
12557	1.08133	2.1
12558	1.08132	1.92363
12559	1.08132	2.1
12560	0.810932	2.1
12561	1.25104	1.04763
12562	0.946131	2.03018
12563	0.946131	1.73081
12564	1.36188	2.1
12565	0.946131	2.1
12566	1.36188	2.1
12567	0.946132	2.1
12568	0.946132	2.09332
12569	0.946132	2.1
12570	0.946132	2.1
12571	0.946132	2.1
12572	0.946132	2.08575
12573	0.946132	2.1
12574	1.25488	2.1
12575	1.32132	0.0644581
12576	0.946126	0.0748892
12577	0.946127	0.0604265
12578	0.946128	0.069379
12579	0.946128	0.0722256
12580	0.946128	0.0693971
12581	0.946128	0.0672604
12582	0.946133	2.1
12583	1.41123	2.1

12584	0.946134	2.1
12585	0.946133	2.1
12586	0.946132	2.03558
12587	0.946133	2.1
12588	0.946132	2.1
12589	1.32163	0.065516
12590	0.946133	0.0678869
12591	0.946133	0.0647991
12592	0.946132	0.0661713
12593	0.946132	0.0518828
12594	0.946132	0.0613411
12595	0.946133	0.0723518
12596	0.946133	2.1
12597	0.946133	2.1
12598	0.946132	2.1
12599	0.946132	2.1
12600	0.946132	2.1
12601	0.946133	2.1
12602	0.946132	2.1
12603	0.94613	0.11867
12604	0.946131	0.111203
12605	0.946128	0.113893
12606	0.946133	0.111012
12607	0.946133	0.0924721
12608	0.946131	0.0855012
12609	0.946131	0.106649
12610	0.946133	0.945631
12611	0.946132	0.47977
12612	0.946132	1.26452
12613	0.946134	0.475542
12614	0.946133	1.48242
12615	0.946133	0.767949
12616	0.946132	0.724867
12617	0.94613	1.8585
12618	0.946128	2.01349
12619	0.946127	1.78097
12620	0.94613	1.70546
12621	0.946131	1.79746
12622	0.946128	1.8083
12623	1.21099	1.92177
12624	0.946132	0.89008
12625	0.946131	1.06591
12626	0.946131	1.06955
12627	0.946133	0.204113
12628	0.946133	0.258765
12629	0.946131	0.385508
12630	0.946132	0.334186
12631	1.34794	0.843602
12632	0.517994	0.804135
12633	0.517965	0.620314
12634	0.946132	1.10525
12635	0.9198	1.80298
12636	0.946132	1.39631
12637	0.9198	1.51139
12638	0.946132	1.53393
12639	0.94613	1.56104
12640	0.946131	0.6561
12641	0.946131	1.57312

12642	0.946131	1.79371
12643	0.946131	1.5714
12644	1.23224	1.14508
12645	0.946132	1.72894
12646	0.946132	1.86984
12647	0.946133	1.66689
12648	0.946132	1.5037
12649	0.946132	1.55797
12650	0.946133	1.64703
12651	1.37511	1.64791
12652	0.946133	0.97403
12653	0.946129	0.962875
12654	0.851104	1.26284
12655	0.946131	0.20534
12656	1.0412	0.432021
12657	1.0412	0.344137
12658	0.851066	1.04882
12659	1.31357	0.0783171
12660	0.946131	0.219075
12661	0.94613	0.132634
12662	0.946131	0.12496
12663	0.94613	0.0679114
12664	0.946131	0.152637
12665	0.946131	0.144807
12666	0.946133	0.710074
12667	1.38149	1.26874
12668	0.946132	1.25749
12669	0.946132	0.76476
12670	0.946133	1.30845
12671	0.946132	1.1713
12672	0.946133	1.23304
12673	0.810944	1.7078
12674	0.810945	2.1
12675	0.810958	2.1
12676	2.65782	2.1
12677	1.35168	2.1
12678	1.08132	1.82165
12679	1.08132	2.1
12680	1.0813	1.93042
12681	0.946113	0.05
12682	0.946119	0.05
12683	0.946118	0.05
12684	0.946118	0.05
12685	0.946119	0.05
12686	0.946131	0.05
12687	0.946106	0.05
12688	0.946132	0.05
12689	0.946132	0.05
12690	0.946128	0.05
12691	0.946131	0.05
12692	0.94613	0.05
12693	0.94613	0.05
12694	0.94613	0.05
12695	1.00099	0.0720094
12696	0.946132	0.11372
12697	1.17876	0.0723419
12698	0.982282	0.082424
12699	0.909981	0.119717

12700	0.946132	0.101939
12701	0.946131	0.0899143
12702	0.946132	1.19668
12703	1.35328	1.31256
12704	0.946132	1.35881
12705	0.946132	1.19348
12706	0.946133	1.31097
12707	0.946132	1.223
12708	0.946133	1.26322
12709	0.946133	0.05
12710	0.946133	0.05
12711	0.946132	0.05
12712	0.946132	0.05
12713	0.946132	0.05
12714	0.946132	0.05
12715	0.946133	0.05
12716	0.900195	0.05
12717	0.946132	0.05
12718	0.900167	0.05
12719	1.41963	0.05
12720	0.900195	0.05
12721	0.946133	0.05
12722	1.35385	0.05
12723	0.946132	1.72993
12724	0.946129	1.87617
12725	0.946129	1.91191
12726	0.946131	1.72016
12727	0.946131	1.93937
12728	1.41585	1.66262
12729	0.94613	1.88547
12730	0.810946	2.1
12731	0.810946	2.1
12732	2.68895	2.1
12733	1.3517	2.1
12734	1.08133	2.1
12735	1.08132	2.1
12736	1.08132	2.1
12737	0.810933	2.1
12738	1.19886	2.1
12739	0.946131	2.1
12740	0.946131	2.1
12741	1.34447	2.1
12742	0.946131	2.1
12743	1.34447	2.1
12744	0.946131	2.1
12745	0.946133	0.0550116
12746	0.946133	0.0593395
12747	0.946132	0.0779015
12748	1.35065	0.0579968
12749	0.946132	0.0787213
12750	0.946133	0.0764271
12751	0.946132	0.0609548
12752	0.946131	0.566599
12753	0.946131	0.735208
12754	0.94613	0.990285
12755	0.946131	0.182995
12756	0.946131	0.217689
12757	0.946131	0.346627

12758	0.946131	0.268633
12759	0.946127	0.146366
12760	0.946131	0.136956
12761	0.946127	0.145072
12762	0.94613	0.115005
12763	0.946131	0.12524
12764	0.946128	0.137224
12765	0.946129	0.127266
12766	0.810946	0.05
12767	0.810947	0.05
12768	0.810959	0.05
12769	2.4391	0.05
12770	1.35168	0.05
12771	1.08132	0.05
12772	1.08132	0.05
12773	1.08131	0.0506048
12774	0.946132	0.05
12775	0.946132	0.05
12776	0.946132	0.05
12777	1.40837	0.05
12778	0.946132	0.05
12779	0.946132	0.05
12780	1.40837	0.05
12781	0.946127	2.1
12782	0.946132	2.1
12783	0.946131	0.419727
12784	0.946127	2.1
12785	0.946131	1.57311
12786	0.946129	1.5372
12787	0.94613	1.73686
12788	0.946133	1.90872
12789	0.946132	1.85085
12790	0.946132	1.83351
12791	0.946132	1.68337
12792	0.946132	1.86283
12793	0.946132	1.61854
12794	0.946133	2.03828
12795	0.946132	0.6619
12796	1.24344	0.132642
12797	0.946132	0.104983
12798	0.946132	0.675999
12799	0.946132	0.27952
12800	1.24344	0.193325
12801	0.946132	0.9624
12802	0.946131	0.137645
12803	0.946128	0.175065
12804	0.946128	0.095841
12805	0.946131	0.196686
12806	1.41232	0.0970763
12807	0.946129	0.0831957
12808	0.94613	0.141596
12809	0.946128	2.1
12810	0.946133	2.1
12811	0.946133	1.75984
12812	0.946131	2.1
12813	0.946131	1.75846
12814	0.946131	2.1
12815	0.94613	2.1

12816	0.946133	0.562419
12817	1.41556	0.399506
12818	0.946133	0.315131
12819	0.946133	0.296177
12820	0.946132	1.02049
12821	0.946132	0.411926
12822	0.946132	0.783754
12823	0.94612	0.42394
12824	1.41843	0.314542
12825	0.946114	0.311377
12826	0.94612	0.28979
12827	0.946119	0.749407
12828	1.41845	0.315114
12829	0.946107	0.624198
12830	0.946129	0.05
12831	0.946129	0.05
12832	0.94613	0.05
12833	0.946132	0.05
12834	0.946126	0.05
12835	1.1509	0.05
12836	0.94613	0.05
12837	0.946131	0.05
12838	0.946132	0.05
12839	0.946132	0.05
12840	0.946132	0.05
12841	0.946132	0.05
12842	1.19263	0.05
12843	0.946132	0.05
12844	0.94613	0.689419
12845	0.946131	0.976881
12846	0.946128	0.756327
12847	0.946133	1.1421
12848	0.946133	1.07575
12849	0.946131	0.838374
12850	0.946131	1.05503
12851	0.946133	1.44383
12852	1.22848	1.73024
12853	1.00336	1.7374
12854	0.827866	1.62054
12855	0.946132	1.77289
12856	0.827865	1.68126
12857	0.946133	1.65133
12858	0.946131	2.1
12859	0.94613	0.616421
12860	0.946127	2.1
12861	1.41498	2.1
12862	0.94613	0.527746
12863	0.946128	1.6332
12864	0.946128	1.25154
12865	0.94613	0.05
12866	0.94613	0.05
12867	0.94613	0.05
12868	1.37358	0.05
12869	0.946129	0.05
12870	0.94613	0.05
12871	1.37357	0.05
12872	0.946111	0.05
12873	0.946118	0.05

12874	0.946118	0.0543322
12875	1.36568	0.05
12876	0.946116	0.05
12877	0.94613	0.0511327
12878	0.946105	0.05
12879	0.946132	0.318193
12880	0.946131	0.205934
12881	0.94613	0.370922
12882	1.23175	0.168603
12883	0.946131	0.157049
12884	0.946131	0.321743
12885	0.946131	0.396029
12886	0.946132	0.058614
12887	0.946126	0.0577425
12888	0.946128	0.0635722
12889	0.946129	0.0595875
12890	0.946129	0.0624183
12891	0.946128	0.0591475
12892	1.25698	0.0605428
12893	0.810943	2.1
12894	0.810944	2.1
12895	2.81171	2.1
12896	1.3517	2.1
12897	1.08132	2.1
12898	1.08133	2.1
12899	1.08132	2.1
12900	0.810932	2.1
12901	0.946132	0.145424
12902	0.946131	0.130396
12903	0.946131	0.164305
12904	0.946132	0.078937
12905	0.946132	0.0940794
12906	0.946132	0.117588
12907	0.946131	0.104716
12908	0.810936	2.07421
12909	0.810935	2.1
12910	2.80914	2.1
12911	1.35167	2.1
12912	1.0813	2.1
12913	1.0813	2.1
12914	1.0813	1.76976
12915	0.810934	2.1
12916	1.23851	2.1
12917	0.946132	2.1
12918	0.946131	2.1
12919	1.40459	2.05471
12920	0.946132	2.1
12921	1.40459	2.1
12922	0.946132	2.1
12923	1.19974	0.122786
12924	0.946131	0.37784
12925	1.19974	0.135821
12926	0.946132	0.332681
12927	0.946132	0.100646
12928	0.946132	0.369199
12929	0.946132	0.187655
12930	0.939141	0.879964
12931	1.14375	1.38875

12932	1.03306	1.24994
12933	0.946132	1.25407
12934	0.946126	1.4109
12935	0.946129	1.37313
12936	0.94613	1.16319
12937	0.946132	0.10997
12938	0.946131	0.12572
12939	0.946132	0.10759
12940	0.946132	0.131247
12941	0.946132	0.106814
12942	0.946132	0.103522
12943	0.946132	0.117966
12944	0.946131	2.1
12945	0.94613	2.1
12946	0.94613	2.1
12947	0.946131	2.1
12948	0.946129	2.1
12949	0.94613	2.1
12950	1.24512	2.1
12951	0.946133	0.0787107
12952	0.946129	0.0695149
12953	0.946131	0.135822
12954	0.946132	0.06593
12955	0.946131	0.0860103
12956	0.946131	0.0726304
12957	0.946131	0.126616
12958	0.946132	0.085896
12959	0.946133	0.102182
12960	0.946132	0.0886789
12961	0.946132	0.1139
12962	0.946133	0.0708872
12963	0.946132	0.0880463
12964	0.946132	0.122019
12965	0.946132	0.91064
12966	1.41392	0.57834
12967	0.946132	0.614387
12968	0.946132	1.03323
12969	0.946132	0.44099
12970	0.946132	0.929912
12971	0.946132	0.802606
12972	0.946132	1.47046
12973	1.24333	1.04953
12974	0.946132	1.10069
12975	0.946133	1.53105
12976	0.946132	0.560082
12977	0.946132	1.51634
12978	0.946133	1.39194
12979	1.30649	0.187597
12980	0.94613	0.217906
12981	0.94613	0.176333
12982	0.946128	0.192688
12983	0.946126	0.244493
12984	0.946127	0.217995
12985	0.946127	0.214208
12986	0.709578	0.313682
12987	0.810943	0.291694
12988	2.53303	0.159921
12989	1.21651	0.161171

12990	0.979951	0.198239
12991	1.08132	0.182825
12992	0.946141	0.302521
12993	0.81096	0.250342
12994	0.946131	0.0835564
12995	0.946131	0.0965322
12996	0.946131	0.0936211
12997	0.946131	0.0650024
12998	0.946131	0.0724805
12999	0.946131	0.0958837
13000	0.946131	0.0793728
13001	0.946132	0.05
13002	0.946129	0.0572919
13003	0.946129	0.0589775
13004	0.94613	0.0550584
13005	1.20634	0.0552232
13006	1.20634	0.0568084
13007	0.946131	0.0559359
13008	0.946133	0.457861
13009	0.946132	0.0732054
13010	0.946132	0.0664348
13011	0.946132	0.407646
13012	0.946132	0.0652664
13013	1.30107	0.109812
13014	0.946133	0.266309
13015	0.810946	2.1
13016	0.810944	2.1
13017	2.73936	2.1
13018	1.35168	2.1
13019	1.0813	2.1
13020	1.08132	2.1
13021	1.08132	2.1
13022	0.810958	2.1
13023	0.946129	2.1
13024	0.946129	2.1
13025	0.946133	1.77668
13026	1.30812	2.1
13027	0.946131	1.89574
13028	0.946131	2.06493
13029	0.946131	2.05838
13030	1.00901	1.68402
13031	0.94613	1.89916
13032	0.94613	1.44222
13033	1.17001	1.7097
13034	0.917259	0.934234
13035	0.94613	1.62396
13036	0.94613	1.66371
13037	0.946126	2.1
13038	0.946127	2.1
13039	0.946131	2.1
13040	0.946127	2.1
13041	0.946131	2.1
13042	0.946129	2.1
13043	1.398	2.1
13044	0.946133	2.1
13045	1.3033	2.1
13046	0.946132	2.1
13047	0.946132	2.1

13048	0.946132	2.1
13049	0.946132	2.1
13050	0.946133	2.1
13051	0.946131	0.483631
13052	0.946131	0.458518
13053	1.09897	0.403185
13054	0.946129	0.750089
13055	0.946131	0.521141
13056	0.94613	0.652594
13057	0.94613	0.490597
13058	0.946132	2.1
13059	0.946131	2.1
13060	0.946131	2.1
13061	0.946132	2.1
13062	0.94613	2.1
13063	0.946131	2.1
13064	1.39858	2.1
13065	1.07117	1.1797
13066	1.08878	1.22611
13067	0.946119	1.24017
13068	1.08878	1.36486
13069	0.946119	1.07073
13070	0.946132	1.19831
13071	1.07116	1.18892
13072	0.946132	0.0654249
13073	0.946132	0.0610259
13074	0.946132	0.0689458
13075	0.946132	0.0544022
13076	0.946132	0.0572036
13077	1.10733	0.061765
13078	0.946132	0.0639999
13079	0.946128	0.0742487
13080	0.946132	0.0648642
13081	0.94613	0.0781359
13082	0.946129	0.059589
13083	0.94613	0.0612911
13084	1.3367	0.0660742
13085	0.94613	0.0716765
13086	0.810943	2.1
13087	0.810944	2.1
13088	2.70234	2.1
13089	1.21653	2.1
13090	1.08132	2.1
13091	0.946155	2.1
13092	0.979951	2.1
13093	0.709578	2.1
13094	1.27174	2.1
13095	0.946129	2.1
13096	0.94613	2.1
13097	1.35117	2.1
13098	0.94613	2.1
13099	1.35117	2.1
13100	0.94613	2.1
13101	0.946133	1.44004
13102	0.946133	1.62736
13103	0.946132	1.92383
13104	0.946133	1.36216
13105	0.946132	2.1

13106	0.946133	1.87674
13107	0.997296	1.97384
13108	0.810943	2.1
13109	0.810944	2.1
13110	0.810945	2.1
13111	2.78833	2.1
13112	1.35169	2.1
13113	1.08132	2.1
13114	1.08132	2.1
13115	1.08132	2.1
13116	0.810946	2.1
13117	0.810943	1.40214
13118	0.810933	1.68149
13119	2.70049	1.76111
13120	1.3517	1.6457
13121	1.08132	2.1
13122	1.08132	0.296192
13123	1.08133	1.62077
13124	0.946127	1.58882
13125	1.35469	1.65929
13126	0.94613	1.64423
13127	0.946131	1.61115
13128	0.946128	1.55418
13129	1.35469	1.73233
13130	0.946129	1.72553
13131	0.946131	0.290829
13132	1.2722	0.232589
13133	0.946132	0.225168
13134	0.946132	0.238615
13135	0.946129	0.326035
13136	1.2722	0.199162
13137	0.94613	0.251982
13138	0.946133	0.0777473
13139	0.946132	0.084406
13140	0.946132	0.2145
13141	0.946132	0.10608
13142	1.41461	0.330114
13143	1.41461	0.090854
13144	0.946133	0.11713
13145	0.946131	1.4398
13146	1.30165	0.68027
13147	0.946132	0.426163
13148	0.946132	1.48909
13149	0.946129	0.854243
13150	1.30165	1.42407
13151	0.946131	1.48484
13152	0.946132	1.87782
13153	0.946129	2.1
13154	0.94613	1.91235
13155	1.39356	2.1
13156	0.946131	1.93443
13157	0.946131	1.97778
13158	0.94613	2.1
13159	0.810946	2.1
13160	0.810947	2.1
13161	0.810934	2.1
13162	2.35003	2.1
13163	1.3517	2.1

13164	1.08132	2.1
13165	1.08132	2.1
13166	1.08133	2.1
13167	0.946131	0.0514171
13168	0.946131	0.0528692
13169	0.946125	0.0628489
13170	0.946127	0.0619915
13171	0.946129	0.0556309
13172	0.946128	0.062138
13173	0.946128	0.056949
13174	0.946131	0.05
13175	0.946131	0.05
13176	0.946131	0.0603898
13177	0.946131	0.0580156
13178	0.946132	0.05
13179	0.946132	0.0590989
13180	0.946131	0.0565266
13181	0.946129	2.1
13182	0.946132	2.1
13183	0.946132	2.1
13184	0.94613	2.1
13185	1.30082	2.1
13186	0.94613	2.1
13187	0.946131	2.1
13188	1.20416	0.05
13189	0.946132	0.0890957
13190	0.946131	0.0677082
13191	0.946132	0.0564902
13192	0.946131	0.05
13193	0.946131	0.0715233
13194	0.946132	0.0648921
13195	1.18711	0.0501059
13196	0.946131	0.0785103
13197	0.946131	0.0687973
13198	0.946132	0.0532805
13199	0.946131	0.05
13200	0.946131	0.0780478
13201	0.946132	0.0672487
13202	0.946133	0.05
13203	0.946133	0.05
13204	0.946132	0.05
13205	1.30212	0.0521441
13206	0.946133	0.0516869
13207	0.946132	0.05
13208	1.30212	0.05
13209	0.946131	0.0528081
13210	0.946131	0.050433
13211	0.946131	0.0671896
13212	0.946132	0.0610707
13213	1.31467	0.0590755
13214	1.31467	0.0526706
13215	0.946131	0.0692334
13216	0.946133	0.05
13217	0.946132	0.05
13218	0.946132	0.0617635
13219	0.946132	0.0549262
13220	1.25124	0.0516792
13221	1.25124	0.05

13222	0.946132	0.0620939
13223	0.946132	2.1
13224	0.946132	2.1
13225	0.946132	2.1
13226	1.36422	2.1
13227	0.946132	2.1
13228	0.946131	2.1
13229	0.946132	2.1
13230	0.946131	1.49572
13231	1.41851	1.71276
13232	0.946132	1.81668
13233	0.946132	1.51827
13234	0.946132	1.71508
13235	0.946132	1.56011
13236	0.946132	1.62099
13237	0.946133	0.0643754
13238	0.946132	0.0655168
13239	0.946132	0.0635732
13240	0.946132	0.0654873
13241	0.946132	0.0627919
13242	1.37794	0.0589942
13243	0.946132	0.0682115
13244	0.946132	2.1
13245	0.946132	2.1
13246	0.946131	2.1
13247	0.946131	2.1
13248	0.946132	2.1
13249	0.946132	2.1
13250	1.4045	2.1
13251	0.946133	2.1
13252	0.946132	2.1
13253	0.946131	2.1
13254	0.946132	2.1
13255	0.946131	2.1
13256	0.946131	2.1
13257	0.946132	2.1
13258	0.946132	2.1
13259	0.946132	2.1
13260	0.946131	2.1
13261	0.946132	2.1
13262	0.946131	2.1
13263	0.946131	2.1
13264	0.946131	2.1
13265	0.946128	2.1
13266	1.31641	2.1
13267	0.946129	2.1
13268	0.94613	2.1
13269	0.94613	2.1
13270	0.94613	2.1
13271	0.94613	2.1
13272	0.946132	2.1
13273	0.946131	2.1
13274	0.946131	2.1
13275	0.946132	2.1
13276	0.946132	2.1
13277	0.946132	2.1
13278	0.946132	2.1
13279	0.946132	2.1

13280	1.41507	2.1
13281	0.946132	2.1
13282	0.946132	2.1
13283	0.946133	2.1
13284	0.946133	2.1
13285	0.946132	2.1
13286	0.946132	2.1
13287	0.946131	2.1
13288	0.946131	2.1
13289	0.946133	2.1
13290	0.946132	2.1
13291	1.32608	2.1
13292	0.946132	2.1
13293	0.94613	1.98315
13294	0.94613	2.07928
13295	0.94613	0.879731
13296	0.946131	2.05251
13297	0.946129	1.52445
13298	0.94613	1.34252
13299	0.94613	1.51047
13300	1.33728	1.95399
13301	0.946131	1.9004
13302	0.94613	1.55057
13303	0.946132	1.96335
13304	0.946132	2.1
13305	0.946131	1.78865
13306	0.946131	1.84438
13307	0.946127	2.1
13308	1.40641	2.1
13309	0.946129	2.1
13310	0.94613	2.1
13311	0.946128	1.87329
13312	0.946129	2.1
13313	0.946129	2.1
13314	0.946129	2.1
13315	0.946128	2.1
13316	0.946133	2.1
13317	1.37467	2.1
13318	0.946131	2.1
13319	0.946131	2.1
13320	1.37467	2.1
13321	0.946131	2.1
13322	0.946131	2.1
13323	0.946132	2.1
13324	0.946132	2.1
13325	0.946129	2.1
13326	0.946131	2.1
13327	1.34912	2.1
13328	0.946132	2.1
13329	0.946133	2.1
13330	0.946133	2.1
13331	0.946132	2.1
13332	0.946132	2.1
13333	0.946133	2.1
13334	0.946132	2.1
13335	0.946127	2.1
13336	0.946131	2.1
13337	0.946132	2.1

13338	0.946131	2.1
13339	0.946127	2.1
13340	0.94613	2.1
13341	1.33607	2.1
13342	0.946117	0.315144
13343	0.946117	1.5656
13344	0.946115	1.4776
13345	0.946124	2.1
13346	0.946119	0.683622
13347	1.32662	1.48157
13348	0.94613	0.537607
13349	0.946119	2.1
13350	0.946114	2.1
13351	0.946118	2.1
13352	0.946114	2.1
13353	0.946118	2.1
13354	0.946104	2.1
13355	0.946129	2.1
13356	0.946132	2.1
13357	0.946133	2.1
13358	0.946133	2.1
13359	0.946133	2.1
13360	0.946132	2.1
13361	0.946132	2.1
13362	0.946133	2.1
13363	0.946133	2.1
13364	0.946132	2.1
13365	0.946132	2.1
13366	0.946133	2.1
13367	0.946132	2.1
13368	0.946132	2.1
13369	0.946132	2.1
13370	0.946133	1.6427
13371	1.30881	1.89031
13372	0.946132	2.1
13373	0.946131	1.63394
13374	0.946131	1.84609
13375	0.946131	1.67133
13376	0.946131	1.76648
13377	0.946128	2.1
13378	0.946128	2.1
13379	0.946132	2.1
13380	0.94613	2.1
13381	0.94613	2.1
13382	0.94613	2.1
13383	1.34652	2.1
13384	0.946133	1.19793
13385	0.946133	1.23035
13386	0.946132	1.82715
13387	0.946133	1.09755
13388	0.946132	2.02901
13389	0.946132	1.58901
13390	0.946132	1.7992
13391	0.946132	0.124248
13392	0.946128	0.0868734
13393	0.94613	0.151003
13394	0.946131	0.05
13395	0.94613	0.159536

13396	0.94613	0.108756
13397	0.94613	0.102429
13398	0.946128	0.548124
13399	0.946132	0.58674
13400	0.94613	0.587785
13401	0.94613	0.476026
13402	0.946131	0.519493
13403	0.94613	0.591315
13404	0.94613	0.531995
13405	0.946129	1.38206
13406	1.32056	1.51428
13407	0.946131	1.55051
13408	0.94613	1.41099
13409	0.946124	1.50475
13410	0.946128	1.43628
13411	0.946127	1.45751
13412	0.946129	1.15985
13413	1.20351	1.52328
13414	0.946132	1.53364
13415	0.946132	1.43871
13416	0.946126	1.52417
13417	0.946129	1.47551
13418	0.946129	1.44896
13419	0.946127	1.99376
13420	0.946127	2.1
13421	0.946131	1.75776
13422	1.25592	1.9961
13423	0.946127	1.79616
13424	0.946129	1.90312
13425	0.94613	1.92337
13426	1.02332	1.75085
13427	0.946133	1.62295
13428	0.946132	1.47693
13429	1.02332	1.62323
13430	0.946132	1.3906
13431	0.96196	1.54855
13432	0.930306	1.54173
13433	0.946133	1.99411
13434	0.946133	2.00973
13435	0.946132	2.1
13436	1.20057	2.05729
13437	0.946132	2.1
13438	0.946133	2.1
13439	1.20057	2.1
13440	0.946128	1.4216
13441	0.946129	1.29422
13442	0.946129	1.27827
13443	1.13245	1.25643
13444	0.946126	1.18537
13445	0.946128	1.27637
13446	0.946129	1.27408
13447	0.946131	1.84458
13448	0.946131	1.92448
13449	0.94613	2.07007
13450	1.25	1.91721
13451	0.946129	2.06788
13452	0.946131	2.1
13453	1.25	2.08261

13454	0.540425	1.9766
13455	0.748493	2.00302
13456	1.46266	2.1
13457	1.20046	2.09919
13458	0.600454	2.01604
13459	1.41283	2.1
13460	0.582567	1.99745
13461	0.946131	1.86967
13462	0.946131	1.88939
13463	0.946132	1.8372
13464	0.946132	1.91919
13465	0.94613	1.83125
13466	0.946131	2.1
13467	1.28439	2.07463
13468	0.946132	0.947104
13469	0.946131	0.730287
13470	0.946131	1.02639
13471	0.946132	0.671443
13472	0.946132	1.19765
13473	1.39335	0.556585
13474	0.946132	0.633786
13475	0.946131	0.22444
13476	0.946131	0.452339
13477	0.94613	0.215532
13478	0.946132	0.20298
13479	0.946131	0.378602
13480	0.946131	0.27219
13481	0.946131	0.287798
13482	0.946131	0.214467
13483	0.94613	0.188187
13484	0.946126	0.247553
13485	0.946131	0.136526
13486	0.946125	0.742695
13487	0.946127	0.149865
13488	0.946128	0.290962
13489	0.946131	0.0524105
13490	0.946131	0.0671872
13491	0.946131	0.0589073
13492	0.946131	0.0503421
13493	0.946131	0.0526307
13494	0.946131	0.0660817
13495	1.39168	0.05
13496	0.946131	0.106259
13497	0.946126	0.0767173
13498	0.946127	0.0892499
13499	0.946131	0.0794386
13500	0.94613	0.11015
13501	1.412	0.0858348
13502	0.946128	0.0958329
13503	0.948901	0.265939
13504	0.943364	0.247749
13505	1.03768	0.245704
13506	0.946132	0.367581
13507	0.946133	0.334105
13508	1.03767	0.346651
13509	0.946132	0.290512
13510	1.00902	0.596087
13511	0.88324	0.448134

13512	0.971584	0.448011
13513	0.946132	0.521173
13514	0.946129	1.19677
13515	0.946131	0.777245
13516	0.971572	0.909586
13517	0.946131	0.0608662
13518	0.946125	0.0542711
13519	1.33587	0.0608267
13520	0.946126	0.065195
13521	0.946127	0.0588847
13522	0.946126	0.0658565
13523	0.946128	0.0633246
13524	0.946132	0.0731717
13525	0.946132	0.067833
13526	1.27512	0.0668432
13527	0.946133	0.0934616
13528	0.946132	0.0756943
13529	0.946133	0.0790813
13530	0.946133	0.0843373
13531	0.946128	0.0755164
13532	0.946133	0.0743428
13533	0.946131	0.0826718
13534	0.94613	0.055255
13535	0.946131	0.0631604
13536	0.94613	0.0705165
13537	0.946131	0.0640478
13538	0.946131	0.0635683
13539	0.946131	0.0619126
13540	0.946131	0.0673579
13541	0.946131	0.0501675
13542	0.946131	0.055042
13543	0.946131	0.0605352
13544	0.946131	0.0562452
13545	0.94613	0.05
13546	0.946131	0.05
13547	0.946131	0.05
13548	0.94613	0.05
13549	0.946132	0.05
13550	0.946131	0.05
13551	1.35316	0.05
13552	0.94613	0.128662
13553	0.94613	0.0871648
13554	0.946128	0.107146
13555	0.946132	0.099733
13556	0.946132	0.121862
13557	1.28727	0.108182
13558	0.94613	0.0898466
13559	0.946131	0.05
13560	0.946132	0.05
13561	0.946132	0.05
13562	1.39502	0.05
13563	0.946132	0.05
13564	0.946132	0.05
13565	0.946132	0.05
13566	0.946125	0.379944
13567	0.946131	0.304076
13568	0.94613	0.29446
13569	0.946129	0.34026

13570	0.946126	0.620077
13571	0.946127	0.456139
13572	1.36173	0.467247
13573	0.946129	0.9453
13574	1.02363	0.448395
13575	0.94613	0.338512
13576	0.946131	0.915899
13577	0.946131	0.401356
13578	1.02363	1.05023
13579	0.946131	1.04127
13580	0.946128	0.05
13581	1.24678	0.05
13582	0.94613	0.05
13583	0.94613	0.05
13584	0.94613	0.05
13585	0.946131	0.05
13586	0.94613	0.05
13587	0.946129	0.05
13588	1.25215	0.05
13589	0.946132	0.05
13590	0.946126	0.05
13591	0.946129	0.05
13592	0.94613	0.05
13593	0.946129	0.05
13594	0.946129	0.410084
13595	0.946133	0.324012
13596	0.946133	0.264138
13597	0.946131	0.33624
13598	0.946131	0.319664
13599	1.29689	0.244671
13600	0.946131	0.298134
13601	0.946128	0.0682341
13602	0.946132	0.0552186
13603	0.946132	0.0614548
13604	0.94613	0.0759044
13605	1.22716	0.056155
13606	1.22716	0.0618926
13607	0.946131	0.0669625
13608	0.946127	0.0882794
13609	0.946131	0.0649854
13610	0.946131	0.074524
13611	0.946128	0.100013
13612	1.30463	0.0666284
13613	1.30463	0.0730309
13614	0.946129	0.0825717
13615	1.29958	0.05
13616	0.946132	0.0516658
13617	0.946132	0.05
13618	0.946132	0.05
13619	0.946132	0.05
13620	0.946132	0.05
13621	0.946133	0.05
13622	1.26201	0.05
13623	0.946133	0.0524113
13624	0.946132	0.05
13625	0.946132	0.05
13626	0.946132	0.05
13627	0.946133	0.05

13628	0.946132	0.05
13629	0.946133	0.0847481
13630	0.946132	0.0690212
13631	0.946132	0.0878337
13632	0.946133	0.0638375
13633	0.946132	0.0658613
13634	1.40427	0.0688857
13635	0.946133	0.081865
13636	0.946132	0.0924805
13637	0.946132	0.0689149
13638	0.946132	0.09387
13639	0.946132	0.0639286
13640	0.946132	0.0666341
13641	1.35123	0.0669441
13642	0.946131	0.0838557
13643	0.946128	0.115595
13644	0.946132	0.0793157
13645	0.946132	0.0933661
13646	0.946129	0.133896
13647	1.22144	0.0809658
13648	1.22144	0.0902242
13649	0.946131	0.108848
13650	0.946127	0.273379
13651	0.946132	0.222791
13652	0.946131	0.21052
13653	0.946127	0.245229
13654	0.946131	0.235845
13655	1.23351	0.201499
13656	0.94613	0.229194
13657	1.18119	0.11229
13658	0.946131	0.158666
13659	0.946132	0.108808
13660	0.946132	0.132295
13661	0.94613	0.166473
13662	0.946131	0.143948
13663	0.946131	0.14325
13664	0.946132	0.220256
13665	0.946132	0.174732
13666	0.946133	0.25892
13667	1.30563	0.149017
13668	0.946132	0.16707
13669	0.946132	0.213292
13670	0.946133	0.274907
13671	1.20256	0.400225
13672	0.946132	1.99743
13673	0.946133	1.65795
13674	0.946132	0.499569
13675	0.946132	0.308867
13676	0.946132	1.79636
13677	0.946133	1.11138
13678	0.94613	1.13151
13679	0.94613	0.7474
13680	0.946128	1.34258
13681	1.33007	0.540294
13682	0.946129	0.452608
13683	0.94613	1.15832
13684	0.946131	1.66382
13685	0.946127	2.1

13686	0.946127	2.1
13687	0.946131	2.1
13688	1.30136	2.1
13689	0.946128	2.1
13690	0.946129	2.1
13691	1.30136	2.1
13692	0.810942	2.1
13693	0.810942	2.1
13694	0.81093	2.1
13695	2.3208	2.1
13696	1.3517	2.1
13697	1.08132	2.1
13698	1.08132	2.1
13699	1.08133	2.1
13700	0.946132	2.1
13701	0.946132	2.1
13702	0.946132	2.1
13703	0.946132	2.1
13704	0.946132	2.1
13705	1.39823	2.1
13706	0.946132	2.1
13707	1.37713	2.1
13708	0.946131	2.1
13709	0.946131	2.1
13710	0.946131	2.1
13711	0.946131	2.1
13712	0.946131	2.1
13713	0.946131	2.1
13714	0.946128	2.1
13715	0.946128	2.1
13716	0.946133	2.1
13717	0.946131	2.1
13718	0.946131	2.1
13719	0.946131	2.1
13720	0.94613	2.1
13721	0.946129	2.1
13722	0.946129	2.1
13723	0.946133	2.1
13724	0.946131	2.1
13725	0.946131	2.1
13726	0.946131	2.1
13727	0.946131	2.1
13728	0.946132	1.71299
13729	1.33694	2.0095
13730	0.946133	2.1
13731	0.946133	1.73168
13732	0.946132	2.05467
13733	0.946132	1.79805
13734	0.946133	1.86389
13735	0.946131	2.1
13736	0.94613	2.1
13737	0.946131	2.1
13738	0.946133	2.1
13739	0.946129	2.1
13740	0.946131	2.1
13741	0.946131	2.1
13742	0.946132	1.55593
13743	1.35943	1.80782

13744	0.946132	2.1
13745	0.946132	1.44717
13746	0.946131	1.6853
13747	0.946132	1.48163
13748	0.946132	1.69414
13749	0.946132	0.05
13750	0.946132	0.05
13751	0.946133	0.05
13752	0.946133	0.05
13753	0.946132	0.05
13754	0.946132	0.05
13755	0.946132	0.05
13756	1.29322	0.05
13757	0.946131	0.05
13758	0.946132	0.05
13759	0.946129	0.05
13760	0.946131	0.05
13761	0.946131	0.05
13762	0.946131	0.05
13763	0.946132	1.83028
13764	0.946132	2.1
13765	0.946131	1.80525
13766	1.41492	0.915152
13767	0.946132	2.1
13768	0.946132	2.08777
13769	0.946131	2.1
13770	0.946132	2.1
13771	0.946132	2.1
13772	0.946132	2.1
13773	1.35028	2.1
13774	0.946132	2.1
13775	0.946132	2.1
13776	0.946132	2.1
13777	0.946132	1.89222
13778	0.946132	2.1
13779	0.946132	2.1
13780	1.32461	2.02189
13781	0.946131	2.1
13782	0.946132	2.0989
13783	0.946132	2.1
13784	0.946131	2.1
13785	0.946131	2.1
13786	0.946131	2.1
13787	0.946132	2.1
13788	0.946129	2.1
13789	0.94613	2.1
13790	1.40369	2.1
13791	0.946132	0.141791
13792	0.946131	0.0976718
13793	0.946131	0.152686
13794	0.979598	0.082459
13795	0.946131	0.0851379
13796	0.946131	0.137226
13797	0.946132	0.156159
13798	0.946131	2.1
13799	1.41897	2.1
13800	0.946132	2.1
13801	0.946132	2.1

13802	0.94613	2.1
13803	0.946131	2.1
13804	0.946131	2.1
13805	0.946131	2.1
13806	0.94613	2.1
13807	0.946132	2.1
13808	0.946132	2.1
13809	0.946129	2.1
13810	0.946131	2.1
13811	1.37791	2.1
13812	0.946127	2.1
13813	0.946131	2.1
13814	0.946131	2.1
13815	0.94613	2.1
13816	0.946128	2.1
13817	0.946129	2.1
13818	1.39384	2.1
13819	0.810945	1.95259
13820	0.810944	1.42674
13821	0.810958	1.49531
13822	2.70852	1.54752
13823	1.35168	1.51345
13824	1.08132	1.78646
13825	1.08132	1.13598
13826	1.0813	1.5384
13827	1.3335	2.1
13828	0.946132	2.1
13829	0.94613	2.1
13830	0.946132	2.1
13831	0.946131	2.1
13832	0.94613	2.1
13833	0.946131	2.1
13834	0.946132	2.1
13835	0.946131	1.94534
13836	0.94613	2.1
13837	1.41192	2.1
13838	0.946132	1.97348
13839	0.946131	2.1
13840	0.94613	2.1
13841	0.946133	1.68304
13842	0.946133	1.02124
13843	0.946132	1.1913
13844	1.39428	0.790892
13845	0.946132	0.209523
13846	0.946132	0.543094
13847	0.946133	1.17615
13848	0.94613	1.49072
13849	0.946129	2.1
13850	0.94613	1.92385
13851	0.946128	1.72443
13852	1.40684	2.1
13853	0.94613	1.80867
13854	0.94613	2.1
13855	0.946131	2.1
13856	0.946131	2.1
13857	0.946131	2.1
13858	0.946131	2.1
13859	1.39021	2.1

13860	0.946131	2.1
13861	0.946131	2.1
13862	0.946131	1.96485
13863	0.946131	1.74645
13864	0.946131	1.35513
13865	0.946131	2.01597
13866	0.946131	2.01393
13867	0.946131	1.28042
13868	0.946131	1.42809
13869	0.810946	2.1
13870	0.810946	2.1
13871	2.69264	2.1
13872	1.35168	2.1
13873	1.0813	2.1
13874	1.08132	2.1
13875	1.08132	2.1
13876	0.810958	2.1
13877	1.16687	2.1
13878	0.946131	2.1
13879	0.946131	2.1
13880	1.34632	2.1
13881	0.946131	2.1
13882	1.34632	2.1
13883	0.946131	2.1
13884	0.946128	0.914789
13885	0.946133	0.422451
13886	0.946133	0.303596
13887	0.946131	0.700121
13888	0.946131	0.332247
13889	1.3564	0.50586
13890	0.94613	0.781552
13891	0.946128	2.1
13892	0.946132	2.1
13893	0.94613	2.1
13894	0.94613	2.1
13895	0.946131	2.1
13896	1.35827	2.1
13897	0.94613	2.1
13898	0.946133	1.10157
13899	0.946132	1.63709
13900	0.946132	1.37385
13901	0.946133	1.56929
13902	0.946133	1.48536
13903	0.946132	1.52722
13904	0.946133	1.58424
13905	0.946132	2.1
13906	0.946132	1.80197
13907	0.946132	0.444195
13908	0.946131	2.1
13909	0.946132	1.43355
13910	0.946132	1.62909
13911	0.946132	1.65326
13912	0.94613	0.41648
13913	0.94613	0.289178
13914	0.946128	0.442069
13915	0.946132	0.247546
13916	0.946132	0.438207
13917	0.94613	0.318509

13918	0.946131	0.310258
13919	0.946126	0.126451
13920	0.946131	0.0845487
13921	0.946127	0.154387
13922	0.946129	0.0734002
13923	0.94613	0.119185
13924	0.946128	0.0896974
13925	0.946128	0.0997345
13926	0.946131	0.0821772
13927	0.946127	0.0813318
13928	0.946127	0.0808413
13929	0.946131	0.0901653
13930	0.946131	0.0706938
13931	0.946128	0.0790046
13932	0.946129	0.0998067
13933	0.94613	0.0897147
13934	0.946131	0.0874846
13935	0.946129	0.0879198
13936	0.946132	0.0942107
13937	0.946132	0.0843646
13938	0.946131	0.0853678
13939	0.94613	0.103554
13940	0.94613	0.109162
13941	0.946131	0.0874804
13942	0.946129	0.099138
13943	0.946132	0.0780348
13944	0.946132	0.106534
13945	1.32827	0.0893904
13946	0.946131	0.101623
13947	0.946132	2.1
13948	0.946132	2.1
13949	0.946131	2.1
13950	0.946131	2.1
13951	0.946132	2.1
13952	0.946131	2.1
13953	0.946131	2.1
13954	0.946132	0.604465
13955	0.946132	2.1
13956	0.946131	0.671319
13957	1.38356	0.248463
13958	0.946133	2.08607
13959	0.946131	0.840669
13960	0.946131	1.38589
13961	0.94613	2.1
13962	0.946131	2.1
13963	0.946131	2.1
13964	0.946128	2.1
13965	0.946133	2.1
13966	0.946131	2.1
13967	1.36918	2.1
13968	0.946131	1.90847
13969	0.94613	2.1
13970	0.946129	2.1
13971	0.946133	2.1
13972	0.946132	2.1
13973	1.3771	2.1
13974	0.946131	2.1
13975	1.30029	1.65321

13976	0.94613	1.28545
13977	0.946129	1.89605
13978	0.946132	1.7231
13979	0.946132	0.125844
13980	0.946131	1.5783
13981	0.94613	0.570621
13982	0.946133	0.05
13983	0.946132	0.0504063
13984	0.946132	0.05
13985	0.946132	0.05
13986	0.946132	0.05
13987	0.946132	0.05
13988	1.21174	0.05
13989	0.810946	2.1
13990	0.810945	2.1
13991	2.63045	2.1
13992	1.35169	2.1
13993	1.08132	2.1
13994	1.08132	2.1
13995	1.08132	2.1
13996	0.810946	2.1
13997	1.1796	2.1
13998	0.946131	2.1
13999	0.946132	2.1
14000	1.31522	2.1
14001	0.946132	2.1
14002	1.31522	2.1
14003	0.946132	2.1
14004	0.946131	2.1
14005	1.40615	2.1
14006	0.946129	2.1
14007	0.946128	2.1
14008	0.946133	2.1
14009	0.946131	2.1
14010	0.94613	2.1
14011	0.810945	2.1
14012	0.810944	2.1
14013	2.80479	2.1
14014	1.35169	2.1
14015	1.08132	2.1
14016	1.08132	2.1
14017	1.08132	2.1
14018	0.810945	2.1
14019	1.35562	2.1
14020	0.94613	2.1
14021	1.35561	2.1
14022	0.946133	2.1
14023	0.946131	2.1
14024	1.40239	2.1
14025	0.946131	2.1
14026	0.946125	0.246831
14027	0.946131	0.39596
14028	0.946127	0.2088
14029	0.946129	0.238353
14030	0.946128	0.417024
14031	0.946129	0.374502
14032	0.946128	0.295698
14033	0.946119	2.1

14034	0.946113	2.1
14035	0.94612	2.1
14036	0.946119	2.1
14037	1.41915	2.1
14038	0.946107	2.1
14039	0.946132	2.1
14040	0.810946	1.92357
14041	0.810943	2.1
14042	0.810934	2.0853
14043	2.31411	2.1
14044	1.35171	2.1
14045	1.08132	1.99292
14046	1.08132	2.1
14047	1.08133	2.05093
14048	0.946127	2.1
14049	0.94613	2.1
14050	0.94613	2.1
14051	0.946131	2.1
14052	0.946126	2.1
14053	0.946128	2.1
14054	0.946128	2.1
14055	0.810944	2.1
14056	0.810947	2.1
14057	0.81096	2.1
14058	2.78019	2.1
14059	1.35168	2.1
14060	1.08132	2.1
14061	1.08132	2.1
14062	1.08131	2.1
14063	0.946133	0.100816
14064	0.946132	0.109632
14065	0.946132	0.108628
14066	0.946133	0.0977234
14067	0.946132	0.114027
14068	1.3495	0.0888466
14069	0.946133	0.12098
14070	0.946133	2.1
14071	0.946132	2.1
14072	0.946132	2.1
14073	1.39529	2.1
14074	0.946133	2.1
14075	0.946132	2.1
14076	0.946133	2.1
14077	0.946133	2.1
14078	0.946132	2.1
14079	0.946132	2.1
14080	0.946132	2.1
14081	0.946132	2.1
14082	0.946133	2.1
14083	0.946132	2.1
14084	0.810946	2.1
14085	0.810945	2.1
14086	2.67861	2.1
14087	1.35169	2.1
14088	1.08132	2.1
14089	1.08132	2.1
14090	1.08132	2.1
14091	0.810946	2.1

14092	1.24616	2.1
14093	0.946132	2.1
14094	0.946132	2.1
14095	1.33931	2.1
14096	0.946132	2.1
14097	1.33931	2.1
14098	0.946133	2.1
14099	0.946133	0.160747
14100	0.946133	0.0543005
14101	0.946132	0.0565328
14102	0.946133	0.0841709
14103	1.3855	0.05
14104	1.3855	0.0539536
14105	0.946133	0.13785
14106	0.946128	2.1
14107	0.946132	2.1
14108	0.946128	2.1
14109	0.94613	2.1
14110	0.946131	2.1
14111	1.40225	2.1
14112	0.94613	2.1
14113	1.40548	0.05
14114	0.946118	0.05
14115	0.946114	0.05
14116	0.946112	0.05
14117	0.946117	0.05
14118	0.946128	0.05
14119	0.946103	0.05
14120	0.94613	2.1
14121	0.946132	2.1
14122	0.946131	2.1
14123	0.946132	2.1
14124	1.37811	2.1
14125	0.946131	2.1
14126	0.946131	2.1
14127	0.946132	2.1
14128	0.946133	2.1
14129	0.946133	2.1
14130	0.946132	2.1
14131	0.946133	2.1
14132	0.946132	2.1
14133	0.946132	2.1
14134	0.946118	2.1
14135	0.946113	2.1
14136	0.946118	2.1
14137	0.946119	2.1
14138	0.946117	2.1
14139	0.946105	2.1
14140	0.946131	2.1
14141	0.946118	0.05
14142	0.946118	0.05
14143	0.946117	0.05
14144	0.946113	0.05
14145	0.94612	0.05
14146	1.41377	0.05
14147	0.946106	0.05
14148	0.946132	1.58008
14149	1.28436	1.38552

14150	0.946131	1.41353
14151	0.946131	1.58258
14152	0.946131	0.5111
14153	0.94613	1.60501
14154	0.946131	1.46541
14155	0.810947	0.394583
14156	0.810946	0.437303
14157	2.68675	0.251205
14158	1.35168	0.292501
14159	1.08132	0.332988
14160	1.08132	0.339051
14161	1.0813	0.424953
14162	0.81096	0.371661
14163	0.946132	0.273092
14164	1.34338	0.23227
14165	0.946132	0.251661
14166	1.34338	0.28053
14167	0.946132	0.305308
14168	1.34337	0.257722
14169	0.946132	0.263812
14170	0.946127	2.1
14171	0.946131	2.1
14172	0.946127	2.1
14173	0.946131	1.99547
14174	0.946131	2.1
14175	0.946128	2.08689
14176	1.30281	2.1
14177	0.946132	0.6256
14178	0.946132	0.384951
14179	0.946132	0.444055
14180	0.946131	0.458899
14181	0.946132	0.612653
14182	1.37973	0.290466
14183	0.946132	0.422955
14184	0.946131	1.25378
14185	1.41798	0.786799
14186	0.946132	0.419494
14187	0.946132	0.817981
14188	0.946129	1.3015
14189	0.946131	0.959431
14190	0.946131	1.29114
14191	0.946118	1.11971
14192	1.41776	0.469941
14193	0.946113	0.466175
14194	0.946119	0.480606
14195	0.946117	1.25131
14196	1.41778	0.496547
14197	0.946105	1.24919
14198	0.946131	1.30277
14199	1.41798	1.3435
14200	0.946132	1.37678
14201	0.946132	1.12686
14202	0.94613	1.3369
14203	1.41799	1.06828
14204	0.946131	1.13326
14205	0.946133	2.1
14206	0.946132	2.1
14207	0.946132	1.68063

14208	0.946132	2.1
14209	0.946133	1.87222
14210	0.946132	2.1
14211	0.946132	2.1
14212	0.946131	2.1
14213	0.946131	2.1
14214	0.94613	1.07015
14215	0.946129	2.1
14216	0.946132	1.65455
14217	0.94613	1.8943
14218	0.946131	2.1
14219	0.94612	1.54968
14220	0.946126	2.1
14221	0.946119	1.59591
14222	0.94612	1.6776
14223	1.40766	1.99346
14224	0.946132	1.27984
14225	0.946107	2.1
14226	0.946128	2.1
14227	1.31213	2.1
14228	0.946129	2.1
14229	0.94613	2.1
14230	0.94613	2.1
14231	0.94613	2.1
14232	0.94613	2.1
14233	0.946127	2.1
14234	0.946131	2.1
14235	0.94613	2.1
14236	0.94613	2.1
14237	1.36653	2.1
14238	0.946128	2.1
14239	0.946128	2.1
14240	0.946127	2.1
14241	0.94613	2.1
14242	0.946131	2.1
14243	0.94613	2.1
14244	0.946128	2.1
14245	0.946128	2.1
14246	0.946129	2.1
14247	0.946133	2.1
14248	1.40081	2.1
14249	0.946132	2.1
14250	0.946132	2.1
14251	0.946133	2.1
14252	0.946132	2.1
14253	0.946133	2.1
14254	0.946128	2.1
14255	1.32282	2.1
14256	0.946129	2.1
14257	0.94613	2.1
14258	0.946131	2.1
14259	0.946131	2.1
14260	0.946131	2.1
14261	0.810945	2.1
14262	0.810943	2.1
14263	0.810933	2.1
14264	2.44097	2.1
14265	1.3517	2.1

14266	1.08132	2.1
14267	1.08132	2.1
14268	1.08133	2.1
14269	0.946131	2.1
14270	0.94613	2.1
14271	0.946133	2.1
14272	0.946133	2.1
14273	0.946128	2.1
14274	0.946131	2.1
14275	1.27676	2.1
14276	0.946126	1.64024
14277	0.946126	2.03468
14278	0.946131	1.54199
14279	0.946127	2.03133
14280	0.94613	0.209521
14281	0.946128	1.06683
14282	0.946128	0.868553
14283	0.946132	0.0693554
14284	0.946133	0.0944485
14285	0.946132	0.0684086
14286	0.946132	0.0971044
14287	0.946132	0.064508
14288	0.946133	0.0756158
14289	0.946132	0.0851188
14290	0.946128	0.0941211
14291	0.946131	0.113157
14292	0.94613	0.0911387
14293	0.946129	0.11626
14294	0.94613	0.0855212
14295	0.94613	0.0894322
14296	0.94613	0.103944
14297	0.946132	0.05
14298	0.946128	0.05
14299	0.94613	0.0613202
14300	0.946131	0.05
14301	0.94613	0.0573657
14302	0.94613	0.0585332
14303	0.946131	0.05
14304	0.94612	0.05
14305	0.946119	0.064816
14306	0.946119	0.0523207
14307	0.946124	0.0610299
14308	0.946119	0.0537389
14309	0.946132	0.0539131
14310	0.946107	0.0527785
14311	0.946128	2.1
14312	0.946133	2.1
14313	0.946131	2.1
14314	0.94613	2.1
14315	0.946131	2.1
14316	1.37869	2.1
14317	0.946131	2.1
14318	1.35703	2.1
14319	0.946132	2.1
14320	0.946132	2.1
14321	0.946132	2.1
14322	0.946132	2.1
14323	0.946132	2.1

14324	0.946133	2.1
14325	0.946133	2.1
14326	0.946133	2.1
14327	0.946132	2.1
14328	1.37017	2.1
14329	0.946133	2.1
14330	0.946132	2.1
14331	0.946133	2.1
14332	0.946114	0.05
14333	0.946119	0.05
14334	0.946119	0.05
14335	0.94612	0.05
14336	0.946119	0.05
14337	0.946132	0.05
14338	0.946107	0.05
14339	0.946131	0.0536948
14340	0.946131	0.0564088
14341	0.946126	0.0690701
14342	0.946127	0.0701869
14343	0.94613	0.0572038
14344	0.946129	0.0706706
14345	0.946128	0.0631979
14346	0.946131	1.47902
14347	0.946131	1.29655
14348	0.946132	1.23484
14349	0.946132	1.43926
14350	0.94613	1.56602
14351	0.946131	1.58088
14352	1.3279	1.55015
14353	0.946132	2.1
14354	1.40932	2.1
14355	0.946132	2.1
14356	0.946132	2.1
14357	0.946132	2.1
14358	0.946131	2.1
14359	0.946132	2.1
14360	0.946131	0.102002
14361	0.946131	0.0989792
14362	0.946132	0.100092
14363	0.946132	0.104314
14364	0.94613	0.130275
14365	0.946131	0.113068
14366	1.26868	0.110404
14367	0.810943	2.1
14368	0.810945	2.1
14369	0.810932	2.1
14370	2.82321	2.1
14371	1.3517	2.1
14372	1.08132	2.1
14373	1.08132	2.1
14374	1.08133	2.1
14375	0.810943	1.02798
14376	0.810944	0.631922
14377	2.63079	1.42967
14378	1.35168	0.856071
14379	1.0813	0.854636
14380	1.08132	0.450811
14381	1.08132	1.94861

14382	0.810957	1.00957
14383	1.17856	0.109642
14384	0.946132	0.829554
14385	0.946128	0.51497
14386	1.3154	1.96592
14387	0.94613	0.704113
14388	1.31539	1.04327
14389	0.94613	1.72057
14390	0.94613	1.76153
14391	0.94613	1.75307
14392	0.946131	1.68211
14393	0.946131	1.79263
14394	0.946129	1.80199
14395	0.94613	2.02546
14396	1.21997	1.96684
14397	0.946131	1.61874
14398	1.25083	1.51077
14399	0.946132	1.4878
14400	0.946132	1.64645
14401	0.946129	1.28876
14402	1.25083	1.7427
14403	0.946131	1.74227
14404	0.946127	1.74427
14405	1.41342	1.17717
14406	0.94613	1.58295
14407	0.946131	1.68583
14408	0.946127	0.459908
14409	0.946129	0.672323
14410	0.946129	1.33669
14411	0.946131	1.49219
14412	1.29149	1.58488
14413	0.946133	2.1
14414	0.946133	1.34005
14415	0.946128	1.82613
14416	0.946131	1.23021
14417	0.94613	1.41409
14418	0.94613	2.1
14419	0.94613	2.1
14420	0.946132	2.1
14421	0.946132	2.1
14422	0.946128	2.05718
14423	0.94613	2.1
14424	1.41022	2.1
14425	0.946132	0.0981527
14426	0.946132	0.0866077
14427	0.946128	0.103646
14428	0.94613	0.0872465
14429	0.94613	0.11167
14430	0.94613	0.0805309
14431	0.946131	0.118677
14432	0.946129	1.23933
14433	1.37758	1.44279
14434	0.946131	1.61402
14435	0.94613	1.25876
14436	0.946124	1.40554
14437	0.946127	1.29665
14438	0.946127	1.35245
14439	0.946129	1.32213

14440	1.38259	1.48391
14441	0.946132	1.56443
14442	0.946132	1.34808
14443	0.946126	1.47034
14444	0.946129	1.38161
14445	0.94613	1.41455
14446	0.946131	0.0572319
14447	0.946132	0.0560244
14448	0.946132	0.0706807
14449	0.946132	0.05
14450	0.946132	0.0635701
14451	0.946132	0.0697523
14452	0.946132	0.0513465
14453	0.946131	0.05
14454	0.94613	0.05
14455	0.94613	0.05
14456	0.946133	0.05
14457	0.946128	0.05
14458	1.25017	0.05
14459	0.946131	0.05
14460	0.946131	2.1
14461	0.946131	2.1
14462	0.946131	2.1
14463	1.3937	2.1
14464	0.946133	2.1
14465	0.946131	2.1
14466	0.94613	2.1
14467	0.946131	1.97597
14468	0.946131	0.716752
14469	0.946131	1.1236
14470	0.94613	0.406149
14471	0.946133	1.97365
14472	0.946132	1.01691
14473	0.946131	0.956771
14474	0.946132	1.51036
14475	0.946132	0.980703
14476	0.946132	0.625103
14477	0.946132	0.685085
14478	0.946131	0.25421
14479	0.946132	0.215646
14480	0.946132	0.285227
14481	0.946129	1.63946
14482	0.94613	2.1
14483	0.94613	1.98302
14484	0.946128	2.1
14485	0.946132	0.343035
14486	0.946131	1.44018
14487	0.94613	1.68481
14488	0.946132	2.1
14489	0.946132	2.1
14490	0.946132	2.1
14491	1.41619	2.1
14492	0.946133	1.8046
14493	0.946133	2.1
14494	0.946132	2.1
14495	0.946132	2.1
14496	0.946132	2.1
14497	0.946132	2.1

14498	1.38244	2.1
14499	0.946132	2.1
14500	0.946131	2.1
14501	0.946132	2.1
14502	0.810946	0.683713
14503	0.810943	1.45331
14504	0.810934	0.751827
14505	2.42741	0.308429
14506	1.35171	0.369175
14507	1.08132	0.462446
14508	1.08132	0.7152
14509	1.08133	0.840082
14510	0.810947	0.655044
14511	0.810943	1.41655
14512	2.44179	0.350383
14513	1.35171	0.4601
14514	1.08132	0.516529
14515	1.08132	0.529747
14516	1.08133	0.572652
14517	0.810934	0.548017
14518	0.946132	1.80419
14519	0.946132	1.75391
14520	0.946133	1.56443
14521	0.946133	1.75177
14522	0.946132	1.95124
14523	0.946132	1.78201
14524	1.12786	1.76309
14525	1.27806	1.43853
14526	0.946132	1.59881
14527	0.946133	1.48103
14528	0.946133	1.61367
14529	0.946132	1.75486
14530	0.946132	1.56932
14531	0.946133	1.67579
14532	0.946132	2.1
14533	0.946132	2.1
14534	0.946132	2.1
14535	1.37098	2.1
14536	0.946133	2.1
14537	0.946132	2.1
14538	1.37098	2.1
14539	0.946125	1.33735
14540	0.946129	1.23916
14541	0.946131	1.44614
14542	0.94613	1.19425
14543	0.946124	1.16026
14544	0.946127	1.20723
14545	1.39514	1.29439
14546	0.946132	2.1
14547	0.946131	2.1
14548	0.94613	2.1
14549	1.39909	2.1
14550	0.946132	2.1
14551	0.946131	2.1
14552	0.94613	2.1
14553	0.946133	2.1
14554	0.946133	1.80089
14555	0.946132	2.1

14556	1.36835	2.1
14557	0.946132	1.87056
14558	0.946133	2.1
14559	0.946132	2.05181
14560	0.946132	2.1
14561	0.946133	2.1
14562	0.946133	2.1
14563	0.946132	2.1
14564	1.39047	2.1
14565	0.946132	2.1
14566	0.946132	2.1
14567	1.33174	0.05
14568	0.946128	0.05
14569	0.94613	0.05
14570	0.94613	0.05
14571	0.94613	0.05
14572	0.94613	0.05
14573	0.946131	0.05
14574	0.946128	0.05
14575	1.36004	0.05
14576	0.946129	0.05
14577	0.94613	0.05
14578	0.94613	0.05
14579	1.36003	0.05
14580	0.94613	0.05
14581	0.946124	2.1
14582	0.946119	2.1
14583	0.946119	2.1
14584	0.946119	2.1
14585	0.94612	2.1
14586	0.946132	2.1
14587	1.29062	2.1
14588	0.946128	2.1
14589	0.946128	2.1
14590	0.946133	2.1
14591	0.94613	2.1
14592	0.946131	2.1
14593	0.946131	2.1
14594	1.30584	2.1
14595	0.946131	2.1
14596	0.946131	2.1
14597	0.946131	2.1
14598	0.946132	2.1
14599	1.39549	2.1
14600	0.946131	2.1
14601	0.946131	2.1
14602	0.913386	1.37193
14603	1.17466	1.18521
14604	1.00473	0.884012
14605	0.946132	1.1951
14606	0.946126	1.40484
14607	0.94613	1.3754
14608	0.946129	1.33509
14609	0.946132	0.840549
14610	1.38894	0.993501
14611	0.946132	0.423208
14612	0.946132	0.377476
14613	0.946132	1.16055

14614	0.946132	0.842846
14615	0.946133	0.78124
14616	0.946128	0.571028
14617	1.23865	0.777543
14618	0.946129	0.270042
14619	0.94613	0.316113
14620	0.94613	0.506187
14621	0.94613	0.367332
14622	0.94613	0.452603
14623	0.946126	0.752738
14624	1.40248	0.910097
14625	0.946129	0.695588
14626	0.94613	0.819832
14627	0.946124	1.17366
14628	0.946127	1.0669
14629	0.946128	0.903654
14630	0.946133	0.526633
14631	1.1391	0.944172
14632	0.946132	1.06269
14633	0.946132	0.435957
14634	0.946132	1.21154
14635	0.946133	0.834342
14636	0.946132	0.985301
14637	1.17156	1.22784
14638	0.946133	1.1766
14639	1.33893	1.19043
14640	0.827864	1.14066
14641	0.995232	1.19633
14642	0.827864	1.17831
14643	0.94613	1.16175
14644	1.21387	0.943069
14645	0.946132	1.05927
14646	0.946131	0.490854
14647	0.94613	0.901558
14648	0.94613	0.998094
14649	0.94613	0.971743
14650	0.94613	1.03498
14651	0.810943	0.158902
14652	0.810945	0.0903098
14653	2.42463	0.109531
14654	1.3517	0.104108
14655	1.08132	0.137975
14656	1.08132	0.144471
14657	1.08133	0.109116
14658	0.810932	0.156068
14659	0.946133	1.84927
14660	0.946132	0.995145
14661	0.946131	1.69231
14662	1.36748	1.56412
14663	0.946133	1.30787
14664	0.946131	1.53389
14665	0.946132	0.289765
14666	1.35856	0.05
14667	0.946131	0.05
14668	0.94613	0.0532836
14669	0.94613	0.05
14670	0.946131	0.05
14671	0.94613	0.0556902

14672	0.94613	0.05
14673	0.946132	2.1
14674	0.946131	2.1
14675	0.946131	2.1
14676	0.946131	2.1
14677	1.41149	2.1
14678	1.41149	2.1
14679	0.946131	2.1
14680	0.946128	2.1
14681	0.946128	2.1
14682	0.946133	2.1
14683	0.946131	2.1
14684	0.946131	2.1
14685	0.946131	2.1
14686	0.94613	2.1
14687	1.41388	0.0915999
14688	0.94613	0.127332
14689	0.946132	0.0889869
14690	0.946132	0.113854
14691	0.946129	0.139905
14692	0.946131	0.11486
14693	0.946131	0.120435
14694	1.02798	0.0860668
14695	0.946132	0.188082
14696	1.02798	0.0924894
14697	0.946133	0.173903
14698	1.14931	0.0871462
14699	0.957746	0.164343
14700	0.93452	0.129171
14701	0.946129	2.1
14702	0.946134	2.1
14703	0.946133	2.1
14704	0.946131	2.1
14705	0.946131	2.1
14706	0.946131	2.1
14707	0.946131	2.1
14708	0.946133	2.03999
14709	0.946132	1.61127
14710	0.946132	1.78797
14711	0.946132	2.03008
14712	1.38773	1.65084
14713	0.946132	1.81454
14714	0.946133	1.70687
14715	0.946131	0.165674
14716	0.946131	0.156512
14717	1.41512	0.155616
14718	0.946133	0.207646
14719	0.946131	0.214266
14720	0.946131	0.209814
14721	0.946131	0.174176
14722	1.35381	1.59832
14723	0.946132	2.1
14724	1.35381	1.6221
14725	0.946133	2.1
14726	0.946132	1.57928
14727	0.946132	2.1
14728	0.946133	2.06927
14729	1.3365	1.62374

14730	0.946132	1.95473
14731	1.3365	1.44334
14732	0.946129	1.83838
14733	0.94613	1.6687
14734	0.946128	1.8769
14735	0.946129	1.72592
14736	0.939895	1.95448
14737	1.14285	2.1
14738	0.952366	2.1
14739	0.946131	2.06813
14740	0.94613	2.1
14741	0.946131	2.1
14742	0.94613	2.1
14743	0.946131	2.1
14744	0.946131	2.1
14745	0.946131	2.1
14746	0.946129	2.1
14747	0.946133	2.1
14748	0.946131	2.1
14749	0.946131	2.1
14750	0.946133	2.1
14751	0.946132	2.1
14752	0.946132	2.1
14753	0.946133	2.1
14754	0.946132	2.1
14755	0.946132	2.1
14756	0.946133	2.1
14757	0.946133	2.1
14758	0.946132	1.73394
14759	0.946132	2.1
14760	0.946133	2.02262
14761	0.946132	2.1
14762	0.946132	2.1
14763	0.946132	1.7137
14764	0.946132	2.1
14765	0.946131	2.1
14766	0.946131	2.1
14767	0.946131	2.1
14768	1.38969	2.1
14769	0.946131	2.1
14770	0.946132	2.1
14771	0.946128	2.0883
14772	0.946133	1.83033
14773	0.946132	1.84585
14774	0.946131	1.84418
14775	0.94613	1.9465
14776	0.946131	1.98033
14777	1.28464	1.95545
14778	0.946119	1.10861
14779	1.37492	1.17873
14780	0.946125	1.18873
14781	0.946119	0.895066
14782	0.946119	1.19289
14783	0.946132	1.08143
14784	0.946107	1.15424
14785	0.810942	2.1
14786	0.810943	2.1
14787	0.709587	2.1

14788	2.64488	2.1
14789	1.21651	2.1
14790	0.946151	2.1
14791	0.979948	2.1
14792	1.0813	2.1
14793	0.946133	1.57528
14794	0.946132	1.83523
14795	0.946132	2.1
14796	0.946133	1.25364
14797	0.946132	1.16971
14798	0.946132	1.26168
14799	1.41134	1.47116
14800	0.946133	2.0052
14801	0.946132	1.66493
14802	0.946133	2.00279
14803	0.946133	1.63502
14804	0.946132	1.34405
14805	0.946133	1.74219
14806	1.31338	1.81564
14807	2.07612	1.11201
14808	0.946102	0.05
14809	0.752448	0.05
14810	1.20617	0.05
14811	0.94613	0.05
14812	0.75242	0.05
14813	0.946131	0.05
14814	0.752419	0.05
14815	0.946132	1.40257
14816	1.25736	1.40781
14817	1.25736	1.3666
14818	0.946132	1.36341
14819	1.08054	1.29261
14820	1.25736	1.32204
14821	0.946132	1.391
14822	0.946128	2.1
14823	1.1896	2.1
14824	1.18959	2.1
14825	0.946131	2.1
14826	1.33271	2.1
14827	0.946105	2.1
14828	0.946156	2.1
14829	0.741946	0.05
14830	1.33587	0.05
14831	0.741947	0.05
14832	1.21954	0.05
14833	0.94613	0.05
14834	0.741946	0.05
14835	1.81347	0.05
14836	1.81347	0.05
14837	0.653322	0.05
14838	1.36935	0.05
14839	0.661882	0.05
14840	1.04753	0.05
14841	0.507464	0.05
14842	0.945844	1.54508
14843	1.59507	2.1
14844	1.04043	1.56421
14845	0.946131	2.1

14846	1.32891	2.01778
14847	0.946131	1.90285
14848	0.946131	1.79893
14849	1.28866	1.49661
14850	1.12682	0.105061
14851	0.851543	0.137267
14852	1.04072	0.0973665
14853	0.946132	0.285234
14854	1.13437	0.0901559
14855	1.04167	0.0977406
14856	0.936652	0.100804
14857	1.258	2.1
14858	0.946131	2.1
14859	0.946132	2.1
14860	1.36968	2.1
14861	0.946132	2.1
14862	1.36968	2.1
14863	0.946131	2.1
14864	2.30046	0.234097
14865	2.82969	0.267722
14866	0.946132	2.1
14867	1.35022	2.1
14868	1.12356	2.1
14869	0.94613	2.1
14870	1.35022	2.1
14871	0.946131	2.1
14872	0.94613	2.1
14873	0.946131	2.1
14874	1.22401	2.1
14875	0.952456	2.1
14876	0.94613	2.1
14877	1.22401	2.1
14878	0.946131	2.1
14879	0.946131	2.1
14880	1.33047	2.1
14881	0.946132	2.1
14882	1.33047	2.1
14883	0.946131	2.1
14884	1.33047	2.1
14885	0.946132	2.1
14886	1.2107	2.1
14887	0.61957	2.1
14888	1.80666	2.1
14889	1.53743	2.1
14890	0.619572	2.03665
14891	1.32547	2.1
14892	1.16166	2.1
14893	1.04074	2.1
14894	0.557621	2.1
14895	0.946128	2.1
14896	1.36607	2.1
14897	0.94613	2.1
14898	1.36607	2.1
14899	1.39416	2.1
14900	0.94613	2.1
14901	0.94613	2.1
14902	1.23969	2.1
14903	0.946131	2.1

14904	0.946131	2.1
14905	1.13537	2.1
14906	1.05719	2.1
14907	0.806848	2.1
14908	1.30251	2.1
14909	0.851526	1.6928
14910	1.14176	2.1
14911	0.93666	1.68291
14912	0.946114	2.1
14913	1.15832	2.0184
14914	0.924624	1.9871
14915	0.967609	1.87013
14916	0.771764	0.149035
14917	1.26652	0.164592
14918	0.946133	0.176533
14919	1.17957	0.0988384
14920	0.771735	0.141671
14921	0.946122	0.169372
14922	0.946133	0.138606
14923	0.924325	1.73081
14924	0.656739	1.80586
14925	0.637876	1.81732
14926	0.530092	1.75861
14927	0.914415	1.72895
14928	0.821311	1.85386
14929	0.630589	1.83441
14930	0.738823	1.8083
14931	0.839109	1.81894
14932	1.55029	0.05
14933	1.13537	1.82699
14934	0.946131	2.1
14935	0.946128	1.9488
14936	1.30162	2.1
14937	0.952476	2.1
14938	1.29289	2.1
14939	0.939783	2.1
14940	0.94612	2.1
14941	0.946131	2.1
14942	1.07998	2.1
14943	0.836985	2.1
14944	1.1604	2.1
14945	0.94613	2.1
14946	0.837016	2.1
14947	0.712461	0.638303
14948	1.36399	0.646401
14949	1.8724	0.437023
14950	1.8724	0.360981
14951	0.623426	1.38447
14952	1.36399	0.544958
14953	1.22089	0.524917
14954	0.946132	0.486239
14955	0.946131	2.1
14956	1.32241	2.1
14957	1.32241	2.1
14958	0.946133	2.1
14959	1.40271	2.1
14960	0.946131	2.1
14961	0.946131	2.1

14962	1.1989	1.68979
14963	0.954106	1.81443
14964	0.820068	1.64258
14965	0.563139	1.74737
14966	1.06448	1.74751
14967	0.892472	1.84953
14968	0.862598	1.70011
14969	0.745851	1.86664
14970	1.13336	1.78153
14971	0.946129	2.1
14972	1.28254	2.1
14973	1.28254	2.1
14974	0.946131	2.1
14975	1.41302	2.1
14976	0.946131	2.1
14977	0.946131	2.1
14978	0.851543	2.07627
14979	1.21961	2.1
14980	0.851484	2.00992
14981	0.851543	2.1
14982	1.15705	2.1
14983	0.946133	2.1
14984	0.909417	2.1
14985	1.13808	1.00334
14986	0.946133	0.163654
14987	0.946131	1.33188
14988	1.30194	0.146123
14989	0.946131	0.265382
14990	1.30194	0.157143
14991	0.946131	0.23466
14992	0.930941	1.50684
14993	1.20185	1.43867
14994	1.02404	1.43503
14995	0.946124	1.59544
14996	1.3563	1.49194
14997	0.946127	1.59786
14998	0.946128	1.52225
14999	0.946116	2.1
15000	1.3144	2.1
15001	1.13088	2.1
15002	0.946118	2.1
15003	1.31438	2.1
15004	0.946105	2.1
15005	0.946131	2.1
15006	1.76972	0.0963845
15007	1.42091	0.0695825
15008	0.946128	0.0920184
15009	0.946101	0.153984
15010	0.863552	0.129887
15011	0.94613	0.0789814
15012	1.21232	0.110189
15013	0.94613	0.150955
15014	1.2137	0.376557
15015	1.23224	1.04963
15016	1.86512	0.482205
15017	1.86512	0.31854
15018	0.580825	1.59927
15019	1.2137	1.09657

15020	0.946133	1.01066
15021	0.580822	0.811832
15022	0.946128	2.1
15023	1.40586	2.1
15024	0.94613	2.1
15025	1.3719	2.1
15026	1.3719	2.1
15027	1.40586	2.1
15028	0.94613	2.1
15029	0.946132	2.1
15030	1.35025	1.78026
15031	1.1794	2.09444
15032	0.946132	0.700063
15033	1.35025	1.62257
15034	0.946131	1.61651
15035	0.946132	1.69823
15036	0.946133	2.1
15037	1.60149	2.1
15038	1.67058	2.1
15039	0.851542	2.1
15040	1.20296	2.1
15041	1.17501	2.1
15042	0.946132	2.1
15043	1.04993	2.1
15044	0.851541	2.1
15045	1.48219	2.1
15046	0.874565	2.1
15047	0.946129	2.1
15048	1.22049	2.1
15049	0.946131	2.1
15050	0.946131	2.1
15051	0.94613	2.1
15052	0.848719	1.97649
15053	1.17195	2.0199
15054	0.946129	1.36429
15055	1.35426	1.53413
15056	0.94613	1.56684
15057	0.94613	1.73883
15058	0.946133	2.1
15059	1.14342	2.1
15060	1.14342	2.1
15061	0.946133	2.1
15062	1.39009	2.1
15063	0.946132	2.1
15064	0.946132	2.1
15065	1.38819	2.1
15066	0.946133	2.1
15067	1.38819	2.1
15068	0.946131	2.1
15069	1.4116	2.1
15070	0.946131	2.1
15071	1.38819	2.1
15072	0.946127	2.1
15073	1.32244	2.1
15074	1.2176	2.1
15075	0.946131	2.1
15076	1.32244	2.1
15077	0.94613	2.1

15078	0.946129	2.1
15079	1.32415	2.1
15080	1.11239	2.1
15081	0.984099	2.1
15082	1.65532	2.1
15083	1.53082	2.1
15084	1.32068	2.1
15085	1.32068	2.1
15086	1.08172	2.1
15087	3.76293	0.05
15088	1.36335	0.05
15089	0.953925	2.1
15090	0.989497	2.1
15091	0.98949	2.1
15092	1.06341	2.1
15093	1.27875	2.1
15094	1.04598	2.1
15095	0.603154	2.1
15096	0.582457	2.1
15097	0.582457	2.1
15098	0.568994	2.1
15099	1.5791	2.1
15100	1.09506	2.1
15101	1.45327	2.1
15102	1.88406	2.1
15103	1.88404	2.1
15104	1.27389	2.1
15105	1.14206	2.1
15106	1.00232	2.1
15107	1.00231	2.1
15108	1.68436	2.1
15109	1.4677	0.05
15110	1.28828	0.05
15111	1.28828	0.05
15112	1.46932	0.05
15113	1.28228	2.1
15114	1.64268	2.1
15115	0.959473	2.1
15116	1.10101	2.1
15117	1.07976	2.1
15118	1.2873	2.1
15119	1.70303	0.121756
15120	1.40066	0.231512
15121	1.5563	0.0624222
15122	1.13727	0.280566
15123	1.03529	2.1
15124	1.11285	2.1
15125	1.03529	2.1
15126	1.28667	2.1
15127	1.32119	2.1
15128	1.44777	2.1
15129	1.44778	2.1
15130	1.61121	2.1
15131	1.09372	0.05
15132	1.17664	0.05
15133	1.17664	0.05
15134	0.872931	0.05
15135	1.02415	2.1

15136	1.28228	2.1
15137	2.14692	2.1
15138	3.90469	2.1
15139	1.00509	2.1
15140	1.28228	2.1
15141	3.32936	0.05
15142	1.1333	0.05
15143	1.11048	2.1
15144	0.979854	2.1
15145	1.65815	2.1
15146	1.00185	2.1
15147	1.84887	2.1
15148	1.59597	2.1
15149	1.78468	2.1
15150	1.87508	2.1
15151	1.82716	2.1
15152	1.33429	2.1
15153	1.34626	2.1
15154	0.797142	2.1
15155	1.00007	1.85974
15156	1.02051	2.1
15157	1.02051	2.02007
15158	1.03373	2.1
15159	1.05786	2.1
15160	0.887631	2.1
15161	0.992773	2.1
15162	1.28462	2.1
15163	2.35274	1.72464
15164	1.82533	1.34409
15165	1.82533	1.58118
15166	1.19496	1.26502
15167	1.28597	0.05
15168	1.09725	2.1
15169	1.60948	2.1
15170	1.20421	2.1
15171	1.16566	2.1
15172	1.88522	2.1
15173	1.81948	0.215662
15174	1.14042	0.177146
15175	1.21558	0.361583
15176	1.56876	0.235701
15177	1.6697	2.1
15178	1.81655	2.1
15179	1.81655	2.1
15180	1.5956	2.1
15181	0.906059	2.1
15182	1.63045	2.1
15183	0.9533	1.68634
15184	1.1475	1.57346
15185	1.72231	2.1
15186	1.94936	1.97183
15187	1.06826	2.1
15188	1.08576	2.1
15189	1.06826	2.1
15190	0.973559	2.1
15191	1.62737	0.05
15192	0.965676	0.05
15193	1.45055	0.0503795

15194	1.17631	0.0516551
15195	1.92535	0.05
15196	1.9503	0.0656904
15197	1.99828	0.05
15198	1.70302	0.0649402
15199	1.65139	0.0513218
15200	1.26784	0.0571681
15201	1.35923	0.0631748
15202	1.70302	0.0667026
15203	1.33033	2.1
15204	1.12481	2.1
15205	1.29452	2.1
15206	1.25399	2.1
15207	1.12481	2.1
15208	0.918358	2.1
15209	1.39332	2.1
15210	1.32368	2.1
15211	1.11712	1.47394
15212	1.07999	1.28782
15213	1.07999	0.927489
15214	1.05619	0.8352
15215	1.1766	2.1
15216	1.35735	2.1
15217	1.42456	0.05
15218	1.089	0.05
15219	1.11081	0.05
15220	2.88521	0.05
15221	1.09754	0.05
15222	1.85479	0.05
15223	1.27922	0.883299
15224	1.03556	0.426053
15225	1.07508	2.07693
15226	1.09795	2.1
15227	0.967672	1.53468
15228	1.07508	1.88365
15229	1.03933	2.1
15230	1.02975	2.1
15231	1.02975	2.1
15232	1.01121	2.1
15233	1.24526	0.05
15234	1.72368	0.05
15235	1.89607	0.05
15236	2.13512	0.05
15237	1.34591	0.25187
15238	1.14721	0.286365
15239	1.15539	2.1
15240	1.90938	2.07878
15241	1.90941	2.1
15242	1.89242	2.1
15243	1.18907	2.1
15244	1.20408	2.1
15245	1.20409	2.1
15246	1.22655	2.1
15247	0.701447	2.1
15248	1.57567	2.1
15249	1.38806	2.1
15250	2.09407	2.1
15251	1.07612	1.63201

15252	0.990019	1.63185
15253	1.15564	2.1
15254	1.01243	1.9237
15255	1.28956	0.05528
15256	1.35146	0.0594871
15257	1.35145	0.0748445
15258	1.22811	0.0818692
15259	1.09662	2.1
15260	1.42486	2.1
15261	1.10106	0.05
15262	1.90814	0.05
15263	1.3946	0.05
15264	1.11926	0.05
15265	0.725839	2.1
15266	0.925068	2.1
15267	0.925074	2.1
15268	1.43282	2.1
15269	1.91756	1.12422
15270	1.74321	1.36679
15271	1.74321	0.691887
15272	1.63413	1.06166
15273	1.9194	0.0574199
15274	1.62246	0.0505072
15275	1.90456	0.0507502
15276	1.06578	0.0504842
15277	1.46019	0.051408
15278	1.17036	0.05
15279	1.70233	1.4589
15280	1.53515	1.19793
15281	2.03494	2.1
15282	1.78441	2.1
15283	1.75992	2.1
15284	1.94351	2.1
15285	1.51057	0.0504132
15286	0.991435	0.05
15287	1.98068	0.0663328
15288	1.64283	0.0620278
15289	2.96126	0.0534097
15290	2.20692	0.0544341
15291	1.95054	0.05
15292	1.21325	0.05
15293	0.996828	0.223663
15294	1.01976	0.197564
15295	1.17129	2.1
15296	1.13664	2.1
15297	1.13664	2.1
15298	1.09627	2.1
15299	2.65123	2.1
15300	3.23062	2.1
15301	3.23062	2.1
15302	3.92038	2.1
15303	1.89019	0.221646
15304	1.79109	0.255108
15305	1.79109	0.185288
15306	1.54197	0.202151
15307	3.41824	2.1
15308	1.93553	0.845958
15309	2.26215	2.1

15310	2.0103	0.696019
15311	1.51569	1.43898
15312	1.01683	1.17095
15313	1.78595	2.1
15314	1.66537	2.1
15315	3.73426	2.1
15316	3.53905	2.1
15317	1.66537	2.1
15318	1.52903	2.1
15319	3.53905	2.1
15320	3.3837	2.1
15321	1.19901	1.33595
15322	1.35002	1.85919
15323	1.35002	0.177175
15324	1.11683	0.28827
15325	1.75326	1.50737
15326	0.712404	0.640544
15327	2.97768	2.1
15328	2.98949	2.1
15329	1.2832	2.1
15330	1.39518	2.1
15331	2.98949	2.1
15332	2.99339	2.1
15333	1.39517	2.1
15334	1.46124	2.1
15335	1.28534	2.1
15336	1.03391	2.1
15337	1.75366	1.82313
15338	1.26247	1.93406
15339	1.13621	2.1
15340	2.43994	2.1
15341	1.47904	0.0514555
15342	1.80085	0.05
15343	1.80085	0.05
15344	1.64656	0.0599637
15345	2.87331	2.1
15346	2.82863	2.1
15347	2.82859	2.1
15348	2.84673	2.1
15349	1.0961	0.0642367
15350	1.39448	0.128453
15351	1.21366	0.12742
15352	1.20143	0.079928
15353	1.85795	0.0762395
15354	1.36768	0.0667702
15355	1.36769	0.0599058
15356	1.45789	0.05
15357	1.32605	0.05
15358	1.27148	0.05
15359	1.70218	0.0527667
15360	1.3063	0.0616996
15361	1.45603	0.0548423
15362	1.89226	0.0785505
15363	1.2742	0.05
15364	2.10112	0.05
15365	1.10748	0.0695162
15366	1.1893	0.0742617
15367	1.04921	2.1

15368	1.22927	2.1
15369	1.22927	2.1
15370	1.46568	2.1
15371	0.811186	2.1
15372	0.792162	2.1
15373	0.792162	2.1
15374	0.758749	2.1
15375	1.03581	2.1
15376	0.737481	2.1
15377	2.24071	2.1
15378	1.34868	2.1
15379	1.47203	1.39311
15380	1.40967	1.41963
15381	1.40967	1.44813
15382	1.27916	1.4342
15383	1.17418	2.1
15384	1.04921	2.1
15385	1.28291	1.13761
15386	1.3213	0.54906
15387	1.46812	0.755317
15388	2.42522	1.07712
15389	1.17879	1.36933
15390	1.28291	1.40824
15391	1.28291	1.38349
15392	1.13438	1.42447
15393	1.75004	2.1
15394	1.78247	2.1
15395	1.78247	2.1
15396	1.76421	2.1
15397	2.25975	0.05
15398	3.66111	0.05
15399	3.6611	0.05
15400	3.44043	0.05
15401	1.13339	2.1
15402	1.13322	2.1
15403	1.13323	2.1
15404	1.20761	2.1
15405	1.34337	0.315776
15406	1.90912	1.15591
15407	1.04399	0.401423
15408	2.00578	1.14001
15409	1.04973	2.1
15410	1.22615	2.1
15411	1.93384	2.1
15412	2.62816	1.66956
15413	1.13127	2.1
15414	1.47642	2.1
15415	2.62816	2.1
15416	3.31755	1.3722
15417	1.47642	2.1
15418	1.80454	2.1
15419	0.964782	2.1
15420	0.949135	2.1
15421	0.949134	2.1
15422	0.949134	2.1
15423	1.10476	1.5426
15424	0.982858	0.197716
15425	1.10938	0.993466

15426	0.982858	0.871477
15427	1.01012	0.05
15428	0.974081	0.05
15429	1.82643	0.05
15430	1.29585	0.05
15431	1.00763	0.423122
15432	1.3294	0.560987
15433	1.3294	0.195555
15434	1.30001	0.7657
15435	1.59151	2.1
15436	1.99443	2.1
15437	1.07369	2.1
15438	1.30584	2.1
15439	1.05071	2.1
15440	0.948676	2.1
15441	1.13842	2.1
15442	1.42884	2.1
15443	1.59019	0.05
15444	1.38212	0.05
15445	1.34102	0.05
15446	1.02663	0.05
15447	0.797011	2.1
15448	0.81301	2.1
15449	0.81301	2.1
15450	0.811187	2.1
15451	0.991869	0.107528
15452	1.23868	0.108077
15453	1.67201	2.1
15454	1.61201	2.1
15455	1.61202	2.1
15456	1.57627	2.1
15457	0.94994	2.1
15458	1.77867	2.1
15459	0.854945	2.1
15460	0.973905	2.1
15461	1.64193	2.1
15462	1.68893	2.1
15463	1.68893	2.1
15464	1.70002	2.1
15465	2.08149	0.328548
15466	1.69456	0.215475
15467	1.67399	0.05
15468	1.83922	2.1
15469	1.91703	2.1
15470	0.967492	2.1
15471	1.13317	2.1
15472	0.980766	2.1
15473	2.06158	0.0500644
15474	0.986498	0.05
15475	1.76289	0.05
15476	1.17248	0.05
15477	1.51794	0.05
15478	1.30266	0.05
15479	1.03165	0.14806
15480	0.947692	0.183286
15481	1.20473	0.127526
15482	0.947693	0.125473
15483	1.54357	2.1

15484	0.980841	2.1
15485	1.34124	0.130372
15486	1.39818	0.435891
15487	1.39818	0.0990539
15488	1.48035	0.623908
15489	1.03932	2.1
15490	1.2065	2.1
15491	1.20651	2.1
15492	1.12717	2.1
15493	1.09483	2.1
15494	0.994293	2.1
15495	0.994293	2.1
15496	0.97356	2.1
15497	0.995252	0.899448
15498	1.66294	1.05053
15499	1.82775	0.493939
15500	1.42762	0.48496
15501	1.04713	0.116218
15502	1.05475	0.10331
15503	1.29002	0.0738854
15504	1.05475	0.0798538
15505	1.17585	2.1
15506	1.39223	2.1
15507	1.39223	2.1
15508	1.71255	2.1
15509	1.56166	0.844485
15510	1.37762	0.432873
15511	1.16858	2.1
15512	1.0361	2.1
15513	1.23966	0.05
15514	1.10718	0.05
15515	1.99957	0.05
15516	0.658392	0.05
15517	1.36904	0.05
15518	1.12524	0.05
15519	1.04321	1.7039
15520	1.04627	1.71387
15521	1.91351	1.19362
15522	1.63379	1.11334
15523	1.79718	1.34522
15524	0.59634	1.33092
15525	0.958257	2.1
15526	1.06457	2.1
15527	0.958255	2.1
15528	1.03933	2.1
15529	1.14195	2.1
15530	1.49438	2.1
15531	1.49438	2.1
15532	1.31259	2.1
15533	1.23036	2.1
15534	1.2016	2.1
15535	1.2016	2.1
15536	1.17129	2.1
15537	1.05724	2.1
15538	1.04291	2.1
15539	1.12341	2.1
15540	2.32525	2.1
15541	3.39038	2.1

15542	2.09374	2.1
15543	1.76185	0.494811
15544	1.7934	0.564543
15545	1.65809	0.566061
15546	1.54852	0.628791
15547	1.38766	2.1
15548	1.28765	2.1
15549	1.26599	2.1
15550	1.21397	2.1
15551	1.28765	2.1
15552	1.20045	2.1
15553	1.21398	2.1
15554	1.15234	2.1
15555	0.953634	0.297458
15556	1.39132	0.0712537
15557	1.17285	2.1
15558	1.21408	2.1
15559	1.21408	2.1
15560	0.982848	2.1
15561	2.00101	2.1
15562	2.00973	2.1
15563	2.20314	2.1
15564	1.89318	2.1
15565	1.23267	2.1
15566	0.947921	2.1
15567	1.18872	2.1
15568	0.740752	2.1
15569	1.92269	0.131426
15570	1.53584	0.0893499
15571	1.53584	0.119073
15572	1.21067	0.0677287
15573	1.40707	0.0649664
15574	1.50437	0.0644251
15575	1.50435	0.0624277
15576	1.45446	0.0650652
15577	1.47954	1.35221
15578	2.08687	1.69354
15579	2.08687	2.07474
15580	1.1233	2.1
15581	1.04025	1.50752
15582	1.36005	1.97382
15583	1.47606	0.111072
15584	1.3407	0.116613
15585	0.758743	2.1
15586	0.716917	2.1
15587	0.716922	2.1
15588	0.672964	2.1
15589	2.40014	2.1
15590	1.96848	2.1
15591	1.96848	2.1
15592	1.53555	2.1
15593	1.06191	2.1
15594	1.29579	2.1
15595	1.29579	2.1
15596	1.71289	2.1
15597	1.78584	2.1
15598	2.15211	2.1
15599	2.00427	2.1

15600	3.41615	2.1
15601	1.66408	2.1
15602	1.73954	2.1
15603	2.91663	2.1
15604	3.65818	2.1
15605	1.93286	2.1
15606	1.08831	2.1
15607	1.31242	0.05
15608	1.11712	0.05
15609	1.26311	0.05
15610	1.31242	0.05
15611	1.10378	2.1
15612	0.968594	2.1
15613	1.48389	0.05
15614	1.49613	0.05
15615	1.04349	1.23257
15616	1.26409	0.666285
15617	1.26409	0.589756
15618	1.05724	1.1372
15619	1.65723	0.129044
15620	1.83116	0.106547
15621	2.06284	0.114288
15622	2.00512	0.0935949
15623	1.83116	0.126324
15624	2.03139	0.100049
15625	2.20564	0.109011
15626	1.90048	0.0866573
15627	0.963392	2.1
15628	1.01346	0.927535
15629	2.98092	2.1
15630	2.70295	2.1
15631	2.70295	2.1
15632	2.48828	2.1
15633	1.63661	2.1
15634	1.64158	2.1
15635	1.64158	2.1
15636	1.6488	2.1
15637	1.17506	0.136026
15638	1.09667	0.124951
15639	2.00539	0.416738
15640	1.09667	0.199223
15641	1.54946	2.1
15642	1.43577	2.1
15643	1.26103	2.1
15644	1.49808	2.1
15645	1.089	0.05
15646	1.22586	0.05
15647	2.35027	0.309333
15648	1.37549	0.188259
15649	3.33706	0.181916
15650	1.69894	0.173518
15651	2.00539	0.215906
15652	1.53798	0.173015
15653	1.28938	2.1
15654	1.20969	2.1
15655	1.20969	2.1
15656	1.15864	2.1
15657	1.7986	2.1

15658	1.75869	2.1
15659	1.58281	2.1
15660	1.7392	2.1
15661	1.30733	1.19646
15662	1.5419	1.29954
15663	1.50883	1.1962
15664	1.85439	1.00623
15665	1.73531	0.874443
15666	1.70225	0.67494
15667	1.22176	0.052986
15668	1.59492	0.0872525
15669	1.59492	0.0735966
15670	2.15542	0.105658
15671	3.24379	2.1
15672	2.64775	2.1
15673	3.16707	2.1
15674	1.93032	2.1
15675	1.50738	2.1
15676	1.24128	2.1
15677	1.24128	2.1
15678	0.994293	2.1
15679	2.90808	0.05
15680	2.45797	0.05
15681	1.85501	0.05
15682	2.90809	0.05
15683	0.892835	1.91823
15684	1.55871	2.1
15685	1.04615	1.20156
15686	1.66643	1.29133
15687	1.70044	2.1
15688	1.80224	2.1
15689	1.70186	1.19484
15690	3.30123	1.80662
15691	1.74789	2.1
15692	1.77446	2.1
15693	1.77901	2.1
15694	1.57995	2.1
15695	1.81518	2.1
15696	2.17716	2.1
15697	2.17716	2.1
15698	2.65123	2.1
15699	2.01899	1.33701
15700	1.77479	1.20586
15701	1.19122	0.544507
15702	1.46724	0.419016
15703	1.70944	0.05
15704	1.08929	0.05
15705	1.68454	0.05
15706	1.08929	0.05
15707	1.51067	1.38745
15708	1.06729	0.979863
15709	1.37762	0.768983
15710	1.13992	0.465872
15711	1.26397	2.1
15712	1.04083	2.1
15713	1.00278	2.1
15714	1.04084	2.1
15715	1.1016	0.0587837

15716	1.23477	0.0717461
15717	1.23477	0.05
15718	1.64985	0.05
15719	1.11117	2.1
15720	1.13006	2.1
15721	1.13006	2.1
15722	1.14721	2.1
15723	1.21946	2.1
15724	1.0186	2.1
15725	1.13178	2.1
15726	1.00133	2.1
15727	1.67046	0.0579321
15728	0.986036	0.196868
15729	1.67046	0.18522
15730	1.34857	1.85948
15731	2.46016	2.1
15732	2.07161	2.1
15733	2.10732	2.1
15734	2.08371	2.1
15735	1.35602	2.1
15736	0.829065	2.1
15737	1.81667	2.1
15738	0.99406	2.1
15739	1.81667	2.1
15740	1.68073	2.1
15741	2.05736	2.1
15742	2.97311	2.1
15743	2.14011	2.1
15744	1.23411	2.1
15745	1.13632	0.050963
15746	1.40862	0.05
15747	1.28055	0.05
15748	1.14305	0.05
15749	1.12035	0.05
15750	1.15483	0.05
15751	1.94099	0.05
15752	1.82126	0.05
15753	1.15481	0.05
15754	0.998957	0.05
15755	2.10078	0.05
15756	1.81617	0.05
15757	1.27581	2.1
15758	0.968536	2.1
15759	1.33916	2.1
15760	1.27581	2.1
15761	1.32784	2.1
15762	1.4259	2.1
15763	1.70377	2.1
15764	1.54231	2.1
15765	3.77569	2.1
15766	3.42809	2.1
15767	1.54231	2.1
15768	1.51298	2.1
15769	3.55866	2.1
15770	2.86136	2.1
15771	1.83301	0.619539
15772	1.26371	0.586123
15773	1.98301	0.681479

15774	2.0609	0.589592
15775	2.26047	0.300935
15776	2.00537	0.259834
15777	1.7913	0.240303
15778	1.48864	0.208011
15779	1.09504	2.1
15780	1.39827	2.1
15781	2.28084	2.1
15782	1.05983	2.1
15783	1.33593	0.05
15784	1.31508	0.05
15785	1.31508	0.05
15786	1.09599	0.05
15787	1.30647	2.1
15788	1.28846	2.1
15789	1.28846	2.1
15790	1.26923	2.1
15791	1.2955	2.1
15792	1.09044	2.1
15793	2.12209	2.1
15794	1.26438	2.1
15795	0.911819	2.1
15796	1.38752	2.1
15797	2.57704	2.1
15798	2.71736	2.1
15799	1.42284	2.1
15800	1.41562	2.1
15801	2.69131	2.1
15802	2.36178	2.1
15803	1.41562	2.1
15804	1.39564	2.1
15805	1.32368	2.1
15806	0.908053	2.1
15807	0.908053	2.1
15808	0.629213	2.1
15809	1.8758	1.9133
15810	1.27362	1.95583
15811	1.3756	1.34527
15812	2.28749	1.61438
15813	1.84577	0.520178
15814	2.17765	0.540407
15815	1.38969	0.488616
15816	1.84578	0.509328
15817	1.32801	0.26211
15818	1.76377	0.236095
15819	1.22845	2.1
15820	1.57766	2.06758
15821	1.57766	2.1
15822	1.39067	2.1
15823	1.15743	0.0552686
15824	1.37721	0.05
15825	1.69055	0.0662242
15826	1.14858	0.0881943
15827	1.38333	2.1
15828	1.10575	1.51637
15829	1.22418	2.1
15830	1.10574	2.1
15831	1.91509	0.138269

15832	1.02001	0.123626
15833	1.03074	0.290603
15834	1.19122	0.218604
15835	1.67887	2.1
15836	1.67597	2.1
15837	1.84984	2.1
15838	1.77775	2.1
15839	1.73571	2.1
15840	1.27921	2.0829
15841	2.11851	1.78967
15842	1.27921	2.06367
15843	1.42486	2.1
15844	1.12503	2.1
15845	1.12503	2.1
15846	1.30116	2.1
15847	0.568995	2.1
15848	0.557348	2.1
15849	0.557348	2.1
15850	0.53907	2.1
15851	0.633575	2.1
15852	1.33756	2.1
15853	1.51074	2.1
15854	1.93384	2.0077
15855	0.655202	2.1
15856	0.731934	2.1
15857	0.731934	2.1
15858	1.09061	2.1
15859	0.966979	2.1
15860	1.31502	2.1
15861	1.31502	2.1
15862	1.72116	2.1
15863	1.70002	2.1
15864	1.67969	2.1
15865	1.67969	2.1
15866	1.62408	2.1
15867	1.01531	2.1
15868	1.66018	2.1
15869	1.01531	2.1
15870	0.886968	2.1
15871	1.22585	0.05
15872	1.20674	0.05
15873	1.20672	0.05
15874	1.02962	0.05
15875	0.956467	2.1
15876	2.00551	2.1
15877	0.553801	2.1
15878	0.617006	2.1
15879	1.26923	2.1
15880	1.23614	2.1
15881	1.23614	2.1
15882	1.16817	2.1
15883	2.14482	2.1
15884	1.72637	2.1
15885	1.09368	2.1
15886	1.26195	2.1
15887	0.760546	2.1
15888	1.84094	2.1
15889	2.62868	2.1

15890	2.79784	2.1
15891	2.79784	2.1
15892	3.00129	2.1
15893	1.27593	1.43857
15894	1.38877	1.62186
15895	1.06486	0.218529
15896	1.2474	0.128373
15897	1.76234	0.05
15898	1.8685	0.05
15899	1.69863	0.05
15900	1.29666	0.05
15901	1.91646	2.1
15902	1.8582	2.1
15903	1.8582	2.1
15904	1.7986	2.1
15905	3.44043	0.05
15906	1.94764	0.05
15907	1.94764	0.05
15908	1.36307	0.05
15909	1.32075	0.05
15910	1.12971	0.05
15911	2.06319	0.05
15912	1.94982	0.05
15913	1.77255	0.05
15914	0.979297	0.05
15915	1.59997	2.1
15916	1.93506	2.1
15917	1.94409	2.1
15918	1.9597	2.1
15919	0.57899	2.1
15920	0.953036	2.1
15921	0.953049	2.1
15922	1.53503	2.1
15923	1.39615	0.0922703
15924	1.06474	0.0962986
15925	0.947564	0.0935138
15926	1.45506	0.109596
15927	1.17799	0.198436
15928	1.21067	0.0750123
15929	1.35945	0.196247
15930	2.05648	0.259679
15931	1.36208	0.224299
15932	2.15229	0.186455
15933	2.89288	0.173013
15934	1.95719	0.189894
15935	2.25169	0.145737
15936	1.52756	0.169458
15937	1.19322	0.342307
15938	1.23146	0.263789
15939	2.45024	0.489311
15940	0.794122	0.29808
15941	1.6343	0.252188
15942	1.34161	0.19248
15943	1.05353	0.555975
15944	1.50795	0.137612
15945	1.05353	0.124865
15946	1.20669	0.129239
15947	1.59289	2.1

15948	0.989543	2.1
15949	1.40588	2.1
15950	0.991427	2.1
15951	0.921822	1.79519
15952	1.46903	0.581871
15953	1.46903	1.99777
15954	1.8214	0.269835
15955	1.39702	2.1
15956	1.25103	2.1
15957	1.25103	2.1
15958	1.10378	2.1
15959	1.71813	2.1
15960	1.39595	2.1
15961	1.386 2.1	
15962	1.13008	2.1
15963	1.31711	0.05
15964	1.70191	0.05
15965	0.893249	0.05
15966	1.42669	0.05
15967	1.23943	2.1
15968	1.1483	2.1
15969	1.04598	2.1
15970	1.1483	2.1
15971	1.00381	0.4874
15972	1.11433	0.192353
15973	0.973501	1.55318
15974	1.0756	1.48999
15975	1.20669	0.142152
15976	1.18508	0.132329
15977	1.18508	0.17449
15978	0.983027	0.133787
15979	1.43687	2.1
15980	1.71059	2.1
15981	1.71059	2.1
15982	1.66762	2.1
15983	2.8415	2.1
15984	2.40555	2.1
15985	1.91546	2.1
15986	1.61385	2.1
15987	2.01569	2.1
15988	1.33183	2.1
15989	1.23218	2.1
15990	1.23999	2.1
15991	1.01335	1.21987
15992	1.12088	1.30885
15993	1.25346	2.1
15994	1.15644	2.1
15995	1.3764	1.56537
15996	1.27558	1.25731
15997	1.2064	2.1
15998	1.15067	2.1
15999	1.01595	2.1
16000	1.15067	2.1
16001	1.41902	2.1
16002	1.30272	2.1
16003	1.30272	2.1
16004	1.19548	2.1
16005	2.99339	2.1

16006	2.98041	2.1
16007	1.46124	2.1
16008	1.42105	2.1
16009	2.98041	2.1
16010	2.86339	2.1
16011	1.42105	2.1
16012	1.42284	2.1
16013	2.31301	0.617971
16014	1.80273	0.619103
16015	2.17765	0.730587
16016	2.31301	0.595053
16017	1.76378	0.266576
16018	2.10275	0.243254
16019	2.10275	0.277257
16020	2.26047	0.271938
16021	0.97541	2.1
16022	1.40863	2.1
16023	1.40863	2.1
16024	1.30751	2.1
16025	1.20143	0.0640973
16026	1.19756	0.0611317
16027	1.43707	0.050836
16028	1.28954	0.0507752
16029	3.34254	0.0629025
16030	1.92916	0.05
16031	1.80089	0.0560028
16032	1.1323	0.0516305
16033	1.47321	0.120279
16034	1.23756	0.0947234
16035	1.87291	2.1
16036	1.53642	2.1
16037	1.53642	2.1
16038	1.5791	2.1
16039	1.0446	2.1
16040	0.992674	2.1
16041	1.12324	0.05
16042	1.29163	0.05
16043	1.29163	0.05
16044	1.15371	0.05
16045	0.996831	0.128959
16046	1.00192	0.141831
16047	1.0019	0.121183
16048	0.958937	0.136605
16049	1.07283	2.1
16050	1.25294	2.1
16051	1.25294	2.1
16052	1.04788	2.1
16053	1.14496	2.1
16054	1.07652	2.1
16055	1.07652	2.1
16056	0.991406	2.1
16057	1.60976	2.1
16058	1.37	2.1
16059	1.37	2.1
16060	1.14195	2.1
16061	1.26593	1.96138
16062	1.75366	2.1
16063	1.57621	0.188903

16064	1.24538	0.177722
16065	0.969118	2.1
16066	1.04147	2.1
16067	0.969118	2.1
16068	1.22615	2.1
16069	0.98084	2.1
16070	1.01843	2.1
16071	1.09435	2.1
16072	1.50876	2.1
16073	1.5976	2.1
16074	1.84322	2.1
16075	1.84324	2.1
16076	1.70377	2.1
16077	1.20709	2.1
16078	2.54729	2.1
16079	0.974716	0.0605059
16080	1.54468	0.0762619
16081	1.39021	0.0572288
16082	1.70303	0.0677219
16083	1.22852	1.49677
16084	1.578	1.24599
16085	1.578	1.56975
16086	1.93852	1.16763
16087	2.2506	2.1
16088	1.39302	2.1
16089	1.5478	2.1
16090	1.2269	2.1
16091	1.70048	2.1
16092	1.45477	2.1
16093	1.30929	2.1
16094	1.15069	2.1
16095	1.00255	2.1
16096	1.05481	0.467077
16097	1.05481	2.1
16098	1.1032	0.47051
16099	0.993888	2.1
16100	1.19294	2.1
16101	1.01121	0.0957689
16102	0.991866	0.0964443
16103	2.20052	1.98422
16104	1.7409	2.1
16105	1.75142	2.1
16106	1.46811	2.1
16107	1.7409	2.1
16108	1.23267	2.1
16109	1.46811	2.1
16110	1.18872	2.1
16111	1.12665	2.1
16112	1.50045	2.1
16113	1.50045	2.1
16114	1.37704	2.1
16115	1.35308	1.22931
16116	1.29983	0.934248
16117	1.29983	1.20656
16118	0.970212	1.00618
16119	1.82272	2.1
16120	1.62784	2.1
16121	1.62784	2.1

16122	1.87691	2.1
16123	3.65294	2.1
16124	2.33374	2.1
16125	2.12888	2.1
16126	1.62229	2.1
16127	1.41496	2.1
16128	1.32707	2.03885
16129	2.18871	1.87963
16130	1.4235	1.13183
16131	1.46015	0.0957748
16132	1.25635	0.0697516
16133	1.45501	0.240378
16134	1.3543	0.160048
16135	1.38139	2.1
16136	1.98159	2.1
16137	2.15868	2.1
16138	4.04308	2.1
16139	1.5362	2.1
16140	1.56328	2.1
16141	1.56327	2.1
16142	1.58163	2.1
16143	1.88987	0.05
16144	1.08761	0.05
16145	0.862154	0.05
16146	1.77688	0.05
16147	1.14238	2.1
16148	1.11691	2.1
16149	1.01537	2.1
16150	1.02923	2.1
16151	0.977425	0.05
16152	1.15211	0.05
16153	2.0815	2.1
16154	1.18621	2.1
16155	1.01485	2.1
16156	1.17054	2.1
16157	1.17054	2.1
16158	1.12665	2.1
16159	1.87213	0.05
16160	1.82244	0.05
16161	1.45039	0.05
16162	1.7226	0.05
16163	1.841	0.05
16164	1.79011	0.05
16165	1.8792	0.05
16166	1.45053	0.050331
16167	1.08947	2.1
16168	1.5008	2.1
16169	1.5008	2.1
16170	1.83186	2.1
16171	1.04159	0.05
16172	1.2489	0.05
16173	1.2489	0.05
16174	1.33592	0.05
16175	1.51973	0.0788661
16176	0.980547	0.0888375
16177	1.51974	0.0967479
16178	1.15991	0.122232
16179	1.34965	2.1

16180	1.2724	2.1
16181	1.2724	2.1
16182	1.38438	2.1
16183	1.02923	2.1
16184	1.06521	2.1
16185	1.06521	2.1
16186	1.0752	2.1
16187	2.03139	0.104627
16188	1.92951	0.0892645
16189	1.74852	0.0834322
16190	1.62772	0.0819663
16191	1.73655	0.132096
16192	1.76809	0.081417
16193	1.62772	0.0841026
16194	1.1432	0.0808974
16195	1.09235	2.1
16196	1.48081	2.1
16197	1.46712	2.1
16198	1.89321	2.1
16199	1.94548	2.1
16200	1.5636	2.1
16201	1.5636	2.1
16202	1.21124	2.1
16203	1.52314	2.1
16204	2.0219	2.1
16205	1.51113	2.1
16206	2.0219	2.1
16207	0.987247	0.255201
16208	1.18347	0.134965
16209	1.89226	0.386916
16210	1.61441	0.146327
16211	1.42884	2.1
16212	1.10961	2.1
16213	1.56991	2.1
16214	1.05337	2.1
16215	1.05337	2.1
16216	1.91547	2.1
16217	1.05931	2.1
16218	1.1729	2.1
16219	1.34526	2.1
16220	1.0593	2.1
16221	1.0856	2.1
16222	1.90854	2.1
16223	1.91674	2.1
16224	1.11345	2.1
16225	1.75067	2.1
16226	1.50022	2.1
16227	1.50022	2.1
16228	1.53619	2.1
16229	1.4748	2.07045
16230	1.74431	1.89424
16231	2.02581	1.7742
16232	1.25288	1.78275
16233	1.01814	0.935377
16234	1.2881	0.647404
16235	1.2881	0.648253
16236	1.54433	0.50869
16237	1.19999	0.540756

16238	1.44056	0.315874
16239	1.02011	0.153045
16240	1.17129	0.214706
16241	1.13775	2.00582
16242	3.34815	2.04384
16243	1.60428	1.8983
16244	1.85928	1.96518
16245	1.75592	2.1
16246	1.25989	2.1
16247	1.75592	2.1
16248	1.71549	2.1
16249	1.37634	2.1
16250	1.64693	2.1
16251	1.64693	2.1
16252	1.64193	2.1
16253	1.18351	0.05
16254	2.19398	0.05
16255	2.16272	0.790666
16256	1.19837	0.720903
16257	1.04973	2.1
16258	1.20149	2.1
16259	1.60574	2.1
16260	1.11164	1.56923
16261	1.87823	0.94463
16262	1.11164	1.70115
16263	0.772882	2.1
16264	1.4714	2.1
16265	2.14242	2.02892
16266	1.12336	2.1
16267	2.8386	1.49831
16268	2.13346	2.1
16269	1.06788	0.0829398
16270	0.947662	0.0823121
16271	1.05297	0.0775172
16272	1.42907	0.0815506
16273	2.17465	2.1
16274	2.18394	2.1
16275	1.14808	2.1
16276	1.21292	2.1
16277	1.21292	2.1
16278	1.27229	2.1
16279	1.20859	0.655583
16280	1.21073	0.747574
16281	1.21073	0.431903
16282	1.22857	1.13837
16283	1.79158	2.1
16284	1.73899	2.1
16285	1.34012	2.1
16286	1.39991	2.1
16287	1.73898	2.1
16288	1.61245	2.1
16289	1.40136	2.1
16290	1.57234	2.1
16291	1.82162	2.1
16292	3.09875	2.1
16293	1.10264	2.1
16294	1.02339	2.1
16295	1.02339	2.1

16296	1.23042	2.1
16297	2.82266	0.79337
16298	1.05698	0.374693
16299	1.96384	0.638861
16300	1.10244	0.48593
16301	0.929061	2.1
16302	1.47007	2.1
16303	0.985303	2.1
16304	1.24554	2.1
16305	1.05993	2.1
16306	2.02074	2.1
16307	2.10106	0.687175
16308	1.18171	0.187508
16309	1.68111	0.594759
16310	1.84226	0.499831
16311	1.82131	0.153883
16312	1.32371	0.0973019
16313	1.41902	0.0889134
16314	1.00205	0.0696202
16315	1.65532	2.1
16316	1.18977	2.1
16317	1.15471	2.1
16318	0.993889	2.1
16319	2.08521	2.1
16320	2.09885	2.1
16321	2.09497	2.1
16322	2.10604	2.1
16323	0.881028	0.934432
16324	1.52828	0.896628
16325	1.40574	0.05
16326	1.09599	0.05
16327	1.45506	2.1
16328	1.50524	2.1
16329	1.50524	2.1
16330	1.54711	2.1
16331	1.41469	2.1
16332	2.40442	2.1
16333	1.40794	2.1
16334	1.89226	2.1
16335	1.31346	2.1
16336	1.51067	2.1
16337	1.31363	2.1
16338	1.11833	2.1
16339	2.3373	2.1
16340	2.19994	2.1
16341	2.19994	2.1
16342	1.09616	2.1
16343	1.63719	2.1
16344	1.52204	2.1
16345	1.52205	2.1
16346	1.40118	2.1
16347	2.5946	2.1
16348	4.07065	2.1
16349	1.33916	2.1
16350	2.05217	2.1
16351	1.67023	2.1
16352	2.05217	2.1
16353	1.59458	0.532996

16354	1.02247	0.443651
16355	1.68108	0.860588
16356	1.03079	0.46639
16357	1.1643	0.145444
16358	1.85795	0.113809
16359	1.70302	0.196261
16360	1.42076	0.15985
16361	1.91351	0.177154
16362	1.01148	0.157402
16363	2.52402	0.05
16364	0.752625	0.05
16365	0.886968	2.1
16366	1.28845	2.1
16367	1.28845	2.1
16368	1.27766	2.1
16369	1.82268	2.1
16370	1.87118	2.1
16371	1.87119	2.1
16372	1.91704	2.1
16373	3.13938	2.1
16374	2.97839	2.1
16375	2.97839	2.1
16376	2.87331	2.1
16377	1.73443	1.44778
16378	1.59457	1.11019
16379	2.01827	2.1
16380	2.04586	2.1
16381	2.04586	2.1
16382	2.00865	2.1
16383	1.19901	2.09414
16384	1.16661	2.1
16385	1.13215	0.274316
16386	1.96256	0.188943
16387	2.34043	0.05
16388	2.04154	0.05
16389	1.58991	0.05
16390	1.45893	0.05
16391	2.56818	0.05
16392	1.70532	0.05
16393	1.17798	0.0565891
16394	1.25796	0.219622
16395	1.25797	0.245612
16396	1.10719	0.251566
16397	1.51067	2.1
16398	1.60707	2.1
16399	2.06946	2.1
16400	3.19218	2.1
16401	2.19965	2.1
16402	1.30521	2.1
16403	2.23684	1.99324
16404	1.43363	2.1
16405	1.51007	2.1
16406	2.46356	2.1
16407	1.90428	2.1
16408	1.13618	2.1
16409	2.13276	0.0694462
16410	0.998345	0.0694731
16411	1.2861	0.0696659

16412	1.20457	0.063801
16413	1.51789	2.1
16414	1.49872	2.1
16415	1.49872	2.1
16416	1.47712	2.1
16417	1.87129	0.0667336
16418	0.949364	0.0822644
16419	1.83142	0.0826644
16420	1.55063	0.12165
16421	1.03298	0.106539
16422	0.980664	0.25686
16423	1.51696	0.069077
16424	1.34927	0.181445
16425	1.70302	0.342616
16426	1.65952	0.247614
16427	1.76658	0.204973
16428	0.986848	0.159165
16429	2.13757	1.48931
16430	1.98142	0.472657
16431	1.98142	1.07194
16432	1.65411	0.511982
16433	1.1323	0.0819732
16434	1.46015	0.0581182
16435	2.15376	1.84698
16436	1.64622	1.87959
16437	0.991867	0.05
16438	1.58074	0.0605796
16439	1.69368	2.1
16440	1.76403	2.1
16441	1.76403	2.1
16442	1.83318	2.1
16443	1.28418	0.410025
16444	1.16851	0.2934
16445	1.1685	0.18504
16446	1.1726	0.155259
16447	1.06455	2.1
16448	1.18773	2.1
16449	0.98511	2.1
16450	1.18774	2.1
16451	1.76476	2.1
16452	1.79825	2.1
16453	1.79825	2.1
16454	0.961895	2.1
16455	1.59589	0.05
16456	2.25975	0.05
16457	2.76339	2.1
16458	3.0551	2.1
16459	1.68761	2.1
16460	1.59561	2.1
16461	2.17257	2.1
16462	1.05845	2.1
16463	1.03512	2.1
16464	2.19011	2.1
16465	1.44859	1.91522
16466	1.35897	0.887188
16467	1.35897	1.54267
16468	1.17822	1.07662
16469	1.13641	2.1

16470	1.18301	2.1
16471	1.18301	2.1
16472	1.25173	2.1
16473	2.41297	2.1
16474	1.36463	2.1
16475	1.54711	2.1
16476	1.5735	2.1
16477	1.5735	2.1
16478	1.58713	2.1
16479	1.69044	0.05
16480	1.57979	0.05
16481	1.57979	0.05
16482	1.14387	0.05
16483	1.04199	2.1
16484	1.96491	2.1
16485	1.04199	2.1
16486	1.46892	2.1
16487	0.983724	2.1
16488	1.70579	2.1
16489	1.88522	2.1
16490	1.23914	2.1
16491	1.79424	2.1
16492	1.52851	2.1
16493	1.04921	2.1
16494	1.11285	2.1
16495	1.25334	1.22239
16496	1.07496	0.258133
16497	1.07497	2.05196
16498	1.34852	0.798703
16499	1.27391	2.1
16500	2.20307	2.1
16501	1.81311	2.1
16502	2.10305	2.1
16503	1.21059	0.0705582
16504	1.44253	0.0879438
16505	1.9194	0.0556783
16506	1.21159	0.0651742
16507	0.947979	2.1
16508	1.01225	1.97361
16509	0.947979	2.1
16510	1.01225	2.1
16511	1.00255	2.1
16512	1.01225	2.1
16513	1.01225	2.1
16514	1.06561	2.1
16515	3.50562	2.1
16516	3.61336	2.1
16517	3.61336	2.1
16518	3.73635	2.1
16519	1.61245	2.1
16520	1.52301	2.1
16521	1.57612	2.1
16522	1.83035	2.1
16523	1.52301	2.1
16524	1.24789	2.1
16525	1.85156	2.1
16526	1.89072	2.1
16527	0.995735	2.1

16528	1.57738	2.1
16529	1.87141	0.0551248
16530	1.73388	0.055156
16531	1.61344	0.0565163
16532	0.922676	0.0526655
16533	1.56614	2.1
16534	0.956294	2.1
16535	1.82589	2.1
16536	1.63719	2.1
16537	1.12399	0.05
16538	1.50103	0.05
16539	1.56976	0.05
16540	1.03567	0.05
16541	1.21776	0.05
16542	1.89708	0.05
16543	1.16323	0.05
16544	1.63533	0.05
16545	2.29397	0.05
16546	3.01596	0.05
16547	1.80145	0.05
16548	1.7666	0.05
16549	1.91621	0.314482
16550	1.10719	0.117303
16551	1.57828	0.62542
16552	1.17105	0.395342
16553	1.15806	2.1
16554	1.31565	2.1
16555	1.31565	2.1
16556	1.46643	2.1
16557	1.47507	2.1
16558	1.87858	2.1
16559	0.958661	2.1
16560	1.87858	2.1
16561	1.44293	0.05
16562	1.43213	0.056618
16563	1.43213	0.05
16564	1.10476	0.0512167
16565	1.03235	1.90281
16566	1.31	0.242789
16567	1.31	1.94412
16568	1.50108	0.820469
16569	1.32215	2.1
16570	1.10611	2.1
16571	1.10611	2.1
16572	0.904978	2.1
16573	1.06486	0.216457
16574	1.18347	0.140544
16575	1.70535	2.1
16576	1.94779	2.1
16577	1.77071	2.1
16578	1.82268	2.1
16579	0.61701	2.1
16580	0.672495	2.1
16581	0.672494	2.1
16582	0.713174	2.1
16583	1.40395	2.1
16584	1.34245	2.1
16585	1.34243	2.1

16586	1.30646	2.1
16587	1.06204	0.23493
16588	0.632691	0.543758
16589	1.75766	0.58195
16590	1.14287	1.69274
16591	1.52101	0.0946665
16592	1.71414	0.0792286
16593	1.86411	0.106892
16594	1.6253	0.0813136
16595	1.04355	0.104743
16596	1.0443	0.0832439
16597	1.34852	1.87257
16598	1.56016	0.191588
16599	1.56016	0.286181
16600	1.03235	0.162363
16601	1.2966	0.0501171
16602	1.82354	0.05
16603	1.2966	0.05
16604	1.67687	0.05
16605	1.19669	1.30997
16606	1.82979	1.33405
16607	1.58972	1.50094
16608	1.09219	1.35175
16609	1.42077	1.38248
16610	1.89226	1.12157
16611	1.19547	2.1
16612	1.10187	2.1
16613	1.10188	2.1
16614	1.01479	2.1
16615	1.81663	2.1
16616	1.81533	2.1
16617	1.83247	2.1
16618	1.51511	2.1
16619	0.983148	2.1
16620	1.7513	2.1
16621	1.85017	2.1
16622	1.26103	2.1
16623	1.23152	2.1
16624	1.65508	2.1
16625	1.23152	2.1
16626	2.15082	2.1
16627	1.57974	2.1
16628	1.07289	2.1
16629	0.81534	2.1
16630	1.51007	2.1
16631	1.00898	0.05
16632	1.18959	0.05
16633	1.32178	0.05
16634	1.25929	0.05
16635	2.17892	0.382491
16636	1.22938	0.349224
16637	3.02933	0.846493
16638	2.34619	0.41716
16639	1.47679	0.127149
16640	1.0819	0.155399
16641	3.10558	2.1
16642	3.31457	2.1
16643	0.985109	2.1

16644	1.10667	2.1
16645	3.31457	2.1
16646	3.54443	2.1
16647	1.10667	2.1
16648	1.25987	2.1
16649	1.77063	1.39561
16650	1.51065	0.94334
16651	1.24688	2.1
16652	1.37652	2.1
16653	1.37652	2.1
16654	1.54168	2.1
16655	1.17042	1.55839
16656	1.13092	1.66337
16657	1.13092	1.82467
16658	1.52938	2.1
16659	1.11787	0.74795
16660	1.00613	2.1
16661	0.781373	0.178075
16662	1.6885	0.191056
16663	2.49439	0.09173
16664	1.42657	0.140601
16665	1.77903	2.1
16666	2.63606	2.1
16667	2.10425	2.1
16668	1.21425	2.1
16669	1.31253	1.43682
16670	1.28418	1.2844
16671	1.28418	1.49188
16672	1.58972	1.49374
16673	0.858948	2.1
16674	1.90196	2.1
16675	1.81024	2.1
16676	1.90194	2.1
16677	1.4263	1.63535
16678	0.947921	1.89803
16679	1.22852	1.06555
16680	1.09194	1.00469
16681	1.60578	0.0750639
16682	1.14721	0.0837017
16683	1.06336	0.114742
16684	1.14721	0.0953585
16685	1.11924	0.05
16686	1.53814	0.05
16687	1.18421	0.05
16688	1.8458	0.05
16689	1.7565	1.96863
16690	1.09344	1.52141
16691	1.09344	1.50706
16692	1.16971	2.01167
16693	1.71465	0.05
16694	1.19412	0.05
16695	1.64985	0.0706792
16696	1.05417	0.0724897
16697	1.2127	0.05
16698	1.48867	0.05
16699	1.27806	0.05
16700	1.21269	0.05
16701	1.37813	1.41948

16702	1.0417	1.41817
16703	1.88626	0.158702
16704	1.14977	0.772032
16705	1.78803	2.1
16706	1.33793	2.09348
16707	1.42266	2.1
16708	1.26865	2.01178
16709	1.09518	2.1
16710	1.36844	2.1
16711	1.20455	2.1
16712	1.36844	2.1
16713	1.22297	1.45131
16714	1.11256	1.61023
16715	1.11256	1.17697
16716	1.03217	1.45533
16717	1.18422	0.05
16718	1.4282	0.05
16719	1.04159	0.05
16720	1.4282	0.05
16721	0.988805	2.1
16722	1.05481	2.1
16723	1.52938	1.55139
16724	1.26593	1.79243
16725	1.53082	2.1
16726	1.09847	2.1
16727	1.75211	2.01144
16728	1.22222	2.1
16729	1.75211	1.07495
16730	1.51592	2.1
16731	1.63413	0.4679
16732	1.26662	0.54213
16733	1.42495	0.306442
16734	1.4356	0.361681
16735	1.29389	1.73682
16736	1.04164	1.97482
16737	1.40574	1.81561
16738	1.04164	1.73511
16739	2.22344	0.551459
16740	1.45945	0.120088
16741	0.987248	0.248405
16742	1.2474	0.144394
16743	0.974702	0.982827
16744	1.43777	0.460151
16745	1.71289	2.1
16746	1.91529	2.1
16747	1.91529	2.1
16748	2.01827	2.1
16749	1.87111	1.32191
16750	1.00864	0.841127
16751	1.26865	1.17826
16752	1.18769	1.31746
16753	1.24167	2.1
16754	1.36133	1.9561
16755	2.28168	2.1
16756	3.98524	2.1
16757	1.36133	2.1
16758	1.69436	1.59257
16759	2.21926	2.1

16760	1.70302	2.1
16761	1.50732	0.0639617
16762	1.42237	0.0659625
16763	1.18659	0.0733732
16764	1.50732	0.077014
16765	1.19785	0.0604017
16766	1.40969	0.0508945
16767	1.28339	0.0726895
16768	1.40882	0.0681938
16769	0.968595	2.1
16770	1.41903	2.1
16771	1.12567	2.1
16772	1.15898	2.1
16773	1.27241	2.1
16774	0.995796	2.1
16775	1.89983	0.05
16776	1.05417	0.05
16777	0.571999	0.05
16778	0.743597	0.05
16779	1.89353	0.05
16780	0.924068	0.05
16781	1.52851	2.1
16782	1.26969	2.1
16783	1.07763	0.226591
16784	1.82301	0.160175
16785	1.07763	0.114441
16786	1.17506	0.117111
16787	1.20045	2.1
16788	1.12486	2.1
16789	1.15234	2.1
16790	1.11598	2.1
16791	1.12486	2.1
16792	1.0491	2.1
16793	1.11598	2.1
16794	1.1047	2.1
16795	1.05902	2.1
16796	1.14361	2.1
16797	1.14361	2.1
16798	1.26851	2.1
16799	1.29389	1.6093
16800	0.988456	1.57983
16801	1.83688	0.05
16802	1.70302	0.05
16803	1.49386	0.05
16804	1.83151	0.05
16805	1.01346	0.803201
16806	0.983607	0.866897
16807	0.983596	2.1
16808	1.08332	1.92437
16809	2.36848	0.109666
16810	2.97066	0.10708
16811	1.12352	0.0806159
16812	2.5311	0.086509
16813	1.09062	2.1
16814	0.962182	2.1
16815	0.962181	2.1
16816	1.2258	2.1
16817	0.974888	2.1

16818	1.06312	2.1
16819	1.73583	0.0559618
16820	1.72399	0.053644
16821	1.724	0.0719441
16822	1.50951	0.068699
16823	1.26755	2.1
16824	1.03824	2.1
16825	1.27875	2.1
16826	1.03824	2.1
16827	1.18902	2.1
16828	1.534	2.1
16829	1.11764	2.1
16830	1.534	2.1
16831	1.52008	2.1
16832	1.34406	2.1
16833	1.34406	2.1
16834	1.2187	2.1
16835	0.963571	1.61004
16836	1.19612	1.28501
16837	0.963571	2.02846
16838	1.00007	2.1
16839	0.903333	2.1
16840	1.12724	2.1
16841	1.23771	2.1
16842	1.17683	2.1
16843	1.20059	0.540964
16844	0.772023	0.287202
16845	1.14749	1.02704
16846	1.2802	1.29199
16847	1.85091	1.88721
16848	2.11193	1.74934
16849	1.2802	0.632221
16850	1.42913	1.05877
16851	2.11193	1.62018
16852	1.27737	1.4443
16853	1.30584	2.1
16854	1.60936	2.1
16855	1.3413	0.104525
16856	1.53336	0.0724807
16857	2.60679	0.0770372
16858	1.3413	0.065568
16859	1.35877	2.1
16860	1.11024	2.1
16861	1.27839	2.1
16862	1.08171	2.1
16863	1.27841	2.1
16864	1.0577	2.1
16865	1.71255	2.1
16866	1.10611	2.1
16867	0.960106	2.1
16868	1.40921	2.1
16869	1.90705	0.130149
16870	2.79564	0.120793
16871	1.45321	0.13306
16872	1.90705	0.123844
16873	0.974718	0.0863275
16874	1.53671	0.0781899
16875	1.69039	0.0809805

16876	1.78229	0.0739196
16877	1.0101	2.1
16878	1.3167	2.1
16879	1.19699	2.1
16880	1.3212	2.1
16881	1.60811	0.0779529
16882	1.43224	0.0774894
16883	1.35401	0.061061
16884	1.17464	0.05
16885	1.27696	2.1
16886	1.05025	2.1
16887	1.05025	2.1
16888	1.18076	2.1
16889	1.37224	1.60312
16890	1.13438	1.58747
16891	1.76788	1.61743
16892	1.73444	1.55046
16893	1.37704	2.1
16894	1.77042	2.1
16895	1.77042	2.1
16896	1.09778	2.1
16897	1.28667	2.1
16898	1.28228	2.1
16899	1.30646	0.468716
16900	1.68867	0.365016
16901	1.30645	1.18939
16902	0.986494	0.795674
16903	1.6253	0.0909206
16904	1.48676	0.0876167
16905	1.48676	0.0889137
16906	1.32974	0.0863897
16907	0.634347	0.05
16908	1.15142	0.05
16909	1.11395	0.05
16910	1.18068	0.05
16911	1.36904	0.05
16912	0.764563	0.05
16913	2.09213	0.05
16914	1.56631	2.1
16915	1.08011	2.1
16916	1.80283	2.1
16917	1.50738	2.1
16918	1.44951	0.116792
16919	1.50102	0.10606
16920	1.50102	0.105392
16921	1.39615	0.101894
16922	1.02653	0.413422
16923	2.98258	0.452529
16924	2.0815	0.05
16925	1.39525	0.05
16926	1.82176	0.05
16927	0.977607	0.05
16928	1.96133	1.82797
16929	1.27189	1.4628
16930	0.826032	1.52658
16931	2.12831	1.48921
16932	1.77798	2.08424
16933	1.02969	1.71602

16934	1.06463	1.69659
16935	1.49219	2.01497
16936	1.10003	2.1
16937	1.21223	2.1
16938	1.00513	1.56502
16939	1.43788	2.1
16940	1.47134	0.88844
16941	0.968249	1.21248
16942	3.22585	0.0589195
16943	3.36933	0.05
16944	3.36932	0.0616644
16945	3.03865	0.05
16946	1.66295	0.203548
16947	1.65583	0.137523
16948	1.65583	0.265424
16949	1.4732	0.121533
16950	1.73704	0.05
16951	1.76476	2.1
16952	2.13345	1.93828
16953	1.55816	2.1
16954	1.55816	2.1
16955	1.02601	2.1
16956	1.08039	2.1
16957	1.05498	1.89989
16958	1.08039	2.1
16959	1.2873	2.1
16960	1.01121	2.1
16961	0.987703	2.1
16962	0.98771	2.1
16963	0.964782	2.1
16964	1.35719	2.1
16965	2.61092	2.1
16966	2.6607	2.1
16967	3.41693	2.1
16968	1.23125	2.1
16969	1.73772	2.1
16970	0.99845	0.295011
16971	1.5176	0.402193
16972	1.13271	0.05
16973	1.332 0.05	
16974	1.63159	2.1
16975	1.61342	2.1
16976	1.03679	2.1
16977	1.19087	2.1
16978	1.97172	2.1
16979	2.68634	2.1
16980	1.19086	2.1
16981	1.50091	2.1
16982	2.84677	2.1
16983	2.77894	2.1
16984	2.77894	2.1
16985	2.74206	2.1
16986	1.3284	2.1
16987	1.83524	2.1
16988	1.65853	2.1
16989	1.56741	2.1
16990	1.00509	2.1
16991	1.45246	2.1

16992	1.26193	2.1
16993	1.45244	2.1
16994	1.61963	0.595527
16995	1.68189	0.282536
16996	1.68189	1.80606
16997	1.49155	1.59274
16998	1.58054	2.1
16999	1.59169	2.1
17000	1.59169	2.1
17001	1.60497	2.1
17002	0.743595	0.05
17003	0.702359	0.05
17004	1.15317	0.05
17005	2.24367	0.05
17006	1.15252	0.05
17007	2.60354	0.05
17008	1.45158	2.1
17009	1.56004	2.1
17010	1.64527	2.1
17011	1.00371	2.1
17012	1.38287	0.335815
17013	0.893024	1.29487
17014	1.23527	1.24124
17015	1.23706	0.541183
17016	2.20993	0.359546
17017	1.54858	1.78
17018	0.798666	0.146289
17019	1.22487	0.13175
17020	1.33158	0.107188
17021	1.85902	0.112242
17022	1.08334	2.1
17023	1.17885	1.9013
17024	1.17885	1.13992
17025	1.07283	1.639
17026	1.30723	0.05
17027	1.62507	0.05
17028	1.62507	0.05
17029	1.08854	0.05
17030	1.59475	2.1
17031	1.58874	2.1
17032	1.58875	2.1
17033	1.35787	2.1
17034	1.04147	2.1
17035	1.15564	2.1
17036	1.78301	2.1
17037	1.90618	2.1
17038	1.71555	2.1
17039	2.13143	2.1
17040	1.10817	2.1
17041	1.032	2.1
17042	1.032	2.1
17043	0.953636	2.1
17044	1.27916	1.43504
17045	1.11938	1.18644
17046	1.11937	1.28139
17047	1.9186	1.17931
17048	1.19967	0.0776682
17049	1.63489	0.0879748

17050	1.00763	0.102244
17051	1.81656	0.106591
17052	1.31814	0.0815678
17053	2.98183	0.0912498
17054	1.20149	2.1
17055	1.29129	2.1
17056	1.29129	2.1
17057	1.37138	2.1
17058	1.23104	0.737729
17059	1.75236	1.26442
17060	1.6	0.303616
17061	1.60565	0.497793
17062	1.60565	0.437277
17063	1.40013	0.529539
17064	1.23042	2.1
17065	1.18278	2.1
17066	1.10853	2.1
17067	1.04724	2.1
17068	1.1766	2.1
17069	1.00319	2.1
17070	1.29435	2.1
17071	1.00319	2.1
17072	1.05655	1.80871
17073	1.01606	1.86809
17074	1.80391	2.1
17075	2.7699	2.1
17076	1.52073	2.1
17077	2.06835	2.1
17078	2.00202	2.1
17079	2.04906	2.1
17080	0.968536	2.1
17081	1.02461	2.1
17082	1.59784	2.1
17083	1.78249	2.1
17084	1.81409	2.1
17085	1.79844	2.1
17086	1.77093	1.99099
17087	2.02356	1.8505
17088	1.32449	2.1
17089	1.10003	2.1
17090	1.16915	0.434043
17091	1.05937	0.337233
17092	1.28096	2.00532
17093	1.34696	1.7134
17094	1.34695	2.077
17095	1.36457	1.79154
17096	1.06478	2.1
17097	1.06239	2.1
17098	1.06239	2.1
17099	1.09629	2.1
17100	0.798209	2.1
17101	1.8532	2.1
17102	2.10112	0.05
17103	2.8704	0.05
17104	2.8704	0.05
17105	2.92457	0.05
17106	1.0752	2.1
17107	1.07745	2.1

17108	1.07745	2.1
17109	1.08911	2.1
17110	1.40182	2.1
17111	1.00133	2.1
17112	1.40182	2.1
17113	1.24509	2.1
17114	2.22243	0.05
17115	1.07303	0.05
17116	1.29736	0.05
17117	1.73584	0.05
17118	2.0815	0.433918
17119	1.07999	0.245466
17120	2.1692	2.1
17121	1.99992	2.1
17122	1.99992	2.1
17123	1.78327	2.1
17124	1.52661	0.05
17125	0.956003	0.05
17126	1.53172	0.05
17127	0.955991	0.05
17128	1.35025	2.1
17129	1.51084	2.1
17130	1.51084	2.1
17131	1.67115	2.1
17132	2.00977	2.1
17133	1.63632	2.1
17134	1.63632	2.1
17135	2.66128	2.1
17136	1.13525	2.1
17137	2.37566	2.1
17138	1.25987	2.1
17139	1.4472	2.1
17140	1.4472	2.1
17141	1.66018	2.1
17142	0.994001	1.33997
17143	1.83151	1.01648
17144	1.83151	0.410132
17145	1.57621	0.361589
17146	1.40915	0.233869
17147	1.24653	1.79627
17148	1.24653	0.242878
17149	1.10609	2.09664
17150	1.61121	2.1
17151	1.74472	2.1
17152	1.74472	2.1
17153	1.0368	2.1
17154	1.04788	2.1
17155	1.28522	2.1
17156	1.28522	2.1
17157	1.54196	2.1
17158	1.23387	2.1
17159	0.896519	2.1
17160	1.60428	2.1
17161	1.08467	2.1
17162	1.56875	0.361294
17163	1.39072	0.226914
17164	1.39073	0.200956
17165	1.6	0.369369

17166	1.01346	2.1
17167	1.06838	1.51484
17168	0.968463	1.53977
17169	1.06837	1.53089
17170	3.22433	0.0643174
17171	2.05993	2.01214
17172	2.01836	0.224172
17173	1.66154	1.96527
17174	1.20871	2.1
17175	1.10586	2.1
17176	1.10586	2.1
17177	1.03853	2.1
17178	1.12076	2.1
17179	1.03899	2.1
17180	1.03899	2.1
17181	0.963973	2.1
17182	0.987132	2.1
17183	1.0894	2.1
17184	2.37823	1.98702
17185	1.75647	1.61983
17186	1.64625	2.1
17187	1.56195	2.1
17188	1.41995	2.1
17189	1.15171	2.1
17190	1.36457	2.1
17191	1.33847	1.71877
17192	1.33847	2.1
17193	0.869601	1.63221
17194	1.42255	2.1
17195	1.3868	2.1
17196	1.3868	2.1
17197	1.33962	2.1
17198	1.05481	2.1
17199	1.08074	2.1
17200	1.08074	2.1
17201	0.857557	2.1
17202	1.13537	1.38675
17203	1.09194	0.924822
17204	1.01179	1.45403
17205	1.13537	1.02701
17206	1.15645	1.88037
17207	1.56409	0.293834
17208	1.56409	0.510439
17209	1.16239	0.911392
17210	1.10746	0.465141
17211	1.47952	1.08422
17212	1.14733	2.1
17213	3.77068	2.1
17214	1.38497	2.1
17215	1.54599	2.1
17216	1.54601	2.1
17217	1.27697	2.1
17218	1.01393	0.635623
17219	1.10558	1.06095
17220	0.995012	0.37982
17221	1.17624	0.733507
17222	1.60497	2.1
17223	1.61588	2.1

17224	1.61587	2.1
17225	1.62182	2.1
17226	1.48866	0.05
17227	1.65977	0.05
17228	1.65977	0.05
17229	1.69044	0.05
17230	0.961892	2.1
17231	1.00659	2.1
17232	1.0066	2.1
17233	1.06226	2.1
17234	1.15171	2.1
17235	1.50853	2.1
17236	1.50853	2.1
17237	1.2955	2.1
17238	1.3204	0.0679498
17239	2.13295	0.05
17240	2.75524	0.05
17241	1.18156	0.05
17242	1.73729	2.1
17243	1.20356	2.1
17244	1.62483	2.1
17245	1.52008	2.1
17246	1.19964	2.1
17247	1.0577	2.1
17248	1.19964	2.1
17249	1.07156	2.1
17250	1.98241	2.1
17251	1.43687	2.05351
17252	1.27863	2.1
17253	1.19915	2.05441
17254	0.97269	2.03349
17255	1.0739	1.80059
17256	1.1427	1.73092
17257	1.22476	1.08728
17258	1.06584	1.84783
17259	1.22626	1.75552
17260	1.31388	2.1
17261	1.16932	2.1
17262	1.28819	2.1
17263	1.0499	2.1
17264	1.55894	2.1
17265	1.78709	2.1
17266	1.75986	2.1
17267	1.764	2.1
17268	1.44801	2.1
17269	1.28186	2.1
17270	1.20797	1.93515
17271	1.06821	2.01873
17272	1.87287	0.519673
17273	2.08014	0.598905
17274	1.63225	0.587139
17275	1.39501	0.653592
17276	1.20132	0.05
17277	1.16182	0.05
17278	1.08848	0.05
17279	1.20132	0.05
17280	1.23475	2.1
17281	1.16661	2.1

17282	2.31317	2.1
17283	2.50145	2.1
17284	1.04367	2.1
17285	2.23875	2.1
17286	0.953632	0.119483
17287	1.38646	0.215666
17288	1.38646	0.253268
17289	1.61963	0.277671
17290	1.62934	1.18729
17291	1.88626	1.53148
17292	1.0775	1.37695
17293	1.119	0.519856
17294	1.08133	0.258454
17295	1.48312	0.22783
17296	1.57125	0.132982
17297	1.74098	0.127619
17298	1.94372	1.89488
17299	1.22237	1.90501
17300	1.18549	1.01451
17301	1.01294	1.99116
17302	1.01294	2.09302
17303	1.2064	2.1
17304	1.14599	2.1
17305	1.00065	2.00851
17306	1.76357	2.1
17307	2.23468	2.1
17308	2.21735	2.1
17309	3.32769	2.1
17310	1.19647	1.18647
17311	1.35307	0.804089
17312	1.19645	0.757771
17313	1.43778	0.502517
17314	1.34412	1.47419
17315	1.1315	2.1
17316	1.1315	0.9661
17317	1.23943	0.859796
17318	1.57829	2.1
17319	1.22537	2.1
17320	1.14357	2.1
17321	1.32772	2.03659
17322	1.4259	2.1
17323	1.34245	2.1
17324	1.47953	2.1
17325	1.70303	2.1
17326	2.21127	0.05
17327	1.47904	0.0620373
17328	1.18793	0.05
17329	2.21126	0.05
17330	1.1032	2.1
17331	1.14986	0.782888
17332	1.14986	2.1
17333	1.19611	0.523037
17334	1.91883	2.1
17335	1.83019	2.1
17336	1.83019	2.1
17337	1.82462	2.1
17338	1.27362	0.135588
17339	1.27421	0.183177

17340	1.27421	0.105128
17341	1.10736	0.243977
17342	1.3003	0.427071
17343	1.31415	1.53204
17344	1.31415	0.544713
17345	1.32201	1.66202
17346	1.21124	2.1
17347	0.81568	2.1
17348	0.81568	2.1
17349	0.551816	2.1
17350	1.67119	2.1
17351	1.63384	2.1
17352	1.63384	2.1
17353	1.63661	2.1
17354	1.48734	2.1
17355	1.52245	2.1
17356	1.52245	2.1
17357	1.50714	2.1
17358	0.996778	0.05
17359	1.56091	0.05
17360	1.56091	0.05
17361	1.90457	0.05
17362	1.1181	2.1
17363	1.06543	2.1
17364	2.66828	2.1
17365	2.76932	2.1
17366	1.06543	2.1
17367	1.01474	2.1
17368	2.76932	2.1
17369	2.86704	2.1
17370	1.29724	0.0938443
17371	1.96938	0.0828592
17372	1.82462	2.1
17373	1.84553	2.1
17374	1.05556	2.1
17375	1.25363	2.1
17376	1.84552	2.1
17377	1.53208	2.1
17378	1.12826	2.1
17379	1.39075	2.1
17380	0.9702	1.25684
17381	1.16481	1.09415
17382	1.08894	1.37052
17383	1.91756	1.46028
17384	1.50865	0.535101
17385	1.06012	0.43168
17386	1.5456	0.0639821
17387	1.37427	0.0640684
17388	1.1211	0.0539126
17389	1.5456	0.0583644
17390	1.33135	0.05
17391	1.07956	0.05
17392	2.23255	0.05
17393	1.21031	0.05
17394	1.52903	2.1
17395	1.35977	2.1
17396	3.3837	2.1
17397	3.20581	2.1

17398	1.35977	2.1
17399	1.15414	2.1
17400	3.20582	2.1
17401	2.07596	2.1
17402	1.6812	0.0894579
17403	1.03939	0.0843398
17404	1.30047	2.1
17405	1.5586	2.1
17406	1.5586	2.1
17407	1.84214	2.1
17408	1.31825	0.58761
17409	1.31923	1.89081
17410	1.31924	0.626209
17411	1.33026	1.76228
17412	1.15818	1.19109
17413	1.17822	1.20063
17414	1.15818	1.72425
17415	1.28096	1.51671
17416	1.06848	0.0801617
17417	1.89639	0.0757068
17418	1.9186	1.32918
17419	1.77065	0.896487
17420	1.59103	0.788902
17421	1.56164	0.452218
17422	1.09697	0.05
17423	2.38719	0.05
17424	2.22123	0.05
17425	2.46059	0.05
17426	1.45506	0.140575
17427	1.03645	0.145904
17428	1.30347	2.1
17429	1.2627	2.1
17430	1.2627	2.1
17431	1.23036	2.1
17432	1.41469	2.1
17433	1.45257	2.1
17434	1.46907	0.05
17435	1.70302	0.05
17436	1.49196	0.05
17437	1.36307	0.05
17438	1.05677	2.1
17439	0.978698	2.1
17440	0.978699	2.1
17441	2.01822	2.1
17442	1.47446	2.1
17443	1.55432	2.1
17444	1.55432	2.1
17445	1.06053	2.1
17446	1.86813	1.94166
17447	1.21892	2.04534
17448	1.53064	1.62881
17449	1.30664	1.6679
17450	1.09629	2.1
17451	1.19244	2.1
17452	1.19244	2.1
17453	1.37635	2.1
17454	1.49358	0.203018
17455	1.57702	0.11509

17456	2.09374	2.1
17457	1.88175	2.1
17458	1.88175	2.1
17459	1.51085	2.1
17460	1.10938	0.176475
17461	1.02478	1.30836
17462	1.33061	0.05
17463	1.13272	0.05
17464	1.75505	2.1
17465	1.78529	2.1
17466	1.6353	2.1
17467	1.59307	2.1
17468	1.60017	0.337103
17469	1.35608	0.393899
17470	1.35608	0.230223
17471	1.04713	0.184257
17472	1.01479	1.87442
17473	1.3868	1.4158
17474	1.3868	1.25938
17475	1.28418	2.1
17476	2.15949	2.1
17477	2.90429	2.1
17478	1.51902	2.1
17479	1.54755	2.1
17480	2.90429	2.1
17481	3.49966	2.1
17482	1.70842	2.1
17483	1.97526	2.1
17484	0.949712	0.0803427
17485	1.92784	0.0778185
17486	1.34093	0.0753511
17487	2.53426	0.0995505
17488	1.92783	0.0745558
17489	3.89373	0.0685015
17490	2.53426	0.0866484
17491	2.35961	0.0845147
17492	1.68108	0.414559
17493	1.15558	0.344499
17494	1.67265	0.320421
17495	1.27608	0.262038
17496	1.42249	2.1
17497	1.39708	2.1
17498	1.39708	2.1
17499	1.39327	2.1
17500	1.97046	0.0763584
17501	1.11688	0.0666887
17502	1.28418	0.92542
17503	1.17302	2.1
17504	1.17302	1.06309
17505	1.04349	2.1
17506	0.979853	2.1
17507	1.26016	2.1
17508	1.18901	2.1
17509	1.26016	2.1
17510	1.22857	0.307709
17511	1.25848	1.10135
17512	1.25848	0.259827
17513	1.2858	1.11072

17514	1.09154	2.1
17515	2.01915	2.1
17516	1.23013	2.1
17517	1.55698	2.1
17518	1.55698	2.1
17519	1.8575	2.1
17520	2.01854	2.1
17521	1.20686	2.05122
17522	1.20684	2.00825
17523	1.77796	2.1
17524	1.46129	2.1
17525	1.04193	2.1
17526	1.10051	2.1
17527	0.983723	2.1
17528	1.1729	2.1
17529	0.98505	2.1
17530	0.985052	2.1
17531	1.03951	2.1
17532	1.03949	2.1
17533	1.17583	2.1
17534	1.42866	2.1
17535	1.97261	2.1
17536	1.73274	2.1
17537	1.53629	2.1
17538	1.98182	2.1
17539	1.72511	2.1
17540	1.42174	2.1
17541	1.44472	2.1
17542	1.72511	2.1
17543	1.45411	2.1
17544	1.44472	2.1
17545	1.43836	2.1
17546	0.962703	0.347375
17547	1.0171	0.796868
17548	1.53336	0.05
17549	1.40274	0.05
17550	1.40272	0.05
17551	1.00213	0.05
17552	1.50892	0.05
17553	1.64984	0.05
17554	1.67464	0.05
17555	1.67481	0.05
17556	1.08212	2.1
17557	1.28095	2.1
17558	1.46956	2.1
17559	1.70303	2.1
17560	1.2925	0.05
17561	1.84716	0.05
17562	1.48676	0.116733
17563	1.48797	0.101818
17564	1.33917	0.0963086
17565	1.22691	0.088962
17566	0.528744	2.1
17567	1.13728	2.1
17568	1.13728	2.1
17569	1.99843	2.1
17570	1.17464	0.05
17571	1.0372	0.05

17572	2.0815	0.05
17573	0.933462	0.05
17574	1.54931	2.1
17575	1.5485	2.1
17576	1.4077	2.1
17577	1.29931	2.1
17578	1.14931	0.129936
17579	2.02223	0.124617
17580	1.04482	0.0938474
17581	0.94971	0.105379
17582	1.54632	1.78274
17583	1.15693	1.78693
17584	1.66763	2.1
17585	1.41238	2.1
17586	1.41238	2.1
17587	1.48068	2.1
17588	1.0171	0.256191
17589	1.1211	0.617027
17590	1.1211	0.232409
17591	1.28418	0.456404
17592	0.993021	2.1
17593	1.764 2.1	
17594	0.993023	2.1
17595	1.05787	2.1
17596	1.90914	0.342718
17597	1.12229	2.09678
17598	1.02025	0.667481
17599	0.585368	2.09448
17600	1.99323	2.1
17601	2.34988	2.1
17602	2.34988	2.1
17603	2.36987	2.1
17604	1.23107	0.0548101
17605	1.90664	0.0683013
17606	1.53496	0.0556975
17607	1.70302	0.0742909
17608	1.98859	2.1
17609	1.61057	2.1
17610	1.79844	2.1
17611	2.10811	2.1
17612	1.4618	2.1
17613	1.96722	2.1
17614	1.95191	2.1
17615	2.13418	2.1
17616	1.50368	0.05
17617	1.61417	0.05
17618	1.15008	0.05
17619	1.19685	0.05
17620	1.90456	0.05
17621	1.90899	0.05
17622	2.08864	0.05
17623	2.10447	0.05
17624	1.16653	2.1
17625	1.23017	2.1
17626	1.0752	1.75203
17627	1.34412	1.4851
17628	1.06226	2.1
17629	1.09252	2.1

17630	1.09252	2.1
17631	0.88579	2.1
17632	1.56178	0.05
17633	1.14305	0.0529772
17634	1.56175	0.0503743
17635	1.08211	0.05
17636	1.32813	2.1
17637	1.32461	2.1
17638	1.32461	2.1
17639	1.32455	2.1
17640	1.42729	0.0989054
17641	1.18716	0.0740915
17642	1.47169	0.185682
17643	1.24687	0.0684439
17644	1.09627	2.1
17645	1.04759	2.1
17646	1.04759	2.1
17647	0.987132	2.1
17648	1.11181	1.84807
17649	1.80662	2.1
17650	1.8066	2.09856
17651	1.06461	1.76286
17652	1.56091	2.1
17653	1.9842	2.1
17654	1.73525	0.140289
17655	1.40557	0.140912
17656	2.26906	0.162319
17657	1.31248	0.226322
17658	1.66832	0.151045
17659	1.32634	0.14137
17660	1.03314	0.05
17661	1.24682	0.05
17662	1.38536	0.05
17663	1.52661	0.05
17664	1.177	1.24881
17665	1.36405	0.735438
17666	1.19837	1.0077
17667	1.17701	0.458751
17668	0.993831	0.0600749
17669	1.0823	0.070951
17670	1.0823	0.0758696
17671	1.01121	0.0861802
17672	1.49653	0.118543
17673	1.89583	0.163384
17674	0.67242	0.116541
17675	1.36047	0.155394
17676	1.00366	0.155035
17677	1.24728	0.159618
17678	1.13387	0.05
17679	1.60867	0.05
17680	1.30572	0.05
17681	1.54982	0.05
17682	0.788592	0.05
17683	1.82961	0.05
17684	1.54982	0.05
17685	1.82961	0.05
17686	1.26062	0.05
17687	1.64002	0.05

17688	1.12035	0.05
17689	1.64002	0.05
17690	1.1673	2.1
17691	1.75367	2.1
17692	0.964034	2.1
17693	1.0305	2.1
17694	1.30896	2.0187
17695	1.52776	2.03207
17696	1.52776	2.1
17697	1.17989	2.1
17698	1.16759	2.1
17699	1.8575	2.1
17700	1.16759	2.1
17701	1.60936	2.1
17702	1.10747	0.05
17703	1.47688	0.0851397
17704	1.40794	2.1
17705	1.95729	1.88722
17706	1.95729	2.1
17707	3.18156	1.8149
17708	1.87598	2.1
17709	1.94343	2.1
17710	1.94343	2.1
17711	2.00551	2.1
17712	1.20067	1.30596
17713	1.70744	1.47305
17714	1.87244	1.35758
17715	1.32075	1.19695
17716	1.64955	2.1
17717	1.44221	2.1
17718	1.31109	2.1
17719	1.14206	2.1
17720	1.60452	1.981
17721	1.12111	1.14116
17722	1.00832	2.1
17723	0.974137	2.1
17724	0.974137	2.1
17725	0.98505	2.1
17726	1.02368	2.1
17727	0.985051	2.1
17728	0.995734	0.694167
17729	1.31993	0.73468
17730	1.31993	0.621744
17731	1.18549	1.9992
17732	1.5176	0.337105
17733	1.33622	0.254639
17734	1.33621	0.284772
17735	1.02782	0.231241
17736	1.66774	2.1
17737	1.85305	2.1
17738	1.85305	2.1
17739	2.07088	2.1
17740	1.78873	2.1
17741	1.47533	2.1
17742	1.23152	2.1
17743	0.99922	2.1
17744	1.48493	2.1
17745	1.1394	2.1

17746	1.19179	2.1
17747	1.64289	2.1
17748	1.47858	2.1
17749	1.89687	2.1
17750	0.972464	2.1
17751	1.79473	2.1
17752	1.06971	2.1
17753	1.21089	2.1
17754	1.12572	2.1
17755	1.30566	2.1
17756	1.30566	2.1
17757	1.52724	2.1
17758	2.32237	0.128736
17759	2.20248	0.110675
17760	2.79564	0.135081
17761	2.32237	0.122529
17762	1.59354	0.0770771
17763	3.098	0.0744165
17764	3.09801	0.0765585
17765	3.45132	0.0753417
17766	1.24001	1.4854
17767	1.41822	1.07765
17768	1.41821	1.92447
17769	1.44859	0.890224
17770	1.34539	2.1
17771	1.06991	2.1
17772	1.18076	2.1
17773	0.978586	2.1
17774	0.999189	0.0516432
17775	2.41076	0.0573769
17776	0.968475	2.1
17777	1.02074	2.1
17778	1.02074	2.1
17779	1.07866	2.1
17780	0.817702	0.0745766
17781	1.5082	0.0797099
17782	1.48376	0.0957059
17783	1.18947	0.0957379
17784	1.10638	2.1
17785	1.86766	2.1
17786	1.19785	2.1
17787	1.34141	2.1
17788	1.34141	2.1
17789	0.991985	2.1
17790	2.35516	0.948282
17791	1.24815	2.00298
17792	1.80454	2.1
17793	1.22633	2.1
17794	0.696255	2.1
17795	1.19379	2.03144
17796	1.14721	2.1
17797	1.16153	2.1
17798	1.16153	2.1
17799	1.17261	2.1
17800	1.20814	2.1
17801	1.54791	2.1
17802	1.54791	2.1
17803	1.60477	2.1

17804	1.29931	2.1
17805	1.19098	2.1
17806	1.19098	2.1
17807	1.13341	2.1
17808	1.00503	1.76384
17809	1.25334	0.353616
17810	1.22655	2.1
17811	1.26282	2.1
17812	1.26282	2.1
17813	0.999489	2.1
17814	1.54197	0.159228
17815	1.17152	0.178016
17816	1.17152	0.179773
17817	1.49627	0.164074
17818	1.36405	0.716969
17819	0.976334	0.584006
17820	1.12495	2.1
17821	1.54096	2.1
17822	1.75263	2.1
17823	1.49114	2.1
17824	0.842998	1.59156
17825	1.60244	1.05315
17826	1.48911	1.33347
17827	2.18685	1.3819
17828	1.75236	1.13777
17829	1.54143	1.64647
17830	1.40013	0.491432
17831	0.946646	0.607694
17832	1.50714	2.1
17833	1.46055	2.1
17834	1.46055	2.1
17835	1.42249	2.1
17836	2.12839	0.0616018
17837	1.75211	0.108382
17838	1.76297	0.139046
17839	1.70736	0.105148
17840	1.19952	0.05
17841	1.56475	0.05
17842	1.74312	0.0951089
17843	1.48973	0.0590753
17844	1.91518	0.193958
17845	1.16534	0.125291
17846	1.53556	2.1
17847	1.56253	2.1
17848	1.56253	2.1
17849	1.58054	2.1
17850	1.27806	0.05
17851	1.44593	0.05
17852	1.44593	0.05
17853	1.3946	0.05
17854	1.34857	0.309902
17855	1.03818	1.966
17856	1.38969	0.204094
17857	1.85548	0.265417
17858	1.03108	0.153364
17859	1.85548	0.173191
17860	1.49346	1.85045
17861	1.83192	2.0413

17862	1.75291	1.55772
17863	1.35435	1.85363
17864	0.921851	0.236278
17865	1.09646	0.217181
17866	1.2183	0.200436
17867	1.36717	0.174012
17868	2.14179	0.100914
17869	1.79805	0.128005
17870	1.3428	0.15209
17871	2.30493	0.146133
17872	1.81545	0.145271
17873	1.92269	0.111635
17874	2.30492	0.242283
17875	1.3663	0.186849
17876	1.22418	2.1
17877	0.956698	1.9829
17878	1.4706	2.1
17879	0.953295	2.1
17880	1.47059	2.1
17881	1.26929	2.1
17882	1.24538	0.115484
17883	1.4468	0.0853644
17884	2.86762	2.1
17885	2.32911	2.1
17886	2.10864	2.1
17887	1.94548	2.1
17888	1.83134	2.1
17889	2.19519	2.1
17890	2.19853	2.1
17891	2.63855	2.1
17892	1.08213	0.0544166
17893	1.46389	0.0599802
17894	1.62235	0.0753627
17895	0.980549	0.0846995
17896	1.08195	1.64572
17897	0.99002	2.05734
17898	1.2269	2.1
17899	1.0838	2.1
17900	0.889491	2.1
17901	1.19587	2.1
17902	1.18801	0.0669427
17903	1.57125	0.0656707
17904	1.47712	2.1
17905	1.45182	2.1
17906	1.45182	2.1
17907	1.42255	2.1
17908	1.17816	0.05
17909	1.00982	0.05
17910	1.21056	0.05
17911	1.17816	0.05
17912	3.80902	0.696283
17913	0.976991	0.570892
17914	1.09962	2.1
17915	1.60976	2.1
17916	1.87029	2.1
17917	2.33806	2.1
17918	2.12549	2.1
17919	2.21472	2.1

17920	2.16221	0.0505672
17921	1.99528	0.05
17922	1.99528	0.05
17923	1.97969	0.05
17924	3.60854	2.1
17925	4.00847	2.1
17926	2.70372	2.1
17927	1.253	2.1
17928	1.65739	2.1
17929	0.994294	2.1
17930	1.36203	2.1
17931	1.00965	2.1
17932	2.33374	2.1
17933	1.36209	2.1
17934	1.64983	0.05
17935	1.36416	0.05
17936	2.19766	0.05
17937	1.36416	0.051433
17938	2.15871	0.479795
17939	1.81454	0.296265
17940	1.54607	0.135386
17941	1.57702	0.297713
17942	0.948748	0.05
17943	1.04522	0.05
17944	1.60994	0.05
17945	1.90382	0.05
17946	1.90382	0.05
17947	1.57999	0.05
17948	1.73905	1.20195
17949	1.26535	1.36921
17950	2.12552	1.29977
17951	1.06891	1.22622
17952	1.13999	1.23829
17953	2.29812	1.24718
17954	2.83329	1.12959
17955	2.16272	0.773575
17956	1.71277	2.1
17957	1.68401	2.1
17958	1.68401	2.1
17959	1.69368	2.1
17960	1.06472	2.1
17961	1.56017	2.1
17962	1.2765	2.1
17963	1.99541	2.1
17964	1.46041	0.05
17965	2.04817	0.05
17966	2.04817	0.05
17967	2.33181	0.05
17968	2.06353	2.1
17969	1.35449	2.1
17970	1.52045	2.1
17971	1.45437	2.1
17972	1.04405	0.05
17973	1.85945	0.05
17974	1.85945	0.05
17975	2.22679	0.05
17976	1.33339	2.1
17977	1.19376	2.1

17978	1.33266	2.1
17979	1.44244	2.1
17980	1.20455	2.1
17981	1.52852	2.1
17982	1.03882	2.1
17983	1.52852	2.1
17984	1.44801	2.1
17985	1.00775	2.1
17986	0.53907	2.1
17987	0.506124	2.1
17988	2.39045	2.1
17989	0.973794	2.1
17990	1.11193	2.1
17991	1.32784	2.1
17992	1.31595	2.1
17993	0.966115	2.1
17994	1.25953	2.1
17995	1.38086	2.1
17996	1.38086	2.1
17997	1.04101	2.1
17998	1.99319	0.0540365
17999	1.23107	0.0569407
18000	3.3606	0.0549494
18001	1.23112	0.0578662
18002	1.40239	2.1
18003	1.26091	2.1
18004	1.26091	2.1
18005	1.05776	2.1
18006	1.23768	0.183059
18007	1.0142	1.63247
18008	1.11435	1.30754
18009	1.01422	0.268759
18010	1.24001	1.20004
18011	1.42186	1.28351
18012	1.42186	1.31343
18013	1.47204	1.3598
18014	1.27361	2.1
18015	1.56642	2.1
18016	1.71676	2.1
18017	2.16815	2.1
18018	1.07855	1.8608
18019	1.07612	1.8186
18020	1.703 0.05	
18021	1.46853	0.05
18022	1.45448	0.0640941
18023	1.27778	0.0676352
18024	0.991986	2.1
18025	1.1192	2.1
18026	1.1192	2.1
18027	1.25953	2.1
18028	2.08506	2.1
18029	1.75162	2.1
18030	1.94523	2.1
18031	1.88405	1.4952
18032	1.15506	0.05
18033	1.1233	0.05
18034	2.09736	2.1
18035	2.98303	2.1

18036	2.71184	2.1
18037	3.51614	2.1
18038	1.18769	2.1
18039	1.24053	2.1
18040	1.24053	2.1
18041	1.45327	2.1
18042	1.26865	2.1
18043	1.1744	1.82787
18044	1.1744	1.91509
18045	1.11712	1.65152
18046	1.15841	0.155136
18047	1.02247	0.190052
18048	1.93649	2.1
18049	1.81191	2.1
18050	1.64718	2.1
18051	1.47521	2.1
18052	1.59654	2.1
18053	1.57823	2.1
18054	1.57823	2.1
18055	1.55768	2.1
18056	1.00665	0.122779
18057	1.28239	0.102108
18058	1.28239	0.13023
18059	1.44951	0.108134
18060	1.22083	2.1
18061	0.957622	2.1
18062	0.887896	2.1
18063	1.27387	2.1
18064	1.00521	1.54333
18065	1.00503	1.37216
18066	1.95844	2.1
18067	1.05042	2.1
18068	2.78662	2.1
18069	2.22399	2.1
18070	2.3356	0.05
18071	1.49543	0.0520253
18072	1.96854	0.05
18073	1.21681	0.05
18074	2.17803	1.72733
18075	1.50998	1.89926
18076	2.35239	1.5071
18077	2.26987	1.42573
18078	1.03824	0.164182
18079	1.51396	0.182015
18080	1.51396	0.121011
18081	1.48693	0.129006
18082	1.08784	1.34543
18083	1.36865	1.25229
18084	0.712405	1.64127
18085	1.08784	1.37567
18086	2.02522	2.1
18087	1.6738	2.1
18088	1.35175	2.1
18089	1.50094	2.1
18090	1.6738	2.1
18091	1.62871	2.1
18092	1.35084	2.1
18093	1.34276	2.1

18094	1.18041	2.1
18095	1.43652	2.1
18096	0.974196	2.1
18097	1.28037	2.1
18098	1.28037	2.1
18099	1.05071	2.1
18100	2.44737	0.05
18101	1.4672	0.05
18102	1.22883	0.05
18103	2.08149	0.05
18104	1.82208	1.53664
18105	1.59826	1.22014
18106	1.59826	0.636821
18107	1.01393	1.25151
18108	1.42757	2.1
18109	0.996775	2.1
18110	1.42757	2.1
18111	1.26397	2.1
18112	1.02461	2.1
18113	1.46129	2.1
18114	1.11833	2.1
18115	1.335 2.1	
18116	2.07087	2.1
18117	1.24041	2.1
18118	1.335 2.1	
18119	1.55398	2.1
18120	1.21171	0.05
18121	1.48589	0.05
18122	1.32886	0.05
18123	1.8027	0.05
18124	1.53515	1.73119
18125	1.60428	1.73845
18126	1.00035	0.05
18127	1.03534	0.302949
18128	1.03535	2.1
18129	1.09962	2.1
18130	0.62132	2.04986
18131	1.21033	2.1
18132	1.30326	2.1
18133	2.6823	2.1
18134	0.991407	2.1
18135	1.29729	2.1
18136	1.29729	2.1
18137	1.16858	2.1
18138	1.66137	0.437118
18139	1.23012	0.289893
18140	1.66139	0.717395
18141	1.41821	0.500384
18142	1.56043	0.390381
18143	1.06848	0.442417
18144	1.56043	0.604646
18145	1.90911	0.574012
18146	0.982847	2.1
18147	1.05863	2.1
18148	1.05863	2.1
18149	1.22352	2.1
18150	1.23019	2.1
18151	1.60509	2.1

18152	1.19122	2.1
18153	1.47769	2.1
18154	2.18868	2.1
18155	1.40984	2.1
18156	3.55197	2.1
18157	3.57227	2.1
18158	3.57227	2.1
18159	3.48926	2.1
18160	1.4375	0.131214
18161	1.11314	0.124034
18162	1.62408	2.1
18163	1.74438	2.1
18164	1.72387	2.1
18165	1.50018	2.1
18166	1.1486	2.1
18167	1.18474	2.1
18168	1.18474	2.1
18169	1.2295	2.1
18170	1.28754	2.1
18171	1.36838	2.1
18172	1.36838	2.1
18173	1.21703	2.1
18174	2.74206	2.1
18175	2.70795	2.1
18176	2.70795	2.1
18177	2.55918	2.1
18178	1.35065	0.770349
18179	1.65411	0.394105
18180	1.48572	0.669803
18181	1.28393	0.526958
18182	1.25265	0.0531752
18183	1.70944	0.05
18184	1.31259	2.1
18185	1.14945	2.1
18186	1.14945	2.06621
18187	0.995725	2.1
18188	1.00307	0.183104
18189	2.05786	0.190063
18190	1.56357	0.11341
18191	1.10907	0.147265
18192	1.27558	1.72753
18193	1.05175	1.61432
18194	1.2685	2.1
18195	1.41369	2.1
18196	1.4137	2.1
18197	1.49654	2.1
18198	1.21489	1.89774
18199	1.14605	1.38093
18200	1.22476	2.1
18201	1.21489	1.15891
18202	2.51472	1.2662
18203	2.52091	1.25682
18204	2.52091	0.91387
18205	1.79171	0.631342
18206	1.03373	2.1
18207	1.04037	2.1
18208	1.04038	2.1
18209	1.03933	2.1

18210	1.74123	0.10751
18211	1.41902	0.0963162
18212	1.89777	0.119483
18213	1.89899	0.135089
18214	1.24728	0.11963
18215	1.30394	0.110727
18216	1.30394	0.0856908
18217	1.16535	0.095374
18218	1.09914	2.1
18219	1.54259	2.1
18220	1.40233	2.1
18221	1.69858	2.1
18222	1.53555	2.1
18223	1.09859	2.1
18224	1.09859	2.1
18225	0.725879	2.1
18226	1.07213	2.1
18227	1.31589	2.1
18228	1.36904	2.1
18229	1.69711	2.1
18230	1.51028	0.05
18231	0.993598	0.0560243
18232	0.61563	0.05
18233	1.26311	0.05
18234	1.19496	2.1
18235	1.65925	2.1
18236	1.65925	2.1
18237	1.48162	2.1
18238	2.75738	1.53725
18239	1.24699	1.48867
18240	4.08729	1.73958
18241	2.35271	1.53412
18242	1.55063	0.135995
18243	1.34563	0.104705
18244	1.34563	0.105986
18245	1.08189	0.0905389
18246	1.09662	0.0670902
18247	2.35961	0.104411
18248	0.986952	0.0604683
18249	0.813251	0.0514471
18250	1.17897	0.05
18251	1.67685	0.05
18252	1.00573	2.00452
18253	1.08542	1.66115
18254	1.00573	2.1
18255	1.12572	2.1
18256	1.05499	2.1
18257	1.00521	1.52435
18258	2.22679	0.05
18259	2.86746	0.05
18260	2.86746	0.05
18261	1.99958	0.05
18262	0.996517	2.1
18263	1.01169	2.1
18264	1.01169	2.1
18265	1.26346	2.1
18266	1.68274	2.1
18267	1.75505	2.1

18268	2.28748	1.9798
18269	1.36555	2.08318
18270	3.47888	0.05
18271	0.692788	0.05
18272	1.0685	0.329108
18273	1.14547	0.386331
18274	1.19017	2.1
18275	1.24976	2.1
18276	1.24976	2.1
18277	1.30682	2.1
18278	2.2153	2.1
18279	1.85457	2.1
18280	2.03267	2.1
18281	1.51042	2.1
18282	1.05619	0.48869
18283	1.05793	0.382729
18284	1.05793	0.29692
18285	1.00763	0.237151
18286	1.77677	2.1
18287	1.02524	2.1
18288	1.02524	2.1
18289	1.18803	2.1
18290	1.68669	2.1
18291	1.71162	2.1
18292	1.556	2.1
18293	1.39523	2.1
18294	0.960288	2.01632
18295	1.24201	2.1
18296	1.24202	2.1
18297	1.3592	2.1
18298	2.14045	0.0777453
18299	1.62054	0.0743688
18300	2.20248	0.107387
18301	2.34699	0.092784
18302	3.45132	0.0684674
18303	3.38241	0.0693785
18304	3.38241	0.0632631
18305	2.96127	0.0629796
18306	2.02612	2.1
18307	2.09351	2.1
18308	2.09351	2.1
18309	2.17465	2.1
18310	1.07445	2.1
18311	1.14808	2.1
18312	1.39067	2.1
18313	1.20958	2.1
18314	1.20958	2.1
18315	1.00382	2.00987
18316	0.986496	0.849525
18317	1.27142	1.17892
18318	1.39857	0.949956
18319	2.26482	1.06853
18320	2.24367	0.05
18321	1.9234	0.05
18322	1.59076	0.05
18323	1.185	0.05
18324	3.86179	0.05
18325	3.0638	0.05

18326	2.7574	0.05
18327	1.83266	0.05
18328	2.59418	2.1
18329	2.23048	2.1
18330	1.39564	2.1
18331	1.36763	2.1
18332	2.23048	2.1
18333	2.02522	2.1
18334	1.36763	2.1
18335	1.35175	2.1
18336	1.16181	0.05
18337	0.97096	0.05
18338	2.05665	0.142414
18339	0.483583	0.146191
18340	0.601928	0.113657
18341	3.88545	0.16328
18342	1.04909	2.1
18343	0.983143	2.1
18344	1.10469	2.1
18345	1.10516	2.1
18346	1.10517	2.1
18347	1.09951	2.1
18348	2.33985	2.1
18349	2.35026	2.1
18350	2.35025	2.1
18351	1.19462	2.1
18352	1.57627	2.1
18353	1.5493	2.1
18354	1.5493	2.1
18355	1.53556	2.1
18356	1.42763	0.393001
18357	1.24682	0.449271
18358	1.24682	0.284836
18359	1.00307	0.375453
18360	1.37392	2.1
18361	1.45408	2.1
18362	1.45408	2.1
18363	1.54249	2.1
18364	1.24474	1.59162
18365	1.07467	1.57638
18366	1.37813	1.55195
18367	1.24474	1.58159
18368	1.06341	2.1
18369	1.09194	2.1
18370	1.09194	2.1
18371	1.11117	2.1
18372	1.1658	2.1
18373	1.93707	2.1
18374	1.93707	2.1
18375	1.47729	2.1
18376	1.25669	2.1
18377	1.07519	2.1
18378	1.65417	2.1
18379	1.50956	2.1
18380	1.37231	2.1
18381	1.27285	2.1
18382	1.23612	2.1
18383	1.1899	2.1

18384	1.08172	2.1
18385	1.20357	2.1
18386	1.5664	2.1
18387	1.7472	2.1
18388	1.48434	2.1
18389	1.23989	2.1
18390	1.58836	2.1
18391	1.38766	2.1
18392	1.23989	2.1
18393	1.26599	2.1
18394	2.56248	2.1
18395	2.08673	2.1
18396	1.87216	2.1
18397	1.60476	0.634806
18398	1.84355	2.1
18399	1.25971	2.1
18400	1.44141	2.05639
18401	1.25521	0.33646
18402	1.19837	0.05
18403	2.31936	0.05
18404	1.38153	2.1
18405	2.41734	2.1
18406	2.70856	2.1
18407	3.48385	2.1
18408	1.89226	1.24761
18409	1.64617	1.23628
18410	1.89112	0.666285
18411	1.23135	0.191439
18412	1.23134	0.325751
18413	1.34927	0.258953
18414	3.60096	0.0539311
18415	1.68103	0.0571348
18416	2.25644	2.1
18417	1.91955	2.1
18418	1.91955	2.1
18419	1.47688	2.1
18420	1.67375	2.1
18421	1.50033	2.1
18422	1.5098	2.1
18423	1.46908	2.1
18424	1.69055	0.05
18425	1.53463	0.05
18426	1.53463	0.05
18427	1.41943	0.05
18428	1.19022	1.82846
18429	1.95981	1.65209
18430	1.15009	1.91038
18431	1.31612	1.8049
18432	1.31611	0.583938
18433	1.10735	0.511668
18434	1.18601	0.0860406
18435	1.3499	0.077236
18436	1.3499	0.0950916
18437	1.29001	0.0810305
18438	1.22083	2.1
18439	1.06191	2.1
18440	1.348	1.28572
18441	1.05758	1.39963

18442	1.34799	1.52778
18443	1.18647	1.58617
18444	2.92457	0.05
18445	1.99182	0.05
18446	2.25313	0.05
18447	1.30859	0.05
18448	1.30682	2.1
18449	1.38957	2.1
18450	1.38957	2.1
18451	1.47446	2.1
18452	0.953638	2.1
18453	1.03408	2.1
18454	1.17885	0.410116
18455	1.3663	0.146012
18456	1.08265	0.307012
18457	1.71948	0.549714
18458	1.29501	0.854725
18459	0.955093	0.524123
18460	0.955081	0.322027
18461	1.88345	0.428555
18462	1.2243	2.1
18463	1.23169	2.1
18464	2.04726	1.15851
18465	0.842869	0.866725
18466	3.18156	2.1
18467	3.60786	1.6754
18468	3.60786	2.1
18469	2.38566	1.45877
18470	1.24526	0.05
18471	1.59019	0.05
18472	1.16776	1.55764
18473	1.05042	2.1
18474	1.17418	2.1
18475	1.05042	2.1
18476	0.949135	2.1
18477	0.973448	2.1
18478	0.973447	2.1
18479	1.02958	2.1
18480	3.57768	2.1
18481	3.04246	2.1
18482	3.04246	2.1
18483	2.47824	2.1
18484	1.35879	2.1
18485	1.10407	2.1
18486	1.14599	2.1
18487	1.10407	2.1
18488	1.87794	1.70038
18489	1.53429	1.66143
18490	1.12717	2.1
18491	1.38634	2.1
18492	1.38634	2.1
18493	1.13127	2.1
18494	1.04546	2.1
18495	1.06007	2.1
18496	1.06007	2.1
18497	1.19785	2.1
18498	1.43652	2.1
18499	1.71741	2.1

18500	1.70921	2.1
18501	1.06994	2.1
18502	0.960106	2.1
18503	1.48077	2.1
18504	1.48077	2.1
18505	2.5946	2.1
18506	2.03045	1.77193
18507	1.76788	1.85611
18508	2.03045	2.09988
18509	0.633098	2.1
18510	1.06756	2.1
18511	1.38667	2.1
18512	0.966979	2.1
18513	1.38669	2.1
18514	1.07156	2.1
18515	0.986095	2.1
18516	1.32813	2.1
18517	0.986095	2.1
18518	1.98697	1.07968
18519	1.12016	1.24407
18520	1.24463	1.41225
18521	1.1967	1.22768
18522	1.30001	0.357541
18523	1.29175	1.03788
18524	1.29175	0.388441
18525	1.3003	1.39367
18526	1.5606	0.05
18527	2.32098	0.05
18528	2.57888	0.05
18529	2.97463	0.05
18530	1.24555	2.1
18531	1.46101	2.1
18532	1.90723	2.1
18533	2.29126	2.1
18534	1.46101	2.1
18535	1.84089	2.1
18536	2.5519	2.1
18537	2.91663	2.1
18538	1.10476	0.05
18539	1.44103	0.05
18540	1.44103	0.05
18541	1.53336	0.05
18542	0.672964	2.1
18543	0.633884	2.1
18544	0.633884	2.1
18545	0.603154	2.1
18546	2.29663	2.1
18547	2.06892	2.1
18548	1.3685	2.1
18549	1.39067	2.1
18550	2.06892	2.1
18551	1.78208	2.1
18552	1.39067	2.1
18553	1.42174	2.1
18554	1.01335	1.53043
18555	1.08195	1.32111
18556	1.86463	2.01577
18557	1.39827	2.02506

18558	0.861515	2.1
18559	2.10074	2.1
18560	2.09511	1.49457
18561	2.04853	1.21336
18562	2.21488	1.13485
18563	1.38287	1.43065
18564	0.974717	0.428052
18565	0.998451	0.370955
18566	1.42281	1.73352
18567	1.82595	1.68873
18568	1.50458	1.13688
18569	1.09408	1.10255
18570	1.23498	1.65512
18571	1.05654	1.62232
18572	1.17261	2.1
18573	1.18058	2.1
18574	1.18059	2.1
18575	1.18907	2.1
18576	2.38998	2.1
18577	2.77437	2.1
18578	1.04546	2.1
18579	1.16817	2.1
18580	2.77437	2.1
18581	2.97768	2.1
18582	1.16817	2.1
18583	1.2832	2.1
18584	1.75766	0.105986
18585	1.4934	1.91254
18586	1.4934	0.227651
18587	1.20324	2.03044
18588	1.07236	0.768328
18589	1.37641	0.450238
18590	1.42913	0.370253
18591	1.93373	0.410142
18592	1.42629	0.378512
18593	1.00636	0.269033
18594	0.933959	1.81489
18595	2.22873	0.689621
18596	2.22873	1.53179
18597	2.13757	0.452412
18598	1.10517	2.1
18599	1.86737	2.1
18600	0.811 2.1	
18601	1.19179	2.1
18602	1.18647	1.67863
18603	0.988453	2.03352
18604	1.12457	0.0991591
18605	1.23851	0.0891325
18606	0.92492	2.1
18607	1.45656	2.1
18608	1.61251	0.623412
18609	1.56696	0.67059
18610	1.90245	0.359069
18611	1.46046	0.349081
18612	1.60329	0.05
18613	1.38247	0.05
18614	1.38247	0.05
18615	1.23198	0.05

18616	1.09518	2.1
18617	1.28159	2.1
18618	0.977777	2.1
18619	1.80778	2.1
18620	1.80777	2.1
18621	1.16655	2.1
18622	1.3515	2.1
18623	0.956573	2.1
18624	1.08022	2.1
18625	1.03437	2.1
18626	1.03437	2.1
18627	0.975409	2.1
18628	1.47369	0.0540934
18629	1.31167	0.0765561
18630	1.01847	0.0526557
18631	2.75562	0.0533864
18632	1.13443	2.1
18633	1.32709	2.1
18634	1.01051	0.190081
18635	0.958947	0.1578
18636	1.7211	2.1
18637	1.922 2.1	
18638	1.922 2.1	
18639	0.991113	2.1
18640	1.00636	0.273315
18641	1.31658	0.299161
18642	1.44627	0.203172
18643	1.03824	0.193401
18644	1.21223	2.1
18645	1.01242	2.1
18646	1.01243	2.1
18647	0.956698	2.1
18648	1.23167	2.1
18649	1.06935	2.1
18650	1.29215	0.05
18651	0.998971	0.05
18652	1.50103	0.05
18653	1.44836	0.05
18654	3.02513	2.1
18655	3.7335	2.1
18656	2.49988	2.1
18657	1.24948	2.1
18658	0.963972	2.1
18659	1.1822	2.1
18660	1.1822	2.1
18661	1.10817	2.1
18662	1.30237	2.1
18663	1.41908	2.1
18664	1.41908	2.1
18665	1.48734	2.1
18666	1.31127	0.173436
18667	1.40822	0.166263
18668	1.40822	0.0936472
18669	1.22176	0.0860252
18670	1.82525	2.1
18671	1.59394	2.1
18672	1.22731	0.05
18673	0.981584	0.05

18674	1.1427	1.13161
18675	1.44091	1.25878
18676	1.49154	1.73856
18677	1.07526	1.57554
18678	1.07527	0.991665
18679	0.96029	1.78274
18680	2.40568	1.5752
18681	3.46486	1.56807
18682	2.417	1.63131
18683	1.52054	1.40072
18684	1.51638	0.131908
18685	1.04786	0.100874
18686	1.51404	0.107398
18687	1.3461	0.08585
18688	1.27696	0.0732832
18689	0.736457	0.0638541
18690	1.16817	2.1
18691	1.04263	2.1
18692	1.13449	2.1
18693	1.50524	2.1
18694	2.1182	2.1
18695	2.49001	2.1
18696	1.11764	2.1
18697	1.39292	2.1
18698	2.49001	2.1
18699	2.76339	2.1
18700	1.39292	2.1
18701	1.68761	2.1
18702	1.25173	2.1
18703	1.34701	2.1
18704	1.02415	2.1
18705	1.34701	2.1
18706	1.332	0.05
18707	0.989764	0.05
18708	0.963167	0.05
18709	1.21056	0.05
18710	1.67375	2.1
18711	1.17965	2.1
18712	1.09154	2.1
18713	1.52314	2.1
18714	1.54433	0.422042
18715	1.81969	0.32904
18716	1.3598	0.237841
18717	1.71041	0.194167
18718	1.70934	0.194332
18719	1.33871	0.162997
18720	1.79485	0.148857
18721	1.51404	0.11149
18722	1.30751	2.1
18723	1.20969	2.1
18724	1.20969	2.1
18725	1.12076	2.1
18726	1.49808	2.1
18727	1.50201	2.1
18728	1.50201	2.1
18729	1.31594	2.1
18730	1.2187	2.1
18731	1.11545	2.1

18732	1.227	2.1
18733	1.09645	2.1
18734	1.05902	0.05
18735	1.41595	1.26016
18736	1.35696	2.1
18737	1.29446	1.94431
18738	0.857957	2.1
18739	1.21125	0.05
18740	2.43115	2.1
18741	2.1151	0.218343
18742	2.0815	1.69572
18743	1.1723	1.9428
18744	1.48035	0.103712
18745	1.59971	0.651952
18746	1.33668	0.200159
18747	1.0961	0.194814
18748	1.46235	2.1
18749	0.882195	2.1
18750	2.46624	2.1
18751	2.72161	2.1
18752	2.96094	2.1
18753	2.25644	2.1
18754	1.25923	0.05
18755	0.976218	0.05
18756	1.10319	0.05
18757	1.82583	0.05
18758	0.888955	2.1
18759	1.30817	2.1
18760	1.30817	2.1
18761	1.60787	2.1
18762	0.988805	2.1
18763	1.54168	2.1
18764	1.19496	1.30086
18765	1.12224	1.61755
18766	1.93597	1.45708
18767	1.31113	1.40439
18768	1.17879	1.54238
18769	1.07468	1.5247
18770	2.0523	2.1
18771	2.41385	2.1
18772	2.41385	2.1
18773	3.0355	2.1
18774	1.36717	0.144836
18775	1.45535	0.113566
18776	1.45535	0.0990637
18777	1.49906	0.0807842
18778	1.37785	2.1
18779	1.04413	2.1
18780	1.06729	1.1723
18781	1.54305	0.81813
18782	1.07444	2.1
18783	1.52724	2.1
18784	1.49151	2.1
18785	1.36814	2.1
18786	1.36814	2.1
18787	1.15541	2.1
18788	1.19379	2.1
18789	1.78657	1.77668

18790	1.6079	2.1
18791	2.59469	1.23885
18792	1.35545	0.814148
18793	1.23602	1.91525
18794	1.23452	2.1
18795	1.56842	2.0665
18796	1.15864	2.1
18797	1.11955	2.1
18798	1.11955	2.1
18799	1.08022	2.1
18800	1.64268	2.1
18801	1.26247	2.1
18802	1.16912	2.1
18803	2.1095	1.26616
18804	1.75549	0.05
18805	1.7491	0.05
18806	1.59833	0.0548024
18807	1.68338	0.0535901
18808	1.14846	0.05
18809	1.06515	0.05
18810	1.2721	0.128812
18811	1.32639	0.108016
18812	1.32639	0.222087
18813	1.19323	0.171353
18814	1.81034	2.1
18815	1.9034	2.1
18816	2.08245	2.1
18817	1.84887	2.1
18818	1.41448	2.1
18819	1.77326	2.1
18820	1.76872	2.1
18821	1.86766	2.1
18822	1.46643	2.1
18823	1.58995	2.1
18824	1.58995	2.1
18825	1.13854	2.1
18826	1.23654	2.1
18827	1.04442	2.1
18828	1.23654	2.1
18829	1.08576	2.1
18830	0.966979	2.1
18831	1.41758	2.1
18832	1.03108	0.0952756
18833	1.94412	0.101531
18834	1.32455	2.1
18835	1.32328	2.1
18836	1.32328	2.1
18837	1.34008	2.1
18838	1.60861	2.1
18839	1.69586	2.1
18840	1.69586	2.1
18841	1.78301	2.1
18842	1.7513	2.1
18843	1.67906	2.1
18844	1.67906	2.1
18845	1.60635	2.1
18846	1.68073	2.1
18847	1.57234	2.1

18848	1.57233	2.1
18849	1.47012	2.1
18850	1.22811	0.137476
18851	1.79683	0.0981789
18852	1.53417	0.109745
18853	1.08732	0.105743
18854	1.8903	2.1
18855	1.20247	2.1
18856	1.12087	1.73498
18857	1.54699	2.08897
18858	1.43635	2.1
18859	1.02368	2.1
18860	1.43635	2.1
18861	1.23007	2.1
18862	1.035 2.1	
18863	1.07889	2.1
18864	3.0355	2.1
18865	3.78805	2.1
18866	2.57183	2.1
18867	1.27835	1.98096
18868	1.29813	0.13018
18869	1.42965	0.100144
18870	1.69832	0.120213
18871	2.18359	0.0935895
18872	1.42964	0.130025
18873	1.65723	0.116166
18874	1.98508	0.118382
18875	2.06284	0.0932489
18876	1.50705	0.063964
18877	1.51153	0.104825
18878	1.36037	0.0790308
18879	1.09767	0.132549
18880	1.70048	2.1
18881	1.15177	2.1
18882	0.713174	2.1
18883	0.763575	2.1
18884	0.763575	2.1
18885	0.797011	2.1
18886	1.01179	1.10484
18887	1.27737	1.15387
18888	3.16531	0.05
18889	1.0141	0.05
18890	1.47354	0.275023
18891	1.62495	0.13228
18892	1.62495	0.0960462
18893	1.39296	0.225014
18894	0.998682	2.1
18895	1.93528	2.1
18896	2.71383	2.1
18897	1.13987	2.1
18898	1.93528	2.1
18899	1.78595	2.1
18900	3.98933	2.1
18901	3.73426	2.1
18902	1.02438	2.1
18903	1.12376	2.1
18904	1.12375	2.1
18905	1.19016	2.1

18906	1.37157	0.130914
18907	1.60814	0.129829
18908	1.70303	0.164121
18909	1.01219	0.146625
18910	1.06757	2.1
18911	1.23475	2.1
18912	1.2295	2.1
18913	1.28014	2.1
18914	1.28014	2.1
18915	1.33339	2.1
18916	1.3201	2.1
18917	1.2679	2.1
18918	1.2679	2.1
18919	1.23504	2.1
18920	1.50108	2.1
18921	1.3715	2.1
18922	1.3715	2.1
18923	1.25075	2.1
18924	1.99222	1.17789
18925	1.4155	0.603202
18926	1.4777	2.1
18927	1.52613	2.1
18928	1.91031	2.1
18929	1.03968	2.1
18930	2.04289	0.225125
18931	0.967192	0.191806
18932	1.03043	0.152182
18933	1.65452	0.235232
18934	1.21564	2.1
18935	1.16938	2.1
18936	2.47056	2.1
18937	2.56853	2.1
18938	1.16938	2.1
18939	1.1181	2.1
18940	2.56853	2.1
18941	2.66828	2.1
18942	1.0465	2.1
18943	1.44709	2.1
18944	1.44709	2.1
18945	1.96491	2.1
18946	1.01976	0.306171
18947	0.961779	0.27192
18948	1.11764	0.295351
18949	0.961779	0.273295
18950	1.21171	0.05
18951	1.35007	0.05
18952	1.03102	0.207196
18953	1.42446	0.230315
18954	1.42446	0.214081
18955	1.03166	0.185823
18956	1.2951	0.096245
18957	1.33575	0.107171
18958	1.33575	0.11331
18959	1.20473	0.124712
18960	1.10049	0.439219
18961	0.99747	0.658072
18962	1.54249	2.1
18963	1.65677	2.1

18964	1.65677	2.1
18965	1.77677	2.1
18966	1.73525	0.142966
18967	1.05689	0.192771
18968	1.26051	0.131856
18969	1.16259	0.126496
18970	1.34009	2.1
18971	1.398 2.1	
18972	1.398 2.1	
18973	1.50437	2.1
18974	2.3245	2.1
18975	1.18237	2.1
18976	1.24509	2.1
18977	1.01595	2.1
18978	3.29047	2.1
18979	2.79168	2.1
18980	1.06561	2.1
18981	1.11077	2.1
18982	1.11077	2.1
18983	0.953927	2.1
18984	1.30509	0.233541
18985	1.04685	0.256644
18986	1.04685	0.191552
18987	0.834593	0.209965
18988	1.0775	0.203293
18989	1.27362	0.18972
18990	1.71762	0.974162
18991	2.5736	0.69286
18992	2.04717	1.10561
18993	1.45521	0.592049
18994	1.33962	2.1
18995	1.27956	2.1
18996	1.27956	2.1
18997	1.20478	2.1
18998	3.65841	0.0543884
18999	1.18656	0.05
19000	1.72272	0.0682632
19001	1.6812	0.0648424
19002	1.09951	2.1
19003	1.08231	2.1
19004	1.08231	2.1
19005	1.06478	2.1
19006	2.16196	1.87935
19007	1.00387	1.637
19008	1.00387	1.14657
19009	0.993999	0.930532
19010	1.04101	2.1
19011	1.27119	2.1
19012	1.27119	2.1
19013	1.51748	2.1
19014	3.00129	2.1
19015	3.27303	2.1
19016	3.27303	2.1
19017	3.60855	2.1
19018	2.22399	2.1
19019	1.71995	2.1
19020	1.54793	2.1
19021	1.227 2.1	

19022	1.07624	2.1
19023	1.264 2.1	
19024	1.19323	1.84496
19025	1.21286	1.62569
19026	1.21286	1.17099
19027	1.20859	1.30885
19028	1.60578	0.108122
19029	1.27778	0.0865275
19030	1.0525	2.06518
19031	1.05758	1.28588
19032	1.0525	1.35508
19033	1.17042	1.49739
19034	1.62536	0.05
19035	3.71662	0.05
19036	1.78169	0.05
19037	1.91434	0.05
19038	2.12331	0.05
19039	1.33406	0.05
19040	1.38437	2.1
19041	1.51829	2.1
19042	1.51829	2.1
19043	1.6697	2.1
19044	0.947114	2.1
19045	1.13335	2.1
19046	1.13335	2.1
19047	1.00965	2.1
19048	1.47521	2.1
19049	1.27679	2.1
19050	1.14911	2.1
19051	0.93841	2.1
19052	1.68437	2.1
19053	1.53822	2.1
19054	1.53822	2.1
19055	1.39702	2.1
19056	0.726453	2.1
19057	0.754101	2.1
19058	0.754101	2.1
19059	0.69776	2.1
19060	0.964033	2.1
19061	1.62397	2.1
19062	1.70302	2.1
19063	1.78637	2.08944
19064	3.68158	0.05
19065	2.11685	0.05
19066	1.33125	2.1
19067	1.15471	2.1
19068	1.0465	2.1
19069	1.33125	2.1
19070	1.17683	2.1
19071	1.25889	2.1
19072	1.25889	2.1
19073	1.34239	2.1
19074	1.40591	0.05
19075	0.79348	0.05
19076	1.04019	0.05
19077	0.999187	0.0591003
19078	1.30218	0.0686235
19079	1.83339	0.0673328

19080	1.88574	0.05
19081	1.5177	0.05
19082	1.23504	2.1
19083	1.19583	2.1
19084	1.19583	2.1
19085	1.14496	2.1
19086	1.62183	2.1
19087	1.62316	2.1
19088	1.62316	2.1
19089	1.62241	2.1
19090	1.02305	0.141829
19091	1.21136	0.134599
19092	0.993832	0.0540691
19093	1.21137	0.0914063
19094	1.12064	2.1
19095	1.24965	2.1
19096	1.24965	2.1
19097	1.41077	2.1
19098	1.00052	0.142204
19099	1.49842	0.112854
19100	1.49843	0.0928472
19101	1.10801	0.0817796
19102	1.54196	2.1
19103	1.18041	2.1
19104	1.73571	1.73273
19105	1.37224	1.84368
19106	1.4375	0.0861282
19107	1.09529	0.0763215
19108	1.44421	0.0633712
19109	1.0953	0.0718472
19110	1.0753	2.1
19111	0.815365	2.1
19112	0.815365	2.1
19113	0.597537	2.1
19114	1.03609	2.1
19115	1.18952	2.1
19116	1.54283	0.196096
19117	1.16719	0.177155
19118	1.16719	0.297385
19119	1.89226	0.22203
19120	2.55919	2.1
19121	2.37492	2.1
19122	2.37492	2.1
19123	2.07435	2.1
19124	1.36016	0.114281
19125	1.70302	0.112472
19126	1.85252	0.126211
19127	1.2111	0.198132
19128	1.62905	2.1
19129	1.49692	2.1
19130	1.49692	2.1
19131	1.40395	2.1
19132	2.19083	2.1
19133	1.30542	2.1
19134	1.58736	2.1
19135	2.16579	2.1
19136	2.0815	2.1
19137	1.17935	2.1

19138	1.53803	2.1
19139	1.09148	1.98307
19140	1.81301	2.1
19141	1.38333	1.76636
19142	1.04412	2.1
19143	1.44437	2.1
19144	1.4675	0.0836111
19145	1.00665	0.0897091
19146	1.50951	0.102992
19147	0.983105	0.0911644
19148	1.80252	0.14537
19149	0.540005	0.106125
19150	1.2545	0.160661
19151	2.14514	0.122135
19152	1.32051	0.094382
19153	1.82302	0.096044
19154	2.71057	2.1
19155	2.51107	2.1
19156	1.34817	2.1
19157	1.3551	2.1
19158	2.51107	2.1
19159	2.29663	2.1
19160	1.3551	2.1
19161	1.3685	2.1
19162	1.51748	2.1
19163	1.23389	2.1
19164	0.901928	0.05
19165	1.70302	0.0564689
19166	1.66787	0.05
19167	1.92535	0.0618132
19168	1.13571	0.210674
19169	1.02836	0.234697
19170	1.55398	2.1
19171	1.54179	2.1
19172	1.89608	2.1
19173	1.07167	2.1
19174	3.3488	0.148552
19175	0.550044	0.129059
19176	0.962877	0.336347
19177	1.00065	0.662884
19178	1.00613	0.351444
19179	1.07497	0.416114
19180	1.00486	0.05
19181	1.00486	0.05
19182	0.726453	2.1
19183	1.32634	0.0694122
19184	1.40008	0.0933236
19185	1.40008	0.0613392
19186	2.42267	0.0589422
19187	1.9216	0.063495
19188	1.28066	0.05
19189	1.79102	2.1
19190	1.18219	0.727636
19191	1.18219	2.00748
19192	0.709123	0.597622
19193	1.07866	2.1
19194	1.15038	2.1
19195	1.15038	2.1

19196	1.24687	2.1
19197	1.83613	2.1
19198	1.99157	2.07934
19199	1.45789	0.05
19200	1.1706	0.05
19201	1.74746	0.05
19202	1.1706	0.05
19203	1.96383	0.05
19204	1.67727	0.05
19205	1.67727	0.05
19206	1.60329	0.05
19207	1.18803	2.1
19208	1.3227	2.1
19209	1.3227	2.1
19210	1.40844	2.1
19211	1.07889	2.1
19212	1.07721	2.1
19213	1.07721	2.1
19214	1.00832	2.1
19215	0.953523	0.766204
19216	1.62875	0.55506
19217	1.07427	0.253677
19218	1.19722	0.204766
19219	1.1972	0.131568
19220	1.1599	0.145289
19221	2.97463	0.05
19222	1.9477	0.05
19223	1.95222	0.05
19224	1.75083	0.05
19225	3.46787	2.1
19226	3.02896	2.1
19227	3.02896	2.1
19228	2.46145	2.1
19229	0.970962	0.05
19230	1.0227	0.05
19231	1.12328	0.05
19232	1.02269	0.05
19233	0.904978	2.1
19234	0.725887	2.1
19235	0.725887	2.1
19236	0.601592	2.1
19237	0.956294	2.1
19238	1.13179	2.1
19239	0.956294	2.1
19240	1.13179	2.1
19241	1.05556	2.1
19242	1.13179	2.1
19243	1.38859	2.1
19244	1.13179	2.1
19245	1.06659	2.1
19246	1.05776	2.1
19247	1.06659	2.1
19248	1.04291	2.1
19249	1.54699	1.66573
19250	1.42307	1.73172
19251	1.5654	1.98494
19252	1.97906	2.1
19253	1.15354	0.05

19254	1.15505	0.05
19255	1.26455	0.0624389
19256	2.50042	0.0547529
19257	2.50042	0.0556383
19258	3.22585	0.05
19259	1.19258	0.157523
19260	1.34562	0.163108
19261	1.49515	0.212784
19262	1.66295	0.128597
19263	1.02395	2.1
19264	1.51067	2.1
19265	1.35821	2.1
19266	2.10671	2.1
19267	1.0305	2.07787
19268	1.07855	2.02256
19269	1.20761	2.1
19270	0.98488	2.1
19271	1.4162	2.1
19272	1.14825	2.1
19273	1.29415	2.1
19274	1.6067	2.1
19275	1.23851	0.1352
19276	1.09651	0.118572
19277	1.60636	2.1
19278	1.53521	2.1
19279	1.53521	2.1
19280	1.46279	2.1
19281	1.28044	0.051699
19282	1.64147	0.0561319
19283	1.18422	0.0583551
19284	1.68208	0.0635121
19285	1.09726	2.1
19286	1.234 2.1	
19287	1.234 2.1	
19288	1.09483	2.1
19289	1.89318	2.1
19290	1.98448	2.1
19291	1.98448	2.1
19292	2.02612	2.1
19293	1.22026	2.1
19294	1.23527	1.86955
19295	1.09044	2.08317
19296	0.993366	1.65143
19297	1.22026	1.99836
19298	0.993368	1.59908
19299	2.61357	2.1
19300	2.48208	2.1
19301	2.48208	2.1
19302	2.62868	2.1
19303	1.85439	1.02275
19304	1.81776	0.633919
19305	1.81776	0.540178
19306	2.1587	0.53508
19307	1.8914	0.681793
19308	2.23961	0.444863
19309	1.49357	0.141635
19310	2.23961	0.42572
19311	1.78014	0.324129

19312	1.67635	0.274556
19313	1.66219	0.256776
19314	1.56036	0.235935
19315	1.14721	0.232601
19316	2.22495	0.329141
19317	1.88509	0.0816359
19318	1.95249	0.0695729
19319	2.11959	0.0664439
19320	1.37427	0.0546387
19321	1.39379	2.06014
19322	1.17614	1.91952
19323	1.39379	2.1
19324	1.14397	1.25691
19325	1.09651	2.1
19326	1.42843	2.1
19327	1.7069	2.1
19328	2.31293	2.1
19329	1.56839	2.1
19330	1.78252	1.7044
19331	1.78254	1.24714
19332	1.09142	2.1
19333	0.99829	0.05
19334	1.09372	0.0525568
19335	1.88175	0.0763225
19336	0.898452	0.05
19337	1.06936	2.1
19338	1.4162	2.1
19339	1.17989	2.1
19340	1.47411	2.1
19341	1.25376	2.1
19342	1.00757	2.1
19343	1.4185	0.18077
19344	1.02011	0.199996
19345	2.22495	0.221411
19346	1.79542	0.207504
19347	1.79542	0.172664
19348	1.19352	0.173977
19349	2.38566	2.1
19350	1.83255	1.68561
19351	1.62667	2.1
19352	1.17879	1.87184
19353	2.79168	2.1
19354	2.28162	2.1
19355	2.28162	2.1
19356	1.90905	2.1
19357	1.40117	2.1
19358	1.28268	2.1
19359	1.41096	2.1
19360	1.22978	2.1
19361	3.20034	2.1
19362	3.42052	2.1
19363	3.42052	2.1
19364	3.50562	2.1
19365	1.46279	2.1
19366	1.3864	2.1
19367	1.3864	2.1
19368	1.3201	2.1
19369	1.35561	0.05

19370	0.974077	0.05
19371	0.967673	0.731839
19372	1.22482	0.51108
19373	1.02305	0.19141
19374	1.22482	0.304794
19375	0.999492	2.1
19376	1.14657	2.1
19377	1.14657	2.1
19378	1.31346	2.1
19379	1.84417	0.05
19380	1.31777	0.05
19381	1.19796	0.05
19382	0.691742	0.05
19383	1.16725	2.1
19384	1.9679	2.1
19385	1.08848	0.05
19386	1.15356	0.05
19387	1.58713	2.1
19388	1.58742	2.1
19389	1.58742	2.1
19390	1.49662	2.1
19391	1.32201	0.563516
19392	1.32097	1.64716
19393	1.32097	0.587469
19394	1.31825	1.74752
19395	1.12028	2.1
19396	1.15852	2.1
19397	1.15852	2.1
19398	1.16239	2.1
19399	1.07624	2.1
19400	1.30047	2.1
19401	1.44847	1.14376
19402	1.55292	1.07451
19403	1.21125	0.05
19404	1.15262	2.1
19405	1.05025	2.1
19406	1.55351	2.1
19407	1.5329	0.05
19408	2.26758	0.05
19409	1.94968	0.05
19410	1.65747	0.05
19411	1.42549	0.0510867
19412	1.57656	0.0507713
19413	2.11157	0.538872
19414	1.56531	0.573933
19415	1.69279	0.387238
19416	1.06204	1.00337
19417	0.947863	1.39336
19418	1.19854	1.51007
19419	0.947862	0.47904
19420	1.31126	0.365503
19421	1.3425	1.67656
19422	1.29976	1.57496
19423	1.05129	0.0743762
19424	1.51026	0.0820923
19425	1.05129	0.0810216
19426	1.2951	0.0930982
19427	1.7155	2.1

19428	1.72196	2.1
19429	1.72196	2.1
19430	1.77867	2.1
19431	0.964755	2.1
19432	1.14142	2.1
19433	1.03766	2.1
19434	0.986723	2.1
19435	1.28072	2.1
19436	1.10609	2.1
19437	1.28072	1.61653
19438	1.16776	2.1
19439	1.61687	2.1
19440	1.60965	2.1
19441	1.60965	2.1
19442	1.59654	2.1
19443	1.26512	2.1
19444	1.23964	2.1
19445	2.07997	2.1
19446	2.60048	2.1
19447	1.23732	2.1
19448	1.3372	2.1
19449	2.62253	2.1
19450	2.47056	2.1
19451	0.92782	0.0937395
19452	1.40335	0.110092
19453	1.26301	0.117147
19454	1.52668	0.118856
19455	0.99818	0.0944228
19456	1.51165	0.110938
19457	1.66213	0.0943479
19458	1.55246	0.0841595
19459	2.31255	2.1
19460	1.34366	2.1
19461	1.90577	2.1
19462	0.969739	2.1
19463	3.4245	2.1
19464	2.8415	2.1
19465	1.97559	2.1
19466	1.82362	2.1
19467	1.48162	2.1
19468	1.23204	2.1
19469	1.23204	2.1
19470	0.95947	2.1
19471	1.05267	1.8407
19472	1.44091	1.45211
19473	1.05267	1.61081
19474	1.5795	1.84967
19475	0.738711	2.1
19476	1.28549	2.1
19477	1.15694	2.1
19478	1.72061	2.1
19479	1.69858	2.1
19480	0.954563	2.1
19481	0.859087	2.1
19482	0.788886	2.1
19483	1.08732	0.135771
19484	1.3357	0.118986
19485	1.3357	0.131349

19486	1.48676	0.106017
19487	2.15079	2.1
19488	2.12414	2.1
19489	2.12414	2.1
19490	2.04664	2.1
19491	1.98859	2.1
19492	0.992159	2.1
19493	1.48064	2.1
19494	1.2163	2.1
19495	1.26337	2.1
19496	1.63159	2.1
19497	1.00366	0.489413
19498	1.65452	0.307936
19499	1.10408	2.1
19500	1.37704	2.1
19501	1.50224	2.1
19502	1.42866	2.1
19503	1.43939	2.1
19504	1.02784	2.1
19505	1.00763	0.100207
19506	1.23746	0.105204
19507	1.23747	0.110254
19508	1.13642	0.125013
19509	1.49906	0.0696272
19510	1.46506	0.0597922
19511	1.46506	0.0575889
19512	1.33061	0.05
19513	1.08166	0.156608
19514	1.01052	0.141901
19515	1.02836	0.165606
19516	1.08166	0.154228
19517	1.25334	0.05
19518	1.23749	0.0624248
19519	1.37501	0.063243
19520	1.47354	0.0914347
19521	1.30167	2.1
19522	1.29897	2.1
19523	1.51042	2.1
19524	1.07684	2.1
19525	1.07684	2.1
19526	0.765121	2.1
19527	1.5535	2.1
19528	1.40602	2.1
19529	1.40603	2.1
19530	1.25669	2.1
19531	1.08276	2.1
19532	1.60948	2.1
19533	1.26929	2.1
19534	1.09847	2.1
19535	0.827082	0.179353
19536	1.15841	0.171053
19537	1.25063	0.396289
19538	1.14397	0.377935
19539	1.25063	1.82947
19540	1.10476	0.228393
19541	1.14872	0.05
19542	1.3659	0.0515236
19543	1.36589	0.05

19544	1.4677	0.0537339
19545	0.590354	2.1
19546	0.655202	2.1
19547	3.48926	2.1
19548	3.32709	2.1
19549	3.32709	2.1
19550	3.13938	2.1
19551	1.24458	0.630872
19552	1.29681	0.665587
19553	1.40915	0.797757
19554	1.17891	1.6522
19555	1.33171	0.05
19556	1.37681	1.81654
19557	1.37681	2.1
19558	1.4516	2.1
19559	2.68241	0.05
19560	1.61031	0.05
19561	1.6488	2.1
19562	1.6585	2.1
19563	1.65848	2.1
19564	1.67061	2.1
19565	1.15152	2.1
19566	1.32548	2.1
19567	1.43677	2.1
19568	1.10264	2.1
19569	1.01472	2.1
19570	0.966964	2.1
19571	2.867 2.1	
19572	2.94897	2.1
19573	1.65815	2.1
19574	1.4502	2.1
19575	1.4502	2.1
19576	1.26755	2.1
19577	1.37138	2.1
19578	1.45026	2.1
19579	1.45026	2.1
19580	1.53446	2.1
19581	0.973502	2.08697
19582	1.08542	2.1
19583	1.13387	0.05
19584	1.71031	0.05
19585	1.08911	2.1
19586	1.10748	2.1
19587	1.21823	2.1
19588	1.25005	2.1
19589	0.657014	2.1
19590	1.38139	2.1
19591	1.01485	2.1
19592	1.26045	2.1
19593	1.12468	2.1
19594	1.26045	2.1
19595	1.89278	0.531485
19596	1.06433	0.331315
19597	1.87861	0.365918
19598	0.967544	0.26135
19599	1.50319	2.1
19600	3.47245	2.1
19601	2.2354	0.827347

19602	1.16701	0.306509
19603	1.3592	2.1
19604	1.3305	1.78424
19605	1.3305	2.1
19606	1.15008	1.43024
19607	1.12457	0.0659112
19608	1.40707	0.0645744
19609	1.01778	2.1
19610	1.35733	2.1
19611	1.01779	2.1
19612	1.12064	2.1
19613	1.30694	0.722147
19614	1.42716	1.45313
19615	1.42717	1.54713
19616	1.04321	1.59491
19617	1.19352	0.153609
19618	1.32634	0.224162
19619	1.14605	1.53798
19620	1.0417	1.49992
19621	1.19676	0.0856216
19622	1.03804	0.0949659
19623	2.09455	0.0990742
19624	1.15338	0.106729
19625	2.21119	2.1
19626	1.26097	2.1
19627	1.88394	1.66843
19628	1.24106	0.738397
19629	2.98143	2.1
19630	2.56248	2.1
19631	1.88394	2.1
19632	1.68703	0.639926
19633	1.5846	2.1
19634	1.94799	2.1
19635	1.20091	2.1
19636	1.40393	2.1
19637	3.09031	2.1
19638	2.90243	2.1
19639	1.27227	2.1
19640	1.32592	2.1
19641	2.90247	2.1
19642	2.71057	2.1
19643	1.32594	2.1
19644	1.34817	2.1
19645	1.4092	2.1
19646	1.44859	2.1
19647	1.52008	2.1
19648	1.401	2.1
19649	1.40099	2.1
19650	1.28936	2.1
19651	1.56002	0.05
19652	1.08811	0.05
19653	1.46041	0.05
19654	1.56002	0.05
19655	1.50524	2.1
19656	1.21304	2.1
19657	1.21304	2.1
19658	0.947115	2.1
19659	1.60665	1.9957

19660	1.85179	1.79485
19661	1.85178	1.79437
19662	2.06277	1.3382
19663	2.17534	2.1
19664	1.23007	2.1
19665	2.17533	2.1
19666	1.66635	1.98123
19667	2.00869	2.1
19668	0.986724	2.1
19669	2.00475	2.1
19670	1.99394	2.1
19671	1.67244	0.135474
19672	1.39604	0.132983
19673	1.39604	0.143236
19674	0.954046	0.123255
19675	0.988863	2.1
19676	1.9864	2.1
19677	1.9864	2.1
19678	3.38645	2.1
19679	1.46932	0.05
19680	1.50847	0.05
19681	1.50848	0.05
19682	1.42705	0.05
19683	1.50108	2.1
19684	1.51621	0.623289
19685	1.94317	1.15544
19686	1.18382	0.157652
19687	2.05091	0.05
19688	1.88593	0.05
19689	1.54299	0.05
19690	0.987265	0.05
19691	1.27766	2.1
19692	1.5178	2.1
19693	1.5178	2.1
19694	1.81518	2.1
19695	1.04626	1.61154
19696	1.31057	1.64574
19697	1.31057	1.60373
19698	1.22297	1.69144
19699	1.67844	2.1
19700	1.62862	2.1
19701	1.62112	2.1
19702	1.87598	2.1
19703	1.17844	2.1
19704	1.37063	2.1
19705	1.37063	2.1
19706	1.68723	2.1
19707	1.99943	0.0706985
19708	1.17631	0.0602224
19709	1.4263	1.9977
19710	1.31023	2.1
19711	1.31023	2.1
19712	1.20871	2.1
19713	1.50356	2.1
19714	1.69311	2.1
19715	1.69599	2.1
19716	1.6801	2.1
19717	1.5843	0.05

19718	0.840233	0.05
19719	1.95358	0.05
19720	1.40216	0.05
19721	1.39078	2.1
19722	1.18948	2.1
19723	1.01606	1.91403
19724	1.18948	2.08324
19725	1.63823	2.1
19726	1.67687	2.1
19727	1.67687	2.1
19728	1.52855	2.1
19729	1.11048	2.1
19730	1.25346	2.1
19731	2.42984	1.45502
19732	3.41731	1.67467
19733	1.2258	2.1
19734	1.2966	2.1
19735	1.2966	2.1
19736	1.37392	2.1
19737	1.41758	2.1
19738	1.35504	2.1
19739	1.35504	2.1
19740	1.30347	2.1
19741	1.50877	2.1
19742	1.11193	2.1
19743	1.40984	2.1
19744	1.54537	2.1
19745	1.71638	2.1
19746	1.77795	2.056
19747	2.07593	2.1
19748	1.93387	2.1
19749	1.9339	2.1
19750	1.75669	2.1
19751	1.80299	0.782312
19752	1.31251	0.402248
19753	1.31249	0.54937
19754	1.13411	0.299016
19755	1.06313	2.1
19756	1.04921	2.1
19757	1.55766	2.1
19758	1.53728	2.1
19759	1.53729	2.1
19760	1.51789	2.1
19761	0.993021	2.1
19762	1.02478	2.1
19763	1.03408	2.1
19764	1.09056	2.1
19765	0.697755	2.1
19766	0.632901	2.1
19767	1.34072	0.110005
19768	1.80841	0.100598
19769	1.80841	0.102094
19770	1.17793	0.0890816
19771	1.75032	1.99663
19772	1.13076	2.1
19773	2.11851	2.1
19774	1.73255	2.0548
19775	1.73255	2.1

19776	1.11181	1.98175
19777	1.43772	2.1
19778	1.67381	2.1
19779	1.52164	2.1
19780	1.60861	2.1
19781	1.30588	0.0535066
19782	1.75241	0.0662694
19783	1.33026	1.86029
19784	0.962705	1.45644
19785	0.99678	0.05
19786	1.30572	0.05
19787	1.41943	0.05
19788	1.2683	0.05
19789	1.14145	0.05
19790	1.01361	0.05
19791	1.29943	2.1
19792	1.3483	2.1
19793	1.34832	2.1
19794	1.83488	2.1
19795	1.71065	2.1
19796	1.66178	2.1
19797	1.39076	2.1
19798	1.37456	2.1
19799	1.66126	2.1
19800	1.562	2.1
19801	1.37456	2.1
19802	1.36065	2.1
19803	1.04026	0.617157
19804	1.51898	0.5058
19805	1.67088	0.195744
19806	1.9726	0.124785
19807	1.74852	1.17132
19808	1.95512	1.50853
19809	1.16165	1.12419
19810	1.06584	0.970245
19811	1.39327	2.1
19812	1.41394	2.1
19813	1.41394	2.1
19814	1.45506	2.1
19815	1.39523	2.1
19816	1.21119	2.1
19817	1.09005	2.1
19818	0.921311	2.1
19819	1.58164	2.1
19820	1.77574	2.1
19821	1.73184	2.1
19822	1.59423	2.1
19823	1.8214	2.1
19824	3.10544	1.28195
19825	1.01243	1.79765
19826	1.30896	1.83116
19827	2.16956	1.44578
19828	1.29608	0.222918
19829	1.38617	1.80226
19830	1.49536	1.69529
19831	2.35239	1.32487
19832	1.24613	1.66012
19833	1.94351	2.1

19834	2.05361	2.1
19835	2.05358	2.1
19836	2.38994	2.1
19837	1.13533	2.1
19838	1.08276	2.1
19839	1.55611	0.132275
19840	1.33957	0.121836
19841	1.33957	0.0912751
19842	1.69011	0.102871
19843	1.5515	0.705799
19844	1.59106	0.636518
19845	1.02958	2.1
19846	1.12266	2.1
19847	1.12266	2.1
19848	1.24925	2.1
19849	1.80223	2.1
19850	1.20242	2.1
19851	2.13665	1.20947
19852	1.3924	1.19539
19853	0.904894	2.04176
19854	1.82633	2.1
19855	1.94138	0.05
19856	1.34009	0.05
19857	1.15096	2.1
19858	0.994292	2.1
19859	1.10101	2.1
19860	1.15096	2.1
19861	1.50438	2.1
19862	1.67825	2.1
19863	1.67825	2.1
19864	1.91894	2.1
19865	2.04664	2.1
19866	1.92063	2.1
19867	1.72857	2.1
19868	1.89757	2.1
19869	1.61843	0.05
19870	1.49431	0.05
19871	2.33925	2.1
19872	1.80683	2.1
19873	1.64257	2.1
19874	1.50108	2.1
19875	2.82561	2.1
19876	2.97847	2.1
19877	2.97848	2.1
19878	3.20034	2.1
19879	0.66055	2.1
19880	1.95919	2.1
19881	1.07976	2.1
19882	1.30237	2.1
19883	1.99943	0.058064
19884	1.31802	0.0598245
19885	1.53336	0.0809989
19886	1.31802	0.0644248
19887	1.27737	1.16907
19888	1.44848	1.19072
19889	2.35499	0.05
19890	1.30041	0.05
19891	2.35499	0.05

19892	1.65381	0.05
19893	0.996083	2.1
19894	1.28754	2.1
19895	0.996081	2.1
19896	1.44859	2.1
19897	1.24763	2.1
19898	1.03697	2.1
19899	0.951273	1.67503
19900	1.60018	1.73723
19901	2.0815	2.1
19902	1.20488	2.1
19903	2.95785	2.1
19904	2.52245	2.1
19905	1.68455	0.0539898
19906	1.12971	0.05
19907	1.1952	0.137882
19908	2.0815	0.179193
19909	1.3963	2.1
19910	1.4934	2.1
19911	1.47973	2.1
19912	1.25559	2.1
19913	1.1233	2.1
19914	1.15009	2.1
19915	1.15009	2.1
19916	0.977775	2.1
19917	1.16482	1.26568
19918	1.29313	1.05454
19919	1.29313	1.14732
19920	1.294 0.961937	
19921	1.48693	0.114136
19922	1.47608	0.124535
19923	1.36005	1.99037
19924	1.47109	2.1
19925	1.47109	2.1
19926	1.3515	2.1
19927	2.48828	2.1
19928	2.32378	2.1
19929	2.32378	2.1
19930	2.1692	2.1
19931	1.03217	0.78531
19932	0.953521	1.20955
19933	1.4515	0.0542048
19934	3.72406	0.0633977
19935	0.963395	2.00533
19936	1.01346	1.55974
19937	1.62241	2.1
19938	1.62027	2.1
19939	1.62027	2.1
19940	1.61687	2.1
19941	1.2593	0.05
19942	0.981586	0.05
19943	0.721961	2.1
19944	0.888955	2.1
19945	1.22031	0.05
19946	1.27566	2.1
19947	2.50066	2.1
19948	1.19814	2.1
19949	1.42278	0.05

19950	2.02657	0.05
19951	1.0756	0.351621
19952	1.10049	0.331185
19953	2.26155	2.1
19954	2.15634	2.1
19955	2.15634	2.1
19956	2.01613	2.1
19957	1.94233	1.71721
19958	1.18127	1.27803
19959	1.36865	0.965214
19960	1.75755	0.781554
19961	2.01613	2.1
19962	1.78958	2.1
19963	1.97995	2.1
19964	1.79198	2.1
19965	1.20841	2.1
19966	1.6519	2.1
19967	1.80207	2.1
19968	1.66774	2.1
19969	1.17935	0.05
19970	1.88071	0.0611786
19971	1.58723	0.0503463
19972	1.7361	0.050585
19973	0.794165	2.1
19974	1.21327	2.1
19975	1.21327	2.1
19976	1.34268	2.1
19977	1.34497	0.05
19978	0.793303	0.05
19979	1.12323	0.05
19980	1.34496	0.05
19981	0.928226	0.0700224
19982	1.89804	0.111613
19983	2.60141	0.05
19984	2.60142	2.1
19985	2.60772	2.1
19986	0.99747	2.1
19987	1.27096	1.38826
19988	1.27096	0.934526
19989	1.03472	1.33851
19990	1.09962	2.1
19991	1.21148	2.1
19992	1.21148	2.1
19993	1.35025	2.1
19994	1.09795	2.1
19995	1.00775	2.1
19996	1.07162	2.1
19997	1.60665	1.99538
19998	1.52984	2.1
19999	1.61179	2.1
20000	1.70057	2.1
20001	1.76063	2.1
20002	1.14547	0.288711
20003	0.964782	0.260548
20004	1.17208	0.261117
20005	0.964769	0.233007
20006	1.04268	2.1
20007	1.57459	2.1

20008	1.57459	2.1
20009	1.32449	2.1
20010	2.07527	1.2861
20011	1.00013	1.29719
20012	0.813711	0.05
20013	1.05677	2.1
20014	1.30664	1.76415
20015	1.96011	1.90829
20016	1.7151	1.62199
20017	1.61309	1.66782
20018	1.18954	2.1
20019	1.05447	2.1
20020	1.05446	0.709028
20021	1.11787	0.686689
20022	1.44437	2.1
20023	1.84739	2.1
20024	1.84739	2.1
20025	1.06203	2.1
20026	1.78391	2.1
20027	1.89267	2.1
20028	1.9212	2.1
20029	2.02997	2.1
20030	1.32051	0.229989
20031	1.47169	0.129974
20032	1.20691	0.0542866
20033	1.72271	0.0538147
20034	1.2858	0.190108
20035	1.31011	1.03784
20036	1.31011	0.131538
20037	1.34124	1.21397
20038	1.294	1.15902
20039	1.03079	0.911267
20040	1.83192	1.99086
20041	1.81979	2.08247
20042	1.81979	2.05789
20043	1.57829	2.1
20044	1.35435	1.81376
20045	1.19023	1.93603
20046	0.991115	2.1
20047	1.27725	2.1
20048	1.38204	2.1
20049	1.48013	2.1
20050	0.983493	2.1
20051	2.26155	2.1
20052	1.02311	0.652005
20053	1.55485	0.511827
20054	1.71033	0.480582
20055	2.07319	0.401509
20056	2.29074	0.947721
20057	1.50822	1.77296
20058	2.08147	2.1
20059	1.1585	2.1
20060	3.74092	1.95136
20061	2.40799	2.1
20062	2.05349	2.1
20063	1.92656	2.1
20064	1.09634	0.159074
20065	1.45502	0.203642

20066	1.17261	0.0934048
20067	1.29383	0.0947641
20068	1.29383	0.0580988
20069	1.47689	0.0674253
20070	1.85717	0.642139
20071	1.53798	0.28345
20072	1.9493	2.1
20073	2.0371	2.09825
20074	1.8519	2.1
20075	1.92119	1.90461
20076	1.00764	0.116093
20077	1.0302	0.109918
20078	1.0302	0.100761
20079	0.90047	0.103373
20080	1.49155	0.169394
20081	2.26403	0.102514
20082	2.06158	0.0566973
20083	1.09506	0.057984
20084	1.9212	1.86631
20085	1.99586	1.6649
20086	1.99586	1.73399
20087	2.07527	1.52974
20088	1.20478	2.1
20089	1.09662	2.1
20090	1.55961	2.1
20091	1.03472	2.1
20092	1.54983	2.1
20093	2.02953	2.1
20094	1.84502	2.1
20095	2.1221	2.1
20096	1.02969	2.00618
20097	2.23523	2.1
20098	1.2377	0.0537388
20099	1.03818	0.085988
20100	1.978 2.1	
20101	1.78159	2.1
20102	1.45762	2.1
20103	1.34234	2.1
20104	2.01624	2.1
20105	2.52072	2.1
20106	1.23013	2.1
20107	2.5593	2.1
20108	3.06464	1.3762
20109	2.63697	1.18573
20110	2.63697	1.31682
20111	1.87111	1.13857
20112	1.04574	0.0539209
20113	2.13428	0.05
20114	1.61715	2.1
20115	1.44137	2.1
20116	1.44867	2.1
20117	1.18283	2.1
20118	1.50584	0.05
20119	1.45466	0.065927
20120	1.4959	0.0549153
20121	1.93489	0.0595418
20122	2.60772	2.1
20123	2.68868	2.1

20124	2.68868	2.1
20125	2.82561	2.1
20126	1.49654	2.1
20127	1.38672	2.1
20128	1.36813	2.1
20129	1.98396	2.1
20130	1.93852	1.48355
20131	2.14237	1.21924
20132	1.44421	0.0546632
20133	1.21749	0.05
20134	1.09572	0.05
20135	1.11081	0.05
20136	2.32363	2.1
20137	1.24375	2.1
20138	3.79242	2.1
20139	3.46787	2.1
20140	1.19854	1.60072
20141	1.35487	1.58369
20142	1.35486	1.69368
20143	1.38616	1.67435
20144	1.67265	0.189899
20145	0.909487	0.150668
20146	2.53562	2.1
20147	1.09564	2.1
20148	2.9068	2.1
20149	3.57307	0.728254
20150	2.50369	2.02102
20151	1.47348	2.1
20152	1.24925	2.1
20153	1.3991	2.1
20154	1.3991	2.1
20155	1.52984	2.1
20156	1.60428	2.1
20157	1.50097	2.1
20158	1.50097	2.1
20159	1.40239	2.1
20160	2.19621	2.1
20161	2.35003	2.1
20162	1.57932	0.05
20163	1.61194	0.05
20164	2.0815	0.05
20165	1.18539	0.05
20166	1.5795	1.47108
20167	1.53064	1.57359
20168	1.89227	0.05
20169	3.01037	0.05
20170	2.08149	2.07622
20171	2.06678	1.93938
20172	1.89226	0.05
20173	3.47888	0.05
20174	1.89226	0.0900017
20175	3.65842	0.0657463
20176	3.88546	0.160395
20177	1.89226	0.272255
20178	1.11239	2.1
20179	1.29133	0.05
20180	0.889309	0.05
20181	0.881934	0.05

20182	1.88406	2.1
20183	0.636946	2.1
20184	0.636945	2.1
20185	1.28073	2.1
20186	1.28075	2.1
20187	0.942007	2.1
20188	1.68436	2.1
20189	1.4677	0.05
20190	0.851542	0.0576871
20191	1.39063	0.05
20192	0.851539	0.0580038
20193	1.11597	0.0565924
20194	1.35403	0.05
20195	0.959473	2.1
20196	1.07976	2.1
20197	0.946129	2.1
20198	0.946129	0.370771
20199	1.38464	1.93548
20200	1.36991	0.821499
20201	0.946131	1.00119
20202	0.94613	1.59291
20203	1.34493	0.403467
20204	1.36991	0.289607
20205	1.36991	0.128135
20206	0.946131	0.242151
20207	0.78392	0.521469
20208	0.80282	0.371224
20209	1.10351	0.497765
20210	0.80846	0.48911
20211	0.838026	0.624377
20212	1.08558	0.424327
20213	0.718203	0.534806
20214	0.501866	0.068279
20215	0.85426	0.455468
20216	1.24249	0.0609413
20217	1.37052	0.0840819
20218	0.851541	0.0654358
20219	1.0092	0.0516445
20220	0.851554	0.0872558
20221	1.49826	0.245672
20222	1.13727	0.158326
20223	0.778149	0.206293
20224	1.20257	0.2617
20225	0.946133	0.213146
20226	0.778149	0.206753
20227	0.77815	0.360998
20228	0.946132	2.1
20229	1.17602	2.1
20230	0.946132	2.1
20231	1.31257	2.1
20232	1.17602	2.1
20233	1.35821	0.087348
20234	1.19993	0.05
20235	0.946131	0.05
20236	0.946131	0.0848032
20237	1.08848	0.102882
20238	1.00509	2.1
20239	1.19629	2.1

20240	0.867593	2.1
20241	0.757921	2.1
20242	0.729116	0.0509714
20243	0.89162	0.0573352
20244	1.16811	0.05
20245	1.3676	2.1
20246	1.2638	2.1
20247	0.946132	2.1
20248	0.946132	2.1
20249	1.2638	2.1
20250	1.00185	2.1
20251	1.60948	2.1
20252	0.602075	2.1
20253	0.942611	2.1
20254	0.946132	2.1
20255	0.942611	2.1
20256	1.11822	2.1
20257	1.21558	0.154638
20258	0.607788	0.230354
20259	0.827029	0.219279
20260	1.00691	0.447649
20261	0.607789	0.216573
20262	0.607775	0.356714
20263	1.23248	1.74774
20264	0.902653	1.97655
20265	0.639143	1.97137
20266	0.851545	1.75284
20267	0.605153	1.85663
20268	1.06826	2.1
20269	1.29365	2.1
20270	1.41232	2.1
20271	0.946131	2.1
20272	1.20854	2.1
20273	0.946131	2.1
20274	0.909005	0.0586261
20275	1.16672	0.0555927
20276	0.810959	0.0589162
20277	0.946131	0.0650145
20278	1.40104	0.0534338
20279	0.946131	0.0647784
20280	1.35403	0.0627596
20281	1.40104	0.0547884
20282	1.92535	0.05
20283	2.14534	0.05
20284	1.77532	0.05
20285	0.851541	0.0586222
20286	1.05585	0.0633782
20287	1.27098	0.0581124
20288	0.870925	0.0625707
20289	1.15547	0.0621839
20290	1.47583	0.0588986
20291	1.35007	0.05
20292	0.740095	0.05
20293	0.644912	0.05
20294	0.819489	0.05
20295	0.983028	0.145498
20296	0.861849	0.05
20297	1.40511	0.05

20298	0.86182	0.05
20299	0.9705	0.05
20300	1.40511	0.05
20301	1.15539	2.1
20302	0.946119	2.1
20303	0.736382	2.1
20304	0.736384	2.1
20305	1.41561	2.1
20306	0.785651	2.1
20307	1.34479	2.1
20308	1.38885	2.1
20309	0.94613	2.1
20310	1.29461	2.1
20311	0.94613	2.1
20312	0.776179	2.1
20313	0.858611	2.1
20314	0.946128	2.1
20315	1.04073	2.1
20316	1.16399	2.1
20317	0.990019	2.1
20318	1.15564	2.08171
20319	1.28956	0.080834
20320	1.35145	0.111863
20321	1.09662	2.1
20322	1.8155	0.05
20323	0.811459	0.05
20324	0.731982	0.05
20325	0.900554	0.05
20326	0.946127	1.11754
20327	0.817066	1.29941
20328	0.817064	1.14749
20329	1.41798	1.11212
20330	0.871606	1.13746
20331	1.30265	0.05
20332	0.849402	1.74419
20333	0.868582	1.60816
20334	0.909137	1.62691
20335	0.807016	1.5347
20336	0.83393	0.679889
20337	0.9034	1.19358
20338	1.53515	1.15568
20339	1.17665	0.302361
20340	0.946132	0.456065
20341	0.946129	0.274763
20342	1.35977	0.254816
20343	1.34254	0.243254
20344	1.14241	0.196966
20345	0.946132	0.291485
20346	0.946132	0.190863
20347	1.27376	0.188231
20348	1.35978	0.177329
20349	1.01976	0.178601
20350	1.23498	1.06122
20351	1.35002	1.43936
20352	1.11683	1.46359
20353	0.88359	0.173155
20354	0.751374	1.19743
20355	0.791714	0.429726

20356	1.6067	2.1
20357	0.923782	2.1
20358	1.44689	2.1
20359	0.94613	2.1
20360	0.946131	2.1
20361	1.5261	2.1
20362	1.26247	2.1
20363	1.80085	0.0640378
20364	1.7693	0.0696248
20365	0.900422	0.0620871
20366	1.31357	0.0747477
20367	0.94613	0.0550953
20368	0.900424	0.0666204
20369	0.900397	0.0702955
20370	1.39448	0.0532253
20371	1.36769	0.05
20372	0.713524	0.0852127
20373	0.713554	0.05
20374	0.946124	0.0568097
20375	1.24979	0.05
20376	1.04765	0.0647506
20377	0.70054	0.05
20378	0.776991	0.05
20379	0.738905	0.05
20380	1.32605	0.05
20381	0.784957	0.05
20382	0.803955	0.05
20383	0.819486	0.05
20384	1.79306	0.052424
20385	1.18275	0.0532371
20386	1.25626	0.0516495
20387	0.936806	0.0517183
20388	0.653124	0.0542265
20389	0.946132	0.0504942
20390	0.653151	0.0560631
20391	1.10748	0.089225
20392	1.95244	2.1
20393	1.40967	1.58448
20394	1.27916	1.56147
20395	0.946132	1.87041
20396	0.946132	2.1
20397	1.30555	2.1
20398	1.38651	2.1
20399	0.946132	2.1
20400	1.23571	0.549148
20401	1.21261	1.31926
20402	1.34225	1.34264
20403	0.946132	1.58502
20404	0.734059	0.64795
20405	0.734084	1.11627
20406	0.78438	0.306698
20407	1.13748	0.548441
20408	0.946131	0.784123
20409	0.78438	0.313085
20410	0.784392	0.462788
20411	0.626495	2.1
20412	0.694635	2.1
20413	0.946125	2.1

20414	0.694632	2.1
20415	1.38521	2.1
20416	0.795469	2.1
20417	0.850877	2.1
20418	1.37098	2.1
20419	0.946131	2.1
20420	0.79547	2.1
20421	0.946132	0.429046
20422	1.30448	0.374872
20423	0.628361	0.135139
20424	0.686964	0.421205
20425	0.493261	0.34097
20426	1.90912	0.589967
20427	0.946127	0.480422
20428	1.36604	0.105694
20429	0.671687	0.0858073
20430	0.946128	0.924703
20431	0.671699	0.345138
20432	1.04973	2.1
20433	0.982858	0.174395
20434	0.974081	0.05
20435	1.69993	0.05
20436	1.00763	0.146188
20437	0.755566	2.1
20438	0.946129	2.1
20439	1.04184	2.1
20440	1.13509	2.1
20441	0.755566	2.1
20442	1.114 2.1	
20443	0.810946	2.1
20444	0.786259	2.1
20445	1.43455	2.1
20446	1.05071	2.1
20447	0.746095	2.1
20448	0.82737	2.1
20449	0.946126	2.1
20450	1.04071	2.1
20451	1.0852	2.1
20452	1.59019	0.05
20453	0.621972	0.05
20454	1.14475	0.05
20455	0.691062	0.05
20456	1.20057	0.05
20457	0.846868	0.05
20458	1.26062	0.05
20459	0.795093	0.05
20460	1.20056	0.05
20461	0.795065	0.05
20462	1.18203	0.05
20463	1.20057	0.05
20464	0.991869	0.0851637
20465	0.841088	2.1
20466	0.946131	2.1
20467	0.869097	2.1
20468	0.946131	2.1
20469	0.841089	2.1
20470	1.77867	2.1
20471	1.99296	0.144603

20472	1.91703	2.1
20473	0.651334	0.0536242
20474	1.31467	0.0508772
20475	0.651332	0.0528098
20476	0.651332	0.05
20477	1.03079	0.0591016
20478	0.808519	0.051062
20479	1.41417	0.05
20480	1.03165	0.153867
20481	1.43577	2.1
20482	1.39053	2.1
20483	1.07006	2.1
20484	0.946132	2.1
20485	0.946133	2.1
20486	1.07006	2.1
20487	1.12717	2.1
20488	0.994293	2.1
20489	0.946132	2.1
20490	1.31912	2.1
20491	0.946131	2.1
20492	1.40571	2.1
20493	1.31912	2.1
20494	1.12292	0.274328
20495	1.07755	0.333279
20496	0.851539	0.279041
20497	0.946131	0.267941
20498	0.929834	0.279926
20499	1.83919	0.442917
20500	1.04713	0.130312
20501	0.929726	0.0829164
20502	1.50985	0.0847792
20503	0.946127	0.082041
20504	1.0227	0.0834253
20505	1.2582	0.0805074
20506	1.1391	0.393086
20507	0.780831	1.05275
20508	0.78086	0.409549
20509	0.946133	0.708163
20510	0.780831	0.46074
20511	1.37762	0.470633
20512	0.946116	2.1
20513	1.22882	2.1
20514	1.40015	2.1
20515	1.40017	2.1
20516	0.946105	2.1
20517	0.553567	0.05
20518	1.19263	0.05
20519	0.946132	0.05
20520	0.553594	0.05
20521	1.19263	0.05
20522	1.18237	0.05
20523	0.946133	0.05
20524	0.978467	0.05
20525	0.56262	0.05
20526	0.946132	0.05
20527	0.562633	0.05
20528	0.900175	0.05
20529	1.04627	1.79071

20530	0.549928	1.19974
20531	1.17765	1.25287
20532	1.11479	1.31282
20533	0.398261	1.38478
20534	0.927608	1.25146
20535	1.38467	1.1841
20536	1.04824	1.08587
20537	1.33893	1.184
20538	1.55775	1.11588
20539	0.816895	1.11452
20540	1.96057	1.26476
20541	1.35798	2.1
20542	1.29262	2.1
20543	0.94613	2.1
20544	0.94613	2.1
20545	1.29261	2.1
20546	1.18067	2.1
20547	0.946131	2.1
20548	0.946132	2.1
20549	1.27809	2.1
20550	1.35799	2.1
20551	1.03933	2.1
20552	1.49438	2.1
20553	0.946132	2.1
20554	1.13537	2.1
20555	1.41666	2.1
20556	1.74237	2.1
20557	0.946132	2.1
20558	1.04291	0.643602
20559	1.63802	2.1
20560	1.24714	2.1
20561	0.842013	2.1
20562	0.810959	2.1
20563	0.767923	0.120838
20564	1.19583	0.11728
20565	0.767921	0.0989121
20566	0.767921	0.0747087
20567	0.961348	0.124787
20568	2.08687	1.84354
20569	0.739729	1.32201
20570	1.04342	1.28508
20571	0.739757	1.26992
20572	0.946129	1.33246
20573	1.04341	1.496
20574	1.38683	0.159997
20575	1.34153	0.136832
20576	0.946129	0.16254
20577	0.946131	0.167516
20578	1.02028	0.160803
20579	1.47606	0.101457
20580	1.20739	0.13261
20581	0.957684	0.102093
20582	0.946116	0.1372
20583	0.851528	0.160484
20584	0.957685	0.151527
20585	1.04504	2.1
20586	0.902938	2.1
20587	0.946132	2.1

20588	1.07566	2.1
20589	1.4558	2.1
20590	1.93286	2.1
20591	1.27241	2.1
20592	0.828671	2.1
20593	0.810933	2.1
20594	1.31335	0.05
20595	1.40305	0.05
20596	0.912698	0.05
20597	0.810946	0.05
20598	1.11608	0.05
20599	1.26409	0.954702
20600	0.946132	0.174385
20601	1.27376	0.177328
20602	0.946132	0.17156
20603	1.03333	0.179941
20604	1.27376	0.172135
20605	0.94613	0.276457
20606	0.812384	0.23995
20607	0.687744	0.196245
20608	0.946132	0.271704
20609	0.687758	0.237374
20610	1.09667	0.179509
20611	1.56977	2.1
20612	0.851544	2.1
20613	1.01136	2.1
20614	0.851481	2.1
20615	0.946131	2.1
20616	0.705409	2.1
20617	1.32449	0.05
20618	1.13508	0.05
20619	0.94613	0.0532329
20620	0.94613	0.05
20621	0.965822	0.053616
20622	1.95301	0.14722
20623	0.851538	0.398041
20624	1.29262	0.347275
20625	0.95417	0.264437
20626	0.851541	0.414341
20627	0.94613	0.357534
20628	1.02727	1.13069
20629	1.37675	1.20742
20630	0.955646	0.918267
20631	0.95114	1.21433
20632	0.851528	1.1669
20633	1.76513	1.33302
20634	0.87155	2.1
20635	0.94613	2.1
20636	0.871519	2.1
20637	0.966631	2.1
20638	1.37791	2.1
20639	1.19122	0.411503
20640	0.818971	0.926533
20641	0.628799	0.464262
20642	0.861027	0.787877
20643	0.800034	0.0619384
20644	0.91846	0.0585663
20645	0.765645	0.0637369

20646	1.13709	0.80263
20647	1.29054	0.999973
20648	0.755336	1.02437
20649	0.755334	0.709273
20650	0.946133	1.30424
20651	0.755336	0.483363
20652	1.14444	0.987448
20653	0.872823	0.957278
20654	0.533645	0.796436
20655	0.946132	0.732173
20656	0.533645	0.456429
20657	1.02311	0.472477
20658	1.00278	2.1
20659	1.1016	0.05
20660	0.629615	0.05
20661	1.12628	0.0590889
20662	0.946131	0.0556807
20663	0.629615	0.05
20664	0.629641	0.05
20665	1.67046	1.57979
20666	1.81667	2.1
20667	1.7145	2.1
20668	0.94613	2.1
20669	1.29322	2.1
20670	1.00365	2.1
20671	0.946131	2.1
20672	1.29322	2.1
20673	1.27581	2.1
20674	1.33916	2.1
20675	1.32784	2.1
20676	1.74787	2.1
20677	2.28084	2.1
20678	2.98092	2.1
20679	0.946132	2.07538
20680	1.2435	2.1
20681	0.946132	2.1
20682	1.4099	2.1
20683	1.2435	2.1
20684	1.26438	2.1
20685	0.745287	2.1
20686	0.696827	2.1
20687	0.696781	2.1
20688	1.41195	2.1
20689	0.946131	2.1
20690	1.8758	1.62452
20691	0.832979	0.820767
20692	0.75054	0.594467
20693	0.810958	0.545877
20694	0.922889	1.18845
20695	0.694844	0.292665
20696	0.694814	0.910557
20697	0.946131	0.439765
20698	0.694844	0.508504
20699	1.02369	2.1
20700	1.4468	0.05
20701	0.946125	0.128515
20702	0.93487	0.0985985
20703	1.23608	0.1763

20704	1.34257	0.0832636
20705	0.851525	0.0962982
20706	1.03298	0.109829
20707	1.22418	2.1
20708	0.946131	2.06624
20709	1.41833	1.91067
20710	1.23452	1.53281
20711	0.946131	0.652371
20712	1.41833	1.6913
20713	1.13077	0.217195
20714	0.942874	1.82401
20715	0.946132	1.78505
20716	0.504366	1.79061
20717	0.593647	2.1
20718	0.898829	1.79064
20719	1.27921	2.06306
20720	1.12503	2.1
20721	1.22539	2.1
20722	1.10332	2.1
20723	0.851541	2.1
20724	0.94613	2.1
20725	0.945109	2.1
20726	0.607962	2.1
20727	0.946133	2.1
20728	0.607961	2.1
20729	0.661956	2.1
20730	1.35048	2.1
20731	1.66018	2.1
20732	0.956467	2.1
20733	0.851542	2.1
20734	0.890734	2.1
20735	0.802455	2.1
20736	1.35943	2.1
20737	1.04798	2.1
20738	1.26426	0.217176
20739	1.3689	0.129984
20740	0.946133	0.171978
20741	0.946133	0.221921
20742	1.09729	0.1954
20743	0.765259	2.1
20744	0.810945	2.1
20745	0.810957	2.1
20746	1.10284	2.1
20747	1.09368	2.1
20748	0.772601	2.1
20749	0.788011	2.1
20750	0.810934	2.1
20751	0.694357	1.4244
20752	1.22641	1.41237
20753	0.946132	1.65434
20754	0.946132	1.18362
20755	0.946133	1.29557
20756	1.42754	1.55195
20757	0.776414	1.29069
20758	0.806525	1.38075
20759	0.575339	1.52217
20760	0.97154	1.25613
20761	0.8217	1.34988

20762	0.838039	1.30335
20763	0.94613	0.916697
20764	0.946132	0.537819
20765	1.39575	0.30811
20766	1.39575	0.428376
20767	1.12818	0.513088
20768	0.946132	1.435
20769	0.946133	0.872165
20770	1.06879	0.415739
20771	1.06879	0.659389
20772	0.970358	0.674425
20773	1.2474	0.199264
20774	1.01919	0.0557632
20775	0.94613	0.065711
20776	1.28952	0.0638712
20777	1.00809	0.0578604
20778	0.849344	0.0587935
20779	0.640304	0.0740632
20780	1.35065	0.0528538
20781	0.946132	0.0597679
20782	0.640333	0.05
20783	0.946132	0.0693196
20784	0.685489	2.1
20785	0.94613	2.1
20786	0.68546	2.1
20787	0.874264	2.1
20788	1.27676	2.1
20789	0.946129	0.0970245
20790	1.09024	0.0880892
20791	0.714563	0.0861057
20792	1.12335	0.0891264
20793	1.07931	0.0880169
20794	1.17799	0.05
20795	1.8795	0.173614
20796	2.15229	0.180227
20797	1.13859	0.456472
20798	0.946132	1.2561
20799	1.11374	0.49486
20800	1.1386	0.644139
20801	0.615713	0.628707
20802	1.19322	0.24573
20803	1.3377	0.191869
20804	0.670801	0.244108
20805	0.94613	0.734468
20806	0.66617	0.445498
20807	1.36173	0.414458
20808	0.980473	0.38768
20809	1.39132	0.230809
20810	0.864653	0.0502323
20811	1.23837	0.05
20812	0.810959	0.05
20813	1.1483	1.73945
20814	1.00381	1.89132
20815	0.946128	1.73224
20816	1.39696	1.50487
20817	0.946131	1.36809
20818	0.946131	1.43449
20819	1.38613	1.65906

20820	0.973501	1.80115
20821	0.946127	1.68278
20822	1.35968	1.72135
20823	0.946131	0.786631
20824	0.946129	1.82205
20825	1.39696	1.78049
20826	1.12088	2.07363
20827	1.41122	2.1
20828	1.14464	0.654161
20829	0.946131	2.1
20830	0.946131	1.9759
20831	1.14464	2.05866
20832	1.33266	1.69048
20833	1.07341	0.977231
20834	0.827287	1.71843
20835	0.946132	1.30921
20836	0.827287	1.72978
20837	1.15644	1.81295
20838	1.27558	0.673347
20839	1.15067	2.1
20840	0.946131	2.1
20841	1.29143	2.06692
20842	1.36281	2.1
20843	1.36281	2.1
20844	0.946131	2.1
20845	1.40863	2.1
20846	1.20143	0.05
20847	1.19755	0.05
20848	0.566153	0.069891
20849	0.946131	0.126075
20850	1.07979	0.05
20851	1.00463	0.109754
20852	0.566153	0.166673
20853	0.768212	2.1
20854	1.32461	2.1
20855	1.13268	2.1
20856	1.13268	2.1
20857	0.946133	2.1
20858	1.53642	2.1
20859	1.5791	2.1
20860	0.726635	2.1
20861	1.2334	2.1
20862	1.29198	2.1
20863	1.29198	2.04598
20864	0.946131	1.95142
20865	0.956585	2.1
20866	1.25294	2.1
20867	0.946133	2.1
20868	1.35287	2.1
20869	1.23877	2.1
20870	0.946131	2.1
20871	1.35287	2.1
20872	1.01843	2.1
20873	3.77569	2.1
20874	0.86707	2.1
20875	0.867066	2.1
20876	1.38639	2.1
20877	1.38639	2.1

20878	0.896168	2.1
20879	0.974716	0.0630151
20880	1.09411	0.0868027
20881	0.663171	0.0686378
20882	0.663171	0.100374
20883	0.946132	0.0938797
20884	0.66317	0.0775328
20885	1.47104	0.0569184
20886	1.13629	0.0657602
20887	0.772342	0.0584842
20888	0.772312	0.070332
20889	0.94613	0.0661588
20890	0.77234	0.0677777
20891	1.43154	2.1
20892	1.37704	2.1
20893	1.40025	2.1
20894	1.3063	2.1
20895	0.946132	2.1
20896	1.04075	2.1
20897	0.946131	2.1
20898	0.946118	2.1
20899	0.750011	1.73891
20900	1.0971	2.01015
20901	0.889667	1.88501
20902	0.709598	1.99894
20903	2.39737	2.1
20904	1.19974	0.0879988
20905	0.94613	0.187549
20906	1.25809	0.0794359
20907	1.19974	0.158056
20908	0.73005	0.274067
20909	1.50764	0.05
20910	1.25809	0.100044
20911	0.946133	0.202309
20912	1.10357	0.120218
20913	1.25809	0.179175
20914	0.628177	0.26775
20915	1.3543	0.05
20916	0.793711	0.0655693
20917	1.18455	0.0603588
20918	1.03966	0.063074
20919	0.946131	0.05
20920	0.767453	0.05
20921	0.612292	0.0507242
20922	0.946131	0.05
20923	1.24128	0.05
20924	1.08761	0.05
20925	0.762059	0.05
20926	1.005 2.1	
20927	1.17054	2.1
20928	1.12665	2.1
20929	0.750368	2.1
20930	0.851542	2.1
20931	0.975436	2.1
20932	0.975442	2.1
20933	0.675367	2.1
20934	1.51973	0.0918316
20935	0.94613	0.112813

20936	1.38954	0.117838
20937	0.946131	0.110395
20938	1.07912	0.1181
20939	1.38954	0.109817
20940	2.0219	2.1
20941	1.18347	0.106821
20942	0.946132	0.563083
20943	0.94613	0.258153
20944	1.00202	0.178866
20945	1.00201	0.202468
20946	0.946132	0.252286
20947	0.946132	0.763304
20948	1.37981	0.355486
20949	1.26874	0.278878
20950	1.26874	0.306728
20951	0.946132	0.339755
20952	2.8687	0.152726
20953	1.42884	2.1
20954	1.05188	2.1
20955	0.625307	2.1
20956	0.810956	2.1
20957	0.526656	2.1
20958	0.526684	2.1
20959	0.724122	2.1
20960	0.946132	2.1
20961	0.526684	2.1
20962	1.05337	2.1
20963	1.0593	2.1
20964	1.47981	2.1
20965	1.74431	1.97974
20966	0.885612	1.96848
20967	0.810946	1.70606
20968	0.845185	1.77459
20969	1.38622	0.659571
20970	0.723194	0.716395
20971	0.554302	1.00148
20972	0.709597	0.633715
20973	0.909507	0.647096
20974	0.912754	0.247281
20975	1.15174	0.192839
20976	0.946131	0.254155
20977	0.912753	0.239639
20978	1.15174	0.226248
20979	1.02011	0.151166
20980	0.792754	1.97675
20981	0.946129	2.1
20982	0.792754	1.95697
20983	1.27575	1.93969
20984	0.94613	1.95978
20985	1.60132	2.07339
20986	0.922455	1.97901
20987	1.18713	1.77852
20988	0.926957	1.81776
20989	1.66525	1.91339
20990	0.946132	2.1
20991	0.851541	2.1
20992	0.961191	2.1
20993	1.27534	2.1

20994	0.851554	2.1
20995	1.0499	2.1
20996	1.01776	0.05
20997	0.97622	0.05
20998	0.810934	0.05
20999	2.6802	0.05
21000	1.31485	0.05
21001	1.09699	0.05
21002	0.681649	0.05
21003	0.790369	0.05
21004	0.946131	0.05
21005	0.838026	1.5565
21006	0.567833	0.806167
21007	0.891492	1.32516
21008	0.986984	0.452964
21009	0.946132	1.35626
21010	1.19028	0.491514
21011	0.986984	0.555447
21012	0.595623	0.651316
21013	0.946131	2.1
21014	1.36502	2.1
21015	1.08109	2.1
21016	0.946129	2.1
21017	1.36503	2.1
21018	0.773897	2.1
21019	0.946133	1.93326
21020	0.613447	2.1
21021	0.613449	2.1
21022	0.946131	2.1
21023	1.11163	2.07918
21024	0.966114	2.1
21025	0.735699	2.1
21026	0.662147	2.1
21027	1.04072	2.1
21028	0.946131	2.1
21029	0.862978	2.1
21030	1.48839	2.02245
21031	0.936141	2.06661
21032	0.946131	2.1
21033	0.936142	2.1
21034	1.40595	2.1
21035	0.946131	2.1
21036	1.05297	0.0692652
21037	3.09035	2.1
21038	1.19952	2.1
21039	0.907198	2.1
21040	0.849144	2.1
21041	0.612469	0.753858
21042	0.551237	0.566129
21043	0.985256	1.10173
21044	0.551209	0.669394
21045	0.85153	1.14998
21046	0.624712	2.1
21047	0.900018	2.1
21048	0.851542	2.1
21049	0.864787	2.1
21050	1.27669	2.1
21051	0.946711	0.549729

21052	0.810947	0.7849
21053	0.841547	0.518891
21054	0.764114	0.736195
21055	1.26692	0.825996
21056	0.946132	0.79704
21057	1.37675	0.433569
21058	1.26692	0.663239
21059	1.37545	0.746714
21060	1.49637	0.131987
21061	0.709598	0.194284
21062	0.751252	0.184289
21063	1.36811	0.173003
21064	0.635625	0.201655
21065	1.31713	0.0706113
21066	1.18977	2.1
21067	0.893377	0.938236
21068	0.707344	2.1
21069	0.946131	2.1
21070	1.20221	2.1
21071	1.20221	2.1
21072	0.707345	2.1
21073	1.26786	2.1
21074	0.988048	2.1
21075	0.751293	2.1
21076	0.632008	2.1
21077	1.51067	2.1
21078	1.13534	2.1
21079	0.946133	2.1
21080	0.946131	2.1
21081	1.25716	2.1
21082	1.3199	2.1
21083	1.09616	2.1
21084	1.36677	1.97679
21085	1.34752	2.09032
21086	0.838041	1.94293
21087	1.67023	2.1
21088	1.02247	0.369589
21089	1.03079	0.380982
21090	0.928982	0.121889
21091	1.13635	0.114317
21092	0.946132	0.133521
21093	0.92898	0.105718
21094	1.13635	0.117865
21095	1.1643	0.085271
21096	0.851541	0.257066
21097	1.39939	0.205076
21098	0.946129	0.266137
21099	1.07775	0.223529
21100	1.14503	0.225601
21101	1.78573	0.149399
21102	1.29827	0.149563
21103	0.872373	0.05
21104	1.01162	0.05
21105	0.83804	0.05
21106	1.48851	0.05
21107	1.47385	0.05
21108	0.843858	0.05
21109	0.938626	0.05

21110	0.89567	0.05
21111	1.59457	1.0797
21112	2.09666	0.141671
21113	0.775132	0.05
21114	0.927005	0.05
21115	0.854224	0.05
21116	0.775142	0.05
21117	0.946106	0.05
21118	1.80784	2.1
21119	2.04593	2.1
21120	1.32282	2.1
21121	0.952138	2.1
21122	0.946132	2.1
21123	0.946132	2.1
21124	0.952138	2.1
21125	1.46988	2.1
21126	0.80465	0.0536356
21127	1.14155	0.0541274
21128	0.810931	0.0548748
21129	1.66203	0.125312
21130	0.674605	0.240004
21131	0.946132	0.273528
21132	1.26706	0.331084
21133	0.674633	0.153972
21134	0.946132	0.539708
21135	0.980664	0.303924
21136	1.34927	0.225292
21137	0.758454	0.108649
21138	0.946133	0.0848406
21139	0.985371	0.076419
21140	1.28632	0.0681293
21141	0.946133	0.154973
21142	1.46868	0.233248
21143	1.7522	0.05
21144	0.737431	2.05628
21145	0.663679	2.06455
21146	1.16541	1.85827
21147	1.3179	1.92978
21148	1.04081	1.9501
21149	0.887866	1.76557
21150	0.806961	1.54012
21151	0.929061	1.69118
21152	1.28418	0.222169
21153	1.38752	0.180812
21154	1.33038	0.119084
21155	0.946132	0.126327
21156	0.946132	0.14576
21157	1.14897	0.141562
21158	1.1685	0.109015
21159	1.18774	2.1
21160	0.961899	2.1
21161	1.48841	2.1
21162	1.79105	2.1
21163	3.51614	2.1
21164	0.682342	2.1
21165	0.783184	2.1
21166	0.946132	2.1
21167	0.868396	2.1

21168	1.21813	2.1
21169	0.848305	1.02529
21170	0.94559	1.11252
21171	0.805691	1.06829
21172	0.752217	2.1
21173	0.946132	2.1
21174	0.839123	2.1
21175	0.946131	2.1
21176	0.752187	2.1
21177	1.96491	2.1
21178	0.520997	2.1
21179	0.946132	2.1
21180	0.73446	2.1
21181	0.946133	2.1
21182	0.520995	2.1
21183	1.88522	2.1
21184	1.8361	2.1
21185	1.11285	2.1
21186	1.25334	1.43561
21187	2.00756	2.1
21188	1.44252	0.0618424
21189	1.00255	2.1
21190	1.64516	2.1
21191	1.12399	0.05
21192	1.13542	0.05
21193	1.09038	0.05
21194	0.746903	0.05
21195	0.672241	0.05
21196	0.871774	0.05
21197	1.73513	0.374233
21198	0.949995	2.1
21199	1.17244	2.1
21200	0.946127	2.1
21201	1.3028	2.1
21202	1.17244	2.1
21203	0.774101	2.1
21204	0.946127	2.1
21205	0.939289	2.1
21206	0.946129	2.1
21207	0.774101	2.1
21208	1.87858	2.1
21209	1.44293	0.05
21210	1.43213	0.05
21211	0.946131	2.1
21212	0.716614	2.1
21213	0.716644	2.1
21214	1.35828	2.1
21215	0.790935	2.1
21216	0.730157	2.1
21217	1.41149	2.1
21218	0.730186	2.1
21219	0.730186	2.1
21220	0.946131	2.1
21221	0.946132	1.41768
21222	0.946132	1.06162
21223	0.990713	0.430039
21224	1.40779	0.625502
21225	0.946132	0.759857

21226	1.06486	0.230248
21227	0.946131	1.31968
21228	0.946133	0.495256
21229	1.40779	0.364415
21230	1.26874	0.402786
21231	0.946131	0.45958
21232	1.02588	0.640988
21233	0.826073	0.0790935
21234	1.41233	0.108185
21235	0.861269	0.0743243
21236	1.04073	0.124588
21237	1.27111	0.142481
21238	1.56016	1.59984
21239	1.60866	0.05
21240	0.94613	0.0551075
21241	0.946129	0.0537576
21242	1.36376	0.05
21243	1.22187	0.0567397
21244	0.94613	0.0577861
21245	1.82581	1.04372
21246	1.19705	1.47067
21247	1.7513	2.1
21248	1.85017	2.1
21249	1.72116	2.1
21250	1.12058	2.1
21251	0.899991	2.1
21252	0.616288	2.1
21253	1.07289	2.1
21254	1.7092	0.677593
21255	0.946132	0.77383
21256	0.946133	0.347703
21257	1.18717	0.267478
21258	1.40029	0.931165
21259	0.946132	0.140018
21260	0.94613	0.109239
21261	1.18547	0.102086
21262	0.960326	0.105508
21263	0.960345	0.0884809
21264	0.942557	0.113275
21265	1.18601	0.0853405
21266	0.946132	0.100053
21267	1.30307	0.0900038
21268	1.08169	0.0918533
21269	1.08169	0.0789159
21270	1.31716	0.101225
21271	1.51065	0.763267
21272	0.877642	0.05
21273	0.617384	0.05
21274	0.575741	0.05
21275	0.972448	0.05
21276	1.11334	0.05
21277	1.13092	2.07448
21278	0.946127	1.76825
21279	1.31724	2.1
21280	1.39751	0.428796
21281	1.39751	1.14617
21282	0.946128	1.28798
21283	0.946132	0.275289

21284	0.713284	0.141057
21285	1.40668	0.299704
21286	1.03547	0.168163
21287	0.713298	0.245666
21288	1.88774	0.146797
21289	0.830164	2.1
21290	1.05211	2.1
21291	0.946119	2.1
21292	0.946107	2.1
21293	1.283 2.1	
21294	2.63609	2.1
21295	1.28418	0.710292
21296	0.946128	1.20951
21297	1.10335	0.924442
21298	0.598377	1.33408
21299	1.18818	0.491086
21300	1.10335	0.935515
21301	1.58972	1.45703
21302	1.90194	2.1
21303	0.946118	2.1
21304	0.946115	2.1
21305	0.950969	2.1
21306	1.36918	2.1
21307	0.950955	2.1
21308	1.4263	2.1
21309	1.11978	1.01466
21310	0.946132	1.07853
21311	0.946133	1.12286
21312	1.37658	1.00292
21313	1.2364	1.04552
21314	1.05496	1.2376
21315	0.946132	1.2321
21316	0.946132	1.28825
21317	1.21858	1.14868
21318	1.37658	1.26769
21319	1.09194	1.2093
21320	1.14721	0.0769881
21321	1.53814	0.05
21322	0.546751	1.457
21323	0.492063	1.51643
21324	1.16889	1.83949
21325	1.00349	1.69097
21326	0.863559	1.52862
21327	1.38102	1.62091
21328	0.597449	1.39195
21329	0.537748	1.27339
21330	1.16297	1.55064
21331	0.999366	1.41515
21332	0.896554	1.37034
21333	0.566962	0.05
21334	0.946129	0.05
21335	0.452729	0.05
21336	0.696552	0.05
21337	0.852524	0.05
21338	1.57992	0.05
21339	1.64985	0.05
21340	0.946128	2.1
21341	1.21847	2.1

21342	0.946129	2.1
21343	1.41131	2.1
21344	1.21847	2.1
21345	1.21269	0.05
21346	1.0417	1.63878
21347	1.15821	1.36691
21348	0.946132	0.490086
21349	0.946132	1.94323
21350	1.37967	0.663163
21351	0.851529	0.965174
21352	1.20455	2.1
21353	1.4282	0.05
21354	1.26593	2.1
21355	0.838026	0.091024
21356	0.985096	0.0815196
21357	0.83804	0.089707
21358	0.822395	0.812162
21359	0.420272	0.347387
21360	0.946131	2.1
21361	1.17461	2.1
21362	0.94613	2.1
21363	1.40225	2.1
21364	1.17461	2.1
21365	1.09847	2.1
21366	1.22222	2.09139
21367	1.41798	0.406114
21368	0.791658	1.1103
21369	0.791656	1.00619
21370	0.946129	0.886275
21371	0.817064	0.806182
21372	0.946114	0.605533
21373	0.75824	0.57483
21374	0.75821	0.687392
21375	1.41778	0.422215
21376	0.791635	0.5886
21377	1.04164	1.77852
21378	0.946131	0.983637
21379	1.11172	0.700142
21380	1.30076	0.39241
21381	1.35721	0.417696
21382	1.11172	0.425723
21383	1.87218	0.139607
21384	0.987248	0.158732
21385	0.729695	0.916321
21386	0.974389	0.239983
21387	1.18002	0.242042
21388	0.822612	0.221048
21389	1.35721	0.24086
21390	0.634295	1.17924
21391	0.634326	1.14892
21392	1.13245	1.36357
21393	1.13245	1.24862
21394	0.937961	1.17072
21395	1.18769	1.13035
21396	0.854949	0.964532
21397	0.967357	1.08751
21398	0.810934	0.779836
21399	3.91082	2.1

21400	0.946132	2.1
21401	0.946132	2.1
21402	1.05383	2.1
21403	1.05383	2.1
21404	0.986498	2.1
21405	1.50732	0.0620586
21406	0.753659	0.0693509
21407	1.22144	0.0826311
21408	1.30463	0.0693986
21409	0.753659	0.0796285
21410	1.30463	0.0762584
21411	0.886189	0.0846663
21412	1.04321	0.080488
21413	0.909928	0.0731398
21414	1.18659	0.0677592
21415	1.19785	0.0523394
21416	1.40969	0.0701592
21417	1.24094	0.0559974
21418	0.946126	0.0623863
21419	1.2929	0.055006
21420	1.24093	0.058819
21421	0.867944	0.0516541
21422	1.41903	2.1
21423	2.47881	0.05
21424	1.12943	0.05
21425	0.764229	2.1
21426	0.764256	2.1
21427	0.94613	2.1
21428	0.94613	2.1
21429	1.1995	2.1
21430	1.83423	2.1
21431	0.634816	2.1
21432	0.877837	2.1
21433	0.94613	2.1
21434	1.04073	2.1
21435	1.06485	2.1
21436	0.94613	0.186589
21437	0.62725	0.155565
21438	0.798212	0.176154
21439	1.33241	0.124183
21440	0.62725	0.160047
21441	1.82301	0.0975203
21442	1.40615	2.1
21443	0.782504	2.1
21444	0.748635	2.1
21445	0.946131	2.1
21446	0.748607	2.1
21447	0.988456	1.90258
21448	1.64833	0.05
21449	1.35657	0.05
21450	1.26155	0.05
21451	0.915755	0.05
21452	0.915755	0.05
21453	0.946132	0.05
21454	1.49386	0.05
21455	1.01346	2.1
21456	0.946131	2.1
21457	0.946132	2.1

21458	1.38651	2.1
21459	1.39523	2.1
21460	0.946132	2.1
21461	1.06312	2.1
21462	0.946131	2.1
21463	0.946129	2.1
21464	1.39523	2.1
21465	1.37372	2.1
21466	0.946131	2.1
21467	1.2929	0.0724607
21468	0.94613	0.103628
21469	1.24448	0.0700375
21470	1.2929	0.0795936
21471	0.862011	0.0708776
21472	1.73583	0.0531001
21473	1.724	0.063616
21474	1.03824	2.1
21475	1.534	2.1
21476	0.725492	1.90636
21477	0.771967	2.08493
21478	0.61731	1.60951
21479	0.475743	1.96802
21480	0.719694	1.6775
21481	1.14878	1.52708
21482	0.946117	1.93235
21483	0.94613	1.93879
21484	0.946105	1.98289
21485	0.482666	1.53863
21486	0.609172	1.54609
21487	0.946131	2.1
21488	1.39509	1.54747
21489	1.07439	2.1
21490	0.946131	2.1
21491	1.39509	2.1
21492	0.963571	1.74812
21493	0.591011	0.325917
21494	1.1357	0.205712
21495	2.11193	1.67879
21496	1.60936	2.1
21497	1.34118	0.0636885
21498	1.27696	0.0597382
21499	0.94613	0.0735715
21500	0.94613	0.0627029
21501	1.04257	0.0544483
21502	1.53336	0.0641384
21503	1.0631	0.0842812
21504	1.04072	0.135588
21505	0.670678	0.0988608
21506	0.709531	0.11717
21507	0.891275	0.120046
21508	0.683583	0.124186
21509	0.902478	0.0944893
21510	0.807671	0.102929
21511	1.11024	2.1
21512	1.08171	2.1
21513	1.16725	2.1
21514	0.946128	2.1
21515	1.13537	2.1

21516	1.40525	2.1
21517	1.28365	2.1
21518	1.04523	2.1
21519	0.960106	2.1
21520	0.946128	2.1
21521	1.23452	2.1
21522	0.946129	2.1
21523	1.3704	2.1
21524	1.23452	2.1
21525	1.16906	1.3966
21526	0.946131	1.64385
21527	0.867246	1.45231
21528	0.867217	1.59293
21529	0.946131	1.49675
21530	1.13438	1.52353
21531	1.28684	1.58659
21532	0.946132	1.73927
21533	0.883935	1.57091
21534	0.883938	2.02484
21535	0.946133	1.59482
21536	1.73444	1.49025
21537	1.21835	2.1
21538	1.28228	2.1
21539	1.8643	0.752435
21540	0.743379	0.088624
21541	1.34921	0.092098
21542	0.743381	0.0936452
21543	0.743381	0.10114
21544	0.946131	0.100098
21545	0.915528	2.1
21546	0.982742	2.1
21547	0.975965	2.1
21548	0.891132	0.05
21549	1.27533	0.05
21550	0.946132	0.05
21551	1.25215	0.05
21552	0.590343	0.05
21553	0.946133	0.05
21554	0.590331	0.05
21555	1.62375	2.1
21556	0.687769	1.29675
21557	1.14374	1.28614
21558	1.20885	0.99312
21559	0.946132	1.32335
21560	1.14374	1.40952
21561	1.09308	0.198517
21562	1.02055	0.206751
21563	0.810934	0.134366
21564	0.917609	1.92087
21565	1.13094	2.06048
21566	0.70959	1.87746
21567	1.11223	2.1
21568	0.83051	1.85941
21569	0.810946	2.03853
21570	0.810959	1.87051
21571	1.5773	0.05
21572	1.96133	1.49127
21573	1.0214	1.48192

21574	0.762783	1.66606
21575	0.838036	1.39143
21576	0.63591	1.77489
21577	0.967411	1.58824
21578	0.946132	2.09477
21579	0.946132	2.05674
21580	1.39699	1.83366
21581	1.61642	1.87456
21582	0.860954	1.53741
21583	0.957411	1.70433
21584	0.660856	1.70001
21585	1.10003	2.1
21586	0.968249	2.1
21587	0.910184	2.08296
21588	1.30029	0.749278
21589	0.754119	1.71878
21590	0.754121	1.96815
21591	0.94613	1.88307
21592	1.08039	2.1
21593	0.810942	0.0845322
21594	1.15153	0.0836524
21595	1.06968	0.0737909
21596	1.38456	2.1
21597	1.04118	2.1
21598	0.838027	2.1
21599	1.04073	2.1
21600	0.754209	2.1
21601	1.332 0.05	
21602	1.26193	2.1
21603	1.15317	0.05
21604	3.86179	0.05
21605	1.56005	2.1
21606	0.683035	0.223842
21607	0.931463	0.198016
21608	0.556019	0.746944
21609	0.929172	0.497459
21610	0.756874	0.14155
21611	1.23527	0.899285
21612	2.05961	1.08051
21613	0.946132	2.1
21614	1.16684	2.1
21615	1.3937	2.1
21616	1.3937	2.1
21617	0.946132	2.1
21618	1.17885	2.1
21619	1.62507	0.05
21620	1.58874	2.1
21621	0.819953	2.1
21622	1.16023	2.1
21623	1.34782	2.1
21624	1.16023	2.1
21625	0.819955	2.1
21626	0.79437	2.1
21627	0.794373	2.1
21628	1.20056	2.04249
21629	1.25 2.09613	
21630	1.20056	2.1
21631	1.35787	2.1

21632	0.946131	2.01113
21633	1.38585	1.99594
21634	0.946128	1.67459
21635	0.946129	1.8406
21636	1.35714	1.97789
21637	1.04147	2.1
21638	0.946133	1.59777
21639	1.30382	1.58248
21640	0.946131	0.881185
21641	0.946131	1.52549
21642	1.38585	1.74798
21643	1.22305	1.29949
21644	1.00763	0.0899518
21645	0.813199	0.140092
21646	0.810948	0.126315
21647	1.15928	0.119171
21648	0.944166	0.0511852
21649	0.94613	0.0595607
21650	0.946133	0.070927
21651	1.4179	0.059642
21652	0.946132	0.0590604
21653	1.36965	0.05
21654	1.05444	0.05
21655	0.690302	0.0608705
21656	0.838038	0.05
21657	0.802821	0.505295
21658	1.40944	0.700629
21659	0.946126	0.950828
21660	0.799998	0.377148
21661	0.800035	0.528019
21662	1.1766	2.1
21663	1.00319	2.1
21664	1.01606	1.8025
21665	0.872158	2.1
21666	1.04884	2.1
21667	0.679494	2.1
21668	0.946131	2.1
21669	1.37609	2.1
21670	0.94613	2.1
21671	0.946131	2.1
21672	1.18486	2.1
21673	0.726287	2.1
21674	0.946131	2.1
21675	0.726286	2.1
21676	1.30318	2.1
21677	1.01712	2.1
21678	0.946132	2.1
21679	1.04404	2.1
21680	1.37017	2.1
21681	1.37017	2.1
21682	0.946133	2.1
21683	0.992084	1.31343
21684	0.946131	0.655549
21685	0.529655	1.96886
21686	0.529686	1.55287
21687	0.946131	0.649785
21688	1.05937	1.58407
21689	1.14622	0.372937

21690	0.946131	0.337892
21691	0.584575	1.97549
21692	0.584575	0.23872
21693	0.946129	0.170026
21694	0.709714	2.1
21695	0.946132	2.1
21696	0.709714	2.1
21697	1.37199	2.1
21698	0.82899	2.1
21699	1.00133	2.1
21700	0.778424	0.05
21701	0.946132	0.05
21702	1.24094	0.05
21703	1.00691	0.05
21704	0.64868	0.05
21705	1.29736	0.05
21706	1.24767	0.379683
21707	1.28953	0.570961
21708	1.34612	1.37614
21709	0.946133	0.758283
21710	1.14187	1.41206
21711	0.946132	1.0917
21712	0.946129	1.53087
21713	1.4099	2.1
21714	0.946131	1.86355
21715	1.34612	1.64731
21716	1.4099	1.77128
21717	0.956003	0.05
21718	1.40572	0.05
21719	3.54443	2.1
21720	0.994001	0.438491
21721	1.03679	2.1
21722	1.08467	2.1
21723	1.56875	0.193528
21724	0.968463	2.1
21725	0.974889	2.1
21726	0.946131	2.1
21727	1.30203	2.1
21728	1.40199	2.1
21729	1.40199	2.1
21730	0.946132	2.1
21731	0.946132	2.1
21732	1.36994	2.1
21733	1.37372	2.1
21734	1.37372	2.1
21735	0.946132	2.1
21736	0.928979	1.56809
21737	0.565509	1.67364
21738	1.60196	0.481911
21739	1.16239	0.194126
21740	1.10746	0.430763
21741	1.15412	2.1
21742	1.15171	2.1
21743	0.946133	2.1
21744	0.940877	2.1
21745	0.940878	2.1
21746	1.41432	2.1
21747	1.04687	2.1

21748	2.75524	0.05
21749	0.946132	0.05
21750	0.766452	0.05
21751	0.766427	0.05
21752	1.36004	0.05
21753	1.06648	0.05
21754	1.62483	2.1
21755	1.0577	2.1
21756	1.39158	2.1
21757	0.94255	2.1
21758	1.05029	2.1
21759	1.03735	1.96541
21760	0.931055	2.1
21761	1.03797	1.99569
21762	1.43687	1.97449
21763	1.28765	1.34551
21764	1.1811	1.47909
21765	0.946132	1.36631
21766	0.946132	1.3906
21767	1.1811	1.40066
21768	0.67805	1.58172
21769	1.50998	1.92083
21770	1.16661	2.1
21771	1.36749	2.1
21772	0.810945	1.00121
21773	1.14745	0.814187
21774	0.865588	0.456842
21775	1.36665	0.214005
21776	0.506964	0.718365
21777	0.506966	1.96055
21778	0.552786	0.108879
21779	0.946133	0.138058
21780	1.21336	0.388435
21781	0.552786	0.0961834
21782	0.552757	2.1
21783	0.653468	0.277659
21784	1.36665	2.00612
21785	1.27922	0.162921
21786	1.08133	0.239077
21787	1.05348	0.114421
21788	0.946132	0.19607
21789	1.19601	0.125724
21790	1.13276	0.128068
21791	0.7856	0.126489
21792	1.57125	0.122848
21793	1.19601	0.233605
21794	0.946131	0.611425
21795	1.34534	0.327921
21796	1.19601	0.267881
21797	0.870489	0.278533
21798	1.49404	2.1
21799	1.35943	1.65289
21800	1.10427	1.89306
21801	0.94613	1.67614
21802	0.946131	1.74835
21803	1.10427	1.75324
21804	1.2064	0.975512
21805	0.946132	1.17841

21806	1.11288	2.1
21807	1.34268	0.126324
21808	1.34268	0.634615
21809	0.946132	0.393296
21810	0.946128	2.1
21811	0.946128	2.08441
21812	1.30835	2.1
21813	1.38463	2.1
21814	0.946129	2.1
21815	1.43778	1.1646
21816	1.23943	1.69924
21817	1.4748	2.1
21818	0.71295	2.1
21819	0.712951	2.1
21820	0.946132	2.1
21821	0.946133	2.1
21822	1.13976	2.1
21823	1.71109	2.1
21824	1.32426	2.1
21825	0.671255	2.1
21826	1.29123	2.1
21827	0.946132	2.1
21828	0.946132	2.1
21829	1.25603	2.1
21830	2.21126	0.0500008
21831	0.892889	2.1
21832	0.946129	2.1
21833	0.892888	2.1
21834	0.92872	2.1
21835	1.34352	2.1
21836	0.622784	0.227071
21837	1.02947	0.113645
21838	0.810957	0.10413
21839	1.91756	1.53347
21840	1.06012	0.87134
21841	1.09569	0.410726
21842	1.5456	0.05
21843	1.1211	0.05
21844	1.21031	0.05
21845	1.03939	0.0842336
21846	0.519697	0.105327
21847	0.933889	0.183382
21848	0.519726	0.168369
21849	1.16699	0.148128
21850	0.933889	0.16744
21851	0.840599	0.0726415
21852	0.94613	0.131707
21853	0.840568	0.102645
21854	1.26868	0.100858
21855	1.16699	0.0987138
21856	1.17822	1.42954
21857	0.922044	0.053771
21858	1.29322	0.0827436
21859	0.946132	0.0654104
21860	0.995178	0.168844
21861	0.922056	0.05
21862	1.76232	0.121364
21863	1.77065	0.804441

21864	1.38149	0.643995
21865	0.959301	1.28421
21866	0.946133	0.608802
21867	0.946132	1.04175
21868	0.959301	1.13253
21869	1.3612	0.823738
21870	0.885314	1.1249
21871	0.885309	0.599931
21872	0.94613	1.14671
21873	0.8853	0.496744
21874	1.56164	0.673577
21875	1.45506	0.123659
21876	1.45257	2.1
21877	0.851541	0.05
21878	1.29018	0.05
21879	0.851541	0.05
21880	1.21087	0.05
21881	1.24979	0.05
21882	1.56983	0.05
21883	1.60151	0.05
21884	1.78952	2.1
21885	0.638164	2.1
21886	0.638163	2.1
21887	1.39754	2.1
21888	1.39754	2.1
21889	0.681589	2.1
21890	1.34993	2.1
21891	1.04525	2.1
21892	0.946133	2.1
21893	1.04072	2.1
21894	0.857805	2.1
21895	1.06053	2.1
21896	1.49066	2.1
21897	1.4185	1.69943
21898	0.934059	1.88907
21899	0.93406	1.70864
21900	0.94613	1.80549
21901	0.93406	1.79338
21902	1.25277	1.83312
21903	1.00792	2.07907
21904	0.609466	1.86323
21905	0.946132	1.95824
21906	0.609463	1.96155
21907	1.30664	1.89013
21908	0.810943	1.66355
21909	1.05741	1.34806
21910	1.3868	2.1
21911	1.15558	0.205297
21912	1.13537	2.1
21913	0.946132	2.1
21914	0.946132	2.1
21915	1.08266	2.1
21916	1.54934	2.1
21917	1.26016	2.1
21918	2.01915	2.1
21919	0.60346	1.8275
21920	0.651529	1.83139
21921	1.15581	2.02354

21922	1.13037	1.81129
21923	1.04071	1.81827
21924	1.77796	1.99639
21925	0.532308	1.73182
21926	0.532308	1.62006
21927	1.15369	1.87736
21928	1.1537	1.70603
21929	0.903288	1.78781
21930	0.804766	2.1
21931	0.804739	2.1
21932	0.946128	2.1
21933	0.946131	2.1
21934	1.16745	2.1
21935	1.04193	2.1
21936	0.98505	2.1
21937	1.40724	0.610775
21938	1.14216	0.425585
21939	0.946131	0.312477
21940	0.946131	0.42374
21941	1.10941	0.411633
21942	0.81268	0.05
21943	0.946131	0.05
21944	1.28673	0.05
21945	1.28673	0.05
21946	0.812681	0.05
21947	1.84716	0.05
21948	1.84716	0.05
21949	0.92361	0.05
21950	0.94613	0.05
21951	0.923582	0.05
21952	1.26227	0.05
21953	0.923581	0.05
21954	0.747394	2.1
21955	1.32582	2.1
21956	0.747365	2.1
21957	0.97587	2.1
21958	1.32582	2.1
21959	1.04182	0.05
21960	0.961743	0.05
21961	0.946126	0.0611694
21962	1.47463	0.0505097
21963	1.12183	0.0532626
21964	0.851552	0.05
21965	1.33177	0.0707028
21966	0.946131	0.109733
21967	1.41512	0.0875354
21968	1.33178	0.0785841
21969	0.946131	0.0726257
21970	0.927735	0.239556
21971	0.97206	0.102208
21972	0.515566	0.163948
21973	0.946129	0.143109
21974	0.515538	0.121705
21975	1.04482	0.083182
21976	0.975535	0.129892
21977	0.522411	0.08808
21978	0.522382	0.144182
21979	0.925346	0.137407

21980	0.575817	0.10413
21981	0.946129	2.1
21982	1.14256	2.1
21983	1.32054	2.1
21984	1.40525	2.1
21985	1.14256	2.1
21986	1.59475	2.1
21987	0.773813	2.1
21988	1.4073	2.1
21989	0.773813	2.1
21990	0.773813	2.1
21991	1.4073	2.1
21992	0.993021	2.1
21993	1.50466	0.0562654
21994	0.851539	0.0749
21995	1.20299	0.0634025
21996	0.946131	0.068833
21997	1.54464	0.0721428
21998	1.20309	0.0655037
21999	2.00701	0.0574838
22000	0.810946	0.0795854
22001	1.07678	0.0775824
22002	0.900128	0.0702105
22003	1.26842	2.1
22004	1.11206	2.1
22005	0.946132	2.1
22006	1.04074	2.1
22007	0.948809	2.1
22008	0.946131	0.05
22009	0.929617	0.05
22010	1.24057	0.05
22011	1.36485	0.05
22012	0.851554	0.05
22013	1.96823	0.05
22014	0.914836	0.05
22015	0.94613	0.0517937
22016	1.38911	0.05
22017	0.914805	0.05
22018	0.946131	0.05
22019	0.946131	0.057527
22020	1.04072	0.05
22021	1.26837	0.05
22022	0.970054	0.0527159
22023	0.923958	0.0539696
22024	1.16653	2.1
22025	1.34412	2.1
22026	0.851541	0.05
22027	1.38645	0.0512552
22028	0.891637	0.051889
22029	0.851487	0.0511947
22030	0.890871	0.0525433
22031	1.56178	0.05
22032	0.946128	0.05
22033	1.26868	0.05
22034	0.946131	0.0891884
22035	1.18789	0.382704
22036	1.26868	0.0920973
22037	1.26868	0.0876769

22038	0.946128	0.0876769
22039	1.18716	0.065998
22040	0.946131	0.114373
22041	0.59358	0.0748472
22042	1.23923	0.116507
22043	1.00024	0.0803132
22044	0.593593	0.0897315
22045	0.946131	0.154616
22046	0.713645	0.122152
22047	1.23175	0.170817
22048	1.23923	0.0895961
22049	0.713633	0.13149
22050	1.24687	0.0623598
22051	1.80662	2.09639
22052	2.15762	2.1
22053	0.780455	2.1
22054	0.946131	2.1
22055	0.780455	2.1
22056	1.40583	2.1
22057	1.39852	2.1
22058	0.565619	2.1
22059	1.1428	2.1
22060	0.663168	0.247454
22061	1.02163	0.194331
22062	1.04072	0.255978
22063	0.663169	0.227747
22064	1.13511	0.2166
22065	1.85277	0.144459
22066	1.17701	0.870194
22067	0.993831	0.0774357
22068	1.0823	0.0825609
22069	0.795761	0.262607
22070	1.24596	0.19623
22071	0.566075	0.265419
22072	0.94613	0.308515
22073	0.56605	0.297426
22074	0.774911	0.05
22075	0.946131	0.0551597
22076	1.24417	0.05
22077	0.774911	0.05
22078	0.946132	0.0506929
22079	1.54982	0.05
22080	1.82961	0.05
22081	1.12035	0.05
22082	0.876835	2.1
22083	0.946131	2.1
22084	0.876836	2.1
22085	1.12968	2.1
22086	1.23536	2.1
22087	2.15082	2.1
22088	0.58365	2.1
22089	0.946133	2.1
22090	0.583653	2.1
22091	0.992097	2.1
22092	1.12968	2.1
22093	1.0305	2.1
22094	0.82613	0.101904
22095	0.879644	0.0897551

22096	1.00688	0.0875549
22097	1.52776	2.1
22098	0.851537	1.5551
22099	1.00851	1.55471
22100	0.946128	1.50192
22101	0.851552	1.55155
22102	1.4895	1.80421
22103	0.761574	2.1
22104	0.946132	2.1
22105	1.01095	2.1
22106	1.01095	2.1
22107	0.761572	2.1
22108	1.8575	2.1
22109	1.19984	0.066532
22110	1.25797	0.0781156
22111	0.946132	0.0950579
22112	0.946132	0.0708867
22113	0.95332	0.113841
22114	0.946131	2.1
22115	0.851542	2.1
22116	1.03172	2.1
22117	1.22889	2.1
22118	1.0313	2.1
22119	1.15977	2.0142
22120	0.918875	1.33348
22121	0.8654	1.96809
22122	0.865374	1.45231
22123	0.946132	1.6565
22124	0.985051	2.1
22125	1.31993	2.00191
22126	1.33622	0.368671
22127	0.959992	0.271147
22128	0.926499	2.1
22129	1.03544	2.1
22130	0.946132	2.1
22131	0.946132	2.1
22132	1.40335	2.1
22133	0.772299	2.1
22134	0.851529	2.1
22135	0.857243	2.1
22136	1.38155	2.1
22137	1.05462	2.1
22138	0.832138	2.1
22139	0.946132	2.1
22140	0.832137	2.1
22141	0.959414	2.1
22142	1.36541	2.1
22143	1.91894	2.1
22144	1.04661	2.1
22145	1.28355	2.1
22146	0.877353	2.1
22147	0.877353	2.1
22148	0.94613	2.1
22149	1.20537	0.0553875
22150	1.09914	0.05
22151	0.94613	0.05
22152	0.499593	0.0533012
22153	0.499606	0.054029

22154	1.0959	0.05
22155	0.838027	0.05
22156	0.883644	0.05
22157	0.95019	0.0868375
22158	1.10638	2.1
22159	0.946132	2.1
22160	1.41744	2.1
22161	1.05842	2.1
22162	0.94613	2.1
22163	1.41744	2.1
22164	0.991985	2.1
22165	3.31755	2.1
22166	1.05556	2.1
22167	0.624075	2.03179
22168	0.814554	1.96985
22169	0.946131	2.01461
22170	0.814554	2.08383
22171	1.31857	2.07123
22172	1.26282	2.1
22173	0.946132	2.1
22174	1.14302	2.1
22175	0.946132	2.1
22176	0.946132	2.1
22177	1.40372	2.1
22178	1.24688	0.154925
22179	0.89889	2.1
22180	1.17286	2.1
22181	0.71741	2.1
22182	0.491096	0.519781
22183	1.05432	0.253344
22184	0.719531	2.1
22185	1.25145	2.1
22186	0.864686	2.1
22187	0.867974	2.1
22188	1.21734	2.1
22189	0.405517	0.842485
22190	0.818878	1.29381
22191	0.502457	0.0598008
22192	1.05868	0.0582329
22193	0.912018	0.0500106
22194	1.40591	0.05
22195	0.957584	0.384735
22196	0.946131	0.57077
22197	1.11612	0.416687
22198	1.3365	0.413958
22199	0.946128	0.626
22200	1.16534	0.120679
22201	1.38969	0.227346
22202	0.891597	1.69991
22203	0.640032	1.83611
22204	0.956758	1.68608
22205	1.3428	0.200248
22206	0.953295	2.1
22207	0.946131	0.142311
22208	1.16167	0.152005
22209	0.94613	0.155286
22210	0.951675	0.149056
22211	1.16167	0.146126

22212	1.24538	0.0809543
22213	1.13482	2.1
22214	1.38775	2.1
22215	0.934061	2.1
22216	0.91408	2.1
22217	1.06099	2.1
22218	0.980549	0.067953
22219	0.946132	0.0837876
22220	1.10321	0.0977654
22221	0.99126	0.0884956
22222	0.991261	0.0959904
22223	1.35732	0.0902567
22224	1.1952	2.1
22225	0.946132	1.73715
22226	0.946132	2.1
22227	1.32819	1.8754
22228	1.30171	1.92228
22229	1.29667	0.068197
22230	1.00982	0.05
22231	0.407589	0.604456
22232	1.18266	1.5822
22233	1.04037	0.549802
22234	0.956694	1.04998
22235	0.552639	0.704474
22236	0.810943	1.75484
22237	1.43144	1.45556
22238	0.918074	1.37031
22239	1.60976	2.1
22240	4.00847	2.1
22241	2.3995	0.05
22242	1.36203	2.1
22243	0.919619	2.1
22244	1.30188	2.1
22245	0.681073	2.1
22246	0.681043	2.1
22247	1.04687	2.1
22248	3.32085	2.1
22249	1.80396	0.05
22250	0.773007	0.201116
22251	0.773034	0.144634
22252	1.22141	0.234237
22253	1.22141	0.236473
22254	0.946131	0.342098
22255	2.02071	0.135414
22256	0.951912	0.05
22257	1.39027	0.054451
22258	0.94613	0.0561563
22259	0.829922	0.05
22260	0.829946	0.05
22261	1.24902	1.39438
22262	2.8333	1.22009
22263	0.946131	0.05
22264	0.929725	0.05
22265	1.1134	0.05
22266	1.17056	0.05
22267	0.929713	0.05
22268	0.749096	2.1
22269	1.31961	2.1

22270	0.870191	2.1
22271	1.04071	2.1
22272	1.1877	2.1
22273	1.03882	2.1
22274	1.44801	2.1
22275	3.55198	2.1
22276	1.11193	2.1
22277	0.663922	2.1
22278	0.663922	2.1
22279	0.946129	2.1
22280	0.946131	2.1
22281	0.97079	2.1
22282	1.36049	2.1
22283	1.06105	2.1
22284	0.92251	2.1
22285	0.94613	2.1
22286	0.922511	2.1
22287	1.04101	2.1
22288	0.810944	0.0595185
22289	0.994109	0.0558599
22290	0.623143	0.0551659
22291	0.864244	0.060706
22292	1.14627	0.0560304
22293	0.64586	0.0552852
22294	0.721422	0.05
22295	0.870885	0.05
22296	0.855175	0.05
22297	0.946131	2.1
22298	1.38157	2.1
22299	1.33266	1.73568
22300	0.946131	2.1
22301	1.38158	2.1
22302	0.946132	2.1
22303	1.3268	2.1
22304	0.946132	2.1
22305	0.946132	2.1
22306	1.38158	2.1
22307	1.42186	1.56515
22308	1.47204	1.57234
22309	1.14665	2.1
22310	0.946132	1.96631
22311	0.94613	2.1
22312	1.30116	2.1
22313	1.32819	2.1
22314	1.25388	2.1
22315	0.887808	2.1
22316	0.946132	2.1
22317	1.23536	2.1
22318	1.34309	2.1
22319	1.07612	2.1
22320	0.728982	0.05
22321	1.3157	0.05
22322	0.946131	0.05
22323	0.686176	0.05
22324	1.3035	0.05
22325	0.686139	0.0501878
22326	0.906806	0.0503742
22327	0.794122	0.05

22328	1.17173	0.0510682
22329	1.8194	0.05
22330	0.851543	2.1
22331	1.19345	2.1
22332	0.946133	2.1
22333	0.946132	2.1
22334	1.56179	2.1
22335	0.620263	2.1
22336	1.30226	2.08899
22337	1.2334	1.93469
22338	1.2334	1.8631
22339	0.946133	1.84091
22340	1.24053	2.1
22341	1.4828	2.1
22342	0.957622	2.1
22343	1.00521	1.59051
22344	1.49463	2.1
22345	1.39621	2.1
22346	1.09861	2.1
22347	0.946131	2.1
22348	0.946131	2.1
22349	1.09861	2.1
22350	2.16987	0.05
22351	0.572182	0.05
22352	1.15802	0.05
22353	0.63574	0.05
22354	1.18897	0.05
22355	0.996733	0.05
22356	1.82402	1.74881
22357	0.969602	1.6143
22358	0.794777	1.73815
22359	1.02626	1.63695
22360	1.51396	0.108389
22361	1.40574	0.174866
22362	1.38683	0.161101
22363	0.946126	0.183277
22364	0.946128	0.16958
22365	1.07138	0.16689
22366	0.946131	2.1
22367	1.41331	2.1
22368	0.94613	2.1
22369	0.94613	2.1
22370	1.27451	2.1
22371	0.974196	2.1
22372	1.2049	0.05
22373	1.42757	2.1
22374	1.02461	2.1
22375	2.07087	2.1
22376	1.92515	1.6751
22377	0.802138	1.67102
22378	0.946128	1.854
22379	0.802107	1.69213
22380	1.23726	1.69166
22381	1.21997	1.69281
22382	0.767577	1.02914
22383	0.946131	1.70201
22384	0.767577	1.54606
22385	1.3279	1.53983

22386	1.23726	1.48826
22387	1.29729	2.1
22388	0.946128	2.1
22389	1.14186	2.1
22390	1.39214	2.1
22391	1.39214	2.1
22392	0.946129	2.1
22393	1.23012	0.311
22394	0.653228	0.0964046
22395	0.946133	0.33634
22396	1.15918	0.236259
22397	1.26893	0.131186
22398	0.653228	0.297182
22399	1.06848	0.403492
22400	0.615121	2.1
22401	0.841488	2.1
22402	0.946131	2.1
22403	1.04071	2.1
22404	0.965717	2.1
22405	1.77893	2.04829
22406	1.11314	0.0890991
22407	1.65411	0.626819
22408	0.85472	0.0588121
22409	1.1604	0.0513521
22410	1.19141	0.0547352
22411	1.19141	0.05
22412	0.94613	0.0571784
22413	1.70944	0.05
22414	1.89082	0.107808
22415	1.05175	1.51725
22416	0.815276	2.1
22417	0.843283	2.1
22418	0.946131	2.1
22419	0.843284	2.1
22420	1.38897	2.1
22421	1.01019	1.15138
22422	1.05197	1.01895
22423	0.810959	1.0669
22424	1.33037	0.0928986
22425	1.3415	0.0638858
22426	0.946118	0.131497
22427	0.946131	0.089418
22428	1.09803	0.0816234
22429	0.930281	2.1
22430	1.3335	2.1
22431	0.784151	2.1
22432	0.78415	2.1
22433	0.946132	2.1
22434	1.6299	0.05
22435	0.995795	2.1
22436	1.84846	1.55892
22437	0.929873	1.99362
22438	0.889245	2.00192
22439	0.808072	1.99833
22440	0.851541	1.81641
22441	1.51956	1.86431
22442	0.94613	1.82205
22443	0.851553	1.84887

22444	0.946787	1.87567
22445	1.15544	1.83369
22446	1.30289	0.0666602
22447	1.28323	0.0675685
22448	0.85024	0.0558804
22449	1.09662	0.0776689
22450	1.67687	0.05
22451	0.946127	2.1
22452	1.40496	2.1
22453	1.22442	2.1
22454	0.946128	2.1
22455	1.40496	2.1
22456	1.00573	2.1
22457	0.636837	2.1
22458	0.988642	2.1
22459	0.573141	2.05064
22460	0.946128	2.07914
22461	0.786538	2.09303
22462	2.28748	1.74001
22463	0.682805	2.1
22464	0.946132	2.1
22465	0.682776	2.1
22466	0.946132	2.1
22467	0.79775	2.1
22468	1.05403	0.05
22469	0.888273	0.05
22470	0.876373	0.05
22471	0.576083	0.05
22472	0.946132	0.05
22473	0.576057	0.05
22474	0.576058	0.05
22475	0.946131	0.05
22476	1.41724	0.361561
22477	1.31678	0.977821
22478	0.946132	1.16259
22479	0.946132	0.933698
22480	1.31678	0.757051
22481	0.935738	2.1
22482	0.946132	2.1
22483	0.935765	2.1
22484	1.32949	2.1
22485	0.946131	2.1
22486	1.02525	2.1
22487	0.946128	2.1
22488	1.41207	2.1
22489	1.04675	2.1
22490	0.946131	2.1
22491	1.41207	2.1
22492	0.946131	2.1
22493	0.946132	2.1
22494	1.41125	2.1
22495	1.41125	2.1
22496	1.27087	2.1
22497	0.924071	0.239845
22498	0.608893	0.185176
22499	0.528907	0.23764
22500	1.78249	0.227304
22501	0.720094	0.211848

22502	0.781667	2.1
22503	0.781638	2.1
22504	1.40756	2.1
22505	1.40756	2.1
22506	0.797635	2.1
22507	1.11946	0.05
22508	1.32068	0.05
22509	0.88757	0.0758941
22510	1.27891	0.0723602
22511	0.810957	0.0732709
22512	0.914656	0.0739187
22513	0.640819	0.0651269
22514	0.745643	0.0738413
22515	0.865108	0.0997122
22516	1.06883	0.0868287
22517	0.806001	0.102443
22518	1.19462	2.1
22519	1.42763	0.356473
22520	1.24682	0.263461
22521	0.78392	0.0692034
22522	1.02874	0.067257
22523	0.884512	0.0661296
22524	1.1658	2.1
22525	0.663026	0.05
22526	1.21174	0.05
22527	0.663056	0.05
22528	1.15968	0.05
22529	1.21174	0.05
22530	0.993976	0.05
22531	0.945495	0.05
22532	0.671202	0.05
22533	1.37312	2.1
22534	1.28499	2.1
22535	0.946133	2.1
22536	0.946132	2.1
22537	1.28499	2.1
22538	1.25081	2.1
22539	0.946132	2.1
22540	0.946132	2.1
22541	1.27985	2.1
22542	1.37312	2.1
22543	0.830638	2.1
22544	1.05358	2.1
22545	1.22209	2.1
22546	1.09874	2.1
22547	0.74758	2.1
22548	2.50011	1.3768
22549	0.6157	0.166179
22550	0.674634	0.17968
22551	0.946128	1.35754
22552	0.674633	0.207778
22553	0.946131	0.355671
22554	1.96192	1.85072
22555	1.31612	0.801246
22556	1.10735	0.451307
22557	0.936676	0.213096
22558	0.808779	0.246665
22559	0.808808	0.271189

22560	1.00245	0.256888
22561	1.26179	0.190892
22562	1.05758	1.9685
22563	1.03408	2.1
22564	0.946132	0.0515365
22565	0.914368	0.05
22566	1.20524	0.05
22567	1.12743	0.05
22568	0.851555	0.05
22569	1.17885	0.177973
22570	0.867881	0.176321
22571	1.00122	0.0987718
22572	0.867883	0.0866635
22573	0.867881	0.0666944
22574	1.19583	0.123283
22575	1.17743	2.1
22576	0.878684	2.1
22577	0.687087	2.1
22578	0.955079	0.320696
22579	0.672977	0.696446
22580	0.735937	0.578632
22581	0.851531	0.727153
22582	0.672957	0.62113
22583	0.923974	0.658654
22584	1.2243	2.1
22585	0.808463	0.595351
22586	0.971078	0.876176
22587	1.06161	0.5374
22588	0.810947	0.958194
22589	0.886074	1.14822
22590	0.786829	0.970184
22591	0.55062	0.816395
22592	0.574458	0.602095
22593	0.946129	0.850513
22594	1.42526	0.540545
22595	1.15962	0.830548
22596	1.24526	0.05
22597	0.94613	0.467722
22598	0.946128	1.34792
22599	1.23279	1.63023
22600	1.38645	1.74446
22601	0.94613	1.0743
22602	1.05042	2.1
22603	0.946128	2.1
22604	0.946129	2.1
22605	1.24286	2.1
22606	1.39659	2.1
22607	0.94613	2.1
22608	1.10407	2.1
22609	1.32809	1.89882
22610	0.993999	1.98514
22611	0.810959	1.88267
22612	0.983643	1.9702
22613	0.869552	2.0713
22614	0.747801	1.86905
22615	1.53429	1.62429
22616	1.13127	2.1
22617	1.38397	2.1

22618	1.08366	2.1
22619	0.946129	2.1
22620	0.946131	2.1
22621	1.08366	2.1
22622	1.06007	2.1
22623	1.79641	2.1
22624	0.758799	2.1
22625	1.28442	2.1
22626	1.06957	2.1
22627	0.946133	2.1
22628	1.28442	2.1
22629	1.76788	1.79336
22630	1.38669	2.1
22631	0.986095	2.1
22632	1.38478	0.360943
22633	0.946132	0.861568
22634	1.24344	0.382248
22635	0.622283	1.73328
22636	1.10335	0.438803
22637	1.24344	0.618196
22638	1.1967	0.466127
22639	0.946131	0.05
22640	0.901005	0.05
22641	1.05751	0.05
22642	1.37771	0.05
22643	0.851527	0.05
22644	1.26643	1.26663
22645	1.08992	1.76191
22646	0.946132	1.44961
22647	0.946132	1.50102
22648	1.08992	1.5028
22649	1.53534	2.1
22650	1.58825	1.86422
22651	1.0447	2.0072
22652	0.593818	1.87333
22653	0.89883	1.96972
22654	0.734088	1.92852
22655	0.998451	0.722809
22656	1.64156	1.03594
22657	1.05654	1.54866
22658	1.37641	0.247347
22659	1.74553	0.260101
22660	1.31112	0.447546
22661	0.74518	0.557138
22662	0.851539	0.443864
22663	0.851554	0.400013
22664	0.851475	0.41183
22665	0.946132	0.05
22666	1.28895	0.05
22667	1.11356	0.05
22668	0.946132	0.05
22669	0.946132	0.05
22670	1.84214	2.1
22671	1.23851	0.0833843
22672	1.25866	0.330487
22673	1.28655	0.74199
22674	0.946131	0.81555
22675	0.946132	0.627314

22676	1.28655	0.496075
22677	1.11929	0.529135
22678	0.946132	0.834968
22679	0.946132	0.442152
22680	1.34254	0.3809
22681	1.25866	0.36054
22682	0.946132	2.1
22683	1.27451	2.1
22684	1.19803	2.1
22685	0.94613	2.1
22686	1.27451	2.1
22687	0.946131	2.1
22688	1.36523	2.1
22689	1.14599	2.1
22690	0.946129	2.1
22691	1.36523	2.1
22692	1.09518	2.1
22693	0.977777	2.1
22694	0.583237	2.1
22695	0.583267	2.1
22696	0.903872	2.1
22697	0.946129	2.1
22698	0.583258	2.1
22699	1.58179	0.056131
22700	0.946131	0.0691654
22701	1.35123	0.065184
22702	0.655837	0.0733806
22703	1.12018	0.0661005
22704	1.35123	0.0634581
22705	0.62967	0.0709823
22706	0.860497	0.0816446
22707	0.816639	0.068809
22708	0.746716	2.1
22709	0.810947	2.1
22710	0.810959	2.1
22711	0.653422	2.1
22712	0.289127	1.9298
22713	0.475716	2.1
22714	0.980651	1.84849
22715	0.946119	2.1
22716	0.946132	2.1
22717	1.08299	2.1
22718	1.34539	2.1
22719	1.28753	0.114489
22720	0.946132	0.192063
22721	0.946132	0.160588
22722	1.19719	0.167942
22723	0.946133	0.164006
22724	0.958947	0.129138
22725	1.3492	0.140388
22726	0.946132	0.220511
22727	0.946128	0.203946
22728	1.22588	0.201717
22729	0.946129	0.194326
22730	0.991113	2.1
22731	1.35966	2.1
22732	0.961 2.1	
22733	0.946129	2.1

22734	0.946131	2.1
22735	0.961001	2.1
22736	0.727096	1.7414
22737	0.810946	1.19143
22738	0.810959	1.53729
22739	1.31658	0.179118
22740	1.23167	2.1
22741	1.44836	0.05
22742	1.50103	0.05
22743	1.23351	0.05
22744	1.35657	0.05
22745	0.851541	0.05
22746	0.93668	0.05
22747	0.946131	0.05
22748	3.7335	2.1
22749	1.1822	2.1
22750	1.31127	0.146726
22751	1.3689	0.11685
22752	1.35822	0.0563377
22753	0.946133	0.0793294
22754	0.946132	0.117031
22755	1.14926	0.100517
22756	1.40822	0.0654327
22757	1.04935	0.140105
22758	0.704112	0.0917743
22759	0.704112	0.126193
22760	0.946131	0.148776
22761	0.704112	0.127389
22762	1.37721	0.05
22763	0.85154	2.1
22764	1.06949	2.1
22765	0.851541	2.1
22766	0.946128	2.1
22767	1.1175	2.1
22768	1.44091	2.06201
22769	0.96029	1.85933
22770	3.46486	1.57038
22771	0.603286	0.110686
22772	0.432283	0.0764391
22773	0.851541	0.0987813
22774	1.09355	0.0762462
22775	0.851534	0.10152
22776	1.04263	2.1
22777	0.851541	2.1
22778	0.965604	2.1
22779	0.94613	2.1
22780	1.23953	2.1
22781	0.869615	2.1
22782	1.02415	2.1
22783	0.983032	2.1
22784	1.35385	1.58387
22785	1.59971	0.0822209
22786	0.915178	2.1
22787	0.962435	2.1
22788	1.3116	2.1
22789	1.38302	2.1
22790	1.13227	2.1
22791	1.25923	0.05

22792	0.988805	2.1
22793	0.611486	1.3834
22794	1.1019	1.35565
22795	1.11124	1.58904
22796	0.716713	1.36249
22797	0.946131	1.43742
22798	1.22297	1.40255
22799	1.12873	1.65492
22800	0.946131	1.67556
22801	0.946132	1.66495
22802	1.3469	1.64259
22803	1.25366	1.70794
22804	1.1611	1.4911
22805	0.946132	1.61236
22806	0.946132	1.51868
22807	1.2854	1.54561
22808	1.3469	1.60274
22809	1.07468	1.49897
22810	1.79317	2.1
22811	1.07444	2.1
22812	1.69436	2.1
22813	0.851539	2.1
22814	0.870925	2.1
22815	0.851541	2.1
22816	0.870951	2.1
22817	1.26097	2.1
22818	1.26247	2.1
22819	0.946132	2.1
22820	1.24041	2.1
22821	0.946132	2.1
22822	0.946132	2.1
22823	1.40274	2.1
22824	0.946131	0.05
22825	0.797945	0.05
22826	1.12988	0.05
22827	1.00681	0.05
22828	0.797932	0.05
22829	0.977483	0.209927
22830	0.943362	0.401058
22831	0.9891	0.201075
22832	1.1703	0.250221
22833	0.663171	0.249149
22834	1.40882	0.103549
22835	1.32639	0.166026
22836	1.24448	0.104083
22837	0.946132	0.159839
22838	1.11015	0.0999419
22839	1.24448	0.123747
22840	0.754768	0.112148
22841	1.13854	2.1
22842	1.23654	2.1
22843	0.714101	0.570727
22844	0.851538	1.38772
22845	0.79261	2.1
22846	1.30598	1.94664
22847	1.05338	1.38865
22848	1.41758	2.1
22849	0.83317	0.10126

22850	1.00058	0.100606
22851	0.810958	0.100125
22852	1.63791	0.140354
22853	1.55116	2.1
22854	1.29735	1.71734
22855	0.945147	2.1
22856	0.94515	1.80108
22857	0.94613	1.92779
22858	0.945149	1.89749
22859	1.02368	2.1
22860	1.12787	2.1
22861	1.04007	2.1
22862	3.78805	2.1
22863	0.816891	0.0669859
22864	0.943996	0.0670455
22865	0.870002	0.0638511
22866	0.564533	0.0712752
22867	0.564536	0.0812475
22868	0.946128	0.213381
22869	0.564535	0.123857
22870	0.946128	0.0976613
22871	1.15177	2.1
22872	1.16806	1.18252
22873	0.999083	1.33699
22874	0.946131	1.19331
22875	0.946131	1.21923
22876	0.999084	1.22733
22877	1.28918	1.21949
22878	1.0383	1.35855
22879	0.946133	1.25057
22880	0.946132	1.26221
22881	1.0383	1.27032
22882	1.27737	1.39259
22883	1.39296	0.256917
22884	0.998682	2.1
22885	3.98933	2.1
22886	1.05328	0.138189
22887	1.23475	2.1
22888	0.900856	1.18801
22889	0.94001	0.953247
22890	0.707748	0.853073
22891	0.707751	0.990709
22892	0.946128	0.863152
22893	0.70775	0.830126
22894	0.996109	0.866334
22895	1.68551	2.1
22896	0.851539	2.1
22897	0.962688	2.1
22898	0.94613	2.1
22899	1.04073	2.1
22900	1.44824	2.1
22901	0.794313	0.308085
22902	0.838031	0.320758
22903	1.11076	0.267647
22904	1.38813	2.1
22905	1.17204	2.1
22906	0.946132	2.1
22907	0.946131	2.1

22908	1.17204	2.1
22909	0.961779	0.246287
22910	1.0685	0.372448
22911	0.810947	1.9511
22912	1.2899	1.62736
22913	0.890404	1.60406
22914	0.909981	2.1
22915	0.810946	1.81105
22916	0.890402	1.78233
22917	0.522844	1.9545
22918	0.593846	1.9465
22919	1.30226	1.75215
22920	1.30226	1.80132
22921	0.946132	1.77824
22922	1.03537	0.055342
22923	1.50035	0.05
22924	0.940356	0.0611953
22925	0.946131	0.0551909
22926	1.03441	0.060591
22927	1.21171	0.05
22928	1.35169	0.0505354
22929	1.32449	0.05
22930	0.946131	0.0546589
22931	0.946131	0.0517684
22932	1.06627	0.0549474
22933	1.03102	0.227876
22934	0.488712	0.05
22935	0.946131	0.05
22936	1.23146	0.05
22937	1.13242	0.05
22938	0.946131	0.05
22939	0.409199	0.05
22940	0.429959	0.05
22941	1.08357	0.05
22942	0.80926	0.05
22943	0.879791	0.05
22944	0.946129	2.07326
22945	1.28828	2.1
22946	0.946131	2.01924
22947	0.946131	2.1
22948	1.35969	2.1
22949	1.10049	1.95735
22950	0.923724	2.1
22951	0.923695	2.1
22952	0.94613	2.1
22953	0.94613	2.1
22954	1.28828	2.1
22955	1.73525	0.139864
22956	0.656239	0.21363
22957	1.2996	0.170821
22958	0.812939	0.234088
22959	0.65624	0.205087
22960	1.2996	0.188998
22961	1.67372	2.1
22962	0.946131	2.1
22963	0.946132	2.1
22964	1.05978	2.1
22965	1.31049	2.1

22966	0.946132	2.1
22967	1.11077	2.1
22968	0.579725	0.31522
22969	0.373532	0.215628
22970	1.13537	0.338195
22971	1.20726	0.250578
22972	0.946131	0.249967
22973	1.45694	0.705989
22974	0.750367	0.0578956
22975	0.946126	0.0628064
22976	0.750365	0.0585231
22977	1.25698	0.0564908
22978	1.35726	0.0577986
22979	1.6812	0.0650474
22980	1.78665	1.95355
22981	1.46568	2.1
22982	1.29606	2.1
22983	1.25298	2.1
22984	0.946115	2.1
22985	0.946129	2.1
22986	1.25296	2.1
22987	1.27778	0.0704275
22988	1.17042	2.08493
22989	1.25858	0.05
22990	0.93106	0.05
22991	0.810959	0.05
22992	1.38905	2.1
22993	1.19216	2.1
22994	0.946131	2.1
22995	0.946131	2.1
22996	1.19216	2.1
22997	1.58339	2.1
22998	0.81094	0.05
22999	0.845129	0.05
23000	0.918535	0.05
23001	0.946132	2.1
23002	0.946132	2.1
23003	1.31904	2.1
23004	1.31904	2.1
23005	1.21275	2.1
23006	1.15471	2.1
23007	1.33125	2.1
23008	0.702954	0.05
23009	0.942973	0.05
23010	0.94613	0.05
23011	0.641772	0.05
23012	0.702942	0.05
23013	0.991865	0.0942634
23014	0.639952	0.05
23015	1.17333	0.05
23016	0.946132	0.05
23017	0.490975	0.05
23018	0.495934	0.155186
23019	1.19904	0.0563758
23020	1.30488	0.0514347
23021	1.02305	0.0901376
23022	1.49842	0.0947892
23023	1.54196	2.1

23024	1.37224	1.73071
23025	1.0953	0.0616824
23026	1.18952	2.1
23027	1.68851	0.279215
23028	0.946131	0.117662
23029	1.12491	0.131614
23030	0.583591	0.124775
23031	1.28727	0.155352
23032	1.12491	0.146712
23033	0.884641	0.421518
23034	0.810944	0.184328
23035	0.917973	0.255304
23036	0.846671	0.2108
23037	0.596968	0.154527
23038	0.716347	0.267565
23039	1.36255	0.0908735
23040	1.11077	2.1
23041	0.946132	2.1
23042	0.946132	2.1
23043	1.26267	2.1
23044	1.26441	2.1
23045	1.85238	2.1
23046	1.36693	2.1
23047	0.616123	2.1
23048	0.481844	2.1
23049	0.709599	2.1
23050	0.815889	2.1
23051	1.1502	2.1
23052	1.63937	1.89993
23053	1.25797	0.0844771
23054	1.22878	0.100402
23055	0.946132	0.113064
23056	0.946132	0.0927515
23057	0.965737	0.118844
23058	1.1893	0.107696
23059	1.22877	0.102568
23060	0.735217	0.12349
23061	0.85154	0.123259
23062	0.851553	0.111067
23063	0.851473	0.127383
23064	1.58916	0.0865918
23065	1.25545	0.079878
23066	0.810942	0.165628
23067	0.919314	0.169222
23068	0.928633	0.143086
23069	2.14514	0.0932499
23070	1.23389	2.1
23071	0.946128	0.059466
23072	0.969504	0.05
23073	0.851539	0.0552599
23074	1.41503	0.0564625
23075	0.958517	0.0518316
23076	0.990615	0.05
23077	1.7943	0.05
23078	0.946133	0.362453
23079	1.27581	0.437714
23080	0.946132	0.379276
23081	1.36362	0.306244

23082	1.36936	0.289012
23083	1.71903	2.1
23084	0.751639	2.1
23085	0.77464	2.1
23086	0.946133	2.1
23087	1.04073	2.1
23088	1.23551	2.1
23089	0.94613	2.1
23090	1.18682	2.1
23091	0.946131	2.1
23092	0.94613	2.1
23093	1.40638	2.1
23094	0.483346	0.205647
23095	0.432527	0.148377
23096	0.946132	0.23448
23097	0.483312	0.191741
23098	0.946132	0.190319
23099	0.878798	0.25444
23100	0.810944	0.249458
23101	1.14604	0.203185
23102	0.946129	0.238735
23103	1.35594	0.197522
23104	0.538378	0.199285
23105	0.946131	0.249747
23106	0.538378	0.232788
23107	1.00065	1.92269
23108	0.962878	0.830576
23109	1.32634	0.0680343
23110	1.28066	0.05
23111	0.785651	2.1
23112	1.20327	2.1
23113	1.47058	2.00664
23114	0.551834	1.70516
23115	1.04072	2.08329
23116	0.506719	1.8644
23117	1.12786	1.92254
23118	1.45789	0.05
23119	0.681532	0.05
23120	1.21087	0.05
23121	0.94613	0.05
23122	0.681534	0.05
23123	1.21087	0.05
23124	1.98618	0.654323
23125	1.71742	1.48235
23126	1.07427	0.228861
23127	1.1972	0.178802
23128	0.933474	0.218514
23129	1.1745	0.257829
23130	0.94612	0.232086
23131	0.933485	0.230708
23132	1.17449	0.227282
23133	0.851541	0.05
23134	1.12774	0.05
23135	0.946129	0.05
23136	0.851486	0.05
23137	1.12517	0.05
23138	1.12328	0.05
23139	1.13179	2.1

23140	1.02438	2.1
23141	1.05776	2.1
23142	1.41273	1.99904
23143	1.1229	2.1
23144	0.875912	2.08513
23145	0.946132	2.1
23146	0.875912	2.1
23147	1.42307	2.1
23148	1.15354	0.05
23149	0.848382	0.05
23150	1.2685	2.1
23151	0.938104	2.1
23152	0.793166	2.1
23153	1.07855	2.1
23154	0.98488	2.1
23155	1.4162	2.1
23156	1.09651	0.113345
23157	1.98502	0.0607068
23158	1.41168	0.164814
23159	1.33879	0.200379
23160	0.946127	0.251064
23161	1.13528	0.165279
23162	0.946128	0.188282
23163	1.09726	2.1
23164	1.22026	1.78126
23165	1.09044	2.1
23166	2.23961	0.434722
23167	1.14721	0.206096
23168	1.17614	2.1
23169	0.615065	2.1
23170	0.946131	2.1
23171	0.778488	2.1
23172	0.94613	2.1
23173	0.615066	2.1
23174	1.78254	2.1
23175	0.898452	0.05
23176	1.47411	2.1
23177	0.59676	0.246081
23178	0.59676	0.174975
23179	0.94613	0.260724
23180	1.13511	0.225315
23181	0.946131	0.203652
23182	0.916275	2.1
23183	0.946132	2.03958
23184	0.916274	1.5191
23185	1.36182	1.84774
23186	0.946131	1.97736
23187	0.857202	0.05
23188	0.941918	0.05
23189	0.943349	0.05
23190	1.2493	0.05
23191	0.946133	0.05
23192	1.01814	0.05
23193	0.927881	0.05
23194	1.11845	0.05
23195	1.2493	0.05
23196	1.11845	0.05
23197	0.946129	0.05

23198	0.967673	0.380885
23199	1.01332	0.05
23200	0.946133	0.05
23201	0.81938	0.05
23202	0.819377	0.05
23203	0.946133	0.05
23204	1.22474	0.103236
23205	1.15321	0.0829537
23206	0.797489	0.0825243
23207	0.946131	0.102462
23208	0.797461	0.086169
23209	1.35328	1.24321
23210	1.03318	1.36317
23211	0.946131	1.27489
23212	0.94613	1.28793
23213	1.03319	1.28767
23214	1.37938	0.864914
23215	1.00512	1.34764
23216	0.946133	1.15051
23217	0.946132	1.21843
23218	1.00512	1.28946
23219	1.5515	1.31575
23220	1.55351	2.1
23221	0.94261	0.05
23222	0.828156	0.05
23223	0.916495	0.05
23224	3.06309	0.05
23225	0.619832	0.05
23226	0.705903	0.05
23227	1.2968	0.05
23228	0.946133	0.05
23229	0.705904	0.05
23230	1.2968	0.05
23231	1.01971	0.05
23232	0.60302	0.05
23233	1.07242	0.469624
23234	1.26426	0.276672
23235	0.946131	0.490254
23236	0.946131	0.51756
23237	0.963109	0.477982
23238	0.947862	0.365844
23239	1.40248	1.47213
23240	0.767143	1.48532
23241	0.767114	1.49656
23242	0.946129	1.50886
23243	0.767143	1.32517
23244	1.22637	1.53206
23245	1.2281	1.46917
23246	0.590909	1.46289
23247	0.490832	1.46332
23248	0.804174	1.50514
23249	0.747368	1.41218
23250	1.05129	0.0661443
23251	1.10609	2.06648
23252	1.3639	0.102297
23253	0.776117	0.0831048
23254	0.752329	0.105639
23255	1.04073	0.140284

23256	0.67713	0.1127
23257	3.4245	2.1
23258	1.18526	2.1
23259	1.2161	1.63325
23260	0.876834	1.83043
23261	0.876831	1.64115
23262	0.946132	1.73968
23263	0.876834	1.72789
23264	0.954563	2.1
23265	0.924132	2.1
23266	1.25075	2.1
23267	1.01652	2.1
23268	0.92413	2.1
23269	1.37579	2.1
23270	0.992159	2.1
23271	1.26092	2.1
23272	0.929435	2.1
23273	0.844958	2.1
23274	0.79598	2.1
23275	0.852559	2.1
23276	0.946131	0.771636
23277	1.28435	0.395458
23278	0.827261	0.596239
23279	0.946128	0.856849
23280	0.827298	0.881311
23281	1.65452	0.17136
23282	1.79929	2.1
23283	0.791947	0.49005
23284	0.902072	1.14588
23285	0.882672	0.496791
23286	1.21703	2.1
23287	0.900338	2.1
23288	1.37712	2.1
23289	0.782591	2.1
23290	0.782562	2.1
23291	0.94613	2.1
23292	1.34749	2.1
23293	0.61359	2.1
23294	1.14397	1.19513
23295	1.02963	0.05
23296	1.36589	0.05
23297	0.946132	0.0641885
23298	1.28644	0.0522364
23299	0.946131	0.0594908
23300	1.40104	0.060499
23301	1.28644	0.0541592
23302	0.810946	0.103148
23303	1.09178	0.0985913
23304	1.26956	0.0842913
23305	0.810943	0.102189
23306	0.885633	0.103606
23307	1.00844	0.0909441
23308	0.825671	0.109276
23309	0.792093	0.100638
23310	0.925037	0.0992599
23311	0.946131	1.89184
23312	0.946131	0.258133
23313	1.18052	0.656169

23314	1.40265	0.565844
23315	0.946132	1.40482
23316	1.17891	0.494324
23317	0.734248	0.05
23318	0.810943	0.05
23319	0.731621	0.05
23320	0.799857	0.315822
23321	0.834507	0.575916
23322	0.810932	0.310273
23323	1.01316	0.05
23324	1.23884	0.05
23325	1.88438	2.1
23326	0.946129	2.1
23327	1.38614	1.97235
23328	1.34245	1.82045
23329	0.946131	1.60171
23330	1.38614	1.80248
23331	0.946132	0.05
23332	0.910733	0.05
23333	1.24726	0.05
23334	1.13742	0.05
23335	0.851555	0.05
23336	1.50368	0.05
23337	0.94613	2.1
23338	1.08998	2.1
23339	1.36899	1.97086
23340	1.36898	2.1
23341	0.946131	2.1
23342	1.26045	2.1
23343	1.06432	0.946206
23344	1.89016	0.351245
23345	0.83514	2.1
23346	1.36653	2.1
23347	1.23426	2.1
23348	0.94613	2.1
23349	0.835112	2.1
23350	1.14567	1.79226
23351	1.12096	1.82651
23352	0.881106	1.82101
23353	1.17787	0.674068
23354	1.29738	2.1
23355	0.940472	1.50733
23356	1.03452	1.73939
23357	0.946131	1.28265
23358	2.7489	1.20307
23359	1.01778	2.1
23360	1.04321	1.4261
23361	1.19352	0.145233
23362	1.16912	1.64065
23363	0.946132	1.50699
23364	0.946132	1.66773
23365	1.40924	1.59934
23366	1.22927	1.7155
23367	2.09455	0.0830073
23368	3.00575	2.1
23369	1.08928	2.1
23370	1.94799	2.1
23371	1.4092	2.1

23372	0.946132	2.1
23373	1.16	2.1
23374	1.01985	2.1
23375	0.946133	2.1
23376	1.16	2.1
23377	1.15371	0.05
23378	1.56002	0.05
23379	1.23007	2.1
23380	1.9992	2.1
23381	0.652605	2.1
23382	0.937903	2.1
23383	0.946132	2.1
23384	0.937902	2.1
23385	1.228	2.1
23386	1.33694	1.97885
23387	1.00191	2.1
23388	0.716814	2.02817
23389	0.94613	2.1
23390	0.716816	2.1
23391	1.70388	1.53968
23392	0.946129	0.154754
23393	1.09362	0.129365
23394	0.946131	0.328884
23395	1.4185	0.261626
23396	1.09362	0.139974
23397	1.31057	1.73623
23398	1.17631	0.0553551
23399	1.19404	0.0607776
23400	0.94613	0.0951205
23401	0.946129	0.0685616
23402	0.99972	0.0749955
23403	0.94613	0.0765503
23404	0.835199	0.05
23405	1.23351	0.05
23406	0.582785	0.05
23407	0.701079	0.05
23408	0.946132	0.05
23409	2.17385	0.05
23410	1.18948	2.07113
23411	1.25346	2.1
23412	1.18586	0.405642
23413	0.880876	0.375465
23414	0.68632	0.816858
23415	0.946118	2.1
23416	0.966936	2.1
23417	0.946117	2.1
23418	1.41636	2.1
23419	1.03798	2.1
23420	0.729178	0.491043
23421	0.721888	0.462958
23422	0.946132	0.533774
23423	0.656286	0.381845
23424	1.13898	0.502654
23425	1.02351	0.0690168
23426	1.48803	0.0856976
23427	0.946131	0.0726857
23428	1.04921	2.1
23429	1.02478	1.50143

23430	0.993023	2.1
23431	1.80841	0.126279
23432	1.29215	0.0946518
23433	0.904206	0.109179
23434	0.904207	0.117465
23435	0.946132	0.124323
23436	0.904207	0.11149
23437	1.07347	2.1
23438	0.555875	1.77397
23439	0.555904	1.80039
23440	1.30841	1.92154
23441	1.30841	1.91826
23442	0.91951	1.96386
23443	1.33026	0.610709
23444	1.43289	0.05
23445	1.15703	1.81191
23446	0.886594	1.95279
23447	1.05129	1.84709
23448	1.08421	0.418819
23449	1.35946	0.250708
23450	0.882955	1.03456
23451	0.946131	0.985548
23452	0.882985	0.719159
23453	0.583501	0.7682
23454	0.532921	0.773678
23455	0.94613	1.84286
23456	0.532892	1.66653
23457	1.23224	1.70545
23458	1.12786	1.6908
23459	0.946131	2.1
23460	1.23732	0.320985
23461	1.40609	2.1
23462	1.40609	1.03477
23463	0.946131	0.865932
23464	0.910672	1.54372
23465	1.08478	1.38709
23466	0.94613	1.09335
23467	0.94613	1.5187
23468	1.30382	1.5531
23469	3.10544	1.46903
23470	0.679509	1.94004
23471	1.15966	1.73453
23472	0.75499	2.01047
23473	1.37511	1.75561
23474	1.01776	1.76098
23475	2.35239	1.34476
23476	0.92406	2.1
23477	0.946118	2.1
23478	0.92406	2.1
23479	1.07979	2.1
23480	1.29604	2.1
23481	1.29897	2.1
23482	0.902645	0.41619
23483	0.729493	1.3019
23484	0.906844	1.08675
23485	0.739518	0.05
23486	1.10564	0.0682499
23487	0.946131	0.05

23488	0.739519	0.0573422
23489	0.739506	0.05
23490	1.79907	0.155092
23491	1.59106	1.14732
23492	1.11067	0.481777
23493	0.795516	1.09451
23494	0.795519	0.75895
23495	0.946132	0.789172
23496	0.795506	0.73823
23497	1.80223	2.1
23498	1.95572	2.0582
23499	1.16237	1.50965
23500	1.07518	2.1
23501	0.696199	1.73947
23502	0.94613	1.8592
23503	0.696199	1.80807
23504	1.6379	0.05
23505	0.946118	0.05
23506	0.851529	0.05
23507	1.08716	0.05
23508	0.973118	0.05
23509	0.851528	0.05
23510	1.15096	2.1
23511	0.65023	0.05
23512	0.94613	0.05
23513	0.650205	0.05
23514	0.650205	0.05
23515	0.811233	0.05
23516	0.778721	1.90763
23517	0.810945	1.53074
23518	0.997518	1.59705
23519	0.838028	0.0708757
23520	0.952893	0.0639106
23521	0.838037	0.0706472
23522	1.58166	0.061551
23523	1.44848	1.36562
23524	1.30041	0.05
23525	1.03697	0.644441
23526	0.946132	1.81149
23527	1.0277	1.67293
23528	0.946131	1.91396
23529	1.00501	1.64717
23530	1.0277	1.78969
23531	1.64988	2.1
23532	0.946131	2.1
23533	0.94613	2.01756
23534	1.27702	2.1
23535	1.55163	2.1
23536	0.897905	2.1
23537	1.12971	0.05
23538	1.35715	0.132469
23539	0.881884	0.648231
23540	0.664007	0.158395
23541	0.663978	0.233172
23542	0.94613	0.209569
23543	0.664006	0.252503
23544	1.48693	0.104989
23545	1.29289	0.0613107

23546	1.32081	0.0725203
23547	0.946119	0.0848876
23548	0.946132	0.0757066
23549	1.02972	0.0827391
23550	0.946131	2.1
23551	0.946127	2.1
23552	1.23513	2.1
23553	1.38154	2.1
23554	0.94613	2.1
23555	1.25933	0.05
23556	0.917954	0.05
23557	0.868589	0.0524106
23558	3.0957	2.1
23559	0.711391	0.05
23560	0.711391	0.05
23561	0.991303	0.05
23562	1.37322	0.05
23563	0.729145	0.05
23564	2.55648	0.05
23565	1.0756	1.85123
23566	0.760299	1.50899
23567	0.763592	1.01246
23568	0.946126	1.42798
23569	0.76359	1.07762
23570	1.35862	1.59134
23571	0.946133	2.1
23572	1.00807	2.1
23573	0.946132	2.1
23574	1.39823	2.1
23575	1.07817	2.1
23576	1.44379	0.405419
23577	1.17934	0.05
23578	1.13451	0.0953413
23579	0.94613	2.1
23580	1.03974	2.1
23581	1.36835	2.1
23582	1.36835	2.1
23583	0.94613	2.1
23584	1.27096	2.1
23585	0.853042	2.1
23586	0.946131	2.1
23587	1.13658	2.1
23588	1.06997	2.1
23589	0.94613	2.1
23590	1.41556	0.326916
23591	1.21708	0.708508
23592	0.94613	0.956624
23593	0.946131	0.622327
23594	1.21708	0.567657
23595	0.964782	0.353831
23596	0.946119	0.725896
23597	1.23447	0.502384
23598	0.946118	0.71556
23599	1.41844	0.429813
23600	1.23446	0.519554
23601	1.1357	0.271671
23602	0.619856	0.05
23603	1.1509	0.05

23604	0.946126	0.05
23605	1.19262	0.05
23606	1.1509	0.05
23607	1.04268	2.1
23608	2.07527	1.12866
23609	1.38259	1.45842
23610	0.980054	1.59647
23611	0.946124	1.47846
23612	0.946127	1.52445
23613	0.980056	1.52282
23614	1.62916	1.80469
23615	0.946131	2.1
23616	1.40571	2.1
23617	0.946131	2.1
23618	1.38885	2.1
23619	1.40571	2.1
23620	1.11787	2.1
23621	0.946129	2.1
23622	1.28831	2.1
23623	1.41498	2.1
23624	1.41498	2.1
23625	0.946131	2.1
23626	0.850964	0.05
23627	0.921299	0.05
23628	1.37358	0.05
23629	0.946132	0.05
23630	0.850964	0.05
23631	0.823988	0.0506111
23632	0.823984	0.0522068
23633	1.36568	0.05
23634	1.36569	0.05
23635	0.921275	0.0518861
23636	1.84739	2.1
23637	1.47169	0.0713467
23638	0.946131	0.24578
23639	0.735844	0.163062
23640	1.11819	0.245295
23641	1.10904	0.116638
23642	0.735831	0.195497
23643	0.946131	2.1
23644	0.946132	2.1
23645	1.23487	2.1
23646	1.3281	2.1
23647	0.946131	2.1
23648	1.72271	0.0528501
23649	0.861359	0.0629282
23650	0.946131	0.089744
23651	0.861357	0.0718147
23652	1.1369	0.0721458
23653	1.26868	0.0709441
23654	0.789173	2.0118
23655	0.789143	2.08827
23656	0.946133	1.83136
23657	1.34386	1.93433
23658	0.789144	1.96182
23659	0.983493	2.1
23660	1.18944	0.440976
23661	1.05121	0.512919

23662	0.777398	0.430821
23663	0.946132	0.933727
23664	0.777423	0.997405
23665	1.09841	0.101796
23666	0.963919	0.0854331
23667	0.474855	0.106168
23668	0.946131	0.106015
23669	0.474855	0.102558
23670	3.40074	2.1
23671	1.29453	2.1
23672	1.01139	0.562165
23673	0.946133	1.54544
23674	1.35381	0.133286
23675	1.18322	1.25034
23676	0.946133	1.67193
23677	1.45502	0.05101
23678	0.970088	1.61697
23679	0.960333	1.64137
23680	0.838036	1.64068
23681	1.30927	0.112313
23682	0.828617	0.120002
23683	0.714795	0.116964
23684	0.946132	0.121872
23685	0.714824	0.113855
23686	0.946131	2.1
23687	0.851542	2.1
23688	1.38204	2.1
23689	1.24512	2.1
23690	1.04086	2.1
23691	0.723573	2.1
23692	0.946131	2.1
23693	0.982454	2.1
23694	0.982454	2.1
23695	0.723544	2.1
23696	1.17261	0.0635007
23697	1.34153	0.0629045
23698	1.41383	0.0597724
23699	0.946132	0.128832
23700	0.946133	0.0705346
23701	1.12818	0.141375
23702	1.29383	0.0956491
23703	0.946133	0.511579
23704	1.0027	0.458065
23705	0.768989	0.327225
23706	0.946133	0.519103
23707	0.768977	0.476609
23708	0.946132	0.881731
23709	1.24333	0.49703
23710	0.928583	0.866751
23711	0.946132	0.903916
23712	0.928583	0.958771
23713	1.53798	0.192597
23714	2.95436	0.0834346
23715	1.30649	0.113041
23716	0.809789	0.191893
23717	0.745744	0.172192
23718	0.745774	0.173016
23719	0.946128	0.162131

23720	1.00777	0.0719633
23721	1.40271	0.0608687
23722	0.768387	0.0783863
23723	0.946132	0.073613
23724	0.922045	0.0731961
23725	0.946132	2.1
23726	1.39021	2.1
23727	1.10046	2.1
23728	0.946132	2.1
23729	1.39021	2.1
23730	2.06158	0.0513092
23731	0.913967	0.365072
23732	1.30107	0.381263
23733	0.946132	0.473355
23734	0.913966	0.10848
23735	1.30107	0.335567
23736	1.64117	2.1
23737	0.514872	1.88944
23738	0.514846	1.71957
23739	1.30811	2.1
23740	1.30812	1.78672
23741	0.888989	1.88444
23742	1.99279	1.9833
23743	0.910271	0.537537
23744	0.838027	0.855141
23745	0.928444	0.549849
23746	1.2377	1.49747
23747	0.851537	2.1
23748	0.899142	2.1
23749	0.810075	2.1
23750	1.3771	2.1
23751	1.03281	2.1
23752	0.719877	2.1
23753	0.901838	2.1
23754	1.01748	2.1
23755	1.12315	2.1
23756	0.64787	2.1
23757	1.173	2.1
23758	0.876903	2.1
23759	0.597337	2.1
23760	1.00291	2.1
23761	0.597309	2.1
23762	1.09897	0.325326
23763	0.946132	0.835716
23764	1.1386	0.302814
23765	1.09897	0.41489
23766	0.596652	0.420807
23767	2.5593	2.1
23768	0.946133	0.386902
23769	1.29172	0.366739
23770	1.11337	0.157046
23771	0.946131	0.368443
23772	0.946131	0.263193
23773	0.96926	1.20024
23774	0.946132	1.18147
23775	0.946132	1.2973
23776	1.07118	1.10348
23777	1.29698	1.14629

23778	1.37867	1.33801
23779	0.946119	1.41964
23780	0.94612	1.53948
23781	1.33154	1.27528
23782	0.946107	1.35873
23783	2.17757	1.34605
23784	0.946131	0.0667365
23785	0.68393	0.0555619
23786	0.68396	0.0596721
23787	1.33671	0.0635939
23788	1.10733	0.0611056
23789	2.31367	2.1
23790	0.989326	1.89304
23791	0.689952	2.1
23792	0.854787	1.80193
23793	3.73635	2.1
23794	1.41489	2.1
23795	1.25544	2.01568
23796	0.946128	2.1
23797	0.946129	2.1
23798	1.13536	2.1
23799	2.10284	1.78428
23800	0.851542	2.1
23801	0.828523	2.1
23802	0.946132	2.1
23803	0.946132	2.1
23804	1.50873	2.1
23805	0.989048	2.1
23806	0.925055	2.1
23807	0.838041	2.1
23808	0.807714	0.760229
23809	0.656831	0.392943
23810	0.997603	0.628027
23811	0.946131	1.52966
23812	1.02602	1.43313
23813	0.946129	1.5313
23814	1.35469	1.28456
23815	1.25083	1.45198
23816	0.851541	0.253839
23817	0.851483	0.254793
23818	0.851539	0.261928
23819	1.19785	0.224501
23820	1.07615	0.216585
23821	0.714507	0.0799275
23822	1.41461	0.0762519
23823	0.714507	0.0787799
23824	0.714507	0.0756453
23825	0.946133	0.0836196
23826	0.946131	1.46527
23827	0.972232	1.32738
23828	0.94613	1.50773
23829	1.25083	0.87903
23830	1.30165	1.31214
23831	1.05332	2.1
23832	0.574768	2.1
23833	1.94541	0.05
23834	0.789633	0.05
23835	0.946127	0.0501507

23836	1.33798	0.05
23837	1.22903	0.05
23838	0.946128	0.054248
23839	0.946128	2.1
23840	1.11882	2.1
23841	0.996487	2.1
23842	0.94613	2.1
23843	1.11883	2.1
23844	0.946127	2.1
23845	1.30082	2.1
23846	1.02608	2.1
23847	0.946128	2.1
23848	1.30081	2.1
23849	1.3507	1.84631
23850	1.19583	0.191867
23851	0.946127	1.75261
23852	0.94613	1.27774
23853	1.19583	1.6417
23854	0.477652	0.05
23855	1.20416	0.05
23856	0.946131	0.05
23857	0.477652	0.05
23858	0.477691	0.05
23859	1.34536	0.05
23860	0.758971	0.0520331
23861	1.25124	0.05
23862	1.31467	0.0501026
23863	0.758971	0.05
23864	1.31467	0.0565778
23865	0.946131	2.1
23866	1.14184	2.1
23867	1.35028	2.1
23868	1.35028	2.1
23869	0.946131	2.1
23870	1.13537	2.08335
23871	0.946132	1.73633
23872	0.946132	2.0853
23873	1.23031	1.86822
23874	1.27555	1.90162
23875	1.53064	1.83705
23876	1.28421	1.27788
23877	1.07707	1.45594
23878	0.946125	1.30913
23879	0.946127	1.34388
23880	1.07707	1.36267
23881	0.946133	0.075697
23882	1.3495	0.0711618
23883	0.946132	0.0811757
23884	1.05704	0.0696961
23885	1.3495	0.0726226
23886	0.946132	0.05
23887	1.37794	0.05
23888	0.946132	0.05
23889	1.01367	0.05
23890	1.37794	0.05
23891	0.928516	2.1
23892	0.946133	2.1
23893	0.928518	2.1

23894	1.14105	2.1
23895	1.26406	2.1
23896	0.797664	2.1
23897	0.946132	2.1
23898	0.797663	2.1
23899	1.34285	2.1
23900	0.929875	2.1
23901	0.946131	2.1
23902	1.21139	0.812707
23903	1.41492	0.910532
23904	1.41492	1.34024
23905	0.946131	0.269215
23906	0.946131	2.1
23907	0.689388	2.1
23908	0.94365	2.1
23909	0.777345	2.1
23910	0.709599	2.1
23911	0.774968	2.1
23912	0.946132	2.1
23913	0.77497	2.1
23914	0.868145	2.1
23915	1.28401	2.1
23916	0.94613	2.07495
23917	1.19655	2.1
23918	1.39428	0.821892
23919	1.39428	2.1
23920	0.946131	2.1
23921	1.1848	2.1
23922	1.07591	2.1
23923	0.946131	2.1
23924	0.946131	2.1
23925	0.874788	2.1
23926	1.35703	2.1
23927	1.35357	2.1
23928	0.946132	2.1
23929	1.26022	2.1
23930	0.946132	2.1
23931	0.946131	2.1
23932	0.966426	2.1
23933	0.946133	2.1
23934	0.946131	2.1
23935	1.3484	2.1
23936	1.26441	2.1
23937	1.2079	2.1
23938	0.946133	2.1
23939	0.946132	2.1
23940	1.2079	2.1
23941	1.2736	2.1
23942	1.25774	2.1
23943	0.946133	2.1
23944	0.851555	2.1
23945	0.96172	2.1
23946	0.946128	2.1
23947	1.06879	2.1
23948	0.94613	2.1
23949	1.32608	2.1
23950	1.06879	2.1
23951	0.946132	2.1

23952	1.32608	2.1
23953	0.946131	2.1
23954	1.40488	2.1
23955	1.32608	2.1
23956	0.94613	1.73004
23957	1.19234	0.694879
23958	0.946129	0.920524
23959	0.94613	1.71514
23960	1.31649	1.76846
23961	0.912059	1.83973
23962	1.04514	1.52836
23963	0.946132	1.52244
23964	0.946132	2.08077
23965	1.34646	2.04023
23966	1.01187	0.35917
23967	1.33728	1.1969
23968	0.761974	0.579714
23969	0.761974	1.13402
23970	0.94613	0.55336
23971	1.40641	2.1
23972	0.955752	0.899414
23973	0.736738	1.94215
23974	0.94613	1.62937
23975	0.736739	1.8198
23976	0.861471	2.1
23977	1.34556	2.1
23978	0.810957	2.1
23979	0.946131	2.1
23980	1.37811	2.1
23981	1.17949	2.1
23982	0.946132	2.1
23983	1.37811	2.1
23984	0.946131	2.1
23985	1.40447	2.1
23986	0.946132	2.1
23987	0.946132	2.1
23988	1.39826	2.1
23989	0.946131	2.1
23990	1.283	2.1
23991	0.94613	2.1
23992	0.946131	2.1
23993	1.40447	2.1
23994	1.20541	2.1
23995	0.946131	2.1
23996	0.946127	2.1
23997	1.33607	2.1
23998	1.41021	2.1
23999	1.01271	2.1
24000	0.946131	2.1
24001	0.920606	2.1
24002	1.13078	2.1
24003	1.60331	2.1
24004	0.946118	1.71205
24005	1.09718	2.1
24006	0.946116	2.1
24007	1.32662	1.87456
24008	1.0972	1.96026
24009	0.94613	1.33901

24010	1.32666	1.77314
24011	0.946128	1.29896
24012	1.31381	0.623998
24013	1.32666	0.724834
24014	0.94612	2.1
24015	0.940748	2.1
24016	1.03561	2.1
24017	1.35111	2.1
24018	0.851527	2.1
24019	0.607156	2.1
24020	0.789158	2.1
24021	0.946129	2.1
24022	1.04071	2.1
24023	1.13144	2.1
24024	0.946133	2.1
24025	1.35663	2.1
24026	0.946132	2.1
24027	0.946133	2.1
24028	1.34237	2.1
24029	0.919455	2.1
24030	1.01143	2.1
24031	0.946128	2.1
24032	0.946131	2.1
24033	1.62796	2.1
24034	1.17796	1.81419
24035	1.23024	2.1
24036	0.946132	1.83325
24037	0.85153	1.94248
24038	0.986867	1.92902
24039	0.889308	2.1
24040	0.94613	2.1
24041	0.889338	2.1
24042	0.946131	2.1
24043	0.889337	2.1
24044	1.18396	1.3298
24045	0.94613	1.18926
24046	0.946125	1.40025
24047	1.39514	1.22337
24048	1.30809	1.34044
24049	0.803322	2.05916
24050	0.925893	1.83508
24051	1.36183	1.57592
24052	0.925892	1.68619
24053	0.946133	1.66999
24054	1.38354	0.106306
24055	1.16009	0.100946
24056	0.946132	0.115319
24057	0.946132	0.0972325
24058	1.01462	0.0755157
24059	1.18854	1.42137
24060	0.888265	1.66548
24061	0.745095	1.53764
24062	0.709597	1.60707
24063	0.936541	1.60106
24064	1.24142	0.54177
24065	1.35059	0.373049
24066	0.946128	0.347488
24067	0.94613	0.466316

24068	1.16623	0.439024
24069	1.24945	0.0966998
24070	1.33035	0.10545
24071	0.946127	0.114005
24072	0.946128	0.0886017
24073	1.13343	0.145376
24074	0.637995	1.45402
24075	1.10455	1.46526
24076	1.22641	1.52087
24077	1.22641	1.46359
24078	0.946132	1.4324
24079	1.3665	0.0981112
24080	1.28507	0.0988213
24081	0.946127	0.112189
24082	0.946129	0.0938993
24083	1.2051	0.134041
24084	0.797374	2.1
24085	0.797376	2.1
24086	1.13113	2.1
24087	1.20057	2.1
24088	1.12049	2.1
24089	0.59387	1.24224
24090	1.13245	1.22515
24091	0.966134	1.32476
24092	0.593842	1.20408
24093	0.946128	1.27001
24094	0.641455	0.774801
24095	0.916507	0.534339
24096	1.04072	1.43201
24097	0.415585	0.602329
24098	0.507244	1.34947
24099	0.678938	2.0731
24100	1.24154	2.06562
24101	1.25	1.90857
24102	1.24154	1.96155
24103	1.25	1.98087
24104	0.851009	1.88431
24105	0.946131	1.98205
24106	0.851539	1.82901
24107	1.21997	1.8073
24108	1.45109	1.83301
24109	0.804877	1.06237
24110	1.00062	0.356973
24111	0.851536	0.931962
24112	0.909673	0.394201
24113	0.946129	0.888193
24114	1.20494	0.122945
24115	1.09088	0.162336
24116	0.887634	0.17849
24117	0.81027	0.0962228
24118	0.900294	0.11607
24119	0.747713	0.0568272
24120	0.975467	0.0582022
24121	0.747712	0.0555063
24122	1.39168	0.05
24123	0.975467	0.0534965
24124	0.94613	0.102046
24125	1.32827	0.106598

24126	0.946128	0.0970718
24127	1.41201	0.105859
24128	1.32827	0.100054
24129	0.981121	0.119578
24130	0.936311	0.196891
24131	1.00715	0.117245
24132	1.15914	0.144296
24133	0.704409	0.134737
24134	0.713444	0.803889
24135	0.954538	0.851809
24136	0.944508	0.675246
24137	0.851165	1.42133
24138	0.946131	1.54694
24139	0.851192	1.32878
24140	0.946131	1.19954
24141	1.3279	1.10071
24142	1.33587	0.0535625
24143	0.946126	0.06054
24144	1.27512	0.057911
24145	1.33587	0.058268
24146	0.748059	0.0528004
24147	1.17451	0.0595395
24148	1.04299	0.0529206
24149	0.851542	0.0642263
24150	0.946131	0.0599681
24151	0.892109	0.0618945
24152	0.946131	0.05
24153	0.851541	0.05
24154	1.2178	0.05
24155	1.2179	0.05
24156	0.946793	0.05
24157	0.709599	0.05
24158	0.987426	0.05
24159	1.39502	0.05
24160	1.13624	0.05
24161	0.73176	0.05
24162	1.36697	0.05
24163	1.33393	0.05
24164	0.946131	0.0505333
24165	0.946132	0.05
24166	1.23845	0.05
24167	0.50702	1.08614
24168	1.06442	0.40608
24169	0.946131	1.10928
24170	1.02363	0.921805
24171	1.13537	0.05
24172	1.25049	0.05
24173	0.94613	0.05
24174	1.14993	0.05
24175	1.38056	0.05
24176	0.946126	0.05
24177	1.24678	0.05
24178	0.684521	0.05
24179	0.946124	0.05
24180	0.684518	0.05
24181	0.851542	0.326304
24182	0.925459	0.282224
24183	0.946131	0.330302

24184	0.851474	0.340281
24185	0.923305	0.324515
24186	1.4055	0.05
24187	1.32077	0.05
24188	0.946127	0.05
24189	1.21757	0.05
24190	0.946129	0.05
24191	0.711214	0.0575139
24192	1.30463	0.0670586
24193	0.711185	0.0629554
24194	0.711185	0.0660495
24195	1.22716	0.0619707
24196	1.29958	0.05
24197	1.26201	0.05
24198	0.568927	0.05
24199	0.568927	0.05
24200	0.946132	0.05
24201	0.851542	0.0782704
24202	1.44968	0.0658649
24203	0.851543	0.0729108
24204	1.35123	0.0761309
24205	1.40427	0.0666819
24206	0.593295	0.0887613
24207	0.946129	0.0950297
24208	1.22144	0.0806599
24209	0.908278	0.102872
24210	1.22144	0.0913708
24211	0.867593	0.219105
24212	1.23351	0.170126
24213	0.946131	0.2186
24214	1.05516	0.216946
24215	1.23351	0.181998
24216	1.15564	0.0698067
24217	0.946131	0.119347
24218	0.766652	0.0821785
24219	0.766681	0.0783109
24220	0.946131	0.097183
24221	0.946131	0.135327
24222	0.897851	0.120677
24223	1.1751	0.158092
24224	1.00273	0.120099
24225	0.851554	0.127088
24226	0.883272	0.105968
24227	1.17111	0.897954
24228	0.851541	0.141293
24229	1.01665	0.175334
24230	0.851555	0.140762
24231	0.946131	0.393032
24232	0.911508	0.246783
24233	1.08054	0.423521
24234	1.33007	0.187765
24235	0.911471	0.313029
24236	1.26787	1.55747
24237	1.16641	1.71935
24238	0.946132	1.51037
24239	0.946133	1.56087
24240	1.16641	1.60575
24241	0.946131	2.1

24242	1.25037	0.638883
24243	1.34733	2.1
24244	1.34733	2.1
24245	0.946131	2.1
24246	0.946131	0.05
24247	1.41377	0.05
24248	0.94613	0.05
24249	1.30541	0.05
24250	1.41377	0.05
24251	1.3473	2.1
24252	1.11804	2.1
24253	0.94613	2.1
24254	0.946131	2.1
24255	1.11805	2.1
24256	1.32068	2.1
24257	0.946131	2.1
24258	0.946128	2.1
24259	1.39384	2.1
24260	1.4181	2.1
24261	1.12913	2.1
24262	0.94613	2.1
24263	0.946129	2.1
24264	1.20328	2.1
24265	1.39385	2.1
24266	1.3335	2.1
24267	1.40987	2.1
24268	0.94613	2.1
24269	1.21474	2.1
24270	0.94613	2.1
24271	0.946132	2.1
24272	1.15203	2.1
24273	1.41192	2.1
24274	1.41192	2.1
24275	0.946132	2.1
24276	0.946131	2.1
24277	1.40684	2.1
24278	1.2597	2.1
24279	0.946131	2.1
24280	1.40684	2.1
24281	0.946131	2.1
24282	1.39113	2.1
24283	1.34049	2.06124
24284	0.946131	1.90626
24285	1.39113	1.96219
24286	0.946132	1.52646
24287	1.31649	0.390916
24288	0.946132	1.17718
24289	0.946132	1.31538
24290	1.34297	1.44799
24291	0.946131	0.365501
24292	1.41766	0.383978
24293	0.954559	0.575173
24294	0.946131	0.436764
24295	0.946131	0.167018
24296	1.25447	0.149532
24297	1.35559	0.0924642
24298	0.946127	0.133558
24299	0.946128	0.174494

24300	1.14874	0.152736
24301	1.33035	0.0890159
24302	1.3665	0.0912104
24303	0.946129	0.105267
24304	0.94613	0.0849248
24305	1.20732	0.124849
24306	0.946131	2.1
24307	0.946131	2.1
24308	1.32949	2.1
24309	1.34921	2.1
24310	0.946131	2.1
24311	0.946132	1.31964
24312	1.25479	1.00932
24313	1.38357	0.294697
24314	1.38357	0.285812
24315	0.946132	0.137807
24316	0.946131	2.1
24317	1.31034	0.396419
24318	1.37127	2.1
24319	1.37127	0.877306
24320	0.946131	1.52545
24321	1.23923	0.138982
24322	1.41169	0.196119
24323	0.94613	0.241567
24324	1.12703	0.137678
24325	0.946131	0.161357
24326	0.946119	2.1
24327	1.41915	2.1
24328	1.02679	2.1
24329	0.946107	2.1
24330	1.41917	2.1
24331	0.946133	2.1
24332	1.36581	2.1
24333	0.946131	2.1
24334	1.36581	2.1
24335	0.946131	2.1
24336	0.946132	2.1
24337	1.04384	2.1
24338	1.34973	2.1
24339	1.34967	2.1
24340	0.851555	2.1
24341	0.946132	0.0605341
24342	1.3855	0.0533375
24343	1.08603	0.0602029
24344	1.08602	0.0511959
24345	1.16234	0.0642633
24346	0.946133	0.0579936
24347	1.29348	0.0512302
24348	1.20111	0.0594772
24349	1.20112	0.0552302
24350	1.3855	0.0614787
24351	0.946128	2.1
24352	1.30798	2.1
24353	0.946129	2.1
24354	1.34828	2.1
24355	1.30797	2.1
24356	1.24932	0.05
24357	1.40549	0.05

24358	0.946117	0.05
24359	1.19615	0.05
24360	0.946106	0.05
24361	0.946131	2.1
24362	1.29926	2.1
24363	1.25144	2.1
24364	0.946131	2.1
24365	1.29925	2.1
24366	0.946132	2.1
24367	1.39826	2.1
24368	0.946133	2.1
24369	0.946133	2.1
24370	1.29926	2.1
24371	0.946119	0.0505686
24372	1.33614	0.05
24373	0.946118	0.05
24374	1.41377	0.05
24375	1.33612	0.05
24376	0.946133	1.71349
24377	1.3933	2.09706
24378	1.34999	1.41236
24379	0.946133	1.61557
24380	1.3933	1.70535
24381	0.946131	2.06948
24382	1.36139	2.1
24383	0.946132	1.81177
24384	0.946131	2.1
24385	1.39329	2.1
24386	1.37449	1.89396
24387	1.27925	0.370654
24388	0.919796	0.682317
24389	1.01176	1.46533
24390	0.94613	1.06261
24391	0.946133	2.1
24392	1.27679	2.1
24393	0.946133	2.1
24394	0.946132	2.1
24395	1.39731	2.1
24396	0.946119	2.1
24397	1.40765	1.89723
24398	1.1981	2.1
24399	0.946131	2.07403
24400	1.40764	2.1
24401	0.946127	2.1
24402	1.3557	2.1
24403	0.946127	2.1
24404	0.946128	2.1
24405	1.13485	2.1
24406	1.4008	2.1
24407	1.1587	2.1
24408	0.946131	2.1
24409	0.946131	2.1
24410	1.1587	2.1
24411	1.32082	0.0785126
24412	1.29215	0.0945582
24413	0.94613	0.106017
24414	0.946131	0.106367
24415	1.02366	0.106392

24416	1.27697	0.0592948
24417	1.2929	0.060996
24418	0.946132	0.0731874
24419	0.946132	0.0590612
24420	1.01451	0.0599066
24421	0.946132	2.1
24422	1.27252	2.1
24423	0.946132	2.1
24424	1.37869	2.1
24425	1.27252	2.1
24426	1.15627	2.1
24427	1.35703	2.1
24428	0.946132	2.1
24429	1.03783	2.1
24430	0.946132	2.1
24431	0.946128	2.1
24432	1.37869	2.1
24433	0.946131	2.1
24434	1.35357	2.1
24435	1.37869	2.1
24436	0.946132	2.1
24437	0.946131	2.1
24438	1.33471	2.1
24439	0.946131	2.1
24440	1.36373	2.1
24441	1.41342	1.17434
24442	1.06832	1.50655
24443	0.946128	1.26488
24444	0.94613	0.469132
24445	1.06832	1.30115
24446	1.26929	2.1
24447	0.946131	2.1
24448	0.946128	2.1
24449	1.41022	2.1
24450	1.37034	2.1
24451	1.37759	1.38793
24452	1.07612	1.54962
24453	0.946126	1.41507
24454	0.946129	1.45022
24455	1.07612	1.46732
24456	0.946131	2.09675
24457	0.94613	0.467197
24458	1.23848	1.10587
24459	1.38897	1.09868
24460	0.946131	1.14866
24461	0.946132	1.18442
24462	0.946133	0.36613
24463	1.34369	0.157666
24464	1.40332	0.531923
24465	0.946132	0.148134
24466	0.946131	2.1
24467	1.24023	2.1
24468	1.38244	2.1
24469	1.38244	2.1
24470	0.946131	2.1
24471	0.946132	2.1
24472	1.33468	2.1
24473	1.41619	2.1

24474	1.41619	2.1
24475	0.946131	2.1
24476	1.1908	1.44726
24477	0.946128	1.35581
24478	0.946126	1.47713
24479	1.26654	1.43948
24480	1.39514	1.57612
24481	0.93608	2.1
24482	1.04105	2.1
24483	1.39551	2.1
24484	1.52471	2.1
24485	1.04072	2.1
24486	1.33174	0.05
24487	0.946128	0.05
24488	0.94613	0.05
24489	1.17801	0.05
24490	0.94613	0.05
24491	0.946132	2.1
24492	0.946132	2.1
24493	1.29065	2.1
24494	1.29065	2.1
24495	0.975266	2.1
24496	0.946131	2.1
24497	1.26025	2.1
24498	0.955601	2.1
24499	0.946131	2.1
24500	1.26025	2.1
24501	0.94613	0.109169
24502	1.23146	0.104474
24503	0.946127	0.105516
24504	1.32827	0.100439
24505	1.23146	0.107827
24506	1.25008	0.401036
24507	1.05018	0.52876
24508	0.94613	0.350577
24509	0.932744	1.09846
24510	1.04207	0.895487
24511	0.946132	1.27112
24512	1.10023	2.1
24513	1.36748	1.2014
24514	1.36748	1.9506
24515	0.946133	1.92395
24516	1.06023	0.05
24517	1.08019	0.05
24518	0.94613	0.05
24519	1.06023	0.05
24520	0.94613	0.05
24521	1.16727	0.05
24522	1.35856	0.0544802
24523	0.946132	0.05
24524	1.16727	0.0662543
24525	0.946132	0.05
24526	0.946133	2.1
24527	0.946131	2.1
24528	1.21691	2.1
24529	1.39558	2.1
24530	0.946131	2.1
24531	1.09905	0.526297

24532	0.946132	1.42343
24533	1.3365	0.449428
24534	1.35381	0.947375
24535	0.946132	1.68797
24536	0.94613	0.715081
24537	0.946128	2.02002
24538	1.40332	1.11503
24539	1.39041	1.6618
24540	0.94613	1.20345
24541	0.946132	2.1
24542	1.3659	2.1
24543	1.39041	2.1
24544	1.39041	2.1
24545	0.946132	2.1
24546	0.840292	2.02606
24547	0.946133	2.03703
24548	0.851541	2.09228
24549	1.28464	2.02341
24550	1.28806	1.99075
24551	1.12591	1.09238
24552	1.05033	1.20765
24553	0.946132	1.13793
24554	0.946132	1.15528
24555	1.13537	1.15165
24556	1.21177	1.50051
24557	0.946132	1.33148
24558	0.946132	1.97049
24559	1.41134	1.28473
24560	1.27079	1.48433
24561	0.946131	0.052623
24562	1.35403	0.0525325
24563	0.94613	0.06829
24564	0.94613	0.0629062
24565	0.946131	0.0522933
24566	0.946131	0.128787
24567	0.946132	0.121832
24568	0.94613	0.347727
24569	1.37052	0.142329
24570	0.946131	0.184584
24571	0.946133	0.0862066
24572	0.946132	0.1725
24573	0.946133	0.116617
24574	1.24249	0.086751
24575	0.946132	0.171691
24576	1.20608	2.1
24577	0.946132	2.1
24578	0.946131	2.1
24579	0.946132	2.1
24580	0.946132	2.1
24581	0.946131	2.1
24582	0.946128	2.1
24583	0.946129	2.1
24584	1.41232	2.1
24585	0.94613	2.1
24586	0.946132	0.0505916
24587	1.32449	0.0525951
24588	0.946131	0.0580595
24589	0.946131	0.054092

24590	1.32449	0.0533915
24591	0.946116	2.1
24592	1.41563	2.1
24593	0.946118	2.1
24594	0.946105	2.1
24595	0.946131	2.1
24596	1.26563	2.1
24597	1.32819	2.03988
24598	0.946131	0.653592
24599	0.946131	2.08324
24600	0.946131	1.75594
24601	0.946131	1.92986
24602	0.946133	1.64057
24603	1.30382	2.1
24604	0.946131	1.69012
24605	0.946131	1.65979
24606	1.30382	1.69189
24607	0.946133	0.05
24608	1.15803	0.05
24609	1.28895	0.05
24610	0.954071	0.05
24611	0.946132	0.05
24612	1.0264	0.830631
24613	0.946132	1.6144
24614	0.946132	1.5092
24615	0.946131	1.55037
24616	0.946132	1.51312
24617	1.35977	0.180976
24618	0.946132	0.277303
24619	0.946129	0.279259
24620	0.94613	0.263618
24621	0.946129	0.239436
24622	0.876631	0.13154
24623	0.946133	0.161307
24624	0.946132	0.125682
24625	0.946132	0.684622
24626	0.876606	0.32199
24627	0.946131	0.0786036
24628	1.24979	0.05
24629	0.94613	0.0603395
24630	0.94613	0.05
24631	0.94613	0.0569418
24632	0.921791	0.05
24633	0.946131	0.05
24634	0.921762	0.05
24635	0.946131	0.05
24636	0.946131	0.05
24637	0.810944	0.067361
24638	0.810945	0.0783474
24639	0.810959	0.0667779
24640	0.946133	0.0866861
24641	1.25797	0.100978
24642	0.946132	0.10999
24643	0.946132	0.0667961
24644	1.25797	0.0915913
24645	1.13748	0.411336
24646	0.851512	0.770027
24647	0.94613	1.33666

24648	1.02373	0.404736
24649	0.946131	0.779498
24650	0.946122	2.1
24651	1.3852	2.1
24652	0.946124	2.1
24653	0.946118	2.1
24654	1.38521	2.1
24655	0.946131	0.318089
24656	0.946131	0.285064
24657	1.30448	0.208542
24658	0.946129	0.384415
24659	0.94613	0.29832
24660	0.946133	2.1
24661	0.94613	2.1
24662	0.946131	2.1
24663	0.946131	2.1
24664	1.36503	2.1
24665	0.810947	0.05
24666	0.810947	0.05
24667	0.810934	0.05
24668	3.34066	0.05
24669	0.946133	0.669642
24670	1.4185	0.13025
24671	0.946132	0.384413
24672	0.946133	0.36876
24673	0.946132	0.367459
24674	0.94613	2.1
24675	0.946128	2.1
24676	1.03925	2.1
24677	0.944743	2.1
24678	0.947518	2.1
24679	0.946132	2.1
24680	1.20573	2.1
24681	0.94613	2.1
24682	0.94613	2.1
24683	1.20573	2.1
24684	1.20056	0.05
24685	0.946132	0.05
24686	0.946131	0.05
24687	0.946131	0.05
24688	0.946131	0.05
24689	0.94613	2.1
24690	0.946128	2.1
24691	1.38302	2.1
24692	0.94613	2.1
24693	0.94613	2.1
24694	0.827866	0.05
24695	0.913552	0.05
24696	0.716526	0.05
24697	0.82784	0.05
24698	1.20634	0.0515544
24699	0.946131	0.05
24700	0.94613	0.05
24701	1.39502	0.05
24702	0.88146	0.0520711
24703	0.946132	2.1
24704	0.946132	2.1
24705	0.946132	2.1

24706	0.946132	2.1
24707	1.39053	2.1
24708	0.946131	2.1
24709	1.40571	2.1
24710	0.946131	2.1
24711	0.946131	2.1
24712	0.946132	2.1
24713	0.94613	0.132035
24714	1.2582	0.192312
24715	0.946127	0.135572
24716	0.946129	0.15685
24717	0.946129	0.153463
24718	0.946129	1.16087
24719	0.946132	1.19
24720	0.946131	0.473028
24721	0.946131	1.23219
24722	1.29054	0.548865
24723	0.810944	0.05
24724	0.810941	0.05
24725	0.810931	0.05
24726	0.946132	1.19677
24727	0.946132	1.16047
24728	0.946132	1.25447
24729	0.946132	1.2116
24730	0.927608	1.10933
24731	0.946128	2.1
24732	0.946133	2.1
24733	0.946131	2.1
24734	0.94613	2.1
24735	1.38813	2.1
24736	0.94613	2.1
24737	0.946128	2.1
24738	0.946132	2.1
24739	1.41666	2.1
24740	0.94613	2.1
24741	0.946118	0.13225
24742	1.34151	0.101078
24743	0.946116	0.136937
24744	0.946131	0.117762
24745	1.34149	0.111422
24746	0.946132	2.1
24747	1.31889	2.1
24748	0.946132	2.1
24749	0.946132	2.1
24750	1.31889	2.1
24751	0.864997	0.05
24752	0.928981	0.05
24753	0.872219	0.05
24754	0.946127	0.685769
24755	0.946131	0.514254
24756	1.37709	0.408261
24757	0.946131	0.651018
24758	0.946129	0.495219
24759	1.21882	0.05
24760	0.938168	0.05
24761	0.938161	0.05
24762	1.06327	0.05
24763	1.33457	0.05

24764	0.946133	1.17064
24765	0.946132	1.15127
24766	0.946133	0.599644
24767	0.946132	1.31567
24768	1.14444	0.497934
24769	1.12628	0.0602907
24770	0.946132	0.0790948
24771	0.946131	0.0816939
24772	1.12628	0.0578973
24773	0.946131	0.0636847
24774	0.946132	2.1
24775	0.946132	2.1
24776	0.946133	2.1
24777	0.946132	2.1
24778	1.29322	2.1
24779	0.946131	2.1
24780	0.946132	2.1
24781	0.94613	2.1
24782	0.946131	2.1
24783	1.28441	2.1
24784	0.946118	2.1
24785	0.946119	2.1
24786	0.946117	2.1
24787	1.28075	2.1
24788	0.946105	2.1
24789	0.810945	2.1
24790	0.810942	2.1
24791	0.81093	2.1
24792	0.946127	2.1
24793	0.946127	2.1
24794	1.28228	2.1
24795	0.946129	2.1
24796	0.946132	2.1
24797	1.14042	2.1
24798	0.946132	2.1
24799	0.946133	2.1
24800	1.14042	2.1
24801	0.946133	0.109412
24802	0.946132	0.0883814
24803	1.28632	0.0739702
24804	1.28632	0.0539524
24805	0.946133	0.0818897
24806	0.946131	1.69363
24807	0.946131	1.60961
24808	0.946131	1.64413
24809	1.31817	1.7236
24810	0.946131	1.60447
24811	1.35048	2.1
24812	0.946132	2.1
24813	0.946132	2.1
24814	0.946132	2.1
24815	0.946131	2.1
24816	0.946131	2.1
24817	1.35943	2.1
24818	0.94613	2.1
24819	0.946132	2.1
24820	0.946131	2.1
24821	0.990713	2.1

24822	0.946132	2.1
24823	0.946132	2.1
24824	0.946133	2.1
24825	0.946132	2.1
24826	0.946133	1.34083
24827	1.01433	1.38926
24828	0.877931	1.47773
24829	0.96573	1.34984
24830	0.946131	1.33992
24831	0.946131	1.32525
24832	0.946133	0.819852
24833	1.39575	0.45953
24834	0.946131	0.552785
24835	0.94613	0.561594
24836	0.810943	0.488114
24837	0.810942	0.512655
24838	0.709589	0.380205
24839	0.924071	0.32821
24840	1.04293	0.246349
24841	1.15747	0.173679
24842	0.946131	0.339526
24843	1.04071	0.292361
24844	0.810946	0.3606
24845	0.810943	0.352809
24846	0.810959	0.295763
24847	0.946128	1.54293
24848	1.39696	1.71695
24849	0.94613	1.38275
24850	0.94613	1.30889
24851	1.39696	1.5464
24852	0.946131	1.33709
24853	0.946131	1.74934
24854	0.946131	1.54798
24855	0.946131	1.45555
24856	1.29735	1.62724
24857	0.946128	1.97337
24858	0.946133	0.51206
24859	0.94613	2.05154
24860	0.946131	1.78207
24861	1.33266	1.20867
24862	0.946131	2.1
24863	0.94613	1.9066
24864	0.946131	2.1
24865	1.40609	2.1
24866	0.946131	2.1
24867	0.946131	2.0108
24868	0.946133	1.99926
24869	0.946129	2.08195
24870	1.23339	2.1
24871	0.946131	1.98699
24872	0.946132	2.1
24873	0.946133	2.1
24874	0.946132	2.1
24875	1.29198	2.1
24876	0.946132	2.1
24877	0.946131	2.1
24878	1.07297	2.1
24879	0.946127	2.1

24880	0.94613	2.1
24881	1.07297	2.1
24882	0.946125	2.1
24883	0.946126	2.1
24884	0.946126	2.1
24885	1.3864	2.1
24886	0.946119	2.1
24887	0.946128	0.100537
24888	1.09411	0.0896222
24889	0.946131	0.125866
24890	0.946131	0.109306
24891	1.09411	0.097982
24892	0.946131	0.0794621
24893	1.25837	0.0708717
24894	0.946131	0.100196
24895	0.946131	0.086242
24896	1.25837	0.0768101
24897	0.810945	2.1
24898	0.810943	2.1
24899	0.810933	2.1
24900	0.94613	0.218019
24901	0.946131	0.731838
24902	0.946132	0.259294
24903	0.946129	0.265126
24904	0.946131	0.529898
24905	0.946131	0.05
24906	1.28911	0.0517437
24907	0.946129	0.057966
24908	0.946133	0.0646807
24909	0.946131	0.0609046
24910	0.94613	0.119822
24911	1.38954	0.117432
24912	0.946132	0.11611
24913	0.94613	0.121786
24914	0.946131	0.12045
24915	0.946131	1.10313
24916	0.946131	0.410319
24917	0.946131	0.312917
24918	1.37981	0.310958
24919	0.946131	0.36448
24920	0.946131	2.1
24921	1.26958	2.1
24922	0.946127	2.1
24923	0.946129	2.1
24924	1.26957	2.1
24925	0.94613	2.1
24926	1.16889	2.1
24927	0.946126	2.1
24928	0.946128	2.1
24929	1.16889	2.1
24930	0.946133	0.32401
24931	1.13511	0.228976
24932	0.946132	0.335702
24933	0.946133	0.277006
24934	0.946132	0.258778
24935	1.27575	1.74333
24936	0.946129	1.97135
24937	0.946131	1.83074

24938	0.94613	2.0009
24939	0.946131	1.90552
24940	0.946131	2.1
24941	0.946128	2.1
24942	1.41712	2.1
24943	1.41712	2.1
24944	0.94613	2.1
24945	0.946129	0.05
24946	0.946132	0.05
24947	0.946131	0.05
24948	0.946131	0.05
24949	0.946131	0.05
24950	0.979045	1.89729
24951	0.946131	1.39253
24952	0.617549	1.9419
24953	0.555844	2.06428
24954	1.04071	1.53927
24955	0.946132	1.96868
24956	0.946132	1.46024
24957	0.946131	2.07021
24958	1.22618	1.71439
24959	0.946132	1.1891
24960	0.946131	2.1
24961	1.13976	2.1
24962	0.946129	2.1
24963	0.946131	2.1
24964	1.13976	2.1
24965	0.810945	0.338504
24966	0.810943	0.619028
24967	0.810932	0.87066
24968	1.25358	1.55268
24969	0.94613	0.541086
24970	0.946128	0.957291
24971	1.25358	1.85205
24972	1.07811	1.33065
24973	0.946129	2.1
24974	1.09197	2.1
24975	0.946131	2.1
24976	0.946131	2.1
24977	1.09197	2.1
24978	0.594912	2.1
24979	0.993703	2.1
24980	1.10173	2.1
24981	0.9916	2.1
24982	0.946131	2.1
24983	0.946128	2.1
24984	0.946131	2.1
24985	0.94613	2.1
24986	0.946129	2.1
24987	0.946132	2.1
24988	1.40335	2.1
24989	0.946133	2.1
24990	0.946133	2.1
24991	1.40335	2.1
24992	0.946132	1.92356
24993	0.946132	2.1
24994	0.946132	1.94387
24995	0.946132	1.96613

24996	1.411	2.0865
24997	0.611675	0.106081
24998	0.946129	0.120554
24999	1.31678	0.0875967
25000	0.946124	0.132671
25001	0.946127	0.0897888
25002	0.946128	0.126064
25003	0.946132	0.449308
25004	0.946131	0.295228
25005	0.946132	0.46746
25006	0.946132	0.341884
25007	0.946132	0.324345
25008	0.810946	0.375819
25009	0.810943	0.395861
25010	0.81096	0.362234
25011	1.39301	0.05
25012	0.946132	0.05
25013	0.625812	0.05
25014	0.946132	0.05
25015	0.625812	0.05
25016	0.810946	1.97941
25017	0.810945	2.09868
25018	0.810931	2.1
25019	0.810943	2.1
25020	0.810944	2.1
25021	0.810958	2.1
25022	0.946131	0.151966
25023	0.946131	0.109939
25024	0.946128	0.180814
25025	0.946129	0.140601
25026	0.94613	0.116876
25027	0.946133	0.197878
25028	0.946133	0.292745
25029	0.98537	0.154403
25030	0.985371	0.0785486
25031	0.946132	0.30063
25032	0.946129	0.276552
25033	1.38752	0.188288
25034	0.946131	0.180354
25035	0.946131	0.225002
25036	1.38752	0.203903
25037	0.946119	0.146531
25038	1.33036	0.0920262
25039	0.946119	0.113776
25040	0.946132	0.113593
25041	1.33035	0.0982077
25042	1.3868	2.1
25043	0.946132	2.1
25044	0.946132	2.1
25045	0.946133	2.1
25046	0.946132	2.1
25047	0.810947	2.1
25048	0.810946	2.1
25049	0.81096	2.1
25050	0.810946	2.1
25051	0.810946	2.1
25052	0.810933	2.1
25053	0.946133	2.1

25054	1.35342	2.1
25055	0.946133	2.1
25056	0.946132	2.1
25057	1.35342	2.1
25058	0.946132	2.1
25059	0.946132	2.1
25060	1.28569	2.1
25061	0.946132	2.1
25062	0.946133	2.1
25063	0.946132	2.1
25064	1.32547	2.1
25065	0.946132	2.1
25066	0.946132	2.1
25067	1.32547	2.1
25068	0.94613	0.05
25069	0.946133	0.05
25070	1.39664	0.05
25071	1.39664	0.05
25072	0.946131	0.05
25073	0.946131	1.99338
25074	0.94613	2.1
25075	0.946132	1.81535
25076	0.946131	2.1
25077	1.41833	2.1
25078	0.946131	0.05
25079	0.946131	0.05
25080	0.946131	0.05
25081	1.26155	0.05
25082	0.946132	0.05
25083	0.810946	2.1
25084	0.810946	2.1
25085	0.810959	2.1
25086	0.94613	1.42869
25087	0.946132	0.580458
25088	1.40779	0.328506
25089	1.40779	0.462679
25090	0.94613	0.592739
25091	0.94613	0.0626008
25092	0.946129	0.0730637
25093	1.36376	0.0614246
25094	1.36376	0.0596486
25095	0.946131	0.0628257
25096	0.946128	2.1
25097	0.946127	2.1
25098	0.946131	2.1
25099	1.40756	2.1
25100	0.94613	2.1
25101	0.946131	2.04785
25102	0.946132	2.1
25103	0.94613	2.0548
25104	0.946131	2.1
25105	1.31947	2.1
25106	0.946131	2.1
25107	0.946131	2.1
25108	0.946129	2.1
25109	0.946131	2.1
25110	1.25768	2.1
25111	0.946132	0.272375

25112	0.94613	0.135758
25113	0.946131	0.131041
25114	0.946131	0.111991
25115	0.946131	0.275951
25116	0.810943	1.63571
25117	0.810942	1.8452
25118	0.81093	1.99504
25119	0.946128	1.17156
25120	0.946133	0.638886
25121	0.946131	1.30793
25122	0.946131	0.831283
25123	0.94613	1.32877
25124	0.946132	1.40227
25125	0.946131	1.19732
25126	0.946132	1.43508
25127	0.946132	1.33025
25128	0.946132	1.49084
25129	0.946119	2.1
25130	0.946119	2.1
25131	1.31523	2.1
25132	0.946132	2.1
25133	0.946107	2.1
25134	0.946133	2.1
25135	0.946132	1.69412
25136	0.946132	2.00115
25137	1.28803	2.1
25138	0.946132	2.1
25139	1.37658	1.15972
25140	0.946132	1.04251
25141	0.946133	1.24126
25142	0.946132	1.06337
25143	0.946132	1.21661
25144	0.946132	1.40253
25145	0.94613	1.40548
25146	0.94613	1.61066
25147	1.15541	1.38163
25148	0.92943	1.49298
25149	0.946126	0.05
25150	0.946129	0.05
25151	1.33849	0.05
25152	1.33849	0.05
25153	0.946129	0.05
25154	0.810946	0.05
25155	0.810944	0.05
25156	0.810959	0.05
25157	1.39027	0.0666615
25158	0.946131	0.0814581
25159	0.946131	0.0910397
25160	1.39027	0.06629
25161	0.946131	0.0710062
25162	1.40924	1.63939
25163	0.946131	1.49901
25164	0.946132	1.67144
25165	0.946132	1.46315
25166	0.946132	1.56133
25167	0.946131	2.1
25168	0.94613	2.1
25169	0.946128	2.1

25170	0.946128	2.1
25171	1.41744	2.1
25172	0.946132	0.917871
25173	0.946128	0.89873
25174	0.94613	1.15633
25175	1.37675	0.88145
25176	0.94613	1.14298
25177	0.946132	1.2847
25178	0.946132	0.386061
25179	0.946132	0.237577
25180	0.946132	0.365001
25181	0.946131	0.367201
25182	0.946132	1.14109
25183	0.946132	1.07811
25184	0.946133	1.28286
25185	1.10003	0.772737
25186	0.946132	1.1849
25187	0.946132	0.0717054
25188	0.946131	0.0788433
25189	0.946132	0.0693895
25190	0.946131	0.0801202
25191	0.946132	0.0735712
25192	0.946132	0.0959425
25193	0.946131	0.0998226
25194	0.946132	0.0855367
25195	0.946132	0.102923
25196	0.946131	0.0940801
25197	1.24094	0.0538274
25198	0.946126	0.0614123
25199	0.946128	0.055439
25200	0.946129	0.0652448
25201	0.946129	0.0552934
25202	1.2929	0.0703395
25203	0.946131	0.0990522
25204	0.946131	0.0783898
25205	0.946131	0.0961168
25206	0.946131	0.0803269
25207	0.810946	0.05
25208	0.810945	0.05
25209	0.810959	0.05
25210	0.810946	1.98562
25211	0.810943	2.00879
25212	0.810933	2.1
25213	0.946128	2.1
25214	1.18315	2.1
25215	0.94613	2.1
25216	0.94613	2.1
25217	1.18316	2.1
25218	0.946126	0.346559
25219	1.3324	0.2389
25220	0.946127	0.371608
25221	0.94613	0.252295
25222	0.946128	0.297637
25223	0.94613	2.1
25224	0.946128	2.1
25225	1.39523	2.1
25226	1.39523	2.1
25227	0.946131	2.1

25228	1.36541	2.1
25229	0.946132	2.1
25230	0.946132	2.1
25231	0.946132	2.1
25232	0.946132	2.1
25233	0.946131	2.1
25234	0.94613	1.67537
25235	0.946132	1.85842
25236	0.946131	0.407701
25237	1.39509	1.69933
25238	0.946132	2.1
25239	0.946132	2.1
25240	1.29319	2.1
25241	0.946132	2.1
25242	0.946133	2.1
25243	0.946132	0.0949994
25244	0.946131	0.163243
25245	0.946132	0.103677
25246	1.01196	0.0927077
25247	0.946132	0.145053
25248	0.946116	2.1
25249	0.946117	2.1
25250	1.38153	2.1
25251	1.38155	2.1
25252	0.946105	2.1
25253	0.946132	1.632
25254	0.946131	1.75178
25255	0.946132	1.58968
25256	1.28684	1.62551
25257	0.946132	1.56101
25258	1.10185	1.85921
25259	0.94613	1.90654
25260	0.946128	2.1
25261	0.94613	2.04795
25262	0.94613	1.9221
25263	0.810947	0.05
25264	0.810945	0.05
25265	0.810932	0.05
25266	0.946119	0.192767
25267	1.16717	0.142515
25268	0.946117	0.191523
25269	0.94613	0.132953
25270	1.16715	0.138688
25271	0.94612	0.472723
25272	0.946119	0.484556
25273	0.946119	0.37476
25274	0.946132	0.237325
25275	1.20882	0.316663
25276	1.07043	1.22648
25277	0.823268	1.14463
25278	0.810959	1.15062
25279	0.946132	1.5781
25280	1.14879	1.34633
25281	0.94613	1.56148
25282	0.946131	1.62286
25283	1.14879	1.21296
25284	1.23837	2.1
25285	0.94612	2.1

25286	0.946118	2.1
25287	0.946131	2.1
25288	0.946105	2.1
25289	0.810946	0.05
25290	0.810946	0.05
25291	0.810959	0.05
25292	0.810947	1.66032
25293	0.810947	0.257832
25294	0.810934	0.307277
25295	0.946128	2.1
25296	0.946131	2.1
25297	0.946128	2.1
25298	0.946129	2.1
25299	0.94613	2.1
25300	0.94613	2.1
25301	0.946131	2.1
25302	0.946131	2.08709
25303	0.946131	2.1
25304	0.946131	2.1
25305	0.946131	1.8399
25306	1.38585	2.1
25307	0.946128	1.88501
25308	0.94613	1.87726
25309	1.38585	1.91531
25310	0.946133	0.157473
25311	0.946133	0.1529
25312	0.946132	0.136189
25313	0.946132	0.160582
25314	0.946132	0.147497
25315	0.918871	0.0562155
25316	0.810248	0.0587544
25317	0.838037	0.0648236
25318	0.946126	0.0730161
25319	0.943647	0.0598069
25320	1.13836	0.0590691
25321	1.03802	0.0516762
25322	0.946129	0.0646811
25323	0.946133	1.37231
25324	0.946132	0.848418
25325	0.946132	2.06865
25326	1.1699	0.757604
25327	0.946132	0.780417
25328	0.946131	2.1
25329	0.946132	2.1
25330	1.37199	2.1
25331	1.37199	2.1
25332	0.946132	2.1
25333	0.946132	1.26413
25334	0.946133	0.854319
25335	0.946133	1.30054
25336	0.946132	0.860756
25337	1.38623	0.706218
25338	0.946132	2.1
25339	0.946132	2.1
25340	0.946132	2.1
25341	0.946132	2.1
25342	1.40098	2.1
25343	1.20787	0.169397

25344	1.41894	0.373456
25345	0.946133	0.308123
25346	1.01777	1.04536
25347	0.946132	2.1
25348	0.946132	2.1
25349	0.946133	2.1
25350	1.40199	2.1
25351	0.946132	2.1
25352	0.810946	1.55595
25353	0.810947	1.76364
25354	0.810934	1.63716
25355	0.946126	1.49364
25356	0.94613	1.47516
25357	0.946125	1.58302
25358	1.40247	1.44787
25359	0.946128	1.483
25360	1.25703	1.69707
25361	0.87821	1.53304
25362	0.878238	1.48475
25363	0.946119	1.77381
25364	0.946132	1.82695
25365	0.946132	0.277042
25366	0.946132	0.284416
25367	0.946131	1.24863
25368	0.946132	0.19279
25369	1.28543	0.28359
25370	0.946115	2.1
25371	1.41638	2.1
25372	0.946116	2.1
25373	0.946104	2.1
25374	0.946129	2.1
25375	0.946132	2.1
25376	1.41432	2.1
25377	0.946132	2.1
25378	0.946132	2.1
25379	0.946132	2.1
25380	0.810947	0.0704683
25381	0.810947	0.0758193
25382	0.709567	0.0729128
25383	0.851542	0.0587803
25384	1.01217	0.05
25385	0.697993	0.05
25386	0.946133	0.05
25387	0.851488	0.05
25388	0.946129	2.06273
25389	0.94613	2.1
25390	1.2254	1.91846
25391	0.946131	2.09301
25392	0.946131	1.98982
25393	0.700785	2.1
25394	0.915893	1.96458
25395	0.838013	1.87656
25396	0.760188	1.54678
25397	1.19601	0.205076
25398	0.946133	0.308466
25399	0.946132	0.313251
25400	0.946132	0.267601
25401	0.946132	0.308541

25402	0.946132	1.89222
25403	0.946133	2.1
25404	0.946132	1.97911
25405	0.946133	2.09667
25406	1.36956	2.1
25407	0.946131	2.1
25408	0.94613	0.181244
25409	0.946133	2.1
25410	1.34268	0.340006
25411	0.946131	0.901202
25412	0.946132	2.1
25413	1.25603	2.1
25414	0.946132	2.1
25415	0.946132	2.1
25416	1.25603	2.1
25417	0.810946	1.42237
25418	0.810943	1.42188
25419	0.810933	1.45772
25420	0.496545	0.421781
25421	0.805767	0.927031
25422	0.936671	0.830652
25423	0.530063	0.826294
25424	1.05954	0.729438
25425	0.810946	0.127766
25426	0.810943	0.12597
25427	0.810959	0.0995184
25428	1.16699	0.114089
25429	0.946131	0.177005
25430	0.946129	0.172414
25431	0.946131	0.14939
25432	0.946131	0.150117
25433	0.810943	1.78854
25434	0.810942	1.85041
25435	0.810933	1.79432
25436	0.946132	1.79353
25437	1.04832	1.77787
25438	0.870714	1.92695
25439	0.957819	1.93144
25440	0.94613	1.8171
25441	1.82931	2.1
25442	0.946132	0.302047
25443	0.946129	0.262978
25444	0.94613	0.327402
25445	0.946131	0.377475
25446	0.94613	0.266776
25447	0.946119	1.13153
25448	0.94612	1.22974
25449	0.94612	0.501556
25450	0.946132	0.967455
25451	1.36118	0.889091
25452	0.810947	0.138752
25453	0.810947	0.108649
25454	0.810934	0.1219
25455	0.946132	2.1
25456	0.946132	2.1
25457	0.946132	2.1
25458	1.39754	2.1
25459	0.946131	2.1

25460	0.946129	2.1
25461	0.946133	2.1
25462	0.946131	2.1
25463	0.946131	2.1
25464	1.41732	2.1
25465	0.946132	1.30886
25466	0.946128	1.79024
25467	0.94613	1.62674
25468	0.94613	1.66858
25469	1.25277	1.73514
25470	0.94613	1.61389
25471	0.946128	1.75529
25472	1.21261	1.36814
25473	0.94613	1.5948
25474	0.94613	1.5513
25475	1.39407	2.1
25476	0.946133	2.1
25477	0.94613	2.1
25478	0.946131	2.1
25479	0.946131	2.1
25480	0.946119	1.64095
25481	0.946119	1.71004
25482	0.946119	1.83742
25483	1.1537	1.75104
25484	0.946106	1.66609
25485	0.946131	2.1
25486	1.11822	2.1
25487	0.946129	2.1
25488	0.946131	2.1
25489	1.11822	2.1
25490	1.46806	0.05
25491	0.498881	0.05
25492	0.996658	0.05
25493	1.2179	0.05
25494	0.991519	0.05
25495	0.946131	0.05
25496	0.946131	0.05
25497	1.28673	0.05
25498	0.946131	0.05
25499	0.946131	0.05
25500	1.33178	0.068904
25501	0.946132	0.0850318
25502	0.946132	0.0794169
25503	0.946132	0.0866667
25504	0.946132	0.0825364
25505	0.946128	0.12573
25506	0.972061	0.0903803
25507	0.946129	0.146009
25508	0.946129	0.133243
25509	0.972059	0.0958579
25510	0.946129	2.1
25511	0.94613	2.1
25512	0.94613	2.1
25513	0.94613	2.1
25514	0.94613	2.1
25515	0.946131	2.1
25516	0.946127	2.1
25517	0.946131	2.1

25518	0.946129	2.1
25519	1.4073	2.1
25520	0.946131	0.0701803
25521	1.05016	0.0671674
25522	0.946132	0.0773176
25523	0.946131	0.0777513
25524	0.946132	0.0695979
25525	0.946128	0.0759749
25526	1.13398	0.0732742
25527	0.946131	0.094523
25528	0.946131	0.0877999
25529	0.94613	0.0770346
25530	0.946131	0.05
25531	0.946131	0.052988
25532	1.38911	0.05
25533	1.38911	0.05
25534	0.946132	0.05
25535	0.946131	0.0591652
25536	0.94613	0.054278
25537	0.946131	0.0610181
25538	0.946131	0.0573615
25539	0.946131	0.0605819
25540	0.946131	0.226781
25541	1.23923	0.167276
25542	0.94613	0.284672
25543	0.946131	0.167543
25544	0.946131	0.185611
25545	1.40511	0.05
25546	0.946131	0.05
25547	0.946131	0.05
25548	0.946132	0.05
25549	0.946131	0.05
25550	1.12968	2.1
25551	0.946132	2.1
25552	0.946132	2.1
25553	0.946131	2.1
25554	0.946132	2.1
25555	0.946132	1.77713
25556	1.4172	2.1
25557	0.94613	1.83839
25558	0.94613	1.84263
25559	1.4172	1.93011
25560	0.946132	2.1
25561	0.946132	2.1
25562	1.31323	2.1
25563	0.946133	2.1
25564	0.946132	2.1
25565	0.946131	2.1
25566	0.94613	2.08474
25567	1.36546	2.1
25568	1.36546	2.1
25569	0.946131	2.1
25570	0.946132	1.83274
25571	0.946133	2.1
25572	0.946132	0.322811
25573	1.36899	1.28312
25574	0.946133	0.80999
25575	0.946131	0.05

25576	0.946132	0.05
25577	1.03543	0.0512242
25578	1.1412	0.0510468
25579	0.941275	0.05
25580	1.28952	0.0907598
25581	0.94613	0.114621
25582	0.946129	0.121534
25583	0.946132	0.0909749
25584	0.94613	0.104285
25585	0.94613	2.1
25586	0.94613	2.1
25587	0.946129	2.1
25588	0.94613	2.1
25589	1.35966	2.1
25590	0.810946	2.1
25591	0.810942	2.1
25592	0.810932	2.1
25593	0.94613	2.1
25594	1.40372	2.1
25595	0.946131	2.1
25596	0.94613	2.1
25597	1.40372	2.1
25598	0.946133	2.1
25599	0.946131	2.1
25600	0.94613	2.1
25601	0.946131	2.1
25602	0.946131	2.1
25603	1.31453	2.1
25604	0.94613	2.1
25605	0.946128	2.1
25606	0.94613	2.1
25607	0.94613	2.1
25608	0.946131	0.0655515
25609	1.12018	0.0617452
25610	0.94613	0.0713797
25611	0.94613	0.0757019
25612	0.94613	0.0604784
25613	0.946131	0.0801212
25614	1.16373	0.0774211
25615	0.946131	0.122738
25616	0.94613	0.0762601
25617	0.946131	0.0777227
25618	0.946126	0.406932
25619	0.946132	0.594619
25620	0.946128	0.438963
25621	0.946129	0.650642
25622	0.946129	0.755011
25623	0.946132	0.0895006
25624	0.946133	0.0912382
25625	0.946132	0.0893721
25626	0.946132	0.0885991
25627	0.946133	0.0935819
25628	0.946132	1.43328
25629	0.946129	1.60029
25630	0.94613	1.39972
25631	1.36399	1.46028
25632	0.946131	1.45495
25633	0.946132	2.1

25634	0.946132	2.1
25635	0.946133	2.1
25636	1.38302	2.1
25637	0.946133	2.1
25638	0.810943	2.1
25639	0.810944	2.1
25640	0.810945	2.1
25641	0.946132	2.1
25642	0.946131	2.1
25643	0.946132	2.1
25644	1.30188	2.1
25645	0.946132	2.1
25646	0.810946	1.1992
25647	0.72985	1.30945
25648	0.810934	1.23195
25649	0.856243	1.05856
25650	0.946129	1.15251
25651	0.946132	1.14762
25652	1.19686	0.869831
25653	0.851527	1.18788
25654	0.946131	2.1
25655	0.94613	2.1
25656	0.94613	2.1
25657	0.94613	2.1
25658	1.31961	2.1
25659	0.946131	2.1
25660	0.946131	2.1
25661	0.94613	2.1
25662	0.946131	2.1
25663	1.3605	2.1
25664	1.30116	2.1
25665	0.946133	1.37812
25666	0.946131	2.1
25667	0.946131	2.04611
25668	0.94613	2.1
25669	0.946132	0.05
25670	1.3035	0.05
25671	0.94613	0.05
25672	0.946131	0.05
25673	0.946131	0.05
25674	0.946132	2.1
25675	1.37869	2.1
25676	1.2051	2.1
25677	0.946132	2.1
25678	1.2051	2.1
25679	0.810946	2.1
25680	0.810945	2.1
25681	0.810946	2.1
25682	1.34623	2.1
25683	0.946131	2.1
25684	0.946131	2.1
25685	1.34623	2.1
25686	1.22993	2.1
25687	1.32299	0.0511225
25688	0.946132	0.066075
25689	0.946132	0.0669609
25690	0.946132	0.0549081
25691	0.946131	0.0651068

25692	0.94613	0.125257
25693	1.40574	0.112869
25694	0.946128	0.141752
25695	0.946131	0.146892
25696	1.40574	0.141138
25697	0.946132	2.1
25698	1.41331	2.1
25699	0.94613	2.1
25700	0.946131	2.1
25701	1.41331	2.1
25702	0.94613	2.1
25703	1.37609	2.1
25704	0.946129	2.1
25705	0.946131	2.1
25706	1.37609	2.1
25707	1.039	1.34507
25708	0.946131	1.80994
25709	0.944514	1.67468
25710	0.946132	1.77026
25711	0.946132	1.71186
25712	1.23726	1.22559
25713	0.946128	1.70651
25714	0.946128	1.62046
25715	0.946129	1.71641
25716	0.94613	1.63942
25717	0.946129	2.1
25718	0.946131	2.1
25719	0.946131	2.1
25720	1.39214	2.1
25721	0.94613	2.1
25722	0.946118	1.96197
25723	0.946118	0.654476
25724	1.41078	0.867008
25725	1.41076	0.445052
25726	0.946131	0.860228
25727	0.946131	0.35794
25728	0.946131	0.290999
25729	0.946132	0.302009
25730	0.94613	0.413608
25731	0.946131	0.309678
25732	0.946132	0.0694976
25733	0.946132	0.0585584
25734	0.946133	0.0521772
25735	1.28924	0.0569202
25736	0.946132	0.0614453
25737	0.946133	0.50419
25738	1.09812	0.332709
25739	0.946132	0.559129
25740	0.946132	0.486286
25741	1.09812	0.535312
25742	0.946132	1.51133
25743	0.946132	1.55102
25744	0.946132	1.48076
25745	0.946132	1.48368
25746	1.15388	1.503
25747	0.946132	0.432671
25748	0.974688	0.25942
25749	0.946132	0.45223

25750	0.946132	0.367305
25751	0.974687	0.426828
25752	1.0644	1.70543
25753	0.946133	1.82763
25754	0.946132	1.71448
25755	0.946133	1.72814
25756	1.26547	1.76446
25757	1.02905	1.44058
25758	0.911537	1.41889
25759	0.838038	1.50187
25760	0.946131	0.0843235
25761	0.94613	0.086315
25762	0.946132	0.0794994
25763	0.946131	0.0745239
25764	1.33922	0.0809149
25765	0.946132	0.0810274
25766	0.946132	0.0644014
25767	1.40926	0.0672694
25768	1.40927	0.0627561
25769	0.946132	0.0617182
25770	0.946129	2.1
25771	0.946131	1.92238
25772	0.946131	1.96202
25773	0.946131	1.61102
25774	1.40496	2.1
25775	0.94613	1.63847
25776	0.94613	1.66347
25777	0.946127	1.59959
25778	0.946128	1.56416
25779	1.38914	1.56499
25780	2.675	0.05
25781	1.22266	0.05
25782	0.946131	0.05
25783	0.946132	0.05
25784	0.946132	0.05
25785	1.22266	0.05
25786	0.946124	0.11652
25787	0.946128	0.0664731
25788	0.946126	0.0680436
25789	1.21428	0.0618894
25790	0.946127	0.099332
25791	0.946131	0.0827823
25792	0.946131	0.17673
25793	0.946132	0.0792197
25794	0.946129	0.0959966
25795	0.940118	0.159641
25796	0.810943	2.1
25797	0.810945	2.1
25798	0.810958	2.1
25799	0.946133	0.0759393
25800	1.11539	0.0710751
25801	0.946132	0.0752968
25802	0.946132	0.0758039
25803	0.946133	0.0763057
25804	0.946133	2.1
25805	1.41195	2.1
25806	0.946133	2.1
25807	0.946132	2.1

25808	0.946133	2.1
25809	1.15968	0.05
25810	0.946131	0.05
25811	0.946131	0.05
25812	0.946131	0.05
25813	0.946131	0.05
25814	0.94613	1.99196
25815	0.946128	2.02907
25816	0.946132	1.70598
25817	0.94613	1.91871
25818	0.94613	1.80897
25819	0.946131	1.77189
25820	1.04211	1.77827
25821	0.850155	1.5734
25822	0.946131	1.70791
25823	0.946131	1.64672
25824	0.946131	0.422765
25825	0.94613	0.298438
25826	0.946129	0.435687
25827	0.946131	0.343985
25828	0.946131	0.326741
25829	1.40193	0.169163
25830	1.19756	0.18976
25831	0.561936	0.227184
25832	0.808778	0.221316
25833	1.19756	0.204474
25834	0.946132	0.05
25835	0.946132	0.05
25836	1.33916	0.05
25837	1.33916	0.05
25838	0.946133	0.05
25839	0.946127	0.170152
25840	0.946127	0.124653
25841	0.946131	0.154439
25842	0.946128	0.103675
25843	0.946129	0.115167
25844	0.589428	0.287298
25845	0.851501	0.162208
25846	0.868924	0.179714
25847	0.877703	0.143981
25848	0.85153	0.16381
25849	0.946132	2.08306
25850	1.25372	2.1
25851	0.946132	2.1
25852	0.946132	2.1
25853	1.25372	2.1
25854	0.946132	1.72777
25855	0.946128	2.09797
25856	0.94613	1.82648
25857	0.94613	1.92014
25858	1.2778	1.97409
25859	0.946118	1.02389
25860	1.35629	0.719198
25861	0.946117	0.995908
25862	0.946131	0.738148
25863	0.946105	0.693392
25864	0.946132	1.78243
25865	0.946132	2.1

25866	1.38646	2.1
25867	1.38646	2.1
25868	0.946132	2.1
25869	0.946128	2.1
25870	0.946128	2.1
25871	1.39659	2.1
25872	1.39659	2.1
25873	0.946129	2.1
25874	0.946132	1.69414
25875	0.946131	1.69271
25876	0.946132	1.69749
25877	0.946132	1.69861
25878	1.15139	1.69188
25879	0.946133	2.1
25880	0.946132	2.1
25881	0.946132	2.1
25882	0.946132	2.1
25883	1.36151	2.1
25884	0.946127	2.1
25885	0.946131	2.1
25886	0.946128	2.1
25887	0.94613	2.1
25888	1.38397	2.1
25889	0.946131	0.237953
25890	1.31112	0.24197
25891	0.946127	0.251732
25892	0.946128	0.257875
25893	1.31112	0.241418
25894	1.29778	0.05
25895	0.883417	0.05
25896	1.32613	0.05
25897	0.94613	0.05
25898	0.94613	0.05
25899	0.946132	2.1
25900	0.94613	2.1
25901	0.946131	2.1
25902	0.946131	2.1
25903	1.36523	2.1
25904	0.946132	0.0799447
25905	1.01653	0.077119
25906	0.946132	0.110797
25907	0.946131	0.101718
25908	0.946131	0.0799315
25909	0.94612	2.1
25910	1.08301	2.1
25911	0.946119	2.1
25912	0.946132	2.1
25913	1.08299	2.1
25914	0.810942	2.1
25915	0.810944	2.1
25916	0.810957	2.1
25917	0.946126	0.226898
25918	0.946132	0.257561
25919	0.946129	0.228384
25920	1.3492	0.211474
25921	0.94613	0.227606
25922	0.946131	0.180743
25923	1.24766	0.165685

25924	0.946128	0.18846
25925	0.946131	0.199437
25926	1.24766	0.192409
25927	0.946132	0.05
25928	0.946131	0.05
25929	0.946131	0.05
25930	1.23351	0.05
25931	0.946132	0.05
25932	0.946131	0.05
25933	0.946132	0.05
25934	0.946131	0.05
25935	1.35657	0.05
25936	0.946131	0.05
25937	0.946132	0.17696
25938	1.3689	0.121443
25939	0.946132	0.158457
25940	0.946132	0.161527
25941	1.3689	0.12551
25942	0.946133	0.105014
25943	1.35822	0.0568322
25944	0.946133	0.0796809
25945	0.946132	0.0851038
25946	1.35822	0.0591129
25947	0.946131	1.10778
25948	0.946131	1.39993
25949	0.946131	1.27255
25950	0.946131	1.24187
25951	1.4101	1.36291
25952	0.810944	1.63319
25953	0.810939	1.63925
25954	0.810955	1.52908
25955	0.946132	2.1
25956	1.37733	2.1
25957	0.946132	2.1
25958	0.946131	2.1
25959	0.946131	2.1
25960	1.32582	2.1
25961	0.946132	2.1
25962	0.946132	2.1
25963	0.946132	2.1
25964	0.946132	2.1
25965	0.946132	2.1
25966	0.946132	2.1
25967	1.31401	2.1
25968	1.31401	2.1
25969	0.946131	2.1
25970	1.3469	1.57297
25971	0.946132	1.71256
25972	0.946132	1.67205
25973	0.946133	1.54885
25974	0.946132	1.58251
25975	0.946126	2.1
25976	0.946127	2.1
25977	0.94613	2.1
25978	0.946128	2.1
25979	1.41206	2.1
25980	0.946127	2.1
25981	1.40109	2.1

25982	0.946128	2.1
25983	0.946129	2.1
25984	1.40109	2.1
25985	1.24448	0.102659
25986	0.94613	0.168143
25987	0.946132	0.125127
25988	0.946129	0.165888
25989	0.946131	0.143018
25990	1.11015	0.154934
25991	0.946133	0.270228
25992	0.946132	0.192466
25993	0.946133	0.252797
25994	0.946132	0.224313
25995	0.946132	2.1
25996	0.946131	2.1
25997	0.946131	2.1
25998	1.38775	2.1
25999	0.946131	2.1
26000	0.946133	1.60124
26001	0.946131	2.1
26002	1.30598	2.1
26003	1.30598	2.1
26004	0.946131	2.1
26005	0.946132	2.1
26006	0.946132	2.1
26007	0.946132	2.1
26008	1.40774	2.1
26009	0.946132	2.1
26010	0.798396	0.05
26011	0.946133	1.10322
26012	0.946128	1.30429
26013	0.946131	1.18325
26014	0.946131	1.2071
26015	1.28918	1.25302
26016	0.946132	0.205175
26017	1.40668	0.118563
26018	0.94613	0.178963
26019	0.94613	0.205287
26020	0.94613	0.331793
26021	0.810946	2.1
26022	0.810945	2.1
26023	0.810933	2.1
26024	0.946131	2.1
26025	1.30338	2.1
26026	0.94613	2.1
26027	1.30339	2.1
26028	0.946131	2.1
26029	0.946127	0.947171
26030	0.87161	0.409989
26031	0.946128	1.04944
26032	0.946129	0.8043
26033	0.827876	0.964254
26034	0.946131	2.1
26035	1.40796	2.1
26036	0.94613	2.1
26037	0.94613	2.1
26038	1.40796	2.1
26039	0.946132	0.856863

26040	0.946132	1.16746
26041	0.946132	0.746561
26042	0.946132	0.572805
26043	1.41724	0.437508
26044	1.34254	0.243982
26045	0.946132	0.421134
26046	0.946132	0.442108
26047	0.946132	0.387871
26048	0.946133	0.35733
26049	0.946133	1.80061
26050	0.946132	1.7643
26051	0.946132	1.71249
26052	1.26501	1.85987
26053	0.946132	1.70609
26054	0.946131	0.0543802
26055	1.35169	0.0552371
26056	0.946131	0.0611312
26057	0.946131	0.0549483
26058	1.35169	0.0546815
26059	0.676844	0.05
26060	0.946131	0.0583662
26061	0.94613	0.0681607
26062	1.13242	0.05
26063	1.13242	0.05
26064	0.946131	0.0616117
26065	0.946128	1.86435
26066	1.28828	2.1
26067	0.946131	1.71039
26068	0.946131	1.9401
26069	1.28828	2.01041
26070	0.946131	0.32405
26071	1.2996	0.261328
26072	0.946133	0.314067
26073	0.946131	0.299579
26074	0.946131	0.268111
26075	0.946132	2.1
26076	1.40638	2.1
26077	0.946131	2.1
26078	0.946132	2.1
26079	1.40638	2.1
26080	2.84909	0.0641799
26081	1.35726	0.0676424
26082	0.946131	0.0785135
26083	0.946131	0.0785003
26084	0.946131	0.0708282
26085	0.946131	0.0748747
26086	1.39852	2.1
26087	0.946132	2.1
26088	0.94613	2.1
26089	0.94613	2.1
26090	0.94613	2.1
26091	0.946126	0.05
26092	0.946131	0.05
26093	0.946128	0.0554901
26094	1.31666	0.05
26095	0.946128	0.05
26096	0.946126	0.0577351
26097	0.946131	0.0524832

26098	0.946129	0.0690205
26099	1.32275	0.05
26100	0.946129	0.0547551
26101	0.946131	2.1
26102	0.946132	2.1
26103	1.41125	2.1
26104	0.946131	2.1
26105	0.946131	2.1
26106	0.946131	2.1
26107	0.946132	2.1
26108	1.31904	2.1
26109	0.946132	2.1
26110	0.946132	2.1
26111	0.946129	0.05
26112	0.946132	0.05
26113	0.94613	0.05
26114	1.02426	0.05
26115	0.9311	0.0506819
26116	0.946128	1.68745
26117	0.946133	1.63111
26118	0.946131	1.61859
26119	1.40092	1.73102
26120	0.946131	1.61974
26121	0.946117	2.1
26122	0.946118	2.1
26123	0.946118	2.1
26124	1.40017	2.1
26125	0.946105	2.1
26126	0.946132	0.162263
26127	1.03547	0.138085
26128	0.946132	0.118151
26129	0.946133	0.16533
26130	0.946132	0.20007
26131	0.946133	2.1
26132	0.946132	2.1
26133	0.946133	2.1
26134	0.946133	2.1
26135	1.36694	2.1
26136	0.946132	0.0985936
26137	1.22878	0.117446
26138	0.946132	0.119521
26139	0.946132	0.086241
26140	1.22878	0.110648
26141	1.11014	0.144747
26142	0.913078	0.150192
26143	1.11015	0.18977
26144	0.49154	0.185292
26145	0.946127	2.1
26146	0.946131	2.1
26147	0.946128	2.1
26148	0.946129	2.1
26149	1.38752	2.1
26150	0.946132	2.1
26151	1.37277	2.1
26152	0.946132	2.1
26153	0.946131	2.1
26154	1.37277	2.1
26155	0.94613	0.412397

26156	1.16834	0.244482
26157	0.946129	0.40805
26158	0.94613	0.321729
26159	0.94613	0.288701
26160	0.946127	0.512137
26161	0.946131	0.345697
26162	1.35594	0.295906
26163	0.946128	0.522231
26164	0.94613	0.371764
26165	0.946128	2.1
26166	0.946128	0.448445
26167	1.38464	1.93299
26168	1.38463	1.07808
26169	0.94613	1.37358
26170	0.946129	1.55235
26171	0.946131	0.434037
26172	0.94613	0.292437
26173	1.39751	0.23767
26174	0.946131	0.789628
26175	1.25837	0.0704553
26176	1.21134	0.0660511
26177	0.700013	0.0768609
26178	0.700041	0.0774492
26179	1.40583	2.1
26180	0.946132	2.1
26181	0.946132	2.1
26182	0.946133	2.1
26183	0.946132	2.1
26184	0.827865	2.1
26185	0.946131	2.1
26186	0.946131	2.04051
26187	0.946131	2.1
26188	0.946131	2.0492
26189	0.946132	0.05
26190	1.21087	0.05
26191	0.94613	0.05
26192	0.94613	0.05
26193	0.946131	0.05
26194	0.94613	0.357609
26195	1.17452	0.263777
26196	0.946132	0.428507
26197	0.94613	0.333692
26198	0.94613	0.419642
26199	0.946119	0.187909
26200	1.27374	0.177904
26201	0.946119	0.194445
26202	0.946131	0.185349
26203	0.946106	0.194956
26204	0.946133	2.1
26205	0.946132	2.1
26206	0.946133	2.1
26207	0.946132	2.1
26208	1.39621	2.1
26209	1.34352	2.1
26210	0.946132	2.1
26211	0.946132	2.1
26212	0.946132	2.1
26213	0.946132	2.1

26214	0.946132	1.64515
26215	0.946128	2.06969
26216	0.94613	1.77916
26217	0.94613	1.86198
26218	1.41273	1.93968
26219	1.23536	2.1
26220	0.946131	2.1
26221	0.946132	2.1
26222	0.946132	2.1
26223	0.946132	2.1
26224	0.946132	2.1
26225	1.41131	2.1
26226	0.946131	2.1
26227	0.946131	2.1
26228	0.946131	2.1
26229	0.946133	0.697863
26230	0.946132	2.07899
26231	0.946133	0.869848
26232	1.41894	0.607385
26233	0.946132	1.23844
26234	0.946128	1.83979
26235	1.4099	2.1
26236	0.946131	1.9531
26237	0.946131	1.98124
26238	0.946131	1.84611
26239	0.810947	2.1
26240	0.810945	2.1
26241	0.810933	2.1
26242	0.946131	0.93425
26243	1.25765	2.065
26244	0.946128	1.62432
26245	0.946129	1.64571
26246	1.25765	1.77451
26247	1.21347	2.1
26248	0.946129	2.1
26249	0.94613	2.1
26250	1.21347	2.1
26251	0.94613	2.1
26252	0.94613	0.05
26253	0.946132	0.05
26254	1.2493	0.05
26255	1.2493	0.05
26256	0.94613	0.05
26257	0.946132	1.20506
26258	0.946128	1.31244
26259	0.946131	1.21993
26260	0.94613	1.24007
26261	1.37938	1.26271
26262	0.946133	0.05
26263	0.946132	0.05
26264	0.946133	0.05
26265	0.946132	0.05
26266	0.946133	0.05
26267	0.946129	0.334485
26268	1.26426	0.241802
26269	0.946131	0.434803
26270	0.946131	0.353837
26271	1.26426	0.266336

26272	0.872447	1.81333
26273	0.946132	1.67224
26274	0.946132	1.92115
26275	0.946132	1.87407
26276	0.946133	1.75903
26277	0.946117	1.40745
26278	0.946116	1.15961
26279	0.946113	1.27835
26280	0.946129	1.40922
26281	1.27211	0.979736
26282	0.94613	0.715809
26283	0.946129	1.66215
26284	1.40029	1.28264
26285	1.40028	1.3992
26286	0.94613	1.28228
26287	0.810942	2.1
26288	0.810943	2.1
26289	0.810932	2.1
26290	1.29521	2.1
26291	0.946129	2.1
26292	0.946131	2.1
26293	1.29521	2.1
26294	1.29521	2.1
26295	0.946132	0.749711
26296	0.946129	0.484485
26297	1.24596	0.421385
26298	0.946131	0.770083
26299	0.946131	0.5857
26300	0.946131	2.1
26301	1.35616	2.1
26302	0.946133	2.1
26303	0.94613	2.1
26304	1.35616	2.1
26305	0.879548	0.521358
26306	1.01272	0.0975001
26307	0.946133	1.19312
26308	0.946133	0.100112
26309	0.946132	0.397822
26310	0.946132	0.823808
26311	0.918009	0.409107
26312	0.91801	1.26916
26313	0.946132	0.272913
26314	0.946132	0.653587
26315	0.946132	0.05213
26316	1.28644	0.0521095
26317	0.946131	0.0634059
26318	0.946131	0.059752
26319	0.946132	0.0547537
26320	0.946131	0.0534222
26321	1.40103	0.0551033
26322	0.946131	0.0668263
26323	0.946131	0.0662718
26324	0.946131	0.0572011
26325	0.946128	0.13452
26326	1.03327	0.108833
26327	0.946129	0.156838
26328	0.946131	0.109208
26329	0.94613	0.124458

26330	0.946132	1.0601
26331	0.946133	0.367075
26332	1.40265	0.33017
26333	1.40265	0.249858
26334	0.946133	0.215264
26335	0.946131	0.05
26336	0.946131	0.0507543
26337	0.946131	0.0504834
26338	0.856655	0.05
26339	0.856616	0.05
26340	0.946132	0.311794
26341	0.946132	0.384997
26342	0.946132	0.814384
26343	1.15844	0.220858
26344	0.946131	0.78911
26345	0.946131	2.1
26346	1.4031	2.1
26347	1.40304	2.1
26348	0.851554	2.1
26349	0.946131	0.05
26350	0.94613	0.0600715
26351	1.24417	0.05
26352	1.24416	0.05
26353	0.946131	0.0516609
26354	0.946132	1.06683
26355	1.20992	1.04921
26356	0.946132	1.06276
26357	0.946132	1.23267
26358	1.20992	1.19334
26359	0.946128	1.80922
26360	0.94613	2.00062
26361	0.94613	1.82124
26362	0.94613	1.84956
26363	1.23426	1.90463
26364	0.810943	0.806428
26365	0.810946	0.118841
26366	0.810933	0.0986657
26367	0.810945	2.1
26368	0.810945	2.1
26369	0.810932	2.1
26370	1.25104	2.1
26371	0.946131	2.1
26372	0.946131	1.98875
26373	1.25104	2.1
26374	1.25104	2.09621
26375	1.25488	2.1
26376	0.946132	2.1
26377	0.946132	2.08023
26378	0.946132	2.1
26379	0.946131	2.1
26380	0.946131	0.063134
26381	0.946126	0.0804537
26382	0.946128	0.0622342
26383	1.32131	0.0675516
26384	0.946128	0.0716397
26385	0.946133	2.1
26386	0.946132	2.04443
26387	0.946132	2.1

26388	0.946133	2.1
26389	1.41123	2.1
26390	0.946132	0.0677155
26391	0.946133	0.0718905
26392	0.946132	0.0869405
26393	1.32163	0.0597435
26394	0.946133	0.076772
26395	0.946132	2.1
26396	0.946132	2.1
26397	1.38651	2.1
26398	1.38651	2.1
26399	0.946132	2.1
26400	0.94613	0.105309
26401	1.3639	0.0980436
26402	0.946128	0.124615
26403	0.946131	0.094291
26404	1.3639	0.0881775
26405	0.946133	1.32533
26406	1.40724	0.603147
26407	0.946132	0.513296
26408	0.946133	0.805933
26409	1.40724	0.707865
26410	1.21099	1.71035
26411	0.946128	1.79339
26412	0.946127	2.03099
26413	0.946128	1.89917
26414	0.946129	1.78081
26415	0.946132	0.302829
26416	1.35946	0.256413
26417	0.946131	1.0896
26418	0.946131	0.469732
26419	1.35946	0.299541
26420	1.34794	0.677354
26421	0.517994	1.71797
26422	0.946132	0.716188
26423	0.946132	1.00293
26424	0.517965	1.42583
26425	1.23224	0.931189
26426	0.94613	1.84241
26427	0.946131	1.63548
26428	0.946131	1.3774
26429	0.946131	1.53542
26430	1.37511	1.52402
26431	0.946132	1.55905
26432	0.946133	1.86929
26433	0.946133	1.71614
26434	0.946132	1.5715
26435	0.851106	0.755539
26436	0.946129	0.685968
26437	0.946131	0.290789
26438	0.946131	1.41267
26439	0.851066	1.12313
26440	0.946131	0.0943406
26441	0.946131	0.171044
26442	0.94613	0.182249
26443	1.31357	0.0921739
26444	0.946131	0.138464
26445	0.946133	1.18271

26446	0.946132	1.29477
26447	0.946133	0.908363
26448	0.946132	1.21689
26449	1.38149	1.21344
26450	0.810944	1.74909
26451	0.810945	1.90904
26452	0.810958	2.09761
26453	0.946118	0.05
26454	0.946118	0.05
26455	1.37835	0.05
26456	1.37837	0.05
26457	0.946106	0.05
26458	0.946128	0.05
26459	0.946131	0.05
26460	1.34831	0.05
26461	1.34831	0.05
26462	0.94613	0.05
26463	0.946132	0.0776771
26464	0.946132	0.132387
26465	0.909981	0.0803639
26466	1.00099	0.0789552
26467	0.946131	0.106217
26468	0.946132	1.14967
26469	0.946133	1.31245
26470	0.946133	1.19638
26471	0.946132	1.21959
26472	1.35328	1.25828
26473	0.946132	0.05
26474	0.946132	0.05
26475	1.35386	0.05
26476	1.35385	0.05
26477	0.946133	0.05
26478	0.946121	0.05
26479	0.946132	0.05
26480	0.900167	0.05
26481	1.01189	0.05
26482	0.946132	0.05
26483	0.946132	2.03271
26484	1.41585	1.74872
26485	0.946131	2.01885
26486	0.946131	1.73766
26487	0.94613	1.91352
26488	0.810946	2.1
26489	0.810946	2.1
26490	0.810933	2.1
26491	1.34447	2.1
26492	0.946131	2.1
26493	0.946131	2.1
26494	1.34447	2.1
26495	1.19886	2.1
26496	0.946132	0.087626
26497	0.946132	0.0589472
26498	0.946132	0.0633558
26499	1.35065	0.0572727
26500	0.946132	0.0804599
26501	0.946131	0.249517
26502	1.3022	0.233093
26503	0.94613	0.836545

26504	0.946131	0.36356
26505	1.3022	0.250577
26506	0.946127	0.130385
26507	1.38683	0.110654
26508	0.946127	0.14018
26509	0.946128	0.13513
26510	1.38683	0.130454
26511	0.810946	0.05
26512	0.810947	0.05
26513	0.810959	0.05
26514	0.946132	0.05
26515	0.946131	0.05
26516	0.946132	0.05
26517	0.946132	0.05
26518	0.946132	0.05
26519	0.946127	1.64491
26520	1.35968	1.92095
26521	0.946131	1.54387
26522	0.946129	1.15869
26523	1.35969	1.75175
26524	0.946133	2.01934
26525	1.35862	1.7908
26526	0.946132	2.08366
26527	0.946132	1.69121
26528	1.35862	1.84886
26529	0.946132	0.844665
26530	0.946132	0.295357
26531	0.946132	1.5911
26532	0.946131	0.180874
26533	0.946132	0.604384
26534	0.946131	0.157388
26535	0.946131	0.133777
26536	0.946128	0.205137
26537	0.946129	0.0675498
26538	1.41232	0.085672
26539	0.946128	2.1
26540	1.29331	2.1
26541	0.946131	2.1
26542	0.946131	2.1
26543	1.29331	2.1
26544	0.946133	0.92519
26545	0.946132	1.01755
26546	0.946132	0.914144
26547	0.946132	0.651316
26548	1.41556	0.412712
26549	0.94612	0.670213
26550	0.946119	0.435186
26551	0.946119	0.682588
26552	0.946132	0.472506
26553	0.946107	0.387188
26554	0.946129	0.05
26555	1.1509	0.05
26556	0.946126	0.05
26557	0.946129	0.05
26558	0.94613	0.05
26559	0.946131	0.05
26560	1.19263	0.05
26561	0.946132	0.05

26562	0.946132	0.05
26563	0.946132	0.05
26564	0.94613	0.851369
26565	1.36939	1.47543
26566	0.946128	0.986079
26567	0.946131	1.19647
26568	1.36939	1.09653
26569	0.946133	1.35293
26570	0.827865	1.74645
26571	0.946132	1.52744
26572	0.946132	1.66697
26573	1.40398	1.70446
26574	0.946127	1.09016
26575	0.946127	2.1
26576	0.94613	0.463879
26577	1.41498	1.63123
26578	0.946128	1.12283
26579	0.94613	0.05
26580	0.946131	0.05
26581	0.946129	0.05
26582	0.94613	0.05
26583	0.94613	0.05
26584	0.946118	0.0540102
26585	0.946119	0.0502856
26586	0.946116	0.05
26587	1.36569	0.05
26588	0.946105	0.0554266
26589	0.946132	0.258458
26590	1.23175	0.192688
26591	0.94613	0.274632
26592	0.946133	0.186102
26593	0.946131	0.210796
26594	1.25698	0.0542012
26595	0.946126	0.0609427
26596	0.946129	0.0609676
26597	0.946128	0.0558997
26598	0.946129	0.0592382
26599	0.810943	2.1
26600	0.810944	2.1
26601	0.810932	2.1
26602	0.946132	0.10861
26603	1.09841	0.0915588
26604	0.946131	0.135908
26605	0.946132	0.117081
26606	1.09841	0.101482
26607	0.810936	1.54241
26608	0.810935	2.08767
26609	0.810934	2.1
26610	1.23851	2.1
26611	0.946132	1.9691
26612	0.946131	2.1
26613	1.23851	2.1
26614	1.23851	2.1
26615	0.946132	0.15801
26616	0.946131	0.401251
26617	0.946132	0.143539
26618	0.946132	0.250545
26619	0.946132	0.453208

26620	0.939141	1.32469
26621	0.953118	1.4563
26622	0.946126	1.04601
26623	0.946129	1.22288
26624	1.03306	1.35081
26625	0.946132	0.107216
26626	1.30927	0.122993
26627	0.946132	0.126939
26628	0.946132	0.104061
26629	1.30928	0.115542
26630	0.94613	2.1
26631	0.946131	2.1
26632	1.24511	2.1
26633	0.94613	2.1
26634	0.94613	2.1
26635	0.946133	0.103175
26636	1.34152	0.0918889
26637	0.946131	0.133041
26638	0.946131	0.0583777
26639	1.34152	0.0586875
26640	0.946132	0.0870392
26641	1.41383	0.105091
26642	0.946132	0.116026
26643	0.946132	0.0585157
26644	1.41383	0.109407
26645	0.946132	1.1158
26646	0.946131	0.742074
26647	1.41392	0.564371
26648	0.946132	1.13362
26649	0.946132	0.816454
26650	0.946132	1.49045
26651	0.946132	1.29947
26652	1.24333	1.02524
26653	0.946133	1.53261
26654	0.946133	1.41541
26655	0.946124	0.211518
26656	0.94613	0.244703
26657	0.946126	0.237516
26658	1.3065	0.193842
26659	0.946127	0.238892
26660	0.810946	0.326352
26661	0.810943	0.240264
26662	0.81096	0.308966
26663	0.946131	0.0737459
26664	1.40271	0.0774648
26665	0.946131	0.0981201
26666	0.946131	0.0811519
26667	1.40271	0.0812854
26668	0.946132	0.0517403
26669	0.94613	0.0577767
26670	0.946131	0.0655597
26671	0.94613	0.0514837
26672	0.946131	0.0603205
26673	0.946133	0.287262
26674	1.30107	0.0621902
26675	0.946132	0.621854
26676	0.946132	0.0893614
26677	0.946133	0.522943

26678	0.810946	2.1
26679	0.810944	2.1
26680	0.810958	2.1
26681	0.946133	1.9004
26682	0.946131	1.89264
26683	0.946131	2.1
26684	1.30812	2.1
26685	0.946131	1.99672
26686	0.94613	1.36231
26687	0.975002	1.59108
26688	0.917259	1.95472
26689	1.00902	1.75824
26690	0.94613	1.15718
26691	0.946131	2.1
26692	0.946127	2.1
26693	1.398	2.1
26694	0.946129	2.1
26695	0.946129	2.1
26696	0.946133	2.1
26697	0.946132	2.1
26698	0.946132	2.1
26699	0.946132	2.1
26700	1.3033	2.1
26701	1.09897	0.310628
26702	0.946131	0.627004
26703	0.946132	0.467825
26704	0.946129	0.58528
26705	0.946131	0.575135
26706	0.946131	2.1
26707	0.946132	2.1
26708	1.39858	2.1
26709	0.946131	2.1
26710	0.946131	2.1
26711	0.946119	1.11524
26712	1.08878	1.14053
26713	0.946119	1.3364
26714	1.0888	1.14339
26715	0.946107	1.29744
26716	0.946132	0.0558579
26717	1.10733	0.0567112
26718	0.946132	0.0638072
26719	0.946131	0.0639579
26720	0.946132	0.0600195
26721	0.946128	0.0614869
26722	1.33671	0.061278
26723	0.94613	0.0699338
26724	0.94613	0.0713973
26725	0.94613	0.0641666
26726	0.810943	2.1
26727	0.810944	2.1
26728	0.810945	2.1
26729	1.35117	2.1
26730	0.946129	2.1
26731	0.94613	2.1
26732	1.35117	2.1
26733	1.27174	2.1
26734	0.946132	2.1
26735	0.946133	2.05314

26736	0.997296	1.77989
26737	0.946133	1.948
26738	0.946132	1.87097
26739	0.810943	2.1
26740	0.810944	2.1
26741	0.810945	2.1
26742	0.810946	1.71111
26743	0.810943	1.83845
26744	0.810933	1.66074
26745	0.946127	1.59356
26746	0.946131	1.58099
26747	0.946128	1.58371
26748	0.94613	1.67922
26749	0.946129	1.69945
26750	0.946131	0.340836
26751	0.946131	0.26834
26752	0.946129	0.351843
26753	0.946131	0.279519
26754	0.94613	0.277313
26755	0.946133	0.0970604
26756	0.946132	0.109458
26757	0.946132	0.0984718
26758	0.946132	0.0883974
26759	0.946133	0.468605
26760	0.946131	1.48468
26761	0.94613	1.18411
26762	0.946129	1.5083
26763	0.946131	1.04571
26764	0.946131	1.48392
26765	0.946132	1.90647
26766	0.946129	2.1
26767	0.94613	2.1
26768	0.946131	2.01972
26769	1.39356	2.1
26770	0.810946	2.1
26771	0.810947	2.1
26772	0.810934	2.1
26773	0.946125	0.0635272
26774	0.946127	0.0626592
26775	1.33798	0.0527531
26776	1.33797	0.05
26777	0.946128	0.0576872
26778	0.946131	0.0504192
26779	0.946131	0.059318
26780	1.23146	0.05
26781	1.23146	0.05
26782	0.946131	0.0514749
26783	0.946129	2.1
26784	0.94613	2.1
26785	0.946131	2.1
26786	0.94613	2.1
26787	1.30082	2.1
26788	0.946131	0.052603
26789	0.946132	0.0618752
26790	0.946131	0.0741108
26791	1.20416	0.0515164
26792	0.946132	0.0618222
26793	0.946132	0.0513473

26794	0.946131	0.0610834
26795	0.946131	0.0728565
26796	1.18711	0.0526345
26797	0.946132	0.0649222
26798	0.946132	0.0527106
26799	0.946132	0.05
26800	0.946133	0.0511656
26801	0.946132	0.05
26802	0.946133	0.0506476
26803	0.946131	0.0721753
26804	0.946132	0.0605854
26805	0.94613	0.0597438
26806	0.946131	0.0526721
26807	0.946131	0.0684297
26808	0.946133	0.0658983
26809	0.946132	0.0525558
26810	0.946133	0.052345
26811	0.946132	0.05
26812	0.946132	0.0615267
26813	0.946132	2.1
26814	0.946132	2.1
26815	0.946132	2.1
26816	1.36422	2.1
26817	0.946132	2.1
26818	0.946131	1.123
26819	0.946132	1.68645
26820	0.946132	1.51567
26821	0.946132	1.55193
26822	1.4185	1.62631
26823	0.946133	0.0693164
26824	1.37794	0.0608221
26825	0.946132	0.0687876
26826	0.946133	0.0613551
26827	0.946132	0.0616589
26828	1.4045	2.1
26829	0.946132	2.1
26830	0.946132	2.1
26831	0.946132	2.1
26832	0.946132	2.1
26833	0.946131	2.1
26834	0.946132	2.1
26835	1.34285	2.1
26836	1.34285	2.1
26837	0.946132	2.1
26838	0.946131	2.1
26839	0.946132	2.1
26840	1.40739	2.1
26841	1.40739	2.1
26842	0.946131	2.1
26843	0.946128	2.1
26844	0.946132	2.1
26845	0.94613	2.1
26846	0.94613	2.1
26847	1.3164	2.1
26848	0.946132	2.1
26849	1.3484	2.1
26850	0.946132	2.1
26851	0.946132	2.1

26852	1.3484	2.1
26853	0.946132	2.1
26854	0.946132	2.1
26855	0.946133	2.1
26856	0.946133	2.1
26857	1.41507	2.1
26858	0.946132	2.1
26859	1.32608	2.1
26860	0.946131	2.1
26861	0.946131	2.1
26862	0.946132	2.1
26863	0.94613	1.46292
26864	1.34646	1.77217
26865	0.946129	1.46392
26866	0.94613	1.0197
26867	1.34646	1.51664
26868	0.946131	1.44498
26869	0.946131	2.1
26870	0.94613	1.91968
26871	1.33728	1.65623
26872	0.946131	1.85908
26873	0.946127	2.1
26874	0.946131	1.86998
26875	0.946128	2.1
26876	0.946129	2.1
26877	1.4064	2.1
26878	0.946133	2.1
26879	0.946131	2.1
26880	0.946131	2.1
26881	0.946131	2.1
26882	0.94613	2.1
26883	1.34912	2.1
26884	0.946131	2.1
26885	0.946129	2.1
26886	0.946131	2.1
26887	0.94613	2.1
26888	0.946132	2.1
26889	1.40447	2.1
26890	0.946132	2.1
26891	0.946133	2.1
26892	1.40447	2.1
26893	1.33607	2.1
26894	0.946131	2.1
26895	0.946127	2.1
26896	0.94613	2.1
26897	0.946129	2.1
26898	0.946117	0.609886
26899	1.32664	1.6743
26900	0.946115	1.60526
26901	0.946105	1.49912
26902	0.94613	0.679515
26903	0.946118	2.1
26904	0.946114	2.1
26905	1.35886	2.1
26906	1.35884	2.1
26907	0.946129	2.1
26908	0.946132	2.1
26909	1.2571	2.1

26910	0.946132	2.1
26911	0.946132	2.1
26912	1.2571	2.1
26913	0.946133	2.1
26914	1.35663	2.1
26915	0.946132	2.1
26916	0.946132	2.1
26917	1.35663	2.1
26918	0.946133	1.5173
26919	0.946129	1.81749
26920	0.946131	1.66051
26921	0.946131	1.67387
26922	1.30881	1.76057
26923	0.946132	2.1
26924	0.94613	2.1
26925	1.34652	2.1
26926	0.94613	2.1
26927	0.94613	2.1
26928	0.946132	2.0543
26929	0.946133	1.76828
26930	1.36183	1.43361
26931	1.36183	1.66809
26932	0.946132	1.80211
26933	0.946132	0.147367
26934	1.38353	0.0797697
26935	0.94613	0.111214
26936	0.94613	0.116347
26937	1.38354	0.0955544
26938	0.946128	0.528666
26939	1.24887	0.424812
26940	0.94613	0.587972
26941	0.94613	0.537901
26942	1.24887	0.550524
26943	0.946129	1.3658
26944	0.946126	1.50386
26945	0.946124	1.39874
26946	0.946128	1.41782
26947	1.32056	1.46746
26948	0.946129	1.30768
26949	0.946128	1.52383
26950	0.946126	1.27428
26951	0.946129	1.40928
26952	1.20351	1.49409
26953	0.946131	1.87247
26954	0.946131	1.87606
26955	0.946127	2.1
26956	1.25591	2.00033
26957	0.94613	1.83673
26958	0.946132	1.52529
26959	0.946133	1.5405
26960	0.946132	1.68697
26961	1.15436	1.52908
26962	0.930306	1.50707
26963	0.946132	2.1
26964	0.946132	2.1
26965	0.946132	1.99598
26966	0.946133	2.1
26967	0.946132	2.1

26968	0.946129	1.29293
26969	0.946132	1.25887
26970	0.946126	1.43527
26971	1.13245	1.2321
26972	0.946129	1.29533
26973	0.94613	2.09553
26974	0.946132	2.08456
26975	0.946129	1.90981
26976	0.946131	2.1
26977	0.946131	2.01068
26978	1.41279	2.05011
26979	1.76254	2.1
26980	0.600454	1.98442
26981	0.858991	2.01042
26982	0.940722	2.041
26983	1.28439	1.80967
26984	0.946131	1.8749
26985	0.94613	1.83943
26986	0.946131	2.04942
26987	0.946131	2.00485
26988	0.946132	1.33266
26989	1.39335	0.536689
26990	0.946132	1.33849
26991	0.946132	0.712114
26992	0.946132	1.29857
26993	1.33879	0.179361
26994	0.946131	0.315059
26995	0.94613	0.517556
26996	1.33879	0.176227
26997	0.946131	0.277332
26998	0.946126	0.271505
26999	0.946131	0.217588
27000	1.37342	0.196374
27001	1.37343	0.166981
27002	0.946128	0.690522
27003	1.39168	0.0510468
27004	0.946131	0.0655589
27005	0.946131	0.0674543
27006	0.946131	0.0549135
27007	0.946131	0.0644231
27008	0.946131	0.108934
27009	1.412	0.0844068
27010	0.946127	0.0926188
27011	0.946128	0.0848918
27012	0.946128	0.0824302
27013	1.1387	0.19392
27014	0.943364	0.356935
27015	0.946132	0.267174
27016	0.946132	0.311159
27017	0.946133	0.295105
27018	1.21085	0.37034
27019	0.88324	0.859542
27020	0.946129	0.865931
27021	0.946131	0.781295
27022	0.946131	0.650129
27023	1.33587	0.0558986
27024	0.946125	0.0630606
27025	0.946129	0.0575484

27026	0.946126	0.0666809
27027	0.946127	0.0568803
27028	1.27512	0.0692046
27029	0.946132	0.0935588
27030	0.946132	0.0993882
27031	0.946133	0.0872307
27032	0.946132	0.0713631
27033	0.946128	0.065435
27034	1.30498	0.0630376
27035	0.946131	0.0757797
27036	0.94613	0.066341
27037	1.30497	0.0658706
27038	0.946131	0.056614
27039	1.25626	0.0551979
27040	0.946131	0.0616232
27041	0.946131	0.0573178
27042	1.25626	0.0583309
27043	0.946131	0.05
27044	0.94613	0.05
27045	1.35316	0.05
27046	0.946131	0.05
27047	0.946131	0.05
27048	0.94613	0.131422
27049	1.28727	0.137165
27050	0.946128	0.0836794
27051	0.946131	0.10841
27052	0.94613	0.0829938
27053	0.946131	0.05
27054	1.39502	0.05
27055	0.946132	0.05
27056	0.946132	0.05
27057	0.946132	0.05
27058	1.36173	0.252002
27059	0.946131	0.484072
27060	0.946126	0.464441
27061	0.946127	0.445231
27062	0.946128	0.376481
27063	0.946129	0.995403
27064	0.946133	0.403794
27065	0.946131	1.11827
27066	0.946131	0.803841
27067	0.946131	1.15372
27068	0.946128	0.05
27069	0.946132	0.05
27070	1.24678	0.05
27071	0.94613	0.05
27072	0.94613	0.05
27073	0.946129	0.05
27074	0.94613	0.05
27075	1.25215	0.05
27076	0.946126	0.05
27077	0.946129	0.05
27078	0.946129	0.406344
27079	1.29689	0.305722
27080	0.946131	0.419429
27081	0.946131	0.336775
27082	0.946131	0.287797
27083	0.946128	0.0615715

27084	0.94613	0.0628053
27085	0.946131	0.0607131
27086	0.94613	0.0649728
27087	0.946131	0.0632553
27088	0.946127	0.0722208
27089	0.946128	0.076579
27090	0.94613	0.0709802
27091	0.946129	0.0784784
27092	0.946129	0.0770642
27093	0.946133	0.05
27094	0.946132	0.05
27095	0.946132	0.05
27096	1.29957	0.05
27097	0.946133	0.05
27098	0.946132	0.05
27099	0.946133	0.05
27100	0.946132	0.05
27101	1.26201	0.05
27102	0.946132	0.05
27103	0.946133	0.0672848
27104	1.40427	0.0668743
27105	0.946132	0.0783142
27106	0.946132	0.0796958
27107	0.946133	0.0683688
27108	0.946132	0.0667208
27109	1.35123	0.0684328
27110	0.946132	0.0816922
27111	0.946132	0.085265
27112	0.946131	0.0682604
27113	0.946128	0.0953918
27114	0.946129	0.09885
27115	0.946131	0.0885368
27116	0.94613	0.104732
27117	0.946131	0.0975745
27118	0.946127	0.267902
27119	1.23351	0.218456
27120	0.946131	0.264929
27121	0.946129	0.244836
27122	0.94613	0.223544
27123	0.946131	0.128091
27124	0.946131	0.177698
27125	0.94613	0.132175
27126	1.18119	0.127713
27127	0.946131	0.152518
27128	0.946132	0.195541
27129	1.30563	0.148979
27130	0.946133	0.219465
27131	0.946132	0.156808
27132	0.946133	0.182467
27133	0.946132	0.323492
27134	0.946132	0.573252
27135	0.946133	1.81426
27136	1.20256	0.423497
27137	0.946133	0.573457
27138	0.94613	1.36567
27139	1.33007	0.641797
27140	0.946128	1.38254
27141	0.946132	0.690324

27142	0.946131	0.865862
27143	0.946131	2.1
27144	0.946131	2.1
27145	0.946128	2.1
27146	0.946129	2.1
27147	0.94613	2.1
27148	0.810942	2.1
27149	0.810942	2.1
27150	0.81093	2.1
27151	0.946132	2.1
27152	1.39823	2.1
27153	0.946132	2.1
27154	0.946131	2.1
27155	0.946132	2.1
27156	0.946131	2.1
27157	0.946131	2.1
27158	0.946131	2.1
27159	1.37713	2.1
27160	0.946131	2.1
27161	0.946133	2.1
27162	0.946131	2.1
27163	1.40594	2.1
27164	1.40594	2.1
27165	0.94613	2.1
27166	0.946133	2.1
27167	0.946131	2.1
27168	1.38155	2.1
27169	1.38155	2.1
27170	0.946131	2.1
27171	0.946132	1.52069
27172	0.946133	1.96996
27173	0.946132	1.74998
27174	0.946132	1.79627
27175	1.33694	1.86369
27176	0.946131	2.1
27177	1.228 2.1	
27178	0.946129	2.1
27179	0.946131	2.1
27180	1.228 2.1	
27181	0.946132	1.3778
27182	0.946132	1.67803
27183	0.946131	1.63008
27184	0.946132	1.49695
27185	1.35943	1.66437
27186	0.946133	0.05
27187	0.946133	0.05
27188	1.02377	0.05
27189	1.02377	0.05
27190	0.946132	0.05
27191	0.94613	0.05
27192	0.946131	0.05
27193	0.946132	0.05
27194	1.29322	0.05
27195	0.946131	0.05
27196	0.946131	2.1
27197	0.946131	0.963105
27198	0.946132	2.1
27199	1.41492	1.99879

27200	0.946131	2.1
27201	0.946132	2.1
27202	0.946131	2.1
27203	0.946132	2.1
27204	1.35028	2.1
27205	0.946132	2.1
27206	0.946132	2.1
27207	0.946132	2.1
27208	0.946131	2.1
27209	1.32461	2.1
27210	0.946132	2.1
27211	0.946131	2.1
27212	0.946132	2.1
27213	1.40369	2.1
27214	0.94613	2.1
27215	0.946131	2.1
27216	0.946132	0.102772
27217	0.979597	0.0937461
27218	0.946131	0.129698
27219	0.946132	0.0917448
27220	0.946132	0.10196
27221	0.946131	2.1
27222	0.946131	2.1
27223	0.94613	2.1
27224	0.946131	2.1
27225	1.41897	2.1
27226	1.37791	2.1
27227	0.94613	2.1
27228	0.946129	2.1
27229	0.946131	2.1
27230	0.946131	2.1
27231	1.39384	2.1
27232	0.946131	2.1
27233	0.946128	2.1
27234	0.946129	2.1
27235	0.946129	2.1
27236	0.810945	1.62854
27237	0.810944	2.08078
27238	0.810958	1.44981
27239	0.946128	2.1
27240	0.946132	2.1
27241	0.94613	2.1
27242	1.3335	2.1
27243	0.946131	2.1
27244	0.94613	2.05745
27245	0.946129	2.1
27246	0.946132	1.53083
27247	1.41192	2.1
27248	0.94613	2.1
27249	0.946132	1.46556
27250	0.946133	1.1421
27251	0.946132	0.364358
27252	1.39428	0.458999
27253	0.946133	0.360094
27254	0.94613	2.05926
27255	0.946128	2.09234
27256	0.946132	1.94554
27257	0.94613	1.86583

27258	1.40684	2.1
27259	0.946131	2.1
27260	0.946131	2.1
27261	0.946131	2.1
27262	0.946131	2.1
27263	1.39021	2.1
27264	0.946131	1.384
27265	1.31649	1.69147
27266	0.946131	1.29424
27267	0.946131	1.25232
27268	1.31649	1.39138
27269	0.810946	2.1
27270	0.810946	2.1
27271	0.810958	2.1
27272	1.16687	2.1
27273	0.946131	2.1
27274	0.946131	2.1
27275	1.16687	2.1
27276	1.16687	2.1
27277	0.946128	0.897579
27278	1.3564	0.401681
27279	0.946131	0.936144
27280	0.946131	0.584262
27281	0.94613	0.802716
27282	0.946128	2.1
27283	1.35828	2.1
27284	0.94613	2.1
27285	0.946131	2.1
27286	0.94613	2.1
27287	0.946133	1.42937
27288	1.36477	1.63949
27289	0.946132	1.61834
27290	0.946132	1.4532
27291	1.36477	1.59755
27292	0.946132	1.63757
27293	1.31857	1.79589
27294	0.946132	1.64373
27295	0.946132	1.65423
27296	1.31857	1.64484
27297	0.94613	0.42866
27298	1.35059	0.286696
27299	0.946128	0.286319
27300	0.94613	0.361422
27301	1.35059	0.307367
27302	0.946126	0.127948
27303	1.35559	0.0840767
27304	0.946127	0.103018
27305	0.946128	0.101254
27306	1.35559	0.0846108
27307	0.946131	0.0849733
27308	1.33035	0.0856751
27309	0.946127	0.0952533
27310	0.946128	0.0611759
27311	1.33035	0.0881873
27312	0.94613	0.0926586
27313	1.3665	0.0901518
27314	0.946129	0.0986091
27315	0.946131	0.0773456

27316	1.3665	0.0910133
27317	0.94613	0.106258
27318	1.32828	0.0888367
27319	0.946129	0.0979135
27320	0.94613	0.0939196
27321	0.946131	0.0898522
27322	0.946131	2.1
27323	0.946131	2.1
27324	1.32949	2.1
27325	1.32949	2.1
27326	0.946131	2.1
27327	0.946131	2.1
27328	0.94613	0.216862
27329	0.946133	2.1
27330	1.38357	0.701352
27331	0.946131	1.44079
27332	0.946131	2.1
27333	0.946128	2.1
27334	1.36919	2.1
27335	0.946131	2.1
27336	0.94613	2.1
27337	0.946131	2.1
27338	1.3771	2.1
27339	0.946129	2.1
27340	0.94613	2.1
27341	0.946131	2.1
27342	0.946131	1.86687
27343	0.94613	1.21185
27344	0.946129	0.382611
27345	1.30029	1.56481
27346	0.94613	0.589156
27347	1.21174	0.05
27348	0.946132	0.05
27349	0.946132	0.05
27350	0.946132	0.05
27351	0.946133	0.05
27352	0.810946	2.1
27353	0.810945	2.1
27354	0.810946	2.1
27355	1.1796	2.1
27356	0.946131	2.1
27357	0.946132	2.1
27358	1.1796	2.1
27359	1.1796	2.1
27360	0.946131	2.1
27361	0.946131	2.1
27362	0.946133	2.1
27363	0.946131	2.1
27364	1.40615	2.1
27365	0.810945	2.1
27366	0.810944	2.1
27367	0.810945	2.1
27368	1.40239	2.1
27369	0.94613	2.1
27370	1.35561	2.1
27371	0.946133	2.1
27372	1.35562	2.1
27373	1.41169	0.197791

27374	0.946131	0.331455
27375	0.946127	0.466462
27376	1.41169	0.203689
27377	0.946128	0.31171
27378	0.946119	2.1
27379	0.946119	2.1
27380	0.946119	2.1
27381	0.946107	2.1
27382	1.41917	2.1
27383	0.810946	1.89807
27384	0.810943	1.93971
27385	0.810934	2.07804
27386	0.946127	2.1
27387	1.1995	2.1
27388	0.946126	2.1
27389	0.946128	2.1
27390	1.1995	2.1
27391	0.810944	2.1
27392	0.810947	2.1
27393	0.81096	2.1
27394	0.946133	0.159619
27395	1.3495	0.09337
27396	0.946132	0.125629
27397	0.946132	0.0951338
27398	0.946133	0.105576
27399	0.946132	2.1
27400	0.946132	2.1
27401	0.946133	2.1
27402	1.39529	2.1
27403	0.946133	2.1
27404	0.946133	2.1
27405	1.28525	2.1
27406	0.946132	2.1
27407	0.946133	2.1
27408	1.28525	2.1
27409	0.810946	2.1
27410	0.810945	2.1
27411	0.810946	2.1
27412	1.33931	2.1
27413	0.946132	2.1
27414	0.946132	2.1
27415	1.33931	2.1
27416	1.24616	2.1
27417	0.946133	0.0832157
27418	0.946133	0.0625069
27419	0.946132	0.102368
27420	0.946132	0.0517728
27421	0.946133	0.0989884
27422	0.946128	2.1
27423	1.40225	2.1
27424	0.946128	2.1
27425	0.94613	2.1
27426	0.94613	2.1
27427	0.946113	0.05
27428	0.946118	0.05
27429	0.946114	0.05
27430	1.40551	0.05
27431	0.946103	0.05

27432	0.94613	2.1
27433	0.946132	2.1
27434	0.946129	2.1
27435	0.946131	2.1
27436	1.37811	2.1
27437	0.946132	2.1
27438	1.39826	2.1
27439	0.946133	2.1
27440	0.946132	2.1
27441	1.39826	2.1
27442	0.946118	2.1
27443	1.28298	2.1
27444	0.946117	2.1
27445	0.946105	2.1
27446	1.283	2.1
27447	0.946118	0.05
27448	1.41375	0.05
27449	0.946117	0.0512055
27450	0.946131	0.05
27451	0.946106	0.05
27452	0.946132	1.58304
27453	0.946129	1.40914
27454	1.28436	1.36336
27455	0.946131	1.59263
27456	0.946131	1.47235
27457	0.810947	0.413031
27458	0.810946	0.42996
27459	0.81096	0.367902
27460	0.946132	0.288741
27461	1.34338	0.254942
27462	0.961435	0.237044
27463	0.961435	0.270882
27464	0.946132	0.26861
27465	1.3028	2.02348
27466	0.946131	2.04652
27467	0.946127	2.1
27468	0.946128	2.1
27469	0.946129	2.07267
27470	0.946132	0.761629
27471	1.37973	0.357133
27472	0.946132	0.794507
27473	0.946132	0.478258
27474	0.946132	0.447576
27475	0.946131	1.30579
27476	0.94613	1.27746
27477	0.946129	1.30605
27478	0.946131	1.25919
27479	1.41798	0.996436
27480	0.946118	1.25289
27481	0.946118	0.905949
27482	0.946117	1.25454
27483	0.946131	1.11363
27484	0.946105	0.934443
27485	0.946131	1.35306
27486	0.946131	1.30837
27487	0.94613	1.35019
27488	0.946131	1.30856
27489	0.946131	1.26947

27490	0.946133	1.93637
27491	1.40274	2.1
27492	0.946133	1.51001
27493	0.946132	2.1
27494	1.40274	2.1
27495	0.946131	1.81953
27496	1.39731	2.1
27497	0.946132	1.53652
27498	0.94613	1.90192
27499	1.39731	2.0974
27500	0.94612	2.1
27501	0.94612	1.72504
27502	0.946119	1.79132
27503	0.946132	1.51281
27504	1.40764	1.97152
27505	0.946128	2.1
27506	0.946132	2.1
27507	0.94613	2.1
27508	0.94613	2.1
27509	1.31213	2.1
27510	0.946127	2.1
27511	0.94613	2.1
27512	0.946127	2.1
27513	0.946128	2.1
27514	1.36653	2.1
27515	0.946127	2.1
27516	1.3557	2.1
27517	0.946128	2.1
27518	0.946128	2.1
27519	1.3557	2.1
27520	0.946133	2.1
27521	0.946132	2.1
27522	0.946133	2.1
27523	0.946132	2.1
27524	1.40081	2.1
27525	0.946128	2.1
27526	0.946133	2.1
27527	0.946131	2.1
27528	0.946131	2.1
27529	1.32282	2.1
27530	0.810945	2.1
27531	0.810943	2.1
27532	0.810933	2.1
27533	1.27676	2.1
27534	0.94613	2.1
27535	0.946128	2.1
27536	0.946131	2.1
27537	0.946131	2.1
27538	0.946131	0.653515
27539	0.946127	0.951983
27540	1.41328	1.2596
27541	1.41327	1.03947
27542	0.946128	0.91731
27543	0.946132	0.0637902
27544	1.32083	0.103882
27545	0.946132	0.096091
27546	0.946133	0.0699653
27547	1.32082	0.0782446

27548	0.946128	0.087495
27549	1.29215	0.117144
27550	0.94613	0.115004
27551	0.94613	0.0874788
27552	1.29215	0.097875
27553	0.946132	0.0504684
27554	1.27696	0.05
27555	0.94613	0.0533386
27556	0.94613	0.0563425
27557	1.27696	0.05
27558	0.94612	0.05
27559	1.29289	0.0722463
27560	0.946119	0.0616911
27561	0.946132	0.0535871
27562	1.29287	0.0538282
27563	0.946128	2.1
27564	1.37869	2.1
27565	0.946131	2.1
27566	0.94613	2.1
27567	0.946131	2.1
27568	0.946133	2.1
27569	0.946132	2.1
27570	0.946132	2.1
27571	1.35703	2.1
27572	0.946133	2.1
27573	0.946132	2.1
27574	0.946132	2.1
27575	0.946133	2.1
27576	1.37017	2.1
27577	0.946133	2.1
27578	0.946119	0.05
27579	0.94612	0.05
27580	1.38583	0.05
27581	1.38585	0.05
27582	0.946107	0.05
27583	0.946126	0.0604904
27584	0.946127	0.0712091
27585	1.22904	0.0562801
27586	1.22904	0.05
27587	0.946128	0.0629509
27588	1.3279	0.812413
27589	0.946131	1.56311
27590	0.94613	1.48733
27591	0.946131	1.54531
27592	0.946131	1.47454
27593	0.946132	2.1
27594	0.946132	2.1
27595	0.946132	2.1
27596	0.946131	2.1
27597	1.40932	2.1
27598	1.26868	0.0807165
27599	0.946131	0.121007
27600	0.94613	0.111696
27601	0.946131	0.106344
27602	0.946131	0.103536
27603	0.810943	2.1
27604	0.810945	2.1
27605	0.810932	2.1

27606	0.810943	2.1
27607	0.810944	0.873643
27608	0.810957	1.05165
27609	1.31539	0.826531
27610	0.946132	2.1
27611	0.946128	0.791225
27612	1.3154	1.75579
27613	1.17856	0.700034
27614	1.21997	1.57316
27615	0.94613	1.83125
27616	0.946129	1.74025
27617	0.94613	1.90032
27618	0.94613	1.82092
27619	0.946131	1.58598
27620	0.946131	1.47843
27621	0.946129	1.59834
27622	0.94613	1.63463
27623	0.946131	1.7344
27624	0.946127	0.799865
27625	0.946131	0.176083
27626	0.946127	1.66817
27627	0.946129	0.94821
27628	1.41342	1.14633
27629	0.946131	0.956671
27630	0.946131	1.60193
27631	0.946128	1.5009
27632	0.946131	1.2448
27633	1.29149	1.3814
27634	1.41022	2.1
27635	0.94613	2.07005
27636	0.946128	2.1
27637	0.94613	2.1
27638	0.94613	2.1
27639	0.946128	0.0777338
27640	0.94613	0.128148
27641	1.27864	0.0732389
27642	1.27864	0.0610157
27643	0.946131	0.100077
27644	0.946129	1.15635
27645	0.946125	1.40742
27646	0.946124	1.25427
27647	0.946127	1.28229
27648	1.37759	1.3521
27649	0.946129	1.28614
27650	0.94613	1.46983
27651	0.946126	1.34116
27652	0.946129	1.36505
27653	1.38259	1.42183
27654	0.946131	0.0588792
27655	1.34118	0.05
27656	0.946132	0.064818
27657	0.946132	0.0621858
27658	1.34118	0.0507988
27659	0.946131	0.05
27660	1.25017	0.05
27661	0.946128	0.05
27662	0.946131	0.05
27663	0.946131	0.05

27664	0.946131	2.1
27665	0.946128	2.1
27666	0.946133	2.1
27667	1.3937	2.1
27668	0.94613	2.1
27669	0.946131	2.1
27670	0.94613	0.662639
27671	1.40404	1.61367
27672	1.40404	0.834273
27673	0.946131	1.21405
27674	0.946132	1.69175
27675	0.946132	0.358015
27676	1.38897	0.310864
27677	1.38897	0.167881
27678	0.946132	0.477231
27679	0.94613	0.91313
27680	0.946128	1.93058
27681	1.40332	1.01015
27682	1.40331	1.35029
27683	0.94613	1.61175
27684	0.946132	1.96476
27685	0.946132	2.1
27686	0.946133	2.1
27687	1.41619	2.1
27688	0.946132	2.1
27689	0.946132	2.1
27690	0.946132	2.1
27691	0.946132	2.1
27692	1.38244	2.1
27693	0.946132	2.1
27694	0.810946	1.55472
27695	0.810943	0.988893
27696	0.810934	0.55674
27697	0.810947	1.47345
27698	0.810943	0.534703
27699	0.810934	0.617882
27700	1.12786	1.66976
27701	0.946132	2.01529
27702	0.946132	1.85094
27703	0.946132	1.77231
27704	0.946133	1.77679
27705	0.946132	1.65006
27706	0.946132	1.73382
27707	0.946132	1.59652
27708	1.27806	1.6987
27709	0.946133	1.62981
27710	0.946132	2.1
27711	0.946132	2.1
27712	0.946133	2.1
27713	0.946132	2.1
27714	0.946133	2.1
27715	1.39514	1.34279
27716	0.946129	1.23821
27717	0.946124	1.3572
27718	0.946127	1.18
27719	0.946127	1.32302
27720	0.94613	2.1
27721	0.946129	2.1

27722	0.946132	2.1
27723	1.3991	2.1
27724	0.94613	2.1
27725	0.946132	1.65348
27726	0.946133	2.1
27727	0.946132	2.09139
27728	1.36835	2.1
27729	0.946132	2.1
27730	0.946132	2.1
27731	0.946132	2.1
27732	0.946133	2.1
27733	0.946132	2.1
27734	1.39047	2.1
27735	0.946132	0.05
27736	0.946128	0.05
27737	0.94613	0.05
27738	1.33174	0.05
27739	0.946131	0.05
27740	0.946128	0.05
27741	0.946131	0.05
27742	0.94613	0.05
27743	0.94613	0.05
27744	0.94613	0.05
27745	0.946119	2.1
27746	0.946119	2.1
27747	1.29064	2.1
27748	0.946132	2.1
27749	0.946107	2.1
27750	0.946133	2.1
27751	0.94613	2.1
27752	1.30584	2.1
27753	0.946131	2.1
27754	0.946131	2.1
27755	0.946131	2.1
27756	0.946132	2.1
27757	0.94613	2.1
27758	0.946131	2.1
27759	1.39549	2.1
27760	0.913386	1.41705
27761	0.978873	1.33194
27762	0.946126	1.39683
27763	0.94613	1.46887
27764	1.00473	1.0799
27765	0.946132	1.08153
27766	0.946133	1.13564
27767	0.946132	0.352487
27768	0.946132	1.11783
27769	1.38894	0.458501
27770	0.946128	0.863387
27771	0.946131	0.55573
27772	0.94613	0.331937
27773	0.94613	0.569917
27774	1.23865	0.316277
27775	0.946126	1.36041
27776	0.946129	1.08793
27777	0.946124	0.862203
27778	0.946127	1.22392
27779	1.40248	0.754224

27780	0.946133	1.07651
27781	0.946132	1.18944
27782	0.946132	0.572634
27783	0.946133	1.06351
27784	1.1391	0.847741
27785	0.827862	1.16053
27786	0.946133	1.12848
27787	0.946131	1.22109
27788	0.946131	1.18411
27789	0.94613	1.14472
27790	0.946128	0.955074
27791	0.946132	1.07798
27792	0.94613	1.09435
27793	1.21388	0.522616
27794	0.94613	1.06202
27795	0.810943	0.146226
27796	0.810945	0.219528
27797	0.810932	0.193136
27798	0.946131	1.83616
27799	0.946131	1.60377
27800	0.946133	1.4006
27801	1.36748	0.991956
27802	0.946132	0.220084
27803	0.946128	0.05
27804	0.946131	0.05
27805	0.94613	0.05
27806	1.35856	0.05
27807	0.94613	0.05
27808	0.946132	2.1
27809	0.946131	2.1
27810	0.946133	2.1
27811	0.946132	2.1
27812	0.946131	2.1
27813	0.946133	2.1
27814	0.946131	2.1
27815	1.39558	2.1
27816	1.39558	2.1
27817	0.94613	2.1
27818	0.946131	0.10688
27819	0.94613	0.147437
27820	0.946129	0.109755
27821	1.41388	0.103736
27822	0.946131	0.126056
27823	0.946133	0.105988
27824	0.946132	0.205989
27825	0.946132	0.108634
27826	0.946133	0.124406
27827	0.93452	0.196079
27828	0.946129	2.1
27829	1.38897	2.1
27830	0.946131	2.1
27831	0.946131	2.1
27832	1.38897	2.1
27833	0.946133	1.83776
27834	0.946132	1.80337
27835	0.946133	1.76774
27836	0.946132	1.57608
27837	1.38773	1.66904

27838	1.41512	0.124916
27839	0.946131	0.207553
27840	0.946129	0.167681
27841	0.946133	0.182823
27842	0.946131	0.183394
27843	0.946132	1.68737
27844	0.946132	2.1
27845	0.946132	1.77488
27846	0.946133	2.1
27847	0.946133	2.1
27848	0.946126	1.60597
27849	0.946132	1.92987
27850	0.946129	1.69148
27851	0.946129	1.88811
27852	0.946129	2.05555
27853	0.939895	2.0473
27854	1.03386	2.1
27855	0.94613	2.04285
27856	0.946131	2.06314
27857	1.03386	2.09439
27858	0.946131	2.1
27859	1.39309	2.1
27860	0.946133	2.1
27861	0.946131	2.1
27862	1.39309	2.1
27863	0.946133	2.1
27864	1.24853	2.1
27865	0.946132	2.1
27866	0.946132	2.1
27867	1.24853	2.1
27868	0.946133	2.03692
27869	1.39699	1.56032
27870	0.946132	1.88827
27871	0.946132	2.1
27872	1.39699	1.58687
27873	0.946132	2.1
27874	0.946131	2.1
27875	0.946133	2.1
27876	0.946131	2.1
27877	1.38969	2.1
27878	1.28464	1.93601
27879	0.946133	2.03505
27880	0.94613	2.07184
27881	0.946131	1.96035
27882	0.946131	1.94448
27883	0.946119	1.10388
27884	0.94612	1.17783
27885	0.946119	1.1191
27886	0.946132	1.1148
27887	1.3749	1.11751
27888	0.810942	2.1
27889	0.810943	2.1
27890	0.810957	2.1
27891	1.41134	1.88395
27892	0.946132	1.23421
27893	0.946132	1.76315
27894	0.946132	1.28
27895	0.946132	1.59951

27896	1.31338	1.92713
27897	0.946132	1.66777
27898	0.946132	1.95986
27899	0.946133	1.73671
27900	0.946132	1.81763
27901	0.946103	0.05
27902	0.752448	0.05
27903	0.946132	0.05
27904	0.946131	0.05
27905	1.20617	0.05
27906	0.946132	1.37113
27907	1.08054	1.1612
27908	1.25736	1.42209
27909	0.946132	1.37213
27910	0.946131	1.37832
27911	0.946128	2.1
27912	1.33275	2.1
27913	1.1896	2.1
27914	0.946131	2.1
27915	1.18962	2.1
27916	1.21955	0.05
27917	0.946131	0.05
27918	1.33587	0.05
27919	0.741946	0.05
27920	1.07387	0.05
27921	0.945844	1.61463
27922	1.3292	2.1
27923	1.59472	2.03274
27924	0.946131	1.75844
27925	1.04043	1.87436
27926	1.13529	0.0898091
27927	0.946132	0.0930563
27928	1.24893	0.105567
27929	1.12675	0.106491
27930	0.936652	0.103272
27931	1.36968	2.1
27932	0.946131	2.1
27933	0.946132	2.1
27934	1.36968	2.1
27935	1.258 2.1	
27936	3.17458	0.141088
27937	0.946132	2.1
27938	1.35022	2.1
27939	1.35022	2.1
27940	0.94613	2.1
27941	1.12356	2.1
27942	0.946131	2.1
27943	1.22401	2.1
27944	1.22401	2.1
27945	0.94613	2.1
27946	0.952455	2.1
27947	1.2107	2.1
27948	0.946132	2.1
27949	1.33047	2.1
27950	0.946131	2.1
27951	0.946132	2.1
27952	0.61957	2.1
27953	0.8636	2.1

27954	0.946132	2.1
27955	1.19294	2.1
27956	0.946128	2.1
27957	1.36607	2.1
27958	0.94613	2.1
27959	1.39416	2.1
27960	1.36607	2.1
27961	1.21474	2.1
27962	0.946131	2.1
27963	0.946131	2.1
27964	1.16288	2.1
27965	1.08542	2.1
27966	0.851526	1.63472
27967	1.30374	2.1
27968	1.01708	2.1
27969	0.946114	1.79326
27970	0.96976	1.89936
27971	0.946132	0.103418
27972	1.49997	0.120418
27973	0.771735	0.113869
27974	0.771764	0.14104
27975	0.946133	0.169526
27976	0.700863	1.82406
27977	1.42222	2.1
27978	0.946131	2.1
27979	0.946128	2.1
27980	1.42509	2.1
27981	1.14298	2.1
27982	0.837014	2.1
27983	0.946131	2.1
27984	1.29426	2.1
27985	0.836985	2.1
27986	0.946129	2.1
27987	1.22089	0.423937
27988	0.946132	1.40486
27989	0.712486	0.425357
27990	0.6234	0.469583
27991	0.946131	2.1
27992	1.40271	2.1
27993	1.32241	2.1
27994	0.946133	2.1
27995	1.32241	2.1
27996	0.769373	1.67193
27997	0.946129	2.1
27998	1.41302	2.1
27999	1.28254	2.1
28000	0.946131	2.1
28001	1.28254	2.1
28002	0.851543	2.03387
28003	1.23056	2.1
28004	1.24356	2.1
28005	0.851543	2.07091
28006	0.909417	2.1
28007	1.13808	0.11897
28008	0.946133	0.523934
28009	0.946131	0.252903
28010	1.13808	1.3694
28011	1.13808	0.246437

28012	0.930941	1.56819
28013	1.37808	1.52787
28014	1.17573	1.29831
28015	0.946124	1.55979
28016	1.02404	1.42134
28017	0.946116	2.1
28018	1.3144	2.1
28019	1.3144	2.1
28020	0.946118	2.1
28021	1.13089	2.1
28022	0.946128	0.0857512
28023	0.946101	0.0779341
28024	1.2606	0.101676
28025	1.26055	0.0803287
28026	0.580823	0.724397
28027	0.946132	1.55783
28028	0.580825	0.850337
28029	1.23224	1.03737
28030	0.946128	2.1
28031	1.3719	2.1
28032	0.94613	2.1
28033	1.3719	2.1
28034	0.94613	2.1
28035	0.946132	1.7713
28036	1.35025	0.390958
28037	1.35025	1.07681
28038	0.946132	1.80354
28039	1.1794	1.69325
28040	0.946133	2.1
28041	1.25598	2.1
28042	1.29249	2.1
28043	1.38154	2.1
28044	0.851541	2.1
28045	1.31508	2.1
28046	1.37558	2.1
28047	0.946129	2.1
28048	0.867663	2.1
28049	0.94613	1.68853
28050	1.35426	1.28133
28051	0.84872	0.615747
28052	0.946129	1.65493
28053	1.17195	1.72551
28054	0.946133	2.1
28055	1.39009	2.1
28056	1.14342	2.1
28057	0.946133	2.1
28058	1.14343	2.1
28059	1.38819	2.1
28060	0.946133	2.1
28061	1.4116	2.1
28062	0.946131	2.1
28063	0.946131	2.1
28064	0.946127	2.1
28065	1.32244	2.1
28066	1.32244	2.1
28067	0.946131	2.1
28068	1.2176	2.1
28069	1.75823	2.1

28070	1.11239	2.1
28071	2.17979	2.1
28072	1.65533	2.1
28073	3.41356	2.1
28074	1.94841	2.1
28075	1.91767	2.1
28076	1.76726	2.1
28077	1.70302	2.1
28078	3.76137	2.1
28079	1.94841	2.1
28080	1.33962	2.1
28081	1.31365	2.1
28082	1.70302	2.1
28083	3.2123	2.1
28084	1.76726	2.1
28085	1.70302	2.1
28086	1.47581	2.1
28087	1.7457	2.1
28088	3.22764	2.1
28089	1.20478	2.1
28090	1.70302	2.1
28091	1.27956	2.1
28092	1.18569	2.1
28093	1.63979	2.1
28094	1.51949	2.1
28095	1.53082	2.1
28096	1.92911	2.1
28097	1.32068	2.1
28098	1.74454	2.1
28099	3.27536	2.1
28100	1.08172	2.1
28101	1.74454	2.1
28102	1.52603	2.1
28103	1.52603	2.1
28104	1.32068	2.1
28105	1.32068	2.1
28106	1.89226	2.1
28107	2.37364	2.1
28108	1.89226	2.1
28109	2.42769	2.1
28110	4.31995	2.1
28111	2.37365	2.1
28112	1.5362	2.1
28113	2.4277	2.1
28114	1.56328	2.1
28115	3.9639	2.1
28116	1.89225	2.1
28117	2.42768	2.1
28118	1.89213	2.1
28119	1.89225	2.1
28120	2.42756	2.1
28121	2.42768	2.1
28122	2.42768	2.1
28123	1.58163	2.1
28124	1.56315	2.1
28125	1.56327	2.1
28126	2.42757	2.1
28127	2.42768	2.1

28128	1.01641	2.1
28129	2.15005	2.1
28130	1.00099	2.1
28131	2.15114	2.1
28132	3.15104	2.1
28133	0.994813	2.1
28134	2.15114	2.1
28135	2.17159	2.1
28136	2.17159	2.1
28137	1.00099	2.1
28138	1.00099	2.1
28139	1.29608	2.1
28140	1.29608	2.1
28141	0.942035	2.1
28142	0.942035	2.1
28143	1.29198	2.1
28144	0.942033	2.1
28145	0.946132	2.1
28146	0.946132	2.1
28147	0.946132	2.1
28148	0.942033	2.1
28149	0.946133	2.1
28150	1.29198	2.1
28151	1.99985	2.1
28152	1.14207	2.1
28153	1.83584	2.1
28154	1.00232	2.1
28155	3.00217	2.1
28156	1.99867	0.05
28157	2.0815	0.05
28158	1.56806	0.05
28159	1.28828	0.05
28160	3.58519	0.05
28161	1.89226	0.05
28162	1.89226	0.0657156
28163	1.89226	0.0573775
28164	1.89226	0.0628553
28165	1.89226	0.0553109
28166	1.89226	0.0611543
28167	1.89226	0.05
28168	1.89226	0.0569453
28169	2.23193	0.05
28170	2.48978	0.064051
28171	4.38204	0.05
28172	1.85293	2.1
28173	1.64279	0.378483
28174	1.76268	2.1
28175	1.54214	0.354261
28176	3.39507	1.04659
28177	1.6428	0.38054
28178	1.17302	1.12308
28179	1.17302	1.9534
28180	1.28419	0.881676
28181	1.54214	0.332057
28182	1.65331	1.02359
28183	1.76268	2.1
28184	1.54214	0.802742
28185	1.65324	2.1

28186	1.42491	0.692194
28187	3.19538	0.788387
28188	0.651043	1.93644
28189	0.651043	1.57528
28190	0.647925	0.567315
28191	0.647925	1.84874
28192	0.521747	0.862318
28193	0.58651	1.02438
28194	0.77107	0.183995
28195	0.712457	0.308202
28196	0.712457	0.425751
28197	0.521747	0.361836
28198	0.58651	0.357032
28199	0.77107	0.523467
28200	1.2873	2.1
28201	1.48751	2.1
28202	1.59659	2.1
28203	1.77596	2.1
28204	3.06326	2.1
28205	1.07976	2.1
28206	1.59659	2.1
28207	1.30237	2.1
28208	1.77723	2.1
28209	2.89897	2.1
28210	1.77723	2.1
28211	1.77596	2.1
28212	1.82614	2.1
28213	1.59659	2.1
28214	2.0055	2.1
28215	1.77596	2.1
28216	2.64523	2.09985
28217	2.73982	0.507222
28218	2.50057	2.04823
28219	2.61606	0.386083
28220	5.26129	1.0934
28221	2.73982	0.276374
28222	2.68987	0.578643
28223	2.61607	0.43871
28224	2.57611	0.626146
28225	5.30593	0.05
28226	1.89226	0.120114
28227	1.89226	0.374257
28228	1.89226	0.124208
28229	2.02178	0.0741639
28230	3.78452	0.117823
28231	1.89226	0.585312
28232	1.89226	0.569934
28233	1.89226	1.69997
28234	1.89226	0.42109
28235	1.89226	1.72113
28236	1.89226	0.317569
28237	2.35204	2.1
28238	1.50108	2.1
28239	1.3715	2.1
28240	2.18469	2.1
28241	3.68577	2.1
28242	2.62514	2.1
28243	2.1847	2.1

28244	2.0487	2.1
28245	2.45213	2.1
28246	4.63682	2.1
28247	1.25075	2.1
28248	2.18469	2.1
28249	1.3715	2.1
28250	1.3715	2.1
28251	2.0487	2.1
28252	2.0487	2.1
28253	1.28667	2.1
28254	1.11285	2.1
28255	1.03529	2.1
28256	1.03529	2.1
28257	1.30711	2.1
28258	1.30711	2.1
28259	2.35053	2.1
28260	1.89225	2.1
28261	1.89225	2.1
28262	2.51216	2.1
28263	4.24278	2.1
28264	1.89225	2.1
28265	1.89225	2.1
28266	1.89225	2.1
28267	1.89225	2.1
28268	3.7845	2.1
28269	1.92025	2.1
28270	1.32119	2.1
28271	1.44777	2.1
28272	1.33834	2.1
28273	1.92025	2.1
28274	1.81082	2.1
28275	1.89226	2.1
28276	2.67272	2.1
28277	2.51218	2.1
28278	2.51218	2.1
28279	1.89226	2.1
28280	1.89226	2.1
28281	1.89226	2.1
28282	1.89226	2.1
28283	1.89226	2.1
28284	1.89226	2.1
28285	3.78452	2.1
28286	1.92027	2.1
28287	1.44778	2.1
28288	1.61121	2.1
28289	2.01555	2.1
28290	3.53147	2.1
28291	1.90727	0.05
28292	2.29851	0.0921346
28293	2.71643	0.0579329
28294	2.17695	0.05
28295	4.47547	0.05
28296	1.90728	0.0746049
28297	1.56339	0.0742595
28298	1.58143	0.05
28299	2.17696	0.0693813
28300	3.74035	0.0746174
28301	1.91368	0.05

28302	2.17696	0.0811265
28303	2.39986	0.0664666
28304	1.75904	0.05
28305	3.936	0.05
28306	1.18793	0.05
28307	2.74744	0.05
28308	1.38716	0.05
28309	2.57465	0.05
28310	3.93537	0.05
28311	1.3148	0.05
28312	2.74744	0.0641325
28313	2.70805	0.05
28314	1.38716	0.05
28315	2.70805	0.0521969
28316	1.38716	0.05
28317	1.20608	2.1
28318	0.975871	2.1
28319	0.946132	2.1
28320	0.946132	2.1
28321	0.946132	2.1
28322	0.946132	2.1
28323	0.975871	2.1
28324	0.975871	2.1
28325	1.20608	2.1
28326	0.946132	2.1
28327	0.946133	2.1
28328	0.975871	2.1
28329	2.8198	2.1
28330	2.29967	2.1
28331	2.09542	2.1
28332	2.63784	1.97768
28333	4.93751	2.1
28334	2.09542	2.1
28335	1.39523	2.1
28336	1.21119	2.1
28337	1.39523	2.1
28338	2.11563	2.1
28339	2.29967	2.1
28340	1.88584	2.1
28341	2.63784	1.83536
28342	2.11262	2.1
28343	1.91132	2.1
28344	4.02153	2.1
28345	1.88584	2.1
28346	1.21119	2.1
28347	1.12606	2.1
28348	1.91132	2.1
28349	3.1225	2.1
28350	2.18077	2.08581
28351	1.78167	1.71177
28352	2.2947	1.43264
28353	1.93139	1.61808
28354	4.07637	1.49537
28355	1.5709	1.43135
28356	1.03235	1.72748
28357	2.05933	1.56134
28358	1.78168	1.36892
28359	1.31	0.286539

28360	1.03235	1.08518
28361	2.2947	1.63826
28362	2.31785	1.91764
28363	2.40627	2.1
28364	2.08288	1.34701
28365	4.37758	1.90259
28366	1.95737	1.71276
28367	1.31	1.69867
28368	2.08289	0.504468
28369	1.50108	0.54089
28370	3.39289	1.14845
28371	2.49676	2.1
28372	2.24781	2.1
28373	2.5276	2.1
28374	2.7352	2.1
28375	5.02436	2.1
28376	2.49676	2.1
28377	2.43549	2.1
28378	2.6398	2.1
28379	2.7352	2.1
28380	5.13656	2.1
28381	1.89226	2.1
28382	2.7352	2.1
28383	1.89226	2.1
28384	2.6398	2.1
28385	1.89226	2.1
28386	2.6398	2.1
28387	2.16843	0.05
28388	1.00185	0.05
28389	1.90039	2.1
28390	3.1547	0.05
28391	2.54064	0.05
28392	2.46752	2.1
28393	1.90039	2.1
28394	2.26044	2.1
28395	4.36791	1.98483
28396	1.65815	2.1
28397	2.48652	2.1
28398	1.65815	2.1
28399	1.9004	2.1
28400	2.21711	2.1
28401	2.5034	2.1
28402	2.37167	2.1
28403	2.59965	2.1
28404	2.62812	2.1
28405	2.40308	2.1
28406	4.99979	2.1
28407	2.50137	2.1
28408	2.59965	2.1
28409	2.62812	2.1
28410	2.5214	2.1
28411	5.12948	2.1
28412	2.62814	2.1
28413	2.4031	2.1
28414	2.43509	2.1
28415	2.65066	2.1
28416	5.06323	2.1
28417	2.62814	2.1

28418	2.52142	2.1
28419	2.53008	2.1
28420	2.65066	2.1
28421	5.15822	2.1
28422	2.0815	2.1
28423	1.92067	2.1
28424	1.59611	2.1
28425	1.89226	2.1
28426	1.62453	2.1
28427	1.92067	2.1
28428	1.89226	2.1
28429	1.89226	2.1
28430	1.89226	2.1
28431	1.89226	2.1
28432	3.78453	2.1
28433	0.946131	2.1
28434	0.946131	2.1
28435	0.929501	2.1
28436	0.910761	2.1
28437	0.9461	2.1
28438	0.9461	2.1
28439	0.910792	2.1
28440	0.910792	2.1
28441	0.946131	2.1
28442	0.929501	2.1
28443	0.910761	2.1
28444	0.946131	2.1
28445	2.0815	2.1
28446	1.81661	2.1
28447	2.1341	2.1
28448	2.0815	2.1
28449	1.86921	2.1
28450	1.81661	2.1
28451	1.89226	2.1
28452	1.89226	2.1
28453	1.89226	2.1
28454	1.89226	2.1
28455	1.89226	2.1
28456	1.89226	2.1
28457	0.946131	2.1
28458	0.946131	2.1
28459	0.949104	2.1
28460	0.9295	2.1
28461	0.946132	2.1
28462	0.946132	2.1
28463	0.929499	2.1
28464	0.929499	2.1
28465	0.946131	2.1
28466	0.949105	2.1
28467	0.946131	2.1
28468	0.929501	2.1
28469	1.66104	2.1
28470	2.34044	2.1
28471	2.08889	2.1
28472	1.33264	2.1
28473	3.67308	2.1
28474	2.0889	2.1
28475	1.08011	2.1

28476	1.08011	2.1
28477	1.33264	2.1
28478	1.87598	2.1
28479	2.12851	2.1
28480	2.27472	2.1
28481	1.89226	1.90804
28482	2.27703	2.1
28483	1.89226	1.8419
28484	4.16699	2.1
28485	1.89226	1.98034
28486	1.89226	1.70425
28487	1.89226	1.89264
28488	1.89226	1.59287
28489	3.78452	1.66704
28490	1.00007	1.96838
28491	1.32316	1.50045
28492	1.29417	1.32192
28493	1.29417	1.91666
28494	1.02051	1.68721
28495	1.02051	1.85741
28496	2.2846	2.1
28497	1.89226	2.09328
28498	2.27703	2.07597
28499	2.27703	2.1
28500	1.89226	1.82769
28501	1.89226	2.1
28502	1.89226	2.1
28503	1.89226	2.03451
28504	1.89226	1.78327
28505	1.89226	0.668699
28506	3.78452	1.52196
28507	1.02051	2.1
28508	1.32316	1.97009
28509	1.03373	1.77266
28510	1.34066	1.69423
28511	2.35689	2.04918
28512	1.703 2.1	
28513	1.89224	2.1
28514	1.52266	2.1
28515	1.89224	2.1
28516	1.23344	2.1
28517	1.53282	2.1
28518	1.89223	2.1
28519	1.89223	2.1
28520	1.89223	2.1
28521	1.89223	2.1
28522	3.78447	2.1
28523	0.796845	2.1
28524	0.796845	2.1
28525	0.812146	2.1
28526	0.812146	2.1
28527	1.08006	2.1
28528	1.02679	2.1
28529	0.551538	2.1
28530	0.528929	2.1
28531	0.551529	2.1
28532	1.08005	2.1
28533	0.528935	2.1

28534	1.0268	2.1
28535	1.5001	2.1
28536	1.70302	2.1
28537	2.0726	2.1
28538	1.70302	2.1
28539	1.99601	2.1
28540	1.70303	2.1
28541	1.89226	2.1
28542	1.89226	2.1
28543	1.89226	2.1
28544	1.89226	2.1
28545	1.89226	2.1
28546	1.89226	2.1
28547	1.17285	2.1
28548	2.16015	2.1
28549	2.23563	2.1
28550	2.23563	2.1
28551	1.10309	2.1
28552	1.10309	2.1
28553	0.946128	2.1
28554	0.946128	2.1
28555	1.21847	2.1
28556	1.2884	2.1
28557	0.94613	2.1
28558	0.94613	2.1
28559	1.21847	2.1
28560	1.21847	2.1
28561	0.946131	2.1
28562	1.21847	2.1
28563	0.94613	2.1
28564	1.2884	2.1
28565	1.83922	2.1
28566	2.5768	2.1
28567	1.83488	2.1
28568	1.82846	0.05
28569	2.43693	2.1
28570	2.43693	2.1
28571	2.36134	2.1
28572	1.81656	2.1
28573	1.86454	2.1
28574	1.6697	2.1
28575	2.55618	2.1
28576	2.36134	2.1
28577	2.55618	2.1
28578	1.9323	2.1
28579	1.9302	2.1
28580	1.81656	2.1
28581	2.66983	2.1
28582	2.55618	2.1
28583	1.89226	1.55934
28584	1.70302	1.67316
28585	1.94935	1.76601
28586	1.70302	1.63525
28587	1.95569	1.5907
28588	1.70302	0.997722
28589	2.82465	2.1
28590	2.41707	2.1
28591	2.24481	2.1

28592	2.68958	2.1
28593	5.10665	2.1
28594	1.41232	2.1
28595	1.34479	2.1
28596	0.946129	2.1
28597	0.946129	2.1
28598	0.94613	2.1
28599	0.94613	2.1
28600	1.41232	2.1
28601	1.41232	2.1
28602	1.34479	2.1
28603	0.94613	2.1
28604	0.946131	2.1
28605	1.41232	2.1
28606	1.86859	0.0517343
28607	1.89226	0.0607794
28608	1.89226	0.0620278
28609	1.89226	0.0565673
28610	1.45875	0.0575752
28611	1.45875	0.05
28612	2.1769	0.440257
28613	2.50259	0.50689
28614	1.94955	0.214877
28615	2.25959	0.242351
28616	4.45214	0.638991
28617	1.89226	1.00946
28618	1.89226	1.04188
28619	1.89226	0.390068
28620	1.89226	1.00077
28621	3.78452	0.421674
28622	1.89226	1.13518
28623	1.89226	1.21688
28624	1.89226	0.922375
28625	1.89226	1.17997
28626	3.78452	1.30519
28627	2.50259	1.05554
28628	2.49445	0.448513
28629	2.25959	0.23539
28630	2.20843	0.699843
28631	4.71102	0.332647
28632	1.94955	0.251154
28633	2.25959	0.18334
28634	1.24689	0.106421
28635	1.79906	0.204407
28636	3.74861	0.146228
28637	1.89226	0.638779
28638	1.89226	0.457254
28639	1.89226	0.178361
28640	1.89226	0.253472
28641	3.78452	0.949689
28642	1.89226	0.432741
28643	1.89226	0.564989
28644	1.89226	0.234729
28645	1.89226	0.288136
28646	3.78452	0.216149
28647	2.25959	0.184923
28648	2.20843	0.30593
28649	1.79906	0.187578

28650	1.98391	0.205419
28651	4.00748	0.262594
28652	1.89226	2.1
28653	1.89226	2.1
28654	1.89226	2.1
28655	1.89226	2.1
28656	3.78452	2.1
28657	1.89226	2.1
28658	1.89226	2.1
28659	1.89226	2.1
28660	1.89226	2.1
28661	3.78452	2.1
28662	1.64718	2.1
28663	1.23085	2.1
28664	1.46028	2.1
28665	1.39564	2.1
28666	1.68372	2.1
28667	1.64718	2.1
28668	1.64718	2.1
28669	1.64981	2.1
28670	1.70303	2.1
28671	1.6991	2.1
28672	3.3199	2.1
28673	1.89226	2.1
28674	1.89226	2.1
28675	1.89226	2.1
28676	1.89226	2.1
28677	3.78452	2.1
28678	1.89226	2.1
28679	1.89226	2.1
28680	1.89226	2.1
28681	1.89226	2.1
28682	3.78452	2.1
28683	0.666701	2.1
28684	0.65896	2.1
28685	0.920797	2.1
28686	0.895441	2.1
28687	0.775871	2.1
28688	0.687493	2.1
28689	0.777393	2.1
28690	0.777393	2.1
28691	0.677856	2.1
28692	0.90095	2.1
28693	0.621435	2.1
28694	0.858124	2.1
28695	1.70303	2.1
28696	1.70731	2.1
28697	1.79663	2.1
28698	1.68783	2.1
28699	3.17337	2.1
28700	1.22724	2.1
28701	2.0021	2.1
28702	1.90427	2.1
28703	1.08698	2.1
28704	3.08907	2.1
28705	2.41689	2.1
28706	2.00209	2.1
28707	1.90427	2.1

28708	2.35245	2.1
28709	4.35454	2.1
28710	1.90427	2.1
28711	0.956294	2.1
28712	0.956294	2.1
28713	1.08698	2.1
28714	1.81488	2.1
28715	1.94556	2.1
28716	1.90427	2.1
28717	2.35245	2.1
28718	2.28979	2.1
28719	1.81488	2.1
28720	4.16733	2.1
28721	1.6272	2.1
28722	1.70302	2.1
28723	1.70303	2.1
28724	1.70302	2.1
28725	1.91167	2.1
28726	1.91167	2.1
28727	1.49063	2.1
28728	1.89226	2.1
28729	1.89226	2.1
28730	1.89226	2.1
28731	1.6272	2.1
28732	1.6272	2.1
28733	2.25988	1.80375
28734	1.89226	0.444579
28735	1.89226	1.47912
28736	2.23152	1.64729
28737	4.12379	0.97476
28738	2.25988	1.86808
28739	1.16361	2.1
28740	1.15853	2.1
28741	2.23153	1.75794
28742	3.39513	2.1
28743	1.89226	1.29714
28744	2.23153	0.848846
28745	2.21022	1.63218
28746	2.23153	1.4058
28747	1.87095	0.578843
28748	1.89226	0.279969
28749	1.16239	2.1
28750	2.23153	1.24964
28751	1.15853	2.03655
28752	1.15853	1.62051
28753	2.21022	2.0438
28754	2.21022	1.59304
28755	1.78381	0.05
28756	1.70302	0.05
28757	1.15453	0.05
28758	1.35007	0.0519102
28759	1.82021	0.05
28760	2.12851	0.05
28761	1.89226	0.0518465
28762	1.89226	0.0626948
28763	1.89226	0.0622918
28764	1.89226	0.072171
28765	1.89226	0.05

28766	1.89226	0.0558569
28767	1.89258	0.05
28768	1.089	0.0501836
28769	1.22586	0.05
28770	1.79877	0.05
28771	2.88777	0.05
28772	1.91564	0.05
28773	1.79877	0.0501901
28774	1.96356	0.05
28775	1.70302	0.0515282
28776	2.37828	0.05
28777	2.04859	0.05
28778	1.89226	0.0505285
28779	1.89226	0.0640017
28780	1.89226	0.0512079
28781	1.89226	0.0672823
28782	3.78453	0.0542384
28783	1.89226	0.05
28784	1.89226	0.0617357
28785	1.89226	0.0524357
28786	1.89226	0.0646544
28787	1.89226	0.05
28788	1.89226	0.0592577
28789	2.28593	2.1
28790	1.89226	2.1
28791	2.2731	2.1
28792	1.89226	1.93825
28793	4.17819	2.1
28794	1.89226	2.1
28795	1.89226	2.08763
28796	1.89226	1.98476
28797	1.89226	1.60509
28798	3.78452	1.77157
28799	1.03933	2.1
28800	1.34355	1.52459
28801	1.35048	1.49499
28802	1.35048	2.1
28803	1.02975	1.55522
28804	1.02975	2.04928
28805	2.25105	2.1
28806	1.89226	2.1
28807	2.27311	2.1
28808	2.27311	2.1
28809	1.89226	2.1
28810	1.89226	2.1
28811	1.89226	2.1
28812	1.89226	2.05188
28813	1.89226	2.06813
28814	1.89226	0.454741
28815	3.78452	1.61667
28816	1.02975	2.1
28817	1.34355	2.00617
28818	1.01121	1.98996
28819	1.33079	1.60161
28820	2.35476	2.1
28821	1.24526	0.05
28822	2.36406	0.05
28823	2.81021	0.05

28824	1.7237	0.05
28825	4.08776	0.05
28826	1.89226	2.1
28827	1.89226	2.1
28828	1.89226	2.1
28829	1.89226	2.1
28830	3.78453	2.1
28831	1.79028	2.1
28832	1.89238	2.1
28833	1.65533	2.1
28834	1.77821	2.1
28835	3.5477	2.1
28836	1.89238	2.1
28837	1.7465	2.1
28838	1.77821	2.1
28839	1.63557	2.04714
28840	3.52795	2.1
28841	1.89226	2.1
28842	1.89226	1.89863
28843	1.89226	2.1
28844	1.89226	2.02481
28845	3.78452	2.1
28846	1.89226	2.1
28847	1.89226	2.1
28848	1.89226	2.1
28849	1.89226	2.1
28850	3.78452	2.1
28851	1.65532	2.1
28852	1.77821	2.1
28853	1.53278	2.1
28854	1.67571	2.1
28855	3.33103	2.1
28856	1.77821	2.1
28857	1.63557	2.1
28858	1.67571	2.1
28859	1.52615	2.1
28860	3.31128	2.1
28861	1.89226	2.1
28862	1.89226	2.1
28863	1.89226	2.1
28864	1.89226	1.87583
28865	3.78452	2.1
28866	2.83129	2.1
28867	1.89226	1.76902
28868	1.89226	2.1
28869	2.6491	2.1
28870	4.54136	2.1
28871	2.83129	2.1
28872	1.47278	2.1
28873	1.3909	2.1
28874	2.6491	2.1
28875	4.12188	2.1
28876	1.89225	2.1
28877	2.65393	2.1
28878	1.89225	2.1
28879	2.69591	2.1
28880	4.58816	2.1
28881	1.91703	2.1

28882	2.00209	2.1
28883	2.65393	2.1
28884	2.65393	2.1
28885	2.69591	2.1
28886	2.00209	2.1
28887	1.89226	2.1
28888	2.69593	2.1
28889	1.93967	2.1
28890	1.89226	2.1
28891	2.74334	2.1
28892	2.69593	2.1
28893	2.74335	2.1
28894	2.0021	2.1
28895	2.76367	2.1
28896	2.69593	2.1
28897	2.06984	2.1
28898	2.0021	2.1
28899	2.24481	2.1
28900	2.58921	2.1
28901	2.68958	2.1
28902	2.68958	2.1
28903	2.11984	2.1
28904	2.11984	2.1
28905	2.11984	2.1
28906	2.58921	2.1
28907	2.46488	2.1
28908	1.98339	2.1
28909	4.5726	2.1
28910	2.7777	2.1
28911	2.58921	2.1
28912	2.46488	2.1
28913	2.70401	2.1
28914	5.29322	2.1
28915	2.17741	2.1
28916	2.54197	2.1
28917	2.54018	2.1
28918	2.18608	2.1
28919	4.72805	2.1
28920	2.17742	2.1
28921	1.58054	2.1
28922	1.59169	2.1
28923	2.18608	2.1
28924	3.76662	2.1
28925	2.54018	2.1
28926	2.18608	2.1
28927	2.19104	2.1
28928	2.5392	2.1
28929	4.72528	2.1
28930	1.60497	2.1
28931	2.18608	2.1
28932	1.59169	2.1
28933	1.59169	2.1
28934	2.19105	2.1
28935	2.19105	2.1
28936	2.58661	2.1
28937	1.06203	2.1
28938	2.7043	2.1
28939	2.21397	2.1

28940	1.55236	2.1
28941	1.06203	2.1
28942	1.89226	1.67621
28943	1.89226	1.55569
28944	1.89226	1.11601
28945	1.89226	1.8185
28946	3.78453	1.53049
28947	1.08195	1.51984
28948	0.990019	2.1
28949	1.31976	1.81493
28950	1.4539	1.31002
28951	2.40171	1.81554
28952	1.89226	1.32258
28953	1.89226	2.02106
28954	1.89226	1.5625
28955	1.89226	0.601174
28956	3.78453	1.47288
28957	1.89226	1.91144
28958	1.89226	1.64777
28959	1.89226	1.49021
28960	1.89226	2.00762
28961	3.78453	1.72087
28962	1.43895	1.99297
28963	1.31976	1.75002
28964	1.70066	1.54639
28965	1.33783	2.1
28966	1.43895	1.84472
28967	1.07612	2.1
28968	0.65988	1.81446
28969	0.850329	1.69235
28970	0.919598	1.19805
28971	0.726952	1.45025
28972	0.788106	1.15124
28973	0.788106	2.1
28974	0.789175	1.96146
28975	0.789175	0.56407
28976	0.850329	1.28951
28977	0.919598	1.29157
28978	0.659879	1.63793
28979	0.726952	1.60371
28980	1.34389	2.1
28981	1.41279	1.97481
28982	1.15564	2.1
28983	1.15564	2.1
28984	1.55998	2.1
28985	1.55998	2.1
28986	1.89226	1.87787
28987	1.89226	1.97846
28988	1.89226	2.1
28989	1.89226	1.6907
28990	1.89226	2.1
28991	1.89226	1.64604
28992	1.55998	2.1
28993	1.41279	2.07047
28994	1.72901	2.1
28995	1.62414	2.1
28996	3.1418	2.1
28997	1.89226	1.96524

28998	1.89226	2.1
28999	1.89226	2.1
29000	1.89226	1.62899
29001	3.78452	2.00977
29002	1.89226	1.59371
29003	1.89226	2.00656
29004	1.89226	1.87286
29005	1.89226	1.86517
29006	1.89226	1.70437
29007	1.89226	1.63619
29008	1.62414	2.1
29009	1.01243	1.97463
29010	1.70932	2.1
29011	1.41279	1.95028
29012	1.30896	2.0062
29013	1.01243	1.88643
29014	1.9719	2.1
29015	1.10725	2.1
29016	1.12411	2.1
29017	1.98691	2.1
29018	3.09601	2.1
29019	1.97189	2.1
29020	2.47932	2.1
29021	2.49092	2.1
29022	1.98691	2.1
29023	4.46282	2.1
29024	1.1486	2.1
29025	1.98691	2.1
29026	1.12411	2.1
29027	1.12411	2.1
29028	2.01602	2.1
29029	2.01602	2.1
29030	2.49093	2.1
29031	1.98691	2.1
29032	2.01601	2.1
29033	2.51651	2.1
29034	4.50694	2.1
29035	1.93147	0.096154
29036	2.51593	0.0626972
29037	1.35146	0.102197
29038	1.35146	0.0951767
29039	2.48665	0.095443
29040	1.90664	0.0770022
29041	1.63979	2.1
29042	1.7457	2.1
29043	1.66046	2.1
29044	1.56558	2.1
29045	3.31128	2.1
29046	0.819895	2.1
29047	0.782792	2.1
29048	0.60239	2.1
29049	0.54831	2.1
29050	0.688347	2.1
29051	0.688347	2.1
29052	0.679857	2.1
29053	0.679857	2.1
29054	0.782792	2.1
29055	0.54831	2.1

29056	0.60239	2.1
29057	0.819895	2.1
29058	1.66046	2.1
29059	1.42485	2.1
29060	1.56558	2.1
29061	1.56558	2.1
29062	1.52995	2.1
29063	1.52995	2.1
29064	2.58101	2.1
29065	2.09803	2.1
29066	2.408	2.00065
29067	1.86893	2.1
29068	4.44994	2.1
29069	2.36062	2.1
29070	1.25104	2.1
29071	1.25104	2.1
29072	1.39703	2.1
29073	1.86893	2.1
29074	2.01492	2.1
29075	2.408	1.95922
29076	1.86893	2.1
29077	2.22575	2.1
29078	1.70677	2.1
29079	4.09468	2.1
29080	0.737605	2.1
29081	0.737605	2.1
29082	0.741302	2.1
29083	0.741302	2.1
29084	0.55189	2.1
29085	0.625517	2.1
29086	0.934468	2.1
29087	0.853389	2.1
29088	0.853389	2.1
29089	0.55189	2.1
29090	0.625517	2.1
29091	0.934467	2.1
29092	2.35053	2.1
29093	1.09615	2.1
29094	1.25998	2.1
29095	2.51216	2.1
29096	3.61051	2.1
29097	1.33636	2.1
29098	1.25609	2.1
29099	0.698455	2.1
29100	0.629995	2.1
29101	0.986092	2.1
29102	0.986092	2.1
29103	0.980263	2.1
29104	0.980263	2.1
29105	1.33636	2.1
29106	0.698455	2.1
29107	0.629995	2.1
29108	1.25609	2.1
29109	1.89226	1.38452
29110	1.89226	1.39907
29111	1.89226	1.41875
29112	1.89226	1.41618
29113	3.78452	1.40354

29114	1.74321	1.39104
29115	1.89226	1.24787
29116	1.91755	1.42815
29117	1.91755	1.32798
29118	1.89226	1.39116
29119	1.89226	1.2765
29120	0.946132	1.34657
29121	0.946132	1.33333
29122	0.949798	1.05702
29123	1.08054	1.25817
29124	1.08054	1.16186
29125	1.08054	1.27976
29126	0.946132	1.33386
29127	0.946132	1.2169
29128	0.946132	1.33076
29129	0.949798	1.12471
29130	1.08054	1.15361
29131	0.946131	1.35087
29132	1.89226	1.38495
29133	1.89226	1.35097
29134	1.89226	1.34102
29135	1.89226	1.33176
29136	1.89226	1.36411
29137	1.89226	1.35295
29138	2.39378	0.0589825
29139	2.29234	0.05
29140	2.29234	0.0506945
29141	2.7933	0.05
29142	1.18422	0.0502687
29143	1.68518	0.0508012
29144	2.79329	0.05
29145	1.89226	0.05
29146	2.29233	0.05
29147	1.89226	0.05
29148	4.18459	0.05
29149	0.857046	0.05
29150	0.857037	0.05
29151	0.94613	0.05
29152	0.94613	0.05
29153	0.851539	0.05
29154	0.851539	0.05
29155	1.0315	0.05
29156	0.952867	0.05
29157	0.851486	0.05
29158	0.952869	0.05
29159	1.03156	0.05
29160	0.851528	0.05
29161	1.61981	2.1
29162	1.93096	2.1
29163	2.00776	2.1
29164	1.62218	2.1
29165	3.62757	2.1
29166	2.00776	2.1
29167	1.68541	2.1
29168	1.83215	2.1
29169	1.62218	2.1
29170	2.21773	2.1
29171	2.00776	2.1

29172	1.89188	0.0640467
29173	1.70303	0.0685413
29174	1.89226	0.0660291
29175	1.72465	0.0636367
29176	1.89226	0.0689317
29177	1.70552	0.0671567
29178	2.48659	0.0734726
29179	2.0815	0.0855661
29180	1.89226	0.0863723
29181	2.08111	0.079697
29182	1.89226	0.0842806
29183	2.08111	0.0821813
29184	2.35332	0.25599
29185	1.83284	0.152061
29186	2.28483	0.172604
29187	2.71955	0.156567
29188	4.63815	0.156934
29189	1.89227	2.1
29190	1.89227	2.1
29191	1.89227	2.1
29192	1.89227	2.1
29193	3.78453	2.1
29194	1.33506	2.1
29195	1.17129	2.1
29196	1.33506	2.1
29197	1.38415	2.1
29198	1.13664	2.1
29199	1.18573	2.1
29200	1.89226	2.1
29201	2.68785	2.1
29202	1.89226	2.1
29203	2.6692	2.1
29204	4.58011	2.1
29205	1.89226	2.1
29206	1.89226	2.1
29207	1.89226	2.1
29208	1.89226	2.1
29209	3.78453	2.1
29210	1.33506	2.1
29211	1.13664	2.1
29212	1.27575	2.1
29213	1.09627	2.1
29214	2.43133	2.1
29215	1.89226	2.1
29216	2.65349	2.1
29217	2.66919	2.1
29218	2.66919	2.1
29219	1.89226	2.1
29220	1.89226	2.1
29221	1.89226	2.1
29222	1.89226	2.1
29223	1.89226	2.1
29224	1.89226	2.1
29225	3.78453	2.1
29226	1.16834	2.1
29227	1.16834	2.1
29228	0.946132	2.1
29229	0.946132	2.1

29230	1.26978	2.1
29231	1.16834	2.1
29232	0.94613	2.1
29233	0.94613	2.1
29234	0.94613	2.1
29235	1.26978	2.1
29236	0.94613	2.1
29237	1.16834	2.1
29238	2.14698	2.1
29239	1.89226	2.1
29240	1.89226	2.1
29241	1.89226	2.1
29242	2.40719	2.1
29243	2.40719	2.1
29244	1.89226	2.1
29245	1.89226	2.1
29246	1.89226	2.1
29247	1.89226	2.1
29248	3.78453	2.1
29249	1.26977	2.1
29250	1.26977	2.1
29251	0.946133	2.1
29252	0.946133	2.1
29253	1.37467	2.1
29254	1.26978	2.1
29255	0.946131	2.1
29256	0.946131	2.1
29257	1.37467	2.1
29258	0.94613	2.1
29259	0.946131	2.1
29260	1.26978	2.1
29261	1.2036	2.1
29262	1.34912	2.1
29263	0.946131	2.1
29264	0.946131	2.1
29265	1.34912	2.1
29266	1.34912	2.1
29267	0.946129	2.1
29268	0.946129	2.1
29269	1.34912	2.1
29270	0.94613	2.1
29271	0.946131	2.1
29272	1.2036	2.1
29273	2.47164	0.401683
29274	1.89019	0.217766
29275	1.79109	0.380689
29276	2.41632	0.2251
29277	4.26273	0.383697
29278	1.54197	0.318473
29279	2.41632	0.185145
29280	1.79109	0.260447
29281	1.79109	0.299535
29282	2.20929	0.210273
29283	2.20929	0.254956
29284	1.70302	1.36457
29285	1.48104	1.56615
29286	1.99365	1.57896
29287	1.32897	1.13172

29288	1.70302	1.58554
29289	1.14034	1.30854
29290	1.70302	1.42573
29291	1.99365	1.3749
29292	1.99297	1.49465
29293	2.36337	0.86712
29294	4.04804	1.40251
29295	1.51569	1.19787
29296	2.49093	0.827528
29297	1.6026	0.879048
29298	1.43229	0.714654
29299	2.0815	1.32777
29300	1.83402	1.08822
29301	2.54665	2.1
29302	2.34044	2.1
29303	2.08889	2.1
29304	2.37653	2.1
29305	4.71697	2.1
29306	2.54665	2.1
29307	2.45212	2.1
29308	2.31012	2.1
29309	2.37653	2.1
29310	4.82865	2.1
29311	2.08889	2.1
29312	2.37653	2.1
29313	2.23366	2.1
29314	1.87598	2.1
29315	4.25251	2.1
29316	2.31012	2.1
29317	2.37653	2.1
29318	2.23367	2.1
29319	2.17384	2.1
29320	4.55037	2.1
29321	0.946131	2.1
29322	0.946131	2.1
29323	1.30338	2.1
29324	1.23186	2.1
29325	0.94613	2.1
29326	0.94613	2.1
29327	1.23187	2.1
29328	1.23187	2.1
29329	0.946131	2.1
29330	1.23186	2.1
29331	1.30338	2.1
29332	0.946131	2.1
29333	1.89226	2.1
29334	2.46373	2.1
29335	2.34725	2.1
29336	2.46373	2.1
29337	1.77579	2.1
29338	1.89226	2.1
29339	2.61901	2.1
29340	2.45899	2.1
29341	2.64067	2.1
29342	2.52708	2.1
29343	5.14609	2.1
29344	2.41795	2.1
29345	2.0815	2.1

29346	2.52708	2.1
29347	1.92277	2.1
29348	4.31368	2.1
29349	2.64067	2.1
29350	2.52708	2.1
29351	2.58638	2.1
29352	2.51039	2.1
29353	5.11346	2.1
29354	2.52708	2.1
29355	2.13642	2.1
29356	2.51039	2.1
29357	2.16633	2.1
29358	4.69341	2.1
29359	1.61028	1.71711
29360	1.65712	0.399676
29361	1.35002	0.250306
29362	1.35002	1.78394
29363	1.88637	0.330677
29364	1.88637	1.99126
29365	2.57663	2.1
29366	2.24227	2.1
29367	2.48354	2.1
29368	2.14317	2.1
29369	4.7198	2.1
29370	2.24227	2.1
29371	1.77353	2.1
29372	2.14317	2.1
29373	1.69956	2.1
29374	3.9167	2.1
29375	2.48353	2.1
29376	2.14317	2.1
29377	2.39282	2.1
29378	1.79824	2.1
29379	4.53599	2.1
29380	2.14317	2.1
29381	1.69956	2.1
29382	1.91427	2.1
29383	1.6347	2.1
29384	3.77787	2.1
29385	1.89226	0.535034
29386	1.89226	0.501256
29387	1.89226	0.235927
29388	1.89226	0.138887
29389	3.78453	0.355049
29390	1.89226	0.396442
29391	1.89226	0.276969
29392	1.89226	0.403663
29393	1.89226	0.175783
29394	3.78452	0.185277
29395	1.89226	0.316939
29396	1.89652	0.473522
29397	1.89226	0.204409
29398	1.89226	1.46109
29399	1.19352	0.3334
29400	1.19352	0.932366
29401	1.89226	0.465243
29402	1.89653	0.193481
29403	1.89226	0.696827

29404	1.89226	1.56267
29405	1.89226	0.115953
29406	1.89226	0.161349
29407	1.89226	0.539116
29408	1.75326	0.514724
29409	1.89226	1.11263
29410	1.12948	0.655507
29411	1.89226	0.348321
29412	1.12948	0.205516
29413	2.61064	2.1
29414	1.89226	2.1
29415	1.89226	2.1
29416	1.89226	2.1
29417	2.6085	2.1
29418	2.6085	2.1
29419	2.6085	2.1
29420	1.89226	2.1
29421	1.89226	2.1
29422	1.89226	2.1
29423	2.62161	2.1
29424	2.62161	2.1
29425	2.16535	2.1
29426	1.44603	2.1
29427	2.79784	2.1
29428	1.84756	2.1
29429	4.01292	2.1
29430	2.65262	2.1
29431	1.03391	2.1
29432	2.20239	2.1
29433	1.74416	2.1
29434	1.49213	2.1
29435	1.03391	2.1
29436	2.29066	2.1
29437	2.49174	2.1
29438	2.56502	2.1
29439	2.33802	2.1
29440	4.85568	2.1
29441	2.39663	2.1
29442	2.49174	2.1
29443	2.56502	2.1
29444	2.45929	2.1
29445	4.96165	2.1
29446	2.56502	2.1
29447	2.33802	2.1
29448	2.37168	2.1
29449	2.59967	2.1
29450	4.9367	2.1
29451	2.56502	2.1
29452	2.45929	2.1
29453	2.50138	2.1
29454	2.59967	2.1
29455	5.0664	2.1
29456	2.27009	1.74515
29457	1.26247	2.1
29458	1.86812	2.0665
29459	2.837	1.82536
29460	4.13822	1.97608
29461	1.89226	2.1

29462	1.89226	2.1
29463	1.89226	2.1
29464	1.89226	2.1
29465	3.78453	2.1
29466	1.75835	2.1
29467	1.72866	2.1
29468	1.65313	2.1
29469	1.64822	2.1
29470	3.3818	2.1
29471	1.72866	2.1
29472	1.58401	2.1
29473	1.64822	2.1
29474	1.52441	2.1
29475	3.25307	2.1
29476	1.89226	2.1
29477	1.89226	2.1
29478	1.89226	2.1
29479	1.89226	2.1
29480	3.78452	2.1
29481	1.89226	2.1
29482	1.89226	2.1
29483	1.89226	2.1
29484	1.89226	2.1
29485	3.78453	2.1
29486	1.65313	2.1
29487	1.64822	2.1
29488	1.55779	2.1
29489	1.57968	2.1
29490	3.23281	2.1
29491	1.64822	2.1
29492	1.52441	2.1
29493	1.57967	2.1
29494	1.474 2.1	
29495	3.10408	2.1
29496	1.89226	2.1
29497	1.89226	2.1
29498	1.89226	2.1
29499	1.89226	2.1
29500	3.78452	2.1
29501	1.86067	1.50346
29502	1.58407	2.1
29503	2.07965	0.455732
29504	1.83123	1.49659
29505	3.66372	1.40093
29506	1.89226	1.45059
29507	1.89226	2.1
29508	1.89226	1.20655
29509	1.89226	2.06368
29510	3.78452	1.73291
29511	1.89226	2.0264
29512	1.89226	2.1
29513	1.89226	1.25686
29514	1.89226	2.1
29515	1.89226	1.41594
29516	1.89226	2.1
29517	1.83123	0.590257
29518	1.03472	2.1
29519	1.91068	1.94687

29520	1.58406	1.83038
29521	1.36133	1.77238
29522	1.03472	1.49532
29523	2.07965	1.32221
29524	1.83123	1.64753
29525	2.28385	1.36714
29526	2.09114	1.62102
29527	4.1708	1.44886
29528	1.89226	1.39085
29529	1.89226	2.07482
29530	1.89226	0.583461
29531	1.89226	1.61045
29532	3.78452	1.51379
29533	1.89226	1.92474
29534	1.89226	2.1
29535	1.89226	0.833141
29536	1.89226	2.1
29537	3.78452	2.03724
29538	1.83123	1.29906
29539	1.36133	2.1
29540	2.09114	0.64452
29541	1.69436	1.80006
29542	3.45248	1.60637
29543	1.89226	2.1
29544	1.89226	2.1
29545	1.89226	2.1
29546	1.89226	2.1
29547	3.78453	2.1
29548	1.53278	2.1
29549	1.67571	2.1
29550	1.40182	2.1
29551	1.55686	2.1
29552	3.07752	2.1
29553	1.67571	2.1
29554	1.52615	2.1
29555	1.55686	2.1
29556	1.38963	1.89645
29557	3.06534	2.1
29558	1.89226	2.1
29559	1.89226	1.98676
29560	1.89226	2.1
29561	1.89226	1.91189
29562	3.78452	2.1
29563	1.89226	2.1
29564	1.89226	2.1
29565	1.89226	2.1
29566	1.89226	2.1
29567	3.78453	2.1
29568	1.40182	2.1
29569	1.55686	2.1
29570	1.24509	2.1
29571	1.38426	2.1
29572	2.78608	2.1
29573	1.55686	2.1
29574	1.38963	2.1
29575	1.2064	2.1
29576	1.2064	2.1
29577	1.38426	2.1

29578	1.56749	2.1
29579	0.810946	2.1
29580	0.810946	2.09991
29581	0.810945	2.1
29582	0.810945	2.1
29583	0.810945	2.1
29584	0.810945	2.1
29585	1.89226	2.1
29586	1.35169	2.1
29587	1.08132	2.1
29588	1.08132	2.01176
29589	1.08132	2.1
29590	1.28418	0.380235
29591	1.59382	0.518809
29592	1.78289	0.688815
29593	1.51021	0.401977
29594	3.10403	0.440968
29595	1.76897	1.89972
29596	1.59382	0.548578
29597	1.78289	0.975518
29598	1.94302	1.44377
29599	3.53684	1.46679
29600	1.89226	0.53308
29601	1.89226	0.845162
29602	1.89226	1.14151
29603	1.89226	0.567955
29604	3.78453	0.659776
29605	1.89227	0.505634
29606	1.89227	0.286647
29607	1.89227	0.300937
29608	1.89227	0.387651
29609	1.89227	0.385937
29610	1.89227	0.459948
29611	1.78286	0.522202
29612	1.51019	0.292843
29613	1.79979	0.338533
29614	2.02511	0.440728
29615	3.5353	0.409514
29616	1.78286	0.692271
29617	1.94299	0.951764
29618	2.17831	1.68339
29619	2.02511	0.552038
29620	3.9681	0.620807
29621	1.89224	0.809496
29622	1.89224	0.398892
29623	1.89224	0.507332
29624	1.89224	0.759161
29625	3.78448	0.652635
29626	1.89224	0.405105
29627	1.89224	0.248433
29628	1.89224	0.278755
29629	1.89224	0.390138
29630	1.89224	0.266851
29631	1.89224	0.387545
29632	1.83198	0.153042
29633	1.0961	0.0655764
29634	1.39448	0.10677
29635	1.99568	0.152966

29636	3.09178	0.129222
29637	1.25503	0.0578512
29638	1.99568	0.118418
29639	2.3661	0.171705
29640	1.70302	0.0900207
29641	3.55833	0.0774835
29642	2.69899	0.081249
29643	1.85796	0.0834245
29644	2.11407	0.0657097
29645	1.36769	0.0617674
29646	3.97203	0.0602844
29647	1.89226	0.0875208
29648	1.89226	0.0961891
29649	1.89226	0.0771955
29650	1.89226	0.0624151
29651	3.78452	0.106109
29652	1.89225	0.0652566
29653	2.63355	0.0714818
29654	2.09529	0.0667524
29655	2.09529	0.0766593
29656	1.89225	0.0695682
29657	1.89225	0.0771592
29658	1.89226	0.0611507
29659	1.89226	0.0693718
29660	1.89226	0.0569385
29661	1.89226	0.0873855
29662	1.89226	0.0559166
29663	1.89226	0.0773705
29664	2.12204	0.0522649
29665	1.89226	0.0657377
29666	2.05798	0.0528116
29667	2.01954	0.0519795
29668	1.70302	0.0546045
29669	1.3865	0.0607043
29670	1.28326	0.657063
29671	1.33326	2.1
29672	1.19052	0.998555
29673	1.25369	2.1
29674	2.53694	1.97226
29675	1.33327	2.1
29676	1.27339	2.1
29677	1.25369	2.1
29678	1.19733	2.1
29679	2.52708	2.1
29680	1.19052	0.796938
29681	1.25369	2.1
29682	1.10938	0.759922
29683	1.17614	2.1
29684	2.36307	1.89517
29685	1.25369	2.1
29686	1.19733	2.1
29687	1.17614	2.1
29688	1.11868	2.1
29689	2.37237	2.1
29690	1.10747	0.0830437
29691	1.47688	0.106385
29692	1.97732	0.0555678
29693	1.62206	0.0705934

29694	3.09894	0.0831881
29695	1.89226	0.110275
29696	1.89226	0.0867238
29697	1.89226	0.0995079
29698	1.89226	0.124722
29699	1.89226	0.066247
29700	1.89226	0.08257
29701	1.62206	0.095206
29702	1.70302	0.0859997
29703	1.22962	0.0993382
29704	1.1893	0.109189
29705	2.15177	0.0773965
29706	2.08121	0.0887864
29707	1.97732	0.0788788
29708	2.08121	0.0801258
29709	1.62206	0.0883411
29710	1.62206	0.119134
29711	2.41464	0.0655723
29712	2.41464	0.0879292
29713	1.89226	0.113343
29714	1.89226	0.137658
29715	1.89226	0.0788913
29716	1.89226	0.0870127
29717	3.78452	0.0912989
29718	0.946133	0.0973291
29719	0.946133	0.10174
29720	0.946132	0.108245
29721	0.946132	0.115654
29722	0.946132	0.104726
29723	0.946132	0.121585
29724	0.946133	0.0811438
29725	0.946133	0.0994513
29726	0.946132	0.0778927
29727	0.946132	0.0959797
29728	0.946132	0.129134
29729	0.946132	0.0990468
29730	1.83157	1.87015
29731	2.19989	1.7251
29732	2.20664	0.762773
29733	1.81487	0.787678
29734	4.03146	1.39425
29735	2.41055	2.1
29736	2.19989	2.1
29737	2.20665	2.1
29738	2.38023	2.0096
29739	4.61044	2.1
29740	2.19989	2.1
29741	2.22558	2.1
29742	2.38023	2.1
29743	2.07065	1.47863
29744	4.42547	2.1
29745	2.19988	1.85013
29746	1.70303	1.54927
29747	1.89939	1.13714
29748	1.86357	0.531102
29749	3.78536	1.34885
29750	1.04921	2.1
29751	1.30711	2.1

29752	1.53076	2.1
29753	1.22927	2.1
29754	2.57997	2.1
29755	1.53076	2.1
29756	1.46568	2.1
29757	1.56056	2.1
29758	1.22927	2.1
29759	1.86206	2.1
29760	1.53076	2.1
29761	1.18353	2.1
29762	2.28593	2.1
29763	1.16979	2.1
29764	2.2731	2.1
29765	3.45571	2.1
29766	0.854776	2.1
29767	0.854776	2.1
29768	0.855641	2.1
29769	0.855641	2.1
29770	1.12552	2.1
29771	1.13655	2.1
29772	0.57216	2.1
29773	0.584893	2.1
29774	0.57216	2.1
29775	1.12552	2.1
29776	0.584893	2.1
29777	1.13655	2.1
29778	1.54462	2.1
29779	2.76309	2.1
29780	1.34869	2.1
29781	2.48573	2.1
29782	4.03035	2.1
29783	1.35328	1.35854
29784	1.0383	1.30946
29785	1.03318	1.46017
29786	0.704834	1.4444
29787	1.0257	1.46124
29788	1.0257	1.36016
29789	1.03241	1.3019
29790	1.03241	1.42979
29791	1.35328	1.42007
29792	1.03319	1.43105
29793	1.0383	1.41397
29794	0.704835	1.42383
29795	2.27126	0.444285
29796	2.77291	2.1
29797	1.81771	1.88179
29798	2.6111	1.79319
29799	4.88236	1.94085
29800	2.77291	2.1
29801	1.89227	2.1
29802	1.89227	1.96772
29803	1.89227	2.1
29804	2.6111	1.89409
29805	2.6111	2.1
29806	2.22061	1.63823
29807	2.41522	1.89391
29808	2.41522	1.04604
29809	2.6111	2.1

29810	1.75765	0.915381
29811	1.95353	2.1
29812	2.27502	0.903713
29813	1.89227	1.60552
29814	1.89227	0.775742
29815	2.8189	0.51908
29816	4.71116	0.54654
29817	1.89226	0.592277
29818	1.89226	1.57655
29819	1.89226	1.35364
29820	1.89226	0.847816
29821	1.89226	1.43516
29822	1.89226	1.23049
29823	1.89226	1.65618
29824	1.89226	1.58337
29825	1.89226	1.63872
29826	1.89226	1.47795
29827	3.78453	1.61
29828	1.89226	1.64506
29829	2.57079	1.3623
29830	2.5932	1.52591
29831	1.89226	1.48408
29832	4.48546	1.48399
29833	0.589396	1.49213
29834	0.641454	1.40717
29835	0.814062	1.56156
29836	0.726518	1.48575
29837	0.675126	1.52596
29838	0.675125	1.46243
29839	0.692847	1.56388
29840	0.692847	1.49577
29841	0.641454	1.44201
29842	0.814062	1.49262
29843	0.726518	1.55235
29844	0.589396	1.51949
29845	1.89226	1.66599
29846	1.89226	1.47793
29847	1.89226	1.60086
29848	1.89226	1.6383
29849	1.89226	1.57296
29850	1.89226	1.62708
29851	1.16906	0.760169
29852	1.2966	1.47936
29853	0.946132	1.47426
29854	0.946132	1.67908
29855	1.16906	1.47321
29856	1.16906	1.26435
29857	0.946132	1.49744
29858	0.946132	1.59548
29859	1.16906	1.48289
29860	0.946131	1.4727
29861	1.2966	1.52323
29862	0.946132	1.59666
29863	0.641455	1.48265
29864	0.567191	1.42269
29865	0.807594	1.60616
29866	0.814062	1.508
29867	0.673735	1.57582

29868	0.673734	1.45314
29869	0.707519	1.4927
29870	0.707519	1.60335
29871	0.807594	1.53087
29872	0.56719	1.57473
29873	0.641455	1.55372
29874	0.814063	1.62106
29875	1.89225	2.1
29876	1.89225	2.1
29877	1.89225	2.1
29878	1.89225	2.1
29879	3.7845	2.1
29880	1.89225	2.1
29881	1.89225	2.1
29882	1.89225	2.1
29883	1.89225	2.1
29884	1.89225	2.1
29885	1.89225	2.1
29886	1.89226	2.1
29887	1.89226	2.1
29888	1.89226	2.1
29889	1.89226	2.1
29890	3.78452	2.1
29891	1.89226	2.1
29892	1.89226	2.1
29893	1.89226	2.1
29894	1.89226	2.1
29895	1.89226	2.1
29896	1.89226	2.1
29897	1.89226	2.1
29898	1.89226	2.1
29899	1.89226	2.1
29900	1.89226	2.1
29901	3.78452	2.1
29902	1.89226	2.1
29903	1.38928	2.1
29904	1.89226	2.1
29905	1.51378	2.1
29906	1.89061	2.1
29907	1.89226	2.1
29908	1.89226	2.1
29909	1.89226	2.1
29910	1.89226	2.1
29911	1.89226	2.1
29912	3.78453	2.1
29913	1.04572	2.1
29914	0.994213	2.1
29915	0.912466	2.1
29916	0.912466	2.1
29917	0.947743	2.1
29918	1.00388	2.1
29919	0.900913	2.1
29920	0.900913	2.1
29921	0.900915	2.1
29922	0.946131	2.1
29923	0.946131	2.1
29924	0.999196	2.1
29925	1.89226	2.1

29926	1.89226	2.1
29927	1.89226	2.1
29928	1.89226	2.1
29929	3.78453	2.1
29930	0.946161	2.1
29931	1.1354	2.1
29932	0.850847	2.1
29933	0.850847	2.1
29934	1.01445	2.1
29935	1.02106	2.1
29936	0.946131	2.1
29937	0.946131	2.1
29938	1.13537	2.1
29939	1.01444	2.1
29940	0.850877	2.1
29941	0.946131	2.1
29942	1.80183	2.1
29943	1.89226	2.1
29944	1.89226	2.1
29945	1.89226	2.1
29946	1.79606	2.1
29947	1.79606	2.1
29948	1.24001	1.41584
29949	1.41822	1.12711
29950	1.95665	1.00219
29951	1.77342	1.38983
29952	3.19666	1.15704
29953	1.95665	1.19948
29954	1.44859	1.02806
29955	1.41821	1.44725
29956	1.41821	1.06944
29957	1.95543	1.142
29958	1.95543	0.980822
29959	1.95665	1.02354
29960	1.77342	1.3689
29961	2.33611	1.26237
29962	2.49694	1.07062
29963	4.29276	1.28382
29964	1.95665	1.26697
29965	1.95544	1.04527
29966	2.4728	0.847958
29967	2.49693	1.2091
29968	4.42945	0.987866
29969	2.24937	2.1
29970	2.49053	2.1
29971	2.16323	2.1
29972	2.36377	2.1
29973	4.61314	2.1
29974	2.49053	2.1
29975	2.58639	2.1
29976	2.36377	2.1
29977	2.47395	2.1
29978	4.95016	2.1
29979	2.45054	2.1
29980	2.36377	2.1
29981	1.92911	2.1
29982	2.181	2.1
29983	4.29288	2.1

29984	2.36377	2.1
29985	2.47395	2.1
29986	2.181 2.1	
29987	2.29881	2.1
29988	4.66258	2.1
29989	1.57885	2.1
29990	1.13339	2.1
29991	1.48594	2.1
29992	1.13322	2.1
29993	2.71207	2.1
29994	1.48596	2.1
29995	1.20761	2.1
29996	1.20761	2.1
29997	1.13323	2.1
29998	1.46568	2.1
29999	1.3913	2.1
30000	1.89226	0.16181
30001	1.89226	0.395366
30002	1.89226	0.307327
30003	1.89226	0.880827
30004	1.89226	0.05
30005	1.89226	0.125563
30006	1.11712	0.304215
30007	0.946129	0.653336
30008	0.686934	0.0542905
30009	0.946133	0.0522378
30010	0.9461	0.301916
30011	1.10729	1.08532
30012	0.686963	0.369707
30013	0.686963	0.053828
30014	1.10945	0.271247
30015	0.80397	0.943888
30016	0.946131	0.29513
30017	0.946131	0.414266
30018	1.89226	2.1
30019	1.89226	1.93324
30020	1.89226	2.1
30021	1.89226	2.1
30022	3.78452	2.1
30023	1.22615	2.1
30024	1.3439	2.1
30025	1.47868	2.1
30026	1.55998	2.1
30027	2.78613	2.1
30028	1.04973	2.1
30029	1.47868	2.1
30030	1.20149	2.1
30031	1.61721	2.1
30032	2.68017	2.1
30033	1.89226	2.1
30034	1.89226	2.1
30035	1.89226	2.1
30036	1.89226	2.07929
30037	3.78452	2.1
30038	0.946133	1.79396
30039	0.946133	2.1
30040	0.946128	2.1
30041	0.946128	2.1

30042	0.946131	1.75771
30043	0.946131	2.1
30044	0.946131	2.1
30045	0.946131	2.03533
30046	0.94613	2.1
30047	0.94613	2.1
30048	0.946131	2.1
30049	0.946131	2.1
30050	0.798126	2.1
30051	0.798126	2.1
30052	0.805719	2.1
30053	0.805719	2.1
30054	0.779992	2.1
30055	0.864507	2.1
30056	0.739338	2.1
30057	0.808607	2.1
30058	0.808606	2.1
30059	0.864506	2.1
30060	0.739339	2.1
30061	0.779993	2.1
30062	0.946128	2.1
30063	0.946128	2.1
30064	1.18959	2.1
30065	1.00304	2.1
30066	1.00304	2.1
30067	1.00304	2.1
30068	0.94613	2.1
30069	0.94613	2.1
30070	0.94613	2.1
30071	1.18959	2.1
30072	1.00304	2.1
30073	0.94613	2.1
30074	2.19163	2.1
30075	1.89226	2.1
30076	2.16541	2.1
30077	1.89226	2.1
30078	4.08389	2.1
30079	1.89226	2.1
30080	1.89226	1.91902
30081	1.89226	2.1
30082	1.89226	1.56535
30083	3.78452	2.1
30084	0.964782	2.1
30085	1.30151	1.51479
30086	1.30318	1.50245
30087	1.30318	2.1
30088	0.949134	1.61269
30089	0.949134	2.1
30090	2.15005	2.1
30091	1.89226	2.1
30092	2.16541	2.1
30093	2.16541	2.1
30094	1.89226	2.1
30095	1.89226	2.1
30096	1.89226	2.1
30097	1.89226	2.07242
30098	1.89226	2.1
30099	1.89226	1.12621

30100	3.78452	1.88705
30101	0.949134	2.1
30102	1.30151	2.05062
30103	0.949134	2.1
30104	1.32143	1.54713
30105	2.25064	2.1
30106	0.982859	0.227279
30107	1.28326	0.601608
30108	1.19052	0.281179
30109	1.19052	0.881428
30110	1.10476	0.212575
30111	1.10476	0.850841
30112	1.37265	0.536328
30113	1.38247	1.36082
30114	1.36636	1.63827
30115	1.3294	0.511363
30116	2.71187	0.91037
30117	1.89226	1.67538
30118	1.89226	0.607486
30119	1.89226	0.632586
30120	1.89226	1.48048
30121	1.89226	0.608291
30122	1.89226	1.49356
30123	1.36636	1.81457
30124	1.3294	0.703472
30125	1.30001	0.753618
30126	1.36416	1.67999
30127	2.69356	1.26274
30128	1.89226	1.85116
30129	1.89226	0.665911
30130	1.89226	0.924005
30131	1.89226	1.8876
30132	1.89226	0.698163
30133	1.89226	1.70353
30134	2.83701	0.21131
30135	1.89226	0.650463
30136	1.89226	1.3271
30137	2.79312	0.262242
30138	4.68539	0.474395
30139	1.44288	2.1
30140	0.948677	2.1
30141	1.84076	2.1
30142	1.42884	2.1
30143	2.78943	2.1
30144	1.89226	2.1
30145	1.89226	2.1
30146	1.89226	2.1
30147	1.89226	2.1
30148	3.78452	2.1
30149	0.909402	2.1
30150	0.909402	2.1
30151	1.3063	2.1
30152	1.56757	2.1
30153	1.00038	2.1
30154	1.5235	2.1
30155	0.946127	2.1
30156	0.946127	2.1
30157	0.946128	2.1

30158	1.26957	2.1
30159	1.00038	2.1
30160	0.946129	2.1
30161	1.17256	2.1
30162	2.2846	2.1
30163	1.1844	2.1
30164	2.28904	2.1
30165	3.469	2.1
30166	0.867886	2.1
30167	0.867886	2.1
30168	0.867277	2.1
30169	0.867277	2.1
30170	1.14296	2.1
30171	1.14452	2.1
30172	0.591766	2.1
30173	0.592199	2.1
30174	0.591766	2.1
30175	1.14296	2.1
30176	0.5922	2.1
30177	1.14452	2.1
30178	1.27408	0.0777492
30179	1.56339	0.102854
30180	1.52522	0.0726801
30181	1.34447	0.0612364
30182	2.90786	0.0704138
30183	1.27408	0.101396
30184	1.01121	0.0870872
30185	0.991868	0.0727152
30186	1.34447	0.0826641
30187	2.35569	0.0845067
30188	0.944818	0.0904247
30189	0.883008	0.088788
30190	0.66957	0.0741827
30191	0.616883	0.0734138
30192	0.755679	0.0675654
30193	0.755679	0.117109
30194	0.791912	0.0827394
30195	0.701943	0.0743333
30196	0.901541	0.0810735
30197	0.634847	0.0807022
30198	0.82781	0.0867883
30199	0.655405	0.0962534
30200	1.73819	2.1
30201	1.70303	2.1
30202	1.70303	2.1
30203	1.70303	2.1
30204	1.94516	2.1
30205	1.94517	2.1
30206	0.94994	2.1
30207	2.07469	2.1
30208	1.08506	2.1
30209	2.0815	2.1
30210	3.12902	2.1
30211	0.946131	2.1
30212	0.946131	2.1
30213	0.946131	2.1
30214	0.946131	2.1
30215	0.946128	2.1

30216	0.946128	2.1
30217	0.946133	2.1
30218	0.946133	2.1
30219	0.946131	2.1
30220	0.946131	2.1
30221	0.94613	2.1
30222	0.94613	2.1
30223	2.51796	2.1
30224	1.64193	2.1
30225	2.51172	2.1
30226	1.68893	2.1
30227	4.20689	2.1
30228	2.51172	2.1
30229	1.70002	2.1
30230	1.70002	2.1
30231	1.68893	2.1
30232	2.48689	2.1
30233	2.4758	2.1
30234	2.27963	0.05
30235	2.21582	0.05
30236	2.60486	0.05
30237	2.5105	0.05
30238	4.79013	0.05
30239	2.21582	0.05
30240	2.02069	0.05
30241	2.5105	0.05
30242	2.28615	0.05
30243	4.53119	0.05
30244	2.60486	0.05
30245	2.5105	0.05
30246	2.55791	0.05
30247	2.47141	0.05
30248	5.06842	0.05
30249	2.5105	0.05
30250	2.28615	0.05
30251	2.47141	0.05
30252	2.23337	0.05
30253	4.74387	0.05
30254	2.06984	2.1
30255	0.967499	2.1
30256	2.12435	2.1
30257	2.0021	2.1
30258	1.08975	2.1
30259	0.967499	2.1
30260	1.89226	0.0746984
30261	1.89226	0.0509915
30262	1.89226	0.05
30263	1.89226	0.055103
30264	1.89226	0.0509463
30265	1.89226	0.0608185
30266	1.89226	2.1
30267	1.89226	2.1
30268	1.89226	2.1
30269	1.89226	2.1
30270	3.78452	2.1
30271	1.89227	2.1
30272	1.89227	2.1
30273	1.89227	2.1

30274	1.89227	2.1
30275	3.78453	2.1
30276	0.55882	2.1
30277	0.696462	2.1
30278	1.07003	2.1
30279	0.937179	2.1
30280	0.818077	2.1
30281	0.818077	2.1
30282	0.815565	2.1
30283	0.815565	2.1
30284	0.696462	2.1
30285	1.07003	2.1
30286	0.93718	2.1
30287	0.55882	2.1
30288	2.53857	2.1
30289	1.87436	2.1
30290	2.27541	2.1
30291	2.27541	2.1
30292	2.14005	2.1
30293	2.14005	2.1
30294	1.89226	2.1
30295	1.89226	2.1
30296	1.89226	2.1
30297	1.89226	2.1
30298	3.78452	2.1
30299	1.89227	2.1
30300	1.89227	2.1
30301	1.89227	2.1
30302	1.89227	2.1
30303	3.78453	2.1
30304	1.39292	2.1
30305	2.14006	2.1
30306	2.41083	2.1
30307	1.68761	2.1
30308	3.80376	2.1
30309	2.53857	2.1
30310	2.14005	2.1
30311	2.41083	2.1
30312	2.82043	2.1
30313	4.9494	2.1
30314	1.90439	0.153316
30315	1.51835	0.151551
30316	1.20473	0.131
30317	0.947693	0.176475
30318	2.85208	0.153018
30319	2.586	2.1
30320	1.46262	2.1
30321	2.14009	2.1
30322	2.78101	2.1
30323	4.72609	2.1
30324	1.89224	2.1
30325	2.78101	2.1
30326	2.586	2.1
30327	2.586	2.1
30328	1.89224	2.1
30329	1.89224	2.1
30330	0.994293	2.1
30331	1.19	2.1

30332	1.09483	2.1
30333	1.09483	2.1
30334	1.06827	2.1
30335	1.06827	2.1
30336	1.89226	2.1
30337	1.89226	2.1
30338	1.89226	2.1
30339	1.89226	2.1
30340	3.78452	2.1
30341	0.946132	2.1
30342	0.946132	2.1
30343	1.31912	2.1
30344	1.3572	2.1
30345	0.946131	2.1
30346	0.946131	2.1
30347	1.31912	2.1
30348	1.31912	2.1
30349	0.946131	2.1
30350	1.31912	2.1
30351	1.35721	2.1
30352	0.946131	2.1
30353	2.24577	0.460995
30354	1.09408	0.476222
30355	1.60239	0.502369
30356	1.09408	0.5148
30357	2.39404	0.432854
30358	2.07382	0.438651
30359	1.70303	0.128062
30360	1.3216	0.176986
30361	1.23104	0.0926848
30362	1.05475	0.139835
30363	2.60408	0.097488
30364	1.89226	0.222657
30365	1.89226	0.229045
30366	1.89226	0.137596
30367	1.89226	0.195645
30368	3.78452	0.274031
30369	1.89226	0.112673
30370	2.3845	0.0959936
30371	2.6875	0.117838
30372	1.89226	0.123839
30373	2.6875	0.084946
30374	1.89226	0.0855561
30375	1.46759	2.1
30376	1.17585	2.1
30377	1.85189	2.1
30378	1.39223	2.1
30379	2.85981	2.1
30380	1.85189	2.1
30381	1.71255	2.1
30382	1.71255	2.1
30383	1.39223	2.1
30384	2.28511	2.1
30385	1.96479	2.1
30386	1.89227	0.593644
30387	1.89227	1.18623
30388	1.89227	1.24632
30389	1.89227	0.459272

30390	1.89227	1.28051
30391	1.89227	0.925421
30392	1.89226	0.774542
30393	1.89226	1.19577
30394	1.89226	1.24231
30395	1.89226	0.39094
30396	1.89226	1.30797
30397	1.89226	0.786834
30398	1.89226	0.779054
30399	1.89226	1.1895
30400	1.89226	1.2138
30401	1.89226	0.388896
30402	1.89226	1.27703
30403	1.89226	0.979396
30404	1.89226	0.469965
30405	2.77788	0.988602
30406	2.21062	1.14719
30407	2.21062	0.376972
30408	1.89226	1.19521
30409	1.89226	0.824889
30410	0.868548	0.396287
30411	0.610104	0.525249
30412	0.796678	0.999191
30413	0.865021	0.359529
30414	1.0883	0.372987
30415	0.904938	0.582512
30416	2.21062	0.42977
30417	1.49548	0.432877
30418	1.2068	1.11178
30419	1.54764	0.497904
30420	0.790005	0.328813
30421	1.89226	2.1
30422	2.67457	2.1
30423	1.89226	2.1
30424	2.56352	2.1
30425	4.56683	2.1
30426	2.67456	2.1
30427	2.20011	2.1
30428	2.56351	2.1
30429	2.11083	2.1
30430	4.78539	2.1
30431	2.56348	2.1
30432	2.1108	2.1
30433	2.45763	2.1
30434	2.00986	2.1
30435	4.56843	2.1
30436	0.946133	0.05
30437	0.946133	0.05
30438	0.946132	0.05
30439	0.946132	0.05
30440	0.946132	0.05
30441	0.946132	0.05
30442	0.946132	0.05
30443	0.946132	0.05
30444	0.946132	0.05
30445	0.946132	0.05
30446	0.946133	0.05
30447	0.946133	0.05

30448	1.45927	1.50475
30449	1.25133	1.81221
30450	1.00082	1.73991
30451	1.41689	1.73975
30452	1.0432	1.62492
30453	1.45927	1.72677
30454	1.41689	1.72369
30455	1.29579	1.86477
30456	1.34874	1.66408
30457	1.25133	1.88684
30458	2.76563	1.99615
30459	0.675555	1.75825
30460	0.674371	1.71716
30461	0.625664	1.72847
30462	0.523133	1.76444
30463	0.676509	1.82438
30464	0.676509	1.83345
30465	0.62471	1.69834
30466	0.62471	1.65647
30467	0.675556	1.64858
30468	0.523132	1.52115
30469	0.674373	1.44027
30470	0.625663	1.64385
30471	1.89226	1.24804
30472	1.89226	1.20227
30473	1.89226	1.09907
30474	1.89226	1.18882
30475	3.78453	1.18184
30476	0.851517	1.28148
30477	0.946133	1.22494
30478	0.946132	1.23344
30479	0.946132	1.24991
30480	0.946133	1.17385
30481	0.851517	1.36754
30482	0.946132	1.2479
30483	0.946132	1.23197
30484	0.851516	1.25405
30485	0.851516	1.1934
30486	0.946133	1.29706
30487	0.946133	1.22462
30488	0.569995	1.24085
30489	0.816895	1.35002
30490	1.33893	1.33332
30491	1.18795	1.25755
30492	1.18795	1.23751
30493	0.569995	1.27649
30494	0.816895	1.37421
30495	1.33893	1.30077
30496	0.859572	1.39307
30497	0.859572	1.26746
30498	1.04936	1.26596
30499	1.04936	1.2039
30500	1.89226	2.1
30501	1.89226	2.1
30502	2.55618	2.1
30503	2.55618	2.1
30504	2.66983	2.1
30505	2.00591	2.1

30506	1.89226	2.1
30507	2.64586	2.1
30508	2.83331	2.1
30509	2.83331	2.1
30510	1.89226	2.1
30511	1.89226	2.1
30512	2.64586	2.1
30513	2.0815	2.1
30514	1.99985	2.1
30515	1.94005	2.1
30516	2.83332	2.1
30517	2.74859	2.1
30518	1.98061	2.1
30519	1.68356	2.1
30520	1.83879	2.1
30521	1.55987	2.1
30522	3.54048	2.1
30523	1.68356	2.1
30524	1.20969	2.1
30525	1.20969	2.1
30526	1.30751	2.1
30527	1.55987	2.1
30528	1.65769	2.1
30529	1.83879	2.1
30530	1.55987	2.1
30531	1.68443	2.1
30532	1.4449	2.1
30533	3.2443	2.1
30534	0.660368	2.1
30535	0.660368	2.1
30536	0.666922	2.1
30537	0.666922	2.1
30538	0.560377	2.1
30539	0.604842	2.1
30540	0.779935	2.1
30541	0.722448	2.1
30542	0.722446	2.1
30543	0.560377	2.1
30544	0.604844	2.1
30545	0.779934	2.1
30546	1.89227	2.1
30547	1.89227	2.1
30548	1.89227	2.1
30549	1.89227	2.1
30550	3.78453	2.1
30551	1.42896	2.1
30552	1.23037	2.1
30553	1.42896	2.1
30554	1.474 2.1	
30555	1.2016	2.1
30556	1.24665	2.1
30557	1.89226	2.1
30558	2.7188	2.1
30559	1.89226	2.1
30560	2.70702	2.1
30561	4.61106	2.1
30562	1.89226	2.1
30563	1.89226	2.1

30564	1.89226	2.1
30565	1.89226	2.1
30566	3.78453	2.1
30567	0.644603	2.1
30568	0.644603	2.1
30569	0.648272	2.1
30570	0.648272	2.1
30571	0.585644	2.1
30572	0.600802	2.1
30573	0.692073	2.1
30574	0.714478	2.1
30575	0.585644	2.1
30576	0.692073	2.1
30577	0.714478	2.1
30578	0.600802	2.1
30579	1.35351	2.1
30580	1.35351	2.1
30581	0.946131	2.1
30582	0.946131	2.1
30583	1.34392	2.1
30584	1.35351	2.1
30585	0.946132	2.1
30586	0.946132	2.1
30587	0.946132	2.1
30588	1.34392	2.1
30589	0.946132	2.1
30590	1.35351	2.1
30591	1.87609	2.1
30592	1.79155	2.1
30593	1.79155	2.1
30594	1.89226	2.1
30595	1.89226	2.1
30596	1.99211	2.1
30597	1.89226	2.1
30598	1.71706	2.1
30599	1.71706	2.1
30600	1.79155	2.1
30601	1.89226	2.1
30602	1.96675	2.1
30603	1.46869	2.1
30604	1.89226	2.1
30605	1.89226	2.1
30606	1.89226	2.1
30607	1.5001	2.1
30608	1.5001	2.1
30609	1.89226	2.1
30610	1.89226	2.1
30611	1.89226	2.1
30612	1.89226	2.1
30613	1.89226	2.1
30614	1.89226	2.1
30615	1.21408	2.1
30616	2.23563	2.1
30617	2.34021	2.1
30618	2.34021	2.1
30619	1.17285	2.1
30620	1.17285	2.1
30621	1.89226	2.1

30622	1.89226	2.1
30623	1.47937	2.1
30624	1.89226	2.1
30625	1.14822	2.1
30626	1.46869	2.1
30627	1.89226	2.1
30628	1.89226	2.1
30629	1.89226	2.1
30630	1.89226	2.1
30631	1.89226	2.1
30632	1.89226	2.1
30633	1.25716	2.1
30634	2.34021	2.1
30635	2.4542	2.1
30636	2.4542	2.1
30637	1.21408	2.1
30638	1.21408	2.1
30639	1.66824	2.1
30640	2.44883	2.1
30641	1.93003	2.1
30642	2.24795	2.1
30643	3.88907	2.1
30644	1.89319	2.1
30645	2.24795	2.1
30646	2.82724	2.1
30647	2.31318	2.1
30648	2.30868	2.1
30649	1.93003	2.1
30650	1.89226	2.1
30651	1.42589	2.1
30652	1.42589	2.1
30653	1.80501	2.1
30654	1.89226	2.1
30655	2.27137	2.1
30656	1.21067	0.0652537
30657	2.39166	0.215009
30658	1.73577	0.0796536
30659	1.73577	0.0678789
30660	1.53584	0.122716
30661	1.53584	0.0895814
30662	1.35025	2.1
30663	2.0599	1.76512
30664	1.51084	2.1
30665	2.27218	0.725894
30666	3.57074	2.0411
30667	2.0599	1.71165
30668	2.51818	2.1
30669	2.27218	0.61372
30670	2.66532	2.07371
30671	4.72522	1.70452
30672	1.67115	2.1
30673	2.27218	1.64075
30674	2.39605	2.1
30675	2.39605	1.54321
30676	1.51084	2.1
30677	1.51084	1.90247
30678	2.27218	1.518
30679	2.66532	2.1

30680	2.39605	0.459735
30681	2.76829	2.05854
30682	5.06137	1.557
30683	2.08684	1.52926
30684	2.36033	1.83179
30685	1.89224	1.46434
30686	1.89224	1.82745
30687	4.25257	1.51498
30688	2.41473	0.138828
30689	1.00307	0.179518
30690	2.01365	0.148925
30691	1.00307	0.194991
30692	3.05113	0.136768
30693	2.04056	0.207311
30694	1.11099	2.1
30695	2.25103	2.1
30696	2.22227	2.1
30697	2.22227	2.1
30698	1.14431	2.1
30699	1.14431	2.1
30700	1.07485	2.1
30701	2.22229	2.1
30702	2.19163	2.1
30703	2.19163	2.1
30704	1.111 2.1	
30705	1.111 2.1	
30706	1.0547	2.1
30707	1.19886	2.1
30708	0.946131	2.1
30709	0.946131	2.1
30710	0.946131	2.1
30711	0.946131	2.1
30712	1.19886	2.1
30713	1.19886	2.1
30714	1.0547	2.1
30715	0.946131	2.1
30716	0.946132	2.1
30717	1.19886	2.1
30718	1.89417	2.1
30719	2.64424	1.19811
30720	1.76585	2.1
30721	2.47695	2.0534
30722	4.37112	2.1
30723	1.89226	2.1
30724	1.89226	2.1
30725	1.89226	2.1
30726	1.89226	2.09846
30727	3.78453	2.1
30728	0.930281	2.1
30729	1.0547	2.1
30730	0.946131	2.1
30731	0.946131	2.1
30732	0.930309	2.1
30733	0.930309	2.1
30734	1.07052	2.1
30735	1.07052	2.1
30736	0.93028	2.1
30737	0.946131	2.1

30738	1.0547	2.1
30739	0.946131	2.1
30740	1.00015	0.87438
30741	1.00015	2.1
30742	1.01918	2.1
30743	1.01918	1.9456
30744	1.17437	1.99295
30745	1.23847	0.668518
30746	0.882926	2.1
30747	0.780859	2.1
30748	0.780859	2.05286
30749	1.17438	2.1
30750	0.882925	2.1
30751	1.23848	2.1
30752	1.89226	2.1
30753	1.89226	1.98738
30754	1.89226	2.1
30755	1.89226	2.1
30756	1.89226	2.1
30757	1.89226	2.1
30758	1.22083	2.1
30759	1.29579	2.1
30760	1.26236	2.1
30761	1.06191	2.1
30762	1.42128	2.1
30763	1.22083	2.1
30764	1.42128	2.1
30765	1.45806	2.1
30766	1.43502	2.1
30767	1.29579	2.1
30768	1.5605	2.1
30769	1.42128	2.1
30770	1.02813	2.1
30771	1.12121	2.1
30772	1.40557	2.1
30773	1.35298	2.1
30774	1.1314	2.1
30775	1.28679	2.1
30776	1.1715	2.1
30777	1.1715	2.1
30778	0.975656	2.1
30779	1.24908	2.1
30780	0.98956	2.1
30781	1.2681	2.1
30782	0.946132	2.1
30783	0.946132	2.1
30784	1.31889	2.1
30785	1.31889	2.1
30786	1.16546	2.1
30787	1.31889	2.1
30788	0.946133	2.1
30789	0.946133	2.1
30790	0.946133	2.1
30791	1.31889	2.1
30792	0.946132	2.1
30793	1.16546	2.1
30794	2.51518	0.05
30795	2.37584	0.05

30796	1.11712	0.05
30797	1.31242	0.05
30798	3.68826	0.05
30799	2.51518	0.05
30800	1.26311	0.05
30801	2.48977	0.05
30802	2.48977	0.05
30803	1.31242	0.05
30804	1.31242	0.05
30805	1.89226	0.969735
30806	1.89226	2.1
30807	1.89226	0.790799
30808	1.89226	2.1
30809	3.78452	1.939
30810	2.04737	0.568595
30811	1.77122	2.1
30812	1.94498	0.735206
30813	1.65174	2.1
30814	3.71621	2.00069
30815	1.41077	2.1
30816	1.29729	2.1
30817	1.77123	2.1
30818	1.77123	2.1
30819	1.65175	2.1
30820	1.29129	2.1
30821	1.89226	2.1
30822	1.89226	2.1
30823	1.89226	2.1
30824	1.89226	2.1
30825	1.89226	2.1
30826	1.89226	2.1
30827	1.89226	0.569785
30828	1.89226	2.1
30829	1.89226	0.794006
30830	1.89226	2.1
30831	3.78451	1.56828
30832	1.94498	0.532747
30833	1.65174	2.1
30834	1.7968	1.08826
30835	1.49831	2.1
30836	3.44329	1.87013
30837	1.49831	2.1
30838	1.29729	2.1
30839	1.49831	2.1
30840	1.65175	2.1
30841	1.16858	2.1
30842	1.32201	2.1
30843	1.89226	2.1
30844	1.89226	2.1
30845	1.89226	2.1
30846	1.89226	2.1
30847	3.78452	2.1
30848	1.89226	0.05
30849	1.89226	0.051571
30850	1.89226	0.050344
30851	1.89226	0.0538478
30852	1.89226	0.05
30853	1.89226	0.05

30854	1.65324	2.08188
30855	1.26409	0.320892
30856	1.42491	0.330452
30857	1.42491	2.08988
30858	1.52268	0.740152
30859	1.52268	2.04994
30860	1.52268	2.1
30861	1.26409	0.791116
30862	1.32697	2.07769
30863	1.05724	0.479104
30864	2.57991	1.75902
30865	1.89226	0.0957542
30866	1.96548	0.0971995
30867	2.0178	0.100637
30868	1.96548	0.0922449
30869	1.94458	0.116568
30870	1.89226	0.105298
30871	1.99366	0.0935667
30872	2.0178	0.0940466
30873	1.89226	0.127877
30874	2.0178	0.0905053
30875	1.75999	0.115203
30876	1.89226	0.0971455
30877	0.946126	2.1
30878	0.946126	2.1
30879	1.28228	2.1
30880	1.19444	2.1
30881	0.946127	2.1
30882	0.946127	2.1
30883	1.19444	2.1
30884	1.19444	2.1
30885	0.946128	2.1
30886	1.19444	2.1
30887	1.28228	2.1
30888	0.946128	2.1
30889	1.89226	2.1
30890	1.89226	2.1
30891	1.89226	2.1
30892	1.89226	2.1
30893	3.78452	2.1
30894	2.14104	2.1
30895	1.89226	2.1
30896	2.19948	2.1
30897	1.89226	2.1
30898	1.89226	2.1
30899	2.19948	2.1
30900	1.89226	2.1
30901	2.38888	2.1
30902	2.23932	2.1
30903	2.38888	2.1
30904	1.74269	2.1
30905	1.89226	2.1
30906	1.89226	2.1
30907	1.89226	2.1
30908	1.89226	2.1
30909	1.89226	2.1
30910	3.78452	2.1
30911	0.946132	2.1

30912	0.946132	2.1
30913	1.07052	2.1
30914	1.07052	2.1
30915	0.946132	2.1
30916	0.946132	2.1
30917	1.05132	2.1
30918	1.07052	2.1
30919	1.05132	2.1
30920	0.946132	2.1
30921	1.07052	2.1
30922	0.946133	2.1
30923	2.40171	2.1
30924	1.89226	2.1
30925	1.89226	2.1
30926	1.89226	2.1
30927	2.42723	2.1
30928	2.42723	2.1
30929	1.89226	2.1
30930	1.89226	2.1
30931	1.89226	2.1
30932	1.89226	2.1
30933	3.78452	2.1
30934	1.63661	2.1
30935	2.3532	2.1
30936	2.33287	2.1
30937	2.33287	2.1
30938	1.64158	2.1
30939	1.64158	2.1
30940	2.38918	2.1
30941	1.89226	2.1
30942	1.89226	2.1
30943	1.89226	2.1
30944	2.40171	2.1
30945	2.40171	2.1
30946	1.89226	2.1
30947	1.89226	2.1
30948	1.89226	2.1
30949	1.89226	2.1
30950	1.89226	2.1
30951	1.89226	2.1
30952	1.64158	2.1
30953	2.3532	2.1
30954	1.6488	2.1
30955	2.37364	2.1
30956	4.002 2.1	
30957	2.54752	0.171316
30958	1.42445	0.225476
30959	1.86697	0.219266
30960	1.86697	0.196271
30961	2.06666	0.2156
30962	2.06666	0.196071
30963	2.50872	0.139118
30964	2.06666	0.198676
30965	1.90439	0.161866
30966	1.51835	0.161976
30967	3.97105	0.16134
30968	0.755161	0.207315
30969	0.755161	0.183938

30970	0.793994	0.155944
30971	0.793994	0.180686
30972	0.712225	0.19867
30973	0.515826	0.185779
30974	1.03333	0.190165
30975	0.759175	0.173367
30976	0.759173	0.188451
30977	0.515827	0.165425
30978	0.712226	0.177092
30979	1.03333	0.180085
30980	1.69633	0.227404
30981	1.89226	0.244358
30982	1.62477	0.217761
30983	1.89226	0.243083
30984	1.62477	0.227408
30985	1.89226	0.244651
30986	1.89226	0.417432
30987	1.89226	0.617428
30988	1.89226	0.564284
30989	1.89226	0.534907
30990	3.78452	0.532448
30991	2.28622	0.226102
30992	1.09667	0.166149
30993	1.17506	0.167008
30994	2.24411	0.204051
30995	1.17506	0.195382
30996	2.24411	0.250492
30997	2.24741	2.1
30998	1.70303	2.1
30999	2.49168	2.1
31000	1.57936	2.1
31001	2.53729	2.1
31002	1.70303	2.1
31003	2.24741	2.1
31004	2.47557	2.1
31005	1.89227	2.1
31006	1.89227	2.1
31007	4.36784	2.1
31008	2.27016	0.05
31009	1.10476	0.050795
31010	1.44103	0.05
31011	1.10476	0.0570895
31012	2.26791	0.05
31013	1.93165	0.0522864
31014	2.27016	0.05
31015	1.89226	0.0580666
31016	1.89226	0.05
31017	2.4065	0.0536862
31018	4.29876	0.0524055
31019	2.27016	0.05
31020	1.44103	0.0509445
31021	1.53336	0.05
31022	2.4065	0.05
31023	3.84753	0.05
31024	2.7542	0.356848
31025	1.89226	0.489519
31026	2.35027	0.431213
31027	1.89226	0.440973

31028	2.35027	0.41621
31029	1.89226	0.426771
31030	2.3301	2.1
31031	2.36071	2.1
31032	2.07629	2.1
31033	2.01105	2.1
31034	4.37176	2.1
31035	1.86512	2.1
31036	2.01105	2.1
31037	2.01105	2.1
31038	2.3301	2.1
31039	1.57459	2.1
31040	1.89364	2.1
31041	2.52671	2.1
31042	2.01105	2.1
31043	1.78251	2.1
31044	1.70302	2.1
31045	3.98362	2.1
31046	1.227	2.1
31047	2.01105	2.1
31048	1.45869	2.1
31049	1.41713	2.1
31050	1.70302	2.1
31051	1.64994	2.1
31052	2.00989	2.1
31053	1.28938	2.1
31054	1.8769	2.1
31055	1.20969	2.1
31056	3.21958	2.1
31057	1.8769	2.1
31058	1.15864	2.1
31059	1.15864	2.1
31060	1.20969	2.1
31061	1.77353	2.1
31062	1.82458	2.1
31063	1.7986	2.1
31064	2.48717	2.1
31065	2.50161	2.1
31066	1.75869	2.1
31067	4.30021	2.1
31068	2.50161	2.1
31069	1.75251	2.1
31070	1.81678	2.1
31071	1.75869	2.1
31072	2.5597	2.1
31073	2.50161	2.1
31074	1.91334	0.578013
31075	1.85439	1.1674
31076	1.91334	0.659827
31077	1.70303	1.18804
31078	1.70303	0.482705
31079	1.49692	1.08613
31080	2.39986	0.0501184
31081	1.89226	0.106781
31082	1.89226	0.0803624
31083	2.50756	0.05
31084	4.39982	0.05
31085	2.39986	0.0907679

31086	1.70303	0.0564267
31087	1.75564	0.0551705
31088	1.75904	0.0993328
31089	2.50272	0.0691578
31090	2.50756	0.0981214
31091	1.89226	0.0921187
31092	2.50756	0.0532854
31093	2.47857	0.0827996
31094	2.50756	0.0873942
31095	1.86327	0.0931486
31096	1.89226	0.0813943
31097	1.22241	0.0823593
31098	1.20827	0.118685
31099	0.772416	0.0678263
31100	0.555208	0.128843
31101	1.004	0.0654146
31102	1.004	0.10305
31103	0.93504	0.127072
31104	0.744364	0.110778
31105	1.2089	0.120293
31106	0.654935	0.122354
31107	0.773927	0.0965414
31108	1.03618	0.104411
31109	1.89227	0.05
31110	1.89227	0.05
31111	1.89227	0.05
31112	1.89227	0.05
31113	3.78453	0.05
31114	1.89226	0.05
31115	1.89226	0.05
31116	1.89226	0.05
31117	1.89226	0.05
31118	3.78452	0.05
31119	1.82643	0.05
31120	2.7084	0.05
31121	2.12654	0.05
31122	2.65614	0.05
31123	1.82643	0.05
31124	2.35603	0.05
31125	1.29385	0.05
31126	0.946133	0.05
31127	1.05492	0.05
31128	0.946132	0.05
31129	1.05492	0.05
31130	1.29385	0.05
31131	0.946133	0.05
31132	0.946133	0.05
31133	1.29385	0.05
31134	1.29385	0.05
31135	0.946132	0.05
31136	0.946132	0.05
31137	0.946132	0.05
31138	1.27329	0.05
31139	1.29385	0.05
31140	0.946129	0.05
31141	1.29385	0.05
31142	1.27328	0.05
31143	0.946131	0.05

31144	0.946131	0.05
31145	1.27328	0.05
31146	1.27328	0.05
31147	0.94613	0.05
31148	0.94613	0.05
31149	1.89226	1.54855
31150	1.89226	1.59256
31151	1.89226	1.16841
31152	1.89226	1.28779
31153	3.78453	1.37207
31154	1.89226	1.47087
31155	1.89226	1.50412
31156	1.89226	1.184
31157	1.89226	1.37766
31158	3.78453	1.28846
31159	1.21488	1.46489
31160	1.14605	1.88746
31161	1.62974	1.34137
31162	1.52399	1.22674
31163	2.84463	1.45954
31164	0.577078	1.22825
31165	0.81251	1.77491
31166	0.821003	1.285
31167	0.543413	1.43167
31168	0.683929	1.2728
31169	0.722433	2.1
31170	0.730674	1.60029
31171	0.730674	1.25532
31172	0.857766	1.24168
31173	0.862119	1.19678
31174	0.600938	1.53489
31175	0.596094	1.31486
31176	1.89226	1.26642
31177	1.89226	1.35192
31178	1.89226	1.05205
31179	1.89226	1.17968
31180	3.78453	1.12492
31181	1.89226	1.23505
31182	1.89226	1.72869
31183	1.89226	1.27461
31184	1.89226	1.17486
31185	3.78453	1.35157
31186	1.62974	1.54556
31187	1.56958	1.70927
31188	2.2362	1.13521
31189	2.11424	1.22768
31190	3.86595	1.26487
31191	2.2362	1.11666
31192	1.85414	1.73433
31193	2.28211	1.03508
31194	1.78367	1.07313
31195	2.2362	1.34866
31196	1.73256	1.62709
31197	0.851539	1.29513
31198	0.851539	1.23191
31199	1.14454	1.95657
31200	0.938988	1.86761
31201	0.851485	1.29729

31202	0.920593	1.68706
31203	0.851541	0.373102
31204	0.851541	1.83366
31205	1.04073	0.914223
31206	1.16342	1.50625
31207	0.825315	1.63369
31208	0.825316	1.38203
31209	1.89226	2.1
31210	1.89226	2.1
31211	1.89226	2.1
31212	1.89226	2.1
31213	3.78453	2.1
31214	2.57016	2.1
31215	1.74789	2.1
31216	2.57016	2.1
31217	2.522	2.1
31218	1.61727	2.1
31219	1.56911	2.1
31220	1.28075	2.1
31221	1.406	2.1
31222	0.946131	2.1
31223	0.946131	2.1
31224	1.28075	2.1
31225	1.00333	2.1
31226	0.94613	2.1
31227	0.94613	2.1
31228	1.041	2.1
31229	1.041	2.1
31230	0.943484	2.1
31231	1.27717	2.1
31232	1.89226	2.1
31233	1.89226	2.1
31234	1.89226	2.1
31235	1.89226	2.1
31236	1.89226	2.1
31237	1.89226	2.1
31238	2.57016	2.1
31239	1.61727	2.1
31240	2.74196	2.1
31241	1.59094	2.1
31242	4.16109	2.1
31243	1.89226	2.1
31244	2.0815	2.1
31245	2.2034	2.1
31246	1.82493	2.1
31247	1.87847	2.1
31248	2.16142	2.1
31249	1.89226	2.1
31250	1.89226	2.1
31251	1.89226	2.1
31252	1.89226	2.1
31253	3.78453	2.1
31254	0.985252	2.1
31255	0.985252	2.1
31256	0.946132	2.1
31257	0.946132	2.1
31258	1.0655	2.1
31259	0.985255	2.1

31260	0.94613	2.1
31261	0.94613	2.1
31262	0.94613	2.1
31263	1.0655	2.1
31264	0.94613	2.1
31265	0.985255	2.1
31266	1.89226	2.1
31267	1.89226	2.1
31268	1.89226	2.1
31269	1.89226	2.1
31270	3.78453	2.1
31271	1.06549	2.1
31272	1.06549	2.1
31273	0.946133	2.1
31274	0.946133	2.1
31275	1.16834	2.1
31276	1.0655	2.1
31277	0.946131	2.1
31278	0.946131	2.1
31279	0.946131	2.1
31280	1.16834	2.1
31281	0.94613	2.1
31282	1.0655	2.1
31283	0.966631	2.1
31284	1.07349	2.1
31285	0.946131	2.1
31286	0.946131	2.1
31287	1.07349	2.1
31288	1.07349	2.1
31289	0.946129	2.1
31290	0.946129	2.1
31291	1.07349	2.1
31292	0.946131	2.1
31293	0.94613	2.1
31294	0.966631	2.1
31295	2.38055	0.97329
31296	1.89226	1.50685
31297	1.89226	1.38671
31298	1.46724	0.446489
31299	3.35949	1.04396
31300	1.42249	2.1
31301	2.05499	2.1
31302	1.39708	2.1
31303	2.09033	2.1
31304	3.45207	2.1
31305	2.05499	2.1
31306	2.57425	2.1
31307	2.51287	2.1
31308	2.47753	2.1
31309	2.09033	2.1
31310	2.05499	2.1
31311	1.39327	2.1
31312	2.09033	2.1
31313	2.16217	2.1
31314	2.16217	2.1
31315	1.39708	2.1
31316	1.39708	2.1
31317	2.16217	2.1

31318	2.57426	2.1
31319	2.73006	2.1
31320	2.73006	2.1
31321	2.09033	2.1
31322	2.09033	2.1
31323	1.89226	0.0772618
31324	1.89226	0.0570114
31325	1.89226	0.0629202
31326	1.89226	0.0757447
31327	3.78452	0.0734187
31328	0.838749	0.05
31329	0.838749	0.05
31330	0.85824	0.0518775
31331	0.85824	0.0554768
31332	0.544644	0.05492
31333	0.854718	0.05
31334	0.842271	0.0526303
31335	1.19141	0.0527708
31336	0.842272	0.0530403
31337	0.544643	0.05
31338	0.854718	0.0552119
31339	1.19141	0.05
31340	1.34214	0.0509103
31341	1.34214	0.05
31342	0.893236	0.0654238
31343	0.893236	0.0633675
31344	0.893265	0.0647285
31345	1.28924	0.0561428
31346	0.946133	0.0519722
31347	0.946133	0.0602113
31348	0.946132	0.0551358
31349	0.893265	0.0645236
31350	0.946133	0.0608532
31351	1.28924	0.0555027
31352	2.7013	0.0570511
31353	1.89226	0.0703909
31354	1.9216	0.0706852
31355	1.9216	0.0572975
31356	1.89226	0.0676943
31357	1.89226	0.054231
31358	1.26397	2.1
31359	1.52995	2.1
31360	1.30116	2.1
31361	1.04084	2.1
31362	2.57079	2.1
31363	1.47013	2.1
31364	2.17459	2.1
31365	2.10668	2.1
31366	1.38328	2.1
31367	3.55787	2.1
31368	2.55006	2.1
31369	2.17459	2.1
31370	2.10668	2.1
31371	2.49168	2.1
31372	4.66626	2.1
31373	0.613622	2.1
31374	0.691639	2.1
31375	1.00105	2.1

31376	1.05334	2.1
31377	0.839874	2.1
31378	0.839874	2.1
31379	0.852814	2.1
31380	0.852814	2.1
31381	0.613622	2.1
31382	1.00105	2.1
31383	1.05334	2.1
31384	0.691639	2.1
31385	1.00105	2.1
31386	1.05334	2.1
31387	1.20844	2.1
31388	1.24584	2.1
31389	1.12717	2.1
31390	1.12717	2.1
31391	1.13462	2.1
31392	1.13462	2.1
31393	1.20845	2.1
31394	1.00105	2.1
31395	1.05334	2.1
31396	1.24584	2.1
31397	2.22558	2.1
31398	1.85981	2.1
31399	2.18943	1.97916
31400	1.89142	0.874133
31401	4.0854	2.1
31402	2.02641	2.1
31403	1.85981	2.1
31404	2.18943	1.95311
31405	1.67346	2.011
31406	3.88622	1.94323
31407	1.85981	0.647387
31408	1.10736	0.365781
31409	1.27421	0.67752
31410	1.67346	0.540616
31411	1.27421	0.42108
31412	1.67346	1.92706
31413	1.85981	1.51969
31414	1.27362	0.997441
31415	1.89142	0.468093
31416	1.27421	0.395415
31417	3.13343	0.525273
31418	1.81893	0.0710542
31419	1.64985	0.0778152
31420	1.23477	0.0597412
31421	2.25405	0.0884056
31422	3.48882	0.0827463
31423	2.23933	0.05
31424	1.89226	0.0739982
31425	1.89226	0.0740471
31426	2.78053	0.070315
31427	1.89226	0.0546673
31428	2.78053	0.05
31429	0.946131	0.0785171
31430	0.946131	0.0777669
31431	0.946132	0.085592
31432	0.946132	0.108693
31433	0.946131	0.116158

31434	0.946131	0.10593
31435	0.946132	0.0800057
31436	0.946132	0.104092
31437	0.946131	0.0804499
31438	0.946131	0.0977274
31439	0.946132	0.072515
31440	0.946132	0.102752
31441	1.89226	2.1
31442	2.48596	2.1
31443	1.89226	2.1
31444	2.51506	2.1
31445	4.40732	2.1
31446	2.48596	2.1
31447	1.63823	2.1
31448	2.51507	2.1
31449	1.67687	2.1
31450	4.1533	2.1
31451	1.89226	2.1
31452	2.51506	2.1
31453	1.92044	2.1
31454	1.89226	2.1
31455	2.54325	2.1
31456	2.51506	2.1
31457	2.51507	2.1
31458	1.5704	2.1
31459	1.70505	2.1
31460	1.67687	2.1
31461	2.54325	2.1
31462	2.51507	2.1
31463	2.0815	2.1
31464	1.70303	2.1
31465	1.70303	2.1
31466	1.80354	2.1
31467	1.98172	2.1
31468	2.09868	2.1
31469	1.27944	2.1
31470	1.79028	2.1
31471	1.13179	2.1
31472	1.65533	2.1
31473	2.93477	2.1
31474	1.89226	2.1
31475	1.89226	2.1
31476	1.89226	2.1
31477	1.89226	2.1
31478	1.89226	2.1
31479	1.89226	2.1
31480	1.98171	2.1
31481	1.89226	2.1
31482	1.89226	2.1
31483	1.89226	2.1
31484	1.65798	2.1
31485	1.65797	2.1
31486	1.00133	2.1
31487	1.65532	2.1
31488	1.53278	2.1
31489	1.53278	2.1
31490	1.13179	2.1
31491	1.13179	2.1

31492	0.810946	2.1
31493	0.810946	2.1
31494	0.810944	2.1
31495	0.810944	2.1
31496	0.810946	2.1
31497	0.810946	2.1
31498	1.89226	2.1
31499	1.35169	2.1
31500	1.08132	2.1
31501	1.08132	2.1
31502	1.08132	2.1
31503	1.99528	0.0529952
31504	1.70303	0.0579589
31505	2.08152	0.0500051
31506	1.70303	0.0651887
31507	2.33877	0.0535618
31508	1.93967	0.0627982
31509	1.89226	0.0586515
31510	1.89226	0.0930952
31511	1.89226	0.0571112
31512	1.89226	0.0588906
31513	3.78452	0.0646096
31514	1.26259	0.0513442
31515	2.17205	0.0536229
31516	2.32468	0.05
31517	2.32468	0.0546222
31518	1.28055	0.05
31519	1.28055	0.0524406
31520	0.946131	0.0566059
31521	0.946131	0.0510653
31522	0.997642	0.0513537
31523	0.997642	0.05
31524	0.946131	0.05
31525	0.946131	0.0601212
31526	0.989846	0.0515549
31527	0.997642	0.05
31528	0.946132	0.05
31529	0.989846	0.05
31530	0.997641	0.0517935
31531	0.946131	0.0538921
31532	1.89226	0.0600815
31533	1.89226	0.0581849
31534	1.89226	0.05
31535	1.89226	0.0755977
31536	3.78452	0.0561686
31537	1.28055	0.05
31538	2.17205	0.0534115
31539	1.14305	0.05
31540	1.92737	0.05
31541	3.3151	0.05
31542	0.954908	0.05
31543	0.9705	0.05
31544	0.946131	0.05
31545	0.946131	0.05
31546	0.946131	0.05
31547	0.946131	0.05
31548	0.9705	0.05
31549	0.9705	0.05

31550	0.954908	0.05
31551	0.946131	0.05
31552	0.946131	0.05
31553	0.9705	0.05
31554	0.835187	0.05
31555	0.954896	0.05
31556	0.946118	0.05
31557	0.946118	0.05
31558	0.835186	0.05
31559	0.835186	0.05
31560	1.06583	0.05
31561	1.06583	0.05
31562	0.835198	0.05
31563	0.946131	0.05
31564	0.954883	0.05
31565	0.946106	0.05
31566	1.89227	2.1
31567	1.89227	2.1
31568	1.89227	2.1
31569	1.89227	2.1
31570	1.89227	2.1
31571	1.89227	2.1
31572	0.885265	2.1
31573	0.885265	2.1
31574	0.871467	2.1
31575	0.871467	2.1
31576	1.11883	2.1
31577	0.787875	2.1
31578	0.996487	2.1
31579	0.637907	2.1
31580	0.996487	2.1
31581	1.11883	2.1
31582	0.787875	2.1
31583	0.637906	2.1
31584	1.89226	2.1
31585	2.39258	2.1
31586	2.39258	2.1
31587	2.54729	2.1
31588	1.89226	2.1
31589	2.04697	2.1
31590	1.89226	2.1
31591	2.0815	2.1
31592	2.0815	2.1
31593	2.48371	2.1
31594	2.0815	2.1
31595	2.51469	2.1
31596	1.00035	0.05
31597	1.55755	0.601935
31598	1.03534	1.52202
31599	1.61333	1.70315
31600	2.59289	0.429005
31601	1.55755	0.601935
31602	1.85217	2.1
31603	1.61333	2.1
31604	1.92875	2.1
31605	3.48629	2.1
31606	1.09962	1.45519
31607	1.61334	0.812226

31608	1.70868	2.1
31609	1.70868	1.81358
31610	1.03535	2.1
31611	1.03535	1.43406
31612	1.61334	0.822982
31613	1.92876	2.1
31614	1.70868	1.64714
31615	2.05274	2.1
31616	3.63744	1.93962
31617	1.82233	0.309428
31618	2.05768	0.806804
31619	1.89226	0.662486
31620	1.65406	0.405822
31621	1.89226	0.641663
31622	1.65406	0.439412
31623	2.26047	0.353985
31624	2.0815	1.1763
31625	1.89226	0.984611
31626	2.01156	0.495035
31627	1.89226	0.936464
31628	2.01156	0.507638
31629	1.89226	2.1
31630	1.89226	2.1
31631	1.89226	2.1
31632	1.89226	2.1
31633	3.78453	2.1
31634	2.70055	2.1
31635	2.28367	2.1
31636	2.56456	2.1
31637	2.33565	2.1
31638	4.84823	2.1
31639	2.28367	2.1
31640	1.5791	2.1
31641	1.96966	2.1
31642	1.09506	2.1
31643	3.37873	2.1
31644	1.89226	2.1
31645	1.89226	2.1
31646	1.89226	2.1
31647	1.89226	2.1
31648	3.78452	2.1
31649	0.94612	2.1
31650	0.94612	2.1
31651	0.946119	2.1
31652	0.946119	2.1
31653	0.946119	2.1
31654	0.946119	2.1
31655	0.946119	2.1
31656	0.946119	2.1
31657	0.946132	2.1
31658	0.946132	2.1
31659	0.946107	2.1
31660	0.946107	2.1
31661	2.56452	2.1
31662	1.57307	2.1
31663	2.10299	2.1
31664	2.1115	2.1
31665	2.52196	2.1

31666	2.52196	2.1
31667	1.89226	2.1
31668	1.89226	2.1
31669	1.89226	2.1
31670	1.89226	2.1
31671	3.78452	2.1
31672	2.37665	2.1
31673	1.89226	2.1
31674	2.28084	2.1
31675	1.89226	2.1
31676	4.26891	2.1
31677	0.946129	2.1
31678	0.946129	2.1
31679	0.946133	2.1
31680	0.946133	2.1
31681	0.946131	2.1
31682	0.946131	2.1
31683	0.946131	2.1
31684	0.946131	2.1
31685	0.946131	2.1
31686	0.946131	2.1
31687	0.946131	2.1
31688	0.946131	2.1
31689	1.7207	0.05
31690	1.65815	0.05
31691	1.33593	0.05
31692	1.31508	0.05
31693	2.99408	0.05
31694	0.676047	0.05
31695	0.676047	0.05
31696	0.684362	0.05
31697	0.684362	0.05
31698	0.547994	0.05
31699	0.702871	0.05
31700	0.829076	0.05
31701	0.657538	0.05
31702	0.657538	0.05
31703	0.547994	0.05
31704	0.829077	0.05
31705	0.702871	0.05
31706	1.9518	2.1
31707	1.30648	2.1
31708	1.28846	2.1
31709	1.92033	2.1
31710	3.22681	2.1
31711	1.89226	2.1
31712	1.89226	2.1
31713	1.89226	2.1
31714	1.89226	2.1
31715	3.78453	2.1
31716	0.946128	2.1
31717	0.946128	2.1
31718	1.10113	2.1
31719	1.09587	2.1
31720	0.946131	2.1
31721	0.946131	2.1
31722	1.09587	2.1
31723	1.09587	2.1

31724	0.946131	2.1
31725	1.09587	2.1
31726	1.10112	2.1
31727	0.94613	2.1
31728	1.26923	2.1
31729	1.92033	2.1
31730	1.28846	2.1
31731	1.28846	2.1
31732	1.89452	2.1
31733	1.89452	2.1
31734	1.89226	2.1
31735	1.89226	2.1
31736	1.89226	2.1
31737	1.89226	2.1
31738	3.78453	2.1
31739	1.89226	2.1
31740	2.19174	2.1
31741	2.15616	2.1
31742	2.19174	2.1
31743	1.85669	2.1
31744	1.89226	2.1
31745	1.89226	2.1
31746	1.89226	2.1
31747	1.89226	2.1
31748	1.89226	2.1
31749	3.78452	2.1
31750	1.89226	2.1
31751	1.89226	2.1
31752	1.89226	2.1
31753	1.89226	2.1
31754	3.78452	2.1
31755	1.51223	2.1
31756	1.41562	2.1
31757	1.5139	2.1
31758	1.42284	2.1
31759	1.6033	2.1
31760	1.51223	2.1
31761	1.51223	2.1
31762	1.25953	2.1
31763	1.38085	2.1
31764	1.6033	2.1
31765	2.89309	2.1
31766	0.946131	2.1
31767	0.946131	2.1
31768	0.946131	2.1
31769	0.946131	2.1
31770	0.946132	2.1
31771	0.946132	2.1
31772	0.946129	2.1
31773	0.946129	2.1
31774	0.946131	2.1
31775	0.946131	2.1
31776	0.94613	2.1
31777	0.94613	2.1
31778	1.89226	2.1
31779	1.89226	2.1
31780	1.89226	2.1
31781	1.89226	2.1

31782	3.78452	2.1
31783	0.707806	2.1
31784	0.697816	2.1
31785	0.823593	2.1
31786	0.801649	2.1
31787	0.74182	2.1
31788	0.74182	2.1
31789	0.757645	2.1
31790	0.757645	2.1
31791	0.823592	2.1
31792	0.697818	2.1
31793	0.707807	2.1
31794	0.801647	2.1
31795	1.52534	2.1
31796	1.6033	2.1
31797	1.64719	2.1
31798	1.38085	2.1
31799	1.64719	2.1
31800	1.38085	2.1
31801	2.67041	2.1
31802	1.89226	2.09713
31803	1.89226	2.1
31804	1.89226	2.1
31805	2.487	2.1
31806	2.487	2.1
31807	2.70097	2.1
31808	1.70303	2.1
31809	1.71163	2.1
31810	2.487	2.1
31811	4.46723	2.1
31812	1.14984	2.1
31813	1.2435	2.1
31814	0.778	2.1
31815	0.697616	2.1
31816	0.967211	2.1
31817	0.967211	2.1
31818	0.973907	2.1
31819	0.973907	2.1
31820	0.697616	2.1
31821	1.14984	2.1
31822	0.778	2.1
31823	1.2435	2.1
31824	2.4158	2.1
31825	1.26438	2.1
31826	1.676	2.1
31827	2.77505	2.1
31828	4.0918	2.1
31829	2.58258	2.1
31830	1.89226	2.1
31831	1.89226	2.1
31832	1.89226	2.1
31833	2.47032	2.1
31834	2.47032	2.1
31835	1.23516	2.1
31836	1.18734	2.1
31837	0.946131	2.1
31838	0.946131	2.1
31839	1.18734	2.1

31840	1.18734	2.1
31841	0.94613	2.1
31842	0.94613	2.1
31843	1.18734	2.1
31844	0.946131	2.1
31845	0.946131	2.1
31846	1.23516	2.1
31847	2.8239	2.1
31848	1.39361	2.1
31849	1.12342	2.1
31850	2.57275	2.1
31851	3.96637	2.1
31852	0.937929	1.57081
31853	0.937929	1.44777
31854	1.39734	1.36029
31855	1.39734	1.27173
31856	1.38914	1.43112
31857	0.94613	1.50228
31858	0.937902	1.46693
31859	0.94613	0.50944
31860	0.94613	1.30237
31861	0.94613	1.38957
31862	1.38914	1.41438
31863	0.937902	1.35613
31864	1.89226	0.242864
31865	2.17765	1.21085
31866	1.84577	1.0863
31867	1.84577	0.478367
31868	1.89226	0.946301
31869	1.89226	0.354005
31870	2.11262	2.05616
31871	1.57766	2.1
31872	2.22689	2.1
31873	1.91132	1.7371
31874	2.19624	2.1
31875	1.79689	0.94705
31876	1.99656	1.66489
31877	1.57766	2.1
31878	1.39067	2.1
31879	1.73802	1.65607
31880	3.31568	2.01613
31881	1.89226	0.237522
31882	1.89226	0.100783
31883	1.89226	0.152255
31884	1.89226	0.530928
31885	1.89226	0.0700285
31886	1.89226	0.195705
31887	1.84936	0.331835
31888	2.0815	0.0751772
31889	2.0815	0.129792
31890	2.47217	0.206378
31891	1.04528	0.0667048
31892	1.62774	0.180813
31893	1.89226	0.169623
31894	1.89226	0.0863291
31895	1.89226	0.10762
31896	1.89226	0.260426
31897	1.89226	0.0717189

31898	1.89226	0.144742
31899	1.69055	0.0565167
31900	2.68518	0.115313
31901	1.69055	0.0601623
31902	2.25055	0.111927
31903	2.39587	0.0526457
31904	3.01801	0.102708
31905	1.89226	0.0907457
31906	1.89226	0.117814
31907	1.89226	0.0682036
31908	1.89226	0.0578646
31909	3.78452	0.0964941
31910	1.24499	2.1
31911	1.70302	2.1
31912	1.41207	2.1
31913	1.10574	1.9309
31914	2.66056	2.1
31915	1.70303	2.1
31916	2.39605	1.87283
31917	2.72674	2.1
31918	2.46904	1.66396
31919	1.84557	2.1
31920	1.67115	1.95937
31921	2.46904	0.369663
31922	2.76829	2.1
31923	2.83666	0.423009
31924	2.83666	1.88058
31925	2.39605	1.29024
31926	2.39605	2.1
31927	1.07323	0.498376
31928	1.07323	2.1
31929	1.06715	2.1
31930	1.18052	1.84033
31931	1.30067	0.852425
31932	1.26542	1.49887
31933	0.914516	2.1
31934	0.839633	2.1
31935	0.936958	1.96955
31936	1.26751	1.96145
31937	0.847764	2.07989
31938	1.35395	2.06898
31939	2.69069	0.329127
31940	1.03074	0.287069
31941	1.74098	0.195762
31942	1.97397	0.262959
31943	3.71495	0.260129
31944	2.44554	2.1
31945	1.89226	2.1
31946	1.89226	2.1
31947	1.89226	2.1
31948	2.40645	2.1
31949	2.40645	2.1
31950	1.89226	2.1
31951	1.89226	2.1
31952	1.89226	2.1
31953	1.89226	2.1
31954	3.78452	2.1
31955	1.82117	2.1

31956	2.55786	2.1
31957	2.51431	2.1
31958	2.51431	2.1
31959	1.85054	2.1
31960	1.84375	2.1
31961	2.38467	2.1
31962	1.89226	2.1
31963	1.89226	2.1
31964	1.89226	2.1
31965	2.44554	2.1
31966	2.44554	2.1
31967	1.89226	2.1
31968	1.89226	2.1
31969	1.89226	2.1
31970	1.89226	2.1
31971	1.89226	2.1
31972	1.89226	2.1
31973	2.02397	2.1
31974	2.55786	2.1
31975	1.77775	2.1
31976	2.61404	2.1
31977	4.33561	2.1
31978	1.89226	1.64197
31979	1.89226	1.62547
31980	1.89226	1.52815
31981	1.89226	1.57672
31982	3.78453	1.64386
31983	1.89226	1.7391
31984	2.80184	1.72078
31985	1.89226	1.65875
31986	1.89226	1.69971
31987	2.03045	1.79761
31988	2.03045	1.81239
31989	2.52263	1.63651
31990	1.27921	1.98171
31991	1.58429	2.03342
31992	1.73571	1.79724
31993	2.37121	1.75112
31994	2.52263	1.64127
31995	0.764974	2.1
31996	0.650577	2.1
31997	0.712428	2.1
31998	0.562516	2.1
31999	0.67264	2.1
32000	0.67264	2.1
32001	0.654851	2.1
32002	0.654851	2.1
32003	0.562515	2.1
32004	0.650579	2.1
32005	0.712427	2.1
32006	0.764976	2.1
32007	1.89227	2.1
32008	1.89227	2.1
32009	1.89227	2.1
32010	1.89227	2.1
32011	3.78453	2.1
32012	1.89226	2.1
32013	1.89226	2.1

32014	1.89226	2.1
32015	1.89226	2.1
32016	3.78453	2.1
32017	1.49248	2.1
32018	1.24959	2.1
32019	1.28211	2.1
32020	1.26432	2.1
32021	1.51027	2.1
32022	1.49248	2.1
32023	1.49248	2.1
32024	1.19017	2.1
32025	1.24977	2.1
32026	1.51027	2.1
32027	2.74225	2.1
32028	1.89226	2.1
32029	1.89226	2.1
32030	1.89226	2.1
32031	1.89226	2.1
32032	3.78453	2.1
32033	1.89226	2.1
32034	1.89226	2.1
32035	1.89226	2.1
32036	1.89226	2.1
32037	3.78453	2.1
32038	0.624796	2.1
32039	0.618472	2.1
32040	0.770147	2.1
32041	0.755132	2.1
32042	0.681445	2.1
32043	0.681445	2.1
32044	0.69216	2.1
32045	0.69216	2.1
32046	0.618472	2.1
32047	0.770147	2.1
32048	0.624796	2.1
32049	0.755132	2.1
32050	1.54029	2.1
32051	1.51027	2.1
32052	1.54029	2.1
32053	1.30682	2.1
32054	1.24977	2.1
32055	1.24977	2.1
32056	0.994293	2.1
32057	2.17159	2.1
32058	2.21149	2.1
32059	2.21149	2.1
32060	0.994814	2.1
32061	0.994814	2.1
32062	0.990481	2.1
32063	2.21149	2.1
32064	2.2671	2.1
32065	2.2671	2.1
32066	0.994293	2.1
32067	0.994293	2.1
32068	2.41579	2.1
32069	1.09847	2.1
32070	1.70302	2.1
32071	2.45069	2.1

32072	3.61169	2.1
32073	2.48689	2.1
32074	1.67969	2.1
32075	1.67969	2.1
32076	1.70002	2.1
32077	2.43826	2.1
32078	2.45859	2.1
32079	2.43827	2.1
32080	1.62408	2.1
32081	1.62408	2.1
32082	1.67969	2.1
32083	2.36729	2.1
32084	2.4229	2.1
32085	2.47199	2.1
32086	1.0823	2.1
32087	1.0607	2.1
32088	2.44276	2.1
32089	3.52506	2.1
32090	2.47199	2.1
32091	1.89226	2.1
32092	1.89226	2.1
32093	2.44277	2.1
32094	4.33503	2.1
32095	1.89226	2.1
32096	1.89226	2.1
32097	1.89226	2.1
32098	1.89226	2.1
32099	3.78453	2.1
32100	1.89226	2.1
32101	1.89226	2.1
32102	1.89226	2.1
32103	1.89226	2.1
32104	1.89226	2.1
32105	1.89226	2.1
32106	1.02986	2.1
32107	2.44276	2.1
32108	1.0607	2.1
32109	1.0607	2.1
32110	2.40055	2.1
32111	2.40055	2.1
32112	1.89226	2.1
32113	2.44277	2.1
32114	2.40056	2.1
32115	2.44277	2.1
32116	1.85005	2.1
32117	1.89226	2.1
32118	1.89226	2.1
32119	1.89226	2.1
32120	1.89226	2.1
32121	1.89226	2.1
32122	3.78453	2.1
32123	1.89226	2.1
32124	1.89226	2.1
32125	1.89226	2.1
32126	1.89226	2.1
32127	3.78452	2.1
32128	2.79502	0.534163
32129	2.63448	2.1

32130	2.68986	0.466949
32131	2.53868	2.1
32132	5.32434	1.25074
32133	2.63449	2.1
32134	2.39282	2.1
32135	2.53869	2.1
32136	2.3085	2.1
32137	4.94299	2.1
32138	2.68986	0.398564
32139	2.53868	2.1
32140	2.5761	0.720571
32141	2.43404	2.1
32142	5.1239	1.18536
32143	2.53869	2.1
32144	2.3085	2.1
32145	2.43405	2.1
32146	2.22575	2.1
32147	4.74255	2.1
32148	1.89226	2.1
32149	1.89226	2.1
32150	1.89226	2.1
32151	1.89226	2.1
32152	3.78453	2.1
32153	1.32391	2.1
32154	1.44951	2.1
32155	1.44951	2.1
32156	1.89226	2.1
32157	1.89226	2.1
32158	2.20203	2.1
32159	0.507656	2.1
32160	0.578945	2.1
32161	1.05568	2.1
32162	0.959007	2.1
32163	0.76264	2.1
32164	0.76264	2.1
32165	0.775313	2.1
32166	0.775313	2.1
32167	0.578945	2.1
32168	1.05568	2.1
32169	0.959007	2.1
32170	0.507656	2.1
32171	1.89226	0.05
32172	2.4065	0.056021
32173	2.36561	0.05
32174	2.4065	0.0590147
32175	1.85138	0.05
32176	1.89226	0.0551298
32177	1.40274	0.05
32178	2.4065	0.0519946
32179	1.53336	0.05
32180	1.53336	0.05
32181	2.36562	0.05
32182	2.36562	0.0514354
32183	0.946115	0.0566175
32184	0.946115	0.05
32185	1.06496	0.05
32186	1.18279	0.0520543
32187	0.946117	0.0555667

32188	0.946117	0.0594114
32189	1.06496	0.05
32190	1.06496	0.05
32191	0.94613	0.0531506
32192	1.06497	0.05
32193	0.946105	0.05
32194	1.18278	0.0533842
32195	1.00213	0.05
32196	2.36559	0.0523361
32197	1.40272	0.05
32198	1.40272	0.05
32199	2.12992	0.0503771
32200	2.12992	0.0516385
32201	0.95646	2.1
32202	2.07201	2.1
32203	1.01808	2.1
32204	2.14876	2.1
32205	3.09009	2.1
32206	1.89226	2.1
32207	1.89226	2.1
32208	1.89226	2.1
32209	1.89226	2.1
32210	3.78453	2.1
32211	1.89227	2.1
32212	1.89227	2.1
32213	1.89227	2.1
32214	1.89227	2.1
32215	1.89227	2.1
32216	1.89227	2.1
32217	2.71886	2.1
32218	1.60497	2.1
32219	1.43935	2.1
32220	2.55151	2.1
32221	4.15648	2.1
32222	2.71886	2.1
32223	1.89226	2.1
32224	1.89226	2.1
32225	2.55151	2.1
32226	4.44377	2.1
32227	1.89226	2.1
32228	1.89226	2.1
32229	1.89226	2.1
32230	1.89226	2.1
32231	3.78453	2.1
32232	1.89226	2.1
32233	1.89226	2.1
32234	1.89226	2.1
32235	1.89226	2.1
32236	3.78453	2.1
32237	1.58942	0.207897
32238	1.92622	0.318584
32239	2.52852	0.279202
32240	2.19457	0.194258
32241	4.12079	0.194876
32242	1.92649	0.166222
32243	1.44426	0.109981
32244	1.87529	0.118558
32245	2.19457	0.172569

32246	3.63883	0.156561
32247	1.87529	0.0894423
32248	2.19457	0.151245
32249	2.7378	0.13094
32250	2.29852	0.0895694
32251	4.49309	0.0896349
32252	1.35976	2.1
32253	1.89452	2.1
32254	1.39616	2.1
32255	1.1423	2.1
32256	1.8996	2.1
32257	1.52068	2.1
32258	1.89226	2.1
32259	1.89226	2.1
32260	1.89226	2.1
32261	1.89226	2.1
32262	1.89226	2.1
32263	1.89226	2.1
32264	1.89226	2.1
32265	2.15617	2.1
32266	2.0953	2.1
32267	2.15617	2.1
32268	1.83139	2.1
32269	1.89226	2.1
32270	1.16817	2.1
32271	1.8996	2.1
32272	1.23614	2.1
32273	1.48338	2.1
32274	1.80218	2.1
32275	2.16263	2.1
32276	1.89226	2.1
32277	1.89226	2.1
32278	1.89226	2.1
32279	1.89226	2.1
32280	1.89226	2.1
32281	1.89226	2.1
32282	1.89226	2.1
32283	2.0953	2.1
32284	2.03074	2.1
32285	2.0953	2.1
32286	1.8277	2.1
32287	1.89226	2.1
32288	1.74455	2.1
32289	2.181	2.1
32290	1.74455	2.1
32291	1.92911	2.1
32292	1.98195	2.1
32293	2.16651	2.1
32294	2.181	2.1
32295	2.29881	2.1
32296	1.98195	2.1
32297	1.80729	2.1
32298	4.28075	2.1
32299	1.74455	2.1
32300	1.98195	2.1
32301	1.52603	2.1
32302	1.76603	2.1
32303	3.51058	2.1

32304	1.76603	2.1
32305	1.94506	2.1
32306	1.76603	2.1
32307	1.98195	2.1
32308	1.86795	2.1
32309	2.08387	2.1
32310	0.984305	2.1
32311	1.9	2.1
32312	2.86619	2.1
32313	1.86189	2.1
32314	3.76189	2.1
32315	1.4621	2.1
32316	1.89226	2.1
32317	2.81374	2.1
32318	1.89226	2.1
32319	3.35437	2.1
32320	0.946133	2.1
32321	0.946133	2.1
32322	0.946132	2.1
32323	0.946132	2.1
32324	0.946132	2.1
32325	0.946132	2.1
32326	0.946133	2.1
32327	0.946133	2.1
32328	0.946132	2.1
32329	0.946132	2.1
32330	0.946132	2.1
32331	0.946132	2.1
32332	1.64268	2.1
32333	1.93673	2.1
32334	2.25284	2.1
32335	1.97548	2.1
32336	3.89552	2.1
32337	1.86206	2.1
32338	1.93673	2.1
32339	2.25284	2.1
32340	2.20075	2.1
32341	4.1149	2.1
32342	2.25284	2.1
32343	2.24781	2.1
32344	1.97548	2.1
32345	1.97548	2.1
32346	2.49676	2.1
32347	2.49676	2.1
32348	2.25284	2.1
32349	2.43549	2.1
32350	2.44467	2.1
32351	2.20075	2.1
32352	2.49676	2.1
32353	2.25284	2.1
32354	2.57928	2.1
32355	2.32606	2.1
32356	2.59522	2.1
32357	2.39022	2.1
32358	4.9695	2.1
32359	2.32606	2.1
32360	1.77677	2.1
32361	2.39022	2.1

32362	2.28669	2.1
32363	4.16699	2.1
32364	2.59522	2.1
32365	2.39022	2.1
32366	2.61901	2.1
32367	2.45899	2.1
32368	5.00923	2.1
32369	2.39022	2.1
32370	2.11575	2.1
32371	2.41795	2.1
32372	2.0815	2.1
32373	4.67085	2.1
32374	1.89226	2.1
32375	2.24723	2.1
32376	1.95405	2.1
32377	1.89226	2.1
32378	2.30902	2.1
32379	2.24723	2.1
32380	1.89226	2.1
32381	1.89226	2.1
32382	1.89226	2.1
32383	1.89226	2.1
32384	3.78453	2.1
32385	0.946131	2.1
32386	0.946131	2.1
32387	1.14643	2.1
32388	1.10673	2.1
32389	1.10673	2.1
32390	1.10673	2.1
32391	0.946133	2.1
32392	0.946133	2.1
32393	0.946131	2.1
32394	1.14643	2.1
32395	1.10673	2.1
32396	0.946131	2.1
32397	1.89226	2.1
32398	2.30902	2.1
32399	1.96635	2.1
32400	1.89226	2.1
32401	2.38311	2.1
32402	2.30902	2.1
32403	1.89226	2.1
32404	1.89226	2.1
32405	1.89226	2.1
32406	1.89226	2.1
32407	1.89226	2.1
32408	1.89226	2.1
32409	0.946131	2.1
32410	0.946131	2.1
32411	1.19156	2.1
32412	1.14643	2.1
32413	1.14643	2.1
32414	1.14643	2.1
32415	0.946133	2.1
32416	0.946133	2.1
32417	0.946131	2.1
32418	1.19156	2.1
32419	0.946131	2.1

32420	1.14643	2.1
32421	1.89226	1.42237
32422	1.89226	1.22669
32423	1.89226	1.30879
32424	1.89226	1.45325
32425	1.89226	1.19193
32426	1.89226	1.27935
32427	1.89226	1.25285
32428	1.89226	1.2434
32429	1.89226	1.09992
32430	1.9645	1.23419
32431	3.78452	1.3041
32432	2.39489	2.1
32433	2.15715	2.1
32434	2.07821	2.1
32435	2.33541	2.1
32436	4.49256	2.1
32437	2.39489	2.1
32438	2.34898	2.1
32439	2.30729	2.1
32440	2.33541	2.1
32441	4.6844	2.1
32442	2.07821	2.1
32443	2.33541	2.1
32444	2.26415	2.1
32445	1.99632	2.1
32446	4.33174	2.1
32447	2.30729	2.1
32448	2.33542	2.1
32449	2.26416	2.1
32450	2.26618	2.1
32451	4.60159	2.1
32452	1.19837	1.6701
32453	2.79151	0.305173
32454	2.25636	0.906117
32455	2.25636	0.62836
32456	1.94072	0.830038
32457	1.94072	0.646441
32458	2.53725	0.0628
32459	1.16699	0.0646891
32460	1.69864	0.0554184
32461	2.0815	0.0679476
32462	3.58273	0.0630853
32463	1.91646	2.1
32464	2.51686	2.1
32465	2.49872	2.1
32466	1.85819	2.1
32467	4.41518	2.1
32468	0.929098	2.1
32469	0.8993	2.1
32470	1.24359	2.1
32471	1.24936	2.1
32472	1.06832	2.1
32473	1.06832	2.1
32474	1.08034	2.1
32475	1.08034	2.1
32476	0.899298	2.1
32477	1.24359	2.1

32478	1.24936	2.1
32479	0.929097	2.1
32480	2.0815	0.0652096
32481	1.89226	0.05
32482	2.0815	0.0608422
32483	1.82811	0.0568035
32484	1.94982	0.0575285
32485	1.71246	0.0516313
32486	2.3256	2.1
32487	1.89226	2.1
32488	1.89226	2.1
32489	1.89226	2.1
32490	2.38467	2.1
32491	2.38467	2.1
32492	1.89226	2.1
32493	1.89226	2.1
32494	1.89226	2.1
32495	1.89226	2.1
32496	1.89226	2.1
32497	1.89226	2.1
32498	1.89763	2.1
32499	2.61405	2.1
32500	2.15035	2.1
32501	2.68796	2.1
32502	1.42219	2.1
32503	1.77775	2.1
32504	2.26306	2.1
32505	1.89226	2.1
32506	1.89226	2.1
32507	1.89226	2.1
32508	2.3256	2.1
32509	2.3256	2.1
32510	1.89226	2.1
32511	1.89226	2.1
32512	1.89226	2.1
32513	1.89226	2.1
32514	1.89226	2.1
32515	1.89226	2.1
32516	1.92887	2.1
32517	2.41915	2.1
32518	2.50142	2.1
32519	2.50142	2.1
32520	1.90388	2.1
32521	1.89763	2.1
32522	1.89224	2.1
32523	1.89224	2.1
32524	1.89224	2.1
32525	1.89224	2.1
32526	3.78448	2.1
32527	2.25315	2.1
32528	1.05843	2.1
32529	2.38995	2.1
32530	1.31541	2.1
32531	3.56856	2.1
32532	0.517552	2.1
32533	0.68548	2.1
32534	1.27675	2.1
32535	1.13076	2.1

32536	0.913611	2.1
32537	0.913612	2.1
32538	0.902633	2.1
32539	0.902633	2.1
32540	0.68547	2.1
32541	1.27673	2.1
32542	1.13078	2.1
32543	0.517559	2.1
32544	1.89226	2.1
32545	1.89226	2.1
32546	1.89226	2.1
32547	1.89226	2.1
32548	3.78453	2.1
32549	0.962936	2.1
32550	0.962936	2.1
32551	0.996141	2.1
32552	0.996141	2.1
32553	0.830609	2.1
32554	0.657714	2.1
32555	1.30136	2.1
32556	1.19499	2.1
32557	1.30136	2.1
32558	0.830609	2.1
32559	1.19499	2.1
32560	0.657713	2.1
32561	1.60057	0.116561
32562	1.39615	0.103387
32563	1.06474	0.0980248
32564	1.39615	0.125827
32565	1.64655	0.0982329
32566	2.04424	0.121889
32567	1.89226	0.100212
32568	1.89226	0.129575
32569	1.89226	0.105887
32570	1.89226	0.125331
32571	1.89226	0.105937
32572	1.89226	0.129835
32573	2.56322	0.0915431
32574	1.89226	0.110307
32575	1.89226	0.0961415
32576	1.89226	0.10767
32577	2.18047	0.0924573
32578	2.18047	0.10315
32579	1.89226	0.597263
32580	1.89226	0.532113
32581	1.89226	0.595555
32582	1.65573	0.438928
32583	1.89226	0.763747
32584	1.65573	0.550811
32585	1.05353	0.396387
32586	1.83158	0.469
32587	1.50282	0.176052
32588	1.20669	0.220846
32589	2.88511	0.232102
32590	1.44807	2.1
32591	1.93592	2.1
32592	1.80703	2.1
32593	1.27806	2.1

32594	3.21398	2.1
32595	2.0815	2.1
32596	1.87465	2.1
32597	1.87465	2.1
32598	1.50566	2.1
32599	2.0815	2.1
32600	1.69263	2.1
32601	1.89226	2.1
32602	1.89226	2.1
32603	1.89226	2.1
32604	1.89226	2.1
32605	1.89226	2.1
32606	1.89226	2.1
32607	1.62632	2.1
32608	0.994294	2.1
32609	0.994294	2.1
32610	1.27806	2.1
32611	1.81921	2.1
32612	2.10776	2.1
32613	2.0815	2.1
32614	1.70303	2.1
32615	1.70303	2.1
32616	1.85749	2.1
32617	1.90451	2.1
32618	2.07724	2.1
32619	1.89226	2.1
32620	1.89226	2.1
32621	1.89226	2.1
32622	1.89226	2.1
32623	1.89226	2.1
32624	1.89226	2.1
32625	2.69773	2.1
32626	1.88129	2.1
32627	2.55196	2.1
32628	2.26469	2.1
32629	4.94367	2.1
32630	1.88129	2.1
32631	1.25889	2.1
32632	1.26614	2.1
32633	1.05914	2.1
32634	2.26469	2.1
32635	1.88129	2.1
32636	2.55196	2.1
32637	2.0588	2.1
32638	2.6536	2.1
32639	2.15287	2.1
32640	4.70484	2.1
32641	0.8516	2.1
32642	0.8516	2.1
32643	0.849002	2.1
32644	0.849002	2.1
32645	0.629445	2.1
32646	0.671197	2.1
32647	1.0294	2.1
32648	1.07644	2.1
32649	1.07644	2.1
32650	0.671197	2.1
32651	0.629446	2.1

32652	1.0294	2.1
32653	2.02115	1.84361
32654	1.18382	1.67303
32655	2.29107	0.273479
32656	1.46903	1.2323
32657	3.47488	1.19291
32658	2.60763	1.64254
32659	2.29107	1.4648
32660	1.82141	1.51742
32661	1.82141	1.60501
32662	1.46903	1.55489
32663	2.29107	1.59696
32664	1.64007	2.1
32665	2.2024	2.1
32666	1.44044	2.1
32667	2.02634	2.1
32668	3.6664	2.1
32669	1.25565	2.1
32670	2.02635	2.1
32671	1.87228	2.1
32672	1.87228	2.1
32673	1.44045	2.1
32674	1.44045	2.1
32675	1.89226	0.05
32676	1.78503	0.052751
32677	1.78503	0.0522089
32678	1.40048	0.0512077
32679	2.0815	0.05
32680	1.62972	0.05
32681	1.27875	2.1
32682	1.1483	2.1
32683	1.40014	2.1
32684	1.40014	2.1
32685	1.04598	2.1
32686	1.04598	2.1
32687	2.63443	1.82142
32688	2.26444	2.0905
32689	2.79392	0.519641
32690	2.4863	2.1
32691	5.12073	1.97657
32692	1.83158	0.670737
32693	1.18508	0.240666
32694	1.20669	0.222761
32695	1.81488	0.381967
32696	1.20669	0.254567
32697	1.81488	0.423247
32698	1.08134	0.25391
32699	1.70303	0.589237
32700	1.24027	0.353602
32701	1.30359	0.25515
32702	1.89939	0.518864
32703	1.70303	0.514663
32704	1.89226	2.1
32705	2.69027	2.1
32706	1.89226	2.1
32707	2.2078	2.1
32708	1.89226	2.1
32709	2.2078	2.1

32710	1.89226	2.1
32711	2.78977	2.1
32712	1.89226	2.1
32713	2.69027	2.1
32714	1.89226	2.1
32715	2.69027	2.1
32716	1.89226	2.1
32717	1.89226	2.1
32718	1.89226	1.95394
32719	1.89226	2.1
32720	3.78452	2.1
32721	1.43687	2.05658
32722	1.71059	2.1
32723	2.53014	2.08178
32724	2.6521	2.1
32725	4.24073	2.1
32726	2.6521	2.1
32727	1.66762	2.1
32728	2.50299	2.1
32729	2.50299	2.1
32730	1.71059	2.1
32731	1.71059	2.1
32732	1.89226	2.1
32733	1.89226	2.1
32734	1.89226	2.1
32735	1.89226	2.1
32736	1.89226	2.1
32737	1.89226	2.1
32738	2.10662	0.213493
32739	2.77417	0.415676
32740	2.78873	1.32321
32741	2.11661	0.28221
32742	4.89078	0.323193
32743	2.10662	0.772456
32744	1.22857	0.0997658
32745	1.25849	1.04484
32746	2.11661	0.698849
32747	3.34518	0.862438
32748	2.78873	1.16228
32749	2.11661	0.216615
32750	2.12475	0.325235
32751	2.79029	0.652266
32752	4.9069	0.360435
32753	1.2858	0.852143
32754	2.11661	0.822749
32755	1.25849	0.537414
32756	1.25849	0.625041
32757	2.12475	0.689382
32758	2.12475	0.805961
32759	1.13537	2.1
32760	1.20714	2.1
32761	0.946129	2.1
32762	0.946129	2.1
32763	0.946131	2.1
32764	0.946131	2.1
32765	1.29521	2.1
32766	1.20713	2.1
32767	1.13537	2.1

32768	0.946131	2.1
32769	0.94613	2.1
32770	1.29521	2.1
32771	1.00533	2.1
32772	0.876055	2.1
32773	0.946128	2.1
32774	0.946128	2.1
32775	0.913908	2.1
32776	0.913908	2.1
32777	1.17081	2.1
32778	0.896413	2.1
32779	0.955905	2.1
32780	0.893715	2.1
32781	1.20327	2.1
32782	0.998544	2.1
32783	1.89226	1.59048
32784	1.89226	1.67806
32785	1.89226	1.08351
32786	1.89226	1.33707
32787	3.78452	1.194
32788	0.540975	1.44998
32789	0.726949	2.0712
32790	0.712198	1.63507
32791	0.506675	1.32675
32792	0.611916	1.3402
32793	0.611916	2.1
32794	0.621708	1.62275
32795	0.621708	1.35915
32796	0.726951	1.29199
32797	0.712196	1.2684
32798	0.506674	1.50324
32799	0.540976	1.51936
32800	1.89226	1.43546
32801	1.89226	2.05007
32802	1.89226	1.7102
32803	1.89226	1.36243
32804	3.78453	1.67092
32805	1.89226	1.28781
32806	1.89226	1.40287
32807	1.89226	1.25826
32808	1.89226	1.70659
32809	1.89226	1.24889
32810	1.89226	2.06973
32811	0.919594	1.30623
32812	0.726949	1.49979
32813	0.898318	1.37636
32814	0.712198	1.49572
32815	0.817559	1.32406
32816	0.817559	2.1
32817	0.814234	1.34811
32818	0.814234	1.58401
32819	0.919597	1.18834
32820	0.898316	1.1492
32821	0.726951	1.54833
32822	0.712196	1.44965
32823	1.79663	1.27047
32824	1.12088	2.01352
32825	1.54699	1.71193

32826	1.17475	1.60651
32827	1.79663	1.85155
32828	1.42439	1.70726
32829	2.28928	1.60419
32830	1.23077	1.51558
32831	1.65458	0.47212
32832	2.66533	1.862
32833	3.94385	1.48465
32834	1.89226	2.1
32835	1.89226	2.1
32836	1.89226	2.1
32837	1.89226	2.1
32838	3.78452	2.1
32839	1.38426	2.1
32840	1.15067	2.1
32841	1.38426	2.1
32842	1.01595	2.1
32843	1.24509	2.1
32844	1.01595	2.1
32845	2.62097	2.1
32846	1.89226	2.1
32847	2.72561	2.1
32848	2.72561	2.1
32849	1.89226	2.1
32850	1.89226	2.1
32851	1.90103	1.91698
32852	1.41902	2.1
32853	1.77249	1.95488
32854	1.30272	2.1
32855	3.20375	2.1
32856	1.77249	1.87574
32857	1.19548	2.1
32858	1.19548	2.1
32859	1.30272	2.1
32860	1.65815	2.03409
32861	1.76539	2.1
32862	2.62161	2.1
32863	1.89226	2.1
32864	1.89226	2.1
32865	1.89226	2.1
32866	2.6446	2.1
32867	2.6446	2.1
32868	2.64459	2.1
32869	1.89226	2.1
32870	1.89226	2.1
32871	1.89226	2.1
32872	2.58257	2.1
32873	2.58257	2.1
32874	1.89226	0.34164
32875	1.89226	1.30774
32876	2.31301	1.19411
32877	2.76309	0.545704
32878	1.89226	1.15469
32879	2.26047	0.545896
32880	1.89226	0.308007
32881	2.31301	1.26682
32882	2.17765	1.15485
32883	2.17765	0.581966

32884	1.89226	1.08151
32885	1.89226	0.490823
32886	2.18516	2.1
32887	1.85196	2.1
32888	1.78595	2.1
32889	2.14606	2.1
32890	3.99802	2.1
32891	2.18516	2.1
32892	2.21189	2.1
32893	2.18937	2.1
32894	2.14606	2.1
32895	4.35796	2.1
32896	1.60735	2.1
32897	2.14606	2.1
32898	2.15714	2.1
32899	1.70302	2.1
32900	3.70055	2.1
32901	2.18937	2.1
32902	2.14606	2.1
32903	2.0927	2.1
32904	2.162	2.1
32905	4.30806	2.1
32906	2.17326	2.1
32907	2.58517	2.1
32908	2.55318	2.1
32909	2.1665	2.1
32910	4.75167	2.1
32911	2.17326	2.1
32912	1.53556	2.1
32913	1.56253	2.1
32914	2.1665	2.1
32915	3.70206	2.1
32916	2.55317	2.1
32917	2.1665	2.1
32918	2.17741	2.1
32919	2.54197	2.1
32920	4.70847	2.1
32921	2.17742	2.1
32922	2.1665	2.1
32923	2.18452	2.1
32924	1.58054	2.1
32925	1.56253	2.1
32926	1.56253	2.1
32927	1.71051	0.05
32928	1.89807	0.0504725
32929	1.19755	0.0631944
32930	1.19755	0.05
32931	2.25632	0.056551
32932	2.25632	0.05
32933	0.876046	0.0947805
32934	1.27293	0.0623141
32935	0.946132	0.147346
32936	0.946132	0.143684
32937	0.946131	0.0847691
32938	0.869312	0.0853643
32939	1.12767	0.125786
32940	0.946132	0.127126
32941	1.22225	0.127542

32942	1.37968	0.052255
32943	0.851555	0.184715
32944	0.851555	0.093111
32945	0.946133	2.0586
32946	0.946133	2.1
32947	0.946132	2.09082
32948	0.946132	1.88335
32949	0.946133	2.0084
32950	0.946133	2.08686
32951	0.946132	2.1
32952	0.946132	2.08708
32953	0.946132	2.1
32954	0.946132	2.1
32955	0.946133	2.06121
32956	0.946133	2.1
32957	1.35152	2.1
32958	0.948936	2.1
32959	1.90166	2.1
32960	1.70303	2.1
32961	2.90858	2.1
32962	1.89226	2.1
32963	1.89226	2.1
32964	1.89226	2.1
32965	1.89226	2.1
32966	3.78452	2.1
32967	1.90166	2.1
32968	1.70303	2.1
32969	2.52454	2.1
32970	2.13919	2.1
32971	3.99529	2.1
32972	1.89226	2.1
32973	1.89226	2.1
32974	1.89226	2.1
32975	1.89226	2.1
32976	3.78452	2.1
32977	1.89226	2.1
32978	1.89226	2.1
32979	1.89226	2.1
32980	1.89226	2.1
32981	1.89226	2.1
32982	1.89226	2.1
32983	2.13918	2.1
32984	0.992674	2.1
32985	2.3397	2.1
32986	1.8023	2.1
32987	1.47769	2.1
32988	0.992674	2.1
32989	1.89226	0.256739
32990	1.89226	0.243299
32991	1.89226	0.345212
32992	1.89226	0.186346
32993	3.78452	0.266055
32994	1.89226	0.2434
32995	2.54752	0.142081
32996	2.63974	0.188944
32997	1.89226	0.178819
32998	4.53201	0.174707
32999	0.498412	0.186888

33000	0.500955	0.171425
33001	0.57955	0.225629
33002	0.563873	0.198163
33003	0.529107	0.188871
33004	0.529107	0.170553
33005	0.53572	0.24437
33006	0.53572	0.177038
33007	0.500956	0.186277
33008	0.579548	0.180178
33009	0.563871	0.189095
33010	0.498414	0.192495
33011	1.89223	0.291558
33012	1.89223	0.1831
33013	1.89223	0.23279
33014	1.89223	0.251528
33015	1.89223	0.228774
33016	1.89223	0.247214
33017	1.28752	0.143693
33018	1.31985	0.174127
33019	0.946119	0.171216
33020	0.946119	0.211029
33021	1.28752	0.174152
33022	1.28752	0.144365
33023	0.946119	0.165739
33024	0.946119	0.313082
33025	1.28753	0.172972
33026	0.946132	0.169943
33027	1.31984	0.188191
33028	0.946107	0.183194
33029	0.500948	0.167955
33030	0.479465	0.158581
33031	0.562335	0.208633
33032	0.579542	0.180661
33033	0.528436	0.174062
33034	0.528436	0.15928
33035	0.530571	0.165397
33036	0.530571	0.219994
33037	0.56234	0.172784
33038	0.479473	0.173934
33039	0.500943	0.181959
33040	0.579533	0.17534
33041	1.89226	2.1
33042	1.89226	2.1
33043	1.89226	2.1
33044	1.89226	2.1
33045	3.78452	2.1
33046	1.50767	2.1
33047	1.14496	2.1
33048	1.50767	2.1
33049	1.58539	2.1
33050	1.07653	2.1
33051	1.15425	2.1
33052	1.89226	2.1
33053	2.43826	2.1
33054	2.43826	2.1
33055	2.48688	2.1
33056	1.89226	2.1
33057	1.94088	2.1

33058	1.89226	2.1
33059	1.89226	2.1
33060	1.89226	2.1
33061	1.89226	2.1
33062	3.78452	2.1
33063	1.50767	2.1
33064	1.07653	2.1
33065	1.41077	2.1
33066	0.991407	2.1
33067	2.49907	2.1
33068	1.21913	2.1
33069	1.21913	2.1
33070	0.946128	2.1
33071	0.946128	2.1
33072	1.21913	2.1
33073	1.18364	2.1
33074	0.946131	2.1
33075	0.946131	2.1
33076	1.18364	2.1
33077	0.946129	2.1
33078	1.21913	2.1
33079	0.94613	2.1
33080	1.89226	2.1
33081	1.89226	2.1
33082	1.89226	2.1
33083	1.89226	2.1
33084	3.78452	2.1
33085	1.93823	2.1
33086	1.60976	2.1
33087	1.93823	2.1
33088	2.20045	2.1
33089	1.36999	2.1
33090	1.63222	2.1
33091	1.38302	2.1
33092	1.38302	2.1
33093	0.946132	2.1
33094	0.946132	2.1
33095	1.27258	2.1
33096	1.38302	2.1
33097	0.946133	2.1
33098	0.946133	2.1
33099	0.946133	2.1
33100	1.27258	2.1
33101	0.946132	2.1
33102	1.38302	2.1
33103	1.89226	2.1
33104	1.89226	2.1
33105	1.89226	2.1
33106	1.89226	2.1
33107	3.78452	2.1
33108	1.93823	2.1
33109	1.36999	2.1
33110	1.70423	2.1
33111	1.14195	2.1
33112	3.08018	2.1
33113	1.27258	2.1
33114	1.27258	2.1
33115	0.946132	2.1

33116	0.946132	2.1
33117	1.17233	2.1
33118	1.27258	2.1
33119	0.946132	2.1
33120	0.946132	2.1
33121	1.17233	2.1
33122	0.946132	2.1
33123	0.946132	2.1
33124	1.27258	2.1
33125	2.70575	2.1
33126	1.89226	1.77212
33127	2.71429	2.1
33128	2.71429	2.1
33129	1.89226	2.1
33130	1.89226	2.08794
33131	0.946133	2.1
33132	0.946133	2.1
33133	1.28713	2.1
33134	1.28713	2.1
33135	0.946131	2.1
33136	0.946131	2.06741
33137	1.28713	2.1
33138	1.36503	2.1
33139	0.946131	2.1
33140	1.36503	2.1
33141	1.28713	2.1
33142	0.946131	2.1
33143	1.89226	2.1
33144	1.89226	1.90246
33145	1.89226	2.1
33146	1.89226	2.1
33147	1.89226	2.1
33148	1.89226	2.1
33149	1.22615	2.1
33150	1.04147	2.1
33151	1.3439	2.1
33152	1.3439	2.1
33153	0.969118	2.1
33154	0.969118	2.1
33155	0.980841	2.1
33156	0.992505	2.1
33157	1.34289	2.1
33158	1.70303	2.1
33159	2.56081	2.1
33160	1.89225	2.1
33161	2.66785	2.1
33162	2.79506	2.1
33163	2.79506	2.1
33164	1.89225	2.1
33165	1.89225	2.1
33166	0.978778	2.1
33167	0.978778	2.1
33168	0.993303	2.1
33169	0.993303	2.1
33170	0.603538	2.1
33171	0.638158	2.1
33172	1.33393	2.1
33173	1.39753	2.1

33174	1.33391	2.1
33175	0.603535	2.1
33176	0.638164	2.1
33177	1.39754	2.1
33178	1.33393	2.1
33179	1.33393	2.1
33180	0.946132	2.1
33181	0.946132	2.1
33182	1.33393	2.1
33183	1.27365	2.1
33184	0.946132	2.1
33185	0.946132	2.1
33186	0.946132	2.1
33187	1.27365	2.1
33188	1.33393	2.1
33189	0.946131	2.1
33190	1.89226	2.1
33191	1.89226	2.1
33192	1.89226	2.1
33193	1.89226	2.1
33194	3.78452	2.1
33195	1.89226	2.1
33196	1.89226	2.1
33197	1.89226	2.1
33198	1.89226	2.1
33199	3.78453	2.1
33200	0.979364	2.1
33201	0.979364	2.1
33202	0.89618	2.1
33203	0.89618	2.1
33204	0.896178	2.1
33205	0.929411	2.1
33206	0.946133	2.1
33207	0.946133	2.1
33208	0.946131	2.1
33209	0.89618	2.1
33210	0.946131	2.1
33211	0.929413	2.1
33212	1.89226	2.1
33213	1.89226	2.1
33214	1.89226	2.1
33215	1.89226	2.1
33216	1.89226	2.1
33217	1.89226	2.1
33218	1.89225	2.1
33219	1.89225	2.1
33220	1.89225	2.1
33221	1.89225	2.1
33222	3.7845	2.1
33223	1.89225	2.1
33224	1.89225	2.1
33225	1.89225	2.1
33226	1.89225	2.1
33227	1.89225	2.1
33228	1.89225	2.1
33229	0.946128	0.143842
33230	0.946128	0.101637
33231	0.946132	0.126108

33232	0.946132	0.0850795
33233	0.94613	0.128641
33234	0.94613	0.155012
33235	0.946129	0.0905422
33236	0.946129	0.0713178
33237	0.94613	0.10903
33238	0.94613	0.11175
33239	0.94613	0.106076
33240	0.94613	0.100805
33241	1.89227	1.27047
33242	1.89227	1.249
33243	1.89227	1.11414
33244	1.89227	1.13778
33245	3.78453	1.14552
33246	1.89226	1.29546
33247	2.43717	1.52085
33248	2.63259	1.33044
33249	1.89226	1.17777
33250	4.52485	1.36579
33251	0.616808	1.19185
33252	0.792277	1.27443
33253	1.04986	1.10063
33254	0.72303	1.20583
33255	0.702675	1.00815
33256	0.76862	1.60327
33257	0.809154	1.32961
33258	0.809154	1.16455
33259	0.808757	1.17949
33260	1.07789	1.03971
33261	0.772568	1.17231
33262	0.598878	1.31433
33263	1.89226	1.25014
33264	1.89226	1.33385
33265	1.89226	1.20378
33266	1.89226	1.14771
33267	3.78453	1.25768
33268	1.36428	1.54997
33269	1.31629	1.3886
33270	0.946132	1.30166
33271	0.946132	1.28222
33272	1.36428	1.22919
33273	1.36428	1.67789
33274	0.946132	1.40141
33275	0.946132	1.43503
33276	1.36428	1.45715
33277	0.946132	1.28122
33278	1.31629	1.41429
33279	0.946132	1.30726
33280	1.578	1.35036
33281	2.85336	1.08328
33282	2.11368	1.02712
33283	2.14375	1.24573
33284	1.93852	1.15241
33285	1.93852	1.36184
33286	2.34806	0.18593
33287	1.42913	0.187853
33288	1.61539	0.316513
33289	2.45322	0.256488

33290	3.88235	0.237024
33291	2.34806	0.127603
33292	1.89226	0.169008
33293	1.89226	0.603136
33294	2.45322	0.552426
33295	4.34548	0.159693
33296	1.89226	0.148907
33297	1.89226	0.114464
33298	1.89226	0.19496
33299	1.89226	0.418708
33300	3.78452	0.281493
33301	1.89226	0.189968
33302	1.89226	0.146964
33303	1.89226	0.262866
33304	1.89226	0.277876
33305	3.78452	0.186622
33306	1.25972	0.133108
33307	2.45322	0.142278
33308	2.58823	0.371777
33309	1.79398	0.29809
33310	4.2472	0.15154
33311	1.89226	0.255241
33312	2.45322	0.110282
33313	2.58823	0.175118
33314	2.45322	0.219179
33315	2.02727	0.0847577
33316	1.89226	0.121583
33317	1.89226	0.0929478
33318	1.89226	0.13818
33319	1.89226	0.206807
33320	1.89226	0.190693
33321	3.78452	0.102021
33322	1.89226	0.14209
33323	1.89226	0.178809
33324	1.89226	0.157578
33325	1.89226	0.0944248
33326	1.89226	0.226784
33327	1.89226	0.145923
33328	1.70048	2.1
33329	2.1572	1.54258
33330	1.45477	2.1
33331	1.93973	1.99086
33332	3.64021	2.1
33333	1.17614	2.1
33334	1.93973	1.92878
33335	1.69661	2.1
33336	1.69661	1.88973
33337	1.45477	2.1
33338	1.45477	1.92296
33339	1.94343	2.1
33340	2.53782	1.92158
33341	2.59903	2.1
33342	2.59903	1.69398
33343	1.87598	2.1
33344	1.87598	1.86071
33345	0.946131	1.06841
33346	0.946131	1.88634
33347	1.26891	2.1

33348	1.26891	0.475623
33349	0.94613	2.02215
33350	0.94613	1.75771
33351	1.26891	1.77383
33352	1.29952	1.57278
33353	1.29952	0.22813
33354	0.946131	0.421464
33355	1.26891	1.56844
33356	0.946131	1.57591
33357	2.00551	2.1
33358	2.59903	1.96478
33359	2.65811	2.1
33360	2.65811	1.84944
33361	1.94343	2.1
33362	1.94343	2.07295
33363	2.65811	1.3478
33364	1.89226	2.1
33365	1.89226	0.607156
33366	1.89226	1.70402
33367	2.59903	1.47619
33368	2.59903	2.05259
33369	1.89227	2.1
33370	1.89227	2.1
33371	1.89227	2.1
33372	1.89227	2.1
33373	3.78453	2.1
33374	1.15471	2.1
33375	0.993889	2.1
33376	1.78919	2.1
33377	1.77325	2.1
33378	2.78308	2.1
33379	1.89226	2.1
33380	1.89226	2.1
33381	1.89226	2.1
33382	1.89226	2.1
33383	3.78453	2.1
33384	1.78919	2.1
33385	1.77325	2.1
33386	2.45969	2.1
33387	2.70835	2.1
33388	4.49753	2.1
33389	0.939314	2.1
33390	0.939315	2.1
33391	1.01133	2.1
33392	1.01133	2.1
33393	0.596471	2.1
33394	1.20813	2.1
33395	0.886619	2.1
33396	1.35417	2.1
33397	1.35417	2.1
33398	1.20813	2.1
33399	0.886621	2.1
33400	0.59647	2.1
33401	0.946131	2.1
33402	0.946131	2.1
33403	0.94613	2.1
33404	0.94613	2.1
33405	0.946133	2.1

33406	0.946133	2.1
33407	0.946128	2.1
33408	0.946128	2.1
33409	0.946131	2.1
33410	0.946131	2.1
33411	0.946131	2.1
33412	0.946131	2.1
33413	1.03939	2.1
33414	1.03939	2.1
33415	0.946132	2.1
33416	0.946132	2.1
33417	0.946132	2.1
33418	0.946132	2.1
33419	1.23851	2.1
33420	1.03939	2.1
33421	1.03939	2.1
33422	0.946132	2.1
33423	0.946133	2.1
33424	1.23851	2.1
33425	0.902503	2.1
33426	0.946129	2.1
33427	0.946132	2.1
33428	0.946132	2.1
33429	0.902472	2.1
33430	0.902472	2.1
33431	1.08736	2.1
33432	0.994093	2.1
33433	0.946133	2.1
33434	0.902504	2.1
33435	1.03939	2.1
33436	0.946132	2.1
33437	1.35306	1.22438
33438	1.29982	1.10807
33439	1.7216	1.02312
33440	1.89183	1.05821
33441	3.07466	1.05796
33442	0.485098	1.08181
33443	0.582401	1.10686
33444	0.649908	1.16201
33445	0.8608	1.09506
33446	0.587739	0.998626
33447	0.58774	1.18452
33448	0.644567	1.08177
33449	0.644567	1.24162
33450	0.58241	1.09651
33451	0.86081	1.01075
33452	0.649898	1.19029
33453	0.485093	1.10687
33454	2.25936	2.1
33455	2.71441	2.1
33456	2.63824	2.1
33457	2.20652	2.1
33458	4.92092	2.1
33459	2.25936	2.1
33460	1.57627	2.1
33461	1.5493	2.1
33462	2.20652	2.1
33463	3.78279	2.1

33464	2.63824	2.1
33465	2.20652	2.1
33466	2.17326	2.1
33467	2.58517	2.1
33468	4.79169	2.1
33469	2.17326	2.1
33470	2.20652	2.1
33471	2.19278	2.1
33472	1.53556	2.1
33473	1.5493	2.1
33474	1.5493	2.1
33475	1.97465	2.1
33476	2.13925	2.1
33477	2.04483	2.1
33478	1.62784	2.1
33479	3.76709	2.1
33480	1.89226	2.1
33481	1.89226	2.1
33482	1.89226	2.1
33483	1.89226	2.1
33484	1.89226	2.1
33485	1.89226	2.1
33486	0.724037	2.1
33487	0.81392	2.1
33488	0.96796	2.1
33489	1.02242	2.1
33490	0.882091	2.1
33491	0.882091	2.1
33492	0.899789	2.1
33493	0.899789	2.1
33494	0.724036	2.1
33495	0.96796	2.1
33496	1.02242	2.1
33497	0.81392	2.1
33498	1.13537	2.1
33499	1.63054	2.1
33500	0.946131	2.1
33501	0.946131	2.1
33502	0.946131	2.1
33503	0.946131	2.1
33504	1.258	2.1
33505	1.63054	2.1
33506	1.22632	2.1
33507	1.22632	2.1
33508	0.946131	2.1
33509	1.258	2.1
33510	1.89226	2.1
33511	1.89226	2.1
33512	1.89226	2.1
33513	1.89226	2.1
33514	1.89226	2.1
33515	1.89226	2.1
33516	0.809739	2.1
33517	0.862674	2.1
33518	1.1995	2.1
33519	1.33899	2.1
33520	1.06324	2.1
33521	1.06324	2.1

33522	0.981932	2.1
33523	1.19563	2.1
33524	0.809727	2.1
33525	1.14092	2.1
33526	1.33902	2.1
33527	0.863494	2.1
33528	2.23742	2.1
33529	1.84902	2.1
33530	1.88088	2.1
33531	2.20392	2.1
33532	4.08644	2.1
33533	1.3592	2.1
33534	1.84902	2.1
33535	1.88088	2.1
33536	1.3305	2.1
33537	1.88088	2.1
33538	1.3305	2.1
33539	1.84902	2.1
33540	1.15008	1.08071
33541	1.3305	2.1
33542	1.70539	2.1
33543	2.9991	2.1
33544	1.84901	2.1
33545	2.0264	2.1
33546	2.20391	2.1
33547	1.70539	2.1
33548	3.87541	2.1
33549	1.03852	2.1
33550	1.38247	1.54298
33551	1.36635	1.6564
33552	1.01537	2.1
33553	2.39784	2.07869
33554	2.0815	2.1
33555	1.91744	2.1
33556	1.91744	2.1
33557	2.06496	2.1
33558	1.89227	2.1
33559	2.03474	2.1
33560	1.89226	1.81506
33561	1.89226	2.1
33562	1.89226	1.81401
33563	1.89226	2.1
33564	1.89226	1.30666
33565	1.89226	2.1
33566	1.36635	1.84164
33567	1.02923	2.1
33568	1.02923	1.98026
33569	1.01537	2.1
33570	1.36416	1.69573
33571	1.3503	2.1
33572	1.89226	2.1
33573	1.89359	2.1
33574	1.89359	2.1
33575	1.91744	2.1
33576	1.89226	2.1
33577	1.91611	2.1
33578	0.946128	2.1
33579	0.946128	2.1

33580	0.946133	1.84456
33581	0.946133	1.55251
33582	0.94613	1.42848
33583	0.94613	2.1
33584	0.946131	2.1
33585	0.946131	1.70562
33586	0.946131	2.1
33587	0.946131	2.1
33588	0.946131	2.1
33589	0.946131	2.1
33590	2.689	2.1
33591	2.0815	2.1
33592	2.72786	2.1
33593	2.48295	2.1
33594	5.02397	2.1
33595	2.72786	2.1
33596	1.86424	2.1
33597	2.7188	2.1
33598	2.08981	2.1
33599	4.81768	2.1
33600	2.08982	2.1
33601	0.986987	2.1
33602	2.08982	2.1
33603	1.87249	2.1
33604	1.01964	2.1
33605	0.906115	2.1
33606	1.44247	2.1
33607	1.43872	2.1
33608	1.17054	2.1
33609	1.17054	2.1
33610	1.43872	2.1
33611	1.26045	2.1
33612	2.0815	0.0500266
33613	2.0815	0.055262
33614	2.0815	0.0564879
33615	1.81468	0.0522455
33616	1.89065	0.0530085
33617	1.55625	0.05
33618	1.89062	0.0502668
33619	1.89224	0.0585427
33620	1.89224	0.0579758
33621	2.27073	0.0539784
33622	1.86856	0.0546475
33623	2.24232	0.05
33624	1.21419	2.1
33625	2.79716	2.1
33626	2.50005	2.1
33627	2.50005	2.1
33628	1.5008	2.1
33629	1.5008	2.1
33630	1.60139	0.05
33631	1.7103	0.05
33632	1.04159	0.05
33633	1.2489	0.05
33634	2.75189	0.05
33635	0.732812	0.05
33636	0.732812	0.05
33637	0.751986	0.05

33638	0.751986	0.05
33639	0.860346	0.05
33640	0.667961	0.05
33641	0.85515	0.05
33642	0.624452	0.05
33643	0.62445	0.05
33644	0.667962	0.05
33645	0.855148	0.05
33646	0.860348	0.05
33647	1.89226	0.14653
33648	2.50872	0.139855
33649	2.77909	0.13668
33650	1.89226	0.165413
33651	2.77909	0.125507
33652	1.89226	0.156895
33653	2.15825	0.1466
33654	2.50872	0.134796
33655	1.90439	0.114515
33656	1.90439	0.16367
33657	2.77909	0.115115
33658	2.77909	0.161143
33659	1.12468	2.1
33660	1.43872	2.1
33661	1.64926	2.1
33662	1.2724	2.1
33663	2.77395	2.1
33664	1.64926	2.1
33665	1.38438	2.1
33666	1.47244	2.1
33667	1.2724	2.1
33668	1.8493	2.1
33669	1.64926	2.1
33670	1.36416	1.93862
33671	1.0652	2.1
33672	1.0652	2.01872
33673	1.02923	2.1
33674	1.37496	1.86892
33675	1.33898	2.1
33676	1.89227	2.1
33677	1.90433	2.1
33678	1.90433	2.1
33679	1.89359	2.1
33680	1.89227	2.1
33681	1.88153	2.1
33682	0.946129	2.1
33683	0.946129	2.1
33684	0.946133	1.93281
33685	0.946133	1.86641
33686	0.946131	1.82913
33687	0.946131	2.1
33688	0.946131	2.1
33689	0.946131	1.81175
33690	0.946131	2.1
33691	0.946131	2.1
33692	0.946131	2.07683
33693	0.946131	2.0883
33694	1.37496	1.97189
33695	1.07519	2.1

33696	1.07519	2.053
33697	1.0652	2.1
33698	1.39725	1.94851
33699	1.38726	2.1
33700	1.89227	2.1
33701	1.9052	2.1
33702	1.9052	2.1
33703	1.90433	2.1
33704	1.89227	2.1
33705	1.8914	2.1
33706	0.946129	2.1
33707	0.946129	2.1
33708	0.946133	1.98721
33709	0.946133	1.90385
33710	0.946131	1.88775
33711	0.946131	2.1
33712	0.946131	2.1
33713	0.946131	1.89732
33714	0.946131	2.1
33715	0.946131	2.1
33716	0.946131	2.1
33717	0.946131	2.1
33718	1.91316	0.0878458
33719	1.89226	0.0952179
33720	1.89226	0.14996
33721	1.89226	0.091263
33722	1.99366	0.0993988
33723	1.99366	0.0885877
33724	1.93083	0.0772211
33725	1.89226	0.222173
33726	1.51379	0.0871924
33727	1.89226	0.0868071
33728	1.53051	0.0995222
33729	1.91316	0.0834419
33730	2.16731	2.1
33731	1.21373	2.1
33732	1.47804	2.1
33733	2.37601	2.1
33734	3.64535	2.1
33735	1.7211	2.1
33736	2.37601	2.1
33737	1.47804	2.1
33738	1.47804	2.1
33739	2.56843	2.1
33740	2.56843	2.1
33741	2.58731	2.1
33742	1.59094	2.1
33743	1.59094	2.1
33744	1.76816	2.1
33745	2.41707	2.1
33746	2.5943	2.1
33747	0.718346	2.1
33748	0.795468	2.1
33749	1.20854	2.1
33750	1.1224	2.1
33751	0.961203	2.1
33752	0.961203	2.1
33753	0.95667	2.1

33754	0.95667	2.1
33755	0.718346	2.1
33756	1.1224	2.1
33757	0.795469	2.1
33758	1.20854	2.1
33759	1.84076	2.1
33760	1.42884	2.1
33761	2.24274	2.1
33762	1.85415	2.1
33763	3.69491	2.1
33764	1.89226	2.1
33765	1.89226	2.1
33766	1.89226	2.1
33767	1.89226	2.1
33768	3.78452	2.1
33769	2.24273	2.1
33770	2.24412	2.1
33771	1.85415	2.1
33772	1.85415	2.1
33773	2.68473	2.1
33774	2.68473	2.1
33775	1.89226	2.1
33776	1.89226	2.1
33777	1.89226	2.1
33778	1.89226	2.1
33779	1.89226	2.1
33780	1.89226	2.1
33781	1.89226	2.1
33782	1.89226	2.1
33783	1.89226	2.1
33784	1.89226	2.1
33785	3.78451	2.1
33786	1.85415	2.1
33787	1.10961	2.1
33788	2.24411	2.1
33789	1.51067	2.1
33790	3.35373	2.1
33791	1.70302	2.1
33792	1.89226	2.1
33793	1.76399	2.1
33794	1.43577	2.1
33795	2.08149	2.1
33796	1.70302	2.1
33797	1.89226	2.1
33798	1.89226	2.1
33799	1.89226	2.1
33800	1.89226	2.1
33801	1.89226	2.1
33802	1.89226	2.1
33803	1.0593	2.1
33804	1.17289	2.1
33805	1.5379	2.1
33806	1.46757	2.1
33807	2.71079	2.1
33808	0.931235	2.1
33809	1.1151	2.1
33810	0.975236	2.1
33811	0.975236	2.1

33812	1.31147	2.1
33813	0.762882	2.1
33814	1.13152	2.1
33815	0.505799	2.1
33816	1.31149	2.1
33817	1.07629	2.1
33818	0.884817	2.1
33819	0.505793	2.1
33820	2.69027	2.1
33821	1.60441	2.1
33822	2.69027	2.1
33823	1.64771	2.1
33824	2.78977	2.1
33825	1.72731	2.1
33826	0.77878	0.05
33827	0.843226	0.05
33828	0.845997	0.05
33829	0.926091	0.05
33830	0.77878	0.05
33831	0.926093	0.05
33832	1.3922	1.73746
33833	1.3922	1.73351
33834	0.872156	1.96649
33835	0.872156	1.9001
33836	0.872157	2.02259
33837	0.946132	1.70474
33838	0.946129	1.65748
33839	1.31822	2.06477
33840	0.94613	1.84024
33841	0.94613	1.88203
33842	1.31823	1.84377
33843	0.872157	1.92186
33844	1.81534	0.769005
33845	1.89226	0.465772
33846	1.59602	0.816093
33847	1.59602	1.07816
33848	1.89226	0.331428
33849	1.89226	0.408941
33850	1.89227	0.368111
33851	1.89227	0.524346
33852	1.89227	0.561721
33853	1.89227	0.370455
33854	3.78453	0.382932
33855	1.89226	0.480045
33856	1.89226	0.261889
33857	1.89226	0.34603
33858	1.89226	0.438669
33859	3.78453	0.469204
33860	2.30348	0.170325
33861	1.4185	0.217359
33862	1.82551	0.252632
33863	1.02011	0.185448
33864	3.24401	0.187975
33865	2.46702	0.164332
33866	1.17129	0.220739
33867	1.82551	0.180158
33868	1.82551	0.192668
33869	1.73525	0.176277

33870	1.73525	0.207671
33871	1.89227	0.421267
33872	1.89227	0.294147
33873	1.89227	0.303561
33874	1.89227	0.331661
33875	1.89227	0.380988
33876	1.89227	0.407371
33877	2.80034	2.1
33878	2.45767	2.1
33879	2.67994	2.1
33880	2.35395	2.1
33881	5.1376	2.1
33882	2.45766	2.1
33883	1.8769	2.1
33884	2.00989	2.1
33885	2.00989	2.1
33886	2.35395	2.1
33887	2.35395	2.1
33888	2.57662	2.1
33889	2.35395	2.1
33890	2.57662	2.1
33891	2.67993	2.1
33892	2.24227	2.1
33893	2.34558	2.1
33894	1.02874	2.1
33895	1.02874	2.1
33896	1.03084	2.1
33897	1.03084	2.1
33898	0.938448	2.1
33899	0.886762	2.1
33900	1.17698	2.1
33901	1.12114	2.1
33902	1.12113	2.1
33903	0.886764	2.1
33904	0.93845	2.1
33905	1.17697	2.1
33906	1.91969	2.1
33907	2.5508	2.1
33908	1.88273	1.83527
33909	2.76609	2.1
33910	4.68578	2.1
33911	2.83423	2.1
33912	1.89226	2.1
33913	2.76609	2.1
33914	1.89226	2.1
33915	4.65835	2.1
33916	1.56685	0.190798
33917	1.11712	0.325816
33918	1.07999	1.42063
33919	1.51448	0.582622
33920	2.6316	1.10399
33921	1.56685	0.0831984
33922	1.67652	0.695712
33923	1.5955	1.43463
33924	1.51448	0.402208
33925	3.19099	0.430491
33926	1.89226	1.3327
33927	1.89226	0.281137

33928	1.89226	0.389405
33929	1.89226	1.62986
33930	3.78453	1.30061
33931	1.89226	0.509176
33932	1.89226	0.380721
33933	1.89226	1.37633
33934	1.89226	1.54268
33935	1.89226	0.193709
33936	1.89226	0.318262
33937	1.0562	0.992204
33938	1.51448	0.209303
33939	1.07999	0.544617
33940	1.07999	0.204619
33941	1.46441	1.23124
33942	1.46441	0.172867
33943	1.5955	1.151
33944	1.51448	0.201977
33945	1.46441	0.504357
33946	1.52909	1.42845
33947	3.04357	0.698459
33948	1.89226	0.26513
33949	1.89226	1.52536
33950	1.89226	1.51552
33951	1.89226	0.429195
33952	3.78453	0.522921
33953	1.89227	0.28237
33954	1.89227	0.245256
33955	1.89227	1.22269
33956	1.89227	1.12074
33957	3.78453	0.955416
33958	1.89994	1.11064
33959	1.70303	0.958372
33960	1.68576	0.29881
33961	1.89608	0.369777
33962	3.8237	0.593981
33963	1.89994	1.64244
33964	2.31116	2.1
33965	1.9386	1.2267
33966	1.88653	1.81923
33967	3.74885	1.74526
33968	2.31117	2.1
33969	2.20665	2.00228
33970	2.09617	1.96949
33971	2.41055	2.1
33972	2.09617	1.96599
33973	2.41055	2.1
33974	1.10777	0.718299
33975	1.0523	0.95359
33976	0.912098	0.812069
33977	0.757533	0.74692
33978	0.89287	1.7248
33979	1.07571	0.325893
33980	0.77485	0.891118
33981	0.98645	0.452839
33982	0.989171	0.294859
33983	0.875089	0.362618
33984	0.979686	0.831822
33985	0.979686	1.50374

33986	2.52442	2.1
33987	1.49998	2.1
33988	2.51692	2.1
33989	1.56957	2.1
33990	4.09399	2.1
33991	1.01298	2.1
33992	1.01298	2.1
33993	1.03079	2.1
33994	1.03079	2.1
33995	0.820964	2.1
33996	0.784786	2.1
33997	1.25898	2.1
33998	1.25846	2.1
33999	1.25898	2.1
34000	0.820964	2.1
34001	1.25846	2.1
34002	0.784786	2.1
34003	1.89226	0.05
34004	1.89226	0.05
34005	2.6297	0.05
34006	1.89226	0.05
34007	3.78452	0.05
34008	1.89226	0.05
34009	1.89226	0.05
34010	1.89226	0.05
34011	1.89226	0.05
34012	1.89226	0.05
34013	1.89226	0.05
34014	2.19397	0.05
34015	1.89226	0.05
34016	2.34112	0.05
34017	2.34112	0.05
34018	1.89226	0.05
34019	1.89226	0.05
34020	2.23366	2.1
34021	1.87598	2.1
34022	1.64716	2.1
34023	2.06048	2.1
34024	3.93645	2.1
34025	2.23367	2.1
34026	2.17384	2.1
34027	2.04818	2.1
34028	2.06048	2.1
34029	4.23432	2.1
34030	1.9855	2.1
34031	2.06048	2.1
34032	1.93163	2.1
34033	1.50737	2.1
34034	3.56785	2.1
34035	2.04818	2.1
34036	2.06048	2.1
34037	2.27465	2.1
34038	1.92264	2.1
34039	3.98312	2.1
34040	2.08149	0.690284
34041	1.89226	1.63419
34042	1.89226	1.52808
34043	1.33153	0.628538

34044	1.68038	1.71356
34045	1.18244	0.898546
34046	1.89226	1.31683
34047	1.89226	1.6485
34048	1.89226	1.56528
34049	1.89226	0.926885
34050	3.78452	1.37422
34051	2.69069	0.539736
34052	1.89226	0.697082
34053	1.89226	0.679771
34054	1.97397	0.567634
34055	1.89226	0.677916
34056	1.97397	0.550034
34057	1.89226	1.64215
34058	2.38056	0.972598
34059	1.89226	1.02727
34060	1.89226	1.40372
34061	3.78452	1.34647
34062	0.904435	2.1
34063	0.904435	2.1
34064	0.925315	2.1
34065	0.925315	2.1
34066	1.13312	2.1
34067	1.08109	2.1
34068	0.70697	2.1
34069	0.696633	2.1
34070	0.70697	2.1
34071	1.13312	2.1
34072	0.696633	2.1
34073	1.08109	2.1
34074	1.45506	2.1
34075	2.26623	2.1
34076	2.38363	2.1
34077	2.38363	2.1
34078	1.41394	2.1
34079	1.41394	2.1
34080	0.946131	2.1
34081	0.946131	2.1
34082	1.13311	2.1
34083	1.13311	2.1
34084	0.946127	2.1
34085	0.946127	2.1
34086	1.13312	2.1
34087	1.19182	2.1
34088	1.19181	2.1
34089	0.946129	2.1
34090	1.13312	2.1
34091	0.94613	2.1
34092	1.89226	0.868285
34093	1.89226	1.94369
34094	1.89226	0.946384
34095	1.89226	1.98708
34096	1.89226	1.59986
34097	1.89226	2.00668
34098	1.05168	2.1
34099	0.696527	2.1
34100	0.907442	1.77856
34101	0.907442	1.92348

34102	0.953145	1.66206
34103	0.782285	2.1
34104	0.773928	2.1
34105	0.773928	1.8514
34106	0.696538	1.90388
34107	0.851554	1.89228
34108	1.04071	1.95238
34109	1.12335	1.9511
34110	1.89226	1.11074
34111	1.89226	0.284086
34112	1.89226	0.534478
34113	1.89226	0.222932
34114	3.78453	0.232079
34115	1.89226	1.06749
34116	1.89226	0.26237
34117	1.89226	0.70899
34118	1.89226	0.211045
34119	3.78453	0.872494
34120	2.50912	0.22326
34121	1.89226	0.161607
34122	1.89226	0.230518
34123	2.63195	0.159289
34124	4.40139	0.156598
34125	2.50912	0.251517
34126	1.3598	0.200921
34127	1.70303	0.208461
34128	2.36874	0.14249
34129	3.82055	0.234469
34130	1.89227	0.266767
34131	1.89227	0.208774
34132	1.89227	0.199792
34133	1.89227	0.132735
34134	3.78453	0.279834
34135	1.89227	0.306607
34136	1.89227	0.176603
34137	1.89227	0.198588
34138	1.89227	0.136719
34139	3.78453	0.1427
34140	1.89226	0.179913
34141	2.67088	0.107668
34142	2.63195	0.10082
34143	2.63195	0.143738
34144	1.89226	0.118171
34145	1.89226	0.192008
34146	1.30202	0.143606
34147	1.28304	0.151983
34148	0.809902	0.138616
34149	0.888801	0.144722
34150	1.13262	0.0989216
34151	0.968276	0.127294
34152	1.0733	0.158179
34153	1.0733	0.13957
34154	1.35419	0.120353
34155	0.801222	0.0944652
34156	0.899732	0.171697
34157	1.23967	0.12302
34158	1.31595	2.1
34159	0.869501	2.1

34160	2.65473	2.1
34161	1.88782	2.1
34162	3.20377	2.1
34163	1.89227	2.1
34164	1.89227	2.1
34165	1.89227	2.1
34166	1.89227	2.1
34167	3.78453	2.1
34168	2.65473	2.1
34169	1.89226	2.1
34170	1.88781	2.1
34171	1.88782	2.1
34172	2.0815	2.1
34173	2.0815	2.1
34174	1.89226	2.1
34175	1.89226	2.1
34176	1.89226	2.1
34177	1.89226	2.1
34178	1.89226	2.1
34179	1.89226	2.1
34180	1.89226	2.1
34181	1.89226	2.1
34182	1.89226	2.1
34183	1.89226	2.1
34184	1.89226	2.1
34185	1.89226	2.1
34186	2.39057	2.1
34187	1.89226	2.1
34188	1.89226	2.05838
34189	1.89226	2.1
34190	2.14243	2.06156
34191	2.14243	2.1
34192	1.89226	0.686506
34193	1.89226	2.1
34194	1.89226	1.73245
34195	1.89226	2.00207
34196	1.89226	1.86691
34197	1.89226	2.1
34198	1.89226	0.920119
34199	1.89226	2.1
34200	1.89226	1.96592
34201	1.89226	2.05769
34202	1.89226	1.832
34203	1.89226	2.1
34204	2.56323	0.0909819
34205	1.36324	0.0886565
34206	1.05296	0.084772
34207	2.18048	0.0922037
34208	3.54372	0.0855769
34209	0.946128	2.1
34210	0.946128	2.1
34211	1.08733	2.1
34212	1.08733	2.1
34213	0.946131	2.1
34214	0.946131	2.1
34215	1.09197	2.1
34216	1.08732	2.1
34217	0.946131	2.1

34218	1.09197	2.1
34219	1.08732	2.1
34220	0.946131	2.1
34221	1.35111	1.63262
34222	1.51327	1.57892
34223	1.64493	1.43924
34224	1.31058	1.66825
34225	2.99605	1.70977
34226	1.8124	1.44222
34227	1.51327	1.63319
34228	1.64493	1.63214
34229	1.31358	1.38354
34230	1.8124	1.61796
34231	1.48105	1.42102
34232	1.64493	1.4996
34233	1.31058	1.64955
34234	1.22297	1.56835
34235	1.70101	1.43911
34236	2.8679	1.45905
34237	1.70101	1.52267
34238	1.99297	1.43784
34239	1.64493	1.50896
34240	1.64493	1.32434
34241	1.99297	1.5676
34242	1.8124	1.14028
34243	2.0815	2.1
34244	1.80258	2.1
34245	1.80258	2.1
34246	1.83506	2.1
34247	2.0815	2.1
34248	2.1212	2.1
34249	2.0815	2.1
34250	1.82129	2.1
34251	1.82129	2.1
34252	1.80445	2.1
34253	2.0815	2.1
34254	2.06092	2.1
34255	1.89224	2.1
34256	1.703 2.1	
34257	1.97839	2.1
34258	1.703 2.1	
34259	1.97402	2.1
34260	1.703 2.1	
34261	1.89226	0.183613
34262	1.89226	0.536517
34263	1.89226	0.401852
34264	2.75351	0.191049
34265	3.78453	0.247456
34266	1.89226	0.417667
34267	3.02887	0.568079
34268	2.03149	0.224754
34269	2.03149	0.445832
34270	1.89226	0.22734
34271	1.89226	0.479666
34272	2.17979	2.1
34273	1.65533	2.1
34274	2.57212	2.1
34275	2.20346	2.1

34276	4.22745	2.1
34277	1.89227	2.1
34278	2.20347	2.1
34279	1.89227	2.1
34280	2.57212	2.1
34281	1.89227	2.1
34282	2.57212	2.1
34283	2.05274	2.1
34284	2.16783	2.1
34285	2.24429	2.1
34286	2.38404	2.1
34287	4.41212	2.1
34288	2.16783	2.1
34289	2.05967	2.1
34290	2.38404	2.1
34291	2.24695	2.1
34292	4.41478	2.1
34293	2.24429	2.1
34294	2.38404	2.1
34295	2.51819	2.1
34296	2.60723	2.1
34297	4.85152	2.1
34298	2.38404	2.1
34299	2.24695	2.1
34300	2.60723	2.1
34301	2.45247	2.1
34302	4.85418	2.1
34303	1.89227	2.1
34304	1.89227	2.1
34305	1.89227	2.1
34306	1.89227	2.1
34307	3.78453	2.1
34308	1.89226	2.1
34309	1.89226	2.1
34310	1.89226	2.1
34311	1.89226	2.1
34312	3.78453	2.1
34313	0.618473	2.1
34314	0.619945	2.1
34315	0.797692	2.1
34316	0.770147	2.1
34317	0.68852	2.1
34318	0.68852	2.1
34319	0.701573	2.1
34320	0.701573	2.1
34321	0.619945	2.1
34322	0.797691	2.1
34323	0.618473	2.1
34324	0.770147	2.1
34325	0.797692	2.1
34326	0.770148	2.1
34327	0.694787	2.1
34328	0.653411	2.1
34329	0.722102	2.1
34330	0.722102	2.1
34331	0.729001	2.1
34332	0.729001	2.1
34333	0.797692	2.1

34334	0.694787	2.1
34335	0.770148	2.1
34336	0.653412	2.1
34337	1.89227	2.1
34338	1.89227	2.1
34339	1.89227	2.1
34340	1.89227	2.1
34341	3.78453	2.1
34342	1.89226	2.1
34343	1.89226	2.1
34344	1.89226	2.1
34345	1.89226	2.1
34346	3.78453	2.1
34347	1.23989	2.1
34348	1.59538	2.1
34349	1.66647	2.1
34350	1.26599	2.1
34351	2.90636	2.1
34352	1.66647	2.1
34353	1.38957	2.1
34354	1.47446	2.1
34355	1.40337	2.1
34356	1.66647	2.1
34357	1.59538	2.1
34358	2.80619	2.1
34359	2.0815	2.1
34360	2.0815	2.1
34361	2.0815	2.1
34362	2.73721	2.1
34363	2.74192	2.1
34364	2.73721	2.1
34365	2.0815	2.1
34366	2.0815	2.1
34367	2.0815	2.1
34368	2.689	2.1
34369	2.69166	2.1
34370	2.62369	2.1
34371	2.19717	2.1
34372	2.60405	2.1
34373	2.20566	2.1
34374	4.80122	2.1
34375	2.19717	2.1
34376	1.37392	2.1
34377	2.20566	2.1
34378	1.45408	2.1
34379	3.65125	2.1
34380	2.60405	2.1
34381	2.20566	2.1
34382	2.57466	2.1
34383	2.21715	2.1
34384	4.8212	2.1
34385	2.20566	2.1
34386	1.54249	2.1
34387	1.54249	2.1
34388	1.45408	2.1
34389	2.21715	2.1
34390	2.12874	2.1
34391	1.60647	2.1

34392	1.31346	2.1
34393	1.82649	2.1
34394	1.51067	2.1
34395	3.13995	2.1
34396	1.89226	2.1
34397	1.89226	2.1
34398	1.89226	2.1
34399	1.89226	2.1
34400	3.78452	2.1
34401	1.89226	2.1
34402	2.6096	2.1
34403	2.8067	2.1
34404	2.8067	2.1
34405	1.89226	2.1
34406	1.89226	2.1
34407	1.82649	2.1
34408	1.3596	2.1
34409	2.17708	2.1
34410	1.70302	2.1
34411	3.40243	2.1
34412	1.89226	2.1
34413	1.89226	2.1
34414	1.89226	2.1
34415	1.89226	2.1
34416	3.78452	2.1
34417	2.27067	2.1
34418	2.43549	2.1
34419	2.63979	2.1
34420	2.41828	2.1
34421	2.27067	2.1
34422	2.04916	2.1
34423	1.30047	2.1
34424	2.04916	2.1
34425	2.27068	2.1
34426	1.5586	2.1
34427	3.57115	2.1
34428	2.27068	2.1
34429	1.84214	2.1
34430	1.80224	2.1
34431	1.5586	2.1
34432	2.51432	2.1
34433	2.27068	2.1
34434	1.63719	2.1
34435	2.15714	2.1
34436	2.07821	2.1
34437	1.52204	2.1
34438	3.67919	2.1
34439	2.07821	2.1
34440	1.40118	2.1
34441	1.40118	2.1
34442	1.52204	2.1
34443	1.99632	2.1
34444	2.11718	2.1
34445	0.946131	2.1
34446	0.946131	2.1
34447	1.06957	2.1
34448	1.06957	2.1
34449	0.946132	2.1

34450	0.946132	2.03769
34451	1.411	2.1
34452	1.06957	2.1
34453	0.946132	2.1
34454	1.411	2.1
34455	1.06957	2.1
34456	0.946132	2.1
34457	1.99875	1.21951
34458	2.57079	0.940728
34459	2.59319	1.52458
34460	1.9105	0.604774
34461	4.59194	1.02066
34462	1.99875	1.27369
34463	1.02247	0.280929
34464	1.07017	0.353501
34465	1.15841	0.377005
34466	1.91051	0.357124
34467	1.99875	0.422229
34468	2.59319	1.47006
34469	1.9105	0.669474
34470	2.33812	1.19902
34471	2.59319	1.32129
34472	1.59457	1.21198
34473	1.59457	1.35803
34474	2.50906	1.03725
34475	2.0467	0.567938
34476	2.03357	0.832828
34477	2.53176	0.469667
34478	4.54263	0.918542
34479	1.294	1.08649
34480	1.66636	0.794517
34481	2.03357	0.741315
34482	1.03079	0.536643
34483	3.32757	0.766427
34484	1.68108	0.547291
34485	2.53176	0.440393
34486	2.36417	0.362394
34487	2.71666	0.308996
34488	1.68108	1.06226
34489	2.03357	0.886619
34490	1.89226	0.159326
34491	2.59302	0.216407
34492	2.2727	0.163178
34493	2.2727	0.169774
34494	1.89226	0.203591
34495	1.89226	0.183439
34496	1.89226	0.228576
34497	1.89226	0.230932
34498	1.89226	0.173906
34499	1.89226	0.123172
34500	3.78452	0.262466
34501	1.89225	0.387319
34502	1.89225	0.094012
34503	1.89225	0.149826
34504	1.89225	0.166754
34505	1.89225	0.14551
34506	1.89225	0.164957
34507	0.934114	0.190325

34508	0.934114	0.124785
34509	0.944544	0.174423
34510	0.944544	0.210344
34511	0.721493	0.120441
34512	0.58215	0.114432
34513	1.2965	0.165274
34514	1.13634	0.113947
34515	1.13635	0.113552
34516	0.582148	0.113485
34517	1.29651	0.164427
34518	0.721491	0.134609
34519	1.89226	0.159695
34520	1.89226	0.1162
34521	1.89226	0.0942477
34522	1.89226	0.0940475
34523	3.78452	0.0886214
34524	0.946131	0.485591
34525	0.946131	0.545581
34526	0.946131	0.421244
34527	0.946131	0.54065
34528	0.946131	0.408871
34529	0.946131	0.44679
34530	0.946131	0.549299
34531	0.946131	0.83856
34532	0.946131	0.455925
34533	0.946131	0.476
34534	0.946131	0.531998
34535	0.946131	0.478572
34536	1.89226	0.0503967
34537	1.89226	0.0531253
34538	2.78602	0.05
34539	1.89226	0.0606561
34540	3.78453	0.05
34541	1.52601	2.1
34542	1.76601	2.1
34543	1.27839	2.1
34544	1.56095	2.1
34545	3.0444	2.1
34546	1.76601	2.1
34547	1.86793	2.1
34548	1.56095	2.1
34549	1.68152	2.1
34550	3.44753	2.1
34551	1.27841	2.1
34552	1.56097	2.1
34553	1.0577	2.1
34554	1.36861	2.1
34555	2.64702	2.1
34556	1.56097	2.1
34557	1.68154	2.1
34558	1.36861	2.1
34559	1.53296	2.1
34560	3.05015	2.1
34561	1.89226	2.1
34562	1.89226	2.1
34563	1.89226	2.1
34564	1.89226	2.1
34565	3.78453	2.1

34566	0.983075	2.1
34567	0.788361	2.1
34568	0.724726	2.1
34569	0.724726	2.1
34570	0.983027	2.1
34571	0.724734	2.1
34572	0.788364	2.1
34573	0.788364	2.1
34574	0.788335	2.1
34575	0.94613	2.1
34576	0.724757	2.1
34577	0.946131	2.1
34578	1.15789	2.1
34579	2.11135	2.1
34580	2.25826	2.1
34581	1.28845	2.1
34582	3.41615	2.1
34583	1.89226	2.1
34584	1.89226	2.1
34585	1.89226	2.1
34586	1.89226	2.1
34587	3.78453	2.1
34588	1.57667	2.1
34589	1.70002	2.1
34590	1.70002	2.1
34591	1.89226	2.1
34592	1.89226	2.1
34593	2.05244	2.1
34594	2.25826	2.1
34595	1.42214	2.1
34596	1.43675	2.1
34597	1.28845	2.1
34598	2.40656	2.1
34599	2.25826	2.1
34600	1.58066	2.1
34601	1.74247	2.1
34602	1.85097	2.1
34603	1.61155	2.1
34604	3.43163	2.1
34605	0.785612	2.1
34606	0.789638	2.1
34607	0.882561	2.1
34608	0.763529	2.1
34609	0.686921	2.1
34610	0.86048	2.1
34611	0.871812	2.1
34612	0.871812	2.1
34613	0.830183	2.1
34614	0.882572	2.1
34615	0.902315	2.1
34616	0.785602	2.1
34617	1.01972	0.05
34618	0.936657	0.05
34619	0.946131	0.05
34620	0.946131	0.05
34621	0.927018	0.05
34622	0.927018	0.05
34623	1.1376	0.05

34624	0.953663	0.05
34625	1.1257	0.05
34626	1.04073	0.05
34627	1.00358	0.05
34628	0.851528	0.05
34629	1.73576	0.113617
34630	2.00244	0.345139
34631	1.17798	0.05
34632	1.25796	0.05
34633	2.99373	0.0504279
34634	2.00244	0.315684
34635	1.10719	0.0514342
34636	2.03236	0.334527
34637	2.03236	0.313206
34638	1.25797	0.0520916
34639	1.25797	0.25358
34640	1.89226	2.1
34641	1.89226	2.1
34642	1.89226	2.1
34643	1.89226	2.1
34644	3.78452	2.1
34645	2.3174	2.1
34646	1.50005	2.1
34647	1.90428	2.1
34648	2.64564	2.1
34649	4.22168	2.1
34650	1.89226	2.1
34651	1.89226	2.1
34652	1.89226	2.1
34653	1.89226	2.1
34654	1.89226	2.1
34655	1.89226	2.1
34656	1.32749	0.0663356
34657	1.70302	0.0700852
34658	2.08149	0.0701335
34659	1.70302	0.0633825
34660	2.19634	0.0732212
34661	1.93083	0.0678307
34662	1.89226	1.76039
34663	1.89226	1.58025
34664	1.89226	1.24154
34665	1.89226	1.88142
34666	3.78453	1.48562
34667	1.89226	1.44884
34668	1.89226	1.7424
34669	1.89226	1.30914
34670	1.89226	1.2608
34671	3.78452	1.45566
34672	2.17499	1.17548
34673	1.79663	1.74057
34674	2.1538	1.24372
34675	1.818	1.37274
34676	2.17499	1.59617
34677	1.83919	2.1
34678	0.898316	1.54558
34679	1.0769	1.38524
34680	0.968276	2.03451
34681	0.773495	1.77826

34682	0.921126	1.84611
34683	0.921126	1.79108
34684	0.92927	1.55145
34685	0.92927	1.79252
34686	1.0769	1.65653
34687	0.968276	1.62032
34688	0.898316	1.66738
34689	0.773495	1.64347
34690	1.89226	1.37799
34691	1.89226	2.00919
34692	1.89226	1.67989
34693	1.89226	1.40648
34694	3.78453	1.69204
34695	1.89226	1.51022
34696	1.89226	1.94564
34697	1.89226	1.1597
34698	1.89226	1.55869
34699	3.78452	1.30866
34700	2.17499	1.56293
34701	2.1538	2.1
34702	2.53574	1.38936
34703	2.54157	1.20703
34704	4.71073	1.53262
34705	1.0769	1.52327
34706	1.26787	1.40628
34707	1.16641	1.73158
34708	0.968276	1.89733
34709	1.11625	1.73819
34710	1.11625	1.68221
34711	1.11989	1.79921
34712	1.11989	1.2857
34713	1.26787	1.55121
34714	1.16641	1.65716
34715	1.0769	1.78047
34716	0.968277	1.71673
34717	1.89226	0.283458
34718	1.89226	0.370009
34719	1.89226	0.372064
34720	1.89226	0.356872
34721	3.78452	0.300397
34722	1.91739	0.220008
34723	2.20045	0.078607
34724	1.79005	0.0981179
34725	2.33802	0.156506
34726	4.25539	0.165287
34727	2.52754	0.14116
34728	1.89226	0.165408
34729	2.69841	0.137324
34730	2.69841	0.184282
34731	1.89226	0.123261
34732	1.89226	0.155335
34733	1.89226	0.361817
34734	1.89226	0.580185
34735	1.89226	0.28389
34736	1.89226	0.820721
34737	3.78452	0.393089
34738	1.93544	0.183215
34739	2.33802	0.245867

34740	1.55063	0.180636
34741	2.1769	0.0969548
34742	3.88865	0.164539
34743	2.20929	0.250778
34744	2.55162	0.170741
34745	2.27807	0.280461
34746	1.48831	0.180279
34747	4.48736	0.190469
34748	2.27807	0.303617
34749	2.72723	0.180209
34750	2.55162	0.235635
34751	2.55162	0.271852
34752	2.43157	0.192129
34753	2.43157	0.238393
34754	2.27807	0.222761
34755	1.51612	0.164772
34756	1.96048	0.160606
34757	1.73022	0.154877
34758	1.70302	0.255724
34759	1.61016	0.239127
34760	2.27807	0.236159
34761	2.08048	0.153668
34762	1.98522	0.170474
34763	2.43157	0.194513
34764	1.83798	0.177595
34765	2.27807	0.206235
34766	1.70302	0.29073
34767	2.68507	0.218013
34768	2.23856	0.247335
34769	2.52174	0.211378
34770	2.35331	0.310478
34771	2.65099	0.264472
34772	1.70303	0.268513
34773	0.986848	0.170866
34774	2.41566	0.172675
34775	1.70303	0.165872
34776	1.88139	0.169204
34777	1.64955	0.169544
34778	1.89226	2.01789
34779	2.34978	1.86209
34780	1.89226	1.98494
34781	2.68773	1.78149
34782	1.89226	2.03694
34783	2.68773	1.84023
34784	1.89226	2.0176
34785	1.89226	1.72864
34786	1.89226	1.69746
34787	1.89226	1.9054
34788	3.78453	1.85776
34789	1.00825	1.51483
34790	1.09847	1.58458
34791	0.939987	1.60364
34792	0.783516	1.74655
34793	0.838041	1.6543
34794	0.838041	1.78763
34795	1.49693	1.8581
34796	1.26602	1.66356
34797	1.10875	1.46061

34798	1.05187	1.95151
34799	1.28743	1.68878
34800	0.946132	1.6689
34801	0.946132	1.73148
34802	0.946132	2.0372
34803	0.946132	1.88925
34804	0.946132	1.57409
34805	1.31823	2.03617
34806	0.946132	1.72264
34807	0.946132	1.86359
34808	0.946131	1.90084
34809	0.946131	2.068
34810	0.946132	1.8307
34811	1.31823	1.90707
34812	1.51688	0.169729
34813	1.79978	0.277013
34814	1.1685	0.176858
34815	1.1685	0.239522
34816	1.85857	0.16825
34817	1.51018	0.27012
34818	0.946116	0.22751
34819	0.946116	0.242472
34820	0.94612	0.142031
34821	0.94612	0.154777
34822	0.946118	0.149279
34823	0.946118	0.22555
34824	0.946118	0.16901
34825	0.946118	0.243619
34826	0.946131	0.182079
34827	0.946131	0.207167
34828	0.946105	0.204318
34829	0.946105	0.202061
34830	1.89224	2.1
34831	1.89224	2.1
34832	1.89224	2.1
34833	1.89224	2.1
34834	1.89224	2.1
34835	1.89224	2.1
34836	1.38678	2.1
34837	1.3349	2.1
34838	0.946119	2.1
34839	0.946119	2.1
34840	1.38678	2.1
34841	1.38678	2.1
34842	0.946119	2.1
34843	0.946119	2.1
34844	1.38676	2.1
34845	0.946107	2.1
34846	0.946132	2.1
34847	1.33491	2.1
34848	1.06455	2.1
34849	1.03932	2.1
34850	1.20649	2.1
34851	1.18773	2.1
34852	2.27105	2.1
34853	1.89226	2.1
34854	1.89226	2.1
34855	1.89226	2.1

34856	1.89226	2.1
34857	1.89226	2.1
34858	1.89226	2.1
34859	1.36918	2.1
34860	1.18774	2.1
34861	1.31144	2.1
34862	1.20651	2.1
34863	1.31144	2.1
34864	1.20651	2.1
34865	2.29066	2.1
34866	1.76476	2.1
34867	1.79825	2.1
34868	2.33801	2.1
34869	4.08891	2.1
34870	1.79825	2.1
34871	2.33801	2.1
34872	2.37168	2.1
34873	1.49325	2.1
34874	4.16993	2.1
34875	1.13641	2.1
34876	1.43213	2.00399
34877	1.44085	2.1
34878	1.18301	2.1
34879	2.61513	2.1
34880	1.89226	2.1
34881	2.23875	2.1
34882	2.23875	2.1
34883	2.10287	2.1
34884	1.89226	2.1
34885	1.75639	2.1
34886	1.89226	2.03362
34887	1.89226	2.1
34888	1.89226	2.06905
34889	1.89226	2.1
34890	1.89226	2.03817
34891	1.89226	2.1
34892	1.44085	2.1
34893	1.25173	2.1
34894	1.25173	2.1
34895	1.18301	2.1
34896	1.4617	2.1
34897	1.39298	2.1
34898	1.89227	2.1
34899	2.23875	2.1
34900	2.42504	2.1
34901	1.89227	2.1
34902	4.13101	2.1
34903	1.89226	2.07164
34904	1.89226	2.1
34905	1.89226	2.01454
34906	1.89226	2.1
34907	1.89226	2.0107
34908	1.89226	2.1
34909	1.89226	2.1
34910	2.41297	2.1
34911	2.70685	2.1
34912	2.70685	2.1
34913	1.89226	2.1

34914	1.89226	2.1
34915	1.89226	2.1
34916	1.89226	2.1
34917	1.89226	2.1
34918	1.89226	2.1
34919	1.89226	2.1
34920	1.89226	2.1
34921	1.89226	2.1
34922	1.89226	2.1
34923	1.89226	2.1
34924	1.89226	2.1
34925	3.78453	2.1
34926	1.89226	2.1
34927	1.89226	2.1
34928	1.89226	2.1
34929	1.89226	2.1
34930	1.89226	2.1
34931	1.89226	2.1
34932	1.69044	0.05
34933	1.57979	0.05
34934	2.27963	0.05
34935	2.21582	0.05
34936	3.85942	0.05
34937	2.21582	0.05
34938	1.30722	0.05
34939	2.02069	0.05
34940	2.02069	0.05
34941	1.57979	0.05
34942	1.57979	0.05
34943	1.67825	2.1
34944	1.89226	2.1
34945	1.89226	2.1
34946	1.89226	2.1
34947	1.91894	2.1
34948	1.91894	2.1
34949	0.946133	2.1
34950	0.946133	2.1
34951	0.946132	2.1
34952	0.946132	2.1
34953	0.946132	2.1
34954	0.946132	2.1
34955	0.946133	2.1
34956	0.946133	2.1
34957	0.946133	2.1
34958	0.946133	2.1
34959	0.946132	2.1
34960	0.946132	2.1
34961	0.942609	2.1
34962	0.942609	2.1
34963	1.329 2.1	
34964	1.329 2.1	
34965	1.32547	2.1
34966	0.94261	2.1
34967	0.946132	2.1
34968	0.946132	2.1
34969	0.946132	2.1
34970	1.32547	2.1
34971	0.946132	2.1

34972	0.94261	2.1
34973	1.55831	1.88142
34974	1.07496	1.96959
34975	1.80327	1.08577
34976	1.34851	1.16275
34977	2.87824	1.28898
34978	2.14346	0.373327
34979	2.77937	0.498783
34980	2.79173	1.35845
34981	2.17129	0.3689
34982	4.95066	0.398245
34983	2.14346	1.15076
34984	1.34124	0.140089
34985	1.39818	0.29334
34986	2.1713	0.751734
34987	3.51253	0.725245
34988	2.79173	0.879974
34989	2.21882	0.347947
34990	2.17129	0.367156
34991	2.17129	0.491652
34992	2.81448	0.433285
34993	2.81448	0.52135
34994	1.10941	0.324282
34995	1.08565	0.389418
34996	0.699089	0.153925
34997	0.740176	0.281554
34998	0.908594	0.171256
34999	0.908594	0.27373
35000	0.91723	0.954516
35001	0.91723	0.158014
35002	1.10941	0.270531
35003	0.740176	0.264561
35004	0.699089	0.223163
35005	1.08565	0.243801
35006	1.89227	0.0662235
35007	1.89227	0.088741
35008	1.89227	0.064805
35009	1.89227	0.0561936
35010	3.78453	0.0754272
35011	2.40032	0.191671
35012	2.19526	0.0787332
35013	1.12907	0.0592149
35014	1.44253	0.13598
35015	3.32433	0.0758799
35016	2.19526	0.0674281
35017	2.71093	0.149709
35018	1.29826	0.0843268
35019	2.0815	0.0590056
35020	4.18911	0.102131
35021	1.89226	0.0699366
35022	1.89226	0.0828081
35023	1.89226	0.05
35024	1.89226	0.0538471
35025	3.78452	0.05
35026	2.30822	0.0603507
35027	1.89226	0.0530893
35028	2.79329	0.05
35029	2.79329	0.05

35030	1.89226	0.0789998
35031	1.89226	0.0951377
35032	0.946131	2.1
35033	0.946131	1.97703
35034	1.38414	2.1
35035	1.38414	1.93156
35036	0.94613	2.1
35037	0.94613	2.1
35038	1.38415	1.99331
35039	1.41833	1.14639
35040	0.946132	2.1
35041	1.41833	2.1
35042	1.38414	2.1
35043	0.946131	2.1
35044	1.89226	2.1
35045	2.6536	2.1
35046	1.89226	2.1
35047	2.72238	2.1
35048	4.61465	2.1
35049	0.946132	2.1
35050	0.946132	2.1
35051	1.38657	2.1
35052	1.38657	2.1
35053	1.38657	2.1
35054	1.36119	2.1
35055	0.946131	2.1
35056	0.946131	2.1
35057	0.946132	2.1
35058	1.38657	2.1
35059	0.946132	2.1
35060	1.36119	2.1
35061	1.89226	2.1
35062	2.4717	2.1
35063	2.5026	2.1
35064	2.4717	2.1
35065	1.92316	2.1
35066	1.89226	2.1
35067	1.89226	2.1
35068	1.89226	2.1
35069	1.89226	2.1
35070	1.89226	2.1
35071	3.78453	2.1
35072	0.946128	2.1
35073	0.946128	2.1
35074	1.3419	2.1
35075	1.3419	2.1
35076	0.946131	2.1
35077	0.946131	2.1
35078	1.36607	2.1
35079	1.3419	2.1
35080	0.946131	2.1
35081	1.36607	2.1
35082	1.3419	2.1
35083	0.94613	2.1
35084	1.89226	2.1
35085	2.50259	2.1
35086	2.53563	2.1
35087	2.50259	2.1

35088	1.92529	2.1
35089	1.89226	2.1
35090	0.946132	2.1
35091	0.946132	2.1
35092	0.946131	2.1
35093	0.946131	2.1
35094	0.946132	2.1
35095	0.946132	2.1
35096	0.946131	2.1
35097	0.946131	2.1
35098	0.946131	2.1
35099	0.946131	2.1
35100	0.946131	2.1
35101	0.946131	2.1
35102	2.0815	2.1
35103	1.86506	2.1
35104	1.86506	2.1
35105	1.82567	2.1
35106	2.0815	2.1
35107	2.03335	2.1
35108	2.0815	2.1
35109	2.0815	2.1
35110	2.0815	2.1
35111	1.88668	2.1
35112	1.83221	2.1
35113	1.57275	2.1
35114	2.34061	0.05
35115	1.89226	0.0506707
35116	2.23254	0.05
35117	1.89226	0.050508
35118	2.23254	0.05
35119	1.89226	0.05
35120	1.8619	0.05
35121	1.89226	0.05
35122	2.34061	0.05
35123	1.89226	0.05
35124	2.34061	0.05
35125	1.89226	0.05
35126	1.89227	0.05
35127	1.89227	0.05
35128	1.89227	0.05
35129	1.89227	0.05
35130	1.89227	0.05
35131	1.89227	0.05
35132	1.89227	0.05
35133	1.89227	0.05
35134	1.89227	0.05
35135	1.89227	0.05
35136	1.89227	0.05
35137	1.89227	0.05
35138	1.06365	0.0516416
35139	0.503305	0.05
35140	0.794912	0.05
35141	1.42752	0.05
35142	1.34826	0.0521241
35143	1.07163	0.052652
35144	0.533113	0.05
35145	0.761277	0.05

35146	0.848403	0.0522294
35147	0.876191	0.0572687
35148	0.963372	0.0525376
35149	0.963372	0.0529025
35150	1.55571	0.05
35151	2.76574	0.0501792
35152	2.92453	0.05
35153	1.70301	0.05
35154	2.33089	0.05
35155	1.06905	0.05
35156	2.28979	2.1
35157	1.81488	2.1
35158	1.63722	2.1
35159	2.22627	2.1
35160	4.04115	2.1
35161	1.98246	2.1
35162	2.15715	2.1
35163	2.22627	2.1
35164	2.22627	2.1
35165	1.63719	2.1
35166	1.63719	2.1
35167	2.1644	0.05
35168	1.903 0.05	
35169	1.23639	0.05
35170	1.42704	0.05
35171	3.61546	0.05
35172	1.89226	0.05
35173	1.89226	0.05
35174	1.89226	0.05
35175	1.89226	0.05
35176	3.78451	0.05
35177	2.61343	0.05
35178	1.89226	0.05
35179	2.08149	0.05
35180	1.74357	0.05
35181	2.08149	0.05
35182	1.74357	0.05
35183	1.63483	0.05
35184	1.8631	0.05
35185	1.8631	0.05
35186	1.70301	0.05
35187	2.0815	0.05
35188	1.94319	0.05
35189	2.0815	0.05
35190	1.89226	0.05
35191	1.89226	0.05
35192	2.05236	0.05
35193	2.23014	0.05
35194	2.41882	0.05
35195	2.14993	2.1
35196	1.87217	2.1
35197	2.34488	2.1
35198	2.5254	2.1
35199	4.39757	2.1
35200	0.949995	2.1
35201	0.630972	2.1
35202	0.936084	2.1
35203	1.17244	2.1

35204	0.922394	2.1
35205	0.922394	2.1
35206	0.88102	2.1
35207	0.88102	2.1
35208	0.949998	2.1
35209	1.17244	2.1
35210	0.936081	2.1
35211	0.630973	2.1
35212	1.89226	2.1
35213	1.89226	2.1
35214	1.89226	2.1
35215	1.89226	2.1
35216	3.78452	2.1
35217	1.89226	2.1
35218	1.89226	2.1
35219	1.89226	2.1
35220	1.89226	2.1
35221	1.89226	2.1
35222	1.89226	2.1
35223	2.34488	2.1
35224	2.5254	2.0931
35225	1.89226	2.09853
35226	1.89226	2.1
35227	4.41766	2.1
35228	1.89226	2.1
35229	1.89226	2.1
35230	1.89226	2.1
35231	1.89226	2.1
35232	3.78452	2.1
35233	1.89226	2.05503
35234	1.89226	2.1
35235	1.89226	2.06644
35236	1.89226	2.1
35237	1.89226	2.1
35238	1.89226	2.1
35239	1.5605	2.1
35240	1.15806	2.1
35241	1.31565	2.1
35242	1.65642	2.1
35243	2.87615	2.1
35244	1.46643	2.1
35245	1.65642	2.1
35246	1.31565	2.1
35247	1.31565	2.1
35248	1.74246	2.1
35249	1.74246	2.1
35250	1.89226	2.1
35251	1.89226	2.1
35252	1.89226	2.1
35253	1.89226	2.1
35254	1.89226	2.1
35255	1.89226	2.1
35256	1.15099	2.1
35257	1.15099	2.1
35258	0.939316	2.1
35259	0.939316	2.1
35260	0.946127	2.1
35261	0.946127	2.1

35262	1.14418	2.1
35263	0.939289	2.1
35264	1.14417	2.1
35265	0.946129	2.1
35266	0.939287	2.1
35267	0.94613	2.1
35268	1.89226	2.1
35269	1.89226	2.1
35270	1.89226	2.1
35271	1.89226	2.1
35272	3.78452	2.1
35273	1.93164	0.0530604
35274	2.64898	0.05
35275	1.43213	0.05
35276	1.43213	0.0590453
35277	2.63206	0.05
35278	2.13254	0.0539217
35279	1.89226	2.1
35280	1.89226	2.1
35281	1.89226	2.1
35282	1.89226	2.1
35283	3.78452	2.1
35284	2.71655	2.1
35285	1.43323	2.1
35286	1.28413	2.1
35287	2.53614	2.1
35288	3.96937	2.1
35289	1.89226	2.1
35290	1.89226	2.1
35291	1.89226	2.1
35292	1.89226	2.1
35293	1.89226	2.1
35294	1.89226	2.1
35295	1.33356	2.1
35296	2.82297	2.1
35297	2.771 2.1	
35298	2.771 2.1	
35299	1.46037	2.1
35300	1.46037	2.1
35301	2.56034	2.1
35302	1.18474	2.1
35303	1.15113	2.1
35304	2.53724	2.1
35305	3.72198	2.1
35306	2.56034	2.1
35307	1.89226	2.1
35308	1.89226	2.1
35309	2.53725	2.1
35310	4.42951	2.1
35311	1.89226	2.1
35312	1.89226	2.1
35313	1.89226	2.1
35314	1.89226	2.1
35315	3.78453	2.1
35316	1.89226	2.1
35317	1.89226	2.1
35318	1.89226	2.1
35319	1.89226	2.1

35320	3.78452	2.1
35321	1.1229	2.1
35322	2.53724	2.1
35323	1.15113	2.1
35324	1.15113	2.1
35325	2.51559	2.1
35326	2.51559	2.1
35327	1.89226	2.1
35328	2.53724	2.1
35329	2.51559	2.1
35330	2.53724	2.1
35331	1.87061	2.1
35332	1.89226	2.1
35333	1.89226	2.1
35334	1.89226	2.1
35335	1.89226	2.1
35336	1.89226	2.1
35337	3.78453	2.1
35338	1.89226	2.1
35339	1.89226	2.1
35340	1.89226	2.1
35341	1.89226	2.1
35342	3.78452	2.1
35343	1.98143	1.36536
35344	2.81559	0.283021
35345	1.65411	0.473222
35346	2.26675	1.10026
35347	4.24818	0.631178
35348	0.810656	2.1
35349	0.810656	2.1
35350	0.821888	2.1
35351	0.821888	2.1
35352	1.1235	2.1
35353	1.07439	2.1
35354	0.535693	2.1
35355	0.509042	2.1
35356	0.535693	2.1
35357	1.1235	2.1
35358	0.509043	2.1
35359	1.07439	2.1
35360	1.10378	2.1
35361	2.247 2.1	
35362	2.27472	2.1
35363	2.27472	2.1
35364	1.07139	2.1
35365	1.07139	2.1
35366	2.08081	2.1
35367	1.40395	2.1
35368	1.34245	2.1
35369	2.00239	2.1
35370	3.40634	2.1
35371	1.89226	2.1
35372	1.89226	2.1
35373	1.89226	2.1
35374	1.89226	2.1
35375	3.78453	2.1
35376	0.946128	2.1
35377	0.946128	2.1

35378	1.13785	2.1
35379	1.11496	2.1
35380	0.946131	2.1
35381	0.946131	2.1
35382	1.11495	2.1
35383	1.11495	2.1
35384	0.94613	2.1
35385	1.11495	2.1
35386	1.13785	2.1
35387	0.946131	2.1
35388	0.975888	2.1
35389	1.00118	2.1
35390	0.671218	2.1
35391	0.653229	2.1
35392	0.825372	2.1
35393	0.825372	2.1
35394	0.829038	2.1
35395	0.829038	2.1
35396	0.975901	2.1
35397	0.653238	2.1
35398	0.671209	2.1
35399	1.00117	2.1
35400	1.89224	2.1
35401	1.89224	2.1
35402	1.89224	2.1
35403	1.89224	2.1
35404	3.78448	2.1
35405	0.946115	2.1
35406	0.946115	2.1
35407	1.11494	2.1
35408	1.10111	2.1
35409	0.946118	2.1
35410	0.946118	2.1
35411	1.10111	2.1
35412	1.10111	2.1
35413	1.10112	2.1
35414	0.94613	2.1
35415	0.946105	2.1
35416	1.11492	2.1
35417	1.04028	0.549394
35418	2.27126	0.391806
35419	2.0815	0.338432
35420	1.82249	1.58608
35421	1.33723	0.408967
35422	1.18684	1.19628
35423	0.812616	0.0863558
35424	0.812616	0.0819867
35425	0.997777	0.0878706
35426	1.08122	0.0861875
35427	0.94613	0.0982651
35428	0.94613	0.0866525
35429	0.812648	0.0834783
35430	0.932779	0.0835707
35431	0.812648	0.0787812
35432	0.94613	0.0716157
35433	0.849335	0.0599613
35434	0.94613	0.0749665
35435	1.80327	1.35162

35436	1.56016	1.22349
35437	1.57731	1.8882
35438	1.34851	1.51727
35439	2.03207	1.58457
35440	1.80327	1.61722
35441	2.03207	1.66438
35442	1.56016	1.61812
35443	2.18076	1.80822
35444	1.78167	1.27233
35445	3.81374	1.59137
35446	1.37597	2.00704
35447	1.60058	2.09863
35448	1.70302	1.362
35449	1.85448	1.43325
35450	3.15028	1.47928
35451	1.60058	1.62211
35452	1.4054	2.1
35453	1.66202	1.2159
35454	1.70302	2.1
35455	3.09979	1.8235
35456	1.70302	1.75277
35457	1.85448	1.74019
35458	2.21317	1.71276
35459	2.0961	1.34994
35460	4.01757	1.53754
35461	1.66202	1.50535
35462	1.70302	2.1
35463	2.09611	0.751514
35464	2.00892	1.99773
35465	3.6468	1.57764
35466	1.2966	0.0513194
35467	2.25495	0.05
35468	1.2966	0.0578482
35469	2.21117	0.05
35470	1.67687	0.0510148
35471	2.6167	0.05
35472	1.89227	0.0757834
35473	1.89227	0.0550269
35474	1.89227	0.0649537
35475	1.89227	0.0989324
35476	3.78453	0.0661327
35477	2.44375	0.0554744
35478	1.89227	0.0678716
35479	2.81853	0.0658491
35480	2.81853	0.0649414
35481	1.89227	0.0580157
35482	1.89227	0.05
35483	1.89226	1.29888
35484	1.75853	1.38984
35485	2.37636	1.33538
35486	2.37636	1.47677
35487	1.89226	0.601741
35488	1.89226	1.36446
35489	1.65814	1.85678
35490	1.10187	2.1
35491	1.10187	2.1
35492	1.19547	2.1
35493	1.56263	1.99006

35494	1.65624	2.1
35495	1.56264	1.71379
35496	1.01479	2.1
35497	1.01479	2.1
35498	1.10187	2.1
35499	1.47561	1.85945
35500	1.5627	2.1
35501	1.89225	2.1
35502	1.89225	2.1
35503	1.89225	2.1
35504	1.89225	2.1
35505	3.7845	2.1
35506	2.54386	2.1
35507	1.91546	2.1
35508	2.46008	2.1
35509	1.82815	2.1
35510	4.37554	2.1
35511	1.91546	2.1
35512	1.04909	2.1
35513	1.82815	2.1
35514	0.983141	2.1
35515	2.8986	2.1
35516	1.89225	2.1
35517	1.89225	2.1
35518	1.89225	2.1
35519	1.89225	2.1
35520	3.78449	2.1
35521	1.89226	2.1
35522	1.89226	2.1
35523	1.89226	2.1
35524	1.89226	2.1
35525	1.89226	2.1
35526	1.89226	2.1
35527	2.46009	2.1
35528	1.7513	2.1
35529	1.82817	2.1
35530	1.82817	2.1
35531	2.38127	2.1
35532	2.38127	2.1
35533	2.55006	2.1
35534	2.64569	2.1
35535	2.60128	2.1
35536	2.49168	2.1
35537	5.13737	2.1
35538	2.51605	2.1
35539	2.64569	2.1
35540	2.60128	2.1
35541	2.48891	2.1
35542	5.1346	2.1
35543	2.60128	2.1
35544	2.49168	2.1
35545	2.41689	2.1
35546	2.55248	2.1
35547	5.04416	2.1
35548	2.60128	2.1
35549	2.48891	2.1
35550	2.45922	2.1
35551	2.55249	2.1

35552	5.04139	2.1
35553	1.89227	2.1
35554	1.89227	2.1
35555	1.89227	2.1
35556	1.89227	2.1
35557	1.89227	2.1
35558	1.89227	2.1
35559	0.96063	2.1
35560	0.96063	2.1
35561	0.958806	2.1
35562	0.958806	2.1
35563	1.28892	2.1
35564	0.749042	2.1
35565	1.17409	2.1
35566	0.630516	2.1
35567	1.17409	2.1
35568	1.28892	2.1
35569	0.630515	2.1
35570	0.749042	2.1
35571	0.92511	2.1
35572	0.92511	2.1
35573	1.19511	2.1
35574	1.19511	2.07079
35575	0.946132	2.1
35576	1.17409	2.1
35577	0.925081	2.1
35578	0.946129	2.1
35579	0.946131	2.1
35580	0.946131	2.1
35581	1.17409	2.1
35582	0.925083	2.1
35583	1.89226	1.6679
35584	1.89226	1.89054
35585	1.89226	1.65108
35586	1.89226	1.76347
35587	1.89226	1.69629
35588	1.89226	1.80239
35589	1.89226	1.79337
35590	1.89226	2.03585
35591	1.89226	1.89614
35592	1.89226	1.99752
35593	3.78453	1.92582
35594	0.946132	2.1
35595	0.946132	2.1
35596	1.28892	2.1
35597	1.28892	2.1
35598	0.946132	2.1
35599	1.28892	2.1
35600	0.946133	2.1
35601	1.17409	2.1
35602	0.946132	2.1
35603	0.946132	2.1
35604	1.17409	2.1
35605	1.28892	2.1
35606	0.625229	2.1
35607	0.890321	2.1
35608	1.21636	2.1
35609	1.13022	2.1

35610	1.07094	2.1
35611	1.03541	2.1
35612	0.942207	2.1
35613	0.942207	2.1
35614	0.898766	2.1
35615	1.26626	2.1
35616	1.0853	2.1
35617	0.600358	2.1
35618	0.719012	2.1
35619	0.719012	2.1
35620	1.17957	2.1
35621	1.17957	2.1
35622	0.719012	2.1
35623	0.952456	2.1
35624	0.946129	2.1
35625	0.946129	2.1
35626	0.946131	2.1
35627	0.952454	2.1
35628	0.946131	2.1
35629	0.719011	2.1
35630	0.94613	2.1
35631	0.94613	2.1
35632	0.94613	2.1
35633	0.94613	2.1
35634	0.946131	2.1
35635	0.946131	2.1
35636	0.946129	2.1
35637	0.946129	2.1
35638	0.94613	2.1
35639	0.94613	2.1
35640	0.94613	2.1
35641	0.94613	2.1
35642	1.1587	2.1
35643	0.973158	2.1
35644	0.750023	2.1
35645	0.536443	2.1
35646	0.84054	2.1
35647	0.84054	2.1
35648	0.854602	2.1
35649	0.854602	2.1
35650	1.1587	2.1
35651	0.750023	2.1
35652	0.973158	2.1
35653	0.536443	2.1
35654	1.89227	1.33945
35655	1.89227	2.1
35656	1.89227	2.1
35657	1.89227	1.88477
35658	3.78453	2.1
35659	1.89226	2.1
35660	1.89226	2.1
35661	1.89226	2.1
35662	1.89226	2.1
35663	3.78453	2.1
35664	1.7632	2.1
35665	1.61149	2.1
35666	1.98749	2.1
35667	2.11615	2.1

35668	3.75068	2.1
35669	1.7632	2.1
35670	1.61149	2.1
35671	1.97733	2.00108
35672	2.11615	2.1
35673	3.74052	2.1
35674	1.89226	2.1
35675	1.89226	1.73239
35676	1.89226	1.23784
35677	1.89226	2.1
35678	3.78453	2.04344
35679	1.89226	2.1
35680	1.89226	2.1
35681	1.89226	2.1
35682	1.89226	2.1
35683	3.78453	2.1
35684	1.98749	2.1
35685	2.11615	2.1
35686	2.43151	2.1
35687	2.27541	2.1
35688	4.41899	2.1
35689	1.97733	1.91221
35690	2.11615	2.1
35691	2.43151	2.1
35692	2.33986	1.99475
35693	4.40883	2.1
35694	2.17892	0.249062
35695	2.80057	1.80276
35696	1.88349	1.27019
35697	2.63062	0.479505
35698	4.80954	0.550949
35699	1.89226	2.1
35700	1.89226	0.90082
35701	1.89226	2.1
35702	1.89226	0.361843
35703	3.78452	1.73517
35704	2.0155	2.1
35705	1.97652	1.02327
35706	1.8247	2.06833
35707	1.80645	0.320159
35708	3.80122	0.860307
35709	1.97652	0.46565
35710	1.79681	1.51619
35711	1.80645	0.319744
35712	1.59244	0.555613
35713	3.56896	0.20802
35714	1.89226	0.603453
35715	1.89226	1.63689
35716	1.89226	0.292462
35717	1.89226	0.587469
35718	3.78452	0.140499
35719	1.89223	2.1
35720	1.89223	0.760017
35721	1.89223	2.1
35722	1.89223	0.326747
35723	3.78447	0.842333
35724	1.82467	2.07643
35725	1.80643	0.40846

35726	1.55159	2.0318
35727	1.53969	0.256314
35728	3.36437	1.02292
35729	1.80643	0.405934
35730	1.59242	1.40371
35731	1.5397	0.29134
35732	1.40538	0.766627
35733	3.13211	0.255463
35734	1.89224	0.526896
35735	1.89224	1.47012
35736	1.89224	0.332748
35737	1.89224	0.649994
35738	3.78447	0.295888
35739	1.37427	0.0607304
35740	2.37086	0.0756201
35741	2.37972	0.0607466
35742	1.94703	0.0796591
35743	2.19299	0.0638008
35744	1.91813	0.0833479
35745	1.07566	2.1
35746	1.12954	2.1
35747	0.946131	2.1
35748	0.946131	2.1
35749	1.12954	2.1
35750	1.12954	2.1
35751	0.946131	2.1
35752	0.946131	2.1
35753	1.12954	2.1
35754	0.946131	2.1
35755	0.946131	2.1
35756	1.07566	2.1
35757	2.25908	2.1
35758	1.89226	2.1
35759	1.89226	2.1
35760	1.89226	2.1
35761	2.37567	2.1
35762	2.37567	2.1
35763	1.37597	1.96244
35764	1.70302	1.49702
35765	1.24162	2.1
35766	1.12219	2.02653
35767	1.37072	2.1
35768	1.70302	2.1
35769	1.37072	2.1
35770	1.70302	1.79112
35771	1.54168	2.1
35772	2.21318	1.74299
35773	3.39238	2.1
35774	1.89226	0.05
35775	1.89226	0.05
35776	1.89226	0.05
35777	1.89226	0.05
35778	3.78452	0.05
35779	1.89227	0.05
35780	1.89227	0.05
35781	1.89227	0.05
35782	1.89227	0.05
35783	3.78453	0.05

35784	1.89226	0.05
35785	1.89226	0.05
35786	1.89226	0.05
35787	1.89226	0.05
35788	3.78453	0.05
35789	1.89226	0.05
35790	2.21654	0.05
35791	2.72007	0.05
35792	2.72007	0.05
35793	1.89226	0.05
35794	1.89226	0.05
35795	0.946128	0.05
35796	0.946128	0.05
35797	0.946132	0.05
35798	0.946132	0.05
35799	0.94613	0.05
35800	0.94613	0.05
35801	0.946131	0.05
35802	0.946131	0.05
35803	0.94613	0.05
35804	0.94613	0.05
35805	0.94613	0.05
35806	0.94613	0.05
35807	1.89226	0.05
35808	1.89226	0.05
35809	1.89226	0.05
35810	1.89226	0.05
35811	3.78453	0.05
35812	1.33457	0.05
35813	0.946131	0.05
35814	1.08045	0.05
35815	0.946132	0.05
35816	1.08045	0.05
35817	1.08045	0.05
35818	0.946132	0.05
35819	0.946132	0.05
35820	1.33457	0.05
35821	1.08045	0.05
35822	0.946132	0.05
35823	0.946132	0.05
35824	1.53347	1.56476
35825	1.13092	2.02928
35826	1.52938	1.8285
35827	1.93568	1.6
35828	3.06285	1.79178
35829	1.89226	0.715581
35830	2.72959	2.1
35831	2.82996	2.1
35832	2.82996	0.891536
35833	1.89226	2.1
35834	1.89226	0.661306
35835	2.7296	2.1
35836	2.57663	2.1
35837	2.48354	2.1
35838	2.48354	2.1
35839	2.82996	2.1
35840	2.82996	2.1
35841	2.63449	2.1

35842	2.7296	2.1
35843	2.39282	2.1
35844	2.39282	2.1
35845	2.48354	2.1
35846	2.72521	2.1
35847	1.89224	2.1
35848	1.89224	1.89224
35849	1.89224	1.86443
35850	1.89224	2.1
35851	3.78447	2.0054
35852	1.89226	2.1
35853	2.10425	2.1
35854	2.5142	2.1
35855	2.5142	2.1
35856	1.89226	2.1
35857	1.89226	2.1
35858	0.946133	1.7027
35859	0.946133	1.88892
35860	0.946128	2.1
35861	0.946128	2.1
35862	0.946131	2.1
35863	0.946131	1.70572
35864	0.94613	1.72979
35865	0.94613	2.1
35866	0.946131	2.08542
35867	0.946131	2.1
35868	0.946131	2.1
35869	0.946131	2.1
35870	1.89226	1.02992
35871	1.89226	1.42858
35872	1.89226	0.840233
35873	1.89226	1.40637
35874	1.89226	0.828028
35875	1.89226	1.42239
35876	1.94446	1.23679
35877	2.50167	1.54215
35878	1.28418	1.22947
35879	1.94446	1.47657
35880	2.05205	1.40659
35881	2.05205	1.47256
35882	1.27835	2.1
35883	1.08341	2.1
35884	0.946132	2.1
35885	0.946132	2.1
35886	0.851542	2.1
35887	0.851542	2.1
35888	0.977356	2.1
35889	1.18368	2.1
35890	1.18378	2.1
35891	0.851555	2.1
35892	0.977346	2.1
35893	0.851476	2.1
35894	1.07369	2.1
35895	2.5813	2.1
35896	1.95053	2.1
35897	1.95053	2.1
35898	1.90196	2.1
35899	1.90196	2.1

35900	1.89226	2.1
35901	1.89226	2.1
35902	1.89226	2.1
35903	1.89226	2.1
35904	3.78452	2.1
35905	1.89224	2.1
35906	1.89224	2.1
35907	1.89224	2.1
35908	1.89224	2.1
35909	1.89224	2.1
35910	1.89224	2.1
35911	1.97004	0.205619
35912	1.8072	1.74707
35913	1.5866	1.74869
35914	1.79478	0.444121
35915	3.60198	0.997986
35916	1.23267	2.1
35917	1.8072	1.61199
35918	1.58661	0.990897
35919	0.947921	2.1
35920	2.75512	2.09392
35921	1.89226	1.74243
35922	1.89226	2.1
35923	1.89226	2.00951
35924	1.89226	2.1
35925	1.89226	1.86342
35926	1.89226	2.1
35927	1.89226	1.84039
35928	1.89226	0.672393
35929	1.89226	0.583921
35930	1.89226	2.00535
35931	3.78452	1.37406
35932	1.4263	1.87998
35933	1.79478	0.512052
35934	1.67651	0.605816
35935	1.79478	1.90954
35936	1.46834	0.632244
35937	1.5866	1.56141
35938	1.89226	0.668523
35939	1.89226	2.02967
35940	1.89226	1.41634
35941	1.89226	1.72075
35942	1.89226	0.80184
35943	1.89226	1.89205
35944	2.23956	1.23845
35945	1.81847	1.35933
35946	2.10991	1.1972
35947	2.75317	1.15956
35948	4.34947	1.24292
35949	1.60578	0.101456
35950	1.94974	0.103025
35951	1.68062	0.139744
35952	1.14721	0.0927848
35953	3.2864	0.100224
35954	1.3946	0.05
35955	1.692	0.05
35956	2.0154	0.05
35957	2.0154	0.05

35958	1.11926	0.05
35959	1.11926	0.05
35960	1.90347	0.05
35961	1.83626	0.05
35962	1.84583	0.05
35963	1.89434	0.05
35964	3.79781	0.05
35965	1.89226	1.58896
35966	1.89226	1.42514
35967	1.89226	1.34278
35968	1.89226	1.50869
35969	3.78452	1.40898
35970	1.20986	1.59799
35971	1.20986	1.52622
35972	0.912635	1.41004
35973	0.912635	1.46833
35974	0.912667	1.35068
35975	1.17637	1.69241
35976	0.946125	1.55034
35977	0.946125	1.63933
35978	0.946128	1.56556
35979	0.912664	1.44745
35980	0.946127	1.66246
35981	1.17637	1.53175
35982	1.89226	0.05
35983	1.65573	0.05
35984	1.65573	0.05
35985	1.89226	0.05
35986	1.89226	0.05
35987	2.12879	0.05
35988	1.89226	0.050368
35989	1.89226	0.0526615
35990	1.89226	0.0621067
35991	1.89226	0.05
35992	1.89226	0.0600175
35993	1.89226	0.05
35994	2.40928	0.05
35995	1.89226	0.05
35996	1.95959	0.05
35997	1.70302	0.05
35998	3.47338	0.05
35999	1.89226	0.05
36000	1.89226	0.05
36001	1.89226	0.05
36002	1.89226	0.0585541
36003	3.78452	0.05
36004	1.64985	0.0816559
36005	2.47846	0.117391
36006	2.25405	0.0889639
36007	1.80768	0.0836266
36008	4.06173	0.080598
36009	1.98846	0.0647512
36010	0.912729	0.0721532
36011	1.05417	0.0602689
36012	1.70303	0.0777099
36013	2.82107	0.0765083
36014	2.47846	0.123836
36015	1.70303	0.128147

36016	2.86918	0.146483
36017	2.05973	0.143635
36018	2.51808	0.11485
36019	1.80768	0.112354
36020	0.946131	0.107935
36021	0.946131	0.0977366
36022	0.946132	0.101423
36023	0.946132	0.15987
36024	0.946131	0.173787
36025	0.946131	0.152931
36026	0.946133	0.103101
36027	0.946133	0.0953633
36028	0.946131	0.127844
36029	0.946131	0.16608
36030	0.946132	0.13967
36031	0.946132	0.148601
36032	0.946133	0.0754329
36033	0.946133	0.0745998
36034	0.946132	0.0977686
36035	0.946132	0.096657
36036	0.946133	0.102564
36037	0.946133	0.0993012
36038	0.946132	0.0748179
36039	0.946132	0.10771
36040	0.946132	0.0776622
36041	0.946132	0.104215
36042	0.946133	0.073569
36043	0.946133	0.0971746
36044	1.89227	0.175681
36045	1.89227	0.202814
36046	1.89227	0.191787
36047	1.89227	0.162517
36048	1.89227	0.230845
36049	1.89227	0.203385
36050	1.1656	2.1
36051	1.21847	2.1
36052	0.836006	2.1
36053	0.806007	2.1
36054	1.00653	2.1
36055	1.00653	2.1
36056	1.01794	2.1
36057	1.01794	2.1
36058	1.1656	2.1
36059	0.806007	2.1
36060	0.836006	2.1
36061	1.21847	2.1
36062	2.82263	2.1
36063	2.3312	2.1
36064	2.25936	2.1
36065	2.71441	2.1
36066	5.0456	2.1
36067	2.25936	2.1
36068	1.61201	2.1
36069	1.57627	2.1
36070	1.61201	2.1
36071	2.29545	2.1
36072	2.3312	2.1
36073	1.21269	0.05

36074	1.48867	0.05
36075	1.68264	0.05
36076	1.9898	0.05
36077	3.17131	0.05
36078	2.0487	2.1
36079	2.45212	2.1
36080	2.31012	2.1
36081	1.94095	2.1
36082	4.39307	2.1
36083	1.02435	2.1
36084	0.970469	2.1
36085	0.625375	2.1
36086	0.567166	2.1
36087	0.796854	2.1
36088	0.796854	2.1
36089	0.79466	2.1
36090	0.79466	2.1
36091	0.970471	2.1
36092	0.567165	2.1
36093	0.625374	2.1
36094	1.02435	2.1
36095	2.31012	2.1
36096	1.94095	2.1
36097	1.4825	2.1
36098	2.17384	2.1
36099	4.11479	2.1
36100	0.946187	2.1
36101	0.803763	2.1
36102	0.581357	2.1
36103	0.510356	2.1
36104	0.745632	2.1
36105	0.745632	2.1
36106	0.734566	2.1
36107	0.734563	2.1
36108	0.896142	2.1
36109	0.510366	2.1
36110	0.946173	2.1
36111	0.552965	2.1
36112	1.89226	1.38203
36113	1.89226	1.44545
36114	1.89226	1.46835
36115	1.89226	1.5713
36116	1.89226	1.36551
36117	1.89226	1.54836
36118	1.50743	1.43593
36119	1.0417	1.63775
36120	1.50743	1.48807
36121	1.62974	1.33432
36122	1.14605	1.32855
36123	1.14605	1.56828
36124	1.89226	1.29174
36125	1.89226	1.50403
36126	1.89226	1.23229
36127	1.89226	1.51537
36128	1.89226	1.26041
36129	1.89226	1.59167
36130	1.89226	1.60669
36131	1.89226	1.46494

36132	1.89226	1.37683
36133	1.89226	1.5125
36134	3.78453	1.45347
36135	1.77186	1.50587
36136	1.35668	1.53114
36137	1.70254	1.4238
36138	1.38756	1.53808
36139	1.77186	1.49137
36140	1.37814	1.64294
36141	0.734857	1.55904
36142	1.00549	1.4864
36143	0.835261	1.30904
36144	1.08357	1.40475
36145	0.97665	1.29849
36146	0.795559	1.95769
36147	0.891385	1.47301
36148	0.891385	1.25004
36149	0.874231	1.30868
36150	1.08356	1.31673
36151	0.734863	1.4323
36152	0.79449	1.45792
36153	1.89226	1.45497
36154	1.89226	0.127286
36155	2.42671	0.240089
36156	1.98783	1.44018
36157	4.31897	1.03763
36158	0.579854	1.20516
36159	0.965582	1.22579
36160	0.634403	1.33592
36161	1.17796	0.465135
36162	1.00592	0.179628
36163	0.797259	2.1
36164	0.905246	1.24491
36165	0.905246	0.453925
36166	0.695715	0.298922
36167	0.637106	0.276222
36168	1.24374	1.11759
36169	0.95617	0.735252
36170	2.0815	0.701025
36171	1.60521	1.63128
36172	1.29556	1.31048
36173	2.32205	1.59104
36174	1.28445	1.14029
36175	2.0815	1.54753
36176	2.3085	2.1
36177	2.21147	2.1
36178	2.3085	2.1
36179	2.39282	2.1
36180	1.98899	2.1
36181	2.0733	2.1
36182	2.3275	2.1
36183	1.63471	2.1
36184	1.98899	2.1
36185	1.56801	2.1
36186	3.62369	2.1
36187	2.3085	2.1
36188	1.98899	2.1
36189	2.22575	2.1

36190	1.90502	2.1
36191	4.21353	2.1
36192	1.90502	2.1
36193	1.56801	2.1
36194	1.90502	2.1
36195	1.98899	2.1
36196	1.49733	2.1
36197	1.58129	2.1
36198	1.89226	2.0287
36199	2.6491	2.1
36200	2.49266	2.1
36201	2.6491	2.07502
36202	1.73582	2.1
36203	1.89226	1.75077
36204	1.31854	2.1
36205	2.6491	2.1
36206	1.3909	2.1
36207	1.3909	2.1
36208	2.49266	2.1
36209	2.49266	2.1
36210	1.89226	1.91221
36211	2.49266	2.1
36212	2.38634	2.1
36213	2.49266	1.92978
36214	1.78595	2.1
36215	1.89226	1.68759
36216	1.23042	2.1
36217	2.49266	2.1
36218	1.31854	2.1
36219	1.31854	2.1
36220	2.38635	2.1
36221	2.38635	2.1
36222	2.83487	2.1
36223	1.89226	2.1
36224	2.594	2.1
36225	2.594	2.1
36226	1.89226	2.1
36227	1.89226	2.1
36228	1.89226	2.1
36229	1.89226	2.1
36230	1.89226	2.1
36231	1.89226	2.1
36232	3.78452	2.1
36233	1.09518	2.1
36234	1.28158	2.1
36235	1.36844	2.1
36236	1.59972	2.1
36237	2.65003	2.1
36238	1.89226	2.1
36239	1.89226	2.1
36240	1.89226	2.1
36241	1.89226	2.1
36242	3.78452	2.1
36243	1.36844	2.1
36244	1.59972	2.1
36245	1.67352	2.1
36246	1.94291	2.1
36247	3.31135	2.1

36248	1.52938	1.78654
36249	1.93568	1.63343
36250	2.36221	1.45928
36251	1.94723	1.9239
36252	3.89158	1.69218
36253	1.94724	1.65176
36254	1.26593	2.07588
36255	1.75367	1.94283
36256	2.4322	1.72608
36257	3.7009	1.88006
36258	2.36221	1.43522
36259	2.43219	1.78336
36260	1.94723	1.67764
36261	1.94723	1.56713
36262	2.8202	1.67559
36263	2.8202	1.55384
36264	2.51604	2.1
36265	2.18533	2.1
36266	2.17228	2.1
36267	2.4889	2.1
36268	4.67423	2.1
36269	2.17228	2.1
36270	1.62183	2.1
36271	1.62316	2.1
36272	1.62183	2.1
36273	2.18666	2.1
36274	2.18533	2.1
36275	2.17227	2.1
36276	2.4889	2.1
36277	2.45922	2.1
36278	2.15686	2.1
36279	4.64576	2.1
36280	2.17228	2.1
36281	1.62316	2.1
36282	1.62241	2.1
36283	2.15686	2.1
36284	3.78002	2.1
36285	1.89226	1.14985
36286	1.89226	1.2816
36287	1.89226	1.03366
36288	1.89226	1.05158
36289	3.78453	1.20042
36290	1.89226	1.13477
36291	1.89226	1.13918
36292	1.89226	1.07759
36293	1.89226	0.377039
36294	3.78452	1.06981
36295	1.89226	1.25104
36296	1.89226	0.747225
36297	1.89226	1.11245
36298	1.89226	1.12526
36299	1.89226	0.997426
36300	1.89226	1.031
36301	1.89226	1.00763
36302	1.89226	1.20634
36303	2.53413	0.273376
36304	1.89226	0.281702
36305	3.78453	0.793939

36306	1.38048	0.44855
36307	1.38048	0.505803
36308	0.490332	0.434684
36309	0.490332	0.395239
36310	0.924679	0.163823
36311	0.946131	1.37
36312	0.946131	1.22197
36313	0.490332	0.105479
36314	0.924678	0.185473
36315	0.490332	0.145145
36316	0.946131	0.564971
36317	0.946131	0.386652
36318	1.89227	0.231314
36319	1.89227	0.717789
36320	1.89227	0.379548
36321	1.89227	1.10669
36322	1.89227	0.252006
36323	1.89227	0.5819
36324	1.30803	2.1
36325	2.55151	2.1
36326	1.43935	2.1
36327	1.43935	2.1
36328	2.34922	2.1
36329	2.34922	2.1
36330	0.946131	2.1
36331	0.946131	2.1
36332	1.27575	2.1
36333	1.17461	2.1
36334	0.94613	2.1
36335	0.94613	2.1
36336	1.17461	2.1
36337	1.17461	2.1
36338	0.946131	2.1
36339	1.17461	2.1
36340	0.946132	2.1
36341	1.27575	2.1
36342	1.89226	2.1
36343	1.89226	2.1
36344	1.89226	2.1
36345	1.89226	2.1
36346	1.89226	2.1
36347	1.89226	2.1
36348	0.946133	2.1
36349	0.946133	2.1
36350	0.946132	2.1
36351	0.946132	2.1
36352	0.946133	2.1
36353	0.946133	2.1
36354	0.946132	2.1
36355	0.946132	2.1
36356	0.946132	2.1
36357	0.946132	2.1
36358	0.946133	2.1
36359	0.946133	2.1
36360	1.07043	2.1
36361	1.17461	2.1
36362	0.654016	2.1
36363	0.5829	2.1

36364	0.870481	2.1
36365	0.870481	2.1
36366	0.887027	2.1
36367	0.887027	2.1
36368	1.07043	2.1
36369	0.5829	2.1
36370	0.654016	2.1
36371	1.17461	2.1
36372	1.89226	2.1
36373	1.89226	2.1
36374	1.89226	2.1
36375	1.89226	2.1
36376	3.78453	2.1
36377	1.89226	2.1
36378	1.89226	2.1
36379	1.89226	2.1
36380	1.89226	2.1
36381	3.78453	2.1
36382	1.26929	2.1
36383	2.19313	2.1
36384	1.09847	2.1
36385	1.69916	2.1
36386	2.96844	2.1
36387	1.84124	2.1
36388	2.24937	2.1
36389	1.69915	2.1
36390	1.75691	2.1
36391	3.94852	2.1
36392	1.53082	2.1
36393	2.04422	2.1
36394	1.53082	2.1
36395	1.69915	2.1
36396	1.92911	2.1
36397	2.09744	2.1
36398	1.39263	1.00107
36399	1.0171	0.412251
36400	1.1211	0.668399
36401	1.46482	0.896518
36402	2.48192	0.791245
36403	1.39263	1.31164
36404	1.57962	1.87927
36405	1.65238	2.1
36406	1.46482	1.3268
36407	3.04443	1.49493
36408	1.89226	1.84958
36409	1.89226	0.643035
36410	1.89226	1.10901
36411	1.89226	1.54416
36412	3.78453	1.47916
36413	1.89227	0.407904
36414	1.89227	0.575816
36415	1.89227	0.834689
36416	1.89227	0.441247
36417	3.78453	0.491907
36418	1.28418	0.529373
36419	1.46482	0.701353
36420	1.1211	0.514747
36421	1.1211	0.867844

36422	1.59382	0.48835
36423	1.59382	0.864529
36424	1.76897	1.99397
36425	1.46482	0.884703
36426	1.59382	1.47395
36427	1.46482	1.80009
36428	1.78138	1.05846
36429	1.65238	1.47486
36430	1.89226	0.809263
36431	1.89226	1.09402
36432	1.89226	0.709655
36433	1.89226	1.38428
36434	1.89226	0.749132
36435	1.89226	1.66871
36436	1.89226	0.668125
36437	1.89226	0.367666
36438	1.89226	0.386297
36439	1.89226	0.514134
36440	1.89226	0.475016
36441	1.89226	0.571285
36442	1.96832	2.1
36443	2.40858	2.1
36444	1.75211	2.05383
36445	2.20797	2.1
36446	4.1607	2.1
36447	1.75211	2.1
36448	2.20797	2.1
36449	1.51592	2.1
36450	2.02064	2.1
36451	3.77275	2.1
36452	2.71793	0.614334
36453	2.67099	0.230252
36454	2.55105	0.337848
36455	2.71297	0.182085
36456	5.26898	0.35546
36457	2.36417	0.572206
36458	2.67098	0.292429
36459	2.55104	0.385356
36460	1.83418	0.207914
36461	4.91522	0.273289
36462	2.55105	0.269625
36463	2.71297	0.197037
36464	2.58032	0.217588
36465	2.28922	0.139952
36466	5.13137	0.180372
36467	2.55104	0.324
36468	2.23787	0.20318
36469	1.67265	0.209152
36470	2.28922	0.170391
36471	4.22369	0.233985
36472	0.851542	1.27156
36473	0.851542	1.26891
36474	0.981419	0.733513
36475	0.854774	0.881827
36476	0.949431	0.832993
36477	0.753763	0.901735
36478	0.946131	1.27838
36479	0.946131	0.979956

36480	1.04073	1.21663
36481	0.981433	0.644715
36482	0.851528	1.2856
36483	0.854828	0.944002
36484	1.89226	1.34043
36485	1.89226	1.288
36486	1.89226	1.2874
36487	1.89226	1.283
36488	1.89226	1.32411
36489	1.89226	1.31937
36490	0.814227	0.805792
36491	0.814227	0.938829
36492	0.946117	0.412436
36493	1.07285	0.420348
36494	0.879132	0.3974
36495	0.923293	0.402828
36496	0.946119	0.975233
36497	0.946119	0.625914
36498	0.946131	0.717846
36499	0.814236	0.334593
36500	0.946106	0.933145
36501	0.870405	0.48207
36502	1.89224	1.29093
36503	1.89224	1.17705
36504	1.89224	1.19406
36505	1.89224	1.2191
36506	1.89224	1.27269
36507	1.89224	1.272
36508	1.29388	1.70787
36509	1.04164	1.81455
36510	1.64834	1.84362
36511	1.82955	1.63421
36512	2.94222	1.79117
36513	1.4934	1.90283
36514	2.41522	1.6627
36515	2.20387	0.84539
36516	2.20387	2.1
36517	1.75765	1.516
36518	1.75765	2.1
36519	2.41522	1.72734
36520	2.77303	2.1
36521	2.20387	2.1
36522	2.58627	2.1
36523	5.00149	2.1
36524	1.22222	1.95038
36525	2.20387	2.09092
36526	1.96831	1.00332
36527	1.96831	2.1
36528	1.4934	1.60671
36529	1.4934	2.1
36530	1.96832	2.1
36531	2.58627	2.1
36532	2.40858	2.09908
36533	2.40858	2.1
36534	2.20387	2.1
36535	2.20387	2.1
36536	1.5605	2.1
36537	1.53775	2.1

36538	1.55398	2.1
36539	1.45806	2.1
36540	1.65642	2.1
36541	1.5605	2.1
36542	0.768876	2.1
36543	0.79033	2.1
36544	0.828211	2.1
36545	0.871233	2.1
36546	0.803843	2.1
36547	0.803843	2.1
36548	0.814698	2.1
36549	0.814698	2.1
36550	0.79033	2.1
36551	0.871233	2.1
36552	0.768876	2.1
36553	0.828212	2.1
36554	1.80252	2.1
36555	2.09715	2.09857
36556	1.9394	2.1
36557	1.50853	2.1
36558	3.60567	2.1
36559	1.96416	1.85385
36560	2.09715	2.02217
36561	1.88484	2.1
36562	1.57916	1.59761
36563	3.67631	2.01222
36564	1.89226	1.96149
36565	1.89226	0.387751
36566	1.89226	1.60137
36567	1.89226	1.69389
36568	3.78453	1.25474
36569	1.89226	2.02857
36570	1.89226	2.1
36571	1.89226	2.1
36572	1.89226	1.71635
36573	1.89226	2.1
36574	1.89226	0.935712
36575	1.5852	2.1
36576	1.50853	2.1
36577	1.2955	2.1
36578	1.44472	2.04032
36579	2.95325	2.1
36580	1.53065	2.1
36581	1.57916	0.958696
36582	1.44472	1.96309
36583	1.78861	1.92466
36584	1.23527	1.95835
36585	1.23527	1.87858
36586	1.89227	0.872214
36587	1.89227	1.39583
36588	1.89227	1.97614
36589	1.89227	0.861975
36590	3.78453	1.29356
36591	1.89226	2.07036
36592	1.89226	2.09929
36593	1.89226	2.1
36594	1.89226	1.9608
36595	1.89226	2.1

36596	1.89226	1.60966
36597	1.89227	1.2673
36598	1.89227	1.14504
36599	1.89227	1.12651
36600	1.89227	1.13929
36601	3.78453	1.12181
36602	0.943708	1.15073
36603	0.752219	1.04091
36604	0.946132	1.09876
36605	0.946132	1.10259
36606	0.946133	0.847539
36607	0.946133	1.3082
36608	0.752218	1.1889
36609	0.752218	1.15979
36610	0.752218	1.1183
36611	0.946132	1.08001
36612	0.946133	1.1723
36613	0.943708	1.1487
36614	0.943992	2.1
36615	0.943992	2.1
36616	1.23507	2.1
36617	1.48209	2.1
36618	1.0384	2.1
36619	1.47953	2.1
36620	0.946128	2.1
36621	0.946128	2.1
36622	0.946129	2.1
36623	1.23293	2.1
36624	1.0384	2.1
36625	0.946129	2.1
36626	1.89226	2.1
36627	2.46586	2.1
36628	2.80218	2.1
36629	2.80218	2.1
36630	1.89226	2.1
36631	1.89226	2.1
36632	1.32455	2.1
36633	1.89128	2.1
36634	1.32328	2.1
36635	1.97299	2.1
36636	3.29754	2.1
36637	1.89128	2.1
36638	2.4255	2.1
36639	1.973 2.1	
36640	2.63807	2.1
36641	4.3985	2.1
36642	1.34008	2.1
36643	1.97299	2.1
36644	2.10766	2.1
36645	2.10766	2.1
36646	1.32328	2.1
36647	1.32328	2.1
36648	2.41055	2.1
36649	2.37491	2.1
36650	2.40754	2.1
36651	2.38023	2.1
36652	4.78546	2.1
36653	2.23743	2.1

36654	2.37491	2.1
36655	2.40754	2.1
36656	2.20392	2.1
36657	4.61234	2.1
36658	2.37491	2.1
36659	2.02641	2.1
36660	2.20392	2.1
36661	2.18943	2.1
36662	4.40132	2.1
36663	2.37491	2.1
36664	2.22558	2.1
36665	2.38023	2.1
36666	2.18943	2.1
36667	4.60049	2.1
36668	1.89226	0.0812235
36669	1.89226	0.15003
36670	1.89226	0.109369
36671	1.89226	0.105422
36672	1.89226	0.104118
36673	1.89226	0.100477
36674	1.03599	0.0792162
36675	0.706698	0.0743992
36676	1.41141	0.0935323
36677	1.17573	0.0844887
36678	1.11957	0.0702545
36679	0.765034	0.0735356
36680	1.05913	0.0669612
36681	1.45054	0.0885951
36682	1.04847	0.0697072
36683	1.04847	0.0819736
36684	1.15323	0.0820894
36685	1.05825	0.0706896
36686	1.89226	0.0996755
36687	1.89226	0.107402
36688	1.89226	0.106441
36689	1.89226	0.0972194
36690	1.89226	0.107758
36691	1.89226	0.0992847
36692	2.22575	2.08389
36693	1.70677	2.1
36694	1.90157	2.03186
36695	1.5534	2.1
36696	3.77914	2.1
36697	1.70678	2.1
36698	0.968594	2.1
36699	0.968594	2.1
36700	1.10378	2.1
36701	1.5534	2.1
36702	1.68859	2.1
36703	2.31429	1.91201
36704	1.5534	2.1
36705	1.90103	2.06562
36706	1.41902	2.1
36707	3.45443	2.1
36708	2.53709	0.05
36709	1.209	0.05
36710	1.05417	0.05
36711	2.6513	0.05

36712	3.70547	0.05
36713	1.02675	0.05
36714	1.16384	0.05
36715	2.22062	0.05
36716	2.48862	0.05
36717	3.38446	0.05
36718	1.89226	0.05
36719	1.89226	0.05
36720	1.89226	0.05
36721	1.89226	0.05
36722	1.89226	0.05
36723	1.89226	0.05
36724	1.89226	1.85403
36725	1.89226	2.1
36726	1.89226	2.1
36727	1.89226	1.92376
36728	1.89226	2.1
36729	1.89226	1.91599
36730	1.89226	1.76127
36731	1.89226	1.99052
36732	1.89226	2.04932
36733	1.89226	1.80372
36734	1.89226	2.02716
36735	1.89226	1.77999
36736	1.89227	1.79081
36737	1.89227	2.00062
36738	1.75032	2.02591
36739	1.75032	1.81905
36740	1.89227	1.99489
36741	1.89227	1.79807
36742	1.89226	0.363375
36743	1.89226	0.514524
36744	1.89226	0.364918
36745	1.89226	0.40516
36746	1.89226	0.424288
36747	1.89226	0.456963
36748	0.946132	0.216178
36749	0.946132	0.210168
36750	0.798215	0.187624
36751	0.848163	0.179894
36752	0.848163	0.210344
36753	0.946131	0.219176
36754	0.946132	0.186003
36755	0.798216	0.178914
36756	0.996105	0.21141
36757	0.996105	0.17096
36758	0.79819	0.187505
36759	0.79819	0.234746
36760	1.07763	0.215852
36761	2.28622	0.200062
36762	2.16109	0.151167
36763	1.17506	0.164082
36764	3.36385	0.179753
36765	1.89226	2.1
36766	1.6663	2.1
36767	1.6663	2.1
36768	1.71706	2.1
36769	1.89226	2.1

36770	1.94302	2.1
36771	1.89226	2.1
36772	1.62801	2.1
36773	1.62801	2.1
36774	1.6663	2.1
36775	1.89226	2.1
36776	1.93055	2.1
36777	1.48734	2.1
36778	1.99609	2.1
36779	1.52245	2.1
36780	2.04137	2.1
36781	3.51854	2.1
36782	1.99609	2.1
36783	2.28563	2.1
36784	2.04136	2.1
36785	2.35019	2.1
36786	4.34628	2.1
36787	1.50714	2.1
36788	2.04137	2.1
36789	2.04945	2.1
36790	2.04945	2.1
36791	1.52245	2.1
36792	1.52245	2.1
36793	2.04136	2.1
36794	2.35019	2.1
36795	2.04945	2.1
36796	2.38681	2.1
36797	4.39964	2.1
36798	2.50924	0.05
36799	1.38305	2.1
36800	2.56918	2.1
36801	1.44535	2.1
36802	3.95222	2.1
36803	1.89226	2.1
36804	1.89226	2.1
36805	1.89226	2.1
36806	1.89226	2.1
36807	3.78453	2.1
36808	1.40615	2.1
36809	1.38851	2.1
36810	0.748607	2.1
36811	0.724468	2.1
36812	1.06362	2.1
36813	1.06362	2.1
36814	1.067 2.1	
36815	1.067 2.1	
36816	1.40615	2.1
36817	0.748607	2.1
36818	0.724468	2.1
36819	1.38851	2.1
36820	2.56918	2.1
36821	1.53377	2.1
36822	1.53706	2.1
36823	1.44535	2.1
36824	2.66088	2.1
36825	2.56918	2.1
36826	1.89226	2.1
36827	1.89226	2.1

36828	1.89226	2.1
36829	1.89226	2.1
36830	3.78453	2.1
36831	1.40239	2.1
36832	1.68466	2.1
36833	1.26091	2.1
36834	1.54641	2.1
36835	2.94881	2.1
36836	1.68466	2.1
36837	1.65324	2.1
36838	1.54641	2.1
36839	1.52268	2.1
36840	3.19965	2.1
36841	1.05776	2.1
36842	1.54641	2.1
36843	1.36595	2.1
36844	1.36595	2.1
36845	1.26091	2.1
36846	1.26091	2.1
36847	1.54641	2.1
36848	1.32697	2.08431
36849	1.52268	2.1
36850	1.52268	2.1
36851	1.36595	2.1
36852	1.36595	2.1
36853	1.18647	1.54264
36854	0.988454	2.06976
36855	1.4867	1.7482
36856	1.63892	1.44624
36857	2.67317	1.86791
36858	1.4867	1.89514
36859	1.63892	1.49563
36860	2.09605	1.38411
36861	1.97645	1.66982
36862	3.58275	1.50018
36863	1.29389	1.8144
36864	1.97645	1.54365
36865	1.4867	1.65265
36866	1.4867	1.81579
36867	1.82955	1.55969
36868	1.82955	1.74995
36869	1.13454	1.49165
36870	0.983594	2.1
36871	1.16809	2.1
36872	1.06836	2.03601
36873	1.08332	2.1
36874	0.983594	1.85516
36875	1.48751	2.1
36876	1.48047	1.8411
36877	1.77596	2.09649
36878	1.75506	1.99629
36879	3.25643	2.08705
36880	1.48047	1.75993
36881	1.25334	1.85857
36882	1.75505	1.91255
36883	1.55831	1.01561
36884	3.03878	1.71601
36885	1.77596	2.1

36886	1.75506	2.1
36887	2.00551	2.1
36888	2.00083	1.70325
36889	3.77679	2.1
36890	1.75506	2.07366
36891	1.80328	1.26614
36892	1.55831	1.82874
36893	1.55831	1.94926
36894	2.00083	1.53512
36895	2.00083	1.71754
36896	2.58627	2.1
36897	2.79047	2.1
36898	2.40858	2.1
36899	2.64748	2.1
36900	5.23375	2.1
36901	1.26755	2.1
36902	1.71717	2.1
36903	1.03824	2.1
36904	1.48901	2.1
36905	2.75656	2.1
36906	1.89226	2.1
36907	1.89226	2.1
36908	1.89226	2.1
36909	1.89226	2.1
36910	1.89226	2.1
36911	1.89226	2.1
36912	1.36541	2.1
36913	1.22156	2.1
36914	0.946132	2.1
36915	0.946132	2.1
36916	1.36541	2.1
36917	1.36541	2.1
36918	0.946132	2.1
36919	0.946132	2.1
36920	1.36541	2.1
36921	0.946133	2.1
36922	0.946132	2.1
36923	1.22156	2.1
36924	1.18901	2.1
36925	1.61149	2.1
36926	1.98748	2.1
36927	1.534 2.1	
36928	3.1765	2.1
36929	1.89226	2.1
36930	1.89226	2.1
36931	1.89226	2.1
36932	1.89226	2.1
36933	3.78452	2.1
36934	1.98748	2.1
36935	1.534 2.1	
36936	2.27541	2.1
36937	1.98748	2.1
36938	1.87436	2.1
36939	1.87436	2.1
36940	2.2988	2.1
36941	2.2604	2.1
36942	2.20831	2.1
36943	2.02802	2.1

36944	4.32683	2.1
36945	2.26041	2.1
36946	1.98957	2.1
36947	2.02803	2.1
36948	1.75021	2.1
36949	4.0176	2.1
36950	2.34607	2.1
36951	2.02803	2.1
36952	1.86795	2.1
36953	1.81448	2.1
36954	3.89597	2.1
36955	2.02803	2.1
36956	1.75021	2.1
36957	1.81448	2.1
36958	1.57887	2.1
36959	3.6069	2.1
36960	0.946131	1.68713
36961	0.946131	2.1
36962	1.1235	2.0156
36963	1.1235	2.1
36964	0.946131	1.82085
36965	0.946131	1.73001
36966	1.1235	2.1
36967	1.13736	2.1
36968	1.13736	2.1
36969	0.946131	2.1
36970	1.1235	2.1
36971	0.946131	2.1
36972	1.89226	2.09407
36973	1.89226	2.1
36974	1.89226	1.55784
36975	1.89226	0.673074
36976	3.78452	1.30487
36977	0.563266	2.1
36978	0.563266	1.76514
36979	0.565606	1.15132
36980	0.565606	2.1
36981	0.647086	2.09191
36982	0.624219	2.03711
36983	0.500035	1.64595
36984	0.481785	1.5742
36985	0.500034	0.660964
36986	0.647087	0.559824
36987	0.624219	1.85938
36988	0.481785	1.72377
36989	0.915788	0.20163
36990	0.915788	0.25237
36991	1.02732	0.606498
36992	1.02732	0.414096
36993	0.660831	0.545413
36994	1.11155	0.206632
36995	0.713295	0.244071
36996	1.26874	0.530383
36997	0.713295	0.350281
36998	0.660831	0.314088
36999	1.11155	0.33287
37000	1.26874	0.318023
37001	1.89226	1.17825

37002	1.85091	0.985388
37003	2.11193	1.43301
37004	1.85091	1.17352
37005	2.15328	0.807537
37006	1.89226	0.663877
37007	0.946131	0.449486
37008	0.946131	0.579764
37009	1.18275	0.573074
37010	1.05596	0.791782
37011	0.946127	0.61312
37012	0.946127	0.334098
37013	1.18275	0.639601
37014	1.18275	0.630045
37015	0.946128	0.584348
37016	1.18275	0.554433
37017	0.946129	0.568326
37018	1.05596	0.563451
37019	1.60936	2.1
37020	2.35846	2.1
37021	2.06424	2.1
37022	2.57651	2.1
37023	4.42269	2.1
37024	1.89226	2.1
37025	1.89226	2.1
37026	1.89226	2.1
37027	1.89226	2.1
37028	3.78453	2.1
37029	1.89226	2.1
37030	1.89226	2.1
37031	1.89226	2.1
37032	1.89226	2.1
37033	1.89226	2.1
37034	1.89226	2.1
37035	1.95053	2.1
37036	2.06424	2.1
37037	1.95053	2.1
37038	1.30584	2.1
37039	2.5	2.1
37040	1.8553	2.1
37041	2.49999	2.1
37042	2.57651	2.1
37043	2.49999	2.1
37044	2.06423	2.1
37045	2.80738	2.1
37046	2.37162	2.1
37047	1.89226	2.1
37048	1.89226	2.1
37049	1.89226	2.1
37050	1.89226	2.1
37051	1.89226	2.1
37052	1.89226	2.1
37053	1.49295	0.109965
37054	1.03824	0.11423
37055	1.51396	0.12926
37056	2.08514	0.126399
37057	3.12338	0.11802
37058	1.49798	0.0685089
37059	2.32017	0.059555

37060	2.68236	0.110437
37061	2.08514	0.0957155
37062	4.4053	0.0733399
37063	1.89226	0.0595402
37064	1.89226	0.0823162
37065	1.89226	0.0883984
37066	1.89226	0.0853476
37067	3.78452	0.0771996
37068	1.89226	0.125943
37069	1.89226	0.135842
37070	1.89226	0.125181
37071	1.89226	0.136249
37072	1.89226	0.118785
37073	1.89226	0.121379
37074	1.51396	0.077733
37075	2.08514	0.0744384
37076	2.55393	0.0917054
37077	2.02901	0.101494
37078	4.11415	0.0822033
37079	1.89226	0.051593
37080	1.89226	0.0504373
37081	1.89226	0.0719514
37082	1.89226	0.0842503
37083	3.78451	0.0542919
37084	1.89226	0.0742374
37085	1.89226	0.108555
37086	1.89226	0.0880203
37087	1.89226	0.0737384
37088	1.89226	0.096507
37089	1.89226	0.0865542
37090	0.946131	0.143263
37091	0.946131	0.164524
37092	0.946132	0.294352
37093	0.946132	0.189124
37094	0.946132	0.215625
37095	0.946132	0.171058
37096	0.946132	0.220063
37097	0.946132	0.276916
37098	0.946132	0.164883
37099	0.946132	0.21066
37100	0.946132	0.222769
37101	0.946132	0.187904
37102	1.89226	2.1
37103	1.89226	2.1
37104	1.89226	2.1
37105	1.89226	2.1
37106	3.78452	2.1
37107	1.28367	2.1
37108	1.82707	2.1
37109	1.11025	2.1
37110	1.6365	2.1
37111	2.92016	2.1
37112	1.82707	2.1
37113	1.8247	2.07688
37114	1.8247	2.1
37115	2.01549	2.1
37116	1.6365	2.1
37117	1.82729	2.1

37118	1.89226	2.1
37119	1.89226	2.09216
37120	1.89226	2.00757
37121	1.89226	2.1
37122	1.89226	2.06357
37123	1.89226	2.1
37124	1.63648	2.1
37125	1.82467	2.1
37126	1.35877	2.1
37127	1.55159	2.01238
37128	3.18345	2.1
37129	1.89223	2.1
37130	1.89223	2.1
37131	1.89223	2.1
37132	1.89223	2.1
37133	3.78447	2.1
37134	1.59972	2.1
37135	1.28037	2.1
37136	1.63702	2.1
37137	1.28158	2.1
37138	1.63581	2.1
37139	1.28037	2.1
37140	1.89226	2.1
37141	1.89226	2.1
37142	1.89226	2.1
37143	1.89226	2.1
37144	1.89226	2.1
37145	1.89226	2.1
37146	1.89226	2.1
37147	1.89226	2.1
37148	1.89226	2.1
37149	1.89226	2.1
37150	3.78451	2.1
37151	1.28037	2.1
37152	1.05071	2.1
37153	1.63581	2.1
37154	1.44287	2.1
37155	2.72324	2.1
37156	1.59972	2.1
37157	1.63581	2.1
37158	1.94291	2.1
37159	2.00118	2.1
37160	3.57872	2.1
37161	1.89226	2.1
37162	1.89226	2.1
37163	1.89226	2.1
37164	1.89226	2.1
37165	3.78452	2.1
37166	0.946131	2.1
37167	0.946131	2.1
37168	0.946127	2.1
37169	0.946127	2.1
37170	0.94613	2.1
37171	0.94613	2.1
37172	0.946128	2.1
37173	0.946128	2.1
37174	0.946128	2.1
37175	0.946128	2.1

37176	0.946129	2.1
37177	0.946129	2.1
37178	0.865056	2.1
37179	0.865056	2.1
37180	0.873224	2.1
37181	0.873224	2.1
37182	0.721437	2.1
37183	0.920378	2.1
37184	0.817903	2.1
37185	1.00059	2.1
37186	1.00059	2.1
37187	0.920376	2.1
37188	0.817904	2.1
37189	0.721437	2.1
37190	3.09157	2.1
37191	1.71255	2.1
37192	2.0815	2.1
37193	1.9918	2.1
37194	2.51364	2.1
37195	2.39396	2.1
37196	2.0815	2.1
37197	1.05051	2.1
37198	2.56724	2.1
37199	1.63991	2.1
37200	3.47929	2.1
37201	1.89226	0.097134
37202	2.2973	0.208135
37203	1.90705	0.174386
37204	1.90705	0.123472
37205	1.89226	0.144478
37206	1.89226	0.0992167
37207	1.89226	0.0924418
37208	1.90705	0.189005
37209	1.45321	0.18289
37210	1.45321	0.128376
37211	1.89226	0.145125
37212	1.89226	0.101002
37213	0.901146	2.1
37214	0.901146	2.1
37215	1.15227	2.1
37216	1.21279	2.1
37217	0.991265	2.1
37218	1.15003	2.1
37219	0.946132	2.1
37220	0.946132	2.1
37221	0.946132	2.1
37222	1.09997	2.1
37223	0.991266	2.1
37224	0.946132	2.1
37225	1.89226	2.1
37226	1.89226	2.1
37227	1.89226	2.1
37228	1.89226	2.1
37229	1.89226	2.1
37230	1.89226	2.1
37231	1.62229	2.1
37232	1.06053	2.1
37233	1.19698	2.1

37234	1.70106	2.1
37235	2.81928	2.1
37236	0.946132	2.1
37237	0.946132	2.1
37238	1.17527	2.1
37239	1.09997	2.1
37240	1.09997	2.1
37241	1.09997	2.1
37242	0.946133	2.1
37243	0.946133	2.1
37244	1.17527	2.1
37245	0.946132	2.1
37246	0.946133	2.1
37247	1.09997	2.1
37248	1.89226	2.1
37249	1.89226	2.1
37250	1.89226	2.1
37251	1.89226	2.1
37252	1.89226	2.1
37253	1.89226	2.1
37254	0.905416	2.1
37255	0.850527	2.1
37256	0.660601	2.1
37257	0.598494	2.1
37258	0.750137	2.1
37259	0.750137	2.1
37260	0.753774	2.1
37261	0.753774	2.1
37262	0.660599	2.1
37263	0.905418	2.1
37264	0.598493	2.1
37265	0.850529	2.1
37266	2.43509	2.1
37267	2.65066	2.1
37268	2.66602	2.1
37269	2.46339	2.1
37270	5.10111	2.1
37271	2.53008	2.1
37272	2.65066	2.1
37273	2.66602	2.1
37274	2.52852	2.1
37275	5.1961	2.1
37276	2.66602	2.1
37277	2.46339	2.1
37278	2.47932	2.1
37279	2.67393	2.1
37280	5.14535	2.1
37281	2.66602	2.1
37282	2.52853	2.1
37283	2.51686	2.1
37284	2.67393	2.1
37285	5.18288	2.1
37286	1.89226	2.1
37287	1.89226	0.637232
37288	1.89226	2.09339
37289	1.89226	0.489552
37290	3.78452	1.65131
37291	1.55161	2.1

37292	1.53972	0.571246
37293	1.32259	2.05879
37294	1.35082	0.303595
37295	2.8623	0.921757
37296	1.53972	0.503822
37297	1.4054	1.39758
37298	1.35083	0.315861
37299	1.24913	0.457504
37300	2.78885	0.363812
37301	1.89226	0.585746
37302	1.89226	1.58113
37303	1.89226	0.488143
37304	1.89226	0.437844
37305	3.78452	0.179894
37306	1.89226	2.06248
37307	1.89226	1.00708
37308	1.89226	2.1
37309	1.89226	0.721598
37310	1.89226	2.1
37311	1.89226	1.07548
37312	1.14599	2.05189
37313	1.35082	0.542372
37314	1.14599	1.4022
37315	1.32259	2.05147
37316	1.19993	0.409156
37317	1.37652	2.06899
37318	1.35083	0.393622
37319	1.24913	1.51707
37320	1.19993	0.58076
37321	1.11787	0.835344
37322	2.44906	0.30514
37323	1.89226	1.10078
37324	1.89226	1.51265
37325	1.89226	0.698266
37326	1.89226	0.404585
37327	3.78452	0.375831
37328	1.51697	0.05
37329	2.57264	0.0780135
37330	1.30203	0.05
37331	2.41701	0.05
37332	3.93398	0.05
37333	1.17464	0.05
37334	2.41701	0.05
37335	2.32462	0.05
37336	2.32462	0.05
37337	1.30203	0.05
37338	1.30203	0.05
37339	2.08149	2.1
37340	1.9839	2.1
37341	1.71398	2.1
37342	1.99182	2.1
37343	3.71121	2.1
37344	2.16061	2.1
37345	2.01399	0.850615
37346	1.99182	2.1
37347	1.83895	2.06103
37348	4.00581	2.09818
37349	1.90444	2.1

37350	1.99182	2.1
37351	1.71717	2.1
37352	1.805	2.1
37353	3.70899	2.1
37354	1.99182	2.1
37355	1.83896	1.54761
37356	1.805	2.1
37357	1.65243	1.60626
37358	3.64425	2.06991
37359	1.18076	2.1
37360	1.27697	2.1
37361	1.05025	2.1
37362	1.27697	2.1
37363	1.25	2.1
37364	1.47671	2.1
37365	1.89226	2.1
37366	1.89226	2.1
37367	1.89226	2.1
37368	1.89226	2.1
37369	3.78452	2.1
37370	2.64124	2.1
37371	1.89226	2.1
37372	1.89226	2.1
37373	1.89226	2.1
37374	2.46904	2.1
37375	2.46904	2.1
37376	1.89226	2.1
37377	1.89226	2.1
37378	2.7408	2.1
37379	2.82858	2.1
37380	4.72084	2.1
37381	1.87875	2.1
37382	1.37704	2.1
37383	1.77042	2.1
37384	2.27801	2.1
37385	3.64916	2.1
37386	1.77042	2.1
37387	2.27801	2.1
37388	2.58523	2.1
37389	1.90178	2.1
37390	4.35565	2.1
37391	1.28667	2.1
37392	1.30711	2.1
37393	1.53077	2.1
37394	1.55635	2.1
37395	2.81743	2.1
37396	1.55635	2.1
37397	1.28228	2.1
37398	1.64268	2.1
37399	1.93673	2.1
37400	3.19903	2.1
37401	1.53077	2.1
37402	1.93673	2.1
37403	1.55635	2.1
37404	1.55635	2.1
37405	1.86206	2.1
37406	1.86206	2.1
37407	1.64892	2.1

37408	1.60532	2.1
37409	1.85282	2.1
37410	1.96716	2.1
37411	3.61608	2.1
37412	1.60531	2.1
37413	1.32414	2.1
37414	1.96716	2.1
37415	1.75823	2.1
37416	3.2913	2.1
37417	1.91219	2.1
37418	1.96716	2.1
37419	2.0815	2.1
37420	2.51396	2.1
37421	4.30843	2.1
37422	1.96716	2.1
37423	2.17979	2.1
37424	1.75823	2.1
37425	1.75823	2.1
37426	2.28541	2.1
37427	2.28541	2.1
37428	1.89226	1.5585
37429	1.89226	1.58086
37430	1.89226	1.57442
37431	1.89226	1.4664
37432	3.78453	1.53956
37433	1.89226	1.62449
37434	1.89226	1.57606
37435	1.89226	1.6155
37436	1.89226	1.57366
37437	1.89226	1.60712
37438	1.89226	1.56599
37439	1.61016	1.57417
37440	1.45303	1.53955
37441	1.62812	1.54715
37442	1.86379	1.524
37443	3.23828	1.53141
37444	1.61016	1.57813
37445	1.78716	1.57549
37446	2.0815	1.46804
37447	1.6774	1.50338
37448	3.54454	1.53765
37449	1.89226	1.59374
37450	1.89226	1.51921
37451	1.89226	1.47694
37452	1.89226	1.50633
37453	3.78453	1.54047
37454	1.89226	1.6479
37455	1.89226	1.57236
37456	1.89226	1.65996
37457	1.89226	1.64541
37458	1.89226	1.55084
37459	1.89226	1.5059
37460	0.807591	1.61847
37461	0.814059	1.52373
37462	1.00217	1.64418
37463	0.931896	1.57558
37464	0.850586	1.60482
37465	0.850586	1.56129

37466	0.8889	1.55681
37467	0.8889	1.60509
37468	0.807592	1.45935
37469	1.00217	1.57828
37470	0.814061	1.58591
37471	0.931894	1.67626
37472	2.0815	1.52118
37473	1.6774	1.60563
37474	2.00435	1.51226
37475	2.36104	1.50286
37476	3.86809	1.49231
37477	1.00532	0.0869338
37478	1.00532	0.100383
37479	1.00183	0.121812
37480	1.00183	0.0939967
37481	1.26377	0.10062
37482	1.34921	0.0861069
37483	0.664872	0.101172
37484	0.743381	0.0955384
37485	0.664872	0.101148
37486	1.26377	0.110131
37487	1.34921	0.102327
37488	0.743381	0.106352
37489	1.26826	0.05
37490	0.946131	2.1
37491	1.02404	2.06073
37492	0.94613	2.1
37493	1.30281	2.09249
37494	0.930941	2.1
37495	0.930941	2.1
37496	1.318	2.09143
37497	1.58161	2.07361
37498	0.94613	2.1
37499	0.94613	2.0244
37500	1.56338	2.07625
37501	1.02404	2.06388
37502	2.81374	2.09527
37503	1.89226	2.1
37504	1.89226	2.1
37505	1.89226	2.1
37506	3.78453	2.1
37507	0.946133	2.1
37508	0.946133	2.1
37509	0.946132	2.1
37510	0.946132	2.1
37511	0.946132	2.1
37512	0.946132	2.1
37513	0.946133	2.1
37514	0.946133	2.1
37515	0.946132	2.1
37516	0.946132	2.1
37517	0.946133	2.1
37518	0.946133	2.1
37519	1.89226	1.87597
37520	1.89226	2.1
37521	1.89226	1.93448
37522	1.89226	2.02368
37523	3.78452	2.04084

37524	1.89226	2.1
37525	1.89226	2.08593
37526	1.89226	2.1
37527	1.89226	1.97286
37528	1.89226	2.1
37529	1.89226	1.97892
37530	0.946133	2.00744
37531	0.946133	2.1
37532	0.946132	2.00004
37533	0.946132	2.1
37534	0.946132	2.1
37535	0.946132	2.1
37536	0.946133	2.1
37537	0.946133	2.01284
37538	0.946132	2.1
37539	0.946132	2.04827
37540	0.946132	2.1
37541	0.946132	2.08331
37542	2.56953	2.1
37543	2.64148	2.1
37544	2.48631	2.1
37545	2.62213	2.1
37546	5.19166	2.1
37547	2.64148	2.1
37548	2.57929	2.1
37549	2.62213	2.1
37550	2.59522	2.1
37551	5.20142	2.1
37552	2.48631	2.1
37553	2.62213	2.1
37554	2.41176	2.1
37555	2.59262	2.1
37556	5.03389	2.1
37557	2.62213	2.1
37558	2.59522	2.1
37559	2.59262	2.1
37560	2.61901	2.1
37561	5.24114	2.1
37562	0.946132	0.05
37563	0.946132	0.05
37564	0.946133	0.05
37565	0.946133	0.05
37566	0.946132	0.05
37567	0.946132	0.05
37568	0.946133	0.05
37569	0.946133	0.05
37570	0.946132	0.05
37571	0.946132	0.05
37572	0.946133	0.05
37573	0.946133	0.05
37574	1.5681	0.05
37575	1.01248	0.05
37576	1.10749	0.05
37577	0.570913	0.05
37578	1.15589	0.05
37579	1.15588	0.05
37580	0.977549	0.05
37581	0.977549	0.05

37582	1.01249	0.05
37583	0.654445	0.05
37584	1.36795	0.05
37585	1.10748	0.05
37586	1.73883	0.05
37587	1.87638	0.05
37588	1.85219	0.05
37589	1.93113	2.1
37590	3.7407	0.05
37591	1.87638	0.05
37592	1.73883	2.1
37593	1.93113	2.1
37594	1.80426	2.1
37595	3.66996	2.1
37596	2.02912	0.134377
37597	1.44951	0.122355
37598	1.50103	0.102851
37599	2.02248	0.116653
37600	3.47199	0.10984
37601	1.89226	0.116745
37602	1.89226	0.137152
37603	1.89226	0.112652
37604	1.89226	0.109681
37605	3.78453	0.118403
37606	2.57881	0.112124
37607	1.89226	0.111211
37608	1.89226	0.114555
37609	1.89226	0.124091
37610	2.6428	0.10392
37611	2.6428	0.114645
37612	1.01379	0.116742
37613	0.985937	0.116243
37614	0.769295	0.109895
37615	0.715547	0.105168
37616	0.853042	0.110659
37617	0.853042	0.137673
37618	0.864117	0.11909
37619	0.864114	0.106649
37620	0.715556	0.112112
37621	1.03047	0.107749
37622	0.731721	0.121953
37623	0.985921	0.12063
37624	1.89227	0.129544
37625	1.89227	0.120409
37626	1.89227	0.102368
37627	1.89227	0.127547
37628	3.78453	0.114298
37629	0.946129	0.116584
37630	0.946129	0.11329
37631	1.28161	0.0983067
37632	1.3214	0.100764
37633	0.94613	0.103794
37634	0.94613	0.127624
37635	1.28161	0.104334
37636	1.28161	0.101676
37637	1.28161	0.0994062
37638	0.94613	0.10468
37639	0.946131	0.109353

37640	1.3214	0.105949
37641	0.946132	0.412911
37642	0.946132	0.378096
37643	0.706045	0.295339
37644	1.16718	0.256536
37645	0.706017	0.284374
37646	0.706017	0.739557
37647	1.4073	0.245149
37648	1.4073	0.221003
37649	0.94613	0.318127
37650	1.16719	0.288326
37651	0.706046	0.389529
37652	0.946131	0.32567
37653	1.89226	0.867662
37654	1.89226	0.581721
37655	1.89226	0.319733
37656	1.89226	1.31912
37657	1.89226	0.302757
37658	1.89226	1.22441
37659	0.946119	0.339818
37660	0.946119	0.334003
37661	0.946115	0.338131
37662	0.946115	0.256915
37663	0.946117	0.34684
37664	0.946117	0.89302
37665	0.946117	0.279609
37666	0.946117	0.233809
37667	0.94613	0.209557
37668	0.94613	0.360502
37669	0.946105	0.456427
37670	0.946105	0.281961
37671	0.946132	1.64176
37672	0.946132	1.55335
37673	0.946131	1.64925
37674	0.946131	1.30737
37675	0.946132	1.64764
37676	0.946132	1.66598
37677	0.946132	1.5314
37678	0.827865	1.49751
37679	0.946132	1.54083
37680	0.946132	1.60176
37681	0.946132	1.54524
37682	0.827865	1.55769
37683	1.89226	1.61631
37684	1.89226	1.5773
37685	1.89226	1.71327
37686	1.89226	1.50775
37687	1.89226	1.80367
37688	1.89226	1.62224
37689	0.93094	1.43066
37690	0.93094	1.44165
37691	1.04649	1.46197
37692	0.614468	1.36584
37693	0.950358	1.35607
37694	0.799012	1.1596
37695	0.682742	1.39076
37696	0.682742	1.44577
37697	1.04073	1.58151

37698	1.15596	1.5259
37699	0.614478	1.54627
37700	0.851524	1.43615
37701	2.22575	2.1
37702	1.90502	2.1
37703	2.10893	2.1
37704	1.80258	2.1
37705	4.01395	2.1
37706	1.90502	2.1
37707	1.40863	2.1
37708	1.49733	2.1
37709	1.49733	2.1
37710	1.80258	2.1
37711	1.80258	2.1
37712	2.10893	2.1
37713	1.68356	2.1
37714	1.80258	2.1
37715	1.80258	2.1
37716	1.98061	2.1
37717	1.98061	2.1
37718	1.80258	2.1
37719	1.40863	2.1
37720	1.68356	2.1
37721	1.30751	2.1
37722	3.11009	2.1
37723	1.35374	1.81086
37724	1.35374	1.74128
37725	0.746096	1.62803
37726	0.746096	1.56359
37727	1.1537	1.63926
37728	0.746095	1.7366
37729	0.946132	1.73362
37730	0.946132	1.59383
37731	0.946132	1.49377
37732	0.746095	1.59575
37733	0.946131	1.53122
37734	1.1537	1.58831
37735	2.15247	2.1
37736	1.70303	2.1
37737	1.41207	2.1
37738	1.43052	2.1
37739	3.22793	2.1
37740	0.940246	2.1
37741	0.728442	2.1
37742	0.650037	2.1
37743	0.539874	2.1
37744	0.694756	2.1
37745	0.694756	2.1
37746	0.776346	2.1
37747	0.656772	2.1
37748	0.733146	2.1
37749	0.563769	2.1
37750	0.66316	2.1
37751	0.899692	2.1
37752	1.21223	2.1
37753	1.22418	2.1
37754	1.43052	2.1
37755	1.43052	2.1

37756	1.24353	2.1
37757	1.46181	2.1
37758	1.89226	0.155204
37759	2.39894	0.0917463
37760	1.89226	0.165278
37761	2.37417	0.0963823
37762	1.89226	0.155514
37763	2.37417	0.0868913
37764	1.12767	0.0924125
37765	1.19947	0.0832053
37766	0.94613	0.224667
37767	0.94613	0.131868
37768	1.12766	0.0906913
37769	0.946128	0.0802129
37770	1.19947	0.11035
37771	0.946132	0.17548
37772	1.19947	0.187832
37773	1.19947	0.0614191
37774	0.94613	0.191564
37775	0.94613	0.0813829
37776	2.22621	0.05
37777	1.14533	2.1
37778	1.1131	2.1
37779	0.88238	2.1
37780	0.868521	2.1
37781	1.01153	2.1
37782	1.01153	2.1
37783	1.00232	2.1
37784	1.00232	2.1
37785	1.14533	2.1
37786	0.882378	2.1
37787	0.868519	2.1
37788	1.11311	2.1
37789	0.910183	2.1
37790	1.07811	1.83425
37791	0.94613	0.543963
37792	0.94613	1.11645
37793	0.910212	1.15972
37794	0.910212	2.1
37795	1.11403	0.654977
37796	1.11403	1.96833
37797	0.910183	1.63099
37798	0.94613	1.8434
37799	0.94613	1.80135
37800	1.07811	1.73778
37801	1.87228	2.1
37802	2.81189	2.1
37803	1.6197	2.1
37804	2.56716	2.1
37805	4.43944	2.1
37806	0.946132	2.1
37807	0.946132	2.1
37808	0.946132	1.04383
37809	0.946132	1.95574
37810	0.946131	2.03049
37811	0.946131	2.1
37812	0.946132	1.20077
37813	0.946132	2.1

37814	0.946132	2.1
37815	0.946132	2.1
37816	0.946132	2.1
37817	0.946132	2.1
37818	1.28724	2.1
37819	2.56716	2.1
37820	2.3014	2.1
37821	2.3014	2.1
37822	1.6197	2.1
37823	1.6197	2.1
37824	0.946132	2.1
37825	0.946132	2.1
37826	0.946132	1.2919
37827	0.946132	2.1
37828	0.946132	2.08129
37829	0.946132	2.1
37830	0.946133	2.1
37831	0.946133	1.77416
37832	0.946133	2.1
37833	0.946133	2.09436
37834	0.946132	2.1
37835	0.946132	2.1
37836	1.08039	2.1
37837	1.24445	2.1
37838	1.2873	2.1
37839	1.48751	2.1
37840	2.53175	2.1
37841	0.946125	1.9364
37842	0.946125	2.1
37843	1.12552	2.1
37844	1.12552	2.1
37845	0.946124	2.1
37846	0.946124	2.1
37847	1.12552	2.1
37848	1.11114	2.1
37849	1.11113	2.1
37850	0.946118	2.1
37851	1.12552	2.1
37852	0.946131	2.1
37853	1.89225	1.88695
37854	1.89225	1.73014
37855	1.89225	2.1
37856	1.89225	2.1
37857	1.89225	1.55277
37858	1.89225	2.1
37859	0.987703	1.84426
37860	1.33078	2.01144
37861	1.31565	1.86541
37862	1.31565	1.88619
37863	1.01121	1.88603
37864	1.01121	2.1
37865	2.19163	2.1
37866	1.89226	2.1
37867	1.89226	2.1
37868	1.89226	2.1
37869	2.22229	2.1
37870	2.22229	2.1
37871	1.89226	2.1

37872	1.89226	1.10136
37873	1.89226	0.685387
37874	1.89226	2.1
37875	1.89226	1.52169
37876	1.89226	2.1
37877	0.57141	2.1
37878	0.57141	1.6646
37879	0.574036	1.98195
37880	0.574036	2.1
37881	0.651591	1.98313
37882	0.657827	1.70327
37883	0.482391	2.06578
37884	0.493855	2.1
37885	0.482391	1.53636
37886	0.651591	1.5249
37887	0.493855	1.97866
37888	0.657828	1.96537
37889	1.88428	2.1
37890	2.03975	2.1
37891	1.97316	2.1
37892	1.8266	2.1
37893	3.86635	2.1
37894	2.23053	2.1
37895	1.42255	2.1
37896	1.3868	2.1
37897	1.8266	2.1
37898	3.24915	2.1
37899	1.97316	2.1
37900	1.8266	2.1
37901	1.77128	2.1
37902	1.91767	2.1
37903	3.74427	2.1
37904	1.77128	2.1
37905	1.8266	2.1
37906	1.77942	2.1
37907	1.33962	2.1
37908	1.3868	2.1
37909	1.3868	2.1
37910	0.944111	2.1
37911	0.944111	2.1
37912	1.26036	2.1
37913	1.16968	2.1
37914	1.03853	2.1
37915	1.16646	2.1
37916	0.946129	2.1
37917	0.946129	2.1
37918	0.94613	2.1
37919	1.25768	2.1
37920	1.03853	2.1
37921	0.946131	2.1
37922	1.82441	0.05
37923	1.33061	0.05
37924	1.13272	0.05
37925	1.60249	0.05
37926	2.95712	0.05
37927	1.82441	0.05
37928	2.08757	0.05
37929	1.81292	0.05

37930	1.60249	0.05
37931	3.63733	0.05
37932	1.81292	0.05
37933	1.332 0.05	
37934	1.60249	0.05
37935	1.60249	0.05
37936	1.5344	0.05
37937	1.5344	0.05
37938	1.10107	2.1
37939	2.51559	2.1
37940	1.1229	2.1
37941	1.1229	2.1
37942	2.49456	2.1
37943	2.49456	2.1
37944	1.89226	2.1
37945	2.51559	2.1
37946	2.49457	2.1
37947	2.51559	2.1
37948	1.87124	2.1
37949	1.89226	2.1
37950	1.89226	2.1
37951	1.89226	2.1
37952	1.89226	2.1
37953	1.89226	2.1
37954	1.89226	2.1
37955	1.89226	2.1
37956	1.89226	2.1
37957	1.89226	2.1
37958	1.89226	2.1
37959	1.89226	2.1
37960	1.89226	2.1
37961	1.89226	2.1
37962	1.23599	2.1
37963	1.24728	2.1
37964	0.550534	2.1
37965	0.541151	2.1
37966	0.893754	2.1
37967	0.893754	2.1
37968	0.894678	2.1
37969	0.894678	2.1
37970	1.23599	2.1
37971	0.541149	2.1
37972	0.550533	2.1
37973	1.24728	2.1
37974	0.946132	2.1
37975	0.946132	2.1
37976	1.23599	2.1
37977	1.24728	2.1
37978	0.946132	2.1
37979	0.946132	2.1
37980	1.23599	2.1
37981	1.23599	2.1
37982	1.23599	2.1
37983	0.946132	2.1
37984	0.946132	2.1
37985	1.24728	2.1
37986	1.89226	2.1
37987	1.89226	2.1

37988	1.89226	2.1
37989	1.89226	2.1
37990	1.89226	2.1
37991	1.89226	2.1
37992	0.946128	2.1
37993	0.946128	2.1
37994	0.946132	2.1
37995	0.946132	2.1
37996	0.94613	2.1
37997	0.94613	2.1
37998	0.946129	2.1
37999	0.946129	2.1
38000	0.94613	2.1
38001	0.94613	2.1
38002	0.94613	2.1
38003	0.94613	2.1
38004	2.58696	0.0781849
38005	2.57073	0.137717
38006	2.12585	0.0798772
38007	2.07607	0.0937677
38008	4.69658	0.0873783
38009	1.89226	0.141184
38010	1.89226	0.1619
38011	1.89226	0.0805502
38012	1.89226	0.175546
38013	3.78452	0.107183
38014	1.89226	0.157528
38015	1.89226	0.283414
38016	1.89226	0.184385
38017	1.89226	0.122108
38018	3.78453	0.161104
38019	2.57074	0.137373
38020	2.32635	0.127291
38021	2.07608	0.123432
38022	1.73623	0.120673
38023	4.30697	0.110946
38024	2.12582	0.0857336
38025	1.51971	0.0821636
38026	1.57255	0.0762298
38027	2.07604	0.0979955
38028	1.62233	0.0716001
38029	2.12582	0.0927727
38030	1.89223	0.0864429
38031	1.89223	0.158882
38032	1.89223	0.103479
38033	1.89223	0.11755
38034	1.89223	0.105219
38035	1.89223	0.121827
38036	0.946119	0.162966
38037	0.946119	0.152613
38038	0.946119	0.105677
38039	0.946119	0.139328
38040	0.946119	0.137595
38041	0.946119	0.167842
38042	0.94612	0.158123
38043	0.94612	0.100872
38044	0.946132	0.139793
38045	0.946132	0.140888

38046	0.946107	0.143335
38047	0.946107	0.141068
38048	0.811456	0.112515
38049	0.811456	0.135222
38050	0.806521	0.081867
38051	0.806521	0.118741
38052	0.868104	0.116824
38053	0.579949	0.138666
38054	0.759859	0.0909935
38055	1.03802	0.105316
38056	0.579957	0.11147
38057	0.759868	0.109048
38058	1.03801	0.112097
38059	0.868093	0.113341
38060	2.7408	2.1
38061	2.82858	2.1
38062	2.57368	2.1
38063	2.52633	2.1
38064	5.3549	2.1
38065	2.7408	2.1
38066	2.31353	2.1
38067	2.14063	2.1
38068	2.52632	2.1
38069	4.83985	2.1
38070	2.57368	2.1
38071	2.52633	2.1
38072	2.3301	2.1
38073	2.36071	2.1
38074	4.88704	2.1
38075	1.86512	2.1
38076	2.52632	2.1
38077	2.14063	2.1
38078	2.14063	2.1
38079	2.3301	2.1
38080	2.3301	2.1
38081	1.86431	2.1
38082	1.89227	2.1
38083	1.68992	2.1
38084	1.68992	2.1
38085	1.89227	2.1
38086	1.89227	2.1
38087	2.0815	2.1
38088	1.89226	2.1
38089	2.04022	2.1
38090	1.86431	2.1
38091	2.07081	2.1
38092	1.89226	2.1
38093	1.91686	2.05009
38094	1.65683	2.1
38095	1.86871	2.1
38096	1.65683	2.1
38097	2.13075	2.1
38098	1.89226	1.80268
38099	1.89226	2.1
38100	1.89226	1.80662
38101	1.89226	1.94693
38102	1.89226	2.1
38103	3.78452	2.06616

38104	0.851541	2.1
38105	0.851541	2.1
38106	0.952803	2.1
38107	0.851573	2.1
38108	0.946129	2.1
38109	0.946129	2.1
38110	0.929184	2.1
38111	0.757482	2.1
38112	0.836274	2.1
38113	0.851528	2.1
38114	0.952816	2.1
38115	1.04073	2.1
38116	1.70303	1.8215
38117	1.70302	2.02906
38118	1.5544	2.1
38119	1.70302	1.89269
38120	1.74957	1.97108
38121	1.91686	1.64447
38122	1.89226	1.78482
38123	1.89226	2.08323
38124	1.89226	2.1
38125	1.89226	1.89148
38126	1.89226	1.95344
38127	1.89226	1.6206
38128	1.89226	2.1
38129	1.89226	2.1
38130	2.03472	2.1
38131	1.89226	2.1
38132	1.99827	2.1
38133	1.85836	2.1
38134	1.00509	2.1
38135	1.55998	2.1
38136	1.83347	2.1
38137	1.45246	2.1
38138	3.01244	2.1
38139	1.23839	2.1
38140	0.884251	2.1
38141	0.946133	2.1
38142	0.946133	2.1
38143	0.884226	2.1
38144	0.884226	2.1
38145	0.946158	2.1
38146	0.946159	2.1
38147	1.23839	2.1
38148	0.946131	2.1
38149	0.946131	2.1
38150	0.884254	2.1
38151	1.89226	2.1
38152	1.89226	2.1
38153	1.89226	2.1
38154	1.89226	2.1
38155	1.89226	2.1
38156	1.89226	2.1
38157	2.1499	2.1
38158	1.45244	2.1
38159	1.83345	2.1
38160	1.83345	2.1
38161	1.87214	2.1

38162	1.87214	2.1
38163	1.89224	2.1
38164	1.89224	2.1
38165	1.89224	2.1
38166	1.89224	2.1
38167	3.78448	2.1
38168	1.49155	2.00414
38169	2.0815	2.1
38170	1.77515	2.01365
38171	1.72377	2.1
38172	3.50659	1.96069
38173	1.69876	0.412167
38174	2.0815	2.1
38175	1.9133	1.65868
38176	1.68189	0.791541
38177	2.17628	2.09824
38178	1.94335	2.1
38179	2.65129	0.05
38180	1.15317	0.05
38181	2.57085	0.05
38182	1.209	0.05
38183	2.57085	0.05
38184	1.209	0.05
38185	1.89226	0.05
38186	1.89226	0.05
38187	1.89226	0.05
38188	1.89226	0.05
38189	3.78452	0.05
38190	2.48861	0.05
38191	1.15252	0.05
38192	2.60515	0.05
38193	2.60515	0.05
38194	1.16384	0.05
38195	1.16384	0.05
38196	1.59903	0.672316
38197	1.26865	0.948687
38198	1.30486	1.77052
38199	1.70302	1.32589
38200	2.98573	1.51254
38201	1.59903	0.403489
38202	1.79478	1.43618
38203	1.79478	1.38778
38204	1.97005	0.296528
38205	1.50365	1.19722
38206	1.64192	1.34722
38207	1.89226	0.301244
38208	1.89226	1.00846
38209	1.89226	1.44457
38210	1.89226	0.626007
38211	3.78453	0.288954
38212	1.89226	1.57531
38213	1.89226	1.06194
38214	1.89226	1.60211
38215	1.89226	0.418827
38216	1.89226	1.50662
38217	1.89226	1.3417
38218	0.761672	0.281556
38219	0.896406	0.439748

38220	0.603509	0.656597
38221	0.574071	0.970273
38222	0.695159	1.49167
38223	0.695159	0.218274
38224	0.762564	1.4462
38225	0.770159	0.613326
38226	0.803021	1.34709
38227	0.572529	1.36087
38228	0.696466	0.45231
38229	0.899733	0.427876
38230	1.66034	0.161702
38231	1.79478	1.08474
38232	1.56685	0.434898
38233	1.68512	1.58785
38234	1.67652	0.997169
38235	1.67652	0.913085
38236	1.89226	1.3651
38237	1.89226	1.08817
38238	1.89226	1.24023
38239	1.89226	0.979566
38240	1.89226	1.25656
38241	1.89226	0.948454
38242	0.946133	0.297082
38243	0.946133	0.389035
38244	0.946132	1.12066
38245	0.946132	1.06155
38246	0.946132	1.35232
38247	0.946132	0.152588
38248	0.946133	1.69023
38249	0.946133	0.580186
38250	0.946133	1.26788
38251	0.946133	1.2993
38252	0.946132	0.321153
38253	0.946132	0.388471
38254	1.80424	2.1
38255	1.45158	2.1
38256	1.9098	2.1
38257	1.56003	2.1
38258	3.36138	2.1
38259	1.90982	2.1
38260	1.71606	2.1
38261	2.15314	2.1
38262	1.70302	2.1
38263	3.89051	2.1
38264	1.96416	1.83847
38265	1.23706	1.22989
38266	1.00053	1.20672
38267	1.38287	0.432487
38268	1.57916	0.814613
38269	1.9615	1.07764
38270	0.827865	0.310863
38271	0.946132	0.210845
38272	0.946132	0.496734
38273	0.946132	0.516815
38274	0.946132	1.19218
38275	0.946132	0.336296
38276	0.946131	1.24104
38277	0.946131	0.578133

38278	0.827865	1.32738
38279	0.946132	0.335306
38280	0.946132	0.280378
38281	0.946132	0.231001
38282	1.89227	2.1
38283	1.89227	2.1
38284	1.89227	2.1
38285	1.89227	2.1
38286	3.78453	2.1
38287	2.3819	2.1
38288	1.58089	2.1
38289	1.58089	2.1
38290	1.6287	2.1
38291	2.33368	2.1
38292	2.3815	2.1
38293	0.946133	2.1
38294	0.946133	2.1
38295	0.946132	2.1
38296	0.946132	2.1
38297	0.946132	2.1
38298	0.946132	2.1
38299	0.946132	2.1
38300	0.946132	2.1
38301	0.946133	2.1
38302	0.946133	2.1
38303	0.946132	2.1
38304	0.946132	2.1
38305	1.89226	2.1
38306	1.89226	2.1
38307	1.89226	2.1
38308	1.89226	2.1
38309	3.78453	2.1
38310	2.7874	2.1
38311	2.33368	2.1
38312	2.71897	2.1
38313	2.27495	2.1
38314	5.05266	2.1
38315	0.970991	2.1
38316	0.970991	2.1
38317	0.956929	2.1
38318	0.956929	2.1
38319	0.732928	2.1
38320	0.790444	2.1
38321	1.16684	2.1
38322	1.13748	2.1
38323	1.13748	2.1
38324	0.732928	2.1
38325	0.790444	2.1
38326	1.16684	2.1
38327	0.946132	2.1
38328	0.946132	2.1
38329	0.946131	2.1
38330	0.946131	2.1
38331	0.946132	2.1
38332	0.946132	2.1
38333	0.946132	2.1
38334	0.946132	2.1
38335	0.946132	2.1

38336	0.946132	2.1
38337	0.946132	2.1
38338	0.946132	2.1
38339	1.10747	0.200453
38340	2.30331	0.338627
38341	1.47954	0.148638
38342	2.63789	0.161032
38343	3.78285	0.212853
38344	1.65215	0.0956081
38345	2.63789	0.136377
38346	2.82465	0.131301
38347	2.82465	0.145465
38348	1.47954	0.101958
38349	1.47954	0.120334
38350	1.13456	2.04487
38351	1.08334	2.1
38352	1.20674	1.23963
38353	1.17885	2.1
38354	2.29008	1.9721
38355	1.20674	1.73003
38356	1.17885	2.1
38357	1.28649	1.37781
38358	1.28516	1.98442
38359	2.4919	1.84011
38360	1.89226	2.1
38361	1.89226	2.1
38362	1.89226	2.1
38363	1.89226	2.09845
38364	1.89226	2.1
38365	1.89226	2.0835
38366	2.50288	1.64784
38367	2.02115	1.82657
38368	2.7717	2.03189
38369	2.29107	1.27557
38370	4.79395	1.77079
38371	1.27339	2.1
38372	1.10817	2.1
38373	1.19733	2.1
38374	1.032	2.1
38375	2.30539	2.1
38376	1.19733	2.1
38377	0.953637	2.1
38378	0.953637	2.1
38379	1.032	2.1
38380	1.11868	2.1
38381	1.19704	2.1
38382	2.01024	1.01627
38383	1.11938	1.0299
38384	1.9186	1.33303
38385	2.76298	0.969387
38386	3.92884	0.894817
38387	0.851541	0.133081
38388	0.851541	0.113026
38389	0.957577	0.105311
38390	0.848952	0.109638
38391	1.08043	0.133491
38392	0.851541	0.140916
38393	0.851484	0.111254

38394	0.957578	0.095421
38395	1.20045	0.127914
38396	0.726289	0.0935847
38397	0.946131	0.110644
38398	0.946131	0.12895
38399	1.50524	2.1
38400	2.38363	2.1
38401	2.49018	2.1
38402	2.49018	2.1
38403	1.45506	2.1
38404	1.45506	2.1
38405	2.49017	2.1
38406	1.89226	2.1
38407	1.89226	2.1
38408	1.89226	2.1
38409	2.38363	2.1
38410	2.38363	2.1
38411	1.54711	2.1
38412	2.49018	2.1
38413	2.56975	2.1
38414	2.56975	2.1
38415	1.50524	2.1
38416	1.50524	2.1
38417	2.56975	2.1
38418	1.89226	2.1
38419	1.89226	2.1
38420	1.89226	2.1
38421	2.49017	2.1
38422	2.49017	2.1
38423	1.89226	0.0915738
38424	1.89226	0.101543
38425	1.89226	0.15294
38426	1.89226	0.0810826
38427	1.89226	0.1533
38428	1.89226	0.0723138
38429	1.89226	0.151851
38430	1.89226	0.136504
38431	1.89226	0.266841
38432	1.89226	0.107539
38433	1.89226	0.268958
38434	1.89226	0.105747
38435	1.89226	0.0752207
38436	1.89226	0.0800599
38437	2.8358	0.055898
38438	1.89226	0.188089
38439	3.78453	0.0610147
38440	1.60565	0.373116
38441	1.89226	1.42227
38442	1.89226	1.2987
38443	1.40013	0.686974
38444	1.89226	1.0282
38445	1.40013	0.685818
38446	1.1766	2.1
38447	1.29435	2.1
38448	1.35735	2.1
38449	1.53509	2.1
38450	2.65169	2.1
38451	0.69701	2.1

38452	0.69701	2.1
38453	0.711303	2.1
38454	0.711303	2.1
38455	0.761138	2.1
38456	0.612149	2.1
38457	0.647174	2.1
38458	0.767548	2.1
38459	0.767548	2.1
38460	0.761139	2.1
38461	0.612149	2.1
38462	0.647174	2.1
38463	1.29764	1.73182
38464	1.29579	1.94538
38465	1.34875	1.86597
38466	1.06717	1.86665
38467	1.29764	1.81295
38468	1.01606	1.78557
38469	0.756635	1.67476
38470	0.648821	1.6934
38471	0.675557	1.86497
38472	0.674374	1.79823
38473	0.74214	1.89022
38474	0.74214	1.73679
38475	0.688868	1.64176
38476	0.688868	1.78144
38477	0.675557	1.61873
38478	0.756634	1.67191
38479	0.648821	1.5568
38480	0.674374	1.6674
38481	1.51327	1.72984
38482	1.48105	1.58282
38483	1.29764	1.74259
38484	1.29764	1.65383
38485	1.48105	1.75134
38486	1.05655	1.59436
38487	1.13537	2.1
38488	1.07326	2.1
38489	0.94613	2.1
38490	0.94613	2.1
38491	0.946132	2.1
38492	0.946132	2.1
38493	1.13537	2.1
38494	1.08927	2.1
38495	1.13537	2.1
38496	0.946131	2.1
38497	0.946131	2.1
38498	0.95947	2.1
38499	1.46892	2.1
38500	1.89226	2.1
38501	1.89226	2.1
38502	1.89226	2.1
38503	1.88031	2.1
38504	1.88031	2.1
38505	1.89226	2.1
38506	1.89226	2.1
38507	1.89226	2.1
38508	1.89226	2.1
38509	3.78453	2.1

38510	2.18254	2.1
38511	1.89226	2.1
38512	2.50005	2.1
38513	1.89226	2.1
38514	4.39231	2.1
38515	1.88031	2.1
38516	2.0815	2.1
38517	2.0815	2.1
38518	1.92078	2.1
38519	2.0815	2.1
38520	1.9218	2.1
38521	1.89227	2.1
38522	1.89227	2.1
38523	1.89227	2.1
38524	1.89227	2.1
38525	1.89227	2.1
38526	1.89227	2.1
38527	1.98693	2.1
38528	1.82857	2.1
38529	2.32	2.1
38530	2.36972	2.1
38531	4.14857	2.1
38532	1.82857	2.1
38533	1.58688	2.1
38534	2.36971	2.1
38535	2.19093	2.1
38536	4.0195	2.1
38537	0.946132	2.1
38538	0.946132	2.1
38539	1.18486	2.1
38540	1.18486	2.1
38541	1.18486	2.1
38542	0.946132	2.1
38543	0.946132	2.1
38544	1.16	2.1
38545	0.946133	2.1
38546	0.946133	2.1
38547	1.16	2.1
38548	1.18486	2.1
38549	1.07798	2.02019
38550	1.08101	1.89813
38551	0.910786	2.1
38552	0.910786	2.1
38553	0.851537	2.1
38554	0.901377	1.89343
38555	1.14652	2.1
38556	1.04072	2.1
38557	0.946129	2.1
38558	0.946129	2.1
38559	1.0019	2.1
38560	1.04263	2.1
38561	1.89226	2.1
38562	1.89226	2.1
38563	1.89226	2.1
38564	1.89226	2.1
38565	3.78452	2.1
38566	1.89226	2.1
38567	2.30146	2.1

38568	1.89226	2.1
38569	2.18412	2.1
38570	4.19372	2.1
38571	2.30146	2.1
38572	1.39962	2.1
38573	2.18411	2.1
38574	1.28765	2.1
38575	3.5891	2.1
38576	1.89226	2.1
38577	1.89226	2.1
38578	1.89226	2.1
38579	1.89226	2.1
38580	1.89226	2.1
38581	1.89226	2.1
38582	1.89226	2.1
38583	1.89226	2.1
38584	1.89226	2.1
38585	1.89226	2.1
38586	3.78452	2.1
38587	2.18412	2.1
38588	1.20045	2.1
38589	1.20045	2.1
38590	1.28765	2.1
38591	2.08808	2.1
38592	2.17528	2.1
38593	0.946131	2.1
38594	0.946131	2.1
38595	0.946128	2.1
38596	0.946128	2.1
38597	0.946127	2.1
38598	0.946127	2.1
38599	0.946131	2.1
38600	0.946131	2.1
38601	0.946129	2.1
38602	0.946129	2.1
38603	0.94613	2.1
38604	0.94613	2.1
38605	1.89226	0.258359
38606	1.89226	1.45466
38607	1.89226	1.75381
38608	1.89226	0.27568
38609	3.78452	0.243996
38610	1.89227	0.459407
38611	1.89227	1.87317
38612	1.89227	0.366122
38613	1.89227	1.43812
38614	1.89227	0.4321
38615	1.89227	1.74692
38616	1.89226	1.4555
38617	1.89226	1.12595
38618	1.89226	0.227064
38619	2.29245	1.17704
38620	3.78452	1.36448
38621	1.89226	1.5498
38622	1.89226	0.155408
38623	1.89226	0.220972
38624	1.89226	1.43934
38625	1.89226	0.267898

38626	1.89226	0.32772
38627	1.28095	1.47862
38628	1.81846	1.49613
38629	2.10991	1.30012
38630	1.34695	1.4841
38631	3.39086	1.44842
38632	2.10991	1.39999
38633	1.36457	1.55703
38634	1.67421	1.61666
38635	1.34695	1.57339
38636	2.43717	1.43538
38637	2.10991	1.51127
38638	2.69541	2.1
38639	1.48572	2.1
38640	2.63553	2.1
38641	1.45385	2.1
38642	4.14926	2.1
38643	2.63553	2.1
38644	1.44207	2.1
38645	1.44207	2.1
38646	1.45385	2.1
38647	2.588 2.1	
38648	2.59978	2.1
38649	1.89227	2.1
38650	1.89227	2.1
38651	1.89227	2.1
38652	1.89227	2.1
38653	3.78453	2.1
38654	1.89226	2.1
38655	1.89226	2.1
38656	1.89226	2.1
38657	1.89226	2.1
38658	3.78453	2.1
38659	2.15929	2.1
38660	1.38276	2.1
38661	1.43617	2.1
38662	1.35614	2.1
38663	2.23932	2.1
38664	2.15929	2.1
38665	2.15929	2.1
38666	2.25168	2.1
38667	2.3558	2.1
38668	2.23932	2.1
38669	4.51509	2.1
38670	1.89226	2.1
38671	1.89226	2.1
38672	1.89226	2.1
38673	1.89226	2.1
38674	3.78453	2.1
38675	1.89226	2.1
38676	1.89226	2.1
38677	1.89226	2.1
38678	1.89226	2.1
38679	1.89226	2.1
38680	1.89226	2.1
38681	2.23932	2.1
38682	1.43854	2.1
38683	1.49173	2.1

38684	1.38276	2.1
38685	2.34829	2.1
38686	2.23932	2.1
38687	2.3558	2.1
38688	2.34829	2.1
38689	2.40685	2.1
38690	2.23932	2.1
38691	2.52332	2.1
38692	2.3558	2.1
38693	1.89226	0.05
38694	1.87361	0.0540448
38695	2.25082	0.05
38696	1.87361	0.057624
38697	2.26947	0.05
38698	1.89226	0.0552122
38699	1.89226	0.055864
38700	1.89226	0.0695114
38701	1.89226	0.0556783
38702	1.89226	0.0850063
38703	1.89226	0.054051
38704	1.89226	0.0783366
38705	2.41314	0.0508192
38706	1.89226	0.0580632
38707	1.89226	0.0505614
38708	1.89226	0.0663576
38709	2.12204	0.0504285
38710	2.12204	0.0629889
38711	1.89226	0.05
38712	2.25082	0.0528209
38713	2.28189	0.05
38714	2.25082	0.0602077
38715	1.92333	0.05
38716	1.89226	0.0573864
38717	1.89226	0.0576659
38718	1.89226	0.0625241
38719	1.89226	0.0554016
38720	1.89226	0.0812876
38721	1.89226	0.0542725
38722	1.89226	0.0720234
38723	2.42521	0.05
38724	1.89226	0.0547745
38725	1.89226	0.05
38726	1.89226	0.0604678
38727	2.41314	0.05
38728	2.41314	0.0574707
38729	1.39725	2.02832
38730	1.07745	2.1
38731	1.07745	2.07621
38732	1.07519	2.1
38733	1.41336	1.98005
38734	1.41111	2.1
38735	1.89226	2.1
38736	1.92039	2.1
38737	1.92039	2.1
38738	1.9052	2.1
38739	1.89226	2.1
38740	1.87708	2.1
38741	0.946128	2.1

38742	0.946128	2.1
38743	0.946133	2.01123
38744	0.946133	1.93209
38745	0.94613	1.93357
38746	0.94613	2.1
38747	0.946131	2.1
38748	0.946131	1.93134
38749	0.946131	2.1
38750	0.946131	2.1
38751	0.946131	2.1
38752	0.946131	2.1
38753	1.41336	2.06702
38754	1.08911	2.1
38755	1.08911	2.1
38756	1.07745	2.1
38757	1.42381	2.04589
38758	1.41215	2.1
38759	1.89227	2.1
38760	1.95833	2.1
38761	1.95833	2.1
38762	1.92039	2.1
38763	1.89227	2.1
38764	1.85433	2.1
38765	0.946129	2.1
38766	0.946129	2.1
38767	0.946133	2.07624
38768	0.946133	1.96919
38769	0.946131	1.96928
38770	0.946131	2.1
38771	0.946131	2.1
38772	0.946131	2.01588
38773	0.946131	2.1
38774	0.946131	2.1
38775	0.946131	2.1
38776	0.946131	2.1
38777	1.41943	2.1
38778	2.74398	2.1
38779	1.18238	2.1
38780	2.53897	2.1
38781	3.9584	2.1
38782	2.74398	2.1
38783	1.89226	2.1
38784	1.89226	2.1
38785	1.89226	2.1
38786	2.53897	2.1
38787	2.53897	2.1
38788	1.89227	1.2141
38789	1.89227	0.689819
38790	1.89227	1.22118
38791	1.89227	0.534242
38792	3.78453	0.769567
38793	1.89226	0.51719
38794	2.08134	0.288956
38795	2.00632	0.289781
38796	2.00632	0.305735
38797	1.89226	0.692013
38798	1.89226	0.641489
38799	1.89227	1.11507

38800	1.89227	0.467022
38801	1.89227	0.972637
38802	1.89227	0.354833
38803	3.78453	0.827712
38804	1.89227	1.17092
38805	1.89227	0.327018
38806	1.89227	0.32388
38807	1.89227	0.489764
38808	1.89227	1.05449
38809	1.89227	1.16918
38810	2.50912	0.289577
38811	1.89226	0.491778
38812	1.89226	0.211777
38813	2.31261	0.310251
38814	2.31261	0.277217
38815	1.89226	0.70426
38816	2.50912	0.331816
38817	1.19999	0.260348
38818	1.44056	0.230555
38819	1.24405	0.218593
38820	2.50912	0.325206
38821	2.31261	0.345009
38822	2.69224	1.5528
38823	2.28373	0.237733
38824	1.94788	1.35281
38825	2.4106	1.18437
38826	4.69434	1.22068
38827	2.69223	1.0994
38828	2.63783	1.74639
38829	2.34737	2.0961
38830	2.4106	1.09573
38831	5.04843	1.72872
38832	1.89226	2.1
38833	1.99753	2.1
38834	1.87366	2.1
38835	1.99753	2.1
38836	1.76839	2.1
38837	1.89226	2.1
38838	1.89226	2.1
38839	1.89226	2.1
38840	1.89226	2.1
38841	1.89226	2.1
38842	3.78452	2.1
38843	0.946132	2.1
38844	0.946132	2.1
38845	1.02744	2.1
38846	1.02744	2.1
38847	0.946132	2.1
38848	0.946132	2.1
38849	1.00989	2.1
38850	1.02744	2.1
38851	1.00989	2.1
38852	0.946133	2.1
38853	1.02744	2.1
38854	0.946132	2.1
38855	1.70302	2.1
38856	1.87366	2.1
38857	1.71618	2.1

38858	1.87366	2.1
38859	1.73105	2.1
38860	1.89226	2.1
38861	1.89226	2.1
38862	1.89226	2.1
38863	1.89226	2.1
38864	1.89226	2.1
38865	1.89226	2.1
38866	1.89226	2.1
38867	1.96624	2.1
38868	1.89226	2.1
38869	1.89226	2.1
38870	1.89226	2.1
38871	2.01977	2.1
38872	2.01977	2.1
38873	1.1032	2.1
38874	1.15114	2.1
38875	1.14986	0.870095
38876	1.19242	2.1
38877	2.301	2.1
38878	1.89226	1.85241
38879	1.89226	2.1
38880	1.89226	0.938546
38881	1.89226	2.1
38882	3.78453	2.1
38883	0.946132	2.1
38884	0.946132	2.1
38885	0.946132	2.1
38886	0.946132	2.1
38887	0.946133	2.1
38888	0.946133	2.1
38889	0.946132	2.1
38890	0.946132	2.1
38891	0.946132	2.1
38892	0.946132	2.1
38893	0.946133	2.1
38894	0.946133	2.1
38895	1.19243	2.1
38896	0.953928	2.1
38897	1.18671	2.1
38898	1.15114	2.1
38899	0.9895	2.1
38900	0.953928	2.1
38901	1.19611	0.91106
38902	1.19242	2.1
38903	1.24693	1.81035
38904	1.24693	2.1
38905	1.14986	1.66058
38906	1.14986	2.1
38907	1.89225	0.984085
38908	1.89225	2.1
38909	1.89225	2.1
38910	1.89225	1.88614
38911	1.89225	2.1
38912	1.89225	2.09237
38913	1.89225	2.1
38914	1.89225	2.1
38915	1.89225	2.1

38916	1.89225	2.1
38917	1.89225	2.1
38918	1.89225	2.1
38919	0.561532	2.1
38920	0.561532	2.1
38921	0.566382	2.1
38922	0.566382	2.1
38923	0.494747	2.1
38924	0.531705	2.1
38925	0.596208	2.1
38926	0.623464	2.1
38927	0.623469	2.1
38928	0.531708	2.1
38929	0.596205	2.1
38930	0.494743	2.1
38931	1.25742	2.1
38932	1.18783	2.1
38933	0.946131	2.1
38934	0.946131	2.1
38935	1.25742	2.1
38936	1.25742	2.1
38937	0.946131	2.1
38938	0.946131	2.1
38939	1.25742	2.1
38940	0.946131	2.1
38941	0.946131	2.1
38942	1.18783	2.1
38943	1.35048	2.1
38944	1.25742	2.1
38945	0.946132	2.1
38946	0.946132	2.1
38947	1.35048	2.1
38948	1.35048	2.1
38949	0.946132	2.1
38950	0.946132	2.1
38951	1.35044	2.1
38952	0.946106	2.1
38953	1.25745	2.1
38954	0.946158	2.1
38955	1.13525	2.1
38956	2.70095	2.1
38957	2.51483	2.1
38958	2.51483	2.1
38959	1.21593	2.1
38960	1.21593	2.1
38961	1.93142	1.26785
38962	2.8053	0.300038
38963	1.559	1.36631
38964	2.60359	0.183215
38965	4.53501	0.435896
38966	2.80531	0.202974
38967	1.89226	0.298982
38968	2.60359	0.301867
38969	1.89226	1.43552
38970	4.49586	0.284412
38971	1.22938	0.567856
38972	2.60359	0.197094
38973	2.37434	1.59365

38974	2.37434	0.661404
38975	1.55901	1.12881
38976	1.55901	0.193377
38977	1.18717	1.23915
38978	1.18717	0.130875
38979	0.946132	0.104827
38980	0.946132	0.474035
38981	0.946133	0.378009
38982	0.946133	1.07265
38983	1.3018	0.109206
38984	1.18717	1.33191
38985	1.18714	0.313102
38986	0.946107	0.369524
38987	1.30183	0.383315
38988	0.946158	0.342822
38989	1.89226	2.1
38990	2.80196	2.1
38991	2.67272	2.1
38992	2.67272	2.1
38993	1.89226	2.1
38994	1.89226	2.1
38995	1.89226	2.1
38996	1.89226	2.1
38997	1.89226	2.1
38998	1.89226	2.1
38999	1.89226	2.1
39000	1.89226	2.1
39001	1.74471	2.1
39002	2.01555	2.1
39003	1.61121	2.1
39004	1.61121	2.1
39005	2.13878	2.1
39006	2.13878	2.1
39007	1.89226	2.1
39008	1.89226	2.1
39009	1.89226	2.1
39010	1.89226	2.1
39011	1.89226	2.1
39012	1.89226	2.1
39013	1.09766	2.1
39014	1.04263	2.1
39015	0.881794	2.1
39016	0.894188	2.1
39017	0.795402	2.1
39018	0.99524	2.1
39019	1.00647	2.1
39020	1.00647	2.1
39021	1.15401	2.1
39022	0.881789	2.1
39023	0.850526	2.1
39024	1.04263	2.1
39025	1.4054	1.99649
39026	1.04788	2.1
39027	1.70302	2.09066
39028	1.41375	2.1
39029	2.82706	2.1
39030	1.70302	1.98378
39031	1.38776	2.1

39032	1.59457	2.1
39033	1.41375	2.1
39034	1.77261	1.7352
39035	1.70302	2.1
39036	1.89226	2.1
39037	1.89226	2.1
39038	1.89226	2.1
39039	1.89226	2.1
39040	3.78453	2.1
39041	2.18042	2.1
39042	1.74738	2.1
39043	2.08433	2.1
39044	1.66347	2.1
39045	3.83171	2.1
39046	1.74738	2.1
39047	1.1181	2.1
39048	1.66347	2.1
39049	1.06543	2.1
39050	2.81281	2.1
39051	1.89226	2.1
39052	1.89226	2.1
39053	1.89226	2.1
39054	1.89226	2.1
39055	3.78453	2.1
39056	1.89227	2.1
39057	1.89227	2.1
39058	1.89227	2.1
39059	1.89227	2.1
39060	3.78453	2.1
39061	2.08433	2.1
39062	1.66347	2.1
39063	1.97092	2.1
39064	1.57431	2.1
39065	3.65864	2.1
39066	1.66347	2.1
39067	1.01474	2.1
39068	1.01474	2.1
39069	1.06544	2.1
39070	1.57431	2.1
39071	1.625 2.1	
39072	1.89226	2.1
39073	1.89226	2.1
39074	1.89226	2.1
39075	1.89226	2.1
39076	1.89226	2.1
39077	1.89226	2.1
39078	1.08467	2.1
39079	1.87228	2.1
39080	1.73305	2.1
39081	1.73305	2.1
39082	1.25565	2.1
39083	1.25565	2.1
39084	0.695361	0.328434
39085	0.799996	0.386313
39086	1.13751	0.572643
39087	1.40945	0.549157
39088	1.40944	0.62999
39089	0.799999	0.282803

39090	1.13751	0.393411
39091	0.695363	0.284163
39092	1.01059	0.333873
39093	1.01059	0.487655
39094	0.926908	0.800071
39095	0.926909	0.278884
39096	1.05481	2.1
39097	1.78069	2.1
39098	1.08074	2.1
39099	1.83284	2.1
39100	2.86143	2.1
39101	1.78069	2.1
39102	2.22044	2.1
39103	1.83284	2.1
39104	2.28696	1.94168
39105	4.06766	2.1
39106	1.07196	2.1
39107	1.64955	2.1
39108	2.02665	2.1
39109	2.0284	2.1
39110	1.08074	2.1
39111	1.08074	2.1
39112	1.64955	2.1
39113	2.28696	2.1
39114	1.92033	2.1
39115	2.1004	2.1
39116	3.74988	2.1
39117	2.83787	1.16508
39118	1.70303	1.52942
39119	1.70303	0.372386
39120	1.83195	0.12879
39121	2.64075	0.772129
39122	2.70231	1.21615
39123	1.2087	2.1
39124	1.52909	1.02118
39125	1.43063	0.952091
39126	1.10586	2.1
39127	2.63494	1.94788
39128	1.89227	2.1
39129	2.10732	2.1
39130	2.10732	2.1
39131	2.2365	2.1
39132	1.89227	2.1
39133	2.02145	2.1
39134	1.89226	1.23266
39135	1.89226	2.1
39136	1.89226	1.89552
39137	1.89226	2.1
39138	1.89226	1.48341
39139	1.89226	2.1
39140	0.51926	2.1
39141	0.552927	2.1
39142	0.691235	0.949195
39143	0.715314	1.1425
39144	0.619684	1.15521
39145	0.619684	2.1
39146	0.624477	2.1
39147	0.624477	1.13875

39148	0.519262	2.06772
39149	0.691234	2.0674
39150	0.715313	2.06579
39151	0.552928	2.05845
39152	1.89226	2.1
39153	1.87321	2.1
39154	1.87321	2.1
39155	1.89658	2.1
39156	2.0815	2.1
39157	2.14789	2.1
39158	0.946128	2.1
39159	0.946128	2.1
39160	0.946133	1.33798
39161	0.946133	1.74597
39162	0.94613	1.20244
39163	0.94613	2.1
39164	0.946131	1.77234
39165	0.946131	2.1
39166	0.946131	1.95505
39167	0.946131	1.94868
39168	0.946131	2.1
39169	0.946131	2.1
39170	1.99297	1.45926
39171	2.19514	0.725477
39172	2.46153	1.27591
39173	2.187	1.07782
39174	4.45451	1.06354
39175	2.0815	1.10519
39176	2.33402	0.676029
39177	2.46154	1.32079
39178	2.83046	0.463887
39179	4.94496	1.0763
39180	2.46153	1.21335
39181	2.187	0.818506
39182	2.27351	1.38038
39183	2.63356	0.491965
39184	4.73504	1.20877
39185	2.46154	1.26061
39186	2.83447	0.409135
39187	2.74513	0.715083
39188	2.5731	1.09132
39189	2.63357	0.598625
39190	2.46154	0.984085
39191	2.80398	2.1
39192	2.60406	2.1
39193	2.73988	2.1
39194	2.57466	2.1
39195	5.34394	2.1
39196	1.89226	1.58446
39197	1.89226	1.34334
39198	1.89226	1.28676
39199	1.89226	1.59412
39200	1.89226	1.32157
39201	1.89226	1.68531
39202	1.89224	1.6378
39203	1.89224	1.33729
39204	1.89224	1.31497
39205	1.89224	1.67511

39206	1.89224	1.34105
39207	1.89224	1.68452
39208	1.40245	1.55122
39209	1.40246	1.67231
39210	0.946117	1.45592
39211	0.946117	1.54326
39212	1.17635	1.38796
39213	1.40246	1.77306
39214	0.946112	1.35404
39215	0.946112	1.69421
39216	0.946127	1.4749
39217	1.17637	1.40789
39218	0.946102	1.66222
39219	1.40244	1.53985
39220	2.53269	2.1
39221	1.70302	2.1
39222	1.56194	2.1
39223	2.17516	2.1
39224	4.19807	2.1
39225	1.89226	2.1
39226	1.89226	2.1
39227	1.89226	2.1
39228	1.89226	2.1
39229	3.78453	2.1
39230	2.57276	2.1
39231	1.89226	2.1
39232	1.89226	2.1
39233	1.89226	2.1
39234	2.32525	2.1
39235	2.32525	2.1
39236	1.15171	2.1
39237	2.17517	2.1
39238	1.41995	2.1
39239	1.41995	2.1
39240	1.80252	2.1
39241	1.80252	2.1
39242	1.89226	2.1
39243	1.89226	2.1
39244	1.89226	2.1
39245	1.89226	2.1
39246	3.78453	2.1
39247	0.946133	2.1
39248	0.946133	2.1
39249	1.16263	2.1
39250	1.04687	2.1
39251	0.946133	2.1
39252	0.946133	2.1
39253	1.04687	2.1
39254	1.04687	2.1
39255	0.946133	2.1
39256	1.04687	2.1
39257	0.946132	2.1
39258	1.16263	2.1
39259	0.682281	1.62678
39260	0.669233	1.72963
39261	1.21859	1.63046
39262	1.31629	1.47291
39263	0.916158	1.3816

39264	0.916158	1.98746
39265	0.971658	1.61683
39266	0.971658	1.58737
39267	0.669234	1.59227
39268	1.31629	1.50238
39269	0.682283	1.6187
39270	1.21858	1.63255
39271	0.669232	1.59622
39272	0.623495	1.66292
39273	1.36428	1.53359
39274	1.31629	1.4253
39275	0.946475	1.37313
39276	0.946476	1.90763
39277	0.99331	1.62047
39278	0.99331	1.56967
39279	1.36428	1.52507
39280	0.623497	1.43397
39281	0.669234	1.55731
39282	1.31629	1.51137
39283	2.02069	0.05
39284	1.46253	0.05
39285	2.51479	0.05
39286	1.87136	0.05
39287	3.89203	0.05
39288	1.46253	0.05
39289	1.38536	0.05
39290	1.38536	0.05
39291	1.05579	0.05
39292	1.73281	0.05
39293	1.46916	0.05
39294	2.51479	0.05
39295	1.87136	0.05
39296	2.23338	0.05
39297	2.15603	0.05
39298	4.55146	0.05
39299	0.822553	0.05
39300	0.822563	0.05
39301	0.842935	0.05
39302	0.842935	0.05
39303	0.744203	0.05
39304	0.675346	0.05
39305	0.874795	0.05
39306	1.00453	0.05
39307	1.00454	0.05
39308	0.782417	0.05
39309	0.675337	0.05
39310	0.858007	0.05
39311	0.946132	1.08452
39312	1.24847	0.973856
39313	0.946132	1.23503
39314	1.16806	1.31255
39315	1.24847	1.04028
39316	1.24847	1.46471
39317	0.946133	0.959813
39318	0.946133	1.26665
39319	0.946133	1.03762
39320	0.946133	1.02986
39321	1.16805	1.16101

39322	1.24847	1.07667
39323	1.2364	1.10917
39324	0.946129	0.957153
39325	0.946133	1.21737
39326	1.24847	1.0878
39327	1.2364	0.976631
39328	1.2364	1.36165
39329	0.946131	0.895572
39330	0.946131	1.51907
39331	0.946131	1.01915
39332	0.946131	0.964306
39333	1.2364	1.27063
39334	1.24847	1.1107
39335	1.89226	1.03453
39336	1.89226	1.29258
39337	1.89226	1.11623
39338	1.89226	1.0734
39339	1.89226	1.17926
39340	1.89226	1.16406
39341	1.89226	1.07645
39342	1.89226	1.07518
39343	1.89226	0.974638
39344	1.89226	1.12207
39345	1.89226	1.04635
39346	1.89226	1.29625
39347	1.69014	1.1019
39348	1.09194	1.10613
39349	1.13537	1.2639
39350	1.13537	1.11835
39351	1.58481	1.09735
39352	1.58481	1.02786
39353	1.58147	1.28127
39354	1.13537	1.12322
39355	1.69015	1.2511
39356	1.01179	1.18267
39357	1.69015	1.08618
39358	1.01179	1.12247
39359	0.946132	1.5464
39360	0.946132	1.16615
39361	1.28543	0.219536
39362	1.07341	1.36713
39363	1.07341	1.52785
39364	1.07341	0.0591718
39365	0.946132	0.381064
39366	0.946132	0.439895
39367	0.946133	0.164323
39368	1.28543	0.689322
39369	1.07341	0.547147
39370	0.946132	1.15613
39371	1.89226	1.49193
39372	1.89226	0.52309
39373	1.89226	0.704316
39374	1.89226	1.97531
39375	1.89226	0.378695
39376	1.89226	2.1
39377	0.805744	2.1
39378	0.988659	2.00366
39379	0.782044	0.60885

39380	0.578223	1.06533
39381	0.77823	1.11654
39382	0.778231	2.1
39383	0.788653	0.552362
39384	0.788653	1.9684
39385	0.988662	1.49192
39386	0.782042	1.41699
39387	0.805745	1.94027
39388	0.578222	1.74083
39389	1.89226	0.561168
39390	1.89226	1.12333
39391	1.89226	1.54937
39392	1.89226	1.92271
39393	1.89226	0.520623
39394	1.89226	2.00244
39395	1.97732	1.92058
39396	1.97819	0.53562
39397	1.56408	0.578309
39398	1.56408	2.04669
39399	2.33986	0.656138
39400	2.33986	2.09631
39401	1.84516	2.1
39402	2.21152	2.1
39403	1.90702	2.1
39404	1.54599	2.1
39405	3.75751	2.1
39406	1.89224	2.1
39407	1.89224	2.1
39408	1.89224	2.1
39409	1.89224	2.1
39410	1.89224	2.1
39411	1.89224	2.1
39412	1.47672	2.1
39413	1.54601	2.1
39414	1.27697	2.1
39415	1.54601	2.1
39416	1.638	2.1
39417	1.90705	2.1
39418	0.946132	2.1
39419	0.946132	2.1
39420	0.946131	2.1
39421	0.946131	2.1
39422	0.946131	2.1
39423	0.946131	2.1
39424	0.946132	2.1
39425	0.946132	2.1
39426	0.946132	2.1
39427	0.946132	2.1
39428	0.946131	2.1
39429	0.946131	2.1
39430	0.946128	2.1
39431	0.946128	2.1
39432	1.32062	2.1
39433	1.4164	2.1
39434	0.946129	2.1
39435	0.946129	2.1
39436	1.32062	2.1
39437	1.32062	2.1

39438	1.32062	2.1
39439	0.94613	2.1
39440	1.41639	2.1
39441	0.946129	2.1
39442	1.98979	0.05
39443	1.65977	0.05
39444	2.21772	0.05
39445	2.21772	0.05
39446	1.48866	0.05
39447	1.48866	0.05
39448	0.973129	0.05
39449	0.973129	0.05
39450	0.980954	0.05
39451	0.980954	0.05
39452	0.845218	0.05
39453	1.13981	0.05
39454	0.829886	0.05
39455	1.10886	0.05
39456	1.10886	0.05
39457	1.13982	0.05
39458	0.845219	0.05
39459	0.829886	0.05
39460	0.946132	2.1
39461	0.946132	2.1
39462	1.3352	2.1
39463	1.41432	2.1
39464	0.946133	2.1
39465	0.946133	2.03493
39466	1.3352	2.1
39467	1.3352	2.1
39468	1.3352	2.1
39469	0.946132	2.1
39470	1.41432	2.1
39471	0.946133	2.1
39472	2.70097	2.1
39473	1.88175	2.1
39474	1.51482	2.1
39475	1.88175	2.1
39476	2.37784	2.1
39477	2.82864	2.1
39478	1.89227	0.0788918
39479	1.89227	0.064816
39480	1.89227	0.05
39481	1.89227	0.0513428
39482	3.78453	0.05
39483	1.89226	0.0515656
39484	2.13295	0.05
39485	2.43203	0.05
39486	2.43203	0.0536769
39487	1.89226	0.05
39488	1.89226	0.0509517
39489	0.946133	0.05
39490	0.946133	0.05
39491	0.946132	0.05
39492	0.946132	0.05
39493	0.946132	0.05
39494	0.946132	0.05
39495	0.946133	0.05

39496	0.946133	0.05
39497	0.946132	0.05
39498	0.946132	0.05
39499	0.946132	0.0533127
39500	0.946132	0.0504433
39501	1.54987	2.1
39502	1.24128	2.1
39503	1.24128	2.1
39504	1.50738	2.1
39505	1.75702	2.1
39506	2.02312	2.1
39507	1.89289	2.1
39508	1.92264	2.1
39509	1.72994	2.1
39510	1.75702	2.1
39511	3.67966	2.1
39512	1.75702	2.1
39513	0.994291	2.1
39514	0.994291	2.1
39515	1.24128	2.1
39516	1.4964	2.1
39517	1.74339	2.1
39518	1.72993	2.1
39519	1.49641	2.1
39520	1.49641	2.1
39521	1.75702	2.1
39522	1.3939	2.1
39523	1.65452	2.1
39524	1.19964	2.1
39525	1.53296	2.1
39526	1.19964	2.1
39527	1.36861	2.1
39528	1.42624	2.1
39529	1.59521	2.1
39530	1.19964	2.1
39531	1.42624	2.1
39532	1.07156	2.1
39533	1.36226	2.1
39534	2.5619	2.1
39535	2.14058	1.3693
39536	1.93569	1.92203
39537	2.36221	1.56507
39538	2.5753	1.40548
39539	4.50279	1.59545
39540	2.14057	1.63431
39541	2.20831	1.89552
39542	2.64725	1.15141
39543	2.57529	1.64076
39544	4.78782	1.52245
39545	2.64725	1.2063
39546	2.57529	1.64059
39547	1.89226	1.29966
39548	1.89226	1.08774
39549	4.53951	1.36717
39550	2.0815	2.1
39551	1.86137	2.1
39552	1.86137	2.1
39553	1.86159	2.1

39554	2.0815	2.1
39555	2.10235	2.1
39556	1.33552	2.1
39557	2.32417	2.1
39558	1.18872	2.1
39559	1.93101	2.1
39560	3.26653	2.1
39561	1.89226	2.1
39562	1.89226	2.1
39563	1.89226	2.1
39564	1.89226	2.1
39565	1.89226	2.1
39566	1.89226	2.1
39567	0.955191	2.1
39568	1.03372	2.1
39569	0.946131	2.1
39570	0.946131	2.1
39571	0.930654	2.1
39572	0.930654	2.1
39573	1.04233	2.1
39574	1.05247	2.1
39575	0.955341	2.1
39576	0.946131	2.1
39577	1.02375	2.1
39578	1.13537	2.1
39579	1.1549	2.1
39580	1.93101	2.1
39581	1.91108	2.1
39582	1.52487	2.1
39583	1.3076	2.1
39584	1.06985	2.1
39585	1.89226	2.1
39586	1.89226	2.1
39587	1.89226	2.1
39588	1.89226	2.1
39589	1.89226	2.1
39590	1.89226	2.1
39591	1.58713	2.1
39592	2.6845	2.1
39593	1.58742	2.1
39594	2.74201	2.1
39595	4.27191	2.1
39596	2.68449	2.1
39597	1.89226	2.1
39598	2.74201	2.1
39599	1.89226	2.1
39600	4.57675	2.1
39601	1.57523	2.1
39602	2.74201	2.1
39603	2.8146	2.1
39604	2.8146	2.1
39605	1.58742	2.1
39606	1.58742	2.1
39607	0.946131	2.1
39608	0.946131	2.1
39609	1.371 2.1	
39610	1.371 2.1	
39611	0.946127	2.1

39612	0.946127	2.1
39613	1.37101	2.1
39614	1.4073	2.1
39615	0.94613	2.1
39616	1.4073	2.1
39617	1.371 2.1	
39618	0.946129	2.1
39619	1.44801	1.95012
39620	1.06821	1.97704
39621	2.00542	1.91331
39622	2.27703	2.04154
39623	3.34524	1.8743
39624	1.70301	0.16371
39625	1.89226	0.241606
39626	1.65809	0.202045
39627	1.89226	0.278133
39628	1.62758	0.116563
39629	1.85744	0.133205
39630	1.89226	0.113456
39631	1.89226	0.272351
39632	1.89226	0.108143
39633	1.89226	0.0960087
39634	3.78452	0.103594
39635	1.89226	0.187692
39636	1.89226	0.116893
39637	1.89226	0.0871485
39638	1.89226	0.11348
39639	3.78452	0.122386
39640	0.851541	0.368455
39641	0.851541	0.350059
39642	0.91193	0.206542
39643	0.824837	0.192876
39644	0.854559	0.260798
39645	0.851534	0.304858
39646	0.81851	0.206761
39647	0.717219	0.205525
39648	0.871743	0.170557
39649	0.837985	0.131864
39650	0.82902	0.254267
39651	0.82902	0.361672
39652	1.89226	0.4305
39653	1.92659	0.0650212
39654	2.03235	0.0768595
39655	1.89226	0.554795
39656	2.03235	0.0626267
39657	1.89226	0.409096
39658	0.585557	0.508459
39659	0.585557	0.252846
39660	1.22612	0.127551
39661	1.09436	0.359755
39662	0.851537	0.537961
39663	0.526987	0.41113
39664	0.983879	0.0767865
39665	1.04072	0.618261
39666	0.585526	0.0570859
39667	0.792325	0.0710389
39668	0.946129	0.405096
39669	0.946129	0.349998

39670	1.68152	2.1
39671	1.81445	2.1
39672	1.68152	2.1
39673	1.86792	2.1
39674	1.65888	2.1
39675	1.84529	2.1
39676	1.81445	2.1
39677	1.57885	2.1
39678	1.65889	2.1
39679	1.48594	2.1
39680	3.23773	2.1
39681	1.68154	2.1
39682	1.65891	2.1
39683	1.53296	2.1
39684	1.56299	2.1
39685	3.24453	2.1
39686	1.56299	2.1
39687	1.48596	2.1
39688	1.56299	2.1
39689	1.65891	2.1
39690	1.46568	2.1
39691	1.5616	2.1
39692	2.68785	2.1
39693	2.08831	2.1
39694	2.66919	2.1
39695	2.1024	2.1
39696	4.79025	2.1
39697	2.08832	2.1
39698	1.15478	2.1
39699	1.15478	2.1
39700	1.09062	2.1
39701	2.10241	2.1
39702	2.03825	2.1
39703	2.65348	2.1
39704	2.1024	2.1
39705	2.12313	2.1
39706	2.12313	2.1
39707	2.66919	2.1
39708	2.66919	2.1
39709	0.813169	2.1
39710	0.813169	2.1
39711	0.825788	2.1
39712	0.825788	2.1
39713	0.612902	2.1
39714	0.577387	2.1
39715	1.0512	2.1
39716	1.06157	2.1
39717	1.06157	2.1
39718	0.612902	2.1
39719	0.577387	2.1
39720	1.0512	2.1
39721	1.27227	2.1
39722	1.91724	2.1
39723	1.32591	2.1
39724	1.99837	2.1
39725	3.24315	2.1
39726	1.89223	2.1
39727	1.89223	2.1

39728	1.89223	2.1
39729	1.89223	2.1
39730	3.78446	2.1
39731	1.89224	2.1
39732	1.89224	2.1
39733	1.89224	2.1
39734	1.89224	2.1
39735	3.78447	2.1
39736	1.91724	2.1
39737	2.321	2.1
39738	1.99838	2.1
39739	2.4149	2.1
39740	4.33214	2.1
39741	1.34817	2.1
39742	1.9984	2.1
39743	2.06238	2.1
39744	2.06238	2.1
39745	1.32593	2.1
39746	1.32593	2.1
39747	1.89226	2.1
39748	1.89226	2.1
39749	1.89226	2.1
39750	1.89226	2.1
39751	1.89226	2.1
39752	1.89226	2.1
39753	1.89226	2.1
39754	1.89226	2.1
39755	1.89226	2.1
39756	1.89226	2.1
39757	3.78452	2.1
39758	1.9984	2.1
39759	2.41494	2.1
39760	2.06239	2.1
39761	2.5194	2.1
39762	4.47732	2.1
39763	1.19901	1.76475
39764	1.16661	2.1
39765	1.56859	2.1
39766	1.61029	1.7326
39767	2.7676	2.1
39768	1.56859	2.1
39769	1.88637	0.718714
39770	1.61029	0.667523
39771	1.61029	2.1
39772	1.856	0.735043
39773	1.856	2.1
39774	1.856	2.04514
39775	1.57916	2.1
39776	1.56859	2.1
39777	1.56859	2.1
39778	1.57916	2.1
39779	1.23475	2.1
39780	0.890924	0.416527
39781	1.07608	0.376966
39782	0.72877	0.69111
39783	0.910106	1.02133
39784	1.00196	1.22804
39785	1.09519	1.02945

39786	0.839967	0.549308
39787	0.719043	0.318136
39788	0.893859	0.350214
39789	0.942408	1.15714
39790	0.873497	0.963013
39791	0.873497	1.84492
39792	1.53046	0.214916
39793	1.89994	0.554643
39794	1.049	0.306768
39795	1.049	0.194971
39796	1.52512	0.210083
39797	1.89994	0.710931
39798	1.65573	1.58424
39799	1.89226	0.519176
39800	1.89226	0.220772
39801	2.73329	1.19585
39802	3.548	1.13358
39803	1.65573	0.13677
39804	2.42671	1.02838
39805	2.73329	0.155346
39806	1.89226	0.154482
39807	2.73329	0.490485
39808	1.89226	0.182505
39809	0.941078	0.568939
39810	1.01599	1.19992
39811	0.818936	0.558134
39812	0.651554	1.20937
39813	0.904729	1.05668
39814	0.665756	0.87154
39815	0.843423	1.68654
39816	0.918649	0.88791
39817	0.855806	1.1843
39818	0.814384	0.218807
39819	0.844437	1.56174
39820	0.844437	1.51522
39821	2.22558	1.96736
39822	1.95785	0.591759
39823	2.07065	1.5206
39824	1.89142	1.54314
39825	2.07065	1.88348
39826	1.89142	1.86517
39827	0.640813	1.20112
39828	0.951654	1.07255
39829	0.797442	0.420094
39830	0.535364	1.12489
39831	0.525269	1.37343
39832	0.620874	0.538374
39833	0.916953	0.858845
39834	0.969374	1.07061
39835	0.788925	1.39811
39836	0.69596	0.282012
39837	0.743154	1.44348
39838	0.743154	1.47707
39839	1.89226	1.96802
39840	1.89226	1.71786
39841	1.89226	1.58609
39842	1.89226	2.01197
39843	3.78452	1.73847

39844	1.32541	1.7149
39845	1.13722	1.61313
39846	0.91103	2.1
39847	0.881123	1.96802
39848	1.03363	2.08149
39849	1.03364	1.78494
39850	1.1505	1.73324
39851	1.1505	1.99093
39852	1.32539	1.89685
39853	1.07662	1.94468
39854	0.881121	1.81856
39855	0.962303	1.81964
39856	1.89226	1.83727
39857	1.89226	2.1
39858	1.89226	2.1
39859	1.89226	1.87234
39860	3.78453	2.0985
39861	1.89226	1.56132
39862	1.89226	1.92776
39863	1.89226	1.84724
39864	1.89226	1.66345
39865	1.89226	1.84319
39866	1.89226	1.68196
39867	1.27606	1.89499
39868	1.07664	1.84253
39869	0.905895	2.1
39870	0.696099	2.1
39871	0.984902	2.1
39872	0.984905	1.92221
39873	1.01878	2.1
39874	1.01878	1.78403
39875	1.27604	2.04738
39876	1.06044	2.03231
39877	1.13192	2.0383
39878	0.696111	2.03715
39879	1.89226	1.95191
39880	1.89226	0.722962
39881	1.89226	0.262371
39882	1.89226	1.98005
39883	1.89226	0.650985
39884	1.89226	1.85463
39885	1.52615	2.1
39886	1.01294	1.00598
39887	1.01294	2.1
39888	1.18549	0.278842
39889	1.38963	1.7478
39890	1.56218	0.727741
39891	1.89226	1.50773
39892	2.40801	1.71074
39893	2.58101	2.0861
39894	2.58101	1.39594
39895	1.89226	2.1
39896	1.89226	0.498184
39897	2.24071	2.1
39898	2.79317	2.1
39899	1.99136	2.1
39900	2.6167	2.1
39901	4.85741	2.1

39902	2.79317	2.1
39903	1.89226	2.1
39904	1.89226	2.1
39905	1.89226	2.1
39906	2.6167	2.1
39907	2.6167	2.1
39908	1.08354	2.1
39909	1.08354	2.1
39910	1.10222	2.1
39911	1.10222	2.1
39912	1.2275	2.1
39913	1.30835	2.1
39914	0.995678	2.1
39915	0.877412	2.1
39916	0.877411	2.1
39917	1.2275	2.1
39918	0.995678	2.1
39919	1.30835	2.1
39920	1.05793	0.61283
39921	1.46441	0.256411
39922	1.0562	0.313553
39923	1.0562	0.272935
39924	1.41787	0.959194
39925	1.41787	0.23477
39926	1.41787	0.358292
39927	1.52909	1.25597
39928	1.46441	0.34703
39929	1.46441	0.951429
39930	1.43063	0.391013
39931	1.43063	0.828387
39932	1.89226	1.38126
39933	1.89226	0.443299
39934	1.89226	0.465542
39935	1.89226	1.84281
39936	3.78453	1.28491
39937	1.89227	1.03138
39938	1.89227	0.843974
39939	1.89227	0.359837
39940	1.89227	0.390667
39941	1.89227	0.987296
39942	1.89227	0.317387
39943	1.00763	0.184535
39944	1.41787	0.360474
39945	1.05793	0.18559
39946	1.05793	0.35606
39947	1.37265	0.686319
39948	1.37265	0.335582
39949	1.37265	0.334595
39950	1.43063	1.10207
39951	1.41787	0.35324
39952	1.41787	0.987042
39953	1.38247	0.388584
39954	1.38247	0.97682
39955	0.946132	1.38422
39956	0.946132	1.50616
39957	0.946132	0.37117
39958	0.946132	0.482433
39959	0.946131	0.429534

39960	0.946131	1.49984
39961	0.946132	1.64702
39962	0.946132	0.812969
39963	0.946132	1.00224
39964	0.946132	1.22019
39965	0.946132	1.04042
39966	0.946132	1.05238
39967	1.89226	0.527271
39968	1.89226	0.579001
39969	1.89226	0.225517
39970	1.89226	0.585732
39971	1.89226	0.264313
39972	1.89226	0.644079
39973	1.19645	1.18286
39974	1.89183	0.925412
39975	1.91106	1.14047
39976	1.35306	1.05354
39977	1.75446	1.12458
39978	1.19645	1.02728
39979	2.37576	1.94071
39980	1.90139	2.1
39981	2.1845	2.1
39982	1.86568	2.1
39983	4.24145	2.1
39984	2.29435	2.1
39985	1.40602	2.1
39986	1.40602	2.1
39987	1.55351	2.1
39988	1.86568	2.1
39989	2.01317	2.1
39990	2.18452	2.1
39991	1.8657	1.7317
39992	2.01399	1.94344
39993	1.70141	2.1
39994	3.87969	2.08515
39995	0.774999	2.1
39996	0.774999	2.1
39997	0.778721	2.1
39998	0.778721	2.1
39999	0.628348	2.1
40000	0.703016	2.1
40001	0.932848	2.1
40002	0.850704	2.1
40003	0.850704	2.1
40004	0.628348	2.1
40005	0.703015	2.1
40006	0.932848	2.1
40007	1.65243	0.792192
40008	1.34412	0.714179
40009	1.45569	1.86389
40010	1.1315	1.85732
40011	2.78393	1.46976
40012	1.48901	2.1
40013	1.805 2.1	
40014	1.48901	2.1
40015	1.71717	2.1
40016	1.60635	2.1
40017	1.83451	2.1

40018	1.805 2.1	
40019	1.65243	0.709551
40020	1.60635	2.1
40021	1.45569	1.78235
40022	3.25879	2.0235
40023	1.27875	2.1
40024	1.60635	2.1
40025	1.27875	2.1
40026	1.48901	2.1
40027	1.40014	2.1
40028	1.6104	2.1
40029	0.715199	1.99935
40030	0.715199	2.1
40031	0.712714	2.1
40032	0.712714	1.08012
40033	0.727843	1.00874
40034	0.619713	1.47663
40035	0.803177	2.1
40036	0.70007	2.1
40037	0.700069	2.1
40038	0.619713	2.1
40039	0.803177	2.07931
40040	0.727844	2.1
40041	1.14357	2.1
40042	2.3506	2.04839
40043	1.14357	2.09682
40044	2.68773	2.00691
40045	1.57829	2.08962
40046	2.68773	1.99298
40047	2.00551	2.1
40048	2.65811	2.064
40049	2.07203	2.1
40050	2.72117	0.831198
40051	4.72667	2.1
40052	0.946131	1.49448
40053	0.946131	2.1
40054	1.32905	2.1
40055	1.32905	0.614973
40056	0.94613	1.59024
40057	0.94613	1.91074
40058	1.32905	1.84343
40059	1.36058	1.7479
40060	1.36058	0.393629
40061	0.946131	0.277394
40062	1.32905	1.71306
40063	0.946131	1.7567
40064	1.21649	0.960569
40065	1.21649	2.1
40066	1.21459	2.1
40067	1.21459	2.1
40068	1.39508	1.77763
40069	1.36057	1.78378
40070	1.03601	2.1
40071	1.07438	2.1
40072	1.07439	2.1
40073	1.39509	2.1
40074	1.036 2.1	
40075	1.36056	2.1

40076	2.79015	0.96784
40077	2.72114	2.1
40078	1.89225	1.49943
40079	1.89225	2.1
40080	1.89225	1.94285
40081	2.72114	1.78531
40082	0.876258	2.1
40083	0.892889	2.1
40084	1.34352	2.1
40085	1.27985	2.1
40086	1.07462	2.1
40087	1.07462	2.1
40088	1.09812	2.1
40089	1.09812	2.1
40090	0.892888	2.1
40091	1.34352	2.1
40092	0.876256	2.1
40093	1.27985	2.1
40094	1.39725	2.03385
40095	1.30029	0.866694
40096	1.31415	1.15461
40097	1.41336	1.98755
40098	2.71366	1.73595
40099	1.89226	1.00722
40100	1.89226	1.9291
40101	1.89226	2.01563
40102	1.89226	1.30271
40103	3.78453	1.69358
40104	0.946133	1.48445
40105	0.946133	1.23937
40106	1.38709	0.404367
40107	1.39437	0.520847
40108	0.946132	0.4113
40109	0.946132	1.0409
40110	1.39437	1.52663
40111	1.39437	0.428808
40112	0.946132	0.798638
40113	1.39437	0.735516
40114	1.38709	0.792102
40115	0.946132	0.826427
40116	0.706681	2.08113
40117	0.711908	2.03471
40118	0.657077	1.00328
40119	0.661003	1.52031
40120	0.684188	1.06777
40121	0.684188	2.03141
40122	0.683496	2.1
40123	0.683496	1.09156
40124	0.711908	1.82639
40125	0.661003	1.88915
40126	0.657076	1.71416
40127	0.706681	1.71903
40128	0.946132	2.05809
40129	0.946132	1.95754
40130	0.946132	1.64015
40131	0.946132	0.992181
40132	0.946132	0.9949
40133	0.946132	1.98195

40134	0.946133	1.64045
40135	0.946133	2.06901
40136	0.946133	1.7938
40137	0.946133	1.83987
40138	0.946132	1.57297
40139	0.946132	1.82962
40140	2.79029	0.479108
40141	1.89227	1.70574
40142	2.78873	0.690644
40143	2.78873	0.87679
40144	1.89383	0.724044
40145	1.89227	0.960824
40146	1.43669	2.1
40147	2.11984	2.1
40148	2.24481	2.1
40149	2.24481	2.1
40150	1.20311	2.1
40151	1.20311	2.1
40152	0.49703	2.1
40153	0.601553	2.1
40154	1.05992	2.1
40155	0.991694	2.1
40156	0.787558	2.1
40157	0.787558	2.1
40158	0.805689	2.1
40159	0.805689	2.1
40160	0.991694	2.1
40161	0.49703	2.1
40162	1.05992	2.1
40163	0.601553	2.1
40164	2.45905	2.1
40165	1.89226	2.1
40166	1.89226	2.1
40167	1.89226	2.1
40168	2.47395	2.1
40169	2.47395	2.1
40170	1.89226	2.1
40171	1.89226	2.1
40172	1.89226	2.1
40173	1.89226	2.05259
40174	3.78452	2.1
40175	1.38427	2.1
40176	2.31203	1.96521
40177	2.28743	1.99184
40178	2.28743	2.1
40179	1.63384	1.97522
40180	1.63384	2.1
40181	2.42723	2.1
40182	1.89226	2.1
40183	1.89226	2.1
40184	1.89226	2.1
40185	2.45905	2.1
40186	2.45905	2.1
40187	0.946131	1.88709
40188	0.946131	2.1
40189	0.946131	2.1
40190	0.946131	2.1
40191	0.94613	2.09004

40192	0.94613	2.1
40193	0.946132	2.1
40194	0.946132	2.1
40195	0.946131	2.1
40196	0.946131	2.1
40197	0.946131	2.1
40198	0.946131	2.1
40199	0.989441	2.1
40200	0.989441	2.1
40201	0.993918	2.1
40202	0.993918	2.1
40203	1.16644	2.1
40204	1.15601	2.1
40205	0.818308	2.1
40206	0.816922	2.1
40207	0.818307	2.1
40208	1.16644	2.1
40209	1.15601	2.1
40210	0.816922	2.1
40211	0.996778	0.05
40212	2.44375	0.05
40213	0.996778	0.0590368
40214	1.92547	0.05
40215	1.56091	0.0510127
40216	2.4896	0.05
40217	1.56091	0.0526249
40218	2.44375	0.05
40219	1.90456	0.0517283
40220	1.90456	0.0538941
40221	2.81853	0.05
40222	2.47487	0.05
40223	1.32143	2.05563
40224	1.43294	2.1
40225	1.37444	1.28724
40226	1.51691	2.1
40227	2.80738	1.93073
40228	1.89226	1.71235
40229	1.89226	2.1
40230	1.89226	1.44549
40231	1.89226	1.81521
40232	3.78453	1.58337
40233	1.89226	2.1
40234	1.89226	2.1
40235	1.89226	1.72063
40236	1.89226	2.1
40237	3.78453	2.1
40238	1.43294	2.09618
40239	1.3268	2.1
40240	1.51691	2.01687
40241	1.44091	2.1
40242	2.87385	2.1
40243	1.37444	2.01634
40244	1.51691	2.1
40245	1.4714	1.36089
40246	1.65434	1.89473
40247	3.02878	1.75252
40248	1.89226	2.03689
40249	1.89226	2.1

40250	1.89226	1.46817
40251	1.89226	1.93424
40252	3.78452	1.73623
40253	1.89226	1.9037
40254	1.89226	2.1
40255	1.89226	1.76226
40256	1.89226	2.1
40257	3.78453	2.1
40258	1.51691	2.1
40259	1.44091	2.1
40260	1.65434	1.7677
40261	1.60647	2.1
40262	3.09526	2.1
40263	1.05013	2.1
40264	1.05013	2.1
40265	0.94613	2.1
40266	0.94613	2.1
40267	1.04959	2.1
40268	1.05014	2.1
40269	0.946129	2.1
40270	0.946129	2.1
40271	0.946131	2.1
40272	1.04958	2.1
40273	0.946131	2.1
40274	1.05013	2.1
40275	1.04959	2.1
40276	1.04959	2.1
40277	0.946131	2.1
40278	0.946131	2.1
40279	1.04765	2.1
40280	1.04959	2.1
40281	0.94613	2.1
40282	0.94613	2.1
40283	0.946131	2.1
40284	1.04765	2.1
40285	0.946131	2.1
40286	1.04959	2.1
40287	1.85744	2.1
40288	1.89226	2.1
40289	1.89226	2.1
40290	1.89226	2.1
40291	1.94839	2.1
40292	1.94839	2.1
40293	0.97419	2.1
40294	0.822054	2.1
40295	0.946123	2.1
40296	0.946123	2.1
40297	1.04202	2.1
40298	0.84898	2.1
40299	0.933307	2.1
40300	0.933307	2.1
40301	0.933285	2.1
40302	1.05018	2.1
40303	0.851528	2.1
40304	0.876787	2.1
40305	1.87528	0.0941073
40306	1.21137	0.0643239
40307	1.57893	0.0612557

40308	1.21137	0.129778
40309	1.81182	0.0634182
40310	1.44426	0.119659
40311	0.993833	0.0552231
40312	1.21137	0.136526
40313	1.57893	0.0787254
40314	1.31774	0.05
40315	2.5291	0.0605191
40316	1.87528	0.0861565
40317	1.57893	0.0558827
40318	1.90728	0.0512807
40319	2.29851	0.0916499
40320	3.87744	0.0816293
40321	1.90728	0.0541295
40322	1.31774	0.0868277
40323	1.56339	0.05
40324	1.31774	0.0842887
40325	1.82458	0.05
40326	1.57893	0.104386
40327	1.89226	1.40112
40328	1.89226	1.47705
40329	1.89226	1.43696
40330	1.89226	1.45463
40331	1.89226	1.43706
40332	1.89226	1.45449
40333	1.89226	1.45773
40334	1.89226	1.48763
40335	1.89226	1.52068
40336	1.89226	1.49196
40337	3.78452	1.5277
40338	1.89226	1.47238
40339	2.17251	1.55553
40340	2.45575	1.47725
40341	2.45575	1.49428
40342	1.89226	1.46703
40343	1.89226	1.47783
40344	1.08625	1.54549
40345	1.22788	1.47894
40346	0.54447	1.4201
40347	0.794861	1.50467
40348	0.967729	1.57232
40349	0.967729	1.55052
40350	0.913386	1.43804
40351	0.913386	1.34752
40352	1.08625	1.53835
40353	0.544469	1.40145
40354	0.794862	1.55763
40355	1.22788	1.52756
40356	0.946128	1.44532
40357	0.946128	1.46276
40358	0.946132	1.46056
40359	0.946132	1.44034
40360	0.94613	1.45896
40361	0.94613	1.45326
40362	0.94613	1.46227
40363	0.94613	1.42177
40364	0.94613	1.42892
40365	0.94613	1.42976

40366	0.94613	1.41868
40367	0.94613	1.4381
40368	0.946127	1.44315
40369	0.946127	1.42942
40370	1.08625	1.41362
40371	0.958779	1.475
40372	1.08625	1.37071
40373	1.08625	1.46391
40374	0.946128	1.43759
40375	0.946128	1.43052
40376	0.946129	1.42136
40377	0.958777	1.43354
40378	1.08625	1.40459
40379	0.946129	1.42493
40380	2.16338	0.0708028
40381	1.94002	0.0542341
40382	1.98836	0.0635482
40383	2.60614	0.0705179
40384	1.5456	0.0506113
40385	2.16338	0.0522066
40386	1.19952	0.05
40387	2.34061	0.05
40388	1.21031	0.05
40389	2.1711	0.05
40390	3.54013	0.05
40391	1.13624	2.1
40392	1.70302	2.1
40393	1.8267	2.1
40394	1.11545	2.1
40395	2.85455	2.1
40396	1.82671	2.1
40397	1.91767	2.1
40398	1.81563	2.1
40399	1.91767	2.1
40400	1.71362	2.1
40401	1.83277	2.1
40402	1.66064	2.1
40403	0.996776	2.1
40404	0.996776	2.1
40405	1.11545	2.1
40406	1.54278	2.1
40407	1.66145	2.1
40408	1.66064	2.1
40409	1.7457	2.1
40410	1.81563	2.1
40411	1.81563	2.1
40412	1.54278	2.1
40413	1.54278	2.1
40414	2.71672	2.1
40415	2.70402	2.1
40416	2.64996	2.1
40417	2.69443	2.1
40418	5.39845	2.1
40419	2.71672	2.1
40420	2.54198	2.1
40421	2.54019	2.1
40422	2.69443	2.1
40423	5.23641	2.1

40424	2.64996	2.1
40425	2.69443	2.1
40426	2.68213	2.1
40427	2.61243	2.1
40428	5.30686	2.1
40429	2.54019	2.1
40430	2.69443	2.1
40431	2.68213	2.1
40432	2.5392	2.1
40433	5.23363	2.1
40434	1.89226	2.1
40435	2.34725	2.1
40436	2.22414	2.1
40437	2.34725	2.1
40438	1.76914	2.1
40439	1.89226	2.1
40440	0.946131	2.1
40441	0.946131	2.1
40442	1.11207	2.1
40443	1.03798	2.1
40444	0.94613	2.1
40445	0.94613	2.1
40446	1.03798	2.1
40447	1.03798	2.1
40448	0.946131	2.1
40449	1.03798	2.1
40450	0.946131	2.1
40451	1.11207	2.1
40452	1.89226	0.203569
40453	1.89226	0.179179
40454	1.89226	0.194934
40455	1.89226	0.165455
40456	1.89226	0.223098
40457	1.89226	0.192337
40458	1.89226	0.214035
40459	1.89226	0.208626
40460	1.89226	0.226939
40461	1.89226	0.178275
40462	1.89226	0.258066
40463	1.89226	0.206414
40464	1.89226	0.125276
40465	1.89226	0.118239
40466	1.89226	0.131616
40467	1.89226	0.114664
40468	1.89226	0.142366
40469	1.89226	0.124471
40470	1.44085	2.1
40471	1.31825	1.10586
40472	1.31923	1.86903
40473	1.31825	2.09628
40474	1.43311	1.54421
40475	1.43213	2.00135
40476	1.89226	1.26007
40477	1.89226	2.02646
40478	1.89226	1.69001
40479	1.89226	2.03821
40480	1.89226	1.34236
40481	1.89226	2.07115

40482	0.946133	1.71792
40483	0.946133	1.3253
40484	1.38969	0.539109
40485	1.39587	0.798538
40486	0.946132	0.575664
40487	0.946132	1.19862
40488	1.39587	1.73562
40489	1.39587	0.588855
40490	0.946132	0.943299
40491	1.39587	0.842254
40492	1.38969	0.986956
40493	0.946132	1.17256
40494	1.44085	2.1
40495	1.31923	1.04773
40496	1.33026	1.86423
40497	1.4617	2.1
40498	2.78093	1.98316
40499	1.89226	1.20094
40500	1.89226	1.99347
40501	1.89226	1.67748
40502	1.89226	2.07239
40503	1.89226	1.38823
40504	1.89226	2.1
40505	0.946133	1.62607
40506	0.946133	1.2013
40507	1.39587	0.722165
40508	1.40724	0.499352
40509	0.946132	0.555687
40510	0.946132	1.05476
40511	1.40724	1.6551
40512	1.40724	0.643838
40513	0.946132	0.859212
40514	1.40724	0.844845
40515	0.946133	1.26947
40516	1.39587	0.904877
40517	1.15818	1.41337
40518	1.81846	1.3336
40519	1.52782	1.22245
40520	1.52782	1.57364
40521	1.28095	1.21771
40522	1.28095	1.45198
40523	2.56709	2.1
40524	1.4349	1.76651
40525	1.4349	2.1
40526	1.75471	2.1
40527	2.29909	2.1
40528	2.61889	2.1
40529	2.5671	2.1
40530	2.82858	2.1
40531	2.57368	2.1
40532	2.29909	2.1
40533	5.12767	2.1
40534	2.29909	2.1
40535	1.09148	1.18664
40536	1.09148	2.1
40537	1.4349	1.97371
40538	1.99776	2.03413
40539	2.34119	2.1

40540	2.57368	2.1
40541	1.99777	2.1
40542	1.99777	2.1
40543	2.29909	2.1
40544	2.36071	2.1
40545	2.66203	2.1
40546	1.89226	1.55224
40547	1.89226	1.67303
40548	1.89226	1.60436
40549	1.89226	1.53535
40550	3.78453	1.55413
40551	0.946128	1.78859
40552	0.946128	1.86911
40553	0.946133	1.69118
40554	0.946133	1.75022
40555	0.946131	1.74026
40556	0.946131	1.84259
40557	0.946131	1.83069
40558	0.946131	1.75574
40559	0.94613	1.84196
40560	0.94613	1.7754
40561	0.946131	1.73078
40562	0.946131	1.79508
40563	1.89226	1.67599
40564	1.89226	1.68103
40565	1.89226	1.53822
40566	1.89226	1.73361
40567	3.78453	1.71381
40568	1.89226	1.71483
40569	1.89226	1.62203
40570	1.89226	1.5276
40571	1.89226	1.6793
40572	3.78453	1.68423
40573	1.89226	1.62771
40574	2.61682	1.82028
40575	1.89226	1.75872
40576	1.89226	1.81205
40577	1.89226	1.61383
40578	1.89226	1.75352
40579	1.89226	0.314493
40580	1.89226	0.217243
40581	1.89226	0.236545
40582	1.89226	0.265841
40583	3.78452	0.303305
40584	1.89226	0.33679
40585	1.89226	0.257061
40586	1.89226	0.259759
40587	1.89226	0.327215
40588	1.89226	0.302597
40589	1.89226	0.310194
40590	1.89227	0.220408
40591	1.89227	0.0868213
40592	1.89227	0.252207
40593	1.89227	0.30547
40594	1.89227	0.0774832
40595	1.89227	0.221527
40596	0.80447	0.291106
40597	0.534241	0.296462

40598	0.995175	0.326594
40599	0.92204	0.121166
40600	0.922042	0.0883192
40601	0.534241	0.179957
40602	0.804468	0.295611
40603	0.995176	0.175495
40604	0.71543	0.351321
40605	0.71543	0.077319
40606	0.813988	0.179703
40607	0.813988	0.156334
40608	0.922045	0.05
40609	0.942899	0.210321
40610	0.946132	0.125838
40611	0.946132	0.104637
40612	1.05169	0.05
40613	0.936661	0.0948082
40614	0.922045	0.0529552
40615	0.946133	0.050954
40616	1.01665	0.056032
40617	1.13008	0.0505346
40618	0.851529	0.05
40619	0.851529	0.05
40620	1.41908	2.1
40621	1.77723	2.1
40622	1.90485	2.1
40623	1.90485	2.1
40624	1.30237	2.1
40625	1.30237	2.1
40626	1.77723	2.1
40627	2.0055	2.1
40628	1.90485	2.1
40629	2.17158	2.1
40630	3.91035	2.1
40631	0.850906	2.1
40632	0.850906	2.1
40633	0.85668	2.1
40634	0.85668	2.1
40635	0.998045	2.1
40636	0.952425	2.1
40637	0.74367	2.1
40638	0.709541	2.1
40639	0.74367	2.1
40640	0.998045	2.1
40641	0.709541	2.1
40642	0.952425	2.1
40643	1.90485	2.1
40644	2.28563	2.1
40645	2.26282	2.1
40646	2.17158	2.1
40647	1.99609	2.1
40648	1.90485	2.1
40649	2.00098	0.146066
40650	1.90462	0.106469
40651	1.60057	0.113322
40652	1.79224	0.137679
40653	3.69686	0.119826
40654	2.65654	0.147752
40655	1.79224	0.10063

40656	2.22332	0.104901
40657	2.22332	0.127777
40658	2.24163	0.11737
40659	2.24163	0.128903
40660	1.89226	0.129606
40661	1.89226	0.122118
40662	1.89226	0.0998463
40663	1.89226	0.126996
40664	3.78452	0.126035
40665	1.89226	0.103892
40666	1.89226	0.212722
40667	1.89226	0.111978
40668	1.89226	0.146207
40669	1.89226	0.113838
40670	1.89226	0.147862
40671	2.24163	0.168647
40672	1.03645	0.131874
40673	1.79224	0.136279
40674	1.79224	0.150933
40675	1.54283	0.156989
40676	1.54283	0.161538
40677	0.946132	0.13221
40678	0.946132	0.134275
40679	0.946129	0.144512
40680	0.946129	0.132858
40681	0.94613	0.11226
40682	0.94613	0.143277
40683	0.946131	0.136287
40684	0.946131	0.153318
40685	0.94613	0.127108
40686	0.94613	0.163629
40687	0.946131	0.136973
40688	0.946131	0.116858
40689	1.89226	2.1
40690	1.89226	2.1
40691	1.89226	2.1
40692	1.89226	2.1
40693	3.78453	2.1
40694	1.52441	2.1
40695	1.30347	2.1
40696	1.52441	2.1
40697	1.58401	2.1
40698	1.2627	2.1
40699	1.3223	2.1
40700	1.89226	2.1
40701	2.72787	2.1
40702	2.72787	2.1
40703	2.74762	2.1
40704	1.89226	2.1
40705	1.91202	2.1
40706	1.89227	2.1
40707	1.89227	2.1
40708	1.89227	2.1
40709	1.89227	2.1
40710	3.78453	2.1
40711	0.56414	2.1
40712	0.561557	2.1
40713	0.527284	2.1

40714	0.541143	2.1
40715	0.527276	2.1
40716	0.631685	2.1
40717	1.52441	2.1
40718	1.00232	2.1
40719	0.784495	2.1
40720	0.842304	2.1
40721	0.721569	2.1
40722	1.36393	2.1
40723	1.36393	2.1
40724	0.946132	2.1
40725	0.946132	2.1
40726	1.36393	2.1
40727	1.3594	2.1
40728	0.946132	2.1
40729	0.946132	2.1
40730	0.946107	2.1
40731	1.35936	2.1
40732	1.36397	2.1
40733	0.946158	2.1
40734	1.89226	2.1
40735	1.89226	2.1
40736	1.89226	2.1
40737	1.89226	2.1
40738	3.78453	2.1
40739	2.20802	2.1
40740	1.05677	2.1
40741	2.30479	2.1
40742	0.978699	2.1
40743	3.18672	2.1
40744	0.94613	2.1
40745	0.94613	2.1
40746	0.669866	2.1
40747	0.669866	2.1
40748	0.681588	2.1
40749	0.669865	2.1
40750	0.946133	2.1
40751	0.946133	2.1
40752	0.94613	2.1
40753	0.681589	2.1
40754	0.946131	2.1
40755	0.669867	2.1
40756	2.73514	2.1
40757	1.78301	2.1
40758	1.90618	2.1
40759	2.83464	2.1
40760	4.64132	2.1
40761	2.73514	2.1
40762	1.89226	2.1
40763	1.89226	2.1
40764	2.83464	2.1
40765	4.6274	2.1
40766	2.03915	2.1
40767	2.26616	2.1
40768	2.23549	2.1
40769	2.01704	2.1
40770	4.2832	2.1
40771	2.03916	2.1

40772	1.55766	2.1
40773	1.53728	2.1
40774	2.01704	2.1
40775	3.5747	2.1
40776	2.23551	2.1
40777	2.01705	2.1
40778	1.99823	2.1
40779	2.21189	2.1
40780	4.22894	2.1
40781	1.51788	2.1
40782	2.01705	2.1
40783	1.53729	2.1
40784	1.53729	2.1
40785	1.99823	2.1
40786	1.99823	2.1
40787	2.588	2.1
40788	1.45581	2.1
40789	1.45581	2.1
40790	1.44207	2.1
40791	2.54949	2.1
40792	2.53574	2.1
40793	0.98641	2.1
40794	0.98641	2.1
40795	1.00371	2.1
40796	1.00371	2.1
40797	0.749992	2.1
40798	0.727904	2.1
40799	1.26221	2.1
40800	1.27474	2.1
40801	1.26221	2.1
40802	0.749993	2.1
40803	0.727904	2.1
40804	1.27474	2.1
40805	1.23105	0.935675
40806	1.35435	1.76952
40807	1.89226	1.60127
40808	1.89226	1.31778
40809	1.89226	1.67428
40810	1.7043	1.48619
40811	1.89226	1.82559
40812	1.89226	1.63783
40813	1.89226	1.11795
40814	1.89226	1.78881
40815	3.78453	1.70468
40816	1.89226	0.807789
40817	1.89226	1.75999
40818	1.89226	1.47181
40819	1.89226	1.45393
40820	1.89226	1.5473
40821	1.89226	1.57003
40822	0.946131	1.04959
40823	1.40945	0.878295
40824	0.94613	1.31759
40825	0.94613	1.58087
40826	0.946129	1.58747
40827	0.946129	1.44105
40828	0.946132	1.66422
40829	1.40945	0.707467

40830	0.94613	1.2757
40831	0.94613	1.40273
40832	0.946131	1.43247
40833	0.94613	1.0239
40834	0.946133	1.33847
40835	0.946133	1.3369
40836	0.946132	1.55656
40837	0.946132	1.68435
40838	0.946132	1.67944
40839	0.946132	1.51036
40840	0.946133	1.53204
40841	0.946133	1.36333
40842	0.946132	1.5184
40843	0.946132	1.53374
40844	0.946133	1.41042
40845	0.946133	1.5152
40846	1.89226	1.45243
40847	1.89226	1.86641
40848	1.89226	1.63438
40849	1.89226	1.57961
40850	1.89226	1.73063
40851	1.89226	1.64301
40852	1.89226	2.1
40853	1.89226	2.1
40854	1.89226	2.1
40855	1.89226	2.1
40856	1.89226	2.1
40857	1.89226	2.1
40858	1.89226	2.1
40859	1.89226	2.1
40860	1.89226	2.1
40861	1.89226	2.1
40862	3.78453	2.1
40863	1.66647	2.1
40864	1.15399	2.1
40865	1.25443	2.1
40866	1.26599	2.1
40867	1.58697	2.1
40868	1.66647	2.1
40869	1.66647	2.1
40870	1.47446	2.1
40871	1.55433	2.1
40872	1.74241	2.1
40873	3.2208	2.1
40874	1.89227	2.1
40875	1.89227	2.1
40876	1.89227	2.1
40877	1.89227	2.1
40878	3.78453	2.1
40879	0.946132	2.1
40880	0.946132	2.1
40881	0.946132	2.1
40882	0.946132	2.1
40883	0.946132	2.1
40884	0.946132	2.1
40885	0.946133	2.1
40886	0.946133	2.1
40887	0.946132	2.1

40888	0.946132	2.1
40889	0.946133	2.1
40890	0.946133	2.1
40891	0.625067	2.1
40892	0.61907	2.1
40893	0.896913	2.1
40894	0.946199	2.1
40895	0.806287	2.1
40896	0.677685	2.1
40897	0.750657	2.1
40898	0.750657	2.1
40899	0.646004	2.1
40900	0.890236	2.1
40901	0.61049	2.1
40902	0.888979	2.1
40903	1.6223	2.1
40904	1.98034	2.1
40905	1.75003	2.1
40906	1.55433	2.1
40907	1.75003	2.1
40908	1.55433	2.1
40909	1.88602	2.1
40910	2.08149	2.1
40911	2.07991	2.1
40912	1.88473	2.1
40913	2.08149	2.1
40914	1.88602	2.1
40915	1.88458	2.1
40916	2.08149	2.1
40917	1.7069	2.1
40918	1.56284	2.1
40919	2.08149	2.1
40920	1.88458	2.1
40921	1.5955	2.1
40922	1.89226	2.1
40923	1.91975	2.1
40924	1.62299	2.1
40925	1.89226	2.1
40926	1.5955	2.1
40927	1.89226	2.1
40928	1.89226	2.1
40929	1.89226	2.00684
40930	1.89226	2.1
40931	1.89226	1.93563
40932	1.89226	2.1
40933	1.89226	2.1
40934	1.89226	2.1
40935	1.89226	2.1
40936	1.89226	2.1
40937	1.89226	2.1
40938	1.89226	2.1
40939	1.62299	2.1
40940	1.89226	2.1
40941	1.71752	2.1
40942	1.44824	2.1
40943	1.89226	2.1
40944	1.62299	2.1
40945	1.89226	1.38056

40946	2.8317	0.783484
40947	1.89226	1.07697
40948	2.64055	0.652799
40949	4.53281	1.32285
40950	2.8317	1.24078
40951	1.83151	0.562595
40952	2.01001	0.84384
40953	2.01001	1.39618
40954	2.64055	0.505188
40955	2.64055	0.997438
40956	0.946132	0.270004
40957	0.946132	0.394806
40958	1.16167	0.299009
40959	1.16167	0.352417
40960	1.16167	0.282024
40961	1.32027	0.270431
40962	0.946131	0.285006
40963	0.946131	0.371551
40964	0.94613	0.257723
40965	1.16167	0.228863
40966	0.946131	0.322878
40967	1.32027	0.30344
40968	2.32335	0.366818
40969	1.83151	0.361475
40970	2.38525	0.34767
40971	2.64055	0.371769
40972	1.57621	0.26918
40973	1.83151	0.280821
40974	2.25677	2.1
40975	1.88158	2.1
40976	1.86856	2.1
40977	2.0815	2.1
40978	1.70303	2.1
40979	1.88158	2.1
40980	2.50381	2.1
40981	1.89227	2.1
40982	2.25677	2.1
40983	2.25677	2.1
40984	1.89227	2.1
40985	1.89227	2.1
40986	1.89226	0.096499
40987	1.50899	0.090651
40988	2.13659	0.0971773
40989	1.50899	0.131743
40990	2.51986	0.0803779
40991	1.89226	0.11555
40992	0.946131	0.133742
40993	0.946131	0.101603
40994	1.06829	0.0893925
40995	0.895375	0.0882727
40996	0.94613	0.0806862
40997	0.94613	0.12848
40998	1.33922	0.0913523
40999	0.895376	0.0858982
41000	0.946131	0.0959888
41001	1.33922	0.100097
41002	0.946131	0.104098
41003	1.06829	0.112792

41004	0.841467	0.377083
41005	0.578428	0.453134
41006	1.18079	0.300357
41007	0.920237	0.489621
41008	0.772317	0.252631
41009	0.856555	0.804254
41010	0.910271	0.372616
41011	0.910271	0.746347
41012	0.975752	0.359452
41013	0.536929	0.283459
41014	0.81952	0.459202
41015	1.15252	0.306545
41016	1.89226	2.1
41017	2.51431	2.1
41018	1.89226	2.1
41019	1.89226	2.1
41020	2.78815	2.1
41021	2.78815	2.1
41022	2.0815	2.1
41023	2.0516	2.1
41024	2.0815	2.1
41025	3.06699	2.1
41026	2.76576	2.1
41027	2.8002	2.1
41028	1.94112	2.1
41029	1.89226	2.1
41030	2.20358	2.1
41031	1.89226	2.1
41032	4.09584	2.1
41033	1.09154	2.1
41034	2.62647	2.1
41035	2.20358	2.1
41036	2.20358	2.1
41037	1.52314	2.1
41038	1.52314	2.1
41039	1.31323	2.1
41040	1.31323	2.1
41041	0.946132	2.1
41042	0.946132	2.1
41043	0.946132	2.1
41044	0.946132	2.1
41045	1.31323	2.1
41046	1.10179	2.1
41047	1.31323	2.1
41048	0.946132	2.1
41049	1.10179	2.1
41050	0.946133	2.1
41051	1.89226	1.5803
41052	1.89226	1.76382
41053	1.89226	1.70233
41054	1.89226	1.8209
41055	1.89226	1.54093
41056	1.89226	1.72351
41057	1.35126	1.86929
41058	1.35126	2.05452
41059	0.903285	1.73756
41060	0.903285	1.78611
41061	1.30841	1.80672

41062	0.903313	2.00335
41063	0.946132	1.81203
41064	0.946132	1.70414
41065	0.946132	1.7098
41066	0.903312	1.78676
41067	1.30841	1.75559
41068	0.946133	1.71689
41069	2.62097	2.1
41070	2.72561	2.1
41071	2.39016	2.1
41072	2.51062	2.1
41073	5.11577	2.1
41074	2.51062	2.1
41075	2.58286	2.1
41076	2.40107	2.1
41077	2.40107	2.1
41078	2.72561	2.1
41079	2.72561	2.1
41080	2.39016	2.1
41081	2.51062	2.1
41082	2.20242	2.1
41083	2.34107	2.1
41084	4.73124	2.1
41085	2.51062	2.1
41086	2.40107	2.1
41087	2.34107	2.1
41088	2.25197	2.1
41089	4.74215	2.1
41090	2.75218	2.1
41091	1.89226	2.1
41092	2.3349	2.1
41093	2.3349	2.1
41094	1.89226	2.1
41095	1.89226	2.1
41096	1.46129	2.1
41097	0.996308	2.1
41098	1.88649	2.1
41099	1.70302	2.1
41100	2.95419	2.1
41101	1.89227	2.1
41102	1.89227	2.1
41103	1.89227	2.1
41104	1.89227	2.1
41105	3.78453	2.1
41106	0.646558	2.1
41107	0.646557	2.1
41108	0.60697	2.1
41109	0.60697	2.1
41110	0.587917	2.1
41111	0.519747	2.1
41112	0.586443	2.1
41113	0.733782	2.1
41114	0.733793	2.1
41115	0.587924	2.1
41116	0.519739	2.1
41117	0.586436	2.1
41118	2.1642	2.1
41119	1.42867	2.1

41120	2.28847	2.1
41121	1.68299	2.1
41122	3.71714	2.1
41123	1.89226	2.1
41124	1.89226	2.1
41125	1.89226	2.1
41126	1.89226	2.1
41127	3.78453	2.1
41128	0.946131	2.1
41129	0.946131	2.1
41130	1.24994	2.1
41131	1.24994	2.1
41132	1.11582	2.1
41133	1.24994	2.1
41134	0.946133	2.1
41135	0.946133	2.1
41136	0.946131	2.1
41137	1.24994	2.1
41138	0.94613	2.1
41139	1.11582	2.1
41140	2.28847	2.1
41141	1.53629	2.1
41142	1.57498	2.1
41143	1.47417	2.1
41144	2.42221	2.1
41145	2.28847	2.1
41146	1.89226	2.1
41147	1.89226	2.1
41148	1.89226	2.1
41149	1.89226	2.1
41150	3.78453	2.1
41151	1.89226	2.1
41152	2.49988	2.1
41153	2.71233	2.1
41154	2.71233	2.1
41155	1.89226	2.1
41156	1.89226	2.1
41157	0.924534	2.1
41158	0.924534	2.1
41159	0.990395	2.1
41160	0.990395	2.1
41161	0.946131	2.1
41162	0.946131	2.1
41163	0.924505	2.1
41164	0.968797	2.1
41165	0.924505	2.1
41166	0.946131	2.1
41167	0.968797	2.1
41168	0.946131	2.1
41169	0.924533	2.1
41170	0.924533	2.1
41171	0.948264	2.1
41172	0.945345	2.1
41173	0.851541	2.1
41174	0.851541	2.1
41175	0.958934	2.1
41176	1.02617	2.1
41177	0.936668	2.1

41178	1.04829	2.1
41179	0.924505	2.1
41180	0.946131	2.1
41181	2.28431	0.64043
41182	1.48035	0.142944
41183	1.59971	0.270227
41184	1.48035	0.329556
41185	2.33818	0.192417
41186	2.21882	0.243044
41187	2.28431	0.325244
41188	1.89226	0.389461
41189	1.89226	0.612756
41190	2.3968	0.300778
41191	4.28906	0.338803
41192	2.28431	0.323385
41193	1.70303	0.151519
41194	1.5428	0.15409
41195	1.59971	0.214286
41196	2.31152	0.218527
41197	2.3968	0.268804
41198	1.99823	2.1
41199	2.21189	2.1
41200	2.18937	2.1
41201	1.979 2.1	
41202	4.19089	2.1
41203	0.999114	2.1
41204	0.9895	2.1
41205	0.758942	2.1
41206	0.749358	2.1
41207	0.874236	2.1
41208	0.874236	2.1
41209	0.874236	2.1
41210	0.874236	2.1
41211	0.9895	2.1
41212	0.749358	2.1
41213	0.758942	2.1
41214	0.999114	2.1
41215	2.18937	2.1
41216	1.979 2.1	
41217	1.95416	2.1
41218	2.162 2.1	
41219	4.14099	2.1
41220	1.95416	2.1
41221	1.49871	2.1
41222	1.47712	2.1
41223	1.49871	2.1
41224	1.9574	2.1
41225	1.979 2.1	
41226	1.68508	0.05
41227	1.64984	0.0598877
41228	2.67711	0.05
41229	2.76407	0.0590515
41230	4.41391	0.0567512
41231	1.84227	0.05
41232	2.76407	0.0539128
41233	2.76407	0.0563431
41234	1.85007	0.05
41235	2.72769	0.053643

41236	1.80812	0.05
41237	1.94516	2.1
41238	1.89226	2.1
41239	1.89226	2.1
41240	1.89226	2.1
41241	1.98853	2.1
41242	1.98852	2.1
41243	1.28095	2.1
41244	2.0815	2.1
41245	2.29123	2.1
41246	2.06833	2.1
41247	1.08212	2.1
41248	0.976848	2.1
41249	1.89226	2.1
41250	1.89226	2.1
41251	1.89226	2.1
41252	1.89226	2.1
41253	1.89226	2.1
41254	1.89226	2.1
41255	1.13537	2.1
41256	1.18233	2.1
41257	0.946132	2.1
41258	0.946132	2.1
41259	0.946132	2.1
41260	0.946132	2.1
41261	0.994264	2.1
41262	1.18233	2.1
41263	1.13537	2.1
41264	0.946131	2.1
41265	0.946132	2.1
41266	0.994264	2.1
41267	0.953204	2.1
41268	0.953204	2.1
41269	0.966515	2.1
41270	0.966515	2.1
41271	1.30583	2.1
41272	1.14561	2.1
41273	0.640475	2.1
41274	0.774103	2.1
41275	1.30584	2.1
41276	0.774101	2.1
41277	0.640474	2.1
41278	1.14562	2.1
41279	0.946131	2.1
41280	0.946131	2.1
41281	0.946131	2.1
41282	0.946131	2.1
41283	0.946128	2.1
41284	0.946128	2.1
41285	0.946133	2.1
41286	0.946133	2.1
41287	0.94613	2.1
41288	0.94613	2.1
41289	0.946131	2.1
41290	0.946131	2.1
41291	0.946132	0.05
41292	0.946132	0.05
41293	0.946131	0.05

41294	0.946131	0.05
41295	0.946131	0.05
41296	0.946131	0.05
41297	0.946132	0.05
41298	0.946132	0.05
41299	0.946131	0.05
41300	0.946131	0.05
41301	0.946132	0.05
41302	0.946132	0.05
41303	0.946131	0.05
41304	0.946131	0.05
41305	0.946131	0.05
41306	0.946131	0.05
41307	0.94613	0.05
41308	0.94613	0.05
41309	0.946132	0.05
41310	0.946132	0.05
41311	0.946131	0.05
41312	0.946131	0.05
41313	0.946131	0.05
41314	0.946131	0.05
41315	2.57882	0.108554
41316	1.48676	0.117586
41317	1.48797	0.103542
41318	2.64281	0.103079
41319	4.12957	0.105862
41320	2.56323	0.0941275
41321	2.64281	0.102067
41322	2.51807	0.0965501
41323	1.36324	0.0930401
41324	1.48797	0.0968123
41325	1.48797	0.0983362
41326	1.04367	2.1
41327	2.42504	2.1
41328	2.65164	2.1
41329	1.49479	2.1
41330	3.91983	2.1
41331	2.15622	2.1
41332	1.70539	2.1
41333	2.02369	2.1
41334	2.39039	2.1
41335	4.17992	2.1
41336	0.966979	2.1
41337	1.7054	2.1
41338	2.0237	2.1
41339	1.31502	2.1
41340	2.99067	2.1
41341	2.02369	2.1
41342	2.65637	2.1
41343	2.39039	2.1
41344	2.39039	2.1
41345	2.29332	2.1
41346	2.29332	2.1
41347	2.0237	2.1
41348	1.72116	2.1
41349	1.58465	2.1
41350	1.31502	2.1
41351	2.29332	2.1

41352	2.0237	2.1
41353	1.0372	0.05
41354	2.32462	0.05
41355	2.2291	0.05
41356	2.2291	0.05
41357	1.17464	0.05
41358	1.17464	0.05
41359	2.68289	0.0521932
41360	1.07805	0.0555072
41361	1.70302	0.05
41362	2.06653	0.0526291
41363	3.5779	0.0520515
41364	1.89226	0.106644
41365	1.89226	0.169454
41366	1.89226	0.135388
41367	1.89226	0.0797204
41368	3.78453	0.110371
41369	1.89226	0.0634148
41370	1.89226	0.0691162
41371	1.89226	0.0698995
41372	1.89226	0.0695673
41373	1.89226	0.0640826
41374	1.89226	0.0634846
41375	1.89225	0.0668407
41376	1.89225	0.0633115
41377	1.89225	0.0656517
41378	1.89225	0.0655597
41379	1.89225	0.0664878
41380	1.89225	0.0667029
41381	0.94613	0.0897538
41382	0.94613	0.113537
41383	0.94613	0.163831
41384	0.94613	0.128137
41385	0.946128	0.106692
41386	0.946128	0.114922
41387	0.946132	0.176634
41388	0.946132	0.0849223
41389	0.94613	0.0997717
41390	0.94613	0.124363
41391	0.94613	0.121667
41392	0.94613	0.095247
41393	1.29417	2.1
41394	1.32732	2.1
41395	1.32316	1.51742
41396	1.34973	2.1
41397	2.65048	2.1
41398	1.89226	1.72103
41399	1.89226	2.1
41400	1.89226	1.59134
41401	1.89226	2.1
41402	3.78453	2.099
41403	1.89226	2.1
41404	1.89226	2.1
41405	1.89226	2.1
41406	1.89226	2.1
41407	3.78453	2.1
41408	1.32732	2.1
41409	1.13006	2.1

41410	1.13358	2.1
41411	1.11117	2.1
41412	1.34973	2.1
41413	1.32732	2.1
41414	1.32316	1.97313
41415	1.34973	2.1
41416	1.34066	1.74192
41417	1.36601	2.1
41418	2.68918	2.1
41419	1.89226	1.99815
41420	1.89226	2.1
41421	1.89226	0.692737
41422	1.89226	2.1
41423	3.78453	2.1
41424	1.89226	2.1
41425	1.89226	2.1
41426	1.89226	2.1
41427	1.89226	2.1
41428	1.89226	2.1
41429	1.89226	2.1
41430	0.624134	2.1
41431	0.624134	2.1
41432	0.624334	2.1
41433	0.624334	2.1
41434	0.56503	2.1
41435	0.573605	2.1
41436	0.674864	2.1
41437	0.683007	2.1
41438	0.683007	2.1
41439	0.573605	2.1
41440	0.565029	2.1
41441	0.674864	2.1
41442	2.16633	2.1
41443	1.40846	2.1
41444	2.13977	2.1
41445	1.40771	2.1
41446	3.57404	2.1
41447	2.13977	2.1
41448	1.29932	2.1
41449	1.29932	2.1
41450	1.40771	2.1
41451	1.98957	2.1
41452	2.09796	2.1
41453	2.6044	0.190267
41454	1.85548	0.273109
41455	1.89226	0.119404
41456	1.89226	0.331017
41457	3.74774	0.210144
41458	1.89226	0.128828
41459	1.89226	0.443417
41460	1.89226	0.158394
41461	1.89226	0.206049
41462	3.78453	0.168625
41463	1.89226	2.1
41464	2.64107	2.1
41465	1.89226	2.1
41466	2.78977	2.1
41467	1.89226	2.1

41468	2.78977	2.1
41469	1.89226	2.1
41470	1.89226	2.1
41471	1.89226	2.1
41472	1.89226	2.1
41473	1.89226	2.1
41474	1.89226	2.1
41475	2.43173	2.1
41476	1.41238	2.1
41477	2.0815	2.1
41478	1.98048	2.1
41479	1.62014	2.1
41480	1.50086	2.1
41481	0.946129	0.428904
41482	0.946129	0.372429
41483	1.27636	0.31712
41484	1.1984	0.233157
41485	0.946131	0.244988
41486	0.946131	0.346941
41487	1.27636	0.449577
41488	1.27636	0.247595
41489	0.946131	0.324866
41490	1.27636	0.318304
41491	0.946131	0.312208
41492	1.1984	0.309699
41493	1.99569	0.16106
41494	2.55272	0.314828
41495	1.77403	0.161048
41496	1.70303	0.194144
41497	3.05772	0.166216
41498	2.55121	0.212857
41499	0.946128	0.311334
41500	0.946128	0.356034
41501	1.38752	0.272704
41502	1.27636	0.196041
41503	0.94613	0.204449
41504	0.94613	0.294022
41505	1.38752	0.366644
41506	1.38752	0.206996
41507	0.946131	0.269912
41508	1.38752	0.268628
41509	0.946131	0.269279
41510	1.27636	0.26508
41511	2.77504	0.246772
41512	1.99569	0.126851
41513	2.29794	0.147429
41514	1.99569	0.194817
41515	2.85497	0.125696
41516	2.55272	0.165992
41517	1.53284	2.1
41518	1.89226	2.1
41519	1.89226	2.1
41520	1.89226	2.1
41521	1.54763	2.1
41522	1.54763	2.1
41523	0.946131	2.1
41524	0.946131	2.1
41525	0.946127	2.1

41526	0.946127	2.1
41527	0.946127	2.1
41528	0.946127	2.1
41529	0.946131	2.1
41530	0.946131	2.1
41531	0.94613	2.1
41532	0.94613	2.1
41533	0.946129	2.1
41534	0.946129	2.1
41535	0.751754	2.1
41536	0.751754	2.1
41537	0.771562	2.1
41538	0.771561	2.1
41539	1.0268	2.1
41540	0.95482	2.1
41541	0.528937	2.1
41542	0.496512	2.1
41543	0.528936	2.1
41544	1.0268	2.1
41545	0.954821	2.1
41546	0.49651	2.1
41547	2.64075	0.232166
41548	1.35181	1.54198
41549	1.35181	0.300824
41550	1.83823	0.261579
41551	2.28373	1.15245
41552	2.88566	1.3995
41553	0.493018	0.932846
41554	0.675903	0.755239
41555	1.14187	0.276033
41556	0.973937	0.0625227
41557	0.821166	0.314091
41558	0.821166	1.23846
41559	0.828674	1.69509
41560	0.828674	0.215258
41561	0.973937	1.28523
41562	0.493018	0.547121
41563	0.675903	0.325981
41564	1.14187	0.359003
41565	1.89226	2.1
41566	2.11667	2.1
41567	1.91184	2.1
41568	1.89226	2.1
41569	2.13625	2.1
41570	2.11667	2.1
41571	1.89226	2.1
41572	1.89226	2.1
41573	1.89226	2.1
41574	1.89226	2.1
41575	3.78453	2.1
41576	0.907729	2.1
41577	0.907729	2.1
41578	1.04459	2.1
41579	0.890955	2.1
41580	0.988181	2.1
41581	0.841856	2.1
41582	0.946133	2.1
41583	0.946133	2.1

41584	1.04072	2.1
41585	1.1064	2.1
41586	0.89099	2.1
41587	0.851541	2.1
41588	1.89225	2.1
41589	2.13623	2.1
41590	1.91044	2.1
41591	1.89225	2.1
41592	2.15442	2.1
41593	2.13623	2.1
41594	1.89225	2.1
41595	1.89225	2.1
41596	1.89225	2.1
41597	1.89225	2.1
41598	1.89225	2.1
41599	1.89225	2.1
41600	0.907723	2.1
41601	0.907723	2.1
41602	1.07536	2.1
41603	1.07814	2.1
41604	0.998499	2.1
41605	1.03575	2.1
41606	0.946126	2.1
41607	0.946126	2.1
41608	1.04072	2.1
41609	1.14025	2.1
41610	0.851528	2.1
41611	0.898284	2.1
41612	1.89226	0.0703337
41613	1.89226	0.140904
41614	1.89226	0.0951308
41615	1.89226	0.0860183
41616	1.89226	0.0876386
41617	1.89226	0.0803466
41618	2.53008	2.1
41619	1.94534	2.1
41620	1.98702	2.1
41621	1.98858	2.1
41622	2.52852	2.1
41623	2.53008	2.1
41624	1.03267	2.1
41625	1.12591	2.1
41626	1.2959	2.1
41627	1.28992	2.1
41628	1.10851	2.1
41629	1.33078	2.1
41630	1.10661	2.1
41631	1.10661	2.1
41632	0.982208	2.1
41633	1.28992	2.1
41634	1.03267	2.1
41635	1.23262	2.1
41636	2.2712	2.1
41637	1.89226	2.1
41638	1.95954	2.1
41639	1.95954	2.1
41640	1.89226	2.1
41641	1.89226	2.1

41642	1.89226	2.1
41643	1.89226	2.1
41644	1.89226	2.1
41645	1.89226	2.1
41646	3.78453	2.1
41647	1.33887	2.1
41648	1.25395	2.1
41649	0.88327	2.1
41650	0.676665	2.1
41651	0.84881	2.1
41652	0.987503	2.1
41653	1.13243	2.1
41654	1.13243	2.1
41655	1.15338	2.1
41656	0.849966	2.1
41657	0.793074	2.1
41658	1.11957	2.1
41659	0.946129	2.1
41660	0.946129	2.1
41661	1.28254	2.1
41662	1.1356	2.1
41663	1.1356	2.1
41664	1.1356	2.1
41665	0.946131	2.1
41666	0.946131	2.1
41667	0.946131	2.1
41668	1.28254	2.1
41669	1.1356	2.1
41670	0.946131	2.1
41671	1.89227	2.1
41672	1.89227	2.1
41673	1.89227	2.1
41674	1.89227	2.1
41675	3.78453	2.1
41676	0.936287	0.05
41677	1.01399	0.05
41678	0.959415	0.05
41679	0.959415	0.05
41680	0.910786	0.05
41681	1.38301	0.05
41682	1.0286	0.05
41683	0.535742	0.05
41684	0.505202	0.05
41685	0.937685	0.05
41686	1.11066	0.05
41687	1.46907	0.05
41688	1.00546	0.0516368
41689	1.01524	0.05
41690	0.94613	0.0631454
41691	0.94613	0.0608799
41692	1.05018	0.0598585
41693	0.914025	0.05
41694	0.913512	0.0640671
41695	0.851542	0.0628988
41696	1.05385	0.0634302
41697	1.05494	0.05
41698	0.91404	0.061393
41699	0.91404	0.0502454

41700	2.03253	2.09933
41701	1.82325	0.406995
41702	1.93938	2.1
41703	1.73756	0.36755
41704	3.77009	1.43412
41705	1.82325	0.34796
41706	1.47562	1.92778
41707	1.73756	0.39115
41708	1.3868	1.67979
41709	3.21317	0.484046
41710	1.93938	2.1
41711	1.73756	0.513633
41712	1.85293	2.1
41713	1.64279	0.379194
41714	3.59049	0.812331
41715	1.73756	0.338377
41716	1.3868	1.74305
41717	1.64279	0.490585
41718	1.28419	1.79914
41719	3.02174	0.557174
41720	1.35048	2.1
41721	1.37999	2.1
41722	1.34355	1.48397
41723	1.37854	2.1
41724	2.72354	2.1
41725	1.89226	1.75132
41726	1.89226	2.1
41727	1.89226	1.45749
41728	1.89226	2.1
41729	3.78452	1.89404
41730	1.89226	2.1
41731	1.89226	2.1
41732	1.89226	2.0913
41733	1.89226	2.1
41734	3.78453	2.1
41735	1.37999	2.1
41736	1.18059	2.1
41737	1.17117	2.1
41738	1.17262	2.1
41739	1.37855	2.1
41740	1.37999	2.1
41741	1.34355	1.94341
41742	1.37854	2.1
41743	1.33079	1.69277
41744	1.3741	2.1
41745	2.71765	2.1
41746	1.89226	1.9045
41747	1.89226	2.1
41748	1.89226	0.870717
41749	1.89226	2.1
41750	3.78453	1.9815
41751	1.89226	2.1
41752	1.89226	2.1
41753	1.89226	2.1
41754	1.89226	2.1
41755	3.78453	2.1
41756	0.640305	2.1
41757	0.640305	2.1

41758	0.643507	2.1
41759	0.643507	2.1
41760	0.590294	2.1
41761	0.594539	2.1
41762	0.689273	2.1
41763	0.687048	2.1
41764	0.687048	2.1
41765	0.594538	2.1
41766	0.590294	2.1
41767	0.689273	2.1
41768	2.01399	1.47996
41769	1.70141	1.77036
41770	1.83896	2.1
41771	1.52482	2.1
41772	3.53881	1.82812
41773	1.70141	2.05772
41774	1.0752	2.1
41775	1.0752	1.96913
41776	1.25669	2.1
41777	1.52482	2.1
41778	1.70632	2.1
41779	1.83896	1.79604
41780	1.52482	1.07975
41781	1.65244	1.68608
41782	1.34412	2.01395
41783	3.17725	1.69852
41784	1.26782	0.0511571
41785	2.90353	0.05
41786	2.54203	0.05
41787	2.41979	0.0589846
41788	1.77007	0.05
41789	1.703	0.0549424
41790	0.946118	0.0661484
41791	0.946118	0.0559344
41792	1.38643	0.0568642
41793	1.38644	0.05
41794	0.946117	0.0607405
41795	0.946117	0.0688146
41796	1.38644	0.0505367
41797	1.15545	0.0587557
41798	0.946131	0.0595625
41799	1.15546	0.0568336
41800	1.38642	0.0579612
41801	0.946105	0.0577898
41802	2.42256	0.209223
41803	2.63056	0.124154
41804	2.43174	0.120205
41805	2.25035	0.111891
41806	4.8543	0.124618
41807	2.58031	0.185596
41808	2.63056	0.145162
41809	2.43174	0.128199
41810	2.32127	0.112548
41811	5.01205	0.140566
41812	2.43174	0.120403
41813	2.25035	0.10241
41814	2.11465	0.0833384
41815	2.13042	0.0856872

41816	4.54639	0.0954896
41817	2.43174	0.12916
41818	2.32127	0.10737
41819	1.92997	0.0836484
41820	2.13041	0.0913757
41821	4.36171	0.102811
41822	1.89227	2.1
41823	1.89227	2.1
41824	1.89227	2.1
41825	1.89227	2.1
41826	3.78453	2.1
41827	0.568724	2.1
41828	0.568724	2.1
41829	0.57678	2.1
41830	0.57678	2.1
41831	0.523796	2.1
41832	0.548137	2.1
41833	0.597368	2.1
41834	0.637877	2.1
41835	0.597367	2.1
41836	0.523796	2.1
41837	0.637877	2.1
41838	0.548137	2.1
41839	1.32674	2.1
41840	1.32674	2.1
41841	0.946132	2.1
41842	0.946132	2.1
41843	1.3195	2.1
41844	1.32674	2.1
41845	0.946132	2.1
41846	0.946132	2.1
41847	0.946132	2.1
41848	1.3195	2.1
41849	1.32674	2.1
41850	0.946133	2.1
41851	1.89227	2.1
41852	1.89227	2.1
41853	1.89227	2.1
41854	1.89227	2.1
41855	3.78453	2.1
41856	1.19473	2.1
41857	0.987132	2.1
41858	0.987132	2.1
41859	1.04759	2.1
41860	1.0894	2.1
41861	1.14986	2.1
41862	1.3195	2.1
41863	1.3195	2.1
41864	0.946132	2.1
41865	0.946132	2.1
41866	1.31185	2.1
41867	1.3195	2.1
41868	0.946132	2.1
41869	0.946132	2.1
41870	1.31185	2.1
41871	0.946133	2.1
41872	0.946132	2.1
41873	1.3195	2.1

41874	1.11256	1.4771
41875	1.70101	1.22785
41876	1.22297	1.47925
41877	1.22297	1.41195
41878	1.7252	1.47751
41879	1.7252	1.39641
41880	1.99298	1.42135
41881	1.70101	1.34893
41882	1.7252	1.48167
41883	2.18701	0.972369
41884	3.71818	1.41853
41885	1.03218	1.38844
41886	1.7252	0.974955
41887	1.11256	1.38017
41888	1.11256	1.25957
41889	1.7457	1.33629
41890	1.7457	1.21436
41891	1.7457	1.41423
41892	2.187	0.751637
41893	1.7252	1.34891
41894	1.7252	1.33133
41895	2.27351	1.08861
41896	2.27351	1.08569
41897	1.95544	0.954945
41898	1.35897	1.58505
41899	1.44859	0.993181
41900	1.44859	1.14955
41901	1.80709	0.973213
41902	1.80709	1.14035
41903	1.80709	0.999448
41904	1.17822	1.21294
41905	1.35897	1.0274
41906	1.35897	1.28902
41907	1.52782	0.989063
41908	1.52782	1.23415
41909	1.11978	1.10333
41910	0.903546	0.994955
41911	0.977719	1.18482
41912	1.2364	1.01249
41913	1.03812	0.971408
41914	1.03812	1.53168
41915	1.05938	0.955198
41916	1.05938	1.36995
41917	1.11978	0.972395
41918	1.2364	0.898268
41919	0.97772	1.12952
41920	0.903546	1.05453
41921	1.80709	1.2492
41922	1.81847	1.17949
41923	1.96029	1.28383
41924	1.52782	1.22027
41925	2.23956	1.13158
41926	1.80709	1.19534
41927	1.89226	0.335683
41928	2.32962	0.210676
41929	2.5992	0.211436
41930	1.89226	0.229891
41931	2.5992	0.316484

41932	1.89226	0.336116
41933	2.55791	0.05
41934	2.47141	0.05
41935	2.13515	0.05
41936	2.10939	0.05
41937	4.66731	0.05
41938	2.47141	0.05
41939	2.23337	0.05
41940	2.10939	0.05
41941	2.03438	0.05
41942	4.34276	0.05
41943	2.13512	0.05
41944	1.71027	0.05
41945	1.71027	0.05
41946	2.10936	0.05
41947	1.60137	0.05
41948	2.00046	0.05
41949	1.71027	0.05
41950	2.03435	0.05
41951	1.71027	0.05
41952	2.10936	0.05
41953	1.72067	0.05
41954	2.11976	0.05
41955	1.177	0.454358
41956	1.36405	0.867304
41957	2.25635	0.86302
41958	2.60151	0.272955
41959	3.6204	0.698788
41960	0.658869	0.0790627
41961	0.781697	0.0907973
41962	0.637037	0.0724716
41963	0.541148	0.0701266
41964	0.654682	0.0650199
41965	0.654682	0.112406
41966	0.641224	0.075894
41967	0.641224	0.0824186
41968	0.637039	0.0682819
41969	0.781696	0.0786452
41970	0.658867	0.0829454
41971	0.541149	0.0819154
41972	1.89226	0.358061
41973	1.59152	0.22931
41974	1.89226	0.229148
41975	1.82125	0.223755
41976	1.89226	0.352236
41977	1.82125	0.333234
41978	2.18967	0.0503543
41979	1.89227	0.0615985
41980	2.72752	0.0669553
41981	2.72752	0.067951
41982	1.89227	0.05
41983	1.89227	0.05
41984	2.18967	0.05
41985	1.92547	0.0682363
41986	1.92547	0.0508229
41987	2.72752	0.05
41988	1.30572	0.05
41989	2.10777	0.05

41990	2.39663	2.1
41991	1.92293	2.1
41992	1.96889	2.1
41993	2.45928	2.1
41994	4.36552	2.1
41995	1.02605	2.1
41996	1.92293	2.1
41997	1.9689	2.1
41998	1.04679	2.1
41999	2.99495	2.1
42000	1.96889	2.1
42001	2.45928	2.1
42002	2.50138	2.1
42003	1.99782	2.1
42004	4.47027	2.1
42005	1.9689	2.1
42006	1.03449	2.1
42007	1.07572	2.1
42008	1.04679	2.1
42009	1.99783	2.1
42010	1.9689	2.1
42011	1.72162	1.13669
42012	1.89186	1.07658
42013	2.29771	1.04951
42014	1.80149	0.590489
42015	4.01933	1.06025
42016	1.16482	1.17569
42017	1.97003	0.911872
42018	1.72162	0.936421
42019	1.72162	1.07494
42020	1.29313	1.04745
42021	1.29313	1.1777
42022	2.29771	1.0939
42023	2.84159	0.425944
42024	2.19622	0.818828
42025	1.97366	0.983817
42026	2.50906	0.943798
42027	2.29771	1.00951
42028	1.29313	1.16197
42029	2.4483	0.764525
42030	2.14223	0.905289
42031	2.14228	1.06369
42032	1.294	1.01722
42033	1.294	1.11203
42034	1.21495	0.05
42035	1.70303	0.05
42036	1.7924	0.05
42037	1.64002	0.05
42038	3.11963	0.05
42039	2.81021	0.05
42040	1.89226	0.05
42041	1.89226	0.05
42042	1.89226	0.05
42043	2.36406	0.05
42044	2.36406	0.05
42045	1.89226	0.05
42046	1.89226	0.05
42047	1.89226	0.05

42048	1.89226	0.05
42049	1.89226	0.05
42050	1.89226	0.05
42051	2.29976	0.05
42052	1.64002	0.05
42053	1.8959	0.05
42054	1.90873	0.05
42055	1.85594	0.05
42056	1.85594	0.05
42057	1.89226	0.05
42058	1.89226	0.05
42059	1.89226	0.05
42060	1.89226	0.05
42061	3.78453	0.05
42062	1.89226	1.53034
42063	2.52823	2.1
42064	2.83441	1.8178
42065	2.83441	2.04157
42066	1.89226	1.66322
42067	1.89226	1.77275
42068	1.04522	1.40614
42069	1.04522	1.40648
42070	1.12831	1.80835
42071	1.18364	2.02271
42072	0.640525	1.4048
42073	0.924882	1.42704
42074	1.27877	1.75543
42075	1.43363	1.41906
42076	0.93474	1.36902
42077	1.58926	1.59597
42078	1.29573	1.72305
42079	0.664247	1.66271
42080	2.02191	2.1
42081	1.89226	2.1
42082	1.89226	2.1
42083	1.89226	2.1
42084	2.58639	2.1
42085	2.58639	2.1
42086	1.16759	2.1
42087	2.11828	2.1
42088	1.60936	2.1
42089	2.35846	2.1
42090	3.52605	2.1
42091	1.89226	2.1
42092	1.89226	2.1
42093	1.89226	2.1
42094	1.89226	2.1
42095	3.78453	2.1
42096	2.82765	0.05
42097	1.89809	0.0905745
42098	2.15971	0.0548078
42099	1.89809	0.0587624
42100	2.29233	0.0521937
42101	2.03071	0.05
42102	1.90664	0.0727636
42103	2.15971	0.05
42104	1.28956	0.0796241
42105	1.28956	0.0675917

42106	2.64414	0.064886
42107	2.02706	0.05
42108	2.82765	0.0720661
42109	1.89226	0.0723202
42110	2.39968	0.0846693
42111	2.39968	0.111367
42112	1.89226	0.0568228
42113	1.89226	0.0506492
42114	0.978643	2.1
42115	0.703968	2.1
42116	0.946129	2.1
42117	0.946129	2.1
42118	0.703939	2.1
42119	0.703939	2.1
42120	0.946159	2.1
42121	0.946159	2.1
42122	0.978642	2.1
42123	0.94613	2.1
42124	0.94613	2.1
42125	0.703968	2.1
42126	1.89226	2.1
42127	2.17309	1.41736
42128	2.17309	2.1
42129	1.47475	1.86101
42130	1.89226	2.1
42131	1.19393	1.78041
42132	1.89226	2.1
42133	1.89226	2.1
42134	1.89226	2.1
42135	1.89226	2.08924
42136	3.78452	2.1
42137	1.17426	2.1
42138	0.978644	2.1
42139	0.946129	2.1
42140	0.946129	2.1
42141	0.946131	2.1
42142	0.946131	2.1
42143	0.978643	2.1
42144	0.978642	2.1
42145	1.17426	2.1
42146	0.946131	2.1
42147	0.978642	2.1
42148	0.94613	2.1
42149	1.89226	2.1
42150	1.89226	1.27192
42151	1.89226	2.08665
42152	2.17309	1.49191
42153	2.56346	2.06672
42154	2.94389	1.68587
42155	0.946131	2.1
42156	0.946131	2.07974
42157	0.946128	2.1
42158	0.946128	2.1
42159	0.946131	2.1
42160	0.946131	2.0763
42161	0.946128	2.1
42162	0.946128	2.1
42163	0.94613	2.1

42164	0.94613	2.1
42165	0.946129	2.1
42166	0.946129	2.1
42167	1.89226	1.01207
42168	1.89226	1.05875
42169	1.89226	1.17536
42170	1.89226	1.06724
42171	3.78453	1.02198
42172	1.89226	1.16324
42173	1.89226	1.03326
42174	1.89226	1.05938
42175	1.89226	1.12594
42176	1.89226	0.990184
42177	1.89226	0.986846
42178	1.89226	0.948106
42179	1.30733	1.1248
42180	1.89226	1.16249
42181	1.92403	1.2083
42182	3.38635	1.08603
42183	1.89226	1.0466
42184	3.02887	1.23962
42185	2.0815	0.988886
42186	1.88422	1.12976
42187	2.0815	1.10105
42188	1.88422	1.20744
42189	1.89226	1.2238
42190	1.70221	1.09871
42191	1.89226	1.16612
42192	1.20068	1.19632
42193	1.89226	1.08165
42194	1.20068	0.970993
42195	1.01393	1.40606
42196	1.20067	0.992742
42197	1.89226	0.909625
42198	2.44958	1.40887
42199	1.01393	0.556176
42200	1.89226	0.436762
42201	1.89226	2.1
42202	1.89226	2.1
42203	1.89226	2.1
42204	1.89226	2.1
42205	1.89226	2.1
42206	1.89226	2.1
42207	1.89226	2.1
42208	1.89226	2.1
42209	1.89226	2.1
42210	1.89226	2.1
42211	1.89226	2.1
42212	1.89226	2.1
42213	1.89226	2.1
42214	1.89226	2.1
42215	1.89226	2.1
42216	1.89226	2.1
42217	1.89226	2.1
42218	1.89226	2.1
42219	1.91161	2.1
42220	2.45782	2.1
42221	1.39653	2.1

42222	2.44652	2.1
42223	4.35812	2.1
42224	2.73092	2.1
42225	1.89226	2.1
42226	1.89226	2.1
42227	1.89226	2.0584
42228	2.44652	2.1
42229	2.44652	1.9466
42230	1.89226	2.1
42231	1.89226	2.1
42232	1.89226	2.1
42233	1.89226	2.1
42234	1.89226	2.1
42235	1.89226	2.1
42236	2.64924	2.1
42237	1.31109	2.1
42238	1.31109	2.1
42239	1.49958	2.1
42240	1.95039	2.1
42241	2.14451	2.1
42242	0.825157	2.1
42243	0.634856	2.1
42244	0.816836	2.1
42245	0.816836	2.1
42246	0.556743	2.1
42247	0.639142	2.1
42248	0.985106	2.1
42249	0.795045	2.1
42250	0.967461	2.1
42251	0.552494	2.1
42252	0.676829	2.1
42253	0.98803	2.1
42254	2.28564	2.1
42255	2.34084	2.02856
42256	2.3502	2.1
42257	2.42908	2.1
42258	4.69104	2.1
42259	2.34084	2.04746
42260	2.18077	2.1
42261	2.42908	2.1
42262	2.2947	1.54279
42263	4.63554	2.08951
42264	2.3502	2.1
42265	2.42908	2.1
42266	2.38681	2.1
42267	2.50438	2.07692
42268	4.85458	2.1
42269	2.42908	2.1
42270	2.2947	1.72745
42271	2.50438	1.92777
42272	2.40627	2.1
42273	4.79908	1.95878
42274	1.89226	1.15565
42275	2.20711	1.87966
42276	1.89226	0.917408
42277	1.89226	1.93947
42278	2.02693	1.03774
42279	2.02693	1.92201

42280	1.2269	2.1
42281	2.20711	1.90371
42282	2.0815	1.91336
42283	1.11506	2.1
42284	3.22109	2.1
42285	0.545802	2.04661
42286	0.547489	2.1
42287	0.85914	1.90993
42288	1.11641	2.03837
42289	0.899974	2.07502
42290	0.849893	2.1
42291	0.747885	2.04996
42292	0.747885	1.81199
42293	0.557184	2.1
42294	0.887039	2.1
42295	1.08948	2.1
42296	0.59815	2.1
42297	0.974139	2.1
42298	1.33385	2.1
42299	1.02368	2.1
42300	1.02368	2.1
42301	1.00833	2.1
42302	1.00833	2.1
42303	1.89226	1.87876
42304	1.89226	0.526253
42305	1.89226	2.07695
42306	1.89226	0.39005
42307	3.78453	0.633237
42308	1.74651	2.1
42309	1.44605	0.288006
42310	1.63557	2.05379
42311	1.31993	0.519411
42312	3.08162	0.63941
42313	1.89226	2.1
42314	1.89226	0.857682
42315	1.89226	1.89943
42316	1.89226	0.903486
42317	3.78452	0.478926
42318	1.63557	2.1
42319	1.31992	0.363529
42320	1.52615	2.1
42321	1.18549	0.837954
42322	2.82106	1.30153
42323	1.89226	0.342481
42324	2.58101	1.91961
42325	2.73797	2.1
42326	2.73797	0.938084
42327	1.89226	2.1
42328	1.89226	0.473515
42329	1.91762	0.232685
42330	1.44354	0.267553
42331	1.33622	0.227864
42332	1.33622	0.197447
42333	2.20248	0.455166
42334	2.20248	0.265029
42335	1.11712	2.1
42336	1.11712	2.1
42337	1.11421	2.1

42338	1.11421	2.1
42339	0.833871	2.1
42340	0.926528	2.1
42341	1.3048	2.1
42342	1.40335	2.1
42343	0.926527	2.1
42344	1.40335	2.1
42345	1.3048	2.1
42346	0.833871	2.1
42347	0.879657	2.1
42348	0.937695	2.1
42349	0.946129	2.1
42350	0.946129	2.1
42351	0.851541	2.1
42352	0.851541	2.1
42353	1.1126	2.1
42354	1.04519	2.1
42355	0.851486	2.1
42356	0.980501	2.1
42357	0.898251	2.1
42358	0.851528	2.1
42359	1.37756	2.1
42360	2.73081	2.1
42361	2.44311	2.1
42362	2.44311	2.1
42363	1.66428	2.1
42364	1.66428	2.1
42365	1.89226	2.1
42366	1.89226	2.1
42367	1.89226	2.1
42368	1.89226	2.1
42369	3.78452	2.1
42370	1.65781	2.1
42371	1.89837	2.1
42372	1.46713	2.1
42373	1.98027	2.1
42374	3.63808	2.1
42375	2.29054	2.1
42376	2.26369	2.1
42377	1.98027	2.1
42378	2.15617	2.03376
42379	4.24396	2.1
42380	1.89226	2.1
42381	1.89226	2.1
42382	1.89226	2.1
42383	1.89226	2.1
42384	3.78452	2.1
42385	0.946131	2.1
42386	0.946131	2.1
42387	0.946128	2.1
42388	0.946128	2.1
42389	0.946129	2.1
42390	0.946129	2.1
42391	0.94613	2.1
42392	0.94613	2.1
42393	0.94613	2.1
42394	0.94613	2.1
42395	0.94613	2.1

42396	0.94613	2.1
42397	0.812186	2.1
42398	0.812186	2.1
42399	0.819781	2.1
42400	0.819781	2.1
42401	0.913532	2.1
42402	0.990134	2.1
42403	0.733564	2.1
42404	0.641833	2.1
42405	0.641832	2.1
42406	0.913533	2.1
42407	0.733564	2.1
42408	0.990134	2.1
42409	0.994262	2.1
42410	0.994262	2.1
42411	0.997354	2.1
42412	0.997354	2.09319
42413	1.00775	1.92065
42414	1.07808	1.99046
42415	0.913533	2.1
42416	0.990135	2.1
42417	0.913532	2.1
42418	1.00775	2.1
42419	0.990134	2.1
42420	1.07808	2.1
42421	0.946131	2.1
42422	0.946131	2.1
42423	0.946128	2.1
42424	0.946128	2.1
42425	0.946128	2.09368
42426	0.946128	1.95211
42427	0.94613	2.1
42428	0.94613	2.1
42429	0.946129	2.1
42430	0.946129	2.1
42431	0.946129	2.1
42432	0.946129	2.1
42433	0.816232	2.1
42434	0.760038	2.1
42435	0.845999	2.1
42436	0.845999	2.1
42437	1.0631	2.1
42438	1.28131	2.1
42439	0.474065	2.1
42440	0.591905	2.1
42441	0.601756	2.1
42442	1.23903	2.1
42443	0.988592	2.1
42444	0.49051	2.1
42445	2.57021	2.1
42446	1.89226	2.1
42447	2.68172	2.1
42448	1.89226	2.1
42449	4.46247	2.1
42450	1.89226	2.1
42451	1.89226	1.85097
42452	1.89226	2.1
42453	1.89226	2.01335

42454	3.78452	2.1
42455	1.12573	2.1
42456	1.65504	1.97738
42457	1.38848	2.1
42458	1.38848	2.1
42459	1.30567	2.1
42460	1.30567	2.1
42461	2.82413	2.1
42462	1.89226	2.1
42463	2.68172	2.1
42464	2.68172	2.1
42465	1.89226	2.1
42466	1.89226	2.1
42467	1.89226	2.1
42468	1.89226	2.1
42469	1.89226	2.1
42470	1.89226	1.73894
42471	3.78452	2.1
42472	1.30567	2.1
42473	1.65504	2.1
42474	1.52724	2.1
42475	1.89249	2.1
42476	3.18228	2.1
42477	2.57386	0.0897575
42478	1.89226	0.192063
42479	1.89226	0.172143
42480	2.66753	0.109932
42481	1.89226	0.140518
42482	2.66753	0.103217
42483	1.89226	0.0849751
42484	1.89226	0.215606
42485	2.2973	0.174587
42486	3.12479	0.117025
42487	1.89226	0.151292
42488	2.57385	0.104919
42489	0.489293	2.1
42490	0.672698	2.1
42491	0.59038	2.1
42492	0.738357	2.1
42493	0.62269	2.1
42494	0.62269	2.1
42495	0.640388	2.1
42496	0.640388	2.1
42497	0.59038	2.1
42498	0.489293	2.1
42499	0.738357	2.1
42500	0.672698	2.1
42501	1.89226	2.1
42502	1.89226	2.1
42503	1.89226	2.1
42504	1.89226	2.1
42505	3.78452	2.1
42506	2.82858	2.1
42507	1.89226	2.1
42508	2.5671	2.1
42509	1.89226	2.1
42510	2.5671	2.1
42511	1.89226	2.1

42512	2.06581	0.05
42513	2.47308	0.05
42514	1.89433	0.05
42515	1.67571	0.05
42516	3.87274	0.05
42517	2.55791	0.05
42518	2.10276	0.05
42519	2.47308	0.05
42520	2.47308	0.05
42521	1.83415	0.05
42522	1.90922	0.05
42523	1.18421	0.05
42524	2.03277	0.05
42525	1.18421	0.05
42526	2.01275	0.05
42527	1.42818	0.05
42528	2.25673	0.05
42529	0.882904	0.05
42530	0.958492	0.05
42531	0.905756	0.05
42532	0.905756	0.05
42533	1.06352	0.05
42534	0.797654	0.05
42535	1.11319	0.05
42536	0.716795	0.05
42537	0.820721	0.05
42538	0.731963	0.05
42539	1.13143	0.05
42540	1.10528	0.05
42541	1.89226	0.05
42542	1.89226	0.05
42543	1.89226	0.05
42544	1.89226	0.05
42545	1.89226	0.05
42546	1.89226	0.05
42547	1.01241	0.0529412
42548	0.703929	0.0526943
42549	0.946133	0.05
42550	0.946133	0.05
42551	1.04072	0.05
42552	1.163	0.0503724
42553	0.639952	0.0568884
42554	0.851543	0.05
42555	0.946132	0.05
42556	0.920761	0.0555797
42557	0.711038	0.0500219
42558	0.711038	0.05256
42559	1.89226	0.05
42560	1.89226	0.05
42561	1.89226	0.05
42562	1.89226	0.05
42563	1.89226	0.05
42564	1.89226	0.05
42565	1.89226	0.05
42566	1.89226	0.05
42567	2.41075	0.05
42568	1.89226	0.05
42569	3.78451	0.05

42570	0.968476	2.09562
42571	1.20674	1.34426
42572	1.13456	1.33652
42573	1.13456	2.1
42574	1.02074	1.4708
42575	1.02074	2.1
42576	1.02074	2.1
42577	1.20674	1.81486
42578	1.07866	1.80886
42579	1.28649	1.44966
42580	2.2854	2.00646
42581	1.94221	0.132035
42582	1.36717	0.122103
42583	1.45535	0.1377
42584	1.93771	0.103617
42585	3.39756	0.130618
42586	1.94222	0.183387
42587	2.25035	0.107641
42588	2.41806	0.12399
42589	2.42256	0.111925
42590	1.93772	0.149094
42591	1.94222	0.128661
42592	1.49906	0.0882747
42593	1.93771	0.0974534
42594	1.45535	0.106012
42595	1.45535	0.116946
42596	1.91484	0.0781645
42597	1.91484	0.0800846
42598	1.91484	0.0841023
42599	2.25035	0.100846
42600	1.93772	0.104395
42601	1.93772	0.109549
42602	2.11465	0.0796502
42603	2.11465	0.0788696
42604	2.61735	0.173138
42605	1.5082	0.157458
42606	2.36481	0.202609
42607	1.58597	0.190937
42608	2.36481	0.167695
42609	1.46653	0.15244
42610	2.36482	0.219657
42611	1.08133	0.253772
42612	1.5082	0.145525
42613	2.01868	0.252125
42614	3.52688	0.256835
42615	0.94613	0.202969
42616	0.94613	0.198468
42617	0.946131	0.256978
42618	0.946131	0.278412
42619	0.946129	0.278148
42620	0.946129	0.239347
42621	0.946132	0.200707
42622	0.946132	0.233056
42623	0.94613	0.240553
42624	0.94613	0.254922
42625	0.946131	0.249292
42626	0.946131	0.243117
42627	2.53725	0.105133

42628	1.89227	0.136805
42629	1.89227	0.137
42630	2.27073	0.13512
42631	1.89227	0.11195
42632	2.27073	0.111275
42633	1.89227	0.213258
42634	1.89227	0.320875
42635	1.89227	0.321731
42636	1.89227	0.276035
42637	1.89227	0.245721
42638	1.89227	0.211798
42639	1.86766	2.1
42640	2.83487	2.1
42641	2.84318	2.1
42642	2.59401	2.1
42643	2.11684	2.1
42644	1.86766	2.1
42645	1.10638	2.1
42646	2.11684	2.1
42647	1.29111	2.1
42648	2.37439	2.1
42649	3.40796	2.1
42650	2.56843	2.1
42651	1.89226	2.1
42652	1.89226	2.1
42653	2.71932	2.1
42654	4.46069	2.1
42655	1.89226	2.1
42656	1.89226	2.1
42657	1.89226	2.1
42658	1.89226	2.1
42659	3.78452	2.1
42660	0.6416	2.1
42661	0.697585	2.1
42662	0.670706	2.1
42663	0.598927	2.1
42664	0.644313	2.1
42665	0.644313	2.1
42666	0.652199	2.1
42667	0.652199	2.1
42668	0.697587	2.1
42669	0.670705	2.1
42670	0.598926	2.1
42671	0.641601	2.1
42672	1.89226	2.1
42673	1.89226	2.1
42674	1.89226	2.1
42675	1.89226	2.1
42676	1.89226	2.1
42677	1.89226	2.1
42678	0.697585	2.1
42679	0.730617	2.1
42680	0.712082	2.1
42681	0.670706	2.1
42682	0.69854	2.1
42683	0.69854	2.1
42684	0.702783	2.1
42685	0.702783	2.1

42686	0.730618	2.1
42687	0.712081	2.1
42688	0.697586	2.1
42689	0.670705	2.1
42690	1.89226	2.1
42691	1.89226	2.1
42692	1.89226	2.04744
42693	1.89226	2.1
42694	1.89226	2.01086
42695	1.89226	2.1
42696	2.0815	2.1
42697	1.12076	2.1
42698	1.38662	2.1
42699	2.01244	2.1
42700	3.41508	2.1
42701	1.89226	2.1
42702	2.54324	2.1
42703	1.91981	2.1
42704	1.89226	2.1
42705	2.57079	2.1
42706	2.54324	2.1
42707	2.54325	2.1
42708	1.94779	2.1
42709	1.76879	2.1
42710	1.73811	2.1
42711	2.5708	2.1
42712	2.54325	2.1
42713	1.89226	2.1
42714	2.57079	2.1
42715	1.91894	2.1
42716	1.89226	2.1
42717	2.59747	2.1
42718	2.57079	2.1
42719	2.5708	2.1
42720	1.82268	2.1
42721	1.79738	2.1
42722	1.77071	2.1
42723	2.59747	2.1
42724	2.5708	2.1
42725	1.89226	1.03077
42726	1.89226	2.1
42727	1.89226	0.908806
42728	1.89226	2.1
42729	3.78451	1.904
42730	2.21582	0.722015
42731	1.94412	2.1
42732	2.13428	0.816074
42733	1.86292	2.1
42734	4.07839	1.87918
42735	1.94412	2.1
42736	1.5854	2.1
42737	1.86293	2.1
42738	1.50767	2.1
42739	3.45179	2.1
42740	1.89226	2.1
42741	1.89226	2.1
42742	1.89226	2.1
42743	1.89226	2.1

42744	3.78452	2.1
42745	1.89226	0.717692
42746	1.89226	2.1
42747	1.89226	0.928614
42748	1.89226	2.1
42749	3.78452	2.02337
42750	2.13428	0.615231
42751	1.86293	2.1
42752	2.04737	0.976133
42753	1.77122	2.1
42754	3.9055	1.77019
42755	1.86293	2.1
42756	1.50767	2.1
42757	1.41077	2.1
42758	1.41077	2.1
42759	1.77123	2.1
42760	1.86813	2.1
42761	0.810943	2.1
42762	0.810943	2.1
42763	0.810945	2.1
42764	0.810945	2.1
42765	0.810958	2.1
42766	0.810958	2.1
42767	1.89226	2.1
42768	1.35168	2.1
42769	1.08132	2.1
42770	1.08132	2.1
42771	1.0813	2.1
42772	0.745979	2.1
42773	0.745979	2.1
42774	0.778781	2.1
42775	0.778781	2.1
42776	0.59549	2.1
42777	0.649658	2.1
42778	0.994784	2.1
42779	0.875102	2.1
42780	0.875102	2.1
42781	0.59549	2.1
42782	0.649658	2.1
42783	0.994784	2.1
42784	1.57887	2.1
42785	1.75021	2.1
42786	1.13341	2.1
42787	1.13341	2.1
42788	1.19098	2.1
42789	1.63644	2.1
42790	2.5761	0.569898
42791	2.43404	2.1
42792	2.44813	0.476405
42793	2.31751	2.1
42794	4.88218	1.18888
42795	2.43405	2.1
42796	2.22575	2.1
42797	2.31751	2.1
42798	2.10893	2.1
42799	4.54298	2.1
42800	2.44813	0.345114
42801	2.31751	2.1

42802	2.30908	0.609006
42803	2.19815	2.09391
42804	4.64628	1.07209
42805	2.31751	2.1
42806	2.10893	2.1
42807	2.19815	2.1
42808	1.98061	2.1
42809	4.30708	2.1
42810	0.94613	2.1
42811	0.94613	2.1
42812	1.40372	2.1
42813	1.40372	2.1
42814	1.40372	2.1
42815	1.37167	2.1
42816	0.946132	2.1
42817	0.946132	2.1
42818	0.946131	2.1
42819	1.40372	2.1
42820	0.94613	2.1
42821	1.37167	2.1
42822	2.80744	2.1
42823	2.06984	2.1
42824	2.83418	2.1
42825	2.74335	2.1
42826	2.16067	2.1
42827	2.06984	2.1
42828	1.077	0.226491
42829	0.809116	0.192653
42830	0.600419	0.289594
42831	0.790277	0.200131
42832	0.927647	0.179707
42833	0.758598	0.177378
42834	0.85749	0.238409
42835	0.85749	0.251475
42836	1.02417	0.199669
42837	0.600411	0.217953
42838	0.751701	0.215598
42839	1.07702	0.193797
42840	1.89226	2.1
42841	1.89226	2.1
42842	1.89226	2.1
42843	1.89226	2.1
42844	1.89226	2.1
42845	1.89226	2.1
42846	1.89226	2.1
42847	1.89226	2.1
42848	1.89226	2.1
42849	1.89226	2.1
42850	3.78453	2.1
42851	1.89226	2.1
42852	1.89226	2.1
42853	1.89226	2.1
42854	1.89226	2.1
42855	1.89226	2.1
42856	1.89226	2.1
42857	0.946131	2.1
42858	0.946131	2.1
42859	0.946131	2.1

42860	0.946131	2.1
42861	0.946133	2.1
42862	0.946133	2.1
42863	0.946128	2.1
42864	0.946128	2.1
42865	0.946131	2.1
42866	0.946131	2.1
42867	0.94613	2.1
42868	0.94613	2.1
42869	2.17159	2.1
42870	2.00083	1.93592
42871	2.21305	2.1
42872	2.00551	2.1
42873	2.20837	2.1
42874	2.00083	2.1
42875	2.20837	2.1
42876	1.80328	1.49012
42877	2.22962	1.93579
42878	2.00083	1.842
42879	2.03207	1.56823
42880	1.80328	1.67805
42881	2.28564	2.1
42882	2.20837	1.93281
42883	2.30406	2.1
42884	2.17159	2.1
42885	2.34084	2.1
42886	2.20837	2.1
42887	1.09523	1.44387
42888	1.09523	1.884
42889	1.09934	2.1
42890	1.09934	2.1
42891	1.01603	1.39639
42892	1.09038	1.89621
42893	1.10419	1.8334
42894	1.17042	2.1
42895	1.17042	1.95748
42896	1.09038	1.93881
42897	1.10419	1.85928
42898	1.01603	1.93811
42899	2.60152	0.280141
42900	0.976334	0.555732
42901	2.22344	0.555877
42902	2.22344	0.336261
42903	1.36405	0.695827
42904	1.36405	0.249013
42905	1.89226	2.1
42906	1.70302	2.1
42907	1.69019	2.1
42908	1.70302	2.1
42909	1.73933	2.1
42910	1.70303	2.1
42911	1.89226	2.1
42912	1.89226	2.02303
42913	1.89226	2.1
42914	1.89226	2.1
42915	3.78452	2.1
42916	1.89226	2.1
42917	1.59036	2.1

42918	1.65683	2.1
42919	1.59036	2.1
42920	1.95873	2.1
42921	1.89226	1.97419
42922	0.946131	1.88418
42923	0.946131	2.04391
42924	0.946127	2.1
42925	0.946127	2.1
42926	0.946127	2.1
42927	0.946127	1.81712
42928	0.946131	2.1
42929	0.946131	2.06227
42930	0.946129	2.1
42931	0.946129	2.1
42932	0.94613	2.1
42933	0.94613	2.1
42934	0.851541	2.1
42935	0.851541	2.1
42936	0.851483	2.1
42937	1.15713	2.1
42938	0.851539	2.1
42939	0.851539	2.1
42940	0.942127	2.1
42941	1.14455	2.1
42942	0.838683	2.1
42943	0.946131	2.1
42944	0.957503	2.1
42945	0.957504	2.1
42946	1.89226	1.67527
42947	1.89226	0.821365
42948	1.89226	1.18579
42949	1.89226	2.1
42950	3.78452	0.72798
42951	1.89226	0.105174
42952	1.89226	0.398313
42953	1.89226	0.480277
42954	1.89226	0.498117
42955	1.89226	0.0983764
42956	1.89226	0.093289
42957	1.89226	0.410162
42958	1.89226	0.358705
42959	1.89226	0.298691
42960	1.89226	0.614333
42961	3.78453	0.291456
42962	1.89226	0.896182
42963	1.89226	2.1
42964	1.89226	2.1
42965	1.89226	2.06201
42966	3.78453	2.1
42967	1.22575	0.471997
42968	1.05438	1.39991
42969	0.946131	1.0708
42970	0.946131	1.18176
42971	0.946129	1.85992
42972	1.22575	0.389677
42973	1.05438	0.557211
42974	0.946132	0.269135
42975	1.05438	0.926832

42976	1.05438	1.41399
42977	0.946131	0.487142
42978	0.946131	1.09229
42979	2.4515	0.399037
42980	1.13726	0.19342
42981	2.10875	0.123563
42982	1.49515	0.175946
42983	2.10875	0.273443
42984	1.49515	0.130227
42985	0.831477	0.0556523
42986	0.747575	0.129284
42987	1.22575	0.19596
42988	1.28174	0.198198
42989	1.28174	0.128171
42990	0.831476	0.197466
42991	1.22575	0.27454
42992	0.747575	0.0966339
42993	1.03561	0.234454
42994	1.03561	0.261217
42995	1.02162	0.0986795
42996	1.02162	0.150582
42997	1.89227	2.1
42998	2.56348	1.60705
42999	1.89227	2.1
43000	2.45151	2.0765
43001	1.89227	2.1
43002	2.45151	1.18535
43003	1.40013	0.695803
43004	1.89226	1.62138
43005	1.89226	1.31393
43006	0.946643	0.623894
43007	1.89226	1.37389
43008	0.946643	0.849785
43009	1.89226	0.060194
43010	1.89226	0.0750534
43011	1.89226	0.0650743
43012	1.89226	0.0775141
43013	3.78453	0.0750759
43014	1.89226	0.110247
43015	1.89226	0.159553
43016	1.89226	0.116512
43017	1.89226	0.234062
43018	3.78453	0.120094
43019	0.946131	0.177499
43020	0.946131	0.156898
43021	0.946128	0.100803
43022	0.946128	0.107296
43023	0.94613	0.167631
43024	0.94613	0.188742
43025	0.946129	0.109961
43026	0.946129	0.103274
43027	0.94613	0.152659
43028	0.94613	0.0856425
43029	0.94613	0.114369
43030	0.94613	0.178293
43031	1.89226	0.05
43032	1.89226	0.0522925
43033	1.17244	0.0530804

43034	1.89226	0.0620461
43035	0.975291	0.0544003
43036	1.57408	0.0534585
43037	0.946132	0.119283
43038	0.946132	0.0707639
43039	0.946131	0.130887
43040	0.946131	0.0813157
43041	0.946132	0.108402
43042	0.946132	0.130665
43043	0.946131	0.0763859
43044	0.946131	0.0812883
43045	0.946131	0.102546
43046	0.946131	0.105657
43047	0.946132	0.0811646
43048	0.946132	0.112849
43049	2.74744	0.0931603
43050	1.89226	0.202074
43051	2.70805	0.124231
43052	1.89226	0.157953
43053	2.70805	0.134675
43054	1.89226	0.178396
43055	2.74744	0.0736303
43056	1.89226	0.1056
43057	2.57465	0.0875451
43058	1.89226	0.166559
43059	4.6397	0.0901073
43060	1.89226	0.252742
43061	1.89226	0.501124
43062	1.89226	0.425431
43063	1.89226	0.678934
43064	3.78452	0.444319
43065	1.89226	0.366531
43066	1.89226	0.847241
43067	1.89226	0.298575
43068	1.89226	0.270613
43069	1.89226	1.01279
43070	1.89226	0.881543
43071	2.16206	0.099187
43072	2.67861	0.327436
43073	3.11996	0.131377
43074	1.70303	0.105391
43075	2.89015	0.113634
43076	1.486 0.0824312	
43077	2.12839	0.0508977
43078	2.83631	0.299606
43079	3.08096	0.0936705
43080	1.84157	0.128552
43081	3.08096	0.163611
43082	1.84157	0.0985001
43083	1.89225	1.77666
43084	1.89225	1.35098
43085	1.89225	1.51412
43086	1.89225	1.65624
43087	1.89225	1.40625
43088	1.89225	1.64018
43089	1.89226	0.808186
43090	1.89226	1.19728
43091	1.89226	1.63503

43092	1.89226	0.593843
43093	3.78452	0.638899
43094	1.89226	0.384646
43095	2.49041	0.343979
43096	1.89226	0.385397
43097	1.89226	0.231466
43098	2.27073	0.209035
43099	2.27073	0.338587
43100	1.09904	0.214363
43101	0.677147	0.109019
43102	1.3365	0.0868933
43103	0.95759	0.402471
43104	0.957591	0.18243
43105	0.677148	0.175795
43106	1.09904	0.250454
43107	1.3365	0.156249
43108	0.996015	0.0925059
43109	0.996015	0.148907
43110	1.01764	0.41896
43111	1.01764	0.210635
43112	2.77349	0.277545
43113	2.72723	0.169655
43114	2.43156	0.317408
43115	2.4564	0.167111
43116	5.20505	0.261304
43117	2.77349	0.254036
43118	2.68508	0.153701
43119	2.33761	0.230726
43120	2.4564	0.163371
43121	5.1111	0.180409
43122	2.43156	0.212791
43123	2.70208	0.148289
43124	1.85779	0.246088
43125	2.10695	0.147588
43126	4.77572	0.160106
43127	2.33761	0.18255
43128	2.70208	0.145031
43129	1.85779	0.213476
43130	1.61597	0.137068
43131	4.19542	0.172049
43132	0.946132	1.65276
43133	0.946132	1.79772
43134	0.746729	1.72116
43135	0.746729	1.86801
43136	0.946132	1.88774
43137	0.915958	1.80272
43138	0.946133	1.63516
43139	0.74673	1.91077
43140	0.946133	1.72587
43141	0.946133	1.72807
43142	0.915958	1.79757
43143	0.746729	1.78197
43144	1.50743	2.1
43145	2.45501	2.1
43146	2.29118	2.1
43147	2.29118	2.1
43148	1.75482	2.1
43149	1.75482	2.1

43150	2.45501	2.1
43151	2.76927	2.03369
43152	2.29118	2.1
43153	2.64523	2.1
43154	5.10024	2.1
43155	1.25559	2.1
43156	2.29118	2.1
43157	2.0815	2.1
43158	2.26387	2.1
43159	1.35668	2.1
43160	1.47667	2.1
43161	2.28481	2.1
43162	2.64523	2.1
43163	2.50057	2.1
43164	2.50057	2.09005
43165	2.29118	2.1
43166	2.29118	2.1
43167	0.946129	0.108308
43168	0.946129	0.093409
43169	1.23929	0.076598
43170	1.15246	0.0949643
43171	0.946131	0.0937364
43172	0.946131	0.128389
43173	1.15246	0.0850536
43174	1.15246	0.101198
43175	0.946131	0.0903057
43176	1.15246	0.105045
43177	0.946131	0.0892945
43178	1.23928	0.102255
43179	0.946128	0.138605
43180	0.946128	0.126456
43181	1.15246	0.108741
43182	0.942814	0.178224
43183	0.946131	0.10491
43184	0.946131	0.143327
43185	0.961347	0.108166
43186	0.942811	0.164077
43187	0.946131	0.119122
43188	0.961347	0.116855
43189	0.946131	0.104603
43190	1.15246	0.136121
43191	2.32334	0.210173
43192	1.57621	0.168546
43193	1.90335	0.146648
43194	1.24538	0.140029
43195	3.47956	0.127846
43196	0.965762	0.0777162
43197	0.965762	0.0889313
43198	1.00245	0.0703301
43199	1.00245	0.0783269
43200	0.92786	0.0888117
43201	0.705297	0.0725075
43202	0.856414	0.0793615
43203	1.28968	0.0713979
43204	1.00306	0.075221
43205	0.705303	0.0722305
43206	1.28967	0.0760379
43207	0.975478	0.0914504

43208	3.05308	2.1
43209	1.76816	2.1
43210	1.76816	2.1
43211	1.69129	2.1
43212	2.58731	2.1
43213	2.51043	2.1
43214	1.89226	0.0720952
43215	2.71464	0.0731322
43216	2.34627	0.0706377
43217	1.89226	0.0864222
43218	2.34627	0.0638932
43219	1.89226	0.0798494
43220	2.34627	0.0697743
43221	2.71464	0.0693668
43222	1.98252	0.0649051
43223	1.98252	0.0895085
43224	1.51026	0.0650997
43225	2.34627	0.076698
43226	2.38698	1.43921
43227	2.35142	2.1
43228	2.60343	1.79437
43229	2.6636	1.42157
43230	4.99041	1.68445
43231	1.07811	2.1
43232	1.1952	2.1
43233	1.17571	1.9359
43234	1.30171	1.77468
43235	1.18322	1.84478
43236	1.18322	2.1
43237	1.18769	2.1
43238	1.18769	1.86638
43239	1.1952	2.1
43240	1.30171	2.1
43241	1.17571	2.1
43242	1.07811	2.1
43243	1.89226	1.7217
43244	2.6636	1.59052
43245	1.89226	0.621463
43246	2.60343	1.61917
43247	1.89226	1.5079
43248	2.60343	2.09616
43249	1.5344	0.05
43250	1.4516	0.05
43251	1.17816	0.05
43252	1.21056	0.05
43253	2.71257	0.05
43254	1.89226	1.8537
43255	1.89226	1.2709
43256	1.89226	1.5586
43257	1.89226	1.60139
43258	3.78452	1.58334
43259	1.89226	1.55639
43260	1.89226	0.431766
43261	1.89226	1.12902
43262	1.89226	0.753114
43263	1.65573	1.28291
43264	1.65573	0.996922
43265	1.89226	1.54049

43266	1.89226	1.61905
43267	1.89226	1.93341
43268	2.64212	1.4477
43269	3.78452	1.58779
43270	1.89226	2.1
43271	1.89226	2.1
43272	1.89226	2.1
43273	1.89226	2.1
43274	3.78452	2.1
43275	2.7367	2.1
43276	2.07947	2.1
43277	2.47319	2.1
43278	1.85057	2.1
43279	4.55267	2.1
43280	2.07948	2.1
43281	1.3294	2.1
43282	1.85057	2.1
43283	1.09962	2.1
43284	3.1791	2.1
43285	1.89226	2.1
43286	1.89226	2.1
43287	1.89226	2.1
43288	1.89226	2.1
43289	3.78453	2.1
43290	0.94613	2.1
43291	0.94613	2.1
43292	0.94613	2.1
43293	0.94613	2.1
43294	0.946128	2.1
43295	0.946128	2.1
43296	0.946132	2.1
43297	0.946132	2.1
43298	0.94613	2.1
43299	0.94613	2.1
43300	0.946131	2.1
43301	0.946131	2.1
43302	1.00875	2.1
43303	1.00875	2.1
43304	1.01676	2.1
43305	1.01676	2.1
43306	0.925282	2.1
43307	0.804877	2.1
43308	1.2366	2.1
43309	1.10023	2.1
43310	0.804878	2.1
43311	1.10023	2.1
43312	1.2366	2.1
43313	0.925284	2.1
43314	1.89226	2.1
43315	1.89226	2.1
43316	1.89226	2.1
43317	1.89226	2.1
43318	3.78453	2.1
43319	1.89226	2.1
43320	1.89226	2.1
43321	1.89226	2.1
43322	1.89226	2.1
43323	1.89226	2.1

43324	1.89226	2.1
43325	1.09766	2.1
43326	1.14842	2.1
43327	1.02909	2.1
43328	1.07659	2.1
43329	1.16718	2.1
43330	0.954189	2.1
43331	1.06653	2.1
43332	1.06653	2.1
43333	1.20738	2.1
43334	1.02909	2.1
43335	1.09767	2.1
43336	0.815951	2.1
43337	2.00793	2.1
43338	1.14048	2.1
43339	1.19086	2.1
43340	1.13723	2.1
43341	2.00793	2.1
43342	2.11772	2.1
43343	1.89226	2.1
43344	1.89226	2.1
43345	1.89226	2.1
43346	1.89226	2.1
43347	1.89226	2.1
43348	1.89226	2.1
43349	1.89226	2.1
43350	1.89226	2.1
43351	1.89226	2.1
43352	1.89226	2.1
43353	1.89226	2.1
43354	1.89226	2.1
43355	1.1779	2.1
43356	1.26166	2.1
43357	1.0875	2.1
43358	1.00397	2.1
43359	1.13286	2.1
43360	1.13286	2.1
43361	1.13277	2.1
43362	1.13277	2.1
43363	1.26166	2.1
43364	1.08749	2.1
43365	1.1779	2.1
43366	1.00396	2.1
43367	1.0875	2.1
43368	1.00397	2.1
43369	0.685749	2.1
43370	0.595432	2.1
43371	0.83976	2.1
43372	0.83976	2.1
43373	0.843169	2.1
43374	0.843169	2.1
43375	1.0875	2.1
43376	0.685749	2.1
43377	1.00397	2.1
43378	0.595432	2.1
43379	1.89226	2.1
43380	1.86443	2.1
43381	2.4988	2.1

43382	2.0815	2.1
43383	2.28173	2.1
43384	1.86443	2.1
43385	1.89226	2.1
43386	1.89226	2.1
43387	1.89226	2.1
43388	1.89226	2.1
43389	1.89226	2.1
43390	1.89226	2.1
43391	0.946131	2.1
43392	0.946131	2.1
43393	0.969491	2.1
43394	0.949105	2.1
43395	0.949103	2.1
43396	0.949103	2.1
43397	0.946133	2.1
43398	0.946133	2.1
43399	0.946131	2.1
43400	0.969492	2.1
43401	0.946131	2.1
43402	0.949105	2.1
43403	1.89226	2.1
43404	2.07428	2.1
43405	1.93464	2.1
43406	1.89226	2.1
43407	2.11667	2.1
43408	2.07428	2.1
43409	0.946132	2.1
43410	0.946132	2.1
43411	0.946131	2.1
43412	0.946131	2.1
43413	0.946132	2.1
43414	0.946132	2.1
43415	0.946132	2.1
43416	0.946132	2.1
43417	0.946132	2.1
43418	0.946132	2.1
43419	0.946132	2.1
43420	0.946132	2.1
43421	1.97992	2.1
43422	1.89226	2.1
43423	1.89226	2.1
43424	1.93898	2.1
43425	1.93898	2.1
43426	1.89226	2.1
43427	2.12475	0.272499
43428	2.79029	0.756521
43429	2.7803	1.51755
43430	2.13052	0.34374
43431	4.92081	0.398065
43432	1.06238	1.25906
43433	1.06526	0.42545
43434	0.642901	0.176743
43435	0.655055	0.960087
43436	0.856391	0.270232
43437	0.856391	0.278235
43438	0.86104	1.35433
43439	0.86104	0.136786

43440	1.06526	0.368469
43441	0.655055	0.342512
43442	0.642901	0.322745
43443	1.06238	0.309104
43444	2.7803	1.15983
43445	2.13053	0.233225
43446	2.14346	0.371947
43447	2.77937	0.74971
43448	4.9099	0.457921
43449	2.14346	0.492085
43450	1.31011	0.128567
43451	1.34124	0.429269
43452	1.31011	0.637213
43453	2.16166	0.167462
43454	2.13053	0.378103
43455	1.89226	2.1
43456	2.58176	2.1
43457	2.01422	2.1
43458	1.89226	2.1
43459	2.70373	2.1
43460	2.58176	2.1
43461	1.89226	2.1
43462	1.89226	2.1
43463	1.89226	2.1
43464	1.89226	2.1
43465	1.89226	2.1
43466	1.89226	2.1
43467	0.946128	2.1
43468	0.946128	2.1
43469	1.38521	2.1
43470	1.38521	2.1
43471	1.38521	2.1
43472	1.35186	2.1
43473	0.946131	2.1
43474	0.946131	2.1
43475	0.94613	2.1
43476	1.38521	2.1
43477	0.946131	2.1
43478	1.35186	2.1
43479	0.946133	2.1
43480	0.946133	2.1
43481	0.946132	2.1
43482	0.946132	2.1
43483	0.946132	2.1
43484	0.946132	2.1
43485	0.946132	2.1
43486	0.946132	2.1
43487	0.946132	2.1
43488	0.946132	2.1
43489	0.946133	2.1
43490	0.946133	2.1
43491	2.33374	2.1
43492	1.89226	2.1
43493	1.89226	2.1
43494	1.89226	2.1
43495	2.60377	2.1
43496	2.60377	2.1
43497	2.6771	0.05

43498	1.36416	0.0509371
43499	1.64983	0.05
43500	1.64983	0.05
43501	2.44131	0.05
43502	2.44131	0.05
43503	2.58321	0.248647
43504	1.89226	0.277345
43505	2.44283	0.135607
43506	2.44283	0.176977
43507	1.89226	0.682485
43508	1.89226	1.03194
43509	2.58321	0.318428
43510	2.44283	0.168283
43511	1.49358	0.116224
43512	1.57702	0.18483
43513	3.93641	0.143695
43514	0.951913	0.0526338
43515	0.81556	0.05
43516	0.946132	0.063501
43517	0.946132	0.0615704
43518	1.04072	0.0620288
43519	1.12182	0.0618119
43520	0.856745	0.05
43521	0.851542	0.05
43522	0.923163	0.0503864
43523	0.923163	0.0696875
43524	1.04304	0.060716
43525	0.85098	0.05
43526	2.70125	1.66725
43527	2.37734	1.91751
43528	2.63495	1.5099
43529	2.30539	1.87159
43530	5.01229	1.87494
43531	1.89226	1.79655
43532	1.89226	2.1
43533	1.89226	1.65018
43534	1.89226	2.1
43535	3.78452	1.6857
43536	1.89226	2.1
43537	1.89226	2.1
43538	1.89226	2.1
43539	1.89226	2.1
43540	3.78452	2.1
43541	2.37734	2.1
43542	1.79346	2.1
43543	2.53594	1.76258
43544	1.70302	2.1
43545	4.27682	2.1
43546	2.63495	1.42962
43547	2.30539	1.58626
43548	2.49445	0.684027
43549	2.12089	1.51481
43550	4.75584	1.29231
43551	1.89226	1.55739
43552	1.89226	1.78768
43553	1.89226	1.38507
43554	1.89226	1.76098
43555	3.78452	1.90599

43556	1.89226	1.81168
43557	1.89226	1.97092
43558	1.89226	2.05422
43559	1.89226	2.1
43560	1.89226	1.63092
43561	1.89226	1.68225
43562	2.53594	1.02426
43563	1.60017	1.73108
43564	1.57079	1.46052
43565	1.70302	1.59544
43566	2.15772	1.30953
43567	2.33299	1.53429
43568	2.09114	2.1
43569	2.34898	2.1
43570	2.30729	2.1
43571	2.06556	2.1
43572	4.41454	2.1
43573	2.09114	2.1
43574	1.59654	2.1
43575	1.57823	2.1
43576	2.06556	2.1
43577	3.6621	2.1
43578	2.30729	2.1
43579	2.06556	2.1
43580	2.03917	2.1
43581	2.26617	2.1
43582	4.33173	2.1
43583	2.03917	2.1
43584	2.06556	2.1
43585	2.045 2.1	
43586	1.55767	2.1
43587	1.57823	2.1
43588	1.57823	2.1
43589	1.89225	2.1
43590	1.09726	2.1
43591	1.61196	2.1
43592	1.28597	2.1
43593	1.28597	0.05
43594	1.45633	2.1
43595	1.45633	2.1
43596	1.9544	0.05
43597	1.61196	2.1
43598	1.45633	2.1
43599	1.53735	2.1
43600	3.14931	2.1
43601	1.89226	2.1
43602	1.89226	2.1
43603	1.89226	2.1
43604	1.89226	2.1
43605	3.78453	2.1
43606	0.810946	2.1
43607	0.810946	2.1
43608	0.810947	2.1
43609	0.810947	2.1
43610	0.810933	2.1
43611	0.810933	2.1
43612	1.89226	2.1
43613	1.3517	2.1

43614	1.08132	2.1
43615	1.08132	2.1
43616	1.08133	2.1
43617	0.851539	2.1
43618	0.851539	2.1
43619	0.972053	2.1
43620	1.05632	2.1
43621	0.94613	2.1
43622	0.94613	2.1
43623	1.0279	2.1
43624	0.94991	2.1
43625	1.0279	2.1
43626	0.946131	2.1
43627	0.865643	2.1
43628	0.94613	2.1
43629	1.89226	2.1
43630	1.89226	2.1
43631	1.89226	2.1
43632	1.89226	2.1
43633	3.78452	2.1
43634	1.03882	2.1
43635	1.91121	2.1
43636	1.41833	2.1
43637	2.34547	2.1
43638	3.32954	2.1
43639	2.3979	2.1
43640	1.89226	2.1
43641	1.89226	2.1
43642	1.89226	2.1
43643	2.0558	2.1
43644	2.0558	2.1
43645	1.89226	2.1
43646	1.89226	2.1
43647	1.89226	2.1
43648	1.89226	2.1
43649	3.78452	2.1
43650	1.05816	2.1
43651	1.05816	2.1
43652	0.842045	2.1
43653	1.05252	2.1
43654	1.36057	2.1
43655	1.14339	2.1
43656	0.873397	2.1
43657	0.585073	2.1
43658	0.873388	2.1
43659	1.15129	2.1
43660	0.69143	2.1
43661	1.1434	2.1
43662	1.93338	2.1
43663	1.85883	2.1
43664	1.51714	2.1
43665	1.85883	2.1
43666	1.54795	2.1
43667	1.89226	2.1
43668	1.89226	2.1
43669	1.89226	2.1
43670	1.89226	2.1
43671	1.89226	2.1

43672	3.78452	2.1
43673	0.844726	2.1
43674	0.844726	2.1
43675	0.960524	2.1
43676	1.16288	2.1
43677	0.851541	2.1
43678	0.851541	2.1
43679	0.929147	2.1
43680	1.15533	2.1
43681	0.844697	2.1
43682	0.946131	2.1
43683	0.851632	2.1
43684	0.946131	2.1
43685	1.89226	2.1
43686	1.70303	2.1
43687	1.74006	2.1
43688	1.70303	2.1
43689	1.99471	2.1
43690	1.93338	2.1
43691	0.946131	2.1
43692	0.946131	2.1
43693	0.946131	2.1
43694	0.946131	2.1
43695	0.94613	2.1
43696	0.94613	2.1
43697	0.946132	2.1
43698	0.946132	2.1
43699	0.946131	2.1
43700	0.946131	2.1
43701	0.946131	2.1
43702	0.946131	2.1
43703	1.58158	2.1
43704	1.89226	2.1
43705	1.89226	2.1
43706	1.89226	2.1
43707	1.68939	2.1
43708	1.68939	2.1
43709	0.946132	0.05
43710	0.946132	0.05
43711	1.12988	0.05
43712	1.30783	0.05
43713	1.30783	0.05
43714	0.946133	0.05
43715	0.946132	0.05
43716	1.12988	0.05
43717	1.30783	0.05
43718	1.30783	0.05
43719	0.946133	0.05
43720	0.946133	0.05
43721	1.89227	0.05
43722	1.89227	0.05
43723	1.89227	0.05
43724	1.89227	0.05
43725	1.89227	0.05
43726	1.89227	0.05
43727	1.89226	0.05
43728	1.89226	0.05
43729	1.89226	0.05

43730	1.89226	0.05
43731	1.89226	0.05
43732	1.89226	0.05
43733	0.946131	0.05
43734	0.946131	0.05
43735	1.30783	0.05
43736	1.37715	0.05
43737	1.37715	0.05
43738	0.946133	0.05
43739	1.30783	0.05
43740	0.946129	0.05
43741	1.37715	0.05
43742	1.37715	0.05
43743	0.946131	0.05
43744	0.946131	0.05
43745	1.17056	0.05
43746	0.591757	0.05
43747	0.522025	0.05
43748	0.929727	0.05
43749	0.929728	0.05
43750	1.17056	0.05
43751	0.591756	0.05
43752	0.522025	0.05
43753	0.889054	0.05
43754	0.889054	0.05
43755	0.803535	0.05
43756	0.803535	0.05
43757	1.49814	2.1
43758	2.37439	2.1
43759	2.63923	2.1
43760	2.63923	2.1
43761	1.29111	2.1
43762	1.29111	2.1
43763	0.946132	2.1
43764	0.946132	2.1
43765	1.18719	2.1
43766	1.18719	2.1
43767	0.94613	2.1
43768	0.94613	2.1
43769	1.1872	2.1
43770	1.31961	2.1
43771	0.946131	2.1
43772	1.31961	2.1
43773	1.1872	2.1
43774	0.94613	2.1
43775	2.49445	0.319122
43776	2.12089	0.974545
43777	2.20843	0.459993
43778	1.82805	0.416797
43779	4.32932	0.651766
43780	1.89226	1.22741
43781	1.89226	1.52565
43782	1.89226	1.0787
43783	1.89226	1.23293
43784	3.78452	0.792074
43785	0.94613	1.28196
43786	0.94613	1.28143
43787	0.946128	0.835404

43788	0.946128	1.46542
43789	0.946131	1.46245
43790	0.946131	1.45659
43791	0.946127	1.3933
43792	0.946127	1.24474
43793	0.946129	1.1578
43794	0.946129	0.985177
43795	0.946129	1.42985
43796	0.946129	1.37311
43797	1.82804	0.284879
43798	1.60017	1.03437
43799	1.87679	0.373691
43800	2.12088	0.463607
43801	1.35608	0.438933
43802	1.60017	0.629501
43803	2.20843	0.230794
43804	1.3216	0.196023
43805	1.44426	0.228782
43806	1.82805	0.288217
43807	1.80399	0.146052
43808	2.20843	0.215168
43809	1.89226	0.378734
43810	1.89226	0.403298
43811	1.89226	0.6965
43812	1.89226	0.657617
43813	1.89226	0.397546
43814	1.89226	0.428012
43815	1.89226	0.511939
43816	1.89226	0.351971
43817	1.89226	0.349906
43818	1.89226	0.312764
43819	1.89226	0.576689
43820	1.89226	0.313092
43821	0.627705	0.111669
43822	0.634941	0.220115
43823	0.629317	0.123926
43824	0.783422	0.153655
43825	0.629328	0.177365
43826	0.448764	0.25767
43827	1.35608	0.261777
43828	1.00805	0.267419
43829	0.814295	0.167093
43830	0.598375	0.234186
43831	1.04461	0.2221
43832	1.09795	2.0353
43833	1.00775	2.1
43834	1.61202	2.0834
43835	1.63419	2.01627
43836	2.64194	1.98203
43837	1.44801	1.83184
43838	1.61201	2.05899
43839	2.35614	1.87077
43840	2.27703	2.004
43841	3.88904	1.96848
43842	2.35615	1.7784
43843	1.63419	1.84575
43844	2.29852	1.75681
43845	1.63419	1.84884

43846	2.27634	1.81695
43847	1.61202	2.02723
43848	0.973793	2.1
43849	2.2671	2.1
43850	2.32981	2.1
43851	2.32981	2.1
43852	0.990481	2.1
43853	0.990481	2.1
43854	1.50877	2.1
43855	1.11193	2.1
43856	2.29667	2.1
43857	1.94158	2.1
43858	3.45035	2.1
43859	2.29667	2.1
43860	1.89227	2.1
43861	1.94158	2.1
43862	1.94158	2.1
43863	1.89227	2.1
43864	1.89227	2.1
43865	2.51379	2.1
43866	1.30026	2.1
43867	1.84502	2.1
43868	2.72099	2.1
43869	4.35882	2.1
43870	1.89226	2.1
43871	2.721	2.1
43872	2.5138	2.1
43873	2.5138	2.1
43874	1.89226	2.1
43875	1.89226	2.1
43876	1.89226	0.0601251
43877	1.89226	0.0658267
43878	2.59101	0.0593165
43879	1.89226	0.0803713
43880	3.78453	0.0605997
43881	0.851541	0.0667171
43882	0.851541	0.0588961
43883	1.42502	0.0624228
43884	0.948679	0.0577526
43885	0.946132	0.067729
43886	0.946132	0.0699835
43887	0.853617	0.0626026
43888	0.853618	0.0555422
43889	0.851475	0.0662081
43890	0.948666	0.0686779
43891	1.166	0.0626707
43892	0.851554	0.0647566
43893	2.51818	2.1
43894	2.60723	2.1
43895	2.66532	2.06865
43896	2.76315	2.1
43897	5.27255	2.1
43898	2.60723	2.1
43899	2.45247	2.1
43900	2.76315	2.1
43901	2.55197	2.1
43902	5.1592	2.1
43903	1.23768	0.201874

43904	1.11433	1.17786
43905	1.32037	1.52614
43906	1.32037	0.569823
43907	1.0142	1.39698
43908	1.0142	0.376643
43909	0.886711	1.22075
43910	1.16806	1.17933
43911	0.999083	1.52269
43912	0.710926	1.39237
43913	0.937757	1.44488
43914	0.937757	1.24172
43915	0.941224	1.13348
43916	0.941224	1.39536
43917	1.16805	1.34136
43918	0.999084	1.42452
43919	0.886709	1.35401
43920	0.710928	1.3485
43921	2.29332	2.1
43922	1.54904	2.1
43923	2.0815	2.1
43924	2.44199	2.1
43925	4.00418	2.1
43926	1.53873	0.578427
43927	1.53422	2.1
43928	1.39661	1.00937
43929	1.42387	2.1
43930	2.9626	1.85806
43931	1.53423	2.1
43932	1.44091	2.1
43933	1.42387	2.1
43934	1.35215	2.1
43935	2.86479	2.1
43936	1.39661	0.947459
43937	1.42387	2.1
43938	1.28325	0.634596
43939	1.33326	2.1
43940	2.70713	1.78738
43941	1.42387	2.1
43942	1.35215	2.1
43943	1.33327	2.1
43944	1.27339	2.1
43945	2.69726	2.1
43946	2.58442	2.1
43947	1.1468	2.1
43948	1.18775	2.1
43949	2.60047	2.1
43950	3.74728	2.1
43951	2.58442	2.1
43952	1.89226	2.1
43953	1.89226	2.1
43954	2.60048	2.1
43955	4.49274	2.1
43956	1.89226	2.1
43957	1.89226	2.1
43958	1.89226	2.1
43959	1.89226	2.1
43960	3.78453	2.1
43961	1.89226	2.1

43962	1.89226	2.1
43963	1.89226	2.1
43964	1.89226	2.1
43965	3.78452	2.1
43966	1.22262	2.1
43967	2.60047	2.1
43968	1.18775	2.1
43969	1.18775	2.1
43970	2.61433	2.1
43971	2.61433	2.1
43972	1.89226	2.1
43973	2.60048	2.1
43974	2.61434	2.1
43975	2.60048	2.1
43976	1.90612	2.1
43977	1.89226	2.1
43978	1.89226	2.1
43979	1.89226	2.1
43980	1.89226	2.1
43981	1.89226	2.1
43982	3.78453	2.1
43983	1.89226	2.1
43984	1.89226	2.1
43985	1.89226	2.1
43986	1.89226	2.1
43987	3.78452	2.1
43988	2.12556	2.1
43989	0.991113	2.1
43990	1.27725	2.1
43991	2.31081	2.1
43992	3.40281	2.1
43993	1.89226	2.1
43994	2.31082	2.1
43995	2.12556	2.1
43996	2.12556	2.1
43997	1.89226	2.1
43998	1.89226	2.1
43999	1.22544	2.1
44000	1.12649	2.1
44001	0.775254	2.1
44002	0.643133	2.1
44003	0.917924	2.1
44004	0.934955	2.1
44005	0.975579	2.1
44006	0.975579	2.1
44007	1.23113	2.1
44008	0.685272	2.1
44009	0.651719	2.1
44010	1.1791	2.1
44011	1.89226	2.1
44012	2.5138	2.1
44013	2.31082	2.1
44014	2.31082	2.1
44015	1.89226	2.1
44016	1.89226	2.1
44017	1.89226	2.1
44018	1.89226	2.1
44019	1.89226	2.1

44020	1.89226	2.1
44021	3.78453	2.1
44022	0.922224	2.1
44023	0.922224	2.1
44024	1.23555	2.1
44025	1.19715	2.1
44026	1.01445	2.1
44027	1.16061	2.1
44028	0.946132	2.1
44029	0.946132	2.1
44030	0.946131	2.1
44031	1.2051	2.1
44032	1.13537	2.1
44033	1.01445	2.1
44034	0.946133	2.1
44035	0.946133	2.1
44036	1.20648	2.1
44037	1.20648	2.1
44038	1.06454	2.1
44039	1.20648	2.1
44040	0.946133	2.1
44041	0.946133	2.1
44042	1.20649	2.1
44043	0.946133	2.1
44044	1.06454	2.1
44045	0.946132	2.1
44046	0.946132	2.1
44047	0.946132	2.1
44048	0.946132	2.1
44049	0.946132	2.1
44050	0.946132	2.1
44051	0.946132	2.1
44052	0.946133	2.1
44053	0.946133	2.1
44054	0.946133	2.1
44055	0.946133	2.1
44056	0.946132	2.1
44057	0.946132	2.1
44058	1.33078	2.09152
44059	1.37409	2.1
44060	1.31565	0.534585
44061	1.37114	2.1
44062	2.68973	2.1
44063	1.89225	1.84629
44064	1.89225	2.1
44065	1.89225	0.611024
44066	1.89225	2.1
44067	3.7845	1.72389
44068	1.89225	2.1
44069	1.89225	2.1
44070	1.89225	2.05798
44071	1.89225	2.1
44072	3.7845	2.1
44073	1.37409	2.1
44074	1.20408	2.1
44075	1.18612	2.1
44076	1.18907	2.1
44077	1.37114	2.1

44078	1.37409	2.1
44079	1.31566	2.06039
44080	1.37115	2.1
44081	1.30318	0.94605
44082	1.37433	2.1
44083	2.68998	2.03559
44084	1.89226	2.07187
44085	1.89226	2.1
44086	1.89226	1.13019
44087	1.89226	2.1
44088	3.78453	1.83516
44089	1.89226	2.1
44090	1.89226	2.1
44091	1.89226	2.1
44092	1.89226	2.1
44093	1.89226	2.1
44094	1.89226	2.1
44095	0.647001	2.1
44096	0.647001	2.1
44097	0.651851	2.1
44098	0.651851	2.1
44099	0.602046	2.1
44100	0.613277	2.1
44101	0.685576	2.1
44102	0.687164	2.1
44103	0.687164	2.1
44104	0.613276	2.1
44105	0.602046	2.1
44106	0.685576	2.1
44107	0.946128	1.72946
44108	0.946128	0.875785
44109	1.15307	2.1
44110	1.19317	2.05165
44111	0.94613	2.04593
44112	0.94613	0.563004
44113	1.15307	1.80699
44114	1.15307	2.1
44115	0.946131	1.85724
44116	1.15307	1.88799
44117	0.946131	1.83288
44118	1.19317	1.82755
44119	1.18278	2.1
44120	2.38635	2.07364
44121	1.23042	2.1
44122	1.23042	2.1
44123	2.30614	2.1
44124	2.30614	2.1
44125	0.946129	1.30968
44126	0.946129	1.25052
44127	1.12994	1.94935
44128	1.15307	1.85902
44129	0.946131	1.85669
44130	0.946131	0.400664
44131	1.12994	1.56058
44132	1.12994	1.8987
44133	1.12994	1.72349
44134	0.946131	1.73096
44135	0.946131	1.63692

44136	1.15307	1.69587
44137	2.25988	2.1
44138	2.30614	1.86067
44139	2.28696	2.1
44140	1.16361	2.1
44141	1.18278	2.1
44142	1.18278	2.1
44143	2.3771	2.1
44144	1.90121	2.1
44145	1.81191	2.1
44146	2.46904	2.1
44147	4.37025	2.1
44148	1.15676	2.1
44149	1.23452	2.1
44150	0.823592	2.1
44151	0.737606	2.1
44152	0.988113	2.1
44153	0.988113	2.1
44154	0.984014	2.1
44155	0.984014	2.1
44156	1.15676	2.1
44157	0.737605	2.1
44158	0.823592	2.1
44159	1.23452	2.1
44160	1.30318	2.1
44161	1.37433	2.1
44162	1.30151	1.34693
44163	1.39067	2.1
44164	2.67584	2.05066
44165	1.89226	1.84293
44166	1.89226	2.1
44167	1.89226	1.3398
44168	1.89226	1.9307
44169	3.78453	1.7023
44170	1.89226	1.8568
44171	1.89226	2.1
44172	1.89226	2.07626
44173	1.89226	2.1
44174	1.89226	2.07965
44175	1.89226	2.1
44176	0.656819	2.1
44177	0.656819	2.1
44178	0.661755	2.1
44179	0.661755	2.1
44180	0.613277	2.1
44181	0.63141	2.1
44182	0.687164	2.1
44183	0.695336	2.1
44184	0.695337	2.1
44185	0.63141	2.1
44186	0.687164	2.1
44187	0.613276	2.1
44188	1.30151	1.93018
44189	1.39067	2.1
44190	1.32143	1.43975
44191	1.43294	2.1
44192	2.73445	2.05347
44193	1.89226	1.89119

44194	1.89226	2.1
44195	1.89226	1.20616
44196	1.89226	2.1
44197	3.78452	1.77787
44198	1.89226	2.01811
44199	1.89226	2.08696
44200	1.89226	2.1
44201	1.89226	2.1
44202	1.89226	2.1
44203	1.89226	2.1
44204	1.43294	2.00262
44205	1.26282	2.1
44206	1.3268	2.1
44207	1.3268	2.1
44208	1.39067	2.1
44209	1.39067	2.1
44210	1.5511	0.112797
44211	1.00665	0.122773
44212	1.28239	0.0959271
44213	1.88631	0.0989448
44214	2.89296	0.103826
44215	1.89227	0.0965864
44216	1.89227	0.133709
44217	1.89227	0.111296
44218	1.89227	0.104063
44219	1.89227	0.121875
44220	1.89227	0.112201
44221	2.08848	0.125407
44222	1.89226	0.121708
44223	1.89226	0.0971318
44224	2.37947	0.117823
44225	4.27173	0.118359
44226	1.01456	0.108366
44227	0.943157	0.107752
44228	0.641198	0.111576
44229	0.724757	0.108763
44230	0.830923	0.113227
44231	0.830923	0.127427
44232	0.836991	0.100043
44233	0.836991	0.114371
44234	1.01456	0.100881
44235	0.724757	0.109078
44236	0.641197	0.111213
44237	0.943157	0.118703
44238	1.89227	0.120617
44239	1.89227	0.135747
44240	1.89227	0.10487
44241	1.89227	0.108149
44242	3.78453	0.111463
44243	0.946129	0.119863
44244	0.946129	0.116762
44245	1.28941	0.115095
44246	1.18974	0.116345
44247	0.94613	0.118154
44248	0.94613	0.12528
44249	1.28941	0.108645
44250	1.28941	0.124306
44251	1.28941	0.111466

44252	0.946131	0.116077
44253	0.94613	0.12171
44254	1.18974	0.126148
44255	0.710636	2.1
44256	0.780249	2.1
44257	0.579033	2.1
44258	0.478812	2.1
44259	0.621822	2.1
44260	0.621822	2.1
44261	0.63724	2.1
44262	0.63724	2.1
44263	0.780251	2.1
44264	0.579032	2.1
44265	0.710637	2.1
44266	0.478811	2.1
44267	1.05499	2.1
44268	1.00521	1.97198
44269	1.24445	2.1
44270	1.23082	1.51781
44271	2.28581	1.9963
44272	1.48751	2.1
44273	1.23083	1.73551
44274	1.4941	2.09166
44275	1.24445	2.06055
44276	1.48047	1.8552
44277	1.23083	2.04628
44278	0.621185	1.49744
44279	0.621185	1.72805
44280	0.620899	1.78458
44281	0.620899	2.03994
44282	0.502515	1.36746
44283	0.626672	1.39379
44284	0.615412	1.49844
44285	0.740234	2.07597
44286	0.740233	1.42624
44287	0.626672	1.43205
44288	0.615413	1.51783
44289	0.502516	1.57235
44290	1.95844	2.1
44291	2.6562	2.1
44292	1.95844	2.1
44293	2.20883	2.1
44294	2.38225	2.1
44295	2.63264	2.1
44296	1.89226	2.1
44297	1.89226	2.1
44298	1.89226	2.1
44299	1.89226	2.1
44300	3.78453	2.1
44301	1.95844	2.1
44302	2.38225	2.1
44303	1.71082	2.1
44304	2.14647	2.1
44305	4.1049	2.1
44306	0.946131	2.1
44307	0.946131	2.1
44308	0.946131	2.1
44309	0.946131	2.1

44310	0.946131	2.1
44311	0.946131	2.1
44312	0.946131	2.1
44313	0.946131	2.1
44314	0.946131	2.1
44315	0.946131	2.1
44316	0.946131	2.1
44317	0.946131	2.1
44318	2.12597	2.1
44319	1.2295	2.1
44320	1.28014	2.1
44321	2.19723	2.1
44322	3.40611	2.1
44323	2.12597	2.1
44324	2.79242	2.1
44325	2.73375	2.1
44326	2.66249	2.1
44327	2.19723	2.1
44328	2.12597	2.1
44329	1.13716	2.1
44330	1.09861	2.1
44331	0.666692	2.1
44332	0.640071	2.1
44333	0.891617	2.1
44334	0.891617	2.1
44335	0.885614	2.1
44336	0.885614	2.1
44337	1.13716	2.1
44338	0.666692	2.1
44339	0.640071	2.1
44340	1.09861	2.1
44341	1.89226	0.0519066
44342	1.70303	0.05
44343	1.89226	0.05
44344	2.64598	0.058717
44345	1.81091	0.05
44346	2.53222	0.0515282
44347	2.32422	0.05
44348	1.89226	0.051435
44349	2.78336	0.0589236
44350	2.78336	0.054782
44351	1.89226	0.05
44352	1.89226	0.05
44353	0.946132	0.05
44354	0.946132	0.05
44355	0.946131	0.05
44356	0.946131	0.055553
44357	0.946132	0.0600097
44358	0.946132	0.0572374
44359	0.946131	0.05
44360	0.946131	0.0542624
44361	0.946131	0.05
44362	0.946131	0.0544419
44363	0.946131	0.0554595
44364	0.946131	0.05
44365	0.946131	0.0585255
44366	0.946131	0.0695339
44367	0.946132	0.060561

44368	0.946132	0.0703719
44369	0.946132	0.0713423
44370	0.946132	0.0703157
44371	0.946131	0.055077
44372	0.946131	0.0548752
44373	0.946131	0.0566884
44374	0.946131	0.0693547
44375	0.946131	0.0690122
44376	0.946131	0.057577
44377	2.24323	1.5532
44378	1.89226	1.76785
44379	2.75021	1.63907
44380	2.75021	1.70669
44381	1.89226	1.34485
44382	1.89226	1.53782
44383	2.30908	0.597686
44384	2.19815	2.09512
44385	2.1609	0.563377
44386	1.83796	2.1
44387	4.35904	1.26033
44388	2.19815	2.09158
44389	2.02268	2.1
44390	2.17869	2.1
44391	1.9559	2.1
44392	2.0815	2.1
44393	1.83424	2.1
44394	2.1609	0.495314
44395	1.88877	2.1
44396	1.98413	0.833063
44397	1.87355	2.1
44398	4.03445	1.47706
44399	1.87355	2.1
44400	2.02268	2.1
44401	1.87355	2.1
44402	2.10488	2.1
44403	1.68443	2.1
44404	1.89448	2.1
44405	0.946131	0.185628
44406	0.946131	0.160325
44407	1.40574	0.182835
44408	1.12373	0.217164
44409	0.946129	0.199905
44410	0.946129	0.18785
44411	1.40574	0.182306
44412	1.40574	0.1754
44413	0.946131	0.174814
44414	1.40574	0.186806
44415	1.12373	0.203995
44416	0.946131	0.185277
44417	2.14277	0.276391
44418	2.81148	0.246985
44419	1.42762	0.250728
44420	1.42762	0.302724
44421	2.96261	0.241645
44422	2.24746	0.277899
44423	1.89226	0.495918
44424	1.89226	0.22345
44425	1.89226	0.2896

44426	1.89226	0.447166
44427	3.78453	0.516961
44428	1.89226	0.281481
44429	1.89226	1.26957
44430	1.89226	0.497966
44431	1.89226	0.0933448
44432	3.78452	0.249379
44433	0.68433	0.12919
44434	0.946127	0.936209
44435	0.543921	0.958676
44436	0.946131	0.0783299
44437	0.946131	1.21711
44438	0.946131	1.1507
44439	0.543918	1.57904
44440	0.543918	0.499671
44441	0.684332	0.325752
44442	0.54392	1.23908
44443	0.946129	0.198618
44444	0.946129	0.599563
44445	1.89226	0.260226
44446	1.08784	1.13029
44447	1.19352	0.574896
44448	1.89226	0.701626
44449	1.19352	1.31465
44450	1.89226	0.906376
44451	0.635711	0.0989994
44452	0.946132	0.459556
44453	0.946132	0.562196
44454	0.56474	0.203119
44455	0.750045	0.403938
44456	1.25657	1.27435
44457	0.635682	0.170797
44458	0.635682	0.342682
44459	0.891954	0.117459
44460	1.32751	0.851913
44461	0.564766	0.318114
44462	0.564766	0.476339
44463	0.858345	0.314973
44464	0.946128	1.09575
44465	0.635712	0.241094
44466	0.946133	0.144774
44467	0.977198	0.40487
44468	0.944521	1.09414
44469	0.635682	0.905056
44470	0.635682	0.099818
44471	1.02924	0.356227
44472	0.705625	1.16558
44473	0.851528	0.0936075
44474	0.851528	1.76773
44475	2.18903	2.1
44476	2.10182	2.1
44477	2.10182	2.1
44478	1.89226	2.1
44479	1.89226	2.1
44480	1.64983	2.1
44481	1.89226	2.1
44482	1.83867	2.1
44483	1.83867	2.1

44484	1.89164	2.1
44485	2.0815	2.1
44486	2.17638	2.1
44487	2.57957	1.43494
44488	2.3506	2.09784
44489	2.69056	0.370065
44490	2.49439	1.72326
44491	5.04116	1.51616
44492	2.3506	1.98187
44493	1.8911	2.1
44494	2.49439	1.48337
44495	2.06689	2.1
44496	4.41748	2.1
44497	2.69056	1.41978
44498	2.49439	1.90488
44499	2.77227	0.802226
44500	2.63443	1.83193
44501	5.32499	1.59009
44502	2.49439	1.68564
44503	2.06689	2.1
44504	2.63443	1.12963
44505	2.26444	2.1
44506	4.70131	2.1
44507	2.46327	2.1
44508	2.54347	2.1
44509	2.52869	2.1
44510	2.16534	2.1
44511	2.82661	2.1
44512	2.46327	2.1
44513	0.946131	2.1
44514	0.946131	2.1
44515	1.27174	2.1
44516	1.27174	2.1
44517	1.27174	2.1
44518	0.94613	2.1
44519	0.946128	2.1
44520	1.4133	2.1
44521	0.946129	2.1
44522	0.946129	2.1
44523	1.27174	2.1
44524	1.4133	2.1
44525	1.42757	2.1
44526	1.7457	2.1
44527	1.66047	2.1
44528	1.7457	2.1
44529	1.45754	2.1
44530	1.54278	2.1
44531	1.42757	2.1
44532	1.52996	2.1
44533	1.66047	2.1
44534	1.66047	2.1
44535	1.26397	2.1
44536	1.26397	2.1
44537	0.968536	2.1
44538	1.02461	2.1
44539	1.57575	2.1
44540	1.62391	2.1
44541	2.59245	2.1

44542	0.946131	2.1
44543	0.946131	2.1
44544	0.946132	2.1
44545	0.946132	2.1
44546	0.946132	2.1
44547	0.946132	2.1
44548	0.946132	2.1
44549	0.946132	2.1
44550	0.946132	2.1
44551	0.946132	2.1
44552	0.946132	2.1
44553	0.946132	2.1
44554	2.23765	2.1
44555	1.62391	2.1
44556	2.22154	2.1
44557	1.57575	2.1
44558	2.2697	2.1
44559	1.62391	2.1
44560	1.89226	2.1
44561	1.89226	2.1
44562	1.89226	2.1
44563	1.89226	2.1
44564	1.89226	2.1
44565	1.89226	2.1
44566	1.89226	2.1
44567	1.89226	2.1
44568	1.89226	2.1
44569	1.89226	2.1
44570	1.89226	2.1
44571	1.89226	2.1
44572	2.2697	2.1
44573	1.46129	2.1
44574	2.25873	2.1
44575	1.62391	2.1
44576	2.09611	2.1
44577	1.46129	2.1
44578	1.89226	2.1
44579	2.07088	2.1
44580	1.89226	2.1
44581	2.31884	2.1
44582	4.21111	2.1
44583	1.89226	2.1
44584	2.31884	2.1
44585	2.11713	2.1
44586	1.89226	2.1
44587	2.54371	2.1
44588	2.31884	2.1
44589	2.54371	2.1
44590	1.24041	2.1
44591	2.55208	2.1
44592	2.31884	2.1
44593	1.23712	2.1
44594	1.00388	2.1
44595	2.72769	0.0519836
44596	2.57507	0.0671123
44597	1.89226	0.05
44598	1.89226	0.0642356
44599	4.46733	0.0552028

44600	1.89226	0.0617774
44601	2.57506	0.0658812
44602	1.89226	0.05
44603	2.30076	0.0634079
44604	1.89226	0.05
44605	2.30076	0.0640718
44606	1.21171	0.05
44607	1.48589	0.05
44608	2.09166	0.05
44609	1.78381	0.05
44610	3.2697	0.05
44611	2.09166	0.05
44612	1.70302	0.05
44613	1.48589	0.05
44614	1.18027	0.05
44615	2.26571	0.05
44616	1.79969	0.05
44617	1.89226	0.05
44618	1.89226	0.0680688
44619	1.89226	0.0648241
44620	1.89226	0.0574134
44621	1.89226	0.064849
44622	1.89226	0.0614518
44623	0.946131	0.0526318
44624	0.946131	0.0828409
44625	0.946128	0.0657781
44626	0.946128	0.0593588
44627	0.946129	0.0658614
44628	0.946129	0.0679659
44629	0.94613	0.0521558
44630	0.94613	0.05
44631	0.94613	0.0535252
44632	0.94613	0.0599022
44633	0.94613	0.0627366
44634	0.94613	0.0586926
44635	1.89226	1.74463
44636	1.89226	1.80153
44637	1.89226	1.90741
44638	1.89226	1.68551
44639	1.89226	1.86124
44640	1.89226	1.66712
44641	1.89226	1.69491
44642	1.89226	1.71841
44643	1.89226	1.83133
44644	1.89226	1.62234
44645	1.89226	1.80966
44646	1.89226	1.61437
44647	1.89226	1.60694
44648	1.89226	1.6397
44649	1.89226	1.73326
44650	1.89226	1.53102
44651	1.89226	1.7313
44652	1.89226	1.53191
44653	1.79103	1.68193
44654	0.968249	2.1
44655	1.44807	2.1
44656	2.17984	0.959188
44657	3.2391	1.98027

44658	1.29735	1.70863
44659	1.08992	1.71156
44660	0.945147	2.1
44661	0.724033	2.1
44662	1.00723	2.1
44663	1.00723	1.80988
44664	1.01416	1.20003
44665	1.01416	2.1
44666	1.29735	1.92326
44667	0.94515	1.93404
44668	0.724034	2.00936
44669	1.08992	2.0342
44670	1.39214	2.1
44671	1.39214	2.1
44672	0.946129	2.1
44673	0.946129	2.1
44674	1.33728	2.1
44675	1.39214	2.1
44676	0.94613	2.1
44677	0.94613	2.1
44678	0.94613	2.1
44679	1.33728	2.1
44680	0.94613	2.1
44681	1.39214	2.1
44682	2.78428	2.1
44683	2.28373	2.1
44684	2.67456	2.1
44685	2.20011	2.1
44686	4.95829	2.1
44687	1.90165	0.170484
44688	2.24206	0.572346
44689	1.66137	0.240654
44690	2.07108	1.68448
44691	3.90343	0.27001
44692	1.89224	0.613742
44693	1.89224	0.633942
44694	1.89224	0.352355
44695	1.89224	0.295907
44696	3.78448	0.275938
44697	2.82159	0.702414
44698	1.89226	0.740007
44699	2.50138	1.72108
44700	1.89226	0.430836
44701	4.39365	0.563178
44702	1.66139	0.163868
44703	1.86399	1.7205
44704	1.56005	0.438973
44705	1.90324	0.729504
44706	3.56463	0.42968
44707	0.946133	0.0873074
44708	0.946133	0.754121
44709	0.946132	0.23493
44710	0.946132	0.985039
44711	0.946133	0.340399
44712	0.946133	0.677113
44713	0.946132	0.213464
44714	0.946132	0.45307
44715	0.946133	0.19688

44716	0.946133	0.272239
44717	0.946132	0.146633
44718	0.946132	0.266206
44719	1.5176	0.866777
44720	1.97964	0.288938
44721	2.32889	0.295962
44722	1.91762	0.412578
44723	3.84649	0.361991
44724	2.3376	0.589266
44725	1.97963	0.445597
44726	2.32889	0.601614
44727	2.58303	0.299007
44728	4.66649	0.473494
44729	2.32889	0.315323
44730	2.20248	0.309158
44731	1.91762	0.231491
44732	1.91762	0.226877
44733	2.5615	0.663103
44734	2.5615	0.343003
44735	2.32889	1.04333
44736	2.58303	0.269651
44737	2.71793	0.429226
44738	2.56149	0.321139
44739	5.04681	0.450106
44740	1.89226	0.413272
44741	2.53785	0.450391
44742	1.89226	0.194361
44743	2.60895	0.357988
44744	1.89226	0.276218
44745	2.60895	0.297351
44746	0.493248	0.13683
44747	1.30448	0.484253
44748	1.26893	0.848817
44749	0.653226	0.314862
44750	0.653227	0.419452
44751	0.493247	1.35796
44752	1.26893	1.00831
44753	1.30448	0.497239
44754	0.832232	0.803535
44755	0.832233	0.775849
44756	0.929944	0.331929
44757	0.929944	0.403838
44758	1.89226	2.1
44759	1.47937	2.1
44760	1.50143	2.1
44761	1.47937	2.1
44762	1.91432	2.1
44763	1.89226	2.1
44764	1.89226	2.1
44765	1.89226	2.1
44766	1.89226	2.1
44767	1.89226	2.1
44768	1.89226	2.1
44769	1.89226	2.1
44770	1.33419	2.1
44771	2.4542	2.1
44772	2.59834	2.1
44773	2.59834	2.1

44774	1.25716	2.1
44775	1.25716	2.1
44776	1.70303	2.1
44777	1.50143	2.1
44778	1.60878	2.1
44779	1.50143	2.1
44780	1.93599	2.1
44781	1.89226	2.1
44782	0.946131	2.1
44783	0.946131	2.1
44784	0.946127	2.1
44785	0.946127	2.1
44786	0.946127	2.1
44787	0.946127	2.1
44788	0.946131	2.1
44789	0.946131	2.1
44790	0.94613	2.1
44791	0.94613	2.1
44792	0.946129	2.1
44793	0.946129	2.1
44794	1.43912	2.1
44795	2.59834	2.1
44796	2.78093	2.1
44797	2.78093	2.1
44798	1.33419	2.1
44799	1.33419	2.1
44800	1.89226	2.1
44801	2.26322	2.1
44802	2.55682	2.1
44803	2.55682	2.1
44804	1.89226	2.1
44805	1.89226	2.1
44806	2.58032	0.215844
44807	2.28922	0.142456
44808	1.88424	0.138355
44809	2.32127	0.126921
44810	4.46456	0.15426
44811	1.67265	0.249545
44812	2.28922	0.177872
44813	1.88424	0.117841
44814	1.11314	0.141173
44815	3.55689	0.144019
44816	1.43751	0.0938483
44817	2.32127	0.121148
44818	1.92998	0.0776929
44819	2.37671	0.0911609
44820	1.43751	0.180897
44821	1.88424	0.174129
44822	2.36729	2.1
44823	1.56714	2.1
44824	1.56714	2.1
44825	1.62408	2.1
44826	2.28373	2.1
44827	2.34067	2.1
44828	0.937268	2.1
44829	0.937268	2.1
44830	0.94636	2.1
44831	0.94636	2.1

44832	0.76004	2.1
44833	0.783572	2.1
44834	1.14187	2.1
44835	1.10006	2.1
44836	1.10006	2.1
44837	0.76004	2.1
44838	0.783572	2.1
44839	1.14187	2.1
44840	1.28753	2.1
44841	1.36838	2.1
44842	1.62617	2.1
44843	1.82857	2.1
44844	3.1161	2.1
44845	1.82856	2.1
44846	1.17996	2.1
44847	1.53417	2.1
44848	1.56618	2.1
44849	1.36838	2.1
44850	1.36838	2.1
44851	1.35065	0.801346
44852	1.42659	0.253432
44853	2.26675	0.548248
44854	2.26675	0.388083
44855	1.62137	0.364376
44856	2.53747	0.531185
44857	1.89226	0.07348
44858	1.89226	0.0579356
44859	1.89226	0.0615009
44860	1.89226	0.0728083
44861	3.78452	0.0695424
44862	1.36569	0.05
44863	1.36569	0.05
44864	0.946131	0.0532601
44865	0.946131	0.059838
44866	1.16041	0.0547326
44867	1.36569	0.05
44868	0.946129	0.0558108
44869	0.946129	0.05999
44870	1.1604	0.05
44871	0.94613	0.0558532
44872	0.946131	0.0519763
44873	1.36569	0.05
44874	2.73139	0.05
44875	1.25265	0.0529407
44876	1.25265	0.0538038
44877	1.64799	0.0513287
44878	2.32081	0.05
44879	2.71615	0.05
44880	0.946133	0.0561166
44881	0.946133	0.0570123
44882	0.946132	0.0698482
44883	0.946132	0.065843
44884	0.946132	0.0612246
44885	0.946132	0.0663637
44886	0.946133	0.0572356
44887	0.946133	0.0619429
44888	0.946132	0.0610838
44889	0.946132	0.0657806

44890	0.946133	0.0631652
44891	0.946133	0.0586857
44892	0.946131	0.0585043
44893	0.946131	0.0606652
44894	0.94613	0.0906025
44895	0.94613	0.0689345
44896	0.946132	0.0800966
44897	0.946132	0.0673568
44898	0.946129	0.0723848
44899	0.946129	0.0854958
44900	0.946131	0.0737879
44901	0.946131	0.0643832
44902	0.946131	0.0621883
44903	0.946131	0.0714598
44904	1.89226	2.1
44905	2.48503	2.1
44906	2.64586	2.1
44907	2.64586	2.1
44908	1.89226	2.1
44909	1.89226	2.1
44910	2.48504	2.1
44911	1.99985	2.1
44912	1.83584	2.1
44913	1.83584	2.1
44914	2.64586	2.1
44915	2.64586	2.1
44916	1.89225	2.1
44917	2.33095	2.1
44918	2.48501	2.1
44919	2.48501	2.1
44920	1.89225	2.1
44921	1.89225	2.1
44922	2.33096	2.1
44923	1.83583	2.1
44924	1.68436	2.1
44925	1.68436	2.1
44926	2.48502	2.1
44927	2.48502	2.1
44928	1.14807	2.1
44929	1.76545	2.1
44930	1.21292	2.1
44931	1.83936	2.1
44932	2.97837	2.1
44933	1.89226	2.1
44934	1.89226	2.1
44935	1.89226	2.1
44936	1.89226	2.1
44937	3.78451	2.1
44938	1.89226	2.1
44939	1.89226	2.01659
44940	1.89226	2.1
44941	1.89226	2.1
44942	3.78452	2.1
44943	1.76545	2.1
44944	2.12608	2.1
44945	1.83937	2.1
44946	2.22529	2.1
44947	3.99074	2.1

44948	0.780222	2.1
44949	0.780222	2.1
44950	0.784871	2.1
44951	0.784871	2.1
44952	0.95863	2.1
44953	0.919681	2.1
44954	0.636145	2.1
44955	0.606461	2.1
44956	0.636144	2.1
44957	0.958631	2.1
44958	0.60646	2.1
44959	0.919683	2.1
44960	1.89226	2.1
44961	1.89226	2.1
44962	1.89226	2.1
44963	1.89226	2.1
44964	3.78452	2.1
44965	1.89226	2.1
44966	1.89226	2.1
44967	1.89226	2.1
44968	1.89226	2.03186
44969	3.78452	2.1
44970	1.03789	2.1
44971	1.03789	2.1
44972	1.04231	2.1
44973	1.04231	2.1
44974	1.16052	2.1
44975	1.11265	2.1
44976	0.958634	2.1
44977	0.919685	2.1
44978	0.958634	2.1
44979	1.16052	2.1
44980	0.919685	2.1
44981	1.11265	2.1
44982	1.43837	2.1
44983	2.57085	2.1
44984	1.39332	2.1
44985	2.58228	2.1
44986	3.96417	2.1
44987	1.89226	2.1
44988	1.89226	2.1
44989	1.89226	2.1
44990	1.89226	2.1
44991	1.89226	2.1
44992	1.89226	2.1
44993	1.89226	2.1
44994	1.89226	2.1
44995	1.89226	2.1
44996	1.89226	2.1
44997	3.78452	2.1
44998	2.57085	2.1
44999	1.89226	2.1
45000	2.58229	2.1
45001	1.89226	2.1
45002	4.46312	2.1
45003	1.32368	2.1
45004	2.58228	2.1
45005	2.55352	2.1

45006	2.55352	2.1
45007	1.39332	2.1
45008	1.39332	2.1
45009	1.89226	2.1
45010	1.89226	2.1
45011	1.89226	2.1
45012	1.89226	2.1
45013	1.89226	2.1
45014	1.89226	2.1
45015	1.89226	2.1
45016	1.89226	2.1
45017	1.89226	2.1
45018	1.89226	2.1
45019	1.89226	2.1
45020	1.89226	2.1
45021	2.55353	2.1
45022	1.89226	2.1
45023	2.58228	2.1
45024	1.89226	2.1
45025	1.89226	2.1
45026	2.58228	2.1
45027	1.40574	1.81456
45028	1.05175	1.62717
45029	1.71613	1.72897
45030	1.99961	1.67315
45031	3.12187	1.6906
45032	1.71613	1.70106
45033	2.50733	1.60968
45034	1.99961	1.8012
45035	1.99961	1.6192
45036	2.25746	1.68356
45037	2.25746	1.55155
45038	2.25746	1.57016
45039	1.89526	1.58411
45040	1.71613	1.71305
45041	1.71613	1.32803
45042	1.89526	1.62986
45043	1.27558	1.3705
45044	1.07536	2.1
45045	1.07536	2.1
45046	1.07034	2.1
45047	1.07034	2.1
45048	0.766879	2.1
45049	0.81527	2.1
45050	1.38896	2.1
45051	1.33043	2.1
45052	0.815264	2.1
45053	1.38895	2.1
45054	0.766884	2.1
45055	1.33044	2.1
45056	1.89225	2.1
45057	1.89225	2.1
45058	1.89225	2.1
45059	1.89225	2.1
45060	1.89225	2.1
45061	1.89225	2.1
45062	1.64059	2.1
45063	1.89225	2.1

45064	1.565	2.1
45065	1.565	2.1
45066	1.89225	2.1
45067	1.89225	2.1
45068	1.89226	2.1
45069	1.89226	2.1
45070	1.89226	2.1
45071	1.89226	2.1
45072	1.89226	2.1
45073	1.89226	2.1
45074	1.67906	2.1
45075	1.89226	2.1
45076	1.6406	2.1
45077	1.6406	2.1
45078	1.89226	2.1
45079	1.89226	2.1
45080	2.18106	1.51451
45081	2.20831	1.99956
45082	2.64725	1.22518
45083	2.63102	1.27374
45084	4.82831	1.3379
45085	2.18106	1.39592
45086	2.09605	2.1
45087	2.5601	1.47866
45088	2.63102	1.34237
45089	4.74116	1.69388
45090	2.64725	1.27953
45091	1.89226	1.35374
45092	1.87603	1.87248
45093	2.63102	1.29235
45094	1.89226	1.5315
45095	2.64725	1.19191
45096	0.946132	1.39742
45097	1.28005	1.40246
45098	0.946132	1.7628
45099	1.31551	1.30267
45100	1.28005	1.44661
45101	1.28005	1.6324
45102	0.946132	1.38726
45103	0.946132	1.42239
45104	0.946132	1.36109
45105	0.946132	1.38586
45106	1.28005	1.45726
45107	1.31551	1.43947
45108	2.0815	2.1
45109	1.59318	2.1
45110	2.0815	2.1
45111	1.40161	2.1
45112	2.49782	2.1
45113	1.68553	2.1
45114	0.819885	2.1
45115	0.935082	2.1
45116	0.867363	2.1
45117	0.867363	2.1
45118	0.715431	2.1
45119	0.902777	2.1
45120	0.738933	2.1
45121	0.980053	2.1

45122	0.936716	2.1
45123	0.917651	2.1
45124	0.775853	2.1
45125	0.74037	2.1
45126	2.78977	2.1
45127	2.38239	2.1
45128	2.78977	2.1
45129	1.96468	2.1
45130	2.64107	2.1
45131	1.80733	2.1
45132	1.80141	2.1
45133	2.28512	2.1
45134	2.28512	2.1
45135	1.8519	2.1
45136	2.64107	2.1
45137	2.20785	2.1
45138	0.946133	1.12512
45139	0.946133	0.712184
45140	0.946132	0.92513
45141	0.946132	0.675559
45142	0.946132	0.954221
45143	0.946132	1.32843
45144	0.946132	0.661871
45145	0.946132	0.625443
45146	0.946132	0.726478
45147	0.946132	1.06926
45148	0.946133	0.70496
45149	0.946133	1.14448
45150	1.89226	0.29231
45151	1.89226	0.291436
45152	1.89226	0.229242
45153	1.89226	0.347198
45154	1.89226	0.237975
45155	1.89226	0.337187
45156	1.89226	0.384112
45157	1.89226	0.434912
45158	1.89226	0.326899
45159	1.89226	0.550166
45160	3.78453	0.428364
45161	1.89226	1.01603
45162	1.89226	0.691319
45163	1.89226	0.460303
45164	1.89226	1.29739
45165	1.89226	0.639333
45166	1.89226	1.12539
45167	2.12925	0.05
45168	2.51397	0.0511796
45169	2.22488	0.05
45170	1.86113	0.0509816
45171	4.3751	0.05
45172	2.82754	0.05
45173	2.51397	0.0506804
45174	2.22489	0.05
45175	2.47291	0.0514078
45176	4.98688	0.05
45177	1.89226	0.0526085
45178	1.89226	0.0708522
45179	1.89226	0.0572697

45180	1.89226	0.0727374
45181	3.78453	0.0687332
45182	1.89226	0.0528649
45183	1.89226	0.071052
45184	1.89226	0.0551365
45185	1.89226	0.0726229
45186	3.78452	0.0574935
45187	0.90848	0.05
45188	1.11244	0.05
45189	0.930566	0.05
45190	0.73385	0.05
45191	0.921298	0.05
45192	0.921298	0.0509636
45193	0.924994	0.05
45194	0.924994	0.05
45195	0.908478	0.05
45196	0.733851	0.05
45197	1.11244	0.05
45198	0.930567	0.05
45199	2.22489	0.05
45200	2.0815	0.05
45201	2.0815	0.05
45202	2.30674	0.05
45203	1.99867	0.05
45204	2.33358	0.05
45205	1.89226	0.0555406
45206	1.89226	0.0710838
45207	1.89226	0.0598827
45208	1.89226	0.0632159
45209	1.89226	0.0578807
45210	1.89226	0.0599723
45211	0.946131	0.0727446
45212	0.946131	0.0669649
45213	0.946128	0.0736585
45214	0.946128	0.0564645
45215	0.946129	0.0709257
45216	0.946129	0.0743574
45217	0.94613	0.0501911
45218	0.94613	0.05366
45219	0.94613	0.0681026
45220	0.94613	0.0546611
45221	0.94613	0.0566403
45222	0.94613	0.0645563
45223	2.28904	2.1
45224	1.89226	2.1
45225	2.2846	2.1
45226	2.2846	2.1
45227	1.89226	1.88386
45228	1.89226	2.1
45229	1.89226	1.87882
45230	1.89226	1.70317
45231	1.89226	2.1
45232	1.89226	2.1
45233	1.89226	1.36054
45234	1.89226	2.1
45235	1.04037	1.7732
45236	1.34066	2.08616
45237	1.34955	1.69368

45238	1.34955	2.01308
45239	1.03373	1.73927
45240	1.03373	2.1
45241	0.946131	1.83117
45242	0.946131	2.1
45243	1.14452	2.1
45244	1.14452	2.1
45245	0.946131	1.94561
45246	0.946131	1.98896
45247	1.14296	2.1
45248	1.14452	2.1
45249	1.14296	2.1
45250	0.946131	2.1
45251	1.14452	2.1
45252	0.946132	2.1
45253	1.89226	1.74686
45254	1.89226	2.01226
45255	1.89226	1.96494
45256	1.89226	2.1
45257	1.89226	0.910298
45258	1.89226	2.1
45259	0.597454	1.75203
45260	0.597454	1.97603
45261	0.597972	1.79043
45262	0.597972	2.1
45263	0.675238	2.06181
45264	0.674776	1.73228
45265	0.519667	1.77587
45266	0.520188	1.83246
45267	0.519667	1.44651
45268	0.675238	1.47074
45269	0.520188	1.7953
45270	0.674777	1.79864
45271	1.89226	0.339666
45272	1.82125	0.223186
45273	1.89226	0.238801
45274	1.84314	0.197708
45275	1.89226	0.45851
45276	1.84314	0.386715
45277	1.89226	0.222093
45278	1.84314	0.240765
45279	1.89226	0.214168
45280	1.65794	0.180378
45281	1.89226	0.258496
45282	1.65794	0.223988
45283	1.348	1.84861
45284	1.43485	2.1
45285	1.79929	1.20776
45286	1.75309	1.49095
45287	3.14728	1.5755
45288	1.348	1.41811
45289	1.63892	1.61971
45290	1.59157	1.87927
45291	1.18647	1.731
45292	1.75309	1.42417
45293	1.348	1.65117
45294	1.79929	1.46222
45295	1.75309	2.1

45296	2.18106	1.34245
45297	2.20831	1.35414
45298	3.98034	1.61424
45299	2.18106	1.30557
45300	1.63892	1.71472
45301	1.75309	1.29617
45302	1.75309	1.90693
45303	2.09605	1.34056
45304	2.09605	1.90572
45305	2.66076	0.124394
45306	1.70302	0.0666247
45307	2.41575	0.0756574
45308	1.70302	0.11999
45309	3.08598	0.0593766
45310	2.3661	0.0900716
45311	1.71053	0.0667438
45312	2.41575	0.09814
45313	1.20143	0.0553759
45314	1.20143	0.0550447
45315	2.2123	0.05
45316	1.66739	0.0570749
45317	2.25633	0.0659334
45318	2.68302	0.0637578
45319	1.71051	0.0534398
45320	1.71051	0.0762936
45321	2.74192	0.05
45322	2.1961	0.05
45323	1.32553	2.1
45324	2.34875	1.87343
45325	2.19139	2.1
45326	2.19139	1.60407
45327	1.56172	2.1
45328	1.56172	0.901148
45329	1.89226	2.1
45330	1.89226	1.89247
45331	1.89226	2.1
45332	1.89226	2.1
45333	3.78453	2.1
45334	2.667	2.1
45335	1.26097	2.1
45336	1.26097	2.1
45337	1.5683	2.1
45338	2.42949	2.1
45339	2.73682	2.1
45340	1.13213	2.1
45341	2.19139	1.72978
45342	2.0815	2.1
45343	1.77615	1.25005
45344	1.37767	2.1
45345	1.19297	0.824425
45346	0.946132	1.93563
45347	0.946132	2.1
45348	0.946131	2.1
45349	0.946131	2.1
45350	0.946131	2.1
45351	0.946131	1.66916
45352	0.946132	2.1
45353	0.946132	2.1

45354	0.946132	2.1
45355	0.946132	2.1
45356	0.946131	2.1
45357	0.946131	2.1
45358	2.02064	2.1
45359	2.3606	2.1
45360	1.47478	2.1
45361	2.22997	2.1
45362	4.25061	2.1
45363	2.36059	2.1
45364	2.56952	2.1
45365	2.22997	2.1
45366	2.48631	2.1
45367	4.79949	2.1
45368	1.84261	2.1
45369	2.22997	2.1
45370	1.65706	2.1
45371	1.78837	2.1
45372	3.88703	2.1
45373	2.22997	2.1
45374	2.48631	2.1
45375	2.19528	2.1
45376	2.41175	2.1
45377	4.64172	2.1
45378	1.6466	2.1
45379	1.89226	2.1
45380	1.35972	2.1
45381	1.35972	2.1
45382	1.89226	2.1
45383	1.89226	2.1
45384	1.89226	2.1
45385	1.89226	2.1
45386	1.89226	2.1
45387	1.89226	2.1
45388	3.78453	2.1
45389	2.44797	2.1
45390	1.07214	2.1
45391	1.31589	2.1
45392	1.1211	2.1
45393	2.44797	2.1
45394	2.25318	2.1
45395	1.95954	2.1
45396	1.89226	2.1
45397	1.6466	2.1
45398	1.6466	2.1
45399	1.89226	2.1
45400	1.89226	2.1
45401	0.946133	2.1
45402	0.946133	2.1
45403	0.946132	2.1
45404	0.946132	2.1
45405	0.946132	2.1
45406	0.946132	2.1
45407	0.946133	2.1
45408	0.946133	2.1
45409	0.946132	2.1
45410	0.946132	2.1
45411	0.946133	2.1

45412	0.946133	2.1
45413	2.44797	2.1
45414	1.36904	2.1
45415	1.70303	2.1
45416	2.37586	2.1
45417	4.00507	2.1
45418	0.631553	0.05
45419	1.24489	0.05
45420	1.11597	0.05
45421	0.496799	0.05
45422	0.872272	0.051483
45423	0.872272	0.05
45424	0.869415	0.05
45425	0.869415	0.05
45426	0.4968	0.05
45427	0.631553	0.05
45428	1.11597	0.05
45429	1.24489	0.05
45430	1.84253	2.1
45431	2.17384	2.1
45432	2.04818	2.1
45433	1.65925	2.1
45434	3.83309	2.1
45435	2.04818	2.1
45436	1.65925	2.1
45437	1.48162	2.1
45438	1.92264	2.1
45439	3.58189	2.1
45440	2.3305	0.127494
45441	1.89226	0.165548
45442	2.52753	0.137304
45443	2.52753	0.22263
45444	1.89226	0.120959
45445	1.89226	0.232442
45446	1.89226	0.193593
45447	1.89226	0.863846
45448	1.89226	0.34632
45449	1.89226	0.944335
45450	1.89226	0.230447
45451	1.89226	0.875587
45452	1.34563	0.143854
45453	2.1769	0.190087
45454	1.94955	0.147349
45455	1.94955	0.46239
45456	1.55063	0.1399
45457	1.55063	0.309206
45458	2.09455	0.101017
45459	1.89226	0.163813
45460	2.3305	0.116612
45461	2.3305	0.172046
45462	1.89226	0.097247
45463	1.89226	0.132616
45464	1.89226	0.149637
45465	1.89226	0.18916
45466	1.89226	0.286051
45467	1.89226	0.173119
45468	1.89226	0.220887
45469	1.89226	0.265021

45470	0.773392	0.106475
45471	0.612765	0.387523
45472	0.79504	0.152108
45473	0.575323	0.166149
45474	1.0819	0.114799
45475	1.06106	0.144855
45476	1.04675	0.109386
45477	0.86858	0.205829
45478	0.796873	0.135028
45479	0.557469	0.156436
45480	0.807663	0.172074
45481	2.2024	2.1
45482	2.34106	2.1
45483	2.02634	2.1
45484	2.20125	2.1
45485	4.36739	2.1
45486	2.34106	2.1
45487	2.25195	2.1
45488	2.20125	2.1
45489	2.13132	2.1
45490	4.47237	2.1
45491	2.02635	2.1
45492	2.22465	2.1
45493	1.68504	2.1
45494	2.0815	2.1
45495	3.92673	2.1
45496	2.22465	2.1
45497	2.13133	2.1
45498	2.0815	2.1
45499	2.23579	2.1
45500	4.44862	2.1
45501	1.89226	1.29457
45502	1.89226	1.40597
45503	1.89226	1.69683
45504	1.89226	1.44145
45505	1.89226	1.54695
45506	1.89226	1.12281
45507	1.93967	0.0533667
45508	1.70302	0.0663981
45509	1.21883	0.0562887
45510	1.70302	0.0659291
45511	1.19889	0.056463
45512	1.70302	0.0653505
45513	0.946131	0.0914473
45514	0.946131	0.0730041
45515	0.946131	0.0637109
45516	0.946131	0.0686417
45517	0.94613	0.0642771
45518	0.94613	0.106246
45519	0.946131	0.0699057
45520	0.946131	0.0695965
45521	0.946131	0.0665161
45522	0.946131	0.0640571
45523	0.946131	0.08432
45524	0.946131	0.0714408
45525	0.883128	0.0618134
45526	0.883128	0.066345
45527	0.827519	0.0527818

45528	0.82752	0.0623514
45529	1.16234	0.0583181
45530	1.19063	0.0765744
45531	0.631294	0.0593398
45532	0.548311	0.06753
45533	0.631293	0.0539067
45534	1.16234	0.0533819
45535	1.19063	0.0655802
45536	0.54831	0.0687461
45537	1.89226	0.056803
45538	1.89226	0.0912223
45539	1.89226	0.106294
45540	1.89226	0.0684176
45541	3.78453	0.0704138
45542	2.33328	0.05
45543	1.87991	0.0516323
45544	1.87991	0.0566486
45545	2.82938	0.05
45546	1.84457	0.0531077
45547	2.79403	0.05
45548	2.82937	0.05
45549	2.70881	0.0525495
45550	1.8799	0.0573671
45551	1.82739	0.05
45552	4.58874	0.05
45553	1.89226	0.101019
45554	1.89226	0.0582253
45555	1.89226	0.0730248
45556	1.89226	0.103715
45557	3.78452	0.0896396
45558	1.17897	0.0531505
45559	1.82739	0.05
45560	1.17897	0.05
45561	1.76199	0.05
45562	1.23885	0.05
45563	1.80989	0.05
45564	0.946131	0.0620148
45565	0.946131	0.0626447
45566	0.946131	0.0828229
45567	0.946131	0.0764906
45568	0.946129	0.0865812
45569	0.946129	0.0616694
45570	0.946133	0.0750333
45571	0.946133	0.0806546
45572	0.946131	0.0771838
45573	0.946131	0.0608852
45574	0.946131	0.0628427
45575	0.946131	0.0678191
45576	0.946126	1.94604
45577	0.946126	2.1
45578	1.24887	2.1
45579	1.24887	2.1
45580	0.946127	2.1
45581	0.946127	2.01778
45582	1.24887	2.1
45583	1.28511	2.1
45584	1.2851	2.1
45585	0.946128	2.1

45586	1.24887	2.1
45587	0.946128	2.1
45588	1.89226	2.1
45589	1.89226	2.04648
45590	1.89226	2.1
45591	1.89226	1.48797
45592	3.78452	1.99347
45593	0.592142	2.1
45594	0.592142	2.1
45595	0.60496	2.1
45596	0.60496	2.1
45597	0.694237	2.1
45598	0.608714	1.85145
45599	0.562863	2.1
45600	0.502864	2.1
45601	0.562863	2.1
45602	0.694237	2.1
45603	0.608714	2.1
45604	0.502864	2.1
45605	1.89226	2.1
45606	1.89226	2.1
45607	1.89226	2.1
45608	1.89226	2.1
45609	3.78453	2.1
45610	1.55779	2.1
45611	1.57968	2.1
45612	1.47186	2.1
45613	1.51725	2.1
45614	3.05154	2.1
45615	1.57967	2.1
45616	1.474 2.1	
45617	1.51725	2.1
45618	1.42895	2.1
45619	3.00863	2.1
45620	1.89226	2.1
45621	1.89226	2.1
45622	1.89226	2.1
45623	1.89226	2.1
45624	3.78452	2.1
45625	1.89226	2.1
45626	1.89226	2.1
45627	1.89226	2.1
45628	1.89226	2.1
45629	3.78453	2.1
45630	1.47186	2.1
45631	1.51725	2.1
45632	1.38859	2.1
45633	1.45332	2.1
45634	2.92519	2.1
45635	1.51725	2.1
45636	1.42895	2.1
45637	1.45332	2.1
45638	1.38414	2.1
45639	2.88228	2.1
45640	1.89226	2.1
45641	1.89226	2.1
45642	1.89226	2.1
45643	1.89226	2.1

45644	3.78452	2.1
45645	1.34497	0.05
45646	1.36747	0.05
45647	1.18919	0.05
45648	2.08149	0.05
45649	1.23556	0.05
45650	2.13702	0.05
45651	1.10106	0.05
45652	2.08001	0.05
45653	1.34497	0.05
45654	2.31605	0.05
45655	1.34497	0.05
45656	2.31605	0.05
45657	2.31605	0.05
45658	2.0815	0.05
45659	1.9419	0.05
45660	2.46665	0.05
45661	4.52922	0.05
45662	1.20349	0.05
45663	2.08149	0.05
45664	1.36948	0.05
45665	1.34737	0.05
45666	2.08149	0.05
45667	2.08149	0.05
45668	0.946132	0.05
45669	0.946132	0.05
45670	1.37715	0.05
45671	0.823199	0.05
45672	1.21598	0.05
45673	0.946132	0.05
45674	1.37715	0.05
45675	0.946132	0.05
45676	1.21598	0.05
45677	0.823198	0.05
45678	0.946133	0.05
45679	0.946133	0.05
45680	1.89226	0.05
45681	1.89226	0.05
45682	1.89226	0.05
45683	1.89226	0.05
45684	3.78453	0.05
45685	1.89227	0.05
45686	1.89227	0.05
45687	1.89227	0.05
45688	1.89227	0.05
45689	3.78453	0.05
45690	0.946132	0.05
45691	0.946132	0.05
45692	1.21599	0.05
45693	1.21599	0.05
45694	0.966749	0.05
45695	0.946133	0.05
45696	1.21598	0.05
45697	0.946132	0.05
45698	0.966748	0.05
45699	1.21598	0.05
45700	0.946133	0.05
45701	0.946133	0.05

45702	0.946133	0.05
45703	0.946133	0.05
45704	1.1134	0.05
45705	1.11555	0.05
45706	1.11555	0.05
45707	0.946132	0.05
45708	0.946132	0.05
45709	1.1134	0.05
45710	1.11555	0.05
45711	1.11555	0.05
45712	0.946132	0.05
45713	0.946132	0.05
45714	0.946133	0.05
45715	0.946133	0.05
45716	1.11555	0.05
45717	0.978467	0.05
45718	0.978468	0.05
45719	0.946133	0.05
45720	1.11555	0.05
45721	0.946132	0.05
45722	0.978468	0.05
45723	0.978468	0.05
45724	0.946132	0.05
45725	0.946132	0.05
45726	0.851542	2.1
45727	0.851542	2.1
45728	1.14168	2.1
45729	1.27897	2.1
45730	1.02817	2.1
45731	1.13536	2.1
45732	0.946131	2.1
45733	0.861207	2.1
45734	0.94613	2.1
45735	0.94613	2.1
45736	0.868751	2.1
45737	1.15181	2.1
45738	0.946132	2.1
45739	0.946132	1.88559
45740	0.946128	2.0475
45741	0.946128	1.63685
45742	0.94613	2.1
45743	0.94613	2.04854
45744	0.946131	1.79297
45745	0.946131	1.68978
45746	0.94613	1.72653
45747	0.94613	2.0223
45748	0.94613	1.82364
45749	0.94613	2.02799
45750	2.53273	0.05
45751	1.89226	0.0501244
45752	1.89226	0.0517026
45753	1.89226	0.0559134
45754	1.89226	0.05
45755	1.89226	0.05
45756	1.89226	2.1
45757	1.89226	2.1
45758	1.89226	2.1
45759	1.89226	2.1

45760	3.78452	2.1
45761	1.89226	2.1
45762	2.54394	2.1
45763	1.89226	2.1
45764	2.42221	2.1
45765	4.43621	2.1
45766	2.54394	2.1
45767	1.62743	2.1
45768	2.42221	2.1
45769	1.5437	2.1
45770	4.08764	2.1
45771	1.89226	2.1
45772	1.89226	2.1
45773	1.89226	2.1
45774	1.89226	2.1
45775	3.78452	2.1
45776	1.89226	2.1
45777	1.89226	2.1
45778	1.89226	2.1
45779	1.89226	2.1
45780	3.78452	2.1
45781	1.89226	2.1
45782	2.30146	2.1
45783	2.42221	2.1
45784	1.89226	2.1
45785	1.89226	2.1
45786	2.42221	2.1
45787	2.30146	2.1
45788	2.42221	2.1
45789	1.39962	2.1
45790	1.39962	2.1
45791	1.5437	2.1
45792	2.44553	2.1
45793	1.89226	2.1
45794	1.89226	2.1
45795	1.89226	2.1
45796	1.89226	2.1
45797	3.78452	2.1
45798	1.62196	2.1
45799	2.00695	2.1
45800	1.95288	2.1
45801	1.70303	2.1
45802	3.53316	2.1
45803	1.80703	2.1
45804	2.00695	2.1
45805	1.92991	2.1
45806	2.00695	2.1
45807	1.85888	2.1
45808	1.93592	2.1
45809	1.89226	2.1
45810	1.89226	2.1
45811	1.89226	2.1
45812	1.89226	2.1
45813	1.89226	2.1
45814	1.89226	2.1
45815	1.89226	2.1
45816	1.89226	2.1
45817	1.89226	2.1

45818	1.89226	2.1
45819	3.78453	2.1
45820	1.95288	2.1
45821	1.50524	2.1
45822	1.83564	2.1
45823	1.70303	2.1
45824	2.00749	2.1
45825	1.84671	2.1
45826	1.62632	2.1
45827	1.82498	2.1
45828	1.92991	2.1
45829	1.92991	2.1
45830	1.47928	2.1
45831	1.47751	2.1
45832	0.946128	2.1
45833	0.946128	2.1
45834	0.946132	2.1
45835	0.946132	2.1
45836	0.94613	2.1
45837	0.94613	2.1
45838	0.946129	2.1
45839	0.946129	2.1
45840	0.94613	2.1
45841	0.94613	2.1
45842	0.94613	2.1
45843	0.94613	2.1
45844	1.89226	2.1
45845	1.89226	2.1
45846	1.89226	2.1
45847	1.89226	2.1
45848	1.89226	2.1
45849	1.89226	2.1
45850	2.27351	1.32407
45851	2.74068	0.447366
45852	2.72278	0.748582
45853	2.63356	1.1267
45854	2.36273	0.674609
45855	2.27351	1.08385
45856	2.36273	1.19007
45857	2.83112	0.397939
45858	2.81212	0.580344
45859	2.74068	0.863163
45860	2.43416	0.574443
45861	2.36273	0.935498
45862	1.89227	1.0296
45863	2.83112	0.380145
45864	2.74069	0.492806
45865	2.74069	0.367845
45866	1.89227	1.22799
45867	1.89227	0.953486
45868	2.40887	2.1
45869	1.4494	2.1
45870	1.52164	2.1
45871	2.47534	2.1
45872	3.93051	2.1
45873	1.89226	2.1
45874	2.47534	2.1
45875	2.40887	2.1

45876	2.40887	2.1
45877	1.89226	2.1
45878	1.89226	2.1
45879	1.60861	2.1
45880	2.47534	2.1
45881	1.52164	2.1
45882	1.52164	2.1
45883	2.55301	2.1
45884	2.55301	2.1
45885	0.946128	2.1
45886	0.946128	2.1
45887	1.23767	2.1
45888	1.2765	2.1
45889	1.23767	2.1
45890	1.23767	2.1
45891	0.946131	2.1
45892	0.946131	2.1
45893	0.946131	2.1
45894	1.2765	2.1
45895	1.23767	2.1
45896	0.946131	2.1
45897	1.89226	2.1
45898	1.87511	2.1
45899	1.87511	2.1
45900	1.95852	2.1
45901	2.0815	2.1
45902	2.22169	2.1
45903	1.89226	2.1
45904	1.89226	2.1
45905	1.89226	2.1
45906	1.89226	2.1
45907	1.89226	2.1
45908	1.89226	2.1
45909	2.0815	2.1
45910	1.80067	2.1
45911	1.80067	2.1
45912	2.05518	2.1
45913	1.89226	2.1
45914	2.16509	2.1
45915	1.87153	2.1
45916	2.65897	2.1
45917	1.58724	2.1
45918	2.38924	2.1
45919	4.26077	2.1
45920	1.89226	2.1
45921	1.89226	2.1
45922	1.89226	2.1
45923	1.89226	2.1
45924	1.89226	2.1
45925	1.89226	2.1
45926	1.89226	1.05827
45927	2.21022	0.590512
45928	2.19769	1.33017
45929	2.21022	0.900679
45930	1.87973	0.321486
45931	1.89226	0.179789
45932	1.18376	1.89172
45933	2.21022	0.72909

45934	1.16239	1.83276
45935	1.16239	0.949802
45936	2.19769	1.83631
45937	2.19769	0.824267
45938	0.946128	0.159997
45939	0.946128	0.215735
45940	1.09362	0.442873
45941	1.09884	0.366385
45942	0.94613	0.419505
45943	0.94613	0.121186
45944	1.09362	0.245881
45945	1.09362	0.281038
45946	0.946131	0.266886
45947	1.09362	0.316032
45948	0.946131	0.149353
45949	1.09884	0.131965
45950	1.19323	1.39249
45951	2.19769	0.394652
45952	1.18376	1.13614
45953	1.18376	0.578607
45954	2.18724	1.19658
45955	2.18724	0.581854
45956	2.0815	2.1
45957	0.922714	2.1
45958	2.0815	2.1
45959	1.84882	2.1
45960	1.20787	2.1
45961	1.08321	2.1
45962	2.6238	0.0760412
45963	1.89226	0.109849
45964	1.89226	0.105751
45965	2.4866	0.0827732
45966	1.89226	0.0935876
45967	2.4866	0.0862044
45968	2.66753	0.0843802
45969	1.89226	0.160472
45970	1.89226	0.14422
45971	2.6238	0.0968612
45972	1.89226	0.120725
45973	2.6238	0.0979275
45974	0.946129	2.1
45975	0.946129	2.1
45976	1.04676	2.1
45977	1.04676	2.1
45978	0.946131	2.1
45979	0.946131	2.1
45980	1.08732	2.1
45981	1.04676	2.1
45982	1.08732	2.1
45983	0.946131	2.1
45984	1.04676	2.1
45985	0.946131	2.1
45986	1.20958	2.1
45987	1.73802	1.56961
45988	1.39067	1.932
45989	1.39067	1.71641
45990	1.50969	1.91514
45991	1.50969	1.79324

45992	1.00382	1.94986
45993	1.50969	0.518996
45994	1.20958	1.9095
45995	1.20958	1.06754
45996	1.32039	1.92531
45997	1.32039	1.44059
45998	2.0815	2.1
45999	2.51396	2.1
46000	2.19826	2.1
46001	2.54175	2.1
46002	4.79599	2.1
46003	2.54174	2.1
46004	2.17978	2.1
46005	2.54174	2.1
46006	2.2854	2.1
46007	2.57212	2.1
46008	2.31578	2.1
46009	2.27103	2.1
46010	2.54175	2.1
46011	2.4255	2.1
46012	2.82251	2.1
46013	4.96725	2.1
46014	2.60614	0.0796815
46015	2.75374	0.0676633
46016	1.94002	0.0526957
46017	2.08392	0.0642664
46018	4.69006	0.0599835
46019	2.75373	0.0758213
46020	2.6875	0.0867276
46021	2.08392	0.0535648
46022	1.99944	0.064496
46023	4.77142	0.0772276
46024	1.1211	0.05
46025	2.08392	0.0601473
46026	1.17787	0.0625299
46027	1.94002	0.0547834
46028	1.32178	0.05
46029	2.08392	0.05
46030	2.08392	0.05
46031	1.99944	0.05
46032	1.32178	0.05
46033	1.25929	0.05
46034	3.34321	0.05
46035	1.22266	0.05
46036	0.779473	0.05
46037	0.94613	0.05
46038	0.94613	0.05
46039	1.04072	0.05
46040	1.1407	0.05
46041	1.10042	0.05
46042	0.851542	0.05
46043	0.942046	0.05
46044	0.942046	0.05
46045	1.04114	0.05
46046	0.826602	0.05
46047	2.37468	2.1
46048	1.89226	2.1
46049	1.89226	2.1

46050	1.89226	2.1
46051	2.27917	2.1
46052	2.27917	2.1
46053	1.13959	2.1
46054	1.09451	2.1
46055	0.946131	2.1
46056	0.946131	2.1
46057	1.09451	2.1
46058	1.09451	2.1
46059	0.94613	2.1
46060	0.94613	2.1
46061	1.09451	2.1
46062	0.946131	2.1
46063	0.946131	2.1
46064	1.13959	2.1
46065	2.52454	1.96527
46066	2.12221	2.1
46067	2.67624	1.78005
46068	2.33865	2.1
46069	4.86319	2.1
46070	1.89226	2.1
46071	1.89226	2.1
46072	1.89226	2.09158
46073	1.89226	2.1
46074	3.78452	2.1
46075	1.89226	2.1
46076	1.89226	2.1
46077	1.89226	2.1
46078	1.89226	2.1
46079	3.78452	2.1
46080	2.12221	2.1
46081	1.47769	2.1
46082	2.33865	2.1
46083	1.62898	2.1
46084	3.81634	2.1
46085	2.67624	2.1
46086	2.37734	2.1
46087	2.33865	2.1
46088	2.33865	2.1
46089	2.70125	2.1
46090	2.70125	2.1
46091	1.89226	2.1
46092	1.89226	2.1
46093	1.89226	2.1
46094	1.89226	2.1
46095	1.89226	1.70132
46096	1.89226	1.80002
46097	1.89226	2.1
46098	1.89226	2.1
46099	1.89226	2.1
46100	1.89226	2.1
46101	1.89226	2.1
46102	1.89226	2.1
46103	2.37733	2.1
46104	1.95927	2.1
46105	1.82516	2.1
46106	1.82516	2.1
46107	2.33865	2.1

46108	2.33865	2.1
46109	2.57466	2.1
46110	2.21715	2.1
46111	2.55653	2.1
46112	2.24775	2.1
46113	4.77367	2.1
46114	0.937095	2.1
46115	0.937095	2.1
46116	0.958028	2.1
46117	0.958028	2.1
46118	0.828386	2.1
46119	0.771244	2.1
46120	1.10858	2.1
46121	1.12388	2.1
46122	1.12388	2.1
46123	0.828386	2.1
46124	0.771244	2.1
46125	1.10858	2.1
46126	2.55653	2.1
46127	2.32606	2.1
46128	2.24775	2.1
46129	2.24775	2.1
46130	2.57928	2.1
46131	2.57928	2.1
46132	0.990481	2.1
46133	0.990481	2.1
46134	1.00094	2.1
46135	1.00094	2.1
46136	0.888385	2.1
46137	0.828386	2.1
46138	1.16303	2.1
46139	1.12388	2.1
46140	1.16303	2.1
46141	0.888385	2.1
46142	1.12388	2.1
46143	0.828387	2.1
46144	0.962524	2.1
46145	0.962524	2.1
46146	0.7976	2.1
46147	0.7976	2.1
46148	0.797628	2.1
46149	0.814 2.1	
46150	0.946125	2.1
46151	0.946125	2.1
46152	0.946118	2.1
46153	0.797623	2.1
46154	0.814006	2.1
46155	0.946131	2.1
46156	1.8978	0.05
46157	2.23892	0.05
46158	1.71469	0.05
46159	4.13672	0.05
46160	2.64136	0.05
46161	2.23892	1.76359
46162	2.37578	1.92792
46163	1.77489	2.1
46164	4.6147	2.09065
46165	1.02284	2.1

46166	0.898796	2.06739
46167	0.948554	2.1
46168	0.948554	2.1
46169	0.878797	2.1
46170	0.796197	2.1
46171	1.09145	2.1
46172	0.963109	2.1
46173	1.12114	2.1
46174	0.782739	2.1
46175	1.11084	2.1
46176	0.850742	2.1
46177	2.48024	2.1
46178	2.405	2.1
46179	2.24585	2.1
46180	2.34795	2.1
46181	4.75295	2.1
46182	1.93794	2.1
46183	2.405	2.1
46184	2.24585	2.1
46185	1.67594	2.1
46186	4.08094	2.1
46187	1.89226	2.1
46188	1.89226	1.99168
46189	1.89226	2.1
46190	1.89226	2.1
46191	3.78452	2.1
46192	1.89226	2.1
46193	1.89226	2.1
46194	1.89226	2.1
46195	1.89226	2.1
46196	3.78452	2.1
46197	2.24585	2.1
46198	2.34795	2.1
46199	2.21155	2.1
46200	2.06626	2.1
46201	4.4142	2.1
46202	2.24585	2.1
46203	1.41498	2.1
46204	1.41498	2.1
46205	1.67594	2.1
46206	2.06625	2.1
46207	2.32721	2.1
46208	1.89226	2.1
46209	1.89226	2.1
46210	1.89226	2.1
46211	1.89226	2.1
46212	1.89226	2.1
46213	1.89226	2.02585
46214	1.89226	2.1
46215	1.89226	2.1
46216	1.89226	2.1
46217	1.89226	2.1
46218	3.78452	2.1
46219	1.89226	0.133639
46220	1.89226	0.206222
46221	1.89226	0.263521
46222	1.89226	0.112431
46223	3.78452	0.1198

46224	1.89226	0.145319
46225	2.53655	0.0766307
46226	1.89226	0.0732039
46227	1.89226	0.0827715
46228	1.89226	0.149531
46229	1.89226	0.156411
46230	1.89227	2.1
46231	1.89227	2.1
46232	1.89227	2.1
46233	1.89227	2.1
46234	1.89227	2.1
46235	1.89227	2.1
46236	0.91339	2.1
46237	0.91339	2.1
46238	1.2158	2.1
46239	1.20629	2.1
46240	1.00473	2.1
46241	1.15749	2.1
46242	0.946132	2.1
46243	0.946132	2.1
46244	0.946133	2.1
46245	1.17513	2.1
46246	1.00473	2.1
46247	0.946132	2.1
46248	1.89226	2.1
46249	1.89226	2.1
46250	1.89226	2.1
46251	1.89226	2.1
46252	1.89226	2.1
46253	1.89226	2.1
46254	1.89226	2.1
46255	2.6066	2.1
46256	2.35026	2.1
46257	2.35026	2.1
46258	1.89226	2.1
46259	1.89226	2.1
46260	2.04056	0.22829
46261	2.77366	0.197267
46262	1.24682	0.194957
46263	1.24682	0.274721
46264	2.93651	0.196642
46265	2.14277	0.250258
46266	2.68524	1.69662
46267	2.81847	1.57809
46268	1.89226	1.55356
46269	1.89226	1.56544
46270	4.57751	1.56639
46271	2.68524	1.67408
46272	1.89226	1.71592
46273	1.89226	1.68002
46274	2.50732	1.61691
46275	1.89226	1.6566
46276	2.50732	1.63165
46277	1.89226	1.56061
46278	1.89226	1.54457
46279	1.89226	1.53587
46280	1.89226	1.65023
46281	1.89226	1.51043

46282	1.89226	1.64995
46283	1.89226	1.71313
46284	1.89226	1.54312
46285	1.89226	1.68244
46286	1.89226	1.66911
46287	1.89226	1.55922
46288	1.89226	1.53232
46289	1.61016	1.54187
46290	1.07467	1.56704
46291	1.24474	1.51232
46292	1.24474	1.65921
46293	1.45303	1.54017
46294	1.45303	1.65948
46295	1.61016	1.54282
46296	1.24474	1.61957
46297	1.61016	1.54647
46298	1.77186	1.5181
46299	1.37813	1.49519
46300	1.37813	1.53081
46301	1.73305	2.1
46302	2.0815	2.1
46303	1.97658	2.1
46304	1.75791	2.1
46305	1.87228	2.1
46306	1.66515	2.1
46307	2.0815	2.1
46308	1.93938	2.1
46309	2.03253	2.1
46310	2.2142	2.1
46311	1.97658	2.1
46312	2.15324	2.1
46313	1.73305	2.1
46314	1.97658	2.1
46315	1.60428	2.1
46316	1.8589	2.1
46317	3.58085	2.1
46318	1.97657	2.1
46319	1.93938	2.1
46320	1.51316	2.1
46321	1.85293	2.1
46322	3.82951	2.1
46323	1.70303	0.0795479
46324	1.96087	0.0995174
46325	1.42308	0.0813502
46326	1.96087	0.124738
46327	1.25023	0.0800256
46328	1.70303	0.0822652
46329	0.946132	0.0947141
46330	0.946132	0.105916
46331	0.946131	0.0860696
46332	0.946131	0.0889215
46333	0.946132	0.0917808
46334	0.946132	0.171479
46335	0.946132	0.0876507
46336	0.946132	0.102313
46337	0.946132	0.093756
46338	0.946132	0.0871507
46339	0.946132	0.104046

46340	0.946132	0.120543
46341	1.89226	0.196179
46342	1.89226	0.338577
46343	1.89226	0.19517
46344	1.89226	0.280768
46345	3.78452	0.240546
46346	1.89226	0.0826999
46347	1.89226	0.0865267
46348	1.89226	0.179796
46349	1.89226	0.273326
46350	1.89226	0.0796416
46351	1.89226	0.0861716
46352	1.89226	0.157197
46353	1.89226	0.108196
46354	1.89226	0.0781908
46355	1.89226	0.232385
46356	1.89226	0.0847239
46357	1.89226	0.242603
46358	1.89226	0.0652449
46359	1.89226	0.0820449
46360	2.48255	0.0604091
46361	1.89226	0.055239
46362	3.78452	0.0632986
46363	1.89226	2.1
46364	2.42758	2.1
46365	1.9209	2.1
46366	1.89226	2.1
46367	2.45622	2.1
46368	2.42758	2.1
46369	2.42758	2.1
46370	1.49987	2.1
46371	1.61028	2.1
46372	1.58164	2.1
46373	2.45622	2.1
46374	2.42758	2.1
46375	0.94613	2.1
46376	0.94613	2.1
46377	1.24298	2.1
46378	1.24298	2.1
46379	1.24298	2.1
46380	1.22811	2.1
46381	0.946131	2.1
46382	0.946131	2.1
46383	0.94613	2.1
46384	1.24298	2.1
46385	0.94613	2.1
46386	1.22811	2.1
46387	2.48596	2.1
46388	2.45622	2.1
46389	1.63823	2.1
46390	1.4304	2.1
46391	1.45739	2.1
46392	2.45622	2.1
46393	2.4169	2.1
46394	2.55249	2.1
46395	2.51125	2.1
46396	2.35245	2.1
46397	4.90494	2.1

46398	2.45922	2.1
46399	2.55249	2.1
46400	2.51126	2.1
46401	2.43226	2.1
46402	4.98474	2.1
46403	2.51125	2.1
46404	2.35245	2.1
46405	2.28979	2.1
46406	2.47228	2.1
46407	4.82473	2.1
46408	2.51126	2.1
46409	2.43226	2.1
46410	2.40668	2.1
46411	2.47228	2.1
46412	4.90453	2.1
46413	2.8045	2.1
46414	1.93707	2.1
46415	2.14087	2.1
46416	2.14087	2.1
46417	2.61595	2.1
46418	2.61595	2.1
46419	1.89226	2.1
46420	1.89226	2.1
46421	1.89226	2.1
46422	1.89226	2.1
46423	1.89226	2.1
46424	1.89226	2.1
46425	1.22368	2.1
46426	1.32339	2.1
46427	0.957125	2.1
46428	0.750074	2.1
46429	1.08438	2.1
46430	1.08438	2.1
46431	1.13094	2.1
46432	0.970797	2.1
46433	1.17568	2.1
46434	0.764392	2.1
46435	0.944286	2.1
46436	1.27523	2.1
46437	1.89226	2.1
46438	1.89226	2.1
46439	1.89226	2.1
46440	1.89226	2.1
46441	3.78453	2.1
46442	2.8239	2.1
46443	1.89227	2.1
46444	2.57276	2.1
46445	2.57276	2.1
46446	1.89227	2.1
46447	1.89227	2.1
46448	1.18139	2.1
46449	1.85195	2.1
46450	1.78595	2.1
46451	1.08172	2.1
46452	2.93367	2.1
46453	1.60735	2.1
46454	1.20356	2.1
46455	0.967034	2.1

46456	1.08172	2.1
46457	1.67324	2.1
46458	1.77293	2.1
46459	1.89226	2.1
46460	1.89226	2.1
46461	1.8213	2.1
46462	1.82129	2.1
46463	2.14171	2.1
46464	2.40192	2.1
46465	0.810943	2.1
46466	0.810943	2.1
46467	0.798469	2.1
46468	0.788336	2.1
46469	0.804015	2.1
46470	0.810945	2.1
46471	1.947	2.1
46472	1.36506	2.1
46473	1.08686	2.1
46474	1.07208	2.1
46475	1.08132	2.1
46476	1.13537	0.863184
46477	1.14754	2.1
46478	0.946131	1.95657
46479	0.946131	2.1
46480	0.94613	1.9404
46481	0.94613	0.903801
46482	1.25104	2.1
46483	1.14754	2.1
46484	1.13537	2.04056
46485	0.946131	2.0576
46486	0.946131	2.1
46487	1.25104	2.09873
46488	0.96542	0.55717
46489	0.922284	2.03505
46490	0.946131	2.1
46491	0.946131	1.98235
46492	0.946131	1.95471
46493	0.946131	0.575811
46494	1.13537	2.1
46495	0.924824	2.1
46496	0.859476	2.01053
46497	0.842305	2.00969
46498	1.25996	1.99917
46499	1.04996	2.02038
46500	1.71007	0.05
46501	2.73283	0.05
46502	2.73283	0.05
46503	1.89226	0.05
46504	1.89226	0.05
46505	1.13262	0.05
46506	0.946131	0.05
46507	0.946131	0.05
46508	0.946131	0.05
46509	0.946131	0.05
46510	0.946132	0.051098
46511	0.946132	0.05
46512	0.946131	0.05
46513	0.946131	0.05

46514	0.946131	0.05
46515	0.946131	0.05
46516	0.946132	0.05
46517	0.946132	0.05
46518	0.946131	0.05
46519	1.36642	0.05
46520	0.946125	0.05
46521	0.946125	0.05
46522	0.946128	0.05
46523	0.946128	0.05
46524	0.946126	0.05
46525	0.946128	0.05
46526	0.946128	0.05
46527	0.946128	0.05
46528	1.36641	0.05
46529	0.946128	0.05
46530	1.89226	0.05
46531	1.89226	0.0529397
46532	1.89226	0.05
46533	1.89226	0.05
46534	1.89226	0.05
46535	1.89226	0.05
46536	2.69784	2.1
46537	2.51651	2.1
46538	2.56999	2.1
46539	2.74623	2.1
46540	5.26783	2.1
46541	2.69784	2.1
46542	2.48718	2.1
46543	2.50161	2.1
46544	2.74623	2.1
46545	5.19945	2.1
46546	1.89226	2.1
46547	1.89226	2.1
46548	1.89226	2.1
46549	1.89226	2.1
46550	3.78453	2.1
46551	0.874264	2.1
46552	1.09879	2.1
46553	0.946131	2.1
46554	0.946131	2.1
46555	1.17062	2.1
46556	1.17062	2.1
46557	0.874293	2.1
46558	0.874293	2.1
46559	1.09879	2.1
46560	0.94613	2.1
46561	0.946131	2.1
46562	0.874265	2.1
46563	1.89226	2.1
46564	1.89226	2.1
46565	1.89226	2.1
46566	1.89226	2.1
46567	3.78453	2.1
46568	0.977382	2.1
46569	1.16424	2.1
46570	0.946133	2.1
46571	0.946133	2.1

46572	1.33012	2.1
46573	1.25589	2.1
46574	0.888498	2.1
46575	0.888498	2.1
46576	1.25375	2.1
46577	0.946131	2.1
46578	0.946131	2.1
46579	0.977384	2.1
46580	1.89226	2.1
46581	2.19757	2.1
46582	2.62906	2.1
46583	1.89226	2.1
46584	1.89226	2.1
46585	2.62906	2.1
46586	1.27944	0.376461
46587	1.27731	1.67177
46588	0.615701	1.41316
46589	0.615701	0.606729
46590	0.615673	0.121036
46591	0.946133	1.83027
46592	0.948264	0.192726
46593	0.946132	0.664945
46594	0.94752	0.165477
46595	0.61706	0.180781
46596	0.946132	0.362497
46597	0.946132	0.695349
46598	2.76604	2.1
46599	1.66728	2.1
46600	1.66728	2.1
46601	1.83036	2.1
46602	2.54515	2.1
46603	2.70823	2.1
46604	2.54515	2.1
46605	1.49958	2.1
46606	1.49958	2.1
46607	1.66728	2.1
46608	2.34465	2.1
46609	2.51235	2.1
46610	1.21128	2.1
46611	1.21128	2.1
46612	0.946131	2.1
46613	0.946131	2.1
46614	0.946131	2.1
46615	0.946131	2.1
46616	1.31401	2.1
46617	1.21128	2.1
46618	1.21128	2.1
46619	0.946131	2.1
46620	0.946131	2.1
46621	1.31401	2.1
46622	1.67374	2.1
46623	2.29586	2.1
46624	1.50033	2.1
46625	2.16598	2.1
46626	3.83972	2.1
46627	1.89226	2.1
46628	1.89226	2.1
46629	1.89226	2.1

46630	1.89226	2.1
46631	3.78452	2.1
46632	1.13537	2.1
46633	1.02188	2.1
46634	0.946132	2.1
46635	0.946132	2.1
46636	0.946132	2.1
46637	0.946132	2.1
46638	1.21128	2.1
46639	1.02188	2.1
46640	1.04672	2.1
46641	1.04672	2.1
46642	1.21128	2.1
46643	0.946132	2.1
46644	0.815411	2.1
46645	0.870464	2.1
46646	0.885437	2.1
46647	0.885437	2.1
46648	1.06395	2.1
46649	1.00483	2.1
46650	0.757125	2.1
46651	0.673956	2.1
46652	0.684461	2.1
46653	1.06979	2.1
46654	0.731403	2.1
46655	1.0559	2.1
46656	1.89226	2.1
46657	1.89226	2.1
46658	1.89226	2.1
46659	1.89226	2.1
46660	1.89226	2.1
46661	1.89226	2.1
46662	1.76897	2.01417
46663	1.76932	2.07099
46664	1.97455	2.1
46665	1.94302	1.79351
46666	3.71233	2.02419
46667	1.83347	2.1
46668	1.76932	2.1
46669	1.97455	2.1
46670	1.76932	2.1
46671	1.76522	2.1
46672	1.55999	2.1
46673	1.89227	2.1
46674	1.89227	2.1
46675	1.89227	2.1
46676	1.89227	2.0144
46677	3.78453	2.1
46678	1.89226	2.07419
46679	1.89226	1.23367
46680	1.89226	1.76525
46681	1.89226	1.9063
46682	3.78452	1.92877
46683	1.97452	2.1
46684	1.94299	1.5817
46685	2.17831	1.84916
46686	2.23195	1.93182
46687	4.17495	2.00208

46688	2.23196	2.07006
46689	2.1499	2.1
46690	1.97452	2.1
46691	1.97452	2.1
46692	2.09088	2.1
46693	1.83345	2.1
46694	0.94612	2.1
46695	0.94612	2.1
46696	0.946119	1.9218
46697	0.946119	1.90226
46698	0.946119	1.92643
46699	0.946119	2.1
46700	0.94612	2.05621
46701	0.94612	2.1
46702	0.946132	2.1
46703	0.946132	2.03692
46704	0.946107	2.1
46705	0.946107	2.1
46706	1.89224	1.29979
46707	1.89224	1.69625
46708	1.89224	1.89233
46709	1.89224	0.936719
46710	3.78447	1.07511
46711	1.70302	0.210731
46712	1.056	0.198361
46713	1.21829	0.391988
46714	1.89906	0.183916
46715	3.07892	0.199552
46716	2.20248	0.203175
46717	1.56498	0.198774
46718	1.46539	0.182315
46719	1.48787	0.167368
46720	2.37625	0.341862
46721	2.37625	0.217727
46722	1.14104	0.165273
46723	1.00675	0.198025
46724	0.700689	0.17854
46725	0.624389	0.189916
46726	0.782149	0.140487
46727	0.940197	0.152122
46728	0.803437	0.181525
46729	0.803437	0.203504
46730	0.995423	0.15994
46731	0.700699	0.135773
46732	0.593895	0.272154
46733	1.00673	0.163402
46734	1.18097	0.168552
46735	1.15839	0.168367
46736	0.995407	0.16831
46737	0.865932	0.167783
46738	1.17299	0.152262
46739	0.957054	0.14842
46740	1.08016	0.191555
46741	1.08016	0.278806
46742	0.995425	0.20631
46743	1.24161	0.131453
46744	0.796771	0.14473
46745	1.15837	0.19907

46746	2.57264	0.05
46747	1.89226	0.0545783
46748	2.41701	0.05
46749	1.89226	0.05
46750	4.30928	0.05
46751	1.69055	0.05
46752	2.39587	0.0657144
46753	1.53463	0.05
46754	2.2053	0.05
46755	3.89585	0.0613342
46756	0.946129	0.05
46757	0.946129	0.05
46758	0.94613	0.05
46759	0.94613	0.0503312
46760	0.946132	0.05
46761	0.946132	0.0909779
46762	0.946126	0.05
46763	0.946126	0.05
46764	0.94613	0.05
46765	0.94613	0.05
46766	0.946129	0.05
46767	0.946129	0.0592977
46768	2.41701	0.05
46769	1.89226	0.05
46770	1.89226	0.05
46771	1.89226	0.05
46772	2.32462	0.05
46773	2.32462	0.05
46774	1.41942	0.05
46775	2.2053	0.05
46776	2.02097	0.05
46777	2.02097	0.05
46778	1.53463	0.05
46779	1.53463	0.05
46780	1.89226	0.05
46781	1.89226	0.05
46782	1.89226	0.05
46783	1.89226	0.05
46784	1.89226	0.05
46785	1.89226	0.05
46786	1.89226	1.63358
46787	1.89226	1.9078
46788	1.89226	1.86212
46789	1.89226	1.72639
46790	1.89226	1.76454
46791	1.89226	1.66047
46792	1.02816	1.55465
46793	1.02816	1.68735
46794	0.595141	1.72873
46795	0.595141	1.7914
46796	0.946132	1.62899
46797	0.595113	1.83054
46798	0.677174	1.76775
46799	0.946132	1.50382
46800	0.946132	1.67528
46801	0.946132	1.68748
46802	0.595113	1.72639
46803	0.677174	1.70814

46804	1.89226	1.57359
46805	1.89226	1.99813
46806	1.89226	1.77301
46807	1.89226	1.64346
46808	1.89226	1.87031
46809	1.89226	1.67467
46810	0.946133	1.54144
46811	0.946133	1.44526
46812	0.946132	1.70875
46813	0.946132	1.75618
46814	0.946132	1.74912
46815	0.946132	1.59679
46816	0.946132	1.61517
46817	0.946132	1.46745
46818	0.946132	1.60239
46819	0.946132	1.67176
46820	0.946133	1.56522
46821	0.946133	1.60019
46822	0.946131	1.69485
46823	0.946131	1.67339
46824	0.946131	1.98921
46825	0.946131	1.96436
46826	0.946128	2.00735
46827	0.946128	1.70122
46828	0.946133	1.98609
46829	0.946133	1.65431
46830	0.94613	1.77332
46831	0.94613	1.88438
46832	0.946131	1.80966
46833	0.946131	1.73935
46834	2.0264	2.1
46835	1.67345	2.05143
46836	2.0264	2.05543
46837	1.70539	1.31771
46838	1.31611	1.8044
46839	1.31611	1.85644
46840	1.89226	0.889537
46841	1.89226	0.636061
46842	1.89226	1.2051
46843	1.89226	0.506154
46844	3.78452	0.585203
46845	1.89226	1.20649
46846	1.89226	0.818108
46847	1.89226	0.946359
46848	1.89226	1.23707
46849	1.89226	0.9693
46850	1.89226	1.2932
46851	1.02645	0.242006
46852	0.810942	0.29078
46853	0.882781	0.319907
46854	0.882781	0.302455
46855	2.80386	0.245824
46856	1.56719	0.293425
46857	1.22499	0.241283
46858	1.08132	0.314916
46859	1.36865	0.288046
46860	0.810957	0.310492
46861	0.810957	0.272505

46862	0.946132	0.520575
46863	0.946132	0.591784
46864	0.946129	0.364901
46865	0.946129	0.414135
46866	0.946131	0.350743
46867	0.946131	0.381246
46868	0.946131	0.596332
46869	0.946131	0.494934
46870	0.94613	0.485059
46871	0.94613	0.514312
46872	0.946131	0.732201
46873	0.946131	0.445197
46874	1.89226	0.693581
46875	1.89226	0.408536
46876	1.89226	0.824803
46877	1.89226	0.418296
46878	3.78453	0.43666
46879	1.30462	0.0986421
46880	1.79906	0.149176
46881	1.01299	0.183937
46882	1.44185	0.131011
46883	1.3499	0.110671
46884	1.79906	0.115376
46885	1.89226	0.115381
46886	1.89226	0.193092
46887	1.89226	0.112718
46888	1.89226	0.158866
46889	1.89226	0.147697
46890	1.89226	0.484384
46891	1.89226	0.172127
46892	1.89226	0.162395
46893	1.89226	0.161309
46894	1.89226	0.191569
46895	1.89226	0.152021
46896	1.89226	0.187483
46897	1.3499	0.103174
46898	1.79905	0.13839
46899	1.29001	0.111098
46900	1.36005	0.143022
46901	1.88294	0.153622
46902	1.79905	0.112092
46903	1.89226	0.109163
46904	2.75374	0.085846
46905	2.60614	0.116891
46906	1.89226	0.118682
46907	2.60614	0.0777738
46908	1.89226	0.0803452
46909	0.946131	0.0939129
46910	0.946131	0.114975
46911	1.37687	0.120978
46912	1.37687	0.101223
46913	0.946131	0.115961
46914	1.34375	0.1135
46915	1.37686	0.101075
46916	0.946128	0.10567
46917	1.37687	0.0926431
46918	1.34375	0.100021
46919	0.94613	0.120769

46920	0.94613	0.116772
46921	1.44247	2.1
46922	1.12665	2.1
46923	1.50044	2.1
46924	1.73651	2.1
46925	2.94291	2.1
46926	1.73652	2.1
46927	1.64926	2.1
46928	1.44247	2.1
46929	1.44247	2.1
46930	1.64926	2.1
46931	1.43872	2.1
46932	1.50044	2.1
46933	1.73651	2.1
46934	2.00955	2.1
46935	1.87875	2.1
46936	3.50999	2.1
46937	1.64926	2.1
46938	1.73652	2.1
46939	2.00955	2.1
46940	1.8493	2.1
46941	3.65881	2.1
46942	1.89226	0.05
46943	2.28188	0.0531679
46944	1.99182	0.05
46945	2.28188	0.06126
46946	1.6022	0.05
46947	1.89226	0.0575354
46948	1.89226	0.0599336
46949	1.89226	0.0643449
46950	1.89226	0.0584497
46951	1.89226	0.0844307
46952	1.89226	0.0551684
46953	1.89226	0.0730518
46954	1.89226	0.0514345
46955	1.89226	0.0537513
46956	2.19578	0.05
46957	1.89226	0.0662207
46958	2.81422	0.05
46959	2.42521	0.0600758
46960	1.89224	0.05
46961	1.99179	0.0535879
46962	1.454	0.051057
46963	1.99179	0.0576807
46964	1.35444	0.05
46965	1.89224	0.0534794
46966	1.89224	0.0589974
46967	1.89224	0.0637097
46968	1.89224	0.0611475
46969	1.89224	0.0792303
46970	1.89224	0.0570961
46971	1.89224	0.0684606
46972	1.703	0.0524197
46973	2.19575	0.0524053
46974	1.9911	0.050676
46975	2.19575	0.062358
46976	1.67811	0.05
46977	1.89223	0.0574735

46978	1.69586	2.1
46979	2.55301	2.1
46980	1.60861	2.1
46981	1.60861	2.1
46982	2.65233	2.1
46983	2.65233	2.1
46984	0.946129	2.1
46985	0.946129	2.1
46986	1.2765	2.1
46987	1.32617	2.1
46988	1.2765	2.1
46989	1.2765	2.1
46990	0.946131	2.1
46991	0.946131	2.1
46992	0.946131	2.1
46993	1.32616	2.1
46994	0.946131	2.1
46995	1.2765	2.1
46996	1.36757	2.1
46997	1.32617	2.1
46998	0.891503	2.1
46999	0.847932	2.1
47000	1.10722	2.1
47001	1.10722	2.1
47002	1.10829	2.1
47003	1.10829	2.1
47004	1.36757	2.1
47005	0.891504	2.1
47006	0.847932	2.1
47007	1.32617	2.1
47008	0.946129	2.1
47009	0.946129	2.1
47010	1.36757	2.1
47011	1.32617	2.1
47012	1.32616	2.1
47013	1.32616	2.1
47014	0.946131	2.1
47015	0.946131	2.1
47016	1.36757	2.1
47017	0.946131	2.1
47018	0.946131	2.1
47019	1.32616	2.1
47020	1.89226	0.0666561
47021	1.89226	0.0505645
47022	1.89226	0.0581135
47023	1.89226	0.0783927
47024	3.78452	0.0581767
47025	2.36336	0.05
47026	1.89226	0.05
47027	2.67831	0.0505961
47028	2.67831	0.05
47029	1.89226	0.05
47030	1.89226	0.05
47031	1.89227	0.0514914
47032	1.89227	0.0851028
47033	1.89227	0.0819414
47034	1.89227	0.0596966
47035	3.78453	0.0644916

47036	1.89226	0.0535312
47037	1.89226	0.0764433
47038	1.89226	0.0858637
47039	1.89226	0.0614395
47040	3.78452	0.0672182
47041	2.36336	0.05
47042	2.0815	0.0550234
47043	2.0815	0.0500532
47044	2.41048	0.05
47045	1.66267	0.05
47046	2.13452	0.05
47047	1.89226	0.0806102
47048	1.89226	0.0628728
47049	1.89226	0.0739156
47050	1.89226	0.053655
47051	1.89226	0.0932564
47052	1.89226	0.0698907
47053	1.89226	0.124517
47054	1.89226	0.109142
47055	1.89226	0.123052
47056	1.89226	0.176965
47057	1.89226	0.085835
47058	1.89226	0.118959
47059	0.946128	0.398507
47060	0.946128	0.172092
47061	1.00122	0.0845521
47062	1.00122	0.0831676
47063	0.94613	0.50118
47064	1.01618	0.0782096
47065	0.946131	0.268766
47066	1.00122	0.0686335
47067	1.00122	0.0713803
47068	1.01618	0.0681301
47069	0.946131	0.244458
47070	0.946131	0.223847
47071	1.89226	2.00171
47072	1.89226	1.75732
47073	1.89226	1.78346
47074	1.89226	2.09337
47075	3.78452	1.87524
47076	1.89226	1.92639
47077	1.89226	2.1
47078	1.89226	2.1
47079	1.89226	1.99703
47080	1.89226	2.1
47081	1.89226	2.00272
47082	1.89226	1.49338
47083	1.89226	1.98797
47084	1.89226	1.92844
47085	1.89226	1.68883
47086	1.89226	1.93152
47087	1.89226	1.69818
47088	1.89226	1.65323
47089	1.89226	2.06362
47090	1.89226	2.01242
47091	1.89226	1.74679
47092	1.89226	1.98924
47093	1.89226	1.75949

47094	2.71279	0.437924
47095	1.4155	0.954168
47096	2.28488	0.651324
47097	1.06122	0.450616
47098	3.70038	0.542437
47099	1.89226	1.09405
47100	1.89226	0.942424
47101	1.89226	0.621323
47102	1.89226	1.25987
47103	3.78452	1.18221
47104	0.851542	1.04502
47105	0.851542	0.84539
47106	0.851484	0.610682
47107	0.865567	0.9394
47108	0.981616	0.935625
47109	0.864767	0.985692
47110	0.851542	0.504374
47111	0.851542	0.851911
47112	0.851477	0.983146
47113	0.851477	0.950841
47114	0.865635	0.872092
47115	0.816472	0.917774
47116	1.68443	2.1
47117	1.44489	2.1
47118	1.54924	2.1
47119	1.34389	2.1
47120	3.02832	2.1
47121	1.44489	2.1
47122	1.03899	2.1
47123	1.03899	2.1
47124	1.12075	2.1
47125	1.34389	2.1
47126	1.42566	2.1
47127	1.54924	2.1
47128	1.34389	2.1
47129	1.44091	2.1
47130	1.25866	2.1
47131	2.7848	2.1
47132	0.573141	2.1
47133	0.573141	2.1
47134	0.575681	2.1
47135	0.575681	2.1
47136	0.481985	2.1
47137	0.519492	2.1
47138	0.671948	2.1
47139	0.629331	2.1
47140	0.629329	2.1
47141	0.481986	2.1
47142	0.519494	2.1
47143	0.671947	2.1
47144	1.89226	0.210392
47145	1.89226	0.525325
47146	1.89226	0.594889
47147	2.31925	0.238189
47148	3.78452	0.458548
47149	1.30916	2.1
47150	1.17426	2.1
47151	0.946129	2.1

47152	0.946129	2.1
47153	0.94613	2.1
47154	0.94613	2.1
47155	1.17426	2.1
47156	1.17426	2.1
47157	1.30916	2.1
47158	0.94613	2.1
47159	1.17426	2.1
47160	0.94613	2.1
47161	2.56346	2.1
47162	1.89226	1.31092
47163	1.89226	2.0663
47164	1.89226	1.45114
47165	2.6074	2.05515
47166	2.6074	1.43674
47167	1.89226	2.1
47168	1.89226	2.1
47169	1.89226	2.1
47170	1.89226	2.05574
47171	3.78452	2.1
47172	1.33064	2.1
47173	1.84121	2.1
47174	0.946129	2.1
47175	0.946129	2.1
47176	0.946131	2.1
47177	0.946131	2.1
47178	1.30916	2.1
47179	1.8412	2.1
47180	1.33064	2.1
47181	0.94613	2.1
47182	1.30916	2.1
47183	0.946131	2.1
47184	1.19283	1.40206
47185	1.19283	1.02136
47186	0.946131	2.1
47187	0.946131	2.04707
47188	0.946132	2.06882
47189	0.946132	1.28282
47190	1.3037	2.1
47191	1.19283	1.18352
47192	1.19283	1.92017
47193	0.946131	1.91864
47194	0.946131	1.88609
47195	1.3037	1.94832
47196	1.89226	2.1
47197	1.89226	2.0492
47198	1.89226	2.1
47199	1.89226	2.1
47200	1.89226	2.1
47201	1.89226	2.1
47202	2.17708	2.1
47203	1.70302	2.1
47204	2.28188	2.1
47205	2.13621	2.1
47206	4.28253	2.1
47207	1.89226	2.1
47208	1.89226	2.1
47209	1.89226	2.1

47210	1.89226	2.1
47211	3.78452	2.1
47212	0.946132	2.1
47213	0.946132	2.1
47214	0.946132	2.1
47215	0.946132	2.1
47216	0.946131	2.1
47217	0.946131	2.1
47218	0.946132	2.1
47219	0.946132	2.1
47220	0.946132	2.1
47221	0.946132	2.1
47222	0.946132	2.1
47223	0.946132	2.1
47224	1.74933	2.1
47225	1.11833	2.1
47226	1.90074	2.1
47227	1.669 2.1	
47228	1.335 2.1	
47229	1.11833	2.1
47230	2.28188	2.1
47231	2.31804	2.1
47232	2.42758	2.1
47233	2.14895	2.1
47234	4.43083	2.1
47235	1.89226	2.1
47236	1.89226	2.1
47237	1.89226	2.1
47238	1.89226	2.1
47239	3.78452	2.1
47240	1.89226	2.1
47241	1.89226	2.1
47242	1.89226	2.1
47243	1.89226	2.1
47244	1.89226	2.1
47245	1.89226	2.1
47246	0.864133	2.1
47247	0.864133	2.1
47248	0.967939	2.1
47249	0.793739	2.1
47250	0.650797	2.1
47251	0.757546	2.1
47252	1.08941	2.1
47253	1.10136	2.1
47254	1.10138	2.1
47255	0.796445	2.1
47256	0.866907	2.1
47257	0.650788	2.1
47258	2.63062	1.60312
47259	1.89226	1.25101
47260	1.89226	1.00707
47261	1.89226	2.1
47262	2.46557	0.685899
47263	2.46557	2.1
47264	1.16777	2.1
47265	1.38859	2.1
47266	1.05042	2.1
47267	1.28909	2.1

47268	2.45686	2.1
47269	1.89227	2.1
47270	1.89227	2.1
47271	1.89227	1.52594
47272	1.89227	2.1
47273	3.78453	2.1
47274	2.15114	2.1
47275	1.89226	2.1
47276	2.15005	2.1
47277	2.15005	2.1
47278	1.89226	2.1
47279	1.89226	2.1
47280	1.89226	2.1
47281	1.89226	1.75799
47282	1.89226	2.1
47283	1.89226	2.1
47284	1.89226	1.63861
47285	1.89226	2.1
47286	0.973447	2.1
47287	1.32143	2.08537
47288	1.37444	2.1
47289	1.37444	2.06734
47290	0.949134	2.1
47291	0.949134	2.1
47292	0.946131	2.1
47293	0.946131	2.1
47294	1.07557	2.1
47295	1.07557	2.1
47296	0.946131	2.1
47297	0.946131	2.1
47298	1.07557	2.1
47299	1.08579	2.1
47300	1.08579	2.1
47301	0.946131	2.1
47302	1.07557	2.1
47303	0.946131	2.1
47304	0.946131	1.59823
47305	0.946131	2.1
47306	0.946131	2.1
47307	0.946131	2.08945
47308	0.94613	1.6288
47309	0.94613	1.73054
47310	0.946131	2.1
47311	0.946131	2.1
47312	0.946131	2.0888
47313	0.946131	2.00055
47314	0.946131	2.1
47315	0.946131	2.1
47316	0.606115	2.1
47317	0.606115	2.1
47318	0.616308	2.1
47319	0.616308	2.1
47320	0.68722	1.6927
47321	0.735699	1.94539
47322	0.514789	2.1
47323	0.486724	2.1
47324	0.514788	2.059
47325	0.735699	2.1

47326	0.486723	2.1
47327	0.687221	2.1
47328	2.33761	1.06617
47329	2.51749	0.399082
47330	2.68092	0.700234
47331	2.58304	0.393799
47332	5.01853	0.80648
47333	2.50906	1.01195
47334	2.51749	0.566495
47335	2.68091	0.954046
47336	2.53176	0.37391
47337	5.18997	0.707564
47338	2.68092	0.777619
47339	2.58304	0.408563
47340	2.71793	0.552842
47341	2.67098	0.262945
47342	5.39885	0.403713
47343	2.68091	1.01563
47344	2.53175	0.342468
47345	2.36417	0.588356
47346	2.67098	0.324915
47347	5.04508	0.64098
47348	2.76309	2.1
47349	1.89226	2.1
47350	1.89226	2.1
47351	1.89226	2.1
47352	2.48573	2.1
47353	2.48573	2.1
47354	1.35879	2.1
47355	1.55161	2.1
47356	1.10407	2.1
47357	1.32259	2.0549
47358	2.68138	2.1
47359	1.89226	2.1
47360	1.89226	2.1
47361	1.89226	2.1
47362	1.89226	2.08448
47363	3.78452	2.1
47364	1.19612	2.06865
47365	1.24694	2.1
47366	1.24844	0.967287
47367	1.29747	2.1
47368	2.49358	2.1
47369	1.89226	2.09648
47370	1.89226	2.1
47371	1.89226	0.53324
47372	1.89226	2.1
47373	3.78453	2.1
47374	1.89226	2.1
47375	1.89226	2.1
47376	1.89226	2.1
47377	1.89226	2.1
47378	1.89226	2.1
47379	1.89226	2.1
47380	0.587463	2.1
47381	0.587463	2.1
47382	0.581977	2.1
47383	0.581977	2.1

47384	0.531709	2.1
47385	0.545972	2.1
47386	0.623469	2.1
47387	0.648733	2.1
47388	0.648733	2.1
47389	0.545971	2.1
47390	0.623469	2.1
47391	0.531709	2.1
47392	1.29417	1.72719
47393	1.29747	2.1
47394	1.27829	2.1
47395	1.24844	1.96606
47396	1.32732	2.1
47397	1.29747	2.06414
47398	1.89226	0.772936
47399	1.89226	2.1
47400	1.89226	1.62398
47401	1.89226	2.1
47402	1.89226	1.7706
47403	1.89226	2.1
47404	1.89226	2.1
47405	1.89226	2.1
47406	1.89226	2.1
47407	1.89226	2.1
47408	1.89226	2.1
47409	1.89226	2.1
47410	0.60346	2.1
47411	0.60346	2.1
47412	0.60086	2.1
47413	0.60086	2.1
47414	0.545971	2.1
47415	0.555587	2.1
47416	0.648733	2.1
47417	0.663661	2.1
47418	0.663661	2.1
47419	0.555587	2.1
47420	0.648733	2.1
47421	0.545971	2.1
47422	1.34066	2.1
47423	1.36601	2.1
47424	1.34955	0.659719
47425	1.37623	2.1
47426	2.71556	2.1
47427	1.89226	1.69775
47428	1.89226	2.1
47429	1.89226	0.641098
47430	1.89226	2.1
47431	3.78452	1.81557
47432	0.946132	2.1
47433	0.946132	2.1
47434	0.946132	2.1
47435	0.946132	2.1
47436	0.946132	2.1
47437	0.946132	2.1
47438	0.946131	2.1
47439	0.946131	2.1
47440	0.946132	2.1
47441	0.946132	2.1

47442	0.946132	2.1
47443	0.946132	2.1
47444	0.631351	2.1
47445	0.631351	2.1
47446	0.632421	2.1
47447	0.632421	2.1
47448	0.573604	2.1
47449	0.580765	2.1
47450	0.683006	2.1
47451	0.688118	2.1
47452	0.688118	2.1
47453	0.580765	2.1
47454	0.683007	2.1
47455	0.573604	2.1
47456	1.34955	1.92437
47457	1.37624	2.1
47458	1.35048	1.20745
47459	1.37999	2.1
47460	2.72954	2.0951
47461	0.946131	2.1
47462	0.946131	1.77279
47463	0.946131	2.1
47464	0.946131	2.1
47465	0.94613	2.1
47466	0.94613	2.1
47467	0.946133	1.51519
47468	0.946133	1.60179
47469	0.946131	2.07876
47470	0.946131	2.07618
47471	0.946132	2.03226
47472	0.946132	1.94015
47473	1.89226	2.1
47474	1.89226	2.1
47475	1.89226	2.1
47476	1.89226	2.1
47477	1.89226	2.1
47478	1.89226	2.1
47479	0.532189	2.1
47480	0.531141	2.1
47481	0.497784	2.1
47482	0.502536	2.1
47483	1.37999	2.1
47484	0.92433	2.1
47485	0.670081	2.1
47486	0.727155	2.1
47487	0.786438	2.1
47488	0.589798	2.1
47489	0.497784	2.1
47490	1.89226	2.1
47491	2.72301	2.1
47492	2.4158	2.1
47493	2.4158	2.1
47494	1.89226	2.1
47495	1.89226	2.1
47496	1.89226	2.1
47497	1.89226	2.1
47498	1.89226	2.1
47499	1.89226	2.1

47500	1.89226	2.1
47501	1.89226	2.1
47502	1.36919	2.1
47503	1.12717	2.1
47504	1.38634	2.1
47505	1.56541	2.1
47506	2.75552	2.1
47507	1.89226	2.1
47508	1.89226	2.1
47509	1.89226	2.1
47510	1.89226	2.1
47511	1.89226	2.1
47512	1.89226	2.1
47513	0.907036	2.1
47514	0.782706	2.1
47515	0.838808	2.1
47516	0.693168	2.1
47517	0.794747	2.1
47518	0.794747	2.1
47519	0.805458	2.1
47520	0.805458	2.1
47521	0.907036	2.1
47522	0.838808	2.1
47523	0.693169	2.1
47524	0.782707	2.1
47525	0.94613	2.1
47526	0.94613	2.1
47527	1.188	2.1
47528	1.28421	2.1
47529	1.18801	2.1
47530	1.18801	2.1
47531	0.946129	2.1
47532	0.946129	2.1
47533	1.28421	2.1
47534	0.94613	2.1
47535	1.188	2.1
47536	0.94613	2.1
47537	1.89226	2.1
47538	1.89226	2.1
47539	1.89226	2.1
47540	1.89226	2.1
47541	1.89226	2.1
47542	1.89226	2.1
47543	1.16817	2.1
47544	1.06007	2.1
47545	1.19785	2.1
47546	1.2832	2.1
47547	2.36602	2.1
47548	0.960106	2.1
47549	2.0073	2.1
47550	1.51754	2.1
47551	2.56883	2.1
47552	3.52484	2.1
47553	1.06756	2.1
47554	1.57913	2.1
47555	1.87532	2.1
47556	1.38666	2.1
47557	2.94287	2.1

47558	2.15622	2.1
47559	1.38668	2.1
47560	1.87534	2.1
47561	1.87534	2.1
47562	1.70539	2.1
47563	1.70539	2.1
47564	0.986095	2.1
47565	1.36226	2.1
47566	1.3331	2.1
47567	1.3331	2.1
47568	1.07156	2.1
47569	1.07156	2.1
47570	0.682081	1.92702
47571	0.68748	1.8344
47572	0.650003	0.693104
47573	0.645873	0.848249
47574	0.666344	0.735307
47575	0.666344	1.81842
47576	0.66161	1.96056
47577	0.66161	0.845299
47578	0.68748	1.24135
47579	0.645873	1.33407
47580	0.650004	1.20078
47581	0.682082	1.2149
47582	0.946132	1.92659
47583	0.946132	1.87001
47584	0.946132	0.863467
47585	0.946132	0.693626
47586	0.946132	0.646062
47587	0.946132	1.8937
47588	0.946133	0.983982
47589	0.946133	1.9546
47590	0.946132	1.27605
47591	0.946132	1.68808
47592	0.946133	1.2993
47593	0.946133	1.47791
47594	1.89227	1.33774
47595	2.79312	0.264527
47596	2.77591	0.475794
47597	2.79312	1.51734
47598	1.87505	0.347281
47599	1.89227	0.711961
47600	0.68748	1.978
47601	0.698626	1.91892
47602	0.645873	0.833712
47603	0.650148	0.939425
47604	0.670561	0.850548
47605	0.670561	1.92957
47606	0.667067	2.01596
47607	0.667067	0.860442
47608	0.698627	1.48648
47609	0.650148	1.55628
47610	0.68748	1.34404
47611	0.645873	1.43794
47612	0.946132	1.96992
47613	0.946132	1.89244
47614	0.946132	1.26721
47615	0.946132	0.973673

47616	0.946132	0.867017
47617	0.946132	1.90657
47618	0.946133	1.47811
47619	0.946133	2.0103
47620	0.946133	1.42558
47621	0.946133	1.37236
47622	0.946132	1.22932
47623	0.946132	1.507
47624	0.946133	1.40781
47625	0.946133	1.01978
47626	1.38709	0.477233
47627	1.38795	0.342628
47628	0.946132	0.342785
47629	0.946132	1.04321
47630	1.38709	1.23007
47631	1.38709	0.386019
47632	1.38709	0.658523
47633	0.946133	0.676469
47634	0.946132	0.578245
47635	1.38795	0.643635
47636	1.70301	0.05
47637	2.07659	0.05
47638	1.89226	0.05
47639	1.89226	0.05
47640	1.76332	0.05
47641	1.76331	0.05
47642	1.89226	0.05
47643	1.89226	0.05
47644	1.89226	0.05
47645	1.89226	0.05
47646	1.89226	0.05
47647	1.89226	0.05
47648	1.89226	0.05
47649	1.89226	0.05
47650	1.89226	0.05
47651	1.89226	0.05
47652	3.78452	0.05
47653	1.89226	0.05
47654	2.12492	0.05
47655	1.89226	0.05
47656	1.89226	0.05
47657	2.07659	0.05
47658	2.07659	0.05
47659	0.94613	0.05
47660	0.94613	0.05
47661	1.17498	0.05
47662	1.32446	0.05
47663	1.32446	0.05
47664	0.94613	0.05
47665	0.946127	0.05
47666	1.17498	0.05
47667	1.32446	0.05
47668	1.32446	0.05
47669	0.946128	0.05
47670	0.946128	0.05
47671	1.89226	2.1
47672	1.86472	2.1
47673	2.11049	2.1

47674	1.89226	2.1
47675	2.08295	2.1
47676	1.86472	2.1
47677	1.89226	2.1
47678	2.08295	2.1
47679	2.14023	2.1
47680	1.89226	2.1
47681	2.33091	2.1
47682	2.08295	2.1
47683	0.809215	2.1
47684	0.809215	2.1
47685	0.810915	2.1
47686	0.810915	2.1
47687	1.09581	2.1
47688	1.08271	2.1
47689	0.520996	2.1
47690	0.537426	2.1
47691	0.520997	2.1
47692	1.08271	2.1
47693	0.537426	2.1
47694	1.09581	2.1
47695	0.79674	2.1
47696	0.79674	2.1
47697	0.799279	2.1
47698	0.799279	2.1
47699	1.07502	2.1
47700	1.0827	2.1
47701	0.508206	2.1
47702	0.520996	2.1
47703	0.508206	2.1
47704	1.07502	2.1
47705	0.520996	2.1
47706	1.0827	2.1
47707	2.15714	2.1
47708	1.70302	2.1
47709	1.78732	2.1
47710	2.02219	2.1
47711	3.98971	2.1
47712	2.0927	2.1
47713	2.162	2.1
47714	2.11563	2.1
47715	2.02219	2.1
47716	4.18419	2.1
47717	1.62483	2.1
47718	2.02219	2.1
47719	1.93617	2.1
47720	1.52008	2.1
47721	3.54227	2.1
47722	2.11563	2.1
47723	2.02219	2.1
47724	2.29218	2.1
47725	2.03975	2.1
47726	4.06194	2.1
47727	2.007	2.1
47728	1.89226	2.1
47729	1.89226	2.1
47730	1.89226	2.1
47731	2.06776	2.1

47732	2.06776	2.1
47733	0.946129	2.1
47734	0.946129	2.1
47735	1.0035	2.1
47736	1.0035	2.1
47737	0.946131	2.1
47738	0.946131	2.1
47739	0.968797	2.1
47740	1.0035	2.1
47741	0.968771	2.1
47742	0.946105	2.1
47743	1.00353	2.1
47744	0.946157	2.1
47745	1.79103	1.82691
47746	2.53286	0.566848
47747	2.60781	2.0192
47748	2.219	1.33642
47749	2.17985	1.54771
47750	1.79103	0.229981
47751	2.219	1.44951
47752	2.66359	1.5332
47753	2.70084	1.92869
47754	2.38698	1.43679
47755	2.53285	1.13691
47756	2.219	1.27902
47757	1.33179	1.39858
47758	0.946128	1.27177
47759	0.946133	1.34078
47760	1.26643	1.5183
47761	1.3318	1.22584
47762	1.3318	1.64046
47763	0.946131	1.28684
47764	0.946131	1.41485
47765	0.946131	1.05837
47766	0.946131	0.917996
47767	1.33179	1.35493
47768	1.26643	1.59944
47769	2.28979	2.1
47770	2.47227	2.1
47771	2.43451	2.1
47772	2.22627	2.1
47773	4.69855	2.1
47774	2.40667	2.1
47775	2.47228	2.1
47776	2.43451	2.1
47777	2.38069	2.1
47778	4.85296	2.1
47779	2.43451	2.1
47780	2.15715	2.1
47781	2.15715	2.1
47782	2.22627	2.1
47783	2.39489	2.1
47784	2.46402	2.1
47785	2.43451	2.1
47786	2.34899	2.1
47787	2.38069	2.1
47788	2.38069	2.1
47789	2.3949	2.1

47790	2.3949	2.1
47791	2.85425	2.1
47792	1.70302	0.561789
47793	1.70302	1.1878
47794	1.52254	0.904314
47795	2.34181	1.47548
47796	2.08499	1.58305
47797	1.89226	1.2866
47798	1.89226	0.955153
47799	1.89226	0.558169
47800	1.89226	0.325932
47801	1.89226	0.439423
47802	1.89226	1.16695
47803	2.34181	1.90307
47804	1.38287	1.27505
47805	1.38287	1.58396
47806	1.84132	0.64879
47807	1.96416	1.34215
47808	2.4907	1.38131
47809	0.931432	1.22551
47810	0.946135	0.129958
47811	0.946133	0.072104
47812	0.946133	0.206605
47813	0.931464	0.436933
47814	0.931464	1.13292
47815	1.15573	0.116564
47816	0.96378	1.45076
47817	0.931434	0.863956
47818	0.946131	0.257879
47819	0.946131	0.161676
47820	1.13808	0.116013
47821	0.946132	1.29029
47822	0.946132	0.517104
47823	0.946132	0.805873
47824	0.946132	0.491379
47825	0.946133	1.27754
47826	0.946133	0.214554
47827	0.946132	1.32353
47828	0.946132	0.683243
47829	0.946133	0.682002
47830	0.946133	1.323
47831	0.946132	0.247193
47832	0.946132	0.294973
47833	1.68074	2.1
47834	2.29459	2.1
47835	2.23361	2.1
47836	1.57234	2.1
47837	3.86693	2.1
47838	2.61243	2.1
47839	2.29458	2.1
47840	2.2336	2.1
47841	2.58234	2.1
47842	4.87692	2.1
47843	0.73506	2.1
47844	0.786166	2.1
47845	1.08729	2.1
47846	1.11679	2.1
47847	0.93134	2.1

47848	0.93134	2.1
47849	0.942113	2.1
47850	0.942113	2.1
47851	0.735065	2.1
47852	1.08729	2.1
47853	1.11679	2.1
47854	0.78616	2.1
47855	1.08729	2.1
47856	1.11679	2.1
47857	1.27502	2.1
47858	1.29116	2.1
47859	1.19259	2.1
47860	1.19259	2.1
47861	1.19923	2.1
47862	1.19923	2.1
47863	1.27503	2.1
47864	1.08729	2.1
47865	1.11679	2.1
47866	1.29115	2.1
47867	0.974715	1.15612
47868	1.43779	0.542033
47869	1.93915	0.524883
47870	1.53908	0.707001
47871	2.91387	0.702377
47872	1.53908	0.406994
47873	0.99845	0.885138
47874	1.5176	1.12392
47875	1.97963	0.310826
47876	3.05668	0.522755
47877	1.93915	0.72974
47878	1.97963	0.437668
47879	1.53908	0.685521
47880	1.53908	0.363418
47881	2.3376	1.08792
47882	2.3376	0.602249
47883	1.89226	2.1
47884	2.59747	2.1
47885	1.91802	2.1
47886	1.89226	2.1
47887	2.62322	2.1
47888	2.59747	2.1
47889	2.59747	2.1
47890	1.87119	2.1
47891	1.84844	2.1
47892	1.82268	2.1
47893	2.62323	2.1
47894	2.59747	2.1
47895	0.94613	2.1
47896	0.94613	2.1
47897	1.32697	2.1
47898	1.32697	2.1
47899	1.32697	2.1
47900	1.31161	2.1
47901	0.946132	2.1
47902	0.946132	2.1
47903	0.94613	2.1
47904	1.32697	2.1
47905	0.946131	2.1

47906	1.31161	2.1
47907	2.62323	2.1
47908	1.91704	2.1
47909	1.90191	2.1
47910	1.87119	2.1
47911	2.65395	2.1
47912	2.62323	2.1
47913	1.89226	2.1
47914	1.89226	2.1
47915	2.15958	2.1
47916	2.15958	2.1
47917	2.46558	2.1
47918	2.19826	2.1
47919	2.46558	2.1
47920	1.89226	2.1
47921	1.89226	2.1
47922	1.89226	2.1
47923	2.61064	2.1
47924	2.61064	2.1
47925	1.81759	0.863936
47926	1.89526	1.08137
47927	2.32219	1.50411
47928	1.881	0.752546
47929	1.81759	1.17721
47930	1.3764	0.499103
47931	1.81759	1.15779
47932	2.57079	0.920474
47933	2.32219	1.54187
47934	2.32219	1.08041
47935	1.99875	1.29571
47936	1.99875	0.376177
47937	0.536182	0.390981
47938	0.579206	0.208477
47939	0.908796	1.08505
47940	0.999375	0.443013
47941	0.732091	0.541782
47942	0.732091	0.227737
47943	0.755911	0.325248
47944	0.755911	0.694175
47945	0.999376	0.426215
47946	0.579206	0.490605
47947	0.908796	0.456344
47948	0.536182	0.450528
47949	0.94613	0.35053
47950	0.94613	0.365302
47951	1.31112	0.41054
47952	1.18275	0.392725
47953	0.946126	0.419382
47954	0.946126	0.254803
47955	1.31112	0.359002
47956	1.31112	0.351178
47957	0.946128	0.364995
47958	1.31112	0.371857
47959	0.946128	0.326752
47960	1.18275	0.321565
47961	2.64617	0.831702
47962	1.27737	0.76049
47963	1.83076	0.914449

47964	1.27737	0.901825
47965	3.03764	0.714794
47966	2.3655	0.618666
47967	1.12991	0.05
47968	1.22375	0.05
47969	1.35931	0.05
47970	1.04971	0.05
47971	1.0419	0.05
47972	1.15237	0.05
47973	1.19593	0.05
47974	1.30306	0.05
47975	1.12937	0.05
47976	1.31934	0.05
47977	1.15203	0.05
47978	1.15203	0.05
47979	2.65227	0.05
47980	1.46041	0.05
47981	2.22712	0.05
47982	2.04816	0.05
47983	2.22712	0.05
47984	2.04816	0.05
47985	1.89226	0.3251
47986	1.89226	2.1
47987	1.89226	1.42219
47988	1.89226	1.19127
47989	3.78452	1.26983
47990	1.24653	1.91355
47991	1.66554	2.1
47992	1.53538	1.0857
47993	1.53538	2.1
47994	1.40915	1.49381
47995	1.40915	2.1
47996	1.66555	2.1
47997	1.75835	2.1
47998	1.53538	2.1
47999	1.65313	2.1
48000	3.31868	2.1
48001	1.89226	2.1
48002	1.89226	2.1
48003	1.89226	1.48848
48004	1.89226	2.1
48005	3.78453	2.1
48006	1.89226	0.758573
48007	1.89226	1.40336
48008	1.89226	0.515044
48009	1.89226	2.1
48010	3.78452	0.835195
48011	0.658778	2.1
48012	0.658778	1.94493
48013	0.661956	1.1629
48014	0.661956	2.1
48015	0.703822	2.1
48016	0.767689	2.1
48017	0.623266	0.980109
48018	0.553046	2.03087
48019	0.703804	2.1
48020	0.55303	2.1
48021	0.623282	1.98499

48022	0.767711	2.00682
48023	1.53538	2.1
48024	1.65313	2.1
48025	1.40765	2.1
48026	1.55779	2.1
48027	3.06078	2.1
48028	1.89227	1.36757
48029	1.89227	2.1
48030	1.89227	2.1
48031	1.89227	2.1
48032	3.78453	2.1
48033	1.10517	2.1
48034	2.16532	2.1
48035	1.94374	2.1
48036	1.94001	2.1
48037	1.37756	2.1
48038	1.37756	2.1
48039	1.7453	0.108173
48040	1.83284	0.129513
48041	2.28483	0.145819
48042	1.77804	0.103301
48043	1.7453	0.173761
48044	1.23851	0.115691
48045	1.7453	0.130823
48046	2.54752	0.118109
48047	2.28483	0.128994
48048	2.28483	0.110079
48049	2.0189	0.14576
48050	2.0189	0.124373
48051	1.12457	0.0920813
48052	2.0189	0.0842932
48053	1.7453	0.09561
48054	1.7453	0.0834657
48055	1.40707	0.105204
48056	1.40707	0.0924723
48057	2.41176	2.1
48058	2.59262	2.1
48059	2.34085	2.1
48060	2.55827	2.1
48061	4.97003	2.1
48062	2.59262	2.1
48063	2.61901	2.1
48064	2.55827	2.1
48065	2.64067	2.1
48066	5.17728	2.1
48067	2.34084	2.1
48068	2.55827	2.1
48069	2.24937	2.1
48070	2.49053	2.1
48071	4.80764	2.1
48072	2.55826	2.1
48073	2.64067	2.1
48074	2.49053	2.1
48075	2.58639	2.1
48076	5.14465	2.1
48077	2.27918	0.05
48078	2.44132	0.0552933
48079	1.89226	0.05

48080	1.89226	0.05
48081	4.33359	0.05
48082	1.89226	0.05
48083	2.44132	0.054151
48084	2.6771	0.0507114
48085	2.6771	0.05
48086	1.89226	0.0531037
48087	1.89226	0.0518973
48088	1.60329	0.05
48089	1.38246	0.05
48090	1.98182	0.05
48091	2.2217	0.05
48092	3.60417	0.05
48093	2.05362	0.05
48094	1.02663	0.05
48095	1.43255	0.05
48096	1.52072	0.05
48097	1.70302	0.05
48098	1.7685	0.05
48099	0.946131	0.05
48100	0.946131	0.05
48101	0.946131	0.05
48102	0.946131	0.05
48103	0.946131	0.0514548
48104	0.946131	0.05
48105	0.946131	0.05
48106	0.946131	0.05
48107	0.946131	0.05
48108	0.946131	0.05
48109	0.946131	0.05
48110	0.946131	0.05
48111	1.89226	0.05
48112	1.89226	0.0591129
48113	1.89226	0.052324
48114	1.89226	0.05
48115	1.89226	0.055754
48116	1.89226	0.0550561
48117	1.92645	0.708211
48118	2.83045	0.476127
48119	1.92225	0.51241
48120	2.01873	0.436269
48121	2.51732	0.931656
48122	2.51732	0.59681
48123	2.51732	0.346392
48124	2.23857	0.370826
48125	1.92645	0.389967
48126	1.92645	0.328135
48127	2.23857	0.640272
48128	1.50865	0.31878
48129	2.02687	2.1
48130	2.39604	2.1
48131	2.29198	2.1
48132	2.73046	2.1
48133	4.68803	2.1
48134	1.21148	1.66376
48135	1.87957	1.8934
48136	2.03555	1.93271
48137	2.03555	1.58142

48138	1.09962	1.8119
48139	1.09962	1.85268
48140	1.92558	0.68619
48141	2.05274	2.1
48142	2.2443	1.71332
48143	2.24429	2.1
48144	1.70869	0.549237
48145	1.70868	2.1
48146	0.818397	0.324665
48147	0.818397	2.1
48148	0.827911	2.1
48149	0.827908	1.88354
48150	1.00418	1.4662
48151	1.02054	1.47953
48152	0.692018	1.99178
48153	0.620899	1.91856
48154	0.65823	1.57934
48155	1.00418	0.420449
48156	0.620899	1.56559
48157	1.0262	1.18425
48158	1.92558	1.75741
48159	2.51818	2.1
48160	1.94816	0.777844
48161	2.01985	2.1
48162	1.85389	0.669588
48163	1.92558	2.1
48164	0.903844	2.1
48165	0.903844	2.1
48166	0.946148	2.1
48167	0.946151	2.1
48168	0.946118	2.1
48169	0.903876	2.1
48170	0.946118	2.1
48171	1.10235	2.1
48172	0.946131	2.1
48173	0.946131	2.1
48174	1.10234	2.1
48175	0.903863	2.1
48176	1.63471	2.1
48177	1.08022	2.1
48178	1.56801	2.1
48179	1.03437	2.1
48180	2.66908	2.1
48181	1.56801	2.1
48182	0.97541	2.1
48183	0.97541	2.1
48184	1.03437	2.1
48185	1.49733	2.1
48186	1.55629	2.1
48187	1.89226	0.0778179
48188	1.89226	0.144545
48189	1.89226	0.0929869
48190	1.89226	0.136793
48191	3.78452	0.138098
48192	0.946131	0.0798757
48193	1.16373	0.0823032
48194	0.946131	0.0901441
48195	0.734637	0.0794005

48196	0.946132	0.0841957
48197	0.946132	0.0948052
48198	1.16373	0.0733825
48199	0.734636	0.0683708
48200	0.946093	0.0839318
48201	0.946093	0.076001
48202	0.734675	0.0745227
48203	0.734675	0.0878886
48204	1.98413	0.704515
48205	1.87355	2.1
48206	1.72774	0.633916
48207	1.67865	2.1
48208	3.60128	1.40825
48209	1.87355	2.1
48210	1.54924	2.1
48211	1.68443	2.1
48212	1.68443	2.1
48213	1.67866	2.1
48214	1.67866	2.1
48215	1.72774	0.417672
48216	1.53422	2.1
48217	1.67865	2.03613
48218	1.67865	0.61848
48219	1.53873	2.02266
48220	1.53873	0.708627
48221	1.67866	2.0645
48222	1.54924	2.1
48223	1.53423	2.1
48224	1.44091	2.1
48225	3.11957	2.1
48226	1.89226	2.1
48227	1.89226	2.01976
48228	1.65573	2.1
48229	1.65573	2.1
48230	1.89226	2.1
48231	1.89226	2.1
48232	1.89226	2.1
48233	1.89614	1.80906
48234	1.7508	2.0395
48235	1.72666	2.09486
48236	1.89226	2.01584
48237	1.89226	2.07873
48238	1.89223	2.1
48239	1.89223	1.7583
48240	1.89223	1.82006
48241	1.89223	2.05636
48242	1.89223	1.86217
48243	1.89223	2.1
48244	1.89224	2.02294
48245	1.89224	1.52211
48246	1.89224	1.70695
48247	1.89224	1.88242
48248	1.89224	1.75199
48249	1.89224	2.03998
48250	2.09773	2.1
48251	2.06623	2.1
48252	2.43268	2.1
48253	2.33713	2.1

48254	1.703	2.1
48255	1.61372	2.1
48256	0.646929	2.1
48257	0.689776	2.1
48258	1.05897	2.1
48259	0.956364	2.1
48260	0.905594	2.1
48261	0.904315	2.1
48262	0.727014	2.1
48263	0.850757	2.1
48264	0.798445	2.1
48265	0.654101	2.1
48266	0.717539	2.1
48267	1.0478	2.1
48268	1.89223	2.1
48269	1.89223	2.1
48270	1.89223	2.1
48271	1.6557	1.90293
48272	3.54794	2.1
48273	0.946118	2.1
48274	0.946118	2.1
48275	0.946118	2.1
48276	0.946118	2.1
48277	0.946117	2.1
48278	0.946117	2.1
48279	0.946119	2.1
48280	0.946119	2.1
48281	0.946105	2.1
48282	0.946105	2.1
48283	0.946131	2.1
48284	0.946131	2.1
48285	1.47671	2.1
48286	1.70303	2.1
48287	1.90705	2.1
48288	1.98945	2.1
48289	1.3454	2.1
48290	1.40353	2.1
48291	1.89226	2.1
48292	1.89226	2.1
48293	1.89226	2.1
48294	1.89226	2.1
48295	1.89226	2.1
48296	1.89226	2.1
48297	2.39438	0.147759
48298	2.69842	0.134645
48299	2.45177	0.191467
48300	2.28563	0.118399
48301	4.84615	0.146525
48302	1.28421	2.1
48303	1.35966	2.1
48304	0.961001	2.1
48305	0.860549	2.1
48306	1.1039	2.1
48307	1.1039	2.1
48308	1.11631	2.1
48309	1.11631	2.1
48310	1.35966	2.1
48311	0.961001	2.1

48312	0.860549	2.1
48313	1.28421	2.1
48314	1.89226	1.66178
48315	1.89226	1.84963
48316	1.89226	1.97364
48317	1.89226	1.58239
48318	3.78452	1.67857
48319	1.48702	1.34768
48320	1.40656	1.269
48321	0.626442	1.6902
48322	0.626442	1.6536
48323	0.626442	1.78726
48324	0.946132	1.17565
48325	1.05489	1.03077
48326	1.05012	1.80016
48327	0.946131	1.58848
48328	0.946131	1.60475
48329	0.626442	1.65982
48330	1.05012	1.61061
48331	1.89226	1.83714
48332	1.97212	1.60221
48333	1.89226	1.56946
48334	1.89226	1.69047
48335	3.78453	1.69928
48336	1.89226	1.94221
48337	1.89226	1.54718
48338	1.89226	1.55804
48339	1.89226	1.73556
48340	3.78452	1.70517
48341	1.89226	1.4367
48342	1.89226	1.61364
48343	1.89226	1.69198
48344	1.89226	1.12375
48345	1.89226	1.74169
48346	1.89226	1.33174
48347	0.946161	1.11538
48348	0.946161	1.48852
48349	0.932156	1.60671
48350	0.932156	1.51569
48351	0.932184	1.5882
48352	0.946132	0.68491
48353	0.946133	1.61051
48354	0.946133	1.18868
48355	0.946132	1.33463
48356	0.946132	1.40926
48357	0.932185	1.44851
48358	0.946133	1.21289
48359	0.946132	1.52113
48360	0.946132	1.18409
48361	0.946132	1.96105
48362	0.946132	1.64072
48363	0.946132	1.61196
48364	0.946132	1.50626
48365	0.946133	1.54399
48366	0.946133	1.6988
48367	0.946132	1.60474
48368	0.946132	1.67
48369	0.946133	1.50806

48370	0.946133	1.55673
48371	1.79397	0.14959
48372	1.00636	0.152613
48373	1.31658	0.208955
48374	2.02924	0.173201
48375	3.0356	0.170731
48376	1.89226	0.17116
48377	1.89226	0.172834
48378	1.89226	0.181401
48379	1.89226	0.18485
48380	1.89226	0.148129
48381	1.89226	0.138712
48382	0.946131	0.257751
48383	0.946131	0.237012
48384	1.24766	0.286881
48385	1.03691	0.302938
48386	0.946129	0.297423
48387	0.946129	0.240308
48388	1.24766	0.274144
48389	1.24766	0.263537
48390	0.946131	0.260377
48391	1.24766	0.276422
48392	1.03691	0.279549
48393	0.946131	0.26202
48394	1.44824	0.113172
48395	2.02924	0.109428
48396	1.7065	0.13496
48397	1.38604	0.168919
48398	2.32017	0.131963
48399	2.32017	0.106765
48400	1.89226	0.10881
48401	1.89226	0.129084
48402	1.89226	0.112225
48403	1.89226	0.0841813
48404	1.89226	0.134506
48405	1.89226	0.111633
48406	0.478349	2.08437
48407	0.612092	2.1
48408	0.606115	2.1
48409	0.506213	2.1
48410	0.550677	2.1
48411	0.550677	2.07849
48412	0.567628	2.1
48413	0.567628	2.1
48414	0.506213	2.1
48415	0.478349	2.1
48416	0.606116	2.1
48417	0.612092	2.1
48418	1.52226	2.1
48419	1.54241	2.1
48420	1.23167	2.1
48421	1.23167	2.1
48422	1.75555	2.1
48423	1.75555	2.1
48424	1.75557	2.1
48425	1.54243	2.1
48426	1.9943	2.1
48427	1.85871	2.1

48428	3.53673	2.1
48429	1.85871	2.1
48430	1.06936	2.1
48431	1.88926	2.1
48432	1.54243	2.1
48433	1.41619	2.1
48434	1.06936	2.1
48435	1.28649	1.86736
48436	1.28516	2.1
48437	1.38986	0.477591
48438	1.42399	1.82254
48439	2.67502	1.7383
48440	1.42399	1.27808
48441	1.07283	2.1
48442	1.46528	2.1
48443	1.28517	1.86421
48444	1.25294	2.1
48445	1.07283	1.93155
48446	1.38986	1.94828
48447	1.42399	2.1
48448	1.52885	1.52238
48449	1.60058	1.54827
48450	2.99043	1.58959
48451	0.715316	2.1
48452	0.715316	1.81103
48453	0.719126	0.817889
48454	0.719126	2.1
48455	0.722447	2.1
48456	0.626469	2.1
48457	0.711995	1.20167
48458	0.800291	1.34187
48459	0.80029	1.91708
48460	0.722447	1.89259
48461	0.711995	2.01829
48462	0.62647	2.00608
48463	2.05632	0.05
48464	1.70303	0.05
48465	2.3923	0.05
48466	2.61277	0.05
48467	1.44836	0.05
48468	1.58184	0.05
48469	1.89226	0.05
48470	1.89226	0.05
48471	1.89226	0.05
48472	1.89226	0.05
48473	3.78453	0.05
48474	1.44091	2.1
48475	1.1822	2.1
48476	1.25866	2.1
48477	1.25866	2.1
48478	1.35215	2.1
48479	1.35215	2.1
48480	1.27338	2.1
48481	1.1822	2.1
48482	1.27338	2.1
48483	1.35215	2.1
48484	1.10817	2.1
48485	1.18694	2.1

48486	0.946132	0.184496
48487	0.946132	0.157557
48488	0.946132	0.121336
48489	0.946132	0.100404
48490	0.946133	0.117568
48491	0.946133	0.161547
48492	0.946132	0.145681
48493	0.946132	0.106368
48494	0.946132	0.153147
48495	0.946132	0.125356
48496	0.946133	0.131696
48497	0.946133	0.150462
48498	2.6875	0.0595885
48499	2.40558	0.0688356
48500	2.1994	0.05
48501	1.70303	0.0596746
48502	4.68305	0.05
48503	0.915523	0.05
48504	0.915523	0.05
48505	0.861057	0.05
48506	0.946796	0.05
48507	0.838652	0.0502501
48508	0.654182	0.05
48509	1.14044	0.05
48510	0.80252	0.05
48511	0.836435	0.05
48512	0.608659	0.05
48513	1.22198	0.05
48514	0.863472	0.055357
48515	2.1994	0.05
48516	0.981586	0.05
48517	1.59619	0.05
48518	1.70303	0.05
48519	1.24816	0.05
48520	1.38523	0.05
48521	2.71141	2.1
48522	1.70302	2.1
48523	2.56527	2.1
48524	1.88649	2.1
48525	2.41279	2.1
48526	1.70302	2.1
48527	1.89226	2.1
48528	1.89226	2.1
48529	1.89226	2.1
48530	1.89226	2.1
48531	3.78453	2.1
48532	1.89226	2.1
48533	1.89226	2.1
48534	1.89226	2.1
48535	1.89226	2.1
48536	1.89226	2.1
48537	1.89226	2.1
48538	2.18867	2.1
48539	0.983723	2.1
48540	2.42263	2.1
48541	1.8014	2.1
48542	1.62992	2.1
48543	0.983723	2.1

48544	2.71141	2.1
48545	1.89226	2.1
48546	2.31157	2.1
48547	2.31157	2.1
48548	1.89226	2.1
48549	1.89226	2.1
48550	1.89227	2.1
48551	1.89227	2.06745
48552	1.89227	2.1
48553	1.89227	2.1
48554	3.78453	2.1
48555	1.89226	2.1
48556	1.89226	2.1
48557	1.89226	2.1
48558	1.89226	2.05721
48559	1.89226	2.1
48560	1.89226	2.06208
48561	1.89226	1.47531
48562	1.89226	1.1607
48563	1.89226	1.80677
48564	1.89226	1.13135
48565	1.89226	1.45329
48566	1.89226	1.01411
48567	1.1427	1.58148
48568	1.14956	2.06572
48569	1.70302	1.14189
48570	1.81267	1.67568
48571	2.88983	1.45146
48572	1.89226	1.12714
48573	1.89226	1.49548
48574	1.89226	1.32766
48575	1.89226	1.18715
48576	3.78453	1.3095
48577	1.89226	1.37456
48578	1.89226	1.20757
48579	1.89226	0.951414
48580	1.89226	1.44339
48581	3.78452	1.15091
48582	0.884197	1.99182
48583	1.1125	1.1431
48584	1.13273	1.27436
48585	0.844557	1.38881
48586	0.981137	1.30925
48587	1.13971	1.22122
48588	0.980432	1.25649
48589	0.980432	1.31035
48590	1.15859	1.16459
48591	1.12206	1.08801
48592	0.898619	1.87098
48593	0.94426	1.23702
48594	2.21016	1.179
48595	1.44091	1.84855
48596	1.98408	1.39625
48597	1.4218	1.26749
48598	2.21016	1.47269
48599	1.64788	1.38465
48600	1.07526	1.38817
48601	1.91531	2.1

48602	1.49155	2.1
48603	1.58551	1.92684
48604	1.49155	1.83366
48605	1.58551	2.1
48606	1.98979	0.05
48607	2.21773	0.05
48608	2.30533	0.05
48609	2.55895	0.05
48610	4.52305	0.05
48611	2.55896	0.05
48612	2.27963	0.05
48613	2.60486	0.05
48614	2.60486	0.05
48615	2.21773	0.05
48616	2.21773	0.05
48617	2.30533	0.05
48618	2.55895	0.05
48619	2.29539	0.05
48620	2.47308	0.05
48621	4.7784	0.05
48622	2.55896	0.05
48623	2.55792	0.05
48624	2.55792	0.05
48625	2.60486	0.05
48626	2.47308	0.05
48627	2.52003	0.05
48628	0.946133	1.92244
48629	0.946133	2.04136
48630	1.20851	1.91495
48631	1.20851	1.82195
48632	0.967411	1.77486
48633	1.20851	1.73074
48634	0.946132	2.1
48635	0.946132	1.99913
48636	0.946132	2.01487
48637	1.20851	1.85076
48638	0.967412	1.85065
48639	0.946133	1.95974
48640	1.89226	1.82865
48641	1.89226	1.65452
48642	1.89226	1.71693
48643	1.89226	1.63129
48644	1.89226	1.89055
48645	1.89226	1.82886
48646	1.89226	2.03096
48647	2.41701	1.90353
48648	2.71724	1.8553
48649	2.71724	1.9055
48650	1.89226	2.08616
48651	1.89226	2.09061
48652	0.946131	1.87361
48653	0.946131	2.02348
48654	0.946128	1.80547
48655	0.946128	1.77172
48656	0.94613	1.60427
48657	0.94613	1.64191
48658	0.94613	1.94813
48659	0.94613	1.97273

48660	0.94613	1.84877
48661	0.94613	1.64477
48662	0.94613	1.75077
48663	0.94613	1.95478
48664	0.851541	0.119442
48665	0.851541	0.140455
48666	0.851481	0.13373
48667	0.997219	0.157282
48668	0.997112	0.106857
48669	0.992853	0.0950256
48670	0.85154	0.0996293
48671	0.85154	0.135621
48672	0.894824	0.0988644
48673	0.894824	0.143643
48674	0.94613	0.15336
48675	0.906485	0.0973699
48676	0.802947	2.1
48677	0.845556	2.1
48678	0.582339	2.1
48679	0.519756	2.1
48680	0.714508	2.1
48681	0.714508	2.1
48682	0.628932	2.1
48683	0.708518	2.1
48684	0.801358	2.1
48685	0.476016	2.1
48686	0.569477	2.1
48687	0.878553	2.1
48688	1.89226	2.1
48689	1.89226	2.1
48690	1.89226	2.1
48691	1.89226	2.1
48692	1.89226	2.1
48693	1.89226	2.1
48694	0.946128	2.1
48695	0.946128	2.1
48696	1.01537	2.1
48697	0.9662	2.1
48698	0.946131	2.1
48699	0.946131	2.1
48700	0.966198	2.1
48701	0.966198	2.1
48702	0.94613	2.1
48703	0.966198	2.1
48704	0.946131	2.1
48705	1.01537	2.1
48706	1.89226	2.1
48707	1.89226	2.1
48708	1.91883	2.1
48709	1.91883	2.1
48710	2.15905	2.1
48711	2.13249	2.1
48712	1.07953	2.1
48713	1.2107	2.1
48714	0.946132	2.1
48715	0.946132	2.1
48716	1.2107	2.1
48717	1.2107	2.1

48718	0.946132	2.1
48719	0.946132	2.1
48720	1.2107	2.1
48721	0.946133	2.1
48722	0.946132	2.1
48723	1.07953	2.1
48724	2.46752	2.1
48725	2.53733	2.1
48726	2.26044	2.1
48727	2.33222	2.1
48728	4.79974	2.1
48729	2.53733	2.1
48730	2.37576	1.97295
48731	2.33222	2.1
48732	2.1845	2.1
48733	4.70798	2.1
48734	2.48652	2.1
48735	2.33224	2.1
48736	2.08149	2.1
48737	1.9839	2.1
48738	4.45623	2.1
48739	2.33223	2.1
48740	2.18452	2.1
48741	2.16061	2.1
48742	2.01399	1.85999
48743	4.34623	2.1
48744	1.4617	2.1
48745	1.34701	2.1
48746	1.34701	2.1
48747	1.25173	2.1
48748	1.5117	2.1
48749	1.41642	2.1
48750	2.65164	2.1
48751	1.89227	2.1
48752	1.89227	2.1
48753	1.89227	2.1
48754	2.42504	2.1
48755	2.42504	2.1
48756	1.89226	2.03665
48757	1.89226	2.1
48758	1.89226	2.1
48759	1.89226	1.91223
48760	3.78452	2.1
48761	1.5117	2.1
48762	1.34701	2.1
48763	1.47001	2.1
48764	1.57961	2.08513
48765	2.92663	2.1
48766	1.89226	2.1
48767	1.89226	1.86551
48768	1.89226	2.08061
48769	1.89226	2.1
48770	3.78452	2.1
48771	0.412773	0.05
48772	0.570839	0.05
48773	0.50721	0.05
48774	0.513638	0.05
48775	0.518805	0.05

48776	0.41278	0.05
48777	1.5344	0.05
48778	0.939282	0.05
48779	0.720051	0.05
48780	0.691767	0.05
48781	0.761152	0.05
48782	1.89226	2.1
48783	1.89226	2.1
48784	1.89226	2.1
48785	1.89226	2.1
48786	1.89226	2.1
48787	1.89226	2.1
48788	1.24618	2.1
48789	2.10585	2.1
48790	1.08134	2.1
48791	1.93032	2.1
48792	3.1765	2.1
48793	1.98474	2.1
48794	2.57777	2.1
48795	1.93031	2.1
48796	2.19041	2.1
48797	3.99251	2.1
48798	1.89226	2.1
48799	1.89226	2.1
48800	1.89226	2.1
48801	1.89226	2.1
48802	3.78452	2.1
48803	1.67374	2.1
48804	2.19042	2.1
48805	1.67374	2.1
48806	2.09769	2.1
48807	2.29586	2.1
48808	2.7198	2.1
48809	1.89226	2.1
48810	1.89226	2.1
48811	1.89226	2.1
48812	1.89226	2.1
48813	1.89226	2.1
48814	1.89226	2.1
48815	0.946132	0.417895
48816	0.946132	0.335848
48817	0.947763	0.358731
48818	0.909304	0.385296
48819	0.946127	0.288648
48820	0.947831	0.248696
48821	0.907673	0.283888
48822	0.907673	0.399338
48823	0.94613	0.296681
48824	0.947834	0.252579
48825	0.907671	0.466469
48826	0.94613	0.256058
48827	0.906519	0.183355
48828	0.906519	0.165352
48829	0.946097	0.19144
48830	0.946097	0.211366
48831	0.947834	0.179524
48832	0.906485	0.150561
48833	0.946131	0.156424

48834	0.946131	0.209371
48835	0.94613	0.282618
48836	0.906485	0.151663
48837	0.946131	0.224994
48838	0.947835	0.206449
48839	2.58638	2.1
48840	2.51039	2.1
48841	2.47395	2.1
48842	2.41539	2.1
48843	5.00178	2.1
48844	2.51039	2.1
48845	2.16633	2.1
48846	2.4154	2.1
48847	2.13977	2.1
48848	4.58173	2.1
48849	2.47395	2.1
48850	2.41539	2.1
48851	2.2988	2.1
48852	2.2604	2.1
48853	4.7142	2.1
48854	2.4154	2.1
48855	2.13977	2.1
48856	2.26041	2.1
48857	1.98957	2.1
48858	4.40497	2.1
48859	2.15721	1.72102
48860	2.31843	0.68445
48861	1.93973	1.99258
48862	2.13243	0.155528
48863	4.25816	0.535333
48864	2.31844	0.389624
48865	2.30908	1.13284
48866	2.13243	0.333032
48867	2.1609	0.41579
48868	4.47934	0.266625
48869	1.93973	1.95146
48870	2.13243	0.280798
48871	1.69661	1.8217
48872	1.9339	0.271433
48873	3.87363	0.754317
48874	2.13243	0.137479
48875	2.1609	0.489318
48876	1.9339	0.305793
48877	1.98414	0.751643
48878	4.0948	0.115489
48879	1.88857	0.05
48880	2.34107	0.05
48881	0.502431	0.05
48882	0.71416	0.05
48883	0.50243	0.05
48884	0.944284	0.05
48885	1.88856	2.1
48886	2.34107	2.1
48887	2.39662	2.1
48888	1.92293	2.1
48889	4.28519	2.1
48890	1.00486	2.1
48891	1.92293	2.1

48892	1.88857	2.1
48893	1.88857	2.1
48894	1.02605	2.1
48895	1.02605	2.1
48896	2.53728	2.1
48897	1.70303	2.1
48898	2.03734	2.1
48899	1.3624	2.1
48900	2.54706	2.1
48901	1.70303	2.1
48902	2.54706	2.1
48903	1.50876	2.1
48904	2.74067	2.1
48905	1.85391	2.1
48906	2.29667	2.1
48907	1.50876	2.1
48908	0.946133	2.1
48909	0.946133	2.1
48910	1.24191	2.1
48911	1.24191	2.1
48912	0.946132	2.1
48913	1.24191	2.1
48914	1.23779	2.1
48915	0.946132	2.1
48916	0.946132	2.1
48917	0.946132	2.1
48918	1.23779	2.1
48919	1.24191	2.1
48920	0.946131	2.1
48921	0.946131	2.1
48922	1.14833	2.1
48923	1.14833	2.1
48924	1.14834	2.1
48925	0.946132	2.1
48926	0.94613	2.1
48927	1.24191	2.1
48928	0.946131	2.1
48929	0.946131	2.1
48930	1.24191	2.1
48931	1.14833	2.1
48932	2.08149	2.1
48933	2.31116	2.1
48934	1.9701	2.00531
48935	2.18328	2.1
48936	4.49563	2.1
48937	2.08149	2.1
48938	2.14059	2.1
48939	1.72377	2.1
48940	2.18328	2.1
48941	3.88931	2.1
48942	2.14059	2.1
48943	2.40755	2.1
48944	2.31145	2.1
48945	2.23743	2.1
48946	2.31145	2.1
48947	2.23743	2.1
48948	2.31117	2.1
48949	2.40754	2.1

48950	2.41055	2.1
48951	2.31145	2.1
48952	2.41055	2.1
48953	2.31145	2.1
48954	2.5615	0.22463
48955	2.37625	0.189127
48956	2.20248	0.176164
48957	2.20248	0.170881
48958	2.65967	0.337848
48959	2.65967	0.250564
48960	2.71793	0.428316
48961	2.65966	0.204204
48962	2.56149	0.176348
48963	2.56149	0.19485
48964	2.71296	0.35957
48965	2.71296	0.292446
48966	1.31528	0.181709
48967	1.32983	0.160433
48968	1.21128	0.181664
48969	1.18812	0.174331
48970	1.24232	0.148326
48971	1.24232	0.151869
48972	1.26109	0.186778
48973	1.26109	0.345442
48974	1.21128	0.209666
48975	1.31528	0.125563
48976	1.18812	0.141361
48977	1.32983	0.207284
48978	2.63056	0.152514
48979	2.58032	0.187364
48980	2.65966	0.120931
48981	2.65966	0.178726
48982	2.58032	0.305091
48983	2.71296	0.277984
48984	1.92039	2.1
48985	1.06226	2.1
48986	1.09252	2.1
48987	1.96052	2.1
48988	3.01291	2.1
48989	1.92039	2.1
48990	2.46338	2.1
48991	2.47522	2.1
48992	2.43508	2.1
48993	1.96052	2.1
48994	1.92039	2.1
48995	0.985949	2.1
48996	0.980261	2.1
48997	0.553625	2.1
48998	0.546262	2.1
48999	0.765671	2.1
49000	0.765671	2.1
49001	0.766539	2.1
49002	0.766539	2.1
49003	0.985949	2.1
49004	0.553625	2.1
49005	0.546261	2.1
49006	0.980261	2.1
49007	1.96052	2.1

49008	2.46338	2.1
49009	1.97189	2.1
49010	1.96052	2.1
49011	2.47932	2.1
49012	2.47932	2.1
49013	0.964608	0.05
49014	0.691524	0.05
49015	1.25462	0.05
49016	0.691524	0.05
49017	1.89226	0.05
49018	1.89226	0.05
49019	1.44894	0.05
49020	2.77655	0.05
49021	1.4237	0.05
49022	2.77701	0.05
49023	2.77701	0.05
49024	1.38847	2.1
49025	1.46031	1.73428
49026	1.65504	1.85902
49027	1.75396	1.63992
49028	3.11535	1.85073
49029	1.89226	1.8196
49030	1.89226	1.73102
49031	1.89226	1.88851
49032	1.89226	1.36584
49033	3.78452	1.63994
49034	1.89226	1.53618
49035	1.89226	2.06857
49036	1.89226	0.593868
49037	1.89226	1.53878
49038	3.78452	1.36537
49039	1.46031	1.47078
49040	1.34851	1.85932
49041	1.75395	1.46712
49042	1.62287	1.52996
49043	3.08318	1.5247
49044	1.65504	2.06652
49045	1.75396	1.93165
49046	1.89249	2.1
49047	1.9857	1.52033
49048	3.64073	1.93626
49049	1.89226	2.09533
49050	1.89226	1.61443
49051	1.89226	1.6344
49052	1.89226	1.53842
49053	3.78452	1.63923
49054	1.89226	1.40357
49055	1.89226	1.79806
49056	1.89226	1.39973
49057	1.89226	1.7529
49058	3.78452	1.55721
49059	1.75395	1.85088
49060	1.62287	1.75575
49061	1.98569	1.32111
49062	1.86067	1.34868
49063	3.60856	1.41005
49064	1.35697	2.1
49065	2.02064	2.1

49066	1.89989	2.1
49067	1.49571	2.1
49068	1.66753	2.1
49069	1.36431	2.1
49070	0.744004	2.1
49071	0.743997	2.1
49072	0.717972	2.1
49073	0.717972	2.1
49074	0.997982	2.1
49075	0.807796	2.1
49076	0.632235	2.1
49077	0.488575	2.1
49078	0.849275	2.1
49079	0.488581	2.1
49080	0.724729	2.1
49081	0.997967	2.1
49082	2.5276	2.1
49083	1.70791	2.1
49084	1.82872	1.38025
49085	1.56362	2.1
49086	2.5276	1.90609
49087	2.24781	2.1
49088	1.37591	2.1
49089	1.23217	2.1
49090	1.00442	1.90246
49091	0.945282	1.13344
49092	1.15446	1.66791
49093	1.09123	2.1
49094	1.21506	2.1
49095	1.21506	1.76677
49096	1.39452	2.00733
49097	1.13108	2.02911
49098	1.27876	2.01952
49099	0.929006	2.01294
49100	1.46505	0.0606087
49101	1.91484	0.0716833
49102	1.49906	0.0711537
49103	1.49906	0.0784206
49104	1.90196	0.0553293
49105	1.90196	0.0578434
49106	1.90196	0.0576201
49107	2.11465	0.077379
49108	1.91484	0.0744186
49109	1.91484	0.0835234
49110	2.04016	0.057513
49111	2.04016	0.0614171
49112	0.912201	0.0505583
49113	0.950978	0.0507976
49114	0.665307	0.05
49115	0.732528	0.055133
49116	0.829512	0.05
49117	0.829512	0.0524225
49118	0.815217	0.05
49119	0.815217	0.05
49120	0.912202	0.05
49121	0.665305	0.05
49122	0.732527	0.059427
49123	0.95098	0.0506807

49124	1.90196	0.0568843
49125	2.08757	0.05
49126	1.9626	0.0534993
49127	2.04016	0.0508558
49128	1.82441	0.05
49129	1.90196	0.05
49130	1.13048	2.1
49131	0.821162	2.1
49132	0.946131	2.1
49133	0.946131	2.1
49134	1.04567	2.1
49135	0.882347	2.1
49136	0.897276	2.1
49137	0.897276	2.1
49138	0.863109	2.1
49139	1.01055	2.1
49140	0.940305	2.1
49141	0.942166	2.1
49142	2.23933	0.05
49143	0.976217	0.05
49144	2.78053	0.05
49145	2.78053	0.05
49146	0.976217	0.0556084
49147	1.65984	0.05
49148	1.54168	2.1
49149	2.01197	2.03993
49150	1.69136	2.1
49151	2.13284	1.77077
49152	3.67452	2.1
49153	0.988805	2.1
49154	1.78069	2.1
49155	1.69136	2.1
49156	1.69136	2.1
49157	1.05481	2.1
49158	1.05481	2.1
49159	1.69136	2.1
49160	2.13284	1.97919
49161	1.78069	2.1
49162	2.22044	2.06359
49163	3.91353	2.1
49164	2.69379	1.48923
49165	2.32219	1.53667
49166	2.25746	1.70997
49167	2.25746	1.44802
49168	2.32219	1.58728
49169	1.89526	1.3318
49170	1.4556	2.1
49171	1.85028	2.1
49172	2.4909	1.93195
49173	2.03799	2.1
49174	2.73495	2.1
49175	1.85028	2.1
49176	2.71938	1.2819
49177	1.85028	2.1
49178	3.36051	2.1
49179	2.73495	2.1
49180	2.47584	2.1
49181	1.85028	2.1

49182	2.47584	2.1
49183	1.04413	2.1
49184	2.1461	2.1
49185	1.74586	2.1
49186	1.44437	2.1
49187	1.04413	2.1
49188	1.89226	2.1
49189	1.89226	1.8991
49190	1.89226	2.1
49191	1.89226	2.1
49192	3.78452	2.1
49193	1.52724	2.1
49194	1.89249	2.1
49195	1.68143	2.1
49196	2.02433	2.1
49197	3.55157	2.1
49198	1.07444	2.1
49199	1.76545	2.1
49200	1.68142	2.1
49201	1.68142	2.1
49202	1.14807	2.1
49203	1.14807	2.1
49204	1.89226	2.1
49205	1.89226	2.1
49206	1.89226	2.1
49207	1.89226	2.1
49208	1.89226	2.1
49209	1.89226	2.1
49210	1.89226	2.1
49211	1.89226	2.1
49212	1.89226	2.1
49213	1.89226	1.88251
49214	3.78452	2.1
49215	1.68143	2.1
49216	2.02433	2.1
49217	1.76545	2.1
49218	2.12608	2.1
49219	3.78978	2.1
49220	2.62871	2.1
49221	1.49149	2.1
49222	1.36814	2.1
49223	2.44761	2.1
49224	3.9391	2.1
49225	1.89226	2.1
49226	1.89226	2.03413
49227	1.89226	2.1
49228	1.89226	2.1
49229	3.78452	2.1
49230	0.851542	2.1
49231	0.851542	2.1
49232	1.00897	2.1
49233	0.931224	2.1
49234	0.838952	2.1
49235	0.838952	2.1
49236	0.956848	2.1
49237	0.942474	2.1
49238	0.946132	2.1
49239	0.838952	2.1

49240	0.878743	2.1
49241	0.946132	2.1
49242	1.15541	2.1
49243	2.44761	2.1
49244	1.36814	2.1
49245	1.36814	2.1
49246	2.19439	2.1
49247	2.19439	2.1
49248	1.89226	2.1
49249	1.89226	2.1
49250	1.89226	2.1
49251	1.89226	2.1
49252	3.78452	2.1
49253	1.89226	2.1
49254	1.67791	2.1
49255	1.5713	2.1
49256	1.67791	2.1
49257	1.78566	2.1
49258	1.89226	2.1
49259	1.89226	0.494793
49260	1.62911	2.1
49261	2.24602	1.86283
49262	1.89226	2.02207
49263	1.98287	1.40645
49264	1.62911	1.68109
49265	1.89226	2.1
49266	1.89226	2.1
49267	1.89226	1.76222
49268	1.89226	2.1
49269	3.78452	2.1
49270	1.26688	2.1
49271	1.11804	2.1
49272	0.852467	2.1
49273	0.693314	2.1
49274	0.977459	2.1
49275	0.977459	2.1
49276	0.982741	2.1
49277	0.982741	2.1
49278	1.26689	2.1
49279	0.852464	2.1
49280	0.693312	2.1
49281	1.11804	2.1
49282	0.946131	2.1
49283	0.946131	1.31044
49284	1.1794	0.698515
49285	1.1794	1.82815
49286	0.991434	2.1
49287	1.1794	1.15648
49288	0.946131	1.21178
49289	0.946131	0.841501
49290	0.946131	2.05933
49291	1.1794	1.90571
49292	0.946131	2.08048
49293	0.991434	1.86908
49294	1.89226	1.42342
49295	1.89226	2.1
49296	1.89226	2.1
49297	1.89226	2.1

49298	3.78452	2.1
49299	2.53377	2.1
49300	1.53443	2.1
49301	2.0815	2.1
49302	2.82979	2.1
49303	4.35462	2.1
49304	1.96192	0.739414
49305	1.23602	2.1
49306	2.16506	1.32883
49307	1.56841	2.03866
49308	3.40108	1.98549
49309	1.89226	2.1
49310	1.89226	2.1
49311	1.89226	1.49474
49312	1.89226	2.1
49313	3.78452	2.1
49314	0.946129	2.1
49315	0.946129	2.1
49316	1.13067	2.1
49317	1.12952	2.1
49318	0.946129	2.1
49319	1.13089	2.1
49320	0.944978	2.1
49321	0.944978	2.1
49322	0.946131	2.1
49323	1.13089	2.1
49324	0.944977	2.1
49325	0.946131	2.1
49326	0.710208	2.1
49327	0.710208	2.1
49328	0.718894	2.1
49329	0.718894	2.1
49330	0.559773	2.1
49331	0.579321	2.1
49332	0.886767	2.1
49333	0.849781	2.1
49334	0.849781	2.1
49335	0.559774	2.1
49336	0.579321	2.1
49337	0.886766	2.1
49338	1.63471	2.1
49339	1.69956	2.1
49340	1.08022	2.1
49341	1.08022	2.1
49342	1.11955	2.1
49343	1.67403	2.1
49344	1.03766	2.1
49345	2.02825	2.1
49346	2.02794	2.1
49347	2.0348	2.1
49348	1.07196	2.1
49349	1.07196	2.1
49350	1.9276	2.1
49351	2.1004	2.1
49352	2.1306	2.1
49353	2.1306	2.1
49354	1.92178	2.1
49355	1.92032	2.1

49356	0.986724	2.1
49357	2.03064	2.1
49358	2.01385	2.1
49359	2.02404	2.1
49360	1.03766	2.1
49361	1.03766	2.1
49362	1.92027	2.1
49363	2.1306	2.1
49364	2.15388	2.1
49365	2.15388	2.1
49366	1.92614	2.1
49367	1.9276	2.1
49368	1.09578	2.1
49369	0.963018	2.1
49370	0.782956	2.00257
49371	0.529693	2.1
49372	0.6994	2.1
49373	0.862532	2.1
49374	0.898605	2.1
49375	0.898605	2.07179
49376	0.776756	2.1
49377	1.14805	2.1
49378	0.966521	2.1
49379	0.617676	2.1
49380	2.67185	2.1
49381	2.099	2.1
49382	2.80548	2.1
49383	2.2727	2.1
49384	4.94455	2.1
49385	1.89226	2.1
49386	1.89226	2.1
49387	1.89226	2.1
49388	1.89226	2.1
49389	3.78453	2.1
49390	1.89226	2.1
49391	1.89226	2.1
49392	1.89226	2.1
49393	1.89226	2.1
49394	1.89226	2.1
49395	1.89226	2.1
49396	0.884801	2.1
49397	0.884801	2.1
49398	0.895199	2.1
49399	0.895199	2.1
49400	0.622774	2.1
49401	0.730501	2.1
49402	1.0495	2.1
49403	1.13635	2.1
49404	1.13635	2.1
49405	0.730503	2.1
49406	1.0495	2.1
49407	0.622775	2.1
49408	1.89226	2.1
49409	1.89226	2.1
49410	1.89226	2.1
49411	1.89226	2.1
49412	1.89226	2.1
49413	1.89226	2.1

49414	1.89226	2.1
49415	1.89226	2.1
49416	1.89226	2.1
49417	1.89226	2.1
49418	1.89226	2.1
49419	1.89226	2.1
49420	2.2727	2.1
49421	1.89586	2.1
49422	1.66913	2.1
49423	1.46101	2.1
49424	2.48082	2.1
49425	2.2727	2.1
49426	1.89226	2.1
49427	1.89226	2.1
49428	1.84641	2.1
49429	1.89226	2.1
49430	1.30868	2.1
49431	1.34118	2.09492
49432	1.89226	1.76791
49433	1.89226	1.9363
49434	1.89226	2.04032
49435	1.89226	1.84332
49436	1.89226	1.85163
49437	1.89226	1.68511
49438	1.89226	1.96502
49439	1.89226	1.62719
49440	1.89226	1.78436
49441	1.89226	1.92112
49442	3.78452	1.82477
49443	0.946132	2.1
49444	0.946132	2.07229
49445	0.946131	2.1
49446	0.946131	2.1
49447	0.946132	2.1
49448	0.946132	1.98262
49449	0.946132	2.1
49450	0.946132	2.1
49451	0.946132	2.1
49452	0.946132	2.1
49453	0.946132	2.1
49454	0.946132	2.1
49455	1.89227	2.01323
49456	1.84641	2.1
49457	2.29517	2.1
49458	1.84641	2.08773
49459	2.34102	2.04793
49460	1.89227	1.85581
49461	1.89226	1.60083
49462	1.89226	1.72165
49463	1.89226	1.79508
49464	1.89226	1.65836
49465	1.89226	1.62823
49466	1.89226	1.0988
49467	1.89226	1.57903
49468	1.89226	1.74482
49469	1.89226	1.79989
49470	1.89226	1.67275
49471	1.89226	1.52376

49472	1.89226	1.06121
49473	1.89226	2.1
49474	1.89226	1.98385
49475	1.89226	2.1
49476	1.89226	1.94943
49477	1.89226	2.08479
49478	1.89226	1.88289
49479	0.851544	0.05
49480	0.757542	0.05
49481	1.13421	0.05
49482	1.04072	0.05
49483	0.946132	0.05
49484	0.946132	0.05
49485	0.841686	0.05
49486	0.834428	0.05
49487	1.13649	0.05
49488	0.937687	0.05
49489	0.841713	0.05
49490	0.841713	0.05
49491	1.89226	0.288037
49492	1.89226	0.301504
49493	1.89226	0.308847
49494	1.89226	0.25475
49495	1.89226	0.333218
49496	1.89226	0.274255
49497	2.31988	1.68916
49498	2.00082	2.1
49499	2.4594	0.670373
49500	2.17638	2.1
49501	4.46022	1.90004
49502	2.00082	2.1
49503	1.41829	2.1
49504	1.61207	2.1
49505	1.43652	2.1
49506	2.17637	2.1
49507	2.00082	2.1
49508	2.4594	1.44905
49509	2.17638	2.1
49510	2.57957	1.16178
49511	2.3506	2.1
49512	4.81	1.77853
49513	2.3506	2.07922
49514	1.57368	2.1
49515	1.89111	1.78709
49516	1.89111	2.1
49517	2.17638	1.6898
49518	2.17638	2.1
49519	1.32813	2.1
49520	1.7237	2.1
49521	1.32461	2.1
49522	1.81656	2.1
49523	3.14468	2.1
49524	1.55132	2.1
49525	2.0815	2.1
49526	1.81656	2.1
49527	2.19826	2.1
49528	3.65818	2.1
49529	0.775951	2.1

49530	0.775951	2.1
49531	0.794601	2.1
49532	0.794601	2.1
49533	0.945641	2.1
49534	0.90828	2.1
49535	0.662302	2.1
49536	0.662273	2.1
49537	0.662274	2.1
49538	0.945639	2.1
49539	0.662304	2.1
49540	0.908279	2.1
49541	1.03062	2.1
49542	1.03062	2.1
49543	1.05054	2.1
49544	1.05054	2.1
49545	1.13551	2.1
49546	1.21275	2.1
49547	0.945641	2.1
49548	0.90828	2.1
49549	0.945641	2.1
49550	1.21275	2.1
49551	0.90828	2.1
49552	1.13552	2.1
49553	1.89226	2.1
49554	1.79883	2.1
49555	1.95341	2.1
49556	1.89226	2.1
49557	1.85998	2.1
49558	1.79883	2.1
49559	1.89226	2.1
49560	1.89226	2.1
49561	1.89226	2.1
49562	1.89226	2.1
49563	3.78453	2.1
49564	0.851541	2.1
49565	0.851541	2.1
49566	0.970398	2.1
49567	0.954365	2.1
49568	0.855356	2.1
49569	0.855357	2.1
49570	0.946133	2.1
49571	0.946133	2.1
49572	1.04072	2.1
49573	0.970397	2.1
49574	0.950003	2.1
49575	0.851538	2.1
49576	1.89226	2.1
49577	1.85998	2.1
49578	1.95296	2.1
49579	1.89226	2.1
49580	1.92067	2.1
49581	1.85998	2.1
49582	0.946133	2.1
49583	0.946133	2.1
49584	0.946132	2.1
49585	0.946132	2.1
49586	0.946132	2.1
49587	0.946132	2.1

49588	0.946132	2.1
49589	0.946132	2.1
49590	0.946132	2.1
49591	0.946132	2.1
49592	0.946133	2.1
49593	0.946133	2.1
49594	1.82152	2.1
49595	1.89226	2.1
49596	1.89226	2.1
49597	2.11152	2.1
49598	1.7472	2.1
49599	1.89226	2.1
49600	2.02687	2.1
49601	2.29199	2.1
49602	1.2734	2.1
49603	1.41077	2.1
49604	1.47381	2.1
49605	2.29199	2.1
49606	1.46889	2.1
49607	2.29199	2.1
49608	1.86766	2.1
49609	2.59401	2.1
49610	4.15965	2.1
49611	1.58995	2.1
49612	1.74246	2.1
49613	1.46643	2.1
49614	1.46643	2.1
49615	1.85097	2.1
49616	1.85097	2.1
49617	1.58995	2.1
49618	1.66584	2.1
49619	1.89214	2.1
49620	1.91787	2.1
49621	3.48208	2.1
49622	1.18474	2.1
49623	1.8144	2.1
49624	1.1486	2.1
49625	1.1486	2.1
49626	2.13071	2.1
49627	2.1257	2.1
49628	2.0815	2.1
49629	2.51651	2.1
49630	1.73681	2.1
49631	2.01601	2.1
49632	2.21406	2.1
49633	2.56998	2.1
49634	1.19995	2.1
49635	1.06256	2.1
49636	0.620677	2.1
49637	0.598084	2.1
49638	0.831426	2.1
49639	0.946056	2.1
49640	0.827692	2.1
49641	0.827692	2.1
49642	1.08958	2.1
49643	0.630132	2.1
49644	0.577547	2.1
49645	1.06104	2.1

49646	2.0815	2.1
49647	2.66249	2.1
49648	2.78966	2.1
49649	2.47108	2.1
49650	2.33858	2.1
49651	2.0815	2.1
49652	1.53735	2.1
49653	1.42278	2.1
49654	1.23654	2.1
49655	1.34678	2.1
49656	2.76955	2.1
49657	1.89226	2.1
49658	1.89226	2.1
49659	1.89226	2.1
49660	1.89226	2.1
49661	3.78452	2.1
49662	1.23654	2.1
49663	1.34678	2.1
49664	1.19	2.1
49665	1.08576	2.1
49666	2.43254	2.1
49667	1.38776	2.1
49668	1.29366	2.1
49669	0.946131	2.1
49670	0.946131	2.1
49671	0.946131	2.1
49672	0.946131	2.1
49673	1.38775	2.1
49674	1.38775	2.1
49675	1.29366	2.1
49676	0.946132	2.1
49677	0.946131	2.1
49678	1.38775	2.1
49679	0.946131	2.1
49680	0.946131	2.1
49681	0.946129	2.1
49682	0.946129	2.1
49683	0.94613	2.1
49684	0.94613	2.1
49685	0.946131	2.1
49686	0.946131	2.1
49687	0.946131	2.1
49688	0.946131	2.1
49689	0.94613	2.1
49690	0.94613	2.1
49691	1.89226	0.167813
49692	1.89226	1.83727
49693	1.89226	0.63312
49694	1.89226	0.582316
49695	1.89226	1.36364
49696	1.89226	1.57605
49697	1.89226	0.543459
49698	1.89226	2.1
49699	1.89226	1.28516
49700	1.89226	1.3913
49701	1.89226	1.15133
49702	1.89226	1.8676
49703	1.89226	0.324583

49704	1.89226	2.00418
49705	1.89226	0.762238
49706	1.89226	0.871312
49707	1.89226	0.648543
49708	1.89226	1.75505
49709	0.92338	2.1
49710	0.92338	2.00496
49711	0.94428	2.02904
49712	0.94428	1.01521
49713	1.19528	2.06984
49714	1.30598	2.1
49715	0.714073	1.34313
49716	0.561679	2.1
49717	1.19528	2.1
49718	0.561678	2.1
49719	0.714072	2.1
49720	1.30598	2.1
49721	2.61197	2.1
49722	1.89226	2.1
49723	1.89226	2.1
49724	1.89226	2.1
49725	2.39056	2.07045
49726	2.39056	2.1
49727	0.946132	2.1
49728	0.946132	2.00641
49729	0.946132	0.518666
49730	0.946132	1.47271
49731	0.946131	1.48418
49732	0.946131	2.1
49733	0.946132	2.00012
49734	0.946132	1.17766
49735	0.946132	1.82553
49736	0.946132	1.77962
49737	0.946132	1.82112
49738	0.946132	1.8148
49739	1.89224	2.1
49740	1.89224	2.1
49741	1.89224	2.1
49742	1.89224	2.1
49743	3.78448	2.1
49744	1.97089	2.1
49745	1.57428	2.1
49746	1.84991	2.1
49747	1.49078	2.1
49748	3.42419	2.1
49749	0.621927	2.1
49750	0.621927	2.1
49751	0.630824	2.1
49752	0.630824	2.1
49753	0.483483	2.1
49754	0.507361	2.1
49755	0.787142	2.1
49756	0.745391	2.1
49757	0.745381	2.1
49758	0.483476	2.1
49759	0.507368	2.1
49760	0.787152	2.1
49761	1.89224	2.1

49762	1.89224	2.1
49763	1.89224	2.1
49764	1.89224	2.1
49765	1.89224	2.1
49766	1.89224	2.1
49767	1.89227	2.1
49768	1.89227	2.1
49769	1.89227	2.1
49770	1.89227	2.1
49771	1.89227	2.1
49772	1.89227	2.1
49773	1.84994	2.1
49774	1.41758	2.1
49775	1.4908	2.1
49776	1.4908	2.1
49777	1.74391	2.1
49778	1.74391	2.1
49779	1.89226	2.1
49780	1.89226	2.1
49781	1.89226	2.1
49782	1.89226	2.1
49783	3.78452	2.1
49784	1.01323	2.1
49785	1.01323	2.1
49786	1.01445	2.1
49787	1.01445	2.1
49788	0.839531	2.1
49789	0.875652	2.1
49790	1.19063	2.1
49791	1.15203	2.1
49792	1.15203	2.1
49793	0.839531	2.1
49794	0.875652	2.1
49795	1.19063	2.1
49796	1.89226	2.1
49797	2.75587	2.1
49798	2.81512	2.1
49799	2.81512	2.1
49800	1.89226	2.1
49801	1.89226	2.1
49802	1.89226	2.1
49803	1.89226	2.1
49804	1.89226	2.1
49805	1.89226	2.1
49806	3.78452	2.1
49807	0.974831	2.1
49808	0.974831	2.1
49809	0.976681	2.1
49810	0.976681	2.1
49811	0.803179	2.1
49812	0.83953	2.1
49813	1.11198	2.1
49814	1.15203	2.1
49815	1.11198	2.1
49816	0.803179	2.1
49817	1.15203	2.1
49818	0.83953	2.1
49819	1.37794	2.1

49820	1.37794	2.1
49821	0.946128	2.1
49822	0.946128	2.1
49823	1.3477	2.1
49824	1.37793	2.1
49825	0.946131	2.1
49826	0.946131	2.1
49827	0.946129	2.1
49828	1.3477	2.1
49829	1.37793	2.1
49830	0.946129	2.1
49831	2.38577	2.05671
49832	1.20247	2.1
49833	1.75182	2.1
49834	2.82546	2.05686
49835	4.1376	2.1
49836	2.82546	2.1
49837	1.89226	1.65952
49838	2.38577	2.06472
49839	2.38577	1.78127
49840	1.89226	1.98181
49841	1.89226	1.78339
49842	1.035	2.1
49843	2.15429	2.1
49844	0.970998	2.1
49845	2.0815	2.1
49846	2.89672	2.1
49847	0.946131	0.103613
49848	0.946131	0.0939773
49849	0.853249	0.109238
49850	1.02715	0.0945323
49851	0.851537	0.11548
49852	0.851537	0.099964
49853	1.02663	0.0896021
49854	1.13139	0.109662
49855	0.946128	0.0969024
49856	0.932039	0.0808696
49857	0.843985	0.0763044
49858	0.946129	0.0946425
49859	1.89226	0.101148
49860	1.86408	0.100599
49861	1.96548	0.0973624
49862	1.86408	0.0926239
49863	1.99366	0.117565
49864	1.89226	0.110609
49865	2.70702	2.1
49866	2.08981	2.1
49867	2.08693	2.1
49868	2.08693	2.1
49869	2.7188	2.1
49870	2.7188	2.1
49871	2.08982	2.1
49872	1.01964	2.1
49873	2.08693	2.1
49874	1.04615	2.1
49875	3.13597	2.1
49876	2.70702	2.1
49877	2.08693	2.1

49878	2.68784	2.1
49879	2.08831	2.1
49880	4.77477	2.1
49881	0.778233	2.1
49882	0.778233	2.1
49883	0.789001	2.1
49884	0.789001	2.1
49885	0.545307	2.1
49886	0.523075	2.1
49887	1.04347	2.1
49888	1.04416	2.1
49889	1.04416	2.1
49890	0.545308	2.1
49891	1.04347	2.1
49892	0.523075	2.1
49893	1.89226	0.0864527
49894	1.89226	0.109006
49895	1.89226	0.177222
49896	1.89226	0.0507008
49897	3.78453	0.0590173
49898	1.39237	0.102681
49899	1.17933	0.485237
49900	0.755796	0.0921274
49901	0.755796	0.313984
49902	0.755767	0.0712566
49903	0.94613	0.655716
49904	1.15918	0.085589
49905	0.94613	0.127626
49906	1.11632	0.0910842
49907	0.92595	0.104478
49908	0.94613	0.0823923
49909	0.94613	0.0888024
49910	1.89226	0.295905
49911	1.89226	0.0735199
49912	1.89226	0.0515063
49913	1.89226	0.0745754
49914	3.78452	0.0639107
49915	0.950067	0.0630571
49916	0.75071	0.0548935
49917	0.906516	0.05
49918	0.906516	0.0516012
49919	0.81586	0.05
49920	0.851541	0.129414
49921	0.962101	0.05
49922	0.851476	0.0591163
49923	0.962105	0.05
49924	0.815808	0.052792
49925	0.851529	0.0805042
49926	0.851529	0.0586662
49927	1.05032	0.0653922
49928	0.972326	0.0653934
49929	0.906515	0.0506334
49930	0.906515	0.0527916
49931	1.12245	0.0527092
49932	1.04072	0.0519219
49933	0.815857	0.0513396
49934	0.851539	0.081858
49935	1.04451	0.0526646

49936	0.815853	0.0733088
49937	0.919795	0.0738781
49938	0.919795	0.0683707
49939	1.5855	2.1
49940	2.14058	2.1
49941	1.9153	2.1
49942	1.79922	2.1
49943	3.72608	2.1
49944	1.58551	2.1
49945	1.24201	2.1
49946	0.960287	2.1
49947	1.79922	2.1
49948	2.82752	2.1
49949	0.679602	2.1
49950	0.621009	2.1
49951	0.899614	2.1
49952	0.940441	2.1
49953	0.679602	2.1
49954	0.94044	2.1
49955	0.621009	2.1
49956	0.899615	2.1
49957	0.794027	2.1
49958	0.794027	2.1
49959	0.785189	2.1
49960	0.785189	2.1
49961	2.14058	2.1
49962	1.79922	2.1
49963	2.23742	2.1
49964	2.23742	2.1
49965	1.78404	2.1
49966	1.88088	2.1
49967	1.15177	2.1
49968	2.0815	2.1
49969	1.93765	2.1
49970	1.78342	2.1
49971	1.39511	2.1
49972	1.28406	2.1
49973	1.97719	2.1
49974	2.50057	2.04359
49975	2.35349	2.1
49976	2.35349	1.99857
49977	2.31686	2.1
49978	2.32857	2.05511
49979	1.93765	2.1
49980	2.35349	2.06188
49981	1.70048	2.1
49982	2.15721	1.7031
49983	4.05397	2.1
49984	0.850388	2.1
49985	0.850388	2.1
49986	0.840019	2.1
49987	0.840019	2.1
49988	1.13736	2.1
49989	1.13852	2.1
49990	0.573661	2.1
49991	0.551891	2.1
49992	0.573661	2.1
49993	1.13852	2.1

49994	0.551892	2.1
49995	1.13736	2.1
49996	0.860203	2.1
49997	0.860203	2.1
49998	0.855758	2.1
49999	0.855758	2.1
50000	1.1423	2.1
50001	1.13852	2.1
50002	0.58628	2.1
50003	0.573661	2.1
50004	0.586281	2.1
50005	1.1423	2.1
50006	0.573661	2.1
50007	1.13852	2.1
50008	1.28918	1.31489
50009	0.999086	1.25634
50010	1.0383	1.4916
50011	0.736019	1.4299
50012	1.00954	1.46865
50013	1.00954	1.32497
50014	1.01566	1.24381
50015	1.01566	1.42117
50016	1.28918	1.4036
50017	1.0383	1.43906
50018	0.999086	1.39874
50019	0.736019	1.40165
50020	2.20797	2.1
50021	2.64748	2.1
50022	2.50375	2.1
50023	2.50375	2.1
50024	2.40858	2.1
50025	2.40858	2.1
50026	2.64748	2.1
50027	2.74744	2.1
50028	2.50375	2.1
50029	2.6521	2.1
50030	5.29958	2.1
50031	2.20797	2.1
50032	2.50375	2.1
50033	2.02064	2.1
50034	2.3606	2.1
50035	4.52439	2.1
50036	2.36059	2.1
50037	2.6521	2.1
50038	2.56952	2.1
50039	2.56952	2.1
50040	2.50375	2.1
50041	2.50375	2.1
50042	2.49439	0.0909284
50043	1.89226	0.171426
50044	2.81337	0.155665
50045	1.89226	0.132389
50046	4.38665	0.123786
50047	1.89226	0.129706
50048	1.89226	0.366742
50049	1.89226	0.363937
50050	1.89226	0.30709
50051	3.78452	0.271683

50052	1.89226	0.171826
50053	1.89226	0.258591
50054	1.89226	0.219456
50055	1.89226	0.271899
50056	3.78452	0.240934
50057	1.93948	0.382151
50058	1.98874	0.205935
50059	1.62494	0.243268
50060	2.14218	0.39556
50061	1.62494	0.0866719
50062	2.14218	0.155768
50063	1.47354	0.240979
50064	1.98874	0.159474
50065	2.01865	0.24947
50066	1.62495	0.130131
50067	2.01865	0.236857
50068	1.62495	0.115069
50069	1.89226	2.1
50070	1.89226	2.1
50071	1.89226	2.1
50072	1.89226	2.1
50073	3.78453	2.1
50074	2.05257	2.1
50075	1.94441	2.1
50076	1.88487	2.1
50077	1.82441	2.1
50078	3.82928	2.1
50079	1.94441	2.1
50080	1.74391	2.1
50081	1.82441	2.1
50082	1.65636	2.1
50083	3.60077	2.1
50084	1.89226	2.1
50085	1.89226	2.1
50086	1.89226	2.1
50087	1.89226	2.1
50088	3.78452	2.1
50089	1.89226	2.1
50090	1.89226	2.1
50091	1.89226	2.1
50092	1.89226	2.1
50093	3.78453	2.1
50094	1.88487	2.1
50095	1.82441	2.1
50096	1.75835	2.1
50097	1.72866	2.1
50098	3.61354	2.1
50099	1.82441	2.1
50100	1.65636	2.1
50101	1.72866	2.1
50102	1.58401	2.1
50103	3.38503	2.1
50104	1.89226	2.1
50105	1.89226	2.1
50106	1.89226	2.1
50107	1.89226	2.1
50108	3.78452	2.1
50109	2.27432	2.1

50110	1.33339	2.1
50111	1.38905	2.1
50112	2.34471	2.1
50113	3.66337	2.1
50114	1.89226	2.1
50115	2.34471	2.1
50116	2.27432	2.1
50117	2.27432	2.1
50118	1.89226	2.1
50119	1.89226	2.1
50120	1.20443	2.1
50121	1.17235	2.1
50122	0.724695	2.1
50123	0.694522	2.1
50124	0.949994	2.1
50125	0.949993	2.1
50126	0.948955	2.1
50127	0.948955	2.1
50128	1.20443	2.1
50129	0.7247	2.1
50130	0.694517	2.1
50131	1.17234	2.1
50132	1.89225	2.1
50133	2.40885	2.1
50134	2.3447	2.1
50135	2.3447	2.1
50136	1.89225	2.1
50137	1.89225	2.1
50138	1.37138	2.1
50139	1.82597	2.1
50140	1.45027	2.1
50141	1.92772	2.1
50142	3.27624	2.1
50143	1.89226	2.1
50144	1.89226	2.1
50145	1.89226	2.1
50146	1.89226	2.1
50147	3.78452	2.1
50148	1.89226	2.1
50149	1.89226	2.1
50150	1.89226	2.1
50151	1.89226	2.1
50152	3.78452	2.1
50153	1.82597	2.1
50154	2.02312	1.74413
50155	1.92772	2.1
50156	2.15888	2.1
50157	3.98485	2.1
50158	1.53446	2.1
50159	1.92772	2.1
50160	2.03975	2.1
50161	2.03975	2.1
50162	1.45027	2.1
50163	1.45027	2.1
50164	1.89226	2.1
50165	1.89226	2.1
50166	1.89226	2.1
50167	1.89226	2.1

50168	1.89226	2.1
50169	1.89226	2.1
50170	1.89226	2.1
50171	1.89226	2.1
50172	1.89226	2.1
50173	1.89226	2.1
50174	3.78452	2.1
50175	1.92772	2.1
50176	2.15888	2.1
50177	2.03975	2.1
50178	2.3111	2.1
50179	4.19863	2.1
50180	1.89226	2.1
50181	1.89226	2.1
50182	1.89226	2.1
50183	1.89226	2.1
50184	3.78452	2.1
50185	1.78376	2.1
50186	1.3201	2.1
50187	1.78376	2.1
50188	1.86102	2.1
50189	1.2679	2.1
50190	1.34517	2.1
50191	1.89226	2.1
50192	2.52442	2.1
50193	1.89226	2.1
50194	2.51691	2.1
50195	4.41667	2.1
50196	1.89226	2.1
50197	1.89226	2.1
50198	1.89226	2.1
50199	1.89226	2.1
50200	3.78452	2.1
50201	1.78376	2.1
50202	1.2679	2.1
50203	1.71839	2.1
50204	1.23504	2.1
50205	3.0188	2.1
50206	1.89226	2.1
50207	2.51795	2.1
50208	2.51691	2.1
50209	2.51691	2.1
50210	1.89226	2.1
50211	1.89226	2.1
50212	1.89226	0.567067
50213	1.89226	1.16324
50214	1.89226	1.25791
50215	1.89226	0.930286
50216	3.78452	1.00863
50217	1.89227	0.707386
50218	1.89227	1.29223
50219	3.26088	1.06346
50220	2.79543	1.26341
50221	2.20733	1.21298
50222	1.89227	1.28049
50223	1.89226	1.20075
50224	1.89226	1.06273
50225	1.89226	0.857804

50226	1.89226	1.22913
50227	1.89226	0.864209
50228	1.89226	1.23953
50229	1.89227	0.651649
50230	2.20733	1.24599
50231	1.46973	1.13791
50232	1.89227	1.06373
50233	1.7848	1.11987
50234	2.20733	1.1062
50235	1.89226	2.1
50236	2.55682	2.1
50237	2.81593	2.1
50238	2.81593	2.1
50239	1.89226	2.1
50240	1.89226	2.1
50241	1.0607	2.1
50242	1.0607	2.1
50243	1.12617	2.1
50244	0.986957	2.1
50245	0.699328	2.1
50246	0.785567	2.1
50247	1.28855	2.1
50248	1.41913	2.1
50249	0.766737	2.1
50250	1.34711	2.1
50251	1.24643	2.1
50252	0.687289	2.1
50253	3.11063	0.355585
50254	1.89226	0.533734
50255	1.96536	0.433805
50256	1.89226	0.458225
50257	1.96536	0.390297
50258	1.89226	0.409298
50259	1.89226	1.34101
50260	1.89226	0.758281
50261	1.89226	1.00976
50262	1.89226	0.852764
50263	3.78452	1.33452
50264	1.89226	0.496254
50265	1.96256	0.233029
50266	1.89226	0.319134
50267	1.89226	0.467239
50268	2.49192	0.444346
50269	2.49192	0.281893
50270	1.85716	0.454177
50271	2.56871	0.289394
50272	1.03043	0.312871
50273	1.03043	0.369963
50274	2.48125	0.217533
50275	1.65452	0.398425
50276	1.89226	2.1
50277	2.09622	2.1
50278	2.09622	2.1
50279	2.08508	2.1
50280	1.89226	2.1
50281	1.88112	2.1
50282	1.04811	2.1
50283	1.04811	2.1

50284	0.946131	2.1
50285	0.946131	2.1
50286	1.05014	2.1
50287	1.04811	2.1
50288	0.94613	2.1
50289	0.94613	2.1
50290	0.946131	2.1
50291	1.05013	2.1
50292	1.04811	2.1
50293	0.946131	2.1
50294	1.398	2.1
50295	2.10766	2.1
50296	2.3018	2.1
50297	2.3018	2.1
50298	1.34008	2.1
50299	1.34008	2.1
50300	1.1509	2.1
50301	1.1509	2.1
50302	0.946132	2.1
50303	0.946132	2.1
50304	0.946132	2.1
50305	0.946132	2.1
50306	1.05383	2.1
50307	1.1509	2.1
50308	1.1509	2.1
50309	0.946133	2.1
50310	0.946132	2.1
50311	1.05383	2.1
50312	0.931141	2.1
50313	0.931141	2.1
50314	0.971946	2.1
50315	0.971946	2.1
50316	1.28569	2.1
50317	1.1509	2.1
50318	0.699	2.1
50319	0.752185	2.1
50320	1.28568	2.1
50321	0.752186	2.1
50322	0.699001	2.1
50323	1.1509	2.1
50324	2.30181	2.1
50325	1.89226	2.1
50326	1.89226	2.1
50327	1.89226	2.1
50328	2.57137	2.1
50329	2.57137	2.1
50330	2.0815	2.1
50331	1.21962	2.1
50332	1.55173	2.1
50333	2.57849	2.1
50334	3.79001	2.1
50335	1.38779	2.1
50336	1.2923	2.1
50337	1.03974	2.1
50338	1.28832	2.1
50339	1.33566	2.1
50340	1.21116	2.1
50341	1.22343	2.1

50342	1.22343	2.1
50343	1.42288	2.1
50344	1.20138	2.1
50345	1.26025	2.1
50346	1.00644	2.1
50347	1.26066	2.1
50348	1.14271	2.1
50349	0.886635	2.1
50350	0.795277	2.1
50351	1.02966	2.1
50352	1.02967	2.1
50353	1.05216	2.1
50354	1.05216	2.1
50355	1.26068	2.1
50356	0.790042	2.1
50357	0.795269	2.1
50358	1.20135	2.1
50359	1.89226	0.642559
50360	1.89226	0.392015
50361	1.89226	0.435503
50362	1.89226	0.330134
50363	1.89226	0.911423
50364	1.89226	0.597851
50365	1.89227	0.392709
50366	1.89227	0.316913
50367	1.89227	0.347637
50368	1.89227	0.273141
50369	1.89227	0.526463
50370	1.89227	0.376317
50371	0.563873	0.246535
50372	0.50988	0.218687
50373	0.598693	0.306316
50374	0.558819	0.259326
50375	0.564855	0.241647
50376	0.564855	0.225822
50377	0.557838	0.291672
50378	0.557838	0.266297
50379	0.563873	0.261568
50380	0.598694	0.250194
50381	0.558819	0.247093
50382	0.50988	0.257959
50383	0.598693	0.297631
50384	0.558819	0.252754
50385	0.572737	0.397206
50386	0.534248	0.324324
50387	0.566788	0.30271
50388	0.566788	0.273339
50389	0.566153	0.355471
50390	0.566153	0.321226
50391	0.598694	0.31816
50392	0.572737	0.311569
50393	0.534248	0.298658
50394	0.558819	0.296937
50395	1.85217	2.1
50396	1.93111	2.1
50397	1.92875	2.1
50398	2.02391	2.1
50399	3.85986	2.1

50400	1.93112	2.1
50401	1.80425	2.1
50402	2.02392	2.1
50403	1.90981	2.1
50404	3.84092	2.1
50405	1.92876	2.1
50406	2.02393	2.1
50407	2.05274	2.1
50408	2.16783	2.1
50409	4.09659	2.1
50410	2.02393	2.1
50411	1.90982	2.1
50412	2.16784	2.1
50413	2.05967	2.1
50414	4.07766	2.1
50415	1.89226	1.83662
50416	1.89226	1.69174
50417	1.89226	1.77726
50418	1.89226	1.76072
50419	3.78453	1.68708
50420	1.89226	1.66262
50421	1.89226	1.79112
50422	1.89226	2.00884
50423	2.44829	1.6283
50424	3.78453	1.80435
50425	1.89226	1.85103
50426	1.89226	1.85909
50427	1.89226	1.8482
50428	2.60452	1.75064
50429	3.78453	1.9319
50430	1.58723	0.05
50431	1.88071	0.0651214
50432	2.97376	0.05
50433	2.13255	0.0552165
50434	4.43982	0.051757
50435	2.20641	0.093878
50436	1.33575	0.136578
50437	2.15824	0.110057
50438	2.15824	0.10821
50439	1.2951	0.111187
50440	1.2951	0.114858
50441	0.825383	0.150051
50442	0.825383	0.128627
50443	0.856104	0.111598
50444	0.856104	0.120829
50445	0.952196	0.143534
50446	0.602363	0.126086
50447	0.667877	0.140532
50448	1.07913	0.112533
50449	0.602363	0.139787
50450	0.667876	0.136815
50451	1.07913	0.136047
50452	0.952197	0.125832
50453	2.57657	2.1
50454	1.44437	2.1
50455	2.65378	2.1
50456	2.25076	2.1
50457	1.84739	2.1

50458	1.44437	2.1
50459	2.0189	0.125421
50460	2.54752	0.128049
50461	2.63975	0.161598
50462	2.31049	0.0987989
50463	4.65865	0.128159
50464	1.40707	0.0877459
50465	1.89605	0.0871093
50466	2.0189	0.0944093
50467	2.0189	0.0792737
50468	1.50438	0.102202
50469	1.50438	0.0854503
50470	2.63971	0.152314
50471	2.38246	0.102236
50472	2.13402	0.117859
50473	2.57503	0.110947
50474	4.77373	0.115636
50475	1.50436	0.086257
50476	1.96803	0.0891243
50477	2.13402	0.108886
50478	1.45446	0.0722602
50479	3.63838	0.0881486
50480	2.30793	0.164292
50481	2.11032	0.143021
50482	1.24082	0.143135
50483	1.05689	0.177271
50484	3.36482	0.161888
50485	1.89226	0.217054
50486	1.89226	0.30504
50487	1.89226	0.321963
50488	1.89226	0.258321
50489	3.78453	0.265484
50490	0.812967	0.204908
50491	0.812967	0.211694
50492	1.00181	0.18842
50493	1.08141	0.185143
50494	0.946132	0.181815
50495	0.812938	0.232856
50496	0.932815	0.226162
50497	1.05577	0.172804
50498	0.812938	0.209531
50499	0.932812	0.205563
50500	0.946133	0.203634
50501	0.946133	0.192206
50502	2.53897	2.1
50503	1.89226	2.1
50504	1.89226	2.1
50505	1.89226	2.1
50506	2.3245	2.1
50507	2.3245	2.1
50508	1.00255	2.1
50509	1.06561	2.1
50510	1.05481	0.993535
50511	1.11077	2.1
50512	2.12042	2.1
50513	1.89226	1.97215
50514	1.89226	2.1
50515	1.89226	1.23827

50516	1.89226	2.1
50517	3.78453	2.1
50518	1.89226	2.1
50519	2.77314	2.1
50520	2.81276	2.1
50521	2.81276	2.1
50522	1.89226	2.1
50523	1.89226	2.1
50524	1.11077	2.1
50525	1.15114	2.1
50526	1.10274	2.1
50527	1.1032	2.1
50528	1.05481	2.1
50529	1.1032	1.93028
50530	1.89226	1.95384
50531	1.89226	2.1
50532	1.89226	0.530839
50533	1.89226	2.1
50534	3.78452	2.1
50535	0.721957	0.385853
50536	0.721957	0.417606
50537	0.946103	0.36995
50538	0.946103	0.284797
50539	0.814237	0.292859
50540	0.721929	0.271166
50541	0.946132	0.455496
50542	0.946132	0.397192
50543	0.721928	0.410046
50544	0.946131	0.487228
50545	0.946132	0.628329
50546	0.814237	0.376898
50547	1.89226	1.24237
50548	1.89226	0.593958
50549	1.89226	0.638848
50550	1.89226	0.68924
50551	1.89226	1.21475
50552	1.89226	1.21556
50553	1.37196	0.372299
50554	2.83556	0.240633
50555	1.5165	0.316783
50556	1.5165	0.377744
50557	2.74923	0.273728
50558	2.74923	0.333075
50559	0.946128	0.551002
50560	0.946128	0.798613
50561	0.827866	0.357244
50562	0.946132	0.465967
50563	0.94613	0.338759
50564	0.94613	0.372608
50565	0.94613	0.828486
50566	0.94613	0.712614
50567	0.94613	0.619023
50568	0.827864	0.751075
50569	0.94613	0.586161
50570	0.94613	1.02265
50571	1.36208	0.276138
50572	2.74923	0.197244
50573	1.37196	0.238952

50574	1.37196	0.267034
50575	2.53256	0.217159
50576	2.53256	0.249466
50577	1.07705	2.1
50578	1.07705	2.1
50579	1.08253	2.1
50580	1.08253	2.1
50581	0.762985	2.1
50582	0.781638	2.1
50583	1.37794	2.1
50584	1.40756	2.1
50585	1.37794	2.1
50586	0.762986	2.1
50587	0.781638	2.1
50588	1.40756	2.1
50589	2.69541	2.1
50590	2.75587	2.1
50591	1.48572	2.1
50592	1.48572	2.1
50593	1.52597	2.1
50594	2.73566	2.1
50595	2.0554	1.68435
50596	1.00387	1.32188
50597	2.01001	1.4318
50598	0.994002	1.19052
50599	3.01389	1.07051
50600	1.89226	2.1
50601	2.41579	2.1
50602	2.12209	2.1
50603	2.12209	2.1
50604	1.89226	2.1
50605	1.89226	2.1
50606	1.89226	2.1
50607	1.89226	2.1
50608	1.89226	2.1
50609	1.89226	2.1
50610	1.89226	2.1
50611	1.89226	2.1
50612	0.88209	2.1
50613	0.899471	2.1
50614	0.651501	2.1
50615	0.533531	2.1
50616	0.724105	2.1
50617	0.711243	2.1
50618	0.692622	2.1
50619	0.692622	2.1
50620	0.890668	2.1
50621	0.738066	2.1
50622	0.778223	2.1
50623	0.531121	2.1
50624	1.89226	2.1
50625	2.77504	2.1
50626	2.4158	2.1
50627	2.4158	2.1
50628	1.89226	2.1
50629	1.89226	2.1
50630	1.89226	2.1
50631	1.89226	2.1

50632	1.89226	2.1
50633	1.89226	2.1
50634	1.89226	2.1
50635	1.89226	2.1
50636	1.70303	2.1
50637	1.39832	2.1
50638	1.51749	2.1
50639	1.68783	2.1
50640	3.24844	2.1
50641	1.89226	2.1
50642	2.38311	2.1
50643	1.98321	2.1
50644	1.89226	2.1
50645	2.47406	2.1
50646	2.38311	2.1
50647	1.89226	2.1
50648	1.89226	2.1
50649	1.89226	2.1
50650	1.89226	2.1
50651	1.89226	2.1
50652	1.89226	2.1
50653	0.946131	2.1
50654	0.946131	2.1
50655	1.25167	2.1
50656	1.19156	2.1
50657	1.19156	2.1
50658	1.19156	2.1
50659	0.946133	2.1
50660	0.946133	2.1
50661	0.946131	2.1
50662	1.25167	2.1
50663	0.946131	2.1
50664	1.19156	2.1
50665	1.89226	2.1
50666	2.47406	2.1
50667	1.99996	2.1
50668	1.89226	2.1
50669	2.58176	2.1
50670	2.47406	2.1
50671	1.89226	2.1
50672	1.89226	2.1
50673	1.89226	2.1
50674	1.89226	2.1
50675	1.89226	2.1
50676	1.89226	2.1
50677	0.946131	2.1
50678	0.946131	2.1
50679	1.32241	2.1
50680	1.25167	2.1
50681	1.25167	2.1
50682	1.25167	2.1
50683	0.946133	2.1
50684	0.946133	2.1
50685	0.946131	2.1
50686	1.32241	2.1
50687	0.946131	2.1
50688	1.25167	2.1
50689	1.65151	2.1

50690	1.92471	2.1
50691	1.81834	2.1
50692	2.13272	2.1
50693	3.74306	2.1
50694	1.92472	2.1
50695	1.9943	2.1
50696	2.13273	2.1
50697	2.24475	2.1
50698	4.16947	2.1
50699	1.81834	2.1
50700	2.13272	2.1
50701	2.02687	2.1
50702	2.39605	2.1
50703	4.21439	2.1
50704	2.13273	2.1
50705	2.24475	2.1
50706	2.39605	2.1
50707	2.54903	2.1
50708	4.6408	2.1
50709	1.10534	2.1
50710	1.22993	2.1
50711	0.946132	2.1
50712	0.946132	2.1
50713	0.946132	2.1
50714	0.946132	2.1
50715	1.22993	2.1
50716	1.22993	2.1
50717	1.10534	2.1
50718	0.946131	2.1
50719	0.946132	2.1
50720	1.22993	2.1
50721	1.47221	2.1
50722	2.14647	2.1
50723	1.95567	2.1
50724	1.95567	2.1
50725	1.71082	2.1
50726	1.71082	2.1
50727	0.946131	2.1
50728	0.946131	2.1
50729	0.946131	2.1
50730	0.946131	2.1
50731	0.946132	2.1
50732	0.946132	2.1
50733	0.946131	2.1
50734	0.946131	2.1
50735	0.946131	2.1
50736	0.946131	2.1
50737	0.946132	2.1
50738	0.946132	2.1
50739	0.998086	2.1
50740	1.09429	2.1
50741	0.946125	2.1
50742	0.946125	2.1
50743	0.90732	2.1
50744	0.90732	2.1
50745	1.14414	2.1
50746	1.16553	2.1
50747	1.05018	2.1

50748	0.907332	2.1
50749	0.99482	2.1
50750	0.851529	2.1
50751	0.80534	2.1
50752	0.80534	2.1
50753	0.812206	2.1
50754	0.812206	2.1
50755	0.895134	2.1
50756	0.977828	2.1
50757	0.736099	2.1
50758	0.639718	2.1
50759	0.639722	2.1
50760	0.89514	2.1
50761	0.736095	2.1
50762	0.977821	2.1
50763	0.946125	2.1
50764	0.946125	2.1
50765	0.946125	2.1
50766	0.946125	2.1
50767	0.946125	2.1
50768	0.946125	2.1
50769	0.946124	2.1
50770	0.946124	2.1
50771	0.946131	2.1
50772	0.946131	2.1
50773	0.946118	2.1
50774	0.946118	2.1
50775	2.22622	0.05
50776	2.43347	0.05
50777	2.34108	0.05
50778	1.24587	2.1
50779	1.21674	2.1
50780	1.14533	2.1
50781	1.11311	2.1
50782	1.17874	2.1
50783	1.17874	2.1
50784	1.18024	2.1
50785	1.18024	2.1
50786	1.14533	2.1
50787	1.24587	2.1
50788	1.21674	2.1
50789	1.11311	2.1
50790	2.43347	2.1
50791	2.34107	2.1
50792	2.39663	2.1
50793	2.49174	2.1
50794	4.8301	2.1
50795	1.86206	2.1
50796	1.41058	2.1
50797	1.70302	2.1
50798	2.42083	2.1
50799	3.68129	2.1
50800	1.70302	2.1
50801	2.43549	2.1
50802	2.20075	2.1
50803	2.50358	2.1
50804	2.04916	2.1
50805	2.33113	2.1

50806	1.07624	2.1
50807	2.12621	2.1
50808	1.57297	2.1
50809	1.60383	2.1
50810	1.30047	2.1
50811	1.30047	2.1
50812	2.33379	2.1
50813	2.56149	1.71704
50814	2.36734	2.1
50815	2.62796	1.84897
50816	4.92883	2.05652
50817	2.5615	1.63203
50818	2.57957	1.6823
50819	2.62796	1.82267
50820	2.69056	1.38675
50821	5.25206	1.56375
50822	2.39322	2.1
50823	2.62796	2.1
50824	2.6849	2.1
50825	2.6849	2.09493
50826	2.36734	2.1
50827	2.36734	2.04612
50828	2.62796	2.1
50829	2.69056	1.7479
50830	2.6849	1.56674
50831	2.77227	1.36074
50832	5.37546	1.56459
50833	1.89226	0.05
50834	1.89226	0.05
50835	1.89226	0.05
50836	1.89226	0.05
50837	1.89226	0.05
50838	1.89226	0.05
50839	1.06974	0.05
50840	1.06974	0.05
50841	1.02299	0.05
50842	1.02299	0.05
50843	1.35299	0.05
50844	1.26227	0.05
50845	0.646252	0.05
50846	0.830464	0.05
50847	0.830463	0.05
50848	1.35299	0.05
50849	1.26227	0.05
50850	0.646252	0.05
50851	2.52454	0.05
50852	1.89226	0.05
50853	2.70598	0.05
50854	1.89226	0.05
50855	4.59824	0.05
50856	1.89226	0.05
50857	1.89226	0.05
50858	1.89226	0.05
50859	1.89226	0.05
50860	1.89226	0.05
50861	1.89226	0.05
50862	1.89226	0.05
50863	1.89226	0.05

50864	1.89226	0.05
50865	1.89226	0.05
50866	1.89226	0.05
50867	1.89226	0.05
50868	1.7666	0.05
50869	2.70598	0.05
50870	2.65378	0.05
50871	2.65378	0.05
50872	1.66093	0.05
50873	1.66093	0.05
50874	2.70598	0.05
50875	1.89226	0.05
50876	1.89226	0.05
50877	1.89226	0.05
50878	2.65377	0.05
50879	2.65377	0.05
50880	0.946132	0.05
50881	0.946132	0.05
50882	0.946131	0.05
50883	0.946131	0.05
50884	0.946131	0.05
50885	0.946131	0.05
50886	0.946132	0.05
50887	0.946132	0.05
50888	0.946132	0.05
50889	0.946132	0.05
50890	0.946131	0.05
50891	0.946131	0.05
50892	1.69858	2.1
50893	2.18238	2.1
50894	2.506	2.1
50895	1.93586	2.1
50896	4.20458	2.1
50897	2.21772	2.1
50898	2.18238	2.1
50899	2.506	2.1
50900	2.59215	2.1
50901	4.72372	2.1
50902	2.50596	2.1
50903	1.93584	2.1
50904	2.16728	2.1
50905	2.7679	2.1
50906	4.67325	2.1
50907	1.53296	2.1
50908	1.56299	2.1
50909	1.42624	2.1
50910	1.52961	2.1
50911	2.98923	2.1
50912	1.56299	2.1
50913	1.46568	2.1
50914	1.52961	2.1
50915	1.51916	2.1
50916	3.08215	2.1
50917	1.36226	2.1
50918	1.52961	2.1
50919	1.36226	2.1
50920	1.42624	2.1
50921	1.55369	2.1

50922	1.61768	2.1
50923	1.52961	2.1
50924	1.51916	2.1
50925	1.55369	2.1
50926	1.64892	2.1
50927	3.07285	2.1
50928	2.13405	0.113119
50929	2.57507	0.114186
50930	2.39438	0.148933
50931	1.93408	0.0954757
50932	4.52843	0.116168
50933	2.13405	0.104622
50934	1.27778	0.0757414
50935	1.2545	0.0891511
50936	1.45448	0.086455
50937	1.93408	0.0853999
50938	2.13405	0.0848433
50939	2.39438	0.136655
50940	1.93407	0.0958823
50941	1.60578	0.11009
50942	1.87985	0.108909
50943	4.00016	0.110469
50944	1.0525	2.0649
50945	1.05758	2.05689
50946	1.43484	1.25143
50947	1.38506	1.70514
50948	2.48734	1.73
50949	1.20455	2.1
50950	1.67352	2.1
50951	1.52852	2.1
50952	2.05164	2.1
50953	3.20203	2.1
50954	1.89226	2.1
50955	1.89226	2.1
50956	1.89226	2.1
50957	1.89226	2.1
50958	3.78452	2.1
50959	1.89226	2.1
50960	1.89226	2.1
50961	1.89226	2.1
50962	1.89226	2.1
50963	1.89226	2.1
50964	1.89226	2.1
50965	2.05165	2.1
50966	1.94291	2.1
50967	2.09398	2.1
50968	1.67352	2.1
50969	2.36337	2.1
50970	1.94291	2.1
50971	1.52851	2.1
50972	2.52049	2.1
50973	2.43433	2.1
50974	2.05164	2.1
50975	1.9112	2.1
50976	1.52851	2.1
50977	1.89226	2.1
50978	1.89226	2.1
50979	1.89226	2.1

50980	1.89226	2.1
50981	3.78451	2.1
50982	1.89226	2.1
50983	1.89226	2.1
50984	1.89226	2.1
50985	1.89226	2.1
50986	3.78452	2.1
50987	2.5205	2.1
50988	2.36337	2.1
50989	2.4605	2.1
50990	2.05165	2.1
50991	2.77222	2.1
50992	2.36337	2.1
50993	1.89226	2.1
50994	1.89226	2.1
50995	1.89226	2.1
50996	1.89226	2.1
50997	1.89226	2.1
50998	1.89226	2.1
50999	1.89226	2.1
51000	1.89226	2.1
51001	1.89226	2.1
51002	1.89226	2.1
51003	3.78452	2.1
51004	1.38905	2.1
51005	1.26166	2.1
51006	1.19216	2.1
51007	1.0875	2.1
51008	1.24393	2.1
51009	1.24393	2.1
51010	1.23261	2.1
51011	1.23261	2.1
51012	1.38905	2.1
51013	1.19216	2.1
51014	1.26166	2.1
51015	1.0875	2.1
51016	1.16233	2.1
51017	1.06028	2.1
51018	0.777395	2.1
51019	0.705508	2.1
51020	0.940018	2.1
51021	0.936465	2.1
51022	0.954216	2.1
51023	0.954216	2.1
51024	1.16125	2.1
51025	0.685505	2.1
51026	1.1157	2.1
51027	0.703534	2.1
51028	1.89226	2.1
51029	1.89226	2.1
51030	1.89226	2.1
51031	1.89226	2.1
51032	1.89226	2.1
51033	1.89226	2.1
51034	1.89226	2.1
51035	1.89226	2.1
51036	1.89226	2.1
51037	1.89226	2.1

51038	1.89226	2.1
51039	1.89226	2.1
51040	2.38433	2.1
51041	1.43456	2.1
51042	1.93881	2.1
51043	2.36952	2.1
51044	4.32313	2.1
51045	1.8493	2.1
51046	1.5927	2.1
51047	1.45535	2.1
51048	1.38438	2.1
51049	1.89657	2.1
51050	1.8493	2.1
51051	2.0815	2.1
51052	1.6697	2.1
51053	2.0657	2.1
51054	1.5927	2.1
51055	2.59749	2.1
51056	2.0815	2.1
51057	2.76597	2.1
51058	1.89226	2.1
51059	1.89226	2.1
51060	1.89226	2.1
51061	2.6126	2.1
51062	2.6126	2.1
51063	2.62513	2.1
51064	1.89226	2.1
51065	1.89226	2.1
51066	1.89226	2.1
51067	2.76598	2.1
51068	2.76598	2.1
51069	2.54665	2.1
51070	2.6126	2.1
51071	2.76598	2.1
51072	2.76598	2.1
51073	2.34044	2.1
51074	2.34044	2.1
51075	2.62514	2.1
51076	2.76598	2.1
51077	2.54666	2.1
51078	2.45212	2.1
51079	5.2181	2.1
51080	2.14063	2.1
51081	1.47521	2.1
51082	1.27679	2.1
51083	1.47521	2.1
51084	2.11511	2.1
51085	2.31353	2.1
51086	0.93256	2.1
51087	1.07032	2.1
51088	0.638396	2.1
51089	0.521342	2.1
51090	0.790674	2.1
51091	0.790674	2.1
51092	0.80098	2.1
51093	0.800981	2.1
51094	0.932535	2.1
51095	0.521328	2.1

51096	1.07034	2.1
51097	0.638414	2.1
51098	1.89226	2.1
51099	1.89226	2.05039
51100	1.89226	2.1
51101	1.89226	2.1
51102	3.78453	2.1
51103	1.89226	2.1
51104	1.9842	2.1
51105	1.89226	2.1
51106	1.89226	2.1
51107	2.79705	2.1
51108	2.79705	2.1
51109	0.469955	2.1
51110	0.791665	2.1
51111	0.989115	2.09179
51112	0.763235	2.1
51113	0.776356	2.08937
51114	0.805461	2.1
51115	0.776759	2.1
51116	0.776759	2.1
51117	0.799151	2.1
51118	0.940869	2.1
51119	0.78449	2.1
51120	0.489635	2.1
51121	1.89226	2.04929
51122	1.89226	2.1
51123	1.89226	1.95028
51124	1.89226	2.1
51125	1.89226	1.94504
51126	1.89226	2.1
51127	2.46058	1.8905
51128	2.28991	2.1
51129	1.8525	2.06424
51130	1.87697	2.1
51131	2.28991	2.03181
51132	1.62397	2.09752
51133	0.946131	0.05
51134	0.974033	0.05
51135	1.31667	0.05
51136	0.946131	0.05
51137	0.885497	0.05
51138	0.885497	0.05
51139	1.0468	0.05
51140	1.04678	0.05
51141	1.31667	0.05
51142	0.974019	0.05
51143	0.946131	0.05
51144	0.946131	0.05
51145	1.89226	0.0559834
51146	1.89226	0.0829917
51147	1.89226	0.0777241
51148	1.89226	0.0692061
51149	3.78452	0.0861473
51150	0.946125	0.0878917
51151	0.946125	0.0856677
51152	0.946131	0.0967792
51153	0.946131	0.10135

51154	0.946128	0.101328
51155	0.946128	0.0910232
51156	0.946128	0.0667466
51157	0.946128	0.0815561
51158	0.946128	0.0975099
51159	0.946128	0.0833152
51160	0.946128	0.0839062
51161	0.946128	0.0863501
51162	0.946131	0.05
51163	0.98453	0.05
51164	1.32276	0.0533941
51165	0.946131	0.05
51166	0.946132	0.05
51167	0.946132	0.05
51168	0.98453	0.05
51169	1.32276	0.05
51170	1.32276	0.05
51171	1.32276	0.05
51172	0.946132	0.05
51173	0.946132	0.05
51174	1.89227	2.1
51175	1.89227	2.1
51176	1.89227	2.1
51177	1.89227	2.1
51178	1.89227	2.1
51179	1.89227	2.1
51180	1.89226	2.1
51181	1.89226	2.1
51182	1.89226	2.1
51183	1.89226	2.1
51184	1.89226	2.1
51185	1.89226	2.1
51186	1.05556	2.1
51187	1.38859	2.1
51188	1.46996	2.1
51189	1.25364	2.1
51190	2.52552	2.1
51191	0.694296	2.1
51192	0.73498	2.1
51193	0.561881	2.1
51194	0.512189	2.1
51195	0.621304	2.1
51196	0.621304	2.1
51197	0.625865	2.1
51198	0.625865	2.1
51199	0.734979	2.1
51200	0.561881	2.1
51201	0.51219	2.1
51202	0.694296	2.1
51203	0.946126	2.1
51204	0.946126	2.1
51205	0.946126	2.1
51206	0.946126	2.1
51207	0.946126	2.1
51208	0.946126	2.1
51209	0.946126	2.1
51210	0.946126	2.1
51211	0.946132	2.1

51212	0.946132	2.1
51213	0.94612	2.1
51214	0.94612	2.1
51215	1.89225	2.1
51216	1.89225	2.1
51217	1.89225	2.1
51218	1.89225	2.1
51219	1.89225	2.1
51220	1.89225	2.1
51221	0.626814	2.1
51222	0.632155	2.1
51223	0.746234	2.1
51224	0.734974	2.1
51225	0.68202	2.1
51226	0.68202	2.1
51227	0.685108	2.1
51228	0.685108	2.1
51229	0.746239	2.1
51230	0.632159	2.1
51231	0.734969	2.1
51232	0.62681	2.1
51233	0.746235	2.1
51234	0.734975	2.1
51235	0.595081	2.1
51236	0.561877	2.1
51237	0.648584	2.1
51238	0.648584	2.1
51239	0.659528	2.1
51240	0.659528	2.1
51241	0.746239	2.1
51242	0.595085	2.1
51243	0.734969	2.1
51244	0.561874	2.1
51245	1.89249	2.1
51246	2.2137	2.1
51247	1.33125	2.1
51248	1.06499	2.1
51249	2.12571	2.1
51250	1.78918	2.1
51251	0.946131	2.1
51252	0.946131	2.1
51253	0.94613	2.1
51254	0.94613	2.1
51255	0.946132	2.1
51256	0.946132	2.1
51257	0.946129	2.1
51258	0.946129	2.1
51259	0.946131	2.1
51260	0.946131	2.1
51261	0.946131	2.1
51262	0.946131	2.1
51263	1.89227	2.1
51264	1.89227	2.1
51265	1.89227	2.1
51266	1.89227	2.1
51267	3.78453	2.1
51268	1.89226	2.1
51269	1.89226	2.1

51270	1.89226	2.1
51271	1.89226	2.1
51272	1.89226	2.1
51273	1.89226	2.1
51274	2.01388	2.1
51275	1.38757	2.1
51276	1.24229	2.1
51277	1.3309	2.1
51278	1.88011	2.1
51279	2.01388	2.1
51280	2.01388	2.1
51281	2.01555	2.1
51282	2.13879	2.1
51283	1.80766	2.1
51284	4.15267	2.1
51285	0.946133	2.1
51286	0.946133	2.1
51287	0.946132	2.1
51288	0.946132	2.1
51289	0.946132	2.1
51290	0.946132	2.1
51291	0.946133	2.1
51292	0.946133	2.1
51293	0.946132	2.1
51294	0.946132	2.1
51295	0.946132	2.1
51296	0.946132	2.1
51297	1.89226	2.1
51298	1.89226	2.1
51299	1.89226	2.1
51300	1.89226	2.1
51301	1.89226	2.1
51302	1.89226	2.1
51303	0.676745	2.1
51304	0.68382	2.1
51305	1.07048	2.1
51306	1.1327	2.1
51307	0.929324	2.1
51308	0.871438	2.1
51309	0.867362	2.1
51310	0.867362	2.1
51311	1.10666	2.1
51312	0.695036	2.1
51313	0.744098	2.1
51314	1.10144	2.1
51315	2.25168	2.1
51316	2.22499	2.1
51317	2.15929	2.1
51318	2.13879	2.1
51319	2.15929	2.1
51320	2.13879	2.1
51321	2.15314	2.1
51322	1.70302	2.1
51323	2.24694	2.1
51324	1.65245	2.1
51325	3.82722	2.1
51326	1.65245	2.1
51327	1.00371	2.1

51328	1.7437	2.1
51329	1.65748	2.1
51330	1.10459	2.1
51331	1.00371	2.1
51332	2.24694	2.1
51333	1.65246	2.1
51334	2.69773	2.1
51335	1.88129	2.1
51336	4.12824	2.1
51337	1.65246	2.1
51338	1.10459	2.1
51339	1.88129	2.1
51340	1.05914	2.1
51341	2.76496	2.1
51342	1.89226	2.1
51343	1.89226	2.1
51344	1.89226	2.1
51345	1.89226	2.1
51346	3.78452	2.1
51347	0.718577	2.1
51348	0.718577	2.1
51349	0.725249	2.1
51350	0.725249	2.1
51351	0.597916	2.1
51352	0.61752	2.1
51353	0.826306	2.1
51354	0.859194	2.1
51355	0.826306	2.1
51356	0.597916	2.1
51357	0.859194	2.1
51358	0.61752	2.1
51359	1.25898	2.1
51360	1.25898	2.1
51361	0.946128	2.1
51362	0.946128	2.1
51363	1.25897	2.1
51364	1.25585	2.1
51365	0.946131	2.1
51366	0.946131	2.1
51367	0.946129	2.1
51368	1.25586	2.1
51369	1.25898	2.1
51370	0.946129	2.1
51371	1.89226	2.1
51372	1.89226	2.1
51373	1.89226	2.1
51374	1.89226	2.1
51375	3.78452	2.1
51376	0.693258	2.1
51377	0.693258	2.1
51378	0.697356	2.1
51379	0.697356	2.1
51380	0.572479	2.1
51381	0.597916	2.1
51382	0.792698	2.1
51383	0.826306	2.1
51384	0.572478	2.1
51385	0.792697	2.1

51386	0.826307	2.1
51387	0.597917	2.1
51388	1.25586	2.1
51389	1.25586	2.1
51390	0.946128	2.1
51391	0.946128	2.1
51392	1.25585	2.1
51393	1.24344	2.1
51394	0.946131	2.1
51395	0.946131	2.1
51396	0.946129	2.1
51397	1.24344	2.1
51398	0.94613	2.1
51399	1.25586	2.1
51400	1.65151	2.1
51401	1.81835	2.1
51402	1.12064	2.1
51403	1.12064	2.1
51404	1.24965	2.1
51405	1.81835	2.1
51406	1.24965	2.1
51407	1.81835	2.1
51408	1.41077	2.1
51409	2.02687	2.1
51410	3.22911	2.1
51411	1.89122	1.90394
51412	1.38776	2.1
51413	2.37014	1.2295
51414	1.80818	2.1
51415	3.6994	2.1
51416	2.15466	1.60234
51417	2.00083	2.1
51418	1.80818	2.08139
51419	1.80818	1.80433
51420	2.31988	2.09173
51421	2.31988	1.66192
51422	1.80818	2.06329
51423	1.18041	2.1
51424	2.00082	2.1
51425	1.43652	2.1
51426	3.18123	2.1
51427	1.89226	1.517
51428	1.89226	1.55625
51429	1.89226	1.52758
51430	1.89226	1.48393
51431	3.78453	1.48725
51432	1.89226	1.6235
51433	1.89226	1.62695
51434	1.89226	1.59333
51435	1.89226	1.50895
51436	3.78452	1.66979
51437	0.80759	1.53904
51438	0.686119	1.58584
51439	0.972147	1.63593
51440	1.00217	1.61347
51441	0.821334	1.77145
51442	0.821334	1.5705
51443	0.866957	1.60934

51444	0.866957	1.5385
51445	0.972145	1.64742
51446	0.686121	2.00742
51447	0.807592	1.69095
51448	1.00217	1.98394
51449	0.972146	1.57412
51450	1.00217	1.54195
51451	1.26654	1.61387
51452	1.1908	1.60553
51453	1.05501	1.58488
51454	1.05501	1.68884
51455	1.10794	1.55762
51456	1.10794	1.477
51457	0.972146	1.42558
51458	1.26654	1.55583
51459	1.00217	1.56791
51460	1.1908	1.67747
51461	1.89226	1.55935
51462	1.89226	1.50886
51463	1.89226	1.5367
51464	1.89226	1.64291
51465	1.89226	1.46219
51466	1.89226	1.53822
51467	0.972147	1.59628
51468	0.867854	1.67637
51469	1.26132	1.56569
51470	1.26654	1.56964
51471	1.0424	1.68985
51472	1.0424	1.68013
51473	1.092	1.50159
51474	1.092	1.62037
51475	1.26132	1.63381
51476	0.867854	2.09073
51477	0.972147	1.72373
51478	1.26654	2.01689
51479	1.4375	0.1249
51480	1.92998	0.101621
51481	1.66953	0.0627399
51482	1.0953	0.0787659
51483	3.10703	0.0728494
51484	1.89226	2.1
51485	1.89226	2.1
51486	1.89226	2.1
51487	1.89226	2.1
51488	3.78452	2.1
51489	2.74034	2.1
51490	2.08808	2.1
51491	2.63859	2.1
51492	2.00343	2.1
51493	4.72667	2.1
51494	0.799825	2.1
51495	0.799825	2.1
51496	0.80211	2.1
51497	0.80211	2.1
51498	0.562428	2.1
51499	0.600223	2.1
51500	1.04404	2.1
51501	1.00171	2.1

51502	1.00171	2.1
51503	0.56243	2.1
51504	0.600225	2.1
51505	1.04404	2.1
51506	0.94613	2.1
51507	0.94613	2.1
51508	0.946128	2.1
51509	0.946128	2.1
51510	0.946127	2.1
51511	0.946127	2.1
51512	0.946131	2.1
51513	0.946131	2.1
51514	0.946129	2.1
51515	0.946129	2.1
51516	0.946129	2.1
51517	0.946129	2.1
51518	1.89226	2.1
51519	1.89226	2.1
51520	1.89226	2.1
51521	1.89226	2.1
51522	3.78452	2.1
51523	2.63859	2.1
51524	2.00343	2.1
51525	2.54388	2.1
51526	1.91548	2.1
51527	4.55407	2.1
51528	1.91548	2.1
51529	2.00342	2.1
51530	1.04909	2.1
51531	1.04909	2.1
51532	1.12486	2.1
51533	1.99124	2.1
51534	0.810942	2.1
51535	0.810942	2.1
51536	0.810942	2.1
51537	0.810942	2.1
51538	1.89226	2.1
51539	1.35169	2.1
51540	1.08131	2.1
51541	1.08132	2.1
51542	1.08132	2.1
51543	0.810944	2.1
51544	0.810944	2.1
51545	1.89226	0.703865
51546	1.89226	2.1
51547	1.89226	0.823952
51548	1.89226	2.1
51549	3.78452	1.68661
51550	1.7968	0.662809
51551	1.49831	2.1
51552	1.59244	0.683282
51553	1.3395	2.1
51554	3.09074	1.87607
51555	1.49831	2.1
51556	1.16858	2.1
51557	1.33951	2.1
51558	1.0361	2.1
51559	2.53442	2.1

51560	1.89226	2.1
51561	1.89226	2.1
51562	1.89226	2.1
51563	1.89226	2.1
51564	1.89226	2.1
51565	1.89226	2.1
51566	1.89223	0.64607
51567	1.89223	2.1
51568	1.89223	1.61382
51569	1.89223	2.1
51570	1.89223	0.742528
51571	1.89223	2.1
51572	1.59241	0.512728
51573	1.18952	2.1
51574	1.33949	2.1
51575	1.33949	1.06585
51576	1.40538	2.1
51577	1.40538	0.612465
51578	1.89226	0.0942914
51579	1.89226	0.112008
51580	1.89226	0.109487
51581	1.89226	0.131583
51582	1.89226	0.129005
51583	1.89226	0.137932
51584	1.89226	2.1
51585	1.89226	2.1
51586	1.89226	2.1
51587	1.89226	2.1
51588	3.78452	2.1
51589	1.89226	2.1
51590	1.89226	2.1
51591	1.89226	2.1
51592	1.89226	2.1
51593	3.78452	2.1
51594	2.01867	2.1
51595	1.46955	2.1
51596	1.58476	2.1
51597	1.38189	2.1
51598	2.22153	2.1
51599	2.01867	2.1
51600	2.01867	2.1
51601	2.52881	2.1
51602	2.21732	2.1
51603	2.21732	2.1
51604	2.22154	2.1
51605	2.22154	2.1
51606	1.89226	2.1
51607	1.89226	2.1
51608	1.89226	2.1
51609	1.89226	2.1
51610	1.89226	2.1
51611	1.89226	2.1
51612	1.89226	2.1
51613	1.89226	2.1
51614	1.89226	2.1
51615	1.89226	2.1
51616	1.89226	2.1
51617	1.89226	2.1

51618	2.22153	2.1
51619	1.63361	2.1
51620	1.77336	2.1
51621	1.46955	2.1
51622	2.52534	2.1
51623	2.22153	2.1
51624	2.34431	2.1
51625	1.62905	2.1
51626	1.49693	2.1
51627	2.19197	2.1
51628	3.82103	2.1
51629	1.89226	2.1
51630	1.89226	2.1
51631	1.89226	2.1
51632	1.89226	2.1
51633	3.78453	2.1
51634	0.946128	2.1
51635	0.946128	2.1
51636	1.22372	2.1
51637	1.17331	2.1
51638	0.946131	2.1
51639	0.946131	2.1
51640	1.17331	2.1
51641	1.17331	2.1
51642	0.946131	2.1
51643	1.17331	2.1
51644	1.22372	2.1
51645	0.94613	2.1
51646	1.04041	2.1
51647	1.09599	2.1
51648	0.748462	2.1
51649	0.701976	2.1
51650	0.896701	2.1
51651	0.896701	2.1
51652	0.901262	2.1
51653	0.901262	2.1
51654	1.04041	2.1
51655	0.701977	2.1
51656	0.748463	2.1
51657	1.09599	2.1
51658	1.89226	2.1
51659	1.89226	2.1
51660	1.89226	2.1
51661	1.89226	2.1
51662	3.78453	2.1
51663	0.946128	2.1
51664	0.946128	2.1
51665	1.17331	2.1
51666	1.13785	2.1
51667	0.94613	2.1
51668	0.94613	2.1
51669	1.13785	2.1
51670	1.13785	2.1
51671	1.13785	2.1
51672	0.94613	2.1
51673	0.94613	2.1
51674	1.17331	2.1
51675	2.50381	2.1

51676	1.89226	2.1
51677	1.89226	2.1
51678	2.73387	2.1
51679	4.39607	2.1
51680	1.80045	2.1
51681	1.99777	2.05929
51682	2.45134	2.1
51683	2.12463	2.1
51684	1.70302	2.1
51685	1.42655	2.1
51686	2.20231	2.1
51687	1.70302	2.1
51688	1.93128	2.1
51689	1.66768	2.1
51690	1.24499	2.02314
51691	1.70302	1.99646
51692	2.55572	0.0940232
51693	1.22811	0.130933
51694	1.82584	0.110269
51695	1.22811	0.0920794
51696	2.62764	0.12267
51697	1.93147	0.10632
51698	2.14513	0.149244
51699	1.89226	0.28357
51700	2.66481	0.153193
51701	1.89226	0.181018
51702	2.66481	0.29457
51703	1.89226	0.324002
51704	2.11649	2.1
51705	1.89226	2.1
51706	1.89226	2.1
51707	1.89226	2.1
51708	2.15426	2.1
51709	2.15426	2.1
51710	2.06776	2.1
51711	1.89226	2.1
51712	1.89226	2.1
51713	1.89226	2.1
51714	2.11649	2.1
51715	2.11649	2.1
51716	1.89226	2.1
51717	1.89226	2.1
51718	1.89226	2.1
51719	1.89226	2.1
51720	1.89226	2.1
51721	1.89226	2.1
51722	0.998134	2.1
51723	0.918904	2.1
51724	0.894708	2.1
51725	0.758742	2.1
51726	0.864276	2.1
51727	0.864276	2.1
51728	0.8926	2.1
51729	0.8926	2.1
51730	0.998134	2.1
51731	0.894709	2.1
51732	0.758742	2.1
51733	0.918904	2.1

51734	1.89226	2.1
51735	1.89226	2.1
51736	1.89226	2.1
51737	1.89226	2.1
51738	1.89226	2.1
51739	1.89226	2.1
51740	1.89226	2.1
51741	1.89226	2.1
51742	1.89226	2.1
51743	1.89226	2.1
51744	1.89226	2.1
51745	1.89226	2.1
51746	1.99627	2.1
51747	1.61047	2.1
51748	2.0815	2.1
51749	2.07569	2.1
51750	3.75063	2.1
51751	0.848077	2.1
51752	1.07748	2.1
51753	0.802498	2.1
51754	0.626808	2.1
51755	0.875552	2.1
51756	0.737527	2.1
51757	0.835602	2.1
51758	0.835602	2.1
51759	0.983245	2.1
51760	0.781108	2.1
51761	0.872306	2.1
51762	0.601497	2.1
51763	2.74745	2.1
51764	2.73988	2.1
51765	2.6521	2.1
51766	2.67792	2.1
51767	5.39198	2.1
51768	2.73988	2.1
51769	2.57466	2.1
51770	2.67791	2.1
51771	2.55653	2.1
51772	5.29641	2.1
51773	2.6521	2.1
51774	2.64148	2.1
51775	2.67792	2.1
51776	2.67792	2.1
51777	2.56953	2.1
51778	2.56953	2.1
51779	2.64148	2.1
51780	2.55653	2.1
51781	2.57929	2.1
51782	2.57929	2.1
51783	2.67792	2.1
51784	2.67792	2.1
51785	1.89226	1.53462
51786	1.89226	1.0612
51787	1.89226	1.94755
51788	1.89226	0.324591
51789	3.78453	0.790657
51790	1.68703	1.56328
51791	2.50385	0.259697

51792	1.45958	0.672516
51793	2.0815	0.179626
51794	3.93806	0.397987
51795	2.50385	0.194033
51796	2.57414	0.480965
51797	2.0815	0.297476
51798	2.39921	1.32785
51799	4.91181	0.275471
51800	1.89226	1.25347
51801	1.89226	0.568394
51802	1.89226	0.270608
51803	1.89226	0.242875
51804	3.78453	0.378972
51805	1.89226	1.95475
51806	1.89226	0.23595
51807	1.89226	1.23991
51808	1.89226	0.993129
51809	1.89226	0.52798
51810	1.89226	1.55931
51811	1.23013	0.352908
51812	2.0815	0.137458
51813	1.90167	0.714233
51814	1.71243	0.407902
51815	1.45887	0.558177
51816	1.31369	0.23179
51817	2.0815	0.246554
51818	2.39921	1.43945
51819	1.7115	0.328858
51820	2.2421	0.486275
51821	4.09829	0.390865
51822	1.89227	0.160056
51823	1.89227	0.300127
51824	1.89227	0.555382
51825	1.89227	0.750291
51826	3.78453	0.103701
51827	2.39697	0.05
51828	1.85594	0.05
51829	1.91161	0.05
51830	2.49867	0.05
51831	4.35461	0.05
51832	1.12035	0.05
51833	1.85594	0.05
51834	1.91161	0.05
51835	1.15483	0.05
51836	3.01077	0.05
51837	1.89226	0.05
51838	1.89226	0.05
51839	1.89226	0.05
51840	1.89226	0.05
51841	1.89226	0.05
51842	1.89226	0.05
51843	1.89226	0.05
51844	1.89226	0.05
51845	1.89226	0.05
51846	1.89226	0.05
51847	1.89226	0.05
51848	1.89226	0.05
51849	0.936516	0.05

51850	1.03496	0.05
51851	1.22607	0.05
51852	1.28058	0.05
51853	1.06317	0.05
51854	1.06317	0.05
51855	1.19735	0.05
51856	1.0813	0.05
51857	0.858168	0.05
51858	1.32278	0.05
51859	0.957674	0.05
51860	1.25178	0.05
51861	1.92659	0.05
51862	0.998957	0.05
51863	0.998957	0.05
51864	1.15481	0.05
51865	1.6031	0.05
51866	1.74459	0.05
51867	0.946119	0.05
51868	0.946119	0.05
51869	0.946118	0.05
51870	0.946118	0.05
51871	0.946118	0.05
51872	0.946118	0.05
51873	0.946119	0.05
51874	0.946119	0.05
51875	0.946131	0.05
51876	0.946131	0.05
51877	0.946106	0.05
51878	0.946106	0.05
51879	0.946118	0.05
51880	0.946118	0.05
51881	0.946119	0.05
51882	0.946119	0.05
51883	0.946118	0.05
51884	0.946118	0.05
51885	0.946119	0.05
51886	0.946119	0.05
51887	0.946131	0.05
51888	0.946131	0.05
51889	0.946106	0.05
51890	0.946106	0.05
51891	2.47164	0.490764
51892	2.83689	0.231483
51893	2.73872	0.517922
51894	2.41632	0.239402
51895	5.21036	0.250417
51896	0.946133	0.399757
51897	0.946133	0.532658
51898	1.41845	0.278204
51899	1.36936	0.441707
51900	1.41845	0.240609
51901	1.41845	0.235597
51902	0.946132	0.530761
51903	0.946132	0.570844
51904	0.946132	0.423717
51905	1.36936	0.277806
51906	1.41845	0.3055
51907	0.946133	0.474506

51908	1.36936	0.176827
51909	1.27581	0.289746
51910	1.20816	0.224447
51911	1.10464	0.437538
51912	1.24454	0.193266
51913	1.24454	0.193602
51914	1.23943	0.327005
51915	1.23943	0.377487
51916	1.10465	0.291812
51917	1.27581	0.262351
51918	1.36936	0.286265
51919	1.20816	0.237149
51920	0.946131	2.1
51921	0.946131	2.1
51922	1.37277	2.1
51923	1.37277	2.1
51924	1.37277	2.1
51925	1.27186	2.1
51926	0.946131	2.1
51927	0.946131	2.1
51928	0.946131	2.1
51929	1.37277	2.1
51930	0.946131	2.1
51931	1.27186	2.1
51932	2.74554	2.1
51933	1.23712	2.1
51934	2.80992	2.1
51935	2.54371	2.1
51936	1.50333	2.1
51937	1.23712	2.1
51938	2.77314	2.1
51939	2.33316	2.1
51940	2.28748	2.1
51941	2.28748	2.1
51942	2.81275	2.1
51943	2.81275	2.1
51944	2.28749	2.1
51945	1.45887	2.1
51946	2.33317	2.1
51947	1.50022	2.1
51948	3.79204	2.1
51949	2.37364	2.1
51950	2.33317	2.1
51951	1.5362	2.1
51952	1.5362	2.1
51953	1.50022	2.1
51954	2.33317	2.1
51955	1.89226	0.637452
51956	1.89226	0.795299
51957	1.89226	0.573158
51958	1.89226	0.770674
51959	1.89226	0.585054
51960	1.89226	0.84795
51961	0.946128	0.347999
51962	0.946128	0.391958
51963	1.0644	0.283161
51964	0.946133	0.338678
51965	1.0644	0.273599

51966	0.94613	0.308652
51967	0.946131	0.418676
51968	0.946131	0.326584
51969	1.0644	0.329383
51970	1.0644	0.33854
51971	0.946131	0.345475
51972	0.946131	0.321709
51973	1.89226	2.1
51974	1.89226	1.31859
51975	1.89226	2.08822
51976	1.89226	0.250042
51977	3.78452	0.884458
51978	1.14599	2.1
51979	1.19993	0.803135
51980	1.00065	2.01824
51981	1.07497	0.232741
51982	2.22096	1.82024
51983	0.546551	1.35613
51984	0.546551	0.725808
51985	0.54987	0.578555
51986	0.54987	1.7348
51987	0.503067	0.496409
51988	0.558937	0.52141
51989	0.599966	0.144938
51990	0.537484	0.272035
51991	0.503067	0.165587
51992	0.537485	0.1779
51993	0.599966	0.296393
51994	0.558936	0.273724
51995	0.946132	0.492109
51996	0.946132	1.57303
51997	0.94613	0.25471
51998	0.94613	1.56007
51999	0.946131	0.504073
52000	0.946131	0.573129
52001	0.946131	0.237082
52002	0.946131	0.192185
52003	0.946131	0.306854
52004	0.946131	0.121824
52005	0.946131	0.497925
52006	0.946131	0.723282
52007	2.19104	2.1
52008	2.5392	2.1
52009	2.53302	2.1
52010	2.19226	2.1
52011	4.73146	2.1
52012	1.09552	2.1
52013	1.09613	2.1
52014	0.802484	2.1
52015	0.807941	2.1
52016	0.95052	2.1
52017	0.95052	2.1
52018	0.952944	2.1
52019	0.952944	2.1
52020	1.09613	2.1
52021	0.807941	2.1
52022	0.802484	2.1
52023	1.09552	2.1

52024	2.533	2.1
52025	2.18531	2.1
52026	2.19224	2.1
52027	2.19224	2.1
52028	2.51603	2.1
52029	2.51603	2.1
52030	1.09266	2.1
52031	1.09612	2.1
52032	0.807935	2.1
52033	0.81091	2.1
52034	0.951897	2.1
52035	0.951897	2.1
52036	0.955134	2.1
52037	0.955134	2.1
52038	0.810915	2.1
52039	1.09266	2.1
52040	1.09611	2.1
52041	0.80793	2.1
52042	2.12741	0.125447
52043	2.79312	0.289108
52044	2.77591	1.26934
52045	2.10714	0.309429
52046	4.90026	0.194051
52047	2.12741	0.188101
52048	1.20859	0.196343
52049	1.21073	1.26631
52050	2.10714	0.6826
52051	3.31573	0.819497
52052	1.38708	0.715961
52053	1.38795	0.372849
52054	1.05331	0.202548
52055	1.05357	0.201414
52056	1.22049	0.314073
52057	1.22049	0.897696
52058	1.22078	1.38526
52059	1.22078	0.131761
52060	1.05331	0.34878
52061	1.38709	0.384403
52062	1.38795	0.209994
52063	1.05357	0.245098
52064	2.10662	0.998569
52065	2.10714	0.161437
52066	2.12499	0.128662
52067	1.22857	1.05561
52068	1.21073	0.355696
52069	1.21073	0.170886
52070	1.82014	0.05
52071	2.08757	0.05
52072	1.81292	0.05
52073	1.67485	0.05
52074	3.63306	0.05
52075	1.82013	0.05
52076	1.21748	0.05
52077	1.29891	0.05
52078	1.4442	0.0554119
52079	1.67484	0.05
52080	1.82013	0.0511961
52081	1.81292	0.05

52082	1.4516	0.05
52083	1.39633	0.05
52084	1.67485	0.05
52085	1.5344	0.05
52086	1.81292	0.05
52087	1.67484	0.05
52088	1.00982	0.05
52089	0.994235	0.05
52090	1.21748	0.05
52091	1.45159	0.05
52092	1.67484	0.05
52093	1.55216	2.1
52094	1.34407	2.1
52095	1.34407	2.1
52096	1.52008	2.1
52097	1.81783	2.1
52098	1.99384	2.1
52099	1.90817	2.1
52100	2.03975	2.1
52101	1.97317	2.1
52102	1.81783	2.1
52103	3.85758	2.1
52104	0.599049	2.1
52105	0.660673	2.1
52106	0.785063	2.1
52107	0.924278	2.1
52108	0.760416	2.1
52109	0.760416	2.1
52110	0.719088	2.1
52111	0.694532	2.1
52112	0.522441	2.1
52113	0.796789	2.1
52114	0.655207	2.1
52115	0.886155	2.1
52116	1.97317	2.1
52117	1.81783	2.1
52118	1.65098	2.1
52119	1.91767	2.1
52120	3.7355	2.1
52121	1.15038	2.00108
52122	1.28649	1.90839
52123	1.38986	2.06895
52124	1.38986	1.96578
52125	1.07866	2.06295
52126	1.07866	2.06189
52127	0.664498	1.30379
52128	0.664498	2.1
52129	0.675122	2.1
52130	0.675122	2.1
52131	0.69493	1.53942
52132	0.764429	1.82845
52133	0.623438	2.1
52134	0.575191	2.1
52135	0.764429	1.82577
52136	0.623438	1.71684
52137	0.575191	2.06295
52138	0.69493	2.04435
52139	1.89226	1.49857

52140	1.89226	0.428706
52141	1.89226	1.85265
52142	1.89226	0.790599
52143	3.78453	0.367435
52144	2.50075	1.90914
52145	2.15276	0.650052
52146	2.24902	1.85022
52147	2.30422	0.610911
52148	4.40178	0.192499
52149	1.70424	2.1
52150	1.34494	2.1
52151	2.15276	1.21669
52152	2.15276	2.1
52153	2.0793	1.01924
52154	1.63078	1.78882
52155	1.89226	0.938623
52156	1.89226	2.1
52157	1.89226	2.1
52158	1.89226	0.473331
52159	1.89226	2.1
52160	1.89226	1.77934
52161	1.89226	1.84744
52162	1.89226	0.928461
52163	1.89226	1.08821
52164	1.89226	0.342284
52165	3.78452	0.267048
52166	2.24902	1.7546
52167	1.91497	0.640196
52168	1.97135	2.1
52169	1.73957	0.457287
52170	3.98859	0.603433
52171	1.73957	0.447034
52172	1.34494	2.1
52173	1.73957	2.1
52174	1.69006	0.523081
52175	1.3126	2.1
52176	1.34848	1.67496
52177	0.946132	0.521724
52178	0.946132	2.1
52179	0.946132	1.65114
52180	0.946132	2.1
52181	0.946132	2.1
52182	0.946132	2.1
52183	0.946131	0.313613
52184	0.946131	0.127969
52185	0.946132	0.947167
52186	0.946132	1.74886
52187	0.946132	1.23668
52188	0.946132	1.8771
52189	1.89226	2.1
52190	1.89226	2.06171
52191	1.89226	2.1
52192	1.89226	2.00972
52193	3.78453	2.1
52194	1.89226	2.05318
52195	2.82624	1.8954
52196	1.89226	2.07337
52197	2.02536	1.87041

52198	1.89226	2.06682
52199	2.02536	1.84449
52200	2.25027	0.05
52201	1.1706	0.05
52202	2.02733	0.05
52203	2.02733	0.05
52204	1.61462	0.05
52205	1.69832	0.05
52206	1.89226	0.05
52207	1.89226	0.05
52208	1.89226	0.05
52209	1.89226	0.05
52210	3.78452	0.05
52211	0.946131	0.05
52212	0.946131	0.05
52213	1.18182	0.05
52214	1.18182	0.05
52215	0.973819	0.05
52216	0.94613	0.05
52217	1.18182	0.05
52218	0.94613	0.05
52219	0.973819	0.05
52220	1.18182	0.05
52221	0.94613	0.05
52222	0.94613	0.05
52223	2.23944	2.1
52224	1.52903	2.1
52225	1.35977	2.1
52226	2.04847	2.1
52227	3.5775	2.1
52228	2.23944	2.1
52229	2.34795	2.1
52230	2.34795	2.1
52231	2.48025	2.1
52232	2.04847	2.1
52233	2.18077	2.1
52234	1.89226	2.1
52235	1.89226	2.1
52236	1.89226	2.1
52237	1.89226	2.1
52238	3.78452	2.1
52239	0.946127	2.1
52240	0.946127	2.1
52241	0.946131	2.1
52242	0.946131	2.1
52243	0.946128	2.1
52244	0.946128	2.1
52245	0.94613	2.1
52246	0.94613	2.1
52247	0.946129	2.1
52248	0.946129	2.1
52249	0.946129	2.1
52250	0.946129	2.1
52251	1.26956	2.1
52252	2.04847	2.1
52253	1.49576	2.1
52254	1.22377	2.1
52255	1.91172	2.1

52256	1.50198	2.1
52257	1.48568	2.1
52258	2.34795	2.1
52259	2.04847	2.1
52260	2.04847	2.1
52261	2.21155	2.1
52262	2.21155	2.1
52263	1.89226	2.1
52264	1.89226	2.1
52265	1.89226	2.1
52266	1.89226	2.1
52267	1.89226	2.1
52268	1.89226	2.1
52269	0.810943	2.1
52270	0.810943	2.1
52271	0.810944	2.1
52272	0.810944	2.1
52273	0.810957	2.1
52274	0.810957	2.1
52275	1.89226	2.1
52276	1.35168	2.1
52277	1.08132	2.1
52278	1.08132	2.1
52279	1.0813	2.1
52280	0.946132	0.05
52281	1.33855	0.0603978
52282	0.946131	0.05
52283	1.38204	0.0628645
52284	0.946131	0.0639837
52285	0.946131	0.063013
52286	1.38204	0.05
52287	1.38204	0.05
52288	0.946131	0.05
52289	0.946131	0.0575254
52290	1.33855	0.05
52291	1.38204	0.061742
52292	1.89226	0.0613628
52293	2.76407	0.0661823
52294	1.89226	0.05
52295	2.72769	0.0644683
52296	1.89226	0.05
52297	2.72769	0.0629741
52298	2.03914	0.05
52299	1.67727	0.05
52300	1.94831	0.05
52301	1.81591	0.05
52302	2.32098	0.05
52303	2.32098	0.05
52304	0.801647	0.05
52305	1.11086	0.05
52306	0.838634	0.05
52307	1.16049	0.05
52308	0.977891	0.05
52309	0.977891	0.05
52310	0.984246	0.05
52311	0.984246	0.05
52312	1.11086	0.05
52313	1.16049	0.05

52314	0.801647	0.05
52315	0.838634	0.05
52316	0.946132	0.05
52317	0.946132	0.0657326
52318	0.946131	0.05
52319	0.946131	0.062632
52320	0.946131	0.0643111
52321	0.946131	0.0630369
52322	0.946132	0.05
52323	0.946132	0.05
52324	0.946131	0.05
52325	0.946131	0.0552993
52326	0.946131	0.0519958
52327	0.946131	0.0607327
52328	0.946131	0.0503669
52329	0.946131	0.0723944
52330	0.946131	0.060744
52331	0.946131	0.05
52332	0.946131	0.0622144
52333	0.946131	0.0624222
52334	0.946131	0.05
52335	0.946131	0.05
52336	0.946131	0.05
52337	0.946131	0.0574435
52338	0.946131	0.0605328
52339	0.946131	0.0581312
52340	2.0815	2.1
52341	1.20787	2.1
52342	1.92277	2.1
52343	1.3227	2.1
52344	3.29401	2.1
52345	2.16633	2.1
52346	2.13642	2.1
52347	1.40846	2.1
52348	1.40846	2.1
52349	1.3227	2.1
52350	2.08058	2.1
52351	0.938106	2.1
52352	0.790258	2.1
52353	0.742975	2.1
52354	0.742975	2.1
52355	0.874389	2.1
52356	1.17364	2.1
52357	0.525948	2.1
52358	0.52513	2.1
52359	0.553732	2.1
52360	0.876996	2.1
52361	0.530954	2.1
52362	1.095 2.1	
52363	1.00833	2.1
52364	1.70608	2.1
52365	1.33385	2.1
52366	1.33385	2.1
52367	1.07722	2.1
52368	1.07722	2.1
52369	0.953523	1.29922
52370	1.7457	0.747239
52371	1.03218	1.1842

52372	1.03218	1.04315
52373	1.79675	1.0825
52374	1.79675	0.943761
52375	1.13676	1.29761
52376	1.18136	0.798301
52377	0.87285	1.03794
52378	0.898375	1.35297
52379	1.03191	0.794116
52380	1.03191	0.601375
52381	1.0223	1.13381
52382	1.0223	1.34971
52383	1.18136	0.987762
52384	0.898374	0.728654
52385	0.87285	0.990252
52386	1.13676	0.898687
52387	2.36273	0.938654
52388	1.46965	0.59771
52389	1.79675	0.504222
52390	1.79675	0.41902
52391	2.43417	1.11143
52392	2.43417	0.851211
52393	1.4714	2.09771
52394	1.65434	2.1
52395	1.62322	1.39868
52396	1.86068	2.00483
52397	3.27756	1.73665
52398	1.89226	2.1
52399	1.89226	2.1
52400	1.89226	1.45205
52401	1.89226	1.64071
52402	3.78453	1.55975
52403	1.89226	1.90984
52404	1.89226	2.1
52405	1.89226	1.53639
52406	1.89226	2.1
52407	3.78453	2.1
52408	1.65434	1.91071
52409	1.60647	2.1
52410	1.86068	1.75464
52411	1.82649	2.1
52412	3.48083	2.1
52413	1.62322	2.09001
52414	1.86068	2.1
52415	1.84368	1.526
52416	2.18862	1.64751
52417	3.81184	1.61938
52418	1.89226	2.1
52419	1.89226	1.98813
52420	1.89226	1.29338
52421	1.89226	1.6915
52422	3.78453	1.64175
52423	1.89226	1.80131
52424	1.89226	2.1
52425	1.89226	1.56579
52426	1.89226	2.1
52427	3.78453	2.1
52428	1.86068	1.85048
52429	1.82649	2.1

52430	2.18862	1.54311
52431	2.1128	2.1
52432	4.01511	2.08882
52433	1.74351	2.1
52434	1.13854	2.1
52435	1.40233	2.1
52436	1.922 2.1	
52437	3.14584	2.1
52438	1.922 2.1	
52439	2.00775	2.1
52440	1.7435	2.1
52441	2.09224	2.1
52442	2.00775	2.1
52443	2.09295	2.1
52444	1.69858	2.1
52445	1.922 2.1	
52446	1.40233	2.1
52447	1.40233	2.1
52448	2.18238	2.1
52449	2.18238	2.1
52450	1.00387	2.1
52451	1.10886	2.1
52452	0.961001	2.1
52453	1.09119	2.1
52454	1.02868	2.1
52455	1.02868	2.1
52456	1.04118	2.1
52457	1.04118	2.1
52458	1.10886	2.1
52459	1.09119	2.1
52460	0.960999	2.1
52461	1.00388	2.1
52462	0.885958	0.260398
52463	0.885958	0.203599
52464	0.875826	0.131297
52465	0.875826	0.309116
52466	0.913965	0.276841
52467	0.598609	0.176995
52468	1.16317	0.158924
52469	0.868115	0.13522
52470	0.868115	0.208582
52471	0.598609	0.21856
52472	1.16317	0.217782
52473	0.913965	0.222643
52474	0.946131	0.346318
52475	0.946131	0.276999
52476	0.946131	0.17104
52477	0.946131	0.14761
52478	0.946131	0.159401
52479	0.946131	0.282149
52480	0.946131	0.392492
52481	0.946131	0.165365
52482	0.946131	0.17879
52483	0.946131	0.182176
52484	0.946131	0.340704
52485	0.946131	0.238426
52486	0.946133	0.05
52487	0.946133	0.05

52488	1.06246	0.05
52489	1.06246	0.05
52490	0.973851	0.05
52491	0.946133	0.05
52492	1.06246	0.05
52493	0.946132	0.05
52494	0.97385	0.05
52495	1.06246	0.05
52496	0.946133	0.05
52497	0.946133	0.05
52498	1.89226	0.05
52499	1.89226	0.05
52500	1.89226	0.05
52501	1.89226	0.05
52502	1.89226	0.05
52503	1.89226	0.05
52504	0.946131	0.05
52505	0.946131	0.05
52506	0.946127	0.05
52507	0.946127	0.05
52508	0.946131	0.05
52509	0.946131	0.05
52510	0.946127	0.05
52511	0.946127	0.05
52512	0.946128	0.05
52513	0.946128	0.05
52514	0.946129	0.05
52515	0.946129	0.05
52516	0.946132	0.05
52517	0.946132	0.05
52518	1.32446	0.05
52519	0.794912	0.05
52520	1.32429	0.05
52521	0.946131	0.05
52522	1.32446	0.05
52523	0.94613	0.05
52524	1.32429	0.05
52525	0.794915	0.05
52526	0.94613	0.05
52527	0.94613	0.05
52528	1.89226	0.05
52529	2.36365	0.05
52530	2.64857	0.05
52531	1.89226	0.05
52532	2.64857	0.05
52533	1.89226	0.05
52534	1.18463	2.1
52535	1.27174	2.1
52536	0.946129	2.1
52537	0.946129	2.1
52538	0.946131	2.1
52539	0.946131	2.1
52540	1.27174	2.1
52541	1.27174	2.1
52542	1.18463	2.1
52543	0.94613	2.1
52544	0.946131	2.1
52545	1.27174	2.1

52546	2.57997	2.1
52547	1.89226	2.1
52548	1.89226	2.1
52549	1.89226	2.1
52550	2.41915	2.1
52551	2.41915	2.1
52552	1.89226	2.1
52553	1.89226	2.1
52554	1.89226	2.1
52555	1.89226	2.1
52556	3.78452	2.1
52557	1.08807	2.1
52558	1.18463	2.1
52559	0.946129	2.1
52560	0.946129	2.1
52561	0.946131	2.1
52562	0.946131	2.1
52563	1.18463	2.1
52564	1.18463	2.1
52565	0.946131	2.1
52566	1.08807	2.1
52567	1.18463	2.1
52568	0.94613	2.1
52569	1.07017	2.1
52570	1.07017	2.1
52571	0.946131	2.1
52572	0.946131	2.1
52573	0.946132	2.1
52574	0.946132	2.1
52575	1.20957	2.1
52576	1.07017	2.1
52577	1.07017	2.1
52578	0.946132	2.1
52579	0.946131	2.1
52580	1.20957	2.1
52581	1.89226	2.1
52582	1.89226	2.1
52583	1.89226	2.1
52584	1.89226	2.1
52585	1.89226	2.1
52586	1.89226	2.1
52587	1.16181	0.05
52588	1.0227	0.05
52589	1.0227	0.05
52590	0.970959	0.05
52591	1.24295	0.05
52592	1.19121	0.05
52593	1.12076	2.1
52594	2.53614	2.1
52595	1.28413	2.1
52596	1.28413	2.1
52597	2.36423	2.1
52598	2.36423	2.1
52599	1.89226	2.1
52600	1.89226	2.1
52601	1.89226	2.1
52602	1.89226	2.1
52603	3.78452	2.1

52604	1.22557	2.1
52605	2.771	2.1
52606	2.73012	2.1
52607	2.73012	2.1
52608	1.33356	2.1
52609	1.33356	2.1
52610	0.998682	2.1
52611	2.36423	2.1
52612	1.12076	2.1
52613	1.12076	2.1
52614	2.13757	2.1
52615	2.13757	2.1
52616	1.89226	2.1
52617	1.89226	2.1
52618	1.89226	2.1
52619	1.89226	2.1
52620	1.89226	2.1
52621	1.89226	2.1
52622	1.13987	2.1
52623	2.73011	2.1
52624	2.70875	2.1
52625	2.70875	2.1
52626	1.22557	2.1
52627	1.22557	2.1
52628	1.89227	2.1
52629	2.79243	2.1
52630	2.6625	2.1
52631	2.6625	2.1
52632	1.89227	2.1
52633	1.89227	2.1
52634	1.89226	2.1
52635	2.55971	2.1
52636	1.89226	2.1
52637	1.89226	2.1
52638	2.68704	2.1
52639	2.68704	2.1
52640	2.22043	2.1
52641	2.38975	1.62996
52642	2.28696	1.85318
52643	2.48682	1.52042
52644	4.67671	1.7532
52645	2.38976	1.41028
52646	2.31988	1.85725
52647	2.48683	1.41175
52648	2.4594	1.63734
52649	4.84915	1.40102
52650	2.28696	2.1
52651	2.48682	1.87903
52652	2.33379	2.1
52653	2.56149	1.44619
52654	4.84845	1.9008
52655	2.48683	1.85055
52656	2.4594	1.78895
52657	2.5615	1.2699
52658	2.57957	1.40245
52659	5.0209	1.37434
52660	1.06659	2.1
52661	1.04291	2.1

52662	1.36595	2.1
52663	1.36595	2.1
52664	1.02761	2.1
52665	1.32697	2.1
52666	0.946132	2.00997
52667	0.946132	1.99206
52668	1.36956	2.1
52669	1.1229	2.1
52670	1.1229	1.97753
52671	1.1229	2.1
52672	0.946132	1.92783
52673	0.946132	2.1
52674	0.946132	2.1
52675	1.36956	2.1
52676	1.1229	2.1
52677	0.946133	2.1
52678	0.946133	1.68524
52679	0.946133	1.67189
52680	0.946128	2.1
52681	0.946128	2.06629
52682	0.946131	1.96841
52683	0.946131	1.71701
52684	0.946131	1.318
52685	0.946131	2.05156
52686	0.94613	1.85292
52687	0.94613	1.843
52688	0.946131	1.86577
52689	0.946131	1.87656
52690	0.966427	1.67765
52691	0.802265	1.77348
52692	0.803714	2.01905
52693	0.808476	1.79869
52694	1.94371	2.0102
52695	1.36851	1.94134
52696	0.853864	2.07784
52697	1.26635	1.83055
52698	1.09085	1.74119
52699	0.857653	1.8762
52700	0.630243	1.86347
52701	1.20132	0.05
52702	1.24295	0.05
52703	1.24295	0.05
52704	1.16182	0.05
52705	1.28465	0.05
52706	1.20351	0.05
52707	0.600658	0.05
52708	0.642323	0.05
52709	0.54424	0.05
52710	0.576779	0.05
52711	0.590985	0.05
52712	0.590985	0.05
52713	0.586452	0.05
52714	0.586452	0.05
52715	0.642323	0.05
52716	0.576779	0.05
52717	0.600658	0.05
52718	0.54424	0.05
52719	1.28463	0.05

52720	1.24294	0.05
52721	1.12328	0.05
52722	1.15505	0.05
52723	2.39798	0.05
52724	1.98431	0.0564673
52725	2.18735	0.0541402
52726	1.98431	0.0548579
52727	1.70303	0.0693498
52728	1.70302	0.108356
52729	1.37788	0.0790247
52730	1.89226	0.0808715
52731	2.37417	0.0746198
52732	1.89226	0.173733
52733	2.18735	0.078405
52734	1.89226	0.148354
52735	2.18735	0.0861379
52736	1.89226	0.28731
52737	1.89226	2.1
52738	1.89226	0.994475
52739	1.89226	1.18727
52740	3.78452	1.11847
52741	1.8613	1.53256
52742	2.03576	2.1
52743	1.61652	0.857189
52744	1.83082	2.1
52745	3.65228	1.85741
52746	2.03576	2.1
52747	2.05257	2.1
52748	1.83082	2.1
52749	1.88487	2.1
52750	3.92064	2.1
52751	1.89226	2.09731
52752	1.89226	2.1
52753	1.89226	1.45902
52754	1.89226	2.1
52755	3.78453	2.1
52756	0.946131	2.1
52757	0.946131	0.68574
52758	0.94613	0.231411
52759	0.94613	1.83867
52760	0.946129	1.19134
52761	0.946129	2.1
52762	0.946132	1.37173
52763	0.946132	0.220484
52764	0.94613	1.03452
52765	0.94613	1.20675
52766	0.946131	0.790447
52767	0.946131	0.864112
52768	0.804708	2.1
52769	0.804708	1.67785
52770	0.815274	2.1
52771	0.815274	0.828171
52772	0.83277	2.1
52773	0.915407	2.1
52774	0.80826	1.61899
52775	0.704574	0.775055
52776	0.832771	2.09091
52777	0.704573	2.1

52778	0.808259	2.00959
52779	0.915409	2.02612
52780	1.83082	2.1
52781	1.88487	2.1
52782	1.66555	2.1
52783	1.75835	2.1
52784	3.55042	2.1
52785	0.810947	1.35618
52786	0.810947	2.1
52787	0.810947	1.24922
52788	0.810947	2.1
52789	0.810934	2.1
52790	0.810934	2.1
52791	1.89227	2.1
52792	1.3517	2.1
52793	1.08132	2.1
52794	1.08132	2.1
52795	1.08133	2.1
52796	1.89226	2.1
52797	1.70303	2.1
52798	1.89226	2.1
52799	2.24712	2.1
52800	2.07363	2.1
52801	1.74617	2.1
52802	1.89226	2.1
52803	1.89226	1.8204
52804	1.89226	1.69643
52805	1.89226	2.1
52806	3.78452	1.91947
52807	1.50108	2.1
52808	1.43894	1.91407
52809	1.8322	1.77722
52810	1.40967	2.1
52811	1.50108	1.96599
52812	1.07854	2.1
52813	1.89226	1.72056
52814	1.89226	2.09563
52815	1.89226	2.07012
52816	1.89226	1.16417
52817	3.78452	1.9156
52818	1.89226	2.1
52819	1.89226	1.95001
52820	1.89226	1.98727
52821	1.89226	2.1
52822	3.78453	2.06851
52823	1.54457	2.1
52824	1.35097	1.9824
52825	1.70754	1.93681
52826	1.29629	2.1
52827	1.54457	2.1
52828	1.0305	2.1
52829	0.731763	2.1
52830	1.00043	2.05441
52831	1.13883	1.64962
52832	0.939026	1.68146
52833	1.04003	1.62616
52834	0.853868	2.1
52835	0.927188	2.08026

52836	0.927188	0.930213
52837	0.861565	1.76993
52838	1.13882	1.80588
52839	0.731769	1.9305
52840	0.89319	1.94938
52841	1.46568	2.1
52842	1.20761	2.1
52843	1.51916	2.1
52844	1.36261	2.1
52845	2.72677	2.1
52846	1.51916	2.1
52847	1.60532	2.1
52848	1.36261	2.1
52849	1.36261	2.1
52850	1.64892	2.1
52851	1.64892	2.1
52852	1.3626	2.1
52853	0.98488	2.1
52854	1.60532	2.1
52855	1.32414	2.1
52856	2.68675	2.1
52857	1.78711	2.1
52858	1.14825	2.1
52859	2.16535	2.1
52860	1.6067	2.1
52861	3.3136	2.1
52862	1.89226	2.1
52863	1.89226	2.1
52864	1.89226	2.1
52865	1.89226	2.1
52866	3.78452	2.1
52867	2.34552	2.1
52868	1.53521	2.1
52869	1.53521	2.1
52870	1.60636	2.1
52871	1.92797	2.1
52872	1.99589	2.1
52873	1.34771	2.1
52874	1.34771	2.1
52875	0.946128	2.1
52876	0.946128	2.1
52877	1.3477	2.1
52878	1.31776	2.1
52879	0.946131	2.1
52880	0.946131	2.1
52881	0.94613	2.1
52882	1.31776	2.1
52883	0.946129	2.1
52884	1.3477	2.1
52885	1.89226	2.1
52886	1.89226	2.1
52887	1.89226	2.1
52888	1.89226	2.1
52889	3.78452	2.1
52890	0.92843	2.1
52891	0.672114	2.1
52892	0.89514	2.1
52893	0.89514	2.1

52894	0.720104	2.1
52895	0.755753	2.1
52896	0.789158	2.1
52897	0.967302	2.1
52898	1.06565	2.1
52899	0.7397	2.1
52900	0.758893	2.1
52901	0.94672	2.1
52902	1.89226	2.1
52903	2.588	2.1
52904	2.588	2.1
52905	2.63552	2.1
52906	1.89226	2.1
52907	1.93978	2.1
52908	1.49347	0.0784023
52909	2.67757	0.113744
52910	1.80061	0.0839154
52911	2.27056	0.0931042
52912	4.07117	0.0810814
52913	1.1016	0.0596476
52914	2.27057	0.0814225
52915	1.49348	0.0926265
52916	1.81893	0.0964229
52917	1.49348	0.0666107
52918	1.81893	0.0610374
52919	1.81893	0.0893014
52920	2.82339	0.137612
52921	2.27057	0.0913966
52922	2.25405	0.0902864
52923	4.52462	0.080038
52924	1.89226	0.0876935
52925	1.89226	0.149742
52926	1.89226	0.144058
52927	1.89226	0.107103
52928	1.89226	0.144894
52929	1.89226	0.124423
52930	1.89226	0.0797025
52931	1.89226	0.114538
52932	1.89226	0.0768651
52933	1.89226	0.084105
52934	3.78453	0.0749703
52935	1.89226	0.141888
52936	1.89226	0.179633
52937	1.89226	0.087845
52938	1.89226	0.189349
52939	3.78452	0.207035
52940	1.89226	0.177282
52941	1.89226	0.366143
52942	1.89226	0.169233
52943	1.89226	0.209214
52944	3.78451	0.15548
52945	1.45633	2.1
52946	1.34678	2.1
52947	1.34678	2.1
52948	1.53735	2.1
52949	1.234	2.1
52950	1.42457	2.1
52951	1.89226	2.1

52952	1.89226	2.1
52953	1.89226	2.1
52954	1.89226	2.1
52955	1.89226	2.1
52956	1.89226	2.1
52957	1.09483	2.1
52958	1.34678	2.1
52959	1.234	2.1
52960	1.234	2.1
52961	1.19	2.1
52962	1.19	2.1
52963	0.946131	2.1
52964	0.946131	2.1
52965	0.946131	2.1
52966	0.946131	2.1
52967	0.946131	2.1
52968	0.946131	2.1
52969	0.946132	2.1
52970	0.946132	2.1
52971	0.946132	2.1
52972	0.946132	2.1
52973	0.946131	2.1
52974	0.946131	2.1
52975	2.82263	2.1
52976	1.89226	2.1
52977	2.71441	2.1
52978	2.71441	2.1
52979	1.89226	2.1
52980	1.89226	2.1
52981	1.1412	2.1
52982	1.1412	2.1
52983	1.14626	2.1
52984	1.14626	2.1
52985	1.28511	2.1
52986	1.34086	2.1
52987	0.992242	2.1
52988	0.946593	2.1
52989	0.992242	2.1
52990	1.34086	2.1
52991	0.946594	2.1
52992	1.28511	2.1
52993	1.18954	2.1
52994	1.18954	2.1
52995	1.21477	2.1
52996	1.21477	2.1
52997	1.41207	2.1
52998	1.34086	2.1
52999	1.01306	2.1
53000	0.992242	2.1
53001	1.01306	2.1
53002	1.41207	2.1
53003	0.992242	2.1
53004	1.34086	2.1
53005	1.09044	2.1
53006	1.44472	1.90698
53007	1.2955	2.1
53008	1.2955	2.1
53009	1.22025	2.1

53010	1.22025	2.1
53011	0.810944	2.02809
53012	0.810944	2.1
53013	0.810946	2.02885
53014	0.810946	0.631947
53015	0.810959	1.96803
53016	0.810959	1.97159
53017	1.89226	1.93486
53018	1.35167	1.99415
53019	1.08132	2.1
53020	1.08132	0.804921
53021	1.0813	1.98653
53022	1.41894	0.480587
53023	1.28953	0.144816
53024	0.946132	0.630672
53025	0.946132	1.94421
53026	0.946133	1.38164
53027	0.946133	0.233273
53028	1.41894	1.59535
53029	1.41894	1.58267
53030	1.28953	0.500315
53031	0.946132	1.22667
53032	0.946132	1.41272
53033	1.41894	0.597095
53034	1.89226	2.1
53035	2.15444	2.1
53036	1.93239	2.1
53037	1.89226	2.1
53038	2.19457	2.1
53039	2.15444	2.1
53040	1.89226	2.1
53041	1.89226	2.1
53042	1.89226	2.1
53043	1.89226	2.1
53044	1.89226	2.1
53045	1.89226	2.1
53046	0.938507	2.1
53047	0.938507	2.1
53048	1.07871	2.1
53049	1.15801	2.1
53050	1.03236	2.1
53051	1.14766	2.1
53052	0.946133	2.1
53053	0.946133	2.1
53054	0.946131	2.1
53055	1.07009	2.1
53056	0.946131	2.1
53057	1.03237	2.1
53058	1.89226	2.1
53059	2.19457	2.1
53060	1.94492	2.1
53061	1.89226	2.1
53062	2.24723	2.1
53063	2.19457	2.1
53064	0.946132	2.1
53065	0.946132	2.1
53066	0.946131	2.1
53067	0.946131	2.1

53068	0.946132	2.1
53069	0.946132	2.1
53070	0.946132	2.1
53071	0.946132	2.1
53072	0.946132	2.1
53073	0.946132	2.1
53074	0.946132	2.1
53075	0.946132	2.1
53076	2.21345	2.1
53077	1.89226	2.1
53078	1.89226	2.1
53079	2.14017	2.1
53080	2.14017	2.1
53081	1.89226	2.1
53082	1.89226	0.362087
53083	1.89226	1.18571
53084	1.89226	0.358268
53085	1.85439	1.17904
53086	2.23961	0.267524
53087	2.19479	0.772292
53088	1.2916	0.353786
53089	1.2916	0.335652
53090	0.946128	0.410128
53091	0.946128	0.312669
53092	1.2916	0.241675
53093	0.946131	0.334273
53094	0.946126	1.13897
53095	1.1198	0.209756
53096	1.2916	0.270509
53097	1.1198	0.71878
53098	0.946128	0.840133
53099	0.946128	0.490289
53100	1.89226	0.68707
53101	2.71262	0.348738
53102	1.89226	0.391356
53103	2.49959	0.675216
53104	4.39185	0.794595
53105	2.71262	0.315274
53106	1.34592	0.374318
53107	2.49959	0.453102
53108	1.14721	0.290346
53109	3.84551	0.30503
53110	0.946131	0.416497
53111	0.946131	0.448279
53112	1.11247	0.306693
53113	1.11247	0.283329
53114	1.24979	0.315977
53115	1.11247	0.384219
53116	0.946129	0.472296
53117	0.946129	0.338809
53118	0.946131	0.353273
53119	1.11247	0.236372
53120	0.946131	0.267852
53121	1.24979	0.321369
53122	0.946131	2.1
53123	0.946131	2.1
53124	0.94613	2.1
53125	0.94613	2.1

53126	0.946132	2.1
53127	0.946132	2.1
53128	0.946129	2.1
53129	0.946129	2.1
53130	0.946131	2.1
53131	0.946131	2.1
53132	0.946131	2.1
53133	0.946131	2.1
53134	2.77816	2.1
53135	1.89226	2.1
53136	1.89226	2.1
53137	1.89226	2.1
53138	3.78453	2.1
53139	1.89226	2.1
53140	1.89226	2.1
53141	1.89226	2.1
53142	1.89226	2.1
53143	1.89226	2.1
53144	1.89226	2.1
53145	1.89226	2.1
53146	1.89226	2.1
53147	1.89226	2.1
53148	1.89226	2.1
53149	3.78452	2.1
53150	1.55698	2.1
53151	1.89226	2.1
53152	1.89226	2.1
53153	1.89226	2.1
53154	1.8575	2.1
53155	1.8575	2.1
53156	1.89226	2.1
53157	1.89226	2.1
53158	1.89226	2.1
53159	1.89226	2.1
53160	1.89226	2.1
53161	1.89226	2.1
53162	1.89227	2.1
53163	1.89227	2.1
53164	1.89227	2.1
53165	1.89227	2.1
53166	1.89227	2.1
53167	1.89227	2.1
53168	1.35509	2.1
53169	2.06238	2.1
53170	2.12244	2.1
53171	2.12244	2.1
53172	1.34817	2.1
53173	1.34817	2.1
53174	0.946126	2.1
53175	0.946126	2.1
53176	0.946131	2.1
53177	0.946131	2.1
53178	0.946127	2.1
53179	0.946127	2.1
53180	0.94613	2.1
53181	0.94613	2.1
53182	0.946128	2.1
53183	0.946128	2.1

53184	0.946128	2.1
53185	0.946128	2.1
53186	1.89226	2.1
53187	1.89226	2.1
53188	1.89226	2.1
53189	1.89226	2.1
53190	1.89226	2.1
53191	1.89226	2.1
53192	1.16742	2.1
53193	1.16742	2.1
53194	1.18122	2.1
53195	1.18122	2.1
53196	1.31745	2.1
53197	1.2597	2.1
53198	1.03119	2.1
53199	1.06122	2.1
53200	1.06122	2.1
53201	1.31745	2.1
53202	1.03119	2.1
53203	1.2597	2.1
53204	1.36849	2.1
53205	2.12244	2.1
53206	2.20092	2.1
53207	2.20092	2.1
53208	1.35509	2.1
53209	1.35509	2.1
53210	1.89226	2.1
53211	1.89226	2.1
53212	1.89226	2.1
53213	1.89226	2.1
53214	1.89226	2.1
53215	1.89226	2.1
53216	1.89226	2.1
53217	1.89226	2.1
53218	1.89226	2.1
53219	1.89226	2.1
53220	3.78452	2.1
53221	1.21731	2.1
53222	1.21731	2.1
53223	1.23412	2.1
53224	1.23412	2.1
53225	1.39021	2.1
53226	1.31745	2.1
53227	1.06122	2.1
53228	1.10046	2.1
53229	1.10046	2.1
53230	1.39021	2.1
53231	1.06122	2.1
53232	1.31745	2.1
53233	2.16503	1.11663
53234	1.78252	1.61165
53235	1.77293	1.63258
53236	1.56839	2.1
53237	2.36957	0.431001
53238	2.16503	2.1
53239	1.89223	1.20388
53240	1.89223	2.1
53241	1.89223	2.07689

53242	1.89223	2.1
53243	3.78447	2.1
53244	2.3696	1.77044
53245	1.78254	1.81331
53246	2.63298	0.30745
53247	2.07312	2.1
53248	4.44272	1.74696
53249	1.89226	2.1
53250	1.89226	2.1
53251	1.89226	1.9131
53252	1.89226	2.1
53253	1.89226	0.898863
53254	1.89226	2.1
53255	1.89226	2.1
53256	1.89226	0.777059
53257	1.89226	2.1
53258	1.89226	0.315784
53259	3.78452	0.820032
53260	2.26369	2.1
53261	2.2168	0.498153
53262	2.15617	2.02666
53263	2.10864	0.248853
53264	4.37234	0.986723
53265	2.2168	0.365545
53266	1.94499	0.678764
53267	1.94499	1.37183
53268	2.04737	0.568214
53269	2.10864	0.155106
53270	2.21103	0.934523
53271	1.89226	1.37968
53272	1.89226	0.516265
53273	1.89226	0.622251
53274	1.89226	0.44353
53275	1.89226	0.70323
53276	1.89226	0.292582
53277	1.89226	2.08135
53278	1.89226	0.853718
53279	1.89226	1.2541
53280	1.89226	2.02318
53281	1.89226	0.427531
53282	1.89226	2.1
53283	1.02995	0.489208
53284	1.02995	2.09628
53285	1.03212	0.717018
53286	1.03212	1.90852
53287	0.988256	0.363965
53288	1.05432	0.244336
53289	1.00775	1.89671
53290	1.07809	1.99021
53291	1.00775	0.820213
53292	0.988257	0.858701
53293	1.07808	1.36745
53294	1.05432	1.1093
53295	2.10864	0.79694
53296	1.94499	1.08438
53297	1.97652	0.402002
53298	1.79681	0.699035
53299	3.9215	0.238765

53300	0.810943	0.493572
53301	0.810943	1.59179
53302	0.810943	0.409686
53303	0.810943	0.452462
53304	0.810958	0.179164
53305	0.810958	0.336281
53306	1.89226	0.71538
53307	1.35168	0.273288
53308	1.08132	0.745351
53309	1.08132	0.55736
53310	1.0813	0.266602
53311	0.657403	0.05
53312	1.35403	0.0547095
53313	1.24038	0.0582097
53314	0.499144	0.065079
53315	1.24039	0.059209
53316	1.35403	0.0708107
53317	0.657402	0.0547654
53318	0.499144	0.05
53319	0.915415	0.05
53320	0.915415	0.0615314
53321	0.937755	0.0553053
53322	0.937755	0.0722481
53323	1.72526	2.1
53324	1.17989	2.1
53325	1.96035	2.1
53326	1.47412	2.1
53327	3.14024	2.1
53328	1.89226	1.8833
53329	1.89226	2.1
53330	1.89226	2.1
53331	1.89226	1.61322
53332	3.78452	2.1
53333	0.946131	1.38533
53334	0.946131	1.58199
53335	1.25764	2.05071
53336	1.25764	2.09761
53337	1.07087	1.39136
53338	1.25765	1.73577
53339	0.946128	1.89887
53340	0.946128	0.749584
53341	0.94613	1.5605
53342	1.25765	1.70276
53343	1.07086	1.73386
53344	0.946129	1.67435
53345	1.96035	2.1
53346	1.32668	2.1
53347	2.29301	2.1
53348	1.70303	2.1
53349	3.56428	2.1
53350	1.89226	2.1
53351	1.89226	1.67472
53352	1.89226	1.78518
53353	1.89226	2.1
53354	3.78452	1.68052
53355	0.897738	0.307624
53356	0.897738	0.319305
53357	0.946102	0.259738

53358	0.946102	0.196126
53359	1.11247	0.242491
53360	0.89771	0.198334
53361	0.94613	0.253845
53362	0.94613	0.337421
53363	0.897709	0.305163
53364	0.946131	0.315886
53365	0.946131	0.393704
53366	1.11247	0.268308
53367	1.89226	2.1
53368	1.89226	2.04437
53369	1.89226	2.1
53370	1.89226	2.1
53371	1.89226	2.1
53372	1.89226	2.1
53373	2.15429	2.1
53374	1.89226	2.1
53375	1.89226	2.1
53376	1.89226	2.1
53377	2.27074	2.1
53378	2.27074	2.1
53379	0.993311	1.47956
53380	0.993311	1.87772
53381	0.904322	2.1
53382	0.904322	2.03521
53383	0.803324	2.1
53384	1.36183	1.46109
53385	0.916274	2.1
53386	0.535808	1.8435
53387	0.535808	1.9058
53388	0.803322	1.91439
53389	0.916276	1.88558
53390	1.36183	1.89099
53391	1.89226	2.1
53392	1.89226	1.92843
53393	1.89226	2.1
53394	1.89226	2.1
53395	1.89226	2.1
53396	1.89226	2.1
53397	2.11955	2.1
53398	2.62097	2.1
53399	1.85542	2.1
53400	2.39016	2.1
53401	4.50971	2.1
53402	2.39016	2.1
53403	2.20242	2.1
53404	2.39016	2.1
53405	1.64008	2.1
53406	1.85542	2.1
53407	1.64008	2.1
53408	1.79667	2.1
53409	1.28267	2.1
53410	1.28267	2.1
53411	1.40117	2.1
53412	1.74514	2.1
53413	1.83994	2.1
53414	0.575914	2.1
53415	0.625292	2.1

53416	0.949144	2.1
53417	0.882782	2.1
53418	0.773697	2.1
53419	0.773697	2.1
53420	0.786497	2.1
53421	0.786494	2.1
53422	0.605477	2.1
53423	0.949148	2.1
53424	0.625291	2.1
53425	0.862374	2.1
53426	1.89226	2.1
53427	2.44918	2.1
53428	2.4616	2.1
53429	2.44918	2.1
53430	1.90468	2.1
53431	1.89226	2.1
53432	1.89226	2.1
53433	1.89226	2.1
53434	1.89226	2.1
53435	1.89226	2.1
53436	3.78453	2.1
53437	0.946128	2.1
53438	0.946128	2.1
53439	1.23631	2.1
53440	1.23631	2.1
53441	0.946131	2.1
53442	0.946131	2.1
53443	1.3147	2.1
53444	1.23631	2.1
53445	1.3147	2.1
53446	0.946131	2.1
53447	1.23631	2.1
53448	0.94613	2.1
53449	1.89226	2.1
53450	2.4616	2.1
53451	2.4717	2.1
53452	2.4616	2.1
53453	1.90237	2.1
53454	1.89226	2.1
53455	0.946132	2.1
53456	0.946132	2.1
53457	0.946131	2.1
53458	0.946131	2.1
53459	0.946132	2.1
53460	0.946132	2.1
53461	0.946131	2.1
53462	0.946131	2.1
53463	0.946131	2.1
53464	0.946131	2.1
53465	0.946131	2.1
53466	0.946131	2.1
53467	0.946128	2.1
53468	0.946128	2.1
53469	1.3147	2.1
53470	1.3147	2.1
53471	0.94613	2.1
53472	0.94613	2.1
53473	1.3419	2.1

53474	1.3147	2.1
53475	1.3419	2.1
53476	0.94613	2.1
53477	1.3147	2.1
53478	0.94613	2.1
53479	1.89226	2.1
53480	1.89226	2.1
53481	1.89226	2.1
53482	1.89226	2.1
53483	3.78452	2.1
53484	2.3333	2.1
53485	1.3864	2.1
53486	1.3864	2.1
53487	1.4628	2.1
53488	1.95763	2.1
53489	2.03403	2.1
53490	1.294 2.1	
53491	1.294 2.1	
53492	0.946128	2.1
53493	0.946128	2.1
53494	1.294 2.1	
53495	1.27474	2.1
53496	0.946131	2.1
53497	0.946131	2.1
53498	0.946129	2.1
53499	1.27474	2.1
53500	1.294 2.1	
53501	0.94613	2.1
53502	1.89226	2.1
53503	1.89226	2.1
53504	1.89226	2.1
53505	1.89226	2.1
53506	1.89226	2.1
53507	1.89226	2.1
53508	0.808088	2.1
53509	0.808088	2.1
53510	0.815623	2.1
53511	0.815623	2.1
53512	0.660053	2.1
53513	0.693199	2.1
53514	0.930512	2.1
53515	0.978817	2.1
53516	0.660053	2.1
53517	0.930513	2.1
53518	0.693198	2.1
53519	0.978817	2.1
53520	1.27474	2.1
53521	1.27474	2.1
53522	0.946128	2.1
53523	0.946128	2.1
53524	1.26221	2.1
53525	1.27474	2.1
53526	0.946131	2.1
53527	0.946131	2.1
53528	0.94613	2.1
53529	1.26221	2.1
53530	0.946129	2.1
53531	1.27474	2.1

53532	0.90207	0.05
53533	0.90207	0.05
53534	0.895719	0.05
53535	0.895719	0.05
53536	0.927855	0.05
53537	1.2493	0.05
53538	0.85723	0.05
53539	0.548484	0.05
53540	0.548485	0.05
53541	0.927855	0.05
53542	0.85723	0.05
53543	1.2493	0.05
53544	1.41319	0.381427
53545	1.22482	0.215204
53546	1.46629	0.260539
53547	1.92622	0.42011
53548	3.15104	0.355115
53549	1.02305	0.147719
53550	1.22482	0.218576
53551	1.80336	0.218269
53552	1.44426	0.14551
53553	2.66908	0.150794
53554	1.3268	2.1
53555	0.999489	2.1
53556	1.44091	2.1
53557	1.14657	2.1
53558	2.4404	2.1
53559	1.89226	2.1
53560	1.89226	2.1
53561	1.89226	2.1
53562	1.89226	2.1
53563	3.78452	2.1
53564	1.89226	2.1
53565	2.28605	2.1
53566	2.43157	2.1
53567	2.43157	2.1
53568	1.89226	2.1
53569	1.89226	2.1
53570	1.60647	2.1
53571	1.44091	2.1
53572	1.31346	2.1
53573	1.31346	2.1
53574	1.14657	2.1
53575	1.44091	2.1
53576	1.89226	2.1
53577	1.89226	2.1
53578	1.89226	2.1
53579	1.89226	2.1
53580	3.78452	2.1
53581	0.946132	2.1
53582	0.946132	2.1
53583	1.3048	2.1
53584	1.3048	2.1
53585	1.3048	2.1
53586	1.21578	2.1
53587	0.946132	2.1
53588	0.946132	2.1
53589	1.3048	2.1

53590	0.946132	2.1
53591	0.946132	2.1
53592	1.21578	2.1
53593	1.89227	0.05
53594	1.89227	0.05
53595	1.89227	0.05
53596	1.89227	0.05
53597	3.78453	0.05
53598	1.6671	0.05
53599	1.87338	0.05
53600	1.6671	0.05
53601	2.02098	0.05
53602	1.38328	0.05
53603	1.73715	0.05
53604	1.89226	0.05
53605	1.89226	0.05
53606	1.89226	0.05
53607	1.89226	0.05
53608	3.78453	0.05
53609	1.6671	0.05
53610	1.38328	0.05
53611	1.35562	0.05
53612	1.01012	0.05
53613	2.67722	0.05
53614	0.819377	0.05
53615	0.647925	0.05
53616	1.17802	0.05
53617	1.33174	0.05
53618	0.98543	0.05
53619	0.98543	0.05
53620	0.994237	0.05
53621	0.994237	0.05
53622	1.17802	0.05
53623	0.647926	0.05
53624	1.33174	0.05
53625	0.819377	0.05
53626	1.27806	0.05
53627	1.68264	0.05
53628	1.44593	0.05
53629	1.878 0.05	
53630	3.15606	0.05
53631	1.878 0.05	
53632	1.9898	0.05
53633	1.878 0.05	
53634	1.68264	0.05
53635	2.30533	0.05
53636	2.10997	0.05
53637	0.814587	0.05
53638	0.814596	0.05
53639	0.82775	0.05
53640	0.82775	0.05
53641	1.0107	0.05
53642	0.91549	0.05
53643	0.741058	0.05
53644	0.714748	0.05
53645	0.714758	0.05
53646	1.03225	0.05
53647	0.704864	0.05

53648	0.915488	0.05
53649	1.90347	0.05
53650	2.06584	0.05
53651	1.878	0.05
53652	1.50236	0.05
53653	2.05749	0.05
53654	2.30533	0.05
53655	2.24475	2.1
53656	1.85871	2.1
53657	2.13711	2.1
53658	2.13711	2.1
53659	1.9943	2.1
53660	1.9943	2.1
53661	1.85871	2.1
53662	1.7871	2.1
53663	1.41619	2.1
53664	1.41619	2.1
53665	2.13711	2.1
53666	2.13711	2.1
53667	2.24475	2.1
53668	2.13711	2.1
53669	2.54902	2.1
53670	2.46327	2.1
53671	4.70802	2.1
53672	1.06913	2.1
53673	1.06913	2.1
53674	1.0821	2.1
53675	1.0821	2.1
53676	0.893552	2.1
53677	1.08267	2.1
53678	1.06855	2.1
53679	1.23163	2.1
53680	1.23163	2.1
53681	1.08267	2.1
53682	0.893551	2.1
53683	1.06855	2.1
53684	1.67046	1.17843
53685	2.4106	0.236569
53686	2.05835	1.46396
53687	2.4106	1.47328
53688	1.59562	0.462755
53689	1.94788	0.241317
53690	2.34737	1.94733
53691	2.4106	0.440656
53692	2.05834	1.55476
53693	1.99655	1.79859
53694	4.40715	1.54762
53695	1.67046	0.253016
53696	2.05835	0.193577
53697	1.588	1.38945
53698	1.34857	1.62705
53699	3.40692	1.09854
53700	1.73802	1.46664
53701	1.89668	1.52632
53702	1.99655	2.01998
53703	1.99655	0.446615
53704	1.79981	1.59654
53705	2.05834	0.364069

53706	1.89226	1.59104
53707	1.89226	1.18076
53708	1.89226	0.802529
53709	1.89226	1.73709
53710	1.89226	1.10126
53711	1.89226	2.1
53712	1.89226	1.49348
53713	1.89226	2.05012
53714	1.89226	1.85354
53715	1.89226	0.642446
53716	3.78453	1.73055
53717	0.719473	2.07292
53718	0.916103	1.88842
53719	1.02744	1.44763
53720	0.850328	1.57751
53721	0.888094	1.4263
53722	0.888094	2.1
53723	0.878337	2.01132
53724	0.878337	0.510832
53725	0.916103	1.58901
53726	1.02744	1.62246
53727	0.719473	1.80623
53728	0.850328	1.84202
53729	1.02744	1.56034
53730	0.850329	1.84682
53731	1.0875	1.20427
53732	0.919597	1.35885
53733	0.975841	1.16728
53734	0.975841	2.1
53735	0.971196	1.13473
53736	0.971196	1.76381
53737	1.02744	1.3294
53738	1.0875	1.22449
53739	0.850328	1.61821
53740	0.919598	1.59852
53741	1.89226	1.90048
53742	1.89226	1.5024
53743	1.89226	1.12322
53744	1.89226	1.51795
53745	3.78453	1.41692
53746	1.89226	1.81573
53747	1.89226	1.34793
53748	1.89226	0.643445
53749	1.89226	1.83963
53750	1.89226	0.679731
53751	1.89226	2.06888
53752	1.11103	1.76202
53753	0.916103	1.96201
53754	1.21177	1.39259
53755	1.02744	1.40334
53756	1.07188	1.32464
53757	1.07188	2.1
53758	1.06659	1.34039
53759	1.06659	1.89371
53760	1.11103	1.62776
53761	1.21177	1.54777
53762	0.916104	1.71098
53763	1.02744	1.777

53764	2.54158	1.23554
53765	2.42354	1.4891
53766	2.17499	1.21249
53767	2.17499	2.01695
53768	2.42354	1.56969
53769	2.05488	2.1
53770	1.89226	0.0532604
53771	1.89226	0.0736495
53772	1.89226	0.0599403
53773	1.89226	0.0712711
53774	3.78453	0.061425
53775	1.89226	0.05
53776	1.89226	0.0642677
53777	1.89226	0.0520617
53778	1.89226	0.0583002
53779	1.89226	0.055432
53780	1.89226	0.0607602
53781	1.89226	0.110396
53782	1.89226	0.130131
53783	1.89226	0.107686
53784	1.89226	0.0716059
53785	1.89226	0.181384
53786	1.89226	0.130029
53787	1.89226	0.108118
53788	1.89226	0.171037
53789	1.89226	0.119461
53790	1.89226	0.201211
53791	3.78452	0.155367
53792	0.802544	0.05
53793	0.946132	0.05
53794	0.946132	0.05
53795	0.787038	0.05
53796	0.787039	0.05
53797	0.787039	0.0554733
53798	0.961637	0.05
53799	0.961637	0.05
53800	0.787038	0.05
53801	0.802544	0.05
53802	0.946132	0.05
53803	0.946132	0.05
53804	1.89226	0.05
53805	1.60509	0.05
53806	1.89226	0.05
53807	1.89226	0.05
53808	1.3905	0.05
53809	1.3905	0.05
53810	0.946132	0.0792709
53811	0.946132	0.056053
53812	0.946131	0.0795108
53813	0.946131	0.0744565
53814	0.946132	0.0743352
53815	0.946132	0.0784639
53816	0.946131	0.0671756
53817	0.946131	0.0715787
53818	0.946131	0.0668982
53819	0.946131	0.0756411
53820	0.946132	0.064844
53821	0.946132	0.0639957

53822	0.946131	0.103039
53823	0.946131	0.108
53824	0.946131	0.124736
53825	0.946131	0.0711379
53826	0.946132	0.102898
53827	0.946132	0.124631
53828	0.946131	0.0787028
53829	0.946131	0.0763748
53830	0.946131	0.108811
53831	0.946131	0.0671143
53832	0.946132	0.078175
53833	0.946132	0.0927242
53834	1.42382	2.1
53835	1.32201	1.07127
53836	1.32096	1.59827
53837	1.42866	2.09035
53838	2.75067	1.92539
53839	1.89226	2.1
53840	1.89226	0.917978
53841	1.89226	1.77691
53842	1.89226	2.01979
53843	1.89226	1.05932
53844	1.89226	1.82021
53845	1.89226	1.78197
53846	2.79029	0.500136
53847	2.7803	0.775917
53848	2.79029	1.57196
53849	1.88227	0.5506
53850	1.89226	0.87309
53851	0.716065	2.09799
53852	0.714333	2.1
53853	0.660483	1.25032
53854	0.659126	1.77218
53855	0.687538	1.38411
53856	0.687538	2.09181
53857	0.685921	2.1
53858	0.685921	1.3047
53859	0.659126	1.92735
53860	0.716065	1.93147
53861	0.660483	1.89716
53862	0.714333	1.89629
53863	1.89226	2.09298
53864	1.89226	1.1228
53865	1.89226	1.77548
53866	1.89226	1.97124
53867	3.78453	1.86735
53868	0.946133	1.16014
53869	0.946133	1.72509
53870	1.39015	0.654657
53871	1.38969	0.462621
53872	0.946132	0.48436
53873	0.946132	0.965517
53874	1.38969	1.65346
53875	1.38969	0.607845
53876	1.38969	0.778512
53877	0.946132	0.832577
53878	0.946133	1.2371
53879	1.39015	0.780528

53880	2.72157	0.0864557
53881	1.22176	0.058171
53882	1.59492	0.0643146
53883	1.22176	0.112367
53884	2.40743	0.0566552
53885	2.03426	0.107447
53886	0.946131	0.150815
53887	0.946131	0.126271
53888	1.36079	0.0860304
53889	1.01713	0.11063
53890	0.94613	0.104974
53891	0.94613	0.135487
53892	1.36079	0.10132
53893	1.36079	0.0788442
53894	0.946131	0.107805
53895	1.36079	0.0981828
53896	1.01713	0.0890871
53897	0.946131	0.103521
53898	1.89226	0.154008
53899	1.89226	0.104411
53900	1.89226	0.105333
53901	1.89226	0.185835
53902	3.78453	0.16592
53903	1.89226	0.106417
53904	1.89226	0.0608943
53905	1.89226	0.0620762
53906	1.89226	0.105574
53907	1.89226	0.0600179
53908	1.89226	0.10702
53909	0.946131	0.154847
53910	0.946131	0.129504
53911	0.946131	0.107594
53912	1.36079	0.0788756
53913	0.94613	0.100346
53914	0.94613	0.131873
53915	0.946133	0.114727
53916	0.946133	0.0809503
53917	0.946131	0.133554
53918	0.946131	0.112456
53919	0.946132	0.117291
53920	1.36079	0.0976207
53921	1.89226	0.106671
53922	1.89226	0.180531
53923	1.89226	0.124312
53924	1.89226	0.135216
53925	1.89226	0.121462
53926	1.89226	0.140869
53927	1.89227	0.112173
53928	1.89227	0.0640631
53929	1.89227	0.0721413
53930	1.89227	0.125551
53931	1.89227	0.0702364
53932	1.89227	0.122081
53933	1.60428	2.1
53934	2.2367	2.1
53935	1.50097	2.1
53936	1.80125	2.1
53937	3.40553	2.1

53938	1.89095	2.1
53939	1.85293	2.1
53940	1.80125	2.1
53941	1.76268	2.1
53942	3.65418	2.1
53943	0.803177	2.1
53944	0.803177	2.1
53945	0.798644	2.1
53946	0.798644	2.1
53947	0.84233	2.1
53948	0.900624	2.1
53949	0.750483	2.1
53950	0.701197	2.1
53951	0.701197	2.1
53952	0.84233	2.1
53953	0.750483	2.1
53954	0.900625	2.1
53955	0.860925	2.1
53956	0.860925	2.1
53957	0.862743	2.1
53958	0.862743	2.1
53959	0.881338	2.1
53960	0.826621	2.1
53961	0.84233	2.1
53962	0.900625	2.1
53963	0.84233	2.1
53964	0.826621	2.1
53965	0.900624	2.1
53966	0.881339	2.1
53967	1.37938	1.20208
53968	1.03319	1.31749
53969	1.00512	1.41942
53970	0.639582	1.43645
53971	1.00463	1.42625
53972	1.00463	1.18308
53973	1.01434	1.15245
53974	1.01434	1.41896
53975	1.37938	1.32803
53976	1.00512	1.4106
53977	1.03319	1.40239
53978	0.639582	1.41534
53979	0.728713	1.02226
53980	0.728713	0.971384
53981	0.73425	1.74414
53982	0.73425	2.1
53983	0.525122	2.1
53984	0.605622	0.328249
53985	0.948901	0.328249
53986	0.857342	0.328249
53987	0.85734	0.314917
53988	0.525123	2.1
53989	0.948899	0.314917
53990	0.605624	0.314917
53991	1.75449	1.01048
53992	1.4378	1.048
53993	1.93916	1.11249
53994	2.22512	0.468224
53995	3.69364	1.01215

53996	1.75448	1.15037
53997	1.89186	0.895081
53998	2.29771	1.00019
53999	2.22512	0.835889
54000	4.05219	1.015
54001	1.93916	1.12557
54002	2.51749	0.394255
54003	2.62357	0.776019
54004	2.22512	0.761719
54005	2.33761	1.00769
54006	1.93916	0.882059
54007	2.29771	1.03421
54008	2.51749	0.535853
54009	2.22512	0.746781
54010	2.22512	0.516573
54011	2.50905	1.0402
54012	2.50905	1.03649
54013	0.87382	0.05
54014	1.33662	0.05
54015	1.20756	0.05
54016	0.890467	0.063841
54017	1.08859	0.0633217
54018	1.08859	0.0623215
54019	1.13084	0.05
54020	1.08793	0.05
54021	1.34722	0.05
54022	1.27184	0.057948
54023	0.917678	0.0552328
54024	0.97821	0.0550996
54025	1.28396	0.0500916
54026	1.70302	0.0686009
54027	2.30076	0.0563714
54028	1.20417	0.0541374
54029	2.57506	0.0581532
54030	2.2656	0.0530691
54031	1.03642	1.98886
54032	1.03642	0.350266
54033	1.07029	0.12477
54034	1.07029	0.511229
54035	1.31531	1.93836
54036	1.23279	0.553398
54037	0.941743	0.202535
54038	0.791399	1.50478
54039	0.791399	0.506722
54040	1.23279	0.482195
54041	0.941744	0.427589
54042	1.31531	0.411833
54043	0.937354	2.00865
54044	0.937354	0.292937
54045	0.954794	0.153726
54046	0.954794	0.509337
54047	1.13563	0.529158
54048	1.23279	1.90985
54049	0.791398	0.244002
54050	0.659359	1.31154
54051	1.13563	0.45562
54052	0.659359	0.486831
54053	0.791399	0.355748

54054	1.23279	0.345631
54055	2.29852	1.4248
54056	1.89227	1.35511
54057	2.14485	1.16813
54058	2.14485	1.48695
54059	1.89227	0.917237
54060	1.89227	1.49097
54061	0.947863	0.884168
54062	1.19854	1.35971
54063	2.16841	0.781356
54064	1.76597	1.146
54065	2.96451	1.15489
54066	1.89227	1.03613
54067	1.89227	1.17405
54068	1.89227	0.679117
54069	1.89227	1.40245
54070	1.89227	0.549656
54071	1.89227	1.39862
54072	2.46489	2.1
54073	1.98339	2.1
54074	1.81667	2.1
54075	2.36914	2.1
54076	4.35253	2.1
54077	2.46488	2.1
54078	2.70401	2.1
54079	2.64996	2.1
54080	2.36914	2.1
54081	5.07315	2.1
54082	1.81667	2.1
54083	2.29459	2.1
54084	2.36914	2.1
54085	2.36914	2.1
54086	1.68073	2.1
54087	1.68073	2.1
54088	2.64996	2.1
54089	2.29458	2.1
54090	2.29458	2.1
54091	2.36914	2.1
54092	2.61243	2.1
54093	2.68698	2.1
54094	1.89226	1.56929
54095	1.89226	1.1078
54096	1.89226	1.53907
54097	1.89226	1.5204
54098	1.89226	1.43324
54099	1.89226	1.46229
54100	1.89224	1.74682
54101	1.89224	1.27788
54102	1.65571	1.2104
54103	1.65571	1.45529
54104	1.89224	1.47576
54105	1.89224	1.5509
54106	1.89224	1.44722
54107	1.89224	0.917676
54108	1.89224	1.31011
54109	1.89224	1.26765
54110	1.89224	1.41525
54111	1.89224	1.27315

54112	1.98252	0.084026
54113	2.20641	0.0893648
54114	1.05129	0.0770095
54115	1.2951	0.112939
54116	3.27762	0.0827475
54117	1.85598	2.1
54118	1.88635	1.58253
54119	2.12346	0.759909
54120	2.10838	2.1
54121	3.97943	1.74932
54122	2.10838	2.07074
54123	1.87532	2.1
54124	1.85597	1.95726
54125	1.85597	2.1
54126	1.87532	2.1
54127	1.57914	2.1
54128	2.12348	1.4615
54129	2.35141	2.04677
54130	2.37191	1.72992
54131	2.10841	1.85806
54132	2.38698	1.59876
54133	2.12348	1.87461
54134	1.07811	2.1
54135	0.937672	2.1
54136	1.17571	2.0061
54137	1.05421	1.4342
54138	1.07087	1.94914
54139	1.07087	2.1
54140	1.06145	2.1
54141	1.06145	1.90166
54142	1.17571	2.1
54143	1.07811	2.1
54144	1.05421	2.1
54145	0.937672	2.1
54146	1.67334	2.1
54147	2.76609	2.1
54148	2.74144	2.1
54149	2.74144	2.1
54150	1.71157	2.1
54151	1.71157	2.1
54152	2.76609	2.1
54153	1.89226	2.1
54154	1.89226	2.1
54155	1.89226	2.1
54156	2.74143	2.1
54157	2.74143	2.1
54158	1.10393	2.1
54159	1.10393	2.1
54160	1.10788	2.1
54161	1.10788	2.1
54162	1.38302	2.1
54163	1.37072	2.1
54164	0.836672	2.1
54165	0.841089	2.1
54166	1.38302	2.1
54167	0.84109	2.1
54168	0.836671	2.1
54169	1.37072	2.1

54170	1.38302	2.1
54171	1.38302	2.1
54172	0.946131	2.1
54173	0.946131	2.1
54174	0.946128	2.1
54175	0.946128	2.1
54176	1.38302	2.1
54177	1.37072	2.1
54178	1.38302	2.1
54179	0.946131	2.1
54180	0.94613	2.1
54181	1.37072	2.1
54182	1.28072	2.1
54183	1.55779	2.1
54184	1.28072	2.1
54185	1.40765	2.1
54186	1.47186	2.1
54187	1.59879	2.1
54188	1.89227	2.1
54189	1.89227	2.1
54190	1.89227	1.73159
54191	1.89227	2.1
54192	1.89227	1.93549
54193	1.89227	2.1
54194	2.80057	1.62182
54195	1.89226	1.80747
54196	1.89226	1.45354
54197	1.89226	2.1
54198	2.63061	0.642452
54199	2.63061	2.1
54200	1.28072	2.1
54201	1.38859	2.1
54202	1.47186	2.1
54203	1.47186	2.1
54204	1.16777	2.1
54205	1.16777	2.1
54206	1.89226	2.1
54207	1.89226	2.1
54208	1.89226	1.94673
54209	1.89226	2.1
54210	1.89226	1.80372
54211	1.89226	2.1
54212	1.83221	2.1
54213	2.0815	2.1
54214	2.0815	2.1
54215	2.08153	2.1
54216	1.86703	2.1
54217	1.87402	2.1
54218	1.86703	2.1
54219	2.08508	2.1
54220	2.08508	2.1
54221	2.29001	2.1
54222	1.89226	2.1
54223	2.08048	2.1
54224	2.7278	0.0962505
54225	1.10909	0.105743
54226	1.50472	0.0908132
54227	1.10909	0.122126

54228	2.69881	0.0881373
54229	2.30319	0.134916
54230	1.23204	2.1
54231	1.92264	2.1
54232	1.48162	2.1
54233	1.48162	2.1
54234	1.72994	2.1
54235	1.72994	2.1
54236	0.864968	2.1
54237	0.696952	2.1
54238	0.61602	2.1
54239	0.479736	2.1
54240	0.66444	2.1
54241	0.66444	2.1
54242	0.680264	2.1
54243	0.680264	2.1
54244	0.696952	2.1
54245	0.479736	2.1
54246	0.61602	2.1
54247	0.864968	2.1
54248	1.89226	0.789679
54249	2.43219	1.7126
54250	1.89226	1.60159
54251	2.82019	1.54018
54252	1.89226	1.58258
54253	2.82019	1.453
54254	1.89226	1.23565
54255	2.83701	1.84795
54256	2.2701	1.76049
54257	2.2701	1.58773
54258	1.89226	1.75531
54259	1.89226	1.60213
54260	1.89226	1.6178
54261	1.89226	1.23776
54262	1.89226	1.48952
54263	1.89226	1.364
54264	1.89226	1.46421
54265	1.89226	1.3534
54266	1.5795	1.62204
54267	1.44091	1.44419
54268	1.05267	1.62764
54269	1.05267	1.39618
54270	1.98408	1.63417
54271	1.98408	1.39089
54272	2.20624	2.1
54273	1.08831	2.1
54274	2.49053	2.1
54275	1.42486	2.1
54276	3.57883	2.1
54277	1.89226	2.1
54278	1.89226	2.1
54279	1.89226	2.1
54280	1.89226	2.1
54281	3.78453	2.1
54282	0.946132	2.1
54283	0.946132	2.1
54284	0.853591	2.1
54285	0.642207	2.1

54286	0.94613	2.1
54287	0.94613	2.1
54288	0.642209	2.1
54289	0.642209	2.1
54290	0.946131	2.1
54291	0.853591	2.1
54292	0.642207	2.1
54293	0.946132	2.1
54294	1.0506	2.1
54295	1.0506	2.1
54296	1.0879	2.1
54297	0.960424	2.1
54298	0.708687	2.1
54299	0.782927	2.1
54300	1.40041	2.1
54301	1.2518	2.1
54302	0.767748	2.1
54303	1.31801	2.1
54304	1.21412	2.1
54305	0.69461	2.1
54306	1.89226	2.1
54307	1.89226	2.1
54308	1.89226	2.1
54309	1.89226	2.1
54310	1.89226	2.1
54311	1.89226	2.1
54312	0.922223	2.1
54313	0.922223	2.1
54314	1.04548	2.1
54315	0.768229	2.1
54316	0.948441	2.1
54317	0.806483	2.1
54318	0.85362	2.1
54319	0.85362	2.1
54320	1.05942	2.1
54321	0.922187	2.1
54322	0.851554	2.1
54323	0.768264	2.1
54324	2.16728	2.1
54325	0.95455	2.1
54326	1.21372	2.1
54327	0.98227	2.1
54328	2.16728	2.1
54329	1.93584	2.1
54330	2.08849	0.12119
54331	1.08732	0.128697
54332	1.3357	0.124616
54333	2.37948	0.112893
54334	3.4668	0.123215
54335	2.57882	0.106235
54336	2.37948	0.119538
54337	2.53055	0.120898
54338	1.48676	0.112915
54339	1.3357	0.121607
54340	1.3357	0.113803
54341	1.89226	2.1
54342	1.89226	2.1
54343	2.17471	2.1

54344	1.89226	2.1
54345	2.60085	2.1
54346	2.26306	2.1
54347	0.946131	2.1
54348	0.946131	2.1
54349	0.946131	2.1
54350	0.946131	2.1
54351	0.94613	2.1
54352	0.94613	2.1
54353	0.946132	2.1
54354	0.946132	2.1
54355	0.946131	2.1
54356	0.946131	2.1
54357	0.946131	2.1
54358	0.946131	2.1
54359	1.89226	2.1
54360	2.17471	2.1
54361	2.08196	2.1
54362	2.17471	2.1
54363	1.79952	2.1
54364	1.89226	2.1
54365	1.89226	2.1
54366	1.89226	2.1
54367	1.89226	2.1
54368	1.89226	2.1
54369	1.89226	2.1
54370	1.89226	2.1
54371	1.86016	2.1
54372	1.89226	2.1
54373	1.89226	2.1
54374	1.89226	2.1
54375	1.84826	2.1
54376	1.84826	2.1
54377	2.50136	2.1
54378	1.99781	2.1
54379	1.99729	2.1
54380	2.5214	2.1
54381	4.49865	2.1
54382	0.51724	2.1
54383	0.496076	2.1
54384	0.998906	2.1
54385	0.998645	2.1
54386	0.742249	2.1
54387	0.742249	2.1
54388	0.752734	2.1
54389	0.752734	2.1
54390	0.998639	2.1
54391	0.496073	2.1
54392	0.517243	2.1
54393	0.998913	2.1
54394	0.998653	2.1
54395	0.994293	2.1
54396	1.26071	2.1
54397	1.26504	2.1
54398	1.12529	2.1
54399	1.12529	2.1
54400	1.12971	2.1
54401	1.12971	2.1

54402	1.26504	2.1
54403	0.994292	2.1
54404	0.998651	2.1
54405	1.26071	2.1
54406	1.89226	2.1
54407	1.89226	2.1
54408	1.89226	2.1
54409	1.89226	2.1
54410	3.78452	2.1
54411	1.89226	2.1
54412	1.89226	2.1
54413	1.89226	2.1
54414	1.89226	2.1
54415	3.78452	2.1
54416	0.675872	2.1
54417	0.675641	2.1
54418	0.943072	2.1
54419	0.898317	2.1
54420	0.775717	2.1
54421	0.775717	2.1
54422	0.79824	2.1
54423	0.79824	2.1
54424	0.675642	2.1
54425	0.94307	2.1
54426	0.675874	2.1
54427	0.898315	2.1
54428	0.943071	2.1
54429	0.898316	2.1
54430	0.998133	2.1
54431	0.918903	2.1
54432	0.922369	2.1
54433	0.922369	2.1
54434	0.939606	2.1
54435	0.939606	2.1
54436	0.943071	2.1
54437	0.998133	2.1
54438	0.898316	2.1
54439	0.918903	2.1
54440	0.946131	2.1
54441	0.946131	2.1
54442	0.946131	2.1
54443	0.946131	2.1
54444	0.946132	2.1
54445	0.946132	2.1
54446	0.946129	2.1
54447	0.946129	2.1
54448	0.94613	2.1
54449	0.94613	2.1
54450	0.946131	2.1
54451	0.946131	2.1
54452	1.89226	2.1
54453	1.89226	2.1
54454	1.89226	2.1
54455	1.89226	2.1
54456	1.89226	2.1
54457	1.89226	2.1
54458	1.35129	2.1
54459	1.88614	2.1

54460	2.01867	2.1
54461	1.38189	2.1
54462	3.36995	2.1
54463	0.943071	2.1
54464	1.00934	2.1
54465	0.998134	2.1
54466	1.10866	2.1
54467	0.992617	2.1
54468	0.992617	2.1
54469	1.01485	2.1
54470	1.01485	2.1
54471	1.00934	2.1
54472	1.10866	2.1
54473	0.943072	2.1
54474	0.998134	2.1
54475	2.67272	2.1
54476	1.61859	2.1
54477	1.70303	2.1
54478	2.52175	2.1
54479	4.5326	2.1
54480	2.00954	2.1
54481	2.27801	2.1
54482	1.87875	2.1
54483	1.87875	2.1
54484	2.37837	2.1
54485	2.37837	2.1
54486	2.00955	2.1
54487	1.78	2.1
54488	2.21813	2.1
54489	1.8493	2.1
54490	2.37838	2.1
54491	2.00955	2.1
54492	2.37838	2.1
54493	2.27801	2.1
54494	2.58523	2.1
54495	2.71597	2.1
54496	4.9636	2.1
54497	2.37838	2.1
54498	2.71597	2.1
54499	1.72709	2.1
54500	2.0815	2.1
54501	2.59749	2.1
54502	2.78034	2.1
54503	0.94613	1.48778
54504	0.94613	1.50932
54505	0.946128	1.38062
54506	0.946128	1.35583
54507	0.94613	1.35386
54508	0.94613	1.3973
54509	0.946128	1.50866
54510	0.946128	1.24749
54511	0.946129	1.46618
54512	0.946129	1.46018
54513	0.946129	1.41256
54514	0.946129	1.40541
54515	1.89226	1.63681
54516	1.89226	1.5576
54517	1.89226	1.70997

54518	1.89226	1.47958
54519	3.78452	1.50908
54520	0.946133	1.11277
54521	0.946133	1.19237
54522	0.946132	0.675361
54523	0.946132	0.804894
54524	0.946132	0.746201
54525	0.946132	0.815658
54526	0.946133	1.2205
54527	0.946133	1.12501
54528	0.946132	1.11094
54529	0.946132	0.375938
54530	0.946132	0.981404
54531	0.946132	0.828061
54532	1.89226	1.46252
54533	2.10495	0.507085
54534	1.89226	0.674929
54535	2.30932	0.803449
54536	1.89226	1.37291
54537	2.30932	1.46926
54538	2.30932	0.600657
54539	1.00366	0.277393
54540	2.10496	0.250076
54541	1.24728	0.289328
54542	3.31298	0.322639
54543	1.87557	2.1
54544	1.07167	2.1
54545	2.03045	2.1
54546	1.25185	2.1
54547	3.10212	2.1
54548	1.89226	2.1
54549	1.89226	2.1
54550	1.89226	2.1
54551	1.89226	2.1
54552	3.78453	2.1
54553	0.851541	2.1
54554	0.851541	2.1
54555	1.12236	2.1
54556	1.05783	2.1
54557	0.870663	2.1
54558	0.951856	2.1
54559	0.946132	2.1
54560	0.946132	2.1
54561	1.04073	2.1
54562	1.1296	2.1
54563	0.776062	2.1
54564	0.851527	2.1
54565	0.859395	2.1
54566	0.859395	2.1
54567	0.870165	2.1
54568	0.870165	2.1
54569	0.625924	2.1
54570	0.714333	2.1
54571	1.0821	2.1
54572	1.01523	2.1
54573	1.0821	2.1
54574	0.714333	2.1
54575	1.01523	2.1

54576	0.625924	2.1
54577	1.89226	2.1
54578	1.89226	2.1
54579	1.89226	2.1
54580	1.89226	2.1
54581	3.78453	2.1
54582	1.89226	2.1
54583	1.89226	2.1
54584	2.23164	2.1
54585	2.67802	2.1
54586	2.03663	2.1
54587	1.89226	2.1
54588	2.42221	2.1
54589	1.61933	2.1
54590	2.80896	2.1
54591	1.87043	2.1
54592	4.29262	2.1
54593	1.89226	2.1
54594	1.89226	2.1
54595	1.89226	2.1
54596	1.89226	2.1
54597	1.89226	2.1
54598	1.89226	2.1
54599	2.80892	2.1
54600	1.87041	2.1
54601	2.67182	2.1
54602	2.30891	2.1
54603	5.07665	2.1
54604	1.89224	2.1
54605	1.89224	2.1
54606	1.89224	2.1
54607	1.89224	2.1
54608	3.78448	2.1
54609	0.946118	2.1
54610	0.946118	2.1
54611	0.946118	2.1
54612	0.946118	2.1
54613	0.946115	2.1
54614	0.946115	2.1
54615	0.94612	2.1
54616	0.94612	2.1
54617	0.94613	2.1
54618	0.94613	2.1
54619	0.946105	2.1
54620	0.946105	2.1
54621	2.3089	2.1
54622	1.02783	2.1
54623	1.98533	2.1
54624	1.76762	2.1
54625	1.24553	2.1
54626	1.02783	2.1
54627	2.2049	0.115562
54628	2.34673	0.110275
54629	1.00763	0.107288
54630	1.23746	0.144125
54631	3.44236	0.112686
54632	1.89226	0.157355
54633	1.89226	0.224138

54634	1.89226	0.196602
54635	1.89226	0.208194
54636	3.78452	0.216324
54637	1.89226	0.231688
54638	1.89226	0.203778
54639	1.89226	0.213311
54640	1.89226	0.300498
54641	3.78453	0.223749
54642	0.891618	0.156728
54643	0.891618	0.147714
54644	0.902158	0.117315
54645	0.902158	0.12909
54646	1.15396	0.148426
54647	0.620408	0.136456
54648	1.17337	0.118096
54649	0.618733	0.147727
54650	0.620408	0.142707
54651	0.618733	0.132462
54652	1.17337	0.137219
54653	1.15396	0.14426
54654	0.69978	0.151533
54655	0.69978	0.141949
54656	0.946102	0.144459
54657	0.946102	0.148981
54658	0.831702	0.161768
54659	0.946129	0.1648
54660	0.699752	0.146454
54661	0.946132	0.13108
54662	0.831703	0.156382
54663	0.699751	0.13361
54664	0.946131	0.135178
54665	0.946131	0.143731
54666	0.831675	0.182094
54667	0.831675	0.178043
54668	0.946163	0.163349
54669	0.864397	0.174424
54670	0.853471	0.213606
54671	0.962623	0.167225
54672	0.748555	0.162055
54673	0.851543	0.167099
54674	0.864714	0.184794
54675	0.760411	0.174132
54676	0.946133	0.167754
54677	0.946133	0.179887
54678	1.89226	0.0828673
54679	1.89226	0.583695
54680	1.89226	0.334817
54681	1.89226	0.078953
54682	3.78452	0.102582
54683	1.89226	0.708354
54684	1.89226	0.407118
54685	1.89226	0.174791
54686	1.89226	0.671413
54687	3.78452	0.439532
54688	0.946132	0.606945
54689	0.946132	0.809494
54690	0.946132	0.159645
54691	0.946132	0.412327

54692	0.946132	0.178803
54693	0.946132	0.614216
54694	0.946133	0.382607
54695	0.946133	0.753727
54696	0.946133	0.196298
54697	0.946133	0.240152
54698	0.946132	0.546269
54699	0.946132	0.544995
54700	1.89226	0.582582
54701	2.39715	0.11051
54702	1.89226	0.110169
54703	1.89226	0.317061
54704	1.89226	0.137485
54705	1.89226	0.331203
54706	0.94613	0.528501
54707	0.94613	0.626396
54708	0.94613	0.310307
54709	0.94613	0.15438
54710	0.946131	0.148625
54711	0.946131	0.548662
54712	0.946129	0.672137
54713	0.946129	0.241175
54714	0.94613	0.209154
54715	0.94613	0.192584
54716	0.94613	0.493533
54717	0.94613	0.449957
54718	2.54204	2.1
54719	2.3148	2.1
54720	2.55328	2.1
54721	2.39646	2.1
54722	4.71126	2.1
54723	1.62611	2.1
54724	2.3148	2.1
54725	2.16986	2.1
54726	1.43745	2.1
54727	3.75225	2.1
54728	1.89226	2.1
54729	1.89226	2.1
54730	1.89226	2.1
54731	1.89226	2.1
54732	3.78453	2.1
54733	1.89226	2.1
54734	1.89226	2.1
54735	1.89226	2.1
54736	1.89226	2.1
54737	3.78453	2.1
54738	2.12125	2.1
54739	2.39642	2.1
54740	2.2941	2.1
54741	1.9933	2.1
54742	4.38972	2.1
54743	1.73784	2.1
54744	1.26892	2.1
54745	1.26892	2.1
54746	1.43743	2.1
54747	1.9933	2.1
54748	2.1618	2.1
54749	0.946118	2.1

54750	0.946118	2.1
54751	0.946118	2.1
54752	0.946118	2.1
54753	0.946119	2.1
54754	0.946119	2.1
54755	0.946118	2.1
54756	0.946118	2.1
54757	0.946131	2.1
54758	0.946131	2.1
54759	0.946106	2.1
54760	0.946106	2.1
54761	1.89224	2.1
54762	1.89224	2.1
54763	1.89224	2.1
54764	1.89224	2.1
54765	3.78448	2.1
54766	2.45922	2.1
54767	2.15686	2.1
54768	2.14259	2.1
54769	2.43225	2.1
54770	4.58911	2.1
54771	1.62027	2.1
54772	2.15686	2.1
54773	1.62241	2.1
54774	1.62241	2.1
54775	2.1426	2.1
54776	2.1426	2.1
54777	2.14259	2.1
54778	2.43225	2.1
54779	2.40667	2.1
54780	2.1281	2.1
54781	4.56035	2.1
54782	1.0713	2.1
54783	1.06405	2.1
54784	0.810136	2.1
54785	0.808433	2.1
54786	0.938508	2.1
54787	0.938508	2.1
54788	0.941223	2.1
54789	0.941223	2.1
54790	1.06405	2.1
54791	0.808433	2.1
54792	0.810135	2.1
54793	1.0713	2.1
54794	1.89226	0.518365
54795	1.89226	0.266967
54796	1.89226	0.443571
54797	1.89226	0.30237
54798	3.78453	0.308585
54799	1.89227	0.312801
54800	1.89227	0.267913
54801	1.89227	0.405054
54802	1.89227	0.223165
54803	3.78453	0.309787
54804	1.19	0.225331
54805	1.12469	0.167101
54806	1.01052	0.195879
54807	1.08166	0.181696

54808	2.20051	0.180938
54809	0.594999	0.223929
54810	0.540832	0.203293
54811	0.514182	0.261897
54812	0.567857	0.209894
54813	0.554259	0.19935
54814	0.554259	0.189267
54815	0.55443	0.22582
54816	0.55443	0.238561
54817	0.540832	0.217092
54818	0.514182	0.195588
54819	0.567857	0.206749
54820	0.594999	0.200755
54821	1.38674	0.26842
54822	0.946132	0.333461
54823	1.36361	0.256157
54824	0.946132	0.308889
54825	1.36362	0.249008
54826	1.36362	0.195319
54827	0.946131	0.336696
54828	0.946131	0.41008
54829	1.38674	0.290733
54830	1.36361	0.2255
54831	0.946132	0.332354
54832	0.946132	0.283743
54833	1.89226	0.314738
54834	2.68508	0.19186
54835	2.77349	0.224854
54836	1.89226	0.248172
54837	2.77349	0.23791
54838	1.89226	0.257319
54839	2.15315	0.0875148
54840	1.96254	0.0747396
54841	1.51377	0.0896002
54842	1.89226	0.10466
54843	1.47799	0.0762132
54844	1.89226	0.0808297
54845	2.49439	0.0974965
54846	1.89226	0.107819
54847	2.15315	0.180647
54848	1.89226	0.215972
54849	2.15315	0.101584
54850	1.89226	0.0953019
54851	1.89226	0.117106
54852	1.89226	0.2788
54853	1.89226	0.219447
54854	1.89226	0.247363
54855	1.89226	0.208556
54856	1.89226	0.252682
54857	1.89226	0.087418
54858	1.89226	0.239233
54859	1.89226	0.0724471
54860	1.89226	0.184393
54861	1.89226	0.0833406
54862	1.89226	0.216942
54863	1.375	0.0649234
54864	2.01865	0.122965
54865	2.12047	0.0682162

54866	1.47354	0.0807422
54867	2.12047	0.0584718
54868	1.47354	0.08085
54869	0.687499	0.05
54870	1.06023	0.0516538
54871	1.08019	0.05
54872	0.604229	0.05
54873	1.08018	0.05
54874	1.06023	0.05
54875	0.687502	0.05
54876	0.60423	0.05
54877	0.80646	0.05
54878	0.806461	0.05
54879	0.858001	0.05
54880	0.858001	0.0581035
54881	2.26414	2.1
54882	1.87342	2.1
54883	1.87342	2.1
54884	1.79667	2.1
54885	2.44045	2.1
54886	2.31924	2.1
54887	2.26414	2.1
54888	2.26616	2.1
54889	2.2355	2.1
54890	2.21858	2.1
54891	4.48474	2.1
54892	2.18516	2.1
54893	1.87344	2.1
54894	2.21859	2.1
54895	2.66233	2.1
54896	1.85196	2.1
54897	2.22236	2.1
54898	2.23551	2.1
54899	2.21859	2.1
54900	2.18516	2.1
54901	2.21189	2.1
54902	4.43049	2.1
54903	1.17171	2.1
54904	2.38924	2.1
54905	2.0815	2.1
54906	2.33348	2.1
54907	1.42851	2.1
54908	1.5959	2.1
54909	1.89226	2.1
54910	1.89226	2.1
54911	1.89226	2.1
54912	1.89226	2.1
54913	3.78452	2.1
54914	2.75425	2.1
54915	1.34366	2.1
54916	1.34366	2.1
54917	1.56512	2.1
54918	2.54186	2.1
54919	2.76332	2.1
54920	1.07751	2.1
54921	2.0815	2.1
54922	1.97259	2.1
54923	1.76455	2.1

54924	1.30191	2.1
54925	1.16461	2.1
54926	0.946131	2.1
54927	0.946131	2.1
54928	0.946129	2.1
54929	0.946129	2.1
54930	0.946131	2.1
54931	0.946131	2.1
54932	0.94613	2.1
54933	0.94613	2.1
54934	0.94613	2.1
54935	0.94613	2.1
54936	0.946131	2.1
54937	0.946131	2.1
54938	1.89226	0.211394
54939	1.89226	0.126943
54940	1.89226	0.0987293
54941	1.89226	0.0959141
54942	3.78453	0.0911104
54943	1.89226	0.157977
54944	1.89226	0.134637
54945	1.89226	0.101808
54946	1.89226	0.0743269
54947	3.78453	0.146292
54948	0.946132	0.0915771
54949	0.946132	0.120112
54950	1.30327	0.0801197
54951	1.33544	0.104477
54952	1.33544	0.0791879
54953	1.33544	0.0739598
54954	0.946132	0.0893206
54955	0.946132	0.0706175
54956	0.946132	0.0708638
54957	1.30327	0.0667506
54958	1.33544	0.0947193
54959	0.946132	0.10414
54960	1.30327	0.0836724
54961	1.33544	0.094905
54962	0.709426	0.0871798
54963	0.790129	0.0918679
54964	1.05886	0.0734894
54965	1.05886	0.0860301
54966	1.03454	0.108487
54967	1.03454	0.0899189
54968	1.30327	0.0811454
54969	0.709426	0.074674
54970	1.33544	0.113359
54971	0.790128	0.0948631
54972	0.946132	0.0740541
54973	0.946132	0.0895914
54974	0.827866	0.0704025
54975	0.946133	0.0896636
54976	0.946132	0.0681188
54977	0.946132	0.0721768
54978	0.946133	0.0719647
54979	0.946133	0.0957083
54980	0.946132	0.0692329
54981	0.827866	0.0926056

54982	0.946133	0.101535
54983	0.946133	0.0931458
54984	1.89227	0.0983178
54985	1.89227	0.0618348
54986	1.89227	0.075173
54987	1.89227	0.0766969
54988	1.89227	0.0691657
54989	1.89227	0.071137
54990	0.946133	0.0640254
54991	0.946133	0.076795
54992	1.21602	0.0610863
54993	1.30327	0.0713017
54994	1.30327	0.0605789
54995	1.30327	0.0594623
54996	0.946132	0.0554706
54997	0.946132	0.0659309
54998	0.946132	0.0559881
54999	1.21602	0.0528394
55000	0.946133	0.0710768
55001	1.30327	0.0667739
55002	1.04227	0.0596843
55003	1.11706	0.0699043
55004	0.968413	0.0549599
55005	0.945482	0.0643032
55006	1.3204	0.065319
55007	1.23215	0.0695851
55008	0.810791	0.0676368
55009	1.38978	0.0578116
55010	1.07728	0.0699302
55011	1.11704	0.0699153
55012	0.608052	0.0702902
55013	1.89226	0.124584
55014	1.89226	0.0765819
55015	1.89226	0.0824498
55016	1.89226	0.10516
55017	3.78453	0.0946401
55018	1.89226	0.0636341
55019	1.89226	0.0690229
55020	1.89226	0.0736138
55021	1.89226	0.0600354
55022	3.78451	0.0662236
55023	2.58476	0.0638712
55024	1.56598	0.0555639
55025	1.55045	0.0614824
55026	1.5124	0.0597956
55027	2.62281	0.0582103
55028	2.58476	0.0569052
55029	2.58477	0.0707406
55030	1.89226	0.0822672
55031	1.89226	0.0853045
55032	1.89226	0.0721242
55033	2.62282	0.081875
55034	2.62282	0.0716811
55035	1.89224	0.0890586
55036	1.89224	0.111729
55037	1.89224	0.138769
55038	1.89224	0.0811661
55039	3.78448	0.0966527

55040	1.89223	0.0794325
55041	1.89223	0.0625403
55042	1.89223	0.0683317
55043	1.89223	0.0665143
55044	1.89223	0.0720883
55045	1.89223	0.0709854
55046	2.62278	0.0664383
55047	1.45053	0.0565523
55048	1.49678	0.0588217
55049	1.56596	0.0609156
55050	2.5536	0.0570419
55051	2.62278	0.059037
55052	1.31139	0.070404
55053	1.2768	0.0650697
55054	0.946118	0.109577
55055	0.946118	0.0830206
55056	1.2768	0.084777
55057	1.2768	0.0729712
55058	0.946116	0.0998536
55059	0.946116	0.0694116
55060	1.27682	0.066764
55061	0.94613	0.0846166
55062	0.946105	0.0842601
55063	1.31138	0.0724038
55064	1.38506	1.49233
55065	1.53348	1.96251
55066	1.17042	1.74197
55067	1.17042	1.66348
55068	1.73669	1.72259
55069	1.73669	1.60321
55070	1.73669	1.56584
55071	1.79928	1.22275
55072	1.38506	1.49065
55073	1.38506	1.68382
55074	1.79928	1.75226
55075	1.43484	1.79306
55076	1.73669	1.43761
55077	1.93568	1.76285
55078	1.53347	1.60826
55079	1.53347	1.62384
55080	2.14058	1.59415
55081	2.14058	1.6074
55082	0.89964	1.20704
55083	1.10415	1.96361
55084	0.868346	1.54695
55085	1.07029	1.82775
55086	0.98687	1.61128
55087	0.98687	2.1
55088	0.985627	1.11079
55089	0.985627	1.78268
55090	1.07029	1.60744
55091	1.10415	1.50503
55092	0.868344	1.60949
55093	0.899642	1.69678
55094	2.40667	2.1
55095	2.1281	2.1
55096	2.11176	2.1
55097	2.38069	2.1

55098	4.50878	2.1
55099	2.11176	2.1
55100	1.61687	2.1
55101	1.60965	2.1
55102	1.61687	2.1
55103	2.12088	2.1
55104	2.1281	2.1
55105	2.11176	2.1
55106	2.34898	2.1
55107	2.34898	2.1
55108	2.38069	2.1
55109	2.09114	2.1
55110	2.12285	2.1
55111	1.05588	2.1
55112	1.04557	2.1
55113	0.804824	2.1
55114	0.798269	2.1
55115	0.92615	2.1
55116	0.92615	2.1
55117	0.927999	2.1
55118	0.927999	2.1
55119	1.04557	2.1
55120	0.798269	2.1
55121	1.05588	2.1
55122	0.804824	2.1
55123	1.43548	0.339188
55124	1.53873	0.787429
55125	1.25063	0.373396
55126	1.39662	1.06933
55127	2.78937	0.301089
55128	1.25063	0.355477
55129	1.39662	1.02752
55130	1.10476	0.297422
55131	1.28326	0.583339
55132	2.53389	0.449339
55133	1.51147	0.05
55134	1.86114	0.0544129
55135	1.86114	0.0510762
55136	2.12926	0.05
55137	1.3659	0.05
55138	1.63401	0.05
55139	1.89226	0.0511305
55140	1.89226	0.0676055
55141	1.89226	0.0612016
55142	1.89226	0.0540085
55143	1.89226	0.0705011
55144	1.89226	0.0705803
55145	1.89226	0.0971181
55146	1.89226	0.13435
55147	2.46286	0.0894748
55148	1.89226	0.164923
55149	3.78452	0.0905284
55150	1.89226	0.143695
55151	1.89226	0.276107
55152	1.89226	0.21042
55153	1.89226	0.201436
55154	1.89226	0.157161
55155	1.89226	0.154155

55156	1.89226	0.107737
55157	1.89226	0.129568
55158	2.28992	0.101357
55159	1.89226	0.16644
55160	3.78452	0.100467
55161	1.13537	0.143384
55162	0.886595	0.50401
55163	0.946131	1.22368
55164	0.946131	0.716753
55165	0.94613	0.553887
55166	0.94613	0.118685
55167	1.25069	1.27604
55168	0.841855	1.12394
55169	1.27436	0.380734
55170	1.06196	0.590638
55171	1.09758	0.70937
55172	0.830306	0.372366
55173	0.826931	2.03341
55174	0.826933	0.390601
55175	0.764631	0.117075
55176	0.764631	0.604136
55177	1.00427	1.88935
55178	0.788034	0.694546
55179	0.726852	0.081165
55180	0.604206	0.882012
55181	0.828475	0.568029
55182	0.604198	0.571949
55183	0.8332	0.43736
55184	1.00428	0.442456
55185	1.89226	0.992694
55186	1.89226	0.412585
55187	1.89226	0.170791
55188	1.89226	1.13379
55189	1.89226	0.464739
55190	1.89226	0.801734
55191	0.768789	2.1
55192	0.849132	0.05
55193	0.666423	2.1
55194	0.688993	1.93958
55195	0.868671	0.05
55196	0.89117	2.1
55197	0.705632	2.1
55198	1.80425	2.1
55199	1.73883	2.1
55200	1.45159	2.1
55201	1.45159	2.1
55202	1.37681	2.1
55203	1.73883	2.1
55204	1.39679	0.05
55205	2.08149	0.05
55206	1.89226	0.05
55207	2.32763	0.05
55208	1.89226	0.05
55209	2.31274	0.05
55210	1.89226	0.05
55211	1.89226	0.0733203
55212	1.89226	0.0598885
55213	1.89226	0.0500144

55214	1.89226	0.0622876
55215	1.89226	0.05
55216	1.89226	0.05
55217	1.89226	0.0614841
55218	1.89226	0.0525164
55219	1.89226	0.05
55220	1.89226	0.0509825
55221	1.89226	0.05
55222	1.95181	0.05
55223	1.89226	0.05
55224	1.89226	0.05
55225	1.89226	0.05
55226	3.78451	0.05
55227	0.946131	0.05
55228	0.946131	0.05
55229	0.946125	0.05
55230	0.946125	0.05
55231	0.946127	0.0500228
55232	0.946127	0.05
55233	0.946128	0.05
55234	0.946128	0.05
55235	0.946128	0.05
55236	0.946128	0.05
55237	0.946128	0.05
55238	0.946128	0.05
55239	1.89226	0.0509407
55240	1.89226	0.0740269
55241	1.89226	0.0607744
55242	1.89226	0.058392
55243	1.89226	0.0613274
55244	1.89226	0.058553
55245	1.89226	1.04507
55246	1.89226	1.04059
55247	1.89226	0.980962
55248	1.89226	0.86083
55249	3.78453	1.01221
55250	1.89226	1.1308
55251	1.89226	0.972265
55252	1.89226	1.0317
55253	1.89226	0.670758
55254	3.78452	0.858552
55255	1.21387	0.749321
55256	0.8164	0.395996
55257	0.946133	0.614385
55258	0.946133	0.766225
55259	0.946102	0.361331
55260	0.946102	0.797327
55261	0.816431	1.08034
55262	0.816431	0.947947
55263	0.816401	0.663552
55264	0.94613	0.454205
55265	0.946131	1.12268
55266	1.21388	0.841633
55267	0.946132	0.481356
55268	0.946132	0.621458
55269	0.946132	0.935505
55270	0.946132	0.66811
55271	0.946132	0.31812

55272	0.946132	0.587384
55273	0.946132	1.01466
55274	0.946132	0.786946
55275	0.946131	0.303936
55276	0.946131	0.695487
55277	0.946132	0.999886
55278	0.946132	0.7436
55279	1.89226	0.944874
55280	1.89226	0.263787
55281	1.89226	0.42372
55282	1.89226	0.878534
55283	1.89226	0.471367
55284	1.89226	0.775459
55285	1.89226	1.00895
55286	1.89226	0.70488
55287	2.15871	0.32426
55288	2.15871	0.872537
55289	1.81454	0.267344
55290	1.54809	0.95184
55291	1.89226	1.03707
55292	1.89226	1.30997
55293	1.89226	1.16568
55294	1.89226	1.04226
55295	3.78453	1.20272
55296	1.89226	1.18544
55297	1.89226	1.00954
55298	1.89226	0.973038
55299	1.89226	1.1318
55300	1.89226	1.2947
55301	1.89226	1.25095
55302	2.29205	1.16401
55303	1.42633	1.16552
55304	1.69015	1.24474
55305	1.69015	1.1422
55306	2.10364	1.10417
55307	2.05344	1.03716
55308	2.29205	1.03592
55309	1.58147	1.26945
55310	2.16529	1.12358
55311	1.56339	1.06396
55312	2.29205	1.26962
55313	1.69015	1.2351
55314	1.89226	1.22555
55315	1.89226	1.12533
55316	1.89226	1.013
55317	1.89226	1.18071
55318	3.78453	1.07778
55319	1.89226	1.14606
55320	1.89226	1.30963
55321	1.89226	1.14624
55322	1.89226	1.08649
55323	3.78452	1.23679
55324	2.29205	1.28618
55325	2.85335	1.00441
55326	2.55409	1.20961
55327	2.04345	1.09857
55328	2.76338	1.1309
55329	2.29205	1.08812

55330	2.29205	1.11148
55331	2.16529	1.27843
55332	2.72418	1.16814
55333	2.76338	0.971062
55334	5.01623	1.22249
55335	1.89227	0.0680425
55336	1.89227	0.0562693
55337	1.89227	0.05
55338	1.89227	0.0507249
55339	3.78453	0.05
55340	2.39588	0.05
55341	2.14918	0.05
55342	2.14918	0.05
55343	2.37907	0.069719
55344	2.20531	0.05
55345	2.4352	0.05
55346	1.89226	0.05
55347	2.64327	0.0656589
55348	2.36175	0.05
55349	1.89226	0.05
55350	4.25402	0.0610179
55351	1.89226	0.05
55352	1.89226	0.05
55353	1.89226	0.05
55354	1.89226	0.05
55355	1.89226	0.05
55356	1.89226	0.05
55357	1.05395	0.05
55358	1.05395	0.05
55359	1.03114	0.05
55360	1.03114	0.05
55361	0.936689	0.05
55362	1.07459	0.05
55363	1.10265	0.05
55364	1.01049	0.05
55365	0.93669	0.05
55366	1.01049	0.05
55367	1.07459	0.05
55368	1.10265	0.05
55369	1.18088	0.05
55370	0.776509	0.05
55371	0.946132	0.05
55372	0.946132	0.05
55373	1.04225	0.05
55374	0.830392	0.0513016
55375	0.931352	0.05
55376	0.931352	0.05
55377	1.13006	0.05
55378	1.04074	0.05
55379	1.0628	0.05
55380	0.851529	0.05
55381	2.40032	2.1
55382	1.89226	2.1
55383	1.89226	2.1
55384	1.89226	2.1
55385	2.38918	2.1
55386	2.38918	2.1
55387	1.89226	2.1

55388	1.89226	2.1
55389	1.89226	2.1
55390	1.89226	2.1
55391	1.89226	2.1
55392	1.89226	2.1
55393	1.6585	2.1
55394	2.37364	2.1
55395	2.39622	2.1
55396	2.39622	2.1
55397	1.6488	2.1
55398	1.6488	2.1
55399	2.44603	2.1
55400	1.89224	2.1
55401	1.89224	2.1
55402	1.89224	2.1
55403	2.40029	2.1
55404	2.40029	2.1
55405	1.89224	2.1
55406	1.89224	2.1
55407	1.89224	2.1
55408	1.89224	2.1
55409	1.89224	2.1
55410	1.89224	2.1
55411	1.01946	2.1
55412	1.01946	2.1
55413	1.02497	2.1
55414	1.02497	2.1
55415	1.21519	2.1
55416	1.19809	2.1
55417	0.835303	2.1
55418	0.82924	2.1
55419	0.835314	2.1
55420	1.2152	2.1
55421	0.829228	2.1
55422	1.19808	2.1
55423	1.91027	2.1
55424	1.05809	2.1
55425	1.00659	2.1
55426	2.06727	2.1
55427	3.11819	2.1
55428	2.37166	2.1
55429	2.06726	2.1
55430	1.66994	2.1
55431	1.67623	2.1
55432	2.40308	2.1
55433	2.40308	2.1
55434	0.960194	2.1
55435	0.939664	2.1
55436	0.531131	2.1
55437	0.503298	2.1
55438	0.729927	2.1
55439	0.729927	2.1
55440	0.733565	2.1
55441	0.733565	2.1
55442	0.960195	2.1
55443	0.531132	2.1
55444	0.503297	2.1
55445	0.939663	2.1

55446	1.20154	2.1
55447	1.21754	2.1
55448	0.960195	2.1
55449	0.939665	2.1
55450	1.07745	2.1
55451	1.07745	2.1
55452	1.07976	2.1
55453	1.07976	2.1
55454	0.960194	2.1
55455	1.21754	2.1
55456	0.939662	2.1
55457	1.20155	2.1
55458	1.89224	2.1
55459	1.89224	2.1
55460	2.08609	2.1
55461	2.08609	2.1
55462	2.09527	2.1
55463	1.90142	2.1
55464	1.65815	2.1
55465	1.71398	2.1
55466	2.2001	2.1
55467	2.23791	2.1
55468	1.4502	2.1
55469	1.4502	2.1
55470	0.793587	2.1
55471	0.793587	2.1
55472	0.792408	2.1
55473	0.792408	2.1
55474	0.952221	2.1
55475	0.858585	2.1
55476	0.725101	2.1
55477	0.633774	2.1
55478	0.633775	2.1
55479	0.858585	2.1
55480	0.952221	2.1
55481	0.725101	2.1
55482	1.5735	2.1
55483	2.56975	2.1
55484	2.63166	2.1
55485	2.63166	2.1
55486	1.54711	2.1
55487	1.54711	2.1
55488	0.946131	2.1
55489	0.946131	2.1
55490	1.28487	2.1
55491	1.28487	2.1
55492	0.946127	2.1
55493	0.946127	2.1
55494	1.28488	2.1
55495	1.31583	2.1
55496	1.31583	2.1
55497	0.94613	2.1
55498	1.28487	2.1
55499	0.946129	2.1
55500	1.0596	2.1
55501	1.0596	2.1
55502	1.06939	2.1
55503	1.06939	2.1

55504	1.34225	2.1
55505	1.31583	2.1
55506	0.793563	2.1
55507	0.786748	2.1
55508	0.793563	2.1
55509	1.34225	2.1
55510	0.786748	2.1
55511	1.31583	2.1
55512	0.946131	2.1
55513	0.946131	2.1
55514	1.31582	2.1
55515	1.31582	2.1
55516	0.946127	2.1
55517	0.946127	2.1
55518	1.34225	2.1
55519	1.31583	2.1
55520	1.34225	2.1
55521	0.946129	2.1
55522	1.31583	2.1
55523	0.946129	2.1
55524	1.89226	2.1
55525	1.89226	2.1
55526	1.89226	2.1
55527	1.89226	2.1
55528	1.89226	2.1
55529	1.89226	2.1
55530	1.89226	2.1
55531	1.89226	2.1
55532	1.89226	2.1
55533	1.89226	2.1
55534	1.89226	2.1
55535	1.89226	2.1
55536	1.26992	2.1
55537	1.75003	2.1
55538	1.79883	2.1
55539	1.26732	2.1
55540	3.06875	2.1
55541	0.875017	2.1
55542	0.899414	2.1
55543	0.811147	2.1
55544	0.85053	2.1
55545	0.851513	2.1
55546	0.851513	2.1
55547	0.859048	2.1
55548	0.859048	2.1
55549	0.899413	2.1
55550	0.85053	2.1
55551	0.811147	2.1
55552	0.875016	2.1
55553	1.89227	2.1
55554	1.89227	2.1
55555	1.89227	2.1
55556	1.89227	2.1
55557	1.89227	2.1
55558	1.89227	2.1
55559	1.89226	2.1
55560	1.89226	2.1
55561	1.89226	2.1

55562	1.89226	2.1
55563	1.89226	2.1
55564	1.89226	2.1
55565	0.633661	2.1
55566	0.642005	2.1
55567	0.93285	2.1
55568	0.899413	2.1
55569	0.764487	2.1
55570	0.764487	2.1
55571	0.776931	2.1
55572	0.776931	2.1
55573	0.642004	2.1
55574	0.93285	2.1
55575	0.899414	2.1
55576	0.633661	2.1
55577	0.932851	2.1
55578	0.899414	2.1
55579	0.905419	2.1
55580	0.85053	2.1
55581	0.886336	2.1
55582	0.886336	2.1
55583	0.897045	2.1
55584	0.897045	2.1
55585	0.93285	2.1
55586	0.905419	2.1
55587	0.899414	2.1
55588	0.850531	2.1
55589	2.39322	2.1
55590	2.74149	2.1
55591	2.70944	2.1
55592	2.6849	1.98489
55593	2.41776	2.1
55594	2.39322	1.78378
55595	2.41776	2.1
55596	2.80992	2.1
55597	2.77256	2.1
55598	2.74149	2.1
55599	2.44882	2.1
55600	2.41776	1.93839
55601	2.80992	2.1
55602	1.89226	1.67915
55603	2.74149	1.97687
55604	2.74149	2.1
55605	1.89226	1.61176
55606	1.89226	2.04795
55607	1.89226	0.0535888
55608	1.89226	0.0536543
55609	1.89226	0.0656346
55610	1.89226	0.05
55611	1.89226	0.0650216
55612	1.89226	0.05
55613	2.38796	0.05
55614	2.0815	0.05
55615	2.0815	0.05
55616	2.49452	0.05
55617	1.55994	0.05
55618	2.14721	0.05
55619	1.89227	0.0586298

55620	1.89227	0.0569134
55621	1.89227	0.0691856
55622	1.89227	0.05
55623	1.89227	0.0730177
55624	1.89227	0.0543075
55625	1.13387	0.05
55626	2.27494	0.05
55627	1.13387	0.058191
55628	2.19489	0.05
55629	1.60867	0.05
55630	2.6994	0.05
55631	1.89226	0.0597508
55632	1.89226	0.0516393
55633	1.89226	0.0575188
55634	1.89226	0.0834614
55635	3.78453	0.0539451
55636	1.42381	2.1
55637	1.10747	2.1
55638	1.10747	2.1
55639	1.08911	2.1
55640	1.42866	2.07288
55641	1.4103	2.1
55642	1.89226	2.1
55643	1.88181	2.1
55644	1.88181	2.1
55645	1.76249	2.1
55646	2.0815	2.1
55647	1.97539	2.1
55648	0.946128	2.1
55649	0.946128	2.1
55650	0.946133	2.1
55651	0.946133	1.92146
55652	0.94613	1.93708
55653	0.94613	2.1
55654	0.946131	2.1
55655	0.946131	2.04972
55656	0.946131	2.1
55657	0.946131	2.1
55658	0.946131	2.1
55659	0.946131	2.1
55660	1.42866	2.1
55661	1.43213	2.1
55662	1.10748	2.1
55663	1.10748	2.1
55664	1.13641	2.1
55665	1.43213	2.1
55666	2.0815	2.1
55667	2.10287	2.1
55668	2.10287	2.1
55669	2.0921	2.1
55670	1.89227	2.1
55671	1.83938	2.1
55672	0.810944	2.1
55673	0.810944	1.95796
55674	0.810945	1.97262
55675	0.810945	2.1
55676	0.810933	2.1
55677	0.810933	2.1

55678	1.89226	2.1
55679	1.3517	2.1
55680	1.08132	2.1
55681	1.08132	2.1
55682	1.08133	2.1
55683	2.33097	2.1
55684	1.53822	2.1
55685	1.53822	2.1
55686	1.68437	2.1
55687	2.17996	2.1
55688	2.32612	2.1
55689	0.99189	2.1
55690	1.18948	2.1
55691	1.19756	2.1
55692	1.19756	2.1
55693	0.978851	2.1
55694	0.926995	2.1
55695	1.26924	1.88975
55696	1.34642	2.1
55697	1.3228	2.1
55698	0.990899	2.1
55699	1.0627	2.1
55700	1.33473	2.1
55701	1.96196	2.1
55702	1.39702	2.1
55703	1.39702	2.1
55704	1.53822	2.1
55705	2.17372	2.1
55706	2.31011	2.1
55707	1.89226	1.92147
55708	1.89226	0.46626
55709	1.89226	1.91996
55710	1.89226	0.526018
55711	3.78453	0.664211
55712	2.38475	2.1
55713	1.73957	0.637375
55714	1.88452	1.92398
55715	1.58695	0.426698
55716	3.6241	0.596292
55717	1.73958	0.671804
55718	1.3126	2.1
55719	1.58695	0.439128
55720	1.14946	2.1
55721	2.88903	1.72917
55722	1.89226	0.455654
55723	1.89226	2.1
55724	1.89226	0.457517
55725	1.89226	2.1
55726	3.78453	1.74293
55727	1.89225	1.96153
55728	1.89225	0.623062
55729	1.89225	1.7641
55730	1.89225	0.824084
55731	3.7845	0.302069
55732	1.88451	1.95934
55733	1.58694	0.224484
55734	1.74649	2.1
55735	1.44604	0.854905

55736	3.33055	0.642471
55737	1.58694	0.272039
55738	0.995727	2.1
55739	0.995727	1.78619
55740	1.14945	1.97904
55741	1.44604	0.465045
55742	1.59976	2.06588
55743	1.89225	0.470395
55744	1.89225	2.1
55745	1.89225	2.1
55746	1.89225	0.843766
55747	1.89225	2.1
55748	1.89225	0.484642
55749	2.01197	2.08314
55750	2.0961	1.63247
55751	2.13283	1.67387
55752	2.25399	1.24467
55753	4.22894	1.58598
55754	2.09611	1.44985
55755	2.37014	1.6649
55756	2.29031	1.6982
55757	2.10084	2.04271
55758	2.25399	1.26254
55759	2.09611	2.00746
55760	2.13283	1.94543
55761	2.25399	1.95649
55762	2.22044	1.97302
55763	2.38975	1.25125
55764	4.52258	1.65616
55765	2.38976	0.293808
55766	2.15466	1.75139
55767	2.31988	0.420462
55768	2.31988	2.03867
55769	2.25399	0.269402
55770	2.25399	2.1
55771	2.58286	2.1
55772	2.29193	0.452446
55773	2.40107	2.1
55774	2.13815	0.225249
55775	4.72101	0.99534
55776	2.29193	0.421976
55777	1.90104	1.96932
55778	2.13815	0.245426
55779	1.77249	1.83298
55780	4.03919	0.542307
55781	2.25197	2.1
55782	2.13815	0.491943
55783	2.00625	1.96757
55784	2.00625	0.394706
55785	2.40107	1.89139
55786	2.40107	1.446
55787	2.13815	0.31087
55788	1.77249	1.90317
55789	2.00625	0.988606
55790	1.65815	2.02284
55791	3.7963	0.671242
55792	2.43417	0.727938
55793	2.06653	0.393852

55794	1.64587	0.37824
55795	1.65284	0.348906
55796	2.46898	0.881139
55797	2.46898	0.666235
55798	2.46895	0.510096
55799	1.89016	0.283606
55800	1.87861	0.298552
55801	1.87861	0.258219
55802	2.47161	0.639456
55803	2.47161	0.478183
55804	0.946132	1.68763
55805	0.946132	1.65246
55806	0.863311	1.79017
55807	1.34768	1.75344
55808	0.851542	1.79204
55809	0.851542	1.58785
55810	1.30451	1.67898
55811	1.45304	1.71957
55812	0.946132	1.70792
55813	1.20992	1.72151
55814	0.855162	1.73498
55815	0.946132	1.69836
55816	1.89226	1.621
55817	1.89226	1.87859
55818	1.89226	1.84413
55819	1.89226	1.65317
55820	1.89226	1.8342
55821	1.89226	1.65783
55822	0.946132	1.52566
55823	0.946132	1.01799
55824	0.946133	1.30325
55825	0.946133	1.34473
55826	0.946132	1.32854
55827	0.946132	0.849284
55828	0.946132	1.02249
55829	0.946132	1.12758
55830	0.946132	1.47352
55831	0.946132	1.31843
55832	0.946133	1.38339
55833	0.946133	1.11421
55834	2.35574	1.96748
55835	1.89226	1.72706
55836	1.89226	2.1
55837	1.89226	1.65519
55838	2.67456	2.1
55839	2.67456	0.576734
55840	0.583505	0.12547
55841	0.761972	0.322309
55842	1.17787	1.60825
55843	1.33728	0.596392
55844	0.965128	1.37498
55845	0.965128	0.173613
55846	0.974714	1.28567
55847	0.974715	1.54206
55848	1.17787	0.671474
55849	0.583506	1.39715
55850	1.33728	0.438757
55851	0.761974	0.497836

55852	1.89226	1.40172
55853	1.89226	0.429946
55854	1.89226	0.512327
55855	1.89226	0.148667
55856	1.89226	0.154496
55857	1.89226	1.11607
55858	1.89226	1.64063
55859	1.89226	0.252821
55860	1.89226	0.701023
55861	1.89226	0.644457
55862	3.78453	0.535122
55863	0.946132	0.138135
55864	0.946132	0.28426
55865	0.946132	0.818743
55866	0.946132	0.580058
55867	0.946133	0.637788
55868	0.946133	0.188094
55869	0.946132	0.775471
55870	0.946132	0.7654
55871	0.946133	0.63148
55872	0.946133	1.40081
55873	0.946132	0.394111
55874	0.946132	1.21079
55875	1.01779	2.1
55876	1.50685	2.1
55877	1.12064	2.1
55878	1.65151	2.1
55879	2.6275	2.1
55880	1.35733	2.1
55881	1.53507	2.1
55882	1.50683	2.1
55883	1.73626	2.1
55884	3.09359	2.1
55885	1.73626	2.1
55886	1.52226	2.1
55887	1.75555	2.1
55888	1.75555	2.1
55889	1.53507	2.1
55890	1.53507	2.1
55891	1.65151	2.1
55892	1.73628	2.1
55893	1.69528	2.1
55894	1.50685	2.1
55895	1.92471	2.1
55896	1.73628	2.1
55897	1.73629	2.1
55898	1.75558	2.1
55899	1.9943	2.1
55900	1.9943	2.1
55901	1.92471	2.1
55902	1.73629	2.1
55903	2.5601	1.38685
55904	1.97645	1.70931
55905	2.09605	1.32768
55906	2.09605	1.65324
55907	2.45853	1.37469
55908	2.45853	1.85526
55909	2.33824	1.68976

55910	1.97646	1.56384
55911	2.45853	1.53899
55912	1.94984	1.73918
55913	2.33824	1.59017
55914	1.82955	1.86957
55915	1.22926	1.70273
55916	0.946128	1.7123
55917	0.946133	1.72027
55918	1.28005	1.43771
55919	1.22927	1.46839
55920	1.22926	1.47302
55921	0.946131	1.57861
55922	0.946131	1.45176
55923	0.946131	1.47207
55924	0.946131	1.4605
55925	1.28005	1.47754
55926	1.22926	1.45945
55927	2.35349	1.98751
55928	2.61606	0.588778
55929	2.48209	1.98585
55930	2.48209	0.563738
55931	2.50057	1.96124
55932	2.50057	0.630988
55933	2.61607	0.493346
55934	2.57611	0.630478
55935	2.4821	1.09065
55936	2.44814	0.363435
55937	5.06421	0.201509
55938	2.15721	1.43409
55939	2.48209	0.747364
55940	2.31844	2.02681
55941	2.31844	0.482584
55942	2.35349	1.99334
55943	2.35349	0.429854
55944	2.44814	0.392503
55945	2.30908	1.06223
55946	2.44814	0.416573
55947	2.31844	0.525618
55948	2.4821	0.348342
55949	2.31844	0.180809
55950	0.91766	0.0792332
55951	0.91766	0.108927
55952	0.912463	0.105701
55953	0.912463	0.107886
55954	1.16525	0.108683
55955	1.26377	0.0945843
55956	0.576692	0.107133
55957	0.664871	0.100833
55958	1.16525	0.0987575
55959	0.576692	0.0994461
55960	0.664871	0.121008
55961	1.26377	0.123411
55962	1.72901	2.1
55963	1.62414	2.1
55964	1.88971	2.1
55965	1.8411	1.77705
55966	3.57011	2.1
55967	1.89226	2.1

55968	1.89226	1.81856
55969	1.89226	1.88898
55970	1.89226	1.80258
55971	3.78452	1.84924
55972	1.89226	1.7637
55973	1.89226	2.1
55974	1.89226	2.06457
55975	1.89226	1.66167
55976	3.78452	2.06286
55977	1.62414	2.1
55978	1.30896	2.08984
55979	1.8411	1.99258
55980	1.52776	2.1
55981	3.15006	2.05843
55982	1.51448	1.95556
55983	1.8411	1.90548
55984	2.02311	2.1
55985	1.99309	1.83684
55986	3.86421	1.89517
55987	1.89226	1.9925
55988	1.89226	1.98953
55989	1.89226	2.1
55990	1.89226	1.94987
55991	3.78452	2.07625
55992	1.89226	2.1
55993	1.89226	1.89539
55994	1.89226	2.05114
55995	1.89226	2.1
55996	3.78452	2.07382
55997	1.8411	2.0833
55998	1.52776	2.1
55999	1.99309	2.1
56000	1.72526	2.1
56001	3.56636	2.1
56002	1.89226	2.1
56003	1.89226	2.07671
56004	1.89226	2.07749
56005	1.89226	2.1
56006	3.78453	2.1
56007	1.0424	2.1
56008	1.12935	2.1
56009	0.929383	2.1
56010	0.929383	2.1
56011	1.24941	2.1
56012	1.12686	2.1
56013	0.946132	2.1
56014	0.946132	2.1
56015	1.12944	2.1
56016	0.851554	2.1
56017	1.05017	2.1
56018	0.929315	2.1
56019	0.898029	2.1
56020	0.973994	2.1
56021	0.946131	2.08823
56022	0.946131	2.1
56023	1.0221	2.1
56024	1.0221	2.1
56025	0.898028	2.07257

56026	0.898028	2.1
56027	0.973994	2.1
56028	0.946131	2.1
56029	0.946131	2.1
56030	0.898029	2.1
56031	1.89226	2.02914
56032	1.89226	2.07546
56033	1.89226	1.99319
56034	1.89226	2.05801
56035	1.89226	1.99071
56036	1.89226	2.05438
56037	1.13537	2.07439
56038	0.879308	2.1
56039	0.946131	2.05141
56040	0.946131	2.08791
56041	1.13537	2.09707
56042	0.85187	2.1
56043	0.94613	2.06913
56044	0.94613	1.97218
56045	1.05777	2.1
56046	1.05777	2.1
56047	0.799318	2.1
56048	0.776452	2.1
56049	0.946116	2.1
56050	0.946116	2.1
56051	1.09531	2.1
56052	1.09531	2.1
56053	0.946118	2.1
56054	0.946118	2.1
56055	1.09135	2.1
56056	1.09531	2.1
56057	1.09134	2.1
56058	0.946105	2.1
56059	1.09532	2.1
56060	0.946131	2.1
56061	0.946129	2.1
56062	0.946129	2.1
56063	1.09137	2.1
56064	1.09137	2.1
56065	0.946131	2.1
56066	0.946131	2.1
56067	1.07713	2.1
56068	1.09137	2.1
56069	0.946131	2.1
56070	1.07713	2.1
56071	1.09137	2.1
56072	0.946131	2.1
56073	0.938916	2.1
56074	0.938916	2.1
56075	0.936995	2.1
56076	1.00447	2.1
56077	0.979819	2.1
56078	1.16821	2.1
56079	1.01263	2.1
56080	0.719167	2.1
56081	1.04536	2.1
56082	1.18901	2.1
56083	0.758057	2.1

56084	0.924111	2.1
56085	1.00532	2.1
56086	1.00532	2.1
56087	0.9925	2.1
56088	0.9925	2.1
56089	1.29322	2.1
56090	1.01985	2.1
56091	1.00365	2.1
56092	0.704601	2.1
56093	1.00365	2.1
56094	1.29322	2.1
56095	1.01985	2.1
56096	0.704602	2.1
56097	2.20011	2.1
56098	1.401	2.1
56099	1.401	2.1
56100	1.52008	2.1
56101	2.11084	2.1
56102	2.22991	2.1
56103	0.854068	2.1
56104	0.854068	2.1
56105	0.851357	2.1
56106	0.851357	2.1
56107	0.644681	2.1
56108	0.700493	2.1
56109	1.0554	2.1
56110	1.00493	2.1
56111	1.00495	2.1
56112	0.64469	2.1
56113	0.700483	2.1
56114	1.05539	2.1
56115	2.46665	0.05
56116	2.22713	0.05
56117	1.85877	0.05
56118	1.8967	0.05
56119	1.56002	0.05
56120	2.22713	0.05
56121	1.89225	2.1
56122	1.89225	2.1
56123	1.89225	2.1
56124	1.89225	2.1
56125	1.89225	2.1
56126	1.89225	2.1
56127	1.89225	2.1
56128	1.89225	2.1
56129	1.89225	2.1
56130	1.89225	2.1
56131	1.89225	2.1
56132	1.89225	2.1
56133	1.284	2.1
56134	1.86569	2.1
56135	1.95398	2.1
56136	1.31091	2.1
56137	3.23798	2.1
56138	0.932844	2.1
56139	0.976992	2.1
56140	0.960128	2.1
56141	0.905413	2.1

56142	0.938531	2.1
56143	0.938531	2.1
56144	0.943874	2.1
56145	0.943874	2.1
56146	0.976985	2.1
56147	0.960122	2.1
56148	0.93285	2.1
56149	0.905419	2.1
56150	1.89227	2.1
56151	1.89227	2.1
56152	1.89227	2.1
56153	1.89227	2.1
56154	3.78453	2.1
56155	1.89226	2.1
56156	1.89226	2.1
56157	1.89226	2.1
56158	1.89226	2.1
56159	1.89226	2.1
56160	1.89226	2.1
56161	0.65546	2.1
56162	0.665451	2.1
56163	1.00694	2.1
56164	0.976998	2.1
56165	0.816261	2.1
56166	0.816261	2.1
56167	0.826189	2.1
56168	0.826189	2.1
56169	1.00694	2.1
56170	0.665451	2.1
56171	0.976998	2.1
56172	0.65546	2.1
56173	2.01555	2.1
56174	2.01388	2.1
56175	1.92027	2.1
56176	1.92027	2.1
56177	2.01388	2.1
56178	1.954 2.1	
56179	2.75466	2.1
56180	1.89226	2.1
56181	1.89226	2.1
56182	2.54931	2.1
56183	4.44158	2.1
56184	2.47917	2.1
56185	1.92772	2.1
56186	1.80682	2.1
56187	2.54931	2.1
56188	4.47702	2.1
56189	0.946132	2.1
56190	0.946132	2.1
56191	1.17602	2.1
56192	1.27466	2.1
56193	0.946132	2.1
56194	0.946132	2.1
56195	1.17602	2.1
56196	1.17602	2.1
56197	0.946132	2.1
56198	1.17602	2.1
56199	0.946131	2.1

56200	1.27466	2.1
56201	2.35204	2.1
56202	2.54931	2.1
56203	2.40783	2.1
56204	1.50108	2.1
56205	1.64256	2.1
56206	1.64256	2.1
56207	1.89226	2.1
56208	1.89226	1.6795
56209	2.31012	1.99586
56210	2.17534	2.1
56211	4.0676	2.07049
56212	2.17533	2.09635
56213	1.89226	1.41051
56214	2.17533	1.80199
56215	1.89226	1.67818
56216	1.66635	1.97445
56217	1.38328	1.81519
56218	1.92027	2.1
56219	2.17597	2.1
56220	2.14619	2.1
56221	2.15388	2.1
56222	1.91087	2.1
56223	1.92027	2.1
56224	1.08622	2.1
56225	1.08622	2.1
56226	0.971184	2.1
56227	0.972761	2.1
56228	1.14845	2.1
56229	1.16321	2.1
56230	0.894769	2.1
56231	0.894967	2.1
56232	0.894775	2.1
56233	1.28563	2.1
56234	1.14844	2.1
56235	1.0167	2.1
56236	1.89226	1.92006
56237	1.89226	2.1
56238	1.89226	2.1
56239	1.89226	1.96207
56240	1.89226	2.1
56241	1.89226	1.97878
56242	0.937933	2.1
56243	0.937933	2.1
56244	1.26192	2.1
56245	1.26192	2.1
56246	0.937903	2.1
56247	1.25372	2.1
56248	0.946132	2.1
56249	0.946132	2.1
56250	0.946133	2.1
56251	1.25372	2.1
56252	0.946132	2.1
56253	0.937902	2.1
56254	0.946132	1.87368
56255	0.946132	1.8243
56256	0.946128	2.1
56257	0.946128	2.1

56258	0.94613	2.1
56259	0.94613	1.82344
56260	0.94613	1.80299
56261	0.94613	2.1
56262	0.946131	2.02176
56263	0.946131	2.03961
56264	0.94613	2.08662
56265	0.94613	2.07921
56266	0.946132	1.92283
56267	0.946132	1.83719
56268	1.2778	2.1
56269	1.00191	2.1
56270	1.00191	1.89111
56271	1.00191	2.1
56272	0.94613	2.1
56273	0.94613	1.90456
56274	0.946131	2.1
56275	1.2778	2.1
56276	1.00191	2.07101
56277	0.94613	2.05812
56278	1.50847	0.05
56279	2.0815	0.05
56280	1.56806	0.05
56281	2.0815	0.05
56282	1.52043	0.05
56283	1.9224	0.05
56284	1.06882	0.05
56285	0.69567	0.05
56286	0.773112	0.05
56287	1.09473	0.05
56288	0.8941	0.05
56289	0.8941	0.05
56290	0.937064	0.05
56291	0.981131	0.05
56292	0.763762	0.05
56293	0.722535	0.05
56294	1.2153	0.05
56295	1.07279	0.05
56296	1.89226	0.0507146
56297	1.89226	0.053832
56298	1.89226	0.05
56299	1.89226	0.05
56300	1.89226	0.06302
56301	1.89226	0.0592134
56302	1.89226	0.05
56303	1.89226	0.0624147
56304	1.89226	0.0533636
56305	1.89226	0.0653406
56306	3.78453	0.0582177
56307	2.51518	0.05
56308	1.89226	0.05
56309	1.89226	0.05
56310	1.89226	0.05
56311	2.37584	0.05
56312	2.37584	0.05
56313	1.25759	0.05
56314	0.946131	0.05
56315	0.946131	0.05

56316	1.24489	0.0569687
56317	0.946131	0.05
56318	0.946131	0.0545395
56319	1.25759	0.05
56320	1.25759	0.05
56321	1.25759	0.05
56322	1.24489	0.05
56323	0.946131	0.05
56324	0.946131	0.0502442
56325	1.08028	0.359487
56326	1.09362	0.249369
56327	0.596616	0.619016
56328	0.606432	0.623893
56329	0.844265	0.737397
56330	0.844265	0.14209
56331	0.855786	0.498587
56332	0.855786	0.536699
56333	1.08028	0.646277
56334	0.606433	0.462215
56335	0.596616	0.489981
56336	1.09362	0.472818
56337	2.837	0.542437
56338	2.16055	0.0925748
56339	2.12741	0.362723
56340	2.79312	1.08813
56341	4.95367	0.206967
56342	2.12741	0.29993
56343	1.21286	0.316
56344	1.20859	0.976293
56345	1.21286	1.3288
56346	2.15628	0.305384
56347	2.16056	0.107285
56348	1.89226	2.1
56349	1.89226	2.1
56350	1.89226	2.1
56351	1.89226	2.1
56352	3.78453	2.1
56353	0.946102	2.1
56354	0.946102	2.1
56355	0.850039	2.1
56356	0.850039	2.1
56357	0.914891	2.1
56358	0.85001	2.1
56359	0.946131	2.1
56360	0.946131	2.1
56361	0.94613	2.1
56362	0.91489	2.1
56363	0.850011	2.1
56364	0.946131	2.1
56365	0.71107	2.1
56366	0.784813	2.1
56367	1.28453	2.1
56368	1.20328	2.1
56369	0.992128	2.1
56370	0.992128	2.1
56371	0.995965	2.1
56372	0.995965	2.1
56373	0.784813	2.1

56374	1.28453	2.1
56375	0.711071	2.1
56376	1.20328	2.1
56377	1.89226	2.1
56378	1.89226	2.1
56379	1.89226	2.1
56380	1.89226	2.1
56381	3.78453	2.1
56382	0.946132	2.1
56383	0.946132	2.1
56384	0.914889	2.1
56385	0.914889	2.1
56386	0.985255	2.1
56387	0.914891	2.1
56388	0.946131	2.1
56389	0.946131	2.1
56390	0.946131	2.1
56391	0.985255	2.1
56392	0.94613	2.1
56393	0.91489	2.1
56394	0.784813	2.1
56395	0.871551	2.1
56396	1.37791	2.1
56397	1.28453	2.1
56398	1.07644	2.1
56399	1.07644	2.1
56400	1.07964	2.1
56401	1.07964	2.1
56402	0.871551	2.1
56403	1.37791	2.1
56404	0.784813	2.1
56405	1.28453	2.1
56406	1.73075	1.7902
56407	2.10582	1.44263
56408	1.89319	1.50422
56409	1.38766	2.03623
56410	3.27777	1.58993
56411	1.08715	1.9613
56412	1.54185	2.0476
56413	1.73074	1.94747
56414	1.73074	2.0984
56415	1.18948	1.73106
56416	1.18948	1.98215
56417	1.89319	1.38177
56418	1.41689	1.98031
56419	1.11619	1.71472
56420	1.38766	1.79172
56421	1.6052	1.59103
56422	1.89319	1.76389
56423	0.647894	1.88829
56424	0.594738	2.02079
56425	0.708445	1.99282
56426	0.770926	1.84798
56427	0.738326	2.02193
56428	0.738326	2.06442
56429	0.680494	1.84947
56430	0.680494	1.65745
56431	0.708443	1.75508

56432	0.647895	1.79412
56433	0.770925	1.84997
56434	0.59474	1.51451
56435	1.89111	2.1
56436	1.06994	2.1
56437	2.06689	2.1
56438	1.37063	2.1
56439	3.13683	2.1
56440	2.06689	2.1
56441	1.39295	2.1
56442	1.56818	2.1
56443	1.37063	2.1
56444	2.26444	2.1
56445	2.06689	2.1
56446	1.49808	2.1
56447	1.50201	2.1
56448	2.57784	2.1
56449	2.58615	2.1
56450	4.08424	2.1
56451	0.946133	2.1
56452	0.946133	2.1
56453	0.946132	2.1
56454	0.946132	2.1
56455	0.946132	2.1
56456	0.946132	2.1
56457	0.946132	2.1
56458	0.946132	2.1
56459	0.946133	2.1
56460	0.946133	2.1
56461	0.946132	2.1
56462	0.946132	2.1
56463	1.89226	2.1
56464	1.89226	2.1
56465	1.89226	2.1
56466	1.89226	2.1
56467	3.78452	2.1
56468	0.977201	2.1
56469	0.977201	2.1
56470	0.980489	2.1
56471	0.980489	2.1
56472	0.657973	2.1
56473	1.20669	2.1
56474	0.751002	2.1
56475	1.29307	2.1
56476	1.29308	2.1
56477	1.20669	2.1
56478	0.751003	2.1
56479	0.657972	2.1
56480	0.946131	2.1
56481	0.946131	2.1
56482	1.29308	2.1
56483	1.29308	2.1
56484	0.946132	2.1
56485	1.29308	2.1
56486	0.946132	2.1
56487	1.28892	2.1
56488	0.946132	2.1
56489	0.946132	2.1

56490	1.28892	2.1
56491	1.29308	2.1
56492	0.946132	2.1
56493	0.946132	1.94979
56494	0.946131	2.1
56495	0.946131	2.1
56496	0.946132	2.1
56497	0.946132	2.07794
56498	0.946132	2.08038
56499	0.946132	1.94307
56500	0.946132	2.06363
56501	0.946132	2.1
56502	0.946132	2.09228
56503	0.946132	2.1
56504	1.89226	2.1
56505	1.89226	2.1
56506	1.89226	2.1
56507	1.89226	2.0502
56508	1.89226	2.1
56509	1.89226	2.00121
56510	0.94613	2.1
56511	0.94613	2.1
56512	1.20669	2.1
56513	1.20669	2.1
56514	1.20669	2.1
56515	0.946132	2.1
56516	0.946129	2.1
56517	1.29307	2.1
56518	0.946131	2.1
56519	0.946131	2.1
56520	1.29308	2.1
56521	1.20669	2.1
56522	1.89226	0.142284
56523	1.89226	0.0826984
56524	1.89226	0.0912791
56525	1.89226	0.123097
56526	3.78453	0.120007
56527	0.946132	0.0648533
56528	0.946132	0.0672049
56529	0.946126	0.0717313
56530	0.946126	0.089996
56531	0.946129	0.0749964
56532	0.946129	0.0689567
56533	0.946129	0.0719392
56534	0.946129	0.0882571
56535	0.946128	0.0684635
56536	0.946128	0.0726842
56537	0.946129	0.0689638
56538	0.946129	0.0715087
56539	0.725277	0.058221
56540	0.588155	0.0563996
56541	1.19403	0.0669143
56542	1.27681	0.0593131
56543	0.918903	0.0605589
56544	0.918903	0.0588974
56545	0.946068	0.0607514
56546	0.946068	0.0572926
56547	1.19404	0.0602863

56548	0.588153	0.0606291
56549	0.725275	0.0595684
56550	1.27682	0.0587317
56551	1.19404	0.07254
56552	1.27682	0.0632606
56553	0.946131	0.100298
56554	0.946131	0.0818383
56555	1.19404	0.0847112
56556	1.19404	0.0728821
56557	0.946129	0.0706051
56558	0.946129	0.0810521
56559	1.19404	0.0764854
56560	0.94613	0.076377
56561	1.27682	0.0685816
56562	0.946131	0.085286
56563	1.89226	0.0835563
56564	1.89226	0.13168
56565	1.89226	0.139811
56566	1.89226	0.097099
56567	1.89226	0.122168
56568	1.89226	0.0760965
56569	1.4263	2.00045
56570	1.67651	0.631041
56571	1.5955	0.803515
56572	1.31022	2.1
56573	2.98674	1.60794
56574	1.18824	2.1
56575	1.28802	2.1
56576	0.946132	2.1
56577	0.946132	2.1
56578	0.946132	2.1
56579	0.946132	2.1
56580	1.28802	2.1
56581	1.28802	2.1
56582	1.18824	2.1
56583	0.946132	2.1
56584	0.946132	2.1
56585	1.28802	2.1
56586	1.89226	0.777181
56587	1.89226	2.1
56588	1.89226	2.1
56589	1.89226	1.01221
56590	3.78452	1.76343
56591	1.5955	1.01292
56592	1.52909	0.940018
56593	1.31022	1.33386
56594	1.31022	2.1
56595	1.2087	2.1
56596	1.52909	2.1
56597	1.11825	2.1
56598	1.18824	2.1
56599	0.946132	2.1
56600	0.946132	2.1
56601	0.946132	2.1
56602	0.946132	2.1
56603	1.18824	2.1
56604	1.18824	2.1
56605	0.946132	2.1

56606	1.11825	2.1
56607	1.18824	2.1
56608	0.946133	2.1
56609	0.810943	2.1
56610	0.810943	2.1
56611	0.810945	1.08787
56612	0.810945	2.1
56613	1.89226	1.09396
56614	1.35168	1.80336
56615	1.08132	1.14986
56616	1.0813	1.9181
56617	1.08132	2.1
56618	0.810959	1.82769
56619	0.810959	1.87272
56620	1.89226	0.410079
56621	1.89226	0.188913
56622	1.89226	0.256312
56623	1.89226	0.384705
56624	3.78453	0.391228
56625	1.89226	0.10042
56626	1.89226	0.138646
56627	1.89226	0.144293
56628	1.89226	0.0868967
56629	1.89226	0.129796
56630	1.89226	0.07692
56631	0.946131	0.0721948
56632	0.946131	0.0772917
56633	0.946126	0.13499
56634	0.946126	0.0995868
56635	0.946128	0.150167
56636	0.946128	0.0917944
56637	0.946128	0.130909
56638	0.946128	0.107963
56639	0.946128	0.100169
56640	0.946128	0.113438
56641	0.946128	0.112412
56642	0.946128	0.0933433
56643	1.89226	0.363094
56644	1.89226	0.12704
56645	1.89226	0.260697
56646	1.89226	0.247363
56647	1.89226	0.18542
56648	1.89226	0.175311
56649	1.89226	0.177566
56650	1.89226	0.278303
56651	1.89226	0.560036
56652	1.89226	0.160527
56653	3.78453	0.160745
56654	1.07176	0.0707006
56655	1.07176	0.0651851
56656	0.946126	0.110697
56657	0.946126	0.0889489
56658	1.07176	0.0746421
56659	0.946129	0.0655947
56660	1.27474	0.0922377
56661	0.94613	0.0988604
56662	1.27474	0.0905958
56663	1.07176	0.0502362

56664	0.94613	0.0664627
56665	0.94613	0.0773458
56666	0.723486	0.0631119
56667	0.94613	0.0565457
56668	0.946126	0.0757534
56669	0.946126	0.0699865
56670	0.946129	0.0698521
56671	0.723484	0.0675266
56672	1.07176	0.0662109
56673	0.946129	0.0582271
56674	1.32398	0.0706213
56675	1.19835	0.0527188
56676	0.723471	0.0632023
56677	0.723471	0.0800068
56678	0.810946	0.316453
56679	0.810946	0.186077
56680	0.810946	0.301203
56681	0.810946	0.244232
56682	0.810933	0.0873681
56683	0.810933	0.250094
56684	1.89226	0.0868467
56685	1.3517	0.19591
56686	1.08132	0.106939
56687	1.08132	0.177758
56688	1.08133	0.210733
56689	2.39923	2.1
56690	1.89226	2.1
56691	1.89226	2.1
56692	1.89226	2.1
56693	2.44606	2.1
56694	2.44606	2.1
56695	1.89226	2.1
56696	1.89226	2.1
56697	1.89226	2.1
56698	1.89226	2.1
56699	1.89226	2.1
56700	1.89226	2.1
56701	1.70302	2.1
56702	2.43041	2.1
56703	2.25275	2.1
56704	2.4747	2.1
56705	1.52079	2.1
56706	1.67063	2.1
56707	2.40644	2.1
56708	1.89226	2.1
56709	1.89226	2.1
56710	1.89226	2.1
56711	2.39923	2.1
56712	2.39923	2.1
56713	0.946131	2.1
56714	0.946131	2.1
56715	0.946131	2.1
56716	0.946131	2.1
56717	0.94613	2.1
56718	0.94613	2.1
56719	0.946131	2.1
56720	0.946131	2.1
56721	0.946131	2.1

56722	0.946131	2.1
56723	0.946131	2.1
56724	0.946131	2.1
56725	1.04938	2.1
56726	1.04938	2.1
56727	1.111	2.1
56728	1.06978	2.1
56729	1.2748	2.1
56730	1.25472	2.1
56731	0.88782	2.1
56732	0.882987	2.1
56733	0.973779	2.1
56734	1.31997	2.1
56735	0.913905	2.1
56736	1.31312	2.1
56737	2.6536	2.1
56738	2.15287	2.1
56739	2.72238	2.1
56740	2.22887	2.1
56741	4.87525	2.1
56742	2.15288	2.1
56743	1.40719	2.1
56744	1.41839	2.1
56745	1.34239	2.1
56746	2.22887	2.1
56747	2.15288	2.1
56748	2.72237	2.1
56749	2.22887	2.1
56750	2.77314	2.1
56751	2.28748	2.1
56752	5.00985	2.1
56753	0.922802	2.1
56754	0.922802	2.1
56755	0.92107	2.1
56756	0.92107	2.1
56757	0.703593	2.1
56758	0.729436	2.1
56759	1.11444	2.1
56760	1.14374	2.1
56761	1.14374	2.1
56762	0.729436	2.1
56763	0.703593	2.1
56764	1.11444	2.1
56765	1.89226	2.1
56766	1.89226	2.1
56767	1.89226	2.1
56768	1.89226	2.1
56769	1.89226	2.1
56770	1.89226	2.1
56771	0.489927	2.1
56772	0.63008	2.1
56773	0.697385	2.1
56774	0.55524	2.1
56775	0.592141	2.1
56776	0.592141	2.1
56777	0.593179	2.1
56778	0.593179	2.1
56779	0.63008	2.1

56780	0.697385	2.1
56781	0.55524	2.1
56782	0.489927	2.1
56783	0.946133	2.1
56784	0.946133	2.1
56785	0.946132	2.1
56786	0.946132	1.89614
56787	0.946133	2.1
56788	0.946133	2.1
56789	0.946132	2.1
56790	0.946132	2.00372
56791	0.946132	2.1
56792	0.946132	2.1
56793	0.946132	2.1
56794	0.946132	2.1
56795	1.89226	2.1
56796	1.89226	2.1
56797	1.89226	2.1
56798	1.89226	2.1
56799	1.89226	2.1
56800	1.89226	2.1
56801	0.805746	2.1
56802	0.63008	2.1
56803	0.881598	2.1
56804	0.697385	2.1
56805	0.749417	2.1
56806	0.749417	2.1
56807	0.753714	2.1
56808	0.753714	2.1
56809	0.881598	2.1
56810	0.805746	2.1
56811	0.63008	2.1
56812	0.697385	2.1
56813	0.881599	2.1
56814	0.697385	2.1
56815	0.805746	2.1
56816	0.626731	2.1
56817	0.755479	2.1
56818	0.755479	2.1
56819	0.752851	2.1
56820	0.752851	2.1
56821	0.881598	2.1
56822	0.805747	2.1
56823	0.697385	2.1
56824	0.626731	2.1
56825	2.65348	2.1
56826	2.12314	2.1
56827	2.63899	2.1
56828	2.13898	2.1
56829	4.76213	2.1
56830	0.763518	2.1
56831	0.765889	2.1
56832	0.84086	2.1
56833	0.84086	2.1
56834	0.632078	2.1
56835	0.597565	2.1
56836	1.08813	2.1
56837	0.985643	2.1

56838	0.982464	2.1
56839	0.66404	2.1
56840	1.0358	2.1
56841	0.598023	2.1
56842	1.29293	2.1
56843	1.29628	2.1
56844	1.19266	2.1
56845	1.19266	2.1
56846	1.07109	2.1
56847	1.13093	2.1
56848	1.34467	2.1
56849	1.35251	2.1
56850	1.34369	2.1
56851	1.12525	2.1
56852	1.28746	2.1
56853	1.13511	2.1
56854	2.19717	2.1
56855	2.16547	2.1
56856	1.37392	2.1
56857	1.37392	2.1
56858	1.2966	2.1
56859	2.11985	2.1
56860	1.89226	2.1
56861	1.89226	2.1
56862	1.89226	2.1
56863	1.89226	2.1
56864	1.89226	2.1
56865	1.89226	2.1
56866	1.65637	2.1
56867	1.41758	2.1
56868	1.65637	2.1
56869	1.74391	2.1
56870	1.35504	2.1
56871	1.44258	2.1
56872	1.40774	2.1
56873	1.40774	2.1
56874	0.946131	2.1
56875	0.946131	2.1
56876	1.40774	2.1
56877	1.38882	2.1
56878	0.946132	2.1
56879	0.946132	2.1
56880	0.946132	2.1
56881	1.38882	2.1
56882	0.946132	2.1
56883	1.40774	2.1
56884	1.89227	2.1
56885	1.89227	2.1
56886	1.89227	2.1
56887	1.89227	2.1
56888	3.78453	2.1
56889	0.603148	2.1
56890	0.600177	2.1
56891	0.558615	2.1
56892	0.580714	2.1
56893	0.558606	2.1
56894	0.67883	2.1
56895	1.65637	2.1

56896	1.07933	2.1
56897	0.842649	2.1
56898	0.905168	2.1
56899	0.774334	2.1
56900	1.38882	2.1
56901	1.38882	2.1
56902	0.946132	2.1
56903	0.946132	2.1
56904	1.38882	2.1
56905	1.37381	2.1
56906	0.946132	2.1
56907	0.946132	2.1
56908	0.946132	2.1
56909	1.37381	2.1
56910	0.946133	2.1
56911	1.38882	2.1
56912	2.3771	2.1
56913	1.9339	2.1
56914	1.70782	2.1
56915	1.9339	2.1
56916	2.42693	2.1
56917	2.83279	2.1
56918	2.34901	0.254639
56919	1.45833	0.400026
56920	1.86695	0.254515
56921	1.03101	0.273316
56922	3.32528	0.289887
56923	1.89226	0.128281
56924	1.89226	0.138708
56925	1.89226	0.0830345
56926	1.89226	0.0697512
56927	3.78453	0.118648
56928	2.71753	0.0824247
56929	1.89226	0.126162
56930	2.76228	0.0669024
56931	2.76228	0.0833048
56932	1.89226	0.139829
56933	1.89226	0.288326
56934	0.946131	0.0844394
56935	0.946131	0.0812328
56936	0.946132	0.0743682
56937	0.946132	0.084149
56938	0.946132	0.138273
56939	0.946132	0.0734973
56940	0.946132	0.109483
56941	0.946132	0.0653829
56942	0.946132	0.0710197
56943	0.946132	0.0742851
56944	0.946132	0.0996893
56945	0.946132	0.0929487
56946	1.89227	0.110923
56947	1.89227	0.0560283
56948	1.89227	0.0714657
56949	1.89227	0.10034
56950	1.89227	0.0504163
56951	1.89227	0.0664553
56952	2.71753	0.0902229
56953	2.37907	0.0520927

56954	2.37907	0.0707605
56955	2.76228	0.103202
56956	2.39588	0.05
56957	2.77909	0.0647674
56958	1.6571	1.17378
56959	1.11682	1.64019
56960	1.47132	1.48703
56961	1.93763	0.156045
56962	3.12842	1.5184
56963	1.93762	0.381443
56964	2.12345	1.46781
56965	1.65709	0.224977
56966	1.65709	1.77312
56967	2.12345	1.28521
56968	1.88635	1.89252
56969	1.47134	1.72739
56970	2.219	0.754838
56971	2.25734	2.03139
56972	1.93765	1.27247
56973	1.79103	1.58063
56974	1.47134	0.513557
56975	1.06174	1.40715
56976	1.19349	1.87638
56977	1.1095	1.44549
56978	0.968827	1.16042
56979	1.07892	0.921429
56980	1.07892	2.1
56981	1.0834	1.48882
56982	1.0834	1.37515
56983	1.1095	1.23345
56984	1.19349	1.16583
56985	0.968825	1.37356
56986	1.06174	1.36193
56987	2.40627	2.1
56988	2.08288	1.53406
56989	2.5444	1.5908
56990	2.29285	1.77184
56991	4.62729	1.62924
56992	2.08289	1.30717
56993	1.65703	1.27636
56994	1.71105	2.1
56995	1.50108	1.55783
56996	2.29286	1.88835
56997	2.08289	1.60033
56998	2.5444	1.7737
56999	2.29285	2.1
57000	2.71429	2.1
57001	2.50288	1.41448
57002	5.04728	2.06846
57003	2.50288	1.35909
57004	2.00927	1.73065
57005	2.02116	1.76722
57006	2.02116	1.98527
57007	2.29286	1.64522
57008	2.29286	1.70336
57009	1.89226	2.1
57010	1.89226	2.1
57011	1.89226	2.1

57012	1.89226	2.1
57013	3.78453	2.1
57014	1.17418	2.1
57015	1.29741	2.1
57016	1.04921	2.1
57017	1.19352	2.1
57018	2.3677	2.1
57019	1.29741	2.1
57020	1.27575	2.1
57021	1.19352	2.1
57022	1.19473	2.1
57023	2.46927	2.1
57024	1.89226	2.1
57025	1.89226	2.1
57026	1.89226	2.1
57027	1.89226	2.1
57028	1.89226	2.1
57029	1.89226	2.1
57030	0.561332	2.1
57031	0.561332	2.1
57032	0.567597	2.1
57033	0.567597	2.1
57034	0.597365	2.1
57035	0.544699	2.1
57036	0.596761	2.1
57037	0.531564	2.1
57038	0.5447	2.1
57039	0.531563	2.1
57040	0.596759	2.1
57041	0.597366	2.1
57042	0.946131	2.1
57043	0.946131	2.1
57044	0.946131	2.1
57045	0.946131	2.1
57046	0.946129	2.1
57047	0.946129	2.1
57048	0.946133	2.1
57049	0.946133	2.1
57050	0.946131	2.1
57051	0.946131	2.1
57052	0.946131	2.1
57053	0.946131	2.1
57054	1.10938	0.6512
57055	1.17614	2.1
57056	1.02478	1.35312
57057	1.09056	2.1
57058	2.19994	2.05416
57059	1.17614	2.1
57060	1.11868	2.1
57061	1.03408	2.1
57062	1.03408	2.1
57063	1.09056	2.1
57064	1.17516	2.1
57065	1.89226	0.552452
57066	1.89226	0.372862
57067	1.89226	0.391742
57068	1.89226	0.306876
57069	1.89226	0.799071

57070	1.89226	0.544304
57071	1.89226	0.302485
57072	1.89226	0.429181
57073	1.89226	0.318303
57074	1.89226	0.257034
57075	1.89226	0.590785
57076	1.89226	0.268398
57077	0.579551	0.231061
57078	0.563873	0.20319
57079	0.611341	0.282846
57080	0.598693	0.241212
57081	0.589856	0.228167
57082	0.589856	0.21101
57083	0.588387	0.266528
57084	0.588387	0.243844
57085	0.57955	0.241597
57086	0.611341	0.230574
57087	0.563873	0.239204
57088	0.598693	0.228682
57089	0.611341	0.274774
57090	0.598693	0.236539
57091	0.586048	0.271103
57092	0.572737	0.294848
57093	0.591824	0.268956
57094	0.591824	0.248939
57095	0.592254	0.318091
57096	0.592254	0.292115
57097	0.611341	0.287502
57098	0.586048	0.264463
57099	0.598693	0.279689
57100	0.572737	0.272235
57101	0.946119	0.346196
57102	0.946119	0.539359
57103	0.946119	0.332337
57104	0.946119	0.482317
57105	0.94612	0.320921
57106	0.94612	0.295765
57107	0.946119	0.519834
57108	0.946119	0.685093
57109	0.946132	0.426205
57110	0.946132	0.390484
57111	0.946107	0.420501
57112	0.946107	0.477778
57113	1.89224	0.349361
57114	1.89224	0.282895
57115	1.89224	0.303529
57116	1.89224	0.228532
57117	1.89224	0.528086
57118	1.89224	0.35466
57119	0.562335	0.208623
57120	0.579543	0.182152
57121	0.59499	0.244636
57122	0.611333	0.213274
57123	0.586619	0.203979
57124	0.586619	0.191168
57125	0.587049	0.234342
57126	0.587049	0.215192
57127	0.594999	0.223218

57128	0.562342	0.219063
57129	0.579535	0.218225
57130	0.611325	0.208482
57131	0.59499	0.234542
57132	0.611333	0.207529
57133	0.567849	0.232685
57134	0.58604	0.250583
57135	0.590978	0.228485
57136	0.590978	0.216534
57137	0.590052	0.263524
57138	0.590052	0.248449
57139	0.567857	0.251876
57140	0.594998	0.24315
57141	0.611325	0.253083
57142	0.586032	0.235869
57143	1.69661	1.81463
57144	1.9339	0.486993
57145	1.39379	1.94241
57146	1.69107	0.275126
57147	3.32768	0.761279
57148	1.9339	0.265953
57149	1.98414	0.777586
57150	1.69107	0.281626
57151	1.72774	0.428284
57152	3.66164	0.25492
57153	1.39379	2.05075
57154	1.69107	0.273672
57155	1.14397	1.66983
57156	1.43548	0.222835
57157	2.82927	1.13534
57158	1.69107	0.153621
57159	1.72774	0.417824
57160	1.53873	0.274288
57161	1.53873	0.809908
57162	1.43548	0.19783
57163	1.62449	1.03953
57164	2.58431	0.0934265
57165	1.34072	0.110843
57166	1.80841	0.0958772
57167	1.34072	0.104635
57168	2.51501	0.10585
57169	2.04732	0.118731
57170	1.89226	0.0953472
57171	1.89226	0.115628
57172	1.89226	0.0956402
57173	1.89226	0.12502
57174	1.89226	0.109004
57175	1.89226	0.127168
57176	0.946131	0.132748
57177	0.946131	0.112417
57178	1.3639	0.0940125
57179	1.1516	0.114194
57180	0.946129	0.121908
57181	0.946129	0.138133
57182	1.36391	0.096378
57183	1.36391	0.0997159
57184	0.946131	0.130054
57185	1.3639	0.106124

57186	1.1516	0.102788
57187	0.946131	0.122251
57188	0.946131	0.110417
57189	0.946131	0.0978902
57190	0.94613	0.12445
57191	0.94613	0.109357
57192	0.946128	0.134664
57193	0.946128	0.103853
57194	0.946133	0.0971128
57195	0.946133	0.125724
57196	0.946131	0.112204
57197	0.946131	0.104266
57198	0.946131	0.122426
57199	0.946131	0.106266
57200	1.89226	1.69439
57201	2.63634	1.97734
57202	1.89226	1.75409
57203	1.83902	1.77136
57204	3.73128	1.71489
57205	1.47059	2.1
57206	2.02928	2.1
57207	1.47059	2.1
57208	1.65706	2.1
57209	1.98628	2.1
57210	2.17274	2.1
57211	2.43618	2.1
57212	2.41176	2.1
57213	1.98627	2.1
57214	2.34084	2.1
57215	4.39803	2.1
57216	1.47059	2.1
57217	1.82453	2.1
57218	1.98627	2.1
57219	1.98627	2.1
57220	1.26929	2.1
57221	1.26929	2.1
57222	1.98627	2.1
57223	2.34084	2.1
57224	1.47264	2.1
57225	2.24937	2.1
57226	4.23564	2.1
57227	1.33026	1.55667
57228	1.51171	2.07801
57229	1.51171	1.74419
57230	1.4617	2.09875
57231	1.35382	1.65486
57232	1.30382	2.1
57233	1.89226	1.11953
57234	1.89226	1.86768
57235	1.89226	1.41888
57236	1.89226	2.0312
57237	1.89226	1.20829
57238	1.89226	2.07257
57239	1.39263	1.48961
57240	0.962704	0.494947
57241	1.0171	0.793395
57242	0.962704	1.23431
57243	1.40822	0.597655

57244	1.35382	0.798445
57245	0.755853	2.1
57246	0.789808	1.97306
57247	0.696315	1.75276
57248	0.676912	1.0698
57249	0.729751	1.13901
57250	0.729751	1.94615
57251	0.722417	2.1
57252	0.722417	1.09702
57253	0.696315	1.89276
57254	0.789808	1.87782
57255	0.755853	1.83337
57256	0.676912	1.81913
57257	0.946132	1.76937
57258	0.946132	1.9444
57259	0.946132	1.38837
57260	0.946132	0.892349
57261	0.946131	0.964147
57262	0.946131	1.74283
57263	0.946132	1.51714
57264	0.946132	1.9453
57265	0.946132	1.54469
57266	0.946132	1.60738
57267	0.946132	1.56851
57268	0.946132	1.52985
57269	0.946133	0.884998
57270	0.946133	0.677691
57271	0.946132	0.458763
57272	0.946132	0.622869
57273	0.946132	0.477511
57274	0.946132	0.65728
57275	0.946133	0.486651
57276	0.946133	0.949897
57277	0.946132	0.668484
57278	0.946132	0.675046
57279	0.946132	0.610984
57280	0.946132	0.634958
57281	1.89227	1.86589
57282	2.53014	1.93618
57283	1.89227	2.04386
57284	1.98241	1.83338
57285	1.89227	2.06113
57286	1.98241	1.86029
57287	1.89226	1.78212
57288	1.89226	2.1
57289	1.89226	1.89424
57290	1.89226	1.91134
57291	1.89226	2.0824
57292	1.89226	2.05923
57293	1.89226	1.65258
57294	1.89226	2.03843
57295	1.89226	1.71051
57296	1.89226	1.75695
57297	3.78452	1.73861
57298	2.45304	1.61284
57299	1.89226	1.84137
57300	1.89226	1.74224
57301	1.89226	1.75033

57302	1.89226	1.62672
57303	1.89226	1.65357
57304	1.89227	1.82431
57305	1.89227	1.83832
57306	1.89227	2.00391
57307	1.89227	1.72593
57308	1.89227	2.01725
57309	1.89227	1.75004
57310	1.89226	1.74751
57311	1.89226	2.1
57312	1.89226	1.87617
57313	1.89226	1.88584
57314	1.89226	2.03399
57315	1.89226	2.02297
57316	2.09871	0.209745
57317	2.71891	0.260452
57318	1.31126	0.191405
57319	1.31126	0.359442
57320	2.55341	0.194069
57321	1.76597	0.792924
57322	1.89226	0.748099
57323	1.89226	1.0828
57324	1.89226	0.285373
57325	1.89226	1.29961
57326	1.89226	0.365762
57327	1.89226	1.38989
57328	0.946128	0.75068
57329	0.946128	0.260664
57330	0.946133	0.237495
57331	0.946133	0.188508
57332	0.946131	0.231783
57333	0.946131	0.362256
57334	0.94613	0.218635
57335	0.94613	0.278711
57336	0.946131	0.275946
57337	0.946131	0.228602
57338	0.946131	0.246177
57339	0.946131	0.289876
57340	2.71891	0.222546
57341	1.89226	0.175055
57342	2.09871	0.150343
57343	2.09871	0.425093
57344	1.89226	0.146939
57345	1.89226	0.323666
57346	1.89227	0.662692
57347	1.89227	0.691197
57348	1.89227	0.288924
57349	1.89227	1.18375
57350	1.89227	0.521335
57351	1.89227	1.33165
57352	0.946129	0.528068
57353	0.946129	0.255369
57354	0.946133	0.207744
57355	0.946133	0.165488
57356	0.946131	0.20075
57357	0.946131	0.340358
57358	0.946131	0.197327
57359	0.946131	0.28128

57360	0.946131	0.256647
57361	0.946131	0.21412
57362	0.946131	0.269112
57363	0.946131	0.227196
57364	2.32462	0.05
57365	1.89226	0.05
57366	1.89226	0.05
57367	1.89226	0.05
57368	2.2291	0.05
57369	2.2291	0.05
57370	0.847669	0.05
57371	0.847669	0.05
57372	0.796966	0.05
57373	0.796966	0.05
57374	0.833552	0.05
57375	1.01049	0.05
57376	0.709709	0.05
57377	0.634147	0.05
57378	0.634148	0.05
57379	0.833549	0.05
57380	0.709711	0.05
57381	1.01049	0.05
57382	1.89226	0.05
57383	1.89226	0.05
57384	1.89226	0.05
57385	1.89226	0.05
57386	1.89226	0.05
57387	1.89226	0.05
57388	1.03611	0.05
57389	0.936655	0.05
57390	0.946132	0.05
57391	0.946132	0.05
57392	0.941918	0.05
57393	0.941918	0.05
57394	1.00308	0.05
57395	1.04117	0.05
57396	1.1406	0.05
57397	1.04074	0.05
57398	0.851529	0.05
57399	1.00311	0.05
57400	1.01814	0.05
57401	1.6671	0.05
57402	1.35561	0.05
57403	1.35561	0.05
57404	1.2683	0.05
57405	1.2683	0.05
57406	0.946128	0.05
57407	0.946128	0.05
57408	0.946129	0.05
57409	0.946129	0.05
57410	0.946132	0.05
57411	0.946132	0.05
57412	0.946126	0.05
57413	0.946126	0.05
57414	0.946129	0.05
57415	0.946129	0.05
57416	0.946129	0.05
57417	0.946129	0.05

57418	2.23338	0.05
57419	1.96	0.05
57420	2.03438	0.05
57421	1.87835	0.05
57422	4.11172	0.05
57423	1.96	0.05
57424	1.52661	0.05
57425	1.87834	0.05
57426	1.53175	0.05
57427	3.40495	0.05
57428	2.03435	0.05
57429	1.65813	0.05
57430	1.65813	0.05
57431	1.87832	0.05
57432	1.72067	0.05
57433	1.94086	0.05
57434	1.65813	0.05
57435	1.40572	0.05
57436	1.87832	0.05
57437	1.87832	0.05
57438	1.31154	0.05
57439	1.53173	0.05
57440	1.86662	2.1
57441	2.0815	2.1
57442	2.0815	2.1
57443	2.0815	2.1
57444	1.87246	2.1
57445	1.87362	2.1
57446	1.0273	2.1
57447	0.996347	2.1
57448	0.946129	2.1
57449	0.946129	2.1
57450	1.04427	2.1
57451	1.04196	2.1
57452	0.910674	2.1
57453	0.910674	2.1
57454	1.04072	2.1
57455	1.10931	2.1
57456	1.03132	2.1
57457	0.933698	2.1
57458	2.63789	0.278856
57459	1.89226	1.19551
57460	2.3033	0.318466
57461	2.3033	0.838538
57462	1.89226	0.324458
57463	1.89226	0.880781
57464	1.89226	1.2809
57465	1.89226	1.42835
57466	1.89226	0.872865
57467	1.89226	1.30325
57468	3.78452	1.34882
57469	1.51898	1.02838
57470	1.36006	1.47039
57471	2.03143	1.45825
57472	2.03143	1.47831
57473	1.04026	1.28511
57474	1.04026	1.3142
57475	0.94613	0.137344

57476	0.94613	0.468041
57477	1.31894	0.143042
57478	1.31894	0.149898
57479	0.94613	0.148316
57480	0.94613	0.427039
57481	1.31894	0.213387
57482	1.41232	0.355965
57483	0.946129	0.240787
57484	1.41232	0.2234
57485	1.31895	0.194657
57486	0.946129	0.274755
57487	1.89226	0.210427
57488	1.89226	1.24607
57489	1.89226	0.180168
57490	1.89226	1.08297
57491	1.89226	0.174827
57492	1.89226	1.1581
57493	1.89804	0.162879
57494	2.03143	0.698294
57495	2.46286	0.431208
57496	2.46287	0.410792
57497	1.51898	0.373571
57498	1.51898	0.359974
57499	1.89226	2.1
57500	1.89226	2.1
57501	1.89226	2.1
57502	1.89226	2.1
57503	3.78452	2.1
57504	1.3864	2.1
57505	1.3864	2.1
57506	0.946132	2.1
57507	0.946132	2.1
57508	1.32905	2.1
57509	1.3864	2.1
57510	0.946132	2.1
57511	0.946132	2.1
57512	0.946132	2.1
57513	1.32905	2.1
57514	0.946133	2.1
57515	1.3864	2.1
57516	2.77279	2.1
57517	1.67883	2.1
57518	1.67883	2.1
57519	1.73415	2.1
57520	2.65811	2.1
57521	2.71343	2.1
57522	0.946131	2.1
57523	0.946131	2.1
57524	0.946128	2.1
57525	0.946128	2.1
57526	0.946127	2.1
57527	0.946127	2.1
57528	0.946131	2.1
57529	0.946131	2.1
57530	0.946129	2.1
57531	0.946129	2.1
57532	0.94613	2.1
57533	0.94613	2.1

57534	1.89226	2.1
57535	1.89226	2.1
57536	1.89226	2.1
57537	1.89226	2.1
57538	3.78452	2.1
57539	1.89226	2.1
57540	2.54394	2.1
57541	2.54394	2.1
57542	2.65811	2.1
57543	1.89226	2.1
57544	2.00643	2.1
57545	2.54394	2.1
57546	2.6581	2.1
57547	1.62743	2.1
57548	1.62743	2.1
57549	1.67883	2.1
57550	2.59533	2.1
57551	1.89226	2.1
57552	1.89226	2.1
57553	1.89226	2.1
57554	1.89226	2.1
57555	1.89226	2.1
57556	1.89226	2.1
57557	1.89226	1.80216
57558	1.89226	1.78406
57559	1.89226	2.07187
57560	1.89226	1.62025
57561	1.89226	2.03938
57562	1.89226	1.58812
57563	1.89226	1.76425
57564	1.89226	1.75082
57565	1.89226	2.03029
57566	1.89226	1.64021
57567	1.89226	1.98232
57568	1.89226	1.55199
57569	1.89226	1.21614
57570	1.89226	0.634458
57571	1.89226	1.7074
57572	1.89226	0.687362
57573	1.89226	1.69151
57574	1.89226	0.664337
57575	2.68537	0.567658
57576	2.22575	2.1
57577	2.47464	0.29676
57578	1.79093	1.90553
57579	4.70039	0.688468
57580	2.81217	2.1
57581	2.47464	0.461102
57582	2.58286	2.03958
57583	2.29193	0.312238
57584	5.0575	0.587885
57585	2.47464	0.386885
57586	2.20365	1.92045
57587	2.29193	0.547013
57588	1.90104	2.01783
57589	4.37568	0.514066
57590	1.89226	1.39462
57591	2.16957	2.00656

57592	2.52823	1.65566
57593	2.52823	1.87961
57594	1.89226	1.51928
57595	1.89226	1.62342
57596	1.36226	2.1
57597	1.55369	2.1
57598	1.33309	2.1
57599	1.62593	2.1
57600	2.98819	2.1
57601	0.802281	2.1
57602	0.802281	2.1
57603	0.835143	2.1
57604	0.835143	2.1
57605	0.824458	2.1
57606	0.92641	2.1
57607	0.776847	2.1
57608	0.812967	2.1
57609	0.812967	2.1
57610	0.926411	2.1
57611	0.776847	2.1
57612	0.824458	2.1
57613	1.33309	2.1
57614	1.62593	2.1
57615	1.32813	2.1
57616	1.7237	2.1
57617	2.95406	2.1
57618	0.814544	2.1
57619	0.875312	2.1
57620	0.910502	2.1
57621	0.910502	2.1
57622	0.886633	2.1
57623	0.975739	2.1
57624	0.792623	2.1
57625	0.840283	2.1
57626	0.796372	2.1
57627	1.06159	2.1
57628	0.81948	2.1
57629	0.933833	2.1
57630	2.63997	2.1
57631	1.29694	2.1
57632	1.28141	2.1
57633	2.62623	2.1
57634	3.92317	2.1
57635	2.63998	2.1
57636	1.89226	2.1
57637	1.89226	2.1
57638	2.62623	2.1
57639	4.5185	2.1
57640	1.89226	2.1
57641	1.89226	2.1
57642	1.89226	2.1
57643	1.89226	2.1
57644	3.78453	2.1
57645	1.89226	2.1
57646	1.89226	2.1
57647	1.89226	2.1
57648	1.89226	2.1
57649	1.89226	2.1

57650	1.89226	2.1
57651	1.25468	2.1
57652	2.62623	2.1
57653	1.28141	2.1
57654	1.28141	2.1
57655	2.60665	2.1
57656	2.60665	2.1
57657	1.89226	2.1
57658	2.62623	2.1
57659	2.60666	2.1
57660	2.62623	2.1
57661	1.87269	2.1
57662	1.89226	2.1
57663	1.89226	2.1
57664	1.89226	2.1
57665	1.89226	2.1
57666	1.89226	2.1
57667	3.78453	2.1
57668	1.89226	2.1
57669	1.89226	2.1
57670	1.89226	2.1
57671	1.89226	2.1
57672	1.89226	2.1
57673	1.89226	2.1
57674	2.30147	1.67487
57675	2.27703	1.98335
57676	1.89227	1.58095
57677	1.89227	1.81807
57678	4.1693	1.74146
57679	1.38617	1.40037
57680	1.24613	1.61129
57681	2.59886	1.36707
57682	2.6238	1.55399
57683	3.86993	1.46572
57684	0.946133	1.68396
57685	0.946133	1.49403
57686	0.946132	1.42812
57687	0.946132	1.99207
57688	0.946133	1.99429
57689	0.946133	1.77845
57690	0.946132	1.48563
57691	0.946132	1.5098
57692	0.946133	1.621
57693	0.946133	1.60323
57694	0.946132	1.56764
57695	0.946132	1.56842
57696	2.21773	2.1
57697	2.59216	2.1
57698	1.68541	2.1
57699	1.68541	2.1
57700	1.84814	2.1
57701	2.38046	2.1
57702	1.89966	2.1
57703	1.70303	2.1
57704	1.2028	2.1
57705	1.29897	2.1
57706	2.02873	2.1
57707	2.19093	2.1

57708	2.75218	2.1
57709	1.70303	2.1
57710	3.10254	2.1
57711	2.24783	2.1
57712	2.56839	2.1
57713	1.70303	2.1
57714	2.56839	2.1
57715	1.08276	2.1
57716	2.24061	2.1
57717	1.67491	2.1
57718	1.60948	2.1
57719	1.08276	2.1
57720	1.89226	1.0684
57721	1.89226	1.17298
57722	1.89226	1.0634
57723	1.89226	1.04434
57724	3.78453	1.0118
57725	1.89226	1.06093
57726	1.89226	1.10138
57727	1.89226	0.917538
57728	1.89226	1.02236
57729	3.78452	1.2361
57730	1.89226	1.08274
57731	2.7535	1.07658
57732	1.89226	1.33253
57733	1.89226	1.90504
57734	1.89226	0.917335
57735	1.89226	1.02768
57736	1.89226	1.09703
57737	1.89226	1.06102
57738	1.89226	0.936172
57739	1.89226	1.26032
57740	1.89226	1.06584
57741	1.89226	1.40157
57742	1.89227	1.23043
57743	1.89227	1.03859
57744	1.89227	0.236089
57745	1.89227	0.873029
57746	3.78453	0.986779
57747	1.89226	1.07918
57748	2.53412	0.560591
57749	1.89226	1.65191
57750	1.89226	0.658973
57751	1.89226	1.05687
57752	1.89226	0.376337
57753	2.21126	0.0582758
57754	1.89226	0.0741559
57755	2.57464	0.0844346
57756	1.89226	0.077884
57757	2.57464	0.0563193
57758	1.89226	0.0566775
57759	1.89226	0.224808
57760	1.89226	0.239114
57761	1.89226	0.669046
57762	1.89226	0.24551
57763	1.89226	0.560679
57764	1.89226	0.192588
57765	1.1418	0.202092

57766	0.653021	0.0581936
57767	1.37282	0.11917
57768	0.931386	0.257745
57769	1.34568	0.308808
57770	1.17669	0.0891176
57771	0.666595	0.0805862
57772	0.786895	0.100489
57773	0.933982	0.145626
57774	1.05351	0.119583
57775	1.00792	0.118133
57776	1.00792	0.0830731
57777	1.89226	2.1
57778	1.89226	2.1
57779	1.89226	2.1
57780	1.89226	2.1
57781	3.78452	2.1
57782	1.89226	2.1
57783	1.89226	2.1
57784	1.89226	2.1
57785	1.89226	2.1
57786	1.89226	2.1
57787	1.89226	2.1
57788	1.65642	2.1
57789	1.30248	2.1
57790	1.38888	2.1
57791	1.25987	2.1
57792	1.70611	2.1
57793	1.65642	2.1
57794	1.65642	2.1
57795	1.81407	2.1
57796	2.19428	2.1
57797	1.92555	2.1
57798	3.8507	2.1
57799	1.89226	2.1
57800	1.89226	2.1
57801	1.89226	2.1
57802	1.89226	2.1
57803	3.78452	2.1
57804	1.89226	2.1
57805	1.89226	2.1
57806	1.89226	2.1
57807	1.89226	2.1
57808	1.89226	2.1
57809	1.89226	2.1
57810	0.705495	2.1
57811	0.809317	2.1
57812	1.1655	2.1
57813	1.09605	2.1
57814	0.983375	2.1
57815	0.797301	2.1
57816	0.906633	2.1
57817	0.906633	2.1
57818	0.85085	2.1
57819	1.16548	2.1
57820	0.705501	2.1
57821	1.00314	2.1
57822	2.19428	2.1
57823	2.31862	2.1

57824	2.27408	2.1
57825	2.45893	2.1
57826	4.46836	2.1
57827	1.89226	1.1533
57828	1.89226	1.23309
57829	1.89226	1.22813
57830	1.89226	1.09975
57831	1.89226	1.26216
57832	1.89226	1.1792
57833	2.0815	1.14671
57834	2.22136	1.14927
57835	1.5515	1.05719
57836	1.86323	0.855303
57837	2.74986	1.24063
57838	3.30238	1.07433
57839	1.89224	0.947828
57840	1.89224	1.12449
57841	1.89224	1.23292
57842	1.89224	0.726049
57843	1.89224	1.26456
57844	1.89224	1.09977
57845	1.89224	1.13523
57846	1.89224	1.20784
57847	1.89224	1.19017
57848	1.89224	1.10285
57849	1.89224	1.24002
57850	1.89224	1.1618
57851	1.17846	0.767802
57852	0.810935	0.952107
57853	0.93347	1.21862
57854	0.93347	1.06046
57855	0.810932	1.16164
57856	0.810932	1.11975
57857	2.22133	1.1001
57858	1.71919	1.00982
57859	1.32629	0.636713
57860	1.0813	1.20395
57861	1.57137	1.08546
57862	2.21149	2.1
57863	1.89226	2.1
57864	1.89226	2.1
57865	1.89226	2.1
57866	2.17159	2.1
57867	2.17159	2.1
57868	0.946131	1.09158
57869	0.946131	2.1
57870	0.946131	2.1
57871	0.946131	2.1
57872	0.94613	1.72531
57873	0.94613	1.84697
57874	0.946132	2.1
57875	0.946132	2.1
57876	0.946131	2.03912
57877	0.946131	2.05914
57878	0.946131	2.1
57879	0.946131	2.1
57880	1.12266	2.1
57881	1.4714	2.04863

57882	1.62322	2.1
57883	1.62322	1.98074
57884	1.02958	2.1
57885	1.02958	2.1
57886	2.2671	2.1
57887	1.89226	2.1
57888	1.89226	2.1
57889	1.89226	2.1
57890	2.21149	2.1
57891	2.21149	2.1
57892	1.89226	2.1
57893	1.89226	1.99637
57894	1.89226	2.1
57895	1.89226	1.86901
57896	1.89226	2.1
57897	1.89226	2.06696
57898	0.736914	2.1
57899	0.736914	2.1
57900	0.822839	2.1
57901	0.822836	2.1
57902	0.791298	1.67524
57903	1.00003	1.95079
57904	0.640254	2.1
57905	0.575379	2.1
57906	0.609001	2.1
57907	1.00005	2.1
57908	0.575371	2.1
57909	0.831908	2.1
57910	1.89226	2.1
57911	1.89226	2.1
57912	1.89226	2.1
57913	1.89226	2.1
57914	1.89226	2.1
57915	1.89226	2.1
57916	1.89226	2.1
57917	1.89226	2.1
57918	1.89226	2.1
57919	1.89226	2.1
57920	1.89226	2.1
57921	1.89226	2.1
57922	0.730617	2.1
57923	0.71052	2.1
57924	0.7329	2.1
57925	0.712083	2.1
57926	0.701051	2.1
57927	0.701051	2.1
57928	0.721552	2.1
57929	0.721552	2.1
57930	0.710521	2.1
57931	0.732898	2.1
57932	0.730619	2.1
57933	0.712081	2.1
57934	0.7329	2.1
57935	0.712082	2.1
57936	0.559601	2.1
57937	0.495993	2.1
57938	0.603718	2.1
57939	0.603718	2.1

57940	0.625174	2.1
57941	0.625174	2.1
57942	0.732899	2.1
57943	0.559601	2.1
57944	0.712082	2.1
57945	0.495993	2.1
57946	0.94613	2.1
57947	0.94613	2.1
57948	0.94613	2.1
57949	0.94613	2.1
57950	0.946131	2.1
57951	0.946131	2.1
57952	0.946129	2.1
57953	0.946129	2.1
57954	0.94613	2.1
57955	0.94613	2.1
57956	0.94613	2.1
57957	0.94613	2.1
57958	1.89226	2.1
57959	1.89226	2.1
57960	1.89226	2.1
57961	1.89226	2.1
57962	1.89226	2.1
57963	1.89226	2.1
57964	1.42104	2.1
57965	1.4658	2.1
57966	1.51223	2.1
57967	1.42283	2.1
57968	2.93327	2.1
57969	0.732899	2.1
57970	0.756115	2.1
57971	0.629763	2.1
57972	0.559601	2.1
57973	0.646134	2.1
57974	0.646134	2.1
57975	0.669582	2.1
57976	0.669582	2.1
57977	0.756115	2.1
57978	0.629763	2.1
57979	0.732899	2.1
57980	0.559601	2.1
57981	2.566 2.1	
57982	1.20242	2.1
57983	2.64084	2.1
57984	2.18297	2.1
57985	1.66029	2.1
57986	1.20242	2.1
57987	0.946133	1.7518
57988	0.946133	1.8473
57989	0.946129	2.1
57990	0.946129	2.1
57991	0.946131	2.1
57992	0.946131	1.71719
57993	0.946131	1.62687
57994	0.946131	2.1
57995	0.946131	1.99225
57996	0.946131	2.05857
57997	0.946131	2.07617

57998	0.946131	2.1
57999	1.89226	0.05
58000	1.89226	0.05
58001	1.89226	0.05
58002	1.89226	0.05
58003	3.78453	0.05
58004	1.03073	0.05
58005	1.03073	0.05
58006	1.04066	0.05
58007	1.04066	0.05
58008	1.20802	0.05
58009	1.32689	0.05
58010	0.883301	0.05
58011	0.744506	0.05
58012	1.20802	0.05
58013	0.744506	0.05
58014	0.883301	0.05
58015	1.32689	0.05
58016	1.20802	0.05
58017	1.20802	0.05
58018	0.946131	0.05
58019	0.946131	0.05
58020	0.94613	0.05
58021	0.94613	0.05
58022	1.20802	0.05
58023	1.32689	0.05
58024	1.20802	0.05
58025	0.946131	0.05
58026	0.946131	0.05
58027	1.32689	0.05
58028	1.89226	0.05
58029	1.89226	0.05
58030	1.89226	0.05
58031	1.89226	0.05
58032	1.89226	0.05
58033	1.89226	0.05
58034	0.946119	0.05
58035	0.946119	0.05
58036	0.946118	0.05
58037	0.946118	0.0521149
58038	0.946118	0.0522126
58039	0.946118	0.05
58040	0.946119	0.05
58041	0.946119	0.05
58042	0.946131	0.05
58043	0.946131	0.05
58044	0.946106	0.05
58045	0.946106	0.05
58046	1.3939	2.1
58047	1.49641	2.1
58048	1.15096	2.1
58049	1.10101	2.1
58050	2.59742	2.1
58051	1.22106	2.1
58052	2.60663	2.1
58053	1.25467	2.1
58054	1.25467	2.1
58055	2.58377	2.1

58056	2.58377	2.1
58057	1.89225	2.1
58058	2.60664	2.1
58059	2.58377	2.1
58060	2.60664	2.1
58061	1.86938	2.1
58062	1.89225	2.1
58063	1.89225	2.1
58064	1.89225	2.1
58065	1.89225	2.1
58066	1.89225	2.1
58067	1.89225	2.1
58068	1.89225	2.1
58069	0.946121	2.1
58070	0.946121	2.1
58071	0.946126	2.1
58072	0.946126	2.1
58073	0.946124	2.1
58074	0.946124	2.1
58075	0.946123	2.1
58076	0.946123	2.1
58077	0.946117	2.1
58078	0.946117	2.1
58079	0.94613	2.1
58080	0.94613	2.1
58081	1.28017	2.1
58082	1.29189	2.1
58083	0.610534	2.1
58084	0.592373	2.1
58085	0.943764	2.1
58086	0.943764	2.1
58087	0.940501	2.1
58088	0.940501	2.1
58089	1.28017	2.1
58090	0.592371	2.1
58091	0.610533	2.1
58092	1.29189	2.1
58093	0.946133	2.1
58094	0.946133	2.1
58095	1.28017	2.1
58096	1.29189	2.1
58097	0.946132	2.1
58098	0.946132	2.1
58099	1.28017	2.1
58100	1.28017	2.1
58101	1.28017	2.1
58102	0.946132	2.1
58103	0.946133	2.1
58104	1.2919	2.1
58105	1.89226	2.1
58106	1.89226	2.1
58107	1.89226	2.1
58108	1.89226	2.1
58109	1.89226	2.1
58110	1.89226	2.1
58111	1.89226	2.1
58112	1.89226	2.1
58113	1.89226	2.1

58114	1.89226	2.1
58115	3.78452	2.1
58116	1.89226	2.1
58117	2.08196	2.1
58118	1.97854	2.1
58119	2.08196	2.1
58120	1.78884	2.1
58121	1.89226	2.1
58122	1.89226	2.1
58123	1.89226	2.1
58124	1.89226	2.1
58125	1.89226	2.1
58126	1.89226	2.1
58127	1.89226	2.1
58128	1.83688	2.1
58129	1.89226	2.1
58130	1.89226	2.1
58131	1.89226	2.1
58132	1.86015	2.1
58133	1.86015	2.1
58134	1.89226	2.1
58135	1.97854	2.1
58136	1.85883	2.1
58137	1.97854	2.1
58138	1.77255	2.1
58139	1.89226	2.1
58140	0.946131	2.1
58141	0.946131	2.1
58142	0.946131	2.1
58143	0.946131	2.1
58144	0.94613	2.1
58145	0.94613	2.1
58146	0.946132	2.1
58147	0.946132	2.1
58148	0.946131	2.1
58149	0.946131	2.1
58150	0.946131	2.1
58151	0.946131	2.1
58152	0.918412	2.1
58153	0.918412	2.1
58154	0.94893	2.1
58155	0.750211	2.1
58156	0.851541	2.1
58157	0.851541	2.1
58158	0.970566	2.1
58159	0.826567	2.1
58160	0.970554	2.1
58161	1.04071	2.1
58162	0.826632	2.1
58163	0.851554	2.1
58164	1.89226	0.05
58165	1.89226	0.05
58166	1.89226	0.05
58167	1.89226	0.05
58168	3.78453	0.05
58169	0.946131	0.05
58170	0.946131	0.05
58171	0.811232	0.05

58172	0.811232	0.05
58173	0.94613	0.05
58174	0.94613	0.05
58175	0.959701	0.05
58176	0.811233	0.05
58177	0.94613	0.05
58178	0.811233	0.05
58179	0.959702	0.05
58180	0.946131	0.05
58181	1.89226	0.05
58182	1.89226	0.05
58183	1.89226	0.05
58184	1.89226	0.05
58185	1.89226	0.05
58186	1.89226	0.05
58187	1.89227	0.05
58188	1.89227	0.05
58189	1.89227	0.05
58190	1.89227	0.05
58191	1.89227	0.05
58192	1.89227	0.05
58193	1.89226	0.05
58194	1.89226	0.05
58195	1.89226	0.05
58196	1.89226	0.05
58197	3.78453	0.05
58198	1.89226	0.05
58199	1.89226	0.05
58200	1.89226	0.05
58201	1.89226	0.05
58202	1.89226	0.05
58203	1.89226	0.05
58204	1.89226	2.1
58205	2.36204	2.1
58206	2.39853	2.1
58207	2.36204	2.1
58208	1.92876	2.1
58209	1.89226	2.1
58210	1.89226	2.1
58211	1.89226	2.1
58212	1.89226	2.1
58213	1.89226	2.1
58214	3.78453	2.1
58215	0.946128	2.1
58216	0.946128	2.1
58217	1.13182	2.1
58218	1.13182	2.1
58219	0.94613	2.1
58220	0.94613	2.1
58221	1.1749	2.1
58222	1.13182	2.1
58223	1.1749	2.1
58224	0.94613	2.1
58225	1.13182	2.1
58226	0.94613	2.1
58227	1.89226	2.1
58228	2.39853	2.1
58229	2.44918	2.1

58230	2.39853	2.1
58231	1.94291	2.1
58232	1.89226	2.1
58233	0.946132	2.1
58234	0.946132	2.1
58235	0.946131	2.1
58236	0.946131	2.1
58237	0.946132	2.1
58238	0.946132	2.1
58239	0.946131	2.1
58240	0.946131	2.1
58241	0.946131	2.1
58242	0.946131	2.1
58243	0.946132	2.1
58244	0.946132	2.1
58245	0.946128	2.1
58246	0.946128	2.1
58247	1.1749	2.1
58248	1.1749	2.1
58249	0.946131	2.1
58250	0.946131	2.1
58251	1.23631	2.1
58252	1.1749	2.1
58253	1.23631	2.1
58254	0.94613	2.1
58255	1.1749	2.1
58256	0.946131	2.1
58257	1.89226	2.1
58258	1.89226	2.1
58259	1.89226	2.1
58260	1.89226	2.1
58261	3.78453	2.1
58262	1.38859	2.1
58263	1.45333	2.1
58264	1.28909	2.1
58265	1.38091	2.1
58266	2.74242	2.1
58267	1.45332	2.1
58268	1.38414	2.1
58269	1.38091	2.1
58270	1.33506	2.1
58271	2.78838	2.1
58272	1.89226	2.1
58273	1.89226	2.1
58274	1.89226	2.1
58275	1.89226	2.1
58276	3.78452	2.1
58277	1.89226	2.1
58278	1.89226	2.1
58279	1.89226	2.1
58280	1.89226	2.1
58281	1.89226	2.1
58282	1.89226	2.1
58283	1.17418	2.1
58284	1.38091	2.1
58285	1.17418	2.1
58286	1.28909	2.1
58287	1.29741	2.1

58288	1.41232	2.1
58289	1.38091	2.1
58290	1.33506	2.1
58291	1.27575	2.1
58292	1.27575	2.1
58293	1.29741	2.1
58294	1.35671	2.1
58295	1.89226	2.1
58296	1.89226	2.1
58297	1.89226	2.1
58298	1.89226	2.1
58299	3.78452	2.1
58300	1.92737	0.0531383
58301	2.40223	0.0519917
58302	1.56178	0.05
58303	1.97605	0.0653083
58304	3.90343	0.0506057
58305	1.89226	0.0563896
58306	1.89226	0.0964731
58307	1.89226	0.0732624
58308	1.89226	0.0684438
58309	3.78452	0.0719759
58310	1.89226	0.0998031
58311	1.89226	0.0785441
58312	1.89226	0.074438
58313	1.89226	0.147223
58314	1.89226	0.0714169
58315	1.89226	0.113717
58316	1.13641	0.0718334
58317	1.13641	0.0650423
58318	1.12763	0.0514682
58319	1.12763	0.0859366
58320	1.29348	0.0785536
58321	1.06293	0.0820442
58322	1.20111	0.0507326
58323	0.988028	0.0662735
58324	0.988028	0.0618237
58325	1.06293	0.0621031
58326	1.29348	0.0567766
58327	1.20111	0.0559708
58328	1.56176	0.05221
58329	1.46387	0.0666082
58330	1.97603	0.0549905
58331	1.97603	0.0551195
58332	1.08211	0.0555865
58333	1.08211	0.0562613
58334	1.89224	0.0704055
58335	1.89224	0.0705012
58336	1.89224	0.0659548
58337	1.89224	0.0865751
58338	1.89224	0.0610533
58339	1.89224	0.0820026
58340	1.89224	0.0923099
58341	1.89224	0.0841078
58342	1.89224	0.072643
58343	1.89224	0.122055
58344	3.78448	0.0892753
58345	0.820185	0.0555836

58346	0.818947	0.069769
58347	0.911041	0.0803997
58348	0.695265	0.0691015
58349	1.46388	0.069105
58350	1.23607	0.0675167
58351	0.927068	0.0674598
58352	1.02691	0.0875766
58353	1.12918	0.0561313
58354	0.846843	0.0676652
58355	0.911039	0.0692119
58356	1.29129	2.1
58357	1.45547	2.1
58358	1.67327	2.1
58359	1.66698	2.1
58360	1.20149	2.1
58361	1.20149	2.1
58362	0.946133	2.1
58363	0.946133	2.1
58364	0.946129	2.1
58365	0.946129	2.1
58366	0.946131	2.1
58367	0.946131	2.1
58368	0.946131	2.1
58369	0.946131	2.1
58370	0.946131	2.1
58371	0.946131	2.1
58372	0.946131	2.1
58373	0.946131	2.1
58374	1.89226	2.1
58375	1.89226	2.1
58376	1.89226	2.1
58377	1.89226	1.94049
58378	3.78452	2.1
58379	0.86875	2.1
58380	0.86875	2.1
58381	0.899002	2.1
58382	0.966245	2.1
58383	0.849364	2.02879
58384	1.01918	2.1
58385	0.764737	2.1
58386	0.89249	2.1
58387	0.764624	2.1
58388	0.967869	2.1
58389	0.808606	2.1
58390	0.864506	2.1
58391	0.773929	2.1
58392	0.773929	2.1
58393	0.781991	2.1
58394	0.836559	2.1
58395	0.983761	2.1
58396	0.834687	2.1
58397	0.680545	2.1
58398	0.640799	2.1
58399	0.70286	2.1
58400	0.935844	2.1
58401	0.629479	2.1
58402	0.823655	2.1
58403	0.946133	2.1

58404	0.946133	2.1
58405	0.946128	2.1
58406	0.946128	2.1
58407	0.94613	2.1
58408	0.94613	2.1
58409	0.946131	2.1
58410	0.946131	2.1
58411	0.946131	2.1
58412	0.946131	2.1
58413	0.946131	2.1
58414	0.946131	2.1
58415	1.89226	2.1
58416	1.89226	2.05382
58417	1.89226	2.1
58418	1.89226	2.1
58419	1.89226	2.1
58420	1.89226	2.1
58421	0.931638	2.1
58422	0.931638	2.1
58423	1.03559	2.1
58424	0.977442	1.99539
58425	0.826487	1.56772
58426	0.993902	2.06926
58427	1.04922	2.1
58428	0.894324	2.1
58429	0.912984	2.1
58430	1.01156	2.1
58431	0.944692	2.1
58432	1.15456	2.1
58433	1.89226	0.0795898
58434	1.89226	0.120269
58435	1.89226	0.157811
58436	1.89226	0.0786111
58437	3.78453	0.0836432
58438	1.89226	0.0754418
58439	1.89226	0.0737716
58440	2.64262	0.0731625
58441	2.64262	0.0635829
58442	1.78052	0.0576277
58443	1.03015	0.0760546
58444	0.999721	0.0652838
58445	0.659011	0.0615226
58446	1.15564	0.10014
58447	1.41388	0.0723102
58448	1.01583	0.0796601
58449	1.01583	0.0656293
58450	1.05706	0.0980835
58451	1.05706	0.0721297
58452	1.15564	0.0722736
58453	0.65901	0.0684562
58454	1.41388	0.0712906
58455	0.999721	0.0680801
58456	1.89226	2.1
58457	1.89226	2.1
58458	1.89226	2.1
58459	1.89226	2.1
58460	3.78453	2.1
58461	2.39022	2.1

58462	1.90734	2.1
58463	2.27634	2.1
58464	1.82666	2.1
58465	4.18368	2.1
58466	1.90734	2.1
58467	1.21564	2.1
58468	1.82666	2.1
58469	1.16938	2.1
58470	3.07672	2.1
58471	1.89226	2.1
58472	1.89226	2.1
58473	1.89226	2.1
58474	1.89226	2.1
58475	1.89226	2.1
58476	1.89226	2.1
58477	1.89227	2.1
58478	1.89227	2.1
58479	1.89227	2.1
58480	1.89227	2.1
58481	3.78453	2.1
58482	2.27634	2.1
58483	1.82666	2.1
58484	2.18042	2.1
58485	1.74738	2.1
58486	4.02372	2.1
58487	1.74738	2.1
58488	1.82666	2.1
58489	1.1181	2.1
58490	1.1181	2.1
58491	1.16938	2.1
58492	1.79865	2.1
58493	1.89226	2.1
58494	1.89226	2.1
58495	1.89226	2.1
58496	1.89226	2.1
58497	1.89226	2.1
58498	1.89226	2.1
58499	1.89226	1.28953
58500	1.89226	1.05142
58501	1.89226	1.17731
58502	1.89226	1.1262
58503	1.89226	1.25446
58504	1.89226	1.15143
58505	0.790736	1.13328
58506	1.08265	1.09489
58507	0.938909	1.31243
58508	0.638683	1.24378
58509	0.858588	1.27733
58510	0.858588	1.12137
58511	0.862743	1.28127
58512	0.862743	1.13416
58513	1.08265	1.21763
58514	0.938911	1.16483
58515	0.790735	1.18481
58516	0.638685	1.1567
58517	1.89226	1.1259
58518	1.89226	1.25983
58519	1.89226	1.21879

58520	1.89226	1.14418
58521	1.89226	1.25513
58522	1.89226	1.1776
58523	1.89226	1.06871
58524	1.89226	1.24082
58525	1.89226	1.1516
58526	1.89226	1.14058
58527	1.89226	1.2146
58528	1.89226	1.21365
58529	1.08265	1.11761
58530	1.36209	1.08025
58531	1.25233	1.18308
58532	0.938909	1.22888
58533	1.14195	1.17352
58534	1.14195	1.10514
58535	1.15904	1.22279
58536	1.15904	1.13478
58537	1.36209	1.15707
58538	1.25233	1.07516
58539	1.08265	1.19274
58540	0.938911	1.1363
58541	1.22077	1.13021
58542	0.915244	1.15229
58543	0.967835	1.24487
58544	0.742495	1.25869
58545	0.78648	1.25924
58546	0.985041	1.15787
58547	0.989098	1.2914
58548	0.989098	1.15831
58549	1.03206	1.22818
58550	0.967897	1.24284
58551	0.915404	1.21508
58552	0.706105	1.20617
58553	2.69662	0.05
58554	1.89226	0.05
58555	2.35499	0.05
58556	1.89226	0.05
58557	4.24725	0.05
58558	1.89227	0.05
58559	1.89227	0.05
58560	1.89227	0.05
58561	1.89227	0.05
58562	3.78453	0.05
58563	2.35499	0.05
58564	1.70302	0.05
58565	1.70302	0.05
58566	1.703	0.05
58567	1.97374	0.05
58568	1.97372	0.05
58569	0.946133	0.05
58570	0.946133	0.05
58571	0.946132	0.05
58572	0.946132	0.05
58573	0.946133	0.05
58574	0.946133	0.05
58575	0.946132	0.05
58576	0.946132	0.05
58577	0.946133	0.05

58578	0.946133	0.05
58579	0.946132	0.05
58580	0.946132	0.05
58581	0.6794	2.1
58582	0.6794	2.1
58583	0.670812	2.1
58584	0.62182	2.1
58585	0.648326	2.1
58586	0.792154	2.1
58587	0.719167	2.1
58588	0.494515	2.1
58589	0.649421	2.1
58590	0.804869	2.1
58591	0.485585	2.1
58592	0.627666	2.1
58593	1.79347	2.1
58594	1.03968	2.1
58595	1.70303	2.1
58596	0.933265	2.06107
58597	2.62068	2.1
58598	1.89226	2.1
58599	1.89226	2.1
58600	1.89226	2.1
58601	1.89226	2.1
58602	3.78452	2.1
58603	1.89226	2.1
58604	2.24604	2.1
58605	2.0554	2.1
58606	2.0554	2.1
58607	1.89226	2.1
58608	1.89226	2.1
58609	1.89226	2.1
58610	1.89226	2.08614
58611	1.89226	2.1
58612	1.89226	2.1
58613	1.89226	2.1
58614	1.89226	2.1
58615	2.0815	2.1
58616	2.64424	1.19544
58617	2.80807	1.96373
58618	3.232 2.1	
58619	1.89417	2.01338
58620	2.18012	2.1
58621	0.946131	2.1
58622	0.946131	1.97766
58623	0.946131	2.1
58624	0.946131	2.1
58625	0.94613	2.1
58626	0.94613	2.05773
58627	0.946132	2.1
58628	0.946132	2.1
58629	0.946131	2.1
58630	0.946131	2.1
58631	0.946131	2.1
58632	0.946131	2.1
58633	1.89227	0.0605188
58634	1.89227	0.086559
58635	1.89227	0.108005

58636	1.89227	0.0659408
58637	3.78453	0.0655941
58638	2.70881	0.055461
58639	2.68687	0.05
58640	2.03046	0.05
58641	1.9682	0.0558443
58642	4.67701	0.0524415
58643	1.08518	0.05
58644	1.08518	0.05
58645	1.09033	0.0556112
58646	1.09033	0.051502
58647	0.842273	0.05
58648	1.19141	0.05
58649	1.34343	0.05
58650	0.9841	0.05
58651	0.9841	0.05
58652	0.842273	0.0506129
58653	1.34344	0.0507972
58654	1.19141	0.05
58655	0.946133	0.0642557
58656	0.946133	0.0630472
58657	0.946132	0.101733
58658	0.946132	0.074126
58659	0.946132	0.0655867
58660	0.946132	0.0647273
58661	0.946132	0.0977869
58662	0.946132	0.0782531
58663	0.946132	0.0615469
58664	0.946132	0.0788984
58665	0.946133	0.0790631
58666	0.946133	0.0648787
58667	1.89227	0.0874247
58668	1.89227	0.0586258
58669	1.89227	0.079937
58670	1.89227	0.0639387
58671	1.89227	0.0710623
58672	1.89227	0.060128
58673	2.03046	0.05
58674	1.9682	0.05
58675	1.23886	0.0562366
58676	1.12971	0.05
58677	3.20706	0.05
58678	1.89226	0.333782
58679	1.89226	1.23855
58680	1.89226	0.248106
58681	1.89226	0.916851
58682	3.78452	0.665906
58683	2.45569	0.151569
58684	1.89226	0.180949
58685	2.22922	0.156292
58686	2.0815	0.434037
58687	1.85074	0.102833
58688	1.70303	0.183365
58689	2.72856	0.15834
58690	1.89226	0.579406
58691	1.89226	0.137976
58692	1.89226	0.2591
58693	3.78452	0.230952

58694	1.89226	0.467996
58695	1.89226	1.09589
58696	1.89226	0.220172
58697	1.89226	1.15566
58698	3.78452	0.656456
58699	0.946131	0.97992
58700	0.946131	0.198305
58701	0.946131	0.595078
58702	0.946131	0.157818
58703	0.94613	0.497621
58704	0.94613	0.721014
58705	0.946132	0.213122
58706	0.946132	0.22122
58707	0.946131	0.310475
58708	0.946131	0.303119
58709	0.946131	0.305733
58710	0.946131	0.326796
58711	2.0595	0.105703
58712	2.58581	0.0823887
58713	1.48694	0.0890377
58714	1.48694	0.0887671
58715	2.60157	0.0841813
58716	2.02901	0.0883768
58717	1.89226	0.088812
58718	1.89226	0.119466
58719	1.89226	0.0990023
58720	1.89226	0.0972604
58721	1.89226	0.100016
58722	1.89226	0.107322
58723	2.04728	0.109566
58724	2.64161	0.0821056
58725	1.47606	0.0975527
58726	1.47606	0.097905
58727	2.63069	0.0925422
58728	2.05947	0.101145
58729	1.89224	0.074862
58730	1.89224	0.11186
58731	1.89224	0.086798
58732	1.89224	0.097432
58733	1.89224	0.0856309
58734	1.89224	0.0841764
58735	0.946114	0.11725
58736	0.946114	0.0885944
58737	0.946118	0.118347
58738	0.946118	0.104988
58739	0.946114	0.124107
58740	0.946114	0.118954
58741	0.946118	0.104023
58742	0.946118	0.0913232
58743	0.946128	0.111921
58744	0.946128	0.113966
58745	0.946103	0.106914
58746	0.946103	0.109112
58747	1.1233	2.1
58748	1.15009	2.1
58749	2.36036	2.1
58750	2.38225	2.1
58751	3.50554	2.1

58752	2.38224	2.1
58753	0.977775	2.1
58754	2.20473	2.1
58755	2.20473	2.1
58756	1.15009	2.1
58757	1.15009	2.1
58758	0.946119	2.1
58759	0.946119	2.09676
58760	1.19111	2.1
58761	1.19111	2.07394
58762	0.946118	2.1
58763	1.19111	2.1
58764	1.18016	2.08161
58765	0.946118	1.77951
58766	0.94613	1.63921
58767	0.94613	2.1
58768	1.18015	2.01856
58769	1.19109	2.06657
58770	0.946118	2.1
58771	0.946118	2.1
58772	1.10235	2.1
58773	1.10235	2.1
58774	0.946118	2.1
58775	1.10235	2.1
58776	0.946115	2.1
58777	1.1911	2.1
58778	0.946129	2.1
58779	0.946129	2.1
58780	1.19109	2.1
58781	1.10234	2.1
58782	1.89226	2.1
58783	2.23932	2.1
58784	2.11568	2.1
58785	2.23932	2.1
58786	1.76862	2.1
58787	1.89226	2.1
58788	1.89226	2.1
58789	1.89226	2.1
58790	1.89226	2.1
58791	1.89226	2.1
58792	1.89226	2.1
58793	1.89226	2.1
58794	2.07838	2.1
58795	1.89226	2.1
58796	1.89226	2.1
58797	1.89226	2.1
58798	2.10264	2.1
58799	2.10264	2.1
58800	1.89226	2.1
58801	2.11568	2.1
58802	1.99753	2.1
58803	2.11568	2.1
58804	1.77411	2.1
58805	1.89226	2.1
58806	0.946128	2.1
58807	0.946128	2.1
58808	0.946133	2.1
58809	0.946133	2.1

58810	0.94613	2.1
58811	0.94613	2.1
58812	0.946131	2.1
58813	0.946131	2.1
58814	0.946131	2.1
58815	0.946131	2.1
58816	0.946131	2.1
58817	0.946131	2.1
58818	0.946132	2.1
58819	0.946132	2.1
58820	1.03919	2.1
58821	1.03919	2.1
58822	0.946131	2.1
58823	0.946131	2.1
58824	1.02744	2.1
58825	1.03919	2.1
58826	1.02744	2.1
58827	0.946132	2.1
58828	1.03919	2.1
58829	0.946132	2.1
58830	1.64834	1.81898
58831	1.82955	1.64278
58832	2.33824	1.63005
58833	2.19301	1.7206
58834	3.98658	1.66928
58835	1.40574	1.85971
58836	2.19301	1.65744
58837	1.64834	1.82449
58838	1.64834	1.7056
58839	1.99961	1.73707
58840	1.99961	1.66991
58841	2.33824	1.61549
58842	2.19301	1.69655
58843	2.68524	1.70491
58844	2.81847	1.5732
58845	5.02349	1.65901
58846	1.99961	1.78572
58847	2.19301	1.61811
58848	2.68524	1.68689
58849	2.50732	1.63531
58850	4.68485	1.65776
58851	1.89226	2.1
58852	1.89226	2.1
58853	1.89226	2.1
58854	1.89226	2.1
58855	3.78453	2.1
58856	1.75142	2.1
58857	2.47026	2.1
58858	1.46811	2.1
58859	2.26652	2.1
58860	4.01794	2.1
58861	1.89226	2.1
58862	1.89226	2.1
58863	1.89226	2.1
58864	1.89226	2.1
58865	1.89226	2.1
58866	1.89226	2.1
58867	1.89226	2.1

58868	1.89226	2.1
58869	1.89226	2.1
58870	1.89226	2.1
58871	1.89226	2.1
58872	1.89226	2.1
58873	0.812082	2.1
58874	0.878759	2.1
58875	0.882782	2.1
58876	0.882782	2.1
58877	1.05572	2.1
58878	1.04037	2.1
58879	0.73849	2.1
58880	0.626857	2.1
58881	0.638677	2.1
58882	1.05581	2.1
58883	0.715695	2.1
58884	1.10492	2.1
58885	1.17058	2.1
58886	1.07863	2.1
58887	1.21113	2.1
58888	1.21113	2.1
58889	1.2811	2.1
58890	1.37319	2.1
58891	1.01788	2.1
58892	0.999492	2.1
58893	1.04352	2.1
58894	1.32112	2.1
58895	1.10492	2.1
58896	1.34699	2.1
58897	1.89226	2.1
58898	1.89226	2.1
58899	1.89226	2.1
58900	1.89226	2.1
58901	1.89226	2.1
58902	1.89226	2.1
58903	1.19813	0.05
58904	1.19813	0.05
58905	2.43432	0.05
58906	1.21716	0.05
58907	0.946124	0.05
58908	1.21715	0.05
58909	0.946118	0.05
58910	2.56627	2.1
58911	1.89226	2.1
58912	1.89226	2.1
58913	1.89226	2.1
58914	2.43434	2.1
58915	2.43434	2.1
58916	1.89227	0.05
58917	1.89227	0.05
58918	1.89227	0.05
58919	1.89227	0.0502005
58920	1.89227	0.05
58921	1.89227	0.05
58922	1.89226	0.0510477
58923	1.89226	0.0640995
58924	1.89226	0.0555173
58925	1.89226	0.05

58926	3.78453	0.0532084
58927	1.89226	0.05
58928	2.0764	0.05
58929	1.89226	0.05
58930	2.81674	0.05
58931	1.89226	0.05
58932	2.81674	0.05
58933	0.946132	0.05
58934	0.946132	0.05
58935	0.946131	0.0593834
58936	0.946131	0.0516019
58937	0.946132	0.0522148
58938	0.946132	0.0504862
58939	0.946132	0.0642307
58940	0.946132	0.05
58941	0.946132	0.05
58942	0.946132	0.0510378
58943	0.946132	0.0550533
58944	0.946132	0.0513014
58945	0.578684	1.56085
58946	0.578684	2.1
58947	0.589487	1.97722
58948	0.589487	2.1
58949	0.625461	1.74125
58950	0.5378	1.82602
58951	0.608714	2.1
58952	0.54271	1.83161
58953	0.608714	1.61246
58954	0.625461	1.56009
58955	0.54271	1.88624
58956	0.537801	1.81531
58957	0.946128	1.52185
58958	0.946128	1.84294
58959	0.946133	1.38449
58960	0.946133	2.1
58961	0.946131	1.42512
58962	0.946131	1.52767
58963	0.946131	1.50433
58964	0.946131	1.68793
58965	0.94613	1.40884
58966	0.94613	1.34601
58967	0.946131	1.54997
58968	0.946131	1.63507
58969	1.21743	2.03562
58970	1.25092	2.04832
58971	1.38847	1.92818
58972	1.46031	1.38378
58973	2.67774	1.77825
58974	0.946128	1.40732
58975	0.946128	1.90511
58976	0.946132	2.1
58977	0.946132	1.40067
58978	0.94613	1.4706
58979	0.94613	1.402
58980	0.94613	1.73866
58981	0.94613	1.5409
58982	0.94613	1.33703
58983	0.94613	1.35163

58984	0.94613	1.62369
58985	0.94613	1.55914
58986	1.89226	1.14978
58987	1.89226	1.54055
58988	1.89226	0.656598
58989	1.89226	2.0319
58990	1.89226	2.1
58991	1.89226	2.01598
58992	0.539566	1.39635
58993	0.534046	2.1
58994	0.577914	1.83226
58995	0.471623	1.79225
58996	1.46031	0.903731
58997	0.945865	1.30214
58998	0.770603	1.16008
58999	0.737201	1.96856
59000	0.714829	1.36457
59001	0.536085	1.65476
59002	0.471617	1.61861
59003	1.89226	1.9255
59004	1.89226	1.50885
59005	1.89226	1.52292
59006	1.89226	1.61899
59007	1.89226	1.72603
59008	1.89226	1.92851
59009	1.89225	1.55626
59010	1.89225	1.08603
59011	1.65572	1.25355
59012	1.65572	1.41932
59013	1.89225	1.47066
59014	1.89225	1.55356
59015	1.89226	1.58391
59016	1.89226	1.49636
59017	1.89226	1.55104
59018	1.89226	1.00629
59019	3.78453	1.60515
59020	1.89226	1.67586
59021	1.89226	1.25707
59022	1.89226	1.30679
59023	1.89226	1.48645
59024	1.89226	1.50216
59025	1.89226	1.7376
59026	1.89225	1.4392
59027	1.89225	0.577311
59028	1.89225	0.899547
59029	1.89225	1.30419
59030	1.89225	1.30427
59031	1.89225	1.45589
59032	0.763592	1.14317
59033	0.763592	1.45776
59034	0.987417	1.36319
59035	0.987417	1.38131
59036	0.763592	0.796303
59037	0.804881	0.640059
59038	0.946126	1.38001
59039	0.946126	1.31232
59040	0.804879	0.699599
59041	0.94613	1.34161

59042	0.946129	1.44019
59043	0.76359	0.892988
59044	1.46055	2.1
59045	2.04945	2.1
59046	2.04587	2.1
59047	2.04587	2.1
59048	1.50714	2.1
59049	1.50714	2.1
59050	2.04587	2.1
59051	2.38681	2.1
59052	2.42082	2.1
59053	2.42082	2.1
59054	2.04945	2.1
59055	2.04945	2.1
59056	0.872964	2.1
59057	0.872964	2.1
59058	0.884806	2.1
59059	0.884806	2.1
59060	1.0275	2.1
59061	1.02293	2.1
59062	0.711243	2.1
59063	0.730273	2.1
59064	0.711244	2.1
59065	1.0275	2.1
59066	0.730273	2.1
59067	1.02293	2.1
59068	2.04587	2.1
59069	2.47753	2.1
59070	2.42994	2.1
59071	2.42082	2.1
59072	2.05499	2.1
59073	2.04587	2.1
59074	0.946132	2.1
59075	0.946132	2.1
59076	1.13078	2.1
59077	1.07817	2.1
59078	0.946132	2.1
59079	0.946132	2.1
59080	1.07817	2.1
59081	1.07817	2.1
59082	0.946132	2.1
59083	1.07817	2.1
59084	1.13078	2.1
59085	0.946132	2.1
59086	1.89226	2.1
59087	1.89226	2.1
59088	1.89226	2.1
59089	1.89226	2.1
59090	1.89226	2.1
59091	1.89226	2.1
59092	1.89226	2.1
59093	1.89226	2.1
59094	1.89226	2.1
59095	1.89226	2.1
59096	1.89226	2.1
59097	1.89226	2.1
59098	1.89226	2.1
59099	1.89226	2.1

59100	1.8173	2.1
59101	2.66885	2.1
59102	2.7438	2.1
59103	2.7438	2.1
59104	1.89226	1.63058
59105	1.89226	0.0567405
59106	1.89226	0.110426
59107	1.89226	0.536755
59108	3.78453	0.773889
59109	1.89226	0.661805
59110	1.89226	0.649917
59111	1.89226	0.615757
59112	1.89226	1.67387
59113	1.89226	0.146899
59114	1.89226	0.666107
59115	1.89226	0.364771
59116	1.97385	0.618671
59117	1.7531	1.28198
59118	1.89226	1.93719
59119	1.7531	0.210485
59120	1.89226	0.473243
59121	0.946131	1.13208
59122	0.840904	0.345459
59123	0.684333	0.0902198
59124	0.94613	0.0825337
59125	0.972337	0.10544
59126	0.946278	1.13335
59127	0.684304	0.0713585
59128	0.684304	0.29799
59129	0.999838	0.150695
59130	0.759605	0.554708
59131	0.851528	0.0616747
59132	0.851528	1.08936
59133	1.89226	0.228945
59134	2.23818	0.402956
59135	1.79771	0.453759
59136	1.89226	1.6953
59137	1.79771	0.0598485
59138	1.89226	0.160299
59139	2.79647	2.1
59140	2.01613	2.1
59141	1.46826	2.1
59142	2.54504	2.1
59143	4.56117	2.1
59144	1.89226	2.1
59145	1.89226	2.1
59146	1.89226	2.1
59147	1.89226	2.1
59148	1.89226	2.1
59149	1.89226	2.1
59150	1.89226	2.1
59151	2.66884	2.1
59152	2.56022	2.1
59153	2.66884	2.1
59154	1.78364	2.1
59155	1.89226	2.1
59156	1.14282	2.1
59157	1.24068	2.1

59158	0.880748	2.1
59159	0.834916	2.1
59160	1.05539	2.1
59161	1.05539	2.1
59162	1.04312	2.1
59163	1.04313	2.1
59164	1.2015	2.1
59165	0.83492	2.1
59166	0.789149	2.1
59167	1.24066	2.1
59168	1.89226	2.1
59169	1.89226	2.1
59170	1.89226	2.1
59171	1.89226	2.1
59172	3.78453	2.1
59173	0.946128	2.1
59174	0.946128	2.1
59175	1.28011	2.1
59176	1.22372	2.1
59177	0.946131	2.1
59178	0.946131	2.1
59179	1.22372	2.1
59180	1.22372	2.1
59181	1.22372	2.1
59182	0.94613	2.1
59183	0.946131	2.1
59184	1.28011	2.1
59185	2.28604	2.1
59186	1.34268	2.1
59187	2.43157	2.1
59188	1.50172	2.1
59189	3.77425	2.1
59190	2.6096	2.1
59191	2.43157	2.1
59192	1.66774	2.1
59193	1.66774	2.1
59194	1.50172	2.1
59195	2.43157	2.1
59196	2.16067	2.1
59197	1.08975	2.1
59198	2.19336	2.1
59199	2.06984	2.1
59200	1.21327	2.1
59201	1.08975	2.1
59202	2.28604	2.1
59203	1.21327	2.1
59204	2.29008	2.1
59205	2.16067	2.1
59206	1.34268	2.1
59207	1.21327	2.1
59208	1.89226	0.382071
59209	1.89226	0.320054
59210	1.89226	0.311198
59211	1.89226	0.163305
59212	3.78452	0.1393
59213	1.89804	0.185429
59214	2.21656	0.239336
59215	1.70537	0.0658622

59216	2.50023	0.0829247
59217	3.8982	0.0851833
59218	1.03136	0.0860979
59219	1.89486	0.093699
59220	0.949364	0.0959205
59221	1.82283	0.0909252
59222	2.84422	0.0768857
59223	1.89226	0.0978783
59224	1.89226	0.099782
59225	1.89226	0.116345
59226	1.89226	0.118149
59227	3.78452	0.19075
59228	0.946132	0.246043
59229	0.946132	0.235938
59230	0.946129	0.241081
59231	0.946129	0.151068
59232	0.94613	0.171542
59233	0.94613	0.554846
59234	0.946131	0.114063
59235	0.946131	0.322992
59236	0.946131	0.4933
59237	0.946131	0.66062
59238	0.946131	0.220977
59239	0.946131	0.801253
59240	1.09466	0.160629
59241	1.09466	0.0919716
59242	0.866512	0.0732252
59243	1.08352	0.0651771
59244	1.19189	0.0610508
59245	1.21883	0.0982333
59246	0.971139	0.14742
59247	0.873213	0.0700355
59248	0.873202	0.0852603
59249	1.00855	0.0549374
59250	0.923735	0.0642358
59251	1.21885	0.07843
59252	1.89225	0.05
59253	2.40019	0.05
59254	1.89225	0.310747
59255	1.89225	0.350102
59256	1.89225	0.230627
59257	1.89225	0.451629
59258	1.89225	0.310747
59259	1.89225	0.497681
59260	2.05088	0.22761
59261	1.89225	0.128123
59262	1.89225	0.114717
59263	1.89225	0.318551
59264	2.03956	0.22761
59265	2.03956	0.350102
59266	1.89226	2.1
59267	2.40021	2.1
59268	2.38473	2.1
59269	2.40021	2.1
59270	1.87679	2.1
59271	1.89226	2.1
59272	1.89226	2.1
59273	1.89226	2.08377

59274	1.89226	2.1
59275	1.89226	2.04425
59276	1.89226	2.1
59277	1.89226	2.1
59278	2.0692	2.1
59279	1.89226	2.1
59280	1.89226	2.1
59281	1.89226	1.98188
59282	2.05089	2.1
59283	2.05089	2.08271
59284	1.89226	2.1
59285	1.89226	2.1
59286	1.89226	2.1
59287	1.89226	2.1
59288	3.78452	2.1
59289	1.89226	2.1
59290	2.50051	2.1
59291	1.89226	2.1
59292	2.30076	2.1
59293	4.39277	2.1
59294	2.50052	2.1
59295	1.70377	2.1
59296	2.30076	2.1
59297	1.54231	2.1
59298	4.04283	2.1
59299	1.89226	2.1
59300	1.89226	2.1
59301	1.89226	2.1
59302	1.89226	2.1
59303	1.89226	2.1
59304	1.89226	2.1
59305	1.89226	2.1
59306	1.89226	2.1
59307	1.89226	2.1
59308	1.89226	2.1
59309	3.78452	2.1
59310	2.07948	2.1
59311	2.30076	2.1
59312	1.3294	2.1
59313	1.3294	2.1
59314	1.54232	2.1
59315	2.29239	2.1
59316	0.810947	2.1
59317	0.810947	2.1
59318	0.810946	2.1
59319	0.810946	2.1
59320	1.89226	2.1
59321	1.35169	2.1
59322	1.08132	2.1
59323	1.08132	2.1
59324	1.08132	2.1
59325	0.810946	2.1
59326	0.810946	2.1
59327	1.34851	1.46864
59328	0.997471	2.1
59329	1.62287	0.527812
59330	1.27096	1.80562
59331	2.62034	1.68042

59332	1.89226	1.83162
59333	1.89226	2.1
59334	1.89226	1.3789
59335	1.89226	2.1
59336	3.78452	1.93525
59337	0.946128	2.1
59338	0.946128	2.1
59339	1.29331	1.96677
59340	1.29331	2.1
59341	1.29331	2.1
59342	1.10699	2.1
59343	0.946131	1.95342
59344	0.946131	2.1
59345	0.94613	2.1
59346	1.29331	2.1
59347	1.10699	2.1
59348	0.946131	2.1
59349	1.62287	1.45885
59350	1.27096	1.84366
59351	1.86067	1.15346
59352	1.58407	1.8357
59353	3.20694	1.65309
59354	1.89226	1.50805
59355	1.89226	2.1
59356	1.89226	0.88006
59357	1.89226	1.84601
59358	3.78452	1.82914
59359	2.62761	0.157135
59360	2.55849	1.03524
59361	2.12261	1.48511
59362	2.22939	1.16995
59363	4.78788	1.16618
59364	0.772522	2.1
59365	0.839588	2.0012
59366	1.31126	0.846439
59367	1.08787	0.414344
59368	1.01318	1.00083
59369	1.01318	2.1
59370	0.900815	2.1
59371	1.10799	0.537947
59372	0.772511	1.64677
59373	1.03473	1.62229
59374	1.31126	1.41504
59375	0.841464	1.49539
59376	1.89227	0.922667
59377	1.89227	2.1
59378	1.89227	1.81476
59379	1.89227	2.1
59380	1.89227	1.43888
59381	1.89227	2.1
59382	1.89226	1.1791
59383	1.89226	1.77081
59384	1.89226	0.618728
59385	1.89226	1.59131
59386	1.89226	0.556995
59387	1.89226	1.50925
59388	2.12261	1.40292
59389	2.22939	0.210869

59390	1.97004	0.344477
59391	1.8072	1.78806
59392	4.03658	0.538734
59393	1.5847	2.1
59394	1.8072	1.11012
59395	2.12262	1.20249
59396	2.12262	2.1
59397	1.23267	1.07447
59398	1.23267	2.1
59399	1.89227	1.20644
59400	1.89227	2.1
59401	1.89227	1.77848
59402	1.89227	2.1
59403	1.89227	1.53401
59404	1.89227	2.1
59405	1.89226	0.246183
59406	1.89226	1.71644
59407	1.89226	1.79518
59408	1.89226	0.511025
59409	3.78452	0.78541
59410	2.49093	2.1
59411	2.67393	2.1
59412	2.47932	2.1
59413	2.47932	2.1
59414	2.67994	2.1
59415	2.67994	2.1
59416	2.49873	2.1
59417	2.67393	2.1
59418	2.67993	2.1
59419	2.69807	2.1
59420	2.49873	2.1
59421	2.51686	2.1
59422	2.49093	2.1
59423	2.67994	2.1
59424	2.69784	2.1
59425	2.51651	2.1
59426	5.18877	2.1
59427	1.24359	2.1
59428	1.24936	2.1
59429	1.33997	2.1
59430	1.34892	2.1
59431	1.28805	2.1
59432	1.28805	2.1
59433	1.2955	2.1
59434	1.2955	2.1
59435	1.34892	2.1
59436	1.24359	2.1
59437	1.24936	2.1
59438	1.33997	2.1
59439	1.15506	1.9862
59440	1.15506	2.1
59441	0.946128	2.1
59442	0.946128	2.1
59443	0.946131	2.1
59444	1.15506	1.98684
59445	1.06997	2.1
59446	0.946128	2.1
59447	1.06997	2.1

59448	1.15506	2.1
59449	0.946129	2.1
59450	0.946129	2.1
59451	1.70608	2.1
59452	2.13994	2.1
59453	1.33384	2.1
59454	1.43635	2.1
59455	3.14243	2.1
59456	2.13994	2.1
59457	2.31012	2.1
59458	1.43635	2.1
59459	1.23007	2.1
59460	3.74646	2.1
59461	2.46361	2.1
59462	1.89226	2.1
59463	1.89226	2.1
59464	1.89226	2.1
59465	2.39045	2.1
59466	2.39045	2.1
59467	1.89226	2.1
59468	1.89226	2.1
59469	1.89226	2.1
59470	1.89226	1.87369
59471	3.78452	2.1
59472	1.52984	2.1
59473	2.25682	1.81889
59474	2.20831	1.80272
59475	2.20831	2.1
59476	1.61178	1.78401
59477	1.61178	2.1
59478	2.47395	2.1
59479	1.89226	2.1
59480	1.89226	2.1
59481	1.89226	2.1
59482	2.46362	2.1
59483	2.46362	2.1
59484	0.946131	1.8197
59485	0.946131	2.1
59486	0.946131	2.1
59487	0.946131	2.1
59488	0.94613	1.93958
59489	0.94613	2.07772
59490	0.946132	2.1
59491	0.946132	2.1
59492	0.946131	2.1
59493	0.946131	2.1
59494	0.946131	2.1
59495	0.946131	2.1
59496	0.982398	2.1
59497	0.982398	2.1
59498	0.931703	2.1
59499	0.97549	2.1
59500	1.11509	2.1
59501	1.10017	1.95293
59502	0.774576	2.1
59503	0.789859	2.1
59504	0.716502	2.1
59505	1.12793	2.1

59506	1.14398	2.1
59507	0.817011	2.1
59508	1.82499	2.1
59509	1.50524	2.1
59510	1.21304	2.1
59511	1.54191	2.1
59512	3.04715	2.1
59513	1.54191	2.1
59514	1.49823	2.1
59515	1.82499	2.1
59516	1.82499	2.1
59517	1.21147	2.1
59518	1.48977	2.1
59519	1.89226	2.1
59520	1.89226	2.1
59521	1.89226	2.1
59522	1.89226	2.1
59523	3.78452	2.1
59524	1.89226	2.1
59525	1.89226	2.1
59526	1.89226	2.1
59527	1.89226	2.1
59528	1.89226	2.1
59529	1.89226	2.1
59530	0.566674	2.1
59531	0.770957	2.1
59532	0.60652	2.1
59533	0.473557	2.1
59534	0.604441	2.1
59535	0.604441	2.1
59536	0.640073	2.1
59537	0.640073	2.1
59538	0.473557	2.1
59539	0.566674	2.1
59540	0.60652	2.1
59541	0.770956	2.1
59542	1.36203	2.1
59543	1.13335	2.1
59544	1.54191	2.1
59545	1.54191	2.1
59546	1.00965	2.1
59547	1.00965	2.1
59548	1.89226	2.1
59549	1.89226	2.1
59550	1.89226	2.1
59551	1.89226	2.1
59552	1.89226	2.1
59553	1.89226	2.1
59554	0.946131	2.1
59555	0.946131	2.1
59556	0.946131	2.1
59557	0.946131	2.1
59558	0.946132	2.1
59559	0.946132	2.1
59560	0.946131	2.1
59561	0.946131	2.1
59562	0.946132	2.1
59563	0.946132	2.1

59564	0.946131	2.1
59565	0.946131	2.1
59566	0.946132	0.05
59567	0.946132	0.05
59568	0.946133	0.05
59569	0.946133	0.05
59570	0.946132	0.05
59571	0.946132	0.05
59572	0.946132	0.05
59573	0.946132	0.05
59574	0.946132	0.05
59575	0.946132	0.05
59576	0.946133	0.05
59577	0.946133	0.05
59578	1.89226	0.05
59579	1.89226	0.05
59580	1.89226	0.05
59581	1.89226	0.05
59582	3.78453	0.05
59583	1.89226	0.05
59584	1.89226	0.05
59585	1.89226	0.05
59586	1.89226	0.05
59587	1.89226	0.05
59588	1.89226	0.05
59589	0.946132	0.05
59590	0.946132	0.05
59591	0.946131	0.05
59592	0.946131	0.05
59593	0.946132	0.05
59594	0.946132	0.05
59595	0.946132	0.05
59596	0.946132	0.05
59597	0.946132	0.05
59598	0.946132	0.05
59599	0.946132	0.05
59600	0.946132	0.05
59601	0.946129	0.05
59602	0.946129	0.05
59603	0.946128	0.05
59604	0.946128	0.05
59605	0.946132	0.05
59606	0.946132	0.05
59607	0.946126	0.05
59608	0.946126	0.05
59609	0.946129	0.05
59610	0.946129	0.05
59611	0.946129	0.05
59612	0.946129	0.05
59613	1.20674	0.05
59614	1.79877	0.05
59615	1.22586	0.05
59616	1.22586	0.05
59617	1.7919	0.05
59618	1.7919	0.05
59619	2.27622	0.05
59620	1.79877	0.0515108
59621	1.7919	0.05

59622	2.27102	0.05
59623	4.06979	0.05
59624	1.89226	0.0526763
59625	1.89226	0.0693686
59626	1.89226	0.05
59627	1.89226	0.0676347
59628	3.78452	0.0650849
59629	1.89226	0.05
59630	1.89226	0.0597828
59631	1.89226	0.0501864
59632	1.89226	0.0638315
59633	3.78453	0.0521222
59634	1.13259	0.05
59635	1.79187	0.05
59636	1.32741	0.05
59637	1.18784	0.05
59638	1.703 0.05	
59639	1.49575	0.0508884
59640	1.46201	0.05
59641	2.27099	0.0512542
59642	1.79187	0.05
59643	1.79187	0.05
59644	2.12923	0.0511736
59645	2.12923	0.0525375
59646	1.89224	0.0503284
59647	1.89224	0.0681649
59648	1.89224	0.0528326
59649	1.89224	0.0678957
59650	3.78447	0.0548244
59651	0.946119	0.0686275
59652	0.946119	0.0599791
59653	0.946118	0.0648958
59654	0.946118	0.0535304
59655	0.946118	0.0647621
59656	0.946118	0.0662628
59657	0.946119	0.0522858
59658	0.946119	0.05
59659	0.946131	0.0610498
59660	0.946131	0.0536064
59661	0.946106	0.0557732
59662	0.946106	0.0579008
59663	2.07526	0.329277
59664	1.89226	0.196063
59665	1.89226	1.15712
59666	2.13445	0.740094
59667	4.02671	0.270777
59668	1.89226	0.238344
59669	1.89226	0.503053
59670	1.89226	0.52793
59671	1.89226	0.195497
59672	1.89226	1.32059
59673	1.89226	0.242689
59674	2.13446	0.40138
59675	1.00013	0.645291
59676	1.1475	1.43434
59677	2.20046	0.57192
59678	3.20059	0.896883
59679	0.946133	0.320428

59680	0.946133	0.376952
59681	1.06722	0.30123
59682	1.10023	0.287881
59683	0.946131	0.396065
59684	0.946131	0.101183
59685	1.10023	0.533475
59686	1.10023	0.230371
59687	0.946131	0.523367
59688	1.10023	0.260966
59689	0.94613	0.22417
59690	1.06723	0.192963
59691	1.89226	0.131552
59692	1.89226	0.284002
59693	1.89226	0.451743
59694	1.89226	0.291111
59695	3.78452	0.346588
59696	1.89226	0.667207
59697	1.89226	0.593727
59698	1.89226	0.83208
59699	1.89226	0.685333
59700	1.89226	0.55534
59701	1.89226	0.434853
59702	1.89226	2.1
59703	1.89226	1.59055
59704	1.89226	2.1
59705	1.89226	1.57316
59706	1.89226	2.1
59707	0.828588	0.05
59708	0.828588	0.05
59709	0.841316	0.05
59710	0.841316	2.1
59711	0.528387	2.1
59712	0.565893	0.05
59713	1.10401	0.05
59714	1.10401	2.1
59715	0.528387	2.1
59716	1.89226	2.1
59717	1.36047	2.1
59718	1.33973	2.1
59719	1.33973	2.1
59720	1.89226	0.05
59721	2.28951	1.58355
59722	1.89226	1.63469
59723	2.80796	1.91252
59724	2.01584	1.89265
59725	2.01584	1.73818
59726	1.89226	1.87455
59727	1.89226	1.72572
59728	1.89226	1.24707
59729	1.89226	1.72127
59730	1.89226	1.67594
59731	1.89226	1.53339
59732	1.89226	1.66727
59733	1.89226	1.52989
59734	2.15223	1.53602
59735	1.30664	1.76976
59736	1.96011	1.73713
59737	2.76516	1.59717

59738	4.11234	1.70092
59739	1.89226	1.1539
59740	1.89226	1.59408
59741	1.89226	1.5725
59742	1.89226	1.44935
59743	1.89226	1.56249
59744	1.89226	1.44592
59745	2.81143	2.1
59746	2.58517	2.1
59747	2.55318	2.1
59748	2.7542	2.1
59749	5.33937	2.1
59750	2.7777	2.1
59751	2.7542	2.1
59752	2.71672	2.1
59753	2.70401	2.1
59754	5.45821	2.1
59755	2.55318	2.1
59756	2.7542	2.1
59757	2.71672	2.1
59758	2.54198	2.1
59759	5.29618	2.1
59760	1.95416	2.1
59761	2.162	2.1
59762	2.11563	2.1
59763	1.91813	2.1
59764	4.08013	2.1
59765	1.95417	2.1
59766	1.47712	2.1
59767	1.45182	2.1
59768	1.91813	2.1
59769	3.39525	2.1
59770	2.11562	2.1
59771	1.91813	2.1
59772	1.50979	2.1
59773	2.03974	2.1
59774	3.95787	2.1
59775	1.85605	2.1
59776	1.91813	2.1
59777	1.88885	2.1
59778	1.42255	2.1
59779	1.45182	2.1
59780	1.45182	2.1
59781	0.94613	2.1
59782	0.94613	1.15398
59783	0.946128	0.619386
59784	0.946128	2.1
59785	0.946127	2.1
59786	0.946127	2.1
59787	0.946131	0.421452
59788	0.946131	0.427095
59789	0.946129	1.50037
59790	0.946129	1.54933
59791	0.946128	1.58181
59792	0.946128	1.86892
59793	0.543052	2.1
59794	0.54608	1.0037
59795	0.602293	0.447919

59796	0.535325	0.661602
59797	1.05446	2.1
59798	0.850496	1.96473
59799	0.699838	1.49093
59800	0.679751	2.1
59801	0.713797	1.47101
59802	0.509794	1.26549
59803	0.602304	0.990883
59804	1.40017	2.1
59805	1.40017	2.1
59806	0.94613	2.1
59807	0.94613	2.1
59808	1.40017	2.1
59809	1.33997	2.1
59810	0.946131	2.1
59811	0.946131	2.1
59812	1.33997	2.1
59813	0.946131	2.1
59814	0.946131	2.1
59815	1.40017	2.1
59816	1.89226	0.0535827
59817	1.89226	0.0685413
59818	1.89226	0.0669518
59819	1.89226	0.0545485
59820	3.78452	0.0575315
59821	0.946133	0.0518057
59822	0.946133	0.0540167
59823	0.946132	0.0717635
59824	0.946132	0.0584171
59825	0.946132	0.0590855
59826	0.946132	0.0624067
59827	0.946133	0.0699184
59828	0.946133	0.0555184
59829	0.946132	0.0520151
59830	0.946132	0.0614773
59831	0.946132	0.0567157
59832	0.946132	0.0574257
59833	1.89224	0.068086
59834	1.89224	0.0585457
59835	1.89224	0.0586361
59836	1.89224	0.0598079
59837	1.89224	0.0664689
59838	1.89224	0.0677274
59839	0.94612	0.0536476
59840	0.94612	0.0550975
59841	0.946119	0.0712536
59842	0.946119	0.0607508
59843	0.946119	0.06078
59844	0.946119	0.0647191
59845	0.94612	0.0599188
59846	0.94612	0.0677828
59847	0.946132	0.061966
59848	0.946132	0.0575775
59849	0.946107	0.0546877
59850	0.946107	0.0655065
59851	2.63939	2.1
59852	1.2862	2.1
59853	1.29891	2.1

59854	2.64419	2.1
59855	3.93039	2.1
59856	2.6394	2.1
59857	1.89226	2.1
59858	1.89226	2.1
59859	2.64419	2.1
59860	4.53646	2.1
59861	1.89226	2.1
59862	1.89226	2.1
59863	1.89226	2.1
59864	1.89226	2.1
59865	3.78453	2.1
59866	1.89226	2.1
59867	1.89226	2.1
59868	1.89226	2.1
59869	1.89226	2.1
59870	1.89226	2.1
59871	1.89226	2.1
59872	1.31998	2.1
59873	1.32209	2.1
59874	0.649456	2.1
59875	0.648474	2.1
59876	0.984995	2.1
59877	0.984995	2.1
59878	0.985573	2.1
59879	0.985573	2.1
59880	1.31999	2.1
59881	0.648472	2.1
59882	0.649455	2.1
59883	1.32209	2.1
59884	0.946133	2.1
59885	0.946133	2.1
59886	1.31999	2.1
59887	1.3221	2.1
59888	0.946132	2.1
59889	0.946132	2.1
59890	1.31999	2.1
59891	1.31999	2.1
59892	1.31999	2.1
59893	0.946132	2.1
59894	0.946133	2.1
59895	1.3221	2.1
59896	0.946132	2.1
59897	0.946132	2.1
59898	0.946131	2.1
59899	0.946131	2.1
59900	0.946132	2.1
59901	0.946132	2.1
59902	0.946131	2.1
59903	0.946131	2.1
59904	0.946131	2.1
59905	0.946131	2.1
59906	0.946132	2.1
59907	0.946132	2.1
59908	0.946128	2.1
59909	0.946128	2.1
59910	0.946132	2.1
59911	0.946132	2.1

59912	0.946131	2.1
59913	0.946131	2.1
59914	0.94613	2.1
59915	0.94613	2.1
59916	0.94613	2.1
59917	0.94613	2.1
59918	0.946131	2.1
59919	0.946131	2.1
59920	1.89226	0.292321
59921	1.89226	0.775523
59922	1.89226	0.739051
59923	1.89226	0.656708
59924	1.89226	0.350362
59925	1.89226	0.360374
59926	1.89226	0.628457
59927	1.89226	1.52719
59928	1.89226	1.11859
59929	1.89226	1.43827
59930	3.78453	1.54006
59931	0.946133	0.479686
59932	0.946133	0.534404
59933	0.946132	0.417276
59934	0.946132	0.376383
59935	0.946133	0.643025
59936	0.946133	0.712378
59937	0.946132	0.521799
59938	0.946132	0.358337
59939	0.946132	0.53368
59940	0.946132	0.656995
59941	0.946132	0.445165
59942	0.946132	0.956535
59943	2.23638	0.17704
59944	1.89226	0.479126
59945	2.66014	0.551856
59946	2.66014	0.557075
59947	1.89226	0.672779
59948	1.89226	0.401967
59949	1.11819	0.126891
59950	1.33007	0.174333
59951	0.660255	0.223259
59952	0.91151	0.17354
59953	0.911511	0.285364
59954	1.33007	0.181323
59955	1.11819	0.106302
59956	0.660255	0.188323
59957	0.985327	0.103309
59958	0.985327	0.186693
59959	1.005	0.22001
59960	1.005	0.228258
59961	1.89226	2.1
59962	1.89226	2.1
59963	1.89226	2.1
59964	1.89226	2.1
59965	3.78453	2.1
59966	1.59533	2.1
59967	2.68571	2.1
59968	1.28291	2.1
59969	2.46974	2.1

59970	4.06506	2.1
59971	2.68571	2.1
59972	1.89226	2.1
59973	1.89226	2.1
59974	1.89226	2.1
59975	2.46973	2.1
59976	2.46973	2.1
59977	1.89226	2.1
59978	1.89226	2.1
59979	1.89226	2.1
59980	1.89226	2.1
59981	1.89226	2.1
59982	1.89226	2.1
59983	1.89226	2.1
59984	1.89226	2.1
59985	1.89226	2.1
59986	1.89226	2.1
59987	1.89226	2.1
59988	1.89226	2.1
59989	0.883594	2.1
59990	0.883594	2.1
59991	0.876484	2.1
59992	0.876484	2.1
59993	1.10442	2.1
59994	1.23487	2.1
59995	0.641457	2.1
59996	0.52521	2.1
59997	1.10442	2.1
59998	0.52521	2.1
59999	0.641457	2.1
60000	1.23487	2.1
60001	0.946131	2.1
60002	0.946131	2.1
60003	0.946131	2.1
60004	0.946131	2.1
60005	0.946131	2.1
60006	0.946131	2.1
60007	0.946131	2.1
60008	0.946131	2.1
60009	0.946131	2.1
60010	0.946131	2.1
60011	0.946131	2.1
60012	0.946131	2.1
60013	2.04016	0.0592801
60014	2.13042	0.0805014
60015	2.11465	0.0754601
60016	2.11465	0.0978252
60017	1.95908	0.0625804
60018	1.95908	0.0694899
60019	1.66953	0.0649578
60020	2.13041	0.0853763
60021	1.95908	0.0585355
60022	2.21952	0.0694445
60023	1.66953	0.0933287
60024	1.92997	0.11205
60025	2.04016	0.0573557
60026	1.95908	0.0627452
60027	1.82014	0.05

60028	2.08757	0.0507214
60029	3.86029	0.0572946
60030	0.834764	0.0674653
60031	0.722102	0.0585322
60032	0.979537	0.058553
60033	0.910065	0.05
60034	0.840053	0.0554058
60035	0.840052	0.0807804
60036	0.861587	0.0580998
60037	0.861587	0.0597081
60038	0.910067	0.0534023
60039	0.722101	0.0520466
60040	0.834763	0.065608
60041	0.979539	0.0628969
60042	1.89226	0.0806605
60043	1.89226	0.0832989
60044	1.89226	0.0852563
60045	1.89226	0.077741
60046	1.89226	0.0894276
60047	1.89226	0.0822712
60048	1.89226	0.0844473
60049	1.89226	0.103391
60050	1.89226	0.126564
60051	1.89226	0.0719191
60052	3.78453	0.0941313
60053	1.89226	0.0632156
60054	1.89226	0.0650237
60055	1.89226	0.0656239
60056	1.89226	0.063156
60057	1.89226	0.0664441
60058	1.89226	0.0649852
60059	1.20691	0.0572901
60060	2.27379	0.0586466
60061	2.58476	0.0593025
60062	1.5124	0.0528979
60063	3.79167	0.0590348
60064	1.89226	0.0958901
60065	2.2738	0.0687898
60066	2.58477	0.066716
60067	1.89226	0.0776502
60068	4.47703	0.0729995
60069	0.952225	1.76828
60070	0.952225	1.89045
60071	0.909865	1.86273
60072	0.909865	1.96895
60073	0.909895	2.039
60074	0.946132	1.81809
60075	0.946132	1.84941
60076	0.915958	1.76903
60077	0.946132	1.84432
60078	0.946132	1.8546
60079	0.909894	1.88645
60080	0.915958	1.90375
60081	0.983493	2.1
60082	2.40055	2.1
60083	1.02986	2.1
60084	1.02986	2.1
60085	2.34217	2.1

60086	2.34217	2.1
60087	1.89226	2.1
60088	2.40056	2.1
60089	2.34217	2.1
60090	2.40056	2.1
60091	1.83388	2.1
60092	1.89226	2.1
60093	1.89226	2.1
60094	1.89226	2.1
60095	1.89226	2.1
60096	1.89226	2.1
60097	1.89226	2.1
60098	1.89226	2.1
60099	1.89226	2.1
60100	1.89226	2.1
60101	1.89226	2.1
60102	1.89226	2.1
60103	1.89226	2.1
60104	1.89226	2.1
60105	0.946133	2.1
60106	0.946133	2.1
60107	1.17109	2.1
60108	1.13078	2.1
60109	0.946132	2.1
60110	0.946132	2.1
60111	1.13078	2.1
60112	1.13078	2.1
60113	1.13078	2.1
60114	0.946133	2.1
60115	0.946132	2.1
60116	1.17109	2.1
60117	1.89226	2.1
60118	1.89226	2.1
60119	1.89226	2.1
60120	1.89226	2.1
60121	1.89226	2.1
60122	1.89226	2.1
60123	2.33963	1.33438
60124	1.89227	0.682832
60125	1.74565	1.03565
60126	1.74565	1.08487
60127	1.89227	0.492953
60128	1.89227	0.651226
60129	1.89227	1.1511
60130	1.89227	0.570487
60131	1.89227	1.36066
60132	1.89227	0.814014
60133	3.78453	0.938622
60134	1.31398	0.474333
60135	1.10392	0.640102
60136	0.796878	0.481075
60137	0.524356	0.434147
60138	0.870614	0.490979
60139	0.945739	0.363535
60140	0.89208	0.589995
60141	0.89208	1.17344
60142	1.23425	0.394772
60143	0.774658	1.05907

60144	1.03182	0.418041
60145	0.557289	0.331194
60146	2.33963	1.32896
60147	1.89226	0.453482
60148	1.89226	1.32655
60149	2.77245	0.640896
60150	4.23189	0.937505
60151	1.89226	1.27495
60152	1.89226	0.539099
60153	1.89226	1.32696
60154	1.89226	1.29625
60155	1.89226	1.01928
60156	1.89226	0.61814
60157	1.89226	0.159403
60158	1.89226	0.16651
60159	1.89226	0.133998
60160	1.89226	0.356223
60161	1.89226	0.123083
60162	1.89226	0.302286
60163	1.89226	0.127442
60164	1.89226	0.114459
60165	1.89226	0.100378
60166	1.89226	0.201293
60167	1.89226	0.0889534
60168	1.89226	0.159339
60169	0.946128	0.113898
60170	0.946128	0.0851396
60171	0.963918	0.0858022
60172	1.37543	0.0698321
60173	0.946129	0.0779568
60174	0.946129	0.107593
60175	1.37542	0.0704978
60176	1.37542	0.0637224
60177	0.94613	0.069303
60178	1.37542	0.0816116
60179	0.963916	0.0751392
60180	0.94613	0.0808843
60181	0.946132	0.144175
60182	0.946132	0.106284
60183	0.946131	0.111754
60184	0.946131	0.0775603
60185	0.946131	0.110219
60186	0.946131	0.141152
60187	0.946132	0.0943529
60188	0.946132	0.0746336
60189	0.946131	0.106092
60190	0.946131	0.110193
60191	0.946131	0.104187
60192	0.946131	0.102652
60193	1.89226	0.168941
60194	1.89226	0.180273
60195	1.89226	0.117659
60196	1.89226	0.283777
60197	3.78452	0.181458
60198	0.946127	0.183603
60199	0.946127	0.114758
60200	0.946131	0.115398
60201	0.946131	0.084376

60202	0.946128	0.112952
60203	0.946128	0.146817
60204	0.94613	0.0947533
60205	0.94613	0.13166
60206	0.946129	0.113422
60207	0.946129	0.113803
60208	0.946129	0.121565
60209	0.946129	0.121183
60210	1.89226	1.57202
60211	1.89226	1.71689
60212	1.89226	2.1
60213	1.89226	1.07135
60214	3.78453	1.16379
60215	1.89226	0.82855
60216	1.89226	0.209845
60217	1.89226	0.378802
60218	1.89226	0.643307
60219	3.78453	0.801014
60220	0.946131	0.450787
60221	0.946131	0.497776
60222	0.946132	1.67173
60223	0.946132	1.19617
60224	0.946132	1.57167
60225	0.946132	0.821001
60226	0.946132	1.59265
60227	0.946132	1.15978
60228	0.946132	1.12541
60229	0.946132	1.56519
60230	0.946132	1.61887
60231	0.946132	0.880918
60232	0.548169	0.247531
60233	0.736603	0.0948032
60234	1.18322	0.347701
60235	1.01139	0.190094
60236	1.01139	0.125159
60237	0.548168	0.152047
60238	0.736603	0.16457
60239	1.18322	0.0671293
60240	0.861589	0.0655235
60241	0.861589	0.365347
60242	0.869798	0.144889
60243	0.869798	0.210223
60244	1.16037	0.199299
60245	0.942012	0.352453
60246	0.942012	0.182571
60247	0.62356	0.260822
60248	0.891932	0.157013
60249	0.900255	0.208008
60250	2.02279	0.259901
60251	1.48677	0.214818
60252	1.54724	0.0935584
60253	0.831456	0.162128
60254	1.19775	0.377891
60255	1.89226	1.64189
60256	2.76291	1.6013
60257	1.89226	1.67505
60258	1.89226	1.61323
60259	3.78452	1.5671

60260	1.89226	0.932927
60261	1.89226	1.3106
60262	1.89226	1.46646
60263	1.89226	1.02904
60264	1.89226	1.35728
60265	1.89226	0.87423
60266	1.89226	1.30052
60267	1.89226	1.15366
60268	1.89226	1.40087
60269	1.89226	1.13401
60270	3.78452	1.34252
60271	1.89226	0.75491
60272	1.89226	1.25115
60273	1.89226	1.34596
60274	1.89226	0.84667
60275	3.78453	1.10638
60276	0.946128	0.647577
60277	0.946128	1.10952
60278	0.946129	1.33144
60279	0.946129	1.20996
60280	0.946132	0.887785
60281	0.946132	1.34583
60282	0.946126	1.28193
60283	0.946126	1.07071
60284	0.946129	1.13478
60285	0.946129	1.18608
60286	0.946129	1.0737
60287	0.946129	0.915456
60288	2.80495	1.26398
60289	1.89225	1.13371
60290	1.89225	1.45493
60291	1.87794	1.42311
60292	1.89225	1.37268
60293	1.87794	1.4598
60294	2.25195	2.1
60295	2.00624	1.4476
60296	2.13132	2.1
60297	1.90239	1.36164
60298	4.13756	0.842334
60299	0.804661	1.92769
60300	0.804663	0.462879
60301	0.889591	0.705613
60302	0.889591	2.0394
60303	0.808324	1.35593
60304	0.761765	1.82415
60305	1.02822	0.147053
60306	0.879624	0.163188
60307	0.879617	0.773078
60308	0.800864	0.686356
60309	0.978023	1.02099
60310	0.808329	0.679747
60311	2.13133	2.1
60312	2.00558	0.680269
60313	2.09316	0.368632
60314	2.09316	2.1
60315	2.03253	0.743035
60316	2.03253	2.1
60317	0.84577	1.74056

60318	0.845767	0.924558
60319	0.843917	0.615474
60320	0.843917	2.03163
60321	0.76177	1.04796
60322	0.719345	1.69251
60323	0.934435	0.10398
60324	0.884197	0.111712
60325	0.934439	0.557147
60326	0.75627	0.56047
60327	0.849424	0.4353
60328	0.76177	0.557562
60329	2.61855	0.109438
60330	1.17793	0.11188
60331	1.42965	0.10316
60332	1.17793	0.0970949
60333	2.50057	0.131278
60334	2.24885	0.128673
60335	0.946133	0.101627
60336	0.946133	0.0964199
60337	1.30928	0.111782
60338	1.12443	0.132662
60339	0.946132	0.138884
60340	0.946132	0.112956
60341	1.30928	0.0947438
60342	1.30928	0.124378
60343	0.946132	0.11605
60344	1.30928	0.112122
60345	1.12443	0.107148
60346	0.946133	0.112375
60347	1.89226	0.104593
60348	1.89226	0.133342
60349	1.89226	0.112458
60350	1.89226	0.108717
60351	3.78452	0.121899
60352	1.89226	0.11072
60353	1.89226	0.0974554
60354	1.89226	0.0993614
60355	1.89226	0.0941662
60356	1.89226	0.114558
60357	1.89226	0.11425
60358	1.89226	0.123301
60359	1.89226	0.118319
60360	1.89226	0.103247
60361	1.89226	0.131367
60362	3.78452	0.110056
60363	0.94613	0.100679
60364	0.94613	0.105829
60365	0.946131	0.098946
60366	0.946131	0.109134
60367	0.946128	0.112531
60368	0.946128	0.0929107
60369	0.946133	0.102672
60370	0.946133	0.122147
60371	0.946131	0.1098
60372	0.946131	0.0956918
60373	0.946131	0.104495
60374	0.946131	0.12548
60375	1.89226	2.1

60376	1.89226	2.1
60377	1.89226	2.1
60378	1.89226	2.1
60379	3.78453	2.1
60380	1.0465	2.1
60381	2.73926	2.1
60382	2.03721	2.1
60383	2.03721	2.1
60384	1.44709	2.1
60385	1.44709	2.1
60386	0.946131	2.1
60387	0.946131	2.1
60388	0.946131	2.1
60389	0.946131	2.1
60390	0.946132	2.1
60391	0.946132	2.1
60392	0.94613	2.1
60393	0.94613	2.1
60394	0.946131	2.1
60395	0.946131	2.1
60396	0.946131	2.1
60397	0.946131	2.1
60398	0.946132	2.1
60399	0.946132	2.1
60400	0.946131	2.1
60401	0.946131	2.1
60402	0.946132	2.1
60403	0.946132	2.1
60404	0.946132	2.1
60405	0.946132	2.1
60406	0.946132	2.1
60407	0.946132	2.1
60408	0.946132	2.1
60409	0.946132	2.1
60410	1.89226	2.1
60411	1.89226	2.1
60412	1.89226	2.1
60413	1.89226	2.1
60414	3.78452	2.1
60415	1.61969	0.120601
60416	1.79374	0.053725
60417	1.29382	0.0794159
60418	1.29382	0.135893
60419	2.05759	0.0543907
60420	2.05759	0.085758
60421	1.89226	0.132257
60422	1.89226	0.0974679
60423	1.89226	0.103583
60424	1.89226	0.172977
60425	1.89226	0.055586
60426	1.89226	0.0817233
60427	1.89226	1.17217
60428	1.89226	1.3971
60429	1.89226	1.21396
60430	1.89226	1.2713
60431	1.89226	1.1406
60432	1.89226	1.20561
60433	1.89226	1.76608

60434	1.89226	1.52605
60435	1.89226	1.66164
60436	1.89226	1.72534
60437	3.78453	1.78476
60438	0.946131	1.48556
60439	0.946131	1.51102
60440	0.946131	1.40544
60441	0.946131	1.46086
60442	0.946131	1.40753
60443	0.946131	1.38167
60444	0.946131	1.47315
60445	0.946131	1.5633
60446	0.946131	1.47292
60447	0.946131	1.49599
60448	0.946131	1.46459
60449	0.946131	1.42342
60450	2.45177	0.179466
60451	2.68507	0.145842
60452	2.3376	0.220417
60453	2.22086	0.136731
60454	4.78937	0.174281
60455	2.45178	0.159495
60456	2.35552	0.114068
60457	1.68062	0.150509
60458	1.81902	0.125813
60459	4.13239	0.13319
60460	2.3376	0.173648
60461	1.55612	0.121254
60462	1.69811	0.128676
60463	2.31527	0.141529
60464	1.79553	0.129707
60465	2.3376	0.145691
60466	1.55613	0.111383
60467	1.06336	0.12255
60468	1.97516	0.122122
60469	1.88966	0.105368
60470	1.06336	0.162329
60471	1.68062	0.105834
60472	1.89226	1.66247
60473	1.91634	1.84085
60474	2.03709	1.95071
60475	1.91634	1.72893
60476	1.83185	1.82509
60477	1.70303	1.06259
60478	1.89226	1.63406
60479	1.89226	1.81064
60480	1.89226	1.94254
60481	1.89226	1.72544
60482	1.89226	1.79112
60483	1.89226	1.10302
60484	1.89226	2.1
60485	2.03472	2.1
60486	2.22183	2.1
60487	2.03472	2.1
60488	2.07936	2.1
60489	1.89226	1.88276
60490	1.89226	1.47483
60491	1.8519	1.52568

60492	1.92119	1.75296
60493	1.8519	1.58103
60494	1.96156	1.1241
60495	1.89226	0.663173
60496	1.89226	1.32627
60497	1.89226	1.62017
60498	1.89226	1.78597
60499	1.89226	1.60353
60500	1.89226	1.1119
60501	1.89226	0.690933
60502	1.89226	1.92115
60503	2.22183	2.1
60504	2.39541	2.1
60505	2.22183	1.97512
60506	2.06585	2.04995
60507	1.89226	1.77286
60508	1.47001	2.1
60509	1.57962	1.93605
60510	1.65238	2.1
60511	1.60502	2.1
60512	3.18464	2.1
60513	1.02415	2.1
60514	1.60503	2.1
60515	1.47001	2.1
60516	1.47001	2.1
60517	1.28228	2.1
60518	1.28228	2.1
60519	0.946133	2.1
60520	0.946133	2.1
60521	0.946132	2.1
60522	0.946132	2.1
60523	0.946132	2.1
60524	0.946132	2.1
60525	0.946133	2.1
60526	0.946133	2.1
60527	0.946132	2.1
60528	0.946132	2.1
60529	0.946133	2.1
60530	0.946133	2.1
60531	1.89226	1.93086
60532	1.89226	2.1
60533	1.89226	2.1
60534	1.89226	1.81059
60535	3.78452	1.98938
60536	1.76897	2.09916
60537	1.60502	2.1
60538	1.65238	1.93667
60539	1.65238	2.1
60540	1.76932	1.8945
60541	1.76932	2.1
60542	1.55998	2.1
60543	1.60503	2.1
60544	1.76932	2.1
60545	1.60503	2.1
60546	1.44657	2.1
60547	1.28228	2.1
60548	0.946133	2.1
60549	0.946133	2.1

60550	0.946132	2.1
60551	0.946132	2.1
60552	0.946132	2.1
60553	0.946132	2.1
60554	0.946133	2.1
60555	0.946133	2.1
60556	0.946132	2.1
60557	0.946132	2.1
60558	0.946132	2.1
60559	0.946132	2.1
60560	0.946128	2.1
60561	0.946128	1.97078
60562	0.946133	1.39035
60563	0.946133	1.80761
60564	0.94613	1.62017
60565	0.94613	1.97719
60566	0.946131	2.1
60567	0.946131	1.89801
60568	0.946131	1.89712
60569	0.946131	1.87494
60570	0.946131	1.96478
60571	0.946131	1.96811
60572	1.85779	0.169921
60573	1.00763	0.136863
60574	1.74874	0.140259
60575	1.74873	0.131004
60576	1.14467	0.186011
60577	1.14466	0.167849
60578	1.85779	0.147368
60579	1.61597	0.121886
60580	1.00053	0.152647
60581	1.03018	0.119896
60582	2.65819	0.127634
60583	1.89226	0.676764
60584	1.89226	0.375084
60585	1.89226	0.690701
60586	1.89226	0.566859
60587	3.78451	0.581512
60588	1.89226	0.606331
60589	1.89226	0.208193
60590	1.89226	0.33098
60591	1.89226	0.355351
60592	1.89226	0.294145
60593	1.89226	0.318374
60594	1.11983	0.173383
60595	0.810945	0.191615
60596	0.913906	0.154162
60597	0.913906	0.180367
60598	2.23303	0.127231
60599	1.66057	0.129999
60600	1.28724	0.141493
60601	1.08132	0.144192
60602	1.49316	0.168264
60603	0.810958	0.171464
60604	0.810958	0.163147
60605	0.827861	0.324397
60606	0.946126	0.356983
60607	0.946132	0.591272

60608	0.946132	0.306985
60609	0.827863	0.363138
60610	0.827863	0.352729
60611	0.946129	0.394061
60612	0.946129	0.393746
60613	0.827863	0.352095
60614	0.946128	0.377366
60615	0.946129	0.304999
60616	0.946129	0.342079
60617	0.974718	0.061382
60618	1.53671	0.0910108
60619	2.80542	0.058869
60620	2.27259	0.0727001
60621	3.8093	0.0669282
60622	1.89226	0.110435
60623	1.89226	0.233129
60624	1.89226	0.092896
60625	1.89226	0.181084
60626	3.78453	0.157409
60627	0.946132	0.103419
60628	0.946132	0.144221
60629	0.946131	0.152137
60630	0.946131	0.110778
60631	0.946132	0.155359
60632	0.946132	0.179438
60633	0.946131	0.0929246
60634	0.946131	0.0943277
60635	0.946132	0.123054
60636	0.946132	0.162121
60637	0.946131	0.121014
60638	0.946131	0.122882
60639	2.80542	0.0711828
60640	1.89226	0.0815337
60641	2.27259	0.0749684
60642	2.27259	0.12636
60643	1.89226	0.0554194
60644	1.89226	0.0827012
60645	1.89226	0.0905954
60646	1.89226	0.213538
60647	1.89226	0.107348
60648	1.89226	0.221268
60649	1.89226	0.0962517
60650	1.89226	0.221591
60651	0.946132	0.203374
60652	0.946132	0.0960201
60653	0.946131	0.179392
60654	0.946131	0.115846
60655	0.946132	0.185238
60656	0.946132	0.189951
60657	0.946131	0.0941978
60658	0.946131	0.107361
60659	0.946131	0.122672
60660	0.946131	0.139617
60661	0.946131	0.112843
60662	0.946131	0.141032
60663	2.68213	2.1
60664	2.58234	2.1
60665	2.58234	2.1

60666	2.61243	2.1
60667	2.67035	2.1
60668	2.70044	2.1
60669	2.68213	2.1
60670	2.53921	2.1
60671	2.53303	2.1
60672	2.67035	2.1
60673	5.20956	2.1
60674	2.67033	2.1
60675	2.58232	2.1
60676	2.55004	2.1
60677	2.64567	2.1
60678	5.228 2.1	
60679	2.53301	2.1
60680	2.64567	2.1
60681	2.64567	2.1
60682	2.67033	2.1
60683	2.51603	2.1
60684	2.54069	2.1
60685	0.907497	2.1
60686	0.907497	2.1
60687	0.926641	2.1
60688	0.926641	2.1
60689	1.14989	2.1
60690	1.10046	2.1
60691	0.695334	2.1
60692	0.684247	2.1
60693	0.695333	2.1
60694	1.14989	2.1
60695	0.684246	2.1
60696	1.10046	2.1
60697	0.946126	2.1
60698	0.946126	2.1
60699	0.946131	2.1
60700	0.946131	2.1
60701	0.946127	2.1
60702	0.946127	2.1
60703	0.94613	2.1
60704	0.94613	2.1
60705	0.946128	2.1
60706	0.946128	2.1
60707	0.946128	2.1
60708	0.946128	2.1
60709	1.89226	2.1
60710	1.89226	2.1
60711	1.89226	2.1
60712	1.89226	2.1
60713	1.89226	2.1
60714	1.89226	2.1
60715	1.42174	2.1
60716	2.29978	2.1
60717	2.41233	2.1
60718	2.41233	2.1
60719	1.39067	2.1
60720	1.39067	2.1
60721	1.89226	2.1
60722	1.89226	2.1
60723	1.89226	2.1

60724	1.89226	2.1
60725	3.78452	2.1
60726	1.89226	2.1
60727	1.89226	2.1
60728	1.89226	2.1
60729	1.89226	2.1
60730	1.89226	2.1
60731	1.89226	2.1
60732	2.41234	2.1
60733	1.89226	2.1
60734	2.29979	2.1
60735	1.89226	2.1
60736	1.89226	2.1
60737	2.29979	2.1
60738	1.09506	0.0631454
60739	2.82921	0.100647
60740	2.53251	0.0616492
60741	2.53251	0.0714777
60742	1.42901	0.0676165
60743	1.42901	0.0712633
60744	0.946132	0.057355
60745	0.946132	0.0644941
60746	0.946128	0.0676839
60747	0.946128	0.0731101
60748	0.94613	0.064946
60749	0.94613	0.0653252
60750	0.946131	0.0846173
60751	0.946131	0.0725035
60752	0.94613	0.0617727
60753	0.94613	0.0727754
60754	0.94613	0.110577
60755	0.94613	0.064705
60756	1.89226	0.0802235
60757	1.89226	0.102646
60758	1.89226	0.427206
60759	1.89226	0.0760219
60760	3.78452	0.105048
60761	2.53251	0.0684901
60762	1.89226	0.0865692
60763	2.82921	0.06793
60764	1.89226	0.0710033
60765	1.89226	0.244639
60766	2.82921	0.454185
60767	1.89226	0.225445
60768	1.89226	0.0558298
60769	1.89226	0.0550416
60770	1.89226	0.0682497
60771	1.89226	0.0640152
60772	1.89226	0.098698
60773	2.53251	0.0676972
60774	1.89226	0.0730697
60775	2.06158	0.0561713
60776	1.89226	0.0589964
60777	4.42477	0.0595564
60778	1.23296	2.1
60779	2.55352	2.1
60780	2.47476	2.1
60781	2.47476	2.1

60782	1.32368	2.1
60783	1.32368	2.1
60784	1.89226	2.1
60785	1.89226	2.1
60786	1.89226	2.1
60787	1.89226	2.1
60788	1.89226	2.1
60789	1.89226	2.1
60790	1.89226	2.1
60791	1.89226	2.1
60792	1.89226	2.1
60793	1.89226	2.1
60794	1.89226	2.1
60795	1.89226	2.1
60796	2.47476	2.1
60797	1.89226	2.1
60798	1.89226	2.1
60799	1.89226	2.1
60800	2.55353	2.1
60801	2.55353	2.1
60802	1.05983	2.1
60803	2.47476	2.1
60804	2.37664	2.1
60805	2.37664	2.1
60806	1.23296	2.1
60807	1.23296	2.1
60808	0.946127	2.1
60809	0.946127	2.1
60810	0.946131	2.1
60811	0.946131	2.1
60812	0.946127	2.1
60813	0.946127	2.1
60814	0.946131	2.1
60815	0.946131	2.1
60816	0.946129	2.1
60817	0.946129	2.1
60818	0.946128	2.1
60819	0.946128	2.1
60820	1.89226	2.1
60821	1.89226	2.1
60822	1.89226	2.1
60823	1.89226	2.1
60824	3.78452	2.1
60825	2.37665	2.1
60826	1.89226	2.1
60827	1.89226	2.1
60828	1.89226	2.1
60829	2.47476	2.1
60830	2.47476	2.1
60831	1.89226	1.04819
60832	1.92119	0.907439
60833	1.99586	1.54786
60834	1.92119	1.43911
60835	1.96693	0.553737
60836	1.89226	0.37062
60837	1.89226	1.00156
60838	1.89226	1.02882
60839	1.89226	1.63387

60840	1.89226	1.3412
60841	1.89226	0.722053
60842	1.89226	0.457609
60843	1.89226	1.72795
60844	2.39541	1.94684
60845	2.57183	2.08264
60846	2.39541	1.81047
60847	2.06868	1.78942
60848	1.89226	1.59606
60849	0.946133	0.513386
60850	0.946133	0.362095
60851	0.997928	0.609005
60852	1.03763	0.632475
60853	0.946131	0.728651
60854	0.946131	0.166182
60855	1.03763	0.404333
60856	1.03763	0.429793
60857	1.03763	0.520883
60858	0.946131	0.508183
60859	0.94613	0.40374
60860	0.99793	0.411402
60861	1.89226	0.281211
60862	1.89226	0.885608
60863	1.89226	0.891908
60864	1.89226	0.347699
60865	1.89226	1.43867
60866	1.89226	0.403702
60867	0.946131	1.31088
60868	0.946131	1.35732
60869	1.36939	1.69863
60870	1.28592	1.85019
60871	0.946129	1.72185
60872	0.946129	0.669601
60873	1.36939	1.44293
60874	1.36939	1.71206
60875	0.946131	1.5339
60876	1.36939	1.55481
60877	0.946131	1.61338
60878	1.28592	1.64391
60879	1.89226	0.125809
60880	1.89226	0.53822
60881	1.89226	0.240918
60882	1.89226	0.12447
60883	3.78453	0.148754
60884	1.89226	0.570665
60885	1.89226	0.155452
60886	1.89226	0.14124
60887	1.89226	0.270126
60888	3.78452	0.187547
60889	1.91005	0.0867126
60890	1.89226	0.525013
60891	2.60215	0.0761988
60892	2.60215	0.0854938
60893	1.89226	0.5827
60894	1.89226	0.448101
60895	0.946132	0.388478
60896	0.946132	0.431066
60897	0.946132	0.176163

60898	0.946132	0.156514
60899	0.946132	0.133466
60900	0.946132	0.297211
60901	0.946132	0.395063
60902	0.946132	0.187834
60903	0.946132	0.17609
60904	0.946132	0.149626
60905	0.946131	0.403672
60906	0.946131	0.328088
60907	1.89226	0.173776
60908	1.89226	0.304326
60909	1.89226	0.374052
60910	1.89226	0.639782
60911	1.89226	0.25281
60912	1.89226	0.269608
60913	0.780483	0.41058
60914	0.78928	0.642737
60915	0.818566	0.31613
60916	0.460387	0.165815
60917	1.82793	0.28444
60918	1.32039	0.447632
60919	0.613889	0.26784
60920	1.0592	0.496815
60921	1.48698	0.366165
60922	0.818555	0.488932
60923	1.11515	0.432147
60924	1.2802	1.12417
60925	2.20046	0.311088
60926	1.1475	0.722567
60927	1.1475	0.277279
60928	2.27062	0.62983
60929	2.27062	0.239414
60930	1.89226	1.05885
60931	2.20046	0.203036
60932	2.27062	0.608955
60933	2.20046	0.485462
60934	1.96242	0.161076
60935	1.89226	0.197442
60936	1.89226	0.230785
60937	1.89226	0.161794
60938	1.89226	0.958088
60939	1.89226	0.422976
60940	3.78452	0.196003
60941	1.89226	0.378213
60942	1.89226	0.398354
60943	1.89226	0.649674
60944	1.89226	0.444075
60945	3.78452	0.480525
60946	1.17403	0.291824
60947	1.13531	0.358206
60948	0.6401	0.333158
60949	0.714566	0.364019
60950	0.915987	0.393414
60951	0.915987	0.14621
60952	0.933891	1.10876
60953	0.933891	0.284048
60954	1.17403	0.364477
60955	0.714566	0.344369

60956	0.6401	0.317047
60957	1.13531	0.271523
60958	0.946133	0.259605
60959	0.946133	0.306831
60960	1.17403	0.214575
60961	1.13531	0.190299
60962	0.946131	0.263442
60963	0.946131	0.13326
60964	1.17403	0.411698
60965	1.17403	0.126329
60966	1.17403	0.234938
60967	0.94613	0.242678
60968	0.946131	0.189454
60969	1.13531	0.158124
60970	1.89226	0.126779
60971	1.89226	0.28823
60972	1.89226	0.207424
60973	1.89226	0.121702
60974	1.89226	0.309005
60975	1.89226	0.148972
60976	0.946131	0.259748
60977	0.946131	0.243414
60978	0.946131	0.317486
60979	0.946131	0.342752
60980	0.946129	0.338716
60981	0.946129	0.169924
60982	0.946133	0.49641
60983	0.946133	0.224112
60984	0.946131	0.287646
60985	0.946131	0.288113
60986	0.946131	0.239457
60987	0.946131	0.246754
60988	2.29413	2.1
60989	1.99332	2.1
60990	1.84774	2.1
60991	2.21582	2.1
60992	4.20915	2.1
60993	0.517359	2.1
60994	0.634469	2.1
60995	0.996661	2.1
60996	0.92387	2.1
60997	0.768098	2.1
60998	0.768098	2.1
60999	0.790242	2.1
61000	0.790242	2.1
61001	0.923871	2.1
61002	0.517359	2.1
61003	0.634469	2.1
61004	0.996661	2.1
61005	1.89226	2.1
61006	1.89226	2.1
61007	1.89226	2.1
61008	1.89226	2.1
61009	3.78453	2.1
61010	0.946132	2.1
61011	0.946132	2.1
61012	0.946131	2.1
61013	0.946131	2.1

61014	0.946132	2.1
61015	0.946132	2.1
61016	0.946131	2.1
61017	0.946131	2.1
61018	0.946132	2.1
61019	0.946132	2.1
61020	0.946131	2.1
61021	0.946131	2.1
61022	1.62708	2.1
61023	2.21582	2.1
61024	2.13925	2.1
61025	2.21582	2.1
61026	1.77117	2.1
61027	1.84774	2.1
61028	0.946132	2.1
61029	0.946132	2.1
61030	0.946131	2.1
61031	0.946131	2.1
61032	0.946132	2.1
61033	0.946132	2.1
61034	0.946131	2.1
61035	0.946131	2.1
61036	0.946131	2.1
61037	0.946131	2.1
61038	0.946131	2.1
61039	0.946131	2.1
61040	1.89226	1.91847
61041	1.89226	1.43186
61042	1.89226	0.966605
61043	1.89226	1.77283
61044	3.78453	1.81338
61045	1.89226	1.2175
61046	1.89226	1.75471
61047	1.89226	1.68242
61048	1.89226	1.95772
61049	1.89226	0.815032
61050	1.89226	1.7404
61051	1.89226	1.0206
61052	1.89226	1.9612
61053	1.89226	0.684251
61054	1.89226	1.93021
61055	1.89226	1.39606
61056	1.89226	2.00174
61057	0.914369	1.82394
61058	0.914369	1.90175
61059	0.810945	2.1
61060	0.810945	1.87417
61061	2.26581	2.1
61062	1.66199	2.1
61063	1.49503	1.91061
61064	1.28816	2.1
61065	1.08133	2.0581
61066	0.810933	2.01909
61067	1.1212	2.02324
61068	1.89227	0.434215
61069	1.89227	0.0939598
61070	1.89227	0.111439
61071	1.89227	0.191095

61072	3.78453	0.132963
61073	1.89226	0.163468
61074	1.89226	0.455447
61075	1.89226	0.273262
61076	1.89226	0.16528
61077	3.78453	0.202755
61078	1.89226	0.522378
61079	1.89226	0.158148
61080	1.89226	0.188975
61081	1.89226	0.485553
61082	3.78453	0.281097
61083	1.89226	0.106777
61084	1.89226	0.442074
61085	1.89226	0.255945
61086	2.25318	0.103974
61087	3.78453	0.131974
61088	1.89226	0.219043
61089	1.89226	0.506798
61090	1.89226	0.220042
61091	1.9637	0.0997929
61092	3.78453	0.133242
61093	1.89226	0.477163
61094	1.89226	0.171235
61095	1.89226	0.164242
61096	1.89226	0.408448
61097	3.78453	0.237866
61098	1.89226	0.197265
61099	1.89226	0.578921
61100	1.89226	0.427539
61101	2.53384	0.198456
61102	3.78453	0.274367
61103	1.55405	1.04644
61104	1.03818	1.60899
61105	1.03818	0.558653
61106	1.34857	0.333621
61107	1.46338	0.285643
61108	1.77377	1.31438
61109	1.86273	0.544487
61110	1.73802	1.22583
61111	1.50969	1.90209
61112	1.46337	1.27396
61113	3.20139	1.40359
61114	1.2377	1.29617
61115	1.46337	0.235664
61116	1.27408	1.42497
61117	1.32039	1.64698
61118	1.50969	1.43481
61119	1.50969	0.719767
61120	1.89226	2.1
61121	1.89226	2.1
61122	1.89226	2.1
61123	1.89226	2.1
61124	1.89226	2.1
61125	1.89226	2.1
61126	1.89169	2.1
61127	1.89226	2.1
61128	1.89226	2.1
61129	1.89226	2.1

61130	1.96624	2.1
61131	1.96624	2.1
61132	2.62871	2.1
61133	1.6201	2.1
61134	1.49149	2.1
61135	1.6201	2.1
61136	2.62559	2.1
61137	2.75419	2.1
61138	0.946129	2.08127
61139	0.946129	2.1
61140	0.946133	2.1
61141	0.946133	2.1
61142	0.946131	2.1
61143	0.946131	2.02848
61144	0.946131	2.1
61145	0.946131	2.1
61146	0.946131	2.1
61147	0.946131	2.1
61148	0.946131	2.1
61149	0.946131	2.1
61150	0.851543	2.1
61151	0.851543	2.1
61152	1.04043	2.1
61153	0.997939	2.1
61154	0.946132	2.1
61155	0.946132	2.1
61156	0.894185	2.1
61157	0.893901	2.1
61158	0.998267	2.1
61159	1.04074	2.1
61160	0.85127	2.1
61161	0.85153	2.1
61162	1.89226	2.1
61163	1.89226	2.1
61164	1.89226	2.1
61165	1.89226	2.1
61166	1.89226	2.1
61167	1.89226	2.1
61168	0.928691	2.1
61169	0.928691	2.1
61170	0.935332	2.1
61171	0.935332	2.1
61172	1.398	2.1
61173	1.14418	2.1
61174	0.479331	2.1
61175	0.719848	2.1
61176	1.398	2.1
61177	0.719848	2.1
61178	1.14417	2.1
61179	0.47933	2.1
61180	2.28835	2.1
61181	1.89226	2.1
61182	1.89226	2.1
61183	1.89226	2.1
61184	2.796	2.1
61185	2.796	2.1
61186	0.946131	2.1
61187	0.946131	2.1

61188	0.946131	2.1
61189	0.946131	2.1
61190	0.946128	2.1
61191	0.946128	2.1
61192	0.946133	2.1
61193	0.946133	2.1
61194	0.946131	2.1
61195	0.946131	2.1
61196	0.94613	2.1
61197	0.94613	2.1
61198	0.946131	2.1
61199	0.946131	2.1
61200	0.946131	2.1
61201	0.946131	2.1
61202	0.946131	2.1
61203	0.946131	2.1
61204	0.946131	2.1
61205	0.946131	2.1
61206	0.946131	2.1
61207	0.946131	2.1
61208	0.946131	2.1
61209	0.946131	2.1
61210	1.89226	2.1
61211	1.89226	2.1
61212	1.89226	2.1
61213	1.89226	2.1
61214	3.78452	2.1
61215	2.05207	0.747563
61216	1.70302	1.79169
61217	1.33794	2.08744
61218	1.98194	1.48445
61219	3.36873	1.7166
61220	2.62761	0.249907
61221	1.98194	1.32583
61222	2.28009	1.64918
61223	2.28009	0.387399
61224	2.22939	1.45062
61225	2.22939	0.323633
61226	1.89226	1.39636
61227	1.89226	0.392035
61228	1.89226	0.834473
61229	1.89226	0.379629
61230	1.89226	1.35667
61231	1.89226	0.520799
61232	1.89227	1.49412
61233	1.89227	1.7176
61234	1.89227	1.94517
61235	1.89227	1.69668
61236	1.89227	1.0348
61237	1.89227	0.430846
61238	0.888355	0.491827
61239	0.990973	1.53436
61240	0.71133	1.74171
61241	0.634324	1.82991
61242	0.806209	1.74787
61243	0.806209	0.238926
61244	0.819087	1.72423
61245	0.819087	1.84997

61246	0.888355	1.72674
61247	0.634324	1.67556
61248	0.71133	1.69956
61249	0.990972	1.66783
61250	1.11469	1.23715
61251	0.985023	0.455676
61252	0.888354	0.412532
61253	0.990972	1.4732
61254	0.994754	1.28308
61255	0.994754	0.51151
61256	1.0083	1.32519
61257	1.0083	0.386399
61258	0.888354	0.365455
61259	0.985024	1.09133
61260	0.990971	0.30187
61261	1.11469	0.149663
61262	0.946132	1.27257
61263	0.946132	0.426239
61264	0.946132	1.17656
61265	0.946132	1.19641
61266	0.946132	1.22692
61267	0.946132	0.378056
61268	0.946133	1.58674
61269	0.946133	0.714095
61270	0.946133	1.167
61271	0.946133	1.19392
61272	0.946132	0.410256
61273	0.946132	0.213556
61274	0.810944	1.34477
61275	0.810944	0.63291
61276	0.810947	1.64339
61277	0.810947	0.209679
61278	1.89227	1.84588
61279	1.35168	1.55695
61280	1.08132	1.64964
61281	1.08131	1.53262
61282	1.08132	0.706987
61283	0.810959	0.669425
61284	0.810959	0.781762
61285	1.70303	2.1
61286	2.03975	2.1
61287	1.99324	2.1
61288	2.17615	2.1
61289	1.40549	2.1
61290	1.53446	2.1
61291	0.946133	2.1
61292	0.946133	2.1
61293	0.946129	2.1
61294	0.946129	2.1
61295	0.946131	2.1
61296	0.946131	2.1
61297	0.946131	2.1
61298	0.946131	2.1
61299	0.946131	2.1
61300	0.946131	2.1
61301	0.946131	2.1
61302	0.946131	2.1
61303	1.89226	2.1

61304	1.89226	2.1
61305	1.89226	2.1
61306	1.89226	2.1
61307	1.89226	2.1
61308	1.89226	2.1
61309	2.17615	2.1
61310	2.3111	2.1
61311	2.50288	2.1
61312	2.50288	2.1
61313	2.03975	2.1
61314	2.03975	2.1
61315	1.76401	2.1
61316	1.95852	2.1
61317	2.32134	2.1
61318	2.12306	2.1
61319	1.8513	2.1
61320	1.70303	2.1
61321	1.89226	2.1
61322	1.89226	2.1
61323	1.89226	2.1
61324	1.89226	2.1
61325	3.78452	2.1
61326	1.89226	2.1
61327	1.89226	2.1
61328	1.89226	2.1
61329	1.89226	2.1
61330	3.78452	2.1
61331	1.2243	2.1
61332	1.2243	2.1
61333	1.24189	2.1
61334	1.24189	2.1
61335	1.37811	2.1
61336	1.25144	2.1
61337	1.08807	2.1
61338	1.17949	2.1
61339	1.17949	2.1
61340	1.37811	2.1
61341	1.08807	2.1
61342	1.25144	2.1
61343	1.89226	2.1
61344	1.89226	2.1
61345	1.89226	2.1
61346	1.89226	2.1
61347	1.89226	2.1
61348	1.89226	2.1
61349	1.89227	2.1
61350	1.89227	2.1
61351	1.89227	2.1
61352	1.89227	2.1
61353	1.89227	2.1
61354	1.89227	2.1
61355	1.89224	2.1
61356	1.89224	2.1
61357	1.89224	2.1
61358	1.89224	2.1
61359	3.78447	2.1
61360	1.89224	2.1
61361	1.89224	2.1

61362	1.89224	2.1
61363	1.89224	2.1
61364	1.89224	2.1
61365	1.89224	2.1
61366	1.89224	2.1
61367	1.89224	2.1
61368	1.89224	2.1
61369	1.89224	2.1
61370	3.78448	2.1
61371	1.89224	2.1
61372	1.703 2.1	
61373	1.89224	2.1
61374	1.72991	2.1
61375	1.07643	2.1
61376	1.36657	2.1
61377	1.44472	2.1
61378	2.41233	2.1
61379	2.51628	2.1
61380	2.51628	2.1
61381	1.42174	2.1
61382	1.42174	2.1
61383	1.89226	2.1
61384	1.89226	2.1
61385	1.89226	2.1
61386	1.89226	2.1
61387	1.89226	2.1
61388	1.89226	2.1
61389	1.89226	2.1
61390	1.89226	2.1
61391	1.89226	2.1
61392	1.89226	2.1
61393	3.78452	2.1
61394	2.51628	2.1
61395	1.89226	2.1
61396	1.89226	2.1
61397	1.89226	2.1
61398	2.41234	2.1
61399	2.41234	2.1
61400	0.996254	2.1
61401	0.996254	2.1
61402	1.01153	2.1
61403	1.01153	2.1
61404	1.28542	2.1
61405	1.25814	2.1
61406	0.719184	2.1
61407	0.72236	2.1
61408	0.719183	2.1
61409	1.28542	2.1
61410	0.722359	2.1
61411	1.25814	2.1
61412	0.946126	2.1
61413	0.946126	2.1
61414	0.946131	2.1
61415	0.946131	2.1
61416	0.946127	2.1
61417	0.946127	2.1
61418	0.94613	2.1
61419	0.94613	2.1

61420	0.946128	2.1
61421	0.946128	2.1
61422	0.946128	2.1
61423	0.946128	2.1
61424	1.89226	2.1
61425	1.89226	2.1
61426	1.89226	2.1
61427	1.89226	2.1
61428	1.89226	2.1
61429	1.89226	2.1
61430	0.946132	2.1
61431	0.946132	2.1
61432	1.25814	2.1
61433	1.25814	2.1
61434	0.946131	2.1
61435	0.946131	2.1
61436	1.28543	2.1
61437	1.25814	2.1
61438	1.28543	2.1
61439	0.946132	2.1
61440	1.25814	2.1
61441	0.946132	2.1
61442	1.89226	1.47001
61443	1.89226	0.735707
61444	1.89226	1.39811
61445	1.89226	1.34573
61446	1.89226	1.07361
61447	1.89226	0.914971
61448	1.89226	1.39215
61449	2.27719	0.633205
61450	1.89226	0.770343
61451	1.89226	0.854772
61452	3.78452	0.793992
61453	1.89225	1.50336
61454	1.89225	1.03687
61455	1.89225	1.29402
61456	1.89225	1.39689
61457	3.78451	1.37683
61458	1.89226	0.590903
61459	1.89226	0.623247
61460	1.89226	1.01649
61461	1.89226	0.382893
61462	3.78452	0.615071
61463	0.946132	0.346958
61464	0.946132	0.630202
61465	0.946132	0.769415
61466	0.946132	0.497979
61467	0.946131	0.401885
61468	0.946131	0.353409
61469	0.946132	0.646867
61470	0.946132	0.37924
61471	0.946132	0.396314
61472	0.946132	0.416784
61473	0.946132	0.446214
61474	0.946132	0.358619
61475	0.946126	0.82359
61476	0.946126	0.557075
61477	0.946132	1.4653

61478	0.946132	1.12088
61479	0.946128	0.645284
61480	0.946128	0.837676
61481	0.946129	1.06202
61482	0.946129	1.3765
61483	0.946129	1.155
61484	0.946129	1.26772
61485	0.946129	1.11158
61486	0.946129	0.914539
61487	1.39858	2.1
61488	1.39858	2.1
61489	0.94613	2.1
61490	0.94613	2.1
61491	0.946132	2.1
61492	0.946132	2.1
61493	1.39858	2.1
61494	1.25002	2.1
61495	1.39858	2.1
61496	0.946131	2.1
61497	1.25002	2.1
61498	0.946131	2.1
61499	2.0815	2.1
61500	1.89226	2.1
61501	1.89226	2.1
61502	2.05298	2.1
61503	2.5593	2.1
61504	2.77667	2.1
61505	0.946133	2.1
61506	0.946133	2.1
61507	0.946132	2.1
61508	0.946132	2.1
61509	0.946132	2.1
61510	0.946132	2.1
61511	0.946133	2.1
61512	0.946133	2.1
61513	0.946132	2.1
61514	0.946132	2.1
61515	0.946132	2.1
61516	0.946132	2.1
61517	1.38908	2.1
61518	1.38908	2.1
61519	0.946132	2.1
61520	0.946132	2.1
61521	0.946132	2.1
61522	0.946132	2.1
61523	1.38908	2.1
61524	1.27965	2.1
61525	1.38908	2.1
61526	0.946132	2.1
61527	0.946133	2.1
61528	1.27965	2.1
61529	0.946133	2.1
61530	0.946133	2.1
61531	0.946132	2.1
61532	0.946132	2.1
61533	0.946132	2.1
61534	0.946132	2.1
61535	0.946133	2.1

61536	0.946133	2.1
61537	0.946132	2.1
61538	0.946132	2.1
61539	0.946133	2.1
61540	0.946133	2.1
61541	1.89227	1.24451
61542	1.89227	1.42458
61543	1.89227	1.21111
61544	1.89227	1.10964
61545	3.78453	1.24121
61546	1.33154	1.34715
61547	1.33154	1.35078
61548	0.946132	1.29903
61549	0.946132	1.24982
61550	1.19095	1.21522
61551	1.33154	1.42435
61552	0.946133	1.3723
61553	0.946133	1.37684
61554	0.946133	1.49313
61555	1.19095	1.25437
61556	0.946132	1.45018
61557	1.33154	1.33522
61558	1.0888	1.22646
61559	1.04745	1.21492
61560	0.946132	1.15268
61561	0.946132	1.1629
61562	1.04745	1.09862
61563	1.04745	1.40834
61564	0.946132	1.38963
61565	0.946132	1.10005
61566	1.04745	1.24169
61567	0.946133	1.11211
61568	0.946132	1.30201
61569	1.0888	1.22613
61570	1.89226	1.48376
61571	1.89226	1.08677
61572	1.89226	1.09518
61573	1.89226	1.23684
61574	1.89226	1.1312
61575	1.89226	1.2651
61576	1.89226	1.52322
61577	1.87592	1.13135
61578	1.87592	1.1851
61579	2.3819	1.28159
61580	1.89226	1.26218
61581	2.39825	1.35899
61582	1.89226	1.0903
61583	2.0949	1.30559
61584	1.88741	1.05583
61585	1.89226	1.05871
61586	1.89226	1.1436
61587	1.88741	1.27234
61588	1.07508	1.62723
61589	1.63419	1.90358
61590	1.63419	1.73725
61591	1.09795	1.88795
61592	1.56137	1.72942
61593	1.02513	2.0297

61594	0.967672	1.11222
61595	1.56137	1.59502
61596	1.56137	1.33659
61597	1.07508	1.53974
61598	1.41319	1.25073
61599	0.926902	1.64743
61600	1.56137	1.50659
61601	1.63419	1.67325
61602	2.29852	1.73709
61603	2.14485	1.48901
61604	3.77904	1.50018
61605	1.41319	0.515642
61606	1.92622	0.836215
61607	1.56137	0.457197
61608	1.56137	1.37903
61609	2.14485	1.39614
61610	1.92622	1.15093
61611	1.89227	2.1
61612	1.89227	2.1
61613	1.89227	2.1
61614	1.89227	2.1
61615	3.78453	2.1
61616	2.56618	2.1
61617	2.08612	2.1
61618	2.48048	2.1
61619	1.99644	2.1
61620	4.5666	2.1
61621	2.08612	2.1
61622	1.30659	2.1
61623	1.99644	2.1
61624	1.25404	2.1
61625	3.34016	2.1
61626	1.89226	2.1
61627	1.89226	2.1
61628	1.89226	2.1
61629	1.89226	2.1
61630	1.89226	2.1
61631	1.89226	2.1
61632	1.89227	2.1
61633	1.89227	2.1
61634	1.89227	2.1
61635	1.89227	2.1
61636	3.78453	2.1
61637	2.48048	2.1
61638	1.99644	2.1
61639	2.39022	2.1
61640	1.90734	2.1
61641	4.38782	2.1
61642	1.90734	2.1
61643	1.99644	2.1
61644	1.21564	2.1
61645	1.21564	2.1
61646	1.25404	2.1
61647	1.94574	2.1
61648	0.810946	2.1
61649	0.810946	2.1
61650	0.810947	2.1
61651	0.810947	2.1

61652	1.89226	2.1
61653	1.3517	2.1
61654	1.08133	2.1
61655	1.08132	2.1
61656	1.08132	2.1
61657	0.810934	2.1
61658	0.810934	2.1
61659	1.89226	2.1
61660	1.89226	1.7112
61661	1.89226	2.04686
61662	1.89226	2.1
61663	3.78453	2.1
61664	1.89226	0.599233
61665	1.89226	0.261642
61666	1.89226	1.39689
61667	1.89226	0.529663
61668	1.89226	0.86736
61669	1.89226	0.256187
61670	0.946131	0.69545
61671	0.946131	0.49211
61672	0.94613	1.10481
61673	0.94613	0.762914
61674	0.946128	0.46507
61675	0.946128	0.454196
61676	0.946133	0.312175
61677	0.946133	0.96882
61678	0.946131	0.672985
61679	0.946131	0.723776
61680	0.946131	0.85982
61681	0.946131	0.517433
61682	1.89226	2.1
61683	1.89226	1.41714
61684	1.89226	2.09799
61685	1.89226	1.93439
61686	1.89226	1.81146
61687	1.89226	1.67078
61688	2.56348	1.65624
61689	1.89226	2.1
61690	1.89226	2.1
61691	2.53234	1.83158
61692	4.45574	1.72726
61693	2.53234	0.230739
61694	1.65583	0.11988
61695	2.56348	0.155135
61696	2.56348	0.469664
61697	1.66295	0.17326
61698	1.65583	0.135593
61699	0.736602	0.23459
61700	0.827916	0.168239
61701	1.26617	0.541552
61702	1.18322	0.10945
61703	0.7366	0.206556
61704	1.18321	0.173897
61705	1.26617	0.104381
61706	0.827918	0.207179
61707	0.99929	0.112921
61708	0.99929	0.426289
61709	1.00348	0.156782

61710	1.00348	0.257977
61711	1.26617	0.501354
61712	1.18322	1.17825
61713	0.946131	2.1
61714	0.946131	1.81027
61715	0.946129	0.977467
61716	1.18321	0.712869
61717	0.946133	2.1
61718	1.26617	2.04632
61719	1.26617	2.0708
61720	1.26617	0.195783
61721	0.946131	2.1
61722	0.946131	1.69725
61723	2.23267	0.0658194
61724	1.89226	0.140523
61725	2.07099	0.107712
61726	1.89226	0.11695
61727	2.07099	0.118439
61728	1.89226	0.105465
61729	0.946132	0.184943
61730	1.35403	0.101441
61731	1.24038	0.104426
61732	0.946131	0.0946846
61733	0.946131	0.165542
61734	0.946131	0.194791
61735	1.24039	0.0990405
61736	1.35403	0.110707
61737	1.24038	0.159652
61738	1.24038	0.157265
61739	0.946131	0.10547
61740	0.946131	0.249792
61741	1.89226	0.343312
61742	1.89226	0.717828
61743	1.89226	0.264464
61744	1.89226	0.276931
61745	1.89226	0.853341
61746	1.89226	0.773824
61747	1.89226	0.197904
61748	1.89226	0.592542
61749	1.89226	0.29272
61750	1.89226	0.418297
61751	3.78452	0.498948
61752	1.56971	0.0533791
61753	2.80086	0.307859
61754	2.59806	0.0907387
61755	1.93489	0.159309
61756	2.59806	0.153525
61757	1.93489	0.129546
61758	0.827467	0.0738521
61759	1.26652	0.110912
61760	1.13679	0.0979302
61761	0.639035	0.103336
61762	1.11547	0.123152
61763	1.30656	0.210124
61764	0.869062	0.0907875
61765	0.696608	0.0693629
61766	1.07205	0.0627153
61767	0.953656	0.1348

61768	0.970744	0.119403
61769	0.970744	0.224158
61770	2.38681	2.1
61771	2.50438	2.1
61772	2.42082	2.1
61773	2.59106	2.1
61774	4.92521	2.1
61775	2.50438	1.99461
61776	2.40627	2.1
61777	2.59106	2.1
61778	2.5444	1.79465
61779	5.04879	2.1
61780	2.42082	2.1
61781	2.70575	2.1
61782	2.64778	2.1
61783	2.59106	2.1
61784	2.47753	2.1
61785	2.42082	2.1
61786	2.70575	2.1
61787	2.5444	1.92815
61788	2.76095	2.1
61789	2.59106	2.1
61790	2.71429	2.1
61791	2.5444	2.1
61792	1.89226	2.1
61793	2.38473	2.1
61794	2.36689	2.1
61795	2.38473	2.1
61796	1.87442	2.1
61797	1.89226	2.1
61798	1.89226	2.1
61799	1.89226	2.1
61800	1.89226	2.1
61801	1.89226	2.1
61802	1.89226	2.1
61803	1.89226	2.1
61804	2.14952	2.1
61805	1.89226	2.1
61806	1.89226	2.1
61807	1.89226	2.1
61808	2.0692	2.1
61809	2.0692	2.1
61810	1.89226	2.1
61811	2.36689	2.1
61812	2.36204	2.1
61813	2.36689	2.1
61814	1.88742	2.1
61815	1.89226	2.1
61816	0.946132	2.1
61817	0.946132	2.1
61818	0.946131	2.1
61819	0.946131	2.1
61820	0.946132	2.1
61821	0.946132	2.1
61822	0.946131	2.1
61823	0.946131	2.1
61824	0.946132	2.1
61825	0.946132	2.1

61826	0.946131	2.1
61827	0.946131	2.1
61828	0.946128	2.1
61829	0.946128	2.1
61830	1.07476	2.1
61831	1.07476	2.1
61832	0.946131	2.1
61833	0.946131	2.1
61834	1.13182	2.1
61835	1.07476	2.1
61836	1.13182	2.1
61837	0.946131	2.1
61838	1.07476	2.1
61839	0.94613	2.1
61840	1.89227	0.251791
61841	1.89227	0.346083
61842	1.89227	0.433901
61843	1.89227	0.312097
61844	3.78453	0.268145
61845	1.89226	0.230978
61846	1.89226	0.268122
61847	1.89226	0.205865
61848	1.89226	0.347419
61849	3.78452	0.255133
61850	1.89226	0.265705
61851	1.89226	0.402559
61852	1.89226	0.382072
61853	1.89226	0.298974
61854	3.78452	0.271336
61855	1.89226	0.340572
61856	1.89226	0.261904
61857	1.89226	0.298686
61858	1.89226	0.393989
61859	1.89226	0.351879
61860	1.89226	0.356409
61861	2.58344	0.492566
61862	2.00267	0.105847
61863	2.14218	0.351755
61864	2.22674	0.543531
61865	2.14218	0.0779109
61866	2.22674	0.201572
61867	2.83531	0.578657
61868	1.56043	0.269266
61869	2.22674	0.468414
61870	1.90912	0.598273
61871	2.22674	0.0708712
61872	1.90912	0.219011
61873	0.780217	0.387547
61874	1.11337	0.367227
61875	1.00133	0.524997
61876	0.804472	0.338804
61877	0.804472	0.301326
61878	0.780217	0.236053
61879	1.00133	0.0924579
61880	1.11337	0.447731
61881	0.856668	0.455358
61882	0.856668	0.495837
61883	0.924883	0.172906

61884	0.924883	0.311048
61885	1.93107	2.1
61886	1.68657	2.1
61887	1.7237	2.1
61888	1.89226	2.1
61889	1.53436	2.1
61890	1.68657	2.1
61891	1.89226	2.1
61892	1.89226	2.1
61893	1.89226	2.1
61894	1.89226	2.1
61895	3.78453	2.1
61896	0.946131	2.1
61897	0.946131	2.1
61898	0.855092	2.1
61899	0.83953	2.1
61900	0.946131	2.1
61901	0.946131	2.1
61902	0.839531	2.1
61903	0.839531	2.1
61904	0.946131	2.1
61905	0.855093	2.1
61906	0.839531	2.1
61907	0.946131	2.1
61908	1.89226	2.1
61909	1.70302	2.1
61910	2.21419	2.1
61911	1.93107	2.1
61912	1.97872	2.1
61913	1.70302	2.1
61914	0.946133	2.1
61915	0.946133	2.1
61916	0.946132	2.1
61917	0.946132	2.1
61918	0.946132	2.1
61919	0.946132	2.1
61920	0.946132	2.1
61921	0.946132	2.1
61922	0.946133	2.1
61923	0.946133	2.1
61924	0.946132	2.1
61925	0.946132	2.1
61926	1.90081	2.1
61927	1.89226	2.1
61928	1.51379	2.1
61929	1.36813	2.1
61930	1.71019	2.1
61931	1.89226	2.1
61932	1.89227	1.36319
61933	1.89227	1.12412
61934	1.89227	1.10896
61935	1.89227	1.34218
61936	1.89227	1.24765
61937	1.89227	1.53133
61938	1.36428	1.39632
61939	1.37869	1.38183
61940	0.946132	1.25244
61941	0.946132	1.33213

61942	1.37869	1.24411
61943	1.37869	1.68397
61944	0.946133	1.39803
61945	0.946133	1.40587
61946	1.37869	1.25884
61947	0.946132	1.29797
61948	1.36428	1.39697
61949	0.946133	1.20357
61950	0.94612	1.24489
61951	0.94612	1.26903
61952	0.946119	1.19782
61953	0.946119	1.22308
61954	0.946119	1.12429
61955	0.946119	1.56039
61956	0.94612	1.2922
61957	0.94612	1.39976
61958	0.946132	1.18382
61959	0.946132	1.14202
61960	0.946107	1.18852
61961	0.946107	1.36504
61962	1.89225	0.05
61963	1.89225	2.1
61964	1.63564	0.05
61965	1.42278	2.1
61966	1.42278	2.1
61967	1.57534	0.05
61968	1.53735	2.1
61969	1.68991	0.05
61970	1.57535	0.05
61971	1.04442	2.1
61972	1.04442	2.1
61973	1.22031	0.05
61974	1.42278	2.1
61975	1.59868	0.05
61976	0.946132	2.1
61977	0.946132	2.1
61978	0.946131	2.1
61979	0.946131	2.1
61980	0.946131	2.1
61981	0.946131	2.1
61982	0.946132	2.1
61983	0.946132	2.1
61984	0.946132	2.1
61985	0.946132	2.1
61986	0.946131	2.1
61987	0.946131	2.1
61988	1.89226	2.1
61989	1.89226	2.1
61990	1.89226	2.1
61991	1.89226	2.1
61992	1.89226	2.1
61993	1.89226	2.1
61994	2.03143	1.62326
61995	1.47111	1.98011
61996	2.28685	1.93709
61997	2.28685	1.99575
61998	1.36006	1.6657
61999	1.36006	1.70525

62000	1.89226	1.36852
62001	1.89226	1.90596
62002	1.89226	1.75136
62003	1.89226	1.85383
62004	1.89226	1.42306
62005	1.89226	1.55439
62006	1.89226	1.76341
62007	1.89226	2.1
62008	1.89226	2.1
62009	1.89226	2.1
62010	1.89226	1.81069
62011	1.89226	2.1
62012	0.902821	2.1
62013	0.902821	2.1
62014	0.889218	2.1
62015	0.889218	2.1
62016	0.675759	2.1
62017	1.05649	2.1
62018	0.735554	2.1
62019	1.14342	2.1
62020	1.14343	2.1
62021	1.05649	2.1
62022	0.735554	2.1
62023	0.675758	2.1
62024	2.46286	0.990781
62025	2.28685	1.56246
62026	2.67843	1.4531
62027	2.67843	1.48415
62028	2.03143	1.27166
62029	2.03143	1.34898
62030	1.89226	1.31213
62031	1.89226	1.47596
62032	1.89226	0.827963
62033	1.89226	0.636758
62034	3.78452	1.46593
62035	0.94613	0.641864
62036	0.94613	1.67007
62037	0.946128	2.01363
62038	0.946128	1.79076
62039	0.94613	2.1
62040	0.94613	1.71835
62041	0.946128	1.25421
62042	0.946128	1.5459
62043	0.946129	0.12241
62044	0.946129	1.74877
62045	0.946129	1.42159
62046	0.946129	2.1
62047	2.67843	1.57884
62048	2.11297	2.1
62049	2.52453	1.96425
62050	2.52453	2.1
62051	2.28685	1.65636
62052	2.28685	1.81212
62053	1.89226	1.35761
62054	1.89226	2.1
62055	1.89226	0.970875
62056	1.89226	2.1
62057	3.78451	2.1

62058	2.39524	0.828616
62059	2.10188	2.1
62060	2.29839	0.926464
62061	2.02242	2.1
62062	4.40028	2.1
62063	1.71839	2.1
62064	1.65261	2.1
62065	2.10189	2.1
62066	2.10189	2.1
62067	2.02243	2.1
62068	1.63893	2.1
62069	0.946132	2.1
62070	0.946132	2.1
62071	0.946129	2.1
62072	0.946129	2.1
62073	0.94613	2.1
62074	0.94613	2.1
62075	0.946131	2.1
62076	0.946131	2.1
62077	0.946131	2.1
62078	0.946131	2.1
62079	0.946131	2.1
62080	0.946131	2.1
62081	1.89226	0.774986
62082	1.89226	2.1
62083	1.89226	1.2173
62084	1.89226	2.1
62085	3.78452	2.07128
62086	2.2984	0.701939
62087	2.02242	2.1
62088	2.21582	1.45619
62089	1.94412	2.1
62090	4.24251	2.04665
62091	1.94412	2.1
62092	1.5854	2.1
62093	2.02243	2.1
62094	2.02243	2.1
62095	1.57431	2.1
62096	1.65261	2.1
62097	0.810943	2.1
62098	0.810943	2.1
62099	0.810945	2.1
62100	0.810945	2.1
62101	1.89226	2.1
62102	1.3517	2.1
62103	1.08133	2.1
62104	1.08132	2.1
62105	1.08132	2.1
62106	0.810933	2.1
62107	0.810933	2.1
62108	1.89226	0.0571511
62109	1.89226	0.0856342
62110	1.89226	0.0733787
62111	1.89226	0.0669314
62112	1.89226	0.069061
62113	1.89226	0.0628306
62114	1.89226	0.0732546
62115	1.89226	0.136635

62116	1.89226	0.0874563
62117	1.89226	0.119871
62118	3.78452	0.118729
62119	1.89226	0.0656689
62120	1.89226	0.100599
62121	1.89226	0.0795053
62122	1.89226	0.0715384
62123	1.89226	0.079893
62124	1.89226	0.0718342
62125	0.946128	0.11814
62126	0.946128	0.0761788
62127	0.946132	0.0795371
62128	0.946132	0.0973307
62129	0.94613	0.121525
62130	0.94613	0.131989
62131	0.946129	0.0866356
62132	0.946129	0.0774676
62133	0.94613	0.0813095
62134	0.94613	0.0975976
62135	0.94613	0.0835839
62136	0.94613	0.0979386
62137	1.89226	0.0626806
62138	1.89226	0.0920778
62139	1.89226	0.0738994
62140	1.89226	0.0714382
62141	1.89226	0.089475
62142	1.89226	0.0888776
62143	2.81478	2.1
62144	1.89226	2.1
62145	2.65557	2.1
62146	1.89226	2.1
62147	4.54783	2.1
62148	1.89226	2.1
62149	1.89226	2.1
62150	1.89226	2.1
62151	1.89226	2.1
62152	3.78452	2.1
62153	2.65557	2.1
62154	1.89226	2.1
62155	1.89226	2.1
62156	1.89226	2.1
62157	2.57997	2.1
62158	2.57997	2.1
62159	0.946131	2.1
62160	0.946131	2.1
62161	0.946128	2.1
62162	0.946128	2.1
62163	0.94613	2.1
62164	0.94613	2.1
62165	0.946129	2.1
62166	0.946129	2.1
62167	0.94613	2.1
62168	0.94613	2.1
62169	0.94613	2.1
62170	0.94613	2.1
62171	1.89226	1.4705
62172	2.27703	1.90439
62173	2.35615	1.94811

62174	2.35615	1.5824
62175	1.89226	1.9524
62176	1.89226	1.57696
62177	0.946132	1.47474
62178	1.17807	2.07389
62179	1.14926	1.44487
62180	0.946132	1.70954
62181	0.946133	1.63665
62182	0.946133	1.69981
62183	1.14926	1.47849
62184	1.14926	1.49326
62185	0.946132	1.5845
62186	0.946132	1.51719
62187	1.17807	1.64786
62188	1.14926	1.5346
62189	2.16841	1.10078
62190	1.35487	1.49336
62191	1.19854	1.37031
62192	1.19854	1.24085
62193	2.46078	1.3642
62194	2.46078	1.33399
62195	0.69308	1.00798
62196	1.3119	1.48397
62197	0.677433	1.54342
62198	1.23039	1.26977
62199	0.978178	1.53417
62200	0.978178	1.53172
62201	0.945294	1.15774
62202	0.945294	1.31449
62203	1.3119	1.42225
62204	1.23039	1.41562
62205	0.693081	1.4188
62206	0.677432	1.42522
62207	1.89226	1.40984
62208	1.89226	1.99141
62209	1.89226	1.91909
62210	1.89226	1.46048
62211	1.89226	1.92128
62212	1.89226	1.46625
62213	0.946132	1.38794
62214	0.946132	2.04598
62215	0.946132	1.21634
62216	0.946132	1.57494
62217	0.946133	1.50629
62218	0.946133	1.59658
62219	0.946132	1.39023
62220	0.946132	1.27313
62221	0.946133	1.44254
62222	0.946133	1.27432
62223	0.946132	1.46585
62224	0.946132	1.41906
62225	2.60637	2.1
62226	1.89226	2.1
62227	1.89226	2.1
62228	1.89226	1.90755
62229	3.78452	2.1
62230	1.89226	1.17612
62231	1.8396	1.82705

62232	1.89226	1.91135
62233	1.89226	1.44509
62234	1.60993	2.08454
62235	1.60993	1.53075
62236	0.946131	1.78307
62237	0.946131	1.95914
62238	0.946128	2.1
62239	0.946128	2.1
62240	0.946131	1.77253
62241	0.946131	2.02851
62242	0.946127	2.1
62243	0.946127	1.90325
62244	0.946129	2.09151
62245	0.946129	2.0773
62246	0.94613	2.09659
62247	0.94613	2.08333
62248	1.89226	2.1
62249	1.89226	1.89002
62250	1.89226	2.1
62251	1.89226	2.1
62252	1.89226	2.1
62253	1.89226	2.1
62254	1.89226	1.05414
62255	1.89226	1.74943
62256	1.89226	1.91314
62257	1.89226	1.26506
62258	1.89226	2.00727
62259	1.89226	1.17868
62260	0.946131	1.7316
62261	0.946131	1.94546
62262	0.946128	2.1
62263	0.946128	2.00752
62264	0.946131	1.71882
62265	0.946131	1.93789
62266	0.946127	1.91292
62267	0.946127	2.1
62268	0.94613	2.04268
62269	0.94613	2.03504
62270	0.946129	2.03843
62271	0.946129	2.0407
62272	0.946133	2.1
62273	0.946133	2.1
62274	1.28153	2.1
62275	1.26781	2.1
62276	0.946132	2.1
62277	0.946132	2.1
62278	1.28153	2.1
62279	1.28153	2.1
62280	0.946132	2.1
62281	1.28153	2.1
62282	0.946133	2.1
62283	1.26781	2.1
62284	1.89226	2.1
62285	1.89226	2.1
62286	1.89226	2.1
62287	1.89226	2.1
62288	1.89226	2.1
62289	1.89226	2.1

62290	2.5844	2.1
62291	1.09563	2.1
62292	1.1468	2.1
62293	1.09563	2.1
62294	2.6142	2.1
62295	2.56304	2.1
62296	0.946126	2.1
62297	0.946126	2.1
62298	1.2922	2.1
62299	1.28152	2.1
62300	0.946126	2.1
62301	0.946126	2.1
62302	1.2922	2.1
62303	1.2922	2.1
62304	1.29221	2.1
62305	0.946132	2.1
62306	0.946119	2.1
62307	1.28151	2.1
62308	1.89225	2.1
62309	1.89225	2.1
62310	1.89225	2.1
62311	1.89225	2.1
62312	1.89225	2.1
62313	1.89225	2.1
62314	0.946121	2.1
62315	0.946121	2.1
62316	0.946126	2.1
62317	0.946126	2.1
62318	0.946124	2.1
62319	0.946124	2.1
62320	0.946123	2.1
62321	0.946123	2.1
62322	0.94613	2.1
62323	0.94613	2.1
62324	0.946117	2.1
62325	0.946117	2.1
62326	1.89226	2.1
62327	1.89226	1.97887
62328	1.89226	1.84731
62329	1.89226	2.1
62330	1.89226	0.947695
62331	1.89226	2.1
62332	1.89226	2.1
62333	1.89226	2.01187
62334	1.89226	1.74532
62335	1.89226	2.1
62336	1.89226	0.914059
62337	1.89226	2.1
62338	1.89226	2.1
62339	2.81281	2.1
62340	2.50369	1.98565
62341	2.50369	2.1
62342	1.89226	2.01013
62343	1.89226	2.1
62344	1.89226	2.1
62345	1.89226	2.1
62346	1.89226	2.1
62347	1.89226	2.1

62348	1.89226	2.1
62349	1.89226	2.1
62350	1.89226	2.1
62351	1.89226	2.1
62352	1.89226	2.1
62353	1.89226	2.1
62354	1.89226	2.1
62355	1.89226	2.1
62356	0.907036	2.1
62357	1.09714	2.1
62358	0.994609	2.1
62359	0.838808	2.1
62360	0.976536	2.1
62361	0.976536	2.1
62362	0.959412	2.1
62363	0.959412	2.1
62364	1.09714	2.1
62365	0.994609	2.1
62366	0.907036	2.1
62367	0.838808	2.1
62368	0.99461	2.1
62369	0.838808	2.1
62370	0.738212	2.1
62371	0.565634	2.1
62372	0.775921	2.1
62373	0.775921	2.1
62374	0.784322	2.1
62375	0.784322	2.1
62376	0.994609	2.1
62377	0.738212	2.1
62378	0.838808	2.1
62379	0.565635	2.1
62380	1.89226	2.1
62381	1.89226	2.1
62382	1.89226	2.1
62383	1.89226	2.1
62384	1.89226	2.1
62385	1.89226	2.1
62386	1.89226	2.1
62387	1.89226	2.1
62388	1.89226	2.1
62389	1.89226	2.1
62390	1.89226	2.1
62391	1.89226	2.1
62392	2.19428	2.1
62393	1.98922	2.1
62394	2.27322	2.1
62395	2.45893	2.1
62396	4.4675	2.1
62397	0.99461	2.1
62398	1.13661	2.1
62399	0.902272	2.1
62400	0.738212	2.1
62401	0.931874	2.1
62402	0.931874	2.1
62403	0.942953	2.1
62404	0.942953	2.1
62405	1.13658	2.1

62406	0.902248	2.1
62407	0.994637	2.1
62408	0.738233	2.1
62409	1.89226	1.9979
62410	1.89226	2.1
62411	1.89226	2.1
62412	1.89226	2.0609
62413	1.89226	2.1
62414	1.89226	1.94767
62415	1.89227	2.02371
62416	2.58246	1.87643
62417	1.89227	2.06881
62418	1.89227	1.93409
62419	3.78453	1.92199
62420	1.89226	2.1
62421	1.89226	2.07631
62422	1.65573	2.1
62423	1.65573	2.1
62424	1.89226	2.1
62425	1.89226	2.1
62426	1.89226	1.76195
62427	1.89226	1.79114
62428	1.89226	1.82715
62429	1.89226	1.81094
62430	1.89226	1.77895
62431	1.89226	1.73829
62432	1.89226	1.74687
62433	1.89226	1.70318
62434	1.89226	1.69064
62435	1.89226	1.74582
62436	1.89226	1.65711
62437	1.89226	1.73147
62438	0.946132	2.08115
62439	0.946132	2.0279
62440	1.01023	1.93591
62441	1.13651	2.1
62442	1.01023	1.91451
62443	0.946133	2.03553
62444	1.0644	2.06423
62445	1.0644	2.08276
62446	0.946132	2.0644
62447	0.946132	2.07467
62448	1.13651	2.08897
62449	0.946132	2.03446
62450	1.89226	1.03609
62451	1.89226	1.63769
62452	1.89226	1.46079
62453	1.89226	0.747974
62454	3.78451	1.10407
62455	1.00934	0.434679
62456	0.741561	0.389792
62457	0.946131	0.602613
62458	0.946131	0.666239
62459	0.946131	0.980813
62460	0.741561	0.308754
62461	1.00934	0.322459
62462	0.946131	0.7358
62463	0.741586	0.466486

62464	0.741586	0.542137
62465	0.946106	0.624316
62466	0.946106	0.41691
62467	1.14895	0.93123
62468	0.957449	1.3047
62469	0.934806	1.55191
62470	0.934806	1.49803
62471	1.13536	0.685205
62472	1.02829	1.63068
62473	0.946129	1.66486
62474	0.946129	1.00897
62475	0.946127	1.45113
62476	0.946127	1.49451
62477	0.946127	1.41893
62478	1.0283	1.47244
62479	1.89226	1.58806
62480	1.89226	1.19291
62481	1.89226	1.05847
62482	1.89226	1.52576
62483	3.78452	1.47506
62484	1.89226	0.530433
62485	1.89226	0.906857
62486	1.89226	0.879924
62487	1.89226	0.473184
62488	3.78453	0.546075
62489	1.89226	0.964697
62490	2.69068	0.54104
62491	1.89226	0.495012
62492	1.89226	0.745562
62493	3.78452	0.716732
62494	1.89226	1.25438
62495	1.89226	1.56808
62496	1.89226	1.56351
62497	1.89226	1.38581
62498	1.89226	1.50902
62499	1.89226	1.22817
62500	1.89226	2.1
62501	1.89226	2.1
62502	1.89226	2.1
62503	1.89226	2.1
62504	1.89226	2.1
62505	1.89226	2.1
62506	1.89226	2.1
62507	1.89226	2.1
62508	1.89226	2.1
62509	1.89226	2.1
62510	1.89226	2.1
62511	1.89226	2.1
62512	0.985109	2.1
62513	1.31144	2.1
62514	1.45864	2.1
62515	1.10667	2.1
62516	2.44375	2.1
62517	0.655718	2.1
62518	0.729316	2.1
62519	0.684594	2.1
62520	0.782707	2.1
62521	0.700849	2.1

62522	0.700849	2.1
62523	0.71306	2.1
62524	0.71306	2.1
62525	0.729318	2.1
62526	0.782705	2.1
62527	0.684592	2.1
62528	0.655719	2.1
62529	0.946128	2.1
62530	0.946128	2.1
62531	0.946133	2.1
62532	0.946133	2.1
62533	0.946131	2.1
62534	0.946131	2.1
62535	0.946131	2.1
62536	0.946131	2.1
62537	0.94613	2.1
62538	0.94613	2.1
62539	0.946131	2.1
62540	0.946131	2.1
62541	1.89226	2.1
62542	1.89226	2.1
62543	1.89226	2.1
62544	1.89226	2.1
62545	1.89226	2.1
62546	1.89226	2.1
62547	0.553334	2.1
62548	0.629936	2.1
62549	0.828211	2.1
62550	0.729319	2.1
62551	0.674025	2.1
62552	0.674025	2.1
62553	0.68523	2.1
62554	0.68523	2.1
62555	0.828211	2.1
62556	0.629935	2.1
62557	0.72932	2.1
62558	0.553334	2.1
62559	0.828208	2.1
62560	0.729316	2.1
62561	0.907037	2.1
62562	0.782707	2.1
62563	0.799048	2.1
62564	0.799048	2.1
62565	0.811867	2.1
62566	0.811867	2.1
62567	0.828209	2.1
62568	0.907035	2.1
62569	0.729318	2.1
62570	0.782706	2.1
62571	2.32981	2.1
62572	1.89226	2.1
62573	1.89226	2.1
62574	1.89226	2.1
62575	2.2671	2.1
62576	2.2671	2.1
62577	1.89226	2.1
62578	1.89226	2.1
62579	1.89226	2.1

62580	1.89226	2.01371
62581	1.89226	2.1
62582	1.89226	2.1
62583	1.3991	2.1
62584	1.81793	2.04113
62585	2.07226	2.1
62586	2.07226	1.96159
62587	1.24924	2.1
62588	1.24924	2.1
62589	0.946131	2.1
62590	0.946131	2.1
62591	1.16491	2.1
62592	1.16491	2.1
62593	0.946131	2.1
62594	0.946131	2.1
62595	1.16491	2.1
62596	1.19523	2.1
62597	0.946132	2.1
62598	1.19523	2.1
62599	1.16491	2.1
62600	0.946131	2.1
62601	1.89226	2.1
62602	1.89226	2.09013
62603	1.89226	2.1
62604	1.89226	1.74849
62605	3.78452	2.1
62606	0.901204	2.1
62607	0.901204	2.1
62608	0.902504	2.1
62609	0.902504	2.1
62610	1.10416	2.02082
62611	1.03613	1.86498
62612	0.764922	2.1
62613	0.699552	2.1
62614	0.764921	2.1
62615	1.10416	2.1
62616	0.699551	2.1
62617	1.03613	2.1
62618	1.89226	1.42307
62619	1.89226	1.5324
62620	1.89226	1.51412
62621	1.89226	1.47237
62622	3.78453	1.53572
62623	1.89226	1.54588
62624	1.89226	1.58646
62625	1.89226	1.55423
62626	1.89226	1.62471
62627	1.89226	1.55991
62628	1.89226	1.62743
62629	0.946131	1.50326
62630	0.946131	1.50312
62631	0.946131	1.44908
62632	0.946131	1.44255
62633	0.946131	1.44923
62634	0.946131	1.50826
62635	0.946131	1.50328
62636	0.946131	1.40324
62637	0.946131	1.42301

62638	0.946131	1.42154
62639	0.946131	1.35593
62640	0.946131	1.43826
62641	1.89226	1.5222
62642	1.89226	1.54694
62643	1.89226	1.52694
62644	1.89226	1.56672
62645	1.89226	1.5235
62646	1.89226	1.56188
62647	0.946127	1.54253
62648	0.946127	1.52688
62649	1.22788	1.5358
62650	1.35469	1.59556
62651	1.35469	1.57613
62652	1.35469	1.60045
62653	0.946128	1.52819
62654	0.946128	1.50805
62655	0.946129	1.53704
62656	1.22788	1.56731
62657	1.35469	1.55259
62658	0.946129	1.51597
62659	1.22788	1.53583
62660	1.35469	1.59557
62661	1.02602	1.45176
62662	0.794861	1.50664
62663	1.15301	1.56936
62664	1.15301	1.59119
62665	1.10089	1.43066
62666	1.10089	1.52007
62667	0.794861	1.44962
62668	1.22788	1.54665
62669	1.02602	1.57036
62670	1.35469	1.55952
62671	1.89226	0.278799
62672	1.89226	1.10164
62673	1.89226	1.50673
62674	1.89226	0.461059
62675	3.78452	0.656946
62676	1.13536	2.1
62677	0.942524	1.12231
62678	0.946131	1.24281
62679	0.946131	0.403418
62680	0.946127	1.26382
62681	0.946127	2.1
62682	1.26282	0.434352
62683	0.917471	1.9935
62684	1.21023	1.60898
62685	1.00851	1.56372
62686	1.17955	1.30655
62687	0.883746	1.29907
62688	1.89226	1.18443
62689	1.89226	2.1
62690	1.89226	2.1
62691	1.89226	2.1
62692	1.89226	2.1
62693	1.89226	2.1
62694	1.89226	0.56685
62695	1.89226	2.1

62696	1.89226	1.19062
62697	1.89226	1.12758
62698	1.89226	1.23047
62699	1.89226	1.8275
62700	0.94613	1.54392
62701	0.94613	0.56763
62702	0.94613	0.103052
62703	0.94613	0.650004
62704	0.946128	0.575838
62705	0.946128	1.74399
62706	0.946131	1.57836
62707	0.946131	0.0565734
62708	0.94613	1.13233
62709	0.94613	1.16984
62710	0.94613	0.727129
62711	0.94613	0.468187
62712	0.883758	2.1
62713	1.03041	1.87001
62714	0.946131	1.2325
62715	0.946131	0.577449
62716	0.851537	1.4318
62717	0.851537	2.1
62718	1.23942	0.674138
62719	1.14906	1.9857
62720	0.851484	1.52434
62721	0.985518	1.15949
62722	1.0156	1.37791
62723	0.851526	1.3083
62724	1.89226	1.29548
62725	1.89226	2.1
62726	1.89226	2.1
62727	1.89226	2.1
62728	1.89226	2.1
62729	1.89226	2.1
62730	1.89226	0.673263
62731	1.89226	2.1
62732	1.89226	1.42848
62733	1.89226	1.61112
62734	1.89226	1.13222
62735	1.89226	1.81654
62736	1.89227	0.241454
62737	1.89227	0.314933
62738	1.89227	0.263038
62739	1.89227	0.259945
62740	3.78453	0.268401
62741	1.89226	0.338468
62742	1.89226	0.272364
62743	1.89226	0.30503
62744	1.89226	0.368494
62745	3.78452	0.362888
62746	0.946132	0.268596
62747	0.946132	0.314032
62748	0.946128	0.352608
62749	0.946128	0.252728
62750	0.94613	0.333452
62751	0.94613	0.243811
62752	0.94613	0.264941
62753	0.94613	0.345493

62754	0.946131	0.281509
62755	0.946131	0.305601
62756	0.94613	0.263026
62757	0.94613	0.273339
62758	1.89226	0.265071
62759	1.89226	0.291716
62760	1.89226	0.265525
62761	1.89226	0.323169
62762	1.89226	0.252719
62763	1.89226	0.32258
62764	1.98874	0.105951
62765	2.33455	0.175718
62766	2.01865	0.268132
62767	2.07024	0.169636
62768	4.32329	0.257267
62769	1.98874	0.16523
62770	2.00266	0.136615
62771	2.14218	0.392642
62772	2.07024	0.350141
62773	3.9914	0.134033
62774	1.00133	0.145717
62775	0.804471	0.0806762
62776	1.03512	0.223617
62777	0.995176	0.223503
62778	0.80447	0.491589
62779	0.995178	0.207283
62780	1.00133	0.275163
62781	1.03512	0.438176
62782	0.880587	0.434351
62783	0.880587	0.0844243
62784	0.959002	0.0972241
62785	0.959002	0.247807
62786	2.33455	0.164131
62787	1.99036	0.176063
62788	2.07024	0.184047
62789	2.58645	0.14012
62790	4.32491	0.0569566
62791	1.89226	1.02702
62792	1.89226	0.739664
62793	1.89226	1.04121
62794	1.89226	0.468398
62795	3.78453	0.516934
62796	1.89226	1.28797
62797	1.89226	0.975035
62798	1.89226	1.24238
62799	1.89226	1.14337
62800	3.78453	1.27947
62801	1.89226	0.548976
62802	1.89226	0.378545
62803	1.89226	0.377263
62804	1.89226	0.489934
62805	1.89226	0.393866
62806	1.89226	0.527083
62807	1.89226	0.418443
62808	2.1523	0.186994
62809	2.53257	0.206474
62810	2.53257	0.199942
62811	1.89226	0.47412

62812	1.89226	0.434476
62813	0.946133	0.457335
62814	0.946133	0.597049
62815	0.946132	0.328847
62816	0.946132	0.372016
62817	0.946132	0.319817
62818	0.946132	0.349402
62819	0.946132	0.636479
62820	0.946132	0.357146
62821	0.946133	0.454552
62822	0.946133	0.484518
62823	0.946132	0.597066
62824	0.946132	0.437289
62825	0.946132	0.773201
62826	0.946132	1.10372
62827	0.946132	0.616022
62828	0.946132	0.882946
62829	0.946132	0.593812
62830	0.946132	0.654637
62831	0.946132	1.14781
62832	0.946132	0.916362
62833	0.946132	0.834835
62834	0.946132	0.783988
62835	0.946131	0.951697
62836	0.946131	0.843012
62837	1.62772	0.0853625
62838	1.70303	0.0835757
62839	1.70303	0.118957
62840	1.70303	0.115088
62841	1.90116	0.086513
62842	1.90116	0.0801224
62843	1.89226	0.0831543
62844	1.89226	0.091028
62845	1.89226	0.373509
62846	1.89226	0.45021
62847	1.89226	0.0875742
62848	1.89226	0.0836978
62849	1.89226	0.133992
62850	1.89226	0.122263
62851	1.89226	0.0968933
62852	1.89226	0.556151
62853	3.78452	0.112319
62854	1.89226	0.206394
62855	1.89226	0.16237
62856	1.89226	0.0965541
62857	1.89226	0.151814
62858	1.89226	0.138828
62859	1.89226	0.365675
62860	0.946132	0.0714137
62861	0.946132	0.0765139
62862	0.946129	0.074531
62863	0.946129	0.0953145
62864	0.94613	0.0746425
62865	0.94613	0.076163
62866	0.946131	0.0859387
62867	0.946131	0.0825878
62868	0.94613	0.557293
62869	0.94613	0.087922

62870	0.946131	0.084157
62871	0.946131	0.585487
62872	1.89226	0.0854114
62873	1.89226	0.101946
62874	1.89226	0.631827
62875	1.89226	0.405288
62876	1.89226	0.0777279
62877	1.89226	0.0722809
62878	1.89226	0.267498
62879	1.89226	1.12847
62880	1.89226	1.16444
62881	1.89226	0.226541
62882	3.78452	0.46273
62883	1.89226	0.826468
62884	1.89226	1.31044
62885	1.89226	0.504216
62886	1.89226	1.45356
62887	1.89226	0.484775
62888	1.89226	1.47186
62889	1.30165	1.02683
62890	0.946127	1.25657
62891	1.09659	0.579512
62892	0.946131	0.657818
62893	1.09659	0.678403
62894	1.09659	1.19309
62895	0.946128	1.8363
62896	0.946128	0.702198
62897	1.30165	0.766569
62898	1.09659	1.00091
62899	0.946129	1.01718
62900	0.946129	1.4637
62901	0.946128	1.01525
62902	0.946128	1.19444
62903	0.946133	0.39067
62904	0.946133	0.653792
62905	0.946131	0.300657
62906	0.946131	1.08412
62907	0.946131	1.17552
62908	0.946131	0.489134
62909	0.94613	0.316377
62910	0.94613	0.383817
62911	0.946131	0.628649
62912	0.946131	0.840844
62913	0.946133	1.35747
62914	0.946133	1.40141
62915	0.946132	0.693658
62916	0.946132	1.06751
62917	0.946132	0.50299
62918	0.946132	1.3354
62919	0.946132	1.44274
62920	0.946132	1.01968
62921	0.946132	1.14307
62922	0.946132	0.918412
62923	0.946133	1.24722
62924	0.946133	1.17863
62925	1.30165	1.2481
62926	0.972228	1.30052
62927	1.09659	0.720012

62928	0.656268	0.620131
62929	1.06217	0.491981
62930	1.06217	1.44274
62931	1.00665	0.874951
62932	1.00665	1.62126
62933	0.97223	0.784277
62934	0.656267	0.80013
62935	1.30165	0.997662
62936	1.09659	1.13571
62937	0.946132	2.00539
62938	0.946132	2.1
62939	1.19895	2.1
62940	1.19895	2.1
62941	0.94613	2.1
62942	0.94613	1.8796
62943	1.39356	2.1
62944	1.19895	2.1
62945	0.94613	2.1
62946	1.39356	2.1
62947	1.19895	2.1
62948	0.946131	2.1
62949	1.89226	1.96161
62950	1.89226	2.1
62951	1.89226	2.1
62952	1.89226	1.9675
62953	1.89226	2.1
62954	1.89226	1.94504
62955	0.946132	1.82992
62956	0.946132	1.87952
62957	0.946128	2.1
62958	0.946128	2.1
62959	0.94613	2.1
62960	0.94613	1.71755
62961	0.94613	1.90865
62962	0.94613	2.1
62963	0.946131	1.97202
62964	0.946131	1.96579
62965	0.94613	2.0717
62966	0.94613	2.02851
62967	1.89226	0.0623896
62968	1.89226	0.0718099
62969	1.89226	0.08251
62970	1.89226	0.0573987
62971	1.89226	0.0829601
62972	1.89226	0.0543423
62973	1.29881	0.05
62974	1.09985	0.05
62975	0.946126	0.0536727
62976	0.946126	0.05
62977	0.851539	0.0535848
62978	1.20421	0.0517727
62979	0.851483	0.05
62980	0.999426	0.05
62981	1.47172	0.0500382
62982	0.999413	0.05
62983	0.851552	0.05
62984	0.851552	0.05
62985	1.89226	0.079365

62986	1.89226	0.0818992
62987	1.89226	0.136691
62988	1.89226	0.0701206
62989	1.89226	0.130671
62990	1.89226	0.0709058
62991	1.89226	0.0665483
62992	1.89226	0.0713485
62993	1.89226	0.0967611
62994	1.89226	0.0659306
62995	1.89226	0.0925324
62996	1.89226	0.0581116
62997	1.33915	2.1
62998	1.99297	2.1
62999	2.05216	2.1
63000	2.60163	2.1
63001	4.04513	2.1
63002	1.89226	2.1
63003	1.89226	2.1
63004	1.89226	2.1
63005	1.89226	2.1
63006	3.78452	2.1
63007	0.946132	2.1
63008	0.946132	2.1
63009	0.946133	2.1
63010	0.946133	2.1
63011	0.946132	2.1
63012	0.946132	2.06709
63013	0.946133	2.1
63014	0.946133	2.1
63015	0.946132	2.1
63016	0.946132	2.1
63017	0.946132	2.1
63018	0.946132	2.1
63019	1.89226	2.1
63020	1.89226	1.88346
63021	1.89226	2.02438
63022	1.89226	2.1
63023	3.78452	2.07432
63024	1.89227	1.91947
63025	1.89227	2.1
63026	1.89227	2.1
63027	1.89227	1.91228
63028	1.89227	2.1
63029	1.89227	2.0119
63030	2.29301	2.1
63031	1.70303	2.1
63032	2.46749	2.1
63033	2.30728	2.1
63034	4.53937	2.1
63035	1.89226	2.05527
63036	1.89226	2.1
63037	1.89226	2.1
63038	1.89226	1.88922
63039	3.78452	2.1
63040	1.89226	1.72069
63041	1.89226	2.1
63042	1.89226	2.1
63043	1.89226	2.08458

63044	1.89226	2.01657
63045	1.89226	1.7357
63046	2.30728	2.1
63047	1.00757	2.1
63048	2.0833	2.1
63049	1.65318	2.1
63050	1.417	2.1
63051	1.00757	2.1
63052	2.46749	2.1
63053	2.0975	2.1
63054	2.79653	2.1
63055	2.44785	2.1
63056	4.91534	2.1
63057	1.89226	2.1
63058	1.89226	2.1
63059	1.89226	2.1
63060	1.89226	2.1
63061	3.78452	2.1
63062	1.89226	1.92612
63063	1.89226	2.1
63064	1.89226	2.1
63065	1.89226	1.97068
63066	1.89226	2.1
63067	1.89226	1.91625
63068	0.970559	2.1
63069	0.970559	2.1
63070	0.979305	2.1
63071	0.979305	2.1
63072	0.708502	2.1
63073	0.901117	2.1
63074	1.04875	2.1
63075	1.22392	2.1
63076	1.22392	2.1
63077	0.901117	2.1
63078	1.04875	2.1
63079	0.7085	2.1
63080	1.89226	1.82596
63081	1.89226	1.53763
63082	1.89226	1.72189
63083	1.89226	1.70019
63084	3.78452	1.8135
63085	1.89226	1.77085
63086	1.89226	1.55588
63087	1.89226	1.86916
63088	1.89226	1.64147
63089	3.78452	1.66618
63090	1.89226	0.822977
63091	1.89226	0.469488
63092	1.89226	0.959236
63093	1.89226	0.634398
63094	3.78453	0.729888
63095	1.89226	1.36445
63096	1.89226	1.36237
63097	1.89226	0.995403
63098	1.89226	0.846882
63099	1.89226	0.910871
63100	1.89226	0.667547
63101	1.89226	1.45831

63102	2.35441	1.44551
63103	1.89226	1.49407
63104	2.30931	0.568724
63105	4.24667	1.16866
63106	1.11612	0.779404
63107	0.946131	0.824313
63108	1.1772	0.8103
63109	0.946131	0.922674
63110	1.17721	0.871221
63111	1.11612	0.829028
63112	0.94613	0.863783
63113	0.94613	1.32354
63114	1.11612	0.820866
63115	1.11612	0.697424
63116	0.946131	1.09149
63117	0.946131	0.777367
63118	1.11612	0.192828
63119	0.582675	0.15635
63120	1.17721	0.156197
63121	0.651969	0.238364
63122	0.651969	0.222601
63123	0.582674	0.18524
63124	1.17721	0.162236
63125	1.11612	0.24836
63126	0.877955	0.148463
63127	0.877955	0.166518
63128	0.881925	0.2596
63129	0.881925	0.214818
63130	1.17721	0.291884
63131	0.651969	0.35964
63132	1.15466	0.263081
63133	0.623642	0.24597
63134	0.623642	0.308465
63135	0.651969	0.184205
63136	1.17721	0.401737
63137	1.15466	0.23623
63138	0.904714	0.246972
63139	0.904714	0.185956
63140	0.901914	0.459736
63141	0.901914	0.285002
63142	1.89226	1.28517
63143	1.89226	0.543843
63144	1.89226	1.55428
63145	1.89226	1.41901
63146	1.89226	0.622313
63147	1.89226	1.52878
63148	1.89226	1.70707
63149	1.89226	0.345854
63150	1.89226	0.552258
63151	1.89226	1.96632
63152	1.89226	0.352658
63153	1.89226	2.06586
63154	2.33986	1.90569
63155	1.97819	0.761816
63156	2.39166	0.265399
63157	2.70141	2.06861
63158	4.73151	0.750526
63159	0.989095	1.37916

63160	1.19583	0.435803
63161	0.851194	0.185824
63162	0.581197	0.236509
63163	0.872704	0.116507
63164	0.872704	1.54935
63165	0.904322	0.34489
63166	0.904322	1.2606
63167	1.19583	0.0575478
63168	0.851193	0.205594
63169	0.989095	1.21129
63170	0.581197	0.33925
63171	1.89226	1.24875
63172	1.89226	1.41459
63173	1.89226	1.68556
63174	1.89226	1.4199
63175	1.89226	1.55758
63176	1.89226	1.52851
63177	1.89226	1.64063
63178	1.89226	0.350125
63179	1.89226	1.18452
63180	1.89226	1.84252
63181	1.89226	0.422925
63182	1.89226	2.01374
63183	2.39166	1.08395
63184	1.70239	1.28703
63185	2.13665	0.356817
63186	2.82684	0.475105
63187	4.52831	1.1279
63188	1.89226	0.0694306
63189	1.89226	0.136379
63190	1.89226	0.10495
63191	1.89226	0.117443
63192	3.78453	0.140136
63193	1.89226	0.0643675
63194	1.89226	0.0692458
63195	1.89226	0.0860064
63196	1.89226	0.061329
63197	1.89226	0.0906162
63198	1.89226	0.0510847
63199	1.89226	0.100407
63200	1.89226	0.112443
63201	1.89226	0.15956
63202	1.89226	0.0868186
63203	1.89226	0.159767
63204	1.89226	0.0750045
63205	1.89226	0.197981
63206	1.89226	0.21623
63207	1.89226	0.399672
63208	1.89226	0.142535
63209	1.89226	0.434054
63210	1.89226	0.133964
63211	1.89226	0.125884
63212	1.89226	0.140544
63213	1.89226	0.241746
63214	1.89226	0.1129
63215	1.89226	0.264493
63216	1.89226	0.0885464
63217	1.5169	0.147839

63218	1.61969	0.082856
63219	1.17262	0.0867509
63220	1.17262	0.139314
63221	1.88868	0.092633
63222	1.88868	0.137483
63223	1.79981	0.228726
63224	1.88868	0.144716
63225	1.5169	0.149926
63226	1.5169	0.21455
63227	2.12891	0.163013
63228	2.12891	0.221542
63229	1.89226	0.204836
63230	1.89226	0.134376
63231	1.89226	0.139411
63232	1.89226	0.211808
63233	3.78452	0.187107
63234	0.946132	0.121931
63235	0.946132	0.136993
63236	0.946132	0.087488
63237	0.946132	0.146789
63238	0.946132	0.0802269
63239	0.946132	0.194775
63240	0.946133	0.088426
63241	0.946133	0.154171
63242	0.946133	0.0965
63243	0.946133	0.113372
63244	0.946132	0.1389
63245	0.946132	0.105811
63246	1.88868	0.143597
63247	2.05759	0.0727593
63248	1.61969	0.0788496
63249	1.61969	0.181558
63250	2.29747	0.0852733
63251	2.29747	0.12922
63252	2.12891	0.210199
63253	2.29747	0.120324
63254	1.88868	0.12512
63255	1.88868	0.171197
63256	2.50894	0.139346
63257	2.50894	0.181669
63258	1.89226	0.11297
63259	1.89226	0.191748
63260	1.89226	0.110199
63261	1.89226	0.164973
63262	1.89226	0.11482
63263	1.89226	0.182599
63264	0.946132	0.120733
63265	0.946132	0.111251
63266	0.946132	0.132857
63267	0.946132	0.0956782
63268	0.946131	0.0731019
63269	0.946131	0.23636
63270	0.946132	0.0772473
63271	0.946132	0.162422
63272	0.946132	0.0873592
63273	0.946132	0.111619
63274	0.946132	0.099233
63275	0.946132	0.17209

63276	2.42758	2.1
63277	2.14895	2.1
63278	2.56975	2.0814
63279	2.28142	2.1
63280	4.71869	2.1
63281	1.89226	2.1
63282	1.89226	2.1
63283	1.89226	2.08897
63284	1.89226	2.1
63285	3.78452	2.1
63286	1.89226	2.1
63287	1.89226	2.1
63288	1.89226	2.1
63289	1.89226	2.1
63290	1.89226	2.1
63291	1.89226	2.1
63292	0.960916	2.1
63293	0.960916	2.1
63294	0.978118	2.1
63295	0.875674	2.1
63296	0.775661	2.1
63297	0.773613	2.1
63298	1.07631	2.1
63299	1.14266	2.1
63300	1.16927	2.1
63301	0.898612	2.1
63302	1.04757	2.1
63303	0.757536	2.1
63304	2.56975	2.02654
63305	2.28142	2.1
63306	2.64384	2.02189
63307	2.40621	2.1
63308	4.97596	2.1
63309	1.89226	2.04279
63310	1.89226	2.1
63311	1.89226	1.68377
63312	1.89226	2.1
63313	3.78452	2.1
63314	1.89226	2.1
63315	1.89226	2.1
63316	1.89226	2.1
63317	1.89226	2.1
63318	1.89226	2.1
63319	1.89226	2.1
63320	1.03328	2.1
63321	1.03328	2.1
63322	1.04321	2.1
63323	1.15424	2.1
63324	0.936183	2.1
63325	0.944491	2.1
63326	1.14266	2.1
63327	1.20517	2.1
63328	1.23323	2.1
63329	0.961268	2.1
63330	1.11215	2.1
63331	0.827247	2.1
63332	1.89226	2.1
63333	1.89226	2.1

63334	1.89226	2.1
63335	1.89226	2.1
63336	3.78453	2.1
63337	2.14647	2.1
63338	2.20589	2.1
63339	1.95567	2.1
63340	2.03224	2.1
63341	4.17871	2.1
63342	2.06035	2.1
63343	1.88452	1.96181
63344	1.88452	2.1
63345	2.26997	2.1
63346	2.03224	2.1
63347	2.42272	2.1
63348	1.89226	2.1
63349	1.89226	1.7533
63350	1.89226	1.24347
63351	1.89226	2.1
63352	1.89226	1.4878
63353	1.89226	2.1
63354	1.89225	2.1
63355	1.89225	2.1
63356	1.89225	2.1
63357	1.89225	2.1
63358	1.89225	2.1
63359	1.89225	2.1
63360	0.952418	2.1
63361	0.952418	2.1
63362	0.95883	2.1
63363	0.95883	2.1
63364	0.946182	2.1
63365	1.01611	2.1
63366	0.895134	2.1
63367	0.977828	2.1
63368	0.89514	2.1
63369	0.946189	2.1
63370	0.977821	2.1
63371	1.01611	2.1
63372	2.03223	2.1
63373	1.88451	2.01161
63374	1.89236	2.1
63375	1.74649	2.08218
63376	3.77687	2.1
63377	0.81094	2.1
63378	0.81094	2.0399
63379	0.810939	2.1
63380	0.810939	2.1
63381	0.810945	2.1
63382	0.810945	2.1
63383	1.89225	1.96507
63384	1.35167	2.1
63385	1.08131	2.1
63386	1.08131	1.84636
63387	1.0813	2.1
63388	1.89226	0.05
63389	1.89226	0.0590462
63390	1.89226	0.0552334
63391	1.89226	0.0557501

63392	1.89226	0.0511643
63393	1.89226	0.0510436
63394	1.01842	0.05
63395	0.936656	0.05
63396	0.946131	0.05
63397	0.946131	0.05
63398	1.11157	0.05
63399	1.04281	0.05
63400	0.925803	0.05
63401	0.925803	0.05
63402	0.851553	0.05
63403	1.11158	0.05
63404	1.05017	0.05
63405	0.925798	0.05
63406	1.89226	0.0513749
63407	1.89226	0.0634178
63408	1.89226	0.0629252
63409	1.89226	0.0603462
63410	1.89226	0.057987
63411	1.89226	0.0534148
63412	1.23504	0.05
63413	1.23504	0.05
63414	0.946131	0.05
63415	0.946131	0.05
63416	1.37358	0.05
63417	1.23504	0.05
63418	0.946129	0.05
63419	0.946129	0.05
63420	1.37358	0.05
63421	0.946131	0.05
63422	0.94613	0.05
63423	1.23504	0.05
63424	1.00261	0.05
63425	1.00261	0.05
63426	1.02952	0.05
63427	1.02952	0.05
63428	0.850964	0.05
63429	0.658553	0.05
63430	1.37358	0.05
63431	1.23504	0.05
63432	1.37358	0.05
63433	0.850965	0.05
63434	1.23504	0.05
63435	0.658553	0.05
63436	0.946133	0.0522404
63437	0.946133	0.05
63438	0.946132	0.0648551
63439	0.946132	0.053604
63440	0.946132	0.0538243
63441	0.946132	0.0545711
63442	0.946133	0.0543145
63443	0.946133	0.0528985
63444	0.946133	0.0540949
63445	0.946133	0.054804
63446	0.946132	0.0570539
63447	0.946132	0.0541486
63448	1.89226	0.0752588
63449	1.89226	0.0632885

63450	1.89226	0.0525802
63451	1.89226	0.0567599
63452	3.78452	0.0659589
63453	1.89226	0.0686627
63454	1.89226	0.0826386
63455	1.89226	0.0578407
63456	1.89226	0.0576544
63457	3.78452	0.0570096
63458	1.89227	0.0878782
63459	1.89227	0.0814692
63460	1.89227	0.0609831
63461	1.89227	0.090157
63462	1.89227	0.0618021
63463	1.89227	0.0654353
63464	0.94613	0.05
63465	0.94613	0.0502235
63466	0.946127	0.05
63467	0.946127	0.0542872
63468	0.94613	0.05
63469	0.94613	0.05
63470	0.946127	0.05
63471	0.946127	0.0514486
63472	0.946128	0.05
63473	0.946128	0.05
63474	0.946128	0.05
63475	0.946128	0.0550487
63476	0.946132	0.05
63477	0.946132	0.0573388
63478	0.946128	0.05
63479	0.946128	0.0647562
63480	0.94613	0.0523829
63481	0.94613	0.053431
63482	0.946131	0.0555846
63483	0.946131	0.0580176
63484	0.94613	0.05
63485	0.94613	0.05
63486	0.94613	0.0656705
63487	0.94613	0.0559813
63488	1.89226	2.1
63489	1.89226	2.1
63490	1.89226	2.1
63491	1.89226	2.1
63492	3.78453	2.1
63493	2.72844	2.1
63494	1.89226	2.1
63495	2.26536	2.1
63496	2.26536	2.1
63497	1.89226	2.1
63498	1.89226	2.1
63499	1.89226	2.1
63500	1.89226	2.09638
63501	1.89226	2.1
63502	1.89226	2.1
63503	1.89226	2.1
63504	1.89226	2.1
63505	1.89226	2.1
63506	1.89226	2.1
63507	1.89226	2.1

63508	1.89226	2.1
63509	1.89226	2.1
63510	1.89226	2.1
63511	2.72844	2.1
63512	2.26536	2.1
63513	2.28367	2.1
63514	1.5791	2.1
63515	4.54903	2.1
63516	0.810945	2.1
63517	0.810945	2.1
63518	0.810943	2.1
63519	0.810943	2.1
63520	1.89226	2.1
63521	1.3517	2.1
63522	1.08133	2.1
63523	1.08132	2.1
63524	1.08132	2.1
63525	0.810933	2.1
63526	0.810933	2.1
63527	1.89227	2.1
63528	1.89227	2.1
63529	1.89227	2.1
63530	1.89227	2.1
63531	3.78453	2.1
63532	2.71897	2.1
63533	2.27495	2.1
63534	2.648	2.1
63535	2.20832	2.1
63536	4.92729	2.1
63537	2.27495	2.1
63538	1.38455	2.1
63539	1.38455	2.1
63540	1.46586	2.1
63541	2.20831	2.1
63542	2.28962	2.1
63543	0.946133	2.1
63544	0.946133	2.1
63545	0.946132	2.1
63546	0.946132	2.1
63547	0.946132	2.1
63548	0.946132	2.1
63549	0.946132	2.1
63550	0.946132	2.1
63551	0.946132	2.1
63552	0.946132	2.1
63553	0.946133	2.1
63554	0.946133	2.1
63555	1.89226	2.1
63556	1.89226	2.1
63557	1.89226	2.1
63558	1.89226	2.1
63559	1.89226	2.1
63560	1.89226	2.1
63561	2.648	2.1
63562	2.08612	2.1
63563	2.08612	2.1
63564	2.20831	2.1
63565	2.56618	2.1

63566	2.68837	2.1
63567	2.08612	2.1
63568	2.20831	2.1
63569	1.30659	2.1
63570	1.30659	2.1
63571	1.38455	2.1
63572	2.16408	2.1
63573	0.810946	2.1
63574	0.810946	2.1
63575	0.810946	2.1
63576	0.810946	2.1
63577	1.89226	2.1
63578	1.3517	2.1
63579	1.08133	2.1
63580	1.08132	2.1
63581	1.08132	2.1
63582	0.810934	2.1
63583	0.810934	2.1
63584	1.89226	1.94527
63585	1.89226	1.66364
63586	1.89226	1.60023
63587	1.89226	1.94612
63588	3.78452	1.71452
63589	1.89226	1.83173
63590	1.89226	2.1
63591	1.89226	2.1
63592	1.89226	1.80922
63593	3.78453	2.07264
63594	1.08733	2.1
63595	0.856615	2.1
63596	1.25964	1.81872
63597	1.12378	1.78608
63598	1.23551	1.78676
63599	0.969894	2.1
63600	0.985977	2.09582
63601	0.985977	1.79075
63602	1.16169	1.86828
63603	1.21072	1.87159
63604	0.765175	1.98384
63605	0.863419	1.97572
63606	2.39934	1.92846
63607	2.44427	1.6138
63608	2.64003	1.56988
63609	2.39077	1.8758
63610	2.39934	1.73951
63611	2.10194	2.01327
63612	1.89226	1.58631
63613	1.89226	1.99359
63614	1.89226	1.85862
63615	1.89226	1.49204
63616	3.78452	1.76995
63617	0.946132	2.08681
63618	0.946132	2.05477
63619	0.946131	1.72879
63620	0.946131	1.80815
63621	0.946132	1.74194
63622	0.946132	2.1
63623	0.946132	2.09264

63624	0.946132	1.84812
63625	0.946132	1.9096
63626	0.946132	1.90973
63627	0.946132	1.92355
63628	0.946132	1.92544
63629	1.19967	1.91587
63630	0.946128	1.84824
63631	0.946133	1.53735
63632	1.32002	1.56368
63633	1.19967	1.76367
63634	1.19967	1.6351
63635	0.94613	1.91485
63636	0.94613	1.33557
63637	0.946131	1.61977
63638	0.946131	1.64589
63639	1.19967	1.71581
63640	1.32001	1.67323
63641	1.89226	1.89983
63642	1.89226	2.1
63643	1.89226	1.11396
63644	1.89226	2.1
63645	3.78451	2.1
63646	2.6402	0.940978
63647	2.28552	2.1
63648	2.50958	1.00907
63649	2.18948	2.1
63650	4.79509	2.1
63651	2.28552	2.1
63652	1.86102	2.1
63653	2.18949	2.1
63654	1.78376	2.1
63655	4.06928	2.1
63656	1.89226	2.1
63657	1.89226	2.1
63658	1.89226	2.1
63659	1.89226	2.1
63660	3.78452	2.1
63661	1.89226	1.0219
63662	1.89226	2.1
63663	1.89226	1.11889
63664	1.89226	2.1
63665	3.78452	2.1
63666	2.50958	0.91838
63667	2.18948	2.1
63668	2.39524	1.01556
63669	2.10188	2.1
63670	4.61146	2.08108
63671	2.18949	2.1
63672	1.78376	2.1
63673	1.71839	2.1
63674	1.71839	2.1
63675	2.10189	2.1
63676	2.16726	2.1
63677	0.810943	2.1
63678	0.810943	2.1
63679	0.810945	2.1
63680	0.810945	2.1
63681	0.810959	2.1

63682	0.810959	2.1
63683	1.89226	2.1
63684	1.35168	2.1
63685	1.08132	2.1
63686	1.08132	2.1
63687	1.0813	2.1
63688	1.89226	1.21506
63689	1.89226	1.53924
63690	1.89226	1.45897
63691	1.89226	1.32505
63692	1.89226	1.44467
63693	1.89226	1.33297
63694	1.28421	1.38125
63695	0.992041	1.27592
63696	1.07707	1.72228
63697	0.789746	1.54407
63698	1.03815	1.66035
63699	1.03815	1.4274
63700	1.03581	1.55823
63701	1.03581	1.3623
63702	1.28421	1.51109
63703	1.07707	1.53986
63704	0.789748	1.45194
63705	0.992038	1.44943
63706	1.89226	1.36561
63707	1.89226	1.63253
63708	1.89226	1.58071
63709	1.89226	1.43193
63710	1.89226	1.57897
63711	1.89226	1.43808
63712	1.89226	1.18045
63713	1.89226	1.46416
63714	1.89226	1.38361
63715	1.89226	1.271
63716	1.89226	1.37863
63717	1.89226	1.27241
63718	0.92236	1.71677
63719	0.655971	1.64905
63720	0.916772	1.69982
63721	0.913834	1.51833
63722	1.18074	1.63154
63723	0.922346	1.65563
63724	2.15414	1.42914
63725	1.53528	1.59116
63726	1.22748	1.65738
63727	1.57442	1.50296
63728	0.874683	1.5979
63729	1.25935	2.1
63730	2.61433	2.1
63731	1.22262	2.1
63732	1.22262	2.1
63733	2.62813	2.1
63734	2.62813	2.1
63735	1.89226	2.1
63736	2.61434	2.1
63737	2.62814	2.1
63738	2.61434	2.1
63739	1.90607	2.1

63740	1.89226	2.1
63741	1.89226	2.1
63742	1.89226	2.1
63743	1.89226	2.1
63744	1.89226	2.1
63745	1.89226	2.1
63746	1.89226	2.1
63747	1.89226	2.1
63748	1.89226	2.1
63749	1.89226	2.1
63750	1.89226	2.1
63751	1.89226	2.1
63752	1.89226	2.1
63753	1.31969	2.1
63754	1.31406	2.1
63755	0.629677	2.1
63756	0.643104	2.1
63757	0.976621	2.1
63758	0.976621	2.1
63759	0.98055	2.1
63760	0.98055	2.1
63761	1.3197	2.1
63762	0.643102	2.1
63763	0.629675	2.1
63764	1.31407	2.1
63765	0.946133	2.1
63766	0.946133	2.1
63767	1.3197	2.1
63768	1.31407	2.1
63769	0.946132	2.1
63770	0.946132	2.1
63771	1.3197	2.1
63772	1.3197	2.1
63773	1.3197	2.1
63774	0.946133	2.1
63775	0.946132	2.1
63776	1.31407	2.1
63777	1.89226	2.1
63778	1.89226	2.1
63779	1.89226	2.1
63780	1.89226	2.1
63781	1.89226	2.1
63782	1.89226	2.1
63783	1.89226	2.1
63784	1.89226	2.1
63785	1.89226	2.1
63786	1.89226	2.1
63787	1.89226	2.1
63788	1.89226	2.1
63789	1.89226	0.0914086
63790	1.89226	0.0738774
63791	1.89226	0.0568742
63792	1.89226	0.067501
63793	3.78452	0.0666884
63794	1.89226	0.0802997
63795	1.89226	0.070102
63796	1.89226	0.0688656
63797	1.89226	0.0743801

63798	1.89226	0.0718667
63799	1.89226	0.0803698
63800	0.946131	0.05
63801	0.946131	0.05
63802	0.946128	0.05
63803	0.946128	0.05
63804	0.94613	0.05
63805	0.94613	0.05
63806	0.94613	0.05
63807	0.94613	0.0534092
63808	0.94613	0.05
63809	0.94613	0.05
63810	0.94613	0.05
63811	0.94613	0.05
63812	1.89226	0.05
63813	1.89226	0.0603538
63814	1.89226	0.0535051
63815	1.89226	0.05
63816	1.89226	0.05
63817	1.89226	0.0545221
63818	0.924614	0.05
63819	0.914275	0.05
63820	0.906003	0.05
63821	0.62479	0.05
63822	2.02733	0.05
63823	1.48332	0.05
63824	0.833106	0.05
63825	1.19364	0.05
63826	1.57482	0.05
63827	1.18103	0.05
63828	0.905988	0.05
63829	1.89226	2.1
63830	1.89226	2.1
63831	1.89226	2.1
63832	1.89226	2.1
63833	3.78452	2.1
63834	1.89226	2.1
63835	1.89226	2.1
63836	1.89226	2.1
63837	1.89226	2.1
63838	1.89226	2.1
63839	1.89226	2.1
63840	0.816805	2.1
63841	0.928516	2.1
63842	1.4045	2.1
63843	1.26268	2.1
63844	1.0881	2.1
63845	1.0881	2.1
63846	1.10309	2.1
63847	1.10309	2.1
63848	0.928518	2.1
63849	1.4045	2.1
63850	0.816807	2.1
63851	1.26267	2.1
63852	1.89226	2.1
63853	2.52535	2.1
63854	1.89226	2.1
63855	1.89226	2.1

63856	2.809	2.1
63857	2.809	2.1
63858	1.89226	2.1
63859	1.89226	2.1
63860	1.89226	2.1
63861	1.89226	2.1
63862	3.78452	2.1
63863	0.946127	2.1
63864	0.946127	2.1
63865	0.946131	2.1
63866	0.946131	2.1
63867	0.94613	2.1
63868	0.94613	2.1
63869	0.946128	2.1
63870	0.946128	2.1
63871	0.946129	2.1
63872	0.946129	2.1
63873	0.946129	2.1
63874	0.946129	2.1
63875	1.89224	2.1
63876	1.89224	2.1
63877	1.89224	2.1
63878	1.89224	2.1
63879	3.78448	2.1
63880	2.39244	2.1
63881	2.27036	2.1
63882	2.25945	2.1
63883	2.27398	2.1
63884	4.52981	2.1
63885	2.27036	2.1
63886	1.97088	2.1
63887	1.86302	2.1
63888	1.84991	2.1
63889	4.12027	2.1
63890	1.89224	2.1
63891	1.89224	2.1
63892	1.89224	2.1
63893	1.89224	2.1
63894	3.78447	2.1
63895	1.89226	2.1
63896	1.89226	2.1
63897	1.89226	2.1
63898	1.89226	2.1
63899	3.78453	2.1
63900	2.25948	2.1
63901	2.33611	2.1
63902	2.05257	2.1
63903	1.94441	2.1
63904	4.20389	2.1
63905	1.92514	2.1
63906	1.84993	2.1
63907	1.94441	2.1
63908	1.74391	2.1
63909	3.79434	2.1
63910	1.89226	2.1
63911	1.89226	2.1
63912	1.89226	2.1
63913	1.89226	2.1

63914	3.78452	2.1
63915	15.1381	1.08757
63916	15.1381	0.981049
63917	15.1381	0.205725
63918	15.1381	1.42115
63919	30.2762	0.615041
63920	15.1381	0.967231
63921	15.1381	1.40829
63922	15.1381	0.739537
63923	15.1381	0.956767
63924	30.2762	1.00473
63925	15.1381	1.9789
63926	15.1381	1.96573
63927	15.1381	1.71615
63928	15.1381	2.07633
63929	30.2762	2.07047
63930	15.1381	0.529246
63931	15.1381	1.14889
63932	15.1381	0.746849
63933	15.1381	0.788045
63934	30.2762	0.647291
63935	1.89225	2.1
63936	1.89225	2.1
63937	1.89225	2.1
63938	1.89225	2.1
63939	3.78449	2.1
63940	1.39909	2.1
63941	1.39909	2.1
63942	0.946123	2.1
63943	0.946123	2.1
63944	1.3291	2.1
63945	1.39908	2.1
63946	0.946126	2.1
63947	0.946126	2.1
63948	0.946118	2.1
63949	1.32909	2.1
63950	0.94613	2.1
63951	1.3991	2.1
63952	3.07801	2.1
63953	1.84322	2.1
63954	1.84322	2.1
63955	1.68788	2.1
63956	2.6582	2.1
63957	2.50287	2.1
63958	1.89225	2.1
63959	1.89225	2.1
63960	1.89225	2.1
63961	1.89225	2.1
63962	3.7845	2.1
63963	1.89226	2.1
63964	1.89226	2.1
63965	1.89226	2.1
63966	1.89226	2.1
63967	3.78452	2.1
63968	1.89226	2.1
63969	2.50051	2.1
63970	2.50051	2.1
63971	2.65822	2.1

63972	1.89226	2.1
63973	2.04997	2.1
63974	2.50052	2.1
63975	2.65822	2.1
63976	1.70377	2.1
63977	1.70377	2.1
63978	1.84324	2.1
63979	2.63998	2.1
63980	0.810947	2.1
63981	0.810947	2.1
63982	0.810946	2.1
63983	0.810946	2.1
63984	1.89226	2.1
63985	1.35169	2.1
63986	1.08132	2.1
63987	1.08132	2.1
63988	1.08132	2.1
63989	0.810946	2.1
63990	0.810946	2.1
63991	1.89226	2.1
63992	1.89226	2.1
63993	1.89226	2.1
63994	1.89226	2.1
63995	3.78453	2.1
63996	0.946164	2.1
63997	0.946164	2.1
63998	0.929842	2.1
63999	0.929842	2.1
64000	0.946132	2.1
64001	0.946132	2.1
64002	1.04101	2.1
64003	0.929874	2.1
64004	0.946132	2.1
64005	0.929874	2.1
64006	1.04101	2.1
64007	0.946132	2.1
64008	1.89226	2.1
64009	1.89226	2.1
64010	1.89226	2.1
64011	1.89226	2.1
64012	3.78453	2.1
64013	1.89226	2.1
64014	1.89226	2.1
64015	1.89226	2.1
64016	1.89226	2.1
64017	1.89226	2.1
64018	1.89226	2.1
64019	1.89226	2.1
64020	1.89226	2.1
64021	1.89226	2.1
64022	1.89226	2.1
64023	1.89226	2.1
64024	1.89226	2.1
64025	0.946131	2.1
64026	0.946131	2.1
64027	0.946131	2.1
64028	0.946131	2.1
64029	0.946132	2.1

64030	0.946132	2.1
64031	0.946131	2.1
64032	0.946131	2.1
64033	0.946131	2.1
64034	0.946131	2.1
64035	0.946132	2.1
64036	0.946132	2.1
64037	1.89226	1.94411
64038	1.89226	1.01601
64039	1.89226	0.717033
64040	1.89226	1.81778
64041	3.78453	0.828342
64042	2.73497	0.625443
64043	1.93823	2.1
64044	1.93823	1.02526
64045	2.20046	2.1
64046	2.42279	1.70843
64047	2.68501	2.1
64048	1.89226	0.820042
64049	1.89226	2.1
64050	1.89226	2.1
64051	1.89226	1.21267
64052	1.89226	2.1
64053	1.89226	0.711114
64054	1.89226	0.828575
64055	1.89226	0.497109
64056	1.89226	0.584876
64057	1.89226	1.76493
64058	1.89226	0.692029
64059	1.89226	0.569092
64060	2.82984	1.78483
64061	2.15276	0.714404
64062	2.15276	1.37739
64063	2.42278	0.060936
64064	2.50074	0.67286
64065	2.77077	0.595939
64066	2.42279	0.481584
64067	1.93823	2.1
64068	1.70423	0.846137
64069	1.70423	2.1
64070	2.15276	1.07951
64071	2.38676	2.1
64072	0.810946	1.44759
64073	0.810946	2.1
64074	0.810944	0.656861
64075	0.810944	0.590906
64076	0.810946	1.6294
64077	0.810946	1.83083
64078	1.89226	2.1
64079	1.35169	1.74914
64080	1.08132	1.50936
64081	1.08132	2.1
64082	1.08132	1.8589
64083	1.89226	2.1
64084	1.89226	2.1
64085	1.89226	2.1
64086	1.89226	2.1
64087	3.78452	2.1

64088	1.89226	2.1
64089	1.89226	2.1
64090	1.89226	2.1
64091	1.89226	2.1
64092	3.78453	2.1
64093	1.04144	2.1
64094	1.04144	2.1
64095	0.938972	2.1
64096	0.938972	2.1
64097	0.969661	2.1
64098	1.03285	2.1
64099	0.946133	2.1
64100	0.946133	2.1
64101	0.946131	2.1
64102	0.969663	2.1
64103	0.946131	2.1
64104	1.03284	2.1
64105	0.946131	2.1
64106	0.946131	2.1
64107	0.946128	2.1
64108	0.946128	2.1
64109	0.946127	2.1
64110	0.946127	2.1
64111	0.946131	2.1
64112	0.946131	2.1
64113	0.94613	2.1
64114	0.94613	2.1
64115	0.946129	2.1
64116	0.946129	2.1
64117	1.89226	2.1
64118	1.89226	2.1
64119	1.89226	2.1
64120	1.89226	2.1
64121	3.78452	2.1
64122	1.89226	2.1
64123	1.89226	2.1
64124	1.89226	2.1
64125	1.89226	2.1
64126	3.78453	2.1
64127	1.89226	2.1
64128	1.89226	2.1
64129	1.85882	2.1
64130	1.85882	2.1
64131	1.93932	2.1
64132	1.97276	2.1
64133	0.94613	2.1
64134	0.94613	2.1
64135	0.946128	2.1
64136	0.946128	2.1
64137	0.946127	2.1
64138	0.946127	2.1
64139	0.946131	2.1
64140	0.946131	2.1
64141	0.946129	2.1
64142	0.946129	2.1
64143	0.946129	2.1
64144	0.946129	2.1
64145	1.89225	2.1

64146	1.89225	2.1
64147	1.89225	2.1
64148	1.89225	2.1
64149	3.7845	2.1
64150	1.0086	2.1
64151	1.09028	2.1
64152	0.946125	2.1
64153	0.946125	2.1
64154	0.916906	2.1
64155	0.916906	2.1
64156	1.13051	2.1
64157	1.1505	2.1
64158	1.11557	2.1
64159	1.04071	2.1
64160	0.851542	2.1
64161	0.991196	2.1
64162	1.89225	2.1
64163	1.89225	2.1
64164	1.89225	2.1
64165	1.89225	2.1
64166	3.7845	2.1
64167	1.89225	2.1
64168	1.89225	2.1
64169	1.89225	2.1
64170	1.89225	2.1
64171	1.89225	2.1
64172	1.89225	2.1
64173	1.89226	2.1
64174	1.89226	2.1
64175	1.89226	2.1
64176	1.89226	2.1
64177	3.78452	2.1
64178	0.890962	2.1
64179	0.952547	2.1
64180	0.946132	2.1
64181	0.946132	2.1
64182	0.851543	2.1
64183	0.851543	2.1
64184	1.11558	2.1
64185	1.06304	2.1
64186	0.994315	2.1
64187	1.04072	2.1
64188	0.900943	2.1
64189	0.851542	2.1
64190	1.89226	2.1
64191	1.89226	2.1
64192	1.89226	2.1
64193	1.89226	2.1
64194	1.89226	2.1
64195	1.89226	2.1
64196	1.89226	2.1
64197	1.89226	2.1
64198	1.89226	2.1
64199	1.89226	2.1
64200	3.78452	2.1
64201	1.89226	2.1
64202	1.89226	2.1
64203	1.89226	2.1

64204	1.89226	2.1
64205	3.78452	2.1
64206	0.782015	2.1
64207	0.929718	2.1
64208	0.946132	2.1
64209	0.946132	2.1
64210	0.782016	2.1
64211	0.782016	2.1
64212	1.06706	2.1
64213	1.11785	2.1
64214	0.782015	2.1
64215	0.946133	2.1
64216	0.878931	2.1
64217	0.946132	2.1
64218	1.89226	2.1
64219	1.89226	2.1
64220	1.89226	2.1
64221	1.89226	2.1
64222	1.89226	2.1
64223	1.89226	2.1
64224	0.946131	2.1
64225	0.946131	2.1
64226	0.946129	2.1
64227	0.946129	2.1
64228	0.946131	2.1
64229	0.946131	2.1
64230	0.94613	2.1
64231	0.94613	2.1
64232	0.946131	2.1
64233	0.946131	2.1
64234	0.94613	2.1
64235	0.94613	2.1
64236	0.827866	2.1
64237	0.946131	2.1
64238	0.946129	2.1
64239	0.946129	2.1
64240	0.94613	2.1
64241	0.94613	2.1
64242	0.94613	2.1
64243	0.94613	2.1
64244	0.827865	2.1
64245	0.94613	2.1
64246	0.94613	2.1
64247	0.94613	2.1
64248	1.89226	2.1
64249	1.89226	2.1
64250	1.89226	2.1
64251	1.89226	2.1
64252	3.78452	2.1
64253	1.89226	2.1
64254	1.89226	2.1
64255	1.89226	2.1
64256	1.89226	2.1
64257	1.89226	2.1
64258	1.89226	2.1
64259	1.89226	2.1
64260	1.89226	2.1
64261	1.89226	2.1

64262	1.89226	2.1
64263	3.78453	2.1
64264	1.89226	2.1
64265	1.89226	2.1
64266	1.89226	2.1
64267	1.89226	2.1
64268	3.78452	2.1
64269	0.744377	2.1
64270	0.541919	2.1
64271	0.652022	2.1
64272	0.544198	2.1
64273	0.764633	2.1
64274	0.673254	2.1
64275	1.89226	2.1
64276	1.31149	2.1
64277	1.05423	2.1
64278	1.02189	2.1
64279	0.870602	2.1
64280	1.89226	2.1
64281	1.89226	2.1
64282	1.89226	2.1
64283	1.89226	2.1
64284	1.89226	2.1
64285	1.89226	2.1
64286	1.89226	2.1
64287	1.89226	2.1
64288	1.89226	2.1
64289	1.89226	2.1
64290	1.89226	2.1
64291	1.89226	2.1
64292	1.89226	2.1
64293	1.89226	2.1
64294	1.89226	2.1
64295	1.89226	2.1
64296	1.89226	2.1
64297	1.89226	2.1
64298	0.815314	2.1
64299	0.815313	2.1
64300	0.714029	2.1
64301	0.782951	2.1
64302	0.810944	2.1
64303	0.782954	2.1
64304	1.89226	2.1
64305	1.30999	2.1
64306	1.08132	2.1
64307	1.03962	2.1
64308	0.95209	2.1
64309	1.89226	2.1
64310	1.89226	2.1
64311	1.89226	2.1
64312	1.89226	2.1
64313	1.89226	2.1
64314	1.89226	2.1
64315	1.89226	2.1
64316	1.89226	2.1
64317	1.89226	2.1
64318	1.89226	2.1
64319	1.89226	2.1

64320	1.89226	2.1
64321	1.89226	2.1
64322	1.89226	2.1
64323	1.89226	2.1
64324	1.89226	2.1
64325	1.89226	2.1
64326	1.89226	2.1
64327	0.871558	2.1
64328	0.713078	2.1
64329	0.756858	2.1
64330	0.703674	2.1
64331	0.838025	2.1
64332	0.862196	2.1
64333	1.89226	2.1
64334	1.33194	2.1
64335	1.10834	2.1
64336	1.06163	2.1
64337	1.01785	2.1
64338	1.89226	2.1
64339	1.89226	2.1
64340	1.89226	2.1
64341	1.89226	2.1
64342	3.78452	2.1
64343	1.89226	2.1
64344	1.89226	2.1
64345	1.89226	2.1
64346	1.89226	2.1
64347	1.89226	2.1
64348	1.89226	2.1
64349	1.89226	2.1
64350	1.89226	2.1
64351	1.89226	2.1
64352	1.89226	2.1
64353	1.89226	2.1
64354	1.89226	2.1
64355	0.958406	2.1
64356	1.1383	2.1
64357	0.865636	2.1
64358	1.10093	2.1
64359	0.838024	2.1
64360	0.975154	2.1
64361	1.89226	2.1
64362	1.38553	2.1
64363	1.10834	2.1
64364	1.11522	2.1
64365	1.15271	2.1
64366	1.89227	2.1
64367	1.89227	2.1
64368	1.89227	2.1
64369	1.89227	2.1
64370	3.78453	2.1
64371	0.946132	2.1
64372	0.946132	2.1
64373	0.946132	2.1
64374	0.946132	2.1
64375	0.946132	2.1
64376	0.946132	2.1
64377	0.946133	2.1

64378	0.946133	2.1
64379	0.946132	2.1
64380	0.946132	2.1
64381	0.946133	2.1
64382	0.946133	2.1
64383	0.719271	2.1
64384	0.774968	2.1
64385	1.28401	2.1
64386	1.17415	2.1
64387	0.960999	2.1
64388	0.960999	2.1
64389	0.988115	2.1
64390	0.988115	2.1
64391	0.774968	2.1
64392	1.28401	2.1
64393	0.719271	2.1
64394	1.17415	2.1
64395	2.77811	2.1
64396	2.34829	2.1
64397	2.56802	2.1
64398	2.52333	2.1
64399	2.56802	2.1
64400	2.52333	2.1
64401	1.89227	2.1
64402	1.89227	2.1
64403	1.89227	2.1
64404	1.89227	2.1
64405	3.78453	2.1
64406	1.89226	2.1
64407	1.89226	2.1
64408	1.89226	2.1
64409	1.89226	2.1
64410	1.89226	2.1
64411	1.89226	2.1
64412	1.89226	2.1
64413	2.77811	2.1
64414	1.89226	2.1
64415	2.56802	2.1
64416	1.89226	2.1
64417	2.56802	2.1
64418	1.89226	1.83457
64419	1.89226	2.1
64420	1.89226	1.64595
64421	1.89226	2.1
64422	3.78452	2.1
64423	2.41802	2.1
64424	1.95764	2.1
64425	1.95764	2.1
64426	2.27727	2.1
64427	2.39311	2.1
64428	2.73961	2.1
64429	0.946132	2.1
64430	0.946132	2.1
64431	0.94613	2.1
64432	0.94613	2.1
64433	0.946131	2.1
64434	0.946131	2.1
64435	0.946131	2.1

64436	0.946131	2.1
64437	0.946131	2.1
64438	0.946131	2.1
64439	0.946131	2.1
64440	0.946131	2.1
64441	1.89226	1.84222
64442	1.89226	2.1
64443	1.89226	2.1
64444	1.89226	1.80741
64445	1.89226	2.1
64446	1.89226	1.62708
64447	2.78856	0.943057
64448	2.28552	2.1
64449	2.28552	1.72035
64450	2.3931	2.1
64451	2.6402	1.5087
64452	2.74778	2.1
64453	2.39311	2.1
64454	1.95764	2.1
64455	2.28552	2.1
64456	1.86103	2.1
64457	4.24316	2.1
64458	1.89226	2.1
64459	1.89226	2.1
64460	1.89226	2.1
64461	1.89226	2.1
64462	1.89226	2.1
64463	1.89226	2.1
64464	1.47412	2.0688
64465	2.18862	1.87598
64466	2.07226	1.52889
64467	2.33645	1.69546
64468	4.26088	1.5988
64469	1.89226	1.55928
64470	1.89226	1.91514
64471	1.89226	1.58306
64472	1.89226	1.82803
64473	1.89226	1.71951
64474	1.89226	2.1
64475	1.89226	1.72636
64476	1.89226	2.1
64477	1.89226	0.309109
64478	1.89226	2.1
64479	3.78453	1.88927
64480	2.18862	1.71257
64481	2.1128	2.1
64482	2.33645	1.52571
64483	2.28188	2.1
64484	4.47051	2.1
64485	2.07226	2.1
64486	2.33645	2.02064
64487	2.20831	1.61553
64488	2.4646	1.42775
64489	4.53686	1.54603
64490	0.946131	1.55444
64491	0.946131	2.1
64492	0.946131	2.1
64493	0.946131	1.58373

64494	0.94613	2.02623
64495	0.94613	1.48245
64496	0.946133	2.03291
64497	0.946133	2.1
64498	0.946132	1.4395
64499	0.946132	1.31642
64500	0.946131	1.93587
64501	0.946131	1.80122
64502	1.89226	1.78773
64503	1.89226	2.1
64504	1.89226	1.22552
64505	1.89226	2.1
64506	3.78453	1.9122
64507	2.33646	1.75998
64508	2.28189	2.1
64509	2.4646	1.07022
64510	2.42758	2.1
64511	4.74648	1.88095
64512	1.89226	2.1
64513	1.89226	2.1
64514	1.89226	2.1
64515	1.89226	2.1
64516	1.89226	2.1
64517	1.89226	2.1
64518	1.89226	2.1
64519	1.89226	2.1
64520	1.89226	2.1
64521	1.89226	2.1
64522	1.89226	2.1
64523	1.89226	2.1
64524	1.89226	2.1
64525	1.89226	2.1
64526	1.89226	2.1
64527	1.89226	2.1
64528	3.78453	2.1
64529	1.89226	2.1
64530	1.89226	2.1
64531	1.89226	2.1
64532	1.89226	2.1
64533	1.89226	2.1
64534	1.89226	2.1
64535	1.89226	2.1
64536	1.89226	2.1
64537	1.89226	2.1
64538	1.89226	2.1
64539	3.78452	2.1
64540	0.877238	2.1
64541	0.877238	2.1
64542	1.24746	2.1
64543	1.04764	2.1
64544	0.964969	2.1
64545	1.1521	2.1
64546	0.946132	2.1
64547	0.946132	2.1
64548	0.946132	2.1
64549	1.12659	2.1
64550	0.964969	2.1
64551	0.946131	2.1

64552	1.89226	2.1
64553	1.89226	2.1
64554	1.89226	2.1
64555	1.89226	2.1
64556	1.89226	2.1
64557	1.89226	2.1
64558	1.89226	2.1
64559	1.89226	2.1
64560	1.89226	2.1
64561	1.89226	2.1
64562	3.78453	2.1
64563	0.868144	2.1
64564	0.992762	2.1
64565	0.946131	2.1
64566	0.946131	2.1
64567	1.07072	2.1
64568	1.07072	2.1
64569	0.868171	2.1
64570	0.868171	2.1
64571	0.992762	2.1
64572	0.946131	2.1
64573	0.946131	2.1
64574	0.868144	2.1
64575	0.946132	2.1
64576	0.946132	2.1
64577	0.946131	2.1
64578	0.946131	2.1
64579	0.946132	2.1
64580	0.946132	2.1
64581	0.946132	2.1
64582	0.946132	2.1
64583	0.946132	2.1
64584	0.946132	2.1
64585	0.946132	2.1
64586	0.946132	2.1
64587	1.89227	2.1
64588	1.89227	2.1
64589	1.89227	2.1
64590	1.89227	2.1
64591	1.89227	2.1
64592	1.89227	2.1
64593	1.89226	2.1
64594	1.89226	2.1
64595	1.89226	2.1
64596	1.89226	2.1
64597	1.89226	2.1
64598	1.89226	2.1
64599	1.98552	2.1
64600	2.22298	2.1
64601	2.22298	2.1
64602	1.89226	2.1
64603	1.89226	2.1
64604	1.54525	2.1
64605	0.810946	2.1
64606	0.810946	2.1
64607	0.810947	2.1
64608	0.810947	2.1
64609	0.81096	2.1

64610	0.81096	2.1
64611	1.89226	2.1
64612	1.35168	2.1
64613	1.08132	2.1
64614	1.08132	2.1
64615	1.0813	2.1
64616	1.89226	2.1
64617	1.89226	2.1
64618	1.89226	2.1
64619	1.89226	2.1
64620	3.78452	2.1
64621	1.7753	2.1
64622	2.39703	2.1
64623	1.90577	2.1
64624	2.03445	2.1
64625	3.82335	2.1
64626	2.39703	2.1
64627	2.47949	2.1
64628	2.26051	2.1
64629	2.36891	2.1
64630	4.76594	2.1
64631	1.89226	2.1
64632	1.89226	2.1
64633	1.89226	2.1
64634	1.89226	2.1
64635	3.78452	2.1
64636	1.89226	2.1
64637	1.89226	2.1
64638	1.89226	2.1
64639	1.89226	2.1
64640	1.89226	2.1
64641	1.89226	2.1
64642	0.883745	2.1
64643	0.973143	2.1
64644	0.988173	2.1
64645	0.988173	2.1
64646	0.941927	2.1
64647	1.02971	2.1
64648	0.854065	2.1
64649	0.82702	2.1
64650	0.847835	2.1
64651	0.981126	2.1
64652	1.02996	2.1
64653	1.10704	2.1
64654	2.28031	2.1
64655	2.26369	2.1
64656	2.26369	2.1
64657	2.36891	2.1
64658	2.30788	2.1
64659	2.42455	2.1
64660	1.89226	2.1
64661	1.89226	2.1
64662	1.89226	2.04344
64663	1.89226	2.1
64664	1.89226	2.08809
64665	1.89226	2.1
64666	2.71406	2.1
64667	1.83244	2.1

64668	1.83244	2.1
64669	2.07567	2.1
64670	2.52044	2.1
64671	2.76367	2.1
64672	1.89226	2.1
64673	1.89226	2.1
64674	1.89226	2.1
64675	1.89226	2.1
64676	3.78453	2.1
64677	1.89226	2.1
64678	1.89226	2.1
64679	1.89226	2.1
64680	1.89226	2.1
64681	1.89226	2.1
64682	1.89226	2.1
64683	2.70714	2.1
64684	2.3148	2.1
64685	2.3148	2.1
64686	2.52044	2.1
64687	2.54204	2.1
64688	2.74768	2.1
64689	2.52044	2.1
64690	2.3148	2.1
64691	1.83244	2.1
64692	1.83244	2.1
64693	1.62611	2.1
64694	2.3148	2.1
64695	0.946131	2.1
64696	0.946131	2.1
64697	0.946131	2.1
64698	0.946131	2.1
64699	0.946131	2.1
64700	0.946131	2.1
64701	0.946131	2.1
64702	0.946131	2.1
64703	0.946131	2.1
64704	0.946131	2.1
64705	0.946131	2.1
64706	0.946131	2.1
64707	1.89226	2.1
64708	1.89226	2.1
64709	1.89226	2.1
64710	1.89226	2.1
64711	3.78453	2.1
64712	1.89226	2.1
64713	1.89226	2.1
64714	1.89226	2.1
64715	1.89226	2.1
64716	1.89226	2.1
64717	1.89226	2.1
64718	1.89227	2.1
64719	1.89227	2.1
64720	1.89227	2.1
64721	1.89227	2.1
64722	3.78453	2.1
64723	1.89226	2.1
64724	1.89226	2.1
64725	1.89226	2.1

64726	1.89226	2.1
64727	3.78453	2.1
64728	0.915781	2.1
64729	0.863524	2.1
64730	0.717056	2.1
64731	0.830182	2.1
64732	0.865094	2.1
64733	0.918003	2.1
64734	1.89226	2.1
64735	1.29933	2.1
64736	1.11215	2.1
64737	1.05227	2.1
64738	0.966269	2.1
64739	1.89226	2.1
64740	1.89226	2.1
64741	1.89226	2.1
64742	1.89226	2.1
64743	1.89226	2.1
64744	1.89226	2.1
64745	1.89226	2.1
64746	1.89226	2.1
64747	1.89226	2.1
64748	1.89226	2.1
64749	1.89226	2.1
64750	1.89226	2.1
64751	1.89226	2.1
64752	1.89226	2.1
64753	1.89226	2.1
64754	1.89226	2.1
64755	1.89226	2.1
64756	1.89226	2.1
64757	1.01725	2.1
64758	0.997048	2.1
64759	0.818657	2.1
64760	0.960724	2.1
64761	0.838013	2.1
64762	1.04859	2.1
64763	1.89226	2.1
64764	1.41754	2.1
64765	1.17625	2.1
64766	1.0793	2.1
64767	1.18822	2.1
64768	2.16402	2.1
64769	1.89452	2.1
64770	1.50084	2.1
64771	2.09288	2.1
64772	3.9874	2.1
64773	2.16402	2.1
64774	2.13925	2.1
64775	2.04483	2.1
64776	2.09288	2.1
64777	4.23212	2.1
64778	1.89226	2.1
64779	1.89226	2.1
64780	1.89226	2.1
64781	1.89226	2.1
64782	3.78452	2.1
64783	1.89226	2.1

64784	1.89226	2.1
64785	1.89226	2.1
64786	1.89226	2.1
64787	1.89226	2.1
64788	1.89226	2.1
64789	1.9824	2.1
64790	2.09288	2.1
64791	1.69889	2.1
64792	1.6817	2.1
64793	2.00695	2.1
64794	2.00695	2.1
64795	1.93592	2.1
64796	2.09288	2.1
64797	2.00695	2.1
64798	2.09288	2.1
64799	1.9589	2.1
64800	2.04483	2.1
64801	0.946128	2.1
64802	0.946128	2.1
64803	0.946132	2.1
64804	0.946132	2.1
64805	0.94613	2.1
64806	0.94613	2.1
64807	0.946129	2.1
64808	0.946129	2.1
64809	0.94613	2.1
64810	0.94613	2.1
64811	0.94613	2.1
64812	0.94613	2.1
64813	0.946131	2.1
64814	0.946131	2.1
64815	0.946131	2.1
64816	0.946131	2.1
64817	0.946131	2.1
64818	0.946131	2.1
64819	0.946131	2.1
64820	0.946131	2.1
64821	0.946131	2.1
64822	0.946131	2.1
64823	0.946131	2.1
64824	0.946131	2.1
64825	1.89226	2.1
64826	2.48083	2.1
64827	2.6968	2.1
64828	2.6968	2.1
64829	1.89226	2.1
64830	1.89226	2.1
64831	1.89226	2.1
64832	1.89226	2.1
64833	1.89226	2.1
64834	1.89226	2.1
64835	3.78453	2.1
64836	1.89226	2.1
64837	1.89226	2.1
64838	1.89226	2.1
64839	1.89226	2.1
64840	1.89226	2.1
64841	1.89226	2.1

64842	1.1017	2.1
64843	1.1017	2.1
64844	1.10605	2.1
64845	1.01647	2.1
64846	0.868255	2.1
64847	0.883424	2.1
64848	1.24624	2.1
64849	1.35473	2.1
64850	0.942231	2.1
64851	1.31464	2.1
64852	0.910064	2.1
64853	1.27147	2.1
64854	1.89226	2.1
64855	1.89226	2.1
64856	1.89226	2.1
64857	1.89226	2.1
64858	3.78453	2.1
64859	1.89226	2.1
64860	1.89226	2.1
64861	1.89226	2.1
64862	1.89226	2.1
64863	1.89226	2.1
64864	1.89226	2.1
64865	1.89226	2.1
64866	1.89226	2.1
64867	1.89226	2.1
64868	1.89226	2.1
64869	3.78453	2.1
64870	1.89226	2.1
64871	2.70015	2.1
64872	1.89226	2.1
64873	2.61064	2.1
64874	4.59241	2.1
64875	2.70015	2.1
64876	2.3372	2.1
64877	2.61064	2.1
64878	1.85184	2.1
64879	4.55198	2.1
64880	1.89226	2.1
64881	1.89226	2.1
64882	1.89226	2.1
64883	1.89226	2.1
64884	1.89226	2.1
64885	1.89226	2.1
64886	1.89227	2.1
64887	1.89227	2.1
64888	1.89227	2.1
64889	1.89227	2.1
64890	3.78453	2.1
64891	1.89226	2.1
64892	2.52194	2.1
64893	2.61064	2.1
64894	2.61064	2.1
64895	1.89226	2.1
64896	1.89226	2.1
64897	1.095 2.1	
64898	1.095 2.1	
64899	1.09189	2.1

64900	1.09189	2.1
64901	0.875334	2.1
64902	0.925919	2.1
64903	1.26097	2.1
64904	1.30532	2.1
64905	1.26097	2.1
64906	0.875334	2.1
64907	1.30532	2.1
64908	0.92592	2.1
64909	1.89226	2.1
64910	1.89226	2.1
64911	1.89226	2.1
64912	1.89226	2.1
64913	1.89226	2.1
64914	1.89226	2.1
64915	1.89226	2.1
64916	1.89226	2.1
64917	1.89226	2.1
64918	1.89226	2.1
64919	3.78453	2.1
64920	1.26097	2.1
64921	1.26097	2.1
64922	0.946132	2.1
64923	0.946132	2.1
64924	1.22176	2.1
64925	1.26097	2.1
64926	0.946132	2.1
64927	0.946132	2.1
64928	0.946132	2.1
64929	1.22176	2.1
64930	1.26097	2.1
64931	0.946132	2.1
64932	2.52194	2.1
64933	1.67381	2.1
64934	1.67381	2.1
64935	1.75067	2.1
64936	2.44352	2.1
64937	2.52038	2.1
64938	1.89226	2.1
64939	1.89226	2.1
64940	1.89226	2.1
64941	1.89226	2.1
64942	1.89226	2.1
64943	1.89226	2.1
64944	1.89227	2.1
64945	1.89227	2.1
64946	1.89227	2.1
64947	1.89227	2.1
64948	1.89227	2.1
64949	1.89227	2.1
64950	1.22176	2.1
64951	1.22176	2.1
64952	0.946132	2.1
64953	0.946132	2.1
64954	1.19095	2.1
64955	1.22176	2.1
64956	0.946133	2.1
64957	0.946133	2.1

64958	1.19095	2.1
64959	0.946132	2.1
64960	0.946133	2.1
64961	1.22176	2.1
64962	2.44352	2.1
64963	1.6287	2.1
64964	1.6287	2.1
64965	1.67381	2.1
64966	2.3819	2.1
64967	2.427	2.1
64968	1.89226	2.1
64969	1.89226	2.1
64970	1.89226	2.1
64971	1.89226	2.1
64972	1.89226	2.1
64973	1.89226	2.1
64974	1.89226	2.1
64975	1.89226	2.1
64976	1.89226	2.1
64977	1.89226	2.1
64978	3.78453	2.1
64979	1.89226	2.1
64980	1.89226	2.1
64981	1.89226	2.1
64982	1.89226	2.1
64983	1.89226	2.1
64984	1.89226	2.1
64985	1.89226	2.1
64986	1.89226	2.1
64987	1.89226	2.1
64988	1.89226	2.1
64989	1.89226	2.1
64990	1.89226	2.1
64991	0.851542	2.1
64992	0.851542	2.1
64993	1.1736	2.1
64994	1.29261	2.1
64995	0.852708	2.1
64996	1.18843	2.1
64997	0.946132	2.1
64998	0.946132	2.1
64999	0.946132	2.1
65000	1.07901	2.1
65001	1.04208	2.1
65002	0.936669	2.1
65003	1.89227	2.1
65004	1.89227	2.1
65005	1.89227	2.1
65006	1.89227	2.1
65007	3.78453	2.1
65008	1.89226	2.1
65009	1.89226	2.1
65010	1.89226	2.1
65011	1.89226	2.1
65012	3.78453	2.1
65013	1.89226	2.1
65014	1.89226	2.1
65015	1.89226	2.1

65016	1.89226	2.1
65017	1.89226	2.1
65018	1.89226	2.1
65019	0.946133	2.1
65020	0.946133	2.1
65021	1.28525	2.1
65022	1.28525	2.1
65023	1.07901	2.1
65024	1.28525	2.1
65025	0.946132	2.1
65026	0.946132	2.1
65027	0.946132	2.1
65028	1.28525	2.1
65029	0.946133	2.1
65030	1.07901	2.1
65031	1.89226	2.1
65032	1.89226	2.1
65033	1.89226	2.1
65034	1.89226	2.1
65035	1.89226	2.1
65036	1.89226	2.1
65037	1.89226	2.1
65038	1.89226	2.1
65039	1.89226	2.1
65040	1.89226	2.1
65041	1.89226	2.1
65042	1.89226	2.1
65043	1.24419	2.1
65044	1.09093	2.1
65045	1.22722	2.1
65046	1.18154	2.1
65047	1.27455	2.1
65048	1.08473	2.1
65049	1.15541	2.1
65050	1.15541	2.1
65051	1.29607	2.1
65052	1.23814	2.1
65053	1.13024	2.1
65054	1.12096	2.1
65055	1.18858	2.1
65056	1.13181	2.1
65057	0.988718	2.1
65058	0.802498	2.1
65059	0.994034	2.1
65060	1.15773	2.1
65061	1.0029	2.1
65062	1.0029	2.1
65063	1.23814	2.1
65064	0.997523	2.1
65065	1.11498	2.1
65066	0.899532	2.1
65067	1.89226	2.1
65068	1.89226	2.1
65069	1.89226	2.1
65070	1.89226	2.1
65071	1.89226	2.1
65072	1.89226	2.1
65073	1.89226	2.1

65074	1.89226	2.1
65075	1.89226	2.1
65076	1.89226	2.1
65077	1.89226	2.1
65078	1.89226	2.1
65079	0.993139	2.1
65080	0.834108	2.1
65081	1.01147	2.1
65082	1.01358	2.1
65083	1.20067	2.1
65084	0.993156	2.1
65085	2.4158	2.1
65086	1.69956	2.1
65087	1.3544	2.1
65088	1.60095	2.1
65089	1.11219	2.1
65090	1.89226	2.1
65091	1.89226	2.1
65092	1.89226	2.1
65093	1.89226	2.1
65094	3.78452	2.1
65095	1.89226	2.1
65096	1.89226	2.1
65097	1.89226	2.1
65098	1.89226	2.1
65099	1.89226	2.1
65100	1.89226	2.1
65101	1.89226	2.1
65102	2.83014	2.1
65103	2.32404	2.1
65104	2.32404	2.1
65105	1.89226	2.1
65106	1.89226	2.1
65107	1.37966	2.1
65108	1.13294	2.1
65109	0.881723	2.1
65110	0.582304	2.1
65111	0.977944	2.1
65112	0.977948	2.1
65113	1.0264	2.1
65114	1.0264	2.1
65115	1.37967	2.1
65116	1.03896	2.1
65117	1.19108	2.1
65118	0.582298	2.1
65119	1.89226	2.1
65120	1.89226	2.1
65121	1.89226	2.1
65122	1.89226	2.1
65123	3.78452	2.1
65124	0.946131	2.1
65125	0.946131	2.1
65126	0.946131	2.1
65127	0.946131	2.1
65128	0.946132	2.1
65129	0.946132	2.1
65130	0.946129	2.1
65131	0.946129	2.1

65132	0.946131	2.1
65133	0.946131	2.1
65134	0.94613	2.1
65135	0.94613	2.1
65136	1.89226	2.1
65137	1.89226	2.1
65138	1.89226	2.1
65139	1.89226	2.1
65140	3.78453	2.1
65141	1.89226	2.1
65142	1.89226	2.1
65143	1.89226	2.1
65144	1.89226	2.1
65145	1.89226	2.1
65146	1.89226	2.1
65147	2.65216	2.1
65148	1.93528	2.1
65149	1.78595	2.1
65150	2.48544	2.1
65151	4.42072	2.1
65152	1.89226	2.1
65153	1.89226	2.1
65154	1.89226	2.1
65155	1.89226	2.1
65156	1.89226	2.1
65157	1.89226	2.1
65158	1.89226	2.1
65159	1.89226	2.1
65160	1.89226	2.1
65161	1.89226	2.1
65162	3.78452	2.1
65163	1.66537	2.1
65164	2.48544	2.1
65165	1.78595	2.1
65166	1.78595	2.1
65167	2.36197	2.1
65168	2.36197	2.1
65169	2.80975	2.1
65170	2.48544	2.1
65171	2.36198	2.1
65172	2.65135	2.1
65173	5.13679	2.1
65174	1.89226	2.1
65175	1.89226	2.1
65176	1.89226	2.1
65177	1.89226	2.1
65178	1.89226	2.1
65179	1.89226	2.1
65180	0.946127	2.1
65181	0.946127	2.1
65182	0.946131	2.1
65183	0.946131	2.1
65184	0.946128	2.1
65185	0.946128	2.1
65186	0.94613	2.1
65187	0.94613	2.1
65188	0.946129	2.1
65189	0.946129	2.1

65190	0.946129	2.1
65191	0.946129	2.1
65192	1.11972	2.1
65193	1.18099	2.1
65194	0.832687	2.1
65195	0.764517	2.1
65196	0.974426	2.1
65197	0.974426	2.1
65198	0.971078	2.1
65199	0.971078	2.1
65200	1.11972	2.1
65201	0.764515	2.1
65202	0.832686	2.1
65203	1.18099	2.1
65204	2.36198	2.1
65205	2.48025	2.1
65206	2.48025	2.1
65207	2.65135	2.1
65208	2.23944	2.1
65209	2.41055	2.1
65210	0.946132	2.1
65211	0.946132	2.1
65212	0.946131	2.1
65213	0.946131	2.1
65214	0.946131	2.1
65215	0.946131	2.1
65216	0.946132	2.1
65217	0.946132	2.1
65218	0.946131	2.1
65219	0.946131	2.1
65220	0.946132	2.1
65221	0.946132	2.1
65222	0.810942	2.1
65223	0.810942	2.1
65224	0.810944	2.1
65225	0.810944	2.1
65226	1.89226	2.1
65227	1.3517	2.1
65228	1.08132	2.1
65229	1.08133	2.1
65230	1.08132	2.1
65231	0.810932	2.1
65232	0.810932	2.1
65233	1.89249	2.1
65234	1.9857	1.76656
65235	2.02433	2.06791
65236	2.15807	1.92784
65237	4.01003	2.02109
65238	1.89226	1.83943
65239	1.89226	1.77921
65240	1.89226	2.1
65241	1.89226	1.38317
65242	3.78452	1.74976
65243	1.89226	1.71247
65244	1.89226	1.83316
65245	1.89226	1.15552
65246	1.89226	1.37488
65247	3.78452	1.30908

65248	1.98569	1.64437
65249	1.86067	1.70939
65250	2.15807	1.84853
65251	2.07965	1.37644
65252	4.06534	1.65473
65253	2.02433	2.1
65254	2.15807	2.1
65255	2.12608	2.1
65256	2.3025	1.69892
65257	4.32683	2.1
65258	1.89226	2.1
65259	1.89226	1.62533
65260	1.89226	1.82305
65261	1.89226	1.71893
65262	3.78452	1.81211
65263	1.89226	1.43834
65264	1.89226	1.60764
65265	1.89226	1.61131
65266	1.89226	1.51312
65267	3.78452	1.50238
65268	2.15807	2.1
65269	2.07965	1.59265
65270	2.3025	1.54287
65271	2.28384	1.56521
65272	4.38215	1.54841
65273	1.89226	2.1
65274	1.89226	0.696377
65275	1.89226	1.8638
65276	1.89226	0.699201
65277	3.78453	1.72154
65278	2.16905	0.488543
65279	2.56369	0.641595
65280	1.88394	1.21519
65281	2.41938	0.447305
65282	4.44763	0.418421
65283	2.56369	0.343356
65284	2.80663	1.1591
65285	2.41938	0.422251
65286	2.69045	0.426269
65287	5.25414	0.350737
65288	1.89226	0.351715
65289	1.89226	0.509822
65290	1.89226	0.632342
65291	1.89226	0.509938
65292	3.78453	0.138089
65293	1.89226	1.60196
65294	1.89226	0.878328
65295	1.89226	2.06883
65296	1.89226	1.45251
65297	1.89226	1.94589
65298	1.89226	0.52718
65299	1.01988	0.725324
65300	1.01988	0.408246
65301	1.03333	0.861984
65302	1.03333	0.843279
65303	1.13811	0.110553
65304	1.20969	0.30081
65305	0.941974	0.882594

65306	0.843515	0.497496
65307	0.843514	0.421043
65308	1.13811	0.577925
65309	0.941973	0.74511
65310	1.20969	0.348154
65311	2.41938	0.190247
65312	2.57414	0.396603
65313	2.57414	1.39532
65314	2.69045	1.23975
65315	2.27623	0.205009
65316	2.39253	1.33069
65317	1.89227	0.633262
65318	1.89227	0.573355
65319	1.89227	0.235803
65320	1.89227	0.260428
65321	1.89227	0.241982
65322	1.89227	0.296435
65323	2.28385	1.68791
65324	2.09114	1.72005
65325	2.46968	1.12567
65326	2.32438	1.34597
65327	4.56082	1.18425
65328	1.89226	1.43346
65329	1.89226	1.71932
65330	1.89226	1.26012
65331	1.89226	1.90421
65332	3.78452	1.57772
65333	1.89226	1.46313
65334	1.89226	2.1
65335	1.89226	1.5869
65336	1.89226	1.88014
65337	3.78452	1.83624
65338	2.09114	0.909669
65339	1.5249	1.85739
65340	2.55684	0.848043
65341	1.86899	1.92966
65342	3.96011	1.61825
65343	2.46968	1.39938
65344	2.32439	1.56858
65345	2.62768	1.67653
65346	2.506	1.46702
65347	4.97568	1.56209
65348	1.89226	1.50471
65349	1.89226	1.91612
65350	1.89226	0.365912
65351	1.89226	1.43986
65352	3.78452	1.40485
65353	1.89226	1.61928
65354	1.89226	1.83233
65355	1.89226	1.64567
65356	1.89226	2.1
65357	1.89226	0.627494
65358	1.89226	2.1
65359	2.55684	1.38008
65360	2.16506	1.82568
65361	1.86901	1.61137
65362	1.86899	1.52896
65363	2.75664	1.48178

65364	2.75662	1.30416
65365	1.89224	0.528211
65366	1.89224	1.17692
65367	1.89224	0.323982
65368	1.89224	2.1
65369	3.78447	0.79557
65370	2.24206	0.718055
65371	2.38862	2.1
65372	2.07107	1.50589
65373	2.23097	2.1
65374	4.47303	1.94222
65375	2.38863	2.1
65376	2.25945	2.1
65377	2.25945	2.1
65378	2.39244	2.1
65379	2.23098	2.1
65380	2.36397	2.1
65381	1.89224	1.12645
65382	1.89224	2.1
65383	1.89224	2.1
65384	1.89224	2.1
65385	1.89224	2.1
65386	1.89224	2.1
65387	1.89226	0.471476
65388	1.89226	2.1
65389	1.89226	1.43174
65390	1.89226	0.740067
65391	1.89226	1.2344
65392	1.89226	1.53054
65393	0.920975	2.1
65394	0.920979	1.70887
65395	1.03015	2.1
65396	1.03015	0.835901
65397	0.992407	2.08448
65398	1.08759	2.1
65399	0.854346	1.66718
65400	0.87241	0.729598
65401	0.872397	1.92604
65402	1.04334	1.91938
65403	1.00965	1.96319
65404	1.0876	1.95427
65405	2.23101	2.1
65406	2.25948	2.1
65407	2.03577	2.1
65408	2.05257	2.1
65409	4.29524	2.1
65410	0.810947	1.28028
65411	0.810947	2.1
65412	0.810947	2.08629
65413	0.810947	1.2708
65414	0.810934	2.08187
65415	0.810934	2.09083
65416	1.89227	2.1
65417	1.3517	2.1
65418	1.08132	2.1
65419	1.08132	2.1
65420	1.08133	2.1
65421	1.89226	2.09819

65422	1.89226	2.1
65423	1.89226	1.79397
65424	1.89226	2.1
65425	1.89226	0.843777
65426	1.89226	2.1
65427	1.89226	2.1
65428	1.89226	2.1
65429	1.89226	2.1
65430	1.89226	2.1
65431	1.89226	2.1
65432	1.89226	2.1
65433	0.879432	2.1
65434	0.879432	2.1
65435	0.900223	1.10524
65436	0.900223	2.1
65437	0.54571	2.1
65438	0.743092	2.1
65439	1.03656	2.1
65440	1.19234	1.20944
65441	1.19234	2.06223
65442	0.743092	2.07582
65443	1.03656	2.1
65444	0.545711	2.1
65445	1.89226	0.551037
65446	2.38467	1.67655
65447	2.69293	0.473639
65448	2.69293	2.1
65449	1.89226	0.214324
65450	1.89226	1.95757
65451	1.89226	1.8968
65452	1.89226	1.95
65453	1.89226	1.06096
65454	1.89226	2.1
65455	3.78452	1.91386
65456	0.946129	2.1
65457	0.946129	2.1
65458	0.946133	1.7255
65459	0.946133	2.1
65460	0.946131	2.1
65461	0.946131	2.1
65462	0.946131	1.62203
65463	0.946131	2.1
65464	0.946131	2.1
65465	0.946131	2.1
65466	0.946131	2.1
65467	0.946131	2.1
65468	1.04846	1.32736
65469	1.04846	2.1
65470	1.05594	0.662997
65471	1.05594	2.06034
65472	0.743092	2.1
65473	0.912061	1.98645
65474	1.19234	0.616622
65475	1.34647	1.50781
65476	0.912061	1.19324
65477	1.34646	1.80475
65478	1.19234	1.88643
65479	0.743093	1.86996

65480	1.89226	1.49185
65481	1.89226	2.1
65482	1.89226	0.971341
65483	1.89226	1.82436
65484	3.78452	1.8805
65485	0.946128	2.1
65486	0.946128	2.1
65487	0.946133	2.1
65488	0.946133	1.68473
65489	0.94613	2.1
65490	0.94613	2.1
65491	0.946131	2.1
65492	0.946131	1.90175
65493	0.946131	2.1
65494	0.946131	2.1
65495	0.946131	2.1
65496	0.946131	2.1
65497	1.89226	1.58188
65498	1.89226	1.96084
65499	1.89226	1.15104
65500	1.89226	1.82382
65501	3.78452	1.59235
65502	1.89226	1.85033
65503	1.89226	1.81646
65504	1.89226	1.69454
65505	1.89226	2.1
65506	1.89226	0.570551
65507	1.89226	2.1
65508	1.89226	2.1
65509	1.89226	2.1
65510	1.89226	1.85773
65511	1.89226	2.1
65512	1.89226	1.38837
65513	1.89226	2.1
65514	0.946132	2.1
65515	0.946132	1.8813
65516	1.17758	0.752511
65517	1.17759	1.93891
65518	1.04514	2.1
65519	1.17758	1.6149
65520	0.946133	0.898767
65521	0.946133	1.53555
65522	0.946107	1.77744
65523	1.17755	1.76518
65524	1.04517	1.81728
65525	0.946158	1.81406
65526	1.89226	2.1
65527	2.10171	2.1
65528	1.95064	2.1
65529	2.10171	2.1
65530	1.7412	2.1
65531	1.89226	2.1
65532	1.89226	2.1
65533	1.89226	2.1
65534	1.89226	2.1
65535	1.89226	2.1
65536	1.89226	2.1
65537	1.89226	2.1

65538	1.89226	2.1
65539	1.89226	2.1
65540	1.89226	2.1
65541	1.89226	2.1
65542	3.78453	2.1
65543	0.946133	2.1
65544	0.946133	2.1
65545	0.946132	2.1
65546	0.946132	2.1
65547	0.946133	2.1
65548	0.946133	2.1
65549	0.946132	2.1
65550	0.946132	2.1
65551	0.946132	2.1
65552	0.946132	2.1
65553	0.946132	2.1
65554	0.946132	2.1
65555	1.70303	2.1
65556	1.95064	2.1
65557	1.74039	2.1
65558	1.95064	2.1
65559	1.69368	2.1
65560	1.89226	2.1
65561	1.89226	2.1
65562	1.89226	2.1
65563	1.89226	2.1
65564	1.89226	2.1
65565	3.78453	2.1
65566	0.946132	2.1
65567	0.946132	2.1
65568	0.946132	2.1
65569	0.946132	2.1
65570	0.946133	2.1
65571	0.946133	2.1
65572	0.946132	2.1
65573	0.946132	2.1
65574	0.946107	2.1
65575	0.946107	2.1
65576	0.946158	2.1
65577	0.946158	2.1
65578	0.946133	2.1
65579	0.946133	2.1
65580	0.946132	2.1
65581	0.946132	2.1
65582	0.946133	2.1
65583	0.946133	2.1
65584	0.946132	2.1
65585	0.946132	2.1
65586	0.946107	2.1
65587	0.946107	2.1
65588	0.946158	2.1
65589	0.946158	2.1
65590	1.89226	2.1
65591	1.89226	2.1
65592	1.89226	2.1
65593	1.89226	2.1
65594	1.89226	2.1
65595	1.89226	2.1

65596	1.89226	2.1
65597	1.89226	2.1
65598	1.89226	2.1
65599	1.89226	2.1
65600	1.89226	2.1
65601	1.89226	2.1
65602	2.5198	2.1
65603	2.45893	2.1
65604	2.27322	2.1
65605	2.27322	2.1
65606	2.63576	2.1
65607	2.63576	2.1
65608	1.24045	2.1
65609	1.11906	2.1
65610	1.13578	2.1
65611	0.916205	2.1
65612	1.12539	2.1
65613	1.08404	2.1
65614	1.08484	2.1
65615	1.08484	2.1
65616	1.22836	2.1
65617	0.919363	2.1
65618	1.16507	2.1
65619	0.924864	2.1
65620	1.89226	2.1
65621	1.89226	2.1
65622	1.89226	2.1
65623	1.89226	2.1
65624	1.89226	2.1
65625	1.89226	2.1
65626	1.89226	2.1
65627	1.89226	2.1
65628	1.89226	2.1
65629	1.89226	2.1
65630	1.89226	2.1
65631	1.89226	2.1
65632	2.6946	2.1
65633	2.63576	2.1
65634	2.5198	2.1
65635	2.5198	2.1
65636	2.8362	2.1
65637	2.8362	2.1
65638	1.3265	2.1
65639	1.24045	2.1
65640	0.983223	2.1
65641	0.954429	2.1
65642	1.06046	2.1
65643	1.10594	2.1
65644	1.1915	2.1
65645	1.1915	2.1
65646	1.31357	2.1
65647	1.09005	2.1
65648	1.29145	2.1
65649	0.995825	2.1
65650	1.89226	2.1
65651	1.89226	2.1
65652	1.89226	2.1
65653	1.89226	2.1

65654	3.78452	2.1
65655	1.89226	2.1
65656	1.89226	2.1
65657	1.89226	2.1
65658	1.89226	2.1
65659	3.78453	2.1
65660	1.91802	2.1
65661	2.27409	2.1
65662	2.48048	2.1
65663	2.22264	2.1
65664	1.91802	2.1
65665	1.66018	2.1
65666	1.13704	2.1
65667	1.24023	2.1
65668	1.22947	2.1
65669	1.31788	2.1
65670	1.23859	2.1
65671	1.23859	2.1
65672	1.23111	2.1
65673	1.23111	2.1
65674	1.24024	2.1
65675	1.31788	2.1
65676	1.13704	2.1
65677	1.22947	2.1
65678	1.89226	2.1
65679	1.89226	2.1
65680	1.89226	2.1
65681	1.89226	2.1
65682	1.89226	2.1
65683	1.89226	2.1
65684	1.89226	2.1
65685	1.89226	2.1
65686	1.89226	2.1
65687	1.89226	2.1
65688	3.78453	2.1
65689	1.05568	2.1
65690	0.959008	2.1
65691	1.32068	2.1
65692	1.24024	2.1
65693	1.15206	2.1
65694	1.15206	2.1
65695	1.14386	2.1
65696	1.14386	2.1
65697	1.05568	2.1
65698	1.32068	2.1
65699	0.959008	2.1
65700	1.24024	2.1
65701	1.32068	2.1
65702	1.24023	2.1
65703	1.31788	2.1
65704	1.4181	2.1
65705	1.31436	2.1
65706	1.31436	2.1
65707	1.3242	2.1
65708	1.3242	2.1
65709	1.32068	2.1
65710	1.4181	2.1
65711	1.24024	2.1

65712	1.31788	2.1
65713	1.89226	2.1
65714	1.89226	0.819128
65715	1.89226	1.8929
65716	1.89226	1.91167
65717	1.89226	1.86945
65718	1.89226	1.96655
65719	1.02925	0.105026
65720	1.16678	0.515617
65721	0.946132	0.561827
65722	0.946132	1.98044
65723	0.935677	0.940879
65724	0.935677	0.169093
65725	1.18902	1.80194
65726	1.21562	1.45219
65727	1.13433	0.42034
65728	1.04071	0.465374
65729	0.851553	0.652417
65730	1.06075	0.668846
65731	1.89226	1.29766
65732	1.89226	0.457478
65733	1.89226	0.561612
65734	1.89226	0.153679
65735	1.89226	0.334608
65736	1.89226	1.36232
65737	1.89226	1.94738
65738	1.89226	0.329195
65739	1.89226	0.829251
65740	1.89226	0.727508
65741	3.78453	0.63677
65742	0.946128	1.31812
65743	0.946128	0.553898
65744	0.946133	0.289038
65745	0.946133	0.453337
65746	0.946131	0.227182
65747	0.946131	0.393931
65748	0.94613	0.531701
65749	0.94613	0.256734
65750	0.946131	1.23263
65751	0.946131	0.232504
65752	0.946131	0.22312
65753	0.946131	0.116683
65754	0.946132	1.40529
65755	0.946132	0.625862
65756	0.946132	1.7408
65757	0.946132	0.930402
65758	0.946132	0.678283
65759	0.946132	0.12189
65760	0.946131	1.22003
65761	0.946131	1.63503
65762	0.946132	0.933573
65763	0.946132	1.15027
65764	0.946132	0.626103
65765	0.946132	0.830636
65766	0.946131	2.00496
65767	0.946131	1.6832
65768	0.946131	1.73256
65769	0.946131	1.10535

65770	0.946131	0.88417
65771	0.946131	1.96668
65772	0.946131	1.08435
65773	0.946131	0.713812
65774	0.946131	1.49822
65775	0.946131	1.49207
65776	0.946131	1.59162
65777	0.946131	1.60085
65778	1.89226	1.97012
65779	1.89226	1.84051
65780	1.89226	1.66282
65781	1.89226	2.1
65782	1.89226	0.844418
65783	1.89226	2.1
65784	1.89226	1.54007
65785	1.89226	1.27791
65786	1.89226	1.42409
65787	1.89226	2.02579
65788	1.89226	1.23848
65789	1.89226	2.1
65790	1.89226	1.94323
65791	1.89226	1.56874
65792	1.89226	1.60522
65793	1.89226	2.1
65794	1.89226	0.562101
65795	1.89226	2.1
65796	1.89226	2.1
65797	1.89226	2.07829
65798	1.89226	0.9263
65799	1.89226	2.1
65800	3.78452	2.0649
65801	0.946131	2.05247
65802	0.946131	1.32675
65803	1.17195	0.510207
65804	1.17195	1.81085
65805	0.958489	2.08169
65806	1.17195	1.59763
65807	0.946129	0.932635
65808	0.946129	1.77209
65809	0.946131	1.62302
65810	1.17195	1.61713
65811	0.958487	1.72363
65812	0.94613	1.73734
65813	2.69656	2.1
65814	2.17516	2.1
65815	2.41885	2.1
65816	2.41885	2.1
65817	2.46476	2.1
65818	2.46476	2.1
65819	2.69657	2.1
65820	2.30233	1.7899
65821	2.30233	2.1
65822	2.59476	2.1
65823	2.46477	2.1
65824	2.7572	2.1
65825	1.89226	2.1
65826	1.89226	0.745083
65827	1.89226	1.70441

65828	1.89226	1.71413
65829	1.89226	1.63865
65830	1.89226	1.82468
65831	1.89226	2.1
65832	1.89226	2.1
65833	1.89226	2.1
65834	1.89226	1.70586
65835	3.78452	2.1
65836	2.46477	2.1
65837	2.17516	2.1
65838	1.80252	2.1
65839	2.09715	2.1
65840	4.27231	2.1
65841	2.46477	2.1
65842	2.09715	2.02738
65843	2.30233	2.1
65844	2.30233	2.06538
65845	1.96416	1.61741
65846	2.09715	1.16543
65847	1.89227	1.98208
65848	1.89227	0.490316
65849	1.89227	1.33862
65850	1.89227	1.59165
65851	1.89227	1.57974
65852	1.89227	1.75332
65853	1.89226	2.1
65854	1.89226	1.64873
65855	1.89226	2.08253
65856	1.89226	2.1
65857	3.78452	2.1
65858	0.946132	2.1
65859	0.946132	2.1
65860	0.946132	2.1
65861	0.946132	2.1
65862	0.946132	2.1
65863	0.946132	2.1
65864	0.946132	2.1
65865	0.946132	2.1
65866	0.946132	2.1
65867	0.946132	2.1
65868	0.946131	2.1
65869	0.946131	2.1
65870	1.89226	2.1
65871	1.89226	2.1
65872	1.89226	2.1
65873	1.89226	2.1
65874	3.78453	2.1
65875	1.89226	2.1
65876	1.89226	2.1
65877	1.89226	2.1
65878	1.89226	2.1
65879	1.89226	2.1
65880	1.89226	2.1
65881	1.89226	2.1
65882	1.63707	2.1
65883	1.63707	2.1
65884	1.38859	2.1
65885	1.89226	2.1

65886	1.64377	2.1
65887	0.94613	2.1
65888	0.94613	2.1
65889	0.94613	2.1
65890	0.94613	2.1
65891	0.946131	2.1
65892	0.946131	2.1
65893	0.946129	2.1
65894	0.946129	2.1
65895	0.94613	2.1
65896	0.94613	2.1
65897	0.94613	2.1
65898	0.94613	2.1
65899	2.15888	2.1
65900	1.99309	2.1
65901	2.16841	2.1
65902	2.16841	2.1
65903	2.02311	2.1
65904	2.02311	1.89099
65905	1.89226	2.1
65906	1.89226	2.07801
65907	1.89226	2.1
65908	1.89226	2.1
65909	1.89226	2.1
65910	1.89226	2.02114
65911	1.89226	2.1
65912	1.89226	2.1
65913	1.89226	2.1
65914	1.89226	2.1
65915	1.89226	2.1
65916	1.89226	2.1
65917	1.99309	2.1
65918	1.96035	2.1
65919	1.72526	2.1
65920	1.72526	2.1
65921	2.16841	2.1
65922	2.16841	2.1
65923	2.15888	2.1
65924	2.16841	2.1
65925	2.3111	2.1
65926	2.35938	2.1
65927	4.51826	2.1
65928	1.89226	2.1
65929	1.89226	2.1
65930	1.89226	2.1
65931	1.89226	1.92543
65932	3.78452	2.1
65933	1.89226	2.1
65934	1.89226	2.1
65935	1.89226	2.1
65936	1.89226	1.84571
65937	3.78452	2.1
65938	2.16841	2.1
65939	1.96035	2.1
65940	2.35938	2.1
65941	2.18493	2.1
65942	4.31973	2.1
65943	1.89226	2.1

65944	1.89226	2.1
65945	1.89226	2.1
65946	1.89226	2.1
65947	3.78453	2.1
65948	1.89226	2.1
65949	1.89226	2.1
65950	1.89226	2.1
65951	1.89226	2.1
65952	3.78452	2.1
65953	0.946158	2.1
65954	0.946158	2.1
65955	0.818509	2.1
65956	0.818509	2.1
65957	0.933975	2.1
65958	0.818539	2.1
65959	0.946128	2.1
65960	0.946128	2.1
65961	0.94613	2.1
65962	0.933974	2.1
65963	0.818537	2.1
65964	0.946129	2.1
65965	0.946131	2.1
65966	0.946131	2.1
65967	0.946131	2.1
65968	0.946131	2.1
65969	0.946132	2.1
65970	0.946132	2.1
65971	0.946129	2.1
65972	0.946129	2.1
65973	0.946131	2.1
65974	0.946131	2.1
65975	0.94613	2.1
65976	0.94613	2.1
65977	1.89226	2.1
65978	1.89226	2.1
65979	1.89226	2.1
65980	1.89226	2.1
65981	3.78453	2.1
65982	1.89226	2.1
65983	1.89226	2.1
65984	1.89226	2.1
65985	1.89226	2.1
65986	3.78452	2.1
65987	1.89226	2.1
65988	1.8706	2.1
65989	1.8706	2.1
65990	1.68112	2.1
65991	2.0815	2.1
65992	1.88715	2.1
65993	0.94613	2.1
65994	0.94613	2.1
65995	0.94613	2.1
65996	0.94613	2.1
65997	0.946131	2.1
65998	0.946131	2.1
65999	0.946129	2.1
66000	0.946129	2.1
66001	0.94613	2.1

66002	0.94613	2.1
66003	0.94613	2.1
66004	0.94613	2.1
66005	1.76401	2.1
66006	2.60983	2.1
66007	2.50461	2.1
66008	2.35897	2.1
66009	1.90965	2.1
66010	1.76401	2.1
66011	1.89226	2.1
66012	1.89226	2.1
66013	1.89226	2.1
66014	1.89226	2.1
66015	3.78452	2.1
66016	1.89226	2.1
66017	1.89226	2.1
66018	1.89226	2.1
66019	1.89226	2.1
66020	1.89226	2.1
66021	1.89226	2.1
66022	1.90965	2.1
66023	2.83834	2.1
66024	2.7538	2.1
66025	2.60983	2.1
66026	2.05361	2.1
66027	1.90965	2.1
66028	1.89226	2.1
66029	1.89226	2.1
66030	1.89226	2.1
66031	1.89226	2.1
66032	1.89226	2.1
66033	1.89226	2.1
66034	1.89226	2.1
66035	1.89226	2.1
66036	1.89226	2.1
66037	1.89226	2.1
66038	3.78452	2.1
66039	2.83834	2.1
66040	1.89226	2.1
66041	2.60983	2.1
66042	2.60983	2.1
66043	1.89226	2.1
66044	1.89226	2.1
66045	1.89226	2.1
66046	1.89226	2.1
66047	1.89226	2.1
66048	1.89226	2.1
66049	1.89226	2.1
66050	1.89226	2.1
66051	1.89226	2.1
66052	1.89226	2.1
66053	1.89226	2.1
66054	1.89226	2.07338
66055	1.89226	2.1
66056	1.89226	2.01349
66057	2.44785	2.1
66058	1.80223	2.1
66059	2.80894	2.1

66060	2.18296	2.1
66061	4.63081	2.1
66062	1.89226	2.1
66063	1.89226	2.1
66064	1.89226	2.1
66065	1.89226	2.1
66066	1.89226	2.1
66067	1.89226	2.1
66068	0.946131	1.88362
66069	0.946131	2.1
66070	0.946127	2.1
66071	0.946127	2.1
66072	0.946131	2.1
66073	0.946131	2.1
66074	0.946128	2.1
66075	0.946128	2.1
66076	0.94613	2.1
66077	0.94613	2.1
66078	0.946129	2.1
66079	0.946129	2.1
66080	1.89226	2.1
66081	1.89226	2.1
66082	1.89226	2.1
66083	1.89226	2.1
66084	3.78452	2.1
66085	1.89226	2.1
66086	1.89226	2.1
66087	1.89226	2.1
66088	1.89226	2.1
66089	3.78453	2.1
66090	2.41083	2.1
66091	2.93937	2.1
66092	1.68761	2.1
66093	1.68761	2.1
66094	1.67248	2.1
66095	2.3957	2.1
66096	1.89226	2.1
66097	1.89226	2.1
66098	1.89226	2.1
66099	1.89226	2.1
66100	1.89226	2.1
66101	1.89226	2.1
66102	1.89227	2.1
66103	1.89227	2.1
66104	1.89227	2.1
66105	1.89227	2.1
66106	1.89227	2.1
66107	1.89227	2.1
66108	1.89224	1.50266
66109	1.89224	0.397393
66110	1.89224	1.371
66111	1.89224	1.42262
66112	3.78448	0.581479
66113	1.89224	2.1
66114	1.89224	1.5285
66115	1.89224	1.99503
66116	1.89224	1.52045
66117	1.89224	1.95912

66118	1.89224	1.71424
66119	1.11152	0.930719
66120	1.29346	1.72154
66121	0.978596	1.97055
66122	0.918324	2.0977
66123	1.06821	1.97919
66124	1.06821	0.622913
66125	0.985654	1.90165
66126	1.15278	2.08193
66127	1.07693	1.89635
66128	0.902432	1.901
66129	0.968939	1.9035
66130	1.34644	1.83181
66131	0.946132	0.397212
66132	0.946132	0.866748
66133	0.946132	0.859937
66134	0.946132	0.775477
66135	0.946131	1.07892
66136	0.946131	0.257299
66137	0.946132	1.36287
66138	0.946132	0.470041
66139	0.946132	0.903548
66140	0.946132	0.695048
66141	0.946132	0.315008
66142	0.946132	0.338139
66143	0.946133	1.61559
66144	0.946133	1.11156
66145	0.946132	1.78699
66146	0.946132	1.92367
66147	0.946132	1.77504
66148	0.946132	0.335167
66149	0.946133	2.1
66150	0.946133	1.43401
66151	0.946132	1.74822
66152	0.946132	1.73023
66153	0.946132	1.30243
66154	0.946132	1.45487
66155	1.89224	2.09245
66156	1.89224	2.1
66157	1.89224	2.1
66158	1.89224	2.1
66159	1.89224	2.1
66160	1.89224	2.1
66161	1.89224	2.1
66162	1.89224	2.1
66163	1.89224	2.1
66164	1.89224	2.1
66165	1.89224	2.1
66166	1.89224	2.1
66167	1.89226	2.1
66168	1.89226	2.1
66169	1.89226	2.1
66170	1.89226	2.1
66171	3.78453	2.1
66172	1.11056	2.1
66173	1.21595	2.1
66174	1.12763	2.1
66175	1.12763	2.1

66176	1.23339	2.1
66177	1.35697	2.1
66178	1.03581	2.1
66179	0.95954	2.1
66180	0.89761	2.1
66181	1.26602	2.1
66182	1.12749	2.1
66183	1.45307	2.1
66184	2.71775	2.1
66185	1.89226	2.1
66186	1.89226	2.1
66187	1.89226	2.1
66188	2.47025	2.1
66189	2.47025	2.1
66190	0.94613	2.1
66191	0.94613	2.1
66192	0.94613	2.1
66193	0.94613	2.1
66194	0.946128	2.1
66195	0.946128	2.1
66196	0.946132	2.1
66197	0.946132	2.1
66198	0.94613	2.1
66199	0.94613	2.1
66200	0.94613	2.1
66201	0.94613	2.1
66202	1.89226	1.86234
66203	1.89226	2.1
66204	1.89226	2.1
66205	1.89226	1.8964
66206	1.89226	2.1
66207	1.89226	1.89397
66208	1.89226	1.80205
66209	1.89226	2.1
66210	1.89226	2.1
66211	1.89226	1.74517
66212	1.89226	2.1
66213	1.89226	1.75357
66214	0.946132	2.1
66215	0.946132	2.1
66216	0.946133	2.1
66217	0.946133	2.1
66218	0.946133	2.1
66219	0.946133	2.1
66220	0.946132	2.1
66221	0.946132	2.1
66222	0.946133	2.1
66223	0.946133	2.1
66224	0.946132	2.1
66225	0.946132	2.1
66226	1.89226	1.87381
66227	1.89226	2.1
66228	1.89226	2.1
66229	1.89226	1.90339
66230	1.89226	2.1
66231	1.89226	1.90844
66232	0.946131	1.80942
66233	0.946131	1.81068

66234	0.946127	2.1
66235	0.946127	2.1
66236	0.946131	1.87383
66237	0.946131	2.1
66238	0.946127	2.1
66239	0.946127	1.92401
66240	0.94613	2.00138
66241	0.94613	2.00015
66242	0.946129	2.03055
66243	0.946129	2.02019
66244	1.94291	2.1
66245	2.00118	2.1
66246	2.36337	2.1
66247	2.41217	2.1
66248	4.35508	2.1
66249	1.89226	2.1
66250	1.89226	2.1
66251	1.89226	2.1
66252	1.89226	2.1
66253	3.78452	2.1
66254	1.89226	2.1
66255	1.89226	2.1
66256	1.89226	2.1
66257	1.89226	2.1
66258	3.78452	2.1
66259	1.06209	2.1
66260	1.06209	2.1
66261	1.05987	2.1
66262	1.05987	2.1
66263	0.920378	2.1
66264	1.12137	2.1
66265	1.00059	2.1
66266	1.20608	2.1
66267	1.20608	2.1
66268	1.12137	2.1
66269	1.00059	2.1
66270	0.920376	2.1
66271	2.77222	2.1
66272	2.41216	2.1
66273	2.77002	2.1
66274	2.36337	2.1
66275	2.81882	2.1
66276	2.41216	2.1
66277	1.89226	2.1
66278	1.89226	2.1
66279	1.89226	2.1
66280	1.89226	2.1
66281	1.89226	2.1
66282	1.89226	2.1
66283	1.89226	2.1
66284	1.89226	2.1
66285	1.89226	2.1
66286	1.89226	2.1
66287	3.78451	2.1
66288	2.41216	2.1
66289	2.24273	2.1
66290	2.81882	2.1
66291	2.68473	2.1

66292	5.09689	2.1
66293	1.89226	2.1
66294	1.89226	2.1
66295	1.89226	2.1
66296	1.89226	2.1
66297	3.78453	2.1
66298	2.17614	2.1
66299	2.65326	2.1
66300	1.93101	2.1
66301	2.58552	2.1
66302	4.58427	2.1
66303	2.65326	2.1
66304	1.89226	2.1
66305	1.89226	2.1
66306	1.89226	2.1
66307	2.58552	2.1
66308	2.58552	2.1
66309	1.89226	2.1
66310	1.89226	2.1
66311	1.89226	2.1
66312	1.89226	2.1
66313	3.78453	2.1
66314	1.89226	2.1
66315	1.89226	2.1
66316	1.89226	2.1
66317	1.89226	2.1
66318	3.78453	2.1
66319	1.93101	2.1
66320	2.58552	2.1
66321	1.46507	2.1
66322	2.52893	2.1
66323	4.45994	2.1
66324	1.26446	2.1
66325	1.26446	2.1
66326	0.946132	2.1
66327	0.946132	2.1
66328	0.94613	2.1
66329	0.94613	2.1
66330	1.29276	2.1
66331	1.26446	2.1
66332	1.26446	2.1
66333	0.94613	2.1
66334	0.94613	2.1
66335	1.29276	2.1
66336	1.89226	2.1
66337	1.89226	2.1
66338	1.89226	2.1
66339	1.89226	2.1
66340	3.78453	2.1
66341	1.89226	2.1
66342	1.89226	2.1
66343	1.89226	2.1
66344	1.89226	2.1
66345	3.78453	2.1
66346	1.83378	2.1
66347	2.52893	2.1
66348	1.75592	2.1
66349	2.49844	2.1

66350	4.28485	2.1
66351	1.24922	2.1
66352	1.24922	2.1
66353	0.946133	2.1
66354	0.946133	2.1
66355	0.946131	2.1
66356	0.946131	2.1
66357	1.26446	2.1
66358	1.24922	2.1
66359	1.24922	2.1
66360	0.946131	2.1
66361	1.26446	2.1
66362	0.94613	2.1
66363	0.946132	2.1
66364	0.946132	2.1
66365	0.946132	2.1
66366	0.946132	2.1
66367	0.946132	2.1
66368	0.946132	2.1
66369	0.946132	2.1
66370	0.946132	2.1
66371	0.946132	2.1
66372	0.946132	2.1
66373	0.946131	2.1
66374	0.946131	2.1
66375	1.89226	2.1
66376	1.89226	2.1
66377	1.89226	2.1
66378	1.89226	2.1
66379	1.89226	2.1
66380	1.89226	2.1
66381	1.0465	2.1
66382	1.0465	2.1
66383	1.06047	2.1
66384	1.06047	2.1
66385	1.25687	2.1
66386	1.24922	2.1
66387	0.87796	2.1
66388	0.857749	2.1
66389	0.857749	2.1
66390	1.25687	2.1
66391	0.877959	2.1
66392	1.24922	2.1
66393	2.49843	2.1
66394	1.89226	2.1
66395	2.51374	2.1
66396	1.89226	2.1
66397	4.406	2.1
66398	1.89226	2.1
66399	1.89226	2.1
66400	1.89226	2.1
66401	1.89226	2.1
66402	1.89226	2.1
66403	1.89226	2.1
66404	1.89226	2.1
66405	1.89226	2.1
66406	1.89226	2.1
66407	1.89226	2.1

66408	1.89226	2.1
66409	1.89226	2.1
66410	1.89226	2.1
66411	1.89226	2.1
66412	1.89226	2.1
66413	1.89226	2.1
66414	1.89226	2.1
66415	1.89226	2.1
66416	1.03835	2.1
66417	1.03835	2.1
66418	1.15388	2.1
66419	0.94267	2.1
66420	0.736433	2.1
66421	0.849239	2.1
66422	1.15014	2.1
66423	1.39058	2.1
66424	0.838639	2.1
66425	1.39056	2.1
66426	1.09399	2.1
66427	0.736444	2.1
66428	1.89226	2.01662
66429	1.89226	2.1
66430	1.89226	2.1
66431	1.89226	2.00817
66432	1.89226	2.1
66433	1.89226	2.03348
66434	0.94613	1.99804
66435	0.94613	2.02293
66436	0.946127	2.1
66437	0.946127	2.1
66438	0.946131	2.1
66439	0.946131	1.95667
66440	0.946126	2.1
66441	0.946126	2.07313
66442	0.946128	2.1
66443	0.946128	2.1
66444	0.946128	2.1
66445	0.946128	2.1
66446	1.89226	2.1
66447	1.89226	1.80638
66448	1.89226	1.76423
66449	1.89226	2.1
66450	3.78453	1.89812
66451	0.946133	1.59075
66452	0.946133	1.50059
66453	0.946128	1.83841
66454	0.946128	1.80366
66455	0.946131	1.7789
66456	0.946131	1.62283
66457	0.946131	0.755453
66458	0.946131	1.76787
66459	0.946131	1.65995
66460	0.946131	1.66639
66461	0.94613	1.71163
66462	0.94613	1.70957
66463	1.89226	1.69822
66464	1.89226	2.0657
66465	1.89226	2.01302

66466	1.89226	1.75589
66467	1.89226	2.01441
66468	1.89226	1.77403
66469	1.89226	1.46018
66470	1.89226	1.79107
66471	1.89226	1.73187
66472	1.89226	1.53376
66473	1.89226	1.71587
66474	1.89226	1.54803
66475	1.89226	1.48672
66476	1.89226	1.8575
66477	1.89226	1.74432
66478	1.89226	1.58858
66479	1.89226	1.74615
66480	1.89226	1.59458
66481	1.89226	1.72048
66482	2.67388	2.1
66483	2.23684	2.02569
66484	2.23684	1.81261
66485	1.89226	2.03861
66486	1.89226	1.82633
66487	1.89226	2.1
66488	1.89226	2.1
66489	1.89226	2.1
66490	1.89226	2.1
66491	1.89226	2.1
66492	1.89226	2.1
66493	1.72197	2.1
66494	2.51374	2.1
66495	2.58004	2.1
66496	2.58004	2.1
66497	1.7155	2.1
66498	1.7155	2.1
66499	2.51374	2.1
66500	1.89226	2.1
66501	1.89226	2.1
66502	1.89226	2.1
66503	2.58003	2.1
66504	2.58003	2.1
66505	0.946132	2.1
66506	0.946132	2.1
66507	0.946132	2.1
66508	0.946132	2.1
66509	0.946132	2.1
66510	0.946132	2.1
66511	0.946132	2.1
66512	0.946132	2.1
66513	0.946131	2.1
66514	0.946131	2.1
66515	0.946132	2.1
66516	0.946132	2.1
66517	1.89226	2.1
66518	1.89226	2.1
66519	1.89226	2.1
66520	1.89226	2.1
66521	3.78453	2.1
66522	1.08262	2.1
66523	1.08262	2.1

66524	1.09674	2.1
66525	1.09674	2.1
66526	1.34652	2.1
66527	1.29002	2.1
66528	0.860984	2.1
66529	0.889338	2.1
66530	1.34652	2.1
66531	0.889338	2.1
66532	0.860983	2.1
66533	1.29002	2.1
66534	1.34652	2.1
66535	1.34652	2.1
66536	0.946133	2.1
66537	0.946133	2.1
66538	0.946131	2.1
66539	0.946131	2.1
66540	1.34652	2.1
66541	1.29002	2.1
66542	1.34652	2.1
66543	0.946131	2.1
66544	0.94613	2.1
66545	1.29002	2.1
66546	1.89226	2.1
66547	1.89226	2.1
66548	1.89226	2.1
66549	1.89226	2.1
66550	1.89226	2.1
66551	1.89226	2.1
66552	1.89226	2.1
66553	1.89226	2.1
66554	1.89226	2.1
66555	1.89226	2.1
66556	1.89226	2.1
66557	1.89226	2.1
66558	0.946131	2.1
66559	0.946131	2.1
66560	0.946131	2.1
66561	0.946131	2.1
66562	0.946131	2.1
66563	0.946131	2.1
66564	0.946131	2.1
66565	0.946131	2.1
66566	0.946131	2.1
66567	0.946131	2.1
66568	0.946131	2.1
66569	0.946131	2.1
66570	1.89226	2.1
66571	1.89226	2.1
66572	1.89226	2.1
66573	1.89226	2.1
66574	1.89226	2.1
66575	1.89226	2.1
66576	1.04103	2.1
66577	1.13849	2.1
66578	0.943014	2.1
66579	0.943014	2.1
66580	0.943591	2.1
66581	0.943591	2.1

66582	1.03757	2.1
66583	1.04098	2.1
66584	1.03761	2.1
66585	0.946131	2.1
66586	0.943041	2.1
66587	0.946131	2.1
66588	0.946128	2.1
66589	0.946128	2.1
66590	0.946133	2.1
66591	0.946133	2.1
66592	0.946131	2.1
66593	0.946131	2.1
66594	0.946131	2.1
66595	0.946131	2.1
66596	0.946131	2.1
66597	0.946131	2.1
66598	0.94613	2.1
66599	0.94613	2.1
66600	0.946132	2.1
66601	0.946132	2.1
66602	0.946132	2.1
66603	0.946132	2.1
66604	0.946132	2.1
66605	0.946132	2.1
66606	0.946132	2.1
66607	0.946132	2.1
66608	0.946132	2.1
66609	0.946132	2.1
66610	0.946131	2.1
66611	0.946131	2.1
66612	1.89226	1.31027
66613	1.89226	1.47304
66614	1.89226	1.3779
66615	1.89226	1.24881
66616	3.78452	1.38508
66617	0.946132	1.49068
66618	0.946132	1.43435
66619	0.946131	1.36017
66620	0.946131	1.39438
66621	0.946132	1.37565
66622	0.946132	1.60772
66623	0.946132	1.49163
66624	0.946132	1.45033
66625	0.946132	1.3905
66626	0.946132	1.41025
66627	0.946132	1.44004
66628	0.946132	1.46753
66629	0.935482	1.51734
66630	1.08999	1.50864
66631	1.24315	1.39987
66632	1.12559	1.40002
66633	1.21343	1.38134
66634	0.889397	1.86043
66635	1.01265	1.36154
66636	1.01265	1.53669
66637	1.07332	1.37269
66638	1.34292	1.40171
66639	0.865649	1.51223

66640	0.840024	1.58803
66641	1.15433	1.41813
66642	1.02858	1.43092
66643	1.34082	1.25407
66644	1.08357	1.24833
66645	1.10938	1.22081
66646	1.33349	1.70119
66647	1.12232	1.45159
66648	1.12232	1.22899
66649	1.21357	1.27726
66650	1.3408	1.27748
66651	1.02859	1.39852
66652	1.24211	1.42247
66653	1.89226	1.40234
66654	1.89226	1.18045
66655	1.89226	1.26325
66656	1.89226	1.47711
66657	1.89226	1.19137
66658	1.89226	1.32372
66659	1.89226	1.33542
66660	1.89226	1.44234
66661	1.89226	1.39609
66662	1.89226	1.50505
66663	1.89226	1.42067
66664	1.89226	1.47143
66665	2.0815	1.49439
66666	2.79029	1.38751
66667	2.36793	1.42065
66668	2.58428	1.56041
66669	2.38161	1.33038
66670	2.59922	1.4465
66671	1.89226	2.1
66672	1.89226	1.79471
66673	1.89226	2.03873
66674	1.89226	2.02137
66675	1.89226	2.04584
66676	1.89226	2.03916
66677	1.89226	1.77034
66678	1.89226	0.609828
66679	1.89226	1.28476
66680	1.89226	1.43246
66681	3.78452	1.5435
66682	1.89226	2.03551
66683	1.89226	1.54666
66684	1.89226	1.83853
66685	1.89226	1.75903
66686	1.89226	1.87177
66687	1.89226	1.78564
66688	0.946132	1.65279
66689	0.946132	1.94808
66690	0.94613	2.1
66691	0.94613	1.97718
66692	0.946131	2.08249
66693	0.946131	1.62685
66694	0.946131	1.93789
66695	0.946131	2.1
66696	0.946131	2.00586
66697	0.946131	2.01566

66698	0.946131	2.00602
66699	0.946131	1.98945
66700	1.85178	1.83162
66701	1.89226	1.40779
66702	2.0815	0.888467
66703	2.27054	1.51827
66704	1.70303	0.634651
66705	1.5217	1.06272
66706	1.89226	2.07575
66707	1.89226	1.5291
66708	1.89226	1.78342
66709	1.89226	1.65064
66710	1.89226	1.92394
66711	1.89226	1.71278
66712	2.58823	0.169715
66713	2.02924	0.190579
66714	1.79398	0.117797
66715	1.79398	0.0819581
66716	2.76708	0.505292
66717	2.76708	0.128825
66718	0.946133	0.165336
66719	0.946133	0.197772
66720	1.29411	0.133915
66721	1.38353	0.0981848
66722	0.946131	0.112605
66723	0.946131	0.134115
66724	1.38354	0.211564
66725	1.38354	0.0933109
66726	0.946131	0.13602
66727	1.38354	0.128771
66728	0.94613	0.121869
66729	1.29411	0.13471
66730	1.89226	0.113651
66731	1.89226	0.0778516
66732	1.89226	0.114364
66733	1.89226	0.173345
66734	3.78452	0.118772
66735	0.946131	0.0968505
66736	0.946131	0.101974
66737	0.946131	0.107531
66738	0.946131	0.117083
66739	0.946129	0.123833
66740	0.946129	0.0710097
66741	0.946133	0.133157
66742	0.946133	0.0961616
66743	0.946131	0.123425
66744	0.946131	0.116898
66745	0.946131	0.0787252
66746	0.946131	0.0840016
66747	1.89226	0.0570457
66748	1.89226	0.0890139
66749	1.89226	0.114791
66750	1.89226	0.0959767
66751	3.78452	0.0657358
66752	0.94613	0.088797
66753	0.94613	0.0804344
66754	0.946131	0.0754381
66755	0.946131	0.0871565

66756	0.946128	0.0901102
66757	0.946128	0.062851
66758	0.946133	0.103517
66759	0.946133	0.0624443
66760	0.946131	0.0939913
66761	0.946131	0.0865083
66762	0.946131	0.0674061
66763	0.946131	0.0661814
66764	0.946129	1.5635
66765	0.946129	1.29341
66766	1.13486	1.13399
66767	0.859753	1.42886
66768	0.851541	1.41275
66769	0.851541	1.03744
66770	1.34346	1.18635
66771	1.26212	1.01161
66772	0.851553	1.12278
66773	1.12403	1.09785
66774	0.851474	0.849411
66775	0.956163	1.05492
66776	0.946132	0.513962
66777	0.946132	0.524775
66778	0.946133	0.59921
66779	0.946133	0.48083
66780	0.946132	0.631804
66781	0.946132	0.460343
66782	0.946133	0.505747
66783	0.946133	0.531343
66784	0.946133	0.503943
66785	0.946133	0.576452
66786	0.946132	0.484644
66787	0.946132	0.509531
66788	1.89226	0.522629
66789	1.89226	0.645888
66790	1.89226	0.733263
66791	1.89226	0.679205
66792	3.78453	0.640634
66793	0.946128	1.56869
66794	0.946128	1.48998
66795	0.946132	1.65169
66796	0.946132	1.50257
66797	0.94613	1.66088
66798	0.94613	1.5729
66799	0.94613	1.60237
66800	0.94613	1.49462
66801	0.946131	1.59471
66802	0.946131	1.60849
66803	0.94613	1.58104
66804	0.94613	1.54207
66805	0.946132	0.32776
66806	0.946132	0.30291
66807	0.946133	0.339647
66808	0.946133	0.290728
66809	0.946132	0.347539
66810	0.946132	0.319128
66811	0.946133	0.302818
66812	0.946133	0.292985
66813	0.946132	0.302555

66814	0.946132	0.331116
66815	0.946132	0.300592
66816	0.946132	0.315156
66817	1.89226	0.383196
66818	1.89226	0.379909
66819	1.89226	0.480228
66820	1.89226	0.334454
66821	1.89226	0.470845
66822	1.89226	0.306104
66823	1.89226	0.89708
66824	1.89226	0.689333
66825	1.89226	0.575341
66826	1.89226	0.7087
66827	1.89226	0.626242
66828	1.89226	0.68405
66829	1.89226	2.1
66830	1.89226	2.1
66831	1.89226	2.1
66832	1.89226	2.1
66833	3.78452	2.1
66834	1.07652	2.1
66835	1.07652	2.1
66836	0.928749	2.1
66837	0.928749	2.1
66838	0.946132	2.1
66839	0.946132	2.1
66840	1.05914	2.1
66841	0.928746	2.1
66842	1.05914	2.1
66843	0.946131	2.1
66844	0.94613	2.1
66845	0.928747	2.1
66846	1.89226	2.1
66847	1.89226	2.1
66848	1.89226	2.1
66849	1.89226	2.1
66850	1.89226	2.1
66851	1.89226	2.1
66852	1.89227	2.1
66853	1.89227	2.1
66854	1.89227	2.1
66855	1.89227	2.1
66856	1.89227	2.1
66857	1.89227	2.1
66858	1.89226	2.1
66859	1.89226	2.1
66860	1.89226	2.1
66861	1.89226	2.1
66862	1.89226	2.1
66863	1.89226	2.1
66864	2.11828	2.1
66865	1.89226	2.1
66866	2.35845	2.1
66867	1.89226	2.1
66868	4.25071	2.1
66869	1.89226	2.1
66870	1.89226	2.1
66871	1.89226	2.1

66872	1.89226	2.1
66873	3.78453	2.1
66874	1.89226	2.1
66875	1.89226	2.1
66876	1.89226	2.1
66877	1.89226	2.1
66878	1.89226	2.1
66879	1.89226	2.1
66880	1.89226	1.09353
66881	1.89226	1.65059
66882	1.65573	1.63125
66883	1.65573	1.26137
66884	1.89226	1.60272
66885	1.89226	1.20702
66886	1.89226	1.22412
66887	1.89226	1.4952
66888	1.89226	1.47101
66889	1.89226	1.37487
66890	1.89226	1.46697
66891	1.89226	1.37288
66892	1.89226	0.94039
66893	1.89226	1.51446
66894	1.89226	1.51814
66895	1.89226	1.25647
66896	1.89226	1.49829
66897	1.89226	1.14119
66898	1.89226	1.25811
66899	1.89226	1.4266
66900	1.89226	1.39418
66901	1.89226	1.29568
66902	1.89226	1.39176
66903	1.89226	1.29387
66904	0.946128	1.30393
66905	0.946128	1.27489
66906	0.946125	1.42606
66907	0.946125	1.42573
66908	0.94613	1.3293
66909	0.94613	1.45025
66910	0.946124	1.42786
66911	0.946124	1.3109
66912	0.946127	1.35499
66913	0.946127	1.36561
66914	0.946127	1.37781
66915	0.946127	1.3685
66916	1.89226	1.32759
66917	1.89226	1.50695
66918	1.89226	1.36413
66919	1.89226	1.27736
66920	3.78452	1.35748
66921	1.89226	1.5991
66922	1.89226	1.58013
66923	1.89226	1.45686
66924	1.89226	1.62243
66925	3.78453	1.57483
66926	1.05926	1.72928
66927	0.891358	2.04403
66928	0.946129	1.61649
66929	0.946129	1.70822

66930	0.946101	1.60795
66931	0.946101	2.1
66932	0.891386	1.62946
66933	0.891386	1.70165
66934	0.891357	1.66306
66935	0.946129	1.65846
66936	0.94613	1.68201
66937	1.05926	1.69866
66938	0.946126	1.45926
66939	0.946126	1.51904
66940	0.94613	1.37476
66941	0.94613	1.44147
66942	0.946131	1.47698
66943	0.946131	1.4754
66944	0.946125	1.51487
66945	0.946125	1.28049
66946	0.946127	1.41124
66947	0.946127	1.3994
66948	0.946128	1.49973
66949	0.946128	1.48667
66950	1.89226	1.47063
66951	1.89226	1.31483
66952	1.89226	1.27338
66953	1.89226	1.57615
66954	1.89226	1.30979
66955	1.89226	1.61801
66956	1.89226	1.55109
66957	1.89226	1.70166
66958	1.89226	1.6098
66959	1.89226	1.73907
66960	1.89226	1.51063
66961	1.89226	1.69292
66962	1.16109	1.82081
66963	1.23243	2.00717
66964	0.659819	1.64126
66965	0.659819	1.72345
66966	0.697965	1.55956
66967	1.00087	2.0967
66968	0.891386	1.80831
66969	0.891386	1.69551
66970	0.946129	1.72033
66971	0.65979	1.63512
66972	0.891357	1.74354
66973	0.946129	1.73967
66974	1.89225	1.33674
66975	1.89225	1.4722
66976	1.89225	1.62663
66977	1.89225	1.31793
66978	1.89225	1.69439
66979	1.89225	1.43222
66980	1.79981	0.255358
66981	2.02514	0.365925
66982	2.33247	0.470166
66983	2.12891	0.250629
66984	4.15405	0.272116
66985	2.48284	1.0972
66986	2.02514	0.426415
66987	2.33247	0.517525

66988	2.02514	0.787774
66989	2.48567	0.480879
66990	2.17834	0.709565
66991	1.89226	0.38786
66992	1.89226	0.568673
66993	1.89226	0.693378
66994	1.89226	0.395914
66995	3.78453	0.436254
66996	1.89227	0.333422
66997	1.89227	0.200928
66998	1.89227	0.208809
66999	1.89227	0.297884
67000	1.89227	0.249231
67001	1.89227	0.315832
67002	2.50894	0.206043
67003	2.70118	0.337401
67004	2.12891	0.213939
67005	2.12891	0.274112
67006	2.7125	0.207258
67007	2.33247	0.291378
67008	1.89226	0.532915
67009	1.89226	0.301828
67010	1.89226	0.318148
67011	1.89226	0.475459
67012	1.89226	0.278646
67013	1.89226	0.419873
67014	0.946133	0.2738
67015	0.946133	0.267554
67016	0.946132	0.178017
67017	0.946132	0.178933
67018	0.946132	0.183
67019	0.946132	0.283238
67020	0.946133	0.206803
67021	0.946133	0.274026
67022	0.946132	0.219816
67023	0.946132	0.204511
67024	0.946132	0.228995
67025	0.946132	0.239871
67026	1.90098	0.105776
67027	1.36463	0.141265
67028	1.56656	0.107491
67029	1.36463	0.133077
67030	2.04386	0.100588
67031	1.89227	0.143649
67032	1.89226	0.120604
67033	1.89226	0.136401
67034	1.89226	0.113128
67035	1.89226	0.142841
67036	1.89226	0.114767
67037	1.89226	0.143415
67038	1.89226	0.150915
67039	1.89226	0.211038
67040	1.89226	0.163278
67041	1.89226	0.229625
67042	1.89226	0.149685
67043	1.89226	0.210929
67044	1.89226	0.155082
67045	1.89226	0.235775

67046	1.89226	0.164397
67047	1.89226	0.209033
67048	1.89226	0.168422
67049	1.89226	0.228157
67050	1.89226	0.0829119
67051	1.70303	0.10643
67052	2.1292	0.0854046
67053	1.70303	0.106509
67054	2.32302	0.0847455
67055	1.90098	0.111624
67056	1.89226	0.101749
67057	1.89226	0.1132
67058	1.89226	0.0919022
67059	1.89226	0.122755
67060	1.89226	0.0997949
67061	1.89226	0.130301
67062	1.89226	0.117813
67063	1.89226	0.138616
67064	1.89226	0.119635
67065	1.89226	0.158906
67066	1.89226	0.111026
67067	1.89226	0.147656
67068	1.89226	0.108491
67069	1.89226	0.128948
67070	1.89226	0.102745
67071	1.89226	0.130427
67072	1.89226	0.105567
67073	1.89226	0.138617
67074	1.89226	1.02622
67075	1.89226	1.8219
67076	1.89226	1.8414
67077	1.89226	1.42565
67078	3.78453	1.238
67079	1.89226	1.85446
67080	1.89226	2.1
67081	1.89226	1.38854
67082	1.89226	2.1
67083	1.89226	1.82741
67084	1.89226	2.1
67085	1.89226	1.81079
67086	1.89226	1.58911
67087	1.89226	1.18971
67088	1.89226	1.69962
67089	3.78452	1.72735
67090	1.89224	1.87438
67091	1.89224	1.37626
67092	1.89224	1.60114
67093	1.89224	1.84437
67094	1.89224	1.20726
67095	1.89224	1.78266
67096	1.89224	1.8872
67097	1.89224	2.1
67098	1.89224	1.95108
67099	1.89224	2.1
67100	1.89224	1.59772
67101	1.89224	2.1
67102	1.31307	2.1
67103	1.31307	2.1

67104	0.888948	1.87736
67105	0.888948	1.8065
67106	1.2559	1.89857
67107	0.888979	2.1
67108	0.946114	2.1
67109	0.946114	1.85043
67110	0.946129	1.81095
67111	0.888989	1.87818
67112	0.946104	1.77807
67113	1.25588	1.86861
67114	1.89224	1.02215
67115	1.89224	1.8367
67116	1.89224	1.31187
67117	1.89224	1.83481
67118	1.89224	1.57896
67119	1.89224	1.9207
67120	1.79375	0.107992
67121	1.47689	0.107528
67122	1.97732	0.0528848
67123	2.26687	0.0769187
67124	3.74375	0.083589
67125	2.05759	0.123812
67126	2.26686	0.0798992
67127	1.79374	0.0919655
67128	1.79374	0.150579
67129	2.49889	0.0617848
67130	2.49889	0.116707
67131	1.89226	0.155325
67132	1.89226	0.1439
67133	1.89226	0.0721137
67134	1.89226	0.124774
67135	3.78452	0.112911
67136	1.89227	0.0805688
67137	1.89227	0.0735348
67138	1.89227	0.116596
67139	1.89227	0.124189
67140	1.89227	0.116078
67141	1.89227	0.104208
67142	1.97732	0.0816864
67143	2.26687	0.104128
67144	2.6607	0.0662971
67145	2.41464	0.0797714
67146	4.68151	0.0839057
67147	1.89226	0.111625
67148	1.89226	0.178183
67149	1.89226	0.0922961
67150	1.89226	0.0882836
67151	3.78452	0.109899
67152	1.89226	0.100978
67153	1.89226	0.0885877
67154	1.89226	0.112155
67155	1.89226	0.174484
67156	1.89226	0.0779836
67157	1.89226	0.105107
67158	1.89226	1.30977
67159	1.89226	1.5556
67160	1.89226	1.41199
67161	1.89226	1.30275

67162	3.78452	1.33213
67163	1.89226	1.38728
67164	1.89226	1.64865
67165	1.89226	1.31804
67166	1.89226	1.61171
67167	1.89226	1.39549
67168	1.89226	1.72359
67169	1.89226	1.58549
67170	1.89226	1.32798
67171	1.89226	1.31584
67172	1.89226	1.54466
67173	1.89226	1.29749
67174	1.89226	1.50167
67175	1.89226	1.38648
67176	1.89226	1.25678
67177	1.89226	1.29721
67178	1.89226	1.51266
67179	1.89226	1.20807
67180	1.89226	1.37196
67181	1.89226	1.37103
67182	2.45282	1.45169
67183	1.89226	1.46153
67184	1.89226	1.62954
67185	1.89226	1.29255
67186	1.89226	1.47679
67187	1.89226	1.2671
67188	1.89226	1.53353
67189	1.89226	1.33577
67190	1.89226	1.30959
67191	1.89226	1.58548
67192	1.89226	1.60402
67193	1.87308	0.0917519
67194	1.88631	0.0950448
67195	1.63908	0.100041
67196	1.70302	0.120845
67197	2.08745	0.0808744
67198	2.16916	0.0908567
67199	2.41019	0.0876664
67200	2.733	0.0772463
67201	2.08121	0.0892563
67202	2.08121	0.114139
67203	2.74362	0.0861094
67204	2.41464	0.0887703
67205	1.89226	0.0865325
67206	1.89226	0.12637
67207	1.89226	0.0817287
67208	1.89226	0.112917
67209	1.89226	0.0891536
67210	1.89226	0.135763
67211	0.946133	0.104788
67212	0.946133	0.0996068
67213	0.946132	0.107917
67214	0.946132	0.107447
67215	0.946132	0.10671
67216	0.946132	0.140973
67217	0.946133	0.0886902
67218	0.946133	0.110133
67219	0.946132	0.0886091

67220	0.946132	0.101082
67221	0.946133	0.10545
67222	0.946133	0.137462
67223	2.02912	0.0942934
67224	2.57016	0.0907978
67225	1.88631	0.0971928
67226	1.88631	0.124341
67227	2.55301	0.09784
67228	2.4102	0.103004
67229	1.89226	0.116161
67230	1.89226	0.13649
67231	1.89226	0.0903943
67232	1.89226	0.0907963
67233	3.78452	0.109621
67234	1.89227	0.117311
67235	1.89227	0.0998724
67236	1.89227	0.108094
67237	1.89227	0.136718
67238	1.89227	0.090201
67239	1.89227	0.104948
67240	1.89226	2.1
67241	1.89226	2.1
67242	1.89226	2.09797
67243	1.89226	2.1
67244	3.78452	2.1
67245	1.2515	2.06842
67246	1.34788	2.1
67247	0.946132	2.1
67248	0.946132	2.1
67249	0.973043	2.1
67250	1.16766	2.1
67251	0.919222	2.0004
67252	1.2159	2.1
67253	0.946132	2.1
67254	0.946132	2.1
67255	1.33746	2.1
67256	1.13537	2.1
67257	1.89226	2.1
67258	1.89226	2.1
67259	1.89226	2.1
67260	1.89226	2.1
67261	1.89226	2.1
67262	1.89226	2.1
67263	1.89226	2.06749
67264	1.89226	2.1
67265	1.89226	2.1
67266	1.89226	2.0749
67267	3.78452	2.09664
67268	1.89227	2.1
67269	1.89227	1.90024
67270	1.89227	2.1
67271	1.89227	2.01123
67272	1.89227	2.1
67273	1.89227	1.99436
67274	0.946132	2.09878
67275	0.946132	2.08419
67276	0.946129	2.1
67277	0.946129	2.1

67278	0.946131	2.1
67279	0.946131	2.1
67280	0.946131	2.1
67281	0.946131	2.08009
67282	0.946131	2.1
67283	0.946131	2.1
67284	0.94613	2.1
67285	0.94613	2.1
67286	1.89226	1.27629
67287	1.89226	1.28027
67288	1.89226	1.25818
67289	1.89226	1.16538
67290	3.78453	1.26995
67291	0.946132	1.38365
67292	0.946132	1.32272
67293	0.946132	1.24905
67294	0.946132	1.27452
67295	0.946132	1.20477
67296	0.946132	1.53737
67297	0.946132	1.34898
67298	0.946132	1.35276
67299	0.946131	1.44341
67300	0.946131	1.26552
67301	0.946132	1.32475
67302	0.946132	1.31456
67303	1.89226	1.33243
67304	1.89226	1.12313
67305	1.89226	1.11243
67306	1.89226	1.21401
67307	1.89226	1.20211
67308	1.89226	1.29862
67309	1.89226	1.31095
67310	1.89226	1.18773
67311	1.89226	1.17122
67312	1.89226	1.19409
67313	3.78453	1.18216
67314	0.96614	1.32516
67315	0.96614	1.35581
67316	0.946131	1.1985
67317	0.946131	1.23634
67318	0.946131	1.24506
67319	0.96614	1.24518
67320	1.37736	1.35392
67321	0.946131	1.33992
67322	1.37736	1.41631
67323	0.96614	1.23952
67324	0.946131	1.32499
67325	0.946131	1.21968
67326	0.946129	1.26397
67327	0.946129	1.21807
67328	0.946128	1.18411
67329	0.946128	1.1973
67330	0.946132	1.1163
67331	0.946132	1.4655
67332	0.946126	1.24075
67333	0.946126	1.22501
67334	0.946129	1.14768
67335	0.946129	1.16952

67336	0.946129	1.15139
67337	0.946129	1.23168
67338	1.89226	0.103987
67339	1.65723	0.115756
67340	1.83117	0.114825
67341	1.65723	0.1021
67342	2.0662	0.13187
67343	1.89226	0.118156
67344	1.89226	0.10687
67345	1.89226	0.129267
67346	1.89226	0.114124
67347	1.89226	0.11004
67348	1.89226	0.124507
67349	1.89226	0.116906
67350	1.89226	0.103348
67351	1.89226	0.130848
67352	1.89226	0.124703
67353	1.89226	0.116311
67354	1.89226	0.125667
67355	1.89226	0.119886
67356	1.89226	0.097338
67357	1.89226	0.107805
67358	1.89226	0.103327
67359	1.89226	0.105714
67360	1.89226	0.112093
67361	1.89226	0.110049
67362	1.89226	0.102018
67363	1.83117	0.112674
67364	1.92807	0.110215
67365	1.83117	0.100349
67366	1.98916	0.123068
67367	1.89226	0.111135
67368	1.89226	0.114368
67369	1.89226	0.123868
67370	1.89226	0.109333
67371	1.89226	0.107815
67372	3.78453	0.115222
67373	1.89226	0.106022
67374	1.89226	0.17136
67375	1.89226	0.208952
67376	1.89226	0.157374
67377	1.89226	0.11963
67378	1.89226	0.11735
67379	1.89226	0.0962677
67380	1.89226	0.105136
67381	1.89226	0.155998
67382	1.89226	0.113374
67383	1.89226	0.109029
67384	1.89226	0.103179
67385	1.88037	0.0891965
67386	1.89227	0.0847858
67387	1.89227	0.0919939
67388	1.89227	0.0931517
67389	1.98599	0.099054
67390	1.98599	0.0944888
67391	1.89226	0.0930083
67392	1.89226	0.0953535
67393	1.89226	0.101619

67394	1.89226	0.214817
67395	1.89226	0.0988616
67396	1.89226	0.101465
67397	1.89226	0.104052
67398	1.89226	0.106274
67399	1.89226	0.113265
67400	1.89226	0.351509
67401	3.78452	0.0902747
67402	1.89226	0.093615
67403	1.89226	0.155902
67404	1.89226	0.123562
67405	1.89226	0.0908571
67406	1.89226	0.126126
67407	1.89226	0.106613
67408	1.90116	0.0892201
67409	1.89227	0.0819489
67410	1.89226	0.0932059
67411	1.89227	0.0982845
67412	1.88036	0.0989031
67413	1.88037	0.0854482
67414	1.89226	0.0911296
67415	1.89226	0.0918435
67416	1.89226	0.11951
67417	1.89226	0.160807
67418	1.89226	0.0944249
67419	1.89226	0.0930763
67420	0.946132	0.121479
67421	0.946132	0.108989
67422	0.946128	0.126621
67423	0.946128	0.132714
67424	0.94613	0.157795
67425	0.94613	0.120465
67426	0.94613	0.104915
67427	0.94613	0.160781
67428	0.946131	0.392634
67429	0.946131	0.73467
67430	0.94613	0.139777
67431	0.94613	0.370435
67432	1.89226	0.108864
67433	1.89226	0.100314
67434	1.89226	0.13008
67435	1.89226	0.135279
67436	1.89226	0.152273
67437	1.89226	0.100649
67438	1.89226	0.689734
67439	1.89226	1.54044
67440	2.35239	1.50045
67441	2.27873	1.10375
67442	1.89226	1.476
67443	1.83301	1.24868
67444	2.68449	1.40527
67445	1.89226	1.81239
67446	1.89226	1.37363
67447	1.89226	1.50775
67448	3.78453	1.39799
67449	0.946132	1.76396
67450	0.946132	1.43069
67451	0.946129	1.24772

67452	0.946129	1.71478
67453	0.946131	1.7194
67454	0.946131	1.60208
67455	0.94613	1.42816
67456	0.94613	1.4798
67457	0.946131	1.55046
67458	0.946131	1.49168
67459	0.946131	1.55373
67460	0.946131	1.60823
67461	1.89226	0.928608
67462	1.89226	1.43589
67463	1.89226	1.49265
67464	1.89226	1.22949
67465	1.89226	1.47405
67466	1.89226	1.26156
67467	1.70303	1.00289
67468	1.89226	1.48471
67469	1.89226	0.719912
67470	1.89226	1.22219
67471	3.78453	0.892717
67472	1.89227	1.45927
67473	1.89227	1.51014
67474	1.89227	1.2095
67475	1.89227	1.65413
67476	3.78453	1.52444
67477	1.89226	1.41687
67478	1.89226	1.81614
67479	1.89226	1.436
67480	1.89226	1.59354
67481	3.78452	1.50288
67482	1.89226	2.03607
67483	1.89226	2.1
67484	1.89226	2.1
67485	1.89226	2.007
67486	3.78452	2.01779
67487	0.946132	1.92172
67488	0.946132	2.03193
67489	0.946129	2.1
67490	0.946129	2.1
67491	0.94613	2.09942
67492	0.94613	1.97812
67493	0.946131	2.1
67494	0.946131	1.99036
67495	0.946131	2.01134
67496	0.946131	2.07517
67497	0.946131	2.04432
67498	0.946131	1.94528
67499	1.89226	2.09663
67500	1.89226	1.87436
67501	1.89226	1.94082
67502	1.89226	2.06482
67503	3.78452	2.06487
67504	1.89226	1.76206
67505	1.89226	2.01616
67506	1.89226	2.08836
67507	1.89226	1.79023
67508	1.89226	2.07598
67509	1.89226	1.78757

67510	1.89227	1.99632
67511	2.48308	1.77658
67512	1.89227	1.78567
67513	1.89227	1.91192
67514	3.78453	1.96137
67515	1.89226	1.85974
67516	1.89226	1.98929
67517	1.89226	2.06556
67518	1.89226	1.84436
67519	1.89226	2.0365
67520	1.89226	1.82105
67521	1.89226	1.94245
67522	1.89226	1.93326
67523	1.89226	1.96972
67524	1.89226	1.94551
67525	3.78453	2.03576
67526	0.946131	1.94782
67527	0.946131	2.01657
67528	0.946131	2.03363
67529	0.946131	2.09902
67530	0.946131	2.03117
67531	0.946131	1.99278
67532	0.946131	1.99939
67533	0.946131	2.02852
67534	0.946131	1.99279
67535	0.946131	2.06973
67536	0.946131	2.01896
67537	0.946131	1.93795
67538	1.23719	2.09508
67539	1.28439	1.96669
67540	0.94613	1.96502
67541	0.94613	2.0351
67542	1.28439	2.07528
67543	1.6431	2.1
67544	0.946129	1.93387
67545	0.946129	1.92258
67546	1.28439	1.89344
67547	0.946131	1.83828
67548	1.21038	1.82224
67549	1.21038	1.92913
67550	1.89226	1.89555
67551	1.89226	1.85454
67552	1.89226	1.89641
67553	1.89226	1.85498
67554	1.89226	1.85847
67555	1.89226	1.83344
67556	1.89226	1.86467
67557	1.89226	1.94134
67558	1.89226	2.02794
67559	1.89226	1.83863
67560	1.89226	1.97431
67561	1.89226	1.80613
67562	1.89226	1.36499
67563	1.89226	0.513607
67564	1.89226	0.628209
67565	1.89226	1.2085
67566	1.89226	1.15864
67567	1.89226	1.39264

67568	0.946128	0.645411
67569	0.946128	1.14836
67570	0.946125	0.301312
67571	0.946125	0.603239
67572	0.94613	0.364363
67573	0.94613	0.493448
67574	0.946124	0.835274
67575	0.946124	1.21074
67576	0.946127	0.427774
67577	0.946127	0.337766
67578	0.946127	0.760047
67579	0.946127	1.15405
67580	1.89226	0.265868
67581	2.78671	0.350101
67582	2.59302	0.300154
67583	2.59302	0.326979
67584	1.89226	0.426339
67585	1.89226	0.419446
67586	1.89226	0.372395
67587	1.89226	0.474765
67588	1.89226	0.297982
67589	1.89226	0.305418
67590	1.89226	0.724898
67591	1.89226	0.69445
67592	1.89225	0.312142
67593	1.89225	0.16258
67594	1.89225	0.406401
67595	1.89225	0.301693
67596	1.89225	0.57614
67597	1.89225	0.230923
67598	0.979536	0.261029
67599	0.816597	0.324531
67600	1.15446	0.247819
67601	1.07423	0.199008
67602	1.08452	0.254449
67603	0.603522	0.230677
67604	1.70302	0.225479
67605	1.29344	0.192171
67606	1.21806	0.212771
67607	0.723758	0.193237
67608	1.37562	0.284493
67609	1.89226	0.0934561
67610	1.92807	0.103423
67611	1.90843	0.10787
67612	1.92807	0.0983188
67613	1.87263	0.109605
67614	1.89226	0.102445
67615	0.946132	0.10011
67616	0.946132	0.103421
67617	0.946131	0.101657
67618	0.946131	0.105453
67619	0.946132	0.10704
67620	0.946132	0.116223
67621	0.946132	0.110719
67622	0.946132	0.103362
67623	0.946132	0.0924386
67624	0.946132	0.098159
67625	0.946132	0.105762

67626	0.946132	0.106677
67627	1.89226	0.112578
67628	1.89226	0.322861
67629	1.89226	0.143014
67630	1.89226	0.117972
67631	3.78452	0.172282
67632	1.89226	0.094032
67633	1.89226	0.135413
67634	1.89226	0.275369
67635	1.89226	0.124946
67636	1.89226	0.100566
67637	1.89226	0.0960955
67638	1.70303	0.0854406
67639	1.90843	0.131869
67640	1.77002	0.0988401
67641	1.90843	0.0881002
67642	1.75709	0.0916402
67643	1.89226	0.0904945
67644	1.89226	0.0980037
67645	1.89226	0.108608
67646	1.89226	0.0916033
67647	1.89226	0.112306
67648	3.78453	0.0957595
67649	1.89226	0.25362
67650	1.89226	0.314884
67651	1.89226	0.106226
67652	1.89226	0.337531
67653	3.78452	0.263885
67654	0.946131	0.122701
67655	0.946131	0.0869692
67656	0.946131	0.0894402
67657	0.946131	0.0903082
67658	0.946129	0.0938839
67659	0.946129	0.133646
67660	0.946133	0.198793
67661	0.946133	0.123472
67662	0.946131	0.113748
67663	0.946131	0.0788912
67664	0.946131	0.105309
67665	0.946131	0.353031
67666	1.89226	0.149205
67667	2.10275	0.517699
67668	1.76377	0.38043
67669	1.76377	0.229159
67670	1.89226	0.296688
67671	1.89226	0.185056
67672	1.89226	0.164887
67673	1.89226	0.603399
67674	2.10275	0.383906
67675	2.55889	0.229886
67676	1.89226	0.334708
67677	2.30274	0.208891
67678	1.89226	0.374206
67679	1.89226	1.30329
67680	1.89226	1.26778
67681	1.89226	0.624965
67682	1.89226	1.23047
67683	1.89226	0.631573

67684	1.89226	0.310462
67685	1.89226	1.2866
67686	1.89226	1.23988
67687	1.89226	0.663196
67688	1.89226	1.19901
67689	1.89226	0.589502
67690	1.89226	0.149984
67691	1.89226	0.636398
67692	1.89226	0.368676
67693	1.89226	0.209809
67694	1.89226	0.320223
67695	1.89226	0.180937
67696	1.89226	0.16479
67697	1.89226	0.622128
67698	1.89226	0.47083
67699	1.89226	0.241878
67700	1.89226	0.411632
67701	1.89226	0.218042
67702	1.89226	0.43878
67703	1.89226	1.29869
67704	1.89226	1.25323
67705	1.89226	0.762289
67706	1.89226	1.23209
67707	1.89226	0.833416
67708	1.89226	1.19974
67709	1.89226	1.26375
67710	1.89226	0.367807
67711	1.89226	0.967808
67712	1.89226	0.397483
67713	1.89226	1.18642
67714	0.946131	0.172084
67715	0.976399	0.155973
67716	1.10124	0.122742
67717	0.946131	0.286062
67718	0.887633	0.266751
67719	0.887633	0.222686
67720	1.16933	0.172469
67721	1.23163	0.14826
67722	0.946131	0.202968
67723	0.946131	0.207838
67724	0.976399	0.206418
67725	1.14992	0.205772
67726	1.89226	0.29713
67727	1.89226	0.935393
67728	1.89226	0.278498
67729	1.89226	0.38791
67730	3.78452	0.270402
67731	1.89226	0.169704
67732	1.89226	0.357331
67733	1.89226	0.147874
67734	1.89226	0.234266
67735	3.78453	0.162582
67736	0.946132	0.113508
67737	0.946132	0.287458
67738	0.946131	0.187956
67739	0.946131	0.255042
67740	0.946131	0.25577
67741	0.946131	0.210755

67742	0.946132	0.106267
67743	0.946132	0.120454
67744	0.946131	0.147197
67745	0.946131	0.191024
67746	0.946132	0.179842
67747	0.946132	0.122197
67748	0.946132	0.351724
67749	0.946132	0.210206
67750	0.946131	0.266455
67751	0.946131	0.448092
67752	0.946131	0.466142
67753	0.946131	0.374884
67754	0.946132	0.234227
67755	0.946132	0.197398
67756	0.946131	0.270658
67757	0.946131	0.377006
67758	0.946132	0.338319
67759	0.946132	0.238896
67760	1.89226	0.429737
67761	1.89226	0.736832
67762	1.89226	0.257313
67763	1.89226	0.805259
67764	3.78452	0.737007
67765	1.89226	1.08633
67766	1.89226	1.07007
67767	1.89226	0.901869
67768	1.89226	0.53713
67769	3.78453	0.992535
67770	1.89226	0.447572
67771	1.89226	1.08322
67772	1.89226	0.926117
67773	1.89226	1.11781
67774	1.89226	0.408897
67775	1.89226	1.06741
67776	1.89225	0.305256
67777	1.89225	0.527581
67778	1.89225	0.493575
67779	1.89225	1.07446
67780	1.89225	0.219487
67781	1.89225	0.790908
67782	1.89226	0.694923
67783	1.89226	0.76657
67784	1.89226	0.627946
67785	1.89226	1.05087
67786	1.89226	0.265388
67787	1.89226	0.569977
67788	1.89226	0.875546
67789	1.89226	0.414341
67790	1.89226	0.190386
67791	1.89226	0.182371
67792	3.78453	0.235482
67793	1.39491	0.284257
67794	1.39491	0.275399
67795	0.924649	0.310232
67796	0.924649	0.341602
67797	0.946131	0.39747
67798	1.37343	0.181405
67799	0.924678	0.10247

67800	0.946131	1.22167
67801	0.924678	0.246293
67802	1.37343	0.187509
67803	0.946131	0.71903
67804	0.946131	0.464225
67805	1.89226	0.490127
67806	1.89226	0.135787
67807	1.89226	0.2271
67808	1.89226	0.401839
67809	1.89226	0.118406
67810	1.89226	0.215674
67811	0.946126	0.0551481
67812	0.975461	0.0560239
67813	0.946132	0.0532105
67814	1.12674	0.0681019
67815	0.946129	0.0706673
67816	0.946129	0.0676804
67817	1.12673	0.0566197
67818	1.12673	0.0543167
67819	0.946129	0.0565261
67820	0.946129	0.0683863
67821	0.975464	0.0653621
67822	1.12673	0.0638088
67823	1.89226	0.0754415
67824	1.89226	0.0697769
67825	1.89226	0.0793334
67826	1.89226	0.0735273
67827	1.89226	0.0745998
67828	1.89226	0.0746087
67829	0.946126	0.0749234
67830	0.946126	0.0769501
67831	0.946131	0.0666462
67832	0.946131	0.106724
67833	0.946129	0.108455
67834	0.946129	0.0949814
67835	0.946128	0.0753793
67836	0.946128	0.0729813
67837	0.946129	0.0802211
67838	0.946129	0.0999069
67839	0.946128	0.090143
67840	0.946128	0.0897218
67841	1.89226	0.0600353
67842	1.89226	0.0896024
67843	1.89226	0.057489
67844	1.89226	0.064885
67845	3.78451	0.0579901
67846	1.89226	0.0777539
67847	1.89226	0.0814707
67848	1.89226	0.063677
67849	1.89226	0.0829798
67850	3.78452	0.0848993
67851	0.946125	0.0742473
67852	0.946125	0.0799618
67853	0.946131	0.0737482
67854	0.946131	0.12232
67855	0.946128	0.126264
67856	0.946128	0.114151
67857	0.946128	0.0835717

67858	0.946128	0.0737759
67859	0.946128	0.0813919
67860	0.946128	0.105239
67861	0.946128	0.0799727
67862	0.946128	0.0981092
67863	1.89226	0.0917659
67864	1.89226	0.117921
67865	1.89226	0.0906016
67866	1.89226	0.0749049
67867	3.78452	0.0887727
67868	1.89226	0.0968703
67869	1.89226	0.105216
67870	1.89226	0.109315
67871	1.89226	0.144026
67872	1.89226	0.0859949
67873	1.89226	0.106489
67874	1.12492	0.14677
67875	1.41201	0.16418
67876	1.12082	0.134215
67877	0.771416	0.128775
67878	1.10725	0.116473
67879	1.10725	0.139537
67880	1.07618	0.253155
67881	1.07618	0.118049
67882	1.12492	0.145713
67883	0.771415	0.189587
67884	1.12082	0.146372
67885	1.41201	0.166243
67886	2.82401	0.106469
67887	1.89226	0.107868
67888	1.89226	0.100921
67889	1.89226	0.128582
67890	2.24983	0.0972484
67891	2.24983	0.11958
67892	0.946133	0.0962001
67893	0.946133	0.105703
67894	0.946129	0.0837635
67895	0.946129	0.0789402
67896	0.946131	0.0744573
67897	0.946131	0.132926
67898	0.946131	0.0963403
67899	0.946131	0.0799659
67900	0.946131	0.0896424
67901	0.946131	0.0876726
67902	0.946131	0.0952237
67903	0.946131	0.100847
67904	0.946132	0.131194
67905	0.946132	0.123626
67906	0.946133	0.111149
67907	0.946133	0.112236
67908	0.946132	0.103371
67909	0.946132	0.177104
67910	0.946133	0.119428
67911	0.946133	0.141754
67912	0.946132	0.11999
67913	0.946132	0.127286
67914	0.946133	0.123402
67915	0.946133	0.120274

67916	1.89227	0.333549
67917	1.89227	0.379189
67918	1.89227	0.392424
67919	1.89227	0.314362
67920	1.89227	0.418659
67921	1.89227	0.336948
67922	1.42982	0.133586
67923	1.19944	0.0963292
67924	0.936311	0.173966
67925	0.936311	0.137574
67926	0.946132	0.143174
67927	1.02993	0.142461
67928	1.41513	0.15522
67929	0.946133	0.167051
67930	0.946133	0.139676
67931	0.946133	0.164504
67932	1.02993	0.165072
67933	1.18181	0.12591
67934	1.89226	0.395692
67935	1.89226	0.52082
67936	1.89226	0.599066
67937	1.89226	0.290232
67938	3.78453	0.405079
67939	1.89226	0.180765
67940	1.89226	0.213391
67941	1.89226	0.225524
67942	1.89226	0.183759
67943	1.89226	0.219428
67944	1.89226	0.180471
67945	1.89226	0.176855
67946	1.89226	0.188408
67947	1.89226	0.200122
67948	1.89226	0.167759
67949	1.89226	0.203099
67950	1.89226	0.171836
67951	0.946131	0.295229
67952	0.946131	0.402073
67953	0.946132	0.54433
67954	0.946132	0.397802
67955	0.946132	0.300881
67956	0.946132	0.345668
67957	0.946132	0.549396
67958	0.946132	0.29892
67959	0.946132	0.337405
67960	0.946132	0.361989
67961	0.946132	0.367181
67962	0.946132	0.319201
67963	1.89226	1.4571
67964	1.89226	1.47618
67965	1.89226	1.54037
67966	1.89226	1.35458
67967	1.89226	1.54734
67968	1.89226	1.40392
67969	1.89226	1.51612
67970	2.22749	0.819911
67971	1.89226	1.01998
67972	1.89226	1.07116
67973	3.78453	1.10227

67974	1.89226	1.43909
67975	1.89226	1.47982
67976	1.89226	1.54739
67977	1.89226	1.32431
67978	3.78453	1.42251
67979	1.89226	1.30195
67980	1.89226	1.27238
67981	1.89226	1.36831
67982	1.89226	0.901178
67983	1.89226	1.42305
67984	1.89226	1.27196
67985	0.94613	1.02237
67986	0.94613	1.40216
67987	0.94613	1.49461
67988	0.94613	1.35244
67989	0.946131	1.04492
67990	0.946131	0.829462
67991	0.946129	1.47894
67992	0.946129	1.28527
67993	0.94613	1.25739
67994	0.94613	1.24647
67995	0.94613	1.25435
67996	0.94613	1.1492
67997	1.89226	0.0900708
67998	1.89226	0.0969027
67999	1.89226	0.0949545
68000	1.89226	0.0894285
68001	1.89226	0.0923833
68002	1.89226	0.0873203
68003	1.89226	0.101867
68004	1.89226	0.148514
68005	1.89226	0.142106
68006	1.89226	0.0786373
68007	3.78452	0.113312
68008	1.89225	0.0665955
68009	1.89225	0.065447
68010	1.89225	0.0644715
68011	1.89225	0.0648149
68012	1.89225	0.0673697
68013	1.89225	0.0685608
68014	1.89226	0.0618419
68015	1.89226	0.0594598
68016	1.89226	0.0622889
68017	1.89226	0.0635331
68018	1.89226	0.0618677
68019	1.89226	0.0636877
68020	0.946132	0.0806846
68021	0.946132	0.102455
68022	0.946132	0.139286
68023	0.946132	0.0988783
68024	0.946131	0.0857379
68025	0.946131	0.0802746
68026	0.946132	0.144784
68027	0.946132	0.0776758
68028	0.946132	0.08315
68029	0.946132	0.093993
68030	0.946132	0.0988312
68031	0.946132	0.0901674

68032	1.89226	0.0838338
68033	1.89226	0.124536
68034	1.89226	0.0872822
68035	1.89226	0.184453
68036	1.89226	0.0768105
68037	1.89226	0.151414
68038	1.89226	0.0934071
68039	1.89226	0.0749882
68040	1.89226	0.0722814
68041	1.89226	0.124778
68042	1.89226	0.0606744
68043	1.89226	0.107459
68044	1.89226	0.0590306
68045	1.89226	0.0704563
68046	1.89226	0.0639257
68047	1.89226	0.0856981
68048	1.89226	0.0522597
68049	1.89226	0.0689414
68050	1.89226	0.0889886
68051	1.89226	0.143761
68052	1.89226	0.0760478
68053	1.89226	0.160949
68054	3.78452	0.100601
68055	1.89226	0.0706338
68056	1.89226	0.0881168
68057	1.89226	0.0684332
68058	1.89226	0.150409
68059	1.89226	0.06477
68060	1.89226	0.121578
68061	1.89226	0.05
68062	1.89226	0.05
68063	1.89226	0.05
68064	1.89226	0.05
68065	3.78453	0.05
68066	1.89226	0.05
68067	2.48862	0.05
68068	1.89226	0.05
68069	2.22062	0.05
68070	1.89226	0.05
68071	2.22062	0.05
68072	1.89227	0.05
68073	1.89227	0.050859
68074	1.89227	0.05
68075	1.89227	0.05
68076	1.89227	0.05
68077	1.89227	0.05
68078	0.946132	0.05
68079	0.946132	0.05
68080	0.946131	0.05
68081	0.946131	0.05
68082	0.94613	0.05
68083	0.94613	0.05
68084	0.946133	0.05
68085	0.946133	0.05
68086	0.946131	0.05
68087	0.946131	0.05
68088	0.946131	0.05
68089	0.946131	0.05

68090	1.89226	0.05
68091	1.89226	0.05
68092	1.89226	0.05
68093	1.89226	0.05
68094	3.78453	0.05
68095	1.89226	0.05
68096	1.89226	0.0540916
68097	1.89226	0.05
68098	1.89226	0.05
68099	3.78453	0.05
68100	1.89226	0.120825
68101	1.89226	0.0780311
68102	1.89226	0.0550881
68103	1.89226	0.111248
68104	1.89226	0.0858688
68105	1.89226	0.129829
68106	1.89226	0.134133
68107	1.89226	0.059791
68108	1.89226	0.0724191
68109	1.89226	0.102729
68110	3.78452	0.101339
68111	1.89226	0.070184
68112	1.89226	0.168276
68113	1.89226	0.104871
68114	1.89226	0.0766736
68115	3.78453	0.0681963
68116	2.57454	0.160355
68117	1.89226	0.141463
68118	1.3663	0.129926
68119	1.89226	0.150198
68120	3.25856	0.13308
68121	1.89226	0.126267
68122	1.89226	0.103598
68123	1.89226	0.102475
68124	1.89226	0.118552
68125	3.78451	0.130694
68126	1.89226	0.0978005
68127	1.89226	0.160087
68128	1.89226	0.101959
68129	1.89226	0.124162
68130	1.89226	0.08332
68131	1.89226	0.0944454
68132	0.810946	0.114225
68133	0.810946	0.112989
68134	0.810946	0.111432
68135	0.810946	0.15875
68136	0.810959	0.112595
68137	0.810959	0.112943
68138	1.89226	0.137557
68139	1.35168	0.135058
68140	1.08132	0.118953
68141	1.08132	0.1287
68142	1.0813	0.136166
68143	1.89226	0.0990923
68144	1.89226	0.328036
68145	1.89226	0.400744
68146	1.89226	0.25174
68147	3.78453	0.155452

68148	1.89226	0.104545
68149	1.89226	0.220829
68150	1.89226	0.0987554
68151	1.89226	0.310877
68152	1.89226	0.0785891
68153	1.89226	0.101842
68154	1.89226	0.149018
68155	1.89226	0.227886
68156	1.89226	0.310663
68157	1.89226	0.138531
68158	3.78452	0.106504
68159	1.89226	0.266381
68160	1.89226	0.271891
68161	1.89226	0.265083
68162	1.89226	0.407045
68163	3.78452	0.322614
68164	2.142	0.443876
68165	1.93949	0.114631
68166	1.39296	0.33104
68167	2.55711	0.502236
68168	1.39296	0.122357
68169	2.55711	0.202703
68170	1.89226	0.167453
68171	2.5571	0.0995946
68172	2.142	0.281684
68173	1.89226	0.428371
68174	4.44936	0.324823
68175	0.946131	0.255645
68176	0.946131	0.17547
68177	1.27855	0.304324
68178	1.29171	0.2809
68179	1.29171	0.322639
68180	0.946127	0.302622
68181	1.27855	0.0973948
68182	0.946131	0.388259
68183	1.29172	0.342337
68184	1.29172	0.203718
68185	0.946129	0.178238
68186	0.946129	0.16973
68187	1.27855	0.232458
68188	1.29172	0.304121
68189	0.969743	0.367888
68190	1.07109	0.353471
68191	1.29172	0.526143
68192	1.07109	0.132596
68193	0.969745	0.107392
68194	1.27855	0.444734
68195	1.10873	0.447915
68196	1.10873	0.378904
68197	1.15273	0.202157
68198	1.15273	0.150452
68199	1.89227	0.0540721
68200	1.89227	0.05
68201	1.89227	0.05
68202	1.89227	0.05
68203	2.1288	0.05
68204	2.1288	0.05
68205	1.89227	0.0661854

68206	1.89227	0.0511217
68207	1.89227	0.05
68208	1.89227	0.0635501
68209	3.78453	0.05
68210	1.89226	0.150106
68211	1.89226	0.212199
68212	1.89226	0.0645597
68213	1.89226	0.06644
68214	3.78453	0.149943
68215	1.89226	0.112536
68216	1.89226	0.0524557
68217	1.89226	0.0512458
68218	1.89226	0.0584573
68219	1.89226	0.05
68220	1.89226	0.0530283
68221	1.94432	0.05
68222	1.89226	0.05
68223	2.07911	0.05
68224	1.89226	0.05
68225	2.07911	0.05
68226	1.89226	0.05
68227	1.89226	0.05
68228	1.89226	0.05
68229	1.89226	0.05
68230	1.89226	0.05
68231	1.89226	0.05
68232	1.89226	0.05
68233	1.89226	0.726977
68234	1.89226	0.699743
68235	1.89226	0.790286
68236	1.89226	0.477102
68237	1.89226	1.0821
68238	1.89226	0.691745
68239	1.89226	0.356959
68240	1.89226	0.292494
68241	1.89226	0.340436
68242	1.89226	0.258123
68243	1.89226	0.460548
68244	1.89226	0.349284
68245	1.86778	0.2574
68246	2.72348	0.2388
68247	1.89226	0.283844
68248	1.89226	0.154043
68249	3.76004	0.260884
68250	1.89226	0.866042
68251	1.89226	0.802826
68252	1.89226	1.29628
68253	1.89226	0.474186
68254	3.78451	0.897904
68255	1.89226	0.378715
68256	1.89226	0.339308
68257	1.89226	0.367137
68258	1.89226	0.27033
68259	1.89226	0.515147
68260	1.89226	0.368427
68261	0.94613	0.212944
68262	0.94613	0.296515
68263	0.94613	0.495412

68264	0.94613	0.25564
68265	0.946131	0.269664
68266	0.946131	0.171866
68267	0.946129	0.316624
68268	0.946129	0.263119
68269	0.94613	0.249587
68270	0.94613	0.27459
68271	0.94613	0.248868
68272	0.94613	0.25889
68273	0.946124	0.434724
68274	0.946124	0.250749
68275	0.94613	1.00602
68276	0.94613	0.412047
68277	0.946128	0.297011
68278	0.946128	0.3264
68279	0.946126	0.887423
68280	0.946126	0.452331
68281	0.946127	0.446631
68282	0.946127	0.423284
68283	0.946127	0.376608
68284	0.946127	0.379067
68285	2.09166	0.05
68286	2.1285	0.05
68287	1.78381	0.05
68288	1.78381	0.0516222
68289	2.47689	0.05
68290	2.47689	0.05
68291	2.09167	0.05
68292	2.26572	0.05
68293	2.73393	0.05
68294	2.4769	0.05
68295	4.74261	0.05
68296	1.89226	0.05
68297	1.89226	0.0717071
68298	1.89226	0.0606725
68299	1.89226	0.0536371
68300	1.89226	0.0659701
68301	1.89226	0.061952
68302	0.946131	0.0533905
68303	0.946131	0.0768394
68304	0.946129	0.0580543
68305	0.946129	0.0675809
68306	0.94613	0.0663327
68307	0.94613	0.0681614
68308	0.946131	0.05
68309	0.946131	0.0508647
68310	0.94613	0.0531933
68311	0.94613	0.0593382
68312	0.946131	0.0562725
68313	0.946131	0.0621078
68314	2.27622	0.0505309
68315	2.66786	0.05
68316	2.1285	0.05
68317	2.1285	0.0518284
68318	2.62461	0.05
68319	2.47689	0.05
68320	1.89226	0.0511212
68321	1.89226	0.0713347

68322	1.89226	0.05
68323	1.89226	0.069177
68324	3.78453	0.0636272
68325	1.89226	0.0505162
68326	1.89226	0.0674076
68327	1.89226	0.0660015
68328	1.89226	0.0729384
68329	1.89226	0.05
68330	1.89226	0.0567383
68331	1.89226	0.328773
68332	1.89226	0.681596
68333	1.89226	0.295012
68334	1.89226	0.871141
68335	1.89226	0.290803
68336	1.89226	0.856661
68337	1.89226	1.32363
68338	1.89226	0.555633
68339	1.89226	0.88547
68340	1.89226	1.33005
68341	3.78452	1.04211
68342	1.89227	0.687436
68343	1.89227	1.26845
68344	1.89227	1.32418
68345	1.89227	0.900598
68346	3.78453	1.19167
68347	0.946127	0.979981
68348	0.946127	0.98804
68349	1.06438	0.43126
68350	0.946131	0.449133
68351	1.06438	0.338302
68352	0.94613	0.808343
68353	0.946128	0.987036
68354	0.946128	0.326629
68355	1.06438	0.399925
68356	1.06438	0.54616
68357	0.946129	0.754937
68358	0.946129	0.681739
68359	1.89226	0.655287
68360	1.89226	1.32161
68361	1.89226	0.93083
68362	1.89226	1.29161
68363	1.89226	0.845733
68364	1.89226	1.27101
68365	0.946133	1.23445
68366	0.946133	1.26874
68367	1.39771	1.22255
68368	0.946132	1.10811
68369	1.39771	1.2099
68370	1.39772	1.27816
68371	0.946133	1.26933
68372	0.946133	0.555151
68373	0.946132	0.836091
68374	1.39772	1.00309
68375	0.946132	0.517579
68376	0.946132	1.04292
68377	1.98182	0.05
68378	2.2217	0.05
68379	2.79889	0.05

68380	2.50097	0.05
68381	4.72267	0.05
68382	2.05362	0.05
68383	1.70302	0.05
68384	2.75109	0.05
68385	2.138 0.05	
68386	2.0815	0.05
68387	1.84751	0.05
68388	0.946131	0.0506171
68389	0.946131	0.05
68390	0.946131	0.05
68391	0.946131	0.05
68392	0.946131	0.052531
68393	0.946131	0.05
68394	0.946132	0.05
68395	0.946132	0.05
68396	0.946131	0.05
68397	0.946131	0.05
68398	0.946132	0.05
68399	0.946132	0.05
68400	1.89226	0.05
68401	1.89226	0.0576822
68402	1.89226	0.05
68403	1.89226	0.0552592
68404	3.78451	0.054233
68405	0.946126	0.05
68406	1.25048	0.05
68407	0.946131	0.05
68408	1.39945	0.05
68409	0.946127	0.05
68410	0.946127	0.05
68411	1.39944	0.05
68412	1.39944	0.05
68413	0.946129	0.05
68414	0.946129	0.05
68415	1.39944	0.05
68416	1.25048	0.05
68417	1.89226	0.05
68418	1.89226	0.0541299
68419	1.89226	0.05
68420	1.89226	0.05
68421	1.89226	0.05
68422	1.89226	0.05
68423	0.946126	0.0570991
68424	0.946126	0.05
68425	0.946131	0.05
68426	0.946131	0.0535484
68427	0.946128	0.0569431
68428	0.946128	0.054435
68429	0.946129	0.05
68430	0.946129	0.05
68431	0.946129	0.05
68432	0.946129	0.05
68433	0.946128	0.05
68434	0.946128	0.0520332
68435	1.89226	0.05
68436	1.89226	0.05
68437	1.89226	0.05

68438	1.89226	0.05
68439	1.89226	0.05
68440	1.89226	0.05
68441	1.01393	0.05
68442	0.775245	0.05
68443	0.946126	0.05
68444	0.946126	0.05
68445	0.78305	0.05
68446	0.78305	0.05
68447	0.946159	0.05
68448	0.929267	0.05
68449	1.14956	0.05
68450	1.04073	0.05
68451	0.851526	0.05
68452	0.704781	0.05
68453	1.89226	0.05
68454	1.89226	0.05
68455	1.89226	0.05
68456	1.89226	0.05
68457	3.78451	0.05
68458	1.89226	0.05
68459	1.89226	0.0556052
68460	1.89226	0.05
68461	1.89226	0.05
68462	1.89226	0.05
68463	1.89226	0.05
68464	1.89226	0.05
68465	1.89226	0.05
68466	1.89226	0.05
68467	1.89226	0.05
68468	1.89226	0.05
68469	1.89226	0.05
68470	1.16317	0.05
68471	1.0292	0.05
68472	0.946126	0.05
68473	0.946126	0.05
68474	0.93562	0.05
68475	0.93562	0.05
68476	1.0418	0.05
68477	1.04181	0.05
68478	1.16317	0.05
68479	0.946129	0.05
68480	1.02919	0.05
68481	0.946128	0.05
68482	0.946131	0.05
68483	0.946131	0.05
68484	0.946126	0.05
68485	0.946126	0.05
68486	0.946129	0.05
68487	0.946129	0.05
68488	0.946128	0.05
68489	0.946128	0.05
68490	0.946129	0.05
68491	0.946129	0.05
68492	0.946128	0.05
68493	0.946128	0.05
68494	1.89226	0.05
68495	1.89226	0.0554007

68496	1.89226	0.05
68497	1.89226	0.05
68498	1.89226	0.05
68499	1.89226	0.0503329
68500	1.89226	0.05
68501	1.89226	0.05
68502	1.89226	0.05
68503	1.89226	0.05
68504	1.89226	0.05
68505	1.89226	0.05
68506	1.89226	0.05
68507	1.89226	0.05
68508	1.89226	0.05
68509	1.89226	0.05
68510	3.78453	0.05
68511	1.89226	0.05
68512	1.89226	0.05
68513	1.89226	0.05
68514	1.89226	0.05
68515	3.78452	0.05
68516	1.89225	0.05
68517	1.89225	0.05
68518	1.89225	0.05
68519	1.89225	0.05
68520	1.89225	0.05
68521	1.89225	0.05
68522	0.946132	0.05
68523	0.946132	0.05
68524	0.946133	0.05
68525	0.946133	0.05
68526	0.946132	0.05
68527	0.946132	0.05
68528	0.946132	0.05
68529	0.946132	0.05
68530	0.946133	0.05
68531	0.946133	0.05
68532	0.946132	0.05
68533	0.946132	0.05
68534	1.89226	0.05
68535	1.89226	0.05
68536	1.89226	0.05
68537	1.89226	0.05
68538	3.78453	0.05
68539	1.13537	0.05
68540	1.09222	0.05
68541	0.946126	0.05
68542	0.946126	0.05
68543	0.946128	0.05
68544	0.946128	0.05
68545	1.16317	0.05
68546	1.13034	0.05
68547	1.30155	0.05
68548	1.08462	0.05
68549	0.992915	0.05
68550	0.807642	0.05
68551	0.946131	0.05
68552	0.946131	0.05
68553	0.946125	0.05

68554	0.946125	0.05
68555	0.946128	0.05
68556	0.946128	0.05
68557	0.946128	0.05
68558	0.946128	0.05
68559	0.946128	0.05
68560	0.946128	0.05
68561	0.946128	0.05
68562	0.946128	0.05
68563	1.89226	0.05
68564	1.89226	0.0546077
68565	1.89226	0.0512128
68566	1.89226	0.0525425
68567	1.89226	0.05
68568	1.89226	0.050358
68569	1.89224	0.05
68570	1.89224	0.05
68571	1.89224	0.05
68572	1.89224	0.05
68573	1.89224	0.05
68574	1.89224	0.05
68575	0.946086	0.05
68576	1.17919	0.05
68577	0.89738	0.05
68578	0.89738	0.05
68579	0.946116	0.05
68580	0.946116	0.05
68581	1.13048	0.05
68582	1.11844	0.05
68583	0.946128	0.05
68584	0.897362	0.05
68585	1.1282	0.05
68586	0.946103	0.05
68587	1.89223	0.05
68588	1.89223	0.05
68589	1.89223	0.05
68590	1.89223	0.05
68591	3.78446	0.05
68592	0.946118	0.0507328
68593	0.946118	0.05
68594	0.946118	0.0534267
68595	0.946118	0.0555841
68596	0.946119	0.0559126
68597	0.946119	0.05
68598	0.946118	0.0506145
68599	0.946118	0.0546596
68600	0.946131	0.0540415
68601	0.946131	0.0534566
68602	0.946106	0.0508735
68603	0.946106	0.0506364
68604	1.89227	0.46848
68605	1.89227	0.97802
68606	1.89227	0.869015
68607	1.89227	0.667736
68608	3.78453	0.634743
68609	1.89226	1.04608
68610	1.89226	0.497751
68611	1.89226	0.487522

68612	1.89226	0.71522
68613	1.89226	0.776407
68614	1.89226	1.01737
68615	1.89226	0.324614
68616	2.59378	0.223007
68617	2.30348	0.221673
68618	2.30348	0.31296
68619	1.89226	0.26647
68620	1.89226	0.401728
68621	1.89226	0.664627
68622	1.89226	0.453211
68623	1.89226	0.444644
68624	1.89226	0.701179
68625	3.78453	0.683378
68626	1.89226	0.604188
68627	1.89226	0.396008
68628	1.89226	0.365015
68629	1.89226	0.608016
68630	1.89226	0.446037
68631	1.89226	0.932443
68632	0.904406	0.225085
68633	1.01667	0.246873
68634	1.11974	0.212989
68635	0.994417	0.276496
68636	1.02013	0.252329
68637	0.628203	0.274446
68638	1.70302	0.293467
68639	1.49898	0.249825
68640	1.14559	0.283587
68641	0.900571	0.246963
68642	1.58886	0.250421
68643	2.81102	0.05
68644	2.3923	0.05
68645	2.05632	0.05
68646	2.43514	0.05
68647	4.82744	0.05
68648	1.89226	0.05
68649	1.89226	0.05
68650	1.89226	0.05
68651	1.89226	0.05
68652	1.89226	0.053258
68653	1.89226	0.05
68654	1.89226	0.05
68655	1.89226	0.0526217
68656	1.89226	0.05
68657	1.89226	0.0558209
68658	3.78451	0.05
68659	2.50803	0.05
68660	1.903	0.05
68661	1.903	0.05
68662	2.19162	0.05
68663	2.09934	0.05
68664	2.39063	0.05
68665	1.00243	0.05
68666	0.731731	0.05
68667	0.876616	0.05
68668	1.00451	0.05
68669	0.986901	0.05

68670	0.986901	0.05
68671	0.887729	0.05
68672	0.887732	0.05
68673	0.876605	0.05
68674	0.769283	0.05
68675	1.00245	0.05
68676	1.18712	0.05
68677	0.946131	0.05
68678	0.946131	0.05
68679	0.946131	0.05
68680	0.946131	0.05
68681	0.946131	0.05
68682	0.946131	0.05
68683	0.946131	0.05
68684	0.946131	0.05
68685	0.946131	0.05
68686	0.946131	0.05
68687	0.946131	0.05
68688	0.946131	0.05
68689	1.89226	0.05
68690	1.89226	0.0536202
68691	1.89226	0.0502475
68692	1.89226	0.0517793
68693	1.89226	0.05
68694	1.89226	0.0505396
68695	1.89226	0.0667069
68696	1.89226	0.106075
68697	1.89226	0.0814259
68698	1.89226	0.089216
68699	3.78453	0.0949537
68700	1.89226	0.109017
68701	1.89226	0.117349
68702	1.89226	0.0933658
68703	1.89226	0.178237
68704	3.78453	0.105575
68705	1.89226	0.0651195
68706	1.89226	0.103958
68707	1.89226	0.0812661
68708	1.89226	0.0809827
68709	1.89226	0.087517
68710	1.89226	0.087772
68711	0.946132	0.0923848
68712	0.946132	0.0694693
68713	0.946131	0.0884847
68714	0.946131	0.0714881
68715	0.946131	0.0904263
68716	0.946131	0.10138
68717	0.946132	0.0663444
68718	0.946132	0.0690721
68719	0.946131	0.0807113
68720	0.946131	0.0712714
68721	0.946131	0.0688914
68722	0.946131	0.0759467
68723	0.946131	0.146449
68724	0.946131	0.0976675
68725	0.946131	0.118724
68726	0.946131	0.0962765
68727	0.94613	0.126788

68728	0.94613	0.152551
68729	0.946132	0.088975
68730	0.946132	0.100096
68731	0.946131	0.104875
68732	0.946131	0.119822
68733	0.946131	0.1094
68734	0.946131	0.109835
68735	1.89226	0.05
68736	1.89226	0.0583586
68737	1.89226	0.0638104
68738	1.89226	0.05
68739	1.89226	0.0600591
68740	1.89226	0.05
68741	1.89226	0.0607511
68742	1.89226	0.0598372
68743	1.89226	0.0559734
68744	1.89226	0.0675202
68745	3.78453	0.0508159
68746	1.89226	0.0642045
68747	1.89226	0.0708106
68748	1.89226	0.10986
68749	1.89226	0.0662691
68750	1.89226	0.0959135
68751	1.89226	0.0606639
68752	0.946132	0.0524444
68753	0.946132	0.05
68754	0.946131	0.0589836
68755	0.946131	0.0570926
68756	0.946132	0.0549399
68757	0.946132	0.05
68758	0.946131	0.05
68759	0.946131	0.05
68760	0.946132	0.0568753
68761	0.946132	0.0584719
68762	0.946131	0.0506266
68763	0.946131	0.0510304
68764	0.946131	0.0605308
68765	0.946131	0.0598137
68766	0.946132	0.0733907
68767	0.946132	0.0779264
68768	0.946132	0.0703761
68769	0.946132	0.0546181
68770	0.946131	0.0592977
68771	0.946131	0.0634617
68772	0.946132	0.0711863
68773	0.946132	0.0702702
68774	0.946131	0.0619762
68775	0.946131	0.0658146
68776	1.89226	0.525688
68777	1.89226	0.731719
68778	1.89226	0.843907
68779	1.89226	0.443682
68780	3.78452	0.461829
68781	1.89226	1.32795
68782	1.89226	1.05266
68783	1.89226	1.25369
68784	1.89226	1.27864
68785	3.78452	1.31615

68786	1.89226	0.958489
68787	1.89226	1.01365
68788	1.89226	1.26498
68789	1.89226	0.609665
68790	3.78452	0.646138
68791	1.89226	0.584908
68792	2.75945	0.235313
68793	1.89226	0.364662
68794	2.35026	0.354048
68795	4.24252	0.607963
68796	0.946131	0.453863
68797	0.946131	0.543587
68798	0.946131	0.323837
68799	0.946131	0.371955
68800	0.946132	0.319026
68801	0.946132	0.359043
68802	0.946129	0.569228
68803	0.946129	0.374208
68804	0.946131	0.462451
68805	0.946131	0.459079
68806	0.94613	0.565233
68807	0.94613	0.448146
68808	1.89226	0.970482
68809	1.89226	1.16396
68810	1.89226	0.830053
68811	1.89226	1.14446
68812	1.89226	0.894432
68813	1.89226	1.23725
68814	0.946129	0.530835
68815	0.946129	0.732682
68816	0.946133	0.667816
68817	0.946133	0.427799
68818	0.946131	0.396071
68819	0.946131	0.41993
68820	0.946131	0.753166
68821	0.946131	0.460966
68822	0.946131	0.620443
68823	0.946131	0.585504
68824	0.946131	0.747388
68825	0.946131	0.551804
68826	1.89226	0.264407
68827	2.35026	0.243086
68828	1.32126	0.215329
68829	1.89226	0.267341
68830	1.77925	0.217573
68831	2.35026	0.263704
68832	1.89226	0.0663379
68833	1.89226	0.121002
68834	1.89226	0.105804
68835	1.89226	0.0796008
68836	1.89226	0.0824797
68837	1.89226	0.06911
68838	1.89226	0.096247
68839	1.89226	0.201879
68840	1.89226	0.134
68841	1.89226	0.184997
68842	3.78453	0.188802
68843	1.89226	0.0799026

68844	1.89226	0.170442
68845	1.89226	0.138088
68846	1.89226	0.113619
68847	1.89226	0.120183
68848	1.89226	0.0947679
68849	0.946131	0.17027
68850	0.946131	0.106575
68851	0.946131	0.113305
68852	0.946131	0.128613
68853	0.946132	0.173136
68854	0.946132	0.185072
68855	0.946131	0.125665
68856	0.946131	0.108039
68857	0.946132	0.0996011
68858	0.946132	0.148768
68859	0.946131	0.11209
68860	0.946131	0.137086
68861	1.89226	0.0752687
68862	1.89226	0.136442
68863	1.89226	0.0930267
68864	1.89226	0.0858727
68865	1.89226	0.143744
68866	1.89226	0.145148
68867	1.89226	0.112155
68868	1.89226	0.181333
68869	1.89226	0.149865
68870	1.89226	0.163831
68871	3.78453	0.170657
68872	1.89227	0.186483
68873	1.89227	0.179988
68874	1.89227	0.166347
68875	1.89227	0.271689
68876	3.78453	0.175861
68877	1.89226	0.102431
68878	1.89226	0.150703
68879	1.89226	0.139003
68880	1.89226	0.137508
68881	3.78452	0.141321
68882	0.946132	0.146995
68883	0.946132	0.119888
68884	0.946131	0.138422
68885	0.946131	0.121853
68886	0.946131	0.149782
68887	0.946131	0.160857
68888	0.946133	0.128568
68889	0.946133	0.106552
68890	0.946132	0.113311
68891	0.946132	0.135895
68892	0.946131	0.111124
68893	0.946131	0.126655
68894	1.89227	0.149524
68895	1.89227	0.217555
68896	1.89227	0.152868
68897	1.89227	0.158776
68898	1.89227	0.199283
68899	1.89227	0.204599
68900	2.20491	0.122108
68901	1.81656	0.120467

68902	1.81656	0.120703
68903	1.89226	0.129222
68904	1.89226	0.103198
68905	1.89226	0.123942
68906	1.89226	0.192108
68907	1.89226	0.2691
68908	1.89226	0.247343
68909	1.89226	0.214123
68910	3.78452	0.217574
68911	1.89226	0.434129
68912	1.89226	0.366838
68913	1.89226	0.339877
68914	1.89226	0.592318
68915	3.78453	0.432959
68916	1.89226	0.364869
68917	1.89226	0.516868
68918	1.89226	0.402661
68919	1.89226	0.481666
68920	1.89226	0.426418
68921	1.89226	0.530841
68922	1.89226	0.207326
68923	2.11032	0.17505
68924	2.30793	0.177201
68925	1.89226	0.195833
68926	2.30793	0.187539
68927	1.89226	0.204849
68928	1.89226	0.286364
68929	1.89226	0.419098
68930	1.89226	0.308166
68931	1.89226	0.359084
68932	1.89226	0.342798
68933	1.89226	0.424092
68934	0.810943	0.379848
68935	0.810943	0.348181
68936	0.810943	0.367067
68937	0.810943	0.32783
68938	0.810933	0.35764
68939	0.810933	0.356271
68940	1.89226	0.285738
68941	1.3517	0.351761
68942	1.08132	0.327203
68943	1.08132	0.40559
68944	1.08133	0.351169
68945	1.89226	0.367878
68946	1.89226	0.178579
68947	1.89226	0.234493
68948	1.89226	0.31147
68949	3.78453	0.327143
68950	1.89226	0.16123
68951	1.89226	0.168275
68952	1.89226	0.221243
68953	1.89226	0.133112
68954	1.89226	0.210818
68955	1.89226	0.122986
68956	0.810945	0.147938
68957	0.810945	0.114252
68958	0.870829	0.145988
68959	0.870829	0.123793

68960	0.990502	0.104866
68961	0.810933	0.122648
68962	2.82777	0.0856347
68963	1.53126	0.117622
68964	1.32076	0.108642
68965	1.201	0.115399
68966	1.08133	0.118408
68967	1.89226	0.219632
68968	1.89226	0.129068
68969	1.89226	0.160747
68970	1.89226	0.153241
68971	1.89226	0.16778
68972	1.89226	0.160025
68973	0.946132	0.192955
68974	0.946132	0.22272
68975	0.946133	0.411888
68976	0.946133	0.256871
68977	0.946132	0.315162
68978	0.946132	0.232359
68979	0.946132	0.279504
68980	0.946132	0.331199
68981	0.946133	0.260606
68982	0.946133	0.218537
68983	0.946132	0.191305
68984	0.946132	0.245844
68985	0.946133	0.10985
68986	0.946133	0.130963
68987	0.946132	0.187192
68988	0.946132	0.216374
68989	0.946132	0.183039
68990	0.946132	0.138323
68991	0.946132	0.166446
68992	0.946132	0.227069
68993	0.946133	0.124717
68994	0.946133	0.157274
68995	0.946132	0.135695
68996	0.946132	0.160982
68997	1.89226	0.132548
68998	1.89226	0.254386
68999	1.89226	0.189075
69000	1.89226	0.190334
69001	1.89226	0.159037
69002	1.89226	0.162956
69003	1.89226	0.200632
69004	1.89226	0.401867
69005	1.89226	0.290143
69006	1.89226	0.36716
69007	3.78452	0.421126
69008	1.89226	0.409368
69009	1.89226	0.44103
69010	1.89226	0.380828
69011	1.89226	0.84109
69012	3.78452	0.371944
69013	1.89226	0.186716
69014	1.89226	0.329623
69015	1.89226	0.215905
69016	1.89226	0.246351
69017	1.89226	0.327917

69018	1.89226	0.337792
69019	0.946128	0.264561
69020	0.946128	0.221842
69021	0.946132	0.238881
69022	0.946132	0.214926
69023	0.946129	0.290523
69024	0.946129	0.317996
69025	0.946131	0.216136
69026	0.946131	0.182015
69027	0.94613	0.259037
69028	0.94613	0.155113
69029	0.946131	0.183223
69030	0.946131	0.222043
69031	0.946129	0.417899
69032	0.946129	0.324191
69033	0.946133	0.348461
69034	0.946133	0.30156
69035	0.946131	0.413601
69036	0.946131	0.445229
69037	0.946131	0.272154
69038	0.946131	0.298711
69039	0.946131	0.398859
69040	0.946131	0.29064
69041	0.946131	0.321616
69042	0.946131	0.373601
69043	1.89226	0.209066
69044	1.89226	0.149372
69045	1.89226	0.156843
69046	1.89226	0.326012
69047	3.78452	0.141941
69048	1.89225	0.0595205
69049	1.89225	0.104617
69050	1.89225	0.110025
69051	1.89225	0.118284
69052	1.89225	0.0568642
69053	1.89225	0.0545052
69054	1.89226	0.102891
69055	1.89226	0.069277
69056	1.89226	0.089343
69057	1.89226	0.127117
69058	3.78452	0.0878057
69059	1.89226	0.148187
69060	1.89226	0.360187
69061	1.89226	0.33415
69062	1.89226	0.244831
69063	3.78453	0.299345
69064	1.89226	0.0952987
69065	1.89226	0.176519
69066	1.89226	0.163509
69067	1.89226	0.141184
69068	3.78452	0.17999
69069	1.22164	0.05
69070	1.22164	0.05
69071	0.806032	0.058492
69072	0.806032	0.05
69073	1.08154	0.0518826
69074	0.946126	0.05
69075	0.946129	0.05

69076	0.806065	0.0627227
69077	0.806063	0.05
69078	1.08155	0.0536264
69079	0.946128	0.05
69080	0.946128	0.0626607
69081	1.28554	0.0581942
69082	1.28554	0.0527794
69083	0.946126	0.0529909
69084	0.946126	0.0801284
69085	1.28554	0.0809724
69086	0.946129	0.052973
69087	1.08155	0.0523266
69088	0.94613	0.0533058
69089	1.08155	0.0549062
69090	1.28554	0.0601877
69091	0.94613	0.0549817
69092	0.94613	0.058598
69093	0.946132	0.127273
69094	0.946132	0.192495
69095	0.946132	0.21453
69096	0.946132	0.285179
69097	0.946132	0.38653
69098	0.946132	0.165855
69099	0.946133	0.155825
69100	0.946133	0.151938
69101	0.946132	0.193961
69102	0.946132	0.202701
69103	0.946133	0.183009
69104	0.946133	0.191262
69105	1.89226	1.2863
69106	1.89226	0.737791
69107	1.89226	1.35718
69108	1.89226	0.840628
69109	3.78453	1.26545
69110	1.89226	1.15674
69111	1.89226	0.584069
69112	1.89226	0.676104
69113	1.89226	0.830515
69114	1.89226	0.75271
69115	1.89226	0.899445
69116	1.89226	0.336113
69117	1.89226	0.228593
69118	1.89226	0.251729
69119	1.89226	0.275814
69120	1.89226	0.276115
69121	1.89226	0.300305
69122	1.89226	0.441255
69123	1.89226	0.27183
69124	1.89226	0.322642
69125	1.89226	0.398991
69126	1.89226	0.348483
69127	1.89226	0.286101
69128	0.946132	0.666623
69129	0.946132	0.776464
69130	0.946132	0.433053
69131	0.946132	0.750959
69132	0.946132	0.459057
69133	0.946132	0.486592

69134	0.946132	0.704121
69135	0.946132	0.627063
69136	0.946131	0.369602
69137	0.946131	0.677575
69138	0.946132	0.541474
69139	0.946132	0.501499
69140	1.89226	0.577192
69141	1.89226	0.31482
69142	1.89226	0.595519
69143	1.89226	0.435598
69144	3.78453	0.46396
69145	0.851541	0.145371
69146	0.72883	0.199922
69147	0.991276	0.209755
69148	0.851485	0.169491
69149	1.00003	0.169717
69150	1.00003	0.140747
69151	0.809788	0.191791
69152	0.809788	0.189053
69153	0.890738	0.193875
69154	0.991264	0.16912
69155	0.851554	0.172902
69156	0.851554	0.184701
69157	0.8628	0.253585
69158	0.78387	0.190738
69159	0.830233	0.239307
69160	0.830233	0.262852
69161	0.808312	0.240548
69162	0.817238	0.16987
69163	1.70303	0.181043
69164	1.24116	0.209084
69165	0.995299	0.168608
69166	1.0989	0.218817
69167	1.02714	0.248828
69168	1.89226	1.2685
69169	1.89226	1.02315
69170	1.89226	0.891066
69171	1.89226	1.96865
69172	3.78451	0.766566
69173	0.946131	1.06911
69174	0.946131	0.443909
69175	0.946131	0.780701
69176	0.946131	0.819975
69177	0.94613	0.701925
69178	0.94613	0.441003
69179	0.946132	0.422251
69180	0.946132	0.516833
69181	0.946131	0.557908
69182	0.946131	0.773379
69183	0.946131	0.540717
69184	0.946131	0.455527
69185	1.89226	0.85022
69186	1.89226	2.1
69187	1.89226	1.88229
69188	1.89226	1.89354
69189	3.78453	2.08788
69190	1.89226	2.1
69191	1.89226	2.1

69192	1.89226	2.1
69193	1.89226	1.91609
69194	1.89226	2.1
69195	1.89226	1.8919
69196	0.946126	0.50676
69197	1.17111	0.17861
69198	1.3393	0.77773
69199	0.946131	0.837469
69200	0.946127	0.857859
69201	0.946127	0.335889
69202	1.3393	0.406182
69203	1.17111	0.419643
69204	1.3393	0.561933
69205	1.3393	0.130541
69206	0.946129	0.393188
69207	0.946129	0.60275
69208	0.946126	2.1
69209	0.946126	1.44083
69210	0.946132	2.1
69211	0.946132	2.1
69212	0.94613	2.1
69213	0.94613	1.94494
69214	0.946129	2.1
69215	0.946129	1.96695
69216	0.946129	0.92351
69217	0.946129	2.1
69218	0.94613	1.93286
69219	0.94613	1.90502
69220	0.946131	0.976903
69221	0.946131	0.767312
69222	0.94613	0.630521
69223	0.94613	0.624692
69224	0.946128	0.980487
69225	0.946128	1.10694
69226	0.946132	0.550192
69227	0.946132	0.609023
69228	0.94613	0.925014
69229	0.94613	0.521499
69230	0.94613	0.664589
69231	0.94613	0.970038
69232	0.94613	1.82932
69233	0.94613	1.63428
69234	0.94613	1.59609
69235	0.94613	1.48564
69236	0.946128	1.81415
69237	0.946128	1.85803
69238	0.946131	1.44071
69239	0.946131	1.36063
69240	0.94613	1.78409
69241	0.94613	1.41711
69242	0.94613	1.53093
69243	0.94613	1.73508
69244	1.89226	1.86597
69245	1.89226	1.6936
69246	1.89226	1.75246
69247	1.89226	2.1
69248	3.78453	1.75926
69249	1.89226	1.30116

69250	1.89226	1.34905
69251	1.89226	0.943894
69252	1.89226	1.30998
69253	1.89226	1.07396
69254	1.89226	1.40306
69255	1.89226	1.22865
69256	1.89226	1.72765
69257	1.89226	1.3662
69258	1.89226	1.69836
69259	1.89226	0.93184
69260	1.89226	1.34719
69261	1.89227	0.456248
69262	2.28622	0.508971
69263	2.16109	0.38683
69264	1.89227	0.396929
69265	4.17849	0.401798
69266	15.1381	0.463774
69267	15.1381	1.03624
69268	15.1381	0.181082
69269	15.1381	0.863927
69270	30.2762	0.77136
69271	15.1381	0.875409
69272	15.1381	1.01272
69273	15.1381	0.173051
69274	15.1381	0.956223
69275	30.2762	0.189952
69276	15.1381	0.912165
69277	15.1381	1.16248
69278	15.1381	0.172912
69279	15.1381	1.28089
69280	30.2762	1.02795
69281	15.1381	0.289385
69282	15.1381	1.11798
69283	15.1381	0.0750498
69284	15.1381	0.892738
69285	30.2762	0.849453
69286	15.138	1.36896
69287	15.138	1.82394
69288	15.138	1.35204
69289	15.138	1.99879
69290	30.276	1.83561
69291	15.138	1.86676
69292	15.138	2.09865
69293	15.138	2.03296
69294	15.138	2.1
69295	30.276	1.93114
69296	15.138	1.65548
69297	15.138	1.54484
69298	15.138	1.39989
69299	15.138	2.03698
69300	30.2759	1.9045
69301	15.138	1.59711
69302	15.138	1.9229
69303	15.138	2.07966
69304	15.138	1.54011
69305	30.2759	1.69795
69306	15.138	2.1
69307	15.138	2.01994

69308	15.138	2.1
69309	15.138	2.09028
69310	30.276	2.09617
69311	15.1381	2.0791
69312	15.1381	2.1
69313	15.1381	2.0805
69314	15.1381	2.1
69315	30.2762	2.1
69316	15.1381	2.1
69317	15.1381	2.1
69318	15.1381	2.1
69319	15.1381	2.1
69320	30.2762	2.1
69321	15.138	1.96097
69322	15.138	2.09417
69323	15.138	2.07626
69324	15.138	2.07591
69325	30.276	2.08183
69326	15.138	2.1
69327	15.138	2.04236
69328	15.138	2.1
69329	15.138	2.1
69330	30.276	2.07474
69331	15.1381	1.61271
69332	15.1381	2.03117
69333	15.1381	1.93741
69334	15.1381	1.87251
69335	30.2762	1.95122
69336	15.1381	1.97672
69337	15.1381	2.09423
69338	15.1381	2.00883
69339	15.1381	2.07395
69340	30.2762	2.00576
69341	15.1381	2.1
69342	15.1381	2.06373
69343	15.1381	2.1
69344	15.1381	2.0754
69345	30.2762	2.1
69346	15.138	0.991911
69347	15.138	0.86387
69348	15.138	0.0661798
69349	15.138	1.16819
69350	30.276	0.703164
69351	1.89226	2.1
69352	1.89226	2.1
69353	1.89226	2.1
69354	1.89226	2.1
69355	3.78453	2.1
69356	1.89226	2.1
69357	1.89226	2.1
69358	1.89226	2.1
69359	1.89226	2.1
69360	1.89226	2.1
69361	1.89226	2.1
69362	1.89226	2.1
69363	1.89226	2.1
69364	1.89226	2.1
69365	1.89226	2.1

69366	1.89226	2.1
69367	1.89226	2.1
69368	1.89226	2.1
69369	1.89226	2.1
69370	1.89226	2.1
69371	1.89226	2.1
69372	3.78453	2.1
69373	1.89226	2.1
69374	1.89226	2.1
69375	1.89226	2.1
69376	1.89226	2.1
69377	1.89226	2.1
69378	1.89226	2.1
69379	0.946127	2.1
69380	0.946127	2.1
69381	0.946131	2.1
69382	0.946131	2.1
69383	0.946131	2.1
69384	0.946131	2.1
69385	0.946128	2.1
69386	0.946128	2.1
69387	0.94613	2.1
69388	0.94613	2.1
69389	0.946129	2.1
69390	0.946129	2.1
69391	1.89226	2.1
69392	1.89226	2.1
69393	1.89226	2.1
69394	1.89226	2.1
69395	1.89226	2.1
69396	1.89226	2.1
69397	1.89226	2.1
69398	1.89226	2.1
69399	1.89226	2.1
69400	1.89226	2.1
69401	3.78452	2.1
69402	1.89226	2.1
69403	1.89226	2.1
69404	1.89226	2.1
69405	1.89226	2.1
69406	1.89226	2.1
69407	1.89226	2.1
69408	0.946129	2.1
69409	0.946129	2.1
69410	0.946133	2.1
69411	0.946133	2.1
69412	0.946131	2.1
69413	0.946131	2.1
69414	0.946131	2.1
69415	0.946131	2.1
69416	0.946131	2.1
69417	0.946131	2.1
69418	0.946131	2.1
69419	0.946131	2.1
69420	0.946132	2.1
69421	0.946132	2.1
69422	0.946132	2.1
69423	0.946132	2.1

69424	0.946132	2.1
69425	0.946132	2.1
69426	0.946133	2.1
69427	0.946133	2.1
69428	0.946132	2.1
69429	0.946132	2.1
69430	0.946133	2.1
69431	0.946133	2.1
69432	1.89226	2.1
69433	1.89226	2.1
69434	1.89226	2.1
69435	1.89226	2.1
69436	3.78452	2.1
69437	1.89226	2.1
69438	1.89226	2.1
69439	1.89226	2.1
69440	1.89226	2.1
69441	1.89226	2.1
69442	1.89226	2.1
69443	0.946129	2.1
69444	0.946129	2.1
69445	0.946133	2.1
69446	0.946133	2.1
69447	0.946131	2.1
69448	0.946131	2.1
69449	0.946131	2.1
69450	0.946131	2.1
69451	0.946131	2.1
69452	0.946131	2.1
69453	0.946131	2.1
69454	0.946131	2.1
69455	0.946132	2.1
69456	0.946132	2.1
69457	0.946132	2.1
69458	0.946132	2.1
69459	0.946132	2.1
69460	0.946132	2.1
69461	0.946133	2.1
69462	0.946133	2.1
69463	0.946133	2.1
69464	0.946133	2.1
69465	0.946132	2.1
69466	0.946132	2.1
69467	1.89226	2.1
69468	1.89226	2.1
69469	1.89226	2.1
69470	1.89226	2.1
69471	3.78453	2.1
69472	0.946128	2.1
69473	0.946128	2.1
69474	0.946132	2.1
69475	0.946132	2.1
69476	0.94613	2.1
69477	0.94613	2.1
69478	0.94613	2.1
69479	0.94613	2.1
69480	0.94613	2.1
69481	0.94613	2.1

69482	0.94613	2.1
69483	0.94613	2.1
69484	0.946127	2.1
69485	0.946127	2.1
69486	0.946131	2.1
69487	0.946131	2.1
69488	0.94613	2.1
69489	0.94613	2.1
69490	0.946128	2.1
69491	0.946128	2.1
69492	0.946129	2.1
69493	0.946129	2.1
69494	0.946129	2.1
69495	0.946129	2.1
69496	0.94613	2.1
69497	0.94613	2.1
69498	0.94613	2.1
69499	0.94613	2.1
69500	0.946131	2.1
69501	0.946131	2.1
69502	0.946129	2.1
69503	0.946129	2.1
69504	0.94613	2.1
69505	0.94613	2.1
69506	0.94613	2.1
69507	0.94613	2.1
69508	1.89226	2.1
69509	1.89226	2.1
69510	1.89226	2.1
69511	1.89226	2.1
69512	3.78453	2.1
69513	1.89226	2.1
69514	1.89226	2.1
69515	1.89226	2.1
69516	1.89226	2.1
69517	1.89226	2.1
69518	1.89226	2.1
69519	1.89226	2.1
69520	1.89226	2.1
69521	1.89226	2.1
69522	1.89226	2.1
69523	1.89226	2.1
69524	1.89226	2.1
69525	0.946131	2.1
69526	0.946131	2.1
69527	0.946131	2.1
69528	0.946131	2.1
69529	0.946132	2.1
69530	0.946132	2.1
69531	0.946129	2.1
69532	0.946129	2.1
69533	0.946131	2.1
69534	0.946131	2.1
69535	0.94613	2.1
69536	0.94613	2.1
69537	1.89224	2.1
69538	1.89224	2.1
69539	1.89224	2.1

69540	1.89224	2.1
69541	3.78448	2.1
69542	1.89224	2.1
69543	1.89224	2.1
69544	1.89224	2.1
69545	1.89224	2.1
69546	3.78447	2.1
69547	1.89223	2.1
69548	2.38995	2.1
69549	2.25315	2.1
69550	2.25315	2.1
69551	1.89223	2.1
69552	1.89223	2.1
69553	1.89224	2.1
69554	1.89224	2.1
69555	1.89224	2.1
69556	1.89224	2.1
69557	1.89224	2.1
69558	1.89224	2.1
69559	1.89226	2.1
69560	1.89226	2.1
69561	1.89226	2.1
69562	1.89226	2.1
69563	3.78453	2.1
69564	1.89226	2.1
69565	1.89226	2.1
69566	1.89226	2.1
69567	1.89226	2.1
69568	1.89226	2.1
69569	1.89226	2.1
69570	1.89226	2.1
69571	2.60272	2.1
69572	2.38998	2.1
69573	2.38998	2.1
69574	1.89226	2.1
69575	1.89226	2.1
69576	0.94613	2.1
69577	0.94613	2.1
69578	0.94613	2.1
69579	0.94613	2.1
69580	0.946131	2.1
69581	0.946131	2.1
69582	0.946129	2.1
69583	0.946129	2.1
69584	0.94613	2.1
69585	0.94613	2.1
69586	0.94613	2.1
69587	0.94613	2.1
69588	1.89226	2.1
69589	1.89226	2.1
69590	1.89226	2.1
69591	1.89226	2.1
69592	3.78452	2.1
69593	1.89226	2.1
69594	1.89226	2.1
69595	1.89226	2.1
69596	1.89226	2.1
69597	3.78452	2.1

69598	1.89226	2.1
69599	1.89226	2.1
69600	1.89226	2.1
69601	1.89226	2.1
69602	1.89226	2.1
69603	1.89226	2.1
69604	0.946132	2.1
69605	0.946132	2.1
69606	0.946132	2.1
69607	0.946132	2.1
69608	0.946131	2.1
69609	0.946131	2.1
69610	0.946132	2.1
69611	0.946132	2.1
69612	0.946132	2.1
69613	0.946132	2.1
69614	0.946132	2.1
69615	0.946132	2.1
69616	1.89226	2.1
69617	1.89226	2.1
69618	1.89226	2.1
69619	1.89226	2.1
69620	3.78452	2.1
69621	1.89226	2.1
69622	1.89226	2.1
69623	1.89226	2.1
69624	1.89226	2.1
69625	3.78452	2.1
69626	0.946129	2.1
69627	0.946129	2.1
69628	0.946133	2.1
69629	0.946133	2.1
69630	0.946131	2.1
69631	0.946131	2.1
69632	0.946131	2.1
69633	0.946131	2.1
69634	0.946131	2.1
69635	0.946131	2.1
69636	0.946131	2.1
69637	0.946131	2.1
69638	0.946132	2.1
69639	0.946132	2.1
69640	0.946132	2.1
69641	0.946132	2.1
69642	0.946132	2.1
69643	0.946132	2.1
69644	0.946133	2.1
69645	0.946133	2.1
69646	0.946133	2.1
69647	0.946133	2.1
69648	0.946132	2.1
69649	0.946132	2.1
69650	1.89224	2.1
69651	1.89224	2.1
69652	1.89224	2.1
69653	1.89224	2.1
69654	3.78447	2.1
69655	1.89223	2.1

69656	1.89223	2.1
69657	1.89223	2.1
69658	1.89223	2.1
69659	1.89223	2.1
69660	1.89223	2.1
69661	1.89224	2.1
69662	1.89224	2.1
69663	1.89224	2.1
69664	1.89224	2.1
69665	3.78447	2.1
69666	1.89224	2.1
69667	1.89224	2.1
69668	1.89224	2.1
69669	1.89224	2.1
69670	3.78448	2.1
69671	1.89226	2.1
69672	1.89226	2.1
69673	1.89226	2.1
69674	1.89226	2.1
69675	3.78452	2.1
69676	1.89226	2.1
69677	1.89226	2.1
69678	1.89226	2.1
69679	1.89226	2.1
69680	1.89226	2.1
69681	1.89226	2.1
69682	1.89226	2.1
69683	1.89226	2.1
69684	1.89226	2.1
69685	1.89226	2.1
69686	1.89226	2.1
69687	1.89226	2.1
69688	0.810946	2.1
69689	0.810946	2.1
69690	0.810946	2.1
69691	0.810946	2.1
69692	0.810933	2.1
69693	0.810933	2.1
69694	1.89226	2.1
69695	1.3517	2.1
69696	1.08132	2.1
69697	1.08132	2.1
69698	1.08133	2.1
69699	1.89226	2.1
69700	1.89226	2.1
69701	1.89226	2.1
69702	1.89226	2.1
69703	3.78453	2.1
69704	1.89226	2.1
69705	1.89226	2.1
69706	1.89226	2.1
69707	1.89226	2.1
69708	3.78452	2.1
69709	1.89226	2.1
69710	1.89226	2.1
69711	1.89226	2.1
69712	1.89226	2.1
69713	1.89226	2.1

69714	1.89226	2.1
69715	0.94613	2.1
69716	0.94613	2.1
69717	0.94613	2.1
69718	0.94613	2.1
69719	0.946131	2.1
69720	0.946131	2.1
69721	0.946129	2.1
69722	0.946129	2.1
69723	0.94613	2.1
69724	0.94613	2.1
69725	0.94613	2.1
69726	0.94613	2.1
69727	1.89226	2.1
69728	1.89226	2.1
69729	1.89226	2.1
69730	1.89226	2.1
69731	3.78453	2.1
69732	1.89226	2.1
69733	1.89226	2.1
69734	1.89226	2.1
69735	1.89226	2.1
69736	3.78452	2.1
69737	1.89226	2.1
69738	1.89226	2.1
69739	1.89226	2.1
69740	1.89226	2.1
69741	3.78452	2.1
69742	1.89226	2.1
69743	1.89226	2.1
69744	1.89226	2.1
69745	1.89226	2.1
69746	1.89226	2.1
69747	1.89226	2.1
69748	1.89226	2.1
69749	1.89226	2.1
69750	1.89226	2.1
69751	1.89226	2.1
69752	3.78452	2.1
69753	1.89226	2.1
69754	1.89226	2.1
69755	1.89226	2.1
69756	1.89226	2.1
69757	3.78452	2.1
69758	0.946128	2.1
69759	0.946128	2.1
69760	0.946133	2.1
69761	0.946133	2.1
69762	0.94613	2.1
69763	0.94613	2.1
69764	0.946131	2.1
69765	0.946131	2.1
69766	0.946131	2.1
69767	0.946131	2.1
69768	0.946131	2.1
69769	0.946131	2.1
69770	0.946132	2.1
69771	0.946132	2.1

69772	0.946132	2.1
69773	0.946132	2.1
69774	0.946131	2.1
69775	0.946131	2.1
69776	0.946132	2.1
69777	0.946132	2.1
69778	0.946132	2.1
69779	0.946132	2.1
69780	0.946132	2.1
69781	0.946132	2.1
69782	1.89226	2.1
69783	1.89226	2.1
69784	1.89226	2.1
69785	1.89226	2.1
69786	3.78452	2.1
69787	1.89226	2.1
69788	1.89226	2.1
69789	1.89226	2.1
69790	1.89226	2.1
69791	3.78452	2.1
69792	0.946129	2.1
69793	0.946129	2.1
69794	0.946133	2.1
69795	0.946133	2.1
69796	0.946131	2.1
69797	0.946131	2.1
69798	0.946131	2.1
69799	0.946131	2.1
69800	0.946131	2.1
69801	0.946131	2.1
69802	0.946131	2.1
69803	0.946131	2.1
69804	0.946132	2.1
69805	0.946132	2.1
69806	0.946132	2.1
69807	0.946132	2.1
69808	0.946132	2.1
69809	0.946132	2.1
69810	0.946133	2.1
69811	0.946133	2.1
69812	0.946132	2.1
69813	0.946132	2.1
69814	0.946133	2.1
69815	0.946133	2.1
69816	1.89226	2.1
69817	1.89226	2.1
69818	1.89226	2.1
69819	1.89226	2.1
69820	1.89226	2.1
69821	1.89226	2.1
69822	0.946128	2.1
69823	0.946128	2.1
69824	0.946133	2.1
69825	0.946133	2.1
69826	0.946131	2.1
69827	0.946131	2.1
69828	0.94613	2.1
69829	0.94613	2.1

69830	0.946131	2.1
69831	0.946131	2.1
69832	0.946131	2.1
69833	0.946131	2.1
69834	1.89226	2.1
69835	1.89226	2.1
69836	1.89226	2.1
69837	1.89226	2.1
69838	1.89226	2.1
69839	1.89226	2.1
69840	1.89226	2.1
69841	1.89226	2.1
69842	1.89226	2.1
69843	1.89226	2.1
69844	3.78453	2.1
69845	1.89226	2.1
69846	1.89226	2.1
69847	1.89226	2.1
69848	1.89226	2.1
69849	1.89226	2.1
69850	1.89226	2.1
69851	0.946127	2.1
69852	0.946127	2.1
69853	0.946131	2.1
69854	0.946131	2.1
69855	0.946131	2.1
69856	0.946131	2.1
69857	0.946127	2.1
69858	0.946127	2.1
69859	0.946129	2.1
69860	0.946129	2.1
69861	0.94613	2.1
69862	0.94613	2.1
69863	0.946131	2.1
69864	0.946131	2.1
69865	0.946131	2.1
69866	0.946131	2.1
69867	0.946133	2.1
69868	0.946133	2.1
69869	0.946128	2.1
69870	0.946128	2.1
69871	0.94613	2.1
69872	0.94613	2.1
69873	0.946131	2.1
69874	0.946131	2.1
69875	1.89226	2.1
69876	1.89226	2.1
69877	1.89226	2.1
69878	1.89226	2.1
69879	1.89226	2.1
69880	1.89226	2.1
69881	1.89226	2.1
69882	1.89226	2.1
69883	1.89226	2.1
69884	1.89226	2.1
69885	3.78452	2.1
69886	1.89226	2.1
69887	1.89226	2.1

69888	1.89226	2.1
69889	1.89226	2.1
69890	3.78452	2.1
[truncated: 429,610 more chars]
